
JOURNAL INFORMATION
==============================
NLM Title Abbreviation: Innov Aging
Journal ID: Innov Aging
NLM Journal ID: 101703706
Journal ID: innovateage

Innovation in Aging

EISSN: 2399-5300
Publisher: Oxford University Press

ARTICLE INFORMATION
==============================
PMCID: PMC10738147
DOI: 10.1093/geroni/igad104
Article ID: igad104
Article version: 1
Subjects: Full GSA 2023 Abstract Book Pdf, AcademicSubjects/SOC02600

Full GSA 2023 Abstract Book PDF

Publication date: 2023 Dec
Electronic publication date: 2023 Dec 21
Volume: 7
Issue: Suppl 1
Issue title: Program Abstracts from The GSA 2023 Annual Scientific Meeting, “Building Bridges > Catalyzing Research > Empowering All Ages”
First page: NP
Last page: NP
Copyright: © The Author(s) 2023. Published by Oxford University Press on behalf of The Gerontological Society of America.
Copyright year: 2023
License: This is an Open Access article distributed under the terms of the Creative Commons Attribution License (https://creativecommons.org/licenses/by/4.0/), which permits unrestricted reuse, distribution, and reproduction in any medium, provided the original work is properly cited.
License URL: https://creativecommons.org/licenses/by/4.0/

Page count: 1234

==============================

INNOVATION IN AGING®

GERONTOLOGICAL SOCIETY OF AMERICA®

Volume 7 • Issue S1

### Innovation in Aging

**Steven M. Albert, PhD, MS, FGSA, Editor-in-Chief**
University of Pittsburgh, USA
E-mail: smalbert@pitt.edu

**Jennifer Tehan Stanley, PhD, FGSA, Deputy Editor-in-Chief**
University of Akron, USA

**Karen J. Jung, MSW, Managing Editor**
The Gerontological Society of America, USA
Email: ia@geron.org

### Associate Editors

**Christine E. Bishop, PhD, FGSA, Associate Editor, Economics of Aging**
Brandeis University, USA
**Julie Blaskewicz Boron, PhD, FGSA, Associate Editor, Technology**
University of Nebraska – Omaha, USA
**Howard B. Degenholtz, PhD, FGSA, Associate Editor, Long-Term Services and Supports**
University of Pittsburgh, USA
**Nancy W. Glynn, PhD, FGSA, Associate Editor, Epidemiology of Aging**
University of Pittsburgh, USA
**Min-Ah Lee, PhD, Associate Editor, Relationships & Well-being**
Chung-Ang University, South Korea
**Amanda N. Leggett, PhD, FGSA, Associate Editor, Dementia and Caregiving**
Wayne State University, USA
**Lena K. Makaroun, MD, MS, Associate Editor, Geriatrics**
VA Pittsburgh Healthcare System, USA
University of Pittsburgh, USA
**Joshua D. Niznik, PharmD, PhD**
UNC Chapel Hill School of Medicine, USA
**Alison Phinney, PhD, RN, Associate Editor, Qualitative Research**
University of British Columbia, Canada
**J. Tina Savla, PhD, FGSA, Associate Editor, Methodology**
Virginia Tech, USA
**Roland J. Thorpe, Jr., PhD, FGSA, Associate Editor, Minority Health/Health Disparities**
Johns Hopkins Bloomberg School of Public Health, USA
**Noah J. Webster, PhD, FGSA, Associate Editor, Health Demography**
University of Michigan, USA

### Editorial Board

**Kathleen Abrahamson, RN, PhD, FGSA**
Purdue University, USA
**Tamara A. Baker, PhD, FGSA**
University of North Carolina – Chapel Hill, USA
**Scott R. Beach, PhD, FGSA**
University of Pittsburgh, USA
**Sara J. Czaja, PhD, FGSA**
Weill Cornell Medicine, USA
**Adam Davey, PhD, FGSA**
University of Delaware – Newark, USA
**Nancy R. Gee, PhD**
Virginia Commonwealth University, USA
**Megan Gilligan, PhD, FGSA**
Iowa State University, USA
**Allen Glicksman, PhD, FGSA**
NewCourtland, USA
**Ellen L. Idler, PhD, FGSA**
Emory University, USA
**Mary Ann Johnson, PhD**
University of Nebraska - Lincoln, USA
**Giyeon Kim, PhD, FGSA**
Chung-Ang University, South Korea
**TsuAnn Kuo, PhD**
Chung Shan Medical University, Taiwan
**Yeonjung Lee, PhD, MSW**
Chung-Ang University, South Korea
**Darren Liu, DrPH, FGSA**
West Virginia University, USA
**Hui Liu, PhD, FGSA**
Michigan State University, USA
**Mariana López-Ortega, PhD, MPP**
National Institute of Geriatrics, National Institutes of Health, Mexico
**Meghan K. Mattos, PhD, RN**
University of Virginia, USA
**Anne Collins McLaughlin, PhD**
North Carolina State University, USA
**David A. Nace, MD, MPH**
University of Pittsburgh, USA
**Lycia Tramujas Vasconcellos Neumann, PhD, MPA**
Highmark Wholecare, USA
**Marcia Ory, PhD, FGSA**
Texas A&M University, USA
**Richard Pak, PhD**
Clemson University, USA
**Karl Pillemer, PhD, FGSA**
Cornell University, USA
**Karen A. Roberto, PhD, FGSA**
Virginia Tech, USA
**Laura P. Sands, PhD, FGSA**
Virginia Tech, USA
**J. Jill Suitor, PhD, FGSA**
Purdue University, USA
**Robert Joseph Taylor, MSW, PhD**
University of Michigan, USA
**Jasmine L. Travers, PhD, RN**
New York University, USA
**Richard Winett, PhD**
Virginia Tech, USA
**Yao Yao, MD**
Peking University, China
**Jeremy B. Yorgason, PhD, FGSA**
Brigham Young University, USA
**W. Quin Yow, PhD**
Singapore University of Technology & Design, Singapore
**Anna Zajacova, PhD, FGSA**
Western University, Canada

### Honorary Editor-In-Chief

**Laura P. Sands, PhD, FGSA** (2017-2020)

### Program, Publications, and Products Committee

**Jennifer Ailshire, PhD, FGSA**
University of Southern California, USA
**Debra Bakerjian, FAAN, FGSA, MSN, NP, PhD**
University of California – Davis, USA
**Kara Dassel, PhD, FAGHE, FGSA**
University of Utah, USA
**Christine Fruhauf, PhD, FAGHE, FGSA**
Colorado State University, USA
**Rita Hu, MSW**
University of Michigan, USA
**Jung Kwak, PhD, MSW, FGSA**
University of Texas at Austin, USA
**Nathan K. LeBrasseur, PT, PhD**
Mayo Clinic, USA
**Judie Lieu, BA**
The Gerontological Society of America, USA
**Karen Lincoln, PhD, MA, MSW, FGSA**
University of California – Irvine, USA
**Elham Mahmoudi, PhD**
University of Michigan, USA
**Brianna Morgan, MSN, CRNP, ACHPN**
University of Pennsylvania, USA
**Tina Newsham, PhD, FAGHE**
University of North Carolina – Wilmington, USA
**Brandt Pence, PhD**
University of Memphis, USA
**Karon Phillips, MPH, PhD, FGSA**
Trust for America’s Health, USA
**Blanka Rogina, PhD, FGSA**
University of Connecticut Health, USA
**Tetyana Shippee, PhD, FGSA**
University of Minnesota, USA
**Mo-kyung Sin, PhD, FGSA**
Seattle University, USA
**Kaipeng Wang, MSW, PhD**
University of Denver, USA

Fellow of the Gerontological Society of America (FGSA)

### PROGRAM ABSTRACTS FROM THE GSA 2023 ANNUAL SCIENTIFIC MEETING

"Building Bridges > Catalyzing Research > Empowering All Ages"

Abstracts are arranged numerically by session and in the order of presentation within each session.
Abstracts are published as received.

### SESSION 1000 (SYMPOSIUM)

Abstract citation ID: igad104.0001

### EDUCATIONAL DISPARITIES IN COGNITIVE DECLINE AND DEMENTIA IN LATER LIFE

Chair: Pamela Herd

According to the most recent (2020) Lancet commission report, one of the most important determinants of Alzheimer's Disease and related dementias (ADRD) is educational attainment. For those aged 80 and older, the risk for dementia is three times lower for those with a college degree compared to those without a high school degree, with even larger educational disparities by ethnicity. Despite these large and persistent gaps, there is much we do not understand about the relationship between education and dementia. Moreover, we have yet to develop effective clinical treatments that substantially slow or reverse the course of dementia. In this context, unpacking the education—ADRD relationship may be key to understanding how to delay ADRD onset. This panel explores how genetic risk, early life conditions, ranging from cognition and the quality of schooling to mid life factors, may mediate and moderate the relationship between education and cognitive decline and dementia in later life. The papers also pay close attention to variance by gender and ethnicity.

Abstract citation ID: igad104.0002

### EARLY LIFE COGNITION, EDUCATION, AND DEMENTIA RISK

Pamela Herd[1], Victoria Williams[2], and Kamil Sicinski[2], *1. Georgetown University, Washington, District of Columbia, United States*, *2. University of Wisconsin-Madison, Madison, Wisconsin, United States*

Educational attainment is one of the strongest predictors of Alzheimer's and Related Dementias (ADRD). Yet, much remains unknown as to how early life factors, particularly early life cognitive function, and early life environments, such as family environments, help explain why education so robustly protects against later life dementia. Is the influence of educational attainment not so much the additional schooling, but rather antecedent factors, like early life cognition and family environments that both build cognitive reserve and predict subsequent educational attainment? In short, is education merely a proxy for these antecedent influences? Consequently, we use the Wisconsin Longitudinal Study, which includes 80 years of prospectively collected data on a sample of 1 in every 3 high school graduates, and a selected sibling, from the class of 1957. Sibling models, and the inclusion of prospectively collected early life cognitive function and educational histories, allows us to explore the role of these early life conditions and the role they play in shaping the relationship between education and ADRD. We find a robust independent influence of education on ADRD risk, early life family environments had little impact on the relationship. Adolescent cognition, however, which we show is influenced by family environments, does partly explain why educational attainment influences later life ADRD risk, though both adolescent IQ and education independently influence the risk for and resilience against dementia.

Abstract citation ID: igad104.0003

### SECONDARY SCHOOL CURRICULAR RIGOR AND COGNITIVE FUNCTION AT AGE 81

Sara Moorman, *Boston College, Boston, Massachusetts, United States*

A current goal is to identify how and for whom years of educational attainment translate into high cognitive function in later life. Recent work has found effects of school quality factors such as teacher experience and racial integration of schools. In this project, I examine secondary school curriculum. Not only is there variation in curriculum across school districts, but also adolescents within a single secondary school select different courses. Curricular opportunities and selections may change the developing brain in lasting ways, and/or may place the student on a path towards future academic and career opportunities that shape risk and resilience. I hypothesize that a rigorous secondary school curriculum positions students to attend college, which in turn promotes lifelong cognitive health. I tested this hypothesis by estimating multilevel structural equation models in data from the Wisconsin Longitudinal Study (WLS), which follows a randomly-selected one-third of all students who graduated from Wisconsin high schools in 1957 (N = 10,317). I created a latent measure of curricular rigor out of participants' highest secondary school math course and number of semesters of English, science, and foreign language. In 2020 (most participants were age 81), participants completed a phone-based cognitive screening (Telephone Interview for Cognitive Status-Modified; TICS-m). Net of school-level factors, parental socioeconomic status, and academic achievement, higher curricular rigor predicted higher cognitive function at age 81. Educational attainment significantly mediated this effect, and a significant direct effect also remained. Both educational attainment and curricular rigor are desirable targets for policy interventions.

Abstract citation ID: igad104.0004

### INTERSECTIONAL AND BIOSOCIAL PATHWAYS TO COGNITIVE FUNCTION AMONG OLDER HISPANICS AND LATINOS

Catherine Garcia[1], and Lauren Brown[2], *1. Syracuse University, Syracuse, New York, United States*, *2. University of Southern California, Los Angeles, California, United States*

© The Author(s) 2023. Published by Oxford University Press on behalf of the Gerontological Society of America.

Hispanics/Latinos are the fastest-growing segment of older adults in the United States (U.S.). They are at high risk for cognitive impairment (CI) and Alzheimer's disease and related dementias (ADRD). While increased research attention has been given to the socioeconomic and cultural factors that influence the prevention, diagnosis, and care provided to Hispanics/Latinos with CI and ADRD, there are still theoretical and methodological gaps in clarifying the mechanisms and pathways that produce ADRD inequities, including life course and multilevel mechanisms. Given the diverse origins of Hispanics/Latinos in the U.S. – in ethnic origin, birthplace, and current residence – it is crucial to understand how these social conditions get "under the skin" that influence cognitive function in older adulthood. Namely, it is important to unpack how different intersectional positions show different relationships with deprivation that influence cognitive and healthy aging. Thus, this study had two main objectives: (1) to examine the mediating role of physiological (dys)regulation between education and cognition, and (2) to understand for which Hispanic/Latino subgroups the interrelated relationships are stronger/weaker for. Correspondingly, we employed multigroup structural equation modeling to address our study objectives. We used data from the Health and Retirement Study (HRS) to discern and assess the effects between education, physiological factors, and cognitive function at baseline and follow-up by Hispanic/Latino subgroups (e.g., foreign-born Mexicans, U.S.-born Mexicans, island-born Puerto Ricans, foreign-born Cubans, etc.). The results from this study underscore the role of structural interventions and community engagement in reducing and eliminating cognitive health disparities among Hispanics/Latinos.

Abstract citation ID: igad104.0005

### STATE-LEVEL EDUCATION CONTEXT AS A MODERATOR OF GENETIC RISK FOR POOR COGNITIVE FUNCTION ACROSS GENDER AND COHORT

Eleanor Kerr[1], Pamela Herd[2], and Katrina Walsemann[1], *1. University of Maryland, College Park, College Park, Maryland, United States*, *2. Georgetown University, Washington, District of Columbia, United States*

APOE, specifically the e4 allele, is the strongest known genetic risk factor for late-onset dementia. Individuals with at least one copy of the e4 allele have a 3-15 times higher risk for dementia, and faster rates of cognitive decline, than those without it. Although education has been shown to moderate this relationship, the quality of U.S. education improved significantly over the first seven decades of the 20th century and saw greater access to educational opportunities among women. In this historical context, it is important to consider if genetic risk for poor cognitive function changes across successive cohorts and if it does so differentially by gender. Consequently, we use genetic and cognition data from the Health and Retirement Study (HRS) linked to state-level data on educational quality available from 1920-1974. Given well-known differences in the effect of APOE genotype on dementia risk based on global and local ancestry, we restrict our sample to non-Hispanic White men (n=5,256) and women (n=6,857) who provided at least one observation on cognitive function from 2006 – 2018. We examine the relationship across three measures of cognition – global functioning, episodic memory, and numeracy. We estimate gender and cohort stratified linear mixed models and determine if states with well-resourced education systems mitigate the genetic risk on cognitive health associated with the e4 allele and compare this relationship across cohorts for men and women. These results can help inform our understanding about how early life education can differentially shape genetic risk for cognitive decline in later life.

### SESSION 1005 (PAPER)

### AGE-FRIENDLY AND AGING IN PLACE

Abstract citation ID: igad104.0006

### AGE-FRIENDLY ENVIRONMENT PROMOTES WELL-BEING IN PEOPLE WITH SUBJECTIVE MEMORY IMPAIRMENT

Odelyah Saad, *Lev Academic Center, Jerusalem, Yerushalayim, Israel*

Objectives Older people with cognitive impairment are more likely to feel lonely and to have lower well-being compared to others (Shankar et al., 2017). One way to overcome difficulties and loneliness is to live in an Age Friendly Environment (AFE) (Gibney et al., 2020). Living in an AFE enables older people to stay active even when function deteriorates (Cramm et al., 2018) and thus- maintain low loneliness and high well-being (Domènech-Abella et al., 2021). Approximately 18%-25% of people aged 60 and over whose objective cognitive function is normal report a subjective cognitive impairment (Röhr et al., 2020; Zullo et al., 2021). They are less active (Parikh et al., 2016) and so, they are lonelier (Montejo Carrasco et al., 2020). The aim of this study was to examine whether living in an AFE enables people coping with subjective memory impairment to maintain higher well-being than those who don't. Methods Applying a correlational study design, 105 community dwelling older adults (mean age 74.5; SD-6.54), 62% of whom were women, were recruited. Hayes' PROCESS macro (Model 7) assessed relationships between socio-demographic data, subjective social support (SS), loneliness, a sense of belonging, AFE, active ageing and negative affect (NA). Results Findings demonstrated a significant indirect association between age-friendly environments and negative affect, which was moderated by subjective memory impairment. Moreover, we found an interaction between AFE and memory.. Those with SMI experienced less loneliness in an AFE In conclusion, AFE is crucial for individuals with SMI to reduce loneliness and negative affect. Conclusion This study is the first to show that when AFE is low, people with subjective memory impairment cope with more loneliness compared to those without it, but when AFE is high then there is no difference in the level of loneliness between people with or without subjective memory impairment. Hence, AFE is especially significant for those coping with subjective memory impairment for reducing loneliness and negative affect. Authorities should strive to create AFE to promote active aging and high WB of older people living in the community.

Abstract citation ID: igad104.0007

### COULD WE CARE LESS? FICTIONAL REPRESENTATIONS OF TIME-RESTRICTED IN-HOME-CAREGIVING IN "DIE PFLEGIONÄRIN"

Eva-Maria Trinkaus, *University of Graz, Graz, Steiermark, Austria*

In this narrative film analysis, I look at the representations of care work and its time restrictions in the German TV series "Die Pflegionärin." In this TV series the lack of adequate time and, thereby, struggle for adequate care in domestic care work becomes visible through ten-minute episodes. Through this representation, both, the perspective of the old patients and their care-needs, as well as the caregiver's perspective and the pressure they have to perform under becomes visible. In this contribution, I will specifically focus on the representation of the relationship between caregiver and patient, and the possibilities of interaction in a restricted period of time that is captured through the way the fictional representation of care is put on screen. Adding to the pressure that the aspect of time creates, the TV series' title "Die Pflegionärin" (transl. "The Carionnaire") is a linguistic blend of carer and legionnaire, and a war metaphor that highlights the struggle of domestic care even further. By allowing the viewers to follow an in-home-caregiver on her everyday tasks 'on the front line' of everyday life, not only the caregiver's perspective, but also the patients' needs become visible. Despite the short air time, rather than providing a quick glimpse into the patient's life, the series provides the viewer with insights into the profession's tasks and duties through the personal, though fictional, stories of patients and nurse and blends the generic conventions of TV series with real-time in-home caregiving.

Abstract citation ID: igad104.0008

### DEVELOPMENT OF AN INTERGENERATIONAL AND AGE-FRIENDLY COMMUNITY: APPLICATION OF THE SOCIAL CAPITAL FRAMEWORK

Youjung Lee, and Jacqueline McGinley, *Binghamton University - SUNY, Binghamton, New York, United States*

In intergenerational programs, people from multiple generations share their talents, build healthy social relationships, and experience improved well-being and social presence and identity (Lee et al., 2021). As a part of age-friendly community initiative in a small city in the Northeast, an intergenerational engagement community project, named "Senior to Senior", involved a multi-year partnership among community agencies, including the local Office for Aging, a university, a high school, a senior center, and a church. As part of this partnership, a community dance was held in May of 2022, where local high school students and older adults participated in a variety of intergenerational activities. This study explored how the principles of social capital are incorporated into the development of intergenerational and age-friendly communities. Using focus groups with community partners (n=6) and surveys with older adult participants (n=29), the research team sought to describe how community agencies collaborate on the development of intergenerational and age-friendly communities. Carpiano's framework of social capital (2008) guided the data analysis and the development of following themes: (a) importance of social capital identification; (b) development of social cohesion among multiple generations; (c) assessment of social antecedent for the development of intergenerational and age-friendly community; and, (d) outcomes of the community events on diverse stakeholders. The findings highlight the importance of understanding the contextual factors of each community and the agencies within, while also elucidating the necessity of stakeholder buy-in for the sustainable development of intergenerational and age-friendly communities.

Abstract citation ID: igad104.0009

### SELF CARE TRAININGS FOR NCD MANAGEMENT IN OLD AGE IN INDIA

Prakash Tyagi, *GRAVIS, Jodphur, Rajasthan, India*

With over 100 million older people, India has the second largest elderly population in the world. Older people in many parts of India, especially in the rural settings live in vulnerable conditions with limited facilities and with an overall poor health status. GRAVIS has been working actively in the Thar Desert of India to address older people's health. It has multi-layered health intervention combining medical services, public health activities and research component. Self-care training is integral part of our work with older people. With help of locally designed contents, GRAVIS team trains older people on healthy ageing. The curriculum includes 10 modules focusing on basics of human body and biology of ageing, Common diseases in old age, prevention of common diseases, management of common diseases and useful information on Government health programmes. Older people in groups of 12-15 attend trainings once in 15 days. To provide service to older people and to take self care education further, GRAVIS also works with a cadre of Village based Health Workers (VHWs). The impact of our self-care training interventions has been quite positive. The trainings are well received within the community. Over 1,500 older people have been trained and over 85% report a significant increase in their knowledge levels. GRAVIS has been able to draw the attention of local health authorities and hopes that the trainings could be scaled up further. Self care and improved health knowledge are critically important for older people to stay healthy, particularly in remote and resource scarce settings.

Abstract citation ID: igad104.0010

### STAKEHOLDERS' PERSPECTIVES ON AN AREA AGENCIES ON AGING SERVING URBAN OLDER ADULTS

Faith Hopp, and Fay Keys, *Wayne State University, Detroit, Michigan, United States*

Area Agencies on Aging (AAAs) in urban areas play a critical role in helping older adults maintain and enhance their quality of life. The Detroit Area Agency on Aging (DAAA), recognizing the importance of involving Detroit area seniors and community stakeholders in long-term strategic initiatives, funded a comprehensive needs assessment of the region's seniors. This study reports findings from an online survey in the winter of 2020, (n=94) aimed at identifying stakeholder views of DAAA programs and services. We used descriptive statistical analyses for closed-ended items and content analysis to identify themes from open-ended questions. Most respondents were either direct service purchase vendors (34.0%) or contractors (22.3%). The highest ranked perceived agency role was information and assistance (mean=2.41; SD=1.02), followed by direct service provision (mean=2.45; SD=1.52), coordination of aging services (mean=2.63; SD=1.54), education (mean=3.40; SD=1.11), and training (mean=4.10; SD=0.99). The most commonly mentioned areas needed for the region to become age-friendly were supportive community services (66.3%), public transportation (44.9%), safety and security (41.6%), available and affordable housing (40.4%), access to healthcare (38.2%), and housing maintenance/modification (24.7%). The most common themes from open-ended responses included outreach, collaboration, and promoting aging in place. These findings suggest the imperative of providing accessible, high-quality services that promote aging in place in urban areas through community outreach and collaboration activities. Detroit area seniors are a vital community resource, and ongoing stakeholder input is needed as part of collaborative efforts to meet their complex emerging health and social needs.

Abstract citation ID: igad104.0011

### VETERAN DIRECTED CARE: A MIXED METHODS STUDY TO INFORM SELF DIRECTED CARE PROGRAM EXPANSION FOR VETERANS AFFAIRS (VA)

Stuti Dang[1], Erin Bouldin[2], Wendy Hathaway[3], Pranjal Tyagi[4], Lauren Penney[5], Orna Intrator[6], and Samer Nasr[7], *1. University of Miami, Miami, Florida, United States*, *2. University of Utah, Salt Lake City, Utah, United States*, *3. James A. Haley VA Medical Center, Tampa, Florida, United States*, *4. South Florida VA Foundation for Research and Education (SFVAFRE), Miami, Florida, United States*, *5. National Institutes of Health, Bethesda, Maryland, United States*, *6. Department of Veterans Affairs, Rochester, New York, United States*, *7. VISN 8, Miami, Florida, United States*

The Department of Veterans Affairs (VA) is shifting from institutional to Home and Community Based Services, such as the Veteran-Directed Care (VDC) program, a self-directed program in which Veterans choose services and employ their providers to avoid or delay long-term institutionalization. The VA plans to expand VDC to all VA facilities by FY2026, but factors affecting VDC expansion are poorly understood. We focused on facilitators and barriers in the seven Veterans Integrated Service Network (VISN) 8 VDC programs between May 2022 and January 2023 to inform program expansion. We used a mixed-methods approach, with surveys followed by semi-structured interviews with VA VDC staff, their partners at Aging and Disability Network Agencies (ADNAs) and VISN 8 leadership, such as lead program coordinators, using the Consolidated Framework for Implementation Research to guide the project rapid analysis techniques. We found substantial variability in the structure, size, and operations of the VDC programs reflecting challenges noted by respondents. VA staff were consistently enthusiastic about VDC's quality benefits for Veterans, however, they recommended additional staff, a dedicated VDC coordinator, and a national guidebook or standard operating procedures to streamline processes. Respondents also noted the need for more program education targeting referring providers. Unique local challenges, like the lack of available caregivers or no local ADNA, and limited local leadership support were other factors noted that could limit expansion. Importantly, Veterans were often overwhelmed by the employer responsibilities they needed to fulfill. Addressing these concerns remains a challenge to VISN 8 and national leadership.

### SESSION 1010 (PAPER)

### ALZHEIMER'S DISEASE AND RELATED DEMENTIAS (SRPP) I

Abstract citation ID: igad104.0012

### ACCESS TO PREFERRED POSTACUTE CARE PATHWAYS FOR PATIENTS WITH ALZHEIMER'S DISEASE AND RELATED DEMENTIAS

Taylor Bucy[1], John McHugh[2], and Dori Cross[1], *1. University of Minnesota School of Public Health, Minneapolis, Minnesota, United States*, *2. Mailman School of Public Health, Columbia University, New York City, New York, United States*

Hospital-to-skilled nursing facility (SNF) transitions are fraught with coordination challenges, motivating selective investments by hospitals to improve transitional care practices with "preferred" (e.g. high-volume) SNF partners. Because these partners have some agency in which patients they admit, it is unclear whether Medicare beneficiaries with social or clinical complexities, such as individuals with Alzheimer's disease and related dementias (ADRD) have equitable access to these preferred SNFs. To answer this question, we use a linear probability choice model to test the differential effect of a SNF's "preferredness" on patient placement for ADRD vs non-ADRD beneficiaries. We use a 1:1 matched sample of ADRD and non-ADRD fee-for-service beneficiaries (N=76,762) to account for other primary factors affecting placement (i.e. discharging hospital, beneficiaries' home address). After controlling for SNF characteristics, the estimated effect of a SNF being "preferred" on likelihood of placement was 12.7% lower for patients with ADRD (0.092 vs 0.103 for non-ADRD; p< 0.001). Simulation results suggest that, as a preferred SNF is assumed to have an increasing percentage of a hospital's discharges, the likelihood of a patient being discharged there grows much faster for non-ADRD patients compared to those with ADRD. Our findings show that ADRD patients may have unequal access to SNFs that are receiving the most investments from hospitals in the form of transitional care improvements. Policymakers should consider systemic investments that benefit all SNF partners, not just the most preferred based on shared discharges, while also weighing the costs of dispersing these investments too broadly.

Abstract citation ID: igad104.0013

### INTERACTION BETWEEN NATIVITY AND NEIGHBORHOOD ON DEMENTIA RISK: FINDINGS FROM TEN YEARS OF NATIONAL US DATA

Roger Wong, and Daniel Soong, *SUNY Upstate Medical University, Syracuse, New York, United States*

Prior research indicates neighborhood disadvantage increases dementia risk. There is, however, inconclusive evidence on the relationship between nativity and cognitive impairment. To our knowledge, our study is the first to analyze how nativity and neighborhood interact to influence dementia risk. Ten years of prospective cohort data (2011-2020) were retrieved from the National Health and Aging Trends Study, a nationally representative sample of 5,362 U.S. older adults aged 65+. Cox regression analyzed time to dementia diagnosis using nativity status (foreign or native-born) and composite scores for neighborhood physical disorder (litter, graffiti, vacancies) and social cohesion (know, help, trust each other), after applying sampling weights and imputing missing data. Average baseline neighborhood physical disorder was significantly higher among foreign-born (0.28) compared to native-born (0.18) older adults [t=-2.4, p=.02]. Average baseline neighborhood social cohesion was significantly lower for foreign-born (3.57) compared to native-born (4.33) older adults [t=5.5, p<.001]. After adjusting for sociodemographic, health, and neighborhood variables, foreign-born older adults had a 51% significantly higher dementia risk (aHR=1.51, 95% CI=1.19-1.90, p<.01). There were no statistically significant interactions for nativity with neighborhood physical disorder or social cohesion. Our findings suggest foreign-born older adults have higher neighborhood physical disorder and lower social cohesion compared to native-born older adults. Despite the higher dementia risk we observed for foreign-born older adults, this relationship was not moderated by either neighborhood physical disorder or social cohesion. Further research is needed to understand what factors are contributing to elevated dementia risk among foreign-born older adults.

Abstract citation ID: igad104.0014

### MAGNIFYING THE VOICE OF A COMMUNITY: ATTITUDES AND BELIEFS AMONG AFRICAN AMERICANS ABOUT DEMENTIA

Jabrina Simmons[1], Victoria Frager[2], Jia Broussard[3], Mikael Anne Greenwood-Hickman[4], Tyler Barrett[4], and Deborah Barnes[3], *1. Northeast Ohio Medical University, Rootstown, Ohio, United States*, *2. University of California San Francisco, San Francisco, California, United States*, *3. University of California, San Francisco, San Francisco, California, United States*, *4. Kaiser Permanente Washington Health Research Institute, Seattle, Washington, United Sta tes*

By 2060, the prevalence of Alzheimer's disease and related dementias (ADRD) in the U.S. is predicted to more than double to 14 million. African Americans (A.A.) 65 and older are 2.5 times more likely to develop Alzheimer's than same-aged White Americans. Furthermore, A.A. are twice as likely to be underdiagnosed, leaving the A.A. community underserved with respect to ADRD. To gain insight into the key drivers of this underdiagnosis, we performed a scoping review of research on attitudes and perceptions held within the A.A. community regarding ADRD, which may impact diagnosis and screening. We searched PubMed, CINAHL, Web of Science, and Black Studies Periodicals Database for studies from 2002 to 2022 using keywords: "Dementia" or "Alzheimer's" and "African Americans," "Ethnicity," "Racial Groups," "Blacks," or "Ethnic and Racial Minorities," and "Health Knowledge, Attitudes, Practice," "Health Services Needs and Demand," or "Perception." We identified 318 studies; 49 met inclusion criteria after title and abstract review and were selected for full manuscript review. We discussed all included manuscripts and identified pertinent themes, noting potential method bias. An identified "knowledge gap" among A.A. about dementia, particularly a belief that dementia is part of normal aging or God's will, was prominent. Other themes included mistrust of the medical community, a desire to maintain everyday family life, the need for culturally appropriate resources, and cultural expectations to provide care despite institutional support obstacles (John Henryism/Superwoman schema). Understanding differences in attitudes and beliefs toward dementia among African Americans may support developing effective strategies to address disparities.

Abstract citation ID: igad104.0015

### SELF-IDENTIFIED STRENGTHS AMONG ADULTS AGING SOLO WITH EARLY DEMENTIA

Jane Lowers[1], Ivree Datcher[2], Dio Kavalieratos[2], Kenneth Hepburn[2], and Molly Perkins[2], *1. Emory School of Medicine, Atlanta, Georgia, United States*, *2. Emory University, Atlanta, Georgia, United States*

People with Alzheimer's and related dementias require increasing assistance with basic life activities, yet one in three lives alone (i.e., is aging solo). Drawing on a strengths-based perspective, this study seeks to identify barriers and facilitators to successful aging among adults who are aging solo with cognitive impairment or early dementia. We conducted semi-structured interviews with 15 adults aging solo with a diagnosis of cognitive impairment or early dementia (age 48-81, mean 69; 80% female; 86% white; 13% black; 7% Hispanic) and used a hybrid inductive/deductive thematic analysis approach to analyze the data. Participants identified self-advocacy, friendship networks, financial resources, and institutions (e.g., assisted living) as potential assets that could address future care needs. Participants described an identity as self-sufficient, fear of being a burden, and worries about exploitation as barriers to help-seeking. Facilitators to care-seeking from friends, neighbors, and others included a belief that helping is rewarding for others, being able to provide reciprocal favors, and framing care-seeking as self-advocacy. Conclusion: Adults aging solo with early dementia face a number of challenges but also exhibit numerous strengths and can use a diversity of skills, attitudes, relationships, and tangible resources to address increasing care needs.

### SESSION 1015 (SYMPOSIUM)

Abstract citation ID: igad104.0016

### NEIGHBORHOODS AS A SPACE AND PLACE FOR HEALTHY AGING: NOVEL APPROACHES AND A VIEW OF THE FUTURE

Chair: Andrea Rosso Co-Chair: Michelle Carlson
Discussant: Yvonne Michael

The importance of neighborhood environments for health outcomes has gained increasing recognition in the past two decades. Both space (physical environment) and place (social environment) have implications for health behaviors and risk for acute events and chronic diseases. This symposium brings together a series of talks that address space (walkability, physical activity destinations) and place (racial and socioeconomic polarization) for falls and fear of falling (Pam Dunlap), physical activity behaviors (Pat Donahue), and blood pressure (Hoda Magid). The talks also advance methodology and conceptualization of space and place through use of novel exposure measurements (e.g., Google StreetView audits, spatial polarization) and more nuanced characterization of outcomes (e.g., considering risk stratification and various behavioral aspects of physical activity). Finally, we end with two talks demonstrating ways to advance methods, including the use of longitudinal data to understand long-term neighborhood exposures in relation to outcomes in older adults (Michael Desjardins) and incorporation of novel measures into existing cohorts (Jana Hirsch). These talks leverage data from a diverse range of studies including long-standing cohorts (CHS, MESA), an intervention trial, and VA nursing home data. Together, these talks further our understanding of how neighborhood environments relate to health at older ages and demonstrate the ways in which the field can advance our conceptualization of space and place as both barriers and promoters of health. Yvonne Michael will lead a discussion with the panel and audience on future directions and methodological needs for the field.

Abstract citation ID: igad104.0017

### ASSOCIATION BETWEEN NEIGHBORHOOD ENVIRONMENT AND FEAR OF FALLING AND FALLS AMONG COMMUNITY-DWELLING OLDER ADULTS

Pamela Dunlap[1], Xiaonan Zhu[1], Jennifer Brach[1], Kyle Moored[2], Alyson Harding[3], and Andrea Rosso[1], *1. University of Pittsburgh, Pittsburgh, Pennsylvania, United States*, *2. Johns Hopkins Bloomberg School of Public Health, Baltimore, Maryland, United States*, *3. University of Minnesota School of Public Health, Minneapolis, Minnesota, United States*

Neighborhood built environment may influence both objective and perceived risk of falling. We examined the association between neighborhood built environmental characteristics and falls, fear of falling (FOF), and gait efficacy among older adults. We used baseline data from a randomized trial that measured falls over the past 12 months, FOF, and Modified Gait Efficacy Scale (mGES). Walkability audits were conducted for participant home addresses using Google StreetView and summarized by two factors: urbanicity and neighborhood quality. Logistic and linear regression were adjusted for age, gender, race, comorbidity index, and body mass index. Analyses were also stratified by past falls and gait speed (< or ≥1.0m/s). We included 249 participants (mean age: 77.4 years; 65% female) with 29.7% reporting a fall over the past year and 40.6% reporting FOF. Mean mGES (0-100) was 85.2±13.7 and 32% participants had gait speed <1.0m/s. In unstratified analysis, greater urbanicity was associated with lower gait efficacy (β=-1.88, p=.039). Among those who had not fallen, greater urbanicity was associated with FOF (β=0.47, p=.012) and greater urbanicity and higher neighborhood quality were associated with lower gait efficacy (β=-2.8, p=.006; β=-2.1, p=.047). Among those with better mobility, urbanicity was associated with lower gait efficacy and FOF (β=-2.4, p=.009; β=0.5, p=.01). There were no significant associations among those who had fallen or with poorer mobility. Neighborhood environmental characteristics may play a larger role in FOF among those who have better mobility and have not yet fallen, as they may have more opportunities to interact with varied environmental conditions.

Abstract citation ID: igad104.0018

### DO NEARBY PHYSICAL ACTIVITY DESTINATIONS ENCOURAGE PEOPLE TO BE F.I.T.T.?

Patrick Donahue[1], Kyle Moored[2], Breanna Crane[2], Emily Richards[1], Michael Desjardins[2], Andrea Rosso[3], and Michelle Carlson[2], *1. Johns Hopkins University, Baltimore, Maryland, United States*, *2. Johns Hopkins Bloomberg School of Public Health, Baltimore, Maryland, United States*, *3. University of Pittsburgh, Pittsburgh, Pennsylvania, United States*

Neighborhood amenities may reduce barriers to engaging in physical activity (PA). However, it is unclear whether proximity to these amenities will indeed promote increased PA. This study examined whether living near PA destinations was associated with greater self-reported PA. The sample included Cardiovascular Health Study participants (N=3,922; mean age=75.3 years) from visit 5 (1992-1993). Quantity of PA destinations (e.g., gyms) within a one-kilometer Euclidean buffer of each participant's home address was provided by the National Establishment Time-Series database. PA over the previous two weeks was self-reported via the Minnesota Leisure Time Activity Questionnaire. The exposure was dichotomized into having at least one PA destination (vs. none) within one kilometer of participants' homes. Outcomes were based on the FITT components of PA outlined by the American College of Sports Medicine: Frequency (activities per week), Intensity (MET-minutes per week), Time (minutes per day), and Type (number of different activities, i.e., variety). Outcomes were modeled separately as five-level ordinal variables considering PA guidelines. Using ordinal logistic regression models adjusting for age, sex, health status, smoking, and body mass index, we found that living within one kilometer of at least one PA destination was associated with significantly increased odds of engaging in higher levels of all four PA components (odds ratios: 1.17-1.20, all Ps < 0.01). After adjusting for social factors (race, education, income), associations were attenuated and no longer significant. When evaluating barriers to PA, social determinants of health should be considered, in addition to proximity of PA destinations.

Abstract citation ID: igad104.0019

### SPILLOVER EFFECTS OF RESIDENTIAL SPATIAL SOCIAL POLARIZATION AND HIGH BLOOD PRESSURE AMONG VA NURSING HOME RESIDENTS

Hoda Abdel Magid, and Michelle Odden, *Stanford University, Stanford, California, United States*

Spatial social polarization (SSP) – the process by which a population within an area diverges across socioeconomic characteristics– has been associated with hypertension. SSP indices measure the extent to which a population is distributed at extremes of privilege and deprivation. The VA nursing home represents an environment where spatially-oriented disparities are expected to be attenuated due to the fact that Veterans have equal access to care. We examined the association between SSP measured at residents' home addresses prior to admission with blood pressure (BP) outcomes in the first four weeks after admission. We evaluated the use of the index of concentration (ICE) of extremes to measure SSP across four socioeconomic domains including race/ethnicity, income, home ownership, and joint race/ethnicity with income at the tract level using 2010 census data. SSP was dichotomized as the first (most polarized and disadvantaged) quintile vs. the 2nd -5th quintiles. The analytic sample included 41,972 long-term care residents aged ≥65 years admitted from 2006-2019. Multilevel mixed-effects regression models were adjusted for individual demographics and chronic conditions. We found Veterans who had resided in the most polarized and disadvantaged quintile (Q1) had a 1.10 (95% 1.01, 1.19) relative risk of high BP compared to those in Q 2-5 for the ICE jointly measuring race/ethnicity and income. We found similar results for systolic BP. In summary, SSP that jointly measures economic and racial/ethnic polarization, may have a spillover effect on health disparities even in a healthcare setting with equal access.

Abstract citation ID: igad104.0020

### IMPROVING LONGITUDINAL STUDIES OF AGING IN GEOSPATIAL HEALTH: AN AGENDA

Michael Desjardins[1], Kyle Moored[1], Emily Murray[2], Gergő Baranyi[3], Matthew Hobbs[4], Sarah Curtis[5], and Michelle Carlson[1], *1. Johns Hopkins Bloomberg School of Public Health, Baltimore, Maryland, United States*, *2. University College London, London, England, United Kingdom*, *3. University of Edinburgh, Edinburgh, Scotland, United Kingdom*, *4. University of Canterbury, Christchurch, Canterbury, New Zealand*, *5. Durham University, Durham, England, United Kingdom*

Recent decades have seen a vast demographic shift in ageing populations worldwide, where over 1.4 billion people will be aged 60 years and older by 2030. Given specific populations may be more vulnerable to medical conditions with ageing, identifying high-risk locations are essential for allocating resources and promoting healthy ageing to mitigate population-level financial- and health-associated burdens. All aspects of public health research on ageing require longitudinal analyses to fully capture the dynamics of outcomes and risk factors such as human mobility, climate change, non-communicable diseases, and endemic, emerging, and re-emerging infectious diseases. Incorporating geospatial approaches in longitudinal health research can facilitate targeted interventions and improve public health policy and decision-making. Furthermore, geospatial approaches can identify where at-risk populations are located and what influences disease risk and exposure, particularly the 'wider determinants' of health across the lifecourse. However, studies in geospatial health are often limited to spatial and temporal cross sections. This generates uncertainty in the timing of exposures and behaviors. Geospatial longitudinal studies can better capture spatiotemporal dynamics of ageing. Here, we outline a research agenda, including key challenges and opportunities of working with longitudinal geospatial health data. Examples include accounting for residential and human mobility, recruiting new birth cohorts, geoimputation, international and interdisciplinary collaborations, spatial lifecourse studies, and qualitative and mixed-methods approaches. As a proof of concept, we present several case studies that better capture longitudinal exposures for the Cardiovascular Health Study cohort, which previously incorporated erroneous spatial indicators of neighborhood-level exposures across baseline and follow-up measures.

Abstract citation ID: igad104.0021

### INCORPORATING NEW MEASURES OF NEIGHBORHOOD SUPPORTS FOR HEALTHY AGING INTO EXISTING COHORTS

Jana Hirsch[1], and Yvonne Michael[2], *1. Dornsife School of Public Health Drexel University, Philadelphia, Pennsylvania, United States*, *2. Drexel University, Philadelphia, Pennsylvania, United States*

Longstanding cohort studies have a wealth of well-characterized sociodemographic, phenotypic, behavioral, genetic, health, and neighborhood longitudinal data, often spanning decades. In addition to original aims, we have an obligation to leverage the current cohort studies to better understand the aging process and neighborhood supports for healthy aging. We used a multi-pronged approach to design and add aging-relevant neighborhood measures to the Multi-Ethnic Study of Atherosclerosis (MESA) during Exam 7 (ongoing since 2022), when the entire cohort had aged to >67 years. In addition to continuing survey measures from historic visits on food environments, safety, social cohesion, trust, and physical activity resources we added novel metrics to the MESA Neighborhood Ancillary questionnaire. These include across environmental domains relevant for physical function (e.g. curb cuts, availability of places to rest, lighting, crosswalk speeds), cognitive function (e.g. places to learn new things, signage), and ability to age in place (e.g. neighborhood changes related to cost or pressures). To complement existing Geographic Information Systems (GIS) data in MESA, we collected and developed new GIS measures similarly related to these three domains: physical function (e.g. senior center density, physical therapist density), cognitive function (e.g. destinations to enrich cognitive function, places to socialize, new greenness measures), and ability to age in place (e.g. age isolation, age-friendly policies). Our ability to leverage decades of data on well-characterized cohorts by complementing existing data with new, neighborhood supports for healthy aging opens novel research opportunities to understand health across the lifecourse.

### SESSION 1020 (SYMPOSIUM)

Abstract citation ID: igad104.0022

### POLICY SERIES: HEALTH AND AGING POLICY FELLOWS: LINKING POLICY AND PRACTICE TO BENEFIT OLDER PEOPLE

Chair: Maureen Henry Discussant: Emily Franzosa

The Health and Aging Policy Fellows program was founded in 2008 after its founders observed a dearth of experts with overlapping experience in policy and aging. The program is creating a growing cadre of leaders to improve the health of older adults by influencing policy at the state and national levels that is joined through an alumni network of more than 150 fellows. The fellowship's goal is to improve the lives of older people across life domains. Past fellows serve as staff members in Congress, in State and Federal Agencies, and in the private sector. The fellowship has helped to launch many policy-focused careers. In this seminar, current and past fellows from medicine, research, social work, and law will share their experiences before, during, and after their fellowship. They will describe what they learned through the training and a placement in a full- or part-time policy role, how viewing health and aging through a policy perspective changed their understanding of systems, and how they think the fellowship will enable them to influence policy to improve the lives of older people in the future. Fellows will also highlight ongoing policy challenges that stop or slow progress toward achieving the goal.

Abstract citation ID: igad104.0023

### AGING AND FOREIGN POLICY: EXPERIENCING THE GLOBAL TRACK OF THE HEALTH AND AGING POLICY FELLOWSHIP

Jonathan Cohen, *University of Southern California, Los Angeles, California, United States*

The Health and Aging Policy Fellowship includes a Global Track that provides fellows with the opportunity to shape global policy or U.S. foreign policy for an aging society. The author, a global health expert and 2022-2023 fellow, sought a fellowship placement with a member of Congress with jurisdiction over U.S. global health policy. Although global health is a significant foreign policy priority for the U.S. government, U.S. global health programs do not include dedicated funding for activities designed to benefit older adults or help development partners adapt to population aging. The result is that US-funded programs on issues from infectious disease to health systems strengthening to disaster relief lack a focus on older adults, despite their many links to this growing population. To help address this policy gap, the author secured a fellowship placement with Senator Benjamin Cardin (D-MD), a senior member of the Senate Foreign Relations Committee (SFRC) and Chair of the SFRC Subcommittee overseeing the State Department, US Agency for International Development, and bilateral development assistance. With his track record of legislation and oversight on issues such as Medicare, health disparities, medical research, oral health, health systems strengthening, and retirement security, Senator Cardin is well positioned to help integrate the needs of older adults into U.S. development assistance. The author used his fellowship year to help advance the global health-related legislative priorities of Senator Cardin and his Health Team, bringing his expertise and perspective on aging to the policy table while gaining invaluable skills in the policy process.

Abstract citation ID: igad104.0024

### CARE PLAN INVENTORY AND ASSESSMENT—A MEANS TO LEARN MORE ABOUT CMS

Maria Carney, MD, *Northwell Health, Manhasset, New York, United States*

The Health and Aging Policy Fellowship (HAPF) provides an orientation on the federal government organization and an introduction to aging policy foundations and agencies. The goal of this orientation is to lead to a growth of a professional network as well as a policy placement opportunity. I will explain the process for choosing a HAPF placement with the Centers for Medicare and Medicaid Services (CMS) and Agency for Health Research and Quality (AHRQ). Using my HAPF experience as an example of how my interest in Age-Friendly Health System work at a large health system led to my placement and personal growth. I will discuss my reasoning in taking part in the Fellowship and the creation of CMS Care Plan Inventory and Assessment. Achieving goal-concordant care is the bedrock of Age-Friendly care. Creating care plans is a means for teams and insurance providers use to create goal concordant care. Measuring goal concordant care is a challenge, but development of care plans would be a potential start. CMS utilizes care planning in programs and in billing codes. My Health and Aging Policy Fellowship placement assessed how care planning is defined and what are the elements in CMS programs and billing codes and if these are aligned with care plan research from AHRQ. My experience, current interests and project development will be the example of how HAPF provided me with a better understanding of CMS programs and how billing code creation affects clinician behaviors and , in turn, patients.

Abstract citation ID: igad104.0025

### TRAINING IN HEALTH AND AGING POLICY MID CAREER—REFLECTING ON A POTENTIAL INFLECTION POINT

Benjamin Springgate, *LSU Health - New Orleans, New Orleans, Louisiana, United States*

The Health and Aging Policy Fellowship (HAPF) trains fellows to understand policymaking with the goal to improve the lives of older people across life domains. Comparatively few mid-career or more senior experts in healthcare or aging have direct experience with federal or state policymaking, or working to inform evidence-based health policy. As a senior physician faculty member at a School of Medicine and School of Public Health, in recent years the author has worked at state and local levels to inform evidence-based health policy. The HAPF offered an opportunity for the author to improve knowledge and understanding of decision-making and policymaking in Washington DC and at the federal level. Fellowship-sponsored training improved fellows' understanding of health policy and potential effectiveness in federal health policy environments. Trainings through HAPF, the American Political Science Association, and Academy Health provided greater breadth and depth of knowledge on roles of various agencies, executive and congressional policymaking, and understanding factors influencing policymaking. This training enhanced the author's readiness for fellowship activities in a U.S. Senate office. Exposure to experts in health and aging opened the door to fellows' participation in efforts to improve opportunities for healthy aging and to advance age-friendly health systems. Work in the U.S. Senate includes exposure to federal, state, non-governmental stakeholders who can work together to influence policy to improve the lives of aging and older people. The author will highlight specific examples of training, policy opportunities, and policy challenges in the context of his fellowship.

Abstract citation ID: igad104.0026

**INSIGHTS FROM A HAPF ALUMNUS ON AGING POLICY ADVOCACY IN SOCIAL WORK**

Nancy Kusmaul, *University of Maryland Baltimore County, Baltimore, Maryland, United States*

"Once a fellow, always a fellow" and "Networks get things done". The Health and Aging Policy Fellows Program has brought together experts in aging for more than ten years for policy education and fellowships, assembling an alumni network of more than one hundred and fifty fellows. Fellow alumni are social work scholars, physicians, nurses, gerontologists, sociologists, and many more. Each of these alumni completed one or more placements in policy making in congress, federal agencies, and advocacy organizations. Some have remained in those spaces while others are academics, state and local officials, and corporate leaders. They continue to advocate for and shape policy in aging in those positions. Told from the perspective of a non-residential program alumnus who is a mid-career faculty member in social work, this presentation will discuss the lessons learned during the fellowship, how the fellowship shaped future policy work, and present insights on how social workers can engage in policy advocacy in aging through the fellowship.

Abstract citation ID: igad104.0027

**IMPROVING THE VALUE OF SERIOUS ILLNESS CARE**

Brystana Kaufman, *Duke University, Durham, North Carolina, United States*

My Health and Aging Policy Fellowship placement with CMMI provides an opportunity to learn about the current policies impacting palliative care payment and process for developing new models. Palliative and hospice care has been one of the fasting growing health services in the U.S. and the VA over the past decade. Although Medicare's hospice benefit requires a 6-months life expectancy, palliative care may be appropriate for seriously ill individuals depending on their clinical and psychosocial needs. Palliative care is an interdisciplinary, team-based approach that focuses on improving quality of life, aligning care with patient preferences, and relieving distressing symptoms for people with serious, life-limiting illness and their families. In practice, palliative consultations, teams and services are highly tailored to each patient and family's needs. The heterogeneity of patient needs, care settings and services creates challenges designing value-based payment strategies. I am working on a team that will assess the feasibility of a value-based strategy for supporting seriously ill beneficiaries' palliative care needs. Current learnings include 1) understanding the CMMI priorities as identified in their strategy refresh in 2021; 2) mapping the design elements that are relevant for palliative care across Medicare Advantage and Traditional Medicare Alternative Payment Models; 3) synthesizing evidence and leveraging my research experience evaluating value-based payment models and outcomes for high cost, high need populations in Medicare and the VA. In addition to key learnings specific to my placement, I will share how the HAP fellowship experience has impacted my research career and opportunities for the future.

### SESSION 1025 (SYMPOSIUM)

Abstract citation ID: igad104.0028

**ADDRESSING THE NEEDS OF AFRICAN AMERICAN BEREAVED DEMENTIA CAREGIVERS**

Chair: Zachary Baker Discussant: Katherine Supiano

Within the next 10 years there will be at least 9 million NEWLY Bereaved Dementia Caregivers, disproportionately affecting African American families due, in part, to dementia prevalence that is twice that of White counterparts. Up to 26% of Bereaved Dementia Caregivers experience pathologically prolonged or exacerbated grief, often referred to as complicated grief or prolonged grief disorder: 2.5x the rate of other chronic disease care contexts, like cancer. The present project used an asset-based approach to assess the needs and resources of African American Bereaved Dementia Caregivers. This project was conducted with community members and university members serving as Co-Principal Investigators. Community PIs lead a grassroots organization that has served Bereaved Dementia Caregivers since 2016. The University PI leads a research team devoted to understanding and improving the experience of Bereaved Dementia Caregivers. The present symposium will discuss the challenges and opportunities offered by this unique collaboration and insights gleaned by a project designed to serve doubly underrepresented groups: African American Bereaved Dementia Caregivers (who sometimes call themselves the "ultra-invisible"). Because this was a project funded through grant dollars, we will also discuss actionable insights that we hope may help others develop fruitful academic-community partnerships. Finally, we will share recruitment strategies for speaking with minoritized populations and insights from engaging gleaned through conversations with African American Bereaved Dementia Caregivers.

Abstract citation ID: igad104.0029

**BUILDING THE COLLABORATIVE COMMUNITY-ACADEMIC TEAM, DESIGNING OBJECTIVES, AND GETTING FUNDED**

Zachary Baker[1], Sheryl Fairbanks[2], Warren Wolfe[3], Robbin Frazier[4], and Ashley Millenbah[2], *1. Arizona State University, Tempe, Arizona, United States*, *2. University of Minnesota, Minneapolis, Minnesota, United States*, *3. Freelance Journalist, Roseville, Minnesota, United States*, *4. University of Minnesota, Center for Healthy Aging and Innovation, Minneapolis, Minnesota, United States*

Having attended the Former Dementia Caregiver Re-Entry Initiative since 2019, its benefits were readily apparent but two limitations were identified: 1) two facilitators (i.e., Wolfe and Fairbanks) cannot serve the millions of Bereaved Dementia Caregivers in the US alone and 2) members have primarily been non-Hispanic White. These realizations inspired efforts to bring the group to more Bereaved Dementia Caregivers. The present community-created model of helping Bereaved Dementia Caregivers deserved community-integrated leadership if that model was to be effectively adapted and disseminated. Our team's ambition is to bring the group to racially and ethnically minoritized Bereaved Dementia Caregivers, broadly. However, we felt that we could most effectively do this in stages. Rather than trying to figure out how to make the model fit for all people, we homed in on African Americans, with whom we had many local ties. We also took a step back (at the behest of Ms. Frazier) to not only ask about how to adapt the model for a need we assumed existed, but also to explore the strengths of African American communities and ask if unmet needs existed. This model had the benefits of intellectual humility, respect for the persons we were working with, and strengthened the value of our funded research (i.e., even if we found there weren't unmet needs, we would be able to learn of strengths that we might be able to share with other communities who do have unmet needs).

Abstract citation ID: igad104.0030

### NOT KNOWING OTHER AFRICAN AMERICAN BEREAVED DEMENTIA CAREGIVERS: SEMISTRUCTURED INTERVIEW RESULTS

Ashley Millenbah[1], Robbin Frazier[2], Sheryl Fairbanks[1], Warren Wolfe[3], and Zachary Baker[4], *1. University of Minnesota, Minneapolis, Minnesota, United States*, *2. University of Minnesota, Center for Healthy Aging and Innovation, Minneapolis, Minnesota, United States*, *3. Freelance Journalist, Roseville, Minnesota, United States*, *4. Arizona State University, Tempe, Arizona, United States*

Semi-structured interviews focused on African American bereaved dementia caregivers and the individual who may serve them in the Twin Cities of Minnesota. The four questions that drove these interviews were linear in nature: 1. What needs exist among African American bereaved dementia caregivers in the Twin Cities? 2. What services exist to help these individuals meet their needs? 3. If unmet needs exist, would the group support model meet those needs? and, 4. If this model is promising, what adaptations might it need to best serve African American bereaved dementia caregivers? Interviews showed that needs exist at points before and after caregiving ends to increase education and destigmatize dementia in African American communities to facilitate understanding, acceptance, and validation during and after caregiving. Support from those who have gone through similar situations is increasingly important both during and after dementia caregiving, but identifying those in similar situations is difficult (few could identify any other African American Bereaved Dementia Caregivers besides themselves). While a culturally specific support group model was of interest to some to create a feeling of comfort, others emphasized that the main importance was a bonding over shared experiences, regardless of cultural background. A former dementia caregiver support group might be held in various locations and hosted by varying individuals, including at a church, hair salon, or even a grocery store. Overall, it was seen as important for there to be a specific meaning, purpose, and goals for a group of African American former dementia caregivers.

Abstract citation ID: igad104.0031

### "AH, A PROGRAM FOR FORMER CAREGIVERS. WHAT A GREAT IDEA! WHY DON'T YOU START SOMETHING?"

Sheryl Fairbanks[1], Warren Wolfe[2], Ashley Millenbah[1], Robbin Frazier[3], and Zachary Baker[4], *1. University of Minnesota, Minneapolis, Minnesota, United States*, *2. Freelance Journalist, Roseville, Minnesota, United States*, *3. University of Minnesota, Center for Healthy Aging and Innovation, Minneapolis, Minnesota, United States*, *4. Arizona State University, Tempe, Arizona, United States*

Sheryl Fairbanks and Warren Wolfe had insight into the experiences of bereaved dementia caregivers, having cared for four parents, two of whom had dementia, who all died within 14 months of each other after 12 years of care. After taking some time to grieve and settling an entire generation's estates, Ms. Fairbanks and Mr. Wolfe sought resources to help them re-engage in life following caregiving. Here they were uniquely qualified to find what resources might exist. Mr. Wolfe had been a newspaper reporter and editor for four decades. Two of these decades were spent covering aging and in 2010 he was a member of the GSA Journalists in Aging Fellows Program. Likewise, Ms. Fairbanks attended GSA in 2015 as a journalist and editor as they contacted experts across the country seeking resources for bereaved dementia caregivers. While many sympathized with the need for such resources, few could be found, and it was suggested that maybe Mr. Wolfe and Ms. Fairbanks ought to create a group to help bereaved dementia caregivers figure out what was next after caregiving. In 2016 they created the Former Dementia Caregiver Re-Entry Initiative, which has helped more than 60 members figure out the next steps for themselves, re-engaging with life after caregiving. They have talked about hundreds of topics, laughed a lot, cried at times, disclosed deep fears, shared small and big discoveries about themselves, and generally have found a firmer footing as they have embarked on this new journey.

Abstract citation ID: igad104.0032

### RECRUITING THE MINORITIZED: AFRICAN AMERICAN BEREAVED DEMENTIA CAREGIVERS

Robbin Frazier[1], Ashley Millenbah[2], Sheryl Fairbanks[2], Warren Wolfe[3], and Zachary Baker[4], *1. University of Minnesota, Center for Healthy Aging and Innovation, Minneapolis, Minnesota, United States*, *2. University of Minnesota, Minneapolis, Minnesota, United States*, *3. Freelance Journalist, Roseville, Minnesota, United States*, *4. Arizona State University, Tempe, Arizona, United States*

Central to this project was recruiting and learning from African American people, who are underrepresented in Bereaved Dementia Caregiver research. Initially this project was conceptualized with the intent of recruiting a wide range of people of African or Caribbean descent, given the geographic presence of large contingents of African Immigrants in the Twin Cities of Minnesota. In talking to our community partners prior to launch, we elected to restrict participation to those who identified as, or identified as serving, African Americans specifically. Our recruitment strategies were largely informed by deep community engagement built through academic, personal, and non-profit partnerships over several years. We also sought to synergize recruitment efforts for this project with another effort targeting augmentations of dementia friendliness in local African American churches. In discussing recruitment strategies we also sought to hear from those who may not be those traditionally heard from. For instance, to include younger individuals, we recruited contacts through our local Alzheimer's Association chapter's Young Champions group. Likewise, because churches are often a major source of African American participant recruitment, we contacted individuals at the Volunteers of America to recruit. Finally, we intentionally broke our recruitment plan into stages (recruiting a handful of individuals at a time) to ensure that participants could nominate those they thought might be most important to hear from within their own communities. This intellectual humility was essential to doing this work properly and maximizing the potential of our results to help African American Bereaved Dementia Caregivers.

### SESSION 1030 (SYMPOSIUM)

Abstract citation ID: igad104.0033

### THE CANNABIS AND OLDER PERSONS STUDY: MOVING ALONG A ROAD FROM ANIMAL MODELS TO STATE CAPITOLS

Chair: Brian Kaskie

The Cannabis and Older Persons Study has examined the increasing use of cannabis among Americans over 60 years old since 2016. This year's symposium presents varied methodological approaches researchers have used to better understand the relationship between age and cannabis use. Barry Setlow examines how cannabis use corresponds with age-associated synaptic dysfunction, neuroinflammation, and tau pathology in animal models (younger v older rats), and how such association may contribute to cognitive aging. Nicole Ennis examines the association between cannabis use and simulated driving performance among adults aged 50 and older focusing on response time, attention, and executive functioning tasks that predict on-road performance. Yan Wang examines short and long-term effects of medical marijuana use among older adults using ecological momentary assessments. Divya Bhagianadh analyzes responses provided by 1398 older persons who completed a special module concerning cannabis use as part of the Health and Retirement Survey and applies a multi-level framework to better understand how cannabis use is shaped by individual attitudes and health status. Fadi Martinos considers how state medical cannabis program eligibility criteria and provider qualifications contribute to cannabis access and use among older adults. Together, these studies reflect how scientific understanding of cannabis use and aging has been informed by the application of multiple disciplinary perspectives, and how researchers use different methodological approaches to identify both desirable and undesirable outcomes associated with cannabis use among older adults. This symposium offers policy makers, program administrators and clinicians empirically-based insights concerning how cannabis use affects older adults.

Abstract citation ID: igad104.0034

### EFFECTS OF ACUTE AND CHRONIC CANNABIS/ CANNABINOIDS ON COGNITIVE FUNCTION ACROSS THE LIFESPAN

Barry Setlow, Sabrina Zequeira, Emely Gazarov, and Jennifer Bizon, *University of Florida, Gainesville, Florida, United States*

Cannabis and cannabinoids such as THC (the major psychoactive component of cannabis) tend to impair cognitive performance, but almost all research on this topic has been conducted in young adults. Given that many aged individuals already exhibit cognitive deficits, it is important to determine how cannabis/cannabinoids affect cognition in this population. We used an animal model (young adult and aged rats) to evaluate the effects of both acute cannabis smoke and chronic oral THC on several tests of cognitive function. Both acute smoke (males only) and chronic THC (both sexes) enhanced working memory in aged rats, but neither affected young adults. In contrast, both regimens tended to impair performance on tests of spatial/episodic memory in an age-independent manner. Results suggest that although cannabis has potential for cognitive benefits in aging, its effects likely depend on both the cognitive domain tested and the manner in which it is administered.

Abstract citation ID: igad104.0035

### THE ASSOCIATION BETWEEN MEDICAL MARIJUANA USE AND DIVIDED ATTENTION ON A SIMULATED DRIVING PERFORMANCE TASK

Nicole Ennis[1], Daniel Dunleavy[2], Katie Kloss[2], Nichole Stetten[3], and Sherrilene Classen[3], *1. Florida State University, Tallahassee, Florida, United States*, *2. Florida State University-College of Medicine, Tallahassee, Florida, United States*, *3. University of Florida, Gainesville, Florida, United States*

Driving is a complex and dynamic task requiring divided attention (DA), awareness of the traffic and surroundings while focusing on critical stimulus. The goal of this project was to examine the relationship between medical marijuana use and divided attention in adults 50 and older during a simulated driving performance task. We recruited adults 50 and older (N=20) pre-exposure to medical marijuana and followed them for the first 3 months of use. Driving performance was examined at baseline (pre-exposure to medical marijuana) and 1-month post medical marijuana exposure During a driving performance identification task that appeared in central or peripheral vision during the simulated drive, DA was assessed as a triangle that appeared on screen at pre-programmed intervals to which participants were required to press a button on the dashboard in response. Results showed that at 1-month post-medical marijuana initiation the 7-day pattern of use could be classified as light, moderate, or heavy. Further, there was a trend of improved performance among medical marijuana users 50 and older with moderate to heavy use having the best performance on the DA. We attribute these findings to the stimulant properties of medical marijuana and improved sleep reported by participants.

Abstract citation ID: igad104.0036

### REAL-TIME HEALTH OUTCOMES AMONG OLDER ADULTS INITIATING MEDICAL CANNABIS FOR CHRONIC PAIN

Yan Wang[1], Kimberly Sibille[1], Zhigang Li[1], Rene Przkora[1], Seigfried Schmidt[1], Margaret Lo[1], Ana Abrantes[2], and Robert Cook[1], *1. University of Florida, Gainesville, Florida, United States*, *2. Brown University, Providence, Rhode Island, United States*

Background: Rigorous data are needed on the short- and long-term effects of medical cannabis (MC) on older adults with chronic pain. In response, this prospective study combines technology-based ecological momentary assessments (EMA) and in-person visits over 12 months to obtain subjective and objective data on MC's effects on older adults.

Method: The study recruits and follows older adults (50 years or older, 50% male) with chronic pain for one year. Some will have initiated MC (MC group) and others will not use MC (comparison group). Target enrollment will be 328 (1:1 ratio for MC and comparison group) participants. Collection of subjective and objective data occurs at in-person visits (baseline & 12 months) and via smartphone- and sensor-based measurement bursts at 1, 3, 6, and 9 months. The EMA data capture detailed MC use patterns and subjective short-term outcomes (e.g., pain intensity rating). These data will be integrated with objective data collected using a wearable sensor-based Fitbit (e.g., physical activity and sleep). Results: Multilevel modeling will examine changes in real-time symptoms measured by EMA and objective, sensor-based behavioral outcomes. Results will include changes in momentary pain intensity rated on 0-100 visual analog, real-time anxiety and depressive symptoms, daily sleep duration and quality, and level of physical activity. Conclusions: Our results will provide evidence on whether older adults who initiate MC will have greater reductions in real-time pain intensity and more improvements in physical and emotional functioning compared to those in the comparison group.

Abstract citation ID: igad104.0037

### CANNABIS USE IN OLDER ADULTS: EVIDENCE FROM THE HEALTH AND RETIREMENT STUDY

Divya Bhagianadh, *Rutgers University, Newark, New Jersey, United States*

Cannabis use among persons over 50 years old has tripled since 2000. Some studies have found negative outcomes associated with increasing use of cannabis use among older adults such as higher rates of injury and emergency department use. Other studies have shown improvements in pain, sleep and other outcomes such as self-reported health and labor force participation. In this study, we identify individual determinants and outcomes related to cannabis use among adults aged 50+ sourcing data from the 2018 cannabis module of the Health Retirement Survey and constructing a longitudinal panel by linking this data with the respondents' 2016 and 2020 data. Among the 1,372 respondents, 10% reported past year use. Among these past year users, 35% were persistent, lifelong users and nearly 25% used solely for medical purposes. Multivariate logistic regression controlling for demographics, health and functional status and individual fixed effects showed that those who reported pain in the last wave had higher odds of reporting cannabis use (OR 1.70, p-value-0.03). Troubled sleep was associated with higher odds of cannabis use (significant at 10%). We did not see any effect of cannabis use on pain in later years or subsequent healthcare utilization (hospital or doctor visits). Our results reflect a nationally representative survey of cannabis use among aging Americans and affirms how motives and outcomes of cannabis use are age sensitive.

Abstract citation ID: igad104.0038

### STATE VARIATION IN CANNABIS REGULATIONS CONCERNING OLDER AMERICANS

Fadi Martinos, *University of Iowa, Iowa City, Iowa, United States*

As of 2022, 21 states have fully legalized cannabis, 23 legalize for medical use only, and 6 states prohibit cannabis use entirely. While previous research has associated discrete aspects of state cannabis regulation with individual outcomes, little is known about the administrative rules most relevant to older persons. We previously have observed how the progressive approach to legalization across the United States, which includes flexible regulation on legal access, medical program eligibility and qualifying conditions, potency limits, care giver autonomy and others has corresponded with increased accessibility and use of cannabis among Americans over 65 years old. In this study, we source data on seven distinct state regulations pertaining to older persons and measured each on a scale reflecting permissiveness relevant to access and use. We used individual item measures to construct aggregated scores of state policies and charted changes within and among these seven state policies from 2016 to 2022. This research advances scientific understanding by capturing finite distinctions among state regulatory approaches most likely to impact older persons and offering reliable time-varying measures to be included in multi-level model formulations.

### SESSION 1035 (SYMPOSIUM)

Abstract citation ID: igad104.0039

### ADVANCE CARE PLANNING IN PRIMARY CARE: ENGAGING ACCOMPANIED OLDER ADULTS WITH COGNITIVE IMPAIRMENT

Chair: Jennifer Wolff Co-Chair: Diane Echavarria
Discussant: Laura Gitlin

Alzheimer's Disease and Related Dementias (ADRD) are among the most profoundly disabling and costly of all health conditions, and persons living with dementia are at heightened risk for high utilization of burdensome and costly end-of-life care. Family caregivers are at the forefront of managing ADRD, however, they are not routinely engaged in primary care discussions about prognosis and the healthcare preferences of the people for whom they care. Advance care planning (ACP) is a longitudinal communication process that supports adults at any age or stage of health in understanding and sharing their personal values, life goals, and preferences regarding future medical care. Early initiation of ACP, communication with the primary care team, and the inclusion of associated caregivers is imperative in ADRD care due to progressive and devastating effects on decision-making capacity connected with the disease. SHARE, a multicomponent communication intervention, engaged 273 patient-family caregiver dyads in a randomized-controlled trial collecting baseline and six-month survey data related to experiences in the primary care setting. 145 patient-caregiver dyads assigned to the intervention received an offer for a facilitated advance care planning conversation. This symposium will highlight 6-month outcomes from caregiver-reported surveys. Attendees will gain a perspective on ACP considerations in a cognitively impaired population including ethical considerations and the factors influencing preparedness for medical decision making for involved family caregivers. Additional attention concerning the quality and content of ACP conversations with patient-caregiver dyads and the development of a fidelity tool for usage with audio-recorded ACP conversations will be presented.

Abstract citation ID: igad104.0040

### PRIMARY OUTCOMES FROM CAREGIVER-REPORTED SURVEYS IN THE SHARE TRIAL

Jennifer Wolff[1], Erin Giovannetti[2], Cynthia Boyd[3], David Roth[1], Talan Zhang[3], Diane Echavarria[1], Valecia Hanna[4], and Daniel Scerpella[5], *1. Johns Hopkins University, Baltimore, Maryland, United States*, *2. MedStar Health Research Institute, Baltimore, Maryland, United States*, *3. Johns Hopkins School of Medicine, Baltimore, Maryland, United States*, *4. Johns Hopkins School of Public Health, Baltimore, Maryland, United States*, *5. Johns Hopkins Bloomberg School of Public Health, Baltimore, Maryland, United States*

This presentation will introduce SHARE, a randomized-controlled trial to proactively engage family caregivers and normalize advance care planning in primary care. The objective of the study is to engage family caregivers in longitudinal interactions with primary care clinicians and stimulate and support ACP discussions in primary care. We focus on persons with mild, moderate, and severe cognitive impairment regardless of clinical diagnosis because of the importance of addressing advance care planning early in the disease trajectory, the under-diagnosis of ADRD, and because of the greater implementation potential of a protocol with broader applicability. We will describe the study design and present on 6-month outcomes to test our hypothesis that as compared with the minimally enhanced control care group, caregivers in the intervention group will report better quality of communication about end-of-life care with primary care clinicians at 6 months (primary outcome).

Abstract citation ID: igad104.0041

### ADVANCE CARE PLANNING FIDELITY TOOL IN THE CONTEXT OF COGNITIVE IMPAIRMENT: THE SHARE TRIAL

John Cagle[1], David Roth[2], Cynthia Boyd[3], Naaz Hussain[4], Ambrym Smith[5], Jenni Reiff[2], and Daniel Scerpella[6], *1. University of Maryland, Baltimore, Baltimore, Maryland, United States*, *2. Johns Hopkins University, Baltimore, Maryland, United States*, *3. Johns Hopkins School of Medicine, Baltimore, Maryland, United States*, *4. Johns Hopkins Community Physicians, Baltimore, Maryland, United States*, *5. MedStar Union Memorial Hospital, Baltimore, Maryland, United States*, *6. Johns Hopkins Bloomberg School of Public Health, Baltimore, Maryland, United States*

2. Conducting high-quality advance care planning (ACP) conversations with persons living with ADRD and their family members is inherently challenging. Differing levels of cognitive function, judgment, ability to engage, and care partner involvement adds to the complexity of such ACP conversations, necessitating flexible fidelity monitoring to accommodate contextual factors. This presentation will focus on the process of measuring ACP fidelity. The SHARE ACP fidelity checklist was developed to measure the protocol adherence during ACP meetings, monitor for areas in which ACP facilitators may require re-training or additional support, and to inform analyses of the impact of the overall exposure to the study intervention. The fidelity checklist items are organized into three overarching domains: Meeting Set-Up, ACP Meeting Topics, and Facilitator Communication Skills. For the present study, a fidelity adjudication team from a pool of 7 raters, listened to and independently rated recorded conversations for protocol adherence. Each checklist item is scored ranging from 0 to 2 (0=not done; 1=needs improvement; or 2=effective) with higher scores indicating greater adherence. Fidelity ratings tended to improve over time, suggesting a facilitator experience effect. The goal to achieve an overall fidelity rating of ≥80% was met in 63% of dyad conversations. Based on subscale scores, project targets were most frequently met on the Communication Skills subscale and less frequently accomplished on the Meeting Set Up subscale. Despite some challenges, preliminary assessments of SHARE intervention fidelity appear promising, and evidence suggests the fidelity checklist is both valid and reliable.

Abstract citation ID: igad104.0042

### EXPLORATION OF PREPAREDNESS FOR MEDICAL DECISION MAKING FOR FAMILY CAREGIVERS OF PEOPLE WITH DEMENTIA

Peiyuan Zhang, and John Cagle, *University of Maryland, Baltimore, Baltimore, Maryland, United States*

As dementia progresses, many family caregivers of people with dementia (PWD) often experience increasing demands of making medical decisions for their loved ones and feel unprepared, causing severe psychological distress. This presentation explores what factors influence preparedness level of family caregivers in medical decision-making for PWD. As part of a larger two-group randomized trial in which 273 PWD-family caregiver dyads received either a control protocol or a multi-component communication intervention including advance care planning (ACP) facilitation, this qualitative study used thematic analysis methods to analyze 10 transcripts resulting from audio-recorded and transcribed ACP conversations with an ACP facilitator, PWD, and family caregivers. Interview duration was over 30 minutes. The major themes identified were: (1) knowledge about PWD's treatment preferences, (2) experience with death and dying, (3) previous decision-making involvement, and (4) spiritual beliefs. Many family caregivers found ACP conversations with PWD about their goals of care to be beneficial in improving their confidence in making medical decisions. A few participants also reported witnessing a loved one's suffering at the end of life or having made care decisions for family before helped them to initiate conversations with PWD about medical wishes, thus enhanced readiness for decision making. Lastly, few caregivers reported belief in God would help them make the "right decisions". Preliminary results highlight that caregivers' understanding of PWD's treatment preferences helps to prepare family caregivers for decision-making, underlining the need for early and culturally appropriate ACP conversations among residents, caregivers, and clinicians.

Abstract citation ID: igad104.0043

### ETHICAL CONSIDERATIONS UNDERLYING ACP FOR ACCOMPANIED OLDER ADULTS WITH COGNITIVE IMPAIRMENT

Martha Abshire Saylor[1], Cynthia Boyd[2], Sydney Dy[3], and Valecia Hanna[3], *1. Johns Hopkins University School of*

*Nursing, Baltimore, Maryland, United States*, 2. *Johns Hopkins School of Medicine, Baltimore, Maryland, United States*, 3. *Johns Hopkins School of Public Health, Baltimore, Maryland, United States*

While clinicians understand the importance of ACP, it is difficult to overcome logistical and ethical challenges in the limited time providers have in primary clinic appointments with older adults with ADRD and other complex co-morbidities. Ethical/moral dilemmas faced by patients, families, and clinicians are reflected in the four principles of biomedical ethics including autonomy, beneficence, nonmaleficence, and justice. While ethical challenges are recognized there is a lack of real-world evidence to guide clinicians or family caregivers in ACP for older adults with ADRD based on the reality of complex patient and family dynamics. Throughout the SHARE trial, facilitated ACP conversations with trained ACP facilitators, older adult patients with cognitive impairment, and their family caregiver were audio recorded and transcribed. The purpose of this presentation is to expand our understanding of best practices for facilitating ACP conversations among persons with ADRD and their family caregivers, guided by medical ethics and the Learning Health Care System Ethics Framework. We draw on the unique dataset of recorded ACP conversations involving older adults with severe cognitive impairment (N=92, average age=88 years), family caregivers, and trained ACP facilitators. We qualitatively coded transcripts for ethical considerations of ACP conversations and conducted the thematic analysis. We will present major themes related to ethical considerations in facilitating ACP conversations among persons with moderate to severe ADRD and their family caregivers. We will provide a discussion framed in balancing patient, family, and provider needs within a Learning Health Care System.

### SESSION 1040 (SYMPOSIUM)

Abstract citation ID: igad104.0044

### CATALYZING NOVEL METHODS OF MEASURING COGNITION BY HARNESSING TECHNOLOGY

Chair: Stacy Andersen Co-Chair: Walter Boot

Early detection of cognitive impairment is essential for enabling access to clinical assessments and treatments and facilitating participation in clinical trials for Alzheimer's disease. By employing smartphones and digital technologies to capture cognitive behaviors with high precision we may be able to detect novel, subtle biomarkers of cognitive impairment during clinic-based assessments, in-home self-administered tasks, and daily activities. In this Collaborative Symposium from the Alzheimer's Disease and Related Dementias and Technology and Aging Interest Groups, we will explore the development of technologies to detect cognitive impairment and discuss the feasibility of obtaining these data in non-traditional settings. The first presentation reports on the use of eye-tracking technology to detect cognitive impairment with high accuracy. The second presentation discusses acoustic features from digital voice recordings of a cognitive screener that are associated with cognitive impairment and increase the screener's specificity. The next presentation describes linguistic features from voice recordings of a memory test that are associated with depression and distinct from those associated with cognitive impairment. The fourth presentation introduces a smartphone application that measures hand grip strength, a known correlate of dementia risk. The symposium concludes with a summary of older adults' attitudes toward wearable technologies to detect cognitive decline and the development of a smart reminder system to promote adherence to home-based assessment. Overall, these presentations highlight ways we can harness technology to capture unique features of cognitive function that increase the accuracy of traditional testing or detect cognitive impairment in novel settings. This is a collaborative symposium between the Alzheimer's Disease and Related Dementias and Technology and Aging Interest Groups.

Abstract citation ID: igad104.0045

### USE OF EYE TRACKING TECHNOLOGY TO DETECT MILD COGNITIVE IMPAIRMENT

Quinn Kennedy[1], Linda Chao[2], and Dorion Liston[3], *1. neuroFit, Carmel, California, United States*, 2. *University of California, San Francisco, San Francisco, California, United States*, 3. *neuroFit, Mountain View, California, United States*

Early detection of dementia is key to helping individuals and their families cope. Use of eye tracking technology to measure eye movements could provide an objective and sensitive measure of cognitive impairment (Hodgson et al, 2019; Wang et al, 2020). In this pilot study, we predicted that eye tracking metrics differ between people with diagnosed MCI and those of healthy controls. Eleven veterans (≥ 55 years) being seen at the San Francisco VA Health Care System who either had a confirmed diagnosis of MCI or had subjective memory complaints and scored lower than a 26 on the MoCA participated. Their results were compared to that of a previously collected control sample (n = 41) (Liston et al, 2017). Participants completed a five-minute visual tracking test with 48 trials based on a classic step-ramp visual tracking paradigm that was administered on a PC computer with a camera and eye tracking capability. The visual tracking test yields 10 z-scored eye tracking metrics that are summarized in a single scalar summary score. Receiver operating characteristics (ROC) were computed and compared to the control sample. The summary score of the MCI group (median: -2.2) differed significantly from the healthy control sample (median: 0.0), which yielded a significant sensitivity of the test to presence or absence of MCI (ROC area = 0.94, p < .001). Although we view the results as preliminary due to the small sample size, results suggest that use of eye tracking technology may be a viable option for MCI detection.

Abstract citation ID: igad104.0046

### DETECTING COGNITIVE IMPAIRMENT FROM DIGITAL VOICE RECORDINGS OF A REPETITION TASK

Stacy Andersen[1], Nicole Roth[2], Seho Park[1], Thomas Perls[3], Stephanie Cosentino[4], Rhoda Au[5], and Paola Sebastiani[6], *1. Boston University Chobanian & Avedisian School of Medicine, Boston, Massachusetts, United States*, 2. *Boston University School of Medicine, Boston, Massachusetts,*

*United States*, *3. Boston University, Boston, Massachusetts, United States*, *4. Columbia University Medical Center, New York City, New York, United States*, *5. Boston University Chobian & Avedisian School of Medicine, Boston, Massachusetts, United States*, *6. Tufts Medical Center, Boston, Massachusetts, United States*

Early detection of Alzheimer's disease is needed but cognitive screeners, used widely in research and clinical settings, suffer from poor sensitivity and specificity. Acoustic features of speech are altered in Alzheimer's disease and may aid detection of cognitive impairment. We sought to test whether acoustic metrics from a cognitive screener differentiate individuals with cognitive impairment from those who are cognitively normal. We extracted 38 acoustic features from digital voice recordings of the Mini-Mental Status Examination repetition task from Long Life Family Study participants using the openSMILE toolkit. Highly correlated features (r>0.9) were removed. Cognitive status (impaired versus cognitively normal) was determined through consensus review of neuropsychological testing and informant interviews. Bayesian Information Criterion-based stepwise regression including 24 acoustic variables and retaining age, sex, education, and MMSE score in all models was used to identify predictors of cognitive status. Acoustic features from preliminary data included 277 participants (mean age 74.8±12.3 years old, 57% females, 34% cognitively impaired due to oversampling). Stepwise selection identified greater loudness (i.e., signal intensity; p<0.001), smaller pitch changes (p=0.007), and lower Harmonics-to-Noise Ratio (i.e., a metric of voice quality; p=0.017) as significantly associated with cognitive impairment. A model with MMSE, demographics, and acoustics had a higher discriminative value for cognitive status (AUC 0.95, sensitivity=0.97, specificity=0.73) than the model without acoustics (AUC = 0.94, sensitivity=0.97, specificity=0.63). Overall, acoustic features from a brief (i.e., 3 second) repetition task were associated with cognitive impairment and added information beyond traditional scoring that increased the specificity of a cognitive screener.

Abstract citation ID: igad104.0047

### LINGUISTIC PROFILES OF PARAGRAPH RECALL PREDICT DEPRESSION IN OLDER ADULTS

Seho Park[1], Nicole Roth[2], Paola Sebastiani[3], Stephanie Cosentino[4], and Stacy Andersen[1], *1. Boston University Chobanian & Avedisian School of Medicine, Boston, Massachusetts, United States*, *2. Boston University School of Medicine, Boston, Massachusetts, United States*, *3. Tufts Medical Center, Boston, Massachusetts, United States*, *4. Columbia University Medical Center, New York City, New York, United States*

Our previous work identified linguistic markers from paragraph recall that predicted cognitive impairment. However, many features were related to psychological constructs, and mood disorders, such as depression, are also associated with cognitive impairment. Therefore, we aimed to identify linguistic markers that are associated with depression and examine their overlap with those for cognitive impairment. We used Linguistic Inquiry Word Count, a natural language processing tool, on Logical Memory transcriptions from the Long Life Family Study. Depression status was positive for participants with a self-reported depression diagnosis or high depressive symptoms (i.e., a CESD-10 score≥10) at testing. We used logistic regression models with Generalized Estimating Equations adjusted by age, sex, education, and within-family relatedness to identify linguistic features associated with depression status (Bonferroni-corrected threshold of p<.001). The sample included 61 participants with evidence of depression and 539 participants without (mean age=72.1±10.9 years, female=63%). A lower percentage of verb use in delayed recall was significantly associated with depression (Odds Ratio [OR]=0.71, CI 0.55-0.93, p=.0001). Additionally, 3 immediate recall features (verbs, OR=0.71; prepositions, OR=1.30; differentiation words, OR=1.30) and 5 delayed recall features (function words, OR=0.68; prepositions, OR=1.50; future tense, OR=0.48; cognitive process words, OR=0.78; past tense, OR=0.74) were significant at a nominal level (p=.009 to p=.035). We identified linguistic features from paragraph recall responses that predict depression and were largely distinct from those associated with cognitive impairment as only two features overlapped. Linguistic analyses of spoken responses may help us understand contributors to test performance and distinguish between cognitive impairment and depression.

Abstract citation ID: igad104.0048

### CHALLENGES AND NOVEL SOLUTIONS TO PROMOTING ADHERENCE TO HOME-BASED COGNITIVE ASSESSMENT

Walter Boot, Ibukun Fowe, Shenghao Zhang, Michael Dieciuc, and Andrew Dilanchian, *Florida State University, Tallahassee, Florida, United States*

New technologies now allow for home-based self-assessment of cognition to promote the early detection and treatment of cognitive decline. Early detection has a multitude of benefits for the individual and for our scientific understanding of cognitive impairments and possible treatments. However, for these solutions to be effective, they will likely require long-term adherence to assessment protocols to detect longitudinal change, and like many health behaviors, adherence can be a major challenge. This presentation will provide an overview of the Adherence Promotion with Person-centered Technology (APPT) project, which aims to develop a tailored, smart reminder system that infers participants' contexts and motivations to provide just-in-time support to facilitate cognitive assessment adherence. Among other studies, this overview will include discussion of a recently completed focus group study (N = 42) that was used to understand motivations and potential barriers to home-based assessment and preferences for adherence support, a recently completed pilot study (N = 44) in which the adherence support system was initially tested and refined, and a series of survey studies (total N > 200) that examined older adults' attitudes toward wearable technologies to detect cognitive decline. The talk will conclude with discussion of an upcoming randomized controlled trial to test the efficacy of the newly developed system with respect to promoting long-term adherence to cognitive assessment via smartphone technologies.

### SESSION 1045 (SYMPOSIUM)

Abstract citation ID: igad104.0049

### ACCESS TO DENTAL CARE AND ORAL HEALTH INEQUITIES AMONG DIVERSE POPULATIONS IN THE UNITED STATES AND CHINA

Chair: Xiang Qi Co-Chair: Xi Chen Discussant: Elisa Ghezzi

Although the oral health status of the world population has improved over the years, structural and societal inequities in dental care and oral health are pervasive with older adults. This symposium discusses findings from three studies that examine structural and societal determinants of dental care and oral health inequities among diverse populations in the US and China. Mao's study investigated correlates of dental care utilization among informal dementia caregivers in the US. Findings demonstrate that spousal caregivers and those who provide intensive care are less likely to visit a dentist. These findings suggest that dementia caregiver support intervention strategies should target these populations to improve dental care-seeking behaviors. Qi's study examined the population attributable fraction of poor oral health on incident dementia in comparison with other modifiable risk factors. This study found that poor oral health, including edentulism and oral health problems, is a significant risk factor for incident dementia. The study highlights the need to integrate dental care as an integral component of a comprehensive approach to promoting cognitive health. Qu's study explored social structural disparities in dental care utilization among adults in China's megacities. This study revealed that social structural factors, such as hukou status, occupational status, and city of residence, are associated with disparities in dental care utilization. Overall, these studies shed light on important factors that impact dental care utilization and oral health. Addressing disparities in dental care utilization and oral health should be a priority in promoting overall health and well-being, particularly among vulnerable populations.

Abstract citation ID: igad104.0050

### POPULATION ATTRIBUTABLE FRACTION OF POOR ORAL HEALTH FOR THE INCIDENCE OF DEMENTIA: A POPULATION-BASED COHORT STUDY

Xiang Qi, and Bei Wu, *New York University, New York City, New York, United States*

Emerging evidence indicates a positive association between suboptimal oral health and increased risks of dementia. Despite the preventable and treatable nature of many oral health problems, oral health has not been considered in the risk factors for dementia that shape public health policy and research priorities. We aimed to quantify and compare the fractions of incident dementia attributable to poor oral health and other modifiable risk factors. The research included a nationally-representative sample of 15,474 cognitively intact adults aged≥50 from the Health and Retirement Study, with the outcome of incident dementia between 2006 to 2016. We estimated the multivariate-adjusted hazard ratio (HR) and population attributable fraction (PAF) of edentulism and nine risk additional dementia factors. Additional analyses were conducted on 1,239 individuals who completed the "Dental Health Module" in 2008, examining oral health problems as an exposure. Edentulism was associated with a higher risk of dementia for both men (HR,1.38; 95%CI, 1.15-1.66) and women (HR, 1.32; 95%CI, 1.16-1.51). The PAF of edentulism for dementia was 4.64%, which exceeded that of hearing impairment (3.58%) and hypertension (2.90%). Oral health problems were also a significant factor for men (HR, 2.01; 95%CI, 1.49-3.39) and women (HR, 1.15; 95% CI, 1.02-1.30), with a PAF of 11.84% among men, the second-largest following less education (22.60%). The substantial estimated proportion of dementia attributed to poor oral health in the aging population, particularly in men, warrants more attention. Dental care should be an integral component of a comprehensive approach to promoting cognitive health.

Abstract citation ID: igad104.0051

### CORRELATES OF DENTAL CARE UTILIZATION AMONG US INFORMAL DEMENTIA CAREGIVERS

Weiyu Mao[1], Bei Wu[2], and Wei Yang[3], *1. University of Nevada, Reno, Reno, Nevada, United States*, *2. New York University, New York City, New York, United States*, *3. University of Nevada Reno, Reno, Nevada, United States*

Informal dementia caregivers tend to report more negative health impacts from the caregiving role, higher levels of unmet needs, and lower levels of service utilization compared to caregivers of persons with other types of illnesses. There remains a gap in knowledge of understanding unique correlates of dental care utilization among informal dementia caregivers. As guided by the Andersen model, this study examined correlates of dental care utilization among U.S. informal dementia caregivers. Data came from the Behavioral Risk Factor Surveillance System collected in 2016, 2018, and 2020 (n=2,931). Respondents were selected when they reported providing regular care or assistance to a friend or family member who has Alzheimer's disease, dementia, or other cognitive impairment disorder. Dental care utilization was dichotomized into "yes" versus "no" within the past year. Caregiving-related characteristics included caregiver role (e.g., spouse, adult child, other relative, and friend), intensity of care, duration of care, and type of care. Other correlates included caregiver characteristics, health behaviors, and physical and oral health needs. Findings from stepwise logistic regression models demonstrate that being a spousal caregiver, providing more than 20 hours of care per week, being male, having high school or less education, being low-income, unemployed, current smoker, having no health insurance, no current alcohol use, or less than adequate sleep was associated with a lower likelihood of vising a dentist within the past year. The findings suggest targeting spousal caregivers and those who provide intensive care to improve dental care-seeking behaviors in dementia caregiver support intervention strategies.

Abstract citation ID: igad104.0052

### SOCIAL STRUCTURAL DISPARITIES IN DENTAL CARE UTILIZATION AMONG ADULTS IN CHINA'S MEGACITIES

Xiaomin Qu[1], Xiang Qi[2], and Bei Wu[2], *1. East China University of Political Science and Law, Shanghai, Shanghai,*

*China (People's Republic)*, *2. New York University, New York City, New York, United States*

This study utilized data from the “2019 New Era and Living Conditions in Megacities Survey” to investigate social structural disparities in dental care utilization among adults aged 18-65 from 10 megacities in China. Logistic regressions were conducted to examine the relationships between social structural factors, such as hukou status, occupational status, migrant status, and city of residence, and dental care utilization. Margins post estimation was used to explore whether the social structural disparities in dental care utilization were associated with changes in oral health status. Results showed that only 22.36% of participants had at least one dental visit per year. Having urban hukou as compared with rural hukou (OR, 1.76; 95% CI, 1.47-2.12), working in state-owned enterprise as compared with in other enterprises (OR, 1.34; 95% CI, 1.14-1.57), and living in more developed cities as compared with in less developed cities (OR, 1.48; 95% CI, 1.27-1.72) were associated with a higher likelihood of regular dental visits. Additionally, participants with urban hukou and those living in more developed cities were more likely to have regular dental visits when their oral health was good, such as when they had full natural teeth. This study highlights social structural disparities in oral health behaviors among adults in China’s megacities. The findings suggest that efforts should be made to improve dental care utilization by promoting public messaging about oral health knowledge, expanding dental care coverage, and improving access to dental care services in China.

### SESSION 1050 (SYMPOSIUM)

Abstract citation ID: igad104.0053

### ALZHEIMER'S DISEASE AND RELATED DEMENTIAS IN A FAMILY CONTEXT

Chair: J Jill Suitor Co-Chair: Megan Gilligan

This symposium considers the ways in which the presence of Alzheimer’s Disease and Related Dementias (ADRD) shapes functional and affective relationships between family members, and raises questions about the future public health implications of ADRD family caregiving. In the first paper, Huo and Kim use dyadic data collected from husbands and wives in which one partner lives with mild-to-moderate cognitive AD to explore the role of relationship quality in caregiver stress. Next, Suitor and colleagues use mixed-methods data from the Within-Family Differences Study to examine how mothers’ cognitive impairment moderates the impact of maternal favoritism/disfavoritism on the psychological well-being of adult sons and daughters. Third, Gilligan, Suitor and Ogle investigate the role of adult children’s care provision in the association between parents’ cognitive impairment and adult sibling tension, also using mixed-methods data from the WFDS. Next, Savla and Roberto use mixed-methods data to explore how extended family caregivers manage their care responsibilities and how care arrangements impact the provision of future care for persons living with ADRD. Finally, Gaugler considers the public health ramifications of family caregiving, summarizing the scientific literature on estimating the future supply of family caregivers, and presenting a research agenda to address the potential family care gap. Taken together, this diverse set of papers highlights the complex ways in which the presence of ADRD shapes patterns of care, relationship quality, and well-being of members of later-life families. This is a collaborative symposium between the Alzheimer's Disease and Related Dementias and Family Caregiving Interest Groups.

Abstract citation ID: igad104.0054

### MATERNAL DIFFERENTIAL TREATMENT AND ADULT CHILDREN'S DEPRESSIVE SYMPTOMS IN THE FACE OF COGNITIVE IMPAIRMENT

J Jill Suitor[1], Megan Gilligan[2], Robert Frase[3], Destiny Ogle[1], and Ranran He[4], *1. Purdue University, West Lafayette, Indiana, United States*, *2. University of Missouri, Columbia, Missouri, United States*, *3. Southern Illinois University, Carbondale, Illinois, United States*, *4. Sociology, West Lafayette, Indiana, United States*

Parental differential treatment has been shown to have a negative impact on adult children’s psychological well-being, particularly among daughters. In this paper, we explore whether mothers’ cognitive impairment (CI) moderates the impact of maternal favoritism/disfavoritism on psychological well-being when children are entering their later years (M=60), and their mothers are in their 80s-90s. We address these questions using mixed-methods data from 297 adult children who participated in wave 3 of the Within-Family Differences Study. Quantitative and qualitative analyses revealed that the effects of perceived favoritism/ disfavoritism were shaped by the combination of child’s gender and mothers’ CI status. Perceptions of disfavoritism, particularly regarding mothers’ disappointment, affected daughters’ depressive symptoms; however, the effect was decidedly muted by mothers’ CI. In the case of mothers’ preferences regarding their future caregivers, the differences by cognitive status were particularly striking. Contrary to expectations, daughters’ perceptions that they were their mothers’ preferred caregivers predicted depressive symptoms when mothers showed no signs of CI, but had no impact when mothers were cognitively impaired. Among sons, depressive symptoms were not affected by mothers’ favoritism or disfavoritism, regardless of mothers’ CI status; a gender difference in the impact of mothers’ appraisals on well-being that we have seen emerge across the 20 years we have followed these families. Qualitative analyses suggest that mothers’ CI reduced daughters’ emotional reactions to their mothers’ differential treatment because they believed that their mothers had less control of their behaviors and less awareness of the negative impact of their appraisals and expectations.

Abstract citation ID: igad104.0055

### CHANGES IN ADULT SIBLING CONFLICT AND OLDER PARENTS' COGNITIVE IMPAIRMENT: THE MODERATING ROLE OF CARE PROVISION

Megan Gilligan[1], J Jill Suitor[2], and Destiny Ogle[2], *1. University of Missouri, Columbia, Missouri, United States*, *2. Purdue University, West Lafayette, Indiana, United States*

The life course concept of “linked lives” emphasizes that the life events of one family member have implications for other family members. Health events have been shown to be particularly salient in later-life families. We considered the consequences of older parents’ cognitive impairment on adult sibling relationship quality. Using mixed methods data from 327 adult children (nested within 96 families) collected as part of the Within-Family Differences Study (WFDS) T3, we examined the role of adult children's care provision in the association between parents' cognitive impairment and adult sibling tension. At T3 of the WFDS, the adult children were on average 60 years old and their parents were on average 90 years old. We conducted lagged multilevel linear regression modeling to account for nonindependence and to control for sibling tension seven years earlier before parents were experiencing cognitive impairment. The results of the quantitative analyses revealed that reports of sibling tension were higher in families in which parents experienced cognitive impairment. Moderation analyses indicated that the association between parents' cognitive impairment and sibling tension was stronger for caregivers than noncaregivers. Qualitative analyses revealed that discrepancies in perceptions of the caregiving situation between caregivers and noncaregivers fueled sibling tension. Noncaregivers often seemed unaware of the caregiving situation or actively disengaged to avoid conflict. Taken together, these findings indicate that sibling tension is particularly high in the context of caring for older parents with cognitive impairment. In particular, adult children who are providing care are at increased risk of sibling tension.

Abstract citation ID: igad104.0056

### WHO'S UP NEXT? EXTENDED FAMILY CAREGIVERS OF PERSONS LIVING WITH DEMENTIA: CURRENT AND FUTURE CARE

Tina Savla[1], and Karen Roberto[2], *1. Virginia Tech, Blacksburg, Virginia, United States*, *2. Virginia Polytechnic Institute & State University, Blacksburg, Virginia, United States*

Various societal trends and expanded family boundaries have led to a broad array of family members involved in caring for persons living with dementia (PLwD). We examined how extended family caregivers (FCG) manage their care responsibilities and how the care arrangement impacts the provision of future care for PLwD. Mixed-method data from 48 extended FCG and 56 nuclear FCG (71% White, 68% female, 63% co-reside with PLwD) revealed that 70% of extended FCG rely on informal helpers to care for PLwD. Spouses and daughters were more often sole caregivers while most sons relied on informal helpers. If no longer able to help, sisters, granddaughters, and nieces more likely foresaw moving PLwD to a care facility, whereas brothers, grandsons, and nephews anticipated another informal helper would assume their care. Extended FCG often expressed dissatisfaction with one or more informal helpers for failing to meet care responsibilities, potentially leading to unmet needs and increased risk for early nursing home placement for the PLwD. The discussion will focus on the attributes of informal helpers of extended FCG that contribute to stable or inconsistent care arrangements for PLwD, which can ultimately affect the well-being of PLwD and FCG.

Abstract citation ID: igad104.0057

### AN AGENDA FOR THE FAMILY CARE GAP: ALIGNING SCIENCE, FAMILY EXPERIENCES, AND PUBLIC HEALTH

Joseph Gaugler, *University of Minnesota, Minneapolis, Minnesota, United States*

When considering the public health ramifications of family caregiving, a major concern is the future supply of family, friends, or other unpaid caregivers (hereafter referred to as family caregivers) of older persons with health needs. This presentation will summarize the state of the science on estimating the future "supply" of family caregivers for older persons, and whether there are structural or social determinants, in addition to long-standing socio demographic trends, which may contribute to the family care gap. In addition, the presentation will outline a potential research agenda on the family care gap that summarizes the potential implications of this phenomenon as well as how it aligns with efforts to elevate family caregiving for older people (particularly those living with dementia) as a matter of public health import.

Abstract citation ID: igad104.0058

### SUPPORT EXCHANGES IN COUPLES COPING WITH EARLY-STAGE ALZHEIMER'S DISEASE

Meng Huo[1], and Kyungmin Kim[2], *1. University of California, Davis, Davis, California, United States*, *2. Seoul National University, Seoul, Republic of Korea*

People living with Alzheimer's disease (AD) typically receive considerable support from their spousal caregivers. Yet, we know little about whether people with early-stage AD may still be able to help their caregivers and how potential support exchanges in these couples may vary depending on the quality of their relationships. We drew on dyadic data from 37 older couples in which one partner lives with mild-to-moderate AD (Mage = 77yrs, 48% male) and the other serves as a spousal caregiver (Mage = 76yrs, 53% female). Couples participated in concurrent interviews during which they each reported on a variety of demographic and relationship characteristics, such as relationship quality, and couple support exchanges. People with early-stage AD perceived their relationships with spousal caregivers as more positive and less negative than did these caregivers. Support did not flow unidirectionally in these caregiving couples, especially not among those in better-quality relationships. Specifically, caregivers who viewed their relationships as more positive/less negative considered it less stressful to provide emotional and practical support to their partners with AD. Patients who viewed their relationships as more positive/less negative provide more frequent emotional and practical support to their caregivers and also found such support provision more pleasant and less stressful. Findings reveal positive contribution that older adults with dementia, who have been solely treated as recipients of care, can make and shed light on the development of dyadic interventions by highlighting the importance of mutually beneficial relationships in early stages of AD.

### SESSION 1055 (PAPER)

### EXPLORING THE CONNECTION BETWEEN SOCIAL NETWORKS AND CAREGIVING

Abstract citation ID: igad104.0059

### COMPARISON OF LONELINESS AND SOCIAL NETWORK BETWEEN OLDER ADULT CAREGIVERS AND NONCAREGIVERS

Ting Yong, Abhijit Visaria, and Rahul Malhotra, *Duke-NUS Graduate Medical School, Singapore, Singapore*

In ageing societies, more older adults are taking up informal caregiving roles. Caregivers, compared to non-caregivers, are found to have poorer psychosocial outcomes such as loneliness and social isolation in various studies. However, most of this research is not specific to older adult caregivers and does not account for selection bias into caregiving. We compare loneliness and social network between older adult caregivers and older adult non-caregivers in Singapore, applying inverse probability weighted regression adjustment (IPWRA), to address selection bias into caregiving, on data from a nationally representative longitudinal study of older adults. Those providing unpaid assistance to any person because of their health condition were considered as caregivers. Loneliness (score: 0-12; higher score=lonelier) and social network (0-60; higher score=greater network) were measured using validated scales. The relationship of caregiving status with loneliness and social network was estimated using IPWRA, controlling for multiple demographic and health characteristics. Of the 2639 respondents, 7.2% were caregivers. Older adult caregivers, versus non-caregivers, were more likely to experience loneliness (average treatment effect [ATE] β-coefficient for loneliness score: 0.328 (95% confidence interval: 0.011-0.645; p-value: 0.042); and odds ratio for being lonely: 1.080 (1.001-1.165); p-value: 0.047). However, they did not differ in terms of their social network (ATE β-coefficient for social network score: 1.456 (-0.405-3.316); p-value: 0.125). Our findings suggest that even with a similar extent of social network, older adult caregivers are at a higher risk of loneliness, i.e., perceived social isolation or relational deficits, compared to non-caregivers.

Abstract citation ID: igad104.0060

### EXAMINING CAREGIVER NETWORK STRUCTURE, COMPOSITION, AND COORDINATION FOR OLDER ADULTS WITH ALZHEIMER'S DISEASE

William McConnell, Katherine Haggar, and María Ortega Hernández, *Florida Atlantic University, Boca Raton, Florida, United States*

Over half of the older adults with Alzheimer's disease and related dementias (ADRD) live in the community with informal caregivers whose unpaid assistance constitutes the majority of their care. Research and interventions targeting caregivers typically limit their focus to one primary caregiver, but many older adults with ADRD rely on a variety of interconnected kin, non-kin, and professional supporters. Little is known about the structure, composition, or consequences of these caregiver networks. To address this gap, we conducted a social network analysis (SNA) of the caregiver networks of 23 older adults with ADRD receiving outpatient services at a Memory Disorder Clinic. SNA data was obtained through detailed social network interviews conducted one-on-one with self-identified primary caregivers. We find that adults with ADRD have an average of 7.7 caregivers in their networks. Quantitative analyses demonstrate that caregiver network properties (including size, density, composition, and care coordination) vary with the context of caregiving (including living situation, level of functional impairment, and use of formal services). An average network includes a roughly even mix of informal caregivers (3.2 members) and professional caregivers (3.5), but they occupy different network positions and fill distinct supportive roles. For example, informal caregivers engage in frequent care coordination, but coordination is comparatively uncommon between formal caregivers. This interdisciplinary research advances beyond the dyadic level of analysis typical of most caregiving research to comprehensively measure caregiver networks. Further, we identify variations in caregiver networks and quantify network-based task-sharing and care coordination among informal and professional caregivers.

Abstract citation ID: igad104.0061

### THE INFLUENCE OF STATIC AND DYNAMIC FACTORS ON LONGITUDINAL PATTERNS OF CAREGIVER NETWORK

Jeong Eun Lee[1], Joseph Svec[2], and Nicholas Cone[1], *1. Iowa State University, Ames, Iowa, United States*, *2. Saint Joseph's University, New York City, New York, United States*

Care networks are defined as the collection of individuals who provide support for older adults with health challenges. Although many older adults receive care from multiple caregivers, with varying intensities of caregiving, little is known about the structure of their care networks and whether or to what extent it changes over time. This study aims to examine the stability of care networks and the predictive effects of static and dynamic factors on patterns of caregiving networks. Using data from the National Health Aging Trends (NHAT) and National Study of Caregiving (NSOC), we examine the static and dynamic intrapersonal structures of care arrangements with regard to systematic continuity across 7 years. We utilize both Latent Profile Analysis and Latent Transition Analysis to identify different types of caregiving arrangements and patterns of transitions over time. Moreover, we use logistic regressions to examine how individual and time-varying factors affect those arrangements and transitions. The results show three groups of caregiving arrangements: 1) intense care with one caregiver, 2) moderate-level care with multiple caregivers, and 3) low-level care with multiple caregivers. Among the groups, moderate-level care with multiple caregivers is the most stable group. Moreover, static factors, such as gender and race, and dynamic factors, such as psychological resilience of care recipients, significantly impacts the probability of transitioning from low-level care to intense care over time. The findings highlight the utility of Latent Transition Analysis in caregiving networks, suggesting that patterns of caregiving outcomes should be understood as process as well as a condition.

### SESSION 1060 (BIOLOGICAL SCIENCES INVITED SYMPOSIUM)

Abstract citation ID: igad104.0062

### THIS IS THE YEAR THAT WAS: THE BEST OF THE JOURNAL OF GERONTOLOGY BIOLOGICAL SCIENCES

Chair: Gustavo Duque

The Journal of Gerontology Biological Sciences (JGBS) is one of the Gerontological Society of America publications. It publishes articles on the biological aspects of aging in areas such as translational gerontology, biomarkers of aging, biochemistry, biodemography, cellular and molecular biology, and geroscience. Biological insights are also published in studies from invertebrates, vertebrates, and mammalian species, including humans. With an impact factor of 6.59, this journal is one of the leading journals in the field. Overall, and although the journal is highly valued by biomedical researchers worldwide, the authors of our top and most impactful papers do not usually have the opportunity to answer the questions from their peers constructively and interactively. In addition, there is a significant number of biomedical researchers that, although members of the Society, do not submit their best results to the journal. This symposium, chaired by the Editor-in-Chief of the JGBS, will comprise six presentations by the authors of the most influential papers of the year (June 2022 to June 2023). The papers will be selected based on their impact via citations and/or social media. Presenters will be chosen following diversity, equity, and inclusion principles. Various topics going from cells to animal models will be covered while highlighting the translational potential of those discoveries. Ten minutes presentation will be followed by 5 minutes of Q&A with ample opportunity to interact.

Abstract citation ID: igad104.0063

**AEROBIC EXERCISE ATTENUATES FRAILTY IN AGING MALE AND FEMALE C57BL/6 MICE AND EFFECTS SYSTEMIC CYTOKINES DIFFERENTIALLY BY SEX**

Susan Howlett, *Dalhousie University, Halifax, Nova Scotia, Canada*

DOI: 10.1093/gerona/glab297

Abstract citation ID: igad104.0064

**FROM MODEL ORGANISMS TO HUMANS, THE OPPORTUNITY FOR MORE RIGOR IN METHODOLOGIC AND STATISTICAL ANALYSIS, DESIGN, AND INTERPRETATION OF AGING AND SENESCENCE RESEARCH**

Steven Austad, *University of Alabama at Birmingham, Birmingham, Alabama, United States*

DOI: 10.1093/gerona/glab382

Abstract citation ID: igad104.0065

**RELATIONSHIP BETWEEN 5 EPIGENETIC CLOCKS, TELOMERE LENGTH, AND FUNCTIONAL CAPACITY ASSESSED IN OLDER ADULTS: CROSS-SECTIONAL AND LONGITUDINAL ANALYSES**

Ilja Demuth, *Charité – Universitätsmedizin Berlin, Berlin, Berlin, Germany*

DOI: 10.1093/gerona/glab381

Abstract citation ID: igad104.0066

**CANAGLIFLOZIN INCREASES INTESTINAL ADENOMA BURDEN IN FEMALE APCMIN/+ MICE**

Justin Korfhage[1], and David Lombard[2], *1. Yale School of Medicine, New Haven, Connecticut, United States*, *2. University of Michigan, Ann Arbor, Michigan, United States*

DOI: 10.1093/gerona/glab254

Abstract citation ID: igad104.0067

**SERUM CONCENTRATIONS OF LOSARTAN METABOLITES CORRELATE WITH IMPROVED PHYSICAL FUNCTION IN A PILOT STUDY OF PREFRAIL OLDER ADULTS**

Peter M. Abadir, *Johns Hopkins University, Baltimore, Maryland, United States*

DOI: 10.1093/gerona/glac102

### SESSION 1065 (PAPER)

### SOCIAL ISOLATION AND LONELINESS I

Abstract citation ID: igad104.0068

**GENTRIFICATION, AGING, AND HEALTH: A NARRATIVE REVIEW**

Dionne Bailey, *University of Minnesota, Minneapolis, Minnesota, United States*

Multiple definitions of gentrification exist, and the lack of consensus about what constitutes gentrification has limited the efforts of this concept's measurement/operationalization as well as an understanding of how gentrification influences outcomes, particularly for older adults. The objective of this literature review is to synthesize existing definitions and measurement approaches to gentrification in studies of older adults and their communities. Another objective is to summarize the health implications of gentrification for older adults. OVID Medline, CINAHL, Academic Search Premier, PsycINFO, and Scopus were searched in order to identify peer-reviewed articles that were published up to August 2022. 'Gentrification' and 'Aging' were the keywords utilized to search for peer-reviewed articles. Post abstract screening, each article was annotated to identify key study characteristics and tabulated to identify patterns and themes. Forty-nine articles were identified and extrapolated. The analysis revealed inconsistencies in the definition of gentrification, which lead to mixed findings related to the health effects of gentrification for older people. Specifically, it was found that there are both positive and negative effects of gentrification. The most consistent findings evident were that minorities were most at-risk during periods of gentrification, particularly Black older adults. It is imperative that a scientific consensus is reached when defining and subsequently operationalizing gentrification. Such refinements will result in more robust and rigorous quantitative and qualitative research that describes the health implications and/or lived experiences of gentrification for older adults.

Abstract citation ID: igad104.0069

**LIVING FOREVERWELL IN RURAL AMERICA: RESULTS FROM A QUALITATIVE EVALUATION OF A YMCA HEALTHY AGING PROGRAM**

Rachel Kappel[1], Alycia Bayne[1], Shena Popat[1], Mabel Frank[1], Seana Hasson[2], and Lindsay Mondick[3], *1. NORC at the University of Chicago, Chicago, Illinois, United States*, *2. YMCA of the USA, Chicago, Illinois, United States*, *3. YMCA of the USA, Eagan, Minnesota, United States*

Older adults benefit from community programs that promote physical and mental health, reduce social isolation, and offer connections to community resources. However, older adults living in rural communities have access to fewer health and social services and experience barriers to participation such as access to transportation. Recognizing these challenges, the YMCA of the USA (Y-USA) expanded ForeverWell, a healthy aging model, to 17 YMCAs ("Ys") serving adults aged 55+ living in rural areas. Developed by the YMCA of the North in Minnesota, ForeverWell is a flexible set of Y activities focused on five dimensions of healthy aging: mind, body, community, spirit, and nature. The goals of ForeverWell are to improve health and well-being, decrease social isolation, and increase community connections among older adults. NORC at the University of Chicago conducted a qualitative evaluation of ForeverWell's expansion in 17 communities in Minnesota, North Dakota, and South Dakota. NORC conducted virtual interviews with 25 Y staff members and 24 program participants to identify facilitators, challenges, and outcomes. NORC transcribed interviews and conducted qualitative content analysis to identify themes. This session describes findings from the interviews. A key facilitator of success was the flexibility of the model. Challenges included staff and financial resources, outreach in rural areas, and transportation access. Ys developed and enhanced older adult programming and created new partnerships. Program participants reported improvements in physical and mental health and strong social connections. ForeverWell is a flexible, holistic healthy aging program that shows promise for older adults living in rural communities.

Abstract citation ID: igad104.0070

### LONELINESS AMONG INVOLUNTARILY CHILDLESS OLDER ADULTS: A LIFE COURSE PERSPECTIVE

Hannelore Stegen[1], Daan Duppen[2], Liesbeth De Donder[2], and Eva Dierckx[2], *1. Research Foundation Flanders (FWO) & Society and Ageing Research Lab (SARLab), Vrije Universiteit Brussel (VUB), Brussels, Brussels Hoofdstedelijk Gewest, Belgium*, *2. Vrije Universiteit Brussel (VUB), Brussels, Brussels Hoofdstedelijk Gewest, Belgium*

To this day, having children is seen as the norm, and the consequences in later life when a child wish is not fulfilled are rarely addressed. Therefore, this study investigates the reasons for involuntary childlessness as well as the relationship with potential feelings of loneliness throughout the life course of older adults. Data were collected through individual life story interviews using the McAdams life story interview (N=15) with involuntary childless adults older than 60 living in Belgium. Using a thematic analysis, the results firstly indicate that respondents expressed several reasons for their involuntary childlessness, such as medical reasons, advancing age, career choices and opportunities, partner status and traumas during the life course. Secondly, involuntary childlessness impacts negatively on individual well-being on the one hand, by experiencing feelings of emptiness and deficiency, but on the other hand, having more free time and flexibility during the life course within the social network is perceived as positive. Thirdly, from the moment people realized they would never have children, feelings of loneliness arose. Also, once peers did have (grand)children, or during holiday seasons (e.g., Christmas Day), loneliness feelings became more prevalent compared to earlier in life. However, feelings of loss and emptiness (e.g., missing someone to pass on norms and values to) prevailed over loneliness feelings. The discussion highlights the extent to which older people pick up where they left off and constitute their lives meaningfully at various levels (i.e., at individual level and regarding social relations) when their child wish was never fulfilled.

Abstract citation ID: igad104.0071

### MATERIAL DEPRIVATION AND LONELINESS AMONG OLDER ADULTS IN HONG KONG

Vivien Foong Yee Tang[1], and Kee Lee Chou[2], *1. The Education University of Hong Kong, Hong Kong, Hong Kong*, *2. The Education University of Hong Kong, Hong Kong, Hong Kong*

Given that poverty as a risk factor on loneliness is understudied, this study examined the relationship between material deprivation and loneliness of older adults in Hong Kong, the mediation effects of social support and social network, and the moderation effects of social support and social engagement. A two-wave data were collected from 1,696 Chinese older adults between 2015 (T1) and 2017 (T2). All Chinese older adults aged 60 years and above, with 53.9% female. Older adults were asked about their level of loneliness, how material deprived they were, social support, social network, social engagement, demographic information, and self-rated health status. The result indicated that older adults who are more materially deprived reported a significantly higher level of loneliness at T2 when controlling for demographic variables and loneliness at baseline. Furthermore, we found that social support and the number of close children were full mediator. A significant moderator was found in neighbourhood collective efficacy and community participation specifically engagement in cultural activities. Hence, our results suggest the need to strengthen the anti-poverty policy measures for materially deprived, develop interventions focusing on improving social support and social network and enhance existing social engagement programmes that promote community participation and neighbour collective efficacy for older adults in Hong Kong.

Abstract citation ID: igad104.0072

### THE LONELINESS OF LOW-INCOME OLDER ADULTS IN A FEDERAL VOLUNTEERING PROGRAM: A MIXED-METHOD NETWORK PERSPECTIVE

Katy (Qiuchang) Cao, *Florida State University, Tallahassee, Florida, United States*

Volunteering is considered an important way to prevent and address loneliness for community-living older adults by fostering social interactions. However, some social network studies conducted outside of the volunteering context suggest that increased interactions among lonely individuals can elevate network members' loneliness through the co-rumination of negative emotions. To understand how and why interactions among older volunteers might protect against loneliness, this study conducted a concurrent mixed-method social network analysis among older volunteers within the Senior Companion Program (SCP) in Columbus, Ohio. These volunteers are 55 years or older, at or below 200% of the federal poverty line, and culturally/linguistically diverse. Qualitative and quantitative data were collected using focus groups and surveys respectively during an SCP monthly in-service training, N=41. A linear network autocorrelation model (LNAM) was constructed to quantify how individuals' loneliness was correlated in SCP. A grounded theory approach informed the qualitative analysis. Mixed-method integration was conducted through a joint display table. LNAM results on the covariates were consistent with previous loneliness studies on diverse older adults. Furthermore, the negative network autocorrelation indicated that less lonely volunteers tended to interact with lonelier volunteers ($\rho = -0.06$, $p < 0.05$). Qualitative results also supported the aforementioned socialization pattern suggestive of altruistic motivations. In other words, the altruistic tendency to interact with other volunteers differing in loneliness may be an important pathway by which volunteering addresses loneliness. Future research shall further examine the mechanism between volunteering and older adults' psychosocial well-being. Findings have implications for maximizing the health and social benefits of volunteering.

### SESSION 1070 (PAPER)

### PLACE, PURPOSE, AND HEALTH

Abstract citation ID: igad104.0073

#### A QUALITATIVE STUDY OF THE WRITINGS OF LONELY OLDER CHRONICALLY ILL ADULTS LIVING IN APPALACHIA

Laurie Theeke, Maritza Dowling, Lashawn Hutto, and Michelle Odlum, *The George Washington University, Washington, District of Columbia, United States*

Loneliness is a well-known growing public health risk that affects a large proportion of our aging population. The perspectives of older adults experiencing loneliness are essential to identify areas of potential support and intervention but have been understudied. We collected writings from older adults who participated in LISTEN intervention studies focusing on belonging, relationships, getting out in the community, challenges of loneliness, and finding meaning. Thirty-three community dwelling elders (81% female; mean age=71.77, SD=10.09 years) with multiple chronic conditions (mean number=2.63, SD=1.55) who reported high loneliness scores in the UCLA Loneliness Scale (mean=52.27; SD=8.302) contributed 50 minutes of writings or recordings (for those who were unable to write). These were accomplished in 10-minute increments during five weekly sessions of LISTEN. Thematic content analysis was conducted on the writings and the transcribed recordings. Five themes emerged: Lost Connections and Self-isolation, Empty Relationships, Life is Upside Down and Can't Be Controlled, Doing Something is Better than Doing Nothing, and Loneliness is Haunting and Relentless. Participants admitted not reaching out to others, worried about being a burden, feeling lonely while with family, and 'not being in the loop'. The downside of life was described as an uncertainty for the future and imminent functional decline. They described how loneliness 'screams' at you and is 'always lurking'. Activities that ameliorated loneliness included painting, reading, crafting, puzzles, television, and faith activities. These findings will inform the refinement of the LISTEN intervention and the design of new interventions for loneliness.

Abstract citation ID: igad104.0074

#### DISSECTING RACIAL DIFFERENCES IN THE PERCEPTION OF DOMAIN-SPECIFIC LIFE SATISFACTION

Timothy Goler[1], and Tirth Bhatta[2], *1. Norfolk State University, Norfolk, Virginia, United States*, *2. University of Nevada, Las Vegas, Las Vegas, Nevada, United States*

This study examines factors contributing to racial differences in domain-specific life satisfaction. Our study offers a major contribution to prior research that has tended to pay greater attention to psychological well-being outcomes such as life satisfaction which do not directly pertain to material circumstances of older adults. Those studies generally report better life satisfaction and fewer depressive symptoms among Black older adults than their White counterparts. Our study is based on 409 randomly selected older adults from Cleveland (mean age 79 and 25% African American). We considered socioeconomic status, stressors such as recent negative life events and coping resources such as religiosity to understand their impact on domain specific life- satisfaction (e.g., satisfaction with neighborhood, housing, and standard of living). Our ordinary least squares regression estimates suggest that, on average, Black older adults reported lower levels of domain-specific life satisfaction ($b=-0.81$, $p< 0.01$) compared to their White counterparts, despite having stronger social support and being more religious. Even though social support and religiosity did not contribute to racial differences in domain specific life satisfaction, the inclusion of education, income, and recent negative life events fully explain such differences. Findings indicate that interrelated factors such as socioeconomic disadvantage and greater life adversities such as negative life events contribute to lower domain life satisfaction among Black older adults. This finding highlights the need for further exploration of factors shaping material-related well-being outcomes for Black older adults. Our study informs future policies addressing racial well-being inequities.

Abstract citation ID: igad104.0075

#### RELIGIOSITY AND SPIRITUALITY DEVELOPMENT ACROSS MID- TO LATER LIFE: AN ACCELERATED LONGITUDINAL DESIGN

Niccole Nelson[1], Raquael Joiner[2], Brandy Martinez[3], and Cindy Bergeman[4], *1. Colorado State University, Fort Collins, Colorado, United States*, *2. University of Southern California, Los Angeles, California, United States*, *3. Durham VAHCS, Durham, North Carolina, United States*, *4. University of Notre Dame, Notre Dame, Indiana, United States*

It is widely accepted that religiosity and spirituality (R/S) increase with age, although the multidimensional nature of R/S complicates this conclusion. Specifically, religiosity entails engagement in communal and independent religious practices, whereas spirituality refers to a sense of communion with divine being(s). Furthermore, religious and/or spiritual individuals may use their faith to carry them through hard times, and thus engage in positive and/or negative religious/spiritual coping. Critically, these R/S facets may follow distinct developmental trajectories across adulthood, and the nature of these trajectories remain unclear. Therefore, in the present study, we followed an accelerated longitudinal design, modeling developmental trends in six R/S facets across ages 45-80. Participants included 768 subjects from the Notre Dame Study of Health & Well-being (NDHWB), a 10-year, multi-cohort, longitudinal study of adult development and aging (Wave 1 Age: Mean= 59.24, SD= 8.65). Using two-level, hierarchical linear modeling, we estimated no change, linear change, quadratic change, and cubic change trajectories across ages 45-80 in communal and independent religious practices, spirituality, positive and negative religious/spiritual coping, as well as composite R/S; cohort effects in these change trajectories were also tested. Communal and independent religious practices, spirituality, negative religious/spiritual coping, as well as composite R/S, followed unique, cubic trajectories across mid- to later life. Positive religious/spiritual coping followed a linear trajectory, although cohort effects precluded the interpretation of developmental change in this R/S facet. Individuals appear to engage with their faith in different ways as they age, meaning extant conclusions about age-graded increases in R/S may be oversimplified.

Abstract citation ID: igad104.0076

### THE CHARM OF COUNTRY LIFE? IMPACT OF RURAL CHILDHOOD RESIDENCE ON PHYSICAL AND MENTAL HEALTH IN LATER LIFE

Danielle Nadorff[1], Angela Duck[2], Crystal Lim[3], and Danielle Fastring[4], *1. Mississippi State University, Mississippi State, Mississippi, United States*, *2. University of Mississippi Medical Center, Jackson, Mississippi, United States*, *3. University of Missouri, Columbia, Missouri, United States*, *4. William Carey University, Hattiesburg, Mississippi, United States*

Background: Most studies of geographic health disparities are focused on adult rural residence. However, previous studies have shown that the residential area in which one grows up during childhood has lasting impacts on adult health. In one of the only studies to date to examine the impact of rural childhood residence on mental health in middle-aged and older adults, Murchland and colleagues (2019) evaluated inequalities by childhood residence and noted elevated depressive symptoms were more common among those living in rural areas compared to those living in non-rural areas. Aims: The current study expands the model proposed by Murchland and colleagues to include further antecedents related to rural childhood residence, and to include multiple outcomes of physical and mental health among middle-aged and older adults. Method: Participants included 4614 individuals aged 40 or older recruited as part of the Midlife in the United States (MIDUS) study. Results: Consistent with Murchland's model, childhood rurality played an important part in middle-aged and older adults' health, despite not having a direct influence. Rurality status was impacted by parental education level and SES during childhood and was associated with the level of education obtained by the participants (and thus their occupation), which played a direct role in their current health status. Mental and physical health had differential predictors. Limitations: The study was limited by its non-diverse sample and self-reported measures. Conclusion: Further research into the impact of childhood rurality on health is needed, utilizing comprehensive self-reported and observed outcome measures.

Abstract citation ID: igad104.0077

### THE MODERATING ROLE OF PURPOSE IN LIFE IN THE RELATIONSHIP BETWEEN DISCRIMINATION AND COGNITION

Heather Farmer, and Alexis Ambroise, *University of Delaware, Newark, Delaware, United States*

Discrimination is a source of chronic stress disproportionately experienced by Black adults that has been associated with worse cognitive outcomes in older adults. Few studies have explored modifiable resilience resources that may protect people from the possible negative impacts of discrimination on cognition. Sense of purpose refers to an individual's perception of future-oriented goals and direction in life, and is a key resource associated with better cognition. However, no studies have explored whether greater purpose may buffer older adults against the negative impacts of discrimination on cognition or whether there are race differences in the stress-buffering impact of purpose on cognition. We use data from 9,775 Non-Hispanic White and 1,687 Black adults in the 2006 to 2016 waves of the Health and Retirement Study (HRS) to explore whether purpose in life moderates the association between everyday discrimination and cognitive functioning and whether this relationship varies by race. Discrimination was measured using the Everyday Discrimination Scale, which assesses how frequently an individual reports unfair and differential treatment (range, 0-5, where higher scores indicate more frequent discrimination). Multilevel linear regression models revealed that frequent discrimination was associated with worse cognition ($b=-0.34$, $p<.001$), controlling for age, gender, race/ethnicity, region, and urbanicity. There was a significant discrimination x purpose interaction ($b=0.16$, $p<.001$), suggesting that purpose may buffer the discrimination-cognition association. However, there was a non-significant discrimination x purpose x race interaction. This research suggests a strong sense of purpose can protect people from experiencing worse cognition when faced with frequent discrimination.

### SESSION 1075 (PAPER)

### DEMENTIA, DEPRESSION, AND AGING

Abstract citation ID: igad104.0078

### AGE DIFFERENCES AMONG WITHIN-PERSON INDICATORS OF STRESS AND DEPRESSION

Jessica Blaxton[1], and Cindy Bergeman[2], *1. Metropolitan State University, Saint Paul, Minnesota, United States*, *2. University of Notre Dame, Notre Dame, Indiana, United States*

Although older adults tend to regulate their emotions and resist stress better than midlife or younger adults (Charles & Carstensen, 2007), research suggests that when older adults do negatively react to stress they may have more difficulty recovering (Charles, 2010). Research reveals that various indicators of stress relate to greater depression – even suggesting that stress causes the depression (van Praag, 2004). Individual perceived stress (PS) as well as perceived stress reactivity (PSR) influence how stress impacts well-being (Folkman et al., 1986). Thus, we examined age differences in the within-person relationships among PS, PSR, and depression, and potential causal determinants of depression with a longitudinal mediation model. We used data from 572 participants in the Notre Dame Study of Health & Well-being (M(age) = 59.77; sd(age) = 14.22) who completed two to four waves of yearly assessments. Sequentially built multilevel models, in which year was nested within person, illustrated that only midlife adults experience an exacerbated effect of within-person fluctuations in PSR on the relationship between within-person PS and depressive levels (gamma41 = -0.004, p < .01). The longitudinal mediation model revealed that PSR at Time 2 mediated the relationship between PS at Time 1 and Depression at Time 3. Findings suggest that older adults illustrate successful emotion regulation strategies at the yearly level --resisting the negative ramifications of years of greater PS and PSR, whereas midlife adults who experience years of greater PSR would particularly benefit from stress management interventions and monitoring of depressive levels.

Abstract citation ID: igad104.0079

### CARE-RESISTANT BEHAVIOR TRAJECTORIES OF PERSONS WITH DEMENTIA IN NURSING HOMES AND DISTAL OUTCOMES IN ORAL HEALTH

Chunhong Xiao, Frank Puga, Carolyn Pickering, Cindy Cain, Maria Geisinger, and Rita Jablonski, *1. The University of Alabama at Birmingham, Northport, Alabama, United States*

Persons with dementia (PWD) who exhibit care-resistant behaviors (CRB) are likely to resist mouthcare and have poor oral health. This study aimed to examine predictors and describe the characteristics of CRB trajectories and their distal outcome in oral health among PWD in nursing homes (NH). Group-based trajectory modeling was used to analyze dynamic changes in CRB intensity over 21 days with two time-points (morning and afternoon) among 75 PWD. Sub-group analysis demonstrated the characteristics of each trajectory group membership. Distal-outcome models explored the association between oral health variation and mouthcare completion to CRB trajectories. The most influential predictors included CRB intensity at baseline, duration of mouthcare, and the number of antipsychotics prescribed. Three distinctive CRB trajectories were identified for both morning and afternoon in the context of mouthcare. Oral health status and the mouthcare completion rates differed by CRB trajectory groups, and worse oral health status was associated with higher level of CRB intensity trajectories. The High-start CRB groups had the worst oral health status and lowest mouthcare completion in both model estimation and pre-post test. Oral health showed improvement across all three trajectory groups. A tailored individual-level strategy based on a daily pattern of CRB intensity may provide the key to developing interventions that find the "sweet spot" between providing optimal mouthcare while minimizing CRB. Results imply that balancing CRB and mouthcare by providing morning-only mouthcare to those with high CRBs may allocate resources to PLWD most in need of mouthcare. Antipsychotics have not shown usefulness in managing CRB.

Abstract citation ID: igad104.0080

### PHYSICAL ACTIVITY AND DEMENTIA PREVENTION: THE ROLE OF HEALTH COGNITIONS AMONG 50+ HEALTHY INDIVIDUALS IN ISRAEL

Offer Edelstein, *Ben-Gurion University of the Negev, Beer-Sheva, HaDarom, Israel*

**Background:** Although dementia cannot be cured, it can be prevented by adopting various health behaviors, including regular physical activity. The current study sought to i) estimate the participation of Israel-born healthy individuals aged 50 years or older in regular physical activity; ii) assess the associations linking Health Belief Model variables (Rosenstock, 1966, 1974) and engagement in regular physical activity. **Method:** This cross-sectional study was conducted in 2021-2022, using online convenience sampling. The study included 328 Israel-born participants aged 50 years or older. Physical activity was assessed by asking whether participants regularly engaged in physical activity (type of activities/times per week/minutes). Cognitive perceptions were assessed using the Motivation to Change Lifestyle and Health Behaviors for Dementia Risk Reduction questionnaire (Kim et al., 2014). **Results:** The average weekly minutes of physical activity was 165.62 (S.D. 176.17), whereas only 43.6% performed 150 minutes of physical activity weekly. A multivariate linear regression indicated that among all of the model's variables, perceived severity ($\beta$=-.204, p<.001), cues to action ($\beta$=.134, p<.001), feminine gender ($\beta$=-.155, p<.01), and low income ($\beta$=-.113,p<.05) emerged as significant predictors of weekly minutes of physical activity. The model explained 14.2% of the variance in the performance of weekly minutes of physical activity [F(7,320)=12.22, p<.001]. **Conclusions:** The current research underlines the role of health cognitions (perceived severity, barriers, and cues for action) regarding engagement in physical activity. The results of the current study might serve as a basis for intervention programs among various target populations.

Abstract citation ID: igad104.0081

### SELF-REPORTED COMPONENTS OF SELF-IDENTITY IN PERSONS LIVING WITH EARLY-STAGE DEMENTIA

Natalie Regier[1], and Valerie Cotter[2], *1. Johns Hopkins University School of Nursing, Baltimore, Maryland, United States*, *2. Johns Hopkins University, Baltimore, Maryland, United States*

The impending loss of "self" is a common concern of persons diagnosed with dementia. However, in contrast to the traditional biomedical model, there is qualitative and quantitative evidence that self-identity persists across stages of the disease. An understanding of the components of self-identity of persons living with early-stage dementia (PLWED) can inform treatment approaches and communication from care partners and providers. Consequently, the aim of the present study is to examine what PLWED identify as the facets of their self-concept. Participants were a purposive sample of 20 community-dwelling PLWED who attended a voluntary health program aimed at facilitating aging-in-place. Focus groups were conducted and recorded on-site at the health program. The interviews were transcribed verbatim from the audio recordings and were analyzed through a conventional content analysis approach. Content analysis of the narratives gleaned from focus group participants revealed six self-identity themes: vocation, family role, personal characteristics, hobbies and interests, religious affiliation, and social networks. Although the deterioration of explicit memory, including autobiographical memory, is a hallmark of dementia, overfocusing on this as the sole component of self-identity contributes to the representation of persons living with dementia as less than whole and lacking "selves." Our findings suggest critical components of self-identity other than memory that should be recognized and supported in persons living with dementia.

### SESSION 1080 (SYMPOSIUM)

Abstract citation ID: igad104.0082

### EMPOWERING ALL AGES THROUGH INTERGENERATIONAL CONNECTION AND LIFELONG LEARNING

Chair: Lisa Hollis-Sawyer Discussant: Laura Donorfio

This symposium will share a variety of pedagogical approaches designed to foster age-inclusivity and intergenerational learning. The authors will share research and outcomes of different educational designs utilizing intergenerational programming and other approaches to engage older and younger adult learners. The first presentation will explore how intergenerational discussions can be used in the classroom to facilitate collaborative learning among older adults, students, and instructors. The second presentation will discuss the development of a course abroad in Scotland called "Global Aging & Age-Friendly Initiatives," aiming to promote intergenerational connection and global age-inclusivity. The third presentation will discuss "Between Islands," an intergenerational virtual contact intervention designed to promote creativity and intergenerational connection between younger and older adults while challenging stereotypes about aging. The fourth presentation will explore the experiences of emeriti faculty in engaging online with Introduction to Gerontology students in a novel intergenerational classroom activity. The final presentation will review intergenerational classroom design guidelines to optimize multiple generations' learning experiences, aging understanding/appreciation of others, and age-inclusivity. The empowerment and benefits of these efforts for the life-long learning of the generations involved will be discussed.

Abstract citation ID: igad104.0083

### INSTRUCTIONAL DESIGN FOR INTERGENERATIONAL AND AGE-INCLUSIVE LEARNING: LESSONS FROM A GERONTOLOGY CLASSROOM

Lisa Hollis-Sawyer, *Northeastern Illinois University (Chicago), Chicago, Illinois, United States*

The paper will present a multi-year research study that examined the learning needs of first-generation younger and older learners in a graduate-level online learning environment. Ageism and related stereotyping will be reviewed as concepts that reflect a potentially biased understanding of all age groups that should be addressed in intergenerational learning activities. Students' perceptions of understanding aging issues and stereotypes were compared pre- and post-course. A model will be presented about designing an age-inclusive, age-friendly online course design utilizing intergenerational learning activities to optimize learning outcomes while reducing ageist assumptions through targeted virtual discussions about diversity perceptions in the classroom—the design of intergenerational engagement discussion and other learning assignment activities in an introductory graduate gerontology course. Implications for creating intergenerational programming will be discussed.

Abstract citation ID: igad104.0084

### CONNECTING THE DOTS: COLLABORATIVE LEARNING AMONG OLDER ADULTS, STUDENTS, AND INSTRUCTORS

Caitlin Reynolds, *North Carolina State University, Raleigh, North Carolina, United States*

Although the aging process is a universal phenomenon, misconceptions regarding late adulthood are prevalent, such as loneliness and dependency, which often lead to negative stereotypes and poor developmental outcomes (Kotter-Grühn, 2015); thus, it is important to minimize these stereotypes in an educational platform. Intergenerational discussions have been shown to be a robust approach in reducing stereotypes (Canedo-García et al., 2017; Golenko et al., 2019); especially if it is used to dismantle "othering" the groups by creating community and collectivist perspectives (D'Antonio, 2020). My goal is to explore how intergenerational discussions can be used in the classroom to facilitate collaborative learning among older adults, students, and instructors. In the Fall 2022 and Spring 2023 semesters, I partnered with the Osher Lifelong Learning Institute (OLLI) to connect undergraduate students with older adults from the community. The course centered around the students' social innovation project- address how current systems may not adequately support older adults and propose a creative solution. The students interviewed the OLLI members throughout the semester about course content (e.g., their awareness of age-related discrimination and barriers in their community). At the end of the semester, students used their accumulated information from both the OLLI members and the course material to propose their social innovation to the OLLI members. The OLLI members asked questions, made recommendations, and highlighted limitations in the students' work. I observed collaborative thinking and sharing across both groups during these presentations. Overall, intergenerational discussions were a valuable tool in the classroom.

Abstract citation ID: igad104.0085

### INTERGENERATIONAL COURSE ABROAD EXPLORES AGE INCLUSIVITY IN SCOTLAND

Andrea June, and Carrie Andreoletti, *Central Connecticut State University, New Britain, Connecticut, United States*

In 2017, Central Connecticut State University (CCSU) joined the Age-Friendly University (AFU) global network. Guided by the AFU framework to promote age inclusivity in higher education, many programs and services at CCSU have been expanded to encourage the participation of older adults in all core activities of the university. The authors have a particular interest in fostering intergenerational learning opportunities, for which there are reciprocal benefits (e.g., Andreoletti & Howard, 2016). With the goals of promoting intergenerational connection and global age-inclusivity, we developed a course abroad to Scotland called Global Aging & Age-Friendly Initiatives. Prior to traveling, students are introduced to theory and research in lifespan development and to the World Health Organization's (WHO) definition of healthy aging and domains of livability aimed at enhancing well-being and longevity. Livability community efforts in the USA are explored so that similarities and differences can be examined while in Scotland. During 9 days of travel, students are exposed to urban and rural areas of Scotland, noting the accessibility of public transportation, the availability of green spaces, the cultural attitude toward older adults, and other domains of livability. They will also hear from local academics and community organizers invested in promoting engagement across the lifespan. In June 2023, eight age-diverse learners will travel with the authors. In addition to providing more detail on the development and implementation of the course abroad, this presentation will share feedback from students and international collaborators with the goal of encouraging other AFUs to develop similar opportunities.

Abstract citation ID: igad104.0086

**BETWEEN ISLANDS—A CREATIVE PRACTICE AND INTERGENERATIONAL CONTACT INTERVENTION**

Ashley Lytle, and Nancy Nowacek, *Stevens Institute of Technology, Hoboken, New Jersey, United States*

Addressing ageism is a serious social issue in need of targeted, empirically based solutions (WHO, 2021). Facilitating intergenerational contact is one promising means of reducing ageism (Lytle et al., 2020). Between Islands were a creative practice and intergenerational contact intervention designed to promote creativity and intergenerational connection between younger and older adults while challenging stereotypes about aging. Over a four-week intervention, younger and older adults meet virtually (on zoom) for an hour each Wednesday to learn, create, and discuss with one another. Before the intervention began, participants received two 2.5 lb blocks of air-drying clay, paints, and paintbrushes. During the four-week intervention, participants engaged in creative practice (designed, sculpted, and decorated two hand-weights out of the provided clay). In addition, participants learned about the history of hand-weights and circuit training and got to know one another through group-based discussion as well as smaller group prompts and breakout rooms. Prior to the start of the intervention, baseline attitudes and stereotyping toward older adults were assessed among the younger adult participants. Attitudes and stereotypes were again measured after the intervention concluded. Younger adults reported a significant decrease in benevolent ageism and intergenerational tension after the Between Islands intervention. In addition, themes that emerged from the open-ended responses illustrated that the intervention challenged younger adults' stereotypes about older adults, encouraged recognition of similarities between younger and older adults, and facilitated the connection between participants.

Abstract citation ID: igad104.0087

**EMERITI PROFESSORS' INTERGENERATIONAL CONNECTION: QUALITATIVE RESEARCH EXPLORING PERCEPTIONS AND SATISFACTION**

Karen Devereaux Melillo, Ramraj Gautam, Lisa Abdallah, and Patcharee Wangwun, *University of Massachusetts Lowell, Lowell, Massachusetts, United States*

UMass Lowell, designated an AFU in 2019, launched AFU Inventory and Climate Surveys in 2020; respondents identified "involving retired faculty in university activities' (AFU Principle 9) as an area for improvement. A follow-up study, guided by the PEACE Model (Levey, 2018), was undertaken to reduce students' negative stereotypes about older adults and promote positive experiences through active engagement with professor emeriti. One study objective was to explore the experiences of emeriti professors' in engaging online with Intro to Gerontology students in a novel intergenerational classroom activity. Emeriti professors met twice for one hour each with a group of 4-5 students. Following the activity, emeriti professors were invited to participate in one-hour qualitative interviews. The purpose was to explore their perceptions of, and satisfaction with, the experience in having engaged online with students. Nine of the 12 emeriti professors agreed to participate. The Zoom interviews were conducted December 2022 and January 2023 by two researchers. Qualitative content analysis coding was carried out individually by four researchers, who then met in pairs of two, followed by all four reaching consensus on identified themes. The findings revealed six major themes: enriching the IGL experiences; passion for teaching and interacting with young people; transition to and life after retirement; impact of COVID on engagement; university role in connecting with and engaging emeriti; and satisfaction with values and ideas expressed in IGL activities. These themes offer guidance in promoting AFU principle 9 through a novel intergenerational activity that benefits emeriti professors, students and universities.

### SESSION 1085 (SYMPOSIUM)

Abstract citation ID: igad104.0088

**INTERDISCIPLINARY PERSPECTIVES ON THE INTERSECTION OF CANCER AND COGNITIVE DECLINE**

Chair: Judith Carroll Co-Chair: Lauren Stratton Discussant: Brent Small

The population of older cancer survivors in the United States is rapidly growing. The long-term cognitive aging of older cancer survivors is not well understood, as large-scale epidemiological studies often show a paradoxical inverse association between cancer history and subsequent dementia; yet cancer survivors often experience cognitive impairment symptoms in the short-term following treatment. Further, the cancer diagnosis and care experiences of older adults experiencing cognitive impairments or dementia warrant further investigation as these are important issues for health care quality and equity amongst older adults experiencing cognitive limitations who are at risk for cancer. The novel findings presented in this interdisciplinary symposium will help to clarify the cognitive aging experiences of older cancer survivors, using innovative data sources. First, Brown will discuss the relationship between cancer and subjective cognitive decline, using the nationally representative Behavioral Risk Factor Surveillance System data. Second, Westrick will present the associations between cancer treatment types and subsequent mortality and cognitive decline for survivors of breast, prostate, and kidney cancer among Medicare beneficiaries in the nationally representative US Health and Retirement Study. Third, also using data from Medicare beneficiaries in the US Health and Retirement Study combined with a mixed-methods physician survey, Mullins will discuss differences in cancer treatment recommendations and receipt for older adults with a pre-existing cognitive impairment who are diagnosed with cancer. Finally, Todorova will take a deeper dive into the potential mechanisms linking cancer treatment with cognitive decline, by examining a biomarker of inflammation and cardiovascular toxicity among patients receiving doxorubicin. This is a collaborative symposium between the Alzheimer's Disease and Related Dementias and Cancer and Aging Interest Groups.

Abstract citation ID: igad104.0089

### RECEIPT OF CHEMOTHERAPY AND COGNITIVE DECLINE AMONG MEDICARE BENEFICIARIES IN THE US HEALTH AND RETIREMENT STUDY

Ashly Westrick[1], Mohammed Kabeto[2], and Lindsay Kobayashi[1], *1. University of Michigan School of Public Health, Ann Arbor, Michigan, United States*, *2. University of Michigan, Ann Arbor, Michigan, United States*

Research on cancer and cognitive aging outcomes has been inconsistent, with clinic-based studies showing short-term cognitive declines after chemotherapy, and population-based epidemiological studies observing inverse associations between most cancers and subsequent cognitive aging. We aimed to determine the effect of chemotherapy on cognitive decline and mortality among Medicare beneficiaries in the population-based US Health and Retirement Study (HRS). Using data from HRS-Medicare linked participants ≥65, we identified 858 incident breast, prostate, and kidney cancer diagnoses from 2000 to 2016. Cognitive function was measured using the modified telephone interview for cognitive status (range 0 to 27). Cancer treatment (chemotherapy only, other treatments, and no treatment) was obtained from Medicare claims. We used joint modeling to simultaneously model the association between cancer treatment with cognition decline and mortality. Of 858 participants, 38.7% had breast cancer, 52% had prostate cancer, and 11% had kidney cancer. The mean age at diagnosis was 72.8 years (SD=7.62). 31.7% (n=272) received chemotherapy only, 39.5% (n=339) received other treatment, and 28.8% (n=247) received no treatment. In pooled analyses, receipt of chemotherapy (vs. no treatment) was not significantly associated with cognitive decline (β: 0.08; 95% CI: -0.59, 0.76) but was significantly associated with mortality (HR: 1.39; 95% CI: 1.04, 1.88). In joint modeling, the overall hazard ratio for the receipt of chemotherapy (vs. no treatment) was 1.38 (95% CI: 1.02, 1.88). We found that receipt of chemotherapy was associated with an increased hazard of mortality, but not cognitive decline, compared to those who received no treatment.

Abstract citation ID: igad104.0090

### MIXED-METHOD PILOT STUDY OF BREAST AND PROSTATE CANCER TREATMENT DIFFERENCES BY COGNITIVE STATUS

Megan Mullins[1], Mohammed Kabeto[2], Paul Abrahamse[2], Sarah Hawley[2], Deborah Levine[2], Lindsay Kobayashi[3], and Lauren Wallner[2], *1. UT Southwestern Medical Center, Dallas, Texas, United States*, *2. University of Michigan, Ann Arbor, Michigan, United States*, *3. University of Michigan School of Public Health, Ann Arbor, Michigan, United Sta tes*

We evaluated the association of cognitive status with treatment for breast and prostate cancer in the US Health and Retirement Study (HRS) linked to Medicare claims (2000-2016), and surveyed physicians at an NCI designated comprehensive cancer center (N= 20, response rate 52%) to explore whether treatment recommendations differ by patient cognitive status. We evaluated the association between cognitive status and treatment receipt among Medicare-eligible HRS participants aged ≥65 whose first and only cancer was breast (n=334) or prostate (n=1,210). Multivariable logistic regression was used adjusting for race, marital status, year, age, activities of daily living, instrumental activities of daily living, education, and comorbid conditions. Physicians were randomized to clinical vignettes presenting a patient with mild cognitive impairment (MCI) or normal cognition and asked their treatment recommendations for early-stage cancer via survey. Women with dementia had 80% lower odds of receiving radiation therapy compared to cognitively normal women (aOR 0.17; 95% CI: 0.03-0.87). Oncologists were less likely to recommend chemotherapy for early-stage breast cancer patients with MCI (vs. normal cognition). Prostate cancer treatment receipt and recommendations did not vary by cognitive status. Notably, 25% of breast oncologists and 40% of urologic oncologists overestimated the risk of dementia among patients with MCI, and both groups were more likely to ascertain caregiver preferences for patients with MCI. These findings suggest there may be differences in cancer treatment recommendations and receipt among patients with MCI compared to those with normal cognition, which may be in part driven by physician overestimation of dementia risk.

Abstract citation ID: igad104.0091

### CANCER SURVIVORSHIP AND SUBJECTIVE COGNITIVE DECLINE IN THE US: RESULTS FROM A NATIONALLY REPRESENTATIVE SAMPLE

Monique Brown[1], Ally Hucek[1], Jingkai Wei[1], Loretta Anderson[2], and Muzi Na[3], *1. University of South Carolina, Columbia, South Carolina, United States*, *2. University of Maryland Baltimore, Baltimore, Maryland, United States*, *3. Penn State, University Park, Pennsylvania, United States*

Cancer may be associated with objective cognitive decline. However, studies examining the link between cancer and subjective cognitive decline (SCD), which occurs earlier during cognitive trajectories, are lacking. In addition, research assessing risk factors for SCD among cancer survivors is scant. Therefore, this study aimed to examine the association between cancer and SCD in the past year, and assess the association between age of diagnosis, cancer treatment, and SCD among cancer survivors. Data were obtained from the 2021 Behavioral Risk Factor Surveillance System (N=15,447). Crude and adjusted logistic regression models, controlling for sociodemographic characteristics, were used to determine the association between cancer diagnosis, age at diagnosis, cancer treatment, and SCD. The association between ever having cancer and SCD in the past year was not statistically significant. However, disparities occurred among those who were cancer survivors. For example, those who were diagnosed with cancer in middle age (aged 35 to 44) had higher odds of SCD compared to being diagnosed at aged 45 and older (adjusted OR (aOR): 2.25; 95% CI: 1.10 – 4.58). Those who were currently undergoing treatment were more likely to have SCD compared to those who completed treatment (aOR: 1.86; 95% CI: 1.06 – 3.27). Respondents who had not started treatment and those who reported treatment was not necessary also had higher odds of SCD compared to those who completed treatment. Findings suggest that age and treatment disparities exist in SCD among cancer survivors. Future studies may include longitudinal observational studies examining cancer-related risks and SCD.

Abstract citation ID: igad104.0092

### PEPTIDOGLYCAN RECOGNITION PROTEIN-1 PREDICTS CHEMOTHERAPY-INDUCED CARDIOVASCULAR DYSFUNCTION

Valentina Todorova, Gohar Azhar, and Jeanne Wei, *University of Arkansas for Medical Sciences, Little Rock, Arkansas, United States*

Doxorubicin (DOX) is an effective anti-cancer agent but may induce cardiotoxicity and cognitive dysfunction through inflammatory mechanisms. Peptidoglycan recognition protein 1 (PGLYRP1), secreted by inflammatory cells such as neutrophils has been elevated in the circulation of patients with cardiovascular diseases such as myocardial infarction and atherosclerosis. We hypothesized that PGLYRP1 might be elevated in cancer patients receiving DOX and will correlate with cardiac or cognitive function. Gene expression of peripheral blood cells and plasma protein concentration of PGLYRP1 were determined by reverse transcription-quantitative polymerase chain reaction (RT-QPCR) and ELISA in 18 breast cancer patients with subclinical cardiotoxicity and in some of the cases with self-reported memory problems in comparison with 22 patients without cardiotoxicity. After adjusting for age and race using multivariate logistic regression, we found a positive correlation between PGLYRP1 log2 fold change of gene expression (0.878 versus 0.534, p=0.05) and protein expression (0.920 versus 0.534, p=0.08) in patients with DOX-induced cardiotoxicity, as evident by left ventricle ejection fraction (LVEF) reduction by >10% versus baseline. Reduced LVEF secondary to DOX, appeared to cause memory impairment similar to vascular cognitive decline. The results from this study suggest that PGLYRP1 could potentially be used as predictive biomarker for DOX-induced cardiotoxicity and Alzheimer's disease related dementias (ADRD). The role of PGLYRP1 in DOX -associated cardiac and cognitive decline in older cancer patients needs further exploration in larger studies.

### SESSION 1090 (SYMPOSIUM)

Abstract citation ID: igad104.0093

### AGING AND DECISION MAKING: THE ROLES OF AFFECTIVE AND SOCIAL CONTEXT

Chair: Julia Nolte Discussant: Kendra Seaman

Age differences in many facets of decision making are well-established. However, a growing literature suggests that the occurrence and strength of these age effects are moderated by contextual factors. The individual talks in this symposium exemplify how age differences in decision making vary by domain, affective intensity, and social distance. First, Nolte and Löckenhoff demonstrate age-related increments in the avoidance of health-based and consumer choices, finding that perceived difficulty, but not affective responses to choice, increase avoidance. Frank and Pachur highlight that affective responses to choice differ across contexts: Younger and older adults exhibit "affect gaps" by making less beneficial, more risk-averse choices in "affect-rich" (health-based) versus "affect-poor" (consumer) contexts. Contrasting monetary and temporal outcomes, Strough, Bruine de Bruin, and Parker illustrate that the tendency to continue failing investments is more pronounced after investing money than time. Moreover, older adults' decreased susceptibility to this "sunk cost bias" is more evident when making choices about their own investments versus others' investments. Lu and Löckenhoff report age differences in how decision makers discount other's financial well-being as social distance increases: Older adults' willingness to share money with others declines more with increasing social distance than is the case for younger adults. Finally, Wild and Löckenhoff demonstrate that across domains, older versus younger decision makers are less willing to consult with distant social partners or experts and consult fewer people fewer times. To identify directions for future research, Seaman will contextualize the five presentations against the extant aging and decision-making literature.

Abstract citation ID: igad104.0094

### TO CHOOSE OR NOT TO CHOOSE: CAN AFFECTIVE OR COGNITIVE MECHANISMS EXPLAIN AGE DIFFERENCES IN DECISION AVOIDANCE?

Julia Nolte[1], and Corinna Löckenhoff[2], *1. Tilburg University, Tilburg, Noord-Brabant, Netherlands*, *2. Cornell University, Ithaca, New York, United States*

Older adults are more likely to avoid decisions, which puts them at risk of experiencing negative consumer or health outcomes. Because the underlying reasons remain unclear, this pre-registered online study assessed the roles of (1) affect (especially regret), (2) subjective difficulty, and (3) cognitive ability in explaining age-related differences in decision avoidance. In addition, half of all participants were randomized to a writing intervention aimed at reducing avoidance tendencies. An adult lifespan sample (N = 432, Mage = 50.94, SDage = 19.53 years, 50% women, 62% Non-Hispanic White) chose between making or avoiding four consumer and health-based decisions. Affect, including regret, was assessed before (pre-decisional), during (peri-decisional), and after making decisions (post-decisional). Subjective difficulty was measured during the decision process. Finally, participants completed assessments of demographic background, socio-emotional experience, health, personality, and cognitive ability. We found that older adults were more likely to avoid decisions (p<.001), but age was unrelated to peri-decisional affect and subjective difficulty. Peri-decisional and post-decisional affect were not consistently related to avoidance, but participants who reported more difficulty were more likely to avoid decisions (p=.002). Covariates and cognitive measures could not explain age-related differences, but lower growth-goal-orientation and stronger maximization tendencies predicted decision avoidance after accounting for age (ps<.05). The intervention was successful at improving peri-decisional cognitive load and peri-decisional affect (ps<.05) but did not reduce decision avoidance. In sum, none of the assessed mechanisms could explain age-related increases in decision avoidance. Across age groups, perceived difficulty but not actual cognitive ability predicted avoidance tendencies.

Abstract citation ID: igad104.0095

### MEDICAL VERSUS FINANCIAL RISKY DECISION MAKING IN OLDER AND YOUNGER ADULTS

Colleen Frank[1], and Thorsten Pachur[2], *1. Center for Vital Longevity, UT Dallas, Dallas, Texas, United States*, *2. Technical University of Munich, Munich, Bayern, Germany*

While risky decision making is often tested using monetary outcomes in the laboratory, many decisions in the real-world elicit considerable levels of affect—for example, when deciding about a medical treatment that has bodily consequences, such as side effects to a medication. Previous research suggests that choices systematically diverge between these more “affect-rich” medical decision problems and economically equivalent “affect-poor” monetary problems, leading to what is called an ‘affect gap’ in risky choice. However, it is unknown to what extent this pattern holds in older adults, who make some of the most consequential medical and financial decisions among the population, and for whom it has been hypothesized that affect may be a more important guide. Thus, in the present, preregistered study, we examined age-related differences in the affect gap of risky decision making. We compared decision quality and risk attitude between affect-rich (medical side effects) and structurally identical affect-poor (monetary losses) decisions in 100 older (aged 65-80, M=69.7) and 100 younger (aged 18-29, M=23.5) adults. In line with previous findings, we found that individuals were more risk-averse (Odds Ratio (OR) = 1.52) and made worse quality decisions (OR = 2.13) for affect-rich, compared to affect-poor, problems and this did not differ between younger and older adults. Follow-up work using computational modeling will reveal if decision strategy differed between age groups. These findings indicate that the affect gap is also present in older adults, which has serious implications for risk communication in medical decision making

Abstract citation ID: igad104.0096

### AGE DIFFERENCES IN SUNK COST DECISIONS FOR ONESELF VERSUS A LOVED ONE

JoNell Strough[1], Wändi Bruine de Bruin[2], and Andrew Parker[3], *1. WVU, Morgantown, West Virginia, United States*, *2. University of Southern California, Los Angeles, California, United States*, *3. RAND Corporation; Pardee RAND Graduate School, Santa Monica, California, United States*

Previous research has established that older adults are less biased by irretrievable prior investments or ‘sunk costs’ when making a decisions about continuing a failing project. Such decisions often have implications for other people, but most research to date has focused on individuals making decisions for themselves. Here, we investigated whether age differences in willingness to discontinue a failing project and start over depended upon the intended beneficiary (self or loved one) as well as the type of investment (money or time). Participants (N=1075, Mage=53.49 yrs; 56.2% Women) from the American Life Panel responded to an online survey and made hypothetical decisions about a failing project. At all ages, when the project was for oneself, individuals were less willing to discontinue it and start over when money (versus time) had been invested, whereas when the project was for a loved one, willingness to start over did not depend on whether money or time had been invested. The association between older age and greater willingness to discontinue a failing project and start over was stronger when projects were for oneself compared to a loved one. Implications of the findings for understanding aging and decision making in a social context, and strategies for reducing sunk-cost bias in people of all ages are discussed.

Abstract citation ID: igad104.0097

### AGE DIFFERENCES IN SOCIAL DISCOUNTING AND CHARITABLE GIVING IN A US SAMPLE

Yi Lu, and Corinna Löckenhoff, *Cornell University, Ithaca, New York, United States*

Social discounting describes the tendency to show less generosity to more socially distant recipients. With respect to age differences, prior studies (Pornpattananangkul et al., 2019; Lin & Zhang, 2020) found that older adults showed more generosity to distant others (i.e., lower social discounting rates) than younger adults. However, prior research relied on small samples, compared extreme age groups, and focused on Asian countries. To examine generalizability, this pre-registered study examined social discounting and charitable giving in a U.S. adult life-span sample (N = 426, age 18-96, Mage = 50.93, SDage = 19.51, 51% female, 61% non-Hispanic White). The social discounting measure was adapted from Jones and Rachlin (2006) and involved five social distances (1, 5, 10, 50, 100) on a 100-point scale (0 = closest, 100 = most distant). For each social distance, participants were asked how much of $100 they would allocate to themselves versus a person at that distance. They made the same decision for their favorite charity. Contrary to previous findings and our predictions, results revealed a significant positive association between age and social discounting rates (p< .01), such that older adults’ generosity declined more with increasing social distance. Age differences remained robust after controlling for demographics. There were no significant age differences in average giving across social distances and in charitable giving, but, regardless of age, charitable giving was higher among those with lower social discounting rates (p< .001). Findings suggest that age differences in social discounting may be sensitive to cultural context.

Abstract citation ID: igad104.0098

**AGE DIFFERENCES IN SOCIAL NETWORK PREFERENCES: IMPLICATIONS FOR DECISION MAKING**

Tess Wild, and Corinna Löckenhoff, *Cornell University, Ithaca, New York, United States*

Many consequential decisions are made in the context of a social network, but it is not clear how lifespan developmental changes may influence the way people integrate others into their decision-making processes. Research shows that, compared to younger adults, older adults perform less exhaustive pre-decisional information searches, have smaller and more close-knit social networks, and place more value on harmonious relationships (e.g., Hess et al., 2015). Drawing on these findings, a pre-registered survey study examined age differences in the size, composition, and utilization of decision-related social networks as well as subjective goals and perceptions regarding joint decisions. An adult lifespan sample (N = 432; M age = 50.9375; SD age = 19.53267, range age = 18-96 years) considered hypothetical decisions involving specific (apartment, car, physician, and insurance plan) and generic (important vs. minor) scenarios. As predicted, linear regressions revealed that compared to younger respondents, older respondents reported that they would consult fewer people fewer times, and they were less likely to recruit distant social partners or professionals (ps<.05). Contrary to predictions, the tendency to prioritize relationship harmony over decision quality was negatively associated with age (p<.05). Further, there was no significant relationship between age and the perceived helpfulness of involving others. Findings remained robust when controlling for scenario type and demographic characteristics (gender, education level, race, income). This indicates that the size, composition, and utilization of decision-related social networks differs by age. Further research is needed to examine potential implications for decision quality and satisfaction.

### SESSION 1095 (SYMPOSIUM)

Abstract citation ID: igad104.0099

**THE ADULT DAY CENTER: UNTAPPED TERRITORY FOR AGING RESEARCH**

Chair: Tina Sadarangani Discussant: Fei Sun

Adult day service centers (ADCs) in the United States are a vital, but overlooked source of community-based long-term care for diverse older adults with multiple chronic conditions, including dementia. The National Institute on Aging has called for more research on ADCs. In this interdisciplinary symposium, we present challenges and opportunities in conducting research in these congregate settings. First, we look at the operating status of ADCs nationally in the aftermath of the COVID-19 pandemic and disparities in access to care using longitudinal data from the National Post-acute and Long-term Care Study. Then we examine the lack of electronic health record utilization and clinical data capture in 4,035 ADCs nationally and discuss strategies to modernize technology in these settings to facilitate data collection. We subsequently present a new database, developed vis a vis an academic/community partnership between the National Adult Day Services Association and researchers, that centralizes and makes data collection feasible in ADCs with limited resources. We will also explore factors supporting intervention research that are unique to ADCs. Finally, we present a protocol adapted for the ADC setting, to test a mobile application that facilitates data collection and enables communication between ADCs, caregivers, and healthcare providers. Our findings show that, while research in ADCs has been limited historically, academic, government, and community partners are working together to spur pragmatic innovations that modernize these settings and better resource them to elevate standards of care.

Abstract citation ID: igad104.0100

**INTEGRATING ADULT DAY CENTERS INTO THE BROADER HEALTH CARE CONTINUUM: THERE MIGHT JUST BE AN APP FOR THAT**

Tina Sadarangani, *New York University, New York City, New York, United States*

CareMOBI is an mhealth application that allows adult day care centers to exchange information with their clients' family caregivers and health care providers. CareMOBI enables adult day center staff, who possesses in-depth knowledge of clients based on their day-to-day observations, to be active members of the care team. This is otherwise challenging in an environment that relies almost exclusively on facsimile and voicemail to communicate with outside providers. CareMOBI was developed to address fragmented communication between adult day centers and outside providers using a user-centered design thinking approach that prioritized the needs of end-users. Results of user-testing, which combined qualitative interviews followed by individual completion of the Technology Acceptance Model questionnaire, show that likelihood of adopting CareMOBI was high among adult day center staff (n=31). CareMOBI was also perceived as having significant clinical value. However, staff expressed a need for training prior to use and expressed confusion over how to embed CareMOBI within their existing workflows. We now present a protocol for implementation that reflects a landscape analysis of resources within ADCs to support technology adoption as well as a stakeholder-led approach to implementation that is responsive to findings from user-testing.

Abstract citation ID: igad104.0101

**ADULT DAY SERVICES DATA COLLECTION AND OUTCOMES: A NATIONAL STRATEGY REALIZED**

William Zagorski, *American Senior Care Centers, Inc., Nashville, Tennessee, United States*

The National Adult Day Services Association (NADSA), in its commitment to advance the development and recognition of adult day services across the United States, has defined a comprehensive set of data points and uniform outcome measures to be utilized throughout the Adult Day Services (ADS) industry. NADSA represents more than 500 unique Adult Day Service Centers (ADSCs) across the country. This project has culminated in the opening of a single national secure data entry portal and repository for the collection of this data free to use for adult day service providers across the country. Prior to its full release in January 2023, the beta testing phase allowed for the utilization of this platform across nearly a dozen states, more than 20 ADSCs and more than 500 ADS participants in a variety of settings. As of January 2023, the system is now available to more than 500 ADSCs serving more than 12,000 participants. This session will describe the data points and outcome measures as well as the uniform tools to be utilized for collection and introduce the data portal to the greater academic community. This longitudinal data will be available to NADSA academic partners to provide greater avenues of research into the benefits of adult day services use to participants, caregivers, payors, and the community.

Abstract citation ID: igad104.0102

**ADULT DAY SERVICES: THE OPTIMAL PLATFORM FOR GROUP INTERVENTION RESEARCH**

Keith Anderson[1], and Holly Dabelko-Schoeny[2], *1. University of Mississippi, Oxford, Mississippi, United States*, *2. The Ohio State University, Columbus, Ohio, United States*

Group intervention research with older adults is fraught with challenges related to access, recruitment, implementation, and sustainability. In this presentation we explore these challenges and offer adult day services (ADS) as an alternative platform to traditional long-term care sites (e.g., nursing homes – NHs, assisted living facilities – ALFs) for group intervention research. Access: NHs and ALFs are often reluctant to engage in research due to concerns with liability and fears that "outsiders" will expose deficits in their services. ADS programs are typically quite receptive to researchers and are eager to learn about innovative ways to improve their services. Recruitment: NHs and ALFs are comprised of individuals living in their private apartments and rooms and recruitment is often a one-on-one, time-consuming process. ADS programs are naturally collective environments where recruitment efforts can occur in a more efficient group format. Implementation: NHs and ALFs often lack adequate physical space and staff support for group research activities – key elements that contribute to successful implementation. ADS programs are specifically designed and staffed for group activities and interventions. Sustainability: The primary focus of NHs and ALFs is on "bed and body" care and long-term buy-in regarding group interventions and activities from facilities and staff is rare. Group programming is central to ADS and there is a much greater potential for buy-in and long-term sustainability. Conclusion: Researchers interested in group interventions should strongly consider ADS as the optimal platform and may find that the ADS setting offers the greatest potential for success.

Abstract citation ID: igad104.0103

**FACTORS ASSOCIATED WITH ELECTRONIC HEALTH RECORD ADOPTION AMONG ADULT DAY SERVICES IN THE US**

Yawen Li[1], Jay Chok[2], Geoffrey Chui[1], Don Roosan[3], and Ken Shultz[1], *1. California State University, San Bernardino, San Bernardino, California, United States*, *2. Keck Graduate Institute, Claremont, California, United States*, *3. Western University of Health Sciences, Pomona, California, United States*

Successful adoption of Electronic Health Records (EHR) is becoming increasingly important for Adult Day Service Centers (ADSC) to function effectively and competently, particularly in care coordination with other healthcare providers. Therefore, in the present study, we assessed the EHR adoption among ADSC and its associated organizational characteristics using the 2018 National Study of Long-Term Care Providers (NPALS). The sample included 4,035 ADSC from a national representative sample. Directors or managers of ADSC completed the survey that included questions on organization characteristics, staffing, practice procedures, services, technology use, etc. Findings reveal that the adoption rate of EHR among ADSC was less than thirty percent and less than twenty percent of ADSC used EHR for health information exchange with either physicians or pharmacists. In addition, we found that an ADSC located near other healthcare providers, with Medicaid authorization and a higher percentage of Medicaid patients, and various computer capabilities such as recording participants' demographic information, clinical notes, medications, service plans, and lab results were related to a higher likelihood of EHR use and EHR exchange. However, possibly due to privacy concerns, the capabilities to record participant problems and view lab results, negatively predict EHR exchange with physicians or pharmacists. The findings are discussed in terms of challenges and strategies to promote EHR adoption in ADSC.

Abstract citation ID: igad104.0104

**NATIONAL POST–ACUTE AND LONG-TERM CARE STUDY: VARIATION IN OPERATING STATUS OF ADULT DAY CENTERS DUE TO COVID-19**

Jessica Lendon[1], Priyanka Singh[2], Zhaohui Lu[2], Christine Caffrey[2], and Manisha Sengupta[2], *1. CDC National Center for Health Statistics, Hyattsville, Maryland, United States*, *2. National Center for Health Statistics, Hyattsville, Maryland, United States*

The 2020 and 2022 National Post-acute and Long-term Care Study (NPALS) captured nationally representative data on ADSCs' operating statuses to mitigate COVID-19 among participants and staff. In 2020/2021, 35% of ADSCs were physically open and only served participants onsite, 32% were physically closed but serving participants at places of residence (i.e. virtually, meal delivery, home-visits), 20% served participants onsite and at places of residence, and 13% were temporarily closed and not serving participants. There was statistically significant variation in operating status by region, policy-relevant organizational and participant-level characteristics, and in the types of services provided. Discussion of results will highlight how NPALS data can be used to assess disparities in ADSC availability during the pandemic. Additional information from the forthcoming 2022 survey data will also be discussed, focusing on potential differences in operating status and ADSC characteristics between the two survey waves.

### SESSION 1100 (SYMPOSIUM)

Abstract citation ID: igad104.0105

**NURSING HOME SOCIAL WORK: CONFLICTING VALUES AND ETHICAL PRACTICE WITH RESIDENTS, FAMILIES, AND STAFF**

Chair: Mercedes Bern-Klug Discussant: Denise Gammonley

People who live in nursing homes and those who spend weeks or months there recuperating from a hip replacement or stroke have a broad range of needs, many urgent and others enduring. Despite the high needs of residents—which have increased over the past decade as resident acuity has increased-- the settings are typically under-staffed both in terms of the number of direct care staff and the preparation of direct care staff (NASEM Report, 2022). Social workers and others in the social service role (i.e., those without a social work education) step into or are tapped to intervene when resident rights conflict with family wishes and/or staff rights and wishes. In this session using findings from both qualitative and quantitative studies, we will describe how and what type of conflicts social workers are involved with and the training they report they need to be better prepared to serve as a resource in this important role.

Abstract citation ID: igad104.0106

### WE CAN WORK IT OUT: NURSING HOME SOCIAL WORK AND CONFLICT MEDIATION

Amy Roberts[1], Nancy Kusmaul[2], and Mercedes Bern-Klug[3], *1. Miami University, Oxford, Ohio, United States*, *2. University of Maryland Baltimore County, Baltimore, Maryland, United States*, *3. University of Iowa, Iowa City, Iowa, United States*

Drawing from a nationally representative survey of social service directors (N=922), this presentation will provide an overview of nursing home staffing trends and describe the ways in which social service staff routinely interact with residents, families, and staff. Social workers are involved in many aspects of care, including the admissions process, planning and providing care, care transitions, and general supportive services. As members of the interdisciplinary team, social workers advocate for resident rights, and support decision-making and care planning in times of family crisis and high conflict situations. Summary statistics will be presented to identify the directors' frustrations with the role – including their perceptions of major barriers to care, and draw attention to the changes that would improve job satisfaction (e.g., higher salary/wage, 51%; lower staff turnover among direct care staff, 47%; and more time to focus on the social and emotional concerns of residents, 45%). The presentation will conclude with an overview of directors' interest in training topics that may help to mediate conflicts among residents, families, and staff. Specifically, we will share how prepared directors feel to train other staff in conflict-resolution skills (e.g., working with the resident/family and team to balance resident self-determination with the nursing home's responsibility to minimize risk), and the use of an ethical framework to guide action.

Abstract citation ID: igad104.0107

### SOCIAL SERVICE DIRECTOR PRIORITIES IN RESOLVING CONFLICTS IN NURSING HOMES

Nancy Kusmaul[1], Amy Roberts[2], and Mercedes Bern-Klug[3], *1. University of Maryland Baltimore County, Baltimore, Maryland, United States*, *2. Miami University, Oxford, Ohio, United States*, *3. University of Iowa, Iowa City, Iowa, United States*

Social workers in nursing homes serve many critical roles such as assessing for residents' unmet psychosocial needs, providing support to residents and families at end of life, and resolving conflicts between residents, families, and staff. In their roles, they often need to balance conflicting priorities and determine whose rights or needs should take priority. Little is known about how these conflicts affect the social workers in their jobs. This presentation will report the results of a content analysis of the answers to two open ended questions, "What do you like about your job?" and "What would you change about your job?" in a nationally representative sample of nursing home social service directors. The results illustrate directors' views of what they value in terms of helping residents, families, and staff navigate change, difficult conversations, and conflicting interests. Social service directors report that their priorities are residents and their families, specifically advocating for resident needs. They also report needing to advocate for the role of social work on the interdisciplinary team. They report that family dynamics are messy, but do not discuss how they prioritize within and between residents, their family members, and nursing home staff. More research is needed to understand the decision making process that social workers employ in decision-making about priorities.

Abstract citation ID: igad104.0108

### SOCIAL WORKERS AS INFORMAL LEGAL INTERMEDIARIES: RESOLVING CONFLICTING RIGHTS IN NURSING HOMES

Angela Perone, *University of California, Berkeley, Berkeley, California, United States*

Conflicting rights arise often in nursing homes among residents, staff, and family. Nursing home social workers sit at a unique nexus of these rights, given their macro, mezzo, and micro-level training. This study employs a multi-method qualitative design with semi-structured staff interviews (n=90) (direct care, mid-level professional, top management), content analysis of long-term care facility policies (n=376), and ethnographic observation of two facilities (n=8 months) for a multi-layered cross-comparative in-depth case study. While social workers represented only a very small number of the overall nursing home workforce, data revealed the overwhelming reliance on social workers to resolve conflicting rights that arose among residents, staff, and family. Certified nursing assistants, nurses, directors, and administrators regularly deferred to social workers via written policies and unwritten practices to resolve a variety of issues, including discrimination concerns by staff, residents, or family, concerns about quality of care and workforce shortage, and concerns about conflicting rights to resident autonomy, dignity, medical decision-making, and safety (e.g., bed rails). Staff at all levels and professions described the emotional labor and unique professional experience that these conflicts required and felt ill-equipped to resolve these issues. While social workers resolved most of these conflicts, they, too, reported feeling ill-prepared for this role and worried about out-of-scope practice. Ultimately, they relied on their social work training in systems-level change, case management, and interpersonal communication to resolve conflicting rights. This presentation will discuss social worker challenges as informal legal intermediaries and opportunities for better support and training.

### SESSION 1105 (SYMPOSIUM)

Abstract citation ID: igad104.0109

### COGNITIVE ASPECTS OF MANAGING STRESS AND UNWANTED EMOTION SUCCESSFULLY IN OLD AGE

Chair: Tammy English

The ability to cope with stressors and manage unwanted emotion is central to health-related outcomes across adulthood. Prominent models of adult development suggest there may be age-related shifts in motivational strivings and accumulated knowledge that help support emotional well-being in old age. At the same time, older adults have cognitive and physical vulnerabilities that may offset some of these strengths. This session will showcase new work leveraging experimental and experiencing sampling methods to examine how people of varying ages and resource levels manage their emotions. These studies provide new insights into how older adults respond to stressors and adverse stimuli in controlled settings and daily life contexts. The first talk centers on how individuals are negatively impacted by cognitive stressors regardless of age, but older adults show more resilience than younger adults after stressor exposure. The second talk focuses on how a cognitive process known as affective labeling (or naming one's emotions) can create emotion regulation difficulties, especially in old age. The third talk examines emotion regulation success and the benefits of instructional support for cognitively normal older adults compared to those with mild cognitive impairment (MCI). The fourth talk extends this work by tracking emotion regulation motives in daily life, showing that cognitively diverse older adults report fewer attempts to manage their emotions, but similar reasons for doing so, compared to relatively younger adults. Together these presentations delineate age-related similarity and differences in emotion regulation, highlighting avenues through which coping capacity may be maintained and improve in late life.

Abstract citation ID: igad104.0110

### EMOTION REGULATION SUCCESS IN A LABORATORY TASK IN YOUNG ADULTS AND COGNITIVELY DIVERSE OLDER ADULTS

Claire Growney[1], and Tammy English[2], *1. Stanford University, Stanford, California, United States*, *2. Washington University in St. Louis, St. Louis, Missouri, United States*

Age is associated with declines in cognitive resources viewed as important for emotion regulation, and older adults with mild cognitive impairment (MCI) are expected to experience difficulty with emotion regulation. The present study examines how providing support through emotion regulation instructions might contribute to improved emotional well-being using different indices of emotion regulation success. Young adults (age 21–34, n = 66) and older adults with MCI (age 70–84, n = 60) and cognitively normal older adults (age 70–84, n = 90) completed a laboratory task in which they viewed high-arousal negative emotional film clips under instructions to regulate prohdonically (a) using any emotion regulation strategy, and (b) using an instructed strategy based on condition: distraction or reappraisal. We examined three indices of emotion regulation success: self-perceived emotion regulation success, self-reported experience of the target emotion, and behaviorally-coded expression of the target emotion. Overall, there were no group differences in experience or expression, but older adults with MCI perceived that they regulated with less success compared with younger adults and cognitively normal older adults. Younger adults reported experiencing less of the target negative emotion on instructed versus self-selected strategy trials, whereas older adults with MCI expressed less of the target negative emotion on instructed versus self-selected strategy trials. Findings suggest that younger adults and older adults with MCI might benefit more from support with regulating emotions compared with cognitively normal adults, who are theorized to have expertise in this domain.

Abstract citation ID: igad104.0111

### HOW OFTEN AND WHY DO PEOPLE MANAGE THEIR EMOTIONS?: EMOTION REGULATION IN HEALTHY AGING AND MILD COGNITIVE IMPAIRMENT

Tabea Springstein, and Tammy English, *Washington University in St. Louis, St. Louis, Missouri, United States*

Prominent theories of adult development suggest that individuals increasingly prioritize emotional goals and social relationships as they grow older. Accordingly, older adults are expected to invest more in maintaining their emotional well-being compared to younger adults. Prior work suggests that older adults may accomplish this goal by structuring their lives in ways that reduce the need to manage unwanted emotion. We tested the hypotheses that (1) older adults regulate their emotions less often in daily life compared to younger adults, and (2) when emotion regulation occurs, older adults are relatively more motivated by pro-hedonic and social concerns. Using experience sampling (7x/day for 9 days), we assessed whether emotion regulation frequency and motives differ between younger adults (N = 70), cognitively normal older adults (N = 88), and older adults with mild cognitive impairment (MCI; N = 60). We found that older adults with and without MCI regulated their emotions less frequently than younger adults, even when controlling for mean levels of positive and negative emotional experience. However, there were largely no group differences in emotion regulation motives (i.e., why people wanted to manage their emotions). Future work is needed to explore how age-related differences in life contexts might contribute to less need for emotion regulation in relatively older adults. Less frequent regulation could be beneficial in terms of helping older adults preserve their more limited cognitive resources. The findings regarding motives add to growing research on aging which suggest maintenance or similarity in many emotion regulation processes across adulthood.

Abstract citation ID: igad104.0112

### EFFECT OF EMOTION NAMING ON EMOTION REGULATION IN YOUNGER AND OLDER ADULTS

Hannah Wolfe, and Derek Isaacowitz, *Northeastern University, Boston, Massachusetts, United States*

Prior research has shown that naming one's emotions before regulation has a detrimental effect on regulation success (Nook et al., 2020). These researchers argued this effect was due to the affect crystallization that occurs after naming an emotion, which in turn makes it harder to modify or regulate that emotion. The aim of the current replication-extension study was to evaluate whether this effect would replicate in an older adult sample. Due to age-related changes in emotion regulation knowledge and behaviors, we hypothesized that age would moderate this effect, such that older adults would not be detrimentally impacted by naming their emotions, but younger adults would. Younger (18-25 years, N=50) and older adults (60+ years, N=50) viewed 80 IAPS images while being told to first 'look' or 'name' their experienced emotion and second to 'look' or 'regulate' their emotions (creating four within-subjects conditions). Unpleasant affect was measured on each trial, analyzed via a 2(age group) X 2(naming: yes/no) X 2(regulating: yes/no) mixed ANOVA. Results suggest a significant three-way interaction; within both age groups, the name-regulate trials were associated with significantly greater unpleasant affect compared to the look-regulate trials, suggesting that naming emotions was detrimental for both age groups. However, contrary to our hypothesis, this effect was larger in older adults (d=.82) than younger adults (d=.41). These findings suggest that affect crystallization may be stronger or faster in older adulthood.

Abstract citation ID: igad104.0113

### AGE DIFFERENCES IN STRESSOR REACTIVITY AND RECOVERY

Alyssa Minton[1], Christian Waugh[2], Jason Snyder[1], Susan Charles[3], Claudia Haase[4], and Joseph Mikels[1], *1. DePaul University, Chicago, Illinois, United States*, *2. Wake Forest University, Winston Salem, North Carolina, United States*, *3. University of California, Irvine, Irvine, California, United States*, *4. Northwestern University, Chicago, Illinois, United States*

Strength and vulnerability integration theory (SAVI; Charles, 2010) posits that age differences in emotional experiences vary based on the distance from an emotionally eliciting event. Before and after a stressor, SAVI predicts that older age is related to motivational strivings that often result in higher levels of well-being. During stressor exposure, however, age effects are predicted to be attenuated or disappear completely. The present study examined how younger (n = 85; M age = 22.56 years) and older (n = 85; M age = 71.05 years) adults reacted to and recovered from a cognitive stressor using repeated positive and negative emotion probes. Results showed that both age groups were negatively impacted by the stressor, and both reported an initial boost in recovery afterward. However, older adults continued to recover across the recovery period compared to younger adults. This work elucidates that older adults are significantly impacted by stress but exhibit a resounding recovery.

### SESSION 1110 (SYMPOSIUM)

Abstract citation ID: igad104.0114

### INTRINSIC HEALTH: TOWARD A FRAMEWORK FOR THE OBJECTIVE STUDY OF THE BIOLOGY OF HEALTH

Chair: Alan Cohen Co-Chair: Martin Picard Discussant: Karen Bandeen-Roche

Many definitions of health have been proposed over the years, but all share the characteristic of being subjective and therefore hard to tie to objective measures of biology. While there are undoubtedly subjective aspects of health, here we propose the notion of "intrinsic health" as the capacity of an organism to maintain its internal, biological dynamic equilibrium. Building on first principles, Cohen and Picard show how intrinsic health emerges from the dynamic interplay of energy, communication, and structure at multiple levels of biological organization. The following three talks then propose novel ways to measure intrinsic health. Khan and Wei show how quantile aberrance – modeling when biomarker values are extreme based on the joint distributions of many biomarkers – can be used to quantify intrinsic health. Liu, Xu, Honfo, and Cohen show how metrics of proteome dynamics reflect communication within the organism and can be extracted to quantify health. Finally, Wang and Pei show how transfer entropy – the synchronization between multiple time series – can be extracted to measure health. While these three metrics are still in preliminary stages of development, they show the potential to measure health based on the multivariate dynamics of our internal systems. Going forward, these and other approaches may be integrated to derive robust, objective metrics of intrinsic health, and could serve as the basis for a novel understanding of how aging emerges from declines in intrinsic health over time.

Abstract citation ID: igad104.0115

### A THEORETICAL FRAMEWORK TO UNDERSTAND INTRINSIC HEALTH

Alan Cohen[1], Martin Picard[2], John Beard[1], Daniel Belsky[1], Linda Fried[1], Kelli Hall[1], Julie Herbstman[1], and Nour Makarem[1], *1. Columbia University, New York City, New York, United States*, *2. Columbia University Irving Medical Center, New York City, New York, United States*

Traditional approaches to measuring health have relied largely on measures of function and capacity, or on subjective assessments. These approaches are important, but they result in a large gap between the biology underlying health and our ability to measure it. Here, we propose a novel concept – intrinsic health – defined as a field-like state emerging from the dynamic interplay of energy, communication, and structure within the organism, which enables robustness, resilience, adaptability, and performance. It reflects the capacity of an organism to maintain its internal dynamic equilibrium. These three pillars – energy, communication, and structure – are derived from first principles, and are the basic ingredients of life. Disruption or optimization in the organized flow of energy and information through biological structures provide a foundation to understand the causes of disease, and the foundation for health, respectively. For each pillar, we discuss available measurements that cover multiple levels of biological organization, empirical evidence linking them to traditional health outcomes, and also the limitations of isolated measurements. This motivates the use of more integrative, time-series data reflecting the dynamic psychobiological interplay among organ systems. We then situate intrinsic health in the context of environmental, behavioral, and cultural factors that affect how it is transformed into "realized health," the health we experience as individuals and populations. The intrinsic health framework can be extended to understand how intrinsic health intersects with aging, disease, and wounds. Finally, we propose a measurement framework to quantify intrinsic health at the individual level.

Abstract citation ID: igad104.0116

### REPRESENTATION LEARNING OF PROTEOME DYNAMICS TO CHARACTERIZE ORGANISMIC COMMUNICATION AND INTRINSIC HEALTH

Molei Liu[1], Bowen Xu[2], Sewanou Honfo[3], Alan Cohen[4], and Martin Picard[5], *1. Columbia Mailman School of Public Health, New York City, New York, United States*, *2. New York University, New York City, New York, United States*, *3. Universitéde Shebrooke, Sherbrooke, Quebec, Canada*, *4. Columbia University, New York City, New York, United States*, *5. Columbia University Irving Medical Center, New York City, New York, United States*

Co-regulation and interactions among organ systems are crucial to maintain health. Therefore, understanding patterns of such organismic communication using proteome dynamics could be an innovative and effective strategy for quantifying intrinsic human health. In this preliminary study, we conducted a case-control study involving six participants with severe genetic mitochondrial disease and six age- and sex-matched healthy subjects. We measured and recorded the salivary proteome of each participant (n=2922 proteins) at 0-, 30-, and 45-minutes post-awakening as well as bedtime (pm), over two days, representing a natural physiological challenge known to involve systems-wide physiological recalibrations. We then analyzed the changes in protein levels across successive time points using a high-dimensional two-sample testing approach (Cai, Liu, and Xia 2013). The protein co-regulation pattern (captured by the covariance matrix of their changes in expression) was significantly different between the healthy and mitochondrial disease participants (p=0.01), thereby linking proteome dynamics with health status. We then implemented Weighted Correlation Network Analysis to identify representative features showing a significant correlation with the subject's health status between pairs of successive time points (0-30, 30-45, 45-pm). The correlation between the extracted feature and the self-rated health was as high as 0.39 (p-value=0.03) for the 30-45 time period. This finding reveals an association between health status and salivary protein co-regulation patterns, and suggests a promising representation learning strategy to quantify the manifestation of health into accessible proteome dynamics.

Abstract citation ID: igad104.0117

### QUANTIFYING INTRINSIC HEALTH USING STRESS-EVOKED INFORMATION FLOW BETWEEN PHYSIOLOGICAL SYSTEMS

Yan Wang[1], Sze Pui Tsang[1], Zain Khan[1], Daniel Malinsky[1], Ying Wei[1], Alan Cohen[1], Martin Picard[2], and Sen Pei[1], *1. Columbia University, New York City, New York, United States*, *2. Columbia University Irving Medical Center, New York City, New York, United States*

As a canonical complex dynamical system, human bodies require interactions between constituent physiological systems to perform functions and maintain health status. Quantifying the dynamic interactions between organ systems using biomarkers collected in the context of psychobiological challenges may provide a novel means of measuring the energy-dependent communication processes that underlie intrinsic health. In this study, we use transfer entropy (TE), an information-theoretic metric, to quantify information flow between three biomarkers (heart rate, blood pressure, and respiration) in 71 participants of the Mitochondrial Stress, Brain Imaging, and Epigenetics (MiSBIE) project. In this stress experiment, both healthy individuals (n=44) and participants with genetic mitochondrial disorders (Mutation n=16, Deletion n=11) were exposed to a standardized 5-minute socioevaluative challenge (i.e., public speaking). Heart rate, blood pressure, and ventilation were collected continuously across four phases (baseline, pre-task, during the challenge, and post-challenge) over a 2-hour period. Using time series constructed from the three biomarkers, we compute six pairwise TE values for each participant in each phase, measuring the direction and strength of the information flow between physiological systems. Across different phases, we identify significant differences in TE values between healthy participants and patients with mitochondrial diseases. For example, during the post-challenge phase, TE from blood pressure to respiration in healthy participants was 19% higher than the deletion group (p=0.038). Our finding highlights the potential use of TE as a computational tool for detecting mitochondrial disorders and, more broadly, quantifying intrinsic health using information transfer between physiological biomarkers.

Abstract citation ID: igad104.0118

### UNDERSTANDING THE DYNAMICS OF INTRINSIC HEALTH USING AGE-DEPENDENT QUANTILE GRAPHICAL MODELS

Zain Khan[1], Daniel Malinsky[1], Alan Cohen[1], Sen Pei[1], Kiros Berhane[1], Martin Picard[2], Daniel Belsky[1], and Ying Wei[1], *1. Columbia University, New York City, New York, United States*, *2. Columbia University Irving Medical Center, New York City, New York, United States*

Human health is a complex and dynamic system. Individual components, such as clinical biomarkers, vary jointly as an ensemble, and appropriate models are needed to study them in the context of health. Network analysis is an effective way to assess and visualize cross-biomarker dependencies, where each node represents a biomarker, and each edge represents the conditional dependency between two biomarkers. We developed a new class of age-dependent quantile graphical models to fully capture the complexity of cross-biomarker dynamics. This model quantifies cross-biomarker dependencies at the boundary (i.e., when biomarkers approach or exceed normal ranges) and differentiates them from those within normal ranges. Further incorporation of age-dependency helps identify biological mechanisms by which humans maintain intrinsic health over their life course. Applying this analysis to the NHANES data (n=60,014), we discovered that cross-biomarker dependencies differed by age and across quantile levels. For participants under 20 years old, at the median quantile we found significant relationships between heart rate (HR) and all other biomarkers: cholesterol, hemoglobin, albumin, systolic blood pressure (SBP), and diastolic blood pressure (DBP) (all p-values < 0.001). However, elderly populations (60+) at the median quantile displayed cholesterol centrality instead—albumin, SBP, DBP, and HR were significantly associated with cholesterol (all p-values < 0.001) while HR was less connected to other biomarkers. At the 0.9 quantile, the <20 group resembled the older age groups in that cholesterol was the more central node. This demonstrates that association levels and centrality patterns varied across age groups and quantile levels.

### SESSION 1115 (SYMPOSIUM)

Abstract citation ID: igad104.0119

### ECONOMICS OF LONG-TERM SERVICES AND SUPPORTS: PRODUCTION, DEMAND, AND COSTS

Chair: Christine Bishop

Economic analysis of the production, demand, and utilization of long-term services and supports (LTSS) can generate insights for public policy. This symposium presents a range of economics approaches to LTSS, with policy implications. The first paper considers in-home care as a production process, estimating the impact of various mixes of informal care and paid formal care on health outcomes. The two types of care, and their combination, contribute to different aspects of self-reported health. Paper Two uses the disruption of the pandemic to examine the productivity of an understudied resource in nursing home care provision: informal care by visiting family and friends. The finding that continuing resident interaction with informal caregivers reduced mortality should inspire future research on this valuable and understudied input into nursing home care. A third paper tracks rising wages for nursing staff wages and increasing use of contract nursing, fueled by workforce shortfalls. These trends have consequences for quality, worker attachment, and costs. An exogenous policy change raising the out-of-pocket price to some consumers of nursing home services in Germany enables the fourth paper to estimate elasticity of demand. Individuals with lower levels of need were more sensitive to rising prices than those with greater needs and fewer alternatives. The fifth paper examines the relationship of an Alzheimer's diagnosis to individual health expenditures in Mexico and for persons of Mexican origin in the US. Cross-national comparisons and urban-rural differences suggest insights into how LTSS for cognitive disabilities may combine with health services in the two countries.

Abstract citation ID: igad104.0120

### HOME-BASED CARE AND PATIENT OUTCOMES: DOES THE DELIVERY TEAM MATTER?

Norma Coe[1], Chuxuan Sun[1], Courtney Van Houtven[2], Anirban Basu[3], and R.Tamara Konetzka[4], *1. University of Pennslyvania, Philadelphia, Pennsylvania, United States*, *2. Duke University School of Medicine, Durham, North Carolina, United States*, *3. University of Washington, Seattle, Washington, United States*, *4. University of Chicago, Chicago, Illinois, United States*

A major challenge facing the U.S. is how to best to meet the growing demand for person-centered, high-quality long-term care in the least restrictive setting possible for community-dwelling older adults who need assistance with daily activities. This paper asks whether who is providing home care impacts patient outcomes. The relative benefits to the care recipient of informal care, formal care, or a combination of the two is largely unknown. A systematic review (Coe et al. 2021) found informal care, either solo or in concert with formal care, tends to lead to improvements in health and well-being of the care recipient compared to formal care alone. However, little of that research can show causation, and most is not based in the U.S. Recipients' utilization of formal and informal care is affected by factors that also may affect outcomes, complicating the estimation of any causal relationship. Using the 2002-2018 Health and Retirement Study (HRS), we use family structure variables as instruments for both formal care and the combination of formal and informal care to estimate the causal impact of the home health care team on self-reported mental and physical health outcomes. Once the endogeneity of the care team is accounted for, having both formal and informal care leads to higher self-reported health and having only formal care leads to worse mental health outcomes, such as higher depression and lower positive affect, compared to informal care alone. Mobility measures seem unaffected by the type of home care provider.

Abstract citation ID: igad104.0121

### EFFECTS OF ESSENTIAL CAREGIVER POLICIES ON COVID-19 AND NON-COVID-19 DEATHS IN NURSING HOMES

R.Tamara Konetzka[1], Nadia Ghazali[2], and Mingyu Qi[2], *1. University of Chicago, Chicago, Illinois, United States*, *2. The University of Chicago, Chicago, Illinois, United States*

Balancing the safety of nursing home residents with their need for social interaction has emerged as a key challenge from the COVID-19 pandemic. Strict bans on nursing home visitation eventually led to growing concern that physical isolation may have unintended harms on nursing home residents. To address this concern, at staggered times between June 2020 and January 2021, 17 states implemented Essential Caregiver policies allowing nursing home visitation by designated family members or friends under controlled circumstances. Using the Nursing Home COVID-19 Public File and other relevant data, we analyze the effects of Essential Caregiver policies on COVID-19 and non-COVID-19 deaths for nursing home residents. To estimate plausibly causal effects, we employ recent innovations in difference-in-differences and event-study estimation. We find that on average, Essential Caregiver policies prevent 0.44 non-COVID-19 deaths, 0.42 COVID-19 deaths, and 0.86 total deaths per 1,000 residents in a two-week period, representing 8 percent, 17 percent, and 11 percent of the sample mean for the three outcomes, respectively. The effects are mainly driven by state policies that are mandatory or implemented without restrictions and are larger for high-quality facilities and highly staffed facilities. Our results suggest that nursing home residents derive substantial benefit from regular social interaction with family members and friends and from the additional care they provide. Our findings support the use and expansion of Essential Caregiver policies to balance resident safety and the need for social interaction for future infectious disease outbreaks.

Abstract citation ID: igad104.0122

### NURSING HOME WORKFORCE CHALLENGES: INCREASED NURSING STAFF LABOR COSTS AND USE OF AGENCY STAFF FROM 2017 TO 2021

John Bowblis[1], Christopher Brunt[2], Huiwen Xu[3], Robert Applebaum[1], and David Grabowski[4], *1. Miami University, Oxford, Ohio, United States*, *2. Georgia Southern University, Statesboro, Georgia, United States*, *3. University of Texas Medical Branch, Galveston, Texas,*

*United States*, *4. Harvard Medical School, Boston, Massachusetts, United States*

Nursing homes face rapidly increasing wages and change their input mix as they face workforce shortfalls. For 15,959 freestanding NHs from 2017-2021, the proportion of hours worked by agency staff and the median wage cost per hour of agency and directly employed nursing staff were calculated from Medicare cost reports and Payroll-Based Journal data. The median hourly wage cost of directly employed staff (wage and fringe) increased 18.1% for RNs (to $41.99 in 2021), 19.8% for LPNs ($32.29), and 27.5% for CNAs ($19.62). The median hourly wage cost of agency staff (amount paid to staffing agency) increased at a faster rate and was substantially higher: 22.8% for RNs ($64.19); 28.3% for LPNs ($52.42), and 34.9% for CNAs ($33.73). The use of agency staff increased, starting from approximately 2% of all nursing staff hours for each of the nursing staff types in Q1 2017 to 5.6% hours for RNs, 9.4% hours for LPNs, and 8.8% hours for CNAs in Q4 2021. These observations have implications for quality and access to nursing home care. High use of agency staff may affect quality of nursing home care, and substantial wage differences between agency and directly-employed staff may affect worker satisfaction and attachment of directly employed workers. Hourly wage costs for all nursing staff have increased more rapidly than nursing home revenues, which only increased 16.6% over the study period; this suggests that, without payment rate increases, nursing homes may reduce labor inputs to contain costs, or may go out of business.

Abstract citation ID: igad104.0123

### THE EFFECT OF GERMANY'S LONG-TERM CARE STRENGTHENING ACT II ON NURSING HOME USE

Freya Diederich, *University of Bremen, Bremen, Bremen, Germany*

In Germany, out-of-pocket costs for nursing home care have substantially increased during the last years. There is an ongoing discussion whether these out-of-pocket costs should be capped. However, a decrease in costs may increase nursing home use and the social costs of nursing home care. This study analyzes how a change in out-of-pocket costs for nursing home care affects nursing home use. We use discontinuities in out-of-pocket costs that arose from Germany's Long-Term Care Strengthening Act II in 2017. The Long-Term Care Strengthening Act II decreased average nursing home care costs for people with higher care needs and increased average costs for people with lower care needs. Long-term care insurance data are analyzed using a regression discontinuity design. There is no significant change in nursing home use among people who enter long-term care with relatively high care needs. However, there is a significant decrease in nursing home use among those who enter long-term care with relatively low care needs. The results are robust regarding different specifications of the model. We conclude that the elasticity of nursing home use depends on the level of care needs. For people with high care needs, nursing home care is often without alternatives, whereas people with lower care needs may substitute nursing home care with informal care. A generous public policy does not necessarily increase demand for nursing home care.

Abstract citation ID: igad104.0124

### THE HEALTH CARE COSTS OF DEMENTIA FOR MEXICANS AND MEXICAN AMERICANS IN MÉXICO AND THE UNITED STATES

Mónika López Anuarbe[1], and Angela Gutierrez[2], *1. Connecticut College, New London, Connecticut, United States*, *2. Ohio University, Athens, Ohio, United States*

Dementia prevalence and costs affect subpopulations differently, generating healthcare and aging disparities. Most research on healthcare costs associated with dementia has overlooked cross-national comparisons among Hispanics in general and Mexicans in particular. This study utilizes the 2012-2018 Mexican Health and Aging Study (MHAS) and Health and Retirement Study (HRS) to assess: 1) relationships between geographic context and healthcare costs (hospitalization, medical, outpatient, prescription, dental); and 2) whether dementia status moderates the geographic context-healthcare costs relationship. We hypothesized that persons with dementia incurred higher healthcare costs in both countries, and that residents in less populated areas had lower healthcare costs than their counterparts. We used multivariable logistic regressions and generalized linear models, accounting for personal, healthcare access, and utilization factors. Results suggest that in 2012 (MHAS n = 15,723), persons with dementia in México had average healthcare costs that were 60.3% greater compared to those without dementia. Individuals with dementia also had a higher probability (11.9% $p = 0.040$) of incurring any healthcare costs in less densely populated areas (under 15,000 persons). In contrast, Hispanics participating in the HRS during that period (n=15,567), 58% of whom were of Mexican origin, did not have higher total healthcare costs if diagnosed with dementia, but incurred larger expenses in rural areas compared to their urban counterparts ($p< 0.001$). These results underscore how geographical context varies across countries and how dementia status can leave some individuals more financially vulnerable than others, affecting their access to healthcare services and overall health.

### SESSION 1120 (SYMPOSIUM)

Abstract citation ID: igad104.0125

### TECHNOLOGY AS A CATALYST FOR SUPPORTING AGING WITH DISABILITY

Chair: Tracy Mitzner

Individuals aging with long-term disabilities may need support to perform and participate in everyday activities associated with well-being and quality of life. Technology solutions have potential to effectively promote autonomy and aging-in-place for this population. This symposium will highlight technology research and development efforts from the Rehabilitation Engineering Research Center on Technologies to Support Aging-in-Place for People with Long-Term Disabilities (RERC TechSAge). TechSAge is dedicated to understanding the needs of, and developing supportive technologies for people aging with long-term vision, hearing, and mobility disabilities. Mumma et al. will present insights from interviews of adults aging with vision loss due to glaucoma and macular degeneration regarding their challenges and response strategies for everyday activities. Lee et al. will discuss findings from interview data exploring how wisdom is manifested from the lived experiences of individuals aging with disability (i.e., hearing, vision, and mobility). Mitzner et al. will describe characteristics of the participant sample from a clinical trial examining the effect of a Tele Tai Chi intervention on physical activity and social connectedness for adults aging with long-term mobility disabilities. Sanford et al. will present the findings from three TechSAge pilot projects focused on the use of technology to support in-home rehabilitation for individuals aging with long-term upper extremity disabilities: DigiHand, KeyStroke, and 3DP for AT. Together these studies illustrate the needs and coping strategies of older adults with disabilities and the potential of technology to support those needs. This is a collaborative symposium between the Lifelong Disabilities and Technology and Aging Interest Groups.

Abstract citation ID: igad104.0126

### METHODS AND BASELINE CHARACTERISTICS FOR THE TECHSAGE TELE TAI CHI CLINICAL TRIAL

Tracy Mitzner, Kara Mumma, and Elena Remillard, *Georgia Institute of Technology, Atlanta, Georgia, United States*

Individuals aging with long-term mobility disabilities encounter a range of significant barriers to participating in physical and social activities, from challenges with transportation, to lack of accessibility and access to appropriate instruction. We developed a Tai Chi telewellness intervention to facilitate both physical and social wellness through accessible, virtual group classes. The intervention translated an in-person, evidence-based tai chi program (Tai Chi for Health Institute's Tai Chi for Arthritis) to be 1) a virtual experience (via Zoom) that includes a social component and 2) accessible and appropriate for those aging with mobility disabilities. We conducted the TechSAge Tele Tai Chi clinical trial to examine the effect of the intervention on physical activity and social connectedness (primary outcomes), as well as exercise self-efficacy, falls efficacy, depression, quality of life, and pain (secondary outcomes) for this population. The participant sample was community-dwelling adults (60-80 years of age) with a self-identified mobility disability (i.e., using a mobility aid or having serious difficulty walking or climbing stairs) for at least 10 years. The presentation will describe the rationale for the study, as well as the study design, methodology, and baseline data for the trial. We will also highlight the user-centered, iterative design approach we used in the design and evaluation of the intervention protocol.

Abstract citation ID: igad104.0127

### UNDERSTANDING EVERYDAY NEEDS OF OLDER ADULTS WITH MACULAR DEGENERATION OR GLAUCOMA

Kara Mumma[1], Elena Remillard[1], Tooba Umar[2], Divya Bhookya[2], and Wendy Rogers[3], *1. Georgia Institute of Technology, Atlanta, Georgia, United States*, *2. College of Applied Health Sciences, University of Illinois Urbana-Champaign, Champaign, Illinois, United States*, *3. University of Illinois Urbana-Champaign, Champaign, Illinois, United States*

Older adults with long-term vision loss may experience challenges in their everyday activities. Different types of vision loss may cause different problems. For example, macular degeneration leads to a loss of foveal vision whereas glaucoma leads to peripheral vision loss. As part of the Aging Concerns, Challenges, and Everyday Solution Strategies (ACCESS) study, we are using a mixed-methods approach to explore the unmet needs of older adults with these two conditions to provide guidance for interventions. We will present insights from 51 older adults (mean age = 69, SD = 5.5) who have had serious difficulty seeing for an average of 42 years (SD = 24.3) due to either glaucoma (n = 28), macular degeneration (n= 19), or both conditions (n = 4). We will present participants' difficulty ratings for a wide range of everyday activities as well as initial themes and illustrative quotes from interview data on challenges and response strategies for their most difficult activities (e.g., shopping, traveling for leisure, dressing). For example, it can be difficult to navigate through a crowded store and read details on labels when shopping. Additionally, flying was described as frustrating and stressful because airports can be unfamiliar and require arrangements (e.g., transportation to/from, guidance to the gate). Moreover, when getting dressed, participants said that they rely on touch to differentiate between clothes; however, color coordination and matching can be very difficult. Data provide insights on unmet needs among people aging with vision loss and illuminate opportunities for technology innovation.

Abstract citation ID: igad104.0128

### EXPLORING WISDOM IN PERSONS AGING WITH DISABILITY

Hye Soo Lee[1], Maya Dye[2], and Wendy Rogers[2], *1. University of Illinois--Urbana-Champaign, Champaign, Illinois, United States*, *2. University of Illinois Urbana-Champaign, Champaign, Illinois, United States*

Wisdom can benefit one's well-being and lead to a more fulfilling life. One pathway is using wisdom as a resource for coping with adversities in life. At the same time, wisdom can be fostered from experiencing adversities in life. The TechSAge ACCESS interview study explored the challenges and coping strategies of individuals aging with disability (i.e., hearing, vision, and mobility), providing us the opportunity to investigate the wisdom fostered from participants' lived experiences. Studying wisdom in a population with disability has at least two practical implications for the intersectional field of aging, disability, and technology. First, we can gain and disseminate the knowledge regarding how to cope with aging with disability, which would benefit individuals with similar disability and their care networks. Second, we can utilize participants' wisdom to guide our research. Focusing on their strengths and coping strategies instead of their weaknesses would offer us additional ways to support individuals aging with disability. There are specific merits to utilizing the ACCESS data for wisdom research. The sample of individuals aging with disability allows us to explore wisdom that manifested as an interaction between a person and a situation. We are exploring participants' answer to the question regarding the advice they would give to others with similar disability as a wisdom assessment task. This is a contribution to the wisdom literature as it is rare to be able to administer assessment tasks that are relevant to participants' lives and interests.

Abstract citation ID: igad104.0129

**IMPROVING REHABILITATION OUTCOMES FOR PEOPLE AGING WITH LONG-TERM HAND IMPAIRMENTS**

Jon Sanford[1], Susan Lee[2], Yi-An Chen[3], Sutanuka Bhattacharjya[2], Emily Buchman[3], Martin Norgaard[4], and Gil Weinberg[5], *1. Dept. of Occupational Therapy, Georgia State University, Atlanta, Georgia, United States*, *2. Georgia State University, Atlanta, Georgia, United States*, *3. Dept. of Occupational Therapy, GA State, Atlanta, Georgia, United States*, *4. Music Education, GA State, Atlanta, Georgia, United States*, *5. School of Music, GA Tech, Atlanta, Georgia, United States*

Despite the importance of hand and upper extremity function in ADL performance and successful aging-in-place, contextual and engaging in-home rehabilitation interventions that promote use, feedback of progress, and long-term monitoring of changes in functional ability among individuals with chronic upper limb deficits are lacking. This presentation will present the findings from three TechSAge pilot projects focused on the use of technology to support in-home rehabilitation for individuals aging with long-term upper extremity disabilities. DigiHand, is a smartphone-based application combined with sensor-embedded objects used in daily activities (e.g., coffee cup) that enables users to self-manage their own rehabilitation, obtain feedback on progress to refine movements, and allow remote monitoring by therapists. Similarly, KeyStroke is an in-home piano training mobile app that provides engaging music exercises to promote long-term rehabilitation and therapists' monitoring of progress of stroke survivors. Finally, 3DP for AT is generating design requirements to support the development of an open-source software tool that would allow assistive technology service providers, who are novice users of 3D printers, to create and fabricate highly customized 3D-printed assistive devices that are more durable, aesthetic, and will better meet the unique needs of people aging with long-term hand impairments.

### SESSION 1125 (PAPER)

### GEROSCIENCE AND EPIDEMIOLOGY IN AGING

Abstract citation ID: igad104.0130

**CHANGES IN ACCELEROMETER MEASURED MOVEMENT BEHAVIORS OVER RETIREMENT: A COMPOSITIONAL DATA APPROACH**

Lawrence Sacco, and Hugo Westerlund, *Stockholm University, Stockholm, Stockholms Lan, Sweden*

Retirement is a significant life transition where individuals change their daily patterns of movement behaviors (i.e. physical activity and sedentary behavior). However, evidence accounting for how individuals re-allocate time from one type of activity to another is lacking, considering that engagement in different activities is co-dependent as time not spent in a given type of activity will be spent in another one. The objective of this paper is to evaluate how relative time spent in movement behaviors (light physical activity, moderate-vigorous physical activity and sedentariness) and sleep change over the retirement transition. Furthermore, the aim is to assess how movement behaviors change differently between genders, manual Vs non-manual work and weekend Vs weekdays. Data was collected from 114 participants (47 men and 67 women; ages: 60 to 73) from the Swedish Retirement Study (SRS) at three timepoints: 6 months before retirement and, 6 and 18 months after retirement. Time-use data on movement behaviors and sleep was collected over a week-long period through thigh-worn accelerometers and wrist-worn actigraphs. Compositional data analysis (CoDA) was used to account for the compositional and finite nature of 24 hour time-use data. Results indicate that small changes in allocation of time between movement behaviors occur during retirement. Changes indicate that after retirement weekdays shows patterns of activity that resemble more patterns typical of pre-retirement weekends. Findings are discussed in relation to implications for physical activity guidelines and the influence of retirement on health.

Abstract citation ID: igad104.0131

**HYPOTHALAMIC MICROSTRUCTURE IN OLDER ADULTS IS ASSOCIATED WITH CORTICAL ATROPHY AND NEUROCOGNITION**

Sandra Aleksic[1], Roman Fleysher[1], Noa Tal[2], Timothy Darby[1], Erica Weiss[3], Nir Barzilai[4], Michael Lipton[5], and Sofiya Milman[1], *1. Albert Einstein College of Medicine, Bronx, New York, United States*, *2. Cedars-Sinai Medical Center, Los Angeles, California, United States*, *3. Montefiore Medical Center/ Albert Einstein College of Medicine, Bronx, New York, United States*, *4. Albert Einstein Clollege of Medicine, Bronx, New York, United States*, *5. Albert Einstein College of Medicine, Bronx, New Jersey, United States*

The hypothalamus is emerging as an important regulator of aging. Hypothalamic inflammation in mice shortens lifespan and accelerates neurocognitive decline. However, the role of the hypothalamus in systemic and neurocognitive aging in humans is unresolved. We studied the cross-sectional relationships between hypothalamic microstructure, cortical atrophy and neurocognition in community dwelling older adults (n=124, median age 78.7 (IQR 74.3-83.4) years, 58% women). Hypothalamic microstructure was assessed using magnetic resonance imaging (MRI)-diffusion tensor imaging (DTI) parameters that included mean diffusivity (MD) and fractional anisotropy (FA), obtained using a novel automated image processing pipeline. Greater MD and lower FA were indicative of compromised microstructural integrity. Neurocognitive assessments included a battery of 11 tests that were used to quantify standardized domain-specific (executive, memory, language, attention and visuomotor) and overall neurocognitive scores. Age was positively associated with hypothalamic MD (beta=0.037 SD per year of age, p=0.005). In linear models adjusted for age, sex, education, white matter hypointensities, and total intracranial volume, hypothalamic MD was negatively associated with cortical thickness in the cingulate, frontal, and parietal lobes (betas -0.07, -0.06, -0.07 respectively, all FDR-adjusted p≤0.01). On the other hand, in a multivariable adjusted model that included cortical thickness, hypothalamic FA was positively associated with the overall and all domain-specific neurocognitive scores (betas ranged 0.14 - 0.18, FDR- adjusted p values< 0.05).

Hypothalamic microstructure is independently associated with cortical atrophy and neurocognition in the aging brain. Longitudinal studies can establish temporality and potential mechanisms by which hypothalamic dysfunction may accelerate systemic and neurocognitive aging in humans.

Abstract citation ID: igad104.0132

### TEMPORAL TRENDS IN INCIDENCE RATE OF DIFFERENT TYPES OF MULTIMORBIDITY AMONG MIDDLE- AND OLD-AGED AMERICANS, 2000–2020

Zhou Yang, Yu-Ming Chen, Hui-wen Xu, and Bei-bei Xu, *Peking University, Beijing, Beijing, China (People's Republic)*

This study aimed to examine secular trends in multimorbidity incidence rates among middle and old aged American adults. Participants aged ≥ 50 years from the Health and Retirement Study (HRS) during 2000-2020 were divided into nine consecutive and overlapping cohorts. Four types of multimorbidity (general multimorbidity, physical multimorbidity, multimorbidity including psychiatric disorders, and cardiometabolic multimorbidity) were identified based on seven self-reported physical diseases and two psychiatric conditions confirmed by validated scales. Generalized estimating equation (GEE) models incorporating a restricted cubic spline were used to examine adjusted nonlinear trends. Joinpoint regression models were adapted to estimate annual percentage changes and determine the contributions of baseline characteristics to the trends. The adjusted general multimorbidity incidence rate (per 1,000 person-years) increased from 76.0 (95% confidence interval(CI) = 71.8-80.4) in 2000-2004 to 112.2 (95% CI = 106.4-118.4) in 2016-2020. Both general multimorbidity and multimorbidity including psychiatric disorders showed a rising trend followed by a period of slight decrease from 2006-2010 to 2010-2014 and then a sustained increase, while physical multimorbidity and cardiometabolic multimorbidity showed a similar trend of increasing till 2006-2010 and then a relative plateau. Race/ethnicity, socioeconomic status, marital status, and dementia were significant contributors to changes in the incidence rate during 2000-2020. The incidence rate of each type of multimorbidity showed different fluctuations among middle and old aged adults in the US from 2000 to 2020. Findings inform clinicians, policymakers, and researchers to develop targeted interventions and provide comprehensive multimorbidity management in community settings.

Abstract citation ID: igad104.0133

### THREE MONTHS OF STRENGTH TRAINING ALTERS LPS-INDUCED IMMUNE RESPONSES IN CIRCULATING PBMC OF OLDER PERSONS

Ivan Bautmans, *Vrije Universiteit Brussel (VUB), Brussels, Brussels Hoofdstedelijk Gewest, Belgium*

BACKGROUND Recently, we showed that 3 months strength training affect 18 canonical pathways related to chronic inflammation in peripheral mononuclear blood cells (PBMC) of older adults. Here we investigate if resistance training improves the stress response of PBMC by mimicking in-vitro an acute infection by lipopolysaccharide (LPS)-challenge. METHODS 14 women aged ≥65 years were randomized into 3 months of either 3×/week intensive strength training (IST: 3×10 rep at 80% 1RM), strength endurance training (SET: 2×30 reps at 40% 1RM) or control (CON: 3×30 sec stretching). Before and after 3 months training, PBMC were isolated and cultured with and without LPS. Prior culture and after 24 hours of culture, RNA was collected from pre-cultured, post-cultured and LPS challenged PBMC's, respectively. Targeted RNA sequencing including 407 inflammation-related genes was performed. Pathway analysis was performed with Ingenuity Pathway Analyses using all 407 genes, a Benjamini-Hochberg p-value < 0.05 and a z-score of ≤-2 or ≥2 were considered as significant. RESULTS Strength training altered 23 pathways in LPS-stimulated PBMC; (IST: 7 upregulated and 2 downregulated, SET: 5 upregulated and 10 downregulated). None of the altered pathways overlapped between IST and SET. The Cytotoxic T Lymphocyte-mediated Apoptosis of Target Cells pathway was enriched oppositely in both training groups (downregulated in IST versus upregulated in SET). CONCLUSIONS Three months IST and SET can induce changes in the inflammatory stress response of PBMC, but by affecting different genes and related pathways. A balanced exercise program altering both training regimes might therefore provide optimal immune adaptations in older persons.

Abstract citation ID: igad104.0134

### TRAJECTORIES OF DISEASES, FUNCTIONAL LIMITATIONS, AND PSYCHOLOGICAL AND COGNITIVE HEALTH OF CENTENARIANS

Erfei Zhao[1], Jennifer Ailshire[2], and Eileen Crimmins[2], *1. University of Southern California, Leonard Davis School of Gerontology, Los Angeles, California, United States*, *2. University of Southern California, Los Angeles, California, United States*

Centenarians are recognized as providing a model of delayed aging. Centenarians are generally healthier throughout their 80s/90s than non-centenarians. However, the extent of the delay in morbidities experienced by centenarians remains unclear as it requires quantifying trajectories of health deterioration with age for both centenarians and their cohort counterparts who did not survive to 100-year-old. We use the Health and Retirement Study, a nationally representative sample, to assess the aging experience of centenarians and non-centenarians in the following domains: diseases, functional limitations, psychological and cognitive functioning. We include data from 1993 to 2018. We include 4,778 respondents born before 1918 as they had the potential to become centenarians during the study period - 201 reached at least 100-year-old and are categorized as centenarians; 4,577 of them died before 100. We use join point regression to determine 1) how many years centenarians were delayed in morbidities onset and 2) whether deterioration is accelerated starting at certain ages. On average, onset of any limitation in activities of daily living (ADL) among centenarians is delayed by about 10 years, instrumental ADLs by 9 years, and diseases by nearly 16 years (if hypertension is not considered). Rates of deterioration are slower, in general, for centenarians; however, starting at the age of 90, centenarians experience accelerated decline in ADL, IADL, functioning, and cognition. Centenarians do not appear to experience decline in psychological well-being whereas non-centenarians do. Our results suggest centenarians generally survive to older ages because they postpone aging; it is not avoidable indefinitely.

### SESSION 1130 (PAPER)

### VULNERABLE POPULATIONS

Abstract citation ID: igad104.0135

### AGEING WELL WITH AUTISM

Laura Brown[1], Rebecca Aitken[2], Katherine Berry[1], and Emma Gowen[1], *1. University of Manchester, Manchester, England, United Kingdom*, *2. The University of Manchester, Manchester, England, United Kingdom*

Autism is characterized by patterns of neurological development that can cause differences in social interactions and sensory processing; restricted and repetitive patterns of behavior; and difficulties in identifying and describing feelings. The highly sociable and unpredictable environments typical of most societies are poorly suited to autistic people*, leading to disadvantage and exclusion. Despite increasing numbers of people aging with autism, little is known about the needs and experiences of middle-aged and older autistic adults. This is important given that autistic people experience higher rates of health and functional difficulties, and express a need for appropriate, accessible forms of support as they age. In this study, we interviewed seventeen autistic adults, aged from 46-72 years, about their views and experiences of aging, and how services could better support them to age well. The transcribed interviews were analyzed using inductive thematic analysis. The findings revealed several potential ways that autism affected people's ability to age well. This included difficulties in accessing formal and informal supports; interactions between autism and health conditions; and negative attitudes and low understanding about autism encountered within services and society. Recommendations for supporting autistic adults to age well included involving autistic people in the design of services, and introducing specialized advocate/coordinator roles or 'one-stop-shop' hubs that could help autistic people navigate services more easily. * Our use of the terms 'autistic people/adults' is in-line with evidence-based guidance for writing about autism research, and is preferred by autistic people to 'person-first' terms like 'person with autism' (https://journals.sagepub.com/pb-assets/cmscontent/AUT/Autism-terminology-guidance-2021-1626860796.pdf)

Abstract citation ID: igad104.0136

### BUILDING CLINICAL JUDGMENT IN THE HEALTH CARE WORKFORCE BY ADDRESSING THE 4MS AND MULTICOMPLEXITY

Margaret Avallone[1], Elyse Perweiler[2], Jennifer DeGennaro[3], and Marilyn Mock[4], *1. Rutgers University-Camden School of Nursing, Camden, New Jersey, United States*, *2. Virtual Health College of Medicine & Life Sciences of Rowan University - Rowan-Virtua School of Osteopathic Medicine, Stratford, New Jersey, United States*, *3. Rowan-Virtua SOM / NJISA, Stratford, New Jersey, United States*, *4. Fair Share Support Services, Inc., Camden, New Jersey, United States*

Since 2019, NJGWEP, Rutgers School of Nursing-Camden and Fair Share Support Services, Inc. partnered in an affordable housing site in an underserved, disadvantaged community to support aging in place. Senior nursing students conduct Resident Health Risk Assessments (RHRAs) using the 4Ms framework to build assessment skills, sharpen clinical judgment, and address the Geriatric 5th M in a multi-complex resident population. Nursing students, working in teams with social service and community health workers, completed 92 RHRAs incorporating the 4Ms (What Matters, Mind, Medication, Mobility). On average, residents had > 3 risk factors related to the 4Ms (M=3.31); 25% +screen for depression, 25% +screen for cognitive impairment, 64% w/ mobility deficits, 83% w/polypharmacy, and 80% on 1 or more high-risk medications. There were 53% w/diabetes, 65% w/hypertension and other biopsychosocial factors related to social determinants of health; 91% could be characterized as multi-complex (the Geriatric 5th M) and in need of services/supports to age in place. The 4Ms framework, with the addition of the 5th M, provides curricular structure for development of clinical judgment through focused assessment, analysis, flagging of key assessment data, prioritization of problems, and developing person-centered plans of care. Interprofessional (IP) case studies of increasing levels of difficulty experienced by affordable housing residents build cultural competence, foster development of critical thinking, clinical reasoning, and, when supplemented by weekly IP case reviews, facilitate shared decision-making and care planning. The process is further supported by a customized electronic database that permits monitoring person-centered recommendations and outcomes over time, gauging impact.

Abstract citation ID: igad104.0137

### DID CHILDHOOD ABUSE PREDICT CARE EXPECTATION FOR FORMAL CARE: THE ROLES OF FAMILY FUNCTIONING AND CARE PREPARATION

Xue Bai[1], Chang Liu[1], Jiajia Zhou[1], and Bo Hu[2], *1. The Hong Kong Polytechnic University, Hong Kong, Hong Kong*, *2. London School of Economics and Political Science, London, England, United Kingdom*

Objectives: The influence of childhood abuse could be lasting and extensive. This study aims to examine the associations between childhood abusive experiences (i.e., physical abuse, emotional abuse, and bully victimization) and ageing adults' care expectation for formal care in later life, as well as the potential mechanism of the associations. Methods: This study used data from the Panel Study of Ageing and Society (PAAS) in Hong Kong, with 5,007 participants aged 50 and over included in the study. Path analyses were performed to examine the direct effects of abusive experiences in childhood on future care expectation and formal care, and the indirect effects through serial mediators of family functioning and care preparation. Results: Ageing adults experiencing more childhood abuse were less likely to report care expectations in the future ( $= -0.051$, $p < 0.01$), and the direct effects ( $= -0.042$, $p < 0.05$) and indirect effects ( $= -0.009$, $p < 0.05$) from the serial mediation were significant. Among those people who reported clear care expectations, they were more likely to choose formal care if they had more abusive experiences in childhood ($= 0.049$, $p < 0.05$), from which the association was largely explained by decreased family functioning and more care preparation ( $= 0.042$, $p < 0.001$). Discussion: This study is the first to connect childhood abuse and late-life care expectation. From a life course perspective, it encourages more considerations regarding care preparation and formal care among people with a history of childhood abuse.

Abstract citation ID: igad104.0138

**LIFETIME REVICTIMIZATION: EVIDENCE FROM THE WISCONSIN LONGITUDINAL STUDY**

Jooyoung Kong[1], Scott Easton[2], and Jason Fletcher[1], *1. University of Wisconsin-Madison, Madison, Wisconsin, United States*, *2. Boston College, Boston, Massachusetts, United States*

Research has documented the increasing prevalence of elder abuse victimization and its devastating impact on the health and well-being of older adults. However, a life-course view of the victimization experiences of older adults has rarely been adopted in the elder abuse literature. The current study examined lifetime links between victimization experiences that span childhood, adulthood, and older adulthood. Specifically, we examined: a) the mediating effect of adverse childhood experiences (ACE) on elder abuse victimization via intimate partner violence (IPV) victimization in middle adulthood, and b) whether this indirect association differs by gender. Data were obtained from the Wisconsin Longitudinal Study (WLS). We analyzed the previous and current victimization experiences of a total of 5,391 older adults in their early 70s and estimated the mediational and moderated mediation models. The key results indicated that a higher ACE score was associated with exposure to IPV victimization in middle adulthood, which was in turn associated with exposure to elder abuse victimization. This indirect association was stronger for women than for men. Regarding specific types of childhood victimization, parental physical abuse, sexual abuse, and witnessing domestic violence significantly predicted elder abuse victimization via IPV. Our results support the phenomenon of lifetime victimization, whereby an individual experiences reoccurring forms of victimization across the life span from childhood to late adulthood. Findings highlight the compelling need for the assessment of cumulative victimization experiences and their impact on elder abuse victims. A life course-based, trauma-informed approach would greatly enhance prevention and intervention services for elder abuse.

### SESSION 1135 (SYMPOSIUM)

Abstract citation ID: igad104.0139

**PRACTICE AND THEORY: THE SIGNIFICANCE OF PARTICIPATING IN CULTURALLY-RELEVANT CRAFTS FOR DIVERSE OLDER ADULTS**

Chair: Carolyn Adams-Price

In the last 25 years, there has been a great increase in interest in the benefits of creative activities for older adults, especially for dementia patients. More recent research (Adams-Price et al., 2018) has identified specific benefits that healthy older adults get from participating in arts activities. However, little research has featured the benefits of traditional craft activities, especially those prominent in different ethnic groups. The focus of this symposium will be on the benefits of traditional crafts for older adults, especially when those crafts are traditional to one's culture and have benefits for identity. The proposed symposium will include papers by Chen and Alcantar on the impact of craft participation on diverse older adults. Kaori Otera Chen will be discussing older women weavers in New York State, and the psychological growth they experience from learning and practicing skills associated with weaving, and from creating beautiful pieces. Sandy Alcantar will be discussing a program that she runs for older Hispanic immigrants that promotes participation in traditional crafts to provide income and connect the immigrants to their culture of origin. Adams-Price will be discussing life-span developmental psychology and the sociocultural theory of creativity from Vlad Glaveanu, and their implications of the theory for discussing the significance of long-term participating in culturally-relevant crafts in later life, singly and in groups. She will tie in examples from diverse cultures, including older rural African American women who quilt.

Abstract citation ID: igad104.0140

**THEORIES OF CREATIVITY AND CRAFTS FOR OLDER PEOPLE**

Carolyn Adams-Price, *Mississippi State University, Mississippi State, Mississippi, United States*

Although gerontologists have increased their discussions of the benefits of creative activities for older adults, some aspects of late life creativity have been discussed very little. Two aspects of late life creativity that have received little attention include 1) long-term participation in a particular creative domain, resulting in learning and the development of skills, and 2) the participation in culturally-meaningful crafts, especially those that connect people or result in a cultural legacy. Referring to examples from diverse cultures, we will discuss how theories of adult development and theories of creativity can be merged into a framework for studying the significance of participation in culturally-significant crafts for diverse older adults. Erikson's theory of adult development and Glaveanu's sociocultural theory of creativity will be used as a framework to explore the meaning and potential of participation in culturally-significant crafts for positive development in late life. We will discuss how practice leads to learning and mastery, how identity benefits from creating culturally-significant crafts, how creating in groups (or with an audience) leads to greater engagement, and how the creation of sharable products can lead to a meaningful legacy for some older creators. Examples from diverse cultures will be included, including from the two previous papers, and from older rural African American women who quilt.

Abstract citation ID: igad104.0141

**CREATIVE ARTS INTERVENTION FOR ISOLATED LATINX OLDER ADULTS TO REDUCE TRAUMA SYMPTOMS**

Sandy Alcantar, *Long Beach Mental Health, Long Beach, California, United States*

Creative arts is an important form of therapeutic psychotherapy to reduce trauma symptoms in migrant older adults by allowing them to explore what they once connected in their country of origin. This therapeutic modality helps to destigmatize mental health, promote health education, and feel accepted, include social connectiveness with individuals who have suffered similar trauma in their country of origin and have migrated to find a safer living environment. Interventions used have been tailored to Spanish Speaking Older adult immigrant clients that are at higher risk of mental health conditions, such as depression, anxiety, and posttraumatic stress disorder. These clients are often hiding in isolation and feel lonely. The main objective was to reduce anxiety due to re-traumatization of symptoms more importantly during pre/post COVID isolation and multiple stressors the country has been going through. For these individuals, their arts are their livelihoods due to their immigration status. It is imperative to encourage our elderly minority community to continue to focus on their crafts i.e., weaving, embroidery, sewing, crochet, cooking, pottery, or any creative arts they pr. This allows the person to have control, autonomy, feel useful and most importantly have a sense of belonging to their community, in a country that they feel displaced from. It is vital to use arts as an option to help our older adults process their life story to support development theories, in this case, Erik Erikson's final stage of psychosocial development, integrity vs despair.

### SESSION 1140 (PAPER)

### LIFE EXPERIENCES: A CLOSER LOOK AT TRAUMA AND RESILIENCE

Abstract citation ID: igad104.0142

### "CAREGIVING MADE ME GRATEFUL": THE UNEXPECTED BLESSINGS OF DEMENTIA CAREGIVING

Jordan Lewis, *University of Minnesota Medical School, Duluth campus, Duluth, Minnesota, United States*

There is a cultural value among Alaska Natives to care for their Elders and this commitment applies to all Elders, regardless of health status or cognitive health. Caregiver duties can lead to stress, isolation, and depression that negatively impact their overall health and wellbeing. Interviews were conducted with 22 Alaska Native caregivers for Alaska Natives with Alzheimer's Disease and Related Disorders in rural and urban communities across Alaska. Interviews lasted approximately 60 min and were transcribed verbatim. We examined the caregivers' experiences of ADRD and the unexpected blessings associated with being a caregiver. Alaska Native caregivers shared the challenges associated with caregiving duties, and the lack of education, awareness, and available resources exacerbated their stress and challenges. As their family member experienced changes related to the ADRD, the blessings took different forms and served different purposes. Spending more time with their family member, remembering family history, engaging in generative behaviors, and strengthening intergenerational relationships were the most frequently discussed blessings. Focusing on these blessings enabled caregivers to continue to find meaning in this role and carry on their cultural values and traditions. This study shifts the focus of caregiving research to highlight the unexpected blessings. The focus on strengths of caregiving and sharing these stories has the potential to provide current and future caregivers to step into this role with a different perspective, or at least not just hear of the challenges and barriers associated with ADRD caregiving.

Abstract citation ID: igad104.0143

### FINANCIAL EXPLOITATION AND MENTAL HEALTH OF HOLOCAUST SURVIVORS: THE MODERATING ROLE OF POSTTRAUMATIC SYMPTOMS

Gali Weissberger[1], Moshe Bensimon[2], and Amit Shrira[1], *1. Bar Ilan University, Ramat Gan, HaMerkaz, Israel*, *2. Bar-Ilan University, Ramat Gan, Tel Aviv, Israel*

Financial exploitation (FE) is associated with devastating emotional, health, and economic consequences. We examined whether anxiety and depressive symptoms associated with self-reported history of financial exploitation (FE) are more pronounced amongst Holocaust survivors, especially those with high-level posttraumatic stress disorder (PTSD) symptoms. A community-based cohort of 137 Israeli older adults born prior to 1945 were included in the study sample. Holocaust survivors ($n = 61$) were participants who reported living in a European country occupied or dominated by Nazi or pro-Nazi regimes between 1939-1945. Groups were further subdivided into survivors with low or high levels of PTSD symptoms (≥31 on the PTSD Checklist; PCL-5). Questionnaires assessed FE history, posttraumatic symptoms (PCL-5), depressive symptoms (PHQ-9), and anxiety (GAD-7). Age, education, self-rated health, and non-Holocaust lifetime adversity were also measured and included as covariates. Hierarchical linear regression models revealed that relationships between FE and depressive and anxiety symptoms were significant only among survivors ($p = 0.005$ and $p = 0.008$, respectively). The interaction between PTSD symptom level group and FE was also significant for both depressive ($p = 0.007$) and anxiety ($p = 0.012$) symptoms, such that survivors with PTSD who reported FE had significantly greater symptoms of depression and anxiety compared to all other groups. Findings suggest that the experience of FE may be particularly impactful amongst survivors who continue to struggle with posttraumatic symptoms related to the Holocaust. Future studies may consider examining whether findings are relevant to other groups with PTSD.

Abstract citation ID: igad104.0144

### POSITIVE ASPECTS OF DEMENTIA CAREGIVING EVOLVE FROM AN INTEGRATIVE STRESS COPING AND EXISTENTIALISM PARADIGMS

Doris S.F. Yu[1], and Sheung Tak Cheng[2], *1. The University of Hong Kong, Hong Kong, Hong Kong*, *2. The Education University of Hong Kong, Hong Kong, Hong Kong*

Family caregiving is the crucial informal care resource to buffer the associated disease burden. Research in this field has focused on promoting effective adaptation of family caregivers of persons with dementia (PWD) by ameliorating their caregiving burden. Little attention is placed on positive aspects of caregiving (PAC). PAC refers to the extent to which caregiving experience is appraised as enriching to one's life, and promoting uplift, personal growth and gratification. This mixed-method study investigated how PAC evolved from the encounter of the family caregivers of PWD. Based on the research evidence about how PAC is shaped, an integrated theoretical model combining the stress coping and existential paradigms were hypothesized to explain the evolvement of PAC. A mixed-method longitudinal study was conducted, and 403 family caregivers of PWD were invited to respond on validated measure for active dementia management strategies, self-efficacy, social support, meaning-making, motivation to care and religiosity. PAC was measured at baseline and 6 months thereafter. Path analysis indicated that PAC was evolved from the self-efficacy derived from active coping with the caregiving experience, with a social support as a significant moderator for such relationship. The use of meaning-focus coping especially in a religious context also optimize this favorable caregiving attribute. The qualitative findings converge with this model, and further elaborate how performance success, self-affirmation, spiritual growth, and relationship solidarity consolidate perceived positivity on the caregiving experience. This study provides important insights on how to advance caregiving supportive care by adding an existential paradigm.

Abstract citation ID: igad104.0145

### RESILIENCE AMONG BLACK FAMILY CAREGIVERS OF PERSONS WITH DEMENTIA

Sheria Robinson-Lane, *University of Michigan School of Nursing, Ann Arbor, Michigan, United States*

Black family caregivers of persons with dementia often experience elevated levels of stress related to lower incomes, chronic health conditions, and care recipient behaviors. Resilience theory suggests that stress may be moderated by various resources and processes, however, few studies have examined factors that may influence emotional resilience among Black family caregivers. Spearman's rank correlation coefficient was used to assess the relationships between adaptive coping, caregiving self-efficacy, and positive affect within a sample of current caregivers (n=20). Self-efficacy in obtaining respite care was significantly correlated with both positive affect (p=.041) and adaptive coping (p=.022). Self-efficacy for managing difficult behaviors was also correlated with positive affect (p=.001). Finally, we identified a significant correlation between body mass index and self-efficacy for controlling upsetting thoughts. Caregiver resilience may be enhanced by providing increased access to respite services and culturally responsive support programs aimed at enhancing positive emotions and affective balance.

### SESSION 1145 (SYMPOSIUM)

Abstract citation ID: igad104.0146

### IDENTIFYING AND PREVENTING RESIDENT-TO-RESIDENT AGGRESSION IN LONG-TERM CARE: EMERGING GLOBAL PERSPECTIVES

Chair: E-Shien Chang Discussant: Laura Mosqueda

Resident-to-resident aggression (RRA) is a widespread form of mistreatment in long-term care facilities, affecting 1 in 5 residents every month. Despite its high frequency and adverse consequences for residents, staff, and family members, RRA remains under-recognized and insufficiently studied. Several reasons exist for this, including challenges in obtaining high-quality data to inform evidence-based prevention and intervention strategies. Improved understanding of RRA aligns with recent national policy imperatives to increase nursing home safety and quality of care. To accelerate knowledge-to-practice translation, this symposium highlights expertise from North America and Asia to review the latest evidence and experiences that characterize and improve RRA. Dr. E-Shien Chang will identify the role of racial and ethnic conflicts in RRA using data from the largest RRA prevalence cohort study of U.S. nursing home residents. Dr. Elsie Yan will examine resident and environment risk factors of RRA in Hong Kong, illustrating the needs for improved dementia care training among direct care workers. Dr. David Burnes will discuss barriers, successes, and lessons learned in replicating and adapting a gold standard U.S. based RRA prevalence study in the unique context of Canada. Dr. Karl Pillemer will describe a novel dissemination model to expand program reach of Improving Resident Relationships in Long Term Care (IRRL), an evidence-based RRA intervention program. Finally, Dr. Laura Mosqueda, as policy expert of long-term care services and discussant, will moderate a discussion on translating RRA research to practice and policy changes within the global contexts of long-term care. This is a collaborative symposium between the Abuse, Neglect and Exploitation of Older Persons and Research in Quality of Care Interest Groups.

Abstract citation ID: igad104.0147

### IDENTIFYING ACUTE PRECIPITANTS AND CONSEQUENCES OF RACIAL/ETHNIC CONFLICTS IN RESIDENT-TO-RESIDENT AGGRESSION

E-Shien Chang[1], Jeanne Teresi[2], Mildred Ramirez[3], Joseph Eimicke[4], Sara Czaja[1], Karl Pillemer[5], Mark Lachs[1], and Tony Rosen[1], *1. Weill Cornell Medicine, New York City, New York, United States*, *2. Columbia University Stroud Center, New York City, New York, United States*, *3. Hebrew Home at RiverSpring Health, Riverdale, New York, United States*, *4. Research Division Of Hebrew Home At Riverdale, New York City, New York, United States*, *5. Cornell University, Ithaca, New York, United States*

Resident-to-resident aggression (RRA) includes negative and aggressive physical, sexual, or verbal interactions between long-term care residents that in a community setting would likely be construed as unwelcome and have high potential to cause physical or psychological distress in the recipient. Though not explored in detail, previous research has shown that ethnic stereotyping may contribute to RRA. Our goal was to examine, for the first time, acute precipitants and consequences of RRA involving racial and ethnic conflicts. The parent study included comprehensive quantitative and qualitative information of 2,011 residents in 10 randomly-selected New York State nursing homes. Our qualitative analyses used an events perspective, focusing on how aggressive situations unfolded. We identified 51 single or repeated incidents of racial and ethnic conflicts during the study observation period. At the facility level, racial and ethnic conflicts were most commonly triggered by congested living spaces. At the relational level, racial and ethnic conflicts were frequently preceded by negative reactions to residents with cognitive impairment. At the individual level, racial and ethnic conflicts were found to be preceded by residents' unmet or unrecognized needs. Unresolved racial and ethnic conflict commonly resulted in aggression escalation and spillover. Our results indicated racial and ethnic conflicts occurred commonly and repeatedly. Such behavior appeared tolerated and normalized, with limited institutional response. As the long-term care population becomes increasingly diverse, recognizing and addressing potential racial and ethnic conflicts becomes more pressing. Targeting racial and ethnic conflicts in RRA for intervention may also improve interactions between residents and frontline workers.

Abstract citation ID: igad104.0148

**IMPLICATIONS OF RESIDENT AND ENVIRONMENTAL CHARACTERISTICS ON RESIDENT-TO-RESIDENT AGGRESSION**

Elsie Yan[1], Daniel W.L. Lai[2], Sheung Tak Cheng[3], Timothy Kwok[4], Vivian Weiqun Lou[1], Habib Chaudhury[5], Karl Pillemer[6], and Mark Lachs[7], *1. THe Hong Kong Polytechnic University, Hong Kong, Hong Kong*, *2. Hong Kong Baptist University, Hong Kong, Hong Kong*, *3. The Education University of Hong Kong, Hong Kong, Hong Kong*, *4. The Chinese University of Hong Kong, Shatin, Hong Kong*, *5. Simon Fraser University, Vancouver, British Columbia, Canada*, *6. Cornell University, Ithaca, New York, United States*, *7. Weill Cornell Medicine, New York City, New York, United States*

This study examines factors associated with resident-to-resident aggression (RRA) in long term care facilities in Hong Kong. A total of 800 personal care worker participated. Participants averaged 42.03 years of age (SD=7.63), were mostly female (92.7%), married (79.1%) and reported an average of 6.28 years of experience in long term care. 96.9 percent of the participants provided care to residents with dementia but 58.9% considered the training they received insufficient. RRA was common: All participants reported having witnessed verbal aggression (100%), 18% disruptive behaviors, 11.8% physical violence, and 3.1% sexual aggression. The following analysis used a subset of 412 participant from 29 long term care facilities where environmental assessments were conducted. General linear model analysis showed that RRA was associated with both resident and environmental characteristics. Victims' neuropsychiatry symptoms, perpetrator male gender and neuropsychiatric symptoms were associated with higher RRA. Separation of residents with and without dementia, inappropriate use of nursing unit for residents as measured by Therapeutic Environment Screening Survey for Nursing Homes (TESS-NH), lack of functional and attractiveness courtyard, less cleanliness of spaces, insufficient lighting and insufficient orientation / cueing as measured by Professional Environmental Assessment Procedure (PEAP) were also associated with higher RRA. There is an urgent need to prevent and intervene RRA in long term care facilities. Improving management of neuropsychiatric symptoms and creating a supportive and accommodating environment could be helpful in this regard.

Abstract citation ID: igad104.0149

**DESIGNING AND IMPLEMENTING A RESIDENT-TO-RESIDENT AGGRESSION PREVALENCE STUDY IN A NEW CONTEXT**

David Burnes[1], Vasuki Shanmuganathan[1], Pamela Baxter[2], Sandra Hirst[3], Adriana Shnall[4], and Lynn McDonald[1], *1. University of Toronto, Toronto, Ontario, Canada*, *2. McMaster University, School of Nursing, Hamilton, Ontario, Canada*, *3. University of Calgary, Calgary, Alberta, Canada*, *4. Baycrest, Toronto, Ontario, Canada*

Resident-to-resident aggression (RRA) is a prevalent issue in long-term care (LTC) settings with serious consequences. Research from the U.S. has found that approximately 20% of residents experience RRA each month and this issue is associated with several physical/mental health morbidities. Very little RRA research has been conducted in LTC settings in Canada. This study sought to estimate the prevalence of RRA in Canadian nursing homes and identify risk factors from different levels of social-ecological influence. Specifically, this study sought to replicate a gold standard RRA prevalence study in the U.S. by selecting a random sample of nursing homes (n = 10) across the province of Ontario and collecting RRA prevalence data using multiple sources/methods, including staff interviews, resident interviews, and direct observation from staff and research assistants. As this study is in early stages of design/implementation, the current presentation will focus on various issues requiring consideration and the challenges faced when setting up a large-scale RRA prevalence study. Specifically, the presentation will focus on the adaptation of existing measurement tools to a new context with unique local language, legal and legislative frameworks, and the development of a sampling strategy requiring considerations related to sample inclusion/exclusion criteria, temporal and geographical logistical concerns, and sample size. We will also discuss new realities and heightened sensitivities involved in conducting LTC-based research and recruiting nursing homes, residents and staff during the COVID era that includes staffing instabilities/shortages and outbreaks. This presentation will assist other researchers around the world in planning/implementing RRA prevalence study designs.

Abstract citation ID: igad104.0150

**A MODEL FOR DISSEMINATING EVIDENCE-BASED RESIDENT-TO-RESIDENT AGGRESSION INTERVENTION PROGRAMS**

Karl Pillemer, and Matthew Luebke, *Cornell University, Ithaca, New York, United States*

In elder mistreatment research, a key problem is the failure to disseminate interventions to broad audiences. This gap prevents most interventions from benefitting victims and improving their quality of life. Interventions are often not disseminated because of a lack funding, staffing, and support from provider organizations. We report on a novel, low-cost dissemination model to circumvent these barriers and expand program reach. The program, Improving Resident Relationships in Long Term Care (IRRL), trains staff to recognize resident-to-resident aggression (RRA) and to learn how to manage it. The training consists of two sessions, (1) recognition and (2) management. A dissemination model was created that included systematic marketing, outreach, and organizational sponsorship. We describe the model, which is replicable for a variety of dissemination efforts: 1) creating an easily accessible manual, supporting documents, and evaluation materials; 2) designing a user-friendly website for distribution; 3) identifying provider associations for program marketing; 4) developing partnerships with LTC organizations for dissemination; 5) providing national informational webinars; 6) offering webinar participants subsequent train-the-trainer sessions and coaching. This plan is feasible with limited expenditures and can be accomplished via the use of student research assistants. Using this model, the IRRL program has gained organizational support and has employed web-based training to reach over 300 providers. The dissemination of the IRRL program offers a potential method for intervention delivery with limited funding. Implications for future dissemination research are discussed.

### SESSION 1150 (SYMPOSIUM)

Abstract citation ID: igad104.0151

### USING COMMUNITY ENGAGEMENT TO DEVELOP, TEST, AND IMPLEMENT INTERVENTIONS FOR DEMENTIA CARE PARTNERS

Chair: Jacqueline Eaton

Interventions for caregivers to persons living with dementia are numerous and target a wide array of outcomes. One challenge limiting the dissemination of research-based interventions is the complexity of translating the intervention models for a variety of community environments and situations. Interventions that plan for future translation and scalability during the development stages have a better chance of quickly and efficiently being adopted by community partners. This symposium describes the methods used and the lessons learned from five research projects that used various forms of community engagement to develop, adapt, and assess feasibility of caregiver interventions, while simultaneously preparing for their translation and scalability: 1) Eaton et al., present the process and benefits gained from partnering with dementia caregivers to iteratively develop the Enhancing Active Caregiver Training (EnACT) intervention. 2) Utz et al., discuss ways to use the RE-AIM implementation framework to guide the development and pilot-testing of the Time for Living and Caring (TLC) respite intervention. 3) Dassel et al., explore the use of community-based participatory methods to adapt a paper-based advance care planning guide to an interactive web-based LEAD (Life-Planning in Early Alzheimer's and other Dementias) intervention. 4) Cotton et al., report on the lessons learned from a formative analysis of their processes used to culturally adapt an intergenerational respite program for older adults within the Latine community. 5) Supiano et al., describe how they engaged and trained nursing home social workers to develop and implement the PreLoss Group Support intervention for dementia family care partners.

Abstract citation ID: igad104.0152

### A COMMUNITY-BASED APPROACH TO REFINING THE ENHANCING ACTIVE CAREGIVER TRAINING (ENACT) INTERVENTION

Jacqueline Eaton[1], Sarah Neller[2], Moroni Fernandez Cajavilca[3], Julene Johnson[4], and Lee Ellington[1], *1. University of Utah, Salt Lake City, Utah, United States*, *2. University of Tennessee, Knoxville, Knoxville, Tennessee, United States*, *3. New York University, New York City, New York, United States*, *4. University of California San Francisco, San Francisco, California, United States*

Several interventions for dementia caregivers target the negative effects of behavioral symptoms. Evidence suggests that actively engaging caregivers in training and preparation improves outcomes, such as reduced caregiver burden, depression, and improved subjective well-being. However, there is little information on the best approaches to optimize active engagement. Enhancing Active Caregiver Training (EnACT) is an arts-based intervention that facilitates active engagement using participant-informed vignettes that portray caregiving experiences. This presentation will describe the process of partnering with dementia caregivers to iteratively develop the EnACT intervention in preparation for a randomized controlled trial (RCT). We conducted three iterative focus groups in partnership with dementia caregivers (n=9). Feedback was incorporated into intervention design and materials following each meeting. During focus group one we reviewed and identified video vignettes for inclusion. In focus group two, we tested intervention activities. In focus group three, participants provided feedback on the facilitator guide developed during this process. Focus groups were audio-recorded, transcribed, and analyzed in three cycles using structural, descriptive, and pattern coding. Across all focus groups, we coded 679 items as what went well and 358 items as needing to change. Intervention revisions focused on narrowing vignette topics, removing confusing components (such as engagement activities that were too burdensome), enhancing accessibility, simplifying instructions, and adding facilitator training. A facilitator guide was developed with four main components: Letter to facilitator, Introduction, Preparation to Begin, and Instructions. Partnering with caregivers improved our ability to enhance acceptability and fidelity in preparation for future testing in a wait-list RCT.

Abstract citation ID: igad104.0153

### USING RE-AIM TO GUIDE INTERVENTION DEVELOPMENT AND PILOT TESTING: CASE STUDY OF THE TLC INTERVENTION

Rebecca Utz, Amber Thompson, Cathi Sparks, Bob Wong, Eli Iacob, Alexandra Terril, and Mike Caserta, *University of Utah, Salt Lake City, Utah, United States*

Research-based interventions are often not fully translated or implemented into health or community settings, even after assessing and establishing feasibility and efficacy during pilot tests and randomized trials. The Time for Living and Caring (TLC) study, an intervention designed to improve respite care for dementia caregivers funded by NIA R01-AG061946, employed the principles of the RE-AIM framework to guide stage-1 research activities related to intervention (re)development and pilot testing, with the hope that implementation could be achieved more quickly and efficiently. RE-AIM is a framework used to guide dissemination and implementation activities by evaluating an intervention in terms of its: Reach to target populations; Effectiveness or efficacy; Adoption by target staff, settings, systems and communities; Implementation consistency, costs and adaptions made during delivery; and Maintenance/sustainment of intervention effects in individuals and settings over time. This presentation will describe the types of data used to evaluate each component of RE-AIM during the study's stage-1 activities. Data include feedback from a community advisory board (n=12), a randomized waitlist-control sample evaluating feasibility and initial efficacy of intervention of the TLC intervention (n=163), and feedback from stakeholders and providers who would be partners in the implementation of the TLC intervention (n=59). Based on stakeholder feedback, our findings support recommending adopting and designing interventions with an eye toward the principles of RE-AIM as best practices to increase the likelihood that interventions developed in the research context can be translated and implemented to those who will benefit from them.

Abstract citation ID: igad104.0154

### LEADING ADVANCE CARE CONVERSATIONS: DEVELOPMENT OF AN INTERVENTION PLATFORM

Kara Dassel[1], Nancy Aruscavage[2], Emily Nachtweih[3], Katherine Supiano[3], Rebecca Utz[3], Eli Iacob[3], and Jordana Clayton[2], *1. University of Utah College of Nursing, Salt Lake Cty, Utah, United States*, *2. University of Utah College of Nursing, Salt Lake City, Utah, United States*, *3. University of Utah, Salt Lake City, Utah, United States*

The LEAD Guide (Life-Planning in Early Alzheimer's and Other Dementias) is an advance care planning guide designed specifically for people with dementia and their care partner. It addresses changes in cognition and goals of care, including values and preferences, for end-of-life care and support across the disease continuum. The goal of using the guide is to create a shared understanding of the care recipient's end-of-life care preferences. We developed an interactive web-based format of the LEAD Guide, an enhanced extension of the original paper guide, for a pilot behavioral intervention. The LEAD Intervention guided community-based Alzheimer's disease and related dementias (ADRD) dyads (N=60 dyads; persons concerned, at risk, or with a dementia diagnosis and their care partners) through advance care planning (ACP) conversations and documentation. The intervention model included the LEAD Guide, interactive instructions, and educational material about ACP in the context of dementia. We developed the LEAD Intervention using an interactive design and beta testing process (including surveys and focus groups) with our community advisory board members. The board members included professional dementia experts, community partners, and ADRD dyads. Board members were enthusiastic about the LEAD Intervention and provided suggested revisions in three areas; technology (navigation of web-based documents, webpage design), personalization (the need to personalize emails and surveys to individual participants), and evaluation (creating simple scaled and open-text questions to obtain feedback on the intervention activities). Creating a feasible, acceptable, and efficacious intervention requires a multi-faceted approach that incorporates input from end users using community-based participatory research methods.

Abstract citation ID: igad104.0155

### AN INTERGENERATIONAL TRAINING PROGRAM TO SUPPORT LATINE ELDERS: LESSONS LEARNED

Quinton Cotton[1], Paul Kissell[2], Elma Johnson[1], Yolima Chambers[3], Manka Nkimbeng[1], and Joseph Gaugler[1], *1. University of Minnesota, Minneapolis, Minnesota, United States*, *2. University of Minnesota School of Public Health, Minneapolis, Minnesota, United States*, *3. Centro Tyrone Guzman, Minneapolis, Minnesota, United States*

Cultural adaptation and tailoring of caregiving interventions has garnered greater attention in part due to high levels of unmet need and poor intervention outcomes among dementia caregivers from racial and cultural groups. Additionally, there is a growing interest in understanding intervention mechanisms. We conducted a formative analysis of the process employed in the design of an intergenerational training program to support elders within the Latine community. A content analysis of memos and stakeholder listening sessions and document review guided this analysis. Framed within a discussion on the continuum of cultural adaptation, we report on lessons learned. Lessons include: 1) embracing community-led design, 2) demonstration of partnership commitment to facilitate trust-building, 3) consistent appreciative inquiry, and 4) demonstration of cultural centeredness within the partnership.

Abstract citation ID: igad104.0156

### ENGAGING WITH NURSING HOME SOCIAL WORKERS TO DEVELOP AND IMPLEMENT CLINICAL INTERVENTION TRAINING

Katherine Supiano[1], Troy Andersen[2], Bradbury Laura[3], and Kimberly Ponce Gonzalez[4], *1. University of Utah, Salt Lake City, Utah, United States*, *2. University of Utah College of Social work, Salt Lake City, Utah, United States*, *3. University of Utah College of Nursing, Salt Lake City, Utah, United States*, *4. University of Utah College of Nursing, Salt Lake city, Utah, United States*

PreLoss Group Support (PLGS) is a 10-session multimodal therapeutic group intervention for dementia family care partners administered by nursing home social workers (NHSWs) to facilitate healthy death preparedness and eventual bereavement. In prior work, we used empirically validated procedures for developing psychosocial interventions, created PLGS participant and facilitator manuals, and implemented and evaluated PLGS in three cohorts of nursing home family care partners using research team LCSWs as facilitators and NHSWs as co-facilitator-trainees. Our present pragmatic trial will determine if telehealth-delivered PLGS reduces risk of poor bereavement outcome in family care partners, when delivered by NHSWs trained in PLGS via telehealth. We systematically engaged with nursing home stakeholders; administrators and NHSWs in rural, urban, and Veteran nursing homes to identify their perceptions of family care partner needs with respect to end-of-life care in residents living with dementia.NHSWs collaborated with our team to develop a full-day clinical education program. We incorporated their suggestions of family "characters" to script into our video modules using professional actors, addressed potential concerns with telehealth delivery of the training and intervention, participant identification and recruitment procedures, and requests for didactic content and interactive case examples of treatment element application. Current NHSWs who attended the full day training completed a 7-item satisfaction scale yielding a satisfaction rate of 95% (highly valuable), with 100% indicating they would recommend training to SWs in other facilities. Sustained collaboration with NH stakeholders optimizes commitment of NHSWs to PLGS participants, intervention delivery and informs later implementation strategies for translation and dissemination.

### SESSION 1155 (SYMPOSIUM)

Abstract citation ID: igad104.0157

### ESPO AND SOCIAL RESEARCH, POLICY, AND PRACTICE SECTION SYMPOSIUM: LAW AS A DETERMINANT OF HEALTH: A SOCIAL JUSTICE LENS TO IMPACTFUL POLICY ENGAGEMENT

Chair: Sara Bybee Co-Chair: Kexin Yu Discussant: Rita Choula

The phrase "social determinants of health" has become a buzzword in health-related research and practice. While the US has long recognized that social determinants such as housing conditions, access to medical care, and neighborhood safety contribute to health outcomes, there has been little attention on more upstream factors. Structural, or political determinants of health are the determinants of the determinants. Political determinants of health refer to laws and policies that can inequitably distribute resources which creates structural barriers to equity for population groups that lack power and privilege. Unfortunately, many current US laws and policies perpetuate health inequities as they were originally written to protect those in power and punish those without power and privilege. Structural stigma and discrimination disproportionately affect historically marginalized and minoritized populations. Laws that disproportionately affect historically marginalized groups need to be identified, systemically tracked and revised if we aim to address health equity. In this symposium, speakers will discuss their research into the health-harming effects of laws and policies that disproportionately affect people of color and older adults. Speaker one will review GSA-related policy and advocacy activities. Speaker two will describe how to use law to address racial disparities in geographic access. Speaker three will discuss housing related laws and policies that perpetuate health inequities for older adults and disproportionately affect people from marginalized groups. This session is intended to fill a gap in current programming by making policy-related discussions more accessible and understandable for individuals who may not have experience in this area.

Abstract citation ID: igad104.0158

### A LIFE COURSE APPROACH TO IDENTIFYING EVICTION LAWS AFFECTING OLDER ADULTS AND HISTORICALLY MARGINALIZED GROUPS

Sara Bybee[1], Sarah Canham[1], Nasser Sharareh[2], Fernando Wilson[3], Nathanael Player[4], and Andrea Wallace[1], *1. University of Utah, Salt Lake City, Utah, United States*, *2. University of Utah Population Health Sciences, Salt Lake City, Utah, United States*, *3. University of Utah Population Health sciences, Salt Lake City, Utah, United States*, *4. Utah State Courts' Self-Help Center, Salt Lake City, Utah, United States*

Alongside population aging, more older adults are becoming at risk of homelessness. Eviction—a type of housing instability in which a landlord initiates legal forced expulsion from a rental property—is one of the main drivers of homelessness. Evictions and homelessness disproportionately affect older adults, individuals of color, women, sexual and gender minority populations, veterans, and those with disabilities. Evictions can have devastating short- and long-term effects on tenants, and these negative consequences may be especially pronounced for individuals with multiple minoritized identities such as older LGBTQ+ adults. Older LGBTQ+ individuals face higher baseline rates of poor mental health, smoking, and health risk behaviors. In this study, key informant interviews (N=5) were utilized to identify state eviction and landlord-tenant laws that disproportionately affect older adults and other historically marginalized groups. Using a life course approach to understanding health disparities as they accumulate across the lifespan, data from LawAtlas—an online database allowing the examination of specific laws across the US—was cross-referenced with data from key informants to identify the following laws which may disproportionately affect older adults and other historically marginalized populations: nuisance property ordinances, source of income discrimination, state fair housing protections, and minimum notice-to-vacate laws. This presentation will describe how intentionally or unintentionally, these laws disproportionately affect older adults and other historically marginalized populations and may lead to negative eviction outcomes, homelessness, and poor physical and mental health. Identifying how such discriminatory laws can contribute to health disparities across the lifespan is necessary to achieve health equity.

Abstract citation ID: igad104.0159

### BEYOND REDLINING: LAW, PROPERTY VALUES, RACIAL INEQUITIES, AND LTSS

Lindsey Smith, *Oregon Health & Science University, Portland, Oregon, United States*

This presentation will provide background regarding the social construction of property values and the interactive role that public and private institutions have played in creating racial disparities in property values. I will discuss known and proposed mechanisms through which this form of structural racism affects access to long-term services and supports (LTSS) and implications for the health of older adults and their care partners, presenting a specific example from my own research, which found that assisted living communities are located in 30% of communities in America. However, among communities with the highest percent Black population in their county, 31% of those located in counties with a low gap in Black-White economic mobility had access to assisted living, while less than 15% of those located within a county with a high Black-White gap in economic mobility had such access. I will describe how policymakers can use law to address racial disparities in geographic access by developing policies that 1) provide financial incentives for private investors to develop assisted living and other forms of LTSS in neighborhoods that otherwise wouldn't see development due to low property values; 2) prioritize the location of new buildings in historically marginalized communities; 3) implement anti-displacement measures; 4) incorporate community input and engagement; and 5) ensure accessibility and affordability. Finally, I will describe how policymakers may consider the various actors and institutions that shape the housing market and how they interact with each other to produce outcomes that may reinforce or challenge structural racism.

### SESSION 1160 (SYMPOSIUM)

Abstract citation ID: igad104.0160

### LATER-LIFE EXPERIENCES OF VETERANS AND THEIR CAREGIVERS

Chair: Janet Wilmoth

Military service is a defining life-course event that is consequential for the well-being of older adults and their caregivers. Although there has been increased attention to veteran status differences in later-life experiences, critical gaps in knowledge persist. This symposium features four presentations that address existing gaps from various perspectives. Kurth and colleagues use data from an online survey of community-dwelling combat veterans. They find that Vietnam Veterans exhibit more PTSD symptoms than veterans in two younger cohorts, and observed cohort differences are explained by military and post-service experiences. Spiro and colleagues examine later-adult trauma re-engagement (LATR) across multiple samples of Vietnam-era veterans and find that there is variation in LATR by gender and warzone experience. London and colleagues analyze data from adults aged 50+ in the 2021 National Health Interview Survey and find that: non-combat and combat veterans have more difficulty remembering/concentrating than nonveterans; hearing difficulty mediates some of the association between military service experiences and remembering/concentrating; and combat experiences moderate the relationship between hearing difficulty and difficulty remembering/concentrating. Haverhals and colleagues assess the Redefining Elder Care in America Program (RECAP) piloted recently in two VA health care centers and find that combat veterans and their caregivers reported shortcomings and challenges associated with program participation even though they were satisfied overall. Collectively, these papers underscore the enduring influence of combat exposure on older adults and the importance of innovative health care service models that are designed to provide support to caregivers. This is an Aging Veterans: Effects of Military Service across the Life Course Interest Group Sponsored Symposium.

Abstract citation ID: igad104.0161

### COHORT DIFFERENCES IN PTSD SYMPTOMS AMONG VIETNAM, PERSIAN GULF, AND POST-9/11 COMBAT VETERANS

Maria Kurth[1], Dakota Witzel[2], Suzanne Segerstrom[3], Soyoung Choun[3], and Carolyn Aldwin[3], *1. Penn State University, University Park, Pennsylvania, United States*, *2. Penn State University, State College, Pennsylvania, United States*, *3. Oregon State University, Corvallis, Oregon, United States*

Research examining long-term effects of military service often focuses on help-seeking Veterans who served in Vietnam, Korea, and WWII. Less is known about Veterans from more recent conflicts or community-dwelling Veterans. We administered online surveys to community-dwelling combat Veterans (N=167; Mage=57.96; 31% women) to examine cohort differences in service appraisals, post-service experiences, and predictors of PTSD symptoms. Chi-square and hierarchical regression were used to test differences in demographics, appraisals of service, post-service experiences, and PTSD symptoms. Among Veterans from Vietnam (n=60), Persian Gulf (n=68), and Post-9/11 cohorts (n=39), only age and educational attainment varied. Vietnam Veterans were older, and Persian Gulf War Veterans more likely to have a college education compared with other eras. There were no cohort differences for military appraisals or post-service experiences. In step one of hierarchical regression, men and those with lower income reported more PTSD symptoms. In step two, including cohort, Vietnam Veterans had more PTSD symptoms than the two younger cohorts, despite age now being inversely correlated with symptoms. In step three, including military and post-service experiences, demographics and cohort were no longer significantly associated with PTSD symptoms. Combat severity, undesirable appraisals, and social support were positively associated with PTSD symptoms, while desirable appraisals were inversely associated with PTSD symptoms. Cohort and demographics may be less important for Veteran mental health than service-related experiences. As Veterans of all eras move through adulthood and into later life, the importance of their miliary experiences must be considered, as it might be a 'hidden' variable of aging.

Abstract citation ID: igad104.0162

### LATER-ADULTHOOD TRAUMA RE-ENGAGEMENT AMONG VIETNAM VETERANS: A COORDINATED ANALYSIS

Avron Spiro[1], Anica Pless Kaiser[2], Brian Smith[3], Jeanne Stellman[4], and Steven Stellman[4], *1. VA Boston Healthcare System, Boston, Massachusetts, United States*, *2. VA Boston Medical Center, Boston, Massachusetts, United States*, *3. Women's Health Sciences Division, National Center for PTSD, Boston, Massachusetts, United States*, *4. Columbia University Mailman School of Public Health, New York City, New York, United States*

Later-Adulthood Trauma Reengagement (LATR) is a framework for understanding the later-life emergence of trauma-related memories among aging veterans. LATR unfolds within the context of aging-related challenges such as retirement, physical decline, and bereavement. We developed a scale to assess LATR, the late-onset stress symptomatology short form (LOSS-SF). In this study, we compare the LOSS-SF scale across multiple samples of Vietnam veterans. Samples were surveyed between 2001 and 2020; all completed the LOSS-SF scale as well as measures of combat exposure and PTSD. We studied three male samples (256 repatriated US prisoners of war [RPW], 260 combat veterans [CV], and 507 deployed American Legion [AL] members) and one sample of 1956 women deployed to Vietnam, mostly nurses [WV]. Across samples, we examined three questions: 1) How prevalent is LATR? 2) Is LATR correlated with combat exposure and PTSD? 3) Using a regression framework, what factors (military characteristics, combat, PTSD) are related to LATR? Prevalence of LATR ranged from 5% to 38%. Mean LATR scores were higher among AL and CV samples than among the RPW and WV. LATR was modestly related to combat exposure (rs = .08 - .36) and moderately related to current PTSD symptoms (rs = .56 - .79). Regressions demonstrated the importance of PTSD to LATR across samples, and combat exposure was related to LATR only for WV. This study indicates that LATR varies across samples differing in gender and warzone experience and suggest further exploration of the overlap between PTSD and LATR among aging Vietnam veterans.

Abstract citation ID: igad104.0163

### NONCOMBAT AND COMBAT MILITARY SERVICE EXPERIENCES, HEARING DIFFICULTY, AND DIFFICULTY REMEMBERING/CONCENTRATING

Andrew London[1], Scott Landes[2], and Janet Wilmoth[2], *1. Syracuse University, Maxwell School of Citizenship and Public Affairs, Syracuse, New York, United States, 2. Syracuse University, Syracuse, New York, United States*

Hearing difficulty is widespread among U.S. veterans. Research documents a strong association between hearing difficulty and a range of cognitive outcomes. Most research that examines cognitive outcomes in older adults does not distinguish veterans from non-veterans or non-combat from combat veterans. We use data on Sample Adults aged 50+ from the 2021 National Health Interview Survey to examine: the associations between military service experiences (non-veteran, non-combat veteran, combat veteran) and difficulty remembering/concentrating (none, some, a lot/cannot do at all) net of sociodemographic controls; the extent to which observed associations are mediated by hearing difficulty (none, some, a lot/cannot do at all); whether military service experiences moderate the association between hearing loss and difficulty remembering and concentrating. This latter hypothesis is motivated by the notion that service-related hearing loss may occur earlier in the life course and therefore be more detrimental to cognitive outcomes. Analyses are weighted; standard errors are adjusted for the complex sampling design. Preliminary results from multivariable ordinal logistic regression models that include sociodemographic controls indicate that: non-combat and combat veterans have significantly more difficulty remembering/concentrating than non-veterans; the associations between non-combat and combat military service experiences and increased difficulty remembering/concentrating are reduced 20% when hearing difficulty is added to the model, but remain statistically significant; hearing difficulty is strongly associated with difficulty remembering/concentrating; and military service experiences moderate the associations between hearing difficulty and difficulty remembering/concentrating. For example, the highest level of hearing difficulty is more strongly associated with difficulty remembering/concentrating among combat veterans than non-combat veterans.

Abstract citation ID: igad104.0164

### CAREGIVER AND VETERAN PERCEPTIONS OF THE REFINING ELDER CARE IN AMERICA PROGRAM (RECAP)

Leah Haverhals[1], Kate Magid[2], Mary Nunnery[3], Chelsea Manheim[3], Jessica Young[4], and Cari Levy[5], *1. Department of Veterans Affairs Rocky Mountain Regional VA Medical Center, Denver, Colorado, United States, 2. Veterans Health Administration, Aurora, Colorado, United States, 3. Rocky Mountain Regional VAMC, Aurora, Colorado, United States, 4. VA Puget Sound Health Care System, Seattle, Washington, United States, 5. University of Colorado, Denver, Colorado, United States*

In 2021, the Veterans Health Administration (VHA) piloted the Redefining Elder Care in America Program (RECAP) at two Department of Veterans Affairs (VA) health care centers. RECAP identifies Veterans at risk for nursing home placement, and coordinators conduct a phone assessment with Veterans or their caregivers to link Veterans to VA services. To identify benefits and challenges of RECAP from Veterans' and caregivers' perspectives, we conducted phone interviews (N=32) from October 2021- May 2022. We analyzed data using a thematic approach, finding that participants appreciated the proactive nature of RECAP, felt supported by RECAP coordinators, and felt the VA cared about them. Caregivers cited RECAP's benefits, such as learning about VA services and having the RECAP coordinator as a point of contact to help them navigate the VA. Caregivers of Veterans who received services reported improvements in caregiver mental health, decreases in caregiver burden, and improvements in Veterans' abilities to remain at home. A few caregivers were uncertain if they could trust the RECAP phone assessment, thinking it was a scam, and two reported challenges contacting the coordinator after the assessment. Several caregivers expressed uncertainty about the timeline for service delivery and next steps to obtain services following the assessment. Caregivers described challenges related to receiving services including Veterans not being eligible for the chosen service and receiving fewer hours of home health care than desired. Overall, participants were satisfied with RECAP. Challenges identified should be addressed to best meet the needs of Veterans and Caregivers.

### SESSION 1165 (PAPER)

### ALZHEIMER'S DISEASE AND RELATED DEMENTIAS (SRPP) II

Abstract citation ID: igad104.0165

### ACCESS TO NEIGHBORHOOD AMENITIES AND RISK OF INSTITUTIONALIZATION AMONG PERSONS WITH DEMENTIA

Yeon Jin Choi, Gillian Fennell, and Jennifer Ailshire, *University of Southern California, Los Angeles, California, United States*

Compared to only 2% of the total population between ages 65 and 84, 15% of persons with dementia (PWD) transition to nursing homes. This may be due to caregiving challenges associated with the behavioral symptoms PWD exhibit and their reduced capacities to perform daily tasks. Access to neighborhood amenities (e.g., parks, food access, libraries, service providers) may reduce the risk of institutionalization by helping PWD maintain their health and independence through the provision of opportunities for physical exercise, nutritious diets, social interaction, and cognitive stimulation while providing their caregivers with supportive services. Using data from the2006-2016 waves of the Health and Retirement Study (HRS), the HRS Contextual Data Resource, and the National Neighborhood Data Archive, this study aimed to identify neighborhood factors that are associated with transition into a nursing home among PWD who lived in the community (N=3,366). A series of logistic regression models were estimated adjusting for sociodemographic characteristics, health status, and neighborhood characteristics. Findings suggest that access to more park areas, healthy food outlets (i.e., grocery stores, supermarkets, supercenters), social amenities (e.g., museums, libraries), and social services for older adults and those with disabilities (e.g., senior centers, adult day care centers, disability support groups) were significantly associated with a lower risk of 2-year institutionalization. Retail stores and home health services were not significantly associated with the risk of institutionalization. These findings emphasize the importance of neighborhood amenities, such as parks and healthy food outlets, in enabling PWD to age in place.

Abstract citation ID: igad104.0166

### CASE SCENARIOS OF CANADIAN MEDIC-ALERT SUBSCRIBERS WITH DEMENTIA WHO GO MISSING

Christine Daum[1], Vanessa Vahedi[1], Emily Rutledge[1], Adebusola Adekoya[1], Antonio Miguel-Cruz[2], and Lili Liu[1], *1. University of Waterloo, Waterloo, Ontario, Canada*, *2. University of Alberta, Edmonton, Alberta, Canada*

With the increasing prevalence of dementia worldwide, it is expected that incidences of getting lost and going missing among persons living with dementia due to wayfinding challenges will also increase. Despite this, there is currently a gap in accessible education materials specific to risk factors and incidents of getting lost and going missing, aimed at first responders and family care partners. Case scenarios can effectively convey the circumstances of recurring events while also accounting for lived experiences. The purpose of this project was to develop case scenarios informed by real-life missing incidents that involve persons living with dementia in Canada. Qualitative description and conventional content analysis were used to analyze summary notes of missing incidents (n=515) obtained from Medic-Alert Foundation Canada hotline database. Summary notes contained: demographics of the missing person, their living situation and circle of support, events leading up to incident, by whom and where they were found, their health condition, and how they were re-united with their care partners. Case scenarios were developed that reflected the common (e.g., going missing while on foot, being found on the street) as well as less common (e.g., going missing while driving, being found seriously injured). Five experts (person living with dementia, care partner, health care professional, support providers) reviewed these scenarios to ensure relevance. Case scenarios can raise awareness of the circumstances encountered during missing incidents. The scenarios can be used by first responders and care partners to learn how to respond to missing incidents and receive support.

Abstract citation ID: igad104.0167

### DEVELOPMENT OF A COMMUNITY-ENGAGED DEMENTIA EDUCATION PROGRAM FOR THE VIETNAMESE AMERICAN COMMUNITY

Christina Miyawaki[1], Kim Nguyen[1], Angela McClellan[2], and Tuong-Vi Ho[3], *1. University of Houston, Houston, Texas, United States*, *2. Baylor University, Waco, Texas, United States*, *3. Texas Woman's University, Houston, Texas, United States*

Despite the life-threatening escape from Vietnam, Vietnamese refugees' health data are scarce. To fill this knowledge gap, we developed the Vietnamese Aging and Care Survey (VACS) and examined the health of community-dwelling older Vietnamese (≥65 years) in Houston, Texas, the 2nd largest Vietnamese-populated metropolitan area in the U.S. The data revealed their adverse health conditions including a high prevalence of cognitive impairment. We proposed a Community-Engaged Dementia Education Program to improve the community's dementia literacy based on the Cultural Exchange Model as a conceptual framework. Using Edwards' 9-stage Community Readiness Model, 14 Vietnamese key informants assessed their current literacy level (Aim 1). To develop dementia educational materials (Aim 2), the informants formed focus-group discussions. We used thematic analysis to analyze the community's understanding of dementia and developed educational materials. Key informants were aged 58 years (mean), Vietnam-born (93%), married (79%), college-educated (72%), women (86%), and full-time employees (79%). They lived in the U.S. for 39 years and in Houston for 30 years. Themes such as the Vietnamese community's low dementia literacy, their perception of dementia as a normal part of aging, and suggestions for introducing dementia conversations to the community emerged. We considered a one-pager as appropriate educational material, and included culturally- and linguistically-appropriate content and its translation, a target population's image, and dementia warning signs encouraging them to see their doctors for cognitive check-ups. Our next steps include utilizing local ethnic social media, disseminating the one-pager, and offering a "Brain Booth" at various Vietnamese health fairs.

Abstract citation ID: igad104.0168

### FUNCTIONAL LIMITATIONS AND ABSENCE OF CARE AMONG OLDER ADULTS LIVING WITH DEMENTIA IN CHINA, ENGLAND, THE EUROPEAN UNION, AND THE UNITED STATES

Zhuoer Lin[1], Yuting Qian[1], Thomas Gill[2], Xiaohui Hou[3], Shanquan Chen[4], and Xi Chen[1], *1. Yale University, New Haven, Connecticut, United States*, *2. Yale School of Medicine, New Haven, Connecticut, United States*, *3. The World Bank, Washington, District of Columbia, United States*, *4. London School of Hygiene & Tropical Medicine, London, England, United Kingdom*

Assistance with daily living is essential for people living with dementia (PLWD) who also have functional limitations. Despite the growing demands for caregiving, the absence of care for PLWD with functional limitations can be large and concerning. Using harmonized longitudinal survey data (2012-2018) on community-dwelling adults aged 50+ from the US, England, Europe (covering 28 countries), and China, we provided the first comparative evidence on the global trends in functional limitations and absence of caregiving for PLWD. We demonstrated three alarming facts in both developing and developed countries:

there was an overall increasing trend in the extent of functional limitations defined as difficulty with basic or instrumental activities of daily living (ADL/IADL) among PLWD; besides, about 1 in 5 PLWD received no care for their ADL/IADL limitations; and most importantly, the above absence of care has not improved over time in all studied countries, regardless of the stages of development. The patterns were evident for both ADL and IADL limitations and for informal and formal care. Our subgroup analysis showed consistency of these three facts, but also highlighted more worrying situations that about 2 in 5 PLWD received no care for their ADL limitations and at least 3 in 5 PLWD received no formal care. We also reported the greater absence of care for more vulnerable populations with less education and living alone. Our estimates highlight the urgency to ensure basic provision of care for PLWD with functional limitations, especially among those who are less educated or live alone.

Abstract citation ID: igad104.0169
### MULTIPLE CHRONIC CONDITIONS AND DISABILITY IN VIETNAMESE OLDER ADULTS IN THE UNITED STATES

Christina Miyawaki[1], Joshua Garcia[1], Kim Nguyen[1], Van Park[2], and Kyriakos Markides[3], *1. University of Houston, Houston, Texas, United States*, *2. University of California, San Francisco, San Francisco, California, United States*, *3. University of Texas Medical Branch, Galveston, Texas, United States*

Asian Americans recorded the highest life expectancy in the U.S. possibly due to selective migration as 86% of them are immigrants. However, the health of refugees such as older Vietnamese has been understudied. Using data from the Vietnamese Aging and Care Survey (VACS) collected in Houston, Texas, the 2nd-largest Vietnamese-populated metropolitan area in the nation, we assessed their chronic conditions, disability, depressive symptoms, and cognitive impairment, and examined the associations between their chronic conditions and disability by comorbidity clusters. Multivariable logistic regression models were used to answer the research questions. The respondents (N=177) were 76 years old (mean), married (62%), and female (58%). They lived in the U.S. for 24 years (mean) and spoke Vietnamese only (92%) in multi-generation (88%) low-income households (90% ≤$25K). They were in fair/poor health (84%) with 2 ADL/4 IADL disability (mean) and ≥3 chronic conditions (60%). Hypertension (79%) and arthritis (54%) were the most common chronic diseases. Respondents with ≥3 chronic conditions had significantly higher ADL (OR=2.77)/ IADL (OR=2.33) disability and cognitive impairment (50%). However, cognitive impairment had the most significant impact on their disability (ADL:2.94-3.75; IADL:5.41-5.79) when combined with most common chronic conditions. Healthcare providers should pay special attention to older Vietnamese' cognitive impairment due to their high prevalence and negative impact on their disability. Culturally and linguistically tailored healthcare services created by policymakers, healthcare professionals, and local social service agencies are recommended for the well-being of refugees who could not come as selected immigrants but migrated to the U.S. for a better life.

### SESSION 1170 (SYMPOSIUM)

Abstract citation ID: igad104.0170
### DIVERSE ENVIRONMENTAL CONTEXTS AND LINKS TO LATER-LIFE WELL-BEING

Chair: Noah Webster Discussant: Markus Schafer

The places older adults spend time have profound impacts on their well-being. As new sources of data and methods become available it is imperative to refine work in this area to guide development of interventions with the precision necessary to address persistent disparities in well-being. This symposium brings together four complementary papers that focus on diverse environmental contexts and well-being using multiple methodological approaches. Perzynski, Berg, and Dalton discuss the potential of Digital Twin Neighborhoods, i.e., digital replicas of real communities, to address health inequalities. They present findings from engagement sessions with community members about priorities, preferences, and concerns with regard to use of digital twins. Sol, Clarke and Zahodne link data from the Detroit-area Michigan Cognitive Aging Project with data from the National Neighborhood Data Archive to examine the association between neighborhood disadvantage and cognitive reserve. They show how this association differs between Black and White participants. Cho, Dunkle, and Smith use data from the Health and Retirement Study (HRS) to examine the association between later life relocation and contact frequency with adult children. They illustrate the importance of proximity and mode of contact in this association. Morris and colleagues also use HRS data to examine the extent to which stressors operating at multiple levels (interpersonal, community, and society) explain racial disparities in memory. They show these contexts together explain 11% of the racial disparity in baseline memory. These papers will be discussed by Markus Schafer who will provide an outlook for future research in this area.

Abstract citation ID: igad104.0171
### DIGITAL TWIN NEIGHBORHOODS FOR PRECISION POPULATION HEALTH: INITIAL RESULTS OF COMMUNITY CONVERSATIONS

Adam Perzynski[1], Kristen Berg[1], and Jarrod Dalton[2], *1. The MetroHealth System, Cleveland, Ohio, United States*, *2. Cleveland Clinic, Cleveland, Ohio, United States*

Neighborhood socioeconomic inequality leads to lower use of preventive services, increased incidence and severity of disease, reduced quality of life, and higher mortality across multiple age groups. To accelerate discovery and prioritize population health strategies, we introduced Digital Twin Neighborhoods (DTNs): digital replicas of real communities that include biological, social, and geographic data and algorithms. We implemented a series of community engagement studio sessions (CES) that aim to elicit and clarify community members' preferences and concerns about DTNs in order to most inclusively and ethically align DTN investigators' research with community priorities. More than 30 participants have attended quarterly sessions. At CES sessions, researchers summarize the DTN framework and then engage with questions from (i) community experts and (ii) scientific experts, and (iii) open dialogue to guide project decision-making. To date, salient community experts' concerns include: potential harms of DTNs for real-life communities; data privacy; selection of spatial indicators; and consequences for vulnerable populations. Suggestions for making DTN work more community-resonant have included: data transparency directly with residents; reconciling data definitions with local meanings of health; recognizing biases in electronic health records; and conducting shared activities within neighborhoods. CES sessions revealed foci for future DTN analyses including: how social isolation and togetherness influence health; identification of spatially defined targets for remediating structural racism; analysis of health-promoting assets' (e.g., green space, churches) differential benefits; and examining how resilience creates practical advantages amidst difficult socioenvironmental circumstances. Conversing with community members, we are now developing implementation plans for each of these areas.

Abstract citation ID: igad104.0172

### ASSOCIATION BETWEEN NEIGHBORHOOD DISADVANTAGE AND COGNITIVE RESERVE IN RACIALLY DIVERSE OLDER ADULTS

Ketlyne Sol, Philippa Clarke, and Laura Zahodne,
*University of Michigan, Ann Arbor, Michigan, United States*

Social determinants of health such as neighborhood disadvantage (NDis) are increasingly implicated in cognitive aging. More study is needed on the role of neighborhoods in cognitive reserve—better-than-expected cognitive function given brain health—and dementia disparities. This cross-sectional study included 98 non-Hispanic Black (NHB) and White (NHW) participants (mean age=62.7; 30% NHB) from the Michigan Cognitive Aging Project, a community-based study of adults transitioning to late life in the Detroit area. Participants' current addresses were geocoded and linked to 2017 measures of NDis at the census tract level from the National Neighborhood Data Archive. NDis is a composite of four indicators of disadvantage for the 26 unique census tracts in the sample. Cognitive reserve was the residual of an episodic memory factor score after regressing out hippocampal volume, cortical thickness across nine Alzheimer's disease signature regions, and education. Linear regression estimated the association between NDis and cognitive reserve, adjusting for age, sex/gender, and race. Analyses were repeated stratifying by race. In the entire sample, NDis was not associated with cognitive reserve (beta= -.08, p=.841), but stratified analyses indicated that more NDis was marginally associated with less cognitive reserve within NHB (beta= -.36, p=.057) but not NHW participants. NDis may contribute to cognitive reserve more strongly among NHB because of racially-patterned risk and resilience factors. Results highlight the need for more research into specific neighborhood factors that facilitate and deplete cognitive reserve in racially diverse older adults. Cognitive reserve may reflect contextual factors in addition to individual factors.

Abstract citation ID: igad104.0173

### CONTACT FREQUENCY WITH CHILDREN FOLLOWING RELOCATION LATER IN LIFE: DO CONTACT MODES AND PROXIMITY TO A CHILD MATTER?

Joonyoung Cho, Ruth Dunkle, and Jacqui Smith, *University of Michigan, Ann Arbor, Michigan, United States*

Contact with children is an important source of support for older adults. It is not clear if relocation and proximity to a child affect in-person communication as well as telephone, email, and social media contact between older parents and children. Using data from the Health and Retirement Study (2014-2018: N=3,332) we examined changes in communication frequencies between parents who either stayed or relocated in the last 4 years and potentially changed their close proximity to a child. We found differential effects of relocation on contact frequency depending on contact modes and proximity. Regarding in-person contact, unadjusted and adjusted linear regressions revealed older adults were less likely to meet up with their children regardless of the distance moved when a child was not nearby. In contrast to stayers, movers with proximity to a child were more likely to meet up with their children (adj. β=0.50, p ≤0.001). We found the same trend for telephone and email contact frequency. Movers with proximity to a child were more likely to have telephone (adj. β=0.17,p=0.034) and email contact (adj. β=0.33, p=0.023). Unlike other types of contact modes, social media contact frequency was not influenced by relocation or child proximity. While older adults tend to constrict their relationships, this trend is not applicable to every older adult. In the social convey model, situational characteristics (e.g., relocation) is considered, and heterogeneity in social relations in later life can be explained.

Abstract citation ID: igad104.0174

### INTERPERSONAL, COMMUNITY, AND SOCIETAL STRESSORS MEDIATE BLACK–WHITE MEMORY DISPARITIES

Emily Morris[1], Jordan Palms[1], Ji Hyun Lee[2], Ketlyne Sol[1], Kiana Scambray[1], Lindsay Kobayashi[3], Jacqui Smith[1], and Laura Zahodne[1], *1. University of Michigan, Ann Arbor, Michigan, United States*, *2. Montana State University, Bozeman, Montana, United States*, *3. University of Michigan School of Public Health, Ann Arbor, Michigan, United States*

Psychosocial factors may contribute to racial disparities in cognitive aging beyond socioeconomic status. Contextual stressors at multiple ecological levels, which may be related to structural racism, disproportionately affect Black older adults in the U.S. and may explain increased dementia risk. This study examined the extent to which interpersonal, community, and societal level stressors independently explained Black-White disparities in memory at baseline and over time. The sample included 16,890 older Black and White adults (Mage=67.98, 22% Black) from the US Health and Retirement Study who completed psychosocial questionnaires and a word list memory task. Interpersonal, community, and societal stressors were operationalized as self-reported everyday discrimination, neighborhood physical disorder, and subjective societal status, respectively. Latent growth curves modeled memory performance over six years. Stressors were modeled simultaneously and allowed to correlate. Covariates included age, sex, education, wealth, and questionnaire year. Compared to White older adults, Black older adults experienced higher discrimination (b = -.004, SE=.001, p<.001), greater neighborhood physical disorder (b = -.012, SE=.002, p<.001), and lower perceived societal status (b = -.003, SE=.001, p=.001), each of which independently mediated the association between Black race and worse initial memory. Together, these stressors explained 11% of the racial disparity in initial memory. There was no racial disparity in memory decline. Individual experiences of stressors across multiple contextual levels contribute to racial disparities in memory, providing evidence that structural racism negatively influences dementia risk among Black older adults. Future research should consider protective factors that buffer against negative impacts of structural racism on health.

### SESSION 1175 (PAPER)

### FAMILY CAREGIVING (SRPP PAPERS)

Abstract citation ID: igad104.0175

### STRATEGIES FOR INVOLVING OLDER ADULTS AND INFORMAL CAREGIVERS IN HEALTH POLICY DEVELOPMENT: A SCOPING REVIEW

Opeyemi Kolade[1], Joshua Porat-Dahlerbruch[2], Rustem Makhmutov[3], Theo van Achterberg[4], and Moriah Ellen[5], *1. Ben-Gurion University of the Negev, Midreshet, HaDarom, Israel*, *2. University of Pittsburgh, Pittsburgh, Pennsylvania, United States*, *3. Martin Luther Universität Halle-Wittenberg, Halle, Sachsen-Anhalt, Germany*, *4. KU Leuven, Leuven, Vlaams-Brabant, Belgium*, *5. Ben Gurion University of the Negev, Beer Sheva, HaDarom, Israel*

Encouraging the active involvement of older adults and informal caregivers in policymaking can lead to cost-effective health and long-term care interventions and enhance the creation of responsive policies. As part of the European Union TRANS-SENIOR program, a scoping review was conducted using the Joanna Briggs Institute's methodology. Published and grey literature was searched. Studies were included if they described the use/evaluation of methods for engaging older adults (65 years and above) and informal caregivers in local, national, or regional health policy development. Analysis was guided by the Multidimensional Framework For Patient And Family Engagement In Health And Health Care. Thirteen engagement strategies were identified from 11 publications meeting the inclusion criteria. Adopting the World Bank's categorization of citizen engagement methods, strategies were categorized as traditional, deliberative, and others. Older adults and informal caregivers are often consulted to elicit opinions for agenda/priority setting. However, their involvement in other stages of the policy cycle: health policy formulation, implementation, and evaluation, was unclear from the literature. The described engagement methods suggested that older adults and their informal caregivers do not often have shared equal power and shared responsibility in policymaking. Findings will guide policymakers in identifying and incorporating engagement strategies to support evidence-informed policymaking processes that can improve health outcomes for older adults/informal caregivers. Future research is needed on the evaluation of involvement methods.

Abstract citation ID: igad104.0176

### NAVIGATING AGING WHILE CAREGIVING: LONGEVITY PLANNING CONSIDERATIONS FOR CAREGIVERS OF ADULT CHILDREN

Lauren Cerino[1], Adam Felts[1], Alexa Balmuth[1], Julie Miller[2], and Joseph Coughlin[1], *1. Massachusetts Institute of Technology, Cambridge, Massachusetts, United States*, *2. AARP, Bethesda, Maryland, United States*

Caregiving is a demanding, complex, and costly role. According to AARP (2020), an increasing population of older adults in the U.S. are providing unpaid care to younger adult family members, with over two million adults in the U.S. providing care for an adult child. Caregivers of adult children experience unique challenges that can affect how they plan for their own longevity, as well as their adult child's longevity. To further explore the experiences of aging caregivers of adult children, the MIT AgeLab conducted an exploratory, qualitative study based on interviews with participants drawn from the MIT AgeLab's CareHive panel. Through a series of interviews conducted in June 2022, three key transition points in the lives of caregivers of adult children were identified. These transitions included the discovery that the child has special needs, the child's transition into legal adulthood, and the aging of the caregiver. The last transition point, focused on the aging of the caregiver, often presents complex implications including balancing one's own care needs with those of their child; navigating difficult family conversations; and preparing for continuation of care after one's own eventual death. Leveraging insights from these interviews, this session will explore complexities within each of the three key transition points, using Schlossberg's Transition Theory as a lens for analysis. The session will also identify resources and support desired by caregivers for support in this transition point, shedding a particular light on important considerations for professionals across multiple sectors and industries.

Abstract citation ID: igad104.0177

### TRENDS IN SUPPORTS FOR FAMILY CAREGIVERS OF OLDER ADULTS THROUGH MEDICAID HOME AND COMMUNITY-BASED SERVICES IN THE US

Katherine Miller[1], Elise Parrish[2], and Johanna Thunell[3], *1. Johns Hopkins Bloomberg School of Public Health, Baltimore, Maryland, United States*, *2. University of Pennsylvania, Philadelphia, Pennsylvania, United States*, *3. University of Southern California, Los Angeles, California, United States*

The aging population in the United States, combined with the dominant preference of older adults to remain in the home and community, has increased the demand for unpaid (family) caregivers - a role often undertaken by spouses. Despite known adverse effects of caregiving on health and economic well-being, few systematic policies financially supporting caregivers exist. Medicaid Home- and Community-Based Services (HCBS) 1915(c) waivers are a primary mechanism for paying family caregivers. Our objective is to systematically document and describe supports for family caregivers through Medicaid HCBS 1915(c) waivers. To accomplish our objective, we scrape data directly from approved waivers (2010-2019) serving adults aged 65+ including, but not limited to: approval date, services provided, limitations of services, allowed payments to relatives and legally authorized representatives, and the unique number of enrollees to be served by each waiver. We then describe training/education and payments available to family caregivers. Approximately 62 waivers across 15 states provide training and education to family caregivers. Waivers across 41 states (n=241) allow for payments to legally authorized representatives through 1915(c) waivers. However, upon examining the restrictions of these waivers, 67.6% of these waivers effectively exclude spouses from receiving payments. Despite growing reliance on family caregivers, supports are not widespread and existing supports reach a limited group of family caregivers. This research further contributes to Medicaid research by creating a database of waiver services; thereby contributing to our understanding to the depth and generosity of HCBS, which can be systematically updated as new waivers are approved.

Abstract citation ID: igad104.0178

### WELFARE REGIME TYPE AND ITS INFLUENCE ON THE FINANCIAL SECURITY OF FEMALE CAREGIVERS IN LATER LIFE

Mengya Wang, *Iowa State University, Ames, Iowa, United States*

Guided by life course theory, this paper examines the effect of early caregiving experience and later life-changing events on the financial security of older women using cross-national data from two panels (2004 and 2014) of the Survey of Health Aging and Retirement in Europe Analyses found that caregiving in midlife and subsequent life-changing events (being divorced or widowhood, changing employment status, and/or declining health) significantly affected women's economic status in later life, Importantly, we found that early caregiving experience intensifies the effects of negative life events, and in turn further deteriorates women's economic security in later life. Tests of welfare regime differences found that women who experienced a marital change (becoming divorced or widowed) in the dual breadwinner model were less likely to experience economic deprivation compared to the other two models. This finding is likely attributable to more gender equality in these countries which makes women more financially independent. Unfortunately, a significant association between welfare regime and caregiving was not found in this study. This study adds to the knowledge about the political, economic and cultural forces that shape the financial security of women in later life. Our findings suggest that public expenditures on long-term care and the healthcare system should continue and/or be augmented to prevent women economic deprivation, especially those who assume caregiving role in their early life. Moreover, the policies and conditions of dual-breadwinner role countries are informative in helping women achieve gender equality.

Abstract citation ID: igad104.0179

### WHAT MATTERS MOST? REACTIONS TO THE RAISE ADVISORY COUNCIL'S NATIONAL STRATEGY TO SUPPORT FAMILY CAREGIVERS

Pamela Nadash[1], Eileen Tell[2], Shan Qu[1], and Maryssa Pallis[1], *1. University of Massachusetts Boston, Boston, Massachusetts, United States*, *2. ET Consulting, Belmont, Massachusetts, United States*

This presentation reports on feedback from advocates and researchers, those working in organizations serving family caregivers, and family caregivers themselves regarding the 2022 National Strategy to Support Family Caregivers. The Strategy was issued by the RAISE Family Caregiving Advisory Council, created under the Recognize, Assist, Include, Support, and Engage (RAISE) Family Caregivers Act (2018). A Request for Information (RFI) published in the Federal Register garnered nearly 600 responses from a wide range of individuals and organizations, providing feedback about top priorities for action, topics or populations that were not adequately addressed, and recommendations for actions that could help meet stated objectives; these comments will prove critical in implementing the National Strategy, as well as in updating and revising it. This presentation sets the progress of the National Strategy in context, reports on the RFI analysis, and describes next steps, highlighting key areas of focus for the federal government, states, and other entities. Priorities included expanding the direct care workforce and increasing access to caregiver training and supports, particularly respite. Other top comments pertained to financial and workplace security for family caregivers - encouraging caregiver-friendly workplaces, paying family members to provide care, and strengthening the Family and Medical Leave Act. Respondents broadly endorsed the Strategy and noted the need for campaigns promoting greater awareness of family caregiving. The findings suggest that there is strong consensus among caregiver advocates regarding the most critical areas for action and the most favored strategies to achieve caregivers' priority goals.

### SESSION 1180 (PAPER)

### SLEEP, SOCIAL CONNECTIONS, AND DISCRIMINATION

Abstract citation ID: igad104.0180

### ASSOCIATION OF SECOND GENERATION EPIGENETIC CLOCKS AND DISCRIMINATION WITH TRAJECTORIES OF CHRONIC HEALTH CONDITIONS

Miriam Mutambudzi[1], Maria Brown[1], and Nai-Wei Chen[2], *1. Syracuse University, Syracuse, New York, United States*, *2. University of Missouri, Columbia, Missouri, United States*

This study aimed to examine whether 2nd generation epigenetic clocks and perceived discrimination as measured by the Everyday Discrimination Scale are associated with differential classification into trajectories of chronic health conditions (CHCs). We used 2014-2020 data from the Health and Retirement Study (HRS) and biological clock data from the 2016 HRS Venous Blood Study (N=3,177). CHC trajectories were constructed using latent class growth models. Multinomial logistic regression analysis examined the association between PhenoAge and GrimAge epigenetic clocks respectively, perceived discrimination, and the constructed trajectories. Participants ($\geq 50$ years) were classified into 4 CHC trajectory classes, graded by risk of increasing mean CHCs, with trajectory class 1 indicating the lowest mean number of CHCs (most favorable) and class 4 the highest (worst), over 3 study waves. Approximately 35.5% Black, 24.9% Hispanic, and 25% White participants reported high discrimination ($p< 0.01$). Compared to Whites, Blacks had increased risk of classification into trajectory class 4 (RRR=1.91, p=0.02 GrimAge and RRR=2.31, $p< 0.01$ PhenoAge). PhenoAge was associated with increased risk of classification into trajectory class 4 for Hispanics (RRR=1.17, 95%CI=1.06-1.28), and modest increases in risk for Blacks (RRR=1.07, 95%CI=1.01-1.15) and Whites (RRR=1.06, 95%CI=1.03-1.09). GrimAge was associated with increased risk of classification into trajectory class

4 for Hispanics (RRR=1.21, 95%CI=1.06-1.38), Blacks (RRR=1.19, 95%CI=1.06-1.33) and Whites (RRR=1.14, 95%CI=1.08-1.20). Despite higher observed rates of discrimination in Blacks, experiences of discrimination increased risk of classification into trajectory class 4 for whites only (RRR=1.59, 95%CI=1.06-2.38). Discrimination findings should be interpreted with caution and future work should account for coping strategies.

Abstract citation ID: igad104.0181

### DAILY ASSOCIATIONS OF SOCIAL INTERACTIONS WITH OBJECTIVE SLEEP MEASURES AMONG OLDER ADULTS

Jee eun Kang[1], Linying Ji[2], Orfeu Buxton[1], and Martin Sliwinski[1], *1. Pennsylvania State University, State College, Pennsylvania, United States*, *2. Montana State University, Bozeman, Montana, United States*

Disturbed sleep is a common problem and can have profound effects on older adults. Social relationships are known to be associated with quantity and quality of sleep. To extend prior research, we examined the associations of daily social interactions and sleep, using objective sleep measures and an ecological momentary assessments (EMA) approach. Participants in the Einstein Aging Study (n=285, Mage=77, range=70-90, 68% women, 46% White, 40% Black) reported their recent social interactions and physical contact four times a day for 16 days via EMA on mobile phones. Objective sleep measures (sleep timing, night sleep time, wake after sleep onset (WASO), sleep maintenance efficiency) were calculated from concurrent wrist actigraphy. Multilevel models controlled for demographics, living arrangement, Instrumental Activities of Daily Living, sleep-disordered breathing, and hypoxemia. Results showed that, on days when older adults had more day-time social interactions than their average, they had earlier sleep timing, shorter night sleep time, and less WASO, but no differences in sleep efficiency. On days when older adults had more physical contact than their average, they had earlier sleep timing and shorter night sleep time but no differences in WASO or sleep efficiency. These micro-longitudinal analyses among older adults suggest that daily social interactions are related to subsequent earlier sleep timing and shorter sleep at night, and can be beneficial for sleep quality. Physical contact seemed to be only associated with the timing and quantity of sleep but not sleep quality. Potential benefits of using an EMA approach in sleep research will be discussed.

Abstract citation ID: igad104.0182

### EVERYDAY DISCRIMINATION AND RACIAL/ETHNIC DISPARITIES IN COGNITIVE FUNCTIONING

Wen-Hua "Zoey" Lai, *Michigan State University, East Lansing, Michigan, United States*

Alzheimer's disease and related dementias are expected to triple from 5 million to 13.7 million by 2050 in the U.S. Black, and Latinx older adults experience poorer cognitive health and a 1.5 to 2 times higher rate of dementia than their white counterparts. Due to the structural racism in the U.S., chronic exposure to everyday discrimination may contribute to cognitive health inequalities, yet limited empirical research examines this association. This study uses parallel linear growth curve modeling to assess the impact of long-term exposure to everyday discrimination on the initial level of cognition (i.e., latent intercept) and change in cognition (i.e., latent slope) among older adults and examines whether such association varies among Black, U.S.-born Latinx, foreign-born Latinx, and white older adults. Using data from the 2006-2012 Health and Retirement Study (N=8,195), these findings show that racial/ethnic minority older adults, especially Blacks, experience greater everyday discrimination and poorer cognitive health than their white peers. Greater exposure to everyday discrimination was associated with lower initial levels of cognitive function and a faster decline in cognitive function. Results from multiple groups analysis further indicate that everyday discrimination shaped cognitive trajectories only for white and Black older adults but not for U.S. -born and foreign-born Latinx older adults. These findings suggest that white and Blacks older adults who experience a higher level of everyday discrimination may be vulnerable to cognitive decline. This study discusses the implication of these unexpected findings and future research directions.

Abstract citation ID: igad104.0183

### FUNCTIONAL LIMITATION: DIFFERENCES BY EPIGENETIC AGING, ETHNICITY, AND EXPERIENCES OF DISCRIMINATION

Maria Brown, and Miriam Mutambudzi, *Syracuse University, Syracuse, New York, United States*

Epigenetic aging provides an opportunity to rethink processes of aging and policy and health interventions to address health disparities across the life course. Previous research has shown that social factors can result in accelerated epigenetic aging, with subsequent unfavorable health outcomes. This cross-sectional study aimed to evaluate the relationship between epigenetic aging and functional and instrumental limitations in White and Hispanic older adults and to explore how recent experiences of perceived discrimination may differentially impact this association. We used 2014 and 2016 Health and Retirement Study (HRS) data for White (n=1072) and Hispanic (n=139) adults aged 50 and older and epigenetic clock data from the 2016 HRS Venous Blood Study. We hypothesized that accelerated epigenetic aging (PhenoAge epigenetic clock regressed on chronological age) would be associated with greater limitations in activities of daily living (ADLs) and instrumental activities of daily living (iADLs), and this impact would differ by ethnicity and perceived discrimination. On average, Hispanics reported significantly more limitations in ADLs (1.10 vs .47, p<.0001) and iADLs (1.22 vs .72, p<.0001), and more experiences of being treated differently in service situations (44.6% vs 33.3%, p=.0084) and of people acting as if they were afraid of them (28.78% vs 22.01%, p=.0738). Epigenetic age was associated with discrimination (b=-1.98, p=.0078), with ADLs in Hispanics (b=-0.04, p=.0812), and with iADLs in Whites (b=.0069, p=.0443). Further studies of the impact of epigenetic aging on functional and instrumental limitations should be longitudinal, utilize larger samples of older adults, and oversample racial and ethnic minorities.

Abstract citation ID: igad104.0184

### LGBTQ-RELATED DISCRIMINATION REDUCES SLEEP QUALITY IN OLDER ADULTS: EVIDENCE FROM VUSNAPS AND UCNETS

Tara McKay, Nathaniel Tran, Lana Trautman, and Kirsty Clark, *Vanderbilt University, Nashville, Tennessee, United States*

Sleep is a critical protective factor for health outcomes with low-quality sleep linked to increased risk of diabetes, cardiovascular disease, and cognitive decline. Theoretical work proposes that minority stress may negatively affect sleep quality for LGBTQ and other minoritized populations. We examine differences in sleep quality across sexual orientation and gender identity and test the relationship between minority stress and sleep quality for LGBTQ adults. Data come from 1) UC Berkeley Social Networks Study (UCNets), a panel study of 50–70-year-olds with an oversample of older LGBTQ adults and 2) the Vanderbilt University Social Networks, Aging, and Policy Study (VUSNAPS), a panel study of older LGBTQ adults aged 50-76. LGBTQ identity includes participants who are transgender and/or gay, lesbian, bisexual, or something else. We estimate Poisson models predicting sleep quality as a function of LGBTQ identity and exposure to LGBTQ-related discrimination. Sleep quality is measured using three items available in both studies: 1) the number of nights per week trouble falling asleep, 2) number of nights per week trouble staying asleep, and 3) number of mornings per week waking well rested. Experiences of LGBTQ-related discrimination in the last year were measured using the Daily Heterosexist Experiences Questionnaire. We find that LGBTQ identity is significantly associated with poorer sleep quality compared to non-LGBTQ peers. Among LGBTQ adults, sleep quality declines as the number and intensity of recent experiences of LGBTQ-related discrimination increases. Poorer sleep quality may be an underlying mechanism exacerbating health disparities across sexual orientation and gender identity.

### SESSION 1185 (PAPER)

### SOCIAL TIES AND SOCIAL SUPPORT

Abstract citation ID: igad104.0185

### CHANGES IN LIVING ARRANGEMENT PREFERENCE AMONG OLDER TAIWANESE: A MULTILEVEL AGE-PERIOD COHORT ANALYSIS

Yao-Chi Shih, *National Taipei University of Nursing and Health Sciences, Taipei City, Taipei, Taiwan (Republic of China)*

Coresidence between older parents and adult children has been a traditional form of living arrangements in East Asia. The majority of older Taiwanese parents still shares the same residence with their adult children. However, recent research shows two contrasting trends: older persons tend to prefer independent living, while younger adults still express their filial responsibilities. Does the difference indicate changes in living arrangement preferences by successive cohort, or does it suggest an age pattern of coresidence preference among people who were growing old? Data from the Taiwan Longitudinal Study on Aging (TLSA) were used to compile an analytic sample aged 50 and older in 1996 with six follow-up interviews to 2019. Four birth cohorts between 1916 and 1953 were identified. The multilevel hierarchical age-period-cohort model (HAPC) were used to examine the effects of age, period, and cohort on potential changes in coresidence preference. Preliminary results suggested that coresidence preference among the post-war birth cohort increased with age, while no clear age patterns were found among other older cohorts. After entering older ages, all birth cohorts converged toward the similar coresidence preference. Findings suggest living with adult children may still be the primary form of living arrangements for older Taiwanese to ensure their sources of care. Despite that realization of coresidence preference would be crucial for older persons' well-being, understanding how coresidence preference changes over time during one's life course can further help us envision the future household structure.

Abstract citation ID: igad104.0186

### COGNITIVE FUNCTION, ACTIVITY MEANINGFULNESS, AND SOCIAL ENGAGEMENT AMONG OLDER ADULTS WITH DEMENTIA

Shinyi Wu[1], Yi-Hsuan Tung[1], and Iris Chi[2], *1. University of Southern California, Los Angeles, California, United States*, *2. USC, Los Angeles, California, United States*

Hess's selective engagement theory (SET) suggests that individuals facing aging-related decline tend to selectively allocate cognitive resources in activity engagement. This selectivity can be mediated or moderated by motivational factors (e.g., activity meaningfulness). Yet, empirical research on SET and older adults with dementia is limited. This study aims to examine how cognitive function and activity meaningfulness affect social engagement among this population. Data is from the 2016 National Health and Aging Trends Study (N= 1,585 participants aged 65+ with dementia). Engagement in four social activities including visiting family or friends (VF), attending religious activities (RA), participating in group activities (GA), and going out for enjoyment (OE) were analyzed in separate multivariate logistic regression models with cognitive function, the corresponding meaningfulness (a mediator and a moderator), and covariates (age, gender, race, living alone, self-rated health, ADL, IADL, and depressive symptoms). Results showed that, of the four social activities, cognitive function was only significantly associated with RA and GA engagement. RA meaningfulness moderates the association between cognitive function and RA engagement, in that high RA meaningfulness shows high RA engagement, while low RA meaningfulness shows low RA engagement and in a negative association with cognitive function. GA meaningfulness partially mediates the relationship between cognitive function and GA engagement. These findings support SET by showing cognitive resources-related selection effects on activity engagement and the roles of activity meaningfulness in selectivity effects. The implication highlights the importance of sustaining both cognitive function and motivational factors to promote social engagement among older adults with dementia.

Abstract citation ID: igad104.0187

### NEIGHBORHOOD SOCIAL TIES AND LONELINESS IN RETIRED AND CURRENTLY WORKING OLDER ADULTS

Jaclyn Keenoy, and Ashley Ermer, *Montclair State University, Montclair, New Jersey, United States*

Peripheral social ties such as coworkers and neighbors have been identified as helpful to protect against loneliness, considering their convenient geographic proximity and ability to contribute diversity into one's social network (Sandstrom & Dunn, 2014). However, the importance of neighborhood social ties for older adults has rarely been investigated by employment status. We anticipated that with an absence of coworkers to serve as peripheral social ties, retirees will rely more heavily on neighborhood social ties to alleviate feelings of loneliness. Using Wave 3 of the National Social Life, Health, and Aging Project, 1,088 currently working and 1,594 retired older adults were compared using path modeling in Mplus (v. 8). We first ran an overall model and found that there was a significant negative association between neighborhood social ties and loneliness among all participants (p=.000). Next, we ran a multigroup model between working and retired older adults. In this model, neighborhood social ties and loneliness were negatively associated for both retired (p=.000) and working (p=.047) respondents. A Wald test revealed that these groups were not significantly different (p=.100). Our results suggest that neighborhood social ties help to alleviate loneliness similarly in both working and retired older adults. This is despite those currently working likely having a network of coworkers as peripheral social ties from their workplaces. These findings contribute to the broader literature on social networks, and particularly advance the social convoy model's understanding of the importance of peripheral social ties, even in older age (Huxhold et al., 2020).

Abstract citation ID: igad104.0188

### ROLE OF PLACE AS A DETERMINANT OF PARENT–ADULT CHILD GEOGRAPHIC PROXIMITY AND CAREGIVING

Stipica Mudrazija[1], Elizabeth Peters[2], and Fernando Hernandez Lepe[2], *1. University of Washington, Seattle, Washington, United States*, *2. Urban Institute, Washington, District of Columbia, United States*

While research has shown that the need for care impacts how close parents and adult children live, the relative importance of various factors shaping parent-child geographic proximity remains unclear, especially as it relates to characteristics of their geographic locations and communities. We explore variation in and determinants of the geographic proximity of older parents and adult children, conditional on parents needing care. Using pooled 2004-2014 data from the Health and Retirement Study on respondents age 65 years or older and their children, alongside detailed county-level contextual information from various sources, we analyze the importance of various determinants of parent-child geographic proximity, including parental health, age, and other personal characteristics, children's personal characteristics, parent-child dyad characteristics, and place-specific characteristics that relate with availability of different health and aging services, housing affordability, and labor market opportunities. Preliminary regression results show that coresidence is substantially less likely in areas with higher availability of nursing care and continuing care options. Among non-coresidents, living in close proximity (distances under 10 miles) is less likely if parent's area has more nursing care options, but more likely if health services that support living in place are available. Additionally, local economic and housing conditions play a role, with higher median household income associated with children living closer to parents and higher cost of rent with living further. Place-specific characteristics are important determinants of parent-child coresidence and proximity, and policymakers and care providers should consider them when determining the most effective ways to support older adults who need care.

Abstract citation ID: igad104.0189

### TRAJECTORIES OF FORMAL SOCIAL PARTICIPATION, GENDER, AND END-OF-LIFE CARE QUALITY AMONG OLDER AMERICANS

Kafayat Mahmoud[1], Jarron Saint Onge[2], and David Ekerdt[2], *1. Boston University, Boston, Massachusetts, United States*, *2. University of Kansas, Lawrence, Kansas, United States*

Limited research has examined the association between formal social participation trajectories and end-of-life care quality. The end of life could be characterized by experiences of heightened feelings of physical and psychological distress, breathlessness, constant hospitalization, and intrusive interventions. Formal social participation may improve the end-of-life care of older adults because they serve as sources of useful information, receipt of emotional support, and improve self-efficacy. This research examines the associations between formal social participation trajectories and proxy ratings of overall end-of-life care quality, and the moderating role of gender. Growth-based trajectory models were used to identify distinct developmental trajectories of formal social participation among older adults in the United States. Findings revealed four social participation trajectory classes among older adults towards the end of life, all with a general tendency to decline across time. Multinomial logistic regression analyses showed that although older adults with higher levels of formal social participation have more positive overall end-of-life care ratings, there are gender differences in these care ratings. Women are less likely than men to chart, by proxy report, positive care ratings at the end of life even though they have higher levels of formal social participation, and these gender differences in end-of-life care rating are explained more by healthcare factors than formal social participation trajectories. These results suggest that both formal social participation and positive interactions with health care at the end of life are more beneficial for older men than women.

### SESSION 1190 (SYMPOSIUM)

Abstract citation ID: igad104.0190

### EVIDENCE-BASED STRATEGIES TO DELIVER AND PROMOTE AGE-FRIENDLY CARE IN HOSPITAL SETTINGS

Chair: Barbara King Co-Chair: Mary Hook Discussant: Blair Golden

The number of adults aged 65 and older will double by 2050. Older adults have more chronic conditions and complex care needs compared to younger adults. To support the health of older adults the Age Friendly Health Systems model was initiated in the US. Age Friendly Care is comprised of 4 core elements (called the 4 M's framework), What Matters, Medication, Mentation and Mobility, aimed to ensure that older adults receive the best care possible and are not harmed by health care systems. This symposium will present several papers describing how components of age friendly care were implemented in hospital settings. Paper 1 will describe strategies used by acute care nursing staff to overcome challenges imposed by COVID to implement a mobility intervention to improve ambulation of older adult patients; Paper 2 will present psychometric findings on an instrument to measure a patient's level of ambulation to promote mobility during a hospital stay; Paper 3 will describe a quality improvement initiative to implement the 4 M's framework on in-patient medical units; and Paper 4 will provide insights on implementing the What Matters component of 4 M care and strategies for promoting patient goals and preferences for care delivery. Age-Friendly Health Systems have the potential to improve care delivery for older adults. The papers presented demonstrate it is feasible to implement 4 M's framework in hospitals, but innovative strategies and reliable tools are needed.

Abstract citation ID: igad104.0191

### INSIGHTS ON THE CLINICAL NURSE ROLE IN IMPLEMENTING WHAT MATTERS FOR OLDER ADULTS IN ACUTE CARE

Murad Taani[1], and Mary Hook[2], *1. University of Wisconsin-Milwaukee, Milwaukee, Wisconsin, United States*, *2. Advocate Health, Milwaukee, Wisconsin, United States*

The Age-Friendly Health System (AFHS) evidence-based 4Ms framework (What Matters, Medication, Mentation, & Mobility) is designed to be implemented in clinical settings to reliably provide high-quality age-friendly care to older adults and reduce harm and hospital-associated complications. The 4M concept of 'What Matters to You' (WMTY) refers to assessing and aligning an individual's specific health outcome goals and care preferences to ensure patient-centered care with a measurable impact on patient experience. While published toolkits support the implementation of WMTY for all disciplines, there is limited guidance and empirical evidence about the implementation and impact of this concept when used by clinical nurses in acute care. Some researchers have identified issues with implementing WMTY with patients who are not able to articulate their wishes or when the question is posed in a granular way. A large healthcare system in the Midwest supported five hospitals to implement the AFHS 4Ms including the WMTY concept but did not observe an improvement in nurse-sensitive patient experience measures. A qualitative study is in progress aimed at gaining an in-depth understanding of the knowledge, perceptions, and experiences of leaders and clinical nurses who have implemented the WMTY concept for older adults in their hospital to evaluate the implementation and impact on patient care. The results gathered by this study will lay the foundation for designing clinical nurse-targeted interventions to successfully implement WMTY and identifying supportive strategies for partnering with patients and their caregivers to ensure that their goals/preferences are fully integrated into care.

Abstract citation ID: igad104.0192

### VALIDATING THE IMPACT OF THE COMPREHENSIVE MOBILITY EVALUATION TOOL USED BY NURSES IN ACUTE CARE

Mary Hook, *Advocate Health, Milwaukee, Wisconsin, United States*

Existing nursing-based mobility tools were developed with clinical and reliability testing with small samples; none focused on predicting assistance needs. The Comprehensive Mobility Evaluation Tool (CMET) was designed using established testing procedures to support nurses to assess all phases of mobilization and predict level of assistance. A prospective descriptive research design was used to test reliability and validate CMET items, scoring, and ability to predict LOA. A randomly selected sample of decisional/English-speaking non-ventilated stable patients were enrolled from four medical/surgical and one critical care units at a large community hospital where CMET was used. Trained data collectors consented patients, completed bedside testing, and distributed patient surveys. Descriptive, inferential, and regression statistics were used for analysis. The representative sample (N=190) had an average age of 67.3 yrs (SD=16.2, 23-101), 50% male, with a robust range of mobility levels. Inter-rater reliability (n=27) yielded an average kappa of 0.94. Six of seven steps contributed significantly to predicting patient LOA with scores that correlated with nurse judgement [r (184) =0.86, p< .0001]. Patients needed time and support to perform testing without assistance for optimal accuracy and benefit. Patients reported CMET was useful (88%) and easy (62%) and identified barriers that limit walking while hospitalized. Study limited by research at a single site. This study represents the initial validation, confirming reliability and benefits of the CMET, a skill-based tool to evaluate mobility status. Providing time and encouragement to perform CMET helps nurses and patients to identify deficits and tailor care to overcome mobility barriers.

Abstract citation ID: igad104.0193

### MOVIN PAST OBSTACLES: BUILDING A CULTURE OF MOBILITY

Sarah Glowinski[1], Mary Hook[1], and Barbara King[2], *1. Advocate Health, Milwaukee, Wisconsin, United States*, *2. University of Wisconsin-Madison, Madison, Wisconsin, United States*

Older adults are at risk for losing their ability to ambulate independently during hospitalization. The "MOVIN" (Mobilizing Older adult patients Via a systems-based Intervention) Study is a cluster-randomized control trial aimed to implement a nursing-led mobility model and monitor the change in nurse behaviors and unit culture before, during and after the 14-week intervention period. The model includes psychomotor training, mobility resources (equipment and ambulation aide), communication tools, ambulation pathways, and support for developing a "culture of mobility" led by nurses.

It was launched on two inpatient nursing units at large urban Magnet quaternary medical center in Wisconsin in July 2021 (Unit 1) and March 2022 (Unit 2). Electronic reports based on documentation were generated to monitor patient ambulation and distances for feedback and celebration. The researchers and unit teams met often to implement and ensure intervention integrity despite increasing care demands and staffing shortages. Unit leaders navigated pandemic-induced obstacles including turnover, shortages, and high patient volumes by refining staff training, streamlining communication, promoting in-room walking, and finding creative ways to celebrate and build a culture within the COVID-19 restrictions. Unit 1 ambulated 75% of patients (20% increase) with distances averaging 47 miles/month (540% increase). Unit 2, with less ambulatory patients, ambulated 73% of patients (18% increase) with distances averaging 23.4 miles/month (680% increase). MOVIN is a promising system-based intervention that supports culture change and promotes nurse-led patient ambulation. Units were able to increase and sustain the quantity and quality of patient walking despite the obstacles imposed by the pandemic.

Abstract citation ID: igad104.0194

**A COLLABORATIVE PILOT FOR AGE-FRIENDLY HOSPITAL CARE**

Kari Hirvela, *UW Health, Madison, Wisconsin, United States*

To ensure older adults receive the best care possible, Age Friendly Health Systems has become a national effort in the US. This initiative is based on 4 core components (called 4 M's framework), What Matters, Medication, Mentation, and Mobility. We piloted a multi-disciplinary care model based on the 4 M's framework to improve healthcare delivery for older adults at a large academic hospital. Using the Plan-Do-Check-Act, a care model was developed. Plan: A multi-disciplinary stakeholder group identified best practices for each of the 4Ms, including: 1) asking and documenting "what Matters most" and mobility; 2) pharmacy review of high-risk medications; 3) creation of 4M provider patient list in electronic health record (EHR); 4) implementation of delirium rounds. Education was provided to hospital champions. An interactive dashboard was created to demonstrate impact. Do: The 4M's were implemented for patients ≥ 65 years old on 2 inpatient units. Check: 4M's interventions was monitored via dashboard. Findings revealed low provider utilization of the patient list template and provider-initiated adaptations to the care plan. Feedback hospital providers was largely positive. Act: Based on feedback, revisions were made within the EHR, and a 4M's patient flyer was created. 73% of older adults received 4M's care on the 2 pilot units, meeting the criteria demonstrating "Committed to Care Excellence" by the Institute of Healthcare Improvement. We were able to effective implement 4 M's care. Age friendly care is an evidence-based model that may improve outcomes for older adults.

### SESSION 1195 (BIOLOGICAL SCIENCES INVITED SYMPOSIUM)

Abstract citation ID: igad104.0195

**BIODEMOGRAPHY OF AGING, CELEBRATING JAMES W. VAUPEL, PHD.**

Chair: Kaare Christensen

James W. Vaupel, PhD (1945 – 2022), was an international leader in demography and aging research and a pioneer in the field of biodemography. He was a highly creative researcher and very entrepreneurial in the development of new interdisciplinary research environments. This symposium highlights a few of the biodemographic research areas that Dr. Vaupel has been a catalyst for. Denmark was Dr. Vaupel's home country during the last third of his life, and Dr. Christensen reports on findings from Danish nationwide genetic-epidemiological studies on twins and the oldest old: familial influence on aging phenotypes, cohort differences in health among the oldest old, associations of early life events with late life health, as well as tongue-in-cheek research on perceived age and teeth. Dr. Alberts discusses the 'invariant rate of aging' hypothesis that was developed by Vaupel and others. She presents comparative analyses from multiple nonhuman primate populations showing that, while life expectancy can continue to improve, we probably can't slow the demographic rate of aging. Dr. Campos presents evidence from a wild baboon population in Kenya that glucocorticoid levels—biological markers of stress responses—are strong prognostic indicators of survival and may be key explanations of life span disparities. Dr. Carey presents key discoveries from large-scale studies involving the Mediterranean fruit fly. These include the slowing of mortality at older ages, context-specific sex mortality differentials, a behavioral biomarker for morbidity, dietary conditions for maximizing lifespan versus reproduction and a stationary population identity whereby life lived equals life left.

Abstract citation ID: igad104.0196

**BIODEMOGRAPHY OF AGING: INSIGHTS FROM TWINS AND THE OLDEST-OLD IN DENMARK**

Kaare Christensen, *University of Southern Denmark, Odense, Syddanmark, Denmark*

The Danish Twin Registry holds health information from surveys and registers on more than 180,000 twins born in Denmark since 1870. Life course studies of the oldest twin cohorts show moderate heritability of lifespan but a substantial genetic influence on physical and cognitive functioning throughout life, i.e., twins are growing old but not growing apart, in terms of health phenotypes. Twins experience substantial growth restriction in the last intrauterine trimester. The generalizability of aging studies of twins hinges on whether this early life exposure has long-lasting health consequences. Generally, after the infant period, twin-singleton health differences are small, and during the 20th century, they have vanished. In the older twin cohorts, sex differences in the association between number of offspring and oral health late in life provided support for the proverb known in many countries: "A tooth per child". Twin studies of biomarkers of aging revealed that perceived age estimated by lay assessors based on photos is a strong biomarker of aging. The 1895, 1905, 1910 and 1915 Danish Birth Cohort Studies of the oldest old were established to study secular trends in the health and functioning of the very old. Among the main findings of the studies are: Exceptional longevity does not lead to exceptional levels of disability; summed over cohorts, more people are living to the highest ages and are functioning better – and this progress is seen across the spectrum of health and functioning – offering hope for continued progress in health and survival among the oldest in society.

Abstract citation ID: igad104.0197

**THE LONG LIVES OF PRIMATES AND THE 'INVARIANT RATE OF AGING' HYPOTHESIS**

Susan Alberts, *Duke University, Durham, North Carolina, United States*

Can we slow the rate of human aging? A decades-long trend toward increasing life expectancy and greater lifespan equality in human populations raises the possibility that we can. But the rate of aging is closely correlated with other traits and may be highly constrained or even relatively fixed within species, according to the 'invariant rate of aging' hypothesis. To gain insight into biological constraints on aging, we use an unprecedented collection of datasets from 39 human and nonhuman primate populations, representing 7 genera distributed across the order Primates. We show that the highly regular linear relationship between life expectancy and lifespan equality reported in humans is recapitulated in other primate genera, and that variation in the rate of aging is several orders of magnitude smaller than pre-adult and age-independent mortality, which are highly variable within genera. Thus, within primate genera, longer life expectancies are associated with fewer early deaths, but not with a lower rate of aging. We also demonstrate that changes in the rate of aging, but not other ageing parameters, can produce striking, species-atypical changes in mortality patterns. Our results support the invariant rate of aging hypothesis, suggesting biological constraints on how much we can slow the human rate of aging.

Abstract citation ID: igad104.0198

**STRESS AND DEATH: GLUCOCORTICOIDS AND SURVIVAL IN A WILD PRIMATE MODEL OF AGING**

Fernando Campos, *University of Texas, San Antonio, San Antonio, Texas, United States*

Chronic exposure to stressors has been linked with a wide range of detrimental effects on health in humans, laboratory animals, and a few wild animal populations. Glucocorticoids (GCs) mediate stress responses, and consequently GC levels are regularly measured in humans and other animals as biological markers of stress. However, no tests in humans or in natural populations of animals have yet established clear connections between environmental stressors, chronically elevated GCs, and shortened lifespan. To fill this gap, we used longitudinal data on wild female baboons to investigate the relationship between GC levels and survival—the single greatest determinant of variation in evolutionary fitness among female baboons. Using 14,173 GC measurements from 242 wild adult female baboons over 1634 female years, we document a powerful link between GCs and survival: females with relatively high current GCs or high lifelong cumulative GCs face an elevated risk of death. A hypothetical female who maintained GCs in the top 90% for her age across adulthood would be expected to lose 5.4 years of life relative to a female who maintained GCs in the bottom 10% for her age. Hence, differences among individuals in HPA axis activity provide valuable prognostic information about disparities in lifespan. Together, our results both support the value of GCs as a window into health, fitness, and aging and suggest that they may mechanistically contribute to the established link between sociality and lifespan.

Abstract citation ID: igad104.0199

**FIVE BIODEMOGRAPHIC PRINCIPLES OF AGING THAT EMERGED FROM RESEARCH ON THE MEDITERRANEAN FRUIT FLY**

James Carey, *University of California, Davis, Davis, California, United States*

Because of its large size, visually-distinct sex differences, abundance in the wild and ease of rearing at both industrial and individual levels, the Mediterranean fruit fly (medfly) is an ideal model organism for conducting research on the demography of aging. I will present details on and implications for what I consider to be the five most interesting and important aging-related discoveries from the NIA-funded research my colleagues and I conducted over the past three decades using the medfly model. These discoveries include (1)Deceleration of mortality at advanced ages; (2) Equivocality of the gender gap due to context-specific sex mortality relationships; (3)Supine behavior as a biomarker of both morbidity onset and time-to-death; (4)A dietary disconnect between maximizing longevity vs maximizing lifetime reproduction; and (5)the life table population identity in which the fraction age x in a stationary population equals the fraction with x years to live. I will end with brief comments about the use of non-conventional model organisms for research on aging in both the laboratory and the field.

### SESSION 1200 (SYMPOSIUM)

Abstract citation ID: igad104.0200

**AGEISM EXPERIENCED AND EXPRESSED: MANIFESTATIONS ACROSS CONTEXTS AND TRANSGRESSORS**

Chair: Alison Chasteen

Ageism was first defined more than fifty years ago, yet its expressions and effects on society continue today. In this symposium, we examine manifestations of ageism across contexts and transgressors in order to determine the effects of ageism in light of a pandemic as well as a post-pandemic world. Important social contexts in which ageism plays a part are examined, such as the workplace and COVID-19 vaccination. As well, efforts to reduce ageism through confrontation are investigated, particularly with respect to reactions to ageist behavior. Providing a window into ageist communication during the pandemic, Bascu and colleagues detail how ageism was expressed in social media regarding policies for COVID-19 vaccination. Delving into the factors that drive older workers' decisions to remain or to leave an organization, both Lagacé et al and Swift investigate the role that perceived ageism plays. Lagacé and colleagues assess how older workers' perceptions of being the target of ageism impacts their well-being, their psychological disengagement, and their intentions to leave their organization. Swift considers how experiences of ageism in the workplace predict older workers' retirement intentions through reduced job satisfaction and lowered intrinsic motivation to work. Chasteen et al. test the impact of confronting ageism on perceptions of transgressors as well as how perceivers use characteristics of an older adult target as a normative cue for determining their reactions to ageist behavior. Taken together, these presentations illustrate the influence of ageism on vital life domains such as healthcare and the labor force.

Abstract citation ID: igad104.0201

### REACTIONS TO PERPETRATORS OF BENEVOLENT AGEISM: WHAT INFLUENCES PERCEPTIONS?

Alison Chasteen[1], Zainab Saleem[1], and Michelle Horhota[2], *1. University of Toronto, Toronto, Ontario, Canada*, *2. Furman University, Greenville, South Carolina, United States*

Ageism against older adults is a longstanding and pervasive problem. Benevolent ageism can be particularly difficult to address, given it is characterized by warmth and condescension. In two studies, we examined the impact of confronting prejudice on perceptions of a young adult perpetrator who expressed benevolent ageism. In both studies, the perpetrator initiated unwanted help to an older person who was either 62 or 82 years old, and the older adult either accepted or politely rejected the unwanted assistance. In both studies, participants felt that rejecting (i.e., confronting) the ageist behavior decreased the likelihood the perpetrator would repeat the behavior. Young adult participants used the age of the older target as a cue for interpreting the perpetrator's intentions, such that they rated the perpetrator's actions as less intending to offend if the older target was 82 than 62 years of age. Moreover, younger adults used the age of the older target to determine whether the perpetrator should be confronted, indicating that confrontation should occur when the target was 62 vs. 82 years old. Taken together, these studies demonstrate that participants of all ages feel that confronting benevolent ageism can be an effective tool for reducing biased behavior. As well, these findings show that the age of the older adult target might be used as a normative cue to interpret the perpetrator's intentions and actions, thus providing a more nuanced picture of what might drive witnesses' responses to ageist behavior.

Abstract citation ID: igad104.0202

### AMBIVALENT AGEISM IN THE WORKPLACE AND ITS IMPACT: EXPLORING PERCEPTIONS OF OLDER WORKERS

Martine Lagacé[1], Ezgi Tasyurek[2], Philippe Rodrigue-Rouleau[2], Caroline Bergeron[2], and Melanie Levasseur[3], *1. Université d'Ottawa, Ottawa, Ontario, Canada*, *2. University of Ottawa, Ottawa, Ontario, Canada*, *3. Université de Sherbrooke, Sherbrooke, Quebec, Canada*

Several countries are currently facing significant labour shortages in different work sectors. One of the solutions being considered to deal with such shortages is the retention of older workers. However, to do so, ageist attitudes and discrimination in the workplace must be countered as well as their negative impacts on older workers' well-being. While previous studies have focused on assessing the impact of hostile ageism in the workplace, less research has been conducted on ambivalent ageism (i.e., stereotypes of fragility and incompetence) in the workplace. This study examines if and to what extent older workers perceive to be the target of ambivalent ageism and how such perceptions impact their well-being, in terms of psychological disengagement, self-esteem, perceived employability as well as intentions to leave their organization. An online, bilingual (French / English) questionnaire was completed by 951 Canadian older workers aged 50 years or more. Preliminary data analysis suggests that ambivalent ageism is negatively associated with perceived employability and self-esteem and positively associated with psychological disengagement and intentions to leave. Further, stratified data analysis by age group suggests that workers aged 62 or older perceive less ambivalent ageism, are less disengaged and have significantly higher self-esteem than workers of younger age groups. Such findings call for the implementation of workplace policies that are age-based inclusive and that account for differential experiences of ageism in the workplace.

Abstract citation ID: igad104.0203

### EXAMINING COVID-19 VACCINE–RELATED AGEISM IN TWITTER DATA

Juanita-Dawne Bacsu[1], Megan O'Connell[2], Allison Cammer[2], Alison Chasteen[3], Sarah Fraser[4], Mehrnoosh Azizi[2], Karl Grewal[2], and Raymond Spiteri[2], *1. Thompson Rivers University, Kamloops, British Columbia, Canada*, *2. University of Saskatchewan, Saskatoon, Saskatchewan, Canada*, *3. University of Toronto, Toronto, Ontario, Canada*, *4. University of Ottawa, Ottawa, Ontario, Canada*

During the pandemic, many high-income countries prioritized older adults for COVID-19 vaccination in an attempt to reduce mortality. This prioritization may have exacerbated ageism and intergenerational conflict, especially with the limited quantity of COVID-19 vaccines. This presentation examines vaccine-related ageism during COVID-19 on social media to inform future vaccination campaigns and policies. Using Twitter, we gathered 1,369 relevant tweets using the Twint application in Python from December 8, 2020 to December 31, 2021. Tweets were assessed using inductive thematic analysis and steps were taken to ensure rigor and trustworthiness. Based on our analysis, four main themes were identified including: i) Blame and aggression; ii) Misinformation and mocking content; iii) Ageist political insults; and iv) Challenging ageism. Our study identified issues of false information, hate speech, and ageist political insults that are contributing to intergenerational conflict. Although some tweets challenged this derogatory messaging and demonstrated intergenerational unity, our findings suggest ageism contributed to COVID-19 vaccine hesitancy among older adults. Accordingly, urgent action is required to challenge vaccine misinformation, counter aggressive ageist content, and support intergenerational unity during the pandemic.

Abstract citation ID: igad104.0204

### AGEISM, JOB SATISFACTION, INTRINSIC MOTIVATION, AND RETIREMENT DECISIONS

Hannah Swift, *University of Kent, Canterbury, England, United Kingdom*

This study examines experiences of ageism in the workplace and how they impact workers' intentions to retire.

Self-report data on experiences of ageism in the workplace, meta-perceptions of older workers, job satisfaction, motivation, and retirement intentions were collected in a sample of 125 UK employees aged 50 and older, alongside other relevant demographic information. In line with previous research, the analysis revealed that the positive relationship between ageism and intentions to retire is mediated by a reduction in job satisfaction. However, additional analysis confirmed that intrinsic motivation also partially mediates the relationship between job satisfaction and intentions to retire. A serial mediation model then established that experiences of ageism at work increased intentions to retire via reductions in job satisfaction, which in turn reduced intrinsic motivation to work. Separate models confirm these relationships remain when the experience of ageism is substituted for negative meta-perceptions of older workers. This research provides further evidence for the need to reduce ageism and negative age stereotypes in organizational settings but also highlights the role of intrinsic motivation as an important indirect pathway through which ageism increases retirement intentions. For the first time, the effects of negative meta-stereotypes on job satisfaction, intrinsic motivation, and intentions are also modelled.

### SESSION 1205 (SYMPOSIUM)

Abstract citation ID: igad104.0205

### AGING IN THE RIGHT PLACE: HOUSING AND SOCIAL SUPPORTS FOR OLDER ADULTS EXPERIENCING HOMELESSNESS

Chair: Anthony Traver Discussant: Holly Dabelko-Schoeny

The Aging in the Right Place (AIRP) framework recognizes that secure housing for older adults should support one's unique vulnerabilities and lifestyles. Applying the AIRP framework to older people experiencing homelessness (OPEH) reveals limited access to the types of living environments and supportive services that promote dignity, health, and inclusion in later life. This symposium aims to use the AIRP framework to examine the places, meanings, and opportunities experienced by diverse groups of OPEH striving to secure housing and promote their well-being. The first paper uses photovoice interviews and thematic analysis to broaden the current conceptualization of AIRP by exploring how feelings, actions, and place inform the meaning of AIRP for older adult residents of one temporary housing program in Vancouver, CA. The second examines the housing and support needs of older adult veterans in Calgary, CA residing in temporary congregate housing through a multi-methods approach involving document reviews, environmental audits, and in-depth interviews. The third paper uses interviews with OPEH and service providers in Columbus, OH to look across the continuum of housing occupied by older adults before, during, and after an episode of late-life homelessness and to investigate the mechanisms that allow OPEH to identify and access the housing they consider optimal. Finally, the discussion positions AIRP for OPEH within the broad context of Age-Friendly ecosystems to highlight the role of coordination between housing providers, government leaders, health services, university researchers, and residents to ensure that AIRP is achievable for precariously housed older persons.

Abstract citation ID: igad104.0206

### PRECARIOUS HOUSING BEFORE, DURING, AND AFTER AN EPISODE OF LATE-LIFE HOMELESSNESS

Anthony Traver, and Holly Dabelko-Schoeny, *The Ohio State University, Columbus, Ohio, United States*

This qualitative study aims to understand how the living environments occupied by older adults before, during, and after an episode of homelessness inform their access to a healthy, stable, and dignified life. Indicators of accommodation, quality, and service integration are explored using the Aging in the Right Place conceptual framework. Through partnerships with a homeless shelter, a meal site, and a mental health outreach team in Columbus, OH, demographic questionnaires and semi-structured interviews were completed with 22 older adults with an episode of late-life homelessness. Nine direct service providers were also interviewed. Interviews were analyzed using the team-based flexible coding method in Nvivo 1.6.1. Results indicated that low or no income in late life forced older adults to occupy a continuum of precarious and low-quality living environments that include shared housing, doubling up, emergency shelters, institutional settings, and living on the land. AIRP indicators are discussed for each. Conflict, death of a support person, mental and behavioral challenges, unit deterioration, rental price increases, and social isolation forced OA down the housing continuum and into homelessness. Sub-optimal conditions interacted with age to exacerbate health conditions, create social isolation, and expose OA to harm. Informal and formal relationships, emergency shelter services, vouchers, and specific behaviors and attitudes were identified as critical for securing affordable and accommodating housing in which to age. Understanding the experiences of OPEH who are striving to occupy their subjective right place to age can help service providers and policymakers meet the unique needs of precariously housed OA.

Abstract citation ID: igad104.0207

### MEANINGS OF AGING IN THE RIGHT PLACE FOR HOUSING INSECURE OLDER ADULTS IN TEMPORARY HOUSING

Atiya Mahmood[1], Sarah Canham[2], Rachel Weldrick[1], Rachelle Patille[1], and Morgan Erisman[2], *1. Simon Fraser University, Vancouver, British Columbia, Canada*, *2. University of Utah, Salt Lake City, Utah, United States*

The concept of aging in place (AIP) is widely acknowledged as the preference of older adults. However, AIP without consideration of the intersections of diversity in later life is critiqued as lacking an equity lens. This gave rise to the exploration of what is needed to age in the right place (AIRP). Building on existing research, we examined the meaning of AIRP to older adults (aged 55+ years) who have experienced homelessness. We conducted photovoice interviews with 11 residents of a temporary housing program in Metro Vancouver (Canada). Using thematic analysis, we investigated the meanings of AIRP based on 1) How one feels, 2) What one does, and 3) Where one lives. Findings demonstrate that meanings of AIRP follow a similar pattern as Maslow's Hierarchy of Needs. That is, when basic needs of shelter are met, participants' considerations of the 'right'

place to live extends beyond affordable housing to include having access to meaningful activities – in urban districts and nature-scapes – enabled by affordable transportation. This consideration includes higher-order factors such as opportunities for meaningful engagement and looking forward to a stable life, being at peace, feeling safe and comfortable in one's home and neighborhood. Our examination of what AIRP means to this group of older adults broadens current conceptualizations. Given the increase of older adults who are homeless it is imperative that policymakers and practitioners be cognizant of meanings of AIRP to enable diverse groups of older adults to thrive in housing that is more than a shelter.

Abstract citation ID: igad104.0208

### AGING IN THE RIGHT PLACE: TEMPORARY SUPPORTIVE HOUSING FOR OLDER MALE VETERANS IN CALGARY, CANADA

Jill Hoselton[1], Alison Grittner[2], and Christine Walsh[1], *1. University of Calgary, Calgary, Alberta, Canada*, *2. Cape Breton University, Sydney, Nova Scotia, Canada*

In 2019 an estimated 1.6% of emergency shelter users in Canada were veterans (1,905 individuals), which is consistent with the proportion of veterans in the general population (1.7%). However, veterans tend to be older, male, and cite an illness or medical condition as a contributing factor to their homelessness, than their non-veteran counterparts. As part of the Aging in the Right Place (AIRP) study, we sought to understand the housing and support needs of older male military veterans (age 50+) living in a congregate temporary supportive housing in Calgary, Alberta. This exploratory, multi-methods study used: (1) de-identified document review, (2) environmental audit, (3) in-depth qualitative key informant interviews with service providers and (4) in-depth qualitative and Photovoice interviews with older shelter residents to understand the shelter needs of older homeless veterans. The data was collected between February and June 2023. Interviews were transcribed, managed with NVivo 1.6.1, and team-based flexible coding was employed on the key informant (n=5) and shelter resident (n=5) interviews to determine older male veterans' shelter needs. In this presentation we share the findings of the study and offer recommendations for shelter and services designed to assist older housing insecure veterans to age in the right place.

### SESSION 1210 (SYMPOSIUM)

Abstract citation ID: igad104.0209

### VARIATION IN ASSISTED LIVING REGULATION: IMPLICATIONS FOR STATE AGENCY OVERSIGHT, PROVIDER KNOWLEDGE, AND PRACTICE

Chair: Lindsey Smith Discussant: Derek Manis

Researchers spend significant time and effort documenting policy, particularly in assisted living, where there are no national regulatory standards, and state governments develop and enforce regulations, leading to significant variation between and within states. Our panel explores the question we often get—do these regulations matter? Do they really impact the structures, processes, and outcomes of care provision?

Abstract citation ID: igad104.0210

### ASSESSING REGULATORY STRINGENCY FOR LICENSED ASSISTED LIVING: A MULTIFACETED APPROACH

Lindsey Smith[1], Jacy Weems[2], Cassandra Hua[3], Sarah Dys[4], Paula Carder[5], and Kali Thomas[6], *1. Oregon Health & Science University, Portland, Oregon, United States*, *2. Brown University School of Public Health, Providence, Rhode Island, United States*, *3. University of Massachusetts Lowell, Lowell, Massachusetts, United States*, *4. Portland State University, Portland, Oregon, United States*, *5. Portland State University Institute on Aging, Portland, Oregon, United States*, *6. Johns Hopkins University, Baltimore, Maryland, United States*

Regulations in long-term care settings aim to ensure quality and accessibility for residents. However, inadequate enforcement can limit their impact on the community. Simply increasing regulations does not always lead to improved care, as it may incentivize facilities to limit admissions. Regulatory stringency encompasses the scope, specificity, and enforcement of regulations. This study examines 10 dimensions of specificity related to medical care regulations in assisted living, focusing on scope and enforcement. We found that regulations related to staffing levels and resident assessments governed almost all assisted living communities, while medication review and administrator certification were the least commonly addressed. The "Residential Care Facilities" license with an "Alzheimer's Dementia Care Unit" certification in Maine had the highest stringency score, though the "Assisted Living Program" license in the same state had one of the lowest. High regulatory specificity and scope scores were not always associated with high enforcement. For example, the "Assisted Living Center" license in South Dakota covered 100% of the scope's dimensions and 75% of the provisions contributing to the specificity score, but had no regulatory enforcement for these rules. Findings suggest that a multifaceted approach to assessing regulatory stringency is crucial for policymakers and researchers to understand regulatory approaches across license types and states. Accounting for differences in licensure is also important for a broad view of regulatory stringency.

Abstract citation ID: igad104.0211

### CIVIL MONETARY PENALTIES GOVERNING ASSISTED LIVING: VARIATION ACROSS AND WITHIN STATES

Jacy Weems[1], Lindsey Smith[2], Cassandra Hua[3], and Kali Thomas[4], *1. Brown University School of Public Health, Providence, Rhode Island, United States*, *2. Oregon Health & Science University, Portland, Oregon, United States*, *3. University of Massachusetts Lowell, Lowell, Massachusetts, United States*, *4. Johns Hopkins University, Baltimore, Maryland, United States*

State survey agencies are responsible for developing minimum requirements for assisted living (AL) communities, including penalties for noncompliance. Civil monetary penalties are a common measure state enforcement agencies use to incentivize regulatory compliance. We searched state statutes and regulations governing licensed AL communities in August 2022 to identify enforceable civil monetary penalties and used descriptive statistics and content analysis to describe regulatory differences within and between states according to license type. Our findings indicate that 48 of 51 states may enforce financial penalties for noncompliance against assisted living communities, though four of these states have one or more license types without applicable monetary penalties. 13 states had differing policies and possible enforceable amounts by license type, so some AL communities are possibly being charged more than others for the same offense within the same state. We found that states took three different approaches to calculating fees, sometimes using multiple approaches depending on the citation and license type. One or more license types in 36 states imposed per-instance fines, per-day fines in 32 states, and per-resident-per-day fines in 3 states. We describe the current landscape of financial penalty enforcement policies for US assisted living communities as well as its implications for state agencies and providers.

Abstract citation ID: igad104.0212

### CONTEXTUALIZING THE RANGE OF MEDICATION-RELATED REGULATORY REQUIREMENTS IN ASSISTED LIVING

Sarah Dys[1], and Lindsey Smith[2], *1. Portland State University, Portland, Oregon, United States*, *2. Oregon Health & Science University, Portland, Oregon, United States*

Prior research has investigated the severity and scope of medication-related deficiency citations; less is known about the specificity of regulatory requirements governing medication-related practices in assisted living (AL). We examine the variation of regulatory specificity for two common policies: medication record review and administration. We coded state requirements for 196 AL licenses linked to 33,246 ALs in the U.S for the presence of specific requirements, weighted for increasing precision (1-4), summed and divided by the maximum value to produce a score ranging from no specificity (0) to maximum specificity (1). Medication record review specificity includes: regularly scheduled review of resident medications generally (1), by AL staff (2), or by a licensed healthcare professional (3). Specificity of staff who administer oral medication includes: direct care staff permitted (1), certified nursing assistant or medication aide required (2), licensed practical/vocational nurse required (3), registered nurse required (4). Most facilities operate under licenses that only require general review of residents' medications (62%) and 32% required medication review by a licensed healthcare provider. Most facilities' licensure requirements permit direct care workers to administer medications (63%) and 16% require a licensed nurse to administer. One in six facilities have requirements that do not address who is permitted or required to administer oral medications. Overall, 36% of facilities operated under regulations that had an overall specificity score ( management and administration) of 0.5 or higher. While most ALs are licensed with low medication policy specificity, understanding regulatory expectations can contextualize and inform medication practices within these settings.

Abstract citation ID: igad104.0213

### ADMISSIONS AND DISCHARGE PRACTICES IN ASSISTED LIVING: THE ROLE OF REGULATORY AND ORGANIZATIONAL CHARACTERISTICS

Cassandra Hua[1], Lindsey Smith[2], Sheryl Zimmerman[3], Gauri Gadkari[4], Philip Slolane[5], Paula Carder[6], Christopher Wretman[7], and Kali Thomas[8], *1. University of Massachusetts Lowell, Lowell, Massachusetts, United States*, *2. Oregon Health & Science University, Portland, Oregon, United States*, *3. University of North Carolina at Chapel Hill, Chapel Hill, North Carolina, United States*, *4. Brown University School of Public Health, Providence, Rhode Island, United States*, *5. University of North Carolina School of Medicine, Chapel Hill, North Carolina, United States*, *6. Portland State University Institute on Aging, Portland, Oregon, United States*, *7. Wretman LLC, Hillsborough, North Carolina, United States*, *8. Johns Hopkins University, Baltimore, Maryland, United States*

This study examined how state regulations and AL organizational characteristics relate to admissions and discharge practices. Using data from a representative sample of 250 AL communities in 7 states and a database of AL regulations, we employed bivariate analysis and multilevel linear probability models to examine regulatory and organizational correlates of AL community admissions and discharge practices for three activities of daily living (ADLs [bathing, getting out of bed, and eating]). There was a pattern between admissions regulation and admissions practices in AL; for example, 38% of communities with license types that had the most flexible admissions regulations allowed residents with feeding limitations to be admitted; conversely, 18% with the least flexible regulations allowed them to be admitted. However, the relationships were not statistically significant in bivariate or multivariate analysis. A higher percent of residents with Medicaid as a primary payer source was associated with a decrease in the probability of admitting ($\beta$ = -0.01, p<.001, 95% confidence intervals [CI]: -0.01, 0.00) and discharging ($\beta$ = 0.01, p=.013, 95% CI: 0.00, 0.01) residents with bathing limitations. Being operated in association with a continuing care retirement community or nursing home was associated with a higher probability of discharging residents who needed assistance with bathing ($\beta$ = 0.14, p=.013, 95% CI: 0.03, 0.26) and more so with feeding ($\beta$ = 0.26, p <.001, 95% CI: 0.15, 0.36). Findings suggest a need to consider whether models of AL and AL practices could better align with the residents' needs.

Abstract citation ID: igad104.0214

### ASSISTED LIVING ADMINISTRATORS' UNDERSTANDING OF REGULATORY REQUIREMENTS IN SEVEN STATES

Paula Carder[1], Kali Thomas[2], Lindsey Smith[3], David Reed[4], Philip Sloane[5], and Sheryl Zimmerman[4], *1. Portland State University Institute on Aging, Portland, Oregon, United States*, *2. Johns Hopkins University, Baltimore, Maryland, United States*, *3. Oregon Health & Science University, Portland, Oregon, United States*, *4. University of North Carolina at Chapel Hill, Chapel Hill, North Carolina, United States*, *5. University of North Carolina, Chapel Hill, North Carolina, United States*

Each state requires assisted living (AL) residences to employ an administrator whose job responsibilities include oversight of resident care, managing staff, and regulatory compliance. This study surveyed administrators of 151 AL residences in 7 states. These AL residences reflect 28 license types (e.g., based on level of care, dementia care services). Administrators were asked whether there were any regulations, policies or procedures that required their AL to do any of 11 health-related activities (e.g., staff able to check vital signs, advance directive on file, reporting change in condition to resident's healthcare provider). Using health services regulatory analysis, we documented the applicable regulations for each of the 151 AL residences, then assessed whether administrator responses were congruent with the licensed requirements. Almost all (95%) administrators of AL residences governed by a licensure requirement regarding change in condition reported this policy in their residence. In contrast, less than half (48%) of administrators who work in an AL in our sample that state law requires to keep an advance directive on file for residents reported that they did not have a policy to do so. Over one-third (37%) of administrators who work in an AL required to have a policy of non-pharmaceutical treatment for residents with dementia said that they did not have this policy in place. Some administrators reported the presence of policies in the absence of state requirements, suggesting that these AL residences exceed minimum requirements. These results vary by AL license type both within and across states.

### SESSION 1215 (SYMPOSIUM)

Abstract citation ID: igad104.0215

### AUTONOMIC ACTIVITY AND THE AGING BRAIN

Chair: Mara Mather

Aging affects both autonomic activity and the brain regions that help modulate autonomic activity. In this symposium, we present new findings on how the relationships between autonomic activity and the brain change in aging. In addition, we demonstrate that modulating autonomic activity can affect the aging brain and emotional and cognitive functions controlled by the brain. Kathy Liu will present research from over 600 participants showing that, unlike younger adults who show the expected positive relationship between heart rate variability (HRV) and brain and behavioral indicators of emotion regulation, older adults showed a negative relationship between HRV and emotion regulation. Julian Thayer will present findings on how blood pressure and total peripheral resistance relate to brain structure. Richard Song will present functional MRI data revealing that the older brain shows less blood oxygen level dependent (BOLD) response to physiological fluctuations than younger brains. Jungwon Min will present findings that random assignment to daily biofeedback to either increase or decrease heart rate oscillations had a large effect on plasma amyloid-β. Mara Mather will present a new theoretical model positing that older brains attempt to compensate for hyperactive peripheral sympathetic activity, and that this ventromedial prefrontal compensatory activity leads to the biases in attention and memory known as the age-related positivity effect. Together, the empirical findings and theoretical perspectives presented in this symposium indicate that the autonomic system exerts important influences over the aging brain and that this provides a significant opportunity for intervening to improve brain health.

Abstract citation ID: igad104.0216

### THE AGING BRAIN: A NEUROVISCERAL INTEGRATION PERSPECTIVE

Julian Thayer, *University of California, Irvine, Irvine, California, United States*

Healthy aging is associated with significant changes in both the brain and the heart. The changes between these, the two most important organs of the body, are linked via the vagus nerve. Using a model of Neurovisceral Integration I will describe how autonomic imbalance and decreased parasympathetic tone in particular may be the final common pathway linking negative affective states and conditions to ill health. The central nervous system network that regulates autonomic balance is closely related and partially overlaps with networks serving executive, social, affective, cognitive, and motivated behavior. This inhibitory cortico-subcortical circuit may structurally as well as functionally, link psychological processes with health-related physiology. When the prefrontal cortex is taken "offline" for whatever reason, parasympathetic inhibitory action is withdrawn and a relative sympathetic dominance associated with disinhibited defensive circuits is released, which can be pathogenic when sustained for long periods. This state is indicated by low heart rate variability (HRV), which is a marker for low parasympathetic activation and prefrontal hypoactivity. In this presentation, I examine the normative changes with aging and the effect that stress may have on how the brain–heart connection changes with age. I will present data linking peripheral physiology to brain structure and function. I conclude by noting that significant sex and ethnic differences exist but that future studies are needed to more fully explicate how they may influence the aging brain and its association with the cardiovascular system.

Abstract citation ID: igad104.0217

### DIFFERENT ASSOCIATIONS BETWEEN HEART RATE VARIABILITY AND EMOTION REGULATION IN OLDER AND YOUNGER ADULTS

Kathy Liu, *University College London, London, England, United Kingdom*

Heart rate variability (HRV), the beat-to-beat variation in heart rate, is proposed to index aspects of self-regulatory processes such as emotion regulation. This is due to overlapping neural regions and networks that contribute to the central autonomic and emotion regulation systems. Paradoxically, increasing age is associated with both improvement in emotion regulation and decline in HRV, and it is unclear whether and how the association between HRV and emotion regulation is influenced by aging. The earliest stages of neurodegenerative processes, such as Alzheimer's disease-related reduction of locus coeruleus (LC) integrity, may also influence the HRV-emotion regulation relationship in older adults, as LC modulates arousal and autonomic activity in response to stress. We pre-registered a study to investigate the cross-sectional relationship between measures of emotion regulation, HRV and

LC MRI integrity in a lifespan sample of cognitively normal healthy adults using the Cambridge Centre for Ageing and Neuroscience (Cam-CAN) dataset, a large population-based cohort aged between 18-88 years (N=678). We hypothesized that any age-related differences in the association between HRV and emotion regulation would be related to reduced LC integrity in older versus younger adults. We found an inverse correlation between resting HRV and measures of emotional reappraisal in older adults, which differed significantly to younger adults, but no evidence of LC influence on this association. Our findings highlight age-related differences in the HRV-emotion regulation relationship which warrant further investigation in future studies.

Abstract citation ID: igad104.0218

### HEART RATE VARIABILITY BIOFEEDBACK MODULATES PHYSIOLOGICAL INFLUENCES ON THE BOLD SIGNAL IN OLDER ADULTS

Richard Song[1], Shiyu Wang[2], Jungwon Min[3], Sarah Goodale[1], Ruoqi Yang[1], Mara Mather[3], and Catie Chang[1], *1. Vanderbilt University, Nashville, Tennessee, United States*, *2. Vanderbilt University, Vanderbilt University, Tennessee, United States*, *3. University of Southern California, Los Angeles, California, United States*

Physiological processes, such as heart rate and breathing, impact the blood oxygen level dependent (BOLD) fMRI signal. While often regarded as noise, BOLD signal fluctuations related to physiological activity may contain valuable information about age-related brain vasculature changes including cerebrovascular arterial stiffening. This study investigates how the dynamics of BOLD responses to physiological activity change with age and in response to heart-rate variability (HRV) biofeedback training, an intervention that affects brain networks implicated in autonomic control. We used the resting-state fMRI and physiological data from the Heart Rate Variability Biofeedback Training and Emotional Regulation Dataset (n = 193). Data were split into younger (18-31 years) and older (54-80 years) age groups. Using least squares projection, we fitted physiological impulse functions to model how variation in end-tidal CO2 and heart rate propagate into BOLD signals. After permutation testing, the physiological component of BOLD signal fluctuations was significantly higher in regions including anterior cingulate cortex, basal ganglia, and white matter regions in younger adults compared to older adults. Following a 5-week HRV biofeedback training aimed to increase heart rate oscillations, areas where the physiological influence on BOLD signals increased in older adults included the prefrontal cortex and thalamus; these effects were primarily driven by greater covariance between BOLD and heart rate. In contrast, physiological effects on BOLD did not significantly change in older adults following HRV biofeedback aimed to decrease heart rate oscillations. Overall, these findings highlight how increasing HRV may improve cerebrovascular health in older adults.

Abstract citation ID: igad104.0219

### POTENTIAL MECHANISMS THROUGH WHICH INDUCING HEART RATE OSCILLATIONS AFFECTS PLASMA AMYLOID BETA LEVELS

Jungwon Min[1], Jeremy Rouanet[2], Steve Cole[3], Kaoru Nashiro[1], Hyun Joo Yoo[1], Christine Cho[1], Elizabeth Head[2], and Mara Mather[1], *1. University of Southern California, Los Angeles, California, United States*, *2. University of California, Irvine, Irvine, California, United States*, *3. University of California Los Angeles, Los Angeles, California, United States*

Alzheimer's disease-related protein amyloid beta (Aβ) plasma levels increase with age, and Aβ40 levels are associated with greater mortality. Most plasma Aβ40 is produced via platelets. Platelet activity is regulated by endothelial cells, which are stimulated by blood flow. Slow paced breathing with a 10-second cycle maximizes heart rate oscillations, which may stimulate endothelial-platelet interactions that affect Aβ production. We investigated this idea by randomizing healthy adults (54 younger and 54 older adults) into two groups with the opposite goals: increasing (Osc+) or decreasing (Osc-) heart rate oscillations. Four weeks of daily practice (20-40 min) resulted in large changes in plasma Aβ40 and Aβ42 levels. We also found that changes in Aβ were not associated with changes in resting-state physiology including heart rate variability. But the Aβ changes were associated with a frequency measure of heart rate oscillations induced during practice, suggesting that the Aβ changes were more driven by blood flow changes during practice. P-selectin is an indicator of endothelial activity. We found a significant intervention effect where the Osc+ intervention increased p-selectin and the Osc- intervention decreased p-selectin in older adults. Changes in p-selectin also showed strong negative correlations with changes in Aβ40 and Aβ42 in older adults. The results suggest that blood flow oscillations caused by high amplitude heart rate oscillations stimulated endothelial cells, which suppressed platelet activity and consequently reduced the production of Aβ in older adults. In follow-up research, we are investigating relationships between plasma Ab changes and changes in brain and cognition.

Abstract citation ID: igad104.0220

### THE EMOTION PARADOX IN THE AGING BODY AND BRAIN

Mara Mather, *University of Southern California, Los Angeles, California, United States*

With age, parasympathetic activity decreases while sympathetic activity increases. Thus, the typical older adult has low heart rate variability (HRV) and high noradrenergic activity. Younger adults with this physiological profile tend to be chronically unhappy and stressed. Yet, with age, emotional experience tends to improve. Why doesn't older adults' emotional well-being suffer as their HRV decreases? To address this apparent paradox, I present the autonomic compensation model. In this model, failing organs and initial phases of Alzheimer's pathology trigger noradrenergic hyperactivity in older adults. Older brains attempt to compensate by increasing autonomic regulatory processes in ventromedial prefrontal (vmPFC) cortex. Age-related declines in nerve conduction reduce the ability of these compensatory attempts to reduce hyperactive noradrenergic activity and increase peripheral HRV. However, despite their muted effects on peripheral autonomic activity, these autonomic compensation attempts bias brain processing in favor of stimuli that tend to increase parasympathetic activity and against stimuli that tend to increase sympathetic activity.

The autonomic compensation model can account for the "age-related positivity effect" in which, compared with younger adults, older adults show attentional and memory biases favoring positive over negative stimuli. Consistent with the autonomic compensation model, the age-related positivity effect is associated with vmPFC activity and functional connectivity and is evident even in early attention processes resistant to goal-directed processing. The autonomic compensation model posits that the aging brain responds to sympathetic hyperactivity in ways that amplify on-going automatic emotion regulation processes and so help maintain emotional well-being.

### SESSION 1220 (SYMPOSIUM)

Abstract citation ID: igad104.0221

### TOWARD A BETTER UNDERSTANDING OF SOCIAL EXCLUSION IN LATER LIFE

Chair: Marja Aartsen Discussant: Michal Myck

Social exclusion (SE) – or the separation of individuals and groups from mainstream society - is a serious problem with adverse health and well-being outcomes and increased societal costs. Older adults form a vulnerable group as they tend to be excluded for longer periods than younger people. A growing body of knowledge suggests that SE is multidimensional, including social, cultural, economic, material, and geographical aspects. There are however noticeable knowledge gaps, of which some are addressed in this symposium. Three presentations are based on large cross-national data sets, the final is a qualitative study on a rarely investigated but relevant social group, i.e., older Roma people. The first presenter shows how childless older adults in Europe feel more excluded than older adults with children and discusses how normative pressures can exclude people from society (data from the European Social Survey, wave 1 and 10). The second presenter explains how depopulation of geographical areas can act as driver of SE through material hardship, low social integration and lacking public infrastructure in the depopulated areas (data derived from SHARE, wave 4 and 6). Based on unique data from Poland and Germany, the third presenter shows how associations between markers of SE and well-being are modified by the macro-social context and characteristics of the individual. By using an intersectional life course perspective, the last presentation indicates how pathways in- and out SE are linked to social and societal processes and the political history of a country in a socially deprived group.

Abstract citation ID: igad104.0222

### COHORT DIFFERENCES IN THE ASSOCIATION BETWEEN CHILDLESSNESS AND SOCIAL EXCLUSION IN OLD AGE

Julia Sauter[1], and Marja Aartsen[2], *1. OsloMet, Oslo, Oslo, Norway*, *2. OsloMet Oslo Metropolitan University, Oslo, Akershus, Norway*

With the progressive institutionalization of Western societies during the first half of the 20th century, socially standardized life trajectories were established, in which certain transitions were expected to be made by a majority of society. These normative transitions, experienced by most individuals at different ages of life, have a temporality implying potential social sanctions for those who carry them out too early, too late or not at all. In earlier born cohorts compared to later born cohorts, the transition to parenthood was an event that was highly valued both socially and institutionally, but a part of society remained childless. This study investigates whether childless people born between 1920 and 1935 were more socially excluded when aged between 60 and 80 than people from the same cohort who transitioned to parenthood. We further examined whether a later life difference in social exclusion between childless people and people with children became smaller in later born cohorts (1940-1955). Gender stratified analyses (N=6441, 54% women) on data from the European Social Survey suggest that, in later life, childless women in the earlier born cohort had less trust in other people and felt less socially included than women who had at least one child. Childless men in the earlier born cohort feel less socially included in later life than childless men in the later-born cohort. This difference persisted for women over the years but became slightly smaller for men suggesting a diminishing importance of children for social inclusion in later life for men, not women.

Abstract citation ID: igad104.0223

### INEQUALITIES IN OLD AGE WELL-BEING: THE ROLE OF REGIONAL DEPOPULATION AND SOCIAL AND MATERIAL CONDITIONS

Martina Brandt[1], and Alina Schmitz[2], *1. TU Dortmund University, Dortmund, Nordrhein-Westfalen, Germany*, *2. TU-Dortmund, Dortmund, Nordrhein-Westfalen, Germany*

Material conditions and social integration are important predictors of wellbeing in old age. It can be expected that older individuals who reside in depopulating areas experience declines in well-being, as their property loses value, their social networks are shrinking, and public infrastructure is lacking. We conduct comparative analyses of Germany and Poland, two countries that show significant differences with regard to the economic and social situation of the older population. We expect that regional depopulation is more detrimental to well-being in Poland, as older individuals rely more strongly on informal support and individual resources in old age. We apply multilevel regression models to investigate the interrelations between overall life satisfaction, social integration, material conditions and regional factors. We combine micro-data from the Survey of Health, Ageing and Retirement in Europe (SHARE) with macro-data on regional characteristics (depopulation, public infrastructure and economic development). Our preliminary analyses suggest that a substantial part of inequalities in old age well-being can be traced back to regional differences in the level of depopulation. Social integration and material conditions are important mediators of this association. Additionally, regional differences in care and health infrastructure are important predictors of well-being – especially for older adults in need of financial and social support. Regional depopulation can be detrimental to well-being in later life due to material hardship, low social integration and lacking public infrastructure. In the next steps, we will investigate whether there are differences at the country level by comparing Germany and Poland.

Abstract citation ID: igad104.0224

### INCOME AND WELL-BEING IN OLD AGE—SYSTEMIC AND REGIONAL FACTORS IN A COMPARISON BETWEEN GERMANY AND POLAND

Monika Oczkowska[1], Ellam Kulati[2], and Michal Myck[3], *1. Centre for Economic Analysis CenEA, Szczecin, Zachodniopomorskie, Poland*, *2. Centre for economic Analysis (CenEA), Szczecin, Zachodniopomorskie, Poland*, *3. Centre for Economic Analysis, CenEA, Szczecin, Zachodniopomorskie, Poland*

We examine the responsiveness of later-life well-being measures to income using unique data, matching regional and individual level information from Germany and Poland. These two neighboring countries still strongly differ both in their welfare system designs, as seen in contrasting public social support as well as the quality of services, and in outcomes among older individuals. For example, the proportion of individuals aged 50+ who declare significant difficulties in making ends meet is only 3% in Germany, and as high as 16% in Poland. While up to 13% of individuals aged 65+ are classified as materially and socially deprived in Poland, the proportion in Germany is only 6%. Granted the importance of contextual factors in determining individual welfare, income is expected to matter less for the well-being of those enjoying higher quality local environments. Our preliminary findings confirm a much stronger relationship between income and well-being in Poland. The relationship is also found to be highly heterogeneous – for instance, it is substantially greater among those in poor health. Individual resources are thus shown to be more strongly related to well-being in conditions wherein individuals cannot rely on support through public services. Surprisingly, in both countries the effect of income on well-being does not significantly change when we control for the different local conditions expected to mediate the relationship. This suggests that the country-wide systemic context matters more for the examined relationship than specific regional environment.

Abstract citation ID: igad104.0225

### PATHWAYS TO OR WITHIN SOCIAL EXCLUSION? AN INTERSECTIONAL LIFE COURSE PERSPECTIVE ON OLDER ROMANIAN ROMA

Laura Tufă[1], Iuliana Precupetu[2], Ionut Anghel[3], and Marja Aartsen[4], *1. University of Bucharest, Bucharest, Bucuresti, Romania*, *2. Research Institute of the University of Bucharest, Bucharest, Bucuresti, Romania*, *3. Research Institute for Quality of Life, Romanian Academy, Bucharest, Bucuresti, Romania*, *4. OsloMet Oslo Metropolitan University, Oslo, Akershus, Norway*

Social exclusion is considered a dynamic process as people can move in and out of exclusion while also experiencing different types of exclusion over their life course. In this paper we set out to understand pathways to social exclusion of older Romanian Roma, a particular population that received little attention in the literature. We aim to understand the lived experiences of social exclusion of Roma with the use of life histories and events calendars and we employ an intersectional life course perspective by looking at the nexus of structural, individual, and relational factors that impacted the pathways of older Roma across their life course. The intersection between ethnicity, gender, education, work, residence/segregation and income is employed in order to understand how disadvantage unfolded across the life course and into late life. The data come from 15 biographical interviews with older Roma living in rural and urban Romania, collected in 2021. Results illustrate how specific structural processes like sedentarization of nomad communities, the fall of the communist regime in 1989 and the retrocession of gold unevenly affected Roma across the intersectional characteristics and transformed into turning points in their lives, while interposing to a little extent with their pathways to social exclusion. De-institutionalized life courses and fast transitions had an important bearing on the ways disadvantage unfolded for older Roma living in segregated communities. Finally, super-linked lives at family and community level, when deprived of outside support, contributed to deep exclusion in later life.

### SESSION 1225 (SYMPOSIUM)

Abstract citation ID: igad104.0226

### THE SOCIAL DETERMINANTS OF BRAIN HEALTH: FROM THE SEARCH FOR MECHANISMS TO RECOMMENDATIONS TO INCREASE EQUITY

Chair: Anja Leist Co-Chair: Graciela Muniz-Terrera

Dementia risk has been established to be partly attributable to modifiable social and behavioral risk factors. However, the biological pathways behind these associations and the effects of intervening on present risk factors to reduce dementia risk have not been fully elucidated. The symposium will present latest research on the social determinants of brain health and dementia. We will explore proposed mechanisms between modifiable risk factors and risk of dementia, and present findings on the modifiability of dementia risk through addressing risk factors. Specifically, the first presentation reports analyses testing possible pathways from socioeconomic status, notably childhood and adulthood indicators such as (parental) education and occupation, to cognitive functioning in later mid-adulthood via indicators of brain damage and white matter connectivity with the Maastricht Study. The second presentation reports empirical analyses testing the possible mediating role of the gut microbiome in the relationships between social and modifiable risk factors and Mild Cognitive Impairment with data of the controls of the Luxembourg Parkinson's Study. The third presentation reports on the associations between hearing loss as a modifiable risk factor and incident dementia in the UK Biobank and investigates the potential to modify hearing loss-associated dementia risk through the use of hearing aids. Lastly, the fourth presentation moves to recommendations for policy and government action. It gives an overview of various fields in which policy and legislation could implement cognitive impact assessments to protect individuals' cognition, with the aim of improving population brain health and health equity. This is a Brain Interest Group Sponsored Symposium.

Abstract citation ID: igad104.0227

### THE ASSOCIATIONS OF SOCIOECONOMIC POSITION WITH STRUCTURAL BRAIN DAMAGE AND CONNECTIVITY AND COGNITION

Anouk Geraets[1], Miranda Schram[2], Sebastian Koehler[2], Jacobus Jansen[2], Martin van Boxtel[2], Annemarie Koster[3], Coen Stehouwer[2], and Hans Bosma[2], *1. University of Luxembourg, Esch-sur-Alzette, Luxembourg, 2. Maastricht University Medical Centre+, Maastricht, Limburg, Netherlands, 3. Maastricht University, Maastricht, Limburg, Netherlands*

Socioeconomic inequalities in the risk for cognitive impairment have been reported, which might partly act through structural brain damage and connectivity. This study investigated the extent to which the association of early-life socioeconomic position (SEP) with later-life cognitive performance is mediated by later-life SEP, and whether the association of SEP with later-life cognitive performance can be explained by structural brain damage and connectivity. We used cross-sectional data from the population-based Maastricht study (n=4,839; mean age 59.2±8.7 years, 49.8% women). Early-life SEP was assessed retrospectively by self-reported poverty and parental education. Later-life SEP included education, occupation, and household income. Participants underwent cognitive testing and 3 T magnetic resonance imaging to measure volumes of white matter hyperintensities, gray matter, white matter, cerebrospinal fluid, and structural connectivity. Multiple linear regression analyses tested the associations between SEP, MRI brain markers and cognition. Structural equation modeling tested mediation. Results indicated that higher SEP was associated with higher grey matter volume, lower white matter volume, and higher structural connectivity. Structural brain damage and lower structural connectivity were associated with lower cognition. Both higher early-life and later-life SEP were associated with higher cognition. The association of early-life SEP with later-life cognition was partly mediated by later-life SES (73.8%). However, the extent to which the association of SEP with cognition could be explained by structural brain damage or connectivity was marginal (up to 5.9%). More research is needed to investigate alternative pathways that can explain the associations between SEP with later-life cognition.

Abstract citation ID: igad104.0228

### EDUCATION AS A RISK FACTOR OF MILD COGNITIVE IMPAIRMENT—THE ROLE OF THE GUT MICROBIOME

Matthias Klee[1], Velma Aho[2], Patrick May[2], Rejko Krueger[2], Paul Wilmes[2], and Anja Leist[1], *1. University of Luxembourg, Esch-sur-Alzette, Luxembourg, 2. Luxembourg Centre for Systems Biomedicine, Esch-Sur-Alzette, Luxembourg*

**Background:** Social determinants of health relate to an individual's risk of MCI and dementia. However, pathways from identified modifiable risk factors such as education are not well understood, yet. While previous findings suggest distinct taxonomic signatures of the gut microbiome in dementia and MCI patients, and further education linked to its composition, we sought to test the possibly mediating role of the gut microbiome in the relationship between education and MCI. Method: Gut microbiome composition was ascertained with 16S rRNA gene amplicon sequencing. MCI classification was based on the Montreal Cognitive Assessment. Education in years was grouped (0-10, 10-16, 16+). Mediation analysis was conducted decomposing direct and indirect effects of education on MCI mediated by gut microbiome diversity (Chao1, Inverse Simpson, Shannon) or individual species at Genus level (ldm-med, permanova-med). Differential abundance analysis across education groups was conducted (ANCOM-BC, DESeq2). Result: After exclusion of participants with PD, below age 50, or with missing data, n=256 participants (n=58 with MCI) of the Luxembourg Parkinson's Study were eligible for analysis (M[SD] Age=64.7[8.3] years). Education (16+ compared to 0-10 years of education) had a natural direct effect of NDE=0.36 (P<.01) on MCI, Chao1 included as mediator. We did not find significant mediation by gut microbiome composition or individual species.

**Conclusion:** Our findings indicate direct effects of education not mediated by the gut microbiome. Taxonomic analysis suggests a signature linked to lower risk of dementia in higher educated individuals. Longitudinal research is needed to investigate associations over time.

Abstract citation ID: igad104.0229

### HEARING LOSS AS A MODIFIABLE RISK FACTOR FOR DEMENTIA IN UK BIOBANK

Jure Mur[1], and Anja Leist[2], *1. The University of Edinburgh, Edinburgh, Scotland, United Kingdom, 2. University of Luxembourg, Esch-sur-Alzette, Luxembourg*

Age-related hearing loss is a predictor of cognitive impairment and dementia. The mechanisms of the observed associations are unclear but could involve depletion of cognitive reserve, the occupation of cognitive resources, and lack of sensory input to the brain. Hearing loss may therefore represent a preventable or treatable causative risk factor for dementia. Indeed, many studies report on lower rates of cognitive decline among hearing aid users when compared to non-users. However, most investigations to date have been observational or with relatively short follow-ups, thus precluding clear interpretations of the underlying mechanism. In our study using UK Biobank data with n=502,386 participants followed up from ~2008 to 2021, we emulate a randomised clinical trial to probe the role of initiating the use of hearing aids in people with conductive hearing loss or with self-reported hearing difficulties in the diagnosis dementia. Diagnosis of dementia was ascertained through hospital and death records, and self-reports in such a way as to increase the positive predictive value. Among participants with hearing loss or hearing difficulties, 2,914 (2.1%) were diagnosed with dementia, compared to 4,982 (1.4%) in those without hearing loss or hearing difficulties. Additional results on the relationship between hearing aid use and dementia, and stratified analyses by sex/gender and ethnicity will be presented. We discuss the modifiability of hearing loss and recommend studies with longer follow-ups to determine the efficacy of hearing loss interventions in reducing the risk of cognitive decline and dementia.

Abstract citation ID: igad104.0230

### PROTECTING COGNITION AND INCREASING EQUITY THROUGH "COGNITIVE IMPACT ASSESSMENTS"

Anja Leist[1], Ariane Bertogg[2], and Skerdi Zanaj[3], *1. University of Luxembourg, Esch-sur-Alzette, Luxembourg, 2. Ludwig-Maximilians-Universitaet Muenchen, Munich,*

*Bayern, Germany, 3. University of Luxembourg, Luxembourg, Grevenmacher, Luxembourg*

Today's societies are characterized by increasing complexity, in which unimpaired cognitive performance is vital to navigate and participate, as well as to retain independence at older ages. Addressing the challenge of improving population brain health has mostly been considered from a dementia risk reduction perspective as identified by the Lancet Commission on dementia. However, risk factors with immediate harm to cognitive functioning, such as shift work or insufficient sleep are currently not explicitly accounted for. As exposure to these risk factors accumulates across the life course, we can expect substantial consequences for later-life brain health. We argue that, in policy and practice, systematic measures should be introduced to protect individuals from harms to their everyday cognitive functioning, and enable them to optimize cognitive performance throughout the life course. On behalf of the international multidisciplinary BRAINHEALTH-POLICY group, we explore the concept of a 'cognitive footprint' of policies with exemplars from current legislation and policy that either implicitly affect cognition, or explicitly address effects on individuals' cognition. We suggest recommendations for systematic pre-intervention 'cognitive impact assessments' in fields such as medicine, where drug trials should include cognition as important secondary endpoint, and work, where labour laws should explicitly account for possibly harmful effects of certain work conditions to cognition. Besides immediate protective effects, we expect 'cognitive impact assessments' to have potential for long-term cognitive benefits on a population level as well as improve brain health equity by enabling individuals to reach optimum cognitive performance, with associated wider health benefits.

### SESSION 1230 (SYMPOSIUM)

Abstract citation ID: igad104.0231

### COGNITION MEASURES AND FINDINGS IN THE NATIONAL SOCIAL LIFE, HEALTH, AND AGING PROJECT

Chair: Linda Waite Discussant: Dawn Carr

Good cognitive function is an important component of health at any age. Certain domains of cognition tend to decline with age and rates of change vary dramatically across individuals and across social groups. This Symposium examines a commonly-used clinical measure of cognition, the Montreal Cognitive Assessment, adapted for survey use (MoCA-SA) and administered in two rounds of the National Social Life, Health and Aging Project (2010 and 2015). It focuses especially identifying and evaluating differential functioning of the MoCA-SA across racial and ethnic groups, across modes of administration of the measure, within intimate dyads and as linked to sensory function. Iveniuk and colleagues examine race difference and find that 7 measures, out of the 18 used in NSHAP's MoCA, formed a scale that was more robust to racial bias and suggest use of this modified measure to compare racial groups. Piedra and coauthors construct an abbreviated MoCA-SA (Spanish version) that compared favorably with the long form MoCA across the different grouping and showed predicative validity with consequential outcomes associated with cognitive decline. Pudelek and colleagues examine mode of assessment. web, phone, and PAPI or in-person interviews and describe an analytic strategy for obtaining a measure comparable across modes. Meiyi Li and Yiang Li find that women's cognitive impairment adversely affects their partner's social connectedness but husband's impairment does not. Zhong et al find that odor identification was associated with some domains of cognition with differences in that association by age, gender, or race, but not by education.

Abstract citation ID: igad104.0232

### RACE/ETHNICITY AND THE MEASUREMENT OF COGNITION IN NSHAP: RECOMMENDATIONS FOR ROBUSTNESS

James Iveniuk[1], Haena Lee[2], Selena Zhong[3], Jocelyn Wilder[3], Gillian Marshall[1], Boyle Patricia[4], Lissette Piedra[5], and Alicia Riley[6], *1. NORC at the University of Chicago, Chicago, Illinois, United States, 2. Sungkyunkwan University, Seoul, Republic of Korea, 3. NORC, Chicago, Illinois, United States, 4. Rush Alzheimer's Disease Center, Chicago, Illinois, United States, 5. University of Illinois at Urbana-Champaign, Urbana, Illinois, United States, 6. UC Santa Cruz, Santa Cruz, California, United States*

In this study, we interrogate measurement of cognition by race, in order to move towards a less-biased and more-inclusive set of measures for capturing cognitive change and decline in older adulthood. We use data from Round 2 (N=3377) and Round 3 (N=4777) of the National Social Life Health and Aging Project (NSHAP), and examine the study's version of the Montreal Cognitive Assessment (MoCA). We employ exploratory factor analyses to explore configural invariance by racial/ethnic group (Non-Hispanic White, Non-Hispanic Black, Hispanic, All else), and then log-likelihood tests of scalar and metric invariance. Using modification indexes we identify items that seem robust to bias by race. We test the predictive validity of the full (18-item) and short (7-item) scales using self-reported dementia diagnosis, Instrumental Activities of Daily Living (IADLs), proxy reports of dementia death, and National Death Index (NDI) reports of dementia death. We found that 7 measures, out of the 18 used in NSHAP's MoCA, formed a scale that was more robust to racial bias. The shortened form predicted consequential outcomes equally well, compared to NSHAP's full MoCA. The short form was also highly correlated with the full form, and displayed lower test-retest correlation between Round 2 and 3. Although sophisticated structural equation modeling techniques could be useful for assuaging measurement invariance by race in NSHAP, the shortened form provides a quick way for researchers to carry out robustness checks – to see if the disparities and associations they document are 'real' or the product of artifactual bias.

Abstract citation ID: igad104.0233

### USING THE MOCA-SA WITH SPANISH-SPEAKING OLDER ADULTS: STRENGTHS, LIMITATIONS, AND CORRECTIONS

Lissette Piedra[1], James Iveniuk[2], Kelly Pudelek[2], Melissa Howe[2], and David Marquez[3], *1. University of Illinois at Urbana-Champaign, Urbana, Illinois, United States*, *2. NORC at the University of Chicago, Chicago, Illinois, United States*, *3. University of Illinois Chicago, Chicago, Illinois, United States*

Exploratory factor analyses using Round 2 and 3 data reveal discrepancies in the MoCA scores of English and Spanish speakers. In this paper, we describe the strengths and limitations of using NSHAP's state-of-art MoCA measure with Spanish speakers and propose a solution to the discrepancies observed between the two linguistic groups. To arrive at our remedy, we calculated modification indexes of Spanish language MoCA items to identify items that remained consistent across ethnoracial language groups. From these items, we constructed an abbreviated MoCA-SAA (Spanish version) that compared favorably with the long form MoCA-SA across the different grouping and showed predicative validity with consequential outcomes associated with cognitive decline.

Abstract citation ID: igad104.0234

### COGNITIVE IMPAIRMENT AND SOCIAL CONNECTEDNESS: A DYADIC ANALYSIS OF OLDER COUPLES

Meiyi Li[1], and Yiang Li[2], *1. University of Wisconsin-Madison, Madison, Wisconsin, United States*, *2. University of Chicago, Chicago, Illinois, United States*

Sociological research extensively identifies social connectedness as an important predictor of cognitive health. However, few examine how social connectedness will in turn be affected by cognitive health, especially in the dyadic context. Using couple data from Waves 2 and 3 of the National Social Life, Health, and Aging Project (NSHAP), this study investigates the gendered dyadic link between cognitive impairment and social connectedness. Results from the actor–partner interdependence models show that women's cognitive impairments negatively affect their socialization. In terms of partner effects, women's cognitive impairments make negative impacts on men's number of friends and neighborhood participation. However, women's social connectedness will not be affected by men's cognitive impairment. Given the interdependence of older couples, this study points to the importance of understanding cognitive impairment in dyadic contexts. Results also call for dyadic interventions to prevent the negative effects caused by cognitive decline in late life.

Abstract citation ID: igad104.0235

### ASSESSING HOW AGE, GENDER, RACE, AND EDUCATION AFFECT THE RELATIONSHIPS BETWEEN COGNITIVE DOMAINS AND OLFACTION

Selena Zhong[1], Kristen Wroblewski[2], Edward Laumann[2], Martha McClintock[2], and Jayant Pinto[2], *1. NORC, Chicago, Illinois, United States*, *2. University of Chicago, Chicago, Illinois, United States*

The associations between cognitive domains and odor identification are well-established, but how sociodemographic variables affect these relationships is less clear; using the survey-adapted Montreal Cognitive Assessment instrument (MoCA-SA), we assess how age, gender, race, and education shape these relationships. First, we used two different methods, cluster analysis and multidimensional scaling, to empirically derive distinct cognitive domains from the MoCA-SA since it is unclear whether the MoCA-SA can be disaggregated into cognitive domains. We then used ordinal logistic regression to test whether these empirically derived cognitive domains were associated with odor identification and how sociodemographic variables modified these relationships. We identified five out of the six theoretical cognitive domains, with the language domain unable to be identified. We found that odor identification was associated with episodic memory, visuospatial ability, and executive function. Stratified analyses by sociodemographic variables reveal that the associations between some of the cognitive domains and odor identification varied by age, gender, or race, but not by education. These results suggest that 1) the MoCA-SA can be used to identify cognitive domains in survey research and 2) the performance of smell tests as a screener for cognitive decline may potentially be weaker in certain subpopulations.

Abstract citation ID: igad104.0236

### MEASURING COGNITION IN NSHAP USING MULTIMODE DATA COLLECTION

Kelly Pudelek[1], Henrique Ochoa Scussiatto[2], L Philip Schumm[2], Kristen Wroblewski[2], Selena Zhong[1], and Meiyi Li[3], *1. NORC at the University of Chicago, Chicago, Illinois, United States*, *2. University of Chicago, Chicago, Illinois, United States*, *3. University of Wisconsin-Madison, Madison, Wisconsin, United States*

The National Social Life, Health, and Aging Project (NSHAP) is a U.S. nationally representative, omnibus study of social relationships and health among home-dwelling older adults. Cognitive function is a key focus, both as an outcome and also because it is implicated in mechanisms by which social relationships affect and are affected by health trajectories. Data collection started in 2005, and respondents have been re-interviewed in 2010, 2015, and 2022–3. Starting in 2010, NSHAP administers a survey-adapted version of the Montreal Cognitive Assessment (MoCA-SA). While Rounds 1–3 were conducted in the home, Round 4 utilized three remote modes (web, phone and PAPI, in that order) in addition to in-home interviews. Respondents were assigned to remote data collection initially if they were judged to be likely to respond based on prior information, otherwise they were assigned to in-home; a random subset of 400 initially assigned to remote were shifted to the in-home group to facilitate examination of possible mode effects on response. Each mode necessarily utilized a slightly different subset of the MoCA-SA items based on feasibility; these were augmented by items from the Rush Alzheimer's Disease Center's (RADC) cognition battery that could be administered via the web or PAPI. This presentation will describe our analytic strategy for harmonizing across modes and generating an overall measure of cognitive function, as well as preliminary results for Round 4. Our approach utilizes a bi-factor item response model calibrated to each mode, and is applicable to data from other studies.

### SESSION 1235 (SYMPOSIUM)

Abstract citation ID: igad104.0237

### CHALLENGES AND OPPORTUNITIES OF (RE) CONNECTING AFTER COVID-19

Chair: Amy Rauer Co-Chair: Katherine Fiori

Although social distancing and other COVID-19 precautions were designed to protect older adults, the resultant disruptions to social connections were considerable. As vaccines became widely available, however, older adults were able to reconnect and resume social activities, a process we refer to in this symposium as social reintegration. The following studies highlight the challenges and opportunities associated with this reintegration process. First, Fuller et al.'s longitudinal, mixed-methods study considers how older adults' coping strategies changed over the course of the pandemic as social distancing precautions were lifted. Building off this paper, Rauer et al. explore how and with whom older adults stayed occupied while reintegrating, and how activity profiles changed across six months. Using the same dataset to investigate the nature of these social interactions and the role of personality, Fiori et al. examine how positive and negative social exchanges and optimism predict changes in older adults' attitudes about how COVID-19 shaped their family life. The role of individual differences is underscored by So et al.'s study of the surprisingly dynamic links between older adults' generativity and their mental health as they reintegrated over six months. The transitory nature of this reintegration period is further highlighted by Marini et al., who found that older adults anticipated a wide variety of stressors and rewards over this time that changed with the emergence of new COVID-19 variants. Together, these papers paint a compelling picture of how older adults navigate times of transition to reconnect to others and establish a "new normal".

Abstract citation ID: igad104.0238

### BURSTING THE BUBBLE? CHANGES IN OLDER ADULTS' LEISURE WITH OTHERS DURING THE PANDEMIC

Amy Rauer[1], Meagan Stewart[1], Katherine Fiori[2], Christina Marini[2], and Jeremy Kanter[3], *1. University of Tennessee, Knoxville, Tennessee, United States*, 2. *Adelphi University, Garden City, New York, United States*, 3. *Child and Family Studies, Knoxville, Tennessee, United States*

After months of social distancing, widespread vaccine availability gave hope to older adults that they could resume social activities. However, as new variants emerged, it was not always clear how safely older adults could connect with others. Thus, the current study examined how older adults' leisure with others changed as they reintegrated using a sample of 136 older adults in the U.S. (Mage = 68, range: 50-91; 69% females; 93% White; 72% partnered; 61% retired; 88% living independently). Participants reported on leisure at three time points (Summer 2021; Fall 2021; Winter 2022) using a modified version of the Pittsburgh Enjoyable Activities Scale (Pressman et al., 2009). Repeated measures analyses revealed that although there were no differences in the total amount of leisure individuals engaged in over time, with whom they did these activities differed both across time and activities. Participants were most likely to do activities with romantic partners at all three waves, followed by activities alone. Leisure with friends and family was least likely. Although leisure alone and with friends remained stable over time, leisure with a romantic partner and with family declined from T2 to T3, when COVID-19 cases were peaking. Interestingly, declines were seen in both more traditionally social activities (e.g., taking trips, sports) and those usually considered more solitary (e.g., computer use, chores). Despite both rising COVID-19 rates and inconsistent rhetoric on social distancing, older adults were still able to do things they enjoyed, though their ability to do so with loved ones appeared to wane.

Abstract citation ID: igad104.0239

### WHO BECAME CLOSER TO FAMILY AFTER THE PANDEMIC?: THE ROLES OF OPTIMISM AND SOCIAL EXCHANGES

Katherine Fiori[1], Amy Rauer[2], and Christina Marini[1], *1. Adelphi University, Garden City, New York, United States*, 2. *University of Tennessee, Knoxville, Tennessee, United States*

Dispositional optimism has been associated with healthy aging, as well as coping with and making meaning from life challenges. Social support plays a similarly positive role in promoting adaptation in late life and helping individuals feel closer to loved ones. During the pandemic, disruptions in older adults' social interactions placed a high demand on both of these potential resources, challenging individuals' ability to derive meaning from these events. Exploring data from a community sample of 136 older adults (M age = 68, range 50-91; 69% females; 93% White) assessed three times over six months during 2021-2022, we examined how optimism and positive and negative social exchanges predicted changes in attitudes about how COVID-19 shaped family life. We found that T1 optimism predicted changes in older adults' feeling closer to their families and appreciating them more at T2 (3 months later), above and beyond T1 positive social exchanges, which were only marginally associated. Older adults who were more optimistic at T1 also reported greater declines in tension and strain in their family ties due to the pandemic at T2 - but not at T3 (6 months later). However, T2 negative social exchanges did predict increased reports of family tension and strain from T2 to T3. Findings suggest that as new COVID-19 variants emerged, benefits of optimism waned, but costs of negative social exchanges increased. These findings illustrate how personality, micro-level context (i.e., social interactions), and macro-level context may work together to shape older adults' views of their families during the pandemic.

Abstract citation ID: igad104.0240

### THE COSTS OF GENERATIVITY FOR OLDER ADULTS' MENTAL HEALTH DURING THE COVID-19 PANDEMIC

Christine So[1], Katherine Fiori[2], Amy Rauer[3], and Christina Marini[2], *1. Adelphi University, Long Island City, New York, United States*, 2. *Adelphi University, Garden City, New York, United States*, 3. *University of Tennessee, Knoxville, Tennessee, United States*

Generativity, the capacity to be productive, caring, and concerned with the well-being of the next generation, promotes positive mental health and posttraumatic growth in later life (Bellizzi, 2004). Generativity may be particularly important in recent years as older adults have faced the pandemic and its aftermath. For example, our previous work showed that generativity was associated with better mental health concurrently during this initial post-vaccine adjustment period (June - September 2021; So et al., 2022). The current study builds upon this work by using longitudinal evidence from a community sample of 95 older adults (M age = 68.4, range 50-91; 68% females; 93% White) to explore whether generativity predicted older adults' anxiety, depressive symptoms, and life satisfaction six months later (December 2021-February 2022). Using structural equation modeling controlling for age, gender, and baseline depressive symptoms, we found that generativity positively predicted depressive symptoms six months later. In contrast, generativity at baseline was not associated with anxiety or life satisfaction six months later. It is possible that older adults who were more generative at baseline (when many still believed that COVID-19 pandemic would end with vaccinations) reported more depressive symptoms six months later when the highly contagious Omicron variant took over. Our findings thus underscore the complexity of the role of generativity in mental health outcomes, with associations sensitive to historical events.

Abstract citation ID: igad104.0241

### FORECASTING THE FUTURE: OLDER ADULTS' ANTICIPATED STRESSORS AND REWARDS IN THE CONTEXT OF COVID-19

Christina Marini[1], Erin Basinger[2], Meagan Stewart[3], Katherine Fiori[1], and Amy Rauer[3], *1. Adelphi University, Garden City, New York, United States*, 2. *University of North Carolina at Charlotte, Charlotte, North Carolina, United States*, 3. *University of Tennessee, Knoxville, Tennessee, United States*

Stress research typically focuses on past/current events as opposed to future ones. We identified future anticipated stressful and rewarding events at 2 timepoints utilizing qualitative data from older adults (N = 103, Mage = 68.11, 68.9% female, 93.2% white) who participated in a study of social reintegration after vaccines were available in the US. COVID-19 cases were typically below 1 million during T1 (Fall 2021), but exceeded 5 million during T2 (Winter 2022), coinciding with the Omicron variant. This afforded the opportunity to examine anticipated stressors and rewards at timepoints with different levels of COVID-19 threat. We used content analysis to identify anticipated stressors and rewards, which fit into 13 categories (Cohen's κ = .86-.96) and examined rates of ambivalence (i.e., same event was a stressor and reward within timepoints) and chronicity (i.e., same stressor or reward was named across timepoints). At T1, the modal stressor was own transition/change (13.6%; e.g., relocating). At T2, it was the pandemic (20.4%; e.g., family member recovering from COVID). Family/friend relationships (e.g., celebrating grandkids' birthdays) was the modal reward at T1 (32%) and T2 (24.3%). A quarter of participants demonstrated ambivalence (T1 = 23.3%; T2 = 28.2%). Stressor and reward chronicity (22.3% and 32%, respectively) were low. Findings highlight variability in the events older adults were concerned and excited about within and across timepoints. Salience of the pandemic as an anticipated stressor ebbed and flowed alongside emerging variants, despite vaccine availability.

Abstract citation ID: igad104.0242

### PATTERNS AND THEMES OF OLDER ADULTS' COPING ACROSS TWO YEARS OF THE COVID-19 PANDEMIC

Heather Fuller, Andrea Huseth-Zosel, Emily Kinkade, Bryce Van Vleet, and Melisa Hajdar, *North Dakota State University, Fargo, North Dakota, United States*

Throughout the COVID-19 pandemic, older adults developed coping strategies to adapt to the necessary social distancing precautions intended to protect from severe illness. However, over time, especially as vaccines became available, their need and ability to adapt and cope shifted. This longitudinal, mixed-methods study investigates whether older adults' perceptions of coping changed across the first two years of the pandemic and how older adults described their experiences with coping at different points during this time. Between April 2020 and June 2022, five waves of interviews were conducted with 76 Midwestern older adults between the ages of 70 and 97. At each timepoint, participants rated their level of perceived coping, quality of life, and stress. They also answered several open-ended questions about their current daily life and experiences during the pandemic. Repeated-measures ANOVAs indicated participants' perceived coping and quality of life significantly increased between the first and final interview. Thematic coding of interview transcripts identified four themes of coping across two years of the pandemic: 1) staying occupied, 2) seeking social support, 3) cultivating an adaptive mindset, and 4) future planning. Theme meaning shifted once vaccines were available, as participants adapted to 'a new normal' lifestyle, gained realistic perspectives, and appreciated their own resilience. Findings suggest older adults had nuanced and shifting coping experiences throughout the initial two years of the pandemic, but overall coped by drawing on life experiences. Our discussion will highlight variability in older adults' coping over time and directions for future study and practice.

### SESSION 1240 (SYMPOSIUM)

Abstract citation ID: igad104.0243

### PREDICTORS OF HOSPICE AND END-OF-LIFE CARE: GEOGRAPHIC LOCATION, PATIENT PORTALS, NURSING HOME CARE, AND HOME HEALTH

Chair: Jennifer Portz Discussant: Karla Washington

Hospice is considered an indicator of high-quality care at end-of-life (EOL) often allowing patients to receive care aligned with their values, in their homes, surrounded by support and loved-ones. However, there is a need to address access barriers to hospice and enroll patients earlier in their disease trajectory. Understanding predictors of hospice use will help identify strategies for addressing hospice inequities, promoting longer hospice stays, and improving overall EOL outcomes. Focusing on research methodologies with large cohorts and national samples, this symposium examines the associations of health care services and inequalities on key EOL outcomes and hospice use. First, Autumn Decker will describe profiles of hospice use and EOL experience by geographic location and race from the National Health and Aging Trends Study. Next, Dr. Jennifer Portz will summarize the association between types of patient portal use and EOL outcomes including hospitalization, advance directives, and hospice use. Dr. Ellis Dillon will follow with a description of hospice use and short hospice length of enrollment for Conneticut's Medicaid decedents, comparing decedents with nursing home stays to those without. Lastly, Dr. Olga Jarrin will present findings from her study of home health care use during the last 3 years of life as a predictor of death with hospice in a Medicare cohort. Our discussant, Dr. Karla Washington, will lead us in a conversation outlining the role of health care services, such as home health, nursing home care, and patient portals, for promoting access to hospice earlier and for all patients.

Abstract citation ID: igad104.0244

### ASSOCIATION BETWEEN PATIENT PORTAL ACTIVITIES AND END-OF-LIFE OUTCOMES

Jennifer Portz[1], Shaowei Wan[1], John Powers[2], and Ted Palen[2], *1. University of Colorado, Aurora, Colorado, United States*, *2. Kaiser Permanente Colorado, Aurora, Colorado, United States*

Examining end-of-life (EOL) patient outcomes associated with patient portal use will help identify benefits and barriers of portal use during EOL care. The primary objective of this retrospective cohort study was to assess the association between EOL outcomes and patient portal use among a cohort (N=6,517) of deceased patients (18+ years old) in the last 12 months of life within Kaiser Permanente Colorado (KPCO). Outcomes including 1) number of hospitalizations in the last 30 days of life, 2) having documented advance directive before death, and 3) dying in hospice were investigated by levels of patient portal use (no use, non-active use, active use without provider, and active use with a provider) for the first six months of the last year of life. A generalized linear model with Poisson distribution, a log link and an offset to account for person-days of follow-up was used to estimate rates of hospitalizations in the last 30 days. Logistic regression was used to estimate advance directive and death in hospice outcomes. Patient portal use appeared to be associated with beneficial EOL outcomes such as advance care planning ($P = 0.0226$) and dying in hospice ($P = 0.0070$). Hospitalizations were greater with higher levels of patient portal use ($P = 0.0492$), suggesting that patients/caregivers may have used patient portals for disease related information and accessing health services during times of need. More research is needed to leverage patient portal use to intentionally engage, educate and guide patients with serious illness and approaching EOL.

Abstract citation ID: igad104.0245

### HOSPICE USE AND END-OF-LIFE EXPERIENCE BY RACE AND GEOGRAPHY: A LATENT CLASS ANALYSIS

Autumn Decker, and Raven Weaver, *Washington State University, Pullman, Washington, United States*

Long existing inequalities near the end-of-life (EOL), including the death experience, have been uncovered because of increased attention during the COVID-19 pandemic. Hospice may be an avenue to equalize EOL experiences. This study aimed to 1) understand the profiles of hospice use and EOL experience in a national representative sample of decedents and 2) uncover how race and/or geographic location predict class membership. We conducted a latent class analysis with covariates using Waves 9-11 (n=1050) from the last month of life interview in the National Health and Aging Trends Study. Based on fit statistics, response probabilities, class separation, interpretability, and fit with theory, a four-class model was ultimately selected. The four latent classes were: well-rounded hospice care; no hospice, goal concordant care; no hospice, poor overall care; and complex hospice care. We found that geographic location was not a significant predictor of latent class membership but being non-White was significantly associated ($p < .001$) with 8.79 times higher odds of being in the no hospice, poor overall care group and 1.85 times higher odds of being in the no hospice, complex care group compared to the reference class (well-rounded hospice care). The current study's findings paint a complex picture of hospice use and EOL experience in a nationally representative sample. Hospice use alone does not make a "good death" experience, particularly for people of color. Rather, there are unique profiles of hospice use and experiences at EOL. Future research should explore the impact of geographic location on the death experience.

Abstract citation ID: igad104.0246

### LESS HOSPICE UTILIZATION AMONG CONNECTICUT MEDICAID DECEDENTS WITH NURSING HOME STAYS BEFORE DEATH

Ellis Dillon[1], Deborah Migneault[2], Noreen Shugrue[3], Dorothy Wakefield[3], Chae Man Lee[4], Doreek Charles[3], Bradley Richards[5], and Julie Robison[6], *1. UConn Health, Farmington, Connecticut, United States*, *2. UCONN Health, Farmington, Connecticut, United States*, *3. University of Connecticut, Farmington, Connecticut, United States*, *4. Univ of CT Health, Farmington, Connecticut, United States*, *5. State of Connecticut, Hartford, Connecticut, United States*, *6. UConn Health, Center on Aging, Farmington, Connecticut, United States*

Hospice use, and length of enrollment of over 7 days are both considered indicators of quality care at the end of life, but the potential impact of nursing home (NH) stays on these outcomes is not well understood. This study analyzes hospice use and short hospice enrollment (0-7 days) for decedents in Connecticut's Medicaid program who died between 2017 and 2020 and had a hospice-appropriate diagnosis. We compare individuals who had a NH stay within 60 days of their death to those who did not. Among 26,264 decedents meeting inclusion criteria, 14,227 (54.2%) had a NH stay within 60 days of death. Overall, 12,368 (47.1%) of all decedents received hospice care prior to death. Patients with a NH stay within 60 days of death were less likely than those without one to use hospice (44.3% vs 50.4%, $p<0.01$) (OR=0.78, (95% CI: 0.74, 0.82), $p<0.01$). Among those receiving hospice care, 4911 (39.7%) had short hospice enrollment. Patients with a NH stay within 60 days of death were 2.4 times more likely to have short hospice enrollment (OR=2.41 (95% CI: 2.24, 2.60), $p<0.01$) compared to those without. These findings suggest a need for further research into why individuals with NH stays are less likely to use hospice care and more likely to have shorter hospice enrollment. Comparing Medicaid decedents with NH stays prior to death to those living in the community may reveal disparities and areas that would benefit from interventions or policy change.

Abstract citation ID: igad104.0247

**THE IMPACT OF HOME HEALTH CARE UTILIZATION ON SUBSEQUENT HOSPICE USE AMONG MEDICARE BENEFICIARIES**

Olga Jarrín[1], Anum Zafar[1], Paul Duberstein[2], Bei Wu[3], Haiqun Lin[4], Hyosin Kim[5], Maria Lopez[1], and S. Marie Harvey[5], *1. Rutgers, The State University of New Jersey, New Brunswick, New Jersey, United States*, *2. Rutgers School of Public Health, Piscataway, New Jersey, United States*, *3. New York University, New York City, New York, United States*, *4. Rutgers, The State University of New Jersey, Newark, New Jersey, United States*, *5. Oregon State University, Corvallis, Oregon, United States*

Home health care may increase the use of hospice among Medicare beneficiaries by providing improved access to care, enhanced care coordination, and earlier identification of the need for hospice. This study evaluated home health care use during the last three years of life as a predictor of death in the care of hospice for a cohort of 2,169,496 Medicare beneficiaries who died in 2019. The cohort included beneficiaries continuously enrolled in Medicare for 5 or more years, aged 40 and older, and living in the U.S. fifty states, District of Columbia, U.S. Virgin Islands, or Puerto Rico at death. Co-variates included acute hospitalizations during the look-back period, age at death, sex, race/ethnicity, comorbidities, insurance, rural/urban area, and state of residence. Overall, 46% of beneficiaries used home health care during the lookback period, and 62% died at home. Among beneficiaries who used home health care, 59% died in the care of hospice, compared to 45% who never used home health care. Beneficiaries who received home health care at any point during the lookback period were more likely to later die at home (OR 1.10) or in the care of hospice (OR 1.07) compared to their counterparts without a history of using home health care. Additionally, persons with dementia had greater odds of home death (OR 1.21) and death with hospice (OR 1.68) compared to their counterparts without dementia. This study provides strong evidence of the value of home health care as a strategy to improve end-of-life care quality for Medicare beneficiaries.

### SESSION 1245 (SYMPOSIUM)

Abstract citation ID: igad104.0248

**INTERGENERATIONAL CONTACT AND THE BENEFITS FOR OLDER ADULTS**

Chair: Xin Zhang Co-Chair: Wanyu Xi Discussant: David Weiss

Intergenerational contact can be in a variety of forms, from day-to-day interaction between family members, such as filial piety expressed by the younger generation, to artificial contact in an on-line game context. No matter what form of intergenerational contact, it is believed that such contact should be beneficial for older adults for many reasons. For example, it can help to reduce feelings of loneliness, stress and provide a greater sense of purpose. It can also help to foster a greater appreciation for differences between generations and create stronger social connections with those of different ages. It can also boost mental health, physical health, and even reduce the risk of depression. In a group of 5 individual presentations, the authors showcased that intergenerational contact could promote life satisfaction in a 5-year-interval (Ayalon & Dikla), reduce ageist attitudes toward older adults in an on-line game context (Xi, Chang, Zhang, & Ayalon), foster positive self-perception of aging (Chang & Zhang; and Tse), as well as enhance health information-seeking behaviors (Zhao, Song, & Song). These results indicated that even from different cultural background (e.g., Chinese or Americans), a positive intergenerational contact, such as providing social support to aged parents, collaborating with partners from different age groups, and even offer kindness actions toward people of a different age group could indeed be beneficial. These studies provided theoretical insights for the intergenerational contact literature, and highlighted important practical implications for potential interventions to facilitate successful aging for older adults.

Abstract citation ID: igad104.0249

**SURROGATE HEALTH INFORMATION-SEEKING FOR OLDER ADULTS: INTERGENERATIONAL SUPPORT IN LIFE TRANSITION CONTEXTS**

Shijie Song[1], Yuxiang Zhao[2], and Xiaokang Song[3], *1. Hohai University, Nanjing, Jiangsu, China (People's Republic)*, *2. Nanjing University, Nanjing, Jiangsu, China (People's Republic)*, *3. Xuzhou Medical University, Xuzhou, Jiangsu, China (People's Republic)*

Due to a lack of experience with technology, many older Chinese rely on their younger family members to search for online health information. This study draws on life transition theory to explore the impact of family-bonded intergenerational support on surrogate health information-seeking (SHIS) during COVID-19. A semi-structured interview was conducted with 28 participants (Mean=32.45, SD=2.36) who had SHIS experiences with their older family members. Data were analyzed using qualitative thematic analysis. The results suggested that intergenerational support in SHIS during COVID-19 included: (1) provision of preventive and diagnostic health information, (2) provision of information on epidemic prevention policies and consultation, (3) guidance on the use of social media and Internet applications for older adults, and (4) communication of emotional support and hedonic information on daily life. Furthermore, intergenerational support in SHIS helps to promote reconstructed health information practices of older adults in life transitions and refine elders' meaning-making.

Abstract citation ID: igad104.0250

**THE EFFECT OF ONLINE IN-GAME INTERGENERATIONAL CONTACT ON AGEISM TOWARD THE OPPOSITE GENERATION**

Wanyu Xi[1], Yuanqing Chang[2], Xin Zhang[2], and Liat Ayalon[3], *1. Nanjing University of Chinese Medicine, Nanjing, Jiangsu, China (People's Republic)*, 2. *Peking*

*University, Beijing, Beijing, China (People's Republic)*, *3. Bar Ilan University, Ramat Gan, HaMerkaz, Israel*

Previous research indicated that online games represent a unique context of intergroup contact but may enhance prejudice toward outgroup players during gameplay. Despite the growing number of older gamers and increasing opportunities of online in-game intergenerational contact, few study have focused on the online in-game intergenerational contact and its effect on ageism. To address such research gap, we designed an online quiz game by manipulating in-game intergenerational contact type (competition vs. cooperation) and game outcome (win vs. loss). 148 young participants (Mage = 22.71, SD = 2.88) and 155 older participants (Mage = 65.4, SD = 4.23) were recruited and randomly assigned into the four experimental conditions to play with a virtual game partner of opposite generation. Positive and negative dimensions of age-stereotype perception, individual's cooperative orientation, and potential covariates were measured. Results showed significant intergenerational contact type × outcome × cooperative orientation effects on the perception of negative old-age-stereotype among younger participants and positive young-age-stereotype among older participants. Younger participants showed significant more negative old-age-stereotypes under competition loss (vs. win) condition, but older participants showed significant more positive young-age-stereotypes under cooperation loss (vs. win) condition. The findings indicated the negative impact of intergenerational competition among younger participants in the online game context, and also suggested the internalized self-ageism among older participants. Moreover, the boundary condition of personal factors revealed, that the effects of in-game intergenerational contact were only significant among those of lower (vs. higher) cooperative orientation. Our study also offered practical implications on designing gamified intergenerational-based intervention.

Abstract citation ID: igad104.0251

### KINDNESS INTERVENTION AND SELF-PERCEPTION OF AGING: AN INTERGENERATIONAL PERSPECTIVE

Dwight Tse[1], and Ewan Cahill[2], *1. University of Strathclyde, Glasgow, Scotland, United Kingdom*, *2. Glasgow Caledonian University, Glasgow, Scotland, United Kingdom*

Performing acts of kindness can enhance benefactors' well-being. However, few studies have examined its impact on older benefactors. This study aimed at evaluating this intervention among older adults and exploring "side benefits" beyond well-being. Specifically, given the negative stereotype on older adults as unproductive and incompetent, we hypothesized that acts of kindness could combat against older adults' internalized negative self-perceptions and enhance their self-concept. We conducted a randomized controlled experiment with 45 participants in the United Kingdom aged 61-80 (73% female). In the experimental condition, for 14 consecutive days, participants recalled up to three acts of kindness they did for others on that day and planned up to three acts of kindness on the next day, as opposed to listing up to three places they were at in the control condition. Surprisingly, our pre-test post-test comparison revealed no significant difference in well-being improvement between the two conditions. Compared to the control group, the experimental group showed an improvement in attitudes toward own aging, especially for their age-related cognitions in the gain (vs. loss) domain. Beneficiaries were mostly family members (34%), followed by community members (25%) and friends/colleagues (21%). For intergenerational acts of kindness, participants reported similar frequencies of younger and older beneficiaries (compared to their age) and provided predominantly emotional (vs. instrumental) support. Taken together, performing acts of kindness may help older adults counteract negative aging stereotypes and see themselves in a more positive lens as productive and competent members of society.

Abstract citation ID: igad104.0252

### MORE FILIAL PIETY FROM ADULT CHILDREN, MORE POSITIVE SELF-PERCEPTION OF AGING IN OLDER PARENTS?

Yuanqing Chang, and Xin Zhang, *Peking University, Beijing, Beijing, China (People's Republic)*

Filial piety, as a central concept in Confucianism, is about the ideas of how children should treat their parents. Previous studies on filial piety have focused on older generation's well-being. However, whether filial piety of adult children could influence their parents' self-perception of aging on a longitudinal dyad basis is largely unknown. Drawing on the Dual Filial Piety Model and the intergenerational contact perspective, we investigated the differential impacts of authoritarian vs. reciprocal filial piety of adult children on older parents' self-perception of aging using a longitudinal parent-child dyads dataset from a large population-based survey (i.e., Chinese Longitudinal Healthy Longevity Survey, CLHLS) from wave 2002 to wave 2005. Self-perception of aging of older parents was measured at two time points, authoritarian and reciprocal filial piety of adult children was measured at wave 2002. Latent change score modeling was performed on a 2387 parent-child dyads sample (Mage = 80.03, SD = 9.86 for parent; Mage = 48.54, SD = 8.10 for child) to test our research hypothesis. Results showed only reciprocal filial piety of adult child at wave 2002 could positively predicted their parents' latent change score of self-perception of aging 3 years later, after controlling for potential covariates. Our study suggested that the effects of adult children's filial piety might have different effects on parents' aging attitude, such that a reciprocal parent-child relationship rather than child's submission to parents could improve parents' attitude toward their own aging.

Abstract citation ID: igad104.0253

### A GENDERED PERSPECTIVE ON INTERGENERATIONAL RELATIONSHIPS IN THE SECOND HALF OF LIFE

Liat Ayalon, *Bar Ilan University, Ramat Gan, HaMerkaz, Israel*

Life satisfaction is defined as the perception of individuals concerning the level of meaning, fulfillment, and satisfaction in their life. It has shown to be an important indicator of older persons' perceived ability to pursue their life goals. The present study evaluated the relationship between older mothers' and fathers' perceptions of their relationship with their children and their life satisfaction four years afterwards.

We relied on the Health and Retirement Study, which is a representative sample of US citizens at the age of 50 and over. In total 1071 continuously married couples participated in both 2006 and 2010 waves and completed the leave behind questionnaire. Relying on path analysis, our findings show that fathers' life satisfaction in 2010 is associated with perceived support from their children in 2006. Mothers' life satisfaction in 2010 on the other hand, is associated with perceived support from their children as well as with lower levels of perceived strain in the relationship with their children in 2006. Results are discussed in relation to gender roles in the second half of life as manifested in intergenerational relationships.

### SESSION 1250 (SYMPOSIUM)

Abstract citation ID: igad104.0254

### EMBRACING THE QUEER ART OF FAILURE IN GERONTOLOGICAL RESEARCH AND EDUCATION

Chair: Sara Bybee Co-Chair: Austin Oswald Discussant: Vanessa Fabbre

Halberstam describes the queer art of failure as a performance of dissidence in which lesbian, gay, bisexual, transgender, and queer plus (LGBTQ+) people willing reject traditional conceptualizations of success. Yet, dominant theoretical frameworks in gerontology are predicated upon notions of success and productivity which may be problematic for understanding the life trajectories of LGBTQ+ people. The use of such heteronormative frameworks has implications for gerontological research in two important ways: 1) Research methods and researchers themselves may be constrained by the normative expectations placed on LGBTQ+ people and 2) Heteronormative frameworks obscure the nuance of LGBTQ+ older adults' lived experiences and may limit important contributions to gerontological knowledge. This symposium applies the queer art of failure to examine LGBTQ+ aging scholarship that deviates from traditional research and education. Speaker one shares experiences from LGBTQ+ individuals facing dementia, using concepts that counter framing dementia as pathology and decline. Speaker two discusses how participants' preferences for receiving research results via found poetry may reflect LGBTQ+ participants' natural inclination to question hegemonic norms. Speaker three describes collaborating with a coalition of LGBTQ+ older adults of color on a participatory action research study, detailing how epistemic tensions shaped the research in unexpected ways. Speaker four discusses how institutional failure led to enthusiasm for LGBTQ+ curriculum development, student mentoring, and knowledge production. These presentations suggest that the inclusion of diverse conceptualizations of success and productivity should inform future aging scholarship, as they may center the experiences of historically marginalized populations such as LGBTQ+ older adults.

Abstract citation ID: igad104.0255

### LIVING WITH DEMENTIA: EXPERIENCES OF LGBTQ PEOPLE WITH DEMENTIA AND SIGNIFICANT OTHERS

Anna Siverskog[1], and Linn Sandberg[2], *1. Södertörn University, Stockholm, Stockholms Lan, Sweden*, *2. Södertörn university, Huddinge, Stockholms Lan, Sweden*

Dementia is commonly conceptualized in biomedical terms as a deterioration of brain function and a subsequent loss of self. However, dementia scholars have increasingly underscored how the self of the person with dementia may be maintained through social interactions in their everyday life. This may pose specific challenges for LGBTQ people who may not be out in care settings due to fear of discrimination and whose chosen family may not be recognized by formal care providers. This paper presents preliminary findings from a qualitative study conducted in Sweden with LGBT individuals living with dementia. The project builds on policy analysis as well as semi-structured interviews with LGBTQ people with dementia, people significant to them such as partners, family and friends, and interviews with frontline care staff and managers within dementia care. The thematic analysis illustrates how experiences of living with dementia as an LGBTQ person vary depending on the progression and type of dementia, the individuals' social support network, how open one is with their sexual orientation or gender identity, living at home or in a dementia care facility, socioeconomics, and other factors. In line with how other ageing scholars have pointed to the potentiality of queer theory to move beyond framing dementia as pathology and decline, concepts of queer time and queer failure are used here to understand the results in ways that disrupt notions of self, temporalities, normativity, subjectivity and what it means to age successfully.

Abstract citation ID: igad104.0256

### PARTICIPANTS' PERSPECTIVES ON RECEIVING RESULTS USING FOUND POETRY: A MIXED-METHODS STUDY

Sara Bybee, Jacqueline Eaton, and Bob Wong, *University of Utah, Salt Lake City, Utah, United States*

Few researchers share study results with participants, often citing a lack of knowledge regarding how to do so. This study explored the dissemination preferences of study participants receiving results in the form of found poetry, developed from dyadic interviews with sexual and gender minority (SGM) and non-SGM couples' regarding their experiences with cancer. Participants (N=24) completed demographic and health questionnaires, a pre-posttraumatic growth-inventory-expanded (PTGI-X), and were randomized to receive the found poem as text, text and audio, audio, or video formats. Participants completed a post-PTGI-X, reported dissemination preferences, and emotions experienced. Open-ended responses demonstrated that using found poetry to disseminate results communicated that participant experiences were understood, fostered introspection, and renewed appreciation for their partner. Only SGM participants (n=3) reported preferring a format other than the one to which they were randomized. The text-only and combined text and audio formats evoked the greatest number of emotions (n=13 each) followed by video (n=4), and audio-only (n=3). Across randomization groups, interest (n=11) was most frequently reported, followed by sadness (n=8), and joy (n=6). Quantitatively, there were no significant changes in participants' PTG—a small sample size may have reduced statistical power—which might be seen as a failure. However, the open-ended responses indicate that innovative approaches to dissemination are acceptable and multiple options may increase accessibility. Only SGM participants preferred an alternate format, potentially reflecting a natural inclination to question the status quo. Including minority perspectives (positive/negative) illuminates the need to offer study participants choices in how and when they receive findings.

Abstract citation ID: igad104.0257

**OVERCOMING TENSIONS IN CULTURALLY RESPONSIVE RESEARCH WITH LGBTQ+ OLDER ADULTS OF COLOR**

Austin Oswald[1], Lujira Cooper[2], and Aundaray Guess[3], *1. University of Washington, Seattle, Washington, United States*, *2. Services and Advocacy for GLBT Elders, New York City, New York, United States*, *3. GRIOT Circle, Brooklyn, New York, United States*

Lesbian, gay, bisexual, transgender, and queer plus (LGBTQ+) older adults of color are underrepresented in gerontological scholarship due to structural factors that limit their research participation. This presentation applies critical reflexivity to weave together the narratives of a gerontological researcher, service provider, and older adult who co-constructed a research project committed to improving the visibility of LGBTQ+ older adults of color in gerontology scholarship. Through the lens of queer failure, we analyze personal narratives documenting our process designing and implementing a participatory action research project to uncover pivotal moments that shifted the course of the research in unexpected, yet critically important ways. Three themes emerged that illuminate key epistemic tensions associated with culturally responsive research with LGBTQ+ older adults of color, specifically navigating cross cultural tensions, intergenerational tensions, and pandemic related tensions. These findings highlight the limitations of traditional research methods which fail to account for historic and contemporary experiences that may shape perceptions of researchers and individuals' willingness to participate in research. Working through these tensions in solidarity contributed to the revision and improvement of traditional methods by developing culturally responsive approaches to research with LGBTQ+ older adults of color. Embodying a participatory, reflexive, and collaborative approach to inquiry has the potential to challenge and transform hegemonic scientific discourse that marginalizes certain communities. This presentation ends by offering implications for research that may support the development of culturally responsive methods for underrepresented communities in which future studies can expand upon, so long as the community is in the lead.

Abstract citation ID: igad104.0258

**FROM FAILURE TO OPPORTUNITY IN QUEERING GERONTOLOGICAL CURRICULUM**

Lauren Bouchard, *Portland State University, Portland, Oregon, United States*

Institutes of higher education vary tremendously in their support of LGBTQ students, which can contribute to unequal educational opportunities and outcomes. The presence of LGBTQ people in education can be inherently divisive, with some states and educational institutions prohibiting the inclusion of LGBTQ-related topics in the curriculum. LGBTQ students who experience discrimination in the classroom and heterosexism in the curriculum may be less motivated to complete program requirements, resulting in lower completion rates. Attrition of these marginalized students can lead to fewer qualified gerontologists who reflect the increasing diversity of the aging population. Additionally, the gerontological curriculum within and across programs may fail to capture nuanced and diverse narratives of resilience and lived experiences due to dominant, heteronormative educational contexts and frameworks. Through the lens of the queer art of failure, the presenter utilizes autoethnography (e.g., analysis of past writing and personal communications) as a queer person in a non-affirming gerontology graduate program. They will discuss how institutional failure lead to enthusiasm for curriculum development, student mentoring, and queer knowledge dissemination. Key findings include the significance of institutional clarity on academic freedom, the necessity of institutional support, and the solidarity of LGBTQ students, faculty, and older adults. The presenter will conclude with their future research directions and strategies to support students and early-career scholars to overcome political, institutional, and what are often felt as personal failures.

### SESSION 1255 (SYMPOSIUM)

Abstract citation ID: igad104.0259

**LONELINESS, SOCIAL ISOLATION, AND HEALTH WITHIN THE CONTEXT OF A GLOBAL PANDEMIC**

Chair: Eileen Graham Co-Chair: Shevaun Neupert
Discussant: Toni Antonucci

Social connectivity is a crucial human experience. The objective experience of being disconnected (i.e., social isolation) and the subjective feeling of being disconnected (i.e., loneliness) are related but distinct experiences that have unique impacts on physical, mental, and cognitive health. The COVID-19 pandemic forced much of the global population into a prolonged state of isolation, and many older adults found themselves abruptly cut-off from important sources of social interaction. The lasting impacts of the pandemic are still unknown. This symposium brings together several projects addressing key questions about longitudinal processes of loneliness and social isolation among middle aged and older adults, both prior to and during the COVID-19 pandemic. Jackson will present results from a coordinated data analysis (CDA) showing the extent to which loneliness, social isolation, and social asymmetry are related to MCI, dementia, mortality, and life expectancy. Rule will discuss the impact of stroke on trajectories of loneliness in a CDA. Zhang and Fingerman will present findings that show how loneliness and social isolation vary among individuals with functional limitations. Zavala will present results from a CDA that explored daily patterns of loneliness during the early months of the COVID-19 pandemic. Lastly, Beck will discuss a CDA that examined trajectories of social asymmetry prior to and during the COVID-19 pandemic. Antonucci will synthesize and discuss each project within the context of broader implications for empowering older adults. In sum, this symposium presents novel evidence showing that loneliness and social isolation in older adulthood are impactful predictors of health.

Abstract citation ID: igad104.0260

### THE IMPACT OF STROKE ON TRAJECTORIES OF LONELINESS

Payton Rule[1], Emily Willroth[1], Eileen Graham[2], Marjorie Nicholas[3], Robin Hattori[4], Tess Thompson[1], and Lisa Connor[4], *1. Washington University in St. Louis, St. Louis, Missouri, United States*, *2. Northwestern University, Chicago, Illinois, United States*, *3. MGH Institute of Health Professions, Boston, Massachusetts, United States*, *4. Washington University School of Medicine in St. Louis, St. Louis, Missouri, United States*

While prior research has identified loneliness as a risk factor for stroke, less is known about whether stroke may increase the risk of loneliness. We examined this question using three large representative panel studies of middle-aged to older adults in the U.S., Europe, and Israel. Specifically, we used harmonized data from the Health and Retirement Study (n=23,834, 10.3% stroke survivors), the English Longitudinal Study of Aging (n=15,779, 5.1% stroke survivors), and the Survey of Health, Aging and Retirement in Europe (n=106,480, 3.6% stroke survivors). Multilevel modeling was used to examine trajectories of loneliness across middle and older adulthood and the impact of stroke on trajectories of loneliness. Across all three samples, loneliness followed a quadratic trajectory, decreasing or remaining stable prior to age 60 and then increasing throughout older adulthood. In individuals who experienced stroke, loneliness increased more sharply after stroke onset. In follow-up analyses conducted within the subsample of stroke survivors, loneliness was higher post-stroke compared to pre-stroke. Moreover, the effect size suggests that the impact of stroke on loneliness trajectories is greater than the impact of normative developmental processes. In this talk we will discuss these results as well as the implications of this work. Specifically, these results highlight the need for loneliness prevention efforts in stroke survivors. Interventions targeting stroke survivors' social participation and autonomy may help prevent or decrease loneliness post-stroke. Additionally, programs aimed at educating stroke survivors' social network on ways to provide effective support may also help promote social connection in this population.

Abstract citation ID: igad104.0261

### A COORDINATED ANALYSIS OF THE ASSOCIATIONS BETWEEN LONELINESS, SOCIAL ISOLATION, AND THE COGNITIVE HEALTHSPAN

Kathryn Jackson[1], Tomiko Yoneda[2], Jing Luo[1], Daniel Mroczek[1], Anthony Ong[3], Andrew Steptoe[4], and Eileen Graham[1], *1. Northwestern University, Chicago, Illinois, United States*, *2. University of California, Davis, Davis, California, United States*, *3. Cornell University, Ithaca, New York, United States*, *4. University College London, London, England, United Kingdom*

Insufficient social connection in older adulthood is a growing public health concern. Current research suggests that loneliness and social isolation are related to consequential health outcomes in later life. There is a large and growing literature suggesting that loneliness and social isolation are associated with increased risk of dementia and mortality, but these outcomes typically have been considered separately and without controlling for competing risk factors. Using multi-state survival models which permit simultaneous estimation of transitions between several states within one model, the current study examined the extent to which loneliness, social isolation, and social asymmetry (defined as the discordance between loneliness and isolation) are associated with transitions between cognitive status categories (non-impaired, mildly impaired, severely impaired) and death. We estimated near-identical models individually across 11 independent long-term longitudinal studies (i.e., ELSA, HILDA, HRS, LISS, OCTO-TWIN, SATSA, SHARE, SHP, SOEP, MAP, MARS) using the coordinated data analysis methodological framework, representing a total sample size of over 140,000, and meta-analysis to provide precise estimates of the effects of loneliness, isolation, and social asymmetry on cognitive healthspan transitions. Preliminary findings suggest that loneliness is associated with increased risk of transitioning from non-impaired to mildly impaired cognitive functioning. Higher loneliness is associated with shorter survival time and fewer years in a healthy cognitive state. Lastly, we found that lower social asymmetry (i.e., social resilience) is associated with longer cognitive health span (more years in NCI). These results provide promising evidence suggesting that poor social relationships are robustly associated with a diminished cognitive healthspan.

Abstract citation ID: igad104.0262

### FUNCTIONAL LIMITATIONS, LIVING ARRANGEMENT, DAILY SOCIAL CONTACTS, AND LONELINESS IN LATE LIFE

Shiyang Zhang, *The University of Texas at Austin, Austin, Texas, United States*

Older adults with functional limitations are at a greater risk of social isolation and loneliness, largely due to difficulties in mobility and compromised social contacts. Lack of social contact throughout the day may contribute to loneliness among older adults with functional limitations, and these associations may differ by living arrangements. This study examined associations between functional limitations, living alone, daily social contacts, and loneliness throughout the day. Community-dwelling older adults aged 65 + (N = 310, Mage = 73.96) from the Daily Experiences and Well-being Study completed a measure of functional limitations in a baseline interview. Then, in ecological momentary assessments (EMA), they reported social contacts and loneliness every 3 hours for 5 to 6 days. Multilevel models revealed a significant interaction between functional limitations and social contacts on loneliness, such that people with higher levels of functional limitations felt less lonely if they had more social contacts in the prior 3 hours, but not for those with lower levels of functional limitations. The three-way interaction between functional limitations, living alone, and loneliness revealed that more social contacts were associated with reduced loneliness only among individuals with functional limitations and who lived with others.

Individuals with functional limitations who lived alone had higher levels of loneliness than those with functional limitations who lived with others. The findings emphasize the vulnerability in social isolation of older adults with functional limitations, especially those who live alone. Yet this vulnerable group may also benefit more from adequate daily social contacts.

Abstract citation ID: igad104.0263

### SOCIAL INTERACTIONS AND LONELINESS AMONG THE ADULT LIFESPAN: A COVID-19 COORDINATED ANALYSIS

Daisy Zavala[1], MacKenzie Hughes[2], Rita Hu[3], Sumbleen Ali[4], Reilly Kincaid[5], and Destiny Ogle[5], *1. Stony Brook University, Stony Brook, California, United States*, *2. Georgia Institute of Technology, Atlanta, Georgia, United States*, *3. University of Michigan, Ann Arbor, Michigan, United States*, *4. University of Connecticut, Storrs, Connecticut, United States*, *5. Purdue University, West Lafayette, Indiana, United States*

The COVID-19 pandemic included physical distancing mandates. Examining responses to isolation across the lifespan during the first year of the pandemic may shed light on age differences in social interactions and loneliness during a time of uncertainty. This coordinated analysis of 2 intensive longitudinal datasets (DCF, EAS) examines social interactions, loneliness, and age during the COVID-19 pandemic. It is a component of a larger, pre-registered coordinated analysis involving traditional longitudinal and cross-sectional studies (https://osf.io/jt4wf). DCF was a diary study of 221 adults ages 21-78 years (M = 48.92, SD = 14.81) who completed online questionnaires every day for 21 consecutive days. DCF participants were drawn from across the USA via MTurk and Qualtrics. Data collection spanned Oct-29-2020 to Nov-18-2020. EAS is an ongoing ecological momentary assessment measurement burst study; data from 82 adults aged 73-96 years (M = 79, SD = 5.41) who completed a 2 week burst of 4 EMA per day between Feb-1-2020 and July-1-2020 were used in this analysis. EAS participants were recruited from registered voter lists in Bronx, NY USA. Separate multilevel models (DCF: 2 level, EAS: 3 level) were conducted with the same covariates and constraints. Between and within individuals, social interactions did not predict loneliness in DCF; older age predicted lower loneliness. In EAS, individuals with more social interactions were less lonely, males reported higher levels of loneliness and relatively younger individuals had lower loneliness following interactions. Results illustrate the value of coordinated analysis for understanding loneliness under pandemic conditions.

Abstract citation ID: igad104.0264

### ALONE TOGETHER: SOCIAL ISOLATION AND LONELINESS ACROSS COVID-19

Emorie Beck[1], Anthony Ong[2], and Eileen Graham[3], *1. University of California, Davis, Davis, California, United States*, *2. Cornell University, Ithaca, New York, United States*, *3. Northwestern University, Chicago, Illinois, United States*

The COVID-19 pandemic has resulted in a worldwide increase in the number of adults who are socially isolated and experiencing high levels of loneliness. Although research has focused on social isolation (the lack of social contact) and loneliness (the perception of being alone), few longitudinal studies have examined isolation and loneliness together in the context of the COVID-19 pandemic. We introduce a novel index of resilience to loneliness (social asymmetry), which captures the degree of congruence or discordance between subjective feelings of loneliness and objective social contact (social isolation). Using seven longitudinal samples (Total N > 75,000) across three continents, we examined trajectories of social asymmetry across the onset of the pandemic using Bayesian multilevel discontinuous growth models. These allowed us to demonstrate differences in level and change of social asymmetry across the onset of the pandemic. Levels and change tended toward resilience over time but did not show significant changes across the onset of the pandemic. However, these results were not consistent across samples, indicating heterogeneity across countries, age groups, and more. We discuss how examining COVID-related changes in social asymmetry advances our understanding of (1) the extent to which broad societal challenges can induce short- and long-term impacts on trajectories of resilience and (2) what factors account for heterogeneity in adaptive responses to adversity.

### SESSION 1260 (PAPER)

### TECHNOLOGY RESEARCH AND APPLICATIONS

Abstract citation ID: igad104.0265

### A GOLD MINE OF TEXT TEXT MINING VERSUS MANUAL CODING METHODS REGARDING QUALITY OF CARE IN LONG-TERM CARE FOR OLDER ADULTS

Coen Hacking[1], Hilde Verbeek[1], Jan Hamers[2], and Sil Aarts[1], *1. Maastricht University, Maastricht, Limburg, Netherlands*, *2. CAPHRI, Maastricht University, Maastricht, Limburg, Netherlands*

In long-term care for older adults, large amounts of text are collected that cover the quality of care, such as transcribed interviews, medical records, and text in electronic health records. Researchers currently analyze textual data manually to gain insights, which is a time-consuming process. Text mining (TM) could provide a solution, as this methodology can analyze large amounts of text automatically. Therefore, this study aims to compare TM to the gold standard of manual coding regarding sentiment analysis and thematic content analysis. Data was collected from interviews with residents (n=21), family (n=20) and care professionals (n=20). TM models were developed and compared to the manual approach. The results of the manual and TM approach were evaluated in three ways: accuracy, consistency and expert feedback. Accuracy showed how similar the approaches are. Consistency showed if an individual approach finds the same themes in similar text segments. Expert feedback was used to show the perceived correctness of the TM approach. The accuracy analysis showed that 81.8% of text segments receive the same sentiment code in both approaches and 83.7% of text segments receive the same thematic codes. Interviews coded by TM had a higher consistency compared to those coded manually. Expert feedback showed that the TM had a limited understanding of the context. However, it also showed certain inconsistencies of manual coding. The current study has shown that TM can be an effective tool for quickly and accurately identifying sentiment and thematic content in large amounts of textual data.

Abstract citation ID: igad104.0266

### ASSESSING USABILITY OF A MEDICATION ADHERENCE SYSTEM FOR PERSONS WITH MILD COGNITIVE IMPAIRMENT

Renato Ferreira Leitao Azevedo[1], Timothy Hale[2], Teresa Warren[2], Eliza Baby[2], Jeannie Lee[3], Kathleen Insel[3], Wendy Rogers[4], and Raksha Mudar[2], *1. University of Illinois Urbana-Champaign, Urbana, Illinois, United States, 2. University of Illinois Urbana-Champaign (UIUC), Champaign, Illinois, United States, 3. The University of Arizona, Tucson, Arizona, United States, 4. University of Illinois Urbana-Champaign, Champaign, Illinois, United States*

Persons with mild cognitive impairment (PwMCI) are at risk of medication nonadherence due to prospective memory deficits. Over time, they are likely to experience accelerated deterioration in cognitive functions and may develop dementia when comorbid treatable health conditions, such as hypertension, are not well managed. A digital therapeutic system called Medication Education, Decision Support, Reminding, and Monitoring (MEDSReM©) has been designed for cognitively normal older adults to support hypertension medication adherence and is currently being tested in a randomized controlled trial. MEDSReM has the potential to support PwMCI with hypertension medication adherence, but it must be optimized specifically for this population. We conducted a cognitive walkthrough to identify usability issues and inform the redesign and optimization of the system for PwMCI. This usability technique is used to evaluate a system from a user's perspective and is typically conducted by subject matter experts. We had 12 individuals with interdisciplinary expertise in cognitive aging, cognitive impairment, hypertension management, nursing, human factors, and health technology participate in the cognitive walkthrough. Usability issues related to figure-ground contrast, text complexity, and flexibility related to decision making were identified. Overall, recommendations included (1) simplifying the user interface (e.g., color contrast/textual cues), (2) eliminating options related to medication taking actions (e.g., snooze, take later) to mitigate memory-related errors, and (3) providing more direct, prescriptive instructions to minimize decision-making demands. Based on these recommendations, MEDSReM is currently being redesigned for testing with PwMCI. Our approach can inform the design of other mobile health tools.

Abstract citation ID: igad104.0267

### HEALTH PROFESSIONALS' ATTITUDES TOWARD TECHNOLOGY AND ARTIFICIAL INTELLIGENCE: AN EXPLORATORY STUDY

Moon Choi, *Korea Advanced Institute of Science and Technology, Daejeon, Republic of Korea*

The growing number of older adults, particularly those living alone, demands innovative health and social care solutions for the aging population. Mobile apps and other technologies are emerging every year to enhance the efficiency and effectiveness of geriatric health professionals' work management. However, limited knowledge exists regarding geriatric health professionals' perceptions of technology. The acceptance of technology is critical because emerging technologies cannot be fully utilized in the field without a positive attitude towards accepting them. The current study aims to investigate geriatric health professionals' perceptions and attitudes towards technology and artificial intelligence (AI). The data were collected from a national survey of health professionals specialized in home visits conducted by community health centers in South Korea (N=1,026). Technology acceptance was evaluated using the Technology Acceptance Model Questionnaire, and study participants were asked about their self-reported knowledge of AI. A series of regression models showed that younger age, higher education level, and male gender were positively associated with higher technology acceptance levels. Regarding AI knowledge, only younger age was positively associated with better AI knowledge. The findings suggest the existence of a cohort difference in perceptions and attitudes towards technology among geriatric health professionals, which warrants further investigation into the underlying mechanism.

Abstract citation ID: igad104.0268

### SOCIAL MEDIA USE AND MENTAL HEALTH AMONG OLDER ADULTS WITH COMORBIDITIES

Long Chen[1], Zuoting Nie[1], Yun Jiang[2], and Rumei Yang[1], *1. Nanjing Medical University School of Nursing, Nanjing, Jiangsu, China (People's Republic), 2. University of Michigan, Ann Arbor, Michigan, United States*

Psychological distress is common and problematic for older adults with comorbidities. Social media has great potential to help older adults engage in meaningful social activities and relationships that might improve mental health. However, little information exists about the actual use and the beneficial effects of the use of social media on mental health among this particular population. Using data from the 2019-2020 Health Information National Trends Survey (HINTS), we aimed to describe the prevalence of social media use, and whether social media use is related to better self-care confidence and thus related to better mental health among U.S. older adults with comorbidities. Data were analyzed using weighted logistic models and mediation analyses that considered the complex survey design with jackknife replications. Variables were assessed by self-reports. The prevalence of social media use increased rapidly from 41% in 2017 to 54% in 2020 among U.S. older adults with comorbidities. Those who were of younger age, women, higher education, or higher income were more likely to use social media, whereas those who were Hispanic or Black were less likely to use social media. Social media use was related to better self-care confidence ($b=0.17$, 95% CI=0.07-0.26, $P< 0.001$) and is further related to better mental health ($b=1.07$, 95% CI=0.86-1.28, $P< 0.001$). Our results suggest that social media is a beneficial supplement to traditional sources of social support for mental health. However, more attention is needed to those who are not only affected by commodities but also socially disadvantaged (e.g., Hispanic or Black with lower education).

Abstract citation ID: igad104.0269

**THE MRI-COMPATIBLE STIMULATION SYSTEM TO CHARACTERIZE THE SUPRASPINAL CONTROL OF GAIT IN AGING: A SYSTEMATIC REVIEW**

Yinglu Hong[1], Dapeng Bao[1], Brad Manor[2], and Junhong Zhou[3], *1. Beijing Sport University, Beijing, Beijing, China (People's Republic)*, *2. Hebrew SeniorLife/Harvard Medical School, Amherst, New Hampshire, United States*, *3. Harvard Medical School/Hebrew SeniorLife, Roslindale, Massachusetts, United States*

The regulation of gait depends upon both peripheral and supraspinal elements. The characterization of these elements can provide insights into how aging and age-related conditions affect gait performance in older adults. Functional magnetic resonance imaging (fMRI) is one widely-used technique to sophisticatedly measure the supraspinal elements. However, people are required to stay motionless during MRI scan, it is thus challenging to characterize the supraspinal control of gait. To overcome this limitation, multiple MRI-compatible devices have been developed to apply the walking-related sensorimotor stimuli. Still, large variance in device design and observation exists, limiting their research applications. We here thus performed a thorough systematic review to comprehensively summarize the progress of this field. Seven electronic databases (e.g., PubMed) were searched. The information on device design, study protocol and neuroimaging observations of twenty-six studies using 13 types of devices was extracted. Specifically, three of these devices can induce somatosensory-only stimuli, eight for motion-only, and two for both types of stimuli. The results demonstrated that using these devices can successfully obtain valid and reliable fMRI data when participants experiencing sensorimotor stimulation. Multiple supraspinal regions, such as the sensorimotor regions, prefrontal cognitive and attention regions, and cerebellum were activated by the sensorimotor stimuli. The evidence level of these observations was still low due to small sample size of participants who were not explicitly screened. It is thus needed to further enhance the performance of these devices and to examine and confirm the findings via studies with more rigorous design (e.g., larger participant sample size).

### SESSION 1265 (SYMPOSIUM)

Abstract citation ID: igad104.0270

**SOCIAL DETERMINANTS OF HEALTH AND DEMENTIA: PUBLIC HEALTH PERSPECTIVES**

Chair: Joseph Gaugler Co-Chair: Lisa McGuire Discussant: Fayron Epps

The U.S. Department of Health and Human Services and its Office of Disease Prevention and Health Promotion define social determinants of health (SDOHs) as "the conditions in the environments where people are born, live, learn, work, play, worship, and age that affect a wide range of health, functioning, and quality-of-life outcomes and risks." Featuring presenters from the three national Building our Largest Dementia Infrastructure (BOLD) Public Health Centers of Excellence as well as other Centers of Disease Control and Prevention Healthy Brain Initiative programs, this symposium will examine how dementia influences, and is influenced by, SDOHs. Specifically, issues that require critical public health action, including prevention, early detection, and family caregiving will be examined within a SDOH framework. Transcending these concerns, this symposium will also feature a presentation that considers the complex associations between health equity, SDOHs, and dementia. We anticipate that this symposium and its presentations will facilitate and advance gerontology's understanding of dementia as a public health imperative, and as importantly, begin to identify how public health at the local, tribal, state, and federal levels can mobilize to address the challenges and opportunities of dementia.

Abstract citation ID: igad104.0271

**THE INTERSECTION OF SOCIAL DETERMINANTS OF HEALTH AND FAMILY CARE OF PEOPLE LIVING WITH DEMENTIA**

Joseph Gaugler[1], Soo Borson[2], Fayron Epps[3], Regina Shih[4], Lauren Parker[5], and Lisa McGuire[6], *1. University of Minnesota, Minneapolis, Minnesota, United States*, 2. *University of Southern California, Los Angeles, California, United States*, *3. Emory University, Atlanta, Georgia, United States*, *4. RAND Social and Behavioral Policy Program, Santa Monica, California, United States*, *5. Johns Hopkins University, Baltimore, Maryland, United States*, 6. *CDC, Atlanta, Georgia, United States*

In this presentation, we will synthesize current research to illustrate the intersection of social determinants of health (SDOHs) and Alzheimer's disease and related dementia (ADRD) caregiving, and outline how public health can support ADRD family caregivers in the U.S. There is an abundance of research that demonstrates family caregivers of relatives living with ADRD experience adverse health outcomes. In addition, family care for persons with ADRD overlaps with SDOHs. Public health actions such as improved surveillance and identification of ADRD caregivers; building and enhancing community partnerships; advancing dementia-capable health care and related payment incentives; and reducing the stigma of dementia and ADRD caregiving can enhance support and services for families. By engaging in these actions, public health practitioners could more effectively address the myriad of challenges facing ADRD caregivers most at risk for emotional, social, financial, psychological, and health disruption.

Abstract citation ID: igad104.0272

**SOCIAL DETERMINANTS OF HEALTH AND THE RISK FOR DEMENTIA**

Matthew Baumgart[1], Kischa Hampton[2], and Chelsea Kline[2], *1. Alzheimer's Association, New York, New York, United States*, *2. Alzheimer's Association, Chicago, Illinois, United States*

Social determinants of health (SDOH) play a role in the risk for dementia in two ways. First, certain SDOH may be direct risk factors for dementia. Second, SDOH contribute to the prevalence of many modifiable risk factors that in turn may elevate dementia risk – and act as barriers to addressing individually modifiable risk factors for dementia. To better understand how SDOH affect the risk for dementia and the role public health has in addressing dementia-related SDOH, the Public Health Center of Excellence on Dementia Risk Reduction (PHCOE-RR), in partnership with Wake Forest School of Medicine, recently synthesized the current state of the evidence. Following that synthesis, the PHCOE-RR conducted (1) a workshop where researchers and public health officials reviewed the science, and (2) a Public Health Roundtable where the public health community discussed the role of public health agencies in addressing dementia-related SDOH. This presentation will review the results of the evidence synthesis and discuss the work of the PHCOE-RR in helping state, local, and tribal public health agencies take action in their communities.

Abstract citation ID: igad104.0273

### THE IMPACT OF SOCIAL DETERMINANTS OF HEALTH ON DETECTING DEMENTIA: REFRAMING HEALTH EQUITY

Joshua Chodosh[1], and Soo Borson[2], *1. NYU Grossman School of Medicine, New York City, New York, United States*, *2. University of Southern California, Los Angeles, California, United States*

Social determinants of health (SDoH) contribute to the incidence of Alzheimer's disease and related dementia (ADRD) and to missed opportunities to reduce the impact of its symptoms. They also play a fundamental role in detection of ADRD, leading to disparities in diagnosis as well as care. Public health practitioners have an outsize role in addressing the intersection between ADRD prevalence and related SDoH, especially for persons from minoritized and under-resourced communities. We will delineate the effects of this intersectionality and the need to reframe dementia detection strategies to promote health equity, reduce related stigma, and repair the mistrust that is integral to care inequities. We will highlight the ongoing efforts of our public health partners who are committed to ensuring that dementia is detected before a crisis occurs and opportunities they offer to support the efforts of our PHCOE partners in dementia prevention and caregiving.

Abstract citation ID: igad104.0274

### DELIVERING BRAIN HEALTH EQUITY TO COMMUNITIES CHALLENGED BY SDOH

Stephanie Monroe[1], Daphne Delgado[2], and Stipica Mudrazija[3], *1. UsAgainstAlzheimer's, Washington, District of Columbia, United States*, *2. UsA2. Center for Brain Health Equity, Washington, District of Columbia, United States*, *3. Urban Institute, Washington, District of Columbia, United States*

Growing evidence suggests that dementia risk can be modified by brain healthy behaviors, including effective management of hypertension, diabetes, and obesity. In fact, the Lancet Commission released new research showing that managing a dozen risk factors could prevent or delay approximately 40 percent of worldwide dementia cases. Under-resourced communities are at a disadvantage in managing these risk factors however, due to systemic inequities in health and education, access to exercise opportunities, and to nutritious food. Social determinants of health (SDOH)—and policies that address them—must be part of the solution to ensuring better brain health for all American families. Ensuring that this hope and opportunity reaches all families regardless of zip code, income, race, or ethnicity will take greater commitment, investment, and strategies centered in health equity in public health and community-based initiatives. UsAgainstAlzheimer's will share what they have done to a) identify, adapt, and disseminate tailored messages to minority-serving communities and b) better equip providers to establish trust and reach those they seek to serve by: 1) better understanding the barriers, opportunities and challenges to reach and build trust in historically underrepresented populations; (2) developing tools, strategies and messages that resonate with these populations; and (3) showing how place matters when it comes to implementing effective public health strategies.

### SESSION 1270 (SYMPOSIUM)

Abstract citation ID: igad104.0275

### BENEFITS AND BARRIERS OF COMMUNITY-ENGAGED RESEARCH AND STRATEGIES FOR STRENGTHENING PARTNERSHIP PRACTICE

Chair: Carrie Leach Co-Chair: Rodlescia Sneed Discussant: Tam Perry

The meaningful engagement of older people in research may help to address their lag behind other groups in health outcomes and low participation in studies. This symposium addresses the critical gap in the aging literature and advances knowledge about community engagement by offering insight from experts on the complexities of partnership and engagement processes that vary by context as well as tools and tactics for overcoming obstacles. The first speaker will present findings and lessons gained from evaluating the needs of a self-organizing community advisory board of Black older adults to increase enrollment in a participant research pool. The second will focus on the challenges of centering a diverse range of partners in the design of a health and wellness program tailored to the needs of formerly incarcerated mid and late-life adults. The third speaker will provide recommendations for researchers interested in working with Indigenous communities and strategies for working collaboratively, and how to overcome relational challenges. The fourth speaker will present findings from a reflective evaluation process of a community-based participatory research partnership that evolved over seven years and discuss how the partnership evaluation tool can enhance partner practice. This symposium concludes with reflections about meaningful and authentic academic-community partnerships in line with supporting and building bridges between aspiring and existing community engaged scholars and older people who are experts on aging.

Abstract citation ID: igad104.0276

### IN THEIR OWN WORDS: DYNAMICS THAT CONTRIBUTE TO SUCCESSFUL AND SUSTAINED PARTNERSHIPS IN CBPR WITH OLDER PEOPLE

Carrie Leach, *Wayne State University, Detroit, Michigan, United States*

Though community-engaged and participatory approaches are increasingly relied on in studies, the inclusion of older adults as co-learners is uncommon. To address this problem, we offer insight into the dynamics that contribute to successful partnerships between researchers and older residents who collaborated in a community based participatory research (CBPR) project in a Mid-western county. Using a CBPR logic model as a guiding framework, we reflected on our process and practices during our collaboration, with three community residents over the age of 65, that unfolded over the course of seven years. We will describe our approach and decisions made that shaped our partnership and present the findings from our reflective process evaluation with our partners, in their own words. We will discuss the utility of the CBPR model in guiding collective reflection as well as the lessons we learned and the dynamics that our partners identified as most salient in contributing to our successful and sustained partnership. We advocate for partnerships with older adults so that their voices are centered as experts on the experience of aging. Centering older adults as experts on the experience of aging in age-related research is one way to ensure that findings can be used to improve the lives of the oldest and most vulnerable members of our society, who stand to gain the most from research outcomes.

Abstract citation ID: igad104.0277

### CHALLENGES OF CONDUCTING COMMUNITY-ENGAGED RESEARCH AMONG JUSTICE-INVOLVED MID- AND LATE-LIFE ADULTS

Rodlescia Sneed, *Wayne State University, Detroit, Michigan, United States*

Formerly incarcerated mid and late-life adults exhibit high rates of chronic disease and disability; however, there is little attention to chronic physical health within programs designed to provide community reentry support. Healthy eating and physical activity are known to reduce chronic disease risk; yet existing physical activity and nutrition programs fail to address the needs of formerly incarcerated mid and late-life adults, who often experience housing insecurity, unemployment, low literacy, and limited access to healthy food. Through a partnership with a community-based reentry program, we designed a health and wellness program tailored to the needs of formerly incarcerated mid and late-adults that included group fitness instructor training, weekly nutrition/fitness classes, and peer coaching. Our team experienced numerous challenges in implementing this program, including low willingness of local community agencies to engage with justice-involved mid and late-life adults, competition with other reentry program activities, individual-level social factors that inhibited participation, and parole/probation restrictions that negatively impacted program attendance. In our session, we will describe how we engaged with a diverse range of partners to address these challenges to ensure program success. There are a number of barriers associated with conducting community-engaged research among formerly incarcerated mid and late-life adults. Our work to date will inform future engagement with this population.

Abstract citation ID: igad104.0278

### CHALLENGES IN THE COMMUNITY-DRIVEN RECRUITMENT OF BLACK OLDER ADULTS TO A HEALTH RESEARCH REGISTRY

Tam Perry[1], Kent Key[2], Elena Flores[3], Vanessa Rorai[4], and Jamie Mitchell[3], *1. Wayne State University, Detroit, Michigan, United States*, *2. Michigan State University, East Lansing, Michigan, United States*, *3. University of Michigan, Ann Arbor, Michigan, United States*, *4. Healthier Black Elders Center, Detroit, Michigan, United States*

Black older adults are underrepresented in health research. Community members representing diverse aging-focused organizations in Flint, Michigan desired more accessible health discoveries, and formed a Community Advisory Board (CAB) in partnership with three local universities. With a grant from the National Institute on Aging (NIH), the CAB sought to promote research engagement through free community health programming and launching a Participant Research Pool (PRP) to connect Black older adults with non-invasive IRB-approved health research at participating universities. Despite extensive recruitment efforts by this academic-community partnership, including monthly virtual health seminars, a new website, tailored print and video promotional materials, a minority aging newsletter, and a community research symposium, the PRP remains severely under-enrolled after a year. An evaluation of recruitment strategies revealed a need for more frequent in-person programming, leveraging the social and professional networks of CAB members, increased intentionality in community partnerships, and more intensive and traditional modes of advertising to reach and recruit the target population. Lessons indicate that Black older adults in the community trust this research endeavor but require tailored and consistent outreach to engage with the participant research pool.

Abstract citation ID: igad104.0279

### FORWARD STEPS AND MISSTEPS: WHAT WE'VE LEARNED THROUGH THE PROCESS OF COMMUNITY-ENGAGED RESEARCH

Jordan Lewis, *University of Minnesota Medical School, Duluth campus, Duluth, Minnesota, United States*

Historically, research with Indigenous populations has disregarded community input, creating mistrust among tribal communities toward researchers, and resulting in communities limiting their involvement in research projects. Today, Indigenous communities are becoming more engaged in the research process, including developing their own tribal review boards, partnering with researchers throughout entire research process, as well as exercising their tribal sovereignty and housing their own data. These efforts have resulted in the development of rigorous Indigenous approval processes, increased engagement of Indigenous people and communities in research, and studies that have direct impacts on those engaged, as well as future generations, as well as protect both the Indigenous communities and the researchers. As a researcher trained to conduct culturally respectful and culturally safe research with Indigenous communities in Alaska, my team and I have gained first-hand experience with tribal research protocols, research processes, and the iterative process of developing relationships, navigating research ethics, as well as ensuring that findings are reflective of the community, its individual members, and the region. Despite our best efforts, research does not go as written in our grant proposals. This presentation will outline the complexities of navigating tribal approval processes in Alaska, highlight some of the challenges we have faced while conducting community-engaged research in Alaska; our lessons learned by doing it incorrectly. We will also share recommendations for researchers interested in working with Indigenous communities and how to work collaboratively with to avoid pitfalls, but also how to get back on track and develop lasting relationships.

### SESSION 1280 (PAPER)

### ALZHEIMER'S DISEASE AND RELATED DEMENTIAS (SRPP) III

Abstract citation ID: igad104.0280

### CARE PARTNERS' ENGAGEMENT IN PREVENTING FALLS FOR COMMUNITY-DWELLING OLDER PEOPLE WITH DEMENTIA

Yuanjin Zhou[1], Clara Berridge[2], Nancy Hooyman[2], and Elizabeth Phelan[3], *1. University of Texas at Austin, Austin, Texas, United States*, *2. University of Washington, Seattle, Washington, United States*, *3. University Of Washington, School Of Medicine, Seattle, Washington, United States*

Care partners adopt various behaviors to manage fall risk for community-dwelling older people with dementia (cd-OPWD). It is unknown if these behaviors reduce falls for cd-OPWD at different levels of fall risk. We linked the data from the 2015 and 2016 National Health and Aging Trends Study (NHATS) and the 2015 National Study of Caregiving (NSOC) (cd-OPWD n=365, 89% aged 75+) to explore this question. The outcome was no fall, one fall, and multiple falls in 2016. We used the same variable in 2015 to indicate fall risk at baseline. We identified 28 items from NSOC to represent dementia care partners' fall risk management (FRM) behaviors based on existing literature and expert review. Using exploratory factor analysis, we identified five categories of FRM behaviors: mobility and safety assistance, health management, accommodations, medical services coordination, and social services coordination. We performed multinomial logistic regression to investigate associations between care partners' FRM behaviors in 2015 and cd-OPWD' falls in 2016 stratified by cd-OPWD's history of falls in 2015, adjusting for selected covariates. For cd-OPWD who had no fall in 2015, mobility and safety assistance was associated with a higher risk of having one fall in 2016; for those who had one fall in 2015, medical services coordination was associated with a lower risk of multiple falls; for those who had multiple falls in 2015, social services coordination was associated with a lower risk of multiple falls. Future research may target care partners' different FRM behaviors based on cd-OPWD's history of falls.

Abstract citation ID: igad104.0281

### FEASIBILITY AND EFFICACY OF LIFE REVIEW DEPRESSION INTERVENTION BY VIDEO-TRAINED FAMILY CAREGIVERS

Christina Miyawaki[1], Angela McClellan[2], Erin Bouldin[3], Cheryl Brohard[1], and Mark Kunik[4], *1. University of Houston, Houston, Texas, United States*, *2. Baylor University, Waco, Texas, United States*, *3. University of Utah, Salt Lake City, Utah, United States*, *4. Baylor College of Medicine, Houston, Texas, United States*

Due to the high prevalence of depressive symptoms and dementia in older Americans, we developed a six-week virtual depression intervention, Caregiver-Provided Life Review (C-PLR) for care recipients (CRs) with early-stage dementia and mild depression and their family caregivers (CGs). We changed the length of CG training iteratively, trained CGs in interviewing and probing skills via video, and CGs delivered life reviews (LR) with CRs at home (N=25 dyads). The objective of the study was to examine the feasibility and efficacy of C-PLR on CRs who were community-dwellers and long-term care facility residents. We collected pre- and post-measures of CRs' depression (primary outcome) and life satisfaction, CGs' burden, positive aspects of caregiving, and CG-CR relationship quality (secondary outcomes) and compared them using paired t-tests. We evaluated if the effect differed by race/ethnicity, residential setting, or living arrangements. CGs were 52 years old (mean), working (64%), college-educated (72%), female (88%), and in good-excellent health (72%). CRs were 81 years old (mean), female (84%), and in poor-fair health (56%). CRs' depression significantly improved following C-PLR ($p< 0.001$), and this effect was found in CRs who were Asian ($p=0.017$), White ($p=0.040$), community-dwelling ($p< 0.001$), lived alone ($p=0.045$), and lived with others ($p=0.002$). Secondary outcomes did not change. This study demonstrated that C-PLR can be successfully taught to CGs via video and effectively reduces CRs' depressive symptoms without increasing CG burden. C-PLR could be implemented broadly to improve symptoms among CRs in community and residential settings, as well as among a diverse population of CRs.

Abstract citation ID: igad104.0282

### IMPLEMENTATION MODELS FOR FOLLOWING UP AFTER A DEMENTIA-RELATED MISSING INCIDENT

Christine Daum, Elyse Letts, Lauren McLennan, Cathy Conway, and Lili Liu, *University of Waterloo, Waterloo, Ontario, Canada*

The rate of persons living with dementia who go missing is a growing concern, especially those who go missing repeatedly. Drawing upon the practices of other populations such as at-risk youth, following up with persons living with dementia and their supports after an incident could reveal contributing factors and also connect people with supports to mitigate the risk for going missing again. In Canada, models for such follow-ups are limited. The purpose of this presentation is to describe two implementation models for following up with persons living with dementia and their care partners after a missing incident has occurred. We conducted 20 individual semi-structured online interviews with police and service providers in Canada and the United Kingdom to understand the scope of and approaches to following up with this population. Generic qualitative description and conventional content analysis were used. We developed a resource guide that describes these approaches and proposes how follow-ups could be consistently employed. Two focus groups with police officers and community service providers (n= 11) were conducted to obtain feedback on the guide. We are now working with community partners in two Canadian provinces to develop implementation models unique to their existing processes, resources, and systems; these models put into practice approaches in the resource guide. Our presentation will showcase the guide and the models developed in collaboration with our partners. These models can guide police organizations, Alzheimer Societies, and communities who support persons living with dementia at risk of going missing.

Abstract citation ID: igad104.0283

### THE MANY FACES OF DEMENTIA IN CANADA: EXAMINING SEX, ETHNICITY, AND AGE OF ONSET IN THE LANDMARK STUDY

Joshua Armstrong, Saskia Sivananthan, and Josée Guimond, *Alzheimer Society of Canada, Toronto, Ontario, Canada*

With the rapidly increasing size of the dementia population, it is important to enhance our understanding of the similarities and differences that are found across the people living with condition. This work takes a closer look at the many faces of dementia in Canada by highlighting the diversity that is found within the national population projections for dementia. To conduct these forecasts, a simulation model was developed using demographic characteristics and risk factors for dementia to estimate the numbers of people living with dementia in Canada from 2020-2050. From the results of the Landmark Study, we will highlight findings related to sex, Indigenous Peoples, ethnic origins, and young-onset dementia. In 2020, 61.3% of dementia diagnoses were in females and this sex ratio is projected to stay constant over the three decades. For Indigenous Peoples of Canada, dementia numbers are estimated to increase by 273% (2020: 10,800; 2050: 40,300). People of Asian origin will increase from 8% of people living with dementia in 2020 to 24% by 2050. The changes are driven both by future immigration, and by people who have already immigrated to Canada in the past few decades. The Landmark Study also projects that there could be over 40,000 people under the age of 65 living with dementia in Canada by 2050.These findings illustrate the changing landscape of people living with dementia in Canada. These demographic characteristics, as well as other distinctions across population groups, profoundly affect the way in which dementia is experienced and their care needs.

Abstract citation ID: igad104.0284

### WORDS OF ADVICE FOR RESEARCHERS: A QUALITATIVE STUDY ON PEOPLE LIVING WITH DEMENTIA'S EXPERIENCES IN RESEARCH

Ha Neul Kim, and Jen Hirsch, *Michigan State University, East Lansing, Michigan, United States*

People living with dementia are the focus of much research, but little is known about their research experiences and how they perceive the world of research. This qualitative study examined the motivations, experiences, and words of advice from people living with dementia regarding their research experiences including both social research and clinical trials. Semi-structured interviews were conducted individually with 7 people, each was interviewed twice for about an hour. Prior to the interview, participants were screened for decisional capacity using a modified UBACC (University of California, San Diego Brief Assessment of Capacity to Consent) to make sure of their understanding of the project. After participants passed the screen, they provided verbal consent. Thematic analysis was conducted to analyze interviews and draw out meaningful themes. Five primary themes emerged: 1) the importance of people living with dementia and their communities in research recruitment, 2) the need for diversity and inclusion in dementia research, 3) the need for a positive attitude of researchers, 4) motivation focused on making contributions and finding a cure for oneself, and 5) the need for balance between showing expertise and explaining with less jargon. The study indicates that there are improvements researchers can make in terms of inclusiveness, listening to people's voices, and engaging people with lived experiences in the research design process, which inspires more participatory research in the dementia research field. This study was supported by the Corey Endowment Award; and the Research Scholars Award.

### SESSION 1285 (SYMPOSIUM)

Abstract citation ID: igad104.0285

### POLICY SERIES: STRATEGIES TO FRAME OUR PUBLIC POLICY WORK

Chair: Patricia D'Antonio Co-Chair: Julie Schoen
Discussant: Hannah Albers

Our words and the way we harness the power of them when advocating for age inclusive policies, like the Older Americans Act (OAA), matters. Since 1965 the OAA has supported our nation with funds to strengthen community social services. It currently authorizes a wide array of service programs through a national network of 56 state agencies on aging, 618 area agencies on aging, nearly 20,000 service providers, 281 Tribal organizations, and 1 Native Hawaiian organization representing 400 Tribes. In 2024, the OAA will be considered for reauthorization. We all have a stake in ensuring it is passed so we all have the infrastructure we require as we age. Join The National Center to Reframe Aging and the National Center on Elder Abuse, the leading organizations for effective framing, in this session to discuss the OAA as an example of how we can apply communication strategies in supporting reauthorization, review framing strategies to advance a more equitable story of aging in America and learn how to apply framing tools to your advocacy communications.

Abstract citation ID: igad104.0286

### STRATEGIES TO FRAME OUR PUBLIC POLICY WORK

Patricia D'Antonio[1], Julie Schoen[2], Hannah Albers[1], and Alycia Claproth[3], *1. The Gerontological Society of America, Washington, District of Columbia, United States*, *2. Keck School of Medicine at USC, Los Angeles, California, United States*, *3. National Center to Reframe aging, Washington, District of Columbia, United States*

Our words and the way we harness the power of them when advocating for age inclusive policies, like the Older Americans Act (OAA), matters. Since 1965 the OAA has supported our nation with funds to strengthen community social services. It currently authorizes a wide array of service programs through a national network of 56 state agencies on aging, 618 area agencies on aging, nearly 20,000 service providers, 281 Tribal organizations, and 1 Native Hawaiian organization representing 400 Tribes. In 2024, the OAA will be considered for reauthorization. We all have a stake in ensuring it is passed so we all have the infrastructure we require as we age. Join The National Center to Reframe Aging and the National Center on Elder Abuse, the leading organizations for effective framing, in this session to discuss the OAA as an example of how we can apply communication strategies in supporting reauthorization, review framing strategies to advance a more equitable story of aging in America and learn how to apply framing tools to your advocacy communications.

Abstract citation ID: igad104.0287

**POLICY SERIES: POSITIVE EXPERIENCES OF AGING AND HEALTH: COMPLEMENTARY EVIDENCE FROM TWO STUDIES**

Julie Ober Allen[1], Valerie Moïse[2], C.J. White[1], and Erica Solway[3], *1. University of Oklahoma, Norman, Oklahoma, United States*, *2. Stephenson Cancer Center at OU Medicine, Oklahoma City, Oklahoma, United States*, *3. University of Michigan, Ann Arbor, Michigan, United States*

Social constructions of aging and associated treatment of older adults are predominantly negative (i.e., ageism) and believed to be harmful for health. Societal beliefs, expectations, and practices linked to old age may also be associated with benefits, such wisdom, respect, greater self-acceptance, more focus on family and chosen activities, and eligibility for age-specific privileges. Positive experiences of aging such as these have not been extensively researched. This study investigated the prevalence of positive experiences of aging and their relationships with health using data from two complementary studies: 5 items focused on positive experiences of aging in a nationally representative sample (2019 National Poll on Healthy Aging (NPHA); Mage=62.6, 54.2% female); and 23 items in a smaller exploratory study (2021-2022 Experiences of Aging in Society (EOA); Mage=71.1, 81.8% female). Associations with health were assessed using multivariable regression. Report of positive experiences of aging was common (NPHA: >65% for all items; EOA: >80% for 20). Most frequently endorsed were being treated with respect, value for one's opinions, wanting to be a good example to younger people, greater acceptance of not work full time and spending time with family (all EOA), and feeling more comfortable with oneself (NPHA and EOA). Positive experiences of aging were associated with better health (NPHA: fewer chronic health conditions (p=.023) and reduced risk of low self-rated physical health, mental health, and depressive symptoms (p-values <.001); EOA: fewer chronic health conditions (p=.004). Increasing positive experiences of aging may be a promising strategy for promoting older adult health and wellbeing.

### SESSION 1290 (SYMPOSIUM)

Abstract citation ID: igad104.0288

**INTEGRATING ANIMAL MODEL, HUMAN SUBJECTS, AND SOCIAL SYSTEMS IN NUTRITION AND AGING RESEARCH**

Chair: Sarah Booth Co-Chair: Haowei Wang Discussant: Roger Fielding

This symposium will bring together speakers who will present cutting-edge research on nutrition and aging from interdisciplinary perspectives using complementary methods. Nutrition plays an important role in understanding the biology of aging, promoting healthy aging, preventing age-related diseases, and supporting aging populations at risks. This symposium will present innovative studies using animal models, population surveys, and nutrition programs to inform future research and promote nutrition security and diet quality. Lee et al. examine sex-specific differences in gut homeostasis and host metabolism in female and male mice fed different rodent diets. Shea et al. and Ardisson Korat et al. focus on dietary quality and patterns in older populations. Using a novel network analysis, Shea et al. examine similarities and differences in the manner in which food groups are consumed in a nationally representative sample from the National Health and Nutrition Examination Survey. Ardisson Korat et al. utilize data from the Nurse's Health Study and evaluate the long-term role of dietary carbohydrate quality in promoting healthy aging. Borth & Whitmire and El-Abbadi et al. investigate food insecurity and nutrition programs among older adults during and after the COVID-19 pandemic. Using data from US Census Bureau's Household Pulse Survey (April 2020 to December 2022), El-Abbadi et al. explore the differences in food insecurity by age-groups, gender, and race and ethnicity. Borth & Whitmire conduct research on congregate nutrition programs to identify successful practices of nutrition programs during the pandemic and discuss challenges and directions in future congregate nutrition programs. This is a Nutrition Interest Group Sponsored Symposium.

Abstract citation ID: igad104.0289

**FOOD INSECURITY AMONG OLDER ADULTS IN THE UNITED STATES DURING THE COVID-19 PANDEMIC**

Naglaa El-Abbadi[1], Zhongqi Fan[2], Amy Yang[3], Ana Ronan[4], Ryan simpson[3], Kimberly Nguyen[3], and Elena Naumova[3], *1. Jean Mayer USDA Human Nutrition Research Center on Aging, at Tufts University, Boston, Massachusetts, United States*, *2. MIT, Cambridge, Massachusetts, United States*, *3. Tufts University, Boston, Massachusetts, United States*, *4. Arizona State University, Glendale, Arizona, United States*

The COVID-19 pandemic severely impacted food access and nutritional security across the United States, and exacerbated disparities in household food insecurity rates particularly among older adults due to potential for reduced food access, mental or physical health limitations, or fixed incomes. This study investigated food insecurity among older adults in the US throughout time periods of the pandemic, exploring differences among population groups for food insecurity risk factors. Data was from the US Census Bureau's Household Pulse Survey, April 2020 through December 2022, weight-adjusted for a nationally representative sample (n=3,884,922). Logistic regression models were constructed to examine the association between older age and self-reported household food insecurity. Reasons given for food insecurity were examined as secondary outcomes. Covariates included demographics, socioeconomic status, household structure, food support programs, and mental health status. Findings showed risk of food insecurity among adults aged 60+ years more than doubled among minority racial groups compared to non-Hispanic White (p<0.001) but was inversely associated with being female compared to male (OR 0.77, p<0.001). Older adults who reported experiencing at least one mental health indicator (anxiety, depression, low interest, or worry) more than half of the week had over five times the odds of being food insecure (OR 5.68, p<0.001). Overall, trends and factors associated with prevalence and reasons for food insecurity were found to vary across age groups, and at different points of the pandemic. Our findings can help elucidate factors to identify older adult groups at greater food insecurity risk.

Abstract citation ID: igad104.0290

**DIETARY CARBOHYDRATE QUALITY IN RELATION TO HEALTHY AGING IN WOMEN**

Andres Ardisson Korat[1], Kyla Shea[2], Paul Jacques[2], Paola Sebastiani[3], Molin Wang[4], Walter Willett[4], and Qi Sun[4], *1. Jean Mayer USDA Human Nutrition Research Center on Aging at Tufts University , Boston, Massachusetts, United States*, *2. Tufts University, Boston, Massachusetts, United States*, *3. Tufts Medical Center, Boston, Massachusetts, United States*, *4. Harvard T.H. Chan School of Public Health, Boston, Massachusetts, United Sta tes*

Our objective was to evaluate the role of dietary carbohydrate quality in healthy aging among women in the Nurses' Health Study (NHS). We included 29,635 NHS participants aged <60 years old in 1984. Total carbohydrate, refined carbohydrate, dietary glycemic index (GI), dietary fiber intake and carbohydrate from fruits, vegetables, whole and refined grains were derived from validated food-frequency questionnaires. Healthy aging was defined as being free from major chronic diseases, having good mental health, and not having any impairments in cognitive or physical function as assessed in the 2014 or 2016 participant questionnaires. We used multivariate logistic regression to estimate the odds ratios (ORs) and 95% Confidence Intervals (CI) for each carbohydrate exposure in relation to healthy aging. A total of 3,731 NHS participants met our criteria for healthy aging. Refined carbohydrate intake was associated with lower odds of healthy aging (ORs[95% CIs] per 5%-calorie increase: 0.94[0.90, 0.97]). Conversely, intakes of carbohydrates from whole grains, fruits, and vegetables were associated with 2% to 6% higher odds of healthy aging for every 1%-calorie increase. Additionally, intakes of total fiber, fruit fiber, vegetable fiber and cereal fiber were associated with 12% to 25% higher odds of healthy aging per 1%-calorie increment. Lastly, the dietary GI was associated with lower odds of healthy aging. In conclusion, intakes of high-quality carbohydrate from fruits, vegetables, whole grains, and dietary fiber in middle adulthood were favorably associated with healthy aging in a large cohort of women, whereas refined carbohydrate intake was unfavorably associated with healthy aging.

Abstract citation ID: igad104.0291

**USING NETWORK ANALYSIS TO EXPLORE DIFFERENCES IN DIETARY INTAKES ACROSS OLDER ADULTHOOD**

Kyla Shea, Gail Rogers, and Kathryn Barger, *Tufts University, Boston, Massachusetts, United States*

Federal nutrition guidance in the Unites States (US) is based on the premise that healthy diets can help delay the onset or progression of age-related disease and disability. Yet, little is known about if or how dietary intakes differ across the older adulthood age span. We used NHANES 2015-2016 and 2017-March 2020 data to examine the dietary intakes of 60-69 year old (n=2079), 70-79 year old (n=1181) and 80+ year old (n=644) adults in the US. Mutual information was used to infer network relationships among all possible pairs of fourteen What We Eat in America food group categories. Based on edge weights, we observed some similarities and some differences in the manner in which food groups were co-consumed across older adulthood. For example, fruit and dairy were co-consumed similarly in the three age groups. Among the 60-69 and 70-79 year olds, protein and vegetables were co-consumed, while fruits were consumed with grains and mixed dishes. However, in the 80+ year olds, vegetables were mostly consumed with fats and oils, but less so with protein, and grains were consumed with dairy, but less so with fruit. Based on a network science approach, the manner in which food groups are consumed appears to change throughout older adulthood. Future studies are needed to evaluate consumption patterns of specific foods among age-defined and other subgroups of older adults and explore potential associations with age-related health outcomes.

Abstract citation ID: igad104.0292

**POSTPANDEMIC SUCCESSES AND CHALLENGES IN REOPENING OLDER AMERICANS ACT CONGREGATE MEALS**

Laura Borth[1], and Meredith Whitmire[2], *1. National Association of Nutrition and Aging Services Programs (NANASP), Washington, District of Columbia, United States*, *2. Matz, Blancato & Associates, Washington, District of Columbia, United States*

The COVID-19 pandemic required Older Americans Act (OAA) congregate nutrition programs to shut down in-person dining but continue serving meals in innovative ways. Re-opening provided a unique opportunity for congregate nutrition programs to continue these innovative changes and/or reinvent how they serve meals. These changes will be crucial for OAA nutrition programs to maintain their relevance, as all adults over age 60 qualify for OAA meals, yet fewer than that attend. To identify successful practices that could be adopted nationally and describe continuing challenges, we conducted surveys, focus groups, and interviews of congregate nutrition programs. Overall, 523 completed the entire survey, nine participated in focus groups, and three were interviewed. Responses came from across 47 states with most (94%) reporting permanently adopting service delivery methods implemented during the COVID-19 pandemic. Overall, nutrition programs described grab-and-go meals having attracted new participants during the pandemic and programs pairing other services with meals to appeal to and retain a wide range of participants. These include medically tailored meals, culturally relevant meals, partnerships with other organizations, and entertainment. These results can be used to strengthen congregate nutrition programs.

Abstract citation ID: igad104.0293

**THE EFFECTS OF AGE AND SEX ON THE GUT MICROBIOME AND GUT HOMEOSTASIS IN MICE**

Jennifer Lee, Kerry Wellenstein, and Gregory Matuszek, *Tufts University, Boston, Massachusetts, United States*

Symbiotic interactions between the gut microbiome and intestinal epithelium are key contributors to regulating gut homeostasis and host metabolism in mice. However, the majority of studies measuring these outcomes have been reported in male and not female mice. Here, we find sex-specific differences in gut homeostasis and host metabolism in conventional C57BL6 chow-fed mice. Functional analyses of the gut metagenomes and metabolomes, gut barrier function, and colonic T-cell populations are different between young and old female and male mice. These data highlight the importance of considering sex as a biological variable in aging rodent studies, which may lead to optimized strategies to improve healthy aging for all.

### SESSION 1295 (SYMPOSIUM)

Abstract citation ID: igad104.0294

### HEARING INTERVENTION TO REDUCE COGNITIVE DECLINE: DESIGN AND FINDINGS OF THE ACHIEVE RANDOMIZED CONTROLLED TRIAL

Chair: Jennifer Deal Co-Chair: Nicholas Reed Discussant: Luigi Ferrucci

Epidemiologic data consistently link age-related hearing loss with accelerated cognitive decline and incident dementia in large, population-based studies. Mechanistic pathways that could underlie this association include the effects of distorted peripheral encoding of sound on cognitive load, changes to brain structure/function, and/or reduced social engagement and increased loneliness. Importantly, these pathways may be amenable to comprehensive hearing rehabilitative treatment including hearing assistive technologies (e.g., hearing aids, other integrated hearing assistive devices) and rehabilitative training. To date, however, there has never been a randomized trial that has investigated whether hearing loss treatment could reduce cognitive and other functional decline in older adults without dementia. The Aging and Cognitive Health Evaluation in Elders (ACHIEVE) trial is a multicenter randomized control trial of 977 cognitively intact adults with untreated, mild-to-moderate hearing loss aged 70-84 years at baseline (2018-19) to determine efficacy of best-practices hearing intervention to reduce cognitive decline over 3 years (Clinicaltrials.gov Identifier: NCT03243422). This session, on behalf of the ACHIEVE Collaborative Research Group, will describe the overall design of the ACHIEVE trial including details about recruitment and implementation of both the hearing (amplification, auditory rehabilitation) and successful aging health education control interventions. We will also present the primary findings of the study – the effect of the hearing intervention vs. control on 3-year cognitive decline – as well as the effect of the hearing intervention on several pre-specified outcomes important for the health of older adults, including social engagement, mood disorders and quality of life.

Abstract citation ID: igad104.0295

### THE ACHIEVE RANDOMIZED TRIAL: EFFECT OF HEARING LOSS INTERVENTION ON COGNITIVE DECLINE IN OLDER ADULTS

Jennifer Deal, *Johns Hopkins University, Baltimore, Maryland, United States*

Hearing loss in older adults is linked to accelerated cognitive decline and incident dementia in population-based observational studies; whether intervention can reduce cognitive decline is unknown. The multi-center NIH-funded ACHIEVE randomized controlled trial is testing efficacy of a best-practices hearing intervention (amplification and rehabilitation) versus a successful aging health education control intervention, on 3-year cognitive decline in 977 dementia-free adults aged 70-84 years with untreated mild-to-moderate hearing loss. This presentation will describe results for the primary outcome of 3-year cognitive decline, measured using a 10-item neurocognitive battery summarized using latent variable methods. Secondary outcomes include (1) domain-specific cognitive declines in memory, language and executive function and (2) time until cognitive impairment defined as a composite of adjudicated dementia or mild cognitive impairment diagnosis, a 3-point drop in the 30-item Mini-Mental State Exam (MMSE) administered in-person, or a 3-point drop in a factor score derived from the 10-item MMSE orientation subscale and 11-item Blessed scale administered over the phone and rescaled to be equivalent to the 30-item MMSE. The trial is ongoing with data closeout March/April 2023; data will be analyzed per the ACHIEVE Coordinating Center statistical analysis plan, approved by the NIA and ACHIEVE DSMB. Although preliminary results are not possible to include here to protect trial integrity, results will be available to present for GSA. ACHIEVE is the first large-scale randomized study to determine if hearing loss intervention reduces cognitive decline. Whether findings are positive or null, this study will have substantial clinical and public health impact.

Abstract citation ID: igad104.0296

### DESIGN, RECRUITMENT, AND BASELINE CHARACTERISTICS OF THE AGING AND COGNITIVE HEALTH EVALUATION IN ELDERS TRIAL

Nicholas Reed, *Johns Hopkins Bloomberg School of Public Health, Baltimore, Maryland, United States*

Hearing loss is highly prevalent among older adults and independently associated with cognitive decline. This presentation will describe the design and baseline characteristics of the first randomized controlled trial to test whether hearing treatment can reduce cognitive decline, the Aging and Cognitive Health Evaluation in Elders (ACHIEVE) trial. This multicenter study was nested within the Atherosclerosis Risk in Communities (ARIC) observational cohort study. The primary outcome is 3-year change in a global cognitive function factor score derived from a comprehensive cognitive test battery. Other prespecified outcomes include loneliness, social network size, depressive symptomatology, health-related quality of life, hospitalization, falls, physical function, and accelerometry-measured physical activity. Cognitively intact, community-dwelling 70-84-year-old participants with adult-onset hearing loss were recruited from the parent ARIC study and de novo from the surrounding communities and randomized 1:1 to the best practices hearing intervention or successful aging health education control intervention. Over a 24-month period, 3004 telephone screenings resulted in 2344 in-person hearing, vision, and cognition screenings and 1294 comprehensive screenings. Among 1102 eligible, 977 were randomized into the trial (median age = 76.4; 53.5%

female; 87.6% White; 53.3% held a bachelor's degree or higher). Participants from the parent ARIC study were recruited much earlier in the process and were less likely to report hearing loss that interfered with their quality of life relative to de novo participants recruited from the community. Minor differences in baseline hearing or health characteristics were found by recruitment method (i.e., ARIC or de novo) or by study site.

Abstract citation ID: igad104.0297

### DESIGN AND IMPLEMENTATION OF THE ACHIEVE HEARING INTERVENTION

Theresa Chisolm, Victora Sanchez, and Michelle Arnold, *University of South Florida, Tampa, Florida, United States*

The ACHIEVE trial required the development of a manualized best-practice hearing intervention designed to meet the needs of older adults with mild-moderate hearing loss. The hearing intervention (HI) was developed using an iterative process including input from a diverse professional panel and a feasibility study to confirm that expected outcomes from a best-practices, evidence-based HI would be obtained. The HI involves use of hearing aids and related technologies along with counselling, coaching and education to support self-management of hearing loss. The HI is delivered in four sessions after the completion of a comprehensive hearing evaluation. Individualized goals are developed using the Client Oriented Scale of Improvement (COSI), which guides selection of hearing aids and other technologies to meet prioritized listening needs and assessment of how well those needs are met. The most frequent COSI listening goal categories included: Conversation in Noise (n= 413; 29%), Conversations in Quiet (n= 296; 21%), and TV/Radio at Normal Volume (n= 280; 20%). Three levels of hearing aid technology differing by the number of signal processing approaches included were: Basic (n=223, 20%), Advanced (n=662, 56%), and Premium (n=283, 24%). Additional hearing assistive technologies included streaming devices, TV connectors, and remote microphones were provided. We discuss how the baseline audiological results and individualized goal setting informed delivery of the HI and how the use of manualization allowed us to maintain excellent treatment fidelity throughout the clinical trial.

Abstract citation ID: igad104.0298

### DESIGN AND IMPLEMENTATION OF THE ACHIEVE SUCCESSFUL AGING HEALTH EDUCATION CONTROL INTERVENTION

Nancy Glynn[1], and Theresa Gmelin[2], *1. University of Pittsburgh School of Public Health, Pittsburgh, Pennsylvania, United States*, *2. University of Pittsburgh, Pittsburgh, Pennsylvania, United States*

Choice of comparison group was essential for participant recruitment and retention for the ACHIEVE Study and necessary to control for key components of the hearing intervention. The Successful Aging (SA) Health Education control intervention followed the protocol and materials developed for the 10 Keys to Healthy Aging (10Keys™), an evidence-based interactive health education program for older adults on topics relevant to chronic disease and disability prevention, previously implemented in other trials. The 10Keys contained up-to-date prevention guidelines based on the current recommendations from leading groups (e.g., United States Preventive Services Task Force, Centers for Disease Control and Prevention). To control for general levels of staff and participant time and attention, participants randomized to the SA control met individually with a 10Keys certified health educator who administered the program every 1-3 weeks, totaling four visits over ~2-3 months. To match the contact schedule with the hearing intervention and promote retention, participants returned for booster sessions semi-annually. During COVID-19 restrictions, we delivered booster sessions by phone. Individually tailored session content included a standardized didactic education component (a 10Keys module) as well as activities, goal-setting, and optional extracurricular enrichment. To further enhance retention and perceived benefit, each session also included a 5- to 10-minute upper body extremity stretching program. We offered the SA control participants the hearing intervention and vice versa at the trial conclusion. Monthly committee calls ensured fidelity and allowed for troubleshooting any participant-related issues. Audio recordings of sessions delivered by health educators were reviewed monthly for quality control.

Abstract citation ID: igad104.0299

### EFFECT OF HEARING INTERVENTION ON SOCIAL FUNCTION, DEPRESSION, AND HEALTH-RELATED QUALITY OF LIFE IN OLDER ADULTS

Alison Huang, *Johns Hopkins Bloomberg School of Public Health, Baltimore, Maryland, United States*

Prior observational studies show associations between hearing loss and worse social and mental health among older adults. Whether hearing loss treatment is an effective intervention is unknown. The Aging and Cognitive Health Evaluation in Elders Study (ACHIEVE) randomized controlled trial (n=977) tests the effect of a best-practices hearing intervention vs. successful aging health education control intervention on pre-specified exploratory outcomes including social isolation (Cohen Social Network Index), loneliness (UCLA Loneliness Scale), depressive symptomology (11-item Center for Epidemiologic Studies Depression scale), and health-related quality of life (RAND-36 Health Survey). Generalized linear mixed effects models test if 3-year change in social and mental health outcomes differ by intervention assignment. Participants have untreated hearing loss and are 70-84 years, 88% White, 54% female, and 53% had a Bachelor's degree or higher. At baseline, prevalence of loneliness was higher among those with moderate or greater (69%) compared to mild (64%) hearing loss. Physical health-related quality of life was lower among those with moderate or greater (mean[standard deviation(SD)]: 44[10]) compared to mild (46[9]) hearing loss. Prevalence of depression (4%), social network characteristics (e.g., mean(SD) social network size: 22[11] members), and mental health-related quality of life (mean(SD): 56[7]) were similar by hearing loss severity. This presentation focuses on hearing intervention effects and results will be available by the time of the conference (data collection ends early 2023). Existing interventions for improving social and mental health are limited in effect size and scalability. Hearing treatment may be an effective intervention with potential for greater widespread implementation.

### SESSION 1300 (SYMPOSIUM)

Abstract citation ID: igad104.0300

### IMPROVING HOSPITAL AND POST-ACUTE OUTCOMES AND EXPERIENCES OF DYADS LIVING WITH DEMENTIA: RESULTS OF THE FAM-FFC TRIAL

Chair: Marie Boltz Co-Chair: Ashley Kuzmik Discussant: Karen Rose

Hospitalizations of persons living with dementia are associated with protracted functional decline, delirium, and behavioral symptoms of distress as well as increased care partner stress. This symposium presents findings from the Family-centered Function -focused Care (Fam-FFC) trial. In Fam-FFC, nurses purposefully empower family care partners in the assessment, decision-making, care delivery, and evaluation of function-focused care during hospitalization and the 60-day post-acute period. The goal is to improve the functional recovery of the patient while increasing family preparedness for caregiving, bridging hospital to post-acute setting. This cluster randomized controlled trial tested the impact of Fam-FFC in 455 dyads of diverse persons living with dementia and their care partners. The first presentation will compare Fam-FFC outcomes for patients and care partners to the attention control group outcomes at discharge, two and six months. The second presentation will report the impact of Fam-FFC upon adverse patient events (falls, emergency department transfers, hospital admissions) in the two arms. Next, the presenter will utilize Fam-FFC data to describe the patient and care partner characteristics that contribute to care partner distress at the time of hospital discharge. The fourth presentation will focus on staff-patient interactions in the Fam-FFC trial, including the influence of clinical factors upon the quality of the interaction. The final paper will report on the trajectory of behavioral symptoms of distress, and associated outcomes, from hospital admission to six-month follow-up. Our discussant, Karen Rose will lead a discussion of unanswered questions, next steps, and implications of this work for policy and practice.

Abstract citation ID: igad104.0301

### PATIENT AND FAMILY PARTNER OUTCOMES OF THE FAM-FFC TRIAL

Marie Boltz[1], Jacqueline Mogle[2], and Ashley Kuzmik[1], *1. Pennsylvania State University, State College, Pennsylvania, United States*, *2. Clemson University, Clemson, South Carolina, United States*

The Fam-FFC trial tested the hypotheses that patients who participated in Fam-FFC would have more return to baseline function, more physical activity, and less delirium, depressive symptoms, and behavioral symptoms of distress, and that care partners would demonstrate increased preparedness for caregiving (primary outcome) and less anxiety, strain, and burden, as compared to participants in the control arm. The likelihood of returning to baseline function across time for Fam-FFC participants was twice that of the control group by the end of 6 months (OR = 2.39, p =.011). Fam-FFC was also associated with fewer behavioral symptoms of distress at six-months (changeintervention = -1.973, SE = .396, p < .0001, d = .467; changecontrol = -.859, SE = .389, p = .028, d = .203); moderate physical activity, depressive symptoms, and delirium severity did not differ by treatment arm. Preparedness for caregiving increased significantly more from 2 months to 6-months (changeintervention=2.4, SE=.439, d=.563; changecontrol=.893, SE=.450, d=.212, overall p = .02), and marginally increased from discharge to six months. (changeintervention= 2.783, SE= 0.44, d=.682; changecontrol=1.695, SE=0.44, d=0.415, overall p =0.08), in the intervention group, with no group differences in anxiety, strain, and burden. Fam-FFC may prevent some of the post-acute functional decline and behavioral symptoms in persons with dementia while increasing FCP preparedness. Future research should focus on sustainability of patient improvements and addressing the chronic stressors associated with the care partner role.

Abstract citation ID: igad104.0302

### DEMENTIA CARE PARTNERS' ANXIETY, DEPRESSION, AND BURDEN AT HOSPITAL DISCHARGE

Ashley Kuzmik, *Pennsylvania State University, State College, Pennsylvania, United States*

At the time of hospital discharge, family care partners are typically planning to deal with the increased care dependency and medically complex needs of the person living with dementia. Consequently, they experience compounded stress to their psychological wellbeing. This study examined patient and care partner factors associated with family care partners' anxiety, depression, and burden when caring for patients with dementia at hospital discharge. The sample consisted of 434 patient and care partner dyads enrolled in the Family centered Function-focused Care (Fam-FFC) study. Multiple regression analyses were conducted to examine factors associated with care partners' anxiety (Hospital Anxiety and Depression subscale anxiety; HADS-A), depression (Hospital Anxiety and Depression subscale depression; HADS-D), and burden (Short Form Zarit Burden Interview; ZBI-12). Care partner anxiety was associated with higher levels of patient behavioral and psychological symptoms of dementia (BPSD; β=.13; p = <.001). Care partner depression was associated with cohabitation (β=1.30; p = <.001), and patient factors including increased BPSD (β=.17; p = <.001) and lower function (β=-.03; p = <.001). Care partner burden was associated with care partner relationship with patient (adult child; β=-.27; p = .02) and higher patient BPSD (β=.67; p = <.001). Findings suggest the need to incorporate the functional and behavioral status and needs of the patient when preparing the care partner for discharge, which may be especially critical if the patient is discharged to the care partner's residence.

Abstract citation ID: igad104.0303

### THE IMPACT OF FAM-FFC UPON ADVERSE OUTCOMES IN HOSPITALIZED PERSONS WITH DEMENTIA

Barbara Resnick, *University of Maryland, Baltimore, Maryland, United States*

The Fam-FCC education and coaching of care partners emphasizes optimizing physical and cognitive function and detection of changes in condition to prevent falls, avoidable transfers to the emergency department and hospital readmissions. Thus, the aim of this study was to examine the impact of Fam-FFC upon the desired outcomes of less transfers to the emergency department, hospital readmissions, and falls. The majority of patients identified as female, with an average age of 81.5 (± 8.4); approximately 64% were White patients and 36% were Black or African American patients. The sample indicated, on average, moderate impairment in cognition (MMoCA=11.7±7.0) and baseline (pre-admission) some impairment in physical function (MBI=78.1±22.6), mild pain (MPAINAD=0.79±1.5) and depression (MCESD=9.1±6.2). There were no group differences in transfers to the emergency department; however intervention participants were approximately 10% less likely to be hospitalized (p<.04) over the study intervention. The total number of falls or transfers to the emergency department did not differ between groups. The findings of this study suggest that Fam-FFC may be implemented without increasing the risk of falls, while possibly decreasing hospitalizations.

Abstract citation ID: igad104.0304

### CLINICAL FACTORS ASSOCIATED WITH THE QUALITY OF INTERACTIONS BETWEEN STAFF AND HOSPITALIZED PERSONS WITH DEMENTIA

Anju Paudel, *Penn State Ross and Carol Nese College of Nursing, State College, Pennsylvania, United States*

The quality of care greatly depends on the quality of interactions between staff and hospitalized older adults with dementia which in turn can be influenced by many factors. This paper examines the clinical factors associated with the quality of interactions between staff and hospitalized older adults with dementia. Following the examination of bivariate associations, we conducted multiple linear regression in a sample of 140 hospitalized older patients with dementia who participated in the final cohort of Fam-FFC. On average, the participants (male = 46.1%, female = 52.9%) were 81.43 years old (SD = 8.29) and had positive interactions with staff. Accounting for 17.5% of the variance in the model, non-pharmacological interventions (b= 0.181; p<.001) and pain (b= -0.201; p<.01) were significantly associated with the quality of staff-patient interactions. To optimize care of hospitalized patients with dementia, staff should be encouraged to use non-pharmacological interventions and prioritize pain management in patients.

Abstract citation ID: igad104.0305

### ASSOCIATION OF BEHAVIORAL AND PSYCHOLOGICAL SYMPTOMS OF DEMENTIA WITH ADVERSE OUTCOMES POSTHOSPITALIZATION

Diane Berish, *The Pennsylvania State University, University Park, Pennsylvania, United States*

In hospitalized persons with dementia, behavioral and psychological symptoms of dementia (BPSD), also known as behavioral symptoms of distress, are associated with increased morbidity, long-term care admissions, and reduced care partner well-being. The Fam-FFC study provided the opportunity to examine the association between BPSD severity and adverse, post-acute outcomes (falls, emergency department transfers, hospital admissions). Logistic regression analysis examined the association of behavioral symptoms (Neuropsychiatric Inventory Questionnaire, NPI) and odds of having a fall, emergency department transfer, and hospital admission, controlling for the intervention, and caregiver and patient characteristics. NPI scores decreased from admission (M = 7.81, SD = 6.12), to discharge (M = 6.4, SD = 5.93), to two months (M = 5.38, SD = 5.91), to six-month follow-up (M = 5.08, SD = 5.94). Higher admission NPI scores were associated with greater odds of experiencing at least one fall (OR = 1.067, p = 0.042) by discharge. Higher discharge NPI scores were associated with higher odds of ED transfer (OR = 1.062, p = 0.006) and at least one hospitalization (OR = 1.06, p = 0.008) after two months. Higher NPI scores at 2-month follow-up were associated with increased odds of ED transfer (OR = 1.062, p = 0.004), experiencing at least one injury (OR = 1.055, p = 0.031), falls (OR = 1.056, p = 0.012), and hospitalizations (OR = 1.065, p = 0.004) at six months. Results underscore the importance of providing interventions to reduce BPSD as a potentially modifiable risk factor to reduce adverse outcomes.

### SESSION 1305 (SYMPOSIUM)

Abstract citation ID: igad104.0306

### MARITAL TRANSITIONS IN MID AND LATE LIFE

Chair: Joan Monin Discussant: Jason Newsom

Marital transitions are common in mid and late life, yet little is known about what makes some middle aged and older adults thrive and others experience hardship through these transitions. This symposium will discuss antecedents, consequences, and moderators of adjustment to marital transitions in mid to late life. Monin and colleagues will address antecedents of divorce/separation, presenting results from a case control study showing that later stage dementia is associated with a lower likelihood of divorce/separation, and certain neuropsychiatric symptoms are associated with a greater likelihood of divorce/separation. In terms of consequences of relationship dissolution, Bourassa and colleagues will present findings from a study across a 20-year period of adulthood, showing that people with more breakups in adulthood have more advanced biological age in midlife. Two studies will discuss moderators of associations between relationship transitions and well-being. Carr and Choi will discuss the experiences of childless older adults who become widowed or divorced, and whether they suffer poorer mental health than their peers with children. They will show that childless widowed men and divorced men who lost a child to death have dramatically higher levels of social loneliness, relative to other men. Finally, Scheffer and colleagues will talk about the transition out of dementia spousal caregiving. They will present findings indicating that although caregivers' emotional well-being and energy improves from moving from being an active caregiver to moving out of the caregiving role, those with lower household income had worse outcomes after caregiving ended.

Abstract citation ID: igad104.0307

### DEMENTIA STAGING, NEUROPSYCHIATRIC SYMPTOMS, AND DIVORCE OR SEPARATION IN LATE LIFE: A CASE CONTROL STUDY

Joan Monin[1], Gail McAvay[2], Emma Zang[3], Brent Vander Wyck[4], Carmen Carrión[4], and Heather Allore[2], *1. Yale School of Public Health, New Haven, Connecticut,*

*United States*, *2. Yale University, New Haven, Connecticut, United States*, *3. Yale Sociology Department, Yale University, New Haven, Connecticut, United States*, *4. Yale School of Medicine, New Haven, Connecticut, United States*

Dementia can be difficult on marriages for many reasons, including but not limited to caregiving burden, loss of intimacy, and financial burden. This study examined the impact of dementia staging and neuropsychiatric behavioral symptoms on the likelihood of divorce or separation for older adult married couples. For this case-control study, we used data from the National Alzheimer's Coordinating Center (NACC) Uniform dataset (UDS) versions 2 and 3. This dataset was from 2007 to 2021 and contains standardized clinical information submitted by NIA/NIH Alzheimer's Disease Research Centers (ADRCs) across the United States (US). This data was from 37 ADRCs. We selected participants who were married or living as married/domestic partners at their initial visit. Cases were defined by a first divorce/separation occurring during the follow-up period, resulting in 291 participants. We selected 5 controls for each married/living as married case and matched on age. Conditional logistic regression estimated the association between overall Neuro Psychiatric Inventory (NPI) score and severity of NPI symptoms with case/control status, adjusted for education, the CDR® Dementia Staging Instrument score, living situation, symptom informant, sex, and race. Separate analyses were conducted for each symptom. Multiple comparisons were accounted for with the Hochberg method. Later stage of dementia was negatively associated with divorce/separation with an adjusted odds ratio. A higher overall NPI score was positively associated with divorce/separation. More severe ratings of agitation/aggression, depression/dysphoria, and disinhibition with divorce/separation were associated with greater odds of divorce/separation. Treating NPI symptoms may be away to protect relationships.

Abstract citation ID: igad104.0308

### JUST THE TWO OF US? THE PSYCHOLOGICAL IMPACT OF MARITAL DISSOLUTION ON CHILDLESS OLDER ADULTS

Deborah Carr[1], and Shinae Choi[2], *1. Boston University, Boston, Massachusetts, United States*, *2. The University of Alabama, Tuscaloosa, Alabama, United States*

An estimated 15% of U.S. adults ages 55+ are childless, with this proportion projected to increase among future cohorts. Of the 15 million childless older adults today, 40 percent are married – challenging the notion that childlessness is the domain of never married persons only. Little is known about the experiences of childless older adults who become widowed or divorced, and whether they suffer poorer mental health than their peers with children. Widowed and divorced persons lack the support of children yet may possess other resources or strengths as they age on their own. We use data from the 2016 and 2018 waves of the Health and Retirement Study (N = 16244) to evaluate whether parental status (childless, any living biological child(ren), stepchild(ren) only, all child(ren) now deceased) moderates the association between marital status (married, cohabiting, divorced/separated, widowed) and mental health (depressive symptoms, and social and emotional loneliness) and whether these patterns differ by gender. Multivariate moderation analyses show that childless widowed men and divorced men who lost a child to death have dramatically higher levels of social loneliness, relative to other men. Childlessness does not moderate the effect of marital status on loneliness or depressive symptoms among women. However, widowed and divorced women with stepchildren only evidence significantly higher depression and emotional loneliness. We discuss implications for understanding late-life mental health, underscoring that childlessness does not uniformly compromise mental health among unmarried persons, and that some parent-child relations (especially step-relations) may be a source of stress rather than solace.

Abstract citation ID: igad104.0309

### RELATIONSHIP DISSOLUTION AND BIOLOGICAL AGING IN MID LIFE

Kyle Bourassa[1], Grace Brennan[2], Avshalom Caspi[2], and Terrie Moffitt[2], *1. Durham VA Medical Center, Durham, North Carolina, United States*, *2. Duke University, Durham, North Carolina, United States*

Several decades of empirical research has shown that people who experience relationship dissolution—such as romantic breakups, divorce, and bereavement—have increased risk for poor health across a range of disease outcomes. However, it remains less clear which physiological pathways might best explain links between relationship dissolution and later health. One candidate mechanism that might help explain this wide range of observed health outcomes is accelerated biological aging, which is theorized to represent a common cause of age-related chronic diseases, disability, and early mortality. The current study examined whether relationship dissolution, measured across a 20-year period of adulthood, was associated with biological age in midlife using participants (n = 910) from the Dunedin Study, a birth cohort assessed over four decades of life. Relationship dissolution was assessed using a count of romantic breakups from age 26 until age 44 from life history calendars. Biological aging was assessed using a midlife aging factor derived from four validated measures of aging—the Pace of Aging, gait speed, BrainAGE, and facial age—assessed at age 45. We found that people with more breakups in adulthood had more advanced biological age in midlife, $\beta = 0.10$, 95% CI [0.04, 0.16], $p = .003$. This association remained when controlling for adversity and poor health in childhood, as well as disadvantaged socioeconomic origins and time spent in romantic relationships, $\beta = 0.09$, 95% CI [0.03, 0.15], $p = .007$. The results suggest accelerated aging may be one physiological mechanism linking relationship dissolution to poor health.

Abstract citation ID: igad104.0310

### LOWER INCOME IN CAREGIVERS PREDICTS LESS EMOTIONAL WELL-BEING IMPROVEMENT AFTER CAREGIVING ENDS

Julian Scheffer[1], Claire Yee[2], Jenna Wells[3], Yuxuan Chen[4], Diana Heath[5], Darius Levan[3], Jennifer Merrilees[6], and Robert Levenson[1], *1. University of California, Berkeley, Berkeley, California, United States*, *2. Mayo Clinic,*

*Scottsdale, Arizona, United States*, *3. University of California Berkeley, Berkeley, California, United States*, *4. Stanford University, Stanford, California, United States*, *5. University of Maryland, College Park, Maryland, United States*, *6. University of California, San Francisco, San Francisco, California, United States*

Lower income in familial caregivers has been shown to associate with increased distress, depression, and burden during active caregiving. However, findings are less clear on whether lower income associates with changes in well-being that occur after caregiving ends. To investigate this question further, we recruited familial caregivers (CGs) of people with neurodegenerative diseases (PWD; N=36). Emotional well-being was assessed using the SF-36 Emotional Well-being and Energy scales both during active caregiving and after caregiving had ended (predominantly due to the PWD's death or transition to institutional care, ~10 years later). Household income was measured after active caregiving had ended. Overall, CG's emotional well-being and energy improved between active caregiving and after caregiving had ended (emotional well-being change: $t(35) = 2.22$, $p = .033$; energy change: $t(35) = 2.88$, $p = .007$). However, this improvement associated with household income, such that when controlling for levels during active caregiving, income associated with worse emotional well-being ($B = -6.72$, $t(33) = -4.56$, $p < .001$) and energy ($B = -8.07$, $t(33) = -4.99$, $p < .001$) in caregivers after caregiving had ended. During the adjustment period after caregiving ends, lower income may limit opportunities for caregivers to take time away from work to mourn the loss of their loved one or address their own health concerns adequately. Thus, low-income caregivers may experience fewer of the benefits associated with these activities. These findings underscore the importance of providing support for lower income caregivers during the critical period after caregiving ends.

### SESSION 1310 (SYMPOSIUM)

Abstract citation ID: igad104.0311

### SENSOR-ENHANCED CARE COORDINATION TO ADDRESS OLDER ADULT HEALTH: LESSONS FROM AN INTERDISCIPLINARY RESEARCH TEAM

Chair: Erin Robinson Discussant: Karla Washington

Researchers at the University of Missouri have a history of developing and testing aging in place (AIP) care coordination models for clinical translation. Vital to this work are in-home, residentially installed sensors to detect early illness and functional decline over time. While much smart home research is focused on older adults living in continuing care retirement communities, our team developed a novel sensor-enhanced care coordination approach for community-dwelling and disabled older adults as part of two ongoing, federally funded projects. For this symposium, we will highlight the Age-friendly Sustainable Smart and Equitable Technologies for Aging in Place (ASSETs for AIP) demonstration program, funded by the Department of Health and Human Services. Our first presentation will overview our interdisciplinary approach to creating this novel care coordination model, with specifics about research team composition, population focus, and methods. Our second presentation will highlight recruitment strategies and share lessons learned from reaching and enrolling this population that often experience residential transitions. Our third presentation will overview the open-source smart home and consumer-grade technologies deployed in this work, and practical considerations for technology installation, support, and sustainability and clinical team training. Our symposium will close with a presentation of selected client case studies that illustrate how clinical care coordinators use sensor data to remotely monitor and empower older adults for behavior change via telehealth consultations. This symposium will help inform other care coordination research, particularly that which uses sensor technologies and telehealth to facilitate aging in place.

Abstract citation ID: igad104.0312

### DEVELOPMENT OF A SENSOR-ENHANCED CARE COORDINATION APPROACH TO FACILITATE AGING IN PLACE

Lori Popejoy[1], Blaine Reeder[2], Erin Robinson[3], Rachel Proffitt[1], Amy Vogelsmeier[1], and Knoo Lee[1], *1. University of Missouri, Columbia, Missouri, United States*, *2. Sinclair School of Nursing, University of Missouri, Columbia, Missouri, United States*, *3. University of Missouri School of Social Work, Columbia, Missouri, United States*

Older adults living in rural areas of the country face unique challenges in accessing health services and community-based supports to facilitate aging in place (AIP). This is especially true for those with health concerns that have required care at a nursing facility. Re-establishing independence after a permanent discharge from a long stay nursing home can be difficult and puts strain on informal care networks. To address this, our team developed a novel approach to help older and disabled adults who were discharged from the Show-Me Home project, which supports clients permanently discharged from the nursing home. The Age-friendly Sustainable Smart and Equitable Technologies for Aging in Place (ASSETs for AIP) is a demonstration project funded by the Department of Health and Human Services. We are an interdisciplinary team of researchers and clinicians who use a strengths-based and client-driven approach to empower older and disabled adults for self-management of their disability, conditions, health, and well-being. Our care coordination team consists of a registered nurse, occupational therapist, and social worker, who assess health-related conditions, set goals, empower change, and monitor progress. For individuals enrolled in ASSETS for AIP, we rely on open-source smart home technologies and consumer-grade wearable devices to monitor activity patterns to identify changes in activity and health. Our care coordination team uses these data as well as client assessments to engage with clients to help them maintain independence. This model shows great promise in addressing the needs of older and disabled adults, thus enabling them to age in place.

Abstract citation ID: igad104.0313

### RECRUITMENT FOR A COMMUNITY-BASED SENSOR TECHNOLOGY PROGRAM: CHALLENGES AND SUCCESSES

Amy Vogelsmeier[1], Ashley Roberts[2], Suzette Bacon[2], Elizabeth Curtis[2], Erin Robinson[3], Blaine Reeder[2], and Lori Popejoy[1], *1. University of Missouri, Columbia, Missouri, United States*, *2. Sinclair School of Nursing, University of Missouri, Columbia, Missouri, United States*, *3. University of Missouri School of Social Work, Columbia, Missouri, United States*

Eligibility criteria for the ASSETs for AIP program included older and disabled adults who were one-year post-discharge from the Missouri's Show-Me Home program and who lived within a designated service area. The service area was comprised primarily of rural Missouri counties within the central I-70 corridor with smaller urban centers retained while larger urban areas were excluded. The goal was to enroll 33 people in year one, with an overall goal to enroll 100 individuals over the three-year project period. A client list was provided to the ASSETs for AIP team based on contact information collected at client discharge from the Show-Me Home Program. Despite diligent efforts, the team encountered several recruitment challenges including: 1) obtaining current contact information for individuals 12 months post-program discharge, 2) working within a limited-service area, and 3) establishing client trust in a new, unknown program upon initial contact. Successful recruitment strategies included 1) partnering with state and local agencies to retrieve up-to-date client contact information, 2) reducing the time from program discharge to facilitate contact and align better with client needs, 3) extending the service area beyond mid-Missouri, and 4) sending out postcard-mailers to potential clients that provided a clear linkage between Show-Me Home program, ASSETs for AIP, and the University of Missouri. A connection to the University was particularly important because the University has a statewide reputation for providing services to our citizens. Within the past 6 months, our enrollment has increased by 225%, with successful recruitment ongoing.

Abstract citation ID: igad104.0314

### PRAGMATIC, SUSTAINABLE, AND COST-FEASIBLE TECHNOLOGIES TO ENABLE REMOTE CARE COORDINATION SERVICES

Knoo Lee[1], Suzette Bacon[2], Elizabeth Conrow[2], Elizabeth Curtis[2], Ashley Roberts[2], and Blaine Reeder[2], *1. University of Missouri, Columbia, Missouri, United States*, *2. Sinclair School of Nursing, University of Missouri, Columbia, Missouri, United States*

The aim of this presentation is to communicate the approach and lessons learned in successfully deploying open source smart home and consumer-grade technologies to enable novel remote care coordination services for older adults as part of the CMS-funded ASSETs for Aging in Place demonstration project. Major barriers to use of technologies that enable service reach to rural or home-bound at-risk populations are technology availability, internet connectivity, cost, configuration, installation, ease of management, and usability and usefulness of clinically relevant data displays for decision-making to support personalized care coordination, coaching, and self-management goals. We have pioneered a reusable, innovative approach and infrastructure through partnership with the State of Missouri Department of Social Services and a non-profit hosting partner that facilitates testing and deployment of such remote services with broad goals of scalability and translation as starting principles. The specific focus of this presentation will describe technology selection, configuration, proof-of-concept testing, use of mobile hot spots to overcome broadband availability in rural areas, team composition and skills, introduction and training of the OT/Nurse/Social Worker teams to technology, resources required to stand up the team and transition to independent technology expertise in the field with mobile, video, and remote messaging tools, and user-centered design engagement of the care coordination and informatics teams to drive dashboard features and visual display developments for new work flows. An overview of the approach, technologies, and practical considerations to support research and development of new technology-enabled services for older adults will be presented.

Abstract citation ID: igad104.0315

### CASE STUDIES OF A SENOR-ENHANCED CARE COORDINATION MODEL TO ADDRESS OLDER ADULT HEALTH

Erin Robinson[1], Elizabeth Heaton Curtis[2], Suzette Mizutani Bacon[2], Ashley Roberts[2], Elizabeth Conrow[2], Lori Popejoy[3], and Amy Vogelsmeier[3], *1. University of Missouri School of Social Work, Columbia, Missouri, United States*, *2. Sinclair School of Nursing, University of Missouri, Columbia, Missouri, United States*, *3. University of Missouri, Columbia, Missouri, United States*

Smart technologies hold enormous potential in the early detection of activity change that may indicate illness or functional decline among older adults. However, little research currently exists on the application of such technologies to community-dwelling older adults. Our program, Age-friendly Sustainable Smart and Equitable Technologies for Aging in Place (ASSETs for AIP) uses open-source smart home technologies and consumer-grade wearable devices to monitor community-dwelling older adults recently discharged from the Show-Me Home program. We employ a novel approach; pairing technology with a care coordination team, comprised of a registered nurse, occupational therapist, and a licensed social worker. This presentation will highlight the function of this team, and how they leverage sensor-generated health information to provide care coordination. Using a clinical interface that displays the sensor-generated health data, this team provides ongoing monitoring of daily activity to identify early changes in routine that may indicate the development of a health problem. As significant changes in health data present, our clinical team works with clients to understand the change and formulate a plan of action. For example, an unexplained spike in heart rate may lead the clinician to discuss recent activity, exertion, and potential modifications in routine for a client. An increase in middle-of-the night bathroom trips may indicate a health change, for the which the client will be coached in what to communicate to their provider. Case studies from our program will be discussed in this presentation, to highlight this model of sensor-enhanced care coordination.

### SESSION 1315 (SYMPOSIUM)

Abstract citation ID: igad104.0316

### TECHNOLOGY TO SUPPORT AGING ADULTS WITH A COGNITIVE IMPAIRMENT

Chair: Sara Czaja Discussant: Lesley Ross

Technology applications can play a key role in enhancing independence and quality of life for aging adults with a cognitive impairment (CI) and offer potential benefits in terms of memory support, cognitive remediation, safety/security, and engagement. Drawing from research conducted by the Center for Research and Education on Aging and Technology Enhancement (CREATE) and the Center for Enhancing Neurocognitive Health, Abilities, Networks, & Community Engagement (ENHANCE) this symposium will highlight examples of existing and emerging technology applications that provide support for aging adults with a CI and discuss challenges and benefits with technology-based approaches. S. Czaja will present findings from a pilot trial that evaluated the benefits of a technology system designed to support information and resource access, and social and cognitive engagement (PRISM-CI) for aging adults with a CI. W. Boot will focus on the unique benefits of virtual reality (VR) for aging adults with and without a cognitive impairment and highlight gaps in knowledge regarding the use of VR to provide meaningful social and cognitive engagement. N. Charness will discuss a project (AUGMENT) that is focused on providing support for mobility for aging adults with a CI due to stroke, MCI, or a traumatic brain injury. W. Rogers will highlight how video technology can provide socially and cognitively engaging activities for aging adults with a CI and present a systematic process to help guide the development of a video intervention for this population. Lesley Ross will lead a discussion of the highlighted topics and areas for future research.

Abstract citation ID: igad104.0317

### PRISM CI: A SOFTWARE SYSTEM TO SUPPORT AGING ADULTS WITH A COGNITIVE IMPAIRMENT

Sara Czaja[1], Francesca Falzarano[1], Darby Lucius-Milliman[2], and Marco Ceruso[1], *1. Weill Cornell Medicine, New York City, New York, United States*, *2. Weill Cornell medicine, New York City, New York, United States*

Maintaining social connections and engaging in activities can become challenging for aging adults with a cognitive impairment (CI), which can result in deleterious cognitive, emotional, and physical health outcomes. This presentation will discuss the PRISM-CI pilot trial, which aims to examine the feasibility and potential efficacy of the PRISM-CI software system on enhancing connectivity and quality of life among a diverse sample of 50 older adults aged 65 and over with a CI. PRISM-CI, adapted from the PRISM system (developed by the Center for Research and Education on Aging and Technology Enhancement) for this population, is intended to support social engagement, memory, and access to resources and information. The sample includes 42 community dwelling individuals aged 65+ (M= 75.5, SD=6.78) with a cognitive impairment. Participants had access to the system for 5 months. We will present data regarding training challenges encountered by our participants, such as the need for more personalized training, and perceptions of usability and the perceived value of the various PRISM-CI features as well as suggestions for improvements (e.g., streaming services such as Spotify, e-Library services). The challenges of implementing PRISM-CI during the COVID-19 pandemic will also be discussed. In addition, preliminary data regarding the impact of the use of PRISM-CI on social isolation and loneliness will be presented. Overall, the findings suggest that use of PRISM-CI was beneficial and enhanced social support and connectivity. We will also discuss how user feedback can be leveraged to inform adaptions to enhance the efficacy and acceptability of technology applications.

Abstract citation ID: igad104.0318

### VIRTUAL REALITY TO SUPPORT THE NEEDS OF OLDER ADULTS WITH AND WITHOUT COGNITIVE IMPAIRMENTS

Walter Boot, *Florida State University, Tallahassee, Florida, United States*

Functional, cognitive, and mobility limitations can interfere with older adults' ability to engage in socially and cognitively enriching activities, which can lead to further decline. Emerging technology solutions, including virtual reality (VR), have the potential to promote engagement in these activities in a way that can account for these limitations, allowing older adults to engage in productive and meaningful activities within their own home. However, an iterative, user-centered design process is necessary to ensure that these solutions reach their potential. This talk provides an overview of a number of recent studies examining the potential of VR to support older adults' engagement in VR interventions aimed at improving mood and social connectivity. The following questions will be asked and answered during this talk, based on recently collected empirical data; What are the potential usability challenges associated with VR interventions?; What are older adults' preferences for different VR experiences?; Can VR provide older adults with meaningful opportunities to engage in social interactions when they are distally located, but co-located in the same virtual space?; To what degree do older adults feel present in the virtual environment?; To what degree are older adults' attitudes toward VR malleable?; If, and under what conditions, is cybersickness a potential barrier? This talk will conclude with a discussion of what is known and what is unknown regarding the use of VR to provide meaningful socially and cognitively engaging experiences for older adults with and without mild cognitive impairment.

Abstract citation ID: igad104.0319

### DESIGNING TUTORIALS TO AUGMENT MOBILITY FOR OLDER ADULTS WITH COGNITIVE IMPAIRMENT

Neil Charness[1], Nicholas Gray[2], Dorota Kossowska-Kuhn[1], Hunui Na[1], and Michael Pervratil[3], *1. Florida State University, Tallahassee, Florida, United States*, *2. Safety National, St. Louis, Missouri, United States*, *3. Psychology Department, Tallahassee, Florida, United States*

The Augmenting User Geocoordinates & Mobility with ENhanced Tutorials (AUGMENT) project aims to support aging adults experiencing navigation difficulties accompanying cognitive impairments due to stroke, mild cognitive impairment, and traumatic brain injury. We describe the process and principles for designing tutorials for Uber and Google Maps, two applications that can support improved mobility for those with navigation difficulties associated with cognitive impairment. Initially we examined predictors of self-reported navigation difficulty in a sample (n=300) of older adults aging normally. In the multiple regression, self-reported memory problems (beta = .35), severity of memory problems (beta=.18), and female gender (beta=.16) were the strongest predictors of wayfinding difficulty in addition to a standardized spatial orientation test (beta=.13). Finding that memory difficulties were strongly related to navigation difficulties even in normal aging implied that tutorials could not make strong demands on retention and helped define the queries for interviews in the needs assessment phase. Next, we interviewed aging adults with cognitive impairment and their care partners about navigation problems, focusing on difficulties using navigation devices and applications. We also presented screenshots of the two target apps and asked simple questions about actions that could be taken. We then designed prototype tutorials using principles such as directing attention to relevant display features by highlighting the features, minimizing extraneous workload, and providing spaced retrieval practice for interactions with the interface. We also describe the iterative design process being used to refine the tutorials and the planned summative test of tutorial efficacy.

Abstract citation ID: igad104.0320

### DEVELOPING CONTENT FOR A VIRTUAL SOCIAL ENGAGEMENT INTERVENTION FOR PERSONS WITH MCI

Wendy Rogers[1], Shraddha Shende[2], Sarah Jones[3], George Mois[4], Elizabeth Lydon[1], and Raksha Mudar[5], *1. University of Illinois Urbana-Champaign, Champaign, Illinois, United States*, *2. Illinois State University, Normal, Illinois, United States*, *3. University of Illinois-Urbana Champaign, Champaign, Illinois, United States*, *4. University of Illinois Urbana Champaign, Champaign, Illinois, United States*, *5. University of Illinois Urbana-Champaign (UIUC), Champaign, Illinois, United States*

Older adults, especially those with mild cognitive impairment (MCI), are at increasing risk of social isolation and loneliness, leading to poorer health outcomes. Video technology has the potential to provide socially and cognitively engaging activities from their homes. We are exploring the benefits of a virtual social engagement intervention in older adults with and without MCI (clinicaltrials.gov NCT05380180). We are using a video-technology called OneClick.chat to present participants with a short presentation followed by conversational breakout groups. One challenge for the intervention development was the selection of content that would be engaging but not too memory demanding and of interest to a broad range of people. We worked with iN2L to design 60 unique events that met predefined criteria: could facilitate conversations, did not rely on episodic memory, covered a range of topics, and reflected a diversity of individuals, identities, cultures, and interests. Events were categorized into five predefined categories: Arts & Culture; Nature, Health, & Wellness; Life Experiences; Science & Technology; and Recreation & Sports. We carefully curated each event to ensure consistency of presentation length, picture type, readability, visibility, number of slides, and audio quality. Presentation format was carefully tested through multiple iterations to ensure the best participant experience. Conversational prompts were designed for each event to stimulate engaging conversations through open-ended questions. During the trial we are gathering data on participants' preferences for different topics that can guide future iterations. Our systematic process of development can guide content design for research on social engagement interventions.

### SESSION 1320 (PAPER)

### SOCIAL CONNECTIONS AND HEALTH

Abstract citation ID: igad104.0321

### MARITAL BIOGRAPHY AND ACCELERATED AGING: EVIDENCE FROM THE HEALTH AND RETIREMENT STUDY

Won Choi[1], Haena Lee[2], and Eileen Crimmins[3], *1. University of Chicago, Chicago, Illinois, United States*, *2. Sungkyunkwan University, Seoul, Republic of Korea*, *3. University of Southern California, Los Angeles, California, United States*

It is well-established that marriage promotes health and longevity. While a large body of research has examined marital status differences in health, no studies have focused on how marital status is linked to biological age. Moreover, the association between marital biography – transitions in and out of marriage across the life course – and biological aging also remains to be explored. Biological age, which refers to the "physiological functioning relative to the average physiological state at a specific chronological age," has been steadily gaining attention as a measure indicative of an individual's health status. In this study, we advance the literature on marital status and health by examining how marital status and marital transitions are associated with biological aging among a representative sample of U.S. older adults. We use an expanded measure of biological age that incorporates 22 biological indicators, more than half of which were not used in previous studies, to better assess how marital experiences shape accelerated aging. Using data from the 2016 Health and Retirement Study, we find that the pace of aging of those who have experienced one disruption is not statistically different from those continuously married. However, for both currently and previously married individuals, the experience of multiple marital disruptions is associated with accelerated aging. Health behaviors account for the association between multiple marital disruptions and accelerated aging for previously married older adults but not for currently married older adults. Psychosocial resources did not mediate the influence of marital biography on accelerated aging.

Abstract citation ID: igad104.0322

### RELATIONSHIPS OF SOCIAL AND COGNITIVE ACTIVITY TO WELL-BEING AND COGNITIVE FUNCTIONING OVER TIME

Hsiao-Wen Liao[1], Li Chu[2], and Laura Carstensen[3], *1. Georgia Institute of Technology, Atlanta, Georgia, United States*, *2. Stanford University, Stanford, California, United States*, *3. Stanford, Stanford, California, United States*

Research on engaged lifestyles points to promising ways to age well. Findings are mixed, however, when different types of social and cognitive activity engagement are examined. The present study, juxtaposing core tenets of socioemotional selectivity theory and the engagement hypothesis, tested the relative importance of social engagement with close partners and engaging in cognitively stimulating activities for older adults' psychological well-being and cognitive performance over time. Of particular interest was the comparison between individuals with high levels of interaction with close social partners yet low levels of cognitive activity engagement and those with low levels of social engagement but high levels of cognitive activity engagement. The sample (N = 1607) was drawn from participants in the Health and Retirement Study who participated in the 2008, 2012, 2016, and 2020 modules. Engagement profiles were created based on the frequency of contact with close social partners outside the household (i.e., children, family members, and friends) and engagement in cognitively stimulating activities (i.e., reading, writing, using computers, cooking, playing word games, and doing hobbies) at baseline. Results of growth curve modeling, controlling for demographic background and baseline performance, showed that, as predicted, older adults with high levels of social interactions and low levels of cognitive activity engagement had superior life satisfaction but poorer performance on delayed recall than their counterparts who had the opposite engagement profiles. We draw on the life-span principle that development involves gains and losses in interpreting the findings.

Abstract citation ID: igad104.0323

### SOCIAL DISCONNECTION, EPIGENETIC AGING, AND MOTORIC COGNITIVE RISK SYNDROME: A CAUSAL MEDIATION ANALYSIS

Xiang Qi[1], Daniel Belsky[2], Yang Yang[3], Susan Malone[1], Yaguang Zheng[1], and Bei Wu[1], *1. New York University, New York City, New York, United States*, *2. Columbia University, New York City, New York, United States*, *3. University of North Carolina at Chapel Hill, Chapel Hill, North Carolina, United States*

Social isolation and loneliness are two different aspects of social disconnection. Motoric cognitive risk syndrome (MCR), a preclinical phase of dementia, is characterized by memory complaints and slow gait. It is unclear how social disconnection relates to MCR and its underlying mechanisms. Using data from Health and Retirement Study 2012-2020, we aimed to 1) examine the association between social isolation and loneliness and MCR and 2) investigate whether accelerated epigenetic aging mediates this association. We measured social isolation and loneliness at baseline (2012-2014) and epigenetic aging in 2016 using three DNA methylation-based measures (GrimAge, PhenoAge, and DunedinPoAm38), and recorded the incidence of MCR in 2016-2020. We used Cox proportional hazard models to investigate the association between social disconnection and MCR, and counterfactual mediation analysis to examine the role of epigenetic aging. Our sample consisted of 3,287 participants without MCR, with 678 developed MCR during follow-up. Our results showed that social isolation was significantly associated with an older GrimAge, a faster aging pace (DunedinPoAm38), and higher risks of MCR (HR, 1.36; 95% CI, 1.10-1.68), while loneliness was associated with faster DunedinPoAm38 aging pace but not MCR (HR, 0.95; 95% CI, 0.79-1.14). Further, GrimAge mediated 16% of the effect of social isolation (Indirect Effect HR, 1.09; 95% CI, 1.03-1.15), and DunedinPoAm38 mediated 11% (Indirect Effect HR, 1.06; 95% CI, 1.02-1.10). Findings suggest that epigenetic aging is a biological mechanism that underlies the association between social isolation and MCR. Further research is necessary to identify strategies to reduce social isolation for dementia prevention.

Abstract citation ID: igad104.0324

### SOCIAL SUPPORT BUFFERS THE EFFECT OF APOE4 GENE ON COGNITION

Ameante Payen[1], James Bateman[2], and Jeanette Bennett[3], *1. UNC Charlotte, Charlotte, North Carolina, United States*, *2. Wake Forest University School of Medicine, Winston-Salem, North Carolina, United States*, *3. University of North Carolina at Charlotte, Charlotte, North Carolina, United States*

APOE4 allele is one of the most robust genetic risks for Alzheimer's Disease (AD). It is associated with an earlier onset and conversion from Mild Cognitive Impairment (MCI) to AD dementia. Several studies have shown epigenetic interactions between the social context and the APOE4 allele's relationship with cognition. Still, explorations of social risk factors for AD in genetically vulnerable populations are limited. This study explored whether the positive effects of social support (SS) on cognition are affected by APOE status in a sample of 115 older adults (n = 72.25, SD = 8.29). SS was measured using the Multidimensional Scale of Perceived Social Support (MSPSS), the preclinical Alzheimer's Cognitive Composite-5 (PACC5) score to assess concurrent cognition, and the APOE polymorphism was determined via genotyping. As hypothesized, SS buffered the effect of APOE4 gene on PACC5 score, $F(1, 107)=5.18$, $p = .02$, $\Delta R2=.02$. Specifically, older individuals who were positive for the allele displayed a significant and positive association between SS and mPACC5 ($b=.19$, $p = .03$). This relationship was not found for those who were APOE4 negative ($b = -.04$, $p =.49$). These results support the need to consider the epigenetic influence of social factors on later-life cognition, especially for those at risk for developing AD.

### SESSION 1325 (BIOLOGICAL SCIENCES INVITED SYMPOSIUM)

Abstract citation ID: igad104.0325

### LATE BREAKING BIOLOGICAL SCIENCES SESSION

Chair: Blanka Rogina

This is a key element specific to the Biological Sciences Section within the Gerontological Society of America annual scientific meeting that allows for very new and unpublished findings to be presented. Due to the rapid advancement of our knowledge, four speakers in this symposium are selected from the submitted late breaking Abstracts. Special emphasis is given to selecting early career faculty, postdoctoral fellows and graduate students.

Abstract citation ID: igad104.0326

### NOVEL MOLECULAR MECHANISMS UNDERLYING THE ASSOCIATION BETWEEN PLCG2, ALZHEIMER'S DISEASE, AND LONGEVITY

Juan Palavicini[1], Juliet Garcia Rogers[2], Henry Miller[3], Susan Weintraub[4], Sarah Hopp[5], *1. The Barshop Institute for Longevity and Aging Studies, San Antonio, Texas, United States, 2. UT Health SA, San Antonio, Texas, United States, 3. Bioinformatics Research Network, Atlanta, Georgia, United States, 4. UT Health SA, San Antonio, Texas, United States, 5. UT Health SA, San Antonio, Texas, United States*

Phospholipase C-gamma-2 (PLCγ2) catalyzes the hydrolysis of the membrane phosphatidylinositol-4,5-bisphosphate (PIP2) to form diacylglycerol (DAG) and inositol trisphosphate (IP3), which subsequently feed into numerous downstream signaling pathways. PLCG2 rare coding variants are associated with both reduced (P522R) and increased (M28L) risk of Alzheimer's disease (AD) and with longevity (P522R). In the brain, PLCG2 is primarily expressed by microglia, where it is proposed to regulate phagocytosis, secretion of cytokines and chemokines, cell survival and proliferation. We analyzed the brains of 3-month-old PLCγ2 knockout (KO), heterozygous (HET), and wild-type (WT) mice using multi-omic and histological approaches. Lipidomic analyses revealed sex-specific changes in total brain PIP2 and DAG content in KOs. Surprisingly, we found that PLCγ2 depletion led to significant decreases in myelin-enriched lipids including cerebrosides, sulfatides and phosphatidylethanolamine plasmalogens. Consistent with our lipidomics results, gene expression profiling revealed sex-specific changes in myelin-related genes. Further, in line with the available literature, gene expression profiling revealed PLCγ2 depletion led to subtle changes on microglia phenotype, suggestive of reduced microglia reactivity. Immunofluorescence confirmed subtle differences in density of microglia and oligodendrocytes in KOs. Exploratory proteomic pathway analyses revealed changes in KO and HET females compared to WTs, with over-abundant proteins pointing to mTOR signaling, and under-abundant proteins pointing to oligodendrocytes. Furthermore, a PLCG2 M28L knockin mouse model displayed phenotypes of accelerated aging, including exacerbated thymic atrophy, increased frailty scores, and impaired spatial memory. In summary, our study unraveled novel putative mechanisms underlying the association between PLCγ2, AD, and longevity (mTOR signaling and myelin homeostasis).

Abstract citation ID: igad104.0327

### CIRCULATING SASP FACTORS ARE ELEVATED IN PATIENTS WITH POSTOPERATIVE DELIRIUM

Allyson K. Palmer[1], Thomas White[1], Elizabeth Atkinson[1], Nathan LeBrasseur[1], *1. Mayo Clinic, Rochester, Minnesota, United States*

Aging, a major risk factor for delirium, is associated with accumulation of senescent cells in many tissues of the body. Senescent cells produce a host of proinflammatory and tissue remodeling factors collectively termed the senescence-associated secretory phenotype (SASP), which are secreted into circulation. The relationship between increased senescent cell burden and risk of delirium is not known. This study aimed to determine whether changes in circulating SASP proteins are associated with increased risk of postoperative delirium in cognitively intact older adults. Participants were recruited among patients scheduled for surgical or transcatheter aortic valve replacement, and blood was obtained at the presurgical visit. SASP factors were measured using commercially available multiplex magnetic bead immunoassays based on Luminex xMAP multianalyte profiling platform and analyzed on a MAGPIX system. Activin A concentration was determined by a Quantikine ELISA kit. Delirium cases were identified by manual chart abstraction, utilizing the problem list, ICD-10 codes, clinical notes, and nursing flowsheets. Eight of 62 (12.9%) of patients within the cohort developed postoperative delirium. Length of stay was significantly longer for patients who developed delirium (10.6 days) versus patients without delirium (6 days). Concentrations of SASP factors Activin A and PAPP-A were significantly higher in plasma of patients with delirium vs. those who did not develop delirium. IL-1 alpha concentration was significantly lower in patients with delirium vs without delirium. Other SASP factors measured were not statistically different between groups. More study is needed to determine whether these changes are directly related to increased senescent cell burden.

Abstract citation ID: igad104.0328

### INVESTIGATING THE CELLULAR AND MOLECULAR EFFECTS OF AGING ON FAT TISSUE IN DROSOPHILA MELANOGASTER

Justice Bell[1], Isaiah Williams[2], Tancia Bradshaw[2], Alissa Armstrong[2], *1. Claflin University, Charlotte, North Carolina, United States, 2. University of South Carolina, Columbia, South Carolina, United States*

Adipose tissue, also known as fat tissue, is an energy reservoir responsible for metabolic homeostasis. Changes in adipocytes during aging have been associated with obesity, insulin resistance, type two diabetes, and cancer. Thus, in response to the obesity epidemic and the United States aging population, the goal of this experiment is to understand the cellular and molecular effects of aging on adipocytes. In doing so, drosophila were aged at 5,10,15, and 20 days and dissected for adipose tissue. We analyzed adipocyte size, lipid droplet composition, and immune response pathway activity. In this experiment we used the immunostaining process to visualize adipocytes across all ages. Cellular and nuclear size were quantified using ImageJ software. Immune response pathways were examined using reverse transcriptase polymerase chain reaction and gel electrophoresis. Expression of antimicrobial peptides were measured and analyzed against a control, RP49. Our results revealed a positive correlation between fly age, adipocyte and nuclear size. Thus, we can associate aging with increased adipocyte size. Various trends in AMP expression were observed; we have contributed this to the variation in function of each AMP. Further directions include characterizing the effect of aging on AMP expression and examining the relationship between adipocyte size and AMP expression in aging.

Abstract citation ID: igad104.0329

### NEURODEGENERATION BIOMARKERS HELP EXPLAIN ASSOCIATION BETWEEN INFLAMMATION AND COGNITIVE DYSFUNCTION

Eric T. Klopak[1], Eileen M. Crimmins[1], *1. University of Southern California, Los Angeles, California, United States*

There is past evidence that immune aging (or immunosenescence) and high systemic inflammation are associated with cognitive dysfunction and ADRD. However, it is unknown whether these immune-related aging processes are associated with neurodegeneration, tauopathy, amyloid accumulation, and/or vascular issues in the brain.

To address this gap, we utilized a set of recently assayed highly innovative markers of neurodegeneration, biomarkers of inflammation, and proportions of naïve lymphocytes (indicative of less immune aging), and cognitive dysfunction (errors on the Telephone Interview for Cognitive Status) in the Health and Retirement Study, a nationally representative sample of US adults over age 50 (N = 3985).

Using structural equation modeling (SEM), we estimated latent factors representing neurodegeneration (neurofilament light chain (NfL), glial fibrillary acidic protein (GFAP), and phosphotau 181 (pTau181)), inflammation (c-reactive protein, IL10, IL1 receptor agonist, IL6, TNF receptor 1, albumin, and percentage of neutrophils), and immune aging (percentages of naïve CD8+ and CD4+ T cells). Results indicate inflammation is associated with neurodegenerative biomarkers ($\beta = .31$, $p < .001$) and with cognitive dysfunction ($\beta = .08$, $p < .001$) after controlling for age, race, gender, education, BMI, smoking, drinking, and immunosenescence. Neurodegenerative biomarkers mediated about half of the association between inflammation and cognitive dysfunction.

It appears that greater systemic inflammation may be associated with brain damage and tauopathy. Interventions focused on reducing systemic inflammation may be successful in reducing neurodegeneration and cognitive dysfunction in older adults.

Abstract citation ID: igad104.0330

### EVALUATING CIRCULATING GEROSCIENCE BIOMARKERS

Nathan Basisty, *National Institute on Aging, Baltimore, Maryland, United States*

### SESSION 1330 (SYMPOSIUM)

Abstract citation ID: igad104.0331

### MAXIMIZING COLLECTIVE IMPACT ACROSS THE AGE-FRIENDLY ECOSYSTEM

Chair: Holly Dabelko-Schoeny Co-Chair: Teri Kennedy

Age-friendly initiatives have the potential to transform communities through inclusive policies and practices that meet the needs of people, families, and communities across ages and abilities. The ecosystem of age-friendly initiatives (communities, universities, public health systems, and health systems) has grown exponentially over the last decade. Despite shared values and principles, each age-friendly initiative largely operates independently with limited opportunities to share knowledge to maximize collective impact and social change. This theoretically-driven symposium provides an overview of each age-friendly initiative, challenges faced, and ideas for innovation within and across each initiative. Dabelko-Schoeny and Sheldon will utilize a macro application of the KAP (knowledge, attitude, practice) theory of behavior change to identify the vast opportunity for synergies and grand challenges across the age-friendly ecosystem. Greenfield will provide an overview of the development of the Age-Friendly Cities and Community Movement, and present ideas regarding challenges and opportunities for the future based on insights from an ongoing, community-engaged study in New Jersey. Montepare will describe the Age-Friendly University initiative and how age-inclusive practices in higher education can extend and enhance ecosystem efforts. Phillips and Wolfe will provide examples of Age-Friendly Public Health Systems including a description of a collective impact approach for state-wide age-friendly policy and practice adoption. Finally, Kennedy will offer approaches and models to advance the adoption and sustainability of age-friendly principles, programs, and policies by leveraging synergies and implementation science. This is an Age-Inclusivity in Higher Education Interest Group Sponsored Symposium.

Abstract citation ID: igad104.0332

### BETTER TOGETHER: STRENTHENING THE AGE-FRIENDLY ECOSYSTEM

Holly Dabelko-Schoeny[1], and Marisa Sheldon[2], *1. The Ohio State University, Columbus, Ohio, United States*, *2. Ohio State University, Columbus, Ohio, United States*

Frameworks to make communities, universities, public health systems, and health services more inclusive and equitable for older adults and people with disabilities have expanded over the last decade. Centering the voices of older adults, these “age-friendly” initiatives have sought social change through building knowledge, changing attitudes, and developing new practices and policies in a variety of settings. Despite the similarities in values and principles across these initiatives, these models of social change have largely operated independently from one another with unique administrative structures, networks, and resources. This presentation will utilize a macro application of the KAP (knowledge, attitude, practice) theory of behavior change to identify the vast opportunity for synergies across the age-friendly ecosystem, strengthening the potential for collective impact and lasting change. Often these initiatives have overlapping and intersecting goals and would benefit from intentional alignment across the initiatives. Modeled after the National Academy of Engineering Grand Challenges and the Grand Challenges for Social Work, we propose the Grand Challenges for the Age-Friendly Ecosystem, areas in need of action and support, for transformational change in our social, educational, service, and built environments to better meet the needs of our aging society.

Abstract citation ID: igad104.0333

### ADVANCING ADOPTION AND SUSTAINABILITY OF AGE-FRIENDLY INITIATIVES THROUGH SYNERGIES AND IMPLEMENTATION SCIENCE

Teri Kennedy, *University of Kansas Medical Center, Kansas City, Kansas, United States*

For communities interested in adopting age-friendly principles, the plethora of age-friendly initiatives (i.e., age-, disability-, and dementia-friendly cities and communities; age-friendly universities, health systems, and public health systems) can be overwhelming. For rural, frontier, underserved, and under-resourced communities, it can be challenging to consider tackling even one such initiative. A major barrier to implementation is the know-do gap between what is known and what gets accomplished in practice. This gap is widened as a result of overly complex models and practices that are difficult to disseminate and scale, difficult to reproduce, or do not apply across settings. The know-do gap can be effectively bridged by focusing on core features common across age-friendly ecosystem initiatives. To successfully advance this work, it is important to consider strategies to maximize the collective impact of age-friendly initiatives and explore opportunities to tailor these initiatives so they can be successfully adapted for and adopted by various settings, contexts, and populations. Using an implementation science approach, three models will be introduced (Strengths-Based Interprofessional Practice and Education (SB-IPE); Micro-, Meso-, Macro- M3: Aligning Your Nexus Through a Systems Lens; and Kennedy Model of Sustainability) to facilitate the adoption and sustainability of evidence-based age-friendly principles, programs, and policies.

Abstract citation ID: igad104.0334

### AGE-FRIENDLY CITIES AND COMMUNITIES: RESEARCH ON LOCAL PRACTICE TO STRENGTHEN THE MOVEMENT'S FUTURE

Emily Greenfield[1], Natalie Pope[2], and Althea Pestine-Stevens[1], *1. Rutgers, The State University of New Jersey, New Brunswick, New Jersey, United States*, *2. Rutgers Social Work, New Brunswick, New Jersey, United States*

The age-friendly cities and communities (AFCC) movement has accelerated a major paradigmatic shift for the field of aging in the 21st century. Launched and spurred by high-profile government agencies and organizations across the globe, this ambitious movement highlights the transformative potential of deliberate, cross-sectoral efforts to improve built, social, and service environments of localities in response to population aging. This presentation will provide a brief overview of the development of a global program of policy and practice on AFCCs, with an emphasis on its emergence in the United States. It will then present insights regarding challenges and opportunities for the future based on findings from an ongoing, longitudinal study of a regional network of AFCC initiatives. The study, designed as a developmental evaluation, explores the perspectives of on-the-ground AFCC leaders on their practice as part of a private philanthropic grantmaking initiative to spur AFCCs in New Jersey. Reflecting on our years of engaged research with this network, the presentation will highlight issues concerning multi-level systems alignment, the promises and pitfalls of public sector leadership, the need for additional practice theories, and greater incorporation of considerations of aging equity and racial justice. The presentation will demonstrate the importance of continuing to leverage the strengths of community-engaged research to better ensure the movement's relevance, reach, and equitable impact across diverse community, organizational, and broader systems contexts.

Abstract citation ID: igad104.0335

### AGE-FRIENDLY CAMPUSES: NEED AND OPPORTUNITY FOR SYNERGY IN THE AGE-FRIENDLY ECOSYSTEM

Joann Montepare, *Lasell University, Newton, Massachusetts, United States*

Historic shifts in age demographics are reshaping societies and challenging institutions of higher education to respond to older, more age diverse populations through new approaches to teaching, research, and community engagement. Student populations are also becoming more age diverse with adults looking to higher education for lifelong learning opportunities to enhance their personal and professional development as they age. This presentation will describe the guiding framework and set of principles provided by the Age-Friendly University (AFU) initiative to help institutions of higher education shape more age-inclusive practices. Age-friendly campuses can also create opportunities to support other initiatives beyond a campus such as those geared toward age-friendly communities, health care systems, and employers. This presentation will also discuss how synergistic AFU partnerships with other age-friendly initiatives can provide mutual support to strengthen and sustain these efforts. Challenges to this end will also be explored. Examples of existing and promising connections in the ecosystem will be offered to demonstrate the value of this synergy.

Abstract citation ID: igad104.0336

### AGE-FRIENDLY PUBLIC HEALTH SYSTEMS IN ACTION

Karon Phillips, and Megan Wolfe, *Trust for America's Health, Washington, District of Columbia, United States*

Trust for America's Health (TFAH) leads the national Age-Friendly Public Health Systems movement, the goal of which is to elevate healthy aging as a core function of state and local departments of health. The nation's public health system has contributed to the increased longevity of the population and there are numerous opportunities for public health to advance healthy aging in collaboration with the health care system, the aging services sector and others. TFAH has worked directly with state and local health departments to test the Age-Friendly Public Health Systems framework and seen first-hand the policy and systems changes that are possible through collective impact. During this symposium, participants will learn about the changes that are occurring in Mississippi and Washington state, where Age-Friendly Public Health practices are being adopted and implemented in collaboration with key partners, including expanding data collection on older adult health, ensuring older adults and caregivers are considered in emergency planning, and working directly with older adults in Tribal communities.

### SESSION 1335 (PAPER)

### ADRD FAMILY CAREGIVING

Abstract citation ID: igad104.0337

### EXAMINING A RANDOMLY ASSIGNED MUSIC PROGRAM ON STRESS AND WELLNESS: CARE PARTNERS OF OLDER ADULTS LIVING WITH DEMENTIA

Catherine Tompkins, Emily Ihara, Shannon Layman, Megumi Inoue, and Gilbert Gimm, *George Mason University, Fairfax, Virginia, United States*

OBJECTIVE: The Mason CARES study examined the effects of a randomly assigned personalized music intervention (Phase II) for care partners of older adults living with dementia after implementation of a 9-week virtual stress management program (Phase I) to reduce stress and increase well-being. **METHODS:** A total of 99 care partners completed the 20-week study across three cohorts in 2022. Baseline data on care partner stress levels and well-being were collected before beginning a 9-week stress management program (Phase I). Care partners were randomly assigned to a 4-week music intervention treatment or control group before the next stage of the intervention (Phase II). Researchers assisted care partners with identifying their family member's favorite music during early adulthood years. MP3 players loaded with the favorite music were mailed to treatment group members. Focus groups were conducted to explore the utilization of personalized music. RESULTS: Nearly half (49%) were spousal care partners, ranging from 35-85 years old with 80% having at least a 4-year college degree, and 62% having an income greater than $70,000. Using ANOVAs to analyze mean stress levels and well-being, no significant differences in stress and well-being were found between the treatment and control groups. CONCLUSION: Despite the lack of statistically significant findings, preliminary analysis of focus group interviews revealed that many care partners benefited from the personalized music intervention. Effectiveness may depend on other factors, such as familiarity with using the technology, past experiences with music, care partner's baseline level of stress, and the older adult's stage of dementia.

Abstract citation ID: igad104.0338

### EXPLORING THE EVERYDAY EXPERIENCES OF FAMILY CAREGIVERS OF PEOPLE LIVING WITH DEMENTIA: A LONGITUDINAL DIARY STUDY

Kishore Seetharaman[1], Lucy Kervin[1], Koushambhi Basu[2], and Jennifer Baumbusch[2], *1. Simon Fraser University, Vancouver, British Columbia, Canada*, *2. University of British Columbia, Vancouver, British Columbia, Canada*

People living with dementia rely largely on the support of unpaid family caregivers to remain living at home in the community. The strain created by this dependence is evidenced by high rates of distress among caregivers of people living with dementia and underscores the importance of access to appropriate formal and informal supports for this group. Providing tailored and meaningful supports and services to families requires a good understanding of the multidimensional and dynamic nature of the caregiving experience. Our study employed longitudinal qualitative methodology to understand how caregivers of people living with dementia were experiencing progression of the disease and shifting support needs over a two year-period. Fourteen family caregivers in British Columbia, Canada, wrote monthly diaries reflecting on their experience of caregiving and navigating supports. Thematic analysis of 137 diary entries shed light on different facets of caregiving experience, including psychological and emotional challenges (e.g., feeling overwhelmed, lacking a sense of control, trouble maintaining sense of self) and difficulties navigating formal care and supports (e.g., lack of access to services and information, lack of flexibility in service delivery). However, accounts also reflected participants' resilience, through a range of coping strategies adopted to resolve problems encountered in caregiving, drawing on emotional and practical support from family, friends, and community. The diaries offer critical perspectives on gaps and inadequacies in formal supports for family caregivers and opportunities to better support the health and wellness of caregivers and recipients through dementia care policy and practice.

Abstract citation ID: igad104.0339

### LOSING PERSONHOOD: EXPERIENCES OF INDIVIDUALS CARING FOR A FAMILY MEMBER WITH DEMENTIA

Rachel Carter[1], Sally Norton[1], Marian Moskow[2], Melanie Bobry[1], Kimberly Van Orden[3], Kathi Heffner[3], and Marsha Wittink[1], *1. University of Rochester, Rochester, New York, United States*, *2. University of Rochester School of Nursing, Rochester, New York, United States*, *3. University of Rochester Medical Center, Rochester, New York, United States*

Over 11 million people in the United States are providing informal care for a person with dementia (PwD). When providing care, family caregivers for a PwD may find their own sense of personhood becomes lost, impacting their social connectedness. This qualitative study characterizes family caregivers' social connection experiences, goals, barriers, and facilitators. We used a criterion-based purposeful sampling strategy to identify 30 family caregivers for PwD ages 50+ who were lonely (n=20) and not lonely (n=10) according to their UCLA loneliness scale short form scores (not lonely = < 5 and lonely = ≥ 6). We recruited selected participants through the Healthy Aging Research Program (HARP) population study. We then conducted individual, semi-structured interviews with each participant and performed a content analysis on all transcribed interviews, utilizing preset codes based on a theoretical frame and developing novel codes until key themes were identified. Participants reported connections between social connectedness—loneliness, disconnection, and feelings of isolation—and the loss or diminishment of their own personhood. When asked about their past and present social activities, the ones caregivers identified as important allowed them to feel connected to others and to themselves, offering either or both: 1) the opportunity to relate to others in ways they could not relate to the PwD; 2) social and emotional engagement that reinforced their sense of personhood. When social activities did not offer either of these, caregivers experienced disconnection. Results indicate potential ways to intervene to mitigate loss of personhood and promote social connectedness in family caregivers.

Abstract citation ID: igad104.0340

### MASON CARES RANDOMIZED STUDY: EFFECTS OF A NINE-WEEK STRESS MANAGEMENT PROGRAM INTERVENTION ON CAREGIVER BURDEN SCORES

Gilbert Gimm, Shannon Layman, Emily Ihara, Megumi Inoue, and Catherine Tompkins, *George Mason University, Fairfax, Virginia, United States*

**OBJECTIVE:** The Mason CARES study examines the impact of a virtual stress management program (Phase 1) and music intervention (Phase 2) on THE stress of family caregivers of older adults with dementia. This study presents Phase 1 findings on 99 participants enrolled in 2022. **METHODS:** Using primary data from 99 participants, we analyzed caregiver stress levels before and immediately after implementation of a 9-week stress management program using Zoom videoconference calls. Caregiver stress was measured using the Zarit Burden Interview (ZBI) score with a range from 0 to 48 (Bédard, 2001). Higher values (ZBI >=17.0) are associated with higher levels of stress. **RESULTS:** Among the 99 caregivers, 49.4% were spouses and 50.6% were non-spouses (e.g., adult children). Mean ZBI scores before and after the 9-week stress busting program intervention across the full sample decreased by 14.7% (from 23.7 to 20.2). Spouses experienced a greater decrease (17.9% from 23.5 to 19.3) in caregiver stress compared to non-spouses (12.1% from 23.9 to 21.0). Reductions in stress levels also occurred for caregivers of older adults with mild cognitive impairment (13.9% from 21.0 to 18.0), moderate dementia (16.5% from 24.1 to 20.1), and advanced dementia (9.7% from 27.7 to 25.0). **CONCLUSION:** Findings show that caregivers had high stress levels at baseline (ZBI score of 17 or more). Spouses reported greater stress improvement compared to non-spouses after the 9-week stress busting program. Also, caregivers of older adults with moderate dementia had greater improvement in stress compared to the mild or advanced dementia sub-groups.

Abstract citation ID: igad104.0341

### PERSPECTIVES OF CAREGIVERS TO BLACK AND HISPANIC PERSONS LIVING WITH DEMENTIA (PLWD) ABOUT END-OF-LIFE COMMUNICATION

Zainab Toteh Osakwe[1], Sasha Perez[2], Michelle Odlum[3], and Ana Stefancic[4], *1. Adelphi University, Garden City, New York, United States*, *2. Icahn School of Medicine at Mount Sinai, New York City, New York, United States*, *3. The George Washington University, Washington, District of Columbia, United States*, *4. Columbia University, New York City, New York, United States*

Black and Hispanic Persons Living with Dementia (PLWD) heavily rely on home healthcare to remain comfortably in their own home, even at the end of life. Despite growth in use of hospice in the U.S., research demonstrates persistent racial differences in utilization of hospice - a key indicator of high-quality end of life care. This is particularly relevant among older Black and Hispanic PLWD who experience additional burdensome interventions at the end of life which often run counter to their goals of care. This study examined perceptions of caregivers to Black and Hispanic PLWD regarding discussions with home healthcare nurses about end-of-life communication and transitions to hospice. We conducted semi-structured interviews with 19 Black and Hispanic family caregivers of PLWD. Interviews were recorded, transcribed, translated and analyzed using conventional content analysis. Results showed that Black and Hispanic caregivers of PLWD do not prioritize aggressive and burdensome care at the end of life, had low expectations of the healthcare system to support their needs and preferences at the end-of-life, and were willing to discuss and accept hospice if discussions were transparent about resources and entitlements; acknowledged the strengths of the PLWD/caregivers; involved positive and respectful communication with families during a home visit; acknowledged respect for their social communities and home environment; sat down during a home visit and recognized the role of religion at the end-of-life. Implications to improve care include incorporating family caregiver discussions, identify social determinants of health, and address the role of religion in future interventions.

Abstract citation ID: igad104.0342

### THE ROLE OF SOCIAL ENTERPRISE ORGANIZATIONS IN SUPPORTING FAMILY CARERS OF OLDER PEOPLE LIVING WITH DEMENTIA

Maria Cheshire-Allen, and Abigail Davies, *Swansea University, Swansea, Wales, United Kingdom*

This paper aims to present findings from a primary qualitative study that explored the role of social enterprise organisations (SEOs) in delivering co-produced support for family carers of older people living with dementia (PLWD). Defined in the UK, as businesses with a social or environmental purpose, SEOs are increasingly recognised as occupying a unique space in the landscape of adult social care delivery. In Wales, devolved legislation requires public bodies to collaborate with SEOs - this provision is intended to create a mixed economy of care with co-produced user-led services. To date, however, there is little firm evidence of SEOs delivering co-produced care solutions within the policy framework in Wales, which can be understood to be particularly permissive of SEO involvement, or of how the sector is involved in the delivery of support for family carers of older PLWD. Findings gathered through five focus groups with a purposive sample of family carers, and SEO care providers in South Wales, show how co-produced family carer support programmes are navigated and enacted, as well as how the individual agency of service providers, PLWD, and their carers are involved in these processes. Adopting an ethics of care approach, and utilising reflexive thematic analysis, key themes identified include competition between and within organisations, parochialism, capacity, and organisational barriers. This study highlights implications for policy and practice related to the rhetoric of co-produced support services for family carers, SEOs' views on delivering that support, and family carers' experiences of navigating and receiving SEO-delivered services.

### SESSION 1340 (SYMPOSIA)

Abstract citation ID: igad104.0343

### FAMILY CAREGIVING DURING THE COVID-19 PANDEMIC FOR OLDER ADULTS WITH DEMENTIA

Chair: Emily Wiemers
Co-Chair: I-Fen Lin

Family caregiving plays a crucial role in meeting the care needs of older adults, especially those living with Alzheimer's disease and related dementias (ADRD). The COVID-19 disrupted an already fragile care landscape by reducing the desirability of nursing home care and the availability of paid caregivers in the home and by destabilizing family caregiving arrangements. Older adult living with ADRD may be at higher risk for destabilized care and more vulnerable to adverse effects from the COVID-19 pandemic’s disruption to the long-term care landscape. This symposium draws on five papers providing qualitative and quantitative evidence of how care arrangements shifted during the COVID-19 pandemic among older adults with ADRD. Patterson and colleagues set the stage for understanding the impacts of the pandemic on family caregiving for older adults with dementia by providing estimates of how many families and households include someone with dementia. Choi and colleagues use a mixed-methods approach to show that family members provided more care during than before the pandemic, especially coresident family members. Lin and colleagues use quantitative evidence to examine how pandemic-specific needs and help from nonresident adult children varied by older adults' cognitive functioning. Leggett and Tsuker examine the consequences of the pandemic for caregiver stress across different ADRD caregiver relationship types. Finally, Wiemers and colleagues extend the focus on unpaid family caregivers to include paid care and show how tradeoffs between paid and unpaid care for older adults with ADRD were affected by pandemic severity.

Abstract citation ID: igad104.0344

### CHANGES IN LONG-TERM CARE ARRANGEMENTS AMONG OLDER ADULTS WITH ADRD DURING THE FIRST YEAR OF THE COVID-19 PANDEMIC

Emily Wiemers[1], Yulya Truskinovsky[2], Amanda Leggett[2], Geoffrey Hoffman[3], and Vicki Freemdan[3], *1. Syracuse University, Syracuse, New York, United States*, *2. Wayne State University, Detroit, Michigan, United States*, *3. University of Michigan, Ann Arbor, Michigan, United States*

This study will examine how COVID-19 affected care needs and immediate long-term care (LTC) outcomes including paid and family care, coresidence with adult children, and nursing home use for older adults living with ADRD. For this study we use the 2018 and 2020 waves of the Health and Retirement Study (HRS), a longitudinal, nationally representative survey of individuals aged 51 and older in the U.S. To assess how long-term care arrangements changed among older adults with dementia during the first year of the COVID-19 pandemic, we first describe the needs and LTC outcomes of older adults living with dementia and examine changes between 2018 and 2020 comparing them to changes among older adults with care needs but no dementia. Then, to identify changes associated with COVID-19 (rather than secular trends due to disease progression) we compare the 2020 LTC outcomes of HRS respondents with care needs who had differential exposure to the severity of the pandemic. We proxy pandemic severity with county level COVID-19 deaths. To understand whether changes in LTC outcomes among older adults with dementia were different from the broader population of high-need older adults, we interact local COVID-19 severity with the presence of dementia in 2018. Preliminary findings indicate a retreat from paid care and increases in coresidence with adult children and receiving unpaid help but no significant differences in LTC outcomes among high need older adults with and without dementia.

Abstract citation ID: igad104.0345

### TIME HELP FROM ADULT CHILDREN DURING THE COVID-19 PANDEMIC: VARIATION BY PARENTS' COGNITIVE FUNCTIONING

I-Fen Lin[1], Emily Wiemers[2], Janecca Chin[1], and Anna Wiersma Strauss[2], *1. Bowling Green State University, Bowling Green, Ohio, United States*, *2. Syracuse University, Syracuse, New York, United States*

Many older adults in the U.S. do not live with adult children but have at least one adult child living nearby. Help from adult children is vital for supporting the health and well-being of older adults. During the COVID-19 pandemic, the need to maintain physical distancing and the fear of infection may cause disruptions in older adults' activities of daily living but the extent to which nonresident adult children provide help with household tasks is largely unknown. This paper uses data from the 2018 and 2020 Health and Retirement Study (HRS) to assess whether adult children responded to their parents' pandemic-specific needs by helping them with shopping for groceries, errands, rides, or chores (i.e., time help). Preliminary analysis shows that parents who had trouble buying food (even with the money) were more likely to receive time help from their nonresident adult children. Not only was time help much more common among older adults with children living nearby than older adults without any child living nearby but adult children living nearby also were more responsive to parents' pandemic-specific needs than children who lived farther away. Additional analysis indicates an overall increase in time help from adult children in response to functional limitations between 2018 and 2020, suggesting that the pandemic may have increased family care more broadly. Because older adults with cognitive limitations may be particularly vulnerable during the pandemic, we will further examine whether pandemic-specific needs and time help from nonresident adult children differed depending on older adults' cognitive functioning.

Abstract citation ID: igad104.0346

### FAMILIES AND DEMENTIA: ESTIMATES AND EXPOSURES

Esther Friedman, Vicki Freedman, and Sarah Patterson, *University of Michigan, Ann Arbor, Michigan, United States*

Roughly 10 to 20% of older adults in the U.S. have dementia, which could have implications for their families and household members, who are likely to be called upon to provide care. Yet, it is not clear how many families and households include someone with dementia. This study provides national level estimates of families and households with an older adult with possible dementia using the 2017 Panel Study of Income Dynamics (PSID). Consistent with prior work, we find that 21.5% of older adults 65+ have possible dementia. Moreover, more than a quarter (26.3%) of households with an adult aged 65+ include an older adult with dementia, and 37.0% of extended family networks (with at least one adult aged 65+) have at least one older adult with dementia. Individual-level stratification of dementia by racial-ethnic group and socioeconomic status translates directly to household- and family-level patterns. For instance, households and families with an older adult who is either Black or Hispanic or does not hold a college degree are more likely to include at least one person with possible dementia. These findings have implications for the extent to which American families and households include someone with dementia, which comes with a risk of serving as a caregiver as well as possible implications for health and well-being. Our findings also establish important baseline estimates that can be measured against future estimates, for instance those post COVID-19.

Abstract citation ID: igad104.0347

### FAMILY DYNAMICS IN CARING FOR PEOPLE WITH DEMENTIA DURING THE COVID-19 PANDEMIC

HwaJung Choi[1], and Amanda Leggett[2], *1. University of Michigan, Ann Arbor, Michigan, United States*, *2. Wayne State University, Detroit, Michigan, United States*

A nuanced understanding of family care dynamics (e.g., change in care availability, care network, and care involvement) is important to assess the coping mechanism in dementia care during the pandemic. We conducted an explanatory sequential mixed methods study. We examined 300 spouses and adult children of people with dementia (PWD) from the National Health and Aging Trend -- Family and Friend, surveyed for July 2020-Feb 2021 (Quantitative analysis), and conducted qualitative analyses based on one-to-one in-depth interviews with an independent sample of 40 family members of PwD (Jan-Dec 2022). Fifty-five percent lived with PwD at the time of the interview (7% moved in with PwD during the pandemic). 35% of the non-coresident family members had fewer in-person contacts with PwD during vs. before the pandemic. More than one in ten of the sample persons lost their jobs during the pandemic. Overall, family members were more likely to provide personal care and instrumental help for PwD during vs. before the pandemic. Caregiving however is more concentrated on coresident family members during the pandemic as in-person visits were limited. Flexible work situations during the pandemic were valued as they facilitated caregiving. Family members who were not directly involved in providing personal care for PwD provided emotional and instrumental support for the primary caregiver. Findings from the study will provide valuable insights into the family dynamics in dementia care during the COVID-19 pandemic, which would inform the development of intervention programs for mitigating the adverse impacts of the pandemic and beyond.

Abstract citation ID: igad104.0348

### ALL IN THE FAMILY: DIFFERENTIAL IMPACT OF COVID-19 BY TYPE OF CAREGIVER FOR PERSONS LIVING WITH DEMENTIA

Amanda Leggett, and Sophia Tsuker, *Wayne State University, Detroit, Michigan, United States*

Existing research points toward higher burden for spousal, and particularly wife, caregivers for persons living with dementia (PLwD). Yet how relationship roles and stressors shifted during the COVID-19 pandemic, where some care dyads were isolated together, or physically distancing, is unknown. The current study explores how the variability in reports of COVID-specific care-related stress (e.g., worry about the PLwD getting COVID-19) and general strain (e.g., caregiver burden) differed among husband, wife, adult child, and other family and non-family, unpaid caregivers. Participants include 100 primary family caregivers for PLwDs (mean age=63.3 years, 75% female) from the COVID STYLE study of which 22 were husbands, 39 were wives, 24 were adult children, and 15 were another relation (e.g., daughter-in-law, friend). ANOVAs and linear regressions (adjusting for cohabitation with the PLwD, dementia severity, and race) were run with each relation as a predictor of COVID-19-specific stress (e.g., sleep quality, isolation) and non-COVID-19-specific well-being (e.g., depressive symptoms, positive affect). The "other" caregiver group showed significantly greater COVID-related financial difficulty of care, worse sleep quality, and isolation, whereas adult children were most worried about the PLwD getting COVID-19 and had greater difficulty in their ability to provide care. Associations largely held in adjusted regressions showing particular contrast between other caregivers and husbands, who fared best. On the other hand, relationships groups did not differ on non-COVID-specific measures of well-being. Non-spousal caregivers reported greater pandemic stress related to caregiving, suggesting an overlooked group who may have provided significant COVID-19 care despite a more distant relationship.

### SESSION 1345 (SYMPOSIUM)

Abstract citation ID: igad104.0349

### ADVANCING FOOD EQUITY AMONG OLDER ADULTS AND ADULTS WITH DISABILITIES IN THE US

Chair: Laura Samuel Co-Chair: Boeun Kim

Older adults and adults with disabilities face challenges related to food insecurity, defined as limited or uncertain access to adequate food due to economic and social conditions. Adults with disabilities have triple the risk of food insecurity than their peers and rates of food insecurity have been rising over the past two decades among older adults and food insecurity is known to be a risk factor for numerous adverse health outcomes. Despite knowledge of disparities, research is needed to identify and test strategies for addressing food insecurity disparities. This symposium will examine mechanisms that may contribute to food insecurity, including local food environments, vision impairment and access to the Supplemental Nutrition Assistance Program, which is the largest food assistance program in the U.S.. National data is used in all three studies to allow population-based inferences to be drawn from study findings. Results have potential to inform our understanding of food equity and advance data-driven policy and programmatic decisions.

Abstract citation ID: igad104.0350

### THE RELEVANCE OF SNAP PROGRAM ACCESSIBILITY FOR FOOD INSECURITY, FOCUSING ON ADULTS WITH DISABILITIES

Laura Samuel[1], Rhonda Wright[1], Varshini Varadaraj[2], and Bonnielin Swenor[2], *1. Johns Hopkins University School of Nursing, Baltimore, Maryland, United States*, *2. Johns Hopkins University, Baltimore, Maryland, United States*

Individuals with disabilities are not only more likely than their peers without disabilities to experience food insecurity, but also encounter disproportionate barriers to Supplemental Nutrition Assistance Program (SNAP) enrollment. This study examined associations between state-level measures of SNAP enrollment accessibility (flexibility, efficiency, and accessibility in the enrollment process; and the presence of either broad-based categorical eligibility or the Combined Application Project) with SNAP participation and food insecurity levels among low-income participants (income ≤185% of the poverty threshold) aged ≥15 years in the 2021 Current Population Survey, both overall

(n=19,895) and among those with disabilities (n=3,484). In multi-level models adjusted for individual and state characteristics, living in a state with broad-based categorical eligibility was associated with higher rates of SNAP participation among all low-income individuals (b= 0.300, 95% CI: 0.096, 0.505). Among those with disabilities, having greater flexibility in SNAP enrollment (b= 0.141, 95% CI: 0.007, 0.276) and living in a state with the Combined Application Project (b= 0.271, 95% CI: 0.017, 0.525) were associated with a higher likelihood of SNAP participation. Living in a state with more efficient SNAP enrollment was associated with lower food insecurity levels (b= -0.073, 95% CI: -0.136, -0.010) among all low-income individuals and having broad-based categorical eligibility was associated with lower food insecurity levels (b= -0.237, 95% CI: -0.451, -0.022) among those with disabilities. These findings highlight the importance of SNAP program accessibility to address food insecurity among all low-income Americans and suggest that enrollment barriers may differ for people with and without disabilities.

Abstract citation ID: igad104.0351

### FOOD DESERT RESIDENCE AND COGNITIVE TRAJECTORIES IN US OLDER POPULATION AGED 65 AND OLDER

Boeun Kim[1], Sarah L. Szanton[1], Roland Thorpe[1], Paris Adkins-Jackson[2], Deidra Crews[1], and Laura Samuel[3], *1. Johns Hopkins University, Baltimore, Maryland, United States*, *2. Columbia University, New York City, New York, United States*, *3. Johns Hopkins University School of Nursing, Baltimore, Maryland, United States*

A food desert is defined as a census tract with limited access to affordable and nutritious foods. Inequitable access to healthy food may contribute to disparities in cognitive health, but little is known about the role of food deserts in cognitive health. We examined association between living in food deserts and cognitive function in older adults 65 years and older. These analyses employed cohort design by linking the US Department of Agriculture 2010 Food Access Research Atlas data to 2011-2021 National Health and Aging Trends Study. Orientation, executive function, and memory were assessed annually. Food desert residence at baseline was determined if more than 500 or 33% residents within a census tract had low income and low access to healthy and affordable foods. Weighted mixed-effects models were fitted among 5270 community-dwelling older adults without dementia at baseline. Mean baseline age was 76.7 (±7.4) years and 10.7% participants (n = 659) lived in food deserts. Black and Hispanic people and individuals with lower educational attainment were more likely to live in food deserts. At the age of 80 years, individuals living in food deserts showed orientation and executive function scores that were -0.26 points (95% CI: -0.48, -0.03) and -0.13 points (95% CI: -024, -0.01) lower than their peers, respectively. Living in food deserts was also associated with 0.05 points faster annual decline (95% CI: -0.09, -0.01) in orientation. Residence in food deserts is associated with worse cognition in older age and could be a driver of disparities in cognitive health.

Abstract citation ID: igad104.0352

### VISION LOSS AND FOOD INSECURITY IN THE NATIONAL HEALTH AND AGING TRENDS STUDY

Louay Almidani[1], Moon Lee[1], Bonnielin Swenor[2], Laura Samuel[3], and Varshini Varadaraj[2], *1. Wilmer Eye Institute, Baltimore, Maryland, United States*, *2. Johns Hopkins University, Baltimore, Maryland, United States*, *3. Johns Hopkins University School of Nursing, Baltimore, Maryland, United States*

Adults with vision impairment (VI) may be at a greater risk of experiencing food insecurity due to economic hardships and difficulty with daily activities. However, the relationship is not well understood. We examined the cross-sectional associations between VI and food insecurity in older community-dwelling US adults among 2,815 National Health and Aging Trends Study participants. VI was measured on categorical (distance acuity VI [> 20/40], near acuity VI [> 20/40], contrast sensitivity impairment [CSI] [<1.55], and any VI (distance, near, or CSI)) and continuous scales (distance and near acuity [per 0.1 logMAR], and contrast sensitivity [per 0.1 logCS units]). Food insecurity was classified based on functional limitations, lack of social support, and/or financial limitations. Overall, older adults with any VI had higher prevalence rates of food insecurity than adults without VI (4.56% vs. 1.90%). In fully-adjusted models, older adults with any VI had double the odds of food insecurity than their peers (OR=2.13, 95% CI: 1.25–3.62). Although no associations were found when assessing other vision measures categorically, continuous scales showed strong associations; worse distance (OR=5.00, 95% CI: 1.85–13.48) and near acuity (OR=2.56, 95% CI: 1.05–6.21, per 0.1 logMAR), and worse CS (OR=2.57, 95% CI: 1.11–5.94, per 0.1 logCS units) were associated with higher odds of food insecurity. These results suggest that older Americans with VI have a higher likelihood of food insecurity, and highlight a need to examine strategies, such as more disability-inclusive policies impacting food access, to improve accessibility and reduce these inequities.

### SESSION 1350 (PAPER)

### SOCIAL DETERMINANTS, SENSORY IMPAIRMENT, AND HEALTH BEHAVIORS

Abstract citation ID: igad104.0353

### ADVERSE CHILDHOOD EXPERIENCES AS A PREDICTOR OF HEALTH BEHAVIORS IN WOMEN

Karina Tavares[1], Cindy Tsotsoros[1], Skye Leedahl[1], and Jessica Tsotsoros[2], *1. University of Rhode Island, Kingston, Rhode Island, United States*, *2. University of Oklahoma Health Sciences Center, Tulsa, Oklahoma, United States*

Individuals who experience adversity during childhood are at a higher risk of negative health outcomes in adulthood. This study aims to identify whether adverse childhood experiences (ACEs) influence health lifestyles throughout adulthood. This was accomplished by collecting data from 233 women aged 18-25 and 65-85 who reported either no ACEs or three or more ACEs. Demographic indicators, the 10-item ACEs questionnaire (Felitti et al., 1998), and the Health Promoting Lifestyle Profile (HPLP-II; Walker, Sechrist, &

Pender, 1987) were examined. ACEs were categorized into three variables: abuse, neglect, and household dysfunction. The HPLP-II was categorized by its six subscales: health responsibility, nutrition, spiritual growth, physical activity, stress management, and interpersonal relations. When comparing the ACEs and no ACEs groups, t-tests revealed significantly different scores for overall HPLP-II and the six subcategories showing that individuals without ACEs have higher scores on health behaviors. A structural equation model was calculated using the three ACE categories and six health domains. Substantial differences were observed in the variance captured for each of the six health behavior measures. Findings indicate that abuse significantly predicts physical activity, stress management, and spiritual growth (β=-.21, -.23, -.20); neglect significantly predicts interpersonal relationships and spiritual growth (β=-.17, -.18); and household dysfunction significantly predicts health responsibility, nutrition, stress management, and interpersonal relations (β=-.20, -.22, -.10, -.17). The present investigation extends research in displaying that ACEs play a significant role in future health behaviors, with household dysfunction being the greatest predictor.

Abstract citation ID: igad104.0354

### ASSOCIATION BETWEEN COUNTY TEMPERATURE AND VISION IMPAIRMENT IN A NATIONALLY REPRESENTATIVE SAMPLE OF OLDER ADULTS

Esme Fuller-Thomson[1], ZhiDi Deng[2], and Elysia G Fuller-Thomson[1], *1. University of Toronto, Toronto, Ontario, Canada*, *2. University of Alberta, Edmonton, Alberta, Can ada*

Several small studies have associated exposure to elevated average temperature with specific vision problems. However, no large-scale studies have examined the relationship between vision impairment and average area temperature in the general population. We conducted a cross-sectional analysis of a large nationally presentative sample of older adults to further explore this relationship through secondary analysis of the American Community Survey (ACS). Data from six consecutive years of the cross-sectional survey were analyzed (2012-2017). The subsample analyzed included community-dwelling and institutionalized older adults aged 65 and older in the coterminous US who lived in the same state in which they were born (n =1,707,333). The question on severe vision impairment was "Is this person blind or does he/she have serious difficulty seeing even when wearing glasses?". Average annual temperature data from the National Oceanic and Atmospheric Administration was combined into a 100-year average and mapped to corresponding US Census Bureau's public use microdata areas (PUMAs) from the ACS. Higher average temperature consistently correlated with increased odds of severe vision impairment across all cohorts (i.e. age, sex, race, income, and educational attainment cohorts). Among all participants, the odds of severe vision impairment increased by 9.2% for every 5°F (2.8°C) elevation in annual average temperature (OR 1.092; 95% CI 1.088-1.097). If the association is found to be causal, the predicted rise in global temperatures could impact the number of older Americans affected by severe vision impairment and the associated health and economic burden.

Abstract citation ID: igad104.0355

### NEIGHBORHOOD CHARACTERISTICS AND INEQUALITIES IN SENSORY HEALTH AMONG OLDER ADULTS IN THE UNITED STATES

Alyssa Goldman[1], and Jayant Pinto[2], *1. Boston College, Boston, Massachusetts, United States*, *2. University of Chicago, Chicago, Illinois, United States*

Sensory function plays a crucial role in older adults' navigation of the environment, maintenance of personal safety, and in quality of everyday life. The residential neighborhood context is a significant social determinant of later life health and health disparities, but it has been understudied with respect to sensory function. We consider that residential neighborhoods are key sites of social and economic resources, social engagement, and environmental stimulation that could influence sensory health and inequalities in sensory impairment. We use data from 2,251 older adults who were interviewed at Rounds 1 and 2 of the National Social Life, Health and Aging Project in a series of longitudinal random effects models that examine how self-reported and objectively measured neighborhood characteristics are associated with self-rated vision and hearing. We find that older adults who reside in census tracts with higher levels of concentrated disadvantage report significantly worse self-rated vision and hearing compared with older adults who live in areas with less concentrated disadvantage. Residing in a more densely populated tract, however, is associated with better self-rated hearing. Black and Hispanic older adults' self-rated hearing benefits significantly more from living in a densely populated tract than does White older adults' self-rated hearing. In cross-sectional models, higher perceptions of neighborhood safety and social ties are associated with better self-rated vision. Our findings prompt a more nuanced understanding of how the social environment affects sensory health and highlight sensory function as a potential underexplored pathway through which neighborhood characteristics shape health disparities in later life.

Abstract citation ID: igad104.0356

### RACIAL DIFFERENCES IN THE ASSOCIATIONS BETWEEN SENSORY FUNCTIONS AND PHYSICAL ACTIVITY INTENSITY

Qun Le[1], Linda Churchill[2], Elizabeth Procter-Gray[1], Anthony Clarke[1], Jie Cheng[1], Danielle LoPilato[1], Lingming Chen[1], and Wenjun Li[1], *1. University of Massachusetts Lowell, Lowell, Massachusetts, United States*, *2. University of Massachusetts, Lowell, Lowell, Massachusetts, United States*

Sensory functions decline in older age. Multisensory training has been shown to improve balance. We analyzed the race differences in the associations of sensory functions with physical activity (PA) intensity. The Healthy Aging and Neighborhood Study enrolled 379 community-dwelling persons aged 65 and over in Central Massachusetts (2018-2019). Data on sensory functions were assessed using self-report questionnaires, range from 0 (very poor) to 4 (very good). PA intensities were measured using waist-worn Actigraphy accelerometers for at least 8 hours per day and at least 5 days including 1 weekend day. PA intensity was categorized using Copeland cutoff points as sedentary (SB), light (LPA), moderate-to-vigorous-intensity PA (MVPA).

Generalized linear models were used to assess the racial differences in the associations of sensory functions with PA intensity, adjusting for age, gender, education, income, general health, comorbidities, activities of daily living (ADL), and instrumental ADL. Average sensory functions of the participants were good to very good (mean±SD: hearing:2.9±1.1, vision:3.4±0.8, touch:3.7±0.6, smell:3.5±0.9, taste:3.6±0.7, balance:3.0±1.0). Two-thirds (66.7%) of participants met the PA guidelines of MVPA (mins/day) SB:465±114, LPA:248±66, MVPA:53±70). Compared to other races, Whites with better touch (b=52.1, SE=23.8) had significantly more SB. Better hearing (b=16.7, SE=7.2), vision (b=32.1, SE=10.5), smell (b=19.3, SE=7.8), taste (b=25.3, SE=9.9) were significantly associated with more LPA. Better vision (RR=1.6, 95%CI:1.1-2.4) and worse touch (RR=0.6, 95%CI:0.4-0.9) were significantly associated with more MVPA. Racial differences are significant in the associations between sensory functions and PA intensity among older adults. The underlying mechanisms should be further investigated to inform future interventions.

### SESSION 1355 (SYMPOSIUM)

Abstract citation ID: igad104.0357

### ADVANCING RESEARCH ACROSS THE ADRD CONTINUUM IN UNDERREPRESENTED POPULATIONS

Chair: Laura Zahodne Co-Chair: Kristine Ajrouch
Discussant: Cerise Elliott

This symposium highlights innovative work advancing ADRD research with African Americans, Latinos, and Arab Americans. Included papers address critical issues along the ADRD spectrum from life course risk factors, through mild cognitive impairment (MCI), to dementia caregiving. Investigating links between the early-life environment and later-life ADRD risk, Dr. Tsotsoros examines cognitive correlates of adverse childhood experiences (ACEs) in two disparate samples, demonstrating the need to characterize risk pathways among older Latina women, who are at increased risk of both ACEs and ADRD. Using nationally representative data, Dr. Esiaka focuses on adult risk factors for ADRD among Black men. She shows that neighborhood physical disorder and poor sleep quality represent key risk factors for worse cognitive health in this critically understudied population. Moving along the ADRD continuum, Dr. Darwish focuses on another severely underrepresented group: Arab American immigrants with MCI. Based on in-depth work with older adults in Lebanon, she details cultural adaptations made in the development of a combined cognitive-behavioral and cognitive rehabilitation intervention for this group. Next, Dr. Bouldin uses nationally representative data on African American, Latino, and non-Latino White older adults with dementia to quantify racial/ethnic differences in the prevalence and correlates of caregiver support service use. Findings highlight both risk and resilience among dementia caregivers from minoritized racial/ethnic groups. Finally, Cerise Elliott from the National Institute on Aging (NIA) will offer perspectives on how disaggregating population subgroups through between- and within-group research designs can advance NIA's goal of understanding and eliminating racial/ethnic inequalities across the ADRD continuum. This is an Alzheimer's Disease and Related Dementias Interest Group Sponsored Symposium.

Abstract citation ID: igad104.0358

### A COMPARISON OF THE IMPACT OF ADVERSE CHILDHOOD EXPERIENCES ON BRAIN HEALTH IN LATINA AND NON-LATINA WOMEN

Cindy Tsotsoros, *University of Rhode Island, Kingston, Rhode Island, United States*

Early-life experiences and social determinants of health influence late-life cognitive performance, cognitive aging trajectories, and risk of Alzheimer's disease and related dementias (ADRD). Research shows adversity during childhood plays a central role in health outcomes later in life, such that more adverse childhood experiences (ACEs) predict higher likelihood of negative physical and mental health outcomes. As ACEs may also predict neurocognitive performance and historically marginalized groups are disproportionately exposed to ACEs, this study aims to enhance diversity in research on ACEs and cognitive aging by clarifying the role of ethnicity. Latinos are 51% more likely to experience ACEs and 1.5x more likely to experience ADRD than their non-Latino counterparts. Data were collected in two studies assessing cognitive functioning in women aged 65-85 who reported no ACEs or 3+ ACEs (54 non-Latinas, Oklahoma; 50 Latinas, Rhode Island). ACEs were measured with the Adverse Childhood Experiences Questionnaire. Cognition was measured using NIH Toolbox Cognition-Battery and Automated Neuropsychological Assessment Metrics. In the non-Latina white sample (Mage=73.2), those with 3+ ACEs exhibited inferior scores on Stroop Interference, p=.050, d=-.44. The ACEs group demonstrated higher scores on List Sorting, p=.003, d=-.78. Small effects were found in Stroop Color, Stroop Color-Word, Cognition Fluid Composite, Flanker, and Cart Sort (d=-.25, -.39, -.29, .24, -.25, respectively). These findings are compared to those within the Latina sample, demonstrating the need to extend research on life course risk factors for ADRD to this underrepresented group, which is at elevated risk of both ACEs and ADRD.

Abstract citation ID: igad104.0359

### DETERMINANTS OF ALZHEIMER'S DISEASE AND RELATED DEMENTIA RISK IN OLDER BLACK MEN

Darlingtina Esiaka, *University of Kentucky, Lexington, Kentucky, United States*

Beyond biological determinants of Alzheimer's disease and related dementias (ADRD), extensive literature also suggests a role for sociocultural determinants—including race, ethnicity, education, neighborhood, and economic environment—in modulating cognitive outcomes. In recent years, research examining sociodemographic disparities in ADRD incidence points to modifiable mechanisms. Important for more personalized dementia prevention is the focus on how social determinants of health interact with lifestyle factors to put people at risk of developing ADRD. However, a historical challenge to research on dementia disparities and prevention relates to stark underrepresentation of Black individuals, particularly Black men. Therefore, this within-group study utilizes data from older Black men to examine social determinants (e.g., neighborhood, home environment, social cohesion), lifestyle factors (e.g., sleep, diet, drinking), and their impact on neurocognitive domains linked to ADRD. Results from 178 Black men, age 70+, from the 2021 National Health and Aging Trends Study show lower presence of neighborhood physical disorders such as vandalism (B=.064, p=0.035) and better sleep quality (B=.022, p=0.036) were associated with better performance on executive functioning tasks. Additionally, ongoing primary data collection (N=100 Black men, age 60+) supports these findings and provides additional insights toward identifying and validating risk/protective factors that predict cognitive outcomes very early in the cognitive aging process among Black men. This work represents a necessary step toward a better understanding of heightened ADRD risk in this understudied group. Results can be used to guide tailored interventions likely to be most effective for mitigating risk and slowing progression in Black men.

Abstract citation ID: igad104.0360

### DEVELOPING A COGNITIVE AND AFFECTIVE INTERVENTION FOR MILD COGNITIVE IMPAIRMENT FOR FOREIGN-BORN ARAB AMERICANS

Hala Darwish, *University of Michigan, Ann Arbor, Michigan, United States*

Cognitive and affective dysfunctions are challenging clinical characteristics of mild cognitive impairment (MCI) and Alzheimer's disease and related dementias (ADRD), which may be more prevalent among foreign-born Arab Americans. One study reported that cognitive limitations were present among 9.7% of foreign-born Arab Americans, compared to 7.4% of US-born non-Hispanic whites, blacks, and Asians. This presentation will describe the design and development of culturally valid interventions targeting cognitive and affective dysfunction in foreign-born Arab Americans with MCI and their caregivers (NIH Stage 1B). Individuals with MCI and their caregivers will receive comprehensive cognitive rehabilitation and behavioral therapies. The cognitive performance will be assessed in Arabic using a culturally valid battery, including a verbal memory Arabic test (VMAT) we developed using a systematic item selection procedure (NIH Stage 1A). During Stage 1A, cultural and psychometric validity evidence for Arabic versions of the Symbol Digit Modalities Test (SDMT), Brief Visuospatial Memory Test-Revised (BVMT-R), and the newly developed VMAT was generated from a sample of 180 healthy Arabic-speaking individuals age 16-80 in Lebanon of whom 45 were 65-80 years, and 63 were retested after 2-3 weeks. Test-retest reliability and criterion-related validity were examined, and regression-based norms were derived. In Stage 1A, we also established the psychometric validity of Arabic measures of anxiety, depression, and fatigue (i.e., Hopkins symptoms checklist-25, modified fatigue impact scale) that will be used in the pilot randomized controlled trial (RCT)-stage 1B. A description of cultural adaptations and preliminary results will be discussed.

Abstract citation ID: igad104.0361

### THE USE OF CAREGIVER SUPPORT SERVICES AND CAREGIVING IMPACT AMONG DIVERSE CAREGIVERS OF OLDER ADULTS WITH DEMENTIA

Erin Bouldin[1], Kaleb Eppich[1], Noah Webster[2], Amanda Leggett[3], and Laura Zahodne[2], *1. University of Utah, Salt Lake City, Utah, United States*, *2. University of Michigan, Ann Arbor, Michigan, United States*, *3. Wayne State University, Detroit, Michigan, United States*

Caregiver support services may improve experiences among caregivers of people with dementia, but racial/ethnic disparities exist in service use. Using data from the 2021 National Study of Caregiving, we measured caregiver service use and caregiving impacts among 470 Black (n=187), Hispanic (n=37), and White (n=246) caregivers of older adults with possible/probable dementia from the National Health and Aging Trends Study. We classified caregivers as using at least one or none of the following services in the past year: caregiver support group, training, or respite care. We calculated the weighted proportion of caregivers reporting caregiver burden (at least one: physical, emotional or financial difficulty), exhaustion, and feeling overwhelmed within each racial/ethnic group. Caregiver service use varied by race/ethnicity (47% of Hispanic, 29% of Black, and 32% of White caregivers). Black and White caregiver service-users were significantly more likely to report a burden than their peers using no service (59% versus 36% of Black, p=0.04, and 90% versus 41% of White, p<0.001, caregivers). There was no significant difference in burden among Hispanic caregivers (59% versus 69%, p=0.65). In each racial/ethnic category, caregivers reported similar prevalence of exhaustion and feeling overwhelmed from caregiving whether they used a service or not. Based on these cross-sectional data, we hypothesize that Black and White caregivers who feel burdened use services more often, and that service use mitigates caregivers' feelings of being exhausted or overwhelmed by caregiving, despite burden. Racial/ethnic differences highlight the need to understand contextual factors that influence caregiver burden and service use.

### SESSION 1360 (SYMPOSIUM)

Abstract citation ID: igad104.0362

### FROM MICRO TO MACRO: PSYCHOSOCIAL AND BEHAVIORAL PREDICTORS OF COGNITIVE AGING ACROSS DAYS AND DECADES

Chair: Jeremy Hamm Co-Chair: Jacqueline Mogle

Healthier cognitive aging is multifactorial, shaped in part by modifiable psychosocial and behavioral predictors. Critically, these predictors exhibit day-to-day and year-to-year fluctuations that could be harnessed to promote better cognitive functioning with age. The present studies thus address short- and long-term differences in psychosocial (perceived control, relationship quality) and behavioral factors (light physical activity, muscle strength) as predictors of normative and non-normative cognitive aging. Cerino et al. use data from the National Study of Daily Experiences to demonstrate how daily perceptions of control over daily stressors relate to episodic memory and executive functioning across age. Jang and Mogle examine the association between daily appraisals of relationship quality and subjective cognitive decline as driven by daily stressful experiences. Hamm et al. use data from the Midlife in the United States Study to investigate how changes in light physical activity across 9-years predict changes in episodic memory and executive functioning beyond more vigorous forms of activity, particularly for older adults. Finally, McGrath et al. use 12-year data from the Health and Retirement Study to investigate a multidimensional measure of maximum voluntary muscle strength as an indicator of elevated cognitive impairment risk among older adults. This symposium thus integrates new research from deeply-phenotyped samples on psychosocial and behavioral factors implicated in cognitive aging and contributes to a more nuanced understanding of how healthier cognitive functioning across short (days) and long (decades) time spans is shaped by beliefs, social dynamics, and behaviors that are amenable to change across the adult lifespan.

Abstract citation ID: igad104.0363

### DOES INCREASING LIGHT PHYSICAL ACTIVITY IN MIDLIFE AND OLD AGE BUFFER AGAINST COGNITIVE DECLINES?

Jeremy Hamm[1], Kelly Parker[1], Margie Lachman[2], Jacqueline Mogle[3], Katherine Duggan[1], and Ryan McGrath[1], *1. North Dakota State University, Fargo, North Dakota, United States*, *2. Brandeis University, Waltham, Massachusetts, United States*, *3. Clemson University, Clemson, South Carolina, United States*

Although it is well established that moderate-to-vigorous physical activity (MVPA) buffers against cognitive declines, less is known about the benefits of lower intensity physical activities that are more feasible, inclusive, and amenable to change in old age. Our study examined the extent to which increases in light physical activity protected against longitudinal declines in cognitive functioning and whether such a relationship become pronounced in old age when opportunities for MVPA are often reduced. We analyzed 9-year data from the national Midlife in the United States Study (n=2,229; age=56±11.13 years) using regression models that assessed whether change in light physical activity predicted corresponding changes in episodic memory and executive functioning for adults across the lifespan. All models controlled for age, sex, socioeconomic status, functional status, and moderate and vigorous physical activity. Beyond more vigorous forms of physical activity, increasing light physical activity predicted less decline in episodic memory (β=.06, p=.010) and executive functioning (β=.15, p<.001) over the 9-year follow-up period. Effect sizes for moderate and vigorous physical activity on episodic memory (βs=.01-.02) and executive functioning (βs=.03) were less than half that observed for light physical activity. Moderation models showed that, for episodic memory, the benefits of increasing light physical activity were pronounced at older ages. Findings provide initial evidence that increasing light physical activity may help slow age-related declines in episodic memory and executive functioning and that its protective influence may become paramount in later life when individuals commonly encounter constraints that can limit their capacity to engage in MVPA.

Abstract citation ID: igad104.0364

### DAILY SOCIAL RELATIONSHIP QUALITY, DAILY STRESS, AND SUBJECTIVE COGNITIVE DECLINE AMONG OLDER ADULTS

Heejung Jang, and Jacqueline Mogle, *Clemson University, Clemson, South Carolina, United States*

Although prior research has shown that social relationships are strongly associated with cognitive function, few studies have explored the link between the quality of daily relationships and subjective cognitive decline (SCD). The present study explores whether the quality of daily relationships is associated with daily stress and how daily stress mediates the relationship between the quality of daily relationships and SCD among older adults. This study uses data from 254 older adults aged 70 or older who completed the Einstein Aging Study. Multilevel mediation analyses were conducted to account for daily measurements nested within individuals. The cognitive change index was used to measure SCD. We tested the indirect effect of daily relationship quality on SCD through daily stress levels. There was a significant positive association between ambivalent (β=.148, p<.001; β=.324, p<.001) and neutral (β=.095, p<.001; β=.297, p<.001) relationships and daily stress levels at both the within- and between-person levels. Between-person daily stress was, in turn, associated with greater SCD (β= .169, p<.05). In particular, there was a significant indirect path from ambivalent relationships to SCD through daily stress. This study contributes to a more detailed understanding of how relationship quality can impact cognition through increased exposure to daily stress. The quality of daily social relationships may be crucial to preserving older adults' cognitive function.

Abstract citation ID: igad104.0365

### COMPOUNDING WEAKNESS IS ROBUSTLY ASSOCIATED WITH COGNITIVE FUNCTIONING

Ryan McGrath, Kelly Parker, and Jeremy Hamm, *North Dakota State University, Fargo, North Dakota, United Sta tes*

Muscle strength has been linked to cognitive functioning, but the utility and discernment of absolute and normalized weakness cut-points for identifying persons at greater odds of poorer cognitive functioning has not yet been examined. We sought to determine whether being weak under multiple definitions was associated with greater odds of cognitive dysfunction. The analytic sample included 17,980 Americans ≥50-years (age: 64.8±10.0 years) from the 2006-2018 waves of the Health and Retirement Study. A handgrip dynamometer measured handgrip strength and the highest recorded measurement was included in the analyses. Sex-stratified absolute, body mass normalized, and body mass index normalized weakness cut-points were used. Individuals were categorized as having weakness under 0, 1, 2, or 3 cut-points. Persons with scores ≤11 on the Telephone Interview of Cognitive Status had any cognitive impairment, while those scoring ≤6 had a severe cognitive impairment. Separate covariate-adjusted multilevel logistic regression models were used for analyses. Persons classified as weak using 2 cut-points had 1.17 (95% confidence interval (CI): 1.02-1.35) greater odds for future any cognitive impairment. However, for those below all 3-weakness cut-points, the magnitude of the odds ratios elevated to 1.68 (CI: 1.44-1.94) and 1.74 (CI: 1.73-2.21) for future any and severe cognitive impairment, respectively. Being below more weakness cut-points increased the odds for cognitive dysfunction, which suggests combining definitions may better identify persons more at-risk for future cognitive impairment. Engaging in modifiable lifestyle behaviors that are conducive to preserving strength and maintaining a healthy body mass during aging may have implications for cognitive function.

Abstract citation ID: igad104.0366

### ASSOCIATIONS BETWEEN DAILY STRESSOR CONTROL AND COGNITIVE FUNCTION

Eric Cerino[1], Brady McClaskey[1], Yesenia Cruz-Carrillo[1], and David Almeida[2], *1. Northern Arizona University, Flagstaff, Arizona, United States*, *2. The Pennsylvania State University, State College, Pennsylvania, United States*

Perceived control is an important psychosocial correlate of cognitive health and aging. Most prior research examining this association has focused on global aspects of control, ignoring influences of more dynamic aspects of specific areas of control, such as control over daily stressors. Using data from the third wave of the Midlife in the United States study and the National Study of Daily Experiences (N=992, Mage=67.67, SD=10.34, 57.20% Female), we examined how control over different types of stressors (arguments, avoided arguments, work stress, home stress, network stress) was associated with cognitive performance. Over eight consecutive days, people reported their perceived control over stressors they had experienced. Participants also completed a telephone-based battery of tests measuring executive function (EF) and episodic memory (EM). Hierarchical regression analyses adjusted for number of daily stressors, age, sex, race, and education. The facilitative role of daily stressor control for cognitive health depended on age and the type of stressor experienced. For EF, greater control over arguments was associated with better EF (Est. = 0.07, SE = 0.03, $p < .05$). For EM, there was a stressor control by age interaction (Est. = 0.01, SE = 0.01, $p < .05$) such that greater control over home stress was associated with better EM among comparatively older adults (Est. = 0.07, SE = 0.03, $p < .05$). Results suggest that perceiving control over daily stressful experiences, especially arguments and home stress, may serve as a psychosocial resource for cognitive health in older adulthood.

### SESSION 1365 (SYMPOSIUM)

Abstract citation ID: igad104.0367

### NUDGING ATTITUDE CHANGE THROUGH EXPERIENCE: UNDERGRADUATE CURRICULAR INNOVATIONS

Chair: Brian Carpenter

Drawing students into gerontology and geriatrics remains a perennial challenge in light of negative stereotypes and ageism deeply entrenched in society. Nonetheless, instructors continue to forge ahead with curricular innovations designed to dismantle stereotypes, promote deeper understanding of the later stage of life, cultivate appreciation for the contributions of older adults, and entice students to consider further academic, extracurricular, and vocational opportunities in the field. This symposium brings together scholars and instructors who have implemented and evaluated course innovations with these goals in mind. The first presentation reviews findings from the Bringing Art to Life program that pairs teams of undergraduate students with persons with dementia attending an adult day services program and engaging in structured art therapy and reminiscence. The next presenter describes positive attitude change about aging among undergraduates in an advanced public health service-learning course. Next is a discussion of how health policy education has been successfully built into an undergraduate adult development and aging course. The fourth presentation summarizes results from a randomized educational intervention to undergraduate students to enhance death education and ensure more equitable access to quality end-of-life services and supports. The final presentation describes innovations in training MSTEM undergraduate students in aging. A Discussant will synthesize themes across presentations and engage session attendees to respond and share their own curricular experiences.

Abstract citation ID: igad104.0368

### BRINGING ART TO LIFE: EXPERIENTIAL LEARNING OUTCOMES WITH GRADUATE AND UNDERGRADUATE STUDENT LEARNERS

Rebecca Allen[1], Keisha Carden[2], Candice Reel[1], and Daniel Potts[3], *1. The University of Alabama, Tuscaloosa, Alabama, United States*, *2. VA Maryland Health Care System, Baltimore, Maryland, United States*, *3. Cognitive Dynamics Foundation, Tuscaloosa, Alabama, United States*

The University of Alabama experiential learning program, Bringing Art to Life (BATL) pairs teams of undergraduate students with persons with dementia attending an adult day services program and engaging in structured art therapy and reminiscence. Graduate students in clinical geropsychology work with an interdisciplinary team and conduct outcomes research focused both on individuals with dementia and student learners. Across seven semesters, we found that students in the BATL course compared with students in either didactic psychology of aging courses or an introductory psychology courses demonstrated improved attitudes towards persons with dementia and increased empathy. Graduate and undergraduate students learned mixed method and community-engaged research methods, producing clinically meaningful research. Experiential learning is essential to the recruitment of a new gerontology workforce.

Abstract citation ID: igad104.0369

### THE IMPACT OF A SERVICE-LEARNING COURSE ON UNDERGRADUATE STUDENT ATTITUDES TOWARD OLDER ADULTS

Britteny Howell, *University of Alaska Anchorage, Anchorage, Alaska, United States*

Undergraduate students often have little contact with older adults, which may result in a lack of interest regarding career options in gerontology or geriatric practice. To combat this problem, the University of Alaska Anchorage's 300-level Public Health for an Aging Society course introduces students enrolled in the Health Sciences Bachelor's program to a variety of gerontological concepts. This service-learning course engages public health and pre-clinical students in the community, providing valuable experience working with local senior agencies to provide a product or service. Students work in interdisciplinary teams and have a variety of community-based projects to select from as part of their coursework. Using the Geriatric Attitudes Scale at the start and end of the semester, this presentation reports on improved student perceptions of older adults as a result of this course. With a sample of 22 undergraduates at the beginning of the semester, scores were relatively high in agreement with positive statements about aging (i.e., "it is society's responsibility to provide care for the elderly"). However, they also report agreement with less positive statements about aging, such as “taking a medical history from elder patients is frequently an ordeal” and “as people grow older, they become less organized and more confused.” At the conclusion of the 15-week course, students report lower agreement with these negative statements about aging as a result of their learning and hands-on experience.

Abstract citation ID: igad104.0370

### INTEGRATING HEALTH POLICY EDUCATION INTO AN UNDERGRADUATE PSYCHOLOGY COURSE

Kelly O'Malley, PhD[1], and Kirsten Graham[2], *1. VA Boston Healthcare System, Boston, Massachusetts, United States*, *2. Southern Utah University, Cedar City, Utah, United States*

Education on health policy processes are lacking in psychology training programs. Psychologists can be helpful to policy making and evaluation processes; however, few have the training, experience, or exposure to health policy necessary to feel comfortable entering into this area. Health policy education was integrated into an existing undergraduate psychology adult development and aging curriculum. Students completed weekly knowledge quizzes, two health policy specific assignments, and a class lecture on the intersection of health policy, aging, and psychology. N = 97 students (Mage = 21.54, SD = 4.94) over six semesters completed pre-post knowledge, skill, and ability assessments. Paired samples t-tests indicated increased knowledge t(96)= -5.89, p < .001, Cohen’s d = .60, and understanding of health policy making t(94)= -5.69, p < .001, Cohen’s d = .58; and ability to critically evaluate health policies t(96)= -3.03, p = .003, Cohen’s d = .31. Students identified psychology t(95)= -5.20, p < .01, Cohen’s d = .53 and psychological research t(95)= -3.71, p < .001, Cohen’s d = .38 as having a greater role in health policy following the course. Students felt they would use the health policy skills and knowledge learned in this course and agreed the policy related topics and assignments were valuable. Integrating health policy education into an undergraduate psychology course was feasible and had a positive impact on student’s knowledge, skills, and abilities in this domain. This format may be appropriate for graduate psychology training and may help build psychologists confidence to participate in health policy activities.

Abstract citation ID: igad104.0371

### ADVANCING DIVERSITY IN AGING: THE AGE-ADAR SCHOLARS PROGRAM AT WVU

Julie Hicks Patrick, Amber Rusch, and Carly Pullen, *West Virginia University, Morgantown, West Virginia, United States*

As the number and racial/ethnic diversity of older Americans, ages 65+ years increases, recruiting, training, and retaining a diverse workforce to support aging-related fields is essential. Supported by an R25 grant from the NIA, the goals of the Appalachian Gerontology Experiences: Advancing Diversity in Aging Research (AGE-ADAR) Scholars Program include training undergraduate MSTEM majors in aging and health disparities research, especially as these topics interact in Appalachia. In this brief presentation, we will review the goals and mechanisms through which these aims are accomplished. We will review both hard outcomes (i.e., conference presentations, publications) and soft outcomes (attitude change, career specialization) among our first two cohorts of Scholars. For example, each Scholar prepares two empirical abstracts, presents a poster on campus, presents one or more posters off-campus, produces a public-facing infographic about health disparities and aging, conducts a literature review, and composes an integrated essay related to how their MSTEM major can help to solve issues of health disparities among older adults in Appalachia. About half of our Scholars have co-authored published manuscripts, as well. Finally, other funding mechanisms to support training the next generation of researchers, scholars, and applied workers in aging will be discussed.

Abstract citation ID: igad104.0372

### AN EDUCATIONAL INTERVENTION FOR UNDERGRADUATE STUDENTS TO PROMOTE AWARENESS OF END-OF-LIFE OPTIONS

Kelly O'Sullivan[1], Autumn Decker[1], Raven Weaver[1], and Cory Bolkan[2], *1. Washington State University, Pullman, Washington, United States*, *2. Washington State University Vancouver, Vancouver, Washington, United States*

Most people, of all ages, do not engage in advance care planning or know about available end-of-life (EOL) choices, despite the personal and societal benefits in an aging society, highlighting the need for educational intervention and outreach. We implemented an online educational intervention to explore accurate understanding of medical-aid-in-dying (MAID; available in ten states) among college students (n=415; Mage=19.6±4.4 years). Pre-intervention, 66% of participants had heard of MAID and 63% supported or strongly supported MAID. Participants were randomized to three conditions: control, testimonial narrative, or testimonial video explaining reasons why individuals pursued MAID (there were no significant differences in opinion by condition). Post-intervention, those in an intervention group were significantly more likely be more supportive of MAID legislation (p=.005); there were no significant differences between narrative or video conditions. Content analysis of open-ended responses revealed the importance of bodily autonomy and having a choice in health care. Participants who expressed neutral or conflicted perceptions wanted to learn more about the law and were concerned about individuals’ mental health when considering MAID. Negative responses reflected concerns about abusing the law and religious-based concerns. More accurate information about MAID and its safeguards is needed. In particular, the undecided group could benefit from selective interventions to improve understanding of MAID and other EOL care options. Future work should focus on education and outreach to individuals, families, and providers about aging and mortality issues to ensure more equitable access to quality EOL services and supports for people across the life span.

### SESSION 1370 (SYMPOSIUM)

Abstract citation ID: igad104.0373

### BOUNCING BACK AFTER SURGERY: QUANTIFYING AND PREDICTING RESILIENCE IN OLDER SURGICAL PATIENTS

Chair: Geeske Peeters Co-Chair: Heather Whitson

During this symposium we will discuss the impact of surgery on older adults physical functioning through a resilience lens. Physical resilience is defined as bouncing back in terms of physical functioning in response to a stressor, i.e. surgery. The first speaker will present data of long term trajectories of physical functioning in older women pre and post hip and knee surgery, and compare these with the trajectories in the general population. Profiles of women with higher or lower resilience to surgery will be discussed. The second speaker will talk about which functional measures, such as grip strength and walk speed, predict recovery in physical daily activities after knee replacement surgery, and the implications of these findings for the development of provocative resilience tests. The third presentation explores the role of biological ageing in fitness for surgery. The final presentation unravels the role of the stressor by exploring variations in types and severity of stressors in patients undergoing knee replacement surgery, and tests the hypothesis that severer stressors are associated with worse outcomes. Together, these presentations give a broad overview of determinants of physical resilience in the context of preparedness for, and recovery from, surgery. We will discuss methods to define stressors, and measure and quantify resilience. Moreover, we will illustrate how this information can inform personalization of care.

Abstract citation ID: igad104.0374

### PHYSICAL FUNCTIONING 12 YEARS BEFORE AND AFTER KNEE OR HIP SURGERY IN OLDER WOMEN

Geeske Peeters[1], Isabel de Munck[2], Leigh Tooth[3], and Rene Melis[1], *1. Radboud University Medical Center, Nijmegen, Gelderland, Netherlands*, 2. *Radboud university medical centre, Nijmegen, The Netherlands, Nijmegen, Gelderland, Netherlands*, 3. *The University of Queensland, Brisbane, Queensland, Australia*

Understanding long-term recovery patterns in the context of ageing-related decline may improve knee or hip surgery outcomes. We examined the impact of hip and knee surgery on long-term trajectories of physical functioning (PF) in older women, and profiled women with higher and lower resilience to surgery. Resilience, defined as bouncing back (PF) after a stressor (hip or knee surgery), was quantified using the expected recovery differential method. Data were from 10,434 women (73-79 yrs) in the Australian Longitudinal Study on Women's Health who completed surveys 2-6 (1999-2011). Adjusted linear mixed models were run to examine the surgery-by-time (-12 to +12 yrs) interaction in association with PF (SF-36 subscale). The difference between observed and expected PF were calculated, with positive/negative values reflecting higher/lower resilience, respectively. PF trajectories differed between women with (n=982) and without (n=8117) hip surgery ($p<0.001$). Among hip surgery patients, the decline was more rapid pre-surgery than post-surgery ($b=-0.7$, $p<0.001$). Women with knee surgery (n=1144) had lower PF than those without surgery (n=7971), but with a slower rate of decline ($p=0.01$). Among knee surgery patients, the rate of decline was similar pre- and post-surgery ($b=-0.3$, $p=0.25$). Both in hip and knee patients, women with higher resilience had fewer comorbidities and symptoms, and were more often physically active and independent in daily activities than those with lower resilience ($p<0.05$). In conclusion, women with and without hip or knee surgery have different trajectories of PF. Women who function better than expected after surgery, tend to have fewer health problems.

Abstract citation ID: igad104.0375

### COMPARISON OF CLINICAL BIOMARKER AND DNAM-DERIVED BIOLOGICAL AGE MEASURES IN AN ELECTIVE SURGERY COHORT

Daniel Parker[1], Shelley McDonald[2], Kathryn Porter Starr[3], Dan Blazer, III[4], E. Shelley Hwang[4], Allan Kirk[4], and Sandhya Lagoo-Deenadayalan[4], *1. Duke University School of Medicine, Durham, North Carolina, United States*, 2. *Geriatrics, Durham, North Carolina, United States*, 3. *Durham VA Health Care System, Durham, North Carolina, United States*, 4. *Surgery, Durham, North Carolina, United States*

Older adults face greater post-operative morbidity due to decreasing biological aging (BA)-associated physical resilience. BA measures may quantify these vulnerabilities. Common BA quantification approaches include measures derived from clinical biomarkers and whole blood DNA methylation. The Duke Surgery One Thousand Patient Cohort is a prospective biospecimen and data repository of patients undergoing elective surgery. Using a subset of this cohort (n=119; 61±13 years of age; 50% female; 21% African American), we sought to determine: 1) the inter-relationship of BA measures; 2) associations of race, sex, BMI, and surgery type with BA; and 3) associations of BA with American Society of Anesthesia (ASA) Classification Score . Clinical biomarker BA measures included Klemera-Doubal method (KDM), homeostatic dysregulation (HD), and PhenoAge. DNAm measures included the DNAm PhenoAge, Hannum Age, and GrimAge. We calculated BA advancement (BAA) as BA minus Chronological Age (CA). We calculated Pearson correlation coefficients and fitted regression models, adjusting for CA, sex, race, and BMI. Overall, participants were biologically older than their CA. Male sex and African American race were associated with greater BAA by most measures . Associations of surgery type with BAA were consistent across measures . Greater BAA by most measures was associated with a higher ASA Score, reflecting higher baseline surgical risk. In conclusion, BA measures using different quantitative approaches showed anticipated associations with demographic factors, surgery type, and surgical risk. Ongoing work will identify BA measures that predict adverse surgical outcomes, and that could be used to identify patients for perioperative prehabilitation.

Abstract citation ID: igad104.0376

### STRESSOR CHARACTERIZATION IN STUDIES OF PHYSICAL RESILIENCE

Karen Bandeen-Roche[1], Qianli Xue[2], Fangyu Liu[3], Ravi Varadhan[4], Brian Buta[1], Charles Brown IV[5], Jeremy Walston[1], and Frederick Sieber[4], *1. Johns Hopkins University, Baltimore, Maryland, United States*, 2. *Johns*

*Hopkins, Baltimore, Maryland, United States*, *3. Johns Hopkins Bloomberg School of Public Health, Baltimore, Maryland, United States*, *4. Johns Hopkins University School of Medicine, Baltimore, Maryland, United States*, *5. School of Medicine, Baltimore, Maryland, United States*

Physical resilience may be considered as rebounding well from physical stressors. Early gerontological consensus has emphasized that resilience properly should be referred to as "resiliencies"—each, specific to a given stressor type and magnitude. Specificities include that one may be resilient to a minor stressor but not a more severe one as well as that one's physiologic state and constellation relevant to determining resilience capacity ("PRC") may better accommodate certain types of stressors than others. Yet, the literature on resilience in older adults has devoted little attention to stressor characterization. In this talk, a conceptual framework for considering the role of stressor variation in studies of resiliency and a process to elicit expert opinion on sources of variation in stressor experience are presented. Both are applied both within the RESiliency in TOtal knee REplacement (RESTORE) study. The conceptual framework distinguishes "exogenous" (potentially randomizable) and "endogenous" (connected to one's PRC) variation in stressor magnitude and type. Elicitation identified anesthesia aspects and surgical technique elements among exogenous features, and perioperative vital indicators, bleeding and pain amelioration among endogenous features. Stressor feature variation was analyzed: this was found to be highest for perioperative vasopressor use, body temperature and postoperative opioid requirements. Analyses describing feature associations with resilience outcomes also will be presented: These examine the validation hypothesis that more severe stressors are associated with worse outcomes. This work is producing measures of stressor magnitude and type suitable for testing the primary RESTORE hypothesis that fitter PRC blunts adverse stressor effects on outcomes.

Abstract citation ID: igad104.0377

### PHYSICAL RESERVE MEASURES AS PREDICTORS OF RESILIENCE IN OLDER ADULTS UNDERGOING TOTAL KNEE REPLACEMENT

Maria Sison[1], Richard Sloane[2], Cathleen Colon-Emeric[1], and Heather Whitson[1], *1. Duke University, Durham, North Carolina, United States*, *2. Duke University Medical Center, Durham, North Carolina, United States*

Measures of physical reserve have been shown to predict resilience following surgery in older adults. The objective of this study was to evaluate the utility of gait speed, hand grip strength, and 3-minute walk distance to predict resilience in older adults scheduled for total knee arthroplasty (TKA), in whom preoperative gait and mobility may be limited by pain or reduced range of motion. We conducted an interim analysis of data from PRIME-KNEE, an ongoing prospective study following participants age ≥60 years undergoing TKA recruited from Duke hospitals. We determined the change in self-reported Lower Extremity Physical Activities of Daily Living (LE PADL) score from pre-surgery baseline to 4 months following TKA in 133 participants (mean age = 72, 59% female). Gait speed, hand grip, and 3-minute walk were collected at baseline. A total of 110 (83%) participants exhibited "resilience," defined dichotomously as any improvement in LE PADL score (occurred in 38 participants) or return to a non-disabled state (occurred in 72 participants). Logistic regression analysis adjusted for age and sex revealed that 3-minute walk distance was significantly associated with resilience at 4 months (OR=1.012 [CI 1.002-1.022]) with an area under the curve of 0.634. The presentation will review the implications of these results and present findings with an updated sample, as well as discuss the need for other predictive measures, such as provocative tests (i.e., tests that measure response to an experimental stressor) and biomarkers.

### SESSION 1375 (SYMPOSIUM)

Abstract citation ID: igad104.0378

### EXPLORING MODELS OF CARE INTEGRATION TO ADDRESS THE NEEDS OF PERSONS AGING WITH HIV

Chair: Abigail Baim-Lance Co-Chair: Gemmae Fix
Discussant: Thomas Laskow

By 2035, the majority of persons living with HIV will be 50 and older. The clinical and social needs of this population have been well documented, including the negative impacts of persistent stigma on quality of life and health outcomes. Responding to such needs requires integrated, patient-centered approaches to care and supportive services. Care integration is defined as the bringing together of two or more previously unlinked services. Care integration is used within and outside of the HIV care settings, but how such models should be designed or adapted for older persons with HIV (OPWH) is less certain given their unique needs. This symposium will explore case studies representing different models of care integration developed within and outside of HIV care settings, their relevance to OPWH, evidence of implementation including known barriers and facilitators, and opportunities to advance such models. Our first presentation describes an embedded geriatrics outpatient consultation service within an HIV care setting in New York City (Condo). Our second presentation focuses on the barriers and facilitators to the integration of substance use treatment into HIV care in Georgia (Bender). Our third and fourth presentations will describe service models developed outside of HIV care, which are now being applied to support OPWH including the Veterans Affairs patient-centered Whole Health approach (Fix) and the CAPABLE program to support aging in place (Smith). With our discussant, we will reflect on these models of integration to enhance care and support systems for this population.

Abstract citation ID: igad104.0379

### INTEGRATING A PATIENT-CENTERED, WHOLE-HEALTH APPROACH WHEN CARING FOR PEOPLE AGING WITH HIV

Gemmae Fix[1], Janet Clark[2], and Allen Gifford[3], *1. US Department of Veteran Affairs, Bedford, Massachusetts, United States*, *2. VA Office of Patient Centered Care & Cultural Transformation, Washington, District of Columbia, United States*, *3. VA Center for Healthcare Organization & Implementation Research, Boston, Massachusetts, United States*

The US Department of Veteran Affairs (VA) is implementing a patient-centered, "Whole Health System of Care" to align care with What Matters Most to patients within their unique circumstances. Implementation is focused on primary care, where most patients receive care. Yet HIV Clinics have historically provided both HIV and primary care because of the complexity of HIV. People living with HIV are now aging and experiencing a constellation of health and life circumstances that would benefit from a Whole Health approach. While there is desire to spread Whole Health beyond primary care, little is known about how to integrate Whole Health into HIV Clinics. We sought to identify Whole Health implementation considerations for HIV Clinics. Our qualitative study design included interviews with vignettes based on our prior work, illustrating themes like aging alone. Findings were mapped to the i-PARIHS implementation framework which includes recipients (HIV providers), context (HIV clinics), and innovation (Whole Health). We interviewed physicians, psychologists, nurses and leadership (N=11) from three HIV Clinics (Northeast, Northwest, South). Participants were largely aware of Whole Health, although few had taken trainings and no clinics currently offered Whole Health services. Many expressed interest incorporating Whole Health into HIV care. They also saw great value, given patients' complexities. Participants described logistical barriers hindering implementation, like awareness of specific resources or how to refer. Only some providers were able to conceptualize a Whole Health clinical encounter- where patients' life circumstances are connected to health. HIV Clinics offer an ideal setting to spread Whole Health.

Abstract citation ID: igad104.0380

### BARRIERS AND FACILITATORS TO INTEGRATED SUBSTANCE USE AND HIV CARE: ADMINISTRATOR AND PROVIDER PERSPECTIVES

Alexis Bender, Kiera Chan, Riley Hunt, and Molly Perkins, *Emory University, Atlanta, Georgia, United States*

Since the beginning of the HIV epidemic, HIV and substance use have been intricately connected. Substance use may cause additional damage to the brain, higher viral loads, low medication adherence, and higher AIDS-related deaths. A potential area to improve access to care and treatment for people aging with HIV and substance use is through integrated care. Yet little is known about the barriers and facilitators to integrated care, especially in practices serving vulnerable populations, such as older adults or PLWH in rural areas. We conducted in-depth interviews with 21 providers (e.g., Physicians, APPs, RNs) and 15 administrators (e.g., clinic managers, care coordinators) across Georgia between January 2021 and November 2022. Interviews explored perceptions about clinic integration and desire, or lack of desire, to increase integration. Data were analyzed using inductive and deductive thematic analysis guided by the Consolidated Framework for Implementation Research. Both groups perceived important benefits to integrated care, especially for older or more vulnerable populations, but the definitions varied across respondents. Providers and administrators agreed that integrated care provided greater access to care with less interruption to patients' lives. Barriers to increasing integration included, but were not limited to, physical space, staffing and budgetary constraints, and limited opportunities for collaboration. Most providers, except those in practices with the highest levels of integration, experienced barriers to getting patients into adequate substance use treatment when needed. Integrated care is vital to treating older adults and vulnerable individuals living with HIV, especially those with co-occurring substance use disorders.

Abstract citation ID: igad104.0381

### CAPABLE: APPLYING AN EVIDENCE-BASED MODEL OF CARE FOR OLDER ADULTS WITH HIV

Jamie Smith[1], Jason Farley[1], Kelly Lowensen[1], Kennedy McDaniel[2], Sarah Szanton[1], and Katherine Ornstein[1], *1. Johns Hopkins University, Baltimore, Maryland, United States*, *2. Johns Hopkins School of Nursing, Baltimore, Maryland, United States*

In the United States, more than half of people living with HIV (OWH) are at least 50 years. Older PWH experience increased comorbidities, earlier onset of geriatric syndromes, and functional decline. These health consequences intersect with complex social and environmental factors, further complicating aging with HIV. Yet initiatives focused on successful aging do not consider the unique needs of older PHWs. Models of care tailored to the individual that addresses both intrinsic (e.g., chronic infections, stress, and nutrition) and extrinsic (socioeconomic status, housing instability, food insecurity, and threats to safety) factors unique to older PWH are needed to improve health and functional outcomes. Community Aging in Place, Advancing Better Living for Elders (CAPABLE), an internationally recognized model of care, is a person-directed program that has been successful in supporting aging in place by reducing functional decline and disability among older adults. Over several months, the participant, occupational therapist, registered nurse, and handy worker collaborate on solutions to improve the participant's bio-psych-functional capacity to function at home. In multiple trials, CAPABLE has demonstrated improvements in independence, safety, self-efficacy, and depression. CAPABLE is associated with healthcare cost reductions, including over $30,000 in Medicare and Medicaid savings. CAPABLE has not been adapted to older PWH, despite being well-positioned to address intrinsic and extrinsic factors affecting adherence, viral suppression, and other health outcomes. This presentation will discuss the first-ever translation of CAPABLE to a cohort of older PWH using human-centered design strategies and discuss initial feasibility trial of older PWH at Johns Hopkins.

Abstract citation ID: igad104.0382

### BRINGING GERIATRIC CARE INTO THE HIV PRIMARY CARE CLINIC: A COMBINED HIV/ GERIATRIC MULTIDISCIPLINARY CLINIC MODEL

Angela Condo[1], Abigail Baim-Lance[2], and Nathan Golstein[3], *1. Icahn School of Medicine at Mount Sinai, New York City, New York, United States*, *2. Veterans Health Administration, Bronx, New York, United States*, *3. Department of Geriatrics and Palliative Care, Mount Sinai, New York, New York, United States*

Persons living with HIV (PLWH) experience accelerated and accentuated aging, developing co-morbidities at earlier ages and with higher frequencies than those without HIV. Older persons with HIV (OPWH) also experience geriatric syndromes. In 2020 we developed a combined HIV/geriatrics multidisciplinary clinic at Mount Sinai Hospital in New York where a geriatrician and multidisciplinary team including a nurse, pharmacist, and social worker are embedded in an HIV primary care clinic. Patients are referred for comprehensive geriatric assessments. We conducted a retrospective chart review after the first 15 months of the program to describe this population of OPWH living in NYC who are referred to a geriatrician. From 9/2020-11/2021, there were 183 appointments scheduled of which 112 (61%) were new patient visits. Sixty-two percent of patients came to their appointment. The average age of patients was 67 years. Fifty-seven percent identified as male. The majority of referrals (50%) were for cognitive assessment followed by mobility assessment (25%), polypharmacy (14%), multimorbidity (8%), advance care planning (1%), and other (2%). Based on this data, we identified a need to improve the show rate to our clinic and implement cognitive screening in OPWH. We have incorporated a community health worker into our interdisciplinary team to assist in this goal and continue to educate patients and providers on geriatric syndromes. A geriatric approach to care is crucial to incorporate into existing HIV clinical practices and we hope to optimize this integration based on the needs of patients and providers.

### SESSION 1380 (SYMPOSIUM)

Abstract citation ID: igad104.0383

### ESPO AND BUTLER-WILLIAMS SCIENTIFIC SYMPOSIUM: CAREER DEVELOPMENT TO PROMOTE DIVERSITY, DISCOVERY, AND AGING

Chair: Jamaine Davis Co-Chair: Brianna Morgan
Discussant: Patricia Jones

The NIA's Butler-Williams Scholars Program and GSA's ESPO Section are united in providing career development opportunities in a manner that promotes leadership, diversity, and inclusivity. This year's theme challenges our emerging scholars to embrace diversity and discovery while thinking—or rethinking—about the perspectives of older adults. Disparities in health associated with race/ethnicity, lived experience, sociocultural and socioeconomic factors, as well as access to and provision of health care are chief concerns of our aging population. GSA's early career professionals and 2022 alumni of the prestigious NIA Butler-Williams Scholars Program address these issues. Dr. Shekinah Fashaw-Walters will discuss her work examining how high-quality providers influence high quality of healthcare, particularly for Black patients. Dr. Sara LaHue will discuss how biological age can be a measure associated with delirium or neuronal inury markers in a cohort of hip fracture patients. Dr. Imilce Rodrigues-Fernandez will present on the importance of identifying pathways that can lead to improvements in stem cell function that can improve age-related tissue degeneration. how both demographics and genetic factors contribute to cognitive performance. The final speaker, Dr. Helena Zomer will present findings on the mechanism by which a hormone can extend healthspan and longevity. The featured talks by rising stars deepen our understanding of the influence of diversity and key discoveries so we can reimagine aging research.

Abstract citation ID: igad104.0384

### STRESS, STEM CELLS, AND THE GUT MICROBIOME—IMPLICATIONS FOR AGING

Imilce Rodriguez-Fernandez, *University of Puerto Rico Rio Piedras, San Juan, Puerto Rico, United States*

Adult somatic stem cells have a crucial role in maintaining the regenerative capacity of tissues throughout the lifespan of the organism. With age comes a reduction in tissue renewal and repair upon injury. Understanding the mechanisms that maintain stem cell function could potentially lead to the development of therapeutic strategies to mitigate age-related tissue degeneration and improve healthspan. Of particular interest is understanding how stem cells deal with different types of stressors such as oxidative stress and bacterial stress. To study this, we use the Drosophila intestinal stem cells (ISCs) as a model system. ISCs are normally quiescent but during stress, they divide asymmetrically to give rise to one stem cell and one differentiated cell: an enteroblast (EB) that will become an Enterocyte (EC) or an enteroendocrine progenitor (EEp) cell that will later become a mature enteroendocrine (EE) cell. ECs have roles in digestion and immune response. EEs have roles in hormones and peptide secretion. ISCs have a crucial role in regenerating lost intestinal cells after bacterial infection, oxidative stress, or normal cell attrition. Thus, ISCs are always sensing the gut environment and respond to cues by activating or repressing proliferation. By studying the function of two important stress-sensing transcriptions namely Nrf2/CncC and HSF1 in ISCs, we have uncovered an unprecedented role of these genes in stem cell identity and the regulation of the gut microbiome. These findings could shed light on the mechanisms altered during aging that contribute to stem cell misdifferentiation and microbial dysbiosis.

Abstract citation ID: igad104.0385

### UNRAVELING NONGENOMIC MECHANISMS BY WHICH 17A-ESTRADIOL EXTENDS HEALTHSPAN AND LONGEVITY

Helena Zomer, Vijay Sirohi, Theresa Medrano, and Paul Cooke, *University of Florida, Gainesville, Florida, United States*

Recent work indicates that exogenous 17α-estradiol (17α-E2), a stereoisomer of 17β-E2, produces unprecedented longevity increases (~20%) in male mice, challenging prevailing assumptions that 17α-E2 is a weak estrogen. However, is unclear how 17α-E2 acts. Therefore, this study aimed to obtain mechanistic insights into 17α-E2 actions in the uterus, the most widely used vivo model system for estrogen action. We hypothesized that 17α-E2 signals through membrane (m)ESR1 to affect target tissues and promote longevity. To test this, we treated pre-pubertal (postnatal day 20) female CD-1 mice with 3 daily IP injections of vehicle or 10 mg/kg of 17α-E2 or 17β-E2 (n=4/group). 17α-E2 treatment did not significantly increase uterine weight – a marker of nuclear (n)ESR1 signaling (20.3 ± 2.1 mg vs. vehicle 15.7 ± 2.0 mg, $p>0.05$), while 17β-E2 strongly increased it to 41.4 ± 1.1 mg ($p<0.05$). Accordingly, histological analysis revealed reduced cell proliferation in 17α-E2- vs. 17β-E2-treated uteri. Both 17α-E2 and 17β-E2 robustly stimulated epithelial p-ERK and p-AKT expression, the active phosphorylated forms of these protein kinases, and western blotting showed a strong and comparable stimulation of EZH2, a critical mediator of estrogen's epigenetic effects, which are linked to mESR1 signaling. In conclusion, our results indicate that despite limited actions through nESR1, 17α-E2 robustly signals through mESR1 to activate uterine protein kinase cascades and produce epigenetic effects. These findings may cause a significant reappraisal of 17α-E2 biological effects and role in normal estrogen physiology and provide critical insights into its unique ability to stimulate longevity.

Abstract citation ID: igad104.0386

### DEFYING THE ODDS: EXCEPTIONAL HOME HEALTH AGENCIES SERVING HIGH PROPORTIONS OF BLACK PATIENTS

Shekinah Fashaw-Walters, *University of Minnesota, Minneapolis, Minnesota, United States*

Healthcare providers with the highest proportions of Black patients are typically assumed to be lower quality, whether they be hospitals, nursing homes, or primary care physicians. However, little research examines the providers that "defy these odds" and rise to the top of the quality ratings. Each year, Medicare home health provides skilled nursing and therapy services to over 3 million homebound older adults with physical and cognitive impairments, and compared to the general Medicare population, Medicare home health patients are more likely to identify as Black. This mixed-methods (QUANT-->QUAL) project uses an assets-based approach as well as the tenets of the Public Health Critical Race Praxis to understand home health providers that are generally assumed to be low-quality providers – that is those who have the highest proportions of Black patients – but instead are high quality providers. This study uses star ratings and the Post-Acute Care Public Use File for home health agencies to characterize and identify agencies with high quality care and a high proportion of Black patients. Publicly available data allows us to characterize the structures and outcomes of these unique home health agencies; while primary data collection (via case studies of exceptional agencies) will allow us to report on their unique processes of care. Data collection and analyses are currently underway and will be completed by May 2023. In 2019, 28% of home health agencies were identified as having high-quality with a high proportion of Black patients, we have selected three agencies for case studies.

Abstract citation ID: igad104.0387

### ACCELERATED BIOLOGICAL AGE IS ASSOCIATED WITH DELIRIUM AND PLASMA NEUROFILAMENT LIGHT IN GERIATRIC HIP FRACTURE

Sara LaHue[1], Joy Youn[2], Matias Fuentealba[3], Vanja Douglas[2], David Furman[3], Julio Rojas[2], Lawren VandeVrede[2], and John Newman[3], *1. University of California, San Francisco, San Francisco, California, United States*, *2. UCSF, San Francisco, California, United States*, *3. Buck Institute, Novato, California, United States*

**Background:** Biological age may be distinct from chronological age. Epigenetic clocks (e.g., PhenoAge) estimate biological age by quantifying changes in DNA methylation (DNAm). Biological age is "accelerated" (AgeAccel) when epigenetic>chronological age. AgeAccel predicts age-related diseases but its association with delirium or neuronal injury markers (e.g., neurofilament light "NfL") is unknown. **Methods:** Adults age 65+ hospitalized for acute hip fracture underwent daily delirium screening with the Confusion Assessment Method Long Form. DNAm status of 850,000 CpG sites was measured in triplicate from pre-op peripheral blood mononuclear cells using Illumina MethylationEPIC arrays. AgeAccel represented the residual of the linear regression model of PhenoAge regressed on chronological age. Plasma NfL was measured in duplicate using Simoa immunoassays. Group differences calculated by T-test. **Results:** Of 12 subjects (enrollment ongoing): mean age 79±8, 75% women, 42% with dementia, 33% were delirious on pre-op blood collection day. Mean AgeAccel was 4.4 years (p=0.02) in delirious vs non-delirious subjects (Fig1A). Those with positive (vs negative) AgeAccel had higher mean NfL (Fig1B, p=0.002). Delirious (vs non-delirious) subjects had higher mean NfL (p=0.004). We found no difference in either AgeAccel or NfL by dementia status. **Conclusions:** In this geriatric hip fracture pilot, accelerated biological age was associated with higher delirium prevalence and NfL. Delirium was also associated with higher NfL. This pilot demonstrates feasibility and utility of measuring biological aging in delirium that warrants study in a larger cohort.

### SESSION 1385 (SYMPOSIUM)

Abstract citation ID: igad104.0388

### HOW CAN DYING, DEATH, LOSS AND BEREAVEMENT BE INCORPORATED IN THE POLICY AND PRACTICE OF AGING IN PLACE?

Chair: Sarah Dury Discussant: An-Sofie Smetcoren

'Aging in place' has been one of the key goals for policymakers in the last 20 years, often translated into policies of deinstitutionalization. Due to the high costs of residential care, the home has re-emerged as a key site for the provision and consumption of care and support. Several critiques of this evolution have been formulated, including the challenges ageing in place might represent for older people, leading to reduced quality of life, and the need to consider low quality of older people's houses, meaning that older people's homes might not be the "best" places to age. However, what has received too little attention in the whole "aging in place" discourse is the end-of-life. This symposium addresses this by illustrating that we cannot talk about ageing in place without talking about dying in place. The authors present research on how dying, death, loss and bereavement might be included in the ageing-in-place policy and practice: Doran sets the stage with a critical reflection of ageing in place and the need to reflect on joining up ageing in place and end-of-life theory and practice. Smetcoren reports on the development of a 'maison de mourance' in a 'co-housing and co-caring' project in Brussels. Dury discusses consolation spaces in Flanders and how they decrease the taboo on death, grief and loss in the neighborhood. And Stegen reports on the caring neighbourhood-movement in Belgium and how some orient themselves to becoming part of a worldwide network of 'compassionate communities'.

Abstract citation ID: igad104.0389
### CONSOLATION SPACES: DO THEY HELP TO MAKE SERIOUS ILLNESS, DEATH, AND LOSS DISCUSSABLE IN THE COMMUNITY?

Sarah Dury, *Vrije Universiteit Brussel, Brussels, Brussels Hoofdstedelijk Gewest, Belgium*

A recent groundswell of movements and organisations is rising in Flanders, Belgium, stimulating engagement with serious illness, death, and bereavement. One result is the huge growth of public consolation spaces. However, it remains largely unknown how consolation spaces are developed, what actions are taken or how they are used. To investigate the development, meaning, their role in a neighbourhood, and the use of consolation spaces, a multi-method design was used. We first examined six different consolation spaces through focus groups. Various stakeholders took part in these, such as volunteers, members of the municipal administration, visitors to the consolation space and care staff. At the same time, an online survey aimed at the use of consolation spaces ran (N=123). Following, the consolation space initiators completed an online survey measuring specific project characteristics, observed outcomes and elements crucial to the realisation of the project (N=still running). Preliminary results indicate that consolation spaces are often created in collaboration with several organisations. The meaning of consolation spaces for people is a space where people seek silence, comfort or to feel connected. Most take or would like to take part in connecting activities linked to the consolation spaces such as consolation walks, reflection moments, planting flower bulbs, etc. People mainly visit this place alone or with a friend and this mainly on an annual basis. The connection with the neighbourhood is present within many consolation spaces. The discussion aims to critically reflect on the findings from the perspective of sustainability and systemic change within neighbourhoods.

Abstract citation ID: igad104.0390
### EXPLORING THE ROLE OF THE COMMUNITY AT THE END OF LIFE: EXPERIENCES FROM A SMALL COMMUNITY-LED END-OF-LIFE PROJECT

An-Sofie Smetcoren, *Vrije Universiteit Brussel, Brussels, Brussels Hoofdstedelijk Gewest, Belgium*

'Care and living in community' (CALICO) is an intergenerational co-housing and co-caring project in Brussels. Within this larger project, a small community-based initiative for palliative and end-of-life care was founded, called Maison Mourance. This initiative aims to mobilize capacity in the broader community to make 'dying-in-place' possible. Given the innovative and grassroots nature of Maison Mourance, the present study wants to understand its vision, functioning, roles of stakeholders involved and the challenges it faces. Therefore a qualitative study was conducted consisting of 10 in-depth interview with several stakeholders and 1 focus group with the steering committee. Their vision centers on creating a homelike environment where people at the end of their lives still feel part of the community. As organisation they want to be nonhierarchical in which each member, formal and informal, is valued and assigned a certain role. Attention is given to the central role of the patient and breaking taboos about dying and death by making it part of the (broader) community. Creating a supportive network of informal and formal actors in palliative care, and thus embedding care in the neighbourhood, is seen as a great added value. Future challenges are financing the project, expanding and keeping the network of volunteers motivated, designing an evaluation plan and selecting evaluation tools. Although this is a promising initiative in terms of community-led palliative care and for making 'dying-in-place' possible, the initiative faces several challenges and threats and thus it will be important to monitor future developments.

Abstract citation ID: igad104.0391
### A CRITICAL REFLECTION ON AGING IN PLACE: INCLUDING END OF LIFE IN AGE-FRIENDLY POLICY

Patty Doran, *The University of Manchester, Manchester, England, United Kingdom*

By taking a critical perspective on the policy objective of ageing in place, this theoretical paper asks where are the end-of-life discussions in age-friendly policies? People do not like to think about "growing old", even less so, perhaps, do they like to talk about end of life and dying. To overcome this, ageing policies have successively been focused on active, successful and healthy ageing. However, there have been increasing calls to examine ageing, and to support ageing in place, from a perspective that acknowledges the heterogeneity of the older population and the challenges faced by diverse groups of older people. The places in which we age, and our experiences of ageing, living and dying, are all influenced by social and political determinants. While separately there is increasing research highlighting inequity in both the ability of older adults to age in place and their experience at end-of-life, there is little literature that suggests how policy that supports ageing in place can also enable better experiences at end-of-life. By adopting a spatial justice lens to the concept of ageing in place, this paper first presents a critical reflection of the places in which we live and age. Second, the paper suggests there are overlapping challenges and opportunities in relation to supporting both ageing in place and end-of-life, therefore there is a need to include greater discussion of end-of-life and dying in age-friendly policy.

Abstract citation ID: igad104.0392
### BREAKING THE TABOO OF SERIOUS ILLNESS, DYING, AND LOSS IN THE COMMUNITY: CARING NEIGHBORHOODS IN BELGIUM

Hannelore Stegen, *Research Foundation Flanders (FWO) & Society and Ageing Research Lab (SARLab), Vrije Universiteit Brussel (VUB), Brussels, Brussels Hoofdstedelijk Gewest, Belgium*

Since the first experiments in 2013 until the launch of 133 Caring Neighbourhoods officially supported by the Minister of Welfare in 2022, Caring Neighbourhoods have become in 10 years time a community-wide movement in Flanders and Brussels. Caring Neighbourhoods aim to support older adults to age well in place: where access to care services is guaranteed, where residents know and help each other, where there are opportunities to meet. The presentation uses insights froms 2 project waves. First, results are presented from the evaluation research of 34 Caring Neighbourhoods in 2020. The project coordinator and a volunteer/community member from each project participated in 1 of the 10 focus groups, using participant-generated photo-elicitation to describe and understand the experienced outcomes and mechanisms of their caring neighbourhood. In evaluating projects it is interesting to see what is going on, what they are doing, what their outputs are. But this presentation will focus on what they were not doing. The question will be raised whether caring neighbourhoods are intended to only provide "small care" and "little help", or to help people with complex needs, in the most difficult moments of life? Second, findings are presented from the current 133 Caring Neighbourhoods and how some of them are trying to respond to issues associated with serious illness, dying, death and loss. The presentation will give insights in actions in how neighbourhoods are encouraged to give more attention to end-of-life topics, and will provide examples on how they are transforming caring neighbourhoods into compassionate neighbourhoods.

### SESSION 1390 (SYMPOSIUM)

Abstract citation ID: igad104.0393

### EXAMINING RELATIONS BETWEEN OLDER ADULTS' HEALTH, WISDOM, AND WELL-BEING DURING TIMES OF HARDSHIP

Chair: Monika Ardelt Discussant: Carolyn Aldwin

This symposium investigates the roles of health and wisdom characteristics as older adults' resources during difficult times and whether ill health might explain lower wisdom during the later years of life. The first two presentations explored COVID-related changes in mental health. Utilizing a national sample of 1258 adults, Park found that acceptance of negative emotions, emotion regulation, and perceived social support at the beginning of the COVID pandemic (mid-April of 2020) predicted middle-aged (36-60 years, Mage=52) and older adults' (61-88 years, Mage=70) lower distress five weeks later. Feng and colleagues analyzed well-being changes in 69 older adults (50-77 years, Mage=59) from before COVID (Time1) to the summer of 2020 (Time2) and 18 months later (Time3). Multiple logistic regressions revealed that greater wisdom and better health at Time2 and higher self-transcendence and better health at Time3 increased the likelihood of being well-adapted across time. Cogdill-Richardson and Bluck examined grief adaptation among 54 older widowed adults (Mage=81). Regression analyses showed that greater wisdom and better physical functioning predicted better grief-related mental health. Whereas good health and wisdom are consistent predictors of well-being in old age, ill health itself is a stressor. Analyzing data of 976 older adults (51-99 years, Mage = 77), Ardelt and Jeste found that the negative association between age and wisdom might be due to ill health that reduces older adults' sense of mastery, which might lessen their motivation for wisdom development. Overall, this symposium demonstrates how health, wisdom, and well-being are interrelated, particularly during times of hardship.

Abstract citation ID: igad104.0394

### WISDOM-RELATED EMOTION REGULATION RESOURCES EXPLAIN MIDDLE-AGED AND OLDER ADULTS' COVID-19-RELATED DISTRESS

Crystal Park[1], Dahee Kim[2], Beth Russell[2], and Michael Fendrich[2], *1. University of Connecticut, Storrs Mansfield, Connecticut, United States*, *2. UCONN, Storrs Manfield, Connecticut, United States*

As the pandemic wrought wide-reaching disruption across the world, younger adults appeared to be faring more poorly than other adults. We hypothesized that younger adults might possess fewer emotion regulation resources and skills, accounting for their relatively high levels of distress. In data gathered from a national sample of 1258 adults, we examined how baseline resources (in mid-April, during initial peak infections) predicted distress (depression, anxiety, PTSD symptoms) five weeks later, when states began initial re-openings. Younger adults (18-35 years; n = 317; mean age = 29.2 years) reported greater distress and less social support, mindfulness, and emotion regulation skills than did middle aged (36-60 years; n = 513; mean age =51.7 years) and older adults (61-88 years; n =428; mean age = 70.1 years). Controlling for stress exposure, younger adults' distress was predicted by impulsivity and lack of perceived strategies while middle-aged and older adults' (lower) distress was predicted by acceptance of negative emotions and emotion regulation abilities; perceived social support was related to lower distress for all groups but mindfulness was unrelated. Results suggest that emotion regulation resources and skills are a promising prevention and intervention focus.

Abstract citation ID: igad104.0395

### THE EFFECTS OF WISDOM AND HEALTH ON NORTH AMERICAN SENIORS' WELL-BEING SINCE COVID-19

Zhe Feng[1], Michel Ferrari[1], Pouria Saffaran[2], Melanie Munroe[3], Asma Shamim[1], and Stephanie Morris[1], *1. University of Toronto, Toronto, Ontario, Canada*, *2. University Of Toronto, Toronto, Ontario, Canada*, *3. Remedy Institute, Toronto, Ontario, Canada*

Public health restrictions necessitated by COVID-19 resulted in significantly reduced social contact for many older people, given their increased risks of infection and developing severe symptoms, even death. While reduced social contact can be a major cause of distress, our recent North American study (n=307) found 3 levels of resilience to the impact of COVID-19 – high ("well-adapted"), average ("getting-by") and low ("struggling") – associated with changes to wellbeing before (Time1), in summer 2020 (Time2), and about 18 months after (Time3). The present study investigates the wellbeing trajectory of 69 older individuals (Mage = 58.83, SDage = 7.21, max. = 77, min. = 50) within that larger sample who reported closely following local physical distancing recommendations. Specifically, it examines how their wellbeing was affected by their country of residence, and self-reported personal wisdom, self-transcendence, and health at Time2 and Time3. Simple logistic regression models suggest that, across Time2 and Time3, higher wisdom and better health were associated with higher likelihoods of being well-adapted vs. just getting-by. Higher self-transcendence at Time3 but not Time2 increases the likelihood of being well-adapted. Multiple logistic regressions with country, personal wisdom, self-transcendence, and health as predictors show that, controlling for all other variables in the model, higher wisdom and better health at Time2, as well as higher self-transcendence and better health at Time3, increase the likelihood of being well-adapted vs. just getting-by. Our findings demonstrate the protective values of personal wisdom, self-transcendence, and health during prolonged periods of isolation and stress for the older population.

Abstract citation ID: igad104.0396

### THE ROLE OF WISDOM IN ADAPTING TO LATE-LIFE SPOUSAL LOSS

Kiana Cogdill-Richardson, and Susan Bluck, *University of Florida, Gainesville, Florida, United States*

Death of one's spouse is normative but one of life's most challenging events (Carr et al., 2001). Wisdom involves compassion, tolerance for uncertainty and reflection, so may be particularly useful in dealing with spousal loss. Past research has related personal wisdom to well-being and life satisfaction in general (Ardelt, 2019). Almost no research, however, has examined wisdom as a resource in the context of bereavement. We (1) expected that higher wisdom would predict better grief adaptation in widowed older adults and, (2) explored whether wisdom effects might be moderated by older adults' physical status. Participants (N = 54, Mage = 81; SD = 7.57, 62.3% female) completed the Brief Wisdom Screening Scale (Glück et al., 2013) and two assessments of grief adaptation: Inventory of Complicated Grief (Prigerson et al., 1995) and Integration of Stressful Life Experiences (Holland et al., 2011). Hierarchical regression, $F(2, 51) = 4.25$, $p < .05$, showed greater wisdom, $\beta = -.27$, $t(52) = -2.061$, $p < .05$ and higher physical functioning ($\beta = -.27$) both predict lower symptoms of complicated grief, with no moderation. A second regression, $F(2,51) = 4.44$, $p < .055$, showed a similar trend with greater wisdom associated with better integration of loss, $\beta = .46$, $t(52) = 1.96$, $p = .055$. Though preliminary, these findings suggest wisdom may serve as a resource for individuals facing loss in later life. Results are discussed in terms of theoretical models (Glück & Bluck, 2013) identifying reciprocal relations between wisdom development and life challenges.

Abstract citation ID: igad104.0397

### DECLINING HEALTH AND SENSE OF MASTERY CAN EXPLAIN THE INVERSE RELATION BETWEEN OLDER AGE AND WISDOM

Monika Ardelt[1], and Dilip Jeste[2], *1. University of Florida, Gainesville, Florida, United States*, *2. University of California, San Diego, San Diego, California, United States*

Previous research found an inverse U-shaped association between age and three-dimensional wisdom with the apex in late midlife. What might explain the negative relation between age and wisdom during the later years of life? We investigated the impact of physical health and mastery, using data from the Successful AGing Evaluation (SAGE) study of 976 adults between the ages of 51 and 99 years (M = 77.23, SD = 12.18). Physical health tends to decline with advancing years and, therefore, might reduce older adults' sense of mastery and control over their life. Ill health and feelings of diminished control, in turn, might lead older adults to focus on improving their health and regaining control and reduce their desire to comprehend the deeper truth about life (cognitive wisdom dimension), look at things from different perspectives (reflective wisdom dimension), and care about others (compassionate wisdom dimension). As expected, results showed that age correlated negatively with subjective health, wisdom, and mastery, while health, wisdom, and mastery were positively correlated. In multivariate regression analyses, age and ill health remained negatively related to mastery even after controlling for education, gender, and race and the positive association with wisdom. By contrast, the negative relations of age and ill health on wisdom became non-significant after mastery was entered into the model. If wisdom declines with age in the later years of life due to ill health that results in a perceived loss of mastery, enabling older adults to maintain their sense of control might offset this decline.

### SESSION 2000 (AWARD LECTURE)

Abstract citation ID: igad104.0398

### MAXWELL A. POLLACK AWARD FOR CONTRIBUTIONS TO HEALTH AGING PRESENTATION AND LECTURE

Chair: Deborah Waldrop

The Maxwell A. Pollack Award for Contributions to Health Aging Lecture will feature an address by the 2022 Pollack Award recipient Nancy Morrow-Howell, MSW, PhD, FGSA, of Washington University in St. Louis. This session will also include the presentation of the 2023 Maxwell A. Pollack Award to recipient Bei Wu, PhD, FGSA, FAGHE of New York University. The Maxwell A. Pollack Award for Contributions to Healthy Aging Award recognizes instances of practice informed by research and analysis, research that has directly improved policy or practice, and distinction in bridging the worlds of research and practice.

Abstract citation ID: igad104.0399

### REFRAMING (MY OWN) AGING: SCHOLARLY AND PERSONAL PERSPECTIVES ON PRODUCTIVE AGING

Nancy Morrow-Howell, *Washington University in St. Louis, St. Louis, Missouri, United States*

Advocacy and scholarship grounded in the productive aging paradigm have evolved over 40 years. From an age-drain to an age-as-asset perspective, productive aging scholars have focused on programs and policies to optimize the vital engagement of older people in paid and unpaid work (working, volunteering, caregiving). I have contributed to this body of work and I have evolved with it. I have changed what I study, how I think about later life, and what I think is important for gerontologists to consider. In this presentation, I will review the development of productive aging literature, including tensions that have existed in the scholarship. Themes of ageism and diversity, equity and inclusion have gained prominence in this work; and I will suggest future directions. I will reflect on how my own aging and experiences have shaped my thinking on productive aging.

### SESSION 2005 (SYMPOSIUM)

Abstract citation ID: igad104.0400

### FAMILY FUNCTIONING: BRIDGING THE RELATION BETWEEN GRANDFAMILY HEALTH AND WELL-BEING

Chair: Nancy Mendoza Discussant: Youjung Lee

In research, it is important to utilize theories to ask and answer questions about a specific phenomenon. In grandfamily research, there is a need to utilize theory as a bridge to understanding the relationship between family functioning and grandfamily health and well-being. In this symposium, four presentations will discuss their work related to grandfamily well-being and how it was guided by theory. In Scott and Nadorff, Bowen's Family Systems theory is used as a framework for examining the extent to which agreement on values and beliefs moderates the relation between intergenerational emotional closeness and well-being in the grandparent generation. Findings demonstrated that affectual solidarity rating and religious ideological differences between grandparents and grandchildren influenced grandparents' well-being. Yancura & Barnett utilized the family life course framework in their study to examine how intergenerational conflict between the grandchildren's parents and the caregiving grandparents predicted stress and depression in grandparent caregivers. Ye and Smith incorporated the family stress model that was developed by Conger and colleagues in their study. Findings suggested parenting practice profiles related differentially to the well-being of caregivers and the emotional and behavioral outcomes of the grandchildren. Musil, Jeanblanc, Zauszniewski, and Burant used McCubbin's Resiliency Model of Family stress to examine how demographics, family demands, problem-solving/coping, resources, and situational appraisals affect depressive symptoms in grandmother caregivers. The inclusion of theory in these studies is significant. Without theory, study findings are nothing more than a pile of findings. Theory helps organize these findings in a meaningful way. This is a Grandparents as Caregivers Interest Group Sponsored Symposium.

Abstract citation ID: igad104.0401

### RELATIONSHIPS MATTERS: INTERGENERATIONAL CONFLICT PREDICTS STRESS AND DEPRESSION IN GRANDPARENT CAREGIVERS

Loriena Yancura, *University of Hawa`i at Manoa, Honolulu, Hawaii, United States*

Literature from both research and applied perspectives suggest that studies consider aspects of grandparent involvement beyond residence and custodial status to examine predictors and correlates of grandparent well-being. These suggestions are supported by a multigenerational family life course framework, which considers lifelong linkages among family and notes that interactions among family members are critically important for well-being. Objective: This study hypothesized that stress and burden in caregiving grandparents (G1) would be predicted by relationship quality with the grandchildrens' parents (G2) when other variables are controlled. Method: Data for these analyses were collected online via Qualtrics Panel Service. Surveys were completed by 312 grandparents who provided care to their grandchildren for at least 20 hours per week.

**Results:** After controlling for grandchild age and grandparent household status (non-resident, skipped generation, multigeneration), stress ($R2=.21$) and depression ($R2= .34$) were positively predicted by non-White race, lower income, and grandparent age. Adding grandchild behavior and grandparent relationship with G2 significantly increased predictive power ($p<.001$) for both stress ($R2=.51$) and ( $R2=.41$) depression.

**Discussion:** These findings suggest that variables beyond residence status are important in understanding the effect of providing care to grandchildren on grandparents' well-being.

Abstract citation ID: igad104.0402

### THE IMPACT OF INTERGENERATIONAL SOLIDARITY ON GRANDPARENT WELL-BEING

Rachel Scott, and Danielle Nadorff, *Mississippi State University, Mississippi State, Mississippi, United States*

Bowen's Family Systems theory provides a framework for examining how values and beliefs are transmitted between family members and the overall potential impact that transmission may have on the well-being of family members. While familial emotional closeness has been shown to predict well-being for older adults, there is still a lack of research investigating the influence of level of agreement on ideology among family members within this relation. Objective: This study examined the impact that level of agreement on religious and political values (consensual solidarity) has on the relation between emotional closeness in intergenerational relationships (affectual solidarity) and well-being in grandparents. Method: Using a sample from the Longitudinal Study of Generations, the current study examined grandparent-adult child (N = 336) and grandparent-grandchild (N = 239) dyads to assess the extent to which agreement on ideological beliefs moderates the relation between intergenerational emotional closeness and well-being in the grandparent generation.

**Results:** Affectual solidarity ratings among the three generations, as well as religious ideological differences between grandparents and grandchildren, were found to influence the well-being of grandparents. Additionally, model fit was found to be excellent for both moderation models between the three generations.

**Discussion:** These findings suggest that emotional closeness is a predominant factor in predicting well-being in grandparents that may not be as heavily influenced by the level of agreement on ideological beliefs as is often assumed.

Abstract citation ID: igad104.0403

### IDENTIFYING DYAD-LEVEL PARENTING PRACTICES AMONG US CUSTODIAL GRANDPARENTS: A LATENT PROFILE ANALYSIS

Minzhi Ye, *Kent State University, Kent, Ohio, United States*

Although half of US custodial grand families contain married grandparents, little is known about the parenting practices of custodial grandfathers (CGF). Based on the Family Stress model and the latent profile analyses, we identified typologies of dyad-level parenting practices among 193 married custodial grandparents (CGP) couples recruited nationally through convenience-based and population-based strategies. Grandmothers (CGM) and CGF were interviewed separately, and four types of parenting practices profiles emerged: (1) Ideal CGM&CGF; (2) Inadequate CGM & Ideal CGF; (3) Ambivalent CGM & High-effective CGF; and (4) High-effective CGM & Inadequate CGF. After controlling for socio-demographics, these profiles related differentially to the psychological well-being of CGF and CGM, as well as to the behavioral and emotional outcomes of grandchildren (CGC). CGF in Profile four reported significantly lower positive affect than those in Profiles one and three, but there were no profile differences regarding CGF depressive symptoms. While CGM in Profile two reported fewer depressive symptoms than the other profiles, there were no differences regarding GM's positive affect. A pattern also occurred whereby CGC from Profile one had significantly fewer externalizing and internalizing difficulties than other profiles. It is essential for future researchers and practitioners to recognize the contribution of CGF to the parenting dynamics in families with married CGP.

Abstract citation ID: igad104.0404

### GRANDMOTHER CAREGIVERS' RESILIENCE, MINDFULNESS, AND RESOURCEFULNESS AND MCCUBBIN'S MODEL OF FAMILY STRESS

Carol Musil, Alexandra Jeanblanc, Jaclene Zauszniewski, and Christopher Burant, *Case Western Reserve University, Cleveland, Ohio, United States*

Grandmothers raising grandchildren face numerous challenges that are reflected in their levels of depressive symptoms and perceived family functioning. Using McCubbin's Resiliency Model of Family Stress, Adjustment and Adaptation, we examine how demographics, family demands (intra-family strains), mediators of problem-solving/coping (resilience, mindfulness and resourcefulness), resources (support) and situational appraisals (stress and reward) affect a) depressive symptoms and b) family functioning. Our participants were a nationwide sample of 300 American grandmothers raising grandchildren who participated in an online research study. Our regression model for general family functioning had an adjusted R2 of .44. Better self-appraisal of family functioning was significantly associated with younger participant age, identifying as white, higher levels of resourcefulness, fewer reported intra-family strains, more subjective social support and instrumental support. Situational appraisals, the problem solving/coping factors of resilience and mindfulness, employment status, and marital status did not significantly predict family functioning. Depressive symptoms, as measured by the CES-D scale, had an adjusted R2 of .46. Participants who were married, had fewer intra-family strains, higher levels of mindfulness and resilience, higher subjective support, and who appraised greater reward from caregiving to grandchildren had lower levels of depressive symptoms. There was no effect from caregiving stress, resourcefulness, employment status, or race. These analyses show the continued utility of McCubbin's model, despite different patterns of predictors of family functioning and depressive symptoms. This further develops our prior work showing that resilience, mindfulness, and resourcefulness are correlated but independent theoretical constructs as they interact very differently with our two outcomes.

### SESSION 2010 (SYMPOSIUM)

Abstract citation ID: igad104.0405

### PSYCHOSOCIAL FACTORS AND NONCOMMUNICABLE DISEASES IN OLDER SUB-SAHARA AFRICANS

Chair: James Muruthi Discussant: Margaret Adamek

Sub-Saharan Africa (SSA) has the fastest-growing proportion of older individuals globally. Concurrently, noncommunicable diseases (NCDs) are a growing burden in the SSA aging population. An essential step towards reducing NCDs is an enhanced understanding of the various diseases and discussing their psychosocial risk factors. This session will provide a unique depiction of the occurrence and measurements of NCDs and their associations with psychosocial factors in aging SSA adults. Dr. Muruthi and colleagues present the psychometric properties of a Swahili-translated Kessler 6 scale in aging Kenyans. Drs Nwakasi and Esiaka discuss HIV health information acquisition among older Nigerian women who are cancer survivors. Dr. Esiaka and colleagues discuss psychosocial determinants of early cancer detection and survivorship among older Nigerian men. Drs. Adamek and Dr. Kotecho will present information about the extent of depression among older adults in Sub-Saharan Africa and an understanding of the factors contributing to high rates of geriatric depression. Findings from this session will help elucidate our understanding of NCDs and related psychosocial factors, a necessary step for developing robust evidence-based interventions to reduce non-communicable diseases (and their effects) among aging people in SSA.

Abstract citation ID: igad104.0406

### HIV KNOWLEDGE AND INFORMATION ACCESS AMONG FEMALE CANCER SURVIVORS IN NIGERIA

Candidus Nwakasi[1], Darlingtina Esiaka[2], and Chizobam Nweke[3], *1. University of Connecticut, Storrs, Connecticut, United States*, *2. University of Kentucky, Lexington, Kentucky, United States*, *3. St George's University, St. George, Saint George, Grenada*

While Nigeria remains heavily burdened by HIV, especially among Nigerian women, there is also a rapidly increasing incidence and prevalence of cancer among aging women in Nigeria. growing. A body of research posits that HIV can be detrimental to cancer survivorship experiences including a worsened health outcome for cancer survivors. Further, HIV-relevant healthy behaviors such as sexual and reproductive health may be limited by a knowledge gap in the relationship between HIV and cancer, thus, increasing the risk of negative health outcomes among cancer survivors. This stresses the importance of HIV prevention and health promotion among cancer survivors in Nigeria, and one of such prevention strategy is the use of HIV health information. This study employed a qualitative descriptive method to assess HIV knowledge and information access among female cancer survivors in Nigeria. Thirty female cancer survivors in Nigeria were recruited and interviewed using a semi-structured interviewing format. Findings based on their responses indicated their knowledge about the connection between HIV and cancer. The themes are comparing HIV and cancer, views on the effect of HIV on cancer, and seeking HIV health information. Our findings highlight the need to utilize tailored public health education interventions for the growing number of cancer survivors in Nigeria to reduce the risk of poor health outcomes in this population.

Abstract citation ID: igad104.0407

### THE ROLE OF SOCIAL DETERMINANTS OF HEALTH ON CANCER RISK BEHAVIORS AMONG OLDER NIGERIAN MEN

Darlingtina Esiaka[1], Candidus Nwakasi[2], and Runcie Chidebe[3], *1. University of Kentucky, Lexington, Kentucky, United States*, *2. University of Connecticut,*

*Storrs, Connecticut, United States*, *3. Miami University, Oxford, Ohio, United States*

Despite the growing cancer incidence and mortality rate in Nigeria, there exists prevalence of imminent and aggressive stage of cancer diagnosis among Nigerian men due to cancer risk behaviors and lack of cancer preventive behaviors such as early screening. This adds to the increasing rates of cancer disparities in Nigeria and Sub-Saharan Africa. Research links social determinants of health (SDOH) to myriad of cancer outcomes including cancer detection and prevention behaviors. To contribute to our understanding of the complexity of cancer risk behaviors, this cross-sectional study aimed to determine the relationship demographic (age, education, employment, income), health (cancer knowledge, cancer beliefs, illness attitudes) and social determinants of health (health insurance, public health infrastructure, rural vs. urban dwelling) have on cancer risk behaviors among older Nigerian men (N=411, age 50+). Results show a significant association between SDOH and cancer risk behaviors. Lack of insurance, absence of health infrastructures, and rural dwelling were correlates of cancer risk behaviors. Also, cancer beliefs, illness attitude, income, and employment had significant relationships with cancer risk behaviors. Our findings point to the detrimental effects of these factors on the fight against cancer disparities. Understanding the mechanisms by which older Nigerian men experience health, requires efforts in identifying constructs that influence their daily-lived experiences and their impact on health-related behaviors. Our data can inform global health actions to eliminate cancer disparities at the individual, organization, and government levels.

Abstract citation ID: igad104.0408

### PSYCHOMETRIC EVALUATION OF A SWAHILI-TRANSLATED KESSLER 6 AMONG OLDER KENYANS

James Muruthi[1], Lucy Maina[2], Elijah Mwega[3], Violet Kagai[4], Ndungu Muruthi[3], and Candidus Nwakasi[5], *1. College of Nursing and Health Professions, Drexel University, Philadelphia, Pennsylvania, United States*, *2. Kenyatta University, Nairobi, Nairobi Area, Kenya*, *3. KARIKA CBO, Nairobi, Nairobi Area, Kenya*, *4. Karika CBO, Nairobi, North-Eastern, Kenya*, *5. University of Connecticut, Storrs, Connecticut, United States*

The six-item Kessler Psychological Distress Scale (K6) has successfully been used to screen psychological distress, albeit among primarily Western dwelling adults. A major advantage of the K6 scale is the capacity to measure a variety of mental health disorders, such as depression and anxiety. However, limited studies have investigated the applicability of the K6 scale to assess psychological distress among aging individuals in Africa. The purpose of the present study is to evaluate the psychometric properties of a culturally adapted (Swahili version) of the K6 in a sample of aging Kenyans. A sample of 376 participants (N = 376; mean age =69.56 years; 73.7% women) was obtained from three regions in Kenya. Factor analyses were used to examine the properties of the translated K6 in aging Kenyans. The correlation between the scale and other health measures were also estimated using structural equation modeling. Factor analyses revealed a single factor model. CFA demonstrated excellent fit (CFI = 0.950; RMSEA = 0.109). Internal consistency was good (alpha =.83) and the scale had acceptable correlations with chronic (r = .15) and physical health (r = .16). Results underlined that the K6 is a one-factor scale that can be used to measure psychological distress in aging Kenyans. These are promising results for health professionals who seek to conduct population studies on psychological health using a brief scale. Future studies should refine these results to maximize the utility of the K6 scale among aging Kenyans.

Abstract citation ID: igad104.0409

### FACTORS CONTRIBUTING TO GROWING DEPRESSION AMONG ELDERS IN SUB-SAHARAN AFRICA

Margaret Adamek[1], and Messay Kotecho[2], *1. Indiana University, Indianapolis, Indiana, United States*, *2. Addis Ababa University, Sadist kilo, Adis Abeba, Ethiopia*

While growing research has addressed physical ailments and communicable diseases in the older adult population in Sub-Saharan Africa, little research has investigated mental health conditions such as depression and anxiety among older Africans. In many African nations, mental health diagnoses are not acknowledged let alone well understood by the general public. Little has been documented about the extent of depression among older adults in Sub-Saharan Africa and the factors contributing to depression among older African adults. This study used secondary sources and a synthesis of recent empirical literature to examine and document the extent of depression among older adults in Sub-Saharan Africa and the contributing factors to geriatric depression. Sources indicate that mental health symptoms of Africans are often considered the work of evil spirits or may be overlooked by families. Available studies report rates of geriatric depression ranging from 37.8% in Ghana, 40% in South Africa, 41.2% in Ethiopia, and up to 44% in Tanzania. Older women in Sub-Saharan Africa have higher rates of depression than older men. Contributing factors to depression include lack of formal education, chronic illness, unstable income, and waning social supports. With an estimated 2 out of 5 older adults in Sub-Saharan Africa experiencing depression, interventions are needed to address the factors contributing to depression among older adults as well as culturally relevant interventions to ameliorate current symptoms. Interventions common in Global North nations such as anti-depressant medication or private therapy may not be appropriate for older African adults struggling with depression.

### SESSION 2015 (PAPER)

### PROTECTIVE ACTIVITIES IN DEMENTIA

Abstract citation ID: igad104.0410

### HEALTH COACHING IMPROVES EVERYDAY COGNITION AMONG INDIVIDUALS AT RISK FOR ALZHEIMER'S DISEASE

Michelle Gray[1], Sally Paulson[2], Joshua Gills[3], Megan Jones[1], Anthony Campitelli[1], Ray Urbina[4], Kelsey Bryk[5], and Jordan Glenn[5], *1. University of Arkansas, Fayetteville, Arkansas, United States*, *2. St. Elizabeth Healthcare, Edgewood, Kentucky, United States*, *3. Rutgers University, Newark, New Jersey, United States*, *4. University of*

*Arkasnas, Fayetteville, Arkansas, United States, 5. Neurotrack, Redwood City, California, United States*

Alzheimer's disease (AD) is the 7th leading cause of death and 6th most burdensome disease among US older adults. With pharmacological treatments for AD being predominantly ill-effective, alternative, non-pharmacological prevention and treatment strategies warrant exploration. Personal health coaching provides individualized strategies designed to improve physical, social, and emotional health leading to positive behavior changes that may improve cognitive ability. The purpose of the present investigation was to examine the effects of a Health Coaching (HC) intervention on cognitive outcomes. Adults (n=182) over 45 years of age were randomly assigned to HC or control arm of a 12-month intervention. Participants (age = 61.9 + 8.4 years) had their cognition (ECog-12) tested at baseline, 16, and 52 weeks. Participants assigned to the HC intervention met with a health coach once per month to establish healthful goals and implementation strategies improving the health and well-being of the participants. The control group received bi-weekly emails including similar information presented during the health coaching sessions. Results revealed a main effect for time for improved ECog-12 scores (p=.04) with a 3.2% improvement in ECog-12 after 52 weeks. Upon further analysis, HC had a significant improvement in cognitive performance after both 16 and 52 weeks, while the control group remained unchanged. Improvement in self-reported cognition was 4.0% (p=.03) and 5.5% (p=.01) in the HC group after 16 and 52 weeks, respectively. These results suggest HC is an effective non-pharmacological prevention strategy for cognitive decline among a group of adults at-risk for developing AD.

Abstract citation ID: igad104.0411

### JOINT ASSOCIATIONS OF PHYSICAL ACTIVITY AND SLEEP DURATION WITH COGNITIVE AGING

Mikaela Bloomberg, Laura Brocklebank, Mark Hamer, and Andrew Steptoe, *University College London, London, England, United Kingdom*

Given the paucity of dementia treatments, identifying contributors to cognitive decline from midlife is important to delay onset of symptoms. Low physical activity (PA) and nightly sleep duration outside 6-8 hours are key interrelated factors thought to contribute to cognitive decline and dementia; how PA and sleep combine to influence cognitive ageing is not well-explored. We used linear mixed models to examine independent and joint associations of PA (low, high) and sleep duration (short [< 6 hours], optimal [6-8], long [>8]) with 10-year cognitive trajectories in cognitively-healthy adults aged ≥50 years from the English Longitudinal Study of Ageing (N=8958). We found low PA (p< 0.001) and suboptimal sleep (p=0.01) were independently associated with worse cognitive performance; short sleep was also associated with faster cognitive decline (p=0.04). At baseline, high PA/optimal sleepers had higher cognitive scores than all sleep categories in the low PA category (e.g. difference high PA/optimal-low PA/short at age 50=0.14 [0.05, 0.24] standard deviations), with no difference in cognitive scores between sleep categories within the high PA category. Differences remained consistent over 10 years of follow-up, except high PA/short sleepers who declined faster than high PA/optimal sleepers, such that scores at 10 years were commensurate with low PA (difference high PA/optimal-high PA/short=0.20 [0.08, 0.33]; high PA/optimal-low PA/short=0.22 [0.11, 0.34]). Baseline cognitive benefits afforded by high PA were insufficient to ameliorate rapid cognitive decline associated with short sleep. While WHO identify PA as a key target for maintaining cognitive health, PA interventions may be ineffective without considering sleep habits.

Abstract citation ID: igad104.0412

### PHYSICAL ACTIVITY AND THE DEVELOPMENT OF DEMENTIA BETWEEN AGES 70 AND 95

Irit Stessman-Lande[1], Jochanan Stessman[2], and Jeremy Jacobs[1], *1. Faculty of Medicine, Hebrew University of Jerusalem, and Hadassah Medical Center, Israel, Jerusalem, Yerushalayim, Israel, 2. Hebrew University of Jerusalem, and Hadassah Medical Center, Israel, Jerusalem, Yerushalayim, Israel*

Whilst the protective effects of physical activity (PA) on cognitive function are well documented, it remains unclear if the preventive effect continues irrespective of advancing age. This study examines the association between physical activity and subsequent dementia between ages 70-95. The Jerusalem Longitudinal Study (1990-2023) prospectively follows a representative community-dwelling cohort born 1920-21, assessed at home visit during 1990,1998, 2005, 2010, 2015 at ages 70, 78, 85, 90, and 95 (n=430, 715, 1136, 660, 492) respectively. Comprehensive assessment included Mini Mental State Examination (MMSE) defining dementia (≤23/30), and PA assessment, dichotomized to Active (PA-A) (>4hours a week) versus Sedentary (PA-S) (< 4 hours weekly). Logistic regression analyses determined Odds Ratios (OR) for developing dementia, adjusted for baseline MMSE, gender, education, depression and BADL. At ages 70, 78, 85, 85, 90, and 95 the frequency of PA-A was 45.9%, 69.7%, 48.7%, 9.5%, 11.9%; and frequency of dementia among PA-A vs. PA-S was 1.7% vs. 5.0%, 1.7% vs. 12.6%, 10.6% vs. 48.5%, 8.6% vs. 35.5%, 18.3% vs. 29.9% (all p< 0.0001 except age 70 due to small numbers) respectively. Adjusted ORs ratios among PA-A at baseline for subsequently developing dementia between ages 78-85, 85-90, 90-95 were: OR 0.44 (95%CI 0.20-0.98); OR 0.52 (95%CI 0.30-0.90); OR 0.37 (95%CI 0.13-1.07) respectively. Our findings show that physical activity is associated with reduced development of dementia throughout follow-up between ages 70-95, and support the hypothesis that there is no upper age-limit to the beneficial effects of PA on cognition.

Abstract citation ID: igad104.0413

### PROTECTIVE EFFECT OF SHINGLES VACCINE ON ALZHEIMER'S DISEASE CAN BE MODULATED BY RISK VARIANT OF NECTIN2 GENE

Svetlana Ukraintseva[1], Hongzhe Duan[2], Rachel Holmes[2], Deqing Wu[2], Igor Akushevich[1], Arseniy Yashkin[1], Konstantin Arbeev[2], and Anatoliy Yashin[2], *1. Duke University, Durham, North Carolina, United States, 2. Social Science Research Institute, Duke University, Durham, North Carolina, United States*

The aging-related decline in immune surveillance was suggested to play a major role in Alzheimer's disease (AD). Adult vaccinations may support the aging immune system beyond prevention of the target disease, and through this provide a protection against AD. Such off-target effects of vaccines may potentially be affected by genetic risk factors for AD. To explore this possibility, we estimated association of the vaccination against shingles (reactivated herpes zoster virus infection) received between ages 65-75 with AD onset at ages 75+ in the Health and Retirement Study (HRS) survey participants, comparing the carriers and non-carriers of rs6859 A allele of NECTIN2 gene (AD risk factor that is also involved in vulnerability to viral infections). We used logistic regression model with covariates including sex, race, education, and smoking status. We found that shingles vaccine was significantly associated with lower odds of AD onset later in life, in the total sample (p-value=0.021; OR=0.55 (0.33, 0.92)). After stratification by genotype, the protective effect of vaccine became stronger in rs6859 A carriers (p-value=0.016; OR=0.44 (0.22, 0.86)), but disappeared in the non-carriers, for both significance and size (p-value=0.81; OR=0.90). We conclude that adult shingles vaccine may reduce chances of AD at older ages, and this effect appears more beneficial for carriers of rs6859 A allele of NECTIN2 gene. Repurposing of existing vaccines may benefit the aging immune system, and be a promising approach to personalized genotype-tailored prevention of AD.

### SESSION 2020 (PAPER)

### HEALTH CARE, PUBLIC HEALTH

Abstract citation ID: igad104.0414

### ASSOCIATIONS BETWEEN HEARING LOSS AND HEALTH CARE UTILIZATION: EVIDENCE FROM NHATS

Emmanuel Garcia, and Nicholas Reed, *Johns Hopkins Bloomberg School of Public Health, Baltimore, Maryland, United States*

We investigated the association between self-reported hearing loss and healthcare utilization among community dwelling older Medicare beneficiaries (age ≥65) who were enrolled in the 2011 cycle of the National Health and Aging Trends Study (NHATS). Participants' survey data were linked to fee-for-service Medicare claims data during the 2011-2016 period. Hearing loss was ascertained using based on participants' hearing aid use, and difficulty hearing in various noisy situations. Those who reported any difficulty hearing or reported hearing aid use were categorized as having hearing loss. Healthcare utilization included: (1) number of inpatient hospitalizations, (2) number of Emergency Department visits, and (3) total number of days spent during hospitalizations per calendar year. Each participant with hearing loss was matched to a participant without hearing loss using a one-to-one propensity score matching to minimize confounding bias. Using generalized estimating equations, we found that among our matched sample those with hearing loss had 21% (Incidence Rate [IR]=1.21; 95% Confidence Interval [CI]= 1.06,1.37) higher rates of emergency department visits, and 48% (IR = 1.48; 95% CI: 1.20,1.82) higher number of days spent in a hospital over the 6-year period from 2011-2016. No association was found between hearing loss and the number of hospitalizations. These finding suggest that older adults with hearing loss may be at risk for increased healthcare utilization. Interventions for hearing loss, such as the use of hearing aids, might reduce healthcare utilization and associated expenditures among older adults in the U.S. Medicare program.

Abstract citation ID: igad104.0415

### CAREGIVER ORAL HEALTH BELIEFS, CONVERSATIONS, AND ACCESS: FINDINGS FROM THE MOTIVATE AT HOME PROGRAM

Jennifer Crittenden[1], Denise O'Connell[2], and Labrini Nelligan[3], *1. University of Maine Center on Aging, Bangor, Maine, United States*, *2. Lunder-Dineen Health Education Alliance of Maine, Bangor, Maine, United States*, *3. Massachusetts General Hospital, Boston, Massachusetts, United States*

The number of caregivers across the nation is growing. A three-way collaboration is examining their knowledge and attitudes related to the connection between oral health and overall health. Currently, little is known about caregiver oral health beliefs and how they relate to oral health care for older adult care recipients (CRs). Survey data from the MOTIVATE at Home program from 135 caregivers/care partners (CPs) across rural Maine were analyzed (age: M = 61 years, SD = 13, range= 25 to 85; 85.2% female). Care partners who felt they have a role in preventing tooth decay were more likely to report an oral health visit in the last year for their CR (M = .75, SD = .438; t(84)= -2.66, p=.009). Caregivers whose CR had not had dental care in the last year were less comfortable talking with dental providers (M = 1.50, SD = .632; t(92.84)= -3.01, p = .003), and non-dental health care providers (M = 1.53, SD =.754; t(94.06) = -2.42, p=.017) about oral health. For CRs who do not have a regular dentist, CPs were less comfortable in speaking with the CR about oral health care (M = 1.58, SD = .751; t(42.13) = -2.23; p=.031). These findings are currently informing the development of an educational intervention for CRs which will: Emphasize the importance of regular oral health visits; Improve understanding of the CP role in promoting oral health; Provide tools for conversations with a range of providers.

Abstract citation ID: igad104.0416

### HOSPICE CLINICIANS' WILLINGNESS TO BE PRESENT DURING MEDICAL AID IN DYING: A CONTENT ANALYSIS OF RATIONALES

Todd Becker[1], John Cagle[1], Cindy Cain[2], Nancy Kusmaul[3], Joan Davitt[1], and Paul Sacco[1], *1. University of Maryland, Baltimore, Baltimore, Maryland, United States*, *2. University of Alabama at Birmingham, Birmingham, Alabama, United States*, *3. University of Maryland Baltimore County, Baltimore, Maryland, United States*

Although clinician presence during medical aid in dying (MAID) may facilitate relief for patients, federal, state, and organizational policy discourage presence. Given the lack of direct examination, we aimed to describe hospice clinicians' willingness to be present during MAID and categorize their rationales. This cross-sectional analysis used Qualtrics data collected from a convenience sample of 75 hospice clinicians. We recruited participants through hospice and palliative care membership associations for physicians, nurses, social workers, and chaplains. A quantitative item asked if participants would be willing to be present with a patient during MAID, given certain safeguards (yes, no, unsure). Participants explained their rationales in an open-ended probe. Erlingsson and Brysiewicz's (2017) four-step content analysis was applied to open-ended rationales grouped under each previously tabulated quantitative response. The categories that we constructed for participants willing to be present (73%) reflected a commitment to patient-centered care, desire to attenuate potential medical complications, and professional responsibility despite personal discomfort. We interpreted responses from those unwilling to be present (16%) through categories of wholesale moral objections to MAID, moral objections specifically to one's participation, and perceived incongruence between MAID and hospice care. We categorized the responses of participants who were unsure (11%) as either competing pressures or fears of personal and professional recourse. Although most participants were willing to be present during MAID, open-ended rationales revealed great nuance within each overarching quantitative response. Participants' reported tensions with themselves, their profession, and society reflect a need for greater professional guidance and support as MAID legalization expands.

Abstract citation ID: igad104.0417

**PHYSICAL AND MENTAL HEALTH STATUS AMONG SANDWICH AND NONSANDWICH CAREGIVERS BY RACE/ETHNICITY**

Christina Miyawaki[1], and Erin Bouldin[2], *1. University of Houston, Houston, Texas, United States*, *2. University of Utah, Salt Lake City, Utah, United States*

Sandwich caregivers are middle-aged adults who care for both a child and an older adult. Using data from the Behavioral Risk Factor Surveillance System Caregiver Module in 50 jurisdictions from 2016-2021, we compared the prevalence of sandwich caregivers and their physical and mental health across racial/ethnic groups. We included 23,853 caregivers aged 45-64 years. Sandwich caregivers (N=2,486) were those who lived with a child (≤18 years) and provided care/assistance to a parent/grandparent with a long-term illness/disability during the past 30 days. The prevalence of sandwich caregiving varied by race/ethnicity, with 18.4% of non-Hispanic Asian, 14.5% of non-Hispanic Black, 11.5% of Hispanic, 10.2% of non-Hispanic White, and 9.5% of non-Hispanic other/multiracial respondents classified as sandwich caregivers. Prevalence ratios (PR) from log-binomial regression models adjusted for age, sex, hours of caregiving, and type of care were used to compare weighted estimates. Sandwich caregivers generally had similar physical health and better mental health compared to non-sandwich caregivers. Compared to non-sandwich caregivers from the same racial/ethnic background, Black sandwich caregivers had a significantly higher prevalence of excellent/very good/good health (PR=1.15) and lower frequent mental distress (PR=0.90), while Asian (PR=0.78) and White (PR=0.91) sandwich caregivers had a significantly lower prevalence of frequent mental distress. Some racial/ethnic groups of sandwich caregivers were in better physical and mental health than non-sandwich caregivers. This could reflect a need for a higher level of physical and mental health to take on a sandwich caregiving role. Caregiver supports should consider sandwich caregivers' unique characteristics and their racial/ethnic backgrounds.

Abstract citation ID: igad104.0418

**WHEN DISASTER STRIKES: ADDRESSING THE UNIQUE NEEDS OLDER ADULTS FACE IN EMERGENCY SITUATIONS**

Amaanali Fazal, Kahir Lalji, and Barbara McMillan, *United Way British Columbia, Burnaby, British Columbia, Canada*

Older adults are more at risk for harm in emergency situations (e.g., pandemics, natural disasters, extreme weather events) and often require more assistance in response and recovery efforts. The COVID-19 pandemic and the increase in climate related emergencies has emphasized the critical need to prepare and respond more effectively to the unique needs of older adults in emergency situations. There is much that community-based seniors serving (CBSS) organizations, emergency management professionals, and local governments can do to develop and implement policies, programs, and practices to effectively support the needs of older adults in emergency preparedness and response. In British Columbia, the COVID-19 pandemic and recent extreme weather events (i.e., forest fires, flooding, and heat waves) have prompted the development of an intentional network strategy for emergency preparedness and response. This innovative approach, which is coordinated by United Way British Columbia, has allowed for increased communication, coordination, collaboration, peer learning, and knowledge translation among CBSS agencies, as well as partner organizations, allied professionals, and other stakeholders. This presentation describes our comprehensive approach to supporting communities to understand and address the specific needs of older adults in emergency planning and management, including: 1) community grants and capacity building; 2) tools and resource development; 3) policies and rights-based strategies; and 4) fostering collaboration between CBSS agencies, emergency management professionals, and all levels of government. Examples are also provided illustrating how these approaches have been utilized in British Columbia to respond to the COVID-19 pandemic and severe weather events.

### SESSION 2025 (SYMPOSIUM)

Abstract citation ID: igad104.0419

**POLICY SERIES: A SECURE RETIREMENT FOR OLDER WOMEN**

Chair: Brian Lindberg Co-Chair: Cindy Hounsell

This session will address policy development related to women and their financial well-being in later life. Panelists will discuss the recently passed SECURE 2.0, the latest programs providing financial literacy to older adults, healthcare workforce related issues, and the role of the Consumer Financial Protection Bureau is protecting older adults from fraud and elder exploitation.

### SESSION 2030 (SYMPOSIUM)

Abstract citation ID: igad104.0420

**ESPO AND BEHAVIORAL AND SOCIAL SCIENCES SECTION SYMPOSIUM: BRIDGES TO ELDER JUSTICE:**

### BUILDING COLLABORATIONS AND COALITIONS TO CATALYZE ELDER MISTREATMENT RESEARCH

Chair: E-Shien Chang Co-Chair: Sara Hackett

Elder mistreatment is a widespread public health problem, affecting 1 in 6 older persons every year. Despite its high frequency and severity, relatively little attention has been paid to ensuring that the findings of elder mistreatment research can be implemented in routine care and clinical practice. Forging meaningful partnerships with health care systems may hold promise to facilitate this translational process, and ultimately improve health outcomes for older persons. In this symposium, we will identify proven approaches and mechanisms to successfully build, maintain, and sustain elder mistreatment research-to-practice partnerships with various health care system stakeholders. Dr. Karl Pillemer will describe best approaches in forging researcher-practitioner collaboration with long-term care providers to implement and disseminate evidence-based resident-to-resident aggression intervention program. Dr. Lena Makaroun will examine lessons learned from four high-impact Veterans Health Administration-led collaborations to accelerate applied elder mistreatment research. Dr. Kristen Lees Haggerty will discuss strategies and successes in developing a novel elder mistreatment screening and response tool for emergency departments nationwide in partnership with the National Collaboratory to Address Elder Mistreatment. Finally, Dr. Zach Gassoumis will describe his experiences with conducting intervention and observational research as well as provide advice to early career scholars looking to build partnerships with health care systems. Taken together, these presentations will shed light on the practice, policy, and research implications in strengthening health care system partnerships to closing the discovery-delivery gap.

Abstract citation ID: igad104.0421

### COLLABORATING WITH LONG-TERM CARE PROVIDERS IN ELDER MISTREATMENT RESEARCH

Karl Pillemer[1], Rhoda Meeador[1], Leslie Schultz[1], Leann Rorick[2], Karen Robson[3], and Jeanne Teresi[4], *1. Cornell University, Ithaca, New York, United States*, *2. Lifespan of Greater Rochester, Rochester, New York, United States*, *3. PA Department of Military and Veterans Affairs, Scranton, Pennsylvania, United States*, *4. Columbia University Stroud Center, New York City, New York, United States*

To study elder mistreatment in institutional settings, collaboration is needed with nursing home and assisted living providers. Studies show that common problems emerge in researcher-practitioner collaboration, including differences in the key areas of orientation, training, nature of evidence, time horizons, and uses of research. Barriers specific to collaboration with. LTC providers also present challenges, including reputational risk from participating in EM studies and demands on administrative and staff time that limit availability for research. We report on a successful model for collaboration with LTC providers, based on the implementation and evaluation of the "Improving Resident Relationships in Long-Term Care" program. This evidence-based program was implemented to prevent and treat resident-to-resident aggression in nursing homes (16 facilities) and assisted living (14 facilities), involving over 2000 residents. Findings revealed a set of replicable elements to obtain LTC provider collaboration on sensitive issues like resident-to-resident aggression: (1) Preparatory efforts to gain commitment of administrative personnel; (2) agreements regarding confidentiality and anonymity; (3) identification of one or more "facility champions," who demonstrate enthusiasm and willingness to advocate for the program; (4) providing payment to participating facilities to cover expenses such as overtime needed to release staff to attend trainings; (5) gaining the support of trusted provider organizations; (6) demonstrating that the program helps meet regulatory requirements; and (7) a procedure to allow for pragmatic modifications to the intervention that allow greater fit with the provider's workflow. Implications for elder mistreatment intervention research are discussed.

Abstract citation ID: igad104.0422

### PROMOTING ELDER JUSTICE IN THE VETERANS HEALTH ADMINISTRATION VIA INTER- AND EXTRADEPARTMENTAL COLLABORATIONS

Lena Makaroun[1], Kathrine Smith[2], Lisa Boris[3], and Jaime Halaszynski[4], *1. University of Pittsburgh School of Medicine, Division of Geriatric Medicine, Pittsburgh, Pennsylvania, United States*, *2. VA Fargo Health Care System, Fargo, North Dakota, United States*, *3. VA Northeast Ohio Healthcare System, Ravenna, Ohio, United States*, *4. VA Butler Health Care System, Butler, Pennsylvania, United States*

The Veterans Health Administration (VHA), the largest integrated health system in the country, provides comprehensive, high-quality care to nearly 19 million Veterans, over half of whom are age ≥60. Older Veterans receiving care in VHA have a high prevalence of most known elder abuse (EA) risk factors, many stemming from their military service. The VHA is a leader in advancing knowledge and care for complex geriatric syndromes, and now leaders in both VHA research and social work are spearheading interdisciplinary collaborations to do the same for EA. In this symposium, we present practical experiences, empirical data and lessons learned from four VHA led collaborations, including: 1) development of the VHA National Social Work Elder Abuse Tiger Team that has worked with VHA researchers to develop and lead a national strategy around EA response; 2) a partnership with the Veterans Benefits Administration to conduct novel research on financial exploitation among Veterans; 3) an education and training collaboration with the National Adult Protective Services Association to demystify the processes and procedures of each organization that has thus far led to 7 presentations to >2,500 attendees; and 4) a relationship with the National Collaboratory to Address Elder Mistreatment to adapt and pilot an EA screening tool for use in VHA emergency departments. Highlighting how the VHA has built bridges both within and outside its organization and the impact this has had on advancing EA research and response will generate ideas for other healthcare systems and those interested in future collaborations with VHA.

Abstract citation ID: igad104.0423

### HEALTH SYSTEM PARTNERSHIPS IN INTERVENTION AND OBSERVATIONAL STUDIES: REFLECTIONS FROM AN EARLY-CAREER SCHOLAR

Zach Gassoumis, *University of Southern California, Los Angeles, California, United States*

The early knowledge base and interventions in the field of elder mistreatment were built by clinician researchers, leading to a natural research-to-practice pipeline. But with more non-clinical researchers steadily entering the field, care must be taken to forge strong research-practitioner partnerships, to three ends: 1) developing clinically informed research and intervention approaches, 2) recruiting clinically relevant samples, and 3) implementing findings into clinical practice. This process can be particularly challenging for early-career non-clinical researchers, who may lack strong partnerships on which to build their research. This presentation extracts lessons for early career scholars by providing overviews of the development of and findings from two studies. The Comprehensive Older Adult Caregiving Supports (COACH) EM prevention program was delivered in partnership with a managed care health system, with referrals received from health system providers. The Better Together study of dementia care groups (i.e., persons living with dementia and their care partners) received referrals from clinicians in mixed managed care/fee-for-service settings, county health facilities, and community partners. Drawing from the principles of community-engaged research, the presentation highlights the relevance of various engagement approaches to elder mistreatment research, including: 1) partnering with senior researchers with existing networks; 2) taking the time to learn the health system's and clinicians' operational systems, pressures, and constraints; 3) honoring practitioner expertise in building research projects and developing research questions; 4) prioritizing meaningful reciprocal engagement, through offering topical or research presentations, funding, co-authorship, etc.; and 5) educating health system administrators about the nuances of conducting elder mistreatment research.

Abstract citation ID: igad104.0424

### THE EMED TOOLKIT: COLLABORATION TO IMPROVE ELDER MISTREATMENT SCREENING AND RESPONSE

Kristin Lees Haggerty[1], Rebecca Stoeckle[1], Kim Dash[2], Gary Epstein-Lubow[2], Randi Campetti[2], Ruthann Froberg[1], and Ellen Tambor[2], *1. Education Development Center, Inc., Waltham, Massachusetts, United States, 2. Education Development Center, Waltham, Massachusetts, United Sta tes*

The National Collaboratory to Address Elder Mistreatment (The Collaboratory) is a group of elder mistreatment (EM) researchers, clinicians, and advocates who came together with the goal of improving elder mistreatment identification and response in hospital emergency departments (ED). Since its inception in 2017, the Collaboratory has prioritized working in close collaboration with health care and aging services providers as well as older adult survivors to develop, test, and disseminate the Elder Mistreatment Emergency Department Toolkit (EMED Toolkit). The EMED Toolkit is a set of streamlined tools designed to improve EM screening and response in hospital EDs and foster improved collaboration between the hospital and community-based services. The EMED Toolkit is comprised of four core components 1) an online staff survey to assess EM practice and drive system change 2) the evidence-based EM Screening and Response Tool designed to efficiently screen all older adult patients for potential mistreatment, 3) online training modules to educate ED staff on elder mistreatment identification and response, including use of the screening tool, and 4) a Community Connections Roadmap designed to help EDs identify and establish connections with community-based services that can support safe discharge and provide ongoing support to older adults experiencing and at risk for mistreatment. In this presentation we will summarize results from a feasibility study testing EMED Toolkit implementation at five hospitals in the US, describe the Collaboratory's approach to disseminating the EMED Toolkit more widely through local, regional, and national systems change initiatives, and share lessons learned from our efforts.

### SESSION 2035 (PAPER)

### ALZHEIMER'S DISEASE AND DEMENTIA (BSS)

Abstract citation ID: igad104.0425

### ADULTS' UNDERSTANDING OF COGNITIVE DECLINE, DEMENTIA, AND ALZHEIMER'S DISEASE

Laura Mehegan, and Chuck Rainville, *AARP, Washington, District of Columbia, United States*

A 2021 nationally representative survey fielded by AARP of adults aged 40 and older (N=3,022) showed that nearly six in ten (58%) believe that cognitive decline is inevitable, and two-thirds (66%) believe they will experience cognitive decline as they age. Nearly half (48%) think it is likely they will get dementia. As a follow up to this research, in 2022, AARP sponsored focus groups (N=31) and in-depth interviews (N=24) to further explore the perceptions of dementia and cognitive decline among individuals aged 40 and older. The main purpose of this research was to evaluate how Americans understand and communicate about dementia and cognitive decline. The results of this research showed personal experience played a greater role than age when it came to participants' concern and understanding about cognitive decline. Worry about cognitive decline increased for those caring for a friend or family members with dementia or Alzheimer's disease. Participants expressed feelings of inevitability of cognitive decline, but not dementia or Alzheimer's disease. Forgetfulness was mentioned by many participants as part of the aging process, but they were clear that dementia and Alzheimer's disease were not inevitable and were not the same as age-related memory lapses. Normal, age-related changes in memory and cognitive function were not well-understood and neither was the distinction between dementia and Alzheimer's disease. The results from these studies suggest the general population needs information on cognitive decline, dementia, and Alzheimer's disease to better understand each and to raise awareness on the proactive steps needed for brain health

Abstract citation ID: igad104.0426

### FEASIBILITY AND EFFICACY OF THE LEAD DEMENTIA ADVANCE CARE PLANNING INTERVENTION

Kara Dassel[1], Eli Iacob[2], Jordana Clayton[3], Nancy Aruscavage[3], Katherine Supiano[2], and Rebecca Utz[2], *1. University of Utah College of Nursing, Salt Lake City, Utah, United States, 2. University of Utah, Salt Lake City, Utah, United States, 3. University of Utah College of Nursing, Salt Lake City, Utah, United States*

Using our dementia-focused advance care planning tool (ACP), the LEAD Guide (Life-Planning in Early Alzheimer's and Other Dementias), we implemented a 9-month NIH Stage-1 behavioral intervention to promote ACP concordance in community-based dementia dyads. Baseline concordance was assessed using individual responses on the LEAD Guide (patient for themselves, care partner as if they were the patient). Dyads compared responses, discussed discrepancies, and the patient revised their responses. Concordance for values (quality of life, burden) and preferences (care location and life-sustaining treatments) across the disease trajectory was calculated using cross-tabs. At baseline, dyadic discordance was less than 10% on quality-of-life measures except for whether the patient would prefer a longer but less satisfying life (23.8%); however, post-conversation, 5.9% of patients changed their responses. About a fifth of the dyads showed discordance regarding financial, emotional, and physical burden (19.0%, 19.1%, and 16.7%, respectively); however, post-conversation, a few patients changed their responses (14.6%, 8.8%, and 5.9%). At baseline, discordance on questions for life-sustaining treatments across the disease trajectory varied; ventilator (now 14.3%, future 26.2%), brain dead (16.7% now and future), feeding tube (30.1% now, 28.6% future), and severe pain (23.8% now, 21.4% future). Following conversations, a few patients' changed their responses on these items (8.8%, 12.1%, 8.8%, and 12.1%). Higher discordance on value questions on burden compared to the quality of life may result from care partners' bias. Fluctuations in concordance regarding life-sustaining treatments in the present versus future states of advanced dementia demonstrate the need to engage in more in-depth care conversations.

Abstract citation ID: igad104.0427

### MOVING TOGETHER: IMPACT OF LIVESTREAM GROUP PROGRAM FOR PEOPLE LIVING WITH COGNITIVE IMPAIRMENT AND CARE PARTNERS

Deborah Barnes[1], Jennifer Lee[2], Margaret Chesney[1], Wolf Mehling[1], Francesca Nicosia[1], Rebecca Sudore[1], Linda Chao[1], and Cynthia Benjamin[2], *1. University of California, San Francisco, San Francisco, California, United States*, *2. Together Senior Health, San Francisco, California, United States*

Moving Together is an online, mind-body, group movement program for people living with cognitive impairment or mild dementia (PLCI/D) and care partners (CPs). We performed a randomized, controlled trial to test the impact of Moving Together on quality of life (primary) or physical, cognitive, social or emotional (secondary) outcomes in PLCI/D or CPs. Study participants were enrolled as dyads and randomly assigned to intervention or waitlist control groups. Participants joined live-streaming classes for 1 hour, 2 days/week, for 12 weeks. Intention-to-treat analyses compared 12-week change between groups. We assessed 603 dyads for eligibility: 382 (63%) declined to participate, primarily due to lack of interest; 123 (20%) were ineligible, primarily due to lack of CP or moderate/severe dementia; and 98 (16%) were eligible and enrolled. Of these, 77 (79%) completed the 12-week assessment and 21 (21%) withdrew, primarily due to health or scheduling issues. PLCI/D were 77±11 years old, 44% women, 22% non-White/Hispanic, 32% Alzheimer's disease, 26% unspecified/mixed dementia, 14% mild cognitive impairment, 10% vascular dementia, and 19% other/unknown causes. CPs were 66±12 years old, 78% women, 31% non-White/Hispanic, 71% spouses, 25% adult children, and 3% paid. Baseline characteristics were similar between groups. Participants who completed the program reported high satisfaction (98% good/excellent). There were no serious adverse events related to study participation. We conclude that Moving Together is a safe program with good retention and high satisfaction. Data collection will be completed in May 2023, and we will present efficacy outcomes at the meeting.

Abstract citation ID: igad104.0428

### PROVIDER PERCEPTIONS OF FACTORS IMPACTING COUPLES' EMOTIONAL DISTRESS AND ADJUSTMENT TO DEMENTIA DIAGNOSES

Sarah Bannon[1], Julie Brewer[2], Nina Ahmad[2], Talea Cornelius[3], Robert Parker[2], Bradford Dickerson[2], Christine Ritchie[2], and Ana-Maria Vranceanu[2], *1. Mount Sinai Hospital, New York City, New York, United States*, *2. Massachusetts General Hospital, Boston, Massachusetts, United States*, *3. Columbia University Irving Medical Center, New York City, New York, United States*

Alzheimer's disease and related dementias (ADRD) are increasingly common conditions that disrupt the lives of persons living with dementia and their spousal care-partners. At the time of ADRD diagnoses, many couples experience challenges that produce emotional distress and relationship strain. At present there are no interventions to address these challenges early after diagnosis to promote positive dyadic adjustment. As the first phase of a larger study, we conducted a qualitative study to elicit and systematically summarize the perspectives of ADRD medical stakeholders (e.g., neurologists, geriatricians, social workers, neuropsychologists, care coordinators) surrounding common factors impacting distress following ADRD diagnoses. Participants (n=15) were recruited from two U.S. academic medical centers and completed a 60-minute focus group or individual interview that was transcribed, deidentified, and analyzed using a rapid data analysis approach to thematic analysis. We identified findings within 3 overarching themes: reactions to diagnosis, changes in relationship roles/dynamics, and insufficient resources. Participants described couples' distress surrounding one partner's denial or lack of insight into the diagnosis and symptoms, experiences of loss and hopelessness, and uncertainty surrounding illness trajectory. They noticed changes in couples' historic roles, social determinants of health, and divergent understandings and reactions to diagnosis as contributors to couples' distress. Finally, providers observed unmet needs surrounding embedded counseling, care-planning, and financial support for current and future care needs. Most providers felt insufficiently prepared to help couples experiencing distress. Our findings underscore the importance of improving resources to address individual, interpersonal, and system-level factors that contribute to couples' adjustment to ADRD diagnoses.

Abstract citation ID: igad104.0429
### USING THE ELECTRONIC MEDICAL RECORD OF NURSING HOME RESIDENTS FOR IMPROVED MEASURE OF BEHAVIORAL SYMPTOMS
Hyunkyung Yun[1], Ellen McCreedy[2], Vincent Mor[2], and Susan Mitchell[3], *1. Brown University, Providence, Rhode Island, United States*, *2. Brown University School of Public Health, Providence, Rhode Island, United States*, *3. Hebrew SeniorLife, Boston, Massachusetts, United States*

Behavioral symptoms as study outcomes are measured by routinely collected data such as the Minimum Data Set (MDS), the standardized assessment of nursing home (NH) residents. However, MDS under-detects neuropsychiatric symptoms in people with dementia, and better identification in this outcome measurement is needed. This work aims to determine if the electronic medical record (EMR) NH data, the Interventions to Reduce Acute Care Transfers (eINTERACT) form, could improve the identification of agitated behaviors (behaviors). 77,936 assessments of the MDS and the EMR of 19,705 long-stay residents with dementia from 322 NHs, conducted between January 2020 and August 2022, were used. MDS behaviors were defined as any verbal or physical behaviors directed at others, resistance to care, or other behaviors (e.g., pacing). eINTERACT behaviors were defined as any general agitation or verbal or physical aggression. 96% of NHs used eINTERACT forms for at least half of their residents. On average, 14.8% (SD 35.6) of residents with a qualifying assessment in a given month had an MDS-documented behavior, and 2.4% (SD 15.2) had an eINTERACT-documented behavior. 1.4% (SD 11.7) had behaviors documented only on the eINTERACT form representing about a 10% increase in detectable behaviors in a given month over MDS alone. Results were robust to stratification by NH-level prevalence of MDS behaviors. Using the eINTERACT forms in addition to the MDS behavioral items increased in detection among long-stay residents with ADRD. There is some indication that greater use of eINTERACT is associated with greater increases in behavior identification.

### SESSION 2040 (SYMPOSIUM)

Abstract citation ID: igad104.0430
### AGING IN PLACE IN THE RURAL SOUTH
Chair: Caitlyn Nix

Aging in place in the rural south involves regionally unique concerns. Understanding the interaction between factors such as geographical isolation, smaller communities, and access to resources is critical for researchers, as results from research in urban areas may not generalize to this population. This symposium will outline regional barriers and protective factors related to aging in place from researchers in a distinctly rural area in the deep south. The symposium will begin with a discussion from Darby Mackenstadt on person-environment fit in a sample of older adults from Mississippi, with an emphasis on predictors of health status such as access to grocery stores and level of education. Carolyn Adams-Price will then review results from qualitative research on older adults' self-reported attachment to home and local communities in rural Mississippi. Finally, Mary Dozier will give an overview of excessive household clutter as a barrier to maintaining functional independence for older adults enrolled in treatment studies for hoarding disorder in the state of Mississippi.

Abstract citation ID: igad104.0431
### GEOGRAPHIC AND DEMOGRAPHIC PREDICTORS OF SELF-RATED HEALTH IN OLDER RURAL AFRICAN AMERICANS
Darby Mackenstadt[1], Carolyn Adams-Price[1], and Sarah Israel[2], *1. Mississippi State University, Mississippi State, Mississippi, United States*, *2. Mississippi State University, Starkville, Mississippi, United States*

Person-Environment (P-E) fit is a major tenet of environmental gerontology and can predict aspects of physical and mental health. Rural older adults may vary greatly in their P-E fit, but their in-group differences have yet to be explored. The current study examines predictors of self-rated health in middle and older adult African Americans in rural Mississippi. Predictors of self-rated health examined included distance from grocery store, age, education, and income. Participants were 47 African Americans (20 males) over 50 (M = 65.2) who were recruited from two rural, adjacent Mississippi Counties and lived at least five miles outside of a micropolitan town. Consistent with literature on health disparities in African American communities, participants on average rated their health as average to low. Results from a multiple regression model indicate that education and distance to grocery stores are significant predictors of subjective health, where more education and being further from a grocery store predicted higher self-rated health. It appears that within this sample, the further from a grocery store they were, the better the P-E fit. This may be due to an increase in gardening or farming, increased necessity for physical activity, or other aspects of this environment that promote healthy behaviors, despite being further from resources. Future research should continue to pursue aspects that add to P-E fit in rural older adults.

Abstract citation ID: igad104.0432
### CULTURAL CONSIDERATIONS AND ATTACHMENT TO PLACE FOR RURAL-DWELLING OLDER ADULTS
Carolyn Adams-Price, *Mississippi State University, Mississippi State, Mississippi, United States*

Mississippi is the only state in the United States where the majority of African Americans live in rural areas. African Americans have lived in rural Mississippi for many generations, despite few resources and health inequities. However, it is a mistake to look at older rural African Americans and see only poverty and illness. Previous research by Rowles and others in Appalachia has indicated that older adults tend to be satisfied with homes in impoverished rural areas. This study examined attachment to home and community in older African Americans living in rural parts of two counties in north Mississippi. Participants were 47 African Americans between 52 and 79 (20 male), with a mean age of 65. Phenomenological analysis for attachment to home yielded 6 themes: home as a peaceful/safe place, legacy/historical roots, attachment to natural features, ownership/I've made it my own, and everyday functionality. The first two themes

(peaceful/safe and legacy/historical roots) were far more prevalent than the theme of everyday functionality; these findings will be discussed in the context of other researchers' findings on attachment to place in later life. However, although our participants have experienced discrimination in the larger environment, home is for most of them a place of safety, warmth, and meaning. Attachment to features of the land is also important.

Abstract citation ID: igad104.0433

**CLUTTER AS A BARRIER TO MAINTAINING FUNCTIONAL INDEPENDENCE**

Mary Dozier[1], and Caitlyn Nix[2], *1. Mississippi State University, Starkville, Mississippi, United States*, *2. Mississippi State University, Mississippi State, Mississippi, United States*

Excessive levels of household clutter can increase fall risk and subsequently prevent older adults from living independently. This may be a particular concern for rural-dwelling older adults, who are likely to have a stronger sense of connection with their physical location. This talk will present data from a comprehensive baseline assessment administered to older adults enrolled in treatment studies for hoarding disorder in rural Mississippi (n = 25; mean age: 67). The majority of participants described themselves as "collectors," (79%) and individuals who considered themselves to be collectors were more likely to have experienced at least one intervention from friends, family, or the community (42% vs. 20%). Thirty-two percent of participants reported they had experienced at least one fall in their home in the previous year and 60% reported they were at least "a little bit" concerned about falling in their home, with 20% indicating that they or someone else had expressed concern specifically about clutter increasing their risk of falling. In addition to fall risk, 28% of participants indicated having at least one health hazard in the home (e.g., mold, insect infestation). Forty-three percent of the sample reported low physical functioning and 50% of the sample indicated a high degree of loneliness. These results highlight the impairment experienced by rural-dwelling older adults with high amounts of clutter. Interventions that target de-cluttering may provide additional utility by also increasing the ability of these older adults to age in place.

### SESSION 2045 (SYMPOSIUM)

Abstract citation ID: igad104.0434

**DATA AND INITIAL EVIDENCE: OCCUPATIONAL CHARACTERISTICS AND LATER-LIFE PHYSICAL AND COGNITIVE HEALTH**

Chair: Dawn Carr

Research on aging is often based on evaluations of health and wellbeing once individuals have already reached later life. However, many of the problems observed are based on experiences that occurred during earlier phases of life. Adults spend approximately 1/3 of their time engaged in paid work, and consequently, occupational exposures are likely to play a critical role in shaping age-related changes over time. Occupational exposures are difficult to study because most studies only collect data on work environments at one point in time or only during the latest phases of one's career. Further, much of the data on occupations is based on subjective experiences, or in association with a small subset of occupational categories. A linkage between the Health and Retirement Study Life History Module Survey (LHMS) and the recent introduction of a new data source measuring occupational characteristics based on the Department of Labor's O*NET data now make it possible to comprehensively evaluate full life histories of work. This symposium provides an overview of this new dataset and how it was created, and provides initial evidence of the important role that occupational histories play in shaping early departure from work due to disability, mid-life metabolic disease risks, and later life cognitive function.

Abstract citation ID: igad104.0435

**LIFE COURSE EXPOSURE TO RISKY WORK ENVIRONMENTS AND EARLY RETIREMENT DUE TO DISABILITY**

Amanda Sonnega[1], Dawn Carr[2], Qize Chen[1], Rebekah Carpenter[2], and Katy (Qiuchang) Cao[2], *1. University of Michigan, Ann Arbor, Michigan, United States*, *2. Florida State University, Tallahassee, Florida, United States*

Jobs that are physically demanding, stressful, and dangerous are typically associated with earlier onset of physical health decline and may be associated with increased risk of early retirement due to disability. Occupations reflect differentiation by race and ethnicity due to structural racism in the educational and judicial systems, and the labor market, with Blacks and Hispanics more likely to occupy jobs that are more physically demanding and dangerous. If such jobs lead to earlier retirement, they may exacerbate wealth disparities. We use new data from the Health and Retirement Study' Life History Mail Survey linked to rich job characteristic data from O*NET to characterize lifetime exposure to risky work environments based on levels of physiological and psychological stress. We evaluate differences by race/ethnicity in exposure to a wide range of risky work environments, explore whether they relate to disability retirement, and explore whether these associations differ by race. We show that there are large racial and ethnic differences in both the likelihood of disability retirement and lifetime exposure to risky work environments. Probit regressions controlling for education and cohort suggest that racial and ethnic differences in disability retirement are partly explained by lifetime exposure to risky work. Our findings may inform modifications to work environments that could improve the lives of workers in "bad jobs" and potentially reduce disparities in disability retirement.

Abstract citation ID: igad104.0436

**RISKY WORK ENVIRONMENTS AND METABOLIC DISEASE: CUMULATIVE CONSEQUENCES OF BAD JOBS IN EARLY AND MID LIFE**

Rebekah Carpenter, *Florida State University, Tallahassee, Florida, United States*

Approximately one in three adults in the United States have a metabolic disease or illness. Metabolic diseases and illnesses are particularly costly for older adults, often resulting from decreased human capital and increased health care-based costs in later life. Social contexts have been hypothesized to be a key contributor to later-life health; however, work environments are often understudied as a critical social factor shaping health over the life course. Utilizing new data from the Health and Retirement Study's Life History Mail Survey linked to occupational work context data from the Occupational Information Network (O*NET), we examine how exposure to dangerous work environments over one's working career relates to the likelihood of developing a metabolic disease or illness between the ages of 50 and 62. Results from logistic regressions suggest that dangerous work environment exposures, such as exposures to contaminants or hazardous conditions, are associated with increased risk of developing metabolic disease and illnesses between ages 50 and 62; however, these associations are dependent upon when in the life course people are exposed to dangerous work contexts and for how long people are exposed to dangerous work contexts. This study speaks to the growing need for understanding the role of occupations as an important social and contextual factor shaping population health. Specifically, findings from this study may better inform the development of social and organizational policies targeted at improving work environments in the US, thereby helping to reduce the risk of developing health-related challenges, including metabolic diseases and illnesses.

Abstract citation ID: igad104.0437

### THE ASSOCIATION BETWEEN JOB COMPLEXITY AND COGNITIVE FUNCTION IN LATER LIFE ACROSS RACIAL/ETHNIC GROUP

Katy (Qiuchang) Cao, Dawn Carr, and Rebekah Carpenter, *Florida State University, Tallahassee, Florida, United States*

The health of older Black and Hispanic populations are disproportionately impacted by cognitive decline later in life. Although cognitive stimulation through work protects against cognitive decline and dementia in later life, Black and Hispanic individuals have less access to cognitively stimulating employment opportunities compared with their white counterparts. This study aims to disentangle the relationship between work complexity and cognitive function later in life across racial/ethnic groups. Using nationally representative data from the Occupation Information Network (O*NET) linked to the Health and Retirement Study' Life History Mail Survey (n=1080), we examine how cumulative exposure to cognitively complex work between ages 25 to 55 is associated with cognitive function at age 70 in regressions controlling for cognitive function at age 55 and relevant socio-demographic measures. A composite measure of work complexity was constructed from making decisions and solving problems, thinking creatively, coaching and developing others, frequency of decision making, and freedom to make decisions. Results from regression analyses suggest that compared with cumulative exposure to less cognitively complex work, cumulative exposure to moderately cognitively complex work was associated with a 0.74 point increase in the cognitive score ($p<0.01$), and cumulative exposure to highly cognitively complex work was associated with a 1.25 point increase ($p<0.01$) in cognitive score. Among those who held moderately cognitively complex jobs, Black individuals experienced fewer cognitive benefits than their white counterparts later in life. Findings have implications for research and interventions addressing disparities in cognitive health among Black and Hispanic populations through employment-related policies and interventions.

Abstract citation ID: igad104.0438

### INTRODUCING A NEW AGE-BASED DATASET FOR EVALUATING OCCUPATIONAL CHARACTERISTICS ACROSS THE LIFE COURSE

Qize Chen[1], Amanda Sonnega[1], Dawn Carr[2], Rebekah Carpenter[2], and Katy (Qiuchang) Cao[2], *1. University of Michigan, Ann Arbor, Michigan, United States, 2. Florida State University, Tallahassee, Florida, United Sta tes*

The connection between early life career choices and later life outcomes is a topic of interest to gerontology researchers, but research has been limited by data availability. In this paper, we present a newly constructed LHMS-HRS-O*NET linked dataset. The 2017 Health and Retirement Study (HRS) Life History Mail Survey (LHMS) includes information on up to 10 jobs that respondents worked for more than a year. We consolidated this employment history with the biennial HRS Core survey data. Then, respondents' occupations from the LHMS were mapped to the O*NET 26.1 Database to add information on their exposure to different work contexts, activities, and etc. The final dataset is a panel at individual-year level with annual occupational exposure from the first job after full-time education until the last job people held before 2017. The data covers over 4,000 individuals in the HRS with all O*NET measures available in O*NET 26.1. This presentation will demonstrate how researchers can use these data to calculate the cumulative occupational exposures for any period of one's career and connect that information to socioeconomic, health, and other outcomes at older ages that are available in HRS. This new data is ideal for studying how full life histories of work are related to differential aging outcomes.

### SESSION 2050 (SYMPOSIUM)

Abstract citation ID: igad104.0439

### JOURNAL OF GERONTOLOGY SOCIAL SCIENCES: FEATURED 2022 EDITOR'S CHOICE ARTICLES

Chair: Jessica Kelley

Social science inquiry on age, aging, and the life course spans many topics and methodologies. This symposium highlights papers that were selected as Editor's Choice articles in the Journal of Gerontology Social Sciences in 2022. These papers highlight methodological innovations, important advancements in our state of knowledge in an area, or emerging issues in the study of aging and older adults. Moen et al. trace the divergence in later-adulthood work trajectories at the intersection of race, gender, and class. Hamler et al. discuss the impact of skin tone on mental health among older Black Americans. Waselmann et al. present an innovation of counting number of days one attended school and whether one lived in the Jim Crow South to help explain Black-White disparities in later-life cognitive function. Falzarano et al. explore cultural differences in orientation toward familialism and its impact on caregiver outcomes. Zimmer et al. examine the linkage between war exposure and later-life frailty among Vietnamese older adults.

Abstract citation ID: igad104.0440
### THE UNEVEN LATER WORK COURSE: INTERSECTIONAL GENDER, AGE, RACE, AND CLASS DISPARITIES
Phyllis Moen[1], Sarah Flood[1], and Janet Wang[2], *1. University of Minnesota, Minneapolis, Minnesota, United States*, *2. University of Michigan, Ann Arbor, Michigan, United States*

We document patterned continuity and change in monthly work attachments and analyze the intersecting effects of age, gender, education, and race/ethnicity. We utilize 16-month panel data from Current Population Survey (2008-2016) to code monthly states: working full-time, long hours, part-time; being self-employed or unemployed; not working due to disability or family care, or retired. Analyses of 346,488 American women and men aged 50–75 years reveal patterned elasticity in the timing and nature of work attachments in the form of six distinctive pathways. Analyses illustrate divergences and disparities: advantages for educated White men, disadvantages for low-educated Black men and women through their early 60s, and intersecting effects of gender, education, and race/ethnicity during the later work course across age groups. We find convergence across social markers by the 70s. This research highlights the importance of intersectional analysis, recasting the gendered work course in later adulthood to reflect its complexities.

Abstract citation ID: igad104.0441
### HOW SKIN TONE INFLUENCES RELATIONSHIPS BETWEEN DISCRIMINATION AND MENTAL HEALTH AMONG OLDER AFRICAN AMERICANS
Tyrone Hamler, *University of Denver, Denver, Colorado, United States*

Skin tone has been identified as an important factor for within-group health disparities. We examine the moderating role of skin tone in the relationship between discrimination, self-rated mental health, and serious psychological distress (SPD) and (b) whether moderated by gender. We used a subsample of African Americans aged 55+ (N = 837) from the National Survey of American Life. Mental health outcomes were SPD and self-rated mental health. Results indicate discrimination was associated with worse self-rated mental health and SPD in the total sample and among women. Skin tone moderated the association between discrimination and SPD among both genders. The effect of discrimination on mental health was stronger among darker-skinned respondents than lighter respondents. Gender-stratified analyses indicated skin tone moderated the association between discrimination and self-rated mental health for men but not women. The negative psychological effects associated with darker complexion indicate one mechanism linking discrimination and mental health.

Abstract citation ID: igad104.0442
### INEQUITY IN SCHOOL ATTENDANCE IN THE JIM CROW SOUTH: IMPLICATIONS FOR BLACK–WHITE DISPARITIES IN COGNITIVE FUNCTION
Katrina Walsemann[1], Stephanie Ureña[2], Mateo Farina[3], and Jennifer Ailshire[3], *1. University of Maryland, College Park, College Park, Maryland, United States*, *2. University of Maryland, College Park, Maryland, United States*, *3. University of Southern California, Los Angeles, California, United States*

Education's contribution to Black–White disparities in cognitive function remains unclear due to data limitations that capture systemic educational inequities in the Jim Crow South. We include differential rates of school attendance across race, years, and states in the Jim Crow South. We linked state-level data on school attendance from the 1919 to 1954 Biennial Surveys of Education to the HRS. Sample restricted to Black and White older adults who attended school in the Jim Crow South in the study period (n = 4,343). We examined total cognitive function, episodic memory, and working memory. Total years of schooling explained 28%–33% of the Black–White disparity in all three outcomes. Differential rates of school attendance explained 41%–55% of the Black–White disparity in these outcomes. Our study highlights the importance of using a more refined measure of schooling for understanding the education–cognitive health relationship.

Abstract citation ID: igad104.0443
### FAMILY MATTERS: CROSS-CULTURAL DIFFERENCES IN FAMILISM AND CAREGIVING OUTCOMES
Francesca Falzarano[1], Jerad Moxley[1], Karl Pillemer[2], and Sara Czaja[1], *1. Weill Cornell Medicine, New York City, New York, United States*, *2. Cornell University, Ithaca, New York, United States*

The increasing number of minority older adults and subsequent increase in minority individuals needed to provide care highlights the need to understand how the caregiving role is experienced and perceived among racial/ethnic minority groups. Familism, a cross-cultural collectivist value, has been examined as a driving mechanism influencing differential outcomes in family caregivers. However, research examining familism in caregiving has yielded mixed results. Guided by the Sociocultural Stress and Coping Model, the purpose of this study is to examine cross-cultural differences in familism and its influence on caregiving outcomes. Baseline data were collected from 243 family caregivers who participated in the Caring for the Caregiver Network randomized controlled trial, a culturally tailored technology-based psychosocial intervention. Results showed that familism was more highly endorsed by Hispanic and African American participants, when compared to Whites, which was subsequently associated with positive aspects of caregiving, depression, and burden. Exploratory analyses among the Hispanic subgroup were conducted to examine how acculturation, operationalized as number of years living in the US, influences familism and found that a longer length of time spent in the US was negatively associated with familism. When dividing our sample into U.S. natives versus non-natives, non-native Hispanic participants reported higher familism compared to natives. Findings indicate that cross-cultural differences in caregiving appraisals may operate as a function of cultural values, which influence one's subjective evaluation of the caregiving role and contribute to more optimal outcomes – highlighting the need for culturally congruent interventions to promote positive caregiving outcomes particularly among racial/ethnic minorities.

Abstract citation ID: igad104.0444

**EARLY-LIFE WAR EXPOSURE AND LATER-LIFE FRAILTY AMONG OLDER ADULTS IN VIETNAM: DOES WAR HASTEN AGING?**

Zachary Zimmer[1], Kim Korinek[2], Yvette Young[2], Bussarawan Teerawichitchainan[3], and Tran Khanh Toan[4], *1. Mount Saint Vincent University, Halifax, Nova Scotia, Canada*, *2. University of Utah, Salt Lake City, Utah, United States*, *3. National University of Singapore, Singapore, Singapore*, *4. Hanoi Medical University, Dong Da, Ha Noi, Vietnam*

We aimed to assess the nature and degree of association between exposure to potentially traumatic wartime experiences in early life and later-life frailty. The Vietnam Health and Aging Study included war survivors in Vietnam, age 60+. Latent class analysis (LCA) is used to construct classes exposed to similar numbers and types of wartime experiences. Frailty is measured using a deficit accumulation approach that approximates biological aging. LCA yields 9 unique wartime exposure classes, ranging from extreme exposure to non-exposed. Higher frailty levels among those with heavy/severe exposures certain combinations of experiences, including intense bombing, witnessing death firsthand, having experienced sleep disruptions during wartime, and having feared for one's life during war. The difference in frailty-associated aging between the most and least affected individuals is more than 18 years. War trauma hastens aging and warrants greater attention toward long-term implications of war on health among vast post-conflict populations across the globe.

### SESSION 2055 (SYMPOSIUM)

Abstract citation ID: igad104.0445

**PSYCHOSOCIAL CORRELATES OF PREVENTION, CARE, AND WELL-BEING AMONG OLDER WOMEN LIVING WITH HIV**

Chair: Thi Vu Discussant: Anna Rubtsova

There are over 4.2 million older adults (ages 50+ years) living with human immunodeficiency virus (HIV) globally. Older women continue to make up a significant proportion of this population. Advances in antiretroviral therapy treatments have allowed HIV+ individuals to live longer, healthier lives. However, compared to all persons living with HIV, women have lower viral suppression rates and are less likely to be retained in care. While older women living with HIV (WLWH) face unique challenges with HIV prevention and care, such as caregiving responsibilities, gender discrimination, and ageism, there is a dearth of research focusing on their experiences. As the population of older WLWH continues to increase, understanding barriers and facilitators to HIV prevention and care among this population is a public health priority. This symposium will provide insight into psychosocial factors that influence HIV prevention, care, and well-being among older WLWH. Our first presentation highlights a qualitative study identifying sources of strength and concerns about aging with HIV among older women. Our second presentation examines social support networks and interpersonal strain in relation to loneliness among older WLWH. Our third presentation highlights the impact of patient-provider communication regarding HIV/AIDs on sexual health communication between older WLWH and their partners. Our fourth presentation examines mental health vulnerabilities and strengths of older WLWH during the humanitarian crisis in Ukraine. Discussant Dr. Anna Rubtsova will contextualize these findings and offer suggestions for future research to enhance well-being of older women across the continuum of HIV prevention and care.

Abstract citation ID: igad104.0446

**EMPOWERING AND ADDRESSING AGE-RELATED CONCERNS AMONG OLDER WOMEN LIVING WITH HIV**

Thi Vu[1], Marielle Quinn[1], Sarah Valeika[2], and Joan Monin[1], *1. Yale University School of Public Health, New Haven, Connecticut, United States*, *2. Yale University, New Haven, Connecticut, United States*

There are currently over 100,000 older (ages 50+ years) women living with HIV/AIDs in the United States. Older women living with HIV (OWLH) face unique psychosocial challenges with HIV prevention and care, including intimate partner violence, gender discrimination, ageism, and caregiving responsibilities. These factors can impede self-care and contribute to increased mental, emotional, and physical burdens. Little is known about the resources OWLH draw upon to help manage psychosocial stressors and their perspectives of aging with HIV. This qualitative study identified sources of strength and self-care among OWLH, as well as their concerns about aging with HIV. We recruited participants through HIV clinics and community support groups. We conducted one-on-one, semi-structured in-depth phone interviews with OWLH. Interviews were recorded and transcribed verbatim. Following a Grounded Theory approach, two coders performed open coding then thematic coding. Participants (N = 23) were on average 60 years old (range 51 – 68) and had been living with HIV for an average of 23.7 years. We found that a desire to self-educate, community activism, and engagement with other OWLH reduced participants' internalized stigma and were sources of empowerment. However, concerns about aging with HIV persists, including pervasive HIV stigma, comorbidities, shrinking caregiver networks, long-term effects of treatment, and lack of HIV research and programs focusing on older women. These findings suggest that while OWLH are empowered agents in their own HIV care journey, structural interventions (e.g., inclusion of older women in HIV trials) are still needed to address the unique concerns of this population.

Abstract citation ID: igad104.0447

**INFORMAL SUPPORT NETWORKS AND LONELINESS IN OLDER WOMEN LIVING WITH HIV**

Jasmine Manalel, and Mark Brennan-Ing, *1. Hunter College - CUNY, New York City, New York, United States*

Older adults with HIV are at increased risk for social isolation and poor well-being. Although older women living with HIV (WLWH) are more likely to be embedded in supportive networks than men, they often report inadequate support in meeting their needs. The function of older WLWH's informal networks, their effectiveness in meeting care needs, and links to well-being are major gaps in the literature. Using a subsample of heterosexual WLWH from The Research on Older Adults with HIV 2.0 Study aged 50 to 77 (Mage = 59; n = 210), we assessed sources of social support, interpersonal strain, and support adequacy, in relation to loneliness. About half the sample reported having ever (i.e., currently or in the past) needed regular assistance due to HIV or other illness, disability, or frailty whereas the rest reported never needed regular assistance. Preliminary regression analyses controlled for age, race, ADLs, partnership, and formal care status. Findings revealed that neither support from family or from friends was associated with loneliness. However, strain from both sources was significantly associated with greater loneliness. In terms of support adequacy, having someone to count on for emotional support was associated with lower loneliness, and needing more emotional support was associated with greater loneliness. Instrumental support (i.e., help with tasks was not significantly associated with loneliness. These findings highlight the importance of considering positive and negative social relations, as well as different dimensions and sources of support. Interventions to improve emotional support resources could help mitigate loneliness among older WLWH.

Abstract citation ID: igad104.0448

### OLDER WOMEN AND HIV: THE IMPACT OF PROVIDER COMMUNICATION OF HIV/AIDS ON CONVERSATIONS WITH NEW SEXUAL PARTNERS

Erin Robinson[1], Geunhye Park[2], and Getrude Nyang'au[3], *1. University of Missouri School of Social Work, Columbia, Missouri, United States*, *2. University of Missouri/ University of Illinois-Chicago, Chicago, Illinois, United States*, *3. University of Missouri - Columbia, Columbia, Missouri, United States*

Older adults account for 50% of people living with HIV and nearly 20% of new HIV infections in the U.S. Inadequate communication with a healthcare provider has been identified as a contributing factor, as stigma surrounding older adults, sex, and HIV/AIDS is persistent. Many older adults report having conversations with their healthcare providers about HIV prevention more routinely in their younger years. To date, however, little is known about provider communication of HIV with older patients. This cross-sectional study aims to help fill that gap. Survey participants (N=427) were asked about the frequency in which their primary healthcare providers talked with them about HIV/AIDS before they turned 50, as well as their future likelihood to discuss HIV/AIDS with a new sexual partner. Participants ranged in age from 50 to 84 years (M=58; SD=6.3), and were predominately white (94%) and female (73%). Regression analysis demonstrated that among women in the sample, provider communication about HIV/AIDS prior to them turning 50 year of age was associated with their future likelihood of talking with new sexual partners about HIV/AIDS ($\beta$=0.151, p<.05). Eighty-seven percent of the sample reported intentions to discuss condoms with a new sexual partner with a mean of 4.89 (SD=1.47, Range=1-6) indicating “Somewhat Agree” and “Agree”. These findings suggest that provider communication of HIV/AIDS is important for risk prevention. Those conversations help guide future decision making among women in particular, even into their later years.

Abstract citation ID: igad104.0449

### MENTAL HEALTH VULNERABILITIES AND STRENGTHS OF OLDER WOMEN WITH HIV DURING THE HUMANITARIAN CRISIS IN UKRAINE

Julia Rozanova[1], Sheela Shenoi[2], Oleksandr Zeziulin[3], Katherine Rich[4], Dan Bromberg[5], Tetiana Kiriazova[3], Alexandra Deac[6], and Valerie Earnshaw[7], *1. Yale University, New Haven, Connecticut, United States*, *2. Yale School of Medicine, New Haven, Connecticut, United States*, *3. Ukrainian Institute of Public Health Policy, Kyiv, Kyyiv, Ukraine*, *4. Harvard Medical School, Boston, Massachusetts, United States*, *5. Yale School of Public Health, New Haven, Connecticut, United States*, *6. King's College London, London, England, United Kingdom*, *7. University of Delaware, Newark, Delaware, United States*

Humanitarian crises impact mental health, yet data lacks among older individuals. We explored how Covid-19 and the Russian invasion has impacted older people with HIV (OPWH, ≥50 years) in Ukraine. We surveyed a longitudinal cohort of 123 OPWH (50% female) during four periods: April-June 2020 (Wave 1), December 2020-February 2021 (Wave 2), December 2021–February 2022 (Wave 3), and July–September 2022 (Wave 4). The primary outcomes were depressive (PHQ9>5) and anxiety symptoms (GAD7>5). Factors associated with these symptoms were assessed using a mixed effects logistic regression model that included gender, time since HIV diagnosis, history of a substance use disorder (SUD), history of another chronic condition, HIV disclosure, living alone, and social support. Logistical regression controlling for SUD assessed factors associated with resilience (BRS). During Wave 4, OPWH were offered free psychological counseling through our project. Across all Waves, women were more likely than men to have mild to severe anxiety and depressive symptoms, but also to have higher resilience, and to accept psychological counseling during the war. Comorbid SUD and other chronic conditions increased likelihood of anxiety, and longer time since HIV diagnosis increased likelihood of depressive symptoms. Unlike women, men showed distress discussing “Social Support” topic during the war. OPWH with SUD had higher resilience across all waves. While female OPWH had more depressive and anxiety symptoms during the crises they have higher resilience, social support, and help acceptance, suggesting bespoke approaches are needed to assist male and female OPWH in humanitarian settings.

### SESSION 2060 (SYMPOSIUM)

Abstract citation ID: igad104.0450

### ALIGNING ASSESSMENTS AND MEASURES OF CAREGIVER NEGLECT WITH INTERVENTION DEVELOPMENT: NEW INSIGHTS AND DIRECTIONS

Chair: E-Shien Chang Co-Chair: David Hancock
Discussant: Alan Stevens

As one of the most common forms of elder mistreatment, caregiver neglect is associated with significant morbidity and mortality. The unique stresses, demands, and challenges associated with family caregiving likely increases the risk of neglect. Addressing the multifaceted nature of caregiver neglect in diverse family caregivers of older persons necessitates alignment of meaningful assessments and methodologies with evidence-based intervention development. Gaps in knowledge that urgently need filling include refining conceptual and operational definitions of caregiver neglect, validating novel measurement approaches, and identifying effective caregiver-centered study engagement and recruitment strategies. To address these gaps, this symposium will draw upon insights from four caregiver neglect studies across community and clinical settings. Dr. David Hancock will characterize prevalence and correlates of caregiver neglect using unmet activities of daily living needs in a sample of dementia patients and their caregivers. Dr. E-Shien Chang will test the impact of varying operational definitions of caregiver neglect on prevalence estimates and associated risk and protective factors. Dr. Tony Rosen will present a novel, validated caregiver neglect severity measure to more accurately assess the impact of neglect on older persons' health and quality of life outcomes. Dr. Farida Ejaz will highlight challenges and successes in identifying and involving family caregivers at risk of neglectful caregiving behaviors to inform intervention. Finally, Dr. Alan Stevens, as policy and advocacy expert for family caregivers, will provide summative comments and recommendations for policy and practice changes to better reduce risk of neglect and improve support for family caregivers.

Abstract citation ID: igad104.0451

### ACTIVITIES OF DAILY LIVING (ADL) NEGLECT IN INDIVIDUALS WITH DEMENTIA

David Hancock, and Sara Czaja, *Weill Cornell Medicine, New York City, New York, United States*

Caregiver (CG) neglect, a form of elder mistreatment, is often measured by examining unmet care needs. A common way to measure unmet needs is to examine if a care recipient has needs for Activities of Daily Living (ADL) or Instrumental Activities of Daily Living (IADL) care needs, and if so, whether those needs are being met. The current study, using data from the Caring for the Caregiver Network Study, describes unmet ADL care needs among persons with dementia. Specifically, we examine how many needs there are, which needs are most common, how bothersome the caregiver finds attending to those needs, and how often those needs go unmet by the caregiver or other family members or friends. Overall, 1 in 10 care recipients experienced neglect as defined by unmet ADL needs. On average, caregivers (n=244) indicated care recipients needed help with 4 ADL care needs (range 0-7). The most common ADL need was needed help bathing, followed by getting dressed, and toileting. Moreover, the CGs generally found these tasks to bother or upset them between "not at all" and "a little." Toileting and bathing were ranked as the most bothersome tasks. Additionally, 10% of the caregivers identified ADL needs which were not being met by either themselves or other family members or friends. The most common unmet need was help with bathing, followed by toileting. Needs were most bothersome and most likely unmet for intimate acts such as bathing and toileting, suggesting possible targets for skill-based intervention development.

Abstract citation ID: igad104.0452

### CHARACTERIZING CAREGIVER NEGLECT IN OLDER PERSONS WITH DEMENTIA: OPERATIONAL DEFINITIONS AND RISK FACTORS

E-Shien Chang[1], Jerad Moxley[1], Karl Pillemer[2], Mark Lachs[1], Tony Rosen[1], and Sara Czaja[1], *1. Weill Cornell Medicine, New York City, New York, United States*, *2. Cornell University, Ithaca, New York, United States*

Neglect by caregivers, a combination of behaviors through acts of omission or commission, is one of the most widespread forms of elder mistreatment. Examining varying definitions and thresholds of caregiver neglect may yield insights to inform intervention development. Our data were drawn from 244 dyads of racially/ethnically diverse dementia caregivers (CG) and care-recipients (CR). Informed by prior research, we developed three definitional thresholds of caregiver neglect: (1) CR's needs were unmet by CG (least restrictive); (2) CR's needs were unmet by CG and there was no additional informal care (moderate restrictive); and (3) CR's needs were unmet by CG and there was no additional informal or formal care (most restrictive). Variables selected for multivariate logistic regression models examining predictors of neglect were based on significance in bivariate analysis ($p< 0.10$). One-week prevalence of caregiver neglect varied according to the three thresholds (43% to 11%). Sociodemographic characteristics of neglect differed across thresholds in bivariate analyses. The caregiver being male (OR=4.1, 95%CI=1.68-10.53) and being African American (OR= 2.12, 95%CI=1.01-4.54) was significantly associated with greater risk of neglect but only in the model predicting the least restrictive neglect. In models predicting moderate and most restrictive neglect, greater CG social support was associated with lower risk of neglect (OR=0.95, 95%CI=0.90-0.99; OR=0.93, 95%CI=0.89-0.98; respectively). Our findings indicate that caregiver neglect was common in our sample and that social support may play a key role in alleviating risk of neglect. Our analysis demonstrates important nuances in predictors of neglect by operationalizing neglect as a multifactorial phenomenon.

Abstract citation ID: igad104.0453

### DEVELOPING A SCALE TO MEASURE NEGLECT SEVERITY: THE HEALTH-RELATED SEVERITY IN ELDER NEGLECT SCALE

Tony Rosen[1], Karl Pillemer[2], David Burnes[3], Terry Fulmer[4], Jeanne Teresi[5], Monika Safford[6], Sara Czaja[1], and Mark Lachs[1], *1. Weill Cornell Medicine, New York City, New York, United States*, *2. Cornell University, Ithaca, New York, United States*, *3. University of Toronto, Toronto, Ontario, Canada*, *4. The John A. Hartford Foundation, New York City, New York, United States*, *5. Columbia University Stroud Center, New York City, New York, United States*, *6. Weill Cornell Medical College, New York City, New York, United States*

Caregiver neglect in older persons can vary dramatically in severity, with differential impact on an older adult's health. Assessing severity is critical for research and clinical practice but has received focus until recently. To address this gap, we developed a scale to describe the health-related severity of elder neglect using an expert consensus method. In development, the experts conceptualized severity as: (1)

the level of risk that neglectful behaviors would cause morbidity or mortality and (2) related timeframe. Additionally, the experts recommended that the scale identify risk for future neglect. The scale was designed iteratively, and, after finalization, we assessed face and construct validity. The final scale was found to have validity. It has 5 levels: not present, not present / potential risk, present / mild, present / moderate, present / severe. Each level has a description to guide assessment. For example, present / mild is described as: "caregiving behaviors not optimal, with potential to create morbidity, but low concern for immediate danger," present / moderate is: "caregiving behaviors with significant potential to create morbidity within the next 4 weeks," and present / severe is "caregiving behaviors creating immediate danger of morbidity or mortality -- insufficient access to shelter, food, medication – with alternative living situation or ED visit / hospitalization recommended." The description of not present / potential risk is: "though neglect not currently occurring, factors present that raise concern for future neglect risk." Assessing neglect severity using this scale may improve understanding of the phenomenon and inform intervention.

Abstract citation ID: igad104.0454

**TESTING AN INTERVENTION TO PREVENT CARETAKER NEGLECT: CHALLENGES IN RECRUITING STUDY PARTICIPANTS**

Farida Ejaz, Samantha Tuft, Courtney Reynolds, and Jessica Bibbo, *Benjamin Rose Institute on Aging, Cleveland, Ohio, United States*

Researchers collaborated with Utah Adult Protective Services (APS) to develop a telephone-based case management intervention to follow-up with both alleged victims and perpetrators of caretaker neglect after their case was determined to be inconclusive and closed by APS. We focused on this group because APS administrative data suggested that reoccurrence of caretaker neglect was common among this cohort. We hypothesized that linking both victims and caretakers with needed community-based services could prevent reoccurrence of caretaker neglect reports over time. In this presentation, we will describe the challenges we faced with recruiting alleged victims and perpetrators of caretaker neglect. In the piloting phase, we approached 87 potential participants; 18 did not meet study criteria, and 35 did not answer or had incorrect or non-working phone numbers. Of the remaining 34, 9 (26%) agreed to participate and one withdrew later during the follow-up phase. Interviewers reported that refusals were related to reading a 6-minute-long consent form over the phone and conducting an hour-long interview that included the research questionnaire and case-management intervention. The feedback led investigators to simplify the study design/protocols and to shorten the questionnaire to focus on critical data elements and the service needs of participants. Data collection is expected to continue across Utah until spring 2024. We will have a clearer sense of whether such adaptations increased response rates. In addition, we learned that research and practice/intervention goals are likely to be different but negotiating trade-offs, and establishing contracts and data-use agreements early on are important for successful partnerships.

### SESSION 2065 (BIOLOGICAL SCIENCES INVITED SYMPOSIUM)

Abstract citation ID: igad104.0455

**PRESIDENTIAL SYMPOSIUM: EXTENDING HEALTHSPAN AND LONGEVITY**

Chair: Blanka Rogina Co-Chair: Rafael de Cabo

Aging is associated with a functional decline in physiological, metabolic, proliferative, and tissue homeostasis leading to deterioration at an organismal level and an increase in risk for disease and death. To prevent or postpone such deterioration, genetic, pharmacological, and nutritional interventions have been successfully utilized, which lead to preserved healthspan and extended longevity. This symposium will discuss new approaches to target aging and the mechanisms underlying interventions that extend healthspan and longevity. The speakers of this symposium include internationally distinguished aging researchers who have dedicated their research to reveal new approaches to extending healthy lifespan. Rafael de Cabo, from NIA, Co-Chair and speaker in this symposium, will talk about the effects of calorie restriction on physiology, metabolism and health in a variety of organisms. Ana Gomes, from Moffitt Cancer Center, will talk about how aging promotes cancer resistance to therapy. Vishwa Deep Dixit, from Yale University, will discuss immunometabolic checkpoints of aging and lessons learned from CALERIE studies in humans. Payel Sen, from NIA, will present her studies on epigenetic mechanisms underlying tissue aging.

Abstract citation ID: igad104.0456

**PROLONGED DAILY FASTING TIMES ARE BEST WHEN COMBINED WITH CALORIE RESTRICTION FOR HEALTH AND SURVIVAL**

Rafael de Cabo, *National Institute on Aging, Baltimore, Maryland, United States*

Emerging new evidence highlights the importance of prolonged daily fasting periods for the health and survival benefits of calorie restriction (CR) and time-restricted feeding (TRF) in male mice; however, little is known about the impact of these feeding regimens in females. Using a carousel feeder system that enables controlling the duration, amount, and timing of food availability, we show that mice on CR had a robust physiological response and decreased progression of malignant neoplasms and severity of inflammatory diseases in the lung compared to TRF. CR is associated with a unique serum metabolomics signature in overnight-fasted animals compared to TRF. The systemic impact of CR and TRF regimens measured from covariation of serum metabolites with energetics and healthspan metrics indicates that daytime (rest-phase) feeding with prolonged fasting periods initiated late in life confers greater benefits when combined with an imposed lower energy intake.

Abstract citation ID: igad104.0457

**AGE-INDUCED SYSTEMIC REPROGRAMMING DRIVES DRUG RESISTANCE IN LUNG CANCER**

Ana Gomes, *Moffitt Cancer Center, Tampa, Florida, United States*

Lung cancer accounts for the largest number of cancer-associated deaths in the United States. While great strides have been made due to the introduction of immunotherapies and targeted therapies against oncogenic drivers, chemotherapies remain the standard of care for the majority of lung cancer patients. However, many patients do not respond to these treatments or relapse following an initial response. We postulate that part of the problem is the lack of consideration in preclinical research and clinical trials of the main driver of lung tumorigenesis, the aging process. Here, we show that the organismal reprogramming that occurs with aging makes lung tumors in an aged host more conducive to withstand chemotherapies and targeted therapies. We traced this to an age-induced chronic accumulation of the stress hormone cortisol in circulation. Mechanistically our data show that chronic accumulation of cortisol drives the upregulation of metallothioneins—small, highly conserved, cysteine-rich metal-binding proteins—through activation of the glucocorticoid receptor in lung cancer cells, which in turn rewire these cells to be refractory to chemotherapies. Together, our work demonstrates a role for age-induced cortisol levels in promoting resistance to standard of care chemotherapies in lung cancer and offers a rationale for using therapeutic agents that block the glucocorticoid receptor to sensitize the average and most vulnerable lung cancer patients, the elderly, to chemotherapy.

Abstract citation ID: igad104.0458

### IMMUNOMETABOLIC CHECKPOINTS OF INFLAMMAGING

Vishwa Dixit, *Yale University, New Haven, Connecticut, United States*

Inflammation is a key hallmark and driver of age-related functional decline. Evidence that innate immune sensor NLRP3 inflammasome links aging to functional decline supports Metchnikoff's original prediction that phagocytes or macrophages drive aging-associated degenerative diseases in an organism. Tissue resident macrophages are key to maintenance of homeostasis, a response that is compromised with age. It remains unclear whether negative energy balance in a host elicited by caloric restriction (CR) impacts mechanisms that control inflammation. The precise identity of factor(s) that link alterations in organismal metabolism to macrophage response are unclear. CR in rodents clearly elicits anti-inflammatory effects, but despite several years of research efforts, the endogenous drivers that couple energy balance to inflammatory cellular quiescence or activation is unknownThe RNA-sequencing analyses of adipose tissues from CALERIE-II study participants identified that CR in humans activates transcriptional programs implicated in mitochondrial metabolism, anti-inflammatory responses, and longevity. Our results show that macrophage derived protein PLA2G7, that is inhibited by CR in humans, reduces inflammaging, protects against thymic involution and protects against metabolic dysfunction. Furthermore, the CR in humans identifies unique regulators of inflammation including a CR-inhibited adipokine called SPARC that controls healthspan. Collectively, our studies establish that CR is relevant to human physiology and immunobiology and offers an important platform to harness immunometabolic checkpoints of inflammation and longevity.

Abstract citation ID: igad104.0459

### EPIGENETIC MECHANISMS OF TISSUE AGING

Payel Sen, *National Institute on Aging, National Institutes of Health, Baltimore, Maryland, United States*

Epigenetic alterations are a hallmark of aging, affecting DNA and histone modifications, 3D genome structure, and gene expression. Our lab studies these changes in various tissues to understand their unique and shared characteristics. In this talk, I will focus on the skeletal muscle, which makes up ~40% of the body weight, contributes to ~30% of resting energy expenditure, and is affected by aging. Aging reduces muscle mass and strength, partly due to the reduced function of resident muscle stem cells (MuSCs). We used an integrative multi-omics approach to profile the transcriptome, epigenome, and 3D genome of MuSCs isolated from young, old, and geriatric mice and comprehensively understand why their regenerative potential diminishes with age. We found an elevated immune response and quiescence exit signature in MuSCs during aging and identified regulatory elements that contribute to these changes. By targeting these networks, we can potentially rejuvenate MuSC function. Overall, we show that the non-coding genome potentiates a functional drift in MuSCs, an otherwise non-immune cell to a more immune function.

### SESSION 2070 (PAPER)

### ALZHEIMER'S DISEASE AND FAMILY CAREGIVING

Abstract citation ID: igad104.0460

### CAREGIVERS WITH MODIFIABLE RISK FACTORS FOR DEMENTIA: LEVERAGING EXISTING PUBLIC HEALTH STRATEGIES

Janelle Gore[1], John Omura[1], Roshni Patel[2], Benjamin Olivari[3], and Lisa McGuire[3], *1. Centers for Disease Control and Prevention, Atlanta, Georgia, United States*, *2. Cyberdata Technologies (CDC Contractor), Atlanta, Georgia, United States*, *3. CDC, Atlanta, Georgia, United States*

Caregivers are more likely to self-report subjective cognitive decline, an early indicator of possible future Alzheimer's disease and related dementias (ADRD), than non-caregivers. Research has identified potential modifiable risk factors for ADRD. To estimate the prevalence of eight modifiable risk factors for ADRD (i.e., high blood pressure, physical inactivity, obesity, type 2 diabetes, depression, current smoking, hearing loss, and binge drinking) among caregivers and non-caregivers, this study analyzed 2021 BRFSS data from 40 jurisdictions that conducted the caregiver module. Among respondents aged ≥18 years (n=232,772), we estimated prevalence of risk factors among caregivers and non-caregivers by selected demographic and health characteristics and the distribution of these characteristics by caregiving status and health condition of care recipient. Significant differences were assessed using t-tests ($p< 0.05$). Overall, caregivers reported significantly higher prevalence of six out of eight modifiable risk factors for ADRD examined (i.e., high blood pressure, obesity, diabetes, depression, current cigarette smoking, hearing loss) compared with non-caregivers, including high blood pressure (38.8% versus 32.6%), depression (25.1% versus 18.4%), and cigarette smoking (17.2% versus 12.1%).

Caregivers of persons living with ADRD reported high blood pressure, diabetes, depression, and hearing loss more often than other types of caregivers. These findings suggest caregivers, especially caregivers of persons living with ADRD, can benefit from existing state-based public health strategies to reduce chronic conditions, such as heart health education and programming. The management of chronic diseases is also beneficial to the brain health of caregivers.

Abstract citation ID: igad104.0461

### CO-DESIGNING A CULTURALLY INFORMED DEMENTIA SYMPTOM SELF-MANAGEMENT MHEALTH APP WITH CARE PARTNERS

Aditi Durga[1], Shih-Yin Lin[1], Daniel David[1], Karen Moss[2], Meghan Reading Turchioe[3], Rupa Valdez[4], and Abraham Brody[1], *1. NYU Rory Meyers College of Nursing, New York City, New York, United States*, *2. The Ohio State University, Columbus, Ohio, United States*, *3. Columbia University, New York City, New York, United States*, *4. University of Virginia, Charlottesville, Virginia, United States*

Over 16 million care partners (CPs) in the U.S. provide more than 17 billion hours of unpaid care for family and friends living with dementia. Yet, significant inequities and access issues exist in assisting CPs to receive high quality support. Thus, this study aimed to tailor our existing evidence-based Aliviado Dementia Care mHealth app for diverse CP users through a user-centered approach to empower CPs with culturally informed knowledge, structure and resources. Based on needs identified by CPs, wireframing was performed. CPs then reviewed the interactive wireframes, were asked to perform various tasks, and provide feedback on any pain points, confusion, usability, and new ideas ("Think aloud"). Eight CPs (3 Black, 1 Asian, 4 White, 1 Hispanic; 38% Female; 29-77 years) from 6 different US states participated in the user testing. CPs found the wireframes easy to navigate and intuitive. Based on CP feedback, changes were made to include inclusive language, reconstruct the dashboard, and add medication alerts and a summary care profile. CPs voiced pleasant surprise that the app was going to have such a substantial feature set, including guiding them through person-centered assessment and care planning of interventions to non-pharmacologically self-manage behavioral and psychological symptoms of dementia and caregiving stress. We thus successfully developed an initial set of wireframes using co-design principles with CPs. CPs appreciated the goal of self-management and activation to work with their primary care providers. Future work will seek to test a functional model with diverse CPs in diverse participants in the community.

Abstract citation ID: igad104.0462

### DEMENTIA DIAGNOSES PREDICT CAREGIVER EDUCATION DISCREPANCIES IN HOSPITAL SETTING

Austin Medlin[1], Catherine Still[2], Nicole Werner[3], and Beth Fields[2], *1. Indiana University, Bloomington, Indiana, United States*, *2. University of Wisconsin-Madison, Madison, Wisconsin, United States*, *3. Indiana University School of Public Health-Bloomington, Bloomington, Indiana, United States*

Many family caregivers report feeling unprepared to provide care for persons living with dementia (PLWD) after their hospital stay. Consequently, PLWD may experience poorer health outcomes and higher service utilization rates. Health systems can provide education to patients and their family caregivers, increasing preparedness, but these practices are less understood. This retrospective data analysis of electronic health records examines the role of diagnostic billing codes in predicting amount of education received by patients and their family caregivers. Our dataset includes 419 unique encounters of hospitalized older adult patients with a documented dementia diagnosis and a listed family caregiver between 1/1/2019 and 8/1/2022. Dementia-related ICD-10 billing codes determined unique patient diagnoses. Encounters were given designations of receiving more vs. less education if the amount of education received was above or below the sample median. Logistic regressions then determined the relationship between diagnostic billing codes and education received while controlling for demographic and contextual characteristics. We found that encounters with diagnostic codes "other degenerative disease of nervous system" and "Alzheimer's disease" were 2.8 and 3.3 times as likely to receive more education, respectively (95% confidence interval = [1.051, 7.347], p = .0394; [1.175, 9.461], p = .0236). Conversely, encounters with the code "other frontotemporal dementia" were 3.3 times as likely to receive less education ([1.150, 9.684], p = .0266). Findings demonstrate certain dementia-related diagnoses predict differences in how likely caregivers are to receive education. Health systems should evaluate for these differences and incorporate new methods to increase caregiving preparedness.

Abstract citation ID: igad104.0463

### TELE-SAVVY@HOME: PRODUCING AND TESTING A FULLY ASYNCHRONOUS ONLINE TELE-SAVVY PROGRAM

Fayron Epps[1], Carolyn Clevenger[2], Melinda Higgins[1], and Kenneth Hepburn[1], *1. Emory University, Atlanta, Georgia, United States*, *2. Nell Hodgson Woodruff School of Nursing, Emory University, Atlanta, Georgia, United States*

Group-based psychoeducational programs like the Savvy Caregiver program and its synchronous/asynchronous online adaptation, Tele-Savvy, have demonstrated that the acquisition of skills, knowledge, and caregiving mastery can ameliorate caregiving stress and enhance the quality of life for persons living with dementia. Many factors, including the constant presence of care recipients, preclude caregivers attending such programs, including synchronous online programs, and limit programs' accessibility and availability. This presentation describes the development of a fully asynchronous online Savvy program (Tele-Savvy@Home; TS@H) and the feasibility, acceptability, and preliminary efficacy of the online program. TS@H employed online continuing education methods to present ~16 hours of content in 46 short interactive segments of 8-15 minutes each. TS@H pilot program participants had no interaction with other participants and didn't engage with a live instructor. Pilot results provided preliminary confirmation of the pilot study's primary and secondary hypotheses regarding positive benefits to caregivers and care recipients. Results from the 29 participants who completed the pilot program and provided follow-up data indicated the program produced moderate to large effects by end of study on enhancing caregiving mastery (d=0.645) and decreasing reported care recipient behavior (d=-0.834) and reduced caregiver reactivity to that behavior (d=-0.732). The program also produced small to moderate benefits (d = -0.25 to -0.49) in the areas of caregiver depression, anxiety, and burden. Caregivers reported the program helped them be less resentful of their caregiver role and to accept the changes in relationship dynamics with their person. These results will inform the next iteration of Tele-Savvy@Home.

### SESSION 2075 (PAPER)

### ENHANCING CAREGIVER SERVICES AND SUPPORTS

Abstract citation ID: igad104.0464

### DEVELOPING A CONCEPTUAL MODEL TO ASSESS ACTIVE ENGAGEMENT WITHIN A DEMENTIA CAREGIVER INTERVENTION

Sarah Neller[1], Moroni Fernandez Cajavilca[2], Julene Johnson[3], Lee Ellington[4], and Jacqueline Eaton[4], *1. University of Tennessee, Knoxville, Knoxville, Tennessee, United States*, *2. New York University, New York City, New York, United States*, *3. University of California San Francisco, San Francisco, California, United States*, *4. University of Utah, Salt Lake City, Utah, United States*

Active engagement is identified as an effective component of psychoeducational interventions for caregivers of persons living with dementia. There are many approaches to active engagement; however, limited assessment of this component within interventions reduces optimization and standardization. To fill this gap and increase transparency, we aimed to strengthen active engagement by developing Enhancing Active Caregiver Training (EnACT), an arts-based intervention designed to help caregivers address the behavioral symptoms of dementia. The intervention uses multi-sensory activities, including interacting with caregiver-informed vignettes, to foster engagement and process caregiving experiences. We partnered with caregivers of persons living with dementia (n=9) to iteratively refine EnACT through six focus groups. The purpose of this presentation is to report the process we took to identify how intervention participants actively engaged with EnACT. Focus group data were analyzed using process coding to identify the patterns of participant engagement with 17 intervention activities. The process codes were mapped to create a diagram of participant engagement for each activity. These diagrams were used to develop the EnACT Cycle of Engagement, a conceptual model illustrating how participants engaged with the intervention. The three-step model illustrates that: 1) antecedents (personal and intervention) contribute to 2) interaction with EnACT through participation, contribution, and application, which leads to 3) EnACT's hypothesized proximal outcomes (capacity to adapt and appraisal of caregiving demands) and distal outcomes (perceived stress and caregiver well-being). The EnACT engagement model will be tested in the forthcoming randomized control trial assessing feasibility and acceptability.

Abstract citation ID: igad104.0465

### INVESTIGATING FORMAL SERVICE UTILIZATION IN CAREGIVERS OF PERSONS LIVING WITH DEMENTIA

Francesca Falzarano[1], Rachel Bloom[2], Megan McCarthy[1], and Francesco Osso[1], *1. Weill Cornell Medicine, New York City, New York, United States*, *2. Fordham University, New York City, New York, United States*

In the United States, no acceptable alternatives exist to the heavy reliance on family caregivers in maintaining the long-term services and supports system. Despite their vital role, caregivers of persons living with dementia (PLWD) are often overlooked in healthcare encounters and their needs remain unaddressed, which can lead to deleterious impacts on the caregiver's health, well-being, and capacity to provide adequate care. Although formal services and resources to assist caregivers of PLWD exist, there is a concerning disconnect between referral and service use uptake – largely due to a siloed, bureaucratic health system in addition to caregivers' lack of knowledge, awareness, applicability, and access. However, the individual and psychosocial factors that influence attitudes toward and utilization of services remain unclear. Thus, the purpose of this study was to examine correlates of formal service use using data from an NIA-funded mixed-methods study of 46 caregivers of PWLD (Mage=55.9, SD=16.3). Across all domains queried (e.g., home health aides, adult day programs) at least 50% of the sample reported lack of use. Caregivers cited concerns regarding lack of trust, safety of the care-recipient, lack of flexibility, and cost as service barriers. Spousal, compared to adult-child, caregivers had more negative perceptions regarding formal services. Further, greater functional dependency, higher pre-loss grief, and lower levels of self-efficacy and preparedness were associated with more negative attitudes toward service use. These results underscore the importance in ascertaining the holistic needs of caregivers to inform more tailored efforts to connect caregivers' unique needs to relevant services.

Abstract citation ID: igad104.0466

### RACIAL AND ETHNIC DIVERSITY IN FAMILY CARE INTERVENTIONS: A CALL FOR ACTION

Naomi Meinertz[1], Jeong Eun Lee[2], and Megan Gilligan[1], *1. University of Missouri, Columbia, Missouri, United States*, *2. Iowa State University, Ames, Iowa, United States*

In the United States, women are disproportionately represented as family caregivers and racial and ethnic minority families experience the challenges of caregiving at greater rates, such as poorer health and psychological distress. Recent research results demonstrate the effectiveness of family care interventions through improvements for caregiver depression, anxiety, and caregiver knowledge; yet most of these programs were developed for and evaluated with White families. The extent to which caregiver support interventions attend to racial and ethnic diversity is inadequate. Limited understanding of intervention results by racial and ethnic subgroups may ultimately further exacerbate disparities across groups by limiting our ability to account for any differences in treatment outcomes. Advances in research on caregiver interventions warrant an examination of the degree to which gender and/or racial differences are considered in the field of caregiver intervention research. Using a combined theoretical lens of symbolic internationalism and gender role theory, we argue for the development of care programs for racially and ethnically diverse family care perspectives. Thus, we conducted a systematic review of caregiver interventions and further explored whether common evidence-based care interventions are efficacious with non-White populations. Additionally, we emphasize the importance of developing targeted intervention programs by reviewing the impact of recently develop programs for specific target populations. This work underscores the gap in current family care interventions and identifies the need for evidence-based family care programs for diverse populations. Our systematic review will further our understanding about disparities in intervention outcomes to develop culturally appropriate interventions.

Abstract citation ID: igad104.0467

### SUPPORT FOR FAMILY CAREGIVERS: WORK STRAIN AND THE IMPACTS OF FORMAL AND INFORMAL SUPPORT

Joseph Svec[1], Natasha Nemmers[2], and Jeong Eun Lee[3], *1. Saint Joseph's University, New York City, New York, United States*, *2. Wayne State University Institute of Gerontology, Detroit, Michigan, United States*, *3. Iowa State University, Ames, Iowa, United States*

In the US, caregiver burnout has significant mental and physical ramifications for an estimated 40 million unpaid caregivers. In addition to higher risks of depression, fatigue, and anxiety, caregivers are often squeezed in the already tenuous balance between work and life. This study seeks to assess whether and to what extent the mental health consequences of caregiver work strain is ameliorated by the presence of additional (informal) family caregivers and formal service use. This study utilizes data provided by the National Study of Caregiving (NSOC) data which is linked with care-recipient information from Health and Aging Trends Study (NHATS) data. We analyze mental health outcomes for caregivers who were present in years 2015 and 2017 (N = 281) using lagged linear regression techniques. We estimate associations of work-strain with mental health issues as well as the moderating effects of informal support (the number of additional caregivers) and utilization of formal support (such as paid service support, formal training, and Medicare funding). Preliminary analyses indicate that the additional informal support mitigates the effect of work strain on mental health. However, formal support corresponds with positive mental health outcomes in the absence of work strain and poorer mental health outcomes when the caregiver experiences work strain. One possible explanation is that formal support is more likely to be used when caregiver burden and isolation is higher, highlighting the importance of timing in service utilization as well as the importance of social and informal resources for employed caregivers.

Abstract citation ID: igad104.0468

### WHAT DOES IT MEAN TO REST? A STUDY OF CAREGIVERS CARING FOR A PERSON WITH DEMENTIA

Sarah Hahn, *Mercy College, Dobbs Ferry, New York, United States*

The number of informal caregivers caring for a person with dementia (PWD) has been increasing steadily over the past decade; more than 11 million Americans provide unpaid care as of 2022 (Alzheimer's Association, 2022). The impact of caregiving is immense; caregivers risk having chronic health issues, poor emotional well-being, and physiological changes (Alzheimer's Association, 2022). Respite care provides a temporary break for the caregiver, but is often underutilized (O' Shea, et al., 2019). Research has found that the responsibilities of caregiving for a PWD, specifically the mental load, is a 24-hour, 7-day position that leaves little room for rest (Chew, et al., 2022). However, while there are several definitions of what rest and respite is in the gerontological literature (O' Shea, et al., 2019), not much is known on the experiences and achievement of rest by caregivers of PWD. This study explored the concept of rest to caregivers of PWD by using phenomenological interviews to understand the meanings attributed to it. Findings indicated three superordinate themes: "roadblocks", "the actuality", and "the ideal", as well as subsequent subthemes.

### SESSION 2080 (SYMPOSIUM)

Abstract citation ID: igad104.0469

### ADVANCING AGE INCLUSIVITY IN HIGHER EDUCATION: INTERGENERATIONAL EXCHANGE AS A PATHWAY TO CAREER READINESS

Chair: Joann Montepare Co-Chair: Kimberly Farah
Discussant: Lisa Borrero

Higher education is becoming more age-inclusive on several fronts and age-friendly teaching and learning strategies are being used in new and novel ways to support a variety of students' educational outcomes. In this AFU-ILRCE collaborative symposium, presenters will discuss ways that intergenerational exchange in the classroom and beyond can help to prepare students for entry into the workforce. To begin, Montepare, Farah, and Frazier will give an overview of the Age-Friendly University (AFU) initiative and discuss how older adults can serve as teaching allies in career-focused classroom activities such as serving as job recruiters in mock interviews. Graf and Terhune will share reflections from focus group data on intergenerational exchanges in the workplace conducted with working and recently retired adults 60+ years of age. They will highlight the utility of such perspectives for informing career training programs for students regardless of major or career path. Mastel-Smith, Kimzey, and Garner will describe Dementia Bootcamp, an online program for high school students that utilized didactic information, experiential activities, presentations by people with dementia, and volunteering at a dementia day program. Focus group findings and how the program has been integrated into a health sciences curriculum also will be presented. Ghazi-Saidi & McKelvey will discuss an innovative experiential learning activity in two core courses of a Speech Language Pathology (SLP) graduate program that prepares future SLPs to use telepractice to provide cognitive health interventions to community older adults. Lisa Borrero, chair of AGHE's Education Resource Development Workgroup (ERDW) will serve as discussant. This is a collaborative symposium between the Age-Friendly University (AFU) Network and Intergenerational Learning, Research, and Community Engagement Interest Groups.

Abstract citation ID: igad104.0470

### BUILDING STUDENTS' CAREER SKILLS THROUGH AGE-FRIENDLY INTERGENERATIONAL CLASSROOM ACTIVITIES

Kimberly Farah[1], Joann Montepare[1], and Charlotte Frazier[2], *1. Lasell University, Newton, Massachusetts, United States*, *2. Lasell University, Auburndale, Massachusetts, United States*

Changing age demographics are reshaping societies and challenging institutions of higher education to consider how they can respond to age-diverse populations through new approaches to teaching, research, and community engagement. As well, institutions are facing a range of challenges as they look to respond to the contemporary needs of students who will be entering an age-diverse workforce. The pioneering Age-Friendly University (AFU) initiative, endorsed by GSA's Academy for Gerontology in Higher Education (AGHE), offers a framework within which institutions can begin to address these issues through more age-friendly programs, practices, and partnerships. Drawing on the AFU principle that advocates for promoting intergenerational learning, this presentation will describe several intergenerational classroom activities that have been developed to support students' success in the workplace – including mock interview sessions, career roundtable discussions, research mentoring efforts, and Careers in Age Week activities. In addition to showing the value of intergenerational exchange to enhance students' career readiness, these efforts demonstrate how older adults can serve as teaching allies and support the educational mission of higher education.

Abstract citation ID: igad104.0471

### PREPARING STUDENTS FOR A MULTIGENERATIONAL WORKFORCE: PERSPECTIVES FROM OLDER WORKERS AND RETIREES

Allyson Graf, and Katherina Nikzad-Terhune, *Northern Kentucky University, Highland Heights, Kentucky, United States*

Workforce diversity can be a major asset for personal, interpersonal, and economic development. Age diversity is prevalent with five generations currently represented in the workforce. Intergenerational tension is common, however, largely fueled by stereotypes centered on age and generation that can infiltrate all workplace processes. Working from the Age-Friendly University framework, our aim was to gather qualitative data to inform the development of a training program for college students, pairing information on age-related bias with opportunities for meaningful intergenerational exchange to mimic workplace interactions. We hosted three community-based focus groups with adults ranging from 60-69 years of age who were either currently employed or recently retired (N = 10) to discuss their intergenerational interactions in the workplace. Participants had an average of 41.06 years of work experience and worked across multiple industries, most commonly administrative work, food services, government, and healthcare. The most common theme reported was the dismissal of participants' work experience by their younger colleagues, which some equated to a lack of respect due to their age, and others painted as a breakdown in communication. Technology was highlighted as exasperating intergenerational tensions. Participants also reported that younger colleagues often approached them for general life advice, which helped to reduce tensions. The willingness to see the value of older adults' wisdom in general, but not specific to the work at hand, has implications for the content and structure of future career-readiness training programs. Recommendations for enhancing positive intergenerational exchanges and reducing generational tensions within the workplace will be discussed.

Abstract citation ID: igad104.0472

### ONLINE DEMENTIA BOOTCAMP FOR HIGH SCHOOL STUDENTS

Beth Mastel-Smith[1], Michelle Kimzey[2], and Jennifer Garner[1], *1. The University of Texas at Tyler, Tyler, Texas, United States*, *2. Texas Christian University (TCU), Fort Worth, Texas, United States*

Caring for people with dementia requires specialized competencies. Previous education programs had a positive effect on college students' dementia knowledge, communication skills, empathy for and attitudes towards people with dementia. To prepare high school students to care for people with dementia – whether it be in a professional or personal capacity - a six session online Dementia Bootcamp was created. Topics included dementia overview, prevention, symptoms, and a framework for understanding them, communication, family support, and evaluation. Information was delivered via short didactic instruction, presentations by people with dementia, and experiential activities such as a virtual dementia simulation. Students met with a mentor, a person with dementia, or volunteered at a dementia day program. Parental consent and student assent were obtained for six students who participated in a focus group. The focus group was audio recorded and an interview schedule was used to understand what students learned, their response to online delivery, and recommendations for future offerings. Data was transcribed and verified, coded for categories and themes developed. Increased dementia awareness and knowledge, confidence in the ability to communicate with someone with dementia, how to prevent dementia, improved cognitive empathy and empathic imagination, and reduced stigma were reported. Online delivery was well received; students recommended longer sessions, more engagement with content and each other, and more information about how dementia affects the brain. The program has been integrated into a high-school health sciences curriculum.

Abstract citation ID: igad104.0473

### INTERGENERATIONAL BONDING: A PATHWAY TO CAREER READINESS IN SPEECH LANGUAGE PATHOLOGY GRADUATE STUDENTS

Ladan Ghazi-Saidi, and Miechelle McKelvey, *University of Nebraska at Kearney, Kearney, Nebraska, United States*

We present a new experiential learning activity for graduate students in two core courses in Speech-Language Pathology. Speech-Language Pathology is a healthcare profession that provides care to people of all ages, including older adults with language, communication, and cognitive disorders. Students typically find these course contents heavy and have limited opportunity to practice the required skills for their future profession. In this multi-faceted program, we provide cognitive intervention to community older adults via telepractice with an intergenerational approach. Students receive real-life interaction to practice the knowledge and skills learned in their academic program. These interactions provide hands-on experience that facilitates better preparation for their future healthcare career. We measure students' perception of competency pre and post-intervention. Additionally, we survey alumni and their supervisors on students' preparedness (required skills) and performance during their clinical internship experience. This intergenerational bonding program creates a fun and enjoyable experience that impacts students' active engagement with older adults. Students report an easier application of academic material, great ownership of learning, and increased motivation to learn new skills. Further, this real-life, interactive experience will impact their career success as future aging healthcare professionals (SLPs).

### SESSION 2085 (SYMPOSIUM)

Abstract citation ID: igad104.0474

### THE POTENTIAL AND PITFALLS OF TELEHEALTH TO SUPPORT AN AGING POPULATION

Chair: Walter Boot

As the population ages, telehealth services are becoming increasingly prevalent and important to help support older adults' aging in place and manage their health at a distance. However, older adults often face barriers such as technology proficiency, physical and cognitive capacity, and lack of technical support to access and use telehealth. Designers and researchers need to design and evaluate telehealth interventions tailored to older adults' needs to ensure these technology-based solutions meet their potential. This session will present a variety of technology-based research exploring the development, evaluation, and implementation of telehealth programs and interventions to better support older adults aging in place. L. Moo will present quantitative and qualitative data on challenges veterans experienced with clinical pharmacists' video visits. L. Adepoju will present factors associated with in-person-only, telemedicine-only, and hybrid healthcare visits among older adults. P. Freddolino will present an evaluation of a telehealth 101 mini-course offered to older adults. F. Abujarad will discuss the development and evaluation of a digital health intervention (VOICES) to increase elder mistreatment identification. Finally, M. Gately will describe a study of person factors and environmental factors on older adults' capacity to participate in video telehealth sessions. Older adult barriers and areas of improvement in telehealth design will be discussed throughout this session.

Abstract citation ID: igad104.0475

### DIRECT OBSERVATION OF OLDER ADULTS VIDEO TELEMEDICINE CHALLENGES

Lauren Moo[1], Maria Venegas[2], Chelsea Hawley[3], Megan McCullough[3], and William Hung[4], *1. Veterans Health Administration, Bedford, Massachusetts, United States*, *2. Veteran Affairs, Bedford, Massachusetts, United States*, *3. VA Bedford Healthcare System, Bedford, Massachusetts, United States*, *4. Veterans Health Administration, New York City, New York, United States*

Video to home has the potential to increase access to clinical services for older adults that face barriers to in-person care and to specialty care such as mobility and transportation challenges. There is evidence that the digital divide has been increasing rather than shrinking for this population. Twenty older cognitively intact Veterans enrolled in Aim 1 of a study of video visits with clinical pharmacists and allowed the study team to observe them in their homes as they prepared for and attempted to connect for the virtual encounter. Technology challenges were encountered in more than 80%, including 60% that required assistance from the in-person study staff, despite these being highly motivated participants. We will share both quantitative and qualitative data regarding the nature of the challenges observed and discuss the implications for geriatric tech equity.

Abstract citation ID: igad104.0476

### COMPARING IN-PERSON ONLY, TELEMEDICINE ONLY, AND HYBRID HEALTH CARE VISITS AMONG OLDER ADULTS IN SAFETY-NET CLINICS

Omolola Adepoju[1], Patrick Dang[1], Maya Singh[1], and Arlette Chavez[2], *1. University of Houston, Houston, Texas, United States*, *2. University of Houston College of Medicine, Houston, Texas, United States*

Introduction: Older adults face challenges in seeking healthcare. This study examined factors associated with in-person only versus telemedicine only versus hybrid healthcare visits among adults 65+ in safety-net clinics.

**Methods:** Data was obtained from a large Texas-based Federally Qualified Health Center (FQHC) network. The dataset included 12,279 appointments for 3,914 unique older adults between March and November 2020. The outcome of interest was a 3-level indicator of telemedicine visits: in-person visits only, telemedicine visits only, and hybrid (In person + Telemedicine) visits during the study period. We used a multinomial logit model adjusting for patient-level characteristics to assess the strength of the relationships.

**Results:** Compared to their white counterparts, Black and Hispanic older adults were significantly likely to have telemedicine only visits vs in-person only visits (Black RR: 0.59, 95% CI 0.41-0.86; Hispanic RR: 0.46, 95% CI 0.36-0.60). However, there were no significant racial and ethnic differences in hybrid utilization (Black RR: 0.91, 95% CI 0.67-1.23; Hispanic RR: 0.86, 95% CI 0.70-1.07).

**Discussion:** Our findings suggest that hybrid opportunities may bridge racial and ethnic disparities in access to care. Clinics should consider building capacity for both in-person and telemedicine opportunities as complementary strategies.

Abstract citation ID: igad104.0477

### PATIENT-FOCUSED TELEHEALTH 101: CONTENT, RESULTS, FEEDBACK, AND PLANS FOR REVISION AND EXPANSION

Paul Freddolino[1], Ha Neul Kim[1], Fei Sun[1], Megan Bentley[1], and Catherine Macomber[2], *1. Michigan State University, East Lansing, Michigan, United States*, *2. Saginaw Valley State University, University Center, Michigan, United States*

A five-part patient-focused Telehealth 101 mini-course was offered one-to-one as part of a technology literacy program for older adults in a rural Midwest community who received home-delivered meals. Participants had already received content on basic digital skills including safety and security, email, internet browsing, videoconferencing, and photo tools. Topics included an overview of material to be covered, types of telehealth (real time encounters, remote patient monitoring, and patient portals), what to expect from providers, how to prepare for telehealth encounters, and a live simulation of a telehealth visit. Subjects rated the sessions positively, and patient engagement in health care (Patient Activation Measure scores) significantly increased after the training. The mini course was also offered virtually on Zoom to groups of older adults at five urban senior centers. Feedback from both settings suggest the need to reduce repetition in the material presented and to include more diversity in the personnel presenting the content, both live and through YouTube videos accessed as part of the content. Revision will also add content on mental health and substance abuse services available through telehealth.

Abstract citation ID: igad104.0478

**FEASIBILITY OF A DIGITAL ELDER MISTREATMENT INTERVENTION FOR COGNITIVELY IMPAIRED OLDER ADULTS**

Fuad Abujarad[1], Chelsea Edwards[2], Judith Neugroschl[3], Ula Hwang[2], and Richard Marottoli[4], *1. Yale University School of Medicine, New Haven, Connecticut, United States*, *2. Yale University, New Haven, Connecticut, United States*, *3. Mount Sinai, New York City, New York, United States*, *4. Yale Medical School, New Haven, Connecticut, United States*

Older adults with cognitive impairments (CI) are at a higher risk for experiencing elder mistreatment (EM) and are less likely to report. Research is limited for self-administrated digital screening in this population, and generally focus on provider-based solutions to increasing EM identification. We developed a digital health intervention, VOICES, to increase EM identification. VOICES combines educational resources, screening, and brief psychoeducational interviewing to encourage older adults to self-report. We recruited participants with CI at a geriatric outpatient center (N=30) and the emergency department (ED) (N=101) to examine feasibility. Most participants could use VOICES independently (Montreal Cognitive Assessment (MoCA) scores ranging from 14-25). 87% of ED participants felt VOICES was appropriate for use in the ED, and 97% felt that the digital coach instructions were easy to understand. Our results suggest that the VOICES is feasible for EM screening in older adults with CI, specifically with MoCA scores ranging from 14-25.

Abstract citation ID: igad104.0479

**INFLUENCE OF PERSON AND ENVIRONMENTAL FACTORS ON OLDER ADULT ENGAGEMENT IN VIDEO TELEHEALTH**

Megan Gately[1], Dylan Waller[2], Matthew Maynard[3], and Lauren Moo[4], *1. VA Bedford Health Care System, Bedford, Massachusetts, United States*, *2. Center to Improve Veteran Involvement in Care (CIVIC), Portland, Oregon, United States*, *3. Veterans Health Administration, Forest City, North Carolina, United States*, *4. Veterans Health Administration, Bedford, Massachusetts, United States*

Rural patients at Veterans Health Administration (VHA) are older and have more complex chronic conditions than Veterans receiving care outside VHA. Rural patients face challenges accessing care, particularly specialty services which are often in urban areas. Though video telehealth can increase access to care by older patients, video was underutilized by this group even during the rapid increase in use of video in response to COVID. The unequal uptake of video telehealth by older patients during a time when there were limited options suggests a gap in our understanding of the factors involved with older adults' utilization of video telehealth. To explore these factors, we interviewed occupational therapy practitioners who were high users of video telehealth (N=27) about their experiences with video telehealth. According to interviews, successful utilization of video telehealth involves consideration of complex factors operating at the person (i.e., patient and clinician) and environmental levels. Person-level factors for the patient and/or family caregiver include technological literacy, sensory impairments, and stress tolerance. Clinician person-level factors include confidence with technology, attitudes towards video (including perceived benefits for clinical care), and specific skills like communication and flexibility. Environmental level factors include the physical setting (e.g., whether the patient/clinician are in a private space), availability of technical assistance for patients and clinicians, caregiver assistance on the patient side, and presence of a device and adequate broadband for patients. Elucidating the network of factors involved with video telehealth may enable sustained integration of video telehealth by rural patients most in need of care.

### SESSION 2090 (SYMPOSIUM)

Abstract citation ID: igad104.0480

**UNTANGLING THE MODEL MINORITY STEREOTYPE: PROMOTING HEALTH EQUITY AMONG ASIAN AMERICAN OLDER ADULTS**

Chair: Bei Wu
Discussant: Yuri Jang

The "model minority" stereotype portrays Asian Americans (AAs) as a monolithic group with common traits, e.g., high levels of education and income, and better health status. Five studies in this symposium used multiple data sources and provided rich information to untangling this stereotype. The first study highlighted racial/ethnic disparities in help-seeking behaviors among Americans with subjective cognitive decline, revealing that AAPIs exhibit the lowest likelihood of seeking help for cognitive problems. The second study examined the degree to which socioeconomic disparity contributes to functional impairment among older AAs relative to other racial/ethnic groups, revealing that AAs fare worst in the burden of functional impairment due to education disparity. The third paper explored the cultural adaptation of the NYUCI for Chinese and Korean dementia caregivers, showing that cultural beliefs have a profound effect on caregivers' views of dementia and caregiving, their use of social and family support, and their understanding of self-care and counseling services. The fourth study assessed factors linked to depressive symptoms among older Southeast AAs, revealing that Vietnamese Americans have significantly more depressive symptoms than Indonesian and Filipino Americans. Resilience and age are negatively related to depressive symptoms, while greater family conflict and inability to speak English fluently are positively associated with depressive symptoms. The fifth study used a large longitudinal survey on older Chinese Americans and demonstrated that higher acculturation levels are associated with a more rapid decline in depression and a slower decline in activities of daily living but are not related to long-term cognitive changes.

Abstract citation ID: igad104.0481

### RACIAL/ETHNIC DISPARITIES IN HELP-SEEKING BEHAVIORS IN OLDER AMERICANS WITH SUBJECTIVE COGNITIVE DECLINE

Xiang Qi, Yaolin Pei, Zheng Zhu, and Bei Wu, *New York University, New York City, New York, United States*

Subjective cognitive decline (SCD) refers to individuals' perceived decline in memory that negatively impacts the daily activities or work. Seeking help for SCD and/or related functional limitations may be beneficial as it can prompts screening and intervention for cognitive impairment. However, racial/ethnic minorities may face disadvantages in help-seeking behaviors due to healthcare access disparities, dementia-related stigma, and cultural differences. To investigate the racial/ethnic differences in help-seeking for cognitive problems among middle-aged and older adults with SCD and/or related functional limitation, we used data from the Behavioral Risk Factor Surveillance System (BRFSS) 2015-2021. The sample included 41,115 adults aged ≥45 with SCD and 20,227 (45.4%) with SCD-related functional limitation. We estimated the prevalence of help-seeking behaviors and conducted a logistic regression analysis accounting for BRFSS survey weights, missing data, state-level clustering, and covariates. Among those with SCD and/or related functional limitation, Asian American and Pacific Islanders (AAPIs) are least likely to initiate communication with their healthcare provider with their cognitive problems. In the multivariate logistic model, AAPIs (OR, 0.45; 95% CI, 0.33-0.61) and non-Hispanic blacks (OR, 0.76; 95% CI, 0.63-0.91) with SCD-related functional limitation were less likely to discuss cognitive problems compared to non-Hispanic whites. Our findings were consistent with studies suggesting that AAPIs with cognitive impairment were less likely to seek for dementia care due to their lower health literacy, limited English proficiency, and dementia-related stigma. Our study emphasizes the need for tailored interventions that promote early patient-provider communication to address racial/ethnic disparities in help-seeking for cognitive problems.

Abstract citation ID: igad104.0482

### SES DISPARITIES IN FUNCTIONAL IMPAIRMENT WITHIN OLDER ASIAN AMERICANS: COMPARISON OF FOUR RACIAL/ETHNIC GROUPS

Katherine Wang[1], Zheng Zhu[2], and Xiang Qi[2], *1. Duke University, Durham, North Carolina, United States*, *2. New York University, New York City, New York, United States*

This study investigated to what extent socioeconomic status (SES) disparities associates with functional impairment within older Asians in comparison with other racial/ethnic groups. Data were from the National Health and Nutrition Examination Survey 2011-2018 that included 3,297 White, 1,755 Black, 1,708 Hispanic, and 730 Asians aged ≥60. Physical functioning was measured by activities of daily living (ADL) or instrumental activities of daily living (IADL). Memory was determined by CERAD-WL, and language fluency was measured by Animal Fluency Tests. We conducted multivariate logistic regressions to examine the association between SES and functioning impairment within racial/ethnic groups and performed seemingly unrelated regressions to compare the regression coefficients across subgroups. For the age- and sex-adjusted prevalence of functional impairment, Asians with ≤high school education had the highest prevalence of memory impairment among all races/ethnicities, and no difference was observed for all racial/ethnic groups with >high school education. After adjustment for key covariates, Blacks and Hispanics had higher odds of ADL/IADL disabilities and cognitive impairment relative to Whites. The odds of ADL/IADL disabilities did not differ with Asians and Whites, whereas Asians were more likely to have language fluency impairment than Whites. Education disparity for ADL disability (OR, 3.42; 95% CI, 2.21-5.29) and memory impairment (OR, 16.87; 95% CI, 8.57-33.21) were largest among Asians compared to Whites, Blacks, and Hispanics. Income disparity for function impairment did not significantly differ across four racial/ethnic groups (all $P > 0.05$). This study indicates that Asians fare worst in the burden of functional impairment due to education disparity.

Abstract citation ID: igad104.0483

### WHAT MATTERS TO CHINESE AND KOREAN DEMENTIA CAREGIVERS: INFORMING THE CULTURAL ADAPTATION OF THE NYUCI-ES PROGRAM

Jing Wang[1], Bei Wu[2], Min Kyoung Johnson[3], I tek Leong[3], Yaolin Pei[2], Cynthia Epstein[4], Soyeon Cho[5], and Mary Mittelman[6], *1. University of New Hampshire, Durham, New Hampshire, United States*, *2. New York University, New York City, New York, United States*, *3. New York University Rory Meyers College Of Nursing, New York City, New York, United States*, *4. NYU Grossman School of Medicine, New York City, New York, United States*, *5. City University of New York, New York City, New York, United States*, *6. NYU Langone HealthNYU Grossman School of Medicine, New York City, New York, United States*

Family caregivers of persons with dementia (PWD) undertake an extensive amount of care and many are challenged by heavy caregiving burden and their own health problems. The New York University Caregiver Intervention (NYUCI) program has demonstrated effectiveness in multiple studies among largely Caucasian samples in improving the wellbeing of caregivers of PWD by decreasing their levels of depression and stress and improving self-reported health. As two large Asian immigrant population in the U.S., Korean and Chinese American caregivers of PWD have unique challenges impacted by their cultural background. However, there are limited studies on what can be done to support them in a culturally sensitive way. The current study aims to inform the cultural adaptation of the program for Chinese and Korean caregivers with multiple chronic conditions by exploring their perspectives to understand their unique challenges, unmet needs, and the best ways to support them in caregiving and engage them in selfcare. We interviewed 14 Chinese and 11 Korean caregivers living in the New York Metropolitan Area. We compared themes mentioned by both groups and found that cultural beliefs profoundly impact their perceptions of dementia and caregiving, their way of utilizing social and family support, and understanding of self-care and counselling services. Caregivers are facing and addressing new challenges during and after the Pandemic, providing insights into how we can use technology and telehealth to assist them in caregiving and coordinating support from others. Our findings provided implications to culturally adapt the NYUCI program to culturally diverse populations.

Abstract citation ID: igad104.0484

### FACTORS ASSOCIATED WITH DEPRESSIVE SYMPTOMS AMONG OLDER SOUTHEAST ASIAN AMERICANS

Kaipeng Wang[1], My Ngoc To[1], Carson De Fries[1], and Bei Wu[2], *1. University of Denver, Denver, Colorado, United States*, *2. New York University, New York City, New York, United States*

Depressive symptoms are prevalent among older adults, particularly those from Southeast Asian American (SEAA) communities. Although SEAAs represent one of the fastest growing minority groups in the U.S, research has given little attention to understanding depression among older SEAAs. This study therefore aimed to examine the factors associated with depressive symptoms among older Southeast Asian Americans. We recruited a total of 251 adults aged 55 and above who identified as Vietnamese American, Filipino American, or Indonesian American via community organizations. Participants completed online or in-person questionnaires. Depressive symptoms were measured with the 10-item Center for Epidemiologic Studies Depression Scale. Items are rated on a 4-point Likert scale, with higher scores indicating more depressive symptoms. We used ordinary least squares (OLS) regression to examine the association between depressive symptoms and various health, psychological, social, demographic, and environmental factors. In general, Vietnamese Americans (mean=1.74, SD=0.65) had significantly more depressive symptoms than Indonesian (mean=1.56, SD=0.60) and Filipino (mean=1.47, SD=0.47) Americans. OLS regression results showed that resilience and age were negatively associated with depressive symptoms, while greater family conflict was significantly associated with more depressive symptoms. Participants who could not speak English fluently had significantly more depressive symptoms than those who could not. Those residing in urban or suburban areas had significantly more depressive symptoms than those living in rural areas. Results contribute to better understanding of the factors that contribute to depression among older SEAAs and inform the strategies for preventing and treating depression in this population.

Abstract citation ID: igad104.0485

### IMPACTS OF ACCULTURATION ON HEALTH OF US OLDER CHINESE IMMIGRANTS

Yanping Jiang[1], Fengyan Tang[2], and Bei Wu[3], *1. Rutgers University, New Brunswick, New Jersey, United States*, *2. University of Pittsburgh, Pittsburgh, Pennsylvania, United States*, *3. New York University, New York City, New York, United States*

Acculturation from Asian- to Western-oriented values and cultural practices is a key aspect of social experience for many Asian Americans. The growing body of literature on this topic shows mixed findings on health impacts of acculturation and is largely relied on cross-sectional studies, preventing to draw definitive conclusions on the health impact of acculturation. The study aimed to examine long-term effects of acculturation on mental, physical, and cognitive health in U.S. older Chinese immigrants. Data were drawn from 2,811 participants (aged from 60 to 104 years) from the Population Study of Chinese Elderly in Chicago study. Acculturation was measured using a 12-item Acculturation Scale assessing language use, ethnic social relations, and media use. Health outcomes included depression assessed using the Patient Health Questionnaire-9, activities of daily living (ADL) assessed using the adapted Katz ADL Scale, and global cognition indexed by a composite score of episodic memory, working memory, executive function, and mental status. Results from latent growth curve models showed that high levels of acculturation were not associated with depression at baseline but predicted a faster decline in depression during the 6-year follow-up. Similarly, acculturation was not related to ADL at baseline but predicted a slower decline over time. Contrarily, acculturation was associated with better global cognition at baseline but was not related to long-term changes in cognition. These results suggest the long-term benefits of acculturation on mental and physical health among U.S. older Chinese immigrants. The health advantages related to acculturation, however, seem not to generalize to cognitive function.

### SESSION 2095 (SYMPOSIUM)

Abstract citation ID: igad104.0486

### CREATING AN AGE-SAVVY WORKFORCE THROUGH GERONTOLOGICAL INTERNSHIPS

Chair: Rona Karasik Co-Chair: Laura Donorfio Discussant: Marilyn Gugliucci

The disconnect between age and work demographics is well-known. Less understood is how to create an age-savvy workforce prepared to meet the diverse needs of a rapidly aging population. To date, considerable research has focused on: (1) Are current and future professionals interested in working with older adults (e.g., King et al., 2013) (2) Does "exposing" current and future professionals to aging content increase their interest in working with older adults (e.g., Gutheil, et al, 2009); and (3) What impact do direct intergenerational experiences have on student attitudes and desires to work with older adults (e.g., Gorelik, et al., 2000)? The results of these studies are mixed, suggesting additional elements be considered, such as the quality and outcomes of the experience. This symposium considers the nature, role, and challenges of internship, practicum, and field work experiences from a range of perspectives. First, Dr. Laura Donorfio introduces the components of internships as they relate to workforce development. Next, Dr. Rona Karasik provides a faculty perspective on intergenerational internships. Third, Dr. Phyllis Greenberg and Jodi Danielson offer insight into the agency and preceptor perspective. Forth, Jessica VanderWerf presents a gerontology student's perspective. Fifth, Camryn Hafner shares the perspective of a student from outside of gerontology – in this case, drama therapy. Finally, moderator and discussant Dr. Marilyn Gugliucci synthesizes the presentation findings and reflects on the promise of gerontological internships to develop professionals both within and outside of gerontology to best meet the demographic reality of the next 30 - 50 years.

Abstract citation ID: igad104.0487

### THE VALUE OF INTERNSHIPS IN CREATING AN AGE-SAVVY WORKFORCE

Laura Donorfio, *University of Connecticut, Waterbury, Connecticut, United States*

As the 65+ population continues to grow, so too does the need for an age-savvy workforce. There is not a field or profession that will not intersect in some way with older adults (Karasik, Donorfio, & Greenberg, 2023). Current work shortages and forecasts, however, raise considerable concern for a sustainable and adequately prepared workforce. With regard to preparation, gerontological field placements, internships, and practicums are becoming increasingly instrumental to workforce development. Both the Gerontology Competencies for Undergraduate and Graduate Education (2020) and AGHE's Gerontology and Geriatric Curricular Standards and Guidelines (2021) strongly recommend internship, practicum, and/or field work experiences for students at all levels. Fostering a successful intergenerational experience, however, can be challenging. In this presentation, key components, core elements, necessary steps, best practices and logistical considerations will be discussed. For example, who are the key players and what are their roles in such experiences? What needs to happen at the programmatic and curricular levels? In addition to exploring the various facets of gerontological field placements, attention will be given to utilizing AGHE's Standards and Guidelines and Gerontology Competencies to help identify several "interactional competencies" that relate to internships, practicums, and field experiences. This session also serves as an introduction to the range of perspectives (student, faculty, community agency) that follow.

Abstract citation ID: igad104.0488

### GERONTOLOGY INTERNSHIPS, PRACTICUM, AND FIELD WORK FROM A FACULTY PERSPECTIVE

Rona Karasik, *Saint Cloud State University, Saint Cloud, Minnesota, United States*

While internships, practicum, and field work tend to be viewed primarily as student and site focused, academic faculty are also important to an internship's success. Faculty roles may include assisting students in selecting and applying for site placements, ensuring that students are a "good fit" and properly prepared for a particular setting, overseeing quality control and assessment, guiding students to reflect on and harvest their learning throughout their internship, and at times, serving as advocate, trouble-shooter and/or mediator (Karasik, Donorfio, & Greenberg, 2023). In addition to student-facing responsibilities, faculty are also often involved in curricular aspects of internships and similar experiences (e.g., setting program parameters, expectations, and student learning goals and objectives), as well employing internship outcomes to academic program assessment (Karasik, 2009). Internship faculty also serve as a liaison between the academic program and community partners. Developing and maintaining community partnerships requires a broad skill-set, including but not limited to knowledge of the local aging network, collegiality, diplomacy, cultural and self-awareness, flexibility, and effective communication. Honing and consistently applying these traits is also essential, as recent studies suggest that at least some community partners perceive faculty involvement in community-based learning to be uneven and/or deficient (e.g., Karasik, 2020; Karasik & Hafner, 2021). Given the many roles faculty play in regard to internships and similar experiences, this presentation explores gerontology-based internships from a faculty perspective and shares the reflections and insight of one faculty member who has served as a gerontology internship coordinator for close to 30 years.

Abstract citation ID: igad104.0489

### WHAT WORKS AND WHAT IS MISSING: A PRECEPTOR AND AGENCY PERSPECTIVE

Phyllis Greenberg[1], and Jodi Danielson[2], *1. St. Cloud State University, St. Cloud, Minnesota, United States*, *2. Good Shepherd Community, Sauk Rapids, Minnesota, United Sta tes*

As has been discussed, the population of older adults is growing at a rapid pace and the need for staff in residential environments has grown to a crisis situation. Agencies are in dire need of staff at all levels from CNAs to nurses, social workers and administrators. There is an opportunity for agencies to help train the new generation of age savvy staff by working with universities to provide hands-on experience such as internships, practicum, and field work. This presentation examines the preceptor and community agency perspective of working with students interested in aging. We explore how to achieve a win/win/win/win experience (student, preceptor-agency/university/older adult). The presenters represent a agency preceptor and a preceptor-faculty member. Key areas for consideration include: 1) Experience/education of students; 2) Time commitment for preceptors/ agencies; 3) Agency and university support for preceptors; 4) Support for students from preceptors, agencies and the university; 5) Flexibility in responsibilities and scheduling between agencies, preceptors, and students; and 6) Ensuring that the experience is valuable to both students and agencies. Presenters will discuss strategies for meeting the needs of students and agencies while at the same time meeting the requirements of the university. These strategies include student involvement in day-to-day activities, as well as projects, teaching moments, providing support to preceptors as well as on-going communication and feedback. The goal is to provide an environment (both agency and university) that fosters growing an age savvy workforce.

Abstract citation ID: igad104.0490

### TRADITIONAL AND NONTRADITIONAL INTERNSHIPS: A GERONTOLOGY STUDENT'S PERSPECTIVE

Jessica VanderWerf, *University of South Florida, Tampa, Florida, United States*

Today's gerontology students are more diverse than ever. Some come to the classroom with little to no experience working with older adults, while others are looking for further education and/or credentials to add to their employment and career opportunities. Both traditional (e.g., nursing homes, senior centers) and non-traditional internship settings (e.g., research, teaching) are examined in regard to providing students with experiences that will enhance their knowledge, career development, and/or future academic goals. As both an undergraduate minor and a Master's level gerontology student, the presenter discusses their own non-traditional internship experiences that helped them to reach their goal of pursuing a doctoral degree in aging studies. Feedback gathered from other students in a variety of internship types is also presented. Finally, lessons learned, finding the right fit, and the overall value of internships and similar experiences is discussed from a student perspective.

Abstract citation ID: igad104.0491

### READY OR NOT: A DRAMA THERAPY STUDENT'S PERSPECTIVE ON WORKING WITH OLDER ADULTS

Camryn Hafner, *New York University, New York City, New York, United States*

Drama therapy has the potential to positively influence the lives of older adults in many facets, i.e., improving self-perceptions of aging (Dokter & Sajnani, 2023), attention, depressive symptoms, and overall quality of life (Lin et al., 2022). But drama therapists may also inadvertently cause harm, reenacting narratives of infantilization and indignity, or reinforcing the socially prescribed roles available to older adults (Dokter & Sajnani, 2023). Training and education may minimize harm and maximize positive outcomes; however, despite growing numbers of drama therapists and drama therapy interns working with older populations, the training of drama therapists minimally reflects this shift. Currently, a total of 800 internship hours, representing work with at least two distinct populations, are required to graduate from New York University with a Master of Arts in Drama Therapy and to qualify for a provisional license. While older adults may represent one of these populations, no courses in aging are required. One elective course, Drama Therapy and Aging: From Midlife to Elderhood, is offered once every two years. In a two-year program, this course may only become available after a student has begun their fieldwork. In this presenter's case, the aging elective was taken concurrently with the start of her fieldwork at an assisted living facility working with persons living with dementia on a secured unit. Benefits and limitations of this student's preparation to provide successful therapeutic experiences for individuals in this environment are considered, as well as the perceived implications for their future career as a drama therapist.

### SESSION 2100 (SYMPOSIUM)

Abstract citation ID: igad104.0492

### SOCIAL NETWORKS: BUILDING RESOURCES FOR DIVERSE AGING FAMILIES

Chair: Kristine Ajrouch Discussant: Toni Antonucci

Social networks provide tangible resources for older adults and their families. Yet, their formation and characteristics may vary across diverse aging contexts. Friedman and colleagues examine multiple dimensions of caregivers' networks using a nationally representative sample (N=2,176). Latent profile analysis identified three classes of caregiving networks: (1) Large, sparse networks with relatively few kin and only some people who assist with caregiving tasks for care recipients; (2) Moderately-sized, somewhat dense, kin-based networks; (3) Small, very dense family networks where caregiving is shared across network members, all of which uniquely influence caregiver well-being. Hu and colleagues evaluate measurement invariance of objective and subjective social isolation between US-born and foreign-born older adults using a regionally representative sample (N=611). Findings show indicators of social network structure and perceived relationship quality may be useful for examining social isolation among older immigrants. Ajrouch and colleagues show distinct dimensions of networks differentially influence older adult's cognitive function. Larger networks and higher proportion of family are positively related while higher contact frequency is negatively related to cognitive function. Using data from the Detroit-based Social Relations Study, Webster and Chin show the extent of similarity in health-related behaviors between older adults and their closest social network members. Analysis of 247 dyads illustrate associations between multiple social network factors (homophily, structure, and relationship quality) and similarity in these behaviors. Together, these papers advance theoretical and methodological approaches to the ways in which social networks serve as a resource across diverse aging contexts.

Abstract citation ID: igad104.0493

### SOCIAL NETWORKS AND COGNITIVE HEALTH IN RACIAL/ETHNIC CONTEXT

Kristine Ajrouch[1], Toni Antonucci[2], Simon Brauer[2], and Laura Zahodne[2], *1. Eastern Michigan University, Ypsilanti, Michigan, United States*, *2. University of Michigan, Ann Arbor, Michigan, United States*

Social networks provide tangible resources for older adults and their families. Yet, their formation and characteristics may vary across diverse aging contexts. Friedman and colleagues examine multiple dimensions of caregivers' networks using a nationally representative sample (N=2,176). Latent profile analysis identified three classes of caregiving networks: (1) Large, sparse networks with relatively few kin and only some people who assist with caregiving tasks for care recipients; (2) Moderately-sized, somewhat dense, kin-based networks; (3) Small, very dense family networks where caregiving is shared across network members, all of which uniquely influence caregiver well-being. Hu and colleagues evaluate measurement invariance of objective and subjective social isolation between US-born and foreign-born older adults using a regionally representative sample (N=611). Findings show indicators of social network structure and perceived relationship quality may be useful for examining social isolation among older immigrants. Ajrouch and colleagues show distinct dimensions of networks differentially influence older adult's cognitive function. Larger networks and higher proportion of family are positively related while higher contact frequency is negatively related to cognitive function. Using data from the Detroit-based Social Relations Study, Webster and Chin show the extent of similarity in health-related behaviors between older adults and their closest social network members. Analysis of 247 dyads illustrate associations between multiple social network factors (homophily, structure, and relationship quality) and similarity in these behaviors. Together, these papers advance theoretical and methodological approaches to the ways in which social networks serve as a resource across diverse aging contexts.

Abstract citation ID: igad104.0494

### SOCIAL NETWORK STRUCTURE AND THE WELL-BEING OF FAMILY CAREGIVERS TO OLDER ADULTS

Esther Friedman[1], James Murphy[2], and David Kennedy[2], *1. University of Michigan, Ann Arbor, Michigan, United States*, *2. RAND, Santa Monica, California, United States*

Personal networks provide social support to both older adults and the family members who care for them, yet we know very little about the different structures of caregiver networks and which are most protective of caregiver well-being. Using a new nationally representative survey of 2,176 individuals who report providing care to a family member or friend age 50 or older with a health condition during the height of the Covid-19 pandemic and give detailed information on their personal networks, we examine caregivers' networks in terms of size, density, proportion kin, and how care is allocated among network members. We also examine the implications of network structure for caregiver well-being including anxiety, depression, and hopefulness. We use latent profile analysis to identify three classes of caregiving networks: (1) Large, sparse networks with relatively few kin and only some people assisting with caregiving tasks for care recipients; (2) Moderately-sized, somewhat dense, kin-based networks; (3) Small, very dense kin networks where caregiving is shared across network members. Caregivers who are male, highly-educated, and providing care for someone with dementia are disproportionately in Class 1. Being in Class 3 is significantly associated with lower caregiver anxiety compared to being in the other two groups, even after controlling for sociodemographic factors, but not with depression and hopefulness. This work has implications for the optimal network structure for facilitating the well-being of family caregivers, and provides a baseline for examining network change and the implications thereof as the impact of the pandemic wanes.

Abstract citation ID: igad104.0495

### SIMILARITY IN HEALTH-RELATED BEHAVIORS WITHIN OLDER ADULTS' CLOSE SOCIAL RELATIONSHIPS

Noah Webster, and Kristi Chin, *University of Michigan, Ann Arbor, Michigan, United States*

Health-related behaviors are socially contagious, spreading through social networks via multiple mechanisms. It remains unclear though which mechanisms are most salient among older adults. Guided by Social Contagion Theory and the Convoy Model of Social Relations we examine similarity in health-related behaviors (exercise, smoking, alcohol consumption) between older adults and their closest social network members and investigate associations with multiple social factors (homophily, network structure, relationship quality). Data are from Wave 3 of the Detroit-based Social Relations Study collected in 2015. Panel respondents nominated people who were close and important in their lives, and up to the first three nominated were interviewed. Analyses were conducted at the dyad-level, and we selected dyads in which at least one member was age 60 or older (n=247 dyads). Generalized estimating equations were used due to some panel respondents being in multiple dyads. Greater homophily was found to be associated with similarity in both exercise frequency and number of alcoholic drinks consumed per week. Specifically, pairs of close social network members who were more similar in self-rated health were significantly more similar in exercise frequency, and those of the same gender and more similar in age were significantly more similar in alcohol consumption. Network structure, specifically greater contact frequency, was associated with significantly more similarity in alcohol consumption, but less similarity in exercise frequency. Findings suggest the salience of multiple aspects of older adults' close social relationships that can be incorporated into behavior change interventions and leveraged to encourage healthy lifestyles in later life.

Abstract citation ID: igad104.0496

### MEASURING SOCIAL ISOLATION AMONG US-BORN AND FOREIGN-BORN OLDER ADULTS

Rita Hu[1], Noah Webster[1], Kristine Ajrouch[2], and Toni Antonucci[1], *1. University of Michigan, Ann Arbor, Michigan, United States*, *2. Eastern Michigan University, Ypsilanti, Michigan, United States*

While older immigrants are at increased risk of social isolation, little research has examined if established measures are equivalent across nativity groups. This study evaluates the measurement invariance of objective and subjective social isolation between US-born and foreign-born older adults. Data are from the Detroit Area Wellness Network (DAWN) study and were collected via a telephone survey from three groups prominent in metro-Detroit: African Americans, Middle Eastern/Arab Americans, and Non-Hispanic White Americans (N=611; Mage = 72.7 [64-98]). The sample was comprised of 29% immigrants. Objective social isolation was measured by network size, household size, and contact frequency with network members. Subjective social isolation was measured by feeling lonely, relationship quality, frequency of arguing, and frequency of conflict. Confirmatory Factor Analysis (CFA) was used to evaluate the dimensional structure, and multiple-group CFA was used to test for measurement variance by immigrant status. The measurement invariance models of objective social isolation showed a good fit after allowing the intercepts of both network and household size to vary in the scalar model. The measurement invariance models of subjective social isolation showed a good fit after allowing the estimate and intercept of loneliness and the intercept of argument to vary in the scalar model. Establishing measurement invariance in both objective and subjective social isolation is essential for valid interpretation of group differences among US- and foreign-born adults in aspects of social isolation. Findings show indicators of social network structure and perceived relationship quality may be useful to examine social isolation among older immigrants.

### SESSION 2105 (SYMPOSIUM)

Abstract citation ID: igad104.0497

### CLIMATE CHANGE AND AGING: FROM VULNERABILITY TO ACTION

Chair: Liat Ayalon Discussant: Karl Pillemer

Older people are particularly susceptible to extreme climate change events. This is attributable to physiological susceptibility, but also to factors such as social isolation, digital exclusion, ageism, and living arrangement. Unfortunately, older people are often not considered in mitigation and adaptation climate change policies contributing to their heightened susceptibility in the face of extreme climate events. Compounding this challenge, it is younger people who are seen as the face of the climate change movement, at times, blaming older people for their (in)action, which has contributed to the current climate situation. Nevertheless, research has shown that older people engage in pro-environmental behaviors and can highly benefit from taking part in climate change activism. The present symposium aims to tackle this topic by discussing the challenges pertaining to older populations occasioned by climate change, and how older populations can engage in being part of mitigation solutions.. The first presentation by Carr and colleagues addresses global population aging and heat exposure with a particular focus on implications for wellbeing and social policy. Delving into the country level, Cherry and colleagues discuss findings concerning long term mental health effects of exposure to a single versus two extreme weather events. Moving to possibly mitigation and adaptation measures, Nikitin and Rupprecht address opportunities associated with exposure to nature, whereas Kohon and colleagues discuss the role that aging and old age capture in climate change policies. The symposium concludes with a theoretical conceptualization by Diehl which reframes aging in the context of climate change. This is a Climate Change and Aging Interest Group Sponsored Symposium.

Abstract citation ID: igad104.0498

### GETTING OLDER ADULTS INVOLVED IN CLIMATE CHANGE INITIATIVES: REFRAMING AGING AS A POTENTIAL APPROACH

Manfred Diehl, *Colorado State University, Fort Collins, Colorado, United States*

In the context of climate change, older adults are often solely portrayed in terms of vulnerability and victimization. Although older adults' vulnerability to the growing effects of climate change and natural disasters is an important topic, this presentation focuses on how to get older adults involved in climate change initiatives and as advocates for the adoption of climate-friendly behavior. The presentation argues that getting the growing population of older adults actively involved in issues of climate change requires moving away from a deficit- and loss-focused view of later adulthood. Instead, a new narrative about aging needs to be adopted, recognizing the many contributions older adults can make to addressing the global climate crisis. These contributions arise out of concerns for the health and well-being of the next generations and draw on older adults' legacy thinking, lifelong experiences, professional expertise, and ability to work in multigenerational contexts. Such a new narrative recognizes and supports older adults as a "natural resource" and important "human capital." The presentation will conclude with a discussion of the challenges to get older adults involved in climate change initiatives and with a description of several exemplary programs that have already generated foundational knowledge in this domain.

Abstract citation ID: igad104.0499

### GLOBAL POPULATION AGING AND HEAT EXPOSURE IN THE 21ST CENTURY: IMPLICATIONS FOR LATE-LIFE WELL-BEING AND POLICY

Deborah Carr[1], Giacomo Falchetta[2], Ian Sue Wing[1], and Enrica DeCian[3], *1. Boston University, Boston, Massachusetts, United States*, *2. International Institute for Applied Systems Analysis, Laxenberg, Salzburg, Austria*, *3. Ca' Foscari University of Venice, Venice, Veneto, Italy*

Climate change has profound consequences for older adults' well-being. Global increases in the frequency, intensity and duration of extreme heat spells pose the most direct threats, given older adults' underlying health conditions and reduced capacity to thermoregulate. Individual-level consequences of heat exposure are well-documented, yet gerontological research has paid less attention to older adults' heat exposure at the population level. World regions with both increasing concentrations of older adults and high temperature extremes are potential hotspots where growing aged populations are at risk of heat-related health threats. We estimate older adults' current and future heat exposure in six global regions (Africa, Asia, Europe, North America, Oceania, South America). Analyses use NASA NEX Global Daily Downscaled Product climate data and country-level projections for the size and distribution of the age 65+ population. We focus on measures of cumulative heat exposure (cooling degree days) and acute exposure to heat extremes (95th percentile of daily maximum temperatures). We project that by 2050, populations aged 65+ globally exposed to >1200 cooling degree days will double. We also project that one-quarter of the aged population worldwide (roughly 340 million) will be living in climates with 95th percentile TMax95 > 99.5 F°, a critical exposure threshold for health. The drivers of this population-level exposure vary by region. Population aging will drive exposure in historically hot regions in Africa and Asia, whereas climate change will drive heat exposure in historically older regions in Europe and North America. These results can inform public policy and local decision-making.

Abstract citation ID: igad104.0500

### EXPOSURE TO NATURE IS RELATED TO GENERATIVITY, ESPECIALLY AMONG ADULTS WITH LIMITED FUTURE TIME PERSPECTIVE

Jana Nikitin, and Fiona Rupprecht, *University of Vienna, Vienna, Wien, Austria*

Theory and research suggest that connection with nature may promote prosocial and environmentally sustainable behaviors. The first objective of the present study was to replicate this finding in an age-heterogeneous sample of N = 115 participants (Mage = 38, SDage = 17, age range 18-85) who reported their exposure to nature up to eight times a day for seven days. We examined the relationship between nature exposure and participants' generativity, i.e., the extent to which they care about future generations and are politically, socially, and environmentally engaged. The second goal of the study was to show that this relationship is particularly pronounced among individuals with a shorter future time perspective that is related to remaining lifetime and associated with the choice of more emotionally significant goals that focus on generativity. We therefore hypothesized that nature experiences would be more strongly associated with generativity among adults whose future time perspective is limited. We found that people who were more exposed to nature in their daily lives also reported higher levels of generativity. This relationship was moderated by future time perspective. As hypothesized, the association was stronger among adults with a limited future time perspective. Contrary to the widely held stereotype that it is primarily younger adults who are concerned about climate and the future of humanity, we showed the opposite: People who perceived their remaining lifetime as limited were the ones for whom nature experiences were more strongly associated with concern for the environment and future generations.

Abstract citation ID: igad104.0501

### MULTIPLE SEVERE WEATHER EVENTS: ARE OLDER ADULTS MORE VULNERABLE?

Katie Cherry[1], Matthew Calamia[1], Emily Elliott[1], Piper Bordes[1], Laura Sampson[2], and Sandro Galea[3], *1. Louisiana State University, Baton Rouge, Louisiana, United States*, *2. Harvard University, Boston, Massachusetts, United States*, *3. Boston University, Boston, Massachusetts, United States*

Hurricanes and floods have psychosocial consequences for directly and indirectly affected individuals. In August of 2016, historic flooding in Baton Rouge, Louisiana resulted in catastrophic damage across a 22-parish (county) region only 11 years after the 2005 Hurricanes Katrina and Rita devastated the US Gulf Coast. In this study, we compared three groups of mostly middle-aged and older adults on the SF-36 Health Survey Questionnaire: (1) non-flooded (controls), (2) single disaster (flooded in 2016), and (3) double disaster (flooded in 2005 and again in 2016). Results indicated that the groups did not differ in self-reported physical health, although the single and double disaster groups reported worse mental health than did the controls. Correlation analyses revealed that age was positively associated with mental health. State hope was positively associated with both physical and mental health. Implications of these findings for reducing vulnerabilities after multiple disaster exposures are considered.

Abstract citation ID: igad104.0502

### CLIMATE CHANGE POLICIES AND OLDER ADULTS: AN ANALYSIS OF STATES' CLIMATE ADAPTATION PLANS

Jacklyn Kohon[1], Bryant Carlson[1], Paula Carder[2], Dani Himes[3], Eiji Toda[1], and Katsuya Tanaka[4], *1. Portland State University, Portland, Oregon, United States*, *2. Portland State University Institute on Aging, Portland, Oregon, United States*, *3. Institute on Aging, Portland, Oregon, United States*, *4. Shiga University, Hikone, Shiga, Japan*

As climate change drives more frequent and intense weather events, older adults face disproportionate impacts, including having the highest mortality rates from storms, wildfires, flooding, and heat waves. State governments are critical in deploying local resources to help address climate change impacts. This policy study analyzes states' climate adaptation plans to assess the methods through which they address the impact of climate change on older adults. This study uses content analysis to analyze available climate change adaptation plans for all U.S. states for strategies designed to increase resilience of older adults to impacts of climate change. Nineteen states have climate adaptation plans, of which 18 describe older adults as a population group with specific health impacts and risks factors. Four categories of adaptation strategies for older adults include communications, transportation, housing and emergency services. State plans vary in terms of the risk factors and adaptation strategies included. To varying degrees, states' climate change adaptation planning address health, social and economic risks specific to older adults, as well as strategies for mitigating those risks. As global warming continues, collaborations between public and private sectors and across regions will be needed to prevent negative outcomes such as forced relocation and other social and economic disruptions as well as disparate morbidity and mortality.

### SESSION 2110 (SYMPOSIUM)

Abstract citation ID: igad104.0503

### EXPLORING THE LINK BETWEEN SLEEP AND DEMENTIA: MULTIFACETED APPROACHES

Chair: Darina Petrovsky Co-Chair: Elizabeth Luth

Sleep and sleep quality have been linked to cognitive function over time. The quality of caregiver sleep can also affect their ability to function and provide support to persons living with dementia. This symposium leverages longitudinal survey data from two large US population-based surveys: the National Health and Aging Trends Study (NHATS) and the National Study of Caregiving (NSOC). This symposium aims to explore the relationship between sleep, cognitive function, and care activities for older adults living with and without dementia and their caregivers. First, Dr. Petrovsky will report findings that examined the relationship between caregiver sleep quality and persons living with dementia status using NHATS and NSOC data. Next, Dr. Esiaka will present cross-sectional findings from examining the relationship between older adult sleep quality, neighborhood disorder, and cognitive function using the NHATS data. Drs. Klingman and Sullivan will present the results of an analysis comparing the relationship between sleep and frequent hospitalization in persons living with dementia and those without dementia. Collectively, these papers will report on findings examining how a potentially modifiable factor, sleep, relates to cognitive function and caregiving.

Abstract citation ID: igad104.0504

### EXAMINING DIFFERENCES IN SLEEP QUALITY AMONG CAREGIVERS FOR PERSONS LIVING WITH AND WITHOUT DEMENTIA

Darina Petrovsky[1], Sophia Geisser[2], and Elizabeth Luth[3], *1. Rutgers University, Jackson, New Jersey, United States*, *2. The University of Alabama, Tuscaloosa, Alabama, United States*, *3. Rutgers University, New Brunswick, New Jersey, United States*

Sleep disturbances among caregivers are linked to increased burden and worse mental health outcomes. Caregivers for persons living with dementia experience more sleep disturbances than non-caregivers. Few studies examine whether sleep quality differs between dementia and non-dementia caregivers. This study analyzes whether dementia caregivers experience worse sleep quality than non-dementia caregivers. Using 2017 National Study of Caregiving, National Health and Aging Trends Study, and time diary interview data, we examined the relationship between caregiver sleep quality ("excellent/very good" versus "good/fair/poor" the previous night) and care recipient's dementia status ("probable" versus "no") for 1,374 caregivers in a series of nested multivariable logistic regression models, controlling for the caregiver and care recipient sociodemographic characteristics, relationship quality, and other controls. Caregivers' mean age was 62.3 years old (SD=14) and about two-thirds were women (68%), non-Hispanic White (69.4%), and married (66%) Thirty-eight percent cared for persons living with dementia. In the bivariate model, being a caregiver for someone living with dementia was associated with significantly lower odds of reporting "excellent/very good" sleep quality (OR: 0.78, 95%CI: 0.62-0.97, p=0.02). This relationship persisted independent of caregiver and care recipient demographic characteristics. However, caregiver perceptions of the quality of their relationship with the care recipient and the benefits of their caregiving role explained the relationship between care recipient dementia status and caregiver sleep quality ratings (AOR: 0.80, 95%CI: 0.63-1.02, p=0.08). Addressing caregiver burden and relationship quality may help improve sleep quality among dementia caregivers and reduce disparities in sleep quality between dementia and non-dementia caregivers.

Abstract citation ID: igad104.0505

### DO SLEEP QUALITY AND SOCIAL COHESION MEDIATE ASSOCIATION BETWEEN NEIGHBORHOOD DISORDER AND COGNITIVE FUNCTIONING?

Darlingtina Esiaka[1], and Elizabeth Luth[2], *1. University of Kentucky, Lexington, Kentucky, United States*, *2. Rutgers University, New Brunswick, New Jersey, United States*

Daily exposure to neighborhoods with high levels of disorder, such as public drinking, graffiti, and vandalism, has been linked to worse cognitive functioning and a higher risk for dementia in older adults. The present study examined the association between neighborhood disorder and cognitive functioning. This study also examined sleep quality and social cohesion as potential mediators of this association using Hayes' (2013) Macro Process via bootstrapping method. The sample included cross-sectional data for 2,529 older adults (1,115 men and 1,482 women, age 70+) using the 2021 National Health and Aging Trends Study. Neighborhood disorder was measured as a 3-item scale capturing physical neighborhood characteristics completed by the interviewer (e.g., trash on sidewalks). The social cohesion scale measured participant agreement with 3 questions about their community (e.g., "People can be trusted"). The sleep quality scale measured participant responses to 3 questions about their sleep in the last month (e.g., taking 30+ minutes to fall asleep). Cognitive functioning was assessed using performance on tasks evaluating memory, orientation, and executive functioning. Results showed greater neighborhood disorder was associated with worse performance on cognitive tasks (B=-0.417; p<0.001). Results of mediation testing indicated that social cohesion was a significant mediator (B=0.224; p<0.0001). Though sleep quality was not a mediator, better sleep quality was associated with better cognitive functioning (B=0.053; p<0.05). Understanding the contribution of neighborhood disorder, sleep quality, and social cohesion to cognitive functioning will inform neighborhood-level interventions that can buffer cognitive decline in older adults aging in place.

Abstract citation ID: igad104.0506

### SLEEP AND FREQUENT HOSPITALIZATIONS AT THE END OF LIFE FOR INDIVIDUALS WITH DEMENTIA

Karen Klingman, and Suzanne Sullivan, *SUNY Upstate Medical University, Syracuse, New York, United States*

Older adults near end-of-life (EOL) are at risk for experiencing non-beneficial hospitalizations, negatively impacting their quality of life. Reasons for frequent hospitalizations include poorly managed symptoms of progressing chronic illnesses, many of which are exacerbated by poor sleep, and may differ depending on presence of dementia. This study characterized sleep for groups with and without frequent (two-or-more) hospitalizations in the final year of life using the National Health and Aging Trends Study. A subgroup of persons living with dementia (PLWD) was also explored. Means for difficulty falling asleep, trouble falling back to sleep, and use of sleep medications were compared for groups with and without frequent hospitalizations via one-tailed t-tests. Association between dementia and frequent hospitalizations was explored via Pearson Chi-Square. Individuals at EOL (n=529, 56% female, 68% white non-Hispanic, average age 80-85, 57% PLWD) were included, and 22% had frequent hospitalizations. Among the entire sample, those with frequent hospitalizations had worse sleep (more trouble falling back to sleep, p=.032, Cohens-d=.2). Sleep was also worse for those without dementia, but with frequent hospitalizations (difficulty falling back to sleep, Cohens-d=.3, p=.02) compared to those without frequent hospitalizations. There was no association between frequent hospitalization and presence of dementia (chi-square=.868 p=.352). However, the PLWD subgroup had worse sleep than those without dementia (difficulty falling asleep Cohens-d=.15, p=.04; use of sleep medications Cohens-d=.26, p=.002), yet sleep did not differ depending on frequent hospitalizations. These results indicate that further investigation into the relationship between dementia, sleep, and frequent hospitalizations at EOL is warranted.

### SESSION 2115 (SYMPOSIUM)

Abstract citation ID: igad104.0507

### CONTRIBUTIONS OF HBCUS TO THE GERONTOLOGY FIELD: DISCUSSIONS OF RELIGION, HEALTH DETERMINANTS, AND BLACK AGING

Chair: Tamara Baker Discussant: Tiffany Washington

Health advancements have allowed older adults to a better quality of life. This however, is not equitably translated to the well-being of older Black adults. To bypass this narrative, actions are need to focus on restructuring fragmented systems that perpetuate health inequities and social injustices. This however cannot be the responsibility of one person, community, or institution, but rather a collective obligation of those willing to service the needs of all older adults. This allows us to not only acknowledge the scholarly work aimed at bettering the lives of our aging communities, but also recognizes the contributions of scholars affiliated with Historically Black Colleges and Universities (HBCUs), through research, policy, community interventions, and scholarly practices. To address this progressive movement, this collection of papers not only acknowledges the contributions of HBCUs to the gerontology discipline, but also to the scientific advancement focusing on the health and well-being of older Black adults. Speaker one will discuss the biopsychosocial determinants of cognitive function of older Black adults. Our second speaker will focus on religious coping and COVID-19 vaccine hesitancy among older Black individuals. Speaker three will highlight SDoH among older adults within Caribbean societies. The final speaker will discuss the impact and contributions of HBCU's on an 'aging' society through data analysis of demographic patterns and historical factors from an Afrocentric and Black perspectives. The discussant will tie together these themes and provide recommendations related to education, scholarly research ventures, particularly as it relates to health outcomes, equity, and collaborating with HBCUs. This is a HBCU Collaborative Interest Group Sponsored Symposium.

Abstract citation ID: igad104.0508

### BIOPSYCHOSOCIAL DETERMINANTS OF COGNITIVE FUNCTION AMONG BLACK OLDER ADULTS

Amy Thierry, *Xavier University of Louisiana, New Orleans, Louisiana, United States*

Black older adults in the United States have twice the risk of being diagnosed with Alzheimer's disease and related dementias than their white counterparts. However, there remains a gap in knowledge of which factors may contribute to this disparity as well as mechanisms that either increase or decrease risk for poor cognitive outcomes, specifically in older Black adults. This presentation will highlight recent interdisciplinary population health research on the biopsychosocial determinants of cognitive function among Black adults 65 years and older in the Health and Retirement Study. Using within-group regression analyses, studies examine associations between multiple measures across neighborhood, psychosocial, and biomarker domains with cognitive function, measured by Telephone Interview for Cognitive Status scores (range: 0-35) which captures memory and overall mental status. We find that high neighborhood disorder and low social cohesion were associated with worse cognitive function, especially for more highly educated Black older adults and those living in urban communities. Greater frequency of experiences of everyday discrimination among Black older adults was also related to lower cognitive function. Volunteering and having a high sense of purpose in life were associated with better cognitive function for older Black men and women, while being physical active was associated with increased cognitive function for older Black women. C-reactive protein, a measure of systemic inflammation, was positively associated with cognitive function. Overall, this body of work demonstrates the need to further critically assess both risk and resilience processes underlying cognitive function among older Black adults towards reducing current cognitive health inequities.

Abstract citation ID: igad104.0509

### "NO FEAR, ALL FAITH": RELIGIOUS COPING AND COVID-19 VACCINE HESITANCY AMONG OLDER AFRICAN AMERICANS

Antonius Skipper, *Georgia State University, Atlanta, Georgia, United States*

While COVID-19 had a devasting impact on many groups across the globe, older African Americans were especially affected by the disproportionate rates of morbidity and mortality associated with the virus. Faced with "double jeopardy" - the nature of being both old and Black - older African Americans were at an increased risk of COVID-19-related infection and death due to their age and factors associated with ethnicity (e.g., access to resources, distrust of healthcare). These risks led many health professionals to prioritize COVID-19 vaccine uptake for older African Americans. However, historically and contemporaneously, African Americans report some of the highest levels of vaccine hesitancy in comparison to other ethnic groups. Many older African Americans use religious coping as a source to alleviate the stress of health threats. Yet, the novelty of COVID-19 contributed to a limited understanding of how and why vaccine hesitant older African Americans used religious coping in response to the pandemic. The present study gathered qualitative data from 22 vaccine hesitant older African Americans to describe the role of religious coping in vaccine hesitancy. Data were analyzed using thematic analysis and inductive coding methods. Analyses identified several salient themes relative to religious coping and COVID-19 vaccine hesitancy, including (1) No Fear, All Faith, (2) Prayer Protects my Peace, and 3) My Faith Justifies my Hesitance. As vaccine uptake remains important for both older adults and African Americans, implications from this study highlight important considerations for navigating the nexus of religion and health with highly religious populations.

Abstract citation ID: igad104.0510

### FROM THESE ROOTS: THE ROLE OF HBCUS IN PREPARING THE NATION'S GERONTOLOGISTS FOR AN AGING SOCIETY

Terrell Brown, *Jackson State University, Jackson, Mississippi, United States*

The aging of the U.S. population is creating an increased need for social workers and other helping professionals with training in gerontology. Estimates indicate that less than 3% of MSW students are enrolled in an aging concentration, as compared to 19% enrolled in children/youth concentrations. The phenomenon of a diverse baby boomer generation joining the ranks of persons age 65 and older has created a plethora of scholarship and curriculum development aimed at readying the aging network for the unprecedented growth of older persons. Social work can make unique contributions to the field of gerontology. This study asserts, however, that social work is not adequately prepared to practice in an increasingly diverse aging society. The social work profession has articulated commitments to acknowledging and affirming how diversity and culture shape the human experience and to developing social workers who can competently engage in research-informed practice and practice-informed research. However, there remains a need in social work education for more widespread use of culturally relevant pedagogies that can help achieve these goals. Informed by both the Afrocentric and Black perspectives, this study presents a content analysis of the curricula infused at two historically Black universities. The nature and extent of the contributions of historically Black universities to social work education is the focus of this article. The scope of this investigation also includes the identification of prominent Black social work educators and discusses the implications of these perspectives for more culturally informed gerontology curricula that promote culturally competent gerontological social workers

Abstract citation ID: igad104.0511

### AGING AND HEALTH IN THE CARIBBEAN REGION: AN OVERVIEW OF THE STATE OF RESEARCH

Nekehia Tamara Quashie, *University of Rhode Island, Kingston, Rhode Island, United States*

The Caribbean region is undergoing rapid population aging. While there is diversity in the pace of population aging among countries within the region, there are some shared population health challenges including the increasing prevalence of chronic non-communicable diseases and the inequalities therein. Relatedly, many Caribbean countries do not have well-established long-term care systems. Therefore, older adults primarily rely on informal care and support mainly from family members, which has implications for the health of older adults and their caregivers. Although Caribbean populations are understudied within gerontology, there is a growing body of research. This presentation provides an overview of the existing state of research on the social determinants of health among older adults within Caribbean societies and discusses directions for future research.

### SESSION 2120 (PAPER)

### INDIGENOUS POPULATIONS

Abstract citation ID: igad104.0512

### CYCLICAL MIGRATION IN ALASKA NATIVE ELDERS AND ITS IMPACT ON ELDERS' IDENTITY AND LATER LIFE WELL-BEING

Jordan Lewis[1], Steffi Kim[2], Zayla Asquith-Heinz[3], and Lena Thompson[4], *1. University of Minnesota Medical School, Duluth campus, Duluth, Minnesota, United States*, *2. University of Alaska Anchorage, Anchorage, Alaska, United States*, *3. University of Minnesota Duluth, Duluth, Minnesota, United States*, *4. University of Minnesota, Duluth, Minnesota, United States*

This paper outlines a unique culturally driven cyclical migration of Alaska Native Elders. This Indigenous cyclical migration is distinct from other previously described mobility observations in that Elders spend extended time in more than one community. We describe the cyclical migration of Alaska Native (AN) Elders and its influence on the Elders' identity, health, and well-being. Employing a life course perspective and social theory of migration, inductive content analysis was employed to identify themes related to Elders' cyclical migration between rural and urban communities and the impact on their identity, health, and well-being. Interviews with 125 AN Elders were conducted across five regions of Alaska: Bristol Bay, Interior, Norton Sound, Aleutian Pribilof Islands, and Southcentral. AN Elders traveled between rural and urban communities to access resources and connections critical to their identities, health, and well-being. Urban Elders maintained a connection to rural villages because they perceive them as healthier places to age based on access to traditional practices, land, and the community. Rural Elders spent extended time in urban settings to access health care services, be closer to family, and to benefit from the lower costs of living. This study builds upon existing migration theories by introducing a cyclical pattern uniquely driven by AN identity, culture, and traditional practices. Findings illustrate how AN communities can support Elders who experience cyclical migration patterns to ensure they age successfully in both locations. Future recommended research should explore cyclical migration patterns among other Indigenous populations with histories of migration.

Abstract citation ID: igad104.0513

### DISCRIMINATION IS ASSOCIATED WITH HIGHER ODDS OF HOSPITALIZATIONS AMONG OLDER AFRICAN AMERICANS

Brittney Lange Maia[1], Bryan James[2], Ana Capuano[2], Francine Grodstein[1], and Lisa Barnes[2], *1. Rush Alzheimer's Disease Center, Chicago, Illinois, United States*, *2. Rush University Medical Center, Chicago, Illinois, United States*

Everyday discrimination—experiences of being treated unfairly based on background characteristics like race—is linked to poor physical and mental health throughout the lifespan. Whether more experiences of discrimination are associated with higher likelihood of being hospitalized in older African Americans has not been explored. Hospitalization can represent both poor health and healthcare access. Participants were community-dwelling African Americans from the Rush Memory and Aging Project or Minority Aging Research Study, longitudinal studies of aging (N=301 with at least 12 months linked Medicare fee-for-service claims; mean age 72.5 years (standard deviation [SD]: 5.7), 79% female). Discrimination was assessed using the Detroit Area Study Everyday Discrimination Scale. Hospitalizations (sub-categorized as elective/non-elective, surgical) were quantified using Medicare claims. Mixed-effects ordinal logistic regression models tested associations between baseline discrimination and subsequent odds of hospitalizations per year (0, 1, 2+). The mean baseline discrimination score was 1.7 (SD: 2.2). Over an average 6.5 years (SD: 4.1), 160 participants had at least 1 hospitalization (respectively, 118, 87, and 127 participants had at least 1 nonelective, elective, or surgical hospitalization). Adjusting for age, sex, education, income, depressive symptoms, and medical comorbidity, more experiences of discrimination were associated with higher odds of hospitalization (odds ratio [OR] per point higher on discrimination score=1.12, 95% CI: 1.03-1.22), and higher odds of nonelective (OR=1.12, 95% CI: 1.01-1.24), but not surgical or elective hospitalizations. Drivers of these associations, which may include preventive healthcare avoidance due to discrimination or poor health due to the chronic stress response to discrimination, should be explored.

Abstract citation ID: igad104.0514

**EXPLORING AI ELDERS' CULTURAL PERCEPTIONS OF DISASTERS AND THE ROLE OF GENERATIVITY IN PREPARING FUTURE GENERATIONS**

Lena Thompson[1], Elana Buch[2], and Sato Ashida[3], *1. University of Minnesota, Duluth, Minnesota, United States*, *2. University of Iowa, Iowa City, Iowa, United States*, *3. University of Iowa College of Public Health, Iowa City, Iowa, United States*

Natural and man-made disasters have a great impact on human health, especially for AI Elders. As keepers of cultural and community knowledge, AIAN Elders also serve as guides on how to best support their communities. This study focuses on a Midwestern Tribe's cultural perceptions of disaster management, specifically being generative, or passing down disaster management knowledge and practices to future generations. In-person semi-structured interviews were conducted with eighteen Elders from the Midwestern Tribe. Recordings were transcribed, de-identified, and coded thematically using an interpretive phenomenological analysis approach. The study received approval from the Tribe's Department of Emergency Management and the interview guide was reviewed and approved by a Community Advisory Board of six individuals. Elders described past experiences with natural and man-made disasters, including what they did before, during, and after disasters. Elders also explained how people cared for one another, including the importance of interconnectedness with all beings, caring for grandchildren, and the role of the Tribal Government in disaster management. Elders also described how culture shaped disaster management practices, such as traditional practices to follow during a storm, and the important role of Elders in teaching communities what to do when disasters happen. Exploring the role of generativity in passing on disaster-related information is one way to learn how Elders' knowledge is valued by the community. Findings suggest that Elders can provide important guidance to Tribal governments and public health systems to determine effective disaster management strategies for AIAN communities.

Abstract citation ID: igad104.0515

**INDIGENOUS CULTURAL GENERATIVE ACTS TO REDUCE GENERATIVE MISMATCH AND IMPROVE HEALTH OF ALL GENERATIONS**

Jordan Lewis, *University of Minnesota Medical School, Duluth campus, Duluth, Minnesota, United States*

The gerontological literature predominantly focuses on aging-related losses and less on the gifts we acquire as we age. Alaska Native Elders have experienced a lifetime of adversity, which persists today, but they have also remained resilient. One characteristics of successful aging among Alaska Native Elders is their commitment and passion for sharing their teachings with the youth. Generativity is concerned with using personal resources to improve the quality of life for future generations. While not commonly used in gerontological social work research, it is a cultural practice among Indigenous Elders. This decade long study has been exploring the concept of successful aging from an Alaska Native perspective, or what it means to age in a good way in Alaska Native communities. Qualitative, in-depth, interviews have been conducted with 154 Elders representing 20 participating communities across the State of Alaska to explore the concept of successful aging and the role of generativity in the aging process. For this presentation, 108 interviews with Alaska Native Elders explored successful aging. This presentation will highlight the critical role generativity plays in Alaska Native Elders' ability age in a good way, how generativity can be adapted to bridge the generative mismatch happening between generations and support each generation to healthy and meaningful lives. This presentation will also explore innovative and culturally responsive ways to teach the youth about aging in a good way and how families and communities can support their Elders to be meaningful engaged in the rapidly changing families in the Arctic.

### SESSION 2125 (SYMPOSIUM)

Abstract citation ID: igad104.0516

**BUILDING BRIDGES TO CONNECT BETWEEN NEEDS, RESOURCES, AND RURAL RESIDENTS**

Chair: Nancy Karlin Co-Chair: Lisa Ann Wiese Discussant: Cassandra Ford

The percentage of older adults living in rural areas is predicted to rise with population aging. This impacts access to resources, creates barriers to effective aging, and promotes a unique aging experience. Consequently, demands are growing for services to support healthy aging in rural regions already experiencing healthcare disparities. This symposium will focus on location variations in resource availability, and unique interventions to promote aging in place. In the first and second presentations, researchers report on differences in current service utilization, satisfaction, and perceived future service needs/use among rural versus frontier-residing older adults in Wyoming, and in Nigeria, respectively. In the third presentation, successes and failures of an interdisciplinary approach to increase Alzheimer's disease and related dementias (ADRD) diagnosis/treatment rates are shared. The fourth group reports the impact of an intergenerational RCT intervention of training older adults in computer literacy and online chair yoga engagement, for decreasing pain levels and cognitive risk. This is a Rural Aging Interest Group Sponsored Symposium.

Abstract citation ID: igad104.0517

**CURRENT AND FUTURE SERVICE USE, SATISFACTION, AND NEED FOR SERVICES AS DEFINED BY RURAL AND FRONTIER OLDER ADULTS**

Nancy Karlin, *University of Northern Colorado, Greeley, Colorado, United States*

This study focused on differences for current service utilization and satisfaction, along with perceived future service use and need for rural versus frontier residing older adults. A sample of older adults (age 65+), stratified using United States government and State of Wyoming definitions for rural (n=70) and frontier (n=72) counties, were interviewed. Frontier respondents reported less education $F(1, 141) = 10.19$, $p = .002$, $\eta 2 = .068$, monthly income $F(1, 141) = 17.03$, $p = .001$, $\eta 2 = .108$ with fewer financial resources $F(1, 141) = 10.32$, $p = .002$, $\eta 2 = .069$, experience with technology $F(1, 140) = 4.53$, $p = .035$, $\eta 2 = .032$, and potential for telehealth $F(1, 140) = 19.27$, $p < .001$, $\eta 2 = .112$, along with fewer computers $F(1, 141) = 11.08$, $p < .001$, $\eta 2 = .084$, and less access to the internet $F(1, 141) = 10.19$, $p = .001$, $\eta 2 = .073$. Differences were evident for recent use of senior nutrition programs and meal sites, dental care, and senior centers along with future use of senior nutrition programs and meal site location, food related services, hearing and vision clinics, and housing needs. Policy concerns have been identified for the funding of future senior housing, medical care, transportation, and food support in frontier and rural areas.

Abstract citation ID: igad104.0518

### BUILDING BRIDGES BETWEEN RURAL PROVIDERS AND RACIALLY/ETHNICALLY DIVERSE RETIRED FLORIDA FARM WORKERS

Lisa Ann Wiese[1], Magdalena Tolea[2], and Joanne Pulido[3], *1. Florida Atlantic University, Boca Raton, Florida, United States*, *2. University of Miami Miller School of Medicine, Miami, Florida, United States*, *3. Palm Beach State College, Lake Worth, Florida, United States*

We report results from a consortium-led randomized controlled clinical trial to optimize rural community health through interdisciplinary detection and care (ORCHID), funded by the Florida Department of Health/Moore Alzheimer's Disease Research Foundation. This project involving providers, nursing students, office staff, community residents and caregivers, targets a health professional shortage area with a 39% poverty rate. Both the control group (56% non-White, n=11) and intervention group ((93% non-White, n=9) received training on screening tools and updated ADRD information using the Florida Department of Health ADRD Resource Guide. The intervention group is participating in an additional four hours of evening online ADRD diagnosis/treatment training geared towards primary care clinicians, available at no-cost through Washington University. Both groups are being offered in-depth cognitive assessments by Adult Gerontological Nurse Practitioners (AGNP) during home visits. Connections to under-utilized local resources are being facilitated by local nursing students. Initial results include that although pre-post total Alzheimer's Disease Knowledge Scores (Carpenter et al., 2009) correlated strongly ($r = .93$; $p = .003$), there was no significant difference in knowledge ($p = .07$) in the control group. There was also no significant increase in ADRD diagnostic rates, and AGNP visits were not requested. Instead, the control group providers report the routine practice of conducting annual cognitive screening in persons over age 65, and referring patients as needed to the nearest neurology providers 30 miles away, which residents do not visit. However, connections to local resources increased by 65%. New ADRD diagnosis/treatment rates in the intervention group will be available August, 2023.

Abstract citation ID: igad104.0519

### UNDERSTANDING THE CURRENT LOCATION DISPARITIES IN ADRD AND ITS DETERMINANTS IN NIGERIA

Oloaigbe (Joyce) Ogidigo[1], Okiemute Okpalefe[2], Kingsley Omeje[3], and Chioma Anosike[4], *1. University of Miami, Miami, Florida, United States*, *2. National Biotechnology Development Agency, Abuja, Federal Capital Territory, Nigeria*, *3. University of Nigeria, Nsukka, Enugu, Nigeria*, *4. University of Nigeria, Enugu, Enugu, Nigeria*

Alzheimer's disease and related dementias (ADRD) is a growing health challenge in low- and middle-income countries, particularly in sub-Saharan Africa. Evidence of geographical variation is associated with increased ADRD incidence, and current research suggests that early-life rural living further increases ADRD risk. However, the scarcity of studies conducted in resource-poor countries limits conclusions. Consequently, the awareness and response to ADRD have been limited. In Nigeria, several rural communities still associate dementia with the normal process of aging, with many patients stigmatized and abandoned in the belief that their condition is beyond any medical intervention. Thus, many of those affected delay seeking medical care and endure poor outcomes. This is exacerbated by the lack of mental health service access in rural areas. This geographic disparity and deeply rooted structural inequalities have posed additional challenges to adequately diagnose and provide care for individuals across the lifespan living in these settings. Hence, population-based data on ADRD incidence in Nigeria is required to estimate its true burden and inform the design of ADRD prevention and management strategies. We report our analysis of the current situation of ADRD in rural Nigeria while exploring future perspectives and directions. For example, heath policies to increase the ratio of mental health professionals per number of patients, mostly in rural areas, foment proactive primary care centers. Ultimately, understanding rural-urban variation in ADRD diagnostic incidence and prevalence will inform needed policies to improve timely management and access to supportive services for older adults in Nigerian and other under-resourced regions.

Abstract citation ID: igad104.0520

### MAINTAINING BRAIN HEALTH VIA TELEHEALTH-BASED CHAIR YOGA FOR RURAL UNDERSERVED OLDER ADULTS

JuYoung Park, Lisa Ann Wiese, and Janet Holt, *Florida Atlantic University, Boca Raton, Florida, United States*

We conducted a randomized control pilot trial in an underserved, racially/ethnically diverse community, to examine the efficacy of (1) computer literacy training provided by high school students meeting rural older adults in their homes, followed by 2) telehealth-based online chair yoga (OCY) or computer brain games (CBG). First, high school students engaged in a "train the trainer" computer skills course using a previously-tested curriculum designed for the target population. Students mentored the older adults in learning computer skills and to access either the OCY ($n = 15$) or CBG ($n=15$), which were randomly assigned. Outcomes measured at pre, post, and three months following the 12-week intervention included computer proficiency, cognitive function, pain levels, and psychosocial well-being. In a linear mixed growth model with random intercepts, there was a significant linear trend in computer-based competency, $t(50, 19) = 2.56$, $p = .013$. Computer proficiency (computer basics, Internet and email use, communication, and calendaring) increased significantly in both the OCY and CBG groups. Importantly, there was a significant linear change in pain by group, controlling for age and living alone, $F(1, 14) = 6.64$, $p = .022$, $\eta 2 = .32$ (large effect size). Chronic pain in the OCY group decreased significantly from baseline to 3-month follow-up (18.89 to 11.35) but increased in the CBG group (14.27 to 17.28). Increases in cognitive function increased pre/post t(29) = -2.98, p = .003 (one-tailed). These results are promising for older adults with limited exercise opportunities, who also face cognitive risk exacerbated by elevated pain levels.

### SESSION 2130 (SYMPOSIUM)

Abstract citation ID: igad104.0521

### EMBEDDING EVIDENCE-BASED CAREGIVER SUPPORT IN COMMUNITY-BASED PROGRAMS: THE ADS PLUS PROGRAM

Chair: Laura Gitlin Co-Chair: Joseph Gaugler Discussant: Kenneth Hepburn

Adult Day Services (ADS) are a community-based option in long-term care, providing a safe, interactive, and managed environment for people with dementia (PWD). ADS also serve racially/ethnically diverse communities, provide respite for family caregivers and help people age-in-place. Nevertheless, research reveals inconsistent benefits of ADS for caregivers of clients and ADS do not typically offer evidence-based caregiver programs as part of routine care. This symposium reports on the results of a novel NIA funded multi-site Hybrid Type I trial with pragmatic elements which evaluated the effectiveness of embedding a caregiver support program, ADS Plus, in ADS and delivered by staff. ADS Plus, provides caregivers with dementia education, stress reduction techniques, validation/support, referrals and linkages, and strategies to address caregiver-identified challenges over 12-months. Reported are the quantitative and cost outcomes, implementation processes, and challenges and lessons learned. Dr. Gitlin will present the main quantitative study outcomes of this multi-site trial involving 34 sites nationally and 234 caregivers, which demonstrated benefits for caregivers and ADS. Next, Dr. Marx will examine implementation findings including session completion and content. Dr. Gaugler will present findings from the qualitative arm examining perceived benefits of ADS Plus by staff and participants. Ms. Prioli will then examine cost savings associated with ADS Plus whereas Dr. Parker will address barriers when partnering with home and community-based services including developing and maintaining partnerships, institutional review board hurdles and staff readiness. To conclude, our discussant, Dr. Kenneth Hepburn, will offer perspectives on moving evidence-based programs into community-based settings such as ADS. This is a Behavioral Interventions for Older Adults Interest Group Sponsored Symposium.

Abstract citation ID: igad104.0522

### OUTCOMES OF THE ADULT DAY SERVICE PLUS (ADS PLUS) PROGRAM: A HYBRID TYPE I MULTISITE TRIAL DESIGN

Laura Gitlin[1], David Roth[2], Katherine Marx[2], Lauren Parker[2], Sokha Koeuth[1], Holly Dabelko-Schoeny[3], Keith Anderson[4], and Joseph Gaugler[5], *1. Drexel University, Philadelphia, Pennsylvania, United States*, *2. Johns Hopkins University, Baltimore, Maryland, United States*, *3. The Ohio State University, Columbus, Ohio, United States*, *4. University of Mississippi, Oxford, Mississippi, United States*, *5. University of Minnesota, Minneapolis, Minnesota, United States*

This paper presents the main outcomes of a cluster randomized hybrid trial (type I) involving 34 adult day services (ADS) in the US. Eighteen sites were randomized to control (ADS as usual); 16 sites provided ADS and caregiver support (ADS Plus). Trained staff met with caregivers onsite to provide dementia education, support/validation, referrals/linkages, and strategies for care challenges and self-care over 12-months. Main outcomes included depressive symptoms (CES-D) and well-being at 6-and 12-months, and attendance of clients (people living with dementia) over 12-months. Of 203 caregivers (Intervention=102; Control=101), 5.9% at 3-months, 12.8% at 6-months, and 22.7% at 12-months were lost-to-follow-up. Caregivers were predominantly female (80.3%), with 76.4% identifying as white/Caucasian, 14.8% Black/African American, and 12.3% Hispanic/Latino. Most (88.2%) had >college education and were 65.0 years old (SD=13.46). For those with 6-month data, 40.4% control and 40.2% ADS Plus caregivers had depressed symptoms (>16 CES-D) at baseline. By 6-months, 43.6% control versus 34.2% ADS Plus caregivers had >16 scores (odds ratio=0.38, p=0.072). By 12-months, after covariate adjustments, ADS Plus caregivers reported reduced total depression scores versus controls (p=0.013) and lower depressed affect scores (p=0.015). Of 18 sites providing 12-month client attendance data, nine intervention sites reported 126.05 days attended versus 78.49 days for nine control sites (p=0.079). Compared to ADS alone, by 12-months ADS Plus improved caregiver mood and increased ADS utilization by 60.6%. Results support ADS staff delivering evidence-based caregiver support to enhance benefits for clients, caregivers and ADS.

Abstract citation ID: igad104.0523

### FIDELITY AND UTILIZATION OF THE ADULT DAY SERVICES (ADS) PLUS PROGRAM

Katherine Marx[1], Laura Gitlin[2], David Roth[1], and Joseph Gaugler[3], *1. Johns Hopkins University, Baltimore, Maryland, United States*, *2. Drexel University, Philadelphia, Pennsylvania, United States*, *3. University of Minnesota, Minneapolis, Minnesota, United States*

The purpose of this study is to examine intervention processes including session completion and content of the ADS Plus program. Interventionists completed an investigator-developed delivery assessment form following completion of each ADS Plus session with participating caregivers. Additionally, information on care challenges addressed and strategies offered in the form of an “ADS Plus prescription were documented. A total of 102 caregivers took part in ADS Plus. There were 954 sessions conducted, an average of 9.4 sessions per caregiver (Range 0-22). The majority of sessions were completed face-to-face (56.8%), or by telephone (40.7%). The average time spent preparing for each session by staff was 15.5 minutes (range 0 -65). Top care challenges selected by caregivers to address in ADS were: (1) Taking care of self (n=34 caregivers), (2) Meaningful activities for the person living with dementia (n=17), and (3) Agitated behaviors (n=13). Most sessions included validation and support of caregivers (91.8%) and information on caregiver self-care (85.5%). Education (49.0%) and skill enhancement for challenge areas (50.1%) were provided in about half of the sessions. Only about a quarter of sessions, (23.9%) included provision of referrals and linkages. At follow-up caregivers reported that the care challenges they sought to address in ADS Plus had gotten better (54.4%) or was eliminated (26.3%). Caregivers of persons living with dementia who attend an ADS can be aided by the addition of the ADS Plus program to address targeted caregiving needs.

Abstract citation ID: igad104.0524

### DEMENTIA INTERVENTION STAFF AND CAREGIVER PERSPECTIVES ON THE DELIVERY OF ADS PLUS

Joseph Gaugler[1], Quinton Cotton[1], Elizabeth Albers[2], Stephanie N. Ingvalson[1], Emily Skalla[3], and Laura Gitlin[4], *1. University of Minnesota, Minneapolis, Minnesota, United States*, *2. University of Minnesota School of Public Health, Minneapolis, Minnesota, United States*, *3. University of Minnesota, Minnesota, Minnesota, United States*, *4. Drexel University, Philadelphia, Pennsylvania, United States*

Interventions targeting persons living with dementia (PLWDs) provide an optimal touchpoint in the service delivery continuum for addressing the growing unmet needs of unpaid/family dementia caregivers. Services such as Adult Day Services (ADS) that generally target PLWDs can be enhanced by embedding an intervention that specifically targets informal caregivers. In a trial evaluating the ADS Plus intervention, open-ended feedback from ADS intervention staff and participants provided insights to improve the administrative, programmatic, and user experience of ADS Plus. Drawing on individual qualitative interviews (n=24), we conducted a thematic analysis revealing insights into the critical implementation factors supporting the effective delivery of ADS Plus. Identified themes included: 1) the relational, or person-centered, nature of the ADS Plus program, 2) the role of learning throughout implementation, 3) the influence of administrative structure on program delivery, and 4) contributors to the acceptability of ADS Plus. Our findings suggest that caregivers draw on multiple supports but that individually tailored supports for dementia care management must address the emotional needs of caregivers through relational intervention models. The effective delivery of ADS Plus was facilitated by perceived intervention benefits to caregivers, robust yet reasonable training for intervention staff, and the opportunity for adaptability in program delivery.

Abstract citation ID: igad104.0525

### COST ANALYSIS OF THE ADULT DAY SERVICES PLUS TRIAL

Laura Pizzi[1], Katherine Prioli[2], Eric Jutkowitz[3], Alexa Molinari[4], and Laura Gitlin[5], *1. ISPOR - The Professional Society for Health Economics and Outcomes Research, Lawrenceville, New Jersey, United States*, *2. Rutgers University, Piscataway, New Jersey, United States*, *3. Brown University, Providence, Rhode Island, United States*, *4. Rutgers University, Picataway, New Jersey, United States*, *5. Drexel University, Philadelphia, Pennsylvania, United States*

We assessed costs associated with ADS Plus vs. control from payer and societal perspectives using trial data. Costs were valued in $US 2021 and included ADS Plus program delivery, direct healthcare utilization (HCU), formal care and social services (FCSS), and indirect caregiver time (CGT). Payer perspective analyses included HCU and FCSS costs, with and without ADS Plus program delivery costs; societal perspective included these plus CGT costs. Analysis consisted of the mean per-dyad difference in change for ADS Plus (n=102) vs. control (n=101) at 6 and 12-months. ADS Plus program delivery costs were $671. At 6-months, comparing ADS Plus to control, there were mean savings in HCU ($79), FCSS ($1,038), and CGT ($6,426). Payer perspective total savings for ADS Plus were $446 and $1,117 with and without ADS Plus program delivery respectively; societal perspective savings were $6,872 and $7,543, with vs. without AD Plus program delivery, respectively. At 12-months for ADS Plus vs. control, HCU savings were $1,686 and FCSS savings were $290. CGT was $76 higher for ADS Plus. Payer perspective savings were $1,305 and $1,976 with and without ADS Plus program delivery respectively; societal perspective savings were $1,228 with ADS Plus delivery and $1,899 without it. ADS Plus costs were lower than control thus representing potential savings from both the payer and societal perspectives. Savings were primarily driven by lower costs in healthcare utilization including inpatient hospitalizations, outpatient visits, and home health aide services. Though favorable, findings were not statistically significant and should be interpreted with caution.

Abstract citation ID: igad104.0526

### HIDDEN BARRIERS TO ENGAGING HOME- AND COMMUNITY-BASED SERVICES IN DEMENTIA CARE RESEARCH

Lauren Parker[1], Katherine Marx[1], Joseph Gaugler[2], and Laura Gitlin[3], *1. Johns Hopkins University, Baltimore, Maryland, United States*, *2. University of Minnesota, Minneapolis, Minnesota, United States*, *3. Drexel University, Philadelphia, Pennsylvania, United States*

The high prevalence of dementia in the United States has resulted in a reliance on home-and community-based services (HCBS) to assist in the provision of care for community-dwelling persons living with dementia. Partnering with HCBS organizations, like Adult Day Services (ADS), is beneficial to disseminating evidence-based dementia care interventions as it is an existing service that can be leveraged to foster relationships between research staff and clients. While benefits exist, challenges in identifying community-partner organization, ethical approval, and community-partner staffing can be unintended barriers. Using the ADS Plus Program as a case-study, this presentation will detail hidden barriers in engaging HCBS partnerships into dementia care research. Specifically, we will discuss barriers to 1) developing relationships to implement a multi-site national-trial, 2) high touch points necessary to engage sites from underrepresented populations, 3) institutional review board requirements (e.g. CiTi training, Federal Wide Assurance Forms), and 4) staff readiness to participate in research. We also offer recommendations based on lessons learned from the ADS Plus Program to enhance the pipeline from engagement with HCBS to participate in research to implementing an evidence-based interventions. Lessons learned from the ADS Plus Program demonstrates that changes are needed to improve the efficiency of the pipeline from community-based participation into dementia care research and implementation of evidence-based interventions.

### SESSION 2135 (SYMPOSIUM)

Abstract citation ID: igad104.0527

### HEARING AND VISION LOSS: EPIDEMIOLOGIC AND INTERVENTION IMPLICATIONS FOR SOCIAL ISOLATION, LONELINESS, AND MOOD DISORDERS

Chair: Alison Huang Co-Chair: Roger O'Sullivan
Discussant: James Lubben

This session will discuss hearing loss and vision impairment as modifiable risk factors for social isolation, loneliness, and mood disorders from both epidemiologic and intervention perspectives. Prevalence of hearing and vision loss is high; two-thirds of older adults have hearing loss and over one-quarter have vision impairment. Hearing loss and vision impairment have been linked to poor cognitive and physical health but remain understudied in the context of social and mental health in large, population-based studies. Research gaps remain in measurement (findings typically based on subjective assessments) and generalizability. This session will investigate associations between objectively measured hearing, vision, and dual sensory loss on social isolation and mood disorders using a sample nationally representative of older adults in the US. This session will also describe potential mechanistic and environmental factors, such as fatigue and exclusion of hearing loss and vision impairment in health care settings that may explain observed associations. Whether treatment of hearing loss and vision impairment is an effective intervention for improving social and mental health is unknown. We will conclude by presenting findings from a randomized controlled trial designed to test whether a hearing intervention consisting of provision of hearing aids and related technologies, counseling, and education could reduce cognitive decline in older adults with untreated hearing loss. We focus on the effect of hearing intervention on social isolation and loneliness, both pre-specified outcomes of this trial. This is a Loneliness and Social Isolation Interest Group Sponsored Symposium. This is a collaborative symposium between the Loneliness and Social Isolation and Sensory Health Interest Groups.

Abstract citation ID: igad104.0528

### VISION LOSS AND PSYCHOSOCIAL FUNCTIONING: WHAT'S THE CONNECTION?

Louay Almidani[1], Rhonda Miller[1], Varshini Varadaraj[2], Aleksandra Mihailovic[1], and Pradeep Ramulu[1], *1. Wilmer Eye Institute, Baltimore, Maryland, United States*, *2. Johns Hopkins University, Baltimore, Maryland, United States*

This study assessed the associations between vision impairment (VI) and psychological and social function in older adults. We conducted a cross-sectional analysis of the National Health and Aging Trends Study, a nationally representative sample of Medicare beneficiaries aged 65 years and older. VI was measured using categorical and continuous scales. Depression and anxiety symptoms were assessed via the Patient Health Questionnaire for Depression and Anxiety (PHQ-4) questionnaire. Social isolation and severe social isolation were defined based on responses to questions on living arrangement, frequency of communication with others, and participation in activities. Results showed that depressive symptoms, social isolation, and severe social isolation were more prevalent in adults with any objective VI measure compared to those without impairment. In adjusted models, objective vision measures were not associated with higher odds of depression or anxiety symptoms, though both outcomes were strongly associated with self-reported VI (OR: 1.82 [95% CI: 1.27 – 2.60], OR: 1.81 [95% CI: 1.30 – 2.53], respectively). In contrast, worse distance and near acuity (per 0.1 logMAR), and worse contrast sensitivity (per 0.1 logCS units) were associated with higher risk of being severely socially isolated (RRR: 3.79 [95% CI: 1.13 – 12.78], 3.26 [95% CI: 1.16 - 9.17], 2.82 [95% CI: 1.11 – 7.18], respectively), however, no associations were found with self-reported VI. This study demonstrates that worse vision is associated with symptoms of depression, anxiety, and social isolation among older US adults. Approaches are needed to enhance the psychological and social well-being of individuals with VI.

Abstract citation ID: igad104.0529

### CONCURRENT VISION AND HEARING IMPAIRMENTS, SOCIAL ISOLATION, DEPRESSION, AND ANXIETY: FINDINGS FROM THE NHATS

Shu Xu[1], Alison Huang[2], Yunshu Zhou[3], and Joshua Ehrlich[3], *1. University of Massachusetts, Boston, Boston, Massachusetts, United States*, *2. Johns Hopkins Bloomberg School of Public Health, Baltimore, Maryland, United States*, *3. University of Michigan, Ann Arbor, Michigan, United States*

Sensory impairments are contributors to poor health for older adults, and the likelihood of having more than one sensory impairment increases with age. There has been little research on population-level associations of visual and hearing function with social and mental health. This study examined the associations between objectively measured hearing and vision impairment, social isolation, depression, and anxiety among older US adults. Using nationally representative data from the 2021 National Health and Aging Trends Study, we conducted cross-sectional analysis among adults aged >70 (n=2,520). Visual impairment (VI) was defined as distance (<20/40), near (<20/40), and/or contrast sensitivity (>1SD below mean) impairment. Hearing impairment (HI) was defined as a better ear pure-tone average >25 decibel hearing level. Respondents with VI and HI were identified as having dual sensory impairment (DSI). Social isolation was assessed by living arrangement, core network size, religious attendance, and social participation. Depressive and anxiety symptoms were measured with the PHQ-2 and GAD-2, respectively. Weighted sample characteristics showed that 25.53% of older adults had DSI. Logistic regression analyses revealed that, compared to those without HI or VI, older adults with DSI were more likely to report social isolation (OR=1.71, 95% CI=1.12-2.60), have depression (OR=4.57, 95% CI=2.34-8.92), and anxiety (OR=2.65, 95% CI=1.36-5.17). Associations with DSI were stronger than for single impairments. All models controlled for age, gender, education, race/ethnicity, marital status, number of chronic conditions. Findings suggested that older adults experiencing DSI are at greater risk of having social isolation and emotional distress.

Abstract citation ID: igad104.0530

### HEARING LOSS AND FATIGUE IN MIDDLE-AGED AND OLDER ADULTS

Kening Jiang[1], Adam Spira[1], Frank Lin[2], Jennifer Deal[3], and Nicholas Reed[1], *1. Johns Hopkins Bloomberg School of Public Health, Baltimore, Maryland, United States*, *2. Johns Hopkins, Baltimore, Maryland, United States*, *3. Johns Hopkins University, Baltimore, Maryland, United States*

Fatigue is associated with worse cognitive and physical functioning, psychosocial wellbeing, and quality of life. Hearing loss may be a risk factor for fatigue, perhaps through greater cognitive demand to compensate for degraded auditory signal. However, prior studies are predominantly in audiology clinical samples, which may be oversampled for individuals experiencing hearing-loss-related fatigue. We conducted a cross-sectional analysis including 3,031 participants from the National Health and Nutrition Examination Survey (NHANES) 2015-18. Fatigue was based on self-reported frequency of feeling tired or having little energy over the past 2 weeks. Hearing loss was defined as better-ear 4-frequency (0.5, 1, 2, 4 kilohertz) pure-tone average >25 decibels hearing level. Multivariable-adjusted multinomial logistic regression was used to model the relative risk ratios (RRR) of reporting a higher vs. reference frequency category of fatigue associated with hearing loss, adjusting for age, sex, race/ethnicity, education, smoking, drinking, occupational and off-work noise exposure, body mass index, comorbidities, and depressive symptoms. In this nationally representative sample of middle-aged and older adults (40-80 years, 48% male, 10% Black), 713 (24%) had hearing loss. When compared to normal-hearing participants, hearing-loss participants were more likely to report fatigue for more than half the days (RRR=2.16, 95% confidence interval [CI]: 1.24, 3.75) and nearly every day (RRR=2.09, 95% CI: 1.24, 3.52) relative to not feeling fatigue at all. Hearing loss is associated with fatigue at the population level. Public health efforts to manage hearing loss might improve fatigue and associated downstream health outcomes.

Abstract citation ID: igad104.0531

### HEALTH CARE SETTINGS: A MISSED OPPORTUNITY TO ADDRESS HEARING LOSS

Margaret Wallhagen, *University of California, San Francisco, San Francisco, California, United States*

**Background:** Identifying hearing loss (HL) within the healthcare system is essential to mitigate its risk for associated negative psychosocial and health conditions, including isolation and depression yet is rarely assessed. This qualitative study sought to gain in-depth understandings of how HL affects communication regarding care preferences within the older adult-partner dyad in the context of chronic illness. Its premise was that older adults with HL may not be able to participate effectively in care discussions and are at risk of not receiving patient centered care. Method: Planned as in-person, pandemic restrictions mandated transition to zoom. 15 older adults (≥60) with HL and 13 partners participated. **Results:** One key story line evolving from the data is "Compulsory Self-Advocacy". Almost uniformly, participants reported that healthcare practitioners "Never Asked" about HL. During one-on-one communication, HL wasn't necessarily brought to the surface and often went unnoted because participants didn't see it as pertinent to issues discussed. Still, they were unsure the practitioner was aware of their HL. Rather, they asked that information be repeated or noted they had trouble hearing or understanding when they realized problems. Reporting their HL didn't generally result in on-going consideration of their specific communication needs. They had to self-advocate to obtain accurate information or bring their partner to appointments to help. Some commented on the value of policies mandating access to notes or written reports. **Conclusions:** Healthcare settings provide unique opportunities to identify HL and initiate interventions that may mitigate its negative effects, including social isolation and loneliness.

Abstract citation ID: igad104.0532

### EFFECT OF HEARING INTERVENTION ON LONELINESS AND SOCIAL ISOLATION IN THE ACHIEVE STUDY

Alison Huang, *Johns Hopkins Bloomberg School of Public Health, Baltimore, Maryland, United States*

Social health interventions in older adults typically have small effect and limited large-scale implementation. Treating hearing loss is a potential intervention, but efficacy is unknown. This study presents baseline associations between hearing loss, loneliness, and social isolation as well as the 3-year effect of a hearing intervention on social health. The Aging and Cognitive Health Evaluation in Elders Study (ACHIEVE) randomized controlled trial (n=977) tests the effect of hearing intervention (provision of hearing aids/related technologies, counseling/education) vs. successful aging health education control intervention on loneliness (UCLA Loneliness Scale) and social network characteristics (Cohen Social Network Index) (pre-specified exploratory outcomes) among older adults with untreated hearing loss. Hearing loss was quantified by better ear, speech-frequency pure tone average (PTA). Speech-in-noise recognition and hearing-related quality of life were also measured. Baseline associations between hearing loss and social health outcomes are assessed by robust Poisson regression adjusted for demographic and health characteristics. Three-year hearing intervention effects are assessed by generalized linear mixed effects models. Participants were 70-84 years, 54% female, and 87% White. At baseline, better speech-in-noise recognition was associated with greater social network characteristics (e.g., larger social network size [IRR: 1.04, 95% CI: 1.00, 1.07]). Worse hearing-related quality of life was associated with higher prevalence of loneliness (PR: 1.15, 95% CI: 1.11, 1.20) and smaller social network size (IRR: 0.96, 95% CI: 0.93, 1.00). Hearing intervention effect results will be available prior to the conference (data collection ends early 2023). Hearing treatment could be an effective social health intervention with greater scalability.

### SESSION 2140 (AWARD LECTURE)

Abstract citation ID: igad104.0533

### THE MARGRET M. AND PAUL B. BALTES AWARD LECTURE AND PRESENTATION

Chair: Shannon Jarrott

The Margret M. and Paul B. Baltes Award Lecture will feature an address by the 2022 Baltes Award recipient, Eric S. Kim, PhD, of the University of British Columbia. This session will also include the presentation of the 2023 Margret M. and Paul B. Baltes Award to recipient Reuben Ng, PhD of the National University of Singapore. The Margret M. and Paul B. Baltes Foundation Award in Behavioral and Social Gerontology recognizes outstanding early-career contributions in behavioral and social gerontology. The award is generously funded by the Margret M. and Paul B. Baltes Foundation.

Abstract citation ID: igad104.0534

**PSYCHOLOGICAL WELL-BEING AND PHYSICAL HEALTH IN THE CONTEXT OF OUR RAPIDLY AGING WORLD**

Eric Kim, *University of British Columbia, Vancouver, British Columbia, Canada*

Our society is rapidly aging. Thus, citizens, researchers, and policymakers alike are seeking ways to re-engineer society to achieve the dual aim of helping our aging population age well and fostering new ways for older adults to deploy the abundance of strengths that accrue with age. Most public health, biomedical, and psychological efforts have focused on reducing harmful risk factors. This approach has contributed greatly to prevention and treatment programs. However, another approach to achieving this dual aim is to expand the focus and evaluate upstream dimensions of psychosocial well-being. This approach might help inform the multidisciplinary and multi-level response efforts we need. In this talk, I will describe a theoretical model, results from a series of studies evaluating associations between a sense of purpose with reduced risk of chronic conditions, and mechanistic biobehavioral processes underlying these associations. This work aims to provide new directions for building a science of resilience and providing new targets for multi-level interventions that we can weave into our daily lives via practices and systems.

### SESSION 2145 (SYMPOSIUM)

Abstract citation ID: igad104.0535

**HOSPITAL-IN-HOME: IMPLEMENTATIONS IN THE VA HEALTH CARE**

Chair: Jennifer Sullivan Co-Chair: Emily Franzosa
Discussant: Dayna Cooper

The Department of Veterans Affairs (VA) Hospital-In-Home (HIH) program delivers patient-centered, acute-level hospital care at home within a single-payer integrated health-care system. Compared to inpatient care, HIH has demonstrated improved patient safety, effectiveness, and patient and caregiver satisfaction. As of 2022, there were eleven HIH sites across the VA, evidence of modest adoption. This symposium presents findings from the first year of a 4-year project that aims to conduct an in-depth inquiry in to the most effective ways to implement, adapt and sustain HIH across the VA. We will present an overview of the VA HIH program, describe one HIH program in-depth, present a birds-eye-view of the national program, present comparisons of sites' implementations in terms of structure, patient characteristics and outcomes, and conclude with an implementation-science framework to examine program adoption, adaptations, successes and challenges. National leadership will provide perspectives on management, planning and policy of Hospital-In-Home.

Abstract citation ID: igad104.0536

**THE BOSTON HOSPITAL-IN-HOME PROGRAM—A LOCAL PERSPECTIVE**

Shivani Jindal, Brittany Pietruszka, Rachel Meears, and James Parris, *VA Boston Healthcare System, Boston, Massachusetts, United States*

The Hospital-in-Home (HIH) model provides patient centered care, may reduce complications and costs. VA Boston Healthcare System (VABHS) planned its HIH program in 2018 and enrolled its first patient in May 2019. HIH provided care to 45 patients in 2019 which grew to 185 in 2022. The team is comprised of 2 clinicians (a hospitalist physician and a physician assistant), a nurse manager, 5 registered nurses (RN), a social worker and a pharmacist. The program practices complementary HIH (admissions from inpatient care) and substitutive care (all other admissions). At this time, majority of the recruitment comes from the inpatient services, followed by heart failure and primary care clinics. The program catchment area is about 25 miles from the West Roxbury campus of VABHS. A hybrid visit approach is taken for clinician visits - initially, the veteran is seen in person or by video by the clinician on admission to the program. Subsequent visits are done with RN assessments in the home and the clinician conducting the visit by telephone or video. In FY 2021, conditions of patients treated by HIH include congestive heart failure (50%), chronic obstructive pulmonary disease (14%), wound care (6%), diabetes (2%) and other conditions (20%). In FY 2022, the average daily census was 15.9 veterans and average visits per day 4.8. The HIH program outcomes include reduction of bed days of acute care in the hospital and reduction in the use of unnecessary medications through deprescribing which may translate to cost avoidance for the medical center.

Abstract citation ID: igad104.0537

**THE DEPARTMENT OF VETERANS AFFAIRS HOSPITAL-IN-HOME PROGRAM: A BIRD'S EYE VIEW**

William Hung[1], Orna Intrator[2], Jennifer Sullivan[3], Rajesh Makineni[4], Jiejin Li[4], and Shubing Cai[5], *1. Icahn School of Medicine at Mount Sinai, New York City, New York, United States*, *2. Department of Veterans Affairs, Rochester, New York, United States*, *3. Center of Innovation in Long Term Services and Supports (LTSS COIN), Providence, Rhode Island, United States*, *4. Canandaigua VA Medical center, Canandaigua, New York, United States*, *5. University of Rochester Medical Center, Rochester, New York, United States*

The Department of Veterans Affairs (VA) Hospital-In-Home (HIH) program offers Veterans the option to receive intensive, institutional-level acute services in their homes. This study presents characteristics of Veterans across 11 HIH programs operating between fiscal years 2021 and 2022. VHA administrative data and Medicare claims were linked to identify HIH stays from HIH visits, extending a stay over time as long as visits were no more than 7 days apart, allowing for intervening ED visits. Two types of HIH stays were identified: complementary HIH (admissions from an acute inpatient stay) and substitutive HIH (admissions from all other locations). HIH patients' sociodemographic characteristics, health conditions, and risk factors are described as well as length of HIH stay (LOS) and discharge destination. We identified 2536 veterans (1,110 complementary and 1,426 substitutive) who had 3,599 HIH stays with 73.9% of Veterans having one stay. Compared to Veterans with complementary HIH, Veterans with substitutive stays were generally younger (67 vs. 72), more likely to be Black (20% vs. 12%), and less sick (probability of 1-year mortality 14% vs 24%). Heart failure, Chronic Obstructive Pulmonary Disease, and COVID were the most frequent admission conditions for both HIH types. Average HIH LOS was 17.3 and 25.8 days for substitutive and complementary models, respectively. About 4% of substitutive and 18% of complementary stays ended in inpatient admission. In the unique VA single-payer, integrated-care system implementations may vary and comparisons of patient characteristics and outcomes can provide the basis for future implementations.

Abstract citation ID: igad104.0538

### LOCAL ADAPTATIONS OF HIH: VARIATIONS IN STRUCTURE AND IMPLEMENTATION OF HIH MODEL ACROSS SIX VA PROGRAMS

Akanksha Samant[1], Sungjae Park[2], Emily Franzosa[3], Jennifer Sullivan[4], Porooshat Dadgostar[5], Derek Darcy[6], and William Hung[3], *1. James J. Peters VA Medical Center, Bronx, New York, United States*, *2. Icahn School of Medicine At Mount Sinai, New York City, New York, United States*, *3. Icahn School of Medicine at Mount Sinai, New York City, New York, United States*, *4. Center of Innovation in Long Term Services and Supports (LTSS COIN), Providence, Rhode Island, United States*, *5. University of Rochester Medical Center, Rochester, New York, United States*, *6. Canandaigua VA Medical center, Canandaigua, New York, United States*

A number of Department of Veterans Affairs (VA) medical centers have adopted the Hospital-In-Home (HIH) model of care to provide at home services to Veterans requiring acute-level hospital care. We aimed to identify similarities and variations in structure and implementation of the HIH model across VHA sites. Of 11 programs, six (55%) used a standardized presentation template to present their specific program characteristics, outcomes and goals at monthly national program meetings. Presentations were transcribed and abstracted into themes by two reviewers. Programs varied in their staffing, services provided, referral sources and clinical outcome metrics. While one site provided in person visits, 3 reported program adaptations such as virtual follow-up visits for COVID patients. Review of clinical outcome metrics also revealed substantial site to site variations including 30-day readmission rates, cost avoidance, bed days or emergency department visits. All sites offered complementary (from hospital) and substitutive (from other than hospital) HIH stays. Substitutive stays varied across referral sources admitting patients from emergency departments, outpatient clinics, home based primary care and inpatient settings. Additionally, variations in catchment area (driving distance), and staffing in the parent stations highlighted differing contexts of HIH programs. We hypothesize that variations among sites may exist due to factors such as the setting and unique needs of the population and facility. An in-depth understanding of the contextual factors that influence implementation can be achieved through interviews with key staff from each site. Future work will examine if such variations are related to program outcomes.

Abstract citation ID: igad104.0539

### VARIATIONS ACROSS 11 DEPARTMENT OF VETERANS AFFAIRS HOSPITAL-IN-HOME PROGRAMS IN 2021–2022

Orna Intrator[1], Shubing Cai[2], Jiejin Li[3], Rajesh Makineni[4], and William Hung[5], *1. Department of Veterans Affairs, Rochester, New York, United States*, *2. University of Rochester Medical Center, Rochester, New York, United States*, *3. Canandaigua VA Medical center, Canandaigua, New York, United States*, *4. University of Rochester, Rochester, New York, United States*, *5. Icahn School of Medicine at Mount Sinai, New York City, New York, United States*

The Department of Veterans Affairs' (VA) Hospital-In-Home (HIH) program provides Veterans with the option to receive acute level services in the comforts of their own homes. A total of 11 HIH programs operated between fiscal years 2021 and 2022. We examined the volume of the programs and average risk of one-year mortality (using the Care Assessment of Needs measure) among each program's participants and characteristics of HIH stays (length-of-stay [LOS] and discharge destination) across program sites and program types (complementary- admissions from hospital and substitutive- other admissions) using VA administrative data linked with Medicare claims. The number of patients served in a year varied between 36 and 680 with 6 programs having <200 patients. Substitutive HIH was the dominant HIH type in 8 programs (>50% of patients) while only one program had clearly more complementary HIH (87%) and only 9 programs had complementary HIH. Average length-of-stay (los) in HIH was 20.5 days (SD=24.4 days, median=13 days), with averages across programs ranging between 3 - 36 days, 6 programs with average los<13 days and 5 programs with average los >19 days. Predicted risk of death within one-year varied: among substitutive HIH, 7 programs' average risk was > 20% and 4 programs' average risk was <15%. Among complementary HIH only one program's risk was <15% and the rest was >20%. Substitutive HIH programs discharged 2-10% of Veterans to hospital, while complementary programs discharged 11-23% to hospital. Interviews with program staff will help elucidate reasons for differences and inform expected outcomes.

Abstract citation ID: igad104.0540

### TRANSLATING SCIENCE INTO PRACTICE: APPLYING IMPLEMENTATION SCIENCE TO STREAMLINE FUTURE HIH ROLL-OUT

Reza Yousefi Nooraie[1], Jennifer Sullivan[2], Lindsey Zimmerman[3], Marlena Shin[4], Emily Franzosa[5], Porooshat Dadgostar[6], Adele Levine[7], and Derek Darcy[8], *1. University of Rochester, Rochester, New York, United States*, *2. Center of Innovation in Long Term Services and*

*Supports (LTSS COIN), Providence, Rhode Island, United States*, *3. National Center for Posttraumatic Stress Disorder, Menlo Park, California, United States*, *4. VA Boston Healthcare System, Boston, Massachusetts, United States*, *5. Icahn School of Medicine at Mount Sinai, New York City, New York, United States*, *6. University of Rochester Medical Center, Rochester, New York, United States*, *7. Boston University School of Public Health, Boston, Massachusetts, United States*, *8. Canandaigua VA Medical Center, Canandaigua, New York, United States*

The delay between establishing evidence-based practice and translating effective practices is well documented. This presentation will focus on the application of implementation science principles to the Department of Veterans Affairs (VA) Hospital-In-Home program using a staged approach. The stages are: 1) assessing current program implementation, 2) measuring organizational readiness with new sites, 3) developing tools and resources to assist new HIH sites, and 4) working with new sites interested in adopting the HIH program using a participatory implementation planning process guided by system dynamics. In this session we will profile our use of theories, models and frameworks including the Consolidated Framework for Implementation Research (CFIR) and Reach Effectiveness Adoption Implementation Maintenance (REAIM) framework. The CFIR Expert Recommendation for Implementing Change (ERIC) Mapping Tool will be introduced as useful to identifying strategies to overcome barriers to implementation. We will apply the participatory approaches for implementation planning, including Implementation Mapping, development of Implementation Logic Models, and Group Model Building to plan for the implementation of HIH in prospective sites. Key implementation science concepts and techniques will be introduced, along with a description of how participatory approaches will facilitate informed planning and stakeholder engagement in future implementation, adaptation, and roll-out of an evidence-based systemic program. Key anticipated outputs include a framework for the core components of HIH intervention, a catalog of important determinants of HIH implementation success, a profile of existing strategies to enhance implementation and sustainability, and a roadmap for the participatory planning of HIH implementation in future contexts.

### SESSION 2150 (SYMPOSIUM)

Abstract citation ID: igad104.0541

### EXPANDING INSIGHTS INTO LATE-LIFE DISABILITY USING OBJECTIVE ASSESSMENTS IN NHATS

Chair: Jennifer Schrack Discussant: Elena Fazio

The National Health and Aging Trends Study (NHATS) is a NIA-funded national panel study that conducts annual in-home interviews with a representative sample of US Medicare beneficiaries ages 65 years and older. Designed as a platform for the scientific study of late life disability trends and trajectories, NHATS fosters research to guide efforts to maximize health, promote independent functioning, and enhance quality of life at older ages. NHATS has collected performance-based measures of physical function since its inception (2011). In 2021 NHATS responded to expanded interest in objective measures of sensory loss and physical activity, by adding measures of vision (visual acuity, contrast sensitivity), hearing (pure tone audiometry), and physical activity (accelerometry). This symposium will introduce these measures and demonstrate their scientific application to answer key research questions related to physical and cognitive health. Dr. Schrack will present an overview of the accelerometry data collection procedures, as well as results by key demographic and health-related features. Dr. Twardzik will examine the association between features of participants' social and physical environments and patterns of accelerometry-based physical activity. Dr. Martinez-Amezcua will examine the association between hearing and phenotypic frailty. Dr. Davoudi will present evidence on the links among sensory and motor function and risk of dementia. Finally, Dr. Wanigatunga will describe the association between measures of hand and leg strength and dementia incidence. Collectively, these presentations will highlight population-level associations of objective measures of health and function with physical and cognitive outcomes to advance the science of disability in older adults.

Abstract citation ID: igad104.0542

### ACCELEROMETER-DERIVED PHYSICAL ACTIVITY IN THE NATIONAL HEALTH AND AGING TRENDS STUDY

Jennifer Schrack[1], Talan Zhang[2], Amal Wanigatunga[3], Vadim Zipunnikov[4], and Vicki Freedman[5], *1. Johns Hopkins University, Baltimore, Maryland, United States*, *2. Johns Hopkins School of Medicine, Baltimore, Maryland, United States*, *3. Johns Hopkins School of Public Health, Baltimore, Maryland, United States*, *4. Johns hopkins Bloomberg School of Public Health, Baltimore, Maryland, United States*, *5. University of Michigan, Ann Arbor, Michigan, United States*

Physical activity (PA) is a modifiable risk factor for disability, chronic conditions, and functional outcomes with aging. Yet the amount of PA in which older adults engage is not well understood, as objective evidence is lacking, particularly among those aged ≥80 years. To address this gap, the National Health and Aging Trends Study (NHATS) implemented wrist-worn accelerometry (Actigraph Centrepoint Insight Watch) in a subset of Round 11 (2021) participants (N=747, 54% women, 40% aged≥80) using a seven-day 24-hour/day protocol. Mean daily PA (total activity counts/day) was examined in those with ≥3 valid wear days by health and demographic factors with analytic survey weights applied. Daily PA averaged 1.67M counts/day, was lower at older ages and varied widely across health and demographic factors, notably: women were more active than men (β=208K), married participants more active than unmarried (β=163K), those with impaired contrast vision were less active than those with normal vision (β=-231K), those with hearing loss were less active than those without hearing loss (β=-233K), and participants with possible or probable dementia were less active than those without dementia (β=-457K) (p<.001 for all). With regards to care and accommodations, those who reported getting help with mobility were less active than those who did not (β=-459K) and those residing in the community were more active than those in (non-nursing home) residential care (β=394K). These data present unique insights into factors that may shape daily

PA in older adults. Future work should examine longitudinal trends and potential interaction with the disablement process.

Abstract citation ID: igad104.0543

### FEATURES OF THE SOCIAL AND PHYSICAL ENVIRONMENT AND ACCELEROMETRY-BASED PHYSICAL ACTIVITY AMONG OLDER ADULTS

Erica Twardzik[1], Vicki Freedman[2], Pablo Martinez-Amezcua[1], and Jennifer Schrack[3], *1. Johns Hopkins Bloomberg School of Public Health, Baltimore, Maryland, United States*, *2. University of Michigan, Ann Arbor, Michigan, United States*, *3. Johns Hopkins University, Baltimore, Maryland, United States*

Social and physical environments shape physical activity (PA) opportunities among older adults. Whether environmental factors are associated with patterning of PA among older adults is less understood. We examined associations of the social and physical environment with accelerometry-based daily PA metrics among older adults. Cross-sectional data from 2021 National Health and Aging Trends Study respondents with non-missing data were included. Using linear regression models with analytic survey weights and adjusted for potential confounders we investigated the associations between four environmental features: social cohesion, neighborhood disorder, broken step into the home, and continuous sidewalks with PA patterns. Three accelerometry-based PA metrics were examined: total activity counts, PA fragmentation, and number of PA bouts. 633 older adults (45% female) were included in our analytic sample. Respondents with a broken step into their home, compared with those without, had lower total activity counts (♌=-170024.3, SE=81530.6, p<.05). Compared to those living in a neighborhood with no disorder, those living in a neighborhood with any disorder had more PA bouts per day (♌=10.5, SE=3.9, p<.01). All other contextual factors examined were not associated with activity counts, fragmentation, or bouts of PA. Greater physical barriers to access the neighborhood may explain why broken steps were associated with less total PA (volume and intensity). Neighborhood disorder was associated with number of PA bouts per day, which may be due to greater reliance on walking for transportation in disordered neighborhoods. Future work should investigate how the neighborhood environment is structured (e.g., walkability) to elucidate these findings.

Abstract citation ID: igad104.0544

### THE ASSOCIATION OF HEARING LOSS AND FRAILTY AMONG OLDER ADULTS IN THE US

Pablo Martinez Amezcua[1], Sahar Assi[2], Wuyang Zhang[2], and Nicholas Reed[1], *1. Johns Hopkins Bloomberg School of Public Health, Baltimore, Maryland, United States*, *2. Johns Hopkins Bloomberg School of Public Health, Epidemiology, Baltimore, Maryland, United States*

Frailty is common in older adults and increases the risk of functional limitations and death. Hearing loss affects ~2/3 of older adults in the U.S. and is associated with poorer physical and cognitive function and social isolation, which may increase the risk for frailty. We aimed to determine if hearing loss is associated with higher odds of being frail or pre-frail versus robust and if hearing aid use is protective. We analyzed data from 2,361 participants from the National Health and Aging Trends Study (NHTAS), a U.S. nationally representative sample of older adults, collected in 2021. Hearing was assessed by pure-tone audiometric testing, and we used a speech-frequency (0.5-4 kHz) better hearing ear's pure-tone average (BPTA) to categorize participants into no hearing loss (BPTA≤25 dB HL), mild (26-40), and moderate or greater (>40) hearing loss. Physical frailty was categorized according to the presence of the frailty phenotype components (unintentional weight loss, exhaustion, low physical activity, weakness, slow walking speed) into robust (0), pre-frail (1-2), and frail (≥3). In our study population (mean age=81, 56% female, 19% Black), there were 1,724 (73%) participants with hearing loss. Using multinomial logistic regression, we found that participants with moderate or greater hearing loss (vs. those without) had higher odds of being frail versus robust (OR=1.82, 95% CI 1.11, 2.99, p-trend=0.016). Hearing loss may be a risk factor for frailty. Future studies on the impact of hearing interventions in preventing and managing frailty will have significant public health and clinical importance.

Abstract citation ID: igad104.0545

### SENSORIMOTOR IMPAIRMENTS AND DEMENTIA: A CROSS-SECTIONAL ANALYSIS OF NATIONAL HEALTH AND AGING TRENDS STUDY

Anis Davoudi[1], Yuri Agrawal[2], Alden Gross[3], Amal Wanigatunga[4], Nicholas Reed[5], Joshua Ehrlich[6], and Jennifer Schrack[1], *1. Johns Hopkins University, Baltimore, Maryland, United States*, *2. Johns Hopkins School of Medicine, Baltimore, Maryland, United States*, *3. Johns Hopkins University Bloomberg School of Public Health, Baltimore, Maryland, United States*, *4. Johns Hopkins School of Public Health, Baltimore, Maryland, United States*, *5. Johns Hopkins Bloomberg School of Public Health, Baltimore, Maryland, United States*, *6. University of Michigan, Ann Arbor, Michigan, United States*

Sensory impairments in vision and hearing are linked to dementia in older adults. However, this association has not been investigated in conjunction with motor function performance. We examined cross-sectional sensory and motor function and dementia status data from 1,765 participants in the National Health and Aging Trends Study (NHATS R11, 2021) (32.7% aged ≥80 years; 55% women) who completed objective physical function and sensory testing. Motor function was defined using grip strength (kg), gait speed (m/s), standing balance, and chair stand speed (stands/sec). Sensory function was defined using hearing (pure tone average for better ear (BPTA) (dB)) and vision (contrast sensitivity (logCS)). Dementia was categorized as possible or probable using NHATS dementia classification. Survey-weighted logistic regression models estimated the likelihood of dementia by impairment in sensory and motor function, adjusted for demographic and medical conditions. 185 (10.5%) participants had possible or probable dementia. In models incorporating both motor and sensory impairments, impaired standing balance (<3 standing balance score in SPPB) and slow walking speed (<0.8m/s) were associated with higher odds of dementia (OR=1.75; 95%CI 1.10-2.79; and 2.39 95%CI 1.37-4.15, respectively), while hearing impairment (BPTA>25dB) and contrast sensitivity (<1.55logCS)

were not associated with higher prevalence of dementia. In this nationally representative sample of older US adults, results suggest motor function is more strongly associated with dementia relative to sensory function among participants able to complete physical function testing. Future research is needed to understand how the associations of sensory and motor function with dementia change from mid-to-late life.

Abstract citation ID: igad104.0546

### MUSCLE STRENGTH AND INCIDENT DEMENTIA IN THE NATIONAL HEALTH AND AGING TRENDS STUDY (NHATS)

Amal Wanigatunga[1], Yidan Lu[1], Francesca Marino[2], Anis Davoudi[3], Ryan Dougherty[3], and Jennifer Schrack[3], *1. Johns Hopkins School of Public Health, Baltimore, Maryland, United States*, *2. Johns Hopkins Bloomberg School of Public Health, Baltimore, Maryland, United States*, *3. Johns Hopkins University, Baltimore, Maryland, United States*

Slower gait speed is linked to dementia risk, but the role muscle strength plays in this relationship is unclear. We examined whether muscle strength measured in the hands and legs is related to dementia risk beyond usual gait speed. We included 3,212 NHATS participants (mean age 75 years, 54% women) followed from 2011-2019 without dementia at baseline. Muscle strength was measured by maximal grip strength (kg) and repeated chair stands (seconds). Incident dementia combined possible and probable dementia defined by a validated NHATS algorithm. Cox regression models were used to estimate longitudinal associations between each baseline muscle strength and incident dementia. Models were adjusted for demographics and medical conditions, and additionally for usual paced gait speed (m/s). Over an average of 5 (SD=3) years, there were 946 (30%) first-time dementia events. For 1 kg higher in grip strength, there was a 2% decrease in incident dementia (HR:0.98; 95%CI: 0.97-0.99;p<0.001), which remained significant after adjustment for gait speed (p=0.005). More time to complete chair stands was positively associated with dementia incidence (HR:1.02; 95%CI:1.01-1.03;p<0.001) but results were attenuated after adjusting for gait speed (p>0.10). All results remained robust after removing events that occurred 1 year from baseline. These findings suggest that grip strength, after accounting for gait speed, is associated with dementia incidence. These results suggest the muscle morphology differences between the hands and legs capture different neuromuscular mechanisms linked to dementia. Replication of these findings and further investigation into potential biological mechanisms are needed.

### SESSION 2155 (SYMPOSIUM)

Abstract citation ID: igad104.0547

### CORTICAL CONTROL OF MOBILITY: MOVING TOWARD CLINICAL OUTCOMES AND INTERVENTIONS

Chair: Andrea Rosso Co-Chair: Qu Tian Discussant: Stephen Kritchevsky

A three-year series of pre-conference workshops at GSA a decade ago brought together leading scientists in the multidisciplinary field of brain, aging, and mobility. Since then, the field has advanced the understanding of motor control as a predominantly subcortical phenomenon to the attentional and cortical control of mobility and has extended the view of cognitive and motor functions as distinct to there being shared causes of cognitive and mobility declines. Emerging studies are further elucidating mechanisms of motor control (Holtzer, Baillargeon), demonstrating relations with important clinical outcomes, including dementia (Tian) and community mobility (Rosso), and exploring novel interventions on clinical and neurophysiological outcomes (Kahya), as demonstrated by the series of talks in this symposium. Roee Holtzer assesses prefrontal cortex efficiency over repeated trials of attention-demanding dual-task walking in older adults with and without multiple sclerosis. Emma Baillargeon explores the heterogeneity of prefrontal responses to walking among older adults and describes the relation of prefrontal response with performance metrics. Qu Tian demonstrates trajectories of gait variability measures over time predict future dementia risk. Andrea Rosso investigates prefrontal activation as a biomarker of gait automaticity and its relation to community mobility. Finally, Melike Kahya demonstrates brain stimulation through transcranial direct current stimulation as a potential intervention to improve standing balance during cognitively challenging tasks and examines the mechanisms of its action through dual-task cost and electroencephalograph (EEG) recordings. Together, these studies will provide an overview of the current understanding of cortical control of mobility and its implications for clinical outcomes and interventions.

Abstract citation ID: igad104.0548

### GAIT AUTOMATICITY AND COMMUNITY MOBILITY OF OLDER ADULTS

Andrea Rosso[1], Emma Baillargeon[1], Kyle Moored[2], Breanna Crane[2], Michelle Carlson[2], Theodore Huppert[1], Jennifer Brach[1], and Caterina Rosano[1], *1. University of Pittsburgh, Pittsburgh, Pennsylvania, United States*, *2. Johns Hopkins Bloomberg School of Public Health, Baltimore, Maryland, United States*

Gait automaticity may decline with age-related impairments in the brain. As subcortical structures supporting gait automaticity decline, the prefrontal cortex (PFC) may provide compensatory function, allowing for maintenance of mobility but with loss of efficiency. Loss of efficiency may reduce ability to navigate complex community environments and restrict community mobility. We assessed the relation between PFC activation during walking in the laboratory with self-reported and objectively measured community mobility in participants aged 65+ (n=42, mean age=76, 60% female) from a randomized trial of a physical activity intervention to improve walking speed. PFC activation was measured by functional near-infrared spectroscopy as change from quiet standing to usual pace walking. Community mobility was measured objectively by 7-day global positioning system recordings of spatial (standard deviation ellipse area (SDEa), maximum distance from home) and temporal (percent time out of home (pTOH)) characteristics. Additionally, step count was recorded from 7-day actigraphy and the Life-Space Assessment (LSA) assessed self-reported mobility. Among participants with complete data at baseline (n=27), higher PFC activation during walking was associated with smaller SDEa (beta=-0.93 (-1.72, -0.15)), less pTOH (beta=-0.45 (-0.78, -0.12)), and lower step count (beta=-346 (-575, -118)) which persisted after adjusting for age, gender, education, or gait speed. PFC activation was not associated with maximum distance or LSA. There were no significant associations at the post-intervention visits (n=39). Greater PFC activation, likely indicating reduced gait automaticity, is related to lower spatial extent, duration, and intensity of community mobility. This association may be mitigated by participation in physical activity interventions.

Abstract citation ID: igad104.0549

### INCREASED TEMPORAL GAIT VARIABILITY OVER TIME IS RELATED TO FUTURE DEMENTIA

Qu Tian, Susan Resnick, Eleanor Simonsick, and Luigi Ferrucci, *National Institute on Aging, National Institutes of Health, Baltimore, Maryland, United States*

Age-related brain changes affect the timing and coordination of gait. High gait variability may stem from impaired brain motor control and has been associated with compromised cognition and cognitive impairment. Whether increased gait variability over time is associated with future MCI or dementia is unknown. In the Baltimore Longitudinal Study of Aging, we used age-, sex-, and race-adjusted linear mixed-effects models to examine whether changes in gait variability were associated with future MCI or dementia in participants aged 65+. Gait characteristics were quantified by 3D motion analysis. Independent of baseline gait speed and compared to cognitively normal participants (n=387), those who developed dementia (n=24) had greater rates of increase in gait cycle time variability, stance time variability, and swing time variability (but not mean performance) before the symptom onset (all p<0.05). Further adjustment for concurrent assessment of visuospatial ability or manual dexterity attenuated these associations. Adjustments for verbal memory, psychomotor speed, attention, or executive function did not attenuate these associations. Rates of changes in these gait variability measures were not statistically different between cognitively normal participants (n=387) and those who developed MCI (n=41). Baseline gait variability measures were not different across groups. These findings suggest that longitudinal trajectories of gait variability, not one-time assessment, provide the additional predictive value of future dementia over and beyond gait speed. Mechanisms may include compromised visuospatial ability or manual dexterity. Future neuroimaging studies are warranted to understand neural substrates connecting increased gait variability and dementia risk.

Abstract citation ID: igad104.0550

### PRACTICE EFFECTS ON BRAIN EFFICIENCY OF WALKING IN AGING AND NEUROLOGICAL DISEASE

Roee Holtzer[1], and Meltem Izzetoglu[2], *1. Yeshiva University, New York City, New York, United States*, *2. Villanova University, Villanova, Pennsylvania, United States*

Evidence provides support for using functional near-infrared spectroscopy (fNIRS) to quantify brain activations, notably in the pre-frontal cortex (PFC), under different walking conditions. Dual-tasks that involve walking impose additional demands on attention resources relative to the single tasks, and Studies using fNIRS revealed increased HbO2 in the PFC in Dual-Task-Walk (DTW) compared to Single-Task-Walk (STW) conditions in several populations. Burst measurement within session, which involves repeated administration of the same task, affords evaluation of short-term practice effects on changes in walking performance and the efficiency of its underlying neural activation. Here, we examined the effects of task (STW vs. DTW) and burst measurement (three counter-balanced repeated trials of each task condition) on walking performance and fNIRS-derived HbO2 in the PFC in older adults with multiple sclerosis (n=80, %female=65%, mean age=64ys) and controls (n=79, %female=65, mean age=69ys). Participants were enrolled in an ongoing cohort study designed to determine cognitive and brain predictors of locomotion in older adults with MS. Results from linear mixed effects models (LMEMs) revealed: 1) higher fNIRS-derived HbO2 in DTW compared to STW (p<.01). 2) fNIRS-derived HbO2 levels declined over repeated trials, notably in DTW (p<.01). 3) gait velocity was slower in DTW compared to STW (p<.01). 4) gait velocity improved over repeated trials (p<.01). The combination of decline in fNIRS-derived HbO2 levels and faster walking over repeated trials under DTW suggests more efficient usage of brain resources to support attention-demanding locomotion in aging and neurological disease.

Abstract citation ID: igad104.0551

### HETEROGENEITY OF PREFRONTAL CORTICAL ACTIVITY DURING WALKING AMONG OLDER ADULTS

Emma Baillargeon, Theodore Huppert, Caterina Rosano, and Andrea Rosso, *University of Pittsburgh, Pittsburgh, Pennsylvania, United States*

Higher prefrontal cortical (PFC) activity during simple walking may indicate neural compensation for impaired gait automaticity. On average, older adults have higher PFC activity during walking than younger adults, but the heterogeneity amongst older adults is understudied. We examined PFC activity during simple and dual-task walking in adults aged 65+ (n=173, mean age=72, 62% female). Participants walked quietly and while reciting every-other-letter of the alphabet (dual-task; 4 repsx15 meters). Change in PFC activity from standing was estimated using functional near-infrared spectroscopy. We grouped participants with significantly increased, significantly decreased, or no change in PFC activity from standing to simple walking (t-test, alpha=0.05). We tested for group differences in PFC activity change from simple to dual-task walking, task performance (gait speed, rate of correct letters), age, and cognitive function (trail-making A/B). PFC activity increased from standing to walking in 52 (30%), decreased in 21 (12%), and did not change in 100 (58%) participants. Those who reduced PFC activity from standing to simple walking (vs no change or increased, respectively) walked faster (mean+/-SD=1.12+/-0.17 vs 1.00+/-0.17 0.99+/-0.18m/s; both p=0.014) and had a greater increase in PFC activity from simple to dual-task walking (t-stat=1.96+/-2.2 vs 0.49+/-1.53 vs -0.26+/-1.43; p=0.008, p=0.0006). Those who increased PFC activity from standing to simple walking had a smaller PFC change (vs no change) from simple to dual-task walking (p=0.005). Age, alphabet performance, and cognitive function did not differ across groups. Differences in PFC activity during simple walking may contribute to differences in task performance and capacity for walking challenges.

Abstract citation ID: igad104.0552

**TRANSCRANIAL DIRECT CURRENT STIMULATION MODULATES BRAIN ACTIVITY DURING DUAL-TASK STANDING IN OLDER ADULTS**

Melike Kahya[1], Natalia Gouskova[2], On-Yee Lo[3], Junhong Zhou[4], Davide Cappon[1], Alvaro Pascual-Leone[2], Lewis Lipsitz[5], and Brad Manor[6], *1. Marcus Institute for Aging Research, Boston, Massachusetts, United States*, *2. Hebrew SeniorLife, Roslindale, Massachusetts, United States*, *3. Hebrew SeniorLife/ Harvard Medical School, Boston, Massachusetts, United States*, *4. Harvard Medical School/Hebrew SeniorLife, Roslindale, Massachusetts, United States*, *5. Hebrew SeniorLife, Boston, Massachusetts, United States*, *6. Hebrew SeniorLife/Harvard Medical School, Amherst, Massachusetts, United States*

In older adults, performing an attention-demanding cognitive task while standing comes with a 'cost' (i.e., decrement) to postural control. Transcranial direct current stimulation (tDCS) designed to facilitate the excitability of the left dorsal-lateral prefrontal cortex (L-DLPFC) and motor cortex (M1) mitigates this dual-task cost (DTC). The effects of tDCS on underlying brain activity measured by electroencephalography (EEG) during dual-tasking, however, is unknown. We hypothesized that tDCS would decrease the DTC of EEG-measured alpha and gamma power, as these frequencies have been linked to attentional processing and their attenuation is believed to reflect more efficient and/or effective postural control. Thirty participants received 20-minutes of tDCS targeting the L-DLPFC and M1, or sham (i.e., control) stimulation, in random order, on two separate visits. Before and immediately after each stimulation, EEG and postural sway area were recorded as participants completed trials of standing with and without verbalized serial subtractions. EEG alpha and gamma power were derived from channels corresponding to the L-DLPFC and M1 areas. The DTC (absolute change from single to dual-tasking) for both EEG and sway outcomes were calculated. tDCS, compared to sham, decreased the DTC to gamma power ($p=0.04$) and sway area ($p=0.04$). No pre-to-post stimulation changes were observed for alpha power ($p=0.42$). Participants who demonstrated a greater decrease in the DTC to gamma power after tDCS exhibited a greater reduction (improvement) in the DTC to sway area ($r=0.66$, $p=0.002$). The results suggest that the tDCS-induced changes in gamma-frequency activity were associated with improved dual-task standing balance in older adults.

### SESSION 2160 (PAPER)

### WORKFORCE (SRPP PAPERS)

Abstract citation ID: igad104.0553

**A COMMUNITY-DRIVEN INITIATIVE TO ENGAGE EMPLOYERS AS ADVOCATES TO SUPPORT WORKING CAREGIVERS**

Kylie Meyer[1], Morgan Zachmeyer[2], Jane Paccione[3], Christina Smith[2], Cynthia Cardenas[4], Dianne Teran[5], and Carol Zernial[2], *1. Case Western Reserve University, Cleveland, Ohio, United States*, *2. WellMed Charitable Foundation, San Antonio, Texas, United States*, *3. San Antonio Area Foundation, San Antonio, Texas, United States*, *4. Good Samaritan Community Services, San Antonio, Texas, United States*, *5. Alzheimer's Association, San Antonio, Texas, United States*

Approximately 62% of family caregivers to older adults living with a chronic illness or disability are employed. Nearly 6 in 10 caregivers report that caregiving has affected their employment in the last year, such as by requiring them to turn down a promotion, reduce work hours, or leave the workforce. Loss of employment income affects caregivers' immediate and long-term financial well-being. To help family caregivers balance employment and caregiving and promote their long-term financial security, in 2021, the Successfully Living and Aging in San Antonio Caregiver/Socialization Workgroup developed a 5-part discussion series to encourage local employers to consider how to better support employees who are caregivers. The series was attended by 75 individuals representing 34 organizations. During initial discussions, attendees identified the importance of institutional culture in affecting workplace policies around caregiving. To help promote supportive workplace cultures, in the third discussion session, participants used a modified nominal group technique to co-develop a 10-point pledge that employers can use support employees who are caregivers. In this presentation, the authors will describe the development of the pledge. Further, we contextualize this initiative in terms of the Shilton Model of Policy Advocacy. By integrating employers into discussions about supporting employees who are caregivers, this local initiative provides an example of how communities can incorporate employers as stakeholder advocates to support policies to help family caregivers remain in the workplace. This work is particularly valuable to advance policy recommendations to promote caregiver financial well-being within the 2022 National Strategy to Support Family Caregivers.

Abstract citation ID: igad104.0554

**HOW WILL AGE INCLUSIVITY SURVIVE IN AN ERA OF ANTI-DEI INITIATIVES?**

Dana Bradley[1], and Laura Allen[2], *1. UMBC, Baltimore, Maryland, United States*, *2. University of Maryland, Baltimore County, Baltimore, Maryland, United States*

For the first time, we have five different generations in the workplace in the United States. Generational diversity in the office is a trend that is not only expected to continue, but it is expected to grow. In that same vein, as companies continue to evolve in the way they work, it will be vital for organizations to understand, embrace and establish a diversity, equity, and inclusion (DEI) strategy in an ongoing effort to help employees of all ages feel like they are respected, valued, understood and belong in their workplace. Recent state court decisions and administrative decisions by governors suggest that DEI strategy implementations may be limited in some states. This paper explores the evolution of age inclusivity as part of DEI policies and practices. Grounded in the work of the United Nations Convention on the Rights of Older Persons, advocates have expanded their work to address ageism and ableism and ensure that society reflects persons'

lives as they age. We reviewed state-level court and administrative decisions regarding DEI initiatives across the US in 2021-23. While findings rarely note age, language in these decisions is written broadly so that outcome is unclear which subsequent court decisions. Our analysis concludes with "best practices" from the corporate sector and higher education, which emphasize systems where people on either end of the age spectrum have a more precise connection point to the decision-makers, each other, and conversations around the age at work. These strategies offer a cautious pathway forward in the US.

Abstract citation ID: igad104.0555

### OLDER ADULT RESEARCH SPECIALISTS SEEK ENCORE CAREERS PROMOTING THE INCLUSION OF OLDER ADULT PEERS IN CLINICAL TRIALS

Kathryn Nearing[1], and Jodi Waterhouse[2], *1. University of Colorado Anschutz Medical Campus, Denver, Colorado, United States*, *2. University of Colorado Anschutz Medical Campus Multi-disciplinary Center on Aging, Aurora, Colorado, United States*

The University of Colorado Multi-disciplinary Center on Aging trains and promotes the hiring of older adults from diverse backgrounds to pursue encore careers as Older Adult Research Specialists (OARS). Health navigation training is the foundation of these research navigators' unique preparation for paid positions that focus on recruiting and retaining older adult peers in clinical trials. Specifically, OARS' roles include: 1) developing effective communication and outreach strategies to engage older adult peers in research; 2) recruiting and consenting participants for clinical trials; 3) supporting retention by identifying barriers to study participation and connecting research participants with needed community-based resources/support; 4) educating research teams on key considerations for including older adults in research; and, 5) catalyzing research innovations to increase representation in, and the relevance and translational potential of, research for improved clinical and community-based practice. OARS, whose average age is 69 (range: 58-80), diversify our research workforce and enhance our capacity to include older adults -- an underrepresented population -- in clinical research. This initiative is advancing our Age-Friendly University work to promote encore careers, foster intergenerational learning, and catalyze research innovation. Ultimately, we aspire to increase the generalizability of research findings to improve healthcare and health outcomes for older adults by promoting the inclusion of older adults in our research workforce and in research studies to enhance the benefits to older adults of new drugs, devices and behavioral health interventions. During this session, we will share our training curriculum, how we created pathways to employment and our program outcomes.

Abstract citation ID: igad104.0556

### PATTERNS OF SELF-EMPLOYMENT AND HEALTH TRAJECTORIES IN LATER LIFE: EVIDENCE FROM CHINA

Yu-Chih Chen, *The University of Hong Kong, Hong Kong, Hong Kong*

Self-employment is a major form of work among older adults, yet most research has focused on its antecedents, with a focus on developed countries. Additionally, the impact of self-employment on health and well-being remains understudied. This study extends the current literature by investigating self-employment in China, a country with a unique self-employment landscape encompassing both agricultural and non-agricultural types. Using data from the 2011-2018 China Health and Retirement Longitudinal Study, this study investigated the impacts of self-employment on physical, mental, and cognitive health among 5,492 older adults. Latent class analysis was used to identify self-employment patterns. Multilevel models were employed to assess the associations between self-employment patterns and health outcomes, adjusting for baseline health conditions. Results showed that three patterns of self-employment were identified: consistently self-employed, wage worker, and switchers who transitioned between self-employment and wage work. Compared to wage workers, self-employed individuals and switchers have lower self-rated health and life satisfaction. No significant differences were found in depressive symptoms and cognitive scores. Notably, such effects were stronger for agriculture self-employment than the non-agriculture self-employment. The study highlights that most older Chinese are pushed into self-employment, which can adversely affect their health outcomes. Structural efforts to support older adults to stay engaged at work, such as age-friendly job redesign, anti-ageism measures in the workplace, or pension reform, would promote health and economic well-being among older adults in China.

Abstract citation ID: igad104.0557

### PSYCHOMETRIC PROPERTIES OF THE CHANGING TALK ONLINE TRAINING PROGRAM EVALUATION SCALE FOR LONG-TERM CARE STAFF

Frances Yang[1], Carissa Coleman[1], Clarissa Shaw[2], Yelena Perkhounkova[3], Maria Hein[4], and Kristine Williams[5], *1. University of Kansas, Kansas City, Kansas, United States*, *2. University of Iowa, Iowa City, Iowa, United States*, *3. University of Iowa, Iowa City, Iowa, United States*, *4. University of Iowa, College of Nursing, Iowa City, Iowa, United States*, *5. University of Kansas Medical Center, Kansas City, Kansas, United States*

The Changing Talk (CHAT) intervention educates long-term care staff about elderspeak and person-centered communication strategies. The online version of CHAT training is a national trial to reduce behavioral symptoms in long-term care residents with Alzheimer's Disease and other dementias. The development and validation of program evaluation scales are important for ensuring valid intervention evaluation. Using item response theory to assess the satisfaction of the CHATO program by staff, we tested for unidimensionality and local independence. Confirmatory factor analysis showed that there was redundancy between two items, based on the best fitting unidimensional model (Root Mean Square Error of Approximation=0.134, Comparative Fit Index=0.999). The IRT parameters showed that the highest discriminating item was the "The information in the course was clearly presented," which also showed the highest range of difficulty parameters for the response choices. Other items in the scale were: "This course enhanced my knowledge of the subject matter," "The information was a good refresher for me," "I can use this training in my job," "The resources listed in the course were useful for my job duties," "The video clips were useful in helping me learn course materials," "I would recommend this course to others," "The learning activities were useful in helping me learn course materials," and "I was satisfied with this course overall." Differential item functioning due to race/ethnic, language, and educational achievement were examined to test for the presence of measurement noninvariance. This program evaluation scale may be utilized in other pragmatic trials as a valid measurement tool.

### SESSION 2165 (SYMPOSIUM)

Abstract citation ID: igad104.0558

### POLICY SERIES: AGING AND HEALTH POLICY UPDATE: A VIEW FROM WASHINGTON

Chair: Brian Lindberg Discussant: Patricia D'Antonio

Leading aging and health policy advocates will present their findings and viewpoints regarding aging and health care policy during a divided Congress. Issues will include behavioral health, social isolation, health care workforce, telehealth, hospice policy, elder justice and nursing home care, home and community-based services, and Medicare and Medicaid. The group will discuss what progress has been made during the 118th Congress and what is still possible in the election year ahead.

### SESSION 2170 (PAPER)

### INTERVENTIONS IN FAMILY CAREGIVING

Abstract citation ID: igad104.0559

### A NOVEL POSTDISCHARGE TELEHEALTH INTERVENTION FOR STROKE CAREGIVERS: IMPLICATIONS FOR IMPLEMENTATION

Jennifer LeLaurin[1], Nathaniel Eliazar-Macke[2], I. Magaly Freytes[3], and Constance Uphold[4], *1. University of Florida College of Medicine, Gainesville, Florida, United States*, *2. U.S. Department of Veterans Affairs, Gainesville, Florida, United States*, *3. NF/SG VHS, Gainesville, Florida, United States*, *4. North Florida/South Georgia Veterans Health System, Gainesville, Florida, United States*

This study investigated the potential for implementation of the RESCUE stroke caregiver telehealth intervention by assessing acceptability, feasibility, and cost. Family caregivers (n=174) of Veteran stroke survivors from eight medical centers were randomized to usual care or intervention. The RN-led intervention involved discharge plan review, problem-solving training, education, and support delivered via one telephone session and eight asynchronous online messaging center sessions. Acceptability was assessed using an adapted Acceptability and Enactment Survey (n=42) and qualitative interviews with a subsample of caregivers (n=16). We used retention data to assess feasibility and calculated cost per participant. Findings indicate the intervention was acceptable to caregivers. Most intervention completers found the intervention very/extremely helpful (90.5%), very/extremely easy to use (78.4%), and resolved most/all caregiving problems (64.3%). Qualitative interviews showed caregivers valued the asynchronous delivery method, personalized nature of the intervention, and ability of the RN to answer medical questions and provide education. More intervention caregivers dropped out compared to usual care (50.0% vs.19.3%, p< 0.001). Intervention dropout was predicted by lower education (p=0.03) and less positive views of caregiving (p=0.01). Cost was $309.98 per intervention completer. Findings support intervention acceptability and highlight the value of RN-led interventions. The relatively low cost and fully remote nature of the intervention support its suitability for widespread implementation. Multiple delivery options and flexible intervention schedules may enhance engagement. The RESCUE intervention provides a model of a low-cost post-discharge telehealth intervention for stroke caregivers that can be adapted for other conditions. Future work should test methods to augment retention.

Abstract citation ID: igad104.0560

### DO PUBLIC HOME CARE CLIENTS "LOSE OUT ON SERVICES" BECAUSE THEY HAVE A FAMILY CAREGIVER?

Janice Keefe[1], Lori Mitchell[2], Jeff Poss[3], and Jasmine Mah[4], *1. Mount Saint Vincent University, Halifax, Nova Scotia, Canada*, *2. Shared Health Manitoba, Winnipeg, Manitoba, Canada*, *3. University of Waterloo, Waterloo, Ontario, Canada*, *4. Dalhousie University, Halifax, Nova Scotia, Can ada*

The public home care (PHC) system in Canada is heavily reliant on family caregivers to enable clients to stay in the community. When caregivers are present, there are concerns formal home care aide (HCA) services are less available. Using data from the Resident Assessment Instrument for Home Care (RAI-HC), we examined caregiver and client characteristics in relation to HCA service amounts. Our cohorts were individuals aged 60+ receiving an initial RAI-HC assessment in 2011-2013, and one subsequent assessment in two provincial jurisdictions: the Winnipeg Regional Health Authority (n=5,251) and Nova Scotia Health (n=5291). We measured weekly HCA hours and constructed a predictive model for hours of care controlling for client need. To identify the most underserved client population, we calculated the ratio between predicted to actual received hours of care ascertaining the lowest client quintile. In both jurisdictions, the odds of being underserviced were higher when the client co-resided or had a spouse. Clients living alone at initial intake were least likely to be underserved. Caregiver characteristics increasing the risk of being underserved in Winnipeg included providing IADLs/ADLs support, caring more than 14 hours per week and being unable to continue. Rurality increased risk of underservice in Nova Scotia. These findings contribute to the debate: is PHC a supplement or substitution for family care? PHC as a supplement to family care was more pronounced in Winnipeg and in rural Nova Scotia. Implications for greater equity across regions is explored using an Equity, Diversity and Inclusion lens.

Abstract citation ID: igad104.0561

### REDESIGNING HOSPITAL WORKFLOW TO INCLUDE FAMILY CAREGIVERS OF PEOPLE LIVING WITH DEMENTIA

Catherine Still[1], Andrea Yahr[1], Kathryn Istvanek[1], Andrea Strayer[2], Nicole Werner[3], and Beth Fields[1], *1. University of Wisconsin-Madison, Madison, Wisconsin, United States*, *2. University of Iowa, Iowa City, Iowa,*

*United States*, *3. Indiana University School of Public Health-Bloomington, Bloomington, Indiana, United States*

Family caregivers of hospitalized people living with dementia (PLWD) report feeling unprepared to fulfill caregiving tasks post hospitalization. Currently, clinicians lack guidance on how to include caregivers in hospital care. This descriptive study used a systems-engineering approach to identify optimal hospital workflows for caregiver inclusion. We conducted 28 hours of direct observations in a large academic hospital with field notes and audio-recorded three hours of interdisciplinary team debriefing meetings. Data analysis was guided by a work system model to map caregiver inclusion workflows using Lucidchart software and identify system barriers and facilitators to caregiver inclusion within each workflow. Results produced workflows of optimal practices for caregiver inclusion during admission, stay, and discharge. Workflow facilitators included healthcare clinicians' willingness/ability to answer questions, caregiver advocacy skills, and presence of medical record fields focused on patient/family education. Barriers included inconsistent documentation, under-utilization of hospital common spaces, and using a passive approach to caregiver inclusion. Comparison of current practices to desired workflows for caregiver hospital inclusion can provide clinicians guidance on how to identify, assess, and educate caregivers. Future research should determine workflow acceptability and effectiveness for improving caregiver preparedness.

Abstract citation ID: igad104.0562

### THE EFFECTS OF PARTNERS' HAPPINESS ON EACH OTHER: AN ACTOR-PARTNER INTERDEPENDENCE MODEL

Elnaz Abaei, and Peter Martin, *Iowa State University, Ames, Iowa, United States*

The study of happiness among older adults has attracted the attention of researchers and practitioners. Happiness is affected by multiple factors, including the relationship with a partner, as they spend more time together in later life. This research aims to evaluate an actor-partner interdependence model, assessing the association of respondent and spouse happiness affecting each other over time. A second aim is to examine the effect of age, gender, education, and whether parents are alive on the respondents' and their spouses' happiness. About 9,570 individuals over 50 years of age from the Health and Retirement Study (HRS) and their spouses were included. Respondents' and their spouses' happiness measured at two time points were included in the analysis, as were age, gender, education, and the parents' living status. Multiple regression analyses in SPSS were computed, and the results demonstrated that past happiness is directly associated with present happiness for both respondents and spouses. Respondents' happiness was also significantly associated with spouses' happiness, as spouses' happiness was associated with respondents' happiness. Furthermore, older age, male gender, and higher education were associated with happiness. Parents' living status was significantly related only to respondents' happiness at the first time point, but not to spouses' happiness. This study helps to understand an actor-partner interdependence model, which highlights partners' influences on each other's subjective well-being. Our exploratory study raises several opportunities for future research regarding the actor-partner interdependence model. More research will be necessary to find further variables affecting happiness among older adults.

### SESSION 2175 (SYMPOSIUM)

Abstract citation ID: igad104.0563

### THERAPEUTIC CANNABIS USE: UNIQUE CONTEXTS FOR OLDER ADULTS

Chair: Pinchas Cohen

Nationwide cannabis use prompts new questions about its therapeutic use. Self-administered cannabis among older adults demands careful study on their treatment outcomes and consumption patterns, in light of a more accessible, legalized cannabis market. Changing sentiments toward the illicit drug urges new research into its viability as a treatment for conditions in older adults. Ailshire uses the 2018-2021 Behavioral Risk Factor Surveillance System (BRFSS) surveys to compare cannabis use by age group. The data, which is representative of 39 U.S. states, indicate that medical marijuana use increases with age. Furthermore, Kaskie's Cannabis and Older Persons Study (COPS) uncovers the influence of age on cannabis use and motives for consumption. The study is grounded in three core objectives: distinguishing senior consumers from other age groups, distinguishing therapeutic and recreational use, and distinguishing life-time and naive cannabis consumers. With the support of the National Cancer Institute, Fahey conducts a cross-sectional study on consumption patterns of cancer patients over the age of 65, analyzing patient reported outcomes of symptom management through cannabis. Finally, Bluthenthal and Cohen perform quantitative, health surveys among 429 community-recruited people who inject drugs (PWID) to understand the potential benefit of a legal cannabis as a substitution for criminalized substances and alcohol. The papers in this symposium provide updated insights into widespread cannabis use among older adults. These findings may help forward conversations about the political and clinical changes that must be made in order to protect the therapeutic needs of older adults amidst a rapidly expanding cannabis industry.

Abstract citation ID: igad104.0564

### PATTERNS OF MARIJUANA USE IN THE US OLDER ADULT POPULATION

Jennifer Ailshire, *University of Southern California, Los Angeles, California, United States*

Marijuana use is increasing in the U.S. population, including among adults 50 and older, but research on population characteristics of marijuana use in middle aged and older adults is limited. We use data from the 2018-2021 Behavioral Risk Factor Surveillance System (BRFSS) surveys, which are representative of U.S. state populations, to estimate prevalence and frequency of marijuana use by age. The data are from 39 states that included the module on marijuana use in the past 30 days (n=466,469). We also examine age patterns in use by legalization status and reasons for use of marijuana. Reported marijuana use is much lower among those ages 50 and older (6.49%) than 18-49 (16.3%). Multivariable regression analyses show that marijuana use declines with age ($p<.001$) and is lower among women ($p<.001$). Marijuana use among older adults is slightly lower in states where it is illegal compared to states that have legalized (4.6% vs. 7.1%, p<.001). Use of marijuana for medical reasons increases with age; nearly two-thirds of adults 80+ who use marijuana report doing so for medical reasons. Adults 80+ also report the highest frequency of use, likely reflecting greater medical need. The most common conditions reported among adults 50+ who use marijuana for medical reasons are arthritis (61%), depression (36%), asthma (20%), and COPD (18%). Our prevalence estimates of use at older ages match those from other national surveys, but our findings suggest older adults are using marijuana largely for medical reasons.

Abstract citation ID: igad104.0565

### CANNABIS USE AMONG OLDER ADULT CANCER PATIENTS: PATTERNS OF USE AND PERCEIVED SYMPTOM MANAGEMENT

Margaret Fahey, *Medical University of South Carolina, Charleston, South Carolina, United States*

Prevalence of cannabis use among U.S. older adults (>65 years) is increasing and is common among cancer survivors of all ages. This National Cancer Institute-funded supplement supported administration of a cross-sectional survey in a state with illegal access to cannabis. This study descriptively examined weighted estimates of cannabis rates (lifetime, post-diagnosis, current) among older patients (>65 years) (N=524; 51% of full sample) with a cancer diagnosis (< two years). Of those endorsing post-diagnosis use, weighted estimates examined patterns of use (timing relative to cancer treatment, reasons for use, primary mode of use) and self-reported impact of cannabis on symptoms (since diagnosis; 5-point Likert scale for 9 symptoms). Almost half (46.0%) of older patients used cannabis in their lifetime, with 18.2% using post diagnosis and 9.8% currently using. Of patients who used cannabis following diagnosis (N=107), approximately half used during cancer treatment (7.0% had not yet started) and most common mode of use was smoking (e.g., joint) followed by edible use. Most common reasons were for pain (44.3%), sleep (43.4%) and recreationally (38.1%). Most common symptoms patients endorsed improved quite a bit, included pain (27.7%), difficulty sleeping (27.3%), and stress, anxiety, depression (23.5%). Overall, few believed cannabis worsened symptoms, with most indicating either improvement or no change. Findings indicate older oncology patients commonly use cannabis to alleviate medical or health symptoms. Oncology care teams require evidence-based recommendations and guidance to inform conversations and shared decision making about cannabis with their older patients, even within a state without a legal cannabis marketplace.

Abstract citation ID: igad104.0566

### CANNABIS USE TRENDS AND INTENTIONS AMONG OLDER PEOPLE WHO INJECT DRUGS IN LOS ANGELES, CA, AND DENVER, CO, 2021–2022

Ricky Bluthenthal[1], and Jonathan Cohen[2], *1. Keck School of Medicine, University of Southern California, Los Angeles, California, United States*, *2. University of Southern California, Los Angeles, California, United States*

One potential benefit of drug legalization is that it might lead to substitution of legal products for criminalized substances. This is particularly true in the case of legal cannabis use by the subpopulation of people who inject drugs (PWID) and thus face substantial risk of physical harm, infectious diseases transmission, and even death especially in criminalized environments. Should substitution occur among older PWID, where other comorbid conditions are more common, the benefits could be even greater. To describe substance use patterns among older people who inject drugs and to examine if older PWID are more likely to use cannabis as a substitute for other substances. We analyze data from quantitative, health surveys collected from 429 community-recruited PWID during 2021/22. Descriptive statistics were used to characterize the drug use patterns among older (50 years of age or more) PWID and multivariate regression to examine if older participants were more likely to substitute cannabis for any other substance.

**Results:** Among the 429 participants, 110 (or 26%) were 50 years of age or older. Older participants reported being significantly less likely than younger participants to use of methamphetamine, fentanyl and cannabis. In terms of cannabis substitution, older participants were significantly more likely to report using cannabis to reduce alcohol use. Risk and benefits of cannabis use vary among subpopulations. Older PWID might benefit from improved access to cannabis when trying to reduce alcohol use. Given the negative consequences of alcohol use, cannabis substitution might have additional benefits for this subpopulation.

Abstract citation ID: igad104.0567

### THE CANNABIS AND OLDER PERSONS STUDY: WHAT WE HAVE LEARNED

Brian Kaskie, *College of Public Health, University of Iowa, Iowa City, Iowa, United States*

The Cannabis and Older Persons Study has examined the increasing use of cannabis among Americans over 60 years old since 2016 reflecting a variety of disciplinary perspectives and methodological approaches. The project team has published a dozen peer reviewed manuscripts and has secured competitive funding swards in California, Colorado, Illinois and Iowa. In this symposium, results from multiple COPS surveys collected from older adults are reviewed with three goals in mind. The first is to demonstrate how older cannabis users are not comparable to younger cannabis users in terms of motives and self-reported outcomes of use. The second is to distinguish therapeutic from recreational cannabis use among older persons by examining survey answers provided by persons with arthritis, cancer, caregivers of persons with Alzheimer's disease, multiple sclerosis and terminal illnesses. The third goal is to differentiate life-time from naïve cannabis users and consider how cannabis may serve as an opioid avoidance or harm reduction strategy. Discussion considers the critical role policy makers, program administrators and clinicians assume in facilitating or hindering cannabis use, and directions for future research.

### SESSION 2180 (PAPER)

### SOCIAL DETERMINANTS, DISPARITIES, AND HEALTH EQUITY IN DEMENTIA

Abstract citation ID: igad104.0568

### ASSOCIATIONS BETWEEN CLINICAL FUNCTIONING AND AD BIOMARKERS AMONG HISPANIC AND WHITE NON-HISPANIC OLDER ADULTS

Miriam Rodriguez[1], Lisandra Mendoza[2], Patricia Garcia[3], Andres Duarte[4], Dilianna Padron[5], Michael Marsiske[6], Jacob Fiala[6], and Ranjan Duara[7], *1. Indiana University Bloomington, Bloomington, Indiana, United States*, *2. Bay Pines VA Health System, Bay Pines, Florida, United States*, *3. Indiana University School of Medicine, Indianapolis, Indiana, United States*, *4. Albizu University, Doral, Florida, United States*, *5. Central Virginia VA Healthcare System, Richmond, Virginia, United States*, *6. University of Florida, Gainesville, Florida, United States*, *7. Mount Sinai Medical Center, Miami Beach, Miami Beach, Florida, United States*

**Objectives:** Hispanics are 1.5x more likely to develop Alzheimer's disease (AD) when compared to White non-Hispanics (WNHs). There is also evidence to support that cognitive performance disproportionately reflects neuropathology among Hispanics and that functional decline is concurrent with the accumulation of AD biomarkers. The current study aimed to examine relationships between AD biomarkers and a functional measure among Hispanic and WNH older adults. It was hypothesized that the functional measure would be strongly related to AD biomarkers among Hispanics.

**Methods:** The modified clinical dementia rating scale (mCDR) was administered in the participants primary language (English or Spanish) to WNH (n=203) and Hispanic (n=258) older adults who were cognitive normal or diagnosed with Mild Cognitive Impairment (MCI) or dementia. Invariance SEM models were used to compare the pattern of relationships between the mCDR and neurocognitive test performance, MRI volumes, and amyloid load adjusting for age, education, ApoE4 status, and intracranial volume.

**Results:** Model fit was good and not significantly worsened by imposing strict structural invariance. Nested model comparisons indicated that regression weights and correlations among measures differed by group, suggestive of moderation by Hispanic status. Among Hispanic participants, sex ($\beta$=-0.17, p<.05) and Amyloid load ($\beta$=0.25, p<.001) significantly predicted mCDR scores. MRI volumes significantly predicted MCDR scores among both Hispanic ($\beta$=-0.51, p<.001) and WNH participants ($\beta$= -0.42, p<.001).

**Conclusions:** Functional measures like the mCDR may better correlate with Amyloid load among Hispanic older adults than among WNHs, while the correlation with MRI volumes may be comparable in both groups.

Abstract citation ID: igad104.0569

### FAITH IN ACTION BRAIN HEALTH PROGRAM: ADDRESSING BRAIN HEALTH EQUITY GAPS IN THE BLACK COMMUNITY

Fayron Epps[1], Mia Chester[1], Janelle Gore[2], Glenna Brewster[1], Mayra Sainz[1], Robbin Frazier[3], Stephanie Monroe[4], and Virginia Biggar[4], *1. Emory University, Atlanta, Georgia, United States*, *2. Centers for Disease Control and Prevention, Atlanta, Georgia, United States*, *3. University of Minnesota, Center for Healthy Aging and Innovation, Minneapolis, Minnesota, United States*, *4. UsAgainstAlzheimer's, Washington, District of Columbia, United States*

Black populations are disproportionately impacted by dementia, being twice as likely to be diagnosed with dementia as White people. To help address brain health equity gaps across the country, a partnership between Alter and UsAgainstAlzheimer's BrainGuide was established to launch, Faith in Action, a campaign and brain health education program to provide culturally appropriate brain health resources to Black faith communities. The campaign makes brain health education free and accessible by integrating the latest information about brain health into a toolkit delivered to faith communities. This presentation will describe the campaign and discuss why faith leaders say brain health is important to Black faith communities. This campaign included the distribution of: a) Fact sheets b) Culturally tailored messaging; c) Church fans, bookmarks, and buttons; and d) A guidebook to integrating brain health messaging. In relation to the importance of brain health, the following 5 themes emerged from faith leaders (N = 33) implementing the Alter program: a) Desire to disrupt statistics; b) Urgency and being proactive; c) Offer valuable intergenerational education programs; d) Establish a level of wellness in underserved communities; and e) Protect older adults. The Faith in Action campaign empowers Black faith communities to foster conversations and educate their community members about brain health. This campaign assists in promoting brain health equity by recognizing the significance of partnering with Black churches, a cornerstone of the Black community. Such partnerships are crucial to increasing equitable access to resources and supportive services, and eradicating dementia-related disparities in the Black community.

Abstract citation ID: igad104.0570

### RELATIONSHIP OF SOCIAL SUPPORT AND SOCIAL ENGAGEMENT WITH COGNITION IN AN ETHNICALLY DIVERSE OLDER POPULATION

Ruth Tappen, Rebecca Koszalinski, and David Newman, *Florida Atlantic University, Boca Raton, Florida, United States*

One quarter of older adults in the U.S. are socially isolated; far more experience subjective loneliness. The potentially negative effects of limited social support and social engagement pose a concern for maintaining the well-being of older individuals, including the effect on cognitive function. To test the hypothesis that cognitive function is associated with social support and social engagement, we employed structural equation modeling (SEM) to analyze data from an ethnically diverse sample (118 African American, 147 Afro-Caribbean, 133 Hispanic, and 235 European American) of 623 community-dwelling older adults age ≥ 65 (173 male, 450 female). Using the model proposed by Cenè et al. (2022), we conceptualized level of social support and engagement as the exposure, emotional state (anxiety, depression, general emotional well-being), and physical function (functional activity, life space, general physical function, and self-rating of health) as mediators and cognitive function as the outcome of interest. Statistically significant direct and indirect effects were found between the three latent variables (emotional state, physical function, and social support and social engagement), indicating that the level of social support and engagement the older individual is related directly and indirectly to cognition ($\beta$=-.75, SE=.49, p<.028; $\beta$=-0.08, SE=0.795, p=0.017). The SEM models showed goodness of fit with $\chi^2$/df=3.001, CFI=0.931, AGFI=0.966, SRMR=0.045, and RMSA=0.058, and explained 35.3% of the variability. These values support the hypothetical model. Results suggest the importance of maintaining existing social support and engagement activities as well as development and testing of approaches to increase social support and engagement in later life.

Abstract citation ID: igad104.0571

### RURAL–URBAN DIFFERENCES IN POSTSURGICAL SERVICE USE AND OUTCOMES AMONG VETERANS WITH DEMENTIA

Heather Davila[1], Yubo Gao[2], Michael Jacobs[3], Katherine Hadlandsmyth[4], Carly Duncan[3], Andrea Strayer[5], Daniel Hall[3], and Mary Vaughan-Sarrazin[2], *1. Iowa City VA Healthcare System, Iowa City, Iowa, United States*, *2. University of Iowa Carver College of Medicine, Iowa City, Iowa, United States*, *3. VA Pittsburgh Healthcare System, Pittsburgh, Pennsylvania, United States*, *4. Iowa City VA Health Care System, Iowa City, Iowa, United States*, *5. University of Iowa, Iowa City, Iowa, United States*

Adults with dementia have worse post-surgical outcomes than those without dementia, including readmission and death. The use of home health or facility-based skilled nursing after discharge may prevent poor outcomes. We know little about rural-urban differences in post-discharge services or outcomes among individuals with dementia. We examined the relationship between post-discharge service use and 30-day readmission or mortality using retrospective data for Veterans Affairs patients aged 65+ with dementia who underwent inpatient surgery between 2013-2019. We used merged VA and Medicare claims to categorize patients as discharged to home without services, home with home health, or skilled nursing/rehabilitation facility [SNF]. We excluded individuals who were transferred to another acute hospital, transferred to hospice, died or were readmitted within 3 days of discharge, or were not enrolled in Medicare. We used multivariable logistic regression to assess associations between rurality, discharge location, and outcomes, while controlling for demographics, frailty, comorbidities, surgical specialty, and surgical complexity. The analytic sample included 4534 Veterans; 48.7% discharged to a SNF, 40.1% to home without services, and 10.4% to home with home health. We did not find rural-urban differences in risk-adjusted readmission or mortality overall. Among those discharged to home (n=2324), receipt of home health was modestly associated with decreased readmission risk overall (OR=0.52; p=0.07), and lower risk of morality among urban Veterans (OR=0.34; p=0.05) but not rural Veterans. Although we did not find rurality disparities in outcomes, further research may identify reasons home health decreases post-surgical mortality among urban but not rural adults with dementia.

Abstract citation ID: igad104.0572

### SEX AND ETHNICITY-RELATED DISPARITIES IN ALZHEIMER'S DISEASE AMONG RESIDENTS OF SKILLED NURSING FACILITIES

Arseniy Yashkin, and Igor Akushevich, *Duke University, Durham, North Carolina, United States*

Residents of nursing homes represent a special subset of the population characterized by increased vulnerability and higher rates of Alzheimer's Disease (AD). Nursing home ethnic composition does not reflect that of the general population with minorities more likely to be concentrated in facilities with serious deficiencies, and to have lower health status at time of admission. Significant ethnic disparities in quality-of-care aspects such as hospitalizations, preventive care, and the management of chronic conditions exist and these patterns extend to sex and ethnicity-specific subgroups of individuals with AD. However, there is a paucity of knowledge on the actual size of sex and ethnicity-related disparities in AD within this high-risk population. In this study we used 100% of all nursing home resident evaluations (N=23,080,518; Male:40.66%; Female:59.34%; White: 77.29%; Black:11.62%; Other:3.80%; Hispanic:4.97%; Asian:1.81%; Pacific Islander:0.15%; Native American Native:0.39%), 1999-2012, from the Minimum Dataset to assess sex and ethnicity-specific differences in age-specific prevalence of AD. Total AD prevalence increases sharply (numbers are prevalence proportions with 95% confidence intervals in parentheses) from 1.79 (1.75-1.84) at age 60 to 13.45 (13.39-13.51) at 85. Notable statistically significant sex and ethnicity-related disparities were present at all ages. For example, at age 85 the prevalence of AD was for: i)Males: 11.34 (11.24-11.44); ii)Females: 14.53 (14.45-14.62); iii)Whites: 13.21 (13.14-13.28); iv)Blacks: 15.32 (15.09-15.55); v)Other/Unknown: 9.75 (9.42-10.07); vi) Hispanics: 18.17 (17.82-18.52); vii)Asians: 11.30 (10.87-11.72); viii)Pacific Islanders: 10.33 (8.65-12.01); ix)North American Natives: 11.78 (10.62-12.94). In depth analysis of the causes, time-trends, and geographic distribution of these disparities is needed.

### SESSION 2185 (SYMPOSIUM)

Abstract citation ID: igad104.0573

### CATALYZING DEMENTIA CARE THROUGH THE LEARNING HEALTH SYSTEM AND CONSUMER HEALTH INFORMATION TECHNOLOGY

Chair: Jennifer Wolff Co-Chair: Danielle Powell

To advance care for persons with Alzheimer’s disease and related dementias (ADRD), real-world health system effectiveness research must actively engage those affected to understand what works, for whom, in what setting, and for how long – an agenda central to Learning Health System principles. The spread of consumer health information technology affords new opportunities to support ADRD caregivers within health care delivery. Patient portals allow patients to view test results and sections of their electronic medical record, perform health management tasks (e.g., scheduling visits, billing, filling prescriptions), access educational materials, and communicate with clinicians using secure messaging. Many care delivery organizations allow patients to share access to their portal account by registering a “care partner” to have their own identity credentials (login/password). Through shared access, patients may select whether and who electronically interacts with clinicians on their behalf, thus, respecting patient preferences while supporting care partners with timely information about patient health and treatments, and a mechanism to electronically navigate health system demands. Little is now known about electronic information sharing preferences and practices of persons with ADRD and their caregivers, the feasibility of deploying novel interventions, or existing initiatives through consumer health information technologies to better support ADRD care and management. This symposium presents early results from projects seeking to increase evidence regarding the role and use of consumer health information technology in ADRD care and management by examining the existing landscape of patient portal use in the context of dementia care from a large integrated academic health system.

Abstract citation ID: igad104.0574

### THE MEDICARE ANNUAL WELLNESS VISIT: POTENTIAL FOR EMBEDDED INTEGRATION OF THE PATIENT AND FAMILY PERSPECTIVE

Danielle Powell[1], MJ Wu[2], Kelly Gleason[3], Stephanie Nothelle[4], Esther Oh[5], Nicholas Reed[6], and Jennifer Wolff[2], *1. University of Maryland, College Park, Maryland, United States*, *2. Johns Hopkins University, Baltimore, Maryland, United States*, *3. Johns Hopkins, Baltimore, Maryland, United States*, *4. Johns Hopkins School of Medicine, Baltimore, Maryland, United States*, *5. Division of Geriatric Medicine, Johns Hopkins University School of Medicine, Baltimore, Maryland, United States*, *6. Johns Hopkins Bloomberg School of Public Health, Baltimore, Maryland, United States*

Patient portals are secure online platforms that provide an opportunity for patients to actively participate in their care by viewing their health information, messaging with clinicians, and completing health risk assessments, like the Medicare Annual Wellness visit (AWV). Using electronic medical record and patient portal (MyChart) data (October 3, 2021-October 2, 2022) from a large integrated academic medical center, we examine uptake of the AWV among Medicare beneficiaries 65+ years with 2 or more primary care visits. We then examine whether responses to the AWV health risk assessment differ by whether patients are registered for and actively use the patient portal. Among 11,378 eligible beneficiaries, 7,194 had an AWV billed (62% female, 69% White, 8.7% dementia, 78% patient portal users). Among those billed, 98% answered at least 1 health risk assessment question +/- 30 days of billing, and 76% completed questions related to all risk assessment themes. Nearly 8 in 10 (79.6%) older adults who completed the AWV were patient portal users. While highly active overall, completion of the AWV and health risk assessment was higher for active patient portal users. Compared to non-active portal users, active users were less likely to report difficulty with ADLs, transportation, chores, mobility issues, vision concerns, living alone, and seeing a dentist. Evaluation of billed AWV alone does not fully characterize completion and care provided for the preventive care visit. Systematic incorporation of the patient portal may aid in integration and dissemination of preventive care.

Abstract citation ID: igad104.0575

### CARE PARTNERS OF PERSONS WITH DEMENTIA USE OF SHARED ACCESS TO THE PATIENT PORTAL

Kelly Gleason, *Johns Hopkins, Baltimore, Maryland, United States*

The patient portal plays a significant and growing role in health system navigation, and most patient portals offer shared access for care partners. Care partners are at the forefront of dementia care, yet little is known about their use of the portal through shared access. We conducted an observational cohort study of patient portal utilization for a 5-year period (10/3/2017-10/2/2022) at an academic health system. The cohort consisted of 49,382 patients ages 65+ with 2+ visits in 24 months. More than half (57.34%) of the cohort was female and white (67.19%) and the average age was 76.56 (SD 8.60). We operationalized diagnosed dementia from ICD10 codes and problem lists. Shared access use was defined as activity happening through "proxy" credentials. We operationalized the patient portal activity metric as the ratio of portal sessions in relation to number of clinical encounters. Older adults with (versus without) diagnosed dementia were similarly likely to be registered for the patient portal (71.20% versus 71.51%, p=0.16) but more likely to have a shared access user (10.41% vs. 3.31%; p<0.001). Among patients with dementia, those who had a shared access user, compared to those who did not, had 341.32 versus 164.33 patient portal sessions, sent 47.56 versus 27.42 messages, and had a 19.22 versus 11.22 patient portal activity metric over the 5-year period (p<0.001 all contrasts). Policy initiatives and organizational efforts that encourage use of shared access may benefit care quality of persons with dementia and their care partners.

Abstract citation ID: igad104.0576

### PATIENT PORTAL MESSAGING IN THE CONTEXT OF DEMENTIA: A FIRST LOOK

Aleksandra Wec[1], Stephanie Nothelle[2], Kelly Gleason[3], Jennifer Wolff[4], and Halima Amjad[5], *1. Johns Hopkins Bloomberg School of Public Health, Baltimore, Maryland, United States*, *2. Johns Hopkins School of Medicine, Baltimore, Maryland, United States*, *3. Johns Hopkins, Baltimore, Maryland, United States*, *4. Johns Hopkins University, Baltimore, Maryland, United States*, *5. Johns Hopkins University School of Medicine, Baltimore, Maryland, United States*

Primary care providers (PCPs) play an integral role in diagnosis and early management of dementia. Secure messaging through the patient portal is increasingly being used by patients to communicate with PCPs. Little is known about the nature of concerns raised by patients with dementia and their care partners when messaging PCPs following dementia diagnosis. We conducted a content analysis of 100 message threads initiated by patients and care partners with PCPs at an academic integrated health system during the twelve months following diagnosis of dementia. Message threads ranged in length from two to eleven messages and included 337 unique messages from 79 distinct accounts, sent between 01/03/2020 and 01/20/2022. Of 100 message threads, 89 were sent by care partners and 11 were sent by patients. Threads often focused on medications. Of 36 threads mentioning medications, 12 discussed dementia-specific medications. Other common dementia-specific topics included behavioral and/or cognitive symptoms of dementia (22/100) and sleep disturbance (8/100). Safety concerns related to falls, wandering, or driving were also common (15/100). Care partners raised issues related to their own needs or wellbeing (e.g., caregiving stress, need for caregiver education) (15/100 threads) and messaged to coordinate care between the PCP and other providers (26/100). Findings demonstrate care partners commonly message on behalf of persons with dementia and use the portal to address dementia-related care needs. Identifying issues raised via messaging offers insight into resources and strategies for PCPs to effectively and efficiently address common concerns after dementia diagnosis, including strategies that leverage the patient portal.

Abstract citation ID: igad104.0577

### PREVALENCE AND TYPES OF PATIENT PORTAL USE AMONG OLDER ADULTS RECEIVING HOME HEALTH CARE

Julia Burdgorf[1], Mingche Wu[2], and Chanee Fabius[2], *1. Center for Home Care Policy & Research, New York, New York, United States*, 2. *Johns Hopkins University, Baltimore, Maryland, United States*

Home health care (HHC) delivers skilled nursing and therapy services in the patient's home and is a valuable source of care for community-living older adults, particularly those living with complex conditions such as dementia. However, lack of visibility into patients' clinical records and difficulty communicating with other providers are perennial challenges to HHC quality. Patient portals—provider-sponsored online applications that allow patients to perform care management tasks including viewing health information and messaging providers—present a promising opportunity to bridge this gap. However, no prior work has examined patterns of portal use among HHC patients. We leveraged a unique dataset containing electronic health record and patient portal utilization data for 10,212 older adults who received HHC between 2017-2019. We found that 25% of HHC patients had an active patient portal prior to HHC and, of those with an active portal, 85% used their portal during HHC. Among those who used their portal during HHC, the most common portal activities were reviewing the clinical record (82%), managing appointments (60%), and seeking provider advice (45%). Compared to community-referred patients, hospital-referred patients were more likely to use their portal to manage appointments or review their clinical record. Compared to patients without dementia, patients with dementia were more likely to use their portal to message their provider but less likely to view their clinical record. Findings suggest that portal users are actively engaging with their portal during HHC, indicating an opportunity to empower patients to facilitate information transfer between HHC and other providers.

### SESSION 2190 (SYMPOSIUM)

Abstract citation ID: igad104.0578

### GSA JOURNAL EDITORS PANEL: WHAT HAPPENS AFTER I PRESS SUBMIT? UNDERSTANDING THE JOURNEY OF A MANUSCRIPT

Chair: Jessica Kelley

You have finished your great manuscript. Formatted it. Wrote the cover letter. But what happens after you press the "Submit" button? This moderated panel of Editors-in-Chief of five of the GSA journals will talk about all aspects of the review process that lead to a final decision. We will cover topics such as how reviewers are selected, top reasons a manuscript may be rejected without review, and review timetables. We will discuss how Editors use the reviews to make a decision, including how they deal with reviewers who may disagree with each other, or a case where an Editor may disagree with a reviewer. Following the moderated discussion, we will have plenty of time for audience questions.

Abstract citation ID: igad104.0579

### EDITOR-IN-CHIEF OF INNOVATION IN AGING

Steven Albert, *University of Pittsburgh, Pittsburgh, Pennsylvania, United States*

Abstract citation ID: igad104.0580

### EDITOR-IN-CHIEF OF THE JOURNALS OF GERONTOLOGY, SERIES A: BIOLOGICAL SCIENCES AND MEDICAL SCIENCES

Lewis Lipsitz, *Hebrew SeniorLife, Boston, Massachusetts, United States*

Abstract citation ID: igad104.0581

### EDITOR-IN-CHIEF OF THE GERONTOLOGIST

Joseph Gaugler, *University of Minnesota, Minneapolis, Minnesota, United States*

Abstract citation ID: igad104.0582

### EDITOR-IN-CHIEF OF THE JOURNALS OF GERONTOLOGY, SERIES A: BIOLOGICAL SCIENCES AND MEDICAL SCIENCES

Gustavo Duque, *McGill University, Montreal, Quebec, Can ada*

### SESSION 2195 (SYMPOSIUM)

Abstract citation ID: igad104.0583

### MUSCLE UP: WHAT AGING LOOKS LIKE FROM THE INSIDE OUT

Chair: Eleanor Simonsick Discussant: Luigi Ferrucci

The Longitudinal Studies Section of the Translational Gerontology Branch of the National Institute on Aging Intramural Research Program has the mission and opportunity to examine aging physiology from the cellular to the full body functional level in model systems and human populations. This symposium presents ongoing research that targets pathologic and normative change in muscle with aging and the significance of these changes for understanding the aging process. Data come from the Baltimore Longitudinal Study of Aging (BLSA) and the Genetic and Epigenetic Signatures of Translational Aging Laboratory Testing (GESTALT) study. In nearly 500 BLSA participants aged 50+, Zenobia Moore evaluates the association between multiple epigenetic age metrics and change over 6 years in mid-femur areas of muscle and subcutaneous and intramuscular fat derived from computed tomography. Qu (Teresa) Tian investigates the relationship between skeletal muscle mitochondrial function and risk of MCI or dementia over 3.3 years in 460 initially cognitively normal BLSA participants and PET and blood biomarkers of AD in available subsamples. In 82 men and women from GESTALT, Stefano Donega examines relationships between physical activity, peak oxygen consumption and muscle oxidative capacity using p31 MR spectroscopy and gene expression, transcript usage and splicing events derived from vastus lateralis biopsies. Using volumetric Focused Ion Beam Scanning Electron Microscopy, Lisa Hartnell shares 3D images of muscle samples at the level of the mitochondrial reticulum and sarcomere from young and old healthy and frail GESTALT participants.

Together, these findings highlight the value of muscle as a window on aging.

Abstract citation ID: igad104.0584

### EPIGENETIC AGE AND MUSCLE CHARACTERISTICS IN BALTIMORE LONGITUDINAL STUDY OF AGING PARTICIPANTS

Ann Zenobia Moore[1], Pei-Lun Kuo[1], Qi Yang[2], Bennett Landman[2], Eleanor Simonsick[1], and Luigi Ferrucci[1], *1. National Institute on Aging, National Institutes of Health, Baltimore, Maryland, United States*, *2. Vanderbilt University, Nashville, Tennessee, United States*

Changes in muscle, including loss of muscle mass and fat infiltration, contribute to loss of muscle strength and subsequent declines in physical function in later life. Measures of body composition are consistently associated with DNA methylation (DNAm) in cross sectional studies. Whether differences in DNAm are informative with respect to longitudinal muscle characteristics has not been fully explored. Here we evaluate the relationship between epigenetic age metrics and computed tomography (CT) derived measures, including areas of muscle, subcutaneous fat, and intermuscular fat, as well as the mean and standard deviation of muscle intensity at mid-femur, in the Baltimore Longitudinal Study of Aging. Participants were at least 50 years old at the evaluation of DNAm (n = 471, 51% female), 61.8% of participants had three or more CT scans and mean follow up time was 6.0 years. Relationships between epigenetic age metrics including Horvath and Hannum DNAm age, PhenoAge, GrimAge and DunedinPACE at the index visit, and the trajectory of muscle composition were evaluated using multivariable mixed effects models adjusted for key demographic characteristics, health behaviors and body size. Epigenetic age metrics at the index visit were negatively correlated with the trajectory of muscle area over follow-up. Higher GrimAge at the index visit was associated with faster loss in muscle area and faster increase in fat areas ($p < .05$). Our analyses suggest that further exploration of DNAm with trajectories of muscle parameters may elucidate novel pathways of muscle dysfunction.

Abstract citation ID: igad104.0585

### MUSCLE MITOCHONDRIAL FUNCTION PREDICTS COGNITIVE IMPAIRMENT AND IS ASSOCIATED WITH PET AND BLOOD BIOMARKERS OF AD

Qu Tian[1], Murat Bilgel[1], Keenan Walker[1], Abhay R. Moghekar[2], Susan Resnick[1], and Luigi Ferrucci[1], *1. National Institute on Aging, National Institutes of Health, Baltimore, Maryland, United States*, *2. Johns Hopkins University, Baltimore, Maryland, United States*

Mitochondrial dysfunction is a hallmark of aging. Whether mitochondrial dysfunction is related to future cognitive impairment and biomarkers of Alzheimer's disease (AD) is unknown. In the Baltimore Longitudinal Study of Aging, we used Cox regression to examine the association of skeletal muscle mitochondrial function (post-exercise recovery rate of phosphocreatine, kPCr) via MR spectroscopy with future MCI or dementia over an average follow-up of 3.3 years among 460 initially cognitively normal participants (mean age=76.8±11.0 years, 56.7% women, 22.4% Black). We also examined associations with PiB-distribution volume ratio (DVR) for β-amyloid (Aβ)(n=115), Flortaucipir (FTP)-standardized uptake value ratio (SUVR) for tau(n=70), and blood biomarkers of AD pathology (Aβ42/Aβ40 ratio,n=299; pTau181,n=505), neuronal injury (NfL) (n=299), and astrocyte-mediated neuroinflammation (GFAP) (n=299) using linear regression. After adjustment for age, sex, and race, each SD higher kPCr (i.e. 0.005) was associated with 45% lower hazards of developing cognitive impairment (19 MCI, 4 dementia) (p=0.04). In the PET sample, each SD higher kPCr was associated with 51% lower odds of being PiB positive(p=0.003). Higher kPCr was specifically associated with lower DVR in precentral and postcentral gyri, superior parietal lobe, and pallidum (FDR-adjusted p<0.05). In samples with blood biomarkers, higher kPCr was associated with lower GFAP(p=0.01) and showed a trend toward significance with lower pTau181(p=0.08). Preserved skeletal muscle mitochondrial function is associated with reduced risk of cognitive impairment and PET and blood biomarkers of AD. Future studies are needed to investigate the role of mitochondrial function of the central nervous system in dementia risk and the progression of AD pathologic change.

Abstract citation ID: igad104.0586

### MULTIPLE MEASURES OF FITNESS ASSOCIATION WITH MUSCLE MRNA SPLICING VARIANTS IN THE GESTALT STUDY

Stefano Donega[1], Nirad Banskota[1], Marta Gonzalez-Freire[2], Ann Zenobia-Moore[1], Ceereena Ubaida-Mohien[1], Ken Fishbein[1], Supriyo De[1], and Luigi Ferrucci[1], *1. National Institute on Aging, National Institutes of Health, Baltimore, Maryland, United States*, *2. Health Research Institute of the Balearic Islands (IdISBA), Palma, Islas Baleares, Spain*

Regular physical activity has been associated with higher skeletal muscle mass and strength as well as better mitochondrial function. Previous studies in master octogenarian athletes found a higher content of mitochondrial- and reduced spliceosome proteins compared to non-athlete age/sex-matched controls. These findings suggest that higher mitochondrial function is associated with down-regulation of the spliceosome. In this study we aimed to detect mitochondrial respiration associations with expression of specific genes and differentially spliced isoforms. We performed RNA-seq from vastus lateralis skeletal muscle biopsies in 82 male/female participants in the GESTALT study (Genetic and Epigenetic Signatures of Translational Aging Laboratory Testing). We correlated gene expression, transcript usage and splicing events with I) self-reported level of physical activity in daily life, II) peak oxygen consumption assessed during an incremental treadmill stress testing, III) muscle oxidative capacity assessed by p31 MR Spectroscopy and IV) mitochondrial-respirometry assessed on permeabilized muscle fibers, using regression models adjusted for age, gender, and batch. Except for IV), all three mitochondrial measures positively correlated with each other, and were downregulated along the Aging axis. We detected 423, 219, 178 and 226 differentially expressed genes (p-value<0.01), respectively for the 4 mitochondrial fitness measures. Gene-set-enrichment analysis (GSEA) detected up-top TCA cycle and mitochondrial respiration kPCr and mito-respirometry detected the highest number of AS events (448 and 412). Dissimilarities among transcriptomic profile associated to these four bioenergetic outcomes provide insight into the biological mechanisms that connect them to mitochondrial function and suggest that mitochondrial function is not the only correlate of movement behaviors.

Abstract citation ID: igad104.0587

**STRUCTURAL DIFFERENCES IN HEALTHY YOUNG AND OLD HUMAN MUSCLE CELLS: APPLYING DEEP LEARNING TO VOLUMETRIC SEM IMAGES**

Lisa Hartnell, *National Institute on Aging, National Institutes of Health, Baltimore, Maryland, United States*

Several studies have shown that muscle strength and mitochondrial function degrade in individuals over time as a function of aging and/or disease. To better understand this process, we examined the relationship between structure and function of muscle in healthy young, healthy old and frail old participants in the Genetic and Epigenetic Signatures of Translational Aging Laboratory Testing (GESTALT) study. Muscle was examined at the level of the mitochondrial reticulum and sarcomere, the basic contractile units of muscle, using volumetric Scanning Electron Microscopy (SEM) imaging. Volumetric SEM imaging using Focused Ion Beam (FIB)-SEM yields isotropic volumes enabling analysis at near membrane resolution and is ideal for mapping the mitochondrial reticulum and structural components of the sarcomere in 3D. The mitochondrial reticulum is a highly organized continuum of individual mitochondria that distribute and provide energy for contraction throughout muscle. We will show our approach to apply deep learning segmentation to volumes of gastrocnemius muscle ranging from 10-20 micrometers cubed collected from young (20-29y) and old (75-89y) participants in GESTALT to quantify differences in the reticulum including fragmentation and degeneration, as well as distribution and number of mitochondria. Preliminary findings suggest a difference with increasing age and more marked structural differences in both sarcomeres and mitochondria associated with frailty. Future work aims to increase the number of individuals sampled and extend the analysis to muscle from middle-aged individuals using biological markers and tests that indicate a functional aging phenotype.

### SESSION 2200 (SYMPOSIUM)

Abstract citation ID: igad104.0588

**AARP PUBLIC POLICY INSTITUTE PRESENTS THE LONG-TERM SERVICES AND SUPPORTS STATE SCORECARD, 2023**

Chair: Susan Reinhard Discussant: Susan Reinhard

This symposium will present and explore in-depth the 2023 Long-Term Services and Supports (LTSS) State Scorecard. With funding from The SCAN Foundation, The Commonwealth Fund, and The John A. Hartford Foundation, this Scorecard is the fifth edition from the AARP Public Policy Institute. The Scorecard ranks states across five key dimensions that comprise a framework for high-performing LTSS system performance: affordability and access, choice of setting and provider, quality and safety, support for family caregivers, and community and integration. In this symposium, speakers will discuss the Scorecard’s history, its most recent findings, and the implications of the COVID-19 pandemic for state LTSS system performance. In addition, the Scorecard explores LTSS equity, and we will discuss how policymakers, practitioners, the private sector, and other actors can further realize equitable systems of supports.

Abstract citation ID: igad104.0589

**THE LTSS STATE SCORECARD AND AGE-FRIENDLY CARE**

Rani Snyder, *The John A. Hartford Foundation, New York City, New York, United States*

Age-friendly care is health care that addresses a person’s unique needs and wants across the lifespan. It is a critical part of the age-friendly ecosystem. Rooted in the essential elements known as the “4Ms” (what Matters, Medication, Mentation, and Mobility), age friendly care is critical to successful delivery systems of health care as well as long-term services and supports. For the first time in its history, the LTSS State Scorecard will measure states as it relates to the presence of Age-Friendly Health System sites. Age-Friendly Health Systems is an initiative of The John A. Hartford Foundation and the Institute for Healthcare Improvement, and helps hospitals, doctor's offices, retail pharmacy clinics, nursing homes, and home-care providers deliver age-friendly care. This portion of the symposium will describe Scorecard findings on Age-Friendly Health Systems and address more broadly the important role of age-friendly care in LTSS.

Abstract citation ID: igad104.0590

**THE LTSS STATE SCORECARD NATIONAL ADVISORY PANEL**

Robert Applebaum, *Miami University, Oxford, Ohio, United States*

Reaching consensus across the field is an important endeavor for a project like the LTSS State Scorecard. Since the Scorecard’s inception in 2008, the AARP Public Policy Institute has engaged a broad range of experts, known as the Scorecard National Advisory Panel (NAP). The NAP is drawn from a broad range of expert stakeholders across private and nonprofit organizations, state agencies, researchers, and the federal government. The NAP has informed virtually all aspects of the Scorecard, including indicator selection, scoring methodology, and the framing and messaging of Scorecard findings. This portion of the symposium will feature remarks and analysis from a long-standing NAP member and expert in the field of long-term services and supports.

Abstract citation ID: igad104.0591

**THE LTSS STATE SCORECARD AND THE ROLE OF STATE POLICY**

Hemi Tewarson, *National Academy for State Health Policy, Washington, District of Columbia, United States*

States play a crucial role in long-term services and supports. State agencies and legislatures have the power to make LTSS and family caregiver supports more (or less) accessible, affordable, and high-quality for the residents they serve. In addition, much of the policymaking during and after the peak of the COVID-19 pandemic took place at the state level. State agencies had the authority to grant providers, consumers, and family caregivers new flexibilities and options as it relates to LTSS, and states have and continue to decide where much of the federal COVID-19 funds (e.g., American Rescue Plan HCBS funding) will ultimately go. This portion of the symposium will present an overview of where states are today with LTSS and how the Scorecard can help both identify and drive system change.

Abstract citation ID: igad104.0592
**THE LTSS STATE SCORECARD AND MULTISECTOR PLANS FOR AGING**
Narda Ipakchi, *The SCAN Foundation, Long Beach, California, United States*

Strong, comprehensive plans are crucial to secure lasting success in state LTSS systems. State Multisector Plans for Aging meet this need and are present and/or under development in more than a dozen states and DC. A Multisector Plan for Aging, a concept led by The SCAN Foundation, is a blueprint that: a) includes planning for 10 or more years; b) is often led by a governor with other executive and legislative leaders; and c) is developed to guide the restructuring of state and local policy, programs, and funding toward aging well in the community. For the first time, the LTSS State Scorecard credits states for having these plans. This portion of the symposium will describe Scorecard findings on Multisector Plans for Aging and address more broadly the important role of these plans to state leadership.

### SESSION 2205 (BIOLOGICAL SCIENCES INVITED SYMPOSIUM)

Abstract citation ID: igad104.0593
**METABOLISM AND AGING**
Chair: Joseph Baur

Reducing energy intake in the absence of malnutrition (caloric restriction) has long been known as the most robust and reproducible way to experimentally increase lifespan in laboratory models. However, our understanding of which metabolites and nutrient-sensing pathways interact with longevity and where they act remains rudimentary, and in many cases, controversial. This session will explore recent findings linking changes in metabolites to the aging process and to organismal health.

Abstract citation ID: igad104.0594
**THE INTERTISSUE COMMUNICATION BETWEEN THE HYPOTHALAMUS AND WHITE ADIPOSE TISSUE IN MAMMALIAN AGING AND LONGEVITY CONTROL**
Shin-ichiro Imai, *Washington University School of Medicine, St. Louis, Missouri, United States*

Recent studies in model organisms have demonstrated that the intertissue communications play a critical role in the regulation of aging and longevity. Our major goal is to understand the systemic regulation of aging and longevity in mammals and translate that knowledge into an effective anti-aging intervention that could make our later lives as healthy and productive as possible (“productive aging”). In mammals, we have previously demonstrated that the intertissue communication between the hypothalamus and adipose tissue, particularly mediated by extracellular vesicles-contained extracellular nicotinamide phosphoribosyltrasferase (eNAMPT), the rate-limiting NAD+ biosynthetic enzyme in mammals, functions to counteract age-associated physiological decline and promote longevity in mice. We have recently found that Ppp1r17-positive neurons in the dorsomedial hypothalamus (DMHPpp1r17 neurons) regulate white adipose tissue (WAT) function, including lipolysis and eNAMPT secretion, through the sympathetic nervous system (SNS), and the feedback loop between DMHPpp1r17 neurons and WAT plays a critical role in the regulation of aging and longevity in mice. Within DMHPpp1r17 neurons, the phosphorylation and subsequent nuclear-cytoplasmic translocation of Ppp1r17, regulated by cGMP-dependent protein kinase (PKG; Prkg1), affect gene expression regulating synaptic function, causing synaptic transmission dysfunction and impaired WAT function. Both DMH-specific Prkg1-knockdown, which suppresses age-associated Ppp1r17 translocation, and the chemogenetic activation of DMHPpp1r17 neurons significantly ameliorate age-associated WAT dysfunction, increase physical activity, decrease age-associated mortality rate, and extend lifespan. Thus, these findings clearly demonstrate the importance of the intertissue communication between the hypothalamus and WAT in mammalian aging and longevity control and provide critical insights into the development of effective anti-aging interventions.

Abstract citation ID: igad104.0595
**TRACING THE COORDINATED CONTROL OF NAD+ HOMEOSTASIS USING C. ELEGANS**
Melanie McReynolds, *Penn State University, University Park, Pennsylvania, United States*

Nicotinamide adenine dinucleotide (NAD+) is a vital coenzyme involved in redox reactions and cellular signaling. Its levels decline with age in humans, mice, and worms, leading to metabolic disturbances associated with age-related diseases. Interestingly, our studies in aged mice indicate that the rate of NAD+ synthesis remains unchanged, ruling out a lack of precursors as the cause of NAD+ decline. Instead, we found that tissues with lower NAD+ concentrations in aged mice exhibit higher fractional turnover, suggesting increased consumption as the primary driver of NAD+ decline during aging. This led us to hypothesize that the loss of NAD+ metabolic robustness and resiliency with age is linked to the hyper-activation and competition of NAD+-consuming enzymes. To investigate the physiological impact of NAD+ consuming enzymes on metabolic robustness, we used genetic tools and mass spectrometry in C. elegans. Surprisingly, we identified novel reproductive and aging phenotypes in mutant animals lacking specific NAD+ consuming enzymes, including Poly ADP-Ribose Polymerase (parp-1 and parp-2), TANKyrase (tank-1), and Toll and Interleukin 1 Receptor (tir-1). These mutants displayed significant reductions in fecundity and extensions in longevity. Additionally, employing High-Performance Liquid Chromatography coupled with Mass Spectrometry (HP-LC-MS), we examined the NAD+ metabolome across different developmental stages in our mutant strains, revealing notable metabolic disruptions associated with the loss of NAD+ consumers. In summary, our findings establish a novel connection between the hyper-activation of NAD+ degradation and the onset of reproductive aging. This research provides valuable insights into the role of NAD+ metabolism and its impact on aging-related processes.

Abstract citation ID: igad104.0596

### HOST-MICROBE METABOLIC INTERACTIONS IN DISEASE AND AGING

Xiaoling Li, *NIEHS, Research Triangle Park, North Carolina, United States*

As a key mediator of environmental influences on cellular functions, metabolism is crucial for cellular adaptation to the environment. Perturbations of metabolic homeostasis by various environmental factors, including diet and lifestyle, are associated with numerous metabolic diseases and contribute to acceleration of aging. To understand how metabolism mediates environmental regulation of cellular functions and how disruption of metabolic homeostasis contributes to pathogenesis of diseases and aging, we have been focused on a nutrient-sensing pathway consisting of NAD+, SIRT1, the most conserved mammalian NAD+-dependent protein deacetylase/deacylase, and protein acylations. Employing genetically modified mice and cultured cells as model systems, we have recently shown that NAD+ and SIRT1 are key mediators of host-microbe metabolic interactions. On one hand, host NAD metabolism is regulated by gut microbiota. On the other hand, SIRT1 age-dependently modulates gut microbiota through regulation of intestinal bile acid metabolism and Paneth cell activation. Furthermore, we identified SIRT1 as a key regulator of methionine metabolism in embryonic stem cells and developing embryos, thereby modulating their sensitivity to methionine restriction, a dietary regimen that protects against metabolic diseases and aging. Intriguingly, we recently demonstrated that dietary methionine restriction impairs anti-tumor immunity through interaction with gut-microbiota. Together, our findings uncover an important role of the NAD+-SIRT1 axis in mediating host-microbe metabolic interactions that influence disease and aging.

Abstract citation ID: igad104.0597

### NAD+ METABOLISM IN MITOCHONDRIA AND MICROBES

Joseph Baur, *Perelman School of Medicine at the University of Pennsylvania, Philadelphia, Pennsylvania, United States*

Nicotinamide adenine dinucleotide (NAD+) is a redox cofactor essential to all living organisms. NAD+ levels fall with age or disease and rise with exercise or caloric restriction. Supplemental NAD+ precursors have beneficial effects on health in rodent models, renewing interest in this molecule and the enzymes that require it. Although NAD+ is present in the mitochondrial matrix and critical to the function of the organelle, the source of mitochondrial NAD+ was only recently elucidated. We identified SLC25A51 as the carrier that is primarily responsible for this activity in cultured cells. Here we show that, like in cells, SLC25A51 is important for establishing the mitochondrial NAD+ pool in vivo. Shifting NAD+ into hepatic mitochondria is sufficient to replicate the effects of supplemental NAD+ precursors on liver regeneration. Thus, the mitochondrial NAD+ pool appears to be responsible for some of the benefits of increasing whole-body NAD+. In a separate series of studies, we have used heavy isotope tracing strategies to show that the popular supplement nicotinamide riboside is metabolized by the microbiome when administered orally to mice. Further studies revealed a surprising role for the microbiome in normal NAD+ metabolism. Host-derived nicotinamide is released into the gut lumen and processed to generate microbial NAD+ as well as nicotinic acid that is reabsorbed by the host. These findings reveal a previously unappreciated cycle by which NAD+ precursors are shared between the host and microbes.

### SESSION 2210 (SYMPOSIUM)

Abstract citation ID: igad104.0598

### INTERDISCIPLINARY WELLNESS CLINICS IN LOW-INCOME SENIOR HOUSING COMMUNITIES

Chair: Sarah Holmes

Low-income older adults may qualify to live in subsidized housing via the Housing and Urban Development (HUD) Section 202 program. Established in 1959, Section 202 is the only HUD program that provides housing exclusively for older adults. More than 1.8 million older adults receive this type of federal housing assistance. Older adults living in low-income senior housing communities are mostly people of color, socioeconomically disadvantaged, and at risk for poor physical and mental health and adverse health outcomes. Additionally, they have limited access to healthcare services and other resources such as internet use, healthy food options, opportunities for physical activity, and safe indoor and outdoor physical environments. Further they are at increased risk of high emergency room utilization and transfer to nursing home settings. The purpose of this project was to develop Interdisciplinary Wellness Clinics for older adults living in low-income senior housing communities and provide direct services including such things as blood pressure monitoring, medication management, hearing evaluation and cerumen removal, foot and nail care, pain management, management of acute medical problems, immunizations, and Medicare Annual Wellness Visits. Interdisciplinary Wellness Clinics are provided monthly to four low-income senior housing communities and serve approximately 500 residents living in these communities. This symposium will provide a review of residents seen and services provided with a focus on findings identified and interventions implemented; a description of the Medicare Annual Wellness services; and a review of approaches used to engage residents in using these services.

Abstract citation ID: igad104.0599

### STRATEGIES FOR ENGAGING LOW-INCOME SENIOR HOUSING RESIDENTS IN INTERDISCIPLINARY WELLNESS CLINICS

Sarah Holmes[1], Jennifer Klinedinst[1], Nicole Brandt[2], and Barbara Resnick[3], *1. University of Maryland School of Nursing, Baltimore, Maryland, United States*, *2. University of Maryland, Baltimore, Baltimore, Maryland, United States*, *3. University of Maryland, Baltimore, Maryland, United States*

Engaging residents in low-income senior housing communities in health and wellness services can be challenging. Despite the significant healthcare needs of residents in senior housing communities, numerous barriers to engaging residents exist including the mistrust of academic or healthcare institutions, stigma about health conditions and treatment, and lack of awareness about services available. To successfully implement Interdisciplinary Wellness Clinics, a participatory approach was used through engagement of stakeholders in the housing communities. The Interdisciplinary Wellness Clinic team worked closely with the Senior Program Manager and the community and regional property managers to establish a plan for implementation. The needs of the communities, day and timing of the clinics that would fit with their schedules, and a plan for ways to invite and engage the residents was developed. Flyers were made by the interdisciplinary team for visits at each community and sign-up sheets for the services provided on any given month. The flyers were posted on bulletin boards within the communities or in a central location for residents to access them by the community property managers. Intermittently, associated with holidays for example, we also held raffles for gifts for anyone who attended the clinic. Regular meetings were held with the property managers at each of the senior housing communities to solicit their input and brainstorm strategies for continued engagement of residents and identify new approaches to increase engagement in wellness clinic services.

Abstract citation ID: igad104.0600

### ANNUAL WELLNESS VISITS (AWV) FOR RESIDENTS IN LOW-INCOME SENIOR HOUSING

Barbara Resnick[1], Jennifer Klinedinst[2], Nicole Brandt[3], and Sarah Holmes[2], *1. University of Maryland, Baltimore, Maryland, United States*, *2. University of Maryland School of Nursing, Baltimore, Maryland, United States*, *3. University of Maryland, Baltimore, Baltimore, Maryland, United States*

Despite the known benefits, less than one-fifth of all eligible Medicare patients receive an Annual Wellness Visit (AWV). Consequently, we brought the AWV to the older residents in low-income senior housing. Across all four communities the residents are invited to receive an AWV during their birthday month via an individual invitation. Invitations are sent to the facility manager who places them in the residents' mailboxes. As per Medicare requirements, the Annual Wellness Visit includes such things as medical and family history, review of providers and/or suppliers, height, weight, blood pressure monitoring, medication review, evaluations of health behaviors, cognition, immunizations, mood, safety, and health screenings. At the end of the review the resident is provided with a set of health goals for the year and a small token birthday gift from the team. Approximately half of those who were invited each month came to the clinic for their Annual Wellness Visit. None of the residents to date who have had a Welcome to Medicare Visit were aware of the opportunity. Goals have focused on better adherence to health behaviors, particularly medication adherence, diet and physical activity, and updating immunizations. Most reported adherence to appropriate age-related screenings, and monitoring of cardiovascular disease and screening for and assessment of diabetes and hypercholesteremia. Lastly, we have been able to encourage the completion of the Maryland Orders for Life Sustaining Treatment among many of the participants who were previously unaware of this form and/or not willing to address it.

Abstract citation ID: igad104.0601

### INTERDISCIPLINARY WELLNESS CLINIC SERVICES PROVIDED TO RESIDENTS IN LOW-INCOME SENIOR HOUSING

Jennifer Klinedinst[1], Barbara Resnick[2], Sarah Holmes[1], and Nicole Brandt[3], *1. University of Maryland School of Nursing, Baltimore, Maryland, United States*, *2. University of Maryland, Baltimore, Maryland, United States*, *3. University of Maryland, Baltimore, Baltimore, Maryland, United States*

To date a total of 15 visits have been made to the four senior housing communities participating in the Interdisciplinary Wellness Clinic program. Flyers are sent to the housing property managers and posted on a community bulletin board so that residents can sign up for services as they want. In addition, residents are free to walk in to the clinic during the hours we are present. At this point in time, a total of 190 residents have been evaluated, averaging about 15 residents per clinic which runs from 1 to 4pm. The majority of residents have been seen once, but 4 have been seen twice, 8 were seen 3 times, and 3 were seen 4 times. The average age of the residents attending the clinics is 77 years old, and the majority are women (91%) and nearly one-third were people of color (30%). During the visits, we have provided blood pressure checks, immunizations (flu, COVID-19 booster, Shingrix), completed Medical Orders for Life Sustaining Treatment, reviewed overall health status and health behaviors (e.g., smoking, exercise, immunizations), completed medication reviews, evaluated hearing and removed cerumen, and evaluated and addressed foot care (skin and nails) and completed Annual Wellness Visits for the residents. A number of emergency home visits and evaluations have also been provided to determine the appropriateness for an emergency room transfer or a need to intervene and call the individual's outside primary care provider.

### SESSION 2215 (SYMPOSIUM)

Abstract citation ID: igad104.0602

### GSA STUDENT CHAPTERS: NEW OPPORTUNITIES FOR US AND INTERNATIONAL HIGHER EDUCATION INSTITUTIONS

Chair: Lauren Bouchard Co-Chair: Marilyn Gugliucci
Discussant: Terri Harvath

The Gerontological Society of America (GSA) Board of Directors approved the formation of a committee to explore the creation of GSA Student Chapters in their December 2020 board meeting. After 13 meetings, a committee wrote a GSA Student Chapter guide outlining policies and procedures for implementation and operation. Next, a GSA Student Chapter pilot project was implemented during the 2022-23 academic year at nine higher education institutions (HEIs) -- eight in the US and one in Portugal. This session will provide the background of (1) how the GSA Student Chapter project began including its design and implementation process, (2) how a pilot GSA Chapter maneuvered bringing together their Sigma Phi Omega Chapter, DreamCatchers, and GSA Student Chapter under a "Gerontology Club" umbrella, (3) how another chapter developed the chapter across multiple campuses, (4) the implementation of a GSA Student Chapter that is housed in a medical school, (5) how a GSA Student Chapter formed in a non-U.S. country, (6) how to connect a student chapter in the U.S. with a chapter that is in another country. A discussant will conclude this session, and there will be time for a question and answer session.

Abstract citation ID: igad104.0603

### GSA STUDENT CHAPTERS: THE BEGINNING AND HOW TO START A CHAPTER AT YOUR INSTITUTION

Marilyn Gugliucci, *University of New England College of Osteopathic Medicine, Biddeford, Maine, United States*

The Gerontological Society of America (GSA) Student Chapter pilot project was approved by the GSA Board of Directors in December 2020. A GSA representative committee was identified where all GSA member groups were represented, including the selection of three ESPO members who applied to participate. Within a year, a GSA Student Chapter Handbook was written by the committee and reviewed by a GSA Student Advisory Panel (ESPO members who applied and agreed to serve). With the support of the GSA leadership and staff, nine higher education institutions (HEIs) volunteered to pilot a GSA Student Chapter (eight in the U.S. and one in Portugal) during the 2022-23 academic year. The first part of this session will provide information on how to create, implement, and operate a GSA Student Chapter at your HEI. The second part of this session will focus on health professions programs and integrating GSA Student Chapters with other national chapters. With a strong support system and mentoring system within ESPO, health professions programs may benefit from having student chapters integrate from various national organizations. These may provide advancement in learning skills, attitudes and knowledge, along with an extensive network of fellow students and practitioners to support their pre-clinical and clinical health professions education. This enhancement for Student Chapters, GSA with other organization chapters provides national and possibly international organizational connections/networking that will serve students throughout their careers.

Abstract citation ID: igad104.0604

### THE UNIVERSITY OF OKLAHOMA GSA STUDENT CHAPTER AND MULTICAMPUS COORDINATION AND COLLABORATION

Keith Kleszynski, *University of Oklahoma Health Sciences Center, Oklahoma City, Oklahoma, United States*

The University of Oklahoma is a public university system comprised of a main campus in Norman, Oklahoma, a health sciences campus in Oklahoma City, a campus in Tulsa, Oklahoma, as well as robust online education programming. The University of Oklahoma GSA Student Chapter launched in late 2022 and is open to student membership from all campuses within the University of Oklahoma system. Student membership recruitment is facilitated by a team of interprofessional faculty whom seek to engage any learner on any campus with an interested in gerontology in its broadest sense. The University of Oklahoma GSA Student Chapter utilizes an umbrella and sub-committee structure to maintain governance. This structure is a result of inter-disciplinary collaboration across campuses, colleges, and among faculty and is replicable in university settings with multiple campuses and diverse sets of learners.

Abstract citation ID: igad104.0605

### THE CREATION OF THE FIRST INTERNATIONAL GSA STUDENT CHAPTER IN PORTUGAL

Giuliana Casanova[1], Lígia Passos[1], Edna Rodrigues[2], and Oscar Ribeiro[1], *1. University of Aveiro, Aveiro, Aveiro, Portugal*, *2. School of Medicine and Biomedical Sciences, Porto, Porto, Portugal*

The Gerontological Society of America (GSA) Student Chapter Pilot Program provides a platform for students in all academic levels to exchange academic ideas, network with industry professionals, and share scientific and educational experiences. Developing an international student chapter based at the University of Aveiro in Portugal was a challenging but fullfiling task, accomplished through the dedication and perseverance of its members. This student chapter, created by and for students, aims to serve as an opportunity for networking, education, research, and community work. We aspire to create a bridge between Portugal and the United States, promoting educational and research opportunities for students and increasing awareness in the field of aging. By sharing the challenges and rewards we encountered during the creation of this chapter, we hope to inspire other universities worldwide to do the same and build an even larger international network. Through this network, students working with older adults can find common ground, share ideas, create connections, and develop new ways to enhance the field of Gerontology worldwide. The GSA student chapter is a unique opportunity that enables students to connect, share knowledge and contribute to the field of Gerontology. Our hope is that this model will be replicated globally, fostering collaboration and advancing our understanding of aging in all corners of the world.

Abstract citation ID: igad104.0606

### OPTIMIZING GSA STUDENT CHAPTER NETWORKING ACROSS CAMPUS AND THE WORLD

Lauren Bouchard, *Portland State University, Portland, Oregon, United States*

The Portland State University (PSU) Gerontological Society of America Student Chapter was formed as a new student organization in 2022. Due to student interest and enthusiasm, this chapter was eager to invest in connections within PSU departments (e.g., public health, social work, and urban affairs) across Portland area campuses (e.g., Oregon Health and Science University) and between other student chapters internationally. Initially, this chapter focused on relationships between undergraduate and graduate students invested in aging research and education on PSU's campus. Once a core group was formed, the chapter recruited members across campus, created an Aging 101 Service event to reach all students, and developed connections with professionals in the regional area. With guidance from the faculty advisor, this chapter reached out to the chapter at the University of Porto, the University of Aveiro in Porto, Portugal, to create a dual chapter networking event with colleagues in Portugal. This presentation will explain the chapter networking and provide suggestions to create regional, national, and international connections.

### SESSION 2220 (PAPER)

### ADULT PROTECTION AND ELDER ABUSE (PAPERS) I

Abstract citation ID: igad104.0607

### FACTORS ASSOCIATED WITH ELDER MISTREATMENT AMONG PERSONS LIVING WITH DEMENTIA

Zach Gassoumis[1], Jeanine Yonashiro-Cho[1], Elizabeth Avent[1], and Laura Mosqueda[2], *1. University of Southern California, Los Angeles, California, United States, 2. Keck School of Medicine of the University of Southern California, Los Angeles, California, United States*

Persons living with dementia (PLWD) are at increased risk of experiencing elder mistreatment (EM), including emotional, physical, or sexual abuse, neglect, or financial exploitation. Because PLWD rely on Care Partners (CP) for varying degrees of support with ADLs/IADLs, factors contributing to EM occurrence may be present in the characteristics of the PLWD, CP, and the environment in which they interact. The present study sought to identify associated risk and protective factors of EM in PLWD-CP care groups. As part of a longitudinal study, survey administration interviews were conducted with 163 care groups consisting of a PLWD and their CP(s) (n=184) to gather data on participant characteristics, current and past relationship quality, current caregiving interactions, and EM occurrence. This analysis used baseline data with responses weighted to control for participation of multiple CPs. Bivariate logistic regression was used to model the likelihood of the PLWD experiencing EM. Nearly a quarter of PLWDs (23.9%; n=39) experienced EM, with emotional abuse (19.0%), neglect (7.4%), and financial exploitation (4.3%) occurring most frequently. Preliminary analyses reveal significant bivariate associations between EM and PLWD alcohol/substance abuse (OR=3.244;CI=1.020-10.320) and educational attainment (OR=1.495;CI=1.077-2.075), and CP symptoms of depression (OR=1.170;CI=1.046-1.308) or anxiety (OR=1.194;CI=1.085-1.315), high caregiving burden (OR=1.099;CI=1.042-1.159), expectations of themselves (OR=1.747;CI=1.064-2.867) and the PLWD (OR=1.730;CI=1.113-2.688), and alcohol/substance abuse (OR=2.163;CI=1.080-4.333). These associations provide a foundation for further evaluation of these characteristics as potential longitudinal risk factors for EM, and provide potential targets for the development of prevention and intervention strategies that are tailored to care groups' characteristics and needs.

Abstract citation ID: igad104.0608

### INTEGRATING PRACTITIONER KNOWLEDGE INTO AN ALGORITHM TO IDENTIFY CASES FOR REVIEW BY AN ADULT MALTREATMENT MDT

Erin Thayer[1], Julia Martinez[2], Lori Mars[3], and Zach Gassoumis[4], *1. University of Southern California - Keck School of Medicine, Los Angeles, California, United States, 2. University of Southern California Keck School of Medicine, Los Angeles, California, United States, 3. USC, Alhambra, California, United States, 4. University of Southern California, Los Angeles, California, United States*

Case review multidisciplinary teams (MDTs) bring together professionals from health, social service, and justice systems to address complex cases of elder abuse, neglect, self-neglect, and maltreatment of younger adults with disability or vulnerability. MDTs are a promising intervention, but MDTs nationwide face challenges identifying cases for review. In partnership with San Francisco Adult Protective Services (APS), we developed an algorithm-driven process to systematically identify APS cases that appear appropriate for MDT review. To construct the algorithm, a retrospective analysis of APS case data was augmented by insights received during six listening sessions held with 48 study partners (MDT members and APS staff). These sessions gauged perspectives on cases' suitability for MDT review. Listening session transcripts were coded using constant comparative analysis, revealing themes of Client, Case Details, and Service Utilization Patterns. Client included physical or cognitive impairment, high-risk of abuse or other harms, need for assistance from multiple service systems, and limited motivation to make changes. Case Details referred to aspects of the abuse such as concurrent self-neglect, client protectiveness of the suspected abuser, client denial of the abuse, and limited evidence of criminal conduct. Service Utilization Patterns were recurrence within APS, attempting multiple solutions, inability to provide services, and referral by medical professionals, fire departments, or paramedics. These topics guided the inclusion of variables in the project's algorithms. This mixed-methods approach provides an example of community-engaged research being applied to develop knowledge and guide the practice of service delivery for vulnerable older adults and other victims of adult maltreatment.

Abstract citation ID: igad104.0609

### PREDICTING CLIENT RETURN TO ADULT PROTECTIVE SERVICES: A SECONDARY ANALYSIS OF RECURRENCE USING NATIONAL APS DATA

Raphael Gaeta[1], Zach Gassoumis[2], and Peter Lovegrove[3], *1. New Editions Consulting, Inc., Falls Church, Virginia, United States, 2. University of Southern California, Los Angeles, California, United States, 3. New Editions Consulting, Charlottesville, Virginia, United States*

APS programs are provided by state and local governments across the U.S. to serve older adults and adults with disabilities facing abuse, neglect, or exploitation ("maltreatment"). The purpose of this analysis was to identify predictors of recurrence, which occurs when clients return to APS for investigation/services after their cases have been closed. The analysis combined several data sources, including the National Adult Maltreatment Reporting System (NAMRS), the first comprehensive, national data reporting system for APS programs. This analysis represents the first study of this kind to use NAMRS data. The main analyses used Bayesian logistic regression with client, case, and state-level APS system characteristics to predict 12-month recurrence in a multi-state model and in separate single-state models. The sample included data on 1,211,360 APS episodes that closed from 2015-2019 in 19 states. These episodes contained information on 946,477 unique APS clients. About one in five clients in this analysis experienced at least one episode of recurrence. Multivariate results revealed several significant predictors of recurrence (e.g., gender, maltreatment type, maltreatment disposition, episode length, case closure reason), with the largest being age (85+ vs. ≤59 AOR=0.876) and self-neglect (AOR=1.132). The findings may be helpful for APS programs to better recognize and serve clients at high risk of returning to APS after their cases close. The findings also highlight the ambiguity surrounding the concept of recurrence in the APS field and the need for further research to determine the circumstances in which recurrence may be considered a negative or a positive outcome.

Abstract citation ID: igad104.0610

### PREDICTORS OF WHETHER SELF-NEGLECT IS VALIDATED BY ADULT PROTECTIVE SERVICES CASE MANAGERS

Samantha Tuft, Farida Ejaz, and Courtney Reynolds, *Benjamin Rose Institute on Aging, Cleveland, Ohio, United States*

The most reported allegation of abuse to Adult Protective Service (APS) agencies nationwide is self-neglect (SN). Researchers collaborated with Oklahoma APS to understand characteristics of community-dwelling clients reported to APS for alleged SN, and examine predictors associated with whether cases were validated by APS case managers. Baseline phone interviews were conducted with 188 clients reported to APS for self-neglect during the COVID-19 pandemic. A logistic regression was run to examine the effects of age, gender, income, race, education, cognitive status, ADL impairment, depression, recent nursing home placements, hospitalizations and falls on the likelihood that participants' allegation of SN was validated. The model was statistically significant, $\chi2(12) = 26.67$, $p = .009$. It explained 24.1% of the variance in case validation and correctly classified 81.8% of cases. Having a history of being in a short-term nursing home and having a history of falls was significantly associated with the increased likelihood of having a validated SN allegation. However, a history of hospitalization was significantly associated with a reduction in the likelihood of having a validated SN allegation. These preliminary findings suggest that perhaps hospital discharge planners may be assisting SN patients to access home-and-community based services (HCBS) early on to address their SN so it is not validated by APS compared to nursing home discharge planners. Implications for practice include educating APS staff on predictors of SN and collaborating with community-based partners like short-term rehabilitation facilities to address SN issues to reduce the likelihood of having a case validated by APS.

Abstract citation ID: igad104.0611

### PREVENTING ELDER MISTREATMENT THROUGH A CAREGIVER-FOCUSED INTERVENTION: THE COACH PROGRAM

Kathleen Wilber[1], Julia Rowan[2], Jeanine Yonashiro-Cho[1], Laura Mosqueda[3], Anthony Hou[4], and Zach Gassoumis[1], *1. University of Southern California, Los Angeles, California, United States*, *2. University of Southern California Keck School of Medicine, Los Angeles, California, United States*, *3. Keck School of Medicine of the University of Southern California, Los Angeles, California, United States*, *4. Kaiser Permanente, Los Angeles, California, United States*

Elder mistreatment has negative implications for individuals, families, communities, and society as a whole, yet little research exists on interventions or prevention approaches. This study reports on the results from the pilot test of an elder mistreatment prevention program. The Comprehensive Older Adult Caregiving Supports (COACH) program was developed as a strengths-based person-centered caregiver support intervention, built on evidence-based prevention approaches used in other types of family violence. In a double-blind randomized controlled trial, family caregivers (n=80) of older adults aged 65 and older who were members of Kaiser Permanente completed surveys at baseline, post-test, and three-month follow-up. The primary outcome was elder mistreatment; additional proximal outcomes were caregiver burden, quality-of-life, anxiety, and depression. All analyses were conducted using nonparametric tests. The treatment group had no mistreatment at 3-month follow-up, a significantly lower rate than the control group (treatment=0%, control=23.08%, p=0.010). Among the proximal outcomes, a significant difference in social quality-of-life was seen at post-test but did not persist to the 3-month follow-up. No further proximal outcome effects were detected. This pilot program offers a promising model to reduce elder mistreatment and its resultant impacts on the health and well-being of both caregivers and the older adults they care for. In helping to ameliorate mistreatment, COACH supports individuals providing crucial care to older adults with chronic health conditions and reduces the burden of addressing this complex condition for an already strained geriatric health and social service delivery system.

### SESSION 2225 (PAPER)

### SOCIAL DETERMINANTS OF HEALTH AND AGING I

Abstract citation ID: igad104.0612

### BIRTHPLACE AND COGNITION AMONG US OLDER ADULTS: EVIDENCE FROM THE HARMONIZED COGNITIVE ASSESSMENT PROTOCOL (HCAP)

Zhuoer Lin, and Xi Chen, *Yale University, New Haven, Connecticut, United States*

Growing evidence shows that place of birth and related circumstances may impose lasting and multiplicative impacts on a host of late-life outcomes; yet how it affects various domains of cognitive function in late-life is less understood. In this paper, we exploit the comprehensive cognitive assessments in the Health and Retirement Study (HRS) Harmonized Cognitive Assessment Protocol (HCAP) and HRS geographic data at birth to determine to what extent birth location measured at the state level is associated with cognitive status in later life for a representative sample of Americans over age 50 (N=3,324). We found that state of birth significantly influenced various cognitive domains later in life (including episodic memory, executive function, attention, visuospatial function, language fluency and orientation); and the regional rankings tended to be highly consistent across the cognitive assessments in each domain. Regression analysis demonstrated that the geographic disparities in cognition across birth states could be partly explained by the late-life differences in socioeconomic statuses. Nevertheless, Shapley decompositions demonstrated that, after accounting for mid-life to late-life factors, state of birth still contributed 20%-30% of the explained variations in cognitive outcomes. Particularly, foreign-born individuals and those born in the southern states tended to score the lowest on average on each of the cognitive tests/domains and can account for most of the explained differences in cognition. These findings suggest that place of birth may have lasting impacts on disparities in cognition later in life. Public policy should play pivotal roles in leveling the playing field in early life.

Abstract citation ID: igad104.0613

### CHILDHOOD INTERPERSONAL TRAUMA AND DEPRESSION OF CHINESE IN MID LATER LIFE: MEDIATION EFFECT OF SOCIAL INTEGRATION

Jiajia Zhou, and Shuai Zhou, *The Hong Kong Polytechnic University, Hong Kong, Hong Kong*

**Objectives:** This study investigates the long-term associations between five types of childhood interpersonal trauma (i.e., physical neglect, emotional neglect, physical abuse, being bullied, exposure to interparental violence) and depression in mid-later life. The mediation roles of social integration in the family (i.e., satisfaction with marriage and children) and broader social contexts (i.e., social participation and perceived social support) are examined in the associations.

**Methods:** This study used two waves (2014 and 2015) of data from the China Health and Retirement Longitudinal Study. The analytical sample included 12,486 participants aged 45 years and older.

**Results:** People who was bullied, physically abused, and witnessed interparental violence during childhood are more likely to develop depression in mid-later years, controlling for socio-demographic characteristics, childhood family socio-economic status, and childhood parental mental health. However, childhood physical neglect and emotional neglect are not directly related to depression in mid- and late-adulthood. The associations between childhood interpersonal trauma and depression were mediated by the satisfaction with marriage, satisfaction with children, and perceived social support, but not social engagement.

**Discussion:** This study calls for attention on the long-term consequences of multiple types of childhood interpersonal trauma on mental health among middle-aged and older adults. It provides a comprehensive understanding of the mechanisms of social integration in bridging early-life interpersonal trauma and depression in mid-later life.

Abstract citation ID: igad104.0614

### DISPROPORTIONATE INCARCERATION AFTER THE 1994 CRIME BILL AND COGNITIVE FUNCTION IN OLDER ADULTS RACIALIZED AS WHITE

Paris Adkins-Jackson[1], Ariana Gobaud[2], Boeun Kim[3], Tiffany Ford[4], Tanisha Hill-Jarrett[5], César Higgins Tejera[6], Lisa Barnes[7], and Jennifer Manly[8], *1. Columbia University, New York City, New York, United States*, 2. *Columbia Mailman School of Public Health, New York City, New York, United States*, 3. *Johns Hopkins University, Baltimore, Maryland, United States*, 4. *University of Illinois at Chicago, Chicago, Illinois, United States*, 5. *University of California, San Francisco, San Francisco, California, United States*, 6. *University of Michigan, Ann Arbor, Michigan, United States*, 7. *Rush University Medical Center, Chicago, Illinois, United States*, 8. *Columbia University Irving Medical Center, New York City, New York, United States*

Structural racism may drive the constellation of determinants that increase racialized disparities in cognitive function. The 1994 Crime Bill is a decades-old federal policy that established an infrastructure in which local governments are incentivized to fund prisons, increase average prison time, and implement adverse policing quotas. Foundational research suggests structural racism via high-contact policing and incarceration for persons racialized as Black, is associated with anxiety and depressed mood among, influencing cognitive function. Minimal research explores whether the intended beneficiaries of structural racism, persons racialized as White, have truly benefitted. We used US county-level incarceration data to compute the Black-White difference in jail and prison population rates between 1995-2005. These data were averaged by decade and linked by county to individual-level cognitive data for participants racialized as White (>50 years) in the Health and Retirement Study (N=13,016). Cognitive performance and cognitive change over time was assessed using a 27-item global cognitive score from the modified Telephone Interview for Cognitive Status for biannual years between 2006-20. A mixed effects model estimated the association of a one-unit change in county-level incarceration on cognitive performance. Greater 10-year Black-White disparities in the jail population were associated with greater cognitive performance among participants racialized as White one year later (b=0.13, 95% CI: 0.02, 0.25). Findings suggest structural racism via disproportionate high-contact policing and incarceration of persons racialized as Black is linked to better cognitive function for older adults racialized as White.

Abstract citation ID: igad104.0615

### SOCIAL DETERMINANTS OF COGNITIVE HEALTH: A SCOPING REVIEW

Lingling Zhang, and Yao Zhang, *University of Massachusetts Boston, Boston, Massachusetts, United States*

Population aging has caused a substantial increase in neurodegenerative disorders, such as Alzheimer's disease (AD), the fifth leading cause of death for people aged 65 and older. It has posed tremendous challenges to families and society. The existing research shows that postponing AD onset by 5 years would reduce the disease prevalence by half. Therefore, to better understand the modifiable risk factors, including social determinants of health (SDOH) for cognitive health, will be helpful for early prevention and resource allocation. SDOH are classified into five domains (healthcare access and quality, education access and quality, social and community context, economic stability, and neighborhood and built environment). There are studies examining the relationship between one or more domains of SDOH and cognitive health, however, to our knowledge, there is no comprehensive review has been done to synthesize the evidence from all domains of SDOH. This literature review aims to map the existing evidences on social determinants of cognitive health to inform future programs and policies. Twenty-nine studies were included in the review through a systematic search of relevant literature published on peer-reviewed journals from four electronic databases (Pubmed, PsychInfo, CINAHL, and MEDLINE) during 2012-2022. The review summarized a broad range of empirical research on how SDOH affect cognition, as well as revealed the racial disparities in cognitive function, which is worth noting. The favorable SDOH conditions such as better healthcare access, education, social engagement, etc. were proved protective factors for cognitive impairment. Race and racial segregation were found to be the opposite.

Abstract citation ID: igad104.0616

### THE EFFECT OF MENTAL HEALTH ON DETROIT RESIDENCE IN OLDER ADULTS DURING THE COVID-19 PANDEMIC

Ivana Vaughn[1], Sean Yaphe[2], Lakshmi Sundaresan[1], Jonathan Freedman[1], Samuel Jesse Weinberg[3], Lois Lamerato[1], and Katarzyna Budzynska[1], *1. Henry Ford Health, Detroit, Michigan, United States*, *2. Clinique L'Actuel, Montreal, Quebec, Canada*, *3. Don Mills Family Health Team, Toronto, Ontario, Canada*

The COVID-19 pandemic had a tremendous impact on global experience of anxiety and depression. Due to social isolation and quarantine, older adults faced unique challenges that disproportionately increase their risk for health outcomes. The purpose of this retrospective study was to examine the relationship between Detroit versus non-Detroit residence and incident mental health disorders. The patient population included older adults (age 65 and older) from a large metropolitan urban health system with patient encounters between December 2018 - June 2021. The primary predictor, Detroit metropolitan status, was based on zip code. Outcome variables, incident depression, anxiety, and composite mental health disorders, were based on screening measures (PHQ-2, PHQ-9 and GAD-7 scores), ICD diagnosis, medical prescriptions, and behavioral health referrals. Fully adjusted logistic regression analysis derived odds ratios of incident outcomes in Detroit versus non-Detroit patients. A total of 5,300 older adult patients were included in this study. Older adults residing in Detroit had significantly reduced odds of incident anxiety compared to non-Detroit residents (OR:0.45; 95%CI:0.26,0.75, p=0.003). Incident depression and composite mental health outcomes also showed protective effects in Detroit patients; however, these outcomes were not statistically significant. Overall, patients residing in Detroit had 55% decreased odds of developing anxiety during the pandemic. Additional research should explore facilitators and barriers to mental health diagnoses during the COVID-19 pandemic for older adults in an urban setting.

### SESSION 2230 (PAPER)

### SOCIAL ISOLATION AND LONELINESS II

Abstract citation ID: igad104.0617

### "YOU BECOME LESS OF A PERSON": BEING UNNEEDED AND THE POLITICAL ECONOMY OF LONELINESS IN LATER LIFE

Ad Maulod, Malcolm Ravindran, and Angelique Chan, *Duke-NUS Medical School, Singapore, Singapore*

Loneliness is a significant public health concern among older persons and refers to the discrepancy between actual and desired forms of social connection. Loneliness is often characterized in terms of (i) lacking companionship, (ii) being left out and (iii) feeling isolated. In comparison, older persons' experience of being unneeded is relatively underexamined as a construct of loneliness. Being unneeded connects to constrains in attaining social recognition and underpins the complex dynamics of social exchange practices. This study explores the relationship between being unneeded and loneliness in later life, focusing on the impact on one's identity and ability to form connections with others. We conducted in-depth interviews with 44 older persons in Singapore with varying experiences of loneliness. Using a life-course perspective, participants narrated their experiences of loneliness across domains of family, friendships, work, and community. Loneliness, attributed to being unneeded, features more prominently in later life and pertains to perceived limited personal mastery, negative ageing perceptions, role-loss, life transitions and declines in health. Experiences of being unneeded is embedded in older persons' beliefs that their self-worth and social recognition is tied to individual strive and diligence– an ideology of meritocracy that has been perpetuated by the Singapore state. Studies have shown how beliefs in meritocracy are associated with lowered likelihood of loneliness among older persons. The Singapore story indicates otherwise – loneliness prevails when older persons with declining resources struggle to find a place in a society which accords worth and recognition to one's productivity and contribution to the nation.

Abstract citation ID: igad104.0618

### ASSESSING BIDIRECTIONAL LONGITUDINAL ASSOCIATIONS BETWEEN SOCIAL RESOURCES AND COGNITIVE DECLINE IN LATE LIFE

Yun Zhang[1], Sean Clouston[2], Stacey Scott[2], and Dylan Smith[2], *1. Columbia University, New York City, New York, United States*, *2. Stony Brook University, Stony Brook, New York, United States*

**Objectives:** To better understand bidirectional longitudinal relationships between social networks and cognitive decline. Method: We examined nine waves of data from the National Health and Aging Trends Study (2011-2019). Our primary outcome was global cognition, consisting of a summation of scores on episodic memory, orientation, and executive function tests. Social network measures included measures of social participation, number and type of social ties, attributes of network alter, and neighborhood markers of social cohesion and disorder. We applied linear mixed models to study associations between time-invariant measures of social cohesion and disorder with cognitive decline over time. We employed dual-outcomes mixed models to examine the bidirectional linkages between time-varying measures of social network structures and cognitive decline. Result: Eligible participants (N = 7,325) were included at baseline and followed for an average of 4.86±3.22 years/waves. Having more friend-ties and a higher percentage of college graduates within a social network at baseline were associated with slower declines in global cognition and episodic memory. Additionally, higher global cognition at baseline was associated with smaller reductions in social network size. Less disorientation at baseline was associated with a slower decline in social participation. Social cohesion and environmental disorder were not associated with cognitive decline.

**Conclusion:** Social networks may improve cognitive health but are also vulnerable to declining orientation. Results suggest that bidirectional longitudinal relationships link social network structure with cognitive decline and inform our understanding of the framework of associations between diverse aspects of social resources and cognitive decline in a longitudinal setting.

Abstract citation ID: igad104.0619

### INTERGENERATIONAL RELATIONSHIPS AND HAPPINESS: MEDIATING ROLES OF PSYCHOLOGICAL RESILIENCE AND COVID-19 ANXIETY

Xue Bai, Shuai Zhou, and Chi-Ko Lee, *The Hong Kong Polytechnic University, Hong Kong, Hong Kong*

The COVID-19 pandemic has largely worsened the well-being of older adults worldwide. Despite recent literature highlighting either social connectedness or psychological responses, whether and how intergenerational relationships influence the emotional well-being of older adults during the pandemic remains unclear. This study aimed to examine the association between intergenerational relationships and happiness among ageing Chinese adults during the pandemic and the mediating roles of psychological resilience and COVID-19 anxiety. A representative survey was conducted from November 2020-January 2021 to collect data from 819 Chinese adults aged 55 or above (52.4% females) in Hong Kong. Path models were used to examine the associations between intergenerational relationships, resilience, COVID-19 anxiety, and happiness. Regression results showed that intergenerational relationship quality was associated with more psychological resilience ($b = 0.21$, $p < 0.001$), lower COVID-19 anxiety ($b = -0.14$, $p < 0.001$), and a high level of happiness ($b = 0.15$, $p < 0.001$). COVID-19 anxiety partially mediated the effects of intergenerational relationship quality on happiness. Moreover, psychological resilience was associated with a higher level of happiness through ameliorated COVID-19 anxiety. This study elucidates that intergenerational relationship quality was positively associated with happiness during the pandemic and the association could be partly explained by enhanced psychological resilience and alleviated COVID-19 anxiety. The findings provide implications that targeted policies and services that strengthen intergenerational solidarity and psychological resilience may be effective in improving emotional well-being in older populations.

Abstract citation ID: igad104.0620

### LONELINESS AMONG OLDER FAMILY CAREGIVERS: A STUDY OF CAREGIVING INTENSITY, TYPE AND LOCATION BASED ON THE CLSA

Lun Li[1], Andrew Wister[2], Yeonjung Lee Lee[3], and Boah Kim[2], *1. MacEwan University, Edmonton, Alberta, Canada*, 2. *Simon Fraser University, Vancouver, British Columbia, Canada*, 3. *Chung-Ang University, Seoul, Republic of Korea*

Caring for family members during aging is a risk factor for loneliness among older caregivers (65 years and older). However, loneliness is less understood than other caregiving outcomes (e.g., burden, depression) in caregiving literature. This study aims to examine loneliness among older caregivers using the second wave of data from the Canadian Longitudinal Study on Aging (2015 to 2018). Based on 6603 older caregivers, linear regression was conducted to examine the relationship between loneliness and caregiving intensity, caregiver type and care location, and ANCOVA was performed to examine the intersection of caregiving intensity and caregiver type, as well as caregiving intensity and care location. A higher level of loneliness is significantly associated with spousal caregivers (vs. non-spousal family caregivers), higher caregiving intensity (vs. lower caregiving intensity), and caregiving to someone living in another household or healthcare institution (vs. in the same household). In addition, spousal caregivers with higher caregiving intensity, and older caregivers to someone in healthcare institutions with higher caregiving intensity are two main risk groups for greater loneliness. The findings contribute to a better understanding of the relationship between loneliness and caregiving situations among older caregivers. Remarkably, more service programs are needed to support older caregiver who support loved ones in healthcare institutions.

Abstract citation ID: igad104.0621

### SOCIAL NETWORK SIZE AND THE TRANSITION TO NONDRIVING

Kellia Hansmann[1], Ronald Gangnon[2], Carolyn McAndrews[3], and Stephanie Robert[2], *1. William S Middleton Memorial Veterans Hospital, Madison, Wisconsin, United States*, 2. *University of Wisconsin-Madison, Madison, Wisconsin, United States*, 3. *University of Wisconsin Madison, Madison, Wisconsin, United States*

Having family and friends available to provide rides is an important coping factor after people stop driving. Social networks may also be a means by which older adults can reduce or stop driving earlier while maintaining their mobility. We examined whether social network size was associated with 3-year changes in driving behavior among U.S. older adults. We analyzed data on community-dwelling drivers in the National Health and Aging Trends Study (n = 5,140; 2015 cohort). We categorized whether participants reported zero, one, or two or more people to talk to about important things (who lived in their county). We used weighted logistic regression models to estimate whether having more same-county people to talk to about important things was associated with incidence of any of four new driving avoidance behaviors (avoiding driving: at night, in bad weather, on highways, or alone) or driving cessation, at three-year follow up, adjusting for biopsychosocial variables. There was no significant association between having two or more same-county people to talk to about important things and the odds of driving cessation at follow up (adjusted Odds Ratio [aOR]: 1.00, 95% Confidence Interval [CI]: 0.58-1.71) compared to drivers who reported having no one to talk to about important things. Similarly, there was no significant association with the incidence of one or more new driving avoidance behaviors (aOR: 1.16 95% 95% CI: 0.91-1.47). Future research should examine whether other aspects of social networks (e.g., type, quality, closer proximity) might play important roles in supporting the transition to non-driving.

Abstract citation ID: igad104.0622

### THE EFFECT OF INTERVENTIONS TO REDUCE SOCIAL ISOLATION AND LONELINESS AMONG FAMILY CAREGIVERS: A SYSTEMATIC REVIEW

Boah Kim[1], Lun Li[2], and Andrew Wister[1], *1. Simon Fraser University, Vancouver, British Columbia, Canada*, 2. *MacEwan University, Edmonton, Alberta, Canada*

Social isolation and loneliness are among the key risk factors for deleterious health and well-being among family caregivers. Interventions to reduce caregiver isolation and loneliness are of great interest as family caregivers are essential in community care, particularly senior care. The current study aims to review the literature on interventions regarding social isolation and loneliness among family caregivers and summarize successful strategies for reducing isolation and loneliness among family caregivers. A systematic literature review was conducted. Electronic databases were searched, and 1130 peer-reviewed journal articles published in English since 2000 were identified. A total of 34 eligible studies (16 quantitative studies, 8 qualitative studies, and 10 mixed methods studies) were included in this review. Findings from qualitative studies (including qualitative components from mixed methods studies) consistently reveal the positive experience of family caregivers in reducing isolation and loneliness. However, findings from quantitative studies add more layers of complexity with considerable evidence of failing to identify the change of isolation or loneliness after attending interventions. The studied caregiver groups, length of intervention, and scales used to measure isolation and loneliness are closely relevant to the effect of the intervention. Besides peer support, mentoring and skill training, technology (Internet-based programs) plays a significant role in facilitating family caregivers to maintain social interaction and connectedness. This review confirms the pleasant impacts of different interventions in reducing caregiver isolation and loneliness, but also calls for more research in identifying changes of isolation and loneliness both short- and long-term among family caregivers.

### SESSION 2235 (SYMPOSIUM)

Abstract citation ID: igad104.0623

### THE EYE OF THE STORM: END-OF-LIFE CARE IN NEW YORK CITY DURING THE COVID-19 PANDEMIC: LESSONS FOR THE FUTURE OF HOSPICE

Chair: Daniel David Co-Chair: Emily Franzosa Discussant: Abraham Brody

Public health containment and mitigation policies in response to the COVID-19 pandemic, including social distancing, infection control, and self-quarantine led to a substantial reduction in transmission of COVID-19. Yet they also brought about significant disruptions in healthcare, particularly care at the end of life. These challenges were amplified in older adults of color and those who contend with differing social determinants of health that impact care outcomes. Hospices played a key role in supporting patients at home throughout the pandemic and yet concomitant with the surge in demand for hospice, there was a gap in hospice delivery, exposing latent vulnerabilities in the capacity to respond to a crisis that demanded a coordinated, synchronized approach. We present the findings of an innovative, mixed methods study funded by the National Institute of Nursing Research that combines qualitative content analysis of linked electronic health records with qualitative stakeholder interviews to gain an in-depth understanding of the challenges of delivering hospice care in NYC during the pandemic. Interviews include the perspectives of nurses, managers, administrators, physicians, medical directors, social workers, and clergy from several healthcare practices. Our presenters address 4 key areas: a) Provider experiences and organizational challenges and successes, b) Challenges in transitioning from primary care to hospice, c) Efforts to provide equity-focused hospice care, and d) The central role of home health aides in end-of-life care. Together, these experiences provide insights into lessons learned and point to new opportunities to design and deliver high-quality hospice care in the future.

Abstract citation ID: igad104.0624

### “BUILDING A PLANE WHILE IT'S TAKING OFF”: PERSPECTIVES OF HOSPICE PROVIDERS FROM GROUND ZERO OF THE COVID-19 CRISIS IN NYC

Daniel David[1], Jonelle Boafo[2], Patricia Kim[3], Laura Moreines[4], Emily Franzosa[3], Dena Schulman-Green[5], Abraham Brody[1], and Melissa Aldridge[3], *1. NYU Rory Meyers College of Nursing, New York City, New York, United States*, *2. Rory Meyers College of Nursing, New York City, New York, United States*, *3. Icahn School of Medicine at Mount Sinai, New York City, New York, United States*, *4. New York University, New York City, New York, United States*, *5. NYU, College of Nursing, New York City, New York, United States*

The provision of care to older adults with serious illness through home hospice visits was extremely challenging for interdisciplinary teams during the COVID-19 pandemic in NYC. We interviewed 20 interdisciplinary providers (nurses, managers, administrators, physicians, medical directors, social workers, and clergy) from three organizations that provided end-of-life care in-person and through telehealth to older adults and their caregivers in NYC. Qualitative content analysis was used to identify themes, categorize findings, and identify important adaptations that were required to provide hospice care during the pandemic. Findings reveal how organizations, providers, patients, and families contended with fear, communicated challenges, and adapted to an ever-changing regulatory landscape. The experience of providing care was markedly different for those who were required to deliver care in person vs. by telehealth. Difficult care choices were made impacting access and the services offered by hospice organizations. Salient themes included: organizational challenges – “building a plane while it’s taking off” (e.g., implementing institutional safety protocols with imperfect COVID-19 information to a wary staff), patient/family challenges – “you aren’t coming in my home” (e.g., inability to enroll patients into hospice without a face-to-face visit), and provider challenges – “some things you can’t do through telehealth.” (e.g., telehealth is a vastly inferior medium to teach isolated family caregivers how to provide end-of-life support to dying family members). These findings provide an opportunity to consider the unique qualities of hospice care, what care can and cannot be effectively delivered through virtual modalities, and what effective adaptations may be sustained long-term.

Abstract citation ID: igad104.0625

### TRANSITIONING TO HOSPICE DURING THE COVID-19 CRISIS: LESSONS FOR CARE COORDINATION AT THE END OF LIFE

Emily Franzosa[1], Patricia Kim[1], Laura Moreines[2], Jonelle Boafo[3], Daniel David[4], Abraham Brody[4], Margaret McDonald[5], and Melissa Aldridge[1], *1. Icahn School of Medicine at Mount Sinai, New York City, New York, United States*, *2. New York University, New York*

*City, New York, United States*, *3. Rory Meyers College of Nursing, New York City, New York, United States*, *4. NYU Rory Meyers College of Nursing, New York City, New York, United States*, *5. Visiting Nurse Service of New York, New York City, New York, United States*

The COVID-19 pandemic caused widespread delays and disruption in end-of-life services. We examined the impact of these disruptions among patients in a large, NYC health system who were referred to hospice and died between March 1, 2020 and March 1, 2021. We linked the electronic health records (EHR) of 124 patients cared for by a geriatric outpatient practice to the hospice EHRs to create longitudinal trajectories of patients' care. We conducted an in-depth qualitative thematic analysis of linked medical and hospice records to examine provider and patient experiences as reflected in the EHR. Our sample (mean age of 91 years) were enrolled in hospice for an average of 173 days, and 81% died at home. We found challenges transitioning from medical to hospice care, including referral and enrollment delays due to infection prevention protocols, communication issues, staffing and equipment shortages (e.g., oxygen, morphine). These disruptions impacted quality of care as patients experienced shortages in personal care services, medical equipment and supplies, and missed home visits due to fear or isolation protocols. Virtual visits helped maintain access to hospice, particularly social work and spiritual care, as did ongoing communication with both the primary care and hospice teams. Our findings demonstrate that while COVID-19 added care transition challenges, new flexibilities and adaptations helped manage disruptions. Retaining these practices, such as reimbursing telehealth social work and spiritual care visits and maintaining ongoing patient contact with trusted primary care providers may help extend emotional support to patients and families during the end of life.

Abstract citation ID: igad104.0626

### IS HOME HOSPICE THE "GREAT EQUALIZER?"—ADDRESSING EQUALITY AND EQUITY DURING THE COVID-19 PANDEMIC

Laura Moreines[1], Jonelle Boafo[2], Emily Franzosa[3], Patricia Kim[3], Dena Schulman-Green[4], Melissa Aldridge[3], Abraham Brody[5], and Daniel David[5], *1. New York University, New York City, New York, United States*, *2. Rory Meyers College of Nursing, New York City, New York, United States*, *3. Icahn School of Medicine at Mount Sinai, New York City, New York, United States*, *4. NYU, College of Nursing, New York City, New York, United States*, *5. NYU Rory Meyers College of Nursing, New York City, New York, United States*

COVID-19 illuminated many of the inequities that exist in the current healthcare system, especially for those who are most vulnerable and facing the end of life. Social determinants of health (SDOH) directly and indirectly influence access to the quality of care received by patients enrolled on the hospice benefit. We interviewed 20 interdisciplinary providers (nurses, managers, administrators, physicians, medical directors, social workers, and clergy) between September 2022 and January 2023 from three healthcare organizations that provided hospice care to older adults and their caregivers in NYC regarding their perceptions of the impact of SDOH on the delivery of hospice care during the pandemic. We then conducted rapid analysis of their responses. Findings emerged around three salient themes: 1. "I provide equal care to everyone" (Some providers recognize SDOH result in inequities within hospice, whereas others perceive hospice as an equalizer of care); 2. "They're going to kill us" (Describing barriers that challenge hospice enrollment and continued access to care); and 3. "We've got to do better" (Addressing inequity through organizational and community programs that address barriers and improve access to hospice). Overall, we learned that SDOH must be addressed in a proactive rather than a reactive way to ensure hospice access. Systemic, rather than individual approaches were needed to overcome existing barriers exacerbated by the pandemic. To this end we will discuss lessons learned and strategies for program development and equitable delivery of home hospice care.

Abstract citation ID: igad104.0627

### THE PROMINENT ROLE OF HOME CARE AIDES IN END-OF-LIFE CARE DURING A PUBLIC HEALTH EMERGENCY AND BEYOND

Patricia Kim[1], Emily Franzosa[1], Laura Moreines[2], Jonelle Boafo[3], Daniel David[4], Abraham Brody[4], and Melissa Aldridge[1], *1. Icahn School of Medicine at Mount Sinai, New York City, New York, United States*, *2. New York University, New York City, New York, United States*, *3. Rory Meyers College of Nursing, New York City, New York, United States*, *4. NYU Rory Meyers College of Nursing, New York City, New York, United States*

Many patients and families rely on paid aides to provide personal care services at the end of life, but the COVID-19 pandemic caused widespread delays and disruption in this care. Our study examined COVID-related care disruptions among patients in a large, New York City health system who died between March 1, 2020 and March 1, 2021. We linked the electronic health records of 124 patients cared for by a geriatric outpatient practice and referred to hospice to the hospice's electronic health records, and conducted an in-depth qualitative thematic analysis. We found many home health aides continued caring for existing patients after hospice enrollment, often in tandem with hospice aides. But, patients and families experienced disruption in both usual and hospice aide services due to patient or aide illness, COVID isolation policies, and the exacerbation of ongoing workforce shortages. Aides experienced emotional strain and escalation of care responsibilities including the provision of more clinically-focused end-of-life tasks (e.g., wound care, oxygen administration) usually performed by hospice nurses. When the hospice team was unable to provide home visits, the aide became the primary source of patient information. Hospice staff supported aides in care tasks and provided emotional and spiritual care, often virtually, to aides and patients' families. Findings highlight the critical role of aides within the care team and its intensification in a crisis, suggesting opportunities to better integrate aides into hospice care teams while providing formal training and support to help aides manage end of life care tasks and emotional demands.

### SESSION 2240 (SYMPOSIUM)

Abstract citation ID: igad104.0628

### SENSORY LOSS IN AGING: SPOTLIGHTING JUNIOR AND NEW GSA INVESTIGATORS IN THE SENSORY HEALTH SPECIAL INTEREST GROUP

Chair: Jennifer Deal Discussant: Heather Whitson

Our senses (hearing, vision, touch, taste, smell) are the means by which our brains perceive our environment, allowing us to engage and connect with our world. Recommendations for maintaining brain health and function with age include maintaining strong social connections and participating in intellectually stimulating mental and physical activities. However, our senses are what allow us to engage in these activities. Sensory loss is common in older adults, with over 2/3 of adults over age 55 experiencing loss in multiple senses. The goal of the Sensory Health Special Interest Group (SIG) is to foster collaboration and dissemination of research and clinical/educational resources for professionals interested in sensory health and the effects of sensory impairments on the overall health and well-being of older adults. This session will meet this goal by highlighting work that demonstrates the importance of sensory health with age conducted by outstanding Sensory Health SIG investigators who are early in their career path or who are new to GSA. We will start by discussing the importance of olfaction for mental health over time in older adults and vision for markers of neurodegeneration. We will then have three presentations that consider multiple risk factors, including sensory loss, for cognitive function. First, we will present on the moderation of the association between hearing loss and dementia by sleep. We will then present on the association between multisensory loss and cognitive test performance, and finally, how the inclusion of sensorimotor measures can improve prediction of subsequent neurodegeneration and cognitive impairment. This is a Sensory Health Interest Group Sponsored Symposium.

Abstract citation ID: igad104.0629

### OLFACTORY DYSFUNCTION AND DEPRESSION TRAJECTORIES IN COMMUNITY-DWELLING OLDER ADULTS

Vidyulata Kamath[1], Kening Jiang[2], Kevin Manning[3], R. Mackin[4], Keenan Walker[5], Willa Brenowitz[4], Eleanor Simonsick[5], and Jennifer Deal[2], *1. Johns Hopkins University School of Medicine, Baltimore, Maryland, United States*, *2. Johns Hopkins Bloomberg School of Public Health, Baltimore, Maryland, United States*, *3. University of Connecticut Health, Farmington, Connecticut, United States*, *4. University of California, San Francisco, California, United States*, *5. National Institute on Aging, National Institutes of Health, Baltimore, Maryland, United States*

Olfactory dysfunction has been linked with all-cause mortality and neurodegenerative disease risk, though less is known about the association between olfaction and late-life depression (LLD). We examined the relationship between baseline olfactory performance and incident significant depressive symptoms and longitudinal depression trajectories in well-functioning older adults from the Health, Aging, and Body Composition study. Older adults (n=2,125, age 71-82, 51% women, 37% Black) completed an odor identification task at Year 3 (our study baseline). Cognitive assessments and depressive symptoms were ascertained across multiple visits over 8 years. We employed discrete time complementary log-log models, group-based trajectory models and multivariable-adjusted multinomial logistic regression to assess the relationship between baseline olfaction and incident depression and longitudinal depression trajectories. Mediation analysis assessed the influence of cognition on these relationships. Individuals with lower olfaction had increased risk of developing significant depressive symptoms at follow-up (hazard ratio [HR]=1.04, 95% CI:1.00, 1.08). Of the three longitudinal depression score patterns identified (stable low, stable moderate, stable high), poorer olfaction was associated with a 6% higher risk of membership in the stable moderate (relative risk ratio [RRR]=1.06, 95% CI:1.02, 1.10) and stable high (RRR=1.06, 95% CI:1.00, 1.12) groups, compared to the stable low group. Poor cognitive status only partially mediated the relationship between olfaction and incident depression severity. Suboptimal olfaction may serve as a prognostic indicator of vulnerability for development of LLD. These findings underscore the need for a greater understanding of olfaction in LLD and the demographic, cognitive and biological factors that influence these relationships over time.

Abstract citation ID: igad104.0630

### MODIFICATION OF THE ASSOCIATION BETWEEN HEARING LOSS AND DEMENTIA PREVALENCE BY SELF-REPORTED SLEEP DISTURBANCES

Kening Jiang[1], Adam Spira[1], Nicholas Reed[1], Alison Huang[1], Frank Lin[2], and Jennifer Deal[3], *1. Johns Hopkins Bloomberg School of Public Health, Baltimore, Maryland, United States*, *2. Johns Hopkins, Baltimore, Maryland, United States*, *3. Johns Hopkins University, Baltimore, Maryland, United States*

Hearing loss and sleep disturbances are both known risk factors for dementia. High prevalence and modifiability in late life make them potential targets for dementia prevention. However, their combined contribution to dementia is unknown. The National Health and Aging Trends Study (NHATS) is a longitudinal panel survey of a nationally representative sample of Medicare beneficiaries. A validated algorithm based on self- or proxy-reported diagnosis, AD8 dementia screening, and cognitive tests was used to classify participants as having probable/possible vs. no dementia. Pure-tone audiometry was added to the 2021 round of NHATS; participants with better-ear 4-frequency (0.5, 1, 2, 4 kilohertz) pure-tone average above 25 decibels were categorized as having hearing loss. Participants reported their frequencies of taking >30 minutes to fall asleep and having trouble falling back asleep and were defined as having each symptom if it was experienced ≥2 nights/week. We used Poisson regression with robust standard errors and included an interaction term between sleep disturbances and hearing loss, adjusting for demographics, smoking, body mass index and comorbidities. Among 2,383 participants aged 71+ years (46% male, 7% Black), hearing loss was cross-sectionally associated with higher dementia prevalence only among participants with sleep disturbances (>30 minutes to fall asleep: prevalence ratio [PR]=1.81, 95% CI:0.99, 3.31; difficulties falling back asleep: PR=1.82, 95% CI:1.02, 3.26). Older adults with both hearing loss and sleep disturbances might represent a high-risk subgroup that needs to be prioritized. With the complex etiology of dementia, a better understanding of the co-occurrence of multiple risk factors is needed.

Abstract citation ID: igad104.0631

### ASSOCIATIONS OF OBJECTIVE MULTISENSORY IMPAIRMENT WITH COGNITIVE IMPAIRMENT: THE ARIC-NCS STUDY

Jason Smith[1], Vidyulata Kamath[2], Honglei Chen[3], Srishti Shrestha[4], Alison Abraham[5], Alden Gross[6], Nicholas Reed[1], and Jennifer Deal[7], *1. Johns Hopkins Bloomberg School of Public Health, Baltimore, Maryland, United States*, *2. Johns Hopkins University School of Medicine, Baltimore, Maryland, United States*, *3. College of Human Medicine, Michigan State University, East Lansing, Michigan, United States*, *4. University of Mississippi Medical Center, Jackson, Mississippi, United States*, *5. Colorado School of Public Health, Aurora, Colorado, United States*, *6. Johns Hopkins University Bloomberg School of Public Health, Baltimore, Maryland, United States*, *7. Johns Hopkins University, Baltimore, Maryland, United States*

Sensory impairments frequently co-occur among older adults. Characterizing the combined impact of a multisensory phenotype on cognitive impairment could help elucidate a potential mode of pathogenesis. We investigated associations between multisensory impairment (co-occurring, objectively-measured hearing loss [pure-tone average ≥25 dB], vision loss [presenting near or distance acuity >0.3 logMAR], anosmia [Sniffin' Sticks test score ≤6], and peripheral nerve damage [≥1 insensate site on left or right foot]) and cognitive impairment (mild cognitive impairment [MCI] or dementia). Our study sample consisted of Black and White adults aged 70+ years in the Atherosclerosis Risk in Communities (ARIC) study; a longitudinal, community-based cohort. We estimated prevalence ratios (PRs) of baseline (ARIC visit 6, 2016-2017) expert-adjudicated MCI/dementia and odds ratios (ORs) of progression to MCI/dementia through ARIC visit 7 (2019) by number of impairments (0 [reference], 1, 2, 3 or more) using Poisson and logistic regression models, respectively. Overall, among 814 participants (age range: 71-93 years, 62% female, 41% Black race), an increasing number of impairments was associated with increased prevalence of baseline MCI/dementia (1 impairment, PR=1.14 [95% CI: 0.67-2.01]; 2 impairments, PR=1.53 [95% CI: 0.87-2.79]; ≥3 impairments, PR=2.26 [95% CI: 1.18-4.38]; p-trend: .007) and increased progression to MCI/dementia (1 impairment, OR=1.47 [95% CI: 0.67-3.49]; 2 impairments, OR=1.64 [95% CI: 0.69-4.12]; ≥3 impairments, OR=5.68 [95% CI: 2.18-15.52]; p-trend: .001). These findings suggest multiple concurrent sensory impairments contribute additively to greater MCI/dementia prevalence, signaling the importance of considering multiple impairments together in studies of the contribution of sensory losses to cognitive impairment.

Abstract citation ID: igad104.0632

### SENSORY AND MOTOR FUNCTIONS IMPROVE LONG-TERM PREDICTIONS OF NEURODEGENERATION AND COGNITIVE DECLINE

Natascha Merten[1], Adam Paulsen[1], Richard Chappell[1], Yanjun Chen[1], Luigi Ferrucci[2], Laura Hancock[1], Sterling Johnson[1], and Carla Schubert[1], *1. University of Wisconsin School of Medicine and Public Health, Madison, Wisconsin, United States*, *2. National Institute on Aging, National Institutes of Health, Baltimore, Maryland, United States*

Pathological changes in dementia often start decades before clinical symptoms making early detection important for interventions. We assessed if midlife sensory and motor functions improve prediction of long-term neurodegeneration as indicated by blood-based biomarker positivity and of cognitive decline and impairment when included in models with the CAIDE or Framingham Risk Score (FRS). This longitudinal study included 1529 (mean age 49yrs) Beaver Dam Offspring Study participants with measured serum neurofilament light chain (NfL) from baseline, 5-, and 10-yr follow-up. We assessed hearing,vision,olfaction, motor, and health history data, and calculated CAIDE and FRS at baseline. Outcomes were neurodegeneration (NfL+; value≥age-specific 97.5%ile), 10-yr cognitive decline (trail-making test B time;10% most decline), and cognitive impairment (neurocognitive case review). Logistic regressions were used to test whether adding baseline sensory and motor functions improves models of 10-yr incidence of NfL+, cognitive decline or impairment compared to models based on CAIDE or FRS alone. There were 115 incident NfL+, 139 cognitive decline, and 240 cognitive impairment cases. The area under the receiver operating characteristics curve (AUROC) for NfL+ prediction improved from 0.68[0.62,0.73] to 0.72[0.66,0.77] (p<0.01) after adding sensory and motor functions to CAIDE-only models. AUROCs also improved for cognitive decline from 0.72[0.68,0.76] to 0.77[0.73,0.81](p<0.001) and impairment from 0.59[0.55,0.63] to 0.65[0.61,0.68] (p<0.001) in CAIDE-only versus CAIDE-sensory-motor models. Improvements were similar in FRS-models. Including midlife sensory and motor functions improved long-term predictions of neurodegeneration, cognitive decline and impairment. Sensory and motor assessments might become relevant for cost-effective and non-invasive screenings to identify high-risk individuals early for future prevention and interventions.

Abstract citation ID: igad104.0633

### ASSOCIATIONS OF VISUAL IMPAIRMENTS AND OCULAR DISEASE WITH BRAIN VOLUMES AND DEMENTIA IN UK BIOBANK

Erin Ferguson[1], Peter Buto[2], Jingxuan Wang Mary Thoma[2], Shea Andrews Kaitlin Casaletto[3], Tom Hoffmann Helene Choquet[2], Kristine Yaffe[4], M Maria Glymour[5], and Willa Brenowitz[6], *1. University of California, San Francisco, San Francisco, California, United States*, *2. University of California San francisco, San Francisco, California, United States*, *3. University of California, San Francisco, California, United States*, *4. UCSF, San Francisco, California, United States*, *5. University of California San Francisco, San*

*Francisco, California, United States*, *6. Kaiser Permanente Center for Health Research, Portland, Oregon, United Sta tes*

Visual impairments and ocular diseases are emerging potentially modifiable risk factors for dementia, however, causal relationships are poorly understood and vision impairment may also impact dementia evaluations. Few studies have examined associations of multiple measures of visual impairments with markers of neurodegeneration. We investigated associations of visual acuity and history of ocular diseases with dementia and brain MRI outcomes. UK Biobank participants aged 40-70 without dementia at assessment (n=496,938) self-reported history of cataract, glaucoma, age-related macular degeneration Subsets had visual acuity exams and refractive error measured by autorefractor (n=117,163) and brain MRIs (n=33,923). In cox proportional-hazard models adjusted for demographics and other health conditions, history of cataracts was associated with higher risk of all-cause dementia (HR:1.19: 95%CI: 1.09-1.31) but other eye conditions were not associated. History of cataract was associated with significantly smaller total gray matter volume (β=-2483 mm3, 95% CI: -4225 to -741) but not an AD brain signature based on cortical thickness. Worse than 20/40 visual acuity was associated with dementia (HR = 1.40, 95% CI: 1.11-1.77) but myopia defined based on refractive error was not, and neither were associated with total or regional volumes on brain MRI. History of cataract and binary 20/40 vision are associated with dementia risk. Our results suggest that cataract, but not myopia, may increase risk of neurodegeneration independent of typical AD, but more investigation is needed.

### SESSION 2245 (SYMPOSIUM)

Abstract citation ID: igad104.0634

### HOW GERONTOLOGY PROGRAMS ARE MEETING CHALLENGES IN HIGHER EDUCATION

Chair: Mary Ann Erickson

Population aging means that gerontology education is increasingly relevant for all students, yet trends in higher education create existential challenges for many gerontology programs, who may not attract robust number of applicants or majors. In this symposium, educators from five different gerontology programs will share their programs' particular challenges as well as a wide variety of strategies they have used to meet these challenges. The first presentation will show how recent losses at Ithaca College, including the Aging Studies major and independent department status, also come with opportunities for collaboration with health science and public health programs. The second presentation will describe the University of North Carolina at Wilmington's approach of combining undergraduate and graduate programs to attract and retain students after being placed on a "low-productivity list" in the not-too-distant past. The third presentation will share how a new Occupational Endorsement Certificate at the University of Alaska Anchorage took the place of the deactivated gerontology minor. Colleagues from the University of Massachusetts Boston will describe their efforts to enhance diversity and grow program enrollment across undergraduate and graduate programs. Finally, the fifth presentation will describe how increased demand for general education courses as well as funding dependent on undergraduate major enrollment created challenges at the University of South Florida. This is a Directors of Aging Centers Interest Group Sponsored Symposium.

Abstract citation ID: igad104.0635

### LEANING INTO COLLABORATION AT ITHACA COLLEGE

Mary Ann Erickson, and Elizabeth Bergman*, Ithaca College, Ithaca, New York, United States*

Ithaca College is a private comprehensive college of about 6,000 students in upstate New York. Its Gerontology Institute (ICGI) was formed in 1992, with a major and a minor in Aging Studies added in 2001. Despite a high profile in the local community and in the field of gerontology, program evaluation consistently revealed important challenges including trouble promoting a gerontology major to high school seniors (especially important at a tuition-dependent school like Ithaca College) and credit-heavy health professions programs where advisors were reluctant to have students add a minor or double major. Institutional forces accelerated by the pandemic led to retrenchment in 2021, including the loss of the major as well as the ICGI being folded into a department of health sciences and public health. In this presentation, we share both details of past challenges as well as the ways in which we are moving forward with a more collaborative model focused on connections within the School of Health Sciences and Human Performance.

Abstract citation ID: igad104.0636

### CHALLENGES TO UNDERGRADUATE AND GRADUATE PROGRAMS AT THE UNIVERSITY OF SOUTH FLORIDA

Debra Dobbs, William Haley, and Nasreen Sadeq,
*University of South Florida, Tampa, Florida, United States*

The University of South Florida has more than 50,000 students. The School of Aging Studies first offered an MA in Gerontology in 1967. We currently offer many undergraduate General Education courses, two undergraduate majors, online graduate certificate and master's programs, and a PhD in Aging Studies. Accountability and addressing university metrics and priorities have become an increasing requirement at USF and elsewhere. We have had great success in our General Education courses, consistently reaching over 5,000 students per year and producing high Student Credit Hour (SCH) numbers, an important metric. Recently, our programs experienced challenges with increased class sizes resulting from higher demand for our general education courses. In several cases, the number of course sections offered and the number of seats in each had doubled. Meeting these demands placed a strain on faculty, instructors, and graduate student assistants. We also face challenges as our university funding formula increasingly focuses on undergraduate major enrollment, and therefore we are focusing on innovative methods to recruit more students at the undergraduate, master's, and PhD level. In this presentation, we will describe some of the strategies that have maximized our successes, and efforts to address challenges in sustaining larger numbers of degree seeking students in our programs. We will include data on SCH, grant funding, publications, citations, and program graduates per year which help us monitor our successes and areas needing improvement.

Abstract citation ID: igad104.0637

**UNDERGRAD AND GRAD COMBINED PROGRAMS: FACILITATING RECRUITMENT THROUGH NEW PATHWAYS**

Tina Newsham, and Elizabeth Fugate-Whitlock, *University of North Carolina Wilmington, Wilmington, North Carolina, United States*

The University of North Carolina Wilmington was reclassified as an R2 institution in 2018. As such, additional focus has been placed on graduate programs and the generation of research. More recently, UNCW's Chancellor Volety followed recommendations from faculty, staff, and students to seek to endorse the Age Friendly University principles. These two accomplishments create ideal conditions for growing the gerontology program, which had been on the low productivity list and considered for dissolution (as has happened with many programs around the country). To facilitate recruitment into the MS in Applied Gerontology, new pathways known as "4 + 1" or "combined programs" were created. By allowing qualified undergraduate students to take a portion of their coursework at the graduate level, students explore their interest in gerontology, become invested in gerontology education, and meet undergrad and graduate requirements simultaneously. Students are then able to complete their MS in Applied Gerontology in as little as one more year. Such programming has created a steady stream of students into the graduate program, which is no longer on the low productivity list. While this approach has many strengths, challenges exist that must be acknowledged. Marketing the program to a now very broad prospective student base requires finesse. For example, marketing that would appeal to an exercise science student may not be what speaks to a psychology student. These challenges also translate to the classroom. Although gerontology is an interdisciplinary field, career goals for students now vary even more widely as do their expectations and academic needs.

Abstract citation ID: igad104.0638

**TEACHING IN THE TUNDRA: LESSONS LEARNED AT THE UNIVERSITY OF ALASKA ANCHORAGE**

Britteny Howell, Rosellen Rosich, and Mikki Easley, *University of Alaska Anchorage, Anchorage, Alaska, United States*

UAA is an open-enrollment, public university of about 12,000 students. The gerontology minor was formed in 2005 as a result of the Geriatric Education Center (GEC). However, the loss of the GEC and most gerontology faculty, coupled with low student enrollment in the minor, led to its deactivation in 2014. Since then, several new faculty have been hired with an interest in gerontology. The Gerontology Faculty Workgroup, instituted by the GEC, remains active and the university now has 4 new courses on aging, including a geriatric Interprofessional Education (IPE) course. This forward momentum in recent years has led to the development of a new Occupational Endorsement Certificate program in gerontology. Training the future workforce and working professionals, the new program is poised to be more sustainable than a minor. In this presentation, we share details of past challenges and current successes within the College of Health.

Abstract citation ID: igad104.0639

**DIVERSIFYING THE PIPELINE OF GERONTOLOGISTS AT THE UNIVERSITY OF MASSACHUSETTS BOSTON**

Edward Miller, Jeffrey E. Stokes, Pamela Nadash, Kathrin Boerner, and ellen Birchander, *University of Massachusetts Boston, Boston, Massachusetts, United States*

The University of Massachusetts (UMass) Boston is a public research university serving approximately 16,000 students. It is the most diverse university in New England and third most diverse university in the nation. UMass Boston prioritizes fostering an anti-racist and health-promoting institutional culture. Consistent with this priority, the Gerontology Department is committed to growing the size and diversity of the professional pipeline in aging services, policy, practice, and research. This presentation describes the multi-pronged approach taken to achieve this objective, while identifying associated facilitators and challenges. Department-wide initiatives include prioritizing work in disparities and a commitment to diversity, equity and inclusion in faculty hiring and implementing curriculum self-assessment and climate survey tools. Doctoral program initiatives include establishing a scholarship in Social Justice and Aging and submitting a training grant application in Health Equity Research. Undergraduate program initiatives include changing the name of the major to "Aging Studies" to make the content clearer and more accessible, proposing a minor in Aging Studies to make it possible for students from across the University to earn a valuable credential, having all courses meet Social and Behavioral requirements in general education (including, in select cases, the Diversity requirement as well), and expanding the number and attractiveness of course offerings (including "Diversity and Aging"). The Department is also implementing an initiative where undergraduates receive paid internships at local-area aging agencies with potential for continued employment post-graduation. Enhancing diversity goes hand-in-hand with growing program enrollment with positive benefits for older adults and their families, caregivers, and communities.

### SESSION 2250 (SYMPOSIUM)

Abstract citation ID: igad104.0640

**AGE-INCLUSIVE STRATEGIES TO ENHANCE LEARNING AND WORKPLACE ENVIRONMENTS IN U.S. HIGHER EDUCATION**

Chair: Nina Silverstein Co-Chair: Lauren Bowen
Discussant: Marla Berg-Weger

This symposium reports on recent research to develop informed strategies to expand age-friendly campus practices related to institutional functions our previous study identified as areas of high-need and broad-impact within an age-friendly campus environment: Teaching and Learning, Personnel, and Student Affairs. The current research aimed to identify barriers and opportunities for age inclusivity as experienced by older campus members (faculty, staff, and students); identify challenges and strategies for implementing age-friendly campus practices; and recommend practical strategies to expand and enhance age-friendly practices. To this end, we drew on data collected from several sources: open-ended responses to the Age-Friendly Inventory and

Campus Climate Survey (ICCS) administered to a sample of 23 U.S. members of the Age-Friendly University (AFU) network; responses from 16 focus groups of faculty, administrators/staff, and students; and 18 experts on a nationally selected Delphi panel of experts in higher education. Based on the focus group data, we generated over 50 strategy recommendations within the academic functions that were then evaluated by the Delphi panelists to assess their efficacy, feasibility, likelihood of implementation, and perceived impact. Silverstein will provide an overview of the research objectives, Bowen will discuss what the open-ended responses revealed about perceptions of campus age-inclusivity, Lin and Xu will describe the study samples, Whitbourne will describe the results of the focus group analyses, and Montepare will discuss the final set of recommendations based on the Delphi panel analyses. Berg-Weger will serve as Discussant.

Abstract citation ID: igad104.0641

### IDENTIFYING CHALLENGES FOR AGE-INCLUSIVE HIGHER EDUCATION: SURVEY RESPONSES BY FACULTY, STAFF, AND STUDENTS

Lauren Bowen, *University of Massachusetts Boston, Boston, Massachusetts, United States*

To ground and contextualize focus group discussions, potential challenges to age-inclusivity were identified from open-ended responses in a survey of faculty, staff, and students at 25 AFU institutions in the United States. First-level coding identified 2,368 responses that indicated participants' perceptions of their campus's efforts to be age-friendly across major function areas of higher education institutions; of these, many responses directly addressed key functions that the current study aims to address: 508 on Teaching and Learning; 260 on Student Affairs; 348 on Personnel. Second-level coding identified particular themes capturing common negative perceptions by faculty, staff, and students within each function area. For example, in teaching and learning, the four common complaints were that classroom practices and environments were not age-inclusive; that accessing appropriate academic support outside the classroom was difficult for older/nontraditional students; and that classes could do more to maximize the benefits of age diversity. As potential challenges to age-inclusivity in higher education, these themes provided the base structure of 8 focus group discussion guides tailored to function area and constituent group: Teaching and Learning (for faculty, staff, and students), Student Affairs (for faculty, staff, and students), and Personnel (for faculty and staff).

Abstract citation ID: igad104.0642

### STUDY METHODOLOGY AND SAMPLE SELECTION TO DEVELOP AGE-INCLUSIVE STRATEGIES

Yan Lin[1], and Shu Xu[2], *1. University of Massachusetts Boston, Boston, Massachusetts, United States*, *2. University of Massachusetts, Boston, Boston, Massachusetts, United States*

Practical, feasible, and impactful age-friendly campus strategies to advance age inclusivity were identified through focus group and Delphi panel methodologies. Sixteen virtual focus groups with 78 participants (41 faculty, 16 students, and 21 staff/administrators; 44% ages 45-64 and 12% 65 and older) were conducted to assess age inclusivity in three institutional functions defined as high impact: teaching and learning, personnel, and student affairs. Facilitators presented participants with questions based on strategies and challenges identified from open-ended survey responses to our previous study. Following similar scripts for each of the three groups, facilitators asked 5 to 6 questions regarding potential strategies to address challenges such as those related to teaching older, age-diverse students; intergenerational tensions and biases; student support services; and incentives for faculty to integrate aging content into their courses. Next, the strategies yielded from this analysis were presented in online surveys to the Delphi panelists to assess their feasibility and impact. These 18 panelists had been selected based on their broad experience in higher education and upper-level administrative knowledge, professional connections, and/or expertise in diversity/inclusion/equity initiatives. In the first round, they evaluated the relative importance of 21 challenges to age inclusivity and their appropriateness, feasibility, and likelihood of implementation. The second-round survey narrowed the list of strategies to those rated as most viable (feasible and appropriate) in the first round and identified priorities among the selected strategies.

Abstract citation ID: igad104.0643

### MEETING THE CHALLENGE OF AGE-INCLUSIVE EDUCATION: FOCUS GROUP PERSPECTIVES

Susan Whitbourne, *University of Massachusetts Boston, Boston, Massachusetts, United States*

Through a series of 16 virtual focus groups involving a national sample of 78 faculty, staff, and students, a set of challenges and related strategies for meeting those challenges were identified based on the ICCS responses from campus constituent groups of faculty, staff, and students. Qualitative analysis of focus group transcripts in the areas of teaching/learning, personnel, and student affairs produced 969 strategy statements that were then collapsed into thematic groupings based on the challenges they were intended to address. Major challenges and their relevant areas included the following: Long-term employees often feel undervalued (personnel), non-traditional age and older students often feel that career services do not meet their needs, and non-traditional age and older students often feel excluded or disregarded in the post-secondary classroom. Focus group members suggested strategies in the area of personnel that included greater recognition of accomplishments of long-term employees. In student affairs, they suggested such strategies as providing more resources for advising and career services. Teaching and learning strategies included incentivizing faculty to develop age-inclusive practices and promoting age-friendly classroom and course offerings. These findings suggest ways for campuses to enhance age-inclusive practices through specific actions, but their feasibility and impact remain necessary to evaluate.

Abstract citation ID: igad104.0644

### IDENTIFYING AGE-FRIENDLY CAMPUS STRATEGIES USING A DELPHI PANEL METHODOLOGY

Joann Montepare, *Lasell University, Newton, Massachusetts, United States*

This presentation will describe the results of a Delphi panel study of 18 experts in higher education who independently assessed age-friendly strategies in three core areas of institutional function: teaching and learning, personnel practices, and student affairs. To begin, experts evaluated the relative importance of 21 common challenges for advancing age inclusivity on U.S. campuses. They then assessed the extent to which 53 potential strategies were appropriate, feasible, and likely to be implemented by campus leaders. In the first round of evaluations, 3 of the 21 challenges were rated as not very important by some experts. On the second round of evaluations, experts assessed why these particular challenges were rated as such. During the first round of reviews, experts also indicated that while 8 strategies were feasible and appropriate to addressing challenges to age inclusivity, they were unlikely to be implemented on campuses. Thus, in the second round, experts were asked why this might have been the case by selecting 2 probable barriers from a list of barriers to adoption. Finally, experts rated the potential impact of all strategies for improving age inclusivity. That is, aside from feasibility or likelihood, the extent to which they thought the strategies could transform age-inclusive environments in higher education. In addition to describing which strategies were most highly recommended, insights will be presented about how the experts viewed various age-friendly challenges and the barriers that hold institutions back from taking up more age-inclusive practices to support older students, faculty, and staff.

### SESSION 2255 (SYMPOSIUM)

Abstract citation ID: igad104.0645

### PREDICTIONS FOR DEMENTIA BURDEN AND POLICY RESPONSES IN CHINA AND UK BY 2050

Chair: Jing Liao Co-Chair: Eric Brunner

The goal of this symposium is to provide a forum to interact with the principal communities, who are developing research on the projection of dementia burden to ageing society and policy responses. It is driven by the UK-China Health and Social Challenges Ageing Project (UKCHASCAP). China's rapid ageing process is occurring at an earlier stage of economic development than many other countries including UK, posing great demands on families, communities and health and care services. Thoughtful comparison of health and social care policy across the contrasting countries will be scientifically illuminating and valuable to policy makers. Preliminary results from four studies will be presented. Two studies based on large ageing cohorts of China and the UK, using multi-state Markov modelling (IMPACT-CAM, IMPACT-BAM) to construct predictions of dementia and related health status (e.g. CVD and physical disability) to 2050. The British model will highlight calculation of dementia incidence trend over time. The following study further used IMPACT-CAM to predict socioeconomic cost of medical, informal and formal care, and quality adjusted life years associated with dementia in China in corresponding years. The fourth study systematically evaluates the effects of implementing long-term care insurance in pilot cities in China, and analyses the main challenges in establishing a nationwide equitable long-term care system for older people. Through this symposium, the panel will provide state-of-art modelling and latest data on the innovative, interdisciplinary and international collaboration for predicting dementia, and provide scientific advices for policy making on healthy ageing in China, the UK and globally.

Abstract citation ID: igad104.0646

### DOWNWARD DEMENTIA TREND GOES INTO REVERSE IN ENGLAND AND WALES? ENGLISH LONGITUDINAL STUDY OF AGING 2002–2019

Yuntao Chen[1], Piotr Bandosz[2], George Stoye[3], Yuyang Liu[4], Sophia Lobanov-Rostovsky[1], Eric French[5], Jing Liao[4], and Eric Brunner[1], *1. University College London, London, England, United Kingdom*, *2. Division of Prevention Medicine & Education, Medical University of Gdansk, Gdansk, Pomorskie, Poland*, *3. Institute for Fiscal Studies, London, England, United Kingdom*, *4. Sun Yat-sen University, Guangzhou, Guangdong, China (People's Republic)*, *5. Faculty of Economics, University of Cambridge, Cambridge, England, United Kingdom*

Recent evidence suggests dementia incidence rates are declining in high-income countries. However, data for the trend after 2010 are scarce. We examined the temporal trend in England and Wales from 2002-2019, considering bias and non-linearity. We used population-based panel data linked to the mortality register across wave 1 (2002-2003) to wave 9 (2018-2019) of the English Longitudinal Study of Ageing. Uniform standard criteria based on cognitive and functional impairment were used to ascertain incident dementia cases. Crude incidence rates were determined in seven overlapping initially dementia-free sub-cohorts followed over four years. We estimated the age- and sex-adjusted trend of dementia incidence with Cox and multi-state models. Restricted cubic splines allowed for potential non-linearity. 19 806 people were included in the study. Crude dementia incidence declined from 2002 to 2008 (8.7 to 7.4 per 1000 person-years), and increased from 2008 to 2019 (7.4 to 10.3 per 1000 person-years). Adjusting for age and sex, and accounting for missing dementia cases due to death, estimated dementia incidence declined by 28.8% from 2002 to 2008 (incidence rate ratio 0.71, 95% CI 0.58-0.88), and increased by 25.2% from 2008 to 2016 (incidence rate ratio 1.25, 1.03-1.54). The higher education group had a sharper decline of dementia incidence from 2002 to 2008, and a smaller increase after 2008. Dementia incidence may not be declining. There was a rebound after 2008 in England and Wales. If the upward dementia incidence trend continues, along with population ageing, the burden on health and social care may be large.

Abstract citation ID: igad104.0647

### GROWING OLD IN CHINA: SYSTEMATIC REVIEW OF LONG-TERM CARE INSURANCE PILOT STUDIES

Sophia Lobanov-Rostovsky[1], Qianyu He[2], Yuntao Chen[1], Natasha Curry[3], Eric Brunner[1], Jing Liao[2], Nina Hemmings[3], and Tishya Venkatraman[1], *1. University College London, London, England, United Kingdom*, *2. Sun Yat-Sen University, Guangzhou, Guangdong, China (People's Republic)*, *3. Nuffield Trust, London, England, United Kingdom*

Between 1970 and 2020, there was a three-fold increase in China’s ≥65 population, compared to less than a doubling in UK. This rapid-ageing demographic has led to a rise in age-related disabilities. At the same time, internal migration and declining fertility have shaken traditional models of care.

An important policy response is to pilot differing long-term care insurance (LTCI) systems, with the aim of establishing equitable care for all. Strengths and limitations of the first set of pilot studies have been identified. In 2020, a second set of 34 pilot studies was introduced. Following PRISMA guidelines, we undertook a systematic review of literature published since introduction of the second pilot phase, to answer the question: 'what are the key challenges to China achieving an equitable nationwide long-term care system for older people?'. Records were eligible for inclusion if published between June 2020 and June 2022 in Mandarin or English. 42 studies (n=16 Mandarin) were included. Four themes emerged: poor quality of service provision, widespread preference for family care, inequitable distribution of cost burden, and varying LTCI eligibility. Key recommendations included increasing salaries to attract and retain staff, mandatory financial contributions from employees and a unified standard of disability with regular assessment. Strengthening support for family caregivers and improving smart care capacity can support preferences to age at home. Our systematic review highlighted significant challenges in provision of equitable care which suits preferences of its users. China's LTCI pilots will provide useful lessons for other middle-income countries with rapidly ageing populations.

Abstract citation ID: igad104.0648

### PREDICTIONS FOR DEMENTIA PREVALENCE IN CHINA BY 2050: A MARKOV MODELING STUDY

Yuyang Liu[1], Yanjuan Wu[1], Yuntao Chen[2], Sophia Lobanov-Rostovsky[2], Piotr Bandosz[3], Jing Liao[1], and Eric Brunner[2], *1. Sun Yat-sen University, Guangzhou, Guangdong, China (People's Republic)*, *2. University College London, London, England, United Kingdom*, *3. Division of Prevention Medicine & Education, Medical University of Gdansk, Gdansk, Pomorskie, Poland*

Dementia brings a heavy burden on older adults, their family and the society at large. Accurate prediction of dementia prevlance is important for preventive strategy for fast- ageing China. The purpose of this study is to predict dementia prevalence in China by 2050, taking into account variations in dementia incidence rates and mortality rates in future. Based on two nationally representative Chinese ageing cohorts (i.e. CLHLS, CHARLS), a ten-state Markov model (IMPACT-CAM), including prevalence, transition probability and mortality rate of dementia and its associated cardiovaiscualr diseases and disability, was constructed to predict dementia prevalence in people aged above 60 years by 2050, with different assumptions on the furture trends of dementia incidence and mortality rates. IMPACT-CAM projected there were approximately 57.9 million (95% uncententiy interval 56.3-59.6 m) people with dementia by 2050, assuming constant incidence rate of dementia and declining mortality rates over coming years. In comparison with this assumption, if the incidence rate of dementia decreasing by 1.0% annually coupled with declining mortality rates, the projected number of dementia cases would be 9.8 million less; if dementia incidence and mortality rates both remained constant since 2022, the projection would be 18.5 million less. The estimated dementia prevlance for people aged 60 years in 2050 were 12.0%, 9.9% and 9.5%, respectively. Alongside the irreversible population ageing trend, the number of people with dementia in China is likely to increase rapidly in the near furture. Nevertheless, effective preventing measures of dementia would restrain the surge substantially.

Abstract citation ID: igad104.0649

### PREDICTIONS FOR DEMENTIA BURDEN IN CHINA BY 2050 AND LONG-TERM CARE INSURANCE POLICY

Yanjuan Wu[1], Yuyang Liu[1], Yuntao Chen[2], Sophia Lobanov-Rostovsky[2], Piotr Bandosz[3], Jing Liao[1], Eric French[4], and Eric Brunner[2], *1. Sun Yat-sen University, Guangzhou, Guangdong, China (People's Republic)*, *2. University College London, London, England, United Kingdom*, *3. Division of Prevention Medicine & Education, Medical University of Gdansk, Gdansk, Pomorskie, Poland*, *4. Faculty of Economics, University of Cambridge, Cambridge, England, United Kingdom*

This study projected the socioeconomic cost and quality adjusted life years (QALYs) lost to dementia in China from 2020 to 2050, and tested robustness of these projections. Using a multi-state Markov model, we projected the number of dementia cases and other health states in China to 2050. Corresponding socioeconomic costs (healthcare, formal care and informal care costs) and QALYs of these health states were forecasted based on China Health and Retirement Longitudinal Study (N=25029) and Chinese Longitudinal Healthy Longevity Survey (N=32628). A series of sensitivity analyses were conducted to test robustness of projections depending on different assumptions. We showed that nearly 2.7% of the age 50+ population had dementia in China in 2020, and the share of total socioeconomic cost per year was 12.2% for those with dementia. Socioeconomic cost and cost of QALYs lost to dementia in 2050 would reach 1189.0 and 807.4 billion US$ respectively. The dominant contributor to socioeconomic costs was informal care costs, while formal care costs covered the smallest share. Furthermore, sensitivity analyses showed that changes to formal care usage would lead to more robust changes in socioeconomic cost of dementia than the unit price of social care. Projected cost would decrease by 13.9% or increase by 34.5% in 2050, depending on different assumptions of time trend of dementia incidence. The socioeconomic cost and cost of QALYs lost to dementia in China are expected to continue their rapid growth into future, with informal care costs being the most significant contributor.

### SESSION 2260 (SYMPOSIUM)

Abstract citation ID: igad104.0650

### MOBILIZING INTERDISCIPLINARY RESEARCH TO ILLUMINATE DIVERSITY ACROSS THE CANCER CARE CONTINUUM

Chair: Sean Halpin Co-Chair: Jessica Krok-Schoen

Cancer diagnoses increase precipitously with age, impacting diverse populations who have different experiences, needs, and resources. In our symposium, we bring together researchers from multiple disciplines to present on the diversity of older adults living with cancer and the ways that health care providers, systems, and researchers are innovating to support them. Carrion presents the knowledge of gender-specific types of cancer among older Latinos/as. Seaman presents research on the use of comprehensive geriatric assessments by oncology and primary care providers who work with older adults. Blackberry discusses the use of age-friendly models of care within cancer care. Halpin discusses the results from a scoping review of cancer therapy decision-making among patients with co-morbid dementia and cancer and their caregivers. Ultimately, we discuss the need for multiple methods to understand the experience of patients from diverse populations and with various cancer diagnoses and innovate in cancer care research.

Abstract citation ID: igad104.0651

### THE ROLE OF AGE-FRIENDLY HEALTH CARE IN OLDER ADULTS WITH CANCER

Irene Darmadi Blackberry[1], Stacey Rich[2], Oliver Hodge[3], Tshepo Rasekaba[1], Nicole Webb[4], and Christopher Steer[5], *1. La Trobe University, Wodonga, Victoria, Australia*, *2. La Trobe University, John Richards Centre, Wodonga, Victoria, Australia*, *3. University Of NSW, Wodonga, Victoria, Australia*, *4. Albury Wodonga Health, Albury, New South Wales, Australia*, *5. Border Medical Oncology and Haematology, Albury, New South Wales, Australia*

It is estimated that 51% of cancer incidence worldwide occurs among adults aged 65 years and older. The care of older adults diagnosed with cancer is complex due to an interplay of biopsychosocial factors, including comorbidities, geriatric syndromes, functional status, nutritional health and socioeconomic characteristics. Working within an age-friendly model of care is important in ensuring the complexity of ageing is taken into consideration, while understanding what matters most to the patient undergoing cancer treatment. Age-friendly models of care such as the 4Ms framework (Mentation, Mobility, Medications, and (what) Matters) are gaining support across the globe, but little is known of the use of age-friendly models of care in cancer care. This scoping review aimed to understand the current state of age-friendly care in cancer care and to highlight how age-friendly models can be implemented in cancer care to improve the experience of treatment for older adults.

Abstract citation ID: igad104.0652

### KNOWLEDGE OF GENDER-SPECIFIC TYPES OF CANCER AMONG OLDER LATINOS/AS

Iraida Carrion[1], Malinee Neelamegam[2], Tania Estapé[3], and Jorge Estapé[3], *1. University of South Florida, Tampa, Florida, United States*, *2. University of North Texas Health Science Center, Fort Worth, Texas, United States*, *3. Fundación contra el cáncer, Barcelona, Catalonia, Spain*

Given the growing population of Latino/a immigrants 60 years and older and the current lack of relevant data, our study aimed to understand the knowledge of gender-specific types of cancer among older Latinos/as to ensure effective prevention, intervention, and cancer care. Using convenience sampling, 200 individuals identifying as Latino/a were surveyed in Tampa, Florida. Results indicate significant differences in men's and women's knowledge of breast cancer and prostate cancer preventative measures. The differences in cancer knowledge among older Latino/a men and women regarding the opposite gender are relevant when providing cancer education to older adults. Providing culturally and linguistically appropriate cancer care and education can help address decision-making, achieve equity, and improve cancer outcomes for older Latinos/as.

Abstract citation ID: igad104.0653

### EXAMINING AWARENESS AND USE OF COMPREHENSIVE GERIATRIC ASSESSMENTS IN CANCER CARE: A QUALITATIVE STUDY

Aaron Seaman[1], Karen Wickersham[2], Julia Rowland[3], Ciaran Fairman[2], James Hebert[2], Cynthia Thomson[4], Rosi Vogel[4], and Daniela Friedman[2], *1. University of Iowa, Iowa City, Iowa, United States*, *2. University of South Carolina, Columbia, South Carolina, United States*, *3. Smith Center for Healing and the Arts, Washington, District of Columbia, United States*, *4. University of Arizona, Tucson, Arizona, United States*

Comprehensive geriatric assessments (CGAs) are critical tools for identifying the needs of older cancer survivors and planning effective treatment and follow-up care. Research on CGA implementation in clinical settings remains limited. We interviewed primary and cancer care providers to better understand current CGA practices, their potential, and the challenges of clinical use. Eight semi-structured interviews, lasting ~60 minutes, were conducted with participants identified through Cancer Prevention and Control Research Network partner listservs. Analysis was conducted iteratively. Providers reported that CGAs were useful and administering them with all incoming patients would be ideal but was not always possible. Instead, they used brief screening tools, which meant CGA domains were inconsistently assessed. Implementation was facilitated by clinic champions, multidisciplinary care teams, and institutional resources and buy-in. Findings from this research will provide potential solutions to addressing barriers to using CGAs related to staff capacity and competing demands, provider experience, and institutional priorities.

Abstract citation ID: igad104.0654

### CO-MORBID DEMENTIA AND CANCER THERAPY DECISION-MAKING: A SCOPING REVIEW

Sean Halpin[1], Gabriel Alain[2], Hui Zhao[3], Mackenzie Fowler[4], Qiuyang Zhang[5], Tamara Cadet[6], Minzhi Ye[7], and Jessica Krok-Schoen[8], *1. RTI, Decatur, Georgia, United States*, *2. The Ohio State University, College of Medicine, Columbus, Ohio, United States*, *3. James Madison University, Harrisonburg, Virginia, United States*, *4. University of Alabama at Birmingham, Birmingham, Alabama, United States*, *5. Tulane University School of Medicine, New Orleans, Louisiana, United States*, *6. The University of Pennsylvania, Philadelphia, Pennsylvania, United States*, *7. Kent State University, Kent, Ohio, United States*, *8. The Ohio State University, Columbus, Ohio, United States*

Co-morbid dementia complicates cancer therapy decision-making in older adults. Our scoping review examined cancer therapy decision-making among older persons with co-morbid cancer and dementia and their caregivers. Pubmed, Embase, CINAHL, PsycINFO, Cochrane Library, Scopus, and Web of Science were searched from 2018-2022, resulting in 8,654 articles screened, with eight meeting the inclusion criteria. Most studies were conducted in the UK (89%) and reported homogeneity in race and geography. Breast (56%) and prostate (45%, with one reported stage) were the most frequent cancers reported. Five studies (56%) reported multiple types of dementia, with two (22%) indicating stages. The studies indicated that communication between patients, caregivers, and clinical teams might alleviate stress caused by worsening health prospects. Future research should consider diverse racial, geographic, cancer types, dementia types, and healthcare stages to improve understanding of these decision-making processes.

### SESSION 2265 (SYMPOSIUM)

Abstract citation ID: igad104.0655

**PHENOTYPES OF COGNITIVE FUNCTION IN THE CONTEXT OF COMORBIDITIES**

Chair: Yaguang Zheng Co-Chair: Weiyu Mao Discussant: Jing Wang

While a growing body of evidence has shown that the prevalence of cognitive impairment varies depending on the population, limited research has explored the complex relationships among different cognitive function domains or changes in cognitive functions in the context of comorbidities (e.g., diabetes and stroke). The objective of this symposium is to address knowledge gaps by demonstrating these complex relationships in the context of comorbidities. Zheng and colleagues discuss the prevalence of mild cognitive impairment and dementia in adults with diabetes using nationally representative data from a large-scale dataset. Zhu et al. explore the inference of changes in cognitive function and relationships among cognitive function domains using a novel phenotypic network approach. Further, Li et al. use a similar phenotypic network approach to identify the dynamic associations across cognitive, physical, psychological function, and quality of life among Chinese patients with acute ischemic stroke. Nan Wang & colleagues discuss factors associated with the perceived risk of Alzheimer's disease and related dementias (ADRD) and how the perceived risk of ADRD is related to cognitive function. Following the paper presentations, Dr. Jing Wang will lead a discussion. The novel finding from this symposium will provide a more comprehensive understanding of how cognition changes over time and how interventions could target multiple domains or be tailored to the varied medical comorbid conditions to improve overall cognitive functions.

Abstract citation ID: igad104.0656

**PREVALENCE OF MILD COGNITIVE IMPAIRMENT AND DEMENTIA IN ADULTS WITH DIABETES**

Yaguang Zheng, Xiang Qi, Zheng Zhu, and Bei Wu, *New York University, New York City, New York, United States*

Diabetes is a risk factor for mild cognitive impairment (MCI) and dementia. However, there is limited evidence on the prevalence of MCI/dementia in individuals with diabetes using a nationally representative sample in the U.S.. We aimed to address this knowledge gap by using the nationally representative data from the 2018 Health and Retirement Study (HRS). The validated 27-point Telephone Interview for Cognitive Status (TICS) was used to assess MCI (TICS 7-12) and dementia (TICS <7). A total of 4192 (28.0%) out of 14,988 adults had self-reported diabetes. The mean age was 66.5±11.4 years, with White (64.5%) and Black (23.1%). Adults with diabetes had a higher prevalence of MCI than those without diabetes (19.9% vs. 14.8%; odds ratio [95% confidence interval] (OR[95%CI]): 1.47 [1.34, 1.61]) and higher prevalence of dementia (5.32% vs. 3.46%; OR[95%CI]: 1.68 [1.42, 1.99]). In those with diabetes, adults aged 65-75 years, and 75 years and older had a higher prevalence of MCI (OR[95%CI]: 1.13 [1.00, 1.27]; 2.36 [2.13, 2.61]) and dementia (OR[95%CI]: 1.60 [1.25, 2.06]; 5.071 [4.14, 6.21]) than those who aged 50-65 years. Black and other races had a higher risk of MCI (OR[95%CI]: 2.14 [1.94, 2.36]; 1.602 [1.41, 1.83]) and dementia (OR[95%CI]: 2.32 [1.93, 2.79]; 1.82[1.43, 2.31]) than White. Our study, using large-scale and nationally representative data, found that adults with diabetes had a higher prevalence of MCI/dementia than those without diabetes. Older adults and racial/ethnic minorities with diabetes need to gain further attention for disease management due to the higher prevalence of MCI/dementia.

Abstract citation ID: igad104.0657

**MECHANISMS OF CHANGE IN COGNITIVE FUNCTION DOMAINS IN OLDER ADULTS**

Zheng Zhu[1], Jessica Zwerling[2], Xiang Qi[1], Yaolin Pei[1], Yaguang Zheng[1], and Bei Wu[1], *1. New York University, New York City, New York, United States*, *2. Albert Einstein College of Medicine, Bronx, New York, United States*

It is crucial to gain a better understanding of these relationships in order to have a more comprehensive understanding of cognitive abilities over time and how interventions can target multiple domains to improve overall cognitive function. This study aimed to investigate the mechanism of changes in cognitive function domains in phenotypic networks. This study used data from the Aging, Demographics, and Memory Study (ADAMS) in Wave A and Wave B. We assessed 12 cognitive function domains. Latent profile transition analysis (LPTA) and cross-lagged panel network model were employed to the dynamic interactions of the 12 cognitive function domains over time in both the deterioration and improvement groups. A total of 252 participants were included in the final analysis. LPTA identified five subgroups and categorized all samples into three main categories: improvement group (n=61), deterioration group (n=54), and no change group (n=137). "D9: psychomotor processing" showed the largest value of outstrength in the deterioration group (r=0.941) and improvement group (r=0.969). The strongest direct positive effect in the deterioration group was "C9: psychomotor processing" -> "C8: attention" ($\beta$=0.39). In the improvement group, the strongest direct positive effect was "C9=psychomotor processing" -> "C7=visual memory" ($\beta$=0.69). Psychomotor processing can affect other cognitive domains, and it plays a crucial role in changes of cognitive function. The paths of psychomotor processing to attention and visual memory were found to be major factors in cognitive deterioration and improvement. Targeting psychomotor processing may lead to the development of more effective and precise interventions.

Abstract citation ID: igad104.0658

### COGNITIVE, PHYSICAL, AND PSYCHOLOGICAL FUNCTION AND QUALITY OF LIFE IN PATIENTS WITH STROKE—A NETWORK ANALYSIS

Juan Li[1], Xiangjing Kong[2], Hanzhang Xu[3], Qin Wang[4], Zhijian Liu[5], Bei Wu[6], and Yanpei Cao[1], *1. Huashan Hospital, Fudan University, Shanghai, Shanghai, China (People's Republic)*, *2. Air Force Hospital of PLA Eastern Theater Command, Nanjing, Jiangsu, China (People's Republic)*, *3. Duke University, Durham, North Carolina, United States*, *4. Outpatient Department, Linyi, Shandong, China (People's Republic)*, *5. Naval Medical University, Shanghai, Shanghai, China (People's Republic)*, *6. New York University, New York City, New York, United States*

This study aimed to identify the dynamic associations across cognitive, physical and psychological function, and quality of life using network analysis among Chinese patients with acute ischemic stroke (AIS). We conducted a cross-sectional study in 2021 that included a total of 636 patients with AIS from three stroke centers in Shanghai, Nanjing, and Linyi, China. Participants completed a complex battery of measures of cognitive function (Montreal Cognitive Assessment, MoCA), physical function (Barthel Index, Modified Rankin Scale), psychological function (Epidemiological Studies Depression Scale, CES-D) and quality of life (short version of Stroke-specific Quality of life scale, SS-QOL). We used the Gaussian Graphical Modeling to estimate the network structure. Cognitive and physical function were important prerequisites to quality of life. Specifically, the most central nodes (strength) in the network were quality of life, attention (cognitive domain), transferring to a chair, caring perineum/cloth at toilet and walking (physical domain). Cognitive function, particularly visuospatial /executive function and attention, and physical (dressing, feeding and drinking, up and downstairs and transferring to a chair) were positively related to quality of life; whereas depressive symptoms and disability were negatively related to quality of life. Factors contributing to quality of life are complex in patients with AIS. Attention, visuospatial /executive function from cognitive function and transferring, caring at toilet and walking from physical function were central nodes to the quality of life network. Interventions that target cognitive and physical function may have a potential to improve quality of life for stroke patients.

Abstract citation ID: igad104.0659

### PERCEIVED RISK OF ALZHEIMER'S DISEASE/DEMENTIAS AND COGNITIVE FUNCTION AMONG US OLDER ADULTS

Nan Wang[1], Hanzhang Xu[2], Jessica West[2], Truls Østbye[3], Bei Wu[4], Ying Xian[5], and Matthew Dupre[2], *1. University of California, Davis, Davis, California, United States*, *2. Duke University, Durham, North Carolina, United States*, *3. Duke University School of Medicine, Durham, North Carolina, United States*, *4. New York University, New York City, New York, United States*, *5. UT Southwestern Medical Center, Dallas, Texas, United States*

To assess factors associated with the perceived risk of Alzheimer's disease and related dementias (ADRD) and how the perceived risk of ADRD was related to cognitive function. We conducted a retrospective cohort study using 5 waves of data from the Health and Retirement Study (2012-2022) that included adults aged 65 years or older with no previous diagnosis of ADRD at baseline. Cognitive function was measured at baseline and over time using a summary score that included immediate/delayed word recall, serial 7's test, objective naming test, backwards counting, recall of the current date, and naming the president/vice-president (range=0-35). Perceived risk of developing ADRD was categorized at baseline as "definitely not" (0% probability), "unlikely" (1-49%), "uncertain" (50%), and "more than likely" (>50-100%). Additional baseline measures included participants' sociodemographic background, psychosocial resources, health behaviors, physiological status, and healthcare utilization. Of 1,457 participants (median age 74 [IQR=63-85] and 59.8% women), individuals who perceived that they would "definitely not" develop ADRD were more likely to be non-Hispanic Black, less educated, and have lower household income than individuals who indicated it was "unlikely" they would develop ADRD. Participants who reported their risks of developing ADRD as "definitely not," "uncertain," and "more than likely" had lower cognitive function than participants who perceived their risk of developing ADRD as "unlikely." Perceived risk of developing ADRD is associated with cognitive function. The (dis)concordance between individuals' perceived risk of ADRD and their cognitive function has implications for increasing public awareness and developing interventions to prevent ADRD.

### SESSION 2070 (SYMPOSIUM)

Abstract citation ID: igad104.0660

### CAREER DEVELOPMENT (K) AWARDS AT NIA: PROGRAMS, PEER REVIEW, AND SETTING YOU UP FOR SUCCESS

Chair: Shoshana Kahana
Discussant: Shoshana Kahana

The National Institute on Aging (NIA), one of the 27 Institutes and Centers (ICs) of the National Institutes of Health (NIH), leads the federal government in conducting and supporting research on aging and the health and well-being of older people. The growing investment in training, and specifically career development awards (K), has resulted in substantial growth at NIA. The purpose of this symposium is to provide an overview of the 1) various types of NIH career development awards and programs; 2) effective strategies for preparing strong career development award applications; and 3) NIA peer review process for career development awards. Providing information to attendees of the various career development awards, as well as the critical factors to understand in the process of the peer review process, can optimize future success.

Abstract citation ID: igad104.0661

### CAREER DEVELOPMENT AWARDS AT NIA/NIH

Maria Carranza, *National Institute on Aging, Bethesda, Maryland, United States*

NIA offers a variety of individual research career development (K) awards, which enable scientists to enhance their careers in biomedical research. Mentored career development (K) awards are effective tools at retaining investigators in research careers and contributing to their subsequent research success. Specifically, those who receive K awards are significantly more likely to have subsequent research publications and to apply for subsequent NIH research awards. In this presentation, Dr. Carranza will review different K programs offered at NIA, including those targeted at postdocs, early career faculty, and more senior faculty, as well as clinician-science focused awards.

Abstract citation ID: igad104.0662

### EFFECTIVE STRATEGIES FOR PREPARING STRONG CAREER DEVELOPMENT AWARD ("K") APPLICATIONS

Jamie Lahvic, *NIH/National Institute on Aging, Bethesda, Maryland, United States*

In this presentation, Dr. Lahvic will present critical policies, best practices, and resources to consider when preparing mentored career development award ("K") applications. Dr. Lahvic will emphasize 1) Starting the process early; 2) Developing an application strategy, including reaching out to relevant program officials, identifying appropriate mentors and collaborators, and evaluating if the career development award will add value to your professional development trajectory; and 3) Understanding the application requirements. Using real, successful and unsuccessful career development applications as examples, Dr. Lahvic will highlight best practices in planning the research and career development strategy, including avoiding proposing an overly ambitious project and ensuring the project is a vehicle towards future independence and is distinct from mentor's research. Knowledge of these practices and available resources to implement them will optimize the application process for individuals.

Abstract citation ID: igad104.0663

### PEER REVIEW PROCESS OF CAREER DEVELOPMENT AWARDS (K) APPLICATIONS AT NIA

Ramona Gianina Dumitrescu, *National Institute on Aging, Bethesda, Maryland, United States*
Bita Nakhai, *National Institute on Aging NIA), National Institute of Health (NIH), Bethesda, Maryland, United States*

In this presentation, Dr. Dumitrescu will take you behind the scenes of what occurs in a peer review meeting of career development award (K) applications at NIA. Some of the mechanics of the peer review process will be discussed, including composition of study groups, general timelines, and how the results of the peer review process are communicated to applicants via Summary Statements. Attendees will understand how reviewers evaluate the 5 core domains that generate a final impact score, including the: 1) candidate's qualifications; 2) the career development plan and career goals & objectives; 3) research plan; 4) mentor(s), collaborators & consultants; and 5) institutional environment & commitment to the candidate. Dr. Dumitrescu will help to de-mystify the peer review process in order help applicants leverage their respective applications for maximum success.

### SESSION 2275 (SYMPOSIUM)

Abstract citation ID: igad104.0664

### PRESIDENTIAL SYMPOSIUM: BUILDING BRIDGES ACROSS THE SCIENCES TO CATALYZE SLEEP DISPARITIES RESEARCH

Chair: Karen Lincoln Discussant: Karen Lincoln

This BSS Presidential Symposium brings together scholars from Sociology, Social and Family Dynamics, Human Development and Family Studies, Occupational Science and Medicine to highlight a multidisciplinary approach to sleep disparities research. Sleep is essential for restoration of healthy brain function, the immune system, and overall physical and emotional well-being. Conversely, sleep deficiencies are associated with a wide range of poor physical and emotional health outcomes, health behaviors and premature mortality. Strong empirical evidence indicates that sleep health is socially patterned, such that minoritized populations are generally more likely to experience sleep disruptions, disorders, and difficulties. These sleep deficiencies are also observed across indicators of socioeconomic status, such as income and educational attainment. Despite documentation of sleep disparities, studies that examine underlying health disparity causal pathways contributing to sleep health disparities are limited. This symposium is aligned with recommended strategies to address sleep health disparities from the National Institutes of Health, including: a) develop and promote integrative research on sleep health disparities, b) investigate the health and social causes and consequences of sleep health disparities, and c) develop multilevel, culturally tailored interventions to address sleep health disparities. By placing people and place at the center of their research, these scholars demonstrate their approach to addressing current research gaps, challenges and opportunities in sleep and health disparities research among middle-aged and older adults that are important to advance this emerging field.

Abstract citation ID: igad104.0665

### SOCIAL DISPARITIES IN SLEEP PATTERNS AMONG US ADULTS IN MID AND OLDER ADULTHOOD

Connor Sheehan, *Arizona State University, Tempe, Arizona, United States*

As the U.S. population ages, understanding social disparities in sleep among older adults is paramount. Even as humans physiologically require sleep, sleep duration decreases, and insomnia increases throughout older adulthood. Further, the implications of sleep related conditions (e.g., heart disease) are elevated in older adulthood. Importantly, these changes in sleep patterns throughout older adulthood are distributed in a manner that reflects broader social inequities, as quality sleep is contingent upon resources that are distributed unequally across society (e.g., time, stress, quiet, comfort, access to quality medical care). The inequities in sleep have profound implications for intergenerational social inequality, well-being, and longevity. Novel results regarding social based sleep disparities by gender, race/ethnicity, and educational attainment among older adults from the Health and Retirement Study and the National Health Interview Survey will be presented. The profound implications of sleep disparities and potential interventions will also be discussed.

Abstract citation ID: igad104.0666

### SLEEP DISTURBANCES IN RACIAL/ETHNIC DIVERSE MIDDLE-AGED AND OLDER ADULTS: FACTORS RELATED TO SLEEP DISTURBANCES

Alyssa Gamaldo[1], Angie Sardina[2], and Jacqueline Mogle[1], *1. Clemson University, Clemson, South Carolina, United*

*States*, *2. UNC Wilmington, Wilmington, North Carolina, United States*

Complaints of poor sleep health is a prevailing U.S. public health issue. Although complaints of sleep disturbances is common in U.S. adults, prior research has indicated rates of sleep disturbances tend to vary across racial/ethnic groups. These cross-racial/ethnic comparison approaches may be limited, however, in identifying specific and unique sociodemographic and health factors related to sleep disturbances within racial/ethnic groups. The current study explored two specific aims. The first aim was to explore racial differences in self-reported sleep disturbances. The second aim was to explore sociodemographic and health factors related to sleep disturbances within racial/ethnic groups. A sample of U.S. adults (N=521; age range=40-92) were surveyed via Qualtrics Software (December 2022). Sleep disturbances was measured by the Patient-Reported Outcomes Measurement Information System (PROMIS) questionnaire. Surveys were also collected on sociodemographic (e.g., age, monthly household income, education years, food insecurity, income changes due to COVID-19) and health (e.g., cognitive function, depressive symptoms, well-being, physical functioning, bodily pain, physical health conditions, and loneliness) factors. A univariate analysis of variance revealed non-significant racial/ethnic group differences in sleep disturbances ($p > .05$). However, bivariate correlations stratified by racial/ethnic groups suggested unique sociodemographic and health factors related to sleep disturbances within each racial/ethnic group. These findings contradict prior research of significant racial/ethnic group differences in sleep disturbances. Conversely, the findings support further investigating unique biopsychosocial factors associated with sleep health within racial/ethnic groups, which may provide meaningful information that may not be detected with cross-racial/ethnic comparison approaches.

Abstract citation ID: igad104.0667

### EXTENDING HOLISTIC, MULTIDISCIPLINARY SLEEP HEALTH INTERVENTION TO OVERLOOKED GROUPS OF OLDER ADULTS

Stacey Schepens Niemiec, *University of Southern California, Los Angeles, California, United States*

Sleep health and overall well-being across the lifespan are inextricably linked. Sleep difficulties are a common occurrence in older adulthood, with disturbed sleep ranking only second to pain as a problematic symptom interfering with older peoples' daily lives. Exacerbating the issue are sleep health disparities such that sleep disturbances disproportionately affect members of under-represented, marginalized, and resource-poor communities. Building, implementing, and mobilizing knowledge from sleep-promoting interventions with potential to effect change on sleep health and quality of life disparities are urgently needed. From an occupational therapy lens, "rest and sleep" is classified as one of nine fundamental "occupations"—other more widely recognized occupations include those such as ADL and work. The Person, Environment, Occupation-Performance (PEOP) Model, an occupation-centric guiding framework that considers multiple layers of dynamic and interacting factors pertinent to individuals' occupational performance, has been applied to sleep intervention. From this model, one can appreciate the myriad facilitators of and barriers to realizing healthy and restorative rest and sleep, directing attention to a wide range of relevant issues such as cultural practices, societal attitudes, and resource scarcity. This presentation will overview the need for addressing inequities in sleep health and extending socio-culturally responsive services to traditionally overlooked subgroups of older people. Examples will be provided of holistic, multidisciplinary intervention research and programming that work to transform the landscape of sleep health disparities in later life.

Abstract citation ID: igad104.0668

### PSYCHOSOCIAL FACTORS AND EARLY MID-LIFE BLACK WOMEN'S SLEEP QUALITY: A CONSIDERATION OF INTERSECTIONAL MEASURES

Christy Erving, *University of Texas at Austin, Austin, Texas, United States*

Beyond an individual behavior, scholars are progressively conceptualizing sleep as embedded in social factors. Extant research establishes that sleep is influenced by experiences based on status characteristics such as gender or race along with psychosocial stressors such as discrimination. Moreover, sleep is important in the context of gender and racial disparities, as Black women experience relatively poorer sleep quality compared to their Black male and White counterparts. Given their relatively poor sleep quality, this presentation will center Black women by examining how psychosocial factors influence their subjective sleep quality in early mid-life, an important life course stage characterized by significant role obligations in personal and professional life domains. This presentation also takes an intersectional approach, examining the sleep implications of two recently developed measures that capture the unique intersectional oppression of Black women: Superwoman Schema and gendered racial microaggressions. To assess the association between these intersectional psychosocial factors and subjective sleep quality, data are drawn from Mechanisms Underlying Stress and Emotions (MUSE) in African-American Women's Health Study, a cohort of 422 Black women (ages 30-46) in Atlanta, GA, USA. Preliminary findings suggest that both Superwoman Schema endorsement (e.g., feeling obligated to help others) and reports of gendered racial microaggressions (e.g., feeling marginalized and silenced in professional settings) are related to poor subjective sleep quality. Moreover, these associations remain even after adjusting for various factors including, but not limited to, depressive symptoms, anxiety symptoms, and social support. Study implications and future research directions will be discussed.

Abstract citation ID: igad104.0669

### SLEEP DISPARITIES AMONG ASIAN AMERICANS: IMPACTS OF DISCRIMINATION AND ACCULTURATIVE STRESS

Lee Sunmin, *University of California Irvine, Irvine, California, United States*

Although Asian Americans have shorter sleep duration and more sleep difficulty compared to Whites or Hispanics, research on Asians remains scarce. 60% of Asians are foreign-born and have high levels of limited English proficiency, which is positively associated with a range of immigrant stressors, such as discrimination and acculturative stress. Importantly, COVID-19 has exacerbated discrimination towards Asian Americans giving rise to 'Asian hate'. In a study using data from the National Epidemiology Study of Alcohol and Related Conditions III (n=1,765), we found that individuals with high discrimination were sleeping 14.4 minutes less (SE: 6.0, p < .05) and had a higher prevalence of sleep difficulty (prevalence ratio (PR): 1.73, 95% Confidence Interval (CI): 1.33-2.24) compared to those with low discrimination. An interaction effect was observed in sleep difficulty by nativity. The association between discrimination and sleep difficulty was stronger among the U.S.-born than the foreign-born. In another study of Chinese and Korean Americans (n=400), Poisson models revealed that greater acculturative stress was associated with a higher prevalence of sleep disturbance (PR: 1.18, 95% CI: 1.06% to 1.31%). Interaction tests indicated that only women had a significant association between acculturative stress and sleep disturbance (PR: 1.30; 95% CI: 1.13 to 1.49), while the association was significant for individuals identifying as "very Asian" (PR: 1.21; 95% CI: 1.08 to 1.35), but not for those identifying as "mostly Asian" or "bicultural/western". Future sleep health research in Asian Americans should consider the critical role of discrimination and acculturative stress.

### SESSION 2280 (PAPER)

### MENTAL HEALTH, SOCIAL DETERMINANTS, AND PSYCHOSOCIAL WELL-BEING

Abstract citation ID: igad104.0670

### CUMULATIVE LONELINESS AND ALL-CAUSE MORTALITY AMONG MIDDLE-AGED AND OLDER ADULTS IN THE UNITED STATES, 1996–2019

Xuexin Yu[1], Tsai-Chin Cho[1], Ashly Westrick[1], Chen Chen[1], Kenneth Langa[2], and Lindsay Kobayashi[1], *1. University of Michigan School of Public Health, Ann Arbor, Michigan, United States*, *2. University of Michigan, Ann Arbor, Michigan, United States*

Recent studies have linked increased mortality to loneliness measured at a single point in time or over a short period of time, omitting the cumulative and dynamic nature of loneliness and its health effects. This study aimed to investigate long-term exposure to loneliness over an eight-year period in relation to subsequent all-cause mortality over 15 years. Data were from 9,032 individuals aged 50+ in the US Health and Retirement Study (HRS) from 1996-2019. Self-reported loneliness (yes; no) was measured biennially from 1996-2004. Cumulative loneliness was defined according to loneliness duration as never, intermittent (1-2 time points), and sustained (3+ time points). Mortality from 2004-2019 was identified from the HRS exit interviews and other sources (e.g., spouse). In the crude analysis, intermittent and sustained experience of loneliness was respectively associated with 137 (95%CI, 104-170) and 288 (95%CI, 233-343) excess deaths per 10,000 person-years, compared to those never feeling lonely. In the fully adjusted Cox proportional hazards model, a longer duration of loneliness was associated with additional 9% (HR=1.09, 95%CI, 1.02-1.17, intermittent vs. never) and 14% (HR=1.14, 95%CI, 1.03-1.27, sustained vs. never) mortality risk, indicating a dose-response relationship (P trend=0.009). Sustained loneliness may be a risk factor for mortality in mid-to-later life, and could be a potential intervention target to increase life expectancy.

Abstract citation ID: igad104.0671

### OPTIMISM MITIGATED THE DETRIMENTAL ROLE OF DEPRESSION AND ANXIETY IN SUBJECTIVE WELL-BEING AFTER CARDIAC SURGERY

Amy Ai, and Veronika Nash, *Florida State University, Tallahassee, Florida, United States*

Cardiac diseases, the primary cause of death among older adults of any races, is comorbid with mental health problems such as depression and anxiety. A recent review article found that cardiovascular health was more consistently associated with optimism and hedonic/subjective well-being than with meaning-laden eudaimonic/psychological well-being. Extensive research has shown the health benefit of dispositional optimism, a personality trait. Few studies, however, have explored the protective effect of optimism the on subjective wellbeing (SWB) one months after cardiac surgery, a life altering procedure in late life. This study estimated the mediating effect of dispositional optimism between preoperative mental health and postoperative SWB. Using a prospective design, we followed 300 patients undergoing cardiac surgery. Mental health was indicated by anxiety and depression, while SWB was indicated by low symptom levels of general distress, based on its definition, one month postoperavely. A confirmatory factor analyses were performed to validate SWB with three subscales. The final solution of a structural equation model (SEM) indicated good fit (Chi-square = 47.55, df = 18, p = 0.000; CFI = 0.95; TLI = 0.92, RMSEA = 0.07, with 90 percent CI from 0.05 to 0.10). Both the CFI (0.96) and TLI (0.93) exceeded the benchmark criterion of .90. The lower CI of RMSEA is under 0.05. Optimism, indicted by two subscales, mediated preoperative mental health and postoperative symptom levels. The finding supports its mitigating role against the damage of depression and anxiety in the SWB of cardiac patients during the critical recovery time – postoperative month.

Abstract citation ID: igad104.0672

### PSYCHOSOCIAL CARE AFTER STROKE AS PROVIDED BY MULTIDISCIPLINARY TEAMS IN STROKE SERVICES

Dagmar van Nimwegen[1], Lisette Schoonhoven[2], Johanna Visser-Meily[3], and Janneke M de Man-van Ginkel[4], *1. University of Applied Sciences Utrecht, Utrecht, Utrecht, Netherlands*, *2. University Medical Center Utrecht, Utrecht University, Utrecht, Utrecht, Netherlands*, *3. UMCU, Utrecht, Utrecht, Netherlands*, *4. Leiden University Medical Center, Leiden, Zuid-Holland, Netherlands*

About 18-31% of all persons with stroke develop one or more psychosocial impairments, such as depression or anxiety. They receive care in stroke services from multidisciplinary teams, consisting of several different healthcare professionals. Currently, it is unclear how psychosocial care is provided within these stroke services, what roles healthcare professionals play in this, and what psychosocial needs persons with stroke experience. Therefore, in this study the following research questions were answered: How do persons with stroke and healthcare professionals experience the current psychosocial care after stroke, and what psychosocial needs do persons with stroke experience? A qualitative study was conducted, which consisted of observations of care moments between 44 persons with stroke and 74 healthcare professionals, and individual interviews with ten persons with stroke. The observations showed that most care moments mainly focus on physical care. Healthcare professionals tend to focus on the care they give at that particular moment. In contrast, persons with stroke asked attention for a broader perspective on the whole rehabilitation process. In addition, the interviews showed that persons with stroke mostly need: clarity about the rehabilitation process, the ability to make their own decisions, a personal approach in stroke care, and to recover as fast as possible. To be able to provide care that fulfills the needs of persons with stroke, awareness about the differences in perspective between healthcare professionals and patients is essential in care programs, possibly leading to the necessity to change such programs to better fit these needs.

Abstract citation ID: igad104.0673

### SLEEP DISORDERS AND RISK OF MID- TO LATE-LIFE SUICIDE ATTEMPT

Amy Byers[1], Emily Dolsen[2], Yixia Li[3], Carolyn Gibson[4], Aoife O'Donovan[5], Thomas Neylan[5], and Randall Kuffel[4], *1. UCSF / San Francisco VA Health Care System, San Francisco, California, United States*, *2. Oakland Behavioral Health Clinic, Oakland, California, United States*, *3. NCIRE, San Francisco, California, United States*, *4. San Francisco VA Health Care System, San Francisco, California, United States*, *5. University of California, San Francisco, San Francisco, California, United States*

Sleep is a compelling target for suicide prevention in later life, as 50% of older adults experience sleep difficulties. The primary objective of the current study was to evaluate whether sleep disorders increase risk of late-life suicide attempt and death by suicide. We examined a nationally based cohort of over 5 million US veterans 50 years and older, who used Department of Veterans Affairs (VA) health services. Veterans with a sleep diagnosis (i.e., sleep apnea, insomnia, hypersomnia, and others) at baseline (October 1, 2011, to September 30, 2013) or earlier (October 1, 2007, to September 30, 2011) were propensity matched 1:1 with patients without these diagnoses based on demographic characteristics and Charlson Comorbidity Index (Total N=1,990,362; 995,181 each group). Secondary analyses examined psychiatric disorders as potential moderators. Fully adjusted models (additionally adjusted for psychiatric disorders) demonstrated that patients with sleep disorders had a 23% increased risk of any suicide attempt (HR 1.23, 95% CI [1.20-1.27]), while risk mitigated for lethality of attempt (HR 0.98, 95% CI [0.92-1.03]). Depression, PTSD, and generalized anxiety disorder were all approximately twice as prevalent among the sleep disorder group. Among veterans with a psychiatric disorder, any sleep disorder diagnosis was associated with a 28% increased risk for suicide attempt, which attenuated among those without a psychiatric diagnosis. In conclusion, sleep disorder diagnoses conferred increased risk for suicide attempt, especially in older adults with psychiatric disorders. These findings suggest that treatment of sleep disorders may mitigate risk of suicide attempts in older vulnerable veterans.

Abstract citation ID: igad104.0674

### SOCIAL DETERMINANTS OF SMOKING CESSATION IN MIDDLE-AGED AND OLDER ADULTS WITH SERIOUS MENTAL ILLNESSES

Heather Leutwyler[1], Erin Hubbard[2], Theodore Bussell[3], Negin Zahedikia[4], Dennys Balestra[1], Margaret Wallhagen[1], and Chizimuzo Okoli[5], *1. University of California, San Francisco, San Francisco, California, United States*, *2. UCSF, San Francisco, California, United States*, *3. University of San Francisco School of Nursing, San Francisco, California, United States*, *4. University of California San Francisco, San Francisco, California, United States*, *5. University of Kentucky, Lexington, Kentucky, United States*

People living with serious mental illnesses (SMI) continue to face a disproportionate burden of tobacco-related prevalence, morbidity, and mortality as compared to those without SMI. The risk of mortality related to cigarette smoking among those with SMI is six-times that for persons without SMI. Tobacco use undermines social determinants of health (SDoH) in general and the social determinants of mental health more specifically. Not only does tobacco use exacerbate mental health symptoms, it further impedes mental health recovery by reducing expendable income, sustaining poverty, hindering food security, and limiting job opportunities. Conversely, stopping tobacco use is associated with improved mental health and, possibly, substance use recovery outcomes. The purpose of this paper is to analyze the social determinants of tobacco smoking cessation as described by adults with SMI. We conducted semi-structured qualitative interviews with 17 middle aged and older adults (mean=52, sd=6.9) with SMI upon completion of a 12-week smoking cessation program. Grounded Theory methodology guided data collection and analysis. Participants described what contributed to their success with smoking cessation or reduction and pointed out how SDoH played a role in the process. Five broad categories of SDoH described were: 1. Support and sense of belonging; 2. Structure and meaningful activities; 3. Employment; 4. Role models; and 5. Access to green space. Our findings illustrate the importance of SDoH in health interventions and are a reminder to consider these SDoH as interventions are tailored to meet the unique needs of people living with SMI.

Abstract citation ID: igad104.0675

### SOMATIC COMORBIDITY PATTERNS IN PEOPLE WITH DEPRESSION: A POPULATION-BASED COHORT STUDY

Hao Luo[1], Davide Vetrano[2], Federico Triolo[3], Alberto Zucchelli[4], Yingyang Zhang[1], Pengcheng Wang[5], Kenneth Man[6], and Francisco Lai[1], *1. The University of Hong Kong, Hong Kong, Hong Kong*, *2. Karolinska Institutet, Solna, Stockholms Lan, Sweden*, *3. Aging Research Center, Solna, Stockholms Lan, Sweden*, *4. Università degli Studi di Brescia, Brescia, Lombardia, Italy*, *5. HKU, Hong Kong, Hong Kong*, *6. University College London, London, England, United Kingdom*

People with depression often present with co-existing somatic conditions, which impact survival and steer patients' clinical management. This study aimed to identify unique patterns of somatic comorbidity in people with depression and examine their associated mortality. We used population-based electronic health records from the Clinical Data Analysis and Reporting System in Hong Kong. People aged 40+ who had an incident depression diagnosis between 2001 and 2015 were included and followed up until 31 December 2018. Latent class analysis was applied, separately by decades, to individuals with depression and at least one comorbid somatic condition. Cox proportional hazard models were used to examine the risk of mortality associated with specific comorbidity patterns, adjusting for sociodemographic and clinical characteristics. We identified 38,889 eligible individuals (mean age: 62.8; 68.7% women). Latent class analysis identified an increasing number of patterns with age (two to seven). Compared with the unspecific pattern, the following patterns were associated with the highest mortality risk in different age groups: cardiometabolic and cerebrovascular diseases in individuals aged 40-49 (hazard ratio [HR] 2.5 [95% CI 1.7-3.7]), cardiometabolic diseases and anemia in those aged 50-59 (HR 3.4 [2.6-4.5]) and 70-79 (2.7 [2.3-3.3]), cardiometabolic, cerebrovascular diseases, and anemia in those aged 60-69 (HR 2.5 [2.0-3.2]), and cardiorespiratory disease in those aged 80+ (HR 2.0 [1.8-2.3]). Specific somatic comorbidity patterns were independently and substantially associated with the risk of mortality. This highlights the importance of taking combinations of somatic conditions into account when tailoring the clinical and care approach to people with depression.

### SESSION 2285 (SYMPOSIUM)

Abstract citation ID: igad104.0676

### CASUAL EFFECTS OF EARLY-LIFE ADVERSITY AND MID/LATE-LIFE BEHAVIORAL INTERVENTION ON THE PACE OF BIOLOGICAL AGING

Chair: Daniel Belsky Discussant: Luigi Ferrucci

Epigenetic clocks have taken gerontology by storm. With the advent of 2nd and 3rd generation epigenetic clocks, including GrimAge and DunedinPACE, the field now has genomic biomarkers of aging that exhibit strong and consistent associations with aging-related morbidity and mortality. But the causes of variation in these powerful indices of aging-related biological changes remain poorly understood. In this symposium, we present four studies at the vanguard of research to elucidate causal drivers of 2nd and 3rd generation epigenetic clocks drawn from four disciplines. From life-course epidemiology, we present a natural-experiment study of the long-term impact of in-utero famine exposure. From economics, we present a quasi-natural experiment study of long-term impacts of early-life exposure to the Great Depression. From psychology, we present results from a randomized controlled trial (RCT) of cognitive behavioral therapy (CBT) for insomnia in older adults. And from geroscience, we present results from an RCT of long-term calorie restriction in healthy, non-obese adults (CALERIE). These studies report novel and important findings that suggest early-life adversity acts to accelerate the pace of biological aging –and— that it may be possible to slow the pace of biological aging in mid- and later-life through behavioral intervention. They also illustrate study designs and methods that can help move the field forward as costs for measurement of epigenetic clocks fall, enabling their introduction into many more cohort and intervention studies.

Abstract citation ID: igad104.0677

### EFFECT OF LONG-TERM CALORIC RESTRICTION ON THE PACE OF BIOLOGICAL AGING IN HEALTHY ADULTS FROM THE CALERIE TRIAL

Daniel Belsky, *Columbia University, New York City, New York, United States*

The geroscience hypothesis proposes that therapy to slow or reverse molecular changes that occur with aging can delay or prevent multiple chronic diseases and extend healthy lifespan. Caloric restriction (CR), defined as lessening caloric intake without depriving essential nutrients, can change molecular processes associated with aging, including DNA methylation (DNAm), and increases healthy lifespan in multiple species. Here we report results of a post-hoc analysis of the influence of CR on DNAm measures of aging in blood samples from the Comprehensive Assessment of Long-term Effects of Reducing Intake of Energy (CALERIE) trial, a randomized controlled trial in which n=220 non-obese adults were randomized to 25% CR or ad libitum control diet for 2 years. We found that CALERIE intervention slowed the pace of aging, as measured by the DunedinPACE DNAm clock, but did not lead to significant changes in PhenoAge- or GrimAge-clock-measured biological age. Treatment effects were small; changes in DunedinPACE suggest a ~3% slowing of the pace of aging. Nevertheless, this small effect may be substantive; in an independent study of older adults, 3% slower DunedinPACE is associated with a 15% lower risk of death. The finding that CR modified DunedinPACE in a randomized controlled trial supports the geroscience hypothesis, building on evidence from small and uncontrolled studies and contrasting with reports that biological aging may not be modifiable. Ultimately, a conclusive test of the geroscience hypothesis will require trials with long-term follow-up to establish effects of intervention on primary healthy-aging endpoints, including incidence of chronic disease and mortality.

Abstract citation ID: igad104.0678

### COGNITIVE BEHAVIORAL TREATMENT OF INSOMNIA SLOWS THE PACE OF BIOLOGICAL AGING: RESULTS FROM AN RCT IN OLDER ADULTS

Judith Carroll, *University of California, Los Angeles, Los Angeles, California, United States*

Insomnia in late life has been linked to accelerated biological aging, presenting a behaviorally modifiable target to slow the pace of aging. We previously demonstrated that treatment of insomnia in older adults using cognitive behavioral therapy for insomnia (CBT-I) reduced the expression of the cellular senescence marker, p16INK4a, in peripheral blood mononuclear cells (PBMC). Next, we tested whether this treatment for insomnia would alter the biological pace of aging, estimated from the DNA methylation derived DunedinPACE, as compared to a sleep education therapy (SET), an active comparator condition. Participants 60+ years old with insomnia were enrolled in a randomized controlled trial and assigned to CBT-I (n=49) or SET (n=45). Epigenetic data were obtained from DNA extracted from blood collected prior to initiation of treatment (baseline) and again 18-24 months later. A mixed linear models adjusting for age, sex, BMI, and education revealed a significant treatment by time interaction (p=0.003). Receipt of CBT-I was associated with a decline in the epigenetic pace of aging, while those receiving SET had an increase in their pace of aging. Findings indicate that a behavioral intervention to improve insomnia was effective at slowing the pace of biological aging among older adults. Future research will be needed to replicate these findings in other age groups and specific populations with heightened risk for accelerated aging.

Abstract citation ID: igad104.0679

**EARLY-LIFE EXPOSURE TO THE GREAT DEPRESSION AND THE PACE OF BIOLOGICAL AGING**

Lauren Schmitz, *University of Wisconsin-Madison, Madison, Wisconsin, United States*

The Developmental Origins of Health and Disease hypothesis proposes that insults to early-life development can result in increased risk for aging-related disease later in life. There is growing interest within gerontology in the potential role such early life exposures play in shaping processes of aging. However, causal research linking early-life exposures to processes of aging in later life is rare. Moreover, there are difficulties in distinguishing the impacts of exposures occurring at different stages of early life development. In this study, we build on a quasi-natural experiment design we developed that leverages state-year variation in economic shocks during the Great Depression to examine the causal effect of environmental exposure in early life on aging outcomes in the US Health and Retirement Study (HRS) (2022 PNAS). Our design allows us to distinguish effects of exposure during different periods of early life development – in-utero, in the first year of life, and later in childhood. In the present study, we apply this framework specifically to study impacts of early-life exposure to the Great Depression on pace of aging in later life. We measure pace of aging using repeated-measures longitudinal functional test and biomarker data and, in parallel, a single-timepoint blood-DNA methylation assessment of the DunedinPACE epigenetic clock through the 2016 HRS assessment wave. We further examine whether impacts of the Great Depression on the pace of aging translate into differences in morbidity and mortality through 2020.

Abstract citation ID: igad104.0680

**IN UTERO EXPOSURE TO THE DUTCH HUNGER WINTER AND THE PACE OF BIOLOGICAL AGING**

Mengling Cheng[1], Bas Heijmans[2], Chihua Li[3], Lambert Lumey[4], Calen Ryan[4], Shuang Wang[4], Jiayi Zhou[4], and Daniel Belsky[5], *1. Swiss Centre of Expertise in Life Course Research, Lausanne, Vaud, Switzerland*, *2. Leiden University Medical Center, Leiden, Zuid-Holland, Netherlands*, *3. University of Michigan Institute for Social Research, Ann Arbor, Michigan, United States*, *4. Columbia University Mailman School of Public Health, New York City, New York, United States*, *5. Columbia University, New York City, New York, United States*

Adults with histories of early-life adversity experience younger age-of-onset for aging-related disease and shorter lifespan, suggesting an accelerated pace of biological aging. To test this hypothesis, we analyzed data from an established natural experiment: in-utero exposure to famine conditions induced by a Nazi blockade of the Southern Netherlands in Winter 1944-5. We analyzed data from the Dutch Hunger Winter Families Study (DHWFS, N=951). DHWFS enrolled survivors of in-utero famine exposure, matched controls born in the same hospitals as the survivors immediately prior to and following the famine period, and siblings of these individuals. Blood samples were collected when the famine survivors were aged 58. We analyzed blood DNA methylation data to measure the GrimAge and PhenoAge 2nd-generation epigenetic clocks and the DunedinPACE 3rd-generation clock. We tested if famine survivors were biologically older and aging faster as compared to controls. We found that famine survivors had faster DunedinPACE pace of aging, as compared with controls, but similar PhenoAge and GrimAge biological ages. In sex-stratified analyses, we found stronger effects of famine exposure in women as compared with men, including for the GrimAge and PhenoAge clocks; among women, famine exposure was associated with faster DunedinPACE and older GrimAge and PhenoAge, whereas in men effect-sizes were near zero. Famine effects were not accounted for by blood-cell composition and were similar for individuals exposed early and later in gestation. Sibling-difference analyses reproduced size and direction of effect estimates. Findings provide natural-experiment evidence for long-term effects of in-utero famine exposure on the pace biological aging.

### SESSION 2290 (SYMPOSIUM)

Abstract citation ID: igad104.0681

**DIVERSE WOMEN'S LIVES: THE STUDY OF BIO-PSYCHO-SOCIAL IMPACT ON DISABILITY WITH AGE**

Chair: Tracie Harrison Co-Chair: Corey Nagel Discussant: Tracie Harrison

In this symposium we examine the extent to which psychosocial interventions might promote healthy aging among women with emotional, mobility, and sensory limitations. A growing literature explains the need for culturally tailored care for men and women with disabilities while also stressing the importance of accommodations when living in inaccessible environments. Creating positive social participation amongst diverse groups based on sex/gender with varying levels of function and types of limitations may require more thought and resources than society is willing to forfeit. In this symposium, we begin by examining these issues among people aging in Mexico with few environmental accommodations in place. Creating recognition for the needs of men compared to women who age into mobility limitations in Mexico begins with identifying their unique individual and environmental risks. Next, we examine the needs of women in acute and long-term care with schizophrenia related emotional limitations. Stigma and neglect may create an environmental barrier to healthy aging in the U.S among this population. Our next study examines how sleep duration and quality can impact levels of depression and fatigue for men and women with sensory limitations, drawing attention to the additive effects of emotional changes on functional limitations. Finally, Taylor's group draws our attention to the work of creating social participation interventions for depression among African American women as they age with severe arthritic pain. We conclude by facilitating a discussion on social and cultural barriers for diverse women aging with functional limitations. This is a Women's Issues Interest Group Sponsored Symposium.

Abstract citation ID: igad104.0682

### RISK FACTORS FOR DISABLEMENT WITH AGING IN CENTRAL MEXICO

Lucia Caudillo-Ortega[1], Shelley Blozis[2], Nancy Blanco-Arroyo[3], and Tracie Harrison[4], *1. Universidad de Guanajuato, Guanajuato, Guanajuato, Mexico*, *2. UC Davis, Davis, California, United States*, *3. The University of Texas at Autin, Austin, Texas, United States*, *4. The University of Arkansas for Medical Sciences, Little Rock, Arkansas, United States*

Health, function, and social participation (e.g., disablement) were studied among community living men and women with mobility limitations living in central Mexico. Psychological (i.e., depression and activity effort), cultural (i.e., attitudes toward assistive devices) and social (i.e., personal resources) variables were tested with the goal to identify differential risk for worse health (Charlson Comorbidity Index) outcomes. We hypothesized significant differences based on psychological, social, and cultural influences. Data was collected using in-person, Spanish language surveys from 2019-2020. Measures were reliable and valid. The sample (N=257) were an average of 70.98 years (std 7.72) with an education of 6.97 years (std 7.22) and onset of functional limitation at 62.5 (std 15.07). Most reported female sex (51%). Using machine learning regression technique, statistically significant interactions were found: (1) Age was an important predictor of health; individuals clustered according to different age groups; those below 65 years had the best health; followed by those between 65 and 70 years, followed next by those between 70 and 75 years, and those over 75 years having the worst health, on average. (2) Age, biological sex, and depression were important predictors of function. Further, for those below 83 years, depression was related to function; specifically, for these individuals, those with depression levels above 18.3 had worse functioning relative to those who depression score was below 18.3. (3) age was an important predictor of social participation. For those with mobility limitations, age, sex, and depression were risks factors for disablement in Mexico.

Abstract citation ID: igad104.0683

### THE LIFE COURSE PERSPECTIVES OF WOMEN AGING WITH SCHIZOPHRENIA: CONCERNS WITH HEALTHY AGING

Veronica Garcia Walker, *UT at Austin, Austin, Texas, United States*

Multiple semi-structured interviews were conducted between August 2021- June 2022 to obtain life course insights of women aging with schizophrenia (N=14). 2 (14%) identified as Hispanic; 5 (36%) as African American, 7 (50%) as non-Hispanic White. Findings: 1) Getting Old Has Made it Worse, indicating decrease in functioning related to aging 2); I Need Meds to Live a Healthier Life, indicating a need for access to health care to address age related health conditions; 3) I'm Afraid to Die, indicating worry expressed as death becomes more imminent; 4) Who's Going to Help an Old Lady Like Me?, indicating a need for assistance to manage health conditions associated with aging. These findings support the need for experts that are considerate, have good communication, are genuine, and trustworthy in gerontology to provide important care for persons to ensure long, high quality lives for people with severe mental illness.

Abstract citation ID: igad104.0684

### GENDER DIFFERENCES IN SLEEP QUALITY, FATIGUE, AND DEPRESSIVE SYMPTOMS AMONG ADULTS OVER 50 WITH TOTAL BLINDNESS

Soyoung Choi[1], and Tracie Harrison[2], *1. University of Illinois at Urbana-Champaign, Urbana, Illinois, United States*, *2. The University of Arkansas for Medical Sciences, Little Rock, Arkansas, United States*

Visual limitations and blindness have been associated with poor sleep quality, increased fatigue, and a higher risk of depressive symptoms in the aging population. In this study, we aimed to investigate sleep quality, fatigue, and depressive symptoms in US adults over the age of 50 with total blindness, and to examine potential gender differences. The study included 88 participants (59 females, mean age 61.34±8.04; 19 males, mean age 62.32±8.17) who completed an online survey providing data on socioeconomic status, comorbidities, sleep quality, fatigue, and depressive symptoms. Descriptive and bivariate analyses were performed using R software and its required packages. While females slept less than males ($t = -2.98$, $p < .05$), there was no significant difference in sleep quality ($t = 1.82$, $p = .08$) or depressive symptoms ($t = 0.51$, $p = 0.62$). Fatigue levels were significantly higher in females than males ($t = 2.53$, $p < .05$). Poorer sleep quality was associated with increased fatigue and severe depressive symptoms in both subgroups. The findings indicate potential gender effects, however, given the small sample size and limitations of the cross-sectional design, further longitudinal research is needed to fully understand gender differences in sleep quality, fatigue, and depressive symptoms among adults over 50 with visual limitations.

Abstract citation ID: igad104.0685

### PARTICIPANT ACCEPTABILITY OF THE DEPRESSION AND PAIN PERSEVERANCE THROUGH EMPOWERED RECOVERY PROGRAM

Catherine Clair[1], Janiece Taylor[2], Rebekah Bhansali[3], Tracie Harrison[4], Anna Beeber[2], and Sarah L. Szanton[2], *1. Johns Hopkins Bloomberg School of Public Health, Baltimore, Maryland, United States*, *2. Johns Hopkins University, Baltimore, Maryland, United States*, *3. Johns Hopkins School of Nursing, Baltimore, Maryland, United States*, *4. The University of Arkansas for Medical Sciences, Little Rock, Arkansas, United States*

Between 2019-2023, the Depression and Pain Perseverance through Empowered Recovery (DAPPER) program was implemented with older African American women living with pain and low mood (N=34) in Baltimore, MD. Of the 21 who completed the intervention, we interviewed 9 women to understand their experiences in the program, their successes, challenges, and areas to improve the program. The average age of the participants was 64.8 (SD: 10.5). Using a qualitative descriptive design, we analyzed the interviews using content analysis. Overall, the main themes related to 1) Changes in Pain Experiences; 2) Influence of Research Study Team; and 3) Value in Resources and Being Listened To. The women described their experience in the DAPPER program positively and the impact that it had on their pain experience. Additionally, participants mentioned how the qualities, knowledge, and perspectives of the study team impacted them: "Respectful," "A good listener," and "Caring." Finally, several of the women mentioned the value of strategies learned, resources provided, and the impact that communicating with the nurses had on them. These findings demonstrate the acceptability of the DAPPER program and highlight the importance of innovative components of intervention and study development (e.g., qualities of team members, budget for purchasing items) that are often not intentionally considered during the research process. Suggestions include adding a group component to the intervention to increase social interactions.

### SESSION 2295 (SYMPOSIUM)

Abstract citation ID: igad104.0686

### A DISCUSSION ON THE STATE OF AFFAIRS ON DIRECT ACTION TO SUPPORT OLDER ADULTS DURING DISASTERS

Chair: Leah Haverhals Discussant: David Dosa

As the frequency and severity of disasters increases, supporting older adults before, during, and after disasters is paramount. Steps toward adequate disaster preparedness include ensuring older adults possess awareness of increased disaster and hazard events and understand their role in preparedness. This symposium will address the current state of affairs in the United States (US) on direct actions to support older adults before, during, and following major disasters. Additionally it will provide context on how continuing effects of climate change impact these efforts. Dr. Lisa Brown will share perspectives and describe efforts of the US National Advisory Committee on Seniors and Disasters. Dr. Sue Anne Bell will discuss the role of implementation science in improving the emergency preparedness in nursing homes. Dr. Lindsay Peterson will describe existing state policies around home and community-based service providers. Dr. Tamar Wyte-Lake will share directions the Department of Veterans Affairs is moving in to best support vulnerable populations, focusing on older Veterans. Dr. Karl Pillemer will address the role of older adults as active agents in climate change prevention and mediation. Dr. David Dosa will act as discussant. These presenters will provide important insights into ongoing efforts while highlighting areas in need of improvement and aligned action, with the hopes that this will generate important discussions well into the future. This is a Disasters and Older Adults Interest Group Sponsored Symposium.

Abstract citation ID: igad104.0687

### NACSD RECOMMENDATIONS FOR OLDER ADULT DISASTER PREPAREDNESS, RESPONSE, AND RECOVERY

Lisa Brown[1], Anna Fisher[2], David Dosa[3], and Sue Anne Bell[4], *1. Palo Alto University, Palo Alto, California, United States*, *2. Hillcrest Health Services, Bellevue, Nebraska, United States*, *3. Brown University, Providence, Rhode Island, United States*, *4. University of Michigan, Ann Arbor, Michigan, United States*

This presentation shares the recommendations drafted by the National Advisory Committee on Seniors and Disasters (NACSD). This federal advisory committee provides advice and guidance to the Assistant Secretary for Preparedness and Response within the U.S. Department of Health and Human Services (HHS) and the HHS Secretary. The NACSD is comprised of seven non-federal members with expertise in geriatric disaster planning, preparedness, response, or recovery and ten federal (non-voting) members who are ex officio representatives from agencies within HHS and other Executive Branch departments. The voting members drafted recommendations intended to address the immediate and persistent challenges in public health emergency preparedness, response, and recovery affecting older adults in the United States. Numerous meetings with federal and non-federal subject matter experts from various disciplines were held to develop the recommendations. The NACSD was also tasked with evaluating issues and programs and providing findings, advice, and recommendations to the Secretary of Health and Human Services to support and enhance all-hazards public health and medical preparedness, response, and recovery activities related to meeting the unique needs of older adults and their families across the entire spectrum of their wellbeing. The NACSD evaluated and provided input concerning the medical and public health needs of older adults related to preparation for, response to, and recovery from all-hazards emergencies; and may provide advice and consultation with respect to State emergency preparedness and response activities relating to older adults.

Abstract citation ID: igad104.0688

### IMPROVING NURSING HOME DISASTER READINESS THROUGH IMPLEMENTATION SCIENCE

Sue Anne Bell[1], Jennifer Inloes[1], Michael Wasserman[2], John Donnelly[3], and Tamar Wyte-Lake[4], *1. University of Michigan, Ann Arbor, Michigan, United States*, *2. Consultant, Santa Clarita, California, United States*, *3. VA QUERI Center for Evaluation and Implementation Resources and HSR&D Center for Clinical Management Research, Ann Arbor, Michigan, United States*, *4. U.S. Department of Veterans Affairs, North Hills, California, United States*

The new investigation on the state of emergency preparedness in nursing homes from the Senate Finance and Aging Committee Chairs is a call to action. As large-scale disasters continue to become increasingly common worldwide, nursing homes, whose residents are more vulnerable to disaster-related health and psychosocial shocks, and their staff, are carrying progressively more responsibility for healthcare readiness practices. Implementation science is a research discipline that seeks to improve uptake of evidence-based practices, such as healthcare readiness planning, and thus has potential to improve nursing home care delivery during and after disasters. The purpose of this review was to describe the limited field of existing evidence-based strategies in the peer-reviewed literature that seek to advance healthcare readiness in the nursing home setting and illustrate how implementation science can better support healthcare readiness planning for nursing homes. We discuss three main themes: 1) implementation science frameworks can strengthen nursing home staff engagement around healthcare readiness; 2) implementation science can support tailoring of emergency preparedness plans to individual nursing homes' unique needs; and 3) implementation science can advance the integration of nursing homes into local, state, and federal healthcare readiness planning initiatives. Finally, research is urgently needed to both generate and disseminate implementation strategies that increase uptake of evidence-based health care readiness practices in the nursing home setting.

Abstract citation ID: igad104.0689

### DISASTER PREPAREDNESS OF COMMUNITY-DWELLING OLDER ADULTS

Lindsay Peterson, *University of South Florida, Tampa, Florida, United States*

Older adults who depend on others for care are especially vulnerable to harm in a disaster. However, the extent to which they are protected depends on the setting in which they live. State and federal regulations require nursing homes and assisted living communities to have preparedness plans that specifically address the needs of their residents. For older adults living at home, however, protections are far less prescriptive, and in some cases lacking altogether, despite the increasing numbers of disabled older adults receiving care in their homes. In this policy-based study, we examined disaster preparedness for community-dwelling older adults in high-disaster states with high populations of older adults. We found that while many of the states required home-based providers to respond to the post-disaster needs of their older adult clients, few took steps to ensure that before the storm they were prepared. In this study, the state of Florida was an exception, with policies calling for service providers to identify and contact at-risk clients before and after an event. The plan further identifies disaster preparedness as a key component of a livable community. Hurricane Ian struck Fort Myers, Florida, in September 2022. Older adults made up a majority of the 148 killed during and immediately after the storm. Many others lost their homes. In the context of this recent event, we will discuss not only the state and local policies, but the practices that affect the health and well-being of community-dwelling older adults faced with a major disaster.

Abstract citation ID: igad104.0690

### VA'S EXPERIENCE IN BUILDING CAPABILITY TO CONDUCT OUTREACH TO VULNERABLE PATIENTS DURING EMERGENCIES

Tamar Wyte-Lake[1], Pete Brewster[2], Terrence Hubert[3], June Gin[4], Darlene Davis[5], Chad Holmes[6], and Aram Dobalian[7], *1. U.S. Department of Veterans Affairs, North Hills, California, United States*, *2. VHA Office of Emergency Management, Martinsburg, West Virginia, United States*, *3. VHA Office of Healthcare Transformation, Washington, District of Columbia, United States*, *4. Veterans Emergency Management Evaluation Center, North Hills, California, United States*, *5. Geriatrics and Extended Care, Veterans Health Administration, Tampa, Florida, United States*, *6. VA Service and Support Center, Denver, Colorado, United States*, *7. Veterans Emergency management Evaluation Center, North Hills, California, United States*

The United States (US) has sustained more than 340 weather and climate disasters since 1980. Older adults with complex medical conditions, increasing in numbers, are typically most severely impacted by such events. A growing population of older, medically complex, individuals residing at home, and an increased frequency of severe natural disasters creates a compounded burden on local communities to reduce potential harm to these community-dwelling members during disaster response and recovery. In response, the Veterans Health Administration (VHA) established the Vulnerable Patient Care, Access, and Response in Emergencies (VP CARE) program to provide standardized data tools and guidance to assist VA medical facilities in conducting outreach to and care coordination of medically frail and older Veterans during major emergencies. VP CARE utilizes geographic information system (GIS) tools to allow outreach prioritization based upon geographic proximity to an event and patient clinical needs. In response to Hurricanes Ian and Fiona, the VP CARE team, with the support of the national VEText team, successfully deployed a new 2-way patient texting communication capability. During an emergency, patients receive a text from VEText, are asked whether they require assistance, and the specific help they need. The new VEText capability helped VA staff reduce outreach call volume by 20% and allowed for triage and prioritization of those indicated as needing assistance to the top of the outreach list. This presentation provides an overview of VP CARE approaches and what VHA has learned through its implementation to assist other healthcare systems with establishing similar capabilities.

Abstract citation ID: igad104.0691

### EXPANDING THE ENGAGEMENT OF OLDER PERSONS IN CLIMATE CHANGE ACTION

Karl Pillemer, and Matthew Luebke, *Cornell University, Ithaca, New York, United States*

Environmental volunteerism and civic engagement have been found to promote physical and mental well-being among older adults as well as helping communities address environmental issues. Despite the growing need for citizen action to address disasters and other climate change effects, however, little is known about how to promote such engagement. At present, few climate change organizations actively recruit or provide specific opportunities for older persons. Academics and policymakers have focused on how older people are particularly susceptible to the impact of climate change. Limited attention has been paid, however, to the ways in which older people can be actively involved on their own behalf in climate change mitigation, including engagement in disaster preparation and response. In this presentation, I review barriers to widespread mobilization of older adults in climate change action, including their lower levels of environmental concern and their systematic lack of access to opportunities for climate change activism. I report on findings from a recent international survey of local and regional organizational efforts to engage older persons in climate change prevention and mitigation. This survey identified successful models for mobilizing older persons to address climate change mitigation, and in particular in low- and middle-resource countries. Recommendations for effective program design to bring older persons more fully into climate change action are presented, as well as ways environmental organizations can expand their focus to include systematically older persons in their activities.

### SESSION 2297 (SYMPOSIUM)

Abstract citation ID: igad104.0692

### POLICY SERIES: BUILDING TRUST, ADDRESSING BURNOUT AND EXPANDING THE DIRECT CARE WORKFORCE; THE 2022 REPORT TO DHSS AND CONGRESS

Chair: Naushira Pandya Discussant: Brian Lindberg

This symposium will review the key points and recommendations of the 2022 report to the Secretary of DHSS and Congress, prepared by the federally appointed Advisory Committee on Interdisciplinary and Community-Based Linkages in a blended didactic and interactive format. Clinician burnout following the Covid-19 pandemic is examined and root causes identified. Erosion of the patient trust in the public healthcare system and the consequences thereof are discussed, enhanced role of Community Health workers as the future primary care workforce is explored to prepare the system to prepare for future challenges.

Abstract citation ID: igad104.0693

### WORKFORCE CHALLENGES DURING COVID-19

Joan Weiss, *Health Resources and Services Administration (HRSA), Rockville, Maryland, United States*

As of October, 2022 nearly 1.1 million individuals in the United States died of COVID-19 with approximately 790,000 being individuals ages 65 and older. This age group accounts for 16% of the total US population but 75% of all COVID deaths to date. Covid-19 has taxed the United States public health system resulting in clinician burnout and a lack of trust in the public health system. The demands of the pandemic physically, psychologically, and logistically overwhelmed many clinicians resulting insomnia, anxiety, depression, and increased risk for substance use and misuse. Burnout at the institutional level is associated with increased medical errors, lower quality of care, and hospital-acquired infections. Compounding the issue of burnout was the lack of public trust that subsequently developed. Facts about COVID-19 competed with misinformation of the disease, false medical cures, contradictory health messages, an overwhelmed health care system in many areas, and conspiracy theories. One solution to these concerns is to increase the use of Community Health Workers (CHC). CHCs are trusted members of the communities they serve and play an essential role in assisting in care, providing factual information, and dispelling misinformation related to health topics such as COVID-19 infection, symptoms, and vaccination. Augmenting this workforce may help the system prepare for future emergencies. This presentation will discuss the recommendations of the Federal Advisory Committee on Interdisciplinary, Community-Based Linkages' latest report "Building Trust, Addressing Burning, and Expanding the Direct Care Workforce".

### SESSION 2300 (PAPER)

### ALZHEIMER'S DISEASE AND RELATED DEMENTIAS AND HEALTH CARE

Abstract citation ID: igad104.0694

### COGNITIVE MISPERCEPTION, DISABILITY, AND MORTALITY AMONG OLDER ADULTS IN 25 COUNTRIES

Zhuoer Lin, Mark Schlesinger, and Xi Chen, *Yale University, New Haven, Connecticut, United States*

Despite a large body of literature on cognitive ability and health, less is known about the health consequences of biased cognitive perception. Using harmonized and nationally representative longitudinal surveys from 25 countries spanning Asia, Europe and the Americas, we document the growing gap between actual and perceived cognitive ability that appears to increase with age, and construct a standardized measure of cognitive misperception. Linking this novel measure with mortality and disability, we model the cognitive misperception – health gradient. Results show that being in the upper quartile of cognitive misperception (i.e., showing heightened overconfidence in cognition) leads to higher mortality rates within 1 year, 3 years, and 5 years. Conditional on survival, being overconfident in cognition also greatly increases the risks of incident disability and frailty, especially for older adults receiving less family support. We identify two possible pathways inducing poorer outcomes: the first stemming from increased risk taking and financial vulnerability; the second associated with suboptimal use of preventive services and declines in health-promoting behaviors. Given the large and profound impacts of cognitive misperception on older adults' health and well-being, more family and social supports, public investment in education and health literacy, and better healthcare access and affordability are needed to increase the timely awareness of cognitive decline.

Abstract citation ID: igad104.0695

### DISPARITIES IN CONTINUITY OF CARE AND USE OF ANNUAL WELLNESS VISITS AMONG OLDER AMERICANS WITH DEMENTIA

Elham Mahmoudi, *University of Michigan Medical School, Ann Arbor, Michigan, United States*

Working on a risk-adjusted, prospective, capitated payment system, Medicare Advantage (MA) plans are incentivized to offer more efficient and coordinated care than traditional Medicare (TM). This study has two aims: (1) compare the continuity of care and use of annual wellness visits between MA and TM enrollees with Alzheimer's disease and related dementia (ADRD), and (2) examine Hispanic-White and Black-White disparities in the continuity of care and annual wellness visits by comparing MA with TM enrollees with ADRD. We used a 20% random sample of TM and MA insurance claims (2018-2019). Participants included individuals 65+, with a diagnosis of ADRD, and two years of continuous enrollment in TM (n=129,177) or MA (n=119,130). We used the Bice-Boxerman Continuity of Care Index to measure continuity of care. Generalized linear models were applied. TM lowered the odds of Black and Hispanic individuals having an annual wellness visit [OR=.80 (95% CI:.79-.82)]. Black and Hispanic TM enrollees had lower odds of annual wellness visits as compared to their Black and Hispanic MA counterparts [OR=.83 (95% CI:78-.88) and OR=.88 (95% CI:.83-.94)], respectively. White, Black, and Hispanic MA enrollees had higher continuity of care than their counterparts in TM (27.4 vs. 24.9; 28.9 vs. 28.1; 32.1

vs. 30.1, respectively). MA enrollees with ADRD had higher care continuity than their TM counterparts. Compared to TM, MA increased the odds of annual wellness visits and reduced Hispanic-White disparity in annual wellness visits. Findings from this study can inform policies promoting preventive and equitable care for patients with ADRD.

Abstract citation ID: igad104.0696

### FINDINGS FROM A PILOT PRAGMATIC CLINICAL TRIAL EMBEDDING CAREGIVER PROGRAMS IN OUTPATIENT SETTINGS

Richard Fortinsky[1], Kenneth Hepburn[2], Carolyn Clevenger[3], Karina Berg[1], Melinda Higgins[2], Tori Pascoe[1], Laura Medders[2], and Vicky Aldrich[1], *1. University of Connecticut, Farmington, Connecticut, United States*, *2. Emory University, Atlanta, Georgia, United States*, *3. Nell Hodgson Woodruff School of Nursing, Emory University, Atlanta, Georgia, United States*

Many interventions targeting family/informal caregivers of community-dwelling persons living with dementia have been tested in clinical trials, but few efficacious interventions have been offered in "real world" settings using embedded pragmatic clinical trial (ePCT) methods. This pilot ePCT engaged outpatient geriatrics and memory care clinics in two health systems to implement two caregiver support programs. We used patient portals in electronic health records (EHR) and caregiver registries to invite caregivers to receive one of two remotely-delivered caregiver support programs: the efficacious partly-synchronous Tele-Savvy (TS) program; or the completely-asynchronous Caregiving During Crisis (CDC) program. Random assignment (3TS:2CDC) was employed. Outcome measures were: self-reported caregiving mastery; perceived stress; and caregivers' reaction to behavioral issues. Measures were collected using self-administered questionnaires sent via patient portals or secure email messaging before randomization and 3 months later (3m). In total, 74 caregivers participated: 59.5% received TS; 73.0% females, 17.6% Black caregivers, with average age 65.5+/-11.8 years. Thirty-four (45.9%) caregivers completed 3m. Compared to the caregivers in CDC, the TS caregivers experienced moderate-to-large effect size improvements in caregiver mastery and frequency of depressive behaviors (mastery: TS Cohen's d=0.65, CDC d=0.00; depressive frequency: TS d=-0.64, CDC d=-0.04); caregivers in both groups reported moderate decrease in stress (TS d=-0.68; CDC d=-0.54). Implementation evaluation findings included: successful EHR buildout to capture and store caregiver-reported outcomes at one site; recruitment challenges due to caregiver difficulties gaining patient portal access and disinterest in randomization; and sub-optimal provider engagement in inviting caregivers to join the study through the EHR.

Abstract citation ID: igad104.0697

### RACIAL/ETHNIC DISPARITIES IN HOSPITAL READMISSION AND FREQUENT HOSPITALIZATIONS AMONG PEOPLE WITH DEMENTIA

Elham Mahmoudi, *University of Michigan Medical School, Ann Arbor, Michigan, United States*

People 65+ must choose between traditional Medicare (TM) and Medicare Advantage (MA). This study has two aims: (1) examine 30-day readmission and frequent hospitalizations in TM vs. MA; (2) examine racial/ethnic disparities in noted outcomes. We used a 20% random sample of TM and MA insurance claims (2018-2019). Participants included individuals 65+, with a diagnosis of Alzheimer's disease and related dementia (ADRD), and two years of continuous enrollment in TM (n=129,177) or MA (n=119,130). Our explanatory variables included age, sex, race/ethnicity, comorbidities, frailty index, continuity of care index, and discharge location. Generalized linear models were applied. There were a higher percentage of Black (13.1% vs 8.2%) and Hispanic (11.9% vs. 7.0%) people in MA vs. TM (p <.001). MA enrollees had lower rates of chronic conditions and were less frail (31.4 vs. 33.6; p< .001), but a higher percentage of them had an annual wellness visit (38.8% vs. 32.3%; p <.001) than TM enrollees. Our matched and adjusted models show that TM increased the odds of readmission [OR=1.16 (95% CI: 1.06-1.26)] and 2+ hospitalizations [OR=1.25 (95% CI: 1.19-1.40)] compared to MA. Hispanic-White disparities in 30-day readmission [OR=1.23 (95% CI: 1.06-1.43)] and 2+ hospitalizations [1.42 (95% CI: 1/31-1.56)] were higher in TM vs. MA. Black people had higher readmission risks [OR=1.18 (95% CI:1.08-1.28)] and frequent hospitalizations [OR=1.26 (95% CI:1.19-1.33)] without any significant difference between MA and TM in Black-White disparities. MA vs. TM enrollees had lower risks of readmission and frequent hospitalizations. Moreover, MA lowered Hispanic-White disparities compared with TM.

Abstract citation ID: igad104.0698

### SOCIAL ISOLATION AND DELAYED DEMENTIA DIAGNOSIS AMONG US OLDER ADULTS

Min Hee Kim, Ruijia Chen, Grisell Diaz-Ramirez, John Boscardin, and M Maria Glymour, *University of California San Francisco, San Francisco, California, United States*

Delayed dementia diagnosis may exacerbate dementia symptoms and reduce opportunities for care arrangements, further harming older adults' health and well-being. Social isolation may increase the risk of delayed dementia diagnosis via a lack of support that prevents seeking help and accessing primary and specialty care for diagnosis. We investigated the association between social isolation and missed/delayed dementia diagnosis using the Health and Retirement Study (2004-2018) linked with the Medicare Claims record. We defined social isolation as having 3 or more affirmative responses to 6 questions about living arrangements, family/friend contact, and social participation. We defined missed/delayed diagnosis as having no clinical diagnosis documented in Medicare in the presence of an algorithm-defined dementia diagnosis in the survey; or having dementia diagnosed more than 3 years after meeting the survey-based algorithmic criterion for dementia. We fitted logistic regression models, adjusting for sociodemographic and health characteristics. Among 2,417 people in our sample (mean age 82.4(SD 5.85); 60% women), 54.7% of older adults experienced social isolation at the time of survey-based dementia onset, 41.5% had missed/delayed diagnosis, and 22.1% died with no dementia diagnosis in their Medicare record. Preliminary results indicate that social isolation was not associated with increased odds of missed/delayed diagnosis (OR 1.09 95% CI 0.90, 1.32). Our findings suggest that social isolation may not be a major driver of healthcare access and system factors pertinent to clinical documentation of dementia. To corroborate findings, we plan to refine our models (e.g., accounting for attrition and selection).

### SESSION 2305 (SYMPOSIUM)

Abstract citation ID: igad104.0699

### CARING FOR PATIENTS IN ACUTE CARE LIVING WITH DEMENTIA: WHERE WE ARE AND WHERE WE NEED TO GO

Chair: Barbara Resnick

There are many challenges to caring for older adults living with dementia when hospitalized including medication management, pain identification and management, behavioral symptoms associated with dementia, and sedentary behavior. To help staff, particularly the nurses providing care to these patients, we developed Function Focused Care for Acute Care using the Evidence Integration Triangle (FFC-AC-EIT) to best address increasing physical activity, pain assessment and management, appropriate use of medications and management of behavioral symptoms associated with dementia. FFC-AC-EIT includes the implementation of four steps: (1) Environment and policy assessments; (2) Education of staff; (3) Establishing patient goals; and (4) Mentoring and motivating of staff, patients, and families. The testing of FFC-AC-EIT is ongoing as a cluster randomized controlled trial that to date has included 365 patients from 10 hospitals randomized to FFC-AC-EIT or FFC-AC-Education only. Eligibility of patients is based on being 55 years of age and older, admitted for a medical reason excluding COVID-19, and demonstrating evidence of dementia. This symposium will address the care and changes that occur during the hospital stay for these participants. Specifically, the following areas will be addressed: psychotropic medication use, participation in function focused care, the impact of physical activity on behavioral symptoms associated with dementia, and treatment of pain. The findings can be used to help guide researchers and clinicians in optimal ways to address the common challenges to care of hospitalized patients living with dementia. Presenters will provide information about future work needed to improve care across each of these areas.

Abstract citation ID: igad104.0700

### PSYCHOTROPIC MEDICATION USE AND CHANGES DURING THE HOSPITAL STAY

Elizabeth Galik, *University of Maryland School of Nursing, Baltimore, Maryland, United States*

The purpose of this study was to build on prior research and describe the current use of psychotropic medications among the first 365 older hospitalized patients living with dementia participating in a study focused on optimizing physical activity [The Function Focused Care for Acute Care Using the Evidence Integration Triangle Study (FFC-AC-EIT)] and consider the impact of current recommendations for decreasing use of psychotropics. It was hypothesized that controlling for age, gender, race, comorbidities, cognition, and hospital site (exposure or not to the Function Focused Care Intervention), that there would be a decrease in use of psychotropic medications from admission to discharge. Age, gender, race, comorbidities, admitting diagnosis, and medications (antidepressants, antianxiety medications, anticonvulsants, dementia drugs, antipsychotics, sedative-hypnotics, and opioids) were obtained at baseline and medications were obtained as well at discharge. To compare change over time generalized estimating equations were used. The participants were mostly female (63%), White (69%), and 83.06 years (SD=7.90) old. There was no treatment effect. Antidepressant, antianxiety, anticonvulsant, dementia medication, sedative hypnotic and opioid use remained unchanged. Antipsychotic medication use increased significantly from 16% to 21% at discharge. Some changes were made across drug groups such as change from haloperidol to second generation antipsychotics to decrease risk of side effects. A more focused deprescribing intervention is needed to best address psychotropic medication use among individuals living with dementia when hospitalized.

Abstract citation ID: igad104.0701

### PHYSICAL ACTIVITY AND IMPACT ON PSYCHOLOGICAL AND BEHAVIOR SYMPTOMS ASSOCIATED WITH DEMENTIA

Brittany Drazich, *University of Maryland Baltimore, Baltimore, Maryland, United States*

The aim of this study was to examine the impact of physical activity during hospitalization on behavioral and psychological symptoms of dementia among older adults, after controlling for covariates (age, gender, race, comorbidities, care interactions, and treatment group) and examine the association of physical and behavioral and psychological symptoms of dementia among hospitalized older adults living with dementia. Data from the first 365 participants in the FFC-AC-EIT study was used. Physical activity was measured over 24 hours and was based on the Physical Activity Survey which included the following activities: walking, wheeling, bathing, dressing, feeding, grooming, and toileting. Behavioral and psychological symptoms associated with dementia were based on the Neuropsychiatric Inventory (NPI). The mean age of participants was 83.18 years, the majority was female (63.6%) and White (69.4%), the mean NPI was 1.28(SD=1.85) and mean activities was 8(SD=5). A total of 16% of individuals performed no activity. The most common activity was eating and the least commonly performed was self-propelling in a wheelchair. A total of 40% engaged in walking and 60% performed some bathing and dressing while hospitalized. After controlling for covariates, physical activity explained an additional 2% of the variance in NPI scores (beta=-.13., p=.05). There was no evidence that engaging patients in physical activity resulted in behavioral symptoms and activity may help to decrease these symptoms. Continued focus is needed to engage older adults living with dementia when hospitalized to engage in routine physical activity by performing activities of daily living.

Abstract citation ID: igad104.0702

### TREATMENT OF PAIN DURING THE ACUTE CARE STAY FOR PATIENTS LIVING WITH DEMENTIA

Marie Boltz, *Pennsylvania State University, State College, Pennsylvania, United States*

The purpose of this study was to describe pain among hospitalized patients living with dementia, the use of pharmacologic and nonpharmacologic treatment, and to compare treatments among those with and without pain. Data from the first 365 participants in the FFC-AC-EIT study was used.

The mean age of participants was 83 (SD=5), the majority was female (65%) and White (67%). Controlling for treatment, between admission to discharge there was a significant decrease in pain from 36% having pain on admission to 31% at discharge (F=5.30, p=.02). At discharge 125 (30%) had pain and 8 (6%) of individuals with pain received no nonpharmacological or pharmacological treatments during the hospital stay. The most frequently used pharmacological intervention was acetaminophen (52%), then tramadol (8%). Comfort measures (49%), physical activity (46%), and communication (38%) were the most common nonpharmacologic approaches. Between those with and without pain there was no difference in use of pharmacologic interventions (F = .38, p =.54) and there was less use of nonpharmacological treatments for those with pain (F=10.24, p=.002). The majority of patients living with dementia were treated for pain, but an ongoing focus is needed to assure optimal pain management for all patients. More attention to use of and evaluation of effectiveness of pharmacologic and nonpharmacologic treatments alone or together are needed for hospitalized patients living with dementia.

Abstract citation ID: igad104.0703

### FACTORS ASSOCIATED WITH PARTICIPATION IN FUNCTION-FOCUSED CARE AMONG HOSPITALIZED PATIENTS LIVING WITH DEMENTIA

Ashley Kuzmik, *Pennsylvania State University, State College, Pennsylvania, United States*

Function focused care is an approach used to increase physical activity in hospitalized older adults living with dementia. This study tested the factors associated with participating in function focused care among patients living with dementia. This was a cross-sectional descriptive study using baseline data from the first 365 participants from the first 10 hospitals in the study entitled, Testing the Effectiveness of Function Focused Care for Acute Care Using the Evidence Integration Triangle. Structural equation modeling was used for model testing. The mean age of the participants was 83.2 (SD=8.0) and the majority were women (64%) and white (69%). Fifteen of the 27 hypothesized paths were significant and explained 25% of the variance in performance of function focused care. Cognition, quality of care interactions, behavioral and psychological symptoms associated with dementia, physical resilience, and pain were all indirectly associated with performance of function focused care through function and/or pain. Tethers, function, and quality of care interactions were all directly associated with function focused care. The x2 / df was 5.37, the normed fit index was .87 and the Root Mean Error Square of Approximation was .12. For hospitalized patients living with dementia the focus of care should be on treating pain and behavioral symptoms, decreasing tethers, and improving quality of care interactions so as to optimize resilience, function and participation in function focused care.

### SESSION 2310 (SYMPOSIUM)

Abstract citation ID: igad104.0704

### EMPOWERING LGBTQ OLDER ADULTS: PROMOTING HEALTH EQUITY AND WELL-BEING ACROSS THE LIFE COURSE

Chair: Hyun-Jun Kim Discussant: Karen Fredriksen-Goldsen

The population is becoming more diverse in terms of sexual orientation and gender identity across all age groups. Lesbian, gay, bisexual, transgender, and queer (LGBTQ) older adults are found to experience health disadvantages over time. However, there is a lack of research on health-related mechanisms, so it is important to understand the unique needs and resources in these underserved communities. This symposium fills this research gap by examining the factors that affect LGBTQ health trajectories and subgroup differences using a life course developmental framework, the Health Equity Promotion Model (HEPM). The HEPM model highlights historical, social, environmental, and cultural influence on health and well-being and varying adaptations of LGBTQ older adults. The symposium draws on data from the first national and longitudinal study, Aging with Pride: National Health, Aging and Sexuality/Gender study, which has been following 2,450 LGBTQ midlife and older adults since 2014. Dr. Nelson will discuss sexual and gender identities and their associations with physical and psychological health-related quality of life. By investigating the interplay between marginalization, health behaviors, and health care access over time, Dr. Fredriksen-Goldsen assesses quality of life over time by gender and generational differences. Dr. Kim will discuss the role of social connectivity and intersectionality in predicting cognitive impairment over time among racially and ethnically diverse LGBTQ older adults. Using a health equity lens, this symposium will shed new light on the ways in which historical and environmental contexts influence human development and how marginalization and resilience shape lives over time. This is a Rainbow Research Group Interest Group Sponsored Symposium.

Abstract citation ID: igad104.0705

### SEXUAL AND GENDER IDENTITY AND THEIR PROPERTIES IN AGING WITH PRIDE: NATIONAL HEALTH, AGING, AND SEXUALITY AND GENDER STUDY

Christi Nelson, Karen Fredriksen-Goldsen, Charles Hoy-Ellis, Meghan Romanelli, and Hyun-Jun Kim, *University of Washington, Seattle, Washington, United States*

Increasing diversity and rapidly evolving sociopolitical context are changing how sexuality and gender identity are experienced, expressed, and measured in contemporary America. Based on longitudinal data from Aging with Pride: National Health, Aging, and Sexuality/Gender Study, we examine sexual and gender identity, differentiation from other related constructs (i.e., desire, behavior, expression, relationships) as well as the associations between properties of sexual and gender identities (e.g., congruence, continua, visibility, centrality, and transformation) and the health and well-being of LGBTQ midlife and older adults (N=2,233), aged 50-102 at baseline. The findings document important differences in current as well as lifetime sexual experiences, desires, and romantic relationships by sexuality and gender identity. Measures incorporating a continuum as opposed to binary response categories better capture the experiences of bisexual, sexually diverse, and transgender older participants as compared to lesbians and gay men. When examining transformation, 7% reported changes to their sexual identity and 3% reported changes to their gender identity over 4 years. Those who changed their identity over time showed a higher level of identity stigma and poorer physical and psychological health-related quality of life. Our findings reveal that heterogeneity and intersectionality are critical to understanding sexual and gender identity. It is important to consider multiple aspects of sexuality and gender (e.g., romantic relationships) that are often overlooked in gerontological and sexuality and gender research. Scholarship on sexual and gender identity development and transformation in later life is needed to better address diversity in the aging population.

Abstract citation ID: igad104.0706

### VIOLENCE, IDENTITY, BEHAVIORS, AND ACCESS: MECHANISMS ACCOUNTING FOR CHANGES IN HEALTH OVER TIME OF LGBTQ OLDER ADULTS

Karen Fredriksen-Goldsen, Hailey Jung, Charles Hoy-Ellis, Austin Oswald, Christi Nelson, and Hyun-Jun Kim, *University of Washington, Seattle, Washington, United States*

As the population of older adults is growing in complexity and diversity, research has established health disparities by sexual and gender identity among older adults. While most research to date has been cross-sectional, this paper utilizes four-wave longitudinal data from Aging with Pride: National Health, Aging and Sexuality/Gender Study (NHAS) to understand the health and quality of life (QOL) of LGBTQ and sexual and gender diverse older adults over time. Mixed models were run to examine: (1) linear changes over time in health and QOL, controlling for background characteristics, (2) effects of key risk and protective factors on these changes, and (3) gender and age group interactions. Increases in violence, discrimination, identity stigma, adverse health behaviors and experiences (e.g., low physical activity, insufficient nutrition, and suicidal ideation), and decreased access to health and care services were related to poorer health, more cognitive and functional impairment, and lower QOL. Men (vs. women) and the Pride (vs. Silenced and Invisible) Generation were impacted to a greater degree by violence and discrimination, and the impacts of insufficient nutrition and suicidal ideation were also greater in the Pride Generation. Cognitive health of men and gender diverse individuals benefited more strongly from healthcare access. This longitudinal study provides important new findings regarding the mechanisms accounting for the accelerated decline in health and QOL over time in these diverse populations. This information is needed to develop tailored interventions that address these mechanisms as well as are responsive to variations among diverse ages, genders, and sexualities.

Abstract citation ID: igad104.0707

### COGNITIVE IMPAIRMENT AMONG OLDER ADULTS WITH INTERSECTING SEXUAL, GENDER, RACIAL, AND ETHNIC MINORITY IDENTITIES

Hyun-Jun Kim, Austin Oswald, Hailey Jung, and Karen Fredriksen-Goldsen, *University of Washington, Seattle, Washington, United States*

Emerging research shows Lesbian, gay, bisexual, transgender, and queer (LGBTQ) older adults experience higher prevalence of cognitive impairment when compared to their heterosexual and cisgender peers of a similar age. The same is true for racial and ethnic minority older adults when compared to their White counterparts. Little is known about the cognitive health of older adults with intersecting sexual, gender, racial, and ethnic minority identities, highlighting a critical gap in gerontological research that requires further investigation. This study utilizes four time-point data from the first longitudinal study of a racially and ethnically diverse sample of LGBTQ older adults. Multilevel mixed models accounting for intersecting sexual, gender, racial, and ethnic identities were applied to examine subgroup differences in cognitive impairment as well as risk and protective factors after controlling for confounding factors. Black and Hispanic LGBTQ minority older adults ($b=5.20$, $p<.01$; $b=4.05$, $p<.01$) experience higher levels of cognitive impairment over time when compared to their White LGBTQ peers. Day-to-day discrimination targeting the confluence of sexual/gender and racial/ethnic minority status ($b=2.78$, $p<.05$) was positively associated with higher cognitive impairment. Community engagement ($b=-.46$, $p<.05$) was negatively associated with cognitive impairment regardless of race and ethnicity whereas network size was exclusively negatively associated with cognitive impairment for Black LGBTQ older adults. The overall findings demonstrate racial and ethnic differences in cognitive impairment among LGBTQ older adults and illuminate the importance of culturally tailored interventions that account for intersectionality, social connectivity, and discrimination to promote cognitive health for specific subgroups.

### SESSION 2315 (SYMPOSIUM)

Abstract citation ID: igad104.0708

### VISUALIZING AGING EQUITY: CROSS-SECTORAL PARTNERSHIPS TO CREATE MAPPING TOOLS FOR SOCIAL CHANGE

Chair: Clara Scher Co-Chair: Emily Greenfield Discussant: Rodney Harrell

This symposium will feature ways in which gerontologists are advancing the development of online, interactive mapping tools on aging. Utilizing GIS mapping technology and leveraging cross-sectoral partnerships, the speakers will present case illustrations of efforts to develop aging-focused mapping tools that aggregate data sources at the municipal, state, and national levels to advance a spatial awareness of supports for older adults across diverse communities. First, as an example from the nonprofit sector, the New York Academy of Medicine will present on IMAGE:NYC, The Interactive Map of Aging. This presentation will describe the utility of this tool for policymakers, planners, researchers, and social service providers. Representing work from within the academic sector, the Rutgers School of Social Work will describe an academic-public sector partnership to adapt the IMAGE:NYC map for two counties in New Jersey. Next, presenters from the University of North Texas will describe their partnership with the City of San Antonio to develop an intelligent demand responsive transportation system for older adults and individuals with disabilities. This project involves the use of GIS mapping tools and qualitative research methods to identify place-based transportation gaps and strategies for improvement. Focusing on the national level, the National Council on Aging will describe its work developing mapping tools, including a map that displays area-level rates in eligibility gaps for three federal benefit programs. With an orientation to aging equity, these tools hold promise for fostering inclusive and healthy communities where older adults of diverse intersectional socio-spatial positions can age well. This is an Environmental Gerontology Interest Group Sponsored Symposium.

Abstract citation ID: igad104.0709

### DEVELOPING AN INTERACTIVE COUNTY MAPPING TOOL TO ADVANCE AGE-FRIENDLY PROGRESS IN NEW JERSEY

Emily Greenfield[1], Clara Scher[1], Karen Alexander[2], Joanne Fetzko[3], Lucas Marxen[4], Althea Pestine-Stevens[1], Davit Topchishvili[5], and Michael Pagan[5], *1. Rutgers, The State University of New Jersey, New Brunswick, New Jersey, United States*, *2. Voorhees Transportation Center, Edward J. Bloustein School of Planning and Public Policy, Rutgers University, New Brunswick, New Jersey, United States*, *3. Somerset County Office On Aging & Disability Services, Somerville, New Jersey, United States*, *4. NJAES Office of Research Analytics, Rutgers University, New Brunswick, New Jersey, United States*, *5. Bergen County Division of Senior Services, Hackensack, New Jersey, United States*

This presentation will describe how a multidisciplinary team at Rutgers, The State University of New Jersey, leveraged a small, internal grant opportunity to partner with leaders of two County government systems to develop a “proof of concept” age-friendly County mapping tool. Based on the Image:NYC mapping tool, the team designed the tool to display the spatial locations of high concentrations of older adults with shared characteristics (e.g., by health status, income) alongside the spatial locations of assets in local communities (e.g., libraries, senior activity centers) at the census tract level within each of the two counties. The presenters will explain how gerontological expertise—in combination with insights, data, and resources from other disciplines and project contributors—were essential to design, produce, and disseminate the map. The presenters will describe how the mapping tool is one component of a growing body of work across the public, private, and academic sectors in New Jersey to advance and support the age-friendly communities movement. The presenters also will reflect on how the age-friendly mapping tool has served to kickstart age-friendly planning and systems change at the county level, with implications for advancing both state- and municipal-level efforts.

Abstract citation ID: igad104.0710

### SAN ANTONIO TRANSPORTATION PLANNING SYSTEM: MAPPING INFRASTRUCTURE CAPACITY AND COORDINATION

Laura Keyes, Sowmya Balachandran, and Simon Andrew, *University of North Texas, Denton, Texas, United States*

Ride Connect Texas and its partnering agencies seek to develop an expanded senior mobility management system supported by a One Call One Click transportation planning system. Its purpose is to enhance on-demand transportation system coordination and the application of technology, providing consumers, service providers, and mobility managers with direct access to comprehensive transportation information. GIS mapping is used to help develop a spatial awareness of the transportation challenges among older people and individuals with a disability and the current system capacity. Interviews with transit, non-transit, and health and human service agencies will reveal factors that influence coordination among providers and opportunities for capacity expansion. Surveys and focus groups of older adults and individuals with disability will show differentiating characteristics of riders and transportation needs and challenges. GIS mapping will allow us to geocode these findings relative to the user’s street address and zip code, illustrating variations in specialized client needs, service availability, travel behavior, and available amenities for basic daily living. Mapping will identify gaps in transportation services, hot zones revealing high user demand and low system capacity, and the ability to reduce fragmentation of service through seamless technology-based coordination. The mapping element informs the measurement of a community’s transportation system capacity for this user group. The value proposition of this tool is its ability to help any community measure its readiness toward developing a comprehensive implementation strategy for coordinated demand response transportation, including resource mobilization strategies, capacity augmentation, phasing of the system, and implementation timeline.

Abstract citation ID: igad104.0711

### THE DEVELOPMENT AND UTILIZATION OF IMAGE:NYC: THE INTERACTIVE MAP OF AGING

Elana Kieffer[1], and Clara Scher[2], *1. The New York Academy of Medicine, New York City, New York, United States*, *2. Rutgers, The State University of New Jersey, New Brunswick, New Jersey, United States*

Online interactive maps are an effective tool to visualize and understand socio-spatial locations of populations layered across the availability of relevant resources. These maps are integral in efforts to advance policies, programs and planning for specific sociodemographic groups. This presentation will describe the development and impact of IMAGE:NYC, The Interactive Map of Aging by The New York Academy of Medicine (NYAM) in partnership with the Center for Urban Research at The Graduate Center at the City University of New York. IMAGE:NYC displays the current and projected population of adults aged 65 and older in New York City with overlays of age-friendly resources, services, and amenities at the neighborhood level. The map draws on several data sources such as the 2016-2020 American Community Survey, local and federal voting patterns, and up-to-date information on health, recreational and housing services. The map has served as an invaluable tool for NYC policymakers, planners, researchers, health care and social service providers. In addition, the IMAGE:NYC model has also been replicated by Rutgers University to create similar maps for two counties in New Jersey. This session will demonstrate the utility of this mapping tool and concrete ways to develop a similar map in other localities.

Abstract citation ID: igad104.0712

### AGING WELL: OLDER ADULTS AND DATA MAPPING

Susan Silberman, *National Council on Aging, Arlington, Virginia, United States*

This presentation will feature how the National Council on Aging (NCOA) has advanced a program of work on data mapping and visualization. It will explain how this work is part of NCOA's mission to improve the lives of millions of older adults, especially those who are struggling. Guided by the idea that how we age should not be determined by gender, color, sexuality, income, or zip code, the mapping tools display area-level data to highlight different aspects of the lives of low-income older adults. This presentation will describe two of NCOA's mapping tools and how they can influence policy and advocacy efforts toward aging equity at the local, regional, state, and national levels. The first tool, released in 2021, displays, in part, area-level access to smartphones and high-speed internet among low-income older adults. This map makes visible the invisible by demonstrating low rates of access even in geo-spatial areas with overall high levels of connectivity. The second tool, to be released in the third quarter of 2023, presents sub-state level data to identify gaps between area-level eligibility for each of three essential benefit programs [Supplemental Nutrition Assistance Program (SNAP)], Supplemental Security Income (SSI), and Medicare Savings Program (MSP)] and area-level enrollment rates. This map demonstrates how socio-spatial positions influence access to these national benefits, and how maps can be used to identify regions and communities to prioritize as part of strategic efforts to improve benefit access and promote aging equity.

### SESSION 2320 (SYMPOSIUM)

Abstract citation ID: igad104.0713

### JGMS EDITOR'S CHOICE ARTICLES OF 2022: BIOLOGIC AND SOCIAL INFLUENCES ON COGNITIVE AND PHYSICAL AGING

Chair: Lewis Lipsitz Discussant: Peggy Cawthon

This symposium will present four 2022 "Editor's Choice" articles from the Journal of Gerontology Medical Sciences that focus on the biological and social influences on cognitive and physical aging. Mercedes Sotos-Prieto and her colleagues, in their article "A Mediterranean Lifestyle and Frailty Incidence in Older Adults: The Seniors-ENRICA-1 Cohort" examine the association between a Mediterranean lifestyle, which included diet, customs, and traditions, and frailty incidence in older adults. Mark Espeland and his colleagues, in "Racial/Ethnic Disparities in Alzheimer's Disease Risk: Role of Exposure to Ambient Fine Particles" used unique data from the Women's Health Initiative Memory Study to investigate this previously unstudied relationship between racial disparities, ambient fine particle exposure, and risk of Alzheimer's. "Trans-Ethnic Meta-Analysis of Interactions Between Genetics and Early-Life Socioeconomic Context on Memory Performance and Decline in Older Americans," written by Jessica Faul and colleagues, examines whether gene regions previously associated with cognitive performance, dementia, and related traits had an interaction with childhood socioeconomic context on memory performance or decline. Lingxiao He and coauthors, in their article, "Prospective Associations of Plasma Growth Differentiation Factor 15 With Physical Performance and Cognitive Functions in Older Adults" examines the relationship between Plasma GDF15, lower-limb physical performance, and global cognitive function in older adults. Our discussant, Peggy Cawthon, will highlight commonalities and lessons learned from these studies.

Abstract citation ID: igad104.0714

### A MEDITERRANEAN LIFESTYLE AND FRAILTY INCIDENCE IN OLDER ADULTS: THE SENIORS-ENRICA-1 COHORT

Mercedes Sotos Prieto[1], Javier Maroto-Rodriguez[1], Mario Delgado-Velandía[1], Rosario Ortola[1], and Fernando Rodriguez-Artalejo[2], *1. University Autonomous of Madrid, Madrid, Madrid, Spain*, *2. Universidad Autónoma de Madrid, Spain, Madrid, Madrid, Spain*

**Background:** Frailty is a geriatric syndrome that entails high risk of hospitalization, disability, and death. While adherence to Mediterranean diet has been associated with lower risk of frailty, the joint effect of diet and lifestyle is uncertain. This study examined the association between a Mediterranean lifestyle (diet, customs, and traditions) and frailty incidence in older adults. **Methods:** We analyzed data from 1 880 individuals aged ≥ 60 from the prospective Seniors-ENRICA-1 cohort. Adherence to the Mediterranean lifestyle was assessed at baseline with the 27-item MEDLIFE index (higher scores representing better adherence), divided into 3 blocks: (1) "Mediterranean food consumption," (2) "Mediterranean dietary habits" (practices around meals)," and (3) "Physical activity, rest, social habits and conviviality." Frailty was ascertained as the presence of ≥ 3 of the 5 Fried criteria: (a) Exhaustion; (b) Muscle weakness; (c) Low physical activity; (d) Slow walking speed; and (e) Unintentional weight loss. Main statistical analyses were performed using logistic regression models, adjusting for the main confounders. **Results:** After a 3.3-year follow-up, 136 incident frailty cases were ascertained. Compared with participants in the lowest tertile of the MEDLIFE score, the OR (95% CI) for frailty was 0.88 (0.58-1.34) for the second tertile, and 0.38 (0.21-0.69) for the third tertile (p-trend = .003). Blocks 1 and 3 of the MEDLIFE score were independently associated with lower frailty risk. Most items within these blocks showed a tendency to reduced frailty. **Conclusions:** Higher adherence to a Mediterranean lifestyle was associated with lower risk of frailty.

Abstract citation ID: igad104.0715

### TRANSETHNIC META-ANALYSIS OF INTERACTIONS BETWEEN GENETICS AND EARLY-LIFE SOCIOECONOMIC CONTEXT ON COGNITION

Jessica Faul[1], Minjung Kho[2], Wei Zhao[3], Kalee Rumfelt[1], Miao Yu[1], Colter Mitchell[1], and Jennifer Smith[4], *1. University of Michigan, Ann Arbor, Michigan, United States*, *2. Seoul National University, Seoul, Republic of Korea*, *3. Institute for Social Research, University of Michigan, Ann Arbor, Michigan, United States*, *4. University of Michigan School of Public Health, Ann Arbor, Michigan, United Sta tes*

Later-life cognitive function is influenced by genetics as well as early- and later-life socioeconomic context. However, few studies have examined the interaction between genetics and early childhood factors. Using gene-based tests

(interaction sequence kernel association test [iSKAT]/iSKAT optimal unified test), we examined whether common and rare variants in 39 gene regions previously associated with cognitive performance, dementia, and related traits had an interaction with childhood socioeconomic context (parental education and financial strain) on memory performance or decline in European ancestry (EA, N = 10,468) and African ancestry (AA, N = 2,252) participants from the Health and Retirement Study. Of the 39 genes, 22 in EA and 19 in AA had nominally significant interactions with at least one childhood socioeconomic measure on memory performance and/or decline; however, only one, SLC24A4 x father's education, remained significant after multiple testing correction (false discovery rate [FDR] < .05). In trans-ethnic meta-analysis, 2 genes interacted with childhood socioeconomic context: MS4A4A x mother's education on memory performance, and SLC24A4 x father's education on memory decline. Both interactions remained significant after adjusting for respondent's own education, apolipoprotein-ε4 allele status, lifestyle factors, body mass index, and comorbidities. For both interactions in EA and AA, the genetic effect was stronger in participants with low parental education. Examination of common and rare variants in genes discovered through genome-wide association studies shows that childhood context may interact with gene regions to jointly impact later-life memory function and decline. Genetic effects may be more salient for those with lower childhood socioeconomic status.

Abstract citation ID: igad104.0716

### ASSOCIATIONS OF PLASMA GDF15 WITH PHYSICAL PERFORMANCE AND COGNITIVE FUNCTIONS IN OLDER ADULTS

Lingxiao He[1], Philipe de Souto Barreto[2], Juan Sánchez Sánchez[3], Yves Rolland[4], Sophie Guyonnet[3], Angelo Parini[5], Alexandre Lucas[5], and Bruno Vellas[3], *1. School of Public Health, Xiamen University, Toulouse, Midi-Pyrenees, France*, 2. *CHU Toulouse: Centre Hospitalier Universitaire de Toulouse, Toulouse, Midi-Pyrenees, France*, 3. *Gérontopôle de Toulouse, Toulouse, Midi-Pyrenees, France*, 4. *Toulouse University Hospital (CHU Toulouse), Toulouse, Midi-Pyrenees, France*, 5. *Institute of Metabolic and Cardiovascular Diseases, Toulouse, Midi-Pyrenees, France*

**Background** Growth differentiation factor 15 (GDF15) has been associated with several age-related disorders, but its associations with functional abilities in community-dwelling older adults are not well studied.

**Methods** The study was a secondary analysis on 1096 community-dwelling older adults (aged 69 to 94 years) recruited from the Multidomain Alzheimer's Preventive Trial. Plasma GDF15 was measured one year after participants' enrolment. Annual data of physical performance (grip strength and short physical performance battery [SPPB]) and global cognitive functions (mini-mental state examination [MMSE] and a composite cognitive score) were measured for four years. Adjusted mixed-effects linear models were performed for cross-sectional and longitudinal association analyses.

**Results** A higher GDF15 was cross-sectionally associated with a weaker grip strength (β = -1.1E-03, 95%CI [-2.0E-03, -1.5E-04]), a lower SPPB score (β = -3.1E-04, 95%CI [-5.4E-04, -9.0E-05]) and worse cognitive functions (β = -2.4E-04, 95%CI [-3.3E-04, -1.6E-04] for composite cognitive score; β = -4.0E-04, 95%CI [-6.4E-04, -1.6E-04] for MMSE). Participants with higher baseline GDF15 demonstrated greater longitudinal declines in SPPB (β = -1.0E-04, 95%CI [-1.7E-04, -2.0E-05]) and composite cognitive score (β = -2.0E-05, 95%CI [-4.0E-05, -3.6E-06]). The optimal initial GDF15 cutoff values for identifying participants with minimal clinically significant decline after one year were 2189 pg/mL for SPPB (AUC: 0.580) and 2330 pg/mL for composite cognitive score (AUC: 0.587).

**Conclusions** Plasma GDF15 is cross-sectionally and longitudinally associated with lower-limb physical performance and global cognitive function in older adults. Circulating GDF15 alone has limited capacity of discriminating older adults who will develop clinically significant functional declines.

Abstract citation ID: igad104.0717

### RACIAL/ETHNIC DISPARITIES IN ALZHEIMER'S DISEASE RISK: ROLE OF EXPOSURE TO AMBIENT FINE PARTICLES

Mark Espeland[1], Diana Younan[2], Xinhui Wang[2], Tara Gruenewald[3], Margaret Gatz[2], Marc Serre[4], William Vizuere[4], and Jiu-Chiuan Chen[2], *1. Wake Forest University School of Medicine, Winston-Salem, North Carolina, United States*, 2. *University of Southern California, Los Angeles, California, United States*, 3. *Chapman University, Orange, California, United States*, 4. *University of North Carolina - Chapel Hill, Chapel Hill, North Carolina, United States*

Whether racial/ethnic disparities in Alzheimer's disease (AD) risk may be explained by ambient fine particles (PM2.5) has not been studied. We conducted a prospective, population-based study on a cohort of Black (n=481) and White (n=6004) older women (aged 65– 79) without dementia at enrollment (1995–98). Cox models accounting for competing risk were used to estimate the hazard ratio (HR) for racial/ethnic disparities in AD (1996–2010) and the association with time-varying annual average PM2.5 (1999–2010) estimated by spatiotemporal model. Over an average follow-up of 8.3 (±3.5) years with 158 incident cases (21 in Black women), the racial disparities in AD risk (range of adjusted HRBlack women=1.85–2.41) observed in various models could not be explained by geographic region, age, socioeconomic characteristics, lifestyle factors, cardiovascular risk factors, and hormone therapy assignment. Estimated PM2.5 exposure was higher in Black (14.38±2.21 μg/m3) than in White (12.55±2.76 μg/m3) women, and further adjustment for the association between PM2.5 and AD (adjusted HRPM2.5=1.18–1.28) slightly reduced the racial disparities by 2%–6% (HRBlack women=1.81–2.26). The observed association between PM2.5 and AD risk was ~2 times greater in Black (HRPM2.5=2.10–2.60) than in White (HRPM2.5=1.07–1.15) women (range of interaction Ps: <.01–.01). We found similar results after further adjusting for social engagement (social strain, social support, social activity, living alone), stressful life events, Women's Health Initiative's clinic sites, and neighborhood socioeconomic characteristics. PM2.5 may contribute to racial/ethnic disparities in AD risk and its associated increase in AD risk was stronger among Black women.

### SESSION 2325 (SYMPOSIUM)

Abstract citation ID: igad104.0718

### BIOSOCIAL PATHWAYS AFFECTING HEALTH AND AGING IN THE HEALTH AND RETIREMENT STUDY

Chair: Eric Klopack Discussant: Helen Meier

Representative population studies, like the Health and Retirement Study (HRS), are gathering large amounts of biomarker and multi-omic data. This presents a unique opportunity to investigate how population-level aging effects is driven by social inequality. This research has the capacity to uncover targets for intervention that could result in extending longevity and healthy life span and reducing inequalities in aging. There has been substantial research and progress in identifying biological processes underlying age-related decline, morbidity, and mortality (the so-called biological hallmarks of aging); however, despite this progress, demographic, social, and psychological factors (the so-called social hallmarks of aging) are substantially associated with cognitive aging and ADRD above-and-beyond genetic and biological factors There is, therefore, a need for research that carefully integrates the social and biological hallmarks of the human aging processes. This symposium highlights research that investigates interactions between biological and social processes that lead to inequalities in health and aging among older adults utilizing recently released data from the HRS Venous Blood Study (a nationally representative sample of US adults over age 55). Included are papers investigating the role of timing of childhood adversity in affecting adult DNA methylation patterns, how childhood parental loss is associated with accelerated epigenetic aging, how socioeconomic status patterns expression of genes related to inflammation and cellular senescence, and how sets of age-related biomarkers can explain sociodemographic inequalities in mortality. These studies contribute to an emerging literature integrating social and biological sciences to better understand inequalities in the aging process.

Abstract citation ID: igad104.0719

### PATHWAYS LINKING PARENTAL DEATH BEFORE 18 TO ACCELERATED EPIGENETIC AGING

Mateo Farina[1], Eric Klopack[2], and Eileen Crimmins[2], *1. University of Texas at Austin, Austin, Texas, United States*, 2. *University of Southern California, Los Angeles, California, United States*

Parental death before 18 is one of the most challenging adverse events that can lead to poor health in later life. Accelerated biological aging may be a key physiological mechanism that underlies this association. Data come from the Health and Retirement Study: the core surveys and the 2016 Venous Blood Study (VBS). We used second- and third- generation epigenetic clocks (GrimAge, PhenoAge, DunedinPace) to evaluate accelerated aging. Parental death before 18 and other covariates were obtained from the core surveys. To assess direct and indirect pathways, we used Structural Equation Modeling with educational attainment, depressive symptoms, and adult health behaviors (smoking, drinking, and BMI) as potential adulthood pathways that link parental death before 18 to accelerated aging. Parental death before 18 was associated with .79 years of accelerated aging. We found evidence of an indirect pathway through adulthood depressive symptoms. We did not find evidence of indirect pathways through educational attainment or adulthood wealth despite their strong negative associations with parental death before 18. Parental death before 18 was associated with accelerated epigenetic aging, which indicates that epigenetic aging may be a a key biological mechanism that links parental death in early life to health in older adulthood. Some of the association may be reduced by addressing mental health challenges that are associated with this traumatic event.

Abstract citation ID: igad104.0720

### DEMOGRAPHIC CORRELATES OF AGING-RELATED GENE EXPRESSION LEVELS AMONG OLDER AMERICANS

Qiao Wu[1], Eric Klopack[1], Jung Ki Kim[1], Jessica Faul[2], Bharat Thyagarajan[3], Steve Cole[4], and Eileen Crimmins[1], *1. University of Southern California, Los Angeles, California, United States*, 2. *University of Michigan, Ann Arbor, Michigan, United States*, 3. *University of Minnesota, Minneapolis, Minnesota, United States*, 4. *University of California Los Angeles, Los Angeles, California, United Sta tes*

Measures of conserved transcriptional response to adversity (CTRA), DNA damage response (DDR), and senescence-associated secretory phenotype (SASP) gene expression can be regulated by social and environmental exposures. The resulting synthesis of proteins can affect aging. To further understand how demographic disparities get "under the skin" and link to health outcomes, we examine associations between (A) age, gender, and race/ethnicity and (B) three gene expression composite scores derived from RNA sequencing data in a nationally representative sample of 2,680 Americans aged 55 and older from the 2016 Health and Retirement Study. We also compare the associations across models using multiple approaches to controlling for blood cell composition. OLS regressions suggest that compared to those aged 55-64, being older is associated with a lower inflammatory sub-score of CTRA (Ages 75-84: $\beta=-0.04$, $p=0.008$; Ages 85+: $\beta=-0.12$, $p<0.001$), lower CTRA (Ages 85+: $\beta=-0.15$, $p<0.001$), higher DDR (Ages 85+: $\beta=0.03$, $p=0.042$), and lower SASP (Ages 75-84: $\beta=-0.05$, $p=0.015$; Ages 85+: $\beta=-0.07$, $p=0.017$) scores. Women have significantly higher inflammatory ($\beta=0.06$, $p<0.001$) and antiviral ($\beta=0.04$, $p=0.002$) sub-scores of CTRA. Compared to non-Hispanic Whites, non-Hispanic Blacks have a higher inflammatory sub-score of CTRA ($\beta=0.04$, $p=0.026$) and a lower SASP score ($\beta=-0.19$, $p<0.001$), and Hispanics have a lower SASP score ($\beta=-0.08$, $p<0.001$). Adjusting for education, drinking, smoking, BMI, and chronic conditions does not change the results much. Overall, using expression levels of the genes that mark major leukocyte subsets to control for blood cell composition does not notably differ from approaches based on complete blood count and flow cytometry.

Abstract citation ID: igad104.0721

### A STRUCTURED APPROACH TO EVALUATING LIFE COURSE INFLUENCES ON DNA METHYLATION AGE ACCELERATION

Jessica Faul[1], Stacey Collins[1], Mateo Farina[2], Eric Klopack[3], Zainab Masood[1], Helen Meier[1], Colter Mitchell[1], and Eileen Crimmins[3], *1. University of Michigan, Ann Arbor, Michigan, United States*, 2. *University of Texas at Austin,*

*Austin, Texas, United States*, 3. *University of Southern California, Los Angeles, California, United States*

Lower socio-economic position (SEP) is associated with increased biological age acceleration measured using DNA methylation (DNAm). Prior studies using cohorts from the US and UK have shown a relationship between social mobility and DNAm age acceleration such that those who experienced upward mobility exhibited slower biological aging relative to continuously advantage groups while those with disadvantaged SEP across the life course experienced the most rapid DNAm age acceleration. However, using a social mobility model, it is not clear to what extent childhood may be a sensitive period for the impact of social disadvantage on DNA methylation or whether an accumulation of disadvantage hypothesis is consistent with the data. In this study, we apply a structured life-course modeling approach (SLCMA), a theory-driven analytical method that compares life course hypotheses of time-dependent exposure-outcome relationships, to determine whether early life is a sensitive period for DNAm modification using data from approximately 4,000 participants of the 2016 Health and Retirement Study (HRS) Venous Blood Study. We hypothesize that childhood is a sensitive period during which exposure to lower socio-economic position results in greater DNAm changes. We compare this to alternative hypotheses including the accumulation of disadvantage, in which the effect of low SEP increases with the duration of exposure, and the recency of disadvantage, in which the effect of low SEP is stronger for exposure in later life.

Abstract citation ID: igad104.0722

### BIOMARKERS HELP CLARIFY HOW SYSTEMIC AGING CONTRIBUTES TO MORTALITY: UTILIZING A MACHINE-LEARNING APPROACH IN HRS

Eric Klopack, and Eileen Crimmins, *University of Southern California, Los Angeles, California, United States*

Research suggests aging is a coordinated physiological decline occurring in multiple systems and at multiple biological levels. However, it is largely unknown how general biological aging and specific systemic aging co-occur and influence one another to affect mortality. There is also emerging interest in understanding how social exposures may differentially accelerate decline in individual physiological systems. We utilize data from the Health and Retirement Study, a nationally representative sample of about 4000 US adults over age 55. We used eXtreme Gradient Boosting (xgboost) in a training subsample to create estimates of mortality risk based on sets of biomarkers representing biological systems (e.g., brain and nervous system, adaptive immune system, cardiovascular system, renal system) as well as cellular aging (using epigenetic clocks and telomere length). These models generated mortality risk scores for each system (e.g., renal functioning using BUN, creatinine, and cystatin C). Mortality was regressed on these scores in a testing subsample to assess the relative impact of each system on mortality risk. Results suggest cellular aging, cardiovascular aging, and age-related metabolic dysfunction contribute most to mortality risk. Aging of these systems also partially mediates the mortality risk associated with age, race/ethnicity, sex, and education. These results suggest aging of specific systems may be differentially affected by sociodemographic exposures and may differentially contribute to mortality risk. This research may help guide interventions focused on reducing inequalities in mortality risk by identifying health risks that would have the greatest impact on mortality at the population level.

Abstract citation ID: igad104.0723

### BMI POLYEPIGENETIC SCORES PREDICT FUTURE CHRONIC CONDITIONS AND CHOLESTEROL

Trey Smith, Thomas Karadimas, Helen Meier, Colter Mitchell, and Jessica Faul, *University of Michigan, Ann Arbor, Michigan, United States*

Body mass index (BMI) is an established risk factor for chronic conditions such as high blood pressure, diabetes, cancer, lung disease, heart condition, stroke, emotional/psychiatric problems, arthritis, and sleep disorders. Poly-Epigenetic Scores (PEGS) have emerged as a promising tool for predicting the risk of chronic diseases. We evaluated the predictive power of BMI PEGS for incident chronic conditions, mortality, and cholesterol levels using data from the Health and Retirement Study, a population representative study of US adults over age 50 years. Three PEGS (Wahl, McCartney, Hamilton) were created from existing epigenome-wide association studies of BMI for participants from the 2016 HRS Venous-Blood study (N=4018). We used element-wise, least-squares regressions to assess the association between each BMI PEG and phenotypes of interest, controlling for demographics, socio-economic status, health-behaviors, and cell type proportions. BMI PEGS were significant predictors of incident chronic conditions in 2020 and these associations were robust to adjustment for covariates. BMI PEGS were also found to be significant predictors of cross-sectional cholesterol levels, a known risk factor for heart disease and stroke. Our study highlights that PEGS can have wide predictive power for future health conditions. These findings can inform personalized interventions for disease prevention and early intervention in the US aging population.

### SESSION 2330 (SYMPOSIUM)

Abstract citation ID: igad104.0724

### TRANSCENDING BOUNDARIES AND BARRIERS: END-OF-LIFE CARE AND QUALITY OF LIFE FOR ASIAN POPULATIONS

Chair: Kaipeng Wang Co-Chair: Fei Sun Discussant: Iraida Carrion

End-of-life care and quality of life are critical issues for older Asian populations, as they face unique cultural, linguistic, and health-related challenges. This symposium will bring together scholars in this field to discuss the latest research and practices for improving end-of-life care planning and quality of life for older Asian populations. The symposium consists of four papers that focus on different Asian ethnic groups. Zhang and colleagues will use survey data to examine the barriers and sociocultural factors of advance care planning among older Chinese Americans. Xu will present a systematic review on non-medical factors that affect quality of life in the last year among older

Chinese. Wang will compare the completion of advance directives and examine its psychosocial factors among older Vietnamese, Filipino, and Indonesian Americans. Using a case study of Somang Society, Lee will discuss how non-profit organizations can navigate social media to provide education and volunteer training to promote advance care planning and healthy aging for older Korean Americans. Collectively, these four papers compare the values, barriers, and resources related to end-of-life care between these groups and propose effective strategies for enhancing end-of-life care planning and quality of life for Asian communities. Findings will inform the development and adaptation of culturally sensitive interventions to promote end-of-life health outcomes for diverse Asian populations. Carrion, the discussant, will address the strengths and limitations of these papers and discuss their contribution to theories, practices, policies, and future research on end-of-life care for minority aging populations.

Abstract citation ID: igad104.0725

### ATTITUDES TOWARD ADVANCE DIRECTIVES AMONG OLDER SOUTHEAST ASIAN AMERICANS

Kaipeng Wang[1], Carson De Fries[1], My Ngoc To[1], Fei Sun[2], Bei Wu[3], and Sue Levkoff[4], *1. University of Denver, Denver, Colorado, United States*, *2. Michigan State University, East Lansing, Michigan, United States*, *3. New York University, New York City, New York, United States*, *4. University of South Carolina, Columbia, South Carolina, United States*

Advance directives (ADs) are legal documents that enable individuals to communicate their end-of-life care preferences in advance. AD completion is associated with decreased likelihood of unwanted medical interventions, healthcare costs, and decisional burden for family members. Compared to older Americans in general, older Asian Americans are less likely to complete ADs. While research focusing on ADs among Asian Americans has grown, few have investigated attitudes toward ADs among older Southeast Asian Americans (SEAAs). The study addresses that gap by examining factors associated with attitude toward ADs among older SEAAs. Using convenience sampling from 12 community partners and snowball sampling, we recruited 251 participants aged 55 and older who self-identified as Vietnamese (n=113), Filipino (n=100), or Indonesian (n=38) Americans. Through online or in-person questionnaires, participants completed the Nolan and Bruder's Advance Directive Attitude Survey, which measures attitudes toward ADs. Responses are rated on a 4-point Likert scale, with higher scores indicating more positive attitudes. In general, Indonesian (mean=3.40, SD=0.55) and Filipino (mean=3.40, SD=0.68) Americans expressed significantly more positive attitude toward ADs than Vietnamese (mean=3.15, SD=0.56) Americans. Results of ordinary least squares regression showed that family cohesion, expectation of intergenerational support, depression, and resilience were positively associated with attitude toward ADs, whereas death anxiety was negatively associated with attitude toward ADs. By enhancing understanding of SEAAs' attitudes towards ADs, these results can inform culturally appropriate interventions to improve AD implementation among SEAAs and contribute to broader awareness of and access to end-of-life care services in this population.

Abstract citation ID: igad104.0726

### PERCEIVED BARRIERS AND SOCIOCULTURAL FACTORS OF ADVANCE CARE PLANNING AMONG CHINESE AMERICAN OLDER ADULTS

Peiyuan Zhang[1], Fei Sun[2], and Jen Hirsch[2], *1. University of Maryland, Baltimore, Baltimore, Maryland, United States*, *2. Michigan State University, East Lansing, Michigan, United States*

Despite documented benefits of advance care planning (ACP), persistent racial and ethnic disparities exist in ACP engagement. This study, guided by a social ecological model, examined perceived barriers and sociocultural factors associated with informal ACP conversations among Chinese American older adults. A purposive sample of 281 community-dwelling older Chinese Americans aged 55 years or older in Arizona and Maryland completed a survey in 2018. The average age of participants was 77.8 (SD = 9.4) and the average number of years living in the U.S. was 24.9 years (SD = 13.4). Most participants were first-generation immigrants (94.7%) and reported Chinese as their primary language (91.8%). About 26.5% of participants had ACP conversations with family. Hierarchical logistic regression results suggest that more perceived barriers were associated with lower odds of ACP conversations (OR = 0.87, $p < 0.01$). Sociocultural factors were associated with greater odds of ACP conversations, including more years in the U.S. (OR = 1.04, $p = 0.02$) and English language proficiency (OR = 11.85, $p = 0.02$). Social support had a significant moderation effect ($b = -.01$, $p < 0.01$), indicating a larger effect of perceived barriers on ACP conversations among those with lower social support. Findings highlighted the importance of language services, translated informational materials, and social support in facilitating ACP discussions among Chinese American older adults. Community-based ACP facilitators who have both language skills and cultural competence may be particularly beneficial in engaging this population. Effective ways to reduce barriers to ACP at various levels are needed.

Abstract citation ID: igad104.0727

### A SYSTEMATIC REVIEW OF NONMEDICAL FACTORS INFLUENCING CHINESE PATIENTS' QUALITY OF LIFE IN THEIR LAST YEAR

Shuo Xu, Chen Zhang, Xinyue Wen, Yixuan Wang, and Ting Xu, *Renmin University of China, Beijing, Beijing, China (People's Republic)*

Many studies have demonstrated that healthcare providers' attention to non-medical issues and involvement in non-medical services can greatly improve the Quality of life (QOL) of dying individuals. Over the past decades, China has made great progress in providing the best possible care for the dying. However, the experiences of Chinese dying persons and their families in the last year of life have not been adequately synthesized. Thus, this systematic review aimed to summarize the last-year experiences of the Chinese dying persons and their families and to identify non-medical factors affecting their QOL. This review followed the PRISMA guideline. Peer-reviewed articles published before November 2022 were searched in both Chinese and English electronic databases. Studies were included if their participants had a life expectancy of one year or less, or family members acting as proxies were asked to recall patients' experiences during their last year of life. The final review included 24 studies.

The findings were classified into four aspects: (1) culture, including filial piety and the importance of family harmony; (2) emotional distress, including feeling like a burden to others, and depressive symptoms; (3) family involvement, including family support and involvement in decision-making; (4) connections to available services, including home-visit nurses and training on caregiving techniques. These findings implied that culturally appropriate non-medical interventions targeted at promoting the mental health of dying individuals, open communication in the family, and connections to available healthcare resources can greatly improve the QOL of dying Chinese persons during their final year of life.

Abstract citation ID: igad104.0728

### HELPING KOREAN AMERICAN OLDER ADULTS THROUGH EDUCATION, RESOURCES, AND VOLUNTEERING: A CASE STUDY OF SOMANG SOCIETY

Jessica Cassidy[1], Hye-Won Shin[2], and Kathy Lee[1], *1. University of Texas at Arlington, Arlington, Texas, United States*, *2. University of California Irvine, Irvine, California, United States*

Many community organizations serving Korean Americans lack culturally or linguistically tailored services for end-of-life care. Subsequently, Korean American older adults often lack understanding of end-of-life care, particularly advance care planning and hospice. Somang Society is a nonprofit organization serving Korean American older adults and their family caregivers with a mission to promote healthy aging, dignity in later life, and a meaningful death. This study details how Somang Society utilizes education, resources, and volunteer programs to increase the awareness of end-of-life issues among Korean American older adults. The organization provides two distinct community education and volunteer trainings regarding (1) end-of-life care planning program and (2) older adults living with dementia and their family caregivers. They provide community education and volunteer trainings in Korean and expect the volunteers to educate others in the community about the importance of healthy aging, dementia, and advanced care planning. As a result, 15,717 Korean American seniors have filed a living will and advance healthcare directives as of February 2023. In addition, Somang Society emphasizes the importance of whole-body donation for training medical students and healthcare professionals. As a result, 2,134 Korean American seniors have donated or registered for the UCI Willed Body Program through Somang Society since 2008. To further disseminate their programming, the organization actively uses social media to educate Korean Americans on these issues. Their YouTube channel analytics indicate the viewers' positive responses and the potential impact technology usage can have for sharing education with this hard-to-reach population.

### SESSION 2335 (BIOLOGICAL SCIENCES INVITED SYMPOSIUM)

Abstract citation ID: igad104.0729

### NEUROSCIENCE AND AGING

Chair: Gregorio Valdez

In this symposium, experts will highlight discoveries that are beginning to bridge the gap between basic neuroscience research and development of therapies to mitigate the ravages of normal aging and age-associated neurological diseases on the nervous system. The symposium will cover cellular, molecular and behavioral approaches to researching aging of the nervous system. It will provide insights about how aging impacts the fate of neurons and their synapses and how age-related changes in cellular maintenance, molecular signaling, and glial function ultimately diminish cognitive and motor function. Dr. Miranda Orr will provide insights about the contribution of senescent cells to age- and disease-associated degeneration of the nervous system. Dr. Julie Andersen will present on how cells in the nervous system use their own organelles to repair damages to avoid dysfunction during aging and in Alzheimer's Disease. Dr. Gregorio Valdez will review recent discoveries revealing the initial cellular and molecular changes that impair voluntary movements during aging. Dr. Scott Pletcher will present evidence that cellular and molecular processes important for feeding, mating and avoiding danger in flies also play key roles in healthy aging of the nervous system. The information provided by the speakers will foster discussion among all attendees about the challenges and opportunities to accelerate translation of discoveries to protect the nervous system from aging and associated diseases.

Abstract citation ID: igad104.0730

### BLUEPRINT TO TARGET THE SPINAL CORD MOTOR CIRCUITRY TO PRESERVE AND RESTORE MOBILITY IN OLD AGE

Gregorio Valdez, *Brown University, Providence, Rhode Island, United States*

Spinal motor neurons are central for the initiation and modulation of all voluntary movements. Within the spinal cord, motor neurons form tens of thousands of excitatory (glutamatergic and cholinergic) and inhibitory (GABAergic and glycinergic) synapses along their dendritic arbor and soma. These synapses contain the information required to execute fine and complex motor commands and are together referred to as the motor circuitry. Once activated, motor neurons drive muscle contraction by releasing neurotransmitters from their axon terminals at neuromuscular junctions (NMJs). Thus, either the death of motor neurons or their disconnection from other neurons are skeletal muscles during aging would undoubtedly compromise motor function. We will show that motor neurons do not die in old female and male mice, rhesus monkeys, and humans. Instead, these neurons selectively and progressively shed excitatory synaptic inputs throughout the soma and dendritic arbor during aging. Thus, aged motor neurons contain a motor circuitry with a reduced ratio of excitatory to inhibitory synapses that may be responsible for the diminished ability to activate motor neurons to commence movements. Additionally, we will show that aged motor neurons present with aberrant changes in genes and molecular pathways with roles in glia-mediated synaptic pruning, inflammation, axonal regeneration, and oxidative stress. I will bring these findings together to suggest that motor neurons at a minimum contribute to the loss of their own synapses with advancing age and discuss possible avenues for therapeutic intervention.

Abstract citation ID: igad104.0731

**SPATIAL PROFILING CELLULAR SENESCENCE IN THE HUMAN BRAIN ACROSS EIGHT DECADES OF LIFE**

Miranda Orr, *Wake Forest University School of Medicine, Winston-Salem, North Carolina, United States*

Neurons with tau-containing neurofibrillary tangles (NFTs) closely correlate with Alzheimer's disease (AD) progression and severity, but do not undergo immediate cell death. Our prior work determined that NFT-bearing neurons undergo a change in cell fate consistent with cellular senescence. CDKN2D/p19 was identified as a key biomarker for identifying senescent neurons in postmortem human AD brain tissue. Studying the interaction between senescent (e.g., p19+/NFT+) neurons and their environments is a necessary next step to understanding how these cells may be impacting their local and synaptically-connected neighboring cells. The goal of this project was to evaluate the expression and subcellular localization of multiple analytes in normal and senescent cells in postmortem mouse and human brains using non-destructive, multi-plex, spatially-resolved profiling methods. Postmortem mouse and human brains were analyzed using NanoString, Inc GeoMx digital spatial profiling (DSP) and CosMx spatial molecular imaging (SMI). Region of interest (ROI) selection included the presence or absence of neuronally expressed AT8 (phosphorylated tau, NFTs) and/or p19 (senescence). NFTs with or without p19 expression were compared to healthy neurons from the same cases. The expression of 86 proteins associated with neuropathology, inflammation, autophagy and cell typing was determined for each ROI and immediate environment using GeoMx. CosMx SMI was used to develop a subcellular expression map of >1000 genes and spatially-resolved cell type map of mouse and human entorhinal cortex and hippocampus. The complete dataset consisted of over 160,000 single cells and ~170 million transcripts, and a spatially-resolved cell type map of mouse brain tissue. Significant differences across biological aging pathways between disease and healthy conditions in both species were identified. Additionally, the results provide insights into which cells are susceptible to senolytic clearing interventions currently underway (NCT04685590).

Abstract citation ID: igad104.0732

**DEVELOPMENT OF MITOPHAGY-INDUCING SMALL MOLECULES AS THERAPEUTICS FOR AGING AND ALZHEIMER'S DISEASE**

Julie Andersen, *Buck Institute for Research on Aging, Novato, California, United States*

Alzheimer's disease (AD) is the leading cause of dementia in those over the age of 65. The underlying molecular basis for the disease remains elusive. Rather than simply viewing individual diseases in isolated silos, researchers have recently turned to study of the contribution of common age-related processes to age-related diseases. One such shared process is loss in mitochondrial function, normally maintained by clearance of defective mitochondria via mitophagy. Mitophagy slows with age and its pre-clinical and clinical elevation has been shown to slow various age-related conditions. An agent which has received considerable attention in this regard is the compound urolithin A (UA). Several labs, including our own, have recently reported neuroprotection in association with its administration in a variety of mouse AD models. As part of an independent small molecule screen carried out by the lab, we identified a second structurally-related compound we named 'mitophagy inducing compound' (MIC). Using C. elegans as an initial validation tool, we showed that both compounds significantly improve mitochondrial function, protect against neuronal cell loss in disease models, and extend lifespan of wildtype animals. These protective effects were found to be translatable to both in vitro and in vivo mammalian models. To identify potential drug targets, we next turned to Cellular Thermal Shift Assay (CETSA). Preliminary data from knockdown of identified targets suggest involvement of a mito-hormetic mechanism which acts to restore overall mitochondrial health. These studies may have important implications for understanding signaling events elicited by these compounds driving their disease-modulating effects.

Abstract citation ID: igad104.0733

**NEURAL STATES ASSOCIATED WITH MOTIVATION AND REWARD MODULATE AGING IN DROSOPHILA**

Scott Pletcher, *University of Michigan Medical School, Ann Arbor, Michigan, United States*

Humans, scientists especially, like to believe that we are in control of our behaviors and that logical, rational thought is the driving force behind them. Even for the most conscious of actions, however, there are serious questions about whether this is true. Economists, for example, are well-versed in the notion that consumer behavior is irrational. Why? Psychologists attribute it to the complex evaluation of internal and external cues, which is often need-based but can be strongly influenced by perceived costs and benefits. Molecular neuroscience has gone a step further in recent years, often using simple model organisms, to provide a well-defined framework for dissecting the causes and consequences of motivated behaviors. Homeostatic needs are as influential in this process as are rewarding experiences, and I will discuss evidence that the neurons, neural circuits, and conserved signaling molecules that influence neural states associated with feeding, mating, danger in flies are also important modulators of healthy aging.

### SESSION 2340 (SYMPOSIUM)

Abstract citation ID: igad104.0734

**TRANSITION AND STABILITY OF PLACE AND AGING IN LATER LIFE: HOUSING AND NEIGHBORHOOD CONTEXTS**

Chair: Frank Oswald Co-Chair: Steven Schmidt Discussant: Malcolm Cutchin

Transition and stability in housing and neighborhood contexts reflect both, a central field of research and policies on place and aging for health and well-being in later life. Studies have demonstrated effects of improving the physical environment, particularly with respect to specific needs, such as for people living with dementia. Moreover, relationships between perceived housing and health and well-being on the one hand and life transitions and health and well-being on the other hand have been addressed. However, research on complex interactions of place and transitions in later life is scarce. This symposium aims to address the dynamic relationship between transition and stability of place and aging in later life in relation to indicators of good ageing. The first contribution by Eriksson and colleagues presents quantitative results on the relationships between perceived housing and changes in health and well-being over time among community-dwelling Swedish older adults. Second, Kylén and colleagues present quantitative data describing which housing preferences are most important when considering relocation in older age. The third contribution by Slaug and colleagues uses a quantitative approach to qualitative interview data from Sweden and Germany, analyzing sequences of and relationships between life transitions over 10 years. Fourth, Chaudhury and colleagues show data that aims to identify spatial and temporal patterns in activities undertaken outside home by people living with dementia, and ways in which the neighborhood-built environment affects their outdoor mobility and social participation. Finally, Malcom P. Cutchin will serve as the session's discussant.

Abstract citation ID: igad104.0735

### RELATIONSHIPS BETWEEN PERCEIVED HOUSING, HEALTH, AND QUALITY OF LIFE: DATA FROM AGES 65–70 IN SWEDEN

Erik Eriksson, Maya Kylén, and Steven Schmidt*, Lund University, Lund, Skane Lan, Sweden*

Theoretical and empirical advances have supported the notion of the importance of the perceived environment, and in particular the housing environment, as we age. For instance, previous research has found significant associations between aspects of perceived housing, such as control beliefs and meaningful aspects of home, and health and wellbeing. These associations have been found for both younger older, and older older, adults. However, much of previous research has been cross-sectional, in particular for younger older adults, leaving the question whether this association remains over time as we age. By using data from a Swedish longitudinal study (N=371, 57 % women) we analyzed associations between Meaning of Home (MOH) and two external subscales of Housing-Related Control Beliefs (HCQ), to a 6-year change in 31 self-reported health symptoms and health-related quality of life, measured by SF-12, using linear regression and modelling for demographic and housing characteristics. Significant results were found between both housing-related control beliefs and meaning of home, to changes in metabolism symptoms (MOH, $R2 = 0.07$, $F(9, 259) = 3.258$, $p = .001$; HCQ, $R2 = 0.072$, $F(9, 281) = 3.494$, $p < .000$ and $R2 = 0.066$, $F(9, 280) = 3.263$, $p = .001$). However, for all other health symptom groups and for health-related quality of life, no significant associations were found. Our results show that while perceived aspects of housing are associated with health at a given point in time, they may not be predictive of future changes in health status.

Abstract citation ID: igad104.0736

### HOUSING ATTRIBUTE PREFERENCES WHEN CONSIDERING RELOCATION IN OLDER AGE

Maya Kylén[1], Björn Slaug[1], Susanne Iwarsson[1], David Dahlgren[1], Jonas Bjork[2], and Magnus Zingmark[1], *1. Lund University, Lund, Skane Lan, Sweden*, *2. Skane University Hospital, Lund, Skane Lan, Sweden*

For the last decades, interdisciplinary researchers have studied housing preferences of various groups in the population. To contribute to knowledge on the needs and preferences of the aging population we have established a cohort (N=1,509) of adults, 55 years or older, who are considering relocation. The baseline survey included questions on the current housing situation and preferences regarding future housing alternatives. The aim was to investigate preferred housing attributes when considering relocation and whether such attributes differed depending on the type of housing participants wanted to move to. Preliminary results show that having a balcony/patio was rated as most important (84%), with significant differences between men and women. Leisure activities and/or social gatherings initiated by the landlord was the least important (1.5%). Differences in preferred attributes were found between those wanting to move to privately-owned housing (e.g., villa), where having a garden (81%) or a view (57%) were the most important attributes, and those wanting to move to an apartment, where having a balcony (86%) or lift (72.6%) were deemed most important. We expect that the longitudinal follow up of the cohort will contribute with new knowledge on housing preferences among people in later life. Such knowledge is important to inform and inspire planners and policymakers to develop more age-friendly communities, including housing options that better meet the needs of this segment of the population.

Abstract citation ID: igad104.0737

### COMBINATIONS AND SEQUENCES OF LIFE TRANSITIONS DURING A 10 YEAR PERIOD AROUND RETIREMENT AGE

Björn Slaug[1], Erik Eriksson[1], Susanne Iwarsson[1], Maya Kylén[1], Frank Oswald[2], Anna Wanka[2], Karla Wazinki[2], and Steven Schmidt[1]*, 1. Lund University, Lund, Skane Lan, Sweden*, *2. Goethe University Frankfurt, Frankfurt am Main, Hessen, Germany*

Our lives can be characterized as periods of stability, alternating with events that bring about change of both practical and psychological nature which require some life adjustment. The scope and depth of such life transitions are related to the kind of event, but also to whether it is perceived as positive or negative, occurs together with other events, addresses an entirely new topic or something that is biographically familiar, comes after a long period of stability or if it is an event in a chain of events occurring shortly after one another. Applying a quantitative approach to qualitative interview data from the Swedish German HoT-age project (N=50; mean age=69; 50% men), life transitions were analysed by means of sequential and configural frequency analysis. During an approximate 10-year period around retirement age, the participants experienced in average 3.7 life transitions and 24% of them more than five. Fifteen different kinds of transitions (retirement, relocation, divorce/separation, new partner, chronic illness etc) were recorded. Besides retirement experienced by almost all (96%), relocation (66%), becoming a grandparent (40%) and chronic illness (40%) were most frequent. The transitions significantly more often (p=0.041) occurring in combination were retirement and relocation. Those who had experienced chronic illness often had a sequence of several events preceding the illness recorded as a transition. Those who found a new partner often had a sequence of events after that. Large variation in sequences and distances between events were found. This study contributes with new knowledge on life transitions around retirement age.

Abstract citation ID: igad104.0738

**THE ROLE OF THE NEIGHBORHOOD-BUILT ENVIRONMENT ON OUTDOOR MOBILITY OF PEOPLE LIVING WITH DEMENTIA**

Habib Chaudhury[1], Lillian Hung[2], Shannon Freeman[3], Mark Groulx[3], Kishore Seetharaman[1], Joey Wong[2], and Cari Randa[1], *1. Simon Fraser University, Vancouver, British Columbia, Canada*, *2. University of British Columbia, Vancouver, British Columbia, Canada*, *3. University of Northern British Columbia, Prince George, British Columbia, Canada*

Dementia-friendly communities (DFCs) aim to foster a supportive, inclusive and empowering environment that promotes equal rights and resources for people living with dementia and their care partners. Central to DFCs is promoting access and navigation of outdoor spaces and destinations in the neighbourhood. While there is a growing interest and uptake of DFCs in policy and practice, there is scarce empirical evidence on the role of the built environment on mobility and navigation for people living with dementia. The "Dementia-inclusive Spaces for Community Access, Participation, and Engagement (DemSCAPE)" study aims to identify spatial and temporal patterns in activities undertaken outside home by people living with dementia, and ways in which the neighbourhood built environment affects their outdoor mobility and social participation. A series of sit-down and video-and-photo-documented walk-along interviews were conducted with 26 participants who are living with (mild to moderate) dementia or mild cognitive impairment in the Metro Vancouver region of British Columbia, Canada. Findings shed light on how people living with dementia understand and navigate the neighbourhood environment, and features that prompt recall of routes, places, and events, and support orientation and wayfinding. Findings also underscore the importance of participants' awareness of barriers and obstacles in their neighbourhood, and how they cope with challenges and demands encountered while walking outside. The study offers planners and designers awareness and insights into the lived experience of navigating the neighbourhood environment with the condition of dementia and guidance on adopting a dementia-friendly and inclusive approach in policy and practice.

### SESSION 2345 (SYMPOSIUM)

Abstract citation ID: igad104.0739

**INNOVATIVE APPROACHES TO ADDRESSING LONELINESS AND PROMOTING AGING IN COMMUNITY**

Chair: Su-I Hou Co-Chair: Li-Fan Liu Discussant: Carol Ma

Research has shown that older adults who are lonely and socially isolated are at increased risk for various negative health outcomes. This symposium brings together leading experts across the globe in the fields of gerontology, public health, and social work to share innovative approaches to addressing loneliness and fostering connections. The symposium will begin with Dr. Ma from the National Singapore University of Social Sciences (SUSS) to share the voice of seniors on digital inclusion for a healthy community in Singapore. Dr. Shapira from Ben-Gurion University of the Negev, Israel, and her research team will discuss a 7-session digital intervention to promote effective coping and alleviate depression and loneliness among older adults in Israel. The symposium will then examine community-based efforts, with Dr. Hou from The University of Central Florida (UCF) in the United States discussing the role of healthcare providers in screening and identifying loneliness and examining communication and comfortability in conducting loneliness screening among older patients. Lastly, Dr. Kuo from Chung-Shan Medical University (CSMU) will introduce the five domains of living a good life and how older adults responded to these topics to prepare themselves for better aging in the community. The presenters will discuss strategies for fostering community-based support networks to address community-dwelling older adults' physical, social, and emotional needs. This global symposium will also examine the role of technology in supporting community-based efforts, including using virtual communication platforms, online resources, and digital tools to promote community engagement and connection. This is an International Comparisons of Healthy Aging Interest Group Sponsored Symposium. This is a collaborative symposium between the Aging Among Asians and International Comparisons of Healthy Aging Interest Groups.

Abstract citation ID: igad104.0740

**LONELINESS AND SOCIAL ISOLATION: TO SCREEN OR NOT? PROVIDERS' COMMUNICATIONS AND COMFORTABILITY**

Su-I Hou, *School of Global Health Management & Informatics, University of Central Florida, Orlando, FL, USA, Oviedo, Florida, United States*

**Purpose:** Research has shown that loneliness and social isolation (SI) can seriously impact older adults' health and well-being. However, it is unknown whether healthcare providers communicate with their older patients about these issues. This pilot study examines providers' communication and comfortability on loneliness screening.

**Methods:** A convenience sample of providers who see older patients were recruited from healthcare and long-term care settings (n=59). Communication about social isolation was adapted from the revised UCLA 3-item Scale. De Jong Gierveld's loneliness 6-item scale was used to assess social and emotional loneliness. Comfortable levels with loneliness screening (4 items) were adapted from UCF researchers' food insecurity screening study.

**Results:** Providers' mean age was 46 years; the majority were females (70%), and 67% were whites, with an average of 17 years of practice experience. About 60% of providers believe loneliness screening should be conducted among older patients during every visit. All the scales showed high internal consistencies with Cronbach's alphas, ranging from .825 to .897. Data showed that communications about loneliness and social isolation were both low, with item means of 2.34 and 2.15, respectively. Overall, providers showed moderate comfort in conducting such screenings (mean=3.72 on a 5-point Likert scale).

**Discussion:** This pilot provides evidence of high reliabilities of these validated measures to assess providers' communication and comfortability to screen for loneliness and SI. Data showed concerning findings on low communication and only moderate comfort among providers. It calls attention to providing provider training for early intervention, particularly among older patients.

Abstract citation ID: igad104.0741

### THE VOICE OF OLDER ADULTS ON DIGITAL INCLUSION FOR HEALTHY COMMUNITY IN SINGAPORE

Carol Ma, *Singapore University of Social Sciences, Singapore, Singapore*

With the rapid ageing population and the experience of Covid-19 pandemic, there is an urgent need to create a digitally inclusive healthy society. Singapore, as a smart nation, has been at the forefront of promoting digital inclusion to all age groups, including older adults. According to the Statista (2022), 88.5% of the population in Singapore are using internet and 56% of those over 60 are smartphone users. The use of digital technology for health promotion holds enormous potential to empower individuals and communities, particularly in the context of healthy and active ageing. The older adults in this study are adept at using the basic function of the smart phone rather than a computer. Some have even attended the national program, "Seniors Go Digital," to learn about basic communication tools, file messaging, video calling, accessing government digital services, using e-payment tools, etc. They have also shared the barriers they face in using the internet, such as declines in vision, cognitive ability, and hearing, as well as the opportunities they have found, such as arrange telemedicine and access to the e-health systems. Besides, they found that the peer support and social networks are very important to support their digital learning journey. Though fear of encountering cheats and scams is common, they can be overcome by building confidence through peer support and self-efficacy. During the presentation, the authors will identify the contextual influences of digital inclusion and its impacts on healthy ageing in Singapore.

Abstract citation ID: igad104.0742

### A DIGITAL INTERVENTION TO PROMOTE EFFECTIVE COPING DURING CRISIS AND ROUTINE AMONG OLDER ADULTS

Stav Shapira[1], Daphna Yeshua-Katz[1], Avital Avigezer[1], Su-I Hou[2], and Orly Sarid[1], *1. Ben-Gurion University of the Negev, Beer Sheva, HaDarom, Israel, 2. School of Global Health Management & Informatics, University of Central Florida, Orlando, FL, USA, Oviedo, Florida, United States*

**Introduction:** We developed and evaluated a short-term group intervention aimed at promoting effective coping and alleviating adverse mental health impacts during the first wave of COVID-19 in Israel. The intervention consists of seven sessions, each including: a guided group discussion, and learning and practicing cognitive-behavioral and mindfulness techniques.

**Methods:** In this pilot randomized controlled trial, we recruited eighty-two community-dwelling older adults who were assigned to either an intervention group (n=64) or a wait-list control group (n=18). Participants completed questionnaires assessing depression, loneliness, subjective health, and social support. We collected measurements before (T0), immediately after participation (T1), 4 weeks after participation (T2), and 1.5 years after participation in the intervention (T3).

**Results:** Our findings revealed statistically and clinically significant reductions in depression in the intervention group, with results maintained at one-month and 1.5-year follow-ups. While loneliness levels also significantly decreased post-intervention, this benefit was not maintained at follow-up measurements. Social support and subjective health did not change significantly. The wait-list control group did not show any overall changes. Participants reported frequently using cognitive reconstruction and positive self-talk techniques at the 1.5-year follow-up. The current degree of technique use/practice significantly predicted depression level at T3.

**Conclusions:** Our intervention presents a simple and easy-to-implement tool with broad relevance beyond the COVID-19 pandemic, as the skills acquired also positively influence mental health outcomes during routine life. This relatively simple model can be effectively utilized by communities globally to help connect lonely and isolated older inhabitants and promote effective coping with various stressful situations.

Abstract citation ID: igad104.0743

### PREPARING FOR OLDER ADULTS TO AGE IN PLACE: COMMUNITY-BASED EFFORTS IN TAIWAN

Tsuann Kuo, *Chung Shan Medical University, Taichung City, Taichung, Taiwan (Republic of China)*

Taiwan's baby-boomers are about to join the super aged society and an action plan was called to increase public awareness of aging-in-place among all community-based senior service sites. A book, entitled How to Live with a Good Life and consisted of 25 topics, was published to guide older adults to prepare themselves in five domains: Healthy living, Social Participation, Recreation and Technology, Health and Long-term care, and Finance and Legal Protection. The purpose of this paper was to test how well the older adults (N=180) responded to the topics. The results showed that older adults responded differently based on age, gender, education and living locations (all $P<0.05$) when it comes to topics that of interests. The top five topics of concerned were: lifelong learning; recreation and assistive devices, nutrition and health, exercise and legal protection. The paper concluded by suggesting ways to let older adults willing to learn and take actions on various aging-in-place topics among the community-based sites.

### SESSION 2350 (SYMPOSIUM)

Abstract citation ID: igad104.0744

### CONTRACT NURSE STAFFING AND NURSING HOMES: ANTECEDENTS AND CONSEQUENCES

Chair: Rohit Pradhan Co-Chair: Robert Weech-Maldonado
Discussant: Jane Banaszak-Holl

Nursing home (NH) quality has been a matter of long-standing policy interest at the federal and state level, as it concerns the health and well-being of one of our most vulnerable populations. Nurse staffing intensity and skill mix have a consequential impact on NH care quality. Unfortunately, NH staffing has been marked by multitudes of challenges including high levels of turnover and absenteeism. In recent years, the utilization of agency or contract nurse staffing has increased as facilities face persistent staffing shortages exacerbated by COVID-19 which has had a disproportionate and devastating impact on NHs. The literature suggests that agency staffing negatively influences the performance of healthcare organizations due to multiple reasons including unfamiliarity with organizational culture, decline in teamwork, and the attendant high costs attached to contract staffing. However, contract nurse utilization practices and its impact on NH care has not been studied adequately, and much of the limited literature relies on datasets with limited understanding of agency staffing. The overarching goal of this symposium is to explore the antecedents and consequences of the increased utilization of contract nurse staffing. As part of the symposium, we include studies examining NH nursing skill mix and intensity, the organizational and market level factors associated with higher levels of contract staffing, and the impact of nurse contract staffing on NH staff turnover, quality, and financial performance. We believe that this series of studies would have important managerial and policy implications. This is a Research in Quality of Care Interest Group Sponsored Symposium.

Abstract citation ID: igad104.0745

### CONTRACT NURSE UTILIZATION AND FACILITY AND COMMUNITY FACTORS

Rohit Pradhan[1], Ganisher Davlyatov[2], Neeraj Dayama[3], and Robert Weech-Maldonado[4], *1. Texas State University, San Marcos, Texas, United States*, *2. University of Oklahoma Health Sciences Center, Oklahoma City, Oklahoma, United States*, *3. Texas Tech University Health Sciences Center, Lubbock, Texas, United States*, *4. University of Alabama at Birmingham, Birmingham, Alabama, United States*

While NHs have long been plagued by nursing staff shortages, the situation has further deteriorated with the COVID 19 pandemic. The NH industry has attempted to address the persistent shortage of nursing staff by increasingly relying on contract nurse staffing. Research suggests that the increased utilization of contract nurses may have negative implications for NH quality and financial performance. However, all NHs are not equally likely to utilize nurse contract staffing. For instance, potentially, NHs with poorer quality of care and financial challenges may be forced to rely more on contract nurse staffing. The primary purpose of this paper is to explore the antecedents---facility and community factors---associated with increased utilization of nurse contract staffing. Employing a pooled cross-sectional observational study design, we extracted secondary data from the PBJ nurse staffing, Nursing Home Compare, Rural-urban commuting area (RUCA) codes, and the American Community Survey (ACS) for the period 2017-2021. To test the relationship between the ratios of contract nurses and facility/community factors, we employed multivariate mixed-effects maximum likelihood regression. We ran three separate regressions for RNs, LPNs, and CNAs. Our results suggest that among facility level factors, NHs with more beds, higher occupancy rates, and higher proportion of Medicare residents were generally more likely to employ contract nurse staffing with some minor differences across the three models (RNs, LPNS, CNAs). Among community factors, location in micropolitan/small town areas, higher poverty rates, and higher education levels were positively associated with nurse contract utilization. Policy and managerial implications are discussed.

Abstract citation ID: igad104.0746

### THE IMPACT OF CONTRACT NURSE UTILIZATION ON NURSING HOME QUALITY

Ganisher Davlyatov[1], Robert Weech-Maldonado[2], Rohit Pradhan[3], Akbar Ghiasi[4], and Justin Lord[5], *1. University of Oklahoma Health Sciences Center, Oklahoma City, Oklahoma, United States*, *2. University of Alabama at Birmingham, Birmingham, Alabama, United States*, *3. Texas State University, San Marcos, Texas, United States*, *4. University of the Incarnate Word, San Antonio, Texas, United States*, *5. Louisiana State University at Shreveport, Shreveport, Louisiana, United States*

Nurse staffing intensity and skill mix have a significant impact on NH quality of care. For instance, higher staffing levels, particularly for registered nurses (RNs), have been associated with better resident outcomes. NHs have increasingly relied on contract nurse staffing to address pressing and persistent nursing shortages. Research suggests that contract nurses may be unfamiliar with institutional policies and procedures or organizational culture. They may require increased supervision, be less participatory in teamwork, and may increase the workload for the permanent staff. The objective of this study is to examine the association between contract nurse utilization and quality of care at US nursing homes. We used secondary data from PBJ nurse staffing, Nursing Home Compare, Healthcare Cost Report Information System (HCRIS) Dataset, and the American Community Survey (ACS) for the period 2017-2021. The dependent variable was CMS quality star rating, and the independent variables were contract nurse to total nurse proportions for each of RNs, LPNs, and CNAs. We employed multivariate random effects ordered logistic regression to test the hypothesis. We found that across all our three models (RNs, LPNs, CNAs), quality star ratings were negatively associated with contract nurse utilization. A 10% increase in contract nurse proportion was associated with a 10% decrease in the odds of reporting a higher quality star rating (OR=0.99, $p<0.001$). Our results suggest the increased utilization of contract nurse staffing may have a negative impact on US NH quality---an important issue that has agitated policymakers for nearly three decades.

Abstract citation ID: igad104.0747

### THE IMPACT OF CONTRACT NURSE UTILIZATION ON NURSING HOME FINANCIAL PERFORMANCE

Akbar Ghiasi[1], Gani Davlyatov[2], Rohit Pradhan[3], and Justin Lord[4], *1. University of the Incarnate Word, San Antonio, Texas, United States*, *2. University of Oklahoma Health Sciences Center, Oklahoma City, Oklahoma, United States*, *3. Texas State University, San Marcos, Texas,*

*United States*, *4. Louisiana State University at Shreveport, Shreveport, Louisiana, United States*

Ensuring the financial viability of NHs is an important policy and managerial concern. The financial sustainability of nursing homes has been threatened in the recent past by high staff turnover, growing competition, low Medicaid reimbursement, and declining occupancy rates. The literature suggests that nurse staffing may have a significant impact on NH financial performance. We examined whether higher ratios of contract nurse use were associated with NHs operating margin. Employing a pooled cross-sectional observational study design, we extracted secondary data from the PBJ nurse staffing, Nursing Home Compare, Healthcare Cost Report Information System (HCRIS) Dataset, RUCA codes, and the ACS for the period 2017-2021. To test the relationship between the ratios of contract nurses and operating margin, we employed multivariate mixed-effects maximum likelihood regression. We ran three separate regressions for RN, LPN, and CNA. Our findings showed that a 10% increase in the contract nurse ratio among RNs, LPNs, and CNAs is associated with a 0.45%, 0.48%, and 0.52% decrease in operating margin, respectively. Our results demonstrate a consistent negative association between the ratio of contract nurses and nursing home operating margin. Our results are consistent with the extant literature with contract nurse staffing said to be significantly more expensive. Considering NHs operate in a financially precarious environment, the increased utilization of contract nurse staffing may pose a further threat to their financial viability, with negative implications for quality and access.

Abstract citation ID: igad104.0748

### NURSE STAFFING CHARACTERISTICS AT US NURSING HOMES: NURSING MIX AND INTENSITY

Gregory Orewa[1], Ganisher Davlyatov[2], Akbar Ghiasi[3], and Robert Weech-Maldonado[4], *1. University of Texas, San Antonio, San Antonio, Texas, United States*, *2. University of Oklahoma Health Sciences Center, Oklahoma City, Oklahoma, United States*, *3. University of the Incarnate Word, San Antonio, Texas, United States*, *4. University of Alabama at Birmingham, Birmingham, Alabama, United States*

NHs have long struggled with lack of appropriate and consistent supply of nursing staff. Recruitment of qualified nursing staff is difficult due to the relatively low pay, supply-demand mismatch, and onerous regulatory and administrative demands. Research suggests that approximately 80% of NHs are understaffed. Despite NHs offering financial incentives to attract staff nurses, the use of contract nurses has been on the rise with 78% facilities reporting using agency nurse staff. However, much of this evidence remains anecdotal or precedes the COVID 19 pandemic, which had a disproportionate impact on the NH industry. The purpose of this research was to explore the overall nursing staffing trends at US NHs from 2017-2022. The study used the CMS Payroll Based Journal (PBJ) data for years 2017-2022. We examined the nursing staffing intensity, the proportion of contract nurses to total nurses, and the proportion of registered nurses (RN) to total nurse staffing. Our results suggest that the RN and licensed practical nurses (LPN) hours per resident day (PRD) stayed relatively consistent through 2017-2022, with a slight increase noted during the COVID-19 pandemic. There was a significant decrease in CNA hours PRD in 2022 compared to 2017. Contract nurse utilization across RNs, LPNs, and CNAs increased in 2022 compared to 2017. Looking at the trends, contract nurse utilization has been consistently increasing, but it may have peaked during the pandemic. RN to total nurse ratio increased in 2022 compared to 2017, though noticeably, the sharpest increase was during the pandemic.

Abstract citation ID: igad104.0749

### EXPLORING THE ASSOCIATION BETWEEN CONTRACT NURSE STAFFING AND NURSE TURNOVER IN NURSING HOMES

Neeraj Dayama[1], Ganisher Davlyatov[2], Rohit Pradhan[3], and Robert Weech-Maldonado[4], *1. Texas Tech University Health Sciences Center, Lubbock, Texas, United States*, *2. University of Oklahoma Health Sciences Center, Oklahoma City, Oklahoma, United States*, *3. Texas State University, San Marcos, Texas, United States*, *4. University of Alabama at Birmingham, Birmingham, Alabama, United States*

NHs face a significant problem with nurse staffing---a critical factor in providing high-quality care to residents. Although contract nurse staffing in NHs may provide a relatively flexible option to address acute nurse shortages, it raises its own challenges including requirement of increased supervision, lower levels of teamwork, and increased workload for the permanent staff. This may lead to disenchantment of the remaining nursing staff, increased turnover---a perfect vicious cycle. Therefore, we examined whether higher ratios of contract nurse utilization were associated with higher nurse turnover rates (RNs, LPNs, and CNAs). Employing a pooled cross-sectional observational study design, we extracted secondary data from PBJ nurse staffing, Nursing Home Compare, Rural-urban commuting area (RUCA) codes, and American Community Survey (ACS) for the period 2017-2021. We employed multivariate mixed-effects maximum likelihood regression to estimate the relationship between the ratios of contract nurses and nurse turnover rates while controlling for organizational/community variables. We ran three separate regressions for RNs, LPNs, and CNAs. Our results suggest that NHs with a high proportion of contract nurses had higher nurse turnover rates for all the three types of staffing (RNs, LPNs, and CNAs). Moreover, NHs with high RN turnover rates had low RN staffing, higher CNA staffing, lower occupancy rates, and higher share of Medicaid residents. High nurse turnover can adversely affect resident outcomes—for instance, due to reduced continuity of care. As such, it is important to closely monitor contract nurse staffing levels and their implications on staffing and resident outcomes.

### SESSION 2355 (SYMPOSIUM)

Abstract citation ID: igad104.0750

### ADVANCING HEALTH EQUITY AMONG OLDER ADULTS THROUGH COMMUNITY ENGAGED RESEARCH

Chair: Rachel O'Conor Co-Chair: Marquita Lewis-Thames

Health equity, the absence of unfair and avoidable differences in health among social groups, has garnered mainstream attention due to recent civil unrest in communities, yet remains an elusive goal for aging health researchers. Community-engaged research is one approach to improve older adult health equity. Community-engaged research involves partnerships between researchers and community organizations, community health centers, and community members to pursue a common goal. To shift the traditional paradigm of investigator-led and -implemented research, community-engaged principles emphasize the co-production and ownership of knowledge. While community-engaged research has a successful track record in facilitating health disparities research, far fewer partnerships have been formed to 1) elevate the voices of older adults and 2) investigate and act on root causes of health disparities in older adults. To advance the practice of community-engaged research focused on older adult health, this symposium presents five community-academic partnerships elevating older adult voices from diverse groups and health conditions while simultaneously describing engagement strategies across the research process. First, Grant outlines processes for leveraging community representatives as partners in developing multilevel interventions for older cancer survivors. Second, Filec describes a multi-year community-engaged research journey that culminated with a study investigating the health impacts of a home repair program. Third, Singh details a user-centered design approach through a community advisory board to develop a telehealth navigation tool. Fourth, Lewis-Thames describes a community-based qualitative assessment capturing telehealth utilization among rural older cancer survivors. Lastly, Oladejo presents the community-driven development of a culturally-tailored dementia caregiving program for Black caregivers. This is a Community-Engaged Research Interest Group Sponsored Symposium.

Abstract citation ID: igad104.0751

### TELEHEALTH UTILIZATION BY RURAL OLDER CANCER SURVIVORS: A COMMUNITY-BASED QUALITATIVE ASSESSMENT

Marquita Lewis-Thames[1], Shawn Trimble[2], and Francesca Rosen[2], *1. Northwestern Feinberg School of Medicine, Chicago, Illinois, United States*, *2. Northwestern University Feinberg School of Medicine, Chicago, Illinois, United States*

In a nationwide survey, rural clinic uptake of telehealth utilization increased by 70% compared to pre-pandemic utilization. Despite advancements in tele-delivered cancer care for rural residents, older adults are slower to engage the technology; yet examinations of the challenges rural older cancer survivors face when incorporating telehealth into complex cancer care are limited. Guided by the Andersen Healthcare Utilization Model, we qualitatively examined barriers and facilitators of telehealth utilization for rural older cancer survivors. Ongoing interviews began in March 2022. We purposively sampled telehealth-using rural older cancer survivors, caregivers of rural older cancer survivors, and healthcare professionals that served this population. The interview guide probed participants regarding telehealth utilization for cancer and survivorship care. We used thematic analysis and deductively coded to the Andersen Model constructs. Preliminary findings are based on six rural older cancer survivors, caregivers, and healthcare professionals. Most participants were Non-Hispanic White, had at least some college, traveled greater than 31 minutes to an oncologist, and used their cell phone or computer to access telehealth. We coded similar perceptions between survivors, caregivers, and healthcare providers. Emergent themes included (1) Telehealth provided critical and necessary services, (2) Existing technological literacy and infrastructure enabled telehealth uptake, (3) Geographic isolation related to the rural environment impaired participant's ability to access telehealth-delivered care, and (4) Real-time provider-patient communication at critical points in participants' care facilitated telehealth utilization. Overall, participants reported that telehealth was useful for overcoming barriers common to rurality and often a suitable strategy to deliver cancer care.

Abstract citation ID: igad104.0752

### LEVERAGING THE COMMUNITY AS PARTNERS TO ADVANCE CANCER RESEARCH IN SOCIALLY DIVERSE OLDER ADULT POPULATIONS

Shakira Grant, *United States Congress, Washington, District of Columbia, United States*

Older Black adults with cancer suffer a “double disadvantage” to their health due to the compounding effects of ageism and racism. This “double disadvantage” further fuels the disparities in cancer-related mortality observed for this population. For example, multiple myeloma (MM) is an incurable and debilitating blood cancer that predominantly affects older Black adults. Despite overall improvements in MM-related survival observed following the introduction of novel therapies, older Black persons with MM continue to die at twice the rate of their White counterparts. This survival gap is largely driven by differential healthcare access and disappears when older Black adults are given equal access to care. Using MM as a chronic disease model, we are partnering with a community advisory board to design and pilot test a community lay navigator program that seeks to address some of the upstream determinants of healthcare access that limit how older Black adults newly diagnosed with MM perceive the need for, seek, reach and engage with the healthcare system. Through our foundational qualitative work focused on older North Carolinians (patients and caregivers) affected by MM, we have identified the following multilevel (individual, interpersonal, and community level) intervention targets: trust, knowledge and health beliefs, availability and adequacy of social support, and patient empowerment. In addition, we are leveraging the resources of an established core (Rapid Case Ascertainment) housed within our Comprehensive Cancer Center and linked to the statewide N.C. Central Cancer Registry to facilitate early identification of eligible participants (within 4-8 weeks of diagnosis) and patient outreach.

Abstract citation ID: igad104.0753

### USING A COMMUNITY ADVISORY BOARD TO DEVELOP A TELEHEALTH CONCIERGE FOR RURAL OLDER CANCER SURVIVORS

Revika Singh[1], Francesca Rosen[2], and Marquita Lewis-Thames[3], *1. Northwestern University, Chicago, Illinois, United States*, *2. Northwestern University Feinberg School of Medicine, Chicago, Illinois, United States*, *3. Northwestern Feinberg School of Medicine, Chicago, Illinois, United States*

Compared to pre-pandemic telehealth utilization, rural telehealth utilization has increased 1700% for some rural healthcare facilities. Yet, rural older adults remain slower to adopt telehealth, partly due to technical knowledge barriers. To identify practical solutions to overcome these navigation barriers for rural older adults, perspectives about telehealth implementation by older adults are necessary but remain a gap in research. To this end, this project adopts a user-centered design approach to develop a relevant, end-user-informed telehealth navigation tool for rural older cancer survivors with cancer-related distress through a community advisory board (CAB). We identified and selected CAB members from May-September 2022 from community-based organizations (CBOs) serving rural counties and zip codes in northern and central Illinois. CBOs were identified through internet search or snowball recruitment. Operating CBOs and interested community members with rural health interests received an email or telephone call about the CAB to review the roles and responsibilities of the CAB and confirm their interests. To date, over 30 CBOs in rural Illinois were identified, contacted, and mapped according to their rural health resources. We conducted two in-person and three virtual orientations. We identified one mental health professional, one older cancer survivor, one community member, three social workers/patient navigators to join the CAB. Preliminary barriers to telehealth utilization identified through ongoing CAB meetings include inadequate/inaccessible internet infrastructure, variable technical literacy, and concerns about trust-building and confidentiality. Ultimately, community advisory boards can be an effective approach in bridging community and academic partners to improve telehealth navigation for older rural adults.

Abstract citation ID: igad104.0754

### IT'S A FAMILY AFFAIR: ADDRESSING THE NEEDS OF BLACK DEMENTIA CAREGIVERS THROUGH COMMUNITY-BASED RESEARCH

Dianne Oladejo[1], Joyce Chapman[2], Tonya Roberson[3], and Rachel O'Conor[1], *1. Northwestern University, Chicago, Illinois, United States*, *2. Far South Chicago Coalition, Chicago, Illinois, United States*, *3. Governors State University, Chicago, Illinois, United States*

Racial disparities exist in dementia diagnosis, co-morbid health conditions, and socioeconomic characteristics between older Black and White adults, compounding caregiving responsibilities for Black families; yet few dementia caregiving programs offer culturally competent, family-centered dementia caregiving programs for Black individuals. In a collaboration between two community-based organizations (CBO) and an academic medical center, a culturally tailored, family-centered, dementia caregiving program for Black individuals on Chicago's Far South Side was proposed. Local CBOs convened community members to co-design a key informant interview guide and conducted 30 interviews among Black dementia caregivers. The caregivers interviewed were primarily caring for parents or grandparents and shared caregiving responsibilities with other family members. Caregivers recommended the program focus on five top concerns related to their caregiving experience: 1) feelings of stress and burden while being committed to their family member with dementia, 2) education on the progression of dementia and discussion of cultural beliefs, 3) personal experiences with racism and intersectional identities, 4) kinship responsibilities and managing dementia-specific changes that occur with loved ones, and 5) a need for respite and local medical and community-based resources. Anticipated barriers to participation in this program included time, an accessible location to members of the far south, and care for family members during participation. Though a community-directed approach, we elevated Black dementia caregiver experiences to better understand the influence that race and culture has on Black caregivers, which will provide the foundation for a Black family-centered dementia caregiving program to build capacity in routine caregiving and reduce caregiver stress.

Abstract citation ID: igad104.0755

### A COMMUNITY-ENGAGED RESEARCH JOURNEY: USING HOME REPAIRS TO PROMOTE HEALTH EQUITY AMONG OLDER ADULTS

Sarah Filec[1], Gail Schechter[2], Mike Laz[2], and Rachel O'Conor[3], *1. Feinberg School of Medicine, Northwestern University, Chicago, Illinois, United States*, *2. Housing Opportunities & Maintenance for the Elderly, Chicago, Illinois, United States*, *3. Northwestern University, Chicago, Illinois, United States*

Homeownership is a primary mechanism to build wealth. The majority of older Chicagoans own their home, but stark racial inequities exist due to discriminatory lending and purchasing practices. Homeownership requires regular maintenance, but low-income older adults are often forced to defer maintenance for financial reasons, which can lead to hazardous living environments, falls, and the need to move. Intuitively, housing conditions play a pivotal role in enabling older adults to continue to live in their home safely, yet few studies have examined the independent benefit of home repairs towards preserving homeownership and improving older adults' health. In 2019, stakeholders from community based organizations (CBO) and academic researchers convened a partnership identifying shared research goals and priorities focused on the impact of housing support services on older adult health. Over the past four years, we formalized a partnership and through iterative conversations with experts in aging and housing, and older adults with lived experience, our team identified a need to evaluate home repairs and their impact on 1) health outcomes, and 2) preserved homeownership and independent living for older adults. Our team iteratively co-developed a pilot study, with all stakeholders (older homeowners, CBOs, researchers) informing the methodology and research questions. In January 2023, we collectively launched our research pilot to evaluate the impact of a home repair program on health outcomes and homeownership preservation among predominantly Black low-income older adults in Chicago. Together we established a trusted partnership that will continue to address root causes of health inequities of older adults.

### SESSION 2360 (SYMPOSIUM)

Abstract citation ID: igad104.0756

### SYNTHESIS AND APPLICATION OF QUANTITATIVE METHODS FOR MODELING CHANGE IN MULTIMORBIDITY AND RELATED HEALTH DOMAINS

Chair: Nicholas Bishop Co-Chair: Corey Nagel Discussant: Ana Quiñones

The longitudinal examination of multimorbidity (≥ 2 chronic conditions) and related health outcomes (e.g., cognition, disability, mortality) has the potential to identify disparities and intervention points in the age-related progression of chronic disease. This symposium will organize discussion of methodological and applied research projects that review and demonstrate the use of longitudinal methods to address critical questions in the study of multimorbidity and related health domains. The lead presentation "Longitudinal Methods for Assessing Progression of Multimorbidity and Related Health Outcomes" will review the use and limitations of several longitudinal methods that can be used to study the progression of multimorbidity and linked health outcomes. Following presentations will demonstrate the application of specific longitudinal methods to key research questions in the study of multimorbidity. "Emerging Cohort Disparities in Multimorbidity in U.S. Adults Entering Early Older Adulthood" presents evidence of cohort disparities in multimorbidity trajectories using mixed-effects regression to estimate age, period, and cohort effects, "Personalized and Typical Concurrent Risk: Joint Models of Recurrent Outcomes and Mortality by Race/Ethnicity" applies joint models of self-rated health, disability, and mortality to examine disparities among minoritized groups, and "Sequence Analysis of Cardiometabolic Multimorbidity and Association With Subsequent Dementia" identifies common patterning of cardiometabolic conditions over a 5-year period and their relationship with prospective dementia classification. This symposium will encourage attendees to apply the presented methods to their own multimorbidity research questions and demonstrate how these methods are being applied to address key gaps in our understating of multimorbidity progression in older adulthood. This is a Measurement, Statistics, and Research Design Interest Group Sponsored Symposium.

Abstract citation ID: igad104.0757

### LONGITUDINAL METHODS FOR ASSESSING PROGRESSION OF MULTIMORBIDITY AND RELATED HEALTH OUTCOMES

Nicholas Bishop[1], Corey Nagel[2], Anda Botoseneanu[3], Heather Allore[4], Jason Newsom[5], David Dorr[6], and Ana Quiñones[7], *1. University of Arizona, Tucson, Arizona, United States*, *2. University of Arkansas for Medical Sciences, Little Rock, Arkansas, United States*, *3. University of Michigan, Ann Arbor, Michigan, United States*, *4. Yale University, New Haven, Connecticut, United States*, *5. Portland State University, Portland, Oregon, United States*, *6. Oregon Health & Science University, Portland, Oregon, United States*, *7. Oregon Health and Science University, Portland, Oregon, United States*

The growing body of longitudinal research on multimorbidity (≥ 2 chronic conditions) has identified that multimorbidity progression in mid and late life is associated with change in other health domains (e.g., mobility limitations) and can have far-reaching effects on person-centered outcomes such as health-related quality of life. Several increasingly accessible quantitative methods can be applied to the longitudinal study of multimorbidity and related health outcomes, but there has been relatively little work describing and synthesizing the merits and limitations of methods available for this purpose. We will discuss the application and limitations of popular longitudinal methods that can be applied to the study of change in multimorbidity and associated health outcomes. We will consider overarching issues in the longitudinal study of multimorbidity including operationalizing multimorbidity, data-related concerns such as measurement invariance, and choice of time scale (i.e., change in age or calendar time). We will then cover methods aligned to four specific research objectives: 1) examining individual change in multimorbidity, 2) identifying population sub-groups that follow similar trajectories of multimorbidity progression, 3) understanding when, how, and why individuals/groups shift to more advanced stages of multimorbidity, and 4) examining the co-progression of multimorbidity with key health domains over time. This work will help establish a common language for assessing longitudinal changes in multimorbidity, inform research that can fill gaps in our knowledge regarding multimorbidity progression and critical periods of change, and support the identification of groups that experience divergent rates and etiological pathways of disease progression.

Abstract citation ID: igad104.0758

### SEQUENCE ANALYSIS OF CARDIOMETABOLIC MULTIMORBIDITY AND ASSOCIATION WITH SUBSEQUENT DEMENTIA

Corey Nagel[1], Heather Allore[2], Anda Botoseneanu[3], Jeffrey Kaye[4], Jason Newsom[5], Nicholas Bishop[6], and Ana Quiñones[4], *1. University of Arkansas for Medical Sciences, Little Rock, Arkansas, United States*, *2. Yale University, New Haven, Connecticut, United States*, *3. University of Michigan, Ann Arbor, Michigan, United States*, *4. Oregon Health & Science University, Portland, Oregon, United States*, *5. Portland State University, Portland, Oregon, United States*, *6. University of Arizona, Tucson, Arizona, United States*

Sequence analysis is used in the social sciences to examine patterns of events occurring across the life course, but there are few examples of its use in multimorbidity research among older adults. We used sequence analysis to identify longitudinal patterns of cardiometabolic multimorbidity over a five-year period among participants in the National Health and Aging Trends Study (N=5,218). Multimorbidity sequences were constructed using self-reported diagnosis of diabetes, heart disease, stroke, and myocardial infarction (MI) assessed annually. Death was included as an absorbing state, yielding a total of 281 distinct sequences. We calculated sequence dissimilarity using optimal matching then used hierarchical clustering to identify seven distinct sequence clusters. The largest cluster (46.2%) was characterized by no baseline cardiometabolic disease and minimal incident disease across the 5-year period. Three clusters were characterized by stable sequences: diabetes (13.1%), heart disease (7.5%), and MI or stroke (7.3%) across the 5-year period. Two clusters exhibited a high rate of incident cardiometabolic disease during the 5-year period, one among persons with no baseline disease (9.6%) and one with rapid accumulation of cardiometabolic multimorbidity (5.3%). Finally, one cluster largely contained persons who died during the study period (11.0%). Compared to those with no baseline and minimal incident cardiometabolic disease, the odds of subsequent dementia were significantly higher among the cluster without prior disease who developed incident cardiometabolic disease (OR= 1.61, 95% CI:1.07,2.43) and the cluster with high cardiometabolic multimorbidity (OR=2.77, 95% CI:1.84,4.18). These findings contribute to our understanding of the impact of cardiometabolic multimorbidity on cognitive health.

Abstract citation ID: igad104.0759

### PERSONALIZED AND TYPICAL CONCURRENT RISK: JOINT MODELS OF RECURRENT OUTCOMES AND MORTALITY BY RACE/ETHNICITY

Heather Allore[1], Gail McAvay[1], Brent Vander Wyk[1], Ling Han[2], and Ana Quiñones[3], *1. Yale University, New Haven, Connecticut, United States*, *2. Yale School of Medicine, New Haven, Connecticut, United States*, *3. Oregon Health and Science University, Portland, Oregon, United States*

The growing burden of multiple chronic conditions documented by a U.S. Department of Health and Human Services initiative noted the importance of studies that 1) "Develop tools to identify and target population subgroups of individuals with multiple chronic conditions who are at high risk for poor health outcomes", and 2) "Improve knowledge about patient trajectories temporally in relation to changes in health status, functional status, and health services use". Expanding upon our methods for jointly modeling multiple outcomes, where each person's shared influence on concurrent outcomes can be captured by using a random intercept term, we demonstrate how to model additional heterogeneity. Based on the estimates from the joint model, the typical concurrent risk (TCR) of each outcome (i.e., probability) can be estimated at the cohort level, providing a method for identifying average longitudinal effects of risk factors useful for healthcare system policies. In contrast, a person-specific effect reflects the probability of each outcome within a defined interval of time, while currently at risk for another non-mutually exclusive outcome (i.e., personalized concurrent risk [PCR]). Chronic conditions and sociodemographic risk factors may partially account for the heterogeneity of a cohort. However, differences in the occurrences and associations among these may differ for minoritized groups. These methods identify persons above (or below) average risk. We expanded these joint models to address truncation of measurement due to death and the interdependence of outcomes at the person-level, while maintaining the Type I error rate. We have made the code publicly available.

Abstract citation ID: igad104.0760

### EMERGING COHORT DISPARITIES IN MULTIMORBIDITY IN US ADULTS ENTERING EARLY OLDER ADULTHOOD

Nicholas Bishop[1], Steven Haas[2], and Ana Quiñones[3], *1. University of Arizona, Tucson, Arizona, United States*, *2. Penn State, University Park, Pennsylvania, United States*, *3. Oregon Health and Science University, Portland, Oregon, United States*

Recent evidence of generational disparities in multimorbidity risk among older adults indicates earlier transitions into multimorbidity and greater disease burden for those born to later cohorts. As costs of managing chronic disease will rise with population aging, it is imperative to identify whether cohort effects in multimorbidity extend to cohorts just entering early older adulthood. We use representative data from the Panel Study of Income Dynamics to model cohort trends in multimorbidity from 1999–2019 in adults who were 50 years of age or older at any observational period (n = 4,975; 40,822 person-observations). Multimorbidity was assessed as a count of nine chronic conditions (stroke, hypertension, diabetes, cancer, lung disease, heart problems, emotional problems, arthritis, mental loss). Weighted Poisson mixed models were used to estimate multimorbidity trajectories adjusted for fixed effects of cohort, linear and quadratic age, and sociodemographic covariates. The model accounted for repeated measures though inclusion of a random effect for within-person error covariance across wave. After adjustment, those born to the Early and Late Baby Boom cohorts (b. 1946–1955 and b. 1956–1964, respectively), as well as those born to the early Gen X cohort (b. 1965–1972), reported significantly greater multimorbidity burden than those born to the late depression-era (b. 1931–1942). These findings extend evidence of cohort effects in multimorbidity in U.S. older adults, indicate these problematic trends apply to U.S. adults entering early older adulthood, and suggest practitioners and policymakers should prepare for an aging population with expanding disease burden.

### SESSION 2365 (SYMPOSIUM)

Abstract citation ID: igad104.0761

### MEASUREMENT MATTERS IN EPIDEMIOLOGIC STUDIES OF SENSORY HEALTH AND AGING

Chair: Jennifer Deal Co-Chair: Bryan James Discussant: Alison Abraham

Valid and reliable measurement is a cornerstone of epidemiologic data collection and analysis. Sensory health is important for maintaining cognitive, physical and social function with age, but multiple measures of sensory health exist, and not all are the same. For example, audiometric hearing loss reflects the cochlea's ability to process sound, while self-report captures an individual's perception of their hearing ability within their social and environmental context. How we measure sensory function has important ramifications for prevalence estimates and results from association studies, and therefore, public health and clinical decision-making. Sensory loss may also have important implications for the measurement of other factors in aging research. For example, neurocognitive tests are administered aurally or on paper, which could present a challenge for older adults with sensory loss. This session, jointly sponsored by the Sensory Health and Epidemiology of Aging Special Interest Groups, focuses on the intersection of measurement and sensory health. We will report on vison and hearing measures in a large, nationally representative study of Medicare Beneficiaries, with a focus on the recent addition of objective measures of vision and hearing loss, and their implications for sensory loss prevalence estimates, particularly for the oldest old. We will also discuss how sensory loss impacts cognitive testing, including best practices for measuring cognitive function in older adults. Finally, we will present on how choice of hearing measure (self-report vs. audiometry) has important ramifications for population attributable fraction estimates, with direct implications for public health intervention for dementia prevention. This is a collaborative symposium between the Epidemiology of Aging and Sensory Health Interest Groups.

Abstract citation ID: igad104.0762

### MEASURING COGNITIVE FUNCTION IN OLDER ADULTS WITH SENSORY LOSS

Jennifer Deal[1], Emma Nichols[2], Alden Gross[3], and Bonnielin Swenor[1], *1. Johns Hopkins University, Baltimore, Maryland, United States*, *2. University of Southern California, Los Angeles, California, United States*, *3. Johns Hopkins University Bloomberg School of Public Health, Baltimore, Maryland, United States*

Neurocognitive batteries are administered orally or on paper, requiring participants to hear and see to participate; yet 55% of Americans aged 60+ years have hearing or vision loss. We will review 3 studies investigating whether sensory loss could bias cognitive testing. First, we used item response theory to test whether cognitive testing is more difficult (biased) for individuals with vs. without sensory loss, controlling for underlying cognitive function using Atherosclerosis Risk in Communities (ARIC) Study and Baltimore Longitudinal Study on Aging data. Although differential item functioning exists for some tests, it is not salient (>1 standard error difference), suggesting that sensory loss may not have a strong impact on measuring cognitive function in well-designed and conducted research studies. Second, we described how audiometric hearing loss impacts completion of neurocognitive testing, and how missing test scores affect estimates of the hearing loss-cognitive test performance association in a cross-sectional analysis of 3,678 adults (72-94 years) in ARIC. Hearing loss was associated with greater missingness on two auditory-only tests (Logical Memory, Digits Backwards) and two written tests (Boston Naming, Trail Making B). Ignoring this missing data underestimated the hearing-cognition association. Finally, we identified cohort studies measuring cognition in older adults through systematic review, and surveyed investigators for how sensory health was addressed in their studies. We found variation in methods used to assess sensory loss, with implications for resource allocation. Using these studies, we will discuss how to best measure cognition in adults with sensory loss, with a focus on inclusion and equity.

Abstract citation ID: igad104.0763

### HEARING LOSS AMONG OLDER ADULTS IN THE NATIONAL HEALTH AGING TRENDS STUDY

Nicholas Reed, *Johns Hopkins Bloomberg School of Public Health, Baltimore, Maryland, United States*

Hearing loss is an important condition that affects healthy aging. However, nationally representative estimates of hearing loss among older adults are limited due to differences in measures and categorization of hearing as well as limited sampling of older adults. The aims of this presentation are to review the meaning of different hearing measures and categorization and to estimate the prevalence of hearing loss by age and demographic covariates of adults over 70 years. The National Health and Aging Trends Study (NHATS) is a large nationally representative panel study of Medicare beneficiaries aged 65 and older with robust oversampling of the oldest old adults that added pure-tone audiometry, the current gold standard for quantifying hearing loss to the 2021 NHATS round 11 protocol. Cross-sectional (n=2620) weighted prevalence estimates by age, sex, race/ethnicity, education, and income were applied to corresponding United States Census Bureau estimates. Using traditional definitions of hearing loss, an estimated 67.9% (22.4 million) of adults over 70 years have at least some degree of hearing loss (37.4% mild, 25.8% moderate, and 4.6% severe). Prevalence is higher among White, male, lower-income, and lower education attainment subpopulations and increases with age such that 97.6% of adults 90 years and older have hearing loss. Application of new World Health Organization categories of hearing loss resulted in an increase in prevalence from 67.9% to 82.7% of adults over 70 years. The near ubiquitous nature of hearing loss in this population raises questions about identifying actionable cut points in future research.

Abstract citation ID: igad104.0764

### NEW MEASURES AND POPULATION PREVALENCE OF VISION IMPAIRMENT IN NHATS

Niranjani Nagarajan, and Joshua Ehrlich, *University of Michigan, Ann Arbor, Michigan, United States*

The National Health and Aging Trends Study (NHATS) is a nationally representative panel study of older U.S. adults that in 2021 (Round 11) added to its annual protocol a suite of objective visual function measures consisting of distance visual acuity, near visual acuity, and contrast sensitivity tests. These measures were developed to approximate clinical gold-standard tests, while allowing for administration on a tablet in participants' homes. The tests were found to have good agreement with clinical gold-standards based on their limits of agreement, correlations, and lack of systematic bias between tests. In the initial 2021 fielding of the NHATS visual function measures, 27.8% (25.5%-30.1%) of U.S. adults over age 70 had one or more visual impairment (VI) (distance VI: 10.3% [8.9%-11.7%]; near VI: 22.3% [20.3%-24.3%]; contrast sensitivity impairment: 10.0% [8.5%-11.4%]), and the prevalence was highest among those who were older, had less education, lower income, and were of Hispanic ethnicity and non-White race. Additionally, the adjusted prevalence ratio for dementia was significantly higher among those with each type of VI compared to those with normal vision (moderate/severe distance VI=1.72 [1.26-2.35]; any near VI=1.40 [1.16-1.69]-; contrast sensitivity impairment =1.31 [1.04-1.66]). Since few other national studies of older adults include objective visual function measures, NHATS now serves as a vital resource for the study of vision epidemiology and the impact of VI and blindness on later-life health outcomes and trajectories.

Abstract citation ID: igad104.0765

### POPULATION ATTRIBUTABLE FRACTIONS FOR THE CONTRIBUTION OF HEARING LOSS ON DEMENTIA RISK ACROSS COHORT STUDIES

Jason Smith[1], Emily Burg[2], A. Richey Sharrett[1], Nicholas Reed[1], Frank Lin[3], Josef Coresh[4], Jennifer Deal[4], and Emily Ishak[5], *1. Johns Hopkins Bloomberg School*

*of Public Health, Baltimore, Maryland, United States*, 2. *University of Wisconsin-Madison, Madison, Wisconsin, United States*, 3. *Johns Hopkins, Baltimore, Maryland, United States*, 4. *Johns Hopkins University, Baltimore, Maryland, United States*, 5. *Columbia University Irving Medical Center, New York City, New York, United States*

Estimates range from 2-8% of dementia cases in the US are attributable to hearing loss. Here, we investigate measurement considerations in quantifying the population attributable fraction (PAF) of dementia from hearing loss to highlight how differences in methodology can underestimate the PAF. We use two cohort studies of older adults in the US: the National Health and Aging Trends Study (NHATS), a nationally-representative sample of older Medicare beneficiaries; and the Atherosclerosis Risk in Communities (ARIC) Study, a large, community-based cohort study with longitudinal follow-up. Each study collects reference-standard hearing measures on participants. We have two specific aims. First, we compare two valid, formula-based approaches to quantifying the PAF. The advantages of quantifying a PAF using a well-characterized, biracial, community-based sample with a longitudinal design are weighed against those of using a nationally-representative sample with a cross-sectional design (moderate or greater hearing loss: PAF in NHATS, 16.9% [95% CI: 4.1-28.7%]; PAF in ARIC, 13.2% [95% CI: 2.2-20.9%]), highlighting the need for longitudinal designs in studies of the PAF from hearing loss. Second, we describe the impact of subjective versus objective measures of hearing loss on the PAF. In ARIC, subjective hearing loss was not associated with incident dementia (hazard ratio <1.0). PAFs of dementia from objective hearing loss were 6- to 8-fold greater than PAFs previously quantified using self-reported hearing measures in population-based studies. Our findings have broad implications for the inclusion of hearing loss as a potentially modifiable risk factor in clinical and public health initiative for dementia prevention.

### SESSION 2370 (SYMPOSIUM)

Abstract citation ID: igad104.0766

### DEMENTIA-FRIENDLY ENVIRONMENTS IN THE COMMUNITY – AN INTERNATIONAL PERSPECTIVE ON CONCEPTS AND INTERVENTIONS

Chair: Karin Wolf-Ostermann Co-Chair: Hilde Verbeek
Discussant: Marie Boltz

Dementia is one of the major age-related societal challenges and causes enormous demands for persons living with dementia (PlwD). Although dementia is one of the major reasons for relocation into a nursing home, most PlwD want to live as long as possible in a familiar domesticity. Therefore, innovative care concepts for community-dwelling PlwD are being developed worldwide as alternative to traditional long-term care settings. Efforts are made to learn more about dementia-friendly environments in order to improve quality of care and quality of life of PlwD. This international symposium will therefore provide four presentations as well as a concluding discussion addressing new concepts for innovative care environments and measuring dementia-friendliness of the built environment as well as nonpharmacological interventions to support high quality person-centred care. The first presentation explores the functions of 24-hour green care farms as alternative to nursing homes and how these can be transferred to other care settings. The second presentation discusses the impact of moving from traditional care environments to innovative care settings for residents and staff. The third presentation presents results of a cluster randomized controlled trial in German shared housing arrangements where a complex nonpharmacological intervention led to significantly less hospital admissions for PlwD. And the final presentation describes an innovative approach to evaluate the wayfinding infrastructure in urban areas for PlwD and to promote dementia-friendliness in urban living structures. The discussant will synthesize the research findings and lead a discussion of research implications and future directions for policy and practice.

Abstract citation ID: igad104.0767

### REDUCING HOSPITAL ADMISSIONS FOR PEOPLE LIVING WITH DEMENTIA—RESULTS FROM THE GERMAN DEMWG-STUDY

Karin Wolf-Ostermann[1], Annika Schmidt[1], Susanne Stiefler[1], Janissa Altona[1], Andre Kratzer[2], Carolin Donath[2], and Elmar Graessel[3], *1. University of Bremen, Bremen, Bremen, Germany*, *2. Universitätsklinikum Erlangen, Erlangen, Bayern, Germany*, *3. Psychiatrische Universitaetsklinik Erlangen, Erlangen, Bayern, Germany*

People living with dementia (PlwD) have a high risk of being admitted to hospital, especially in advanced stages of dementia – with often severe negative consequences. The DemWG-study therefore aimed to reduce the number of hospital admissions for long-term care dependent PlwD by a complex intervention - including motor and cognitive training for PlwD and education of caregivers and general practitioners. By means of a cluster randomised longitudinal design, data on hospital admissions as well as quality of life, risk of falls, challenging behaviours and cognitive functioning were assessed in 97 shared-housing arrangement (SHA) in Germany with a total of 341 PlwD at baseline and after a 6 months intervention. Results of the of longitudinal data analysis showed a significant decline in hospital admissions for the intervention period and less challenging behaviours. Quantitative and additionally qualitative data were also collected 12 and 18 months after baseline. Caregivers perceived improved social interaction, which in turn has a positive effect on living together in the SHA. Based on the results and in light of the urgent need for easy to implement non-pharmacological and/or psychosocial therapy approaches recommendations for better care and support can be derived and the intervention can be implemented in everyday practice.

Abstract citation ID: igad104.0768

### WHAT CAN OTHER DEMENTIA CARE SETTINGS LEARN FROM GREEN CARE FARMS? AN EXPLORATION OF THEIR FUNCTIONS AND FORMS

Katharina Rosteius, Bram de Boer, Gijs Steinmann, and Hilde Verbeek, *Maastricht University, Maastricht, Limburg, Netherlands*

Green Care Farms (GCFs) are an innovative long-term care alternative in dementia. They aim to better meet residents' needs by integrating nature and animals into daily life. This study explores the functions of GCFs and possible forms of realizing them in practice. A multiple case study using ethnographic methodology was conducted on two sites: one original GCF established for seven years and one regular nursing home redesigned and recently opened aiming to apply GCF characteristics. Participatory observations (n=58), interviews (n=64), a focus group, an environmental assessment and expert panels were conducted. Six functions of GCFs were identified, crucial for the successful execution of their innovative vision in practice: providing opportunities for purposeful activities, stimulating residents, creating a community, sharing responsibilities, integrating the vision in all actions and continuously transforming. These functions may take several forms, which could be implemented in regular care, taking into account the specific local context.

Abstract citation ID: igad104.0769

### EXPERIENCES OF STAFF MEMBERS RELOCATING FROM A NURSING HOME TO AN INNOVATIVE LIVING ARRANGEMENT FOR OLDER ADULTS

Mara Brouwers[1], Bram Boer[2], Wim Groen[3], and Hilde Verbeek[2], *1. Maastricht university, Maastricht, Limburg, Netherlands*, *2. Maastricht University, Maastricht, Limburg, Netherlands*, *3. Amsterdam UMC, Amsterdam, Noord-Holland, Netherlands*

More innovative living arrangements are developed within long-term care, leading to an increase in relocations. Relocating within long-term care is a long pending and intensive undertaking, especially for staff members. The combination of relocating and achieving a successful culture change might be a daunting task for staff members. Given the increase in the number of relocations and the fast development of innovative living arrangements, it is key to expand our knowledge concerning the impact such an undertaking has. The research question is therefore: 'How is the relocation from a regular to an innovative living arrangement experienced by staff members?' To answer this research question, 41 semi-structured interviews with staff members of several nursing homes undergoing a relocation were conducted. These nursing homes had to undergo a relocation to an innovative living arrangement. Findings indicate that a relocation process within long-term care is an intensive and stressful undertaking. Staff members often describe the relocation process as 'stressful', 'chaotic', and 'intense'. Furthermore, although staff members are mostly positive about the new physical environment, they experience an array of barriers when trying to implement a new way of working. They define two key elements for successfully relocating and implementing a new way of working. First, clear communication and receiving sufficient information throughout the relocation process. Second, proper preparation for implementing a new way of working and coaching on the job. It is evident that relocating to an innovative living arrangement is a large undertaking that requires careful planning.

Abstract citation ID: igad104.0770

### EVALUATING WAYFINDING INFRASTRUCTURES FOR PEOPLE LIVING WITH DEMENTIA—THE 360 DEGREE MAPS STUDY DENHB

Janissa Altona[1], Henrik Wiegelmann[2], Emily Mena[2], Julia Misonow[2], Benjamin Schuez[2], and Karin Wolf-Ostermann[2], *1. University of Bremen, Institute of Public Health and Nursing Research, Bremen, Bremen, Germany*, *2. University of Bremen, Bremen, Bremen, Germany*

Recent studies point to the importance of the built environment for the cognitive and social health of community-dwelling people living with dementia. One key aspect is wayfinding infrastructure, especially in urban areas. However, currently there are no clear indicators of wayfaring infrastructure and no standardized assessment instruments. Here, we provide such an instrument with the potential for online digital assessment as the result of the German DenHB-study. Based on an umbrella review and further research, we first developed a questionnaire to evaluate local wayfinding structures which are sectioned in different thematic blocks like general streetscape, signage, public transportation, buildings etc. Second, we conducted a cross-sectional validation study in the urban area of Bremen, Germany using Apple Maps Street View (2022) with a 360-degree viewing angle at more than 3,000 randomly selected geo coordinates within urban districts with a high proportion of older residents (>65 years). The results document supportive as well as inhibiting wayfinding structures from the perspective of people living with dementia and can also form a basis for a later automated digital evaluation of wayfinding structures. Hence, providing easy to obtain new insights helps to support the development of actions to promote the dementia-friendliness of urban living structures.

### SESSION 2375 (PAPER)

### LONG-TERM CARE AND WORKFORCE

Abstract citation ID: igad104.0771

### ASSESSING STAFF PERCEPTIONS OF WORKPLACE SAFETY IN NURSING HOMES: ITEM SET DEVELOPMENT AND TESTING

Katarzyna Zebrak[1], Naomi Yount[1], Joann Sorra[2], Laura Gray[1], Jessica Kirchner[1], and Joanne Campione[3], *1. Westat, Rockville, Maryland, United States*, *2. Westat, Westat, Maryland, United States*, *3. Westat, Durham, North Carolina, United States*

Workplace safety is important for advancing resident safety and eliminating harm to both nursing home staff and residents. The goal of this study was to develop and test survey items to assess how the organizational culture in nursing homes supports workplace safety for staff. After conducting a literature review and background interviews with nursing home staff and workplace safety experts, we identified important areas of workplace safety culture (i.e., workplace hazards; moving/transferring/lifting residents; inappropriate resident behavior toward staff; interactions among staff; supervisor/management support for workplace safety; workplace safety reporting; and, work stress/burnout) and drafted survey items to assess each area. These draft items were administered with the AHRQ Surveys on Patient Safety Culture (SOPS) Nursing Home Survey to staff in 48 U.S. nursing homes. We conducted psychometric analysis on data from 2,468 respondents. Confirmatory factor analysis results and internal consistency and site-level reliability coefficients were acceptable for the 18 survey items grouped into 6 composite measures. All composite measures were significantly correlated ($p < 0.05$) with each other and with the overall rating on workplace safety, indicating adequate conceptual convergence among survey measures. On average, 33% of respondents reported symptoms of burnout. Higher burnout was significantly correlated with lower job satisfaction (rs=-0.42) and greater intent to leave the nursing home (rs=0.37). Nursing homes and researchers can use the Workplace Safety Supplemental Item Set to assess nursing home staff burnout and the dimensions of organizational culture that support staff safety, and to identify both strengths and areas for improvement.

Abstract citation ID: igad104.0772

### CARE FOR OLDER ADULTS: WHAT SKILLS DO HEALTH CARE EMPLOYERS VALUE IN COMMUNITY COLLEGE STUDENTS?

Runcie Chidebe[1], Phyllis Cummins[1], Donnette Narine[2], and Takashi Yamashita[3], *1. Miami University, Oxford, Ohio, United States*, *2. University of Maryland Baltimore, Baltimore, Maryland, United States*, *3. University of Maryland, Baltimore County, Baltimore, Maryland, United States*

Community colleges (CCs) play an important role in educating healthcare workers engaged in caring for aging populations with greater healthcare and long-term care needs. Healthcare employers and nursing home leaders are obligated to ensure that care provided by their employees meets quality standards, is patient-centered and free from psychological and physical harm. To increase the employability of CC students and understand the skills valued by employers in improving care for older adults, we explored the role literacy, numeracy, and problem-solving skills play in CC student success in the workplace. Using a qualitative descriptive design, we conducted semi-structured interviews with CC administrators (n=9), faculty (n=6), and employers (n=10) who hire healthcare workers (certified nursing assistants, licensed practical nurses (LPNs), and registered nurses (RNs)) educated at CCs. Our findings show that communication skills are highly valued, especially the ability to communicate on the level of the nursing home resident or hospital patient, along with their family members. Numeracy skills, such as measuring medication dosages, critical thinking skills, and problem-solving skills, including conflict resolution, emotional intelligence, and compassionate care are valued by employers. Employers reported that LPNs and RNs have limited leadership and supervisory skills and there is a need to use innovative mechanisms (e.g., case studies, online courses) to improve those skills. Serving on CC advisory councils, clinical rotations and employers' input into program development were reported as beneficial strategies. Findings from this project will inform both CC administrators and employers regarding skills that are valued in caring for older adults.

Abstract citation ID: igad104.0773

### CONTINUING CHALLENGES IN STATE EFFORTS TO IMPROVE DIRECT CARE WORKER WAGES

Denise Tyler[1], Melissa Hunter[2], Kristie Porter[2], Olga Khavjou[3], Marie Squillace[4], Judith Dey[5], and Iara Oliveira[6], *1. RTI International, Hancock, Maine, United States*, *2. RTI International, Durham, North Carolina, United States*, *3. RTI International, Raleigh, North Carolina, United States*, *4. Office of the Assistant Secretary for Planning and Evaluation, Washington, District of Columbia, United States*, *5. US Department of Health and Human Services, Washington, District of Columbia, United States*, *6. Office of Behavioral Health, Disability, and Aging Policy (BHDAP), Washington, District of Columbia, United States*

Direct care workers (DCWs) receive low wages and some states have tried to improve their wages through policy changes. This mixed methods study examined the effect of state wage policies on changes in DCW wages. We also conducted 38 interviews with national experts and stakeholders across six states to determine the results of these policy changes and continued barriers to improving DCW wages. Results show some states that implemented policies to improve the wages of DCWs since 2009 reduced the gap between DCW wages and the wages of other entry-level workers, yet the gap was still substantial in many states. Experts and state stakeholders repeatedly cited consistent funding of wage policies through Medicaid reimbursement rate increases as important to effectively increasing and sustaining higher wages. Interviewees recommended that funding increases be continual, rather than requiring annual reauthorization and noted it was particularly challenging when funds were inadequate to support annual wage increases. Interviewees described Medicaid reimbursement rates as a barrier when rates did not keep pace with market trends. Interviewees also recommended professionalizing the field, increasing the respect afforded these workers, and developing ways to increase the pipeline of workers as additional ways to improve direct care work. Until there is meaningful policy change, states will continue to struggle with barriers to improved wages for DCWs which results in a disproportionate number of DCWs relying on public benefits and difficulties with recruitment and retention.

Abstract citation ID: igad104.0774

### HOW CARE WORKERS' TURNOVER INTENTION IS ASSOCIATED WITH WORKPLACE VIOLENCE?: THE ROLE OF STRESS AND JOB SATISFACTION

Sung Hyun Ko, and Yeonjung Lee, *Chung-Ang University, Seoul, Republic of Korea*

This study examines the impact of workplace violence experienced by long-term care workers from the care recipients on their turnover intention and the mediating effect of work-related stress and job satisfaction in the relationship between workplace violence and turnover intention. Compared to the unfair experiences of abuses experienced by care recipients, care workers' experiences have been relatively under-explored. Adopting the affective events theory as a theoretical framework and using the Korean National Long-Term Care Survey in 2019, the current analysis focuses on 2,427 care workers, excluding social workers, nurses, and occupational/physical therapists. The results of structural equations modeling and effect decomposition show a mediating effect of job satisfaction, indicating that the experience of workplace violence decreased the job satisfaction of care workers, resulting in increased turnover intention. On the other hand, workplace violence did not have a statistically significant indirect effect on turnover intention through work-related stress. Findings provide several implications for developing an inclusive and supportive policy and practice for care workers as well as the long-term care system in general in South Korea. Support programs and policies can be targeted toward the care workers who have experienced workplace violence and are less satisfied with their job. These can be done not only by highlighting the importance of training and education for care workers to help prevent workplace violence but also by providing a safe work environment, an appropriate compensation system, and the fair opportunities for promotion.

Abstract citation ID: igad104.0775

### RECRUITING AND RETAINING NURSES AND FRONTLINE CARE WORKERS IN LONG-TERM CARE: THE REACH REALIST REVIEW

Reena Devi[1], Kirsty Haunch[1], Sonia Dalkin[2], Natalie King[1], Angela Bate[2], Karen Spilsbury[1], Claire Goodman[3], and Liz Jones[4], *1. University of Leeds, Leeds, England, United Kingdom*, *2. Northumbria University Newcastle, Newcastle, England, United Kingdom*, *3. University of Hertfordshire, Hertfordshire, England, United Kingdom*, *4. National Care Forum, Nottingham, England, United Kingdom*

**Introduction:** increasing demand for care, high staff turnover and low numbers of new staff are causing significant staff shortages in the long-term-care (LTC) sector. Solutions need to 1) take the heterogeneity of organisational and staff characteristics into account, and 2) make use of the large volume of literature describing the reasons staff join and stay in LTC work. Our research aimed to develop strategies which attract, recruit and retain Registered Nurses and frontline care workers in LTC; explain how and why strategies are effective, for which staff, the conditions needed, and the costs involved.

**Method:** This realist review involved (i) co-developing strategies and initial programme theories, (ii) systematically searching the evidence to test IPTs and develop theoretical propositions, and (iii) validating and refining propositions with stakeholder groups.

**Results:** IPTs around the following strategies were rated as important for attracting, recruiting and retaining staff in LTC, and taken forward to the testing phase of our review: rewarding and recognising staff, offering flexible working, career development opportunities, enhanced salary package, welcoming and inducting staff, listening to and providing staff feedback, developing caring work environments and effective staff interviewing. Leadership, caring cultures, teamwork, resources and adequate staffing levels are important conditions which enable these strategies lead to positive outcomes.

**Discussion:** this is the first study to use a realist approach to developing strategies focused on attracting, recruiting and retaining the LTC workforce. Our findings will be relevant at an international level. Acknowledgement: we thank Karen Winterburn and Edna Feenan for their important input throughout the project. They have both provided care home family perspectives, and have been involved throughout the development and execution of the project.

### SESSION 2380 (SYMPOSIUM)

Abstract citation ID: igad104.0776

### KUAKINI HHP CENTER FOR TRANSLATIONAL RESEARCH ON AGING: LATEST FINDINGS FROM MODEL ORGANISMS TO HUMANS

Chair: Bradley Willcox Co-Chair: Richard Allsopp
Discussant: Peter Martin

Kuakini Medical Center (Kuakini) was funded by NIH in late 2019 to create an interdisciplinary Hawai'i-based, Center of Biomedical Research Excellence (COBRE), for translational research on aging. This Center is building upon Kuakini's five-decades of prior NIH-funded research. These resources include clinical data from the 58-year ongoing Kuakini Honolulu Heart Program (HHP), Honolulu-Asia Aging Study, HHP Offspring Study and a large biorepository with over 500,000 biological samples. The Center's overarching aim is to increase infrastructure for collaborative aging research in Hawaii. The first step is to grow the Center's faculty by hiring and mentoring research project leaders (RPLs) from diverse disciplines to become independent, R01-funded, investigators on aging. Our first RPL has graduated after obtaining R01-funded status. His project utilizes novel CRISPR methods to i) improve the safety and efficacy of delivering potentially therapeutic genes (such as FOXO3) to the mouse genome, and ii) test whether temporal enhancement of FOXO3 expression improves healthy aging in this mouse model - both key steps for potential translation to human clinical therapies. This work will be highlighted in the Program Overview session followed by current RPL findings. These findings include novel findings on a potential relation between FOXO3 genotype and stroke dynamics in elderly Japanese-American males; venous congestion in the brain and its potential impact on cognitive function; new findings on the Okinawan longevity phenomenon, among other interesting findings related to healthy aging. Supported by NIGMS 5P20GM125526 and NIA R01AG027060.

Abstract citation ID: igad104.0777

### KUAKINI COBRE CONTRIBUTIONS FROM LONG-LIVED OKINAWA PREFECTURE: THE FIRST BLUE ZONE

D. Craig Willcox[1], Richard Allsopp[2], Makoto Suzuki[3], Michio Shimabukuro[4], Moritake Higa[5], Philip Davy[6], Bradley Willcox[7], and Trevor Torigoe[8], *1. Okinawa International University, Ginowan, Okinawa, Japan*, *2. University of Hawaii, Honolulu, Hawaii, United States*, *3. Okinawa Research Center for Longevity Science, urasoe, Okinawa, Japan*, *4. Fukushima Medical University, Fukushima, Fukushima, Japan*, *5. Tomishiro Central Hospital, Tomigusuku, Okinawa, Japan*, *6. Kuakini medical center, Honolulu, Hawaii, United States*, *7. Kuakini Medical Center, Honolulu, Hawaii, United States*, *8. University of Hawaii, honolulu, Hawaii, United States*

The Okinawa-Hawaii connection has a long history. The first group of immigrants to Hawaii from Okinawa arrived in 1899, and descendants of these immigrants make up approximately 20% of the Kuakini Honolulu Heart Program (HHP) cohort. Research in Okinawa, and among the Okinawan community in Hawaii, has provided many insights into the subtle differences in lifestyle and genetics among these populations. In order to gain further insight into the lifestyle and genetic factors associated with healthy aging and longevity, the Kuakini COBRE has built a strong collaborative team of researchers from Okinawa and initiated a number of research projects, as well as cross-Pacific training opportunities for COBRE junior investigators (Research Project Leaders). This presentation will outline new insights into healthy aging and longevity that have been discovered through this collaborative relationship. Our findings highlight the intricate relationship between FOXO3 and telomere length change with age, and include the first validated longevity gene variant that shows an association with negligible loss of telomere length with age in humans. This finding reveals that reduced telomere attrition with age may be a key mechanism for the longevity-promoting effect of the FOXO3 genotype. This presentation will also discuss sex differences that play a role in how, and under what conditions, FOXO3 exerts its role in healthy aging. Finally, future collaborative projects involving mitochondrial health and aging will be introduced.

Abstract citation ID: igad104.0778

### FOXO3 LONGEVITY GENOTYPE AND CEREBROVASCULAR DISEASE

Kazuma Nakagawa, *Kuakini Medical Center, Honolulu, Hawaii, United States*

The influence of the FOXO3-longevity associated genotype on cerebrovascular disease (CVD) is little studied. There are reports of a protective effect against stroke mortality in some populations. But these findings have not been widely replicated, nor have potential mechanisms been identified. This presentation will present recent, novel insights on the relation of FOXO3 genotype to CVD. We studied a cohort of over 800 American men of Japanese ancestry from the Kuakini Honolulu Heart Program (KHHP) and Kuakini Honolulu-Asia Aging Study (KHAAS). This population-based dataset included brain autopsy data and age-adjusted prevalence of various types of CVD, including intracerebral hemorrhage (ICH) and cerebral microinfarcts (CMI). Logistic regression models, adjusted for age at death, cardiovascular risk factors, FOXO3 and APOE-ε4 genotypes, among other risk factors were utilized to determine the predictors of both outcomes. FOXO3 genotype did not directly modify risk for ICH or CMI. However, the FOXO3 longevity genotype had significant interaction (protective) effects against hypertension-associated ICH and CMI. These data suggest that the longevity-associated FOXO3 G-allele mitigates the impact of hypertension on the risk of ICH and CMI. Further research is needed in other populations.

Abstract citation ID: igad104.0779

### PREVALENCE OF CEREBRAL VENOUS DRAINAGE STENOSES ON RETROSPECTIVELY ANALYZED CTA FOR STROKE TRIAGE

Ferdinand Hui, *Kuakini Medical Center, Honolulu, Hawaii, United States*

Cerebral venous congestion (CVC) has been identified as a hypothetic cause of cognitive impairment. Prior work has estimated that 15-30% of the population may have jugular venous narrowing, a potential cause of CVC, based on reviews of CT angiography (CTA) performed at a large academic medical center. However, few comprehensive studies exist. Therefore, for the current work, we reviewed fifty CTAs that were obtained for ischemic stroke triage and thrombectomy planning at a comprehensive stroke center in Hawaii (Queens Hospital, Honolulu HI). These brain CTAs were analyzed for arterial and venous variants and other abnormalities that might place the patient at increased risk for arterial injury and/or venous congestion, both of which may result in cognitive decline. The CTAs were analyzed retrospectively for every known level of venous and arterial stenosis, and CVC-associated pre-morbid symptoms were analyzed. Preliminary data support the hypothesis that venous anatomical anomalies may be a risk factor for cognitive impairment. CVC may be an under-diagnosed contributor to cognitive impairment, particularly in older patients.

### SESSION 2385 (SYMPOSIUM)

Abstract citation ID: igad104.0780

### INTERDISCIPLINARY APPROACHES TO EPIDEMIOLOGY AND PREVENTION OF FALLS IN OLDER ADULTS

Chair: Wenjun Li Discussant: Wenjun Li

Prevention of falls is important to independent living and good health in older age. This symposium brings together five interdisciplinary studies on falls among community-dwelling older adults. The first presentation examines the relationship between multisite pain and fear of falling among older adults with dementia. The second discusses the differential effects of anxiety on indoor vs. outdoor falls. The cohort study found that anxiety was associated with higher rate of indoor but not outdoor falls. The third investigates racial differences in association of accelerometer measured physical activity with indoor and outdoor falls. The study found that a 30-minutes increase in moderate-to-vigorous intensity physical activity was associated with 25% lower rate of indoor falls and 42% higher rate of outdoor falls among non-White but not White participants, an interesting example of effect modification by race and location of falls. The fourth reports findings of a cross-sectional survey of older adults in Thailand which observed high prevalence of falls, and substantial differences in fear of falling, daily activities and quality of life between fallers and non-fallers, and between persons with and without fear of falling. The fifth reports rural-urban differences in circumstances and correlates of falls from the same study in Thailand. The study found higher fall rates, higher level of fear of falling and worse general health in urban than rural residents, highlighting the need for considering urban-rural differences in falls prevention. Together, these studies demonstrate the complexity and promises of interdisciplinary approaches to falls research.

Abstract citation ID: igad104.0781

### ASSOCIATION BETWEEN PAIN AND FALL WORRY AMONG COMMUNITY-DWELLING OLDER PEOPLE WITH DEMENTIA IN THE UNITED STATES

Yuanjin Zhou[1], Tatiana Sadak[2], Nayanika Ghosh[1], and Elizabeth Phelan[3], *1. University of Texas at Austin, Austin, Texas, United States*, *2. University of Washington,*

*Seattle, Washington, United States*, 3. *University Of Washington, School of Medicine, Seattle, Washington, United States*

Previous studies have found that pain is associated with fall worry among community-dwelling older adults. However, both pain and fall worry are poorly understood and under-addressed among community-dwelling older people with dementia (cd-OPWD). We thus sought to examine the association between pain and fall worry among this vulnerable subgroup. We used data from the 2015 National Health and Aging Trends Study (analytic sample: cd-OPWD who were self-interviewed, n=1103; mean age: 79; age range: 65-107). The number of pain sites in the prior month was assessed by presenting the cd-OPWD with a card listing common pain sites (e.g., back, knees). Past-month fall worry was assessed by two questions, "did you worry about falling down" and "did this worry ever limit your activities". Fall worry was endorsed by 34%; activity-limiting fall worry was endorsed by 39% of those with fall worry. Twenty percent reported a single site of pain, and 53% reported multisite pain. Those who endorsed fall worry were more likely to have a higher number of pain sites, and those who reported activity-limiting fall worry were more likely to report knee, hand, or leg pain. In multinomial logistic regression analyses, adjusting for sociodemographic and health characteristics, a higher number of pain sites was significantly associated with activity-limiting fall worry. These findings suggest pain and fall worry are common among cd-OPWD and can be elicited directly from them. Fall prevention for cd-OPWD should prioritize pain management to mitigate activity-limiting fall worry, since activity limitation increases the risk of falls.

Abstract citation ID: igad104.0782

### THE ASSOCIATION OF ANXIETY WITH INDOOR AND OUTDOOR FALLS AMONG COMMUNITY-DWELLING OLDER ADULTS

Lingming Chen[1], Elizabeth Procter-Gray[1], Linda Churchill[2], Meng Zhang[1], Qun Le[1], Sarah Berry[3], Marian Hannan[4], and Wenjun Li[1], 1. *University of Massachusetts Lowell, Lowell, Massachusetts, United States*, 2. *University of Massachusetts, Lowell, Lowell, Massachusetts, United States*, 3. *Hebrew Rehabilitation Center, Boston, Massachusetts, United States*, 4. *Harvard Medical School, Boston, Massachusetts, United States*

One in three older adults aged 65 years or greater fall at least once each year. The effects of psychosocial factors on falls remain to be fully understood. This study examined the association of anxiety with subsequent indoor and outdoor falls among older adults. The prospective cohort study enrolled 359 community-dwelling older adults aged 65-95 years old in central Massachusetts (2018-2020). Anxiety at baseline was measured by the Beck Anxiety Inventory. Falls were reported on monthly falls calendars, and when a fall was reported, the circumstances were collected via telephone interview. Negative binomial models were used to estimate the effect of anxiety on indoor and outdoor falls, separately. Models were adjusting for sociodemographic, physical health (BMI, bodily pain, comorbidities), depression, stress, functional status (Activities of Daily Living, chair standing test), physical activity, drinking, smoking, the fear of falling and ever fallen in the past year. Overall, the rates of indoor falls and outdoor falls were 41 per 100 person-years and 46 per 100 person-years, respectively. Anxiety was a statistically significant predictor of indoor falls; for every one point increased in Beck Anxiety Inventory, there was a 6% increase in the incident rate of indoor falls after adjusting for covariables listed above (IRR (95% CI): 1.06 (1.01-1.11)). Anxiety was not significantly associated with outdoor falls (IRR (95% CI): 0.98 (0.93-1.02)). In conclusion, anxiety was associated with higher rate of indoor falls but not outdoor falls. Future falls prevention should consider the differential impact of anxiety on indoor and outdoor falls.

Abstract citation ID: igad104.0783

### RACIAL DIFFERENCES IN ASSOCIATION OF OBJECTIVELY MEASURED PHYSICAL ACTIVITY WITH INDOOR AND OUTDOOR FALLS

Qun Le[1], Linda Churchill[2], Kevin Kane[1], Lingming Chen[1], Scott Crouter[3], Sarah Berry[4], Marian Hannan[5], and Wenjun Li[1], 1. *University of Massachusetts Lowell, Lowell, Massachusetts, United States*, 2. *University of Massachusetts, Lowell, Lowell, Massachusetts, United States*, 3. *The University of Tennessee Knoxville, Knoxville, Tennessee, United States*, 4. *Hebrew Rehabilitation Center, Boston, Massachusetts, United States*, 5. *Harvard Medical School, Boston, Massachusetts, United States*

Fall-related injury and hospitalization rates have been steadily increasing by 2% and 4% per year. Physical Activity Guidelines advise older adults to remain physically active to promote muscle strength, bone health, and balance. The Healthy Aging and Neighborhood Study enrolled 379 community-dwelling persons aged 65 years or older in Central Massachusetts (2018-2020). Participant physical activity (PA) levels were measured using waist-worn Actigraphy accelerometers for at least 8 hours per day for 5 or more days including 1 weekend day. Average daily PA intensities were categorized according to minutes of sedentary (SB), light (LPA), and moderate-to-vigorous-intensity PA (MVPA) using Copeland cutoffs. Falls, including the circumstances of the fall, were ascertained using monthly falls calendars and follow-up telephone interviews. Negative Binomial Regressions were used to estimate the associations of PA with rates of indoor and outdoor falls. Models were run overall and by race, with all models adjusting for age and gender. Mean age was 73.71(SD:6.41) years, 58.31%(n=221) were female, and 35.62%(n=135) were non-White. There was no significant difference in SB, LPA, and MVPA by race. White race was associated with higher rates of outdoor falls (RR(95% CI)=2.73 (1.51, 4.93)). Every 30 more minutes of MVPA was significantly associated with 25% lower rate of indoor falls (RR(95% CI)=0.74 (0.58, 0.97)) and 42% higher rate of outdoor falls (RR(95% CI)=1.42 (1.04, 1.95)) among non-White but not White (p for interaction=0.03, 0.07, respectively). Socioeconomic factors may explain the differences observed by race. Thus, future investigations should consider socioeconomic when examining indoor and outdoor falls.

Abstract citation ID: igad104.0784

### FALLS, FEAR OF FALLING, DAILY ACTIVITIES, AND QUALITY OF LIFE IN THAI COMMUNITY-DWELLING OLDER ADULTS

Jaroonrat Rodniam[1], and Ladda Thiamwong[2], *1. Panyapiwat Institute of Management, Sattahip, Chon Buri, Thailand*, *2. University of Central Florida, Orlando, Florida, United States*

This study aimed to examine the prevalence of falls, fear of falling (FoF), activities of daily living (ADL) and quality of life (QoL) in community-dwelling older adults in Thailand. A cross-sectional survey of 433 community-dwelling older adults was conducted. We used a simple random sampling method to recruit urban and rural adults aged 60 years and older from 23 districts in Thailand. The measurements included demographics, fear of falling, history of falls, QoL, the modified Barthel ADL, chronic diseases, and medications. Data were analyzed by using both descriptive and inferential statistics. Among the participants, 45.3% reported having at least one fall in the past year, and 80% reported high level of fear of falling. Compared to non-fallers and persons without fear of falling, fallers and persons with fear of falling were more likely to have at least one chronic disease, hypertension and stroke. Fallers and persons with fear of falling were more likely to take more medications or anti-hypertensive drugs. Daily activities including eating, bathing, dressing, toileting, and walking differed significantly between non-fallers and fallers ($p< 0.01$ for all), and between persons with and without fear of falling ($p< 0.01$). Quality of life measures also differed significantly between fallers and non-fallers ($p < 0.01$). In conclusion, fallers and persons with fear of falling were more likely to have poorer health, more medications, lower quality of life, and different daily activity patterns. Future interventions should consider the differences between fallers and non-fallers and persons with and without fear of falling.

Abstract citation ID: igad104.0785

### URBANIZATION, FALLS, AND FEAR OF FALLING IN COMMUNITY-DWELLING OLDER ADULTS IN THAILAND

Ladda Thiamwong[1], Rui Xie[1], Sirinart Tongsiri[2], Boon Peng Ng[1], Joon-Hyuk Park[1], Jaroonrat Rodniam[3], Wenjun Li[4], and Elizabeth Eckstrom[5], *1. University of Central Florida, Orlando, Florida, United States*, *2. Mahasarakham University, Mahasarakham, Maha Sarakham, Thailand*, *3. Panyapiwat Institute of Management, Sattahip, Chon Buri, Thailand*, *4. University of Massachusetts Lowell, Lowell, Massachusetts, United States*, *5. Oregon Health & Science University, Portland, Oregon, United States*

Nearly half of the Thai population lives in urban areas without retirement pensions. Urbanization may increase physical inactivity, falls, and other non-communicable diseases. Little is known about the urban-rural differences in falls and fear of falling among community-dwelling older in Thailand. We surveyed a random sample of 433 persons who were: i) aged 60 years and older; and ii) had no cognitive impairment (Chula Mental Test ≥ 16/36). The participants had the mean age was 70.40 (± 7.53) years, included 62.9 % female, 22% having poor balance, 85% having limits in doing vigorous physical activity, 71% having limits in performing moderate physical activity, and 63.5% having pain interfere with normal activities. About half had financial problems sometimes, and 68 % reported living with a spouse and children. 30% had hypertension and took antihypertensive medication. 16% felt tired most of the time, and 11% felt nervous all the time. We found that urban older adults had a higher fall rate than rural older adults (52.8% vs. 40.4%, $p =.034$). Urban older adults also had a higher level of fear of falling than rural older adults (43.9% vs. 28.4%, $p = .000016$). Additionally, urban older adults had significantly worse general health than rural older adults ($p =.0046$). There were no significant differences in balance performance and fall self-efficacy scores. It is important to recognize and identify fall risk factors among urban older adults and provide access to systematic community-based fall prevention programs incorporating screening and tailored interventions based on those risk factors.

### SESSION 2390 (SYMPOSIUM)

Abstract citation ID: igad104.0786

### ENGAGING IN ARTISTIC AND CREATIVE OPPORTUNITIES TO HELP UNDERSTAND THE AGING EXPERIENCE

Chair: Alexander Bishop Co-Chair: Melinda Heinz
Discussant: Victor Fung

This symposium provides an overview of artistic and creative engagement methodologies to understand aging experiences. The first presentation examines a collaborative effort involving a researcher and filmmaker to film, edit, and produce a documentary about Irish Men's Sheds to increase awareness of diversity within the dissemination of narrative stories. The second presentation focuses on drawing, investigating how young children socially and cognitively represent perceptions of human longevity and of the concept of what constitutes "old." Children were asked questions regarding what it means to be "old" and what they might look like at 100 years, and to draw a self-portrait image. Findings demonstrating internal working models consistent with socially-learned attitudes, beliefs, and stereotypes of aging. The third presentation explains digital narrative gerontology and a life story project of the older adult's life created by younger and older adults. Thematic analysis revealed improved well-being, reduced generational stereotypes, and feelings of loneliness and isolation. The fourth presentation details an intergenerational project involving university students and older adults with cognitive impairment. Participants engaged in a six-week, telephone-based reminiscence experience and then co-created a storyboard/script to create a digital story. Thematic analysis demonstrated six themes: Family, religion and purpose in life, loves and hates, career/work, stress/coping, and major life turning points. The final presentation details music as a therapeutic intervention to improve older adults' quality of life and foster social connections, self-understanding and expression. Observations from working with older adults in a variety of environments and preferred music styles will also be discussed.

Abstract citation ID: igad104.0787

### CREATING A DOCUMENTARY CAPTURING THE NARRATIVES OF MEN ATTENDING IRISH MEN'S SHEDS

Melinda Heinz[1], and Laura Gleissner[2], *1. Upper Iowa University, Fayette, Iowa, United States*, *2. University of Northern Iowa, Cedar Falls, Iowa, United States*

How do we engage more individuals to consider and care about issues impacting older adults? Diversifying the mediums of how older adult stories are told offers one solution. Filmmaking is one medium where the narratives of older adults can be captured. Seeing and hearing directly from older adults speak about the issues that affect them provides a distinct way to understand experiences of aging. I interviewed men in County Limerick about how Men's Sheds promoted purpose and meaning in the lives of older men. After transcribing each interview, I partnered with a filmmaker to help create a documentary to tell the story of how Men's Sheds create an inclusive atmosphere for older adult men to continue feeling useful in older adulthood. This presentation will explain how researchers who conduct interviews with older adults are uniquely positioned to collaborate with filmmakers to help tell the stories of older adults.

Abstract citation ID: igad104.0788

### DRAWING AS A METHOD TO DETECT SOCIO-COGNITIVE PERCEPTIONS OF HUMAN LONGEVITY IN EARLY CHILDHOOD

Alexander Bishop, Larissa Callaway-Cole, and Hailey Grimmett, *Oklahoma State University, Stillwater, Oklahoma, United States*

The aim of this investigation was to understand how young children socially and cognitively perceive human longevity through drawing. Data for this study were collected from young children, ages 3 to 6, enrolled in Pre-K and Kindergarten classrooms through a university-supported early childhood education center. Investigators first conducted brief open-ended interviews to gauge participant understanding of the concept of being "old." Participants were then asked to draw a self-portrait image of what might look like at 100 years of age. Finally, participants were asked to describe their picture as well as share a story regarding the self-portrait image they had crafted. Images were decoded and analyzed relative to depicted content, pattern and position, and color preferences. Findings highlight reliance on internal working models which appear to highlight socially emergent and learned attitudes, beliefs, and stereotypes about growing old. Further discussion relative to underlying indication of emotional development will be highlighted.

Abstract citation ID: igad104.0789

### DIGITAL NARRATIVE GERONTOLOGY AS BRIDGES BETWEEN GENERATIONS TO IMPROVE WELL-BEING, LEARNING, AND SHARED EXPERIENCES

Béatrice Crettenand Pecorini, and Emmanuel Duplàa, *University of Ottawa, Ottawa, Ontario, Canada*

Elders are an essential society component for the collective success of the demographic transition, for social inclusion and the fight against ageism (Berrut, 2020). Ageism - discrimination by age, is a very present concern. One way to respond to this challenge is to build bridges between generations in society; intergenerational programs can reduce the perception of stereotypes about other generations (Barbosa et al., 2021; Pentecouteau & Eneau, 2017), strengthen intergenerational solidarity, and help develop capital and social cohesion (Topping, 2020). Based on the theories of lifelong learning and intergenerational learning, our research engages digital narrative gerontology (Crettenand Pecorini, 2019). We have organized several meetings in pairs (elder – young) where each pair created a digital narrative from the oral narration of the elder life story, supported by the multimedia skills that the young adult acquired during the workshop we have set up. From individual and pairs semi-directed interviews, logbooks, debriefings, and digital narrative, based on case study thematic analysis, our preliminary results confirm the results obtained in 2019: improved general well-being, reduction in the perception of generation stereotypes, reduction in feelings of loneliness and isolation, strengthening intergenerational solidarity, satisfaction to rediscover one's life or the projection of one's life stimulated by example, new knowledge acquisition, as well as the pride of artifact creation and its sharing. This innovative project can be applied in schools, colleges, and universities as well as in community centers and seniors' residences.

Abstract citation ID: igad104.0790

### DIGITAL STORIES FROM AN INTERGENERATIONAL, TELEPHONE-BASED REMINISCENCE PROGRAM

Noelle Fields[1], Ling Xu[1], Brooke Troutman[2], Kathryn Daniel[3], Megan Westmore[4], and Jessica Cassidy[3], *1. The University of Texas at Arlington, Arlington, Texas, United States*, *2. Air Force Academy, Air Force Academy, Colorado, United States*, *3. University of Texas at Arlington, Arlington, Texas, United States*, *4. University of Texas at Arlington School of Social Work, Arlington, Texas, United States*

Reminiscence strategies combined with an intergenerational approach may yield social and mental health benefits for older adults with cognitive impairment. There is also evidence to support the use of digital storytelling (DST) with persons with cognitive impairment combined with intergenerational programs. DST typically involves the production of a two-to-five-minute audio-visual clip combining text, images, music, photographs, voice-over narration, and other audio. However, there are few studies that combine reminiscence, DST, and an intergenerational approach with persons with cognitive impairment. The current study is a thematic analysis of DST products created by university students in collaboration with older adults with cognitive impairment (N = 27) as part of a larger project examining how intergenerational reminiscence using DST may improve the social and emotional well-being of persons with memory loss. After the completion of a structured, six-week, telephone-based reminiscence, the younger and older adults co-created a storyboard and script as well as selected photos and music to accompany the narrative in the DST. An interactive team approach using the phases of thematic analysis of the DST products resulted in six themes: 1) family, 2) religion and purpose in life, 3) loves and hates, 4) career/work, 5) stress and coping, and 6) major life turning points. Sub-themes included childhood and early adulthood, family legacy, faith, volunteering, travel, music, and resilience. Findings from the study extend current research by examining intergenerational connections, reminiscence, and the use of DST with persons with memory impairment. We close with recommendations for practice and research.

Abstract citation ID: igad104.0791

**MUSIC AND QUALITY OF LIFE IN OLDER ADULTS**

Lisa Lehmberg[1], and Victor Fung[2], *1. University of Massachusetts Amherst, Amherst, Massachusetts, United States*, *2. University of South Florida, Tampa, Florida, United States*

Research studies have indicated that music is an important contributor to older adults' quality of life. In this segment, the presenter will share overarching observations from their original research and the literature regarding music making, listening, creating, and activities involving movement to music in relation to facets of quality of life, such as identity, social connections, enjoyment, and self-understanding and expression. These studies were done in a range of contexts (e.g., retirement communities and senior centers), formats (small and large groups, participatory and presentational), and groups that engage in different musical styles (e.g., classical and vernacular). Music has been shown to be critical to the quality of life of those who participate. However, additional research is needed with diverse populations, including those who are homebound, homeless, in supervised care (e.g., assisted living, nursing homes, or hospice care), as well as older adults of varying ages and national origin.

### SESSION 2395 (SYMPOSIUM)

Abstract citation ID: igad104.0792

**VALUING AND SUPPORTING THE INVISIBLE WORKFORCE OF FAMILY CAREGIVERS**

Chair: Susan Reinhard

The 2023 update to the Valuing the Invaluable series estimates the economic value of unpaid, family caregiving in America was $600 billion in 2021. This estimate continues a 25-year trend of increasing economic value for family caregiving, which fills an essential role in the fractured LTSS system. This work also details the diversity of family caregivers and their care experiences, the challenges that caregivers face juggling care with work and child care, and the impact of direct care worker shortages on family caregivers. This symposium showcases the national and state estimates of the economic value of caregiving, details who caregivers are today, what challenges they face, and what policies and practices can support and recognize them and the vital care they provide. Presenter 1 shares an overview of the methodology used in this report and the national and state estimates of the economic value of caregiving. Presenter 2 discusses the increasing generational, racial and ethnic, and sexual identity and orientation diversity of family caregivers today and the challenges specific to different communities of caregivers. Presenter 3 highlights promising policies and practices to support caregivers and details recommendations for building on those promising policies and practices. This is a Family Caregiving Interest Group Sponsored Symposium.

Abstract citation ID: igad104.0793

**ESTIMATING THE ECONOMIC VALUE OF FAMILY CAREGIVING AT A NATIONAL AND STATE LEVEL**

Selena Caldera[1], Susan Reinhard[1], Ari Houser[2], and Rita Choula[1], *1. AARP, Washington, District of Columbia, United States*, *2. AARP Public Policy Institute, Washington, District of Columbia, United States*

The 2023 update to the Valuing the Invaluable series estimates the economic value of unpaid, family caregiving in America at $600 billion in 2021. This is the latest estimate in almost 20 years of work tracking the economic value of family caregiving in the U.S. The Valuing the Invaluable series of reports uses a meta-analysis approach drawing on various surveys of family caregivers to produce estimates at the state and national levels of the numbers of caregivers, the hours of care they provided, the average economic value of that care per hour, and the total economic value of all caregiving provided in that year. This presentation will detail the methodology for this meta-analysis, the 2021 national estimate of the economic value of caregiving, and variation in state-level estimates of the economic value of caregiving. While this estimated economic value of caregiving seeks to be comprehensive, it does not account for opportunity costs tied to forgoing work or scaling back work because of caregiving, out-of-pocket costs of caregivers, or foregone retirement savings of caregivers. These limitations will be discussed and the direction they offer for future work understanding the full value of unpaid caregiving contributions to the LTSS system and the state and federal budgets.

Abstract citation ID: igad104.0794

**IF YOU KNOW ONE CAREGIVER, YOU KNOW ONE CAREGIVER: UNDERSTANDING THE DIVERSITY OF US CAREGIVING EXPERIENCES**

Rita Choula[1], Susan Reinhard[1], Selena Caldera[1], and Ari Houser[2], *1. AARP, Washington, District of Columbia, United States*, *2. AARP Public Policy Institute, Washington, District of Columbia, United States*

For many years, the image of a family caregiver has been of a white woman in her late 40s providing care to her much older mother. The shifting demographics of the U.S. population have lead to a new understanding of the heterogeneity of family caregiving experiences and arrangements. Delays in having children and increasing longevity have led to an increase in caregivers sandwiched between care for someone under 18 at home and an older family member or friend and the share Gen Z and Millennial caregivers. The increasing racial and ethnic diversity and acceptance and visibility of persons identifying as LGBTQ has been reflected in the population of caregivers. Each of these communities of family caregivers have distinct care experiences and face unique challenges providing that essential care to family and friends with chronic, disabling, or serious health conditions. This presentation explores the diversity of the U.S. population of family caregivers and their unique care experiences detailed in the Valuing the Invaluable: 2023 Update. An overview of existing survey data on caregivers and quantitative and qualitative research into distinct caregiver experiences will provide attendees with a nuanced understanding of who family caregivers are and what they and their care recipients experience. In summary, the range of challenges these different caregivers face and positive experiences will be discussed.

Abstract citation ID: igad104.0795

**PROMISING POLICY AND PRACTICE SUPPORTING FAMILY CAREGIVERS AND RECOMMENDATIONS FOR THE FUTURE**

Susan Reinhard[1], Selena Caldera[1], Ari Houser[2], and Rita Choula[1], *1. AARP, Washington, District of Columbia,*

*United States*, *2. AARP Public Policy Institute, Washington, District of Columbia, United States*

There have been significant federal and state policy developments since the 2019 update of Valuing the Invaluable. Many inroads have been made in recognizing and supporting family caregivers, but additional steps can be taken at the federal and state levels, and even in the private sector. The National Strategy to Support Family Caregivers, released in September 2022, lays out a unified approach in five organizing goals to achieving this improvement in support for family caregivers. The policy and practice recommendations highlighted in the Valuing the Invaluable: 2023 Update reflect the National Strategy goals. In this presentation, recent policy changes will be summarized that support caregivers and their care recipients in the health care and LTSS systems, which provide financial relief for caregiving, and help working caregivers stay in the workforce. Then the increasing visibility of the family caregiving experience in media and the arts will be explored. This visibility is essential for bringing broader attention to all aspects of caregiving, normalizing the care experience, and eliminating stigma around disability and dementia. Lastly, policy recommendations for building on the National Strategy and bolstering support for family caregivers and care recipients will be detailed for discussion.

### SESSION 3000 (AWARD LECTURE)

Abstract citation ID: igad104.0796

### THE CLARK TIBBITTS AWARD AND HIRAM J. FRIEDSAM AWARD LECTURES AND PRESENTATIONS

Chair: Dana Bradley

The Clark Tibbitts Award Lecture will feature an address by the 2023 award recipient Edward Schneider, MD of the University of Southern California. AGHE's Clark Tibbitts Award was established in 1980 and named for an architect of the field of gerontological education. The award is given each year to an individual or organization that has made an outstanding contribution to the advancement of gerontology and geriatrics education. The Hiram J. Friedsam Award Lecture will feature an address by the 2023 award recipient Harvey Sterms, PhD, FGSA of the University of Akron. Hiram J. Friedsam was the professor, co-founder, and director of the Center for Studies in Aging and dean of the School of Community Service at the University of Northern Texas. Dr. Friedsam was an outstanding teacher, researcher, colleague, and mentor to students, faculty, and administrators, as well as a past president of AGHE. The purpose of this award is to recognize those who emulate Dr. Friedsam's excellence in mentorship.

Abstract citation ID: igad104.0797

### CREATING INTERNET-BASED DISTANCE LEARNING IN GERONTOLOGY

Edward Schneider, *University of Southern California, Los Angeles, California, United States*

Dr. Schneider was Associate Director and then Deputy Director of the National Institute on Aging from 1980 through 1986. Dr. Schneider helped to create the Geriatric Leadership Academic Award. This award funded geriatric positions in many medical schools including Harvard, Johns Hopkins, Duke the University of California at Los Angeles, and the University of Tennessee. These awards led the creation of Geriatric Divisions at numerous American medical schools. After he became Dean of the Leonard Davis School of Gerontology, Dr. Edward Schneider with Maria Henke created the nation's first Internet based Master's Degree program in Gerontology. This program has graduated over 1000 students since then.

Abstract citation ID: igad104.0798

### YOU ARE ONLY MY STUDENT FOR A LITTLE WHILE; YOU ARE MY COLLEAGUE FOR LIFE

Harvey Sterns, *The University of Akron, Akron, Ohio, United States*

The philosophy of you are my student for a little while-you are my colleague for life was shared with me by my major professor Paul Baltes. This has been my approach over my 50+ year career. I have had a number of significant mentors and have had many great students. In the spirit of this award, I will share my understanding of mentorship.

### SESSION 3005 (SYMPOSIUM)

Abstract citation ID: igad104.0799

### PRESIDENTIAL SYMPOSIUM: FRAMING, BRIDGING, AND TRAVERSING SOCIAL DETERMINANTS OF HEALTH, DISPARITIES, AND INEQUITIES IN OLDER ADULTS

Chair: Deb Bakerjian Discussant: Mo-kyung Sin

In alignment with the theme of this year's conference, Building Bridges>Catalyzing Research>Empowering All Ages, this symposium focuses on research that frames, bridges, and transverses various constructs of the social determinants of health, intersectional frameworks, and inequities that impact older adults at various times in life from mid to older adulthood through death. We start with a framework for how to consider social and structural conditions in which people live and the cumulative effects that an individual's life course might impact their trajectory with Alzheimer's disease and other related dementias and their families. Continuing on with the theme of family, dementia, and caregivers, we delve into the differences in caregiver burden across and within gender and racial/ethnic groups, looking at the linkage with financial, emotional, and physical burdens. From there, we go on to explore whether there is an association between negative family interactions and mental health outcomes and discuss how those outcomes might vary by race and ethnicity. Building upon several concepts in these first three papers, we will discuss how one can evaluate the intersectional impact of race and gender on end-of-life outcomes including pain, anxiety/depression, patient autonomy, and overall care quality. Our last speaker will share intriguing, and somewhat unexpected findings related to social inequities on health from midlife to death and the impact of early exposure to socioeconomic disadvantages might have on disparities. Given the importance of reducing inequities to improve health outcomes, this HS section Presidential Symposium is sure to challenge and enhance our thinking.

Abstract citation ID: igad104.0800

### A FRAMEWORK FOR CONSIDERING SOCIAL AND STRUCTURAL DETERMINANTS OF HEALTH IN ALZHEIMER'S DISEASE RESEARCH AND CARE

Shana Stites, *University of Pennsylvania, Philadelphia, Pennsylvania, United States*

Social and structural determinants of health (SSDoH) refer to the conditions in which individuals live, work, and age. SSDoH can have cumulative effects across the life course that can have an impact on outcomes for persons with Alzheimer's disease and Alzheimer's disease related dementias (AD/ADRD) and their family members. In our two-part presentation, we first describe the theoretical and empirical underpinnings of core SSDoH constructs in AD/ADRD research and practice. We review empirical support for the specific SSDoH that we include and highlight their relevance in the care of older adults. The core set of SSDoH that we focus on have been identified as being important across a broad range of socially and culturally heterogeneous populations and we walk through the strengths and limits of this. We also report results from data obtained during a pilot test of these measures in the UPenn Alzheimer's Disease Research Center (ADRC), which offer empirical support of their cultural appropriateness. We outline a rationale for the consideration of SSDoH in aging and AD/ADRD research, policy, and practice. In the second part of our presentation, we offer a case example of how sex, gender, and sexual orientation operate as core SSDoH in the healthy, aging population and in the care of older adults. In our case example, we also overview definitions, clinical considerations, and policy applications.

Abstract citation ID: igad104.0801

### DIFFERENCES IN CAREGIVING BURDEN ACROSS THE INTERSECTIONALITY OF RACE AND GENDER

Ruotong Mona Liu[1], Iris Chi[2], and Shinyi Wu[3], *1. Oregon Health & Science University, Portland, Oregon, United States*, *2. USC, Los Angeles, California, United States*, *3. University of Southern California, Los Angeles, California, United States*

Caregivers may be at different risks of various types of burdens by virtue of their gender and racial/ethnic status. This study explored the differences in caregiving burdens among caregivers to people with dementia across the intersectionality of race and gender. Using Round 5 (conducted in 2015) and Round 7 (conducted in 2017) of the National Study of Caregiving and National Health and Aging Trends Study data, the study examined differences in caregiver burdens across and within different gender and racial/ethnic groups, within the realms of financial, emotional, and physical burdens. The sample consisted of 1,206 caregivers who provided services to Medicare beneficiaries. Logistic regressions were performed to assess the three types of burdens each subgroup was experiencing. Results indicated that within the intersectionality framework, compared to White female caregivers, Black male caregivers were 3.3 times (95% confidence interval [CI] 1.77-6.22) more likely to experience financial burden, and Black female caregivers were 54% (95% CI 0.28-0.76) less likely to experience physical burden. Surprisingly, compared to White female caregivers, all the other groups were 37% (95% CI 0.41-0.95) to 71% (95% CI 0.15-0.56) less likely to have emotional burden. The findings highlighted that Black male caregivers are experiencing financial burden and White female caregivers are experiencing emotional burden disproportionately. To develop effective interventions and programs for dementia caregivers, a special focus should be put on monitoring the differences in the types of burdens that the above-mentioned population subgroups experience.

Abstract citation ID: igad104.0802

### THE MODERATING ROLE OF RACE AND ETHNICITY BETWEEN NEGATIVE FAMILY INTERACTIONS AND MENTAL HEALTH AMONG OLDER ADULTS

Fei Wang[1], Ann nguyen[2], Karen Lincoln[3], Weidi Qin[4], and Tyrone Hamler[5], *1. Case Western Reserve University, Cleveland, Ohio, United States*, *2. Jack, Joseph, and Morton Mandel School of Applied Social Sciences, Case Western Reserve University, Cleveland, Ohio, United States*, *3. University of California, Irvine, Irvine, California, United States*, *4. University of Michigan, Ann Arbor, Michigan, United States*, *5. University of Denver, Denver, Colorado, United States*

Studies generally show that negative social interactions are detrimental to mental health for older adults. Furthermore, empirical evidence suggests that negative interactions may function differently in relation to mental health across racial/ethnic groups given their unique life circumstances and social conditions. This study examines whether the association between negative family interactions and mental health outcomes varies by race and ethnicity. Samples of older African Americans, Caribbean Blacks, and non-Latino Whites aged 55 and older were drawn from the National Survey of American Life (N = 1,439). Mental health variables included depressive symptoms, any lifetime disorder according to The Diagnostic and Statistical Manual of Mental Disorders, fourth edition (DSM-IV), and number of lifetime DSM-IV disorders. Regression models were used to test the study aim. Analyses indicated that negative interactions with family were positively associated with all 3 mental health outcomes. Several racial/ethnic differences emerged. The association between negative family interactions and depressive symptoms was stronger among Whites than African Americans. While negative family interactions were positively associated with number of disorders among Caribbean Blacks, negative interactions were unrelated to number of disorders among African Americans. This study demonstrates the racial and ethnic differences in diverse aging populations and the importance of recognizing the heterogeneity of the Black American population in minority research. Clinical practice should focus on reducing negative family interactions, and future research should examine whether psychosocial resources (e.g., stress appraisals, neighborhood social cohesion) can attenuate the association between negative family interactions and mental health for older African Americans.

Abstract citation ID: igad104.0803

### THE INTERSECTIONAL IMPACT OF RACE AND GENDER ON END OF LIFE

Zainab Suntai[1], Hyunjin Noh[2], Lewis Lee[3], Gregg Bell[4], Megan Lippe[5], and Hee Yun Lee[4], *1. Baylor University, Waco, Texas, United States*, *2. University of Alabama,*

*Tuscaloosa, Alabama, United States*, 3. *School of Social Work, The University of Alabama, Tuscalossa, Alabama, United States*, 4. *The University of Alabama, Tuscaloosa, Alabama, United States*, 5. *University of Texas Health Science Center, San Antonio, Texas, United States*

Over the past few decades, research on quality of life at the end of life has proliferated, with an increased focus on issues of diversity. However, few studies have considered the impact of multiple disadvantaged identities, including the combination of race and gender. This study assessed the intersectional impact of race and gender on four end-of-life outcomes: pain, anxiety/depression, patient autonomy, and overall care quality. Data were derived from the combined 2012 to 2020 last-month-of-life interviews conducted by the National Health and Aging Trends, which is an annual longitudinal panel survey of Medicare beneficiaries aged 65 and older. Multivariate logistic regression models were used to test the association between the race/gender intersection and each of the four outcomes. Results showed that Black women were the most likely to be in pain, most likely to have a lack of autonomy in decision-making, and the least likely to have excellent or good care at the end of life. White women were the most likely to have had anxiety/depression, followed by Black women, Black men, and then White men. Across all four outcomes, White men were the most likely to have had favorable outcomes, confirming the theory of intersectionality. These results point to a significant disparity in quality end-of-life care for Black women, who have double-jeopardy as a result of their membership in two vulnerable groups. Thus, there is increased need for practice, policy, and research interventions to attain equitable end-of-life care for all individuals.

Abstract citation ID: igad104.0804

### SOCIAL INEQUITIES AND THE IMPAIRMENT PARADOX IN LATER LIFE

Michal Engelman[1], and Heide Jackson[2], 1. *University of Wisconsin-Madison, Madison, Wisconsin, United States*, 2. *University of Maryland, College Park, Maryland, United States*

Research on health across the life course consistently documents widening racial and socioeconomic disparities from childhood through adulthood, followed by stabilization or convergence in later life. This pattern appears to contradict expectations set by cumulative (dis)advantage (CAD) theory. Informed by the punctuated equilibrium perspective, we examine the relationship between midlife health and subsequent health change and mortality and consider the impact of earlier socioeconomic exposures on observed disparities. Using the Health and Retirement Study, we characterize the functional impairment histories of a nationally representative sample of 8464 older adults between 1994 and 2016. We employ nonparametric and discrete outcome multinomial logistic regression to examine the competing risks of mortality, health change, and attrition. As expected, exposures to disadvantages are associated with poorer functional health in midlife and mortality. However, we find that a higher number of functional limitations in midlife is negatively associated with the accumulation of subsequent limitations for White men and women and for Black women, and the impact of educational attainment, occupation, wealth, and marriage on later-life health differs across race and gender groups. We conclude that the observed stability or convergence in later-life functional health disparities is not a departure from the dynamics posited by CAD, but rather a result of the differential impact of racial and socioeconomic inequities on mortality and health at older ages. Higher exposure to disadvantages and a lower protective impact of advantageous exposures lead to higher mortality among Black Americans, a pattern which masks persistent health inequities later in life.

### SESSION 3010 (SYMPOSIUM)

Abstract citation ID: igad104.0805

### MEASURING QUALITY IN LONG-TERM SERVICES AND SUPPORTS THROUGH PERSON-REPORTED OUTCOMES: VALUE OF NCI DATA

Chair: Tetyana Shippee Co-Chair: Stephanie Giordano

Long-Term Services and Supports (LTSS) include medical, social, and personal care services that people may need to perform activities of daily living due to physical or cognitive impairments. Measuring the quality of LTSS is essential for improving the care and outcomes of people with disabilities and older adults and ensuring that services meet consumer needs. Several frameworks have been developed to measure LTSS quality, including the National Quality Forum's LTSS Framework. Yet, few data sources are available to measure person-reported quality in HCBS. This symposium will provide an overview of National Core Indicators (NCI) as a unique data source to address this gap. It also includes presentations from researchers from organizations in two states who utilize NCI data – Minnesota and Massachusetts – who will share their perspectives about the value of the data, results from empirical analyses, and how they use and plan to use the data to inform policy. Researchers from the Lurie Institute for Disability Policy will present findings on the association between person-centered planning and key person-reported outcomes in Medicaid HCBS. Researchers from the University of Minnesota School of Public Health and Brown University will share findings from national NCI data on unmet needs among older adults by dementia status and race/ethnicity. They will also share results of analyses in MN, linking person-reported HCBS plan quality and healthcare utilization, using claims data. Collectively, this symposium will address the gap in measurement of HCBS quality nationally and identify key directions for policy and future research.

Abstract citation ID: igad104.0806

### MEASURING UNMET NEEDS FOR COMMUNITY-DWELLING CONSUMERS OF HOME- AND COMMUNITY-BASED SERVICES USING NCI-AD DATA

Tetyana Shippee[1], Taylor Bucy[2], John Mulcahy[1], and Eric Jutkowitz[3], 1. *University of Minnesota, Minneapolis, Minnesota, United States*, 2. *University of Minnesota School of Public Health, Minneapolis, Minnesota, United States*, 3. *Brown University, Providence, Rhode Island, United States*

Rebalancing efforts have focused on helping more people age in the community rather than institutional settings. Yet, we know little of whether actual services being offered meet consumer needs. HCBS provides intermittent services and supports to consumers and variability can create increased reliance on informal caregivers, contributing to unmet need. This can be especially true for consumers living with Alzheimer's disease and Alzheimer's disease and related dementias (AD/ADRD), whose needs may fluctuate and intensify as functional and/or cognitive impairments advance. This presentation examines unmet need among a national sample of older adult (65+) consumers of publicly funded HCBS, using data from the National Core Indicators - Aging and Disability (NCI-AD) survey from 2016-2019. We restrict our sample to community-dwelling older adults, where community-dwelling includes: own or family house/apartment, senior living apartment/complex, group home, adult family home, foster, or host home. Our results showed that services that were most commonly used were home-based services (57%), with at least half of the consumers using two different services to support their needs. Factors associated with wanting more services included being a woman, living alone, having a physical disability, and a diagnosis of AD/ADRD. Older adults with AD/ADRD desired more services than those without AD/ADRD, with additional desired services for caregiver supports and transportation. Our findings speak to the need to measure and improve person-centered quality of publicly-funded HCBS for older adults, with particular attention to the needs of those living with AD/ADRD.

Abstract citation ID: igad104.0807

### USING NATIONAL CORE INDICATORS—AGING AND DISABILITIES—FOR QUALITY, POLICY, AND SYSTEM TRANSFORMATION

Stephanie Giordano, *Human Services Research Institute, Cambridge, Massachusetts, United States*

National Core Indicators Aging and Disabilities (NCI-AD™), uses survey data to support State Medicaid, aging, and disability agencies to conduct surveys with individuals receiving publicly-funded long-term services and supports and measure and track their own outcomes and performance using reliable data collection methods and tools. Established in 2015, NCI-AD is now in use by 22 states nationwide. The NCI-AD portfolio includes the flagship survey – the Adult Consumer Survey (ACS) and the State of the Workforce—Aging and Disabilities (SotW-AD) survey. The ACS collects data directly from older adults and people with physical disabilities to assess the outcomes of services provided to individuals. Indicators address key areas of concern including service planning, rights, community inclusion, choice, health and care coordination, safety and relationships. In 2022, NCI-AD piloted the SotW-AD survey with five participating states. A first of its kind for the aging and disabilities field, this new effort collects and aggregates statewide information about wages, benefits, and turnover of the direct service workforce—as reported by the agencies that employ them. The SotW-AD will launch to all AD states in summer 2023. This session will provide an overview of the NCI-AD program, the type of data collected from each survey, and how state-level and research partners have utilized the data sets.

Abstract citation ID: igad104.0808

### THE ASSOCIATION BETWEEN PERSON-CENTERED PLANNING AND PERSON-REPORTED OUTCOMES IN HOME- AND COMMUNITY-BASED SERVICES

Natalie Chong[1], and Joseph Caldwell[2], *1. The Heller School for Social Policy and Management at Brandeis University, Waltham, Massachusetts, United States*, *2. Lurie Institute For Disability Policy, Brandeis University, Waltham, Massachusetts, United States*

Person-centered planning (PCP) allows home and community-based service (HCBS) users to plan and coordinate services and supports according to their preferences and needs. The extent to which HCBS systems provide high-quality PCP and evidence for the relationship between PCP and user outcomes is limited. This study examines HCBS user characteristics related to PCP and the association between PCP and both unmet need and community living. We used the 2018-2019 National Core Indicators-Aging and Disability survey's Person-Centered Planning Module, collected among adult Medicaid HCBS users in 12 states (n = 6,924). Independent variables included two general PCP measures (one on decision-making and another on preferences and choices being reflected in service plans) and a PCP fidelity scale to assess the extent to which users' most recent service planning meeting was person-centered. Outcomes included two measures of unmet need for services and three measures of community living (i.e., participation, control, and satisfaction). We examined user characteristics associated with PCP and used logistic regression models to assess the relationship between PCP and outcomes. About 85% of users reported they were fully involved in decision-making, 77% reported their preferences and choices were reflected in their service plan, and 57% had their most recent service planning meeting coded as "high fidelity" on the PCP fidelity scale. Each marker of high-quality PCP was consistently related to lower likelihood of unmet need and greater likelihood of experiencing each community living outcomes. Findings suggest high-quality PCP is important for meeting HCBS users' needs and improving community living outcomes.

Abstract citation ID: igad104.0809

### ASSOCIATION OF MANAGED CARE ORGANIZATION HCBS QUALITY AND HEALTH CARE UTILIZATION

Eric Jutkowitz[1], John Mulcahy[2], Peter Huckfeldt[3], Christopher Frenier[4], and Tetyana Shippee[2], *1. Brown University, Providence, Rhode Island, United States*, *2. University of Minnesota, Minneapolis, Minnesota, United States*, *3. University of Minnesota School of Public Health, Minneapolis, Minnesota, United States*, *4. Yale, New Haven, Connecticut, United States*

Providing high quality home and community-based services (HCBS) to older adults is a priority, but surprisingly little is known about the association between HCBS quality and client health care utilization. We linked data from the 2018 wave of the National Core Indicators-Aging and Disabilities (NCI-AD) Adult Consumer Survey for participants in the State of Minnesota enrolled in the Elderly Waiver (n=1,449) to their Centers for Medicare and Medicaid Services (i.e., claims data). We attributed respondents in our sample to 1 of 8 managed care organizations (MCOs) responsible for managing the health and HCBS of beneficiaries. For each MCO, we calculated overall plan level HCBS quality using a validated approach based on items in NCI-AD with subdomains of care experience, security and inclusion. Plan quality was the independent variable in a series of multivariate regressions that controlled for county and beneficiary characteristics. Outcomes (yes/no for any utilization in year) included: 1) hospitalizations, 2) emergency department, 3) home health, 4) durable medical equipment, 5) personal care assistance, 6) transportation, 7) case management, 8) home services, and 9) adult day. Quality was positively associated with case management utilization, but no other measure. As the market share of the highest quality plan in a county increased, all beneficiaries (regardless of MCO) in the county used more durable medical equipment, case management services, and adult day care. In conclusion, MCO quality may be driven by client encounters with plans via case managers. A next step is to determine the causal relationship between quality and outcomes.

### SESSION 3015 (SYMPOSIUM)

Abstract citation ID: igad104.0810

### INFLUENCES OF CULTURAL VALUES ON THE INDIGENOUS DEMENTIA CAREGIVERS' EXPERIENCE

Chair: Steffi Kim Co-Chair: Jordan Lewis

This symposium will outline and discuss the unique circumstances and challenges of American Indian, Alaska Native, and Native Hawaiians and Pacific Islander dementia caregivers. Dementia is one of the top causes of death worldwide, with Alzheimer's disease currently being the ninth leading cause of death among American Indian and Alaska Native people (AI/AN). One in three American Indians and Alaska Natives over 65 will receive a dementia diagnosis in the next 25 years. Yet, we know very little about caregivers' experiences in these communities. Presenters will present on how cultural influences shape caregiver's experiences within American and Alaska Native, Native Hawaiians and Pacific Islanders. The second presenter will discuss the need for culturally safe dementia caregiver resources based on recommendations from Alaska Native caregivers. The third presenter will examine decision-making processes of dementia caregiver involved in the use of governmental and tribal dementia care resources within the ONEIDA Nation to improve utilization of existing services.

Abstract citation ID: igad104.0811

### CULTURAL INFLUENCES ON AMERICAN INDIAN AND ALASKA NATIVE DEMENTIA CAREGIVERS

Steffi Kim[1], and Lexie Jackson[2], *1. University of Alaska Anchorage, Anchorage, Alaska, United States*, *2. Pacific University, Forest Grove, Oregon, United States*

Associated with aging, Alzheimer's disease and related dementias (ADRD) are likely having a disproportionate effect on North American Indigenous communities due to longer life expectancies and high rates of chronic diseases. Despite missing prevalence data on ADRD in for Native Elders, it is known that one in five American Indians and Alaska Natives experience subjective cognitive decline and one in four adults are currently caregivers. Similarly, Native Hawaiians and Pacific Islanders (NHPI) are twice as likely to have dementia compared to non-Hispanic Whites. Yet, little is known about the journey of dementia caregivers within American and Alaska Native, Native Hawaiians and Pacific Islanders (NHPI) and how their unique cultural influences shape their caregiving experiences within their communities. We will present the voices from the transcripts of three 3 different qualitative data sets that include 93 American Indians and Alaska Natives, and six Native Hawaiian/Pacific Islander caregivers and elders about their personal experiences with taking care of family or community members with dementia and approaches to care. in their communities. The presentation focuses on the influences on cultural values on the caregiving experience, caregiver access to resources, and generative moments throughout caregiving. Results identify similarities among these groups but also distinct variations, which emphasize the importance of linguistic and culturally tailored research, interventions, and policies. Based on our findings, we outline culturally specific recommendations to improve the caregiving experience for each of these Indigenous groups.

Abstract citation ID: igad104.0812

### ONEIDA HELPING ONEIDA: DECISION MAKING ABOUT SERVICE USE BY ONEIDA NATION FAMILY CAREGIVERS

Mary Wyman[1], Haley Buerger-Cole[2], Nickolas Lambrou[2], Joseph Perzynski[3], Sacheen Lawrence[2], Debra Miller[4], Florence Petri[5], and Jordan Lewis[6], *1. Madison VA/Univ Wisconsin, Madison, Wisconsin, United States*, *2. University of Wisconsin, Madison, Wisconsin, United States*, *3. Madison VA Hospital, Madison, Wisconsin, United States*, *4. Oneida Comprehensive Health, Madison, Wisconsin, United States*, *5. Oneida Nation of Wisconsin, Madison, Wisconsin, United States*, *6. University of Minnesota Medical School, Duluth campus, Duluth, Minnesota, United States*

Native American communities are disproportionately impacted by Alzheimer's disease and related dementias and are more likely to identify as family caregivers compared to other racial groups. Caregiver resources such as educational, supportive, and respite services have been shown to decrease burden and improve health outcomes. However, government and tribal services to support caregivers are underutilized. Using a CBPR approach, within the framework of an existing partnership between the Oneida Nation of Wisconsin and the Wisconsin Alzheimer's Disease Research Center, all phases of research were conducted with Oneida partners. We used a mixed-method approach to gain a better understanding of the cultural and community-related factors impacting resource utilization by Native American dementia caregivers. Ten caregivers of older adults with memory loss completed surveys followed by in-depth interviews. Caregivers were aged 38-69 years, 90% female, and living on or near the Oneida reservation; time caregiving varied from <1 to >10 years. Most care recipients had not received a formal diagnosis of memory disorder. Interviews revealed barriers to service use including low awareness, complexities of service funding structures, and care recipient preferences. Perceptions of alignment of traditional cultural values with service use were nuanced and varied. Our findings underline the multiple factors impacting Native families coping with dementia and contribute to the scant literature on ADRD caregiving among Native Americans. Results can inform public health initiatives to address ADRD disparities and improve outcomes for Indigenous persons with ADRD.

Abstract citation ID: igad104.0813

### ALASKA NATIVE DEMENTIA CAREGIVERS AND THE INCREASING NEED FOR CULTURALLY SAFE RESOURCES

Jordan Lewis[1], Steffi Kim[2], Zayla Asquith-Heinz[3], and Lena Thompson[4], *1. University of Minnesota Medical School, Duluth campus, Duluth, Minnesota, United States, 2. University of Alaska Anchorage, Anchorage, Alaska, United States, 3. University of Minnesota Duluth, Duluth, Minnesota, United States, 4. University of Minnesota, Duluth, Minnesota, United States*

With the rate of dementia increasing among Alaska Native people and the tribal health system ill-equipped to meet the challenges associated with dementia care, there is an urgent need to better understand cultural perspectives on dementia caregiving and what states and communities can do to support their caregivers. Despite the fact caring for others is a value and cultural practice for Indigenous communities, and caregivers tend to report fewer challenges and barriers related to care, they still experience barriers and challenges. Guided by Kleinman's explanatory model of illness, a qualitative, the aim of this study was to gain insight on Alaska Native caregiver experiences in caring for someone living with dementia in communities across Alaska. 21 interviews with Alaska Native dementia caregivers in rural and urban communities were completed. Despite being innovative and resourceful in providing care, caregivers reported the high cost of care, inadequate training of health care providers, and the paucity of culturally safe caregiver training, education, and resources are especially concerning and need to be addressed in a timely fashion. Caregivers in this study recommended the development of basic educational materials to be aware of dementia, its stages, and the available supports, as well as caregiver support and respite services. The recommendations from this study focus on outreach and education for caregivers to improve their access to education, training, and support to reduce isolation, increase their dementia knowledge, expand their awareness of ancillary resources, and inform help-seeking decisions.

### SESSION 3020 (PAPER)

### DEMENTIA CAREGIVING

Abstract citation ID: igad104.0814

### CAREGIVING INTERACTIONS OF FORMAL CAREGIVERS AND PERSONS WITH DEMENTIA: A SYSTEMATIC REVIEW OF MEASURES

Anju Paudel[1], Wen Liu[2], Ying-Ling Jao[3], Thakshila Dasanayake[4], Yo-Jen Liao[5], and Nahida Akter[5], *1. Penn State Ross and Carol Nese College of Nursing, State College, Pennsylvania, United States, 2. The University Of Iowa, Iowa City, Iowa, United States, 3. Pennsylvania State University, State College, Pennsylvania, United States, 4. The Pennsylvania State University, State College, Pennsylvania, United States, 5. Penn State University, State College, Pennsylvania, United States*

A psychometrically sound assessment of caregiving interactions is essential to collect valid information about the interactions, and to establish interventions to optimize the interactions in dementia care. This review aims to identify measures of caregiving interactions between formal caregivers and persons with dementia and evaluate their psychometric properties. We conducted a systematic review of studies published by February 2023 in four databases— PubMed, PsychINFO, CINHAL, and Web of Science. COnsensus-based Standards for the selection of health status Measurement INstruments (COSMIN) checklist was chosen for quality appraisal of selected studies. Our search yielded 818 scholarly records and identified five eligible instruments (published in six scholarly records). Three measures assessed interactions between formal caregivers and persons with dementia from both caregiver and person with dementia's perspectives while one evaluated from the caregiver perspective and the other from person with dementia's perspective. Additionally, three measures assessed interactions based on observations while other two were based on interviews. Preliminary results showed the Communication Behavior in Dementia (CODEM) as a promising measure with some excellent psychometrics but it needed further testing with larger sample and assessment of responsiveness to change. The remaining four measures (IC-Communication-SR, Coding Scheme for Mealtime Interactions, Montreal Evaluation of Communication Questionnaire for use in Long-Term Care (MECQ-LTC), and Barnards Feeding Scale) had concerns related to low internal consistency, low reliability, and lack of adequate psychometric tests. Overall, this review identified gaps in current measures, suggesting a need to establish psychometrically sound tools to assess and optimize caregiving interactions in dementia care.

Abstract citation ID: igad104.0815

### EARLY WARNING SIGNS AND CRISES IN ADRD/AD: VIEWS FROM LATINO/AFRICAN AMERICAN CAREGIVERS AND PROVIDERS

Melinda Kavanaugh, *University of Wisconsin - Milwaukee, Milwaukee, Wisconsin, United States*

Incidence of Alzheimer's disease and related dementias (ADRD) in Latino and African American populations is significantly greater than for non-Hispanic Whites. Latino and African American caregiver families rely on family for care, including children and youth, and access formal care systems less frequently and at later stages compared to non-Latino White families. Yet we know little about what precipitates the eventual move to formal care. This study sought to understand whether African American and Latino families living with ADRD were able to identify Early Warning Signs (EWS) and potential crises in persons living with ADRD and explore differences in how families and health care professionals perceive EWS and crises. Semi-structured interviews were conducted with health care professionals (n = 26), Latino and African American adult (n = 36), and youth caregivers aged 10 – 19 (n = 31). Participants listed eight EWS (cooking, getting lost, forgetfulness, emotional/anger, repeating, bringing up the past) and defined Crisis as an event or series of events that led to seeking formal care, included getting lost, bad falls, and death of a loved one. Adults felt frustration, even at the beginning of EWS while youth found humor in the care. There were differences in stories shared within the same caregiver family; youth recognized EWS but reported that adults would not talk about it. Health care professionals identified EWS but believed families would be unable to identify EWS. This potential misperception needs further attention to reduce communication barriers around the care planning for this population.

Abstract citation ID: igad104.0816

### HOME CARE DYADIC COMMUNICATION PATTERNS OF FAMILY CAREGIVERS AND PERSONS LIVING WITH DEMENTIA: SEQUENTIAL ANALYSIS

Sohyun Kim[1], Wen Liu[2], and Kristine Williams[3], *1. University of Texas at Arlington, Arlington, Texas, United States*, *2. The University Of Iowa, Iowa City, Iowa, United States*, *3. University of Kansas Medical Center, Kansas City, Kansas, United States*

Examining temporal relationships of communication patterns between family caregivers and persons living with dementia (care recipients) is useful to determine which caregiver communication patterns are more effective in engaging care recipients and prioritize the use of such communication patterns. This study examined temporal relationships between antecedent family caregiver communication and subsequent care recipient communication during 75 in-home care video observations. A 5-second timed-window analysis was conducted to examine the likelihood of the relationships between dyadic communication patterns. Joint frequency and conditional probability statistics demonstrated that care recipients used the same communication patterns at differing frequencies during and within a 5-second window following each communication pattern by family caregivers. Dyadic communication patterns yielded likelihood probabilities with an effect size greater than chance (Yule's Q = 0.31-1.00). Care recipient's engaging body language most likely occurred after the caregiver's facilitative body language preceded (Yule's Q = 0.31-.1.00). Care recipient's relevant communication and verbal response most likely occurred after the caregiver's simplified message preceded (Yule's Q = 0.55-1.00). Specific caregiver facilitative communication exhibited subsequent care recipient engaging communication. Future research can expand on how characteristics, types, and frequencies of caregiver communication would impact care recipient engaging communication.

Abstract citation ID: igad104.0817

### THE PUBLIC HEALTH IMPLICATIONS OF DEMENTIA CAREGIVING IN THE US: FINDINGS FROM THE 2021 BRFSS

Joseph Gaugler[1], Raza Lamb[2], Ben Denno[3], Matthew Baumgart[3], Hawking Yam[1], and Lisa McGuire[4], *1. University of Minnesota, Minneapolis, Minnesota, United States*, *2. Alzheimer's Association, Chicago, Illinois, United States*, *3. Alzheimer's Association, New York, New York, United States*, *4. CDC, Atlanta, Georgia, United States*

To further elevate dementia caregiving as a public health imperative, representative data that highlights more local variations in dementia caregiving is essential, as such data can better target and tailor services as well as advance policy innovations at the state level. We empirically examined state variations in dementia caregiving in the U.S. by analyzing data from the Behavioral Risk Factor Surveillance System (BRFSS) among the 39 states that used the Caregiver Module in 2021. Prevalence of hypertension among dementia caregivers ranged from a low of 28.7% (CI 16.7-44.8) in South Dakota to a high of 53.6% (CI 43.9-63.1) in Arkansas. The mean hypertension prevalence rate in the 39 states for dementia caregivers was 42.0%, compared with a mean hypertension prevalence rate for non-dementia caregivers of 38.2%. Close to half (44.2%, CI 31.8-56.6) of dementia caregivers in Michigan provide care for 40 or more hours per week. In 35 of the 39 states, at least one-third of dementia caregivers provide care for 20 or more hours per week; in comparison, at least one-third of non-dementia caregivers provide care for 20 or more hours per week in only 7 of the 39 states. The findings illustrate how intensity and the health implications of dementia care vary across states in the U.S. and can help inform more localized programmatic and policy targets to address the public health implications of dementia care.

### SESSION 3025 (SYMPOSIUM)

Abstract citation ID: igad104.0818

### THE STATE OF THE DIGITAL DIVIDE

Chair: Walter Boot

Over the past few decades, the age-related digital divide has narrowed. For example, since the year 2000 in the United States, the percentage of older adults (65+) online has increased dramatically from 14% to 75%. However, compared to the 96% of individuals 50 to 64 years of age online, this still represents a substantial divide. Similar trends are observed in terms of social media usage and smartphone adoption, and even for older adults who own a smartphone, on average, smartphone proficiency can be substantially lower relative to younger people. This divide can put older adults at a substantial disadvantage and lock them out of the many benefits of existing and emerging technologies. This session will tackle issues surrounding the age-related digital divide. S. Cotten will provide a broad overview of the digital divide and theoretical perspectives that can help explain it. J. Chung will describe how the digital divide impacted social connectivity during the pandemic in low-income communities. S. Pace will discuss African American older adults, ICT use, and loneliness. S. Zhang will discuss attitudinal contributions to technology use and adoption relevant to the digital divide. Finally, W. Boot will discuss technology access and adoption and intersectionality and will discuss potential solutions to close or eliminate the gap in technology adoption and proficiency between younger and older people.

Abstract citation ID: igad104.0819

### BRIDGING THE DIVIDE: REACHING THE POTENTIAL OF TECHNOLOGY TO ENHANCE OLDER ADULTS' WELL-BEING AND QUALITY OF LIFE

Walter Boot, *Florida State University, Tallahassee, Florida, United States*

Existing and emerging technologies hold great promise with respect to improving the lives of older adults, in particular, by supporting and enhancing their independence, productivity, health, safety, social connectivity, and quality of life. Unfortunately, although differences in technology use and adoption between younger and older adults have declined over the past decades, barriers still exist that fully prevent the promise of technology from being realized to achieve these aims. This presentation will provide an overview of the current state of the intersection of aging and technology (nationally and internationally), with special attention to factors such as diversity, disability, and geographic region. Next, I will discuss approaches available to reduce the digital divide. These include changing the person (e.g., changing attitudes and proficiency) and changing the technology (human factors engineering). I will then discuss how a user-centered design process is crucial for the success of technology-based interventions. Without placing the user at the center of the design process, many novel technology solutions will likely fail to reach their potential. Finally, I will conclude with a discussion of if and when the age-related digital divide might close entirely by projecting changes in demographics, technology proficiency, technology proliferation, and technology change into the future.

Abstract citation ID: igad104.0820

### CHALLENGING IDEAS OF CONSTANT CONNECTION AND DIGITAL INCLUSION

Shelia Cotten, and Amy Schuster, *Clemson University, Clemson, South Carolina, United States*

1We live in a world where we are constantly connected to devices. This constant digital connection is where many individuals communicate and exchange social support with social ties. For most, this results in being online and connected to devices multiple times each day. Older adults have been slower to adopt and use emerging technologies. This presentation challenges the concept of digital inclusion by focusing on older adults and their technology use. We provide an overview of technology usage by different age groups using existing national level data. Next, we utilize life course and aging theoretical perspectives to help articulate that while a constant connection to devices may be normative for younger age groups, this may not, and perhaps should not, be the case for older adults. We conclude with a discussion of the social construction of digital inclusion and emphasize the significant variation that exists in this construct.

Abstract citation ID: igad104.0821

### RAPID DIGITAL TRANSFORMATION EXPERIENCES FOR SOCIAL CONNECTEDNESS AMONG LOW-INCOME OLDER ADULTS

Jane Chung[1], Tracey Gendron[1], Jodi Winship[2], Rachel Wood[3], Natalie Mansion[4], and Pamela Parsons[1], *1. Virginia Commonwealth University, Richmond, Virginia, United States*, *2. Virginia Center on Aging, Virginia Commonwealth University, Richmond, Virginia, United States*, *3. Bon Secours Mercy Health System, Cincinnati, Ohio, United States*, *4. Virginia Commonwealth University College of Health Professions, Richmond, Virginia, United States*

Digital technology has become critical for making social connections and providing social support during COVID-19. However, social isolation challenges disproportionately affected older adults living alone in low-income communities due to a lack of internet access, digital technology ownership, and limited capital. We conducted a mixed-methods study to examine the role of digital technology in this population to keep socially connected during the national lockdown (N=25; 76% African American). Of the participants, 36% reported social isolation, and 36% reported loneliness. Both quantitative and qualitative data illustrate differential experiences of technology use for remaining socially connected. The qualitative data from focus group interviews show that participants were forced to adapt to the isolated situation quickly by learning about new ways of connecting with others through technology. This enabled a long-overdue adoption of digital technologies among individuals who had experienced digital exclusion before the pandemic. Despite the high interest in digital technology-enabled social connections, concerns about its surveilling nature, the possibility of over-dependence on technology, and negative technology experiences due to unreliable functions or lack of tech support made them hesitant to adopt new technology. Future research should highlight the need to include older adults in all stages of technological solution development that recognize the sociocultural context of use with a focus on growth-oriented opportunities for older people.

Abstract citation ID: igad104.0822

### PREDICTING OLDER ADULTS' CONTINUED COMPUTER USE AFTER INITIAL ADOPTION

Shenghao Zhang, and Walter Boot, *Florida State University, Tallahassee, Florida, United States*

Digital technologies have the potential to help older adults in various aspects of their lives, but older adults need to adopt and keep using the new technologies to reap the benefits. Previous research on digital divide mainly focuses on adoption and acceptance of new technologies, and little is known about what might influence actual use and disuse after this initial stage. The current study modeled changes in constructs related to computer use after initial computer adoption and examined whether these changes predict continued use. We used data from the computer arm (N = 150, MAge = 76.15) of a 12-month field trial examining the potential benefits of computer use in older adults. Individual differences identified in the technology acceptance literature (perceived usefulness, ease of use, computer interest, computer self-efficacy, computer anxiety, quality of life, social isolation, and social support) were measured before (baseline), during (month 6), and after the intervention (posttest). Univariate and bivariate latent change score models examined changes in each predictor and their potential causal relationship with use. Results demonstrated large inter-individual differences in the change patterns of individual difference factors examined. Change in perceived usefulness, perceived ease of use, and computer interest, computer self-efficacy, and computer anxiety were correlated with but not predictive of change in use. Our findings demonstrate the limitation of popular constructs in technology acceptance literature in predicting continued use after initial adoption and point out important gaps in knowledge to be targeted in future investigations.

Abstract citation ID: igad104.0823

### HOW TECHNOLOGY IMPACTS LONELINESS: PERSPECTIVES OF AFRICAN AMERICAN OLDER ADULTS

Sarah Pace, *University of South Carolina, Columbia, South Carolina, United States*

The use of information and communication technologies (ICT) has been found to decrease loneliness among older adults by enabling communication with friends and family. Over 25% of older adults experience loneliness, and having a larger social network can reduce this risk (Chawla et al., 2021; Dahlberg et al., 2022). Despite the success of using ICTs and the growing ubiquity of ICTs among people of varying social demographic backgrounds there remains a digital divide, which refers to inequality in terms of access to ICT based on race, age, economic status, or other factors. While ICT use is becoming increasingly common among older adults, few studies have included African American older adults, who are less likely to have access to high-speed internet and computers than their White counterparts. However, African American adults are more likely to use text messages, video and voice calls, email, and social media to stay connected. The proposed research aims to explore the perspectives of African American older adults on how ICT impacts loneliness. Semi-structured interviews will be used to collect data from a purposive sample of about 10 people who meet specific selection criteria. Data will be analyzed using thematic analysis. The results may be useful for gerontological practitioners interested in promoting ICT use as a way to enhance the social well-being of African American older adults. The study could lead to the development of interventions that increase technology adoption and use, ultimately reducing loneliness among African American older adults.

### SESSION 3030 (SYMPOSIUM)

Abstract citation ID: igad104.0824

### POLICY SERIES: LET'S CONNECT: LINKING RESEARCH TO POLICY IN FAMILY CAREGIVING

Chair: Lauren Bangerter Co-Chair: Michael Wittke
Discussant: Fawn Cothran

There is a need in the field of family caregiving to bridge the gap between academic research and policy translation by providing interdisciplinary caregiving researchers the skills and training they need to ensure their research is relevant to today's policy discussions on family caregiving at the state and national levels. Quality research rests on sound institutional processes and methodological practices. This session will explore opportunities to elevate caregiving research for advocacy and policy audiences and how to inform policymakers about cutting-edge caregiving research across disciplines - particularly by advancing goal 5 within the National Family Caregiver Strategy - which calls for a stronger research and data collection infrastructure. We will offer caregiving researchers the best approaches for integrating policy research and implementation science approaches into their work as well as tactics to help sharpen their skills in messaging caregiving research for policy audiences, writing for a variety of audiences and platforms, and presenting data for policy and advocacy audiences. Presenters will cover topics such as facilitating additional research by public and private institutions by standardizing, expanding, and improving data collection by federal agencies; improving data collection on family caregiver health conditions and risk factors; improving data collection on family caregivers of people with multiple conditions; constructing additional channels on the local level that can gather detailed patient and family caregiver experience data to inform public health programming and policy development; and disaggregating data on diverse family caregiver populations to address the lack of segmentation and intersectionality in family caregiver research.

Abstract citation ID: igad104.0825

### LEVERAGING A BEHEMOTH: TRANSLATING VA FAMILY CAREGIVER RESEARCH TO POLICY AND PRACTICE

Ranak Trivedi, *Stanford University, Stanford, California, United States*

The VA has been a pioneer in research, practice and policy that supports the needs of caregivers. The VA HSR&D funded Elizabeth Dole Center of Excellence in Veteran and Caregiver Research, of which Dr. Trivedi is a site PI and part of the core leadership, is a testament to this investment in research, as are several scientific projects. The VA also provides support for caregivers by providing an array of services (e.g., hotline; caregiver training programs; stipends) and has deployed 2000+ staff nationally to connect caregivers to VA and non-VA services. The VA has also invested in rigorous evaluations of existing practice and policy with the eye on refining and improving. Dr. Trivedi will provide an overview of her work within the VA that focuses on developing behavioral intervention to target the collective stress coping needs of Veteran-caregiver dyads. With the lens of implementation science, she will share her work identifying barriers and facilitators of accessing home and community-based services, and describe how these insights are being used to inform VA practice and policy.

Abstract citation ID: igad104.0826

### TARGETING AND APPROACHING LEGISLATURES TO SUPPORT FAMILY CAREGIVING: LESSONS LEARNED

Beth Fields, *University of Wisconsin-Madison, Madison, Wisconsin, United States*

The Caregiver Advise, Record, and Enable (CARE) Act has passed in 45 states and requires hospitals to ask patients if they have a family caregiver, notify that family caregiver about the patients upcoming discharge, and educate family caregivers how to provide the care the patient will need after discharge. Early research demonstrates that this Act has favorably influenced hospital and patient outcomes such as quality indicators and family caregiver confidence. Despite this research and widespread support, this Act has yet to be passed in Wisconsin. This presentation will report on lessons learned from a systematic method for targeting and approaching Wisconsin state legislatures within the Assembly Committee on Health, Aging, and Long-term Care. Interviews, fieldnotes, in-person visits, and telephone and email correspondences with legislatures were conducted to learn their perspectives on advancing family caregiving policy. Lessons learned from this systematic method for targeting and approaching legislatures included the timing of interactions matter, the use of concise language maximizes understanding, the mode of delivery impacts response rate, and the transformation of family caregiving research to infographics increases uptake. Findings from this work can be used to elevate family caregiving support by bridging the evidence to policy gap.

Abstract citation ID: igad104.0827

### A NOVEL NATIONAL FAMILY CAREGIVER ADVOCACY TRAINING PROGRAM: LESSONS LEARNED

Heidi Donovan, *University of Pittsburgh, Pittsburgh, Pennsylvania, United States*

The 2022 National Strategy to Support Family Caregivers recommended 24 legislative or policy changes to improve the national caregiving landscape. With basic training, unpaid family caregivers (CG) can be powerful stakeholder advocates to drive policy change. In collaboration with national caregiver advocacy organizations, the University of Pittsburgh National Rehabilitation Research and Training Center on Family Support hosted a Family Caregiver Advocacy Program (FCAP) with the educational objectives of increasing CG motivation, self-efficacy for, and participation in policy advocacy. FCAP is a series of virtual workshops for family caregivers on caregiving-related policies, how laws are written and passed, how caregiver advocates can inform the policy-making process, how to contact and meet with legislators, and how to tell their story to advocate for change. Caregivers (n=68) enrolled in the inaugural FCAP workshops participated over a 3-month period to develop and implement group-selected advocacy products. FCAP participants ranged from 20-29 to 70+ years old, were primarily women (88%) caring for individuals who are aging (65%), have a physical disability (41%), have an intellectual / developmental disability (27%), and / or have a mental illness (27%). This presentation will report on the CG products and outcomes and plans for future iterations of FCAP based on lessons learned.

### SESSION 3035 (PAPER)

### GLOBAL AGING (BSS)

Abstract citation ID: igad104.0828

### EXAMINING SOCIOCULTURAL RESILIENCE FACTORS IN WIDOWHOOD AND COGNITIVE FUNCTION AMONG OLDER CHINESE IMMIGRANTS

Yura Lee[1], and Yanping Jiang[2], *1. University of Wisconsin-Milwaukee, Milwaukee, Wisconsin, United States*, *2. Rutgers University, New Brunswick, New Jersey, United States*

Loss of a spouse is one of the most stressful life events in later life and often entails significant physical and mental health outcomes for widowed individuals. Although there is growing evidence on widowhood and cognitive function, existing studies have shown mixed results. Little is known about resilience factors that may attenuate the adverse effect of widowhood on cognition among older Asian immigrants. This study explored potential moderators (i.e., social support, acculturation, leisure activities), which may contribute to resilience in the association between widowhood and cognitive function among older Chinese immigrants. The study sample included 2,515 adults aged 60 or older who completed two waves (2011–2013 and 2013–2015) of the Population Study of Chinese Elderly in Chicago. Cognitive function was measured by global cognitive function and episodic memory. Widowhood group was categorized into three groups based on the marital status at two waves: 1) married, 2) recently widowed and 3) previously widowed. Linear regression analyses were conducted with interaction terms. Our results show that social support moderated the relationship between widowhood and global cognitive function, and acculturation moderated the relationship between widowhood and episodic memory. Specifically, the findings indicate that adverse effect of widowhood on cognitive function is more pronounced at lower levels of social support and acculturation. This implies buffering roles of social support and acculturation in cognitive health among older Chinese immigrants who experience widowhood. Providing supportive programs and interventions to increase social support and acculturation is suggested to promote cognitive function in this population.

Abstract citation ID: igad104.0829

### FORGING A PATH TO DEVELOPING AGE-FRIENDLY CITIES AND COMMUNITIES IN GHANA: ISSUES AND RECOMMENDATIONS

Samuel Asante[1], and Grace Karikari[2], *1. Northeastern State University, Broken Arrow, Oklahoma, United States*, *2. University of North Dakota, Grand Forks, North Dakota, United States*

The World Health Organization in 2007 launched the Age-friendly Cities and Communities (AFCC) initiative in an effort to make cities around the world more attuned to the needs of older adults. While AFCC initiatives have mostly been undertaken in developed societies around the world, developing countries appear to be excluded in this effort, as there is little to show for in the quest to creating AFCC. One of such countries is Ghana, where the proportion of older adults is expected to increase exponentially in the next decade, thereby heightening the need for AFCC. Utilizing a multiple case study approach, this paper synthesizes the procedures and findings of 16 case studies from around the world published on the eight domains of WHO's Age-friendly Cities Framework, to make a case for developing AFCC in Ghana. Five important considerations were identified as fundamental to the effort to developing AFCC in Ghana: 1. Strengthening traditional views/perceptions on aging and older adults; 2. Involving key players in age-friendly efforts; 3. Responding to the complex interplay of needs and demands of older adults; 4. Designing policies and social programs to stimulate and enable cities and communities in Ghana to become age-friendly; 5. Developing validated instruments to assess the age-friendliness of cities and communities in Ghana. Designing and implementing AFCC require long-term commitment and devoting considerable resources to the cause. The paper concludes by discussing a culturally-appropriate model and policy recommendations to facilitate the move toward building age-friendly communities and age-inclusive cities in Ghana.

Abstract citation ID: igad104.0830

### YOU JUST LEARN WHAT TO HOLD ON TO AND WHAT TO LET GO: NARRATIVES OF MINDFUL AGING IN SOUTH ASIAN AMERICAN OLDER ADULTS

Mushira Khan[1], and Ajla Basic[2], *1. Mather Institute, Evanston, Illinois, United States*, *2. Mather, Evanston, Illinois, United States*

Mindful Sustainable Aging (MSA) is a multidimensional concept incorporating elements from four prominent gerontological theories – activity theory, disengagement theory, successful aging theory, and gerotranscendence theory (Nilsson et al., 2015). Informed by mindfulness practices, MSA is a holistic approach to supporting healthy aging in the social, physical, mental, and spiritual dimensions of life (ibid.). Using MSA as a theoretical framework, this qualitative study explores mindful aging in community-dwelling South Asian American older adults – one of the fastest-growing yet relatively understudied racial/ethnic minority groups in the US. In-depth, semi-structured interviews were conducted with 32 South Asian Americans aged 50+ (18 male, 14 female; mean age=63.5 years). Thematic analysis of the interview data showed that aging was accompanied by significant modifications such as adjusting to everyday life after the death of a spouse, perceived loss of role and status in the family, reduced finances, and limited mobility due to emergent health concerns. Yet, participants expressed an overall sense of peace and gratitude, embracing aging as an "inevitable" and "natural" process of discovery and acquiring wisdom. Participants tried to remain connected to their social networks through messaging and video calling apps like WhatsApp, volunteered at local places of worship like temples and mosques, and helped their adult children with childcare and household chores. Limited availability of culturally-appropriate opportunities for active aging was a key barrier to participants' sense of well-being. These findings suggest that culturally-sensitive, mindfulness-based interventions like gentle yoga or bilingual guided meditations may enhance subjective well-being in this population sub-group.

### SESSION 3040 (SYMPOSIUM)

Abstract citation ID: igad104.0831

### BUILDING BRIDGES, CATALYZING RESEARCH, EMPOWERING ALL AGES: A CHAT WITH NIA SENIOR LEADERSHIP

Chair: Amy Kelley Discussant: Amy Kelley

The National Institute on Aging (NIA) at the National Institutes of Health, Department of Health and Human Services, is the federally designated lead agency on aging research and supports research to understand the nature of aging and to extend the healthy, active years of life. Over the last decade, NIA's budget has increased substantially. In keeping with NIA's scientific mission, funding includes significant investments in Alzheimer's disease (AD) and AD-related dementias research, as well as in non-AD research. This symposium will provide a forum for exploration of the implications of the NIA budget for the general research community. NIA's senior staff will discuss research priorities and programs supported by the Institute. A question-and-answer session will follow these remarks on current funding and future priorities and research directions of NIA.

Abstract citation ID: igad104.0832

### OVERVIEW OF NIA BUDGET

Richard Hodes, *National Institutes of Health (NIH), Bethesda, Maryland, United States*

Dr. Hodes will discuss the NIA budget, overall research priorities of the Institute, and key programs and initiatives.

Abstract citation ID: igad104.0833

### NIA DIVISION OF AGING BIOLOGY

Ronald Kohanski, *National Institute on Aging, Bethesda, Maryland, United States*

Dr. Kohanski will discuss research priorities for the NIA Division of Aging Biology. Dr. Kohanski will also be available for small group discussion.

Abstract citation ID: igad104.0834

### NIA DIVISION OF BEHAVIORAL AND SOCIAL RESEARCH

Lisbeth Nielsen, *National Institute on Aging, Bethesda, Maryland, United States*

Dr. Nielsen will discuss research priorities for the NIA Division of Behavioral and Social Research. Dr. Nielsen will also be available for small group discussion.

Abstract citation ID: igad104.0835

### NIA DIVISION OF GERIATRICS AND CLINICAL GERONTOLOGY

Basil Eldadah, *NIA, Bethesda, Maryland, United States*

Dr. Eldadah will discuss research priorities for the NIA Division of Geriatrics and Clinical Gerontology. Dr. Eldadah will also be available for small group discussion.

Abstract citation ID: igad104.0836

### NIA DIVISION OF NEUROSCIENCE

Jennie Larkin, *National Institute on Aging, Bethesda, Maryland, United States*

Dr. Larkin will discuss research priorities for the NIA Division of Neuroscience. Dr. Larkin will also be available for small group discussion.

Abstract citation ID: igad104.0837

### NIA DIVISION OF EXTRAMURAL ACTIVITIES

Kenneth Santora, *NIA, NIH, Bethesda, Maryland, United States*

Dr. Santora will discuss the work of the NIA Division of Extramural Activities. Dr. Santora will also be available for small group discussion.

Abstract citation ID: igad104.0838

### NIA OFFICE OF SPECIAL POPULATIONS

Patricia Jones, *NIH, NIA, Bethesda, Maryland, United States*

Dr. Jones will discuss the work of the NIA Office of Special Populations. Dr. Jones will also be available for small group discussion.

### SESSION 3045 (SYMPOSIUM)

Abstract citation ID: igad104.0839

### THE AGING NETWORK AS A CATALYST FOR COMMUNITY GERONTOLOGY AND APPLIED RESEARCH

Chair: Traci Wilson Discussant: Emily Greenfield

The Older Americans Act (OAA) established Area Agencies on Aging (AAAs) fifty years ago as local community-based organizations responsible for planning, coordinating, funding, and delivering nutrition and other supportive services which allow older adults to remain living in their homes and communities. Local control and decision-making, mandated by the OAA, has allowed AAAs to adapt to the evolving needs and demands of their communities The centrality of their role was never more apparent than during the COVID-19 pandemic. As national and societal circumstances have resulted in increased social isolation, housing insecurity, and public health concerns for older adults, AAAs have adapted and expanded beyond traditional OAA services to address the social and health needs of an increasingly diverse older adult population. The Community Gerontology framework provides a useful structure to understand the evolution and impact of AAAs and the broader Aging Network on the health of older adults. Using data from the 2022 National Survey of AAAs and a new qualitative study on Community Care Hubs, academic and Aging Network-based researchers will illustrate the many partnerships and pathways of mutual influence between AAAs and the micro and macro levels to describe how Aging Network services are expanding provision of services for health care partners, public health initiatives, and new housing and homelessness services and partnerships to address emerging needs. The symposium will conclude with an illustration of how applied researchers and Aging Network organizations partnered on research-informed practice to innovate and strengthen services for at-risk older adults.

Abstract citation ID: igad104.0840

### THE ROLE OF AREA AGENCIES ON AGING IN PUBLIC HEALTH VACCINATION CAMPAIGNS: FINDINGS FROM A NATIONAL SURVEY

Traci Wilson, *USAging, Washington, District of Columbia, United States*

When COVID-19 vaccinations became available, AAAs played a critical role in ensuring that older adults could receive the lifesaving vaccine. AAAs built on their existing service expertise and vast partnership networks to quickly implement vaccine outreach and education, scheduling support, transportation and other supportive services to attend the appointment, and age-friendly and culturally appropriate vaccination clinics. The CDC recognized AAAs' early contributions and awarded $50 million to support continuation of these activities. In early 2023, the Aging and Disability Networks received an additional $75 million from ACL, awarded to USAging's Aging and Disability Vaccination Collaborative, to fund local efforts across the United States to increase vaccine uptake among older adults and people with disabilities, targeting individuals from rural, minority, and disadvantaged backgrounds. This paper describes how AAAs were uniquely positioned to assume this expanded public health role and specific characteristics that contributed to their readiness. Data from the 2022 National Survey of Area Agencies on Aging (n=457 AAAs, 74% response rate) describe new and expanded AAA activities and partnerships that support public health efforts. We will discuss implications for community partnerships to improve public health and look ahead to measuring the impact of these vaccine promotion activities.

Abstract citation ID: igad104.0841

### RESPONDING TO THE HEALTH CARE ENVIRONMENT: DEVELOPMENT OF CBO NETWORKS TO ADDRESS SOCIAL DETERMINANTS OF HEALTH

Beth Blair[1], Suzanne Kunkel[2], Abbe Lackmeyer[2], Robert Graham[2], and Marisa Scala-Foley[1], *1. USAging, Washington, District of Columbia, United States*, *2. Miami University, Oxford, Ohio, United States*

AAAs, with their wide array of community partnerships and ability to respond to local needs and context, are an important connection between social and health care. Recent research describes the impact of AAA services and partnerships on population health, and AAAs are increasingly contracting with health care systems and payers to provide a variety of supportive services that enable older adults to live at home and in the community. To better meet the needs of health care partners, and to maximize their service reach and impact, AAAs and other community-based organizations (CBOs) are increasingly forming networks to contract with health care entities. Data from the 2021 CBO-Health Care Contracting Survey shows that 44 percent of AAAs are part of a network that provides contracted services to health care entities, but little is known about how these networks are formed and sustained. Findings from a new qualitative study on CBO networks, which consisted of 23 semi-structured interviews with network leads (also known as community hubs) and member organizations, describe how AAAs and other CBOs are developing networks and contracting with health care entities. This session will share findings from the qualitative study to describe the opportunities and ongoing challenges of network participation. Networks allow CBOs to serve more people and contract with health care partners to provide integrated care, but ongoing challenges include lack of data infrastructure and financial viability for members that may have fewer clients to serve. This session will also discuss the policy implications of these findings.

Abstract citation ID: igad104.0842

### THE INCREASING INVOLVEMENT OF AAAS IN ADDRESSING THE HOUSING NEEDS AND HOMELESSNESS OF OLDER ADULTS

Robert Graham[1], Isha Karmacharya[1], Traci Wilson[2], and Molly French[2], *1. Miami University, Oxford, Ohio, United States*, *2. USAging, Washington, District of Columbia, United States*

While services addressing the housing needs and homelessness of older adults are not a core service of the OAA, the Aging Network is adapting to the levels of growing housing insecurity facing older adults. The need for affordable and accessible housing has increased in recent years. In the 2019 National Survey of AAAs, 30% of agencies reported a desire for training and technical assistance on better addressing housing/homelessness issues; by 2022 that interest increased to 46% of agencies.

Further, 81% of AAAs said that they are now providing one or more housing/homelessness programs and services. AAA involvement in housing and homeless programs and services may range from providing home modifications, supportive housing, or even owning senior housing. Community partnerships play a critical role in meeting the housing needs of older adults. This presentation shows how AAAs have adapted to address these needs, including programs, services, community partnerships, and advocacy efforts.

Abstract citation ID: igad104.0843

### THE AGING NETWORK AND CLIMATE RESILIENCE

Holly Dabelko-Schoeny[1], Smitha Rao[1], Marisa Sheldon[2], and White Katie[3], *1. The Ohio State University, Columbus, Ohio, United States*, *2. Ohio State University, Columbus, Ohio, United States*, *3. Central Ohio Area Agency On Aging, Columbus, Ohio, United States*

Originally established to support planning and services related to community-based supports, Area Agencies on Aging (AAA) are well-positioned to lead cutting edge innovations driven by local community needs and changing socio-environmental contexts. This presentation illustrates a partnership between the Central Ohio Area Agency on Aging (COAAA) and the Age-Friendly Innovation Center at The Ohio State University focused on advancing research-informed practice in response to the realities of aging in a warmer world. This project included three main phases. First, safety and emergency preparedness data were collected from individuals aged 50 and older living in central Ohio through a random sample survey (N=1417), revealing 25% of non-white residents (compared to 21% white) did not have or know they have 3-day emergency supplies, food, and medicine, and 84% of residents in urban areas did not have access to an alternative source of power in event of outages. Second, a community conversation identifying priorities and needs related to the rising number of extreme weather events was held with COAAA leadership, community organizations, and residents. Third, targeting locations of high density of older adults at highest risk, the last phase of this work focused on the development of a tool to reduce harm for older adults living in affordable housing in partnership with COAAA embedded Service Coordinators. This project illustrates how AAAs can partner with applied researchers to innovate and strengthen services within communities and build evidence to address emerging community needs across the aging network.

### SESSION 3050 (SYMPOSIUM)

Abstract citation ID: igad104.0844

### MULTIDIMENSIONAL AND INTERSECTIONAL APPROACHES TO ACCESSIBLE AND HEALTHY AGING IN DIVERSE POPULATIONS

Chair: Catherine Garcia

Aging is a complex process that affects different individuals in different ways. To ensure that healthy aging is accessible for all individuals, it is crucial to consider the unique needs of diverse populations through multidimensional and intersectional approaches. Multidimensional approaches recognize that aging is a complex process and that many different factors shape how individuals experience it. Intersectional approaches to aging consider individuals' multiple identities and social locations, creating unique experiences and needs around aging. When these two approaches are combined, it is possible to create accessible and healthy aging for individuals from all backgrounds. The five presentations in this symposium center on two large and diverse racial and ethnic groups in the U.S. – Vietnamese and Puerto Rican older adults – groups underrepresented in studies of aging. Using data from the Vietnamese Aging and Care Survey (VACS), Miyawaki and colleagues examine the association between disability, chronic disease, and depression. The next set of presentations uses longitudinal data from the Puerto Rican Elderly Health Conditions Project (PREHCO). Quashie and colleagues explore whether neighborhood socioeconomic status moderates the relationship between living arrangements and cardiometabolic disease. Thompson and colleagues examine the relationship between hurricane stressors and cognition. Guinn and colleagues examine whether social support mediates the relationship between hurricane-related stressors and depression. Ballard and colleagues examine how hurricane-related stressors influence perceived stress. Overall, the results from these investigations show a need for authentic community and stakeholder engagement to develop, implement, and create sustainable interventions to promote healthy aging.

Abstract citation ID: igad104.0845

### ASSOCIATIONS BETWEEN DISABILITY, CHRONIC DISEASES, AND DEPRESSIVE SYMPTOMS IN VIETNAMESE OLDER ADULTS

Christina Miyawaki[1], Joshua Garcia[1], Luis Medina[1], Kim Nguyen[1], Oanh Meyer[2], and Kyriakos Markides[3], *1. University of Houston, Houston, Texas, United States*, *2. University of California at Davis, Davis, California, United States*, *3. University of Texas Medical Branch, Galveston, Texas, United States*

After the fall of Saigon in 1975, several waves of Vietnamese immigrants/refugees migrated to the U.S. currently comprising 2.2 million individuals, the 4th-largest Asian-origin subgroup in the U.S. Despite the large number and their traumatic, adverse lifelong experiences, health research on this population is scarce. To fill this gap, we developed the Vietnamese Aging and Care Survey and collected health data on older Vietnamese in Houston, Texas, the 2nd-largest Vietnamese-populated metropolitan area in the nation. The purpose of this study was to examine the associations between their physical disability, chronic diseases, and depressive symptoms. Respondents (N=210) were 76 years old (mean), married (63%), and female (56%) with 8 years of education. They lived in the U.S. for 25 years (mean) and spoke Vietnamese only (88%) in multi-generation (80%) low-income households (91%, ≤$25K). They self-rated their health as fair/poor (80%) with ≥1 chronic condition (76%). Those with higher depressive symptoms were less likely to be married ($p=0.004$) and have more chronic diseases ($p=0.004$) compared to those without depressive symptoms. Regression analyses showed significant associations between arthritis ($\beta=2.73$), liver disease ($\beta=6.24$), ADL disability ($\beta=0.72$), and higher depressive symptoms. Limited control over their basic function (ADL disability) would restrict their daily lives, and mobility issues and pain (arthritis and liver disease) might impact them psychologically. While older Vietnamese may leverage their multi-generation living condition, and receive tangible support with ADL disability within their families, healthcare professionals should connect them with culturally and linguistically relevant adult daycare centers that provide social and exercise opportunities.

Abstract citation ID: igad104.0846

### NEIGHBORHOOD SOCIOECONOMIC POSITION, LIVING ARRANGEMENTS, AND CARDIOMETABOLIC DISEASE AMONG OLDER PUERTO RICANS

Nekehia Tamara Quashie[1], Catherine Garcia[2], Gabriella Meltzer[3], Flavia Andrade[4], and Amilcar Matos-Moreno[5], *1. University of Rhode Island, Kingston, Rhode Island, United States*, *2. Syracuse University, Syracuse, New York, United States*, *3. Columbia University Mailman School of Public Health, New York City, New York, United States*, *4. University of Illinois--Urbana-Champaign, Urbana, Illinois, United States*, *5. Pennsylvania State University, State College, Pennsylvania, United States*

Cardiometabolic diseases are among the leading causes of mortality worldwide and are increasingly prevalent in rapidly aging populations such as Puerto Rico. Neighborhood socioeconomic position (SEP) and living arrangements are increasingly recognized as important determinants of cardiometabolic health but have not been examined within Puerto Rico. Using longitudinal data from the Puerto Rican Elderly Health Conditions Project (Waves I 2002/03, and II 2006/07), linked with 2000 Census data for neighborhood-level conditions, this study examined the association between neighborhood SEP, living arrangements, and risk of cardiometabolic conditions among island-dwelling Puerto Ricans aged 60 and older, who lived in the same residence across both waves (N=2,769). Findings show that residence in a socioeconomically advantaged neighborhood was positively associated with reporting having one cardiometabolic condition at baseline, but neighborhood SEP was unrelated to developing cardiometabolic conditions at follow-up. Living without a partner - alone, with children, or with others - was negatively associated with reporting having and developing cardiometabolic conditions relative to living with a partner. Living arrangements significantly modified the relationship between neighborhood SEP and cardiometabolic conditions. Compared to living with a partner, living alone was associated with lower risk of reporting having one condition, and living with children was associated with lower risk of developing one cardiometabolic condition when living within a socioeconomically advantaged neighborhood. Living arrangements are more salient to cardiometabolic health than neighborhood SEP. Social services focused on household composition and familial support are needed to identify older Puerto Ricans potentially at risk of underdiagnosed chronic conditions.

Abstract citation ID: igad104.0847

### THE RELATIONSHIP BETWEEN HURRICANE STRESSORS AND COGNITION IN PUERTO RICAN OLDER ADULTS

Sheila Thompson[1], Victoria Guinn[1], Erin Ballard[2], Michael Crowe[3], and Lisa Brown[1], *1. Palo Alto University, Palo Alto, California, United States*, *2. University of Alabama at Birmingham, Birmingham, Alabama, United States*, *3. University of Alabama-Birmingham, Birmingham, Alabama, United States*

Acute and chronic stress exposure negatively impact cognition in older adults. There is little research on this relationship within the context of an acute disaster. This presentation explores the relationship between perceived stress, hurricane stressors, subjective cognition, and cognition in Puerto Rican older adults post-Hurricane Maria. 613 Puerto Rican older adults completed Wave 3 measures for the longitudinal PREHCO study. Perceived stress was measured via a 4-item version of the Perceived Stress Scale. Hurricane stressors were classified as three factors: Home Damage, Family/Friends Network, and Work/Health. Subjective cognition was measured by asking the participant to rate their memory based on a 5-point Likert scale. Cognition was measured via the Telephone Interview for Cognitive Status assessment and the Minimental Cabán. Analyses included two multiple linear regressions with cognitive status and cognitive functioning as the outcomes, respectively, while controlling for age, depressive symptoms, education, gender, and race. There was a significant relationship between higher concern for others post-hurricane and worse generalized cognitive functioning ($p<0.05$, $t=-2.04$). Higher levels of cognitive status were significantly associated with better perceptions of memory function ($p= 0.015$, $t=-2.43$). Yet higher levels of perceived stress were significantly associated with worse perceptions of memory function ($p<0.001$, $t=7.037$). Older adult Puerto Ricans presented with worse cognitive functioning when they were worried about their loved ones' well-being. Even those who maintained an intact cognitive status believed their memory was worse when they were experiencing more stress. This highlights the importance of differentiating the impact of stress on cognitive ability from subjective cognition.

Abstract citation ID: igad104.0848

### HURRICANE STRESSORS AND BUFFERS IN RELATION TO DEPRESSIVE SYMPTOMS AMONG OLDER ADULTS IN PUERTO RICO

Victoria Guinn[1], Sheila Thompson[1], Erin Ballard[2], Michael Crowe[3], and Lisa Brown[1], *1. Palo Alto University, Palo Alto, California, United States*, *2. University of Alabama at Birmingham, Birmingham, Alabama, United States*, *3. University of Alabama-Birmingham, Birmingham, Alabama, United States*

The present study examines the role of religiosity and social support on depressive symptoms and the moderating role of hurricane stress related to friends and family following Hurricane Maria in a sample of Puerto Rican older adults. Data was collected from the Puerto Rican Elder: Health Conditions (PREHCO) study. The present study consists of 613 participants in Wave 3 of the longitudinal study. Depressive symptoms were measured using the Geriatric Depression Scale (GDS). Social support was measured using the Lubben Social Support Network scale. Religiosity and hurricane stress related to friends and family were measured using survey items developed by PRECHO investigators. Religion items include church attendance and the helpfulness of religion for health conditions. A linear regression was conducted. Controlling for education and days since the hurricane, church attendance

(b=0.642, p<.001), and social support (b=-0.098, p<.001) predicted depressive symptoms. When hurricane stressors related to family and friends were added to the model, church attendance (b=629, p<.001) and social support (b=-0.098, p<.001) remained significant predictors, and hurricane stressors predicted depressive symptoms (b=-0.378, p=.018). Helpfulness of religion was not a significant predictor of hurricane stress. Church attendance and social support are important coping strategies for Puerto Rican older adults. Increased social support and frequent church attendance buffer the development of depressive symptoms even when hurricane-specific stress is present. Future research may examine if church attendance and social support buffer the development of depressive symptoms when other non-social hurricane stressors are present.

Abstract citation ID: igad104.0849

### ASSOCIATIONS BETWEEN HURRICANE-RELATED STRESSORS AND PERCEIVED STRESS IN A SAMPLE OF PUERTO RICANS

Erin Ballard[1], Victoria Guinn[2], Sheila Thompson[2], Ross Andel[3], Lisa Brown[2], and Michael Crowe[4], *1. University of Alabama at Birmingham, Birmingham, Alabama, United States*, *2. Palo Alto University, Palo Alto, California, United States*, *3. University of South Florida, Tampa, Florida, United States*, *4. University of Alabama-Birmingham, Birmingham, Alabama, United States*

This project examines the relationship between hurricane-related stressors and perceived stress using data from the latest (third) wave of the Puerto Rican Elder: Health Conditions (PREHCO) study. A total of 617 PREHCO survivors, age 78 and older, were asked questions about their experience with stressors related to hurricane Maria. We assessed self-reported fright felt immediately after the hurricane as a primary stressor. A list of secondary stressors was examined using a principal components analysis (PCA) that yielded three components related to concern over home damage, family/friends, and employment/health. Demographic factors (age, education, race, sex) and length between the hurricane and interview date (conducted 2021-2022) were included in analyses. The majority (97%) of our sample was in Puerto Rico at the time of the hurricane. Approximately 41% of participants reported being "somewhat" or "very" frightened following hurricane Maria and over half of participants reported stressors like home repairs, damage to their neighborhood, and concern about bill payment. Regression analyses revealed higher reported fright immediately following hurricane Maria was associated with higher levels of perceived stress at the time of interview (b=.53, p<.001). In addition, all three secondary stressor components were significantly associated with higher perceived stress in separate models (home damage: b=.44, p<.001; family/friends: b=.40, p<.001; employment/health: b=.31, p<.05). Being female was associated with higher perceived stress across each of the regression models. Results suggest long-lasting relationships between hurricane-related stressors and perceived stress several years later, further implicating the role of these stressors on long-term mental and physical functioning.

### SESSION 3055 (SYMPOSIUM)

Abstract citation ID: igad104.0850

### A PUBLIC HEALTH APPROACH TO BRAIN HEALTH: THE HBI ROAD MAP SERIES

Chair: Shelby Roberts Discussant: Diane Ty

In 2005, congressional support led to the creation of the Healthy Brain Initiative (HBI) and the longstanding collaboration between the Alzheimer's Association and the Centers for Disease Control and Prevention to advance understanding of and support for cognitive decline as a central part of public health practice. Since then, HBI partners have worked together to implement public health strategies that promote brain health, address dementia and help support caregivers. The Healthy Brain Initiative Road Map Series guides this effort. The first HBI Road Map was released by CDC and the Alzheimer's Association in 2007 and was the first framework for viewing cognitive health and dementia as a public health issue. The second HBI Road Map was released in 2013 and expanded the role of state and local public health departments and partners in improving cognitive health. The third HBI Road Map, published in 2018, continued to chart a course for state and local public health departments and their partners by aligning actions with the essential services of public health. The HBI Road Map for Indian Country, published in 2019, is the first public health guide focused on dementia in American Indian and Alaska Native communities. The release of the fourth HBI Road Map in 2023 continues to advance the field and prepare public health departments to better address health equity and build capacity as new treatments become available. This symposium highlights successes of the Road Map Series and the development of the 4th edition of the HBI Road Map.

Abstract citation ID: igad104.0851

### THE HEALTHY BRAIN INITIATIVE ROAD MAP CATALYZES ACTION AMONG STATE, LOCAL, AND NATIONAL PARTNERS

Meghan Fadel[1], Peter Holtgrave[2], Carissa Beaty[3], and Shelby Roberts[1], *1. Alzheimer's Association, Chicago, Illinois, United States*, *2. National Association of County and City Health Officials (NACCHO), Washington, District of Columbia, United States*, *3. Emory University, Atlanta, Georgia, United States*

The Healthy Brain Initiative Road Map Series (Road Map) has achieved measurable success in spurring action in health departments across the country. Evaluation data from the Alzheimer's Association shows all 50 states have implemented actions from the 2018-2023 Road Map and the majority of states completed at least one action in each of the four domains of the Road Map. The Healthy Brain initiative has also sponsored initiatives to accelerate the implementation of Road Map actions. The projects described are separate from the CDC's BOLD Program Awards for state and local health departments. The Road Map Strategist Initiative supports local health departments and tribal organizations. It has funded 16 agencies over the past two years to implement Road Map actions in their communities. A coordinated campaign to increase implementation and utilization of the BRFSS caregiving and subjective cognitive decline modules has near nationwide uptake, and, in 2023, a new project, Data for Action, launched in four states to support the use and dissemination of BRFSS and other data. Together, these projects improve assessment, planning and measurement of community health outcomes related to brain health. The 4th edition of the HBI Road Map has an accompanying evaluation tool offering common measures for state and local health departments to track their success as they implement the new actions. This session will explore the past impact of Road Map actions and opportunities for future measurement.

Abstract citation ID: igad104.0852

### BUILDING SUSTAINABILITY IN PUBLIC HEALTH ALZHEIMER'S DISEASE PROGRAMS

Lisa McGuire[1], and Heidi Holt[2], *1. CDC, Atlanta, Georgia, United States*, *2. Centers for Disease Control and Prevention, Atlanta, Georgia, United States*

While public health experts cannot yet say how to prevent Alzheimer's disease and related dementias (ADRD), public health has a role in slowing ADRD through the implementation of risk reduction strategies, early diagnosis, and better education and training of front-line health care professionals. Efforts to enhance the sustainability of programs, with changes to systems, environments, and policies enhances uptake and increases the lifespan of these interventions, as well as facilitates continuity and integration of efforts across jurisdictions. This approach includes multiple partners from a wide-range of sectors. This presentation will describe public health approach to ADRD including increasing awareness and action on dementia risk reduction, early detection, and coordination with aging services including supporting dementia caregivers. We will discuss innovative approaches of achieving sustainability, including identifying approaches that seek to reach entire populations for widespread and sustained impact utilizing examples from BOLD Programs to develop widespread public health infrastructure.

Abstract citation ID: igad104.0853

### MULTISECTOR COLLABORATION DRIVES THE DEVELOPMENT OF THE FOURTH HEALTHY BRAIN INITIATIVE ROAD MAP

Kristen Clifford[1], James Appleby[2], Talyah Sands[3], Juan Rodriguez[4], and John Shean[4], *1. Alzheimer's Association, Austin, Texas, United States*, *2. The Gerontological Society of America, Washington, District of Columbia, United States*, *3. Association of State and Territorial Health Officials, Arlington, Virginia, United States*, *4. Alzheimer's Association, Chicago, Illinois, United States*

Partnerships and collaboration across sectors played a critical role in the development of the fourth edition of the Healthy Brain Initiative Road Map (Road Map). The public health community, leaders in related fields, and people living with dementia contributed to the development and review. The Alzheimer's Association and CDC invited 20 national leaders from public health, aging, academia, health care and health systems to guide the development of the fourth HBI Road Map as a part of the Leadership Committee. Their contributions included reviewing data, setting the overall direction of the review and leading five workgroups, composed of 68 subject-matter experts, who recommended actions on Risk Reduction of Cognitive Decline; Timely Detection, Diagnosis and Management of Cognitive Impairment; Dementia Caregiving; Health Equity; and Community Linkages. Health departments offered input through facilitated listening sessions and an open input period. Their feedback emphasized the importance increased information on health equity and partnerships in the Road Map. The Leadership Committee also advised continued partner engagement through the review process. This translated to review by the Alzheimer's Association, CDC subject matter experts, external health equity experts, the Leadership Committee, workgroup members, the Healthy Brain Initiative Collaborative, the Alzheimer's Association Early Stage Advisory Group and ultimately CDC leadership. This presentation will explore important themes that emerged throughout this process that shaped the design and evolution of the HBI Road Map series to its most recent iteration, as a guiding document with health equity and multi-sector collaboration action at its core.

### SESSION 3060 (SYMPOSIUM)

Abstract citation ID: igad104.0854

### A HISTORICAL-CONTEXTUAL PERSPECTIVE ON GRANDPARENTS' ACTIVITIES WITH GRANDCHILDREN

Chair: Jill Juris Discussant: Amy Rauer

Grandparenting is one of the most positive roles for many older adults, yet there is limited understanding of what activities grandparents and grandchildren actually engage in together or how these may vary based on both context (e.g., residential grandparenthood, grandparents' marriage) and historical events (e.g., COVID-19). Drawing from multiple theoretical frameworks and methodological approaches, this symposium begins with Flood providing a broad view of time grandparents and grandchildren spend together using large-scale population-level data from IPUMS. Illustrating how such activities may vary based on context, Stephan and Chan provide insights into how activity engagement varies by caregiver status and whether activity engagement with grandchildren is related to grandparents' perceived roles. Extending this examination of context to consider specific processes, Juris and Zvonkovic will then describe the process of family leisure among grandparent couples by examining how both individuals in a couple experience family leisure with their grandchildren. Providing a historical perspective on these experiences, Fruhauf and colleagues reveal the challenges of raising grandchildren in the context of COVID-19 and the importance of engaging in different and new activities with their grandchildren during this time. Finally, as discussant, Rauer will draw upon Bronfenbrenner's socioecological framework to illustrate how grandparenting is situated within a complex network of interdependent systems and dynamic contexts. Together, this symposium will highlight the implications of grandparenting for catalyzing development and enhancing well-being across multiple generations.

Abstract citation ID: igad104.0855

### LARGE-SCALE POPULATION-LEVEL DATA FOR STUDYING GRANDPARENTING IN THE UNITED STATES

Sarah Flood, *University of Minnesota, Minneapolis, Minnesota, United States*

The questions we can answer about grandparenting in the United States are shaped by the availability of data. This presentation will review previous research on trends and sociodemographic variation in grandparenting in the United States based on large-scale population-level data from IPUMS (www.ipums.org). It will highlight the various ways that researchers have measured families that include grandparents (e.g., three-generation families, grandfamilies). It will also discuss the limitations of these data, specifically the focus on coresidence, for studying grandparents. The presentation will then review research on grandparents using the American Time Use Survey, the only data in IPUMS that enables analyses of grandparents time spent with both their resident and non-resident grandchildren. These analyses will focus on the leisure activities that grandparents share with their grandchildren and how this varies by living arrangements and sociodemographic characteristics.

Abstract citation ID: igad104.0856

### GRANDPARENTS' ACTIVITY ENGAGEMENT WITH GRANDCHILDREN AS A FUNCTION OF CAREGIVER STATUS

Abigail Stephan[1], and Athena Chung Yin Chan[2], *1. Clemson University, Clemson, South Carolina, United States*, *2. Texas Tech University, Lubbock, Texas, United States*

Though the grandparent population varies across virtually all sociodemographic characteristics, caregiver status (i.e., whether grandparents are raising grandchildren), may be most salient in how grandparents engage in activities and perceive their roles in relation to their grandchildren. Informed by the bioecological process-person-context-time model, this mixed-methods study explores how activity engagement varies by caregiver status and whether activity engagement with grandchildren is related to grandparents' perceived roles. A convenience sample of 86 mid-life and older adults (Mage=65.12 years, range=42-82) completed an online survey with open- and closed-ended items related to their grandparenting experience. 22% of grandparents self-identified as grandparent caregivers (GC), or those who have been raising their grandchildren for six months or longer; the remaining 78% self-identified as non-caregiving grandparents (NCG). Comparative analyses did not yield statistically significant results, revealing GCs and NGCs largely engaged in similar activities with their grandchildren. However, activity engagement intervals differed by group (i.e., daily basis for GCs vs. weekly or monthly basis for NGCs), and a greater proportion of NCGs reported digital activity engagement with their grandchildren. Both GCs and NGCs identified their roles as emotional supporters to their grandchildren, though GCs also identified with instrumental roles (e.g., surrogate parent, anchor, financial supporter). These findings shed light on the connection between self-reported activity engagement and subjective role perceptions across just one sociodemographic characteristic—caregiver status—and suggest both groups share a similar grandparenting foundation, with an additional, "dual-roles" component for GCs. Implications for future research and practice will be discussed.

Abstract citation ID: igad104.0857

### HOW GRANDPARENT COUPLES ENGAGE IN FAMILY LEISURE WITH GRANDCHILDREN

Jill Juris[1], and Anisa Zvonkovic[2], *1. Appalachian State University, Boone, North Carolina, United States*, *2. University of Georgia, Athens, Georgia, United States*

There is limited understanding of how grandparent couples negotiate time with grandchildren. Family leisure is often purposive by being planned, facilitated, and executed by parents or grandparents to achieve goals such as improved interaction, communication, and family cohesion. Moving beyond studies of counting time in leisure, few studies of family leisure have included how people experience leisure as couples. Guided by a life course approach, this qualitative study provides insight to an understanding of the process of family leisure among grandparent couples by examining how both individuals in a couple experience family leisure with their grandchildren. Grandparent couples (n=10) ranged in age from 60 to 75 years old (M=66.8, SD=5.01). The grandparents interviewed were not providing custodial care to their grandchildren. Participants completed individual interviews that were then analyzed at the couple level. Through constructivist grounded theory, this study developed a process model of grandparents' decision-making that unfolded into four options for engaging in family leisure with grandchildren. The four options included: wanting a grandparent fix, going with the flow when invited by children, hosting grandchildren in their homes, and meeting expectations for family gatherings. Grandparent couples often described more than one option of family leisure based on their life course dimensions (i.e., individual, family, and sociohistorical). Implications for application in the fields of recreation and family sciences will be discussed.

Abstract citation ID: igad104.0858

### "EXPECT THE UNEXPECTED": CUSTODIAL GRANDPARENTS' PERSPECTIVES ON RAISING GRANDCHILDREN DURING COVID-19

Christine Fruhauf[1], Deborah Whitley[2], Susan Kelley[2], Andrea Smith[3], Youjung Lee[4], Heidi Tseng[1], and Bert Hayslip[5], *1. Colorado State University, Fort Collins, Colorado, United States*, *2. Georgia State University, Atlanta, Georgia, United States*, *3. Western Michigan University, Ada, Michigan, United States*, *4. Binghamton University, State University of New York, Binghamton, New York, United States*, *5. University of North Texas, Denton, Texas, United States*

Managing COVID-19 demands may exacerbate caregiving responsibilities of custodial grandparents raising grandchildren. This presentation aims to discuss qualitative data obtained from 14 custodial grandmothers who participated in virtual focus groups to understand their parenting experiences in conjunction with meeting COVID-19 demands. Participants were recruited from a larger sample (N=145, age range 30-79 years, Mage = 61.4 years, SD = 8.5) of custodial grandparents who completed a quantitative survey on COVID-19 stressors. Guided by McCubbins and Patterson's Family Stress and Adaptation model, three researchers conducted open coding of the focus group data. The analysis revealed distinguishing patterns related to managing challenges of grandchild caregiving brought on by COVID-19. Some grandparents discussed the demands of COVID-19 as difficult, but not as challenging as the day-to-day experiences of raising grandchildren. This was reflected when grandparents discussed raising grandchildren with special needs in the pandemic environment, advocating with schools to adjust virtual learning expectations, managing custody cases and social service home visits while 'protecting' grandchildren and themselves from COVID-19 exposure. Grandparents stated they did not change their parenting style to manage COVID-19 demands, yet they discovered a stronger, internal commitment to raising grandchildren. Grandparents reflected when isolating they enjoyed engaging in different and new activities with their grandchildren while becoming closer to them as a result. Findings suggest that challenges of raising grandchildren were heightened in the context of COVID-19 demands. Although grandparents experienced losses during the pandemic, they also reflected on gains related to their resourcefulness and resiliency in managing stressors.

### SESSION 3065 (SYMPOSIUM)

Abstract citation ID: igad104.0859

### RACIAL DISPARITIES IN OCCUPATIONAL EXPOSURES AND LATER-LIFE HEALTH

Chair: Amanda Sonnega Discussant: Theresa Andrasfay

Racial and ethnic health disparities remain a persistent and urgent social problem. Researchers are beginning to explore the role of work experiences, both good and bad, in attenuating or exacerbating health disparities. The recent availability of the entire life course experience of work in the Health and Retirement Study (HRS) Life History Mail Survey (LHMS) has spurred innovative research on the role of occupational exposures on health disparities in later life. Linking these data to the information available in the Occupation Information Network (O*NET) provides a rich characterization of lifetime occupational exposures. Sheftel et al. use work histories from the LHMS to study the role of racial segregation in occupations on later life dementia. They show that complex work with data during working ages may be protective against dementia at older ages, potentially contributing to the differentials in dementia prevalence by race/ethnicity. Park et al. similarly use data on lifetime work histories, to investigate the role of accumulated years of physically demanding work (PDW) in young and middle adulthood on the number of functional limitations at age 60. They show that cumulative PDW is strongly associated with functional limitations at age 60 and accounts for a part of the racial/ethnic gap in functional limitations at age 60. Both papers will discuss policy implications of findings. Andrasfay will provide critical commentary and discussion.

Abstract citation ID: igad104.0860

### RACIAL/ETHNIC DISPARITIES IN HEALTH: THE ROLE OF PHYSICALLY DEMANDING WORK

Sung Park[1], Anne Pebley[2], Noreen Goldman[3], Borianna Pratt[3], and Mara Sheftel[4], *1. University of Massachusetts Boston, Boston, Massachusetts, United States*, *2. University of California, Los Angeles, Los Angeles, California, United States*, *3. Princeton University, Princeton, New Jersey, United States*, *4. Penn State University, State College, Pennsylvania, United States*

Work is an important contributor to racial/ethnic health disparities. Because older-age functional limitations are associated with accumulated physical wear and tear, investigating work-related mechanisms that differentially expose Black and Hispanic Americans to difficult work conditions over time is an important step towards understanding these disparities. Using data on lifetime work histories, this study investigates the role of accumulated years of physically demanding work (PDW) in young and middle adulthood on the number of functional limitations at age 60 (FL60). It also assesses whether cumulative PDW accounts for the observed racial/ethnic differences in FL60 among U.S.-born Black, Hispanic, and White respondents. Results show cumulative PDW is strongly associated with FL60 and that it accounts for a part of the racial/ethnic gap in FL60 in the presence of extensive control variables. The findings illustrate the importance of work as a dynamic process that ultimately influences the health of a diverse, aging population. Policy implications are addressed.

Abstract citation ID: igad104.0861

### RACIAL/ETHNIC COGNITIVE HEALTH DISPARITIES: THE ROLE OF OCCUPATIONAL COMPLEXITY AND OCCUPATIONAL STATUS

Mara Sheftel[1], Noreen Goldman[2], Anne Pebley[3], Borianna Pratt[2], and Sung Park[4], *1. Penn State University, State College, Pennsylvania, United States*, *2. Princeton University, Princeton, New Jersey, United States*, *3. University of California, Los Angeles, Los Angeles, California, United States*, *4. University of Massachusetts Boston, Boston, Massachusetts, United States*

High levels of labor market segregation mean that Black and Latino workers may not have the same exposure to jobs involving complex work as their White counterparts, an aspect of work that appears to be protective of older adult cognition. The association between cognitive disparities and variation in exposure to occupational complexity by race/ethnicity remains understudied. Using work histories constructed from the Health and Retirement Study (HRS) life history data we analyze the role of occupational complexity in the development of dementia at older ages and address a conjecture that complexity reflects occupational status. Findings highlight that: (1) occupations involving complex work with data during working ages may be protective against dementia at older ages, potentially contributing to the differentials in dementia prevalence by race/ethnicity, and (2) occupational complexity reflects occupational status. This research increases understanding of the implications of labor market segregation for cognitive health disparities by race/ethnicity.

### SESSION 3070 (BIOLOGICAL SCIENCES INVITED SYMPOSIUM)

Abstract citation ID: igad104.0862

### STEM CELLS HOMEOSTASIS AND AGING

Chair: Leanne Jones Co-Chair: Emmanuelle Passegue

Adult (tissue) stem cells support tissue homeostasis and repair throughout the life of an individual. Numerous changes occur with age that result in altered stem cell behavior and reduced tissue maintenance and regeneration. Changes can be cell autonomous including changes in cell cycle progression, decreased bioenergetic efficiency, increased DNA damage, and epigenetic alterations. In addition, poorly understood changes to the local and systemic environments occur that result in decreased stem cell activity or alterations in commitment or differentiation potential. This symposium will cover the impact of aging on multiple adult stem cell systems, including hematopoietic stem cells (HSCs), intestinal stem cells (ISCs), and neural stem cells.

Abstract citation ID: igad104.0863

### AUTOPHAGY COUNTERS INFLAMMATION-DRIVEN GLYCOLYTIC IMPAIRMENT IN AGING HSCS

Emmanuelle Passegue, *Columbia University, New York City, New York, United States*

Abstract citation ID: igad104.0864

### RESPONSE OF NEURAL STEM CELLS TO DEVELOPMENTAL, HOMEOSTATIC AND AGING CUES

Michael Bonaguidi, *University of Southern California, Los Angeles, California, United States*

Abstract citation ID: igad104.0865

### CHRONIC IFNΓ-DRIVEN INFLAMMATION IMPAIRS INTESTINAL TOLERANCE AND REGENERATION IN AGING

David Castillo-Azofeifa, *Genentech, Inc., South San Francisco, California, United States*

The intestine's remarkable regenerative capacity decreases with aging as the stem cellniche interaction is impaired. Beyond defects in the stem cell-niche interaction, there is a direct correlation between age-related changes in immunity and increased susceptibility to infections. However, how the aging immune system affects the regenerative capacity of the intestine to challenges such as infections is unclear. Here, we infected young and aged mice with Citrobacter rodentium, a model that activates the immune response and causes epithelial damage. Young mice successfully healed, while old mice succumbed to infection. In the aged infected intestines, we identified elevated basal levels of interferon γ (IFNγ) that significantly increased in lymphoid cells, CD4+, and CD8+ T cells. Additionally, aged post-infected intestines exhibited dramatic epithelial barrier disruption, partly due to a decrease in Lgr5+ stem cells and an imbalance of cell differentiation towards goblet cell production, raising the question if the aging intestine is more susceptible to infection due to persistent exposure to IFNγ. To test the response of young and aged intestinal epithelium to continuous IFNγ exposure, we treated young and aged organoids with IFNγ. We found that old organoids undergo massive apoptotic cell death as early as 24 hours post-IFNγ treatment. In contrast, young organoids were unaffected, demonstrating that IFNγ drives the age-related impairment of intestinal healing. Although a short-term boost of IFNγ is necessary for intestinal regeneration in adults, our findings indicate that prolonged IFNγ activation associated with aging can impede intestinal repair.

### SESSION 3075 (PAPER)

### ADULT PROTECTION AND ELDER ABUSE (PAPERS) II

Abstract citation ID: igad104.0866

### AGING AND MASS MARKETING FRAUD: EVIDENCE ON REPEAT VICTIMIZATION USING PERPETRATOR DATA

Marguerite DeLiema[1], Lynn Langton[2], M Daniel Brannock[3], and Edward Preble[3], *1. University of Minnesota, Twin Cities, Saint Paul, Minnesota, United States*, *2. RTI International, Washington, District of Columbia, United States*, *3. RTI International, Research Triangle Park, North Carolina, United States*

Fraud complaint data indicates that adults in their 70s and 80s experience significantly greater fraud losses than middle aged and young adults, however older adults are less likely to self-report victimization overall. One explanation for older adults' relatively higher financial losses may be that they experience a greater incidence of repeat victimization. In this study, we examine the relationship between age and repeat mass marketing fraud using a novel dataset of nearly one million US mail fraud victims. The data come from four mail fraud enterprises that were investigated by the US Postal Inspection Service and that ran overlapping scams between 2000 and 2018. The perpetrators collected information on victims (name, address, age) and how often they paid money in response to scam solicitations. Datasets were merged, cleaned, and deduplicated. We use a Prentice-Williams-Peterson adaptation of a Cox proportional hazards model to estimate the effect of age on the likelihood of experiencing a subsequent victimization, controlling for the number of prior victimizations. Compared to victims in their 50s, victims aged 70-79 and 80-89 had a 9% greater risk of experiencing another victimization, whereas victims in their 20s had a 24% lower risk of experiencing another victimization. Logistic regression analyses indicate that adults in their 70s and 80s are significantly more likely than those younger than 50 to experience victimization by multiple types of mail scams. This is the first study to use perpetrator data, rather than self-report, to show that older adults face a greater risk of repeat fraud victimization.

Abstract citation ID: igad104.0867

### DIGITAL DANGERS: TECHNOLOGY-FACILITATED ABUSE AMONG ADULTS AGE 50+

Alycia Bayne, Elizabeth Mumford, Caroline Lancaster, and Jackie Sheridan-Johnson, *NORC at the University of Chicago, Chicago, Illinois, United States*

Older adults (age 50+ years) are adopting new technologies to increase their independence, prevent social isolation, and help them to age in place. However, digitalization introduces new risks for technology-facilitated abuse (TFA). TFA is any form of online abuse including threats, identity theft, financial fraud, among other forms of victimization. Limited research has explored TFA in general population samples among older adults in the U.S. With support from the U.S. Department of Justice, NORC at the University of Chicago conducted a survey of TFA experiences in a nationally representative sample of n=1,011 U.S. adults aged 50 years and older. Latent class analyses were applied to understand the pattern of older adults' exposure to ten different forms of TFA, resulting in three classes distinguished by the number of different forms of TFA experienced. This session describes findings from the study, such as socio-economic characteristics associated with TFA profiles, the respondents' relationship to the perpetrator, behaviors taken in response to TFA, and resulting harms. Among key findings are that six in 10 community-dwelling adults age 50+ have experienced at least one form of TFA in their lifetime. The most common forms of TFA experienced were financial abuses and identity theft. Different demographic groups of older adults experienced different victimization patterns. For example, those who identify as LGBQA were at a much higher risk of experiencing TFA. This session concludes with research and education opportunities for preventing TFA across multiple levels among older adults.

Abstract citation ID: igad104.0868

### POWER OF ATTORNEY FACTORS AND CONDITIONS INFLUENCING ELDER FAMILY FINANCIAL EXPLOITATION

Jennifer Crittenden[1], Julie Bobitt[2], Katelyn Galladay[3], and Virginia Vincenti[3], *1. University of Maine Center on Aging, Bangor, Maine, United States*, *2. University of Illinois Chicago, Chicago, Illinois, United States*, *3. University of Wyoming, Laramie, Wyoming, United States*

Approximately 16% of older adults worldwide experience some form of elder abuse including elder family financial exploitation (EFFE), the unlawful transfer and theft of funds and property perpetrated by a family member. Power of Attorney (POA) arrangements, when constructed and executed properly, can be critical tools for protecting against such exploitation. This national research study examined family reports of EFFE using the Bronfenbrenner Ecological Framework to classify risk factors within the POA arrangement. Interviews were carried out with 31 family members within 24 families that had experienced EFFE. Using constructivist grounded theory analysis methods, the following themes related to risk factors were noted across EFFE stories about professionals involved in the EFFE situations: 1) Conflict of interest between attorneys and POA agents, 2) A perceived lack of competence of consulting attorneys; and 3) A lack of transparency around POA arrangements. Policy and education implications include the need to create legal requirements for transparency reporting and/or registration for POAs, strengthening legal ethics and EFFE training, and providing public education to families about the POA process and how to locate appropriately trained legal professionals.

Abstract citation ID: igad104.0869

### SAFE AND SENSATIONAL: ASSESSING THE SECURE AND PRIVATE ONLINE DATING WORLD FOR OLDER ADULTS

Naheem Noah, and Sanchari Das, *University of Denver, Denver, Colorado, United States*

Online dating applications have become a popular way for people worldwide to meet potential partners, including older adults aged 65 and above. However, using these applications involves revealing personally identifiable information (PII), which raises privacy and security concerns, particularly for older adults who are often the targets of cyberattacks. To address this issue, this paper conducted a literature review of 38 peer-reviewed publications on online dating security and privacy, with a focus on older adults. The review found that while 10 papers evaluated older adults' cybersecurity issues, and two papers explored mobile security management with adults, no papers explicitly focused on older adults and dating applications. To fill this research gap, this paper conducted an application-level security and privacy analysis of ten popular online dating applications, including Tinder, Grindr, Match, Bumble, OkCupid, SilverSingles, Harmony, EliteSingles, Christian Mingles, and LDS Singles. The study revealed that these applications have concerning data security practices that may affect the privacy and security of users, especially older adults. The paper concludes by providing actionable recommendations for secure and privacy-preserving user experience. For instance, online dating applications should implement secure authentication mechanisms, such as two-factor authentication, and use end-to-end encryption to protect users' data. Additionally, the applications should enable users to control their data and allow them to choose the information they want to share with others. Moreover, the dating apps should provide clear and concise privacy policies that are easy to understand and readily available to users.

### SESSION 3080 (PAPER)

### COGNITION (BSS PAPERS) I

Abstract citation ID: igad104.0870

### DAILY SLEEP QUALITY AND EXECUTIVE FUNCTIONING PERFORMANCE ACROSS TWO WEEKS IN INDIVIDUALS APPROACHING MID LIFE

Tina Vo[1], Anqing Zheng[2], Elizabeth Muñoz[3], Sally Wadsworth[2], Martin Sliwinski[4], and Chandra Reynolds[2], *1. University of California Riverside, Riverside, California, United States*, *2. University of Colorado Boulder, Boulder, Colorado, United States*, *3. The University of Texas at Austin, Austin, Texas, United States*, *4. Pennsylvania State University, State College, Pennsylvania, United States*

Sleep quality (SQ)-cognition associations are prevalent across aging, with stronger associations observed in midlife. Whether these associations persist within micro-level timescales (e.g., day-to-day) is unclear, particularly in individuals approaching midlife. Burst data from a subsample of the Colorado Adoption/Twin Study of Lifespan behavioral development and cognitive aging (CATSLife1) who participated in the ambulatory smartphone substudy (N=440, Mage=35.78 range=28.07-51.32, %Female=57.7%) were utilized to examine associations between self-reported daily SQ and cognitive performance, measured up to three times a day for 14 days, while accounting for age, sex, and educational attainment. There was a small positive age effect on average SQ (e.g., d=.18 comparing 36 versus 46 year-olds). Separable between-person and within-person time varying covariate effects were extracted for SQ to capture individual differences in typical levels versus variations from one's typical pattern. The between-person SQ was individually significant and positive (B=0.023, se=0.009, p=0.019) suggesting better Stroop accuracy for each unit increase in SQ. Daily differences from one's average SQ did not additionally predict daily Stroop performance suggesting the salience of general quality. Estimates suggest higher accuracy for individuals who report higher average SQ (d=.19, average SQ versus +2SD above average SQ). However, there were no Age by SQ effects on Stroop accuracy. Additional SQ aspects (e.g., sleep latency and sleep maintenance) are explored. Examining sleep quality-cognition associations across micro-level timescales are salient to discern whether and how effects accumulate and contribute to performance differences over larger timescales in individuals approaching midlife.

Abstract citation ID: igad104.0871

### DYADIC LONELINESS, AGE, AND COGNITIVE FUNCTIONING AMONG MID-LIFE AND OLDER BLACK COUPLES

Jeffrey E. Stokes[1], and Heather Farmer[2], *1. University of Massachusetts Boston, Boston, Massachusetts, United States*, *2. University of Delaware, Newark, Delaware, United States*

Cognitive health is a growing concern for the older Black population, whose risk for dementia is about twice that of White older adults. Moreover, close relationships, such as marriage, are critical for well-being in later life. Within older adults' marriages, loneliness has been linked with individuals' own and their partners' mental, physical, and cognitive health. Yet little research has examined these relationships within the older Black population, and even less work has situated Black older adults within a relational, dyadic context. Lastly, research is needed on the circumstances under which partners' loneliness may be influential for cognition, including potential variation by individuals' age or their own level of cognitive functioning. This study used longitudinal dyadic data from the Health and Retirement Study (2010-2016; n=1,270 participants from 635 couples) from both partners in opposite-sex midlife and older married couples where at least one partner reported being Black or African American. Lagged dependent variable (LDV) structural equation modeling (SEM) addressed our research questions. Results indicated that (1) loneliness was not associated with participants' own cognitive functioning 4-years later, yet (2) husbands' loneliness was associated with worse cognitive functioning 4-years later for wives who had high baseline cognitive functioning themselves, and (3) wives' loneliness was associated with worse cognitive functioning 4-years later only for oldest-old husbands. Findings indicate that loneliness has dyadic consequences for cognitive functioning among older Black couples, but that context is crucial for determining which older partners are at greatest risk of experiencing harmful cognitive repercussions from a partner's loneliness.

Abstract citation ID: igad104.0872

### EARLY-LIFE SOCIOECONOMIC POSITION AND LATER-LIFE COGNITIVE FUNCTIONING: A META-ANALYSIS

Hanamori Skoblow[1], Francisco Palermo[1], and Christine Proulx[2], *1. University of Missouri, Columbia, Missouri, United States*, *2. University of Vermont, Burlington, Vermont, United States*

A life course perspective on cognitive aging suggests that experiences early in life may be linked to later performance on cognitive tests. A growing literature shows that childhood socioeconomic position (SEP) is positively associated with later-life cognitive functioning. To identify the strength of this association and potential moderating variables, we used meta-analytic techniques to identify the magnitude of the association between early-life (aged 0-17) SEP and later-life (aged 50+) cognitive functioning. After systematically searching PsycINFO, Scopus, and ScienceDirect, 30 studies representing 39 samples and 223,381 participants met the inclusion criteria. Extracted data included participant and study design characteristics, construct operationalization and measures, and effect sizes. The results of the meta-analysis revealed a significant correlation between childhood SEP and later-life cognitive functioning, whether measured as an overall effect size (r = .179, 95% CI = [.156, .201]), global performance (r = .206, 95% CI = [.157, .255]), verbal episodic memory (r = .160, 95% CI = [.132, .188]), verbal fluency (r = .203, 95% CI = [.168, .237]), or processing speed (r = .146 (95% CI = .108, .184]), but not inhibition (r = .146, 95% CI = [-.150, .418]). Subgroup analyses revealed that, in some domains, the correlation was weaker with an older mean sample age. Correlations did not vary by study design (i.e., prospective or retrospective) or journal impact factor. These results provide important evidence for the promotion of policies that buffer children from economic hardship, as one's cognitive performance in later life begins far earlier than older adulthood.

Abstract citation ID: igad104.0873

### INCARCERATION HISTORY AND ITS ASSOCIATION WITH COGNITION AND ISOLATION AMONG OLDER BLACK AMERICANS

Eskira Kahsay[1], Rodlescia Sneed[2], Chuwen Zhong[3], and Briana Mezuk[1], *1. University of Michigan, Ann Arbor, Michigan, United States*, *2. Wayne State University, Detroit, Michigan, United States*, *3. School of Public Health, University of Michigan- Ann Arbor, Ann Arbor, Michigan, United States*

Incarceration is a risk factor for adverse health and disproportionately affects Black Americans. Little is known about its implications for later life functioning. This study investigated the associations between incarceration and social isolation and cognition among older Black Americans. Sample included African American and Caribbean Black respondents aged 50+ from the National Survey of American Life, 2001-2003 (N=1,561; 12.6% with incarceration history). Lifetime incarceration history (yes/no) was defined as having been in jail, prison, or juvenile detention. Continuous cognitive score was measured using the WHO Disability Assessment Schedule-II, with higher scores indicating higher cognitive difficulty. Binary isolation was assessed by combining two indicators of frequency of contact and feelings of closeness, for friends and family, using a 4-point Likert scale. Multivariable weighted linear and logistic regressions were fit for the associations between cognitive score and social isolation, respectively. All models adjusted for age, sex, ethnicity, poverty, education, marital status, and drug use; model for cognition further adjusted for mental health and traumatic experiences and models for isolation further adjusted for discrimination. Moderation by nativity (U.S. versus foreign born) was assessed using interaction. History of incarceration was associated with worse cognition ($\beta$ = 0.09, 95% CI: -1.48, 1.66) and higher odds of social isolation for frequency (AOR: 1.48 95% CI: 0.89, 2.46) and closeness (AOR: 1.05, 95% CI: 0.52, 2.15). Results not moderated by nativity. Findings suggest contexts surrounding incarceration, like social supports, may be important engagement points for successful transitions and promotion of psychological and cognitive health in this population.

Abstract citation ID: igad104.0874

**NEUROFILAMENT LIGHT CHAIN (NFL) AND COGNITIVE PERFORMANCE INCONSISTENCY IN ADULTS APPROACHING MID LIFE**

Chandra Reynolds[1], Elizabeth Muñoz[2], Donald Evans[3], and Andrew Smolen[1], *1. University of Colorado Boulder, Boulder, Colorado, United States*, *2. The University of Texas at Austin, Austin, Texas, United States*, *3. University of Colorado - Boulder, Boulder, Colorado, United States*

Neurofilament light chain (NfL) indexes axonal integrity, where higher NfL values correlate with lower cognitive performance. However, associations of NfL with variability in performance and earlier in the lifespan are unclear. We evaluated inconsistency in trial-level performance within three speed or spatial tasks and dispersion across nine verbal, spatial, speed, and episodic memory tasks in participants from the Colorado Adoption/Twin Study of Lifespan behavioral development and cognitive aging (CATSLife1). Individuals were tested at 28-49 years (M=33.1, SD=4.9). Quanterix Simoa assays of plasma NfL (pNfL, log-transformed), cognitive test scores, and sociodemographic covariates were available for up to 1144 individuals. Multilevel regression analyses accounted for sibling relatedness and sociodemographic covariates. Above average pNfL coupled with higher age were associated with greater inconsistency across trials for the WAIS-III Digit Symbol subtest (d=.15 per 3 years of age increase, p=0.037) adjusting for performance, and with reduced performance across age (d=-.27, p=0.014). Moreover, higher pNfL was associated with higher dispersion among nine specific cognitive ability tasks across age, adjusting for general cognitive ability (GCA) factor scores (d=.156, p=0.037). Building on our earlier report of plasma NfL and GCA, higher pNfL was associated with greater dispersion in performance across a battery of tests. Moreover, higher pNfL was associated with poorer overall speed performance, which was also observed for trial level inconsistency even when adjusting for performance level. Hence, at equal levels of ability, within-task inconsistency and dispersion across tasks may be associated with plasma neurofilament light chain in early- to mid-adulthood.

Abstract citation ID: igad104.0875

**PERCEIVED WORKPLACE AGEISM AND RACIAL/ ETHNIC DISPARITIES IN COGNITIVE FUNCTIONING AMONG OLDER WORKERS**

Duygu Basaran Sahin[1], Frank Heiland[2], Na Yin[3], and Ryan Smith[2], *1. RAND Corporation, Brooklyn, New York, United States*, *2. Baruch College, New York, New York, United States*, *3. Baruch College, CUNY, New York City, New York, United States*

Research suggests that discrimination contributes to racial/ethnic disparities in health, but little is known about the role of perceived workplace ageism in this phenomenon. Workplace discrimination threatens a person's livelihood, self-esteem, and identity. Older workers from racial/ethnic minority groups may experience both race- and age-based discrimination. Using data from the Health and Retirement Study, we investigate whether workplace ageism is related to older workers' cognition and whether the link differs by race/ethnicity. We find that workers who perceive ageism in their workplaces tend to do worse on standard cognition tests than those who do not. For example, the immediate and delayed word recall performance is 13-14% of one SD lower among individuals who report that co-workers pressure older workers to retire before age 65. The relationships are stronger (more negative) for Hispanic workers, but there is no evidence that they differ between Non-Hispanic Black and Non-Hispanic White workers.

### SESSION 3085 (PAPER)

### SOCIAL STATUS AND RETIREMENT ACROSS AGE AND NATIONS

Abstract citation ID: igad104.0876

**ASSOCIATION BETWEEN SUBJECTIVE SOCIAL STATUS AND PHYSICAL FRAILTY AMONG OLDER ADULTS IN INDIA**

T Muhammad[1], and Manacy Pai[2], *1. International Institute for Population Sciences, Mumbai, Maharashtra, India*, *2. Kent State University, Kent, Ohio, United States*

Research on the association between subjective social status (SSS) and physical frailty is critical to crafting interventions that can combat functional decline, prolong physical vitality, and sustain quality of life. We assessed the association between SSS and physical frailty and the mediation of this association by perceived everyday discrimination and ill-treatment among older adults in India. Data come from the Longitudinal Aging Study in India with a sample of 31,464 older adults age 60 years and above. Physical frailty was assessed using an adapted version of the frailty phenotype developed by Fried and colleagues. SSS was assessed using the Macarthur scale. Multivariable logistic regression along with KHB (Karlson–Holm–Breen) method was employed to examine the associations and the mediation effects. The prevalence of physical frailty was 30.65% and those with lowest SSS had higher prevalence of physical frailty (42.06%). After adjusting for a number of confounders, odds of physical frailty were significantly lower among those with a high SSS in comparison to those with low SSS, and the variance explained by the SSS was higher than that explained by household consumption quintiles. The association between SSS and physical frailty was mediated by perceived everyday discrimination (percent mediated: 8.20) and experiences of ill-treatment (percent mediated: 9.32). The findings underscore that when examining the association between socioeconomic status and physical frailty, it is important to consider SSS given that perceptions of one's social standing likely reflect the less apparent psychosocial components associated with it.

Abstract citation ID: igad104.0877

**BRIDGE JOBS AS PATHS TO WORKING LONGER: DO GENDER AND RETIREMENT IDENTITIES MATTER?**

Yun taek Oh[1], and Phyllis Moen[2], *1. University of Michigan, Ann Arbor, Michigan, United States, 2. University of Minnesota, Minneapolis, Minnesota, United States*

Leaving a full-time career job happens not only by completely exiting the labor force but also by having bridge jobs. When choosing to have bridge jobs, some workers perceive themselves as partially or completely retired while others are not. This study first categorizes bridge jobs into three types: switching occupations, leaving employers, and reducing work hours to part-time. Then, this study uses the Health and Retirement Study to investigate how the self-perceived retirement status in bridge jobs affects the timing of complete withdrawal from the labor force by using two-way fixed-effects event study regression. The results show that a significant proportion of older workers consider themselves partially or completely retired when leaving employers or reducing work hours but do not when switching occupations. The results also show the effects of bridge jobs on the complete withdrawal are different by the self-perceived retirement status and gender. Switching occupations reduces the probability of retirement for male workers who do not consider it as retirement while it increases the probability of retirement for female workers who consider it as retirement. Leaving the employer reduces the probability of retirement for male workers who consider these bride jobs as retirement while it does not for female workers. These findings imply the importance of self-perception of retirement in labor force participation and how it differs by the types of bridge jobs.

Abstract citation ID: igad104.0878

### CONSTRUCT VALIDITY OF FRABONI SCALE OF AGEISM IN A CHINESE-MAJORITY SAMPLE FROM SINGAPORE

Yuanyuan Cao, Jie Xin Lim, Ringo Ho, and Yin-Leng Theng, *Nanyang Technological University, Singapore, Singapore*

The 29-item Fraboni Scale of Ageism (FSA; Fraboni et al., 1990) was constructed to measure three dimensions of ageism (Antilocution, Discrimination, and Avoidance) using samples from Canada. The factor structure of the FSA has been challenged in recent studies using US samples (Rupp et al., 2000) and Chinese samples (Fan et al., 2020), resulting in alternative factor structures. The current study aimed to test the different factor structures proposed in these past studies with a sample from Singapore, a Chinese-majority multicultural country. Data from 311 individuals, aged between 21- and 55-year-old were collected. They completed the 29-item FSA using a six-point agreement scale. Confirmatory factor analysis was used to test the goodness-of-fit of the aforementioned factor structures. The results indicated that none of three models provided a good fit to the data. A follow-up exploratory factor analysis with parallel analysis suggested a 3-factor structure. Thirteen out of the 29 items were found to have at least one salient cross-loading after Geomin rotation. These findings suggest that the samples likely interpreted and responded to the items differently resulting in different item-factor configurations, implying the impact of regional culture on the construct validity of FSA. Researchers aiming to quantify ageism in their population using FSA are advised to examine the FSA factor structure prior to using it.

Abstract citation ID: igad104.0879

### CORRELATES OF RETIREMENT EXPECTATIONS AMONG BABY BOOMERS IN US AND SOUTH KOREA: APPLICATIONS OF TREE-BASED METHODS

Linh Dang, and Briana Mezuk, *University of Michigan, Ann Arbor, Michigan, United States*

75 million US Baby Boomers are expected to retire by 2030. Retirement decisions are a result of a complex interplay of multiple psychosocial factors, including individual (demographics, health characteristics), family (marital status, spousal characteristics), work-related factors (job characteristics, workplace environment), and cultural contexts surrounding retirement. However, traditional regressions of retirement expectations limit the inclusion of a large set of factors and their interactions. This study employed decision trees and random forests to explore the salient psychosocial correlates of retirement expectations among Baby Boomers in two countries that differ in retirement norms and policies, US and South Korea. Sample included adults aged 52-65 from the 2016 wave of the Health and Retirement Study (n=1,192) and the Korean Longitudinal Study of Aging (n= 766). Expectation (probability) of working full-time for the next 5-10 years was modeled continuously (range: 0-100). Prediction models of expectations included 52 psychosocial factors relating to individual, family, and work characteristics. Mean expectations were higher in Koreans than US adults (71.4% vs. 36.7%). Age, self-reported probability of living to age 75, and work hours strongly predicted expectations in both US and Korean adults. While type of employment, Social Security or private pension benefits, and employment tenure were important predictors in US adults, flexible work arrangements were important in Koreans. Differences in features predicting retirement expectations between the US and Korean adults suggest opportunities for country-specific programs and policies aimed to promote workplace environment and pension benefits for successful retirement planning in the Baby Boom generation.

### SESSION 3090 (SYMPOSIUM)

Abstract citation ID: igad104.0880

### FINANCIAL EXPLOITATION OF OLDER ADULTS: INTERDISCIPLINARY PERSPECTIVES ON NEW DIRECTIONS

Chair: Julie Miller

Financial exploitation of older adults is a topic that has drawn increased public recognition in recent years. Despite advances in research, services, products, and policies working to prevent financial exploitation of older adults and support victims, interdisciplinary perspectives are needed to strengthen interventions. This symposium will bring together scholars and practitioners to address the issue of financial exploitation from a variety of disciplines, all united in their mission to detect and prevent financial exploitation of older adults and to support victims. The first two presentations in the symposium will highlight conceptual understandings of financial exploitation of older adults, including a revisionary model of financial exploitation of older adults that includes emerging cognitive, cultural, and other contextual factors (Presentation 1) and a study of financial vulnerability and mental health as they are associated with financial exploitation, particularly when perpetrated by trusted others (Presentation 2). The remaining presentations will describe research from cross-sector, cross-industry, interventions. Presentation 3 will describe study findings about a helpline for concerned persons of exploitation victims that dually offers services directed to primary victims of financial exploitation. Presentation 4 will highlight a study of case characteristics associated with a new adult protection law designed to protect older and vulnerable adults experiencing exploitation. Presentation 5 will describe a study of financial professionals' experiences with, and attitudes toward, financial exploitation of aging clients. Findings from all five presentations will point to next steps for research and intervention across multiple disciplines, including social work, law and policy, healthcare, and financial services.

Abstract citation ID: igad104.0881

### FINANCIAL EXPLOITATION IN OLDER AGE: A REVISIONARY MODEL

Duke Han, *University of Southern California, Los Angeles, California, United States*

Financial exploitation can have a devastating impact on the independence and wellbeing of older adults, yet the reasons why some older adults experience financial exploitation remain elusive. Recent work informed by the fields of neuropsychology, neuroscience, behavioral economics, and epidemiology has increasingly demonstrated links between financial vulnerability in older age and serious health outcomes such as cognitive decline and incident Alzheimer's Disease. Because of this, research on financial exploitation in older age has progressively been made a public health priority, and this has led to a substantial expansion in knowledge on the topic. Despite this beneficial growth in understanding, gaps in knowledge, misconceptions, and ageist viewpoints continue to persist. To address these, this presentation will (1) discuss the potential role of age-associated cognitive changes in financial exploitation, (2) review neuroimaging findings from our group and others relevant to understanding possible brain changes involved, and (3) highlight cross-cultural, interpersonal, and other considerations that need prioritization in future research endeavors. In conclusion, we will present a revisionary model of financial exploitation in older age which considers not only the potential vulnerabilities of the older adult, but also the persons committing the financial exploitation and the contextual factors that may contribute to poorer outcomes. This revisionary model can more effectively inform interventions for the prevention and mitigation of financial exploitation in older age.

Abstract citation ID: igad104.0882

### TRUST AND BETRAYAL TRAUMA IN OLDER ADULT FINANCIAL EXPLOITATION

LaToya Hall[1], Peter Lichtenberg[1], and Jennifer Gomez[2], *1. Wayne State University, Detroit, Michigan, United States*, *2. Boston University, Boston, Massachusetts, United States*

Despite a growing body of literature on financial exploitation (FE) and older adults, considerably less research has focused on sub-populations of older adults' experiences with substantiated financial exploitation, particularly exploitation by trusted others. A 2023 study conducted through Wayne State University's Successful Aging through Financial Empowerment (SAFE) program used a sample of 95 clients ages 55 and over to examine factors associated with participants' experiences of substantiated FE perpetrated by trusted others. The SAFE program was created in early 2017 to provide no cost assistance to older adults addressing the financial fall out associated with being financially exploited. The study used betrayal trauma theory (BTT) as a conceptual framework in an attempt to understand how financial vulnerability and mental health are associated with FE committed by trusted others. The study used a cross-sectional design to investigate group differences among 32 (33.7%) older adults who were victims of financial exploitation by trusted others and 63 (66.3%) older adults who were victims of financial exploitation perpetrated by strangers. The group of older adults who were victims of FE by trusted others had lower functional ability scores, higher stress and financial exploitation vulnerability scores and lost more money on average than those victimized by strangers. These results suggest older adults who have been victimized by trusted others are more vulnerable and need more assistance with functional tasks than those victimized by strangers. The present study also supports the notion that BTT provides a valuable framework for understanding vulnerability to various types of FE.

Abstract citation ID: igad104.0883

### THE ELDER ABUSE HELPLINE FOR CONCERNED PERSONS (CPS): HOW SERVICES FOR CPS IDENTIFY AND MITIGATE FE AND ABUSE

Lisa Rachmuth, *Weill Cornell Medicine, New York City, New York, United States*

Elder abuse can profoundly impact older adults who experience it. Historically, little is known, however, about another group of individuals in the lives of older adults: non-abusing family members, friends, and neighbors, referred to as "Concerned Persons" (CPs). Through the Elder Abuse Helpline for Concerned Persons (the Helpline), a program at Weill Cornell Medicine, the CPs receive supportive counseling for themselves and referrals to ongoing services for themselves and the Older Adults who are abused. Financial exploitation and abuse often go unrecognized by many. Previous research on the first year of data from Helpline calls indicated that CPs might be critical in enabling support and help-seeking for elder abuse victims, and may be an essential intervention target group to help promote help-seeking behaviors of Older Adult Financially exploited victims. This presentation will highlight findings from a recent study involving secondary analysis of de-identified Helpline data that a) explored characteristics of CPs, alleged victims/abusers, as well as types and circumstances surrounding alleged abuse; and b) characterized the range of services and interventions requested and provided to concerned persons. Primary questions of interest included ones such as, "What are CP's relationships to alleged victims/abusers?; How do the patterns, types, and circumstances surrounding alleged abuse vary across characteristics of CPs and their relationships to alleged victims/abusers?; What are the common concerns of CPs and the type of services requested by CPs?" Findings from this study improve our current understanding of CPs in the lives of alleged elder abuse victims and CPs' help-seeking behaviors.

Abstract citation ID: igad104.0884
### TEMPORARY ACCOUNT HOLDS TO PROTECT VULNERABLE ADULTS AT RISK OF FINANCIAL EXPLOITATION

Marti DeLiema[1], Maruska Sommers[2], and Carrie Binder[2], *1. University of Minnesota, Minneapolis, Minnesota, United States*, *2. University of Minnesota School of Social Work, Saint Paul, Minnesota, United States*

Elder financial exploitation presents a serious challenge to law enforcement and adult protective services (APS) agencies working to prevent losses. The goal of this project is to understand case characteristics associated with a new adult protection law in Minnesota–Chapter 45A–that authorizes temporary holds of suspicious transactions and withdrawal requests for up to 5 weeks to protect older and vulnerable adults experiencing exploitation. Most states have recently passed similar laws, but none have systematically collected data on when temporary holds are applied and for whom, what investigation takes place, and how interventions impact vulnerable and older adults' financial safety. Analyzing administrative data on Chapter 45A, we found that 286 financial exploitation cases were referred for investigation and temporary holds in 2022 (mean= 23 cases per month) with approximately $3M in funds at risk of further losses. Temporary holds were implemented in 25% of the referred cases. More than 2/3rds of Chapter 45A cases involved fraud perpetrated by strangers compared to fewer cases of financial abuse by friends and family. There is substantial under-reporting of cases involving Black, Hispanic, American Indian, Asian/Asian Pacific Islander, and immigrant older and vulnerable adults relative to the size of these groups in Minnesota. More research is needed to determine why minority clients are not receiving equal access to Chapter 45A intervention, and how to remedy these disparities in case referrals. Greater outreach and education may help improve knowledge of the Chapter 45A reporting processes among financial institutions, law enforcement, and APS agencies that serve these populations.

Abstract citation ID: igad104.0885
### FINANCIAL EXPLOITATION OF OLDER CLIENTS: EXPLORING FINANCIAL PROFESSIONALS' ROLES AND EXPERIENCES

Lauren Cerino[1], Julie Miller[2], Alexa Balmuth[1], Adam Felts[1], and Joseph Coughlin[1], *1. Massachusetts Institute of Technology, Cambridge, Massachusetts, United States*, *2. AARP, Bethesda, Maryland, United States*

Research shows that financial exploitation of older adults can be difficult to detect and underreported (Deane, 2018; Sullivan-Wilson & Jackson, 2014). Financial exploitation of older adults is both prevalent and costly, with estimated annual losses in the billions (Wood & Lichtenberg, 2017). Initiatives such as AARP's BankSafe training program demonstrate that the financial services industry can prevent and address cases of financial exploitation (DeLiema & Deevy, 2016). But it is worth exploring how financial professionals perceive their own ability to support older adults in cases of financial exploitation. The MIT AgeLab conducted an exploratory study to examine financial professionals' experiences with, and preparedness for, addressing financial exploitation against aging clients and their families. Data are inclusive of a sample (N=30) of financial professionals who completed an online questionnaire in April 2022. Participants' encounters with financial exploitation in their work were wide-ranging in frequency and nature. Most participants reported that they felt well-prepared to support a client who was experiencing financial exploitation. Despite high levels of individual preparedness, participants often felt that the financial industry's policies and services could better address elder financial exploitation. Results from this study suggest that, although financial professionals feel well-prepared as individuals to address financial exploitation, other entities—especially the financial services industry—could instill more confidence by increasing attention to the topic, implementing training, and providing resources. With additional knowledge, training, and resources, financial professionals may be able to more confidently address situations of financial exploitation.

### SESSION 3095 (SYMPOSIUM)

Abstract citation ID: igad104.0886
### PRECARITY AND WORK IN LATER LIFE: EXPERIENCES OF OLDER WORKERS IN UNCERTAIN TIMES

Chair: Cal Halvorsen Co-Chair: Christina Matz Discussant: Julie Miller

Employees later in their careers often earn the highest income of their lives, enabling them save for retirement while living in relative comfort. Yet many workers do not experience this late-career economic boost and instead face age bias and discrimination, difficulty finding and keeping work, and social and economic inequalities. Using an intersectionality lens, these difficulties often lead to disparities by race, ethnicity, gender, and immigration status, highlighting the varied socioeconomic experiences of older adults. The economic and social effects of the COVID-19 pandemic have often exacerbated these disparities. This symposium will highlight the varied experiences of late-career workers since the advent of the pandemic, with an emphasis on lower income workers and disparities by race and ethnicity. Dr. Choi-Allum will describe the results from a nationally representative survey of 2,000 workers ages 40 and older by AARP, highlighting how perceived age discrimination and the sense of a weak economy is driving uncertainty in finding and keeping paid work. Dr. Carr will identify racial and ethnic disparities in pandemic-related job disruption and its associated financial setbacks using Health and Retirement Study data. Dr. Halvorsen will describe the experiences of older, lower-income, and often immigrant job seekers within the federally funded Senior Community Service Employment Program in Massachusetts. And Dr. Matz will outline research with older adults who live in public housing on how social resources obtained partly through work may buffer financial strain's impact on health. To conclude, Dr. Miller will place the four studies into the broader socioeconomic context. This is an Aging Workforce Interest Group Sponsored Symposium.

Abstract citation ID: igad104.0887
### EXPERIENCES AND WELL-BEING OF LOW-INCOME WORKERS IN THE SENIOR COMMUNITY SERVICE EMPLOYMENT PROGRAM

Cal Halvorsen[1], Patrick Lai[2], Elizabeth Howard[3], Karen Lyons[1], Sara Moorman[1], and Christina Matz[1], *1. Boston College, Boston, Massachusetts, United States*, *2. Boston College School of Social Work, Boston, Massachusetts, United States*, *3. Boston College Connell School of Nursing, Boston, Massachusetts, United States*

The Senior Community Service Employment Program (SCSEP) is the only federal job-training program specifically for older workers. SCSEP enrolls unemployed older workers with incomes at or below 125% of the federal poverty level into on-the-job and classroom-based training. SCSEP participants often struggle to secure unsubsidized work, as they experience, on average, more than 3 barriers to employment (e.g., low literacy levels, disability, limited English proficiency). While some outcome metrics are tracked (e.g., job attainment, median unsubsidized wages), we know relatively little about participants' health, well-being, and training experiences. In response, and in partnership with the Massachusetts Executive Office of Elder Affairs, we fielded a survey on the multidimensional health, well-being, and experiences of participants throughout Massachusetts. Fielded in the spring and summer of 2022 and in six languages, a total of 91 SCSEP participants took the survey with an age range of 57 to 82. Almost half (44%) spoke a language other than English at home, of which Cantonese and Vietnamese were the most common. Respondents generally reported being in moderately good health (only 3% reported "excellent" health) with at least one chronic health condition (86%), and two-thirds (66%) reported reduced social activities due to the COVID-19 pandemic. Less than one-quarter (22%) reported having money left over at the end of the month, but that SCSEP itself improved their finances, social engagement, and self-confidence. To conclude, we will offer reflections on the importance of tracking additional characteristics of SCSEP participants and engaging in respectful community-based work with diverse older populations.

Abstract citation ID: igad104.0888

### LOW-INCOME, MARGINALIZED OLDER WORKERS: FINANCIAL STRAIN, HEALTH, AND MENTAL HEALTH

Christina Matz[1], Rebekah Levine Coley[2], Samantha Teixeira[3], and Patrick Lai[3], *1. Boston College, Boston, Massachusetts, United States*, *2. Boston College Lynch School of Education, Boston, Massachusetts, United States*, *3. Boston College School of Social Work, Boston, Massachusetts, United States*

Social inequalities over the life course shape later life opportunities and outcomes in important ways. Research on paid work in later life has not always captured (and has sometimes mischaracterized) the variety and complexity of lived experiences in later life—in particular for low-income workers, workers of color, women, and others marginalized due to their social position. Marginalized older workers often face greater financial instability, limited interpersonal power, and reduced access to benefits like health insurance and paid leave, all of which can lead to increased risk of early retirement, poor health/well-being, and early mortality. Trend statistics and other aggregated data often obscure how the most marginalized older adults are faring in the aging and work literature and there is a need for more research that centers these voices to inform social change, particularly in the wake of the COVID-19 pandemic. The current study aims to fill this gap by drawing on data collected in 2022/2023 among residents of a large public housing development in New England who are age 40 and older (current N=166; expected N=250). Analyses explore relationships between social resources, financial strain, health, and well-being by work status and age. Preliminary analyses suggest that certain forms of social support can buffer the negative effect of financial strain on mental health (but not physical health) among older adults living in poverty and in poor housing conditions. Implications for interventions aimed toward older marginalized adults who are working or looking for work will be discussed.

Abstract citation ID: igad104.0889

### A WEAK ECONOMY AND AGE DISCRIMINATION DRIVES JOB UNCERTAINTY AMONG OLDER WORKERS

Lona Choi-Allum, *AARP, Washington, District of Columbia, United States*

Older workers have experienced disruptions in the workplace over the past five years, with recent studies showing that a reduction in hours as the most commonly cited reason. Not surprising, the COVID-19 pandemic was a primary contributor, leading to reduced hours or leaving a job involuntarily (laid off or fired). The AARP Value of Experience survey sought to understand the experiences of 2,000 workers ages 40 and older, weighted to be nationally representative by gender, race/ethnicity, age, income, and other variables. Results showed that a weak economy is the main driver of why older workers anticipate losing their job within a year. Those under 50 (37%) are just as likely as those ages 50+ (34%) to say that they could lose their job due to a weak economy. When asked how confident those in the labor force are in finding a new job within three months, one in four workers say they are not confident while nearly half of those who are looking for a job say the same. Those age 50+ (29%) are more likely than those under age 50 (14%) to report low confidence in finding a similar job within three months. The main reasons for low confidence include age discrimination and a weak economy. Workers ages 50+ (40%) are twice as likely as those under age 50 (19%) to say age discrimination is a factor in not being able to find another similar job. This study highlights the perceived difficulties that older workers face in continued employment.

Abstract citation ID: igad104.0890

### RACIAL AND ETHNIC DISPARITIES IN THE EFFECTS OF COVID-19 ON EMPLOYMENT DISRUPTION AND FINANCIAL PRECARITY

Dawn Carr[1], Amanda Sonnega[2], Rebekah Carpenter[1], Katy (Qiuchang) Cao[1], and Qize Chen[2], *1. Florida State University, Tallahassee, Florida, United States*, *2. University of Michigan, Ann Arbor, Michigan, United States*

The COVID-19 pandemic disproportionately influenced minoritized individuals, with significantly higher rates of infection and death. One reason for this higher rate is hypothesized to relate to occupational environments of older minoritized populations had going into the pandemic, which also make them more likely to experience disruption in employment due to factors such as illness, and consequently, more financial consequences of the pandemic than their white counterparts. The current project evaluates race and ethnicity differences in disruptions in employment among older workers during the pandemic, and the extent to which those disruptions were related to the inability to pay monthly bills such as rent/mortgage, utilities, medical bills, food, or debt-related payments. Using data drawn from the Health and Retirement Study, including a COVID-19 survey collected in September 2021, this study evaluates older workers who were employed prior to the start of the pandemic to evaluate differences by race-ethnicity in whether and for what reasons they experienced job disruptions, and the extent to which these disruptions were associated with an inability to pay monthly bills. We further evaluate the extent to which being unable to pay monthly bills is moderated by pre-pandemic financial vulnerabilities. Results indicate that Black and Hispanic older people experience more significant financial consequences of job disruptions, and that pre-pandemic financial vulnerabilities significantly moderate the relationship between race-ethnicity and COVID-related financial consequences of job disruptions. To conclude, the policy-implications of these results will be discussed, particularly potential factors that may decrease financial vulnerability during retirement among minoritized populations.

### SESSION 3100 (SYMPOSIUM)

Abstract citation ID: igad104.0891

### EXAMINING THE ROLE OF AGE STEREOTYPES FOR DEVELOPMENT IN CONTEXT

Chair: M. Clara P. de Paula Couto Co-Chair: Maria Wirth

This session will focus on age stereotypes, that is, beliefs individuals hold about older adults and the aging process. Two types of age stereotypes will be addressed: those related to how older adults are assumed to be (i.e., descriptive stereotypes) and those related to how older adults should be (i.e., prescriptive stereotypes). Age stereotypes have been shown to be incorporated into older adults' self-concepts (via stereotype internalization) and to influence their development in old age by shaping their expectations, motivations, and behaviors (stereotype embodiment). Considering the relevance of age stereotypes for developmental outcomes, the talks in this symposium will address how they shape individual aging and to what extent age stereotypes and their effects on development are context dependent. The first two presentations focus on cross-cultural differences in age stereotypes. de Paula Couto et al. examine prescriptive age stereotypes across different countries and investigate underlying factors that may influence their differential endorsement. Fung and Fung investigate cultural and age-related differences in the internalization of age stereotypes. The developmental consequences of negative age stereotypes will be addressed by Kornadt et al., whereas their malleability and specificity will be examined by Wirth et al. The last talk by Degen et al. will focus on the fear of loneliness in older adults, and how it affects their motivation to engage in behaviors that can prevent being lonely in old age.

Abstract citation ID: igad104.0892

### "OLDER ADULTS SHOULD...": CROSS-NATIONAL DIFFERENCES IN ENDORSEMENT OF PRESCRIPTIVE AGE STEREOTYPES

M. Clara P. de Paula Couto[1], Helene Fung[2], Sylvie Graf[3], Thomas M. Hess[4], Shyhnan Liou[5], Jana Nikitin[6], and Klaus Rothermund[1], *1. Friedrich Schiller University Jena, Jena, Thuringen, Germany*, *2. The Chinese University of Hong Kong, Hong Kong, Hong Kong*, *3. Czech Academy of Sciences, Brno, Moravskoslezsky kraj, Czech Republic*, *4. NC State University, Raleigh, North Carolina, United States*, *5. National Cheng Kung University, Tainan, Tainan, Taiwan (Republic of China)*, *6. University of Vienna, Vienna, Wien, Austria*

Normative expectations about how older adults should behave are known as prescriptive age stereotypes (or "prescriptive views of aging," PVoA). Previous research has shown that endorsement of PVoA varies across age groups but has not yet examined the variability of PVoA endorsement across countries. Considering that context may influence the endorsement of PVoA, we investigated differences in endorsement reported by an international sample of adults (N = 2,902) from the Aging as Future study covering the age range from 40 to 90 years in five countries: Czechia, Germany, Hong Kong, Taiwan, and the United States (US). We focused on endorsement of two types of PVoA, that is, disengagement (older adults should make way for the younger generation), and activation (older adults should remain socially engaged). Overall, participants reported a stronger endorsement of activation compared to disengagement (i.e., a focus on activation). Replicating previous studies, compared to young and middle-aged adults, older adults more strongly endorsed PVoA. Most importantly, cross-national differences emerged, indicating that overall endorsement of PVoA (averaged across activation and disengagement) was the strongest in Taiwan and the weakest in Czechia and Germany. However, the focus on activation (vs. disengagement) was the highest in the US followed by Hong Kong and Germany, and then by Czechia and Taiwan. Cross-national differences in the belief that the State should support older adults, in cultural values, and in views of own aging predicted the focus on activation and partially explained differences in the strength of this focus between countries.

Abstract citation ID: igad104.0893

### AGE STEREOTYPE INTERNALIZATION ACROSS AGE AND CULTURES

Nicole Long Ki Fung, and Helene Fung, *The Chinese University of Hong Kong, Hong Kong, Hong Kong*

The stereotype embodiment theory suggests that people internalize age stereotypes when they become self-relevant. This study explored the relationship between age and age stereotype internalization across cultures. We recruited adults aged 30 to 99 years from Hong Kong (N=524, Mage= 62.49), the United States (N=492, Mage= 57.90), Germany (N=790, Mage=63.38), Czechia (N=617, Mage=62.05), and Taiwan (N=660, Mage=60.78). Participants rated their self-perception of aging and attitude towards older adults. Across all the items, we calculated an intra-personal correlation score between self-perception of aging and attitude towards older adults. This index serves as a proxy of age stereotype internalization. Among all cultures, Hong Kong (r=0.57) and Taiwan (r=0.58) have the highest levels of internalization, followed by Czechia (r=0.47) and Germany (r=0.45). The United States (r=0.40) has the lowest level of internalization. The relationship between internalization and age varied across cultures. In Germany, there was a cubic relationship between internalization and age (p <.001). Internalization decreased with age till 52 years old, then it went up with age till 79 years old and dropped again. In Taiwan, we observed a quadratic relationship between internalization and age (p <.001). Internalization increased with age till the age of 64, then started decreasing. In Czechia, internalization increased with age linearly (p <.001). In Hong Kong and the United States, internalization was not associated with age. Our findings suggest that the internalization of age stereotypes into the self depends on cultures and age. More cross-cultural and longitudinal studies should be conducted to understand the age stereotype internalization process.

Abstract citation ID: igad104.0894

### NEGATIVE AGE STEREOTYPES TEMPORARILY MAKE YOU FEEL YOUNGER BUT MAKE YOU FEEL OLDER OVER TIME

Anna E. Kornadt[1], David Weiss[2], M. Clara P. de Paula Couto[3], and Klaus Rothermund[3], *1. University of Luxembourg, Esch-sur-Alzette, Luxembourg, 2. Martin-Luther-University of Halle-Wittenberg, Halle, Sachsen-Anhalt, Germany, 3. Friedrich Schiller University Jena, Jena, Thuringen, Germany*

Negative age stereotypes have negative, assimilative effects on the subjective aging experience due to internalization processes, but positive contrast effects are reported as well, reflecting dissociation and downward comparisons. Our aim was thus to compare short-term and long-term consequences of age stereotypes on the subjective aging experience, to test the hypothesis that contrast effects are visible cross-sectionally, whereas internalization processes are observed when considering more long-term effects. We assessed age stereotypes and subjective age in a sample of N=459 participants (initial age range 30 – 80 years) from the aging-as-future project (Lang et al., 2022) across three consecutive measurement occasions spanning a longitudinal interval of 10 years. Short-term and long-term effects were estimated with latent change-score models by assessing effects of age stereotypes on the intercepts (cross-sectional) and on the slopes (longitudinal) of subjective age, respectively, while also taking current self-views into account. Age stereotypes had opposite effects on subjective age depending on the time frame. A cross-sectional contrast effect was found (B = -.17, SE = .05, p < .001), whereas longitudinal effects were assimilative in nature (B = .36, SE = .18, p = .04). Our findings support the time-dependent nature of effects of age stereotypes on the subjective aging experience. Negative age stereotypes can temporarily lead to a significant younger subjective age, indicating dissociation from one's age group and downward comparison. In the long run, however, negative (positive) age stereotypes become internalized into the self-views of older people and are linked to an older (younger) subjective age.

Abstract citation ID: igad104.0895

### MANIPULATING THE ENDORSEMENT OF PRESCRIPTIVE VIEWS OF AGING—SPECIFICITY AND INTERNALIZATION

Maria Wirth, M. Clara P. de Paula Couto, and Klaus Rothermund, *Friedrich Schiller University Jena, Jena, Thuringen, Germany*

Prescriptive views of aging are beliefs about how older adults should be and behave. The two most prominent views entail that older adults should remain active and contribute to society (active aging) and that they should withdraw from important positions (altruistic disengagement). Prescriptive views of aging set societal standards for age (in)appropriate behaviour and many older adults internalize these norms so much that they appear completely natural to them. This study investigated the malleability of endorsing prescriptive norms using social consensus information: 367 participants (50–87 years) were asked to indicate whether the general public agreed or disagreed with altruistic disengagement or active aging in the health or social domain. Subsequently, participants were presented with (fictitious) social consensus feedback which was either in line with participants' estimation of social consensus or not (random assignment). Our results indicate that presenting social consensus feedback affected the endorsement of prescriptive views of aging. Learning that a majority of others supported (dis)agreement with a prescriptive age norm led to (lower) higher endorsement of this norm. This effect was specific to the norm and domain for which consensus feedback was presented. Thus, activation and disengagement are represented as separate norms in a domain-specific fashion. Endorsing prescriptions for older adults, in general, led participants to also more strongly endorse this norm for themselves. Our findings attest to the complexity and specificity of prescriptive views of aging and offer important insights for designing interventions.

Abstract citation ID: igad104.0896

### IS THERE A WORRY ADVANTAGE? THE ROLE OF FEAR OF LONELINESS IN OLD AGE IN LONELINESS PREVENTIVE ACTIVITIES

Nora Degen[1], Yaeji Kim-Knauss[2], and Frieder Lang[2], *1. Friedrich-Alexander-Universität Erlangen-Nürnberg, Nürnberg, Bayern, Germany, 2. Friedrich-Alexander-Universität Erlangen-Nürnberg, Nuremberg, Bayern, Germany*

While worries are generally known to be aversive, behavioral theories have also addressed potential advantages of worrying throughout life. Drawing on this notion, the present study focused on the fear of loneliness in old age and its beneficial effects on individuals' engagement in loneliness preventive activities. Data were collected from 702 German adults (aged 22-96) who had participated in at least two out of five measurement occasions with a time interval of two years. Fear of loneliness in old age was measured using a single item (i.e., how much do you fear being lonely in old age?) and loneliness preventive activities were measured as a mean score of five social activities specifying the very intention to prevent loneliness in old age (e.g., I actively pursued contacts with friends and acquaintances in order not to be lonely in old age). Based on longitudinal multilevel regression models, we found that at the between-person level old age, female gender, and better health were independently associated with higher average loneliness preventive activities. At the within-person level, participants reported more preventive activities on occasions when they were more feared for the loneliness in old age. Such benefits of fear were particularly reported on occasions when individuals reported less state loneliness at that time. Findings indicate that aging-related worry serves as a motivator for loneliness preventive activities, particularly in situations in which individuals felt socially well connected.

### SESSION 3105 (SYMPOSIUM)

Abstract citation ID: igad104.0897

### INTIMATE RELATIONSHIPS, SOCIAL ISOLATION, AND COGNITION

Chair: Michal Engelman Co-Chair: Sara Moorman

This symposium considers the cognitive impacts of a spectrum of social relationships, including both intimate partnerships and, in contrast, social isolation. Drawing on rich data from the Wisconsin Longitudinal Study (WLS), the Health and Retirement Study (HRS), the National Social Life, Health, and Aging Project (NSHAP), and the Mexican Health and Aging Study (MHAS), the papers showcase empirical linkages between older adults' cognitive health with a set of exposures including marital status and relationship characteristics, social activities, and a lack of social connections. We begin with papers that examine the impact of particular marital relationship attributes and social behaviors on cognition and health. One paper employs the WLS to explore the impact of concordance and discordance in marital couples' reports of similarity with and closeness to their spouse on cognition, while the second draws on NSHAP to consider the cognitive consequences of various domains of couples' sexuality. The third paper uses the HRS to examine couples' co-trajectories of physical, mental, and cognitive health, focusing particularly on how a spouse's cognitive decline influences the other partner's subsequent health trajectory. The fourth paper shifts our attention to variation by marital status in the cognitive impacts of leisure activities via data from MHAS, while the fifth focuses specifically socially-isolated older adults in the HRS and highlights variation in the relationship between social isolation and dementia by race and ethnicity. The symposium will conclude with a discussion of future directions for research on the cognitive consequences of relationships, social connections, and social isolation.

Abstract citation ID: igad104.0898

### MARITAL CLOSENESS, SPOUSAL SIMILARITY, AND COGNITION: THE GENDERED IMPACT OF DISCORDANT PERCEPTIONS

Yue Qin[1], Sara Moorman[2], and Michal Engelman[1], *1. University of Wisconsin-Madison, Madison, Wisconsin, United States*, *2. Boston College, Boston, Massachusetts, United States*

Many older adults are in long-term marriages, and an emerging literature examines the impact of marital attributes on later-life cognitive function. While both spouses' perceptions of the marital relationship may influence health outcomes in the couple, most research only captures one partner's report. Using unique data on 2,791 heterosexual married couples from the Wisconsin Longitudinal Study, we analyze the dyad's evaluations of their similarity and closeness in 2004 and the impact of concordance or discordance in these marital attributes on cognitive health in 2020, when participants were approximately 80 years old and cognitive variation is more manifest. Results from OLS regressions reveal notable gender differences. Compared with dyads in which both spouses reported being "very similar," women in marriages with discordant views on the partners' similarity had significantly lower cognition in 2020 (wife gave the poorer rating: b=-0.74, p<.05; husband gave the poorer rating: b=-0.84, p<.05). Men's cognitive function was not influenced by assessments of similarity in the couple. Furthermore, compared with dyads in which both spouses reported being "very close," men in marriages with discordant views on the partners' closeness had significantly lower cognition in 2020 (woman gave the poorer rating: b= -0.88, p < .05). Women's cognitive function was unrelated to dyadic reports of closeness. Our results suggest that within-couple discordance in assessment of marital closeness and spousal similarity – more than marital attributes themselves – influences later-life cognitive function for women and men in varied ways.

Abstract citation ID: igad104.0899

### SEXUAL FREQUENCY AND COGNITIVE DOMAINS: EVIDENCE FROM THE NATIONAL SOCIAL LIFE, HEALTH, AND AGING PROJECT

Shen Shannon, *Hope College, Holland, Michigan, United States*

This research draws upon the life course perspective to examine how sexual frequency is related to different cognitive domains among community-dwelling, partnered older adults. I analyzed data from 1,526 respondents across two rounds of the National Social Life, Health, and Aging Project (NSHAP). I tested six cognitive domains: temporal orientation, visuospatial skills, attention, language, executive function, and short-term memory. I ran cross-lagged models to test for reciprocal relationships between sexual frequency and cognitive domain performance. Age-stratified results indicate that more frequent sex was related to better performance on temporal orientation, executive function, attention, and short-term memory domains over time for adults aged 75-90, with no significant results for adults aged 62-74. There was limited evidence that cognitive domain performance was related to later sexual frequency for the 75-90 age group. The results highlight nuanced pathways by which sexuality is tied to the health of older adults. Understanding which cognitive domains may be maintained through more frequent sex is informative for health care practitioners who consider sexual activity as a behavior to prevent cognitive impairment.

Abstract citation ID: igad104.0900

### IMPACT OF HUSBAND'S DEMENTIA: INNOVATIVE METHODS TO STUDY HEALTH PROFILES AND CO-TRAJECTORIES BETWEEN COUPLES

Yan Zhang[1], Yiyang Yuan[2], Maria Roche-Dean[3], Richard Gonzalez[4], and Irving Vega[5], *1. East Carolina University , Greenville, North Carolina, United States*, *2. University of Massachusetts Chan Medical School, Worcester, Massachusetts, United States*, *3. Western Michigan University, Kalamazoo, Michigan, United States*, *4. University of Michigan, Ann Arbor, Michigan, United States*, *5. Michigan State University, Grand Rapids, Michigan, United States*

Traditional life course studies often focus on how one predictor influences one health outcome that may not be able to capture the interdependence of two or more health trajectories very well.

**Objectives:** (1) demonstrate an innovative method to create a 3-dimensional health profile cube that presents physical, mental, and cognitive health and the changing trajectories over time; (2) display how a woman's health profile may change due to the onset of their male partner's dementia; and (3) visualize how couple's co-trajectories of health profiles may vary by their age, race/ethnicity, and household wealth.

**Methods:** We focus on married and partnered couples, drawing longitudinal data from the Health and Retirement Study (2000-2016; n=3,578). Physical, mental, and cognitive health were respectively measured by functional limitation, depression symptoms (CESD-II), and modified version of the Telephone Interview for Cognitive Status (TICS). We use vector autoregression (VAR) models to analyze these multivariate paths in the same visual representation as the original data.

**Results:** The approach provides both an analytic framework and a visualization tool that depicts data and model in the same spatial representation to permit assessment of model fit and model comparison. This study advances the traditional life course studies by representing underlying processes as a multidimensional time vector of health outcomes, which better reflect the interdependent co-trajectories of health outcomes and the essence of the life course conceptual framework.

**Discussion:** This paper provides a blueprint for studying complex health profiles or trajectories.

Abstract citation ID: igad104.0901

### LEISURE ACTIVITIES, MARITAL STATUS, AND COGNITIVE FUNCTIONING IN LATER LIFE IN MEXICO

Maria Monserud, *University of Houston, Houston, Texas, United States*

Prior research indicates that leisure activities can shape cognition in later life. However, little is known about whether the implications of specific group and individual leisure activities for older adults' cognitive functioning can be contingent on marital status and gender and whether physical and mental health can make a difference in the associations between leisure participation and cognition. Moreover, research on the role of these factors in older adults' cognitive functioning in Mexico has been scarce. Using data from the most recent wave (2018) of the Mexican Health and Aging Study, a nationally representative sample of Mexicans aged 50+, this study examines the implications of group and individual leisure activities for cognition among married and unmarried older men and women in Mexico while considering their physical health (i.e., vision, hearing, chronic conditions, and ADL and IADL limitations) and depressive symptoms. The results suggest that not only group-based but also individual activities can be predictive of cognitive functioning among older Mexicans. The findings also reveal some marital status and gender differences and similarities in the effect of certain leisure activities on cognition. Additionally, the results demonstrate that experiencing physical health challenges and depressive symptoms can prevent older Mexicans from deriving cognitive benefits from some leisure activities. Yet, despite poorer physical and mental health, certain leisure activities can still lead to better cognitive functioning in this population group. The insights from this study can be useful for the development of preventive strategies and intervention programs to improve cognitive health in later life.

Abstract citation ID: igad104.0902

### SOCIAL ISOLATION AND RISK OF DEMENTIA: DO RACE, ETHNICITY, AND GENDER MATTER?

Zhiyong Lin[1], and Hui (Cathy) Liu[2], *1. University of Texas at San Antonio, San Antonio, Texas, United States*, *2. Purdue University, West Lafayette, Indiana, United States*

Social isolation has emerged as a significant predictor of dementia risk in later life. However, our understanding of how this association may vary across race-ethnicity and gender remains limited. To address this gap, we used longitudinal data from the Health and Retirement Study (2000-2016) to examine the link between social isolation and dementia risk among older adults, separately for non-Hispanic Whites, non-Hispanic Blacks, and Hispanics. We also explored gender variations in this association within each racial-ethnic group. Our results indicate that social isolation increases the risk of dementia for both non-Hispanic Whites and Blacks, even after controlling for basic sociodemographic characteristics. However, social isolation does not appear to be a significant predictor of dementia risk among Hispanics, suggesting that cultural and social factors may play a key role in the association between social isolation and dementia risk across racial/ethnic groups. Further, we found few significant gender variations in the association between social isolation and dementia, except for a stronger effect of severe isolation among Black men compared to Black women. Economic resources and health-related factors were identified as significant mediators of the relationship between social isolation and dementia risk for both non-Hispanic Whites and Blacks. Our study highlights the importance of addressing social isolation as a potential risk factor for dementia among older adults, particularly for those in vulnerable groups. The findings underscore the need for targeted interventions to reduce social isolation and promote social engagement in diverse communities to mitigate dementia risk.

### SESSION 3110 (SYMPOSIUM)

Abstract citation ID: igad104.0903

### ROOTS TO GROW AND WINGS TO FLY: EDUCATING MEDICINE AND PHARMACY TRAINEES ON VIONE MEDICATION DEPRESCRIBING METHODOLOGY

Chair: Saraswathy Battar Co-Chair: Michael Silverman

An aging world population translates to more chronic illnesses, more medications with potential and relevant cumulative burdens such as non-compliance, adverse outcomes, prescription cascades, confusion, compromised patient safety and quality of life standards. VIONE stands for the 5 filters that categorize medications as Vital, Important, Optional, Not indicated, Every medication has an indication. This is an electronic health record methodology currently implemented in 122 VA facilities across the nation. Physicians and pharmacists review medications, prioritize medications that merit deprescribing interventions, deprescribe through shared decision making and monitor outcomes. In the VA system, within 7 years, we successfully deprescribed over 1.4 million inappropriate medications impacting over 600,000 veterans, used by over 14000 medical providers, with an annualized cost savings of ~ 132 million US dollars. We incorporate VIONE deprescribing education while training our pharmacy residents, medical students, residents and fellows rotating at our VA Medical Center through case based, bed side, didactic and team based multi media learning strategies . The engagement, feedback, demonstrated formal and informal responses remain very promising. Capturing the attention of trainees in their formative years deepens their roots as they spread their wings into practicing safe medication prescribing practices that are current, cutting edge, evidence based and empowering as we train the trainers. We envision medication deprescribing to be part of future academic core curriculum. Our Interdisciplinary team will present compelling data, experiences and outcomes stemming from education of trainees regarding safe medication optimization and deprescribing practices utilizing VIONE methodology that won national and international acclaim.

Abstract citation ID: igad104.0904

### PLANNED CESSATION OF POTENTIALLY INAPPROPRIATE MEDICATIONS: TRAINEE EXPERIENCES FROM LONG-TERM CARE PROGRAMS

Kimberly Dickerson[1], and Jennifer Quellhorst[2], *1. Central Arkansas Veterans Healthcare System, Little Rock, Arkansas, United States*, *2. WPB VAMC, West Palm Beach, Florida, United States*

Polypharmacy is a global concern. There is no universal consensus on definition. VIONE is an innovative, end user friendly, portable, simple methodology that is easy to teach, adapt, customize across the continuum of care, in any clinical setting, for any medication. Trainees demonstrate immense eagerness, ability and potential to benefit from exposure to medication optimization through both safe prescribing and safe deprescribing practices. Given the good fortune of long term care settings, trainees follow the patients and directly engage in implementing methodical safe deintensification of potentially inappropriate medications through shared decision making. Trainees address prescription cascades and health literacy issues. Trainees also track outcomes to deprescribing, are made aware of the data and intervene as needed. Overall - this has been a very professionally rewarding and personally enriching experience. We have case reports with specific timelines, medications, doses, duration, rationale, responses and data analytics that illustrate the merits of engaging trainees early on in their careers to safe medication optimization and deprescribing practices. Data tracked includes number of patients impacted, number of medications deprescribed, dose decreased, safer alternatives prescribed, number of users, cost savings, location, medications deprescribed etc. that are updated daily with VIONE score cards and dashboards utilizing medical informatics and corporate data warehouse data - thus proving the rich granularity, transparency and data integrity.

### SESSION 3115 (SYMPOSIUM)

Abstract citation ID: igad104.0905

### GIS/GPS METHODOLOGIES TO MEASURING OUTDOOR MOBILITY, ENVIRONMENTAL CONTEXT, AND HEALTHY AGING

Chair: Christina Roecke Co-Chair: Minxia Luo Discussant: Jeffrey Kaye

There has been increasingly more interest in using GIS/GPS methodologies in healthy aging research, but insufficient consideration of the latest developments in this area. This symposium showcases recent studies using GIS/GPS methodologies to measure outdoor mobility and environmental context across different countries, including one long-term longitudinal study and three micro-longitudinal studies. Specifically, Lum and colleagues examine a four-year survey from over 2,000 older adults in Hong Kong. They show that public open space (including its accessibility, its number of leisure facility types, and social interactions happened therein) was not only associated with functional ability cross-sectionally, but also with the decline of functional ability over time. Perchoux and colleagues collect seven days data from 471 older adults in Luxembourg. They show that built environments (such as higher street connectivity and lower density of parking lots) were associated with lower total sedentary time. Luo and colleagues examine data from a 15-day study with 109 older adults in Switzerland. They show that characteristics of neighborhood walkability (including higher street connectivity, more access to places for walking and cycling, and lower mixture of land use) strengthens the positive associations between daily out-of-home mobility and daily working memory performance. Giannouli and colleagues present their project of a mobile app for early detection of mobility impairments. Through two one-week studies, they demonstrate the reliability, validity, and usability of their app as a tool to assess walking speed and life-space mobility of older adults. Finally, Jeffrey A. Kaye will discuss all papers from a healthy aging perspective.

Abstract citation ID: igad104.0906

### UNDERSTANDING THE LONG-TERM EFFECTS OF PUBLIC OPEN SPACE ON OLDER ADULTS' FUNCTIONAL ABILITY AND MENTAL HEALTH

Terry Lum[1], Yuqi Liu[2], Yingqqi Guo[3], Shiyu Lu[4], On Fung Chan[1], Cheryl Chui[1], Hung Chak Ho[1], and Yimeng Song[5], *1. The University of Hong Kong, Hong Kong, Hong Kong*, *2. South China University of Technology, Guangzhou, Guangdong, China (People's Republic)*, *3. Hong Kong Baptist University, Hong Kong, Hong Kong*, *4. City University of Hong Kong, Kowloon, Hong Kong*, *5. The Hong Kong Polytechnic University, Hong Kong, Hong Kong*

Little is known about how public open space (POS) environment quality and vitality influence older adults' functional ability and mental health over time. POS vitality refers to the capacity of POS to accommodate a variety of users and activities. We undertook a four-year longitudinal survey of 2081 older adults in Hong Kong to investigate longitudinal relationships between POS environment quality, POS vitality, functional ability and mental health. We applied environment quality evaluation and space use behavior observation to collect data on the environment quality and vitality of POSs within the 200-m buffer area of participants' residences. POS environment quality attributes included the number of leisure facility types, accessibility, shade, and bench quality. POS vitality attributes comprised the diversity of users and activities. We used the Chinese Lawton Instrumental Activities of Daily Living Scale to measure older adults' functional ability and the Geriatric Depression Scale (15-item)

to evaluate mental health. We applied latent growth curve models to analyze the longitudinal associations. Accessibility to POS and social interactions among users in POS were related to better functional ability and mental health among older adults at baseline. The number of leisure facility types, and social interactions among users in POS led to a slower decline in functional ability over time. However, there were no significant associations between POS and mental health over time. These findings have theoretical implications for the healthy aging research framework and practical insights for planning policies using POS as an intervention tool to facilitate older adults' healthy aging.

Abstract citation ID: igad104.0907

**OUT-OF-HOME MOBILITY, DAILY WORKING MEMORY, AND NEIGHBORHOOD WALKABILITY IN HEALTHY OLDER ADULTS**

Minxia Luo[1], Eun-Kyeong Kim[2], Robert Weibel[1], Mike Martin[1], and Christina Roecke[1], *1. University of Zurich, Zurich, Zurich, Switzerland*, *2. Luxembourg Institute of Socio-Economic Research, Luxembourg, Diekirch, Luxembourg*

Out-of-home mobility could enhance cognitive performance, but environmental contexts — such as neighborhood walkability — may influence the associations. This study examined daily out-of-home mobility in relation to daily cognitive performance in healthy older adults and the moderator effects of neighborhood walkability therein. Participants wore a custom-built mobile GPS sensor („uTrail") and completed smartphone-based working memory assessments for 15 days, and responded to the baseline Neighborhood Environment Walkability Scale. Analyses included 947 days' data from 109 Swiss older adults aged 65 to 89 years. Out-of-home mobility (i.e., out-of-home time, maximum distance from home) were extracted from the GPS data. Multilevel models showed that daily out-of-home time and daily maximum distance from home were not associated with daily working memory performance across the study sample, but the associations were moderated by neighborhood walkability. Out-of-home time and maximum distance from home were positively (vs. negatively) associated with working memory performance in participants who lived in neighborhood with higher (vs. lower) street connectivity and more (vs. less) access to places for walking and cycling. Additionally, maximum distance from home were positively (vs. negatively) associated with working memory performance in participants in neighborhood with lower (vs. higher) mixture of land use. To conclude, spending more time out of home and travelling longer distance away from home could be beneficial for daily cognitive performance of older adults who lived in well-connected and higher-walkable and -bikeable neighborhood. Travelling longer distance could also benefit those who lived in a neighborhood with a lower mixture of land use.

Abstract citation ID: igad104.0908

**USABILITY AND PSYCHOMETRIC PROPERTIES OF THE MOBITEC-GP APP FOR REAL-LIFE MOBILITY ASSESSMENT**

Eleftheria Giannouli[1], Eun-Kyeong Kim[2], Cheng Fu[3], Robert Weibel[3], Alexandros Sofios[3], Arno Schmidt-Trucksäss[4], Andreas Zeller[4], and Timo Hinrichs[4], *1. ETH Zurich, Zurich, Zurich, Switzerland*, *2. Luxembourg Institute of Socio-Economic Research, Luxembourg, Diekirch, Luxembourg*, *3. University of Zurich, Zurich, Zurich, Switzerland*, *4. University of Basel, Basel, Basel-Stadt, Switzerland*

Modern technologies such as the Global Navigation Satellite System and accelerometers which are integrated in every smartphone enable tracking mobility patterns over time, which in turn enables early detection of mobility impairments. Aim of the MOBITEC-GP project was to develop a mobile app which measures walking speed and spatial mobility under everyday conditions. Reliability and validity of the application was tested first (Study 1). The applicability in everyday life and the acceptance by older adults (Study 2) was also investigated. In study 1, participants underwent several supervised walking speed assessments as well as a 1-week life-space assessment during two assessment sessions 9 days apart. Fifty-seven older adults (47.4% male, mean age= 75.3 years) were included in the study. The app showed moderate to excellent test-retest reliability and validity of walking speed measurements of 50 meters and above and of most 1-week life-space parameters, including life-space area, time spent out-of-home, and action range. Study 2 revealed a good usability of the app (mean System Usability Scale score=77.18) among older adults (N=60 with ≥2 chronic conditions) for a 1-week usage of the app including both, a 1-week life space assessment and at least one gait speed measurement in a 30-minute stroll. The MOBITEC-GP app is a reliable, valid and usable tool for the assessment of real-life walking speed (at distances of 50 metres and above) and life-space parameters of older adults. Future studies should look into technical issues more systematically in order to avoid invalid measurements.

### SESSION 3120 (SYMPOSIUM)

Abstract citation ID: igad104.0909

**MUSICAL THEATER AND MEANING MAKING IN A RETIREMENT COMMUNITY**

Chair: James Powers Discussant: Robyn Stone

"I think I've had it up to here with pickleball…" Thus begins the first song in "Movin", an original musical written, produced, and performed by members of a continuing care retirement community (CCRC), which addresses themes ranging from dementia, death and widowhood to finding community, purpose, and late-life love. The idea began in informal conversation between two former university colleagues, Philip Sloane (a geriatrician, GSA member, and former CCRC board member) and Hugh Tilson (a CCRC resident, aged 82) about describing the aging experience through song. Hugh gathered a group of fellow CCRC residents, none of whom had professional songwriting or theater experience but several of whom had musical backgrounds or an interest in theater. Meeting regularly over nearly two years, they envisioned, created, and then produced an original musical around the theme of "freshman year in a retirement community." The final production included 9 cast members, 12 original songs developed by five lyricists and two composers, many production-RELATED volunteers, and a narration to link the songs together. This symposium will describe the process, present and illustrate aging-related themes from the production, place the musical in the context of existing research about meaning-making and the arts in later life, and conclude with a discussion by a senior leader in a major senior housing organization. Presentation about development and production of the musical will include videos of rehearsals, songs from the production, and interviews with performers. Sponsored by the Humanities, Arts, and Cultural Gerontology Advisory Panel

Abstract citation ID: igad104.0910

### DEVELOPMENT AND PRODUCTION OF AN ORIGINAL MUSICAL WITHIN A RETIREMENT COMMUNITY

Philip Sloane, *University of North Carolina, Chapel Hill, North Carolina, United States*

In 2021-23 residents of a continuing care retirement community (CCRC) conceptualized, wrote, produced, and presented an original musical about life in a senior community. This presentation will describe that process, illustrating it with a video presentation by the 83-year-old CCRC resident who organized and coordinated the process, video clips of rehearsals and of interviews with the songwriters and performers, and video performances of four songs from the musical. Among the gerontological issues to be highlighted include: deciding which song topics the community would deem acceptable and unacceptable to address in the production; accommodating the physiological limits of the cast (e.g., reduced vocal ranges with age) and audience (e.g., hearing impairment and limited bladder capacity); and adjusting to absences due to illnesses, medical appointments, family events, and travel among the organizers and performers. A portion of this presentation will center around four original songs written and performed by seniors as part of the musical: “Rules of the Road” (about the informal “do’s and don’ts” of living in a retirement community); “The Ladies of Lavender Lane” (about groups of widows who have banded together for support); “The Rhythm of our Lives” (about how the community deals with the high frequency of resident deaths); and “I’m Just a Little Worried about Eddie” (about early dementia). The development of each song will be summarized, including the process by which the song went from idea to production, and a video of the song during a performance.

Abstract citation ID: igad104.0911

### COMMUNITY-BASED MUSICAL THEATER AND MEANING-MAKING IN LATER LIFE

Justine McGovern, *Lehman College, Bronx, New York, United States*

This presentation will situate the impact of participating in artistic performances for the first time in later life in relevant scholarship. A growing body of research indicates that older adults benefit from active engagement in the arts, and that this can reduce loneliness, depression and isolation, and improve subjective well-being and quality of life. Benefits include strengthening social connections and a sense of belonging, as well as defining and asserting a sense of self. These outcomes can have a positive impact on physical health as well – older adults engaged in the arts experience lower rates of memory loss and balance issues, heart disease and other health challenges than peers not actively engaged with the arts or passively receiving arts. Participation in performance arts can also improve communication across generations and support processing of loss. Senior centers and community centers with senior members rarely, however, position engagement in performing arts such as musical theater front and center. Programming in visual arts (e.g., painting) and traditional arts (e.g., crochet) tends to take precedence, and this can be detrimental to center members for whom participation in performing arts can be transformative. The presentation will explore meaning-making processes embedded in arts performance and argue for promoting performance opportunities among older adults, as well as suggest ways to replicate the model in other settings.

### SESSION 3125 (SYMPOSIUM)

Abstract citation ID: igad104.0912

### ACCELERATED AGING IN MID LIFE: BEHAVIORAL, EMOTIONAL, AND PHYSIOLOGICAL MARKERS OF AGING

Chair: Maayan Agmon

The aging process starts at midlife and is multifactorial, involving behavioral, emotional, and biological pathways. Most studies to date have focused on either older ages, when age-related diseases already are present, or a single mechanism, thus missing the full scope of the aging process. The proposed symposium aims to fill this gap by highlighting the many-sided nature of aging among midlife adults, especially the contributions of behavioral, emotional, and biological markers. We will discuss how physical capacity, sensory modulation, and exposure to violence are associated with biological age, reflecting the true state of the body and the diversity of aging. Understanding these relationships can both illuminate early markers of the aging process and support the design of tailored interventions to reduce the prevalence of accelerated aging. The symposium will present the latest research on the combined markers to gain insight into normal and accelerated aging. Professor Agmon will give an overview of newly developed behavioral markers that quantify the aging process, such as the photogrammetry-based analysis of posture and physical capacity battery. Roy Tzemah-Shahar will discuss the relationships between physical capacity metrics and rate of aging, Merav Asher will explicate the association between one's sensory profile and the rate of aging, and Khalil Iktilat will share how exposure to violence explains the accelerated aging of midlife Muslims. Finally, we will discuss the need for more research on midlife adults and the potential benefits of early interventions.

Abstract citation ID: igad104.0913

### PHYSICAL CAPACITY AS MARKER FOR RATE OF AGING IN MID LIFE

Roy Tzemah-Shahar[1], and Maayan Agmon[2], *1. University of Haifa, Ramat-Gan, HaZafon, Israel*, *2. University of Haifa, Haifa, HaZafon, Israel*

Biological age captures the heterogeneity of aging by providing an estimation for rate of aging. As an alternative to the widely investigated laboratory biomarkers of aging, behavioral functional markers of physical capacity may offer a feasible alternative for identifying individuals at risk for negative aging trajectory. We aimed to examine the associations of different functional tests both together and as a composite score of physical capacity with rate of aging. We conducted a cross-sectional study, with midlife adults (age ~45) reporting being able to perform leisure physical exercise. Biological age was estimated using the Klemera-Doubal method and a set of physiological biomarkers; rate of aging, ΔAge, was defined as the difference between age and estimated biological age. Physical capacity was measured using a testing battery of 15 functional tests linked with six physical capacity domains. 116 participants completed the testing battery. For women, better results in terms of strength, flexibility, cardiorespiratory fitness, and balance tests were negatively correlated with ΔAge (r=0.38-0.29, p<0.05); for men, beyond these tests, agility was also negatively correlated with ΔAge (r=0.27-0.59, p<0.05). A sex-standardized composite score of physical capacity was negatively associated with ΔAge after controlling for chronological age, smoking, and education (r=-0.437, p=0.007; r=-0.491, p<0.001 for women and men respectively). The suggested physical capacity battery offers a functional assessment for ΔAge. Higher physical capacity metrics correlate with smaller ΔAge, corresponding with younger biological age. Measuring physical capacity may help to assess aging trajectory and offer a suitable behavioral intervention goal.

Abstract citation ID: igad104.0914

### LOW SENSORY RESPONSIVENESS IS ASSOCIATED WITH ACCELERATED AGING IN MID LIFE

Merav Asher, and Maayan Agmon, *University of Haifa, Haifa, HaZafon, Israel*

The rate of aging is affected by genetic, behavioral, and emotional mechanisms; one possibly additional mechanism is the ability of the nervous system to sense and react appropriately to stimuli. This ability, called sensory responsiveness, may be a factor in shaping the aging mechanism and could be used as a marker of aging. The aim of this study was to examine the association between sensory responsiveness levels and rate of aging in midlife. A total of 97 participants born between 1974-1976 (age 45.63±0.67 at end point, 46% women) were included. Biological age was estimated at age ~32 and ~45 using the Klemera-doubal method and a set of biomarkers. Rate of aging was calculated by the difference in estimated biological age scores divided by passing years, reflecting temporal change in biological age. Sensory responsiveness was evaluated using the Sensory Responsiveness Questionnaire Scale, identifying high or low sensory responsiveness subtypes. We found that low sensory responsiveness was positively associated with rate of aging while controlling for chronological age, social engagement, and physical performance (linear regression, r=0.211; p=0.038). High sensory responsiveness subtype was not significantly associated with rate of aging (linear regression, r=-0.02; p=0.843). To conclude, low sensory responsiveness subtype may have a significant role in identifying the rate of aging in midlife. These findings highlight the importance of sensory responsiveness evaluations as a marker for rate of aging and the need for interventions focusing on sensory responsivity difficulties, as a mean of supporting healthier aging.

Abstract citation ID: igad104.0915

### THE LINK BETWEEN VIOLENCE EXPOSURE AND BIOLOGICAL AGING IN MID-LIFE MUSLIMS IN ISRAEL

Khalil Iktilat, and Maayan Agmon, *University of Haifa, Haifa, HaZafon, Israel*

Trauma and accumulated chronic stress throughout life are associated with negative health outcomes; more specifically, exposure to violence has been linked to an accelerated aging state. In Israel's Arab sector, rates of violence have been steadily rising for the past several years. Homicide rates, relative to population size, are seven times higher among Arabs. We aimed to determine the effects of exposure to violence on estimation of biological age as a measure of rate of aging within the ethnic minority of Muslims in Israel. We have conducted a cross-sectional study that included 375 participants age 50.5±6.9 years, of which 61% women. Biological age was estimated using the Klemera-Doubal method and physiological biomarkers; exposure to violence was measured using the Screen for Adolescent Violence Exposure questionnaire followed by a principal component analysis. The average total score of violence exposure was 1.83±0.36 (range 1-5, 1 corresponds with lower exposure). We found a positive and significant relationship among biological age, the "direct evidence of violence" component, and the "evidence of severe violence in the immediate environment" component (r = 0.119, p<0.05; r = 0.111, p<0.05, respectively). These results demonstrate that exposure to violence negatively affects health and accelerates aging within Israel's Muslim minority. Comprehensive, culturally- and gender-adapted programs are required to reduce violence and address its contribution to the negative aging trajectory in this population.

### SESSION 3130 (SYMPOSIUM)

Abstract citation ID: igad104.0916

### CHALLENGES OF VERY LATE LIFE: PHYSICAL AND MENTAL HEALTH, CAREGIVING, AND AGING PERCEPTIONS

Chair: Daniela Jopp Co-Chair: Kathrin Boerner

The very old are the fastest growing population in developed countries, yet research on this age group remains limited. There are, however, specific challenges of very old age that differ from those in earlier late life phases. Very old individuals and their families may thus have unique needs and may require adapted services. In this symposium, we address selected challenges associated with reaching very old age, including physical and mental health issues, as well as implications of both receiving and providing care. Ribeiro and colleagues report on centenarians' health and morbidity profiles using the Portuguese census, by comparing individuals aged 100 years old in 2011 and 2021. Jopp and colleagues present findings from the first national Swiss centenarian study on centenarians' health, cognition, and well-being. Gomes da Rocha and colleagues investigate depressive symptoms in Swiss centenarians during the COVID-19 pandemic, showing notably elevated scores. Kim and colleagues focus on aging perceptions and their correlates among dyads of very old parents and their old children from South Korea, highlighting the impact of the care experience for both dyad members. Gallagher and colleagues examine how old children of very old parents with dementia manage behavioral and psychological symptoms through specific strategies, offering insights on how to better support family care givers of very old dementia patients. In sum, this symposium offers novel insights on specific challenges encountered in very old age, by the individual and their families, and proposes potential pathways towards better understanding and handling of such challenges.

Abstract citation ID: igad104.0917

### HEALTH PROFILE OF PORTUGUESE CENTENARIANS FROM 2011 TO 2021: AN UPDATE BASED ON NATIONAL CENSUS

Oscar Ribeiro[1], Lia Araujo[2], Constanca Paul[3], and Laetitia Teixeira[4], *1. University of Aveiro, Aveiro, Aveiro, Portugal*, *2. Cintesis, Porto, Porto, Portugal*, *3. ICBAS, Portugal, Porto, Porto, Portugal*, *4. School of Medicine and Biomedical Sciences, University of Porto, Porto, Porto, Portugal*

According to the Portuguese National Census, the number of centenarians increased from 1526 (82.1% women) in 2011 to 2801 (82.0% women) in 2021. Along with a description of main sociodemographic characteristics, this paper provides an updated health profile of this population based on their perceived difficulties in six functional domains of basic actions (seeing, hearing, walking, cognition, self-care, and communication) as assessed by a set of questions developed by the Washington Group on Disability Statistics that is consistent with the International Classification of Functioning, Disability, and Health (ICF). Sex differences are also presented, as well as comparisons between data from Census 2011 and Census 2021. Most centenarians show major constraints in their mobility and personal care (76.3% cannot/have great difficulties in walking/climbing stairs, and 72.8% cannot/have great difficulties in bathing/dressing), and a better outcome is reported in communication (64.2% report no/reduced difficulty for understanding/being understood). This profile globally follows the pattern observed in 2011, but with fewer centenarians reporting such level of difficulties. The percentage of centenarians reporting no/reduced difficulties in seeing and hearing increased from 32.6% and 27.7% in 2011, to 54.4% and 44.8% in 2021, respectively. In both censuses, women presented a higher percentage of difficulties in all health-related variables. Globally, this comparative perspective shows improvements in the perceived health profile of the later born cohort of Portuguese centenarians and features the continued need for addressing gender-specific and gender-sensitive interventions that acknowledge older women's care needs.

Abstract citation ID: igad104.0918

### VULNERABILITY AND RESILIENCE IN SWISS CENTENARIANS: FINDINGS FROM THE SWISS100 PHONE STUDY

Daniela Jopp[1], Stefano Cavalli[2], Francois Herrmann[3], Armin von Gunten[4], Mike Martin[5], Kim Uittenhove[1], and Justine Falciola[6], *1. University of Lausanne, Lausanne, Vaud, Switzerland*, *2. University of Applied Sciences and Arts of Southern Switzerland, Manno, Ticino, Switzerland*, *3. Geneva University Hospitals, Thônex, Geneve, Switzerland*, *4. Lausanne University Hospital, Prilly, Vaud, Switzerland*, *5. University of Zurich, Zurich, Zurich, Switzerland*, *6. Geneva University Hospital, Thônex, Geneve, Switzerland*

Altfhough every second child born after the year 2000 is expected to become a centenarian, little is known about what characterizes life at age 100. This is also the case for Switzerland, one of the countries with the highest life expectancy world-wide. In this presentation, we will report findings from SWISS100, the first nation-wide centenarian study in Switzerland. During the COVID-19 pandemic, we conducted a telephone study with centenarians and a family member as proxy informant, to investigate specific characteristics and challenges of centenarians. Centenarians were identified through the national address registry. A total of 171 centenarian cases were included in the SWISS100 Phone Study, including reports from 96 centenarians and 102 proxy respondents (mostly children). Centenarians were on average 102 years old, with a range of 100 to 110 years; 75% were women and 25% were men. One third of the centenarians had received basic education only, one third had completed an apprenticeship and about one third had higher education. Most of them lived in institutions (63%). Of those living in private, about half lived alone, one fourth lived with a child, and 12% still lived with a spouse. The majority was widowed. About 85% had children. Over half of the centenarians reported good to excellent subjective health; at the same time, over 70% of the centenarians indicated to often experience health restrictions. Those still able to communicate had high well-being and notable psychological strengths. In sum, findings demonstrate vulnerability but also psychological resilience in Swiss centenarians.

Abstract citation ID: igad104.0919

### DEPRESSIVE SYMPTOMS IN CENTENARIANS DURING THE COVID-19 PANDEMIC: FINDINGS FROM THE SWISS100 STUDY

Carla Gomes da Rocha[1], Armin von Gunten[2], Justine Falciola[3], Kim Uittenhove[4], Stefano Cavalli[5], Francois Herrmann[6], Mike Martin[7], and Daniela Jopp[4], *1. HES-SO Valais-Wallis, Lausanne, Vaud, Switzerland*, *2. Lausanne University Hospital, Prilly, Vaud, Switzerland*, *3. Geneva University Hospital, Thônex, Geneve, Switzerland*, *4. University of Lausanne, Lausanne, Vaud, Switzerland*, *5. University of Applied Sciences and Arts of Southern Switzerland, Manno, Ticino, Switzerland*, *6. Geneva University Hospitals, Thônex, Geneve, Switzerland*, *7. University of Zurich, Zurich, Zurich, Switzerland*

Depressive symptoms (DS) are prevalent among older adults (WHO, 2021). However, data on DS appear to be scarce in centenarians. In addition, DS may have been aggravated by the COVID-19 pandemic, especially in centenarians who may have been more susceptible to the negative consequences of pandemic restrictions. Thus, as part of the SWISS100 study (Jopp et al., 2023), we measured DS in centenarians living in Switzerland during the COVID-19 pandemic. Randomly selected centenarians from across the country and their proxies were invited to participate in a telephone interview between December 2020 and June 2022. The study sample was composed of 171 centenarians, with a mean age of 101.8 (SD=1.7) years; 128 (74.9%) participants were female and 63 (36.8%) lived at home. DS were assessed via five selected items of the Geriatric Depression Scale (Sheikh and Yesavage,

1986). The mean DS score was 1.6 (SD=1.6). Considering a cut-off ≥2 (Brañez-Condorena et al., 2021), 75 (43.9%) centenarians were screened positive for possible depression. Further analysis indicated that women had a higher level of DS, and that nursing home residents were more depressed than community-dwelling centenarians. Thus, our study suggests that almost half of the centenarians may have presented with relevant DS during the pandemic in Switzerland, which is notably higher than in other studies. DS in centenarians should be screened systematically, not less so in the context of a health crisis. Depressive symptomatology in the very old is highly relevant for successful professional care and needs further investigation to develop best practice.

Abstract citation ID: igad104.0920

### SELF-PERCEPTIONS OF AGING AMONG VERY OLD PARENTS AND THEIR OLD CHILDREN IN KOREA

Kyungmin Kim[1], Joohyun Kim[1], Joohong Min[2], Yijung Kim[3], Kathrin Boerner[4], Daniela Jopp[5], and Gyounghae Han[1], *1. Seoul National University, Seoul, Republic of Korea*, *2. Jeju National University, Jeju, Republic of Korea*, *3. The University of Texas at Austin, Austin, Texas, United States*, *4. University of Massachusetts Boston, Boston, Massachusetts, United States*, *5. University of Lausanne, Lausanne, Vaud, Switzerland*

Research has documented the health consequences of how individuals view their own age-related changes and aging (i.e., self-perceptions of aging) in later life. However, less is known about how individuals' self-perceptions of aging are shaped by their intergenerational experiences. This study aims to examine very old parents' and their old children's self-perceptions of aging within the dyads—where parents and children age together. Utilizing data from 105 dyads of parents (aged 81+) and their children (aged 65+) in South Korea, we examined how parents' and children's health status and care experiences are associated with their self-perceptions on aging. On average, parents reported less positive self-perceptions of aging than their children; parents' self-perceptions of aging were positively correlated with their child's self-perceptions of aging. A path model found that care experiences within the parent-child dyads were significantly associated with self-perceptions of aging for both parents and children—such that more caregiving burden was associated with less positive self-perceptions of aging for children and higher levels of satisfaction with received care from the child were associated with more positive self-perceptions of aging for parents. However, health status was significant only for parents; that is, parents with fewer functional limitations in activities of daily living tended to show more positive self-perceptions of aging. The findings highlight the interdependence of self-perceptions of aging within the parent-child dyads in very late life, suggesting that care experiences within the prolonged relationship constellation should be considered to enhance older adults' views on age-related gains and loss.

Abstract citation ID: igad104.0921

### CARING FOR A VERY OLD PARENT WITH DEMENTIA: STRATEGIES EMPLOYED TO MANAGE BEHAVIORAL AND PSYCHOLOGICAL SYMPTOMS

Elizabeth Gallagher[1], Kathrin Boerner[1], Yijung Kim[2], Kyungmin Kim[3], and Daniela Jopp[4], *1. University of Massachusetts Boston, Boston, Massachusetts, United States*, *2. The University of Texas at Austin, Austin, Texas, United States*, *3. Seoul National University, Seoul, Republic of Korea*, *4. University of Lausanne, Lausanne, Vaud, Switzerland*

Population aging has given rise to an increasingly common new group of caregivers, older adult children caring for their very old parents. These caregivers often find themselves balancing several other roles and responsibilities on top of caring for their parent, such as navigating their own health issues, caring for grandchildren, or transitioning into retirement. When a parent has dementia, this presents an additional challenge to caregivers, especially given that most persons with dementia exhibit behavioral and psychological symptoms of dementia (BPSD). The goal of the present research was to examine the range of strategies older caregivers employ in their daily lives in response to parents' BPSD, using applied thematic analysis. Specifically, we sought to identify and describe the different caregiver strategies for managing BPSD and examine how these strategies are connected, including whether certain strategies co-occur. We conducted in-depth interviews with 100 caregivers (aged 65+) caring for parents with dementia (aged 90+) as part of the Boston Aging Together Study. Results revealed seven strategies used by child caregivers in managing BPSD—including "setting the record straight," "going along with it," distracting and redirecting, ignoring, telling "fiblets" or lies, medicating their parent to alleviate symptoms, and altering their physical environment. The most frequently co-occurring strategies employed by child caregivers included telling "fiblets" or lies as well as distracting and redirecting. This study has the potential to inform future caregiver interventions aimed at effectively addressing BPSD and improving quality of life for older child caregivers and their very old parents with dementia.

### SESSION 3135 (SYMPOSIUM)

Abstract citation ID: igad104.0922

### INTRAINDIVIDUAL VARIABILITY ACROSS ADULTHOOD: DIVERSITY, VARIETY, AND ADHERENCE AS KEY INDICATORS OF DAILY LIFE

Chair: Rachel Koffer Co-Chair: Soomi Lee Discussant: Susan Charles

Alongside long-term change, lifespan theories highlight the importance of examining intraindividual variability (the fluctuations that occur in daily and momentary life) as an indicator of dynamic characteristics, like plasticity or rigidity. The present symposium demonstrates five unique characterizations and implications of variability in daily life across the adult lifespan. Paper 1 demonstrates that greater social activity variety is linked to lower mortality risk over 11 years, and this effect is stronger for older adults. Paper 2 finds that consistent adherence to health behaviors in daily life is linked to better cognitive function in midlife adults. Paper 3 finds that middle-aged adults experience greater diversity in types of positive events in daily life (positive event diversity) compared to older adults, and positive event diversity is associated with higher positive affect. Paper 4 establishes that positive affect with high mean levels and high variability (fragile high positive affect) is associated with greater odds of depression 10 years later. Lastly, Paper 5 demonstrates that although the spread of stressor types experienced in daily life (stressor diversity) declines with age across both cohorts, the types of stressors experienced in modern life differ from those experienced 18 years prior. Together, findings demonstrate how variability in daily life changes across age and has implications for health and well-being of older adults. Dr. Susan Charles will critically discuss these contributions to our understanding of aging in the context of Strength and Vulnerability Integration Theory and provide considerations for understanding the complexity of daily life across the lifespan.

Abstract citation ID: igad104.0923

**THE GOOD OLD DAYS?: LATER-BORN COHORTS REPORT DIFFERENT STRESSORS IN DAILY LIFE COMPARED TO EARLIER-BORN COHORTS**

Rachel Koffer, *Arizona State University, Phoenix, Arizona, United States*

In addition to the total amount of exposure to stressors in daily life, the types of stressors experienced (e.g., work stress, arguments), and the relative spread of the stressor types experienced (stressor diversity) is key to emotional and physical well-being across adulthood and aging. While daily stress processes and their links to health and well-being have been studied extensively, historical changes in daily stress processes have been largely unexplored. To examine secular trends in the types of daily stressors experienced across adulthood, we use data from two independent cohorts of the National Study of Daily Experiences surveyed 18 years apart (1995/1996 cohort: n = 1,499 adults aged 20 to 74 years; 2013/2014 cohort: n = 782 adults aged 25 to 75, stratified by age and gender to match the 1995-96 cohort). General linear models are used to test cohort differences in total stressor exposure, exposure to specific types of stressors, and stressor diversity, all controlling for age, income relative to cohort, education relative to cohort, gender, and chronic conditions. While the 2013/2014 cohort experience higher exposure to stressors in daily life than the 1995/1996 cohort, the cohorts experience the same stressor diversity, which decreases with older age. The specific stressor types experienced differ by cohort: adults in 2013/2014 report more arguments, avoided arguments, and work stressors, but less discrimination stressors and "other" stressors than in 1995/1996. These results highlight the sociohistorical embedding of daily stressor experiences. Sociodemographic differences and implications for future research and clinical practice are discussed.

Abstract citation ID: igad104.0924

**PARTICIPATION IN DIVERSE SOCIAL ACTIVITIES IS ASSOCIATED WITH LOW MORTALITY RISK**

Sangha Jeon[1], Nicholas Turiano[2], and Susan Charles[1], *1. University of California, Irvine, Irvine, California, United States*, *2. West Virginia University, Morgantown, West Virginia, United States*

Social activity is often associated with better physical health outcomes. Yet, less is known about specific qualities of social activity beyond its frequency, such as whether engagement in diverse social activities (social activity variety) is important for health. The current study assessed whether social activity variety, which was calculated as the number of different types of social activity, relates to mortality risk beyond non-social activity variety and how its relationship varies depending on age groups. Using data from the Health and Retirement Study (HRS), we included 5,559 adults who completed a questionnaire on social activity participation in 2008 and whose mortality status was recorded in 2019. We also examined whether age was related to the relationship between social activity variety and mortality risk. Cox proportional hazard model analyses revealed that those with higher social activity variety were more likely to survive over the following 11 year than those with low social activity variety. This result remains robust when adjusting for health status and non-social activity variety. The association of social activity variety with mortality risk is significant across all age, but it is stronger in older adults compared to young adults. Findings suggest that social activity variety is beneficial for longevity beyond non-social activities and health status across adulthood.

Abstract citation ID: igad104.0925

**GREATER ADHERENCE TO DAILY HEALTH BEHAVIORS RELATE TO BETTER COGNITIVE FUNCTION**

Fumiko Hamada[1], and Soomi Lee[2], *1. University of South Florida, Tampa, Florida, United States*, *2. The Pennsylvania State University, University Park, Pennsylvania, United States*

Healthy life habits cannot be formed overnight; therefore, adherence to optimal sleep, physical activity, and healthy eating behaviors is vital. This paper examined the relationship between the adherence rate of the three key health behaviors (i.e., sleep, physical activity, and healthy diet) and cognitive function in adulthood. Data were retrieved from Midlife in the United States third wave (MIDUS 3) Project 2, National Study of Daily Experiences (n=1,061). For eight consecutive days, participants reported nightly sleep duration, daily amount of physical activity, and daily consumption of fast food. Adherence rates of health behaviors were calculated by the sum of having optimal sleep ($6 < \text{sleep hours} \leq 9$), any intensity of physical activity (60 min.≤), and not eating fast food (yes/no) across the study period. Participants also completed a Brief Test of Adult Cognition by Telephone (BTACT). A series of general linear models examined the associations of the three adherence variables with the overall BTACT cognition score adjusting for sociodemographic and health covariates. Greater adherence rates of optimal sleep ($\beta$=0.02; SE=0.01; p=0.01), physical activity ($\beta$=0.03; SE=0.01; p<0.01 ), and not eating fast food ($\beta$=0.02; SE=0.01; p=0.02) were each and associated with higher BTACT score. Among the three adherence variables, adherence to physical activity was the most important for cognition independent of other health behaviors. Consistent associations were found for executive function, but not episodic memory. These findings suggest that adults with greater adherence to a healthy lifestyle have better cognitive function, and consistency of healthy behaviors is vital for cognitive health.

Abstract citation ID: igad104.0926

**POSITIVE EVENT DIVERSITY AND AGE: TESTING CURVILINEAR ASSOCIATIONS**

Patrick Klaiber[1], Patrick Hill[2], David Almeida[3], Anita DeLongis[4], and Nancy Sin[4], *1. Tilburg University, Tilburg, Noord-Brabant, Netherlands*, *2. Washington University in St. Louis, St. Louis, Missouri, United States*, *3. The Pennsylvania State University, State College, Pennsylvania, United States*, *4. University of British Columbia, Vancouver, British Columbia, Canada*

Prior research has found that older adults report more frequent positive events compared to younger and middle-aged adults. It remains unknown, however, to what extent age is related to experiencing positive events spread across different positive event types (i.e., positive event diversity, PED). We therefore examined age differences in PED and its affective well-being correlates across three large daily diary datasets: wave 2 (N = 1919) and the Refresher sample (N = 744) of the National Studies of Daily Experiences, and the Coping with the COVID-19 Outbreak Study (N = 1392). We used Shannon's Entropy to quantify PED. There was a curvilinear association between PED and age, indicating that PED was higher among middle-aged compared to younger and older adults. On a zero-order basis, PED was correlated with higher positive affect and uncorrelated with negative affect. However, the pattern of results changed when considering positive event frequency as a moderator. Among people with high positive event frequency, PED was linked to higher negative and lower positive affect, but not associated with either positive or negative affect at low levels of positive event frequency. These relationships did not differ by age. This study suggests that middle-aged adults experience the widest range of positive event types, perhaps reflecting the diverse set of social roles that middle-aged adults often fulfill (e.g., worker, parent, partner). In addition, there might be negative consequences to experiencing a lot of positive events across very different contexts which may indicate social role strain.

Abstract citation ID: igad104.0927

### REVISITING POSITIVE AFFECT: THE ROLE OF AFFECT MEAN LEVELS AND VARIABILITY ON DEPRESSION IN MID AND LATER LIFE

Sun Ah Lee[1], Dahlia Mukherjee[2], and David Almeida[3], *1. The Pennsylvania State University, University Park, Pennsylvania, United States*, *2. Penn State College of Medicine, Hershey, Pennsylvania, United States*, *3. The Pennsylvania State University, State College, Pennsylvania, United States*

Recent research suggests that affect dynamics (e.g., variability) combined with mean levels are important predictors of psychological and physical health outcomes. This study explores the joint effect of affect levels and variability on depression across 10 years among middle-aged and older adults. We used the data from the second and third waves of Midlife in the United States (MIDUS) study. During telephone interviews, daily positive (PA) and negative affect (NA) were assessed across eight consecutive evenings. Daily affect mean levels were calculated by averaging daily affect across interview days, and daily affect variability was calculated as the individual's standard deviation (iSD). Depression status was assessed using the Composite International Diagnostic Interview-Short form at baseline and 10-year follow-up. The final analytic sample includes 1,499 adults aged 55 years old on average (age range = 34-83; 55.7% female; 93% white). Logistic regression analysis was performed to test the interaction effect of daily affect mean levels and variability on depression. We found a significant interaction effect between PA mean levels and variability on depression status at follow-up. For individuals with high PA levels, higher PA variability was associated with higher odds of developing depression, after adjusting for depression and anxiety disorder at baseline. For NA, we only found main effects where higher NA variability was associated with higher odds of depression at follow-up. Findings suggest fragile high PA (i.e., variable high PA) may be a significant predictor of depression, highlighting the need for understanding PA as multidimensional construct.

### SESSION 3140 (SYMPOSIUM)

Abstract citation ID: igad104.0928

### SPIRITUALITY AND RELIGIOSITY IN BRAZILIAN AND AMERICAN OLDER ADULTS, CHARACTERISTICS AND NURSING CARE APPROACH

Chair: Angelo Jose Bós Discussant: Amelia Gallagher

This symposium aims to understand two aspects of spirituality and religiosity: the importance of religiosity for older adults, in a population-based study, and a proposal approach for nursing care in cancer patients: Lift the Spirit approach. In the Brazilian study, we will show that religiosity is significantly associated with sex, race, level of education, perceived health status and non-transmittable chronic diseases, including cancer. The results evidence the important role of religious faith in the Brazilian older adult's health. We concluded that religion and spiritual approaches should always be considered for older adults. In the American study, we will show that preliminary findings suggested Lift the Spirit was feasible, acceptable, and positively impacted knowledge, skills, and self-efficacy. The innovative combination of synchronous and asynchronous online delivery of the intervention and data collection methods has implications for research, education, and clinical practice. Lift the Spirit delivered on an entirely online format is widely accessible and convenient to researchers, educators, and practitioners, as well as participants and students. Equipping nurses to conduct spiritual histories through an intervention like Lift the Spirit is crucial to overcoming a significant barrier to providing spiritual care.

Abstract citation ID: igad104.0929

### INTERVENTION DEVELOPMENT USING PALLIATIVE CARE CLINICAL PRACTICE GUIDELINES CONCERNING SPIRITUALITY

Monica Beck[1], and Amelia Gallagher[2], *1. University of Alabama in Huntsville, Huntsville, Alabama, United States*, *2. The University of Alabama in Huntsville, Huntsville, Alabama, United States*

Nurses may be reticent to engage older adult cancer patients in spiritual histories despite evidence of improved health outcomes citing lack of education, skills, and time as barriers. Few interventional studies target these barriers using the Clinical Practice Guidelines for Quality Palliative Care as a framework for providing spiritual care excellence. Lift the Spirit, an enhanced educational communication intervention, integrated the guidelines for assessing and respecting patient and family spiritual beliefs, provider self-reflection, and spiritual screening and assessment using a validated instrument. The mixed-methods pilot study aimed to assess the feasibility and acceptability of Lift the Spirit. A purposive sample of nurses (n = 17) viewed an educational module, role-played conducting spiritual histories using the Faith, Importance, Community, Address (FICA) © instrument, and completed debriefing interviews. Pre/post outcome measures of Spiritual History Knowledge and Spiritual History Self-Efficacy were analyzed using a Wilcoxon paired signed-rank test. Debriefing interview data were content analyzed. The Theoretical Framework of Acceptability was used to content code and identify acceptability-related content. There were significant differences between pre and post knowledge and self-efficacy scores, respectively (Wilcoxon Signed-Ranks: Z = -3.18, p = 0.002; and Z = -3.20, p = 0.001). Qualitative findings supported the feasibility and acceptability of Lift the Spirit; participants found the FICA© instrument and role-playing especially helpful. Preliminary findings suggested Lift the Spirit was feasible, acceptable, and positively impacted knowledge, skills, and self-efficacy. The utility of an intervention like Lift the Spirit to improve older adult cancer patient outcomes was demonstrated.

Abstract citation ID: igad104.0930

### FAITH IS SIGNIFICANTLY ASSOCIATED TO SOCIODEMOGRAPHIC AND CLINICAL CHARACTERISTICS IN BRAZILIAN OLDER ADULTS

Yolanda de Souza, Angelo Jose Bós, and Mariana Salecker, *Pontifical Catholic University of Rio Grande do Sul, Porto Alegre, Rio Grande do Sul, Brazil*

Religiosity is a strong factor related to health in older adults, which could positively affect physical and mental health. We aimed to observe the association between sociodemographic and clinical factors and the religiosity level in older adults. This is a secondary analysis of the Brazilian Older-adults Health Longitudinal Study (ELSI-Brazil) run by Fiocruz Institute in 2015 using a national sample of older adults (60+ year-old) population. The dependent variable was “How much the religious faith give meaning to your life?” and the independent were: sociodemographic (age-group, sex, race, marital status, and scholarly) and clinical (auto perception of health and non-transmittable chronic diseases diagnose - NTCD) characteristics, tested by Chi-Square with p<0.05 significant level. The total sample accounted for 4,669 participants, 4,080 (87.4%) answered that faith gives a lot of meaning to their lives. This percentage was significantly higher in women (91.2% vs 81.8% in men, p=0.003), indigenous race (95%, p=0.003), lower scholarly (p<0.001) and better auto perception of health (p=0.021). NTCD significantly related to higher importance of religiosity were hypertension (88.3%), dyslipidemia (90.0%), angina (93.9%), arthritis (90.5%), osteoporosis (91.0%), depression (88.7%), and cancer (90.4%). Participants with Diabetes (86.1%), pneumopathies (85.9%), and Parkinson (86.7%) had significant lower levels of religiosity. Religiosity had a significant association with sex, race, scholarly, health auto perception and NTCD, including cancer. The results evidence the important role of religious faith in the Brazilian older adult’s health. We concluded that religion and spiritual approach should always be considered to older adults.

Abstract citation ID: igad104.0931

### HOW IMPORTANT IS IT TO PRAY AND MEDITATE FOR BRAZILIAN OLDER ADULTS? SOCIODEMOGRAPHIC AND CLINICAL CHARACTERISTICS

Mariana Salecker[1], Yolanda de Souza[1], and Angelo José Bós[2], *1. Pontifical Catholic University of Rio Grande do Sul, Porto Alegre, Rio Grande do Sul, Brazil, 2. IGG-PUCRS, Porto Alegre, Rio Grande do Sul, Brazil*

Praying and meditating are considered spiritual expressions, but little is known about this importance among older adults. Therefore, this abstract aim to understand the frequency and the sociodemographic and clinical characteristics related to the importance given by the Brazilian older adults to praying and meditating. This is a secondary analysis of the transversal data of the Older Adults Health Longitudinal Study, made by Fiocruz in 2015. The dependent variable is: “How much daily praying and meditating is important to you?” Independent variables included: sex, age, educational level, marital status, race, and self-perception of health. The relation between the variables were tested by Chi-Square, considering p<0.05 significant. Praying and meditating everyday were important in 87.6% of the participants. Higher percentages were noticed between the ages of 60 to 69 years old (88.8%) and the ages 80 to 89 years old (88.1%, p<0.001), women (91.6%, p<0.001), widows (90.8%, p<0.001), indigenous (89.1%, p<0.001), with primary school complete (90.5%, p<0.001) and with regular auto perception health (88.4%, p=0.051). We conclude that there is higher than expected frequency of Brazilian older adults who consider important praying and meditating. They are more likely to be women, widow, indigenous, younger, less educated and have regular self-perception of health.

Abstract citation ID: igad104.0932

### EXPLORING FACILITATORS AND BARRIERS TO CONDUCTING SPIRITUAL HISTORIES IN OLDER ADULTS

Monica Beck[1], and Amelia Gallagher[2], *1. University of Alabama in Huntsville, Huntsville, Alabama, United States, 2. The University of Alabama in Huntsville, Huntsville, Alabama, United States*

Nurses can relieve spiritual suffering in older adult cancer patients through spiritual histories, yet may be reticent to engage in spiritual conversations. The qualitative aim of Lift the Spirit, a pilot feasibility and acceptability study, was to explore factors affecting cancer nurses' perceptions of the barriers and facilitators to conducting spiritual histories in clinical practice. Lift the Spirit, an enhanced educational communication intervention, was designed to equip nurses with the knowledge, skills, and attitudes to conduct spiritual histories. A purposive sample of nurses (n = 17) independently engaged in an online multimedia course, Spiritual Assessment in Clinical Practice, then synchronously role-played conducting a spiritual history in dyads (nurse/patient), engaged in a skills performance, and participated in Debrief Interviews. Qualitative data were collected during Debriefing Interviews using the Debrief Interview Guide. Interpretive description guided the qualitative content analysis of Debrief Interview data. Structural coding was used to sort data into barriers and facilitators on the first pass. Next, within each structural code, data fitting the definition of barrier and facilitators were inductively descriptively coded. Participants described barriers and facilitators at the levels of institution/profession (lack of education and training), self (vulnerability, perceived riskiness), and patient (cultural difference) that were similar to barriers already noted in the literature. Facilitators included feeling equipped and supported and having external cues as reminders. The degree of angst, fear, and vulnerability expressed towards conducting spiritual histories was surprising. Equipping nurses with the knowledge, skills, and attitudes is essential to reduce spiritual suffering in older adults.

### SESSION 3145 (AWARD LECTURE)

Abstract citation ID: igad104.0933

### JOSEPH T. FREEMAN AWARD AND EXCELLENCE IN REHABILITATION OF AGING PERSONS AWARD PRESENTATIONS AND LECTURES

Chair: Kirsten Corazzini

The Joseph T. Freeman Award lecture will feature an address by the 2023 Freeman Award recipient Joe Verghese, MD, FGSA of the Albert Einstein College of Medicine. The Joseph T. Freeman Award is a lectureship in geriatrics awarded to a prominent clinician in the field of aging, both in research and practice. The award was established in 1977 through a bequest from a patient's estate as a tribute to Dr. Joseph T. Freeman. The Excellence in Rehabilitation of Aging Persons Award lecture will feature an address by the 2023 Excellence in Rehabilitation Award recipient Brad Manor, PhD of Hinda and Arthur Marcus Institute for Aging Research. The Excellence in Rehabilitation of Aging Persons Award is designed to acknowledge outstanding contributions in the field of the rehabilitation of aging individuals.

Abstract citation ID: igad104.0934

### MOTOR PATHWAYS TO DEMENTIA

Joe Verghese, *Albert Einstein College of Medicine, Bronx, New York, United States*

Emerging evidence indicates that non-cognitive manifestations such as gait impairments occur early during dementia. The Motoric Cognitive Risk (MCR) is a pre-dementia syndrome characterized by slow gait and cognitive complaints. MCR is a simple and easily accessible clinical dementia risk assay that does not require cognitive testing. MCR has incremental predictive validity for Alzheimer's disease and related dementias over its individual cognitive and motoric components. Recent studies from across the world have helped to elucidate the epidemiology, biology, and brain substrates of MCR; paving the way for future interventions to prevent progression to dementia.

Abstract citation ID: igad104.0935

### TRANSCRANIAL ELECTRICAL STIMULATION AS A TOOL TO UNDERSTAND AND ENHANCE MOBILITY IN OLDER ADULTS

Brad Manor, *Hebrew SeniorLife/Harvard Medical School, Amherst, Massachusetts, United States*

Standing and walking are complex cognitive-motor tasks that rely on the function of numerous brain networks. This reliance upon supraspinal elements of the motor control system increases with age and many age-related diseases, especially when an individual must navigate unfamiliar environments and/or simultaneously perform cognitive tasks like talking, reading signs, or making decisions. Noninvasive transcranial electrical stimulation (tES) can safely and selectively induce both acute and longer-term changes in brain network function. It thus enables cause-and-effect study of the motor control system and moreover, holds promise as a therapeutic strategy to counteract age- and disease-related alterations in standing, walking, and mobility. The purpose of this talk is to introduce the fundamentals of tES, describe the effects of tES on the neural control of mobility in older adults, and finally, discuss current limitations and related avenues for future research and development within this rapidly-growing field of study.

### SESSION 3150 (SYMPOSIUM)

Abstract citation ID: igad104.0936

### PRESIDENTIAL SYMPOSIUM: EDUCATIONAL NEEDS AND STRATEGIES ACROSS GSA: BUILDING BRIDGES WITHIN THE SOCIETY

Chair: Tina Newsham

Discussant: Joann Montepare

Across GSA member groups, we teach, train, and mentor in a variety of ways and for different purposes. Education is a tie that connects us across the Society. In this AGHE Presidential Symposium, representatives from the different GSA membership sections will discuss the educational needs of their members and share strategies they employ with their students, employees, mentees, and others to ensure the best preparation of the workforce. Researchers, care providers, educators, advocates, and others must be well-prepared to address the opportunities and demands of an aging society. Tamar Shovali, from Behavioral and Social Sciences, will present empirically supported approaches to intergenerational communication and training to foster relationships between aging individuals and their communities. Biological Sciences member LaDora Thompson will highlight current educational efforts in translating geroscience discoveries into new medical advances. Health Sciences member Matt Peterson will present strategies, challenges and opportunities learned from a currently funded health sciences mentoring program. Social Research, Policy, and Practice member Nancy Kusmaul will discuss efforts to shape policy to enhance geriatrics and gerontology education. AGHE Chair Joann Montepare will serve as discussant, exploring how AGHE can build more bridges by integrating educational efforts in several unique ways. Collectively, presenters in this session will highlight the connections and opportunities for collaboration across GSA member groups.

Abstract citation ID: igad104.0937

### POLICY CONSIDERATIONS FOR GERONTOLOGY AND GERIATRICS CONTENT IN HEALTH PROFESSIONAL EDUCATION

Nancy Kusmaul, *University of Maryland Baltimore County, Baltimore, Maryland, United States*

Health professions educators in fields such as nursing, medicine, and social work struggle to prepare practitioners for the needs of growing numbers of older adults. In 2008, the National Academies of Science Engineering and Medicine identified that fewer than 5% of physicians choose to specialize in geriatrics, and even fewer in geriatric psychiatry, leading to significant shortages in mental health care for older adults. While other health professions such as clinical social workers or psychiatric nurse practitioners could potentially fill this gap, the story is the same in social work (4%) and nursing (1%). These numbers have improved marginally over the last fifteen years, but growing numbers of older adults have exacerbated shortages. In these professions, and in others, the model has been to have geriatrics/gerontology as a specialty, rather than integrated into the curriculum. Yet the interest in this specialization is not there and practitioners will have more exposure to this population than they might think. Members of the Social Research Practice & Policy section of GSA consider how we can shape policy to enhance geriatrics and gerontology education, and where AGHE can support health professions education (nursing, medicine, social work, etc.) and interdisciplinary health professional education in gerontology.

Abstract citation ID: igad104.0938

**ADAPTING TO THE HEALTH RESEARCH MENTORING LANDSCAPE: THE ENHANCING RESEARCH FOR ONLINE LEARNERS (ENROLL) PROGRAM**

Matthew Peterson, *University of North Carolina Wilmington, Wilmington, North Carolina, United States*

Engaging students in mentored research has contemporary challenges, including virtual environments and student disengagement. This provides an opportunity to develop novel methods to attract and support students in health research, with the goal of encouraging a career in the health sciences. The overarching objective of EnROLL is to attract, support, and engage on-line and on-campus learners in faculty-led, interdisciplinary health science research. The specific aims of EnROLL are, 1) to develop a focused program and curriculum in faculty-led research to engage on-line graduate and underserved undergraduate students in health research, 2) to develop and implement an effective model of research mentor training for dissemination, and 3) to assess the value in the program as a model to disseminate for engaging students in faculty-led health research. Now in the second year of funding, attracting students into the mentoring structure has been the biggest challenge. To address this challenge, novel strategies for student recruitment and engagement have been implemented. A stipend-supported scholars program and novel intergenerational events are two examples. Engaging external institutional partners has also yielded differing perspectives and collaborations. This program's recruitment challenges have yielded new programmatic strategies and unforeseen opportunities. The need for creative and adaptable programming will be highlighted and discussed.

Abstract citation ID: igad104.0939

**BUILDING BRIDGES TO BETTER INTERGENERATIONAL RELATIONSHIPS**

Tamar Shovali, *Eckerd College, St. Petersburg, Florida, United States*

Intergenerational relationships are necessary to prepare the workforce for careers in the field of gerontology. Intergenerational interaction and reflection have been shown to result in valuable skills, such as positive attitudes toward older adults, increased knowledge of aging and needs of older adults, genuine relationships with older adults and a greater appreciation for older generations, greater comfort with the idea of themselves aging, and high satisfaction when embedded into course curriculum. Intergenerational contact in the structure of undergraduate courses also has reciprocal benefits, such as recognizing commonalities, building appreciation and trust, and creating a sense of belonging between generations that can be lasting. Building on empirical evidence, an intensive three-week intergenerational relationships course for first year students using intentional integration with general education goals, while also training students in basic understanding of aging is described here. Students collaborated with the college's Academy of Senior Professionals, a unique community of retired older adults engaged in lifelong learning, to develop a college-wide intergenerational event. Considerations, outcomes, and lessons learned will be reviewed.

Abstract citation ID: igad104.0940

**TRAINING THE NEW SCIENTISTS OF AGING: BUILDING ON GEROSCIENCE OPPORTUNITIES**

LaDora Thompson, *Boston University, Boston, Massachusetts, United States*

Geroscience is focused on improving health and well-being by beneficially altering or reversing the cellular and molecular changes that take place as we age. These changes, uncovered by the biology of aging research, can contribute to a progressive loss of function and the onset of frailty and many diseases prevalent at older ages. In the 21st century there is a need for increased engagement with researchers and clinicians across multiple disciplines to translate these geroscience discoveries more effectively into new medical advances. This presentation will highlight efforts to increase awareness and understanding of these geroscience discoveries to stakeholder groups and to build a more diverse and interdisciplinary geroscience workforce, including researchers, clinicians, and members of the public. To date, there are creative education programs aimed at enhancing and expanding broader awareness of geroscience research (e.g., skills development, hands-on research experiences, formal curriculum informed in the science of aging, engagement activities dealing with ethical, economic, political and societal ramifications of extending human lifespan, development of shared methods, networking with experts in the geroscience field, materials and outreach programs on the topic of geroscience), which target basic, translational, clinical researchers and members of the public. Lastly, this presentation will outline the activities within the Translational Rehabilitation in Geroscience Initiative and the Translational Geroscience Network, a collaboration of researchers looking at clinical interventions that target fundamental mechanism of aging to delay, prevent or treat age-related diseases and disabilities as a group, instead of one at a time, enabling more-rapid translation of therapies.

### SESSION 3155 (PAPER)

### SEX AND GENDER DIFFERENCES

Abstract citation ID: igad104.0941

### ASSESSING THE PSYCHOMETRIC PROPERTIES OF THE GERIATRIC SUICIDE IDEATION SCALE IN MIDDLE-AGED AND OLDER MEN

Marnin Heisel[1], and Gordon Flett[2], *1. University of Western Ontario, London, Ontario, Canada*, *2. York University, Toronto, Ontario, Canada*

Middle-aged and older men have high rates of suicide, necessitating risk detection. We developed the Geriatric Suicide Ideation Scale (GSIS; Heisel & Flett, 2006) as an age-specific, multidimensional suicide risk assessment tool. The GSIS has shown strong psychometric properties in clinical, community, and residential samples (see Heisel & Flett, 2016), yet research has lagged investigating its utility with middle-aged and older men. The purpose of the present study was thus to assess the psychometric properties of the GSIS administered to 82 men, 55 years and older (M=63.3, SD=4.6 years), who participated in a meaning-centered psychological intervention group for those concerned about or struggling with the transition to retirement (Heisel et al., 2020). Psychometric analyses included investigation of participant response characteristics, internal consistency, and construct validity. Findings demonstrated acceptable internal consistency for GSIS totals ($\alpha$ =.88) and for its Suicide Ideation, Death Ideation, Loss of Personal and Social Worth, and Perceived Meaning in Life subscales ($\alpha$ =.62-.81). Positive associations between the GSIS and negative psychological factors (depression, anxiety, hopelessness, loneliness, perceived lack of mattering to others, and history of suicidal behavior; r =.30 to .51) and negative associations with positive factors (life satisfaction, psychological well-being, perceived support, and meaning in life; r = -.21 to -.51) supported its construct validity. These and other findings will be discussed in the broader context of upstream population level approaches to suicide risk detection and prevention. (Note: this abstract was accepted for the 2022 GSA meeting, but we had to withdraw due to family loss)

Abstract citation ID: igad104.0942

### GENDER DIFFERENCES IN ANXIETY AMONG OLDER ADULTS: THE MEDIATION ROLE OF OUT-OF-HOME ACTIVITY

Lingming Chen[1], Elizabeth Procter-Gray[1], Linda Churchill[2], Annabella Aguirre[1], Jie Cheng[1], Qun Le[1], Scott Crouter[3], and Wenjun Li[1], *1. University of Massachusetts Lowell, Lowell, Massachusetts, United States*, *2. University of Massachusetts, Lowell, Lowell, Massachusetts, United States*, *3. The University of Tennessee Knoxville, Knoxville, Tennessee, United States*

Research shows gender differences in anxiety. Given that men and women have different levels of out-of-home activity and out-of-home activity is associated with anxiety, this study examines if gender differences in anxiety are mediated by out-of-home activity among older adults. The prospective cohort study enrolled 303 women and men aged 65 to 95 years old in central Massachusetts (2018-2020). Anxiety was evaluated by the Beck Anxiety Inventory. The out-of-home activity was measured by the percentage of daily step counts outside the home, which was objectively assessed by accelerometer and Global Positioning System devices. Data were analyzed by using mediation analysis, controlling demographic (age, race/ethnicity), physical health (self-rated health, comorbidities), and mental health (depressive symptoms, perceived stress). Results showed that women reported significantly greater scores in anxiety and did less out-of-home activity than men. Furthermore, out-of-home activity was significantly associated with anxiety. With the bootstrapping procedure, a significant indirect effect of gender on anxiety through out-of-home activity was shown (indirect effect: 0.025; 95% CI: 0.002-0.078), which explained 12% of the total effect (total effect: 0.214; 95% CI: 0.032-0.396). In conclusion, out-of-home activity partially mediates gender differences in anxiety. This study indicates that increasing out-of-home activities may help reduce anxiety and gender differences in anxiety among older adults.

Abstract citation ID: igad104.0943

### NINE-YEAR ASSOCIATION OF OPTIMISM AND COGNITIVE FUNCTIONING IN A COHORT OF AGING MEN

Victoria Marino[1], Samsuk Kim[2], Avron Spiro[3], and Lewina Lee[4], *1. Boston University Chobanian & Avedisian School of Medicine, Boston, Massachusetts, United States*, *2. The VA Boston Healthcare System, Boston, Massachusetts, United States*, *3. VA Boston Healthcare System, Boston, Massachusetts, United States*, *4. Boston University School of Medicine, Boston, Massachusetts, United States*

Higher levels of optimism have been linked to better physical and emotional health, and growing evidence suggests optimism may also protect against cognitive impairment and decline. However, our understanding of its prospective associations with cognitive functioning is limited. We examined 9-year associations of optimism with cognitive functioning among 793 men from the VA Normative Aging Study. Optimism was assessed via the Minnesota Multiphasic Personality Inventory-2 in 1986 (baseline: AgeM = 60.1, SD=7.1). Cognitive functioning was measured at participants' earliest cognitive testing occasion between 1993 and 1996. We considered both global cognition (MMSE) and three domains of cognitive functioning: executive function (verbal fluency via Animal Naming Test, working memory via WAIS-R Digit Span Backward), short-term memory (immediate and delayed CERAD Word List Recall), and visuospatial ability (CERAD and VMI spatial copying, NES-2 Pattern Comparison). Analyses used ordinary least squares regression adjusted for childhood socioeconomic status, and demographics and major diseases at baseline. Optimism was not associated with global cognition. However, higher optimism was associated with better verbal fluency (B=0.48, SE=0.19, 95% CI .11–.86, p=.01) and immediate word list recall (B=0.33, SE=0.15, 95% CI .04–.62, p=.02), and weakly associated with delayed word list recall (B=0.14, SE=0.07, 95% CI -.01–.28, p=.06). Optimism was not associated with visuospatial ability or working memory performance. Our findings suggest that optimism could be particularly important for verbally mediated aspects of executive functioning and memory in aging men. Optimism may be a valuable intervention target to promote healthy cognitive aging.

### SESSION 3160 (SYMPOSIUM)

Abstract citation ID: igad104.0944

### ADVANCES IN GLOBAL AGING RESEARCH FROM CROSS NATIONAL COMPARISONS

Chair: Emma Nichols Discussant: Jennifer Ailshire

Harmonized surveys on aging populations around the world provide an unprecedented opportunity to advance our understanding of the factors that promote healthy aging. The papers in this symposium use existing and newly available harmonized data from the NIA-funded Gateway to Global Aging to examine how patterns of physical and cognitive aging vary across countries and to identify potential modifiable risk factors for slowing aging-related decline. Jayakody et al. use data on 21 countries to identify risk factors for Motoric Cognitive Risk (MCR), a preclinical predictor of dementia, that are both country-specific and universal. Knapp et al. also investigate potential modifiable risk factors for poor cognitive health in 31 countries and identify the factors that are universally associated with higher risk across countries. These two studies provide novel insights into how we can harness studies across multiple countries to better understand which factors of aging are universal and which are context dependent. The next two studies examine patterns and risks through comparison of neighboring countries. Focusing on Mexico and Brazil, Gomes Goncalves et al., determine the extent to which rurality and education confer risk of cognitive impairment in the two largest countries in Latin America. Finally, Fermín-Martínez et al. develop a new measure to capture age-related functional decline that focuses on anthropometric information available in aging surveys, and they validate their measure in older adults in the United States and Mexico. The papers in this symposium show how cross-national comparisons are being used to advance our understanding of aging.

Abstract citation ID: igad104.0945

### A CROSS-NATIONAL COMPARISON OF RISK FACTORS ASSOCIATED WITH THE MOTORIC COGNITIVE RISK (MCR) SYNDROME

Oshadi Jayakody, Helena Blumen, and Joe Verghese, *Albert Einstein College of Medicine, Bronx, New York, United States*

The Motoric Cognitive Risk (MCR) syndrome predicts risk of Alzheimer's disease and vascular dementia. Cross-national comparisons of risk factors for MCR are important for identifying region-specific factors to design regionally tailored preventive interventions. We examined risk factors associated with MCR in 20,468 adults aged ≥65 years from the U.S Health and Retirement Study (HRS), English Longitudinal Study of Ageing (ELSA), Mexican Health and Aging Study (MHAS), China Health and Retirement Study (CHARLES), Harmonized Diagnostic Assessment of Dementia for the Longitudinal Aging Study in India (LASI-DAD), Survey of Health, Ageing and Retirement in Europe (SHARE) and Brazilian Longitudinal Study of Aging (ELSI-Brazil). MCR was defined as the presence of cognitive complaints and slow gait with no mobility disability and dementia. Associations between demographic [sex, education], medical [hypertension, diabetes, Parkinson's, heart disease, stroke, obesity, depression, multiple falls], sensorimotor [grip strength, hearing] and behavioral factors [smoking, sedentariness, sleep quality], and prevalent MCR were examined using logistic models. Despite some overlap, cohort-specific risk factors for MCR were identified with relative risk ratios ranging as: 0.4-2.8 for education and multiple falls in HRS, 2.3-7.4 for poor hearing and sedentariness in ELSA, 2.3-2.6 for poor grip and diabetes in MHAS, 1.9-2.2 for depression and sedentariness in CHARLES, 0.5-5.3 for education and stroke in LASI-DAD, 0.3-2.4 for education and depression in SHARE and 2.4-4.0 for multiple falls and stroke in ELSI-Brazil. In this cross-sectional, cross-national comparison of 21 countries, MCR associated risk factors were region-specific, laying the foundation for tailored interventions to prevent MCR and subsequently dementia.

Abstract citation ID: igad104.0946

### RURAL–URBAN DISPARITIES IN COGNITIVE PERFORMANCE IN BRAZIL AND MEXICO

Natalia Gomes Goncalves[1], Jaqueline Avila[2], Laiss Bertola[3], Alejandra Obregon[4], Cleusa Ferri[3], Rebeca Wong[5], and Claudia Suemoto[6], *1. University of Sao Paulo Medical School, Sao Paulo, Sao Paulo, Brazil*, *2. University of Massachusetts Boston, Boston, Massachusetts, United States*, *3. Federal University of Sao Paulo, Sao Paulo, Sao Paulo, Brazil*, *4. University of Texas Medical Branch, Galveston, Texas, United States*, *5. UTMB, Galveston, Texas, United States*, *6. Universidade de São Paulo, São Paulo, Sao Paulo, Brazil*

Latin America (LA) has the highest dementia burden globally among people 60 years or older, with a prevalence of 8.5%. However, there is little evidence of the disparities in cognitive performance in rural and urban areas in LA. We use nationally representative data from the two largest countries in LA, Brazil and Mexico, to assess if rurality is associated with cognitive impairment, and assess how education and country modify this relationship. The sample included 9,412 participants from the Brazilian Longitudinal Study of Aging (ELSI) and 14,779 participants from the Mexican Health and Aging Study (MHAS). Participants were classified as cognitively impaired or not impaired using cognitive performance scores and a regression-based approach. Logistic regression models were used to estimate the association between rural/urban residency and cognitive function. The modifying effect of education and country was investigated by adding an interaction term in the fully adjusted model. ELSI participants had a mean age 62.5±9.8 years, 54% were women, 13.3% were illiterate, and 6% had cognitive impairment. MHAS participants from had a mean age 64.8±9.7 years, 55% were women, 18% were illiterate, and 5% were cognitively impaired. In Mexico, those living in rural areas were more likely to have cognitive impairment than those in urban areas (OR=1.43, 95%CI=1.20;1.71), but this difference was not seen in Brazil (OR=1.10, 95%CI=0.87;1.36). The three-way interaction of rurality with country and education showed that rural residents of Mexico had lower odds of cognitive impairment compared to rural residents of Brazil with the same level of education (p<0.001).

Abstract citation ID: igad104.0947

### A CROSS-COUNTRY ANALYSIS OF COGNITION AND ASSOCIATED RISK FACTORS

David Knapp, Arie Kapteyn, Alessandro Giambrone, and Tabasa Ozawa, *University of Southern California, Los Angeles, California, United States*

The widely cited Lancet Commission (Livingston et al., 2017) concluded that a third of dementia cases may be preventable through appropriate interventions targeting what they refer to as modifiable risk factors. These risk factors have been widely studied individually, but rarely investigated collectively and across many countries. We analyze the cross-country consistency of relationships between these modifiable risk factors and cognition using an internationally comparable set of aging studies in 31 countries including the United States, England and Europe. Cross-country differences in culture, policies, economy, and other collective experiences lead to significant variation in lifecycle outcomes, including dementia onset and modifiable risk factors. We find a limited number of robust relations: education, depression, and hearing show clear, consistent associations with our cognition measure, the sum of immediate and delayed recall. The evidence for other factors, including obesity, smoking, diabetes, and hypertension is weaker and becomes almost non-existent when correcting for multiple hypotheses testing. The inconsistent relationship across countries between these risk factors and cognition suggests the lack of a causal mechanism leading to cognitive decline – a necessary condition for these risk factors to be modifiable and effective targets for policy interventions aimed at controlling the prevalence and cost of dementia.

Abstract citation ID: igad104.0948

### CROSS-COUNTRY VALIDATION OF ANTHROPOMETRIC AGE (ANTHROPOAGE) AS A MEASURE OF BIOLOGICAL AGING

Carlos Fermin-Martinez[1], Omar Yaxmehen Bello-Chavolla[2], Neftali Antonio-Villa[3], Luisa Fernandez-Chirino[4], and Daniel Ramirez-Garcia[5], *1. School of Medicine, National Autonomous University of México (UNAM), Mexico City, Distrito Federal, Mexico*, *2. Instituto Nacional de Geriatría, Mexico City, Distrito Federal, Mexico*, *3. Instituto Nacional de Cardiologia Ignacio Chavez, Mexico City, Distrito Federal, Mexico*, *4. Instituto Nacional de Geriatria, Mexico City, Distrito Federal, Mexico*, *5. Facultad de Medicina, Universidad Nacional Autonoma de Mexico, Mexico City, Distrito Federal, Mexico*

Aging occurs at unique rates at an individual and population-level. In contrast to chronological age (CA), biological age (BA) is better at capturing functional decline and risk of age-related diseases, disability, and death. Nonetheless, it is still unclear how this phenomenon changes across adults from different races/ethnicities. We recently developed a BA metric using anthropometric measurements (AnthropoAge) in participants from NHANES-III. Here, we aim to validate AnthropoAge across countries and to determine whether it displays population-specific patterns using harmonized cohorts from the U.S. and Mexico from the Gateway to Global Aging Data Team, including the Mexican Health and Aging Study and the Health and Retirement Study. We found that AnthropoAge is a robust predictor of overall mortality risk in both American (AUROC=0.797; 95%CI 0.786-0.808) and Mexican (0.776; 0.740-0.812) adults, and this was consistent across multiple cutoffs of time. Notably, we found that the prediction improved in participants aged 50-75 years in comparison to older adults aged 75-100 years. AnthropoAge was also associated with number of comorbidities and disability in activities of daily living independently of CA. Finally, we observed marked changes of AnthropoAge and its acceleration across the years in both countries, with a significant interaction with Mexican ethnicity (beta for interaction: 0.2034; p=5.94E-05). AnthropoAge is a robust and reproducible measure of BA, however, new studies are required to decipher the heterogeneity of anthropometric aging in specific ethnicities and age categories.

### SESSION 3165 (SYMPOSIUM)

Abstract citation ID: igad104.0949

### PUBLISHING ON A GLOBAL SCALE IN GERONTOLOGY: A VIEW FROM THE EDITORS' DESKS

Chair: Edward Miller Co-Chair: Elizabeth Simpson
Discussant: Nicholas Castle

Global aging has proceeded at an unprecedented and accelerating rate. The aging of the population creates both opportunities and challenges for older adults, their families, and society. Importantly, there is substantial variation in the effects of and response to global aging both within and across nations depending, in part, on prevailing cultural expectations and values, political and economic imperatives, and social and demographic characteristics. Thus, while some regions and countries have responded with innovative policies and programs to better enable the growing cohort of older adults to remain active and engaged in the community, other regions and countries have struggled with their response or barely begun to plan for the rising population of older adults. This symposium assembles editors at five leading gerontological journals to demonstrate the role that peer-reviewed scholarship can play in disseminating knowledge that informs gerontological research, policy, and practice internationally. The editors include: Edward Alan Miller, PhD, Journal of Aging & Social Policy; Kyungmin Kim, PhD, Research on Aging; Julie Robison, PhD and Debra Dobbs, PhD, Journal of Applied Gerontology; Julie Patrick, PhD, International Journal of Aging and Human Development; and Sandra Torres, PhD, Ageing & Society. Each presenter will review the scope, content, and focus of their journals and the role and opportunities for international scholarship. Nicholas Castle, PhD, International Editor at The Gerontologist, will serve as discussant.

Abstract citation ID: igad104.0950

### INTERNATIONAL PUBLISHING OPPORTUNITIES AT THE JOURNAL OF AGING & SOCIAL POLICY

Edward Miller[1], Elizabeth Simpson[1], Michael Gusmano[2], and Pamela Nadash[1], *1. University of Massachusetts*

*Boston, Boston, Massachusetts, United States*, 2. *Leigh University, Bethlehem, Pennsylvania, United States*

Policymakers, practitioners, and researchers need a balanced, thoughtful, and analytical resource to meet the challenge of global aging at a rate that's historically unprecedented. The Journal of Aging & Social Policy (JASP), which was founded in 1989, serves this role by drawing contributions from an international panel of policy analysts and scholars who assume an interdisciplinary perspective in examining and analyzing critical phenomena that affect aging and the development and implementation of programs for older adults from a global perspective. Study settings extend beyond the United States to include Europe, the Middle East, Australia, Latin America, Asia, and the Asia-Pacific rim. This presentation will document the scope, content, and focus of JASP, including the rise of international submissions, which now account for approximately half of articles published. Opportunities for publishing in JASP will be discussed; so too will strategies for navigating the peer-review process successfully.

Abstract citation ID: igad104.0951

### RESEARCH ON AGING: THE INTERNATIONAL VIEW FROM THE EDITORS' DESKS

Kyungmin Kim[1], Jeffrey Burr[2], and Changmin Peng[3], *1. Seoul National University, Seoul, Republic of Korea*, 2. *University of Massachusetts Boston, Boston, Massachusetts, United States*, 3. *University of Massachusetts, Boston, Boston, Massachusetts, United States*

We review the scope, content, and focus of the peer-reviewed journal, Research on Aging (SAGE), publishing its 45th volume this year. We will discuss how scholarship produced from researchers around the globe has changed over the years. Data on submissions, acceptance rates, and the important role of an international editorial board will be presented. The review process will be described, along with suggestions on how to increase chances of success when submitting original research. Although Research on Aging is sometimes considered to focus primarily on social gerontology, the scope has widened considerably, with manuscripts in aging studies published from such fields as economics, psychology, demography, public health, and public policy, as well as from sociology and social work, among others disciplines. One of several special issues forthcoming in the journal will be described to demonstrate the possibilities for international impact.

Abstract citation ID: igad104.0952

### THE JOURNAL OF APPLIED GERONTOLOGY: AN INTERNATIONAL PERSPECTIVE

Debra Dobbs[1], and Julie Robison[2], *1. University of South Florida, Tampa, Florida, United States*, 2. *UConn Health, Center on Aging, Farmington, Connecticut, United States*

The mission of applied gerontology is to bridge science and practice to benefit the health and well-being of older people, their families, their communities, and other contexts. The Journal of Applied Gerontology features original investigations or meta-analyses/systematic reviews with significant clinical, policy, and/or practice implications. This presentation will provide insights from the Journal of Applied Gerontology and its commitment to publish and disseminate scholarship with international application. Following an overview of the growing internationalization of peer-reviewed submissions to the Journal on a variety of topics and from a range of perspectives, the presentation will highlight opportunities to apply gerontological scholarship to aging contexts worldwide. Concluding comments will examine how outlets for dissemination and authors themselves can better position their work to enhance their influence on aging in an international context.

Abstract citation ID: igad104.0953

### PUBLISHING WITH THE INTERNATIONAL JOURNAL OF AGING AND HUMAN DEVELOPMENT

Julie Hicks Patrick, *West Virginia University, Morgantown, West Virginia, United States*

For more than 50 years, under the leadership of four editors and two publishers, The International Journal of Aging and Human Development (IJAHD) has featured multidisciplinary scholarship related to aging processes and older adults. With the publication of eight issues a year and over 1000 pages of scientific content, the IJAHD places emphasis upon psychological and social studies of aging and the aged. However, the Journal also publishes research that integrates observations from other disciplines that illuminate the "human" side of gerontology. A more recent focus includes midlife development, as well. About half (47%) of the publications in the IJAHD are from international colleagues. IJAHD is delighted to support new investigators as they navigate dissemination. This presentation will discuss tips for both international and US-based scholars for ensuring timely reviews and positive decisions for manuscript submissions, including such areas as key words, suggesting unbiased reviewers, formatting, writing mechanics, clearly-articulated methods, and a sound theoretical basis.

Abstract citation ID: igad104.0954

### PUBLISHING IN AGEING & SOCIETY: TOP 10 TIPS FROM THE EDITOR-IN-CHIEF

Sandra Torres, *Uppsala University, Uppsala, Uppsala Lan, Sweden*

With 43 volumes to its name (and coming out with 12 issues a year), Ageing & Society is an interdisciplinary and international journal devoted to advancing the understanding of ageing, and the circumstances of older people, in their socio-economic and cultural contexts. Committed to publishing original and high-quality research papers that substantially contribute to ongoing debates in social gerontology, this journal welcomes submissions that use different theoretical and methodological approaches as long as they aim to advance research, policy and practice and encourage the exchange of ideas across the broad audience of multidisciplinary academics and practitioners working in the field of ageing. By bringing specific attention to what Ageing & Society looks for in the hundreds of submissions that are sent to the journal, and the routines that the team of associate editors utilize, this presentation aims to give attendees valuable insights into how to write a paper if they wish to submit it to this journal.

### SESSION 3170 (PAPER)

### FAMILY DYNAMICS

Abstract citation ID: igad104.0955

### A MICRO CAREGIVING INITIATION: LATE-LIFE REMARRIAGE OF OLDER WIDOWED ARAB MUSLIM MEN IN ISRAEL

Chaya Koren[1], and Hanan Ali Morshed[2], *1. University of Haifa, Haifa, HaMerkaz, Israel*, *2. University of Haifa, Haifa, HaZafon, Israel*

New caregiving solutions are increasingly in demand within families undergoing rapid modernization processes such as the Arab Muslim family in Israel. According to patriarchal collectivist values, caregiving for aging men is provided by women within the extended Arab Muslim family. Modernization within the Arab Muslim society has enabled women to acquire higher education and increased employment opportunities outside the household. Therefore, older Arab Muslim widowers in need for caregiving can depend less on their daughters or daughter's in-law and as such seek other solutions. The aim of this presentation is to describe and examine the caregiving role of late life remarriage for older Arab Muslim widowers. Using a phenomenology framework, semi-structured qualitative interviews for understanding the experience of late life remarriage and its meaning were conducted with 14 Arab Muslim widowers who remarried between age 70 and 80, to never married middle-aged women. Findings describe and illustrate caregiving as a primary motivation for late life remarriage, reasons for caregiving as motivation to remarry and the wife's role as located between caregiver and spouse. Conclusions discuss the meaning of late life remarriage as a micro caregiving solution, dealing with modernization processes alongside preserving patriarchal collectivist values and gender-based roles.

Abstract citation ID: igad104.0956

### PERCEIVED STRAIN ON THE SPOUSAL RELATIONSHIP AMONG MARRIED CUSTODIAL GRANDPARENTS WITHIN THE PARENTING ROLE

Eunbea Kim[1], Jeong Eun Lee[1], and Gregory Smith[2], *1. Iowa State University, Ames, Iowa, United States*, *2. Kent State University, Kent, Ohio, United States*

Providing primary care for grandchildren is known to have a significant impact on custodial grandparents' well-being. For married custodial grandparents, although their caregiving burden may spill over to marriage which can lead to negative psychological outcomes, it has rarely been examined. We examined how custodial grandparents couples' perceived stress in the spousal relationship due to parenting (PSSR) is associated with their psychological outcomes mutually using Actor Partner Interdependent Model. We further tested the moderating effect of coping strategies on the association between PSSR and psychological outcomes. Participants are grandparents couples providing full-time care to their grandchild in the absence of the grandchildren's biological parents (N_dyad = 193). We found that one's PSSR was associated with one's higher depressive symptoms for both grandmothers and grandfathers. Regarding anxiety symptoms, only grandmothers' PSSR was positively related to both their own and their spouses' anxiety level.In terms of coping strategies, we found that a higher level of problem-focused coping was related to higher levels of anxiety for both grandmothers and grandfathers when grandmothers experienced higher PSSR. A higher level of emotional coping was related to higher level of depressive symptoms in both grandfathers and grandmothers when they experienced a higher level of PSSR. A higher level of emotional coping was related to both grandfathers and grandmothers' higher level of anxiety when grandmother's PSSR was higher. Our research adds to the existing body of literature by emphasizing how emotional and problem-focused coping can moderate the connection between increased PSSR and psychological outcomes among custodial grandparents.

Abstract citation ID: igad104.0957

### RECIPROCITY AMONG CARE PARTNER DYADS AND ITS INFLUENCE ON OUTCOMES IN DEMENTIA FAMILY CAREGIVERS

Francesca Falzarano, and Sara Czaja, *Weill Cornell Medicine, New York City, New York, United States*

Dementia family caregiving is a heterogenous experience influenced by multidimensional inter- and intra-personal factors. The interdependence between the well-being of caregivers and care-recipients, and that feelings of mutuality and shared involvement in care responsibilities by both the caregiver (CG) and care-recipient (CR) influence family caregiver outcomes. This presentation will present findings from an analysis that examined the influence of caregiver/care-recipient demographic characteristics, care intensity (functional status, medical/nursing tasks), degree of cognitive impairment, and CR depression and quality of life on CG outcomes including burden, self-efficacy, depression, and positive aspects of caregiving. Baseline data were drawn from 99 family caregiver-care-recipient dyads who participated in the Care Partners intervention, a non-pharmacological intervention for individuals with Alzheimer's disease and their caregivers. Results showed that CG difficulty in performing medical/nursing tasks, greater distress related to the CR's functional impairment, CR's degree of cognitive impairment, lower levels of mutuality, and lower CR ratings of quality of life and depression were significantly related to higher CG burden, depression, and lower CG self-efficacy. Alternatively, being a male CG and greater CG distress related to performing medical/nursing tasks and assistance with activities of daily living was associated with lower positive aspects of caregiving. Having a female CR and reporting higher levels of mutuality and preparedness was subsequently associated with more positive appraisals of caregiving. These findings underscore the importance of reciprocal interactions and the need to target both members of the dyad in interventions aiming to improve quality of life and well-being in dementia care partners.

Abstract citation ID: igad104.0958

### RETRAUMATIZATION WHEN AN ADULT CHILD CARES FOR THEIR PARENTAL PERPETRATOR THROUGH SERIOUS ILLNESS OR THE END OF LIFE

Jaime Goldberg[1], and Jooyoung Kong[2], *1. University of Wisconsin-Madison , Madison, Wisconsin, United States*, *2. University of Wisconsin-Madison, Madison, Wisconsin, United States*

Current trauma-related literature does not readily include the experiences of the caregiving population. While the caregiving literature does recognize that caregivers experience trauma, it has not yet explored when the caregiver's source of trauma is the care recipient. To begin to address this gap, this research explores the retraumatization adult survivors of parental childhood maltreatment may experience when they become caregivers for their parental perpetrator during serious illness or the end of life. Participants were recruited through purposeful sampling techniques, via social media and word-of-mouth. Using a phenomenological approach, 22 semi-structured, virtual interviews were conducted; 13 participants identified retraumatization as part of their caregiving experience. Focused content analysis revealed three subthemes under the umbrella of retraumatization: 1) reminders of past abuse; 2) re-experiencing abusive behaviors; and 3) physical contact with the body of the former abuser. Results suggest that memories of past abuse, ongoing abusive behavior, and proximity to the parental perpetrator's body evoke feelings of fear/anxiety, worthlessness, shame, anger, and disgust in participants, reminiscent of the feelings they experienced in childhood. Taking on the roles/responsibilities of caregiving triggered these feelings unexpectedly, as the adult children were now interacting with their parent more frequently and in new/more intimate ways. These findings invite health and mental health professionals to have heightened awareness of and sensitivity to this often-overlooked population and use trauma-informed assessment and intervention techniques to support caregivers' health and well-being. Results can inform future research at the nexus of caregiving and trauma.

Abstract citation ID: igad104.0959

### WHY DO I CARE? MOTIVATIONS OF SURVIVORS OF PARENTAL CHILDHOOD HARM WHO ARE CARING FOR THEIR PARENTAL PERPETRATOR

Jaime Goldberg[1], and Jooyoung Kong[2], *1. University of Wisconsin-Madison , Madison, Wisconsin, United States, 2. University of Wisconsin-Madison, Madison, Wisconsin, United States*

Adult child survivors of parental childhood maltreatment may remain in relationship with their parental perpetrator across the life course and even take on the caregiving role when their parent becomes older and/or ill. Yet, becoming a caregiver for a parent who was harmful is associated with increased depressive symptoms, heightened stress responses, and maladaptive coping for the caregiver. Given that survivors of parental childhood maltreatment may find caregiving to be detrimental to their mental health and well-being, it is crucial to better understand why an adult child would choose to engage in caregiving for their parental perpetrator. This qualitative research, grounded in Intergenerational Solidarity Theory, examines the motivations to participate in parental caregiving through serious illness or the end of life for survivors of childhood parental harm. Based on the phenomenological perspective, in-depth semi-structured interviews were conducted with 22 adults ages 24-70. Participants were recruited through word-of-mouth and social media. Content analysis revealed five themes: 1) obligation and ambivalence; 2) differentiating from the abuser; 3) "a second chance;" 4) belief systems that influence caregiving; and 5) financial incentives. This information heightens awareness of the experiences of this often-overlooked population of caregivers and invites gerontological researchers and practitioners to consider the complexity in their decision to participate in their perpetrator's care. Gaining a better understanding of the lived experiences of adult children who are caregivers for their parental perpetrator and their motivations for participating in such care can inform the development of trauma-informed assessment and intervention strategies tailored to this population.

### SESSION 3175 (SYMPOSIUM)

Abstract citation ID: igad104.0960

### POLICY SERIES: THE MOVING FORWARD NURSING HOME COALITION: NURSING HOME REFORM AND THE RESIDENTS' GOALS, PREFERENCES, AND PRIORITIES

Chair: Tara McMullen Co-Chair: Tetyana Shippee
Discussant: Howard Degenholtz

The 2022 National Academies of Sciences, Engineering, and Medicine (NASEM) report called for nursing facilities to provide "care aligned to the individual's goals and preferences." Missing has been an efficient process to uncover the individual's detailed goals, preferences, and priorities (GPPs). GPPs are a care planning process that translates into actionable interventions, supported by quality measurement to determine whether the care provided aligns with those GPPs, and health information technology to collect GPPs efficiently and to determine if the care provided is concordant with the individual's GPPs. A diverse set of experts representing the Moving Forward Nursing Home Quality Coalition are collaborating to develop an integrated approach to these shortcomings and will discuss these issues in detail including new approaches to the identification and documentation of GPPs for use in a modified care planning process, the status of health information technologies (HIT) processes being developed to support these changes and the development of new quality measures. While the Moving Forward Coalition is focused on nursing facilities, the approaches being developed here to improve care and to standardize the vocabulary for GPPs are applicable to all care settings. Three committees from the Moving Forward Nursing Home Coalition will present work focused on the development of a standardized GPP care planning process to enhance the identification, documentation, communication, and implementation of resident GPPs, the measurement of the care planning process to support process improvement, and the creation of a HIT-enabled process to measure the concordance of care provided with an individual's GPPs. This is a collaborative symposium between the Aging Workforce, Hospital Elder Life Program, Measurement, Statistics, and Research Design, Systems Research in Long Term-Care, and Technology and Aging Interest Groups.

Abstract citation ID: igad104.0961

### MEASURING GOALS, PRIORITIES, AND PREFERENCES: REFORM EFFORTS FOR TRANSPARENCY AND EMPOWERMENT

Tara McMullen[1], and Anna Fisher[2], *1. U.S. Department of Veterans Affairs , Sykesville, Maryland, United States*, 2. *Hillcrest Health Services, Bellevue, Nebraska, United States*

Longstanding evidence suggests that the goals, preferences, and priorities (GPP) of individual nursing facility residents may not be adequately addressed during episodes of care. Aligning care with residents' GPP represents an opportunity to improve outcomes and reduce disparities associated with race, ethnicity, gender orientation, and other characteristics of the resident population. The 2022 National Academies of Sciences, Engineering, and Medicine (NASEM) report recommended improvement in quality measures under the domain of resident and family experience, along with reporting of these measures on Care Compare to improve shared decision-making and transparency. The subcommittee on quality improvement and measurement is focused primarily on measures of GPP as a useful addition to CMS' plans to expand the Quality Reporting Program (QRP) measure set, which was signaled in the recently issued Skilled Nursing Facility Prospective Payment System (SNF PPS) rule. In this presentation, committee members will discuss the identification and testing plan for one or more resident experience of care measures that assess the experience of care specific to processes of care and the resident's GPP. This presentation will also discuss the development of a care concordant measure, assessing the relationship between the care provided to the resident and the resident GPPs.

Abstract citation ID: igad104.0962

### IDENTIFYING, DEVELOPING, AND IMPLEMENTING CARE PLANS CONSISTENT WITH GOALS, PRIORITIES, AND PREFERENCES

Tonya Roberts[1], Laci Cornelison[2], Tetyana Shippee[3], Mary Lou Ciolfi[4], Susan Ryan[5], and Penny Cook[5], *1. University of Wisconsin, Madison, Wisconsin, United States*, *2. Kansas State University, Manhattan, Kansas, United States*, *3. University of Minnesota, Minneapolis, Minnesota, United States*, *4. University of Maine Center on Aging, Bangor, Maine, United States*, *5. Center for Innovation, Linthicum, Maryland, United States*

NASEM's first recommendation to reform nursing home (NH) care is to deliver comprehensive, person-centered, equitable care that ensures residents' health, quality of life, and safety, promotes autonomy, and manages risks. The NH Code of Federal Regulations requires person-centered care (PCC). However, while there are existing structures (i.e. MDS 3.0) to support care planning clinical needs, most NHs lack tools and procedures to systematically identify and provide care that aligns with resident goals, priorities, and preferences (GPPs). This is especially true for GPPs that reflect personhood and the human desire for autonomy. Our subcommittee aims to change the culture of care planning in NHs to integrate residents and families in co-developing holistic, person-centered care plans that centralize residents' GPPs in ways that promote and support personhood. Our subcommittee will develop and test a) a standardized GPP care planning process that includes enhanced approaches to identification, documentation, communication, and implementation of resident GPPs; b) a repository of tools to support identification and documentation of resident GPPs; and c) training to prepare staff to understand and recognize resident GPPs. We will collaborate with other Moving Forward Committees to develop approaches to evaluating and measuring quality in care planning with inclusion of health information technology . When completed, the proposed work will provide evidence of successful and feasible quality improvement approaches to care planning resident GPPs that can augment existing federal requirements and can inform future policy changes or guidance to surveyors.

Abstract citation ID: igad104.0963

### THE MOVING FORWARD NURSING HOME COALITION: A CALL TO ACTION TO SUPPORT NURSING HOME REFORM

Alice Bonner[1], Isaac Longobardi[2], Tara McMullen[3], Tetyana Shippee[4], Terry O'Malley[5], Tonya Roberts[6], and Gregory Alexander[7], *1. Institute for Healthcare Improvement (IHI), Westborough, Massachusetts, United States*, *2. Moving Forward: Nursing Home Quality Coalition, Washington, District of Columbia, United States*, *3. U.S. Department of Veterans Affairs , Sykesville, Maryland, United States*, *4. University of Minnesota, Minneapolis, Minnesota, United States*, *5. Massachusetts, United States*, *6. University of Wisconsin, Madison, Wisconsin, United States*, *7. Columbia University School of Nursing, New York City, New York, United States*

The Moving Forward Nursing Home Quality Coalition advances the goals outlined by the 2022 National Academies of Sciences, Engineering, and Medicine (NASEM) report aimed at improving nursing home (NH) quality. Over two years, Coalition committee members – along with a network of national leaders, nursing home residents, and the general public – will develop, test and promote action plans that will improve the way the United States finances, delivers, and regulates care in Nursing Homes. The Coalition is comprised of a steering committee and working committees that align with and advance the seven NASEM goals including; 1) advancing delivery of person-centered care; 2) ensuring a well-prepared and compensated workforce; 3) increasing financial transparency and accountability; 4) creating a rational and robust financing system; 5) designing more effective and responsive quality assurance; 6) expanding quality measurement and continuous quality improvement; and 7) adopting health information technology. Coalition committees have engaged in three of four phases of their two-year work plan including convening stakeholders to prioritize and develop action plans to test and promote change. Throughout the work, committee collaboration has ensured action plans are refined and expanded in synergistic ways to shape state and national policy. This presentation will provide an overview of the broader Coalition work and give context for the other presentations which provide an example of collaborative work leading to proposed recommendations for critical policy and regulatory change around person-centered care planning.

Abstract citation ID: igad104.0964

### MOVING FORWARD TOWARD DEPLOYING HEALTH INFORMATION TECHNOLOGY IN ALL US NURSING FACILITIES

Gregory Alexander[1], Christina Caraballo[2], Maria Moen[3], Cynthia Morton[4], and Terry O'Malley[5], *1. Columbia University School of Nursing, New York City, New York, United States*, *2. HIMSS, Chicago, Illinois, United States*, *3.*

*Richardson, Texas, United States*, *4. ADVION, Washington D.C., District of Columbia, United States*, *5. Massachusetts, United States*

The Moving Forward Coalition established a health information technology (HIT) committee to develop action plans to advance the adoption of HIT in all nursing facilities. Currently, more than 70% of nursing facilities in the US have some HIT, although the levels of utilization vary greatly. Few facilities, however, have successfully implemented the capabilities required to survive in a Value Based Payment (VBP) model which Medicare will mandate by 2030. Fewer still have HIT that collects the resident's goals, preferences, and priorities (GPPs) efficiently and in detail despite current CMS requirements for person-centered care. To the best of our knowledge, no nursing facilities possess an HIT-enabled capability to determine whether the care provided aligns with the individual's GPPs. In this presentation, members of the HIT Committee of the Moving Forward Coalition will discuss the rationale for adopting the following three HIT priorities to realize the target goals of the Coalition and will review progress made to date: 1) develop a benchmarking tool that enables nursing facilities to understand and plan for the HIT capabilities needed to meet upcoming regulatory requirements and for successful participation in a VBP model, 2) create a taxonomy of GPPs and an HIT-based process to collect them, and 3) develop and test an HIT-enabled process to measure the concordance of care provided with an individual's GPPs. Although the Moving Forward Coalition and the NASEM report focus on nursing facilities, the HIT tools being developed here will benefit all sites of care.

### SESSION 3180 (SYMPOSIUM)

Abstract citation ID: igad104.0965

### MEASURING AND IMPLEMENTING PERSON-CENTERED LONG-TERM CARE: GLOBAL PERSPECTIVES

Chair: Tonya Roberts Co-Chair: Franziska Zuniga
Discussant: Michael Lepore

Person-centered care (PCC) is internationally recognized as an essential strategy for achieving high quality care and quality of life for older adults who need long-term care (LTC). Despite international endorsement, our understanding of PCC is largely derived from research and practice in western countries. It is less clear whether PCC practices and benefits are generalizable on a global scale. PCC may vary structurally across healthcare contexts and cultures where values, experiences, and needs of older adults differ. Understanding how to culturally adapt or extend the concept, measurement, and implementation of PCC is needed to better inform care and improve outcomes for a diverse and international population of older adults. The purpose of this symposium is to present theory and research across healthcare settings and from countries across multiple continents to comparatively analyze the cultural implications of PCC concepts and discuss the implications for implementation, measurement, and research. The first presentation will contextualize the concept of PCC from an international perspective, summarizing and comparing what is known about PCC from existing literature. The second presentation will compare barriers and facilitators of PCC implementation during mealtimes in three European countries. The third will highlight variations in PCC definitions from qualitative research conducted with providers in middle- and low-income countries. The fourth presentation will report on the development and implementation of a system of PCC and LTC in Brazil. The final presentation will use secondary data to compare the measurement and LTC needs of family caregivers across the US and China. This is a Common Data Elements for International Research in Residential Long-term Care Interest Group Sponsored Symposium.

Abstract citation ID: igad104.0966

### PERSON-CENTERED CARE: SETTING THE INTERNATIONAL SCENE FROM A CONCEPTUAL PERSPECTIVE

Tonya Roberts[1], and Annica Backman[2], *1. University of Wisconsin, Madison, Wisconsin, United States*, *2. Umea University, Umea, Vasterbottens Lan, Sweden*

Over 142 million older persons worldwide need assistance with meeting basic needs, often requiring some level of long-term support and services. Person-centered care (PCC) is internationally recognized as an essential strategy for achieving high quality care and quality of life for older adults who need long-term care. Rather than focusing only on disease management or preventing morbidity, PCC results in positive outcomes for older people and their caregivers by focusing care on the whole person in their unique environment and aligning care with what is holistically meaningful to older people. Although PCC has become the gold standard for care of older adults, its widespread implementation has led to theoretical, conceptual, and practical inconsistencies that make it challenging to compare or evaluate its application or effectiveness globally. This is particularly problematic given our understanding of PCC is largely derived from research and practice in western cultures. Despite positive outcomes, the wide spectrum of person-centered interventions introduces variation which has made global comparisons difficult and ability to identify needs for cultural adaptation largely absent. To be able to compare PCC internationally, and reflect on potential needs for cultural adaptation, more conceptual clarity of the concept of PCC, its measurement, and implementation is needed. The purpose of this presentation is to review the use, value, and implementation of PCC concepts in international literature and present possible future directions for extending PCC to be more culturally inclusive for a global older adult population.

Abstract citation ID: igad104.0967

### RESIDENT EXPERIENCES OF PERSON-CENTRED CARE AT MEALTIMES IN LONG-TERM RESIDENTIAL CARE: A RAPID ETHNOGRAPHY

Megan Davies[1], Franziska Zuniga[2], Hilde Verbeek[3], and Sandra Staudacher[4], *1. Medical Faculty, University of Basel, Basel, Basel-Stadt, Switzerland*, *2. University of Basel, Basel, Basel-Stadt, Switzerland*, *3. Maastricht University, Maastricht, Limburg, Netherlands*, *4. Universität Basel, Basel, Basel-Stadt, Switzerland*

Poor nutrition is a common ongoing problem in long-term residential care, often resulting in reduced quality of life. Previous research has concluded that the content of the meal, dining environment, service style and general atmosphere all add to the mealtime experience, suggesting that person-centred mealtimes are optimal. However, knowledge about which elements of person-centred care can be achieved in a mealtime setting in a given context is currently lacking. As part of the TRANS-SENIOR research network, rapid ethnographies were conducted across multiple sites (including interviews, observations and informal conversations), in long-term residential care homes in the UK, Switzerland and the Netherlands between October 2020 and December 2021. During analysis and interpretation of data, different themes were developed where either successfully achieved or missed opportunities for person-centred moments were observed. We observed differences between the long-term care homes in as much as the setting could be considered, resident choice was implemented, residents were enabled, care was individualized in the communal setting and how much the person of the residents was known with their past and present. In the presentation we will discuss the possibility to provide person-centred care during mealtimes in view of differences between staff approaches, the overall environment (size of dining area, seating arrangements etc.), allocation of staff resources and country-specific rules and regulations. We see an interplay of factors in place for mealtimes to be moments of participatory choice, interaction, independence, and dignity.

Abstract citation ID: igad104.0968

### MEASURING AND IMPLEMENTING PERSON-CENTERED, RESIDENTIAL LONG-TERM CARE: BRAZILIAN CONTEXT

Patrick Wachholz[1], Michael Lepore[2], and Franziska Zuniga[3], *1. Faculdade de Medicina de Botucatu - Universidade Estadual Paulista (Unesp), Botucatu, Sao Paulo, Brazil*, *2. University of Maryland School of Nursing, Baltimore, Maryland, United States*, *3. University of Basel, Basel, Basel-Stadt, Switzerland*

Older adults represent most users in the Brazilian Unified Health System (SUS), which provides at least one service to 57.7% of the national older population. Despite Brazilian legislation ensuring the rights of older adults, health inequities are evident, including barriers to access prevention services, weaknesses and inequities in the distribution of human resources trained to meet the needs of older adults, and several vulnerabilities to the social determinants of health that undermine healthy aging prospects. Family members provide most care, and informal caregivers are neither paid nor receive financial support. The proportion of older adults living with disability has grown significantly over the last decade, and approximately two-thirds of the care-dependent population in Brazil are adults over 65 years of age. Institutional long-term care (LTC) represents a growing part of the Brazilian older population, but several factors challenge the implementation of person-centered care (PCC) in residential LTC. Although 73% of Brazilian families are covered by primary care provided at SUS, only 56.3% of older people in Brazil are registered in the Family Health Strategy, and most people living in LTC facilities do not have access to primary care. The purpose of this presentation is to contextualize how the dissemination and implementation of a PCC culture and the definition of a national conceptual perspective are affected by the immaturity of the Brazilian LTC sector, the lack of coordinated long-term care policies, and ageism in the healthcare sector.

Abstract citation ID: igad104.0969

### UNDERSTANDING PERSON-CENTERED CARE FOR OLDER PEOPLE IN SIX DEVELOPING COUNTRIES

Jing Wang[1], and Barbara Bowers[2], *1. University of New Hampshire, Durham, New Hampshire, United States*, *2. University of Wisconsin-madison, Madison, Wisconsin, United States*

Person-centered care (PCC) is a widely acknowledged and encouraged approach to quality care for older adults residing in residential care facilities. However, current understandings of PCC are mainly based on research and practice in western, first world nations. The lack of research on PCC in developing countries leads to uncertainty over whether and how current understandings of PCC apply to cultures and contexts in non-western, developing countries. To enhance care and outcomes for diverse populations, it is crucial to comprehend and culturally adapt PCC understandings and approaches. Therefore, we conducted interviews with researchers and practitioners from six countries in various stages of development to identify elements that form the core of high-quality care for older people and how they relate to Western notions of PCC. We gained valuable insights into how cultural differences, population health priorities, political conflict, and limited resources impact their understanding and implementation of PCC. In addition to variations in the language of PCC, some first world assumptions about PCC do not resonate well in developing countries. One example is the emphasis on individualism, a basic element of PCC. The role of the older person in decision making and focus on family and community priorities reflects both cultural and economic differences between first world, western nations and non-western, developing countries. The findings can serve as a valuable starting point for the development of a globally applicable measurement for the quality of care.

Abstract citation ID: igad104.0970

### ASSESSING CAREGIVING SUPPORT NETWORKS FOR PERSONS WITH DEMENTIA IN A LONG-TERM CARE CONTEXT IN CHINA AND THE US

Hanzhang Xu[1], Matthew Dupre[1], Scott Lynch[1], Truls Østbye[2], and Bei Wu[3], *1. Duke University, Durham, North Carolina, United States*, *2. Duke University School of Medicine, Durham, North Carolina, United States*, *3. New York University, New York City, New York, United States*

Informal caregivers are vital to the provision of care for persons living with dementia (PLWD). Although PLWD often receive care from multiple sources, little is known about the broad caregiving support networks that PLWD have. Furthermore, the structure of caregiving support networks is likely influenced by the availability, accessibility, and affordability of LTC services. Therefore, caregiving support networks in China and in the U.S. are likely to differ. In this study, we identified the major types of caregiving support networks that PLWD have using the Health and Retirement Study and China Health and Retirement Longitudinal Study. Results from latent class analysis show that both countries have three major types of caregiving networks, but they differ in the proportion of each country's population that follow each type. Unlike conventional secondary data studies that often only focus on existing data, we are working with WE-THRIVE Consortium to further gain a comprehensive understanding of the dementia care context in China and in the U.S. Through an expert panel survey, we will assess the scope, services, needs, funding, and ownerships of LTC in each country. We will then link the data from expert survey to the two national aging studies to contextualize the caregiving support networks in persons with dementia. This new way of linking existing individual-level with country-level data is directly responsive to the priority research areas identified by the National Academy of Medicine and will shed light on which caregiving support networks need more external formal support to promote person-centered dementia care.

### SESSION 3185 (SYMPOSIUM)

Abstract citation ID: igad104.0971

### BUILDING BRIDGES, CATALYZING RESEARCH, EMPOWERING ALL AGES: NIA SESSION FOR EARLY-CAREER RESEARCHERS

Chair: Amy Kelley Discussant: Amy Kelley

The National Institute on Aging (NIA) at the National Institutes of Health, Department of Health and Human Services, supports biomedical and behavioral research with a lifespan focus. NIA research seeks to understand the basic processes of aging, improve prevention and treatment of diseases in later life, and improve the health of older persons, in addition to an emphasis on Alzheimer's disease and related dementias. NIA supports a variety of training and career development opportunities for students, junior faculty, early-career investigators, and emerging scholars. In this session, we will provide an overview of NIA-funded research, followed by a presentation on funding mechanisms and strategies to consider when applying for extramural grants, particularly for early-career scientists at multiple career stages. Finally, attendees will be able to join any number of breakout group discussions (led by extramural NIA training staff and program officials) to discuss programs, initiatives, mechanisms, and/or policies specific to various career paths.

Abstract citation ID: igad104.0972

### OVERVIEW OF NIA MISSION AND RESEARCH

Richard Hodes, *National Institutes of Health (NIH), Bethesda, Maryland, United States*

Dr. Hodes will provide an overview of NIA's structure and mission, in addition to discussing research foci from across the Institute's scientific divisions.

Abstract citation ID: igad104.0973

### HOW TO GET AN NIA GRANT

Kenneth Santora, *NIA, NIH, Bethesda, Maryland, United States*

Dr. Santora will provide an overview of the NIA application process, funding opportunities, and relevant policy changes for early-career researchers.

### SESSION 3190 (SYMPOSIUM)

Abstract citation ID: igad104.0974

### WHO CARES? RETENTION STRATEGIES FOR DIRECT CARE WORKERS

Chair: Robert Applebaum Co-Chair: Leah Janssen
Discussant: Robert Applebaum

More than 30 years ago the Gerontological Society sponsored a congressional briefing about a growing problem; concerns about the adequacy and quality of the direct care workforce in long-term care. In 2002 the Robert Wood Johnson Foundation sponsored the Better Jobs, Better Care demonstration, in response to this continued challenge. Today recruiting and retaining high quality workers in the long-term services system has moved beyond a challenge to an all-out crisis. The initial paper of the symposium presents recent retention and work culture data highlighting todays' hurdles, collected from more than 80% of long-term care facilities across Ohio. The second presentation provides a comprehensive review of state-level initiatives and innovations aimed at improving recruitment and retention for direct care workers (DCWs) in both facility-based long-term care and home and community-based long-term services across the country. A third paper focuses on retention-promoting factors from the perspective of providers and DCWs working in high-performing long-term care facilities in Ohio. In-depth interviews with 37 providers and DCWs revealed an overarching theme of the importance of work culture, highlighting the modifiable, intangible aspects for promoting DCW retention. The final paper introduces a tip sheet with practical strategies for providers who are seeking to retain direct care workers. Symposium findings will include both practical implications for long-term care providers and ideas for future studies. The direct care workforce crisis is the result of an array of challenges, which means that there will not be one solution, but rather many ideas will be needed.

Abstract citation ID: igad104.0975

### PRACTICAL STRATEGIES FOR RETAINING DIRECT CARE WORKERS

Leah Janssen, *Scripps Gerontology Center / Miami University, Oxford, Ohio, United States*

Direct care workers (DCWs), such as state-tested nurse aides, personal care aides, and home care aides are a critical component of the long-term services system. While there is consensus in both the research and practice community on the challenges faced, the plan for a long-term solution continues to be developed. The final paper of this symposium is based on a review of state and national initiatives and interviews with high-performing long-term services and support providers, and culminates into a tip sheet of practical strategies for providers who are seeking to recruit and retain DCWs. The tip sheet is organized in relation to the symposium theme of work culture, which includes elements of creating a welcoming and respectful work environment, employing a participative leadership model, and opportunities for DCWs to anonymously assess work culture. It also discusses staffing ratios that are critical to recruiting and retaining workers, as well as an examination of career ladders, mentorship opportunities, and strengthening training standards. DCW economic well-being (e.g., financial incentives such as wages, bonuses and benefits) and community impact are also explored. The priority of this section is to help providers gain insights on establishing a work culture that is more receptive to DCWs' needs.

Abstract citation ID: igad104.0976

### PROVIDERS AND DIRECT CARE WORKERS' PERCEPTIONS OF RETENTION

Isha Karmacharya[1], Leah Janssen[2], and Bailee Brekke[1], *1. Miami University, Oxford, Ohio, United States*, *2. Scripps Gerontology Center / Miami University, Oxford, Ohio, United States*

Direct care worker (DCW) retention remains a persistent challenge for long-term services and supports (LTSS). Multiple studies have identified key factors for worker retention, including worker training and empowerment, career advancement opportunities, compensation, and communication. However, few studies include multiple perspectives on understanding work culture from both providers and DCWs, which is essential for developing effective retention strategies that address the needs and concerns of different stakeholders. This qualitative study explores elements of work culture through semi-structured in-depth interviews with 21 providers and 16 DCWs from 12 high-performing LTSS facilities and agencies across Ohio. The facilities/agencies were selected based on state-level family satisfaction and staff retention data. Three major elements of work culture emerged as important for DCW retention: 1) a family-like approach, 2) working conditions, and 3) worker empowerment. DCWs value a family-like approach in the workplace, which included relationships with clients and their supervisors. Favorable working conditions comprised freedom of choice, participative leadership, and effective communication that promoted retention. DCWs experienced empowerment through recognition and appreciation, coupled with financial incentives and career advancement opportunities. These findings suggest simple modifiable changes to work culture that improve worker retention in LTSS. This study urges policymakers to consider these implications for addressing the ongoing challenge of DCW retention.

Abstract citation ID: igad104.0977

### STATE-LEVEL INITIATIVES FOR DIRECT CARE WORKERS

Bailee Brekke, *Miami University, Oxford, Ohio, United Sta tes*

As problems surrounding recruitment and retention of direct care workers continue to worsen, changes have been made at both the state and national level to accommodate for these challenges. This paper provides a literature review of various state-level initiatives and innovations aimed at improving recruitment and retention strategies for direct care workers in both facility-based long-term care and home and community-based long-term services across the country. Research revealed three themes, 1) strategies to improve wages and benefits, 2) education and training, and 3) community support. To improve wages and benefits, many states increased compensation and benefits for DCWs with the implementation of wage pass-throughs, minimum wage increases, and earmarked funds. Education and training efforts were identified as another way to enhance recruitment and retention efforts for the direct care workforce, such as through credential and certification opportunities for workers, as well as standardizing current training requirements. Lastly, increasing community support was identified through advocacy and research efforts specifically aimed at understanding and supporting DCWs. This study seeks to inform policymakers and providers of current initiatives that can be used to address the ongoing workforce crisis.

Abstract citation ID: igad104.0978

### STRATEGIES USED BY OHIO'S NURSING HOMES TO RETAIN CERTIFIED NURSING ASSISTANTS: DO THEY WORK?

Oksana Dikhtyar[1], and Ian Matt Nelson[2], *1. Scripps Gerontology Center, Miami University, Oxford, Ohio, United States*, *2. Miami University, Oxford, Ohio, United States*

A low retention rate of certified nursing assistants (CNA) in nursing homes (NHs) is a known problem; it drives up facilities' operating costs and negatively impacts the quality of care provided. The COVID-19 pandemic exacerbated this problem and left facilities scrambling to retain workers. Using data from the 2021 Ohio Biennial Survey of Long-Term Care Facilities, this study examines strategies implemented by facilities to improve retention of their CNAs. Facilities have tried to implement a variety of workplace environment changes such as allowing staff teams to manage schedule and financial benefit strategies such as longevity wage increase to improve their CNA retention. The statewide average NH retention rate was 64% for CNAs ranging between 0 and 100%. Three groups were constructed from the retention data: high or rate of 75% or higher (N = 220), medium or between 50% and 75% (N = 254), and low or under 50% (N = 145). We found that a higher proportion of low-retention facilities implemented most of these strategies compared to facilities with high and medium CNA retention rates. At the same time, we found that a higher proportion of administrators in the high retention group knew all their CNAs by name compared to the low-retention group (50.0% vs 41.7%). These findings suggest that environmental and financial strategies may not have much of an impact on retention rates, but making people feel respected and appreciated could.

### SESSION 3195 (SYMPOSIUM)

Abstract citation ID: igad104.0979

### RECENT ADVANCES IN ASSESSING AGE DIFFERENCES IN AFFECT DYNAMICS

Chair: Maria Wirth Co-Chair: Mai Mikkelsen Discussant: Susan Charles

Affective experiences are inherently dynamic and short-term changes in affect offer important insights into well-being. Changes in the processes underlying affect dynamics, namely, affect reactivity and regulation, are often invoked as explanations for age-related stability or enhancement of well-being. However, previous evidence for age differences in affect dynamics is inconsistent. In this symposium, we leverage theory-based approaches and cutting-edge statistical tools to gain a deeper understanding of how individuals manage daily affect. Wirth and colleagues investigated age differences in affect reactivity and regulation, using a formalized theoretical approach to affect dynamics. Their results indicated complex, multidirectional age differences in reactivity and regulation. Neupert and Graham focused on the idea that perception of time remaining in life plays a key role in age differences in affect dynamics. Feeling closer to death was related to lower positive and higher negative affect. These relations were particularly pronounced in older adults. Wolfe and colleagues investigated age-differential associations between cognitive capacities and affect regulation preferences. Their results indicated that cognitive capacities affected regulation preferences mostly in middle-aged rather than older adults. Mikkelsen and colleagues related affect regulation patterns to well-being in an age-diverse sample. Their findings indicated that older adults exhibit less variation in regulation strategy use and that regulation effectiveness was less important for well-being in older adults. The discussion will center on the value of considering age-related similarities and differences in affect dynamics, age-relevant factors for successful emotion regulation, and the contexts that might influence affect dynamic processes.

Abstract citation ID: igad104.0980

### AGE DIFFERENCES IN AFFECT DYNAMICS: APPLYING A FORMALIZED THEORETICAL APPROACH

Maria Wirth[1], Andreas Voss[2], and Klaus Rothermund[1], *1. Friedrich Schiller University Jena, Jena, Thuringen, Germany*, *2. University Heidelberg, Heidelberg, Baden-Wurttemberg, Germany*

Emotional aging research is dominated by the idea of age-related improvements that result from shifts in motivation. Socioemotional selectivity theory (SST) proposes that as individuals age, they increasingly favor emotion-related goals and savor positive but avoid negative emotions. Previous age-comparative studies on everyday emotional experience typically were descriptive or studied the processes underlying emotional experience in isolation. We aimed at a more holistic approach to test hypotheses derived from SST regarding age-related differences in general emotional dispositions (i.e., anchoring), emotional reactivity, and emotion regulation by using a comprehensive computational approach. We applied our Model of Intraindividual Variability in Affect (MIVA) to data on everyday emotional experiences in an age-diverse sample (N = 378, age range 14 – 86 years). Parameter estimations were carried out within a Bayesian framework. Our results provide partial support for predictions derived from SST. Consistent with SST, affect elicited by pleasant events was down-regulated less by older adults and affect elicited by unpleasant events was down-regulated more by older compared to younger adults. Inconsistent with SST, anchoring showed a negative age-related trend, indicating a more positive general affect disposition in younger, not older adults. Reactions to pleasant events showed no age-related differences. Reactivity to unpleasant events was highest in midlife and lower for younger and older adults. We discuss the broader implications of our approach for understanding emotional development across the lifespan.

Abstract citation ID: igad104.0981

### DAILY SUBJECTIVE NEARNESS TO DEATH AND AFFECTIVE DYNAMICS

Shevaun Neupert, and Lyndsey Graham, *North Carolina State University, Raleigh, North Carolina, United States*

A core tenet of Socioemotional Selectivity Theory is that perception of time remaining in life plays a key role in the selection and pursuit of emotion regulation goals. The current study examined this idea within a daily diary framework, where subjective perceptions of nearness to death were reported on consecutive days. Age differences in the within-person coupling between daily subjective reports of nearness to death and daily affective dynamics were tested. A 14-day daily diary study of 440 adults (M age = 65, range 50-85) in the U.S. was conducted where participants reported on their subjective nearness to death, positive affect, and negative affect each day. Multilevel models were conducted separately for positive and negative affect. Being older and feeling farther away from death on average were each associated with higher levels of positive affect. Daily negative affect was predicted by both within-person and between-person constructs. On days when people reported feeling subjectively closer to death than their own average, they also experienced an increase in negative affect. A significant interaction revealed that this effect was particularly strong among older adults (aged 65+). These results suggest that daily manifestations of perceptions of limited time remaining in life are salient for daily affective dynamics, and that this salience is particularly pronounced among older adults relative to those in midlife.

Abstract citation ID: igad104.0982

### ASSOCIATIONS BETWEEN COGNITIVE RESOURCES AND EMOTION REGULATION TACTICS IN AN ADULT LIFESPAN SAMPLE

Hannah Wolfe, Marissa DiGirolamo, and Derek Isaacowitz, *Northeastern University, Boston, Massachusetts, United Sta tes*

The current study investigated how trait-level cognitive capacity relates to emotion regulation tactic preferences in everyday life in adulthood and old age. 51 younger adults (ages 18-39), 53 middle-aged adults (ages 40-59), and 55 older adults (ages 60+) completed measures of working memory and verbal fluency, as well as 21 days of experience sampling. On each survey, participants indicated if they had regulated since the last survey and if so, what emotion regulation strategies they used and how they implemented that strategy through specific emotion regulation tactics. Each strategy laid out by the process model (situation selection, situation modification, attentional deployment, reappraisal, response modulation) could be implemented in three specific ways (called "tactics"): positivity-upregulating, negativity-downregulating, or negativity-upregulating. For example, a person may indicate they used situation selection and then would be asked whether they chose to (a) seek out a positive situation, (b) leave a negative situation, and/or (c) enter a negative situation. Acceptance was also included as a fourth tactic type but was not categorized under any strategy. Proportions of tactics used in each instance was calculated. Acceptance use was significantly negatively correlated with working memory performance; however, this association appeared to be driven primarily by middle-aged adults. Negativity-downregulation was positively correlated with numerous cognitive capacity measures in middle-aged adults. In sum, individual differences in cognitive resources appear to play the strongest role in tactic preferences in midlife rather than old age.

Abstract citation ID: igad104.0983

### THE EFFECT OF AGE ON EMOTION REGULATION PATTERNS IN DAILY LIFE: FINDINGS FROM AN EXPERIENCE SAMPLING STUDY

Mai Mikkelsen, Mia O'Toole, Emma Elkjær, and Mimi Mehlsen, *Aarhus University, Aarhus C, Midtjylland, Denmark*

The aim of the present experience sampling study was to examine how age affects emotion regulation patterns and their impact on well-being in daily life. A sample of 406 adults (age range: 18-81, 62.8 % female) were prompted five times a day for seven days to rate their momentary emotions, emotion regulation strategy use, and emotion regulation strategy effectiveness. Based on these ratings, indicators of emotion regulation variability and differentiation were calculated. Well-being outcomes included daily positive and negative emotions as well as symptoms of anxiety and depression assessed at baseline. The findings revealed reduced emotion regulation variability with age and a negative association between emotion regulation variability and well-being. There was no effect of age on emotion regulation effectiveness or differentiation. Emotion regulation effectiveness was associated with more positive and less negative daily emotions, and this association was stronger for young adults than older adults. The findings may indicate that older adults rely on a smaller set of effective emotion regulation strategies that are adapted to their specific context and resources, leading to reduced variability in emotion regulation and ultimately greater well-being. Concerning emotion regulation effectiveness, the findings suggest that effectiveness is less important for well-being in daily life for older adults, possibly because well-being is determined by other factors (e.g., less frequent and more predictable stressors) in older age.

### SESSION 3200 (SYMPOSIUM)

Abstract citation ID: igad104.0984

### OPPORTUNITIES AND CHALLENGES IN LARGE QUALITATIVE DATASETS IN GERONTOLOGICAL RESEARCH

Chair: Angela Perone Co-Chair: Pamela Saunders

Qualitative research focuses on in-depth data to understand people, processes, and phenomena. It often involves smaller datasets that do not intend to produce generalizable results but instead illuminate nuances, counterexamples, or underrepresented voices. Qualitative data can produce theory and allow for in-depth analyses. Recent methodological developments have created opportunities for large mixed methods projects to produce voluminous amounts of qualitative data that can and sometimes are shared as secondary datasets. The growing use of big data has also produced new possibilities for large qualitative datasets. Additionally, longitudinal and comparative qualitative research can produce significant data. This symposium presents four studies that examine opportunities and challenges in large qualitative datasets in gerontology. The first article uses multiple qualitative datasets to examine how long-term care staff balance resident rights of safety and autonomy. The second article uses multiple qualitative methods in a larger mixed methods Friendship Study to examine meaningful connections among people living with dementia in long-term care. Through a multi-site community university partnership, the third study extracts qualitative data from over 500 patient records to examine experiences of terminally ill hospice patients from residential homes for the dying. The fourth study examines nearly ten years of qualitative data from over 20 communities to examine successful aging from an Alaska Native perspective. Altogether, these four studies illuminate diverse strategies for identifying qualitative data, using data software, managing data, addressing ethical dilemmas, and employing research teams for coding and analyzing large qualitative datasets in gerontological research. This is a Qualitative Research Interest Group Sponsored Symposium.

Abstract citation ID: igad104.0985

### RESIDENT RIGHTS: BALANCING SAFETY AND AUTONOMY IN LONG-TERM CARE

Angela Perone, *University of California, Berkeley, Berkeley, California, United States*

Long-term care regulations require staff to keep residents safe while also respecting their autonomy. Building on street-level bureaucracy theory (Lipsky, 2010), which focuses on how front-line workers use discretion to implement ambiguous policies, this study examines how staff at various levels (floor staff, mid-managers, top management) balance these rights. This study employs a multi-method qualitative extended comparative case study of three levels of staff in two long-term care facilities (independent, corporate structure). Data includes in-depth semi-structured staff interviews (n=80), participant observation (n=8 months), and review of facility policy documents (n=376) and legal data (i.e., laws and regulations). Autonomy and safety presented conflicting rights for staff in three situations: fall prevention, food intake/refusal, and medication management. Staff exercised discretion via interpersonal conversations, documentation, and organizational lenses/resources and used interprofessional team approaches across staff hierarchies, especially among floor staff and mid-managers. Even when upper-managers sought external guidance (e.g., attorneys/risk managers), they deferred to the discretion of floor staff and mid-managers to communicate and implement decisions. Team approaches across staff hierarchy were less common when conflicting rights arose in medication management. Differences in organizational structure became relevant only when conflicting rights regarding autonomy and safety arose for food intake. This presentation will discuss the study's strategies for employing coding teams and data software to analyze large datasets across diverse methodological approaches. It will also discuss ethical dilemmas with sharing large qualitative datasets as secondary data of populations deemed "vulnerable" by IRB.

Abstract citation ID: igad104.0986
### METHODOLOGICAL CONCERNS FROM A MIXED-METHODS STUDY OF FRIENDSHIP IN PERSONS WITH DEMENTIA LIVING IN LTC

Pamela Saunders[1], and Kate de Mederios[2], *1. Georgetown University, Washington, District of Columbia, United States*, *2. Miami University, Oxford, Ohio, United States*

This paper will examine the methodological issues that arose as part of the Friendship Study (de Mederios, Saunders, Doyle, Mosby, 2012). These issues range from tracking to integrating data as well as ethical considerations of multiple participants living with dementia and their legally authorized representatives. The Friendship Study investigated 'friendships' in people with dementia living in long-term care. We used a mixed-methods approach that emphasized qualitative methods over quantitative ones, with integration during the analysis stage. Specifically, we investigated friendships among 31 assisted living residents with moderate to advanced dementia. The Friendship Study was based on the growing literature on social interactions in dementia settings. 'Social interactions' describe communicating, verbally and non-verbally, at least once with another person while 'friendship' suggests a deeper, more meaningful connection that may include reciprocity, intimacy, and shared trust. From our qualitative analyses, we found that friendships among residents were characterized by voluntary participation and accommodation in conversation, and recognition of the uniqueness of the other. Further quantitative results revealed no correlation between memory test scores, or demographic characteristics (except gender) and friendship dyads identified by staff. Staff's perceptions of residents' friendships were not supported through observation. This paper will explore the variety of qualitative methods employed (e.g., ethnographic observation, semi-structured interviews, transcription, coding) and outline how these data were collected, managed, and synthesized. Best practices will be provided.

Abstract citation ID: igad104.0987
### COLLABORATIVE RESEARCH WITH A MIXED-METHOD DATASET USING DECEDENT DATA

Kelly Melekis[1], and Carol Weisse[2], *1. University of Vermont, Burlington, Vermont, United States*, *2. Union College, Schenectady, New York, United States*

This presentation will present opportunities and challenges in building and sharing a mixed method dataset of terminally ill hospice patients from residential homes for the dying (social model hospice). As nonprofit, community-based settings, residential homes for the dying document care decisions using paper files and handwritten notes, and files range in size based on length of stay (with some files being hundreds of pages in length). Through a multi-site community-university partnership, we have scanned over 500 patient records, extracted quantitative data, and de-identified data for qualitative analysis. We will share the challenges of data management and organization, extraction, and de-identification. We will also discuss ethical issues related to working with decedent data, community-engaged research, and multi-site collaboration. Based on our experiences with qualitative data analysis of resident files, we will share strategies for extracting qualitative data from decedent records, using qualitative data analysis software, and sharing data for collaborative research.

Abstract citation ID: igad104.0988
### THE CHALLENGES OF LARGE QUALITATIVE DATASETS AND MEETING THE EXPECTATIONS OF INDIGENOUS COMMUNITIES AND FUNDERS

Jordan Lewis, *University of Minnesota Medical School, Duluth campus, Duluth, Minnesota, United States*

Literature on Alaska Native Elders and how they subjectively define a successful older age is limited. The lack of a culturally specific definition often results in the use of a generic definition that portrays Alaska Native Elders as aging less successfully than their White or non-Native counterparts. However, there is very little understanding of the diverse array of successful aging experiences and how this data will translate to other racial and ethnic minority populations, as well as advance the field of successful aging. This decade long study research the concept of successful aging from an Alaska Native perspective, or what it means to age well in Alaska Native communities and the lessons Elders. Qualitative, in-depth, interviews have been conducted with 154 Elders representing 20 participating communities to explore the concept of successful aging and the role of their community in the aging process. This study highlights the four (6) elements of successful aging, or "Eldership:" emotional wellbeing, spirituality, community engagement, Native ways of life, generativity, and physical health. These elements serve as the foundation of how communities define who is an Elder and what is important when considering who has aged successfully or not. This presentation will discuss challenges of analyzing large datasets to inform future studies, the development of a theory and measure of successful aging, as well as how to balance the needs of Indigenous community partners and the funders.

### SESSION 3205 (PAPER)

### IMPROVING ASSESSMENT, EDUCATION, AND TRAINING

Abstract citation ID: igad104.0989
### DEVELOPMENT AND TESTING OF THE PC-MET (PERSON-CENTERED MEASURE EVALUATION TOOL)

Sheryl Zimmerman[1], Benjamin Mast[2], Cynthia Port[3], Kimberly VanHaitsma[4], and Sam Fazio[5], *1. University of North Carolina at Chapel Hill, Chapel Hill, North Carolina, United States*, *2. University of Louisville, Louisville, Kentucky, United States*, *3. Self-employed, Bloomington, Indiana, United States*, *4. Penn State University, State College, Pennsylvania, United States*, *5. Alzheimer's Association, Chicago, Illinois, United States*

Despite the importance of person-centered measurement -- and that numerous research instruments are now available to measure person-centeredness -- there are no tools to evaluate the extent to which existing research instruments themselves are developed consistent with principles of person-centeredness. The PC-MET (Person-Centered Measure Evaluation Tool) was developed to fill that gap. Based on literature and theory, the PC-MET assesses the process of instrument development and the content of the instrument itself based on six overarching criteria of person-centeredness: co-creation, accommodation, incorporation, biopsychosocial-cultural components, pragmatism, and systemic focus. The inter-rater reliability of the PC-MET was evaluated based on instruments recommended for use in relation to the Alzheimer's Association 2018 Dementia Care Practice Recommendations (DCPR). Independent raters evaluated two measures within each DCPR area using the PC-MET. Agreement was excellent (kappas>0.81) across all PC-MET criteria with the exception of the criteria assessing biopsychosocial-cultural components (where agreement was moderate; kappa=0.42) and pragmatism (where agreement was substantial; kappa=0.68). Raters provided feedback on the PC-MET and agreed or strongly agreed that the tool is easy to understand and score; the six PC-MET criteria are relevant and complete; and the PC-MET could improve the person-centeredness of dementia measurement instruments. The PC-MET has broad potential use, including to critique the extent to which existing instruments are person-centered, to promote efforts to make existing measures more person-centered, and to inform the development of new person-centered measures. This work was supported by the NIA through a grant to the Alzheimer's Association and by the LINC-AD Research Steering Committee.

Abstract citation ID: igad104.0990

### EXPLORING THE POTENTIAL OF CHATGPT FOR ASSESSING DELIRIUM SYMPTOMS IN OLDER ADULTS WITH COGNITIVE IMPAIRMENT

Yong Kyung Choi[1], and Shih-Yin Lin[2], *1. University of Pittsburgh, Pittsburgh, Pennsylvania, United States*, *2. NYU Rory Meyers College of Nursing, New York City, New York, United States*

Delirium is a common and serious geriatric syndrome, especially in older adults with cognitive impairment. Accurate caregiver assessment and reporting of delirium in community-dwelling older adults can help healthcare providers diagnose and treat delirium more effectively. Yet, caregivers often lack confidence in their ability to accurately assess and report delirium. An automated method to detect the presence and symptoms of delirium could potentially boost caregiver confidence and assist with more accurate delirium assessment and reporting. This study explored the use of ChatGPT, a generative AI based on the Large Language Model (LLM), to detect the presence and symptoms of delirium. We trained ChatGPT using a series of prompts specifically designed to enhance its understanding of the domain knowledge and the scoring mechanism of a caregiver-centered, seven-item delirium assessment, the Sour Seven. The trained ChatGPT model was asked to identify and score various delirium symptoms in five previously validated geriatric case vignettes using the Sour Seven scale. Each case was repeated three times to test for reliability. The ChatGPT assessment results were compared against those of human experts. The preliminary analysis showed that items 3, 6 and 7 were scored correctly in all cases, but mixed results were found on items 1, 2, 4, and 5, mostly because the AI applied a single symptom statement to multiple items. Overall, the results indicate that generative AI has the potential to accurately capture and represent complex delirium symptoms in natural language, which could assist caregivers in accurately identifying and reporting delirium symptoms.

Abstract citation ID: igad104.0991

### FAMILY QUIZZING BEHAVIOR DURING IN-HOME DEMENTIA CARE: A SEQUENTIAL BEHAVIORAL ANALYSIS WITH VIDEO CASE STUDIES

Carissa Coleman[1], Paige Wilson[2], and Kristine Williams[3], *1. University of Kansas, Kansas City, Kansas, United States*, *2. The University of Kansas, West Palm Beach, Florida, United States*, *3. University of Kansas Medical Center, Kansas City, Kansas, United States*

Quizzing is an understudied communication behavior used by care partners during in-home dementia care interactions. The aim of this study was to characterize and describe the behavior, identify types, characterize care situations, and determine how people living with dementia react to quizzing. Forty video observations of in-home interactions were coded to determine relationships between care partner quizzing and the person with dementia's reactions and analyzed using sequential behavioral analysis. Ten case studies with high instances of quizzing were examined for interpersonal context. Quizzing was defined as a series of rapid questions by the care partner that most often appeared to test the memory or knowledge of the person with dementia. Instances of quizzing that included long-term and short-term memory checks were most likely to elicit a negative reaction from the person with dementia (resistiveness, distress, and apathy). Long-term memory check always preceded resistiveness and distress (CP=1.0) and apathy preceded long-term memory check short-term memory check (CP = 0.71, 0.21). Quizzing used for distraction or reminiscing appeared to elicit positive responses from the person with dementia. Distraction and mutual reminiscing always followed cooperation (CP=1.0). Case study results indicated the care partners seemed to be testing the abilities of the person with dementia; possibly to alleviate or reverse dementia symptoms. Additional research is warranted to understand the use of quizzing during in-home care. The use of behavioral coding, sequential analysis, and case study examination can provide evidence of communication best practices to develop communication training for dementia care dyads.

Abstract citation ID: igad104.0992

### MENTAL HEALTH FIRST AID TRAINING: EVIDENCE OF ITS EFFECTIVENESS FOR MENTAL HEALTH PROMOTION AMONG OLDER ADULTS

Iftekhar Amin, *University of North Texas at Dallas, Dallas, Texas, United States*

**Background:** About 15% of adults aged 60 and over have a mental health disorder. However, stigma and lack of knowledge of mental health resources act as barriers for them to seek professional help. Mental Health First Aid (MHFA) is an evidence-based program that teaches participants how to identify, understand and respond to signs of mental illnesses and substance use disorders. The Older Adults version of this training focuses on the unique experiences and needs of older adults. This study evaluated the effectiveness of a Substance Abuse and Mental Health Services Administration (SAMHSA) funded MHFA training program among older adults. Method: Data were collected from MHFA training sessions delivered to 27 staff and 36 residents at two assisted living facilities from January to March 2023. A

quasi-experimental, pre-post design was used. Two outcomes mental health literacy and stigma were measured at the pretest, posttest, and 4 months after the the training. A paired sample t-test was performed to test the differences between the pre- and post-test.

**Results:** Statistically significant improvements in self-confidence in promoting help-seeking and reduction in stigmatizing attitudes were observed among participants in post-tests and 4-month tests. Participants reported increased knowledge in how to provide initial support to someone who may be mental health crisis and help connect them to the appropriate care.

**Conclusion:** Results indicate MHFA is likely to improve mental health awareness and reduce stigma among older adults. This study adds new evidence to the literature on the MHFA program's effectiveness among older adults and their caregivers.

### SESSION 3210 (PAPER)

### MINORITY AND DIVERSE POPULATIONS (PAPERS) I

Abstract citation ID: igad104.0993

### A POINT OF NO RETURN: A QUALITATIVE ANALYSIS ON RETURN MIGRATION AND AGING OF JAPANESE INDIVIDUALS LIVING IN NEW YORK

Itsuko Toyama[1], and Taeko Nakashima[2], *1. St. Andrew's University, Osaka, Kyoto, Japan*, *2. Nihon Fukushi University, Mihama, Aichi, Japan*

Our previous quantitative research studies have revealed that many older Japanese individuals living in overseas communities maintain hope of receiving culture-oriented care or harbor aspirations of returning to Japan to live out their final days. In this study, we analyzed the qualitative data of a free-answer question on anxieties about aging from a quantitative research study on older Japanese individuals living in Greater New York in 2018. We aimed to clarify the qualitative characteristics of anxieties among three groups: those who had decided to stay in the United States ("abiding"), those who had decided to return to Japan ("returning"), and those who had not yet decided ("undecided"). In the quantitative study, 2,057 questionnaires were distributed, with an overall return rate of 29.7%. The respondents were divided into three groups based on their answers regarding their intentions for the location of their final residence: the United States, Japan, or undecided. We performed the KJ method (affinity diagram) to visualize the differences and commonalities of their anxieties. The following is a summary of the findings: (1) Although most of the respondents are economically advantaged and healthy, "ambiguous" anxieties about aging in New York arise in all three groups; (2) All three groups express uneasiness about supporting themselves after paying medical and nursing care costs; (3) Those in the "abiding" group express loneliness due to the societal relationship vulnerability in New York; (4) Those in the "returning" group worry about the additional burden of undergoing legal procedures and re-adjusting to the current Japanese society.

Abstract citation ID: igad104.0994

### NIMHD INVESTMENTS IN RESEARCH ON OLDER ADULTS EXPERIENCING HEALTH DISPARITIES: 2018–2022

Aaron Ogletree[1], Michelle Doose[2], and Yewande Oladeinde[2], *1. National Institutes of Health, Bethesda, MAryland, United States*, *2. National Institutes of Health, Bethesda, Maryland, United States*

The National Institute on Minority Health and Health Disparities (NIMHD) is the nation's leading institute on the health of populations experiencing health disparities. These populations include individuals with low socioeconomic status (SES), those living in rural areas, sexual and gender minorities (SGM), and racial and ethnic minorities. Despite the need to advance research on these populations, particularly in old age when health disparities may be most salient, there has been no analysis of NIMHD funding on older adults to date. This portfolio analysis synthesized and described NIMHD's investments in research on older adults using data from fiscal years 2018 through 2022. The Research, Condition, and Disease Categorization system was used to identify funded projects in the area of Aging; manual review confirmed project eligibility. In-depth project characteristics were extracted and topic areas evaluated. Findings demonstrate that NIMHD funded a total of 98 unique research projects focused on older adults. Of these, 47% focused on the etiology of health disparities, 38% on interventions, and 15% on methods and measurement. The most specified population was racial and ethnic minorities (91%), followed by individuals with low SES (21%), rural older adults (11%), and SGM older adults (8%). Projects focused on diagnostic or clinical care (16%), care coordination (6%), caregivers (6%), and shared decision-making (4%) were limited. Findings highlight opportunities for future research to advance health care and reduce health disparities for the growing proportion of older adults from populations experiencing health disparities.

Abstract citation ID: igad104.0995

### RACE/ETHNICITY DISPARITY FOR PEOPLE WITH LTSS NEEDS IN CALIFORNIA: DISABILITY, FINANCE, AND HEALTH AND WELL-BEING

Lei Chen, *UCSF Philip R. Lee Institute for Health Policy Study, San Francisco, California, United States*

Older adults and people with disabilities have high needs for Long-Term Services and Supports (LTSS). Disability status, financial difficulties, and health and well-being are likely not uniformly experienced by all people with LTSS needs. Subgroups of older adults and people with disabilities, such as racial/ethnic minorities, may be at significant risk of experiencing financial difficulties and stress about making ends meet. This study used the first cycle of data (2019-2020) from the California Long-Term Services and Supports (CA-LTSS) survey, merged with select data from the California Health Interview Survey (CHIS) (N = 2,030). Multivariate Regressions and Conditional Process Analysis (CPA) were applied to test the hypothesized relationships. Findings show that black/African American and Asian participants with LTSS needs were more likely to have cognitive impairments. Black/African American participants with LTSS needs were more likely to report difficulties in activities of daily living. Racial/ethnic minorities with LTSS needs experienced diverse and more financial difficulties on average than their white counterparts. Asian participants with LTSS needs reported worse psychological distress than other racial/ethnic groups. American Indians and Alaska Natives with cognitive impairments were more likely to have more financial difficulties, and those with difficulty in instrumental activities of daily living were more likely to report worse self-rated health. This study moves beyond the typical white/black disparities analyses and focuses on diverse racial/ethnic groups. We need to address the financial needs of various racial/ethnic groups of people with LTSS needs and target policies and service programs to meet their mental health needs.

Abstract citation ID: igad104.0996

**RACIAL AND ETHNIC DISPARITIES IN NEIGHBORHOOD SOCIOECONOMIC CHARACTERISTICS ASSOCIATIONS WITH VISCERAL FAT**

Shawna Follis[1], and Jeremey Shropshire[2], *1. Stanford University, Stanford, California, United States*, *2. Morehouse University, Atlanta, Georgia, United States*

Visceral adipose tissue (VAT) has a more significant role in the pathophysiology of cardiometabolic diseases than subcutaneous adipose tissue (SAT). Our previous findings suggest VAT represents an important pathway through which racial disparities in the social environment are biologically embodied. To understand the role of structural racism in this pathway, this paper investigated racial disparities in neighborhood socioeconomic status (NSES) and VAT. We hypothesized that NSES is inversely associated with VAT and VAT/SAT ratio over 6 years and associations differ by race and ethnicity. Abdominal VAT and SAT area (cm2) were measured at years 0, 3, and 6 using dual-energy X-ray absorptiometry in a subsample (n=11,020) of the Women's Health Initiative (age 50-79). The NSES Z-scores were obtained from the US Census and American Community Survey based on participant's census tract residence. We evaluate racial and ethnic disparities in the exposure and outcome using ANOVA, and associations between NSES with VAT using mixed effects models. We found that NSES and VAT area differed by race and ethnicity. American Indian and Alaska Native (AIAN), Black, and Latina groups were significantly (P< 0.01) more likely to live in neighborhoods with NSES Z-scores below the population mean (Z-score=0) compared with White and Asian races. AIAN, Latina, and Black women had the highest VAT (220 cm2, 185 cm2, 177 cm2, respectively). Findings support the hypothesis that associations between NSES and VAT differ by race and ethnicity. Results from mixed effects models are expected to provide longitudinal evidence that structural determinants are associated with VAT.

Abstract citation ID: igad104.0997

**RACIAL AND NATIVITY DIFFERENCES IN ADVERSITY PROFILES AMONG MIDDLE-AGED AND OLDER ADULTS**

Man Guo, Yi Wang, and Kara Carter, *University of Iowa, Iowa City, Iowa, United States*

Cumulative adversity theory explains how distribution of resources and stress accumulate over time, resulting in divergent later life outcomes. Focusing on the nexus of race and nativity, this study examined profiles of adversity (i.e., the overall typological patterns of adversity) and their mental health implications in four groups of middle-aged and older adults: native-born whites, native-born ethnic minorities, foreign-born whites, and foreign-born ethnic minorities. Data were from the 2018 psychosocial assessment of the HRS (N = 5,534). Latent class analysis (LCA) with multigroup analyses were employed to identify typological patterns of ten adversity indicators and to compare the latent structures and class prevalence across the race/nativity groups. Regressions were used to examine the associations between adversity profiles and three outcomes: depression, pessimism, and life satisfaction. adversity profiles emerged: low adversity (60.05%), low human capital (15.69%), socially marginalized (13.67%), and neighborhood adversity (10.59%). These adversity profiles were structurally the same for the four race/nativity groups, but the class prevalence varied across the groups. Overall, having low adversity profile was associated with the best mental health outcomes. However, the mental health implications of each adversity profile varied by outcomes and by race and/or nativity. Findings highlight the importance of examining heterogeneity in adversity and its mental health implications among disadvantaged populations and the need of developing tailored programs to address unique needs of different minority populations.

### SESSION 3215 (BIOLOGICAL SCIENCES INVITED SYMPOSIUM)

Abstract citation ID: igad104.0998

**REPRODUCTIVE AGING**

Chair: Yousin Suh

Reproductive aging is a major health, personal and societal issue, but ovarian aging has received limited scientific attention. Ovarian aging has been known to influence diverse health outcomes in women including lifespan, cardiovascular disease, metabolic syndromes, neurodegenerative disorders and various types of cancer. Yet, the mechanisms behind this broad implication remain elusive. This session will explore recent insights into the molecular, cellular and genetic basis of ovarian aging and new intervention targets to maintain reproductive health and later health outcomes in our aging female population.

Target audience:

This inter-disciplinary session is designed to educate basic scientists, physician-scientists, clinicians, and a general interest audience of providers or industry representatives interested in understanding the mechanisms of ovarian aging and its sequalae.

Abstract citation ID: igad104.999

**OVARIAN AGING: A TARGET FOR GEROPROTECTION IN WOMEN**

Yousin Suh, *Columbia University, New York City, New York, United States*

The ovary is the first organ to undergo early-onset aging in the human body, affecting both fertility and overall health in women. However, the biological mechanisms underlying human ovarian aging have been poorly understood. To understand the molecular, cellular, and genetic basis of ovarian aging in humans, we performed integrative single-nuclei multi-omics analyses of young and reproductively aged human ovaries. Our analysis reveals coordinated changes in the regulatory landscapes across all cell types in the ovary during aging, characterized by transcriptomic and chromatin accessibility signatures of the canonical "Hallmarks of Ageing". By performing integrative analyses of our single-nuclei multi-omics data with genetic variants associated with age at natural menopause from genome-wide association studies (GWAS), we demonstrate a global impact of functional genetic variants on changes in gene regulatory networks across ovarian cell types. Our work provides a comprehensive multimodal landscape of human ovarian aging and mechanistic insights into inherited variation influencing the timing of menopause. Our results raise the hope that geroprotectors targeting the "Hallmarks of Aging" may be used to delay ovarian aging, thereby promoting reproductive health and extending healthspan and longevity in women.

Abstract citation ID: igad104.1000

### GENOMIC ARCHITECTURE OF FEMALE REPRODUCTIVE AGING

Anna Murray, *University of Exeter, Exeter, England, United Kingdom*

Female reproductive lifespan is characterised by declining ovarian reserve, but the underlying mechanism is poorly understood. Reproductive ageing is a multifactorial trait with both genetic and environmental modifiers. Our research uses genome-wide analyses to identify the genes and biological processes that govern female reproductive ageing. We have identified 290 genetic loci with relatively common variants (>0.1%) that are associated with timing of natural menopause, in a meta-analysis of >200,000 women in the ReproGen consortium. In combination these common variants explain around 12% of the variation in menopause timing. Women in the top 1% combined genetic risk centile for earlier menopause are approximately 5x more likely to have menopause before 40 years. A high genetic risk for earlier menopause in mothers, but not fathers, is also associated with increased de novo mutation rate in offspring. We assessed the role of rare (MAF<0.1%) protein-coding variants on ovarian function in data from 104,733 females in the UK Biobank using gene burden association tests. We identified nine genes with associations that passed our multiple testing threshold. Protein-truncating variants in ZNF518A were associated with earlier menopause (effect size 5.61 years, P=1.2x10-10), with an effect larger than for any previously reported gene or variant. Deleterious variants in SAMHD1 were associated with >1 year later menopause and were also a germline risk factor for cancer predisposition in males and females. Understanding the genetics of reproductive ageing is not only important for fertility, but also has implications for broader age-related health outcomes, such as cancer, diabetes and osteoporosis.

### SESSION 3220 (PAPER)

### SOCIAL DETERMINANTS OF HEALTH AND AGING II

Abstract citation ID: igad104.1001

### ASSOCIATIONS BETWEEN REVERSED CHILDHOOD EXPERIENCE AND LATE-LIFE COGNITIVE IMPAIRMENT: THE CASE IN CHINA

Yalu Zhang, *Stony Brook University School of Social Welfare, Stony Brook, New York, United States*

Exposure to violence, parental incarceration, household substance abuse and some other reversed childhood experience is associated with some negative impacts in one's late life in some countries, such as difficulties in maintaining interpersonal relationships, being more likely to experience depressive symptoms, and performing certain physical functions. However, the impacts of reversed childhood experiences, such as starvation, unmet needs of healthcare services, and loss of parents, are still unknown. Using nationally representative data, China. Longitudinal Aging Social Survey, and the hierarchical cluster-based partial least squares regression, this study found a notably high prevalence of reversed childhood experience regarding childhood hunger and unmet healthcare needs. In China, 65% of the older adults aged 60 and above had experienced falling asleep with hunger, 83.1% think that their healthcare needs were not met in childhood, and 10.26% lost one or both parents before 10 years old. The regression analysis results show that compared to those who did not have experience falling asleep with hunger, those who had such experience were more likely to have a declined cognitive function by 33.8% (p< 0.001). Additionally, the loss of parents also significantly caused poorer cognitive function by 44.2% (p< 0.001). However, such a causality relationship was not detected among those who experienced unmet healthcare needs. These findings suggest that early prevention and interventions are needed among older adults with adverse childhood experience.

Abstract citation ID: igad104.1002

### DIFFERENCES IN MEDICARE RESOURCE USE BY ENGLISH PROFICIENCY

Lilly Estenson[1], Eric Roberts[2], and Mireille Jacobson[1], *1. University of Southern California, Los Angeles, California, United States*, *2. University of Pennsylvania, Perelman School of Medicine, Philadelphia, Pennsylvania, United States*

Medicare is complicated and coverage decisions can be complex. Accordingly, the Centers for Medicare & Medicaid Services (CMS) provides a hotline, handbook, and website to help Medicare beneficiaries understand their benefits and coverage options. In this study, we used 2016-2018 Medicare Current Beneficiary Survey data and linear probability models to assess whether the likelihood of CMS Medicare resource use differed between beneficiaries with English proficiency and those with limited English proficiency (LEP). Our primary outcome was an indicator of having used at least one CMS resource. We considered English proficiency in both speaking and reading. Limited English-speaking proficiency or, separately, limited English-reading proficiency signified that the beneficiary reported speaking or reading English

"not well" or "not at all." Among a nationally representative sample of noninstitutionalized Medicare beneficiaries, 5.7% had limited English-speaking proficiency, 8.7% had limited English-reading proficiency, and 67.0% had used a CMS resource. Adjusting for health and social factors, age, language spoken at home, county-level Medicare Advantage characteristics, and state and year fixed effects, beneficiaries with limited English-reading proficiency were 16.7 percentage points less likely to have used a CMS resource compared to beneficiaries who reported reading English "well" or "very well" ($p < .001$). Limited English-speaking proficiency was not associated with CMS resource use. These findings have implications for federal initiatives to improve language access in Medicare, particularly efforts to reduce literacy barriers. Differences in Medicare coverage choice and benefits utilization among beneficiaries with LEP may be partially explained by differential access to or engagement with written CMS Medicare information.

Abstract citation ID: igad104.1003

### EXTREME HEAT VULNERABILITY AMONG OLDER ADULTS: A MULTILEVEL RISK INDEX FOR PORTLAND, OREGON

Jacklyn Kohon[1], Katsuya Tanaka[2], Dani Himes[3], Eiji Toda[1], Paula Carder[4], and Bryant Carlson[1], *1. Portland State University, Portland, Oregon, United States*, *2. Shiga University, Hikone, Shiga, Japan*, *3. Institute on Aging, Portland, Oregon, United States*, *4. Portland State University Institute on Aging, Portland, Oregon, United States*

Extreme heat is an environmental health equity concern disproportionately impacting low- income older adults and people of color. Exposure factors, such as living in rental housing and lack of air conditioning, and sensitivity factors, such as chronic disease and social isolation, increase mortality risk among older adults. Older persons face multiple barriers to adaptive heat mitigation, particularly for those living in historically temperate climates. This study measures two heat vulnerability indices to identify areas and individuals most vulnerable to extreme heat and discusses opportunities to mitigate vulnerability among older adults. We constructed two heat vulnerability indices for the Portland, Oregon metropolitan area: one using area scale proxy measures extracted from existing regional data and another at the individual scale using survey data collected following the 2021 Pacific Northwest Heat Dome event. These indices were analyzed using principal component analysis (PCA) and Geographic Information Systems (GIS). Results indicate that the spatial distribution of areas and individuals vulnerable to extreme heat are quite different. The only area found among the most vulnerable on both indices has the largest agglomeration of age- and income-restricted rental housing in the metropolitan area. Due to spatial variations in heat-related risk at the individual and area scales, measures addressing heat risk should not be spatially uniform. By focusing resources on older adult individuals and areas in particular need of assistance, heat risk management policies can be both highly efficient and cost-effective.

Abstract citation ID: igad104.1004

### OLDER ADULT FALLS IN THE COMMUNITY: DOES UNSAFE HOME ENVIRONMENT HAVE A RISK ROLE?

Debasree Das Gupta[1], Uma Kelekar[2], and Sidney Turner[3], *1. Utah State University, Logan, Utah, United States*, *2. Marymount University, Arlington, Virginia, United States*, *3. Fors Marsh Group LLC, Falls Church, Virginia, United Sta tes*

Hazardous environmental conditions are frequent in older adult homes and are linked to a majority of the older adult falls in the community. However it is unclear whether it is the presence/absence of home hazards per se, or the interaction between unsafe home conditions and physical functioning that is of salience to older adults' fall risk. To address this gap, we conduct a mediation analysis using the Karlson–Holm–Breen methodology to estimate: 1) whether home disorder (peeling/flaking paint, pests: cockroaches/rodents, broken furniture/lamp, unsafe flooring/tripping hazards, room clutter) and unsafe bathroom features (absence of bathtub/shower stall, grab bar, seat in shower/tub, raised toilet) adversely associated with subsequent fall risk of older adults, and 2) what proportion of those influences were mediated through limitations in daily and instrumental activities of daily living (ADL/IADL). Using a nationally representative large sample of Medicare beneficiaries (≥65 years) living in the community who were respondents in the 2018-2019 National Health and Aging Trends Study (NHATS; n=2,599), we examined baseline home environment in 2018 and its relation with subsequent falls in 2019, after controlling for covariates. Results indicate while home disorder had both a direct (adjusted Odds Ratio [aOR]: 1.11, 95% Confidence Interval [CI]:1.01,1.22) and an indirect (aOR: 1.01; 95% CI: 1.00,1.02) effect on subsequent falls through limitations in ADL and IADL, unsafe bathroom features did not significantly predict subsequent fall risk of older adults. Given these findings, addressing home disorder through policy/housing assistance initiatives and educational programs highlighting home environmental safety would be essential.

Abstract citation ID: igad104.1005

### PARADOXICAL FINDINGS AND OTHER SURPRISES IN PROVIDING STREET MEDICINE TO UNSHELTERED HOMELESS OLDER ADULTS

Alexis Coulourides Kogan[1], Anuva Mittal[2], Enya Lowe[3], and Corinne Feldman[3], *1. University of Southern California, Los Angeles, California, United States*, *2. Keck School of Medicine of USC, Los Angeles, California, United States*, *3. University of Southern California, Keck School of Medicine of USC, Los Angeles, California, United States*

Older adults constitute the fastest-growing segment of the homeless population in the US. Previous research has found older homeless adults to have more chronic conditions, greater odds of physical disability, and experience accelerated aging compared to housed populations. Little is known about the role of street medicine in providing care and support to aged and aging patients experiencing unsheltered homelessness. The purpose of this study was to elicit the perspective of clinicians and unsheltered homeless patients on aging and managing serious illness. We conducted interviews with patients receiving street medicine and their clinicians. Interviews were guided by a semi-structured protocol, audio recorded, and transcribed verbatim. Data were analyzed following a thematic analysis approach. Eight clinicians and eight patients were interviewed. On average, clinicians were 41 years old, white (50%), and female (50%) with 1-16yrs street medicine experience. Patients were commonly male (63%), had 3+ chronic conditions (100%), and aged 56 years. Thematic analysis revealed four major paradoxical themes around: End-of-life, shelter, durable medical equipment, and community. The clinician and patient perspective on aging and managing serious illness while living outside offer insight into the significant and paradoxical challenges each face when balancing restrictions from the healthcare system, social services, and patient/self needs. Results highlight constraints placed on clinicians and patients by the rigid healthcare system and its incompatibility with the unique circumstances of unsheltered older adults. Findings from this study suggest actionable strategies that hold implications for policy and practice to better meet the needs of unsheltered homeless older adults.

### SESSION 3225 (SYMPOSIUM)

Abstract citation ID: igad104.1006

### BIOLOGICAL AND SOCIAL DETERMINANTS OF LATE-LIFE DISABILITY AND ITS IMPLICATIONS FOR AGING WORKFORCE

Chair: Yuan Zhang Discussant: Kate Duchowny

As the population continues to age, the number of people living with disability is increasing. Disability prevention is crucial for independent living and quality of life for older adults. Identifying biological, individual, and social factors that can delay the disablement process has significant policy and public health implications. This symposium comprises four papers examining critical determinants of old-age disability and the implications for the aging workforce. These papers use data from nationally representative biosocial surveys and cover a range of biological and social factors, including infection, immune aging, gender, social relationships, and work environment. First, Duchowny and Noppert will present findings on the association between advanced immune aging and physical disability in men and women. Their results suggest that immunosenescence may be a critical factor underlying gender differences in disability. Second, He will present her work on estimating the fraction of disability attributable to Cytomegalovirus (CMV) infection, highlighting the potential benefits of a vaccine for CMV in preventing disability. Third, Zhang will present results from a longitudinal study examining the pathways among various aspects of social relationships, physical limitations, and disability risk. Finally, Abrams will present educational disparities in health-induced work limitations and the importance of work characteristics in explaining such disparities. Together, the four studies presented in this symposium will enhance our understanding of determinants of the disablement process and provide valuable insights that can inform policies and interventions aimed at reducing disability and maintaining the productivity and well-being of older adults.

Abstract citation ID: igad104.1007

### CELLULAR IMMUNE AGING AND PHYSICAL DISABILITY: RESULTS FROM THE HEALTH AND RETIREMENT STUDY

Grace Noppert, and Kate Duchowny, *University of Michigan, Ann Arbor, Michigan, United States*

Given long-standing inequities in physical disability, we leveraged data from the nationally-representative Health and Retirement Study to investigate gender differences in the association between advanced immune aging and physical disability. Among women, we found a one standard deviation increase in the CD8+:CD4+ ratio, a marker of advanced immune aging, was associated with a 24% greater prevalence of ADL disability (PR= 1.24, 95% CI: 1.23, 1.25). No association was observed among men. Results suggest that among women, advanced immune aging may serve as a salient risk factor in shaping physical disability.

Abstract citation ID: igad104.1008

### ESTIMATING THE EFFECTS OF A POTENTIAL VACCINE FOR CYTOMEGALOVIRUS ON DISABILITIES AMONG OLDER ADULTS IN THE US

Yuelin He[1], Jessica Faul[2], Kate Duchowny[2], Chihua Li[1], Rebecca Stebbins[3], and Grace Noppert[2], *1. University of Michigan Institute for Social Research, Ann Arbor, Michigan, United States*, *2. University of Michigan, Ann Arbor, Michigan, United States*, *3. King's College London, London, England, United Kingdom*

Cytomegalovirus (CMV) infection has been identified as contributing to the development several aging-related diseases, such as disabilities. However, the development of CMV vaccines has been slow, particularly of those targeting older individuals. With few studies that have investigated the potential effects of a CMV vaccine on disabilities among older adults, we aim to study such effects on mobility disabilities by tasks. We use longitudinal data from the Health and Retirement Study (HRS) from 2016 to 2020 to quantify what proportion of disabilities could be prevented in the United States with a viable CMV vaccine. The analytic sample includes N=5,855 individuals aged 65 or above. Under the assumption that a hypothetical vaccine would shift CMV levels from seropositive to seronegative, we construct a binary indicator based on the continuous IgG antibody measure of serum CMV. Using a novel logistic regression-based approach that allows adjustment for continuous age, we estimate attributable fractions (AF) to seropositive CMV levels for five dichotomous outcomes about difficulty with activities of daily living (ADLs), including walking across a room, dressing, bathing, eating, getting in and out of bed, and using the toilet, and stratify the analyses by sex and race/ethnicity. The findings will implicate the potential impact of a vaccine for CMV on mobility disability in older adults, laying the groundwork for future studies to examine the impact of vaccines on disabilities at different time points with varying efficacy levels among the elderly.

Abstract citation ID: igad104.1009

### MULTIFACETED LINKS BETWEEN SOCIAL RELATIONSHIPS AND THE DISABLEMENT PROCESS IN LATER LIFE

Yuan Zhang, *Columbia University, New York City, New York, United States*

While a negative association between social relationships and physical limitations or disability has been reported persistently, the possible reciprocal relationships and pathways through which social relationships affect physical function and disability remain to be explored. This study used longitudinal data from the US Health and Retirement Study (HRS) and the National Social Life, Health, and Aging Project (NSHAP) to examine the multifaceted links between different dimensions of social relationships and physical limitations/disability among older adults in the US. We used Structural Equation Models to explore pathways between social relationships and physical limitations in the HRS and longitudinal residual change models to examine the effects of social relationships on changes in physical limitations between two waves in NSHAP. Results show that for men, greater social integration may lower subsequent physical limitations, whereas, for women, social support may prevent declines in physical function. For both genders, greater physical limitations and disability significantly lower subsequent social integration. This work tested the potential causal benefits of the social relationship hypothesis and selection processes and incorporated different aspects of social relationships. As such, our findings advance the explanations of the association between social relationships and later-life health and help better understand the determinants of the disablement process.

Abstract citation ID: igad104.1010

### QUANTIFYING CONTRIBUTIONS OF WORK CHARACTERISTICS TO EDUCATIONAL DISPARITIES IN HEALTH-INDUCED WORK LIMITATIONS

Leah Abrams[1], and Lisa Berkman[2], *1. Tufts University, Medford, Massachusetts, United States*, *2. Harvard Center for Population and Development Studies, Harvard T.H. Chan School of Public Health, Cambridge, Massachusetts, United States*

While public policies are incentivizing longer working lives, poor health and inhospitable working conditions pose challenges to working longer, especially for Americans with low educational attainment. This analysis sought to quantify how poor health and inhospitable working conditions each contribute to educational disparities in work disability in mid life and old age. Using the Health and Retirement Study (2006-2016), we showed that educational disparities in reporting "any impairment or health problem that limits the kind or amount of paid work" in ages 51-80 have been profound and persistent over time. Blinder-Oaxaca three-fold decomposition revealed that distributions of income and employer insurance made the largest contribution to explaining different rates of work limitations among respondents with versus without high school degrees, followed by work characteristics (physical job demands, insufficient hours) and health conditions (diabetes, lung disease). Comparing respondents with high school versus college degrees, distributions of health conditions mattered most (high blood pressure, lung disease, heart disease, stroke), followed by health behaviors (smoking, drinking). Health-induced work limitations are often used as a health outcome, but we found that work characteristics explained 57% of the disadvantage of those without a high school degree and 44% of the disadvantage of high school compared to college graduates. Therefore, improving work environments holds promise for reducing educational disparities in mid-to-late-life disability.

### SESSION 3230 (PAPER)

### STRESS, RACIAL AND ECONOMIC DISPARITIES, AND HEALTH

Abstract citation ID: igad104.1011

### EARLY-LIFE CIRCUMSTANCES AND RACIAL DISPARITIES IN COGNITION AMONG US OLDER ADULTS

Zhuoer Lin[1], Justin Ye[1], Heather Allore[1], Thomas Gill[2], and Xi Chen[1], *1. Yale University, New Haven, Connecticut, United States*, *2. Yale School of Medicine, New Haven, Connecticut, United States*

There are marked racial disparities in cognitive outcomes between non-Hispanic Black (Black) and non-Hispanic White (White) older adults in the US. Existing studies concentrate on exploring mid-life to late-life risk factors, yet the early-life circumstances through which the racial gaps may arise remain under-explored. Using a wide spectrum of early-life factors assembled from the Health and Retirement Study, including demographics, SES, health, trauma, family relationships, and genetics, we investigated how racial gaps in cognitive score and cognitive impairment among older Americans were tied to racial differences in early-life circumstances. 9,015 (7,381 White, 1,634 Black) Americans aged 50 and older were included in the analysis. Using Blinder-Oaxaca-Decomposition, we showed that Black participants generally had less favorable early-life circumstances than White participants, especially in early-life residence, education level, quality and experience. We demonstrated that overall, differences in early-life circumstances explained up to 61.9% of the racial gaps in cognitive score, and 80.9% of the racial gaps in cognitive impairment between White and Black participants. Disentangling racial gaps by individual factors, we found that differences in the level and quality of education contributed the most. Years of education and school racial segregation respectively accounted for 22.7-24.8% and 26.0%-42.8% of the gaps in cognitive outcomes. Early-life educational experience and place of birth/residence additionally contributed to the disparities. However, childhood trauma, health, and genetic factors were not significant contributors. These findings suggest that exposure to less favorable early-life circumstances is associated with clinically meaningful and statistically significant racial gaps in cognition, which warrants targeted interventions.

Abstract citation ID: igad104.1012

### LONGITUDINAL CHANGES IN DAILY STRESS REACTIVITY AND FUNCTIONAL HEALTH ACROSS 20 YEARS OF ADULTHOOD

Jonathan Rush[1], Emily Willroth[2], Eric Cerino[3], Jennifer Piazza[4], Susan Charles[5], Daniel Mroczek[6], and David Almeida[7], *1. University of Victoria, Victoria, British Columbia, Canada*, *2. Washington University in St. Louis, St. Louis, Missouri, United States*, *3. Northern Arizona University, Flagstaff, Arizona, United States*, *4. California State University, Fullerton, Fullerton, California, United States*, *5. University of California, Irvine, Irvine, California, United States*, *6. Northwestern University, Chicago, Illinois, United States*, *7. The Pennsylvania State University, State College, Pennsylvania, United States*

The daily within-person association between stress exposure and negative affect (i.e., stress reactivity) has been shown to be predictive of a number of adverse health outcomes (e.g., inflammation, chronic conditions). These findings typically rely on a single burst of daily assessments. Little is known about the influence of long-term changes in daily stress reactivity across adulthood and its association with changes in health outcomes. The present study examined whether longitudinal changes in daily within-person associations between stress and affect over 20 years predicted long-term changes in functional health. We used measurement burst data from the National Study of Daily Experiences subsample (N=2,880) embedded within the MIDUS longitudinal study. Three measurement bursts were separated by ten years, with each containing daily measures of stress and affect across eight consecutive days, yielding 33,942 days of data across 20 years of adulthood. Functional health was measured by basic and instrumental activities of daily living (BADL; IADL) at three measurement waves spanning 20 years. Multilevel structural equation models were fit to simultaneously model short-term within-person associations between stress and affect (i.e., stress reactivity) at Level 1; long-term changes in stress reactivity at Level 2; and the association between changes in stress reactivity and changes in functional health at Level 3. Changes in stress reactivity predicted changes in both BADLs and IADLs across 20 years (estimate=0.518, SE=0.153, p=.016; and estimate=0.656, SE=0.200, p<.001, respectively). Individuals who increased more in their stress reactivity across the 20 year period also showed greater increases in their functional health limitations.

Abstract citation ID: igad104.1013

### RACIAL–ETHNIC DISPARITIES IN STRESS EXPOSURE AND THE ONSET OF LATER-LIFE LIMITATIONS IN ACTIVITIES OF DAILY LIVING

Madison Sauerteig-Rolston, and Kenneth Ferraro, *Purdue University, West Lafayette, Indiana, United States*

This study investigates the relationship between stress exposure – including cumulative stress burden and domain-specific stressors – and the onset of activities of daily living (ADL) limitations in later life and examines whether there are racial-ethnic and nativity differences in this relationship. Examining data from the Health and Retirement study (HRS), we apply logistic regression and Weibull accelerated failure-time models to examine associations between stress and baseline ADL limitations as well as stress exposure and age of onset of ADL limitations over a decade. Interaction tests between stress and race-ethnicity and nativity were employed to understand how stress exposure uniquely influences ADL limitations among White, Black, US-born Hispanic, and foreign-born Hispanic older adults. Black, US-born Hispanic, and foreign-born Hispanic adults were more likely to develop an ADL limitation at a younger age compared to White adults (β = -0.06; β = -0.07; β = -0.09; respectively). Cumulative stress burden, childhood traumatic events, adult financial strain, everyday discrimination, and major lifetime discrimination were associated with an earlier onset of ADL limitation among White, Black, US-born Hispanic and foreign-born Hispanic adults. Further, US-born Hispanic adults that experienced the same amount of childhood traumatic events as White adults experienced an earlier transition to ADL limitation by a factor of 0.65 or in about 35% of the time. Reducing racial-ethnic and nativity disparities in ADL limitations in later-life will require efforts to adjudicate the long lasting historical and structural disadvantages that contribute to differential stress exposure across the life course.

Abstract citation ID: igad104.1014

### SOCIOECONOMIC DISPARITIES IN THE MEDICARE HOSPICE CARE INDEX (HCI) INDICATORS

Michael Plotzke[1], and Thomas Christian[2], *1. Abt Associates, St. Louis, Missouri, United States*, *2. Abt Associates, Cambridge, Massachusetts, United States*

The Centers for Medicare & Medicaid Services' Hospice Care Index (HCI) measures quality using ten claims-based indicators characterizing hospice service provision. We examine socioeconomic disparities amongst those indicators for hospice users using Medicare claims data from Federal Fiscal Years 2020-2021. We estimate ten ordinary least squares regression models where the dependent variables are each of the ten indicators and the independent variables are provider- and beneficiary-level characteristics. To examine socioeconomic disparities, we also include indicators for beneficiary race/ethnicity, dual eligibility for Medicaid status, urban/rural status, and an area deprivation index. The model with all control variables showed the smallest difference between the socioeconomic variables. For example, the average probability of a Black beneficiary having a least an eight-day gap between nursing visits is 60.3% versus 58.9% (p-value < 0.01) for a white beneficiary. If we only control for socioeconomic variables and no hospice or provider-level controls, we find larger differences, with the average probability of a Black beneficiary having a least an eight-day gap between nursing visits equal to 62.4% versus 58.7% (p-value < 0.01) for a white beneficiary. The results varied by the HCI indicator as well as the socioeconomic variable being examined. These findings suggest that two otherwise similar beneficiaries from different socioeconomic groups are, on average, treated equally by the same hospice. Instead, socioeconomic disparities for some indicators result from how beneficiaries sort into hospices, where the lowest quality hospices are more likely to treat the most disadvantaged.

### SESSION 3235 (SYMPOSIUM)

Abstract citation ID: igad104.1015

### CREATING STRUCTURES FOR SUSTAINABILITY OF EVIDENCE-BASED DEMENTIA CAREGIVER PROGRAMS

Chair: Carey Wexler Sherman Discussant: Joseph Gaugler

Ensuring sustainable impact for broad-based evidence-based programs (EBPs) serving caregivers for persons living with dementia in the U.S. requires systemic maintenance of programmatic fidelity. This symposium aims to assist decision makers, aging and public health networks, and intervention developers to anticipate and deploy effective strategies and resources required to implement EBP interventions and sustain them. A series of four papers describes precursor studies and activities leading to the development of a structure aimed at ensuring that one such EBP, the Savvy Caregiver® (Savvy), can be faithfully sustained by sponsoring organizations. The first paper presents results from in-depth qualitative interviews of key staff (n=25) in nine organizations delivering Savvy. Findings highlight organizational, structural, and regional collaborations that contributed to sustained EBP implementation with fidelity. The second paper describes an online program to train Savvy interventionists. Informed by the first paper's findings a fully asynchronous multi-media professional continuing educational interventionist training program was developed in collaboration with the educational design team at Emory's School of Nursing. The third paper presents results of the online trainer program evaluation conducted among 76 interventionists from 32 organizations in eight states. High scores on content comprehension (95-99%) and participant feedback document benefits of self-paced learning, interactive materials and quizzes, and self-reflective exercises. The final paper describes a platform (Savvy Systems, LLC) created to ensure

Savvy's long-term dissemination, programmatic integrity and sustained use. The single platform provides for authorized content dissemination, leader training, enhanced referrals, and copyright compliance through licensing. Discussant will offer policy and practice implications. This is a collaborative symposium between the Alzheimer's Disease and Related Dementias and Family Caregiving Interest Groups.

Abstract citation ID: igad104.1016

### BUILDING AN ONLINE TRAINING PROGRAM FOR SAVVY FACILITATORS

Kenneth Hepburn[1], Carey Wexler Sherman[2], John Hobday[2], Adarsh Char[3], and Lai Reed[1], *1. Emory University, Atlanta, Georgia, United States*, *2. Savvy Systems, LLC, Minneapolis, Minnesota, United States*, *3. Full Tilt Ahead, LLC, Norcross, Georgia, United States*

Scaling evidence-based interventions to implementing organizations not linked to the development source poses difficulties in maintaining program fidelity. Program facilitator training must ensure consistency of training approach and content material across organizations. A training program must be able to certify that trainees can demonstrate a detailed understanding of the basic curriculum intent, learning objectives, core principles, mechanism of action, and operational details of the intervention. This presentation will describe the development of an online program to train interventionists to facilitate the Savvy Caregiver Program (SCP), a multi-session, group-based psychoeducational intervention grounded in Social Cognitive Theory that helps family caregivers develop the knowledge, skills, and sense of competency and confidence (mastery) important for effective caregiving. In collaboration with the educational design team at the Emory School of Nursing, we developed a prototype, fully asynchronous professional continuing educational interventionist training program. In seven components, the program employed embedded video, text, interactive interfaces, and short self-correcting quizzes to teach core principles, essential facilitator skills, and the dynamics of each of the six SCP sessions. At the end of each segment, learners were required to provide reflections on segment content; they were required to attain a score of at least 80% in order to advance to the next segment (retries were allowed). Based on feedback from the 35 interventionists who participated in the prototype training program, we added additional video assets to further illustrate methods for facilitating the program, further developed the internal quizzes and improved course navigation mechanisms.

Abstract citation ID: igad104.1017

### ESTABLISHING THE EFFECTIVENESS OF THE ONLINE SAVVY FACILITATOR TRAINING PROGRAM

Carey Wexler Sherman[1], Kenneth Hepburn[2], John Hobday[1], and Lai Reed[2], *1. Savvy Systems, LLC, Minneapolis, Minnesota, United States*, *2. Emory University, Atlanta, Georgia, United States*

A total of 76 individuals took part in the revise training program we developed following prototype testing. These individuals were affiliated with 32 organizations in eight states. Each individual was scored on the observation/reflection provided at the end of each training session. Fifty-one percent of trainees received scores between 95-99% across all assessments; only 8.1% received summed scores less than 90%. In only 4 cases, was an individual required to repeat the observation/reflection exercise. Fifty-six trainees responded to a post-training evaluation survey that sought their Likert scale assessment (1=Strongly Disagree; 5=Strongly Agree) of their experience with elements of the online program and their sense of preparedness to lead SC program. The clarity and completeness of the program, overall, received a 4.40 rating, and the ratings for individual segments ranged from 4.40 to 4.5. Trainees gave the quizzes and assessments a 4.40 rating as learning aids. Trainees rated their overall preparedness to lead the SCP at 4.1. Trainees appreciated that the course was self- paced. They indicated particular benefit from the embedded videos, trainer manual, and the interactive materials and quizzes.

Abstract citation ID: igad104.1018

### STRATEGIES FOR MAINTAINING FIDELITY AND SUSTAINABILITY IN EVIDENCE-BASED PROGRAM DELIVERY

Kate Gordon[1], Carey Wexler Sherman[2], and Kenneth Hepburn[3], *1. Splaine Consulting, Silver Spring, Maryland, United States*, *2. Savvy Systems, LLC, Minneapolis, Minnesota, United States*, *3. Emory University, Atlanta, Georgia, United States*

Federal agencies and advisory councils emphasize the need to expand delivery of evidence-based programs (EBPs) to meet the needs of the increasing millions of family members and friends providing long-term, complex care to persons living with dementias in the U.S. (Alzheimer's Association, 2022). Achieving lasting value and impact of such implementations requires systematic and replicable programmatic fidelity and sustainability to ensure caregivers' broad access and positive outcomes, and the significant investments of providers, stakeholders, and funders are maintained. Unfortunately, a paucity of reported evidence on such strategies continues to limit the full benefit of most intervention efforts. (Hailemariam, M., Bustos, T., Montgomery, B. et al. 2019). To inform the larger RO1 effort described in the previous two papers, we recruited nine organizations from diverse regions that deliver Savvy Caregiver® to discern the key strategies employed to ensure program fidelity and sustainability. Semi-structured interviews were conducted with organization directors (n=9) and Savvy coordinators and group leaders (n=23). Thematic content analyses of transcribed transcripts yielded salient findings on fidelity best practices, including: Assuring EBP coherence with an organization's mission; proactively integrating fidelity into interventionists' training; and integration of fidelity monitoring strategies. Regarding structural practices supporting sustainability, findings included: Engaging leadership buy-in of Savvy as an anchor program; establishing succession plans for seamless program delivery; creation of local collaborative partnerships for long-term recruitment or in-kind contributions; establishment of infrastructure to secure ongoing state and federal financial support. Findings highlight effective, accessible, and achievable strategies for ensuring long-term benefits for all EBP implementation efforts.

Abstract citation ID: igad104.1019

CREATING A UNIFIED NATIONAL STRUCTURE FOR SAVVY'S DISSEMINATION AND SUSTAINABILITY

John Hobday, and Carey Wexler Sherman, *Savvy Systems, LLC, Minneapolis, Minnesota, United States*

Intervention efforts, including established evidence-based programs (EBP), often can't support or monitor programmatic fidelity or sustainability when external funding ends. Delivery can drift substantially or cease altogether. This paper describes the development of an authorized entity dedicated to sustain and scale Savvy Caregiver® with fidelity that was highly informed by efforts described in previous papers. The initial stage involved collaborating with the Offices of Technology Commercialization at University of Minnesota (primary copyright holder) and Emory University (lead adaptation institution). Inter-Institutional Agreements (IIA) outlined guidelines for copyright and trademark compliance, research, fidelity management, and content updates. Next, Savvy Systems, LLC, was launched to document current availability and reach of Savvy and manage the training and certification of Savvy interventionists and distribution curricula and materials through a single portal (www.savvycaregiver.com). Fidelity and essential program delivery skills are now assessed and supported via a new Savvy Fidelity Certification Exam and fidelity webinars. Finally, a flexible licensing plan for organizations and researchers ensures legal use of and access to updated authorized Savvy materials. Creation of the first national database of Savvy sites, featured on the website and the Best Practices Caregiving EBP portal (www.benrose.org), enables direct referrals and other support for Savvy sites and caregivers. Taken as a whole, this type of structure is expected to play an instrumental role in supporting the long-term fidelity, viability, and expansion of Savvy Caregiver. Adaptation and application of such processes may inform other translational and EBP efforts to ensure programmatic integrity and sustained use over time.

### SESSION 3240 (SYMPOSIUM)

Abstract citation ID: igad104.1020

CULTURALLY RESPONSIVE INTERVENTIONS FOR OLDER ADULTS: FROM DEVELOPMENT TO ADAPTATION

Chair: Manka Nkimbeng Co-Chair: Quinton Cotton
Discussant: Lauren Parker

Culture is an important aspect of health and healthcare because it influences perceptions and health seeking behaviors, but many health interventions are developed without consideration of the cultural needs or do not include the voices of the of the target population. Yet culturally responsive interventions have been proven to be more effective than generic interventions. Opportunely, there is a growing understanding of the need for culturally responsive interventions but limited understanding of the process. This symposium will offer an overview of the process of developing culturally responsive interventions. It will present three unique projects that are in different stages and on different levels on the continuum of culturally responsive interventions and programs. African MADE is a culturally tailored dementia education program that was developed with the African immigrant community, while "Community-Organization Based Intervention Planning for Older Latine Adults: Lessons Learned" describes the co-development process employed in the design of an intergenerational respite program for older adults within the Latine community. Finally, we will present findings of a feasibility study of an evidence based intervention that was adapted for African American women with pain and depression.

Abstract citation ID: igad104.1021

THE MAKING OF AFRICAN MADE: MEMORY AND DEMENTIA EDUCATION BY AND FOR THE AFRICAN IMMIGRANT COMMUNITY

Manka Nkimbeng[1], Truphosa Aswani[2], Wynfred Russel[2], Tetyana Shippee[1], and Joseph Gaugler[1], *1. University of Minnesota, Minneapolis, Minnesota, United States*, *2. African Career Education And Resources Inc. (ACER), Brooklyn Park, Minnesota, United States*

African immigrants are a fast-growing segment of the U.S. Black population but dementia literacy and care needs of this population are not fully understood. A dementia care assessment in the community highlighted dementia education as a high priority need in the African immigrant community in Minnesota. This presentation will describe the collaborative process between the research team and a community project advisory board (PAB) to develop a culturally tailored dementia education program and booklet. Following several meetings and reviews, the PAB approved an interview guide that was used to conduct 10 key informant interviews (with leaders including priest, executive directors etc.) and three community conversations (30 participants). This data was used to identify important cultural dimensions for incorporation into the education program. Themes identified were need for respect, attitudes, beliefs and challenges related to dementia care. Identification and debunking of dementia and dementia care myths were also highlighted. The resulting educational content has been presented at 3 community events/organizations with many more scheduled in spring 2023. The African community in Minnesota is very receptive of the education program with many individuals noting that it truly captures African immigrant cultural norms and values. The collaborative process between the research team and the advisory board could serve as a model for creating culturally tailored dementia education programs in other communities. Additionally, successful implementation of this education program has implications for improving dementia care among African immigrants and reducing health disparities in this population.

Abstract citation ID: igad104.1022

EMBRACING CULTURE AND DESIGNING AN INTERGENERATIONAL TRAINING PROGRAM TO SUPPORT LATINE ELDERS

Quinton Cotton[1], Paul Kissell[2], Elma Johnson[1], Yolima Chambers[3], Manka Nkimbeng[1], and Joseph Gaugler[1], *1. University of Minnesota, Minneapolis, Minnesota, United States*, *2. University of Minnesota School of Public Health, Minneapolis, Minnesota, United States*, *3. Centro Tyrone Guzman, Minneapolis, Minnesota, United States*

Although there have been efforts to promote health equity and increase representational diversity in research, there is a gap in our understanding on how to integrate cultural considerations into the intervention design process. Dementia caregiving interventions too frequently lack prioritization and integration of community-directed and culturally appropriate inputs during intervention planning. Consequently, intervention fit among racial and ethnic populations is sometimes culturally incongruent and intervention acceptability in community settings is weakened. We conducted a formative analysis drawing from research team member memos, document review, World Cafes with Latine community residents, and group interviews with Latine organizational staff to understand the intervention development process of an intergenerational dementia training program to support elders within the Latine community. The design process integrated iterative periods of listening through data gathering, informing by applying learned information to the developing program, and affirming through validation. Our analysis demonstrates the need for a broad range of skills and capacity to facilitate effective intervention design (language mastery among key research team members, program design skills, listening, relationship building).

Abstract citation ID: igad104.1023

### FEASIBILITY AND ACCEPTABILITY OF A DEPRESSION AND PAIN INTERVENTION WITH OLDER AFRICAN AMERICAN WOMEN

Janiece Taylor[1], Catherine Clair[2], Laura Gitlin[3], Karen Bandeen-Roche[1], Melissa deCardi Hladek[4], Tiffany Riser[5], Roland Thorpe[1], and Sarah L. Szanton[1], *1. Johns Hopkins University, Baltimore, Maryland, United States*, *2. Johns Hopkins Bloomberg School of Public Health, Baltimore, Maryland, United States*, *3. Drexel University, Philadelphia, Pennsylvania, United States*, *4. Johns Hopkins School of Nursing, Baltimore, Maryland, United States*, *5. John Hopkins School of Nursing, Baltimore, Maryland, United States*

Older African American women experience social determinants of health that put them at risk for experiencing comorbid pain and depressive symptoms. The purpose of our study was to tailor a preexisting evidence-based depression intervention to include pain and test it in older African American women. We conducted a randomized waitlist control study with 21 frail or pre-frail, African American women, 50 years of age and older with pain and depressive symptoms. The average age of the participants was 64.8 (SD: 10.5), average pain intensity was 7.0 (SD: 1.9) out of 10, and average PHQ-9 depressive symptoms score was11.9 (SD: 4.0). Effect sizes at 12 weeks post intervention were -1.05 for depressive symptoms indicating a substantial decrease in depressive symptoms. We did not see a significant change in pain intensity but identified changes in pain behavior scores. The women described the intervention as beneficial and provided suggestions for future iterations.

### SESSION 3245 (SYMPOSIUM)

Abstract citation ID: igad104.1024

### NEW DIRECTIONS IN RESEARCH ON THE DYNAMICS OF PURPOSE IN LIFE AND COGNITIVE AGING

Chair: Angelina Sutin

Purpose in life is the feeling that one's life is goal oriented and has direction. There is converging, replicated evidence that having a greater sense of purpose in life is associated with better cognitive aging outcomes, including less cognitive decline across middle and older adulthood and lower risk of incident dementia. Emerging evidence also suggests that cognitive function may likewise support purpose in life. This symposium highlights recent advances on the dynamics between purpose in life and cognition across varying time scales and populations. Kim will describe the association between purpose in life and the maintenance of better cognitive function across six years in older adulthood and moderators of this association. Nelson and Bergeman will describe how cognitive function supported purpose in life through the uncertainty of the Covid-19 pandemic and moderators of this association. Pfund and colleagues will report on the bidirectional associations between purpose and cognition in older adulthood across four longitudinal samples using a coordinated data analytic approach. Finally, Sutin and colleagues will report on an ecological momentary assessment study that found that in moments when participants felt more purposeful, they had faster processing speed. Collectively, these talks emphasize the dynamic and often bidirectional relation between purpose in life and cognitive function. This work will help stimulate research to better understand purpose and cognitive health and how to leverage purpose in life for healthier cognitive aging outcomes.

Abstract citation ID: igad104.1025

### HAVING A SENSE OF PURPOSE IN LIFE AS A PROTECTIVE FACTOR FOR COGNITIVE DECLINE IN LATER ADULTHOOD

Giyeon Kim, *Chung-Ang University, Seoul, Republic of Korea*

The purpose of this symposium presentation is to discuss the role of having a sense of purpose in life in protecting against cognitive decline in later adulthood. The speaker presents recent findings on purpose in life as a protective factor for cognitive decline from a nationally representative sample of older adults. First, the speaker presents evidence that older adults with a purpose in life showed slower trajectories of cognitive decline during the 6-year period and that purpose in life was a significant moderator of age- and race/ethnicity-cognitive decline associations in later adulthood. Second, the speaker presents evidence that a significant moderating role of purpose in life in the relation of widowhood to total cognition and fluid intelligence, but not in the widowhood-crystallized intelligence relation and that the significant moderating role of purpose in life was found only for those who were female, White, and more educated. Lastly, the speaker discusses implications for research and practice, as well as directions for future research on educational programs to improve purpose in life among older adults from diverse backgrounds.

Abstract citation ID: igad104.1026

### PURPOSE IN LIFE ACROSS THE COVID-19 PANDEMIC: THE EFFECTS OF COGNITIVE ABILITY, AGE, AND GENDER

Niccole Nelson[1], and Cindy Bergeman[2], *1. Colorado State University, Fort Collins, Colorado, United States*, *2. University of Notre Dame, Notre Dame, Indiana, United States*

In March of 2020, the World Health Organization declared COVID-19 a pandemic. Almost immediately, 95% of the US population was under stay-at-home orders, resulting in widespread uncertainty about the future, low community engagement, and social isolation. These effects may have been detrimental to maintaining a sense of purpose in life across this period, which has been linked to several health and wellness factors, including cognitive ability. Although previous work has indicated that higher cognitive ability predicts a stronger sense of purpose in life, the relationship between cognitive ability and changes in purpose across this universal, chronic stressor is unknown. The present study, therefore, examined the effects of cognitive ability, age, and gender on trajectories of purpose in life across the COVID-19 pandemic. Participants included 206 individuals (Age Range=45-88, M=69.04, SD=9.29) who participated in a cognitive assessment before the pandemic, as well as the multi-wave Notre Dame Study of Health & Well-being-COVID Study (NDHWB-CS) from September 2020-February 2022. Two-level, multilevel modeling tested the effects of total, fluid, and crystalized cognitive abilities, as well as age and gender, on trajectories of purpose in life. Results indicated that greater total and fluid cognitive abilities were associated with higher initial levels of purpose in life. Furthermore, although crystalized, fluid, and total cognitive abilities were each associated with change in purpose across the pandemic, there were age and gender differences in these effects. Theoretical and practical implications regarding the relationship between cognitive ability and sense of purpose in the context of stress will be discussed.

Abstract citation ID: igad104.1027

### RECIPROCAL RELATIONSHIP BETWEEN SENSE OF PURPOSE AND COGNITIVE CHANGE IN OLDER ADULTHOOD: A COORDINATED ANALYSIS

Gabrielle Pfund[1], Bryan James[2], Patrick Hill[3], Jason Hassenstab[3], Payton Rule[3], and Emily Willroth[3], *1. Northwestern University, Chicago, Illinois, United States*, *2. Rush University Medical Center, Chicago, Illinois, United States*, *3. Washington University in St. Louis, St. Louis, Missouri, United States*

Sense of purpose (i.e., the extent to which one has personally meaningful goals and directions guiding them through life) is a consistent promoter of healthy aging for older adults. Unfortunately, while individuals benefit from having a high sense of purpose as they age, many people's sense of purpose declines during older adulthood. Some evidence suggests that these declines may in part be explained by health declines experienced later in life. With sense of purpose being a powerful predictor of cognitive functioning in older adulthood, the current project evaluates whether this relationship may be more reciprocal in nature. Using a coordinated data analytic approach, this work investigates how sense of purpose and cognitive functioning promote each other and change together as older adults age. Longitudinal data comes from the Health and Retirement Study (N=15,497; 4 waves over 12 years), Memory and Aging Project (N=1,700; up to 15 waves over 15 years), Minority Aging Research Study (N=950; up to 12 waves over 12 years), and Wisconsin Longitudinal Study (N=22,334; 3 waves over 18 years). Based on a series of random intercept cross-lagged panel models, initial results indicate that sense of purpose change precedes cognitive functioning change and vice versa, when accounting for between-person and concurrent within-person associations. Moreover, bivariate latent growth models show that longer-term change in sense of purpose co-occurs alongside changes in cognitive functioning. In this talk, we will discuss how promoting sense of purpose can support successful cognitive aging, and the repercussions of cognitive decline for sense of purpose.

Abstract citation ID: igad104.1028

### MOMENTARY ASSOCIATIONS BETWEEN PURPOSE IN LIFE AND COGNITIVE FUNCTION IN EVERYDAY LIFE

Angelina Sutin[1], Martina Luchetti[2], Alyssa Gamaldo[3], Jacqueline Mogle[3], Hephzibah Lovett[1], Justin Brown[1], Martin Sliwinski[4], and Antonio Terracciano[5], *1. Florida State University College of Medicine, Tallahassee, Florida, United States*, *2. Florida State University, College of Medicine, Tallahassee, Florida, United States*, *3. Clemson University, Clemson, South Carolina, United States*, *4. Pennsylvania State University, State College, Pennsylvania, United States*, *5. Florida State University, Tallahassee, Florida, United States*

Purpose in life has been associated consistently with healthier cognitive aging outcomes, including better cognitive function and lower risk of incident dementia. Most within-person research on purpose and cognition has focused on cognition measured over years, which has been essential to identify the long-term protective benefits of purpose. The present research shrinks the timescale to momentary assessments over days to address whether purpose operates in the moment to support healthier cognitive function in everyday life. Participants from the Couples Healthy Aging Project (N=303) completed smartphone-based momentary assessments of purpose and short cognitive tasks three times a day for eight days. In moments when participants felt more purpose driven than their average, they had faster processing speed (b=-1.240, SE=0.194; p<.001) and less variability in processing speed (b=-0.437, SE=0.135; p<.001). These associations were independent of person, temporal, and contextual factors and practice effects. Momentary purpose was unrelated to visual memory performance. Similar patterns were found for other purpose-related items (momentary engagement, motivation) but not for hedonic emotions (momentary positive affect). These results suggest that feeling more purpose-driven in the moment supports faster processing speed in daily life. This work demonstrates that the positive association between purpose in life and better cognitive function operates in daily life and may be one mechanism through which purpose is associated with better long-term cognitive outcomes.

### SESSION 3250 (SYMPOSIUM)

Abstract citation ID: igad104.1029

### PREPARING TO MEET THE NEEDS OF OLDER ADULTS: WORKFORCE, TRAINING, AND EDUCATION ISSUES

Chair: Grace Caskie

Due to projected growth in the older adult population in the United States, those working in healthcare and education will encounter increasing numbers of older adults. This population shift presents challenges and opportunities for individuals working in these settings to be ready to meet the needs of older adults. In this symposium, we highlight research on several issues critical to preparing the workforce in healthcare and education for older adults' specific needs. Poon's paper addresses geriatric behavioral health workforce needs identified by a survey of licensed behavioral health providers. Several barriers to providing care to older adults are identified by these providers. Rodriguez et al. investigated the clinical practice experiences and challenges from ten years' worth of alumni of the VA Geriatric Scholars program. This training program provides a model for improving clinical practice with older adults. Hollis-Sawyer examines older students' first experiences taking a course in an online teaching setting and provides specific suggestions to maximize their learning environment. Patterson et al. present an extensive scoping review of end-of-life training for medical students that identified several themes that can direct efforts to improve end-of-life care for older adults. Finally, Caskie et al. address health biases and age biases in doctoral psychology trainees' anticipated clinical work with depressed older adult clients of varying health statuses and provide guidance to psychology programs for improving training. This symposium aims to provide those working in healthcare and educational settings with ways to address workforce preparedness to meet the needs of older adults.

Abstract citation ID: igad104.1030

### EFFECT OF OLDER ADULT HEALTH STATUS AND AGE ON TRAINEES' PERCEPTIONS OF CLINICAL WORK WITH OLDER ADULT CLIENTS

Grace Caskie, Benjamin Johnson, and Eve Root, *Lehigh University, Bethlehem, Pennsylvania, United States*

The growing older adult population in the US will result in more psychologists providing services to older clients. Yet, most psychology doctoral programs lack specific training on mental healthcare for older adults, which allows ageism and healthism to remain unchallenged. To examine how age and health status affect trainees' clinical perceptions, we used a 2 age X 3 health repeated-measures experimental design. Doctoral trainees (N=219; age=21-58) in clinical and counseling psychology were randomly assigned to the 71-year-old or 81-year-old condition. Trainees then read three randomly-ordered clinical vignettes depicting clients with depressive symptoms that varied health condition (healthy, recent Alzheimer's diagnosis, heart disease). Trainees provided clinical ratings for each client. Regardless of age, relative to the client with a recent Alzheimer's diagnosis, the healthy client was perceived as more likely to develop an effective therapeutic relationship, and both the healthy client and client with heart disease were considered more appropriate candidates for therapy. For the 71-year-old client, prognosis, competence, and comfort did not differ between the healthy client and the client with heart disease, but both were rated significantly better than the client with Alzheimer's. For the 81-year-old client, competence and comfort treating were significantly lower for the client with Alzheimer's as compared to both the client with heart disease and the healthy client, but trainees also expressed less comfort and competence treating the client with heart disease than the healthy client. Doctoral psychology programs should include training addressing trainees' clinical knowledge and biases to ensure competent care for older adults.

Abstract citation ID: igad104.1031

### OVERVIEW OF THE NEBRASKA GERIATRIC BEHAVIORAL HEALTH WORKFORCE SURVEY

Cecilia Poon, *Nebraska Medicine/ UNMC, Omaha, Nebraska, United States*

According to the US Census, more than 16% of Nebraska's population is 65 and older. Many older Nebraskans reside in rural communities. Although clinicians in existing state-wide workforce surveys endorse working with older adults, it is unclear how frequently they are involved in geriatric mental health. To identify the needs of those who are providing mental health services to older Nebraskans, the Behavioral Health Education Center of Nebraska (BHECN) conducted an online survey of eligible licensed practitioners from August to December 2022. This presentation highlights the background characteristics of 193 respondents, nearly 40% of whom came from rural areas. Two-thirds identified either as licensed mental health therapists (38%), licensed clinical social workers (26%), or licensed psychologists (14%). More than a quarter did not provide direct clinical services to older adults. On average, respondents estimated devoting 18% of their time working with older adults. Respondents listed insurance restrictions, lower reimbursement rates, and limited specialized geriatric mental health training opportunities as major reasons that had kept them from serving older clients more frequently. These findings support BHECN's ongoing efforts to further develop training and support, and advocate for potential policy changes to enhance the state's geriatric behavioral and mental health workforce.

Abstract citation ID: igad104.1032

### THE FOUR C'S OF TEACHING OLDER LEARNERS: IMPLICATIONS FOR THE WORKPLACE AND CAREER WORK–LIFE EXTENSION EFFORTS

Lisa Hollis-Sawyer, *Northeastern Illinois University (Chicago), Chicago, Illinois, United States*

From Spring 2017 through Spring 2022, a sample of older (age 40 and above) undergraduate and graduate students who were also first-time online students (n = 86) from a very diverse student population at Northeastern Illinois University were analyzed on various motivational and performance indices regarding fully online course performance (e.g., student expectations, receptivity to on-line modules, time management skills). A comparative analysis of the two student samples yielded some interesting "universal" course design issues across education levels, as well as by cultural background and gender differences. Specific fully online design issues to be discussed as are how to create the clearest "face-to-face" representation of the classroom experience via a course learning management system. Effective design issues will be discussed in creating a supportive, fully online course environment for older online course students. General design issues to be discussed are how to create a well-structured "face-to-face" representation of the classroom experience for a diverse older student population with equally diverse learning needs/issues, a supportive fully online learning environment for "first-time" online course students, and tailored, adaptive learning environment to meet the dynamic needs of students (undergraduate, graduate) over the progress of an e-course. The presentation will present and discuss the four C's in promoting a positive and enriched learning environment for diverse "non-traditional" adult learners faced with online learning in different contexts with implications for learning in the workplace for adaptive career work-life extension.

Abstract citation ID: igad104.1033

### EXAMINING THE GERIATRIC SCHOLARS PROGRAM: IMPACT ON CLINICAL PRACTICE AMONG VA PSYCHOLOGISTS

Rachel Rodriguez[1], Priyanka Mehta[2], Jeffrey Gregg[3], Josea Kramer[4], Ana Alfaro[5], and Christine Gould[5], *1. Durham VA Health Care System, Durham, North Carolina, United States*, *2. VA Palo Alto Health Care System, Palo Alo, California, United States*, *3. Durham VA Medical Center, Durham, North Carolina, United States*, *4. VA Greater Los Angeles GRECC, Los Angeles, California, United States*, *5. VA Palo Alto Health Care System, Palo Alto, California, United States*

Since 2014, the Geriatric Scholars Program – Psychology Track (GSP-P) has offered VA post-licensure psychologists with little-to-no prior geropsychology training an opportunity to enhance their knowledge and skills to better serve rural-dwelling older Veterans. This is achieved through an intensive course on geropsychology competencies, completion of a geriatric mental health focused quality improvement project and optional advanced learning components. A survey of GSP-P alumni was conducted to assess the long-term impact of participation on clinical practice with older Veterans and overall job satisfaction. Out of 148 alumni, 40 (27%) responded to the survey. Most alumni reported working within the home based primary care setting (45%), with the others working in a range of inpatient and outpatient settings. Utilizing a retrospective post-pre design, practice change was evaluated using a measure comprised of 12 items related to clinical practices/behaviors with older Veterans (e.g., "Incorporated a caregiver/family member in the assessment of an older Veteran.") both before and after participating in the GSP-P. A paired samples t-test, $t(29) = -2.91$, $p = .007$, demonstrated a significant difference in clinical practice pre to post engagement with GSP-P, with participants reporting more frequent age-adapted clinical practices/behaviors post GSP-P participation. Sensitivity analyses showed a moderate effect size $d = -.53$ (95% CI: -.91, -.144). Qualitative responses reflected alumni growth in confidence and satisfaction in working with older Veterans, as well as more utilization of age-adapted evidenced based assessment and intervention. Overall, this evaluation shows that targeted training can improve psychologists' clinical practice with older Veterans.

Abstract citation ID: igad104.1034

### DEATH AND DYING EDUCATION IN US MEDICAL SCHOOLS: A SCOPING REVIEW

Logan Patterson[1], Autumn Decker[2], Raven Weaver[2], Cory Bolkan[3], Angelique King[1], and Anna Roman[1], *1. Washington State University, Spokane, Washington, United States*, *2. Washington State University, Pullman, Washington, United States*, *3. Washington State University Vancouver, Vancouver, Washington, United States*

With an aging population, knowledge about death, dying, loss, grief, and end-of-life (EOL) care is an essential skill for most physicians. However, EOL education has been minimally incorporated into medical school curricula in the United States (U.S.); there is a significant need to better prepare medical students for the unique challenges of improving patient wellbeing at end-of-life. To understand how death and dying topics have been incorporated into U.S. medical school education, we conducted a systematic scoping review, using PRISMA guidelines, of published research on death education for medical students between 2010 through 2022. An initial search in EMBASE and PubMed yielded 3,443 citations; 265 articles met the initial inclusion criteria (e.g., research-based, EOL education specific), with 97 articles selected for full-text review. Overall, we found significant variation in the type and content of EOL training in U.S. medical schools. Using a competency-based medical education framework that reflects tangible knowledge, skills, and attitudes as a guide to ensure proper EOL training, a deductive coding approach yielded several themes including: reliance on brief, skills-based workshop interventions and limited longitudinal integration into curricula; general research on student attitudes and wellbeing; and an emphasis on needs assessments highlighting gaps in evidence-based EOL training interventions. Using these findings, we offer applied considerations for teaching EOL related skills in U.S. medical schools. In turn, this work contributes to the enhancement of health across the lifespan and might ultimately lead to a society where a "good death" is not an exception, but a reality.

### SESSION 3255 (SYMPOSIUM)

Abstract citation ID: igad104.1035

### AGETECH AND THE ETHICAL IMPERATIVE

Chair: Charlene Chu Co-Chair: Jennifer Boger Discussant: Andrew Sixsmith

"AgeTech" refers to technological trends in e-health, robotics, artificial intelligence (AI), and mobile technologies" as a way to improve the health, well-being, independence, and social participation of older people. This symposium provides an overview of the imperative to integrate ethics into the design and development of technology, from start to finish. The first talk presents reflections from older adult members of two AgeTech groups: AGE-WELL's Older Adults and Caregivers Advisory Committee, and the Research Group of Seniors 411, which will outline the ethical issues and ageism experienced by older adults. To further examine these issues, the second presentation explores a framework for how ageist attitudes can be encoded and perpetuated as digital ageism, both in AI and other technologies, and highlights a need for broader community inclusion. The third paper explores the challenges of virtual community inclusion, highlighting the challenges and benefits of digital participation in a post-COVID world. A health equity focus will be applied when highlighting the unique needs of seldom-heard and underserved older people in virtual spaces to enhance social inclusion. The last paper will highlight the "ethical by design" approach that will encourage and support culture change within AgeTech research and industry. This symposium will contribute to a more ethical, and inclusive design of technologies to support healthy aging.

Abstract citation ID: igad104.1036

**DIGITAL AGEISM AND ITS IMPLICATIONS FOR OLDER ADULT INCLUSION**

Charlene Chu, *University of Toronto, Toronto, Ontario, Canada*

"Digital ageism" is a new form of ageism that is embedded into technology and AI systems. Building on the World Health Organization's recently published policy brief entitled "Ageism in AI for Health", our work draws attention to digital ageism referring to the nexus of ageism (discrimination or bias related to age) that is mediated and perpetuated by artificial intelligent (AI) systems and technologies. This paper presents a conceptual framework for identifying various cycles of injustice that perpetuate ageism into our digital spheres. This framework also identifies key contributors that need to be addressed in order to break these cycles. We present the results of a scoping literature review, informed by Arksey and O'Malley, to identify the mechanisms in which digital ageism can be introduced as demonstrated in the literature. This work contributes to foundational knowledge about age-related biases in AI and how they might be encoded or amplified in AI systems. This work sheds light on the need for ethical thinking and other approaches to better include older people, and ensure technology benefits the aging population.

Abstract citation ID: igad104.1037

**OLDER ADULTS' PERSPECTIVES ON ETHICAL ISSUES RELATED TO AGETECH**

Olive Bryanton[1], Gerry Dragomir[2], Jim Mann[3], and Marjorie Moulton[4], *1. University of Prince Edward Island, Charlottetown, Prince Edward Island, Canada*, *2. AGE-WELL NCE, Toronto, Ontario, Canada*, *3. Community Engagement Advisory Network, Vancouver, British Columbia, Canada*, *4. AGE-WELL, Victoria, British Columbia, Canada*

Most funding programs in the AgeTech sector emphasise the involvement of the end user in research and innovation activities in order to strengthen relevance, appropriateness and real-world impact. However, this engaged approach is still a work in progress and often remains a matter of tokenism with older adults. Involving older adults is seen as key when creating ethically appropriate and inclusive AgeTech for their age group. This paper based on the reflections from members of two AgeTech groups: AGE-WELL's Older Adults and Caregivers Advisory Committee and the Research Group of Seniors 411 in Vancouver, BC. This paper highlights some ethical problems inherent in AgeTech, particularly how ageist assumptions can be built into technology-based healthcare, including the ongoing challenges of community participation, and presents a more radical agenda of older adults at the forefront of setting research priorities and shaping the development and implementation of AgeTech. Central to this is the building of community capacity; training to facilitate older adults' engagement in the research process: mobilizing expertise within the community to address local needs; developing mechanisms for connecting older adults and researchers in an integrated knowledge mobilization process. The paper reflects on how this approach can have a significant impact on the lives of older people, and address the digital divide that marginalizes them. Remaining challenges include how to sustain older adults' participation in innovation initiatives, and providing hard evidence to demonstrate that a more engaged approach has tangible benefits and impact in terms of developing new products and services.

Abstract citation ID: igad104.1038

**INCLUSIVE AGING AND DIGITAL PLACE-MAKING IN AGETECH: THE INCLUDEAGE PROJECT**

Judith Sixsmith, and Mei Fang, *University of Dundee, Dundee, Scotland, United Kingdom*

This presentation will address the challenges of virtual community inclusion for older people, with a focus on seldom-heard and under-served groups, such as those with intellectual and developmental disabilities (IDD) and those who identify as lesbian, gay, bi, trans + (LGBT+). Given that the COVID-19 pandemic has amplified the digital divide, further excluding marginalized social groups, the aim of this ESRC funded IncludeAge Project is to explore how virtual spaces can be made more inclusive to enhance social inclusion and support health equity. The presentation will consist of two key elements. Firstly, current discourses and definitions of 'community' and 'inclusion' in digital spheres will be discussed through findings from a scoping review. Secondly, the presentation will introduce new and innovative ways of digital participatory mapping working alongside older people with IDD and those who identify as part of the LGBT+ community using ArcGIS digital storymapping. This approach offers a unique and powerful way of including under-served groups in the design and development of inclusive AgeTech. The presentation will further highlight the challenges and benefits of digital participation in a post-COVID world, particularly for those who experience exclusion. It will demonstrate the importance of involving under-served groups in the design and development of digital spaces to enhance social inclusion and support health equity. To conclude, we will discuss how these new ideas might be used to shape government policy and organizational practices when it comes to the future of inclusive AgeTech research, design, and development with and for older people.

Abstract citation ID: igad104.1039

**LEVERAGING AN "ETHICAL BY DESIGN" APPROACH TO CREATE MORE INHERENTLY RESPONSIBLE TECHNOLOGY TO SUPPORT AGING**

Jennifer Boger, *University of Waterloo, Waterloo, Ontario, Canada*

Responsible technologies are technologies that appropriately support the needs, abilities, and values of the people using them. While many aspects of responsible technology are ubiquitous, there are a multitude of considerations that are specific to older adults. These age-related aspects must be properly understood and incorporated into the technology development and selection process if technology is to be inclusive of and useful to older adults. However, as many of these aspects are subjective, qualitative, and/or dynamic, developers often consider them to be too abstract, undefined, or difficult to address, resulting in their omission from the technology creation process. In this presentation we will discuss how we can leverage the concept of 'Ethical by Design' to drive the change in thinking and doing that is required to enable developers to engage with age-related concepts in technology design, development, and evaluation. Concepts such as user-centered design, values-based engineering, brave spaces, and co-creation will be presented as some of the ways we can directly access lived experiences and translate them into the creation of technologies that are more inclusive of older adults.

### SESSION 3260 (SYMPOSIUM)

Abstract citation ID: igad104.1040

### STRONGER TOGETHER: GLOBAL COMPARISONS OF THE USE OF CITIZEN SCIENCE TO CREATE AGE- AND DEMENTIA-FRIENDLY COMMUNITIES

Chair: Annie Robitaille Co-Chair: Kimberly Campbell Discussant: Linda Garcia

The active involvement of older people in the development of age-friendly environments has been strongly recommended. However, participatory methods with older adults remain underutilised even though older people may be more qualified to recommend what makes an age-friendly space. Using a citizen science approach in research involving older adults presents a key opportunity to have them be active participants and for researchers to obtain meaningful insight. Using this approach, older adults are viewed as co-designers. They are involved in the research process from beginning to end - research design, development of tools, data collection, analysis and knowledge dissemination. This symposium will highlight some of the work done in Canada, Australia and the United Kingdom to make communities more age-friendly using a citizen science approach. Special attention will be given to how the approach might be adapted to citizen scientists who live with dementia. After an introductory overview of the citizen science approach, the use of this method in studies including persons living with dementia and their care partners will be discussed, both in general terms and as an application to air travel. One of the presentations will also address the use of citizen science in examining population ageing and urbanization. Our symposium will conclude with a discussion of future policy, practice, and research direction.

Abstract citation ID: igad104.1041

### SENIOR CITIZEN SCIENCE FOR AGE-FRIENDLY COMMUNITIES

Helen Barrie, *University of South Australia, Adelaide, South Australia, Australia*

This presentation explores the use of citizen science with older participants; highlighting the potential for this underutilized methodology to create better, data driven, outcomes for age friendly cities and communities. Local neighborhoods have major impacts on older people's mobility, autonomy and independence, and quality of life. The WHO has developed clear guidelines for achieving age-friendly cities or communities. Design and delivery of quality public spaces that meet the age friendly guidelines should promote health and well-being, social engagement with others, and engagement with the environment but little data is currently collected about how older people perceive or use public spaces. Daily experiences, often over long periods of time, mean older residents have acquired intimate first-hand knowledge of their neighborhoods, and thus, may be more qualified than experts to assess the age friendly qualities of neighborhood public spaces. A citizen science approach addresses this need for a deeper understanding of how public spaces are used and viewed by older residents. Through multiple citizen science projects over the past five years, we have co-designed and trialed a 'senior' citizen science approach to auditing and evaluating the age friendliness of public spaces. Our research shows that citizen science allows for richer, deeper qualitative, quantitative and longitudinal data that incorporates lived experience perspectives; produces better project designs and outcomes; develops a cohort of older people interested and engaged in research, and engages and trains people to apply a critical 'age friendly' perspective to their neighborhoods and communities enabling them to be future change agents.

Abstract citation ID: igad104.1042

### ASSESSMENT OF AGE AND DEMENTIA FRIENDLINESS OF OTTAWA COMMUNITIES AS PERCEIVED BY PERSONS WITH DEMENTIA AND CARE PARTNERS

Kimberly Campbell[1], Annie Robitaille[1], Linda Garcia[1], Michael Mulvey[1], Helen Barrie[2], Cat van Es[3], Ana Blanco[3], and Alexandra Chiareli[1], *1. University of Ottawa, Ottawa, Ontario, Canada*, *2. University of South Australia, Adelaide, South Australia, Australia*, *3. The Dementia Society of Ottawa and Renfrew County, Ottawa, Ontario, Canada*

In Canada there are over 500,000 persons living with dementia with prevalence estimates reaching as high as 912,000 by the year 2030. Given that age is the strongest known predictor of dementia and the fact that our population is ageing, there is an urgent need to create communities that promote older adults (including those living with dementia) reaching their maximum potential and feeling welcomed and included while ageing in place. The aim of our study was to determine the utility of a tool developed using a citizen science approach with persons living with dementia and their care partners to determine how they perceive the age- and dementia-friendliness of their neighbourhoods (where they live, work, conduct business and socialise). Ten participants were recruited for a pilot study which took place over a six-week period. The project designed and tested an audit tool, accessed via a smart phone/tablet, that allowed data to be collected quickly and in real time. This audit tool also allowed participants to upload quantitative and qualitative responses (including photos of locations being audited). Participants were trained to become citizen scientists in a series of workshops where they also collaborated with researchers to develop the audit tool. During the data collection period, citizen scientists audited locations/spaces that they visited during their day and submitted their responses using the app. Our findings present a case for increased inclusion of older adults, including those living with dementia, in research and intervention programs that target the promotion of age- and dementia-friendly communities.

Abstract citation ID: igad104.1043

### USING TRANSDISCIPLINARY CO-PRODUCTION TO GAIN INSIGHTS INTO SPATIAL JUSTICE AND AGE-FRIENDLY CITIES

Patty Doran, *The University of Manchester, Manchester, England, United Kingdom*

Globally, a popular policy approach to address the two interrelated challenges of population ageing and urbanisation has been to adopt the World Health Organization's Age-Friendly City framework, a model that can help to identify and address barriers to the well-being and participation of older people. This paper reports early findings from the 'Ageing in Place in Cities' project. The research was conducted from a critical gerontology perspective and embedded a spatial justice perspective by centring the concepts of equity, diversity and co-production in our underpinning theoretical framework. Discussing findings from seven case study cities (all who were early adopters of the Age-Friendly Cities framework), this paper discusses how a transdisciplinary co-production approach has been used to gain greater understanding of age-friendly initiatives in each city. Using mixed methods, we first developed descriptive profiles for each city using both census and other local demographic data to explore how the populations have changed over time, and narratives and policy documents detailing the initiatives used to support ageing in place. Second, we conducted semi-structured interviews with a variety of actors in each city to examine how the cities delivered age-friendly change. We integrated the data using a comparative case study approach to draw out insights across the cities. Our findings reveal the role of key leaders and stakeholders in age-friendly cities and will be of interest to policy makers and academics interested in population ageing and urban change. Further, our transdisciplinary co-production methods present a novel approach to conducting age-friendly research.

Abstract citation ID: igad104.1044

### EVALUATION OF THE DEMENTIA FRIENDLINESS OF AIR TRAVEL—A CANADIAN PILOT STUDY

Linda Garcia, Alexandra Chiareli, Annie Robitaille, Michael Mulvey, and Valentina Primossi, *University of Ottawa, Ottawa, Ontario, Canada*

An earlier initiative involving various stakeholders in the air travel industry, disability advocates, researchers, and individuals living with dementia and their travel partners identified five key areas to reduce the challenges faced by travellers living with cognitive limitations: there was a need for information and awareness on the topic, a discussion on disclosure and identification, a readjustment of processes and spaces, a need for formal training and standardized processes across jurisdictions. Next, focussing on current and planned initiatives for reducing these obstacles, the research team determined what might be available to consumers living with dementia and their families at Canadian airports. This symposium presentation will focus on the design and benefits of a citizen science approach for addressing the identified obstacles and the accessibility of the stated airport initiatives through the lens of passengers with dementia and their travel partners. Moreover, a prototype tool, adapted from the citizen science tool used in other presentations in this symposium, that includes data from the findings of both the stakeholder group and the airport analyses symposium will be presented and discussed in terms of its appropriateness and usefulness to evaluate the dementia-friendliness of air travel.

### SESSION 3265 (SYMPOSIUM)

Abstract citation ID: igad104.1045

### WHO WORKS LONGER IN LATER LIFE? KEY VARIATIONS IN WORKING LIFE EXPECTANCY IN INTERNATIONAL PERSPECTIVE

Chair: Brian Beach Discussant: Hugo Westerlund

In the context of population aging, policymakers are eager to encourage and enable longer working lives, assuming this will lead to increased tax revenue and reduced demand on social security and health care systems. Simultaneously, individuals who work longer will gain additional income and may experience the psychosocial benefits that come from workplace engagement. However, many factors drive early labor market exit, and blanket policy strategies to extend working lives often ignore the nuances that shape the length of real working lives. Such policies have emerged partly from increased life expectancies, although these measures fail to capture subgroup differences in the capacity to continue working. They also do not account for often discontinuous labor market trajectories, particularly around retirement. As such, scholars have turned to more refined methodologies and concepts like working life expectancy (WLE) to better inform policies that actively aim to increase retirement ages. This symposium highlights an ongoing program of work by the IDEAR network, which examines WLE using longitudinal studies of aging in Europe. The symposium features results from Sweden and England, estimating WLE using multi-state models. Unlike other approaches to estimate life expectancy employing the Sullivan method, this approach allows for reversible transitions between work and non-work, reflecting the dynamic and complex nature of modern labor market transitions and exits. The symposium further presents how WLE varies according to key determinants, including socioeconomic status, healthy lifestyles, and unpaid caregiving, highlighting the policy implications that emerge from recognizing inequalities and variations in WLE across vulnerable subgroups.

Abstract citation ID: igad104.1046

### ESTIMATION OF WORKING LIFE EXPECTANCY: APPLICATION OF A MULTISTATE MARKOV MODEL

Holendro Singh Chungkham, *Stress Research Institute, Stockholm University, Stockholm, Stockholms Lan, Sweden*

Longer life expectancy in Western countries has led to concerns about sustainable workforces amid the rising costs of securing aging populations. Retirement age is no more a good indicator of the end of the working life, because many older workers move in-and-out of workforce. A more useful measure is working life expectancy (WLE), which is the expected number of remaining years from a given age that a person will work. This measure considers transitions across different employment states. The method adopted for estimation of WLE is along the same line to estimate health expectancy (HE) based on cross-sectional data without considering transitions in different states of work over time. A better approach is based on multi-state survival models considering transitions among states over time using longitudinal data. These models assume a Markov Process to estimate transition probabilities. WLE is the weighted sum of duration of stay in a state weighted by the transition probabilities. The method adopted in IDEAR consortium is a multistate survival model assuming a first-order Markov process in continuous time to estimate transition probabilities. Transition probabilities are estimated by the msm package. This model takes care of interval censoring where the exact dates of transitions from one state to another is not known. WLEs are estimated by using the package elect (van den Hout, 2019) incorporating age as a time dependent variable by using a Gompertz model instead of the exponential model. The method is demonstrated to estimate WLE using Swedish Longitudinal Occupational Survey of Health (SLOSH).

Abstract citation ID: igad104.1047

### SOCIOECONOMIC STATUS AND WORKING LIFE EXPECTANCY IN SWEDEN

Robin Högnäs, *Stress Research Institute, Stockholm, Stockholms Lan, Sweden*

Longer life expectancy and fertility decline have increased concerns about the security of old-age pensions. Raising retirement ages is one strategy to offset rising costs, though the option to retire varies considerably by socioeconomic status (SES) and sex. The level of variation in SES may depend the measure used. Also, many workers now transition into retirement slowly, e.g., move from full- to part-time work. Thus, retirement age may not sufficiently capture how long people work. Working life expectancy (WLE)—the expected average number of years worked—better measures total working life. We use data from the Swedish Longitudinal Occupational Survey of Health (SLOSH) from 2008 to 2020 (n=4,940 people age 50+ and n=74,093 person-observations) to examine WLE by SES. We estimate a three-state multistate model (i.e., working, not working, dead) and a four-state model (working part-time, working full-time, not working, and dead); both assume a continuous-time first-order Markov process. Models are sex-stratified models, cross-classified by: 1) occupation; 2) education; 3) income. We find that professionals work full-time 1 year more than routine workers, regardless of sex. The low educated work full-time 1 year less than the highly educated. In our weighted three-state model, where part-time work contributed ½ of full-time work, the difference increased to 1.14 and 1.05 years, respectively. Our unweighted three-state model showed slightly larger education differences. Findings suggest that WLE differs by SES, regardless of sex, and the differences are greater by education than occupation. This has implications for extending working life policies.

Abstract citation ID: igad104.1048

### BEHAVIOR-RELATED RISK FACTORS AND WORKING LIFE EXPECTANCY IN ENGLAND

Paola Zaninotto, *University College London, London, England, United Kingdom*

Over the past decades, the UK has introduced pension and labour market reforms that aimed at increasing the length of working life among the older working population. Behaviour-related risk factors such as low physical activity level, alcohol consumption, smoking as well as obesity are associated with labour market participation and early retirement. However, the extent to which the co-occurrence of these behaviour-related risk factors predict how long people stay in employment (working life expectancy) has not yet been investigated. We used a large nationally representative sample of older people in England participants of the English Longitudinal Study of Ageing (ELSA), from 2002 to 2018. We applied multistate life table models to repeated measures to estimate sex-specific working life expectancy from the age of 50. We considered the following behaviours: smoking, daily alcohol consumption, being physically inactive and being obese. At the age of 50, men with no behaviour-related risk factors could expect to work on average 11 years (95%CI:10.96; 11.44) longer (9 for women, 95%CI:8.98;9.44), whereas men with 2+ behaviour-related risk factors could expect to work on average 9.2 additional years (95%CI:9.19; 9.85) (7.4 for women, 95%CI:7.3; 7.8). Men and women engaging in one behaviour-related risk factor had slightly higher lower working life expectancy than those not engaging in any behaviour-related risk factors.

Abstract citation ID: igad104.1049

### WORKING LIFE EXPECTANCY AND UNPAID CAREGIVING: HOW WORKING LIVES DIFFER ACROSS CAREGIVER GROUPS

Lawrence Sacco[1], and Brian Beach[2], *1. Stockholm University, Stockholm, Stockholms Lan, Sweden*, *2. University College London, London, England, United Kin gdom*

Informal or unpaid caregiving (i.e. unpaid and untrained help and support to an ill relative or acquaintance) is becoming more prevalent as a result of population ageing and policies promoting ageing in place. Informal or unpaid caregiving is most common in mid-to-later life and can generate conflict with individuals' participation in paid work at a time when working lives are being extended in several countries. Few studies have examined the impact of unpaid caregiving on working life as a whole. The current study examines how unpaid caregiving affects working life expectancy (WLE) among people aged 50 or older. Measuring paid work through WLE allows engagement in paid work in later life to be examined accounting for the fact that retirement transitions have become more complex due to the diversity of retirement pathways (e.g. un-retirement, phased retirement). Furthermore, using longitudinal data to measure WLE in the context of unpaid caregiving over several years enables us to differentiate among frequent caregivers and sporadic spells of caregiving. Data from the English Longitudinal Study of Ageing (ELSA) and the Swedish Longitudinal Occupation Survey of Health (SLOSH) were used to evaluate how WLEs are affected among individuals who provided unpaid care. We highlight the impact of different trajectories in unpaid caregiving on WLE, distinguishing caregivers according to intensity (hours and frequency per week) as well as provision of care over time.

### SESSION 3270 (SYMPOSIUM)

Abstract citation ID: igad104.1050

### IMPLEMENTATION AND EFFECTIVENESS OF VIRTUAL REALITY PROGRAMS IN LONG-TERM CARE SETTINGS

Chair: Marie Savundranayagam Discussant: Kristine Williams

Virtual reality (VR) is an immersive technology that allows users to experience a computer-generated simulation of a three-dimensional environment. It is used in healthcare for education and training purposes, as therapeutic interventions, and for social engagement. However, there are few studies exploring the use of VR in long-term care settings. The purpose of this symposium is to share lessons learned on the implementation and effectiveness of VR programs in long-term care settings. All studies use the Consolidated Framework for Implementation Research to guide their exploration of the facilitators and barriers to implementation. They use qualitative methods, such as interviews, focus groups, and ethnographic observation, to collect data on the experiences of staff and residents participating in the programs. Study one highlights the critical need to engage decision makers to assess organizational readiness prior to creating an implementation strategy. Study two showcases the iterative process involved in translating an in-person, person-centered communication training into a VR training program for frontline healthcare workers. Study three features the benefits and challenges of using VR with older residents. Study four explores staff perspectives on facilitators and barriers to adopting VR in long-term care. The studies highlight the potential benefits of VR in enhancing communication skills, social engagement, and support for residents' personhood. They also identify factors for successful implementation, such as staff champions, sufficient technological infrastructure, and organizational support for infrastructure and human resources. The studies offer valuable insights for researchers and practitioners working to implement novel VR interventions in long-term care settings.

Abstract citation ID: igad104.1051

### ORGANIZATIONAL READINESS FOR A VIRTUAL REALITY TRAINING PROGRAM CALLED BE EPIC-VR

Marie Savundranayagam[1], Annette Schumann[2], Adriana Sagrak[3], Grace Norris[3], Allison Chen[2], Jennifer Campos[4], and Joseph Orange[1], *1. Western University, London, Ontario, Canada*, *2. Western University - Sam Katz Community Health & Aging Research Unit, London, Ontario, Canada*, *3. Sam Katz Community Health & Aging Research Unit, London, Ontario, Canada*, *4. University of Toronto, Toronto, Ontario, Canada*

An essential first step to implementing virtual reality programming in home care and long-term care settings is to assess organizational readiness by determining factors that enhance the likelihood of its successful implementation. Be EPIC-VR is one such virtual reality program that supports dementia-specific, person-centered communication training for frontline healthcare workers. Guided by the Consolidated Framework for Implementation Research (CFIR), the current study aimed to identify factors influencing Be EPIC-VR's implementation in home care and long-term care settings. Semi-structured interviews were conducted with nine managers from home care and long-term care settings in Canada. Transcripts from these interviews were analyzed using the Framework Analysis' five-step ongoing, iterative process: familiarization, identifying a thematic framework, indexing, charting, and mapping/interpretation. Textual data were open-coded and organized deductively (using CFIR's preset codes) and inductively (for emergent codes) into themes and subthemes. Four themes emerged as factors contributing to organizational readiness including 1) openness to virtual reality as a training tool, 2) staffing and training logistics, 3) organizational culture supporting staff development, and 4) external pressures for organizational sustainability. These findings will guide how the Be EPIC-VR implementation team collaborates with organizational decision makers to ensure that Be EPIC-VR is a good fit for those organizations, to prepare for its implementation, and to optimize the likelihood of success. The study findings offer valuable insights for researchers and practitioners working to implement new virtual reality interventions in home care and long-term care settings.

Abstract citation ID: igad104.1052

### BE EPIC-VR: TRANSLATING AN IN-PERSON, PERSON-CENTERED COMMUNICATION TRAINING PROGRAM INTO VIRTUAL REALITY

Marie Savundranayagam[1], Allison Chen[2], Grace Norris[3], Annette Schumann[2], Simran Kaur[4], Jennifer Campos[5], and Joseph Orange[1], *1. Western University, London, Ontario, Canada*, *2. Western University - Sam Katz Community Health & Aging Research Unit, London, Ontario, Canada*, *3. Sam Katz Community Health & Aging Research Unit, London, Ontario, Canada*, *4. Western University-Sam Katz Community Health & Aging Research Unit, London, Ontario, Canada*, *5. University of Toronto, Toronto, Ontario, Canada*

Be EPIC is a dementia-specific, person-centered communication training program for frontline healthcare workers that uses simulations with trained actors, reflection, and feedback. Scaling Be EPIC is limited by resource and methodology demands of recruiting and training actors and consistency of delivery across sites. Virtual reality (VR) is a promising solution to the problem of scaling Be EPIC because it provides interactive, realistic, and consistent simulations. However, there is limited research on implementing VR to train frontline healthcare workers. Guided by the Consolidated Framework for Implementation Research, the current study used an effectiveness-implementation hybrid design to compare simulation experiences of Be EPIC-VR with Be EPIC-in person and real-world clinical encounters. The study also explored factors that can influence the future implementation of Be EPIC-VR in long-term care home settings during the pre-implementation period. Six frontline healthcare workers who completed the Be EPIC in-person version with trained actors also completed the same assessment simulation in VR, followed by an interview. Thematic analyses revealed two themes that contributed to the simulation's realism: the immersive nature of the virtual environment and the accuracy of the avatar's visual, verbal, and behavioral characteristics. Moreover, the factors influencing the successful implementation of VR simulations in long-term care home settings include sufficient technological infrastructure and workplace personnel who can assist with Be EPIC-VR implementation. The findings highlight the potential to scale Be EPIC using VR and provides insights into the iterative process involved in translating an in-person training into a VR training program.

Abstract citation ID: igad104.1053

**USING VIRTUAL REALITY IN LONG-TERM CARE TO REDUCE SOCIAL ISOLATION**

Lillian Hung[1], Joey Wong[1], Mona Upreti[2], and Winnie Kan[1], *1. University of British Columbia, Vancouver, British Columbia, Canada*, *2. UBC IDEA lab, Vancouver, British Columbia, Canada*

Virtual Reality (VR) has become increasingly accessible for older adults, providing opportunities for interventions that address loneliness and social isolation in long-term care. However, the effectiveness of VR programs can be influenced by various factors, such as the backgrounds, preferences, and capacities of the target population. This qualitative study investigates the acceptability and feasibility of a recreational VR program for social engagement in two Canadian long-term care homes since January 2023. The study involved 20 residents (with various levels of cognitive and physical impairments) who participated in weekly VR group sessions facilitated by staff. Ethnographic observation and video-recorded conversational interviews were conducted with residents during the VR sessions. We also conducted 10 focus groups with 20 staff members. Four patient partners were involved as co-researchers in the team. We performed the thematic analysis with patient partners. We identified three themes: (1) storytelling builds residents' sense of self, (2) positive emotions persist even when the video is forgotten, and (3) VR empowers resident-resident and staff-resident connections. The findings demonstrate that using VR in long-term care settings is feasible and acceptable for older adults with different cognitive and physical impairments. VR programs have the potential to enhance social engagement and support residents' personhood as a meaningful activity by improving inclusion, social engagement, comfort, and recognition of their identity. Future research could explore the long-term impact of VR experiences in addressing social isolation and loneliness among older adults in long-term care.

Abstract citation ID: igad104.1054

**FACILITATORS AND BARRIERS TO IMPLEMENTING A VIRTUAL REALITY PROGRAM IN LONG-TERM CARE**

Lillian Hung[1], Joey Wong[2], Mona Upreti[3], Winnie Kan[2], Alisha Tumar[3], Sonia Hardern[4], Jim Mann[5], and Christine Wallsworth[6], *1. Vancouver General Hospital, Vancouver, British Columbia, Canada*, *2. University of British Columbia, Vancouver, British Columbia, Canada*, *3. UBC IDEA lab, Vancouver, British Columbia, Canada*, *4. Vancouver Coastal Health, Vancouver, British Columbia, Canada*, *5. Community Engagement Advisory Network, Vancouver, British Columbia, Canada*, *6. Vancouver, British Columbia, Canada*

To successfully implement virtual reality (VR) programs for long-term care (LTC) residents, it is essential to consider contextual factors. However, current research does not explore the LTC staff's perspectives on implementing VR in their workplaces. This qualitative study aimed to fill this gap by exploring the facilitators and barriers to adopting VR in LTC, guided by the Consolidated Framework for Implementation Research (CFIR). We applied a Collaborative Action Research (CAR) approach, which involved three phases: (1) Reflect and Plan, (2) Act and Adapt, and (3) Evaluate. Ten focus groups were conducted with 20 staff in two Canadian long-term care homes. Thematic analysis was performed collectively with the team, including researchers, trainees, and patient and family partners. Our findings suggest that implementing a VR program in LTC requires readiness and capacity for implementation within the care home. Key factors that enabled implementation were staff champions, perceived benefits, and ease of use of the equipment. However, there were also barriers, such as limited resources, including Internet infrastructure, limited adaptability to meet local needs, and relative priority and staff workload. To overcome these barriers, our results indicate a need for organizational support for infrastructure and human resources. In addition, future research can evaluate the potential impact of facilitating residents' VR sessions on staff's job satisfaction and the involvement of residents' families/caregivers as well as volunteers during the sessions to reduce staff hesitancy and workload.

### SESSION 3275 (SYMPOSIUM)

Abstract citation ID: igad104.1055

**THE BENEFITS OF POSITIVE SOLITUDE IN LATE LIFE**

Chair: Yuval Palgi Co-Chair: Ehud Bodner Discussant: Dikla Segel-Karpas

Positive solitude is the capacity to choose spending time by yourself in a positive manner. This session provides a wide glance on the advantage of this capacity at the second half of life. The session will try to present new theoretical perspectives regarding this capacity. The lectures will give an opportunity to follow the directions in which research in this new field is being developed. The first lecture by Palgi will describe the theoretical background for the study of positive solitude, and will provide new findings from the positive solitude scale that was lately develop by the authors. The second presentation by Bodner will describe how emotional regulation through music moderates the relationship between mindfulness and positive solitude. The third lecture by Zambrano Garza is contributing to the understanding of solitude in the interpersonal domain. Based on dyadic diary study, demonstrates that more voluntary solitude was associated with more positive affect of the partner and more negative solitude was related to more negative affect of partner. The forth lecture by Segel-Karpas will focus on the contribution of positive solitude and loneliness on negative aspects of mental health. Finally, the fifth presentation, by Jennifer Lay, will show use natural language processing to identify solitude experiences from older adults' reports. We will conclude the session by discussing future research directives for the implementation of positive solitude in the field of gerontology, such as the development of interventions that may enhance the tendency for positive solitude in old age.

Abstract citation ID: igad104.1056
### TIME BY YOURSELF: THEORETICAL BACKGROUND AND FINDINGS FROM THE NEW POSITIVE SOLITUDE QUESTIONNAIRE

Yuval Palgi[1], Dikla Segel-Karpas[1], Sharon Ost Mor[1], Yaakov Hoffman[2], and Ehud Bodner[3], *1. University of Haifa, Haifa, Hefa, Israel*, *2. Bar Ilan University, Ramat Gan, HaMerkaz, Israel*, *3. Bar Ilan University, Tel Aviv, Tel Aviv, Israel*

The present study describes the development of a new questionnaire for measuring positive solitude. First we will discuss the conceptualization of positive solitude, and refer to the contradictions that exist in the literature regarding this concept. We will then present the theoretical background on which we based our questionnaire, and some empirical findings regarding its utility. We will describe the 9-item tool and its relationships to positive and negative measures of well-being and mental health. Furthermore, we will discuss findings from a Holocaust survivors study showing the role of positive solitude on posttraumatic symptoms. We will conclude this presentation by discussing several future directives, some of which that will be discussed in other presentations of this symposium.

Abstract citation ID: igad104.1057
### EMOTION REGULATION THROUGH MUSIC AND MINDFULNESS ARE RELATED TO POSITIVE SOLITUDE

Ehud Bodner[1], Yuval Palgi[2], and Noa Bachman[2], *1. Bar Ilan University, Tel Aviv, Tel Aviv, Israel*, *2. University of Haifa, Haifa, Hefa, Israel*

Mindfulness and emotion regulation through music listening are skills that share some attributes with the skill of positive solitude (PS; defined as an inner choice to dedicate time to a meaningful, enjoyable activity or experience managed by oneself, with or without the presence of others). Nevertheless, little is known about their relationship with PS in the second half of life. Hence, we recruited a convenience sample of community-dwelling adults in the second half of life (N = 123; M = 68.63, SD = 10.99), who completed self-report measures of demographics, emotion regulation through music, mindfulness, and PS. A hierarchical linear regression demonstrated significant positive associations between emotion regulation through music listening and PS, and between mindfulness and PS. Moreover, age moderated the relationship between mindfulness and PS. This relationship was found to be positive and significant only among older adults. These findings support the study's hypotheses and emphasize the contribution of the current research to developmental research on PS in the second half of life.

Abstract citation ID: igad104.1058
### THE MODERATING ROLE OF POSITIVE SOLITUDE IN THE RELATIONS BETWEEN LONELINESS AND DEPRESSIVE SYMPTOMS

Dikla Segel-Karpas[1], Yuval Palgi[1], Sharon Ost Mor[1], Yaira Hamama Raz[2], Mayan Shacham[2], Menachem Ben-Ezra[2], and Lee Greenblatt Kimron[2], *1. University of Haifa, Haifa, Hefa, Israel*, *2. Ariel University, Ariel, HaMerkaz, Israel*

Positive solitude, the choice of being alone to engage in meaningful inner or physical, spiritual, mental, or cognitive activity/ experience, was recently suggested as a stand-alone phenomenon differentiated from loneliness and negative solitude. As loneliness was previously found to have adverse implications for mental health, the present study examined whether the ability to engage in positive solitude can moderate the harmful effect of loneliness on depressive symptoms. The sample consisted of 520 community dwelling older adults in Israel aged 68-87 (Mage=72.66). Participants answered an online questionnaire through a survey company (Ipanel) assessing their background characteristics, depressive symptoms, loneliness, and positive solitude. The results show that loneliness was positively associated with depressive symptoms, whereas positive solitude was negatively associated with depressive symptoms. Furthermore, positive solitude moderated the relationship between loneliness and depressive symptoms, such that higher levels of positive solitude weakened this association. The findings indicate that positive solitude may serve as a buffering factor for mental health among older adults by augmenting coping with the adverse outcomes of loneliness. The results provide insight for tailoring future treatment interventions focusing on positive solitude to enhance mental health among older adults.

Abstract citation ID: igad104.1059
### SOLITUDE LINKS WITH INDIVIDUAL AND RELATIONAL WELL-BEING: EVIDENCE FROM DYADIC DAILY LIFE ASSESSMENTS

Elizabeth Zambrano Garza[1], Rachel Murphy[1], Maureen Ashe[1], Kenneth Madden[1], Denis Gerstorf[2], Christiane Hoppmann[1], and Theresa Pauly[3], *1. University of British Columbia, Vancouver, British Columbia, Canada*, *2. Humboldt University of Berlin, Berlin, Berlin, Germany*, *3. Simon Fraser University, Vancouver, British Columbia, Canada*

Solitude, defined as physical aloneness or the absence of social interactions, has been associated with both positive and negative outcomes. Older adults spend a significant amount of their waking time by themselves (exacerbated during the pandemic), but they may be better equipped to regulate the negative emotions that are often linked with being alone. Additionally, solitude may provide a balance between social connectedness and autonomous needs. Using pandemic, end-of-day daily diary data across 10 days from 136 older Canadian adults and a close other of their choice (59% spouses, M = 66.49 years, SD = 13.26 range: 18-87 years, 88% White, 62% women), this project aims to examine associations of actor and partner solitude with relationship quality and daily affect. Multilevel models revealed that participants who reported more voluntary solitude experienced more daily positive affect and those who reported more negative solitude experienced both more negative affect and less positive affect at both daily and overall levels. We also found daily partner effects such that more voluntary solitude of the partner was associated with less actor negative affect and negative solitude of the partner being associated with less actor positive affect and more negative affect over and above actor effects. Finally, when partners reported more voluntary solitude, participants reported higher relationship support. Findings shed light on the benefits and drawbacks of how solitude is experienced in older adulthood, highlighting the influence social partners affect quality and relationship satisfaction.

Abstract citation ID: igad104.1060

**ALONE WITH MY THOUGHTS: USING NATURAL LANGUAGE PROCESSING TO DISTINGUISH LONELY FROM CALM ALONENESS**

Jennifer Lay, *University of Exeter, Exeter, England, United Kingdom*

Time spent alone is common in daily life, and particularly in old age; it may at times be experienced as lonely, and at times as calming (positive solitude). The use of machine learning to interpret human language (e.g., natural language processing or NLP) has been growing in psychology, and NLP has been used to distinguish experiences of loneliness from positive solitude in social media data (e.g., tweets). How effective might such NLP techniques be for classifying naturally-occurring aloneness experiences in older adults' everyday lives? The present study uses 1,546 thought samples collected from 133 adults aged 18-85 (M = 49.6 years; 73% female) at moments when they were alone over a 1-3 week period; participants reported their current thoughts (open-ended) and affective states (lonely, calm) at each assessment. Support vector machines were then used to classify moments of aloneness as being lonely (vs. not lonely) and calm (vs. not calm) based on the words participants used when describing their thoughts. When compared against participants' actual reported affective states, these classifications achieved up to 67% accuracy for "lonely" and 79% accuracy for "calm". "Lonely" thought reports included the word "work" more frequently, whereas "calm" thought reports more frequently included the words "reading" and "think/thought", suggesting individuals engage in quiet or contemplative activities during positive solitude. Age differences therein are also examined. Findings suggest that NLP may be a useful tool for identifying older adults at risk of loneliness (and its negative health implications) by using brief reports of their everyday thoughts.

### SESSION 3280 (SYMPOSIUM)

Abstract citation ID: igad104.1061

**ACTIVE ENGAGEMENT AFTER RETIREMENT**

Chair: Dannii Yeung Co-Chair: Edwin Chung

Retirement has been shown to accelerate the declines in physical and mental health (Dave et al., 2008) and cognitive functioning (Celidoni et al., 2017; Xue et al., 2018) due to the identity disruption and removal of a cognitively simulating environment after leaving the workforce. This necessitates a need to systematically uncover factors that could potentially buffer the negative impacts brought by retirement. This symposium aims to identify the types of self-initiated activities that can maintain post-retirement well-being through research conducted in Hong Kong and Japan. Specifically, the first paper from Chung reveals that retirees engaging in postretirement work exhibit lower life satisfaction but higher cognitive functioning than those without work. Similarly, the second paper from Katagiri also shows that work has negative impacts on life satisfaction, but such effect is buffered by civic participation. The third paper from Ho suggests that the beneficial effects of ICT use on personal mastery is more prevalent in retirees than in non-retirees. The fourth paper from Lin discloses that participation in cognitively demanding volunteering activities is associated with better cognitive and psychological functioning. These four presentations unveil various types of post-retirement activities that are beneficial to retirees, including re-engagement in the workforce, the use of technology for leisure and financial purposes, and participation in cognitively stimulating volunteering. Given that most of the pre-retirees do not have much planning on social life arrangement, findings of this symposium provide practical implications to improve psychological adjustment to this critical life event.

Abstract citation ID: igad104.1062

**THE EFFECTS OF POSTRETIREMENT WORK ON WELL-BEING OF RETIRED ADULTS**

Edwin Chung, Gloria Lin, and Dannii Yeung, *City University of Hong Kong, Hong Kong, Hong Kong*

There has been evidence that a growing number of retirees are reentering the labor force (DeSilver, 2016; Greenwald et al., 2017), possibly due to financial needs and seeking continuity in their preretirement job (Burkert & Hockfellner, 2017). However, the effects of post-retirement work on retirees' well-being remain under-examined. This cross-sectional study thereby aims to examine the effects of post-retirement work in a sample of 768 younger and 530 older Hong Kong Chinese retirees (Mage = 65.1, SD = 2.72, range = 60 – 69, and Mage = 75.2, SD = 4.50, range = 70 – 95, respectively). Participation in post-retirement work, life satisfaction, cognitive functioning and hand-grip strength were measured. The results of two-way between-subject robust ANOVAs demonstrated that retirees engaging in post-retirement work exhibited lower life satisfaction (Q = 5.82, p = .02) but higher cognitive functioning (Q = 5.29, p = .02) than those without post-retirement work. Furthermore, as compared with younger retirees, older retirees engaging in post-retirement work exhibited higher cognitive functioning (Q = 3.66, p = .05) and stronger hand-grip strength (Q = 4.21, p = .04). These results remain significant even after controlling for sex, education, socioeconomic status, perceived health, and financial control. The findings of this study thus reveal the beneficial effects of post-retirement work on cognitive and physical health, but not on life satisfaction. The negative effect of post-retirement work on subjective well-being will be discussed in relation to the unsatisfactory working environment or employee benefits received by older workers in Hong Kong.

Abstract citation ID: igad104.1063

**GOOD FOR HEALTH, BAD FOR SUBJECTIVE WELL-BEING: EFFECT OF CONTINUING WORK ON JAPANESE OLDER ADULTS**

Keiko Katagiri, *Kobe University, Kobe, Hyogo, Japan*

Recently, the labor force rate of Japanese older adults has been increasing. The white paper of Japan's Cabinet Office shows that half of the people in their late 60s are employed in 2022. Previous literature has indicated that work has positive effects on health and cognitive functions; however, the work environment is not age-friendly. Most companies set the mandatory retirement age at 60, after which people must work under a worse labor contract. This study aims to explore the effect of work on Japanese older adults' subjective well-being and to examine whether social participation and civic activity can buffer work's negative effects. Random sampling survey was conducted in 2019 on people living in the Tokyo and Hyogo prefectures. The response rate was 43.0% (N = 1,063, aged 50–74). Analyses of covariance were conducted on well-being, depression, anxiety, loneliness, and life satisfaction. We found that work has negative effects on anxiety and life satisfaction, with civic activity moderating the negative effect on life satisfaction. Gender differences were observed regarding anxiety and loneliness. Women showed higher anxiety, and men showed higher loneliness. Regarding age difference, people in their late 60s exhibited the lowest depression and those in their late 50s higher anxiety. It is argued that Japan's third agers live in more difficult work circumstances than ever before, and it is thus necessary to explore age-friendly labor conditions for older adults.

Abstract citation ID: igad104.1064

### THE EFFECTS OF ICT USE ON PERSONAL MASTERY: RETIREMENT STATUS MATTERS

Alvin Ho, Daisy Li, and Dannii Yeung, *City University of Hong Kong, Hong Kong, Hong Kong*

Internet and technology use have provided middle-aged or older adults with a new arena to regain control over their personal and social lives (McMellon & Schiffman, 2002) and improve their well-being (e.g., Sala et al., 2020; Shufford et al., 2021; Yeung et al., 2022). Still, whether such effects depend on retirement status has not been thoroughly examined. A total of 2391 middle-aged and older participants provided their retirement status (retired or not), and their use of information and communication technology (ICT) for different purposes (social, leisure, financial, and medical) and level of personal mastery were assessed. Four regression analyses were performed to test the moderating effect of retirement status on the relationship between each type of ICT use and personal mastery. Significant interaction was found between retirement and ICT use for leisure ($\beta = .002$, $p = .046$), financial ($\beta = .003$, $p = .009$), and, marginally, medical purposes ($\beta = .002$, $p = .059$), but not for social purpose ($\beta = .000$, $p = .520$). The beneficial effect of the use of ICT on personal mastery was shown to be either exclusive (for medical purpose) or significantly stronger (for leisure and financial purposes) for participants who were retired. This suggests that encouraging middle-aged and older adults to perform ICT-related activities may be particularly crucial when they are retired, presumably due to their capability to compensate for the loss of work-related control and identity.

Abstract citation ID: igad104.1065

### BENEFITS OF VOLUNTEERISM FOR RETIREES: TYPES OF VOLUNTEERING MATTER

Gloria Lin, Edwin Chung, and Dannii Yeung, *City University of Hong Kong, Hong Kong, Hong Kong*

A recent study has disclosed that the effects of volunteering vary as a function of types of volunteering activities (Lam et al., 2021). Volunteering is one of the major social participations among retirees. Therefore, we aim to investigate which types of volunteering activities are beneficial to the well-being of retirees. A total of 719 older Hong Kong Chinese adults (Mage = 66.31, SD = 4.18, range = 60 – 80) who were retired within ten years were invited to take part in this study. Their participation in instrumental and cognitively demanding volunteering activities, life satisfaction, depressive symptoms, and cognitive functioning were assessed. In the current sample, 58.97% of the retired persons did not volunteer; and the remaining engaged in instrumental volunteering (10.15%), cognitively demanding volunteering (26.43%), or both types (4.51%). In addition, the results of ANCOVAs revealed that the retirees engaging in cognitively demanding volunteering had better cognitive functioning than those who did not volunteer at all (adj. Mdiff = .78, p = .03), while the retirees engaging in instrumental volunteering exhibited more depressive symptoms compared with those who engaged in cognitively demanding volunteering (adj. Mdiff = 2.04, p = .02) and those who did not volunteer at all (adj. Mdiff = 1.91, p = .02). These results remained significant even after controlling for sex, education, socioeconomic status, and perceived health. Our findings suggest that cognitively demanding volunteering activities should be promoted to maintain the psychological and cognitive well-being of retired persons.

### SESSION 3285 (SYMPOSIUM)

Abstract citation ID: igad104.1066

### LONG-TERM PAID CARE WORKFORCE: SHORTAGE, BURNOUT, TRAINING, TURNOVER, WHAT'S NEXT?

Chair: Ann Rhodes Discussant: Anne Martin-Matthews

Although the long-term care workforce has struggled historically with staff satisfaction, safety, and retention, the pandemic triggered increased turnover and higher levels of staff burnout. As a result, many residential care organizations are using agency staff at unprecedented levels. Given that continuity of care is central to high-quality person-centered care, and the workforce should have access to a physically and psychologically safe environment, it is imperative that the gerontological research and practical community understand the changing conditions experienced by this workforce in crisis and offers solutions at the workplace, industry, and policy levels. In this symposium, authors explore different facets of paid long-term care workforce to stimulate thought and discussion. One paper contrasts the well-being of the workforce to that of the residents during COVID-19. A second paper presents workforce management capabilities and strategies and posits essential questions for the capacity of management and leadership needs of the workforce. Two papers highlight the struggles that are presented as paid long-term care relies increasingly on contract, short-term workers. Paper three discusses how the use of the contract workforce does not support the provision of person-centered care for conditions like dementia. Paper four uses payroll-based journal data to delve into short and long-term use of contract workforce and discusses the nature of contract staffing, and considers how contract staffing may be disruptive to both the direct care worker and the nursing home environment. The discussant will draw out the policy and practice implications arising from the papers. This is a collaborative symposium between the Aging Workforce and Paid Caregiving Interest Groups.

Abstract citation ID: igad104.1067

### SPECIAL FOCUS FACILITIES AND COVID-19: THE IMPACT ON STAFF AND RESIDENT HEALTH

Ann Rhodes[1], Thomas Caprio[2], Tracey Gendron[1], and Leland Waters[1], *1. Virginia Commonwealth University, Richmond, Virginia, United States*, *2. University of Rochester, Rochester, New York, United States*

The Special Focus Facility (SFF) program is an intensive, quality improvement intervention for 88 of the lowest-quality nursing facilities in 49 states. An additional 435 candidate facilities (SFFc) have similar quality problems but do not receive the SFF quality intervention. In the calendar year 2020, a total of 50 SFFs, and 197 SFFc were enrolled for all 12 months. These nursing facilities were compared on COVID-19 outcomes (Case Fatality Rate, staff cases, resident cases) to randomly selected 5-star nursing facilities in the same state for a total sample size of N=494. After controlling for case-mix hours per resident day resident (Case-Mix staffing) and size of nursing facility SFF and SFFc had significantly lower staff confirmed COVID-19 cases in 2020 as compared to 5-star facilities, adjusted R2 =.25, F (2, 4217)=358.2,p=<.005. Despite the lower staff COVID-19 rates, a higher resident Case Fatality Rate was observed, R2 =.013, F (4, 358) =15.42, p=<.005. Although both SFF and SFFc had lower total staff cases, there was a slightly higher rate of reported staff COVID-19 deaths in SFF and SFFc. However, this difference was not significant. R2 =.01, F (4, 4127)=12.32,p=.859. It is important to note that 2020 was the onset of the COVID-19 pandemic and, therefore, a time of extreme disruption from normalcy for residents and staff. However, these findings are a reminder that quality interventions should be designed to consider the linkages between staff and resident well-being and longevity in order to protect both residents and the workforce.

Abstract citation ID: igad104.1068

### FOSTERING CONSISTENT AND ENGAGING CARE FOR PEOPLE WITH DEMENTIA IN LONG-TERM CARE

Jennifer Morgan[1], Candace Kemp[1], Andrea Hill[1], and Alexis Bender[2], *1. Georgia State University, Atlanta, Georgia, United States*, *2. Emory University, Atlanta, Georgia, United States*

Maintaining a stable and qualified direct care workforce in long-term care organizations has become increasingly difficult as shortages intensify, turnover increases, and the use of agency staff remains at high levels. Efforts to improve this situation need to include high quality training that is incorporated into onboarding, ongoing continuing education, and infused into the culture of the organization. Using data from our ongoing NIA-funded study, “Meaningful Engagement and Quality of Life among Assisted Living Residents with Dementia,” we describe the competencies used by direct care workers (DCWs) with exceptional capacity, or ability and resources to engage people with dementia, and the factors that promote or limit the development and use of these competencies. Focal residents with dementia (N=66) were followed at eight diverse assisted living communities for one year and 53 DCW were interviewed across three waves of data collection. Interview transcripts and fieldnotes from more than 2000 hours of observation were analyzed using a grounded theory approach. Organized by four engagement approaches: knowing the person, connecting with and meeting people where they are, being in the moment, and viewing all encounters as opportunities, we identified competencies and the multiple intersecting factors which impact meaningful engagement and competency development including community staffing, consistent assignment, scheduling, DCW training, DCW tenure, and community resources. Implications for practice, which emphasize the elaboration of specific competencies, have the potential to inform the development of DCW training, the promotion of supportive work environments, and social policy that support person-centered dementia care.

Abstract citation ID: igad104.1069

### ESSENTIAL CAPABILITIES FOR MANAGING THE LONG-TERM CARE WORKFORCE: EVIDENCE FROM AN INTERNATIONAL SCOPING REVIEW

Philip Taylor, *Federation University Australia, Ballarat, Victoria, Australia*

Amid ongoing concerns about meeting the staffing needs of long-term care providers, arguably exacerbated by the COVID-19 pandemic, there is a need to better understand the skills and capabilities required of managers in the sector. Internationally, the management of the long-term care workforce has been somewhat neglected by both the research literature and recent enquiries into the sector. There is fundamental agreement that well-managed staff provide better care. The sector’s leadership and its workforce have been under scrutiny for some time and the context of COVID-19 has shone further light on their limited ability to respond to crises. It is recognised that operational deficiencies in terms of the organization and characteristics of the sector’s workforce as well as its socio-cultural positioning have contributed to sub-optimal functioning. This project collated evidence and recommendations that could inform policymaking among long-term care providers as well as policy and decision makers. An evidence review of relevant international gray and academic literature was undertaken and examples of successful models of management practice across a range of dimensions were identified and written up as vignettes. The paper reports key information regarding the building of capabilities in long-term care workforce management, including: current and future workforce considerations, value proposition, onboarding of staff, career development and retention. Recommendations regarding essential management capabilities in the long-term care workforce are set out with the intention of providing a basis for developing innovative and novel methods for improving and monitoring the quality and impact of management on the workforce.

Abstract citation ID: igad104.1070

### EXAMINING THE USE AND IMPACT OF CONTRACT CERTIFIED NURSING ASSISTANT STAFFING IN NURSING HOMES

Kezia Scales[1], Laurie Hailer-O'Keefe[2], Laura Wagner[3], and Lina Stepick[1], *1. PHI, New York City, New York, United States*, *2. UCSF Healthforce Center, San Francisco, California, United States*, *3. University of California, San Francisco, San Francisco, California, United States*

Certified nursing assistants (CNAs) provide the majority of hands-on care in nursing homes, and adequate CNA

staffing is associated with better resident and staff outcomes. However, low wages and overall poor job quality make adequate CNA staffing particularly challenging. There is evidence that nursing homes have increased reliance on contract staffing to fill staffing shortages, though the full scope of this trend for CNAs remains unclear and the impacts on care quality are still unknown. This study addresses this gap by examining staffing levels and patterns for fully employed versus contract CNAs in nursing homes over the past five years and assessing how these staffing trends are associated with quality outcomes for nursing home residents. Specifically, this session will present analysis of data from the Centers for Medicare and Medicaid Services (CMS) Payroll-Based Journal daily nurse staffing files, CMS nursing homes data archive, LTCFocus data, community transmission of COVID-19 data, and unemployment data to demonstrate how staffing patterns among fully employed versus contract CNAs varied from 2017 to 2022 according to facility-level characteristics, local market conditions, and pandemic-related factors. We also examine how CNA staffing levels and patterns affected care quality for nursing home residents. These findings will be presented with policy and practice recommendations for strengthening and stabilizing CNA nursing home staffing.

### SESSION 3290 (SYMPOSIUM)

Abstract citation ID: igad104.1071

### PRESIDENTIAL SYMPOSIUM: HIGHLIGHTING EMERGING PROFESSIONALS WHO BUILD BRIDGES, CATALYZE RESEARCH, AND EMPOWER ALL AGES

Chair: Brianna Morgan Co-Chair: Rita Hu Discussant: Jacqui Smith

There are many ways to carve a path forward in aging research, policy, and practice. Sometimes the career possibilities are overwhelming, leaving us unsure of how best to proceed. As aging professionals, we know that there is much to learn from those who have gone before us. Therefore, in the ESPO Presidential Symposium we highlight four emerging professionals who are trailblazing diverse and fulfilling career paths in aging by building bridges, catalyzing research, and empowering all ages. Speakers include Drs. Cal Halvorsen, Lindsay Kobayashi, Jamie Justice, and Glenna Brewster Glasgow. Dr. Jacqui Smith will serve as the discussant, synthesizing main findings, emphasizing positive outcomes, and exploring challenges. Starting with identifying the "So What?" of his career, Dr. Cal Halvorsen will explore how his practice roots in policy and advocacy led to his current approach of merging scholarly pursuits and public communication to make an impact beyond the walls of academia. Next, moving to cross-national aging research, Dr. Lindsay Kobayashi will discuss the opportunities and challenges of combining diverse cross-national cohorts to identify policy-relevant and context-specific modifiers of health risks for older adults. Next, Dr. Jamie Justice will focus on her career in translational geroscience and share a translational framework for aging outcomes trials. Finally, from an interdisciplinary approach to conducting research with families living with cognitive impairment in the US and the Caribbean, Dr. Glenna Brewster Glasgow will reflect on practical strategies to develop and maintain collaborative relationships with researchers and stakeholders locally, nationally, and internationally.

Abstract citation ID: igad104.1072

### FINDING THE "SO WHAT?": DEVELOPING A MEANINGFUL CAREER THAT MAKES AN IMPACT

Cal Halvorsen, *Boston College, Boston, Massachusetts, United States*

As a foundation to my social work scholarship, I consistently ask myself a question that my advisor once posed to me: "So what?" In other words, why and to whom does your research matter, and what are you going to do with it? In this presentation, I will show how my scholarship is framed by my practice experience working in Washington, DC for a think tank and advocacy organization. I will describe my approach to merging traditional academic pursuits with public communication and dissemination activities, including publishing in open-access journals, writing op-eds and reports, and pursuing organizational and community collaborations. I will provide specific examples of my collaborative research with local, state, and national organizations, including how we discuss potential research projects and reach mutually beneficial agreements. Lastly, I will describe how I blend this exciting and personally meaningful approach with more established expectations for tenure-track professors.

Abstract citation ID: igad104.1073

### CATALYZING CROSS-NATIONAL AGING RESEARCH: OPPORTUNITIES, CHALLENGES, AND STRATEGIES FOR EARLY CAREERISTS

Lindsay Kobayashi, *University of Michigan School of Public Health, Ann Arbor, Michigan, United States*

Cross-national comparisons in aging research allow for the triangulation of evidence on health outcomes across diverse populations and contexts. They allow researchers to identify policy-relevant and context-specific modifiers of health risks faced by older adults. Catalyzing cross-national comparison studies is a challenge, as researchers need to carefully consider social, cultural, political, socioeconomic differences across populations and make sure that local partners are appropriately represented and involved. This presentation will describe experiences in building a research career focused on understanding healthy cognitive aging from a global perspective, using data from diverse high-, middle-, and low-income settings. Opportunities, challenges, and strategies for early career researchers interested in catalyzing cross-national aging research will be discussed. These include the importance of advocacy for research on aging in contexts outside of the United States, theoretical and methodological approaches to cross-national research, and building diverse interdisciplinary collaborations.

Abstract citation ID: igad104.1074

### HARNESSING NETWORKS IN TRANSLATIONAL GEROSCIENCE RESEARCH

Jamie Justice, *Wake Forest University School of Medicine, Winston-Salem, North Carolina, United States*

Understanding and addressing age-related chronic diseases and disability requires a "root cause" approach: to look beyond the individual diseases to biological aging as the shared driver of multiple conditions. This goal is the foundation of an emerging discipline, geroscience. Geroscience has fostered new research on the basic biology of aging with rapid development of biomarkers of biological age, and therapeutics that target aging biology advancing to clinical trials. This presentation will provide a translational framework for early-career investigators who seek to evaluate biological processes underlying aging, and test promising interventions targeting these processes from animal models to clinical trials. This includes: 1) developing outcomes and biomarker frameworks for aging outcomes trials, and 2) establishing functional consequences of cellular senescence in older adults; and 3) creating opportunities to grow a diverse inter-disciplinary research program in geroscience through professional organizations, scientific networks, and NIA-supported research centers.

Abstract citation ID: igad104.1075

**BUILDING BRIDGES TO CONDUCT RESEARCH WITH FAMILIES LIVING WITH COGNITIVE IMPAIRMENT IN THE US AND THE CARIBBEAN**

Glenna Brewster, *Emory University, Atlanta, Georgia, United States*

My program of research aims to tailor and/or develop individual or dyadic behavioral and/or psychoeducational interventions to improve sleep and quality of life for persons living with cognitive impairment and their care partners. In order to conduct this research, it has been important to collaborate with colleagues in other disciplines and work beyond the borders of the United States. During this presentation, I will reflect on my research experience thus far using that to share practical strategies I used to develop and maintain collaborative relationships with researchers and stakeholders, and lessons learned from working within research teams locally, nationally and internationally.

### SESSION 3295 (PAPER)

### EDUCATION, TRAINING, AND EMPLOYMENT I

Abstract citation ID: igad104.1076

**A COMPARATIVE STUDY OF GRIEF SUPPORT AND BURNOUT AMONG NURSING HOME STAFF**

Frances Hawes[1], and Shuangshuang Wang[2], *1. University of Wisconsin-Eau Claire, Eau Claire, Wisconsin, United States, 2. Southwest Jiaotong University, Chengdu, Jiangsu, China (People's Republic)*

A half-million older adults die in U.S. nursing homes (NHs) each year. However, only a few studies have been conducted on the experiences of the nursing home (NH) staff who provide this care. This study collected data from 555 NH workers in Fall 2022 and examined how their experiences with grief support affected burnout. Grief support was measured by the Grief Support Health Care Scale. Burnout was measured by the Maslach Burnout Inventory, which includes 3 subscales: exhaustion, depersonalization, and personal achievement. Findings indicate that 95% of sampled workers were exposed to at least one death at work; yet 31% percent of the sample never received any formal grief training. Examining the comparative dimensions of burnout, the highest percentage of workers with exhaustion and depersonalization ratings were RNs, nurse practitioners, and physicians, followed by CNAs. The lowest ratings of personal accomplishment were by direct support workers (maids/janitors housekeeping and laundry food service workers). Controlling for NH and individual characteristics, multivariate logistic regression showed that compared to workers who received low grief support, those who received high grief support were more likely to report high personal achievement and low feelings of depersonalization (i.e., impersonal response to residents). This research provides valuable information about the importance of grief support for nursing home workers to reduce feelings of burnout. In addition, findings indicate that there is a difference in grief support and burnout between different categories of nursing staff and there is a need to provide increased grief support to frontline workers.

Abstract citation ID: igad104.1077

**PERSPECTIVES OF GERIATRIC FELLOWS TOWARD SCHOLARLY ACTIVITY**

Sadaf Milani[1], Adeeb Ahmed[2], Shilpa Rajagopal[1], and Mukaila Raji[3], *1. University of Texas Medical Branch, Galveston, Texas, United States, 2. University of Texas Medical Branch at Galveston, Galveston, Texas, United States, 3. The University of Texas Medical Branch, Galveston, Texas, United States*

Previous research conducted nearly two decades ago found that almost half of geriatricians published during their fellowships. About 17% of these geriatricians also indicated a need for further training in scholarly activity. However, it is unclear what barriers and facilitators to participating in scholarly activity geriatric fellows currently face. Our objective was to assess perceived barriers, facilitators, and attitudes toward scholarly activity among current geriatric fellows in ACGME-accredited programs. We conducted a survey of current geriatric fellows from September 2022 to November 2022. An online survey was emailed to geriatric fellows and geriatric program coordinators, when geriatric fellow emails were not publicly available, to assess fellows' perceived barriers, facilitators, and attitudes toward scholarly activity. We used descriptive statistics to assess geriatric fellows' perceptions of scholarly activity. Our sample (n=36) was mostly women (61.1%). The most common scholarly activities fellows planned to participate in during fellowship included quality improvement projects (80.6%), case report/series (44.4%), or original research studies (44.4%). The most common perceived major barriers reported were the short duration of fellowship (69.4%) and lack of training to conduct scholarly activity (58.3%). Lastly, about 44.4% of fellows reported being extremely or very enthusiastic and 50% reported being somewhat enthusiastic about participating in research during their fellowship. Most fellows reported plans for at least one type of scholarly activity and reported at least being somewhat enthusiastic about participating in research, however, barriers were identified. A follow-up email survey will be distributed at the completion of fellowship to assess changes in fellow perspectives.

Abstract citation ID: igad104.1078
### SKILLSETS FOR FUTURE-READY OLDER WORKERS: SKILLS GAPS AND LEARNING BARRIERS IN SINGAPORE
Vered Seidmann, Yuanyuan Cao, Yuezhong Liu, Ringo Ho, Andy Khong W. H., Dion Goh, and Yin-Leng Theng, *Nanyang Technological University, Singapore, Singapore*

Population ageing in Singapore is on a rapid rise as approximately 25% of Singaporeans will be aged 65 or over by 2030. The employment rate of older workers aged 55 and above rose from 24.3% in 2014 to 26.4% in 2021 (MOM , 2022). Older workers are at greater risk of skills mismatch. To enhance employability, governments, and international organizations explore appropriate responses to shifting skills, employability and training. This paper presents results of the qualitative phase in a two-year project that aimed to identify emerging skills, skills gaps, and training barriers among older persons in the Singaporean workforce and provide recommendations to policy makers to better support upskilling and reskilling among employees aged 55 and over. The project included 132 participants (29 age 45-54, 53 age < 55, 35 managers and 15 training providers) from three sectors: Retail, Food Services, and Precision Engineering. The qualitative data revealed skills mismatch resulting from the implementation of technological innovation in daily tasks. Learning barriers among older workers included accessibility to training, mindset and attitudes towards reskilling, fear of technology and training applicability. Recommendations to policy makers included practical solutions to enhance accessibility to course lists and career coaches; identify opinion leaders among older workers and company managers; closer collaboration between employers (inhouse training) and training providers for better follow-up and needs assessments; and utilize Andragogy theory for training design.

### SESSION 3300 (PAPER)

Abstract citation ID: igad104.1079
### EDUCATIONAL APPROACHES TO FACILITATE LEARNING IN GERONTOLOGY AND GERIATRICS COURSES

Presenters in this session share teaching approaches designed to foster engagement and reduce barriers to students engaging in gerontology and geriatrics educational opportunities.

Abstract citation ID: igad104.1080
### PILOT TESTING OF NEWLY DEVELOPED OPEN EDUCATIONAL RESOURCES FOR AN AGING AND MENTAL HEALTH COURSE
Jocelyn McGee, Madison Ambrose, Sinai Wood, and Chris Zakrzewski, *Baylor University, Waco, Texas, United States*

The cost of secondary-level education is higher than ever in the United States. With the costs of tuition, housing, meal plans, and other necessary expenses, economically vulnerable students have difficulty locating affordable textbook options for their courses. In addition to finding affordable options, it is challenging to locate low-cost and credible up-to-date resources that best prepare students for the fields they wish to pursue after graduation. Furthermore, within these resources, the perspectives of diverse and underrepresented scholars are often overlooked. With an ever-increasing and diversifying United States, these areas need to be addressed within gerontological education to provide the best care for older populations. In this presentation, we detail our process of developing open education resources (OER) for an Aging and Mental Health course from a positive psychology framework integrating diverse perspectives and state of the art research, results from our first pilot test of the materials with a group of 25 students, and how we are going about adapting this course into an interactive Pressbook which will be available in the future at no-cost to the field.

Abstract citation ID: igad104.1081
### PUBLIC HEALTH CURRICULUM: DEMENTIA CAREGIVING AS A PUBLIC HEALTH CHALLENGE AND OPPORTUNITY
Eva Jackson[1], Tara Redd[2], Elma Johnson[3], Dionne Bailey[3], Maya Koffski[3], Shelby Roberts[1], and Joseph Gaugler[3], *1. Alzheimer's Association, Chicago, Illinois, United States*, *2. Rollins School of Public Health, Emory University, Atlanta, Georgia, United States*, *3. University of Minnesota, Minneapolis, Minnesota, United States*

A new, free, one-hour, interactive, online module, Dementia Caregiving as a Public Health Challenge and Opportunity, is designed for public health students, educators and professionals. This course approaches caregiving for people with Alzheimer’s disease and other dementias from a population-based perspective , while remembering that these are the individual stories and lives of millions of people today. Dementia caregiving is a public health priority because of its prevalence; its profound influence on individuals, families, communities, and healthcare systems; and its disproportionate effects on subgroups of individuals. A number of public health actions can help to address the population effects of dementia caregiving. There remains a gap in public health curricula that features dementia and dementia caregiving, and this module represents an educational innovation to introduce key concepts in dementia caregiving for students and others. This module defines dementia caregiving, specifies why dementia caregiving is a public health issue, and reviews evidence-based programs for assisting dementia caregivers. Public health examples and case studies are used to support and assess learner understanding. This module was developed for the Alzheimer’s Association by the Emory Centers for Public Health Training and Technical Assistance with support from the Building our Largest Dementia Infrastructure (BOLD) Public Health Center of Excellence on Dementia Caregiving, and represents a novel strategy to elevate dementia caregiving as a matter of public health concern.

Abstract citation ID: igad104.1082
### PUSHING BACK AGAINST AI: USING UNGRADING TO PROMOTE CRITICAL THINKING AND INTRINSIC MOTIVATION
Jenny Inker, Catherine MacDonald, Michael Forder, Shannon Arnette, and Leland Waters, *Virginia Commonwealth University, Richmond, Virginia, United Sta tes*

Ungrading is an innovative pedagogical practice in which instructors give frequent, detailed feedback on students' work and/or encourage self-evaluation rather than assigning them points or letter grades. Ungrading has been shown to increase students' intrinsic motivation to learn, their willingness to take intellectual risks, and the quality of their thinking. Prior to the introduction of advanced artificial intelligence (AI) writing in academia, grades were shown to be a motivating factor for cheating. Ungrading removes the pressure of grades, thus making the use of AI and other services including paper writing and test answer sharing less enticing. Ungrading could be key to pushing back against the potential for the advent of artificial intelligence tools, which have many universities "alarmed." Educators at Virginia Commonwealth University College of Health Professions, supported by their Director of e-Learning, planned and piloted ungrading in one undergraduate and two graduate gerontology courses. Twenty-five students completed pre and post-surveys asking for their reactions to ungrading, if their views had changed as a result of the ungrading pilot, as well as their perceptions of traditional grading schemes. They also completed scaffolded assignments with multiple opportunities for receiving in-depth instructor feedback, rather than grades, at various points during the semester. Faculty journaled their ungrading experience in order to recognize how it can be a disruptive, and at times, uncomfortable practice and as a result, educators can better guide themselves and students through the transition. This presentation will include an Implementation Guide for gerontology faculty who wish to implement ungrading.

Abstract citation ID: igad104.1083

### VIRTUAL REALITY FOR PALLIATIVE CARE COMMUNICATION TRAINING: A NOVEL DISSEMINATION STRATEGY

Kei Ouchi, *Brigham and Women's Hospital/Harvard Medical School, Boston, Massachusetts, United States*

Towards the end of life, 75% of older adults visit the emergency department (ED). During acute health decompensation, most older adults with serious illness (predicted survival of less than one year) do not have advance directives and are at risk of receiving care inconsistent with their goals. To deliver patient-centered care, palliative care communication skills are essential, especially during the shared decision-making for cardiopulmonary resuscitation (i.e., code status conversations). Facilitated by skilled faculty and trained actors playing the role of a seriously ill older adult in acute health decompensation, the traditional palliative care communication training combines role play and individual feedback to practice communication skills. These evidence-based, palliative care trainings have demonstrated to improve communication skills in trained clinicians with enduring skills 12-15 months later. However, these trainings take a minimum of four hours with highly trained faculty and professional actors to role play, which hinders wider dissemination. To improve nationwide dissemination of evidence-based practices in code status conversations, we have leveraged virtual reality technology to allow clinicians to experience in-person, palliative care communication training. In collaboration with a virtual reality company, a 360-degree view, virtual reality training content has been filmed in a real hospital environment. Using the same evidence-based, palliative care communication contents, clinicians in training can now experience the role play in a virtual reality environment in one hour instead of four. By leveraging virtual reality technology, evidence-based palliative care communication training can now be disseminated with much less institutional investment (e.g., training time, faculty resources, etc.).

### SESSION 3305 (SYMPOSIUM)

Abstract citation ID: igad104.1084

### INNOVATIVE VOLUNTEER PROGRAMS TO SUPPORT FAMILY CAREGIVERS AND PERSONS LIVING WITH DEMENTIA

Chair: Noelle Fields Co-Chair: Ling Xu Discussant: Allison Gibson

There is a continued need for accessible and cost-effective community-based services and supports for family caregivers and persons living with Alzheimer's disease and related dementia (ADRD). Utilizing trained volunteers may offer a scalable approach to filling gaps in ADRD care and support. This symposium will provide evidence for utilizing trained volunteers in innovative interventions designed for family caregivers and persons living with dementia. The first paper presents findings from the Senior Companion Program Plus, an intervention using lay providers to deliver a culturally informed psychoeducational intervention with African American ADRD family caregivers. The second paper highlights the Faith Care Family Project which utilizes trained church volunteers from predominantly African American churches to reach African American ADRD family caregivers. The third paper explores the PorchLight Project, a dementia-capable training and respite delivered by volunteers for older people living in the community throughout Minnesota and in select regions of North Dakota. The fourth paper presents findings from an intervention using trained, experienced Chinese dementia caregivers to be volunteer mentors to newer dementia caregivers in the same ethnic community. The final paper presents findings from a telephone-based, intergenerational reminiscence intervention with college student volunteers and persons living with cognitive impairment that incorporates digital storytelling. The symposium will conclude with a critical reflection on the empirical contributions needed to advance scholarship on leveraging volunteers to meet the needs of family caregivers and persons living with ADRD.

Abstract citation ID: igad104.1085

### EXAMINING THE PRELIMINARY EFFECTIVENESS OF THE SENIOR COMPANION PROGRAM PLUS FOR AFRICAN AMERICAN CAREGIVERS

Ling Xu[1], Noelle Fields[1], Ishan Williams[2], Joseph Gaugler[3], Alan Kunz Lomelin[4], Daisha Cipher[1], and Gretchen Feinhals[5], *1. The University of Texas at Arlington, Arlington, Texas, United States*, *2. University of Virginia, Charlottesville, Virginia, United States*, *3. University of Minnesota, Minneapolis, Minnesota, United States*, *4. University of Texas at Arlington, Arlington, Texas, United States*, *5. The Senior Source, Dallas, Texas, United States*

**Objectives:** A culturally informed, peer-led, lay provider model, the Senior Companion Program (SCP) Plus, was implemented to decrease caregiving burden/stress and improve coping skills, and social support for African American ADRD caregivers. This study reported the preliminary effectiveness of this intervention.

**Methods:** An explanatory sequential mixed methods design was used in this study and a randomized control trial was conducted for the SCP Plus intervention among participants in three sites (N = 20). A sub-sample of participants (n = 7) consented to a qualitative interview about their experiences with the intervention. The Wilcoxon signed-rank tests, Friedman tests, and one-way repeated measures ANOVA were conducted for quantitative analyses. Thematic analysis was used for the qualitative interviews.

**Results:** Results demonstrated that knowledge of AD/dementia (KAD) and preparedness for caregiving were significantly improved for all senior companions in the intervention group. Results also showed that caregivers in the intervention group reported significantly decreased caregiving burden, as well as increased KAD, satisfaction with social support, and positive aspects of caregiving. Themes from the qualitative interviews included: learning new skills about caregiving, gaining knowledge about ADRD, and benefits for the dyad. Discussions: Findings from this study implied that SCP Plus was a promising model for African American family caregivers as it benefits both the SC volunteers and the African American ADRD family caregivers.

Abstract citation ID: igad104.1086

### THE FAITH CARE FAMILY PROJECT: A PILOT STUDY PROTOCOL

Ishan Williams[1], Noelle Fields[2], Ling Xu[2], and Fayron Epps[3], *1. University of Virginia, Charlottesville, Virginia, United States*, *2. The University of Texas at Arlington, Arlington, Texas, United States*, *3. Emory University, Atlanta, Georgia, United States*

The use of lay providers/trained volunteers, to address ADRD caregiver needs may provide a scalable and culturally congruent approach for bridging gaps in ADRD care, particularly for African American families. Research examining the church as a platform for raising awareness about ADRD suggests that churches are well-suited to providing support and services to families affected by ADRD. This research protocol describes the development and testing of the Faith Care Family Project (FCF), a lay provider intervention for African American family caregivers in a church setting to address caregiver burden, coping skills, and social support. Guided by the revised sociocultural stress and coping model, an explanatory sequential mixed methods design will be utilized to test the efficacy of the FCF intervention with a sample of 18 church volunteer-family caregiver dyads. Specifically, the study addresses four aims: 1) How do church members and pastors perceive the key components of the FCF and what are their recommendations for implementation? 2) Do the church volunteers gain knowledge of ADRD after the 6-hour, FCF training? 3) Do family caregivers report more knowledge of dementia, less caregiver stress and improved well-being, more social support, and better coping skills after they participate in the FCF? and 4) What are the experiences of participants with the FCF? Data are collected at baseline, mid-intervention, post- intervention, 3 months-post intervention and through qualitative interviews. These evaluation activities will allow us to determine the applicability of the FCF as an effective, lay provider intervention for African American ADRD family caregivers.

Abstract citation ID: igad104.1087

### LAUNCHING THE PORCHLIGHT PROJECT

Joseph Gaugler[1], Elizabeth Albers[2], Katie Louwagie[1], Noelle Fields[3], Eric Jutkowitz[4], Manka Nkimbeng[1], Tetyana Shippee[1], and David Roth[5], *1. University of Minnesota, Minneapolis, Minnesota, United States*, *2. University of Minnesota School of Public Health, Minneapolis, Minnesota, United States*, *3. The University of Texas at Arlington, Arlington, Texas, United States*, *4. Brown University, Providence, Rhode Island, United States*, *5. Johns Hopkins University, Baltimore, Maryland, United States*

The Porchlight Project (PLP) is a 5-year study designed to evaluate the real-world efficacy of dementia capable training and respite delivered by volunteers for older people living in the community throughout Minnesota and in select regions of North Dakota. Volunteer regions managed by the Lutheran Social Services of Minnesota (LSS-MN) throughout Minnesota and North Dakota were randomly assigned to receive the dementia training and volunteer support of PLP or the usual care control condition with standard volunteer training. The purpose of this presentation is to describe the process involved in establishing the PLP within LSS-MN's routine programming for volunteers and embedding key outcome measures within LSS-MN's standard data collection process for volunteers, their clients, and their clients' caregivers. Various challenges to PLP integration included: human subjects research categorization for an evaluation such as the PLP; achieving survey response rate goals; and establishing a baseline data collection interval prior to volunteer training completion. Strategies to proactively address these challenges to promote successful embedding of the program include creation of a PLP coordinator position within LSS-MN, who is responsible for the day-to-day programming of the project, as well as the development of a robust, yet flexible protocol. The flexibility allows for navigating LSS-MN programmatic activities, such as changes to the LSS-MN internal database, fluctuation in staffing and/or volunteers, revisions to survey follow-up procedures and correspondence, and consideration for those who will not complete training (e.g. non-English speakers, etc.). This presentation will also feature baseline descriptive data from the evaluation.

Abstract citation ID: igad104.1088

### A PILOT STUDY OF A PEER MENTORING SUPPORT INTERVENTION FOR CHINESE AMERICAN DEMENTIA CAREGIVERS

Jinyu Liu[1], Ethan Siu Leung Cheung[2], and Yifan Lou[3], *1. Baylor University, Waco, Texas, United States*, *2. University of Utah, Salt Lake City, Utah, United States*, *3. Yale University, New Haven, Connecticut, United States*

**Objectives:** Although many older Chinese Americans are expected to need intensive care because of cognitive impairment, a large gap exists in development of culturally sensitive interventions to reduce stress among caregivers in Chinese

American communities. Informed by the sociocultural stress and coping model, we developed and tested a peer mentoring program. In the intervention, experienced Chinese caregivers are recruited and trained to be volunteering mentors, who provide three-month mentoring support to newer caregivers in the same ethnic community.

**Methods:** A pilot randomized controlled trial was conducted among 38 Chinese American caregivers in New York City. During the three-month intervention, the 19 caregivers in the intervention group were paired with and weekly supported by 8 mentors via telephone calls. The control group received educational materials on caregiving skills and self-care. Three outcome variables, caregiving competence, loneliness, caregiver burden and depressive symptoms, were measured at the baseline, the 3-month follow-up and the 6-month follow-up.

**Results:** T-test results show no significant differences in any outcome measures at the baseline between the two groups. Compared to the control group, the intervention group had larger reduced scores in loneliness at the 3-month follow-up (p=0.047) and caregiver burden (p=0.057) and depressive symptoms at the 6-month follow-up (p=0.057). We did not identify any significant differences in caregiving competence changes identified between the intervention and control groups.

**Conclusion:** The pilot results indicate the potential effectiveness of empowering existing human resources of experienced caregivers in the same ethnic community in reducing stress of Chinese American dementia caregivers.

Abstract citation ID: igad104.1089

### INTERGENERATIONAL REMINISCENCE AND DIGITAL STORYTELLING: EXPLORING THE EXPERIENCE OF COLLEGE STUDENT VOLUNTEERS

Noelle Fields[1], Ling Xu[1], Brooke Troutman[2], Kathryn Daniel[3], and Allyson Miles[3], *1. The University of Texas at Arlington, Arlington, Texas, United States*, *2. Air Force Academy, Air Force Academy, Colorado, United States*, *3. University of Texas at Arlington, Arlington, Texas, United States*

Research suggests that reminiscence combined with an intergenerational approach may yield significant psychosocial benefits for older adults with Alzheimer's disease and related dementia (ADRD). Through intergenerational reminiscence, older persons may pass on their life experience and life lessons to younger generations. Intergenerational contact and relationships between older and younger adults can prevent stereotypes or negative attitudes from forming, or counter existing ones. Additionally, outcomes from intergenerational programming include the younger generation's ability to build relationships with older adults and have positive changes in attitudes toward aging. The current paper is part of a larger telephone-based intervention that used intergenerational reminiscence with digital storytelling in sample of university student volunteers and community-dwelling older adults with ADRD. University student volunteers were provided with training before the intervention about the myths and facts about ADRD, communicating with persons with ADRD, and how to create a digital story. Using Rapid and Rigorous Qualitative Data Analysis (RADar), a research team examined the weekly written reflections of the student volunteers (N = 27) during the 10-week pilot study. The analysis yielded 6 themes: positive connections, resilience, life lessons, empathy, collaboration, and growth. Many students reported increased confidence in talking with older adults and shared an interested in remaining connected after the study ended. Few students reported technological challenges with the DST. Findings from the study extend the existing literature related to volunteer-based interventions for older adults with ADRD and highlight the potential of involving younger adults in new approaches to ADRD research and practice.

### SESSION 3310 (SYMPOSIUM)

Abstract citation ID: igad104.1090

### EAST MEETS WEST: INNOVATIVE APPROACHES TO GERONTOLOGY EDUCATION AND TRAINING

Chair: Megumi Inoue Co-Chair: Takashi Amano
Discussant: Nancy Morrow-Howell

The global population is aging at an increasingly fast pace compared to past generations. Although the magnitudes and rates of aging in the United States and Japan differ, the drastic change in population structure has resulted in common challenges for both countries. One challenge is preparing people and society for living a long life while promoting well-being in old age for everyone. Combating ageism, delivering accurate information about aging, and encouraging people to have a balanced view of the aging process are all important topics for living in an aging society. Therefore, gerontology education and training plays a critical role. In this symposium, we will present examples of current efforts and research findings on aging education and training in both the United States and Japan. The first presenter will discuss their efforts on promoting gerontology education through redesigning communities with interdisciplinary collaboration in Japan. The second presenter will explain gerontology curriculum and degree programs in Japan. The third presenter will introduce an international gerontology education course designed for United States graduate students. The last presenter will report findings from a scoping review on intergenerational activities in higher education. Implications for future gerontology education will also be discussed.

Abstract citation ID: igad104.1091

### GERO-EDUCATION IN JAPAN: COMMUNITY RE-DESIGNING TO ACHIEVE HEALTHY AGING AND WELL-BEING

Katsuya Iijima, *The University of Tokyo, Tokyo, Tokyo, Japan*

Japan has achieved the world's highest level of life expectancy and is currently experiencing a super-aging society. Because of this, we have to take steps towards achieving a healthy aging society with evidence-based policies and longevity with happiness (well-being). In addition, it is necessary to integrate digitalization with information and communications technology through more than just the conventional healthcare measures such as going into the local community to increase opportunities for people from all generations to connect and interact. We also have to realize a true "population approach" with the aim of building a community where residents can easily engage in community-based activities centered on self-help and mutual aid. It is an urgent task to create a place for activities such as lifelong education and multi-generational exchanges as well as to build a comprehensively integrated community care system that allows people to live with peace of mind when they need long-term care. Thinking about planning the 100-year life, the super-aging society has had consequences for the healthcare and social care of older people in Japan, leading to societal innovations that promote health in older people. To extend the healthy life expectancy and achieve a bottom-up sense of community resilience and activity, an interdisciplinary gerontological collaboration with multi-stakeholders is indispensable, with collaborators such as municipal governments, industries, professional staffs, academic researchers (including universities, young career researchers, and students), and citizens. This session will introduce our efforts to promote gerontology education through redesigning the community with comprehensive knowledge.

Abstract citation ID: igad104.1092

### EXPANDING EDUCATIONAL OPPORTUNITIES IN GERONTOLOGY IN JAPAN

Noriko Tsukada[1], Nobuhiro Maeda[2], and Katsuya Iijima[3], *1. Nihon University, Setagata-city, Tokyo, Japan*, *2. NLI Research Institute, Chiyoda-ku, Tokyo, Japan*, *3. The University of Tokyo, Tokyo, Tokyo, Japan*

There are two universities that offer Gerontology curriculum and confer Gerontology degrees in Japan, including J.F. Oberlin University and The University of Tokyo. J.F. Oberlin University started its Master's program in 2002 and its doctoral program in 2004. The University of Tokyo endowed a Gerontology Research Division in 2006 and started its cross-departmental program in Gerontology in 2008. The endowed Gerontology Research Division was elevated to the Institute of Gerontology (IOG) in 2009. From April 2014 through March 2021, the Gerontology program, known as the Global Leadership Initiative for Age-Friendly Society (GLAFS) at The University of Tokyo, was funded by the Ministry of Education, Culture, Sports, Science and Technology. Today, The University of Tokyo now funds the program which was renamed World-leading Innovative Graduate Study Program in Gerontology: Global Leadership Initiative for Age-Friendly Society (WINGS-GLAFS). This paper first presents a brief history of Gerontology-related events in Japan, including foundational education proposals of The Science Council of Japan, then introduces Japan's leading Gerontology education programs offered by J.F. Oberlin University and The University of Tokyo in detail. Finally, it presents other gerontology related activities including the Gerontology Literacy Test started by the IOG in 2013.

Abstract citation ID: igad104.1093

### DEVELOPMENT OF AN INTERNATIONAL GERONTOLOGY EDUCATION COURSE BETWEEN THE US AND JAPAN

Takashi Yamashita[1], Flavius Lilly[2], and Mio Kamijo[2], *1. University of Maryland, Baltimore County, Baltimore, Maryland, United States*, *2. University of Maryland, Baltimore, Baltimore, Maryland, United States*

Sociocultural aspects of gerontology education have received growing attention in recent decades, as globalization and population aging resulted in more diverse older populations across global communities. Accordingly, gerontologists must equip with cultural and international competencies for their future careers, regardless of the fields, such as health care, long-term care, research, and education. This presentation outlines a development and case study (2023) of an international gerontology education course designed for graduate students in the U.S. After the preparation sessions, students take a faculty-led two-week trip to Japan, which is known as a super-aging society with nearly 30% of the population is age 65 years and older, and visit multiple aging-related sites including government agencies, research and education organizations, local communities, industries (e.g., robotics) and cutting-edge programs (e.g., health care research incubation program). The presentation offers three components. First, the academic objectives, such as the enhancement of cultural competencies and cross-national perspectives in gerontology, are described. Second, the practical course development process, including curriculum construction, planning trips, professional networking, student recruitment, funding source, coordination between two countries, and outcome assessment strategies, are illustrated. Finally, using the course evaluation data (n = 13 graduate students from 9 aging-related programs) and qualitative data (journals and field notes), positive outcomes (e.g., increased interest in working with socio-culturally diverse older adults) and challenges (e.g., funding international travel; scheduling difficulty; language barrier) are evaluated.

Abstract citation ID: igad104.1094

### INTERGENERATIONAL LEARNING TO ADDRESSING AGEISM IN HIGHER EDUCATION: SCOPING REVIEW FINDINGS

Michelle Hand, Catherine Tompkins, Emily Ihara, Megumi Inoue, Hyun Kang, and Kaitlyn Harvey, *George Mason University, Fairfax, Virginia, United States*

Intergenerational learning strategies can increase wellness, both among students and older community members, while challenging ageism on college and university campuses. Still, existing studies on the mutual benefits and effectiveness of intergenerational learning strategies for addressing biases among college and university students are limited and as such, are continuing to emerge. Thus, a scoping review was conducted to explore current research on intergenerational learning in higher education, the potential relevance of such strategies to reduce ageism and better prepare future service providers, and to explore current implications for education, practice, policy, and research. Included in this scoping review were peer-reviewed and grey sources based on primary research on what is known about intergenerational learning strategies in higher education and their potential impacts on ageism. Excluded were sources not based on primary research or without clear implications for relevant education, practice, policy or research. In total, 313 articles were identified; 67 of these were screened in for full text reading, and 37 were initially screened in for analysis, after which another 8 were excluded, resulting in 29 cases for thematic analysis. Key themes surround intergenerational service learning and creative expression, attitudes toward older adults, perceptions on aging, mutual benefits of intergenerational learning (e.g., cultural learning and integration), and remaining needs for research and education, through integrating service learning opportunities into existing programs and courses in higher education. The results of this scoping review will be further discussed, along with future implications for research, practice, policy and education focused on reducing ageism.

### SESSION 3315 (SYMPOSIUM)

Abstract citation ID: igad104.1095

### POLICY SERIES: GSA CONGRESSIONAL UPDATE

Chair: Patricia D'Antonio Co-Chair: Brian Lindberg

This popular annual session will provide cutting-edge information on what the 118th Congress has and has not accomplished to date, and what may be left for end of the First Session. Speakers will discuss key issues such as ending of public health emergency, Social Security, Medicare, Medicaid, and the Older Americans Act.

### SESSION 3320 (SYMPOSIUM)

Abstract citation ID: igad104.1096

### POLICIES TO OUTCOMES: MULTILEVEL CONSIDERATIONS FOR THE ASSISTED LIVING WORKFORCE

Chair: Sarah Dys Co-Chair: Cassandra Hua Discussant: Kezia Scales

Assisted living (AL) staff mix, levels, training requirements, regulatory enforcement, and career opportunities vary widely across license types, companies, and states. ALs represent the largest care providers for people living with dementia across the U.S. In most states, ALs must have enough qualified, trained staff to sufficiently meet the needs of their resident populations. While the use of interventions to address appropriate staffing levels and workforce retention is increasing, little is known about how the variation in the AL workforce relates to resident and staff experiences or manifests from policies governing these settings. This symposium uses a socioecological framework to examine AL-specific workforce variation. Presentations focus on national variation in staff training requirements, workforce recruitment and retention, trends in staffing levels, how staffing levels can relate to resident outcomes, and direct care staff experiences with stress and coping in long-term care communities. Findings emphasize similarities and differences in workforce composition and experience between states and across different types of facilities. From national comparison of state regulations to facility contexts to individual resident and staff experiences, this panel embraces multilevel contextual understanding of how policies, practices, and outcomes can intersect across AL and residential care environments. This is an Assisted Living Interest Group Sponsored Symposium.

Abstract citation ID: igad104.1097

### ASSISTED LIVING DIRECT CARE WORKFORCE TRAINING REQUIREMENTS BY LICENSE TYPE

Lindsey Smith[1], Sarah Dys[2], Cassandra Hua[3], Jacy Weems[4], and Kali Thomas[5], *1. Oregon Health & Science University, Portland, Oregon, United States*, *2. Portland State University, Portland, Oregon, United States*, *3. University of Massachusetts Lowell, Lowell, Massachusetts, United States*, *4. Brown University School of Public Health, Providence, Rhode Island, United States*, *5. Johns Hopkins University, Baltimore, Maryland, United States*

Assisted living (AL) residents and families often assume that care staff have clinical certifications or specialized training. We analyzed direct care worker training requirements for each AL license type across all 50 states and DC, using health services regulatory and content analyses. Across all licenses, we found the most common required training topics specific to care provision were resident rights, providing assistance with daily living, identifying and alleviating safety risks for residents with cognitive impairment, medication management and non-pharmacological interventions, nutrition and hydration, preservation of resident dignity, recognizing and reporting changes in a resident's condition, understanding resident actions and behavior as communication, and coping with difficult behaviors. We found the most common approach—accounting for the regulations governing 22% of licensed AL communities in the U.S.—was licenses that require between 1 and 10 hours of initial and annual training, but do not have a detailed list of training topics. Only three states (Arizona, Michigan, and New Hampshire) have no training requirements for any unlicensed direct care workers, while 20 states have at least one license without requirements regarding training hours or topics that must be covered. We found that 10 states have a license that requires more than 10 hours of initial and annual training and specifies a detailed list of training requirements. Interestingly, four of these states (New Mexico, Pennsylvania, Virginia, and Washington) have these requirements for all license types, but three of these states (North Carolina, Illinois, and DC) also have a license with no training requirements.

Abstract citation ID: igad104.1098

### ANALYSIS OF OHIO'S ASSISTED LIVING DIRECT CARE WORKFORCE

Katherine Kennedy[1], Cassandra Hua[2], and Matt Nelson[3], *1. Providence VA Medical Center, Providence, Rhode Island, United States*, *2. University of Massachusetts Lowell, Lowell, Massachusetts, United States*, *3. Miami University, Oxford, Ohio, United States*

Direct care workers (DCWs) in Ohio's licensed residential care facilities (RCFs) provide the most assistance with activities of daily living to individuals with disabilities. DCWs include medication aides, personal care aides, and nurse aides. In 2017, 41% of Ohio AL administrators reported high DCW recruitment issues and 36% high DCW retention problems (8+ on 10-point scale). Understanding how RCFs with no or little recruitment/retention challenges compare to other RCFs on strategies used, training hours, wages, benefits, resident composition, and RCF characteristics can identify potential strategies to increase recruitment/retention. This study uses the 2019 Ohio Biennial Survey of Long-Term Care Facilities (n=540 RCFs) to compare RCF strategies to recruit and retain DCWs by measures of recruitment/retention problem-severity and their 3-year retention rates among full-time (FT) and part-time (PT) DCWs. In 2019, 65% of Ohio RCF administrators reported recruitment issues at the median or greater (8+). Similarly, 62% of RCFs experienced retention issues at the median or more (7+). On average, 37% of FT DCWs worked in the RCF for 3 years or more and 21% among PT DCWs (ranges: 0%-100%). 28.24% of RCFs reported high 3-year retention among FT DCWs (60-100%) and 26.51% reported high 3-year retention among their PT DCWs (30%-100%). RCFs with less serious recruitment challenges report providing more hours of training for newly employed DCWs before they provide resident care (34.54 vs. 29.60, t=-2.7, p<.01). Individual retention strategies did not show relationships with high retention. Results indicate pervasive challenges with DCW retention and recruitment that require new approaches.

Abstract citation ID: igad104.1099

### TRENDS IN COMMUNITY-BASED CARE STAFFING LEVELS IN OREGON: DIFFERENCES BY FACILITY CHARACTERISTICS

Sarah Dys[1], Ozcan Tunalilar[1], and Paula Carder[2], *1. Portland State University, Portland, Oregon, United States, 2. Portland State University Institute on Aging, Portland, Oregon, United States*

The COVID-19 pandemic re-centered the workforce crisis across long-term care settings in the U.S. Variation in regulatory requirements, industry practices, staff training, and lack of longitudinal data introduces complexity in understanding workforce trends specific to assisted living and residential care (AL/RC). Pooling five waves of the Oregon Community-Based Care study, we examine trends in AL/RC care staff levels and associations with facility characteristics: license type, Medicaid census, profit status, and rurality. From 2017-2022, 548 unique facilities participated in the study, 55% contributing to four to five waves. The estimated average care staff-to-resident ratio was 0.79 (median= 0.67), increasing between 2017 (0.74) and 2022 (0.87). The average staffing level (care hours per resident per day; hours:minutes) was 3:28 (median= 2:58). We estimated ordinary least squares regression models with robust standard errors, controlling for facility-level characteristics and year. On average, staffing levels were higher by 1:38 in memory care (MC) and 0:42 in nonprofit settings. Compared to non-Medicaid settings, overall staffing levels were about 1:00-1:30 lower in mixed-payer settings, and 2:00 higher in settings providing specialty care (e.g., behavioral health, bariatric) at increased Medicaid reimbursement rates. Licensed nursing staff levels were slightly higher in MC, nonprofit, and special contract Medicaid settings. Certified nursing/medication aide levels were 0:30 higher in nonprofit settings. There were no differences in caregiver levels by profit status or rurality, though mixed-payer setting levels were lower by 0:32-0:44. Reduced occupancy during the pandemic resulted in higher staffing levels, though findings suggest continued disparities in staff mix by facility characteristics.

Abstract citation ID: igad104.1100

### STAFFING LEVELS AND INJURY-RELATED EMERGENCY DEPARTMENT VISITS IN OHIO

Cassandra Hua[1], Matt Nelson[2], Portia Cornell[3], Elizabeth White[4], and Kali Thomas[5], *1. University of Massachusetts Lowell, Lowell, Massachusetts, United States, 2. Miami University, Oxford, Ohio, United States, 3. Providence VA Medical Center, Providence, Rhode Island, United States, 4. Brown University, Providence, Rhode Island, United States, 5. Johns Hopkins University, Baltimore, Maryland, United States*

Assisted living (AL) residents with dementia are at increased risk of injuries compared to residents without dementia. Nursing home research indicates that changes in staffing levels are related to quality, including rate of injuries among residents. We leveraged novel data from a survey administered to AL communities in Ohio from 2007-2015 linked to administrative claims of Medicare beneficiaries with dementia to explore the relationship between staffing levels and injuries among AL residents with dementia. We examined the relationship between changes in each type of staffing level (RN, LPN, and aide hours per resident day [HPRD]) and injury-related emergency department use among AL residents with dementia. We adjusted for demographic characteristics, AL characteristics, and fixed effects for year and AL. Our sample included 13,929 fee-for-service Medicare beneficiaries with dementia (141,749 person-months) from 472 AL communities. Staffing levels increased slightly over time; median aide HPRD were 0.93 in 2007 and 1.10 in 2015. Median RN HPRD were 0.00 in 2007 and 0.04 in 2015. Median LPN HPRD increased from 0.29 to 0.41. Using linear regression modeling, we did not detect a statistically significant relationship between staffing levels (RN, LPN, or aide) and injury-related emergency department visits among AL residents with dementia. Within-AL changes in staffing levels may have no effect on rates of ED visits. However, it is possible that we did not observe enough within-AL variation to detect an association, or that unobserved differences in resident acuity associated with staffing levels biased our finding toward the null.

Abstract citation ID: igad104.1101

### STRESS AND COPING AMONG NURSING ASSISTANTS AND PERSONAL CARE AIDES IN LONG-TERM CARE

Lea Efird-Green[1], Philip Sloane[2], Christine Lathren[1], Krista Perreira[1], Karen Bluth[1], and Sheryl Zimmerman[1], *1. University of North Carolina at Chapel Hill, Chapel Hill, North Carolina, United States, 2. University of North Carolina, Chapel Hill, North Carolina, United States*

Care in nursing homes and assisted living communities is largely provided by direct caregivers (nursing assistants [NAs] and personal care aides [PCAs]), who are >50% minoritized, 20% immigrant, 90% female, 87% without a college degree, and often in poverty. The well-being of direct caregivers is critical to quality of care, but research aimed at improving well-being is hampered because existing measures of stress and coping translate poorly to their backgrounds and work-related experience. To address this gap, we designed and conducted research aimed at describing the range of coping approaches used to address work-related stress, gathering data on a diverse sample of 391 direct caregivers from 9 nursing homes and 4 assisted living communities in 3 states, of which 75% were racially or ethnically minoritized and 21% were immigrant – mirroring the national workforce. Results indicated that 78% of respondents often felt stressed at work, with 30% screening positive for depression and 28% for anxiety, with some caregivers experiencing both (21%). However, working with residents and their families, while stressful (76%), was considered less stressful than workload (90%) and lack of support (83%); in fact, 89% of caregivers said that caring for residents makes them feel appreciated. Coping strategies were diverse, but the most common includedresilient coping (93%), mindfulness (89%), recognizing common humanity (84%), self-kindness (83%), and planning (69%). This presentation will address differences in coping based on demographic characteristics, as well as the relationship between types of coping strategies and reasons for staying in a direct caregiver position.

### SESSION 3325 (SYMPOSIUM)

Abstract citation ID: igad104.1102

### THE AGING PROFESSOR OF GERONTOLOGY: MINING THE PLATINUM

Chair: Laura Donorfio Co-Chair: Lyn Holley Discussant: Becca Levy

This symposium explores evidence of the subtle, pernicious influence of unconsciously internalized ageist stereotypes on the engagement, job performance, perception of self and life satisfaction of older persons who are teachers of Gerontology in higher education. Unsolicited responses to the Tibbitts Award Lecture at the 2022 Conference of the Gerontological Society of American inspired this symposium and comments that indicated many older teachers of Gerontology in institutions of higher learning had become more aware of how internalized ageism and the ageism of others influences one's pedagogy of aging. This includes one’s gerontological imagination, self-disclosure, thoughts about retirement, and organizational self-esteem. Learning to accept one's own aging, personal growth, and activities to maintain professional competence are challenges to continuing in one’s role. This symposium captures the reflections of four established Gerontologists about these issues and how they shape careers, especially late life work. Results of a scoping literature review and some case studies of topics relevant to breaking the age code are reported. Topics to be covered include: the intersection of personal aging and aging pedagogy, career self-management of late life work, assessing and dealing with career competence and incompetence in late life work, and the Role of Age Friendly Environments. The discussant will capture the impact of ageism on these topics, and how working on beliefs about aging can influence and improve late life work and empower a productive lifespan.

Abstract citation ID: igad104.1103

### HOW CAN WE ADVANCE THE FIELD OF GERONTOLOGY BY LEVERAGING OUR LIVED EXPERIENCES AS GERONTOLOGISTS AND GERIATRICIANS?

Laura Donorfio, *University of Connecticut, Waterbury, Connecticut, United States*

Reflecting on my development as a professor of gerontology has become a normative part of my mid-life career. Through this reflection, I have identified 4 stages of career progression, none of which were planned nor anticipated. I have labeled the stages: 1. Content and Logistics; 2. Confidence and Innovation; 3. Generativity and Meaning; and 4. Being the Other. This last stage, which I presently float in and out of, has made me very aware that my teaching about aging has intersected with my personal aging and unexpectedly, has shaped my personal pedagogy in profound ways--increased empathy, sensitivity, and social awareness. I am living the subject matter I thought I intimately knew. I am the other. Has my teaching been inauthentic? Hypocritical? Ageist? Aging and ageism are now personal. Others have written about this mental transition or awakening (Brody, 2010; Cohen et al., 2022; Greenberg, 2022; Pruchno, 2017; Scheidt, 2017), but very little scholarship has been directed towards this phenomenon. O’Neill (2016) also brings attention to this shares, “Although geriatric medicine is widely perceived as an advocate for older people, it is striking how little scholarship has been directed towards the attitudes and personal beliefs of geriatricians toward ageing and, in particular, towards their own future narratives.” This presentation will draw from the existing literature to frame this emergent 4th stage of career progression and begin the discussion about how this insight can be leveraged to advance the fields of gerontology/geriatrics and teach the next generation of scholars.

Abstract citation ID: igad104.1104

### CAREER COMPETENCE IN LATE LIFE WORK

Lyn Holley, *University of Nebraska Omaha, Omaha, Nebraska, United States*

Career Competence in Late Life Work For most professionals, ageing manifests gradually – perhaps a practically imperceptible “slowing” of thought occurs sooner and sooner in the semester, perhaps a stiffness in joints or dozens of other cues from our gerontology lectures and texts; “natural” phenomena that signal we should be thinking of retirement. Self perceptions accumulate, their weight discouraging attempts to continue to teach energetically and creatively. Post-docs, pre-tenure faculty, and financially-challenged Deans withdraw, and the “voice in your head” agrees you are not what you were and should turn to figuring out how you can best spend the next ten to thirty years as your connectedness and career competence continue to dwindle. Meanwhile, the need for experienced faculty expands, and support to fund unemployed older adults edges nearer to financial collapse. A review of the literature on these phenomena suggests that excising the embedded ageist stereotypes is likely to unleash and focus energy on continuing to learn and contribute to society. Many colleges already have begun to provide support for late life learning, and to design opportunities for retirees and other older adults to contribute to the higher learning enterprise that enhance rather than shrink available “slots”, and expand capacity for research and education.

Abstract citation ID: igad104.1105

### CAREER SELF-MANAGEMENT OF LATE LIFE WORK

Harvey Sterns, *The University of Akron, Akron, Ohio, United States*

The choice to retire can be gradual or sudden depending on the planning, the opportunities presented, and crises encountered. Issues of self-management will be examined focusing on the challenges faced by older faculty members seeking to continue working. Present and future older academics are increasingly pressed to take active involvement and responsibility for career management. A multidimensional model for self-management of career and retirement will be presented.

Abstract citation ID: igad104.1106

**DON'T STOP BELIEVING – MAKING AGE MATTER**

Joann Montepare, *Lasell University, Newton, Massachusetts, United States*

As educators and scholars in the field of aging, many of us have seen how ageism operates in nuanced and powerful ways on many fronts and coming from different directions. In this presentation I will use examples from my personal experiences in higher education to describe how age biases can rear their heads and how they have guided me to respond. Beginning with my experiences as a junior faculty member in the 1980s when lifespan developmental approach was reshaping the traditional view of aging, I will share how attitudes about "age doesn't matter" prevailed and warranted brave and creative responses. Reshaping courses from the inside out to integrate aging became my mission and passion. Whether it a traditional developmental psychology class, a core social psychology class, or a specialized class on face perception – aging could be shown to matter. Fast forward 40 years and much as come to fruition, as seen in the response to the Age-Friendly University (AFU) initiative that I have been helping to build with AGHE leaders, GSA colleagues, and AFU partners. However, there is much more work to be done as we see ourselves being the older adult in our courses about whom we have discussed and the older faculty member in an organizational structure built around people of a certain age. However, we know that if we "Don't stop believin', hold on to that feelin" we can continue to pave the way for age inclusivity in even more impactful ways.

### SESSION 3330 (SYMPOSIUM)

Abstract citation ID: igad104.1107

**THE QUALITY OF NURSING CARE PROVIDED TO PATIENTS LIVING WITH DEMENTIA: FROM COMMUNICATION- TO FUNCTION-FOCUSED CARE**

Chair: Barbara Resnick

Although shortages in nursing abound across all levels of care, it is important that the little time nurses have to engage with patients living with dementia while hospitalized be optimal. The Function Focused Care for Acute Care intervention helps nurses interact with patients living with dementia in a way that engages them in physical activity during all care interactions. This includes such things as walking a patient to the bathroom instead of using a commode chair or bedpan, role modeling for patients and having them wash their face and upper body, walking patients in the hallway when agitated, or doing hand over hand feeding and bathing if the patient is totally dependent. Function focused care provides staff with ways in which to interact with patients living with dementia to be more positive and encouraging with regard to activity and engagement versus neutral or negative. Positive interactions are beneficial in that they encourage participation and engagement in care, neutral interactions are brief and indifferent and provide no encouragement, and negative interactions restrict patients often due to inappropriate safety concerns or for the convenience of the health care team. This symposium will provide an overview of the way in which nurses provide function focused care to patients living with dementia when hospitalized, the quality of care interactions provided and the interventions used in our Function Focused Care work that have helped to improve the percentage of time function focused is provided and the quality of care interactions.

Abstract citation ID: igad104.1108

**PERFORMANCE OF FUNCTION-FOCUSED CARE BY NURSING STAFF**

Elizabeth Galik, *University of Maryland School of Nursing, Baltimore, Maryland, United States*

To help acute care nurses learn to provide function focused care during care interactions the Function Focused Care intervention is implemented on nursing units. The Function Focused Care intervention is based on the Social Ecological Model, Social Cognitive Theory and the Evidence Integration Triangle and includes four components: (1) Development of a stakeholder team; (2) Education; (3) Development of functional care plans; and (4) Mentoring and motivating. Our nurse interventionist provides function focused care education as per the preference of the setting. Following education, direct observation during care interactions is done and the nurse interventionist provides recommendations related to the approach being used or provides positive reinforcement if function focused care is provided. The observation tool used to evaluate function focused care by the nursing staff includes 19 different care interactions (e.g., bathing, dressing, transferring). Guidelines are provided for what is and is not function focused care. In the first four treatment sites, at 2 months post implementation the nurses provided function focused care during 81% of the of care interactions observed, by 6 months this increased to 85% and by 10 months this increased to 88%. There was also a decline in the number of care interactions in which function focused care was not provided. The findings suggest that nurses can provide function focused care even during times of significant shortages and that continued oversight and education can help improve the use of this approach.

Abstract citation ID: igad104.1109

**QUALITY OF CARE INTERACTIONS IN ACUTE CARE**

Rachel McPherson, *University of Maryland, Baltimore, Maryland, United States*

Care interactions are verbal or nonverbal exchanges between staff and patients and promote quality of care and optimize outcomes for hospitalized patients living with dementia. Care interactions vary regarding quality and are differentiated between positive interactions (e.g., interactions that provide beneficial companionship or appropriate care communication for residents), neutral interactions (e.g., brief, indifferent interactions), or negative interactions (e.g., interactions that restrict residents due to safety concerns or interactions that are restrictive for no justified reason). Patients exposed to negative care interactions, particularly those that are living with dementia, are at risk for negative outcomes such as increased resistiveness to care, anxiety, depression, and apathy. The Quality of Care Interactions

Survey (QuIS) is an observation measure used to evaluate interactions between staff and patients based on definitions of positive social, positive care, neutral, negative protective, or negative restrictive interaction. Scores range from 0 to 7, with higher scores indicating more positive care interactions between staff and residents. Nursing interactions with the first 365 patients in the FFC-AC-EIT study indicated that on admission the majority of the care interactions were positive (82%), half were neutral (50%), and a small percentage were negative protective (7%) and negative restrictive (6%). By discharge, there was a decrease in neutral interactions to 47%, negative protective to 6% and negative restrictive to 5%. Ongoing oversight and education is needed to decrease neutral and negative interactions between staff and patients using techniques provided in our function focused care intervention.

Abstract citation ID: igad104.1110

### WHAT DO NURSES NEED TO IMPLEMENT FUNCTION-FOCUSED CARE IN THE HOSPITAL?

Marie Boltz, *Pennsylvania State University, State College, Pennsylvania, United States*

The intervention, FFC-AC-EIT, is a theoretically based intervention that includes the implementation of four steps: (1) Environment and policy assessments; (2) Education of staff; (3) Establishing patient goals; and (4) Mentoring and motivating of staff, patients, and families. The nurse interventionist works with a unit-based stakeholder team to develop function-focused goals for the unit and an action plan to meet the goals, while considering barriers to implementation. Based on content analysis of field notes we will describe stakeholder goals established, action plans, and barriers and facilitators to meeting goals in five hospital units. Goals focused on improving staff competency in increasing mobility and physical activity in persons with dementia, integrating mobility assessment and plans into workflows, and improving patient outcomes including decreased falls and less use of constant observation. Common barriers included unstable staffing, inconsistent prioritization among staff and managers, family attitudes, and lack of operating procedures and resources to provide function-focused care. Facilitators at the unit level included administrative support, nurse managers being actively involved in all aspects of planning and oversight, engagement of families, effective communication strategies, involvement of nursing assistants, interdisciplinary collaboration, and access to necessary supplies and equipment. Key implementation strategies included multiple approaches to staff training and communication, the use of champions, clear operating procedures, hardwiring assessment and plans into the electronic record, the use of a feedback loop, and alignment of functional-focused care with other hospital and unit quality initiatives.

Abstract citation ID: igad104.1111

### THE ASSOCIATION BETWEEN RESIDENT ENGAGEMENT AND QUALITY OF CARE INTERACTION AMONG ACUTE CARE PATIENTS WITH DEMENTIA

Rachel McPherson, *University of Maryland, Baltimore, Maryland, United States*

Care interactions are essential to patients with dementia. The purpose of the study was to describe the quality of staff-patient care interactions among acute care (AC) patients with dementia and to test whether the quality of staff-patient care interactions varied by resident level of engagement in the interaction. Specifically, it was hypothesized that controlling for age, cognition, pain, behavior, and function, actively engaged patients would have more positive care interactions with staff compared with passively engaged patients. This secondary data analysis used baseline data from the Function Focused Care for Acute Care (FFC-AC- EIT), an ongoing intervention study. The sample included 286 patients. Descriptive statistics and a multiple linear regression were conducted. The majority of interactions that occurred were positive care ($n = 225$, 79%). Actively engaged patients had significantly more positive interactions compared to passively engaged patients ($b = 1.49$, $p < .001$). Patients with more pain ($b = -0.26$, $p < .001$) and behavioral symptoms ($b = -0.12$, $p < .05$) had significantly poorer care interactions. Passively engaged patients or patients with pain and behavioral symptoms may be at risk to receive poor quality care interactions. Findings from this study will inform future interventions and training for AC staff to promote positive care interactions among patients with dementia. Managing pain, managing behavior, and supporting resident engagement in care will likely help increase the frequency of positive care interactions in future work.

### SESSION 3335 (PAPER)

### WORKFORCE AND HEALTH ISSUES IN CARE OF OLDER ADULTS

Abstract citation ID: igad104.1112

### DOES WORKFORCE ROLE IMPACT LEARNING IN NURSING HOME EDUCATION? EVIDENCE FROM THE CHATO NATIONAL TRIAL

Clarissa Shaw[1], Kristine Williams[2], Maria Hein[3], Yelena Perkhounkova[4], Carissa Coleman[5], and Frances Yang[5], *1. University of Iowa, Iowa City, Iowa, United States*, *2. University of Kansas Medical Center, Kansas City, Kansas, United States*, *3. University of Iowa, College of Nursing, Iowa City, Iowa, United States*, *4. University of Iowa, Iowa City, Iowa, United States*, *5. University of Kansas, Kansas City, Kansas, United States*

Nursing home residents rely on a variety of staff to meet their physical and psychosocial needs. Our communication intervention for nursing home staff—entitled CHATO—is currently being tested in a pragmatic clinical trial nationwide. All staff, regardless of role, are encouraged to participate to create a culture of person-centered communication. We analyzed differences in learning outcomes, and completion and satisfaction, among five staff roles including 274 aides (CNA, CMA), 180 nurses (LPN, RN), 52 administrators, 42 professional staff (social work, speech-, physical-, occupational-therapy), and 134 other non-care staff (dietary, custodial, transport). We compared changes in knowledge of effective person-centered communication, measured by (1) scores on the Changing Talk Scale (CHATS) and (2) ratings of video-based communication appropriateness, communication effectiveness, recognizing person-centered, and elderspeak communication. At baseline, aides and non-care staff had significantly lower CHATS scores than professional staff (p<.05). All roles demonstrated similar and significant knowledge gain with mean score increases ranging from 17.8-24.6 (p's <.001). Similar patterns of changes were observed for communication ratings. The overall completion rate for enrolled staff was 82.6%, of whom only 6.2% reported dissatisfaction. The completion and satisfaction outcomes did not vary by role. These findings suggest that CHATO can be a useful education program for enhancing person-centered communication across different staff roles. Although baseline knowledge was higher in certain roles, the knowledge gains, completion rates, and satisfaction ratings did not vary by staff role. It is important to remember that all staff, not just direct care staff, benefit from education in nursing homes.

Abstract citation ID: igad104.1113

### INCLUSION OF HEARING AND VISION IMPAIRMENTS IN COGNITIVE TRAINING INTERVENTIONS WITH OLDER ADULTS

Francesca Marino[1], Kening Jiang[1], Jason Smith[1], Diefei Chen[2], Jennifer Deal[3], George Rebok[3], and Alison Huang[1], *1. Johns Hopkins Bloomberg School of Public Health, Baltimore, Maryland, United States*, *2. Johns Hopkins, Baltimore, Maryland, United States*, *3. Johns Hopkins University, Baltimore, Maryland, United States*

Cognitive training intervention implementation and efficacy should be evaluated among representative samples, including those at highest risk of cognitive decline. Hearing and vision impairments are prevalent among older adults and confer an increased risk of cognitive decline/dementia. Whether cognitive training interventions enroll and are designed to include this important subgroup is unknown. A scoping review of PubMed and PsycINFO was conducted to examine the inclusion of older adults with hearing and/or vision impairment in cognitive training interventions. Eligible articles included English-language cognitive training randomized controlled trials and a study population that was cognitively unimpaired, aged ≥55-years, and community-dwelling. Among the 130 articles in the review, 103(79%) were cognitive training and 27(21%) were multimodal interventions that included cognitive training. Over half the trials systematically excluded participants with hearing and/or vision impairment (n=60, 58%). Few studies measured and reported participant hearing and/or vision impairment (cognitive: n=16, 16%; multimodal: n=3, 11%) or reported incorporating low hearing/vision accessibility features into intervention design (cognitive: n=7, 7%; multimodal: n=0, 0%). Greater representation of older adults with hearing and/or vision impairment in cognitive training interventions is needed and can be achieved by strengthening measurement and reporting of participant hearing and vision, minimizing barriers to participation by increasing accessibility in recruitment and intervention design, and intentional inclusion of older adults with hearing and/or vision impairment. With greater representation of older adults with hearing and/or vision impairment, studies can better evaluate group-specific differences in training effect and begin to tailor interventions to potentially maximize benefit.

Abstract citation ID: igad104.1114

### THE ASSOCIATION OF COVID-19 OUTBREAKS AND OTHER FACTORS WITH NURSING HOME RESIDENTS' QUALITY OF LIFE

Matthias Hoben[1], Emily Dymchuk[2], Sube Banerjee[3], and Carole Estabrooks[2], *1. York University, Toronto, Ontario, Canada*, *2. University of Alberta, Edmonton, Alberta, Canada*, *3. University of Plymouth, Plymouth, England, United Kingdom*

Nursing home (NH) residents' Quality of life (QoL) is an important goal of care. However, it is understudied, particularly during the COVID-19 pandemic. Our objective was to examine whether COVID-19 outbreaks, care aide emotional exhaustion, and lack of resident access to geriatric professionals were associated with NH residents' QoL. In this cross-sectional study (Jul-Dec 2021), we purposefully selected 9 NHs in Alberta, Canada based on their COVID-19 exposure (no or minor/short outbreaks vs repeated or extensive outbreaks). Using a validated questionnaire (DEMQOL-CH), we assessed dementia-specific QoL of 689 residents from 18 care units through video-based interviews with care aides. Independent variables included COVID-19 outbreak in the NH in the last 2 weeks (health authority records), proportion of care aides on a care unit with high emotional exhaustion scores (9-item short form Maslach Burnout Inventory), and resident access to geriatric professionals (validated facility survey). We ran mixed-effects regression models, adjusted for facility and care unit characteristics (validated facility and care unit surveys), and resident covariates (Resident Assessment Instrument – Minimum Data Set 2.0). COVID-19 outbreaks within two weeks of the data collection (β=0.189, 95% confidence interval [CI]: 0.058;0.320), higher proportions of emotionally exhausted care aides on a care unit (β=0.681, 95%CI: 0.246;1.115) and lack of access to geriatric professionals (β=0.216, 95%CI: 0.003;0.428) were significantly associated with poorer resident QoL. Policies aimed at reducing infection outbreaks, better supporting care staff, and increasing access to geriatric specialists, may help to mitigate negative effects of COVID-19 NH residents' QoL.

Abstract citation ID: igad104.1115

### VARIATION IN USE OF HOSPICE AIDES BY RESIDENTIAL SETTING

Jennifer Reckrey, Karen Mckendrick, and Melissa Aldridge, *Icahn School of Medicine at Mount Sinai, New York City, New York, United States*

Hospice care frequently includes visits from hospice aides, but need for hospice aide care may be different for those in the community compared to settings like assisted living or nursing homes where personal care is routinely provided. The objective of this longitudinal cohort study is to identify factors associated with hospice aide visits (any versus none) and frequency of hospice aide visits (proportion of routine hospice cays with an aide visit) in different residential settings (community, assisted living, and nursing home). We identified Medicare beneficiaries enrolled in the Medicare Current Beneficiary Survey (MCBS) who died between 2010-2019 (n=1,915). We used self-reported survey responses, Medicare claims data, and Medicare Provider of Service data to compare decedent hospice care experience across residential settings and estimated multivariable models to compare factors associated with hospice aide visits among residential settings. Hospice aide visits were least common in the community setting (64.4% vs 76.6% vs 72.6% with any hospice aide visits in community, assisted living, and nursing home respectively, p=0.002). In models adjusted for sociodemographic, clinical/functional, and hospice factors, neither hospice aide visits nor the proportion of routine hospice days with hospice aide visits varied significantly by residential settings. Future work to understand when and why hospice aides are utilized and how hospice aides collaborate with families and existing care teams in will help ensure that hospice care is appropriately tailored to individual care needs in all residential settings.

### SESSION 3340 (SYMPOSIUM)

Abstract citation ID: igad104.1116

### AN INTERNATIONAL PERSPECTIVE ON OPTIMIZING FUNCTION AND ACTIVITY IN GERIATRIC CLIENTS ACROSS ALL SITES OF CARE

Chair: Sandra Zwakhalen Co-Chair: Silke Metzelthin
Discussant: Barbara Resnick

With an increasing age, people are at risk for functional decline and care dependency. Frail older people in any site of care –at home, in nursing homes and in acute care– are supported by nursing staff to optimize their functioning, remain active, and maximize their independence. However, nursing staff often tend to work in a task-oriented manner and take over tasks unnecessarily which may deprive older people's remaining abilities and adversely impact their life quality. Nursing staff require support to improve their activity supportive behavior in practice. This symposium presents the results of several international studies from primary care, home care, nursing home care and acute care, which aim to improve nursing staff behavior and –in turn– client outcomes. First, Prof. Galik will present the results of a randomized controlled theoretically-based trial testing the 'Function-Focused Care' approach in which over 350 patients participated. Dr. Vluggen will present the results of a cluster-randomized trial conducted in Dutch nursing home care in which the effectiveness of the 'SELF-program' is evaluated, including over 250 nursing staff and 200 patients. Dr. Metzelthin will discuss the feasibility of the SELF-program, which has been subjected to a thorough pilot-study in Dutch home care. Last, Prof. Graff will discuss the experiences of people with dementia and caregivers after following a coaching-based psychosocial program for primary care (SOCAV). The symposium is chaired by Dr. Vluggen and discussed by Prof. Resnick who has a considerable track record in intervention studies focusing on optimizing function and activity in geriatric clients.

Abstract citation ID: igad104.1117

### EFFECTIVENESS OF THE "SELF-PROGRAM" IN DUTCH NURSING HOME CARE: RESULTS OF A MULTICENTER CLUSTER-RANDOMIZED TRIAL

Stan Vluggen[1], Silke Metzelthin[2], Sandra Zwakhalen[3], Michel Bleijlevens[3], Janneke M de Man-van Ginkel[4], and Getty Huisman - De Waal[5], *1. Zuyd Hogeschool University of Applied Sciences, Kerkrade, Limburg, Netherlands*, *2. Maastricht University, Care and Public Health Research Institute, Maastricht, Limburg, Netherlands*, *3. Maastricht University, Maastricht, Limburg, Netherlands*, *4. Leiden University Medical Center, Leiden, Zuid-Holland, Netherlands*, *5. Radboud University Medical Center, Nijmegen, Gelderland, Netherlands*

For frail older people it is evident to maintain their functional abilities and independence. Nursing staff are in a key position to encourage older people's independence and stimulate their engagement in functional activity. However, nurses tend to take over tasks frequently thereby depriving older people's remaining abilities. Clearly nurses need support to optimize their activity encouragement behavior. The Function-Focused Care based 'SELF-program' was developed based on lessons learned and implications from previous programs. SELF is an interactive, tailored, holistic and theory-grounded training program consisting of 7 seven sessions spread over a period of 3 months which aims to improve nurses' activity encouragement behavior and in turn client's self-reliance in daily activities. The program was subjected to a cluster-randomized trial in Dutch nursing home care which aimed to evaluate the program's effectiveness. The MAINtAIN and GARS-5 questionnaires were applied to assess the effectiveness on nurses' activity encouragement behavior and clients' self-reliance. 28 wards from three care organizations across the Netherlands were recruited and randomized to the SELF-program or care as usual. 287 nurses and 241 geriatric clients participated. Measurements took place at baseline, directly after implementing the SELF-program (three months) and 9 months after baseline. Mixed linear regression demonstrated that nurses' activity encouragement behavior significantly improved (p=.004; ES=.05). At client level, no effects were found (p=.09; ES=.13). However, a trend towards slower decline in self-reliance was observed for those clients allocated to the SELF-program condition. The SELF-program was effective in improving nurses' behavior, however, effects were not translated to client populations.

Abstract citation ID: igad104.1118

### INCREASING ACTIVITY-STIMULATING BEHAVIOR IN DUTCH HOME CARE: FEASIBILITY OF THE SELF-PROGRAM

Silke Metzelthin[1], Lotte Hermens[2], Getty Huisman - De Waal[3], Stan Vluggen[4], Janneke Man - van Ginkel[5], and Sandra Zwakhalen[2], *1. Maastricht University, Care and Public Health Research Institute, Maastricht, Limburg, Netherlands*, *2. Maastricht University, Maastricht, Limburg, Netherlands*, *3. Radboud University Medical Center, Nijmegen, Gelderland, Netherlands*, *4. Zuyd Hogeschool University of Applied Sciences, Kerkrade, Limburg, Netherlands*, *5. Leiden University Medical Center, Leiden, Zuid-Holland, Netherlands*

The concept of aging-in-place has become increasingly popular worldwide, including the Netherlands. However, as individuals age, their ability to perform activities of daily living often declines leading towards a need for homecare. Unfortunately, staff often unnecessarily take over care tasks and thereby potentially accelerate older adults' functional decline. SELF is an interactive, tailored, holistic and theory-grounded training program consisting of 7 sessions spread over a period of 14 weeks, which aims to change staffs' behavior towards more activity stimulating behavior. The program was evaluated in a cluster-randomized trial in Dutch nursing homes. However, its feasibility in the homecare setting was not yet known. Therefore, a feasibility study in one Dutch homecare team was conducted using a mixed-methods approach combining quantitative (i.e. checklist, logbooks, attendance forms and questionnaires) and qualitative data (i.e. focus group interviews) among program participants, team manager, coaches and trainers. Data was collected based on the three main pillars of the MRC framework: implementation, mechanisms of impact and contextual factors. In total, 24 staff members participated in the study, who delivered care to 145 clients. The SELF-program was largely implemented as intended, resulting in positive changes in staff knowledge, self-efficacy, and skills. However, contextual factors influenced the implementation of activity stimulating behaviour in practice. While reporting in electronic patient records and manager support were identified as facilitators, resistance from clients, bad expectation management by hospitals and GPs and time constraints acted as barriers. Overall, the SELF-program was feasible in Dutch homecare and only small program modifications are needed.

Abstract citation ID: igad104.1119

### FUNCTION-FOCUSED CARE FOR ACUTE CARE USING THE EVIDENCE INTEGRATION TRIANGLE: IMPACT OF PATIENT CARE ACTIVITIES

Elizabeth Galik[1], Barbara Resnick[2], and Marie Boltz[3], *1. University of Maryland School of Nursing, Baltimore, Maryland, United States*, *2. University of Maryland, Baltimore, Maryland, United States*, *3. Pennsylvania State University, State College, Pennsylvania, United States*

The benefits of physical activity during hospitalization are particularly relevant for older adults living with dementia, as these individuals are at the greatest risk for functional decline, delirium, behavioral symptoms associated with dementia, increased length of stay, adverse events increased readmissions and institutionalization post discharge. One effective way to engage these patients in physical activity is through implementation of a function focused philosophy of care. Function focused care helps nurses to evaluate patient's underlying physical capability and motivate them to participate in all care activities. The Function Focused Care for Acute Care Using the Evidence Integration Triangle study is a randomized controlled theoretically based trial that includes four components include: (1) Development of a Stakeholder Team; (2) Education of Staff; (3) Development of a Function Focused Care careplan; and (4) Mentoring and Motivating of Staff and Patients. Those randomized to control were exposed to education about Function Focused Care only. To date a total of 10 hospitals and 365 patients living with dementia have participated in the study. The mean age is 82, and the majority are female (62%) and white (72%). Although not statistically significant, there is evidence of improvement in the number of care interactions in which patients participated in the treatment versus control group. Cognition, quality of care interactions, behavioral and psychological symptoms associated with dementia, comorbidities, physical resilience, tethers and pain were all indirectly associated with performance of function focused care through performance of activities of daily living and/or pain.

Abstract citation ID: igad104.1120

### BEHAVIORAL CHANGES OF NURSING STAFF AFTER A COACHING-BASED PSYCHOSOCIAL PROGRAM SUPPORTING SELF-DIRECTION IN PLWD

Maud Graff, Phebe Das, Gebrich Douma, Lieve Roets, and Hanneke Donkers, *Radboud University Medical Center, Nijmegen, Gelderland, Netherlands*

The psychosocial person-centred intervention "SOCAV-in-primary-care" (SOCAV-PC) offers training and reflective peer-coaching to nursing staff and training and person-centred care-planning to caregivers. SOCAV-PC aims to support persons with dementia (PwD) to maintain/improve their self-direction and daily functioning in meaningful activities with support of their caregivers. This study presents the experiences of the PwD, and changes in attitudes and behaviour of caregivers and nursing staff when following SOCAV. Spread over nine months, caregivers and nursing staff were trained and coached in the person-centred approach by a trained SOCAV-peer coach. Content analysis was performed using qualitative data derived from (1) intervention and coaching diaries from nursing staff, (2) coaching diaries of peer coaches, (3) focus groups among nursing staff, and (4) individual interviews with PwD and their caregivers. PwD felt heard and respected in making their own choices. Caregivers became more aware of the influence of their own behaviour on the mood of the PwD, and started trying to convert their controlling behaviour, meant to provide safety, into supporting wishes and needs of PwD. Among nursing staff, an ongoing process of positive changes in attitudes and behaviours towards person-centred care emerged: from acting on intuition and automatisms, towards learning to reflect on the behaviour of PwD and their own actions. Moreover, they gained a better understanding of the concept of self-direction, and discovered the power of conversation and observation through a more open attitude and behavioural change. Reflective coaching is an essential tool to enhance person-centred attitudes and behaviour among nursing staff.

### SESSION 3345 (SYMPOSIUM)

Abstract citation ID: igad104.1121

### THE NIH STAGE MODEL AND STUDY EXAMPLES OF EARLY STAGES IN DEMENTIA CARE INTERVENTION DEVELOPMENT RESEARCH

Chair: Debra Dobbs Discussant: Hongdao Meng

The NIH's Stage Model provides a common language to facilitate discussion of intervention development research. According to NIH Stage Model (2022), "an examination of the mechanisms of behavior change or the principles underlying an intervention is encouraged by the NIH Stage Model in every Stage of behavioral intervention development." The five papers in this symposium focus on early-stage intervention development by refinement, adaptation and pilot testing (Stage 1) related to dementia and palliative care (PC) clinical trials. The first paper is a cluster trial of a PC education program for nurses (N=23) in 10 assisted living communities that tested the mechanism of staff self-efficacy in increases in advance care planning discussions with family members of residents living with dementia. The second paper is a clinical trial that tested the feasibility of Companion Robots in live-alone Korean-American older adults (N=30) to determine which features help improve health behaviors (e.g. medication adherence, daily exercises). The third paper is a single-site clinical trial pilot study developed with stakeholders which tested the feasibility of a palliative care community health worker support program for hospitalized minoritized persons living with dementia and their caregivers. The fourth paper adapts an evidence-based e-PainSupport intervention for use with dementia caregivers to report patient pain and administration of analgesics. The fifth paper is a protocol to test the feasibility and acceptability of a culturally sensitive caregiver peer support program (Pair2Care) in current (n=15) and trained former (n=15) African-American dementia caregivers. The discussant will highlight ways to improve clinical trial mechanisms.

Abstract citation ID: igad104.1122

### AN EDUCATIONAL PROGRAM IN ASSISTED LIVING TO INCREASE STAFF SELF-EFFICACY IN PROVISION OF DEMENTIA PALLIATIVE CARE

Debra Dobbs[1], Hongdao Meng[1], Jessica Yauk[1], Harleah Buck[2], and William Haley[1], *1. University of South Florida, Tampa, Florida, United States*, *2. University of Iowa, Iowa City, Iowa, United States*

Advance care planning among persons with dementia, their family members and staff in assisted living (AL) communities can facilitate meeting care preferences. In a recent NIH Stage 1a feasibility cluster randomized trial in AL communities (N=118 residents with dementia, 23 staff; k=10 ALs). The Palliative Care Education in Assisted Living for Dementia Care Providers (PCEAL-DCP) intervention showed an increase in documentation of ACP discussions (e.g., completion of advance directives, having goals of care discussions with family) in the treatment group from 12% to 51% compared to control group (8% to 23%) from baseline to six-months (effect size 0.86, Cohen's d statistic). The current study is an NIH Stage 1b pilot using the same sample to test staff self-efficacy as a mechanism and if staff self-efficacy mediates the effects of increased documentation of ACP discussions. The measure of staff self-efficacy includes two self-reported survey items at pre- and post-intervention (baseline and one-month) that asks them how much they agree with their ability to provide care for terminally ill persons and terminally ill persons with dementia (completely agree, somewhat agree, completely disagree). Descriptive results indicate a larger increase for the treatment AL staff from baseline to one month for the percentage who completely agreed with both items. For the item specific to providing care for terminally ill persons with dementia, the treatment group was 47% baseline to 84.6% one-month compared to 66% baseline and 50% control group. Results for mediation between staff-self efficacy and ACP will be discussed.

Abstract citation ID: igad104.1123

### EFFECTS OF COMPANION ROBOTS ON DEPRESSION AND SOCIAL ISOLATION AMONG KOREAN AMERICAN OLDER ADULTS

Othelia Lee, *University of North Carolina at Charlotte, Charlotte, North Carolina, United States*

**Objectives.** During the COVID-19, older immigrants in low resource areas who are already excluded from much of social life, had to distance themselves even further, deepening their social isolation. Lack of stimulation can be particularly detrimental, as it influences mood and increases loneliness. Selected for cost-effectiveness and interactive features, we introduced the Companion Robot (CR) system. Preliminary studies conducted in South Korea found statistically significant improvement in quality of sleep and cognition among low-income older patients living alone with multiple chronic conditions and cognitive impairment. Method. Thirty CRs were deployed to solo-living Korean American older adults. We followed up with them after 3 months. In this Stage 1A clinical trial, we tested feasibility, usability and acceptability of CRs, investigating which features of CRs will help improve health behaviors such as medication adherence and daily exercises. Robotic Social Attitude (RoSAS) are assessed with a generic 18-items questionnaire (Krägeloh et al., 2019).

**Results.** The primary clinical outcomes measured at pre- and post-intervention were medication adherence and reduction of depression, social isolation and disabilities. The user-friendly doll-shaped design of CRs helped participants feel comfortable interacting with the CR on their own. Exposure to individualized CR-delivered content, including quizzes for brain training and inspirational messages, combined with consistent interaction with CR appeared to be beneficial for cognitive function.

**Discussion.** Since very few participants had reported diagnosis of dementia, we need to screen for undetected dementia using the Modified Mini-Mental Status Exam. The next stage is to implement further cultural adaptation for the U.S. participants.

Abstract citation ID: igad104.1124

### CO-DESIGNING A STAGE 1B FEASIBILITY STUDY OF INPATIENT PALLIATIVE COMMUNITY HEALTH WORKER SUPPORT

Jung Kwak[1], Elizabeth Kvale[2], Sarah Mills[3], and Snehal Patel[3], *1. University of Texas at Austin, Austin, Texas, United States*, *2. Baylor College of Medicine, Houston, Texas, United States*, *3. UT Austin Dell Medical School, Austin, Texas, United States*

Persons living with dementia (PLWD) are 2-3 times more likely than their non-dementia peers to be hospitalized annually and experience burdensome care transitions and treatments at the late stage of dementia. Minoritized PLWDs are significantly more likely to be hospitalized and experience frequent transitions between care settings, and also less likely to access palliative care. There is a pressing need to develop better models of care transition support for PLWDs and their family caregivers. Proactive palliative care can reduce unnecessary hospitalization and improve outcomes for PLWDs and family caregivers. Community health workers (CHW) can effectively bridge cultural gaps between vulnerable patients/families and formal care systems, and provide care navigation and coaching support. We are currently conducting a single-site randomized controlled pilot trial study to co-design with key stakeholders (Stage 1a), implement and assess (Stage 1b) the acceptability and feasibility of a palliative CHW support for hospitalized PLWDs and caregivers. To date, we have conducted a needs assessment with

34 PLWD/caregiver dyads, key informant interviews with 4 palliative care providers and 2 CHW and social care experts, and multiple stakeholder meetings with hospital frontline providers and CHW groups to develop intervention components and research implementation plans that address language and cultural barriers and healthcare provider and system level barriers experienced by PLWD and caregivers from minoritized and financially vulnerable backgrounds. In this presentation, we will describe stakeholder engagement and co-design process outcomes and preliminary findings on intervention fidelity, participant recruitment and retention, and PLWD/caregiver outcomes.

Abstract citation ID: igad104.1125

### BARRIERS AND FACILITATORS IN USING THE E-PAINSUPPORT INTERVENTION FOR ADRD PATIENTS AND CAREGIVERS

Masako Mayahara, *Washington University in St. Louis, St. Louis, Missouri, United States*

Pain is one of the most common comorbidities of ADRD and is experienced by 45.8% of ADRD patients. The major barrier to effective pain management is poor adherence to pain management regimens, due in part to caregiver lack of knowledge and lack of self-efficacy in administering analgesics. Pain in ADRD patients is associated with depression, wandering, agitation, and aggression. Digital applications may facilitate pain management by: (1) delivering education to increase knowledge and self-efficacy, (2) expediting pain reporting to nurses, and (3) improving adherence to pain management protocols. e-PainSupport is a self-administered, digital pain management application. It has three elements: (a) Educational Module, (b) Patient Pain Record, and (c) Pain Summary for Nurses. The efficacy of the app is currently being tested in a randomized controlled trial funded by the National Institutes of Health. The preliminary findings support the efficacy of e-PainSupport in reducing patient pain intensity and increasing caregivers' self-efficacy and knowledge. However, the current intervention relies on patients' self-reporting of pain and thus is not appropriate for all ADRD patients. Therefore, the purpose of this study is to explore facilitators for and barriers to using e-PainSupport for ADRD patients and caregivers. The data will be collected through post-intervention, semi-structured interviews of nurses and caregivers with patients in the e-PainSupport condition. Findings will inform future adaptations of e-PainSuport for ADRD patients and caregivers.

Abstract citation ID: igad104.1126

### PEER SUPPORT FOR CAREGIVERS OF AFRICAN AMERICANS LIVING WITH DEMENTIA: A PHASE 1 BEHAVIORAL INTERVENTION PROTOCOL

Karen Moss[1], Mary Beth Happ[1], Abraham Brody[2], Celia Will[1], Alai Tan[3], Seuli Bose Brill[1], and Karen Bullock[4], *1. The Ohio State University, Columbus, Ohio, United States*, *2. NYU Rory Meyers College of Nursing, New York City, New York, United States*, *3. The Ohio State University College of Nursing, Columbus, Ohio, United States*, *4. Boston College, Boston, Massachusetts, United States*

African American caregivers of older adults living with Alzheimer's disease or a related dementia are at high risk for physical, spiritual, and psychosocial challenges. Peer mentorship, a relationship-centered person-to-person approach may reduce healthcare decision-making burden among African American caregivers through cultural tailoring by promoting oral traditions, personal contact, and storytelling. The purpose of this Stage 1 intervention study is to use a stakeholder-informed approach in further developing and pilot testing the co-created Peer Support for Caregivers of African Americans Living with Alzheimer's Disease and Related Dementias (Pair2Care), a culturally sensitive caregiver peer support program. Pair2Care feasibility and acceptability will be evaluated in current (n=15) and trained former (n=15) African American Alzheimer's and related dementia caregivers. Pair2Care will be deemed feasible and acceptable by evaluating eligibility criteria, recruitment and retention data, protocol adherence, satisfaction, and feedback on program appropriateness for broader dissemination. Preliminary intervention outcomes of caregiver anxiety, depression, quality-of-life, social support, self-efficacy for surrogate decision-making, and intent to use palliative care or hospice services will be assessed to evaluate intervention effectiveness. At 6 months as compared to baseline, current caregivers will report less anxiety and depression, improved quality-of-life, social support, and self-efficacy for surrogate decision-making, as well as intent to use palliative care or hospice services as part of healthcare and advance care planning decision- making. Pair2Care poses an innovative strategy with the potential to address advance care planning and healthcare decision-making, a significant health disparity problem and thereby promote health equity.

### SESSION 3350 (SYMPOSIUM)

Abstract citation ID: igad104.1127

### EMPOWERING PEOPLE OF ALL AGES BY PROMOTING POSITIVE VIEWS OF AGING AMONG PK-12 STUDENTS

Chair: Tina Newsham Discussant: Lisa Borrero

Beliefs about aging and older adults form early in life and persist. People who hold positive views of aging rather than internalized ageism live, on average, 7.5 years longer. Communities where older adults retain meaningful engagement and are respected experience greater longevity and better social and physical well-being among people of all ages. Having positive interactions with older adults increases likelihood of pursuing careers working with older people. Promoting positive, accurate views of aging benefits individuals (of all ages) but is most effective when implemented early in life. Children, however, are rarely intentionally taught about aging and learn primarily negative stereotypes through media such as cartoons, books, and fairy tales. In this symposium, presenters will share projects aimed at disrupting the perpetuation of ageist stereotypes through intentional work with young children to promote positive views of aging. Laura Donorfio will present an aging education project developed for the Connecticut school system via an Administration on Aging grant. Next, Elizabeth Bergman will present two intergenerational case study projects involving elementary school-aged children, college students, and elders. Edward Miller will then the describe and sharing findings from an ongoing intergenerational tutoring intervention, begun during the early stages of the COVID-19

pandemic, wherein older adults provide free, online evidence-based tutoring to bi-lingual kindergartners. Finally, Tina Newsham and Cynthia Hancock will share research on a toolkit developed for preschool-2nd grade teachers to introduce information about aging and centenarians as a way to celebrate the 100th day of school. Lisa Borrero will serve as discussant. This is an Intergenerational Learning, Research, and Community Engagement Interest Group Sponsored Symposium.

Abstract citation ID: igad104.1128

### AGING EDUCATION IN THE UNITED STATES: WHERE WE ARE AND HOW DID WE GET HERE?

Laura Donorfio, *University of Connecticut, Waterbury, Connecticut, United States*

Aging education at the precollege level is relatively new in the United States, making its debut in the literature in the 1960s (Myers, 1977). The first White House Conference on Aging (WHCoA) in 1961, centered on the need for aging education and recommended it be integrated into curricula in public schools, higher education institutions, and libraries (McGuire, 2017). At the 1971 WHCoA it was noted that such educational efforts were indeed rare and again recommended that aging and life cycle education be mandatory at all levels of education, programs be developed to train teachers about aging, and older citizens be used as school volunteers (McGuire, 2017). Since then, aging curricula and intergenerational programming have made their way slowly and insufficiently into education at both the pk-12 and college levels. Interestingly, more recently, it seems more for-profit organizations are investing in aging education and training versus educational institutions to educate their workforce to better meet the needs of their older consumer base to increase their profit margins. Beyond profit margins, what is the heuristic value of aging education and why is it increasingly important and needed not only in the United States, but worldwide? This paper will kick off the symposium and set the stage with a retrospective account of what factors have propelled aging education in the United States and what we have learned to move aging education forward at both the pk-12 and higher education levels.

Abstract citation ID: igad104.1129

### EXPLORING NEW WAYS TO IMAGINE OUR FUTURE THROUGH INTERGENERATIONAL CASE STUDY PROJECTS IN ELEMENTARY SCHOOL

Elizabeth Bergman, *Ithaca College, Ithaca, New York, United States*

Aging education is largely absent from the PK-12 curriculum. When students do encounter messages about aging and older adults it is generally through such activities as dressing “like a 100-year-old” to celebrate the 100th day of school which promotes the internalization of ageist stereotypes starting at a young age. Motivated by a desire to push back against these ageist messages in school, the presenter partnered with elementary school educators and school administrators to develop and deliver two intergenerational case study projects involving elementary school-aged children, undergraduate students, and older adults. The presenter will highlight objectives, activities, and outcomes of the Let’s Dream Together and Ages & Stages in Our Community projects and provide suggestions for aging educators interested in similar initiatives.

Abstract citation ID: igad104.1130

### “I DON'T KNOW WHO GETS MORE OUT OF IT”: OLDER ADULTS' EXPERIENCES WITH THE ONLINE INTERGENERATIONAL TUTORING PROGRAM

Edward Miller[1], Jessica Hoffman[2], James Hermelbracht[1], Jeffrey Burr[1], and Jan Mutchler[1], *1. University of Massachusetts Boston, Boston, Massachusetts, United States*, *2. Northeastern University, Boston, Massachusetts, United States*

Due to pandemic-related academic and social stressors experienced by children and social isolation and loneliness experienced by older adults, a team of school psychologists and gerontologists collaborated with older adults to develop the Online Intergenerational Tutoring Program. Conceived in fall 2020, the program is now in its third year of implementation. The program addresses a service delivery gap in schools because older adult volunteers expand schools' capacity to implement reading instruction with students in need of support. Older adults share their time and talents with young children and children benefit from the care, attention, and instruction from older adults. Tutors meet with students after school 3-4 times per week over a 6–8-week period over Zoom; each of the 24 sessions last 30 minutes. Evaluation data show the program is socially valid, tutors deliver instruction with accuracy, students have high engagement, and there are literacy gains among students.

Abstract citation ID: igad104.1131

### CELEBRATING CENTENARIANS IN ELEMENTARY SCHOOLS: PROMOTING POSITIVE VIEWS OF AGING AMONG YOUNG CHILDREN

Cynthia Hancock[1], Tina Newsham[2], Daniel Alston[1], Elizabeth Fugate-Whitlock[2], and Katherina Nikzad-Terhune[3], *1. UNC Charlotte, Charlotte, North Carolina, United States*, *2. University of North Carolina Wilmington, Wilmington, North Carolina, United States*, *3. Northern Kentucky University, Highland Heights, Kentucky, United States*

Upon learning that some teachers celebrate the 100th day of school by asking young children to dress up “like a 100-year-old,” the presenters questioned what ideas about aging are reinforced by such an activity. Social media images reveal that many of these activities lead to stereotypical presentations of older people. Additionally, the “fun” activity of dressing like a centenarian teaches children that it is okay to have fun at the expense of another segment of the population and reinforces the belief that older people are frail, weak, confused, and out of touch. Partnering with experts in early childhood and elementary education, the presenters created a toolkit to offer accurate information about centenarians, aging, and ageism and to give teachers options for celebrating the 100th day of school that reinforce important academic content, address prekindergarten to second-grade learning standards/outcomes, and support age-inclusivity. The presenters will share data from a pilot study of the curriculum and suggest next steps for advancing aging education among very young learners.

### SESSION 3355 (BIOLOGICAL SCIENCES INVITED SYMPOSIUM)

Abstract citation ID: igad104.1132

### INTER-ORGANELLE CROSSTALK IN THE REGULATION OF AGING AND LONGEVITY

Chair: Meng Wang

In eukaryotic cells, specific functions are compartmentalized into various organelles that are evolutionarily conserved across species. Many of these organelles play crucial roles in regulating cellular homeostasis and organismal health. Lysosomes, mitochondria, lipid droplets, and endoplasmic reticulum (ER) are highly metabolic active and support the metabolic balance of lipids, amino acids, carbohydrates, and nucleotides. These organelles also regulate signal transduction, transcriptional responses, and epigenetic dynamics, and contribute to the control of lifespan and healthspan in a variety of organisms. Importantly, these organelles do not act in isolation, instead exhibit complex intercommunication at structural, metabolic and signaling levels. The dysfunction of these organelles and their intercommunications have been associated with aging and age-related pathologies. This symposium aims to encourage innovative studies on the role of organelle interactions and communications in regulating aging and longevity. Speakers have been invited to share their new findings in this research topic. We will discuss how lysosomal metabolism couples with lysosomal signaling to regulate organelle crosstalk, tissue communication, and organism lifespan; how ER and mitochondria communicate through calcium signaling and promote longevity; how lipid droplets play a novel role in remodeling carbohydrate metabolism and define the metabolic balance in supporting organism health; and how lysosomal activity, mitochondrial function and cytosolic proteostasis orchestrate to support cellular homeostasis.

Abstract citation ID: igad104.1133

### LYSOSOMAL SIGNALS IN LONGEVITY REGULATION ACROSS THE SCALE

Meng Wang, *Janelia Research Campus, Ashburn, Virginia, United States*

Lysosomes are key organelles in the cell that constitute an acidic subcellular environment and contain approximately 60 different types of hydrolytic enzymes. With the aid of these acidic hydrolytic enzymes, lysosomes are highly metabolically active and can digest various macromolecules delivered through endocytosis, phagocytosis, and autophagy. Moreover, lysosomes function as a "signaling hub" that integrates metabolic inputs, organelle interaction, and longevity control. Our studies discovered a pro-longevity lysosomal acidic lipase that activates a lysosome-to-nucleus retrograde lipid signaling pathway in Caenorhabditis elegans, and in turn revealed the critical role of this lysosomal lipid signaling in promoting oxidative stress tolerance and lipid catabolism through tuning mitochondrial activities. Furthermore, we discovered that this lysosomal lipase induces the release of lipid messengers from peripheral metabolic tissues, which act on neurons and lead to the up-regulation of the neuropeptide signaling pathway by activating a nuclear hormone receptor. More recently, lysosome-specific proteomic profiling has revealed that AMPK is specifically recruited to the lysosome upon lysosomal lipolysis and mediates the longevity effect, suggesting a previously unknown interaction between AMPK and lysosomal lipid signaling. Together, these findings highlight the crucial role of lysosomal signals in actively coordinating organelle crosstalk and tissue communication to improve longevity and promote healthy aging.

Abstract citation ID: igad104.1134

### ER CALCIUM SIGNALING COORDINATES MITOCHONDRIAL DYNAMICS AND METABOLIC FUNCTION TO PROMOTE LONGEVITY

Kristopher Burkewitz, *Vanderbilt University, Nashville, Tennessee, United States*

Inter-organelle communication is a critical determinant of cellular metabolic homeostasis, and defects in the interactions between organelles are increasingly correlated with age-dependent diseases. Despite these promising links, exploration of how inter-organelle signaling pathways can be targeted to promote healthy organismal aging is a nascent area of research. We have established a foundation in C. elegans to investigate how the communication between endoplasmic reticulum (ER) and mitochondria, two of the most central metabolic hubs in cells, controls the aging process. Combining electron microscopy, live-imaging and biochemical approaches, we provide evidence that C. elegans forms ER-mitochondrial contacts and that through molecular mechanisms conserved to mammals, ER calcium flux potently stimulates mitochondrial bioenergetics. Furthermore, genetic manipulation of the ER inositol triphosphate receptor (InsP3R) reveals that ER calcium signaling promotes mitochondrial homeostasis at multiple other levels, including mitochondrial gene expression and dynamics. Building on this foundation, we now reveal that ER calcium signaling is essential for longevity in contexts of mitochondrial reprogramming. Surprisingly, InsP3R-stimulated mitochondrial bioenergetics do not appear to be the key mechanism of longevity in these contexts, but rather the underappreciated roles of the ER in tuning mitochondrial gene expression and morphology. Our results thus highlight the InsP3R as a potent and central regulator of diverse mitochondrial properties and reveal that the connections between ER and mitochondria are deeper and more complex than current models suggest. Overall, we reveal that inter-organelle communication between ER and mitochondria is an essential mechanism of longevity in paradigms involving mitochondrial reprogramming.

Abstract citation ID: igad104.1135

### DEFINING METABOLIC BALANCE BETWEEN SUGAR AND FAT METABOLISM, AND THEIR ROLES IN DEVELOPMENT AND FERTILITY"

Mike Henne, *University of Texas Southwestern Medical Center , Dallas, Texas, United States*

Animals store excess nutrients in two major forms: triglycerides (TGs) held within lipid droplets (LDs) and carbohydrates in the form of glycogen. These nutrient reserves can serve specialized roles in animal development and homeostasis, but how they uniquely contribute to animal growth, bioenergetics, aging, and fertility is poorly understood. Furthermore, it is unclear how loss of one nutrient storage pool influences the other, and whether metabolic remodeling can occur to bypass loss of fat or carbohydrates. Here, we utilize Drosophila as a genetically tractable model system to dissect the tissue-specific contributions of fat and glycogen storage in animal development and fertility. We define metabolic remodeling in carbohydrate metabolism that occurs in Drosophila models nearly devoid of fat. Intriguingly, we find that loss of specific LD subpopulations in the Drosophila fat body lead to metabolic remodeling of carbohydrate metabolism, as well as alterations in female fertility and development. Our work indicates functional crosstalk and active sensing between fat and carbohydrate pools, and indicates that animals can re-wire nutrient storage pathways to facilitate development.

Abstract citation ID: igad104.1136

### INTERDEPENDENCE OF CYTOSOLIC PROTEOSTASIS, LYSOSOMAL ACIDIFICATION, AND MITOCHONDRIAL FUNCTION

Chuankai (Kai) Zhou, *Buck Institute for Research on Aging, Novato, California, United States*

Loss of proteostasis, mitochondrial dysfunction, and lysosome defects are conserved hallmarks of aging. One common consequence of cytosolic proteostasis stress is the formation of protein aggregates that are attached to the mitochondrial outer membrane. It remains unknown why cytosolic protein aggregates are attached to the mitochondria. Here we show that Tom70, a conserved receptor for mitochondrial import, moonlights to nucleate the aggregation of cytosolic proteins through some of the newly synthesized mitochondrial proteins. As the cytosolic accumulation of these aggregate-nucleating mitochondrial proteins causes proteostasis stresses, cells balance their import and synthesis through a TOM70-FKH1/2 pathway to regulate the biogenesis of mitochondrial proteins. The reduction of Tom70 during aging causes age-dependent mitochondrial defects in the biogenesis of mitochondrial proteins and attachment of cytosolic protein aggregates, both of which can be rescued by overexpressing TOM70. Additionally, we observed an unexpected rescue of vacuole/lysosome dysfunction in the cells with mitochondrial function preserved during aging. Further investigation revealed a novel conserved mitochondria-to-vacuole/lysosome axis of organelle crosstalk. Our results estabslih novel connections among different cellular compartments related to three hallmarks of aging.

### SESSION 3360 (PAPER)

### CAREGIVING AS TOLD BY CAREGIVERS

Abstract citation ID: igad104.1137

### CAREGIVERS' PERCEPTION DISCREPANCY IN THE CAREGIVING CONTEXT: THE IMPACT ON PSYCHOLOGICAL WELL-BEING

Yulri Kim[1], Jeong Eun Lee[1], Joseph Svec[2], and Natasha Nemmers[3], *1. Iowa State University, Ames, Iowa, United States*, *2. Saint Joseph's University, New York City, New York, United States*, *3. Wayne State University Institute of Gerontology, Detroit, Michigan, United States*

Guided by the social exchange perspective, caregiving involves a reciprocal exchange in which caregiver perceptions influence a bevy of positive and negative outcomes. Positive perceptions correspond with enhanced psychological well-being (Ejem et al., 2018; Call et al., 1999). However, those who perform high levels of care but feel undervalued may experience poorer psychological well-being compared to those who perform lower levels of care but feel appreciated (Nah et al., 2022). The matrix of outcomes, contingent on the balance of performance and appreciation, highlights how perception discrepancies in the caregiving context can explain variations in psychological well-being among caregivers. In this study, we examine the psychological well-being of caregivers in four distinct groups based on their caregiving activities (high/low) and their perceived appreciation (high/low) of their efforts from care recipients. We analyze 2,096 informal caregivers who were included from Round 5 of the National Study of Caregiving (NSOC). Utilizing an analysis of covariance, the results indicate that high activity caregivers with low perceived appreciation had poorer psychological well-being than other groups. Additionally, caregivers with low caregiving activities and high perceived appreciation have higher psychological well-being than caregivers with high care activity and high perceived appreciation. These findings highlight the significance of perceived appreciation from care recipients for improving psychological well-being of caregivers.

Abstract citation ID: igad104.1138

### CAREGIVING EXPERIENCES OF ADULT DAUGHTERS WITHOUT SIBLINGS IN UFA, RUSSIA

Polina Ermoshkina, and Eva Kahana, *Case Western Reserve University, Cleveland, Ohio, United States*

This study is focused on the informal caregiving experiences of a unique group of women: only children of single mothers from Ufa, a metropolitan city in the Ural Mountains. Twenty middle-aged (M=40.75, SD=3.43), college-educated women participated in a two-hour, semi-structured interview. The average age of care recipients (mothers) was 65.3 (SD=3.85). Since the retirement age in Russia is 55 for women and 60 for men, all care recipients were retired. Inductive thematic analysis revealed three themes: incongruence of mothers' needs with the micro-environment, aging in the "cement box," and distrust of outsiders. Previous studies found that person-environment congruence is a determinant of older adults' well-being. A discrepancy between older adults' needs and preferences and environmental presses can become a source of chronic stress. This study found that both microenvironment (apartments) and larger environmental units (apartment buildings and neighborhoods) were incongruent with the needs of both older women and their caregivers. The majority of the housing stock in Russia is from the Khrushchev era: five-story buildings with no elevators or nine-story buildings with one small elevator. Older adults are trapped in their apartments due to steep stairs and a lack of elevators. Furthermore, adult daughters had to deal with conflicting cultural values and norms of care including the daughter's duty and moral obligation to care for the aging parents and their own work-related and personal needs. Mothers' dissatisfaction, in spite of daughters' best efforts, resulted in unsatisfactory caregiving efforts and frustration by both caregivers and care receivers.

Abstract citation ID: igad104.1139

### EXPLORING THE LIVED EXPERIENCES OF BLACK INFORMAL CAREGIVERS: A SCOPING REVIEW

Ivana Alexander, *University of Maryland Baltimore, Baltimore, Maryland, United States*

Understanding the unique caregiving experiences of Black informal caregivers, most of whom are women, and how they are influenced by the historical and sociopolitical context of the Black experience in the America is critical to building an effective care infrastructure that is inclusive. Gaps in existing literature include a paucity of studies that explore in-group differences in the caregiving experiences of Black informal caregivers as well as a dearth of qualitative studies that center their voices and lived experiences. This scoping review explored current representations of the Black informal caregiving experience in qualitative literature. It sought to address the heterogeneity in the Black caregiver experience through amplification of caregivers' voices. Specifically, it included studies that focused on varied informal caregiving arrangements which exist for Black informal caregivers who are caring for chronically or terminally ill adults. Overall, 65 studies were selected for full-text review. After data characterization of the full-text articles, nine studies remained and were included in the analysis. Four broad themes were identified: (1) cultural beliefs, (2) religion and spirituality, (3) systemic barriers (healthcare system and care structures), and (4) caregiver health and well-being (physical and mental health). Findings underscore the need for an intersectional lens in exploring the implications of the Black informal caregiving experience. Their cultural and religious/spiritual identities are linked to the historical experience of Black people in the United States. Their caregiving experiences often involve cultural obligations and expectations that create tension between fulfilling caregiver roles and providing for their own health and well-being.

Abstract citation ID: igad104.1140

### HOW DO FORMER DEMENTIA CAREGIVERS STRUCTURE HIGH- AND LOW-POINT NARRATIVES?

Tara Matta-Singh[1], Shubam Sharma[2], Talha Ali[3], Amanda Piechota[4], Anissa Abboud[4], Terri Fried[5], Joan Monin[4], and Emily Mroz[5], *1. University of South Florida, Tampa, Florida, United States*, *2. Kennesaw State University, Kennesaw, Georgia, United States*, *3. Tufts University, Medford, Massachusetts, United States*, *4. Yale School of Public Health, New Haven, Connecticut, United States*, *5. Yale School of Medicine, New Haven, Connecticut, United States*

Former caregivers of people living with dementia often develop and structure narratives of their lived experiences as caregivers in terms of subjective high and low points. We utilized thematic analysis to examine narratives of these high and low points in interviews with 32 former caregivers of a parent living with dementia. Across both high and low points, caregivers often structured their narratives using three types of "elaboration," that is, points not necessary to recall a factual event, but which help convey deeper meaning. Caregivers who utilized comparisons contrasted their caregiving experiences with other individuals or caregiving situations to help make sense of their own experience. When participants made connections to earlier life, they linked caregiving to prior experiences such as growing up with their parents. Shifting the focal point occurred where caregivers moved the narrative towards or away from a certain positive or negative storyline, unprompted. We discuss how caregivers who utilize elaborations adaptively (e.g., to tell a fuller story that reinforces how their caregiving relationship is beneficial) may come to better make sense of their experience which can alleviate some distress. We also discuss how elaborations that emphasize incompetency or promote focus on difficult moments may threaten the caregivers' sense of well-being, leading to psychological harm. We conclude by introducing concrete narrative strategies that can help former caregivers use elaborations in adaptive ways, such as comparing difficult caregiving experiences to other difficulties that they have overcome to re-write narratives surrounding personal incompetency in caregiving.

Abstract citation ID: igad104.1141

### THE IMPACT OF GRANDCHILD PROBLEMS ON CORESIDENTIAL GRANDMOTHERS' SITUATIONAL APPRAISALS

Alexandra Jeanblanc, and Carol Musil, *Case Western Reserve University, Cleveland, Ohio, United States*

The lives of coresidential grandmothers and their families are often complicated by grandchild health, behavioral, and educational problems Using data from a nationwide sample of 342 American grandmothers living with and raising grandchildren, we examine the effect of grandchild physical, behavioral, and educational problems on co-residential grandmothers' appraisals of: how positive their living situation is for themselves and for their grandchildren and their self-appraised stress and reward related to living with and caring for grandchildren. Participants lived with 1-7 grandchildren under the age of 18 (mean=1.82, SD 1.04), and grandchild problems were dichotomized on the basis of whether they were reported for any co-residential grandchild. We ran independent t-tests comparing grandchild problems against participants' responses to the 4 situational appraisals (measured on a 10 point visual analog scale). The presence of grandchild health problems did not affect grandmother's appraisal of their own living situation or their caregiving reward, but was significantly associated with a lower mean appraisal of how positive their living situation was for their grandchildren (8.39vs8.80) as well as higher reported caregiving stress. Grandchild behavioral problems were significantly associated with lower appraisals of grandmother's own living situation (6.69vs7.53), grandchild living situation (8.45vs8.91), lower caregiving reward (7.36vs8.31)and higher caregiving stress (8.09vs6.81). Educational problems were significantly associated with lower appraisals of grandmother's own living situation (6.70vs7.34), grandchild living situation (8.18vs8.93), lower caregiving reward (7.41vs8.06)and higher caregiving stress (8.18vs7.06). These analyses show the complex interaction between grandchild problems and grandmother perceptions of their own and their grandchildren's' lives.

### SESSION 3365 (PAPER)

### LONG-TERM CARE (SRPP PAPERS) I

Abstract citation ID: igad104.1142

### A SPOTLIGHT ON ADAPTATION: PREIMPLEMENTATION OF MONTESSORI-BASED APPROACHES IN VA LONG-TERM CARE

Caroline Madrigal[1], Whitney Mills[2], Camilla Pimentel[3], Christine Hartmann[3], A Lynn Snow[4], Cameron Camp[5], and Michelle Hilgeman[4], *1. VA Boston Healthcare System, Boston, Rhode Island, United States, 2. VA Providence Healthcare System, Providence, Rhode Island, United States, 3. VA Bedford Healthcare System, Bedford, Massachusetts, United States, 4. Tuscaloosa VA Medical Center, Tuscaloosa, Alabama, United States, 5. Center for Applied Research in Dementia, Solon, Ohio, United States*

Effectively adapting evidence-based interventions for nursing home (NH) implementation is a critical, yet under-examined, component of improving care quality. Montessori-based activity programming (MAP) is an evidence-based intervention that promotes person-centered care, engages persons living with dementia, and mitigates distress behaviors. Currently, there is sparse examination of MAP in Department of Veterans Affairs NHs (i.e., Community Living Centers, CLCs). We report on the use of the Framework for Reporting Adaptations and Modifications-Enhanced (FRAME) to track adaptations made to MAP – providing a pre-implementation exemplar for NH clinicians and implementation scientists. Qualitative and quantitative data were collected across two phases (i.e., pre-implementation and pilot implementation) at eight VA CLCs between 2017-2019. We used an iterative, rapid content analytic approach to triangulate findings across data sources (e.g., advisory panel, staff interviews, training evaluations, field notes, fidelity assessments) and identify needed adaptations for the CLC setting and population. More than 300 frontline VA CLC staff participated in qualitative interviews and/or provided feedback on quantitative staff training. Thirty-six adaptations were made. Most adaptions occurred during the pre-implementation phase, were reactive, focused on training/evaluation, and involved researchers, intervention developers, and practitioners. All were fidelity-consistent with MAP. The most common goal across adaptations was increased reach/engagement of the intervention. CLCs and community NHs can use findings to support intervention adaptation, and adapt and implement MAP to improve meaningful engagement for persons living with dementia and other residents. Future research should further evaluate and standardize FRAME for diverse users of complex interventions.

Abstract citation ID: igad104.1143

### COMPARING COVID-19 CASES AND MORTALITY RATES: THE GREEN HOUSE VERSUS THE TRADITIONAL NURSING HOME IN THE UNITED STATES

Yuchi Young[1], Virgile Barnes[2], Taylor Perre[3], Wan-Hsiang Hsu[4], Ashley Shayya[5], and Thomas O'Grady[6], *1. University at Albany, Rensselaer, New York, United States, 2. SUNY Albany, School of Public Health, Albany, New York, United States, 3. Home Care Association of New York State, Albany, New York, United States, 4. University at Albany, Albany, New York, United States, 5. University at Albany School of Public Health, Rensselaer, New York, United States, 6. State University of New York at Albany, Albany, New York, United States*

Introduction: The COVID-19 pandemic has posed significant challenges to nursing home (NH) care. However, Green Houses (GH), with their small size and unique care model, may offer a solution for infection control and COVID-19 prevention. This study aims to compare COVID-19 case and mortality rates between GHs and traditional NHs in the United States.

**Methods:** Secondary data were used to compare COVID-19 case and mortality rates among three groups: GHs (n=19), traditional small NHs (n=266), and traditional large NHs (n=2932) located in 10 US states from June 2020 to September 2022. Poisson regressions using generalized estimating equations (GEE) were utilized to quantify differences in COVID-19 case and mortality rates while controlling for average age, gender, and ADL score.

**Results:** The study population had a mean age of 73.38 and was primarily female (57.8%) and white (78.2%). The GEE results indicated that small and large NHs have significantly higher relative risks (RR) of COVID-19 cases (RR = 1.61; 95% CI 1.25-2.08 and RR = 1.75; 95% CI 1.36-2.24, respectively) compared to GHs. For COVID-19 mortality rates, there is an increased risk in large NHs compared to GHs (RR = 1.67; 95% CI 1.01-2.77), but no difference between GHs and small NHs.

**Conclusion:** After adjusting for age, gender, and ADL disability, GHs have lower COVID-19 incidence and mortality rates than traditional NHs. These differences may be attributable to GHs having fewer beds, offering more resident privacy, and having a higher certified nursing assistant-to-resident ratio compared to traditional NHs.

Abstract citation ID: igad104.1144

### FINANCIAL OUTCOMES ASSOCIATED WITH COVID-19 IN NURSING HOMES

Gregory Orewa[1], Robert Weech-Maldonado[2], Sue Feldman[2], David Becker[2], Ganisher Davlyatov[3], and Justin Lord[4], *1. University of Texas, San Antonio, San Antonio, Texas, United States, 2. University of Alabama at Birmingham, Birmingham, Alabama, United States, 3. University of Oklahoma Health Sciences Center, Oklahoma City, Oklahoma, United States, 4. Louisiana State University at Shreveport, Shreveport, Louisiana, United States*

Financial stability and well-being are critical for the success of any organization. COVID-19 put nursing homes (NH) in a financial bind, affecting their costs and revenues and mounting pressure on them despite government intervention. NHs have been one of the weak links in our care continuum for the U.S. healthcare system, and the pandemic caused havoc in the industry. Given the pandemic impact on NH operating cost and revenue, this study examined the level to which financial performance in NHs deteriorated. Six different datasets were merged for 2018–2021: Medicare cost reports, Payroll-Based Journal, NH Compare, Area Health Resource File, HHS Provider Relief Fund, and CDC COVID-19 Data Tracker. The data was modeled using random-effects regression. Dependent variables were operating revenue per patient day, operating cost per patient day, and operating margin. Independent variable was pre- and post-COVID-19. Organizational, community-level, and COVID-19-related variables were used as controls. Results suggest that the impact of COVID-19 was associated with an increase in operating cost per patient day ($\beta$=56.64, $p< 0.05$), and a decrease in operating margin ($\beta$=-8.04, $p< 0.05$), but the associated operating revenue per patient day ($\beta$=26.78, $p< 0.05$), did not increase at the rate at which the operating cost increased. The increase in operating cost per patient day compared to the operating revenue per patient day was double. Given the negative impact of the COVID-19 pandemic on NH's financial performance, policymakers should monitor the financial health of NH. Further deterioration in financial performance may result in NH closures and reduced access to long-term care.

Abstract citation ID: igad104.1145

### NURSING HOME CLOSURES DURING THE COVID-19 PANDEMIC

Kelly Hughes[1], Micah Segelman[2], Kristie Porter[3], Miranda Diaz[1], and Iara Oliveira[4], *1. RTI International, Research Triangle Park, North Carolina, United States*, *2. RTI International, Washington, District of Columbia, United States*, *3. RTI International, Durham, North Carolina, United States*, *4. Office of Behavioral Health, Disability, and Aging Policy (BHDAP), Washington, District of Columbia, United States*

Nursing homes experienced unprecedented financial challenges during the COVID-19 pandemic, raising concerns about closures. This study identified closures from 2020-2021 using certification surveys and examined characteristics associated with closures using multivariate analysis. We interviewed nursing home industry experts and providers about pandemic-related challenges and policies affecting closure. Closures did not increase in 2020 and 2021 relative to 2011-2019. However, nursing homes with more staff shortages and COVID-19 infections were more likely to close. Additional characteristics associated with closure include smaller size, lower occupancy rate, and fewer Medicare residents. Interviews noted the following financial challenges: costly contract staffing, increased PPE and COVID-19 testing costs, reduced occupancy rates, and increased internal resources needed to implement new infection guidance. Interviews suggested that strategies to mitigate staffing shortages and federal and state monetary assistance may have prevented closures in 2020 and 2021, but continuing attention to closures is needed in 2022 and 2023 as funding ends.

Abstract citation ID: igad104.1146

### PROMISES AND CHALLENGES OF IMPLEMENTING TELEHEALTH IN LTC FACILITIES: LESSONS FROM A MULTISITE PILOT PROJECT

Mengzhao Yan, Yujun Zhu, and Kathleen Wilber, *University of Southern California, Los Angeles, California, United States*

In response to the COVID-19 pandemic, the demand for telehealth implementation has expanded in long-term care (LTC) facilities. To better understand the promises and challenges of implementing telehealth, we evaluated a pilot project in which five LTC facilities in California implemented telehealth from September 2021 to May 2022. Employing a mix-method approach, we used a diverse set of evaluation tools (i.e., documenting 37 telehealth visits, holding 8 monthly learning sessions, conducting 36 check-in interviews, and tracking monthly COVID-19 cases) to examine how telehealth was adopted, used, and perceived among residents, administrators, and health practitioners. From a descriptive analysis of quantitative data and a thematic analysis of qualitative data, we found that telehealth demonstrated the capability to treat a variety of conditions; prevented nearly 20% of transfers to emergency departments; residents indicated a high degree of comfort with telehealth; and healthcare practitioners perceived that telehealth could improve care delivery. In addition to benefits, several barriers were identified including initial concerns about the quality of visits, required upfront costs, time constraints, difficulties in coordinating different platforms in the healthcare system, uncertainty about payment parity with in-person care, and reduced staffing related to COVID-19. Based on our evaluation, we propose six strategies to help implement telehealth in LTC facilities: recognize sources of resistance to change; recognize the need for initial investment; understand and address contextual issues; prepare and pave the way for staff buy-in; identify a champion; and ensure sufficient and ongoing training and support.

### SESSION 3370 (SYMPOSIUM)

Abstract citation ID: igad104.1147

### THE LANDSCAPE AND OUTLOOK FOR CONTINUED WORK LATER IN LIFE IN THE 2020S

Chair: Kevin Cahill Co-Chair: Benjamin Shaw Discussant: Brian Beach

For the first time in more than three decades, older workers' labor force participation rates are no longer increasing. Even more remarkable is that this trend contrasts with changes among younger workers, who have experienced increases in labor force participation in the wake of the Covid-19 pandemic. A narrative that has existed for more than two decades—that older workers have been the exception to reductions in labor force participation generally—may be coming to an end. A question then is, in the 2020s, will older workers who exited the labor force during the pandemic return? This symposium explores the new landscape that older workers face. The first paper addresses work-related trade-offs and the extent to which older workers assess the benefits and costs of continued work later in life. The second paper explores how pension eligibility triggers a new stage of the life course, in which older workers can leverage their newfound financial gains to improve work conditions or, alternatively, to exit the labor force. The third paper explores, specifically, how the Covid pandemic changed the workplace for older workers. The fourth paper rounds out the symposium by exploring the relationship between income and health in recent years in both the United States and in China. The flexibility of the U.S. labor force enables fairly abrupt shifts in workforce dynamics in response to macroeconomic changes and other events. The Covid-19 pandemic was one such shift. This symposium provides insight into the post-pandemic labor market for older workers. This is an Economics of Aging Interest Group Sponsored Symposium.

Abstract citation ID: igad104.1148

HOW MUCH IS WORKPLACE FLEXIBILITY WORTH TO OLDER WORKERS? AN ANALYSIS OF MONETARY AND NONMONETARY TRADE-OFFS

Kevin Cahill[1], Michael Giandrea[2], Joseph Quinn[1], and Loretta Platts[3], *1. Boston College, Boston, Massachusetts, United States*, *2. U.S. Bureau of Labor Statistics, Washington, District of Columbia, United States*, *3. Stockholm University, Stockholm, Sweden*

To what extent are older workers willing to forgo wages to obtain a flexible work arrangement when transitioning from career employment later in life? Older workers generally, and pension-eligible workers in particular face a plethora of trade-offs when it comes to continued work later in life. In this paper, we examine the components of older workers' total compensation and explore how the mix of compensation changes as workers transition out of the labor force. Specifically, we focus on the relationship between monetary compensation (wages, fringe benefits) and workplace flexibility (phased retirement, hours flexibility), and explore the degree to which workers, by their choices, are willing to accept lower wages in return for increased flexibility on their job. To estimate this relationship, we use longitudinal, biennial data on thousands of older Americans representing four different cohorts available from the nationally-representative Health and Retirement Study (HRS). The HRS began in 1992 and, therefore, contains 30 years of follow-up data for the oldest cohort. For each respondent, we construct individual work histories and examine individuals' evolving wage and hours arrangements from career employment to complete labor force withdrawal. Using multivariate techniques, we find that, all else equal, individuals experience a modest 5.7 percent decline in hourly compensation in return for a 20 percent reduction in hours worked. The findings from this paper can inform policymakers interested in continued work later in life about the degree to which older workers are willing to forgo the financial benefits of work in return for flexible schedules.

Abstract citation ID: igad104.1149

POSTPENSIONABLE AGE AS A DISTINCT CAREER STAGE

Loretta Platts[1], and Kevin Cahill[2], *1. Stockholm University, Stockholm, Sweden*, *2. Boston College, Boston, Massachusetts, United States*

This paper presents a new, two-phase model of the late career. Drawing on life course theory, we assert the importance of age-norms for ordering the timing of people's lives in countries with universal old-age pension systems. Three age boundaries may govern the transition to retirement: social security rules, employment rules and social norms. Upon reaching these boundaries: (1) old-age pension receipt provides a reliable income easing breadwinner pressures, (2) compulsory retirement may push older people out of the workforce, and (3) social expectations encourage participation in leisure rather than paid work. Once old-age pensions ease or eliminate bread-winning pressures, financial necessity no longer drives most of these oldest workers. Further, these working pensioners are defying social conventions calling on them to retire from paid work. Post-pensionable-age or "phase II" jobs tend to be more flexible and rewarding, and less stressful than those held by younger workers. Intriguingly, not only do these workers have quite distinctive motivations to work, they have unique ways of subduing the potential burdens of working. However, most research lumps together workers over pensionable age (in phase II) with workers still in their fifties (in phase I) who are fully exposed to labor market risks. This paper presents theoretical arguments and empirical evidence to support the idea that post-pensionable-age work is a discrete career phase economically, subjectively, and in terms of leverage with employers. It explores likely geographical and socio-economic limits to this two-phase model of the life course.

Abstract citation ID: igad104.1150

EFFECTS OF COVID-19 PANDEMIC ON OLDER WORKERS' LABOR MARKET AND RETIREMENT DECISIONS

Siavash Radpour, *Stockton University, Galloway, New Jersey, United States*

Several studies have documented the increase in retirement since the onset of the Covid-19 pandemic. I contribute to this literature by answering two key questions that show the pandemic excess retirement is caused primarily by demand-side factors: first, using the monthly Current Population Survey (CPS) data on employment and retirement of older workers and CPS's panel structure, I explore if older workers left their jobs and the labor force voluntarily as a part of "the great resignation". I find that very few workers quit their jobs voluntarily, and most retirements were preceded by involuntary job loss and unemployment. Second, using the Health and Retirement Study (HRS) with its rich data on wealth, employment, and the special Covid-19 related questions included in 2020 HRS (Wave 15), I examine if older workers who experienced job loss during the pandemic retired because they were financially prepared for retirement. I find that among the small group of older workers who quit their jobs because of the pandemic, the majority did not have adequate retirement assets and their decision is more likely to be based on their health status and vulnerability during the pandemic.

Abstract citation ID: igad104.1151

THE EFFECT OF INCOME SECURITY IN OLD AGE ON HEALTH OUTCOMES: A COMPARISON BETWEEN CHINA AND THE US

Heng Wu, *Dalhousie University, Halifax, Nova Scotia, Can ada*

This paper examines the social determinants of health, particularly the social, political, and economic context of socioeconomic status and health outcomes. Many studies on the political economy of health have highlighted the effects of welfare states on health and health inequalities, typically using welfare state regimes as proxies for social policies. Few research studies have described the association between frailty phenotype and financial wellbeing, however, particularly considering the three-legged stool of retirement income security (public pensions, private pensions, and personal savings/assets) across different welfare state regimes. This study investigates the effects of old-age income disparities on frailty among older adults in the United States and China. Specifically, this paper examines the associations between different sources of old-age income (public and private pensions, personal savings and assets, earnings, workplace subsidies, worker's compensation, household subsidies, and unemployment insurance benefits) and the five-item frailty phenotype from the RAND Health and Retirement Study (HRS) and the China Health and Retirement Longitudinal Study (CHARLS). The findings reveal that health outcomes measured by the Fried's frailty phenotype vary considerably by country and income type.

### SESSION 3375 (SYMPOSIUM)

Abstract citation ID: igad104.1152

### RISE: A COMMUNITY-BASED ELDER ABUSE AND SELF-NEGLECT RESPONSE INTERVENTION ADDRESSING A CRITICAL SYSTEMS GAP

Chair: David Burnes Discussant: Karl Pillemer

Knowledge of effective interventions for elder abuse and self-neglect (EASN) is limited. Adult Protective Services (APS) represents the primary agency responsible for receiving reports and investigating suspected cases of EASN in the US. However, APS lacks a distinct, conceptually informed intervention phase to support EASN cases. Based on theory, research, and consultations with stakeholders, RISE was designed to address this intervention gap within overall EASN response systems. Informed by ecological-systems, relational, and client-centered perspectives, RISE is a community-based EASN intervention that integrates core modalities (motivational interviewing, restorative justice, teaming, supported decision making) demonstrating evidence and/or promising results in EASN and other domains. The intervention operates at Relational, Individual, Social, and Environmental levels of ecological influence. Specifically, RISE works with both older adult victims and others, including alleged harmers, their relationships, and strengthens the social supports surrounding them. RISE began as a pilot in two Maine counties, was expanded to the entire state, has been used in over 450 cases, was written into Maine's 2023 budget, is now being implemented and tested in New Hampshire and Toronto, Canada, and is being expanded to the criminal justice system. This symposium will describe RISE's development and conceptual underpinnings (presentation 1), findings on implementing "teaming" (social support), an intervention modality (presentation 2), a qualitative evaluation of RISE from the perspective of APS caseworkers (presentation 3), evidence of RISE reducing EASN recidivism (presentation 4), and case studies of implementing RISE (and its restorative justice modality) in the criminal justice diversion context (presentation 5). This is an Abuse, Neglect and Exploitation of Older Persons Interest Group Sponsored Symposium.

Abstract citation ID: igad104.1153

### RISE: A CONCEPTUAL MODEL OF INTEGRATED AND RESTORATIVE ELDER ABUSE AND SELF-NEGLECT INTERVENTION

David Burnes[1], M.T. Connolly[2], Erin Salvo[3], Patricia Kimball[4], Geoff Rogers[5], and Stuart Lewis[6], *1. University of Toronto, Toronto, Ontario, Canada*, *2. University of Southern California, Los Angeles, California, United States*, *3. Department of Health and Human Services, Augusta, Maine, United States*, *4. Elder Abuse Institute of Maine, Brunswick, Maine, United States*, *5. City University of New York, New York City, New York, United States*, *6. Dartmouth College, Lebanon, New Hampshire, United States*

Despite a growing number of elder abuse and self-neglect (EASN) cases nationwide, community-based EASN response programs such as Adult Protective Services (APS) lack a conceptually driven, defined, prolonged intervention phase to address these complex situations. This presentation provides a conceptual overview and developmental description of RISE, a community-based model of EASN intervention that works alongside APS or other systems that interact with older adults who are at risk of or experiencing EASN. RISE was developed in consultation with APS caseworkers and supervisors, as well as practitioner and research stakeholders and experts from numerous sectors in Maine and nationwide, building bridges among varying stakeholders. Informed by ecological-systems, relational, and client-centered perspectives and adapting evidence-based or promising modalities from other fields (including motivational interviewing, restorative justice, teaming, supported decision making, goal attainment scaling, and engagement), the RISE model intervenes at levels of the individual older adult victim and others, including the alleged harmer, their relationship, and the systems of informal and formal support surrounding the victim-harmer dyad. The RISE model addresses an intervention gap in response systems to better meet the wishes and needs of EASN victims and others in their lives, leading to more sustainable outcomes. RISE has fostered new research partnerships between researchers and community advocates, who have become both research allies and contributors. RISE supports people with cognitive impairment to make their own decisions. RISE also empowers both older people and younger people in their lives, motivating change, and strengthening and restoring the relationships among them.

Abstract citation ID: igad104.1154

### USING A "TEAMING" (ENHANCED SOCIAL SUPPORT) APPROACH IN THE CONTEXT OF THE RISE ELDER ABUSE AND SELF-NEGLECT INTERVENTION

Andie MacNeil[1], M.T. Connolly[2], Erin Salvo[3], Patricia Kimball[4], Geoff Rogers[5], Stuart Lewis[6], and David Burnes[1], *1. University of Toronto, Toronto, Ontario, Canada*, *2. University of Southern California, Los Angeles, California, United States*, *3. Department of Health and Human Services, Augusta, Maine, United States*, *4. Elder Abuse Institute of Maine, Brunswick, Maine, United States*, *5. City University of New York, New York City, New York, United States*, *6. Dartmouth College, Lebanon, New Hampshire, United States*

Our understanding of what intervention strategies are effective in improving the well-being of older adults experiencing elder abuse and self-neglect (EASN) is severely limited. However, data consistently demonstrate that social support is a protective factor. As a component of a larger community-based EASN intervention, RISE, this study examined the use of a method called "teaming," a wrap-around approach to establish sustained formal and informal social supports surrounding victims and alleged harmers in EASN cases. Qualitative interviews and a focus group were conducted with the original pilot cohort of RISE "advocate" caseworkers (n = 4). A descriptive phenomenological approach involving two independent assessors was used to code transcripts into themes. Three domains were identified: (1) team and support forming process, which describes the development of a supportive network based on each client's needs; (2) techniques, which refers to the specific strategies advocates utilized to promote collectivity and shared responsibility around the client; and (3) implementation challenges, which discusses the difficulties advocates encountered when using teaming with people experiencing EASN. The experiences of advocates suggest that teaming is a beneficial approach to support the individualized needs of each client, and to promote improved and sustainable case outcomes for clients. This study represents the first in-depth exploration of teaming in the context of EASN intervention.

Abstract citation ID: igad104.1155

### A QUALITATIVE EVALUATION OF RISE FROM THE PERSPECTIVE OF ADULT PROTECTIVE SERVICES CASEWORKERS

Geoff Rogers[1], Andie MacNeil[2], M.T. Connolly[3], Erin Salvo[4], Patricia Kimball[5], Stuart Lewis[6], and David Burnes[2], *1. City University of New York, New York City, New York, United States*, *2. University of Toronto, Toronto, Ontario, Canada*, *3. University of Southern California, Los Angeles, California, United States*, *4. Department of Health and Human Services, Augusta, Maine, United States*, *5. Elder Abuse Institute of Maine, Brunswick, Maine, United States*, *6. Dartmouth College, Lebanon, New Hampshire, United States*

Our understanding of effective elder abuse and self-neglect (EASN) response interventions is limited. Adult Protective Services (APS), the primary agency responsible for responding to EASN, lacks a coherent, conceptually driven, prolonged intervention phase. Informed by ecological-systems, client-centered, and relational perspectives and adapting evidence-based modalities from other fields, the RISE intervention addresses this systems gap and compliments and augments APS services. Based on a three-year pilot project involving a partnership between RISE and Maine APS, the current study conducted a qualitative evaluation of RISE, from the perspective of APS caseworkers (n=14) who worked with RISE. The purpose of this evaluation was to understand RISE strengths, areas for improvement, and qualities of the RISE/APS partnership. Findings suggest APS workers perceive that RISE benefits clients, complements the scope and nature of APS, enhances APS caseworker well-being, and reduces repeat APS cases. Further APS/RISE collaboration and clarification on RISE role responsibilities and referral eligibilities represent areas of growth. This study provides preliminary evidence for RISE as a community-based EASN intervention in partnership with APS

Abstract citation ID: igad104.1156

### EFFECT OF RISE ON REPEAT ELDER ABUSE AND SELF-NEGLECT INVESTIGATIONS BY ADULT PROTECTIVE SERVICES

Stuart Lewis[1], Andie MacNeil[2], M.T. Connolly[3], Erin Salvo[4], Patricia Kimball[5], Geoff Rogers[6], and David Burnes[2], *1. Dartmouth College, Lebanon, New Hampshire, United States*, *2. University of Toronto, Toronto, Ontario, Canada*, *3. University of Southern California, Los Angeles, California, United States*, *4. Department of Health and Human Services, Augusta, Maine, United States*, *5. Elder Abuse Institute of Maine, Brunswick, Maine, United States*, *6. City University of New York, New York City, New York, United States*

Adult Protective Services (APS) is the primary agency responsible for investigating Elder Abuse and Self-Neglect (EASN) allegations in the US. The harms of EASN are well established; however, APS lacks a conceptually derived, evidenced-based intervention phase. RISE is a community-based EASN intervention designed to complement and augment APS that provides enhanced services and a longer intervention phase. The objective of this study was to test whether exposure to the APS/RISE collaboration reduced the case outcome of recurrence (repeat investigations) compared to usual APS only services. This study was based on a retrospective observational design (n = 1947) in two randomly selected counties of Maine where RISE was available to provide enhanced services to persons referred from APS. Analysis used an extended regression endogenous treatment Probit model using APS administrative data to predict case recurrence. Between 2019 and 2021, 154 cases participated in RISE and 1793 received usual APS only services. 49% of cases referred to RISE had a complex history with 2 or more prior substantiated allegations versus 6% for those receiving usual APS only services. After accounting for the non-random treatment assignment, RISE significantly lowered the likelihood of recurrence compared to persons receiving usual care provided by APS (probability of recurrence reduced by 0.55 for the Average Treatment Effect on the Treated and 0.26 for the Average Treatment Effect). A reduction in recurrence carries important implications for APS costs, resources, and workflow. It may also serve as a proxy indicating a reduction in re-victimization and harm for EASN victims.

Abstract citation ID: igad104.1157

### USING RISE IN CASES DIVERTING DEFENDANTS IN ELDER ABUSE SCENARIOS OUT OF THE CRIMINAL SYSTEM AND INTO TREATMENT

M.T. Connolly[1], Page Ulrey[2], Patricia Kimball[3], Geoff Rogers[4], Stuart Lewis[5], and David Burnes[6], *1. University of Southern California, Los Angeles, California, United States*, *2. King County Prosecuting Attorney's Office, Seattle, Washington, United States*, *3. Elder Abuse Institute of Maine, Brunswick, Maine, United States*, *4. City University of New York, New York City, New York, United States*, *5. Dartmouth College, Lebanon, New Hampshire, United States*, *6. University of Toronto, Toronto, Ontario, Canada*

Criminal justice system interventions are a common response to elder abuse, neglect and exploitation (EA), but there is severely limited understanding of their efficacy in reducing EA or improving the well-being of older adults. A goal attainment scaling feasibility study revealed that APS clients' goals often included getting help (like substance use or mental health treatment) for someone else, often family members harming them. Some APS and RISE clients reported reluctance to seek help or report harm, fearing that involvement of the criminal justice system might result in them losing control over their lives, losing a caregiver, being forced into a facility, or legal punishment of someone they care about. Given these findings, the RISE team engaged in a lengthy outreach and planning phase with state and local prosecutors, elder victim service providers, restorative justice experts, and researchers, to design a novel intervention, using RISE in conjunction with drug court, to divert cases involving substance using defendants alleged to have mistreated an older person. This session will describe the extensive planning and design process and provide a case study analysis, which suggests that a combination of drug court's in-patient treatment for the defendant, services for the victim, and RISE's focus on restorative justice approaches that repair breached relationships reduced harm and benefited the older person, the defendant, and their families. Implementation of RISE in partnership with the criminal justice system carries important implications for the integration of restorative justice practices in some EA cases.

### SESSION 3380 (PAPER)

### SUCCESSFUL AGING AND THE GOOD LIFE

Abstract citation ID: igad104.1158

### "DO IT ANYWAY"—RESIDENT-DRIVEN ENGAGEMENT TO MAINTAIN MEANINGFUL LIFE IN CONTINUING-CARE RETIREMENT COMMUNITIES

Xiaoli Li, *Southern Illinois University, Carbondale, Illinois, United States*

As the aging population continues to grow, the demand for Continuing Care Retirement Communities (CCRCs) is increasing. However, residents in CCRCs often face social isolation and loneliness, which can negatively impact their health and well-being. To combat these issues, activities and engagement have been proposed as potential solutions. However, the voices of those vulnerable to social isolation are seldom heard. This study aimed to explore the perspective of residents on how resident-driven engagement in CCRCs may maintain a meaningful life. A qualitative research approach was used, consisting of semi-structured interviews with 26 residents in CCRC. Thematic analysis identified five key themes related to resident-driven engagement: a sense of community, turning lemons into lemonade, doing it anyway, pink letters, and committees and clubs. The "do it anyway" attitude emerged as a prevalent theme, emphasizing the importance of pushing past challenges and limitations to pursue activities that bring joy and meaning to residents' lives. The findings suggest that resident-driven engagement can lead to a sense of purpose, belonging, and fulfillment in CCRCs. The research provides insights into how communities can foster an environment that supports such engagement. By prioritizing resident-driven activities and empowering residents to take control of their lives, CCRCs can promote the well-being and quality of life of their residents.

Abstract citation ID: igad104.1159

### NEIGHBORHOOD CONTEXT AND QUALITY OF LIFE OF OLDER ADULTS IN EASTERN NEPAL: A CROSS-SECTIONAL STUDY

Krishna Sapkota[1], Saruna Ghimire[2], and Aman Shrestha[2], *1. Miami University, Oxford, Ohio, Oxford, Ohio, United States*, *2. Miami University, Oxford, Ohio, United States*

Introduction: Increased life expectancy has resulted in population aging and subsequently entails addressing the needs of older adults. Shifting family structures from multi-generational to nuclear have had a significant effect on the older adults' livelihood and their ways of interaction with community members. This research aims to comprehend the relationship between neighborhood context and older adults' quality of life (QoL) in Nepal.

**Methods:** A total of 847 non-institutionalized older adults (≥60 years) from eastern Nepal were interviewed in 2021. The QoL was measured by13-item version of the Older People's Quality of Life questionnaire (OPQOL-brief), and each item was measured on a 5-point Likert scale. The OPQoL mean score < 3 was recoded as low, and ≥3 was high QoL. Ethnic diversity, monthly income, connection with family, friends, and neighbors, cultural connection, residential instability, and accessibility were measured to see the effect of neighborhood context. Multivariable logistic regression assessed the relationship between neighborhood context and QoL.

**Results:** More than 20% of older adults reported low QoL. Participants engaged in occupation [OR=2.63], not consuming tobacco [OR=1.78], living in communities with higher ethnic diversity [OR=1.03], frequently in contact with family [OR=4.77] and neighbor [OR=13.79], and stable residence [OR=5.43] had significant associations with better QoL.

**Conclusion:** In this study, neighborhood context was a determinant of better QoL in older adults. Therefore, engagement in income generation activities, and strengthening families and neighborhood ties bring good QoL among older adults.

Abstract citation ID: igad104.1160

### SUCCESSFUL AGING AMONG OLDER PERSONS IN VIET NAM

Yasuhiko Saito[1], Nguyen Vu[2], and Linh Dang[2], *1. Nihon University, Chiyoda-ku, Tokyo, Japan*, *2. Institute of Population, Health and Development, Hanoi, Ha Noi, Viet nam*

The present study aims to explore the prevalence and correlates of successful ageing among Vietnamese older persons. Data used for this study is from the baseline survey of the Longitudinal Study of Ageing and Health in Viet Nam (LSAHV). LSAHV is a nationally representative survey of older persons aged 60 and above in 2018-19 with 6,050 respondents. Face-to-face interviews were conducted using structured questionnaires and tablets. Rowe and Kahn's definition was used to study successful ageing among older persons in Viet Nam. Study sample size was 4,370 older persons. Logistic regression was employed to examine the association of sociodemographic and lifestyle factors with successful aging among older persons. The prevalence of Vietnamese older persons meeting criteria of successful ageing was 16.2% at the time of baseline survey. The percentage of older persons reporting no major diseases, no disability, high cognitive function, high physical function, and engagement with life was 35.0%, 88.5%, 55.1%, 77.9%, and 79.7%, respectively. Logistic regression analysis showed that older persons who were younger, male, having higher education level, and often doing physical exercise were likely to be successful agers. The prevalence of successful ageing was quite low among Vietnamese older people. More health interventions, successful ageing guidelines, and health care services should be provided for older persons to improve successful ageing status. It is necessary to study successful ageing to improve well-being of older persons becaise Viet Nam is currently experiencing a rapid population ageing.

Abstract citation ID: igad104.1161

**THE MATCH/MISMATCH IN PERCEPTIONS OF SUCCESSFUL AGING AMONG OLDER BURUNDIAN REFUGEES IN THE US**

Jonix Owino[1], and Heather Fuller[2], *1. Sacred Heart University, Stratford, Connecticut, United States*, *2. North Dakota State University, Fargo, North Dakota, United States*

Perceptions of successful aging may be impacted by unique lived experiences such as migration, adaptation, and traumatic life experiences. While there exist studies on successful aging among immigrant communities, research studies on successful aging among older refugees, who flee due to life threatening situations, are sparse. The present qualitative study aimed to shed light on the match or mismatch that may exist between older refugees' perceptions of successful aging and their perceptions of their lived experiences, as well as sex variations in those perceptions. Twenty-one Burundian refugees, aged 50+, were recruited from an upper Midwest community to participate in in-depth interviews. The qualitative interviews were analyzed using thematic content analysis. Themes denoting a match in aging refugees' perceptions of success aging and lived experience included: wisdom/ability to give advice (i.e., guidance especially to younger individuals), experiencing peace and security, and fostering family relationships. A mismatch was highlighted in themes related to work experiences (i.e., the challenge of engaging in jobs not related to their prior skills), lack of social connections, and communication difficulties. Gender differences were apparent in that women emphasized shifts in work-related gender roles while men emphasized shifts in dependency-related gender roles. In many ways, aging refugees' unique backgrounds and experiences seemed influential for their match/mismatch in successful aging perceptions and experiences. These findings highlight the importance for host communities to better understanding how refugees perceive and experience successful aging to best inform the implementation of effective interventions for aging refugees.

### SESSION 3385 (SYMPOSIUM)

Abstract citation ID: igad104.1162

**ESPO AND ACADEMY FOR GERONTOLOGY IN HIGHER EDUCATION SECTION SYMPOSIUM: UTILIZING TECHNOLOGY IN THE ADVANCEMENT OF OLDER ADULT EDUCATION**

Chair: Yan-Jhu Su Co-Chair: Janelle Fassi Discussant: Christine Fruhauf

TThis symposium intends to echo one of the core values of the GSA 2023 Annual Scientific Meeting — empowering all ages. Online lifelong learning has become more common in the 21st century. The speed of technological development far exceeds people's imagination. The speed at which technology develops is shown to create a widening gap between older and younger populations, known as the digital divide. Introducing technologies into intergenerational learning environments remains an important step to bridging the generational digital divide. In accordance with the AGHE gerontological education competencies, these authors will describe how older adults utilize technology. The first speaker will discuss the effectiveness of a virtual intergenerational program that addressed experiences of ageism for older and younger participants. The second speaker will introduce user-centered design principles and innovative tools that facilitate virtual Information Communications Technology (ICT) support and training for older people with limited abilities in using technology. The third speaker will illustrate concrete practice through international cases from different intergenerational technology integration projects. The fourth speaker will present key elements from the University of Rhode Island (URI) Engaging Generations Cyber-Seniors Program internship and describe student experiences enrolled in the program. The last speaker will discuss the benefits and challenges of technology use among older learners and state the experience of leading older learners using technology in Taiwan. This is a Geriatric Education Interest Group Sponsored Symposium.

Abstract citation ID: igad104.1163

**LIFELONG LEARNING FOR THE OLDER POPULATION—TECHNOLOGY WITH THE OLDER LEARNERS**

Yan-Jhu Su, and Andrew Alberth, *University of Massachusetts Boston, Boston, Massachusetts, United States*

Advances in digital information technology and population aging are two hallmarks of the 21st century. Modern students are increasingly dependent on digital technology in their education. However, how to make digital technology less exclusive to young students remains an important issue. The COVID-19 pandemic has also popularized remote learning and the use of communication software among all ages. Teaching remotely has broadened access to education for all ages. Therefore, how to use technology to help older people achieve the goal of lifelong learning remains critical. This presentation will review the literature to explore the relationship between technology and older adults, as well as share the author's experience leading older adults to use technology for online learning in Taiwan. The key takeaways from this presentation will help educators understand the benefits and challenges of technology use among older adults, and highlight ways to bridge the digital divide between older and younger learners in the classroom.

Abstract citation ID: igad104.1164

**THE EFFECTIVENESS OF A VIRTUAL INTERGENERATIONAL ACTIVITY FOR REDUCING YOUNGER AND OLDER ADULTS' AGEISM**

Janelle Fassi, *University of Massachusetts Boston, Boston, Massachusetts, United States*

This presentation will discuss results from a study that examined whether a virtual intergenerational activity was feasible and effective for reducing ageism among older

(OA) and younger adults (YA) during the COVID-19 pandemic. OA (n = 16) and YA (n = 15) completed a virtual intergenerational activity, and analysis was conducted for a subsample (n = 5 OA, n = 10 YA) who completed pretest-posttest ageism surveys. Paired samples t-tests showed no significant ageism change in attitudes toward OA. OAs' ageist attitudes toward YA (p = .012) were significantly reduced. Open-ended responses revealed several themes (e.g., challenging ageist stereotypes), which contrasted with statistical findings among the YA sample. Preliminary evidence demonstrated the feasibility of this activity. Further, OA and YA gained greater respect and understanding for each other as indicated by the open-ended responses. However, a larger sample size is needed to better determine efficacy. Nonetheless, the key takeaways from this presentation will help educators and program organizers alike explore when and how a virtual intergenerational activity can bridge gaps between older and younger adults.

Abstract citation ID: igad104.1165

### APPLYING EVIDENCE-BASED PRACTICES TO INTERGENERATIONAL TECHNOLOGY-SUPPORTED LEARNING OPTIMIZES MUTUAL BENEFIT

Shannon Jarrott, *The Ohio State University, Columbus, Ohio, United States*

Numerous intergenerational programs support varied goals using technology. Technological competence may be the desired outcome or the means for achieving outcomes in such programs. For example, Cyber Seniors operates in many settings with digital mentors providing technology training to more than 11,000 older adults. This and other technology-supported intergenerational programs have been delivered in-person and remotely with considerable success. Now that researchers have demonstrated feasibility and acceptability of intergenerational technology-supported learning, they may want to address the potential for such programs to be one sided. In the typical scenario, the youth bestows their technological expertise upon the older learner, but the pair can mentor each other to achieve additional goals. Application of evidence-based intergenerational strategies can optimize mutual benefit of technology-supported learning programs. In this paper, specific practices are illustrated with international examples from diverse intergenerational programs that incorporate technology. The author's and others' research linking practice with outcomes reveals that humanistic practice strategies (e.g., supporting equal group status in the contact setting) ensure that youth and older adults build relationships as well as technical skills while they learn from each other.

Abstract citation ID: igad104.1166

### HOW AN INTERGENERATIONAL TECHNOLOGY INTERNSHIP MODEL BUILDS CAREER READINESS AND AGE FRIENDLINESS

Skye Leedahl, Kristin Souza, and Emma Pascuzzi, *University of Rhode Island, Kingston, Rhode Island, United States*

This presentation will describe the key elements of the University of Rhode Island (URI) Engaging Generations Cyber-Seniors Program internship and discuss results from an assessment study that examined post-survey data related to student experiences. Past research has shown that this program is successful in reducing ageism in student participants. In this study, we examined questions from two scales about youth empowerment and participation in planning that corresponded to the Career Readiness Competencies employers seek in graduates today as determined by the National Association of Colleges & Employers (NACE) and measures for building age-friendliness. Survey findings (n=112) showed that the areas in which students stated the most impact were: effort, problem solving, identity exploration, goal setting, and time management. This corresponds to improvements in the NACE Career Readiness Competencies of career & self-development, professionalism, equity & inclusion, and leadership. Related to age-friendliness, scores were particularly high for questions about students speaking out and educating people about older adulthood, telling people about how older people can be digitally included, feeling confident about teaching older adults to use technology, and having ideas about how to improve digital support for older people. The key takeaway from this presentation will be for educators to identify a way in which student experiences can be assessed for growth in career readiness and building age-friendliness.

Abstract citation ID: igad104.1167

### TECHNOLOGY AND OLDER ADULTS: BEST PRACTICES FOR ENHANCING USABILITY, ENGAGEMENT, AND COMMUNICATION

Francesca Falzarano, *Weill Cornell Medicine, New York City, New York, United States*

The massive growth in population aging has coincided with a time of continued fast-paced innovations in technology. Technology can be leveraged to support health, well-being, and quality of life in older adults with health, mobility, and cognitive issues and is a means of maintaining social engagement in later life. However, for technology to achieve its intended purpose, older adults must have access to and recognize the value of such products. Despite the sheer size of the older adult population, the field of Information Communications Technology (ICT), has long overlooked the perspectives of older adults with regard the design of ICT applications. ICT systems that fail to consider the diverse capabilities and attitudes of older adult users are likely to alienate a significant, growing segment of the population and will likely be underutilized. This presentation will review best practices to enhance older adults' usability and engagement with technology, with an emphasis on user-centered design principles as well as innovative tools facilitating virtual ICT support and training (e.g., TeamViewer software) to provide older adults who have limited to no technology proficiency with the skills needed to navigate its use. Key takeaways from this presentation will include a greater understanding of the importance of design principles to enhance usability and adoption of ICT systems among older adults; means of increasing access and adoption of ICT applications; and how user feedback can be leveraged to inform adaptions to enhance the efficacy and acceptability of technology applications, and ultimately foster psychosocial well-being in older adults.

### SESSION 3390 (SYMPOSIUM)

Abstract citation ID: igad104.1168

### MOVEMENT AND HEALTH: NOVEL INSIGHTS INTO PHYSICAL ACTIVITY, ENERGY EXPENDITURE, AND MOTOR FUNCTION

Chair: Jennifer Schrack Co-Chair: Amal Wanigatunga
Discussant: Nancy Glynn

Physical activity, energy expenditure, and motor function are well-established predictors of health and longevity with aging. Traditionally, most research using these measures has focused on speed of movement and intensity of activity. More recently, interest has grown in ways to refine these movement-related measures—and the methods used to analyze them—to improve predictive and discriminatory power for older adult health outcomes. This symposium will focus on measures derived from traditional in-lab assessments of motor function as well as free-living accelerometry, and their novel application across a variety of large U.S. cohort studies. Ms. Marino will present analyses on the links among heart rate variability, physical activity, and cognitive health in the Atherosclerosis Risk in Communities (ARIC) study. Dr. Dougherty will present the association of fine and gross motor function with MRI-derived brain volumes in the Baltimore Longitudinal Study of Aging (BLSA). Dr. Davoudi will present evidence on the link between physical activity quantities and variability with dementia in the National Health and Aging Trends Study (NHATS). Dr. Schumacher will compare measures of absolute and relative physical activity intensity with incident cardiovascular outcomes and all-cause mortality in the Objective Physical Activity and Cardiovascular Health (OPACH) Study. Finally, Dr. Wanigatunga will describe objectively measured physical activity quantities and patterns by self-reported fatigue levels and HIV status in the Multicenter AIDS Cohort Study (MACS). Collectively, these presentations will highlight novel ways to utilize motor function, energy expenditure, and physical activity data to advance prevention science and promote healthy aging. \ This is an Epidemiology of Aging Interest Group Sponsored Symposium.

Abstract citation ID: igad104.1169

### FREE-LIVING HEART RATE VARIABILITY/PHYSICAL ACTIVITY AND COGNITIVE FUNCTION AND DEMENTIA IN ARIC

Francesca Marino[1], Hau-Tieng Wu[2], Mary Rooney[3], Elsayed Soliman[4], Jennifer Deal[5], Amal Wanigatunga[6], Jennifer Schrack[5], and Lin Yee Chen[7], *1. Johns Hopkins Bloomberg School of Public Health, Baltimore, Maryland, United States*, *2. Duke University, Durham, North Carolina, United States*, *3. Johns Hopkins University Bloomberg School of Public Health, Baltimore, Maryland, United States*, *4. Wake Forest University School of medicine, Winston-Salem, North Carolina, United States*, *5. Johns Hopkins University, Baltimore, Maryland, United States*, *6. Johns Hopkins School of Public Health, Baltimore, Maryland, United States*, *7. University of Minnesota School of Medicine, Minneapolis, Minnesota, United States*

Low heart rate variability (HRV) and physical activity (PA) are associated with cognitive dysfunction in clinical settings. Yet, associations with these measures among older adults in the free-living environment remain unexplored. We hypothesize higher free-living HRV and PA are associated with higher cognitive test scores and less likelihood of mild cognitive impairment (MCI)/dementia. This study includes 1,590 ARIC participants aged 72-94 years who wore the Zio XT Patch for 14-days to simultaneously capture ECG and accelerometry, from which we derived HRV as the standard deviation (SDNN) or root mean squared successive difference (rMSSD) of normal RR intervals and PA as total mean amplitude deviation (TMAD; higher values represent more movement). Linear or ordinal regression models were fitted to estimate cross-sectional associations between log transformed HRV or PA with latent-variable derived global and domain-specific cognitive factor scores or adjudicated MCI/dementia, respectively. Participants were on average 79 years, 57% female, and 32% Black. There were no significant associations between either HRV measure and cognition. After adjusting for demographic and medical covariates, 1-unit higher in log TMAD was associated with a 0.30-point higher global factor score (95% CI: 0.16-0.44), 0.38-point higher executive function factor score (95% CI: 0.22-0.53), 62% lower odds of MCI (95% CI: 0.22-0.67) and 75% lower odds of dementia compared to normal cognition (95% CI: 0.08-0.74). In summary, free-living PA, but not HRV of RR intervals, is associated with cognitive and dementia status. Further investigation of other objective HRV measures and directionality of associations between PA and cognition is needed.

Abstract citation ID: igad104.1170

### SHARED AND DISTINCT ASSOCIATIONS OF MANUAL DEXTERITY AND GROSS MOTOR FUNCTION WITH BRAIN ATROPHY

Ryan Dougherty[1], Hang Wang[1], Alden Gross[2], Jennifer Schrack[1], Yuri Agrawal[3], Eleanor Simonsick[4], Susan Resnick[4], and Qu Tian[4], *1. Johns Hopkins University, Baltimore, Maryland, United States*, *2. Johns Hopkins University Bloomberg School of Public Health, Baltimore, Maryland, United States*, *3. Johns Hopkins School of Medicine, Baltimore, Maryland, United States*, *4. National Institute on Aging, National Institutes of Health, Baltimore, Maryland, United States*

Poor motor function is associated with brain volume and cognitive impairment. Less is known about the associations among motor domains and brain atrophy and whether associations are affected by cerebrovascular burden and/or physical activity. We analyzed data from 726 Baltimore Longitudinal Study of Aging participants (mean age 70.6±10.1 years, 56%women, 27%Black), 525 of whom had repeat MRI scans over 5.0±2.1 years. Two motor domains were operationalized as latent variables. Manual dexterity using the Purdue Pegboard and gross motor function using grip strength and comprehensive physical performance testing. Associations between the latent variables and cortical and subcortical brain volumes of interest were examined using latent growth curve modeling, adjusted for demographics, white matter hyperintensities, and physical activity. Both higher manual dexterity and gross motor function were cross-sectionally associated with smaller ventricular volume and greater white matter volumes in the frontal, parietal, and temporal lobes (all p<.05). Higher manual dexterity was also cross-sectionally associated with greater parietal gray matter ( =0.14;95%CI:0.05,0.23), hippocampal ( =0.10;95%CI:0.01,0.20), postcentral gyrus ( =0.11;95%CI:0.01,0.20), and occipital white matter ( =0.10;95%CI:0.01,0.21) volumes, and better gross motor function with greater temporal gray matter volume ( =0.16;95%CI:0.05,0.26). Longitudinally, higher manual dexterity and gross motor function were associated with lower temporal white matter and occipital gray matter atrophy (all p<0.05). Greater manual dexterity was also associated with a slower rate of ventricular enlargement ( =-0.17;95%CI:-0.29,-0.05) and less atrophy of occipital white matter ( =0.39;95%CI:0.04,0.71). Among cognitively normal middle- and older-aged adults, manual dexterity and gross motor function exhibited shared as well as distinct associations with brain atrophy over time.

Abstract citation ID: igad104.1171

### PHYSICAL ACTIVITY AND DEMENTIA IN THE NATIONAL HEALTH AND AGING TRENDS STUDY (NHATS)

Anis Davoudi[1], Patrick Donahue[1], Michelle Carlson[2], Ryan Dougherty[1], Amal Wanigatunga[3], Vicki Freedman[4], and Jennifer Schrack[1], *1. Johns Hopkins University, Baltimore, Maryland, United States*, *2. Johns Hopkins Bloomberg School of Public Health, Baltimore, Maryland, United States*, *3. Johns Hopkins School of Public Health, Baltimore, Maryland, United States*, *4. University of Michigan, Ann Arbor, Michigan, United States*

Individuals living with dementia exhibit disrupted sleep/wake rhythms; however, whether daily physical activity volume and patterns are worse among those living with dementia is not well established. We examined the cross-sectional association between dementia classification and accelerometry-derived patterns of daily physical activity in NHATS. Wrist-worn accelerometry was collected using the Actigraph Centrepoint Insight Watch in a subset of Round 11 (2021) participants. Dementia status, including possible and probable dementia, was determined using the NHATS dementia algorithm. We characterized daily physical activity patterns using: most active 10-hours/day (M10), time of M10, least active 5-hours/day (L5), time of L5, relative amplitude (RA), intra-daily variability (IV), inter-daily stability (IS), and activity fragmentation using the active-to-sedentary transition probability (ASTP). We used separate linear regression models to study the association between dementia and each actigraphy feature, adjusting for relevant demographic features including age, gender, race, BMI, and education. 721 participants had ≥3 days of valid accelerometry data, dementia status, and complete covariate data (40% aged ≥80 years, 54% women). Compared to cognitively normal participants, those living with dementia averaged fewer activity counts/day (beta: -382K counts/day p<0.0001), lower M10 (beta: -261K counts, p<0.0001), lower IS (beta: -0.04; p= 0.0066), and higher ASTP (beta: 7.1%; p<0.0001). Participants with and without dementia did not differ in terms of IV, L5, or RA (p>0.05 for all). These results suggest important differences in volume and patterns of daily movement by dementia status. Future studies should investigate whether similar differences exist in earlier stages of cognitive impairment to enhance preventative efforts.

Abstract citation ID: igad104.1172

### ASSOCIATIONS OF RELATIVE INTENSITY OF PHYSICAL ACTIVITY WITH INCIDENT CARDIOVASCULAR OUTCOMES AND MORTALITY

Benjamin Schumacher[1], Michael LaMonte[2], Chongzhi Di[3], Humberto Parada Jr.[4], Steven Hooker[4], John Bellettiere[5], Eleanor Simonsick[6], and Andrea LaCroix[5], *1. University of Pittsburgh School of Public Health, Pittsburgh, Pennsylvania, United States*, *2. University at Buffalo - SUNY, Buffalo, New York, United States*, *3. Fred Hutchinson Cancer Center, Seattle, Washington, United States*, *4. San Diego State University, San Diego, California, United States*, *5. University of California, San Diego, San Diego, California, United States*, *6. NIA IRP, Baltimore, Maryland, United States*

For an older individual, the energy expended (absolute intensity) during a specific activity (e.g., casual walk) may be closer to their maximal effort (higher relative effort) than for a younger individual. We compared the associations of relative and absolute intensity of physical activity (PA) with all-cause mortality and incident major cardiovascular disease (CVD). Accelerometer-measured PA in the Objective Physical Activity and Cardiovascular Health (OPACH) Study (mean age 78.5±6.7) was used to estimate daily hours of absolute light intensity PA (LPA) and moderate-to-vigorous PA (MVPA). Accelerometer-estimated metabolic equivalents (METs) in each 15-second epoch were divided by two maximal MET capacity estimates. These percent maximal effort metrics were categorized and aggregated into daily hours of relative LPA and MVPA. Cox proportional hazards models estimated the associations of a one-hour daily increase in absolute and relative PA with the two outcomes. On each PA measurement scale, an increase in either intensity category reduced the risk of both outcomes. A one-hour increase in absolute LPA reduced the risks of both outcomes by 12%, and a one-hour increase in absolute MPVA reduced the risk of death and CVD by 45% and 27%, respectively. On the relative scale, LPA was more strongly associated with both outcomes than MVPA. Increasing absolute MVPA was more strongly associated with the outcomes than increasing relative MVPA. The PA intensity paradigm should keep shifting towards recommendation of more movement, regardless of intensity, and placing greater emphasis on relative light intensity activities to reduce the risks of death and incident major CVD.

Abstract citation ID: igad104.1173

### LINKING SELF-REPORTED FATIGUE TO OBJECTIVELY MEASURED PHYSICAL ACTIVITY IN MEN WITH AND WITHOUT HIV

Amal Wanigatunga[1], Sohyeon Kwon[1], Frank Palella[2], Susan Koletar[3], Stephen Gange[1], Todd Brown[4], Joseph Margolick[1], and Jennifer Schrack[5], *1. Johns Hopkins School of Public Health, Baltimore, Maryland, United States*, *2. Northwestern Feinberg School of Medicine, Chicago, Illinois, United States*, *3. Ohio State University School of Medicine, Columbus, Ohio, United States*, *4. Johns Hopkins School of Medicine, Baltimore, Maryland, United States*, *5. Johns Hopkins University, Baltimore, Maryland, United States*

Fatigue, a symptom commonly associated with reduced physical activity, is difficult to measure among people living with HIV (PLWH), a population with the potential for comorbidity. We examined the association between self-reported fatigue and objectively measured physical activity by HIV serostatus across three commonly used fatigue measures. Self-reported fatigue and accelerometry data were collected cross-sectionally from 699 men (mean age 59 years; 57% PLWH) enrolled in the Multicenter AIDS Cohort Study. Linear regression was used to estimate differences in total daily physical activity (min/day) by self-reported fatigue measured by three separate assessments: 1) "unable to get going"; 2) "felt everything was an effort"; and 3) "work/activity difficulty due to physical health". Models adjusted for age, BMI (kg/m2), and HIV serostatus, and interactions between HIV serostatus and each fatigue assessment were tested. Fatigue prevalence, defined as "unable to get going" (43%), "felt everything was an effort" (39%), or "work/activity difficulty" (20%) and total daily physical activity (7.4 hours active/d) appeared similar by HIV status. Men reporting "unable to get going" and "work/activity difficulty" were estimated to have an adjusted average of 21 (SE=8) fewer active min/day (p=0.01) and 35 (SE=10) fewer active min/day (p=0.001) than those without not reporting either, respectively. There was an interaction between HIV+ serostatus and "work/activity difficulty" (interaction beta: -54 active minutes/day, p=0.003). No association was detected with "felt everything was an effort" (p=0.16). Among the three questions assessed, "work/activity difficulty" was identified as a potentially useful measure of physical activity limitation in men living with HIV.

### SESSION 3395 (SYMPOSIUM)

Abstract citation ID: igad104.1174

### RESEARCHERS BEWARE: CAUTIONARY TALES OF FRAUDULENT RESEARCH PARTICIPANTS AND WHAT TO DO ABOUT THEM

Chair: Justine Sefcik Discussant: Harleah Buck

This symposium presents a series of cases where gerontological researchers identified fraudulent participants and bots engaging in their studies. These presentations describe the complex nature of participants misrepresenting themselves and being creative to enroll in studies for financial incentives. Dr. Sefcik shares how a snowball sample led to participants enrolling in a study and misrepresenting themselves during virtual qualitative interviews. Dr. Boon illuminates how Facebook recruitment led to bot responses and steps taken to identify if participants were real. Dr. Frechman reveals how recruitment on social media platforms and email distribution lists led to bot attacks of the study survey. Dr. Carpenter explains a study involving a multi-methods approach in which a bot completed several surveys and an interviewee gave nonsensical responses. Dr. Wallace explains two types of fraudulent activity occurring, the first with bots completing an online survey and the second with deception during interviews. All presenters discuss their experiences of suspecting fraudulent research participation, approaches on how they verified participants, action steps to address misrepresentation, processes put in place to uphold the integrity of their studies, and tips to mitigate future fraud. This is a Nursing Care of Older Adults Interest Group Sponsored Symposium.

Abstract citation ID: igad104.1175

### WHEN SNOWBALL SAMPLING LEADS TO AN AVALANCHE OF FRAUDULENT PARTICIPANTS

Justine Sefcik[1], Zachary Hathaway[2], and Rose Ann DiMaria-Ghalili[3], *1. Drexel University College of Nursing and Health Professions, Philadelphia, Pennsylvania, United States*, *2. Drexel University, Philadelphia, Pennsylvania, United States*, *3. College of Nursing and Health Professions, Drexel University, Philadelphia, Pennsylvania, United States*

The scientific literature has examples of how fraudulent respondents who gainfully access studies for financial incentives negatively affect the integrity of research results. However, there have been less examples shared of participant misrepresentation in qualitative research, the implications, and how to prevent deception. This session entails the presentation of a qualitative study where we believe participants misrepresented themselves during an interview for the financial incentive. For the study, we sought caregivers for individuals with a diagnosis of Alzheimer's disease or Alzheimer's disease related dementia with a history of chronic leg wounds. One of our recruitment efforts was through ResearchMatch. After one ResearchMatch interviewee completed the interview, he reached out to the project manager and asked if the study flyer could be shared with others that were "dealing with similar issues". This resulted in an uptick of people being interested in study participation. Several of these participants were scheduled to complete individual interviews on one day with an experienced qualitative researcher. Clues indicating potentially fraudulent participants were vague and illogical responses to questions, and similar nonsensical responses shared across interviews. Once potential fraud was detected, interviews were paused for the following interview day so the study team could regroup and none of the interviews were used for data analysis. The lessons learned and steps for researchers to take to help reduce the number of fraudulent individuals participating in interviews will be shared. This includes two-step screening where the study staff interacts with the potential participants through methods other than over email.

Abstract citation ID: igad104.1176

### USING PARADATA AND MANUAL REVIEW TO DETECT AUTOMATED ONLINE RESEARCH SURVEY PARTICIPATION

Jeffrey Boon[1], and Cathy Maxwell[2], *1. Vanderbilt University Medical Center, Nashville, Tennessee, United States*, *2. Vanderbilt University, Nashville, Tennessee, United States*

With an increase in health research recruitment on social media, there is also an increase in prevalence of software known as bots auto-enrolling in online studies. Despite efforts to prevent automated fraudulent enrollment, bots have become more sophisticated with some able to bypass a CAPTCHA and generate false email addresses. This session describes such fraudulent enrollment in one study and our efforts to detect it. For an online study of family caregivers of people with dementia, we recruited via family caregiver groups on Facebook. Potential participants could proceed to the online study and immediately answer screening questions and complete the study. A CAPTCHA was used to screen non-human respondents, but we detected rapid enrollment compared with other methods, raising suspicions. Paradata generated in the process of survey administration and unusual email addresses flagged suspected bot responses. To screen the responses, we reviewed the time to complete surveys compared with known human responses. We also noted email addresses that consisted of long alphanumeric usernames not common with most human respondents. After screening for suspicious respondents, we attempted to contact the email addresses of borderline cases to verify they were real respondents. Only one of 157 (0.006%) were determined to be a real respondent. Lessons learned from this experience include using paradata as part of data quality monitoring, using survey design methods to elude automated bots, and the value of researchers interacting with participants during enrollment.

Abstract citation ID: igad104.1177

### ATTACKED BY BOTS AND HACKED BY BAD APPLES: LESSONS LEARNED NAVIGATING RESEARCH PARTICIPANT FRAUD

Erica Frechman[1], Harleah Buck[2], and Cathy Maxwell[3], *1. Atrium Health Wake Forest Baptist, Winston-Salem, North Carolina, United States*, *2. University of Iowa, Iowa City, Iowa, United States*, *3. Vanderbilt University, Nashville, Tennessee, United States*

In a time of emerging technologies for participant recruitment and data collection, researchers need proven and comprehensive strategies to protect research integrity. Incorporation of proactive, rather than reactive, methods to prevent fraudulence will mitigate additional stressors and headaches associated with fraud and fraud detection. This session presents a case which describes a researcher's experience detecting, navigating, problem-solving, and then managing participant fraud that occurred twice in a multi-methods study. Participants were recruited from a senior center and two YMCA sites using social media platforms and email distribution lists which linked them with an online survey. After the initial study launch, a "bot attack" with "bot fast" deception within the study survey caused the study to shut down. The researcher worked with the study team, community sites, and IRB to detect potential causes of fraud and implement protective factors to ensure quality control and deter the bot system. Unfortunately, after the second study launch, the survey was once again successfully attacked. This time the researcher chose to work with the study team in order to create a step-wise rigorous approach to verifying accurate data and participants, while working to maintain trust of the community sites and real participants. Finally, to mitigate further fraud, the researcher pivoted to the use of ResearchMatch to ensure recruitment goals and trustworthy data. Within this presentation, the researcher will share lessons learned in survey fraud, proactive strategies to prevent fraudulence, communication methods interacting with IRB, study sites, and participants, and maneuvering perils involved in manuscript preparation.

Abstract citation ID: igad104.1178

### INCENTIVES + SOCIAL MEDIA RECRUITMENT + MINIMAL SUBJECT INTERACTION = POTENTIAL FOR FRAUD!

Joan Carpenter[1], Casey Jackson[2], and Liza Behrens[3], *1. University of Maryland School of Nursing, Baltimore, Maryland, United States*, *2. University of Maryland, Baltimore, Maryland, United States*, *3. Pennsylvania State University, State College, Pennsylvania, United States*

Social networking technologies create opportunities to recruit a more diverse and representative research sample. However, social media recruitment strategies with low researcher interaction for screening brings threats to data integrity, especially when incentives are involved. During a recent study designed to ascertain direct care nursing home staffs' perceptions of risk during the COVID-19 pandemic, we experienced fraudulent research participants. We aimed to recruit, enroll, and collect survey and semi-structured interview data from N=30 participants between June 2021 through March 2022. Participants were compensated $25. Initial recruitment efforts targeted Facebook listservs for licensed and unlicensed nurses, social workers, and activity directors. Research staff provided participants who screened eligible for the study via email a link to initiate consent and complete the survey electronically. Four months into study recruitment, study staff discovered during an interview that one participant was unable to answer questions in the correct context as a direct care worker and responses were nonsensical. Additional quality control measures confirmed that "bots" had completed surveys (N=8). The fraudulent data were removed from the study database and corrective actions implemented into screening to rule out ineligible participants/bots including screening for repetitive language and grammatical errors in email conversations, verifying eligibility through phone communications, and adding a Captcha verification prior to electronic survey consent. Our experiences highlight the importance of creating study protocols that include social media recruitment with safeguards to fraudulent participants. This is especially important for investigators attempting to recruit hard to reach populations and participants who wish to remain anonymous.

Abstract citation ID: igad104.1179

### ARE YOU A REAL PARTICIPANT? EXPERIENCES WITH FRAUDULENT PARTICIPATION ACROSS TWO FUNDED STUDIES

Cara Wallace, *Trudy Busch Valentine School of Nursing, Saint Louis University, St. Louis, Missouri, United States*

While offering financial incentives for participation in research studies is common, less discussed is the potential impact of fraudulent participation for financial gain. Survey and data management software provide safeguards for fraud detection but enabling detection does not automatically weed out fraudulent responses. Few resources provide guidelines for identifying or managing fraudulent participation. Thoughtful processes are important, while also being mindful of normal research behaviors which might get flagged as suspicious, for example due to low literacy skills. This presentation discusses experiences across two studies with adults living with serious illness and/or their family members and two different types of fraudulent participation:

online surveys and studies requiring personal interaction. Study one is a pilot instrument validation study collecting online survey data. Recruitment occurred locally through listservs and a research participation database from a large university hospital. Study two is a mixed-methods longitudinal design where survey and interview data are collected via phone or video calls. Participants for this study were recruited nationwide using multiple strategies, including social media. Both offer time-saving insights for dealing with fraudulent participation in research. For example, study one provides tips for planning how to manage spam/bots prior to data collection, including setting up filters to automatically weed out identified spam, creating automatic messaging included in the survey in the event it needs to be paused, and identifying potential problematic patterns in data that bypasses fraud detection. Study 2 emphasizes the importance of creating strong verification procedures for study eligibility when relying on participant reporting.

### SESSION 3400 (SYMPOSIUM)

Abstract citation ID: igad104.1180

### CONNECTING COMMUNITIES: ROLES OF AGING ATTITUDES, PLACE ATTACHMENT, AND PSYCHOLOGICAL WELL-BEING

Chair: Su-I Hou Co-Chair: Li-Fan Liu

As the global population ages, communities must find innovative ways to support older adults. This symposium focuses on the intersections of aging, community, and culture, particularly emphasizing the importance of place attachment, aging attitudes, psychological well-being, and engagement in promoting healthy aging. Leading experts from social gerontology, psychology, and public health across the globe will explore the complex relationships between aging, community, and culture. Drs. HM Chen from Texas A&M University–Central Texas (TAMCT) and Hou from the University of Central Florida (UCF) from the United States will start the symposium with a systematic literature review on culture and aging in the community among Chinese older adults. Drs. JJ Chen and Liu from the National Cheng-Kung University (NCKU) will examine the mediating effect of psychological place attachment, how older adults perceive their living environments, and (positive versus negative) attitudes towards aging and psychological well-being. Dr. Hou from UCF will further assess aging attitudes (engagement versus enjoyment) among older adults participating in neighborhood lunch versus lifelong learning programs to discuss the implications of tailored aging service development. Finally, Drs. Lin and Fung from The Chinese University of Hong Kong (CUHK) will discuss the association between activity diversity and psychological well-being among community-dwelling older adults in Hong Kong. Through interdisciplinary dialogue and sharing of lessons learned, this global symposium promotes a deeper understanding of the roles of aging attitudes, place attachment, culture, and psychological well-being among older adults to foster innovative approaches to promoting healthy aging and community engagement. This is an Aging Among Asians Interest Group Sponsored Symposium. This is a collaborative symposium between the Aging Among Asians and International Comparisons of Healthy Aging Interest Groups.

Abstract citation ID: igad104.1181

### ENGAGEMENT OR ENJOYMENT? ATTITUDES OF OLDER ADULTS IN GROUP LUNCH VERSUS. LIFELONG LEARNING PROGRAMS

Su-I Hou, *School of Global Health Management & Informatics, University of Central Florida, Orlando, FL, USA, Oviedo, Florida, United States*

Purpose: This study examines aging attitudes among older adults who participated in the neighborhood lunch (NLP) and lifelong learning (LLP) programs.

**Methods:** Older adults from an NLP and an LLP in the same county in Florida participated. An aging attitudes measurement originally developed by German researchers was used.

**Results:** A total of 193 older adults participated, 43% from NLP. The mean age was 73.2 (SD=7.78) years. Data showed that LLP older adults endorsed higher on "would like to have responsibilities," "have a task," "do unpaid volunteer work," and "help others" (p<.001). NLP participants, on the other hand, endorsed higher on "no longer have to contribute to society," "want to enjoy life," and "finally want to rest and relax" (all p<.001). Exploratory factor analyses (EFAs) using principal component extraction methods and varimax rotation with Kaiser normalization showed a clear balanced 2-factor pattern, with total variance explained 60% (30% each). These two factors were consistent with the theory representing engagement versus enjoyment aging attitudes. Reliabilities of the two-factor scales were satisfactory, with Cronbach's alpha of .764 (CITC ranged: .457 ~ .660) for the engagement scale (4-item) and.634 (CITC ranged: .379~.499) for the enjoyment scale (3-item).

**Discussion:** Current findings showed that LLP participants endorsed higher engagement, while NLP participants endorsed higher enjoyment towards aging. This tested aging attitudes scale consists of 7 items with two clear theory-consistent factors among community-dwelling older adults. Results can help researchers quickly assess aging attitudes of diverse groups of older adults for tailored program and service development.

Abstract citation ID: igad104.1182

### A SYSTEMATIC REVIEW ON AGING IN THE COMMUNITY AND CULTURE AMONG CHINESE OLDER ADULTS

Hao-Min Chen[1], and Su-I Hou[2], *1. Texas A&M University-Central Texas, Killeen, Texas, United States*, *2. School of Global Health Management & Informatics, University of Central Florida, Orlando, FL, USA, Oviedo, Florida, United States*

Chinese older adults face many barriers to healthy aging in community, such as increased financial burden, threatened place security, limited accessibility to medical services, language and transportation difficulties, daily housework and repairs challenges, etc. This systematic review aimed to examine and report the results current scholars have published in journal articles in the last decade (2010-present) on the influence of culture on Chinese older adults aging in community. Combination terms of "aging in community or aging in place," "Chinese," and "culture or cultural or ethnicity or identity or values" were used when searching in the four databases: CINAHL Complete; ERIC; MEDLINE; and PsycInfo. Researcher examined abstracts of all journal articles retrieved using the above criteria and further determined their relevance for inclusion. A total of 38 journal articles were included. Findings suggest that aging in community reflects a changing policy tendency from institution-based to community-centered care which better responds to the needs of the growing Chinese senior population. Family care remains older adults' preferences. However, individual, community, and larger contextual factors can be influenced, such as the number of adult children in the household, education level, residential area, accessibility to healthcare facilities, perceived cultural values of filial piety, state-owned enterprise reformation, and the one-child policy in China. Policymakers are encouraged to promote older adults' personalized care plans; comprehensive health insurance coverage; constructive social/community environments (e.g. community parks or community open spaces for age-friendly activities); home care support, or smart home technologies for regular health monitoring.

Abstract citation ID: igad104.1183

### THE MEDIATING EFFECT OF PLACE ATTACHMENT ON ATTITUDE TOWARD AGING AND PSYCHOLOGICAL WELL-BEING

Jia-Jen Chen, and Li-Fan Liu, *National Cheng Kung University, Tainan City, Tainan, Taiwan (Republic of China)*

Psychological well-being has become a social challenge for vulnerable older adults and age-friendly cities/communities has been a key aging policy focusing on environmental factors to enhance well-being in Taiwan. However, how older adults perceive their living environments and whether it is important when advocating older adults' positive attitudes toward aging leading to psychological well-being remains unknown. One of the place attachment dimensions is psychological attachment which has attracted attention in planning for aging in place and age-friendly communities (Aliakbarzadeh et al., 2021; 2022). This study aims to investigate whether psychological attachment mediated the relationship between attitudes toward aging and psychological well-being among community-dwelling older adults in Taiwan. A faced-to-faced interview of 306 individuals aged 50 and over living in the community were interviewed. The Cronbach's alpha of all scales was over 0.70 which showed good reliability. The associations and mediation effects were tested using structural equation modeling (SEM) and the model fitted the data adequately. After controlling for sex and age, the SEM findings demonstrated that the lower level of negative attitudes toward aging were significantly and positively associated with well-being (direct effect = 0.16, $p < 0.05$). Furthermore, the lower level of negative attitudes toward aging was positively related with place attachment and consequently predicted better psychological well-being (indirect effect = 0.048, $p = 0.034$; total effect = 0.209, $p = 0.003$). We suggest that place attachment should be monitored in the policy of age-friendly communities while improving older adults' positive attitudes toward aging leading to a better sense of well-being.

Abstract citation ID: igad104.1184

### A DIVERSE LIFE IS A BETTER LIFE? THE ASSOCIATION BETWEEN ACTIVITY DIVERSITY AND PSYCHOLOGICAL WELL-BEING IN HONG KONG

Zhixuan Lin, and Helene Fung, *The Chinese University of Hong Kong, Hong Kong, Hong Kong*

The debate between the devotedness and evenness of activity participation has long existed. On the one hand, the theory of selective optimization with compensation (Baltes & Baltes, 1990) argues that successful aging depends on concentrating one's participation on fewer domains of activities. On the other hand, the social integration perspective (Rosow, 1967) posits that diversity indicates extensive knowledge and a sense of purpose, which contributes to well-being. A previous study found a positive relationship between activity diversity and older adults' psychological well-being in a U.S. sample (Lee et al., 2018). However, it might not be the case for people living in extremely hot weather as diverse activities might increase the risk of exposure to the heat. The current study examines their relationship in the hot summer months in Hong Kong, a tropical Asian city. Community-dwelling participants aged 60 years or above (N = 344, Mage = 67.15, SDage = 5.26) completed surveys 3 times each day over 10 days, reporting their activities, positive and negative affect, meaning and engagement of each activity, and loneliness. The regional heat-risk index was acquired from Hua et al. (2021). Results showed that activity diversity negatively correlated with positive affect, meaningfulness and engagement of daily activities, and positively correlated with loneliness. Heat risk did not moderate the relationship, but it correlated with less positive and more negative affect. Our findings suggest that Hong Kong older adults benefit from focused rather than broad participation in activities.

### SESSION 3405 (SYMPOSIUM)

Abstract citation ID: igad104.1185

### LEVERAGING NEIGHBORHOOD EXPOSURES AS MODIFIERS OF INDIVIDUAL RISK FACTORS FOR COGNITIVE DECLINE AND DEMENTIAS

Chair: Michelle Carlson Co-Chair: Andrea Rosso
Discussant: Gina Lovasi

There are numerous ways in which neighborhood environmental factors have been shown to contribute to adverse health. More remains to be done to link long-term neighborhood exposures, such as social and physical activities, to modifiable individual risk factors for cognitive decline and dementia. Individual interventions are usually small in scale and conducted in those with higher socioeconomic status (SES). Additionally, increases in healthy behaviors, such as walking, have been difficult to promote and sustain. Each of these risk factors is known to be influenced by environmental features. This symposium will explore the roles of neighborhood-level social (nSES, affluence, cohesion, discrimination) and physical (air pollution) factors as drivers of cognitive aging and dementia across multiple longitudinal studies of community-based aging. Studies span urban and rural communities, including the Health ABC Study, the Cardiovascular Health Study, and the Think Phresh study. These studies are important to evaluating the role of neighborhood features on cognitive aging by expanding beyond cross-sectional data and by studying residentially stable older adults most likely to be affected by their local context. Those neighborhood factors that impact individual activity, cognitive aging, and Alzheimer's disease risk will be further discussed for differences by race/ethnicity.

Abstract citation ID: igad104.1186

### NEIGHBORHOOD AFFLUENCE AND RISK OF INCIDENT DEMENTIA: ROLE OF PHYSICAL ACTIVITY DESTINATIONS

Kyle Moored[1], Michael Desjardins[1], Breanna Crane[1], Patrick Donahue[2], Parveen Garg[3], Andrea Rosso[4], Gina Lovasi[5], and Michelle Carlson[1], *1. Johns Hopkins Bloomberg School of Public Health, Baltimore, Maryland, United States*, *2. Johns Hopkins University, Baltimore, Maryland, United States*, *3. University of Southern California Keck School of Medicine, Los Angeles, California, United States*, *4. University of Pittsburgh, Pittsburgh, Pennsylvania, United States*, *5. Dornsife School of Public Health, Philadelphia, Pennsylvania, United States*

Neighborhood affluence encompasses the degree of high educational, occupational, and wealth concentration within residential communities. Highly affluent neighborhoods may reduce dementia risk in part by providing amenities that promote physical activity. We examined associations between neighborhood affluence, and specifically the quantity of nearby physical activity (PA) destinations (e.g., gyms), with incident dementia. Participants were 2,942 adults ≥65 years old from the Cardiovascular Health Cognition Study. Baseline neighborhood measures included 1) a 1990 Census-derived affluence index (percent with college education, percent with professional/managerial occupation, median home value) and 2) the count of PA destinations from the 1990 National Establishment Time Series (NETS) database that fell within a 1-km Euclidean buffer around the home. A total of 469 dementia cases (240 Alzheimer's, 208 mixed/vascular, 21 other) were clinically adjudicated from neurological/neuropsychological examinations (Mean=5.3±1.8 follow-up years). In Cox proportional hazards models adjusted for clinic site and individual demographics (age, gender, race, marital status), each 1-SD higher neighborhood affluence was associated with 15% reduced hazard of all-cause dementia (HR=0.85, 95%CI: 0.77-0.94), largely driven by mixed/vascular subtype (HR=0.73, 95%CI: 0.64-0.86), but not Alzheimer's subtype (HR=0.93, 95%CI: 0.81-1.07). Further, participants with ≥2 (versus no) nearby PA destinations had 37% reduced hazard of specifically mixed/vascular dementia (HR=0.63, 95%CI: 0.41-0.99). After also adjusting for individual health, income, and education, neighborhood affluence remained protective against mixed/vascular dementia (HR=0.77, 95%CI: 0.65-0.90), but number of PA destinations was no longer associated. Neighborhood affluence may reduce dementia risk via improved vascular health and operate through additional mechanisms beyond nearby PA amenities.

Abstract citation ID: igad104.1187

### NEIGHBORHOOD SOCIOECONOMIC STATUS AND CHANGES IN COGNITIVE FUNCTION IN OLDER ADULTS: FINDINGS FROM HEALTH ABC STUDY

Greta Jianjia Cheng[1], Sara Godina[2], Erica Fan[3], and Andrea Rosso[4], *1. University of Pittsburgh, Pittsbugrh, Pennsylvania, United States*, *2. University of Pittsburgh School of Public Health, Pittsburgh, Pennsylvania, United States*, *3. University of Pittsburgh, School of Public Health, Pittsburgh, Pennsylvania, United States*, *4. University of Pittsburgh, Pittsburgh, Pennsylvania, United States*

Neighborhood socioeconomic status (NSES), an overall marker of neighborhood condition, may influence multiple aspects of health and well-being in older adulthood, including cognitive function, though results regarding neighborhood and cognition have been largely cross-sectional. We examined the association between NSES and changes in cognitive function using 610 participants from the Pittsburgh site of the Health, Aging, and Body Composition (Health ABC) Study, a longitudinal cohort of community-dwelling, Black and white adults aged 70 and older. Changes in cognitive function were measured annually over a 6-year follow-up (2006-2012) using the Modified Mini-Mental State Examination (range: 0-100, 3 visits on average per person). Using baseline home address, NSES was calculated for census tracts by summing z-scores of six 2010 census variables representing wealth/income, education, and occupation, and divided into tertiles. Multivariate mixed-effect linear regression models assessed associations, adjusting for individual-level sociodemographic and health-related covariates. In the fully adjusted model, the lowest NSES tertile (vs. the highest) was not associated with baseline cognitive function (β: -0.99, 95% CI: -3.86, 1.87) but was associated with a faster rate of decline in cognitive function (β: -1.54, 95% CI: -2.67, -0.41). No significant differences in baseline cognitive function or rates of change were observed in the middle (vs. the highest) NSES tertile. There was no evidence of effect modification by race. The results indicate that exposure to adverse neighborhood socioeconomic environments may accelerate cognitive decline in late life. The findings add to existing literature concerning the role of environments in the context of aging.

Abstract citation ID: igad104.1188

### TOXIC CHEMICAL EMISSIONS AND DEMENTIA RISK: RESULTS FROM THE CARDIOVASCULAR HEALTH COGNITION STUDY

Breanna Crane[1], Kyle Moored[1], Patrick Donahue[2], Anne Corrigan[1], Frank Curriero[1], Andrea Rosso[3], Gina Lovasi[4], and Michelle Carlson[1], *1. Johns Hopkins Bloomberg School of Public Health, Baltimore, Maryland, United States*, *2. Johns Hopkins University, Baltimore, Maryland, United States*, *3. University of Pittsburgh, Pittsburgh, Pennsylvania, United States*, *4. Drexel University Dornsife School of Public Health, Philadelphia, Pennsylvania, United States*

Air pollution is a modifiable risk factor for dementia. Yet, studies on specific forms of air pollution (e.g., toxic chemical emissions from industrial facilities) and dementia risk are underexplored. We examined associations between toxicity-weighted concentrations of industrial pollution and dementia outcomes among a large, multi-site cohort of older adults. Participants (n=2,770) were ≥ 65 years old (M=75.3, SD=5.1 years) from the Cardiovascular Health Cognition Study. Toxicity-weighted concentrations were estimated using the Risk Screening Environmental Indicator (RSEI) model which incorporates total reported chemical emissions with toxicity, fate, and transport models. Estimates were aggregated to participants' baseline census tract, averaged across 1988-1992, and log10-transformed. Dementia status was clinically adjudicated in 1998-1999 (M=5.2 person-periods) by subtype (all-cause, Alzheimer's, vascular, mixed). We assessed whether RSEI-estimated toxicity-weighted concentrations were associated with: 1) odds of prevalent dementia and 2) incident dementia risk by subtype. After adjusting for individual and neighborhood-level covariates, a 1-unit increase in log10-toxicity was associated with a 34% increase in the odds of prevalent dementia (OR=1.34, 95% CI: 1.01, 1.78). In discrete-time survival models, each 1-unit increase in log10-toxicity was associated with a 65% increased hazard of vascular dementia (HR=1.65, 95% CI: 1.04, 2.61) but was not significantly associated with all-cause, Alzheimer's disease, or mixed dementia. Living in regions with higher toxic air emissions was associated with greater odds of prevalent dementia and a heightened risk of incident vascular dementia. Results suggest that the RSEI model may be a novel screening tool for examining associations between toxic chemical releases and dementia risk.

Abstract citation ID: igad104.1189

### UNFAIR TREATMENT, SOCIAL COHESION, AND COGNITION IN BLACK ADULTS LIVING IN URBAN NEIGHBORHOODS

Erica Fan[1], Tamara Dubowitz[2], Wendy Troxel[2], Andrea Weinstein[3], Tiffany Gary-Webb[4], Bonnie Ghosh-Dastidar[2], Jennifer Manly[5], and Andrea Rosso[3], *1. University of Pittsburgh, School of Public Health, Pittsburgh, Pennsylvania, United States*, *2. RAND Corporation, Pittsburgh, Pennsylvania, United States*, *3. University of Pittsburgh, Pittsburgh, Pennsylvania, United States*, *4. University of Pittsburgh, Graduate School of Public Health, Pittsburgh, Pennsylvania, United States*, *5. Columbia University Irving Medical Center, New York City, New York, United States*

Exposure to discrimination has been associated with poorer cognitive function but this association may be influenced by broader social factors, such as neighborhood social cohesion. We investigated the associations between unfair treatment, social cohesion, and cognitive function in 234 participants ≥50 years, recruited from urban, primarily Black neighborhoods in Pittsburgh, PA. Neuropsychological assessments were conducted in 2018 to create six cognitive domain z-scores, adjusted for age, sex, and education level. Social cohesion was assessed in 2013 and 2018. Change in social cohesion scores over time categorized participants into four groups: consistently high, consistently low, increasing, and decreasing scores. Unfair treatment was assessed via questionnaire in 2018. Adjusted generalized linear models assessed associations between unfair treatment and cognition. Unfair treatment was significantly associated with executive function (β=-0.041, p=0.01) and marginally associated with language (β=-0.030, p=0.07). Associations persisted with change in social cohesion in the models and we found that social cohesion moderated the association between unfair treatment and executive function (p=.06). In those with consistently low social cohesion, more unfair treatment was associated with worse executive function (β=-0.085, p=0.02). Both increasing and decreasing social cohesion showed a similar negative association between unfair treatment and executive function at a trend level (β=-0.122, p=0.08 and β=-0.082, p=0.10 respectively). There was no association for those with consistently high social cohesion. Individuals experiencing unfair treatment and consistently low levels of neighborhood social cohesion had lower executive function. These findings highlight the importance of intervening at multiple levels and addressing both interpersonal discrimination and structural sources.

### SESSION 3410 (SYMPOSIUM)

Abstract citation ID: igad104.1190

### PALLIATIVE AND END-OF-LIFE CARE FOR OLDER ADULTS WITH EXPERIENCES OF HOMELESSNESS

Chair: Kelly Melekis Discussant: Kelly Melekis

Although palliative and end-of-life (PEOL) care offers many benefits to those with life-limiting illness, unhoused older adults face significant barriers in accessing these types of care. This symposium examines disparities in PEOL care, emerging services for disadvantaged older adults, and recommendations for capacity-building within healthcare, housing, and aging services. The first paper is an exploratory study of programs serving the PEOL care needs of unhoused adults with serious illnesses. The second paper is a mixed methods study that explores provider perspectives on approaches and challenges to addressing the PEOL care needs among unhoused individuals. The third paper uses the CBPR method photovoice to highlight the experiences of terminally ill patients accessing social model hospice care, a free option to facilitate access to hospice for unhoused older adults. In addition to well-established disparities in the prevalence of serious illness and access to palliative care among people with experiences of homelessness, limits and barriers to holistic support remain once housed. The fourth paper uses secondary data analysis of the Research and Supportive Care at Later-life for Unhoused People (RASCAL-UP) study to describe current practices, perceived barriers, and recommendations in permanent supportive housing for older residents with complex comorbidities. The final paper, also using secondary data analysis of the RASCAL-UP study, addresses the role of skilled nursing for palliative care patients with histories of homelessness. Together, findings illuminate challenges to providing PEOL care for unhoused older adults and those with prior experiences of homelessness, as well as opportunities to improve access to care. This is a Hospice, Palliative and End-of-Life Care Interest Group Sponsored Symposium.

Abstract citation ID: igad104.1191

### EMERGING PALLIATIVE CARE INTERVENTIONS FOR HOMELESS ADULTS IN 10 US CITIES

Arden ODonnell, *Boston University, Boston, Massachusetts, United States*

America's adult homeless population is growing older; its median age is now 50 years of age. Due to harsh living conditions of chronic homelessness, these individuals often experience geriatric conditions in their 50s and 60s and face premature mortality. Yet, little is known about palliative care for this population. A 2017 systematic review of scientific journals revealed only six palliative care intervention studies for homeless adults. Anecdotal evidence, however, suggests community responses are emerging. Focusing on the 10 U.S. cities with the largest adult homeless populations, this exploratory study had two objectives: 1) through a web-based search of the "grey literature," the identification of organizations developing or adapting programs to serve the palliative care needs of homeless adults; and 2) through in-depth zoom interviews with directors, gaining an understanding of the programs' core aspects as well as their successes and challenges in development, implementation, and sustainability. The 18 interviewed agencies fell into two distinct service categories, healthcare/palliative organizations and homelessness/housing organizations. Identified modifications included targeted advanced care planning (ACP) groups, the development of an ACP repository for unhoused persons, rapid re-housing for terminally ill patients, harm reduction strategies, and hospice options in shelters. Common themes were the challenges of working across multiple systems and the lack of dedicated resources and funding for initiatives to support the population's multiple and complex needs. The discussion highlights the need for greater organizational resources and flexibility in both the healthcare and homeless systems to promote value-based-goal concordant care for homeless adults.

Abstract citation ID: igad104.1192

### "AN INHUMANE LACK OF RESOURCES": PERSPECTIVES ON PALLIATIVE AND HOSPICE CARE FOR UNHOUSED OLDER ADULTS

M. Pilar Ingle[1], Asia Cutforth[1], and Elise Matatall[2], *1. University of Denver, Denver, Colorado, United States*, *2. Elevated Denver, Denver, Colorado, United States*

Palliative and end-of-life (PEOL) care offer many benefits to those with life-limiting illness; however, older unhoused individuals face barriers in accessing these types of care. The purpose of this study was to explore healthcare and social service providers' perspectives on approaches and challenges to addressing PEOL needs among unhoused individuals in Colorado. A descriptive, convergent mixed-methods design was used, including an online survey and semi-structured interviews. Participants were eligible if they worked in healthcare or social services in Colorado and provided direct care/services to adults. Guided by the Behavioral Model for Vulnerable Populations, the survey assessed satisfaction with care provided and identification of challenges in providing care along predisposing, enabling, and need contexts. Interviews focused on deeper accounts of provider experiences. Thematic analysis was used to evaluate qualitative data. Sixty-seven providers completed the survey, and 17 participated in interviews. Only 30% were satisfied with care provided to unhoused individuals with life-limiting illness within their organization. Lack of informal care support, mental illness, substance use, complex care needs, and lack of community resources were most frequently identified in the survey as challenges in providing care. Interviews highlighted person-centered and holistic approaches to care, as well as challenges of providing complex care in the context of an inhumane lack of resources. Tailored PEOL interventions, including street-based palliative care and social model hospice homes, were discussed as possible solutions. This study highlights humanizing approaches within structural challenges to providing PEOL care for unhoused individuals, and highlights potential opportunities to improve access to care.

Abstract citation ID: igad104.1193

### OPPORTUNITIES AND BARRIERS IN INTEGRATING PALLIATIVE CARE SERVICES INTO PERMANENT SUPPORTIVE HOUSING

Ian Johnson[1], Anthony Traver[2], and Michael Light[3], *1. University of Texas- San Antonio, San Antonio, Texas, United States*, *2. The Ohio State University, Columbus, Ohio, United States*, *3. University of Washington Palliative Care Training Center, Seattle, Washington, United States*

Despite well-known inequities in rates of serious illness and premature mortality among people experiencing homelessness, as well as the unique biopsychosocial and spiritual care needs of people with homelessness histories, there is a lack of translational research focused on palliative care service delivery within permanent supportive housing. This presentation will present data from the Research, Action, & Supportive Care at Later-life for Unhoused People (RASCAL-UP study) to describe the current practices, perceived barriers, and recommendations in providing support to older permanent supportive housing residents during serious illness and end-of-life. Through a thematic analysis of interviews with direct care providers (n=30) across multiple care settings, themes were identified: 1) the importance of on-site relationships; 2) cross-sector collaboration barriers; 3) interdisciplinary communication barriers within permanent supportive housing teams; 4) environmental and spatial considerations; and 5) missing levels of care between supportive housing and higher levels of medical care. From these results, we put forth recommendations for translational research, supportive housing policy, and capacity-building efforts for interdisciplinary and cross-sector healthcare and housing teams.

Abstract citation ID: igad104.1194

### INTERROGATING THE SKILLED NURSING STAYS OF HOMELESS AND SERIOUSLY ILL OLDER ADULTS

Namrata Mukherjee[1], Ian Johnson[2], and Michael Light[3], *1. University of Tennessee, Knoxville, Knoxville, Tennessee, United States*, *2. University of Tennessee, Knoxville, Tennessee, United States*, *3. University of Washington Palliative Care Training Center, Seattle, Washington, United States*

The culture change movement has fostered an intentional move away from restrictive ethos of care in hospitals, post-acute rehabilitation, and skilled nursing. However, for the millions in the United States who are uninsured, Medicaid-insured, or dual-eligible are less likely to access 'deinstitutionalized' healthcare environments. This presentation presents findings from a constructivist grounded theory study, Research and Supportive Care at Later-life for Unhoused People (RASCAL-UP), on the role of skilled nursing in the experiences of palliative care patients with histories of homelessness. Through provider interviews (n=30), field observations (n=12), and longitudinal chart documentation of a subset of homeless palliative care patients with stays in skilled nursing (n=13), this presentation will reflect on major findings: (1) barriers to skilled nursing entry; (2) successes and challenges in transitioning into skilled nursing residence; (3) acts of resistance to (re)institutionalization; and (4) discharge options and outcomes. Results showcase a need to fill the residential service gap between supportive housing and skilled nursing care and policy change that incentivizes consumer-directed practices for all.

Abstract citation ID: igad104.1195

### USING PHOTOVOICE TO UNDERSTAND SOCIAL MODEL HOSPICE FROM RESIDENTS' PERSPECTIVES

Francine Jensen, *Utah Valley University, Orem, Utah, United States*

Individuals who experience homelessness and are diagnosed with six months or less to live are disadvantaged at a critical time of life. Many people choose to die at home using hospice services supported by family or close friends, but those without stable housing are unable to access hospice, demonstrating inequitable access at the end of life. For some, social model hospice (SMH) care is being provided free of charge by communities to increase access to hospice services for these individuals. The literature about SMH is growing, but currently missing are the voices of those receiving this care and its impacts on their end-of-life experiences. Using photovoice, a community-based participatory method, three residents shared their first-person perspectives using disposable cameras to document meaningful aspects of care, both positive and negative, and helped researchers analyze themes. Until this study, photovoice had not been used with people experiencing homelessness at the end of life. We found participants eager to take part in all aspects of photovoice as their health permitted. Their perspectives documented and informed the humanistic side of SMH care, illuminating the value of respect, care, autonomy, meeting physical needs, and forming supportive relationships; few criticisms were offered. Consistent with photovoice aims, this method is a powerful research tool involving a disadvantaged group who provided visual commentary on what is working and what could improve at one SMH location. Findings can inform policy decisions for communities interested in starting SMHs and help functioning SMHs make programmatic decisions to support vulnerable residents.

### SESSION 3415 (SYMPOSIUM)

Abstract citation ID: igad104.1196

### THE ESSENTIAL ROLE OF PAID CAREGIVERS IN HOME-BASED DEMENTIA CARE

Chair: Jennifer Reckrey Discussant: Joanne Spetz

Paid caregivers (e.g., home health aides, other home care workers) provide essential support to the rapidly growing number of people with dementia living in the community. This symposium presents innovative findings on the role of paid caregivers in home-based dementia care though exploration of the clinical, family, and care-delivery contexts in which paid caregivers work. First, Reckrey et al describe the scope of paid caregiving in dementia using nationally representative data examining factors associated with receipt of paid care among older adults with overlapping dementia and non-dementia serious illness. Next, we examine mechanisms of delivering paid care: Osakwe et al explore paid caregiver perceptions about the use of telehealth in home health and identify paid caregivers concerns about routine use of this technology in care of people with dementia, and Gordon-Wexler et al explore family caregiver perspectives on navigating Medicare and Medicaid insurance for people with advanced dementia and the challenges they experience coordinating paid care. Finally, we explore how paid caregivers work with other care team members. Quach et al discuss multidisciplinary perspectives about how paid caregivers support person-centered and relationship-centered care to family dementia caregivers, while Fabius et al examine information sharing practices within home care agencies and describe differences and similarities in information sharing needs for people with and without dementia. Taken together, these presentations highlight the important ways that paid caregivers work with providers and families within the care delivery system and identify ways to better support and integrate these essential workers into dementia care teams. This is a Paid Caregiving Interest Group Sponsored Symposium.

Abstract citation ID: igad104.1197

### THE ROLE OF DEMENTIA AND SERIOUS ILLNESS IN RECEIPT OF PAID CARE

Jennifer Reckrey[1], Hannah Kleijwegt[1], Amy Kelley[2], and Katherine Ornstein[3], *1. Icahn School of Medicine at Mount Sinai, New York City, New York, United States*, *2. National Institute on Aging, National Institutes of Health, Bethesda, Maryland, United States*, *3. Johns Hopkins University, Baltimore, Maryland, United States*

Little is known about receipt of paid care for individuals living at home with dementia and other non-dementia serious illnesses. This retrospective cohort study sought to characterize receipt of paid care among individuals with impairment in self-care activities and/or other manifestations of serious illness in the context of family caregiving and socioeconomic status. Data from participants ≥65 years enrolled in the Health and Retirement Study between 1998-2018 with new-onset need for help in ≥1 self-care task was linked to fee-for-service Medicare claims (sample n=2,521). We found that those who received paid care experienced higher healthcare utilization than those without paid care. Receipt of paid care was highest among those with multiple manifestations of serious illness: 41.7% of those with self-care impairment, dementia, and non-dementia serious illness received ≥40 hours of paid care per week. When present, paid care accounted for 54% of total caregiving hours. In multivariable models, those with Medicaid were more likely to receive paid care ($p<0.001$), but those in the highest income quartile received more hours of paid care ($p=0.05$.) Those with non-dementia serious illness were more likely to receive paid care ($p<0.001$), but those with dementia received more hours of paid care ($p<0.001$). The role of paid caregivers grows as people experience multiple manifestations of serious illness and high care hours are common among those with dementia. Future work should explore how paid caregivers can collaborate with families and healthcare teams to improve the health and well-being of the seriously ill living at home.

Abstract citation ID: igad104.1198

### "CAREGIVING IS TEAMWORK..." INFORMATION SHARING IN HOME CARE FOR OLDER ADULTS WITH DISABILITIES IN THE COMMUNITY

Chanee Fabius[1], Aleksandra Wec[2], Martha Abshire Saylor[3], Jamie Smith[1], Joseph Gallo[1], and Jennifer Wolff[1], *1. Johns Hopkins University, Baltimore, Maryland, United States*,

2. *Johns Hopkins Bloomberg School of Public Health, Baltimore, Maryland, United States*, 3. *Johns Hopkins University School of Nursing, Baltimore, Maryland, United States*

Direct care workers provide assistance with daily activities to persons with disabilities. Direct care workers are well positioned to recognize and share information about care gaps and changes in function and behaviors but are often viewed as unskilled and excluded from interdisciplinary care team interactions. We conducted 11 semi-structured interviews with administrators (n=5), nurses (n=3), and direct care workers (n=3) from home care agencies ("residential service agencies" in Maryland) to understand information sharing between direct care workers, family caregivers, and clinicians in home care delivery for older adults with disabilities, including those living with dementia. Thematic analysis was completed by the research team. Leveraging Systems Engineering Initiative for Patient Safety (SEIPS) 2.0 model, a human factors framework, respondents highlighted aspects of information sharing across (1) work systems, (2) processes, and (3) dementia-related adaptations. Work system characteristics relevant to information sharing included the utilization of electronic management systems and patient portals to communicate within agencies and with clinicians and using multiple methods to gather information about client goals and preferences (e.g., assessments, client profiles). Process characteristics include collaborations between families and residential service agency staff to deliver optimal care. Direct care workers did not report differences in coordinating care for persons living with dementia, however they reported dementia-related adaptations like an increased need to establishing and sustaining strong relationships with family members and communicating with clinicians during medical visits. Findings highlight the care demands experienced by direct care workers and support calls to better coordinate information sharing between interdisciplinary care teams.

Abstract citation ID: igad104.1199

### PERCEPTIONS OF HOME HEALTH AIDES (HHA) CARING FOR PERSONS LIVING WITH DEMENTIA (PLWD) ABOUT ADOPTION OF TELEHEALTH

Zainab Toteh Osakwe[1], Michelle Odlum[2], Amarilis Céspedes[3], and Sasha Perez[4], 1. *Adelphi University, Garden City, New York, United States*, 2. *The George Washington University, Washington, District of Columbia, United States*, 3. *Columbia University School of Nursing, New York City, New York, United States*, 4. *Icahn School of Medicine at Mount Sinai, New York City, New York, United States*

Home healthcare agencies serve a large and growing population of Persons living with Dementia (PLWD). The COVID-19 pandemic led to rapid adoption and use of telehealth services in the delivery of home healthcare, allowing patients to visit with home healthcare clinicians remotely using virtual technology. Yet, concerns remain about feasibility of telehealth for PLWD on HHC, who largely depend on home health aides to communicate their care concerns with the health care teams. We interviewed 25 HHAs to PLWD between June and August 2020 about their experiences with telehealth as part of a larger study focused on HHA informed care plans for PLWD. Interviews were recorded, transcribed, translated and analyzed using conventional content analysis. We interviewed 25 HHAs employed by 4 unique home healthcare agencies. HHAs had a mean age of 49.8 (± 9.1), 24 (97%) female, 11 (44%) Black, 12 (48%) Hispanic. Twelve HHAs were interviewed in Spanish. The main themes identified were: (1) preference for telehealth platforms that include video conferencing to enhance human interactions, (2) preference for telehealth as a supplement, not substitute, for nursing visits, (3) additional burden of using the tablet or computer while care for PLWD and (4) concerns about inadequate training needed to use telehealth. HHAs expressed concerns about the need to not substitute in person home visits with Telehealth. As home healthcare agencies consider best approaches to integrate telehealth into their care delivery models, communication with HHAs remains key to develop strategies that prioritize the care needs of PLWD.

Abstract citation ID: igad104.1200

### "IT SHOULDN'T BE LIKE THIS": FAMILY CAREGIVERS NAVIGATING INSURANCE FOR FAMILY MEMBERS WITH DEMENTIA

Mikayla Gordon-Wexler, Deborah Watman, and Jennifer Reckrey, *Icahn School of Medicine at Mount Sinai, New York City, New York, United States*

Family caregivers of individuals with dementia who are dually eligible for Medicaid and Medicare must navigate the health insurance landscape to meet the complex medical and home care needs of their family members. The goal of this study is to explore how family caregivers make decisions around insurance and how these choices affect care. Semi-structured interviews were conducted from June-January 2022 with 15 family caregivers of dually eligible individuals with dementia enrolled in home-based primary care in New York City. Interviews were analyzed using both deductive and inductive thematic coding. Participants described how social workers and other professionals helped navigate insurance choices, but no centralized system for obtaining information existed. Once insurance plans were set-up, participants described advocating on behalf of their family member within the constraints of current insurance plans rather than considering alternatives due to high perceived burden of changing plans. Obtaining adequate home care hours (which frequently required multiple appeals) was a particular challenge. Caregivers did not see integrated Medicare-Medicaid plans as providing any inherent benefit to navigating insurance. This study highlights the barriers facing family caregivers as they navigate insurance choices and the limited support available. These challenges may contribute to resistance to changing plans even when gaps in needed coverage exist, especially gaps related to inadequate home care. Robust professional support for family members both immediately after loved one's dementia diagnosis and as the disease progresses could alleviate burden on caregivers and increase their capacity to get their family members needed medical and home care.

Abstract citation ID: igad104.1201

### PAID CAREGIVERS PROVIDE SOCIAL SUPPORT TO FAMILY CAREGIVERS OF OLDER ADULTS WITH DEMENTIA

Emma Quach[1], Emily Franzosa[2], Lauren Moo[3], and Christine Hartmann[4], *1. VA Bedford, Bedford, Massachusetts, United States*, *2. Icahn School of Medicine at Mount Sinai, New York City, New York, United States*, *3. Veterans Health Administration, Bedford, Massachusetts, United States*, *4. VA Bedford Healthcare System, Bedford, Massachusetts, United States*

Family caregivers support older adults with dementia to age in place but often experience caregiver strain, though sharing the care with paid caregivers could reduce such strain. We investigated the potential synergy between family caregivers and paid caregivers. In summer 2021, we conducted 14 interviews with family caregivers (6), home care workers (3), and social workers who work with family caregivers and home care agencies (5). We audio-recorded, transcribed, and thematically analyzed our interviews. Our analysis revealed 4 types of support family caregivers received from paid caregivers: 2 centering on the family caregiver as an individual ("person-centered") and 2 on the relational role the individual holds as a family caregiver ("relationship-centered"). (1) Family caregivers felt positive, emotional bonding with paid caregivers, describing them as "part of the family". (2) Family caregivers valued instrumental aid from paid caregivers, paradoxically for tasks that are beyond their formal responsibilities, e.g., feeding pets. (3) Family caregivers recognized and valued expert personal care provided to their loved ones by highly experienced paid caregivers. (4) Family caregivers valued paid caregivers' sharing information about their loved ones and their living situations, especially when family caregivers lived separately from their loved ones. Paid caregivers may reduce or prevent family caregiver strain by providing person- and relationship-centered to family caregivers. Home care agencies should foster the relationship between paid caregivers and family caregivers.

### SESSION 3420 (SYMPOSIUM)

Abstract citation ID: igad104.1202

### OUT OF THE MEDICINE CABINET: NONPHARMACOLOGICAL APPROACHES TO IMPROVE COGNITION AND ASSOCIATED FUNCTIONS

Chair: Ashley Curtis Discussant: Constance Visovsky

There is a lack of pharmacological agents for improving cognition, and the negative impact of polypharmacy on cognitive and physiological functioning in aging adults is well established. This has prompted investigation of nonpharmacological interventions aimed at improving cognition and associated functions. This symposium will provide a comprehensive evaluation of several of these promising evidence-based behavioral approaches. Paper 1 will describe several computerized cognitive training programs for improving cognition and sleep in middle-aged and older adults. Paper 2 will evaluate short-term complex music training versus music listening on cognitive function in older adults. Paper 3 will describe the impact of cognitive behavioral and virtual reality treatments for insomnia on sleep, cognition, arousal, and mood in several primarily middle-aged and older adult populations with chronic insomnia. Paper 4 will examine physical differences in gait function of older adult breast cancer survivors, providing insight into fall risk indicators, and targets for behavioral intervention. Paper 5 will describe results from a randomized clinical trial evaluating a mindfulness-based stress reduction for improving self-reported and objective cognition as well as fatigue. As Discussant, Dr. Constance Visovsky will integrate research findings and discuss areas for future research investigation. In sum, this symposium will provide unique insight into the impact of alternative approaches 'beyond the medicine cabinet' for improving not only cognition, but other areas of associated functioning in mid-to-late life. Thus, this session will help to inform interventions that can be delivered online and in the community to help mitigate risk and/or delay onset of cognitive decline.

Abstract citation ID: igad104.1203

### COGNITIVE TRAINING APPROACHES TO IMPROVE COGNITION AND ASSOCIATED FUNCTIONS IN MID TO LATE LIFE

Ashley Curtis[1], Amy Costa[1], Madison Musich[2], Chloe Rzeppa[3], and Christina McCrae[1], *1. University of South Florida, Tampa, Florida, United States*, *2. University of Missouri-Columbia, Columbia, Missouri, United States*, *3. University of Missouri, Columbia, Missouri, United Sta tes*

Cognitive Training (CT) has yielded inconsistent benefits in non-patient populations. Despite known cognition/sleep associations, limited studies have investigated CT's impact on sleep. We examined cognitive and associated outcomes across three CT programs in older adult (OA) insomnia patients [with/without mild cognitive impairment (MCI)] and middle-aged adults (MA) with generalized anxiety disorder (GAD). In Study 1, OAs with insomnia (Mage=67.9±8.0; 16 women/8 men) completed 6-week CT (Nintendo DS-Big Brain Academy; 60 mins/day, 3x/week) and waitlist control (WLC) in a cross-over design. At baseline and post-CT/post-WLC, participants completed NIH toolbox-cognitive tasks. In Study 2, MAs with GAD (Mage=49.8±4.0; 29 women) underwent 8-week CT (Cognifit, n=14) or Trivia Training (TT, n=15), 45 mins/day, 3x/week and completed pre/post Inquisit-cognitive assessments. In Study 3, OAs with insomnia and MCI (Mage=67.6±8.0; 10 women/3 men) completed 8-week CT (Cognifit; 45 mins/day, 3x/week) and pre/post Cambridge Brain Sciences-cognitive and polysomnography-sleep assessments. In Study 1, proportional RAVLT-verbal memory improvement was larger [t(23)=2.01, p=.028] following CT(17%) versus WLC(9%). Similarly, proportional Flanker-inhibition improvement was larger [t(23)=1.71, p=.05] following CT(2%) versus WLC(-.3%). In Study 2, CT improved Posner-attention orienting more than TT [F(1,27)=5.25, p=.03]. In Study 3, CT improved Digit-span-working memory [t(12)=-1.84, p=.045], reduced number of awakenings [t(12)=2.70, p=.01), increased %REM [(t(12)=-1.77,p=.05], and showed trending improvement for spatial planning/reasoning [t(12)=1.70, p=.057] and %Lighter-sleep [t(12)=1.62,p=.066]. CT benefits non-trained cognition (transfer effects) in mid-to-late life individuals with insomnia, GAD and MCI, and shows promise for improving sleep in MCI. Follow-up in larger samples investigating mechanisms underlying CT effects (domain-specific training, sleep improvement) is warranted.

Abstract citation ID: igad104.1204

### SHORT-TERM MUSIC TRAINING ENHANCES INHIBITION IN AGING

Jennifer Bugos, *University of South Florida, Tampa, Florida, United States*

Music training was shown to benefit inhibition in adults; however, little is known about the benefits of specific types of music training and the dosages necessary to demonstrate gains in older adults. Based on the ADAM model, professional musicians use predictive temporal extrapolation to anticipate and perform coordinated bimanual movements. We applied this model to beginning adult musicians by isolating the effects of bimanual coordination when compared with non-motor standardized music interventions. We evaluated the effects of two standardized music programs (piano training, music listening instruction) on inhibition in novice adults after four-week intervals. Older adults (N=18) were recruited and randomly assigned to a manualized piano program (Keys to Staying Sharp) or music listening instruction (Music Listening Today, standard protocol). Participants completed a battery of standardized measures including measures of inhibition (Flanker, Cued Color Word Stroop) at pretesting and at each of the three follow-up intervals: 4 weeks, 8 weeks, and 12 weeks. Results of a Repeated Measures Group X Time ANOVA for the first four months of training showed reduced error rates, F(1, 14)=6.48, p=.023, and faster reaction times, F(1, 14) =5.27, p=.038 for piano training as compared to music listening instruction. Increased differences were prominent after eight-weeks of training for the piano group as compared to music listening. Data suggests that music training interventions while manualized are not created equal. Benefits from complex music performance are evident after short-term manualized music interventions. Short-term bimanual music interventions may increase cognitive performance beyond music listening instruction due to cognitive load.

Abstract citation ID: igad104.1205

### COGNITIVE BEHAVIORAL AND VIRTUAL REALITY TREATMENTS FOR INSOMNIA (CBTI AND IVR): OFF-LABEL IMPACT ON COGNITION

Christina McCrae, Melanie Stearns, and Ashley Curtis, *University of South Florida, Tampa, Florida, United States*

Chronic insomnia (CI) is common in mid-to-late life and associated with hyperarousal and impaired cognition and mood. CBTi improves sleep and may also improve cognition, arousal and mood but evidence for these latter outcomes is limited. We examined these outcomes across 3 studies with different delivery platforms (telehealth, digital, VR), dosages (2 sessions, 4 sessions), and (primarily) mid-to-older CI populations (fibromyalgia, dementia caregivers). Study 1 compared 8-week CBTi vs sleep hygiene in women with fibromyalgia (n=43, Mage=44.45). Study 2 piloted 4-session web-based CBTi in caregivers (n=5, Mage=62.40). Study 3 piloted 4-session iVR (n=18, Mage=36.45). Participants completed 2-week daily diaries pre/post treatment (sleep onset latency-SOL; wake after sleep onset-WASO; total sleep time-TST) and Insomnia Severity Index-ISI. Other measures included: study 1(arousal/heart rate variability-RMSDNN), studies 1 and 3(Wisconsin Card Sort Test-WCST, Stroop), study 2(Cognitive Failures Questionnaire-CFQ, Beck Depression Inventory-BDI-II, Perceived Stress Scale). Group x time interactions (study 1) and within-group pre/post differences were examined. CBTi improved sleep across studies (ps<.05). Study 1 found and study 3 trended toward improved cognitive flexibility (WCST %perseverative errors-t(14)=2.65, p=.019 and t(10)=1.76, p=.055, respectively). Study 1 found improved attention and processing speed [Stroop reaction time(RT)-congruent trials-t(14)=2.59, p=.023], inhibition (Stroop RT-incongruent trials-t(14)=2.43, p=.031), and arousal [increased RMSDNN; F(1,41)=4.87, p=.03]. Study 2 found improved subjective cognition-CFQ (t(4)=2.44, p=.04), arousal-RMSDNN (t(4)=2.09, p=.05), and depression-BDI-II (t(4)=2.89, p=.02). CBTi improved sleep, cognition, arousal and mood in mid-to-older CI populations. Research using randomization, active controls, and follow-ups is needed to delineate temporality and explore sleep's mechanistic contribution to cognition and other "off-label" outcomes.

Abstract citation ID: igad104.1206

### GAIT PARAMETERS IN OLDER BREAST CANCER SURVIVORS WITH PERSISTENT CHEMOTHERAPY-INDUCED PERIPHERAL NEUROPATHY

Constance Visovsky, *University of South Florida-College of Nursing, Tampa, Florida, United States*

Chemotherapy-induced peripheral neuropathy (CIPN) results from chemotherapy for breast cancer treatment. CIPN symptoms persist causing significant loss of functional abilities and increased risk of falls. This study aimed to assess the spatiotemporal gait parameters in breast cancer survivors with CIPIN using an IMU system. We hypothesized that spatiotemporal gait parameters will differ between patients with persistent CIPN as compared to normative values. Thirty-two women who completed taxane-based chemotherapy for breast cancer and who have symptomatic neuropathy >6 months following completion of chemotherapy. Gait was assessed using seven Inertial Measurement Unit (IMU) sensors positioned at the sacrum, upper and lower leg, and foot. Participants completed a 2-minute walk at a comfortable walking pace. The outcome variables assessed included gait speed, single limb support, stride length, gait cycle time, stance percent, swing percent, double support percent, and cadence. Mean age 65(10) years, gait speed was 0.95(0.2) meters/second, single limb support 37.9(2.2) % of gait cycle, stride length 1.1(0.2) meters, gait cycle time 1.2(0.1) seconds, stance percent 62.1(2.2)% GC, swing percent 37.9(2.2)% GC, double support percent 23.9(4.3)% GC, and cadence 104.6(12.3) steps/min. Gait parameters assessed in this study showed an excellent test-retest reliability (r >0.86; ICC > 0.90). This is the first study to assess gait parameters in older breast cancer survivors with CIPN. Breast cancer survivors with CIPN showed reductions in all gait parameters. Reduced gait speed and stride length are critical indicators of fall risk for women with CIPN. Our study confirmed differences in spatiotemporal parameters between patients with persistent CIPN.

Abstract citation ID: igad104.1207

**MINDFULNESS-BASED STRESS REDUCTION (MBSR): TREATMENT FOR COGNITIVE IMPAIRMENT POST CHEMOTHERAPY (CT)**

Cecile Lengacher, and Richie Reich, *University of South Florida, Tampa, Florida, United States*

The purpose of this R01 trial was to evaluate the efficacy of MBSR(BC) compared to the Breast Cancer Education Support program(BCES) or Usual Care(UC) regimens for improvement in cognitive functioning among breast cancer survivors(BCS) who received (CT), or CT and radiation. **Methods.** BCS (212) were randomized to MBSR(BC) (n=91) or BCES(90) or UC (n = 31) with MBSR(BC) and BCES participants attending a 6-week program. Measures of neuropsychological and perceived cognitive performance, psychological and physical symptoms were collected at baseline, 6, 12, and 26 weeks. Linear mixed models assessed the MBSR(BC) effects over time. Characteristics at baseline were tested as moderators of MBSR(BC) program effects. **Results.** 212 BCS had a mean age of 57, 73% were White, non-Hispanic, 78% received CT and radiation; and 22% CT alone. All BCS improved across the study on all measures. For most measures, cognitive and symptom improvement effects followed the hypothesized pattern favoring MBSR(BC), followed by BCES and UC groups. Effect sizes were generally small, particularly for measures of cognition. The largest week 26 effect sizes were observed in the physical symptom fatigue, where the MBSR(BC) group improved with a 0.45 Cohen's d effect over BCES and 0.65 over UC. Type of CT, radiation treatment, time since treatment, and informal and formal practice techniques of MBSR(BC) moderated the effects of MBSR(BC). **Conclusions.** Although effects were modest, BCS receiving the MBSR(BC) and BCES groups improved in objective and subjective cognitive functioning and symptoms compared to UC. Longer follow-up studies are needed to determine sustained effects.

### SESSION 3425 (PAPER)

### AGEISM AND PERCEPTIONS OF AGING

Abstract citation ID: igad104.1208

**AGEISM IS NOT A CATCH-ALL: THE INTERRELATEDNESS OF AGEISM AND ABLEISM**

Tracey Gendron[1], Camp Alyssa[1], Amateau Gigi[1], Mullen Mia[2], Jacobs Kristen[3], Jenny Inker[1], and Sarah Marrs[1], *1. Virginia Commonwealth University, Richmond, Virginia, United States*, *2. Leading Age National, Washington, District of Columbia, United States*, *3. Leading Age National, Washinton, District of Columbia, United States*

Ageism represents discrimination based on age, while ableism represents discrimination toward people with disabilities. Given the natural occurrence of physical decline accompanying aging, it is essential to explore how prejudice toward disability intersects with fear about aging. 913 individuals responded to a survey on ageism and ableism. Multiple regression with internalized ageism as the criterion variable was significant F(7, 705) = 81.71, p <.001. Overall, the regression model explained 44.8% of the variance in internalized ageism with significance for age (t(705) = -5.45, p < .001), relational ageism (t(705) = 11.72, p < .001), affinity for older people (t(705) = -2.77, p = .006), ableism for physical disability (t(705) = 6.88, p < .001), and ableism for cognitive disability (t(705) = -2.86, p = .004), Multiple regression with relational ageism as the criterion variable was significant F(7, 705) = 88.12, p <.001. Overall, the regression model explained 46.7% of the variance in relational ageism with a significant for affinity for older people (t(705) = -3.80, p < .001), internalized ageism (t(705) = 11.72, p < .001), relational ableism (t(705) = 6.73, p < .001), ableism for cognitive disability (t(705) = 2.15, p = .007), and ableism for sensory disability (t(705) = -2.78, p = .006). Examining the intersection between ageism and ableism represents the next critical juncture to increasing our understanding of developing effective anti-ageism interventions that address the root anxieties influencing attitudes about aging.

Abstract citation ID: igad104.1209

**DYADIC PROFILES OF COUPLES' SELF-PERCEPTIONS OF AGING: IMPLICATIONS FOR MENTAL HEALTH**

Meng Huo[1], and Kyungmin Kim[2], *1. University of California, Davis, Davis, California, United States*, *2. Seoul National University, Seoul, Republic of Korea*

The way older adults perceive their own aging processes influences their mental health, but we know little about how this occurs in a dyadic context, where spouses' perceptions and health are often intertwined. The current study aims to identify dyadic profiles of self-perceptions of aging (SPA) in couples and examine how certain dyadic profiles are associated with each partner's mental health over time. A pooled sample of 3,850 heterosexual couples aged 50+ in the Health and Retirement Study (2012/2014–2016/2018) rated positive and negative SPA and provided data on demographic characteristics, couple relationships, and health. Latent profile analysis revealed four profiles of couples' SPA: similarly positive (32%), similarly negative (10%), wife positive–husband negative (38%), and wife negative–husband positive (20%). Physical health and marital quality differentiated couples and predicted profile membership. Couples with similarly positive SPA reported fewer depressive symptoms over time than did couples in other profiles; couples with similarly negative SPA fared worse mental health. We observed a gender difference when comparing the two opposite profiles, such that wives in the wife positive–husband negative profile reported increasingly fewer depressive symptoms than wives in the wife negative–husband positive profile. Husbands in those two profiles did not differ in mental health. This study adds to the emerging literature that advocates for an interpersonal approach to SPA. Findings revealed risk and resilience in couples as they experience and perceive aging and inform dyadic interventions that aim to protect older adults' mental health in the long run.

Abstract citation ID: igad104.1210

**NEGATIVE AGING STEREOTYPES: FEAR OF DEPENDENCY EFFECTS ON ANXIETY AND DEPRESSION IN MIDDLE AND OLDER ADULTS**

Darby Mackenstadt, and Carolyn Adams-Price, *Mississippi State University, Mississippi State, Mississippi, United States*

Negative stereotypes of aging tend to depict older adults as frail or a burden on society. According to Stereotype

Embodiment Theory, adults who believe negative aging stereotypes tend to internalize these thoughts, and have been found to experience negative physical and mental outcomes, in later life. One aspect of these internalized stereotypes is the fear of being dependent on other people, which highlights the older adults' worry of being seen as incompetent or child-like. Participants were aged 50 to 82-years-old (M=58.5), and primarily White (87%) women (62%). This study explored the relationship between Fear of Dependency Scale (FoD) and anxiety and depression, evaluating gender, age, and physical health as moderators. FoD was related to higher ratings of depression and anxiety in women, with age and physical health acting as moderators. When reporting a high fear of dependency, middle-aged women and women reporting poor physical health had the highest reported anxiety and depression. The early impact of FoD on women is intriguing. FoD was positively associated with higher ratings of depression for men but not anxiety, with only physical health acting as a moderator. Men with high FoD scores and poor physical health reported the highest levels of depression. It appears the negative views of aging can affect individuals, even before they reach older adulthood, and especially if they have poor perceptions of their physical health. Having negative perceptions of aging can negatively impact individuals as they age, and some groups may be more at risk for these outcomes than others.

Abstract citation ID: igad104.1211

### PERCEIVED AGE DISCRIMINATION IN THE SECOND HALF OF LIFE: AN EXAMINATION OF AGE, PERIOD, AND COHORT EFFECTS

Liat Ayalon[1], and Octavio Bramajo[2], *1. Bar Ilan University, Ramat Gan, HaMerkaz, Israel, 2. Autonomous University of Barcelona, Cerdanyola del Valles, Catalonia, Spain*

The present study aimed to examine variations in perceived age discrimination in the second half of life. Perceived age discrimination is different from objective age discrimination as it depends on respondents' recognition, acknowledgement, and willingness to report the discriminatory event. We adopt a comprehensive approach which examines whether perceived age discrimination varies by age (chronological time from birth), period (when data were collected), or cohort (a group of people with shared life events experienced at a similar age) across gender and ethnic origin. Relying on psychosocial data from Health and Retirement Survey between 2006 and 2018. Our findings show that perceived age discrimination increases with age. In addition, older cohorts of women are more likely to report perceived age discrimination, whereas White women born around 1940 are less likely to report perceived age discrimination. White men, in contrast show a slight increase in perceived discrimination. Results are discussed from an intersectional perspective, which considers societal life course events.

### SESSION 3430 (AWARD LECTURE)

Abstract citation ID: igad104.1212

### AGHE FACULTY AWARD PRESENTATIONS AND LECTURES

Chair: Dana Bradley

The AGHE Faculty Award Presentations and Lectures will feature an address by the 2023 recipient Lisa Borrero, PhD, FAGHE of the University of Indianapolis. The Distinguished Faculty Award recognizes persons whose teaching stands out as exemplary, innovative, of impact, or any combination thereof. The Rising Star Early-Career Faculty Award Lecture will feature an address by 2023 co-recipient Nasreen Sadeq, PhD of the University of Southern Florida. Co-recipient Berenice Benayoun, PhD, of the University Southern California will present their lecture at a later date. The Rising Star Early-Career Faculty Award acknowledges new faculty whose teaching and leadership stand out as influential and innovative.

Abstract citation ID: igad104.1213

### TRANSLATING AGING: REACHING STUDENTS ACROSS DISCIPLINES AND GEOGRAPHY

Lisa Borrero, *University of Indianapolis, Indianapolis, Indiana, United States*

What does it mean to translate gerontological concepts among groups of very different students? How can this be optimized for online graduate education? Even within a field that is itself interdisciplinary, it can feel challenging to meaningfully convey key information about aging when students are already firmly established in their own, unique disciplines and are learning from a distance. This lecture will reflect on ways in which gerontology instructors can connect a wide array of graduate students to the universality of aging, promote the relevance of aging to their chosen field, and, hopefully, engender a deeper interest in working with older adults. From the importance of maintaining social and cognitive presence in the online classroom to offering experiential engagement with course content, we can communicate about aging in purposeful and novel ways to a variety of students – and reap the benefits of learning from their expertise in return.

Abstract citation ID: igad104.1214

### IN THEIR OWN WORDS – HOW STUDENT RESPONSES TO OPEN-ENDED QUESTIONS CAN IMPROVE GERONTOLOGY EDUCATION

Nasreen Sadeq, *University of South Florida, Tampa, Florida, United States*

In gerontology education, we know that there are many benefits to assigning students open-ended questions. They allow students to think more critically and can lead to a better understanding of course concepts. Student responses can also help us identify gerontology topics that are not well understood or need reinforcement, as well as topics that can generate interesting in-class discussions. Examining students' answers can help us fill the gaps in current gerontology education and improve our teaching practices. In this lecture, I will share how I have used content analysis to examine students' responses from several assignments, and how I have modified lectures and assignments in response. In addition, I will share how I have added some of these assignments to our general education gerontology courses to help students challenge ageist beliefs and acquire an appreciation for gerontology.

### SESSION 3435 (SYMPOSIUM)

Abstract citation ID: igad104.1215

### CAREGIVING FOR OLDER ADULTS: EXPLORING THE IMPLICATIONS FOR FAMILIAL RELATIONS, DYNAMICS, AND FRIENDSHIP

Chair: Arthur Chia Co-Chair: Rahul Malhotra

In this symposium, we discuss about various aspects of caregiving for older adults who require care because of chronic illness, frailty, and/or long-term disability. Their needs take many forms and so do the demands and concerns of their caregivers, comprising family members and close friends. Under these trying circumstances, relationships are put to the test; shared sensibilities and understandings built over decades of marital and family life begin to unravel, and friendships fray. Thus, caregiving has profound implications for the family as well as non-family members who provide care. Caregiving itself is marked by varying intensities expressed in anguished voices of loss, frustration, and anger but they also convey a deep sense of responsibility, gratitude, and love. There is much that caregiving could offer in terms of the potential for deeper meaning, connection, satisfaction, and goodness. Hence, the papers in this symposium will examine how caregiving responsibilities are distributed among family and non-family members; analyze the implications of caregiving on the notion of home/homeliness for caregivers; account for the meaning of caregiving for a parent or older family member as diverse expressions of filial piety; and explore the issues and challenges around caregiving for older adults with cognitive impairment including dementia from the perspectives of family and non-family members who are caregivers. Collectively, the papers highlight the relational and reciprocal nature of caregiving, and in which intimate relational and cultural processes shape not only the quality and experiences of caregiving but also foundations of the family and notions of home.

Abstract citation ID: igad104.1216

### INFRASTRUCTURES OF CAREGIVING: RECONSTITUTING HOMELINESS AND FAMILY IN SINGAPORE

Arthur Chia[1], and Rahul Malhotra[2], *1. Duke-NUS Graduate Medical School, Singapore, Singapore*, *2. Duke-NUS Medical School, Singapore, Singapore*

Caregiving comprises concrete everyday tasks that are built-on and revolve around the home, yet few studies have looked at caregiving as practice(s) that shape the home as a place of/for care. We examined the socio-material arrangements of people, activities, and things that enable caregiving at home, asking how caregiving is orchestrated, sustained, and delivered at home, and how the home is in turn re-constituted by caregiving. In-depth interviews were conducted with 40 family caregivers, aged 55-85 years, caring for their spouse and/or parent(s). Relations, activities, and things that enabled caregiving were analyzed through the theoretical lens of "care infrastructure". Caregiving is enacted through a network of people and things – including family members, live-in hired helpers, medical and social workers; technologies like surveillance cameras and strategically placed mirrors at home to monitor care recipients; televisions turned-on 24/7; assistive devices such as wheelchairs and ramps, and placement of hospital beds at home, etc. – that change living arrangements and routines. Care tasks, responsibilities, and decisions are marked by negotiations, tensions, and compromises between people and with things. Caregivers appropriate, adapt, and improvise in situations of uncertainty, conflict, and impossible demand. We conclude that new socio-material arrangements of people, activities, and things that emerge because of caregiving change/disrupt the notion of home as a place of safety, familiarity, and comfort for caregivers. Understanding the emplacement/displacement and interconnection of things, activities, and people sheds light on how the home shapes the effects of caregiving and vice versa.

Abstract citation ID: igad104.1217

### PATTERNS OF FAMILY CAREGIVING FOR OLDER ADULTS: THE ROLES OF PRIMARY AND SECONDARY CAREGIVERS

Rahul Malhotra, Jeremy Lim-Soh, Pildoo Sung, and Ha-Linh Quach, *Duke-NUS Medical School, Singapore, Singapore*

Family members are often front-line carers for older adults. With research studies focusing largely on the primary family caregiver, less is known about how caregiving responsibilities are shared between family members. Using latent class regression, we described the prevalence of distinct patterns of family caregiving for older adults, in terms of who (division of assistance between the primary caregiver and secondary caregivers) provides what assistance (three types: with (a) activities of daily living; (b) health and social services; and (c) other socio-emotional needs), and their correlates. Primary family caregivers of 277 older adults (75 years and older) with functional limitations in Singapore, were interviewed in 2019-2020. Three patterns of family caregiving were identified. The 'Shared-Diverse' (43%) pattern typically involved multiple family caregivers providing all three types of assistance. The 'Shared-Informal' (27%) pattern also typically involved multiple family caregivers but no provision of assistance with health and social services; such care-recipients were more likely to be female (versus the Shared-Diverse pattern). The 'Solo-Diverse' (30%) pattern typically involved the primary family caregiver solely providing all three types of assistance; such caregivers were less likely to be working. Primary family caregivers in the Solo-Diverse pattern, versus the Shared-Diverse pattern, had higher depressive symptoms. The predominance of caregiving patterns involving multiple family members highlights the need for expanding family caregiving research, policies, and programs beyond primary family caregivers. The findings also underscore the importance of solo family caregivers, whose employment ability and mental health may be affected by their caregiving duties.

Abstract citation ID: igad104.1218

### THE "GOOD" CAREGIVER: DOES FILIAL PIETY NECESSITATE SELF-SACRIFICE?

Ad Maulod, Sasha Rouse, Nur 'Atiqah Lee, and Rahul Malhotra, *Duke-NUS Medical School, Singapore, Singapore*

Family caregiving policies that amplify the enactment of 'filial piety' – respect and care towards parents, grandparents and older persons – are especially pronounced in Asian societies, where caring for older family members is culturally expected and appraised yet is associated with stress and burden. Numerous studies have shown that fulfilment of filial piety contributes to better psychological outcomes for caregivers while mismatched filial expectations contribute to poorer psychological outcomes. However, few studies acknowledge how filial care varies according to individual resources and socio-cultural contexts. We seek to understand how family caregivers negotiate their self-determination through filial care, which contributes to positive and negative caregiving experiences. We conducted in-depth interviews with 34 caregivers of older persons with functional limitations in Singapore, purposefully selected based on varying positive and negative caregiving experiences. For caregivers with predominantly negative caregiving experiences, filial care was seen as an act of sacrifice and expectation of duty, with low autonomy to exercise self-determination. In comparison, caregivers with predominantly positive caregiving experiences described filial care as an act of sincere love cultivated through close relationships with their care-recipient, and aligned to their self-determination. Strong family support was also evident in the latter. Treating filial piety as a 'natural desire to care for parents' obscures efforts required to amplify positive caregiving experiences while normalisation of self-sacrifice and suffering hinders caregivers from expressing burden and accessing support. A nuanced understanding of how caregivers negotiate filial care can inform policy interventions to empower caregivers and care-recipients and enhance intergenerational relationships.

Abstract citation ID: igad104.1219

### FEELING ALONE TOGETHER: LONELINESS IN OLDER ADULTS WITH COGNITIVE IMPAIRMENT AND THEIR CAREGIVERS WITH LOW MASTERY

Pildoo Sung[1], Jeremy Lim-Soh[2], and Angelique Chan[2], *1. Hong Kong Baptist University, Hong Kong, Hong Kong*, *2. Duke-NUS Medical School, Singapore, Singapore*

Loneliness can be contagious because lonely people tend to share their loneliness with others. According to this perspective, loneliness in older persons with cognitive impairment (PCI) may beget loneliness in their family caregivers. However, not all caregivers of lonely PCI experience loneliness. This may be because caregivers have buffering resources that mitigate the contagion of loneliness. Nevertheless, empirical evidence on whether and how PCI and caregiver loneliness are related is lacking. Therefore, we examined the association between PCI loneliness and caregiver loneliness, and the moderation role of caregiver mastery on this association. We used dyadic data from 135 PCI and their family caregivers in Singapore. Descriptive statistics and bivariate correlation showed that PCI reported higher levels of loneliness than their caregivers, and PCI and caregiver loneliness were weakly related. Multivariable regression showed that PCI loneliness was not associated with caregiver loneliness, taking other covariates into account. Nevertheless, we found that the interaction between PCI loneliness and caregiver sense of mastery was significant, such that PCI loneliness was significantly associated with caregiver loneliness when caregivers had low mastery. In conclusion, lonely PCI may share their loneliness with their caregivers, and this may lead to caregiver loneliness if caregivers have low mastery. Tailored interventions should be designed to reduce loneliness among PCI and their caregivers and improve caregiver mastery as a protective factor against the spread of loneliness between PCI and caregivers.

Abstract citation ID: igad104.1220

### IT'S OVERWHELMING FOR EVERYBODY: EXPERIENCES OF DEMENTIA CAREGIVERS WHO AREN'T CLOSE FAMILY

Jane Lowers[1], Ivree Datcher[2], Michelle Delk[3], Dio Kavalieratos[2], Molly Perkins[2], and Kenneth Hepburn[2], *1. Emory School of Medicine, Atlanta, Georgia, United States*, *2. Emory University, Atlanta, Georgia, United States*, *3. Emory Rol, Atlanta, Georgia, United States*

One in three people with Alzheimer's or other dementias lives alone, without a spouse/partner or nearby children (i.e., is aging solo). Friends, neighbors, and extended family often assist with tasks that preserve independence, but most dementia caregiving literature, and theory, to date focuses on spousal or parent/child dyads for whom reciprocity, indebtedness and self-interest are common motivators. This study seeks to address this knowledge gap. Design: Semi-structured interviews with caregivers of adults with cognitive impairment or dementia who were not the caregivers' spouses or parents. Hybrid inductive/deductive thematic analysis incorporating social exchange and stress-coping theories.

**Results:** We interviewed 11 caregivers (100% female; age 54-85, mean 71; 91% white, 9% black; 27% friend; 27% church congregant; 27% sibling or in-law; 18% neighbor). Caregivers described altruism, empathy, and, in cases of extended family, duty as motivators to provide care for acquaintances with dementia. Caregivers who themselves lacked close family identified with their care recipient's vulnerability and the possibility of needing similar care someday. Non-family caregivers described feeling unprepared and unqualified to make legal, financial, and guardianship decisions and looked to care recipients' distant family, health care providers, and religious leaders to guide decisions that require balancing care recipients' safety and autonomy, such as moving to a nursing home.

**Conclusion:** Dementia caregiving outside spouse or parent/child dyads can produce practical and emotional challenges for caregivers who are not immediate family. These caregivers may benefit from guidance about practical and ethical decisions in the absence of immediate family decision makers.

### SESSION 3440 (PAPER)

### RACE, ETHNICITY, AND NATIVITY

Abstract citation ID: igad104.1221

### A CONVERGENT PARALLEL MIXED METHODS ANALYSIS OF SUCCESSFUL AGING IN CHINESE AMERICANS: DOES ACCULTURATION MATTER?

Mushira Khan[1], Ezer Kang[2], and Chien-Ching Li[3], *1. Mather Institute, Evanston, Illinois, United States*, *2. Howard University, Washington, District of Columbia, United States*, *3. Rush University, Chicago, Illinois, United States*

Chinese Americans comprise the largest Asian origin group aged 65 years and older (26%), yet little is known about how aging, particularly in the context of acculturation, is experienced within this group. Further, although immigration scholarship underscores a robust, positive relationship between successful aging and acculturation, how major lifecourse events shape perceptions of aging well in Chinese Americans is not well-understood. Using an intersectional lifecourse perspective, this convergent parallel mixed methods study presents integrated findings from a cross-sectional survey (N=98; 58.2% female; mean age=64.4 years) and qualitative interviews (N=32; 53% female; mean age=59.4 years) with community-dwelling Chinese Americans 50+, about 80% of whom were foreign-born. Survey measures included the Short Acculturation Scale, the Successful Aging Scale, self-reported health, and demographic variables. Quantitative data were analyzed using multiple regression analysis. No significant association was found between acculturation and successful aging, even after controlling for length of stay and sociodemographic variables. Thematic analysis of qualitative data revealed feelings of ambivalence and alienation. Several participants shared that despite having lived in the US for decades, they felt like they "no longer belonged", feared for their safety, and felt a "sense of betrayal" after the recent increase in Anti-Asian hate crimes. Many were planning to return to their home country, where they believed it would be easier to age successfully. These findings suggest that important turning points in the lifecourse trajectory, such as experiences of racism, can negatively influence perceptions of aging in Chinese Americans, regardless of degree of acculturation and length of stay.

Abstract citation ID: igad104.1222

### ALZHEIMER'S DISEASE AND RELATED DEMENTIAS DIAGNOSES IN THE US BY RACE, ETHNICITY, AND NATIVITY

Tiffany Kindratt[1], Laura Zahodne[2], Kristine Ajrouch[3], and Florence Dallo[4], *1. University of Texas at Arlington, Arlington, Texas, United States*, *2. University of Michigan, Ann Arbor, Michigan, United States*, *3. Eastern Michigan University, Ypsilanti, Michigan, United States*, *4. Oakland University, Rochester, Michigan, United States*

Timely clinical diagnosis of ADRD is important for intervention, resource allocation, and mitigating safety concerns. While studies have examined ADRD diagnoses disparities by race/ethnicity, few include its intersection with nativity. Our aims were to: 1) estimate the odds of diagnosed ADRD among US- and foreign-born racial/ethnic groups compared to US-born White older adults and 2) compare US- and foreign-born older adults within each racial/ethnic group. We linked 2000-2017 National Health Interview Survey (NHIS) and 2001-2018 Medical Expenditure Panel Survey (MEPS) data (65+ years; n=38,033). Race/ethnicity and nativity were measured using NHIS data. Diagnosed ADRD was determined using ICD-9 (290/294/331/797) or ICD-10 (F01/F03/G30/G31) billing codes created from self-reported ADRD diagnoses during MEPS household interviews. Covariates were measured using MEPS data. US-born Black (OR=1.74; 95%CI=1.48-2.05), Hispanic (OR=1.62; 95%CI=1.14-2.29) and foreign-born Hispanic (OR=1.63; 95%CI=1.24-2.15) older adults had higher odds of diagnosed ADRD compared to US-born White older adults after adjusting for age and sex. Results were no longer significant among either Hispanic group after adjusting for education, health insurance, and access to care. However, after adjusting for education, health insurance, access to care, and chronic conditions, US-born Black older adults, but not foreign-born Black older adults, had 1.54 times greater odds (95%CI=1.27-1.87) of diagnosed ADRD compared to US-born White older adults. There were no statistically significant differences in ADRD diagnosis by nativity within each racial/ethnic minoritized group. Findings highlight the need for including nativity in studies comparing racial/ethnic groups to Whites to fully capture the burden of ADRD among US-born Black older adults.

Abstract citation ID: igad104.1223

### ASSOCIATIONS BETWEEN SEDENTARY AND PHYSICAL INACTIVITY ON QUALITY OF LIFE IN THAI MIDDLE AGE AND OLDER OFFICE WORKERS

Katika Akksilp[1], Yot Teerawattananon[2], and Cynthia Chen[1], *1. National University of Singapore, Singapore, Singapore*, *2. Health Intervention and Technology Assessment Program, Muang Nonthaburi, Nonthaburi, Thailand*

Sedentary behaviour and physical inactivity negatively affect health-related quality of life in all age groups. Moreover, the ageing population needs even more sophisticated health promotion programmes to tackle the increasing prevalence of non-communicable diseases. This study aims to estimate the effects of sedentary behaviour and physical inactivity on health-related quality of life among middle-age and older office workers in Thailand. The analyses used secondary data from the Physical Activity at Work cluster-randomised trial. Objective physical activity data were collected using the ActiGraph™ accelerometer. Health-related quality of life was collected using EuroQol's EQ-5D-5L. Sedentary was defined as spending more than nine hours per day in sedentary behaviours. Physically inactive was defined as spending less than 150 minutes of moderate-intensity equivalent activity per week. Tobit regression was used to compare utility indices. Being physically active alone was associated with a higher EQ-5D value (0.0746; 95%CI -0.0142 to 0.163) compared to the inactive. Also, non-sedentary participants had higher EQ-5D value (0.0548; 95%CI -0.0121 to 0.122) compared to sedentary, although not statistical significance. In addition, participants who were both physically active and not sedentary had a significant difference in the EQ-5D value of 0.0700 (95%CI 0.00187 – 0.138) compared to inactive and sendentary participants. This is the first study in Thailand to explore the association between sedentary behaviour, physical inactivity on health-related quality of life in middle-age and older population. It highlights the importance of incorporating health-related quality of life assessment for Thai physical activity promotion programmes, building a foundation for economic evaluation studies.

Abstract citation ID: igad104.1224

### RACIAL DIFFERENCES IN THE ASSOCIATION BETWEEN SENSE OF CONTROL AND SOCIAL SUPPORT: A CROSS-LAGGED PANEL ANALYSIS

Eunbea Kim, Gina Lee, and Jeong Eun Lee, *Iowa State University, Ames, Iowa, United States*

While it is widely acknowledged that having a sense of control and social support are crucial elements in promoting both physical and mental health among older adults, the relationship between these two factors and how they operate in diverse racial/ethnic settings is still unclear. To address this gap, we examined the bidirectional relationship between sense of control (constraints and mastery) and support received from three different resources (spouses, children, and friends) using cross-lagged panel regression analyses. Participants are White and Black Americans from four waves of Health and Retirement Study (N = 7,846 White; 944 Black). We found racial differences in the cross-over effects between social support and sense of control. In terms of constraints, while White Americans showed negative bidirectional associations between constraints and support from all resources across four waves, such associations were not observed in Black Americans. Additionally, positive bidirectional associations between mastery and support from all resources were found among White participants, whereas Black Americans showed only spill-over effects for mastery and support received from spouse and children across four waves. Notably, Black Americans' support received from friends positively predicted their mastery across four waves. These findings underline the importance of considering racial/ethnic differences in understanding the complex interplay between social support and sense of control. Future studies should illuminate the causal relation and examine differential mechanisms in White and Black American older adults.

Abstract citation ID: igad104.1225

### WORKING THROUGH IT: EMPLOYMENT DISCRIMINATION, LABOR FORCE OUTCOMES, AND MENTAL HEALTH AMONG OLDER BLACK AMERICANS

Lauren Brown[1], Leah Abrams[2], and Yuan Zhang[3], *1. University of Southern California, Los Angeles, California, United States*, *2. Tufts University, Medford, Massachusetts, United States*, *3. Columbia University, New York City, New York, United States*

We examine lifetime experiences of employment discrimination as one pathway by which racism impacts Black older adults' well-being. We use data from the Health and Retirement Study's (HRS) leave behind questionnaire to characterize lifetime experiences of being unfairly fired, not hired, or not promoted among Black older adults (N=2,948) and test associations of employment discrimination with labor force status at age 62, job satisfaction among those working, and depressive symptoms. Employment discrimination was commonly reported by Black older adults, especially among men and those with any college education. Employment discrimination was not associated with employment status but was associated with job dissatisfaction (OR=2.00, p=0.001) and depressive symptoms (Beta=0.34, p< 0.001). Findings demonstrate the detrimental impact of employment discrimination on Black older adults' work experiences and mental health, while highlighting the limitations of HRS's discrimination items. This research points to racism in the workplace as an obstacle to healthy aging for Black adults in America.

### SESSION 3445 (SYMPOSIUM)

Abstract citation ID: igad104.1226

### EXAMINING OPPORTUNITIES TO ALIGN WITH THE UNIFYING GOAL OF HELPING CAREGIVERS: A STRENGTH-BASED MODEL

Chair: Jeong Eun Lee Co-Chair: Naomi Meinertz
Discussant: Tina Savla

Caregiving is a complex and multifaceted experience, yet it is often addressed from a deficit model. Recent research indicates that strength-based interventions or programs from person- centered approach demonstrate improvements in caregivers' experiences. The purpose of this symposium are is to highlight a collection of studies that each bring a unique perspective to family the care issues by, reporting improved caregiver psychosocial factors contributing to caregiver outcomes and strength or person centered approach to enhance caregiver outcomes. The first session will demonstrate the effectiveness of a person-centered caregiver intervention, Powerful Tools for Caregivers (PTC), in reducing depressive symptoms, improving self-efficacy, and utilizing emotion management skills. The second session will share a baseline data from a live online intervention, Chronic Grief Management Intervention-Video (CGMI-V), and identifying intervention targets by examining caregivers' feelings of role captivity and grief. The third presentation will introduce the results of a pilot intervention , called "Building a Bridge," which that improved caregivers' knowledge and access to community resource. The final session will introduce a program based on the Acceptance and Commitment Therapy (ACT) model that reduces caregiver burden for community-dwelling parents and grandparents raising grandchildren or children in the community setting. Together, these presentations underscore the benefit of using strength-based models to relieve caregiver burden of caregivers. Savla , the discussant, will integrate key points from these interventions while and addressing considerations for future research to optimize outcomes for caregivers. This is a Mental Health Practice and Aging Interest Group Sponsored Symposium.

Abstract citation ID: igad104.1227

### POWERFUL TOOLS FOR CAREGIVERS: THE ESTABLISHED BENEFITS OF A PERSON-CENTERED CARE INTERVENTION

Naomi Meinertz, *University of Missouri, Columbia, Missouri, United States*

Family caregivers account for 89% of caregivers in the United States, yet few evidence-based family care programs focus specifically on the caregiver's needs rather than the care receiver. The challenges that caregivers experience, such as increased depression symptoms and feelings of burden, impede their ability to fulfill their role as care partner and can lead to lasting health and financial issues. Intervention literature underscores the importance of using person-centered models to incite behavior change, yet many caregiver interventions do not address the caregiver's needs and instead approach caregiving from a practical perspective to caregiving such as establishing a power of attorney or educating caregivers about dementia. Powerful Tools for Caregivers (PTC)

is a six-week program designed to provide caregivers with the skills to address their unique care challenges. Within in-person or virtual sessions, caregivers are provided with the skills to improve caregiver self-efficacy, emotional management, self-care, and resource access and knowledge. The study integrates empirical findings and demonstrates the effectiveness of PTC in improving caregiver well-being in each focus area. Findings also highlight the benefits of the group-based activities as well as improved depressive symptoms and reduced caregiver stress and burden. This research also demonstrates the same effectiveness of delivery format (in-person or virtual mediums) and location (rural or urban settings). The success of PTC sheds light on the need for more programs to approach family care through the lens of a person-centered model and emphasizes the benefit of evidence-based programs.

Abstract citation ID: igad104.1228

### FIVE REALITIES: A TYPOLOGY OF CAREGIVERS OF PERSONS WITH DEMENTIA AND THEIR INTERVENTION OUTCOMES

Sato Ashida[1], Freda Lynn[2], Lena Thompson[3], Maria Donohoe[1], Haley Schneider[2], and Kristine Williams[4], *1. University of Iowa College of Public Health, Iowa City, Iowa, United States*, *2. University of Iowa, Iowa City, Iowa, United States*, *3. University of Minnesota, Duluth, Minnesota, United States*, *4. University of Kansas Medical Center, Kansas City, Kansas, United States*

Caregivers of persons living with dementia report wide-ranging lived experiences, including feelings of burden and frustration but also positive outcomes. This study uses novel survey data to describe distinct types of caregiving experiences from a holistic perspective. We then explore the extent to which responses to a caregiver intervention are contingent on experience type. Using baseline data from a randomized trial, we use the k-means clustering algorithm on eight caregiver perceptions (adversity: burden, anxiety, network unmet-expectations, and network stress; positivity: positive aspects of caregiving, self-efficacy accessing services, caregiving preparedness, and network support) to identify experience types. Differential responses to intervention at 3-months by typology were assessed using OLS regression methods. Eighty-one caregivers were partitioned into five latent classes: Thriving (low adversity, high positivity); Deeply Struggling (high adversity, low positivity); Struggling but feeling prepared; Struggling and unprepared; No Plans (unprepared but not anxious). Attrition was highest among struggling groups and lowest among thrivers. Caregivers who are "Struggling but feeling prepared" ($p=0.01$) or "Struggling and unprepared" ($p=0.03$) showed significant increase in self-efficacy to use community services at 3-months. The latter group also showed a significant increase in knowledge about community services ($p=0.03$). Network stress at 3-months increased significantly more for the control than intervention group ($p=0.03$); by cluster, group differences were most pronounced among "Deeply struggling" ($p=0.04$) and "No Plans" ($p=0.05$). Types of caregiving experiences may determine caregiver participation in programs and their response to programs intended to support caregivers. Research and practice should focus on identifying strategies that fit caregiver experiences.

Abstract citation ID: igad104.1229

### ADRD CAREGIVERS' ROLE PERCEPTION AND MENTAL HEALTH AFTER LTC-PLACEMENT OF FAMILY MEMBERS

Hyejin Kim[1], Olimpia Paun[2], Jessica Bishop-Royse[2], Ben Inventor[2], Masako Mayahara[3], Sarah Ailey[2], and Louis Fogg[4], *1. Rush University College of Nursing, Chicago, Illinois, United States*, *2. Rush University, Chicago, Illinois, United States*, *3. Washington University in St. Louis, St. Louis, Missouri, United States*, *4. University of Illinois at Chicago, Chicago, Illinois, United States*

The Chronic Grief Management Intervention-Video (CGMI-V) clinical trial tested the effects of an 8-week live online intervention for caregivers who placed their family members with ADRD in LTC on caregivers' mental health outcomes (grief, depression and anxiety symptoms, positive states of mind). This cross-sectional study used baseline data ($n=95$) from the CGMI-V. We performed hierarchical linear regression to examine the main effects of caregiving role perceptions on caregivers' levels of grief, depression, anxiety symptoms, and positive states of mind, controlling for potential covariates. At baseline, perceived caregiver role captivity was the most significant factor of outcome variables, followed by feelings of guilt and loss. Increased feelings of role captivity were linked to sum scores of grief as measured with the Marwit-Meuser Caregiver Grief Inventory. These findings indicate that increased feelings of role captivity were associated with increased feelings of loss and loss of interpersonal connections. Higher levels of perceived role captivity were associated with more depressive and anxiety symptoms. Caregivers with lower levels of perceived role captivity had higher levels of positive states of mind. The findings confirmed the need for interventions targeting caregivers' feelings of role captivity, loss, and guilt related to caregiving obligations post-LTC placement of family members with ADRD to reduce caregivers' grief, depression, and anxiety and to increase their positive states of mind. These baseline findings are preliminary. Future presentations will report the effects of the CGMI-V intervention on caregivers' grief and mental health, including weeks 8 and 24 follow-up data.

Abstract citation ID: igad104.1230

### ACCEPTANCE AND COMMITMENT THERAPY INTERVENTION FOR PARENT CAREGIVERS: A STRENGTH-BASED APPROACH

Miranda Frederick, Jeong Eun Lee, and Amie Zarling, *Iowa State University, Ames, Iowa, United States*

ACT has emerged as a behavioral change approach that has been applied to address a wide range of challenges. Empirical research has shown that ACT can deliver positive long-term effects across various populations, including caregivers. Using six core principles of ACT, we have revised an acceptance and commitment therapy program (ACT) for custodial grandparents and their grandchildren. Our intervention program emphasized the importance of acceptance and promoting resilience among participants. This approach is particularly valuable for this population as the focus of the program is to strengthen their competencies, life skills and emotional resilience. The program consists of a web based ACT program with online coaching meetings across four sessions delivered for each age group. This program is unique in the sense that it utilizes both individual and group session techniques to facilitate the learning process. The main purpose of the program is to promote effective coping strategies, to reduce parenting stress among grandparents and to increase life skills (i.e., decision-making, proactivity) among children. Preliminary findings suggest that participating in ACT programs could help custodial, foster and biological parents improve self-efficacy, emotional well-being, higher self-confidence, social competence, lower depressive symptoms, and parenting distress, thereby leading to positive outcomes such as improved mental health and higher resilience.

### SESSION 3450 (PAPER)

### HEALTH DISPARITIES I

Abstract citation ID: igad104.1231

### "WHEN WE ARRIVED IN THIS COUNTRY, WE WERE ALREADY VERY OLD": HEALTH AND AGING IN WISCONSIN'S HMONG REFUGEE COMMUNITY

Michal Engelman[1], Maichou Lor[1], Mai Xiong[1], Tou Lee[2], and Casper Vang[1], *1. University of Wisconsin-Madison, Madison, Wisconsin, United States*, *2. University of Madison - Wisconsin, Madison, Wisconsin, United States*

The Hmong arrived in Wisconsin as refugees following wars in Vietnam and Laos, and are the state's largest Asian American group. They have a disadvantaged socioeconomic profile and face high rates of trauma-linked mental illness. Due to limited English proficiency, low literacy, and a lack of familiarity with and trust in research, the Hmong are underrepresented in existing data sources, which fold them into a pan-ethnic Asian-American group considered exceptionally successful. To address this gap, the Diversity, Inclusion, and Aging in the Midwest: Opportunities for New Directions with Wisconsin's Hmong Community (DIAMOND-Hmong) project has engaged Hmong older adults in a research study designed to document their unique life experiences and social determinants of health. Drawing on semi-structured life history interviews with 40 older Hmong men and women in Wisconsin, we describe participants' experiences during wars, forced relocations, and refugee camp spells, as well as the challenges and rewards of migrating to and aging in the U.S. Using a constructionist grounded theory approach, we show that participants' narratives link life-course hardships with physical and mental health challenges, generating a historically and culturally-specific delineation of trauma as both individual and collective experiences. Our analysis situates individual trauma within broader geopolitical and institutional circumstances and demonstrates that familial and communal ties – and their absence – are sources of both tension and resilience in this population. Our findings point to the importance of a trauma-informed approach to health assessment for the Hmong, and the role of memory and storytelling in their healing process.

Abstract citation ID: igad104.1232

### ADVERSE EFFECTS OF INTERNET USE ON SUBJECTIVE SOCIAL STATUS AND DEPRESSION SYMPTOMS AMONG CHINESE OLDER ADULTS

Aruhan Mu, *Huazhong University of Science and Technology, Wuhan, Hubei, China (People's Republic)*

Internet use has been found to be associated with decreases in depressive symptoms among older adults. However, past evidences may have had publication bias due to the negative consequences of internet use demonstrated in other age groups. Thus, this study aims to investigate the adverse effect of older adults' internet use on depressive symptoms and its mechanism. The inverse subjective social status (SSS) in depressive symptoms is a well-established research finding. Based on this, we hypothesize that older adults' internet use could expand their reference group and exposing them to more ageist content, thus reducing their SSS which in turn increases depressive symptoms. We examined how older adults' internet use influences SSS and how SSS mediates the effect of internet use on depressive symptoms in China. The data were from wave 3 (2016, T1), 4 (2018, T2), and 5 (2020, T3) of the China Family Panel Studies, a nationally representative survey. The longitudinal mediation analysis included 3,237 participants who were aged 60 and above at T1 (wave 3) and were followed through waves 4 and 5. As expected, Internet use at T1 predicted lower levels of SSS at T2. Lower levels of SSS at T2 predicted higher levels of depressive symptoms at T3. This medicating effect was significant. This time-varying mediation which appeared to be explained by the relative deprivation and ageism following internet use among older adults. This study theorizes and provides evidence for previously overlooked psychosocial pathway through which internet use among older adults produces negative consequences.

Abstract citation ID: igad104.1233

### ASSOCIATION OF CHILDHOOD EXPOSURE TO SCHOOL RACIAL SEGREGATION WITH LATE-LIFE COGNITION AMONG AMERICAN ADULTS

Zhuoer Lin[1], Yi Wang[1], Thomas Gill[2], and Xi Chen[1], *1. Yale University, New Haven, Connecticut, United States*, *2. Yale School of Medicine, New Haven, Connecticut, United States*

Racial segregation may contribute to enduringly worse health outcomes and aging. Prior research mainly focuses on residential segregation, yet the long-term effects of school segregation and the effects for different racial/ethnic groups are largely unknown. Linking measures of primary school segregation using administrative data with a nationally representative population survey in the US, we examine how childhood exposure to school racial segregation shapes the late-life cognitive function. 23,752 non-Hispanic White (White), 6,364 non-Hispanic Black (Black) American adults aged 50 and older were identified from the Health and Retirement Study; and state-level school dissimilarity indexes in late 1960s were linked to participants' latest wave of cognitive assessment using childhood residence. Multivariate regression analyses demonstrated that exposure to White-Black school segregation had strong, negative effects on cognitive outcomes for both White and Black participants. One standard deviation increase in dissimilarity index was associated with lower cognitive score (White: = -0.16 [95%CI, -0.22, -0.11]; Black: = -0.42 [95%CI, -0.53, -0.30]) and more cognitive impairment (White: Odds Ratio (OR) = 1.10 [95%CI, 1.06, 1.13]; Black: OR = 1.21 [95%CI, 1.14, 1.27]) adjusting for age and sex. Educational attainment and other socioeconomic characteristics explained most associations (40-70%), and the effects remained significant even after accounting for a rich set of late-life factors. Overall, these findings suggest that childhood exposure to school segregation has long-lasting effects on late-life cognition for both White and Black Americans. Educational policy and reform should be promoted to reduce school racial segregation within and between school districts to address health inequities.

Abstract citation ID: igad104.1234

**RELIGIOSITY, RELIGIOUS BELIEFS, AND COGNITIVE STATUS AMONG BLACK AND WHITE MEN WITH SIMILAR INCOMES**

Marino Bruce[1], Bettina Beech[2], Gillian Marshall[3], Nicole Phillips[4], Harlan Jones[4], Corinne Pettigrew[5], and Roland J. Thorpe[5], *1. University of Houston/Tilman J. Fertitta Family College of Medicine, Houston, Texas, United States*, *2. University of Houston, Houston, Texas, United States*, *3. NORC at the University of Chicago, Chicago, Illinois, United States*, *4. University of North Texas Health Science Center, Fort Worth, Texas, United States*, *5. Johns Hopkins University, Baltimore, Maryland, United States*

Black men experience multiple stressors linked with cognitive impairment and have higher risks for Alzheimer's Disease and related dementias than their White peers. Yet, few studies have focused on coping resources. Religious beliefs and institutions are thought to have health benefits; however, no study to our knowledge has examined religious practices or beliefs and their association with cognitive impairment disparities among Black and White men. The purpose of this study was to examine the association between religious practices and/or beliefs and cognitive status among Black and White men in the Health and Retirement Study (HRS). Data were drawn from Black and White men who reported incomes at or below $50,000 and completed the Core and Leave Behind Questionnaires in the 2016 HRS. The primary outcome was any cognitive impairment, a dichotomous variable derived from a modified version of the Telephone Interview for Cognitive Status (TICS). Religious service attendance, private prayer frequency, and religious beliefs were the primary independent variables. The proportion of Black men was 24.2% and had a larger proportion of individuals with any cognitive impairment than their White peers (47.2% vs 29.4%). Results from race-stratified Modified Poisson Regression models indicated that occasional religious service attendance was beneficial for White men (PR=0.68, CI:0.52-0.89) while private prayer (PR=0.71, CI:0.52-0.98) and religious beliefs (PR=0.90, CI:0.83-0.97) were inversely related to any cognitive impairment among Black men. Full-scale studies of Black men with robust religiosity measures are needed to further examine the association between religious factors and cognitive functioning among this group.

### SESSION 3455 (SYMPOSIUM)

Abstract citation ID: igad104.1235

**POLICY SERIES: PUBLIC HEALTH AND AGING POLICY: EXPERIENCE OF 2023 GSA SUMMER POLICY INTERNS**

Chair: Patricia D'Antonio Discussant: Brian Lindberg

The Gerontological Society of America (GSA) is home to an established summer policy internship program. Aimed at emerging scholars, these professional development opportunities are named in memory of Kathryn Hyer, MPP, PhD, FGSA, FAGHE, and Greg O'Neill, PhD, who were policy scholars and long-time GSA members. Interns participate in an 8-week in person experience in Washington, DC. Interns will share how their experiences have impacted their research and their future career goals in gerontology.

Abstract citation ID: igad104.1236

**INSIDE PAHPA: A GSA POLICY INTERN'S JOURNEY TO THE INTERSECTION OF POLICY AND PREPAREDNESS**

Alisha Thompson[1], Bailee Brekke[2], Christina Mu[3], and Patricia D'Antonio[4], *1. LSU, Baton Rouge, Louisiana, United States*, *2. Miami University, Oxford, Ohio, United States*, *3. University of South Florida, Tampa, Florida, United States*, *4. The Gerontological Society of America, Washington, District of Columbia, United States*

Public health and safety are facing increasingly severe threats from natural disasters, infectious diseases, and emergencies caused by human actions, both intentional and unintentional. These incidents now impact a larger number of people and cover broader geographical areas compared to the past. Chief among those most affected are older people, as highlighted during the COVID-19 pandemic and other crises. Addressing these disparities is crucial, and the federal government plays a pivotal role in tackling this issue. Much of my research up to this point has focused on preparedness among older people for emergencies that threaten public health. My experience as a GSA Summer Policy Intern has significantly influenced my understanding of the relationship between public health emergencies and aging policy. Through witnessing the legislative process at the national level, monitoring resource allocation in the federal budget, and attending Congressional hearings, I gained valuable insights into the complexities of this field. My perspective was further shaped by closely monitoring, synthesizing, and disseminating information about the 2023 Reauthorization of the Pandemic and All-Hazards Preparedness Act (PAHPA). Additionally, I provided valuable feedback on the relevance of this bill to the interests of GSA and contributed to comments on a bipartisan discussion draft related to this legislation. Equipped with this newfound insight and understanding, I am confident that I will be a more effective advocate and scholar in the field of aging policy.

Abstract citation ID: igad104.1237

**ALZHEIMER'S DISEASE AND OPPORTUNITY FOR POLICY CHANGE**

Bailee Brekke[1], Christina Mu[2], Alisha Thompson[3], and Patricia D'Antonio[4], *1. Miami University, Oxford, Ohio, United States*, *2. University of South Florida, Tampa, Florida, United States*, *3. LSU, Baton Rouge, Louisiana, United States*, *4. The Gerontological Society of America, Washington, District of Columbia, United States*

Amidst the introduction of breakthroughs to slow the progression of Alzheimer's Disease, hesitation remains among people living with dementia and their care partners. Currently, over 7 million Americans are impacted by Alzheimer's Disease and another 11 million Americans provide unpaid care for someone living with the disease. Despite the immense efforts made to develop treatments for those diagnosed, barriers in accessibility remain. As a GSA policy intern, I attended multiple coalition meetings centered around advocating for equitable access to new treatment options. I also analyzed legislation aimed at improving early detection, delaying disease progression, and improving access to treatments for people with Alzheimer's disease and related dementias (ADRD). While following the work of agencies including, the Centers for Medicare and Medicaid Services (CMS), I found that noteworthy progress has been made in terms of coverage expansion, but GSA and other organizations will need to continue to advocate for those older adults who have been left out of the equation. It is important to recognize the considerable progress that has been made and the immense efforts in support of people affected by this disease and their care partners; however, it is evident that Alzheimer's Disease must remain at the forefront of bipartisan policy initiatives to ensure that legislation is passed, and these barriers are alleviated.

Abstract citation ID: igad104.1238

### HEALTH EQUITY, INTERSECTIONALITY, AND CLIMATE CHANGE: PUBLIC HEALTH CONSIDERATIONS FOR RESEARCH

Christina Mu[1], Alisha Thompson[2], Bailee Brekke[3], and Patricia D'Antonio[4], *1. University of South Florida, Tampa, Florida, United States*, *2. LSU, Baton Rouge, Louisiana, United States*, *3. Miami University, Oxford, Ohio, United States*, *4. The Gerontological Society of America, Washington, District of Columbia, United States*

With the rapidly growing aging population, we are faced with an increasingly diverse population of older people. In the 2023 MedPac Report, the Commission found that health disparities by race/ethnicity and income persist. Black Medicare beneficiaries and people with low-income subsidies are at higher risk of poor health outcomes (e.g., increase hospitalization, readmission, and emergency department visits) compared to non-Hispanic White, Asian, and higher income beneficiaries. In the coming years, the aging population will be faced with novel challenges that threaten individuals' pursuit of health, well-being, and life. The focus of this presentation will be to discuss three emerging research areas including health disparities, intersectionality, and climate change. All are distinctly important and interconnected with health research more broadly. As a GSA summer policy intern, I researched legislation and synthesized literature related to these topics. For instance, I monitored and reviewed the Pandemic All Hazards Preparedness Act, Survival Aid for Emergencies Through Medicare Act, and MedPac recommendations to promote health equity. Additionally, I reviewed and contributed to GSA sign-on letters for congressional legislation. Within my own research focused on the joint associations of sleep and pain, this internship experience has provided new insights in line with my dissertation, which also seeks to uncover differences by age, sex, and race/ethnicity, as well as understand how contextual factors may influence the individual's health. Looking forward, researchers may have vested interested in contributing to gaps in knowledge regarding health disparities and climate change.

### SESSION 3460 (SYMPOSIUM)

Abstract citation ID: igad104.1239

### BUILDING INTERDISCIPLINARY COLLABORATIONS ACROSS THE AGING CONTINUUM: ROLE OF CENTERS ON AGING

Chair: Tetyana Shippee

Interdisciplinary collaborations and team science are essential in advancing research across the aging continuum. This involves bringing together experts from different disciplines to work together to address complex topics related to aging. Yet, despite the value of building interdisciplinary collaborations, multiple barriers often exist, that include finding the time to connect, developing shared goals by faculty dispersed across different departments, and developing a commitment to learning from each other and having open communication. This symposium will review multi-pronged strategies to build interdisciplinary collaboration in aging as part of the Center for Healthy Aging and Innovation at the University of Minnesota. In particular, we will discuss the formation of Special Interest groups (SIGs), which have become one key way to encourage interdisciplinary collaborations. SIGs are member driven and are meant to encourage collaboration via joint publications, presentations and grant applications as well as other shared initiatives agreed upon by the group (e.g., resource identifications, book club). Each special group has its own sense of community and draws on members from different disciplines. The symposium will include presentations from four of the seven SIG chairs, focused on the Financial Decision Making SIG, Alzheimer's Disease and Related Dementias SIG, Transportation SIG, and Aging and Chronic Disease Management SIG. Importantly, the symposium includes perspectives from faculty at different career ranks as well as PhD students.

Abstract citation ID: igad104.1240

### HARNESSING COMPLEMENTARY RESEARCH SKILLS TO ADVANCE RESEARCH ON FINANCIAL DECISION MAKING

Marti DeLiema, and Laura Hemmy, *University of Minnesota, Minneapolis, Minnesota, United States*

Financial decision making comprises a set of cognitive skills including problem-solving, critical thinking, and an understanding of key financial facts that help people make informed decisions about their money. Research indicates that financial decision making is one of the earliest areas of cognitive functioning to be affected by aging and by aging-related neurological disease. The Financial Decision Making Special Interest Group (SIG) has brought together several interdisciplinary researchers from social work and neuropsychology to advance our understanding of financial decision making trajectories in older adults, and how these trajectories impact their risk of harmful financial outcomes. Members bring different skills to the collaboration--cognitive assessment, working with clinical populations, and deep knowledge of the effects of poor decision making on financial security and risk of fraud and exploitation. In this presentation, SIG

members will share how they overcame challenges to develop a collaborative research agenda, including building membership and identifying samples in which to pilot measures of financial decision making.

Abstract citation ID: igad104.1241

### BUILDING SCIENTIFIC COLLABORATIONS IN ALZHEIMER'S DISEASE RESEARCH: THE ADRD SPECIAL INTEREST GROUP

Joseph Gaugler[1], Dana Urbanski[1], Hawking Yam[1], Zachary Baker[2], Emily Merkel[3], Stephen Waring[4], and Kerry Sheets[5], *1. University of Minnesota, Minneapolis, Minnesota, United States*, 2. *Arizona State University, Tempe, Arizona, United States*, 3. *University Of Minnesota, Minneapolis, Minnesota, United States*, 4. *Essentia Institute of Rural Health, Duluth, Minnesota, United States*, 5. *Hennepin Healthcare, Minneapolis, Minnesota, United States*

Since the advent of the National Alzheimer's Project Act, an unprecedented increase in federal funding to advance the science of dementia and dementia care has occurred. The Alzheimer's Disease and Related Dementia (ADRD) Special Interest Group (SIG) of the University of Minnesota's Center for Healthy Aging and Innovation aims to build and enhance scholarly collaborations in ADRD research between faculty, post-doctoral fellows, and graduate students across the University of Minnesota and beyond. Given the challenges of identifying mutual areas of scientific interest across disciplines, the ADRD has utilized several strategies to identify potential points of engagement, including a crosswalk procedure to create a matrix of members' interests, methodological expertise, and available data resources; a member-led journal club; and a dashboard to track collaborative process. Although early in its development, the ADRD SIG aims to facilitate these initial partnerships to result in peer-reviewed publications and, eventually, team science-based project in ADRD.

Abstract citation ID: igad104.1242

### FORMING AGING RESEARCH TEAMS THROUGH SPECIAL INTEREST GROUPS: THE GRADUATE STUDENT EXPERIENCE

Dana Urbanski, Kamakshi Lakshminarayan, Maritza Steele, Farah Baig, Adrianna Rivera-Leon, Michelle Berning, and Joseph Gaugler, *University of Minnesota, Minneapolis, Minnesota, United States*

The study of aging is inherently interdisciplinary, requiring formation of collaborative research teams that span a variety of fields and disciplinary backgrounds. This team approach is essential for answering complex, multifaceted questions about human aging; however, it may serve as a significant barrier to entry for graduate students interested in obtaining training and expertise in aging research. By and large, many graduate students are highly centralized in single academic departments and lack sufficient resources and opportunities to gain interdisciplinary research training and experience. The University of Minnesota Center for Healthy Aging and Innovation (CHAI) has created Special Interest Groups (SIGs) to facilitate team science and interdisciplinary collaboration by providing infrastructure and community for faculty, post-doctoral fellows, and—importantly—graduate students from across campus who have shared interests in aging. Here, we discuss a research project developed and organized by CHAI's Aging and Chronic Disease Management SIG which has drawn substantial graduate student engagement. Together, eight graduate students representing four University of Minnesota colleges are working with faculty to conceptualize and complete a systematic literature review on caregiving for older adults with hypertension. Drawing on this example, we share early lessons learned as well as key challenges and opportunities for engaging graduate students in interdisciplinary aging research through SIGs. Additionally, we highlight student experiences and perspectives participating in CHAI SIGs, along with recommendations for recruitment and inclusion of graduate students in aging research teams.

Abstract citation ID: igad104.1243

### FOSTERING COLLABORATION TO BENEFIT OLDER DRIVERS

Laura Hemmy[1], Nichole Morris[1], and Kyle Shelton[2], *1. University of Minnesota, Minneapolis, Minnesota, United States*, 2. *University Of Minnesota, Minneapolis, Minnesota, United States*

Access to transportation is a fundamental contributor to independence and quality of life, and for many independent driver status is a seminally important part of agency and self-concept. Driving is a lifelong skill utilizing a variety of physical, sensory and cognitive abilities, but also influenced by the community in which one lives, built infrastructure, vehicle design and engineering, social and family networks, and driver insight. Because older drivers are at increased risk for negative crash outcomes, generating knowledge about how to promote safe driving in the older population, when to consider retirement from driving, and compensatory sources of transportation, are of the utmost importance. Faculty and trainees with interest in these questions come from disparate and often unconnected parts of academic research institutions. The University of Minnesota (UMN) Center for Healthy Aging Innovation (CHAI) Special Interest Group (SIG) structure, in collaboration with the UMN Center for Transportation Studies (CTS), has served as a platform for connection. SIG members will share how the opportunity to connect across disciplines, schools and departments has led to new collaborative work to promote safe driving in older adults, including a state funded intervention study.

### SESSION 3465 (SYMPOSIUM)

Abstract citation ID: igad104.1244

### HEALTH AND AGING AMONG LATIN AMERICAN OLDER ADULTS: FINDINGS FROM STUDIES IN BRAZIL, CHILE, COLOMBIA, AND MEXICO

Chair: Margarita Maria Osuna Discussant: Jennifer Ailshire

Aging in Latin America is occurring rapidly, in a context of high levels of poverty, low education and limited knowledge on the health conditions of older adults. This symposium is focused on heath-disparities found in some of Latin America's largest middle-income countries, Mexico,

Brazil, Chile, and Colombia. The symposium explores various health-related topics associated with aging, that may have significant implications for longevity, overall health, and disease risk. The papers in this symposium examine a variety of health-related dimensions and disparities among older Latinos that include major chronic health conditions, such as diabetes, hypertension, cardiovascular diseases and cognition. Using data from the Brazilian Longitudinal Study of Aging, Farina examines the relationship between childhood conditions and older adult health. Using the Mexican Health and Aging Study (MHAS) and the Health and Retirement study (HRS), Cantu compares cognitive measures and their association with gender in Mexico and the US. Using mortality data, Sandoval studies how healthy life expectancy varies among the largest indigenous group in Chile compared to non-indigenous populations. Lastly, Osuna uses data from the Colombian Survey of Health, Well-Being, and Aging to examine how diseased life expectancy varies from self-reported data compared to measured data among older Colombians. Results underline what populations in Latin American have increased risk of experiencing poorer health outcomes and disparities that may exist. The findings highlight the importance of understanding the conditions under which Latin American older adults are aging and the implications this can have in the future.

Abstract citation ID: igad104.1245

### SELF-REPORTED VERSUS DIAGNOSIS-BASED DISEASED LIFE EXPECTANCY AMONG OLDER COLOMBIANS

Margarita Maria Osuna[1], Eileen Crimmins[2], and Jennifer Ailshire[2], *1. University of Southern California Leonard Davis School of Gerontology, Los Angeles, California, United States*, *2. University of Southern California, Los Angeles, California, United States*

The number of older adults in Colombia will soon double and it is essential to understand the disease burden in this population. Disease prevalence is often estimated from medical diagnoses, which can underestimate disease in populations with less health care access and low health literacy. We compare differences in diseased life expectancy (DLE) using self-reported vs. measured markers for diabetes and hypertension using data from the 2015 health and wellbeing survey of older adults in Colombia (SABE-Col), a nationally representative survey of Colombians ages 60+. Respondents reported whether they had ever been told by a doctor that they had either diabetes or hypertension. Using direct measures of glucose and blood pressure and clinical cut-points, we determined which respondents were considered to have diabetes or hypertension. We used the Sullivan method to estimate life expectancy with and without disease. Data from self-reports alone generally suggest lower DLE than from both self-reported and measured disease statuses. At age 60, women have a total life expectancy of 20.56 years. We find a lower DLE for hypertension using self-reported data (12.66 years) than measured (13.24 years). Conversely, reported diabetes is similar (4.18 years) to the measured (4.28 years) DLE. Men, at age 60, have a total life expectancy of 18.04 years. DLE for hypertension using self-reports is lower (8.78 years) than measured (9.61 years). Similarly, DLE for diabetes is lower for self-report (2.98 years) than measured (3.04). Our findings show the burden of disease in this rapidly aging population.

Abstract citation ID: igad104.1246

### GENDER DIFFERENCES IN INFORMANT REPORTS OF COGNITIVE FUNCTIONING IN MEXICO AND THE US

Phillip Cantu, *UTMB, Galveston, Texas, United States*

**Background:** Increasingly, researchers are attempting to measure cognitive functioning in large population-based surveys using both direct measurement and informant reports, but little work examines how gender affects measurements. For example, the role of gender in the division of labor is different in the U.S. and Mexico so questions relating to household chores will overestimate functional impairment in Mexico relative to the U.S. Method: Data from the U.S. Health and Retirement Study (HRS) and the Mexican Health and Aging Study (MHAS) will be used to examine disparities in how informants report cognitive change, measured by the total Community Screening Instrument for Dementia (CSI′D') score, are related to gender of participants and informants. Result: Female participants in Mexico have higher CSID scores than men but not in the U.S. Conversely, female informants report higher CSID scores than male informants in the U.S. but not in Mexico. **Conclusion:** Further analysis is needed to determine if national differences in the association of gender and reports of cognitive impairment are due to differences in how informants report impairment.

Abstract citation ID: igad104.1247

### EARLY-LIFE ORIGINS OF LATER-LIFE HEALTH AMONG OLDER BRAZILIANS: DIFFERENCES IN ASSOCIATIONS BY HEALTH CONDITION

Mateo Farina, *University of Texas at Austin, Austin, Texas, United States*

**Background:** A rich body of literature has linked poor childhood health to adverse adult health outcomes in later life. This association, however, has largely been studied in high-income countries. Less attention has been given to other country contexts where childhood exposures and adulthood socioeconomic conditions are vastly different. Brazil represents an important context due to the significant growth in older adult population, significant rise in life expectancy, and rapid industrialization that occurred since the 20th century. Method. Data come from the ELSI-Brazil Study, a nationally representative sample of older Brazilians. Logistic regression was used to predict the association between childhood conditions (socioeconomic adversity and poor health) and adult health conditions (heart conditions, heart failure, diabetes, and stroke). **Results.** Poor child health was positively associated with stroke, heart disease, and heart failure, but was not associated with diabetes or high blood pressure. Poor child socioeconomic status was associated with diabetes and stroke but not associated with heart conditions, heart failure, or high blood pressure. **Discussion.** The importance of childhood conditions for later life remained important in Brazil, but questions remain as to why certain conditions were found to be significant while others were not. Differences may stem from larger contextual changes such as the impact of globalization on food choices during adulthood for these cohorts. This study highlights the significance of contextual and conditional factors in comprehending the multifaceted dynamics underlying the association between early life conditions and later life health.

Abstract citation ID: igad104.1248

### ETHNIC DIFFERENCES IN HEALTHY LIFE EXPECTANCY IN CHILE

Moises Sandoval[1], Marcela Alvear Portaccio[2], and Cecilia Albala[3], *1. University of Chile, Santiago, Region Metropolitana, Chile*, *2. Departamento Administrativo Nacional de Estadística - DANE, Bogotá, Distrito Capital de Bogota, Colombia*, *3. INTA, Universidad de Chile, Santiago de Chile, Region Metropolitana, Chile*

Socioeconomic and sex differences in life expectancy (total and healthy) in Chile have been well established. However, the existence of ethnic differences in population longevity is unknown. Thus, we estimate total, healthy, and unhealthy life expectancy among Mapuche (largest indigenous group) and non-Mapuche older adults (≥60 years) in Chile. We estimated abbreviated mortality tables (using indirect demographic methods) and with the EDES health prevalence we applied Sullivan's method. Disability was defined as the unhealthy state, evaluated through the methodology suggested by Albala et al. (2004). Results show that at age 60 the Mapuche have a life expectancy 7 years lower (26.4 vs. 18.9 years respectively). Although differences diminish with age, Mapuches always have lower total life expectancy. In addition, we detected that the proportion of disability-free life years is higher for non-Mapuche (1.5, 2.0 and 3.7 times higher at 60, 75 and 90 years respectively). In contrast, Mapuches expect to live a higher fraction of their life with disability compared to non-Mapuche (e.g., 84.1% vs. 62.9% at age 80). This is the first study addressing inequities in healthy life expectancy between the Mapuche ethnic group and non Mapuche population, reflected in lower life expectancy, lower healthy life expectancy and higher disabled life expectancy in Mapuche when compared with no Mapuche population.

### SESSION 3470 (PAPER)

### AGING AND COGNITION (PAPERS) I

Abstract citation ID: igad104.1249

### ASSOCIATIONS BETWEEN ENVIRONMENT AND COGNITIVE FUNCTIONING FOR OLDER ADULTS: A CASE STUDY OF SINGAPORE

Yuezhong Liu[1], Chek Hooi Wong[2], Ringo Ho[1], and Yin-Leng Theng[1], *1. Nanyang Technological University, Singapore, Singapore*, *2. Research for Impact, Singapore, Singapore*

This study aims to examine the interplay between the intrinsic capacity of older adults and built environmental characteristics, and how these factors influence their outside daily life gait speed (DGS) in neighbourhoods. We collected and analyzed 33 participants who travelled a total of 2,428 kilometres and took a combined 1,428,793 steps outside of their homes over a period of 220 days in Singapore. Mini-Mental State Examination (MMSE) cut-offs (accounting for education attainment) were used to distinguish between those with cognitive impairment (CI) and those without cognitive impairment (Non-CI). The mean (SD) age of the participants was 69.2 (7.14), and 21 (64%) of them were female. 14 (42%) participants were identified as having cognitive impairment. A high Gross Plot Ratio was found to be associated with a lower DGS for both CI (b=-0.03, t = 0.19, $p < .01$) and Non-CI (b=-0.03, t = 0.16, $p < .01$). The DGS of individuals with CI was generally slower compared to Non-CI, with the greatest difference observed in business areas where the Non-CI group walked at a speed twice as fast. Individuals with CI had a slower DGS and required more adaptation to the environment. They also experienced lower quality of life-space interaction and relied more on vehicular travel from the density and frequency of activity points. Creating an environment that requires less attention and reduces cognitive load may help promote safe walking among individuals with CI and compensate for their reduced ability to perceive environmental risk.

Abstract citation ID: igad104.1250

### DEPRESSIVE SYMPTOMS AND SUBJECTIVE COGNITIVE FUNCTION IN OLDER ADULTS WITH MULTIMORBIDITY

Irina Mindlis[1], and Tracey Revenson[2], *1. Weill Cornell Medicine, New York City, New York, United States*, *2. City University of New York, New York City, New York, United States*

Among older adults with multimorbidity (MM), depression has been associated with poorer cognitive function and vice versa; however, evidence for this temporal association is limited. Further, while the relationship between depression and cognitive function is commonly studied with dementia, less is known about the relation of depression to cognitive impairment (SCI) in older populations without dementia. We compared bidirectional models of the relationship between depressive symptoms and SCI among community-dwelling older adults with MM. Adults ≥ 62 years with MM (n = 107) recruited through a national health volunteer registry completed validated scales twice, six months apart, assessing chronic illnesses, SCI (PROMIS Cognitive Function Short Form 4a), and depressive symptoms (Geriatric Depression Scale 15). Regression analyses examined two models of depressive symptoms and SCI over time (comparing causal direction), adjusting for age, gender, and number of diseases. Participants were 63-87 years old and living with multiple illnesses (M = 4.2; range: 2-9); 21% reported moderate-to-severe depressive symptoms. Depressive symptoms at the first time point significantly predicted SCI six months later after controlling for baseline SCI ($\beta = -.335$; $p < .001$), such that greater depressive symptoms were associated with poorer SCI in adjusted six-month models. In comparison, SCI at baseline was not a significant predictor of depressive symptoms after controlling for initial depressive symptoms ($\beta = -.057$; $p = .53$) in adjusted models. Among older adults with MM, depressive symptoms may be a risk factor for future SCI or a prodromal symptom of cognitive impairment.

Abstract citation ID: igad104.1251

**MID-LIFE FINANCIAL STRESS AND COGNITIVE AND PHYSICAL PROBLEMS IN OLDER AGE: THE ROLE OF POTENTIALLY MODIFYING FACTORS**

Ingemar Kåreholt[1], Charlotta Nilsen[2], Deborah Finkel[3], and Shireen Sindi[4], *1. Jönköping University, School of Health and Welfare, Jönköping, Jonkopings Lan, Sweden*, 2. *Institute of Gerontology, Jönköping, Jonkopings Lan, Sweden*, 3. *Jönköping University, Jönköping, Jonkopings Lan, Sweden*, 4. *Karolinska Institutet, Solna, Stockholms Lan, Sweden*

Financial stress is an important source of chronic stress and has been associated with cognitive and physical impairments. This study investigates whether midlife financial stress is associated with the combination of cognitive and physical impairment, the role of potentially modifiable factors, and sex differences. **Methods:** The Cardiovascular Risk Factors, Aging, and Dementia population-based cohort study from Finland was used (n=1497) (baseline collected 1972-1987, mean age 50 years). There were two late-life re-examinations (mean total follow-up 25 years). Midlife financial stress was measured using two questions on financial situation. Cognitive impairment was based on six cognitive domains. Physical impairment was self-reported. Potential modifying factors investigated were smoking, alcohol, physical activity, cohabiting/not, non-manual work, and sleep disturbances. Sex differences were investigated. We used path analyses with full information maximum likelihood estimation. **Results:** Among women and men, midlife financial stress associated with cognitive impairment, physical impairment and their combination. Smoking and sleep disturbances mediated associations between financial stress, physical impairment, and combined impairments. Among men: manual/non-manual work mediated the association to cognitive impairments; cohabitation mediated to cognitive impairment; financial stress was associated with cognitive impairment only among smokers and stress had a stronger association to physical and combined impairments among non-drinkers. Among women, sleep seems to have role in the association between financial stress and cognitive impairment. **Conclusions:** Midlife financial stress associates with late-life impairments, and lifestyle/sociodemographic factors may modify these associations. Sex differences were observed. Interventions promoting healthier lifestyle and psychosocial factors may buffer against the deleterious role of financial stress.

Abstract citation ID: igad104.1252

**THE TELEPHONE INTERVIEW FOR COGNITIVE STATUS (TICS): LATENT STRUCTURE OF COGNITION IN DOD-ADNI VIETNAM WAR VETERANS**

Larry Tupler[1], and Igor Akushevich[2], *1. Duke University Medical Center, Durham, North Carolina, United States*, 2. *Duke University, Durham, North Carolina, United States*

The Telephone Interview for Cognitive Status (TICS) is a convenient, user-friendly neuropsychological tool developed to correlate with the MMSE and screen for dementia. Typically, thresholding supports diagnostic inferences despite the arbitrary assignment of individual item contributions to the score. Furthermore, although partially validated with World War II veterans, little subsequent psychometric work with aging military cohorts exists. We therefore deconstructed and analyzed the TICS dataset from the DoD-ADNI study recruitment for neuroimaging of Vietnam War veterans with traumatic brain injury (TBI) and/or posttraumatic stress disorder (PTSD) compared with deployed controls (N=1,828; mean age 69.4±4.5 years). Both disorders constitute putative risk factors for Alzheimer's disease and related dementias. SAS statistics revealed raw and standardized Cronbach's alpha with and without inclusion of missing values in the mid .70 range of internal-consistency reliability, with a dispersion of 0.55%–99.29% items incorrect. Exploratory factor analysis applying the Oblimin rotation of van den Berg et al. (2012; MEDLINE UI: 22384819) yielded results similar to that study of healthy civilians analyzing TICS section totals assigned a priori (e.g., temporal orientation, list learning). However, neuropsychological component processes were more subtly differentiated with our enrichment of analyses with deconstructed individual items, e.g., a factor structure reflecting primary versus secondary memory and speech-language. We discuss the advantage of differentiating this proposed latent structure of the TICS in achieving more precisely characterized neuropsychological domains of cognitive impairment underlying specific neurodegenerative and trauma-related dementias. We later apply these results to the longitudinal examination of cognitive aging, TBI, and PTSD in this cohort.

### SESSION 3475 (SYMPOSIUM)

Abstract citation ID: igad104.1253

**FATIGABILITY, FITNESS, AND CHRONO-NUTRITION: RESULTS FROM THE STUDY OF MUSCLE, MOBILITY AND AGING (SOMMA)**

Chair: Nancy Glynn Co-Chair: Stephen Kritchevsky
Discussant: Jennifer Schrack

The Study of Muscle, Mobility and Aging (SOMMA) is a new longitudinal cohort study conducted at the University of Pittsburgh and Wake Forest University School of Medicine, coordinated by the San Francisco Coordinating Center, with management of biological specimens by the Translational Research Institute at AdventHealth. SOMMA aims to provide the foundation for discoveries in the biology of human aging, mobility and other age-related phenotypes. SOMMA enrolled 879 older women and men; mean age 76.3±5.0 years (range 70-94); mean walking speed 1.04±0.20 m/s; 59.2% women, and 15.8% identify as other than Non-Hispanic White. Our symposium will introduce novel baseline SOMMA findings on key measures related to fatigability, fitness, and chrono-nutrition. First, Dr. Schumacher will share results on the association between perceived physical and mental fatigability and cognitive function. Next, to begin to decipher how skeletal muscle energetics impact aging phenotypes, Ms. Gay will examine the relation between skeletal muscle energetics (ATPmax and maximal complex I&II supported oxidative phosphorylation [maxOXPHOS]) and age-related physical fatigability. Our next two presentations will focus on cardiorespiratory fitness (VO2peak) measured by gold standard treadmill cardiopulmonary exercise testing

(CPET), which declines with age. Ms. Moffit will explore whether a usual-paced 400m walk can predict VO2peak, and Dr. Erickson will examine the association between CPET measured VO2peak and circadian functions derived from rest-activity-rhythms extracted from wrist-worn accelerometers. Lastly, Dr. Farsijani will explore chrono-nutrition behaviors and their association with muscle health (i.e., D3-creatine, leg power). Discussant, Dr. Jennifer Schrack will provide insight to SOMMA's novel work.

Abstract citation ID: igad104.1254

### ASSOCIATIONS OF PERCEIVED PHYSICAL AND MENTAL FATIGABILITY WITH COGNITIVE PERFORMANCE

Benjamin Schumacher[1], Caterina Rosano[2], Yujia (Susanna) Qiao[2], Andrea Rosso[2], Peggy Cawthon[3], Stephen Kritchevsky[4], and Nancy Glynn[1], *1. University of Pittsburgh School of Public Health, Pittsburgh, Pennsylvania, United States*, *2. University of Pittsburgh, Pittsburgh, Pennsylvania, United States*, *3. California Pacific Medical Center, Research Institute, San Francisco, California, United States*, *4. Wake Forest School of Medicine, Winston Salem, North Carolina, United States*

Greater perceived fatigability has been associated with neurological diseases, but we do not know whether there are associations with cognition among at-risk older adults. At baseline, SOMMA participants completed the Pittsburgh Fatigability Scale (PFS) Physical and Mental subscales (each range 0–50; higher scores = greater fatigability; clinically meaningful increment=4-point physical, 3-point mental) and four cognitive function assessments [Digit Symbol Substitution Test (DSST), Montreal Cognitive Assessment (MoCA), Trail Making Test Part B (TMT-B), and California Verbal Learning Test-Second Edition, Short Form (CVLT-II SF)]. Linear regression quantified associations between PFS subscales and cognitive assessment scores adjusting for site, age, sex, race, education, marital status, and history of stroke, cancer, heart failure, and lung disease. In the 873 participants (59.2% women; age 76.3+/-5.0 years; 85% White), 54% had greater physical fatigability (PFS Physical≥15) and 23% had greater mental fatigability (PFS Mental≥13). Prevalence of cognitive impairment was 2% moderate (MoCA 10-17) and 0% severe ( <10). After adjustments, for each 4-point higher PFS Physical score participants had 0.8 fewer correct DSST items [Beta coefficient and 95% confidence interval: -0.8 (-1.2, -0.4); n=866] and 2.0 seconds slower TMT-B time [2.0 (0.1, 3.8); n=835]. Associations were similar for each 3-point higher PFS Mental score [DSST: -0.7 (-1.1, -0.4) and TMT-B time: 2.5 (1.0, 4.1)]. Neither PFS subscale was associated with MoCA or CVLT. Our results suggest that higher perceived physical and mental fatigability scores may both be indicative of cognitive impairment, particularly in processing speed.

Abstract citation ID: igad104.1255

### MITOCHONDRIAL ENERGETICS AND PERCEIVED PHYSICAL FATIGABILITY SEVERITY IN OLDER ADULTS

Emma Gay[1], Yujia (Susanna) Qiao[2], Adam Santanasto[2], Bret Goodpaster[3], Philip Kramer[4], Russell Hepple[5], Steven Cummings[6], and Nancy Glynn[1], *1. University of Pittsburgh School of Public Health, Pittsburgh, Pennsylvania, United States*, *2. University of Pittsburgh, Pittsburgh, Pennsylvania, United States*, *3. AdventHealth, Orlando, Florida, United States*, *4. Wake Forest University School of Medicine, Winston-Salem, North Carolina, United States*, *5. University of Florida, Gainesville, Florida, United States*, *6. California Pacific Medical Center Research Institute, San Francisco, California, United States*

It has been hypothesized that lower skeletal muscle energetics may contribute to onset of age-related perceived fatigability. Consistent with this idea, previous research found that maximal ATP production (ATPmax) was associated with higher perceived exertion (i.e., fatigability) measured in older adults with the Borg Scale at the end of a slow walking task. We extend this work to SOMMA examining skeletal muscle energetics (ATPmax and maximal complex I&II supported oxidative phosphorylation [maxOXPHOS]) with perceived physical fatigability using the Pittsburgh Fatigability Scale (PFS, range 0-50, higher=greater). PFS captures fatigue perception anchored to activities of varying intensity and duration. We assessed ATPmax in vivo by 31P magnetic resonance spectroscopy following an acute knee extensor exercise bout. maxOXPHOS was quantified ex vivo in permeabilized fiber bundles by high-resolution spectroscopy. Participants (N=721 with PFS and ≥1 energetics measure) were aged 76.4±5.0 years, 56.7% women, 86.7% White. Lower ATPmax (r=-0.21) and maxOXPHOS (r=-0.29) correlated with higher PFS scores (both p<0.001). Linear regressions adjusted for age, sex, technician, and total daily accelerometry-derived physical activity revealed that each one SD lower ATPmax (0.15 mM/sec) was associated with 1.15 (95% CI: 0.50, 1.79) higher PFS scores; each one SD lower maxOXPHOS (18.5 pmol/(sec*mg)) was associated with 1.79 (95% CI: 1.14, 2.45) higher PFS scores. Impaired skeletal muscle energetics may be implicated in the etiologic pathway of age-related fatigability. Next steps will examine the longitudinal association between changes in skeletal muscle energetics and age-related fatigability.

Abstract citation ID: igad104.1256

### ESTIMATING CARDIORESPIRATORY FITNESS USING A USUAL-PACED 400 M–LONG DISTANCE CORRIDOR WALK IN OLDER ADULTS

Reagan Moffit[1], Peggy Cawthon[2], Bret Goodpaster[3], Frederico Toledo[4], Daniel Forman[5], Barb Nicklas[6], Anne Newman[7], and Nancy Glynn[1], *1. University of Pittsburgh School of Public Health, Pittsburgh, Pennsylvania, United States*, *2. California Pacific Medical Center, Research Institute, San Francisco, California, United States*, *3. AdventHealth, Orlando, Florida, United States*, *4. University of Pittsburgh, School of Medicine, Pittsburgh, Pennsylvania, United States*, *5. University of Pittsburgh, Pittsburgh, Pennsylvania, United States*, *6. Wake Forest School of Medicine, Winston Salem, North Carolina, United States*, *7. University, Pittburgh, Pennsylvania, United States*

Cardiopulmonary exercise testing (CPET) is the gold standard to measure cardiorespiratory fitness (CRF), assessed as peak oxygen consumption (VO2peak), yet is not always feasible in older adults due to cost, safety, and participant burden. Usual-paced 400-m Long Distance Corridor

Walk (LDCW), a measure of mobility, may be an alternative to traditional CPET. We examined whether usual-paced 400-m LDCW was associated with VO2peak among older adults using cross-sectional data in SOMMA. Participants (N=820, age = 76.2±4.9, 58% women, 86% White) completed a usual-paced 400-m LDCW (seconds) and a treadmill CPET using a modified symptom-limited protocol at baseline. VO2peak (mL/kg/min) was defined as the highest 20-second average O2 consumption achieved during CPET. Mean VO2peak was 20.2±4.8 mL/kg/min and mean usual-paced 400-m LDCW time was 388.8±69.7 seconds. Usual-paced 400-m LDCW time was moderately inversely correlated with VO2peak (r=-0.52, p<0.0001). Potential covariates considered for the step-wise linear regression included age, sex, body mass index (BMI), Borg Rating of Perceived Exertion (RPE, range 6-20) at end of usual-paced 400-m LDCW, min/week moderate-vigorous physical activity (MVPA, CHAMPS questionnaire), 4-m gait speed, and Short Physical Performance Battery (range 0-12). The final parsimonious model included usual-paced 400-m LDCW time, age, sex, BMI, RPE, and MVPA. Every 30-second higher usual-paced 400-m LDCW time was associated with a 1.98 mL/kg/min lower VO2peak (p < 0.001), explaining 30.5% of the 55.7% total variance in VO2peak. Usual-paced 400-m LDCW was a strong estimator of CRF (VO2peak) after adjustment, providing an inexpensive and safer alternative among older adults across ranges of physical function and fitness.

Abstract citation ID: igad104.1257

### CARDIORESPIRATORY FITNESS IS ASSOCIATED WITH CIRCADIAN BEHAVIORAL PARAMETERS OF PHYSICAL ACTIVITY IN OLDER ADULTS

Melissa Erickson[1], Terri Blackwell[2], Theresa Mau[3], Samaneh Farsijani[4], Lauren Sparks[1], Nancy Glynn[5], Steven Cummings[6], and Karyn Esser[7], *1. AdventHealth, Orlando, Florida, United States*, *2. California Pacific Medical Center, San Francisco, California, United States*, *3. San Francisco Coordinating Center, San Francisco, California, United States*, *4. University of Pittsburgh, Pittsburgh, Pennsylvania, United States*, *5. University of Pittsburgh School of Public Health, Pittsburgh, Pennsylvania, United States*, *6. California Pacific Medical Center Research Institute, San Francisco, California, United States*, *7. University of Florida, Gainesville, Florida, United States*

Cardiorespiratory fitness (VO2peak) declines with age, and this may be due in part to weakened circadian functions. Rhythmic patterns of rest-activity behavior over 24h represents a novel feature of physical activity, indicative of circadian behavior. Whether VO2peak relates to rest-activity rhythms (RAR) is unknown. We determined cross-sectional associations between VO2peak and RAR in SOMMA (N=714, Age: ≥70 yrs). Activity data from wrist-worn accelerometry (ActiGraph GT9X) were collected in one-minute epochs, over 8, 24h periods. An extension to the traditional cosine model was used to map RAR to activity data, calculating these parameters: strength of the rhythm (amplitude); robustness of rhythm (pseudo-F statistic); and timing of peak activity (acrophase). RAR parameters were expressed as quartiles. Linear models examining associations of RAR and VO2peak were adjusted for potential confounders (age, race, height, comorbidities and lifestyle). Interactions of RAR predictors with sex were examined. Associations between VO2peak and rhythmic amplitude appears to differ by sex (p-interaction=0.07). Among men, VO2peak was positively associated with amplitude (adjusted mean Q1: 21.3 vs. Q4: 23.9, p-trend=0.005)-even after adjusting for moderate-to-vigorous physical activity levels (MVPA)-although associations were more modest and not significant in women. Those with higher VO2peak had an earlier acrophase (time of peak activity) (adjusted mean: Q1 <1:30PM: 21.0 vs. Q4>3:09PM 19.3, p-trend <0.0001) and no sex interaction was observed. There were no associations between the robustness of RAR and VO2peak. Analyses reveal that better cardiorespiratory fitness relates to features of circadian behavior independent of MVPA, supporting emerging research that circadian-based interventions promote healthy aging.

Abstract citation ID: igad104.1258

### THE RELATIONSHIP BETWEEN CHRONO-NUTRITION BEHAVIORS AND MUSCLE HEALTH IN OLDER ADULTS

Samaneh Farsijani[1], Ziling Mao[1], Peggy Cawthon[2], Stephen Kritchevsky[3], Nancy Glynn[4], Frederico Toledo[5], and Anne Newman[6], *1. University of Pittsburgh, Pittsburgh, Pennsylvania, United States*, *2. California Pacific Medical Center, Research Institute, San Francisco, California, United States*, *3. Wake Forest School of Medicine, Winston Salem, North Carolina, United States*, *4. University of Pittsburgh School of Public Health, Pittsburgh, Pennsylvania, United States*, *5. University of Pittsburgh, School of Medicine, Pittsburgh, Pennsylvania, United States*, *6. University, Pittburgh, Pennsylvania, United States*

Chrono-nutrition affects body composition via circadian clock entrainment, yet its impact on muscle health is overlooked. Here, we determined cross-sectional associations between chrono-nutrition behaviors, muscle mass (in kilogram by D3-creatine), and leg power (in Watts by Keiser) in 828 older adults (76 ± 5 years) in SOMMA. We extracted first and last intake time from 24-hr food recalls, and defined eating window as the time between first and last food/beverage intake. Chrono-nutrition's relationships with muscle mass and power were assessed by linear regression models, adjusting for age, sex, race, energy intake, weight, height, physical activity, education, health status, study site, and marital status. Average eating window was 11 ± 2 h/d; first and last intake time were at 8:00 ± 1:00 and 19:00 ± 1:00, respectively. Eating window and last intake time were associated with muscle mass (b ± SE: 0.20 ± 0.09; 0.30 ± 0.11, respectively, P < 0.05). Longer eating window was associated with higher leg power (4.23 ± 2.14; P = 0.049). Our study found that longer eating duration is associated with higher muscle mass and power in older adults. This finding contradicts the reported benefits of intermittent fasting in promoting muscle health which has been mostly supported by non-human studies. Our findings highlight the importance of meal timing and circadian rhythms in promoting health in older adults. The impact of chrono-nutrition on health requires further investigation in different age groups and clinical settings.

### SESSION 3480 (SYMPOSIUM)

Abstract citation ID: igad104.1259

### COGNITIVE IMPAIRMENT, FALLS, AND DEMENTIA: NOVEL APPROACHES FOR EARLIER DETECTION AND MORE EFFECTIVE INTERVENTION

Chair: Kieran Reid Co-Chair: Helena Blumen Discussant: Joe Verghese

This symposium will discuss novel digital approaches for the remote assessment of cognitive status and detection of fall risk in older adults. We will then describe several emerging digital exercise, ballroom dancing, and other non-invasive intervention strategies targeted for vulnerable older populations at increased risk for falling, or developing cognitive impairment and dementia. Collectively, we posit that these innovative detection and intervention strategies hold significant potential as more effective and scalable approaches for identifying cognitive impairment, preventing falls, and mitigating cognitive decline in high-risk older adults. In presentation 1, Roque et al. examines the utility of ecological momentary cognitive assessment and its potential as a digital biomarker of cognitive impairment in community-dwelling older adults. In presentation 2, Mahoney et al. discusses the development and clinical potential of a novel digital health app, CatchU®, that remotely captures visual-somatosensory integration performance to detect fall propensity in older adults. In presentation 3, Bajdek et al. describes the feasibility of a 12-week digitally delivered home-based fall prevention exercise program for older adults with elevated fall risk. In presentation 4, Blumen et al. report preliminary efficacy and mechanistic findings from a randomized trial of social ballroom dancing in older adults at increased risk for dementia. In presentation 5, Reid et al. describes the feasibility of a real-world community-level physical activity intervention for older adults with motoric cognitive risk syndrome. The discussant, Dr. Verghese, will integrate the key findings from these studies, discuss their theoretical and methodological contributions, and consider future research directions.

Abstract citation ID: igad104.1260

### REVOLUTIONIZING COGNITIVE ASSESSMENT: THE POWER OF DIGITAL BIOMARKERS AND ECOLOGICALLY VALID METHODS

Nelson Roque[1], Martin Sliwinski[2], Mindy Katz[3], and Cay Anderson-Hanley[4], *1. Pennsylvania State University, University Park, Pennsylvania, United States*, *2. Pennsylvania State University, State College, Pennsylvania, United States*, *3. Albert Einstein College of Medicine, Bronx, New York, United States*, *4. Union College, Schenectady, New York, United States*

Digital technologies have allowed neuropsychologists to utilize process-based approaches to assess cognitive function and change. Traditional methods of neuropsychological assessment involve obtaining single scores (often composites) to represent cognitive function. However, modern process-based techniques involve the use of digital data collection to acquire insights into both the manner and reasoning behind an individual's performance. The present study will share insights into the task development and validation of active and passive digital cognitive assessments from two longitudinal studies of aging: (1) a study of non-dementia participants (N=322), ultra-brief mobile cognitive tests were administered up to six times per day for two weeks; and (2) a neuro-exergaming study (N=31) enrolling patients with MCI and their co-residing study partner, where participants used a study-provided tablet to complete a neuro-exergaming protocol and digital cognitive assessments (e.g., digital trailmaking and eStroop). We analyzed both traditional and process-based metrics in two studies, including mean response time and patterns of inaccuracy. Study one found significant correlations between MCI status and metrics for survey completion time, working memory, and processing speed. Study two found weak to moderate correlations between cognitive function measures from digital and paper-based neuropsychology. Our findings suggest that remote self-administered digital measures can detect subtle cognitive changes. The event-level data, which is collected through a process-based approach, has enormous untapped potential for discovering and confirming novel digital biomarkers of cognitive health and progression that can be monitored beyond clinical settings.

Abstract citation ID: igad104.1261

### CATCHU: A NOVEL VISUAL-SOMATOSENSORY INTEGRATION TOOL FOR PREDICTING FALLS

Jeannette Mahoney[1], Claudene George[2], and Joe Verghese[1], *1. Albert Einstein College of Medicine, Bronx, New York, United States*, *2. Montefiore Medical Center, Bronx, New York, United States*

Ability to integrate information across sensory systems is a vital aspect of human functioning in the real world. While multisensory integration effects are measured across a wide array of populations using various sensory pairings and different neuroscience techniques, a gold standard for quantifying multisensory integration was seemingly lacking. We recently established a standardized protocol for administering and calculating behavioral multisensory integration effects across diverse clinical populations and age-ranges. Our previous work has linked the magnitude of visual-somatosensory integration (measured behaviorally using simple reaction time tasks) to important cognitive (attention) and motor (balance, gait, and falls) outcomes in healthy and cognitively impaired older adults. Given the paucity of extant translational multisensory integration tools in clinical settings, we recently developed a novel digital health app grounded in over 15 years of multisensory research. CatchU® (Before You Fall…) quantitatively measures visual-somatosensory integration performance, while considering medical comorbidities, to detect one's fall propensity. Our goal is to facilitate the identification of patients who are at increased risk of falls and promote physician-initiated falls counseling in routine practice (e.g., annual wellness, sick, or follow-up visits). This will thereby raise awareness of falls and foster physician efforts to alleviate disability, promote independence, and improve the well-being of older adults, all while aiming to reduce the significant societal cost of falls in older adults and others at high risk.

Abstract citation ID: igad104.1262
**FEASIBILITY OF A DIGITAL FALL PREVENTION EXERCISE PROGRAM FOR AT-RISK OLDER ADULTS**
Nicole Bajdek[1], Nancy Latham[1], Mary Dishaw[1], Shannon Farrell[1], Alisha Williams[2], Naomi Hachen[2], and Kieran Reid[3], *1. Brigham and Women's Hospital, Boston, Massachusetts, United States*, *2. Best Buy Health, Inc., Richfield, Minnesota, United States*, *3. Brigham and Women's Hospital/Harvard Medical School, Boston, Massachusetts, United States*

Falls have serious consequences and are a leading cause of disability, institutionalization, and mortality for older adults. While exercise programs with in-person supervision reduce the occurrence and risk of falls, there are accessibility and adherence barriers associated with older adults' participation in such programs. Digitally delivered home-based exercise interventions utilizing wearable technology and mobile applications may be an alternative fall prevention strategy for many vulnerable older adults. We evaluated the feasibility of a progressive 12-week, digitally delivered fall prevention exercise program in 23 at-risk older adults who reported ≥ 2 falls or ≥ 1 injurious fall in the past year, or a fear of falling (mean age: 76.6±6 yrs; BMI: 28.9±9 kg/m2; baseline short physical performance battery (SPPB) score: 8.9±3; 73.9% females). The structured exercise program was delivered via a tablet device and consisted of moderate-intensity strength and balance training with an embedded educational component (3x/wk) and walking exercise. A study interventionist provided participants with regular motivational coaching telephone calls throughout the program. Overall program adherence rates were high (≥ 80%) with minimal safety concerns. Preliminary efficacy findings revealed trends for improvements in fear of falling, cognitive performance, gait speed and overall SPPB score. The present study shows that a digitally delivered, home-based exercise program is feasible for older adults with elevated fall risk. Additional studies are warranted to confirm the efficacy of this highly scalable fall prevention strategy which could have important benefits for large populations of at-risk older adults.

Abstract citation ID: igad104.1263
**NONINVASIVE INTERVENTIONS IN HEALTHY AND AT-RISK OLDER ADULTS: THE CASE FOR SOCIAL DANCING**
Helena Blumen, *Albert Einstein College of Medicine, Bronx, New York, United States*

Physical and cognitive activities can induce neuroplasticity and lead to modest improvements on cognition in healthy and at-risk older adults. Yet, many unanswered questions remain – including what type of physical, cognitive, or combination of physical and cognitive activities, are most beneficial, and to whom (e.g., healthy, or at-risk older adults). This single-blind randomized clinical trial (RCT; NCT03475316) contrasted 6 months of social ballroom dancing with 6 months of treadmill-walking. Twenty-five dementia-at-risk older adults (mean age = 76.45 years; 66% women; 48% White/Caucasian; 24% Black/African American; 20% Hispanic/Latino) were randomized between June 2019 and March 2020. Sixteen participants completed this RCT prior to early study termination due to COVID-19 (8 in each study arm). Dementia-at-risk was defined as a memory impairment screen score of ≥3 to ≤6, and/or and AD-dementia screening interview of ≥1. A composite measure of executive function – generated from digit-symbol-substitution, flanker interference, and walking-while-talking tasks – improved following both social dancing and treadmill-walking. Yet, social dancing generated greater improvements than treadmill-walking on the digit symbol substitution task. No intervention-related differences were observed in brain activation. Less hippocampal atrophy, however, was observed in the social dancing arm than in the treadmill-walking arm. These preliminary findings partly support the hypothesis that social dancing may promote neuroplasticity and benefit cognition to a greater extent than traditional physical activities. This is presumably because social dancing is not only a physical demanding activity, but also a socially and cognitively challenging activity. Yet, these findings need to be confirmed in larger-scale RCTs

Abstract citation ID: igad104.1264
**FEASIBILITY OF A COMMUNITY-LEVEL PHYSICAL ACTIVITY STRATEGY FOR OLDER ADULTS WITH MOTORIC COGNITIVE RISK SYNDROME**
Kieran Reid, *Brigham and Women's Hospital/Harvard Medical School, Boston, Massachusetts, United States*

The motoric cognitive risk syndrome (MCR) is a predementia syndrome characterized by slow walking speeds and subjective memory complaints. To date, the feasibility of a community-based physical activity (PA) program for improving outcomes in MCR has not been examined. To address this knowledge gap, we conducted a translational randomized controlled trial (RCT) of 24-weeks of PA or a healthy aging education (HE) control intervention delivered within the existing infrastructure of an urban senior center in Greater Boston. A senior center employee was trained to administer the multimodal group-based PA program that included moderate-intensity aerobic walking, strength, flexibility and balance training. A total of 79 older adults attended the senior center for a screening visit of whom 25 met the MCR criteria and were randomized to PA or HE (mean age: 74.4 ± 7 yrs; BMI: 32.4 ± 7 kg/m2; 85% female; 3MSE score: 92.4 ± 7; gait speed: 0.52 ± 0.1 m/sec; SPPB score 4.8 ± 1.9). Due to COVID-19 the study was terminated early, however participants successfully completed baseline and follow-up study assessments that included objective tests of physical function and an iPad-based cognitive testing battery. Participants could appropriately adhere to the study interventions (overall attendance rate: PA: 69.3% vs. HE:70.1% at study termination). No study-related adverse events occurred. Trends for improved executive cognitive performance were exhibited in PA compared to HE. Our study provides important preliminary information to aid the design of larger-scale RCTs to preserve the independence of vulnerable older adults with MCR in community-based settings.

### SESSION 3485 (SYMPOSIUM)

Abstract citation ID: igad104.1265
**SPIRITUAL NEEDS OF AGING: BRIDGING RESEARCH AND PRACTICE TO IMPROVE HEALTH OUTCOMES IN OLDER ADULTS**

Chair: Katherine Britt Co-Chair: Chad Federwitz
Discussant: Jill Hamilton

Accumulating research reports that spiritual and religious practices are associated with better mental and physical health and support successful aging. Spiritual and religious practices often include the exploration of meaning and purpose in life, connectedness, inner peace, belonging, contentment, and near-end-of-life completion, all of which are tied back to health and health outcomes. However, these experiences and related positive outcomes may not always be meaningfully explored or considered. Social distancing and isolation studies have emphasized the importance of supporting the spiritual needs of older adults for connectedness and meaning. Less is known about how to support these spiritual needs in practice and how to provide spiritual care. This symposium will explore the current status of practices, research, and tools that address the spiritual needs of older adults, particularly in connection to health concerns and quality of life. A focus on current practices towards spiritual care for older adults and future implications regarding research and practice will also be considered. Recommendations for future gerontological inquiry into the importance of spiritual care and diverse approaches within gerontological practice will be highlighted and discussed. Dr. Zhao will describe the spiritual needs of persons living with dementia (PLWD). Dr. Epps will present the effectiveness of a culturally relevant and faith-based home toolbox for Black caregivers of PLWD. Dr. Lekhak will highlight the role of compassionate love in mediating the relationship between meditation practice and mental health. Dr. Britt will present religious attendance associations with better sleep quality in older adults to support cognitive function. This is a Religion, Spirituality and Aging Interest Group Sponsored Symposium.

Abstract citation ID: igad104.1266

### RELIGIOUS ATTENDANCE AND SLEEP QUALITY IN US OLDER ADULTS

Katherine Britt[1], Fanghong Dong[2], and Nancy Hodgson[3], *1. The University of Pennsylvania, Philadelphia, Pennsylvania, United States*, *2. University of Pennsylvania, Philadelphia, Pennsylvania, United States*, *3. University of Pennsylvania School of Nursing, Philadelphia, Pennsylvania, United Sta tes*

Sleep is an important proponent of memory, learning, and cognitive processing with changing patterns throughout the life course; poor sleep contributes to cognitive decline and dementia risk. Spiritual and religious activities are associated with better cognitive health, yet the biological mechanism is not well understood. This study aims to investigate whether religious and spiritual activity is associated with sleep quality in U.S. adults. Data were drawn from a U.S. longitudinal survey of a national probability sample, Midlife in the United States (MIDUS), from MIDUS Wave 2 (2004-2006), and MIDUS refresher (2011-2014). We examined cross-sectional associations between self-reported religious service attendance measured with 1 item (high, low religious attendance) and sleep quality measured with the Pittsburgh Sleep Quality Index (PSQI) (n=1,168) across three adult age groups (early, middle, late). We adjusted for sex, education, marital status, BMI, and smoking. Higher religious service attendance was significantly associated with better sleep quality among older adults (aged 60 – 80 years) (B=-1.02, p=0.011), while associations in young (aged 20-40 years) (B=-0.76, p=0.087) and middle-aged adults (aged 40-60 years) (B=-0.54, p=0.061) were marginal. Religious service attendance may contribute to better sleep quality amongst older adults by providing a sense of purpose and reducing feelings of helplessness and a sense of loss of control associated with aging. In addition, religious attendance is associated with decreased depressive symptoms and anxiety, possibly contributing to greater spiritual well-being and promoting better sleep. Longitudinal studies are needed to examine these associations over time.

Abstract citation ID: igad104.1267

### AN INTEGRATIVE LITERATURE REVIEW ON SPIRITUAL NEEDS FROM THE PERSPECTIVE OF OLDER ADULTS LIVING WITH DEMENTIA

Hui Zhao[1], Augustine Boateng[2], Francesca Ezeokonkwo[1], Chad Federwitz[3], Fayron Epps[4], and Katherine Britt[5], *1. James Madison University, Harrisonburg, Virginia, United States*, *2. University of Pennsylvania, Philadelphia, Pennsylvania, United States*, *3. Western Colorado Community College/Colorado Mesa University and Pitkin County Senior Services, Grand Junction, Colorado, United States*, *4. Emory University, Atlanta, Georgia, United States*, *5. The University of Pennsylvania, Philadelphia, Pennsylvania, United States*

Spirituality is a part of successful aging, promoting better sleep, well-being, and cognitive function. Persons living with dementia (PLWD) are reported to have greater needs related to their spirituality (spiritual needs) compared to persons without dementia; however, support for spiritual needs is minimally present or absent in dementia care. Unmet spiritual needs may lead to depression, anxiety, and greater physical pain; thus, addressing the spiritual needs of PLWD is essential to support well-being and inform personalized care. Our integrative literature review explored characteristics of spiritual needs of PLWD and how to support them. ATLA Religion, CINAHL, PsychInfo, PubMed, and SocIndex were searched between 2000 and 2022, resulting in five original research articles meeting inclusion criteria focusing on spiritual needs of PLWD, including PLWDs' perspectives. Most studies (n=4) were qualitative design, conducted worldwide in various settings, and included mild to moderate stage dementia. Components of spiritual needs covered the importance of connection and meaning for a sense of peace to support their identity and values. Connection to elements of spirituality included religion, meaningful relationships, nature, art, and self. Meaning was connected to past sources and experiences, supported by spiritual and religious activities. A sense of peace is supported through meaningful activities and religious rituals to help them rely on their faith through fear and memory loss. Support for spiritual needs may facilitate meaningful engagement for PLWD, leading to a sense of peace. Healthcare providers may utilize complementary, low-cost holistic care to promote personhood, values, and well-being among PLWD.

Abstract citation ID: igad104.1268

### FAITH-BASED HOME ACTIVITY TOOLBOX FOR BLACK FAMILIES AFFECTED BY DEMENTIA

Fayron Epps[1], Katie Pierson[1], Mayra Sainz[1], and Janelle Gore[2], *1. Emory University, Atlanta, Georgia, United States*, *2. Centers for Disease Control and Prevention, Atlanta, Georgia, United States*

The purpose of this study is to explore the effectiveness of a culturally relevant and faith-based home toolbox, Faith-HAT, for Black caregivers of people living with dementia (PLWD). Caregivers face high levels of stress, anxiety, caregiver burden, and depression, and engaging in religious activities often serves as a protective factor to promote better mental health. Despite the significant role of religiosity among caregivers, few resources exist for religious Black caregivers. A toolbox was developed to improve the physiological health of Black caregivers and provide caregivers and PLWD with meaningful activities to engage in. Participants were recruited from the Atlanta Metropolitan Area (n=16). Caregivers all cared for a family or friend experiencing moderate to severe dementia and had a history of participating in religious activities. Over 6 weeks, the caregiver and PWLD engaged in Faith-HAT, selecting different activities to complete three times a week. Journal entries were made a total of 12 times (at the beginning and end of each week), and caregivers met online with a research team member for weekly updates and baseline, midline, and endline interviews. Preliminary results suggest engaging with the toolbox has reconnected the caregiver and PLWD, cultivated religious connectedness, and supported continuation of caregiving activities. This faith-based, family-oriented, person-centered approach is geared towards enhancing the quality of life for families affected by dementia and draws on powerful Christian principles from within the black community to achieve this goal.

Abstract citation ID: igad104.1269

### MANAGING ANXIETY DURING THE COVID-19 PANDEMIC: THE SIGNIFICANCE OF MEDITATION AND COMPASSIONATE LOVE

Nirmala Lekhak[1], Tirth Bhatta[1], Eva Kahana[2], and Joel Snyder[1], *1. University of Nevada, Las Vegas, Las Vegas, Nevada, United States*, *2. Case Western Reserve University, Cleveland, Ohio, United States*

**Background & Significance:** Prior research has consistently established the role of meditation as a coping resource to mitigate the adverse impacts of stressful life situations on mental health. Yet, our understanding of mechanisms that transmit the positive effects of meditation on mental health among older adults is limited. Specifically, little research has examined the impact of meditation on positive emotions such as compassionate love and its subsequent effect on later-life psychological well-being. Purpose: We rely on Fredrickson's broaden-and-build theory of positive emotions to assess the role of compassionate love in mediating the relationship between meditation practice and mental health. **Methods:** Using data from our nationwide web-based survey (n=1,861), we conducted a mediation analysis to assess the indirect effect of meditation on mental health outcomes (depressive symptoms measured with a 10-item Center for Epidemiologic Studies Depression scale and anxiety measured with a 6-item PROMIS Emotional Distress-Anxiety Scale) via compassionate love. **Results:** Our ordinary least squares regression (OLS) model estimates suggest that older adults who practiced meditation (compared to those who did not) had significantly higher feelings of being loved (b = 0.11, p<0.05) and those who experience more love had lower symptoms of depression (b = -2.10, p<0.001) and anxiety (b = -0.99, p<0.001). We documented significant indirect effects of meditation (via compassionate love) on depressive symptoms (b = -0.23, p<0.05) and anxiety (b = -0.11, p<.05). Conclusion/ Implications: Our study underscores the need for contemplative interventions that can foster compassionate love to improve mental health among older adults.

### SESSION 3490 (PAPER)

Abstract citation ID: igad104.1270

### IMPROVING DEMENTIA DETECTION IN PRIMARY CARE: PROCESS EVALUATION FOR THE ERADAR BRAIN HEALTH TRIAL

Deborah Barnes[1], Mikael Anne Greenwood-Hickman[2], Clarissa Hsu[3], Leah Karliner[4], Deborah King[5], Sei Lee[1], Rebecca Coley[2], and Sascha Dublin[5], *1. University of California, San Francisco, San Francisco, California, United States*, *2. Kaiser Permanente Washington Health Research Institute, Seattle, Washington, United States*, *3. Kaiser Permanent Washington Health Research Institute, Seattle, Washington, United States*, *4. University of California San Francisco, San Francisco, California, United States*, *5. Kaiser Permanente Washington, Seattle, Washington, United States*

The eRADAR Brain Health Study is an ongoing embedded, pragmatic trial testing whether targeted screening improves detection and diagnosis of dementia in primary care clinics at Kaiser Permanente Washington and the University of California, San Francisco. The validated eRADAR algorithm, which uses electronic health record data to calculate a risk score for undiagnosed dementia, identifies high-risk patients age ≥65. Participants are randomized at the primary care physician (PCP) level to receive usual care or an invitation to a brain health visit for functional and cognitive assessment. We assessed patient acceptance (% completed brain health visit) and satisfaction (responses to post-visit survey) in patients enrolled to date. Of 575 high-risk patients invited, 157 (27%) agreed and 116 (20%) completed a brain health visit. The primary reason for declining the visit was lack of interest. Those who completed the visit had a mean (SD) age of 83.4 (5.1) years; 70 (60.34%) were women; 82 (70.7%) attended with a care partner. Seventeen (15%) had results consistent with dementia and were referred to their PCP for further evaluation. Fifty-five (47%) had results consistent with mild cognitive impairment (MCI) and were counseled to follow up with their PCP in 1 year. Analysis of the first 40 survey responses indicated high satisfaction: 33 (83%) agreed the visit was helpful and 36 (90%) did not feel upset/concerned by the visit. This suggests that, although acceptance was low, those who participated had high rates of possible dementia or MCI and reported high satisfaction with the targeted screening process.

Abstract citation ID: igad104.1271
ALZHEIMER'S DISEASE AND RELATED DEMENTIAS (HS)

### RELATIONSHIP BETWEEN CEREBRAL MICROINFARCTS AND DEMENTIA BY GENDER AMONG COMMUNITY-BASED OLDER ADULTS

Mo-kyung Sin[1], Yan Cheng[2], Jeffrey Roseman[3], Edward Zamrini[4], and Ali Ahmed[5], *1. Seattle University, Seattle, Washington, United States*, *2. George Washington University, Washington, District of Columbia, United States*, *3. University of Alabama at Birmingham, Birmingham, Alabama, United States*, *4. Irvine Clinical Research, Irvine, California, United States*, *5. Washington DC Veterans Medical Center, Washington, District of Columbia, United States*

Cerebral microinfarcts are common in older adults and associated with cognitive impairment. Less is known about sex-related variation in the relationship between microinfarcts and dementia in older adults, the examination of which was the objective of this study. This case-control study was based on 727 participants (308 men, 419 women) in the Adult Changes in Thought (ACT) autopsy data. Microinfarcts (presence, number, and location) were ascertained by blinded board-certified neuropathologists, and dementia diagnoses (yes, no) were made by the ACT Consensus Diagnosis Conference per DSM-IV criteria. Multivariable logistic regression analyses were conducted to examine the relationship. Microinfarcts were present in 356 (49%) participants (144 had one; 211 ≥2, 257 cortical, 216 subcortical), and 381 (52.4%) had dementia. More participants with dementia had ≥2 microinfarcts, although there was no difference in the distribution of a single microinfarct. Adjusted OR (95% CI) for dementia associated with any microinfarct was 1.36 (0.97–1.91). Respective ORs (95% CIs) in women and men were 1.45 (0.91–2.30) and 1.24 (0.75–2.06; p for interaction, 0.34). Adjusted OR (95% CI) for dementia associated with cortical and subcortical microinfarcts were 1.19 (0.83–1.69) and 1.65 (1.14–2.38), respectively, without any evidence of heterogeneity (p for interaction, 0.96 and 0.55, respectively). The presence of subcortical microinfarcts, but not cortical, had a significant association with dementia in older adults, but there was no evidence of a sex-related heterogeneity in that association. Future studies need to examine strategies to delay and manage vascular risk factors contributing to subcortical microinfarcts.

Abstract citation ID: igad104.1272

### RELATIONSHIPS BETWEEN CEREBRAL VASCULOPATHIES AND MICROINFARCTS IN COMMUNITY-BASED OLDER ADULTS

Mo-kyung Sin[1], Yan Cheng[2], Jeffrey Roseman[3], Edward Zamrini[4], and Ali Ahmed[5], *1. Seattle University, Seattle, Washington, United States*, *2. George Washington University, Washington, District of Columbia, United States*, *3. University of Alabama at Birmingham, Birmingham, Alabama, United States*, *4. Irvine Clinical Research, Irvine, California, United States*, *5. Washington DC Veterans Medical Center, Washington, District of Columbia, United States*

Cerebral microinfarcts are associated with cognitive impairment and dementia. Less is known about the relationships of various vasculopathies (arteriolosclerosis and cerebral amyloid angiopathy (CAA) with microinfarcts. This case-control study examined the associations of the vasculopathies with microinfarcts in 842 older adults in the Adult Changes in Thought (ACT) autopsy data. Arteriolosclerosis was assessed by histological examination using the H&E stain; CAA was assessed using the Congo red histochemical stains; and microinfarcts was ascertained by blinded board-certified neuropathologists. Multivariable-adjusted logistic regression models were used to estimate odds ratios (ORs) and 95% confidence intervals (CI) for the presence of cerebral microinfarcts associated with the two cerebral vasculopathies. Cerebral microinfarcts were present in 417 (49.5%) participants. Mild, moderate, and severe arteriolosclerosis was present in 13.2%, 43.9%, 29.7% of participants with microinfarcts, and 28.9%, 40.0%, and 12.5% of participants without microinfarcts, respectively. ORs (95% CIs) for microinfarcts associated with moderate and severe arteriolosclerosis were 2.15 (1.45-3.17) and 4.59 (2.88-7.33), respectively. Mild, moderate, and severe CAA was present in 18.0%, 17.5%, and 3.6% of participants with microinfarcts, and 18.1%, 16.5%, and 2.4% of participants without microinfarcts, respectively. ORs (95% CIs) for microinfarcts associated with moderate and severe CAA were 0.97 (0.65-1.44) and 1.37 (0.59-3.21), respectively. Cerebral microinfarcts were common in older adults and were associated with a higher prevalence of arteriolosclerosis, but not with CAA. Our study underscores the need for additional studies to better understand this complex relationship to develop interventions for primary prevention of cognitive impairment.

Abstract citation ID: igad104.1273

### SARCOPENIA AND MOBILITY DYSFUNCTION AS INDICATORS OF INCREASED ADRD RISK IN ASYMPTOMATIC DISEASE

Magdalena Tolea[1], Lilah Besser[2], and James Galvin[2], *1. University of Miami Miller School of Medicine, Miami, Florida, United States*, *2. University of Miami, Miami, Florida, United States*

Sarcopenia, the age-related loss of muscle mass and function, affects up to 30% of older adults. In addition to being an established component of frailty and predictor of mobility dysfunction, sarcopenia is associated with cognitive decline and increased risk of cognitive impairment, with ~70% individuals with late-stage dementia having sarcopenia. In this study, we investigated whether sarcopenia differentiates high- vs low- ADRD risk among 141 community-dwelling, older adults who were healthy controls (HC) or had mild cognitive impairment (MCI) (65% female; mean Age=70±11yr; mean Educ=16±3yr). Using the Brain Health Platform (BHP) developed at the Comprehensive Center for Brain Health, we categorized participants based on measures of resilience, vulnerability, cognitive performance as: Low-risk HC (36.9%); High-risk HC (12.8%), and MCI (50.4%). Amyloid positivity increased across risk groups (20% in Low-risk HC; 33.3% in High-risk HC; 39.1% in MCI). Prevalence of sarcopenia was 26.7% and increased by ADRD risk (12.2% vs 18.8% vs 39.1%, p=0.004). The MCI group had lower flexibility compared to both the low-risk (p=0.024) and high-risk HC (p=0.039) groups. Timed Up and Go (TUG) score increased with risk group (p< 0.001MCI vs Low-risk

HC; p=0.005MCI vs High-risk HC). Both mobility measures (i.e., flexibility, TUG) showed good discrimination for MCI vs Low-risk HC [AUC=0.77flexibility; AUC=0.78TUG] as well as for MCI vs High-risk HC [AUC=0.71flexibility; AUC=0.73TUG). Findings suggest that sarcopenia and other markers of mobility dysfunction have the potential to identify asymptomatic individuals at increased risk of ADRD, who could benefit from early intervention.

Abstract citation ID: igad104.1274

**SERUM SOLUBLE ALPHA-KLOTHO AND COGNITIVE FUNCTIONING IN OLDER ADULTS AGED 60 AND 79**

Song Ge[1], Fanghong Dong[2], Chong Tian[3], and Jingkai Wei[4], *1. University of Houston-Downtown, Houston, Texas, United States*, *2. Hebei University, Baoding, Hebei, China (People's Republic)*, *3. Huazhong University of Science and Technology, Wuhan, Hebei, China (People's Republic)*, *4. University of South Carolina, Columbia, South Carolina, United States*

**Objectives:** We aimed to examine the association of serum soluble alpha-klotho with cognitive functioning among older adults using a nationally representative sample of U.S. older adults. Method: A total of 2,173 U.S. older adults aged 60-79 years in the National Health and Nutrition Examination Survey 2011 to 2014 were included. Serum soluble alpha-klotho was measured in the laboratory. Cognitive function was measured using the Consortium to Establish a Registry for Alzheimer's Disease Word Learning subtest (CERAD-WL) immediate and delayed memory, the Animal fluency test (AFT), and the Digit Symbol Substitution Test (DSST). Test-specific and global cognition z-scores were calculated based on sample means and standard deviations. Multivariable linear regression models were applied to examine the association of quartiles of serum soluble alpha-klotho with test-specific and global cognition z-scores. Subgroup analysis was conducted by sex. The following covariates were included in the analysis- age, sex, race/ethnicity, education, depressive symptoms, smoking status, body mass index (BMI), physical activity, stroke, prevalent coronary heart disease, total cholesterol, and systolic blood pressure.

**Results:** Serum soluble alpha-klotho level in the lowest quartile was associated with lower z-scores for DSST (beta [β] =-0.13, 95% confidence interval [CI]: -0.25, -0.01). For subgroup analysis, serum soluble alpha-klotho level in the lowest quartile was associated with lower z-scores for DSST (β=-0.16, 95% CI: -0.32, -0.003) and global cognition (β=-0.14, 95% CI: -0.28, -0.01) among female participants. No significant association was found among male participants.

**Conclusions:** Lower serum soluble alpha-klotho level was associated with poorer cognitive functioning among older women.

Abstract citation ID: igad104.1275

**THE MINI-MENTAL STATE EXAMINATION IN A CHINESE POPULATION: RELIABILITY, VALIDITY, AND MEASUREMENT INVARIANCE**

Bjoern Andersson[1], and Hao Luo[2], *1. University of Oslo, Oslo, Oslo, Norway*, *2. The University of Hong Kong, Hong Kong, Hong Kong*

The Mini-Mental State Examination (MMSE) is a widely used screening tool for cognitive impairment and dementia in many cultures and regions. However, the properties of the MMSE in the Chinese context have not been evaluated with large population-representative samples. It is also unclear whether item properties of the MMSE differ across age and education subgroups. We study the psychometric properties of the MMSE using data from 8974 respondents aged 60 years or older in the 2018 Chinese Health and Retirement Longitudinal Study (CHARLS). Using item response theory (IRT) for the ordinal data, we confirm a three-dimensional factor structure for the MMSE in CHARLS in accordance with the underlying theoretical framework and establish acceptable model-data fit (SRMSR = 0.036). Total scores and subscale scores of the MMSE correlated positively with each other and correlated negatively with ADL (r = -0.19), IADL (-0.30), and CESD scores (-0.25). The reliability of the total MMSE scores was estimated at 0.78, indicating moderate measurement precision in the study population. Multiple group IRT analysis found that item characteristics and item functioning of 5 items differed by age group and 14 items differed by educational level. This analysis provided evidence on violations to measurement invariance of MMSE items, pointing to a need to account for item-level differences in measurement properties primarily with respect to education level when using the MMSE in CHARLS and, more generally, in Chinese populations. In addition, revised cognitive measures are advised to be used in CHARLS to improve the precision of measurement.

### SESSION 3495 (BIOLOGICAL SCIENCES INVITED SYMPOSIUM)

Abstract citation ID: igad104.1276

**EMERGING MR IMAGING AND SPECTROSCOPY APPLICATIONS FOR AGING RESEARCH IN MODEL ORGANISMS**

Chair: Rafael de Cabo Co-Chair: Mustapha Bouhrara

Advances in quantitative magnetic resonance imaging and spectroscopy (MRI/MRS) methodology have enabled probing tissue microstructure and function with exquisite specificity and sensitivity in human and model systems. Using this technology to examine changes in tissue microstructure and function in aging or pathology has the potential to provide a window into the underlying age-related diseases' mechanisms, and to nominate MR biomarkers for longitudinal assessment and intervention. Water molecules within biological tissues undergo interactions with their environment through nuclear relaxation, magnetization transfer, chemical exchange, and diffusion. These processes are sensitive to underlying local tissue properties such as density, microstructure, temperature, acidity, composition, and geometry. Through use of combinations of magnetic fields and radiofrequency pulse formalisms, MRI/MRS provides unique sensitivity and specificity to probe these mechanisms in all biological tissues. Advances in acquisition strategies, hardware designs, computational analyses, and signal modeling have positioned MRI/MRS as powerful emerging noninvasive modalities to studying biological tissue to differentiate normal from abnormal cell-level processes. These techniques are based on multicomponent relaxometry or diffusion, magnetization transfer, high-dimensional imaging or spectroscopy, susceptibility imaging, cerebral functioning, etc. However, their integration in clinical trials and investigations is still limited. This session will provide an overview about these emerging MRI/MRS techniques, their value and application in studying aging. The overarching goal is to generate further interest within the scientific community, disseminate and integrate these mature and unique noninvasive approaches in research settings and routine clinical protocols to advance our understanding of aging processes.

Abstract citation ID: igad104.1277

### IMAGING MICROSCOPIC NEUROPATHOLOGY OF ALZHEIMER'S DISEASE USING MRI: FROM MODEL SYSTEMS TO HUMAN IN VIVO

Dan Benjamini, *NIA, Baltimore, Maryland, United States*

Microstructural magnetic resonance imaging (MRI) techniques are unique among neuroimaging modalities because they probe tissue features at the micron scale that are invisible using other methods. This class of MRI methods promises to address the unmet needs for neuroimaging in Alzheimer's disease (AD) and related dementias: (1) early detection of subtle cellular and protein alterations and (2) delineation of comorbid pathologies. In particular, diffusion MRI is sensitive to alterations in cellularity and other microstructural changes, and relaxometery MRI is sensitive to chemical composition and macromolecular content. Benefiting from both modalities, multidimensional MRI is a new and emerging modality that maximizes chemical and microstructural information by probing relaxation and diffusion mechanisms simultaneously. This approach is ideal for detecting AD pathology, namely beta-amyloid plaques and Tau tangles, as well as comorbid pathologies, because of the microstructural and compositional alterations they induce in the affected regions. In order to actualize the promise of this method, a bridge must be built between the pathologic changes that define AD and detectable multidimensional MRI signatures. Our strategy starts with a ``bottom-up'' approach that first uses radiologic-pathologic correlation in postmortem human tissue to elucidate the associations between specific cellular and macromolecular alterations in AD and their unique multidimensional MRI signatures. Building on that knowledge, we can then adapt this technology towards clinical in vivo application and complete the journey from bench-to-bedside.

Abstract citation ID: igad104.1278

### MULTI-COMPONENT RELAXOMETRY FOR STUDY OF AGING

Shannon Kolind, *University of British Columbia, Vancouver, British Columbia, Canada*

While relaxation characteristics of MRI are used regularly for conventional, qualitative imaging, more information can be extracted by fitting multiple water components to the measured signal. Parameters including the myelin water fraction or T2 of intra/extracellular water can provide information about myelin development and degeneration, or inflammation and edema. Multiple approaches to both acquisition and analysis have emerged. This talk will provide an overview of some recent advancements and uses in aging.

Abstract citation ID: igad104.1279

### COMBINED USE OF NOVEL MR IMAGING AND FLUID BIOMARKERS REVEAL DETERMINANTS OF MYELIN AND AXONAL LOSS WITH AGING

Keenan Walker, *National Institute on Aging, National Institutes of Health, Baltimore, Maryland, United States*

Cerebral white matter damage is a feature of Alzheimer's disease, vascular dementia, and other neurodegenerative conditions, yet little is known about the molecular underpinnings of key features of white matter pathology. In a series of studies we have used targeted assays and large-scale proteomics platforms to determine how biomarkers of Alzheimer's disease pathology (Aß42/40 ratio; pTau181), neuronal injury (NfL), reactive astrogliosis (GFAP), and other disease processes relate to state-of-the-art diffusion-based MRI measures of brain myelin content and axonal density in cognitively unimpaired older adults. Using data from the Baltimore Longitudinal Study of Aging (BLSA) and the GESTALT study, we measured plasma biomarkers using the Quanterix Simoa assay (targeted measurement of 4 proteins) and the SomaScan proteomic platform (untargeted measurement of 7000 proteins). Additionally, participants underwent 3T MRI, from which measure of myelin content (defined by myelin water fraction [MWF]) and axonal density (defined by neurite density index [NDI]) were derived. We found an association between higher plasma GFAP and lower myelin content in temporal brain regions. Additionally, higher levels of NfL and GFAP were associated with lower total brain axonal density. Using untargeted proteomics, we identified a set of 155 plasma proteins that were associations with myelin content ($P < 0.05$). Using these findings, we applied machine learning to construct a plasma protein-based predictor of brain myelin content, which we plan to validate in a cohort of individuals with multiple sclerosis: a condition characterized by demyelination.

Abstract citation ID: igad104.1280

### ASSESSING AGE-RELATED CHANGES IN SKELETAL MUSCLE PERFUSION AND MICROVASCULAR FUNCTION WITH MRI

David Reiter, *Emory University, Atlanta, Georgia, United States*

MRI affords a wide variety of non-invasive and quantitative approaches for characterizing tissue perfusion, each providing distinct strengths and weaknesses. Skeletal muscle exhibits a tremendous range of metabolic activity with up to a 50-fold increase in oxygen consumption between resting state and heavy exercise. Even at rest, perfusion of skeletal muscle is a dynamic process where blood flow delivery to capillary beds fluctuates and is regulated by a host of cellular, metabolic, and neurovascular inputs. This talk will present an overview of current and emerging MRI methodologies used for characterizing skeletal muscle perfusion and microvascular function. Strengths and weaknesses of each methodology will be presented with emphasis on studies in aging and age-related disease.

Abstract citation ID: igad104.1281

### QUANTITATIVE RELAXOMETRY APPROACHES TO CHARACTERIZING BRAIN AGING: APPLICATIONS ACROSS THE LIFESPAN

Doug Dean, *University of Wisconsin, Madison, Wisconsin, United States*

Quantitative magnetic resonance imaging (qMRI) aims to measure meaningful physical or chemical parameters that are sensitive to and that can be used to characterize the underlying tissue microstructure. The quantitative nature of these data suggest that derived measures are ideally reproducible within and across different sites, allowing qMRI data to be more readily compared across different anatomical regions, among different subjects, and across time. The longitudinal (T1) and transverse (T2) relaxation times are fundamental to the measured MRI signal and, in the brain, highly sensitive to several anatomical changes, including changes of free water, iron content, and brain myelination. Thus, measurement of these relaxation times can be used to characterize the underlying tissue microstructure and can provide insight into the underlying processes of brain development and aging. As such, there is growing interest in using relaxometry-based techniques for a broad range of clinical and research applications. In this presentation, I will discuss recent advancements in quantitative relaxometry approaches and describe how these techniques are being used to gain a better understanding of the highly dynamic and nonlinear mechanisms that support brain development and aging.

### SESSION 3500 (SYMPOSIUM)

Abstract citation ID: igad104.1282

### SLEEP, MENTAL HEALTH, AND COGNITION ACROSS DIVERSE ADULT POPULATIONS

Chair: Soomi Lee
Co-Chair: Christopher Kaufmann
Discussant: Adam Spira

Sleep is often impaired in late life, raising concerns about the effect of poor sleep on mental and cognitive health in aging individuals. This symposium brings together five rigorous studies that show how poor sleep may be an underrecognized risk factor for mental health and cognitive outcomes in the second half of life. Specifically, this symposium showcases data from a variety of study cohorts (including Midlife in the United Study, Einstein Aging Study, and Study of Muscle, Mobility, and Aging), using diverse indicators of subjective and objective sleep. Paper 1 focuses on healthcare workers (HCWs), a group critical for the delivery of quality patient care, and examines both individual and joint associations of poor sleep and pain with mental health in HCWs vs. non-HCWs. Paper 2 investigates the association between EEG-measured sleep and depressive symptoms in older adults. Paper 3 explores the relationship between intra-individual variability of sleep and mild cognitive impairment (MCI) and identifies gender differences in these associations. Paper 4 establishes the associations of actigraphy-measured sleep and circadian rest-activity rhythms with multiple domains of cognition in older adults. Paper 5 examines the effect of latent dose/duration patterns of common sleep medications on development of Alzheimer's disease and related dementias. The discussant, Dr. Adam Spira, will integrate key findings from these studies, discuss their contributions to the literature, and consider opportunities for future research. This is a Sleep, Circadian Rhythms and Aging Interest Group Sponsored Symposium. This is a collaborative symposium between the Alzheimer's Disease and Related Dementias, Geroscience, and Health Behavior Change Interest Groups.

Abstract citation ID: igad104.1283

### LONGITUDINAL USE TRAJECTORIES OF SEDATIVE-HYPNOTICS AND RISK FOR DEMENTIA AMONG MEDICARE BENEFICIARIES

Christopher Kaufmann[1], Bobby Jones[2], Grace Hsin-Min Wang[2], Deanna Fernandes[3], Ronald Shorr[4], and Wei-Hsuan Lo-Ciganic[2], *1. University of Florida College of Medicine, Gainesville, Florida, United States*, *2. University of Florida College of Pharmacy, Gainesville, Florida, United States*, *3. University of Florida, Gainesville, Florida, United States*, *4. Geriatric Research, Education and Clinical Center, North Florida/South Georgia Veterans Health System, Gainesville, Florida, United States*

Research suggests use of sedative-hypnotic medications (Benzodiazepines [BZDs; e.g., diazepam] and non-BZD hypnotics [e.g., zolpidem]) to be associated with incident Alzheimer's disease and related dementias (ADRD). We identified longitudinal dose and duration use patterns of these medications and their associations with ADRD risk. This retrospective cohort study used 2016-2018 claims data from a 15% national sample of fee-for-service Medicare beneficiaries and included beneficiaries aged ≥65 years initiating use of a) BZDs only, b) non-BZDs only, or c) BZDs+non-BZDs concurrently. We excluded beneficiaries with ADRD diagnosis 6 months before and 12 months after first BZD/non-BZD prescription date. Based on average weekly diazepam milligram equivalent dose (low: <15, moderate: 15-30, high: >30), we used group-based multi-trajectory models to identify distinct trajectories of BZD/non-BZD use over 12 months after initiation (i.e., trajectory period). We estimated risk of time to first occurrence of ADRD after trajectory period associated with these trajectories using inverse propensity score weighted Cox proportional hazards models. Among 131,574 beneficiaries (mean age=74 years, 68% female, 89% White), we identified 11 trajectories (4 BZD only [78% of cohort], 4 non-BZD only [18%], and 3 concurrent users [4%]). Compared to beneficiaries with low-dose discontinuing non-BZD only use, trajectories associated with an increased risk of ADRD included: stable moderate-dose BZD use with concurrent stable low-dose non-BZD use (HR=2.28, 95%CI=1.62-3.21) and high-dose declining BZD only use (HR=1.57, 95%CI=1.26-1.96). Results highlight a need to assess not only patterns of BZD and non-BZD use alone, but also concurrent use, when considering risk for ADRD.

Abstract citation ID: igad104.1284

### INDIVIDUAL AND JOINT ASSOCIATIONS BETWEEN SLEEP AND PAIN ON MENTAL HEALTH: DIFFERENCES BY WORK STATUS AND OCCUPATION

Christina Mu[1], and Soomi Lee[2], *1. University of South Florida, Tampa, Florida, United States*, *2. The Pennsylvania State University, University Park, Pennsylvania, United States*

Researchers have posited that healthcare workers (HCWs) are at greater risk of sleep and pain concerns due to their demanding work schedules and stressful workplace environment. Currently, more research is needed to understand the joint associations between sleep and pain and differences by work status and occupation type. This study examined the individual and joint associations between sleep and pain on mental health symptoms by workers (vs. non-workers) and HCWs (vs. non-HCWs). Data came from the Midlife in the United States (MIDUS) study (Time 1: 2004-2005; Time 2: 2013-2019). At baseline, 2,894 participants were working (2,640 were not working; n=294 HCWs). Sleep quality, sleep duration, number of pain locations, and pain interference were assessed. Over time, as sleep quality worsened, sleep duration shortened, and the number and interference of pain increased, then depressive and anxiety symptoms increased across all groups. The individual associations of poor sleep or more pain with depressive and anxiety symptoms were stronger among non-workers compared to workers. The relationships between poor sleep or more pain with depressive symptoms was stronger among HCWs compared to non-HCWs, but weaker or not statistically significant when anxiety symptoms was the outcome. There was evidence that the combination of poor sleep characteristics and greater pain exacerbated anxiety symptoms among all groups. Findings show differences in mental health as functions of sleep and pain by work status and HCW status. Evidence of the joint associations of sleep and pain support the interrelated nature of sleep and pain.

Abstract citation ID: igad104.1285

### SLEEP AND DEPRESSIVE SYMPTOMS IN COMMUNITY-DWELLING OLDER ADULTS: FINDINGS FROM AMBULATORY SLEEP EEG

Jing Huang[1], Adam Spira[2], Miranda McPhillips[3], Russell Calderon[1], Mengchi Li[1], Yeabsira Tufa[1], and Junxin LI[1], *1. Johns Hopkins University, Baltimore, Maryland, United States*, *2. Johns Hopkins Bloomberg School of Public Health, Baltimore, Maryland, United States*, *3. The University of Pennsylvania, Philadelphia, Pennsylvania, United States*

There is limited and inconsistent evidence on the association between electroencephalography (EEG) measured sleep and depressive symptoms among community-dwelling older adults. This study investigated the cross-sectional association between EEG-measured sleep and depressive symptoms in 72 community-dwelling older adults without dementia [Age> 65 years; Montreal Cognitive Assessment (MoCA)>17], using baseline data from a randomized clinical trial (NCT03959202). Sleep and depressive symptoms were measured using two-night in-home sleep EEG (Sleep Profiler™) and the Geriatric Depression Scale (GDS-15). Multiple linear regression analyses were conducted with each sleep parameter as the primary predictor and GDS score as the outcome; models were adjusted for age, sex, race, education, marital status, chronic conditions, and MoCA score. Several sleep variables were associated with depressive symptoms (GDS score), including a higher percentage of sleep stage N1 (b = 0.11, 95% confidence interval [CI]: 0.02 – 0.20) and N2 (b = 0.04, 95% CI: 0.00 – 0.08), a lower percentage of N3 sleep (b = -0.04, 95%CI: -0.08 – -0.01), greater wake after sleep onset [b = 0.01, 95%CI: 0.00 – 0.02], and more awakenings ≥ 90s/hour (b = 0.87, 95%CI: 0.21–1.53). Neither total sleep time nor sleep efficiency were associated with GDS score. In conclusion, we found that more lighter (stage N1, N2) sleep, less deep (N3) sleep, and more fragmented sleep, measured by EEG, were associated with more depressive symptoms among community-dwelling older adults without dementia. Sleep architecture and fragmentation may play more important roles than sleep duration in relation to depression.

Abstract citation ID: igad104.1286

### GENDER DIFFERENCES IN ASSOCIATIONS OF SLEEP INTRAINDIVIDUAL VARIABILITY AND MILD COGNITIVE IMPAIRMENT

Yuqi Shen[1], Linying Ji[2], Jennifer Graham-Engeland[1], Carol Derby[3], and Orfeu Buxton[1], *1. Pennsylvania State University, State College, Pennsylvania, United States*, *2. Montana State University, Bozeman, Montana, United States*, *3. Albert Einstein College of Medicine, Bronx, New York, United States*

Previous studies suggest that cognitive function in older adults is related to sleep, with females more vulnerable to poor sleep. Little is known about the relationship between cognition and intraindividual variability of sleep (IIV). We examined the cross-sectional relationship between sleep IIV and mild cognitive impairment (MCI) by gender using data from the Einstein Aging Study. We hypothesize that individuals with MCI have a greater IIV in daily sleep characteristics, and these associations will be stronger for females. Eighty-nine men (age = 76.81±4.73, 61% White, 28% Black, 27% MCI) and 197 women (age = 76.60 ± 4.93, 40% White, 46% Black, 29% MCI) were included in the analysis. Individuals completed 2 weeks of actigraphy (mean ± SD = 15.7 ± 1.1 days), and the Montreal Cognitive Assessment, and MCI was based on the Jack/Bondi criteria. Sleep IIV was estimated as within person standard deviation of nighttime sleep duration, 24-hour sleep duration, wake after sleep onset, and nighttime sleep midpoint. Linear regression models evaluated associations between sleep IIV and MCI status separately by gender, controlling for BMI, age, ethnicity/race, nocturnal hypoxemia, Oxygen Desaturation Index ≥15. Among women, having MCI was associated with greater IIV in nighttime sleep midpoint (b=0.19, p=.002), and in 24-hour total sleep duration (b=13.10, p = .047) with a similar trend in nighttime sleep duration (p = .050). No significant associations were found among men. Findings suggest the importance to keep considering gender in work to understand how sleep variability relates to MCI status in older adults.

Abstract citation ID: igad104.1287

### OBJECTIVE SLEEP AND CIRCADIAN REST–ACTIVITY RHYTHMS AND MULTIPLE DOMAINS OF COGNITIVE FUNCTION IN OLDER ADULTS

Katie Stone[1], Haley Barnes[1], Terri Blackwell[1], Nancy Glynn[2], Karyn Esser[3], Anne Newman[4], Steven Cummings[5], and Peggy Cawthon[6], *1. California Pacific Medical Center, San Francisco, California, United States*, *2. University of Pittsburgh School of Public Health, Pittsburgh, Pennsylvania, United States*, *3. University of Florida, Gainesville, Florida, United States*, *4. University, Pittburgh, Pennsylvania, United States*, *5. California Pacific Medical Center Research Institute, San Francisco, California, United States*, *6. California Pacific Medical Center, Research Institute, San Francisco, California, United States*

Evidence suggests that sleep and circadian rest-activity rhythms (RAR) play a key role in dementia and Alzheimer

Disease (AD) pathology. Studies with objective measures of sleep and RAR and multiple domains of cognition remain limited. We studied 820 older adults enrolled in the Study of Muscle, Mobility and Aging (SOMMA). Actigraphs were worn for one week, and processed for sleep-wake measures including total sleep time (TST) and sleep efficiency (SE); and parametric (acrophase, pseudo-F) and non-parametric (interdaily stability [IS], intradaily variability [IV], and relative amplitude [RA]) 24-hour RAR variables. Cognitive assessments included global cognition (the Montreal Cognitive Assessment), executive function (Trails B test), memory (California Verbal Learning Test-short form; and psychomotor performance (Digit-Symbol Coding Test). Adjusting for age, sex, clinic site and education, we examined adjusted mean cognitive test scores by categories of sleep and RAR exposures. Mean age was 76 years, 58% were female, and 15% were non-white. Higher RA, reflecting a more robust 24-hour RAR, was associated with better cognition on all tests (p for trend <0.05). Reduced SE was related to lower cognitive function (psychomotor performance, global cognition, memory; p<.05). There were no associations with TST and any cognitive measures examined. SE and RAR were variably associated with both global and specific domains of cognition in older men and women. The RA in particular was strongly related to multiple domains of cognition related to AD, and may be useful in identifying individuals at risk. Additional analyses will explore sex differences and longitudinal associations.

### SESSION 3505 (SYMPOSIUM)

Abstract citation ID: igad104.1288

### MAINTENANCE OF HEALTH BEHAVIOR CHANGE: CHALLENGES, IMPLICATIONS, AND FUTURE DIRECTIONS

Chair: Jaime Hughes

Health behavior change is critical for healthy aging. Evidence-based programs are available to support a variety of health behavior changes in older adults, including programs for activity, diet, sleep, and medication adherence. Health behavior change programs are typically guided by one or more theoretical models or frameworks describing common constructs related to behavior change. Examples of such constructs include intention, motivation, self-efficacy, and self-regulation. To date, much focus has been on how such constructs relate to initiating and/or achieving behavior change. Although some models specify anticipated outcomes of behavior change over time, maintenance remains largely overlooked. This symposium presents a series of presentations exploring concepts and challenges related to maintenance of health behaviors in older adults. Together, these presentations will explore the definitions and characteristics of maintenance as described in the scientific literature, review maintenance-related trends within physical activity interventions, and discuss findings from stakeholder-engaged sessions designed to identify gaps within the theoretical and scientific literature describing maintenance. The session will conclude with recommendations for future research. This is a Health Behavior Change Interest Group Sponsored Symposium.

Abstract citation ID: igad104.1289

### DEFINING AND CHARACTERIZING MAINTENANCE: RESULTS FROM A LITERATURE REVIEW

Rahma Ajja[1], Anna Thorpe[2], Bridget Smeekens[2], and Jaime Hughes[3], *1. Wake Forest University School of Medicine – Winston-Salem, North Carolina, Winston Salem, North Carolina, United States*, *2. Wake Forest University School of Medicine, Winston-Salem, North Carolina, United States*, *3. Wake Forest School of Medicine, Winston-Salem, North Carolina, United States*

Leading models of health behavior change define maintenance as sustainment of the target behavior over time. Although behavior change interventions for physical activity have shown to be effective in older adults, maintenance of activity levels after an active intervention period is rarely examined. This literature review aimed to identify the following: (1) frequency with which maintenance is described and measured in the scientific literature; (2) intervention techniques utilized to promote maintenance; and (3) common barriers and facilitators to maintenance. Systematic searches of electronic databases (Embase, CINAHL, PubMed) identified 20 eligible studies (conducted in United States, single component physical activity intervention with 1+ follow-up period(s) beyond post-treatment assessment). Six studies reported maintenance in the title and only 8 included a theoretical model or framework [Social Cognitive Theory (N=5), Theoretical Domains Framework (N=1), Attribution Theory (N=1), Three-Level Hierarchical Framework (N=1)]. No studies defined maintenance. The majority of studies (N=16) provided only 1 follow-up after the post-treatment assessment (range: 4 weeks – 18 months). One-half of studies described specific intervention strategies to support maintenance, including goal-setting, personalized feedback, and social support. Six studies described barriers, including costs, aversion to activity or environment, low motivation, time constraints, and self-efficacy. Only two studies reported facilitators to maintenance, including knowledge, confidence, self-efficacy, and social support. These findings highlight the gap in health behavior scientific literature addressing maintenance of physical activity behaviors following structured interventions for older adults.

Abstract citation ID: igad104.1290

### MAINTAINING WALKING THROUGH WALK ON!: A COMMUNITY-BASED WALKING PROGRAM FOR OLDER ADULTS

Barb Nicklas[1], Elizabeth Chmelo Kemp[2], Michelle Gordon[2], Jaime Hughes[3], Rahma Ajja[4], and Justin Moore[2], *1. Wake Forest School of Medicine, Winston Salem, North Carolina, United States*, *2. WFSM, Winston Salem, North Carolina, United States*, *3. Wake Forest School of Medicine, Winston-Salem, North Carolina, United States*, *4. Wake Forest University School of Medicine – Winston-Salem, North Carolina, Winston Salem, North Carolina, United States*

The NIA-funded LIFE study showed that a walking-based intervention prevents mobility disability, even in those with compromised walking ability. However, increases in activity were not maintained following cessation of the intervention, suggesting novel approaches and accessible opportunities are needed to provide continued support for this population to maintain increased walking. We previously reported high feasibility and acceptability from a pilot of a supervised, group-based, and fee-for-service walking program (Walk On!) for adults with mobility challenges. The program focuses on walking stamina, and takes place two days/week for 12 weeks. Walk On! was offered for a total of ten consecutive sessions from late 2017 until March 2020 at a local church. The majority of attendees were >75 years (71%), female (65%), and had low levels of physical function (usual gait speed=0.79±0.16; 31% used an assistive device). Of the 49 unique (first-time) attendees who registered for Walk On!, only 18 (37%) did not re-register for at least one subsequent session. Thus, 63% (n=31) registered for at least 2 sessions, 51% (n=25) for at least 3 sessions, 20 (41%) for at least 4 sessions, and 15 (31%) for at least 5 of the 10 sessions. Mean attendance at scheduled group walking days for all attendees was 77%±21% for all attendees and did not decrease substantially for those attending multiple sessions. The safe, social, and accessible community-based Walk On! program may be one approach to sustain walking for older adults with mobility challenges. Future research will focus on measuring individual characteristics associated with maintenance.

Abstract citation ID: igad104.1291

### PHYSICAL ACTIVITY AND WEIGHT LOSS MAINTENANCE IN OLDER ADULTS: LONG-TERM FOLLOW-UP DATA FROM FIVE RANDOMIZED TRIALS

Denise Houston[1], Rebecca Neiberg[2], Shyh-Huei Chen[1], Michael Miller[1], Dalane Kitzman[1], W Rejeski[3], Steve Messier[3], and Barb Nicklas[4], *1. Wake Forest University School of Medicine, Winston Salem, North Carolina, United States*, *2. Wake Forest University School of Medicine, Winston-Salem, North Carolina, United States*, *3. Wake Forest University, Winston Salem, North Carolina, United States*, *4. Wake Forest School of Medicine, Winston Salem, North Carolina, United States*

Intentional weight loss (WL) in older adults is controversial as it may accelerate age-related muscle and bone loss, even if weight is regained. Current WL guidelines recommend physical activity (PA) for the maintenance of WL. However, whether participants who maintain WL have higher PA following randomized controlled trials (RCTs) of WL and PA is unknown. We examined PA among older adults from five WL and PA RCTs. Participants were invited to return for a follow-up visit 5-15 yrs after RCT completion (mean, 9.1 yrs). Among the 632 participants alive at long-term follow-up, 326 completed a follow-up visit (mean±SD age and BMI at randomization, 67.5±4.2 yrs and 33.8±4.9 kg/m2; 70% women). Weight change from baseline to long-term follow-up was assessed and participants categorized as weight gainers (>3%, 16%), weight maintainers (±3%, 32%), small weight losers (3-<10%, 28%), and large weight losers (>10%, 25%). Self-reported (CHAMPS) and objective (ActivPAL worn for 7 days) PA were assessed at long-term follow-up. Differences in PA by weight change groups were examined using mixed effects models adjusted for age, sex, race, WL assignment, time and study (as a random effect). There were no differences in self-reported PA by weight change group (all p's>0.0.08). Objectively-measured steps per day (p=0.06) and stepping time (p=0.04) were marginally significant between groups, with large weight losers having greater values compared to weight gainers (mean: 4915 vs. 3790 steps/day and 70.8 vs. 55.1 minutes/day, respectively; p<0.05). Among those who returned at long-term follow-up, objectively measured PA was greater among large weight losers.

Abstract citation ID: igad104.1292

### MAINTENANCE OF HEALTH BEHAVIOR CHANGE: RECOMMENDATIONS FOR FUTURE RESEARCH

Jaime Hughes[1], Aliza Randazzo[2], and Rahma Ajja[3], *1. Wake Forest School of Medicine, Winston-Salem, North Carolina, United States*, *2. Wake Forest University School of Medicine, Winston-Salem, North Carolina, United States*, *3. Wake Forest University School of Medicine – Winston-Salem, North Carolina, Winston Salem, North Carolina, United States*

Modifying poor health behaviors can enhance function, improve quality-of-life, and reduce morbidity and mortality among older adults. However, achieving and sustaining behavior change can be difficult. A growing body of research suggest that initiation and maintenance are unique constructs within health behavior change; each requiring unique skills and each associated with unique outcomes. Although some leading health behavior change models (e.g., Transtheoretical Model of Change) include maintenance as a construct, maintenance is often overlooked in the scientific literature. As part of a NIA Research Centers Collaborative Network (RCCN) pilot award, this project utilized a series of qualitative activities to explore the concept of maintenance and identify future recommendations for advancing the field. Three unique listening sessions were conducted with clinician-researchers (1 group) and community-based service organizations (2 groups). An interdisciplinary expert think tank was then convened to reflect on key findings. All sessions were conducted virtually; transcripts were reviewed using a priori themes with additional themes generated by an open coding approach. Major themes included the following: (1) To date, there is no consensus definition of maintenance nor is there agreement on maintenance across behaviors. (2) Few behavior change models consider how health behaviors must shift to accommodate for age-related changes in cognition and/or physical function. (3) Existing funding mechanisms and timelines may not allow sufficient follow-up periods to examine maintenance over time. (4) Although the terms maintenance and sustainment are used interchangeably, community leaders emphasized the importance of sustaining health promotion programs in order to enable maintenance to occur.

### SESSION 3510 (PAPER)

### EPIDEMIOLOGY AND COGNITIVE IMPAIRMENT/DEMENTIA

Abstract citation ID: igad104.1293

### AGE-SPECIFIC POPULATION ATTRIBUTABLE FRACTIONS FOR THE IMPACT OF HYPERTENSION ON INCIDENT DEMENTIA

Jason Smith[1], A. Richey Sharrett[1], James Pike[2], Rebecca Gottesman[3], Pamela Lutsey[4], B. Gwen Windham[5], Josef Coresh[2], and Jennifer Deal[2], *1. Johns Hopkins Bloomberg School of Public Health, Baltimore, Maryland, United States*, *2. Johns Hopkins University, Baltimore, Maryland, United States*, *3. NINDS/ NIH, Bethesda, Maryland, United States*, *4. University of Minnesota, Minneapolis, Minnesota, United States*, *5. UMMC-The MIND Center, Jackson, Mississippi, United States*

The population attributable fraction (PAF) of dementia in United States attributable to hypertension is estimated at 7-9%. However, prior studies of the PAF might underestimate hypertension's impact on dementia risk, given specific design limitations. Additionally, prior work neither accounted for changing prevalence/effects of hypertension with age nor considered early vs. later occurring dementia. Here, we quantified PAFs of dementia by age 80 from hypertension measured at midlife and late-life using individual-level data from the Atherosclerosis Risk in Communities Study. Incident dementia was determined by adjudicated review, telephone interviews, informant interviews, hospitalization records, and death certificates. We used measures of systolic and diastolic blood pressure (SBP, DBP) to define BP categories: normal BP (SBP < 120 and DBP < 80), elevated BP (SBP 120-129 and DBP < 80), stage 1 hypertension (SBP 130-139 or DBP 80-89), and stage 2 hypertension (SBP ≥140 or DBP ≥90); we also defined an overall non-normal BP value (SBP ≥120 or DBP ≥80). We generated hazard ratios (HRs) of 32-year incident dementia from Cox regression models that adjusted for demographic and clinical characteristics, and stratified by age at BP ascertainment (45-54, 55-64, 65-74). We then used the HRs to quantify PAFs of dementia from hypertension. We estimated that 15-20% of dementia cases by age 80 were attributable to non-normal BP. The strongest PAFs (12-21%) were from stage 2 hypertension. Current estimates of the dementia risk attributable to hypertension may be low. Targeting hypertension from midlife through early late-life could sizably reduce dementia risk.

Abstract citation ID: igad104.1294

### PREDICTING DEMENTIA DIAGNOSIS FROM COGNITIVE FOOTPRINTS IN ELECTRONIC MEDICAL RECORDS USING MACHINE LEARNING

Jiayi Zhou, Hao Luo, Wenlong Liu, and Huiquan Zhou, *The University of Hong Kong, Hong Kong, Hong Kong*

The cognitive footprint theory suggests that a person's risk of dementia is affected by activities and events (footprints) across the lifespan. This study aimed to develop a clinical algorithm for predicting dementia diagnosis from cognitive footprints in electronic medical records (EMRs) using machine learning. Population-based EMRs from the Clinical Data Analysis and Reporting System in Hong Kong were employed. We included all patients with dementia diagnosed at 65+ between 2000 and 2018. Controls without dementia were matched (1:1) by age, sex, and index date. The prediction accuracy of a standard logistic regression model was compared with five machine learning models (LASSO, Random Forest, Multilayer perceptron, XGBoost, and LightGBM), using a wide range of established and exploratory risk factors identified at mid- ( < 65 years) and late-life (65+ years). A total of 261,116 individuals (59.3% female; mean age at index date [SD]: 82.70 [8.54]) with and without dementia were included. Compared with the logistic model (area under the curve [AUC]: 0.60), the predictive and diagnostic accuracy of dementia improved substantially in machine learning models. The Multilayer perceptron and LightGBM models showed comparable performance with the same test AUC of 0.720. LASSO (AUC: 0.765) and Random Forest (AUC: 0.768) showed a slightly worse performance. Antipsychotic drugs, education, antidepressants and head injury in late life were the top 4 important predictors in all models. The machine learning-based algorithm for predicting dementia can be used to identify patients with an increased likelihood of dementia to allow precise and timely primary interventions.

Abstract citation ID: igad104.1295

### RISK OF ALZHEIMER'S DISEASE AND RELATED DEMENTIA IN PATIENTS WITH SLOW PROGRESSIVE CANCERS

Igor Akushevich[1], Arseniy Yashkin[1], Vinit Nalawade[1], and Julia Kravchenko[2], *1. Duke University, Durham, North Carolina, United States*, *2. Duke University School of Medicine, Durham, North Carolina, United States*

Evidence is accumulating that individuals with cancer diagnoses exhibit Alzheimer's disease (AD) and related dementia (ADRD) risk profiles that differ from the general population of U.S. older adults. In this study we used SEER-Medicare data to compare the relative risk of AD/ADRD between individuals with slow-progressive cancers and the non-cancer general population. The study cohort included individuals age 65+ (N=2,023,054) with a primary diagnosis (1999-2017) of one of nine slow progressive cancers (breast, colorectal, prostate, uterine, kidney, ovarian, and urinary bladder cancers, as well as lymphomas and melanoma) and no clinical record of AD/ADRD prior to cancer diagnosis. This cohort was then matched by age to a comparable non-cancer population (N=1,142,641). The hazard ratios of AD/ADRD for each cancer compared to the non-cancer cohort were evaluated individually in 29 age-specific groups for each cancer type. All cancers had similar patterns of dependence for post-cancer AD/ADRD risks. We found that the presence of cancer was associated with higher risk of AD/ADRD at age at diagnosis 65-75; the relative risks decline with age at diagnoses becoming protective at advanced ages. Furthermore, for any given age at diagnosis the relative risk of AD/ADRD (i.e., cancer vs. non-cancer) also declines with time. Detailed discussion of possible causes of these effects including cancer treatment, genetic variation, possible trade-off effects, common risk and protective factors, possibly lower administration and adherence of AD/ADRD diagnostic procedures for individuals with cancer, and the roles of competing risks (first of all due to death cases) is presented.

Abstract citation ID: igad104.1296

### THE ASSOCIATION OF A NOVEL PHYSICAL ACTIVITY METRIC WITH COGNITION AND RISK OF DEMENTIA

Yurun Cai[1], Junhong Zhou[2], Qu Tian[3], Amal Wanigatunga[4], Eleanor Simonsick[3], Susan Resnick[3], Luigi Ferrucci[3], and Jennifer Schrack[5], *1. University of Pittsburgh, Pittsburgh, Pennsylvania, United States*, *2. Harvard Medical School/ Hebrew SeniorLife, Roslindale, Massachusetts, United States*, *3. National Institute on Aging, National Institutes of Health, Baltimore, Maryland, United States*, *4. Johns Hopkins School of Public Health, Baltimore, Maryland, United States*, *5. Johns Hopkins University, Baltimore, Maryland, United States*

Physical activity (PA) is associated with cognitive function and Alzheimer's disease and related dementias (ADRD). Although associations between continuously measured PA and ADRD have been assessed, multiscale entropy (MSE), which quantifies dynamics of physiological systems over multiple time scales, has not been used to quantify daily activity rhythms in older adults by cognitive status. We examined the association of this novel metric of PA complexity with cognitive function, mild cognitive impairment (MCI), and dementia in participants of the Baltimore Longitudinal Study of Aging. A total of 615 older adults (mean age=73.9±11.3 years, 54.5% women) completed a 7-day wrist-worn accelerometer assessment between 2015-2019. Global cognitive function was measured using the Mini-Mental State Examination (MMSE). Mild cognitive impairment (MCI) or dementia was diagnosed based on Petersen criteria and Diagnostic and Statistical Manual of Mental Disorders, respectively. Unadjusted logistic regression models showed that participants in the lowest tertile of complexity had 2.31 times the odds of low MMSE score (≤26) compared to those in the highest tertile (odds ratio [OR]=2.31, 95% confidence interval [CI]=1.09-4.89). This association lost significance after adjusting for age, sex, race, and education years. The lowest tertile of complexity was also associated with 2.58 times the odds of MCI/dementia diagnosis, adjusting for demographics (OR=2.58, 95% CI=1.06-6.31). These results suggest that lower complexity of accelerometry-detected movement is associated with poorer cognitive function and greater risk of MCI/dementia. Future longitudinal studies are warranted to examine whether altered complexity of daily activity rhythms may act as a preclinical indicator of ADRD.

Abstract citation ID: igad104.1297

### THE RELATIONSHIP BETWEEN 10-YEAR CHANGES IN COGNITIVE ABILITY AND SUBSEQUENT MORTALITY: FINDINGS FROM THE ACTIVE

Diefei Chen[1], Alden Gross[2], Sherry Willis[3], and George Rebok[4], *1. Johns Hopkins, Baltimore, Maryland, United States*, *2. Johns Hopkins University Bloomberg School of Public Health, Baltimore, Maryland, United States*, *3. University of Washington, Seattle, Washington, United States*, *4. Johns Hopkins University, Baltimore, Maryland, United States*

Cognitive ability and cognitive decline have been linked with mortality in older adults. However, little was understood about the role of cognitive interventions on mortality outcomes in this population. Using twenty-year follow-up data from the Advanced Cognitive Training for Independent and Vital Elderly (ACTIVE) trial, we examined the association between cognitive change and mortality risk, and the effect of ACTIVE cognitive training (memory, reasoning, and speed of processing) on mortality risk. Mortality was ascertained through linkage to the National Death Index database. To model time to death as a function of cognitive change and training effect, we used shared growth-survival models with simultaneously estimated latent intercepts and slopes as predictors. Among the 2802 participants, 2021 died on or before the year 2019 (72.1%). Both higher baseline level and slower decline in global cognition were associated with lower hazards of all-cause mortality after adjusting for covariates (HR = 0.68, 95% CI 0.58, 0.79; HR = 0.42, 95% CI 0.40, 0.44, respectively). We did not observe any significant effects of ACTIVE cognitive training in memory, reasoning, or speed of processing on all-cause mortality. Our findings demonstrated the association between the trajectory of cognitive change and mortality among older adults, independent of cognitive training interventions. More work is needed to identify relevant timing as well as modalities of non-pharmaceutical interventions that can promote healthy longevity.

### SESSION 3515 (PAPER)

### SOCIAL AND TECHNOLOGICAL SUPPORTS FOR AGING

Abstract citation ID: igad104.1298

### AGE AND COHORT TRENDS OF RACIAL/ETHNIC DIFFERENCE IN RELIGIOUS PARTICIPATION AMONG MIDDLE-AGED AND OLDER AMERICANS

Jingwen Liu, *University of Maryland, College Park, College Park, Maryland, United States*

Religious involvement is an important way of maintaining social connectedness for older Americans. While large quantities of studies have explored age and cohort effects of religious participation separately, less is known about racial/ethnic disparities. Applying growth curve modeling to the 2004-2020 waves of the Health and Retirement Study (N=134,734 person-years), the current study examines how religious attendance changes across the life course and among recent birth cohorts, as well as how exposure to immigration policy regimes (IPR) shapes Hispanic immigrants' religious participation behaviors. Results suggest an overall reversed U-shape age trajectory with lower average levels and faster declines among more recent cohorts. Compared to White older adults, Black and Hispanic populations attend religious activities more frequently, but their attendance declines at faster rates in later life, leading to minimal White-Nonwhite differences in the oldest ages. However, Hispanic immigrants display significantly less decline in religious participation among more recent cohorts relative to Whites, although no similar trend is found between White and Black/native Hispanic older adults. Further analyses of Mexican Hispanic immigrants suggest that longer exposure to restricted IPR (since the Immigration Reform and Control Act of 1986, IRCA) is associated with increased religious attendance, but less restricted IPR (between 1964 and 1985) is associated with decreased religious participation net of age and cohort effects. These findings emphasize the importance of understanding the civic engagement experiences of Hispanic immigrants against the context of immigration policy regimes.

Abstract citation ID: igad104.1299

### FUNSAT: A FUNCTIONAL SKILLS TRAINING PROGRAM

Sara Czaja[1], Philip Harvey[2], and Peter Kallestrup[3], *1. Weill Cornell Medicine, New York City, New York, United States, 2. University of Miami Miller School of Medicine, Miami, Florida, United States, 3. i-Function, Miami, Florida, United States*

Performance of everyday activities is often challenging for older adults, especially for aging adults with a cognitive impairment (CI), which threatens independence and quality of life. We developed a novel computer-based functional skills assessment and training (FUNSAT) program, which includes simulations of everyday technology-based tasks (e.g., online banking, prescription refill, online shopping) important to everyday living. This presentation will present preliminary findings from our study that is evaluating the FUNSAT program with aging adults with and without a cognitive impairment. The sample includes 153 adults (83 with a CI, 130 females and 23 males) ranging in age from 60-92 yrs. Initially, participants completed the FUNSAT assessment and then engaged in the FUNSAT training program for up to 24 hours over 12 weeks at home after an initial in-person training. The non-cognitively impaired participants completed the FUNSAT training alone. The participants with a cognitive impairment were randomized into one of two conditions; FUNSAT training alone, or Cognitive Training (3 weeks) followed by FUNSAT training. We also evaluated transfer of training using ecological momentary assessment (EMA). Our preliminary findings indicate that participants irrespective of cognitive status are demonstrating significant gains in performance following training on the training tasks. Further, they are reporting an increase in confidence with respect to performing the tasks in daily living and the EMA data suggest transfer of training to other technology-based tasks. We will also discuss adherence to the home training protocol, which has important implications for implementation of the program beyond the research setting.

Abstract citation ID: igad104.1300

### INTENTION TO USE WEARABLE ROBOTS FOR INDEPENDENT LIVING : THE ROLE OF CULTURE AND PERSUASIVE HEALTH COMMUNICATION

Clio Yuen Man Cheng, and Vivian Weiqun Lou, *The University of Hong Kong, Hong Kong, Hong Kong*

Soft wearable robots are emerging assistive technologies that can prolong independent living among older adults experiencing progressive muscle loss, such as sarcopenia. In order to fulfil the wish of aging-in-place and relieve caregiver burden, it is essential to increase wearable robots acceptance through persuasive health communication strategies. This study draws on the integration of the senior technology acceptance model, health communication, and cultural value orientation to predict the intention to use wearable robots among older adults. A 2 (message framing: gain- versus loss-framed) x 2 (temporal framing: distal- versus proximal-framed) randomized experiment was conducted with 154 older adults aged 50 to 74 years, using a self-administered cross-sectional from December 2022 to March 2023 in Hong Kong. A hierarchical multiple regression analysis was conducted with six blocks of variables. Demographic variables including age, gender, and sarcopenia potential were not significantly associated with intention to use. Horizontal individualism is significantly associated with intention to use (p=.011). Also, horizontal collectivism is significantly associated with intention to use (p=.018). Vertical collectivism interacts with persuasive health communication to predict intention to use among older adults (p=.041). The final model (F(12,141) = 3.699, p<.001, R2=.24) which included cultural value orientations showed significant improvement from the first model which only included age and gender. Overall, the final model accounted for 23.9% of the variance. This study is the first to predict the intention to use wearable robots among older adults and provide valuable insights on future policy development to promote aging-in-place, with the aid of wearable robots.

Abstract citation ID: igad104.1301

### USING TIMEBANK TO IMPROVE VOLUNTEERING AMONG OLDER PEOPLE: A QUASI-EXPERIMENTAL STUDY

Shiyu Lu[1], Cheryl Chui[2], and Terry Lum[2], *1. City University of Hong Kong, Kowloon, Hong Kong, 2. The University of Hong Kong, Hong Kong, Hong Kong*

Timebanking, a system in which people earn time credits through volunteering and exchange earned credits for goods and services, can potentially promote late-life volunteering. However, evidence to support this innovation remains limited. This paper reports the findings of a quasi-experimental study on a timebanking program implemented in six districts in Hong Kong between January 2021 and August 2022. Three districts were assigned as the experimental districts, while others were assigned as the control districts. We recruited 116 older people in the three experimental districts to join the timebank group and 114 older people in three control districts to join the comparison group. Both groups were offered opportunities to volunteer in community elderly centres within their districts (e.g., home visits or phone calls to older people with disabilities). The timebank group could exchange their earned time credit for rewards (e.g., dining coupons and cookies offered by social enterprises), while no reward was offered for the comparison group. Their volunteering hours were recorded using a tailored mobile phone application, and their volunteering intentions were assessed at baseline, during the timebank intervention (7th month), and post-timebank intervention (13th month). After controlling for covariates (e.g., age, years of volunteering experience) using linear mixed models, the timebank group had significantly more volunteering hours (adjusted mean difference [AMD]=1.37, 95% CI: 0.20-2.54, p=0.021) and higher volunteering intention (AMD=0.51, 95% CI 0.29-0.82, p=0.001) than the comparison group. This is the first quasi-experimental study to provide evidence that timebank is a promising and effective approach to promoting late-life volunteering.

### SESSION 3520 (SYMPOSIUM)

Abstract citation ID: igad104.1302

### THE ROLE OF PSYCHOSOCIAL AND LIFESTYLE FACTORS IN PROMOTING COGNITIVE HEALTH

Chair: Emily Willroth

This symposium will highlight the role of psychosocial and lifestyle factors in cognitive health. Across four talks, we consider a wide range of psychosocial and lifestyle factors including personality, stress, physical activity, and nutrition, and a wide range of cognitive health outcomes including subjective and objective cognitive performance, clinical diagnoses of cognitive status, and cognitive healthspan. First, Dr. Patrick Hill will share findings on links between Big Five personality traits and the degree of concordance between subjective and objective cognitive functioning. Second, Dr. Tomiko Yoneda uses longitudinal data to investigate personality trait predictors of transitions between cognitive states (no cognitive impairment, mild cognitive impairment, dementia) and death, as well as personality effects on cognitive healthspan (i.e., years without cognitive impairment) and total longevity. Third, Nicole Stuart used ecological momentary assessment and activity trackers to investigate links among daily stress, daily physical activity, and daily cognitive performance. Finally, Dr. Julia Sheffler will discuss how we can apply our knowledge of psychosocial and lifestyle factors in cognitive health to inform the development of interventions. She will share quantitative and qualitative findings from proof-of-concept (N=9) and pilot (N=58) studies of a nutrition intervention in older adults at risk for dementia, and she will discuss implications for lifestyle intervention development more broadly. Together, this symposium showcases how a wide range of methods can be employed to reveal the importance of psychosocial and lifestyle factors for promoting healthy cognitive aging.

Abstract citation ID: igad104.1303

### PERSONALITY AND WORD RECALL AS INTERACTIVE PREDICTORS OF PERCEIVED MEMORY ABILITY

Patrick Hill[1], Gabrielle Pfund[2], Patrick Cruitt[3], Isaiah Spears[1], Sara Norton[1], Ryan Bogdan[1], and Thomas Oltmanns[1], *1. Washington University in St. Louis, St. Louis, Missouri, United States*, *2. Northwestern University, Chicago, Illinois, United States*, *3. Minneapolis VA Health Care System, Minneapolis, Minnesota, United States*

Cognitive gerontology research requires consideration of both actual performance and perceptions of performance. While subjective memory perceptions are positively associated with memory performance, these correlations typically are modest in magnitude. Therefore, researchers have noted the need to consider potential moderators of this association, to better understand whether certain people show weaker or stronger linkages between performance and perceptions. The current study leveraged personality (NEO Big Five), memory performance (i.e., word recall), and perceptions of memory ability (i.e., metamemory in adulthood scale and subjective memory complaints) data from the St. Louis Personality and Aging Network (SPAN) study (n = 774, mean age: 71.52 years). Personality traits were more associated with subjective than objective memory, and the most consistent associations were for extraversion (r = -.20 for complaints, r = .26 for metamemory) and conscientiousness (r = -.26 for complaints and r = .33 for metamemory). Moreover, extraversion moderated associations between word recall and both memory capacity and complaints. Results suggested that associations between word recall and the two subjective indicators were weaker for those adults higher in extraversion, relative to adults who scored lower on extraversion. One possible interpretation is that extraverted older adults may construct their cognitive self-perceptions using information from their social interactions more than objective cues. These findings highlight the need to determine how personality traits influence which sources of information older adults employ when thinking about their cognitive ability, which in turn may explain their choices for activity engagement.

Abstract citation ID: igad104.1304

### THE IMPORTANCE OF PERSONALITY TRAITS FOR COGNITIVE HEALTHSPAN

Tomiko Yoneda[1], Tristen Lozinski[2], Eileen Graham[3], Andrea Piccinin[4], and Graciela Muniz-Terrera[5], *1. University of California, Davis, Davis, California, United States*, *2. University of Regina, Regina, British Columbia, Canada*, *3. Northwestern University, Chicago, Illinois, United States*, *4. University of Victoria, Victoria, British Columbia, Canada*, *5. Ohio University, Athens, Ohio, United States*

The existing literature consistently finds that personality traits are associated with dementia and with risk of mortality. However, few studies have simultaneously examined the impact of traits on dementia while accounting for death as a competing risk, which is critical as dementia and mortality are dependent outcomes and the likelihood of each increases alongside older age. In response, data were drawn from the Rush Memory and Aging Project (N=1954; baseline mean age=80 years; 74% female; up to 23 annual assessments). We used multi-state survival models to investigate whether traits (conscientiousness, extraversion, and neuroticism) were associated with transitions between cognitive status categories (No Cognitive Impairment, NCI; Mild Cognitive Impairment, MCI; dementia) and death. Further, multinomial regression models estimated cognitive healthspan (i.e., years without cognitive impairment) and total longevity. Adjusting for demographics, depressive symptoms, and APOEε4, personality traits were most important in the transition from NCI to MCI; higher conscientiousness was associated with a ~22% decreased risk of transitioning from NCI to MCI (HR=0.78, 95%CI=0.72, 0.85), whereas higher neuroticism was associated with a ~12% increased risk of transitioning from NCI to MCI (HR=1.12, 95%CI=1.04, 1.21). Life expectancy analyses suggested that participants who were higher in conscientiousness were estimated to live longer without cognitive impairment compared to individuals lower in conscientiousness. Findings were similar, though less pronounced, for individuals higher in extraversion or lower in neuroticism. Importantly, none of the traits appeared to be important for total longevity. Together, these results highlight the importance of personality traits, particularly conscientiousness, for maximizing cognitive healthspan.

Abstract citation ID: igad104.1305

### DAILY STRESS AND SAME-DAY SUBJECTIVE MEMORY: AGE DIFFERENCES AND PHYSICAL ACTIVITY AS A BUFFER

Nicole Stuart[1], Jin Wen[1], Patrick Klaiber[2], Anita DeLongis[1], Eli Puterman[1], and Nancy Sin[1], *1. University of British*

*Columbia, Vancouver, British Columbia, Canada*, 2. *Tilburg University, Tilburg, Noord-Brabant, Netherlands*

Growing research indicates that daily stress is associated with poor same-day memory performance, but it is unclear whether this relationship varies by age and whether moderate-to-vigorous physical activity (MPVA) might offset the within-person link between stress and memory in daily life. Ecological momentary assessment data were collected from adults aged 25-88 across British Columbia, Canada. For 14 days, participants (N = 249) wore a tri-axial physical activity monitor, reported stressor occurrence and perceived stressfulness of the events in mobile surveys 4 times per day, and rated their memory at the end of each day. Multilevel modeling was run to evaluate daily perceived stressfulness as a predictor of subjective memory, with same-day MVPA engagement (activity counts >570 counts/60s) and age (years) entered as moderators. At the within-person level, on days when stressors were perceived as more stressful than usual, subjective memory was rated as relatively worse (b = -0.037, CI[-0.061, -0.017], p < .001). MVPA did not buffer against the within-person associations of daily stress with subjective memory, nor did age (p > .05). Future work could examine the mechanisms that might explain the link between stress and memory, for example cortisol, as well as the associations of different intensity and forms of physical activity on stress across age groups.

Abstract citation ID: igad104.1306

### LESSONS LEARNED DEVELOPING A NUTRITIONAL ADHERENCE INTERVENTION FOR DEMENTIA PREVENTION: A QUALITATIVE ANALYSIS

Julia Sheffler[1], Juhi Patel[2], Dimitris Kiosses[3], and Sylvie Naar[2], *1. Florida State University College of Medicine, Tallahassee, Florida, United States*, *2. Florida State University, Tallahassee, Florida, United States*, *3. Weill Cornell Medicine, New York City, New York, United States*

Lifestyle interventions show considerable promise in delaying the onset and reducing risk for Alzheimer's disease and related dementias (ADRD). Unfortunately, scalable interventions that address issues of long-term adherence have not been adequately developed or disseminated. Especially important for this effort is iterative evaluation and adaptation of interventions using participant feedback throughout the development process. Using the NIH ORBIT model for behavioral intervention development, our team has established the proof-of-concept (N=9) and pilot tested (N=58) a Mediterranean ketogenic nutrition (MKN) adherence program for older adults at risk for ADRD. This program incorporates motivational interviewing strategies and cognitive behavioral skills training to enhance uptake and long-term adherence to MKN. For each of these pilots, participants completed exit interviews at the conclusion of the 6-week, group intervention and 3-months post-intervention. Qualitative and quantitative data on the acceptability of the program, as well as detailed participant feedback about their experience and recommendations for improvements were collected. While quantitative data demonstrate high acceptability, thematic analysis of qualitative interviews demonstrate a range of recommendations for enhancing and refining important components of the intervention that will be necessary for future scaling and dissemination. We discuss important themes, future directions of this work, and implications for lifestyle intervention development more broadly.

### SESSION 3525 (SYMPOSIUM)

Abstract citation ID: igad104.1307

### EXAMPLES OF ENGAGING HARD-TO-REACH POPULATIONS IN CLINICAL RESEARCH

Chair: Evan Plys
Discussant: Melissa Gerald

Individuals, communities, and settings that are systematically and historically under-represented in research are sometimes referred to as "hard-to-reach" populations. Investigators often face additional challenges to engaging hard-to-reach populations, as research commonly occurs in settings or situations where clinical science has not historically taken place. The perspectives of hard-to-reach participants are vital for moving toward health equity. Yet, investigators must be equipped with specific strategies and tools to conduct high-quality clinical research with hard-to-reach populations. In this symposium, a diverse panel of speakers will present examples of research with various hard-to-reach patient populations and settings. The first discusses research targeting socioeconomically disadvantaged short-stay skilled nursing residents and their care-partners. This presentation highlights challenges and solutions for partnership formation, budgeting, recruitment, and engaging community advisors in skilled nursing facilities. The second centers on research with Hispanic families caring for an elder living with dementia. This presentation highlights challenges and solutions for recruitment as well as language translation and cultural adaptation of study materials. The third discusses research with incarcerated older adults and those receiving treatment from outpatient substance use disorder clinics. This presentation highlights challenges and solutions for recruitment, assessment, and adapting protocols in these settings. The final discusses research with adults receiving Hospice care for advanced cancer. This presentation highlights practical, ethical, and cultural considerations for engaging participants in their final months of life. We conclude with a broader discussion of approaching clinical research with hard-to-reach populations, with the ultimate goal of inspiring investigators to enhance the reach of their science.

Abstract citation ID: igad104.1308

### BUILDING RESILIENCE IN SKILLED NURSING (BRISK): A CASE OF COMMUNITY-ENGAGED RESEARCH IN SKILLED NURSING FACILITIES

Evan Plys[1], Christopher Lyons[2], and Ana-Maria Vranceanu[2], *1. Massachusetts General Hospital/ Harvard Medical School, Boston, Massachusetts, United States*, *2. Massachusetts General Hospital, Boston, Massachusetts, United States*

Older adults receiving post-acute rehabilitation care in a skilled nursing facility (SNF) commonly present with high physical, psychological, and social needs. Psychosocial stressors and associated consequences (e.g., poor transitions, re-hospitalizations) may be magnified among families with limited financial resources. The current study aimed to co-develop a dyadic psychological intervention tailored to meet the unique psychosocial needs of socioeconomically disadvantaged patients and their care-partners during the transition from SNF to home. In this presentation, we describe the process of partnering with SNFs and forming a community advisory board (CAB) to meet the objectives of the current study. First, we discuss practical and regulatory considerations related to identifying facility partners (e.g., facility chains, physician groups) and recruiting patient-care-partner dyads in partner facilities. Second, we highlight strategies to incentivize potential SNF partners as well as historically and systematically under-represented patient participants, including budgetary considerations, recruitment strategies, sustained contact after discharge, and clinical service provision. Third, we discuss the recruitment, formation, and role of a CAB with diverse perspectives, including patients, family care-partners, SNF staff, policy makers and advocates. Overall, this presentation will generate discussion on practical strategies for building and maintaining mutually beneficial research partnerships with SNFs and similar hard-to-reach healthcare settings (e.g., long-term care facilities, home health agencies).

Abstract citation ID: igad104.1309

### CHALLENGES AND OPPORTUNITIES FOR RECRUITMENT AND RETENTION OF OLDER HISPANICS FOR CLINICAL RESEARCH

Rafael Samper-Ternent, *UTHealth Houston, Houston, Texas, United States*

Older Hispanics represented 9% of the population over 65 years of age in the USA in 2019. That number is expected to increase to 21% by 2060. Representation of older Hispanics in clinical research is not proportional to the projected increase of older Hispanics. I will discuss the lessons learned on recruitment and retention of older Hispanics and their family caregivers from a large multi-site pragmatic clinical trial I am a part of and a small pilot project I am co-leading. The pragmatic trial compares a health-system-based approach and a community-based approach to dementia care. For this pragmatic trial, 2,176 dyads of persons living with dementia and their family caregivers were recruited. More than 20% of participating dyads belong to underrepresented racial and ethnic groups and 8.8% identified as Hispanic. The pilot project tests the cultural adaptation of the Patient Priorities Care (PPC) approach for older Hispanics. For the pilot project, we recruited 5 older Hispanics with multiple chronic conditions (MCC) and 20 Hispanics with MCC and dementia. I will describe the recruitment strategies used in each study, the barriers we faced, and the approaches we think worked best for the recruitment of Hispanics. I will also reflect on the challenges we currently face to conduct pragmatic trials that include older Hispanics and the opportunities for improvement.

Abstract citation ID: igad104.1310

### RECRUITING SAMPLES OF OLDER ADULTS FROM VULNERABLE GROUPS

Lisa Barry, *UConn Center on Aging, Farmington, Connecticut, United States*

Since 2000, the number of incarcerated persons age ≥50 in the U.S. grew 280% and those in this age group with substance use disorders (SUDs) grew from 1.7 to 5.7 million. Yet, these groups, which comprise some of the most medically vulnerable persons in society, are difficult to recruit to research studies. We successfully recruited and retained 171 incarcerated persons age ≥50 in a cohort study. We will discuss how recruitment required strong partnership with the state corrections system, how we had to be cognizant of measures that were meaningful in the context of the unique environment (prison activities of daily living), and how measures were modified (when life gets hard I just want to escape). As we continue to recruit persons ≥50 seeking SUD treatment, we will discuss the usefulness of snowball recruiting and balancing decisions regarding modification of inclusion criteria while maintaining integrity of the original research question.

Abstract citation ID: igad104.1311

### TECHNIQUES FOR OVERCOMING CHALLENGES TO RESEARCH WITH INDIVIDUALS NEARING THE END OF LIFE

Ellis Dillon[1], Meghan Martinez[2], Martina Li[2], Alyssa Hernandez[2], Su-Ying Liang[2], and Manali Patel[3], *1. UConn Health, Farmington, Connecticut, United States*, *2. Sutter Health, Palo Alto, California, United States*, *3. Stanford University, Stanford, California, United States*

People living with advanced disease and nearing the end of their lives are often omitted in research on healthcare and the lifespan. This omission occurs for practical, ethical, and cultural reasons. Drawing on examples from studies on individuals receiving home hospice care, and individuals with advanced cancer and their caregivers, we will review (1) practical, ethical, and cultural challenges to doing this research, (2) techniques to increase access, recruitment, and participation through developing partnerships, flexibility and resource allocation, and (3) research team training and support. Practical challenges include the unpredictability of illness and symptoms leading to cancellations and increased attrition, and increased time and resources needed to recruit, consent, and travel to community residences. Ethical and cultural challenges include concerns about "vulnerability," and stigma around directly discussing dying. Techniques to facilitate successful inclusion of these populations include developing partnerships with community organizations, clinical teams, and patient or family advocates, justifying and allocating sufficient resources and flexibility in study design and word choices to reflect the spectrum of understanding and experiences, and additional training and support for research staff. Other techniques to include this population include use of secondary data, reconsidering research exclusion criteria, and surveying/interviewing family members and other caregivers. Hard to reach populations are often overlooked, yet they are vital to ensuring full representation of lived experience. Science benefits from developing strategies to include these populations in research.

### SESSION 3530 (SYMPOSIUM)

Abstract citation ID: igad104.1312

### BEST PRACTICES FOR STUDYING OLDER MIGRANTS IN CONTEXT

Chair: Ashley Ritter Co-Chair: Allen Glicksman Discussant: Robert Schrauf

Understanding health outcomes of the older migrant (ages 60+) requires insight into the perceptions of the older person and considering the impact of the environment in which the older person lives. The four papers on this panel address these issues, each using a different methodology. Bilecen's study, using qualitative methods, examines the impact of the wording of questions on the ways in which older Turkish migrants in the Netherlands discuss loneliness. The paper helps us understand both perceptions of older migrants and the influence of methodology used on the types of responses collected. Diederich's paper on Cuban migrants to the United States uses census data to investigate the impact of changes in the environment of arrival at different points in time on care arrangements later in life. She demonstrates the value and limitations of using this type of data. Torres uses a survey of the scientific literature to ask questions about the impact of bigotry on the well-being of older immigrants and whether lack of attention to this topic means there is an important missing piece in our understanding of the experience of the older migrant. Glicksman et. al. use clinical data to describe interactions with the formal health and social service systems by persons of limited English proficiency, an opportunity to understand both actual service use and the benefits and limitations of using clinical data for this purpose. Schrauf's response will discuss both the findings and the relation of methods to goals in the study of older migrants. This is an International Aging and Migration Interest Group Sponsored Symposium.

Abstract citation ID: igad104.1313

### "GOD ALONE EXISTS IN SOLITUDE": INTERVIEWING OLD MIGRANTS ABOUT LONELINESS

Basak Bilecen, *University of Groningen, Groningen, Groningen, Netherlands*

Current research emphasizes the need for a more critical reflective approach to studying older migrants, with particular attention paid to methodological and ethical challenges during fieldwork. One of the key methodological challenges researchers face is selecting appropriate questions, as the questions asked can influence the insights gained into the phenomenon under study. This is because researchers gain an understanding of the phenomenon based on the questions they ask. Previous research on the well-being of older migrants has primarily focused on their family relationships, health status, social participation into activities, and vulnerabilities. However, an important question to understand the well-being of older migrants is whether and to what extent they experience loneliness. This study involved conducting 20 semi-structured interviews with first-generation Turkish migrants over the age of 65 living in northern Netherlands. The findings suggest that the phrasing of questions plays a significant role in both how participants interpret the questions and their responses. During the interviews, while loneliness was generally considered a negative issue, some participants approached it from a religious perspective, leading to reflection on more fundamental existential questions, which in turn led to denial.

Abstract citation ID: igad104.1314

### IMMIGRANT EXPERIENCES AND CARE ARRANGEMENTS LATER IN LIFE: EVIDENCE FROM THE MARIEL BOATLIFT

Freya Diederich, *University of Bremen, Bremen, Bremen, Germany*

The role of public policy in older immigrants' use of and access to services has mainly been regarded with respect to its legal framework. However, the way public policies are framed and societies' perceptions of immigrant groups can additionally influence immigrants' experiences, with long-lasting effects on needs and services use later in life. Still, the importance of these mechanisms has rarely been analyzed. This study uses the Mariel boatlift in 1980 as a case study to assess the relationship between circumstances that surround an immigrants' cohort and their care arrangement later in life. Cuban immigrants from the Mariel boatlift were perceived in a different way by the federal government and the general population than Cuban immigrants who arrived previously. Based on data from the American Community Survey (2008-2021), care arrangements of older Cubans who arrived in 1980, who are limited in daily activities, and who reside in the Miami area are compared to care arrangements of Cubans that immigrated before. Regression models are estimated and individual characteristics are accounted for. Compared to Cubans who arrived before 1980, Cubans of the Mariel boatlift are more likely to live in the community than in an institutional setting. However, among those living in the community, Cubans of the Mariel boatlift reveal a higher risk of living alone. The results suggest that Cubans of the Mariel boatlift are at higher risk for unmet needs. It is discussed how circumstances that surround immigrants' cohort of arrival shape immigrants' services needs and use.

Abstract citation ID: igad104.1315

### RACISM IN RESEARCH ON OLDER MIGRANTS AND ETHNO-RACIAL MINORITIES: INSIGHTS FROM TWO SCOPING REVIEWS

Sandra Torres, *Uppsala University, Uppsala, Uppsala Lan, Sweden*

The study of ethno-racialized minorities used to be primarily the domain of American social gerontologists. Meta-analyses of this scholarship have shown that this research has so far been characterized by two sets of practices: it engages in racialization but vaguely acknowledges racism, and it seems inequality-obsessed but justice-oblivious. Some gerontologists have therefore argued that the scholarly imagination that informs inquiries on older ethno-racialized minorities needs to be expanded, and that one way to do this could entail taking cues from European migration scholars' inquiries on aging and old age. These scholars place the social position that is migrancy (rather than ethnicity and/or race) at the center of their scholarly imagination. This presentation presents the results of a scoping review of peer-reviewed literature published between 1990-2022 that focuses specifically on how the literature on older migrants engages with racism (since literature on older ethno-racialized minorities was in focus in another scoping review; 1998-2017). The review shows that although scholarship on older migrants recognizes that these axes of oppression could have an impact in their life-courses, empirical inquiries on the ways in which older people experience such oppression are still quite rare. The presentation will juxtapose the results of both scoping reviews in order to argue that our failure to inquire into older migrants', and older ethno-racialized minorities' experiences of racism impedes us from furthering our understanding of how the social positions that are ethnicity, race and migrancy impact (on their own and together) people's experiences of aging and old age.

Abstract citation ID: igad104.1316

### THE USE OF AMBULATORY VERSUS EMERGENCY SERVICES AMONG SENIOR ADULTS WITH LIMITED ENGLISH PROFICIENCY

Allen Glicksman[1], Adam Davey[2], Steven Albert[3], Ari Friedman[4], Philip Lai[5], Lauren Ring[6], Misha Rodriguez[7], and Michael Liebman[8], *1. NewCourtland, Haverford, Pennsylvania, United States*, *2. University of Delaware, Newark, Delaware, United States*, *3. University of Pittsburgh, Pittsburgh, Pennsylvania, United States*, *4. University of Pennsylvania, Philadelphia, Pennsylvania, United States*, *5. Philadelphia Senior Center, Philadelphia, Pennsylvania, United States*, *6. Private, Philadelphia, Pennsylvania, United States*, *7. APM, Philadelphia, Pennsylvania, United States*, *8. IPQ Analytics Inc, Kennett Square, Pennsylvania, United States*

Senior adults who are immigrants with limited English proficiency may have difficulty accessing health and social services. However, identifying patients with LEP in large databases such as insurance claims has historically been challenging. We overcome this with two strategies. First, we conducted focus groups among older Spanish and Chinese speakers. Based on the findings we hypothesize that 1) we can expect distinct patterns of service use associated with a common language and 2) Chinese speakers are more likely than Spanish speakers to use ambulatory services and less likely to use emergency services because Chinese speakers feel more confident in their ability to navigate formal systems. Next, we obtained data on Spanish and Chinese speakers aged 60 and older and living in Philadelphia from a regional health information exchange, including routinely collected data on patients' primary language and English proficiency. We are using the data to test our hypotheses regarding these specific LEP populations. The data will be used as pilot for a study to further investigate attitudes and perceptions of formal care providers in these two LEP communities.

### SESSION 3535 (SYMPOSIUM)

Abstract citation ID: igad104.1317

### DESIGN, DELIVERY, AND EVALUATION OF DYADIC INTERVENTIONS WITH OLDER ADULTS

Chair: Karen Lyons Discussant: Kalisha Bonds Johnson

With increasing evidence of the interpersonal context of health and illness, there is growing interest in interventions that target the needs of the family care dyad. Yet, there are often specific challenges to the design, delivery and evaluation of dyadic interventions that are not often disseminated. This session includes four papers that will discuss the benefits (but also challenges and lessons learned) of four different dyadic interventions targeted at older adults and their care partners in both cognitive impairment and chronic illness contexts. First, Dr. Rashelle Hoffman and colleagues will present results from focus groups with rural MCI care dyads that inform the adaptation of an existing efficacious telerehabilitation physical activity intervention (TPAB). Second, Dr. Glenna Brewster and colleagues will discuss recruitment and delivery challenges, and the benefits of a pilot behavioral sleep intervention for adults with cognitive impairment and their care partners. Third, Dr. Lyndsey Miller and colleagues will report the feasibility, acceptability and preliminary efficacy of a program to support preparation for future care needs and improved well-being in care dyads living with dementia. Finally, Dr. Karen Lyons and colleagues will present findings from a pilot communication-based intervention for couples living with heart failure. The presentation will discuss the individual versus dyadic benefits of the program and challenges related to recruitment and retention. Speakers and Discussant, Dr. Kalisha Bonds Johnson, will share challenges and best-practices in designing, delivering, and evaluating interventions to optimize the health and well-being of the older adult care dyad.

Abstract citation ID: igad104.1318

### ADAPTING A PHYSICAL ACTIVITY INTERVENTION FOR RURAL AREAS: PEOPLE WITH MCI AND CARE PARTNER DYADS

Rashelle Hoffman[1], Shinobu Watanabe-Galloway[2], Catherine Riffin[3], and Joan Monin[4], *1. Creighton University, Omaha, Nebraska, United States*, *2. University of Nebraska Medical Center, Omaha, Nebraska, United States*, *3. Weill Cornell Medicine, New York City, New York, United States*, *4. Yale School of Public Health, New Haven, Connecticut, United States*

Rural populations face an increased risk of Alzheimer's Disease (AD) compared to urban populations. Physical inactivity is a modifiable risk factor of AD and a prevalent issue for people with mild cognitive impairment (MCI) that can be addressed remotely with telerehabilitation. However, rural older adults differ from urban-dwelling as they often view physical activity as physical labor and have unique barriers (e.g., low social support). Involving a care partner for social support and improving physical activity is essential for people with MCI. The telerehabilitation physical activity behavioral (TPAB) intervention involves weekly discussions about self-monitoring physical activity with a FitBit, setting goals, and discussing personal barriers/facilitators and has been shown to be feasible and efficacious to increase daily step count in other older adult populations. Evidence is lacking for the TPAB's use in people with MCI living in rural settings and their care partners (patient + care partner dyads). Results from the first phase of our study include three focus groups (four people per group) from a Nebraska rural community including medical providers, care partners, and older community-dwelling adults to identify ways to adapt the study design and TPAB intervention for rural patient + care partner dyad implementation. Discussion will focus on feasibility (Person-Based Approach) and adaptation (FRAME framework) of the TPAB intervention for implementation in rural communities. The results of this project will lead towards using telehealth technology to overcome detrimental geographical impacts on health (i.e., living in rural communities) to advance the care for patient + care partner dyads.

Abstract citation ID: igad104.1319

### DELIVERING AND EVALUATING A VIRTUAL DYADIC BEHAVIORAL SLEEP INTERVENTION

Glenna Brewster[1], Jordan Pelkmans[2], and Kenneth Hepburn[1], *1. Emory University, Atlanta, Georgia, United States*, *2. Emory University, Nell Hodgson Woodruff School of Nursing, Atlanta, Georgia, United States*

Disturbed sleep is prevalent among persons living with cognitive impairment and dementia and their care partners.

While there are few interventions for care partners to manage their sleep problems there is a paucity of interventions where both the person living with cognitive impairment and their care partner work together to improve their sleep outcomes. The aim of this presentation is to discuss how we recruited participants and delivered and evaluated caregiver and person living with cognitive impairment outcomes of a 4-week videoconferencing delivered cognitive behavioral therapy for insomnia intervention. Recruitment is time intensive but feasible. In general, both members of the dyad reported a reduction in their insomnia scores. Care partners supported the person living with cognitive impairment with completing the diaries. Qualitatively, care partners didn't report much difficulty with completing the diaries. Dyads reported enjoying the relaxation and the pleasant activities. Among those who had pets, it was difficult to follow the instructions regarding not having them in bed. They also had difficulty with the cognitive therapy logs. Most persons living with cognitive impairment did not use the cognitive therapy logs. This pilot study of a 4-session videoconferencing-delivered cognitive behavioral therapy for insomnia intervention resulted in positive outcomes for persons living with cognitive impairment and/or dementia and their care partners and qualitative feedback provided ways to tailor the intervention for a larger study.

Abstract citation ID: igad104.1320

### THE READYR PROGRAM FOR WELL-BEING IN DEMENTIA: PILOT STUDY AND SIX-MONTH FOLLOW-UP RESULTS AMONG SPOUSE–PARTNER DYADS

Lyndsey Miller[1], Joel Steele[2], Shirin Hiatt[1], Jeffrey Kaye[1], and Carol Whitlatch[3], *1. Oregon Health & Science University, Portland, Oregon, United States*, *2. University of North Dakota, Grand Forks, North Dakota, United States*, *3. Benjamin Rose Institute on Aging, Cleveland, Ohio, United States*

The READyR Program assesses and targets everyday routines of care dyads (older adults with cognitive impairment and their co-habiting partners) using in-home sensors and video-conference sessions in order to understand future care needs, and promote greater well-being. This study aimed to evaluate the feasibility, acceptability, and pilot test of efficacy for READyR. Fifteen dyads (96% married) were enrolled in the 6-month study, and randomized to immediately join READyR (n=8 dyads) or to complete READyR after an educational control condition (n=7 dyads). Feasibility was high: 100% of enrolled dyads completed ≥1 session; 80% completed all sessions. Acceptability was also high: responding participants (n=26) reported that READyR helped them express their care values and understand their care needs (92%), that they were comfortable sharing their views (92%) and doing so with their partner present (96%). Hearing and visual impairments were reported to interfere with online sessions for 4 and 6 participants, respectively, but 93% of participants reported that the digital components were easy to use and understand. After receiving the READyR sessions, awareness of future care needs among care partners increased (d = 0.85) and relationship strain for both dyad members decreased (d = 0.58–0.82), when compared to changes among participants in the active control group. No effect was observed on participants' quality of life, mood, or care-related strain at post-test or at 6 month follow-up. As a remote dyadic intervention, READyR is feasible, acceptable, and may help dyads decrease strain in the care relationship and prepare for future care needs.

Abstract citation ID: igad104.1321

### ACCEPTABILITY AND PRELIMINARY EFFICACY OF THE TAKING CARE OF US INTERVENTION FOR COUPLES LIVING WITH HEART FAILURE

Karen Lyons[1], Carol Whitlatch[2], Amanda Vest[3], Jenica Upshaw[3], Stacy Hutton Johnson[4], Anna Walters[1], and Christopher Lee[1], *1. Boston College, Boston, Massachusetts, United States*, *2. Benjamin Rose Institute on Aging, Cleveland, Ohio, United States*, *3. Tufts University School of Medicine, Boston, Massachusetts, United States*, *4. Canopy Consultants Inc., Arlington, Massachusetts, United States*

There are over 1 million hospital admissions and 3 million emergency room visits for heart failure (HF) annually in the USA. Despite significant impact of HF on both members of the care dyad, there are few interventions focused on optimizing the mental and relational health of the dyad. The Taking Care of Us© (TCU) intervention is a communication-based program for the couple living with HF. Sessions focus on facilitating greater shared appraisal, communication, collaborative illness management, confidence, and support between partners. Thirty seven couples were randomized to TCU (18 couples) or an educational control (19 couples). Both programs were delivered virtually over two months. Adults with HF (AwHF) were, on average, 69.9 (SD=9.6) years old and predominantly male (70%). Eighty one percent of couples participated in at least one session (attrition was relatively even across conditions and primarily due to poor health and partner drop-out). Twenty-four couples (65%) completed follow-up surveys. The majority of TCU participants were satisfied with the program (AwHF 82%; care partners 100%) and felt they understood their partner's needs better (AwHF 91%; care partners 91%). TCU care partners felt the relationship had improved (91%) and felt more confident managing HF (91%). Depressive symptoms decreased in TCU AwHF (ES=.60) compared to the active control group. TCU couples also reported increases in collaboration (ES=.50-.70) and communication (ES=.35-.50). Discussion will focus on the individual versus dyadic benefits of the TCU program, recruitment and retention challenges, and qualitative feedback from participants regarding strengths of the program and areas for improvement.

### SESSION 3540 (SYMPOSIUM)

Abstract citation ID: igad104.1322

### ORAL HEALTH INTERVENTIONS AND TREATMENTS FOR OLDER ADULTS WITH COGNITIVE IMPAIRMENT AND DIABETES

Chair: Stephen Shuman Co-Chair: Bei Wu Discussant: Michele Saunders

Older adults with cognitive impairment or diabetes have a higher risk of poor oral health. It is therefore critically important to develop effective intervention strategies to improve oral health for these vulnerable populations. In this session, we have included three studies that aim to achieve this goal using different strategies. The first study used stakeholder engaged processes to develop an app that provides information and guidance to caregivers (i.e., paid and family caregivers) to support their oral care provision for older adults with dementia. Findings from this study show that the app was well-accepted and provided an easily accessible tool for caregivers during care provision. The second study aimed to improve oral hygiene outcomes (plaque index and gingival index) for individuals with mild cognitive impairment (MCI) or mild dementia (MD). In this study, the authors identified and compared active behavior change techniques (BCT) used in a care partner-assisted oral health intervention for individuals with MCI and MD using a qualitative, secondary analysis of coaching sessions with care partners. Findings from this study provide insight into the mechanisms of change in individuals' behaviors using these interventions. The third study was a systematic review aiming to explore the age differences in the effect of multi-component periodontal treatments on oral and metabolic indicators among individuals with periodontitis and diabetes. This study identified 115 eligible trials. This study found that the effect of multi-component periodontal treatments on periodontal outcomes and HbA1c may be more effective among younger populations. This is an Oral Health Interest Group Sponsored Symposium.

Abstract citation ID: igad104.1323

### AGE DIFFERENCES IN THE EFFECTS OF PERIODONTAL TREATMENTS ON ORAL AND METABOLIC HEALTH IN PEOPLE WITH DIABETES

Zheng Zhu, Xiang Qi, Yaguang Zheng, Yaolin Pei, and Bei Wu, *New York University, New York City, New York, United States*

**Background:** This systematic review aims to explore the age differences in the effect of multi-component periodontal treatments on oral and metabolic indicators among individuals with periodontitis and diabetes.

**Methods:** Six databases were searched from database inception to August 2022. Trials reporting the effects of multi-component periodontal treatments on oral and metabolic indicators among participants aged 18 and above with periodontitis and diabetes were included. We used bivariate and multivariate meta-regression models to examine the association between age and treatment effect size. The primary outcomes were changes in probing depth (PD), clinical attachment level (CAL), and hemoglobin A1c (HbA1c).

**Results:** A total of 115 trials (119 articles) met inclusion criteria. We found that multi-component periodontal treatment significantly reduce PD (g=0.929, 95% CI [0.689-1.169], I2=94.1%), CAL (g=0.879, 95% CI [0.669-1.089], I2=92.1%), and HbA1c (g=0.603, 95% CI [0.443-0.763], I2=87.5%). A significant decreasing trend was observed in the effect size for PD (P for trend = 0.020) and CAL (P for trend = 0.028) as age increases. Results from multivariate meta-regression showed that age was associated with smaller effects of PD (β [standard error] = -0.123 [0.041], P=0.004) and CAL (-0.159 [0.055], P=0.006). Compared to their younger counterparts, the effect size for HbA1c was smaller among participants aged 55 and older (-0.792 [0.322], P=0.017).

**Conclusions:** The effect of multi-component periodontal treatments on PD, CAL, and HbA1c may be more effective among younger populations. Our study suggests that tailored interventions are needed to improve oral health and diabetes management for individuals with periodontitis and diabetes.

Abstract citation ID: igad104.1324

### BEHAVIOR CHANGE TECHNIQUES IN PERSONS WITH MCI AND MD AND THEIR CARE PARTNERS: A REPORT OF AN ORAL HEALTH INTERVENTION

Ashley Bryant[1], Youngmin Cho[2], courtney caiola[3], Donald Bailey[4], Brenda Plassman[4], Bei Wu[5], and Ruth Anderson[2], *1. The University of North Carolina at Chapel Hill, Chapel Hill, North Carolina, United States*, *2. University of North Carolina at Chapel Hill, Chapel Hill, North Carolina, United States*, *3. East Carolina University, Greenville, North Carolina, United States*, *4. Duke University, Durham, North Carolina, United States*, *5. New York University, New York City, New York, United States*

Older adults with cognitive impairment are likely to experience a decline in oral health and hygiene, which can have profound negative impacts such as cavities, gum disease and tooth loss. Poor oral health is also linked to adverse health outcomes including diabetes, cardiovascular disease, and aspiration pneumonia. Despite these factors, inadequate oral hygiene practices provided by formal and informal care partners may contribute to poor oral hygiene. In this study, we aimed to improve oral hygiene outcomes (plaque index and gingival index) for individuals with mild cognitive impairment (MCI) or mild dementia (MD) by fostering behavior changes among care partners trained to assist them. We identified and compared active behavior change techniques (BCT) used in a care partner-assisted oral health intervention for individuals with MCI and MD using a qualitative, secondary analysis of coaching sessions with care partners (n=17 dyads: 10 dyads for MCI, 7 dyads for MD). Frequently used BCTs in both MCI and MD groups were prompts and cues, instruction on how to perform the behavior, review behavioral goal, and problem solving. Common BCTs were found across both MCI and MD groups that provide clarity on the active components for behavior change. Social support-unspecified emerged for MCI group and credible source emerged for MD group. In this intervention, BCTs were focused on oral health, however BCTs can assist in addressing multiple types of behavior change. Findings from this study provide insight into the mechanisms of changes in individuals' behaviors using these interventions.

Abstract citation ID: igad104.1325

### A TOOL TO SUPPORT FAMILY AND PAID CAREGIVERS PROVIDE DAILY ORAL CARE TO PERSONS LIVING WITH DEMENTIA

Sato Ashida[1], Tim Beachy[2], Maria Donohoe[1], Haley Schneider[2], Hianca Pinho[1], Haley Wilson[1], and Leonardo Marchini[3], *1. University of Iowa College of Public Health, Iowa City, Iowa, United States*, *2. University of Iowa, Iowa City, Iowa, United States*, *3. Case Western Reserve University, Cleveland, Ohio, United States*

Persons living with dementia often receive daily oral care from family caregivers or paid caregivers such as nurse aides. However, these caregivers report limited knowledge about and confidence in providing daily oral care. This study used stakeholder engaged processes to develop an app that provides information and guidance to caregivers to support their oral care provision. Contents to support oral care for persons living with dementia were developed through a collaboration between the College of Dentistry and College of Public Health based on prior research. In-depth interviews were conducted with key stakeholders including dental professionals, family caregivers, nursing home administrators, and nurse aides. Participants identified many benefits of this support tool to improve their oral care provision. Dental professionals confirmed the accuracy and importance of the information and provided additional suggestions. Family caregivers appreciated the detailed information and guidance on how to provide daily care, how to identify oral health problems, and what to do if they identify problems. Photos especially helped caregivers identify the nature of the problem to determine action steps. For paid caregivers, the app served as a good reminder and reference guide. Nursing home administrators wanted to use the app as a training tool to standardize and improve the overall care. The app was well-accepted and provided an easily accessible tool for caregivers during care provision. Additional qualitative data and pilot test results will be presented.

### SESSION 3545 (SYMPOSIUM)

Abstract citation ID: igad104.1326

### USING INTENSIVE REPEATED MEASUREMENT TO EXAMINE THE AFFECTIVE CONSEQUENCES OF NEGATIVE RELATIONSHIP EXPERIENCES

Chair: Dakota Witzel Discussant: Kelly Cichy

Across midlife and older adulthood, daily relational contexts provide a lens through which daily experiences shape health and well-being. How daily relational contexts inform health and well-being is complex, with buffering and exacerbating effects of the relationship context on well-being reported in past work. One mechanism through which daily relational experiences influence health and well-being is through changes in emotions. The current series of talks examine how daily relational experiences (interpersonal stress, marriage and loneliness, perceived social isolation, caregiving stress) influence emotional well-being using innovative intensive repeated measurement designs. First, using eight days of daily diary data from the National Study of Daily Experiences, Dakota Witzel will consider the protective effects of perceived control on affective reactions to interpersonal daily stressors on the same day and the following day. Second, using a pre-registered, 14-day ecological momentary assessment design, Jin Wen will discuss the potential implications of affective reactions to interpersonal daily stressors on subsequent sleep quality. Third, using 14 days of ecological momentary assessments from the Einstein Aging Study, Karina Van Bogart will explore the moderative influences of marriage and cognitive impairment on the effects of daily emotional loneliness on intrusive thoughts. Finally, using a 14-day daily diary design, Frank Puga will present on the implications of daily caregiver stress and perceived social isolation on perceived emotional control. Kelly Cichy will serve as discussant and summarize the theoretical and methodological contributions of these studies for enhanced understanding of the dynamic interplay between relational experiences and emotional well-being in daily life.

Abstract citation ID: igad104.1327

### DAILY STRESSOR CONTROL AND AFFECTIVE REACTIONS: THE UNIQUE ROLE OF INTERPERSONAL STRESS

Dakota Witzel[1], Eric Cerino[2], Zachary Taylor[2], and David Almeida[3], *1. Penn State University, State College, Pennsylvania, United States*, *2. Northern Arizona University, Flagstaff, Arizona, United States*, *3. The Pennsylvania State University, State College, Pennsylvania, United States*

Perceived control over daily stressors varies across life and stressor domains, but little is known about the function of perceived control as a protective resource for the impact of daily stressors on affective well-being. Using the third wave of the National Study of Daily Experiences (NSDEIII; N=1,263, Mage=62.62, 57.20% women), we examined how stressor control across domains (interpersonal stress, work and home overloads, network stressors) was associated with both negative and positive emotions on the same-day (i.e., reactivity) and next-day (i.e., residue). Over eight consecutive days, participants reported their daily negative and positive emotions, as well as exposure to and control over stressful experiences. After adjusting for age and gender, two-level multilevel models revealed a protective patterning of stressor control for dampened affective reactions to daily stressors driven by stressor control domains specific to interpersonal interactions (ps<.05). Days when people perceived more control over their arguments or avoided arguments were related to less negative and positive affective reactivity (smaller increases/decreases in negative/positive affect, respectively) compared to days with less perceived control. Perceived control over daily work stressors and home stressors were associated with dampened negative and positive affective reactivity, respectively. No associations between perceived stressor control and affective residue were significant. Results underscore the import of perceived stressor control for interpersonal stressors and the utility of perceived control as a protective factor for the effects of arguments and avoided arguments. Individuals may aim to leverage additional resources to feel more in control of their own daily arguments and avoided arguments.

Abstract citation ID: igad104.1328

### TIFFS, TOSSES, AND TURNS: EFFECTS OF AFFECTIVE REACTIVITY TO INTERPERSONAL STRESSORS DURING THE DAY ON NIGHTLY SLEEP

Jin Wen[1], Patrick Klaiber[2], and Nancy Sin[1], *1. University of British Columbia, Vancouver, British Columbia, Canada*, *2. Tilburg University, Tilburg, Noord-Brabant, Netherlands*

**Background:** Sleep has been recognized as an antecedent as well as a consequence of daytime stress. However, less research has compared the role of different stressor types on same-night sleep in the context of daily life. Interpersonal stressors may be particularly important, given that social stressors to elicit greater stress responses than other forms of stressors. This study tested the hypothesis that following days when a person exhibits greater negative affect (NA) reactivity to stressors (versus on days with less NA reactivity), sleep quality will be lower. This link between affective reactivity to stressors and subsequent sleep was expected to be more pronounced for interpersonal stressors versus non-interpersonal stressors. **Methods:** In this pre-registered study, 252 adults in British Columbia, Canada (ages 25 to 87y; 68% women; 64% White) completed mobile surveys 5x/day for 14 days to assess daily stressors and NA. Self-reported sleep quality was assessed in morning surveys. Multilevel-models tested daily stressors (interpersonal, non-interpersonal, or no stressors), daily NA (averaged across the day), and their interaction as predictors of subsequent sleep quality, controlling for prior-night sleep quality and sociodemographics. **Results:** Daily NA and stressor occurrence independently predicted poorer subsequent sleep quality. Stressor type moderated the relationship between NA and sleep quality, such that this association was stronger for interpersonal compared to non-interpersonal stressors. **Discussion:** The findings suggest that encountering interpersonal stressors may be particularly impactful to one's subsequent sleep. Future studies should consider investigating potential mechanisms that may underlie this association, such as pre-sleep cognitive, emotional, and physiological arousal.

Abstract citation ID: igad104.1329
### DAILY FACTORS ASSOCIATED WITH EMOTIONAL DYSREGULATION AMONG DEMENTIA CAREGIVERS
Frank Puga[1], Danny Wang[2], Natashia Bibriescas[3], Abigail Poe[1], Loreli Alvarez[4], Rita Jablonski[1], and David Vance[1], *1. University of Alabama at Birmingham, Birmingham, Alabama, United States*, *2. The Pennsylvania State University, University Park, Pennsylvania, United States*, *3. University of Texas at Austin, Austin, Texas, United States*, *4. The University of Alabama at Birmingham, Birmingham, Alabama, United States*

Family dementia caregivers experience increased burden and stress that can negatively impact emotion regulation and overall well-being. Further, a lack of social connection may contribute to emotion dysregulation among dementia caregivers. The purpose of this study was to examine relationships between daily caregiving stress, social isolation, emotion regulation, and depression in a sample of community-dwelling dementia caregivers. Participants (N=30) completed a baseline survey and a series of daily diaries over a 14-day period (n=323 data points) that asked about day-to-day caregiving experiences. Data were analyzed using mixed-level modeling. Higher than average stress related to behavioral symptoms of dementia was associated with an increase in the odds of experiencing feelings of losing control over one's emotions (OR=1.08, 95% CI [1.01, 1.15], p<0.05). Similarly, higher perceived social isolation on a given day was associated with an increase in the daily odds of feeling a loss of control over one's emotions (OR=1.28, 95% CI [1.00, 1.27], p<0.05). Further, feeling a loss of control over one's emotions was associated with an increase in the daily odds of depression-related symptoms (OR=3.39, 95% CI [1.43, 8.08], p<0.01). These findings highlight the importance of addressing the emotional well-being of family dementia caregivers, particularly in the context of stress related to behavioral symptoms of dementia and social isolation. Caregiver support programs and interventions that focus on improving emotion regulation and reducing social isolation may help promote the mental health and well-being of dementia caregivers.

Abstract citation ID: igad104.1330
### THE ROLE OF MILD COGNITIVE IMPAIRMENT AND MARITAL STATUS ON LONELINESS AND INTRUSIVE THOUGHTS IN DAILY LIFE
Karina Van Bogart[1], Christopher Engeland[2], Dakota Witzel[1], and Jennifer Graham-Engeland[3], *1. Penn State University, State College, Pennsylvania, United States*, *2. Pennsylvania State University, University Park, Pennsylvania, United States*, *3. Pennsylvania State University, State College, Pennsylvania, United States*

Loneliness is associated with maladaptive cognitions (e.g., rumination and intrusive thoughts) among young adults, but less is known about this association among older adults. Experiencing loneliness and intrusive thoughts throughout daily life may disrupt emotion regulation and increase risk for cognitive decline during later life. Moreover, social and cognitive factors, including marital status and mild cognitive impairment (MCI), may be protective or exacerbating factors that moderate these associations. For example, having close relationships (e.g., being married) is associated with better mental health and overall well-being for older adults and intrusive thoughts have been associated with poorer cognitive performance. We used multilevel modeling to examine the association between momentary loneliness and intrusive thoughts among older adults and whether MCI and marital status moderated this association. Participants were 316 diverse older adults without dementia (40% Black; 13% Hispanic, mean age = 77.45 years, 67% women) living in the Bronx, NY who completed ecological momentary assessments 5 times daily for 14 consecutive days. There was a significant three-way interaction ($\beta$ = -0.175, $p < 0.05$) such that the association between momentary loneliness and intrusive thoughts was strongest for those with MCI who were not married and weakest for those with MCI who were married. Covariates included race/ethnicity, years of education, age, and gender. These findings suggest that for those who have MCI, being married may be a protective factor against moments of loneliness and intrusive thoughts. For those who are not married, tailoring interventions to accommodate MCI status may be worthwhile.

### SESSION 3550 (SYMPOSIUM)

Abstract citation ID: igad104.1331
### NOVEL APPROACHES AND OUTCOMES TO INVESTIGATE RECOVERY AND MOBILITY IN HIP FRACTURE PATIENTS
Chair: Carl-Philipp Jansen

Hip fracture is the most frequent non-intentional injury of older persons leading to hospital admission in North America and Europe. Failure to fully recover and regain pre-fracture function and mobility is considered the single most important disability symptom experienced by hip fracture patients; severe detrimental effects on older adults' physical, mental, and psychosocial health are reported. Being able to move back and live independently at home is seen as one of the most important aspects of recovery. However, at present, mere survival and care home admission are still the predominant reported outcomes in the recovery and rehabilitation phase. Only limited attention has been given to outcomes such as days at home or mobility performance in everyday life. At the same time, aspects of mobility recovery grounded in patients' experience have not been addressed appropriately so far. Against this background, this symposium brings together research endeavours from Europe and North America, presenting novel approaches to investigate and disentangle trajectories of hip fracture recovery in older adults. In the first two talks, healthy days at home are investigated as central outcome after hip fracture with two different foci: the contribution of cognitive impairment to sex-based differences in recovery, and pre- vs. post-fracture development. The last two talks investigate mobility in hip fracture patients, again with two foci: results of real-world sensor-based mobility performance are presented and compared against patient-reported outcomes, and a conceptual framework of walking was developed based on a systematic review on patients' walking experience.

Abstract citation ID: igad104.1332

### IMPACT OF SEX AND ADRD ON DAYS SPENT AT HOME AFTER HIP FRACTURE AMONG OLDER MEDICARE BENEFICIARIES

Denise Orwig[1], Heather Mutchie[2], Ann Gruber-Baldini[1], Abree Johnson[2], Jay Magaziner[3], and Jason Falvey[1], *1. University of Maryland School of Medicine, Baltimore, Maryland, United States*, *2. University of Maryland, Baltimore, Maryland, United States*, *3. University of Maryland Baltimore, Baltimore, Maryland, United States*

Men recovering from hip fracture tend to have poorer health and outcomes compared to women. Alzheimer's disease or related dementias (ADRD), which is differentially recognized between the sexes, is one potential factor contributing to these differences. Data were drawn from 2010-2017 Medicare fee-for-service beneficiaries aged 65 years and older who survived hospitalization for hip fracture. Our primary outcome was days alive and at home (DAAH) which subtracts from 365 the number of days in a hospital and/or rehabilitation facility, nursing home, emergency department or hospital observation, and days not alive (index fracture admission day to date of death, if applicable). Multivariable Poisson regressions were adjusted for demographics, injury severity, chronic disease burden, and hospital-level fixed effects, to model the association between DAAH and ADRD in the 12 months post hip fracture, with an interaction term for sex and ADRD status. Among survivors, males with ADRD spent a mean of 160.7 DAAH compared to 228.4 for males without ADRD, 177.8 for females with ADRD and 248.0 for females without ADRD. In adjusted analyses, males without ADRD spent 8.2% fewer DAAH compared to females (rate ratio (RR)=0.92, 95% CI 0.92-0.92). This sex difference increased significantly when comparing those living with ADRD, with males spending an additional 3.3% fewer DAAH (interaction RR=0.96, 95% CI 0.96-0.97). Males spend fewer DAAH after hip fracture than females and males with ADRD have the fewest DAAH. This suggests that cognitive impairment may be a small but significant contributor to sex-based differences observed during hip fracture recovery.

Abstract citation ID: igad104.1333

### DAYS SPENT AT HOME BEFORE AND AFTER HIP FRACTURE AMONG MEDICARE BENEFICIARIES LIVING WITH DEMENTIA: A COHORT STUDY

Jason Falvey[1], Chixiang Chen[2], Abree Johnson[2], Kathleen Ryan[2], Michelle Shardell[1], Haoyu Ren[3], Lisa Reider[4], and Jay Magaziner[5], *1. University of Maryland School of Medicine, Baltimore, Maryland, United States*, *2. University of Maryland, Baltimore, Maryland, United States*, *3. University of Maryland Baltimore County, Baltimore, Maryland, United States*, *4. Johns Hopkins Bloomberg School of Public Health, Baltimore, Maryland, United States*, *5. University of Maryland Baltimore, Baltimore, Maryland, United States*

Hip fracture is a disabling event experienced disproportionately by older adults with Alzheimer's disease or related dementias (ADRD). Understanding how to better prognosticate outcomes for this population could drive more tailored clinical decision making. Our objective was to identify distinct trajectories of health before a hip fracture and determine their influences on post-fracture recovery trajectories and 1-year mortality rates among older adults with Alzheimer's disease or related dementias (ADRD). We conducted a cohort study of 16,576 community-dwelling Medicare beneficiaries living with ADRD who experienced hip fracture between 2010 and 2017. We used latent mixture modeling to evaluate trajectories of days at home assessed monthly up to 180 days prior to hospitalization, and their associations with post-fracture days at home and 1-year mortality. Before a hip fracture, 3 distinct trajectories were identified: Robust (n=14,980, 90.3%), Impaired but Improving (n=809, 5.3%), and Impaired and Declining (n=787, 4.7%). Membership in the Impaired and Declining pre-fracture trajectory was strongly associated with membership in less favorable post-fracture recovery trajectories and 65% higher 1-year mortality rate (hazard ratio 1.65, 95% confidence interval 1.45-1.87) as compared to those in the Robust trajectory group. Similar albeit weaker associations were observed between membership in the Impaired but Improving pre-fracture trajectory and post-fracture outcomes. Overall, our results indicate that pre-fracture days at home trajectories are highly associated with post-hospitalization outcomes among older adults with ADRD and could be used to guide care management decisions.

Abstract citation ID: igad104.1334

### BEYOND STEP COUNTING: SENSOR-BASED EVALUATION OF HIP FRACTURE PATIENTS' MOBILITY IN THE REAL WORLD

Carl-Philipp Jansen[1], Jochen Klenk[1], Hubert Blain[2], Beatrix Vereijken[3], Jorunn Helbostad[3], and Clemens Becker[1], *1. Robert-Bosch-Hospital Stuttgart, Stuttgart, Baden-Wurttemberg, Germany*, *2. Centre Hospitalier Universitaire de Montpellier, Montpellier,*

*Aquitaine, France*, *3. Norwegian University of Science and Technology, Trondheim, Nord-Trondelag, Norway*

After a hip fracture, rehabilitation starts with the primary aim to get older persons back on their feet and mobile again. Multidisciplinary hospital treatment is suggested as the first essential step for optimization of care immediately after the fracture, followed by subacute exercise interventions to further improve mobility. However, reported outcomes of rehabilitation vary widely geographically and within regions and most often consist of repeated physical capacity measures without knowledge of the actual mobility behaviour of patients after hospital care. Evidence emerges that small changes in physical capacity (e.g., gait speed) coincide with larger changes in physical activity (e.g., number of steps) during the first six months post-fracture. In a pooled longitudinal analysis of n=785 hip fracture patients (mean age: 83.4 years, SD=6.1) from Germany and Norway, we found daily walking duration trajectories to increase until week 28 post surgery, and that 50% of the peak mobility was achieved within the first four weeks. Still, in order to better understand the underlying mechanisms of mobility performance and to improve rehabilitation measures, in-depth data on patients' real-world mobility beyond walking volume is needed. Therefore, in the Mobilise-D project, a novel sensor algorithm is used to quantify patients' real-world mobility with higher granularity and more extensive information. Baseline data on physical capacity measures, sensor-based mobility outcomes, and patient-reported outcomes of mobility and function of n= 454 (mean age: 78.1 years, SD=9.2) hip fracture patients is presented.

Abstract citation ID: igad104.1335

### LISTENING TO THE PATIENTS' VOICE: A CONCEPTUAL FRAMEWORK OF HIP FRACTURE PATIENTS' WALKING EXPERIENCE

Laura Delgado-Ortiz[1], Ashley Polhemus[2], Alison Keogh[3], Lynn Rochester[4], Anja Frei[2], Milo Puhan[2], and Judith Garcia-Aymerich[5], *1. ISGlobal, Barcelona, Catalonia, Spain*, *2. Zürich University, Zürich, Zurich, Switzerland*, *3. University College Dublin, Dublin, Dublin, Ireland*, *4. Newcastle University, Newcastle upon Tyne, England, United Kingdom*, *5. IS Global, Barcelona, Catalonia, Spain*

Walking is crucial for an active and healthy ageing, but it changes with age and in the presence of diverse health conditions, including hip fracture and frailty. So far, conceptual frameworks of walking have not included the impact of these conditions and individuals' lived experiences on their walking. Thus, we aimed to identify and synthesize evidence describing the walking experience from the perspective of individuals recovering from a hip fracture, as well as from the perspective of individuals living with highly prevalent walking-impairing conditions of diverse etiology. We conducted a systematic review and meta-ethnography, following appropriate guidance. Out of 2,552 unique records, 117 (8.5% hip fracture) were deemed eligible for the meta-ethnography. We identified seven themes that explain the experience of walking: (1) becoming aware of the walking experience, (2) the walking experience as a link between individuals' activities and sense of self, (3) the physical walking experience, (4) the mental and emotional walking experience, (5) the social walking experience, (6) the context of the walking experience, and (7) behavioral and attitudinal adaptations resulting from the walking experience. We proposed a framework that describes the interplay between these themes, providing a conceptualization of walking that is grounded in the experiences of individuals recovering from a hip fracture or living with other walking-impairing health conditions, and that may be used to set priorities and improve patient centricity in clinical practice, research and public health initiatives.

### SESSION 3555 (SYMPOSIUM)

Abstract citation ID: igad104.1336

### GETTING BETTER OR GETTING WORSE: MARKERS OF LONGEVITY ACROSS COHORTS AND COUNTRIES

Chair: Peter Martin Co-Chair: Yasuyuki Gondo Discussant: Oscar Ribeiro

The expansion of life expectancy and the lengthening of the human life span are extraordinary achievements observed over the last century. Among those accomplishments is the increasing number of centenarians in many countries. However, it is not clear whether new cohorts of centenarians are in better health when compared to earlier born cohorts of centenarians. The purpose of this symposium is to compare centenarian cohorts across different countries. Two centenarian cohorts each from Hong Kong, Japan, and the United States are included in the analyses. Results provide different perspectives across countries. The Hong Kong Centenarian Studies compared current centenarians to those who participated at an earlier time and noted that proxies in the second study rated centenarians in worse health when compared to centenarian self-ratings. The Kyotango study assessed living conditions, care need level, ADL, IADL, and cognitive function by gender and noted cognitive improvement for men. Finally, later cohorts of the U.S. Health and Retirement Study were in worse health when compared to the earlier born cohort. The results provide an indication for policy makers and practitioners who will prepare for a larger number of centenarians in need of additional support. Although some improvement for later born cohorts is noticeable, it is obvious that more care provision will also be needed as centenarians around the world will survive with more health conditions. Additional research should evaluate centenarians in other parts of the world.

Abstract citation ID: igad104.1337

### INTRODUCING CENTENARIAN STUDIES IN AN AGING WORLD

Peter Martin, *Iowa State University, Ames, Iowa, United States*

Centenarian studies have come of age. With the first comprehensive centenarian studies conducted more than 35 years ago, there are now centenarian databases in the United States, in Europe, and in Asian countries. The major focus of these studies has been on genetic and family longevity factors, on support systems, personality, and on health behaviors. Another emphasis has been on various health components, such as physical health, mental health, frailty, and psychological well-being. In recent years, a second generation of centenarian studies has been initiated, comparing earlier born cohorts with later born cohorts. Among the first studies conducting a cohort comparison, the Danish centenarian studies, the Georgia centenarian studies, and the Tokyo centenarian studies showed inconsistent results. Whereas some studies indicated that later born cohorts enjoyed higher levels of functioning (e.g., Denmark, U.S. Georgia), other studies showed poorer performance (e.g., Japan). The current symposium sheds additional light on cohort comparisons with recently collected data from the second Hong Kong Centenarian Study, the Kyotango centenarian cohort, and the Health and Retirement Study. This presentation summarizes major findings from centenarian studies conducted around the world and highlights activities of the International Consortium of Centenarian Studies (ICC).

Abstract citation ID: igad104.1338

### IMPROVING COGNITIVE FUNCTION IN THE NEW COHORT—BUT ONLY FOR MEN. FINDINGS FROM THE KYOTANGO CENTENARIAN STUDY

Yasuyuki Gondo, and Xinyu Zhang, *Osaka University, Suita, Osaka, Japan*

The Kyotango Centenarian Cohort Study was established with the aim of tracing long-term changes in the overall functioning of centenarians. This study started in 2014 in Kyotango City. Since then, new centenarians and those who were expected to be 100 years old the following April were invited to the study every year in September. Eligible centenarians were invited by the city, and centenarians and their families agreed to participate to answer questions either by mail or face-to-face interviews, according to their preference. For the non-participants, anonymized living conditions and care need levels were provided by the city. The number of centenarian candidates varied by year (24-55). However, the number of woman centenarians had increased through the 8-year surveyed period (31 and 42 for women, 10 and 9 for men in 2014 and 2021). On average, every year 40 new centenarians were invited and 80% of them participated in the survey. Among the functional variables, cognitive function was estimated by proxies, which had a high correlation with MMSE. No clear improving trend for nursing care level in Japanese long-term care insurance, as measured by activities of daily living and cognitive function rating, was observed. Meanwhile, an improving trend for cognitive function was observed among men but not among women. This might be caused by the gender difference in the survival ratio that frail women were more likely to survive. Future research should investigate possible reasons for a cohort improvement in Japanese centenarian men, and cognitive intervention programs should particularly target women.

Abstract citation ID: igad104.1339

### PROFILES OF TWO COHORTS OF NEAR-CENTENARIANS AND CENTENARIANS: FINDINGS FROM HONG KONG CENTENARIAN STUDY 1 AND 2

Bobo Hi-po Lau, *Hong Kong Shue Yan University, Hong Kong, Hong Kong*

Hong Kong launched its first centenarian study in 2011 when there were just over 3,600 centenarians, and subsequently, the second phase in 2021/22 when the population of centenarians tripled. Both studies investigated the multidimensional well-being of local older adults of Chinese ethnicity aged 95 or above (near centenarians and centenarians, NCCs). A comparison of the two cohorts of community dwelling NCCs who lived with their family (2011: N=77; 2021/22: N=151) indicates a substantial drop in sensory functions (2011 vs 2021/22: intact vision: 94.8% vs 40.4%; intact hearing: 93.5% vs 53.6%), social activities (weekly or more: 48.1% vs 40.4%), outdoor activities (weekly or more: 75.3% vs 45.3%), and independence in activities of daily living (ADL) (no. of dependent task: 0.79 vs 2.79). Although the prevalence of comorbidities was comparable (Charlson age-adjusted comorbidity index: Mean = 2.7 vs 3.5), the frailty and pre-frailty was considerably more common in the 2021/22 cohort (55.3%; 39.0%) than the 2011 cohort (50.6%; 23.4%). We also found disparate predictors for health evaluations. Among the 2011 cohort, being male and having better psychological well-being were related to more favorable assessments of health compared to their peers. Among the 2021/22 cohort, favorable peer-compared health assessments were related to fewer ADL dependent tasks, better psychological well-being, higher age and being female. Despite the impact by COVID on the 2021/22 cohort, our findings alert policy makers and practitioners worldwide to the growing number of community-dwelling adults of advanced age with considerable personal care and health needs.

Abstract citation ID: igad104.1340

### COMPARING EARLIER- VERSUS LATER-BORN CENTENARIAN COHORTS FROM THE HEALTH AND RETIREMENT STUDY

Gina Lee, and Peter Martin, *Iowa State University, Ames, Iowa, United States*

The current study aimed to compare the health status of earlier and later born centenarian cohorts. Using the data from the Health and Retirement Study, we selected those who survived to at least 98 years and divided them into earlier (n=189) and later (n=304) cohorts. The birth year of the earlier cohort ranged from 1890 to 1906, and the birth year of the later cohort ranged from 1907 to 1920. We first recoded variables to examine health status when all participants were at least 98 years old. To explore differences in health status between earlier vs. later cohorts, we computed t-tests/$\chi 2$ difference tests with activities of daily living, depressive symptoms, self-report of health and health change (SRHC), BMI, current smoking, drinking, cognition, and a number of diseases. There were significant differences in depressive symptoms, SRHC, high blood pressure, and current drinking between the earlier and later centenarian cohorts. The later cohort had significantly higher blood pressure, more depressive symptoms, worse SRHC, and later cohorts had a higher proportion of participants who currently consumed alcohol. Finally, the results of two separate regression analyses predicting depressive symptoms indicated that no demographic or health variables of the earlier cohort significantly predicted depressive symptoms, but years of education and current diagnosis of cancer significantly predicted depressive symptoms for the later cohort. Overall, the later born cohort had a worse health status compared to the earlier born cohort. This findings highlights the need for healthcare providers to pay attention to psychological health among cancer-diagnosed centenarians.

### SESSION 3560 (SYMPOSIUM)

Abstract citation ID: igad104.1341

### COMMUNITY COLLABORATOR AND OLDER ADULT EXPERIENCES WITH INTERVENTIONS DELIVERED BY OLDER ADULTS

Chair: Nicole Crawford Co-Chair: Amber Gum Discussant: Lawrence Schonfeld

This symposium will include researchers, collaborators from healthcare and social service organizations, and older adults serving as volunteer coaches or peer educators; speakers will describe their experiences conducting two randomized controlled trials involving behavioral interventions for older adults delivered by older adults. The first trial is a NIMH-funded three-site RCT for community-dwelling older adults with symptoms of depression, in which participants receive behavioral activation delivered by a masters-level clinician or a streamlined version ("Do More, Feel Better") delivered by an older adult volunteer. This trial involves close collaboration with senior centers and an Area Agency on Aging (AAA). The second trial is a PCORI-funded RCT to reduce health disparities for Black/African-American and Hispanic/Latino older adults after medical hospitalization, in which participants receive the evidence-based Care Transitions Intervention (CTI), CTI plus a peer educator intervention, or usual care. This trial involves close collaboration with three regional hospital systems and an AAA. First, research investigators will describe the background, rationale, and methodology of each trial. Second, two representatives from the collaborating organizations (one from each trial) will present about their experiences collaborating on these trials – benefits of collaborating, challenges and strategies for overcoming challenges, and recommendations for other researchers engaged in similar community-based research. Third, two representatives from the older adult volunteers and peer educators (one from each trial) will present about their experiences collaborating on these trials – benefits of collaborating, challenges and strategies for overcoming challenges, and recommendations for other researchers engaged in similar community-based research.

Abstract citation ID: igad104.1342

### RANDOMIZED CONTROLLED TRIAL OF LAY-DELIVERED INTERVENTION FOR DEPRESSED OLDER ADULTS

Amber Gum[1], Kyaien Conner[2], Nicole Crawford[3], Patrick Raue[4], and Jo Anne Sirey[5], *1. University of South Florida, Tampa, Florida, United States*, *2. University of Pittsburgh , Pittsburgh, Pennsylvania, United States*, *3. The University of South Florida, Tampa, Florida, United States*, *4. University of Washington, Seattle, Washington, United States*, *5. Weill Cornell Medicine, Ithaca, New York, United States*

The first speaker will describe the background, aims, methods, and progress of an ongoing NIMH collaborative R01, three-site RCT. Across three sites (University of Washington, Cornell Weill Medicine, and University of South Florida), 288 depressed older adults will be enrolled, randomized to receive 9 sessions of BA from clinicians (e.g., social worker or mental health counselor) or 9 sessions of DMFB from a trained older adult volunteer coach, and assessed on primary outcomes (Behavioral Activation for Depression Scale [BADS], Hamilton Rating Scale for Depression [Ham-D]) and other outcomes at baseline, 3-, 6-, 9-, 24-, and 36-weeks. Clinician BA training and certification involves two 2-hour training workshops including didactics, demonstration, and role play; followed by delivering the intervention with one "certification" client reviewed and approved by external raters. Coach DMFB training and certification follow a similar process, except that training is longer (4 2-hour workshops) with more didactics, demonstrations, and role plays. After training, clinicians and coaches participate in regular weekly supervision, and all client sessions are audio-recorded, with at least one session per client randomly selected for independent review. This trial involves close collaboration with representative of a county lead aging service agency, numerous senior centers and organizations, and an Area Agency on Aging (AAA) for recruitment, outreach events, engagement of clinicians and volunteer coaches, and study dissemination. Across sites, 24 clinicians and 23 volunteers have been trained and certified, and 124 participants have been enrolled (as of February 15, 2023).

Abstract citation ID: igad104.1343

### RANDOMIZED CONTROLLED TRIAL OF LAY-DELIVERED INTERVENTION FOR OLDER ADULTS DISCHARGED FROM THE HOSPITAL

Kyaien Conner[1], Stephanie Kip[2], and Amber Gum[2], *1. University of Pittsburgh , Pittsburgh, Pennsylvania, United States*, *2. University of South Florida, Tampa, Florida, United States*

The second speaker will describe the background, aims, methods, and progress of a PCORI-funded RCT to reduce rehospitalization for Black/African American and Hispanic/Latino older adults after medical hospitalization. Across three regional hospitals, 402 older adults living with chronic illness (e.g., cardiovascular disease, diabetes, hypertension etc.,) will be enrolled while inpatient at one of our three partnering hospitals and randomized to receive one of three interventions in this comparative effectiveness trial: (1) the evidence-based Care Transitions Intervention (CTI), (2) CTI plus a peer educator intervention (CTI+Peer), or (3) usual care. Participants are assessed at 4 timepoints (baseline, 30 days, 90 days and 6 months) to examine differences in the primary outcomes of 30-day all cause hospital readmissions and emergency department visits. Secondary outcomes including quality of life, self-efficacy managing chronic disease, functioning, perceived social support and mortality. CTI Coaches and Peer educators participate in rigorous trainings which includes both didactic and interactive components to ensure interventionists are able to deliver the intervention with fidelity to the models. After training, CTI Coaches and Peer Educators participate in regular supervision meetings. They receive weekly virtual supervision check-ins from USF study staff and participate in a monthly group-based supervision in person. This trial involves close collaboration with leaders and coordinators at each hospital and an Area Agency on Aging (AAA) for recruitment, engagement of study interventionists and study dissemination. Across sites, 18 CTI Coaches and 11 Peer Educators have been trained and certified, and 145 participants have been enrolled (as of 3/8/2023).

Abstract citation ID: igad104.1344

### OLDER ADULT VOLUNTEER AND PEER EDUCATOR EXPERIENCES COLLABORATING ON INTERVENTION RESEARCH

Enid Rubio, and Odell Allen, *University of South Florida, Tampa, Florida, United States*

The final two speakers will present on their experiences, benefits, and challenges of collaborating on both trials described previously as an older adult volunteer coach or peer educator. One speaker is a DMFB Coach trained and certified in the DMFB intervention for depressed older adults, who delivered the intervention to five English-speaking older adults and is in the process of delivering the intervention to five Spanish-speaking older adults. One speaker is a Peer Educator for the hospital trial, trained and certified in a peer educator intervention for older adults after hospitalization, to support their transition and self-management at home. Each speaker will discuss their experiences delivering their respective interventions, including an overview of the training, certification, and supervision processes, as well as benefits and challenges. Benefits include the satisfaction of helping others, connecting with other coaches and peer educators, and learning technical skills and the intervention. Challenges have included technical issues related to virtual delivery, which are being overcome through technical assistance from the study teams; balancing the expectations of the program with other personal responsibilities, which are managed through discussion with supervisors and the study team being as flexible as possible, such as with scheduling; and occasional challenges with clients, which are addressed through regular and ad hoc supervision. The speakers will end with recommendations for other study teams that plan to work with older adults delivering behavioral interventions.

Abstract citation ID: igad104.1345

### AGING SERVICE AND HOSPITAL ADMINISTRATORS' EXPERIENCE COLLABORATING ON INTERVENTION RESEARCH

Mary Jo McKay[1], and Jason Wilson[2], *1. Hillsborough County Aging Services, Brandon, Florida, United States*, 2. *USF Health, University of South Florida, Tampa, Florida, United States*

The next two speakers will present on the methods, benefits, and challenges of collaboration on the two RCTs described previously. One speaker is Nutrition and Wellness Manager for an aging service lead agency that oversees the county's senior centers and nutrition sites. She and her team meet monthly with the study team for the depression trial and coordinate regularly to facilitate recruitment, intervention, outreach, and dissemination activities. One speaker is Division Chief for an academic medical center's Division of Emergency Medicine, where he oversees collaboration on the hospital trial. Each speaker will discuss benefits of collaboration, including expanding services offered by their organization to older adults, enhancing relationships with other community organizations, and contributing to an evidence base that could impact nationwide service delivery. Each speaker will also discuss challenges that have arisen during the two trials and strategies being used to address challenges. One challenge has been recruitment. For the depression trial, the collaborators meet monthly to review recruitment progress and identify new strategies, such as a countywide phone line, connecting with other aging service organizations, and attending in-person events. For the hospital trial, research coordinators have been embedded into each hospital with primary responsibilities for using EHR and working with hospital staff to identify and enroll participants. As a result, both trials are meeting recruitment targets thus far. Other issues that have benefited from close collaboration involve COVID-19 adaptations, technical support to older adults participating in the interventions virtually, and engaging older adult volunteers and peer educators.

### SESSION 3565 (SYMPOSIUM)

Abstract citation ID: igad104.1346

### FUNCTIONAL WELLNESS AMONG OLDER ADULTS WITH HIV/AIDS: BARRIERS AND FACILITATORS TO HEALTH AND LONGEVITY

Chair: Erin Robinson Co-Chair: Molly Perkins Discussant: Mark Brennan-Ing

In the United States, older adults make up over half of all people living with HIV/AIDS (PLWH). Today, this population is living longer and healthier lives due to advancements in HIV treatment. However, older adults living with HIV (OALWH) present a unique set of challenges as they face aging-related health considerations that intersect with their HIV status. This symposium brings together leading researchers in the field of HIV and aging, to highlight the complexity of this topic and present innovative research that examines the overall health and well-being of OALWH. Our first presentation is a qualitative inquiry into barriers to successful aging that are shared between older women and men living with HIV/AIDS, as well as differences. Our second presentation used a latent health profile model to identify subgroups of PLWH who had been discharged from a short-term skilled nursing facility (SNF). The goal of this research was to identify risk for institutional admission following their SNF stay. Our third presentation examined age-varying cognitive impairment among a sample of PLWH, people who use methamphetamines, and meth-negative/seronegative individuals to determine whether social relationships meditate the complex associations between meth use and cognitive impairment for PLWH and without HIV. And finally, our fourth presentation examined perspectives on weight change and body image among PLWH and the extent to which age and gender have an impact on those attitudes. Findings from this symposium demonstrates the complexity of what it means to be an OALWH and the various considerations for their well-being. This is a HIV, AIDS and Older Adults Interest Group Sponsored Symposium.

Abstract citation ID: igad104.1347

### BARRIERS TO SUCCESSFUL AGING AMONG OLDER WOMEN AND MEN LIVING WITH HIV

Anna Rubtsova[1], Tonya Taylor[2], Gina Wingood[3], Ighovwerha Ofotokun[4], Deborah Gustafson[5], David Vance[6], Dilip Jeste[7], and David Moore[8], *1. Emory University, Rollins School of Public Health, Atlanta, Georgia, United States*, *2. SUNY Downstate Medical Center, Brooklyn, New York, United States*, *3. Columbia University, New*

*York City, New York, United States*, *4. Emory University School of Medicine, Atlanta, Georgia, United States*, *5. SUNY Downstate, Brooklyn, New York, United States*, *6. University of Alabama at Birmingham, Birmingham, Alabama, United States*, *7. University of California, San Diego, San Diego, California, United States*, *8. University of California at San Diego, San Diego, California, United States*

This study identified and compared barriers to successful aging (SA) among older women and men living with HIV. Participants were recruited through 1) the Women's Interagency HIV Study (WIHS), Atlanta and Brooklyn sites; and 2) the HIV Neurobehavioral Research Program at the University of California, San Diego. Our sample included 31 participants living with HIV: 17 women and 14 men, ages 52 to 73 years, 58% African American. We conducted semi-structured interviews, exploring SA with HIV. The interviews (~90 minutes long) were audio recorded, fully transcribed, and imported into MAXQDA software for analysis. Thematic and comparative analyses were conducted within a social-constructivist framework. We uncovered several themes related to SA barriers that were shared by women and men: challenging chronic conditions, stigmas, substance use, and lack of resources. We also found differences. While men stressed dreading old age and loneliness, women emphasized stress and 'giving up' as barriers to SA.

Abstract citation ID: igad104.1348

### LATENT HEALTH PROFILES ASSOCIATED WITH INSTITUTIONAL ADMISSION AFTER SNF STAY AMONG MEDICARE PLWH

Brianne Olivieri-Mui[1], Sandra Shi[2], Ellen McCarthy[2], Gahee Oh[2], and Dae Hyun Kim[3], *1. Northeastern University, Boston, Massachusetts, United States*, *2. Hebrew Senior Life, Roslindale, Massachusetts, United States*, *3. Hebrew SeniorLife, Roslindale, Massachusetts, United States*

**Background:** Health profiles may help identify PLWH at risk for institutional admission after a short-term skilled nursing facility (SNF) stay.

**Methods:** We estimated rates of institutional admission in one-year follow-up after discharge from a SNF stay (<100 days) among Medicare FFS beneficiaries with HIV (2014-2019). Latent profile analysis identified subgroups based on prevalent conditions one year prior to discharge including 8 indices: mental health (MH range 0-6), substance use (SU 0-4), cardiovascular conditions (CV 0-9), sensory (SE 0-4), musculoskeletal (MU 0-10), pulmonary (PU 0-2), learning disabilities (LD 0-9), and other chronic conditions (OCC 0-11). Cox models estimated the association between latent profiles and time to institution admission (hospital, nursing home), adjusting for demographics, dual eligibility, HIV treatment; censoring included death and study end.

**Results:** The 618 PLWH studied were male (73%), with mean age 60 (standard deviation [sd] 11). Two latent health profiles were: CV+OCC+MH (n=77), median values CV=3, OCC=2, MH=1; healthy (n=541), median 0 for all. Compared to the healthy group, the CV+OCC+MH group had similar days spent in an institute (60 days [96] vs 66 days [96]), and similar hazards for institutional admission in adjusted models (HR:1.09, 95% CI [0.81-1.46]) in one-year after SNF discharge. HIV treatment for ≥80% of the time prior to discharge had lower hazards (HR:0.76 [0.59-0.98]) and dual eligibility had higher hazards (HR:1.32 [1.09, 1.61]) of returning to an institutional setting.

**Conclusions:** Latent profiles had similar risk for institutional admission in one-year after discharge from a SNF stay, despite having significantly different contributing factors.

Abstract citation ID: igad104.1349

### AGE-VARYING COGNITIVE IMPAIRMENT AND SOCIAL SUPPORT AMONG PEOPLE AGING WITH AND WITHOUT HIV

Alexis Bender[1], Regine Haardörfer[1], Molly Perkins[1], David Moore[2], and Hannah Cooper[1], *1. Emory University, Atlanta, Georgia, United States*, *2. University of California at San Diego, San Diego, California, United States*

There is compelling but limited evidence that the combination of HIV, methamphetamine (meth) use, and aging form a deleterious trio that undermines cognitive functioning. Currently, a gap exists in our understanding of the intertwining relationships among drug use, HIV, and cognitive function across biological ages and over time. Interventions to reduce meth use and support brain health among aging PWH are vital and improving social relationships is a promising area of intervention. Cross-sectional and limited longitudinal studies indicate that positive social relationships and social support are associated with better cognitive function among healthy older populations. This association has received limited attention among PWH who use drugs beyond adolescence and young adulthood. This study leverages NIH's prior and ongoing investments in research to develop a new analytic cohort by combining participants from 23 studies at the HIV Neurobehavioral Research program. Enrollment in this cohort spans from 2009-2021. At baseline, our sample consists of 862 people with a mean age of 52 (range 18-81). Most participants are male (73%), White (74%), and over half (61%) are living with HIV. Using a life course perspective and time-varying estimation modeling, we examine age-varying cognitive impairment and variation in cognitive function across age by HIV status, meth use, and social relationships. First, we examine how cognitive impairment changes over time for the full sample then examine the differing transitions/turning points among those aging with and without HIV and meth use. Lastly, we show how positive and negative social relationships alter these trajectories among each group.

Abstract citation ID: igad104.1350

### THE IMPACT OF AGING ON ATTITUDES TOWARD WEIGHT CHANGE AND BODY IMAGE AMONG ADULTS LIVING WITH HIV

Jennifer Kaufman[1], Yiyi Wu[2], Jasmine Manalel[3], Ethan Fusaris[4], Arlene Correa[5], Jerome Ernst[4], and Mark Brennan-Ing[6], *1. Brookdale Center for Healthy Aging, Hunter College, City University of New York, New York City, New York, United States*, *2. Hunter College, City University of New York, New York City, New York, United States*, *3. Hunter College, New York City, New York, United States*, *4. Amida Care, New York, New York, United States*, *5. Brown University, Providence, Rhode Island, United*

*States*, *6. Hunter College, CUNY, New York City, New York, United States*

Some newer HIV antiretroviral therapy regimens have been associated with weight gain in populations at risk for obesity. This study examined perspectives on weight change and body image among people living with HIV and the extent to which age and gender have an impact on those attitudes. We conducted interviews with 61 individuals in a Medicaid managed care plan. The age range was 23 to 65 years; 49% were aged 50 or older. The sample was 56% non-Hispanic Black and 31% Hispanic. About half were cisgender men, 36% were cisgender women, and 13% were transgender/nonbinary individuals. Many participants had weight gain they associated with HIV medications. While experiences varied widely, we found that older and younger participants offered some different perspectives. Younger people, including transgender individuals, more often welcomed weight gain, associating it with better health. Participants aged 50 and older sometimes saw weight gain as a natural part of aging, and spoke of acceptance, whereas younger participants tended to focus on their appearance. Among those with unwanted weight gain, older participants expressed concerns about its negative effect on mobility and comorbid health conditions. Some older women in particular struggled with improving their diets. Healthcare providers should consider these different perspectives and the various challenges individuals face associated with age when advising patients with HIV about weight management. For providers working with older patients with HIV, specific considerations of how increased weight affects mobility and risk for comorbid conditions are warranted.

### SESSION 3570 (PAPER)

### TECHNOLOGY, DRIVING, AND AGING IN PLACE

Abstract citation ID: igad104.1351

### AGING-IN-PLACE TECHNOLOGY: PROGRAMMING TO ADDRESS SOCIAL CONNECTEDNESS AND MENTAL HEALTH

Keith Morgen[1], and Peter Jacob[2], *1. Centenary University, Hackettstown, New Jersey, United States*, *2. Jewish Family Service of Central New Jersey, Elizabeth, New Jersey, United States*

According to the National Academies of Sciences, Engineering, and Medicine, the drastic increase of older adult social isolation from the pandemic has contributed to premature death, dementia, anxiety, and suicide. Jewish Family Service of Central New Jersey (JFSCNJ) clients corroborated this phenomenon with 65% meeting the diagnostic criteria for moderate to severe older adult depression or anxiety in 2020. In 2022, JFSCNJ established the Fig Tree Virtual Senior Center, a dynamic program that focused on older adults as they readjust to a post-pandemic world. By addressing social isolation through a Person-Centered Trauma Informed and biopsychosocial approaches, various wellness programs were provided in-home via the use of virtual video programming. Sixteen older adults (age range 68-95 years-old) were assessed on anxiety and depressive symptoms and social connectedness both prior to and six months following the start of the video programming intervention. Paired samples t-testing found a significant reduction in self-reported anxiety (p=.01) and depressive (p=.05) symptoms after video programming engagement at six-months follow-up. Path analysis using bootstrapping with bias corrected confidence intervals found amount of video programming with a positive and significant indirect effect on both increased social connectedness and improved well-being. Implications for homebound older adult social engagement and well-being programming will be discussed.

Abstract citation ID: igad104.1352

### GENDER AND AGE DIFFERENCES IN ACCEPTANCE OF ADVANCED DRIVER ASSISTANCE SYSTEMS: INSIGHTS FROM OLDER AUSTRALIANS

Abigail Hansen[1], Kim Kiely[1], Tuki Attuquayefio[2], Diane Hosking[3], Michael Regan[4], Ranmalee Eramudugolla[1], Lesley Ross[5], and Kaarin Anstey[1], *1. University of New South Wales, Sydney, New South Wales, Australia*, *2. UNSW, Sydney, New South Wales, Australia*, *3. National Seniors Australia, Canberra, Australian Capital Territory, Australia*, *4. University of NSW Sydney, Sydney, New South Wales, Australia*, *5. Clemson University, Clemson, South Carolina, United States*

Advanced Driver-Assistance Systems (ADAS) are in-vehicle technologies that promise to improve driver safety and may help support older drivers to drive safer for longer, however there is little research examining acceptance and use of ADAS among older adults. This study investigated age and gender differences in attitudes to ADAS and use of ADAS. We conducted an online survey of 1330 drivers aged 65 years or older (M=72, SD=6.9, 23% women) in partnership with National Seniors Australia. ADAS use was self-reported, classifying respondents in to users (96%) and non-users (4%). ADAS acceptance was measured by the Partial Automation Acceptance Scale. Linear regression estimated gender and age differences in acceptance. Logistic regression estimated differences in ADAS use by gender, age and acceptance. Older age was associated with higher levels of trust in ADAS ($\beta$ = 0.032, p = 0.04) and lower perceived ease of use ($\beta$ = -0.02, p = 0.02). Women were less likely to report ADAS use ($\beta$ = -0.99, p = 0.008) but had higher levels of trust ($\beta$ = 0.86, p = 0.001) than men. After adjusting for age and gender, positive attitudes towards ADAS ($\beta$ = 0.12, p = 0.003) and lower perceived risk ($\beta$ = 0.22, p = 0.046) were associated with higher levels of ADAS use. Consideration of the gender and age differences may inform future vehicle design, and older drivers may benefit from education on the risks and benefits of ADAS to aid in the acceptance and consequent use of ADAS.

Abstract citation ID: igad104.1353

### LIVING WITH A ROBOT AT HOME: THE COMPLEXITY OF LIVING WITH ASSISTIVE ROBOTS LABRADOR AND DOUBLE IN EVERYDAY LIFE

Lillian Hung[1], Donna Case[2], Haopu Ren[1], Nathan Velazquez[3], and Olga Petrovskaya[4], *1. University of British Columbia, Vancouver, British Columbia, Canada*, *2. University of Michigan Flint, Flint, Michigan, United States*, *3. University of Michigan-Flint, Flint, Michigan, United*

*States, 4. University of Victoria, Victoria, British Columbia, Canada*

The potential for assistive robots to support older adults' independence and social connections requires careful consideration of their implications in everyday use. This study investigates the use of two assistive robots, Labrador and Double, in older adults, guided by Actor-Network Theory (ANT). Labrador (a delivery robot) assists with medication management, meals, laundry, house cleaning to support independence, while Double (a teleprence robot) enables virtual social visits. ANT offers a way to understand how the robots interact with different actors, such as older adults, family members, staff, and the environment in which they operate. We applied a qualitative approach to explore how users construct meanings, use, and make sense of the robots in their everyday contexts. Semi-structured interviews and ethnographic fieldwork were conducted with participants to generate data. Reflexive thematic analysis was performed, and three themes emerged: (1) the human-robot relationship, (2) the robot's agency, and (3) ethical implications. The findings suggest that having the robots in everyday life is a process of constant negotiation with the people, practice, and the robot. The study highlights the challenges and opportunities associated with the implementation of robots to improve quality of life in senior care. While there is a fear that assistive robots will dehumanize caring practices, our study shows that they have the potential to foster innovative user-technology relationships, which requires further research.

Abstract citation ID: igad104.1354

### PRELIMINARY EFFICACY OF CARFREEME™-DEMENTIA: A DRIVING RETIREMENT PROGRAM FOR FAMILIES IN THE US

Colleen Peterson[1], Stephanie N. Ingvalson[2], Robyn Birkeland[2], Katie Louwagie[2], Maya Koffski[2], and Joseph Gaugler[2], *1. University of Michigan, Ann Arbor, Michigan, United States*, *2. University of Minnesota, Minneapolis, Minnesota, United States*

Driving retirement can be a disruptive decision for people with Alzheimer's disease and related dementias (AD/ADRD) and their families. Retiring drivers with AD/ADRD may lose their sense of independence and connection to community. Family members can experience relationship stress convincing persons with dementia to stop driving and feel strained taking on transportation responsibilities. CarFreeMe™-Dementia (CFM™-D) is an individualized psychosocial driving retirement program for families who are planning for, implementing, or adjusting to driving retirement. A small feasibility study of the program in the U.S. indicated that participants found it acceptable and useful for navigating driving retirement. Phase 2 of this study, enrolling 17 care partners, 16 retiring drivers, and 17 dyads, evaluated the preliminary efficacy of CFM™-D to provide emotional support and increase driving retirement readiness. Data from baseline, 3-, and 6-month surveys and an exit semi-structured interview were collected. Interviews confirmed that CFM™-D provided emotional support and practical preparation for driving retirement. Chi-square analyses comparing baseline to 6-month follow-up data showed fewer caregivers reached thresholds for concern on scales of strain ($p = .019$) and isolation ($p = .001$). Retiring drivers reported reductions in loneliness at 6-months ($p = .017$) and improved Assessment of Readiness for Mobility Transition scores at 3- and 6-month follow-ups ($p = .003$, $p = .014$, respectively). Findings have informed intervention delivery improvements for a future randomized trial evaluating whether CFM™-D is an efficacious tool for addressing the psychosocial complexities of driving retirement amongst people with AD/ADRD and their family.

Abstract citation ID: igad104.1355

### SERIAL TRICHOTOMIZATION TO IDENTIFY UNSAFE DRIVERS: UPDATE FROM A PROSPECTIVE STUDY

Michel Bedard[1], Sacha Dubois[2], Hillary Maxwell[2], Stephanie Schurr[3], Bruce Weaver[1], and Arne Stinchcombe[4], *1. Lakehead University, Thunder Bay, Ontario, Canada*, *2. St. Joseph's Care Group, Thunder Bay, Ontario, Canada*, *3. St. Joseph's Care Group, Thunder Bay, Ontario, Canada*, *4. University of Ottawa, Ottawa, Ontario, Canada*

Identifying older adults who may be unsafe to drive remains a difficult task. Gibbons et al. (2017, American Journal of Occupational Therapy) used serial trichotomization based on five cognitive tests to determine if drivers should: 1) continue driving, 2) undergo further evaluation, or 3) stop driving. The tests included the Trail-making-tests A and B (TMT-A/B), the clock-drawing test (CDT), the Motor-Free Visual Perception Test (MVPT), and the Montreal Cognitive Assessment (MoCA). Gibbons et al. relied on dual cut-off values to achieve 100% sensitivity and specificity (within their sample) to reduce false positives and false negatives that arise from using these tests in stand-alone fashion. We used the Gibbons et al. cut-off values prospectively on a cohort of 293 drivers (mean age = 66, SD = 14) referred for driving evaluations at a chronic care and rehabilitation hospital. Each driver completed the five tests. Trained occupational therapists (OTs) provided a recommendation to continue driving, undergo further evaluation, or stop driving. We examined congruence between the tests and the OTs recommendations. Weighted Kappas ranged from a low of .03 (95% CI = -.01 to .08) for the CDT, to a high of .53 (95% CI = .45 to .61) for the TMT-B. Using the same cut-offs, and serial trichotomization, the congruence with the final recommendations was moderate (k = .57, 95% CI = .49 to .66). These results remind us of the variability inherent in stand-alone cognitive tests and even within a serial trichotomization framework.

### SESSION 3575 (AWARD LECTURE)

Abstract citation ID: igad104.1356

### IRVING S. WRIGHT AWARD, VINCENT CRISTOFALO AWARD, AND TERRIE FOX WETLE AWARD PRESENTATIONS AND LECTURES

Chair: Steven Austad

The Irving S. Wright Award of Distinction Lecture will feature an address by the 2023 recipient Rafael De Cabo, BS, FGSA of National Institute on Aging, National Institutes of Health. The Vincent Cristofalo Rising Star Award in Aging Research lecture will feature an address by the 2023

recipient Ming Xu, PhD of UConn Health. The Terrie Fox Wetle Award lecture will feature an address by the 2023 recipient Claire Ankunda, MD, MPH. MSc of Icahn School of Medicine at Mount Sinai. These awards are given by the American Federation for Aging Research, Inc.

Abstract citation ID: igad104.1357

### CALORIC RESTRICTION AND AGING: FROM CALORIES TO FASTING TIMES

Rafael de Cabo, *National Institute on Aging, National Institutes of Health, Baltimore, Maryland, United States*

The caloric content and manipulations of nutrient composition have defined our research focus over decades, leading to the formulation of ideal diets and supplements for every phase of life. In the last few years, the control of meal size, frequency, and timing have become a powerful strategy to ameliorate and postpone disease onset and delay aging. Emerging evidence highlights the importance of prolonged fasting periods for the health and survival benefits of calorie restriction (CR) and time-restricted feeding (TRF) across multiple species. Furthermore, there is new evidence of the potential combinatorial use of these dietary interventions with established therapeutical approaches to enhance outcomes, particularly in anticancer treatments.

Abstract citation ID: igad104.1358

### A P21-CRE MOUSE MODEL TO MONITOR AND MANIPULATE P21-HIGHLY-EXPRESSING SENESCENT CELLS IN VIVO

Ming Xu, *UConn Health, Farmington, Connecticut, United States*

The role of senescent cells has been implicated in various tissue dysfunctions associated with aging, obesity and other pathological conditions. We generated a p21-Cre mouse model, containing a p21 promoter-driving inducible Cre, enabling us to examine p21Cip1-highly expressing (p21high) cells, a previously unexplored cell population exhibiting several characteristics typical of senescent cells. By crossing p21-Cre mice with different floxed mice, we managed to monitor, sort, image, eliminate or modulate p21high cells in vivo. We showed that p21high cells can be induced by various conditions, and percentages of p21high cells varied from 1.5% to 10% across different tissues in 23-month-old mice. Intermittent clearance of p21high cells improved physical function in 23-month-old mice. p21-Cre mouse model might be a valuable and powerful tool for studying p21high cells to further understand the biology of senescent cells in aging.

Abstract citation ID: igad104.1359

### HOW MEDICARE ADVANTAGE IS SHAPING THE CARE OF OLDER ADULTS WITH SERIOUS ILLNESS

Claire Ankuda, *Icahn School of Medicine at Mount Sinai, New York City, New York, United States*

The Medicare Advantage (MA) program currently insures half of Medicare beneficiaries, and yet there are major gaps in our understanding of how it is impacting older adults, particularly those with serious illness and at the end of life. This population is particularly vulnerable to lapses in care quality in MA given their complex health care needs and challenges navigating MA cost-control mechanisms such as prior authorizations and narrow networks. I will present a framework to consider how MA is influencing care delivery and outcomes for older adults, a review of what we know about the impact of the dramatic growth of MA plans, and emerging data on the experience of older adults with serious illness in MA. This session will highlight best-practices regarding use of claims and population data to evaluate the MA program.

### SESSION 4000 (SYMPOSIUM)

Abstract citation ID: igad104.1360

### PRESIDENTIAL SYMPOSIUM: IMPACT OF DISCRIMINATORY POLICIES ON LGBTQ+ OLDER ADULTS' HEALTH AND WELL-BEING AND HOW TO COMBAT THEM

Chair: Jung Kwak Discussant: Karen Fredriksen-Goldsen

Despite some advances in LGBTQ+ rights over the past few decades, many states lack legal protections against discrimination based on sexual orientation or gender identity, and the current political climate raises concerns about future challenges to LGBTQ+ rights. This symposium explores the implications of discriminatory policies on the health, housing, and economic well-being of LGBTQ+ older adults, and solutions for promoting empowerment, equity, and inclusion throughout the care continuum, from long-term services and supports to end-of-life care. The first presentation describes a complex paradox in which sexual and gender minority (SGM) older adults avoid formal services due to fear of discrimination and poor treatment despite significant health disparities and a lack of informal support, discusses opportunities to improve access and health through education and awareness, and elucidates implications for policy and research. The second presentation describes geographic variations in access to, and receipt of gender-affirming surgery among transgender and gender-diverse Medicare beneficiaries using national Medicare data and discusses policy implications. The third paper focuses the role of spirituality and faith in the lives of LGBTQ+ older adults and reports findings from a mixed-methods study on how involvement in faith communities shape their end-of-life care perceptions and preparation. The fourth presentation describes barriers and facilitators to communicating with SGM hospice patients and caregivers about their sexual orientation and gender identity and recommendations for developing communication training to promote authentic end-of-life care for this population. Karen Frederickson-Goldsen as discussant will provide policy and practice implications of these important works.

Abstract citation ID: igad104.1361

### RECEIPT OF GENDER-AFFIRMING SURGERY AMONG MEDICARE BENEFICIARIES

Em Balkan[1], Gray Babbs[2], Theresa Shireman[2], Jaclyn Hughto[2], and David Meyers[2], *1. Brown University School of Public Health, Providence, Rhode Island, United States*, *2. Brown University, Providence, Rhode Island, United States*

While transgender and gender diverse (TGD) people may never seek treatment related to their gender identity, many require access to gender-affirming treatments, including surgery. Starting in 2014, Medicare began covering gender-affirming surgery (GAS) on a case-by-case basis. In this study, we use national Medicare data to compare receipt of GAS by beneficiary characteristics. We classified TGD beneficiaries using an algorithm that combines diagnosis and procedure codes. Our study compared characteristics for all TGD identified beneficiaries and those who received GAS using a population-averaged logistic model using a generalized estimating equation (GEE) adjusting for dual status, age group, race/ethnicity, original reason for entitlement, and CMS region. Our sample accounted for 49,945 observations for 11,162 individuals, with a total of 290 identified records of GAS. In our adjusted analysis, we found that TGD beneficiaries in certain parts of the country had lower odds of GAS compared to their counterparts. Compared to CMS Region 1 (which includes Connecticut, Maine, Massachusetts, New Hampshire, Rhode Island, and Vermont), those living in Region 3 (Delaware, District of Columbia, Maryland, Pennsylvania, Virginia, and West Virginia), Region 4 (Alabama, Florida, Georgia, Kentucky, Mississippi, North Carolina, South Carolina, and Tennessee), and Region 6 (Arkansas, Louisiana, New Mexico, Oklahoma, and Texas) had a significantly lower odds of receiving GAS. Access to gender-affirming care may be influenced by CMS regional contractors, as well as state-by-state variation in Medicaid coverage for gender-affirming care. Further research is needed to explain why beneficiaries may have lower access to GAS to inform policy recommendations.

Abstract citation ID: igad104.1362

### LGBTQ AGING PARADOX: NEEDING WHILE AVOIDING LONG-TERM SERVICES AND SUPPORTS

Rajean Moone, *University of Minnesota, Minneapolis, Minnesota, United States*

A growing body of literature recognizes the significant health disparities experienced by sexual and gender minority (SGM) older adults including higher rates of chronic conditions, mental illness, and disabilities in comparison to their peers. Simultaneously SGM older adults are more likely to age alone and not have a friend or family caregiver to provide support. At the same time SGM older adults express hesitation and fear in accessing formal long term services and supports (LTSS). One study of trans older adults noted that suicide was a preferred option to a nursing home. This paper will outline a complex paradox: Many SGM older adults (1) experience significant health disparities which may require additional support; (2) lack informal support thus needing to rely on formal LTSS; and (3) avoid formal services due in large part to fear of discrimination and poor treatment. Opportunities to mitigate the paradox through education and awareness as well as implications for policy and research will be discussed.

Abstract citation ID: igad104.1363

### FAITH, SOCIAL SUPPORT, AND END-OF-LIFE CARE PREPARATION AMONG LGBTQ+ OLDER ADULTS

Nik Lampe[1], Harry Barbee[2], Nathaniel Tran[3], and Tara McKay[3], *1. University of South Florida, Tampa, Florida, United States*, *2. Johns Hopkins University, Baltimore, Maryland, United States*, *3. Vanderbilt University, Nashville, Tennessee, United States*

Faith communities can provide older adults support as they plan for end-of-life care, but how this support unfolds for lesbian, gay, bisexual, transgender, and queer (LGBTQ+) people is uncertain. Although many LGBTQ+ people experience marginalization within faith communities, they also report experiences of acceptance and affirmation. This mixed-methods study investigates how LGBTQ+ older adults' involvement in faith communities shape their end-of-life care perceptions and preparation. First, we analyze panel data from the Vanderbilt University Social Networks, Aging, and Policy Study (N=1,256) to assess the relationship between faith community involvement and levels of social support. We find that 63% of older LGBTQ+ respondents identify with a religious affiliation, and 31% attend religious services several times a year or more. Attending religious services several times a year are more is associated with having a spouse in the household (11.4 vs 7.1%, p<.05) and reporting larger personal networks (11.7 vs 9.9 individuals; p<.001). Service attendance is also associated with having support when seeking advice about important decisions and having social support from friends. In the second portion of our study, we analyze data from in-depth interviews with 47 LGBTQ+ older adults to understand how faith communities influence preparation for end-of-life care. Many interviewees described their faith communities as guides that help them select reliable healthcare providers and formulate their end-of-life care plans. Overall, these findings reveal important ways that faith communities can foster social support and promote optimal end-of-life care among older LGBTQ+ adults.

### SESSION 4005 (SYMPOSIUM)

Abstract citation ID: igad104.1364

### BUILDING BRIDGES ACROSS AGE-FRIENDLY ELEMENTS GUIDED BY THE 4MS FRAMEWORK

Chair: Darina Petrovsky Co-Chair: Anne Mitchell

The 4 Ms framework is a set of evidence-based elements of high-quality care delivered to older adults. These include What Matters, Mobility, Medication, and Mentation. By following these principles and practicing age-friendly older adults are more likely to experience good quality care that addresses their needs. This symposium aims to build bridges across age-friendly elements that focus on the 4 Ms framework by highlighting the research of four nurse scientists. Representing the "What Matters" element, advance care planning and palliative care interventions aim at understanding and aligning care with older adults' care preferences. In the first presentation, Dr. Rahemi will present findings of health disparities across sociodemographic groups in advance care planning using data from the Health and Retirement Study. In the second presentation, Dr. Carpenter will report on findings from a pilot pragmatic clinical trial that examined the feasibility, acceptability, and preliminary effectiveness of a primary palliative care intervention among nursing home residents with serious illness. Under the element of "Mobility", Dr. Sefcik will present findings from a qualitative descriptive study aimed at discovering how the COVID-19 pandemic affected the outdoor activities of senior living community-residing older adults. In the fourth presentation,

Ms. Coates presentation examines the last two elements of the framework - Medication and Mentation. She will report on findings from a longitudinal case-control observational study using data from the National Health and Aging Trends Study examining differences in the frequency of unpleasant symptoms experienced by older adults with polypharmacy and/or dementia.

Abstract citation ID: igad104.1365

### ADVANCE CARE PLANNING ACROSS SOCIODEMOGRAPHIC CHARACTERISTICS AND COGNITION LEVELS

Zahra Rahemi[1], and Swann Adams[2], *1. Clemson University, Clemson, South Carolina, United States*, *2. University of South Carolina, Columbia, South Carolina, United States*

Older adults from underrepresented and minority groups have higher rates of chronic disease and cognitive impairment and lower rates of advance care planning and comfort care at the end of life. This study examined advance care planning in U.S. older adults across different sociodemographic characteristics and cognition levels. Secondary data analyses were conducted using the 2014 Health and Retirement Study. Logistic regression models were run with each outcome variable (having living will, durable power of attorney for healthcare [DPOAH], and both the living will and DPOAH) stratified by cognition (dementia/impaired cognition versus normal cognition). Prediction variables included race, ethnicity, rurality, marital status, gender, education, age, everyday discrimination, social support, and loneliness. Of the 17,698 respondents, 77.8% had normal cognition and 22.2% had a diagnosis of dementia or impaired cognition. Black and Hispanic participants and younger individuals with lower levels of education were less likely to have a living will or both DPOAH and living will in place. Race and ethnicity were significant predictors of DPOAH among the normal cognition group but not in the dementia group. Rurality significantly predicted having a living will and both DPOAH/living will for the normal cognition group but not the dementia/impaired cognition group. This study provides evidence of the need to understand advance care planning disparities among older adults with multiple marginalized identities and different cognition levels. The findings are important for examining advance care planning regarding what matters to diverse older adults as they age and confront health and cognition decline.

Abstract citation ID: igad104.1366

### OUTCOMES OF A PILOT PRAGMATIC TRIAL IMPLEMENTING PALLIATIVE CARE IN NURSING HOME CARE

Joan Carpenter[1], Laura Hanson[2], Nancy Hodgson[3], Amy Jackson[4], Merve Gurlu[5], Shijun Zhu[1], and Mary Ersek[6], *1. University of Maryland School of Nursing, Baltimore, Maryland, United States*, *2. University of North Carolina at Chapel Hill, School of Medicine, Chapel Hill, North Carolina, United States*, *3. University of Pennsylvania School of Nursing, Philadelphia, Pennsylvania, United States*, *4. University of Maryland, Baltimore, Maryland, United States*, *5. University of Maryland, Baltimore, Baltimore, Maryland, United States*, *6. University of Pennsylvania, Philadelphia, Pennsylvania, United States*

Palliative care that aligns treatments with preferences for people living with serious illness in nursing homes (NHs) can improve quality of life. Given limited access to specialty palliative care in NHs, enhanced primary palliative care is needed. We conducted a pilot pragmatic clinical trial to establish feasibility, acceptability, and preliminary effectiveness of an NH primary palliative care intervention. Nurse practitioners, employed by each intervention site, attended a 5-hour virtual course, one-day intensive workshop, and monthly 1-hour facilitation sessions to enhance palliative care practices. We recruited residents living with serious illness and palliative care needs from 12 NHs in the MidAtlantic US. Using a nonequivalent group design, participants were clustered by site and assigned to intervention or control group in a 2:1 (respectively) ratio. Our primary outcome was the Palliative Outcome Scale Version 2 (POSv2, range 0-40). Of 318 residents screened, 119 met inclusion criteria, and 45 consented for participation (n=35 intervention; n=10 control). Participants (mean age=86) were mostly White (98%), female (76%), and able to make their own decisions (58%). Clinician intervention fidelity and participant acceptability were high. At 21 days, quality of life did not differ between control (POSv2=8.79) and intervention groups (POSv2=10.03; p=0.72). We conclude that an NH primary palliative care intervention is feasible and acceptable; effectiveness to improve quality of life may depend upon the successful implementation of a highly tailored intervention and longer-term follow-up.

Abstract citation ID: igad104.1367

### OUTDOOR ACTIVITY ENGAGEMENT PRIOR TO AND DURING THE COVID-19 PANDEMIC IN SENIOR LIVING COMMUNITIES

Justine Sefcik[1], Elizabeth Euiler[1], Annalisa Na[2], Sherry Greenberg[3], Danielle Katsev[4], and Rose Ann DiMaria-Ghalili[5], *1. Drexel University College of Nursing and Health Professions, Philadelphia, Pennsylvania, United States*, *2. Drexel University, Philadelphia, Pennsylvania, United States*, *3. Monmouth University, Marjorie K. Unterberg School of Nursing & Health Studies, West Long Branch, New Jersey, United States*, *4. Drexel Univeresity, Philadelphia, Pennsylvania, United States*, *5. College of Nursing and Health Professions, Drexel University, Philadelphia, Pennsylvania, United States*

There is growing evidence regarding the benefits of spending time outdoors. However, it is less understood if the COVID-19 pandemic has deterred or increased older adults' outdoor leisure activities when they reside in senior living communities. This study aimed to discover how the pandemic affects senior living community-residing older adults' outdoor activities. Older adults 65 and older were recruited through ResearchMatch and university partnerships. A qualitative descriptive approach was taken with semi-structured individual interviews conducted over Microsoft Teams, telephone, or in-person. A conventional content analysis was employed. Interviews were conducted in 2021-2022 with 4 females and 3 males from suburban Pennsylvania. While there were indoor restrictions and regulations (e.g., mask wearing when around others), the outdoor environments were less regulated and routine outdoor activities continued during the pandemic. Facilitators to outdoor activities were: 1) senior living campus features (e.g., paved walking paths, outdoor recreational sport areas), 2) structured outdoor activities (e.g., bocce ball and golfing tournaments facilitated by staff), and 3) access to transportation (e.g., to drive or visit other locations). A barrier to outdoor activities was: needing assistance from staff to exit buildings due to decreased mobility. This study illuminates that amenities and structured activities at senior living facilities promoted mobility in outdoor activities for those who preferred going outside. Findings inform design features that encourage mobility and outdoor activities among older adults. Policy implications include supporting senior living communities with less resources for maintaining campuses and neighborhoods, which could contribute to promoting outdoor activities and health equity.

Abstract citation ID: igad104.1368

### POLYPHARMACY AND THE SYMPTOM EXPERIENCE OF OLDER ADULTS WITH AND WITHOUT ALZHEIMER'S DISEASE AND RELATED DEMENTIAS

Martha Coates[1], Leslie McClure[1], Margaret Finley[2], Justine Sefcik[3], Laura Gitlin[1], and Rose Ann DiMaria-Ghalili[4], *1. Drexel University, Philadelphia, Pennsylvania, United States*, *2. Drexel Univeresity, Philadelphia, Pennsylvania, United States*, *3. Drexel University College of Nursing and Health Professions, Philadelphia, Pennsylvania, United States*, *4. College of Nursing and Health Professions, Drexel University, Philadelphia, Pennsylvania, United States*

Older adults, including those living with Alzheimer's disease and related dementias (ADRD), experience polypharmacy (PPY) (>5 medications daily) due to multiple chronic conditions. High symptom burden in older adults with ADRD and multimorbidity is well known. However, there is a gap in knowledge regarding symptoms experienced by older adults with and without ADRD who experience PPY. To address this gap, we conducted a longitudinal case-control observational study utilizing Rounds 6 to 9 (2016 to 2019) of the National Health and Aging Trends Study (NHATS) datasets. PPY was categorized by the number of medications from Round 6. We categorized individuals into four groups based on presence of ADRD and/or PPY (PPY and ADRD, PPY only, ADRD only, no PPY/ADRD) (N=2052). We identified symptoms associated with PPY (hopelessness, anxiety, general pain, stomach pain, breathing problems, weight loss, balance problems, fatigue, insomnia) in each group over each time point. Among those with ADRD (11.8%), over half experienced PPY (52.3%). Older adults with PPY and ADRD experienced a mean of 4.85 symptoms over all time points compared to 3.52 (ADRD only), 2.79 (no PPY/ADRD) and 3.93 (PPY only) (p=<0.001). Symptom frequencies in the PPY and ADRD group remained higher than all other groups across all four time points. Understanding the impact of medications on older adult's quality life is a goal of the 4Ms framework. Future research will examine the symptoms associated with PPY and ADRD, physical function and health outcomes to inform medication management for older adults with dementia.

### SESSION 4010 (SYMPOSIUM)

Abstract citation ID: igad104.1369

### WHO, HOW, AND WHEN TO PREPARE FOR CARE? PREPAREDNESS AMONG CAREGIVERS OF PERSONS LIVING WITH DEMENTIA

Chair: Emily Mroz Co-Chair: Nicole Crawford Discussant: Joan Monin

Our health systems rely on family caregivers to provide critical support for over six million persons living with dementia (PLWD) in the US. Caregivers, however, often report low preparedness for care roles, promoting negative outcomes for themselves and their care recipients. As the number of PLWD grows, preparing close others to perform and thrive in caregiving roles is a public health priority. This symposium provides insights on who, how, and when to prepare for the care of a PLWD. First, Falzarano et al. will discuss the individual, psychosocial, and contextual factors influencing caregivers' use of supportive services, and implications for self-efficacy and preparedness. Mroz et. al. will introduce the concept of 'experienced' caregivers of PLWD, those who have been caregivers previously, and describe ways to address persisting psychological tolls and low confidence from prior care roles to prepare these caregivers for current care roles. Fox et al. will describe interaction between preparedness and family dynamics, showcasing differences in these relations across dementia- and non-dementia care roles. Vu et al. will describe how caregiver engagement in a health priorities identification process can better prepare caregivers to make values-based medical decisions on behalf of the PLWD. Crawford et al. will describe the influence of burden on caregivers' emergency preparedness. Takeaways from these papers and Joan Monin's discussion collectively respond to priorities outlined by the NIA IMPACT Collaboratory's Lived Experience Panel, offering direction on best practices and needed initiatives to bolster preparedness in this caregiver population.

Abstract citation ID: igad104.1370

### PREPARING "EXPERIENCED" CAREGIVERS FOR FUTURE CARE: PERSPECTIVES FROM FORMER CAREGIVERS OF PARENTS WITH DEMENTIA

Emily Mroz[1], Amanda Piechota[2], Talha Ali[3], Tara Matta-Singh[4], Anissa Abboud[2], Shubam Sharma[5], Terri Fried[1], and Joan Monin[2], *1. Yale School of Medicine, New Haven, Connecticut, United States*, *2. Yale School of Public Health, New Haven, Connecticut, United States*, *3. Tufts University, Medford, Massachusetts, United States*, *4. University of South Florida, Tampa, Florida, United States*, *5. Kennesaw State University, Kennesaw, Georgia, United States*

Providing care for more than one care partner across adulthood is becoming increasingly common, resulting in a growing number of 'experienced' caregivers (those who have been caregivers before and are anticipating or engaging in caregiving roles again). Caregivers have opportunities to develop caregiving skills and experience positive and negative psychological consequences that persist after care roles have ended. Little is known about how these factors shape preparedness in the large and growing group of former adult-child caregivers of parents living with dementia (PLWD) who anticipate future caregiving roles. We conducted a qualitative study of 32 interviews with midlife former caregivers of now-deceased PLWD. Consensual Qualitative Research and grounded theory techniques guided study design and analysis. Analyses demonstrated that preparedness for future caregiving roles was shaped by personal narratives from former caregiving roles across two dimensions: insights and confidence. All caregivers described insights (i.e., caregiving self-conduct, care systems and resources, and relating with a care partner) that contributed to their preparedness. Though some former caregivers also described a positive (i.e., boosted or sustained) sense of confidence, others described a diminished (i.e., restricted or impeded) sense of confidence. Findings suggest that for some former caregivers, past experiences offer cumulative advantages; for others, past experiences may contribute to apprehension towards, or rejection of, future caregiving roles. Entering new roles with diminished confidence may have negative consequences for caregivers' and care partners' wellbeing. This presentation will conclude with suggestions for resources that address both insight-based and confidence-based preparedness and are tailored to experienced caregivers.

Abstract citation ID: igad104.1371

### FORMAL SERVICE USE BARRIERS AND ITS IMPLICATIONS ON PREPAREDNESS IN DEMENTIA FAMILY CAREGIVERS

Francesca Falzarano, and Megan McCarthy, *Weill Cornell Medicine, New York City, New York, United States*

Dementia family caregivers are critical to sustaining the long-term services and supports system in the US, yet the identification-assessment-referral pipeline to connect caregivers to needed resources remains siloed and complex. The use of formal services can serve as a mechanism to foster preparedness, resiliency, and enhance positive aspects of the caregiving role and ameliorate against adverse care-related outcomes. Despite resounding sentiment among caregivers expressing their need for relevant services, formal service utilization remains strikingly low. A greater understanding is needed regarding the multidimensional factors, stemming from within and beyond the direct care environment, influencing perceived ability to carry out care responsibilities. Thus, the purpose of this study was to examine the individual, psychosocial, and contextual characteristics associated with preparedness and self-efficacy among 46 dementia family caregivers (Mage=55.9, SD=16.3) who participated in a mixed-methods study examining facilitators and impediments influencing service utilization. Overall, 65.9% of participants reported receiving no formal preparation for their care responsibilities. Results showed that age, spousal kinship, longer duration of care, financial stress, more difficulty accessing formal services, and more negative service experiences were associated with lower feelings of perceived preparedness and self-efficacy. Findings underscore the need for more holistic, person-centered assessment to equip caregivers with the needed, relevant resources to enhance their ability to provide effective care.

Abstract citation ID: igad104.1372

### DEMENTIA CAREGIVERS, BURDEN, AND EMERGENCIES

Nicole Crawford[1], Bruce Levin[2], Amber Gum[2], Dinorah Martinez Tyson[1], Anthony Masys[2], and Makeba Huntington-Symons[3], *1. The University of South Florida, Tampa, Florida, United States, 2. University of South Florida, Tampa, Florida, United States, 3. Alzheimers Association, Florida Gulf Coast Chapter, Clearwater, Florida, United States*

Caregivers of people living with dementia face multiple barriers, including social isolation, mental health problems, limited time for themselves, and limited support services. Navigating emergencies, including hospitalization, financial loss, and natural disasters, can exacerbate the burden that dementia caregivers face. This study examined dementia caregivers' perspectives on burden, emergencies, and emergency preparedness. Semi-structured interviews were conducted with a predominantly female sample of fourteen dementia caregivers living in Florida. Data were analyzed using thematic analysis methods to define codes and identify themes. Results showed all dementia caregivers identified the term burden as having a negative connotation about their experience and the person living with dementia. Dementia caregivers predominantly stated that they cared for their family members out of love and a sense of responsibility. Findings also showed individual and caregiving experiences (e.g., being a single mom or having a previous caregiving role) prepared dementia caregivers to define and encounter emergencies. Understanding caregivers' perspectives of burden, emergencies, and emergency preparedness is essential to developing evidence-based interventions and support services that aim to mitigate the adverse effects of emergencies on caregivers. Implications of using preferred terminology and recommendations for providing person-centered emergency planning will be discussed.

Abstract citation ID: igad104.1373

### FAMILY CAREGIVERS' REFLECTIONS ON FAMILY SUPPORT AND PREPAREDNESS FOR END-OF-LIFE CAREGIVING

Aimee Fox[1], Christine Fruhauf[2], and Julia Sharp[2], *1. Utah Valley University, Utah, United States, 2. Colorado State University, Fort Collins, Colorado, United States*

Family caregivers often report not having the knowledge or skills to provide care needed at their care receiver's end of life (EoL), leaving them vulnerable to emotional, psychological, physical, and financial stress. These stressors may be due to increasingly complex caregiving responsibilities, difficult medical decisions, and anticipatory grief. Studies have also found a higher incidence of adverse effects among dementia caregivers. Few studies have examined how family support is associated with preparedness for EoL caregiving. Thus, the purpose of this study was to explore family caregivers' feelings of preparedness and family-member support for EoL caregiving, comparing responses between dementia care providers with all other caregivers. A sample of family caregivers were recruited to complete an online, self-report survey. Open-ended questions were included to explore caregivers' feelings of preparedness and family support. Of all participants, 206 family caregivers responded to the open-ended questions. Nearly 40% (n=79) of these caregivers were providing care to a family member with Alzheimer's disease or related dementia. Qualitative methods were used to analyze data, develop a coding scheme, and identify themes. Findings suggest that most family caregivers are providing care without adequate help, support, or communication from other family members. This lack of support seems to leave dementia caregivers feeling less prepared and more frustrated than other caregivers. Our findings highlight the importance of family communication about advance care planning and caregiving responsibilities to promote family caregiver preparedness and well-being, and effective care and quality of life for an individual at the end of their life.

Abstract citation ID: igad104.1374

### PERSONS LIVING WITH DEMENTIA AND MULTIPLE CHRONIC CONDITIONS IDENTIFYING HEALTH PRIORITIES WITH CARE PARTNERS

Thi Vu[1], Emily Mroz[2], Kizzy Hernandez-Bigos[3], Denise Chow[1], Rafael Samper-Ternent[4], Kalisha Bonds Johnson[5], Mary Tinetti[2], and Joan Monin[6], *1. Yale University School of Public Health, New Haven, Connecticut, United States*, *2. Yale School of Medicine, New Haven, Connecticut, United States*, *3. Yale University School of Medicine, New Haven, Connecticut, United States*, *4. UTHealth Houston, Houston, Texas, United States*, *5. Emory University, Atlanta, Georgia, United States*, *6. Yale School of Public Health, New Haven, Connecticut, United States*

Many older persons living with dementia (PLWD) also live with multiple chronic conditions (MCCs). Care partners of PLWD will increasingly need to make care decisions for PLWD, but the health care priorities of PLWD with MCCs may vary. The Patient Priorities Care Health Priorities Identification Process is an evidenced-based tool to support older adults with MCCs to identify and communicate their health priorities. In this pilot study, we adapted this tool to engage care partners of PLWD as active listeners while the PLWD discussed their healthcare goals and priorities. We recruited dyads consisting of a care partner and a care recipient in the early stages of dementia. We assessed feasibility and acceptability of going through the process together through open-ended questions. Two coders used thematic analysis to examine their responses. Five dyads (N=10) participated in the pilot study. The PLWD ranged in age from 70-83 years and had a range of three to eight chronic conditions. Care partners ranged from 39-78 years of age. The PLWD had no issues completing the process with their care partners present and enjoyed having their care partner present. Care partners valued engaging as active listeners and had a better understanding of the PLWD's health care priorities. Engagement in this process also helped them recognize that the PLWD is proactive in their own health goals. Involving care partners in a healthcare priorities identification process is a valuable, feasible, and acceptable way to prepare care partners to be care proxies.

### SESSION 4015 (SYMPOSIUM)

Abstract citation ID: igad104.1375

### HOW ARE OLDER PEOPLE FARING POST-PANDEMIC? EVIDENCE FROM THE ENGLISH LONGITUDINAL STUDY OF AGING

Chair: Giorgio Di Gessa Co-Chair: Paola Zaninotto
Discussant: Paola Zaninotto

This session provides insights into the experiences of older people in England post-COVID-19 pandemic. Evidence suggests that stay-at-home and lockdown measures during the pandemic impacted several aspects of people's lives with detrimental consequences for their wellbeing and mental health, particularly among older people. Little is known, however, on whether and to what extent there has been a return to 'normal life' since COVID-19 restrictions were lifted. Using data from the English Longitudinal Study of Ageing (ELSA) this symposium aims to provide new evidence on the experiences of older English people since the successful and rapid COVID-19 vaccination rollout and the easing of restrictions in England through the second half of 2021. ELSA is an ongoing longitudinal biennial survey representative of individuals aged 50 and over in private households that has collected data before the pandemic (with the most recent pre-COVID-19 data collected in 2018/19), during the pandemic (on two separate occasions in June/July and November/December 2020), and post-COVID-19 pandemic (with interviews taking place between October 2021 and March 2023). Exploiting the richness of this dataset, this symposium will present the most up-to-date picture of how older people are faring post-pandemic. Results will focus on mental health and wellbeing, social isolation and loneliness, employment and financial status, as well as social participation (including volunteering, caring, and grandparental childcare).

Abstract citation ID: igad104.1376

### SOCIAL ENGAGEMENT AMONG OLDER PEOPLE IN ENGLAND SINCE THE COVID-19 PANDEMIC.

Giorgio Di Gessa, *University College London, London, England, United Kingdom*

Volunteering, providing grandparental childcare, and provision of family care were quite common activities pre-pandemic among older people. However, during the pandemic, older people in particular were strongly advised to stay indoors, to work from home, and to avoid or at least limit interactions with people outside of their immediate household, including grandchildren and friends. As a result, evidence from the English Longitudinal Study of Ageing (ELSA) showed dramatic changes in the level of engagement in these socially productive activities. About 10% of grandparents stopped looking after grandchildren altogether during the first 9 months of the pandemic, with 22% reporting an overall decrease in the amount of grandchild care provided. Similarly, among those who were volunteering or caring for someone outside their household pre-COVID-19, almost two thirds decreased or stopped altogether engaging in these activities, with overall negative consequences for their mental health and wellbeing. To date, though, little is known on whether, in emerging from the current pandemic and as restrictions are lifted, social engagement among older people has resumed pre-COVID-19 pandemic levels and whether this is related to a rebound also in emotional wellbeing. Using data from ELSA collected in 2021/23 allows for an exploration of changes in grandparental childcare provision, caring, and volunteering since the COVID-19 pandemic, with a focus on levels of engagement among those older people who had stopped altogether these activities during the first months of the COVID-19 pandemic

Abstract citation ID: igad104.1377

**MENTAL HEALTH AND WELLBEING ACROSS THE COVID-19 PANDEMIC AMONG OLDER PEOPLE IN ENGLAND**

Brian Beach, and Paola Zaninotto, *University College London, London, England, United Kingdom*

Numerous studies have identified declines in mental health and psychosocial wellbeing over the course of the COVID-19 pandemic across the world and in different age groups, including older people. A key question for researchers, health practitioners, and policymakers alike is whether the influence of the pandemic on these outcomes is temporary or more long-lasting. The lived experience of the pandemic, such as lockdowns, likely played a role in shaping mental health and wellbeing, but recovery is not always immediate following the removal of such negative stimuli. Our recent work using the English Longitudinal Study of Ageing (ELSA) identified that depression, anxiety, and quality of life demonstrated statistically significant worsening trajectories between ELSA Wave 9 (2018/19) and through two assessments during the pandemic, i.e. June/July and Nov/Dec 2020. Because depression and anxiety are also common neuropsychiatric symptoms among people with dementia or mild cognitive impairment, we also examined differences according to cognitive function, finding a convergence among cognitive function groups in the risk for poor mental health through the pandemic. This paper expands on this work to incorporate ELSA Wave 10, the first survey assessment after the easing of key pandemic-related restrictions and health crisis measures, to assess how older people are faring since the acute phase of the pandemic in terms of mental health and wellbeing. It highlights key subgroup dynamics, including cognitive function group, that are associated with patterns of recovery in mental health and wellbeing to pre-pandemic levels and those linked to more long-lasting adverse experiences.

Abstract citation ID: igad104.1378

**SOCIAL WELL-BEING OF OLDER PEOPLE IN ENGLAND SINCE THE PANDEMIC: DID ALREADY-ISOLATED OLDER ADULTS FARE WORSE?**

Claryn Kung, *University College London, London, England, United Kingdom*

The COVID-19 pandemic and actions to reduce transmission (including stay-at-home orders and workplace closures) have had an impact on actual and perceived social isolation and support across all age groups. There was a clear increase in social isolation during the pandemic, especially among older adults, potentially due to the heightened risks of complications and mortality if infected with the novel coronavirus. In England, employing data from the nationally representative English Longitudinal Study of Ageing and using pre- (2018/19) and during pandemic data (June/July 2020 and November/December 2020), we found that overall, older adults who were already experiencing social isolation prior to the pandemic (isolated group) experienced a larger rise in loneliness and drop in social contact during the pandemic than older adults who were previously socially connected (non-isolated group). Results are robust even when we account for the fact that respondents socially isolated (including those not married and with infrequent contact with relatives and friends) felt lonelier and less supported even before the pandemic, and when we adjust for demographic and socioeconomic characteristics. Expanding on this work with post-pandemic data collected between October 2021 and March 2023, this paper describes loneliness, social contact, and social support pre-, during, and post-COVID-19 pandemic and investigates whether and to what extent trends in social well-being continued to diverge between the isolated and non-isolated groups.

Abstract citation ID: igad104.1379

**EMPLOYMENT AND FINANCIAL STATUS BEFORE AND AFTER THE COVID-19 PANDEMIC AMONG OLDER PEOPLE IN ENGLAND**

Jingmin Zhu, *University College London, London, England, United Kingdom*

The COVID-19 pandemic has negatively affected society and the economy worldwide. By the end of 2021, most legal COVID-19 restrictions were lifted in England, pushing society into a post-pandemic phase. However, it remains unclear whether the post-pandemic world has convalesced, particularly with regard to socioeconomic circumstances among older people. According to the English Longitudinal Study of Ageing (ELSA), the percentage of employment among older people aged 50 years and above living in England decreased dramatically from 24.9% in 2018/9 to 17.6% in June/July 2020 and 19.8% in November/December 2020. Preliminary results from data collected between October 2021 and November 2022 suggest that post-pandemic the percentage of older people in paid work bounced back to pre-pandemic levels. Nevertheless, the post-pandemic financial status was worse than that before the pandemic. Whereas approximately 22% of respondents were worried about the future financial situation during the pandemic, this prevalence increased to nearly 30% in 2021/2. Moreover, there is an indication that some groups (including those with low education; those in routine and manual occupations; and those living alone) fared worse in the post-pandemic times. Overall, despite some evidence suggesting that the employment of English older people has bounced back to pre-COVID-19 pandemic levels, their financial situation seems to have deteriorated, most likely reflecting also the current rising cost of living and financial crisis in the UK.

### SESSION 4020 (PAPER)

### SOCIAL CONNECTIONS, TECHNOLOGY, AND SOCIAL ISOLATION

Abstract citation ID: igad104.1380

**LONELINESS AND OLDER ADULTS: RESULTS FROM A COMMUNITY-BASED INTERVENTION**

Catherine Macomber[1], and Paul Freddolino[2], *1. Saginaw Valley State University, University Center, Michigan, United States*, *2. Michigan State University, East Lansing, Michigan, United States*

The number of older adults identifying themselves as lonely has increased during the last two years as they have struggled with the impact of the pandemic on their perceptions of relationships with others. Recent research has shown that the negative health implications of loneliness are similar to those found with smoking or obesity. Most current interventions continue with past practice by adding people to engage with the older adult, although research suggests that while this intervention addresses social isolation, it does not address loneliness directly. Information regarding the development and implementation of a loneliness intervention with older adults based on the Health Beliefs Model in one rural mid-west county will be presented. Participants were recipients of home-delivered meals and had volunteered for a project that provided peer-to-peer instruction to improve digital literacy. In addition to technology training sessions, participants received a weekly contact from a separate volunteer as a friendly 'check-in.' Beginning in the fourth week, brief 5-10 minute coaching sessions about loneliness were included in these weekly contacts. The presentation will include details of the loneliness content, and describe training and support provided to the volunteers. Interviews were conducted with participants at baseline, approximately three months, and at the end of contact with each participant. Results showed no statistically significant change in loneliness, but participants reported they saw potential usefulness for the content with family and friends and potentially themselves in the future. They also provided helpful suggestions for revision. Plans for revision will be presented.

Abstract citation ID: igad104.1381

### PEER-BASED CHECK-IN SUPPORT AND PERSONAL AGENCY IN TANGIBLE INTERFACE DESIGN FOR REDUCING ISOLATION IN OLDER ADULTS

Pallabi Bhowmick, and Lesa Huber, *Indiana University Bloomington, Bloomington, Indiana, United States*

About one in four older adults in the US are socially isolated, an issue worsened by the COVID-19 pandemic. The aims of a 2-week diary study and field deployment of a peer-based tangible check-in system with 16 participants aged 65 to 87 were to understand how older adults check-in with others, who they check-in with, what difficulties they face, and how technology can help them stay connected. Findings show that perceived check-in behavior varies from actual check-in behavior. Although participants listed family as their strongest relationships forming the closest social circle, 75% of the participants had up to third-quarters of their communication with friends as compared to all other relationships. Secondly, passive check-in and monitoring from family members make older adults non-participatory recipients of care and takes away human agency. The check-in system allowed participants to actively check-in on their peers. Participants found the technology non-stigmatizing and bonded with each other quickly. Finally, older adults prefer tangible interfaces, such as buttons and physical controls over digital interfaces, because they do not require fine motor skills or visual acuity. Tangible systems can provide familiar, intuitive, and accessible systems by leveraging everyday physical objects as user interfaces. Study participants found the tangible check-in system easy and enjoyable to use. Such tangible peer-based systems improve social connectedness, address ageism by restoring agency, meet accessibility and aesthetic needs, thereby encouraging technology adoption. They also empower older adults to maintain their independence, instill in them a strong sense of community, and improve quality of life.

Abstract citation ID: igad104.1382

### SINGLE-TIE CORE NETWORKS AMONG OLDER EUROPEANS: A POSITION OF PRECARITY AND LONELINESS?

Haosen Sun[1], Jina Lee[2], and Markus Schafer[3], *1. UNR School of Public Health, Reno, Nevada, United States*, *2. University of Toronto, Toronto, Ontario, Canada*, *3. Baylor University, Waco, Texas, United States*

The problems of late-life social isolation and loneliness prompt significant concern. Individuals who have core personal networks limited to a single connection may be especially susceptible to loneliness, particularly when that connection is no longer available. The present research considers: 1) how prevalent such single-tie networks are and who is most likely to embed in them; 2) whether older adults in single-tie networks are more lonely than people with more expanded network forms; and 3) whether such networks put people at highest of loneliness in the event of network member loss. Using ego-centric network data from Waves 4 (2011) and Wave 6 (2015) of the Survey of Health, Ageing, and Retirement in Europe (SHARE), we conduct lagged dependent variable logistic regressions. Results show that a total of 28.2% of older Europeans rely on a single person as an important personal tie. Among those maintaining a single-tie network, spouses are the most common choice (62.4%), followed by a child (15.2%), a relative (8.5%), and a non-relative (13.8%). Factors significantly associated with holding different types of single-tie networks include age, gender, education, financial pressure, rural residence, participation in social activities, and grandparental roles. Child-only networks are significantly associated with greater levels of loneliness compared to multi-tie networks. Meanwhile, the loss of a partner as the only connection is associated with significantly increased loneliness, even after considering possible network additions. Future research should investigate how to better protect older adults in precarious network settings, especially during network losses.

Abstract citation ID: igad104.1383

### SOCIAL PARTICIPATION AND DEPRESSION AMONG HONG KONG OLDER ADULTS: THE MODERATING ROLE OF DIGITAL ENGAGEMENT

Chang Liu, Xue Bai, and Chi-Ko Lee, *The Hong Kong Polytechnic University, Hong Kong, Hong Kong*

Depression is a significant public health issue among older adults. Active social participation is crucial for older people to promote mental well-being. Previous studies mainly focused on the impacts of offline social activities on older people's mental health, despite that digital participation has substantially changed their patterns of social participation in the context of Covid-19 pandemic. Extending the use of Activity Theory to digital context, this study investigated how social participation in three types of activities (i.e., formal, informal and solitary activities) and digital engagement independently and jointly influenced mental health among Hong Kong older adults. Data from 5,007 participants were drawn from the baseline survey of the Panel Study of Active Ageing and Society, a biannual longitudinal survey conducted with a representative sample of ageing Chinese adults aged 50 years and above in Hong Kong. Exploratory factor analysis, ordinary least squares regression analysis and mediation analysis were conducted. The results showed that participation in informal and solitary activities was related to less depressive symptoms, while participation in formal activities was related to more depressive symptoms but higher life satisfaction. Although the main effect of digital engagement on depressive symptoms was not significant, more digital engagement reduced the negative effect of formal activities and decreased the effect of informal activities in reducing depressive symptoms. The findings have implications for policy-making and intervention development to encourage and balance social and digital participation among ageing populations.

Abstract citation ID: igad104.1384

### STABILITY AND CHANGE IN SOCIAL ISOLATION PROFILES OVER TIME AMONG OLDER ADULTS

Pildoo Sung[1], and Angelique Chan[2], *1. Hong Kong Baptist University, Hong Kong, Hong Kong*, *2. Duke-NUS Medical School, Singapore, Singapore*

This study examined transitions in social isolation profiles over time and factors associated with them, using longitudinal data from 1,305 older adults in Singapore. While prior studies have investigated social isolation profiles that combine objective measures of social disconnectedness and subjective feelings of loneliness, less is known about the stability and change in these profiles over time. We utilized random-intercept latent transition analysis to identify social isolation profiles and their transition patterns over time. Multivariable regression then examined whether health and sociodemographic characteristics were associated with specific transitions of interest. The empirical analyses yielded three key findings. First, at baseline, about 10% of older adults were "disconnected and lonely," while 30% were "disconnected but less lonely." The remaining respondents were "connected and less lonely." Second, at follow-up, 44% retained their baseline profiles, while 56% transitioned into different profiles. Third, older adults with lower socioeconomic status were more likely to transition from the "connected and less lonely" profiles to the disconnected and less lonely profile. Older adults with declining mental health were more likely to remain in or transition into the "disconnected and lonely" profile. In summary, approximately half of the older Singaporeans were socially disconnected, and one in five of them perceived loneliness occasionally or more often. Socioeconomically disadvantaged older adults were more likely to experience social disconnectedness over time, but this does not necessarily accompany loneliness. Rather, it is declining mental health that leads to both social isolation and loneliness, which calls for tailored interventions.

### SESSION 4025 (PAPER)

### FAMILY CAREGIVING (HS)

Abstract citation ID: igad104.1385

### EFFECTS OF ENGAGEMENT IN PHYSICAL ACTIVITY BY INFORMAL CAREGIVERS ON THEIR QUALITY OF LIFE

Shamirah A'Azman[1], Pildoo Sung[2], and Rahul Malhotra[1], *1. Duke-NUS Medical School, Singapore, Singapore*, *2. Hong Kong Baptist University, Hong Kong, Hong Kong*

Informal caregivers are more likely to have poorer health and wellbeing than non-caregivers. Studies have shown the health benefits of engagement in physical activity (PA) among caregivers. However, little is known about whether and how caregiver engagement in PA promotes or protects caregiver wellbeing. We examined (1) the association of caregiver engagement in PA with caregiver quality of life (QoL) and (2) the moderating effect of caregiver engagement in PA on the relationship between caregiving stressors and QoL. We used data on 278 care-recipient/caregiver dyads from the baseline interview of a longitudinal study of caregivers of older adults in Singapore. Multivariable regression first examined whether caregiver engagement in PA (assessed by number of days in a week with at least 30 minutes of PA, self-reported) was associated with physical, psychological, social, and environmental domains of QoL. Subsequently, interaction terms between caregiving stressors (care-recipient physical, memory, behavioral, and mood impairments) and caregiver engagement in PA were introduced and tested. We found that caregiver engagement in PA had a positive association with psychological, social, and environmental domains of QoL. Furthermore, caregiver engagement in PA mitigated the negative association between care-recipient mood impairment and caregiver QoL in the physical and social domains. Our findings suggest that QoL of informal caregivers of older adults may be improved or protected by their regular engagement in PA, especially if their care-recipients have mood impairments. Providers serving caregivers should encourage caregivers to engage in PA and equip them with the necessary support to do so.

Abstract citation ID: igad104.1386

### GENDER DIFFERENCES IN CAREGIVER BURDEN AMONG FAMILY CAREGIVERS OF PERSONS WITH DEMENTIA: A SYSTEMATIC REVIEW

Thitinan Duangjina[1], Thanakrit Jeamjitvibool[1], Chang Park[1], Rebecca Raszewski[2], Valerie Gruss[1], and Cynthia Fritschi[3], *1. College of Nursing, the University of Illinois Chicago, Chicago, Illinois, United States*, *2. The University of Illinois Chicago, Chicago, Illinois, United States*, *3. UIC College of Nursing, Chicago, Illinois, United States*

Family caregiving for individuals with dementia is associated with high levels of burden. Caregiver burden is likely contextual and related to sociocultural norms, including those for gender. We conducted a systematic review and meta-analysis to examine gender differences in caregiver burden among family caregivers of persons with dementia and to compare such gender differences across cultures.

After searching five databases—CINAHL, Medline/PubMed, EMBASE, PsycINFO, and Sociological Abstracts, we included relevant articles published in English through December 2022. Two independent reviewers extracted study data and assessed the risk of bias. Forty-three studies were included, and from those, 17 effect sizes were extracted for meta-analysis. We found that family caregivers providing homecare were mainly female and adult children of care recipients. Most studies used the Zarit Burden Interview to measure caregiver burden. Random-effects models revealed moderate effects of gender, with higher burden in female than in male family caregivers (d = 0.41, 95% confidence interval: 0.32 to 0.50, p<.05). However, we found that only 20% of Asian studies reported effects of gender on caregiver burden, while more than half the U.S. (83.33%) and European (56.41%) studies reported gender effects. We conclude that, due to sociocultural norms, female Asian caregivers may more readily accept their caregiving role than females in other regions. Thus, future studies should include measures of cultural expectations related to caregiving. Also, researchers should more extensively explore gender-specific sources of caregiver burden before creating gender-specific caregiver interventions and social policy recommendations for alleviating the burden of female caregivers.

Abstract citation ID: igad104.1387

### HIGHER STRESS AMONG ETHNIC MINORITY ADULT-CHILD CAREGIVERS COMPARED TO SPOUSAL CAREGIVERS IN DEMENTIA CARE

Jung-Ah Lee[1], Eunae Ju[2], Julie Kim[2], Eilleen Sabino-Laughlin[2], and Seunghye Hong[3], *1. University of California-Irvine, Irvine, California, United States*, *2. University of California, Irvine, Irvine, California, United States*, *3. University of Hawaii at Manoa, Thompson School of Social Work & Public Health, Manoa, Hawaii, United States*

Ethnic minority family caregivers of persons with dementia (PWD) suffer from negative health conditions resulting from continuous care responsibility for PWD. While adult-child caregivers have additional family care and job responsibilities, cultural and linguistic needs in ethnic minority families further compound caregiving challenges. Little research examines ethnic minority caregivers' health statuses. This study aimed to understand the emotional health status and dementia care experiences of minority caregivers, between spouses and adult-children. The study collected cross-sectional survey data on perceived stress and utilizations of caregiving resources: Alzheimer's agencies, state-funded in-home supportive service, adult-day service, support groups, and caregiving education. Recruitment focused on diverse communities with special emphasis on minority caregivers who speak Spanish, Vietnamese, or Korean in California. Seventy-six caregivers participated in surveys in their respective language; 42% Korean, 38% Vietnamese, 20% Spanish; 49% spouses/partners vs 51% adult-children/grandchildren; 78% females; 55% PWD with Medicaid; Mean English Proficiency (EP) of 3/5(1.3). Sixty-four percent of adult-child caregivers were employed (full-time or part-time) compared to 5% of spousal caregivers (P<.05). Adult-child caregivers showed higher stress compared to spousal caregivers (P<.05) despite no differences in PWD's activities of daily living (ADL) scores and instrumental ADL scores. Although EP of adult-child caregivers was higher than spousal caregivers (P<.05), both caregivers underutilized care resources and showed little awareness of Alzheimer's agencies. Findings demonstrated higher caregiving stress of adult-child caregivers compared to spousal caregivers of PWD among minority groups. Tailored interventions with appropriate language and cultural aspects should consider the caregiver's relationship to the PWD.

Abstract citation ID: igad104.1388

### RURAL–URBAN DIFFERENCES IN INFORMAL CAREGIVER RESOURCES, CAREGIVING INTENSITY, AND HEALTH-RELATED QUALITY OF LIFE

Steven Cohen[1], Neelam Ahmed[2], and Mary Greaney[1], *1. University of Rhode Island, Kingston, Rhode Island, United States*, *2. Brown University School of Public Health, Providence, Rhode Island, United States*

Rural-urban health disparities are pervasive among older adults and their caregivers. Rural areas in the US have a disproportionately high population of older adults, have reduced access to services, and are more reliant on family and friends for care. Research examining rural-urban disparities for informal caregivers is limited. There is a critical need to understand how caregiving intensity, access to resources, and caregiver health-related quality of life (HRQoL) differ by rural-urban status. Study objectives were to explore rural-urban differences in caregiving resources, intensity, and HRQoL. Data were abstracted from Waves 1 and 5 of the National Study of Caregiving. Rural-urban status was based on Rural Urban Continuum Codes. Generalized linear models, accounting for complex sampling, were used to explore associations between rural-urban status and caregiver resource utilization, caregiving intensity, and HRQoL. Compared to urban caregivers, rural caregivers were significantly more likely to be female (OR 1.19, 95%CI 1.12-1.26), older (beta=1.13 years, 95%CI 0.70-1.55), have obesity (OR 1.25, 95%CI 1.17-1.32), report poor/fair health (OR 1.08, 95%CI 1.01-1.16), and higher numbers of comorbidities (beta=0.18, 95%CI 0.13-0.22). Compared to urban caregivers, rural caregivers were significantly less likely to attend support groups (OR 0.55, 95% CI 0.46-0.65), but more likely to have caregiver training (OR 1.46, 95%CI 1.33-1.62). They assisted with fewer instrumental activities of daily living (beta=0.54, 95%CI 0.44-0.65), although there was not a significant difference for activities of daily living. Findings can facilitate the translation and adaption of policies, programs, and interventions to support and address the unique needs of rural caregivers.

Abstract citation ID: igad104.1389

### THE IMPACT OF COVID-19 ON RELATIONSHIPS BETWEEN CARE TEAMS AND FAMILY CAREGIVERS IN CONGREGATE CARE

Matthias Hoben[1], Emily Dymchuk[2], Stephanie Chamberlain[2], and Anna Beeber[3], *1. York University, Toronto, Ontario, Canada*, *2. University of Alberta, Edmonton, Alberta, Canada*, *3. Johns Hopkins University, Baltimore, Maryland, United States*

Family/friend caregivers are essential in the quality of care and life of residents living in congregate care (nursing homes and assisted living). However, the COVID-19 pandemic and related public health measures substantially changed the way family/friend caregivers could be involved in resident care. It also changed the dynamic of the relationships between care teams and family/friend caregivers. While research has explored these impacts, much of the research has focused on the family/friend caregiver perspective. Our objective was to explore the impact of COVID-19 and related public health measures on these relationships from the perspective of care teams. Using videoconferencing, we conducted 9 focus groups and 2 semi-structured interviews (total of 37 participants) with managers/administrators of 6 congregate care settings and their teams (care aides, nurses, recreation and rehabilitation staff, educators). The themes we identified reflect (1) pressures care teams experienced from family/friend caregivers, (2) conflicts between care teams and family/friend caregivers, (3) support care teams received from family/friend caregivers, and (4) how care teams supported family/friend caregivers. The COVID-19 pandemic disrupted long-standing relationships between care teams and family/friend caregivers, negatively impacting care teams, family/friend caregivers, and residents. However, care teams also reported encouraging examples of successful collaboration and support from family/friend caregivers. Involving family-friend caregivers in decision making was particularly critical. Learning from these promising practices will be critical to improving preparedness for future public health crises, as well as quality of resident care and life in general.

Abstract citation ID: igad104.1390

### UNPAID CAREGIVERS' PROCESS OF COLLABORATING DURING OLDER ADULT HOSPITAL TO HOME TRANSITIONS

Daniel Liebzeit, Olivia Geiger, Saida Jaboob, and Samantha Bjornson, *The University of Iowa, Iowa City, Iowa, United States*

Unpaid/family caregivers often provide support critical to older adult hospital to home transitions, but commonly lack time and preparation for their role. There is limited evidence regarding important collaboration and resources for caregivers during the transition. The objective of this grounded theory study was to examine caregivers' process of collaborating with others during older adult hospital to home transitions. Interviews were conducted with unpaid caregivers of adults aged 60 and older and recently discharged from a medical/surgical unit at a large Midwestern teaching hospital, were audio-recorded, and transcribed verbatim. Data were analyzed using open, axial, and selective coding. Caregiver participants (N = 16) included spouses (n = 8), friends (n = 4), children (n = 3), and siblings (n = 1) of an older adult transitioning from hospital to home, were mostly female (n=14), and all were white, non-Hispanic. A conceptual model was developed which illustrates participants' process of collaborating with a caregiving team, providers, networks, and other resources to support themselves and their older person. The process began with the participants taking on and identifying their role, and ultimately led to either supporting their older person's progress towards independence or providing long-term caregiver support. Conditions, including finances, caregiver health, expectations, proximity, and availability, impacted the collaborative process. Consequences varied based on participants' approach to caregiving, such a team versus individual approach. Implications include opportunities to promote active engagement of caregivers in the transition process and facilitation of a team-based approach to supporting the older person and caregivers.

### SESSION 4030 (SYMPOSIUM)

Abstract citation ID: igad104.1391

### EVALUATING AND ADDRESSING ASPECTS OF SOCIAL HEALTH AMONG AGING POPULATIONS

Chair: Allison Marziliano Discussant: Thomas Cudjoe

The physical and psychological aspects of human functioning have traditionally been at the heart of gerontological research. In recent years, however, social health, including such concepts as social isolation, social support and loneliness, have received increasing attention. This symposium describes research in this developing area, starting with a description of the evaluation of disparities in social health and culminating with an overview of the development process for interventions to improve poor social health. First, Victor and Taylor, in their respective presentations, focus on identifying disparities (racial, ethnic, gender, marital status, household composition) in social health across older adults in two geographic regions (United Kingdom, United States). Next, Marziliano describes the iterative process of developing 6 brief (~5 minute) videos to administer an adaptation of Meaning-Centered Psychotherapy for the purpose of increasing meaning and reducing loneliness in caregivers of persons with Alzheimer's Disease (AD) or AD-related dementias (ADRD). Development of this intervention consisted of three phases: preparatory work among the study team, interviews to collect stakeholder feedback with 15 AD caregivers, and construction of the web-based tool. Lastly, guided by Intervention Mapping, the Health Belief Model and Social Cognitive Theory, Thomas describes the process of developing an educational video and website to address social isolation among homebound older adults. This process incorporated multiple steps: a needs assessment during two focus groups; literature review; pilot of educational materials; and finally, implementation and evaluation of the intervention.

Abstract citation ID: igad104.1392

### THE DEVELOPMENT OF RELOAD-C, A WEB-BASED PLATFORM TO REDUCE LONELINESS IN CAREGIVERS OF PERSONS WITH ADRD

Allison Marziliano[1], Allison Applebaum[2], Edith Burns[3], Marzena Gieniusz[4], Jessica Mongelli[1], Priya Patel[1], Huma Babar[1], and Michael Diefenbach[1], *1. Northwell Health, Manhasset, New York, United States*, *2. Memorial Sloan Kettering Cancer Center, New York City, New York, United States*, *3. Zucker School of Medicine at Hofstra/Northwell, Manhasset, New York, United States*, *4. Hofstra-Northwell, New Hyde Park, New York, United States*

There is a need for effective evidence-based interventions to reduce loneliness in caregivers of persons with Alzheimer's Disease and Related Dementias (ADRD). Interventions to reduce loneliness may be strengthened by incorporating concepts from Meaning-Centered Psychotherapy (MCP), effective in increasing meaning in life in patients with advanced cancer and their caregivers. Our central hypothesis is that concepts from MCP can be adapted for caregivers of persons with ADRD to reduce their loneliness through increasing their sense of meaning and purpose in life. Our purpose is to describe the development of RELOAD-C (REducing LOneliness in Alzeheimer's Disease-Caregivers), a web-based platform that delivers MCP concepts, adapted for ADRD caregivers, via 6 brief videos, 7 virtual peer group meetings, and written content. The development of RELOAD-C occurred in 3 stages: 1) Preparatory Work/Adaptation of scripts from 6 existing MCP videos. 2) Stakeholder Involvement. N=15 AD/ADRD caregivers underwent individual interviews to provide expert feedback on the scripts. 3) Development of RELOAD-C. In collaboration with web specialists, RELOAD-C is created by integrating adapted MCP videos, links to virtual peer groups to discuss MCP concepts, and written MCP content. Input from stakeholders indicated: 1) preference for terminology (e.g., "caregiver" over "care partner"); 2) removing MCP content that urges caregivers to engage in complex conversations with the care recipient; 3) providing an explanation of ambiguous concepts (e.g., "unfinished business"); and 4) re-ordering the delivery of the MCP concepts. RELOAD-C is the product of multiple rounds of review by a multidisciplinary team and ADRD caregivers as expert stakeholders.

Abstract citation ID: igad104.1393

### RACIAL DIFFERENCES IN LONGITUDINAL TRAJECTORIES OF SOCIAL ISOLATION AND LONELINESS

Harry Taylor, *University of Toronto, Toronto, Ontario, Canada*

The purpose of this study was to determine if there are racial differences in the longitudinal trajectories of social isolation and loneliness among older adults. Data come from the Health and Retirement Study Leave Behind Questionnaire, waves 2006-2016. Social isolation was operationalized as a social network index and loneliness was operationalized by the UCLA-3 item loneliness scale. Race was operationalized by White, Black, and Hispanic. I also adjusted for age, gender, education, household income, and employment status. Racial differences in longitudinal trajectories were determined by multilevel models with survey weights included by the HRS. In unadjusted models, I found Hispanic older adults were less likely to be socially isolated compared to White and Black older adults; furthermore, White older adults were less likely to be lonely compared to Black and Hispanic older adults. In fully adjusted models, White older adults had greater social isolation in comparison to Black and Hispanic older adults, and Black older adults had greater isolation compared to Hispanic older adults. For loneliness, Black older adults were found to be lonelier compared to Hispanic older adults, but not White older adults. This study is one of the first to examine racial differences in longitudinal trajectories of social isolation and loneliness. I find race is a salient factor that may influence objective isolation and feelings of loneliness among older adults. Given the established relationship between isolation, loneliness, and health, these findings are useful for identifying potential racial groups at higher risk for these conditions and informing future interventions.

Abstract citation ID: igad104.1394

### DISPARITIES IN LONELINESS AMONG OLDER ADULTS IN THE UK: AN EVIDENCE OVERVIEW AND CRITICAL EVALUATION

Christina Victor, *Brunel University-London, Uxbridge, England, United Kingdom*

We propose that there are three elements to healthy ageing: physical, mental and social. As a concept social health is less well researched although there is a significant literature on specific aspects of this concept such as loneliness or isolation. Researchers in the USA and Europe focussing on inequality in later life health outcomes have predominantly emphasised inequalities in physical and/or mental health. It is only comparatively recently that attention has been given to addressing disparities in aspects of social health because of the wide-spread stereotype that 'all older adults' are lonely and isolated. UK studies consistently demonstrate that specific individual characteristics such as gender, marital status, household composition, physical/mental health, ethnicity and socio-economic status are associated with higher risk of loneliness. Interventions for loneliness are rarely effective. We critique this evidence base on three grounds. First analysis of loneliness disparities focuses on individual characteristics rather than considering an intersectional approach that addresses the complexity and multiplicity of characteristics and identities that each of us embody and how these are associated with loneliness and other social health outcomes. Second research evidence is predominantly cross-sectional with some longitudinal studies. We argue that to fully understand loneliness and other late life social health disparities we need to adopt a lifecourse approach. Thirdly analysis of disparities in loneliness emphasises individual characteristics rather than adopting an ecological perspective which incorporates these, the effects of time as well as macro-level factors such as cultural norms and meso neighbourhood type, urban design and community cohesion.

Abstract citation ID: igad104.1395

### USING INTERVENTION MAPPING TO DEVELOP RESOURCES TO PROMOTE SOCIAL ENGAGEMENT WITH HOMEBOUND OLDER ADULTS

Kali Thomas[1], Kristen Smith[2], Jennifer Bunker[2], and Kim Gans[2], *1. Johns Hopkins University, Baltimore, Maryland, United States*, *2. Brown University, Providence, Rhode Island, United States*

Homebound older adults are at increased risk of social isolation. Drivers who deliver meals to 800,000+ homebound older adults yearly are uniquely positioned to address social isolation through their conversations and daily interactions. The objective of this study was to develop and evaluate resources to educate meal-delivery drivers about social isolation and promote driver-client social engagement. We used Intervention Mapping (IM), a six-step theoretical- and evidence-based planning approach, to develop this intervention. First, we conducted a needs assessment through two focus groups with drivers and 12 interviews with subject matter experts (Step 1). Our literature review revealed the behavior outcomes, determinants, methods, and strategies to target (Steps 2&3). We piloted the intervention materials with stakeholders, meal-delivery drivers, and subject matter experts (Step 4). Our needs assessment indicated that drivers receive no training or education on social isolation. Drawing on the Social Cognitive Theory and Health Belief Model, we developed a 2.5 minute educational video and website with resources for workers who engage with socially isolated homebound older adults. The video and website included the following change methods and strategies: tailoring, modeling, chunking, cuing, raising, framing, and using imagery to influence behaviors. Feedback from drivers and experts on the resources was positive. The presenter will discuss the results implementing and evaluating the intervention (Steps 5&6) with meal-delivery drivers in Mississippi through an online survey. Our findings indicate the benefits of using an iterative, step-by-step intervention development framework to organize and structure an intervention to address social isolation among homebound older adults.

### SESSION 4035 (PAPER)

### AGING AND COGNITION (PAPERS) II

Abstract citation ID: igad104.1396

### BRAIN HEALTH TOGETHER: DEVELOPING AND PILOT-TESTING AN ONLINE GROUP PROGRAM TO REDUCE DEMENTIA RISK

Deborah Barnes[1], Coles Hoffmann[2], Margaret Chesney[1], Henry Brodaty[3], Linda Chao[1], Paul Tang[4], Rebecca Sudore[1], and Patricia Turo[2], *1. University of California, San Francisco, San Francisco, California, United States*, *2. Together Senior Health, San Francisco, California, United States*, *3. University of New South Wales, Sydney, New South Wales, Australia*, *4. Stanford University, Stanford, California, United States*

Growing evidence suggests that multi-domain interventions can slow cognitive decline and reduce dementia risk in older adults. However, most programs have been in-person and/or had relatively long intervention periods (1 to 2-years). The primary goal of this study was to develop and pilot-test Brain Health Together, a 12-week, virtual-group program to reduce dementia risk through targeting modifiable risk factors in older adults who report cognitive decline. Initial content was developed with input from an external Science Advisory Board. 7 individuals who had been participating in online group movement classes for the previous 2 years were invited to pilot-test and provide feedback on Brain Health Together content and structure. In addition to the movement classes, the pilot program included weekly 1-hour group brain health education classes and 30-minute individual brain health coaching sessions for 12 weeks. The first 4 education classes included general content. Based on participant feedback, subsequent classes provided in-depth content on a single brain health risk factor each week, including expert videos and facilitated discussion. Before their first coaching session, participants completed a brief survey about their current lifestyle behaviors and medical conditions related to brain health. This was used to generate an individualized brain health report that summarized areas with low, moderate or high opportunity for change. Coaching sessions focused on setting SMART (Specific, Measurable, Achievable, Realistic, Timely) goals to address modifiable risk factors. After 12 weeks, participants reported more exercise, less loneliness, and longer sleep duration. We are currently enrolling for a larger clinical trial.

Abstract citation ID: igad104.1397

### NOBODY CAN DRAG ME DOWN: AGING AS A DRAG PERFORMER

Laura Donorfio, Kathleen Henneberry, and Brian Chapman, *University of Connecticut, Waterbury, Connecticut, United States*

This presentation will detail the findings of an exploratory, qualitative research study examining the intersection of aging and drag performance. Even though drag queens have achieved significant levels of visibility in mainstream entertainment and have become increasingly present topics of discussion in news outlets, modern media, and even political landscapes, they are an understudied and underrepresented population (Knutson et al., 2020; O'Brien, 2018). Older drag queens have yet to be studied in the social science literature (Henneberry et al., 2022). "Drag Queens possess unique characteristics as a subpopulation of the lesbian, gay, bisexual, and transgender (LGBT) community that warrant exploration" (Knutson, Koch, Sneed, & Lee, 2018). This research aims to understand and represent the relatively understudied population of drag queens in academic literature while drawing attention to the specific sub-population of older adults in the profession. A semi-structured interview protocol and thematic analysis was utilized to understand the unique perspectives of self identified drag performers over the age of 50 (n=15; ages 54-94). Examining drag artistry and expression through the lens of aging has revealed themes of drag as a form of armor, ageism within the community, and shifts in social perceptions of drag performers. This presentation will introduce the term "dragism" in an attempt to explain the discrimination against drag queens within the LGBTQ+ community, as well as the interplay of both dragism and ageism for the participants of this study (Donorfio et al., 2022).

Abstract citation ID: igad104.1398

### PERSONALITY AND PLASMA BIOMARKERS OF REACTIVE ASTROGLIOSIS (GFAP) AND NEURONAL INJURY (NFL)

Antonio Terracciano[1], Keenan Walker[2], Yang An[2], Martina Luchetti[3], Abhay R. Moghekar[4], Angelina Sutin[3], Luigi Ferrucci[2], and Susan Resnick[2], *1. Florida State University, Tallahassee, Florida, United States*, *2. National Institute on Aging, National Institutes of Health, Baltimore, Maryland, United States*, *3. Florida State University, College of Medicine, Tallahassee, Florida, United States*, *4. Johns Hopkins University, Baltimore, Maryland, United States*

Personality traits are associated with cognitive functioning and risk of Alzheimer's disease and related dementias. To understand the biological mechanisms of these associations, it is essential to examine the amyloid (A), tau (T), and neurodegeneration (N) components of the ATN framework. A recent meta-analysis found that neuroticism and conscientiousness are associated with amyloid and tau.

However, less is known on whether personality traits are associated with markers of neurodegeneration. This study examines whether personality traits are concurrently related to plasma glial fibrillary acidic protein (GFAP), a marker of reactive astrogliosis, and neurofilament light (NfL), a marker of neuronal injury. Cognitively unimpaired participants from the Baltimore Longitudinal Study on Aging (N=786; age: 22-95) were assayed for plasma GFAP and NfL and completed the Revised NEO Personality Inventory (NEO-PI-R), which measures five domains and 30 facets of personality. Neuroticism (particularly vulnerability to stress, anxiety, and depression facets) was associated with higher GFAP and NfL. Conscientiousness was associated with lower GFAP. Extraversion (particularly positive emotions, assertiveness, and activity facets) was related to lower GFAP and NfL. These associations were independent of demographic, behavioral, and health covariates and not moderated by age, sex, or APOE genotype. The personality correlates of astrogliosis and neuronal injury tend to be similar, are found in individuals without cognitive impairment, and point to potential neurobiological underpinnings of the association between personality traits and neurodegenerative diseases.

### SESSION 4040 (PAPER)

### HEALTH AND SOCIAL INTERVENTIONS I

Abstract citation ID: igad104.1399

### A WHOLE-HEALTH MODEL OF CARE TO PROMOTE SOCIAL AND RACIAL JUSTICE

Anna Faul, Sam Cotton, Pamela Yankeelov, and Barbara Gordon, *University of Louisville, Louisville, Kentucky, United States*

Whole health models of care aim to coordinate care across sectors, address the needs of older adults with multiple chronic conditions, and promote health equity. The FlourishCare team will present data on the effectiveness of a whole health model, the FlourishCare Model (FCM), serving 159 older adults with multiple chronic conditions, using a two-level longitudinal design and hierarchical linear modeling to test a multilevel growth model. The results showed significant growth in total FlourishCare Index (FCI) scores over time for individual health behaviors, healthcare access, and social determinants. Biological determinants showed significant growth only for rural patients. The FCM was able to support patients with lower education attainment to improve at a higher rate than those with higher education attainment, both for the total score on the FCI and for healthcare access. The FCM was also able to support rural patients at a higher rate than urban patients to gain access to healthcare, and it supported Hispanic patients the most in improving their social determinants of health. The study demonstrates the importance of a whole health approach to care, which focuses on what matters most to patients, particularly older adults who place importance on quality-of-life outcomes. The study also shows how a whole health approach can address health equity by accelerating the flourishing of rural, Hispanic patients with lower educational attainment, promoting social and racial justice.

Abstract citation ID: igad104.1400

### MUSIC IN THE EVERYDAY LIFE OF PEOPLE LIVING WITH DEMENTIA

Joseph Kotarba[1], and Amanda Couve[2], *1. Texas State University, San Marcos, Texas, United States*, *2. Heritage Creek Assisted Living Center, San Antonio, Texas, United States*

A common approach to dementia care is the clinical application of music to improve cognition, memory, quality of life, etc. Although the literature on this work continues to expand, relatively little scholarly attention has been given to understanding common, everyday life experiences of music among those living with dementia. The present paper is derived from the qualitative research informing "Music in the Course of Life" (Routledge, 2023). Following the symbolic interactionist/existentialist paradigm, we reconfigured the life course model of organizing aging by seeing life course as a concept of the self that can be observed in various situations, that are not necessarily linearly ordered, in everyday life. We observed music as it is presented and performed in residential facilities, at home, in hospitals and in hospice. The self experience can be portrayed as "Being," "Becoming," and "Been There" in the case of many seniors. The primary value of music for dementia care is not predicated on eliciting music from memory or from regenerating cognition. The pleasures of music are exceptionally situational so that the elderly person need not have any prior exposure to or experience with music that now is positive affectively, cognitively and/or socially. The meaningful music situation--especially at end of life--is composed of significant others providing the music, attendees singing or humming along with the music, a caretaker to orchestrate the situation.

Abstract citation ID: igad104.1401

### PET OWNERSHIP AND LONGITUDINAL CHANGE IN PSYCHOLOGICAL ADAPTATION – EVIDENCE FROM THE BLSA

Erika Friedmann[1], Nancy Gee[2], Eleanor Simonsick[3], Barbara Resnick[1], Merve Gurlu[4], Ikmat Adesanya[5], and Soyeon Shim[5], *1. University of Maryland, Baltimore, Maryland, United States*, *2. Virginia Commonwealth University, Richmond, Virginia, United States*, *3. NIA IRP, Baltimore, Maryland, United States*, *4. University of Maryland, Baltimore, Baltimore, Maryland, United States*, *5. University of Maryland Baltimore, Baltimore, Maryland, United States*

Successful aging depends in part on maintaining psychological adaptation. Research examining the relationship of pet ownership (PO) or human-animal interaction (HAI) to human health supports contributions to psychological status, mostly in populations with diseases or disabilities. We examine the contributions of PO to maintaining psychological adaptation among generally healthy community-dwelling older participants in the Baltimore Longitudinal Study of Aging (BLSA). Participants (N=637, age M=68.3, SD=9.6 years, pet owners N=149) completed assessments of anxiety (Perceived Stress Scale-10), depression (Center for Epidemiological Studies Depression), happiness (1-10 rating) and psychological wellbeing (SF-12 Mental Component Score) every 1-4 years and a 10-year PO history. Linear mixed models with random intercepts were used to examine changes in psychological adaptation outcomes over up to 13 years (M=7.5, SD=3.6) according to time-varying PO. Overall anxiety decreased (b=-0.0360,se=0.0104,p=0.001) and depression increased (b=.099141, se=0.015837, p< 0.001) with aging. Psychological wellbeing (b=0.00055, se=0.00119, p=0.644) and happiness (b=-.00405,se=0.0206,p=0.844) did not change. PO moderated changes in anxiety (b=0.05 8452,se=0.024376,p=0.017), happiness (b=-0.101373,se=0 .04779,p=0.034), and psychological wellbeing (b=-0.0055 41,se=0.002764,p=0.045), but not depression (b=-0.01134, se=.037065,p=0.769). Anxiety and happiness improved in pet owners and deteriorated in non-owners. Psychological wellbeing deteriorated faster for pet owners than non-owners. This study provides important longitudinal evidence that PO may promote some aspects of psychological adaptation among generally healthy community-dwelling older adults by moderating age-related declines in psychological adaptation later in life. It also highlights differences in psychological responses to animal assisted interventions in residents of care facilities from changes during the life-courses of generally healthy community-residing older adults.

Abstract citation ID: igad104.1402

### TESTING MULTICOMPONENT DIGITAL THERAPIES TO PROMOTE HEALTHIER LIFESTYLE BEHAVIORS IN OLDER ADULTS: A PILOT STUDY

Lorraine Evangelista[1], Andrew-Thomas Reyes[2], Dieu-My Tran[2], and Reimund Serafica[3], *1. University of California Irvine, Irvine, California, United States, 2. University of Nevada, Las Vegas, Las Vegas, Nevada, United States, 3. UNLV, Las Vegas, Nevada, United States*

Multicomponent digital interventions to encourage healthy diet and physical exercise in older persons at risk for cardiovascular disease are understudied. We compared the feasibility and effectiveness of Get FIT vs. Get FIT+, two digital-based therapies, to encourage healthier lifestyle behaviors in this high-risk population. Fifty-four older adults (66.6 ± 5.9 years, 61% females, 61% married, 50% Asians, 39% Whites) were randomized to Get FIT (n=24), which included a health app, an activity tracker, and one 45-minute behavioral counseling session on healthy eating and exercise, or Get Fit+ (n=30), which added three months of health coach-personalized text messaging. We assessed retention, engagement, satisfaction, diet, physical activity, weight, cardiometabolic risks, and psychological well-being pre-and post-intervention. At three months, both groups had a 100% retention rate. Get FIT+ participants expressed high satisfaction, with 92% reporting that they would recommend Get FIT+. Individuals in both groups increased their physical activity and reduced their caloric consumption from baseline to three months. However, these changes were significantly greater in the intervention group (p .0001). Get Fit+ participants averaged a three-pound weight loss, whereas their counterparts averaged a three-pound weight gain (p .0001). Get FIT+ individuals had significantly lower systolic and diastolic blood pressure, triglycerides, total cholesterol, and HgbAa1C than Get FIT participants from baseline to three months. Both groups showed similar psychological well-being over time. Our findings imply that the Get Fit+ intervention may be more effective in lowering cardiometabolic risk in this cohort, although longer-term trials are needed.

### SESSION 4045 (PAPER)

### PREDICTING FUTURE OUTCOMES I

Abstract citation ID: igad104.1403

### AGE AND STRESSOR-RELATED NEGATIVE AFFECT IN OLDER ADULTS: CROSS-SECTIONAL AND LONGITUDINAL EVIDENCE

Giselle Ferguson[1], Daisy Zavala[2], Samantha Corley[3], and Stacey Scott[3], *1. Stony Brook University, The State University of New York, Stony Brook, New York, United States, 2. Stony Brook University, Stony Brook, California, United States, 3. Stony Brook University, Stony Brook, New York, United States*

Most daily stress studies show stressor-related increases in Negative affect (NA), but older adults may show smaller increases due to emotion-regulation skills. However, most of this work is based upon single burst diary or ecological momentary assessment (EMA) studies which are functionally cross-sectional age comparisons; longitudinal analyses are needed to reveal whether individuals change as they age. In this secondary analysis of data from a measurement burst study of older adults in Bronx, NY (N=305; Mage=76.98), we examined how age interacted cross-sectionally and longitudinally with the effect of stressors on NA. Between 2017-(pre-pandemic) 2020, participants completed up to three 14-day EMA bursts. Five times daily, they reported current NA and whether a stressor had occurred since the last assessment. In a 4-level multilevel model accounting for demographics, Neuroticism, and stressors, NA was significantly higher with reports of stressors, including at the moment-level, day-level, and person-level. Regarding aging's role in average NA for non-stressor moments, there were not significant cross-sectional age differences, but longitudinally across three bursts, NA increased significantly within-persons. We did not find evidence for cross-sectional age differences nor longitudinal change in the stressor-NA slope. Although neither cross-sectional age differences nor longitudinal change was found in the stressor-NA slope, we found significant longitudinal increases in non-stressor NA as individuals aged. Our results illustrate the value of measurement burst designs for disentangling differences between cross-sectional and longitudinal effects in daily processes. Further research on aging-related differences in affect and stress would benefit from these approaches.

Abstract citation ID: igad104.1404

### DAILY EMOTION REGULATION AND AFFECT ACROSS WORK AND FAMILY CONTEXTS IN MID LIFE

Madeline Nichols, and Kelly Chandler, *Oregon State University, Corvallis, Oregon, United States*

Emotion regulation is an important skill for adults to use in interpersonal situations; however, limited research has examined whether emotion regulation strategies vary by context or the potential influences these strategies have on daily affect. We examined the frequency of emotion regulation strategies used in work and family contexts and the extent to which these strategies predicted daily positive and negative affect (PA; NA). Middle-aged adults (N=27, Mage=60.15, SD=3.77; Female=91.67%) completed 7 daily diary surveys.

In both contexts, adults most often reported modifying situations to be more positive and using situational approaches. At work, adults also frequently expressed their emotions; at home, they reported modifying situations to be less negative. Preliminary multilevel model results indicated that NA was significantly higher on days when individuals reported distracting themselves from work situations (Est.=7.36, p<.01), suppressing emotions at work (Est.=3.29, p<.01), or expressing emotions around family (Est.=4.41, p<.01) compared to days when they did not use these strategies; conversely, on days when individuals distracted themselves from family situations, they reported lower NA (Est. =-2.80, p=.03) than on days they did not. On average, people who modify family situations to be more positive report higher PA (Est.=6.73, p<.01) than those who do not. Our results suggest that there is daily variability in emotion regulation strategy use and consequential impacts on affectual wellbeing by context. Understanding which emotion regulation strategies yield the most benefit in different contexts may inform interventions targeting emotion regulation as a modifiable behavior to promote health and wellbeing in later life.

Abstract citation ID: igad104.1405

### INVESTIGATING RELATIONSHIPS BETWEEN MUSIC REWARD AND EMPATHY ACROSS THE ADULT LIFESPAN

Naomi Adjei[1], Amy Belfi[2], and Janelle Beadle[3], *1. University of Nebraska Omaha, Omaha, Nebraska, United States*, *2. Missouri S&T, Rolla, Missouri, United States*, *3. University of Nebraska at Omaha, Omaha, Nebraska, United States*

Music can evoke positive mood and even enhance empathy towards others. Understanding the mechanisms supporting music-based enhancements in emotion are essential for developing interventions for older adults with chronic conditions, such as Alzheimer's disease. Previous studies have primarily focused on younger adults and have shown that trait empathy can modulate responses to music through engaging empathy-related brain networks. However, little is known about the specific relationship between music reward and trait empathy, especially across the adult lifespan. In the present study, we hypothesized that there would be a positive relationship between music reward and empathy. The sample included healthy adults 19-86 years (N=74; Mage=54.36; 62.2% female; Medu=16.2). Participants completed the Barcelona Music Reward Questionnaire (BMRQ), a 20-item questionnaire assessing music reward/appreciation and the Empathy Quotient Inventory (EQ), a 40-item questionnaire measuring multidimensional empathy (i.e., cognitive empathy, emotional reactivity, and social skills). We conducted a linear regression investigating the degree to which empathy predicted significant variance in music reward (age and gender were covariates of no interest). The overall model was significant (p=.007) and there was a significant, positive relationship between the EQ total score and the BMRQ total score (p=.004). Exploratory analyses demonstrated that the BMRQ total had positive correlations with cognitive (p=.006) and emotional empathy (p=.004). In summary, music reward was positively associated with empathy in this adult lifespan sample, adding to the literature focused on younger adulthood. Future research is needed to examine relationships between empathy and other aspects of music experience across the adult lifespan.

Abstract citation ID: igad104.1406

### SELF-RATINGS OF HEALTH BY AGE AND BIRTH COHORT: FOR WHOM DO THEY PREDICT MORTALITY?

Ellen Idler, and Emily Dore, *Emory University, Atlanta, Georgia, United States*

Global self-rated health as a predictor of mortality has become one of the most reliable outcomes in population-based follow-up studies. However, few studies focus on age or birth cohort differences in the mortality predictiveness of self-ratings of health. Personal knowledge of health accumulates throughout the life course, leading to the hypothesis that self-rated health would be most predictive in old age or in earlier birth cohorts. However, the pronounced effect of self-rated health on mortality means that those with the higher mortality risk of poor self-rated health in earlier adulthood would be less likely to survive to old age, potentially diminishing the effect through selective survival. In this paper we test for potential variations in the effects of age and birth cohort on the association between self-rated health and mortality in a large sample of adults aged 25-101 in the Panel Study of Income Dynamics (N=6437) followed from 1999-2019. We ask if self-rated health -- net of sociodemographic factors, physical and mental health conditions, and health risk behaviors – is equally predictive of mortality in three age groups spanning young, middle, and late adulthood, and in four birth cohorts. Fully adjusted models show that self-rated health was the most predictive for the group aged 41-64, and for those born 1946-1980, and that there were nearly null effects for those aged 25-40 or born 1981-1994. Thus, the effects of self-rated health on mortality vary by age and cohort, in ways that support both knowledge-based and selective survival interpretations of the association.

### SESSION 4050 (SYMPOSIUM)

Abstract citation ID: igad104.1407

### DEMYSTIFYING IMPACTS OF NEIGHBORHOOD ENVIRONMENT ON HEALTH OF OLDER CHINESE AMERICANS

Chair: Yanping Jiang Discussant: Fengyan Tang

Residential segregation is a key social determinant of health that disproportionately affects racial/ethnic minority and immigrant populations. The health impact of residential segregation and subjective neighborhood environment, however, is poorly understood among older Asian Americans. Findings from the limited existing studies on this topic exclusively relied on cross-sectional data and/or masked distinctive differences across Asian subgroups due to the usage of aggregated data. The purpose of this symposium is to provide a better understanding of health impacts of residential segregation and subjective neighborhood environment among older Chinese Americans, the largest Asian subgroup in the U.S. Findings presented in this symposium were from the Population Study of Chinese Elderly in Chicago (PINE) study, a longitudinal study of community-dwelling older Chinese immigrants living in greater Chicago.

Session 1 describes key characteristics of neighborhood, sociodemographic, and health behaviors of older Chinese immigrants living in segregated neighborhoods. Session 2 moves beyond to determine unique and joint effects of residential segregation and neighborhood socioeconomic status on 8-year change in cognitive functioning. Session 3 focuses on mental health by investigating main and indirect effects of residential segregation on depression via social processes. Session 4 tests the interactive effects of social support with residential segregation on cognitive functioning and depression. Lastly, Session 5 demonstrates how subjective neighborhood environment, directly and indirectly, affects depression via coping. In sum, findings provide innovative and important evidence to facilitate a better understanding of health implications of living in segregated neighborhoods among older Chinese Americans.

Abstract citation ID: igad104.1408

### NEIGHBORHOOD SEGREGATION AND CHARACTERISTICS OF OLDER CHINESE IMMIGRANTS LIVING IN GREATER CHICAGO

Yanping Jiang[1], Fengyan Tang[2], and Dinesh Mendhe[3], *1. Rutgers University, New Brunswick, New Jersey, United States*, *2. University of Pittsburgh, Pittsburgh, Pennsylvania, United States*, *3. Rutgers University New Brunswick, Piscataway, New Jersey, United States*

Neighborhoods profoundly influence health of older adults, with those living in more affluent neighborhoods tending to be happier and healthier. However, which neighborhoods to live, is shaped by pull and push factors, particularly among racial/ethnic minorities that disproportionally live in segregated neighborhoods characterized by social and economic deprivation. Residential segregation, however, is not well-examined among older Asian Americans. This study aimed to close this gap by investigating neighborhood, sociodemographic, and health behavior characteristics among older Chinese immigrants living in segregated neighborhoods. Data were drawn from 3,097 participants from Population Study of Chinese Elderly (PINE). Residential segregation was operationalized using Index of Concentrations at the Extremes (ICE), and the tertiles of ICE scores were used to categorize neighborhoods into four groups, ranging from neighborhoods extremely segregated toward Chinese speakers to segregated toward English-only speakers. Results showed that, compared to neighborhoods segregated toward English-only speakers, neighborhoods extremely segregated toward Chinese speakers had lower neighborhood socioeconomic status and cohesion but higher neighborhood disorder. Also, participants in neighborhoods segregated toward Chinese speakers were more likely to be unmarried but less likely to live alone, receive less education, and live in the U.S. longer. These participants also reported lower acculturation and racial discrimination, were more likely to smoke, and had lower adoption of preventive health practices than those living in neighborhoods segregated with English-only speakers. Findings highlight challenges and opportunities among older Chinese immigrants living in segregated neighborhoods and provide insights into existing mixed findings on health impacts of residential segregation among this understudied population.

Abstract citation ID: igad104.1409

### NEIGHBORHOOD SEGREGATION, SOCIOECONOMIC STATUS, AND COGNITIVE FUNCTIONING AMONG OLDER CHINESE IMMIGRANTS

Fengyan Tang[1], Yanping Jiang[2], Ke Li[3], and Andrea Rosso[1], *1. University of Pittsburgh, Pittsburgh, Pennsylvania, United States*, *2. Rutgers University, New Brunswick, New Jersey, United States*, *3. University of New Hampshire, Durham, New Hampshire, United States*

The fast-growing population of older Chinese immigrants and their segregated residence highlight the importance of understanding the role of neighborhood context in cognitive health. The segregation-cognition association is equivocal based on a limited number of studies among Hispanic and Asian Americans. To close the knowledge gap, this study examined the associations of neighborhood segregation and socioeconomic status (NSES) with cognitive functioning among older Chinese immigrants. Four waves of cognitive performance tests were conducted in the Population Study of Chinese Elderly in Chicago (2011-2019) and linked to the 2010-2014 American Community Survey estimates of neighborhood contexts. NSES was a summary z-score of six census variables of education, income/wealth, and occupation. Neighborhood segregation was measured by the Index of Concentrations at the Extremes (ICE) that simultaneously assesses Chinese and English language use within a given census tract. There were 170 census tracts in the present sample of 2,044 participants. Latent growth curve models with adjusted cluster robust standard errors were estimated. On average, cognitive functioning declined over time ($b= -0.07$, $p<.001$). After adjusting for individual-level predictors, living in high-NSES neighborhoods was associated with slower cognitive decline ($b=0.003$, $p=0.04$). ICE was not associated with cognitive functioning, but boosted the protective effect of high NSES on cognitive decline ($b=0.006$, $p=0.05$). Neighborhood socioeconomic advantage was related to slower cognitive decline among older Chinese immigrants, especially among those living in neighborhoods with more English speakers or less segregation. This finding suggests complex associations between neighborhood context and cognitive health among Chinese immigrants.

Abstract citation ID: igad104.1410

### RESIDENTIAL SEGREGATION AND DEPRESSIVE SYMPTOMS IN OLDER CHINESE IMMIGRANTS: MEDIATING ROLE OF SOCIAL PROCESSES

Ke Li[1], Fengyan Tang[2], Yanping Jiang[3], and Andrea Rosso[2], *1. University of New Hampshire, Durham, New Hampshire, United States*, *2. University of Pittsburgh, Pittsburgh, Pennsylvania, United States*, *3. Rutgers University, New Brunswick, New Jersey, United States*

Older Chinese immigrants are particularly at risk for depression due to acculturative stress and language barriers. Residential segregation with respect to language use may play an important role in mental health of historically marginalized immigrant populations. Previous research provided mixed evidence about the segregation effect among older Latino and Asian immigrants. Guided by a model of social processes, we examined the direct and indirect effects of residential segregation on depressive symptoms via multiple mechanisms of acculturation, discrimination, social network, social support, social strain, and social engagement. Four waves of depressive symptoms were assessed in the Population Study of Chinese Elderly (2011-2019, N=1,970), and linked to the 2010-2014 American Community Survey estimates of neighborhood context. Residential segregation was measured by the Index of Concentrations at the Extremes that simultaneously assesses Chinese and English language use within a given census tract. Latent growth curve models with adjusted cluster robust standard errors were estimated after controlling for individual-level factors. Residents of segregated Chinese-speaking neighborhoods had fewer baseline depressive symptoms but a slower decline rate than those living in segregated toward English-only speaking neighborhoods. Racial discrimination, social strain, and social engagement partially mediated the association between segregation and initial level; social strain and social engagement also partially mediated the association with decline in depressive symptoms. This study demonstrates the importance of residential segregation and social processes in shaping mental well-being among older Chinese immigrants and suggests potential mechanisms to alleviate mental health risks.

Abstract citation ID: igad104.1411

**SOCIAL SUPPORT, RESIDENTIAL SEGREGATION, AND HEALTH OF OLDER CHINESE AMERICANS**

Yiming Ma[1], Yanping Jiang[1], Stephanie Bergren[2], and Fengyan Tang[3], *1. Rutgers University, New Brunswick, New Jersey, United States*, *2. Rutgers, the State University of New Jersey, New Brunswick, New Jersey, United States*, *3. University of Pittsburgh, Pittsburgh, Pennsylvania, United States*

The limited but increasing body of research on residential segregation among U.S. older Asian Americans shows inconsistent findings on the health impact of residential segregation. These mixed findings suggest that the relationship between residential segregation and health may depend on some individual characteristics. This study aimed to investigate the moderating effect of perceived social support on the relationship between residential segregation and the two health outcomes: depressive symptoms and cognitive function among older Chinese Americans. Data were drawn from 3,097 older Chinese immigrants from the PINE Study in 2011-2013. Residential segregation was measured by the Index of Concentrations at the Extremes (ICE) at census tract level, which captured the concentration of Chinese-only speakers and English-only speakers at extreme. The social support measurement was the perceptions of social support provided by spouse, family, and friends. Depressive symptoms were measured by Patient Health Questionnaire (PHQ-9). Cognitive function was assessed by Mini-Mental State Examination, executive function, working memory, and episodic memory measures. Results showed a positive and significant moderating impact of social support on the relationship between ICE and depressive symptoms ($\beta=0.086$, $p=0.034$). Specifically, living in neighborhoods segregated towards Chinese-speaking was associated with fewer depressive symptoms at high, but not low levels of perceived social support. There were no moderation effects of perceived social support on the relationship between ICE and cognitive function. These results indicate the protective effect of living in neighborhoods segregated towards Chinese-speaking on depression, but only when older Chinese immigrants have high levels of perceived social support.

Abstract citation ID: igad104.1412

**NEIGHBORHOOD AND DEPRESSION AMONG US CHINESE OLDER IMMIGRANTS: THE MEDIATION EFFECTS OF COPING**

Yi Wang[1], Man Guo[1], Jinyu Liu[2], Yifan Lou[3], and Kara Carter[1], *1. University of Iowa, Iowa City, Iowa, United States*, *2. Baylor University, Waco, Texas, United States*, *3. Yale University, New Haven, Connecticut, United States*

Studies have shown that neighborhood environment shapes older Americans' aging experience and health. However, it remains largely unknown whether and how neighborhood environment influences the well-being of older Asian Immigrants. Guided by the neighborhood stress process model, this study aims to investigate (a) the associations between neighborhood environmental stressors and depression among Chinese older immigrants and (b) the potential mediation effects of intrapersonal (sense of mastery and sense of hopefulness) and interpersonal coping (social engagement) resources in such associations. This study analyzed data collected from 2,801 Chinese older immigrants in the greater Chicago area. Structural equation modeling with bootstrap resampling was used to fit path models on neighborhood environmental stressor, intra- and interpersonal coping resources, and depression. Findings showed that neighborhood social disintegration and physical disorder were associated with more depressive symptoms directly and indirectly via lower intra- and interpersonal coping resources. Specifically, older immigrants living in neighborhoods with greater social disintegration reported lower sense of mastery and social engagement, which in turn were associated with more depressive symptoms (partial mediation). Older immigrants living in neighborhoods with greater physical disorder reported lower sense of hopefulness and mastery, which subsequently were associated with more depressive symptoms (full mediation). The findings showed that neighborhood environmental stressors are risk factors for mental health of Chinese older immigrants, and coping resources may serve as pathways of the associations. The findings have implications for practice as they are helpful to inform the development of tailored psychosocial interventions to enhance coping resources in older immigrants.

### SESSION 4055 (SYMPOSIUM)

Abstract citation ID: igad104.1413

**THE IMPORTANCE OF ROMANTIC PARTNERS FOR HEALTH—SHARED TIME, DAILY ACTIVITIES, AND COUPLE DYNAMICS**

Chair: Martina Luchetti

Research has shown that social relationships, particularly with a spouse or romantic partner, affect health, for better or worse. More work is needed to better understand relational dynamics within couples and their cascade effects on health throughout the life course. This symposium brings together four empirical studies that examine the importance of romantic relationships for multiple aspects of health, from biological markers (i.e., inflammation) to mental health and social connection, using diverse methodologies, from macro (long-term) and micro (short-term) longitudinal assessments to dyadic analysis. First, Dr. Stokes and colleagues examine marital status in the Health and Retirement Study and find that transitions, such as becoming widowed (as compared to remaining married), heighten inflammation and potentially harm health. Second, Dr. Karakose and colleagues examine perceptions of time spent together within married couples and find that time together is protective against stress for both partners, and against depressive symptoms for wives. Third, Dr. Luchetti and colleagues examine momentary, phone-based assessments of loneliness within romantic couples and find loneliness in males to affect subsequent loneliness in their female partners. Finally, Dr. Nikitin and colleagues examine possible sources of loneliness within couples and find an association between low-quality daily activities with partners and momentary/daily loneliness. These studies focus on adults across the lifespan, including middle adulthood, which is a critical yet under studied period of life and regarded as a window of opportunity for interventions to improve health. Results highlight the importance of couple dynamics for the health and well-being of aging adults.

Abstract citation ID: igad104.1414

### MARITAL STATUS TRANSITIONS AND INFLAMMATION DURING MID AND LATER LIFE: EVIDENCE FROM THE HEALTH AND RETIREMENT STUDY

Jeffrey E. Stokes[1], Heather Farmer[2], and Sarah Patterson[3], *1. University of Massachusetts Boston, Boston, Massachusetts, United States*, *2. University of Delaware, Newark, Delaware, United States*, *3. University of Michigan, Ann Arbor, Michigan, United States*

Research has increasingly utilized inflammatory markers to understand how social factors influence health, yet little research to date has examined marital status, or–crucially–later-life changes to marital status in relation to inflammation. Further, few studies have examined variation in such associations by gender, race/ethnicity, or age. We used data from the 2016 Health and Retirement Study (HRS) Venous Blood Study (n=9,933), which captures five inflammatory markers: C-reactive protein (CRP), Interleukin-6 (IL-6), Interleukin IL-10 (IL-10), Interleukin-1 receptor antagonist (IL-1Ra), and tumor necrosis factor alpha (TNF). Information concerning marital status came from the 2014 and 2016 waves of HRS, to ascertain stability of and changes to marital status over 2 years. Interactions were examined between marital status/transition groups and participants' gender, age, and race/ethnicity. Overall, results were consistent across outcomes. Across 4 of 5 outcomes, the Newly Widowed and Never Married exhibited higher inflammation than the Continuously Married. Further, relationships between Continuously Widowed status and higher CRP and IL-6, and between Newly Widowed status and higher IL-1Ra, were present only for men. The association of Continuously Widowed status with higher CRP and IL-6 weakened with age, as did the effect of Newly Married status on IL-6. No clear patterns were identified concerning interactions with race/ethnicity. Findings indicate that marital transitions may have potential to harm physiological health. Negative consequences of widowhood for inflammation were also greater for men than for women, yet these harms may be attenuated at older ages when widowhood is an "on-time" rather than an "off-time" event.

Abstract citation ID: igad104.1415

### ASSOCIATIONS BETWEEN SHARED TIME AND MENTAL HEALTH IN MARRIED COUPLES

Selin Karakose[1], Thomas Ledermann[2], Angelina Sutin[1], and Antonio Terracciano[2], *1. Florida State University College of Medicine, Tallahassee, Florida, United States*, *2. Florida State University, Tallahassee, Florida, United States*

Spending time with the partner has been found to be beneficial in many ways for both the relationship and the individuals. However, little is known about the associations between time spend together and the partner's mental health. The present study examined the dyadic associations between time spent with spouse and mental health, particularly depressive symptoms, anxiety, and stress. Using data from 145 married couples from Turkey (M wives = 39.84 years, M husbands = 42.97), we investigated whether time spent with the spouse is associated with the mental health of both the individual and their spouse. Actor-Partner Interdependence Model (APIM) analyses indicated that both husbands' and wives' perception of time spent together was associated with lower stress. Additionally, for wives, time spent together was negatively associated with their own depression. These findings revealed that spending time together can help to cope with stress and have a positive effect on wives with elevated depressive scores.

Abstract citation ID: igad104.1416

### IS LONELINESS CONTAGIOUS? AN ECOLOGICAL MOMENTARY ASSESSMENT WITHIN ROMANTIC COUPLES

Martina Luchetti[1], Fiona Rupprecht[2], Jana Nikitin[2], Alyssa Gamaldo[3], Jacqueline Mogle[3], Thomas Ledermann[4], Martin Sliwinski[5], and Angelina Sutin[5], *1. Florida State University, College of Medicine, Tallahassee, Florida, United States*, *2. University of Vienna, Vienna, Wien, Austria*, *3. Clemson University, Clemson, South Carolina, United States*, *4. Florida State University, Tallahassee, Florida, United States*, *5. Pennsylvania State University, State College, Pennsylvania, United States*

Although marriage has been found to be protective against feeling lonely, loneliness might also be 'contagious' and spread from one individual to another, particularly within close relationships. Feelings of loneliness tend to correlate within spouses and are associated with relationship strain and lower perceived support from the partner. Despite interest in loneliness among married couples, little is known about how loneliness might affect romantic partners, especially in everyday life. In this study, we examined momentary states of loneliness within middle-aged and older couples, and how these states affect each partner over the course of days. Data were from 137 opposite-sex couples (274 participants), between 40 and 70 years old, who completed phone-based assessments of loneliness on a scale from 0to 100, three times a day, for eight consecutive days. Data were modeled using a combination of dynamic structural equation modeling and actor partner interdependence modeling. For both couple members, auto-regressive associations indicated that loneliness states carried over from moment to moment for both partners. In addition, loneliness of the female partner at time t was associated with loneliness of the male partner at the previous assessment (time t - 1), but not vice versa. Partner effects were in part moderated by relationship quality (strain, perceived support). The results provide insight into how loneliness functions within couples in everyday life contexts and suggest that loneliness might have dyadic implications for the health and well-being of aging couples.

Abstract citation ID: igad104.1417

### QUALITY OF DAILY ACTIVITIES AND LONELINESS IN A ROMANTIC RELATIONSHIP

Jana Nikitin, Fabio Selovin, and Fiona Rupprecht, *University of Vienna, Vienna, Wien, Austria*

Romantic relationships can be a source of both the most positive and the most negative experiences. Thus, partners can feel fully integrated or lonely in a romantic relationship, depending on the quality of the relationship. Cross-sectional and longitudinal studies have shown that loneliness (defined as a subjectively perceived deficiency in a person's social relationship) is more pronounced in low-quality relationships. Little is known about daily sources of loneliness within a couple. To explore this question, we investigated whether low-quality activities in a partnership can lead to feelings of loneliness in daily situations. To examine this question, we analyzed data from 72 individuals (M age = 43 years, range = 18 - 82 years; 64% female) who provided 576 measurement occasions on seven consecutive days in which they were involved in activities with their romantic partners. Participants indicated how they experienced the activity in each situation (positively or negatively) and how lonely they felt. Results showed that participants who reported lower-quality activities with their partner reported higher levels of daily loneliness (47% explained variance); and on occasions when participants reported lower-quality activities than usual, they also reported higher levels of loneliness (25% explained variance). Moreover, individuals who reported higher levels of loneliness during the 7-day assessment benefited less from high-quality activities with their partner in terms of reduced loneliness. Thus, loneliness not only results from, but also contributes to, how people experience daily activities with their partners. Gender and age had no effect on these associations, suggesting generalizability of the results.

### SESSION 4060 (SYMPOSIUM)

Abstract citation ID: igad104.1418

### OLDER ADULTS WITH MULTIPLE CHRONIC CONDITIONS FROM VULNERABLE POPULATIONS: THE SUCCESS OF PATIENT PRIORITIES CARE

Chair: Rafael Samper-Ternent Discussant: Lee Jennings

As older adults in the USA grow more diverse, equitable translation of knowledge and best practices to vulnerable groups is essential. Older adults with multiple chronic conditions (MCC) benefit from care focused on the health priorities of individuals. Health priorities include: 1) core values that provide meaning to life; 2) specific, actionable, and realistic health goals that reflect these values; and 3) care preferences that include what individuals are able and willing to do (or not) to achieve their goals. For vulnerable older adults, adverse life experiences provide context to their values. A diverse panel will review the Patient Priorities Care (PPC) approach and describe how PPC has been successfully adapted at different institutions. The first presentation will describe PPC and the importance of identifying values, goals, and health preferences for older adults with MCC. The presenter will show that PPC improves outcomes among older adults, their families, and healthcare providers. The second presentation will describe the implementation of PPC in the VA system. The presenter will describe the impact of PPC and efforts to disseminate it. The third and fourth presentations will describe the cultural adaptation of PPC for older African Americans and older Hispanics respectively. The presenters will describe the adaptation process and discuss the barriers they faced, and the lessons learned from each population group. The panel will conclude by providing suggestions on how to disseminate and increase the adoption of PPC to identify what matters most for diverse older adults.

Abstract citation ID: igad104.1419

### ADAPTATION OF THE PPC APPROACH FOR HISPANIC OLDER ADULTS

Rafael Samper-Ternent[1], Gilberto Perez[1], Melissa deCardi Hladek[2], and Carolina Fonseca[3], *1. UTHealth Houston, Houston, Texas, United States*, *2. Johns Hopkins School of Nursing, Baltimore, Maryland, United States*, *3. Harvard Medical School, Boston, Massachusetts, United Sta tes*

Older Hispanics have a high prevalence of MCC, contributing to a higher risk for dementia. As a result, there is a rapid increase in the number of young Hispanics assuming caregiver roles. The caregiver role is a cultural expectation among Hispanics and caregiving by the family is preferred over professional caregiving. There is limited information on how the unique context of Hispanics impacts the dyadic interactions between older adults and their care partners. There is a gap in the literature on how health values among Hispanics frame their healthcare goals and preferences and whether care partner involvement impacts the identification of these goals and preferences. There is also a limited number of evidence-based interventions that can help shift the current healthcare paradigm from clinical-guideline-based care to patient-centered care for underrepresented older adults. Thus, we used the Heuristic Framework to culturally adapt PPC for Hispanics with MCC and cognitive impairment living in Texas and embed it in two Geriatrics Clinics. We will describe the adaptation and implementation process and share findings from a pragmatic pilot study with 25 older Hispanics that participated in the PPC approach. We will share the outcomes from this pilot and the feedback received from patients, caregivers, and clinicians on the implementation of PPC at these clinics. We will conclude by sharing the current plans to expand the outreach of PPC for diverse older adult populations.

Abstract citation ID: igad104.1420

### PATIENT PRIORITIES CARE: THE PAST, THE PRESENT, AND THE FUTURE

Aanand Naik, *University of Texas Health Science Center at Houston, Houston, Texas, United States*

Healthcare based on patient goals can improve the care of older adults, particularly those with MCC. Prior studies have identified five healthcare values that guide how patients establish healthcare goals and make medical decisions: 1) Self-sufficiency, 2) Enjoyment, 3) Connection, 4) Balancing quality and length of life, 5) Engagement in care. The Patient Priorities Care (PPC) approach is an evidence-based approach that allows patients to identify their healthcare values, or what matters most to them, and identify one thing, the most important goal they want to address based on their healthcare preferences. We have demonstrated that PPC is feasible and results in less burdensome care, including fewer medications and referrals to specialists. In the VA system, we have also shown that PPC helps clinicians recommend home and community services that are aligned with the patient's priorities. To enhance its relevance for Veterans, African Americans, and Hispanics, a deliberate effort to adapt PPC has been used at different institutions. We believe that evidence-based interventions like PPC can help address health disparities by: 1) improving communication between healthcare providers and patients with different beliefs, race, ethnicity, and culture; and 2) aligning prevention and timely treatment with the health priorities of older adults with MCC. We will review what we have learned from PPC thus far. We will then describe current efforts to implement PPC in a more effective and sustainable manner and disseminate it in diverse healthcare systems. Finally, we will share our vision moving forward and opportunities for collaboration and growth.

Abstract citation ID: igad104.1421

**PATIENT PRIORITIES CARE FOR VETERANS**

Marcia Mecca, *VA Connecticut/Yale School of Medicine, West Haven, Connecticut, United States*

People receiving care in the Veterans Affairs (VA) health system are older, have greater socioeconomic disadvantage, and more chronic conditions than those receiving healthcare outside of VA. PPC is well-suited for older adults with multiple chronic conditions and aligns with the VA's Whole Health movement. A small VA PPC pilot study demonstrated that those receiving PPC compared to usual care had fewer medications added and more home and community services aligned with priorities. VA primary care is a cornerstone and hub for veteran care utilizing a team-based approach. A two-site randomized controlled trial exploring the implementation of PPC in VA primary care is currently recruiting veterans. The early phase of the project involved semi-structured formative assessment interviews with key stakeholders to obtain feedback and perspectives on PPC implementation in VA primary care. Interviews were audio recorded, transcribed verbatim, and analyzed using thematic analysis. Concerns elicited were time constraints, follow up and documentation of changes in priorities over time, managing patient expectation, communication between primary care and specialty care, as well as how PPC would impact quality metrics. In response to these findings, the patient priorities note template was shortened and streamlined, and the study communications workflow was adapted to align with clinician preferences. Succinct templated language for care alignment was crafted and shared with study providers. Education for clinicians highlighted ways in which priorities could be used to optimize communication. Study procedures were added to identify quality metric exclusions. The study will explore how PPC impacts veteran care and experience.

Abstract citation ID: igad104.1422

**FORMATIVE EVALUATION TRIAL OF THE "MY HEALTH PRIORITIES" PROGRAM IN SOUTHERN OLDER AFRICAN AMERICANS: THE BHIP STUDY**

Deborah Ejem, *University of Alabama at Birmingham, Birmingham, Alabama, United States*

Older African Americans (AA) with multiple chronic conditions (MCCs) living in the Deep South are less likely to have access to early palliative care (PC) despite experiencing higher symptom burden and healthcare use, and poorer communication around goals of care. This disparity in PC use may partly be due to a lack of culturally-responsive care practices that effectively activate AAs with MCCs to identify their own values and priorities for end-of-life care. We conducted a formative evaluation of the web-based, self-directed "My Health Priorities" Identification Program to determine cultural acceptability and feasibility of use among AAs with MCCs in a primary care setting. We are now recruiting 20 AA patient with MCC and caregiver dyads from UAB Kirklin Primary Care Clinic. Recruited dyads will complete the "My Health Priorities" program and participate in semi-structured acceptability interviews. Patients and caregivers will also complete pre- and post-test measures of perception of care, treatment burden, shared decision-making, and communication exchange. Preliminary findings from six dyads suggest that the current program lacks spirituality-specific content. Dyads also expressed that the exemplar character included in the program was not relatable or representative. Overall, participants stated that the program was useful in helping them to think about and articulate their healthcare values and priorities. The findings from the research will directly inform a small-scale pilot grant that will assess the acceptability, feasibility, and potential efficacy of a values solicitation and operationalization intervention for AAs with MCCs and caregivers.

### SESSION 4065 (BIOLOGICAL SCIENCES INVITED SYMPOSIUM)

Abstract citation ID: igad104.1423

**COMPARATIVE TRANSCRIPTOMIC OF LONGEVITY**

Chair: Vera Gorbunova

Transcriptome analysis provides a nuanced view into the changes that occur in cells and tissues. Transcriptome changes rapidly and reproducibly in response to physiological influences and environmental insults. Recent years have seen an exponential increase in transcriptome data at bulk, single cell and spatial resolution that allows insights into the mechanisms and regulatory pathways of aging and longevity. In this session Drs. Gorbunova (University of Rochester) and Gladyshev (Harvard Medical School) will discuss comparative transcriptomics of longevity across species with diverse lifespans that revealed unique signatures of longevity and the integration of transcriptome and proteome data. Dr. Gladyshev will discuss development of transcriptomic clocks of measuring biological aging. Dr. Artyomov will discuss single-cell resolution approaches to reveal aspects of immune aging in humans, and Dr. Palovics will present the use of transcriptomics to understand rejuvenating effects of heterochronic parabiosis.

Abstract citation ID: igad104.1424

**COMPARATIVE TRANSCRIPTOMICS AND PROTEOMICS ACROSS SPECIES**

Vera Gorbunova, *University of Rochester, Rochester, New York, United States*

Mammals differ more than 100-fold in maximum lifespan. Here, we conducted comparative transcriptomics on 26 species with diverse lifespans. We identified thousands of genes with expression levels negatively or positively correlated with a species' maximum lifespan (Neg- or Pos-MLS genes). Neg-MLS genes are pri- marily involved in energy metabolism and inflammation. Pos-MLS genes show enrichment in DNA repair, microtubule organization, and RNA transport. Expression of Neg- and Pos-MLS genes is modulated by interventions, including mTOR and PI3K inhibition. Regulatory networks analysis showed that Neg-MLS genes are under circadian regulation possibly to avoid persistent high expression, whereas Pos-MLS genes are tar- gets of master pluripotency regulators OCT4 and NANOG and are upregulated during somatic cell reprogramming. Pos-MLS genes are highly expressed during embryogenesis but significantly downregulated after birth. This work provides targets for anti-aging interventions by defining pathways correlating with longevity across mammals and uncovering circadian and pluripotency networks as central regulators of longevity. I will also discuss integrating this transcriptome and proteome data.

Abstract citation ID: igad104.1425

### STRATEGIES OF LIFESPAN REGULATION WITHIN AND ACROSS SPECIES

Vadim Gladyshev, *Harvard Medical School, Boston, Massachusetts, United States*

Lifespan varies within and across species, but the general principles of its control remain unclear. We conducted multi-tissue RNA-seq analyses across mammalian species, identifying longevity signatures and examining their relationship with transcriptomic biomarkers of aging and established lifespan-extending interventions. Signatures of long-lived species were positively correlated with age-related changes and enriched for evolutionarily ancient essential genes. Conversely, lifespan-extending interventions counteracted aging patterns and affected younger, mutable genes enriched for energy metabolism. The identified biomarkers revealed new longevity interventions. We further developed a transcriptomic clock that quantifies progression through lifespan and reports the effects of longevity interventions. Overall, this work uncovers universal and distinct strategies of lifespan regulation within and across species and provides tools for quantifying aging and discovering longevity interventions.

Abstract citation ID: igad104.1426

### IMMUNE AGING AT SINGLE-CELL RESOLUTION

Maxim Artyomov, *Washington University in St. Louis, St. Louis, Missouri, United States*

I will discuss how single-cell resolution approaches reveal aspects of immune aging in tissues and in circulation. We will discuss comparison of immune aging in mouse tissues, accumulation of GZMK+ CD8 T cells and their human counterparts that accumulate in blood. We will discuss analysis of scRNA-seq profiling of PBMC along the healthy aging (317 samples from 25 to 85yo) and emerging subpopulation and antigen receptor changes during aging.

Abstract citation ID: igad104.1427

### ATLAS-SCALE SINGLE-CELL DATA SCIENCE REVEALS SIGNATURES OF AGING AND REJUVENATION

Robert Palovics, *Stanford University, Stanford, California, United States*

The use of recent high-throughput transcriptomics technologies allows us for the first time to study aging at an unprecedented granularity and temporal resolution. This talk aims to provide insights into aging and heterochronic parabiosis, a model for systemic rejuvenation, by studying an atlas-scale single-cell dataset encompassing >100k cells across 20 organs and 50 cell types in young and aged mice as well as in parabionts. We will explore the age mimicking and age reversing effects of parabiosis within each cell type, identify parabiosis mediated rejuvenation signatures that appear throughout the whole body, and present a structural overview of aging and parabiosis across different organs.

### SESSION 4070 (SYMPOSIUM)

Abstract citation ID: igad104.1428

### LIVING WELL WITH MILD TO MODERATE DEMENTIA. FINDINGS FROM THE IDEAL PROGRAM: A BRITISH COHORT STUDY

Chair: Linda Clare Co-Chair: Christina Victor

IDEAL (Improving the Experience of Dementia and Enhancing Active Life) is a British-based cohort study that commenced in 2014 and recruited 1545 people with dementia and 1277 carers, following them for 3 waves at yearly intervals. IDEAL-2 continued to follow those who remained in the study for a further 3 waves and incorporated 205 additional participants as part of an enrichment cohort. A key research and policy goal is to understand the factors that promote or inhibit 'living well' for people with dementia and their carers, defined as the best achievable state of physical, mental and social health. In this symposium we examine how living well outcomes vary longitudinally and with social (minority ethnic status) factors. Two papers focus on quality of life of people with dementia longitudinally. Our first paper shows that for most study participants quality of life scores remained stable. For those with declining quality of life key associated factors include demographic characteristics, cognition, and psychological health including depression. Our second paper has a clinical dimension and demonstrates that, beyond the point of diagnosis, quality of life does not vary according to dementia subtype. Our third paper focuses on social health using social isolation as our measure. Isolation increased over time and was higher in carers than people with dementia. Our final paper demonstrates that, compared with their white British counterparts, minority ethnic carers report higher stress and role captivity and lower relationship quality while people with dementia have lower quality of life and higher loneliness.

Abstract citation ID: igad104.1429

### LONGITUDINAL FACTORS ASSOCIATED WITH LIVING WELL IN PEOPLE WITH MILD TO MODERATE DEMENTIA

Linda Clare[1], Laura Gamble[2], Anthony Martyr[3], and Fiona Matthews[2], *1. University of Exeter Medical School, Exeter, England, United Kingdom*, *2. Newcastle University, Newcastle upon Tyne, England, United Kingdom*, *3. University of Exeter, Exeter, England, United Kingdom*

Understanding the factors associated with quality of life can help us become more effective in optimizing the potential for people with mild-to-moderate dementia to 'live well'. Identifying individuals at risk of declining quality of life could help to target support more appropriately. We used longitudinal data from 1749 individuals with mild-to-moderate dementia participating in the IDEAL cohort. Assessments took place on entry and at 12, 24, 48, 72 and 96 months. Using mixed effects modelling we examined change in quality of life, as measured by QoL-AD, since time of diagnosis, and looked at time-invariant and time-varying measures and their association with decline in quality of life at 6 years. Mean quality of scores life were stable over our six year follow up period. However, we were able to identify some individuals whose quality of life declined by varying amounts over this period. A larger decline was associated with being male and older, and those with a decline in quality of life had worsening cognitive function and increasing depression in the years following diagnosis. Our results show that quality of life does not decline for most people following a diagnosis of dementia. However, for those that did decline we were able to identify key factors associated with this decline. This model allows us to investigate further factors that may influence living well in people with dementia, and identify, at diagnosis, those most 'at risk' of decline and identify potential remediating factors that could inform potential intervention to mitigate this decline.

Abstract citation ID: igad104.1430

### DEMENTIA SUBTYPES AND LIVING WELL IN PEOPLE WITH MILD TO MODERATE DEMENTIA. LONGITUDINAL FINDINGS OVER SIX YEARS

Linda Clare[1], Anthony Martyr[2], Laura Gamble[3], and Fiona Matthews[3], *1. University of Exeter Medical School, Exeter, England, United Kingdom*, *2. University of Exeter, Exeter, England, United Kingdom*, *3. Newcastle University, Newcastle upon Tyne, England, United Kingdom*

The heterogeneity of symptoms across dementia subtypes has important implications for clinical practice and dementia research. Variations in subtypes and associated symptoms may influence capability to 'live well' for people with dementia. In this paper we investigate the potential impact of dementia subtypes on the quality of life of people with dementia. We used longitudinal data from 1749 individuals with mild-to-moderate dementia participating in the IDEAL and IDEAL-2 studies. Assessments took place on entry and were followed up at 12, 24, 48, 72 and 96 months. We examined change in quality of life post diagnosis and associations with dementia subtypes using mixed effects models. Slightly more than half of participants , 52.4%, had a diagnosis of Alzheimer's disease, 10.2% vascular dementia and 19.8% mixed Alzheimer's and vascular dementia. Rarer subtypes of dementia, frontotemporal, Parkinson's type, Lewy bodies accounted for 17.6% of participants . Dementia subtype had the greatest associations with quality of life at the time of diagnosis. Compared to individuals with Alzheimer's disease, those with Parkinson's disease dementia (-4.89, 95% CI: -6.60 to -3.417) and dementia with Lewy bodies (-4.90, 95% CI: -6.38 to -3.42) had relatively poorer quality of life at baseline. The difference in quality of life for people with vascular dementia (-1.70, 95% CI: -3.55 to -1. 35) and mixed Alzheimer's and vascular dementia (-1.70 95% CI:-2.47 to -0.92) was less marked. There was little change in quality of life longitudinally and no difference in quality-of-life trajectories by dementia subtype

Abstract citation ID: igad104.1431

### SOCIAL HEALTH IN PEOPLE WITH DEMENTIA AND THEIR CARERS. PRELIMINARY FINDINGS FROM THE IDEAL PROGRAM

Christina Victor[1], and Isla Rippon[2], *1. Brunel University-London, Uxbridge, England, United Kingdom*, *2. Brunel University London, Uxbridge, England, United Kingdom*

WHO defines health as a state of complete physical, mental, and social wellbeing. There is an extensive literature examining physical and mental health in later life and, typically, measures of healthy ageing focus upon physical and/or mental health. In the IDEAL dementia research programme we investigated social health outcomes because of their importance in understanding the experience of 'living well' for people with dementia. Using data from the IDEAL cohort study, we examined the prevalence and predictors of social isolation in 1,052 people with mild-to-moderate dementia and 928 caregivers who were followed up at 12 and 24 months. Social isolation was assessed using the six-item Lubben Social Network Scale where a score of less than 12 suggests participants are at risk of social isolation. Linear mixed models showed that both people with dementia and caregivers experienced increased social isolation across the two-year period. At baseline 29% of people with dementia were categorised as being socially isolated compared with 14% of caregivers. For both people with dementia and caregivers, loneliness and depression were associated with greater social isolation whilst increased cultural engagement mitigated the impact of social isolation. For people with dementia cognition, the number of green and blue spaces nearby and higher trust in the local community were also important factors. Interventions aimed at increasing cultural engagement and interaction with blue and green spaces may go some way to reducing social isolation.

Abstract citation ID: igad104.1432

### LIVING WELL? A MATCHED ANALYSIS OF MINORITY ETHNIC AND WHITE BRITISH PEOPLE WITH DEMENTIA AND THEIR CARERS

Christina Victor[1], Catherine Quinn[2], Fiona Matthews[3], Claire Pentecost[4], and Laura Gamble[3], *1. Brunel University-London, Uxbridge, England, United Kingdom*, *2. Bradford University, Bradford, England, United Kingdom*, *3. Newcastle University, Newcastle upon Tyne, England, United Kingdom*, *4. University of Exeter, Exeter, England, United Kingdom*

The increasing heterogeneity of the older population is reflected in the UK in an increasing number of those with dementia and carers drawn from minority ethnic groups. Data from the IDEAL dementia study is used to compare 'living well' among people with dementia and carers from ethnic minority groups with matched white British peers. We used a cross-sectional case-control design. Outcomes for both groups were quality of life, life satisfaction, wellbeing, loneliness and social isolation and, for carers, measures of stress, relationship quality, role captivity and caring competence. Our sample consisted of 20 people with dementia and 15 carers who self-identified as being of Indian, Pakistan, Bangladeshi, Black African, Black Caribbean or mixed ethnicity who were matched with 60 white British participants with dementia and 45 white British carers. People with dementia from minority ethnic groups had poorer quality of life (-4.74, 95%

CI: -7.98 to -1.50) and higher loneliness (1.72, 95% CI: 0.78 to 2.66) scores whilst minority ethnic carers had higher stress (8.17, 95% CI: 1.72 to 14.63) and role captivity (2.00, 95% CI: 0.43 to 3.57) and lower relationship quality (-9.86, 95% CI: -14.24 to -5.48) than their white British peers. People with dementia from minority ethnic groups experience lower quality of life and caregivers higher stress and role captivity and lower relationship quality. Confirmatory research with larger samples is required to facilitate analysis of the experiences of specific minority ethnic groups and examine the factors contributing to these disadvantages.

### SESSION 4075 (SYMPOSIUM)

Abstract citation ID: igad104.1433

### WORKFORCE EDUCATION, TECHNOLOGY, AND EVALUATION TO REDUCE HEALTH DISPARITIES IN A PROVIDER SHORTAGE STATE

Chair: Ji Yoo Co-Chair: Peter Reed Discussant: Laurie Kim

State of Nevada has been a provider shortage area and ranks 50th state of primary care providers per capita in 2022. As a result, Medicare spends highest spending per capita and highest 30-day readmission rate of vulnerable and older adults, i.e., individuals with Alzheimer's Disease Related Dementias. To respond these challenges, geriatric workforce education program (GWEP) was launched in 2019. Along with interdisciplinary education of healthcare providers, GWEP overarched to an innovative technology, i.e., digital health and machine learning methodology aiming at mitigating bias in healthcare and predicting events, i.e., nursing home placement and designating power of attorney timelines that are useful for planning advance care in primary care. GWEP team evaluates the impact of these education and technology implementation impact on healthcare delivery efficiency, i.e., healthcare cost. in real-world payment system. This is a collaborative symposium between the Community-Engaged Research, Geriatric Education, and Patient/Person Engagement in Research Interest Groups.

Abstract citation ID: igad104.1434

### BRIDGE GERIATRIC WORKFORCE EDUCATION CURRICULUM BETWEEN 4M COMPETENCIES AND INTERDISCIPLINARY EDUCATION

Ji Yoo[1], Jay Shen[2], Ian choe[3], maryam Tabrizi[4], and Iulia Ioanitoaia-Chaudhry[5], *1. Kirk Kerkorian School of Medicine at UNLV, Las Vegas, Nevada, United States*, *2. School of Public Health, UNLV, Las Vegas, Nevada, United States*, *3. Optum Nevada, Las Vegas, Nevada, United States*, *4. University of Nevada in Las Vegas, Las Vegas, Nevada, United States*, *5. VA Southern Nevada Healthcare System, North Las Vegas, Nevada, United States*

In a provider shortage area, education priority strategy is more practical rather than structure investment priority strategy. In aims of establishing Age-Friendly Community in this state where primary care provider number per capita ranks 50th state, 4Ms (What Matters, Mobility, Medication, Mentation) based interdisciplinary education across medicine, dental, pharmacy, nursing, social work, physical therapy were developed and implemented. We evaluated the impact of 4Ms application on healthcare providers education by measuring served patient outcomes (1) CMS defined Merit Incentive Payment System, patient satisfaction, and healthcare cost and (2) education outcomes of healthcare providers using 4M education competencies. We adopted STEPPS interprofessional communication skill toolkit and applied simulation training with standardized patients.

Abstract citation ID: igad104.1435

### EVALUATE THE IMPACT OF INTERDISCIPLINARY EDUCATION AND TELEHEALTH PRIMARY CARE: QUALITY AND EFFICIENCY

Jay Shen[1], Ji Yoo[2], and Pearl Kim[1], *1. School of Public Health, UNLV, Las Vegas, Nevada, United States*, *2. Kirk Kerkorian School of Medicine at UNLV, Las Vegas, Nevada, United States*

In a provider shortage area, an Interprofessional Education and Telehealth Primary Care (IPETC) for geriatrics workforce was developed in 2020. To analyze the impact of IPETC on quality and efficiency of care, we measured quality outcomes defined by the CMS defined quality measures - dementia caregiver education/referral and advance care plan, and efficiency outcomes by estimating healthcare cost savings from reducing hospitalization. Fifty eight community-dwelling adults aged 67 to 94 with mild to moderate dementia were selected in an urban safety-net primary care clinic. Propensity - demographics and level of physical impairment was matched. Main outcomes were (A) hospitalization-related healthcare cost estimates from the State Inpatient Dataset using the ICD-10 codes of principal diagnosis and hospital length of stay between January-December 2021. Twenty nine patients were cared by primary care providers who received the IPETC;29 patients were by those who did not have the IPETC; (2) CMS quality measures were compared between 2019 (baseline) and 2021. An average cost-saving of $22,135 per patient was observed among those treated by the healthcare providers who received the IPE/telehealth curriculum training than those treated by the providers without the training. CMS quality measures improved from 15.6% to 51.0% in dementia caregiver education/referral; 10.8% to 42.8% in documented advance care plans. Innovative interdisciplinary education and telehealth primary care has shown improvement of quality and efficiency of dementia care in a provider shortage area.

Abstract citation ID: igad104.1436

### TECHNOLOGY-ASSISTED CARE PLANNING IN PRIMARY CARE OF A PROVIDER SHORTAGE AREA

Mingon Kang[1], Laurie Kim[2], and Ji Yoo[2], *1. Howard Hughes College of Engineering at UNLV, Las Vegas, Nevada, United States*, *2. Kirk Kerkorian School of Medicine at UNLV, Las Vegas, Nevada, United States*

We applied faireness algorithm to identify and mitigate bias in healthcare system. We also developed DeepPlan soft that can assist primary care providers, patients and caregivers with providing real-time risk prediction system, for example, discussing nursing home placement and designation of power of attorney. Our DeepPlan was built using cloud database system than can be exchangeable in secure way between patients and primary care providers. We linked DeepPlan use to workforce education and now we are ready for presenting education and patient clinical outcomes of using DeepPlan in primary care in a provider shortage area.

### SESSION 4080 (SYMPOSIUM)

Abstract citation ID: igad104.1437

### EPIDEMIOLOGIC GEROSCIENCE INNOVATIONS AT THE NEXUS OF REPRODUCTIVE AGING AND WOMEN'S HEALTH

Chair: Michelle Shardell

Signs of reproductive aging in women usually begin in women's 30s and co-occur with declining circulating estrogen concentrations and irregular menstrual cycle onset. Menopause, defined by cessation in menstruation for ≥1 year, occurs on average at age 51 years, but about 1% experience menopause before age 40. Menopause and reproductive aging contribute to multiple health conditions such as certain cancers; however, a less studied condition is the genitourinary syndrome of menopause (GSM). GSM signs and symptoms include vulvovaginal atrophy, vaginal dryness, urinary tract infections, and more. Additionally, menopause leads to increased risk of osteoporosis, dementia, and other aging-related conditions. Given the multifactorial implications of reproductive aging in women's health, an interdisciplinary geroscience-guided framework for epidemiology of aging is needed to identify biological contributors and consequences of reproductive aging to develop healthspan-enhancing interventions. In this symposium, we present five new developments across multiple stages of reproductive aging, consistent with meeting theme "Building Bridges. Catalyzing Research. Empowering All Ages". Among women aged 35-60 years, we assess the role of cervical "inflamm-aging" in GSM; diabetes and vaginal dysbiosis associations with GSM; and vaginal microbial and metabolic signatures of urinary conditions/dysfunction. Among women aged ≥65 years, we examine associations of post-hip fracture sex hormones with physical recovery and links between age and type of menopause with Alzheimer's disease. Building on the 2021 Journals of Gerontology Series A special issue on intersections of reproductive and aging biology, we discuss opportunities to advance reproductive aging and women's health research by combining molecular assessment with rigorous epidemiology. This is a collaborative symposium between the Epidemiology of Aging, Geroscience, and Women's Issues Interest Groups.

Abstract citation ID: igad104.1438

### ASSOCIATIONS OF URINARY CONDITIONS WITH VAGINAL MICROBIOTA AND METABOLITES ACROSS REPRODUCTIVE STAGES

Michelle Shardell[1], Sarah Robbins[1], Joanna-Lynn Borgogna[2], Christina Stennett[1], Carl Yeoman[2], Anne Burke[3], Jacques Ravel[4], and Rebecca Brotman[1], *1. University of Maryland School of Medicine, Baltimore, Maryland, United States*, *2. Montana State University, Bozeman, Montana, United States*, *3. Gynecology and Obstetrics, Baltimore, Maryland, United States*, *4. University of Maryland School of Medicine, Baltimore, Maryland, United States*

The genitourinary syndrome of menopause describes signs/symptoms of the female genitourinary tract, including urinary symptoms/conditions, resulting from declining estrogen in menopause. Menopause is also associated with declining abundance of Lactobacillus spp. in the vagina. Vaginal and urinary microbiota are highly concordant, where low abundance of certain Lactobacillus spp. have demonstrated associations with urinary symptoms/conditions. The vaginal microenvironment also includes microbially- and host-produced metabolites that may impact genitourinary health. Thus, we aimed to identify vaginal microbial and metabolic signatures of urinary conditions. In a two-year cohort study comprising 476 participants aged 35-60 years who contributed 1,153 semiannual person-visits, 15.2% (27/178) postmenopausal and 10.4% (31/298) non-postmenopausal participants self-reported a history of urinary conditions (overactive bladder, recurrent urinary tract infection[UTI], or cystitis). Among postmenopausal participants, history of urinary conditions was associated with higher covariate-adjusted odds of L. gasseri-dominated (OR[Odds Ratio]=4.29, 95% Confidence Interval[CI]=1.31-14.88) versus L. crispatus-dominated communities. Significant metabolites were enriched for the lipids sphingomyelins and metabolites involved in glycolysis ($P<.05$). Urinary conditions were associated with joint microbiota-metabolite profiles characterized by higher sphingomyelins, pyruvate, and genera Varibaculum and Escherichia; and lower dihydroxyacetone phosphate, nicotinamide, Gardnerella vaginalis, and genus Fusobacterium ($P<.001$). In contrast, among non-postmenopausal participants, urinary conditions did not significantly relate to microbial community ($P>.10$); however, sphingomyelins remained significantly enriched ($P<.05$). Signatures among postmenopausal participants include known UTI pathogen E. coli and metabolites that may facilitate and fuel its growth. Among non-postmenopausal participants, urinary conditions more strongly related to metabolic profiles than microbiota. Investigation of these signatures in incident/recurrent urinary conditions is needed.

Abstract citation ID: igad104.1439

### CERVICAL "INFLAMM-AGING" AND GENITOURINARY SYNDROME OF MENOPAUSE: A GEROSCIENCE CASE-CONTROL STUDY

Christina Stennett[1], Patti Gravitt[2], Rebecca Brotman[1], and Michelle Shardell[1], *1. University of Maryland School of Medicine, Baltimore, Maryland, United States*, *2. Center for Global Health, National Institutes of Health, Rockville, Maryland, United States*

The genitourinary syndrome of menopause (GSM) is a reproductive aging condition that describes signs and symptoms of the female reproductive tract associated with declining estrogen that women experience during menopause. Up to 84% of menopausal women experience signs and symptoms of GSM, including vaginal dryness and vulvovaginal pain. "Inflamm-aging" is a chronic aging-related elevation of proinflammatory markers that has been linked with multiple aging-related health conditions, but little geroscience research has examined cervical inflammation and GSM. To fill this gap, we carried out a case-control study (N=133) of cisgender women aged 35-60 years, where women with clinical signs of GSM (cases) were matched with up to 3 controls based on age and vaginal microbiota composition, which are known to track with reproductive stage. Participants' cervical secretion samples were assayed to quantify immune marker concentrations. Samples with at least three out of seven predetermined proinflammatory cytokines and chemokines (IL-1ß, IL-8, IL-23, MIP-1α, MIP-1ß, IP-10, RANTES) in the highest quartile were classified as having a proinflammatory state. Of 133 participants, 35 (17/47 cases, 18/86 controls) had a proinflammatory state. A proinflammatory state was associated with higher odds of GSM in conditional logistic regression additionally adjusted for education and body mass index (aOR=2.39; 95% CI: 1.05-5.46). Findings demonstrate that participants with GSM signs had higher odds of a proinflammatory ("inflamm-aging") state, independent of multiple characteristics, including vaginal microbiota. Additional geroscience research is needed to identify pathways connecting immune markers with GSM to discover interventions that may slow the pace of genitourinary aging.

Abstract citation ID: igad104.1440

### ASSOCIATION OF DIABETES AND VAGINAL MICROBIOTA WITH THE GENITOURINARY SYNDROME OF MENOPAUSE

Sarah Robbins[1], Michelle Shardell[1], Amber Beitelshees[2], Ryan Miller[1], Jacques Ravel[3], Simeon Taylor[1], and Rebecca Brotman[1], *1. University of Maryland School of Medicine, Baltimore, Maryland, United States*, *2. University of Maryland, Baltimore, Baltimore, Maryland, United States*, *3. University of Maryland School of Medicine, Baltimore, Maryland, United States*

The genitourinary syndrome of menopause (GSM) comprises urogenital signs and symptoms associated with estrogen deficiency during menopause. For many, decreased estrogen reduces vaginal Lactobacillus species abundance, a state associated with adverse urogenital outcomes. For these reasons, vaginal microbiota in women's reproductive aging is an emerging topic of interest in geroscience research. The risk of diabetes also increases with menopause; however, few studies have jointly assessed diabetes and vaginal microbiota with GSM. Therefore, we assessed the relationships between diabetes, vaginal microbiota and GSM in a study of 811 participants aged 35-60 recruited to a 2-year observational cohort with follow-up every 6 months. Diabetes was defined as self-reported diabetes medications, history, or serum glycated albumin >16.5%. There were 22 and 251 postmenopausal participants with and without diabetes, respectively. Mixed-effects logistic regression quantified the association of vaginal microbiota with GSM outcomes among those with and without diabetes. After adjustment for covariates, low-Lactobacillus vaginal microbiota states were associated with lower odds of vulvovaginal atrophy (VVA) (aOR=0.23, 95% CI: 0.04-1.33) among postmenopausal participants with diabetes; however, this finding was not statistically significant. In contrast, among postmenopausal participants without diabetes, low-Lactobacillus microbiota was significantly associated with higher odds of VVA (aOR=1.63, 95% CI: 1.07-2.47). Diabetes significantly modified the association between vaginal microbiota and VVA among postmenopausal individuals (p=.032). Findings were similar for clinician-assessed vaginal dryness. These results suggest that the relationship between vaginal microbiota and GSM may differ by diabetes status. Larger studies are needed to further understand the relationships between diabetes, vaginal microbiota and GSM.

Abstract citation ID: igad104.1441

### THE ROLE OF REPRODUCTIVE AGING FOR ALZHEIMER'S DISEASE AND RELATED DEMENTIAS IN THE CARDIOVASCULAR HEALTH STUDY

Alexis Reeves[1], Oscar Lopez[2], Michelle Shardell[3], and Michelle Odden[4], *1. Stanford University; School of Medicine, Stanford, California, United States*, *2. University of Pittsburgh, Pittsburgh, Pennsylvania, United States*, *3. University of Maryland School of Medicine, Baltimore, Maryland, United States*, *4. Stanford University, Stanford, California, United States*

Most US individuals with ADRD are women. As early as midlife, Black individuals have nearly double the ADRD risk than Whites. Menopause occurs in midlife, accompanied by declines in potentially neuro-protective and cardio-protective hormones. Earlier menopause possibly means earlier exposure to decreased sex hormones, which increases ADRD risk. Surgical menopause via reproductive surgeries causes a sudden loss of hormones and could be a modifiable ADRD risk factor. We used data from 1,977 women prospectively followed for an average of 7.5 years, to determine if menopausal type (natural/surgical) and pre-mature menopause (<40 years) are associated with adjudicated-ADRD. Participants self-reported age of natural menopause and/or surgery (hysterectomy and/or bilateral-oophorectomy), hormone-therapy, education, and race (Black/White). Adjudication for ADRD was conducted by neurologist/psychiatrist committee review using neuropsychological tests, neurological examinations, medical records, physician questionnaires, and proxy/informant interviews. Mean age was 78, 15% were Black, 25% had less than a high-school education, 18% had ADRD, 13% had pre-mature menopause, and 42% surgical menopause. Black women had higher prevalence of pre-mature (22% versus 12%) and surgical (54% versus 29%) menopause versus White women. Pre-mature menopause was associated with a non-significant higher hormone use-adjusted hazard of ADRD (HR=1.09 [0.95-1.25]), findings were attenuated after adjustment for education (HR=1.08 [0.94-1.24]) and race (HR=1.01 [0.88-1.17]). Surgical menopause was associated with higher hormone use-adjusted hazard of ADRD (HR=1.12 [1.02-1.24]), findings were similar after adjustment for education (HR=1.12 [1.01-1.23]) and attenuated after adjustment for race (HR=1.03 [0.93-1.14]). Further work examining menopause, ADRD, and contributions to Black-White disparities are warranted.

Abstract citation ID: igad104.1442

### SEX HORMONES ARE ASSOCIATED WITH BONE DENSITY AND PHYSICAL FUNCTION IN WOMEN RECOVERING FROM HIP FRACTURE

Anne Cappola, *University of Pennsylvania School of Medicine, Philadelphia, Pennsylvania, United States*

Testosterone and estrogen have anabolic effects, and alterations could affect recovery from hip fracture. We hypothesized that differences in sex hormones during the year after a hip fracture would be associated with bone mineral density (BMD) and physical function. This analysis included 149 women aged 65 years or older enrolled in the Baltimore Hip Studies 7th cohort. Testosterone and estradiol by LC/MS/MS and sex hormone binding globulin were assayed at four time points post hip fracture (within 15 days of admission, and at 2, 6, and 12 months) on fasting morning samples. Longitudinal regression models accounting for clustering by subject were constructed to examine the relationship between each hormone and BMD and short performance physical battery (SPPB) score over time. Mean age was 81 years, BMI was 25.6 kg/m2, and 92% were white. Testosterone levels were low in women at all time points (median 10-12 ng/dL), as were estradiol levels (median 3-5 pg/mL). In adjusted models, increases in total and free estradiol during recovery were associated with improvement in BMD, whereas total testosterone, but not free testosterone, was associated with improvement in SPPB. These analyses support physiologically relevant relationships between higher estradiol levels and improvement in BMD and higher testosterone levels and improvement in physical function during the year after hip fracture, even at the low levels of sex hormones found in post-menopausal women. These findings suggest that differences in reproductive aging that affect sex hormone production may have clinical effects on recovery from a sentinel event, long after menopause.

### SESSION 4085 (SYMPOSIUM)

Abstract citation ID: igad104.1443

### BUILDING BRIDGES ACROSS THE GENERATIONS: RESEARCH ON DIVERSE APPROACHES FOR INTERGENERATIONAL CONNECTIONS

Chair: Amy Eisenstein Discussant: Ernest Gonzales

Intergenerational bonds are a unique and critically important set of social connections that have powerful impacts on families and communities, as well as the older and younger people themselves. America is increasingly age-segregated, so that naturally occurring, cross-generational contacts are not as common as they have been in previous eras. Research from the FrameWorks Institute and others suggest that ageist language can further divide the generations, create a sense of "othering," and contribute to the isolation and minimization of older adults. Conversely, studies also show that meaningful intergenerational programming, which brings to light the strengths of both generations, may reduce social isolation and loneliness and enhance the physical and mental well-being of older adults. This symposium will bring together three research projects each exploring diverse approaches to intergenerational connections. The first paper will present lessons learned from a study of digital communication between grandparents and grandchildren during the COVID-19 pandemic, and the implications this shift in communications has on the psychological well-being of older grandparents. The second paper will present findings regarding the physical and psychosocial outcomes experienced by older adults who provide intergenerational tutoring in elementary school settings. The final session will highlight the process of integrating implementation science frameworks into a multistage study design aimed to assess the implementation of an intergenerational technology program within higher education. The session will wrap-up with a discussion on how these projects fit within the broader scope of intergenerational programs, and will highlight gaps and further opportunities for research in this field.

Abstract citation ID: igad104.1444

### HEALTH AND WELL-BEING OUTCOMES OF THE OASIS INTERGENERATIONAL TUTORING PROGRAM

Peter Sun, and Nancy Morrow-Howell, *Washington University in St. Louis, St. Louis, Missouri, United States*

The Oasis Intergenerational Tutoring Program is a national program that connects older adult tutors with elementary children in Grades K-3. We are conducting an ongoing project to assess multiple health and well-being outcomes associated with this tutoring program, especially among four sub-groups: first-time tutors, male tutors, tutors who are caregivers, and tutors who report being lonely. In year one of our project, we have collected data from 329 older adults who tutored children in the 2021-2022 school year. We found that a larger proportion of tutors who felt lonely reported improvements to their health, compared to tutors who did not feel lonely. We also found that first-time tutors experienced larger increases in at least two areas of health (physical, emotional, and cognitive), compared to repeat tutors. When we asked tutors about their experience tutoring during the pandemic, major themes included a sense of purpose in life, increased social engagement, and the negotiation of risks and benefits (e.g., remote vs. in-person tutoring). An upcoming two-year follow-up study will enable us to compare our findings with the Health and Retirement Study, a nationally representative sample of older adults. Findings from this project will inform programs across the nation and policies that seek to harness the power of intergenerational bonds.

Abstract citation ID: igad104.1445

### IMPLEMENTATION FRAMEWORKS IN ACTION: A QUALITATIVE STUDY OF INTERGENERATIONAL TECHNOLOGY IN HIGHER EDUCATION

Jill Juris[1], Rachel Scrivano[2], Skye Leedahl[3], and Shannon Jarrott[4], *1. Appalachian State University, Boone, North Carolina, United States*, *2. Boston College, Boston, Massachusetts, United States*, *3. University of Rhode Island, Kingston, Rhode Island, United States*, *4. The Ohio State University, Columbus, Ohio, United States*

Cyber-Seniors is an intergenerational technology program that bridges the digital divide by training younger people to assist older adults with using technology. While higher education is an ideal setting for utilizing this program for service learning or student internship opportunities, there are often challenges to implementing this program in a higher education setting. In this study, implementation factors must be studied to improve sustainability and increase the potential to scale-up the use of Cyber-Seniors programming within higher education. This study is guided by the dynamic sustainability framework (Chambers et al., 2013) with the goal to investigate prominent contextual factors of implementation that permit and prohibit program sustainability. The study will also identify needed adaptations to Cyber-Seniors' implementation, which will be documented using Stirman and colleagues' (2019) Framework for Reporting Adaptations and Modifications-Enhanced (FRAME). In this presentation, we will illustrate the process of infusing implementation science frameworks into a multistage qualitative study designed to allow for in-depth understanding of experiences implementing Cyber-Seniors programming in higher education and designing an adapted checklist for program implementation. We will describe how the implementation science frameworks have been used to design the research instruments, protocols, and the analysis plan. The presentation will conclude with next steps for the research project. A key takeaway from this presentation will be for attendees to gain an understanding of how to infuse implementation science frameworks when designing, implementing, and evaluating research programs and interventions.

Abstract citation ID: igad104.1446

**DIGITAL COMMUNICATION AS COMPENSATION FOR INFREQUENT IN-PERSON CONTACT WITH GRANDCHILDREN DURING THE PANDEMIC**

Xiaoyu Fu[1], Woosang Hwang[2], and Merril Silverstein[1], *1. Syracuse University, Syracuse, New York, United States*, *2. Texas Tech University, Lubbock, Texas, United States*

Maintaining interactive relationships with grandchildren represents an important and rewarding aspect of family life for grandparents. The goal of this study is to explore stylistic patterns of intergenerational contact with grandchildren including digital communication (texting, e-mail, video call, and social media interaction) and how they were associated with older grandparents' psychological well-being during the COVID-19 pandemic. Using data from the 2021-22 survey of the Longitudinal Study of Generations (LSOG), the analysis applied latent class analysis to identify communication styles with grandchildren among 264 grandmothers and 174 grandfathers. We identified four communication styles for both grandmothers and grandfathers. Life satisfaction was relatively low and no different among grandparents with a detached style (little contact of any type) and those with a digitally connected style (little in-person contact but some digital contact). However, fully connected grandparents (digital contact and in-person contact) had significantly better life satisfaction than detached grandparents. Results suggest that digital communication with grandchildren did not compensate for the lack of face-to-face contact with grandchildren but complemented this more traditional form of contact. We conclude that digital contact with grandchildren provides added value above and beyond in-person contact for the psychological well-being of older grandparents.

### SESSION 4090 (SYMPOSIUM)

Abstract citation ID: igad104.1447

**DOES BECOMING FRAIL MEAN THE END? SOCIAL, LIFESTYLE, AND HEALTH SURROGATES OF RESILIENCE IN THE MOST VULNERABLE**

Chair: Chenkai Wu Discussant: Qianli Xue

Frailty is a prevalent geriatric syndrome characterized by reduced reserve to stressors and increased vulnerability to adverse outcomes. Despite many efforts, reversing the frail status remains difficult, primarily due to a lack of understanding of its etiology. Intervention efforts have primarily focused on improving functions and alleviating symptoms instead of targeting frailty-causing biology and treating the syndrome. Although no magic pill exists for treating frailty, becoming frail doesn't mean the end of life. Frail individuals are a socioeconomically, behaviorally, and clinically diverse group. Identifying socioeconomic, lifestyle, and health features that could mitigate the frailty-induced health risks and promote resilience is critical for guiding patient-centered management of frailty. This symposium has a collection of four studies focusing on different ways to promote health and reduce risks among the most vulnerable. The first study suggested that adherence to a healthy lifestyle may provide an excellent opportunity to decrease the risk of adverse outcomes among the frail, especially those living in an unfavorable social environment. The second study found frailty to be associated with higher healthcare expenditure only among persons with poor self-rated health, suggesting individuals reporting good health are resilient to frailty-induced health risks. The third study found that physical frailty and self-rated health were overlapping but distinct constructs. Good self-rated health was predictive of low mortality among frail individuals, and the association was stronger among the frail than the non-frail. In the fourth study, intrinsic capacity, assessed by a validated, multidimensional tool, provided prognostic value for healthcare utilization beyond multi-morbidity.

Abstract citation ID: igad104.1448

**IS FRAILTY THE END? ASSOCIATION OF LIFESTYLES AND SOCIAL ENVIRONMENT WITH DEATH AMONG THE FRAIL**

Junhan Tang, *Duke Kunshan University, Kunshan, Jiangsu, China (People's Republic)*

Despite many efforts, reversing the frail status among older adults remains difficult. However, frailty does not mean the end of life. Adherence to a healthy lifestyle and a desirable social environment may reduce the risk of adverse outcomes among the frail. We examined whether adherence to a healthy lifestyle was associated with lower all-cause mortality and the joint association of lifestyles and social environment with mortality among the frail. Data were from UK Biobank; 15,594 physically frail middle-aged and older adults were included. Frailty was assessed by Fried's physical frailty phenotype approach. We created a composite healthy lifestyle score using four lifestyle factors: smoking, alcohol consumption, physical activity, and diet. We used 17 social factors to construct a polysocial score for measuring the social environment. We used the Cox model to estimate the association of each lifestyle factor and the composite lifestyle score with mortality among the entire sample and by polysocial score (low, intermediate, and high level). We also evaluated the joint association of lifestyle and polysocial scores with mortality. Frail participants with a healthy level of smoking, physical activity, diet, and composite lifestyle score had a 40%, 33%, 15%, and 34% lower hazard of death than those with an unhealthy level, respectively. We found a joint effect of a healthy lifestyle and social environment on mortality. Adherence to a healthy lifestyle may provide an opportunity to decrease the risk of adverse health outcomes among the frail, especially among those living in an unfavorable social environment.

Abstract citation ID: igad104.1449

### CAN GOOD SELF-RATED HEALTH MITIGATE THE FRAILTY-INDUCED HEALTH CARE EXPENDITURE? EVIDENCE FROM CHINA

Haiyu jin, *University of Southampton, Southampton, England, United Kingdom*

As the pathophysiology of frailty remains unknown, intervention efforts have primarily focused on improving functions and alleviating symptoms instead of treating frailty-causing biology. Frail individuals are not a clinically homogeneous group; further risk stratification may identify subgroups more resilient to health risks. Self-rated health (SRH), a simple measure of an individual's overall perception of overall health, might be a surrogate of resilience among the frail. This work aims to determine the joint association of frailty and SRH with healthcare expenditure. Data were from the China Health and Retirement Longitudinal Study. Persons were classified as non-frail, prefrail, and frail. SRH was classified as poor (including very poor) and good (fair/good/very good/excellent). Healthcare expenditure was classified into outpatient, inpatient, and self-treatment. The Cox model was used to address the study's aims. All analyses were adjusted for age, sex, residence, education, income, insurance, smoking, and chronic conditions. Although frailty was positively correlated with poor SRH, 36.0% of the frail reported good health and 26.6% reporting poor health were deemed non-frail. Frailty and SRH were independently associated with higher odds of incurring and higher average expenditures. Among persons with poor SRH, prefrailty and frailty were associated with higher odds of incurring the cost and higher average expenditures than the non-frail, while no association was found between frailty and healthcare expenditure among those with good SRH. Frailty was associated with higher healthcare expenditure only among persons with poor SRH. Frail individuals reporting good health are resilient to frailty-induced health risks.

Abstract citation ID: igad104.1450

### DISENTANGLE THE RELATION BETWEEN FRAILTY, SELF-RATED HEALTH, AND DEATH: IMPLICATIONS FOR RESILIENCE

Chenkai Wu[1], and Qianli Xue[2], *1. Duke Kunshan University, Kunshan, Jiangsu, China (People's Republic)*, *2. Johns Hopkins, Baltimore, Maryland, United States*

Frail older adults represent a heterogeneous group. Identifying factors predictive of adverse outcomes among this group could advance our understanding of ways to withhold the devastating effects of frailty. Self-rated health (SRH) is a valuable health measure among the general population. However, it is unknown whether SRH remains predictive among the frail. We examined the joint association of frailty and SRH with mortality and assessed the association between SRH and death among the frail. Data were from the UK Biobank, a large-scale cohort study comprising 0.5 million individuals. At baseline, participants were assessed for physical frailty (non-frail, prefrail, and frail) and SRH (excellent/good, fair, and poor). The Poisson model was used to address the study's aims. Age, sex, education, marital status, and smoking were adjusted. Of 488,590 participants, 3.2% were frail, and 4.3% reported poor health. Although frail participants were more likely to report poor health, 19.7% of the frail reported excellent/good health, and 15.9% of the non-frail reported fair or poor health. SRH was strongly associated with death among the non-frail, prefrail, and frail subgroups. Among frail participants, reporting fair and excellent/good was associated with a 17% and 25% lower hazard of death. The association between SRH and death was stronger among the frail than the non-frail, although the interaction term was insignificant. Future investigation into the determinants of positive SRH despite being frail could provide new targets for intervention to improve resilience and risk screening of downstream outcomes in the most vulnerable.

Abstract citation ID: igad104.1451

### HEALTH BEYOND DISEASE IN OLD AGE: RELATIONSHIP OF INTRINSIC CAPACITY WITH HEALTH CARE UTILIZATION

Yafei Si, *UNSW Sydney, Sydney, New South Wales, Australia*

In contrast to the traditional disease-centered approach, intrinsic capacity (IC) is a fine-grained and comprehensive measure of overall health. It plays an essential role in the healthy aging model proposed by the World Health Organization. However, how IC contributes to healthcare utilization and medical expenditures beyond the presence of chronic conditions in old age is not well understood. Data were from the China Health and Retirement Longitudinal Study. We used a validated, multidimensional tool (locomotor, cognitive, psychological capacity, sensory capacity, and vitality) for assessing IC at baseline (Wave 2011). Outcomes include outpatient and inpatient use, and their expenditure measured in Wave 2013. We used logistic and linear regression models to identify the association of IC with healthcare utilization, adjusting for demographics, lifestyles, and multi-morbidity (the presence 2+ out of 14 chronic conditions). We included 14,887 participants, and equally categorized them into three groups (low, intermediate, and high IC). Compared with those with a low IC, older adults with an intermediate level or high level of IC had a significant reduction of outpatient use (OR=0.80, 95% CI=0.72-0.90; OR=0.61, 95% CI=0.53-0.69) and inpatient use (OR=0.69, 95% CI=0.58-0.81; OR=0.58, 95% CI=0.49-0.70), after adjusting for demographics, lifestyles, and multi-morbidity. We did not find a significant association between IC and healthcare expenditure. Assessment of IC for older adults provides valuable information for healthcare use beyond chronic conditions. Assessment of IC in routine clinics may help screen for older adults with high healthcare utilization, which could optimize the allocation of healthcare resources and improve patient-centered care.

### SESSION 4095 (SYMPOSIUM)

Abstract citation ID: igad104.1452

### ENHANCING CARE FOR OLDER VETERANS WITH COMPLEX PHYSICAL, COGNITIVE, AND PSYCHOLOGICAL COMORBIDITIES

Chair: Jeffrey Gregg
Co-Chair: Christine Gould
Discussant: Michele Karel

Older veterans present in healthcare settings with more diagnoses than younger veterans and non-veteran older adults. Older veterans are also more likely to present with co-occurring physical, cognitive, and psychological conditions that are difficult to assess and treat. This is further complicated by the fact that many older veterans reside in rural areas without easy access to specialty care and, even in non-rural settings, there is a dire shortage of providers with geriatric expertise both within and outside of the Veterans Health Administration (VHA) system. The current symposium highlights promising findings from a variety of VHA programs aimed at enhancing the quality and reach of care provided to older veterans with complex needs. First, Dr. Bamonti will present mixed-methods results from her work in the development of Step-Cognitive Behavioral Therapy, a novel virtual intervention for older veterans with COPD. Second, Ms. Carlson will describe program evaluation outcomes from a project aimed at expanding geriatric mental health services via telehealth to older veterans with complex needs residing in rural areas. Third, Dr. Peeples will discuss factors affecting the implementation of the VA Behavioral Recovery Outreach Team model, which is aimed at facilitating successful discharge from inpatient settings and preventing rehospitalization for older veterans. Finally, Dr. Gregg will describe qualitative findings examining a component of the Geriatric Scholars Program for Psychologists, a national post-licensure training program for psychologists treating older rural veterans with complex needs. Dr. Karel, National Director of VHA Geriatric Mental Health, will close with discussion and highlight future directions.

Abstract citation ID: igad104.1453

### FACILITATING GROWTH VIA INDEPENDENT LEARNING PLANS FOR PSYCHOLOGISTS TREATING OLDER VETERANS WITH CHRONIC ILLNESS

Jeffrey Gregg[1], Rachel Rodriguez[2], Priyanka Mehta[3], Josea Kramer[4], and Christine Gould[5], *1. Durham VA Medical Center, Durham, North Carolina, United States*, *2. Durham VA Health Care System, Durham, North Carolina, United States*, *3. VA Palo Alto Health Care System, Palo Alo, California, United States*, *4. VA Greater Los Angeles GRECC, Los Angeles, California, United States*, *5. VA Palo Alto Health Care System, Palo Alto, California, United Sta tes*

The VA Geriatric Scholars Program-Psychology Track (GSP) is a national post-licensure geropsychology training program for VA psychologists caring for rural-dwelling older veterans with complex physical, cognitive, and psychological comorbidities. In 2019, GSP developed an advanced workshop model to invest in the continued growth its alumni. The workshop focused on enhancement of geropsychology competencies in treating older veterans with chronic mental and physical illnesses (e.g., PTSD, dementia, diabetes). To facilitate ongoing and individually tailored learning following the workshop, participants developed and executed Independent Learning Plans (ILPs) during a 6-month window with consultation from an experienced geropsychologist. Upon completion, participants submitted final reports and responded to three prompts assessing overall experience, usefulness, and future plans. We examined these qualitative data using a rapid analysis approach with summary templates. Fourteen of 19 participants (73.7%) submitted both final reports and responses to the prompts. All 14 respondents endorsed clinical and/or personal usefulness of the ILPs, with emerging themes including increased confidence, enhanced skills for use with patients with complex needs, and career advancement. Regarding overall experience, most participants noted positive outcomes, including feelings of fulfillment, and noting that ILPs were feasible and learning resources were accessible. A minority (35.7%) identified meaningful barriers most often associated with not having enough time. Future directions included plans to learn new assessment/treatment approaches, read relevant literature, and pursue additional career advancement (e.g., board certification in geropsychology). Overall, our findings suggest ILPs are a viable way to enhance psychologists' experience and effectiveness in treating older veterans with complex needs.

Abstract citation ID: igad104.1454

### EVALUATING THE BEHAVIORAL RECOVERY OUTREACH (BRO) TEAM MODEL: TEAM MEMBERS' PERSPECTIVES

Amanda Peeples[1], Elizabeth MacDonald[2], and Kathleen Matthews[3], *1. Corporal Michael J. Crescenz VA Medical Center, Philadelphia, Pennsylvania, United States*, *2. VHA Serious Mental Illness Treatment and Evaluation Center, Ann Arbor, Michigan, United States*, *3. VHA VISN 23. , Des Moines, Iowa, United States*

The VA's Behavioral Recovery Outreach (BRO) Team model seeks to address the unmet needs of aging Veterans with complex behavioral, psychological, neurocognitive, and medical concerns. Consisting of a core team of a social worker, nurse, and psychologist, BRO teams collaborate with the broader VA Community Living Center or inpatient team on behavioral assessment and treatment planning to help stabilize the Veterans' behaviors and ready them for discharge to the community. Then, upon discharge the BRO Team provides ongoing behaviorally focused transitional support services to help ensure the Veterans' sustained stability in the community and prevent unnecessary rehospitalizations. While dissemination efforts have demonstrated positive clinical outcomes for the Veterans served, little was known about the factors that support or hinder successful implementation of this complex program. This paper presents results from qualitative interviews with ten members from seven BRO Teams (out of ten active teams at the time) undertaken in late 2021 as part of a larger BRO program evaluation. The BRO Team model, including the goals and core components of BRO, are defined. Determinants of successful BRO implementation and team operations, including staffing, inter-departmental relationships, model clarity, and support, are described. BRO Team members' views of the impact on and response of BRO recipients (BRO-enrolled Veterans, family members, and VA and community care partners), such as helping family members understand dementia, assistance with navigating the VA system, and support with behavioral health care planning and modification leading up to and following transitions to community care, are also detailed.

Abstract citation ID: igad104.1455

### THE LIVED EXPERIENCE OF COPD AND EMOTIONAL DISTRESS IN US VETERANS: A MIXED-METHOD STUDY

Patricia Bamonti, Grace Rose, Stella Park, Amy Silberbogen, Marilyn Moy, and Jennifer Moye, *VA Boston Healthcare System, Boston, Massachusetts, United States*

Behavioral interventions targeting low physical activity (PA) and comorbid emotional distress are critically needed to address gaps in the management of chronic obstructive pulmonary disease (COPD). A mixed-methods study was conducted to guide the development of Step-Cognitive Behavioral Therapy (Step-CBT), a virtual behavioral intervention under study integrating PA promotion with CBT for those with COPD. Semi-structured interviews with U.S. Veterans with COPD at the VA Boston Healthcare System explored the lived experience of COPD and emotional distress. Participants completed the Patient Health Questionnaire-9 [PHQ-9], Beck Anxiety Inventory [BAI]), and wore an accelerometer (ActiGraph wGT3X-BT) for 14 days to measure objective PA. Thematic analysis was used to code responses and identify themes. Participants (n=22; 90.9% male, age 68.0±6.0 years; 96% White) were assessed. PHQ-9 indicated moderate depression severity (M=11.3± 5.3) and BAI scores indicated moderate anxiety severity (M=26.5±9.4). Average daily step count was (M=6,611±4,799). In response to questions eliciting participants' experience of emotional distress, participants described Emotions, Behaviors, and Cognitions associated with distress that interacted with COPD and PA (e.g., "I've gotta catch my breath where before I could just do up the yard with no problem… so that gets me depressed"). When asked about engaging in daily PA in the context of emotional distress, three themes emerged: Emotion Regulation and Coping (e.g., healthy behavioral coping, maladaptive coping, etc.), Factors that Motivate (i.e., routine/schedule, etc.), and Social Context. Findings illuminate linkages between emotional distress and PA that will guide the creation of vignette-based treatment modules in the development of Step-CBT.

Abstract citation ID: igad104.1456

### CONNECTING AGING RURAL VETERANS WITH TELE-GERIATRIC MENTAL HEALTH: REFERRING PROVIDERS' SURVEYED PERSPECTIVE

Chalise Carlson[1], Marika Humber[1], Ranak Trivedi[2], Amanda Peeples[3], Althea Lloyd[4], and Christine Gould[1], *1. VA Palo Alto Health Care System, Palo Alto, California, United States*, *2. Stanford University, Stanford, California, United States*, *3. Corporal Michael J. Crescenz VA Medical Center, Philadelphia, Pennsylvania, United States*, *4. VISN 7. Clinical Resource Hub, VA Atlanta Health Care System, Decatur, Georgia, United States*

Providing comprehensive, individualized care to older Veterans with complex needs is particularly challenging in rural areas. Geriatric mental health specialists are scarce but needed for care including psychiatric medication and behavior management, consultation on complex comorbidities, cognition, and caregiver support and education. Tele-geriatric mental health services were initially developed in one VA regional telehealth hub and then expanded to three more regional telehealth hubs. Referring providers (N = 37) to tele-geriatric mental health services during a 1-year period were surveyed via REDCap. Providers came from varied settings in the 4 VA regions with 76% serving rural Veterans. Referring providers included prescribers (N = 26; pharmacists, physicians, physician assistants, nurse practitioners and advanced practice nurses) and non-prescribers (N = 11; psychologists, social workers). Most worked in Patient Aligned Care Teams, mental health clinics, or Community Living Centers and reported geriatric training and comfort with geriatric patients. When asked about their perception of the impact of the services, most providers believed that patients (91.9%) and care teams (94.6%) benefitted from the telehealth services and would recommend the services (94.6%). Most followed through on recommendations (83.7%) and believed the service increased access to geriatric mental health (89.2%). Open-ended feedback included gratefulness for providing care to aging rural Veterans lacking resources. These services are appreciated by providers and may facilitate access to specialized mental health care particularly in rural areas. Having access to specialists to assist with the care of patients with complex needs may ease the care coordination needed by referring providers.

### SESSION 4100 (SYMPOSIUM)

Abstract citation ID: igad104.1457

### ENGAGE UNDERREPRESENTED GROUPS IN TECHNOLOGY AND AGING RESEARCH: LIVED EXPERIENCE AT GEOGRAPHICALLY DIVERSE SITES

Chair: Jeffrey Kaye Co-Chair: Zach Beattie

Minoritized and marginalized older adults face multifaceted barriers that could prevent them from participating in aging research, including but not limited to not having access to research opportunities, time and transportation constraints, lack of trust with health care providers and researchers, and language barriers. When the research projects involve technology, whether as a tool for engagement or recruitment, intervention delivery, or outcome monitoring and assessment, additional challenges exist in recruiting underrepresented groups. The technology adoption gap has been widely reported. Minoritized and marginalized older adults are less likely to have internet access or feel less confident in using technology compared to their more privileged counterparts. This symposium introduces the lived experience and lessons learned from engagement and recruitment of underrepresented populations at multiple Alzheimer's Disease Research Centers. The first presentation presents outreach and recruitment efforts at the Oregon Outreach, Recruitment & Education Core. The authors will discuss strategies and benefits to engage people of underrepresented backgrounds in the research workforce. The following two presentations focus on recruitment efforts for installing an in-home monitoring technology platform for diverse older adult populations, including African Americans in Chicago, IL, and Hispanic/Latino immigrants in San Antonio, TX. The fourth presentation describes the response, retention, and enrollment rate of an online dementia research registry and the effort to adapt the registry and build capacity in recruiting Asian American immigrants with limited English proficiency. Sharing successes and lessons learned at multiple sites will contribute to developing more effective recruitment strategies for future aging research involving technology.

Abstract citation ID: igad104.1458

### RESPONSE AND RETENTION OF AN ONLINE RESEARCH REGISTRY: ADAPTATIONS MADE FOR RECRUITING ASIAN AMERICANS

Kexin Yu, Sarah Gothard, Emily Tupper, Ona Golonka, Allison Lindauer, and Aimee Pierce, *Oregon Health & Science University, Portland, Oregon, United States*

Challenges in recruiting research volunteers, especially racial/ethnic minority older adults, have been documented as one of the most significant obstacles to developing effective treatments for Alzheimer's Disease and Related Dementia (ADRD). To address the need for recruitment and raise awareness about ADRD research, the Layton Aging and Alzheimer's Disease Research Center built the online dementia research registry, Alzheimer's Comprehensive Treatment Network of Oregon and Washington (ACTNOW). Individuals aged 18 and above are eligible to join, and prospective participants self-initiated enrollment into the registry. As of March 2023, there are 497 participants (mean age 63.9, 71.6% female, 74% had bachelor's or above degree, 12% residing in a rural area) registered in ACTNOW, with an average follow-up period of 1.8 years (SD=1.2, Max=5). To date, 20 research projects have used ACTNOW for their recruitment, and 93% of registry volunteers have been referred to at least one requesting study. The current ACTNOW participants are predominantly non-Hispanic White (92.4%). We will describe ACTNOW's infrastructure expansion to improve the registry's capacity for engaging and recruiting older Asian American adults with limited English proficiency. We tested the feasibility of using translated informed consent forms and questionnaires, reconciling data collected in different languages with existing data structures, and the effectiveness of community outreach sessions. Developing the capacity to recruit underrepresented populations for future ADRD research projects can be critical to addressing health disparities in dementia awareness, prevention, diagnosis, and care. Standardizing communications with all registry stakeholders, both researchers and volunteers, helps engagement and accurate recruitment efforts.

Abstract citation ID: igad104.1459

### RECRUITMENT SUCCESSES AND CHALLENGES OF EMPLOYING IN-HOME SENSOR TECHNOLOGY WITH OLDER AFRICAN AMERICAN ADULTS

Shayla Calloway[1], Journie Raulston[2], and Lisa Barnes[1], *1. Rush University Medical Center, Chicago, Illinois, United States*, *2. Rush University, Chicago, Illinois, United States*

Older African Americans are under-represented in clinical research studies of aging. Many previous studies have shown results of in-home monitoring technologies to collect objective data for aging in place but few studies include minoritized populations. The CART platform uses home-based sensor technology to unobtrusively assess activity in the home and was embedded in homes of participants from the Minority Aging Research Study (MARS), an ongoing longitudinal study of cognitive decline in older African Americans. This presentation will highlight efforts to engage, recruit, and retain older African American participants in Chicago, with a particular focus on challenges and lessons learned. Recruitment consisted of presentations and mailings to potentially eligible MARS participants based on pre-specified inclusion criteria. Of 153 participants who met initial eligibility criteria, 36 refused before consent, 34 were ineligible after screening, and we enrolled 76 non-Hispanic African Americans participants (80% women; mean age = 80.4 (6.3) years); mean education = 16 (2.8) years; 78% without cognitive impairment). Enrolled participants were slightly younger, more likely to be female, and had higher education. There was no difference in cognitive impairment between those who enrolled compared to those who were ineligible after screening. Since enrollment, 11 have withdrawn and 7 are no longer active. The most common challenges include privacy concerns, broadband internet issues, emerging medical conditions, or changes in residency or resident occupancy. These results demonstrate that older African Americans participating in an ongoing longitudinal study can be recruited and retained in an ancillary study that involves home-based sensor technology.

Abstract citation ID: igad104.1460

### CONECTAR: A MINDSET TO INCREASE HISPANIC ENGAGEMENT IN DIGITAL TECHNOLOGY RESEARCH

Vanessa Young[1], Luis Serrano-Rubio[1], Carlos Gaona[1], Floyd Jones[1], Sudra Seshadri[1], Jeffrey Kaye[2], Zach Beattie[2], and Mitzi Gonzales[3], *1. University of Texas Health Science Center at San Antonio, San Antonio, Texas, United States*, *2. Oregon Health & Science University, Portland, Oregon, United States*, *3. UT Health San Antonio, San Antonio, Texas, United States*

In-home digital technology affords the opportunity for more precise and ecologically valid assessments of daily activity engagement. However, concerns about loss of privacy can hinder recruitment, especially within communities already underrepresented in research. Literature on effective, culturally tailored recruitment strategies is limited. Herein, we describe strategies employed for an in-home digital technology study with 100% Mexican American representation. Hispanic participants were recruited from the greater San Antonio area. Direct recruitment strategies conducted in both English and Spanish included community outreach events, a participant repository, cross-enrollment with other research studies, and by word of mouth. Mass recruitment strategies included flyer dissemination, newsletters, and digital announcements through community organizations (i.e., Salud America!) and national (i.e., Research Match) and institutional websites. Between September 2022 and February 2023, 31 participants were enrolled out of 154 individuals who were contacted. Of these, 29% were recruited from community events, 22.6% from word of mouth, 19.4% from cross-enrollment, 12.9% from the research repositories, and 16.1% from mass recruitment strategies. The most common reasons for non-enrollment included lack of interest (n=49), screen failure (n=29), and privacy concerns (n=12).Direct recruitment efforts achieved higher rates of enrollment, while mass media campaigns yielded lower responses and resulted in more unsuccessful contact attempts. Understanding the relationship between social networks and research beliefs may be particularly relevant given the

Hispanic culture's collectivist and family-oriented nature. Hence, social network theories, combined with a diffusion of innovation framework, may be advantageous for increasing trust and may consequently improve representation of diverse groups in research.

Abstract citation ID: igad104.1461

### BUILDING COMMUNITY RELATIONSHIPS FOR MORE MEANINGFUL ENGAGEMENT WITH OLDER BLACK ADULTS

Patrice Fuller, Charles Fennell, Allison Lindauer, and Raina Croff, *Oregon Health & Science University, Portland, Oregon, United States*

Black people are grossly underrepresented in clinical trials, especially in Oregon, where only 2% of the population is Black. The Oregon Alzheimer's Disease Research Center's (OADRC) Outreach, Recruitment, and Engagement (ORE) Core aims to correct this disparity by promoting research participation, fostering retention, and supporting quality experiences for Black Oregonians. Essential to this goal has been having an ORE Core that is 50% Black, leaders experienced in Black-centered cognitive health research, and a team that demonstrates commitment to community partnerships. The ORE Core engages a Community Outreach Specialist (COS) whose efforts reflect our Core's value of "service before sign-up." The COS differs from a traditional "recruiter" in that the focus is on community relationship-building, rather than recruitment. This can only be supported by first serving the community (e.g., joining community boards, volunteering) before asking community members to sign-up for research. The OADRC promotes research participation by supporting studies that focus exclusively on older Black health. The African American Dementia and Aging Project, supported by a valuable community health partnership, aims to understand lifestyle factors affecting cognitive health. The Sharing History through Active Reminiscence and Photo-imagery Study (SHARP) aims to improve brain health through a culturally celebratory framework centering the Black experience. As a result, SHARP has sustained high retention rates and increased Black participation in Center research. We discuss the role of ORE Cores, the role, values, and reflections of our COS, and examine how Center studies foster sustained community relationships, ultimately for more meaningful engagement within the Black community.

### SESSION 4105 (SYMPOSIUM)

Abstract citation ID: igad104.1462

### APPLIED METHODS FOR HEALTH EQUITY AND END-OF-LIFE OUTCOMES RESEARCH USING MEDICARE DATA

Chair: Olga Jarrín Co-Chair: Hyosin Kim

This symposium brings together four related and complementary projects on the development of new data sources and methods to analyze the new data to study health equity and end-of-life outcomes research. The first abstract presents the development of the Care Setting Trajectory Database, which integrates data from various CMS data sources for Medicare beneficiaries to create a longitudinal care experience for older adults across healthcare settings. The second abstract describes the creation of a new race/ethnicity variable to augment the Medicare administrative data for minority populations using harmonized, self-reported race/ethnicity data. The third abstract describes the development of harmonized datasets to study geriatric syndromes' prevalence, cost, and health outcomes over time. The fourth abstract identifies and predicts place of care trajectories during the last three years of life among Medicare beneficiaries, identifying dual trajectories of inpatient/institutional care and skilled home healthcare/home hospice. Finally, the fifth abstract includes an application of the care setting trajectory database and our new, harmonized, self-reported race/ethnicity variable to evaluate the impact of home health care on later use of hospice and place of death in a large cohort of Medicare decedents. This symposium introduces innovative approaches for harmonizing and merging CMS data sources for Medicare beneficiaries. The methodological advances described in this symposium aim to support health services researchers and policymakers to generate and evaluate research improving care coordination, continuity, and quality of life for older adults with serious illnesses.

Abstract citation ID: igad104.1463

### DEVELOPMENT OF THE MEDICARE BENEFICIARY CARE SETTING TRAJECTORY DATABASE

Olga Jarrín[1], Anum Zafar[1], Hyosin Kim[2], and Haiqun Lin[3], *1. Rutgers, The State University of New Jersey, New Brunswick, New Jersey, United States*, *2. Oregon State University, Corvallis, Oregon, United States*, *3. Rutgers, The State University of New Jersey, Newark, New Jersey, United States*

Despite national efforts to shift from fragmented to value-based/coordinated care, there is a lack of understanding of older adults' longitudinal aging experience across diverse healthcare settings in their last years. To improve patient care and gain insights into areas for improvement in care coordination and management, it is crucial to have evidence about older adults' longitudinal care experience and healthcare transitions. This study outlines the development of the Care Setting Trajectory File, which combines information on service dates and discharge destinations from various Medicare administrative, assessment, and claims data. The data sources include MedPAR, Hospice, Minimum Data Set (MDS), MDS-Swing Bed (SB), Outcome and Assessment Information Set (OASIS), and the Inpatient Rehabilitation Facility Patient Assessment Instrument (IRF-PAI). The Care Setting Trajectory File contains a day-level summary of care settings, including inpatient hospice, inpatient-short stay, inpatient-long stay, inpatient-SNF, inpatient-VA, inpatient, rehab facility, nursing home, home health, hospice home, and home. This day-level file can then be utilized at various levels of granularity, i.e., monthly, quarterly, or yearly periods, for further analysis. For illustration, the file construction method is detailed for a cohort of Medicare decedents who died in 2019. Examples of how this analytic file can be used to generate important insights for policymakers and healthcare providers on improving care coordination, continuity, and quality of life for older adults with serious illnesses will be discussed.

Abstract citation ID: igad104.1464

### HARMONIZATION OF SELF-REPORTED RACE/ETHNICITY TO ENHANCE HEALTH EQUITY RESEARCH USING MEDICARE DATA

Anum Zafar[1], Olga Jarrín[1], Hyosin Kim[2], Soko Setoguchi[1], Marie Harvey[2], Bei Wu[3], Maria Lopez[1], and Haiqun Lin[4], *1. Rutgers, The State University of New Jersey, New Brunswick, New Jersey, United States*, *2. Oregon State University, Corvallis, Oregon, United States*, *3. New York University, New York City, New York, United States*, *4. Rutgers, The State University of New Jersey, Newark, New Jersey, United States*

Collection of valid race and ethnicity data is critical to understanding and promoting health equity. However, current administrative data often misclassifies members of the racial/ethnic minority groups. To address this issue, the Research Triangle Institute (RTI) developed an imputed race variable, which improves on administrative race data for Hispanic and Asian beneficiaries, but still has limitations. This study compares the RTI race with our new, harmonized, self-reported race/ethnicity data collected during Medicaid enrollment and post-acute and long-term care assessments. Out of 2,322,071 Medicare beneficiaries aged 18 and older who died in 2019 in the U.S., self-reported race/ethnicity was available for 75.1% (1,745,327) who formed the study population. Compared to the RTI race variable, our new race variable categorized more Medicare beneficiaries as non-Hispanic white (79.5% vs. 77.9%) and Asian American/Pacific Islander (2.5% vs. 2.3%), a similar percentage as American Indians/Alaska Native (0.5% vs. 0.5%), and slightly fewer as Black (11.1% vs. 10.9%) and Hispanic (7.2% vs. 6.5%). Using our new race variable as the gold-standard, RTI race had good validity (k=0.81), however sensitivity was highest for non-Hispanic white (96.5) and Black (95.8) beneficiaries, and lower for Hispanic (87.2), Asian American/Pacific Islander (76.2), and American Indian/Alaska Native (58.7) beneficiaries. These findings are consistent with prior work, comparing RTI race with self-reported race from a single data source. Our study provides empirical evidence to support the U.S. Office of the Inspector General's recommendations to the Centers for Medicare and Medicaid Services to incorporate self-reported race/ethnicity data available to reduce error and bias.

Abstract citation ID: igad104.1465

### HARMONIZATION OF CHRONIC CONDITIONS WAREHOUSE DATA FOR OUTCOMES RESEARCH ON GERIATRIC SYNDROMES

Suzan Ahmad[1], Hyosin Kim[2], Soko Setoguchi[3], Fred Kobylarz[3], Anum Zafar[3], Maria Lopez[3], Haiqun Lin[1], and Olga Jarrín[3], *1. Rutgers, The State University of New Jersey, Newark, New Jersey, United States*, *2. Oregon State University, Corvallis, Oregon, United States*, *3. Rutgers, The State University of New Jersey, New Brunswick, New Jersey, United States*

Geriatric syndromes such as cognitive impairment, falls, depression, urinary incontinence, pressure ulcers, and polypharmacy are common conditions and syndromes that affect older adults. Studying geriatric syndromes in Medicare beneficiaries may provide important insights for improving health outcomes and quality of life. As a result of the Improving Medicare Post-Acute Care Transformation Act of 2014, it is now easier to compare standardized geriatric assessment data (including information about geriatric syndromes) collected in post-acute and long-term care settings with corresponding data from claims, encounter, Part D, and durable medical equipment files. However, to our knowledge, methods to link and harmonize post-acute and long-term care assessment data (available for 75% of decedents) are yet to be utilized in the health services research community. The goal of this project is to develop harmonized datasets that can be used to identify geriatric syndromes and study their prevalence, cost, health outcomes, and service delivery factors over time. This work will allow our team and other researchers to fully leverage these data for use in predictive algorithms and artificial intelligence and machine learning (AI/ML) research. The project uses the five most recent years of data available from the Centers for Medicare and Medicaid Services (CMS) Chronic Conditions Data Warehouse. Data sources include Medicare and Medicaid claims, encounter, MedPAR, Hospice claims, Minimum Data Set (MDS), MDS-Swing Bed (SB), Outcome and Assessment Information Set (OASIS), Inpatient Rehabilitation Facility Patient Assessment Instrument (IRF-PAI), Pharmacy (Part D), and Durable Medical Equipment (DME).

Abstract citation ID: igad104.1466

### IDENTIFYING AND PREDICTING PLACE OF CARE TRAJECTORIES DURING THE LAST THREE YEARS OF LIFE

Haiqun Lin[1], Anum Zafar[2], Soko Setoguchi[2], and Olga Jarrín[2], *1. Rutgers, The State University of New Jersey, Newark, New Jersey, United States*, *2. Rutgers, The State University of New Jersey, New Brunswick, New Jersey, United States*

Aging in place is a goal for most older adults, however in presence of advanced illness and/or Alzheimer's disease and related dementia (ADRD) institutional placement may be needed. The number of days in each care setting in each quarter of the last three years of life was determined through linkage of several Medicare datasets. Using group-based modeling we examined place of care trajectories during the last three years of life among a 10% sample of Medicare beneficiaries who died in 2018 (n=199,828), providing a pre-COVID19 baseline for future studies, to identify dual trajectories of inpatient/institutional care, and skilled home healthcare/home hospice. Nine distinct trajectory classes and their associated sociodemographic and clinical characteristics were identified. The first three classes were characterized by healthcare use concentrated in the last three months of life (comprising 29.1%, 20.5%, and 9.1% of the sample); the next three classes were characterized by low use of inpatient/institutional care with long-term use of skilled home healthcare/hospice (14.2%, 8.2%, and 4.8%); and the remaining inpatient/institutional classes characterized by low use of skilled home healthcare/hospice with long-term use of inpatient/institutional care (4.8%, 4.9%, and 4.6%). The beneficiaries in the remaining sample are assigned to a place of care trajectory class using the parameter estimates derived from the 10% sample without further model fitting. We investigate the prediction utility of risk factors in multiple domains of demographic, clinical, and social determinants of health. Our findings may help to inform care preference during last period of life understanding the associated factors.

Abstract citation ID: igad104.1467

**APPLYING THE CARE SETTING TRAJECTORY DATABASE TO STUDY END-OF-LIFE OUTCOMES IN PERSONS WITH DEMENTIA**

Hyosin Kim[1], Olga Jarrín[2], Paul Duberstein[3], Bei Wu[4], Haiqun Lin[5], Anum Zafar[2], S. Marie Harvey[1], and Maria Lopez[2], *1. Oregon State University, Corvallis, Oregon, United States*, *2. Rutgers, The State University of New Jersey, New Brunswick, New Jersey, United States*, *3. Rutgers School of Public Health, Piscataway, New Jersey, United States*, *4. New York University, New York City, New York, United States*, *5. Rutgers, The State University of New Jersey, Newark, New Jersey, United States*

This study highlights an application of the care setting trajectory database and our new, harmonized, self-reported race/ethnicity variable to evaluate the impact of home health care on later use of hospice and place of death in a large cohort of Medicare decedents. This study evaluated home health care use during the last 3 years of life as a predictor of death in the care of hospice for a cohort of 895,526 Medicare beneficiaries with dementia who died in 2019. The cohort was limited to beneficiaries continuously enrolled for at least 5 years, who were age 40 and older, and who lived in the U.S. fifty states, District of Columbia, or Puerto Rico at death. Co-variates included number of acute hospitalizations during the last 3 years of life, age at death, sex, race/ethnicity, comorbidities, insurance, rural/urban area, and state of residence. 54.5% of the cohort used home health care at any point during the last 3 years of life. Compared to beneficiaries who did not use home health care, those who received home health care were more likely to die at home (64.5% vs. 60%, OR 1.08) or in the care of hospice (65% vs. 58%, OR 1.05). Among beneficiaries who died with hospice, use of home hospice was more prevalent for those who had previously used home health care (53% vs. 49%). This study provides compelling evidence of the value of home health care as a strategy to improve end-of-life care quality among Medicare beneficiaries living with dementia.

### SESSION 4110 (SYMPOSIUM)

Abstract citation ID: igad104.1468

**INTERPROFESSIONAL HEALTH CARE: INTEGRATING AUDIOLOGY AND GERIATRICS IN INTERNATIONAL SETTINGS**

Chair: Michelle Arnold Co-Chair: Jennifer Deal Discussant: Kathleen Pichora-Fuller

Hearing is fundamental to communication and important for quality of life and overall health of older adults. Despite a potentially large role in how patients engage in healthcare, its importance is underrecognized in healthcare settings. Decreased patient-provider communication and poor treatment understanding may drive decreased satisfaction with healthcare services and avoidance of routine medical care, resulting in poorer health outcomes for older adults with hearing loss. Additionally, despite a high prevalence of hearing loss, less than 20% of those who could potentially benefit from hearing healthcare use hearing aids, potentially exacerbating sequalae of hearing loss in healthcare settings. Amidst growing recognition of the need for interprofessional healthcare collaboration, this session will focus on how audiologists and hearing specialists may partner with other healthcare providers (pharmacists, nurses, physicians) to address hearing needs in primary care, specialty care and hospital settings. We will include perspectives from the United Kingdom, the United States and Canada. We will describe factors that facilitate and impede communication between pharmacists and patients with hearing loss in the community pharmacy. We will also present results of a mixed-methods study from an interdisciplinary team (gerontology, public health, audiology, and otolaryngology/ear nose throat (ENT)) to inform primary care/ENT physicians about preferences for hearing treatment among Black women in the United States. Within a hospital setting, we will present how audiologist-nurse partnerships impact delirium. We will conclude with a discussion of how policy for hearing aid coverage impacts primary care providers recommendations for hearing healthcare in the United States.

Abstract citation ID: igad104.1469

**INTERDISCIPLINARY MANAGEMENT OF OLDER ADULTS WITH HEARING LOSS: POLICY MATTERS**

Michelle Arnold, *University of South Florida, Tampa, Florida, United States*

Hearing aids are an efficacious approach for treating hearing loss yet are underutilized in the US due to high out-of-pocket costs. Lack of hearing aid insurance coverage is one source of the breakdown in the interdisciplinary management of older adults with hearing loss, causing hesitation among primary care providers to refer patients to audiology for treatment. Recently, access and affordability of hearing aids for adults has gained traction, with several states mandating private insurance coverage of hearing aids. The purpose of this study was to understand state-level variability in private insurance hearing aid mandates and to quantify yearly averages of the share of privately insured adults aged <65. We conducted a longitudinal policy surveillance of state statutes for mandates through January 2023. Policy data were then combined with individual-level age, insurance, state, and year variables from American Community Survey and Medical Expenditure Panel Survey Insurance Component to estimate the share of privately-insured adults covered by a mandate from 2008-2022. We identified 25 states and 1 territory with effective private insurance hearing aid mandates. There was substantial variability in these mandates, including in exceptions, maximum age eligibility, allowable frequency of benefit use, and total coverage reimbursement amount. Between 2008-2022, we estimated that <10% of privately-insured adults lived in a state with coverage. We conclude that hearing aid mandates are covering a small but growing share of Americans. A federal mandate would be the fastest way to ensure access, but states can improve access by adopting exception-free mandates with limited utilization management.

Abstract citation ID: igad104.1470

**AN INTERDISCIPLINARY APPROACH TO ADDRESSING HEARING LOSS IN THE INPATIENT SETTING TO IMPROVE COMMUNICATION**

Nicholas Reed, *Johns Hopkins Bloomberg School of Public Health, Baltimore, Maryland, United States*

Nearly half of all adults over 60 years of age have hearing loss which is associated with decreased satisfaction with care, higher risk of readmission, and longer length of stay. These associations may be mediated by the barriers hearing loss places on patient-provider communication. There is a paucity of sustainable programs focused on addressing hearing loss among older adults in the inpatient. An interdisciplinary collaborative team comprising audiologists, geriatricians, and nurses developed and implemented a quality initiative to address hearing loss in the inpatient setting in two medical inpatient units at Johns Hopkins Bayview Medical Center that started with offering 16 training session slots to raise awareness of hearing loss and teach technologic, environmental, and communication considerations for adults with hearing loss. Over a 3-month period, 543 of 644 admitted patients were screened for hearing loss using a sustainable model built into the admission process. Those without hearing loss (52.6%) received no intervention, those with mild loss (31.3%) received signage in the room reminding providers of training, and those with moderate or greater loss (16.1%) received signage and an amplifier. At discharge, 79% and 81.6% of those with mild and moderate hearing loss indicated improved communication during their current stay relative to previous stays. Notably, a larger proportion of those without hearing loss (52.9%) also indicated improved communication. A post study survey among 18 nurses suggested themes of moderate (33%) desiring more training and overwhelming perceptions (94.4%) the program didn't interfere with other duties and made communication with patients easier.

Abstract citation ID: igad104.1471

**KNOWLEDGE, ATTITUDES, AND BELIEFS ABOUT HEARING HEALTH CARE OF OLDER BLACK WOMEN SEEKING HEARING HELP IN ENT SETTINGS**

Julia Toman, Charity Lewis, Victora Sanchez, and Michelle Arnold, *University of South Florida, Tampa, Florida, United States*

There are documented barriers to access and use of healthcare services faced by older Black adults in the US. Black women are more likely to report negative past experiences with the healthcare system and as a result have inherent mistrust of providers. Black women are also less likely to receive referrals for specialty and allied health care, but it is unknown if this trend is apparent for hearing healthcare. The purpose of the current study was to investigate the experiences of older Black women seeking help for hearing loss in an otolaryngology (ENT) clinic setting. Our interdisciplinary team consisting of experts in gerontology and Black women's health, public health, audiology, and otolaryngology conducted a mixed methods study which included (1) semi-structured interviews (n=15) and (2) online administration of a validated hearing beliefs questionnaire (n=86). Interview themes uncovered included experiences with racism in healthcare, the role of religion in help-seeking, and the importance of seeking treatment for hearing loss. Triangulation of findings revealed consistency between themes and questionnaire results, including little to no expressed stigma surrounding hearing aid use and little resistance to hearing healthcare or hearing loss treatment. Our results suggest that primary care and ENT should feel confident in referring older Black women who are seeking help for hearing loss to audiology for treatment options. We will also discuss other known facilitators and barriers to serving this population in an ENT setting.

Abstract citation ID: igad104.1472

**AGE-RELATED HEARING LOSS IN COMMUNITY PHARMACY: BARRIERS AND FACILITATORS TO COMMUNICATION**

Penny Lewis[1], Shanice Thomas[2], Jane Griffiths[2], Denham Phipps[2], Gabrielle Saunders[2], and Christopher Todd[2], *1. The University of Manchester, Manchester, England, United Kingdom*, *2. University of Manchester, Manchester, England, United Kingdom*

Pharmacists have an important role in primary care, communicating with people and ensuring safe and appropriate medication use. However, inadequate communication is a barrier to the delivery of effective care for people with hearing loss. This study sought to explore factors that facilitate and impede communication with people with age-related hearing loss (presbycusis) in the community pharmacy, in order to identify solutions to improve these interactions. Online semi-structured interviews with people with age-related hearing loss (presbycusis), older people (>50 years old) without hearing loss, and online focus groups and interviews with community pharmacists were conducted. Data were analysed using the framework method. Sixteen people with age-related hearing loss, three older people without hearing loss and eight community pharmacists took part. Participants described a multitude of environmental barriers to communication and person-centred pharmaceutical care such as heavy workload, lack of privacy, noise levels and Covid-19 safety measures. There was a perception among participants that their hearing loss is not relevant to the community pharmacy setting and that more could be done to signify that a pharmacy recognises the needs of those with hearing loss, furthermore, participants discussed their limited interaction with pharmacy personnel. There were varying perceptions about communication and levels of awareness among pharmacists about the key facilitators to communication. Greater interdisciplinary collaboration to develop and implement strategies/adaptations tailored to the needs of people with hearing loss would support the engenderment of hearing-friendly community pharmacies and the safe use of medicines.

### SESSION 4115 (SYMPOSIUM)

Abstract citation ID: igad104.1473

**FROM CELLS TO SYSTEMS**

Chair: Raya Kheirbek Co-Chair: Kenzie Latham-Mintus

Individuals who have been incarcerated may experience chronic pain due to various reasons, such as physical injuries sustained during their time in prison, untreated chronic medical conditions, or addiction to prescription medications. The pain may persist into late life, resulting in reduced quality of life, increased healthcare costs, and limited access to end of life care and hospice services. This issue requires collaboration among experts in pain management, geriatrics, and palliative care to develop effective interventions that address the unique needs of this population. Suicide among veterans is another pressing issue that requires an interdisciplinary approach. Incarcerated veterans may face numerous challenges upon release, including limited access to healthcare and social support, economic instability, and the stigma associated with a criminal record. These factors may contribute to increased risk of suicide. Addressing this issue requires a collaborative effort among experts in mental health, social work, and criminal justice to develop comprehensive programs that provide support for veterans transitioning from incarceration to civilian life. Being imprisoned can have lasting negative health consequences that persist throughout a person's lifetime. Incarcerated individuals may face limited access to quality end-of-life care and hospice services, resulting in increased suffering during their final days. The experience of incarceration can also lead to social isolation and stigma, exacerbating mental health issues and further decreasing the quality of life at the end of life. Effective interventions are needed to address the unique healthcare needs of incarcerated individuals, particularly as they approach the end of life

Abstract citation ID: igad104.1474

### PROFILES OF OLDER INCARCERATED PERSONS WHO ATTEMPT SUICIDE FOLLOWING RELEASE

Lisa Barry[1], Yixia Li[2], and Amy Byers[3], *1. UConn Center on Aging, Farmington, Connecticut, United States*, *2. NCIRE, San Francisco, California, United States*, *3. UCSF / San Francisco VA Health Care System, San Francisco, California, United States*

Release from incarceration is a strong risk factor for suicide. Yet little is known regarding factors associated with suicide attempt among older adults who reentered the community following incarceration. Leveraging a national cohort of U.S. veterans age ≥50 released from incarceration between FY2007 and FY2018 (N=30,584), we conducted a 1:3 matched case-control study and compared demographic and comorbidity factors between those who attempted suicide (cases, N=643) and those with no suicide attempt (controls, N=1,929) up to 13 years post-release. Comorbidity profiles were determined using latent class analysis. As compared with controls, cases had higher proportions of chronic illnesses (e.g., chronic lung disease [35.2% vs. 19.6%], diabetes [32.2% vs. 19.4%], Hepatitis C [24.9% vs. 9.6%]); depression (75% vs. 28.7%); substance use disorders (SUDS) (83.0% vs. 39.4%); and other serious mental illnesses (SMI) (e.g., schizophrenia [34.2% vs. 13.2%], psychotic illness [39.5% vs. 12.5%]). We found four distinct latent clusters among cases (entropy statistic = 0.80): (1) “Minimal Comorbidity” (18%); (2) “PTSD/SUD” (35%); (3) “High Psychiatric/SMI” (32%); and (4) “High Medical/Psychiatric Comorbidity” (15%), and four clusters among controls (entropy statistic = 0.84): (1) "Minimal Comorbidity" (50%); (2) "SMI" (8%); (3) "Tobacco dependence/SUD" (33%); and (4) "High Medical/Psychiatric Comorbidity" (9%). Compared with controls, fewer cases were likely to have the “Minimal Comorbidity” profile (18% vs. 50%), whereas more were likely to have profiles with SMI (32% vs. 8%). Establishing comorbidity profiles of older persons reentering the community after incarceration may help determine who is more likely to die by suicide, thus informing prevention efforts.

Abstract citation ID: igad104.1475

### THE HIDDEN TOLL OF INCARCERATION: EXPLORING THE LINK BETWEEN IMPRISONMENT HISTORIES AND PAIN AMONG OLDER ADULTS

Yulin Yang[1], Gabriel Lutz[2], Chixiang Chen[3], and Raya Kheirbek[2], *1. University of California, San Francisco, San Francsico, California, United States*, *2. University of Maryland School of Medicine, Baltimore, Maryland, United States*, *3. University of Maryland, Baltimore, Maryland, United States*

Objective: Incarceration is linked to poor health outcomes across the life course. However, little is known whether and to what extent incarceration histories shape pain in later life. This study aims to examine the link between incarceration histories and pain outcomes among middle-aged and older adults in the United States.

**Methods:** We used data from a nationally representative sample of community-dwelling adults aged 50+ in the 2012-2018 biennial waves of the US Health and Retirement Study (HRS) to estimate pain trajectory in U.S. older adults with and without a history of incarceration. We fit generalized estimating equation (GEE) models adjusting for socio-demographic covariates and early life experience to estimate the risk of reporting moderate-to-severe pain and related limitation.

**Results:** We included 12,394 respondents aged 68.76 on average (SD= 10.34), 59% female, and 7% with incarceration histories (n= 870). Persons with incarceration histories have a greater risk of reporting moderate-to-severe pain (PR=1.38, 95% Confidence Interval [CI]: 1.24, 1.53) and pain-related limitation (PR=1.50, 95% CI: 1.34, 1.67) even after adjusting for socio-demographic covariates and early life experience.

**Discussion:** In a nationally representative sample of older adults (with or without incarceration history), our study demonstrates an independent association between a history of incarceration and pain in later life. Our findings highlight the far-reaching impact of incarceration and the need for developing optimal pain management strategies to reduce the burden of disabling symptoms. Interventions should prioritize socioeconomically vulnerable groups, who may have the least access to pain treatment in later life.

Abstract citation ID: igad104.1476

### EXAMINING A PERSON-CENTERED MODEL OF COMMUNITY SUPERVISION FOR OLDER ADULTS

Victoria Helmly, *Georgia State University, Atlanta, Georgia, United States*

Though research in aging and the criminal legal system is growing, there is still limited data on how the carceral system impacts this population. In this presentation, I will discuss my dissertation research on older adults on parole and probation (community supervision). This qualitative research study examines both the experiences of older adults under community supervision and the experiences of the officers who supervise this population. This data is collected through semi-structured interviews with community supervision officers and older adults under supervision. In addition to learning about the unique experiences of older adults impacted by the criminal legal system, this research aims to better understand a "person-centered model of community supervision" in the Southeastern United States. The interviews inquire about how this model is implemented, perceived, and understood by the participants. This research aims to inform policy and practice of community supervision and stimulate additional research in this area. In this presentation, I will discuss the study's relevance to Gerontology, the research methodology, and the findings from the participant interviews. I will also discuss how this research can inform practice and future studies.

Abstract citation ID: igad104.1477

### A TALE OF TWO EXPERIENCES: NAVIGATING END-OF-LIFE CARE WITH A HISTORY OF INCARCERATION

Gabriel Lutz[1], Yulin Yang[2], Chixiang Chen[3], and Raya Kheirbek[1], *1. University of Maryland School of Medicine, Baltimore, Maryland, United States*, 2. *University of California, San Francisco, San Francsico, California, United States*, 3. *University of Maryland, Baltimore, Maryland, United States*

**Objectives:** The negative health effects of being imprisoned are well-documented throughout one's lifetime, but the impact of incarceration on end-of-life experience is not fully understood. Thus, this study aims to explore the relationship between a person's history of incarceration and their end-of-life experience

**Methods:** The Health and Retirement Study, a survey of Americans aged 50 and above that is nationally representative, provided the data for this study, which involved a secondary analysis of existing information. The dataset included individuals who had participated in the HRS but had deceased between 2012 and 2018.

**Results:** Among 1710 respondents aged 78 (SD10 years) on average at baseline, there are 53% female, and 8% with incarceration history (n=139). We found incarceration history to be positively associated with caregiving and lower risk of dying alone OR 1.84 (95% CI: 0.64-5.21) higher chance of using hospice OR 1.16 ( 95% CI: 0.72- 1.87) and dying at home, but lower levels of quality of life at the end of life OR -0.44 (95% CI: -1.88, 1.01) and utilization of palliative care services OR 0.49 ( 95% CI: 0.21, 1.12).

**Conclusion:** Our study, which included representative sample of older adults from across the country has revealed an association between a person's past imprisonment and their end-of-life experience . The study highlights the pervasive effects of incarceration and underscores the importance of developing effective palliative care strategies for early identification of the needs to alleviate suffering and improve quality of life for this vulnerable population.

### SESSION 4120 (PAPER)

### BIOLOGY OF AGING RESEARCH

Abstract citation ID: igad104.1478

### BIOLOGICAL MARKERS OF FALLS IN OLDER ADULTS WITH MILD COGNITIVE IMPAIRMENT

Frederico Pieruccini-Faria[1], Canan Okuyan[2], Yanina Sarquis-Adamson[1], Surim Son[3], Munira Sultana[4], and Manuel Montero-Odasso[4], *1. Gait and Brain Lab/ St. Josephs Hospital/Lawson Research Institute/Parkwood Institute, London, Ontario, Canada*, 2. *Sakarya University, Academy of Applied Sciences, Antakya, Sakarya, Turkey*, 3. *Western University, London, Ontario, Canada*, 4. *University of Western Ontario, London, Ontario, Canada*

Mild Cognitive Impairment (MCI) nearly doubles the risk of falls in older adults. While the association between cognition and falls risk is relatively well understood, no study has explored how biological markers of organic integrity contribute to falls risk in MCI. This study aimed to identify biological markers that could be associated with an increased rate of incidental falls in MCI. A total of 142 participants with MCI (aged 74.3 ± 6.1 years;75% women) with blood tests performed at baseline in Gait and Brain Cohort Study were analyzed. Participants were followed up to 7 years (mean of 28 ± 22.1 months), and falls were reported during the follow-up period(70% fell). Eighteen biological markers extracted from blood serum were dichotomized into low and moderate-to-high levels based on tertile cut-offs. Negative binomial regression model was used to estimate the incident rate ratio (IRR) of falls for biological markers, adjusted for age, sex, global cognition, gait speed, total follow-up duration, and history of falls in the past 12 months at baseline. Results showed that low Parathyroid Hormone (PTH) level (IRR=1.73; 95%CI=1.07, 2.79; p=0.02) and moderate-to-high Interleukine-8 (IL-8) level (IRR= 1.81; 95%CI 1.05-3.14; p=0.03) were associated with an increased rate of falls during the follow-up. Moderate-to-high IL-8 level was also associated with an increased rate of falls with injuries (IRR=2.07; 95%CI=1.05, 4.08; p=0.03). This suggests that in addition to cognitive deficits, impaired calcium metabolism caused by low PTH level and inflammatory processes affecting internal organs may increase falls risk in older adults with MCI.

Abstract citation ID: igad104.1479

### CROSS-TISSUE COMPARISON OF AGING BIOLOGY MEASURES AS NOVEL BIOMARKERS FOR HEALTHY AGING

Reem Waziry, *Columbia University, New York City, New York, United States*

We compared the role of aging biology biomarkers measured in saliva (N=5,808) and blood (N=9,934) as novel biomarkers for prevention of stroke and heart disease in a sample of older adults (aged 50 and older) from the Health and Retirement Study (HRS). Individuals who consented for blood and saliva draw in the HRS 2016 Venous blood and 2008 Telomere length studies were included. Measures in saliva included telomere length. Measures in blood included DNA methylation and physiology biomarkers. The main outcomes included stroke, heart disease and disease-free aging. Cases and disease-free adults were matched on age and sex using a probit link and propensity scores. No association was observed between saliva-based telomere length with stroke or heart disease. In contrast, blood-based biomarkers of aging biology showed strong associations with stroke and heart disease. Biologically older individuals had 3- and 2.5-times higher odds of stroke based on DNA methylation Grim Age (aOR=3.00, 1.87, 4.82, P< 0.001) and Physiology-based Phenotypic Age (aOR=2.50, 1.57, 3.98, P<0.001).

Individuals who were biologically older had 3.6- and 2.3-times higher odds of heart disease according to physiology-based Phenotypic Age (aOR=3.64, 95% CI 2.68, 4.94, p < 0.001) and DNA methylation Grim Age (aOR=2.36, 95% CI 1.73, 3.23, P<0.001). Findings of the present study suggest that saliva-based telomere length and blood-based DNA methylation and physiology biomarkers likely capture different aspects of individual aging and vary in their precision and sensitivity as novel biomarkers for prevention of stroke and heart disease in older adults.

Abstract citation ID: igad104.1480

### PREDICTING THE RISK FACTORS OF HYPERTENSION AMONG INDIAN OLDER POPULATION: A MACHINE LEARNING APPROACH

Ayushi Das, *International Institute for Population Sciences (IIPS), Mumbai, India, Mumbai, Maharashtra, India*

Hypertension, often known as high blood pressure, is a disorder in which the pressure inside the blood arteries remain consistently high. It is a common chronic illness that often occurs along with other morbidities. Risk prediction of hypertension further contributes to the detection, development of interventions, and treatment. The current study used different machine learning models to identify the high-risk factors of developing hypertension using a large-scale survey dataset LASI. A sample of 58,757 collected through the survey aged 45 and above used for model training and testing. We identified 35 risk factors from the literature and then applied feature selection methods to identify only the important ones. Based on the selected features, three models were built i.e., random forest, decision tree, and logistic regression, and the hyper-parameters were optimized on the training set using 10-fold cross-validation. The performance of the models was measured using area under the curve value, precision, recall, and F measure. The random forest classifier outperformed the rest of the two algorithms with an AUC score of 0.64, a precision of 0.65, a recall score of 0.57, and an F1 score of 0.61. Body Mass Index, diabetes, age, type of work, parents having hypertension, and multimorbidity are the primary risk factors for hypertension. The results can be further applied to make algorithms to predict hypertension among an older individuals based on their demographic, health and behavioral factors. Predictive models for hypertension can aid in determining the degree of community interventions required, ensuring a favorable outcome.

Abstract citation ID: igad104.1481

### RETINAL AGING IN COMPANION DOGS

Freya Mowat[1], Michele Salzman[1], Natascha Merten[2], and Callie Rogers[1], *1. University of Wisconsin-Madison, Madison, Wisconsin, United States*, *2. School of Medicine and Public Health, University of Wisconsin, Madison, Wisconsin, United States*

Companion dogs cohabit with humans in approximately 45% of US households. They experience similar environmental exposures and lifestyles to their human companions, and have shown sensory, cognitive, and neurological decline with aging. The relationship between age and retinal neurologic function in dogs has not been described. The aim of this study was to determine associations between age and retinal full-field electroretinographic measures in companion dogs, an important translational model species for human neurologic aging. Healthy adult companion dogs with no ophthalmic abnormalities were included (n=54, 18 male, 36 female, median weight 25.7kg, median age 89 months). Unilateral full-field light-adapted and dark-adapted electroretinography (ERG) was performed using a handheld device, with mydriasis and topical anesthesia. Standard least squares effect leverage models were used with log transformed data to determine the cross-sectional effect of age, sex, and bodyweight on ERG amplitudes and peak times. Age and sex were significantly associated with ERG outcome variables, more consistently with ERG amplitude than peak time; age had the largest and most significant effect leverage on outcomes (significantly affecting 3/9 peak time and 8/10 amplitude outcome variables). Younger and female dogs had higher ERG amplitudes. Body weight had no significant effect on any outcome. Our finding that aged companion dogs have reduced ERG amplitudes and prolonged peak times mirror ERG findings in aging humans. Because dogs have a shorter lifespan compared with humans, they may act as a sentinel species to monitor for emerging risk factors for age-related neurologic decline.

Abstract citation ID: igad104.1482

### TRAJECTORIES OF C-REACTIVE PROTEIN OVER THE MENOPAUSE TRANSITION: THE STUDY OF WOMEN'S HEALTH ACROSS THE NATION

Samar El Khoudary[1], Meiyuzhen Qi[1], Xirun Chen[1], Gail Greendale[2], Arun Karlamangla[3], and Karen Matthews[1], *1. University of Pittsburgh School of Public Health, Pittsburgh, Pennsylvania, United States*, *2. UCLA School of Medicine, Los Angeles, California, United States*, *3. University of California, Los Angeles, Los Angeles, California, United States*

Inflammation is a mechanistic pillar of aging contributing to the pathogenesis of several common medical conditions. Numerous in-vitro and animal experiments support a link between estrogen decline over the menopause transition (MT) and inflammation. Whether the MT per-se is associated with increased inflammatory load is not obvious. Using longitudinal data from 1,459 (11,373 observations) women (at baseline: 42-52 years old; underweight/normal 39.6%, overweight 28%, obese 32.4%; White 44.5%, Black 27.8%, Chinese 10.7%, Japanese 12.1%, Hispanic 4.9%) with a known final-menstrual-period (FMP) date and at least 3-measures of high sensitivity C-reactive protein (hs-CRP) spanning between 10 years before to 14 years after FMP, we characterized trajectories of hs-CRP and their associations with premenopausal obesity and race/ethnicity. Observations indicating acute inflammation were deleted and data were censored at first use of hormone therapy. Group-based trajectory modeling adjusted for time-varying smoking-status and steroids/statin use was utilized to identify trajectories. LOESS plots and piecewise linear mixed-effects models were used to determine if trajectories were linear or had inflection points around FMP. Three distinct hs-CRP trajectories were identified: low-rise (low-level significantly increased 2.5Yrs after FMP; 26.6%), medium-stable (remained stable across MT; 41.7%), and high-decline (high-level significantly declined at FMP; 31.7%). White women were more likely to show the medium-stable trajectory, Black and Hispanic the high-decline, and Chinese and Japanese the low-rise trajectory. Premenopausal obesity predicted the high-decline trajectory, underweight/normal predicted the low-rise, while overweight predicted the medium-stable trajectory. Heterogeneous patterns of hs-CRP over the MT exist and are related to women's race/ethnicity and premenopausal obesity.

### SESSION 4125 (PAPER)

### EFFORTS TO IMPROVE AGING SERVICES AND HEALTHCARE PROVIDER TRAINING AND DELIVERY

Abstract citation ID: igad104.1483

### BUILDING A STRATEGIC PARTNERSHIP: TURNING CHALLENGES INTO OPPORTUNITIES

Pamela Elfenbein, *University of North Georgia, Oakwood, Georgia, United States*

The University of North Georgia's Institute for Healthy Aging (IHA) and the Georgia Department of Human Services, Division of Aging Services (DAS), have partnered to address critical workforce needs in the program areas of Elder Abuse, Neglect and Exploitation and Public Guardianship. Working together they have aligned requirements a new University System of Georgia degree, the Nexus degree, with the educational and experiential requirements of employment in these DAS program areas. The University System of Georgia's Nexus Degree, a new academic credential and the first new degree program in the United States since the 1890s when the associate's degree was added, was designed to help more Georgians access careers in high demand areas. Creation of this degree is in direct response to talent demand analysis with employers in high demand career areas. The Division of Aging Services faces many challenges that hamper finding and retaining the right workers for the difficult but rewarding roles in Adult Protective Services and Public Guardianship. This presentation will outline the DAS needs and UNG academic program, and how working together to meet the needs of at-risk elders, a direct pipeline to employment has been developed. This paper will discuss and provide insight into this unique collaboration and newly created training approach.

Abstract citation ID: igad104.1484

### DEVELOPMENT AND EVALUATION OF AN INTERPROFESSIONAL LEADERSHIP IN HEALTH CARE CERTIFICATE

Bronwyn Keefe, Craig Slater, and Karen Jacobs, *Boston University, Boston, Massachusetts, United States*

Leaders of healthcare teams have a critical role in coordinating services to deliver high-quality care to the patients and communities they serve. Leading teams can be challenging in the context of complex healthcare systems, increasing costs, resource limitations, and workforce issues. We created the Interprofessional Leadership in Healthcare Certificate for health professionals who lead, or aspire to lead, interprofessional teams. Learners complete courses relating to interprofessional collaboration, effective communication, mentoring and supervision, business acumen, and contemporary leadership models. The live classroom sessions use Project ECHO® to facilitate technology-enabled, collaborative learning. Participants completed an evaluation survey that included questions about course learning objectives, engagement in learning activities, application of content to professional practice, and interprofessional learning. The survey included open-ended questions about the most helpful aspects of each course and suggestions for changes. Evaluation data for each of the courses were collated across four cohorts. Participants represented over 10 healthcare professions. There was over 90% agreement that content met all of the learning objectives for each course. There was over 90% agreement that participants learned from other professions and engaged in interprofessional collaboration. The qualitative data indicated that learners found that the content helped them to be more intentional with their collaborative and leadership practices in their workplaces. The certificate program had a positive impact on participants' knowledge, skills, and workplace practices relating to interprofessional collaboration and leadership. This may be attributable to intentional interprofessional collaboration in both program development and learning experiences and the use of the project ECHO model.

Abstract citation ID: igad104.1485

### EVALUATING A MICROLEARNING INTERVENTION ABOUT NUTRITIONAL CARE FOR OLDER PEOPLE TARGETED AT NURSES

Debbie ten Cate[1], Jeroen Dikken[2], Roelof Ettema[1], Lisette Schoonhoven[3], and Marieke Schuurmans[4], *1. Utrecht University of Applied Sciences, Utrecht, Utrecht, Netherlands*, *2. The Hague University of Applied Sciences, The Hague, Zuid-Holland, Netherlands*, *3. University Medical Center Utrecht, Utrecht University, Utrecht, Utrecht, Netherlands*, *4. Dutch Healthcare Authority, Utrecht, Utrecht, Netherlands*

Nurses play a pivotal role in the care of older people with malnutrition or who are at risk. However, research shows that they lack the motivation to give adequate nutritional care. To change this motivation, an educational intervention targeted at nurses would be desirable. To optimize learning efficacy, we developed a microlearning intervention and conducted a first evaluation. The aim of this study was to evaluate the microlearning intervention on reaction, learning and behavioral change among nurses. The evaluation was assessed with focus groups, a questionnaire, participant observations and patient records. The data was both qualitatively and quantitatively analyzed. Two focus groups were held with a total of seven nurses. They mostly mentioned positive experiences, stated that their knowledge about nutritional care increased and that their behavior in daily practice changed. Of the 306 participants, who received the intervention, 94 filled in the questionnaire of which 66% was satisfied with the microlearning intervention and over 69% agreed that they learned something new. Five nurses were observed while giving care to a total of twelve older care recipients. No clearly detectable changes in behavior during care moments or in reporting in patient records were observed. Nurses had a mainly positive experience with the microlearning intervention. They confirmed learning and changing behavior in daily practice. However, from observations this behavioral change was not visible. The intervention may be potentially useful in education for (future) nursing professionals.

With the microlearning intervention, nurses may be better equipped to provide nutritional care to older people.

Abstract citation ID: igad104.1486

### PLANNING FOR OLD AGE BY GRADUATES OF A MASTER'S GERONTOLOGY PROGRAM IN ISRAEL

Ella Cohn-Schwartz[1], Doron Sagi[2], and Lian Meiry[3], *1. Ben Gurion University, Haifa, HaZafon, Israel*, *2. College of Law and Business, Ramat Gan, Israel (CLB), Maitar, HaDarom, Israel*, *3. Faculty of Health Sciences at Ben-Gurion University of the Negev, Beersheba, HaDarom, Israel*

The master's programs in gerontology in Israel were founded in 1999 in response to the challenges brought by extended life courses and the accompanying accelerated growth of welfare and health services for the aged. Since its beginning, thousands of students graduated from the programs and occupy central positions in the gerontological field. However, no empirical data exists on the extent of the involvement by graduates in employment in the field of aging. The current study aims to provide a first-time assessment of the experiences of graduates in Israel following their studies, as well as examine the level of preparedness to old age among graduates, since their work and study of older adults might make their own future old age more salient. We collected data using online surveys from 197 graduates of the gerontology programs. Participants were asked about their work experience in the gerontology field and about the extent to which they prepare for old age. The findings showed that most graduates (80%) currently work in the field of aging, and are overall pleased with the jobs. However, most of the graduated (68%) also reported difficulties in finding employment. Logistic regressions showed that those who currently work in aging and who graduated before less years were also less likely to make preparations for needing nursing care in old age and talking with relatives about their death. Thus, working and studying about aging could be related to being less likely to make preparations for old age.

### SESSION 4130 (PAPER)

### FACTORS IMPACTING HEALTH IN OLDER ADULTS

Abstract citation ID: igad104.1487

### COVID-19–ASSOCIATED HOSPITALIZATIONS AMONG ADULTS AGES ≥75 YEARS—COVID-NET, OCTOBER 2022–FEBRUARY 2023

Christopher Taylor[1], Kadam Patel[2], Kyle Openo[3], H. Keipp Talbot[4], and Fiona Havers[5], *1. Centers for Disease Control and Prevention (CDC), Atlanta, Georgia, United States*, *2. Centers for Disease Control and Prevention, Atlanta, Georgia, United States*, *3. Emory University, Division of Infections Diseases, Atlanta, Georgia, United States*, *4. Vanderbilt University Medical Center, Nashville, Tennessee, United States*, *5. CDC, Atlanta, Georgia, United States*

Older adults are at increased risk for COVID-19-associated hospitalizations and other severe outcomes. Recent hospitalizations among adults ages ≥75 years were described using data from CDC's COVID-19-Associated Hospitalization Surveillance Network (COVID-NET), a population-based surveillance network of >300 acute-care hospitals across 14 U.S. states. Percentages were statistically weighted as necessary to represent the underlying population of the surveillance catchment area, unless noted. From October–December 2022, population-based hospitalization rates among adults ages ≥75 years were the highest of all age groups and ≥3 times as high as rates among the next oldest age group, adults ages 65–74 years. During that same period, adults ages ≥75 years comprised 42% (unweighted) of the 13,721 laboratory-confirmed adult hospitalizations identified in COVID-NET. Among a stratified random sample of adults ages ≥75 years (N=391), the most common underlying medical conditions present upon admission included chronic lung disease (72%), cardiovascular disease (65%), neurologic disorders including dementia (45%), and diabetes (36%). Among adults ages ≥75 years, 9% had received the updated booster and 74% were vaccinated but had not received an updated booster dose. Adults ages ≥75 years comprise an increasingly disproportionate share of COVID-19-associated hospitalizations with the majority of recent hospitalizations occurring among those who have not received an updated COVID-19 booster dose. Underlying medical conditions can increase the risk for hospitalization and other severe outcomes and are common in this group. Receipt of updated COVID-19 bivalent booster doses and early antiviral treatment to prevent hospitalization are essential for this age group.

Abstract citation ID: igad104.1488

### EVALUATION OF AMINO ACIDS LEVELS OF HOSPITALIZED PATIENTS WITH DELIRIUM

Gohar Azhar, Shakshi Sharma, Regina Gibson, Amanda Pangle, Robert Wolfe, and Jeanne Wei, *University of Arkansas for Medical Sciences, Little Rock, Arkansas, United States*

Delirium is an acute and often fluctuating change in cognitive status characterized by disorientation, disorganized thought processes and changes in perceptual and psychomotor function. The common precipitating etiologies of delirium include any acute illnesses, infections, hospitalizations, fluid or electrolyte imbalance, or medications. Neurotransmitters are synthesized from amino acids. We hypothesized that regardless of the clinical reason for delirium, a sudden imbalance of plasma amino acids might alter the level of neurotransmitters and contribute to delirium. We measured amino acids in hospitalized patients 60 or older, both genders, with delirium secondary to any infection (n=13) for a period of 3 days and compared them with healthy control, non-delirious group (n=13). Delirium was evaluated twice daily using the Confusion Assessment Method (CAM) test. Plasma amino acids were analyzed using LC-MS. Our results showed that level of isoleucine was significantly higher in delirious patients on day 1 (64.26 %) as compared to healthy control (43.25%, $p < 0.01$). The level of tryptophan was slightly lower on day 1 as compared to control (31.13 % vs 36.80%, p=NS). However, the tryptophan/isoleucine ratio on day 1 was significantly lower in delirious compared to healthy controls (49.96% vs 85.54 %, $p < 0.01$). The tryptophan/isoleucine rose on day 2 of delirium but was still significantly lower compared to the healthy control (59.0% vs 85.54%, p < 0.01). Our results suggest that significant alterations in the levels and ratios of amino acids, in particular, tryptophan and isoleucine might disrupt the synthesis of neurotransmitters and hence contribute to delirium.

Abstract citation ID: igad104.1489

### METABOLIC SYNDROME AND POOR SELF-RATED HEALTH AS RISK FACTORS FOR PREMATURE EMPLOYMENT EXIT

Katharina Runge[1], Sander van Zon[2], Kène Henkens[3], and Ute Bültmann[2], *1. Netherlands Interdisciplinary Demographic Institute (NIDI), University Medical Center Groningen (UMCG) - University of Groningen, The Hague, Zuid-Holland, Netherlands*, *2. University Medical Center Groningen, Groningen, Groningen, Netherlands*, *3. Netherlands Interdisciplinary Demographic Institute (NIDI), The Hague, Zuid-Holland, Netherlands*

Poor self-rated health (SRH) is a well-established risk factor for premature employment exit through unemployment, work disability, and early retirement. However, it is not clear whether the premature employment exit risk associated with underlying cardio-metabolic health conditions is fully captured by poor SRH. This study examines the metabolic syndrome (MetS), an early-stage risk factor for cardiovascular disease and type two diabetes mellitus, as a risk factor for premature employment exit while controlling for poor SRH. We analyzed data from N=54,108 middle-aged and older Dutch workers (40-64 years) from five waves of the Lifelines Cohort Study and Biobank. MetS components were based on physical measures, blood markers, and medication use. SRH and employment states were self-reported. The associations between MetS, SRH, and premature employment exit types were analyzed using competing risk regression analysis. During follow-up (4.3 years), MetS was identified as an independent risk factor for unemployment (adjusted subdistribution hazard ratio (SHR): 1.13, 95% CI: 1.03, 1.25) and work disability (adjusted SHR: 1.35, 95% CI: 1.13, 1.61) when adjusted for poor SRH, chronic diseases, and sociodemographic factors. MetS was not associated with early retirement. To conclude, poor SRH did not fully capture the risk for unemployment and work disability associated with MetS. Workers with MetS might thus not be aware of their cardio- metabolic health related risks for premature employment exit. More awareness about MetS as a risk factor for premature employment exit is needed among workers, employers, and occupational health professionals. Further, MetS prevention might help to prolong working lives.

Abstract citation ID: igad104.1490

### RACE AND SEX DIFFERENCES IN RELATIONS OF DIABETES BIOMARKERS WITH PHYSICAL FUNCTIONING BEFORE DIABETES ONSET

Tasneem Khambaty[1], Ashley Splain[1], Leslie I. Katzel[2], Michele K. Evans[3], Alan B. Zonderman[3], and Shari R. Waldestein[1], *1. University of Maryland, Baltimore County, Baltimore, Maryland, United States*, *2. Baltimore VA Medical Center, Baltimore, Maryland, United States*, *3. National Institute on Aging, National Institutes of Health, Baltimore*

It remains unclear if declines in physical functioning linked to Type 2 diabetes (T2DM) occur even before T2DM onset. We examined the longitudinal associations of T2DM biomarkers with upper and lower extremity strength among adults without T2DM at baseline. Participants were 1,572 African American (AA) and White adults (M(SD) age at baseline = 47.3 (9.7) years, 55% female, 53% AA) from the Healthy Aging in Neighborhoods of Diversity across the Life Span (HANDLS) study, assessed on up to 3 time points between 2004-2017. Participants provided blood samples for measurement of fasting glucose and glycosylated hemoglobin (HbA1c), and completed dominant and nondominant handgrip strength assessment, and a chair stands task to assess upper and lower extremity strength, respectively. Linear mixed effects regression models estimating relations of HbA1c on age-related change and adjusting for race, sex, literacy, and poverty status revealed significant four-way interactions of HbA1c, race, sex, and age (B = -.2.59, t(2853) = -2.0 p = 0.04), and to a lesser extent of fasting glucose, race, sex, and age (B = -.07, t(2863) = -1.84 p = 0.06) on change in the chair stands task, but not on handgrip strength (ps > 0.29). Among younger AA and White men, and younger White women, increasing HbA1c was related to worse lower extremity strength. However, among AA women, and older White women, increasing HBA1c was related to better lower extremity strength. Our findings suggest differential relations of worse glucose regulation on physical functioning amongst African American and White men and women.

Abstract citation ID: igad104.1491

### WOMEN SUPPORTING WOMEN: PEER SUPPORT GROUP FOR OLDER WOMEN WITH METASTATIC BREAST CANCER IN NIGERIA

Runcie Chidebe[1], Agha A. Agha[2], Onyedikachi Nwakanma-Akanno[3], Chika Okem akwiwu[4], Candidus Nwakasi[5], Simeon Aruah[6], Darlingtina Esiaka[7], and Phyllis Cummins[1], *1. Miami University, Oxford, Ohio, United States*, *2. University of Nigeria Nsukka, Nsukka, Enugu, Nigeria*, *3. SMILE With Me Foundation, Lagos, Lagos, Nigeria*, *4. The Psych Lounge, Hoschton, Georgia, United States*, *5. University of Connecticut, Storrs, Connecticut, United States*, *6. National Hospital Abuja, Abuja, Federal Capital Territory, Nigeria*, *7. University of Kentucky, Lexington, Kentucky, United States*

Metastatic breast cancer (mBC) is incurable and devastating to all cancer patients. In Nigeria, the proportion of patients with stage III or IV/metastatic breast cancer at diagnosis is 98%. Although many younger mBC patients live for years, older mBC patients have poorer prognosis, more physical symptoms, poorer access to palliative care, and experience higher social isolation. Also, most older Nigerian women diagnosed with mBC experience psychological challenges during and after their treatment, with higher possibility of abscondment from treatment. While peer support groups (PSG) have become increasingly beneficial for improving the quality of life (QoL) of these older women, there is paucity of literature exploring the benefit of these groups and how they provide support to cancer patients in Nigeria. Using a qualitative research approach and interpretative phenomenological analysis, we explored the experiences of older Nigerian women (n=8) living with mBC who belong to a PSG. Our finding shows that for older women living with mBC, seeing peers with similar lived experiences inspire them to keep living, being hopeful, and experience a reduction in death anxiety. PSG provides a culture of love, peer support, socialization and strengthened connections. In addition to pain and inability to walk (mBC linked disabilities) being recurring physiological challenges for many older women with mBC, forced retirement/inability to work, death anxiety, financial burden, and limited access to cancer care are some of the socio-psychological challenges they face. Therefore, the establishment of PSG in cancer treatment centers in Nigeria would improve QoL and reduce abscondment from treatment.

### SESSION 4135 (AWARD LECTURE)

Abstract citation ID: igad104.1492

### DONALD P. KENT AND ROBERT W. KLEEMEIER AWARD LECTURES

Chair: James Nelson

The Donald P. Kent Award lecture will feature an address by the 2022 Kent Award recipient Nancy R. Hooyman, PhD, FGSA, of the University of Washington. The Kent Award is given annually to a member of The Gerontological Society of America who best exemplifies the highest standards of professional leadership in gerontology through teaching, service, and interpretation of gerontology to the larger society. The Robert W. Kleemeier Award lecture will feature an address by the 2022 Kleemeier Award recipient Sheryl Zimmerman, PhD, FGSA, of the University of North Carolina at Chapel Hill. The Kleemeier Award is given annually to a member of The Gerontological Society of America in recognition for outstanding research in the field of gerontology.

Abstract citation ID: igad104.1493

### CARE JUSTICE FOR UNPAID AND UNDERPAID CARE WORKERS

Nancy Hooyman, *University of Washington, Seattle, Washington, United States*

Scholarship on older women, family caregiving, feminist gerontology, community-based services and policy change intersected throughout my career. As a social worker, care of older adults has always been a matter of social justice. My focus now is on care justice for those who perform the critical work of long-term services and supports: unpaid family caregivers and underpaid home care workers. This lecture will examine the interrelationships among both types of care laborers, who often face structural constraints on their decision to care and whose work is devalued, marginalized and invisible. Inequities for care workers by race, immigrant status, and sexual orientation are rooted in systemic racism, sexism, xenophobia, and homophobia. This lecture presents the components of a care justice framework, informed by feminism, intersectionality, and care theory, of fundamental structural changes to value collectively the essential work of care, ensure meaningful choice to care, and reduce inequities in care labor.

Abstract citation ID: igad104.1494

### UNIVERSITY KENAN DISTINGUISHED PROFESSOR AND CO-DIRECTOR, PROGRAM ON AGING, DISABILITY, AND LONG-TERM CARE

Sheryl Zimmerman, *University of North Carolina at Chapel Hill, Chapel Hill, North Carolina, United States*

The concept of person-centeredness -- simply stated, knowing the person in a holistic perspective and meeting the individual's needs and preferences -- has become so ubiquitous in health and supportive care that it borders on being a cliché. More so, it is often not reflected in national surveys and databases; considered in relation to the capacity of varying systems of care; forward-thinking in terms of emerging realities; or dissected in reference to some of the nuanced and implicit issues responsible for bringing it to the forefront of national attention. This Kleemeier Award lecture will reconstruct the concept of person-centeredness based on literature review, questionnaires, interviews, forums, and think tank meetings with diverse experts. It is supported by National Institute of Aging through a grant to the Alzheimer's Association's LINC-AD.

### SESSION 4140 (SYMPOSIUM)

Abstract citation ID: igad104.1495

### OLDER ADULTS' USE OF HOME AND COMMUNITY-BASED SERVICES: INDIVIDUAL ATTRIBUTES AND LOCATION DISPARITIES

Chair: Karen Roberto Co-Chair: Tina Savla

Home and Community-Based Services (HCBS) are designed to support older adults with functional and cognitive limitations. Yet, use of services is low, gaps exist in service provision, and available services do not always meet the person's need. This symposium focuses on idiosyncrasies surrounding HCBS use. Nah and colleagues identify individual (income, ADL limitations, informal support) and county-level (poverty and public spending on services) factors associated with HCBS use among persons living with dementia in rural Appalachia. Shippee and colleagues' examination of services received/desired indicates that older adults who are Black/other race/ethnicity were more likely to receive ADS; individuals living with AD/ADRD consumed personal care services at the highest proportion. Cheng's and Li's assessment of generosity in supporting HCBS reveals that while overall state support for HCBS has increased, Medicaid and OAA programs expenditures for services varies substantially across states. Fortinsky and colleagues' examination of risk factors for emergency department use among older adult Medicaid HCBS users shows use was associated with high school education, more severe depressive symptoms, and more chronic conditions; non-Hispanic Asian individuals had the lowest likelihood of use. Konetzka and Wang provide national evidence that use of Medicaid HCBS influences Medicare post-acute care utilization among the dually enrolled. Collectively, the symposium highlights the importance of taking into consideration individual variations in care needs, local and state support for HCBS, and disparities in healthcare and service use influencing older adults' use of HCBS, and advances understanding of intersecting micro- and macro-level effects on service availability, use, and outcomes.

Abstract citation ID: igad104.1496

### INDIVIDUAL AND COUNTY-LEVEL FACTORS OF SERVICE USE BY DEMENTIA FAMILY CAREGIVERS IN RURAL APPALACHIA

Suyoung Nah[1], Tina Savla[2], and Karen Roberto[3], *1. Virginia Polytechnic Institute and State University, Blacksburg, Virginia, United States*, *2. Virginia Tech, Blacksburg,*

*Virginia, United States*, 3. *Virginia Polytechnic Institute & State University, Blacksburg, Virginia, United States*

Formal services have been shown to provide health benefits for care recipients and family caregivers. Despite prior efforts, it is still unclear which factors play a role in the decisions to use formal services by family caregivers living in under-resourced rural regions. Drawing upon the Andersen model of health service use, this study examined individual and county-level factors' associations with the utilization of support services (e.g., homemaker, respite) and personal services (e.g., home health nurse, personal care aide) by dementia family caregivers residing in rural Appalachia (N = 163). Results of multilevel models revealed that caregivers with higher income and care recipients with more limitations in activities of daily living tended to use more support and personal services. Greater informal support from family or friends was associated with less utilization of support services, but not personal services. Caregivers residing in counties with higher poverty and lower public spending on support services for older adults were more likely to use support services. Conversely, caregivers living in counties with more home health agencies were more likely to utilize personal services. These findings suggest that caregivers living in rural regions may depend more on informal support for meeting basic care needs of their relative living with dementia. However, if the care recipient requires more specialized care, living in resource-rich counties increases their chances of receiving appropriate care. Discussion will focus on strategies to allocate funds and resources to expand care coverage to vulnerable individuals such as people living with dementia in rural regions.

Abstract citation ID: igad104.1497

### UNMET NEED AMONG A NATIONAL SAMPLE OF OLDER ADULT HCBS USERS

Tetyana Shippee[1], Taylor Bucy[2], John Mulcahy[1], and Eric Jutkowitz[3], 1. *University of Minnesota, Minneapolis, Minnesota, United States*, 2. *University of Minnesota School of Public Health, Minneapolis, Minnesota, United States*, 3. *Brown University, Providence, Rhode Island, United States*

Long-term services and supports delivered in the home or community (home and community-based services) continue to be favored by consumers and policymakers alike. Given that HCBS is largely state-directed, there is considerable heterogeneity in program design and eligibility, and no standardized tool by which quality is measured. In the absence of the widespread use of standardized and uniform measures of HCBS quality, unmet need is poorly understood. We examined the relationship between services received and desired as a means of predicting unmet need among a national sample of older adult (65+) consumers of publicly funded HCBS (e.g., Medicaid, PACE, OAA) using data from the National Core Indicators - Aging and Disability survey. Our sample included 13,459 older community-dwelling adults, of which 60.0% were White, 23.2% were Black, and 17.0% of another race/ethnicity. Roughly 15% of older adults reported having AD/ADRD (54% White, 20% Black). Older adults with AD/ADRD were more likely to use personal care services (59.6%), followed by delivered meals (21.3%), and homemaker services (18.6%) compared to those without AD/ADRD. Adults with AD/ADRD desired more caregiver support services and transportation services vs what they were already receiving. Of 10% already receiving caregiver support services, approx. 9% desired additional support. Of 9.6% receiving transportation services, 7.3% desired additional support. We found variation in HCBS received and desired among older adults by both AD/ADRD status and race/ethnicity, suggesting gaps in unmet needs for these groups.

Abstract citation ID: igad104.1498

### STATE GENEROSITY IN HOME- AND COMMUNITY-BASED SERVICES (HCBS): VARIATIONS AND IMPACT ON DEMENTIA OUTCOMES

Zijing (Flora) Cheng, and Yue Li, *University of Rochester Medical Center, Rochester, New York, United States*

To meet the needs of community-living older adults and reduce overall medical spending, home- and community-based services (HCBS) provide assistance and care for persons with significant physical and cognitive limitations. Together, Medicaid long-term services and supports (LTSS) coverage and the Title III program under the Older Americans Act (OAA) of 1965 support the majority of the HCBS for older adults living in the U.S. with low-income and/or disabilities. We evaluated state variations in generosity of supporting HCBS in recent years and how these variations affect the healthcare outcomes of community-living older adults and socio-economic and racial disparities in dementia outcomes. State HCBS generosity from the perspectives of program participation and intensity of services was defined by the exploratory and the confirmatory factor analysis with data from multiple sources, including state reports from the National Association of State Budget Officers. State data was linked to a nationally representative sample of older adults who enrolled in the Health and Retirement Study in 2020. Analyses revealed that overall state supports for HCBS increased over time in recent years while state Medicaid expenditures for institutional long-term care remained largely stable. State supports through both Medicaid and the OAA programs for HCBS varied substantially and persisted in recent years. Discussion will focus on instrumental variable analyses that determine the causal impact of state variations in HCBS generosity on health outcomes (i.e., unmet need; hospitalization) for persons living with dementia persons.

Abstract citation ID: igad104.1499

### EMERGENCY DEPARTMENT USE AMONG OLDER ADULTS RECEIVING MEDICAID HOME- AND COMMUNITY-BASED SERVICES

Richard Fortinsky[1], Julie Robison[2], Dorothy Wakefield[1], James Grady[1], David Steffens[3], Deborah Migneault[4], and Chae Man Lee[5], 1. *University of Connecticut, Farmington, Connecticut, United States*, 2. *UConn Health, Center on Aging, Farmington, Connecticut, United States*, 3. *University of Connecticut School of Medicine, Farmington, Connecticut, United States*, 4. *UCONN Health, Farmington, Connecticut, United States*, 5. *Univ of CT Health, Farmington, Connecticut, United States*

Medicaid-funded home and community-based services (HCBS) for older adults aim to prolong independent living despite physical and cognitive disabilities. Medicaid-eligible HCBS populations are racially and ethnically heterogeneous, with multiple chronic conditions. Clinical threats to continued independent living among older adult HCBS recipients include acute illnesses, injuries, and exacerbations of chronic conditions, which could trigger a pathway of emergency department (ED) visits, hospitalization, and long-term nursing home admission. Despite its importance in the pathway from independent living, little is known about the rate of, or risk factors for, ED use in this target population. Accordingly, we determined predictors of ED use in a statewide cohort (N=5,651) in Connecticut's Medicaid HCBS program for adults age >65, who were clinically assessed using a uniform tool between April 2020-March 2021. ED use, (0 vs 1+ visits), was ascertained over 12 months following clinical assessment using Medicare and Medicaid claims data. Predictor variables were racial and ethnic group, primary language, age, sex, educational attainment level, dementia diagnosis or moderate-severe cognitive impairment, depressive symptom severity, and Charlson comorbidity index. Cohort members were 76% female, 30% Hispanic, 15% non-Hispanic Black, 4% non-Hispanic Asian, 33% age >85. Forty-one percent had 1+ ED visits. Multivariate logistic regression analyses revealed that ED use was associated with (adjusted odds ratios $p<0.05$): high school education, more severe depressive symptoms, and more chronic conditions. Non-Hispanic Asian individuals had lowest ED visit likelihood. ED use among older adults receiving Medicaid HCBS might be reduced if depressive symptoms and multiple chronic conditions are optimally managed.

Abstract citation ID: igad104.1500

### THE ROLE OF MEDICAID HOME- AND COMMUNITY-BASED SERVICES IN USE OF MEDICARE POST–ACUTE CARE

R.Tamara Konetzka, and Sijiu Wang, *University of Chicago, Chicago, Illinois, United States*

Medicaid-funded long-term services and supports are increasingly provided through home and community-based services (HCBS) to promote continued community living. While an emerging body of evidence examines the direct benefits and costs of HCBS, there may also be unexplored synergies with Medicare-funded post-acute care (PAC). This study aims to provide empirical evidence on how the use of Medicaid HCBS influences Medicare PAC utilization among the dually enrolled. Using national Medicare claims, Medicaid claims (TAF), MDS, and OASIS data from 2014 to 2017, we estimated the relationship between prior Medicaid HCBS use and PAC utilization in a national sample of duals with qualifying index hospitalizations. We used inverse probability weights to create balanced samples on observed characteristics and estimated multivariable regression with hospital fixed effects and extensive controls. We also conducted stratified analyses for key subgroups and tested for interactions between HCBS use and three measures of states' HCBS generosity. We found HCBS use was associated with a 5.2 percentage-point increase in use of any Medicare-funded PAC, a 7.2 percentage-point increase in home health use, a 2.1 percentage-point decrease in institutional PAC, and a 1.95-day reduction of skilled nursing facility length of stay. In other words, use of Medicaid-funded HCBS was associated with a shift in Medicare-funded PAC use toward home-based settings. These findings were stronger in states with more generous HCBS funding. As we found that use of Medicaid-funded HCBS may alter choices about Medicare-funded PAC use, this synergy with HCBS should be considered by policymakers when contemplating further HCBS expansions.

### SESSION 4145 (SYMPOSIUM)

Abstract citation ID: igad104.1501

### ESPO AND HEALTH SCIENCES SECTION SYMPOSIUM: BUILDING BRIDGES: LEVERAGING SOCIAL CONNECTIONS AND ENVIRONMENTS TO IMPROVE HEALTH IN LATER LIFE

Chair: Kyle Moored Co-Chair: Katherine Britt Discussant: Thomas Cudjoe

The COVID-19 pandemic has renewed focus on social connections as fundamental contributors to health across the lifespan. Social connections have been studied in numerous ways (e.g., social network size, daily social interactions, engagement in social activities) and may facilitate health via multiple, dynamic biopsychosocial mechanisms. In line with this year's theme, we review novel insights from early-career scholars into how "building bridges" with others throughout life may promote mental and cognitive health in later life. Further, we emphasize how bridging disciplines (e.g., social and neurocognitive sciences) is vital to understanding how social environments may "get under the skin" to impact health. Presentations will span multiple social-ecological levels (community, institutional, interpersonal). Beginning at the community/institutional level, Dr. Moored will discuss how the quantity of neighborhood social destinations contributes to GPS measures of community mobility in older adults. Dr. Britt will then describe how religious participation, a specific social institutional activity, is associated with an inflammatory biomarker predictive of cognitive impairments. The remaining talks will then report on daily social experiences. Dr. Ng will highlight links between daily support exchanges and life satisfaction in older adults and how these relationships differ by marital status. Extending to the caregiving context, Dr. Puga will present on how role captivity and daily changes in social isolation contribute to mood symptoms in family caregivers of individuals living with dementia. Our Discussant, Dr. Cudjoe, will provide a critical review in the context of new directions for research on social connections and environments.

Abstract citation ID: igad104.1502

### ASSOCIATIONS BETWEEN NEIGHBORHOOD SOCIAL DESTINATIONS AND GPS-DERIVED COMMUNITY MOBILITY OF OLDER ADULTS

Kyle Moored[1], Breanna Crane[1], Pamela Dunlap[2], Michelle Carlson[1], and Andrea Rosso[2], *1. Johns Hopkins Bloomberg School of Public Health, Baltimore, Maryland, United States*, *2. University of Pittsburgh, Pittsburgh, Pennsylvania, United States*

Neighborhood social destinations may encourage greater out-of-home travel (i.e., community mobility) and support independent functioning in later life. We examined associations between quantity of destinations that promote social interactions (e.g., arts/recreation, restaurants, stores, civic/

religious institutions) and objective, GPS-derived community mobility. Participants were 146 adults ≥65 years old (Mean=77.0±6.5 years, 68% women) at baseline of a randomized trial to improve walking coordination. Participants carried a GPS device that passively collected real-time location data for 5-7 days. Spatial extent of community mobility was quantified using median standard deviational ellipse area (SDEa, km2) and maximum distance from home (mDOH, km). Duration of community mobility was characterized using percentage of time spent out-of-home (pTOH). Community mobility measures were stratified into tertiles due to positive skew. We used National Establishment Time Series (NETS) classifications to derive a total social destination count at the Census tract-level (Mean=48.9±35.7 social destinations per tract). Ordinal logistic regressions were adjusted for demographics (age, gender, race, education), device, season, and number of comorbidities and were clustered on Census tract. Each 1-SD greater destination count was associated with 46% greater odds of being in a higher tertile of pTOH (OR=1.46, 95% CI: 1.08-1.97, p=.014). Number of social destinations was not associated with SDEa or mDOH (all p's>.05). Nearby social destinations may encourage greater duration, but not necessarily spatial extent, of community travel. Future work utilizing GPS can objectively measure activity in community social spaces to assess its benefits for cognitive and mental health in later life.

Abstract citation ID: igad104.1503

### ASSOCIATION OF RELIGIOUS ATTENDANCE, RELIGIOSITY/SPIRITUALITY, AND C-REACTIVE PROTEIN IN MIDDLE TO OLDER ADULTS IN THE U.S.

Katherine Britt[1], Augustine Boateng[1], Joshua Sebu[2], Hayoung Oh[3], Ruby Lekwauwa[4], Lauren Massimo[1], and Benjamin Doolittle[4], *1. The University of Pennsylvania, Philadelphia, Pennsylvania, United States*, 2. *University of Cape Coast, Cape Coast, Western, Ghana*, 3. *Georgetown University, Washington, District of Columbia, United States*, 4. *Yale University School of Medicine, New Haven, Connecticut, United States*

Elevated levels of C-reactive Protein (CRP), a biomarker of systemic inflammation, are associated with a higher risk of cognitive decline and dementia. Religious attendance and religiosity/spirituality are associated with better cognitive health, yet the underlying physiological mechanisms are not well understood. We examined the association between religious service attendance, religiosity/spirituality, and CRP levels in a nationally representative sample of U.S. adults aged 50 years and older (n=6,652) from the Health and Retirement Study from 2006 to 2014. The mean age was 70.7(9.32) years, 84.4% were White, 58.4% were female, and mean CRP levels were 3.98 (8.50) ug/mL. Utilizing the generalized estimating equation (GEE), a significant negative association was found between religious service attendance and religiosity/spirituality with average CRP levels, even after controlling for age, sex, education, marital status, race/ethnicity, income, body mass index (BMI), and health conditions (diabetes, cancer, hypertension) [B: -0.39, 95% CI:-0.76,-0.03, p<0.034 and B: -0.04, 95% CI:-0.07,-0.01, p<0.01]. Age, income, and education were negatively associated with CRP levels, while being female, Black/African American, having diabetes, cancer, hypertension, and BMI were positively associated with CRP levels. Findings contribute to our understanding of a possible mechanism by which religious service attendance and religiosity/spirituality influence cognitive health through reduced systemic inflammation. Interventions are needed to sustain religious attendance supporting religiosity/spirituality for adults who find religion/spirituality important, with the potential to reduce the risk of cognitive decline and dementia.

Abstract citation ID: igad104.1504

### DAILY SUPPORT EXCHANGES WITH FRIENDS AND PSYCHOLOGICAL WELL-BEING IN LATE LIFE

Yee To Ng[1], William Chopik[2], and Karen Fingerman[3], *1. Institution of Social Reseach, Ann Arbor, Michigan, United States*, 2. *Michigan State University, East Lansing, Michigan, United States*, 3. *The University of Texas at Austin, Austin, Texas, United States*

Support exchanges are crucial to older adults' well-being. Functionalist and identity theories suggest that individuals engage in support exchanges with social ties that are consistent with their identities/roles to maximize their well-being. This study examined support exchanges with friends in daily life and tested the link between support exchanges with friends and daily well-being, particularly how this link varied between married and unmarried older adults. Adults aged 65+ (N = 292, Mean age = 73.72, 60% married) provided information about their demographics and social partners. Across five days, they completed surveys about support exchanges with friends (i.e., receiving or giving advice, practical support, and emotional support, respectively) and daily life satisfaction at the end of each day. Multilevel logistic models revealed that unmarried older adults were more likely to receive all types of support from friends and also more likely to provide advice and emotional support (non-tangible support) to friends than married older adults. Support provision was unrelated to life satisfaction. On days when older adults received advice from friends, they reported increased life satisfaction than on days that they did not (within-person association). This within-person association was stronger among unmarried than married older adults. Patterns of findings remained unchanged after adjusting for support exchanges with other social ties. Findings highlight the role of marital status in older adults' daily support exchanges. Unmarried older adults may compensate for the lack of a spouse with friends and are more reactive to daily support exchanges than married older adults.

Abstract citation ID: igad104.1505

### ROLE CAPTIVITY, DAILY SOCIAL ISOLATION, AND THE MENTAL HEALTH OF DEMENTIA CAREGIVERS

Frank Puga[1], Danny Wang[2], Natashia Bibriescas[3], Abigail Poe[1], Loreli Alvarez[4], Rita Jablonski[1], and David Vance[1], *1. University of Alabama at Birmingham, Birmingham, Alabama, United States*, 2. *The Pennsylvania State University, University Park, Pennsylvania, United States*, 3. *University of Texas at Austin, Austin, Texas, United States*, 4. *The University of Alabama at Birmingham, Birmingham, Alabama, United States*

Family members providing informal care to individuals living with Alzheimer's disease and related dementias (ADRDs) have an increased risk of poor mental and cognitive health. Social connection has been proposed as an intervention target to support the health and well-being of ADRD caregivers; however, little is known about daily variation in social connection, contextual factors that impact social connection, and its association with caregiver mental health experiences. Thus, the purpose of this study was to examine the relationship between role captivity and daily social isolation (i.e., lack of social connection) among dementia caregivers, as well as associations between social isolation and depression and anxiety-related symptoms. A sample of community-dwelling ADRD caregivers (N=30) completed a baseline survey followed by 14 days of daily diaries (n=323 data points). Participants were asked about role captivity, social isolation, and daily depression and anxiety-related symptoms. Data were analyzed using mixed-level modeling. Role captivity reported at baseline was significantly associated with daily social isolation (B=0.82, 95% CI [0.68, 0.96], p <0.001). Further, daily social isolation was significantly associated with both depression (B=0.69, 95% CI [0.61, 0.77], p <0.001) and anxiety (B=0.55, 95% CI [0.48, 0.62], p <0.001) related symptoms reported on a given day. The findings from this study contribute knowledge on ecologically valid interventions that target daily experiences of social isolation and support the mental health of dementia caregivers. Such interventions are critically important given the proposed links between mental health, social isolation, and cognitive health in later life.

### SESSION 4150 (SYMPOSIUM)

Abstract citation ID: igad104.1506

### RESEARCH TO STRENGTHEN, INNOVATE, AND TRANSFORM AGE-FRIENDLY COMMUNITY PRACTICE

Chair: Kathy Black

There is much research being conducted to better understand and advance age-friendly community practice. This symposium presents research from leading age-friendly researchers and practitioners across the United States. Drs. Black and Oh provide an analysis of the nation's sectoral efforts based on progress reported by the age-friendly communities. Drs. Hernandez and Coyle will describe the research and community engagement that led to the development of an aging equity conceptual framework and examples of how it is being operationalized in the City of Boston. Drs. Greenfield and doctoral student Pope will present on a scoping review of studies in the U.S. and Canada on the range of ways in which the public sector participates in age-friendly community efforts. Drs. Coyle and Oh and doctoral students Gleason and Somerville present on a study that explored factors inhibiting communities from officially joining the age-friendly network. Dr. Perry reports on efforts to elevate the voice of older adults on social justice issues pertaining to aging in place in the domain of housing. Individual abstracts provide further detail on each study's methods and findings.

Abstract citation ID: igad104.1507

### EXPLORING SECTORAL REACH IN AGE-FRIENDLY COMMUNITIES

Kathy Black[1], and Patricia Oh[2], *1. University of South Florida,Sarasota-Manatee, Sarasota, Florida, United States*, *2. UMaine Center on Aging, Bangor, Maine, United States*

There is increasing interest in better understanding the sectoral reach of age-friendly community practice. Action across a wide variety of actors is central to achieving age-friendly societal change according to the World Health Organization and an explicit goal for governmental participation in its Global Network of Age-Friendly Cities and Communities. However, there is limited knowledge regarding the scope of sectoral reach by age-friendly communities. We used qualitative inquiry to assess sectoral efforts reported by American age-friendly communities that completed a five-year cycle of participation (n=40). We employed directed content analysis using a priori indicators by sectoral actors: public (i.e., government), private (i.e., business), and civil society including nonprofit organizations and volunteers. We classify sectoral actions by type (i.e., intersectoral, multisectoral) and by clustered domain community foci (i.e., built, social, service). Our study identifies the extent and types of sectoral actors and actions reported by age-friendly communities with the greatest efforts reported in the public sector, and while similarly distributed across all the domains, slightly more efforts were noted in the built environment. We also found greater intersectoral efforts (i.e., explicitly working towards shared goals) than multisectoral (i.e., not necessarily in collaboration on shared goals), particularly across government. While our study substantiates the breadth of actions towards age-friendly change, additional research is needed to examine the ways in which the public and other sectoral actions are further linked to outcomes in communities in the United States and in other countries.

Abstract citation ID: igad104.1508

### PUBLIC SECTOR INVOLVEMENT IN AGE-FRIENDLY CITIES AND COMMUNITIES: A SCOPING REVIEW

Emily Greenfield[1], Natalie Pope[2], Laura Keyes[3], and Elizabeth Russell[4], *1. Rutgers, The State University of New Jersey, New Brunswick, New Jersey, United States*, *2. Rutgers Social Work, New Brunswick, New Jersey, United States*, *3. University of North Texas, Denton, Texas, United States*, *4. Trent University, Peterborough, Ontario, Canada*

The global age-friendly cities and communities (AFCC) movement has long centered the involvement of the public sector, calling on high-ranking authorities to commit to improving the built, social, and service environments of their localities. However, there is little understanding of how the public sector is involved in actual practice. To address this gap, we conducted a scoping review of peer-reviewed articles published since 2010, with a focus on studies in Canada and the United States, to synthesize the evidence on the ways in which the public sector has been involved with AFCC work. Our review identified four primary themes. The first theme describes the variety across studies in descriptions of the overall positioning of the public sector in AFCC efforts, with public sector actors ranging from leaders to partners to targets for advocacy. The second theme addresses activities that are initiated by a public administration that targets age-friendly improvements for that administration. The third theme encompasses activities that help to connect a public administration with, as well as influence, outside actors, primarily the private sector and government administrations at other systems levels. The final theme elucidates the various ways in which the public sector is involved with AFCC

assessment, evaluation, and research. We discuss the implications of our findings for practice, policy, and continued empirical research. We also discuss theoretical implications for AFCC efforts in terms of distinguishing "whole of government" from "whole of society," grassroots, and planning-specific approaches.

Abstract citation ID: igad104.1509

### ALTERNATIVE PATHS TO AN AGE-FRIENDLY COMMUNITY

Shayna Gleason[1], Patricia Oh[2], Caitlin Coyle[3], and Ceara Somerville[3], *1. University of Massachusetts, Boston Gerontology, Boston, Massachusetts, United States*, *2. UMaine Center on Aging, Bangor, Maine, United States*, *3. University of Massachusetts Boston, Boston, Massachusetts, United States*

While the age-friendly community movement has seen tremendous progress in the past decade, with over 700 U.S. member communities in the Network of Age-Friendly States and Communities (NAFSC), many more U.S. communities have not yet joined the network. Joining NAFSC can spur activity to improve local conditions for older residents, as well as allow communities access to informational resources and contacts from peer communities that have undertaken similar initiatives. A better understanding of the barriers preventing communities from joining NAFSC, the hesitancy about joining, and the age-friendly work being done outside of NAFSC is critical to extending age-friendly work nationally. The present study explored factors that inhibit communities from joining NAFSC. We conducted 12 semi-structured virtual interviews with community leaders in Maine and Massachusetts whose communities are not NAFSC members but are actively working on aging-related issues. Participants were recruited through the researchers' professional networks. We intentionally cultivated diversity of community size during recruitment. Transcripts and interview notes were coded thematically. Resulting themes included a lack of knowledge/misconceptions about what joining NAFSC requires, a perception that there are few benefits to joining, political concerns, insufficient personnel capacity, other financial priorities (e.g., children's programming, COVID relief), and a desire to retain local control over aging-related initiatives. Recommendations include clearer communication of NAFSC membership requirements and benefits, additional technical assistance to communities, and loosening of reporting requirements for small communities.

Abstract citation ID: igad104.1510

### "OUR NATION'S DEMOCRACY IS AT A CRITICAL CROSSROAD": OLDER ADULTS TAKING ACTION AND LEADING COMMUNITY ENGAGEMENT

Tam Perry[1], Vanessa Rorai[2], Sarah Whitney[2], Wilma Stringer[2], Henry Swift[2], James Bridgforth[2], and Patricia Mullin[2], *1. Wayne State University, Detroit, Michigan, United States*, *2. Healthier Black Elders Center, Detroit, Michigan, United States*

Launched by older Black adults from the Healthier Black Elders Center (HBEC) of the Michigan Center for Urban African American Aging Research, Critical Crossroads was created to engage older Detroiters in community conversations, advocacy, and strategies around issues of social injustice. Critical Crossroads provides older adults a space to discuss what concerns them at both national and local community levels, share their personal impact stories and experiences, and learn how these issues connect to social determinants of health. One important feature of this program has been attention to concerns of housing and its impact on older adults. This presentation will highlight two Critical Crossroads housing related segments (e.g., CDC moratorium on eviction, applying for FEMA aid for flooding). This presentation will share the This presentation will share the development of this community-led initiative, program goals, and lessons learned.

Abstract citation ID: igad104.1511

### ADVANCING AGING EQUITY AMONG OLDER BOSTONIANS

Adriana Hernandez, and Caitlin Coyle, *University of Massachusetts Boston, Boston, Massachusetts, United States*

More than five years after the City of Boston started its journey towards becoming an Age and Dementia Friendly community, the initiative and its collaborators have been working to ensure that the initiative and its many contributions are equally realized by all Bostonians. Drawing on the concept of aging equity--that all older adults, despite their life course histories, have equitable access to programs and services that help them age well. To achieve this means removing obstacles to accessing community features that support healthy aging, establishing social and civic engagement opportunities, ensuring safe environments, establishing access to healthcare, and disseminating knowledge of available supports and services. This presentation will describe the research and community engagement that led to the development of an aging equity conceptual framework and examples of how it is being operationalized in the City of Boston. For example, we will share the ways in which outreach and communication strategies have been altered to more equitable practices including efforts to build trust, engage in cultural translation of programs and services, and consider new channels of information sharing.

### SESSION 4155 (SYMPOSIUM)

Abstract citation ID: igad104.1512

### EAST MEETS WEST: INTERVENTIONS, PRACTICE MODELS, AND POLICIES TO SUPPORT FAMILY CAREGIVERS IN DIVERSE CULTURES

Chair: Fei Sun Co-Chair: Juanjuan Sun Discussant: Jiehua Lu Discussant: Tracie Harrison

This East Meets West symposium consists of six studies that examined innovative interventions, practice models and policies designed to support family caregivers of older adults in the U.S., Taiwan, and mainland China. The first two papers both used case management interventions to assist family caregivers with a focus on caregiver depression. The paper from the U.S. found that more difficulties of performing caregiving tasks and less caregiver preparation contributed to higher levels of depression, and the paper from Taiwan found that caregivers who received the case management service reduced depression at four-month and six-month follow-up assessments. The third paper discussed a "time travel" paradigm developed to guide service design and delivery for persons with dementia at different stages. The fourth paper used the state of Michigan as a case to examine service gaps in the current aging service network, and identifies an urgent need for statewide policy renovations to support family caregivers. The fifth and sixth papers both used large secondary data analyses to examine the effects of piloted policy and practice efforts on family caregiver outcomes. One study found that the long-term care insurance programs have resulted in positive caregiver outcomes including reduced caregiving financial cost, time, and physical and psychological burden. The other study found the use of home-based care services reduced adult children caregiver burden and did not affect the relationship between older adults and adult children caregivers. After individual presentations are done, two discussants will summarize shared themes in these papers and provide critical feedback.

Abstract citation ID: igad104.1513

### CAREGIVER WELL-BEING OUTCOMES IN A STROKE TRANSITIONS TRIAL: THE EFFECTS OF CAREGIVER BURDEN AND PREPARATION

Amanda Woodward, *Michigan State University, East Lansing, Michigan, United States*

The Michigan Stroke Transitions Trial (MISTT) was an open, pragmatic randomized trial which tested the effectiveness of a 60-day in-home social work case management for patients and caregivers transitioning home after hospitalization for a stroke. This secondary analysis explores the effect of caregiver burden and preparation on caregiver outcomes. The analytic sample is the 169 caregivers who participated in MISTT. Dependent variables are the Bakas Caregiver Outcomes Scale (BCOS) and the Patient Health Questionnaire-9 (PHQ9) at 90 days after discharge home. Key predictors include the Preparedness for Caregiving Scale and the Oberst Caregiving Burden Scale which measures caregivers' perception of burden in relation to the both the time and difficulty of caregiving tasks. Analyses that controlled for outcomes at 7 days post discharge as well as caregiver age, marital status, and education were conducted using Stata v.16. Results indicate that caregivers who perceived caregiving tasks as more difficult had worse outcomes as measured by the BCOS. In addition, there was a significant interaction between task difficulty and age such that the effect of task difficulty on BCOS declined with age. In addition, depression was associated with greater task difficulty and less preparation. This study contributes to a growing body of work related to the complexity of transitions of care and has implications for how we support caregivers as their care recipient moves from inpatient to outpatient care.

Abstract citation ID: igad104.1514

### THE USE OF THE "TIME TRAVEL" PARADIGM OF ALZHEIMER'S DISEASE FOR FAMILY CARE PARTNER EMPOWERMENT

Christopher Johnson[1], Roxann Johnson[2], and Fei Sun[3], *1. Texas State University, San Marcos, Texas, United States, 2. Aging Consultants Inc, Austin, Texas, United States, 3. Michigan State University, East Lansing, Michigan, United States*

This study describes how family carers are taught the Time Travel model of Alzheimer's Disease (AD). As persons with AD experience cognitive, emotional, social, physical and functional time travel, families can learn to join them on their journey. Stage models (e.g., Reisberg and associates FAST and GDS) of AD clarify how changes through time occur using stage markers. Yet the disease is non-linear. Persons with AD don't travel neatly in stages but fluctuate in recall of names, faces and events. They migrate in a non-linear downward spiral fluctuating back and forth through time, revisiting people, places, events and traumas of their distant past. Time Travel apps and other life history data are available to provide real person-centered care that's time appropriate. Families are trained to connect in the approximate time frame in which the person has traveled. Hence, care partners are taught time appropriate communication and interventions (e.g., activities, pictures, music etc.) to respond to challenging behaviors. The Models use with family carers empowers them to validate their loved ones' approximate time frame they are in. Family carers are inspired to become "best friends" of AD persons by understanding how this cognitive disability works. When a lady with travels to age 20 in her mind but sees an 80-year-old face in the mirror, she demands the person leave the bathroom. This behavior is puzzling to families. Time Travel model explains how this works driving care planning, redesigns (e.g., mirrors) and activities, and supports validation therapy, "Best Friends" and other pedagogy.

Abstract citation ID: igad104.1515

### A CRITICAL EXAMINATION OF AAA SERVICES AND SUPPORTS FOR CAREGIVERS IN MICHIGAN: POLICY AND PRACTICE IMPLICATIONS

Joan Ilardo, and Angela Zell, *Michigan State University, East Lansing, Michigan, United States*

To identify the service gaps for family caregivers in Michigan, this study examined the perceptions of stakeholders from the 16 area agencies on aging (AAA). An online questionnaire was administered to assess a point-in-time picture of current caregiver assessments and services in 2020. Fifteen AAAs responded to see definitely or probably an increase in the number of caregivers in their catchment areas. Fifteen AAAs currently purchase or contract for respite care services. Fourteen provide some type of direct caregiving resources. Twelve utilize staff to develop and monitor care plans. While some provided caregiver training, none directly provided supportive services. AAAs anticipated rapidly increasing needs for caregiver supports and services. While each provided a different array of services, they anticipated that demand would continue to increase given a rising aging population in Michigan. Results were used to craft a proposal to establish a network of regional caregiver resource centers. Recommendations in the study include creating a task force of stakeholders to identify standardized service definitions, measures and reporting requirements; create a no-wrong-door, multi-prong approach for caregivers to access programs; provide respite care through contracts and referrals to community-based organizations; create a network of trainers to disseminate evidence-based best practices to AAAs and contractors; use untapped resources such as AARP and Alzheimer's Association; explore forming local healthcare systems and provider collaborations to identify caregivers and triage them. Next steps are to pursue funding from the legislature and other sources to create regional caregiver resource centers across the state.

Abstract citation ID: igad104.1516

**THE BENEFITS OF LONG-TERM CARE INSURANCE FOR FAMILY CAREGIVERS: EVIDENCE FROM CHINA**

Dan Tang[1], and Qing Hu[2], *1. Renmin University of China, Beijing, Beijing, China (People's Republic)*, *2. Renmin University, Beijing, Beijing, China (People's Republic)*

This paper aims to evaluate the impacts of the long-term care insurance (LTCI) program on family caregivers of older adults with functional limitations in mainland China. The study used data collected from 1,658 LTCI beneficiaries or those in the LTCI application process in 16 pilot Chinese cities in 2021. Descriptive statistics, chi-square tests, and multiple regression analyses were used to analyze the data. The results indicate that LTCI beneficiaries are more likely to receive institutional care than non-beneficiaries (27.8% vs. 16.8%), reducing the responsibilities and potential burden of family caregivers. LTCI beneficiaries are also more likely to receive social care services than non-beneficiaries (35.9% vs. 26.7%). Family caregivers of LTCI beneficiaries spend less time per day on care than non-beneficiaries (15.45 vs.16.59 hours), and they are less likely to delay their work due to caregiving (41.4% vs. 60.7%). Additionally, LTCI has a positive impact on various aspects for family caregivers, with over 40% reducing their caregiving time, 55.0% reducing care service costs, 68.5% feeling that LTCI has reduced their physical burden, and 77.9% indicating that their psychological burden has been lessened. The study concludes that the establishment of long-term care insurance nationwide should be promoted as the pilot sites have seen significant positive effects on family caregivers. LTCI should also consider more direct support to family caregivers.

Abstract citation ID: igad104.1517

**THE IMPACT OF HOME-BASED CARE SERVICES ON ADULT CHILDREN AS CAREGIVERS OF OLDER ADULTS IN CHINA**

Juanjuan Sun[1], Yuxin Chen[2], and Jiayin Tian[2], *1. Institute of Gerontology, Beijing, Beijing, China (People's Republic)*, *2. Renmin University, Beijing, Beijing, China (People's Republic)*

The family has always been the primary resource for elder care support in China, and adult children are the main caregivers of older parents. In the background of rapid development of home-based care services for older adults, this study aims to explore the impacts of the home-based care services on the adult children as caregivers for their older parents. Based on a national representative sample of aged 60 years or older (N=8674) from the China Longitudinal Aging Social Survey (CLASS) in 2018 and 2020, this study used propensity score matching difference-in-differences method to analyze the data. Firstly, we found that home-based care services for older adults could reduce the care burden of family caregivers by sharing part of the instrumental support effectively for the elderly. Secondly, this substitution effect is more significant among the families with more dependable and vulnerable older adults. Finally, the use of home-based care services appeared not to affect the intergenerational relationships between older parents and their adult children caregivers. Home-based care services may relieve the burden of family caregivers by providing instrumental care support for older adults. Therefore, it is very necessary to accelerate the implementation of an elder care programs in community to benefit the older and younger generations in Chinese family.

Abstract citation ID: igad104.1518

**THE EFFECTIVENESS OF A CASE MANAGEMENT PROGRAM FOR FAMILY CAREGIVERS OF PERSONS WITH MCI OR EARLY STAGE DEMENTIA**

Yi-Chen Chiu[1], Li-Min Peng[2], Min-Chia Chen[3], Chiu-Mei Tai[4], Pei-Kwei Tsay[5], Hsui-Li Huang[6], and Wen-Chuin Hsu[7], *1. Chang Gung University, Guishan District, Taoyuan, Taiwan (Republic of China), 2. Hsin Sheng Junior College of Medical Care and Management, Taiwan (Republic of China), 3. Ching Kuo Institute of Management and Health, Keelung, Taiwan (Republic of China), 4. Catholic Mercy Hospital, Hsinchu, Taiwan (Republic of China), 5. Chang Gung University, Taoyuan, Taiwan (Republic of China), 6. National Taipei University of Nursing and Health Sciences, Taipei, Taipei, Taiwan (Republic of China), 7. Taoyuan Chang Gung Memorial Hospital, Taoyuan, Taiwan (Republic of China)*

This study examined the effectiveness of a case management program for family caregivers of persons with mild cognitive impairment, early to mild dementia. Using a quasi-experimental study design, we recruited family caregiver and care recipient dyads from memory clinics in Taiwan. We randomly assigned the dyads into experimental groups (n= 38 dyads) or control groups (n= 33 dyads). The experimental group participants received case manager services over a four-month period, while those in the control group received general health education. Data were collected at three time points: baseline, the 4th and 6th months for both groups. The results of the generalized estimating equation revealed that in the fourth month, the experimental group exhibited a declined depression ($\beta = -3.74$, $p < .001$) and increased use of social resources ($\beta = 1.014$, $p = .045$). In the sixth month, the depression of caregivers declined further ($\beta = -3.186$, $p = .002$), whereas their use of social resources showed no significant difference. This study illuminates the feasibility and efficacy of using a case management model to support caregivers encountered in memory clinics. Findings are promising to suggest the need to call for more education and supports available at clinics to prepare and assist caregivers who have a relative of at the early stage of cognitive impairment or dementia.

### SESSION 4160 (SYMPOSIUM)

Abstract citation ID: igad104.1519

**POLICY SERIES: INTERDISCIPLINARY PUBLIC POLICY DISCUSSION SESSION**

Chair: George Taffet

Discussant: Brian Lindberg

This interactive session is an interdisciplinary look at the impact of social isolation and loneliness policy issues as we age. This session, organized by the GSA Public Policy Committee, will provide both GSA section leadership and attendees an opportunity to have an open dialogue on important public policy issues of significance in the field of aging. The panelists represent the six sections of GSA: ESPO, BS, BSS, SRPP, HS, and AGHE.

### SESSION 4165 (SYMPOSIUM)

Abstract citation ID: igad104.1520

### STRENGTHENING CAREGIVING NETWORKS: PROMISING PRACTICES TO SUPPORT PAID AND FAMILY CARE TEAMS IN THE HOME

Chair: Emily Franzosa Discussant: Katherine Ornstein

Older adults and people with disabilities often rely on both family and paid caregivers to live independently in the community. But while paid and family caregivers work closely together in the home, these groups are often siloed in research and policy. Research describing the structure and work of caregiving teams is limited, as are efforts to better support them in providing person-centered care. In this symposium, we present innovative interdisciplinary research that highlights the vital relationship between paid and family caregivers and how this influences the care they provide. We begin with two studies focusing on the structure and function of older adults' formal and informal care networks. First, Franzosa et al present results from a care mapping project which found care networks were often far more expansive than what is captured in medical records. Reckrey et al describe the need to provide family-centered home care that reflects the nuanced and individualized care that these teams provide, based on a study of interviews with families and paid aides. Next, we describe results of two promising interventions to support family and paid care in the home. Weidenbacher et al describe a community health worker-led navigation program connecting family caregivers of seriously ill veterans to supportive resources, and Scales and King describe a training for home care workers and family members to facilitate collaborative problem solving and person-centered care. Taken together, these studies broaden our understanding of care teams in the home and highlight promising opportunities to support team-based home care. This is a collaborative symposium between the Family Caregiving and Paid Caregiving Interest Groups.

Abstract citation ID: igad104.1521

### VISUALIZING VETERANS' CARE: UNDERSTANDING THE BREADTH OF OLDER VETERANS' PAID AND FAMILY SUPPORT NETWORKS

Emily Franzosa[1], and Imran Ali[2], *1. Icahn School of Medicine at Mount Sinai, New York City, New York, United States*, *2. The New Jewish Home, New York City, New York, United States*

Many older veterans rely on a complex network of paid and unpaid caregivers to age safely at home. Medical records often only capture a primary family caregiver, giving medical teams a limited picture of veterans' supports at home as well as plans for contingency care. We tested a novel visual tool, care mapping, to better understand the scope of veterans' formal and informal support. We created collaborative care maps with 10 dyads/triads of veterans age 65+ eligible for VA-paid home care and their primary paid and family caregivers. We compared the maps to veterans' electronic medical records (EMRs) to explore care network structure, function and adequacy. Care maps identified substantially more care partners than EMRs, including functional, medical, socioemotional and spiritual supports such as family, paid aides, neighbors, Veterans' groups, faith communities, local businesses (pharmacies, food delivery), and social media. Veterans, family and paid caregivers described working together closely, with aides serving as a "third arm" to fill care gaps. Care was often mutual, as veterans described financial and emotional support they provided to family members, aides and their families, and friends. Participants also described these networks as dynamic and fluid, changing based on care needs and care partners' availability and skills. Identifying the range of support Veterans receive from family, paid aides, and their community may better help medical teams understand often overlooked community supports and relationships, identify care gaps and tailor home and community-based services where they are most effective and needed.

Abstract citation ID: igad104.1522

### PARTNERING TO IMPROVE CARE: STRENGTHENING THE FAMILY CAREGIVER AND HOME CARE WORKER RELATIONSHIP

Kezia Scales, Jessica King, and Emily Dieppa, *PHI, New York City, New York, United States*

Family caregivers and home care workers provide essential daily care for millions of older adults and individuals with disabilities across the United States—and together they constitute the backbone of home and community-based services (HCBS). In many cases, these unpaid and paid caregivers work closely together and share common knowledge, skills, experiences, and challenges. Yet the overlap and relationship between them remains relatively understudied. This session will describe the importance of strengthening the family caregiver/home care worker dyad and discuss promising developments in research, policy, and practice. We will then highlight a pilot training intervention designed to improve the shared knowledge and interpersonal competencies of family caregivers and home care workers. The intervention— Partnering to Improve Care: Direct Care Workers and Families—was developed by PHI (a national nonprofit research, public education, and consulting organization) in partnership with the Florida Pioneer Network, Home Instead, and Florida State University through the North and Central Florida Geriatrics Workforce Enhancement Partnership (GWEP). Informed by the PHI Coaching Approach®, the intervention consists of five parallel online training modules for family caregivers and for home care workers that utilize adult learner-centered teaching methods and reflect input from home care stakeholders. The modules portray common scenarios that family caregivers and home care workers face and aim to build communication and relationship skills that facilitate collaborative problem-solving and the provision of person-centered care. We will present key findings on the feasibility and acceptability of this intervention, discuss next steps in this research, and highlight broader policy and practice recommendations.

Abstract citation ID: igad104.1523

### FEASIBILITY OF A COMMUNITY-BASED NAVIGATOR SUPPORT PROGRAM FOR CARE PARTNERS OF SERIOUSLY ILL VETERANS

Nathan Boucher[1], Hollis Weidenbacher[2], Madeleine Eldridge[2], Kimberly Johnson[3], Courtney

Van Houtven[4], Karen Steinhauser[5], and Kelli Allen[2], *1. Veterans Health Administration/Duke University, Durham, North Carolina, United States*, *2. Durham VA Health care System, Durham, North Carolina, United States*, *3. Duke University, Durham, North Carolina, United States*, *4. Duke University School of Medicine, Durham, North Carolina, United States*, *5. Duke/Durham VA, Durham, North Carolina, United States*

We examined the feasibility and satisfaction of a short-term Veterans Health Administration (VHA)-based care partner support intervention delivered by trained navigators during COVID-19 through a single-arm interventional cohort feasibility study of a telephone-based care partner navigation intervention. Navigators (n=9) received the standard state curriculum online from a local community health worker training program plus an additional eight weeks of online role-specific training by the study team. Subsequently, navigators contacted care partners (n=30) of seriously ill veterans weekly via telephone over a 12-week period to assess social and practical needs, and connect care partners to appropriate community-based or VHA resources. All navigators (9 female; 1 male) and 17% of care partners were African-American (83% were Caucasian or other). Care partners were 90% female and 10% male; age range 41 to 80. In this telephone-based support program delivered to care partners by standard-trained navigators across 12 counties, care partners were overall very satisfied ($\bar{x}$=4.69 out of 5, sd =.77) with their navigator's responsiveness and social support throughout the 12-week course of the study. Navigators' confidence increased over time ($\bar{x}$=8.2 out of 10 (sd=1.14) to $\bar{x}$=8.78 (sd=.97)), while their self-assessed effectiveness ratings lowered over time as they encountered real-life scenarios ($\bar{x}$=8.5 (sd=1.43) to $\bar{x}$=7.89 (sd=.93)). Navigators documented successful connections to services in the VHA and in care partners' communities. Using remote navigators with minimal but standard training is a feasible and acceptable model of care partner support.

Abstract citation ID: igad104.1524

### THE NEED FOR FAMILY-CENTERED HOME CARE IN HOME-BASED DEMENTIA CARE

Jennifer Reckrey[1], Deborah Watman[1], and Emma Tsui[2], *1. Icahn School of Medicine at Mount Sinai, New York City, New York, United States*, *2. CUNY Graduate School of Public Health, New York, New York, United States*

Family caregivers, paid caregivers, and geriatricians frequently work together to care for people with dementia living at home, yet little is known about the paid caregiver role in home-based dementia care. We conducted repeated, one-on-one, semi-structured interviews with the care team (i.e., family caregiver, paid caregiver, geriatrician) of 9 people living at home with dementia over 6 months to explore multiple perspectives on paid caregivers' role in care over time. In all, 29 unique respondents participated in 75 interviews that were analyzed using the framework method of analysis. Results revealed nuanced and highly individual care arrangements where paid caregiver roles in care varied significantly, though changes to this role over time were minimal. Families were the key players in defining the paid caregivers' role regardless of home care agency involvement or source of paid caregiver payment (i.e., Medicaid-funded vs. privately paid) and geriatricians frequently deferred to family caregivers to determine how they should engage with paid caregivers. While paid caregivers often described their emotional connection to their clients, family caregivers and geriatricians rarely described this as an important aspect of paid care. To meet the nuanced care needs of people with dementia, home care needs to be centered around the unique needs of both people with dementia and their family caregivers. Rather than provide prescriptive standards for home care, family-centered home care should facilitate improved communication and clear expectation setting to be sure that care best meets the unique care needs of people with dementia living at home.

### SESSION 4170 (SYMPOSIUM)

Abstract citation ID: igad104.1525

### PRAGMATIC IMPLEMENTATION OF AN ADVANCE CARE PLANNING INTERVENTION: FINDINGS FROM SHARING CHOICES

Chair: Daniel Scerpella
Co-Chair: Jennifer Wolff
Discussant: Joseph Gaugler

Advance care planning (ACP) is defined as a key task in ambulatory care for patients to share values, goals, and preferences for future medical care. Ideally ACP is revisited throughout the course of serious illness and engages family in discussions with clinicians. Most interventions, however, have not targeted primary care as a setting for ACP but have instead targeted a specific illness, conversation or setting such as the inpatient hospital or nursing home. Additionally, family is often not included in ACP discussions. This symposium will discuss critical components of the SHARING Choices cluster-randomized pragmatic trial, including the setup, monitoring, and analysis of this intervention within two primary care health systems located in the Baltimore-Washington DC metropolitan corridor. Each presenter will discuss key efforts conducted during the trial that highlight challenges, lessons learned, and novel approaches of embedding an evidence-based intervention within usual care settings targeting older adults 65 years of age and older. Preliminary analyses indicate that the ACP intervention was highly effective for increasing the likelihood of having advance directives in the patients' electronic health records within 1-year of intervention initiation (odds ratio = 2.88, $p < 0.001$). Attendees of this symposium will take away relevant insights for implementing effective ACP and communication interventions within primary care settings as well as supporting outcome data. Our discussant will provide thoughts on the implications of these results for current primary care organizations and in the field of ACP and communication for older adults including those with dementia.

Abstract citation ID: igad104.1526

### PRELIMINARY FINDINGS OF THE SHARING CHOICES INTERVENTION ON PRIMARY OUTCOME MEASURES

Jennifer Wolff[1], Talan Zhang[2], Erin Giovannetti[3], Sri Rebala[3], and David Roth[1], *1. Johns Hopkins University, Baltimore, Maryland, United States*, *2. Johns Hopkins School of Medicine, Baltimore, Maryland, United States*, *3. MedStar Health Research Institute, Baltimore, Maryland, United States*

A cluster randomization approach was applied to 55 primary care practices across two health systems. A 1:2 randomization design resulted in 19 practices being randomized to the SHARING Choices intervention condition and 36 practices randomized to a usual care control condition. The primary outcome variables include documentation of an advance directive (AD) or medical orders for life-sustaining treatment in the electronic health record (EHR) within 12 months of exposure to the intervention for all patients and receipt of potentially burdensome care within 6 months of death for patients who die with a serious illness diagnosis. At the time of this abstract submission, preliminary data on AD documentation in the EHR have been analyzed for one of the two health systems. In that health system, among 8,350 patients in intervention practices, AD documentation in the EHR increased from 19.2% to 25.1% one year later, compared to increases from 15.7% to 17.9% among 19,179 patients in control practices. This markedly greater increase for patients in intervention practices was highly significant statistically in a multilevel logistic regression model that took patient clustering within practices into account (adjusted odds ratio = 2.25; 95% confidence interval = 2.01, 2.51; $p < 0.001$). The SHARING Choices intervention, therefore, more than doubled the odds of having an AD added to the EHR within one year. Preliminary findings on this outcome from the other health system and other outcomes from both health systems will be analyzed in the months ahead and presented at the GSA meeting in November.

Abstract citation ID: igad104.1527

### USING SIMULATION TO ASSESS THE FIDELITY OF ADVANCE CARE PLANNING IN THE CONTEXT OF A PRAGMATIC TRIAL

Valerie Cotter[1], Danetta Sloan[2], Daniel Scerpella[2], Jennifer Wolff[1], and Kelly Smith[3], *1. Johns Hopkins University, Baltimore, Maryland, United States*, *2. Johns Hopkins Bloomberg School of Public Health, Baltimore, Maryland, United States*, *3. University of Toronto, Toronto, Ontario, Canada*

There is growing recognition of the importance of and challenges to maintaining fidelity in pragmatic randomized clinical trials. Simulations using standardized patients are a high-fidelity, low-stake, non-threatening opportunity to evaluate knowledge, skills, and competencies associated with high-quality healthcare delivery. We created standardized patient scenarios grounded in the Respecting Choices First Steps™ Advance Care Planning (ACP) curriculum to assess embedded trial ACP facilitators. Scenarios included simulations representing one-on-one encounters with a patient, and with a patient-family dyad. A standardized encounter observation checklist was used to assess and score relevant skills and behaviors including encounter set-up, ACP topics, and general communication. Each item was scored on a scale from not-done (0) to effective (2) with lower scores indicating lower fidelity. Six facilitators with varied backgrounds (social work, nursing, lay persons) each completed the two simulation scenarios. Group average domain scores across all six facilitators were moderately high. ACP Setup scoring averaged 75.5%; ACP Topics were 72.0%; and Communication were 77.4%. The lowest group scoring was observed in the coverage of ACP Topics (72%). The highest group average was observed in Communication skills at 84.9%. Lower individual scores were observed across all domains for staff who were newly hired at the time of the simulation exercise. Simulation using standardized patients and caregivers allowed investigators to monitor the fidelity of ACP communication to the trial design and provided targeted opportunities for improvement that were not readily available through usual care.

Abstract citation ID: igad104.1528

### PRAGMATIC IMPLEMENTATION OF AN ADVANCE CARE PLANNING INITIATIVE: MOVING KNOWLEDGE INTO ACTION

Kelly Smith[1], Jessica Colburn[2], Daniel Scerpella[3], Maura McGuire[4], Kathryn Walker[5], and Jennifer Wolff[6], *1. University of Toronto, Toronto, Ontario, Canada*, *2. Johns Hopkins, Baltimore, Maryland, United States*, *3. Johns Hopkins Bloomberg School of Public Health, Baltimore, Maryland, United States*, *4. Johns Hopkins Community Physicians, Baltimore, Maryland, United States*, *5. MedStar Health, Columbia, Maryland, United States*, *6. Johns Hopkins University, Baltimore, Maryland, United States*

Primary care is an important setting for patient and family-centered communication with older adults given the high frequency of interactions, longitudinal trusted relationships, and patient preferences for advance care planning (ACP). Increasing ACP conversations and documentation of advance directives in primary care is a focus of quality-of-care initiatives and is particularly relevant for older adults, including those with dementia. The SHARING Choices ACP program evaluation was designed to examine the fidelity of implementation with the two partner health systems. SHARING Choices includes a mailing (introductory letter, an agenda-setting checklist, a blank advance directive, and information about shared-portal access for family) sent two weeks prior to scheduled primary care visits for patients aged 65 and above along with access to an ACP facilitator in each practice. Embedding SHARING Choices within primary care practices involved a multi-step process, including onboarding of health system partners, facilitator identification and training, tailored onboarding, practice champion engagement, and practice orientation and ongoing support. Of 23,220 candidate patients, 17,931 outreach attempts by phone (77.9%) and the patient portal (22.1%) were made by ACP facilitators and 645 conversations occurred. Most conversations (94.8%) were less than 45 minutes in duration. Study findings reinforce the value of adaptable study design; co-designing workflow adaptations with frontline staff; adapting implementation processes to fit the operational and organizational priorities of the health system.

Abstract citation ID: igad104.1529

### MULTILEVEL PERCEPTIONS OF IMPLEMENTING SHARING CHOICES: A QUALITATIVE STUDY

Martha Abshire Saylor[1], Valecia Hanna[2], Sydney Dy[2], and Danetta Sloan[3], *1. Johns Hopkins University School of Nursing, Baltimore, Maryland, United States*, *2. Johns Hopkins School of Public Health, Baltimore, Maryland, United States*, *3. Johns Hopkins Bloomberg School of Public Health, Baltimore, Maryland, United States*

Reviews have found that ACP interventions alone, including those in primary care, improve advance directive documentation but have not shown effectiveness for person-centered or end-of-life outcomes. One potential reason is that these interventions may not be sufficiently comprehensive and do not address the wide scope of population, communication and systems issues needed to improve outcomes. We conducted semi-structured interviews to explore experiences with the SHARING Choices intervention through the perspectives of key stakeholders involved in the trial. We recruited all embedded ACP facilitators; the clinicians and practice managers from primary care practices from both participating health systems; and patients (and/or family) who participated in the intervention. Tailored interview guides addressed how implementation occurred and evolved, acceptability and key enablers/barriers/adjustments in implementation, and the use and perceived impact of different elements of the intervention. A multidisciplinary team generated a coding scheme based on these topics of interest and used thematic analysis to identify, analyze, describe, and report emergent themes from responses across interview groups. We interviewed 40 participants (all 9 facilitators, 11 patients and families, and 20 clinicians and administrators). We will present major themes and differences across interview groups, and how this informs the quantitative results of the pragmatic trial.

### SESSION 4175 (PAPER)

### EDUCATION, TRAINING, AND EMPLOYMENT II

Abstract citation ID: igad104.1530

### PERCEIVED WORKPLACE AGEISM AND OLDER WORKERS' LABOR FORCE TRANSITIONS DURING THE GREAT RECESSION

Duygu Basaran Sahin, *RAND Corporation, Brooklyn, New York, United States*

Previous research suggests that workplace ageism increased during the Great Recession (GR). During the GR, despite having lower unemployment rates compared to younger workers, older workers took considerably longer to find jobs once they were unemployed. Yet, no study has investigated the potential role of workplace ageism in older workers' labor force outcomes. This study examines the role of perceived workplace ageism in older workers' labor force transitions during the GR using longitudinal data from the Health and Retirement study. I conduct a multi-period analysis and compare how the association of perceived workplace ageism with labor force transitions changed during the GR compared to pre and post-recession periods. Results from multivariate logistic regressions show that only in the GR period, both measures of perceived workplace ageism – pressure to retire before age 65 and preference for young in promotions – were associated with a higher likelihood of employment instability ($b= 0.336$ $p< 0.05$ and $b= 0.340$ $p< 0.05$), net of demographic, socioeconomic, financial, job-related and health-related factors. Moreover, older workers perceiving workplace were less likely to retire and thereby more likely to be unemployed ($b= -0.667$ $p< 0.05$). In contrast, perceived workplace ageism had no association with reducing or increasing the hours worked per week. In sum, this study provided the first individual-level evidence on the impact of perceived workplace ageism on older adults' labor force transitions during the GR. Findings can be used to motivate additional studies on older workers' experience during the Covid19 crisis.

Abstract citation ID: igad104.1531

### UNDERSTANDING RESOURCE PROFILES OF OLDER LEARNERS TO PROMOTE RECURRING ENGAGEMENT IN LEARNING

Pildoo Sung[1], Ad Maulod[2], Angelique Chan[2], and Rahul Malhotra[2], *1. Hong Kong Baptist University, Hong Kong, Hong Kong*, *2. Duke-NUS Medical School, Singapore, Singapore*

Understanding characteristics of recurring learners among older adults is important for designing targeted strategies to promote lifelong learning. Studies have shown that older adults with human, social, and psychological resources are more likely to engage in learning. However, less is known about the interplay of these resources in motivating older learners to remain engaged in learning. Using longitudinal data from 515 older learners, aged 50 years and above, collected in Singapore in 2017-2018, we examined distinct resource profiles among older learners and their impact on recurring engagement in learning. Latent profile analysis explored (1) clusters of individuals with distinct resource profiles at baseline, comprising varying levels of human (education), social (civic attitudes, civic behaviors, and loneliness), and psychological resources (motivation to learn and control/autonomy/self-realization/pleasure), and (2) whether the resource profiles were associated with recurring engagement in learning (defined as attending another course within six months from baseline). Empirical analyses identified four resource profiles: (1) educated, resourceful (42.2% of older learners), (2) less educated, less resourceful (24.5%), (3) less educated, resourceful (20.0%), and (4) educated, less resourceful (17.0%). 'Educated, less resourceful' learners had a lower likelihood of recurring engagement in learning than 'educated, resourceful' learners. In summary, older adult learners are likely to possess varying levels of human, social, and psychological resources, which predict their recurring engagement in learning. Interventions helping older learners enrich their civic and psychological resources may facilitate their continued engagement in learning.

Abstract citation ID: igad104.1532

### UNDERSTANDING THE NEW NIH DATA SHARING REQUIREMENTS: WHY THEY IMPROVE THE QUALITY OF RESEARCH

James McNally, *University of Michigan, Ann Arbor, Michigan, United States*

In 2021, NIH began a long period of planning, drafting, and requesting public comment to create a new data-sharing policy, beginning with a Request For Information (RFI). The new policy, beginning January 25, 2023, will apply broadly to all scientific data produced with NIH funding. NACDA and (ICPSR), drawing upon our expertise and understanding of best practices for data sharing, have been working actively to develop tools and resources to assist NIH grant recipients in complying with federal data-sharing expectations with minimal disruptions to their research. This presentation will provide a detailed overview of the new data-sharing requirements and how to incorporate them into the required data-sharing plan. We will offer examples of successful data-sharing plans, identifying an appropriate repository, budgeting for the data preparation process, and creating informed consent documents consistent with the spirit of the NIH Data Sharing requirements. In addition to explaining the new data-sharing policy, the presentation will discuss the rationale behind the requirements and how they contribute to data equity using the FAIR principles. By increasing access to federally funded data, we contribute to a multidisciplinary approach that enhances cross-disciplinary communication and a broad understanding of different ways of knowing when working collaboratively with other researchers. Team Science has facilitated substantive discourse between qualitative and quantitative specialists. Multidisciplinary research increasingly represents a critical aspect of overall knowledge creation. Finally, the presentation will illustrate how data-sharing increases the impact of studies and enhances the credentials of researchers who engage in data-sharing.

### SESSION 4180 (SYMPOSIUM)

Abstract citation ID: igad104.1533

### THE EXPERIENCE OF AGING IN DAILY LIFE IN CROSS-CULTURAL CONTEXTS

Chair: Shevaun Neupert Co-Chair: Yuval Palgi

Subjective views of aging capture the many ways that people acknowledge, contemplate, and reflect on aging. These views are related to a wide range of health and well-being indicators including emotional well-being, mental health, chronic health conditions, memory functioning, and even life expectancy. However, most of what we know about subjective views of aging and health is based on W.E.I.R.D. (Western, Educated, Industrialized, Rich, Democratic) samples. The Subjective Aging within Global Everyday ecological Studies (Subjective AGES) consortium was built to address this problem by collecting parallel daily diary data from the United States, Turkey, Germany, United Kingdom, and Israel. Neupert will provide an overview of the consortium and present findings from the U.S. data, focusing on the interaction of daily stressors and uplifts for subjective aging. Can will demonstrate the deleterious effects of daily stressors on subjective aging within the Turkish data. Kornadt will show the beneficial effects of daily uplifts on subjective aging within the German data. Tse will demonstrate that doing personally meaningful and flow-conducive activities predicts feeling younger in the United Kingdom data. Shrira, Palgi, and Neupert will show cultural differences and similarities between Israeli Jews and Arabs and then present cross-country differences comparing the Israeli sample to the American sample. The talk will end by synthesizing the findings from each talk and pointing to future directions for the consortium. Collectively, these findings point to the importance of taking a cultural perspective in the daily relationships between subjective aging and well-being.

Abstract citation ID: igad104.1534

### THE SYNERGISTIC EFFECT OF STRESSORS AND UPLIFTS FOR DAILY SUBJECTIVE AGING IN THE US

Shevaun Neupert, *North Carolina State University, Raleigh, North Carolina, United States*

Exposure to daily stressors is consistently associated with reduced health and well-being, including feeling subjectively older. Experiencing uplifts, however, are typically associated with increased health and well-being, but have not yet been examined with respect to subjective aging. The current study tested daily stressors and uplifts as unique as well as interactive effects for daily subjective age and daily ageist attitudes. A sample of 440 U.S. adults aged 50-85 participated in a 14-day daily diary study, reporting on their subjective age, ageist attitudes, daily stressors, and daily uplifts each day. All variables demonstrated significant within-person variability, with ICCs ranging from .68 (uplifts) to .75 (subjective age). Daily stressors and daily uplifts were each uniquely predictive of daily ageist attitudes, where daily stressors were positively associated and daily uplifts were negatively associated with daily ageist attitudes. For daily subjective age, there was a synergistic effect of daily stressors and daily uplifts. On days with no stressors, daily uplifts were not associated with daily subjective age. However, on days with stressors, increases in daily uplifts were associated with significant decreases in subjective age. These findings underscore the multidimensionality of subjective age constructs and will be situated within cross-cultural comparisons.

Abstract citation ID: igad104.1535

### DOES STRESS MAKE YOU FEEL OLDER? A DAILY DIARY STUDY OF SUBJECTIVE AGING, STRESS, AND UPLIFTS IN TURKIYE

Reyyan Can[1], and Shevaun Neupert[2], *1. North Carolina State University, Raleigh, North Carolina, United States, 2. North Carolina State University, Raleigh, North Carolina, United States*

Subjective aging, how one perceives their age, is associated with psychological, emotional, and social well-being. Adults' subjective perceptions of their age are malleable and can fluctuate on a daily basis, influenced by various life events such as experiencing stress or uplifts. We conducted a 14-day daily diary study to assess how subjective age and ageist attitudes are predicted by daily stressful events and uplifts in Turkey/Turkiye. 68 Turkish participants aged 50 to 90 years (M= 57) responded to subjective age, ageist attitudes, stress, and uplifts questionnaires every day. Multilevel model analyses indicated that individuals perceived themselves as emotionally and physically older on days when they encountered more stressors and fewer uplifting events. Additionally, on those days, participants reported that they looked and behaved older than their chronological ages. Uplifts corresponded negatively to ageist attitudes; individuals who experienced more uplifting events during the day had fewer negative judgments of aging. Taking age as a subjective construct, these results suggest that having fewer stressors in daily life changes people's feelings about their aging process. In addition, involvement in uplifts can buffer ageist attitudes on a daily basis.

Abstract citation ID: igad104.1536

**DAILY EXPERIENCES OF UPLIFTS, STRESSORS, AND SUBJECTIVE VIEWS ON AGING IN GERMANY**

Anna E. Kornadt, *University of Luxembourg, Esch-sur-Alzette, Luxembourg*

People's subjective experience of aging can vary from day to day and is related to other experiences in people's daily lives. While stressors have been previously investigated as predictors of subjective aging, less research has taken the role of positive events into account. We were thus interested in the relationship of daily stressors and uplifts on different indicators of daily subjective aging in later life. In a preliminary sample of N = 42 German adults aged 52 - 75 years, participants completed up to 14 daily-diary questionnaires on subjective age (single item and multidimensional) and ageist attitudes, daily stressors and uplifts. All variables showed significant within-person variability, with ICCs ranging from .24 (stressors) to .87 (ageist attitudes). Uplifts were related to daily subjective age: On days with more uplifts, people felt younger. Stressors were not related to subjective age and no relation was found with ageist attitudes for uplifts and stressors. Findings will be contextualized in a cross-cultural sample.

Abstract citation ID: igad104.1537

**FEELING YOUNGER TODAY? THE ROLE OF MEANINGFUL AND FLOW-CONDUCIVE ACTIVITIES**

Dwight Tse, *University of Strathclyde, Glasgow, Scotland, United Kingdom*

Given the conceptualization of subjective age being a construct that varies daily, one question naturally follows is whether doing particular activities may be associated with daily subjective age. Specifically, extrapolating from flow and vital engagement theory, we hypothesize that activities that are personally meaningful or flow-conducive (activities that are conducive to flow experience, a psychological state of simultaneously intense concentration and enjoyment) may be associated with older adults' feeling younger on the day. In a 14-day diary study, participants residing in the United Kingdom reported their subjective age at the end of the day. They also identified the activity on which they spent most time during the day and reported their flow experience and perceived meaningfulness in the activity. With the initial data from a sample of N = 69 adults (age range: 50 to 83) who completed at least 7 daily surveys, we found that higher-than-usual (i.e., within-person) levels of both activity meaningfulness and flow experience were associated with feeling younger. These effects were robust even after controlling for daily stressors and uplifts. Interestingly, there was an interaction between within-person activity meaningfulness and flow experience, such that the effect of flow experience was stronger in activities with lower (vs. higher) levels of meaningfulness. Our findings provide preliminary support that doing personally meaningful and flow-conducive activities may be associated with feeling younger on the day, underscoring the possibility of activity-level behavioral change that influences subjective age.

Abstract citation ID: igad104.1538

**WITHIN- AND BETWEEN-COUNTRY DIFFERENCES IN DAILY ASSOCIATION OF VIEWS OF AGING AND NEGATIVE AFFECT**

Amit Shrira[1], Yuval Palgi[2], and Shevaun Neupert[3], *1. Bar Ilan University, Ramat Gan, HaMerkaz, Israel*, *2. University of Haifa, Haifa, Hefa, Israel*, *3. North Carolina State University, Raleigh, North Carolina, United States*

Few studies found cultural differences, both across and within countries, in the association between subjective views of aging (VoA) and psychological concomitants. However, a question remains whether such cultural differences exist on a daily level. We therefore examined cultural differences in daily covariation of VoA and negative affect (NA) within an Israeli sample (comparing Israeli Jews to Arabs) and between countries (comparing the Israeli sample to an American sample). The Israeli sample included 75 participants (age ranged 50 to 88, 36% Arabs). The American sample included 77 participants (age ranged 50 to 82). Participants reported subjective age, subjective accelerated aging, ageist attitudes, and NA across 14 days. Within-person variability ranged from 28% (ageist attitudes) to 52% (NA) of the total variance in the Israeli sample, and 26% (subjective age) to 38% (subjective accelerated aging) in the American sample. On days respondents felt younger, felt to be aging slower, or had less ageist attitudes, they also reported lower NA. Cross-level interactions showed that the associations of subjective age and subjective accelerated aging with NA were significantly stronger among Israeli Arabs (versus Israeli Jews) and significantly stronger in the Israeli sample (versus the American sample). The daily covariance between ageist attitudes and NA remained similar across cultural groups. These findings attest to the importance of taking a cultural perspective on daily relationships between VoA and psychological concomitants. Our talk will discuss potential explanations for the abovementioned cultural differences and similarities and will point to future directions in our Subjective AGES project.

### SESSION 4185 (PAPER)

### COGNITION (BSS PAPERS) II

Abstract citation ID: igad104.1539

**AGING AND NUMERACY: A META-ANALYSIS**

Ryan Best, and Farima Sadeqi, *West Virginia University, Morgantown, West Virginia, United States*

Numeracy, the ability to competently make use of numbers and numerical information, is a unique cognitive ability associated with diverse positive outcomes across the lifespan. Often included as a covariate in studies of cognitive aging, there is some scattered evidence of lower numeracy in older adults when compared to younger adults. Importantly, one longitudinal study found evidence of a quadratic decline in numeracy starting in later middle-age. The current study systematically reviews the literature on aging and numeracy as measured by one of three frequently implemented objective measures: the Lipkus numeracy scale (Lipkus et al., 2001),

Weller et al.'s Rasch-based numeracy scale (2013), and the Berlin Numeracy Test (Cokely et al., 2012). Effect sizes were calculated from two types of studies: those including age as a categorical extreme group comparison variable or as a continuous variable from adult lifespan samples. Hedge's g was computed as the effect size in the former studies and Pearson's r was calculated as the effect size for the latter. For comparison across studies in a single random-effects model, Pearson's r effect sizes were mathematically transformed into Hedge's g's. Type of sample (extreme group vs. adult lifespan) and age range were included in a second mixed-effects model to account for heterogeneity in effect sizes. Initial results show lower numeracy in older adults, but reduced effect size estimates in lifespan samples compared to those using extreme group comparisons. The implications for using linear estimates of effect size for the quadratic relationship between age and numeracy are discussed.

Abstract citation ID: igad104.1540

### HOW'S YOUR MEMORY? CONVERGENCE OF AMBULATORY COGNITIVE PERFORMANCE AND RETROSPECTIVE PERFORMANCE REPORTS

Anqing Zheng[1], Tina Vo[2], Elizabeth Muñoz[3], Sally Wadsworth[1], Martin Sliwinski[4], and Chandra Reynolds[1], *1. University of Colorado Boulder, Boulder, Colorado, United States*, *2. University of California Riverside, Riverside, California, United States*, *3. The University of Texas at Austin, Austin, Texas, United States*, *4. Pennsylvania State University, State College, Pennsylvania, United States*

Calibration accuracy, the agreement between individuals' perceived performance and actual performance, plays a critical role in self-regulated learning, knowledge monitoring, and goal maintenance. However, it is unclear how calibration accuracy operates at the micro-level timescales, especially among individuals approaching midlife. We investigated the convergence between perceived cognitive performance and the trajectory of momentary assessments collected repeatedly over 14 days. We analyzed the data of 440 participants (Mage = 35.78, %Female = 57.7%) from the Colorado Adoption/Twin Study of Lifespan Behavioral Development and Cognitive Aging (CATSLife1), who completed spatial working memory tasks administered via smartphones up to three times a day. On average, participants' working memory improved across the 14-day period ($\beta$=-.014, SE=.001, p<.001), with lower scores indicating more accurate placement and better performance. Moreover, we observed less day-to-day variability in working memory performance ($\beta$=-.010, SE=.001, p<.001). The trajectory of working memory performance was consistent with participants' evaluations of cognitive performance after completing the study: those perceiving improvement in their performance tended to perform better overtime (r = .25, SE = .046) and showed less variability in momentary assessments (r = -.28, SE = .046). Participants' self-reported performance was also positively correlated with their working memory intercept at the start of the study (r = .34, SE=.045). Exploratory analyses then examined the associations between momentary calibration accuracy and other personality characteristics (e.g., openness). Our findings suggest that understanding the dynamic interplay between calibration accuracy and cognitive performance could shed light on individual differences during periods of cognitive maintenance versus decline.

Abstract citation ID: igad104.1541

### PERSPECTIVE-TAKING DECLINE IN OLDER ADULTS WITH AND WITHOUT SUBJECTIVE COGNITIVE DECLINE

W. Quin Yow, Nina Ye, and Xiaoqian Li, *Singapore University of Technology and Design, Singapore, Singapore*

Subjective cognitive decline (SCD), a self-reported decline in cognitive function without objective cognitive decline, is an early risk marker of pathological aging (Jessen et al., 2020). Healthy older adults with SCD(+) were found to have lower objective cognition and increased rates of cognitive decline compared to those without SCD(-) (Morrison & Oliver, 2023). Cognitively-healthy older adults (OA) had shown a decline in perspective-taking abilities compared to younger adults (YA). However, little research has examined whether self-report SCD plays a role in such age-related declines. We examined age-related changes in perspective-taking abilities in OA with and without SCD vis-à-vis YA. Participants were 25 YA (Mage=22.44, range=20-27), 27 SCD- (Mage=68.67, range=60-77), and 20 SCD+ (Mage=66.55, range=60-72). OA were categorized as SCD-/SCD+ based on SCD-Questionnaire (Rami, 2014). SCD- and SCD+ groups were comparable in general cognition (Montreal Cognitive Assessment; p=.91). Participants completed the "owl" task assessing visual perspective-taking (VPT) and the "director" task assessing cognitive perspective-taking (CPT). Kruskal-Wallis tests revealed that YA outperformed OA (Zs>3.52, ps<.001) in VPT, with no significant difference between SCD- and SCD+ (Z=1.27, p=.21). Surprisingly, both YA and SCD+ were better than SCD- in CPT (Zs>2.34, ps<.04), and SCD+ did not differ significantly from YA (Z=0.02, p=.99). Our results suggest that while visual perspective-taking may decline with age regardless of SCD, cognitive perspective-taking may be preserved in older adults with SCD. SCD(+) may signal a hyperawareness of cognitive states in self and others, which in turn stimulate better cognitive perspective-taking. Implications of SCD on social cognition will be discussed.

Abstract citation ID: igad104.1542

### THE CURRENT AND RETROSPECTIVE COGNITIVE RESERVE (2CR) SURVEY AND ITS RELATIONSHIP WITH COGNITIVE AND MOOD MEASURES

Stephen Aichele[1], Erika Borella[2], Paolo Ghisletta[3], and Elena Carbone[2], *1. Colorado State University, Fort Collins, Colorado, United States*, *2. University of Padova, Padova, Veneto, Italy*, *3. University of Geneva, Geneva, Geneve, Switzerland*

Cognitive reserve (CR) is often assessed with surveys spanning demographic, lifestyle, and socio-behavioral variables. The role of both past and current life experiences on CR has, however, rarely been examined. We developed the Current and Retrospective Cognitive Reserve (2CR) survey to assess classical CR proxies (socio-economic status, engagement in leisure and social activities) and other dimensions of potential importance (family engagement, religious/spiritual activity) both currently (CRc; in later adulthood) and retrospectively (CRr; as recalled from younger adulthood). We administered the 2CR, measures of general cognitive functioning, working memory (WM), vocabulary, fluid reasoning, and depressive symptoms (DS) to 235 community-dwelling Italian adults (ages 55-90 years). We used exploratory and confirmatory factor analyses to examine the 2CR latent structure, and we estimated correlations of its dimensions with cognitive abilities and DS. Analyses confirmed a three-level factor structure with two global CR factors (CRc and CRr) at the top level, dimensional CR factors (socio-economic status, family engagement, leisure activity, social engagement, and religious/spiritual activity) at mid-level, and observed items at the lowest level. Item-factor representations partially differed across CRc and CRr. Both CRc and CRr were positively correlated with measures of intelligence, WM, and DS. Associations with measures of intelligence were comparatively stronger for CRr, whereas associations of WM and DS were slightly stronger for CRc. The 2CR can be considered a reliable survey for assessing CR proxies within a multidimensional, "life stage-dependent" framework insofar as CRc are CRr closely related but also differently associated with intelligence, WM, and DS.

### SESSION 4190 (PAPER)

### LONG-TERM CARE (SRPP PAPERS) II

Abstract citation ID: igad104.1543

### CORPORATE PERSPECTIVES OF A NATIONAL NURSING HOME PRAGMATIC TRIAL AND FACTORS AFFECTING IMPLEMENTATION FIDELITY

Jacy Weems, Ellen McCreedy, Grace Wittenberg, and Rosa Baier, *Brown University School of Public Health, Providence, Rhode Island, United States*

Implementation fidelity is concerning for embedded pragmatic clinical trials (ePCTs), where effectiveness is evaluated in real-world settings instead of a structured clinical environment. Healthcare facilities paused ePCT research activities during the COVID-19 pandemic to address related staffing issues and infections. We examined a 54-nursing-home ePCT implementing Music & Memory, a nonpharmacological intervention using music to improve quality of life for residents living with dementia. Our qualitative study describes factors influencing implementation fidelity across four nursing home corporations. The interview guide assesses corporate perceptions of implementation barriers, facilitators, and participation incentives. Themes were organized within the Conceptual Framework for Implementation Fidelity's potential modifying factors: 1) context, 2) participant responsiveness, 3) intervention complexity, 4) facilitation strategies, 5) quality of delivery, and 6) recruitment. Interviewees (n=4) lead corporate-wide dementia initiatives. 1) Context: COVID-19-related capacity issues and local "champion" turnover impacted success. 2) Participant responsiveness: Developing playlists strengthened staff-resident relationships, although time-consuming and requiring staff competency. Residents experienced decreased agitation while staff satisfaction increased. 3) Intervention complexity: Brown's research team adapted processes to save staff time. 4) Facilitation strategies: The relationship with Brown promoted problem-solving. 5) Quality of delivery: The ePCT was an effective, non-pharmacological approach addressing dementia symptoms. 6) Recruitment: Corporate leadership makes decisions regarding individual nursing homes' recruitment in research. Financial incentives did not drive participation, although some voiced appreciation for offsetting time and resources invested. Recommendations for future efforts included in-person champion training and site-specific quality improvement efforts. Researchers maintaining relationships with corporate and local leadership promotes sustainable engagement.

Abstract citation ID: igad104.1544

### FROM COMFORT TO PROFITABILITY: RELATIONSHIP BETWEEN CARE QUALITY AND FINANCIAL PERFORMANCE IN US HOSPICES

Ganisher Davlyatov[1], Mengying He[2], Deepthi Padakandla[1], and Robert Weech-Maldonado[3], *1. University of Oklahoma Health Sciences Center, Oklahoma City, Oklahoma, United States*, *2. California State University, Los Angeles, California, United States*, *3. University of Alabama at Birmingham, Birmingham, Alabama, United States*

Hospice care is a critical aspect of end-of-life care in the United States, providing comfort and support to individuals with life-limiting illnesses. Quality of care is a key concern for hospice facilities, as it can impact both patient outcomes and financial sustainability. Poor quality of care can lead to negative patient experiences, increased hospitalizations, and even legal issues. It can also result in financial penalties or reduced reimbursement from payers. However, there is limited research on the relationship between quality of care and financial performance in hospice facilities. Employing a cross-sectional observational design, we examined the relationship between Hospice and Palliative Care Composite Process Measure (lagged by 1 year) and the total margin of hospice facilities across the United States. We used secondary data from Nursing Home Compare, Healthcare Cost Report Information System Dataset, and the American Community Survey for the period 2017-2021. We found a positive association between quality process measures and financial performance in hospice facilities after controlling for organizational and community factors that may influence financial performance, such as facility size and location. Specifically, a 10-unit increase in quality measure was associated with a 2% increase in total margin. The findings of this study have important implications for the management and financing of hospice facilities. Investments in quality improvement initiatives may lead to improved financial performance. Policymakers should consider these findings when designing payment systems for hospice care, and administrators of hospice facilities should prioritize quality improvement initiatives to ensure both high-quality care and financial viability.

Abstract citation ID: igad104.1545

### REVISITING THE TEACHING NURSING HOME: PRAGMATIC EVALUATION IN A REAL-WORLD SETTING

Howard Degenholtz, *University of Pittsburgh, Pittsburgh, Pennsylvania, United States*

Revisiting the Teaching Nursing Home (RTNH) is a renewal of the Teaching Nursing Home model pioneered in the 1980s, which found that creating partnerships between schools of nursing (SONs) and nursing homes (NHs)

improved clinical outcomes. In its first two years, the RTNH initiative has fostered partnerships between three SONs and four NHs in Pennsylvania, and is planning to expand. The program is implementing the Institute for HealthCare Improvement Age-Friendly Health System "4Ms" quality improvement framework through an online learning network. The 4M quality improvement framework focuses on four evidence-based elements: Mobility, Mentation, Medication, and What Matters. In addition, the participating SONs are incorporating the 4Ms into curricula and clinical rotations. The evaluation will examine both implementation and outcomes for SONs and NHs. Measures include student competency, staff satisfaction and burnout, clinical process of care and resident outcomes. While some resident level measures can be extracted from the Minimum Data Set (MDS), others (deprescribing, rehabilitation goals, preference sensitive care) require access to the electronic medical record (EMR). The goal of this presentation is to describe the methodology for measuring the wide range of outcomes and to examine the performance of a synthetic comparison group to study overall quality using CASPAR data. We selected 1,764 similarly sized, non-profit NHs and conducted bivariate, and propensity score analysis to determine if the participating NHs were different on any observable characteristics. The findings suggest that the participating NHs were not statistically significantly different in terms of overall quality rating, staffing, or inspections.

Abstract citation ID: igad104.1546

### THE EFFECT OF PATIENT-DRIVEN PAYMENT MODEL REIMBURSEMENT POLICY CHANGE ON NURSING HOMES

Gregory Orewa[1], Robert Weech-Maldonado[2], Ganisher Davlyatov[3], Sue Feldman[2], David Becker[2], and Justin Lord[4], *1. University of Texas, San Antonio, San Antonio, Texas, United States*, 2. *University of Alabama at Birmingham, Birmingham, Alabama, United States*, 3. *University of Oklahoma Health Sciences Center, Oklahoma City, Oklahoma, United States*, 4. *Louisiana State University at Shreveport, Shreveport, Louisiana, United States*

To promote more value in skilled nursing facility spending in the US, CMS modified the way it pays for nursing homes (NH) services on October 1, 2019. The new Medicare reimbursement methodology, the Patient Driven Payment Model (PDPM), moves away from the traditional system focused on therapist minutes to a value-based focused on resident clinical characteristics. Using contingency theory, this study explores NH responses to PDPM by: 1) examining whether NH's therapist and nursing staffing patterns are aligning with the policy incentives; and 2) whether the adjustments are resulting in improved financial performance. Seven different datasets were merged for 2017– 2021: Medicare cost reports, Payroll Based Journal, NH Compare, Area Health Resource File, HHS Provider Relief Fund, CDC NH COVID-19 public file, and CDC COVID-19 Data Tracker. The data was modelled using random-effects regression, and we tested for potential moderation effect of staffing on the relationship between PDPM and financial performance. Dependent variables were nursing and therapy staffing intensity, while the independent variable was PDPM for model 1. Dependent variable was operating margin, moderator variable was staffing intensity, and independent variable was PDPM for model 2. Organizational, community level, and COVID-19 related variables were used as controls. Results suggest that PDPM was associated with an increase in RN and LPN staffing intensity, but a decrease in Physical Therapist, Physical Therapist Assistant, and Occupational Therapist Assistant staffing intensity ($p < 0.05$). PDPM had a positive impact on financial performance moderated by RN and PT staffing intensity ($p < 0.05$). Policy and managerial implications are discussed.

Abstract citation ID: igad104.1547

### UTILIZATION OF ELDER CARE AMONG PEOPLE LIVING WITH DEMENTIA IN SWEDEN: A REGISTER-BASED STUDY

Atiqur sm-Rahman[1], Bettina Meinow[2], Lars-Christer Hydén[3], and Susanne Kelfve[1], *1. Linköping University, Norrköping, Ostergotlands Lan, Sweden*, 2. *Stockholm Gerontology Research Center, Stockholm, Stockholms Lan, Sweden*, 3. *Department of Culture and Society (IKOS), Linköping, Ostergotlands Lan, Sweden*

The growing number of people living with dementia (PlwD) implies an increase in the demand for eldercare at different stages of the disease. This study aims to investigate the utilization of eldercare among people with and without dementia in Sweden during the last five years of life and what social-background factors influence the use of eldercare. Data were derived from four linked Swedish national registers comprising all decedents aged 70+ in Sweden as of November 2019 (n=6294). The primary outcome variable was the utilization of eldercare (no care, homecare, residential care). Following the study sample retrospective from death, data analysis was performed using multinomial and linear logistic regression models. Results showed that (1) nearly a quarter of all PlwD did not use any eldercare, primarily people who were newly diagnosed with dementia and living with partners; (2) three out of four PlwD used residential care in the last years of life; and (3) age, gender, and cohabitation status were important social-background factors determining utilization of eldercare for PlwD. This study provides unique insight that many Swedes with a dementia diagnosis do not receive any eldercare and that the utilization of eldercare increases with time since dementia diagnosis. We suggest more research to investigate why a substantial part of PlwD does not have any eldercare at all and what the policy implications of this might be.

### SESSION 4195 (PAPER)

### MINORITY AND DIVERSE POPULATIONS (PAPERS) II

Abstract citation ID: igad104.1548

### DIMINISHED RETURNS OF PARENTAL EDUCATION ON COGNITION: EVIDENCE FROM THREE POPULATION STUDIES IN THE US

Addam Reynolds[1], and Emily Greenfield[2], *1. University of Southern California, Los Angeles, California, United States*, 2. *Rutgers, The State University of New Jersey, New Brunswick, New Jersey, United States*

Mounting evidence suggests that the protective effects of one's own higher socioeconomic status (SES) on health is diminished among minoritized racial/ethnic groups in the United States (U.S.) with respect to outcomes such as mortality, hospitalization and mental health. Extending this area of research to childhood SES and later life outcomes, this study examines whether the protective effects of higher parental education on cognition is diminished among Black adults relative to their non-Hispanic White peers. Using data from the Health and Retirement Study, the Midlife in the U.S. Study, and the National Social Life, Health and Aging Project, I examine whether associations between parental education and two measures of cognition (episodic memory and global cognition) are moderated by race (non-Hispanic White or Black) using a random-effects individual participant data meta-analysis approach. I find evidence for a small, but robust, protective effect of higher parental education on both episodic memory and global cognition among White adults. Among Black adults, there was no association between parental education and neither episodic memory nor global cognition. This study provides evidence that the protective effect of higher parental education on cognition is not the same across racialized populations, consistent with the theory of Minority Diminished Returns. As scholars continue calls for life course-oriented efforts to reduce racialized cognitive disparities, it is important to consider early-life risk and protective factors in the context of structural racism.

Abstract citation ID: igad104.1549

### DISPARITIES IN ELECTION, ACCESS, AND OUTCOMES IN MEDICARE END-OF-LIFE CARE: A NATIONAL STUDY

Thomas Christian[1], Michael Plotzke[2], Mariana Sarango Cancel[1], Catherine Hersey[1], and Zinnia Harrison[3], *1. Abt Associates, Cambridge, Massachusetts, United States, 2. Abt Associates, St. Louis, Missouri, United States, 3. Abt Associates, Inc., Rockville, Maryland, United States*

We examined whether end-of-life care racial disparities persist even within groups with similar geographic access and health care options. We reviewed calendar year (CY)2021 fee-for-service Medicare claims to determine if a beneficiary ever: elected hospice, had an end-of-life care conversation with their physician, and/or received advanced care planning services. We obtained beneficiary characteristics from Medicare administrative records and used Consumer Assessment of Health Providers and Systems Hospice star ratings to identify poorer quality hospices (one- or two-stars out of five). As a measure of the quality-of-care processes beneficiaries received, we calculated Hospice Visits in the Last Days of Life (HVLDL). Using logistic regression, we calculated Adjusted Odds Ratios (AORs) and 95% Confidence Intervals (CIs) to characterize the association between race/ethnicity and end-of-life services utilization. Relative to white decedents, beneficiaries were less likely to elect hospice if they were black [AOR 0.67 95% CI 0.65-0.69], Asian [AOR 0.62 95% CI 0.59-0.66] or Hispanic [AOR 0.76 95% CI 0.72-0.81]. Additionally, non-white beneficiaries more often received care from poorer quality hospices than the white beneficiaries residing in their same zip code (black AOR 1.26 95% CI 1.21-1.31, Asian AOR 1.21 95% CI 1.14-1.29, Hispanic AOR 1.16 95% CI 1.09-1.24). In such poorer quality hospices, non-white beneficiaries have lower HVLDL rates (white 48.5%, black 40.3%, Asian 42.6%, Hispanic 45.8%). We found no substantial racial/ethnic disparities in recorded advance care plans or end-of-life conversations. CMS should continue to monitor trends in hospice utilization and ensure all beneficiaries receive equal and adequate care.

Abstract citation ID: igad104.1550

### HOW DO OLDER ADULTS COPE WITH INTERSECTIONAL EXPERIENCES OF AGEISM AND RACISM?

Andrew Steward[1], Yating Zhu[2], Carson De Fries[2], Annie Zean Dunbar[3], Miguel Trujillo[4], and Leslie Hasche[2], *1. University of Wisconsin-Milwaukee, Milwaukee, Wisconsin, United States, 2. University of Denver, Denver, Colorado, United States, 3. University of Denver Graduate School of Social Work, Denver, Colorado, United States, 4. Utah Division of Multicultural Affairs, Salt Lake City, Utah, United States*

The World Health Organization recently launched a global campaign to end ageism due to its widespread, insidious negative health impacts on older adults. Similarly, racism is a key driver of health disparities and has been declared a public health crisis by jurisdictions around the United States. While a strong literature summarizes coping theory in general as well as beneficial and maladaptive coping mechanisms among older adults, limited research describes how older adults cope with experiences of ageism and racism, particularly through an intersectional lens. The aim of this qualitative, phenomenological study was to understand how older adults cope with intersectional experiences of ageism and racism. Twenty adults 60+ residing in the U.S. Mountain West who identified as Black, Hispanic/Latino(a), Asian-American/Pacific Islander, Indigenous, or White participated in a one-hour, semi-structured interview. A team of five coders engaged in an inductive coding process through independent coding followed by critical discussion. Peer debriefing, member checking, and an audit trail enhanced credibility. Nine themes were identified: 1) resistance, 2) exhaustion, 3) acceptance/'let it go,' 4) avoidance, 5) increased awareness through aging, 6) help others, 7) educate others, 8) self-determination/defy stereotypes, and 9) healthy lifestyle. These themes exemplify problem-focused and emotion-focused, as well as individual and collective coping strategies. Novel findings include how older adults may cope with ageism and racism through helping peer older adults and via increased awareness through aging. Practitioners should recommend coping strategies which combine collective and problem-focused responses to ageism and racism such as resistance, helping others, and educating others.

Abstract citation ID: igad104.1551

### IMPLICIT AND EXPLICIT SPIRITUALITY IN THE LIVES OF TRANSGENDER AND GENDER-EXPANSIVE OLDER ADULTS

Vanessa Fabbre[1], Courtney Taylor[2], Stef Sloan[3], and Eleni Gaveras[4], *1. Washington University in St. Louis, St. Louis, Missouri, United States, 2. Brown School of Social Work, Washington University in St. Louis, St. Louis, Missouri, United States, 3. University of Kansas, Lawrence,*

*Kansas, United States*, *4. Washington University in St Louis, St. Louis, Missouri, United States*

Religion and spirituality for transgender and gender expansive people (whom we refer to collectively as trans) are complicated by mainstream religions' history of stigmatizing and marginalizing sexual and gender minorities. We conducted an interpretive content analysis of biographical interviews with 88 trans older adults from across the United States, applying six tenets of spiritual psychotherapy to their life narratives. Our findings suggest that some trans older adults' spirituality is experienced both implicitly and explicitly. Implicit spirituality reflects the ways in which meaning, purpose, and connection in one's life are nurtured with respect to one's gender identity. Explicit spirituality reflects the process of consciously renegotiating one's spiritual beliefs and religious practices to validate one's gender identity and place in society. This knowledge is potentially helpful for gerontological practitioners who seek to nurture trans people's spirituality and well-being as they age.

Abstract citation ID: igad104.1552

**MAPPING MENTAL HEALTH PATHWAYS AMONG BISEXUAL WOMEN, MEN, AND NONBINARY INDIVIDUALS IN MID AND LATER LIFE**

Sarah Jen[1], Hyun Kang[2], Hyun-Jun Kim[3], Olivia Lafountain[1], and Karen Fredriksen-Goldsen[3], *1. University of Kansas, Lawrence, Kansas, United States*, *2. George Mason University, Fairfax, Virginia, United States*, *3. University of Washington, Seattle, Washington, United States*

The health-related pathways of bisexual individuals are most often compared to those of lesbians and gay men or examine participants of different genders separately. However, little is known regarding how the health-related pathways of bisexual individuals of diverse genders compare to one another, particularly in mid- to later life. This analysis examines identity-related and social factors which impact the mental health outcomes of midlife and older bisexual women, men and non-binary individuals and compares health-related pathways across genders. Analyses examined data from the National Health, Aging, and Sexuality/Gender Study (NHAS), including bisexual women (96), men (102), and non-binary individuals (16) ages 50+. The study utilized structural equation modeling to investigate direct and indirect associations between gender and mental health via sexual identity-related (victimization, negative identity perception, and outness) and social factors (LGBTQ community engagement). For both men and women, negative identity perception was associated with being less out, particularly among women. Bisexual men who reported more victimization also reported more negative identity perception. However, negative identity perception was associated with worse mental health only among women. Across genders, victimization negatively impacted mental health and identity outness increased community engagement. Community engagement mediated the relationship between identity outness and mental health among men. Findings indicate distinct mental health pathways across genders. Thus, mental health interventions might also differ. While LGBTQ community engagement might be particularly beneficial to bisexual men, bisexual women might struggle more with identity perception. Future research will benefit from larger subsamples, particularly among non-binary individuals.

### SESSION 4200 (BIOLOGICAL SCIENCES INVITED SYMPOSIUM)

Abstract citation ID: igad104.1553

**SENESCENCE NEW FRONTIERS - SENNET CONSORTIUM**

Chair: Laura Niedernhofer Co-Chair: Viviana Perez Montes

Senescence is a cell fate triggered by stress, activating signaling cascades that stably arrest the cell cycle, thereby preventing mutagenesis and cancer. Senescent cells are critical for wound healing and other physiological processes, during which they secrete a host of factors that remodel tissue and attract immune cells, which are responsible for removing the senescent cells. However, with age and immune dysfunction, senescent cells accumulate and are established to play a causal role in aging and most age-related diseases, largely through their secretome that drives chronic sterile inflammation. Senescent cells have proven tractable as drug targets, potentially affording novel geroscience approaches to treat a variety of age-related diseases, including Alzheimer's disease. To optimize this opportunity much more must be learned about senescent cells. This is a challenge as there are no specific biomarkers to detect or isolate senescent cells. Furthermore, senescent cells are anticipated to be highly heterogeneous based on cell lineage, the cause of senescence, their secretome, and tissue context. This challenge sparked the creation of the SenNet Consortium, an NIH Common Fund effort involving hundreds of scientists across the globe charged with creating a 4D atlas of senescent cells in 18 healthy human tissues across human lifespan, using mice and perturbations to inform the mapping. The speakers in this session will define the goals of SenNet, the scientific opportunities and challenges, the novel technologies applied, and the anticipated deliverables including large public datasets and innovative search and visualization tools.

Abstract citation ID: igad104.1554

**SENNET CONSORTIUM: OPPORTUNITIES AND CHALLENGES**

Laura Niedernhofer, *University of Minnesota, Minneapolis, Minnesota, United States*

The Cellular Senescence Network (SenNet) Program was established in 2021 by the NIH Director's Office Common Fund with the goal of comprehensively identifying and characterizing senescent cells across human lifespan in numerous organs. Mapping senescent cells in mice was added in 2022 to leverage genetic and pharmacologic approaches to drive, report, and ablate senescence in vivo. Senescent cells are implicated in driving most if not all age-related chronic diseases as well as accelerated aging caused by chronic diseases and their treatments (e.g., cancer chemotherapy). Furthermore, therapeutics specifically targeting senescent cells (senotherapeutics) have been developed and are currently being tested in clinical trials for multiple disease and geriatric endpoints. Thus, identifying and characterizing senescent cells throughout human aging and disease states has the potential to significantly impact human health. The SenNet Consortium is a highly collaborative network of scientists across the globe jointly benchmarking tools, technologies, and data analytic approaches, and aligning their activities with other NIH Common Fund initiatives, such as HuBMAP. While mapping senescent cells holds great biomedical promise, there are challenges such as the absence of specific biomarker to identify senescent cells, the appearance of senescence under physiological and pathological conditions, and little knowledge of what drives senescence in vivo. Analogous to the Human Genome Project, to achieve SenNet goals, innovative tools and technologies for single cell and spatial tissue analysis must be developed. Products of SenNet, in addition these new approaches, will include common nomenclature to define senescent cells, novel biomarkers of senescent cells, and publicly accessible databases and 4D atlases of senescent cells in 18 human and murine organs that enables 4D visualization.

Abstract citation ID: igad104.1555

**SINGLE-CELL AND SPATIAL OMICS FOR MAPPING CELLULAR SENESCENCE IN HEALTH, AGING AND DISEASE**

Rong Fan, *Yale University, New Haven, Connecticut, United States*

NIH SenNet consortium aims to dissect the heterogeneity of senescent cells (SnCs) and map their impact on the microenvironment at a single cell resolution and in the spatial tissue context, which requires the implementation of an array of omics technologies to comprehensively identify, characterize, and spatially profile SnCs across tissues in humans and mice. These technologies are broadly categorized into two groups –single cell omics and spatial mapping. To achieve single cell resolution and overcome the scarcity of SnCs, high-throughput single-cell and single-nucleus transcriptomic techniques have become a mainstay tool for surveying tens of thousands of cells to identify transcriptional signatures in rare cell populations, enabling discovery of potential new SnC biomarkers. Novel single cell mass spectrometry methods are developed for unbiased discovery of proteomic signatures of SnCs. A hallmark of SnCs is the senescence-associated secretory phenotype (SASP), which requires the use of proteomics, secretomics, metabolomics and lipidomics, especially SASP-associated extracellular vesicles, for comprehensive characterization of SAPS. High resolution molecular and cellular imaging of gene expression (e.g., MERFISH) or protein markers (e.g., CODEX) is critical for the study of SnCs in the large-scale tissue context. NGS-based spatial omics sequencing is poised to bridge the gap to realize both genome scale and cellular resolution in mapping SnCs in tissue. Novel technologies such as Seq-Scope and Pixel-Seq developed within SenNet further enabled subcellular resolution. SenNet investigators also developed spatially resolved epigenome and multi-omics sequencing techniques to link transcriptional or proteomic phenotype of SnCs to epigenetic mechanism. Further integration with high-resolution imaging makes spatial omics the crucial linchpin in connecting mechanistic underpinnings and molecular signatures with morphological features and spatial distribution. All these are critical for the construction of a map of SnCs and associated niches in the native tissue environment implicated in human health, aging, and disease, which is one of the main goals of the SenNet consortium.

Abstract citation ID: igad104.1556

**BUILDING A HUMAN REFERENCE ATLAS**

Andreas Bueckle, *Indiana University, Bloomington, Indiana, United States*

The Human Reference Atlas (HRA, https://humanatlas.io) is a comprehensive, high-resolution, three-dimensional atlas of all the cells in the healthy human body. The HRA provides standard terminologies and data structures for describing specimens, biological structures, and spatial positions linked to existing ontologies. In this talk, we will present a high-level overview of the major components of the HRA--including 67 anatomically correct 3D Reference Objects for 29 organs and 31 Anatomical Structure, Cell Types, and Biomarker (ASCT+B) Tables--and the tools to explore, use, author, and review the HRA--including the Registration User Interface, the Exploration User Interface, the ASCT+B Reporter, and the HRA Organ Gallery in virtual reality. We welcome experts and practitioners to join the monthly WG meetings (sign up at https://iu.co1.qualtrics.com/jfe/form/SV_bpaBhIr8XfdiNRH), to explore and contribute to this effort, and to provide feedback on the evolving HRA from diverse perspectives.

### SESSION 4205 (SYMPOSIUM)

Abstract citation ID: igad104.1557

**FINDINGS FROM NSHAP: SOCIAL CONNECTEDNESS, HEALTH INDICATORS, MEDICATION EFFECTS, AND PREDICTING MORTALITY**

Chair: Lissette Piedra Discussant: Amelia Karraker

The National Social Life, Health, and Aging Project's broad range of both social measures, and objective and self-reported health measures enable detailed analysis of the intersections between these fundamental aspects of older adults' lives. The papers in this symposium explore various aspects of these topics from different angles. The first explores employment as an important form of social participation, establishing that full-time employment among respondents is associated with better cognitive function, and less ADL and IADL difficulties. The second examines how social isolation affects men and women differently. Social Networks are the focus of the third paper and compare family to friendship ties. Using NSHAP's unique medication log, Wilder examines sleep disturbances and the prevalence of respondents taking medications with somnolence as an adverse event, demonstrating the need for more research into how this might affect older adults' health and well-being. Li uses NSHAP data to develop machine learning models to predict 10-year mortality of older adults in the US which perform with better accuracy than logistic regression.

Abstract citation ID: igad104.1558

**EMPLOYMENT AS A FORM OF SOCIAL PARTICIPATION AMONG OLDER ADULTS: LINKS TO COGNITIVE AND FUNCTIONAL HEALTH**

Peilin Yang[1], Linda Waite[2], and Ashwin Kotwal[3], *1. University of Michigan Ann Arbor, Ann Arbor, Michigan, United States*, *2. University of Chicago, Chicago, Illinois, United States*, *3. University of California San Francisco, San Francisco, California, United States*

Within the active aging literature, studies on social participation and health concur that people who are better socially integrated and engage in social activities tend to have better physical, mental, and cognitive health. This study revisits the literature by aiming to address three primary knowledge gaps in prior literature: 1) we explicitly examine the change within the 5-year-interval in cognition, activities of daily living (ADL), instrumental activities of daily living (IADL) associated with social participation five years prior; 2) we examine diversity in participation's association with not only cognitive function, but also ADL and IADL, which we lack knowledge of; 3) we conceptualize employment in later life as a kind of social participation, a part of older adults' lives that is overlooked in the social participation literature. We also examine whether the relationship between social participation and cognition, ADL, and IADL is the same for men and women, and for those employed and those not employed. The study finds that neighborhood participation at a high level indicates worse cognitive, ADL, and IADL outcomes 5 years later, and a higher level of neighborhood participation is more indicative of worse cognitive outcomes for men than for women. Full-time employment predicts better cognitive, ADL, and IADL outcomes 5 years later. We also find evidence that full-time work creates a stronger buffer against cognitive decline, developing ADL difficulties, and IADL difficulties, even among older adults who socialize with family and friends, participate in the community, and the neighborhood at a high level, respectively.

Abstract citation ID: igad104.1559

### THE RELATIONSHIP OF SOCIAL ISOLATION TO SELF-NEGLECT AMONG OLDER ADULTS: RESULTS OF A NATIONAL SURVEY

Ashwin Kotwal[1], Rebecca Mullen[2], Irena Cenzer[1], Jason Burnett[3], Carla Perissinotto[1], and Louise Hawkley[4], *1. University of California San Francisco, San Francisco, California, United States*, *2. University of Colorado, Aurora, Colorado, United States*, *3. University of Texas Health Science Center at Houston, Houston, Texas, United States*, *4. NORC at the University of Chicago, Chicago, Illinois, United States*

Self-neglect among older adults is characterized by inattention to hygiene and one's immediate living conditions, and may reflect unmet needs from social relationships. We therefore determined if social isolation was associated with self-neglect and how the association differed by gender. We used data from the National Social Life, Health, and Aging Project (NSHAP) Wave 3 (2015), a nationally-representative survey of 3,677 community-dwelling older adults. Social isolation was determined using a 12-item scale assessing household contacts, social network interaction, and community engagement. Self-neglect was assessed in-person and included 1) body neglect (lowest quintile of bodily self-presentation related to clothes and hygiene) and 2) household neglect (lowest quintile of household building condition, cleanliness, odor and clutter). Logistic regression was used to determine the adjusted probability of self-neglect by social isolation, and interaction terms with gender. Results indicated the association between social isolation and self-neglect differed by gender (p-values for interaction: body neglect: 0.02, household neglect: 0.20). Among women, social isolation was associated with a higher risk of body neglect (social isolation: 26% vs no isolation: 14%, p=0.001) and household neglect (23% vs 17%, p=0.05). For men, social isolation was not associated with body neglect (27% vs 23% p=0.2) or household neglect (23% vs 22%, p=0.8). In summary, social isolation was associated with body and household neglect among women, but was not associated with neglect among men. Future work should investigate mechanisms for gender differences and interventions to address or prevent self-neglect through enhancing social connectedness.

Abstract citation ID: igad104.1560

### LOCAL FAMILY AND FRIEND TIES AND THEIR RELATIONSHIP TO SOCIAL SUPPORT AND STRAIN AMONG OLDER ADULTS

Won Choi, *University of Chicago, Chicago, Illinois, United States*

Family members and friends who live nearby are likely valuable sources of support for older adults. At the same time, local family and friend ties may also be a source of strain as spatial proximity to close ties can generate more intense interactions. Using data from Round 3 (2015-2016) of the National Social Life, Health, and Aging Project (NSHAP) (N=3,615), this study examines how local family and friend ties reported in older adults' social network roster are associated with instrumental and emotional support and social strain among community-dwelling older adults aged 50 and older. Results from ordered logistic regression models show that having a local friend tie is associated with higher levels of instrumental and emotional support from friends and lower levels of instrumental and emotional support from family. Having a local family tie, on the other hand, is associated with higher levels of instrumental support from family and lower levels of emotional support from friends. Having a local family tie is not related to emotional support from family or instrumental support from friends. Results also indicate that having a local friend tie increases the odds of reporting that friends make too many demands (i.e., higher friend strain) whereas having a local family tie is not a predictor of family strain. Together, results suggest that spatial proximity to friends and, to a lesser degree, family members are linked to how older adults experience social support and strain.

Abstract citation ID: igad104.1561

### USE OF PRESCRIPTION MEDICATIONS WITH SOMNOLENCE AS A POTENTIAL ADVERSE EFFECT AMONG OLDER ADULTS IN THE UNITED STATES

Jocelyn Wilder, *NORC, Chicago, Illinois, United States*

Over half of community-dwelling older adults experience sleep disorders, with approximately 40% reporting somnolence or/and excessive daytime sleepiness, associated with an increased risk for cognitive impairment and premature mortality. The use and concurrent use of prescription medication with somnolence as an adverse effect may be an overlooked contributor to this growing problem. This study aims to characterize the use or concurrent use of medications with somnolence as an adverse effect and to assess associations between their use and the prevalence of somnolence (excessive daytime sleepiness or sleep duration of ≥9 hours) using data from the National Social Life, Health, and Aging Project (NSHAP) 2010-2011 (N=3,338). Adjusted prevalence was estimated from multivariable logistic regression models adjusted for socio-demographic measures and the number of medications without somnolence as an adverse effect. The estimated prevalence of somnolence was 54% for those reporting use of 3 or more medications with somnolence as an adverse effect vs. 36.5% for those not using such medications. The use of prescription medications with somnolence as a potential adverse effect was prevalent. Concurrent use of medications with somnolence as an adverse effect was associated with a higher prevalence of somnolence. These findings demonstrate the need for more research to understand the impact of concurrent use of medications with similar adverse side effects on OAs' health and well-being.

Abstract citation ID: igad104.1562

### PREDICTING 10 YEAR MORTALITY WITH MACHINE LEARNING: EVIDENCE FROM NATIONAL SOCIAL LIFE, HEALTH, AND AGING PROJECT

Yiang Li, *University of Chicago, Chicago, Illinois, United States*

The current academic literature extensively used linear logistic models with social, behavioral, and psychological status to predict mortality. However, few address the interdependency of predictors and the imbalance of targets that adversely bias the results. Using the National Social Life Health and Aging Project (NSHAP), we developed two machine learning models predicting the 10-year mortality of older adults in the US. We first used tree-based algorithms of Decision Tree (DT) that account for the interdependency of the social features and decide the splitting nodes and thresholds using entropy gain conditional on the previous splitting predictor to discern disposition status. Second, we used the Fuzzy Support Vector Machine (FSVM) that regards every sample as a node in high-dimensional vector space and splits the nodes with an optimum plane by finding the best linear combination of features to get an optimum prediction accuracy. Additionally, FSVM addresses the target imbalance problem by conducting a more delicate classification of samples with close predicted probabilities of being alive and deceased. Compared to the accuracy rates achieved by the Logistic Regression, our algorithms perform better on the entire population and the population near the class boundary. We also discussed the social and demographic characteristics of the cases whose disposition statuses were either wrongly predicted as deceased or alive by our algorithms. The findings serve important purposes for public health practitioners in accurately understanding the risk and protective factors of mortality in aging.

### SESSION 4210 (SYMPOSIUM)

Abstract citation ID: igad104.1563

### EFFECTS OF DISCLOSING DIAGNOSES AND BIOMARKER RESULTS FOR ALZHEIMER'S DISEASE AND RELATED DISORDERS

Chair: Takashi Amano Discussant: Brian Carpenter

Early diagnosis of mild cognitive impairment (MCI) and Alzheimer's disease and related dementias (ADRD) has been recognized as a key strategy to improve health-related outcomes. However, given that these conditions are highly stigmatized, receiving a diagnostic label or knowing about biomarkers related to MCI or ADRD may have a profound impact on the person's life. As the impacts of disclosure of diagnostic information related to MCI and ADRD on the well-being of people living with these conditions are not well understood, this symposium attempts to address this knowledge gap. The first presenter will describe a study that examined racial/ethnic variations in the effects of diagnostic labeling of ADRD on social aspects of the person's life. The second presenter will discuss findings from a systematic review of the effects of ADRD and cognitive impairment on social engagement. The third presenter will present the findings of the study on the effect of amyloid PET results disclosure on health-related behaviors of people with MCI. The fourth presenter will describe the results of an online vignette study on the outcomes of a preclinical Alzheimer disease diagnosis. The fifth presenter will discuss the use of blood biomarkers to aid in the diagnosis of ADRD in primary care (PC). At the end of the presentation, the discussant will highlight implications for future research and policy development to alleviate negative impacts and maximize the positive impacts of a diagnostic label of MCI and ADRD.

Abstract citation ID: igad104.1564

### RACIAL AND ETHNIC VARIATIONS IN THE EFFECTS OF KNOWING ABOUT ONE'S DIAGNOSTIC LABEL OF DEMENTIA ON SOCIAL SUPPORT

Takashi Amano[1], Cal Halvorsen[2], Seoyoun Kim[3], Addam Reynolds[4], Clara Scher[5], and Yuane Jia[1], *1. Rutgers University, Newark, New Jersey, United States*, *2. Boston College, Boston, Massachusetts, United States*, *3. Texas State University, San Marcos, Texas, United States*, *4. University of Southern California, Los Angeles, California, United States*, *5. Rutgers, The State University of New Jersey, New Brunswick, New Jersey, United States*

Theoretically, knowing about one's diagnostic label of dementia may increase a chance to receive necessary social support. However, empirical evidence especially regarding the racial/ethnic variations in the effects of diagnostic labeling on provision of social support is lacking. Data from the Health and Retirement Study (HRS, 2000-2018) were utilized to examine variations in the effects of knowing about a diagnostic labeling. A total of 7,192 person-year observations who had dementia were included in the analysis. Knowledge about a diagnostic label of dementia was measured by asking whether a doctor told the person that he/she had dementia. Formal and informal types of received social support were measured. Regression analyses with inverse probability weighting were performed. A moderating role of race/ethnicity was examined by conducting subgroup analyses. Knowing about a diagnostic label significantly increased likelihood of having more informal (OR = 1.58, $p < 0.001$, 95% CI = [1.34, 1.86]) and formal support (b = 0.09, p < 0.001, 95% CI = [0.05, 0.13]). The significant effect of a diagnostic label on formal support was not observed among non-Hispanic Black (b = 0.07, p = 0.086, 95% CI = [-0.01, 0.15]) or Hispanic (b = 0.07, p = 0.152, 95% CI = [-0.03, 0.16]) individuals. Results suggest that knowing about a diagnostic label of ADRD may increase informal and formal social support. However, the beneficial impacts may not be salient for non-Hispanic Black and Hispanic individuals. Strategies to maximize the beneficial effects of diagnostic labeling should be identified to address racial/ethnic disparities.

Abstract citation ID: igad104.1565

### INFLUENCES OF HAVING COGNITIVE IMPAIRMENT OR DEMENTIA ON SOCIAL ENGAGEMENT AMONG OLDER ADULTS: A SYSTEMATIC REVIEW

Clara Scher[1], Takashi Amano[2], Addam Reynolds[3], and Yuane Jia[2], *1. Rutgers, The State University of New Jersey, New Brunswick, New Jersey, United States*, *2. Rutgers University, Newark, New Jersey, United States*, *3. University of Southern California, Los Angeles, California, United States*

Studies have suggested that promoting and maintaining social engagement are positively associated with various well-being outcomes in later life. One notable barrier to sustaining social engagement in later life is development of cognitive impairment (CI) and/or Alzheimer's disease and related dementias (ADRD). This study aims to synthesize existing literature exploring the impact of CI and ADRD on social engagement among older adults. We conducted a search of studies written in English language in the following databases: PsychInfo, PubMed, MEDLINE, CINAHL, and The Cochrane Library. Inclusion criteria included: observational studies that were conducted outside of residential long-term care facilities, included social engagement as an outcome variable, and examined the impact of cognitive impairment and/or ADRD on social engagement. Eighteen studies were included for qualitative synthesis. The majority of studies used author-developed scales to measure social engagement. Three utilized formal clinical diagnosis to measure cognitive impairment and ADRD. Twenty unique social activities were included, such as volunteering, attending community groups, and religious services. Four primary themes emerged regarding the relationship between CI/ADRD and social engagement: decreased levels of social engagement, reduced variety of social activities, lowered motivation, and heterogeneity in social engagement. Results suggest that people with CI/ADRD have reduced social engagement, but the evidence is limited due to the inconsistent use of measures for social engagement and CI/ADRD. Future research is needed to develop valid and reliable measures of social engagement that can be used among individuals living with CI/ADRD.

Abstract citation ID: igad104.1566

### EFFECT OF AMYLOID PET RESULTS DISCLOSURE ON HEALTH-RELATED BEHAVIORS IN PERSONS WITH MILD COGNITIVE IMPAIRMENT

Yan Wang[1], Dianxu Ren[2], Scott Roberts[3], and Jennifer Lingler[2], *1. School of Nursing University of Pittsburgh, Pittsburgh, Pennsylvania, United States*, *2. University of Pittsburgh, Pittsburgh, Pennsylvania, United States*, *3. University of Michigan, Ann Arbor, Michigan, United States*

Mounting evidence supports the clinical utility of amyloid PET. However, whether patients use knowledge of their amyloid status to alter health behaviors remains unclear. We describe health-related actions taken by Mild Cognitive Impairment patients following amyloid PET disclosure (n=34) vs. a comparison group (n=37). Patients were 92% non-Hispanic white, 59% male, 73±8.61 years old with 16.25±2.49 years of education. Over 12 months of follow-up, all participants reported at least one behavior change from baseline on a 14-item health behavior questionnaire. Amyloid positive patients reported the most behavior changes (mean=4.67±1.83) while amyloid negative patients reported the fewest (mean=3.72±1.58). Across groups, no significant differences were observed in: lifestyle, vitamin/supplement use, stress reduction activities, cognitive stimulation, or advance directive completion. Amyloid negative participants were significantly less likely than controls to consider long-term care insurance (63.6% vs. 89.2%; P=.025), and to endorse changes classified as "other" (36.4% vs. 64.9%; P=0.037). After adjusting for education level, gender, and MMSE, logistic regression showed that amyloid negative patients were 74% less likely than controls to report "other" changes (OR=0.26, 95% CI [0.08, 0.85], P=0.025), and 78% less likely to consider long-term care insurance (OR=0.22, 95% CI [0.06, 0.86], P=0.03). Qualitative analysis of supplemental interviews with scan group participants revealed "other" activities to include changes in employment, driving, and residential status, and engagement in other non-medical activities (pursuing bucket lists and mending strained relationships). Health-related behavior changes following amyloid PET disclosure may differ by scan result and encompass actions to enhance not only cognition but quality of life.

Abstract citation ID: igad104.1567

### AN ONLINE VIGNETTE STUDY TO EXAMINE THE OUTCOMES OF A PRECLINICAL ALZHEIMER'S DISEASE DIAGNOSIS

Matthew Picchiello[1], Matthew Wynn[2], and Brian Carpenter[1], *1. Washington University in St. Louis, St. Louis, Missouri, United States*, *2. University of California: San Francisco Memory & Aging Center, San Francisco, California, United States*

Preclinical Alzheimer disease (AD) is characterized by abnormal levels of amyloid beta and tau proteins in the brain of a cognitively normal person. There are potential risks and benefits of communicating biological marker (biomarker) risk information for AD using the preclinical AD label. The current study used a vignette methodology to evaluate older adults' reaction to their hypothetical risk for developing AD. Community dwelling cognitively intact older adults (n = 300, M age = 64.1) completed an online survey, randomized to disclose biomarker results and risk information (with or without the preclinical disease label) pertaining either to heart disease or AD. Participants reported on their perceptions of risk and behavioral intentions based on the Health Belief Model. A series of ANCOVAs and moderated mediation models support the idea that, across disease conditions, a preclinical label does not influence perception of the disease or behavioral intentions. Both groups indicated a similar and proactive intention to engage in more health-promoting behaviors and plan for the future. Results also highlight differences in individual perceptions of AD versus heart disease such that participants in the AD condition perceived their risk information as implying a more severe condition, perceived fewer benefits to knowing their risk, and reported lower self-efficacy about doing anything to address that risk. Despite these perceptions, older adults who received risk information for AD maintained interest in undertaking behavioral changes that may improve their quality of life. These findings have implications for the development of empirically supported disclosure processes for preclinical AD.

Abstract citation ID: igad104.1568

### FEASIBILITY AND ACCEPTABILITY OF USING PLASMA BIOMARKERS FOR DIAGNOSING ALZHEIMER'S DISEASE IN PRIMARY CARE

Nicole Fowler[1], Kristen Swartzell[2], Dustin Hammers[3], Jared Brosch[3], James Murray[4], and Deanna Willis[1], *1. Indiana University School of Medicine, Indianapolis, Indiana, United States*, *2. Indiana University School of Medicine, IU Center for Aging Research, Indianapolis, Indiana, United States*, *3. Indiana University, Indianapolis, Indiana, United States*, *4. Davos Alzheimer's Collaborative, Wayne, Pennsylvania, United States*

Blood-based biomarkers (Aβ, P-tau, neurofilament light) are clinically available to aid in the diagnosis of Alzheimer's disease and related dementias (ADRD). However, no research has examined the use of blood biomarkers to aid in the diagnosis of ADRD in primary care (PC). Our study will test feasibility and acceptability of implementing blood-based biomarkers for ADRD in PC. Participants include: all PC patients ≥65 years presenting to one of six PC clinics between 6/1/22 and 5/31/23 who score cognitively impaired on the Linus Health Digital Clock and Recall (DCR™), and PC providers (PCPs) of these patients. Patients will view a decision guide about biomarkers and complete the Concerns about Alzheimer's Disease Dementia Scale, and the Future Time Perspective Scale and the Impact of Events Scale. These measures and the PHQ-9 and GAD-7 will be repeated within 2 weeks of results disclosure. PCPs will receive training on biomarker disclosure techniques and best practices. To date 9 PCPs have consented to provide the biomarker results disclosure and 11 have declined. Following completion of PCP consent (n=100), a total of 200 patients who failed the DCR are eligible to be approached for consent. By November 2023, we anticipate that 150 patients will have completed biomarker testing, and we will have examined the biomarker results in the context of patient neuropsychological and clinical data, comorbidities, and sociodemographic information. This study will provide information regarding feasibility and utility of ADRD biomarkers in PC and a preliminary analysis of biomarker results compared with the patients' clinical profiles.

### SESSION 4215 (SYMPOSIUM)

Abstract citation ID: igad104.1569

### FINDING COMMON GROUND: IMPROVING RESEARCHER-PRACTITIONER COLLABORATION IN ADULT MALTREATMENT RESEARCH

Chair: Marguerite DeLiema Discussant: Kathleen Wilber

The work of adult protection is inherently multidisciplinary–involving health care professions, social services agencies, law enforcement, criminal and civil justice systems, and the broader aging services network. Researchers seeking to develop and evaluate screening tools and interventions for elder abuse and neglect must cultivate partnerships with state and local agencies that are responsible for adult protection. In this symposium, early career researchers will share the unique challenges they have faced when collaborating with adult protective services (APS), healthcare organizations, and other state agencies on research to improve adult maltreatment outcomes. First, Reynolds and colleagues will summarize three collaborative projects involving a large healthcare system in Texas and APS agencies in Oklahoma. They will share how the research team adapted protocols to pandemic-related disruptions to data collection, and how they created new systems to standardize documentation. Liu interviewed state agencies in Montana and analyzed transcripts using thematic analysis. She will share the challenges and strategies that surfaced and relate to improving state-level collaboration in addressing the needs of mistreatment victims and survivors. DeLiema will present on planning for and navigating administrative barriers to cross-sector collaborations, including contracts and legal agreements that are part and parcel to university-government research partnerships. Last, Burnes will introduce the RISE project and share how he and his colleagues managed expectations to co-create a new outcomes measurement tool with adult protection workers. Reaching consensus necessitated honesty, openness, humor, and patience, but ultimately strengthened the collaboration to yield a more valid outcomes measurement assessment.

Abstract citation ID: igad104.1570

### ENHANCING COLLABORATION FROM ADULT PROTECTIVE SERVICES TO PROVIDERS WORKING WITH MISTREATMENT VICTIMS/SURVIVORS

Pi-Ju Liu, Wei- Lin Xue, and Zachary Hass, *Purdue University, West Lafayette, Indiana, United States*

Adult Protective Services (APS) is the frontline agency that receives, investigates, and provides services and/or referrals to victims/survivors of adult abuse, neglect, and exploitation. However, APS cannot resolve adult mistreatment alone, and must collaborate with healthcare, social, legal, and other community-based providers to address the needs of victims/survivors. Partnering with Montana APS and healthcare, legal, social and aging services providers, AARP, American Indian Health, local law enforcement, judge, attorneys, Medicaid Fraud Control Unit, ombudsman, and Certification and Licensing Bureaus, our goal is to examine how state agencies can work together to reduce mistreatment and systems burden. Twenty-two interviews were conducted with state agencies to inquire about collaboration challenges and strategies, what has worked well with APS, and what can be improved. Thematic analysis was used to characterize barriers and solutions for collaboration, and social network analysis was used to map out the collaboration network.

We found that information sharing is a boon for collaboration among agencies using integrated information systems, but becomes a barrier when information sharing is limited by statute. State agencies also identified other agencies they have worked with to address mistreatment and reflected on challenges and strategies in working with these other professionals. Challenges for state agencies to work together center around lack of funding and service shortages, especially during the pandemic. Strategies to improve collaboration involve agency cross-training to remove silos. These interviews offer rich insight to guide practitioners across agencies to work together at the state-level to reduce mistreatment and its recurrence.

Abstract citation ID: igad104.1571

### A RESEARCHER–PRACTITIONER COLLABORATION ON MEASURING SUCCESS IN ELDER MISTREATMENT INTERVENTION

David Burnes[1], Patricia Kimball[2], Polly Cox[2], Kathryn Harnish[2], Carol Ayoob[2], M.T. Connolly[3], Geoff Rogers[4], and Stuart Lewis[5], *1. University of Toronto, Toronto, Ontario, Canada*, *2. Elder Abuse Institute of Maine, Brunswick, Maine, United States*, *3. University of Southern California, Los Angeles, California, United States*, *4. City University of New York, New York City, New York, United States*, *5. Dartmouth College, Lebanon, New Hampshire, United States*

One of the major barriers to conducting elder mistreatment intervention research is a lack of understanding on how to measure outcomes of success. Within the context of the RISE project, this presentation will describe a highly collaborative process between researchers and RISE practitioners (supervisor and advocates) to develop a measurement instrument that meets validated research standards as well as the needs of practitioners who work directly on elder mistreatment cases and are responsible for administering the tool. Informed by ecological-systems, relational, and client-centered perspectives, RISE is a conceptually driven, evidence-based, community-based elder mistreatment response program that works with older adults at risk of or experiencing EM, as well as their alleged harmers, their relationship, and their surrounding social support systems. This presentation will describe the process of developing an appropriate measurement tool through weekly meetings over a 6-month period, from a starting point characterized by widely discrepant instrument expectations across the researcher-practitioner dyad toward an ending that achieved mutual agreement. This process required high levels of honesty, openness, patience, and humor among members. The final version of the measurement tool will be presented, which measures intervention outcome constructs of older adult social support, self-efficacy, life satisfaction, and perceived stress, as well as practitioner-client working alliance.

Abstract citation ID: igad104.1572

### PARTNERING TO ADDRESS ADULT MALTREATMENT: HARD-EARNED LESSONS AND WISDOM GAINED BY APPLIED RESEARCHERS

Courtney Reynolds, Samantha Tuft, and Farida Ejaz, *Benjamin Rose Institute on Aging, Cleveland, Ohio, United States*

We focus on three projects in which applied researchers collaborated with community-based and government partners to address adult maltreatment. In our first study with Texas, we partnered with a large healthcare system and Adult Protective Services (APS). Two APS workers served as consultants to clinicians and 529 patients were reported to APS. Researchers struggled with quantifying case notes on the consultations, pointing to the importance of creating a systematic method to document case notes for future studies. In the second grant, the same partners evaluated a case management intervention to prevent self-neglect among at-risk, adult primary care patients. A total of 477 randomly selected participants completed a baseline interview. Lessons learned included revising language in how to discuss self-neglect, particularly in consent forms and becoming more flexible in where to conduct interviews. Other challenges encountered included the time and effort it took to develop data use agreements that allowed us to access APS administrative data. Our third study with Oklahoma APS was conducted in the midst of a pandemic, requiring us to change study protocols from home to telephone-based interviews. Despite these challenges, we interviewed 188 clients over the phone. Drawing on the experiences across all three projects, we will discuss challenges – from contracting to IT issues, to recruitment and data collection – and how each one has presented an opportunity for growth and wisdom that can be applied to future research activities.

Abstract citation ID: igad104.1573

### SO MANY STAKEHOLDERS, SO MANY CONTRACTS!

Marguerite DeLiema, *University of Minnesota, Twin Cities, Saint Paul, Minnesota, United States*

Elder financial fraud is a growing challenge for adult protective services (APS), financial institutions, and state and federal law enforcement agencies that seek effective interventions to protect older adults from continued financial loss. One solution is temporary account holds that prevent suspicious withdrawals from an older or vulnerable adult's account while investigation and additional interventions are put in place. With funding from the Administration for Community Living, this project seeks to understand the outcomes of temporary account holds on elder safety and financial autonomy in Minnesota. We first worked to improve communication between APS and other Minnesota state agencies to ensure that cases are properly referred and that protection activities are coordinated to achieve the best outcomes for the older adult. Like most collaborative research projects that involve university researchers and state agencies, significant legal agreements were necessary. Multiple subawards and cost share agreements were drafted, data use agreements were created, and IRB approvals from multiple entities were sought. In this presentation, Dr. DeLiema will share lessons learned about how researchers can better prepare for the administrative burden and delayed starts associated with completing these legal administrative processes. She will describe common areas of conflict and competing interests between state agencies and universities around data privacy and ownership, strategies for finding common ground, and possible workarounds to fulfill project aims. Dr. DeLiema will also highlight how to "pitch" a collaboration to community stakeholders using a value proposition that outlines the benefits of the project to the stakeholder.

### SESSION 4220 (SYMPOSIUM)

Abstract citation ID: igad104.1574

### OLDER ADULTS AND CAREGIVERS AS PARTNERS IN PATIENT- AND FAMILY-CENTERED EDUCATION

Chair: Jennifer Severance Co-Chair: Diana Cervantes

Patient and family-centered education is an approach to the planning, delivery, and evaluation of curricula that is grounded in mutually beneficial partnerships among patients, families, students, and educators. Engaging patients and families in genuine partnerships through health professions education can broaden the student's perspectives of health to include social, economic, and environmental factors while helping them recognize unique individual dimensions, such as cultural context, capacities, and values, that influence health. While patient-centeredness is a widely recognized component to high-quality healthcare, partnering with older adult patients and caregivers is a less recognized standard in health professions education. This symposium examines the role of older adults and caregivers in co-creating geriatric and gerontology education with a HRSA-funded Geriatric Workforce Enhancement Program (GWEP) to promote the knowledge, skills, and attitudes necessary for patient-centered clinical practice. First, presenters will share educational initiatives involving older adults as mentors and subject matter experts for health professions students and student teams. This is followed by projects engaging and empowering older adults and caregivers as advocates for quality care and advisors for educational improvements. Finally, presenters will explore evaluation tools and strategies to collect input and ensure the patient's voice remains at the center of education planning, delivery, and improvement. Presenters discuss methods of adapting these models to a local context as well as strategies and opportunities to incorporate the input and feedback from older adults and caregivers into health professions education.

Abstract citation ID: igad104.1575

### COMMUNITY ADVISORY COUNCILS: AN ENGAGEMENT STRATEGY FOR PATIENT-CENTERED LEARNING

Jennifer Severance, *University of North Texas Health Science Center, Fort Worth, Texas, United States*

Advisory councils provide a forum for patients and families to partner with health care systems and share experiences, perspectives, ideas, feedback, and recommendations that can improve both the patient experience and health care outcomes. Research shows that patient partnerships fostered through advisory councils can also positively impact health professions education and training by supporting patient-centered learning. With a goal to promote collaboration among community stakeholders in the delivery of geriatrics education and training, a Geriatric Workforce Enhancement Program formed a Community Advisory Council. A project team developed the council goals, structure, application and recruitment processes, and facilitation methods. Over two years, a total of 27 older adults, caregivers and community advocates participated in the advisory council both online and in-person. Council members completed an orientation session and met four times over a twelve-month period. At each meeting, faculty prepared two to four open-ended discussion questions regarding knowledge, skills, and attitudes relevant to the nursing home workforce, student education, continuing education, and community outreach. Responses were collected through paper surveys and online forms. Thematic analysis of qualitative responses identified common themes that were shared with education and clinical leadership to inform and improve learning experiences, health care processes, and council engagement. Presenters will describe the council's structure and composition as well as recruitment, facilitation, and evaluation methods, and lessons learned. This project demonstrates a replicable model of evaluating education that involves older adults, families and community in co-creating quality learning and patient experiences.

Abstract citation ID: igad104.1576

### OLDER ADULT VOLUNTEERS AS MENTORS IN GERIATRIC EDUCATION

Janice Knebl, *UNTHSC, Fort Worth, Texas, United States*

Health professions education that involves older adults has been shown to be effective in improving students' clinical skills and attitudes towards older adults. With the goal of introducing healthcare students to geriatric care through first-hand experiences, the "Seniors Assisting in Geriatrics Education (SAGE)" Program involves older adult volunteers age 65+ in virtual person-centered health promotion visits with teams of three to four students. Through an application process, mentors are selected and receive orientation materials before visits with their assigned team. Students represent eight disciplines: dietetics, medicine, nursing, pharmacy, physical therapy, physician assistant studies, social work, and speech language pathology. Students practice basic clinical skills and screening techniques while working as a team and receiving feedback and input from their mentor. In 2022, we surveyed older adult volunteers to evaluate their mentor experience using open-ended and Likert style questions on a scale from strongly agree to strongly disagree. Survey responses (n=99) show that most mentors agreed students collaborated well (94%), health promotion topics for each visit were beneficial (83% to 85%), and they were satisfied with their SAGE experience (96%). Mentors identified opportunities for improvement related to student and mentor preparation, in-person versus online visits, and establishing rapport between mentors and teams. Results were used to enhance mentor training and engagement and to create community partnerships for recruitment and mentor diversity. By prioritizing recruitment of and training for volunteers, older adults can actively and effectively participate in meaningful educational experiences with health professions students.

Abstract citation ID: igad104.1577

### ADVANCING NURSING STUDENTS' DEMENTIA KNOWLEDGE THROUGH ENGAGEMENT WITH PEOPLE LIVING WITH DEMENTIA

Michelle Kimzey, *Texas Christian University (TCU), Fort Worth, Texas, United States*

The numbers of people living with dementia are overwhelming. Dementia education is important to prepare nursing students to care for this population. To better understand the reality of dementia, we incorporated an expert by experience in teaching a pilot course on dementia. This training was offered to senior nursing students as an elective in their final semester of undergraduate work. Our goal was to teach students about dementia, what it's like to live with dementia, and actions they can take to support people living with dementia in nursing homes and other settings. The course incorporated clinical experiences at a community memory care facility and small group discussions co-led by a person with dementia and a nursing instructor. Students also participated in an adult day program by leading activities and interacting with people with dementia. Eleven students participated in the pilot course. At program completion, students were invited to share their experiences in a focus group. Qualitative analysis of student feedback identified the benefits of the pilot, including reduced stigma of dementia, increased empathy of those living with dementia, and improved skills in explaining the impact on those diagnosed with dementia. Students appreciated the clinical experiences because it allowed them opportunities to apply their knowledge of dementia and experience the individuality of dementia. This pilot empowered nursing students during their final semester to appreciate, provide care, and support the needs of the people living with dementia.

Abstract citation ID: igad104.1578

### EVALUATING PROGRAMS WITH OLDER ADULTS AND CAREGIVERS AS PARTNERS IN PATIENT EDUCATION

Sara Murphy, *University of North Texas Health Science Center, Fort Worth, Texas, United States*

The Geriatrics Workforce Enhancement Program at the University of North Texas Health Science Center has several programs where patients, older adults, caregivers and healthcare stakeholders are incorporated directly into the educational experience or provide feedback to inform the design of educational programs. In addition to the program design, there are evaluation considerations related to incorporating these important stakeholders to best capture the impact of this group. The goal of evaluation research has become more and more important in program design, especially with interdisciplinary programs and programs involving multiple stakeholder groups. Evaluation research is the assessment of outcomes and information that will inform future decision making. These stakeholders are important partners and have an important impact on program design and research outcomes, which is something that should be measured along the way. The University of North Texas Health Science Center Community Advisory Council, Infection Control Advocate and Resident Education (ICARE), Dementia Education, and Seniors Assisting in Geriatric Education are programs that will be included in this evaluation discussion. For these projects, we included evaluation components to keep the patient's voice at the center of education planning, delivery, and improvement. This presentation will discuss the evaluation of impact in these programs, discuss barriers to measurement, and report early results. One key finding is the lack of systematic measurement tools available and the need for additional tools that can be used across settings where patients are part of the education program design.

Abstract citation ID: igad104.1579

### INFECTION CONTROL ADVOCATE AND RESIDENT EDUCATION (ICARE): PERSON-CENTERED INFECTION PREVENTION FOR QUALITY CARE

Diana Cervantes, *University of North Texas Health Science Center, Fort Worth, Texas, United States*

Applying infection prevention and control (IPC) measures while ensuring person-centered care (PCC) remains a major challenge in nursing homes. PCC focuses on resident rights to engage and empower nursing community residents, families, advocates, and staff to deliver quality care. To address this gap, The Infection Control Advocate and Resident Education (ICARE) program was developed for nursing home residents and families. ICARE is a step-based program which begins with educating participants on IPC and resident rights through seven web-based modules. The program encourages support and collaboration of nursing home advocate organizations including the long-term care ombudsman program, quality care organizations, and public health departments to assist residents and families in becoming more engaged and empowered in quality care beginning with infection prevention and control. Utilizing ICARE program materials, participants progress to engagement by learning how quality care is provided in their nursing home community, with the goal of empowering residents to actively participate in quality care improvement through quality improvement projects. The ICARE Program assists nursing home advocate organizations to identify opportunities to incorporate elements of infection control, quality care, and resident rights in current procedures and activities. The overall program design and theory of the ICARE program will be presented to provide a foundation for the creation of similar programs.

### SESSION 4225 (SYMPOSIUM)

Abstract citation ID: igad104.1580

### THE ROLE OF CONTEXTUAL FACTORS IN ADULT DEVELOPMENT AND AGING

Chair: Gizem Hueluer Co-Chair: Takeshi Nakagawa
Discussant: Hans-Werner Wahl

Individual development is embedded in multiple environmental systems, ranging from broader distal cultural and historical contexts to more proximal residential, family, and partnership contexts. The goal of this symposium is to showcase research studying the role of distal and proximal contextual factors in the development and aging of key domains, including health, cognitive function, social relationships, and emotional experiences. Nakagawa et al. examine cultural differences in the associations of positive affect and loneliness with longevity using data from prospective surveys in the United States (1986–2005) and Japan (1987–2006). Hülür et al. use 35-year longitudinal data (1977-2012) from 3,482 participants of the Seattle Longitudinal Study to examine associations between perceived residential neighborhood characteristics and trajectories of cognitive aging. Kobayashi et al. study cohort and gender differences in trajectories of social contact and social participation and the effects of being married and living with a child using data from the National Survey of the Japanese Elderly (1987-2021). Meier et al. will present data from a study on emotional co-regulation in younger and older couples' daily lives focusing on situational factors (e.g., psychological availability) within romantic relationships that can shape daily emotional experiences and relationship quality. The discussion by Hans-Werner Wahl will focus on how these approaches contribute to our understanding of processes of context dynamics and how they can be utilized to promote the maintenance of health and function in aging.

Abstract citation ID: igad104.1581

### DO POSITIVE AFFECT AND LONELINESS PREDICT LONGEVITY ACROSS CULTURES? FINDINGS FROM THE UNITED STATES AND JAPAN

Takeshi Nakagawa[1], Gizem Hueluer[2], and Andrea Horn[3], *1. National Center for Geriatrics and Gerontology, Obu, Aichi, Japan*, *2. University of Bonn, Bonn, Nordrhein-Westfalen, Germany*, *3. University of Zurich, Zurich, Zurich, Switzerland*

Prior studies have revealed the beneficial effects of positive affect and the harmful effects of negative affect on health and longevity. The majority of the evidence comes from Western countries, but cross-cultural studies suggest that the relationship between affect and health varies by culture. In this preregistered secondary analysis (https://osf.io/ke5pr), we investigated variations in the links of positive affect and loneliness with longevity across cultures. Data were derived from comparable surveys in the United States (1986–2005) and Japan (1987–2006). We identified case-matched American and Japanese samples based on age and sex (n = 1,663 per country, mean age = 70 years). Positive affect (2 items), negative affect (2 items), and loneliness (1 item) were measured using five available items from the Center for Epidemiological Studies Depression Scale. The measurement model of positive and negative affect was invariant across cultures. The affect scores were standardized based on each sample. Cox proportional hazard models assessed the associations of positive affect and loneliness with mortality. We used multiple imputation for missing data. Greater positive affect was associated with reduced mortality risk across cultures (hazard ratio [95% confidence interval] = 0.955 [0.914–0.998]) after controlling for negative affect and demographic and health factors. Culture did not moderate the association. Additional adjustment for behavioral factors attenuated the hazard ratio to statistical non-significance. Loneliness and negative affect were not associated with mortality. Our findings suggest that the health benefits of positive affect are primarily similar across cultures.

Abstract citation ID: igad104.1582

### PERCEIVED NEIGHBORHOOD CHARACTERISTICS AND COGNITIVE AGING: FINDINGS FROM THE SEATTLE LONGITUDINAL STUDY

Gizem Hueluer[1], Sherry Willis[2], Warner Schaie[2], and Denis Gerstorf[3], *1. University of Bonn, Bonn, Nordrhein-Westfalen, Germany*, *2. University of Washington, Seattle, Washington, United States*, *3. Humboldt University of Berlin, Berlin, Berlin, Germany*

Lifespan developmental perspectives widely acknowledge that cognitive development and aging is shaped by an individual's social, historical, and geographic context. The residential neighborhood is the most immediate geographic context of an individual. In this study, we used within-person longitudinal data from the Seattle Longitudinal Study to examine how cognitive aging trajectories in five primary mental abilities are associated with perceived characteristics of one's residential neighborhood. 3,482 participants (aged 19 thru 100 years, 54% women) contributed a total of 8,021 observations over up to 35 years. Longitudinal trajectories were examined across age and analyses additionally controlled for sex, education, and income. Our findings showed that cleanness of air and availability of trees and open spaces were indeed associated with higher levels of most primary mental abilities at age 60. In addition, cleanness of air was associated with less pronounced cognitive aging for two of the five primary mental abilities and availability of trees and open spaces was associated with less pronounced cognitive aging for four of the five primary mental abilities. In contrast, noise, closeness to transportation, and feelings of safety showed only few associations with trajectories of cognitive aging, whereas familiarity with neighbors was associated with lower levels of most primary mental abilities at age 60. Taken together, the findings of this study demonstrate that characteristics of an individual's neighborhood are related to trajectories of performance-based measures of cognitive aging over and above indicators of socio-economic status. We will discuss potential mechanisms underlying these associations and implications for future research.

Abstract citation ID: igad104.1583

### DOES LIVING WITH A FAMILY AFFECT SOCIAL CONTACT/PARTICIPATION? VARIATIONS ACROSS BIRTH COHORTS AND GENDER IN JAPAN

Erika Kobayashi, *Tokyo Metropolitan Institute for Geriatrics and Gerontology, Tokyo, Tokyo, Japan*

Changing family structures and values might have affected social relationships outside the household in a different way for men and women. This study clarified birth cohort and gender differences among older Japanese in terms of (a) trajectories of social contact and participation and (b) effects of living with a family on them. Data were obtained from ten waves of the National Survey of the Japanese Elderly (1987-2021) for 5,855 individuals who commenced participation by 2012. Quadratic growth models were examined as a function of age at time t (≥63 years) for the frequency of contact with children living apart, with friends and neighbors, and participation in community groups, whose intercept and slope were predicted by gender and birth cohorts (C1:1902-13, C2:1914-25, C3:1926-37, C4:1938-49). Whether effects of being married and co-residing with a child would differ by gender and cohorts was also examined, controlling for socioeconomic and health status. The results showed that the level of contact/participation at age 75 increased in women in recent cohorts. The frequency for friends and groups declined with age after the mid-70s across cohorts. C4 showed a steeper decline in the child/friend contact, indicating the

COVID-19 influence. Living with a family generally impeded social contact, except that married men contacted non-coresident children more frequently than their unmarried counterparts. The negative effect of living with a child was greater in women and the earliest birth cohort. Thus, social isolation could matter not only for older adults living alone, but also for those living with a family.

Abstract citation ID: igad104.1584

### EMOTIONAL CO-REGULATION IN YOUNGER AND OLDER COUPLES' DAILY LIVES: THE ROLE OF PSYCHOLOGICAL AVAILABILITY

Tabea Meier[1], Zilla Huber[2], Mike Martin[2], and Andrea Horn[2], *1. Northwestern University, Chicago, Illinois, United States*, *2. University of Zurich, Zurich, Zurich, Switzerland*

Close relationships represent an important context for aging. Romantic partners can provide important resources for day-to-day individual and relational functioning by regulating their emotions together. Emotional co-regulation attempts are, however, not always successful and may depend on situational factors such as psychological availability, i.e., an individual's current ability to be open and attentive to their partner's needs. The present study examined emotional co-regulation in the daily lives of younger (N=62; 18-33 years) and older (N=56; 57-87 years) couples, focusing on the particular role of psychological availability. In a 21-day experience-sampling study, both participating partners reported their momentary emotional experiences, perceived psychological availability, relationship quality and (co)-regulation strategies three times a day. Results from dyadic multilevel modeling revealed that older and younger participants did not differ in the strategies they used to regulate their own and their partners emotions, but older couples showed greater emotional synchrony (i.e., coordinated changes between partners' emotional experiences) throughout the study. When partners experienced greater psychological availability, they used more adaptive (e.g., positive humor, responsive touch) and fewer maladaptive (e.g., ruminative brooding) emotion regulation strategies. They also experienced more positive emotions and greater relationship quality when they were more psychologically available, and these associations were particularly evident among younger couples. The findings suggest that older partners were more attuned to each other's emotions, but that their co-regulation depended less on psychological availability. This potentially speaks to more automatic co-regulation among older couples and opens the door for further investigations of situational factors that impact successful co-regulation.

### SESSION 4230 (SYMPOSIUM)

Abstract citation ID: igad104.1585

### FRIENDSHIP MATTERS: UNDERSTANDING THE LINK BETWEEN FRIENDSHIP AND HEALTH IN LATER LIFE

Chair: Emily Lim Discussant: Katherine Fiori

Friendship matters in later life, as friends provide social support to older adults. Yet, current social relationships and health literature focuses primarily on family relationships. This symposium aims to highlight studies that explore the associations among friendship, health, and health behaviors, with a focus on individual differences. First, using the Health and Retirement Study (HRS), Lim and Burr demonstrate a non-linear relationship between the number of friends and loneliness, with a possible threshold effect for men but a more complex relationship for women. In their investigation of the moderating role of friendship on the association between widowhood and cognition using the HRS, Zhang et al. also find interesting gender differences where greater friendship strain is associated with a faster cognitive decline for widowed men, but not women. Ng and Birditt also uncovers important individual differences, this time in the association between friendship and mental health among Black and White dementia caregivers. They find that emotional support from friends is linked to lower levels of anxiety and depression, but primarily for White caregivers. Mimbs et al. extend these studies to focus on health behaviors, addressing links between interactions with family and friends and hurricane preparedness and evacuation likelihood. Using an online survey of older Floridians, they show that more interactions with friends are associated with greater preparedness and evacuation likelihood. Our discussant, Katherine Fiori, will discuss how these findings can extend the literature on friendship and health in later life, with a focus on the roles of individual differences and context.

Abstract citation ID: igad104.1586

### THE ASSOCIATION BETWEEN NUMBER OF FRIENDS AND LONELINESS FOR OLDER MEN AND WOMEN USING THE HEALTH AND RETIREMENT STUDY

Emily Lim, and Jeffrey Burr, *University of Massachusetts Boston, Boston, Massachusetts, United States*

Loneliness is a significant health risk. Older adults are susceptible to loneliness and friendship is a source of support, potentially alleviating loneliness in later life. Few studies examined the association between friendship and loneliness among older adults and whether gender moderates this association. We examined the association between friendship and loneliness for older men and women longitudinally with data from the Health and Retirement Study (2006, 2010, 2014, 2018). Our sample included 7,257 community-dwelling adults aged 50 and above (M=68.05, SD=10.38). Using multilevel linear modeling, we examined the effects of number of friends, including quadratic and cubic terms for number of friends. After controlling for covariates, our results showed having more friends is negatively associated with loneliness ($\beta$=-0.21, p<.001) and for the quadratic friends term ($\beta$=0.01, p<.001), and the cubic friends term ($\beta$=-0.00, p<.01) on loneliness. While the number of friends is also negatively associated for both men ($\beta$=-0.15, p<.001) and women ($\beta$=-0.30, p<.001), there were significant effects of the quadratic ($\beta$=0.04, p<.001) and cubic friends terms on loneliness for women ($\beta$=-0.00, p<.001). For men, only the quadratic friends term was significant ($\beta$=0.02, p<.001) but not the cubic friends term. These results show there is a non-linear relationship between number of friends and loneliness, with a possible threshold effect for men and a more complex relationship for women. Our findings have implications for friendship in later life, indicating that social interventions may need to be tailored differently for older men and women to promote friendships and potentially reduce loneliness.

Abstract citation ID: igad104.1587

### WIDOWHOOD AND COGNITIVE DECLINE: THE ROLES OF FRIENDS AND GENDER

Zhenmei Zhang[1], Hui (Cathy) Liu[2], and Ning Hsieh[1], *1. Michigan State University, East Lansing, Michigan, United States*, *2. Purdue University, West Lafayette, Indiana, United States*

Widowhood has been identified as a risk factor for cognitive decline and dementia. Yet less is known about the protective and exacerbating factors of cognitive decline following widowhood. This study examines the roles of friendship characteristics in moderating the negative effect of spousal loss on cognition for older Americans. Drawing on data from the 2006-2018 Health and Retirement Study, we aim to answer two questions: 1) whether friendship network and quality moderate the association between spousal loss and cognitive decline in late life; 2) whether the moderating effect of friendship characteristics on cognition differ for men and women. Cognitive trajectories were assessed every other year from 2006 to 2018 (in total 7 waves). Friend characteristics were assessed in the 2006 psychosocial questionnaire and included the number of close friends, contact frequency, and support and strain from friends. The analytical sample comprised 3,636 respondents aged 50 and older who were married in 2006 and participated in the psychosocial questionnaire. Among the final sample, 370 lost spouses between 2006 and 2010, and 3,266 remained married throughout the observation period. Preliminary results from latent growth curve models showed that several characteristics of friendship including contact frequency and strain from friends are significantly associated with cognitive function at baseline. Furthermore, strain from friends interacted with widowhood status and gender to shape cognitive trajectories. Specifically, higher friendship strain was associated with a faster cognitive decline for the widowed but not for the married a pattern observed only among men but not women

Abstract citation ID: igad104.1588

### FRIENDSHIP NETWORKS AND HEALTH AMONG BLACK AND WHITE DEMENTIA CAREGIVERS

Yee To Ng[1], and Kira Birditt[2], *1. Institution of Social Researach, Ann Arbor, Michigan, United States*, *2. Institute for Social Research, University of Michigan, Ann Arbor, Michigan, United States*

Social networks and support protect against health risks associated with dementia caregiving. Yet, little is known about the unique role of friends in the dementia caregiving context, as most prior caregiving studies do not differentiate between family and friends when examining social networks and support. The experience of racism and stronger familial values among Black individuals may affect their friendship networks and the implications of friends for health. This study examines racial differences in the number of friends in close social networks, contact frequency and support from friends, and links with mental health among Black and White dementia caregivers. A total of 98 dementia caregivers (63% White Americans, 90% female, Meanage = 62.66) completed baseline interviews in which their social network and mental well-being were assessed. Findings revealed that White caregivers reported more friends in their close networks than Black caregivers. However, there were no racial differences in contact frequency with network friends, or the frequency of receiving practical and emotional help from friends in assisting patients with dementia. Furthermore, receiving emotional support from friends was linked to lower levels of anxiety and depression, and these effects were greater among White caregivers. Together, this study provides a better understanding of racial disparities in friendship networks among dementia caregivers and their implications for mental health.

Abstract citation ID: igad104.1589

### WHAT ROLE DO SOCIAL RELATIONSHIPS PLAY IN HURRICANE SURVIVAL?

Hope Mimbs, Anne Barrett, Jessica Noblitt, Cherish Michael, and Brianna Soulie, *Florida State University, Tallahassee, Florida, United States*

Despite the disproportionately high hurricane mortality rates for older adults, we know little about how hurricane preparedness and evacuation decisions are influenced by social relationships, specifically interaction with family and friends. Using data from an online survey of Floridians aged 50 and older that was conducted between December 2020 and April 2021 (n=3,797), we examine the association between frequency of interaction with family and friends and two hurricane survival measures: hurricane preparedness and likelihood of evacuation. We found that interacting more frequently with friends predicts greater hurricane preparedness, as well as a greater likelihood of evacuation. We also found that interacting more frequently with family members predicts a greater likelihood of evacuation. Our study highlights yet another benefit of social relationships: Interacting more often with friends and family can save lives during emergencies.

### SESSION 4235 (SYMPOSIUM)

Abstract citation ID: igad104.1590

### DYADIC HEALTH SCIENCE IN GERONTOLOGY: RECENT ADVANCES AND INTERDISCIPLINARY PERSPECTIVES

Chair: Lynn Martire Co-Chair: Karen Lyons Discussant: Christine Proulx

Close others impact health in profound ways, and many of the critical pathways can only be understood by examining the dynamics that emerge between two people. This symposium will present new theoretical and methodological advances in dyadic health science, and invite discussion of differing perspectives on the scope of dyadic research. Dr. Wilson will overview the essential components of rigorous dyadic health research and explain the range of approaches used to define dyadic research, setting the stage for the cutting-edge work described in the subsequent presentations. Drs. Novak and Yorgason will discuss the importance of matching research questions with dyadic data and how to best model congruence/similarity, difference scores, accuracy or bias, and idealization between partners. Dr. Pauly will describe a recently proposed developmental-contextual model of couple synchrony, and present findings from research using a smartwatch and smartphone system (DyMand) that captures dyadic interactions. Dr. Polenick will present findings from research examining effects of incongruent and discordant health conditions within older couples on depressive symptoms. Dr. Christine Proulx, an internationally recognized scholar of close relationships in late life, will offer perspective on these advances and critical future directions for the field. This is a Dyadic Research on Health and Illness Across the Adult Lifespan Interest Group Sponsored Symposium.

Abstract citation ID: igad104.1591

### NEW OPPORTUNITIES FOR ADVANCING DYADIC HEALTH SCIENCE IN GERONTOLOGY

Stephanie Wilson[1], Joshua Novak[2], Jeremy Yorgason[3], and Lynn Martire[4], *1. Southern Methodist University, Dallas, Texas, United States*, *2. Auburn University, Auburn, Alabama, United States*, *3. Brigham Young University, Provo, Utah, United States*, *4. Penn State University, State College, Pennsylvania, United States*

As dyadic health science enters a golden age, important conceptual, theoretical, and technical challenges remain. Based on a forum review published in The Gerontologist by a group of dyadic scholars, this presentation will provide an overarching framework for understanding the essential components of robust dyadic health research, and the wide array of approaches used within the field. Each component of the framework will be discussed in relation to supporting empirical examples. The presentation will close by identifying crucial challenges and considerations that coincide with important future directions for the field.

Abstract citation ID: igad104.1592

### MATCHING DYADIC RESEARCH QUESTIONS WITH DYADIC ANALYTIC MODELS

Joshua Novak, *Auburn University, Auburn, Alabama, United States*

Examining shared and common experiences between dyads affords opportunities to answer interesting research questions using dyadic data. Emerging dyadic methodology has expanded the types of research questions researchers can ask and answer. This session aims to equip researchers with knowledge and resources for grant writing, when planning new data collection projects, or when conducting secondary data analyses. Reviewing both cross-sectional and longitudinal methods, we will walk attendees through several types of research questions and match them with appropriate dyadic analytic strategies. These include the Actor-Partner Interdependence Model (APIM), the Common Fate Model, the Dyadic Growth Curve Model (and ALT Model), the Common Fate Growth Curve Model, the Random Intercept Cross-lagged Model, as well as various extensions. We will address accounting for non-independence in dyadic models, along with how to explore substantive dyadic processes. We will review ways that different approaches conceptualize, assess, and examine within and between dyad comparisons. We will also consider ways to address self and other reports of the same construct, such as health congruence/concordance or discordance/difference, as well as perceptions of accuracy or bias, enhancement, and idealization between dyads. Attendees will learn of resources available to learn more about the methods addressed in this session.

Abstract citation ID: igad104.1593

### A DEVELOPMENTAL–CONTEXTUAL MODEL OF COUPLE SYNCHRONY: CENTRAL TENETS AND EMPIRICAL APPLICATION

Theresa Pauly[1], Denis Gerstorf[2], Hans-Werner Wahl[3], Janina Lüscher[4], Urte Scholz[5], and Christiane Hoppmann[6], *1. Simon Fraser University, Vancouver, British Columbia, Canada*, *2. Humboldt University of Berlin, Berlin, Berlin, Germany*, *3. Heidelberg University, Heidelberg, Baden-Wurttemberg, Germany*, *4. Swiss Paraplegic Research, Nottwil, Luzern, Switzerland*, *5. University of Zurich, Zurich, Zurich, Switzerland*, *6. University of British Columbia, Vancouver, British Columbia, Canada*

Most current models of social dynamics and health focus on individuals; our developmental-contextual model of couple synchrony (CoSynch) proposes a uniquely dyadic approach and deliberately moves to a N + 1 perspective. CoSynch emphasizes the importance of between-person synchrony for shaping development and health, which refers to the way in which physiological states and health behaviors fluctuate in sync between romantic partners. Synchrony is proposed to follow a u-shaped curve across adulthood, with younger and older couples showing greater synchrony than middle-aged couples, and with greater diversification of synchrony in very old age. We assume that synchrony is shaped by the closeness and shared contexts of couples, and is correlated with individual and dyad characteristics (e.g., attachment). Prior research has linked synchrony to perspective taking and effective collaboration. Thus, synchrony is assumed to be important for supportive interactions in couples managing chronic disease. As an empirical illustration, we apply the CoSynch model to analyze 992 daily life assessments from 11 Swiss couples in which one partner had Type II diabetes. Specifically, we used a smartphone and smartwatch system (DyMand) that captured daily social contexts and heart rate synchrony. Couples showed small to moderate interconnections in their heart rate fluctuations, which tended to be stronger when engaged in conversation with the partner. We will discuss the utility of using wearables to better understand health-relevant social developmental dynamics. Insights from collecting such data could be used to inform future interventions using technology to promote healthy aging and adaptive disease management.

Abstract citation ID: igad104.1594

### CHRONIC CONDITION DISCORDANCE WITHIN OLDER COUPLES: IMPLICATIONS FOR HEALTH AND WELL-BEING

Courtney Polenick[1], and Kira Birditt[2], *1. University of Michigan, Ann Arbor, Michigan, United States*, *2. Institute for Social Research, University of Michigan, Ann Arbor, Michigan, United States*

Nearly half of U.S. adults aged 45-64 and over 80% of those 65 and older have two or more potentially debilitating chronic conditions such as arthritis, diabetes, and heart disease. Chronic condition discordance (i.e., the extent that two or more conditions have non-overlapping self-management requirements) increases care complexity and can make multiple chronic conditions more difficult to manage. Most research on multimorbidity and chronic condition discordance focuses on individuals, however older adults with chronic conditions often have a spouse or partner with chronic health problems that further complicate self-care. Our dyadic concordance model of multimorbidity posits that chronic condition discordance at the individual level (i.e., discordance in two or more chronic conditions within individuals) and the couple level (i.e., discordance in two or more chronic conditions between spouses) has adverse implications for the psychological well-being and self-management behaviors of both partners. We discuss key findings that support this model using longitudinal data from the Health and Retirement Study, which include the implications of individual-level and couple-level chronic condition discordance for depressive symptoms, physical activity, alcohol use, and global and health-related perceived control over time. We then describe how these findings inform clinical care and interventions to improve the health and well-being of couples living with multiple chronic conditions. Finally, we propose future directions to build on and expand the dyadic concordance model of multimorbidity and its use in understanding how older adults manage multiple chronic conditions within the context of their close relationships.

### SESSION 4240 (SYMPOSIUM)

Abstract citation ID: igad104.1595

### RESEARCH ON SUPPORT FOR ELDER CARE IN DIFFERENT CULTURES AND SETTINGS

Chair: Lin Jiang Discussant: Ning Zhang

This cross-culture symposium consists of four studies that examined the interventions, patterns, technology, and challenges for elder care across settings in the U.S., China, and other countries. The first two papers focus on caregivers for patients with Alzheimer's disease and related dementia (ADRD). The paper from the U.S. fills that gap by examining the information and communications technology utilization in relation to mental health among Mexican American informal caregivers for persons with ADRD. The findings suggest a need to explore further different types of ICT and their effects on Mexican American informal caregivers' mental health. To help t reduce social isolation and loneliness, we may consider choosing an appropriate ICT based on their situation and culture. The paper from China highlights ADRD informal caregivers' needs and struggles, which were ignored by the Chinese public. Also, the paper examined a model for healthcare professionals at inpatient and outpatient clinics to provide support to caregivers medically and emotionally. The third paper is a systematic review summarizing the latest evidence regarding the importance of using technology among people with dementia and their caregivers as an intervention. The fourth paper broadens our horizon by using a multinational comparison perspective to analyze research on pattern recognition and innovation of home-based care under the change of family structure in China. After individual presentations are done, one discussant will summarize shared themes in these papers and provide critical feedback.

Abstract citation ID: igad104.1596

### MENTAL HEALTH AND TECHNOLOGY UTILIZATION AMONG MEXICAN AMERICAN INFORMAL CAREGIVERS FOR PATIENTS WITH ADRD

Lin Jiang[1], Adelaido Garcia-Andres[2], Cassandra Sarmiento[3], Lubna Nasr[4], Isis Macias Moreno[4], Romeo Escobar[1], Denise Longoria[1], and John Gonzalez[5], *1. The University of Texas Rio Grande Valley, Edinburg, Texas, United States*, *2. Universidad Autonoma de Nuevo Leon, San Nicolas de los Garza, Nuevo Leon, Mexico*, *3. Teen health center, Galveston, Texas, United States*, *4. Knapp UTRGV Family Medicine Residency, Mercedes, Texas, United States*, *5. The University of Texas Rio Grande valley, Edinburg, Texas, United States*

**Background:** The number of Latinos with Alzheimer's disease and related dementia (ADRD) is expected to rise to 1.3 million by 2050. Mexican Americans with ADRD rely heavily on unpaid informal care. The use of information communication technology (ICT) among caregivers is associated with significant improvements in mental health and social support. However, current ICT studies focus mainly on White American caregivers. This study fills the gap. Method: From November 2021 to November 2022, we collected 115 surveys, of which 11 were collected from in-person interviews at a clinic and 104 were collected online. The survey included the UCLA Loneliness Scale version 3, the Center for Epidemiologic Studies Depression (CES-D), and the Online Social Support Scale. **Results:** The participants, aged from 18 to 82 and have cared for their family members with ADRD for two years on average. About 70.4% of them use the internet daily. The results indicated that the more time spent on Instagram, YikYak, and first-person shooter online games, the higher depression the participants had. Also, the more first-person shooter online games they use, the higher is their loneliness. Other social media use is not related to loneliness or depression. **Conclusion:** This study suggests that it is necessary to explore an appropriate internet-based ICT for Mexican American informal caregivers to reduce their loneliness and depression. Future research needs to examine their feelings and reflections on using internet-based ICT and discern how their caregiver's role and Latino culture affect the utilization of technology.

Abstract citation ID: igad104.1597

### SUPPORTING FAMILY CAREGIVERS OF OLDER PATIENTS WITH DEMENTIA: A PSYCHOEDUCATIONAL MODEL IN SHANGHAI

Jing Nie[1], Yang Yang[2], and Xia Li[3], *1. Shanghai Jiaotong University, Shanghai, Shanghai, China (People's Republic)*, *2. Shanghai AI Care Co Ltd., Shanghai, Shanghai, China (People's Republic)*, *3. Shanghai Mental Health Center, Shanghai Jiaotong University School of Medicine, Shanghai, Shanghai, China (People's Republic)*

In China, where community-based health and social supports designated for persons living with dementia (PLWD)

and caregivers are few and scarce, doctors at the neurological and geropsychiatric clinics and hospitals are expected to provide psychoeducation programs for family caregivers. Geropsychiatric doctors, in particular, championed the efforts to provide counseling, education, and support to family caregivers when they are not able to handle the psychological and behavioral problems of PLWD. This case study examines the preliminary efficacy of the efforts of the geropsychiatric unit in the largest mental health center in Shanghai to provide education, empowerment, and supports to family caregivers of dementia. A total of 40 dementia caregivers were interviewed to identify their unmet service needs when their PLWD visited the clinic or were admitted to the inpatient unit. Most caregivers reported a knowledge deficit in dementia treatment and care and expected to receive training and support to improve their understanding and skills. In line with their needs, the researcher developed a program facilitating the dialogue between medical staff and caregivers. The dialogue occurs once a week, includes the medical staff and up to 6 family caregivers, and often last for 90 minutes. Each week focuses on a different topic covering the medical procedures, diagnosis and treatment, person-centered care for the PLWD, self-care for the caregiver, and transition care needed for community living. Caregivers who received these sessions indicated great satisfaction with this program, increased confidence, and decreased worries and anxieties.

Abstract citation ID: igad104.1598

### A SYSTEMATIC REVIEW OF EFFECTIVENESS OF TECHNOLOGY INTERVENTIONS FOR PEOPLE WITH DEMENTIA AND INFORMAL CAREGIVERS

Ling Xu[1], and Lingya Zhao[2], *1. The University of Texas at Arlington, Arlington, Texas, United States*, *2. Capital University of Economics and Business, Beijing, Beijing, China (People's Republic)*

**Objectives:** Most people with dementia live at home and get care from informal caregivers. To support people with dementia and informal caregivers, a series of technology interventions have been introduced and implemented. The aim of this study was to evaluate effectiveness of recent technology interventions. Method: A systematic literature search was carried out in 14 online databases and Google Scholar, covering articles published during January 2005 and January 2020. Over three hundred articles were identified, of which 23 met the inclusion criteria and were included in the review. Meta-analyses for characteristics of included studies were conducted. **Results:** Most studies demonstrated that technology interventions had various benefits for both people with dementia and their informal caregivers living in the community. For informal caregivers, these interventions tend to improve their psychological conditions and provide useful help when caring for people with dementia. For people with dementia, these interventions have potential to improve their performance and mental conditions, and keep them safe at home, which could delay institutionalization and save financial costs. **Conclusion:** Technologies can be used independently by people with dementia, to help them in positive psychological condition, and to maintain their basic social interaction should be developed. Technology interventions should be developed and selected according to the progression of dementia. More assessment studies using randomized controlled trails should be conducted, so that we can evaluate extant technology interventions more rigorously.

Abstract citation ID: igad104.1599

### PATTERN RECOGNITION AND INNOVATION OF HOME-BASED CARE IN CHINA: FROM THE PERSPECTIVE OF MULTINATIONAL COMPARISON

Hong Mi[1], and Yuxia Wu[2], *1. Zhejiang University, Hangzhou, Zhejiang, China (People's Republic)*, *2. Ningbo University of Engineering, Ningbo, Zhejiang, China (People's Republic)*

The development trend of family structure’s miniaturization and nucleation in China and the overlapping occurrence of population aging and associated frailty and morbidities have created an urgent need for innovation in the home care service model. In this paper, home-based care is regarded as a dynamic pension method. Based on the analysis of dynamic changes in population structure and family structure, this paper uses multi-stage microsimulation technology to scientifically predict the evolution of the older adult population's distribution state and multi-health state. This approach leads to early identification of the risks of various home-based care models in the process of family structure changes. Based on this, with reference to the home-based care model cases in China, Japan, Europe, and the United States, this paper proposes the innovation of the multi-health state and the " integrated care at home" model for households with an aging resident and brings up the optimization plan of home-based care service for accelerating the aging process. Implications for policies and practices to support family members to realize this optimized home care model will be discussed.

### SESSION 4245 (SYMPOSIUM)

Abstract citation ID: igad104.1600

### IMAGINING THE FUTURE OF AGETECH

Chair: Andrew Sixsmith

The intersection of global population aging and technological trends in e-health, robotics, artificial intelligence, and mobile technologies has led to the emergence of “AgeTech” as a way to improve the health and well-being of older people. While this might be seen as an almost deterministic process of technological change, the visioning, development and implementation of technology is essentially a creative and socially-situated process. This requires us to understand AgeTech as a socio-technical imaginary, examining the intersecting spheres of social action and technological development. This idea of technological development as a creative process, runs counter to an idea of technology in purely rational and instrumental terms and brings into play cultural, political and behavioural factors. Our symposium examines the socio-technical imaginary of AgeTech in three papers: Paul Higgs and Chris Gilleard discuss Fourth Ageism and the Technological Imaginary of Long-term care and argue that the problematizing of aging present in current thinking needs to be challenged at the conceptual stage of technological development. Andrew Sixsmith et al will present on A Global AgeTech Agenda, with a particular focus on how AgeTech development and innovation should connect with global initiatives such as the UN's Strategic Development Goals. Mei Lan Fang et al will take an Ethical Perspective on AgeTech to discuss some of the complex challenges around the creation and use of technologies and argue that culture change is needed within the research and innovation communities to develop ethically grounded AgeTech. This is a Technology and Aging Interest Group Sponsored Symposium.

Abstract citation ID: igad104.1601
### A GLOBAL AGETECH AGENDA
Andrew Sixsmith[1], Sally Fowler-Davis[2], Sadiq Bhanbhro[2], and Rebecca White[1], *1. Simon Fraser University, Vancouver, British Columbia, Canada*, *2. Sheffield Hallam University, Sheffield, England, United Kingdom*

AgeTech is increasingly seen as a way to support and enhance the health and independence of older people. However, AgeTech research and innovation agendas overwhelmingly focus on the needs of older people in developed countries and have so far failed to recognise that population aging is a global-level trend that intersects with other "megatrends", such as climate change, urbanisation and international migration. Drawing on an environmental scan of the current and emerging AgeTech sector, this paper argues that technology research, development and innovation needs a global agenda that engages with initiatives such as the UN's Strategic Development Goals and Decade for Healthy Ageing. It is also important to situate AgeTech in the wider debate on global disparities. The ongoing impact of colonial legacy (afterlife colonization) continues to reduce life chances, leads to poor health, and is present in everyday relationships between people and institutions at global and local levels. This is particularly apparent in the unequal impact of climate change on people and places, but as yet, the discussion of these issues is notably absent in the narratives surrounding AgeTech. This misses significant opportunities for global reciprocity in health, co-development of useful and appropriate technologies, and more sustainable approaches to innovation (e.g opportunities for frugal innovation), The paper highlights the potential role of older people as active participants in technological change, for example for mitigating climate change and its unequal impact on the Global South

Abstract citation ID: igad104.1602
### FOURTH AGEISM AND THE TECHNOLOGICAL IMAGINARY OF LONG-TERM CARE
Paul Higgs, and Chris Gilleard, *University College London, London, England, United Kingdom*

Many high-income countries are pre-occupied with the rising number of older people needing long term care and the consequent demand for a labour force to carry out these tasks. In many other sectors of the economy the problem of labour supply is increasingly being met by the introduction of sophisticated technology. Seeking to extend this strategy to the long-term care sector (LTC) has led to research and development in various care technologies. This paper examines the imaginaries fuelling such efforts, as distinct from the outcomes of such research. One such imaginary is that of a fourth age, a state of frailty and dependency that requires others to carry out instrumental and related activities of daily living to those deemed to have fallen under its shadow. This creates a set of contrasting goals, between on the one hand the desire to deliver LTC in a way that maximises the dignity and integrity of the persons receiving LTC and on the other the desire to reduce demands on the workforce. While the former stresses the maximisation of the humanity of LTC the latter is often motivated by the reduction of the (human) labour inputs involved. These dilemmas will arguably become acute if such various technological imaginaries become realities. This paper argues that forms of implicit fourth ageism present in current thinking need to be challenged at the conceptual stage of this technological imaginary.

Abstract citation ID: igad104.1603
### TRANSDISCIPLINARY WORKING FOR CULTURE CHANGE IN ETHICAL AGETECH
Charlene Chu[1], Judith Sixsmith[2], Mei Lan Fang[2], and Jennifer Boger[3], *1. University of Toronto, Toronto, Ontario, Canada*, *2. University of Dundee, Dundee, Scotland, United Kingdom*, *3. University of Waterloo, Waterloo, Ontario, Canada*

This presentation proposes a new way of understanding ethical culture in AgeTech as a dynamic and changing set of ethical research, design, and developmental processes and practices. By focusing on dynamics of ethical performance and the values, beliefs, and expectations that underpin them, this approach emphasizes ongoing ethical negotiation of all stakeholders involved in the research, design, and development of technology, rather than as a checklist at the research outset. Transdisciplinary working (TW) is proposed as an effective means to implement ethical processes and practices. By involving diverse stakeholders in the development of shared aims and objectives, ethical considerations become detached from the domain of researchers and become a more inclusive stakeholder negotiation. TW acknowledges that the development of new technologies cannot be separated from the people who design and use them; and the social practices, social norms, and social meanings in which they are steeped. The co-creation of socio-technical AgeTech systems is a key strategy for creating culture-change in ethical working environments. By involving diverse stakeholders in dynamic ethical processes across the research pathway, meaningful involvement of all stakeholders can be ensured, resulting in the effectiveness and relevance of AgeTech. Subsequently, technologies that are more responsive to the needs and desires of older people, carers, and professionals, and better reflect the complex ethical landscape in which they operate are created. Overall, this presentation offers a new perspective on ethical culture in AgeTech, one that involves diverse stakeholders in the creation of socio-technical systems that are both effective and ethical.

### SESSION 4250 (SYMPOSIUM)

Abstract citation ID: igad104.1604

### LOCKED IN SUFFERING: UNLOCKING HOPE AND IMPROVING CARE FOR ELDER INMATES: A CALL FOR ACTION

Chair: Raya Kheirbek Co-Chair: Kenzie Latham-Mintus

Our program will focus on several key areas, including care for hospitalized inmates, the importance of providing comprehensive care for all prisoners as mandated by 8th amendment, and the importance of identifying inmates facing end of life who would benefit from compassionate release. We will begin with a discussion on the challenges faced by elder inmates and address their unique physical, psychosocial and spiritual needs. With a growing population of aging prisoners, it is critical that we examine the healthcare and social services required to ensure that they receive adequate care. Next, we will explore the differences in healthcare provision for inmates versus non-inmates who are hospitalized. The presentation will examine the acute exacerbation of chronic disease, ethical considerations and legal requirements involved in providing care to both populations. We will also discuss the importance of interdisciplinary care for incarcerated individuals, which requires collaboration among medical providers, mental health professionals, social workers, chaplains and other specialists. Our panel of experts will share their experiences and provide insights on how this approach can improve health outcomes and reduce recidivism rates after release . Finally, we will hear from advocates who are working to ensure that all prisoners receive adequate healthcare and social services, They will share strategies for improving access to care, reducing health disparities, and addressing the unique needs of incarcerated elders Our symposium provides a unique opportunity to explore and share insights on how we can work together to improve the health and wellbeing of incarcerated individuals.

Abstract citation ID: igad104.1605

### HOSPITALIZATIONS OF OLDER INCARCERATED ADULTS: HOW ARE THEY DIFFERENT?

Farah Kaiksow, *University of Wisconsin School of Medicine and Public Health, Madison, Wisconsin, United States*

By 2030, over one-third of the US prison population will be ≥55 years old. In addition to their biological age, incarcerated patients are at increased risk of geriatric conditions due to accelerated aging. As these individuals age, so will their need for community hospitalizations, which already account for 20% ($1.5 billion annually) of state correctional health spending. To date, there has been very limited data on these hospitalizations. This descriptive study provides the largest amount of information yet, comparing 10 years of data on male incarcerated patients (N=4,401 admissions) to a cohort of nonincarcerated males (N=22,150) from a tertiary care center. Incarcerated patients were more likely to be ≤55 than non-incarcerated (53.9% vs. 20.7%) and have shorter lengths of stay (4.7 vs. 5.8 days). The most common diagnosis for both groups was cardiac-related (ICD-10 Chapter 9), while mental health diagnoses (ICD-10 Chapter 5) were the second most common for incarcerated patients but not in the top five for non-incarcerated. Incarcerated patients had higher Charlson Comorbidity Index scores, with incarcerated patients having higher scores beginning at age 50 than non-incarcerated ≥80. Surprisingly, the one-year mortality rate for incarcerated patients was almost half that for non-incarcerated (56/1,000 vs. 101/1,000); one possible explanation for this disparity is that the community health system data on incarcerated patients may be incomplete. This study supports the theory of accelerated aging among incarcerated individuals and highlights the need for improved data on which to base policy changes to improve the care of this vulnerable population.

Abstract citation ID: igad104.1606

### VOICES FROM THE INSIDE: MEDICAL TREATMENT FURLOUGH IN THE LARGEST STATE PENITENTIARY IN US: LESSONS LEARNED

Jolie Harris, *Louisiana State University Health Science Center New Orleans, New Orleans, Louisiana, United Sta tes*

As part of the criminal justice reform to reduce prison population, the State of Louisiana undertook an initiative to explore a medical treatment furlough project. The plan was to transition long-term inmates from the prison to long-term care (LTC)). Angola, the largest maximum security prison in the U.S, had over 1000 prisoners 60 years or older. Eighty percent of the prison population is serving a life sentence. Prisoners with significant health and mobility issues were to be considered to be released to off-site facilities with parole supervision. We present lessons learned from exploring all aspects of a possible partnership between a Louisiana long-term care company and the correction department including: conducting site visits at Angola and the long-term care facility; gathering best practice data; exploring a referral process and acuity classification tool; exploring the role of the parole officer; and examining the prison population against the regulations for long-term care facilities from the perspective of the prisoners and current residents. Additional considerations were the impact on the local community and on-going support for services provided, along with staffing, staff training, and security and safety of the facility. After multiple discussions with representative from the Louisiana Department of Health we found the obstacles were too great to implement the program. Exposure to the challenges facing the corrections department and examining how to implement the Act as written was a lesson in the value of viewing an issue from multiple perspectives.

Abstract citation ID: igad104.1607

### COMPASSIONATE RELEASE OF DYING INMATES: A STEP TOWARD SOCIAL JUSTICE IN THE CRIMINAL JUSTICE SYSTEM

Gabriel Lutz[1], Yulin Yang[2], and Raya Kheirbek[1], *1. University of Maryland School of Medicine, Baltimore, Maryland, United States*, *2. University of California, San Francisco, San Francsico, California, United States*

Mr. John is a 60-year-old incarcerated male with advanced stage IV lung cancer that had metastasized. He was admitted to the hospital with significant pain and failure to thrive. Several lines of chemotherapy failed him, and he desired to be at home with his family as he approached the end of life. Unfortunately, he was unable to leave the hospital except to be back to prison. He was continually shackled to bed with 2 police officers in his room. The case highlights the need to advocate for compassionate release, that allows for the early release of terminally ill inmates who are not a danger to society, and offers tools and steps to help release hospitalized inmates to the care of their families. To obtain compassionate release, the first step is to identify eligible inmates with a terminal illness and a life expectancy of less than six months, which must be confirmed by a medical evaluation. The second step involves filing a petition for compassionate release with the appropriate authority, including medical records, a statement from the treating physician, and a plan for the inmate's release and care in the community. The third step entails advocating for the inmate's release, which may involve working with advocacy groups, contacting elected officials, and raising public awareness. By granting compassionate release to dying inmates, we can provide them with the opportunity to die with dignity and spend their final moments with their loved ones, which is a fundamental aspect of social justice.

### SESSION 4255 (PAPER)

### CIVIC ENGAGEMENT

Abstract citation ID: igad104.1608

### ASSET-BASED COMMUNITY DEVELOPMENT FOR DEMENTIA-FRIENDLY COMMUNITIES (ABCD FOR DFC): A PILOT PROJECT IN HONG KONG

Cheryl Chui[1], Shiyu Lu[2], On Fung Chan[1], and Terry Lum[1], *1. The University of Hong Kong, Hong Kong, Hong Kong*, *2. City University of Hong Kong, Kowloon, Hong Kong*

There is little understanding about how a dementia-friendly community can be constructed in East Asian societies, particularly in geographically remote areas characterized with majority older adult residents and limited external support. A pilot project using an asset-based community development (ABCD) approach in constructing a dementia-friendly community in Hong Kong was implemented. Informed by the theoretical tenets of ABCD, the purpose of this paper is to examine the effects of this pilot project in enabling older adult residents and other community stakeholders to better respond to challenges resulting from growing prevalence of dementia. Multiple stakeholder interviews were conducted with older adult residents, church leaders, district councillors, and social workers. Participants were asked to share the strengths and challenges of this ABCD pilot project in building a dementia-friendly community. Data generated from interviews were analyzed using thematic analysis. Four key preliminary themes were identified, including on the individual level: enhanced dementia literacy, and enacted proactive behaviour; and on the community level: strengthened bridging social capital, and improved community caring capacity. Uncertain long-term financial and professional support posed challenges to participants. Nevertheless, findings underscore the importance of departing from traditional service delivery models, to one that infuses ABCD approaches in constructing a dementia-friendly community. Mobilising existing human and social capital in the community, and embedding older adults in co-creating solutions are key to improve dementia-related interventions. We contribute to ongoing theoretical and practice discussions on the intersections between population aging and employing community-based solutions to create a more dementia-friendly society.

Abstract citation ID: igad104.1609

### STRATEGIES FOR OLDER ADULT AND INFORMAL CAREGIVER INVOLVEMENT IN HEALTH POLICY DEVELOPMENT: STAKEHOLDERS' VIEWS

Opeyemi Kolade[1], Joshua Porat-Dahlerbruch[2], Saritte Perlman[3], Theo van Achterberg[4], and Moriah Ellen[5], *1. Ben-Gurion University of the Negev, Midreshet, HaDarom, Israel*, *2. University of Pittsburgh, Pittsburgh, Pennsylvania, United States*, *3. Ben Gurion University of the Negev, Beersheba, Israel*, *4. KU Leuven, Leuven, Vlaams-Brabant, Belgium*, *5. Ben Gurion University of the Negev, Beer Sheva, HaDarom, Israel*

Older adults and informal caregivers' involvement in policy-making processes can enhance the creation of responsive policies and the provision of better health and well-being services. However, current involvement literature is more focused on involvement in research and healthcare decision-making. This research aims to explore stakeholders' perspectives on approaches for the involvement of older adults and informal caregivers in health policy development. A qualitative study using semi-structured interviews was conducted within the European TRANS-SENIOR consortium and included policymakers, researchers, and advocacy organizations in nine countries. Participants were included if they had experience with older adult/informal caregiver involvement in health policy development. A total of eighteen interviews were conducted with nine representatives from older adult/informal caregiver advocacy organizations, five policymakers, and four researchers. Identified themes were: 1) diversity of involvement approaches, 2) impact of involving older adults and informal caregivers in health policy development, and 3) facilitators and barriers of involvement. Findings indicated that older adult and informal caregiver involvement vary in nature and intensity and that the actual effect of older adult and informal caregiver involvement in health policy development is difficult to assess. There is no one-size-fits-all approach for involving older adults and informal caregivers in health policy development; stakeholders can focus on exploring and implementing context-specific tailored strategies. Our study findings provide evidence that can be useful to decision-makers to involve older adults and informal caregivers in health policy development.

Abstract citation ID: igad104.1610

### THE URBAN AGING RESIDENT'S COALITION: A CASE STUDY IN LATE-ADULTHOOD COMMUNITY ACTIVISM

Kathy Wright[1], Pamela Shields[2], Karen Moss[1], Katie White[3], Judith Tate[1], and Karen Rose[1], *1. The Ohio State University, Columbus, Ohio, United States*, *2. African American Men's Wellness Organization, Columbus, Ohio, United States*, *3. Central Ohio Area Agency on Aging, Columbus, Ohio, United States*

The COVID-19 global pandemic, police brutality, and threats to voting rights retraumatized African American older adults, who lived during civil rights, causing feelings of powerlessness. Older adults can be equipped to regain agency and control through community activism. We provide a case study of an older African American activist's (Ms. P) journey to create the Urban Aging Resident's Coalition (UARC)—an affiliation of the National African American Men's Wellness Agency. UARC was founded in 2019 to improve mental and physical health for urban older adults. Post-pandemic, Ms. P along with teenage volunteers, held monthly educational sessions that included interactions with local police and voting rights education. To facilitate a non-confrontational experience, Ms. P had the police bring Golden Retriever therapy dogs and a community support person. To increase relatability for voter education, Ms. P formed a panel of retired legislators, the first African American state senator who developed the Ohio Office of Minority Health, a younger state representative, and the former CEO of the Urban league to hold an open forum on voting rights and redistricting. Nearly 60 older adults attended the monthly educational sessions. The community activist addressed those traumas experienced by older adults in a safe and encouraging social environment. Participants reported feeling more comfortable interacting with the police. Older adults increased their awareness of the importance of voting to elect leaders that represent them and their ideas. This model, to engage minoritized groups to regain agency and power, can be a template for other communities.

Abstract citation ID: igad104.1611

### VOLUNTEERING AND CHANGES IN CARDIOVASCULAR BIOMARKERS: LONGITUDINAL EVIDENCE FROM THE HEALTH AND RETIREMENT STUDY

Seoyoun Kim[1], Cal Halvorsen[2], and Sae Hwang Han[3], *1. Texas State University, San Marcos, Texas, United States*, *2. Boston College, Boston, Massachusetts, United States*, *3. University of Texas at Austin, Austin, Texas, United States*

A growing body of research shows that volunteering is beneficial for those served, the volunteers, and the larger communities. However, major challenges remain that hinder the practical implications for volunteer activity as a public health intervention, including potential selection effects, lack of longitudinal studies that adjust for baseline characteristics, and a paucity of studies that consider multiple physical health outcomes in a single model. Using data from the 2006-2016 waves of the Health and Retirement Study (N=18,847) and an outcome-wide approach, the current paper evaluated if changes in volunteering between 2006/2008 (t0) and 2010/2012 (t1) were associated with seven cardiovascular disease biomarkers four years later (2014/2016, t2). Further, these models were adjusted for demographic factors, socioeconomic status, health behaviors, chronic conditions, baseline biomarkers, and baseline volunteering. Inverse probability treatment weights (IPTW) were included for the selection into volunteering and multiple imputation was conducted for missing data. Compared to non-volunteers, volunteering more than 200 hours a year was associated with a lower risk for clinically high diastolic blood pressure. Increased volunteering effort (change from 1-99 hours at t0 to >100 hours at t1) was associated with a lower likelihood of clinically high systolic and diastolic blood pressure levels. Sustained high volunteering (>100 hours at both t0 and t1) was associated with lower diastolic blood pressure. The current study adds to the evidence of the health benefits of volunteering for adults 50 and older by inferring a potential causal link between high-intensity volunteering and reduced blood pressure.

### SESSION 4260 (PAPER)

### EPIDEMIOLOGY AND AGING

Abstract citation ID: igad104.1612

### CHANGES IN D3CR MUSCLE MASS, WALKING SPEED, GRIP STRENGTH, AND CHAIR STANDS PERFORMANCE IN THE OLDEST OLD MEN

Peggy Cawthon[1], Li-Yung Lui[2], Megan Hetherington-Rauth[2], Kristine Ensrud[3], Jane Cauley[4], Adam Santanasto[4], William Evans[5], and Eric Orwoll[6], *1. California Pacific Medical Center, Research Institute, San Francisco, California, United States*, *2. California Pacific Medical Center, San Francisco, California, United States*, *3. University of Minnesota, Minneapolis, Minnesota, United States*, *4. University of Pittsburgh, Pittsburgh, Pennsylvania, United States*, *5. University of California, Berkeley, Berkeley, California, United States*, *6. OHSU, Portland, Oregon, United States*

One tenet in sarcopenia research is that strength is lost substantially more quickly than muscle mass with age. This assertion is mostly supported by data that uses indirect proxies for muscle mass, such as lean mass from dual energy x-ray absorptiometry (DXA). We posit that changes in direct estimates of muscle mass, such as muscle mass determined by d3-creatine dilution (D3Cr muscle mass), will be similar in magnitude to changes in strength and physical performance. In men who completed the Year 14 and Year 20 visits (N=220, Year 14 mean age: 82.9 yrs) of the MrOS study, we measured change in D3Cr muscle mass (kg), walking speed (m/s), grip strength (kg), and five repeat chair stand performance (stands/10 seconds). Over an average of 6.1 years of follow-up, men had extensive declines in D3Cr muscle mass (-3.29 kg, -12.8%), grip strength (-5.4 kg,-15.1%), walking speed (-0.23 m/s, -17.8%), and chair stands performance (-1.0 stands/10 seconds,-9.0%). Annualized percent change was of similar magnitude for D3Cr muscle mass (-2.2 %/year), grip strength (-2.5 %/year), walking speed (-2.9%/yr); with slightly lesser decline in chair stands performance (-1.5%/year). Change in D3Cr muscle mass was correlated with change in grip strength (r=0.22, p<.05) but not with change in walking speed or chair stand performance. Our finding that changes in muscle mass and strength were of similar magnitude and intercorrelated suggest that muscle mass itself has a more important role in loss of strength with age than has been previously considered, at least in the oldest-old men.

Abstract citation ID: igad104.1613

### DEVELOPMENTAL ORIGINS OF EXCEPTIONAL HEALTH AND SURVIVAL: A FOUR-GENERATION COHORT STUDY

Matthew Keys[1], Pernille Larsen[1], Dorthe Pedersen[1], Alexander Kulminski[2], Mary Feitosa[3], Mary Wojczynski[3], and Kaare Christensen[1], *1. University of Southern Denmark, Odense, Syddanmark, Denmark*, *2. Duke University, Durham, North Carolina, United States*, *3. Washington University School of Medicine, St. Louis, Missouri, United States*

Little is known about the early life health trajectories associated with longevity, due to the length of follow-up required for such studies. In the Long Life Family Study (LLFS) we have identified 659 Danish long-lived sibships and their descendants across three generations and followed them through national civil and health registries. We have previously shown lower adult mortality and morbidity in both offspring and grandchildren generations. Here we compared perinatal, infant, and maternal health outcomes of third-generation grandchildren (n = 5637) and also fourth-generation great-grandchildren (14,908) born into these long-lived families between 1973-2018, to matched controls from the general population. Hazard ratios (HRs) and odds ratios (ORs) were estimated by Cox proportional hazards and conditional logistic regression models. A general pattern of reduced risk was observed in the grandchildren across a range of perinatal and infant health outcomes, most notably in infant mortality (HR = 0.53, 95% CI [0.36, 0.77]), preterm birth (OR = 0.82, [0.72, 0.93]), and low birth weight (OR = 0.83, [0.76, 0.90]). These associations were robust to further adjustment for parental education levels. Negligible infant survival advantage was observed in the great-grandchildren (HR = 0.90, [0.70, 1.17]). However, there were signals of benefit across some other perinatal outcomes, although much fewer and weaker in magnitude than in the grandchildren. Our findings suggest that phenotypic longevity may have developmental origins as early as the perinatal period, and that this effect persists over at least three generations.

Abstract citation ID: igad104.1614

### EPIDEMIOLOGY OF THE INVERSE COMORBIDITY OF DEMENTIA AND CANCER: THE CHALLENGE OF SELECTIVE SURVIVAL BIAS

Michelle Shardell[1], Alan Rathbun[2], Ann Gruber-Baldini[1], Jack Guralnik[1], Justine Golden[1], Dimitrios Kapogiannis[3], and Eleanor Simonsick[3], *1. University of Maryland School of Medicine, Baltimore, Maryland, United States*, *2. University of Maryland, Baltimore, Baltimore, Maryland, United States*, *3. National Institute on Aging, National Institutes of Health, Baltimore*

Observational studies have consistently found dementia to be inversely related to cancer, where presence of cancer predicts lower dementia risk, and presence of dementia predicts lower cancer risk. This “inverse comorbidity” relationship is of interest as an avenue to potentially identify novel intervention targets to develop for both conditions. However, it is unknown whether inverse comorbidity reflects underlying physiological mechanisms or is explained by selective survival bias. Specifically, persons with cancer are more likely to die before dementia onset than otherwise similar persons without cancer. Thus, we aimed to assess the robustness of inverse comorbidity between dementia and cancer to statistical methods that vary in how they address selective survival bias. Four discrete-time modeling methods were applied to data from the Health, Aging and Body Composition Study (n=3075; age: 70-79 years; 41.7% black; 51.5% women): (1) generalized estimating equations (GEE) assuming all missingness to be completely at random; (2) weighted independence estimating equations partly conditioned on being alive that only weight data missing for non-mortality reasons; (3) multiply-weighted GEE weighting data for all sources of missingness; and (4) marginal structural models that additionally adjust for covariates using inverse propensity score weights. Covariates included: demographics, APOEε, lifestyle characteristics, and health conditions. Methods (1)-(3) produced almost identical results: (1) Risk Ratio(RR)=0.81, 95% Confidence Interval(CI)=0.69-0.95; (2) RR=0.81, 95%CI=0.69-0.94; and (3) RR=0.81, 95%CI=0.69-0.94. Results for method (4) were slightly attenuated: RR=0.85, 95%CI=0.72-0.99. Inverse comorbidity was consistently observed and robust to statistical handling of missing data/mortality. Future research aims to identify mechanisms of inverse comorbidity.

Abstract citation ID: igad104.1615

### THE U-SHAPED RELATIONSHIP BETWEEN DAILY ENERGY INTAKE AND ALL-CAUSE MORTALITY IN US ADULTS

Samaneh Farsijani[1], Ziling Mao[1], and Anne Newman[2], *1. University of Pittsburgh, Pittsburgh, Pennsylvania, United States*, *2. University, Pittburgh, Pennsylvania, United States*

The association between low energy intake and longevity observed in non-human studies has not been confirmed in large-scale human investigations. Therefore, we determined the associations between daily energy intake and all-cause mortality in US adults. We included 33,094 US adults (age>19 years) from the National Health and Nutrition Examination Survey (2003-2018). Total energy intake was obtained from 24-hour food recalls and mortality was determined through December 2019 using the National Death Index. Survey-weighted multivariable Cox proportional hazard models were used to calculate all-cause hazard ratios (HR) across categories of energy intake. HR models were stratified by age, sex, race, and BMI, while controlling for potential covariates. During a median follow-up of 96 months, 4,162 participants died. Low energy intake (< 1,000 kcal/day) was associated with higher mortality (HR=1.53 [95% CI 1.26-1.86]) vs. the reference category (2,000-2,500 kcal/d) independent of age, sex, race, BMI, and income. Similarly, low energy intake was associated with higher mortality risk (P< 0.05) in middle-aged (40-64y) and older (≥65y) adults, men, women, and Whites. We observed the same negative associations between energy intake and mortality in normal-weight (18.5-24.9 kg/

m2), over-weight (25.0-29.9), and obese (≥30.0) individuals (P< 0.05). The only exception was in Blacks, where lower energy intake was associated with lower mortality (HR=0.75 [0.57-0.99]). Higher energy intake (≥4,000 kcal/d) was also associated with higher mortality, though HRs were not significant, except in young (20-39y), women, and normal weight adults. Our result showed a U-shaped relationship between total energy intake and mortality, like the observed relationship between BMI and mortality.

### SESSION 4265 (PAPER)

### FACTORS IMPACTING SLEEP AND AGING

Abstract citation ID: igad104.1616

### ACTIGRAPHIC MEASURES OF SLEEP AND METABOLIC SYNDROME AMONG OLDER ADULTS

Diefei Chen[1], Yiwei Yue[1], Sharmin Hossain[2], Jill Rabinowitz[1], Amal Wanigatunga[1], Luigi Ferrucci[3], Eleanor Simonsick[3], and Adam Spira[1], *1. Johns Hopkins Bloomberg School of Public Health, Baltimore, Maryland, United States*, *2. Maryland Department of Health, Linthicum Heights, Maryland, United States*, *3. NNational Institute on Aging, National Institutes of Health, Baltimore*

Self-reported sleep disturbances are associated with obesity and diabetes, but little is known about links between objectively measured sleep and metabolic syndrome (MetS) in later life. We investigated associations between actigraphic sleep parameters and MetS in 406 participants (53.5% women, 20.9% Black) aged 72.8±10.1 years at baseline (range: 50-96) in the Baltimore Longitudinal Study of Aging. Participants completed wrist actigraphy (6.6±1.0 nights) and were classified as having MetS if they had ≥3 of the following: waist circumference ≥102 cm for men, ≥89 cm for women; high-density lipoproteins < 40 mg/dL in men, < 50 mg/dL in women; triglycerides >150 mg/dL; high blood pressure ≥130/85 mmHg; fasting blood glucose ≥100 mg/dL. Overall, 37 participants had MetS at baseline and 27 participants developed MetS over 2.3±2.2 years of follow-up. Cross-sectionally, compared to participants with total sleep time (TST) < 6 hours, those with 6-8 hours TST had lower odds of MetS (OR=0.28, 95%CI 0.13,0.61); higher sleep efficiency (SE) was associated with lower odds of MetS (OR=0.65, 95%CI 0.48,0.89) and longer wake bout length (WBL) was associated with higher odds of MetS (OR=1.47, 95%CI 1.07,2.01). Excluding 37 participants with MetS at baseline, TST >8 hours was associated with faster increases in the likelihood of developing MetS (OR=1.62, 95% CI 1.03, 2.55). Findings link intermediate TST and higher SE to lower odds of MetS, and longer WBL to higher odds of MetS in older adults cross-sectionally; TST >8 hours was associated with higher odds of incident MetS. Further studies are needed to understand mediators of sleep-MetS associations.

Abstract citation ID: igad104.1617

### ASSOCIATIONS OF NIGHT AND DAY SLEEP WITH COGNITIVE PERFORMANCE IN SHIFT WORKERS: PRELIMINARY RESULTS

Jinyoung Kim[1], Soonhyun Yook[2], Stephanie Nam[1], Hosung Kim[2], Mohammad Hesam Alavi[1], Joanna Oliver[1], and Diane Lim[3], *1. University of Nevada, Las Vegas, Las Vegas, Nevada, United States*, *2. University of Southern California, Los Angeles, California, United States*, *3. Miami VA Healthcare System, Miami, Florida, United States*

Emerging evidence suggests that night shift workers are at higher risk of severe cognitive impairment, mainly because of the repeated disruption of the sleep–wake schedule and the resulting poor sleep (e.g., less deep sleep or shorter sleep time). However, questions remain about how daytime and nighttime sleep characteristics correlate with cognitive performance in shift workers. The purpose of this study is to examine the association of objective sleep quality, measured on workdays and days off, with cognitive performance, determined by the psychomotor vigilance task (PVT), in shift workers compared to day workers. We included eight night shift and ten day shift workers from an ongoing pilot study in this analysis. Participants undergo multiple home sleep studies with electroencephalograms on their workdays or days off. Sleep quality measures include total sleep time (TST), percentage of deep sleep stage in TST, and wake time after sleep onset (WASO). Night shift workers showed longer TST and shorter WASO during their nighttime sleep on days off compared to day shift workers. In night shift workers, longer deep sleep on days off was significantly correlated with faster mean reaction time to a visual stimulus (better cognitive performance) on the PVT (r = -.944, p = .016), after controlling for age. However, longer deep sleep during their daytime sleep was associated with slower reaction time (r = .756, p = .049). Further studies are necessary to elucidate differential impacts of daytime and nighttime sleep on the risk of future cognitive impairment in night shift workers.

Abstract citation ID: igad104.1618

### OBSTRUCTIVE SLEEP APNEA RISK AND LONGITUDINAL TRAJECTORY OF FUNCTIONAL HEARING LOSS OVER EIGHT YEARS OF FOLLOW-UP

Kening Jiang[1], Adam Spira[1], Nicholas Reed[1], Frank Lin[2], and Jennifer Deal[3], *1. Johns Hopkins Bloomberg School of Public Health, Baltimore, Maryland, United States*, *2. Johns Hopkins, Baltimore, Maryland, United States*, *3. Johns Hopkins University, Baltimore, Maryland, United States*

Hearing loss prevention is important for physical, cognitive and psychosocial health among older adults. Obstructive sleep apnea (OSA) might cause functional hearing loss through ischemic damages to the cochlea or central auditory processing, but there is a lack of longitudinal evidence. The National Health and Aging Trends Study (NHATS) is a longitudinal panel survey of Medicare beneficiaries aged 65+ years. STOP-Bang is a validated screening tool for OSA risk and a subsample of NHATS round 3 participants responded to a modified version (age>50, male, high blood pressure, body mass index >35 kg/m2, snore loudly, observed apnea, tiredness; range 0-7, elevated risk if ≥3). Functional hearing loss at follow-up (rounds 3-11) was defined as self-reported deafness, hearing aid use, or inability to hear well enough to use the telephone or have conversations in a room with the television or radio on. We used generalized estimating equations logistic regression with inverse probability attrition weighting, adjusting for demographics and comorbidities. Among 1,433 participants, elevated OSA (58%) was associated with higher odds of hearing loss at baseline (odds ratio:1.50, 95% confidence interval[CI]:1.08, 2.10). Both OSA groups had significant annual increases in odds of functional hearing loss (Low:1.07, 95% CI:1.01, 1.13; Elevated:1.12, 95% CI:1.07, 1.18) during the first 4 years. Afterwards, the low OSA risk group continued to have significant increase in odds of functional hearing loss but not the elevated OSA risk group. OSA might be a novel risk factor for hearing loss. Screening and treating OSA might be important for hearing loss prevention.

Abstract citation ID: igad104.1619

### WHAT CAN WE LEARN FROM THE LINK BETWEEN SLEEP AND GAIT ON THE AGING PROCESS?

Maayan Agmon[1], Roy Tzemah-Shahar[2], and Tamar Shochat[1], *1. University of Haifa, Haifa, HaZafon, Israel*, *2. University of Haifa, Ramat-Gan, HaZafon, Israel*

Falls are a major problem among older adults; and older adults with sleep complaints are at a four-fold risk of falls compared with those without sleep complaints. Recent studies demonstrate comorbidity between gait and sleep, which are two essential, modifiable, and age-sensitive functions. Yet, most studies were conducted on older adults, whereas studies on midlife adults are lacking. Such studies can open a window to better understanding of the aging process. We aim to explore the link between sleep quality and gait among community-dwelling midlife adults. We recruited 103 midlife adults (age 59.3±7). Gait was evaluated with the cognitive Dual-Task (DT) paradigm. Gait speed and variability were assessed with the APDM mobility lab. Sleep quality was evaluated objectively using one-week actigraphy and subjectively using the Pittsburg Sleep Quality Index (PSQI). We found that age was significantly associated with earlier bedtime (p=0.007), lower sleep efficiency (p=0.01) and increased wake after sleep onset (p=0.01). Sex differences were found for objective and subjective sleep duration (actigraphy and PSQI, p=0.002 and p< 0.001, respectively), with women sleeping significantly more than men. No sex differences were found for sleep efficiency. A significant main effect was found for actigraphy-based sleep duration (short/intermediate/long) on gait speed, such that gait speed was slower in long compared to short and intermediate sleepers under DT only (p=0.018). To conclude, a negative association between sleep quality and gait speed under DT was found, showing adverse health outcomes for long versus short and intermediate sleepers in midlife.

### SESSION 4270 (AWARD LECTURE)

Abstract citation ID: igad104.1620

### THE M. POWELL LAWTON AWARD LECTURE AND PRESENTATION

Chair: Tiffany Washington

The M. Powell Lawton Award Lecture will feature an address by the 2022 recipient Jon Pynoos, PhD, FGSA, FAGHE, of the University of Southern California. This session will also include the presentation of the 2023 M. Powell Lawton Award to recipient. The M. Powell Lawton Award is presented annually to an individual who has made outstanding contributions from applied research that has benefited older people and their care. The Lawton Award is generously funded by the Polisher Research Institute of Abramson Senior Care.

Abstract citation ID: igad104.1621

### HOUSING AND HOME MODIFICATION FOR AGING IN PLACE: PERSPECTIVES, PROGRESS, AND FUTURE DIRECTIONS

Jon Pynoos, *University of Southern California, Los Angeles, California, United States*

Aging in place remains a high priority of older adults. However, the home environments in which they reside often have hazards and lack supportive features, putting them at risk of falls and making it difficult to carry out daily activities. People who need home modifications the most (i.e., vulnerable populations) are the least likely to have them. In response to the needs of the growing population of older adults, there has been notable progress in the field. Now, with an increasing recognition of the impact of housing on health, there are new opportunities to move the field forward. Highlighting Powell Lawton's contributions as a foundation, this lecture will: 1) review major developments in research, service delivery, policy, and consumer education, and 2) discuss recommendations for action that practitioners, researchers, policymakers, and advocates could undertake to realize Lawton's vision of home environments that support aging for all.

### SESSION 4275 (PAPER)

### MEANINGFUL MODERATORS AND PATHWAYS

Abstract citation ID: igad104.1622

### DETERMINANTS OF INFORMAL CARE TRAJECTORIES IN ENGLAND

Giorgio Di Gessa[1], and Christian Deindl[2], *1. University College London, London, England, United Kingdom*, *2. TU Dortmund University, Dortmund, Nordrhein-Westfalen, Germany*

In the context of an ageing population, combined with long-standing challenges in the delivery of formal social care for older people, unpaid caregivers play a key role in promoting the quality of life of older people and their extended families and ensuring that needs for care and support are met. Although many studies have provided snapshots of informal care provision, few so far have examined longitudinal patterns of informal care provision among older people. This paper aims to describe caregiving trajectories in later life and to examine how socioeconomic, demographic, and health characteristics of older adults relate to these patterns (including needs and enabling factors). Using six waves of the English Longitudinal Study of Ageing, we conduct latent trajectory analysis to cluster people's diverse trajectories into a finite number of groups. The intensity of informal care provision is also considered when identifying longitudinal trajectories. Preliminary results show five distinct trajectories of informal care provision (including those who never provide informal care, those who provide sporadic informal care, those with increasing and decreasing commitment to care provision, and those who care throughout). Gender, age, health, income, living arrangements, and family compositions all relate to long-term trajectories of informal care, with younger married women in good health more likely to provide informal care throughout. Support should be provided to this group of caregivers, given that engagement in long-term caregiving might be detrimental to mental health.

Abstract citation ID: igad104.1623

### DIFFERENCE IN TIME SPENT CAREGIVING BETWEEN CAREGIVERS OF IMMIGRANT AND NONIMMIGRANT OLDER ADULTS

Zainab Suntai[1], and Joana Okine[2], *1. Baylor University, Waco, Texas, United States*, *2. University of Alabama, Tuscaloosa, Alabama, United States*

Each year, the number of informal caregivers in the United States continues to rise, in conjunction with the increasing aging population. Concurrently, the number of immigrants in the United States had progressively increased over the past few decades, leading to an intersection of three pivotal issues: caregiving, aging, and immigration. While there are several studies targeting caregiving in the United States, fewer studies have aimed to understand the experiences of caregivers of older immigrants. This study examined differences in time spent caregiving between caregivers of older immigrants compared to caregivers of older non-immigrants. Data were taken from Round 11 (2022) of the National Study of Caregiving, a nationally representative study of informal caregivers caring for an older adult with functional limitations. Chi-square tests were used to test bivariate associations, and a multivariate logistic regression model was run to determine the relationship between and older adult's immigration status and time spent caregiving by their caregiver. Results showed that caregivers of older immigrants were significantly more likely to report that they provided care every day, compared to caregivers of non-immigrants. These results point to potential cultural implications for immigrant families, who are generally less likely to use formal caregiving avenues such as nursing homes, and less likely to use additional services such as home health aides. Implications for researchers, policymakers, and practitioners are discussed.

Abstract citation ID: igad104.1624

### DIFFERENTIAL EFFECTS OF ADULT CHILDREN'S HEALTH PROBLEMS ON PRIMARY AND SECONDARY CAREGIVING FOR PARENTS

Catherine Stepniak[1], J Jill Suitor[1], and Megan Gilligan[2], *1. Purdue University, West Lafayette, Indiana, United States*, *2. University of Missouri, Columbia, Missouri, United States*

Adult children often serve as primary and secondary caregivers to their aging parents as they face greater needs for assistance. However, it could be expected that the flow of caregiving support from adult children to parents might be disrupted if adult children are facing their own serious health challenges. Surprisingly, the literature provides no clear picture of the way in which children's health moderates their provision of care to older parents. In the present study, we shed new light on this question using quantitative and qualitative data collected from 156 mothers ages 75-85 regarding assistance provided by their 586 adult children as part of the Within-Family Differences Study. Additionally, we will explore if there are differences based on timing of the children's health problems, the types of health problems (physical health vs. psychological health), and gender of the adult children. Quantitative analyses showed that adult children with serious health problems were just as likely as their healthy siblings to be a primary or secondary caregiver to their mothers. Qualitative data revealed the complex emotional and practical cost-benefit analyses mothers and their adult children with health problems engaged in to maintain reciprocal support exchanges. Our findings underscore the importance of using mixed-method approaches to understand the ways in which the provision of care to parents is shaped by adult children's own health status.

Abstract citation ID: igad104.1625

### SUBJECTIVE AGE MODERATES THE RELATIONSHIP BETWEEN GLOBAL COGNITION AND SUSCEPTIBILITY TO SCAMS

Gali Weissberger[1], Aaron Lim[2], Laura Mosqueda[3], Annie Nguyen[2], Laura Fenton[2], and Duke Han[2], *1. Bar Ilan University, Ramat Gan, HaMerkaz, Israel*, *2. University of Southern California, Los Angeles, California, United States*, *3. Keck School of Medicine of the University of Southern California, Los Angeles, California, United States*

Understanding factors that increase susceptibility to scams in old age is of critical importance. Cognitive functioning has been demonstrated to be an important factor in predicting financial exploitation and scam risk. There is also evidence that subjective age, or one's perceived age relative to chronological age, may be associated with financial exploitation risk. This study examined the interactive effect of subjective age on the relationship between global cognition and susceptibility to scams. Sixty-five participants underwent an assessment of global cognition (Mini-Mental State Examination; MMSE), reported their subjective age, and responded to a self-report questionnaire on scam susceptibility. A main effect of global cognition on scam susceptibility was found ($b = -0.029$, $ß = -0.280$, $p = 0.028$); there was no main effect of subjective age on scam susceptibility ($p = 0.819$). An interaction between cognition and subjective age was found, such that the negative association between cognition and scam susceptibility increased as subjective age increased ($b = -0.185$, $ß = -0.311$, $p = 0.016$). Examination of conditional effects demonstrated that the relationship between cognition and scam susceptibility was not significant amongst those with subjective ages below one standard deviation (SD) of the mean, but was significant for those whose subjective ages fell around or above the mean (within 1SD, $b = -0.03$, $SE = 0.01$, $p = 0.03$; +1SD, $b = -0.06$, $SE = 0.02$, $p = 0.001$). Findings suggest that individuals with older subjective ages may be particularly vulnerable to the negative effects of lower cognition on scam susceptibility.

### SESSION 4280 (SYMPOSIUM)

Abstract citation ID: igad104.1626

### THE IMPACT OF COVID-19 ON RESIDENTS AND FAMILY/FRIEND CAREGIVERS IN ASSISTED LIVING HOMES

Chair: Matthias Hoben Co-Chair: Colleen Maxwell
Discussant: Anna Beeber

Healthcare reforms have neglected assisted living (AL) and nursing homes (NHs) for decades, setting them up for the excessive rates of death and suffering during COVID-19. Research before and during the pandemic has primarily focused on NHs, largely overlooking AL. AL is rapidly expanding, caring for people with similar vulnerabilities as those in NHs, yet is less regulated, offers fewer services, has lower staffing/skill mix levels and requires significant family/friend involvement in care. This symposium presents a program of research (COVCARES, COVID-19 and the Care of Assisted living Residents), aiming to understand how the pandemic has impacted AL resident, family/friend, and facility outcomes, and how resident outcomes compare between NHs and AL. Our research started over a decade ago with the first population-based cohort study comparing AL and NHs in Canada. Our current research includes repeated surveys (10/2020–04/2021 and 07/2021–09/2021) with family/friend caregivers and AL facilities, and population-based clinical and health administrative data (2017-2021) from AL and NH residents in Western Canada. Five presentations will report on the design/methods/goals of COVCARES (#1), the impact of the pandemic on family/friend involvement in AL resident care (#2), impacted of the pandemic on psychotropic drug prescriptions in AL (#3), and the association of AL home preparedness for and response to the pandemic on resident pain (#4) and loneliness (#5). Our discussant (Anna Beeber) will highlight similarities and differences between AL and NHs, similarities and differences in both settings between the US and Canada, and how policymakers can account for these differences.

Abstract citation ID: igad104.1627

### COVID-19 AND THE CARE OF ASSISTED LIVING RESIDENTS: THE COVCARES PROGRAM OF RESEARCH

Matthias Hoben[1], and Colleen Maxwell[2], *1. York University, Toronto, Ontario, Canada*, *2. University of Waterloo, Waterloo, Ontario, Canada*

This presentation describes the goals, design and methods of the COVID-19 and the Care of Assisted living Residents (COVCARES) program of research. Our goals were to address critical knowledge gaps related to how COVID-19 impacted assisted living (AL) residents, family/friend caregivers, and homes, and to compare how outcomes differed between AL and nursing homes (NH). Partnering closely with provincial Alzheimer Societies, family caregiver advocates, and health policy decision makers, we conducted two interconnected studies: The first was a prospective cohort study, surveying family/friend caregivers of AL residents and directors of care of AL homes in the Canadian provinces of Alberta and British Columbia twice (10/2020–04/2021 and 07/2021–09/2021). 673 caregivers and 104 AL homes responded to our first survey. Of those, 386 caregivers and 78 AL homes submitted a second survey. The second was a population-based retrospective cohort study (01/2017–12/2021), using clinical and health administrative data of all AL and NH residents in Alberta. Data included Resident Assessment Instrument (RAI) records, acute care and emergency department admission data, prescription medications, data on COVID-19 infections, and vital statistics of 23,355 AL residents and 41,583 NH residents. Analyzing each of the aforementioned data sets individually, and linking family and facility surveys, as well as resident and facility survey data, we provide unique insights into the impacts of the COVID-19 pandemic on AL settings. The presentations in this symposium will share selected key findings from our program of research.

Abstract citation ID: igad104.1628

### CAREGIVER INVOLVEMENT AND CONCERNS WITH RESIDENT CARE IN ASSISTED LIVING BEFORE AND DURING COVID-19

Matthias Hoben[1], Hana Dampf[2], David Hogan[3], Kyle Corbett[2], Kim McGrail[4], Lauren Griffith[5], Andrea Gruneir[2], and Colleen Maxwell[6], *1. York University, Toronto, Ontario, Canada*, *2. University of Alberta, Edmonton, Alberta, Canada*, *3. Univeresity of Calgary, Calgary, Alberta, Canada*, *4. University of British Columbia, Vancouver, British Columbia, Canada*, *5. McMaster University, Hamilton, Ontario, Canada*, *6. University of Waterloo, Waterloo, Ontario, Canada*

Family/friend caregivers are essential in promoting assisted living (AL) residents' health/well-being, but their involvement was restricted during the COVID-19 pandemic. Care needs in AL are similarly complex as in nursing homes, but fewer staffing resources and services are available. Caregiver involvement and concerns with care of AL residents before and during the COVID-19 pandemic is a critical knowledge gap. This prospective cohort study collected online surveys from caregivers to AL residents in Western Canada. Surveys assessed socio-demographics, ways of visiting or communicating with residents, involvement in care tasks, concerns with resident physical/mental health, perceived lack of resident access to care services, and whether caregivers felt well informed/involved with resident care. Our first survey assessed outcomes in the three months before and after pandemic wave 1 (03/2020), our second survey assessed outcomes in pandemic wave 2 (11/2020 -02/2021). Based on 386 responses, in-person visits dropped significantly in wave 1 (91.5% to 30.6%) and so did caregiver involvement in nearly all care tasks (e.g., toileting: 7.5% to 2.6%, social/recreational activities: 73.6% to 25.7%). While these rates increased in wave 2, most did not return to pre-pandemic levels. Caregiver concerns increased in wave 1 (e.g., concerns about residents' depressed mood: from 21.3% to 50.3%) and stayed high in wave 2. Concerns were particularly high (e.g., >20% higher for depressed mood) among caregivers who did not feel well informed/involved with resident care. Continued caregiver involvement in resident care and communication with caregivers is key to mitigating caregiver concerns and supporting caregiver and resident well-being.

Abstract citation ID: igad104.1629

### ASSOCIATION OF ASSISTED LIVING HOMES PREPAREDNESS AND RESPONSES TO THE COVID-19 PANDEMIC WITH RESIDENT LONELINESS

Rashmi Devkota[1], Colleen Maxwell[2], Hana Dampf[1], Shovana Shrestha[1], and Matthias Hoben[3], *1. University of Alberta, Edmonton, Alberta, Canada*, *2. University of Waterloo, Waterloo, Ontario, Canada*, *3. York University, Toronto, Ontario, Canada*

Loneliness has been an ongoing problem among Assisted living (AL) residents and has worsened during the COVID-19 pandemic. Loneliness is associated with an increased risk of mental and physical illness, cognitive decline, suicidal behavior, and all-cause mortality. However, the impact of the pandemic on AL residents' loneliness is poorly understood. We surveyed 42 AL homes in Alberta to understand whether resident characteristics and homes' pandemic preparedness and response to the pandemic were associated with resident loneliness. The surveys reflected pandemic waves 1 (Mar-Jul 2020) and 2 (Nov 2020-Feb 2021) and were linked to Resident Assessment Instrument (RAI) records of residents who lived in these homes during these periods. Using generalized estimating equation models, we assessed whether resident characteristics, residents' social relationships, and home preparedness for and responses to the pandemic were associated with resident loneliness (measured as present or absent based on RAI items). Our sample included 1,828 residents (wave 1: 890, wave 2: 938). Almost 13% of the residents reported loneliness (wave 1: 11.2%, wave 2: 14.2%). Depressive symptoms (odds ratio [OR]=2.73, 95% CI, 1.9-3.9) daily/excruciating pain (OR=1.97, 95% CI, 1.4-2.8), and cognitive impairment (OR=0.38, 95% CI, 0.2-0.6) were significantly associated with loneliness. Caregiver availability, hours of caregiver help, and home preparedness were not associated with loneliness, but more communication with caregivers (OR=2.19, 95% CI, 1.1-4.2) and same or improved staff morale (OR=0.61, 95% CI, 0.4-0.9) were. Improving staff morale and communication with caregivers is crucial in addressing resident loneliness.

Abstract citation ID: igad104.1630

### ASSOCIATION OF ASSISTED LIVING HOMES PREPAREDNESS FOR AND RESPONSES TO THE COVID-19 PANDEMIC WITH RESIDENT PAIN

Shovana Shrestha[1], Colleen Maxwell[2], Hana Dampf[1], Rashmi Devkota[1], and Matthias Hoben[3], *1. University of Alberta, Edmonton, Alberta, Canada*, *2. University of Waterloo, Waterloo, Ontario, Canada*, *3. York University, Toronto, Ontario, Canada*

The COVID-19 pandemic severely disrupted care processes in assisted living (AL) and negatively affected resident outcomes associated with resident pain (e.g., mobility problems, depression). Pain has severe consequences, including hopelessness, insomnia, depression, poor quality of life. However, we lack research on how the pandemic affected AL resident pain. To address this gap, we linked surveys from 42 AL homes in Alberta, reflecting pandemic waves 1 (Mar-Jul 2020) and 2 (Nov 2020-Feb 2021), to the Resident Assessment Instrument (RAI) records of 1,828 residents (wave 1: 890, wave 2: 938) who lived in these homes during these periods. Using generalized estimating equation models, we assessed whether resident characteristics, physical and occupational therapy received, home preparedness for and responses to the pandemic were associated with resident pain (measured as at least moderate daily pain or pain of excruciating intensity, based on the RAI pain items). Over 19% of the residents reported pain (wave 1: 19%, wave 2: 19.1%). Resident characteristics associated with pain were cognitive impairment (OR=0.4, 95% CI: 0.3-0.6), loneliness (OR=1.8, 95% CI: 1.3-2.6), arthritis (OR=2.1, 95% CI: 1.6-2.8), fractures (OR=1.97, 95% CI: 1.4-2.9), polypharmacy (OR=1.82, 95% CI: 1.3-2.6) and use of analgesics (OR=1.76, 95% CI: 1.3-2.3). Home preparedness and physical and occupational therapy received were not associated with pain, but more communication with family/friend caregivers (OR=0.41, 95% CI: 0.2-0.8) was. Effective communication of family/friends with residents may promote better management of residents' pain. Further, longitudinal studies examining resident and AL home characteristics and its impact on residents' pain are needed.

Abstract citation ID: igad104.1631

### COVID-19 AND PSYCHOTROPIC DRUG USE IN ASSISTED LIVING RESIDENTS: VARIATION BY WAVE AND SETTING TYPE

Colleen Maxwell[1], Hana Dampf[2], Wajd Alkabbani[1], Cecilia Cotton[1], Jean-Michael Gamble[1], David Hogan[3], Andrea Gruneir[2], and Matthias Hoben[4], *1. University of Waterloo, Waterloo, Ontario, Canada*, *2. University of Alberta, Edmonton, Alberta, Canada*, *3. Univeresity of Calgary, Calgary, Alberta, Canada*, *4. York University, Toronto, Ontario, Canada*

The onset of COVID-19 was associated with significant, albeit modest, increases in the use of psychotropics and opioids in nursing home residents. Little research exists on whether similar trends occurred among older residents of publicly funded assisted living (AL) homes, a growing and poorly investigated setting. We examined the impact of pandemic wave (1 to 4) and setting type (dementia designated spaces [AL-D] vs other [AL-O]) on prevalent antipsychotic, antidepressant, benzodiazepine, opioid and anticonvulsant use in AL residents from Alberta, Canada. Using linked population-based clinical and health administrative databases, we conducted a repeated cross-sectional study of quarterly medication prevalence from January 2018 to December 2021. Log-binomial GEE models estimated prevalence ratios (PR) for 4 waves (vs 2018-19 historical months) and setting (AL-D vs AL-O) and period-setting interactions. On March 1, 2020, there were 2,874 AL-D and 6,611 AL-O residents in our cohorts (mean age 82.4 vs 79.9 years and 93.5% vs 42.6% with dementia, respectively). Antipsychotic prevalence increased during waves 2-4 for both settings but this increase was significantly greater for AL-D than AL-O in later waves (e.g., AL-D: PR 1.21, 95%CI 1.14-1.27; AL-O: 1.12 (1.07-1.17) for March-May 2021 vs 2018-19). For both settings, there was a significant but modest increase in antidepressants but a decrease in benzodiazepines during several waves. No pandemic effect was observed for opioids in either setting. The AL resident and home characteristics associated with these medication trends, concerns about medication risks (particularly for dementia care settings), and consequent health outcomes for residents require further study.

### SESSION 4285 (SYMPOSIUM)

Abstract citation ID: igad104.1632

### THE NEXUS OF AGING AND MIGRATION: THE CURRENT STATE OF KNOWLEDGE AND WHERE GERONTOLOGICAL INQUIRIES OUGHT TO HEAD

Chair: Sandra Torres Co-Chair: Alistair Hunter Discussant: Allen Glicksman

Research on older ethno-racialized minorities (which comes primarily from North America) has dominated ethno-gerontological debates over the past few decades. In this research, ethnicity and race have been primarily viewed as social positions that place older people at risk of social exclusion. Migrancy, however, has yet to receive adequate attention from social gerontologists, despite the centrality of migration and aging for demographic change globally in recent decades. When migrancy is considered by gerontologists, it is not uncommon for us to think of the vulnerability trope. The highly-charged political debates in which the social exclusion and deservingness of migrant populations have been at stake have undoubtedly contributed to this state of affairs. From the field of migration studies, some have argued that the nexus of aging and migration offers new lenses from which the intersections of ethnicity, race, migrancy and old age can be approached. This symposium brings together contributors to an international network that has been working to survey the current state of knowledge at the aging-migration nexus and where future research ought to head. This symposium will argue that the study of older migrants has far-reaching implications for the economy, public policy, societal cohesion and individual wellbeing, and that – as such – this is a theoretically-profuse angle of investigation for social gerontology.

Abstract citation ID: igad104.1633

### ETHNICITY IN RESEARCH ON OLDER MIGRANTS: HOW THE SCHOLARSHIP CONCEPTUALIZES IT AND WHERE WE NEED TO HEAD

Sandra Torres, *Uppsala University, Uppsala, Uppsala Lan, Sweden*

A recently completed scoping review of peer-reviewed articles on older ethno-racialized minorities has shown that although conceptual and theoretical advancements have been made in the field of ethnicity and race, the social gerontological literature on these minorities continues to regard ethnicity in essentialist ways, conflates ethnicity with race and seldom operationalizes what these social positions mean to aging and old age. The impact of ethnicity is, in other words, almost always inferred in this research, which is why the field has been deemed to be characterized by data-richness and theory-poorness. Over the past three years, researchers working on the nexus of aging and migration have surveyed the current state of knowledge on older migrants (focusing on migrancy, rather than ethnicity and/ or race). One of these inventories is a meta-analysis of how research on older migrants conceptualizes ethnicity, and the manner in which this social position is presumed to impact on migrants' old age- and aging-related experiences. At stake has also been the assessment of whether this literature differs from the scholarship on older ethno-racialized minorities, and the ways in which that scholarship conceptualizes ethnicity. This presentation offers insight into what this inventory has found out. In focus are the current state of affairs about how ethnicity is approached in research on older migrants, and the launching of a theoretically-informed and empirically-astute research agenda for the study of ethnicity in gerontology.

Abstract citation ID: igad104.1634

### SOCIAL RELATIONS AND OLDER MIGRANTS

Kristine Ajrouch[1], Toni Antonucci[2], and Rita Hu[2], *1. Eastern Michigan University, Ypsilanti, Michigan, United States, 2. University of Michigan, Ann Arbor, Michigan, United States*

This paper elaborates on how the key life course tenets of timing and context represent important factors that influence older adult social relations following the act of migration. The timing of migration may yield specific social relations. For instance, migrating later in life is likely to result in more homogenous and restricted networks compared to migrating as a young adult. Reasons for migration (political instability, family reunification, or socio-economic aspirations) as well as host country factors such as immigrant policies and receptivity infer important contexts in which social relations emerge. The process of movement from one country to another, and the circumstances around those movements, frame the migrant experience and its effects on social relations. Beyond timing and context, there are also demographic and cultural factors that influence the experience. For instance, gender, race and SES indicate social positions in systems of stratification that can influence and structure the people with whom one has ties. Further, cultural factors such as country of origin and language not only signal the ease with which host society incorporation occurs, but also inform the acculturation and meaning-making processes upon which the formation and function of social relations rest. In sum, the concepts found in the life course perspective provide a useful starting point to demonstrate that the effects of migration on older adults' social relations occur within various influential contexts.

Abstract citation ID: igad104.1635

### ACCESS AND USAGE OF CARE AMONG OLDER MIGRANTS: CHALLENGES AND OPPORTUNITIES

Mushira Khan, *Mather Institute, Evanston, Illinois, United States*

Equitable access to care is critical to support healthy aging in underserved groups. Recent global demographic trends such as the rapid aging of the population in developing and developed countries, the growing proportion of migrants from the Global South to the Global North over the past 50 years, and increasing health inequalities between and within countries reflect a continued need to address the underlying pathways and causal mechanisms that determine access to and usage of care among migrants. This paper reviews the current state of knowledge on the micro-, meso-, and macro-level determinants that intersect and shape access to and usage of care services and programs among older migrants. It makes the case that foreign-born older adults are more likely to experience challenges accessing services and programs due to cultural and language barriers and factors related to their socio-economic status, race/ethnicity, gender, and time since immigration. Next, the paper provides a deeper exploration of the interface of structure and agency and an interrogation of how access and usage of care among older migrants may be conditioned, not simply by individual socio-economic circumstances and life course events, but also by macro-level factors like the distribution of power relationships and neoliberal public policies. The paper concludes with a call for sustained efforts to acquire a more profound understanding of individual-level, socio-economic and socio-political forces that influence access and usage of care. Finally, it provides directions for future research with the ultimate goal of supporting healthy aging among older migrants.

### SESSION 4290 (SYMPOSIUM)

Abstract citation ID: igad104.1636

### PRAGMATIC CLINICAL TRIALS TO IMPROVE DEMENTIA CARE: IMPLEMENTATION, ADAPTATION, AND OUTCOMES

Chair: Kali Thomas Discussant: Joseph Gaugler

Pragmatic clinical trials test the effectiveness of interventions in “real-world” settings. One challenge with conducting such research in non-controlled environments is exposure to unpredictable events: in the case of the studies here, the COVID-19 pandemic. This session shares the implementation experiences and findings from four pragmatic clinical trials designed to improve dementia care and describes how the studies were impacted by and adapted to respond to the pandemic. The first speaker discusses a successful, team-based approach for adapting and implementing an intervention to improve sleep for nursing home residents with dementia. The second speaker presents the modifications made to implementation strategies between two trials of a personalized music intervention delivered to nursing home residents with dementia. The third speaker discusses findings from a mixed-methods evaluation with frontline staff at VA Community Living Centers enrolled in a stepped-wedge cluster randomized trial of Montessori-based approaches to improve dementia care. The fourth speaker shares findings from a pilot trial comparing two modes of meal delivery to homebound older adults with dementia. The discussant situates these adaptations and findings in the context of lessons learned by other trialists working to improve care provided to people living with dementia.

Abstract citation ID: igad104.1637

### RESULTS FROM A PILOT TRIAL COMPARING MODES OF MEAL DELIVERY TO HOMEBOUND OLDER ADULTS WITH DEMENTIA

Kali Thomas[1], Jennifer Bunker[2], Ellen McCreedy[3], Michelle Hilgeman[4], Emily Gabois[3], and Roee Gutman[2], *1. Johns Hopkins University, Baltimore, Maryland, United States*, *2. Brown University, Providence, Rhode Island, United States*, *3. Brown University School of Public Health, Providence, Rhode Island, United States*, *4. Tuscaloosa VA Medical Center, Tuscaloosa, Alabama, United States*

Home-delivered meals promote independence among homebound older adults. However, it is unclear which mode of meal delivery delays nursing home placement among homebound older adults with dementia. We conducted a pilot multisite, two-arm, pragmatic clinical trial in May 2021-May 2022 in which participants with dementia were randomized to receive either: 1) a lunch-time meal delivered 5 times per week or 2) 10 frozen meals that were mailed to participants every two weeks. We used the Minimum Data Set to examine time from randomization to nursing home placement (primary outcome). Our analytic sample included 225 individuals with self-reported dementia on waiting lists at three Meals on Wheels programs in Florida and Texas. 121 participants were randomized to daily-delivered meals and 104 to frozen, drop-shipped meals. At 6 months from randomization, 9(7%) who received daily-delivered meals were admitted to a nursing home, compared to 15(14%) who received frozen, drop-shipped meals p-value=0.14,95%CI(-0.02,0.16). The adjusted log hazard ratio of nursing home placement was -0.65(SE=0.42)p-value=0.12). While this pilot was not adequately powered to detect statistically significant differences between arms, results suggest that even with social distancing requirements and a reduction in the number of deliveries because of the COVID-19 pandemic, there is a trend toward lower nursing home placement among participants with dementia who received daily-delivered meals compared to frozen, drop-shipped meals.

Abstract citation ID: igad104.1638

### PRAGMATIC IMPLEMENTATION OF A BEHAVIORAL INTERVENTION PRE- AND POSTPANDEMIC: A TALE OF TWO TRIALS

Ellen McCreedy[1], Aleena Dewji[2], Laura Dionne[1], Vincent Mor[1], and James Rudolph[3], *1. Brown University School of Public Health, Providence, Rhode Island, United States*, *2. Brown University, School of public Health, Providence, Rhode Island, United States*, *3. Brown University, Providence, Rhode Island, United States*

We describe two embedded pragmatic trials (ePCTs) of a personalized music intervention for nursing home (NH) residents with ADRD conducted pre-COVID (2019) and 2021 (COVID). Between trials, the implementation strategy was modified to incorporate learnings from the first trial and to address COVID-related restrictions. 54 intervention NHs (27 from Trial 1, 27 from Trial 2) comprised the sample. We compare the trials using the Framework for Implementation Fidelity (FIF) criteria: coverage (number of residents who received the intervention); duration (dose of music received); and frequency (how often nursing staff used the music with the residents). Adherence was worse in Trial 2 (post-COVID) versus Trial 1 (pre-COVID). On average, 12.7 (SD: 3.6) residents were exposed per NH in Trial 1 and 7.5 (SD: 5.5) in Trial 2. Median minutes of music per day was 27.1 (SD: 23.9) minutes in Trial 1 and 2.5 (SD: 5.2) minutes in Trial 2. A greater proportion of exposed residents had nursing staff administer music in Trial 1 (frequency; 0.4 (SD:0.3)) than Trial 2 (0.2 (0.3)). Despite changes to increase nursing engagement and clinical targeting, adherence to intervention was dramatically lower in Trial 2 than in Trial 1. While it is impossible to completely disentangle changes to the implementation strategy from the lingering effects of COVID, it is clear that the NH environment fundamentally changed during the pandemic. With that change comes the need to re-evaluate strategies for implementation and incentivizing participation

Abstract citation ID: igad104.1639

### "SMALL BITES" QUALITY IMPROVEMENT: A SLEEP INTERVENTION FOR RESIDENTS WITH DEMENTIA IN THE NURSING HOME "NEW NORMAL"

A Lynn Snow[1], Megan McCullough[2], Robert Morgan[3], Rosa Baier[4], Ellen McCreedy[4], Kathy Richards[5], Liam Fry[6], and Christine Hartmann[7], *1. University of Alabama, Tuscaloosa, Alabama, United States*, *2. University of Massachusetts Lowell, Lowell, Massachusetts, United States*, *3. University Of Texas Health Science Center, Houston, Texas, United States*, *4. Brown University School of Public Health, Providence, Rhode Island, United States*, *5. University of Texas at Austin, Austin, Texas, United*

*States*, *6. University of Texas at Austin, The, Austin, Texas, United States*, *7. VA Bedford Healthcare System, Bedford, Massachusetts, United States*

Together with three nursing home (NH) corporations, we co-designed and adapted a "small bites" approach to pragmatic clinical trial (PCT) implementation that was successful even during the pandemic height. Our pilot PCT to improve sleep for nursing home residents with dementia began with the start of the pandemic, with its increased resident acuity and resident care burden, increased workforce shortages and instability, and need for increased leadership involvement. Using co-design principles, we reviewed all procedures with our first NH and identified ways to reduce staff burden by "bite-sizing" the as much implementation protocol as much as possible (e.g., serial 5-minute staff trainings). We implemented revised procedures in all three pilot NHs. To identify adaptations, we used a modified version of the Framework for Reporting Adaptations and Modifications-Enhanced (FRAME) to code meeting minutes, researcher fieldnotes about adaptations, and 35 group interview transcripts with the leadership/champion teams. We also coded for feasibility, acceptability, and sustainability. We used an iterative, rapid content analytic approach to qualitative analyses. Most adaptations occurred in the pre-implementation phase, although some occurred in the pilot implementation phase. Adaptations concerned reducing staff burden, such as moving from in-person visits to a fully virtual approach that used 30-minute once-a-week project meetings and streamlining how staff completed measures. The resulting intervention package worked in all corporations on all levels: feasibility, outcome, and sustainability. Necessity being the mother of invention, we landed on a better process than originally planned, one that may be valuable for the wider nursing home research community.

Abstract citation ID: igad104.1640

### NURSING HOME STAFF UNDERSTANDING AND CONFIDENCE IMPLEMENTING MONTESSORI APPROACHES AFTER TRAINING

Michelle Hilgeman[1], Katherine Kennedy[2], Sylvia Haigh[3], Amy Mochel[3], Teddy Bishop[1], Kimberly Curyto[4], and Whitney Mills[5], *1. Tuscaloosa VA Medical Center, Tuscaloosa, Alabama, United States*, *2. Providence VA Medical Center, Providence, Rhode Island, United States*, *3. Providence VAMC, Providence, Rhode Island, United States*, *4. Center for Integrated Healthcare, VA Western NY Healthcare System, Batavia, New York, United States*, *5. VA Providence Healthcare System, Providence, Rhode Island, United States*

Staff understanding and confidence delivering non-pharmacological interventions are critical for improving outcomes related to behavioral distress in dementia. We conducted a mixed methods evaluation with frontline staff (nursing, recreation therapists, etc.) at 8 VA Community Living Centers enrolled in a stepped-wedge cluster randomized trial of Montessori-based approaches. Training and data collection were conducted remotely and surveys were shortened to reduce participant burden. Normalization Process Theory (NPT, May et al., 2022) guided questions and analysis. Staff completed N=21 qualitative interviews, N=307 surveys (1-month pre-training and during baseline), and N=906 post-training evaluations. Results revealed 83%-87% passed post-training knowledge exams. NPT surveys reflected Montessori was not a normal part of staff work currently (M=5.58 and 6.35 on a 10-point scale); though they believed it would become a normal part of their work in the future (M=7.11 and 7.68). After training, Montessori was rated as more familiar (M=5.15 to M=6.55, t=-3.12, p=.002), and significantly more staff reported that their role included Montessori delivery/oversight X2=13.26(2), p=.001. Content analysis was used to examine qualitative transcripts for the NPT domain Coherence (e.g., comprehension, self-efficacy, internalization). Three themes emerged: 1) ability to describe Montessori principles, 2) familiarity with Montessori approach, and 3) willingness to try it. All participants were able to describe Montessori, though some reported conditional self-efficacy/confidence. Representative quotes reflect challenges and successes in understanding and early application of the intervention in VA CLC environments. Considerations for pragmatic trial designs amid fluctuating COVID precautions are also discussed (e.g., nursing shortages, implementation and evaluation discrepancies, and site-level variability).

### SESSION 4295 (SYMPOSIUM)

Abstract citation ID: igad104.1641

### POLICY SERIES: ARE WE THERE YET? MEASURING PROGRESS IN ACHIEVING POLICY GOALS FOR FAMILY CAREGIVERS

Chair: Pamela Nadash Discussant: Rani Snyder

The RAISE Family Caregiving Advisory Council, created under the Recognize, Assist, Include, Support, and Engage (RAISE) Family Caregivers Act (2018) has been tasked to support the Secretary of Health and Human Services in developing a national family caregiving strategy, published in 2022 as its National Strategy to Support Family Caregivers. One key concern identified by advocates and others is the accountability question: how can we hold policymakers at all levels of government accountable for achieving the policy goals articulated in the National Strategy? This symposium discusses that issue by first providing an overview of a key mechanism for state accountability, AARP's State Scorecard on Long-Term Services and Supports, which identifies support for family caregivers as one of its four dimensions. Susan Reinhard, PhD, Senior Vice President at AARP and Director of its Public Policy Institute, will describe the history, goals, and future of the Scorecard's family caregiver component. Salom Teshale, PhD, from the National Academy for State Health Policy will describe how states are focusing their energies in current actions supporting family caregivers, while Mike Wittke, MPA, of the National Alliance for Caregiving will discuss how federal actions might be tracked and assessed. Drawing on research supporting the RAISE Advisory Council, Eileen J. Tell, MPH will suggest additional dimensions that could be integrated into the assessment of progress. Rani Snyder, MPA, of the John A. Hartford Foundation will act as discussant.

Abstract citation ID: igad104.1642

### THE ROLE OF THE LTSS SCORECARD IN PROMOTING STATE ACCOUNTABILITY FOR CAREGIVER POLICY

Susan Reinhard, *AARP, Washington, District of Columbia, United States*

In 2011, the AARP produced its first LTSS Scorecard in “Raising Expectations: A State Scorecard on Long-Term Services and Supports for Older Adults, People with Physical Disabilities, and Family Caregivers”. This presentation discusses the history, background, and goals of the Scorecard, which examines four key dimensions of state LTSS system performance: affordability and access; choice of setting and provider; quality of life and quality of care; and support for family caregivers. This presentation focuses on the extent to which support for family caregivers was a focus of that first report and how that focus has changed over time. In addition, the presentation will consider the extent to which the Scorecard has proved an effective policy tool in holding states accountable for progress in supporting family caregivers. Has the Scorecard achieved its stated goal to “to spark conversations, galvanize broad-based coalitions, and focus stakeholders’ attention on the factors that most directly impact consumers and their families”? What has AARP learned through its experience of developing and administering the Scorecard, and what changes are being considered with respect to the dimensions pertaining to family caregivers? The presentation will conclude by noting synergies with the work of the RAISE Family Caregiver Advisory Council and the priorities indicated in its National Strategy to Support Family Caregivers.

Abstract citation ID: igad104.1643

### IMPLEMENTING A STAKEHOLDER AND POLICY MAP TO TRACK FEDERAL CAREGIVER POLICY ACTIVITY

Michael Wittke, *National Alliance for Caregiving, Washington, District of Columbia, United States*

A top priority for family caregiver policy should be a focus on accountability and transparency regarding federal actions identified within the National Strategy to Support Family Caregivers. It is crucial to have accountability and transparency as the National Strategy is being updated and refined over the course of time that occurs between the release of the initial strategy and the publishing of the next version of the strategy. Increased accountability and transparency are especially important as stakeholders continue to champion and build on the foundation that has been established in the process of creating the 2022 National Strategy to Support Family Caregivers. Establishing outcomes measures will also inform how the stakeholder community may shift their priorities and operations to align with successes and challenges outlined in an updated National Strategy. To ensure that accountability and transparency are at the forefront of public interest, and as agency leadership communicates the intended outcomes of their critical contributions to America’s family caregivers, it is essential to establish a process to communicate the meaningful impact regarding federal actions on this population. As the National Strategy is being examined for future updates, NAC has facilitated a systematic process to map the actions that federal agencies have identified as core priorities and track their progress toward implementation. That mapping process will be revealed and analyzed during this presentation.

Abstract citation ID: igad104.1644

### THE NATIONAL STRATEGY TO SUPPORT FAMILY CAREGIVERS: A STATE-LEVEL PERSPECTIVE ON IMPLEMENTATION

Salom Teshale, *The National Academy for State Health Policy, Washington, District of Columbia, United States*

The 2022 National Strategy to Support Family Caregivers includes action steps that a wide range of non-federal organizations, including states, can take to support family caregivers. States have a variety of ways that they can approach supporting family caregiver policy via action steps, including but not limited to strengthening access to services, collaborating among agencies and organizations to coordinate activities, and tracking caregiver data to help ensure services are meeting caregiver needs. Several states are taking the lead in promoting use of caregiver assessment tools. States can monitor whether initiatives and activities are achieving their goals of supporting caregivers and care recipients through tracking the implementation of the state’s actions to support family caregivers. Drawing on the National Academy for State Health Policy’s role in supporting states through its Resource and Dissemination Center, this presentation will highlight ranges of action steps in the National Strategy that states can take to support family caregivers; note frameworks that states can consider using to track implementation of action steps; and highlight states’ initiatives aligning with action steps.

Abstract citation ID: igad104.1645

### USING THE LTSS STATE SCORECARD TO MEASURE PROGRESS ON THE 2022 NATIONAL STRATEGY TO SUPPORT FAMILY CAREGIVERS

Eileen Tell[1], and Pamela Nadash[2], *1. ET Consulting, Belmont, Massachusetts, United States*, *2. University of Massachusetts Boston, Boston, Massachusetts, United States*

Through the Recognize, Assist, Include, Support, & Engage (RAISE) Family Caregivers Act of 2018, the RAISE Family Caregiver Advisory Council was created. Since 2019, the Council has directed in-depth information-gathering and research efforts to develop recommendations and an implementation plan for addressing our critical family caregiving crisis. The Council recently released the 2022 National Strategy to Support Family Caregivers, which highlights nearly 350 actions the federal government will take to support family caregivers in the coming year and more than 150 actions that can be adopted at other levels of government and across the private sector to build a system to support family caregivers. Integrating measures of improvement with regard to family caregiver support, across the domains identified in the National Strategy into the LTSS State Scorecard will provide a tool by which progress toward the RAISE goals can be measured. Currently, the Scorecard focuses on indicators specific to Supporting Working Family Caregivers. While these are critical, our research team has identified at least 10 additional indicators that could be included and that would address other caregiver support domains such as adequacy of direct care workforce, integration of family caregivers into care plans, direct pay to family caregivers, and more. In this session we will identify the indicators, explain how they relate to RAISE goals and discuss methods for including them in the Scorecard.

### SESSION 4300 (PAPER)

### HEALTH DISPARITIES II

Abstract citation ID: igad104.1646

### ORAL HEALTH EQUITY, BELIEFS, PRACTICES, PERCEPTIONS AMONG OLDER DETROIT RESIDENTS

Mark Luborsky[1], Jocie Osika[1], Kimberly Shay[1], Mariam Shilbayeh[2], Anisa Anisa Simoni[3], Candace Ziglor[4], Divesh Byrappagari[4], and Judith Jones[4], *1. Wayne State University, Detroit, Michigan, United States*, *2. University of Detoit Mercy Dental School, Detroit, Michigan, United States*, *3. University of Detroit Mercy, Detoit, Michigan, United States*, *4. University of Detroit Mercy School of Dentistry, Detroit, Michigan, United States*

Oral health disparities are widespread among older and disadvantaged Detroit residents. While preventable, poor oral health is strongly associated with adverse health and social outcomes and increased risk of cognitive impairments. To address limitations in access and knowledge among older Detroit persons, the University of Detroit Mercy Oral Health Equity project is providing free drop-in clinics offering screenings and basic care at multiple local community centers. However, we need person-centered knowledge about the reasons for an individual's current oral health status and to inform strategies to increase service uptake. We conducted interviews with attendees onsite after their services to learn oral health perceptions, wants, and practices. Findings are reported from a sample, n=62, of predominantly African American, median age 81, female 69%, 50% over two years without a dental visit, and 61% fair to poor self-reported oral health. Analyses used topic, theme, pattern, and critical case content analytic methods. Constructs comprising four key domains were identified: (a) Oral health care exists at the intersection of forces featuring societal, political, historical, local urban place, and economic dimensions beyond individual-level health and behaviors; (b) lifespan pathways into current oral health and care practices, including wishes for future oral health status; (c) barriers and resources to oral health care access and practices; and (d) personal knowledge gaps regarding signs and mechanisms of oral health. Discussion will focus on strategies to reduce disparities and increase utilization, community and person-level knowledge gaps, and training dental students to engage older disadvantaged persons effectively.

Abstract citation ID: igad104.1647

### PREMATURE MORTALITY IN LGBT VERSUS NON-LGBT VETERANS: THE ROLE OF HEALTH RISK FACTORS AND SOCIAL DETERMINANTS OF HEALTH

Sherry Beaudreau[1], Julie Lutz[2], Marcela Otero[3], Allison Warren[4], and Joseph Goulet[4], *1. VA Palo Alto/ Stanford University School of Medicine, Palo Alto, California, United States*, *2. VA Palo Alto Health Care System, Palo Alto, California, United States*, *3. Max-Plank Institute for Social Law and Social Policy, Munich, Bayern, Germany*, *4. Yale School of Medicine, New Haven, Connecticut, United States*

Lesbian, Gay, Bisexual, and Transgender (LGBT) adults experience substantially higher rates of mental health conditions and other health risk factors than non-LGBT adults, but there is limited reseach on how these conditions increase risk for premature mortality. The current study examined this issue among middle-aged and older Veterans ages 40-99 who used Veterans Administration healthcare services from 01OCT2009 to 30SEP2019 (N = 845,122). Earlier age of death by suicide, overdose, or all-causes was hypothesized for LGBT (n = 675,639 ) vs. non-LGBT (n = 675,639) Veterans, and health factors and social determinants of health were predicted as key risk factors of earlier age of mortality. As hypothesized, LGBT Veterans had significantly higher rates of death by suicide, overdose, and all-causes among younger age groups than non-LGBT Veterans. Adjusted risk ratios indicate a significant contribution of mental health conditions (e.g., anxiety/depression), medical comorbidity, pain, smoking history, and military sexual trauma accounting for differential mortality rates by age in LGBT and non-LGBT participants. Social determinants of health, such racial/ethnic minority status and housing instability, were also significant contributors. Findings suggest the need for Whole Health interventions for LGBT Veterans to manage multiple health risk factors contributing to increased mortality risk. Suicide prevention is critical for the many middle-aged and older adults who die from suicide each year, and for LGBT Veterans especially, beginning targeted suicide prevention efforts earlier in middle age may prevent suicide deaths in later life. Lastly, findings implicate subgroups of Veterans who may particularly benefit from outreach and services.

Abstract citation ID: igad104.1648

### WHO GETS RESOURCES ACROSS THE LIFESPAN? A COORDINATED DATA ANALYSIS OF PERSONALITY AND SOCIOECONOMIC CONDITIONS

Stephen Antonoplis[1], Olivia Atherton[2], Eileen Graham[1], and Daniel Mroczek[1], *1. Northwestern University, Chicago, Illinois, United States*, *2. University of Houston, Houston, Texas, United States*

From schooling in early life to employment, income, and generating wealth in adulthood, people must navigate socioeconomic conditions throughout their entire life. Personality traits have been shown to predict how people navigate socioeconomic conditions, and a number of open questions remain. In this project, we address several of these questions, including 1.) do stable, between-person trait levels or within-person fluctuations matter for socioeconomic outcomes?, 2.) do traits matter across the lifespan for socioeconomic outcomes or only in specific age periods?, and 3.) do periods of economic recession change the relationship between traits and socioeconomic outcomes? To answer these questions, we examine the prospective associations between personality traits and socioeconomic conditions across the lifespan, using eleven longitudinal datasets. Participants were born from 1880–2000 and aged 5–102; myriad ages and generations were present for all major U.S. military and economic events of the last 50 years. The samples were racially diverse, containing datasets focused on African American, Asian American, and Pacific Islander experiences, and several contemporary nationally representative datasets. Initial results show, for income for instance, that 1.) between-person levels, not within-person fluctuations, matter, and 2.) between-person levels matter across all ages. Openness to Experience, Conscientiousness, and Extraversion predicted earning a higher yearly income; Neuroticism predicted earning a lower yearly income. Effects sizes for traits were comparable to that for parental education. Our results shed light on how people navigate social structures across the lifespan.

### SESSION 4305 (PAPER)

### SOCIAL DETERMINANTS OF HEALTH AND AGING III

Abstract citation ID: igad104.1649

### GENDERED PATHWAYS TO FINANCIAL CAPABILITY: IMPLICATIONS FOR PHYSICAL, MENTAL, AND FINANCIAL HEALTH IN LATER LIFE

Yu-Chih Chen[1], and Sicong Sun[2], *1. The University of Hong Kong, Hong Kong, Hong Kong*, *2. The University of Kansas, Lawrence, Kansas, United States*

Financial capability, comprising financial literacy, access, and behavior, can influence an individual's ability to effectively use financial resources, thus impacting their health and well-being. However, studies have predominantly focused on financial literacy and overlooked a more comprehensive measure of financial capability. Additionally, there is limited knowledge on the role of gender in these associations. This study investigated how gender may moderate the links between financial capability and health. The study recruited 1,109 community-dwelling adults (aged 45+) through an online survey and employed multivariate linear and logistic regression to examine the gender differences in the associations between financial capability and physical (perceived health and mobility limitations), mental (life satisfaction and depression), and financial (retirement worry and financial satisfaction) health. Results showed that financial access and behavior, compared to financial literacy, had a more significant influence on determining health outcomes. Gender differences in financial capability were also identified through simple slope analyses, with financial literacy being more important for men's self-rated health and life satisfaction, while financial behavior was more critical for women. Additionally, although financial access was not related to retirement worry among men, it was significantly associated with lower retirement worry among women. Findings suggest that gender-specific pathways to financial capability may lead to health disparities. Policies and programs aimed at improving population's health and well-being, particularly for women, should target not only financial literacy but also strengthen financial inclusion and encourage responsible financial behavior.

Abstract citation ID: igad104.1650

### LOWER SOCIOECONOMIC STATUS PREDICTS NEGATIVE PREFERENCES FOR PALLIATIVE CARE

Molly Nowels[1], Elissa Kozlov[1], David Nowels[2], and Paul Duberstein[1], *1. Rutgers School of Public Health, Piscataway, New Jersey, United States*, *2. University of Colorado School of Medicine, Aurora, Colorado, United States*

With an aging US population, more people than ever live with serious illnesses. Although palliative care (PC) can improve outcomes in serious illness, there are inequities in PC utilization. People with low socioeconomic status (SES), men, and Black and Hispanic people are less likely to receive and benefit from PC services. Despite these established demographic differences in PC utilization, there is a dearth of relevant survey research on preferences for PC in the general population. To address this gap, we surveyed a random sample of 1,500 NJ adults. Respondents were given a brief definition of PC and asked to indicate how likely they would be to schedule, attend, and routinely attend PC visit(s) if they were diagnosed with a serious illness. Predictors included in logistic regression modeling were SES indicators (income, educational attainment, insurance status, employment status), gender, and race/ethnicity. Data were weighted to be representative of the population of NJ. Modeling results revealed that lower income and lower educational attainment were associated with significantly lower odds of endorsing willingness to schedule, attend, and routinely attend PC visits in the event that one would become seriously ill. Unexpectedly, there were no gender or race/ethnicity differences in preferences for PC. These findings highlight the importance of public health education for what PC is and its benefits for an aging population, especially among those with lower SES. Future research efforts are needed to understand discrepancies in reported PC preferences versus real-world PC utilization for men and Black and Hispanic individuals.

Abstract citation ID: igad104.1651

### NO PENSION, NO HAPPINESS?—FINANCIAL SUPPORT EXPECTATION IN THE OLDEST-OLD AGE AND DEPRESSIVE SYMPTOMS

Chengming Han, and Nan Zhou, *Case Western Reserve University, Cleveland, Ohio, United States*

Objective. This paper aims to explore the effect of financial support expectation on depressive symptoms among the oldest-elderly. Method. Data were drawn from the China Health and Retirement Longitudinal Study (CHARLS) 2018. The analytical sample included 10641 respondents who were older than 45. Financial support expectations refer to whom they would reply on when they cannot work, which includes four categories: children, themselves (savings or commercial life insurance), pensions, and others. Linear regression models were employed after controlling for pension, health insurance and urban-rural household registration (hukou).

**Results.** More than half of the sample reported that they would rely on their children when they became too old to work. Those who reported that they would rely on themselves (b=-1.72, p=0.000) or pensions (b=-1.23, p=0.000) reported lower levels of depressive symptoms compared to those who would rely on their children. When pension and health insurance were controlled for, only those who would rely on themselves presented lower level of depressive symptoms. Pension and health insurance (except rural health insurance) mediated the association between the financial support expectation and depressive symptoms. However, the hukou status inhibited the mediating effect of pension and health insurance.

**Conclusion.** Financial uncertainty is a potential threat in the oldest-old age. Reducing the urban-rural inequality in pension and health insurance would help reduce the negative effects of financial uncertainty.

Abstract citation ID: igad104.1652
### PREVALENCE AND DISPARITIES OF VERY LOW-INCOME OLDER ADULT RENTERS IN US METROPOLITAN AREAS

Anthony Traver, and Holly Dabelko-Schoeny, *The Ohio State University, Columbus, Ohio, United States*

Income eligibility for federal housing subsidies is based on HUD calculations of area median income (AMI) and family size. Frequently used measures of poverty and income do not reflect the geographic economic variations on which HUD eligibility is based. By integrating Current Population Survey (CPS) data for metropolitan-dwelling people in 2000, 2010, and 2020 with HUD yearly 50% AMI thresholds (very low-income (VLI)), this study aims to provide a novel estimate of the number of metropolitan older adult (OA) (62+) VLI renters and to compare their growth rate, rental assistance use, race, and health with other population groups. Sample weights are used to produce national estimates of four metropolitan OA subgroups: VLI renters, VLI owners, non-VLI renters, and non-VLI owners. Results show that between 2000 and 2020, the total number of metropolitan OAs increased by an estimated 84%, OA VLI renters increased by an estimated 74%, and OA VLI renters without rental assistance increased by an estimated 71%. Across all years, metropolitan VLI OA renters were more likely to be non-white, disabled, report poorer health, and receive rental and utility assistance. Approximately two-thirds of metropolitan VLI OA renters did not receive any form of rental assistance across time points. VLI OA renters without rental assistance face significant affordability challenges as metropolitan rental prices rise faster than most retirement incomes. Communities should proactively link their growing OA VLI renter populations with financial assistance and supportive services to reduce their risk of housing precarity in late life.

### SESSION 4310 (PAPER)

### FRAILTY

Abstract citation ID: igad104.1653
### ASSOCIATION BETWEEN MULTIPLE FRAILTY INDICES AND CVD MORTALITY

Benjamin Seligman[1], Saadia Qazi[2], Manas Rane[3], Sarah Preis[4], Ariela Orkaby[5], J. Michael Gaziano[6], and Jane Driver[7], *1. UCLA School of Medicine, Los Angeles, California, United States*, *2. Brigham and Women's Hospital, Boston, Massachusetts, United States*, *3. West Roxbury VA Medical Center, West Roxbury, Massachusetts, United States*, *4. Boston University School of Public Health, Boston, Massachusetts, United States*, *5. Harvard Medical School, Boston, Massachusetts, United States*, *6. Brigham and Women's Hospital / VA Boston, Boston, Massachusetts, United States*, *7. VA Boston Healthcare System, Boston, Massachusetts, United States*

Introduction Frailty, a syndrome of physiologic vulnerability, increases cardiovascular disease (CVD) risk. Which frailty tool is ideal for risk stratification remains unclear. We calculated three frailty scores from the Million Veteran Program (MVP) and examined their association with mortality and CVD in older Veterans. **Methods** Participants were from MVP - a large, contemporary Veteran cohort study - and aged ≥50 years at baseline (2011-2018). The frailty scores used were: MVP-FI (36-item questionnaire deficit accumulation frailty index), VA-FI (31-item EHR index), and modified Study of Osteoporotic Fractures (mSOF; a physical frailty score). MVP-FI and VA-FI scores of ≤0.10 were robust, 0.11-0.20 pre-frail, and ≥0.21 frail; mSOF scores of 0 were robust, 1 pre-frail and ≥2 frail. Primary outcomes were all-cause and CVD mortality. Secondary outcomes were incident stroke, myocardial infarction (MI), and heart failure (HF). Cox regression was used to evaluate the association of frailty with outcomes. Results Among 190,688 participants, mean age was 69 ±9, 94% were male. By MVP-FI, 29% were robust, 42% pre-frail, and 29% frail. Hazard ratios (HR, 95% CI) for all-cause mortality were 1.66 (1.61-1.72) and 3.05 (2.95-3.16) for pre-frailty and frailty, respectively. For CVD mortality, HRs were 1.76 (1.65-1.88) and 3.65 (3.43-3.90) for pre-frailty and frailty. Hazards of stroke, MI, and HF also increased with greater frailty. VA-FI and mSOF yielded concordant results. Conclusion Irrespective of measure, frailty is associated with increased all-cause mortality and CVD event risk. Clinicians and researchers may consider the most convenient tool for available data to incorporate frailty into practice.

Abstract citation ID: igad104.1654
### DEVELOP AND EXTERNALLY VALIDATE A RISK PREDICTION MODEL FOR SCREENING COGNITIVE FRAILTY IN OLDER ADULTS

Wenting Peng, Yuqian Luo, Cen Mo, Kehan Liu, and Minhui Liu, *Central South University, Changsha, Hunan, China (People's Republic)*

Cognitive frailty, the combination of physical frailty and mild cognitive impairment, is a growing public health concern in aging populations. We aimed to develop and validate a risk prediction model for screening cognitive frailty in community-dwelling older adults without probable dementia (aged ≥ 65 years). We used Year 2011 data from National Health and Aging Trends Study (NHATS), with participants randomly divided into the training set (N=4,222) and internal validation set (N=2,111). We used Year 2015 data of NHATS as the external validation set (N=3,380). Cognitive frailty was assessed with the Fried phenotypic criteria and cognitive performance in three domains (memory, orientation and executive function). Independent risk factors were screened by multivariate logistic regression analysis. Model performance was assessed by discrimination (area under the curve [AUC]) and calibration (Hosmer-Lemeshow test). The final model included 13 key predictors (age, gender, education, smoke, walking for exercise, vigorous activity, self-rated health, depressive symptoms, balance impairments, arthritis, hospitalization, activities of daily living, and instrumental activities of daily living score). The model showed good discrimination with an AUC of 0.92 (95% CI: 0.90-0.93) for the training set, 0.92 (95% CI: 0.89-0.95) for the internal validation set, and 0.88 (95% CI: 0.85-0.91) for the external validation set. The Hosmer-Lemeshow test yielded P values of 0.10, 0.92, and 0.85 for the three sets, respectively, suggesting good calibration. Our risk prediction model has the potential to screen older adults at risk for cognitive frailty, enabling early interventions to prevent or delay cognitive decline and physical disability.

Abstract citation ID: igad104.1655

### HIGH-INTENSITY WALKING EXERCISE REDUCES FRAILTY AMONG PREFRAIL AND FRAIL OLDER ADULTS

Margaret Danilovich[1], Daniel Corcos[2], Ying Cheung[2], David Conroy[3], and Lauren Balmert Bonner[2], *1. CJE SeniorLife, Chicago, Illinois, United States*, *2. Northwestern University, Chicago, Illinois, United States*, *3. Penn State University, State College, Pennsylvania, United States*

Exercise is the most recommended therapeutic intervention for frailty, yet the precise exercise prescription remains elusive. The purpose of this study was to use a cluster randomized controlled trial to evaluate the impact of exercise intensity on frailty in older adults. We tested a high (HIW) vs. low (LIW) intensity walking intervention among n=165 pre-frail and frail residents from n=12 independent living communities. The 3x/week, 4 month supervised intervention targeted >70% heart rate reserve for HIW participants and < 50% heart rate reserve for LIW participants. The primary outcome was frailty category improvement as assessed by the SHARE-FI. We measured usual and fast gait speed, 6-minute walk test, Timed Up and Go, SPPB, Berg Balance, Falls Efficacy, and PROMIS Global Health. Participants were on average 79 years of age, 79% female, and 75% white. We analyzed outcomes based on a modified intention-to-treat principle using a linear or generalized linear mixed model with fixed arm, baseline outcome measure score and random cluster effects. We found increased odds of improving at least one SHARE-FI frailty category in the HIW group (OR=2.91 [reference group=LIW], 95% CI 1.19,7.13, p=0.02, N=103). Among secondary outcomes, there was a significant difference in change in usual gait speed (mean difference = 0.078, 95% CI = 0.026, 0.131, Hochberg adjusted P-value = 0.0357), as well as 6-minute walk test (mean difference = 160.72, 95% CI = 69.2, 252.2, Hochberg adjusted P-value = 0.0075). Results suggest that older adults should engage in more intensive/higher heart rate cardiovascular exercise to reduce frailty.

Abstract citation ID: igad104.1656

### JOINT TRAJECTORIES OF MULTIMORBIDITY AND FRAILTY AMONG OLDER AMERICANS

Zhou Yang, Yan Luo, Yu-Ming Chen, Hui-wen Xu, and Beibei Xu, *Peking University, Beijing, Beijing, China (People's Republic)*

Little is known about how health status considering both multimorbidity and frailty develops over time. This study aims to investigate joint trajectories of multimorbidity and frailty and examine their associated factors among older American adults.We used data from the National Health and Aging Trends Study (2011–2019). Fourteen chronic conditions were ascertained each year. Frailty was measured annually using the Fried frailty phenotype. Group-based trajectory models were fitted to identify joint trajectories of multimorbidity and frailty. Multinomial logistic regression model was adapted to examine the association of baseline characteristics with the trajectories. We identified six distinct joint trajectories of multimorbidity and frailty, including four concordant and two discordant trajectories. Compared with the Newly-developing condition and Constant non-frailty group, increasing age, low education, dual Medicare-Medicaid status, former or current smoker, mild or severe insomnia symptoms, and bothersome or activity-limiting pain were significantly associated with an increased likelihood of belonging to the other five groups with higher levels of multimorbidity or frailty (all P< 0.05). Multiple distinct joint trajectories of multimorbidity and frailty offer complementary evidence to the heterogeneous patterns in aging, as well as inform clinicians, policymakers, and researchers to develop targeted interventions and provide comprehensive care management in community settings.

Abstract citation ID: igad104.1657

### PLASMA NEUROFILAMENT LIGHT CHAIN IS ASSOCIATED WITH PHYSICAL FRAILTY IN OLDER ADULTS WITHOUT DEMENTIA

Junxin LI, Jessica Gill, Jing Huang, Daniel Waszczuk, and Sarah L. Szanton, *Johns Hopkins University, Baltimore, Maryland, United States*

Evidence suggests a link between brain biomarkers of neurodegeneration and physical function and frailty. However, the association of neurodegenerative biomarkers in the plasma with physical frailty in older adults remains unelucidated, as current evidence is limited and inconsistent. We tested the association between plasma neurofilament light chain (NFL), a plasma neurodegeneration biomarker, and physical frailty in a sample of community-dwelling older adults. We used baseline demographics, frailty, and plasma NFL data collected from 97 participants in a randomized controlled trial in community-dwelling, sedentary older adults without dementia [Montreal Cognitive Assessment (MoCA)>17]. Physical frailty was assessed using a composite measure of weakness, slow walking speed, unintentional weight loss, exhaustion, and low physical activity. Participants' frailty levels were categorized into robustness, pre-intermediate frailty, and frailty. Multiple linear regression and ordinal logistic regression models adjusting for age, sex, race, education, and comorbidities were conducted to test the associations of NFL with frailty and levels of frailty. The sample was 70.0 ± 6.0 years old, with 80% being females, 72% having intact cognition, and 28% having mild cognitive impairment (MCI). In the adjusted models, higher plasma NFL was associated with higher frailty scores ($\beta$=0.16, 95%CI [Confidence Interval] = [0.06,0.26]) and a higher likelihood of being in a higher level of frailty group (odds ratio=1.28, 95% CI =1.02, 1.61). In summary, plasma NFL was associated with greater frailty in our sample of non-demented older adults. Plasma NFL may be a sensitive and promising neurodegenerative biomarker for physical frailty in older adults without dementia.

### SESSION 4315 (SYMPOSIUM)

Abstract citation ID: igad104.1658

### LIVING WITH DEMENTIA: AN INTERDISCIPLINARY COMMUNITY-BASED EDUCATION SERIES TO EMPOWER CARE PARTNERS

Chair: Erica Husser
Discussant: Marie Boltz

Approximately 75% of persons living with dementia receive essential day-to-day direct care and support from informal, unpaid care partners who receive little education or guidance from health care professionals on how to negotiate day-to-day challenges associated with living with dementia. This symposium describes the development, implementation, and impact of a comprehensive, four-part Living with Dementia community education program focused on dementia care featuring an optional Memory Café, developed collaboratively by the Penn State College of Medicine and College of Nursing for the purpose of empowering care partners. The first presentation will describe the educational content and instructional strategies, designed by an interdisciplinary team (physician, nurse, and long-term care dementia specialist), and based on the Medicare Cognitive Assessment and Care Plan Services visit and using evidence-based curricula. The second presentation describes the concurrent and collocated intergenerational respite program (Memory Café) for persons living with dementia (PLWD) that enabled care partners to attend the education. Health care students were paired with a PLWD and during the two-hour session each pair engaged in Opening Minds though Art™ (OMA), reminiscence, and music therapy; outcome data includes PLWD pre- and post-mood assessments. The third presentation provides overall evaluation data from the 4-part education series. Respondents were asked about the overall impacts of the program, the skills they learned, the usefulness of the education now and into the future, and what additional support would be most helpful. Our discussant will address the implications of our findings for practice, policy, and future program development.

Abstract citation ID: igad104.1659

### MULTIMETHOD COMMUNITY-BASED DEMENTIA CARE EDUCATION TO EMPOWER CARE PARTNERS AND IMPROVE WELLNESS

Rollin Wright[1], Janice Whitaker[2], and Peg Chabala[3], *1. Penn State Health at Hershey Medical Center, Hershey, Pennsylvania, United States*, *2. Nese College of Nursing, Penn State University, State College, Pennsylvania, United States*, *3. Penn State University, University Park, Pennsylvania, United States*

Inspired by the Medicare Cognitive Assessment and Care Plan Services visit, we describe an Age-Friendly 4Ms curriculum developed to empower informal dementia-care-partners to understand dementia, develop skills to communicate and work with people living with dementia (PLWD), and plan for the future with dementia. Curriculum was developed by an interdisciplinary team and included the integration of evidence-based approaches to care, such as the Positive Approach® to Care model. Through 4 interactive workshops (Understanding Dementia-related Brain Changes, Understanding Behaviors as a Form of Communication, Resources for Dementia Caregivers, Planning for the Future with Dementia) we introduced and had care partners process and practice material to help them navigate everyday challenges faced by PLWD with the aim of assisting them to find opportunities to help improve quality of life with the disease for both themselves and their PLWD. We reached a total of 146 attendees in 3 rural and suburban communities in Central Pennsylvania. Fifty-nine (40.4%) people attended 1 session, 39 (26.7%) people attended 2 sessions, 22 (15.1%) people attended 3 sessions, and 26 (17.8%) people attended all 4 sessions. Across all four sessions, more than 80% of attendees reported knowledge and skill gains, and most attendees reported meeting session objectives. Final evaluation results suggest attendees wanted a way to stay informed, stay connected, and remain supported by the program.

Abstract citation ID: igad104.1660

### MEMORY CAFÉ PROVIDES INTERGENERATIONAL ENGAGEMENT OPPORTUNITIES FOR LWD CARE-PARTNERS' LOVED ONES

Janice Whitaker[1], Diane Berish[2], Jenny Rank[3], Meghan McGraw[3], and Erica Husser[4], *1. Nese College of Nursing, Penn State University, State College, Pennsylvania, United States*, *2. The Pennsylvania State University, University Park, Pennsylvania, United States*, *3. Penn State University, University Park, Pennsylvania, United States*, *4. Penn State University, State College, Pennsylvania, United States*

To facilitate attendance by care partners attending the Living with Dementia (LWD) education sessions, an optional Memory Café was available at the same location. We describe the design and implementation of the Memory Café, which included evidence-based programming to engage and provide socialization for the older adults. Basic health, safety, person-centered, and care preference information was collected at registration to ensure quality supervision and support during the Memory Café. The Café was facilitated by interprofessional staff and included Opening Minds through Art (OMA), an intergenerational no-fail art-making experience for persons with neurocognitive disorders ("artists") that fosters creative self-expression and engagement for persons living with dementia (PLWD). Facilitators also integrated reminiscence, music, and movement therapies during the two-hour LWD education session. Nineteen community dwelling PLWD attended an average of two of the four Memory Café sessions, 26 participants were recruited from a care home at one location, and 124 volunteers were paired with PLWD for OMA and attended at least one Memory Café (volunteers included undergraduate nursing students, graduate students, Penn State faculty and staff, and community members). Fifty-one pre- and post-mood assessments were completed by the 19 community dwelling OMA artists (using a 5-point Likert scale of faces, from frowning to smiling, to indicate how they felt in the moment) and data showed mood improved by an average of .67 points (standard deviation = .95). Care partner evaluation data was positive; 8 (22%) reported that having the Memory Café option made it possible for them to attend LWD.

Abstract citation ID: igad104.1661

### LIVING WITH DEMENTIA: COMMUNITY-BASED CARE PARTNER EDUCATION EVALUATION RESULTS

Erica Husser[1], Rollin Wright[2], Janice Whitaker[3], Diane Berish[4], Olivia Rubio[5], and Donna Fick[1], *1. Penn State University, State College, Pennsylvania, United States*, *2. Penn State Health at Hershey Medical Center, Hershey, Pennsylvania, United States*, *3. Nese College of Nursing, Penn State University, State College, Pennsylvania, United States*, *4. The Pennsylvania State University, University Park, Pennsylvania, United States*, *5. Penn State University, University Park, Pennsylvania, United States*

A mixed methods overall series evaluation was administered at session four in each location to assess preparedness, perceived increase in caregiver self-efficacy and wellness, development and implementation of skills, and overall program value. Fifty-five participants (73% response rate) completed the evaluation; 44 (80%) were care partners of a person living with dementia (PLWD). Twenty-four (44%) participants attended all four sessions, 9 (16%) attended three, 8 (15%) attended two, and 11 (20%) attended one. Those who attended all four sessions were more likely to strongly endorse that they felt better prepared (96% vs. 75%; X2 = 3.7, p = 0.05). On a 5-point scale (1 = Strongly disagree, 2 = Disagree, 3 = Neither agree/disagree, 4 = Agree, 5= Strongly agree), 38 (85%) reported agree or strongly agree to improved preparedness to meet the needs of their PLWD, and 41 (93%) reported improved confidence as a care-partner. The top 5 communication skills learned: Using fewer words (89%); Slowing down speech (89%); Focusing on the relationship (86%); Ending an encounter with thank you (86%); and Going with the flow (84%). Content analysis of qualitative data suggest care-partners' new understanding and improved communication skills were having a positive influence on the pace and quality of daily life and reported less conflict and improved mood; challenges persisted with activities of daily living, such as showering and toileting. Programs like LWD that provide care partners education, skill development and support for self-care can improve daily life for care-dyads living with dementia.

### SESSION 4320 (SYMPOSIUM)

Abstract citation ID: igad104.1662

### DISCRIMINATION AND MINORITY STRESS IN OLDER ADULTHOOD: NEW DIRECTIONS FOR PSYCHOLOGICAL RESEARCH

Chair: Megan Wilson

The experience of marginalization can be harmful to individuals, in part because of increased experiences of discrimination (cite) and stress related to minority identities (cite). While older adults do tend to report experiencing less discrimination, past work suggests that older adults may be more impacted by experiences of discrimination compared to younger adults. Considering older adults from marginalized groups specifically, these individuals may be particularly susceptible to negative effects of discrimination related to age and other group identities that may harm their well-being and health. Given the wide-ranging negative implications of experiences of discrimination for older adults in particular, there is a need to examine potential strategies for combatting these negative effects. Thus, this symposium will examine the roles of discrimination and marginalization across aspects of health and well-being, and how these effects may differ for different marginalized groups. In addition, the symposium will discuss potential strategies and tools to help reduce harmful effects of discrimination and marginalization on older adults' lives. First, Danielle McDuffie will present work on bereavement and gratitude for Black adults. Next, Megan Wilson will discuss nuances in the relationship between discrimination and purpose and their implications for health. Next, Lydia Ong will examine the effects of daily discrimination on cortisol reactivity. Finally, Michael Vale will discuss minority stress and loneliness for sexual minority individuals.

Abstract citation ID: igad104.1663

### DISCRIMINATION IN OLDER ADULTHOOD: UNDERSTANDING THE IMPACT ON PURPOSE IN LIFE

Megan Wilson, Isaiah Spears, Sara Norton, Ryan Bogdan, and Thomas Oltmanns, *Washington University in St. Louis, St. Louis, Missouri, United States*

Experiences of discrimination negatively impact individuals across a variety of outcomes, and may similarly impact individuals' sense of purpose, meaning the feeling that one has meaningful goals and direction in life (Ryff et al., 2003). However, counter to a deficit perspective on marginalization, research also suggests that discrimination may be less negatively associated with sense of purpose for minority groups (Wilson & Hill, 2023a). One possible reason is that members of minority groups may be more likely to find purpose through discrimination (Wilson & Hill, 2023b). Thus, the current study examined a sample of 1630 older adults (Mean Age = 73.08) in order to understand the relationship between discrimination and sense of purpose, and moderators of this association. Results indicate that discrimination is negatively associated with sense of purpose. Contradicting past work, this effect did not differ for racial minority group members. However, we do find that individuals with higher education show a less negative association between discrimination and sense of purpose, suggesting education as a potential pathway to overcome discrimination in the pursuit of meaningful life goals. Moreover, while discrimination was negatively associated with health, sense of purpose moderated this relationship, suggesting a potential for sense of purpose to serve as a resilience factor to protect individuals from harmful effects of discrimination. Findings will be discussed in terms of how we may help older adults foster a sense of purpose and life direction, and in terms of the broader implications on older adults' well-being and health.

Abstract citation ID: igad104.1664

### AGE DIFFERENCES IN EVERYDAY DISCRIMINATION AND CORTISOL DYNAMIC RANGE IN DAILY LIFE

Lydia Ong[1], Patrick Klaiber[2], Jin Wen[1], Nicole Stuart[1], and Nancy Sin[1], *1. University of British Columbia, Vancouver, British Columbia, Canada*, *2. Tilburg University, Tilburg, Noord-Brabant, Netherlands*

Extensive work has linked everyday discrimination with poorer health. However, research testing age differences in this relationship is limited. The current study examined everyday discrimination and cortisol dynamic range, and whether the association differed by age. Cortisol dynamic range is believed to reflect physiological responsiveness to external demands such as stressors, with a compressed range indicating less responsiveness. Participants were 250 community-based adults living in British Columbia (ages 25-88, mean=46 years, 64% White, 68% women). At baseline, participants completed the Everyday Discrimination Scale, followed by four days of at-home saliva collection 3x per day: upon waking, 30 minutes post-waking, and before bed. Cortisol dynamic range was calculated as the log-cortisol peak minus log-cortisol nadir across the four days. Multiple regression controlled for race/ethnicity, education, gender, medications, average length of waking day, and depressive symptoms. Results showed a negative association between age and cortisol dynamic range, such that older age was associated with a smaller range (b=-0.01, SE=0.003, p<0.001). There was no main effect of everyday discrimination on cortisol dynamic range. However, there was a significant interaction between age and everyday discrimination. Among younger adults, more frequent everyday discrimination was associated with a smaller cortisol dynamic range. In contrast, among middle-aged adults, more frequent everyday discrimination was associated with a larger range (b=0.37, SE=0.08, p<0.001). There was no significant association between discrimination and cortisol dynamic range among older adults. Findings indicate differing patterns in discrimination and cortisol dynamic range between younger, middle-aged, and older adults.

Abstract citation ID: igad104.1665

**MINORITY STRESS AND LONELINESS IN A LIFESPAN SAMPLE OF SEXUAL MINORITIES**

Michael Vale, *Sacred Heart University, Fairfield, Connecticut, United States*

Marginalized individuals often experience social isolation (i.e., loneliness), which is a risk factor for diminished health, especially for older sexual minorities (i.e., LGB+) who were socialized in a period with less social recognition and overt stigmatization (i.e., minority stress). Although it is known that minority stressors contribute to loneliness, little is known about the contextual factors that predict this relationship, such as age/cohort. First, we examined cross-sectional baseline data from a lifespan sample of sexual minorities (N=333, ages 18-90) to investigate the relationship between seven different minority stressors and loneliness. Compared to their younger peers, older sexual minorities reported lower levels of loneliness and minority stress; however, age moderated the links between internalized homonegtaivity (B=.01, p<.01), anticipated stigma (B=.02, p<.01), and family-directed stigma (B=.01, p<.05) on loneliness, respectively. Secondly, daily experiences of minority stress and levels of loneliness were examined across a 21-day period (N=1,878 days) from a subsample of the baseline study (N=112, ages 19-79). There was higher daily loneliness on days when participants concealed their identity and had higher internalized homonegativity. Conversely, loneliness was lower when participants disclosed their identity. We further explored lagged-effects and found that loneliness was significantly higher following high internalized homonegativity (B=-.02, p<.001) from the prior day and that this change was moderated by age. These findings underscore the importance of considering age and cohort when examining minority stress. They also highlight the need for interventions that address minority stressors in this population to prevent poorer well-being and higher levels of loneliness.

Abstract citation ID: igad104.1666

**IT'S GRATEFULNESS: THE ROLE OF GRATITUDE ON ADVERSE OUTCOMES AMONG BEREAVED BLACK ADULTS**

Danielle McDuffie, *Durham VAMC, Durham, North Carolina, United States*

The Black population has been found to experience more bereavement than other racial/ethnic groups. Accompanying loss for Black populations is often other stressful factors such as racial trauma, childhood trauma, and comorbid depression. However, despite these hardships, one of the most prevalent strengths in the Black population is their engagement in religious faith as a means of coping. Gratitude, a major factor in Positive Psychology, shares many tenets with religious beliefs; however, the utility of gratitude has yet to be fully investigated within a Black bereaved population. This study sought to identify whether gratitude moderated the relationship between experienced negative life events (i.e., racial trauma; RT, adverse childhood experiences; ACEs) and depression in a sample of bereaved Black middle to older adults. 103 Black adults aged 45+ (33% female) were administered items assessing depression, gratitude, RT, and ACEs as part of a larger bereavement study. ACEs and RT both significantly predicted depression (p=.009, p=.008, respectively), and there was a significant conditional effect of gratitude on depression, reflecting differences in the endorsement of depression across levels of ACEs and RT. Accordingly, with the significant role of gratitude amongst the present sample, there is evidence for utilizing a strengths-based, Positive Psychology informed approach in the clinical care of Black adults experiencing bereavement, particularly in the context of impactful and racially traumatic events. Additionally, a fruitful direction for future research and policy is addressing the role of adverse traumatic events on Black bereavement.

### SESSION 4325 (SYMPOSIUM)

Abstract citation ID: igad104.1667

**LONGITUDINAL CHANGES IN WELL-BEING ASSOCIATED WITH AGING ATTITUDE, COGNITIVE STATUS, AND WIDOWHOOD TRANSITION**

Chair: Lydia Li

This symposium brings together four studies that employ national data collected from older Americans to examine longitudinal changes in older adults' cognitive, social, and psychological health. The first study focuses on the role of self-perception of aging in memory decline and social mechanisms linking aging perception and memory. It found that negative perceptions of aging predicted memory decline 8 years later and that loneliness and social isolation were pathways. The second study is concerned with neuropsychological changes before the onset of dementia. Using latent growth modeling, it found that the trajectory of depression increased over time in a quadratic fashion before the onset of incident dementia. Further, there were racial/ethnic group differences in levels of depression. Using fixed-effect linear regression, the third study reports that changes in cognitive status were associated with changes in social isolation in older adults. Specifically, it found that transitioning from cognitive intact to cognitive impairment status was associated with increased social isolation. The fourth and final paper examines changes in psychological well-being related to the transition to widowhood. It found that during the year right after the spouse died, older adults experienced increased depressive symptoms and reduced positive well-being. But adaptation is evident—the surviving spouse returned to preloss levels of psychological well-being around two years after the death. Together, the four studies show that attitude, cognitive status, and life course transition shape the dynamics of cognitive, social, and psychological health in later life.

Abstract citation ID: igad104.1668

### EFFECTS OF SELF-PERCEPTIONS OF AGING ON COGNITIVE HEALTH: MEDIATING ROLE OF SOCIAL ISOLATION AND LONELINESS

Rita Hu[1], Lydia Li[1], Toni Antonucci[1], Ruth Dunkle[1], Jacqui Smith[1], and Alexandra Crampton[2], *1. University of Michigan, Ann Arbor, Michigan, United States*, *2. Marquette University, Milwaukee, Wisconsin, United States*

Cognitive decline is one of the most common age-related stereotypes. The stereotype embodiment theory suggests that negative age stereotypes can be internalized through socialization and become negative self-perceptions of aging (SPA). However, the potential mediating role social relationships play between SPA and cognitive health remains unclear. Using three-wave data spanning eight years (2008/2010 – 2016/2018) from the Health and Retirement Study, this study aims to examine (1) SPA's long-term effects on changes in cognitive health; and (2) the mediating role of social isolation and loneliness (N = 3,569, Mage = 72.30). SPA was measured by an 8-item scale. Cognitive health was measured by immediate and delayed word list recall. Social isolation was measured using network size, network diversity, frequency of contact with networks, and frequency of social participation. Loneliness was measured using the 11-item UCLA Loneliness scale. We controlled for baseline measures of the dependent variables and health-related and sociodemographic covariates. We found that negative SPA at baseline was associated with memory decline eight years later ($b = -0.03$, $SE = 0.02$, $p < 0.05$). The path analysis revealed that social isolation and loneliness were mediators between SPA and cognitive health, with social isolation accounting for 15% and loneliness accounting for another 32% of the total effects. The results suggest that social isolation and loneliness are pathways by which SPA exerts adverse effects on memory among older adults. Negative SPA should be a target for intervention. In addition, social relationship building could alleviate the impact of SPA on cognitive health.

Abstract citation ID: igad104.1669

### TRAJECTORIES OF DEPRESSIVE SYMPTOMS BEFORE THE ONSET OF DEMENTIA: EXAMINING RACIAL/ETHNIC DISPARITIES

Ji Hyun Lee[1], Amanda Cook Maher[2], Jordan Palms[2], Stipica Mudrazija[3], William Chopik[4], and Laura Zahodne[2], *1. Montana State University, Bozeman, Montana, United States*, *2. University of Michigan, Ann Arbor, Michigan, United States*, *3. Urban Institute, Washington, District of Columbia, United States*, *4. Michigan State University, East Lansing, Michigan, United States*

Biological and cognitive changes can often be detected up to 16 years prior to a formal diagnosis of Alzheimer's disease (AD). Psychological symptoms of AD (e.g., depression) may also start to develop during this prodromal phase. Depressive symptoms may be an early marker of neurodegeneration and/or a reaction to early cognitive decline. Higher levels of depressive symptoms could create additional burdens for individuals with preclinical AD and their families. Unfortunately, little is known about the trajectory of depression during the preclinical phase of AD. Using the Langa-Weir method for algorithmic classification, a sample of incident dementia cases (n=2168) was identified from longitudinal data from the Health and Retirement Study (2000-2018). Depressive symptoms were measured with eight dichotomous items from the Center for Epidemiologic Studies Depression (CES-D). Latent growth curve models estimated the trajectory of depressive symptoms over up to 10 waves prior to dementia classification, and disparities across racial/ethnic groups were examined. Results show that depressive symptoms increased slightly over time throughout the preclinical phase of AD, following a quadratic trajectory. Older Hispanic adults reported consistently higher levels of depressive symptoms across timepoints, followed by older non-Hispanic Black and older non-Hispanic White adults. These findings highlight the importance of examining the psychological changes occurring during the preclinical phase of dementia for understanding the disease progression of AD and racial/ethnic disparities in psychological burden. The implications of using algorithmic dementia classifications in studying cognitive race/ethnic disparities will also be discussed.

Abstract citation ID: igad104.1670

### THE IMPACT OF CHANGES IN COGNITIVE FUNCTIONING ON SOCIAL ISOLATION

Jay Kayser, and Lydia Li, *University of Michigan, Ann Arbor, Michigan, United States*

This study examines if transitioning from normal to impaired cognitive functioning is associated with changes in social isolation among community-dwelling older adults without cognitive impairment at baseline. This study uses nine waves (2011-2019) of data from the National Health and Aging Trends Study (NHATS). The sample (N=8,245) was representative of Medicare beneficiary at baseline. Social isolation was measured using a seven-item index that captures a range of social activities and network characteristics. Cognitive status was assessed through self-report of having Alzheimer's disease, the Dementia Screening Interview (AD8), and cognitive testing. Based on pre-determined criteria, participants were grouped as either cognitively intact or impaired. Fixed effect linear modeling with robust standard errors was used to examine the impact of transitioning from intact to impaired cognitive functioning on social isolation. At baseline 5,575 Participants were cognitively intact. At each successive wave, between 12% and 16% of the sample was cognitively impaired. After controlling for time-varying covariates, transitioning from intact to cognitive impairment was associated with a statistically significant increase in social isolation (β=.18, SE=.03, p <.001). Gender and educational attainment did not impact this association. These findings underscore the need for policies and interventions to increase social connection among older adults, particularly during early stages of cognitive impairment.

Abstract citation ID: igad104.1671
**DYNAMICS OF PSYCHOLOGICAL WELL-BEING ACROSS THE TRANSITION TO WIDOWHOOD**
Lydia Li, Rita Hu, Jay Kayser, and Joonyoung Cho,
*University of Michigan, Ann Arbor, Michigan, United States*
The present study examines within-person changes in psychological well-being (PWB) associated with the transition to widowhood. We analyzed 9-wave data collected annually from a probability sample of Medicare beneficiaries. The analyzed sample included married respondents at baseline (N=3,711). Five hundred ten respondents, 208 men and 202 women, became widowed in the survey period. We created variables to indicate widowhood status, the time before and the time since widowhood. Depressive symptoms measured by the PHQ9 and positive well-being assessed by a 6-items scale (e.g., My life has meaning and purpose) were dependent variables. Fixed-effect linear regression, which uses only within-person change over time in the panel data, was used. Results show that immediately after the spouse's death, depressive symptoms increased and positive well-being decreased. There is a curvilinear relationship between time since widowhood and both depressive symptoms and positive well-being. Specifically, after the widowhood transition year, depressive symptoms began to decline, positive well-being began to increase, up to about four years after widowhood when depressive symptoms slightly rose and positive well-being slightly declined. Men and women have similar patterns. The findings suggest that right after spousal loss is challenging for older adults; some may need counseling and extra support. About two years after the spouse's death, they return to the pre-loss level of PWB and continue improving until about four years after widowhood. Improvement in depressive symptoms and positive well-being shows older adults' resilience and potential for personal growth after adversity.

### SESSION 4330 (SYMPOSIUM)

Abstract citation ID: igad104.1672
**EXCLUSION FROM MULTIDIMENSIONAL CIVIC ENGAGEMENT OF DIVERSE OLDER ADULTS: A PAN-EUROPEAN PERSPECTIVE**
Chair: Fredrica Nyqvist Discussant: Sarah Dury
Despite decades of research on social exclusion, the understanding of the intersection between ageing and exclusion from civic engagement remains limited, both theoretically and empirically. Moreover, the research on civic engagement, including volunteering or political participation, has received unequal attention in research and the study of multidimensional civic engagement has scarcely been addressed. This symposium therefore brings together qualitative and quantitative research on multidimensional civic engagement in later life, as a part of the pan-European CIVEX-project, devoted to critically assess multidimensional civic engagement of diverse and potentially marginalized groups of older adults in a European context. The objective is to explore the main dimensions of exclusion from multidimensional civic engagement in later life and older adults' experiences of such exclusion. Toon Vercauteren starts with a study using data from the European Quality of Life Survey (EQLS) in 33 European countries to examine multidimensional civic engagement of older adults in Europe and its key risk and support factors. Bas Dikmans will use a life course perspective to study features of exclusion from civic engagement in disadvantaged neighbourhoods in Belgium, using data collected within the international CIVEX-project. Subsequently, Fredrica Nyqvist will present results based on EQLS data on social capital and political participation in younger as well as older adults. Sandra Torres will close the symposium with a presentation on civic engagement amongst older migrants based on interview data collected in Belgium, Finland, Spain and Sweden.

Abstract citation ID: igad104.1673
**MULTIDIMENSIONAL CIVIC ENGAGEMENT OF OLDER PEOPLE: A LATENT CLASS ANALYSIS**
Toon Vercauteren[1], Marina Näsman[2], Fredrica Nyqvist[2], Dorien Brosens[1], Rodrigo Serrat[3], and Dury Sarah[1], *1. Vrije Universiteit Brussel, Brussels, Brussels Hoofdstedelijk Gewest, Belgium*, *2. Åbo Akademi University, Vaasa, Pohjanmaa, Finland*, *3. University of Barcelona, Barcelona, Catalonia, Spain*
Civic engagement of older people remains an understudied topic in gerontological research, focusing for the biggest part on formal activities, such as volunteering and political engagement. This research proposes a more inclusive definition referring to multidimensional civic engagement which includes associational engagement, formal and informal volunteering, digital engagement, and formal and informal political engagement. Additionally, research fails to address whether older people combine different civic activities or are only engaged in one. Using EQLS data (2016) collected in 33 European countries, this research examined the multidimensional civic engagement of older people and whether they combine civic activities. Descriptive analysis was used to map the multidimensional civic engagement of those over 65 years (n = 9,031). Latent class analysis was conducted to create profiles among those who are civically engaged to study whether activities are combined and to explore the socio-structural and social capital resources of each profile (n = 6,142). The results indicate that over two-thirds of older Europeans are engaged in minimally one civic activity. Five profiles can be differentiated, ranging from low diversity profiles to a high diversity profile regarding the chance of engaging in a multitude of civic activities in later life. The results clearly show that having more resources enables diverse civic engagement in later life. This study appropriately appreciates the multidimensional civic engagement of older people and identifies the resources that must be considered when promoting multidimensional civic engagement.

Abstract citation ID: igad104.1674

### EXCLUSION FROM CIVIC ENGAGEMENT IN TWO DISADVANTAGED NEIGHBORHOODS IN BRUSSELS, BELGIUM: A LIFE COURSE PERSPECTIVE

Bas Dikmans[1], Liesbeth De Donder[2], Dury Sarah[3], and Rodrigo Serrat[4], *1. Vrije Universiteit Brussels, Brussels, Brussels Hoofdstedelijk Gewest, Belgium*, *2. Vrije Universiteit Brussel (VUB), Brussels, Brussels Hoofdstedelijk Gewest, Belgium*, *3. Vrije Universiteit Brussel, Brussels, Brussels Hoofdstedelijk Gewest, Belgium*, *4. University of Barcelona, Barcelona, Catalonia, Spain*

Although research into older people's civic engagement has come a long way in the last decades, there are still some blind spots on this scholarship. Notably, the influence of neighbourhood features on in- or exclusion from civic activities, as well as the multifaceted nature of civic engagement and the dynamics of such processes across the life course have been scarcely addressed. Therefore, this research focuses on the life course trajectories of older people in two socioeconomically disadvantaged neighborhoods in Brussels, Belgium, in order to understand their inclusion and exclusion from multidimensional forms of civic engagement, including formal volunteering, informal helping behaviours, memberships in associations, political participation and digital civic engagement. We conducted forty semi-structured interviews supported by life diagrams that focused on the changes of in- and exclusion from multidimensional civic engagement throughout the life course. The life stories of these older residents are thematically and narratively analyzed. The outcome of this study underlines the importance of a life course perspective in understanding older people's experiences of inclusion and exclusion from multidimensional civic engagement. The findings will be used for informing policy recommendations to foster multidimensional civic engagement for older people living in disadvantaged communities.

Abstract citation ID: igad104.1675

### DOES SOCIAL CAPITAL ENHANCE POLITICAL PARTICIPATION? A MULTILEVEL ANALYSIS OF YOUNGER AND OLDER ADULTS IN EUROPE

Fredrica Nyqvist[1], Mikael Nygård[1], Rodrigo Serrat[2], and Marina Näsman[1], *1. Åbo Akademi University, Vaasa, Pohjanmaa, Finland*, *2. University of Barcelona, Barcelona, Catalonia, Spain*

Political participation encompasses institutionalised activities such as attending meetings of a political organisation as well as non-institutionalised activities including contacting politicians, signing petitions or boycotting. Both forms of activities tend to be dependent upon birth cohort, political socialisation, and period effects like changes in the welfare state design. Furthermore, it is also connected with other domains of civic engagement as well as trust. Social capital theory focuses on social connections and roles fostering civic engagement, including political participation, however, the relationship between social capital and political participation might look different for different types of political activities. The aim of this study was, therefore, to test the importance of social capital for different types of political participation in younger and older adults by analysing cross-national data from the European Quality of Life Survey (EQLS) in 2016. Data was collected in 33 European countries and includes more than 36,000 individuals aged 18 and over. Multilevel regression analysis was used to explore individual-level and country-level social capital on different measures of institutionalised and non-institutionalised political participation in younger and older adults. The results showed that a third of the sample participated in non-institutionalised activities whereas 11 percent participated in institutionalised activities. Both forms of political participation were lower among older adults as compared to younger age groups. While social capital was identified as an important predictor of political participation, it was also shown that these associations were conditioned by larger cultural, political and welfare institutional contexts.

Abstract citation ID: igad104.1676

### CIVIC ENGAGEMENT AMONG OLDER MIGRANTS IN EUROPE: EXAMPLES FROM FOUR EUROPEAN COUNTRIES

Sandra Torres[1], Pernilla Ågård[2], Emilia Häkkinen[3], Bas Dikmans[4], Inma Peiro-Milan[5], and Rodrigo Serrat[5], *1. Uppsala University, Uppsala, Uppsala Lan, Sweden*, *2. Uppsala University, Dept. of Sociology, Uppsala, Uppsala Lan, Sweden*, *3. Åbo Academy University, Vasa, Pohjanmaa, Finland*, *4. Vrije Universiteit Brussels, Brussels, Brussels Hoofdstedelijk Gewest, Belgium*, *5. University of Barcelona, Barcelona, Catalonia, Spain*

Scholarship on old age social exclusion has identified civic engagement as a research area that deserves empirical attention. Research on older people's civic engagement has in turn pointed out that experiencing life-course deficits in social capital, and/ or having had a life-course that does not resemble the continuity that social gerontology takes for granted, could put people at risk of becoming civically excluded in old age. These are two of the starting points for the European project known as CIVEX, which aims to study the life-course trajectories of civic engagement in groups of older people that have not yet received enough empirical attention. One of these groups is constituted by those who have migrated to the countries in which they are now based as adults, and whose life-course may therefore be more characterized by discontinuity rather than continuity. Departing from 60 life-course qualitative interviews with older migrants that are based in Sweden, Belgium, Spain and Finland, this presentation will draw attention to the ways in which these older people define civic engagement and the manner in which they describe how their migratory life-courses have impacted their civic engagement. The presentation will argue that there is theorizing potential embedded on the migratory life course, and that this could expand the scholarly imagination on old age social exclusion in general, and civic engagement in particular.

### SESSION 4335 (BIOLOGICAL SCIENCES INVITED SYMPOSIUM)

Abstract citation ID: igad104.1677

### AGING AND CIRCADIAN CLOCK

Chair: Jennifer Hurley

Circadian rhythms are highly conserved, 24-hour, oscillations that tune physiology to the day/night cycle, enhancing fitness by ensuring that appropriate activities occur at biologically advantageous times. Disruption of proper circadian timing negatively impacts organismal fitness, making understanding the mechanism underlying circadian regulation over cellular physiology critical to appreciating a fundamental rule of life on earth. As we age, our bodies circadian rhythms change due to stress, chronic disruption of our clocks, neurodegeneration, and a host of other reasons, which can have a profound effect on our systems. Therefore, understanding the aging circadian clock is important to promote longevity and healthy aging. In this session, we will investigate some of the research going on that links the clock to aging. Topics will include the investigation of the role of the clock in timing immunometabolic regulation in the context of inflammation and Alzheimer's disease, the optimization of the timing of exercise in the effort to maintain homeostasis and decrease risk, the connection between aging and the reduction of the number of rhythmically expressed genes and the weakening of circadian control, and the effect of dietary restriction on the circadian clock. The take home message of this session will be the importance of factoring daily time into research, preventative measures, and treatment regimens, to maximize overall health as we age.

Abstract citation ID: igad104.1678

### CIRCADIAN TIMING OF THE IMMUNOMETABOLIC RESPONSE AFFECTS ALZHEIMER'S DISEASE PATHOLOGY

Jennifer Hurley, *Rensselaer Polytechnic Institute, Troy, New York, United States*

Though the predominant model of the biological mechanisms underlying ADRDs is based on the beta-amyloid (Aβ) hypothesis, evidence suggests that inflammation from resident immune cells is also a key component of the development of ADRD pathophysiology. A major biological regulator of inflammation is the circadian clock, the 24-hour molecular timekeeper that broadly regulates gene expression throughout mammalian tissues to synchronize biology to the Earth's day/night cycle. Concordantly, chronic disruption of the circadian clock (CD) increases the risk for and severity of ADRDs. Our research has revealed that the circadian clock times metabolism, with the clock timing mitochondrial division which in turn times the response of mammalian macrophages to ADRD pathological factors. We show that subunits of the enzyme NOX2, a driver in the creation of reactive oxygen species (ROS) that uses metabolites to impart inflammation, are driven to oscillate in a 24 hr rhythm by the circadian clock in macrophages and microglia. Notably, oscillations in these NOX2 subunits are dysregulated when an organism undergoes CD, leading to an increase in the levels of ROS released by macrophages and microglia and elevating overall levels of inflammation. Given the apparent interconnection between inflammation, circadian rhythms, and ADRDs, an understanding of the circadian timing of the immune system could be exploited to develop treatments that alleviate ADRD symptoms, contribute to a potential cure, and prevent a population-wide increase in neurodegeneration due to a societal surge in CD.

Abstract citation ID: igad104.1679

### AGE-ASSOCIATED DECLINE IN TISSUE CIRCADIAN CLOCK FUNCTION

Karyn Esser, *University of Florida, Gainesville, Florida, United States*

Cellular circadian clocks direct a daily transcriptional program that supports homeostasis and resilience. Emerging evidence has demonstrated age-associated changes in circadian functions. To define age-dependent changes at the systems level, we profile the circadian transcriptome in the hypothalamus, lung, heart, kidney, skeletal muscle, and adrenal gland in three age groups. We find age-dependent and tissue-specific clock output changes but these changes exhibit tissue specific patterns. In general, aging reduces the number of rhythmically expressed genes (REGs), indicative of weakened circadian control. These REGs are enriched for the hallmarks of aging, adding another dimension to our understanding of aging. We determined there were tissue specific changes in the phase distribution of REGs with the hypothalamus and skeletal muscle tissues of the old mice showing a large reduction with REG peak expression at only one phase of the day. Analyzing all expressed genes within a tissue for differences at four distinct times of day identified unique clusters of differentially expressed genes (DEGs). These outcomes identify non-circadian but temporally distinct age associated changes in gene expression. This analysis extends the landscape for understanding aging and highlights the impact of aging on circadian clock function and temporal changes in gene expression.

Abstract citation ID: igad104.1680

### DIETARY RESTRICTION AND THE TRANSCRIPTION FACTOR CLOCK DELAY EYE AGING TO EXTEND LIFESPAN IN DROSOPHILA MELANOGASTER

Pankaj Kapahi, *Buck Institute for Research on Aging, Novato, California, United States*

Many vital processes in the eye are under circadian regulation, and circadian dysfunction has emerged as a potential driver of eye aging. Dietary restriction (DR) is one of the most robust lifespan-extending therapies and amplifies circadian rhythms with age. We have previously shown that lifespan extension upon DR requires the presence of circadian clocks for maximal benefits. Herein, we demonstrate that dietary restriction extends lifespan in Drosophila melanogaster by promoting circadian homeostatic processes that protect the visual system from age- and light-associated damage. Altering the positive limb core molecular clock transcription factor, CLOCK, or CLOCK-output genes, accelerates visual senescence, induces a systemic immune response, and shortens lifespan. Flies subjected to dietary restriction are protected from the lifespan-shortening effects of photoreceptor activation. Inversely, photoreceptor inactivation, achieved via mutating rhodopsin or housing flies in constant darkness, primarily extends the lifespan of flies reared on a high-nutrient diet. Our findings establish the eye as a diet-sensitive modulator of lifespan and indicates that vision is an antagonistically pleiotropic process that contributes to organismal aging.

Abstract citation ID: igad104.1681

### RUN FOR ANTI-AGING: EXERCISE TIMING SPECIFIES METABOLIC RESPONSES

Shogo Sato, *Texas A&M University, College Station, Texas, United States*

Exercise has been widely recognized as an effective non-pharmacological intervention to delay aging and reduce the onset of chronic metabolic diseases such as type 2 diabetes and cardiovascular diseases. The metabolic response to exercise is influenced by factors such as frequency, intensity, duration, and modality. However, the role of the timing of exercise in activating metabolic pathways has remained an outstanding question. The circadian clock allows the organism to adapt to the cyclic environmental changes imposed by the earth's rotation, thereby placing temporal boundaries on physiological functions, including metabolic responses to exercise. Our recent studies performed unbiased high-throughput metabolomics of 8 different metabolic tissues in response to a single-bout acute treadmill exercise at different times of the day in mice, revealing that daily timing of exercise is a critical variable for the metabolic impact on different metabolic tissues. This indicates that timing is key to unlocking the benefits of exercise on metabolic health and anti-aging. Therefore, optimizing exercise outcomes following when to exercise is essential to establishing personalized exercise interventions aiming at health improvement and reducing the risk of chronic diseases associated with aging. Our ongoing research is studying the time-of-day-dependent metabolic responses to exercise in aged mice compared to young mice. The ultimate goal is to identify the best time for exercise to promote longevity and healthy aging.

### SESSION 4340 (SYMPOSIUM)

Abstract citation ID: igad104.1682

### CONNECTING THE DOTS: SUPPORTING OLDER ADULTS LIVING WITH HIV-ASSOCIATED NEUROCOGNITIVE DISORDERS

Chair: Elliott Weinstein Co-Chair: Daniel Jimenez
Discussant: Hetta Gouse

Although people living with HIV are living longer than ever before, significant HIV-related physical and mental health disparities remain among older adults living with HIV (OALWH) due to issues related to advanced aging. HIV-associated neurocognitive disorders (HAND) are becoming increasingly common and have the potential to negatively impact the cognitive, motor, and psychological well-being of OALWH. However, more research on both predictors of HAND and interventions to support cognitively impaired OALWH is needed. This symposium offers insights along the spectrum of intervention development. Two presentations will focus on best identifying predictors of HAND among groups most at-risk up while the other three discuss tailoring and evaluating interventions to alleviate HAND-related symptoms within diverse samples of OALWH from across the country. The first presentation centers on how cerebrospinal fluid markers of Alzheimer's-related pathology can disentangle diagnosing HAND and amnestic mild cognitive impairment among OALWH in Southern California while the second highlights the role of grip strength in predicting frailty and cognitive impairment among OALWH living in South Florida. The third presentation assesses the feasibility, acceptability, and preliminary intervention effects of a physical activity intervention on cognition adapted for Latino OALWH in South Florida while the fourth examines qualitative feedback from several focus groups on adapting cognitive remediation group therapy as a hybrid group intervention for OALWH with cognitive concerns living in two Canadian provinces. Finally, the last presentation reviews findings of a 2-year randomized clinical trial examining speed of processing training on everyday functioning among OALWH in the U.S. Deep South.

Abstract citation ID: igad104.1683

### HEALTH PROMOTION TO PREVENT AND REDUCE COGNITIVE DECLINE IN LATINOS LIVING WITH HIV/AIDS

Elliott Weinstein[1], Marcela Kitaigorodsky[2], Victoria Behar-Zusman[1], and Daniel Jimenez[3], *1. University of Miami, Miami, Florida, United States*, *2. Florida Neuro-Health, Hollywood, Florida, United States*, *3. University of Miami Miller School of Medicine, Miami, Florida, United States*

Older Latinos living with HIV (LWH) are at increased risk for earlier onset of aging-related cognitive decline. HIV-related cognitive impairments are observed in several domains including memory, reasoning/executive functioning, and speed of processing. Depression, a known neurocognitive risk, has been reported at five times the level among older Latinos LWH than in the general population, and older Latinos LWH are more likely to be sedentary, and not as actively engaged in pursuing changes in physical activity compared to their non-Latino White counterparts. Thus, this is a population that is particularly vulnerable to cognitive decline due to multiple risk factors. The aim of this study is to assess feasibility, acceptability, and preliminary intervention effects on cognition of the Happy Older Latinos are Active (HOLA) health promotion intervention. Thirty Latinos living with HIV with a mean age of 61.6 years (SD=6.1) were enrolled in a pilot single-arm trial. Participants were assessed at two time points on measures of cognitive and psychosocial functioning as well as biomarkers of cognition. In 7 months, we met our enrollment target with <5% of eligible participants refusing participation. Participants attended over 70% of sessions and 3 participants were lost to follow up. These results indicate that HOLA is an innovative health promotion program that is uniquely tailored to address the multiple concerns that are prevalent in this community in a non-stigmatizing and culturally acceptable manner.

Abstract citation ID: igad104.1684

### CEREBROSPINAL FLUID MARKERS OF ALZHEIMER'S-RELATED PATHOLOGY RELATE TO AMCI AMONG PEOPLE WITH HIV

Judith Lobo[1], Erin Sunderman[2], Ben Gouaux[1], Scott Letendre[1], Laura Campbell[1], Mark Bondi[1], and David Moore[3], *1. University of California San Diego, San Diego, California, United States*, *2. San Diego State University, San Diego, California, United States*, *3. University of California at San Diego, San Diego, California, United States*

Older people with HIV (PWH) are at-risk for Alzheimer's disease (AD) and its precursor, amnestic mild cognitive impairment (aMCI). Identifying aMCI among PWH is challenging because memory impairment is also common in HIV-associated neurocognitive disorders (HAND). To investigate the utility of CSF AD pathology markers in differentiating aMCI from HAND, we assessed how these markers differentially relate to aMCI versus HAND status among PWH. Participants included 74 PWH (Mean age=48 [SD=8.5]; 87.4% male, 56.5% White) from the National NeuroAIDS Tissue Consortium who completed a neurocognitive test battery assessing seven cognitive domains and had CSF biomarker data for Aβ42, p-tau181, p-tau181/Aβ42 ratio, Neopterin and NfL. aMCI was defined as impairment (<1.0 SD below normative mean) on ≥2 memory outcomes among learning, delayed recall and recognition with at-least one recognition impairment required. HAND was defined as impairment (<1.0 SD below normative mean) in ≥2 cognitive domains. Separate linear regression models adjusting for demographic and HIV-related characteristics were used to examine how individual biomarkers relate to diagnostic status. Fifty-eight percent of participants were diagnosed with HAND, 50.5% were diagnosed with aMCI. CSF p-tau/Aβ42 ratio levels were higher (indicative of greater pathology) in PWH with versus without aMCI (β=.363, p=.001), but did not differ by HAND status. No other AD biomarker significantly differed by group status. Results indicate that the CSF p-tau181/Aβ42 ratio relates specifically to an aMCI-like profile among PWH, and, thus, may contribute to disentangling aMCI from HAND and informing the need for further diagnostic procedures.

Abstract citation ID: igad104.1685

### COGNITIVE IMPAIRMENT, FRAILTY, AND GRIP FORCE GENERATION IN PEOPLE LIVING WITH HIV

Kaitlyn Dillon[1], Bonnie Levin[2], and Roger McIntosh[1], *1. University of Miami, Miami, Florida, United States*, *2. University of Miami miller school of medicine, Miami, Florida, United States*

People living with HIV (PLWH) face an earlier onset and higher rates of geriatric syndromes, including frailty and cognitive impairment. Grip force, an index of frailty, is sensitive to cognitive decline, central nervous system integrity, and may independently predict adverse health outcomes. The current study evaluates frailty, weakness, and cognitive impairment, as well as the neural correlates of grip force as they predict cognitive impairment. PLWH aged 55+ (N = 26; M = 61.9, SD = 4.7; 53.8% female; 76.9% Black) completed an fMRI task in which they were asked to generate 0%, 20%, 50%, and 80% of their maximum force, a neuropsychological battery, and a physical assessment. Cognitive impairment was indexed using the Global Deficit Score (GDS). Per Fried's Frailty Phenotype, 3.8% of the sample was robust, 57.7% was pre-frail, and 38.5% was frail. Impairment in memory significantly predicted GDS, controlling for age (B = 0.3, t = 4.9, p < .001). Robust frailty status significantly predicted GDS, controlling for age (B = -1.1, t = -2.4, p < .05). Age was also a significant predictor of GDS, controlling for frailty status (B = -0.1, t = -2.7, p < .05). Weakness, as indexed by grip strength, predicted frailty (F = 4.5, p < .05). Additional analyses will be conducted to examine activation in brain regions germane to force generation during the fMRI task as a predictor of GDS. To our knowledge, this is the first study to examine neural correlates of grip force generation in PLWH.

Abstract citation ID: igad104.1686

### ADAPTING COGNITIVE REMEDIATION GROUP THERAPY AS A HYBRID INTERVENTION FOR PEOPLE WITH HIV AND COGNITIVE CONCERNS

Andrew Eaton[1], Jenny Hui[2], Marvelous Muchenje[2], Kate Murzin[3], Soo Chan Carusone[4], francisco Ibáñez-Carrasco[2], Nuelle Novik[5], and Sharon Walmsley[6], *1. University of Regina, Saskatoon, Saskatchewan, Canada*, *2. University of Toronto, Toronto, Ontario, Canada*, *3. Realize, Toronto, Ontario, Canada*, *4. McMaster University, Hamilton, Ontario, Canada*, *5. University of Regina, Regina, Saskatchewan, Canada*, *6. University Health Network, Toronto, Ontario, Canada*

Cognitive impairment is a significant health issue for people aging with HIV/AIDS. With pharmacological treatment lacking, psychosocial group therapies may best help people aging with HIV and cognitive challenges cope with symptoms. The COVID-19 pandemic demonstrated how in-person group therapies need adaptation for hybrid or online delivery. Peer-led focus groups discussed adapting cognitive remediation group therapy (CRGT) as a hybrid or online intervention. CRGT combines mindfulness-based stress reduction and brain training activities. Purposive sampling recruited people aging with HIV (40+) who self-identified cognitive concerns and resided in two Canadian provinces. Content analysis was employed on transcripts by 7 independent coders. Ten, two-hour focus groups were conducted between August and November 2022. Participant (n=45) demographics included age (M=53.22, SD=7.62) gender (45% women, 42% men, 13% trans/non-binary), sexuality (42% gay, 40% heterosexual, 18% other), ethnicity (45% white, 33% black, 13% Indigenous, 9% mixed-race), and employment (33% employed, 67% retired/disability), and all were retained in care. Overall, participants responded favourably to CRGT's modalities and preferred a hybrid model blending in-person and online interactions. Preferred intervention facilitators were peers and mental health professionals. Knowledge of HIV's impacts on cognitive health, including HIV-associated neurocognitive disorder, was very low despite high reports of cognitive concerns (e.g., trouble remembering, impaired attention, difficulty problem-solving). Given the aging of the HIV population in Canada and the United States, increasing support will be required to improve quality of life. This presentation will discuss a hybrid CRGT adaptation, alongside considerations for how COVID-19 has impacted intervention research.

Abstract citation ID: igad104.1687

### A TWO-YEAR RCT EXAMINING SPEED OF PROCESSING TRAINING ON EVERYDAY FUNCTIONING IN ADULTS WITH HAND IN THE US DEEP SOUTH

David Vance[1], Pariya Fazeli[2], Andres Azuero[1], Sarah Khalidi[2], Jennifer Frank[2], Virgina Wadely[2], James Raper[2], and Caitlin Pope[3], *1. University of Alabama at Birmingham, Birmingham, Alabama, United States*, *2.*

University of Alabama-Birmingham, Birmingham, Alabama, United States, 3. University of Kentucky, Lexington, Kentucky, United States

Approximately 44% of people living with HIV (PLWH) experience HIV-associated neurocognitive disorders (HAND). Cognitive training approaches, such as speed of processing (SOP) training, may reduce the detrimental impact of HAND on everyday functioning. In this experimental design study, 216 participants 40 and older with HAND or borderline HAND (82% Black) were randomized to one of three groups: 1) 10 hours of SOP training (n=70); 2) 20 hours of SOP training (n=73), or 3) 10 hours of Internet Navigation Control Training (a contact control group; n=73). Participants completed several everyday functioning measures at baseline, posttest, and year 1 and year 2 follow ups. Everyday functioning measures included: 1) Modified Lawton and Brody Activities of Daily Living (ADL) Questionnaire; 2) Timed Instrumental Activities of Daily Living (TIADL) Test; 3) Patient's Assessment of Own Functioning (PAOFI); 4) Medication Adherence Questionnaire (MAQ); and 5) Medication Adherence Visual Analog Scale (VAS). Linear mixed-effect models and generalized estimating equation models were fitted to estimate between group differences at all follow-up time points. At follow-up timepoints, those in the 10-hour and 20-hour training groups performed better on medication adherence measures (MAQ and VAS) than those in the control group, with effects (Cohen's d) ranging from 0.13 to 0.41 for MAQ and 0.02 to 0.43 for VAS. In conclusion, SOP training improved some indicators of everyday functioning, specifically medication adherence; however, the therapeutic effects diminished over time. This is an interesting finding given the importance of medication adherence in viral suppression and overall survival in PLWH.

### SESSION 4345 (SYMPOSIUM)

Abstract citation ID: igad104.1688

### THE ROLE OF COMMUNITY COLLEGES IN TRAINING HEALTH CARE WORKERS FOR AN AGING POPULATION

Chair: Phyllis Cummins Discussant: Philip Taylor

The aging population in the U.S. has resulted in an increased need for healthcare workers in multiple health settings (e.g., home healthcare, nursing homes, hospitals, medical laboratories). Community colleges play an important role in educating healthcare workers that are critical to an aging population. Many of these occupations focus on home healthcare, empowering adults to remain at home rather than in a long-term care setting. This session will focus on novel ways that community colleges are effectively addressing workforce needs in multiple settings, including hospitals, home healthcare, long-term care, and medical laboratories. A panel of community college and university gerontology and education professionals representing GSA's Community College and the Aging Workforce Interest Groups will share the innovative ways in which they are addressing the needs of an aging population. Yamashita and Narine will discuss the current and future demand for employees in healthcare occupations, especially those in long-term and home healthcare settings, and will focus on careers that do not require a bachelor's degree. Velazquez's presentation will focus on the impact COVID had on training healthcare workers in healthcare related occupations and how one community colleges adapted their curriculum. Cummins et al. will discuss a study that focused on the role literacy, numeracy, and problem-solving skills play in community college students' success in the classroom and the healthcare workplace, focusing on employer interviews. Finally, Kaur will discuss the role Hillsborough Community College plays in training medical laboratory scientists and in providing employees for critical medical services to an aging society This is a collaborative symposium between the Aging Workforce and Community College Interest Groups.

Abstract citation ID: igad104.1689

### HEALTH CARE EMPLOYERS' PERSPECTIVES ON SKILLS REQUIRED FOR STUDENT SUCCESS IN THE WORKPLACE

Phyllis Cummins[1], Runcie Chidebe[1], Rita Karam[2], and Jenna Kramer[3], *1. Miami University, Oxford, Ohio, United States, 2. RAND Corporation, Santa Monica, California, United States, 3. RAND Corporation, Arlington, Virginia, United States*

Community colleges (CCs) play a critical role in educating healthcare workers that are important to an aging population. This study investigated the role literacy, numeracy, and problem-solving skills play in CC student success in the classroom and the workplace. Semi-structured virtual and in-person interviews were completed with CC administrators (n=9) and faculty (n=6), along with employers (n=10) who hire students from CCs. We focus on healthcare employers (i.e., home healthcare, nursing homes and hospitals) and the skills they value along with skills that are lacking in CC hires. Examples of health care occupations filled by CC students include certified nursing assistants, qualified medication aides, licensed practical nurses (LPNs), and registered nurses (RNs). Employers reported working closely with CCs, including providing opportunities for clinical rotations, serving on program advisory councils, and providing input into program development. Numeracy skills, such as measuring medication dosages, critical thinking skills, and problem-solving skills, including conflict resolution, are important and expected by employers. Communication skills are also highly valued, especially the ability to communicate on the level of the nursing home resident or hospital patient, along with their family members. Employers noted that LPNs and RNs sometimes have limited leadership and supervisory skills and remarked they are working with CC faculty and administrators to identify mechanisms (e.g., role playing, case studies) to improve those skills. Findings from this project will inform both CC administrators and employers regarding skills that are necessary to succeed as students and healthcare workers to meet the needs of an aging population.

Abstract citation ID: igad104.1690

### ROLES OF COMMUNITY COLLEGES IN TRAINING THE HEALTH CARE WORKFORCE IN THE AGING SOCIETY

Takashi Yamashita, *University of Maryland, Baltimore County, Baltimore, Maryland, United States*

As population aging advances, the need for health, medical and long-term, care necessarily increases. On one hand, certain occupations mostly with routine tasks are likely to be automated by computers and technology over time. On the other hand, the previously estimated job automation risks clearly suggest that health care occupations are less likely to be automated, compared to other industries. For example, while 12-45% of health care occupations could be replaced by technology, over 80% of simple food preparation and entry-level customer service is projected to be automated within a few decades. Thus, the demand to train the health care workforce for the aging population will continue to be high, despite the advancement in computers and technology. Certain health care occupations such as licensed practical nurses, nurse assistants, medical technicians, and home health aides, do not require bachelor's degrees. Considering the current and projected health care workforce shortage, education and training of the future health care workforce is a critical issue when preparing for growing older populations in the U.S. This study triangulated data on health care occupations, literature on workforce development, and emerging trends on aging-related health and long-term care needs, and critically analyzed the role of community colleges to meet the demands for health care workforce in the U.S. Results showed that the history, geographic locations, and existing health care programs position community colleges as one of the most effective and efficient health care workforce development institutions. Specific examples of health care professional training programs are also evaluated.

Abstract citation ID: igad104.1691

### MEDICAL LABORATORY SCIENTISTS' ROLE IN THE CARE OF AN AGING POPULATION

Tripat Kaur, *Hillsborough Community College, Tampa, Florida, United States*

Medical Laboratory Scientists (MLS) are a group of highly skilled professionals in the healthcare industry who are responsible for working on specimens and providing results that are accurate in diagnosing medical disorders and monitoring treatments. These heroes work behind the scenes and face a critical shortage of personnel required to meet the needs of an ageing population. The number of clinical tests has increased 388% since 1993, from 12,683 to nearly 62,000. In addition, the Centers for Disease Control reports that for the >500 million primary care patient visits each year, one out of three patient encounters result in the ordering of at least one laboratory test. To meet these high testing volumes medical and clinical laboratories are highly automated with instrumentation utilized in all aspects of testing to improve the turnaround time for reporting results. Programs that offer medical laboratory science programs often must prepare the students for this highly technical field. To assist in the shortages that the laboratories faced, 6 years ago, BayCare Health System approached Hillsborough Community College Health Science division about setting up a post-baccalaureate medical laboratory science program. The program has now had approximately 100 graduates who have jobs around the Tampa Bay hospitals. Educational institutions face challenges in meeting current needs because of the lack of visibility of the medical laboratory science profession, a drop in the total enrollment in colleges over the last 5 years, and a rise in the national median age of the current population.

Abstract citation ID: igad104.1692

### IMPACTS OF COVID-19 ON TRAINING HEALTH CARE WORKERS AT EAST LOS ANGELES COMMUNITY COLLEGE

Janice Velazquez, *East Los Angeles College, Monterey Park, California, United States*

The Pandemic changed how community colleges prepare the next generation of healthcare workers, and they are slowly adjusting to remain relevant in the current environment. How community colleges train their students will determine how well their communities will be cared for, which is why this topic is important. Some of the strategies implemented to fulfill the East Los Angeles' College (ELAC) workforce demands following COVID are: aligning basics skills with some of the courses; providing career counseling, including mock interviews; opportunities to branch out and transfer in two years; connecting students with services to address their basic needs including mental health. Faculty are also required to stay current, and they need support from their local Districts and national collaborators. Some completed specialized training in humanizing curriculum and transforming faculty learning programs to improve undergraduate STEM faculty's instructional practice. Moreover, the ELAC faculty felt that developing a humanities course tailored to Health and Human Services could be a great start to connect current needs with our academic programming. Faculty are revising and collecting content to address recent changes impacting our society and how we operate in the fast-changing work environment. After the Pandemic, the use of technology is more common and accepted. This change facilitates interactions, and this is a contributing factor to the program development process. For example, the advisory questionnaire is now available electronically, and therefore expecting more participation. Faculty have more variety and options for training to improve content development and delivery strategies.

### SESSION 4350 (SYMPOSIUM)

Abstract citation ID: igad104.1693

### IMPLEMENTATION OF A COMMUNITY WALKING PROGRAM FOR OLDER ADULTS WITH MOBILITY CHALLENGES—WALK-ON!

Chair: Barb Nicklas Co-Chair: Jaime Hughes
Discussant: Kathryn Porter Starr

Clinical trials in controlled settings show walking interventions improve function and reduce mobility loss in older adults. In the NIA-funded LIFE study, participants with more compromised function derived the most benefit, yet improvements were not sustained. Based on this research, clinicians often prescribe walking for older patients. However, few communities provide accessible opportunities for those with limited function to engage in safe, ability-appropriate, walking. Additionally, no evidence-based National Council on Aging exercise programs emphasize ambulation over longer distances in supervised and social settings. In collaboration with community partners, we developed and initiated

Walk On!, a group-based, facilitator-led program designed to improve walking endurance, balance, confidence, and social connectedness in older adults with mobility challenges. In addition to walking, the program incorporates balance, gait, and flexibility components, and follows a safe progression based on individual ability. Sessions take place for one-hour, twice/week, over 12 weeks. The program is currently offered at four sites in Winston-Salem and Charlotte, NC (two churches-one primarily black membership and one primarily white, a YWCA, and a community fitness center). This symposium provides an overview of Walk On! and initial implementation. Both quantitative and qualitative program data on feasibility, satisfaction, and effectiveness for improving physical and psychosocial outcomes will be presented. Based on these early data, we believe there are countless underserved older adults who may be socially isolated due to limited mobility, yet are willing and would benefit from incorporating more ambulation in a safe and accessible environment.

Abstract citation ID: igad104.1694

### SATISFACTION WITH WALK ON! FROM PROGRAM CLIENTS AND LEADERS

Rahma Ajja[1], Elizabeth Chmelo Kemp[2], Jaime Hughes[3], Lauren Van Laethem[4], Kimberly Welsh[5], Tami Guerrier[4], Mark Hirsch[4], and Barb Nicklas[6], *1. Wake Forest University School of Medicine – Winston-Salem, North Carolina, Winston Salem, North Carolina, United States*, *2. WFSM, Winston Salem, North Carolina, United States*, *3. Wake Forest School of Medicine, Winston-Salem, North Carolina, United States*, *4. Atrium Health Carolinas Rehabilitation, Charlotte, North Carolina, United States*, *5. Atrium Health Carolinas rehabilitation, Charlotte, North Carolina, United States*, *6. Wake Forest School of Medicine, Winston Salem, North Carolina, United States*

This presentation demonstrates the high degree of satisfaction with Walk On! from qualitative survey data from both clients and program leaders. As part of the curriculum, each attendee completes a survey asking about satisfaction around location, day/time, length of program, and changes in walking abilities. The survey also includes an opportunity for open-ended responses and suggestions for improvement. Overall, surveys showed a very high degree of satisfaction with the program with 80-100% of clients indicating they are “Very Much” satisfied with the program components. All open-ended responses were positive and examples include: “Very pleased with the instruction and environment”; “Encouraged me to walk at home and neighborhood”; “This program gave me energy to walk better and has changed my life”; “Enjoyed the fellowship with others”; and “I now walk with more confidence”. Leaders also complete a satisfaction survey upon completion of their Leader Training program and after leading a Walk On! program. A majority of the leaders “Strongly Agreed” or “Agreed “that: The length of the training was appropriate (83%); Course materials were explained well (100%); Course materials were easy to use (100%); I feel prepared to lead Walk On! (67%). Leader statements include: “I loved everything about this program. It was not just a walking program to me. I had social, cognitive and physical growth” and “This program afforded me the opportunity to have a real collaborative, immersive experience with elders.” Together, these positive findings support the need to scale and spread WalkOn! into additional community settings.

Abstract citation ID: igad104.1695

### EFFECTS OF THE WALK ON! PROGRAM ON PHYSICAL FUNCTION IN COMMUNITY-DWELLING OLDER ADULTS

Judy Foxworth[1], Allison Heinrich[2], Kynleigh Holley[3], Caleb Bauguess[3], Amanda Deputy[3], Lindsay Ludwig[3], Jo Ann Coco-Ripp[1], and Barb Nicklas[4], *1. Winston-Salem State University, Winston-Salem, North Carolina, United States*, *2. WFSM, Winston Salem, North Carolina, United States*, *3. WSSU, Winston Salem, North Carolina, United States*, *4. Wake Forest School of Medicine, Winston Salem, North Carolina, United States*

The objective of this study was to determine the effects of the Walk On! program on pain, strength, gait speed, gait endurance, and balance in community-dwelling, African American older women. Data presented are from those who could ambulate greater than 200 feet independently and who completed one 11-week session of the Walk On! program offered at a local church. Participants walked laps around a large multi-purpose room and completed balance challenges and leg strengthening exercises. The participants completed a battery of physical functional tests including gait speed (self-selected and fast speed), 6-minute walk distance (6MWT), 5 sit to stand time (STS), timed up and go (TUG), and single leg stance (SLS). Participants who completed both the pre- and post-tests and at least 75% of the intervention sessions were included in the analysis (n=14). Paired sample t-test was used to determine significant differences in the physical function tests with alpha level set at 0.05. The mean age of the those who completed the study was 75.8 ± 7.3 years. Both the TUG ($p = .018$) and pain scores ($p = .009$) significantly improved. There was no significant improvement in other physical function measures. Older African American women who participated in a bi-weekly supervised walking program had improvements in functional mobility and reduced pain. Further research is necessary to generalize these results for all older adults.

Abstract citation ID: igad104.1696

### IMPACT OF THE WALK ON! COMMUNITY WALKING PROGRAM ON PSYCHOSOCIAL ATTRIBUTES OF OLDER AFRICAN-AMERICAN WOMEN

Jo Ann Coco-Ripp[1], Allison Heinrich[2], Kasual Maxwell-Crudup[3], Kaliya Hill[3], and Judy Foxworth[1], *1. Winston-Salem State University, Winston-Salem, North Carolina, United States*, *2. WFSM, Winston Salem, North Carolina, United States*, *3. WSSU, Winston Salem, North Carolina, United States*

Walking in a structured community setting can improve function for older adults with functional limitations. In collaboration with others, recreation therapy (RT) and physical therapy (PT) students and faculty implemented the Walk On! program at a community church. Twenty-four African American females initially started the 11-week program and agreed to engage in the research aspect by completing pre/post-tests focused on physical function and quality of life, loneliness, and a geriatric depression scale.

The hour-long biweekly intervention included warm up/cool down stretching, music, group motivation, walking around the large multipurpose room, and various balance activity stations interspersed. PT and RT students as well as faculty and community partners provided leadership and encouragement. Results were analyzed for 14 participants who completed all tests, as well as 75% of sessions. Data for the Geriatric Depression Scale and UCLA Loneliness Scale were analyzed using the Wilcoxon Signed Rank test. No statistical differences between pre/post-test scores were found. Visual inspection of GDS scores showed four participant scores increased slightly, meaning reporting more elements of depression, and one score decreased by 50%, indicating a drop from depression to not an issue. No changes were found in the other nine scores. The Loneliness Scale had slight changes for five participants with two increasing and three decreasing slightly. On the SF-36 Health Survey, Vitality increased or fatigue decreased (p=0.009) and Mental health or wellbeing increased (p=0.041). Further research on the psychosocial aspects of the Walk On! program in other populations will be conducted.

### SESSION 4355 (SYMPOSIUM)

Abstract citation ID: igad104.1697

### LONG-TERM IMPACT OF A LIFESTYLE INTERVENTION: THE ACTION FOR HEALTH IN DIABETES (LOOK AHEAD) TRIAL AND FOLLOW-UP

Chair: Denise Houston Co-Chair: Mark Espeland
Discussant: Sara Espinoza

Mid-life weight loss and maintenance is often recommended as a means to improve health later in life, however its long-term benefits have not been rigorously established. The Look AHEAD randomized controlled trial compared an intensive lifestyle intervention (ILI) designed to induce and maintain a 7% weight loss through caloric restriction and increased physical activity with a control condition of diabetes support and education (DSE) in 5,145 individuals aged 45-76 years with type 2 diabetes and overweight/obesity over up to 11 years of follow-up beginning in 2001. Compared to DSE, ILI had greater initial improvements in cardiovascular disease risk factors; however, there were no overall differences in the incidence of the trial's primary and secondary cardiovascular outcomes between the ILI and DSE groups. ILI did, though, have a positive impact on diabetes control and complications, depression, physical health–related quality of life, and health care use and costs. Subsequently, the Look AHEAD trial transitioned to an observational study in 2012 to assess the long-term benefits and potential harms of ILI, which continued for 10 years. This symposium will describe the design of the Look AHEAD trial and transition to the Look AHEAD Aging study, the most recent phone-only observational follow-up begun in 2022, and the long-term impact of the lifestyle intervention on sleep (Spira and colleagues), cognitive resilience (Espeland and colleagues), multi-morbidity and frailty assessed by the deficit accumulation model (Simpson and colleagues), and medical care utilization and costs (Davis and colleagues).

Abstract citation ID: igad104.1698

### LOOK AHEAD SLEEP: STUDY DESIGN AND BASELINE CHARACTERISTICS

Adam Spira[1], Suzanne Bertisch[2], Haiying Chen[3], Jingzhong Ding[4], Susan Redline[5], Marie-Pierre St-Onge[6], and Kathleen Hayden[3], *1. Johns Hopkins Bloomberg School of Public Health, Baltimore, Maryland, United States*, *2. Brigham and Women's Hospital, Harvard Medical School, Boston, Massachusetts, United States*, *3. Wake Forest University School of Medicine, Winston-Salem, North Carolina, United States*, *4. Wake Forest University, Winston Salem, North Carolina, United States*, *5. Harvard Medical School, Boston, Massachusetts, United States*, *6. Columbia University Irving Medical Center, New York City, New York, United States*

Older adults with obesity and type 2 diabetes (T2D) are at elevated risk for mild cognitive impairment (MCI) and dementia. Compared with younger persons, older adults are more likely to have sleep-disordered breathing (SDB), which is also common among adults with T2D and obesity and is associated with higher risk of dementia. The Look AHEAD Sleep (LA-Sleep) study investigates links of SDB and circadian rest/activity rhythms with cognitive performance, MCI, and dementia in older adults with T2D and obesity/overweight enrolled in Look AHEAD Aging (LA-A). LA-Sleep participants completed cognitive measures at baseline and were randomized to sleep measurement in the first (2022-2023) or second year (2023-2024). Sleep measures include assessments of SDB (WatchPAT), circadian rest/activity rhythms (ActiGraph) and daily sleep diary. Of those who enrolled in LA-A, 62.8% consented to LA-Sleep. To date in 203 participants have provided data from WatchPAT and 107 provided actigraphy data. Participants were a mean (±SD) age of 76.1±5.4 y, had a mean BMI of 34.2±6.7 kg/m2, 56.7% identified as female, and 75.4% were Non-Hispanic White, 12.3% Non-Hispanic Black, and 6.9% Hispanic. Approximately 90.2% had >high school education. Of participants with obesity in 2018-2020, 27.9% (41/147) had an apnea hypopnea index (AHI) of ≥15 to <30/h (moderate SDB) and 20.4% (30/147) had ≥30 events/h (severe SDB). Mean actigraphy-estimated total sleep time was 7.1±1.4 h, and mean wake after sleep onset was 62.5±31.0 min/night. Among adults with T2D and overweight/obesity, a high proportion have SDB and sleep fragmentation, potentially increasing risk for dementia.

Abstract citation ID: igad104.1699

### LONG-TERM ASSOCIATION OF AN INTENSIVE LIFESTYLE INTERVENTION ON GLOBAL COGNITIVE FUNCTION

Mark Espeland[1], Kathleen Hayden[1], Stephen Rapp[1], Bonnie Sachs[1], Debbie Pleasants[1], Debbie Booth[1], Rena Wing[2], and Rebecca Neiberg[1], *1. Wake Forest University School of Medicine, Winston-Salem, North Carolina, United States*, *2. Miriam Hospital, Providence, Rhode Island, United States*

The long-term effects of mid-life changes in lifestyle on later life cognitive function have not been rigorously assessed among individuals with type 2 diabetes. The Look AHEAD

Aging study examined differences in standardized composite cognitive function scores in 1367 individuals, currently aged 64 to 95 years, who had enrolled an average of 20 years previously in a randomized controlled trial to compare a 10-year multidomain intensive lifestyle intervention (ILI) to a condition of diabetes support and education (DSE). The composite averaged standardized scores from the telephone administration of tests chosen to capture the individual cognitive domains of memory, attention/processing speed, executive function, and global cognitive function. At enrollment into the Look AHEAD Aging study there was no overall difference between intervention groups in cognitive test scores: mean [SE]: -0.01 [0.03] ILI versus 0.00 [0.04] DSE (p=0.76). However, among participants with overweight at Look AHEAD enrollment, those assigned to ILI had mean [95%CI] of 0.33 [0.08, 0.57] better global cognitive function scores than those assigned to DSE. For those initially with obesity, there was no relative intervention benefit: mean [95%CI] -0.08 [-0.18, 0.03] (interaction p=0.003). Benefits for those with overweight appeared to be strongest for tests of memory. ILI should be recommended to individuals with type 2 diabetes and overweight to provide long-term resilience against cognitive aging. The cognitive benefits we have reported earlier for individuals with overweight during intervention delivery endure for a decade following the cessation of the intervention.

Abstract citation ID: igad104.1700

### LONG-TERM ASSOCIATION OF AN INTENSIVE LIFESTYLE INTERVENTION ON FRAILTY AND MULTIMORBIDITY

Felicia Simpson[1], Mark Espeland[2], Joni Evans[3], Karen Johnson[4], Judy Bahnson[3], Denise Houston[5], Mia Yang[3], and Lynne Wagenknecht[6], *1. Winston-Salem State University, Winston-Salem, North Carolina, United States*, *2. Wake Forest University School of Medicine, Winston-Salem, North Carolina, United States*, *3. Wake Forest School of Medicine, Winston-Salem, North Carolina, United States*, *4. University of Tennessee Health Science Center, Memphis, Tennessee, United States*, *5. Wake Forest University School of Medicine, Winston Salem, North Carolina, United States*, *6. Atrium Health/Wake Forest Baptist, Winston-Salem, North Carolina, United States*

Multidomain lifestyle interventions may slow aging as captured by deficit accumulation frailty indices (FIs) and multimorbidity. We previously reported that the 10-year Look AHEAD multidomain intensive lifestyle intervention (ILI) that focused on caloric restriction, increased physical activity, improved diet, and cardiometabolic risk factor monitoring, significantly slowed the progression of both FI and a multimorbidity index during intervention delivery. We assessed whether these benefits endured after ILI delivery was terminated and the degree to which these benefits may be interrelated. The Look AHEAD Aging study enrolled N=1606 individuals, ages 63-94 years, who had been assigned a median of 20 years earlier (i.e., 10 years after ILI was terminated) to either the Look AHEAD ILI or its control condition of diabetes support and education (DSE). At the original Look AHEAD randomization, they had a mean (SD) 39-component FI score of 0.128 (0.056) and mean (SD) 9-component multimorbidity index of 1.63 (1.01), both balanced across intervention groups. When enrolled in the Look AHEAD Aging study, participants who had been assigned to ILI, compared with those assigned to DSE, averaged 6% lower FI scores (p<0.0001). They had also accrued 0.14 fewer additional age-related chronic diseases from baseline (p<0.0001). Relative benefits associated with ILI for these indices were similar for both genders and across the full cohort's age range. Increases in FI and multimorbidity were highly correlated (r=0.55; p<0.001), similarly in both arms. Multidomain lifestyle interventions can result in persistent long-term benefits in two common markers of aging.

Abstract citation ID: igad104.1701

### EFFECT OF AN INTENSIVE LIFESTYLE INTERVENTION IN ADULTS WITH TYPE 2 DIABETES ON LONG-TERM HEALTH CARE SPENDING AND USE

Ian Davis[1], Christopher Frenier[2], Peter Huckfeldt[3], Ann Harada[4], Nicholas Pajewski[5], Mark Espeland[5], and Dana Goldman[1], *1. University of Southern California, Los Angeles, California, United States*, *2. Yale University, New Haven, Connecticut, United States*, *3. University of Minnesota School of Public Health, Minneapolis, Minnesota, United States*, *4. Leonard D. Schaeffer Center for Health Policy & Economics, Los Angeles, California, United States*, *5. Wake Forest University School of Medicine, Winston-Salem, North Carolina, United States*

This study investigates the impact of the Look AHEAD intensive lifestyle intervention (ILI) on long-term health care spending. We investigated self-reported health care use and spending from 2002-2012 (during the intervention) and 2013-2019 (after the intervention). Lasso regressions, trained in Medicare data, were used to predict outpatient, inpatient, prescription drug, and total healthcare spending based on self-reported health care use. Other outcomes included self-reported outpatient visits, unique prescription drugs, and inpatient hospitalizations. Regressions comparing spending and use outcomes for the ILI and diabetes support and education (DSE) control groups were adjusted for baseline demographic characteristics, clinical status, and study site. We included 3957 participants (1997 ILI, 1960 DSE) who had at least one interview during the post-intervention period. Similar to prior work, we found that the ILI group had lower inpatient, outpatient, and prescription drug spending relative to the DSE group during the intervention period, resulting in lower total spending (adjusted difference: -$715: [95% CI: -$1178 to -$252]; P<0.001). However, most of the intervention's effects on spending and use did not persist beyond the intervention period, except for prescription drug spending (adjusted difference: -$189: [95% CI: -$364 to -$14]; P=0.03) and unique prescriptions (adjusted difference: -0.3: [95% CI: -0.5 to -0.1]; P<0.001). Our results imply that an intensive lifestyle intervention can reduce health care spending for adults with type 2 diabetes, but interventions may need to be continued to maintain spending reductions.

### SESSION 4360 (SYMPOSIUM)

Abstract citation ID: igad104.1702

### EXPLORING THE UTILIZATION OF MERIT-BASED INCENTIVE PAYMENT SYSTEM (MIPS) PROGRAMS IN WORKFORCE DEVELOPMENT

Chair: Sam Cotton Co-Chair: Anna Faul

Measuring patient outcomes is a critical component of the HRSA funded Geriatric Workforce Enhancement Programs (GWEPs), which aim to improve the quality of care for older adult patients. In 2019, GWEPs were tasked with aligning patient outcomes with the CMS Merit-based Incentive Payment System (MIPS) program, which incentivizes quality care and patient outcomes. Collecting and using MIPS data effectively can be a challenging task, particularly when done across multiple levels of care. To address these challenges, the National Association of Geriatric Education Centers (NAGEC) created a workgroup to develop advocacy and solutions for patient outcomes. This symposium will discuss the utilization of MIPS as performance metrics across three GWEPs, the challenges in reporting these outcomes, and implications for future policy changes. This symposium's primary objective is to explore how GWEPs can effectively measure patient outcomes, despite the challenges involved in collecting and utilizing MIPS data. By discussing these challenges and proposing potential solutions, this symposium aims to improve the effectiveness and sustainability of GWEPs, ultimately leading to better outcomes for older adult patients. Overall, measuring patient outcomes is critical for ensuring that older adult patients receive high-quality care that is tailored to their individual needs and preferences. Despite the challenges involved in collecting and utilizing MIPS data, our symposium provides an opportunity to explore potential solutions and advocate for policies that support improved patient outcomes in geriatric care.

Abstract citation ID: igad104.1703

### ADDRESSING THE CHALLENGES OF MIPS WITHIN THE GWEP PROGRAM: A CALL TO CO-CREATE MEANINGFUL WAYS TO SHOW IMPACT

Anna Faul, *University of Louisville, Louisville, Kentucky, United States*

To address the challenges that unique challenges that the GWEP programs had in collecting and using MIPS data to show meaningful and quality work with older adult patients, we created a collaboration between NAGEC and the GWEP MIPS workgroup. Our primary goal was to develop a collective understanding of how GWEPs perceived MIPS as main outcomes. To better understand the collective challenges, a survey was created to examine the diversity in the sites across all GWEPs where MIPS data was collected and to quantify the types of challenges that GWEPs faced in collecting this type of patient data. From this survey we found the following issues were most prominent: 1.) Most GWEPs had primary care sites that had a difficult time pulling date from their electronic health records; 2.) Providers at participating primary care sites struggled to enter MIPS datapoints into the EHRs; 3.) Some of the participating primary sites did not collect MIPS data; 4.) Some of the participating primary care sites do not have the time to provide us with MIPS data in a timely fashion; and 5.) Some of our participating primary care sites are different than the traditional primary care sites (eg. skilled nursing facility, hospice, emergency departments), making MIPS measures irrelevant. From this survey, we were able to develop a solution focused statement to present to HRSA and to start the conversation about the difficulties and challenges in collecting MIPS data.

Abstract citation ID: igad104.1704

### REPORTING MERIT-BASED INCENTIVE PAYMENT SYSTEM LIKE DATA IN A UNIQUE AGE-FRIENDLY HEALTH CARE SETTING

Leland Waters, and Patricia Slattum, *Virginia Commonwealth University, Richmond, Virginia, United States*

The Richmond Health and Wellness Program (RHWP) offers screening, education, care coordination, and resources as an enhancement to primary care for older adults in five low-income apartment buildings and in a community center. Participants may receive primary care through multiple healthcare systems in the region that do not have a common electronic medical record (EMR). To track age-friendly workflows to the Health Resources Services Administration (HRSA) in the domains of advance care planning, falls prevention, and high-risk medication use, case note documentation in a REDCap database is used to generate Merit-based Incentive Payment System (MIPS)-like data. We report on advance care plans developed, fall risk screenings and risk reduction interventions implemented, high-risk medications screened, and referrals to primary care for high-risk medication deprescribing, including risk reduction with opioid use. When reporting MIPS data to HRSA, we include a disclaimer for RHWP data defining the numerator and denominator used for the MIPS-like measure calculation. For example, when reporting advance care plan completion, we include "RHWP is a care coordination site and care is not provided at the site. The population includes adults aged 55+ or people (of any age) with disabilities in low-income congregate living facilities and all adults participating in RHWP at the Virginia Commonwealth University Health Hub." The numerator indicates the number of clients with advanced care plans created, and the denominator indicates the total number of clients seen during the reporting period. This presentation will provide strategies to meet reporting requirements when EMR records are not available.

Abstract citation ID: igad104.1705

### LINKING MERIT-BASED INCENTIVE PAYMENT SYSTEM (MIPS) MEASURES TO AGE-FRIENDLY HEALTH SYSTEMS

Sara Murphy[1], Jennifer Severance[1], Sarah Ross[1], Kate Taylor[1], and Janice Knebl[2], *1. University of North Texas Health Science Center, Fort Worth, Texas, United States*, *2. UNTHSC, Fort Worth, Texas, United States*

IHI's Age-Friendly Health Systems initiative provides a framework for older adult primary care focused on the 4Ms: Mentation, Mobility, Medications, and What Matters which addresses the need for an integrated system of care. Additionally, the Centers for Medicare & Medicaid Services have implemented merit-based incentivized payment system (MIPS) measures to promote provider practices that improve healthcare quality. MIPS measures and age-friendly care practices may be improved through training, which may lead to better patient outcomes. To integrate the 4Ms to improve care and patient safety, the Center for Older Adults at the University of North Texas Health Science Center standardize workflows and increase the number of annual wellness visits (AWV). A standardized template in the electronic medical record was created, questions in the patient registration software were added to identify eligible patients, and three different types of AWVs were standardized. Training courses were developed and completed by providers. The percentage of patients who receive an AWV is measured on an annual basis. Learnings have been integrated into training courses. Between July 2020 to June 2021, 297 healthcare professionals completed online training modules, with post-survey ratings showing helpful content with learning objectives being met. Provider support through training and incorporating MIPS measures and the Age-Friendly 4Ms framework into the AWV can support consistent outcome measurement and improve quality care for older adults.

### SESSION 4365 (SYMPOSIUM)

Abstract citation ID: igad104.1706

### RURAL HEALTH, FAMILY CAREGIVING, AND DEMENTIA: CHARACTERISTICS AND NEEDS ACROSS POPULATIONS

Chair: Caroline Stephens Co-Chair: Rebecca Goodwin Discussant: Katherine Ornstein

The rural population in the US is aging faster, more likely to live alone, and has more comorbidities than the overall aging population. Despite the greater need for long-term care services, rural residents may experience the most barriers to formal services (hospital, home health) and availability of family caregivers. Unfortunately, there is a dearth of research on aging and end-of-life care in rural areas. This symposium includes 4 presentations focused on rural health, family caregiving and dementia and the characteristics and needs across these diverse populations. The first presentation examines rural disparities in use of family and formal caregiving for older adults with disabilities using data from the Health and Retirement Study. The second presentation describes the unmet services needs among people with dementia and their caregivers living in rural Appalachia, and highlights contextual factors that shape service access and utilization. The last two presentations use data from the Utah Population Caregiving Database to examine the characteristics of families of nursing home (NH) residents who die with dementia and of those who die in rural NHs. Understanding the unique characteristics and needs of these underserved populations will help inform rural health practice and policy considerations to better serve this population, especially as we continue to see changes in the hospital and NH landscape across the US. Moreover, it is critical that even as federal and state policies rebalance long-term care from institutional settings to home- and community-based settings, the unique needs of those living in more rural areas are considered.

Abstract citation ID: igad104.1707

### RURAL DISPARITIES IN THE USE OF FAMILY AND FORMAL CAREGIVING FOR OLDER ADULTS WITH DISABILITIES

Katherine Miller[1], Katherine Ornstein[2], and Norma Coe[3], *1. Johns Hopkins Bloomberg School of Public Health, Baltimore, Maryland, United States*, *2. Johns Hopkins University, Baltimore, Maryland, United States*, *3. University of Pennslyvania, Philadelphia, Pennsylvania, United States*

As federal and state policies rebalance long-term care from institutional settings to home- and community-based settings, reliance on formal (paid) and family (unpaid) caregivers for support at home nationally has increased in recent years. Yet, it's unknown if use of formal and family care varies by rurality as a disproportional share of the aging population reside in rural areas. Using the Health and Retirement Study, we describe patterns in receipt of combinations of formal and family home care by rurality among community-dwelling adults aged 65+ with functional limitations from 2004-2016. Compared to older adults in urban areas, older adults in rural areas are more likely to receive any family care than those in urban areas and less likely to receive any formal care (2.4 and -4.4 percentage points respectively, $p<0.05$). From 2004 to 2016, a growing proportion of older adults in rural areas receive care from family caregivers exclusively while a decreasing proportion receive care from formal caregivers exclusively. When examining older adults in urban areas, we find the opposite trend — a growing proportion of urban adults rely exclusively on formal care and a decreasing proportion rely exclusively on family care. We find that national estimates of sources of caregiving and their trends mask significant heterogeneity in uptake by rurality. Understanding how older adults in rural areas are, or are not, receiving home-based care compared to their urban peers and how these patterns are changing over time is the first step to informing supports for family and formal caregivers.

Abstract citation ID: igad104.1708

### UNMET SERVICE NEEDS AMONG RURAL APPALACHIAN CAREGIVERS OF PEOPLE WITH ALZHEIMER'S AND DEMENTIA

David Russell[1], Christina Miyawaki[2], and Erin Bouldin[3], *1. Appalachian State University, Boone, North Carolina, United States*, *2. University of Houston, Houston, Texas, United States*, *3. University of Utah, Salt Lake City, Utah, United States*

Risk factors for Alzheimer's disease and related dementias (ADRD) are higher in rural areas than urban areas, and home- and community-based services (services) are less accessible. This study sought to identify unmet services needs among people with ADRD and their caregivers living in rural Appalachia, and highlight contextual factors that shape service access and utilization. We interviewed 22 caregivers and persons with ADRD living in Western North Carolina between August 2021 and April 2022 with recruitment assistance from local agencies and organizations. Themes were identified across domains of our conceptual model informed by theories of health services use, family caregiving, and sociocultural contexts. Family caregivers communicated multiple unmet needs, foremost of which included fragile and insufficient support systems or imbalances between family and paid caregiving support, a lack of clarity about their services options, and feeling behind in preparing for the future. In the presence of unmet care needs, family caregivers made decisions (or not) to use services in aiding them with caring for persons with ADRD. These decisions were shaped by illness-level (e.g. ADRD symptoms and progression), predisposing (e.g. work experiences), and enabling factors (e.g. family support). The sociocultural contexts in which family caregivers and the persons they cared for with ADRD were embedded within, including their culture, beliefs and family norms, also shaped perceptions and use of services. These contexts should be better understood and considered when developing and implementing acceptable services for people with ADRD in rural areas.

Abstract citation ID: igad104.1709

### DYING WITH DEMENTIA IN NURSING HOMES: A POPULATION-BASED PERSPECTIVE OF DECEDENTS AND THEIR FAMILIES

Caroline Stephens[1], Rebecca Goodwin[2], Eli Iacob[1], Michael Hollingshaus[1], Ken Smith[1], Djin Tay[1], Rebecca Utz[1], and Katherine Ornstein[3], *1. University of Utah, Salt Lake City, Utah, United States*, *2. National Library of Medicine, NIH & University of Utah College of Nursing, Salt Lake City, Utah, United States*, *3. Johns Hopkins University, Baltimore, Maryland, United States*

Families play a critical role in person-centered end-of-life (EOL) care for nursing home (NH) residents with dementia. Many NH residents, however, do not have any family to advocate for care, which may contribute to poorer EOL outcomes. Little is known about the characteristics of families of persons with dementia who die in a NH despite the importance of family at EOL. This study aimed to describe the size and composition of first-degree families (FDF) of Utah NH residents with dementia who died 1998-2016 (n=18,842). Using the Utah Population Caregiving Database, we linked NH decedents with dementia to their FDF (n=54,174; spouses=11.3%; children=57.6%; siblings=30.7%) and compared characteristics of those with and without FDF. Compared to NH decedents with FDF (78.5%), those without (21.5%) were more likely to be older (median 87.3 vs 85.9), female (64.7% vs 57.1%), non-white/Hispanic (10.3% vs 3.4%), less educated (<9th grade; 42.2% vs 33.4%); die in a rural/frontier NH (24.8% vs. 23.2%); and less likely to have 2+ hospitalizations in the last 6 months of life (14.3% vs. 16.5%, all p<.001). Findings highlight social determinants of health that may increase the risk of poor EOL outcomes in an already vulnerable population. Understanding the nature of FDF relationship type on EOL outcomes for NH residents with dementia is an important next step in clarifying the role of families and understanding the supports they may need.

Abstract citation ID: igad104.1710

### WHO DIES IN A RURAL NURSING HOME WITH AND WITHOUT FAMILY? EVIDENCE FROM THE UTAH POPULATION DATABASE

Rebecca Goodwin[1], Katherine Ornstein[2], Eli Iacob[3], Michael Hollingshaus[3], Ken Smith[3], Djin Tay[3], Rebecca Utz[3], and Caroline Stephens[3], *1. National Library of Medicine, NIH & University of Utah College of Nursing, Salt Lake City, Utah, United States*, *2. Johns Hopkins University, Baltimore, Maryland, United States*, *3. University of Utah, Salt Lake City, Utah, United States*

Rural nursing homes (NHs) are an increasingly important focus for policy makers given the lack of available post-acute and long-term care services in these areas. Families often play a critical role in filling this void in services and supporting NH residents at the end of life (EOL). No population-based studies to date, however, have documented the characteristics of families of persons who die in rural NHs. This study aimed to describe the size and composition of first-degree families (FDF) of Utah NH residents who died in rural/frontier NHs 1998-2016 (n=10,567). Using the Utah Population Caregiving Database, we linked rural/frontier NH decedents to their FDF (n=31,118; children=53.7%; siblings=33.8%; spouses=11.3%; parents=1.1%) and compared characteristics of those with and without FDF. Compared to rural/frontier NH decedents with FDF (77.5%), those without (22.5%) were more likely to be older (median 85.7 vs 84.8), female (61.3% vs 56.8%), Hispanic or Non-White Non-Hispanic (6.8% vs 3.7%), and less educated (<9th grade; 45.9% vs 37.4%), all p<0.001. There were no differences in diagnosis of dementia, Charlson Comorbidity Index, or hospitalizations in the last 6 months of life. Persons who live and die in rural NHs without FDF may be in double jeopardy, if you will, given the lack of family support and few to no options for care. Understanding the impact of family, or lack thereof, on rural NH resident EOL care trajectories is an important next step.

### SESSION 4370 (SYMPOSIUM)

Abstract citation ID: igad104.1711

### CONTEXTUAL FACTORS THAT SHAPE EXPERIENCES OF SEXUAL INTIMACY AMONG LATER-LIFE COUPLES

Chair: Jeremy Yorgason Co-Chair: Stephanie Wilson
Discussant: Hui (Cathy) Liu

Sexuality is an important aspect of romantic dyadic relationships. Although people experience physical changes with age that result in changes in sexual interactions, the sexual aspect of couple relationships remain important. Research examining sexuality among later life couples is maturing, allowing investigators to explore more nuanced questions about couple contexts that shape sexual experiences. The current group of papers address some of these important contextual factors that can impact the couple sexual relationship, including sexual motives among same and different sex couples, personality characteristics and how they relate to sexual interactions, how couples' sexual interactions are affected by transitions such as menopause, and how sleep patterns among caregivers are related to sexual interactions. Presenters in this session draw upon national studies of older adults including NSHAP, MIDUS, and HARP. They used advanced statistical modeling to explore their questions. Findings suggest that self-determined sexual motives among same and different sex couples were associated with better marital quality and lower depression. The menopause transition, along with relationship transitions in later life, were associated with sexual interactions. Conscientiousness and neuroticism were associated with sexual pleasure and emotional satisfaction, along with preferences regarding physical touch within the relationship. Among caregivers, sleep duration was less important; sharing a bed with a care-recipient partner was associated with physical pleasure; and napping was negatively associated with sexual quality. In summary, various factors impact the sexual interactions of older couples in different ways. Findings from these papers given nuanced insight into a number of important contextual factors.

Abstract citation ID: igad104.1712

### SEXUAL MOTIVES, MARITAL QUALITY, AND DEPRESSIVE SYMPTOMS OF MID-LIFE SAME- AND DIFFERENT-SEX COUPLES

Hye Won Chai[1], Susanna Joo[2], Jihye Lee[3], and Debra Umberson[1], *1. University of Texas at Austin, Austin, Texas, United States*, *2. BK21. Symbiotic Society and Design, Seoul, Republic of Korea*, *3. Yonsei University, Seoul, Republic of Korea*

Self-determined sexual motives, or self-endorsed reasons for having sex, is a significant determinant of psychological health with notable gendered patterns. However, previous studies mostly focused on young adult different-sex couples and fell short of exploring the mechanisms through which self-determined sexual motives are associated with psychological health. Using dyadic data from the Health and Relationships Project (HARP) on 124 gay, 171 lesbian, and 124 straight midlife married couples, this study examined the mediating role of marital quality in the association between self-determined sexual motives and depressive symptoms. Actor-partner interdependence mediation model (APIMeM) was used for analyses. Results showed significant mediating effects of individual's own marital quality for all couple types (i.e., gay, lesbian, straight couples), such that individuals with higher levels of self-determined sexual motives reported better marital quality, which in turn was associated with their lower levels of depressive symptoms. For partner effects, same patterns emerged for lesbian and gay couples wherein individuals' own marital quality mediated the association between their spouse's sexual motives and their own depressive symptoms. However, there were gendered patterns within straight couples. For women, their own marital quality mediated the association between their partner's sexual motives and their own depressive symptoms. For men, their spouse's marital quality mediated the association between their partner's sexual motives and their own depressive symptoms. These findings suggest marital quality as a useful intervention point to enhance the psychological health of midlife couples in same-sex and different-sex marriages and also highlight the salience of gendered dynamics within straight couples.

Abstract citation ID: igad104.1713

### EFFECTS OF MENOPAUSE AND MARITAL TRANSITIONS ON SUBJECTIVE SEXUAL SELF-EFFICACY AMONG MIDDLE-AGED AND OLDER ADULTS

Melissa Ferguson, and Yin Liu, *Utah State University, Logan, Utah, United States*

Circumstances in aging including menopause, physical decline, widowhood, divorce and remarriage may influence sexual experiences and well-being in later life. While marital transition can affect both men and women in middle and older life, menopausal transition is especially salient for aging women. This study aims to examine how subjective sexual self-efficacy changes over time and whether marital transitions and menopausal transitions moderate the trajectories. Participants completed two waves of the Midlife in the United States (MIDUS) surveys (N = 1002), ranging from 40 to 84 years of age at baseline (M = 57.44; SD = 10.17) with over half being female (54%, n = 538). Factor analysis confirmed a one-factor model with a five-item subjective sexual self-efficacy construct. Over 10 years, 12% of participants experienced marital transitions, and 29% of women reporting menstrual status experienced menopause transition. Growth curve analysis was used the examine the research questions.

Abstract citation ID: igad104.1714

### DYADIC EFFECTS OF PERSONALITY AND SEXUAL BEHAVIOR IN OLDER ADULTHOOD

William Chopik, *Michigan State University, East Lansing, Michigan, United States*

Personality traits are associated with important life, work, and relational outcomes across the lifespan. Although personality has been implicated in models of relationship functioning, there has been relatively little research matching individual and partner personality characteristics to the frequency, quality, and variety of sexual experiences, particularly in older adulthood. The current study examined individual and spousal effects of the Big Five personality traits on sexual outcomes among 953 older opposite-sex couples followed from 2010/2011 to 2015/2016 as part of the National Social Life, Heath, and Aging Project (NSHAP; Mage = 70.60, SD = 7.92; 74.2% White, 12.2% Hispanic, 11.1% Black, and 2.5% other). Individual and spousal conscientiousness was associated with more frequent dyadic and solitary sexual activity and being part of a physically pleasurable and emotionally satisfying relationship. Those with higher levels of neuroticism and those married to spouses high in neuroticism expressed a lower preference for physical/sexual touch and cuddling, were less likely to report having a physically pleasurable and emotionally satisfying relationship, and were more likely to avoid having sex because of physical problems (and report more pain during sex). Longitudinal analyses suggested that most of these associations wavered in strength over time. The findings are discussed in the context of how relationship processes might mediate the link between personality characteristics and sexual outcomes or whether sexual outcomes promote relationship-enhancing characteristics depending on people's personalities.

Abstract citation ID: igad104.1715

### SLEEP AND SEXUALITY AMONG CAREGIVERS AND THEIR PARTNERS

Christine Proulx[1], Hanamori Skoblow[2], and Amy Rauer[3], *1. University of Vermont, Burlington, Vermont, United States*, *2. University of Missouri, Columbia, Missouri, United States*, *3. University of Tennessee, Knoxville, Tennessee, United States*

Older adult caregivers have increased risk of disturbed sleep, which is associated with declines in sexual frequency. Spousal caregivers engage in less sexual activity than caregivers of non-spouses. We examined whether the associations between sleep behaviors (duration, napping frequency, feeling rested) and couples' sexual frequency, quality, and pleasure were similar among dyads in which one spouse was providing care to a spouse (n = 146) or a parent or other care recipient (n = 211). Using Wave 2 of the NSHAP and actor-partner interdependence models, we found significant differences (p <.01) between dyads. For all spouses, only frequency of sharing a bed was related to frequency of sexual activity in the previous 12 months. Sleep duration was not associated with any outcomes. In both groups, frequency of sharing a bed was positively associated with reports of physical pleasure (b's ranged from .20 to .50). In spousal caregiver couples, caregivers' greater napping frequency was negatively associated (b = -.29, p <.01) with their partner's reports of a physically pleasurable relationship, and their reports of feeling rested were positively (b = .17, p < .01) associated with their own reports of pleasure. For couples in which the caregiver was caring for a non-spouse, there were actor effects for caregivers' and noncaregivers' napping frequency on lower reports of sexual relationship quality (b = .19 and .49, respectively), and a partner effect for non-caregivers (b = .25). These results point to the importance of understanding the context of caregiving, sleep behaviors, and sexual relationships.

### SESSION 4375 (SYMPOSIUM)

Abstract citation ID: igad104.1716

### THE PROMISE OF DIGITAL INCLUSION FOR OLDER ADULTS: BUILDING BRIDGES FOR UNDERSERVED OLDER ADULTS IN TWO STATES

Chair: Skye Leedahl Co-Chair: Kathleen Wilber Discussant: Namkee Choi

Digital inclusion refers to activities completed to ensure that all individuals, especially those who are underserved, have access to and are able to utilize technology and needed support. This includes affordable, robust internet service; devices; digital literacy training; technical support; and applications/online content (National Digital Inclusion Alliance, 2022). Recognizing digital exclusion among older adults as a major public health and social equity concern, particularly during the pandemic, two states (California and Rhode Island) partnered with their respective state units on aging to offer technological devices (e.g., iPads) and intergenerational support to underserved older adults who speak multiple languages across the states. The university-based programs worked with their local Areas Agencies on Aging (CA) and senior centers (RI) through the State units on Aging to recruit and support older participants. This symposium will describe findings from three research projects within these large, ongoing research initiatives and discuss some of the lessons learned for conducting this type of applied research. The first paper by Marnfeldt and colleagues describes factors associated with digital readiness among participants. The second paper by Leedahl and colleagues examines predictors of digital competence among those interested in the program. The third paper by Tsotsoros and colleagues examines the influence of technological growth on various social well-being measures.The fourth paper by Batista-Malat and colleagues describes the strengths and barriers in working with community partners when completing applied research.

Abstract citation ID: igad104.1717

### PREDICTORS OF DIGITAL COMPETENCE AMONG OLDER ADULTS INTERESTED IN AN INTERGENERATIONAL TECHNOLOGY PROGRAM

Skye Leedahl, Cindy Tsotsoros, Alexandria Capolino, and Josie Santilli, *University of Rhode Island, Kingston, Rhode Island, United States*

The Rhode Island Office of Healthy Aging initiated a digiAGE collaborative. As a major part of this initiative, the University of Rhode Island launched the Engaging Generations: Cyber-Seniors digiAGE iPad Program and research project. We analyzed participants who took part in the study in 2021 and 2022 (N=324; age range=50-92; 77.2% female, 22.5% male; 65.1% White, 16.4% Hispanic, 11.4% Black, 4.9% Native American or Alaska Native, 1.2% Asian, 1.9% Multiple races; 83.6% English, 15.4% Spanish as primary language). In this study, we examined demographic characteristics (e.g., income, marital status, employment status, primary language, living alone status, education, gender, race/ethnicity, and Wifi access), and social well-being characteristics (e.g., depression, social isolation, loneliness, quality of life) as predictors of digital competence using structural equation modeling. Overall, this model showed excellent fit ($\chi 2(53, N=324)=104.29$, p<.05, with CFI = .96, TLI = .92, RMSEA = .06,CMIN/df = 1.97). The model indicated several direct and indirect effects. For indirect effects, income status predicted depressive symptoms, depressive symptoms predicted quality of life, and quality of life predicted digital competence. For direct effects, having access to a wireless internet connection within the home and education level predicted digital competence (Adj. R Square = 0.112). As technology becomes increasingly integrated into our daily lives, examining predictors of digital competence among older adults is important to identify whom to target for digital inclusion initiatives and how to provide support appropriately.

Abstract citation ID: igad104.1718

### CROSSING THE DIGITAL DIVIDE: ASSESSING DIGITAL READINESS AMONG UNDERSERVED OLDER ADULTS

Sheila Salinas Navarro, Mengzhao Yan, Sindy Lomeli, and Kathleen Wilber, *University of Southern California, Los Angeles, California, United States*

The California Department of Aging launched the "Connecting, Health, Aging, & Technology (CHAT) Program, "aimed at bridging the digital divide and mitigating social isolation among older adults by providing an iPad device and technology training. Because providing a device does not guarantee uptake, we examined factors associated with technology adoption among research participants (n=1,219). We used a global "digital readiness" scale, and two open-ended questions to examine potential device uptake. We ran multivariate regressions with explanatory independent variables including demographic characteristics (age, gender, race/ethnicity, living arrangement, and education), health indicators (self-rated health and mental health status) and the intention to adopt technology against the global readiness question. The analysis showed that digital readiness was associated with age and education, and Asian or Pacific Islander older adults were more ready than non-Hispanic Whites. Further, digital readiness was negatively associated with mental health but positively associated with self-rated health. Digital readiness was also associated with an increased intention to adopt technology. A content analysis of the open-ended questions in Atlas.ti informed the regression analyses and revealed participants' strong desire for digital self-efficacy, informed citizenship, lifelong learning, a proactive desire to improve quality of life, and the desire to foster connection to family and friends. Our research highlights the personal and multifaceted nature of technology adoption among older adults, and the complexity of fostering digital inclusion on a large scale.

Abstract citation ID: igad104.1719

### CHANGES IN TECH USE PREDICTING SOCIAL WELL-BEING FOR INTERGENERATIONAL PROGRAM PARTICIPANTS

Cindy Tsotsoros, Skye Leedahl, Melanie Brasher, Alexandria Capolino, and Josie Santilli, *University of Rhode Island, Kingston, Rhode Island, United States*

Previous research has shown that technology-based interventions show promise for reducing social isolation and loneliness. Less research has examined quality of life outcomes for these programs or interconnectedness of these outcomes when examining pre/post-change. This study examined the connections between technology use and knowledge with loneliness, social isolation, and quality of life before and after participation in the program. The final sample (N=324) was rather diverse regarding work and living status (for example, 67.5% retired; 67.8% lived alone; 70.1% had less than $30,000 income; 40.2% had a high school education or less). Using structural equation modeling, this study examined pre- and post-data to identify to what extent technology usage and knowledge (frequency of iPad use, digital competence) predicted an individual's change in loneliness, social isolation, and quality of life. This final model showed excellent model fit ($\chi 2(148, N=324)=247.01$ $p<.05$ with CFI = .95, TLI = .93, RMSEA = .05, CMIN/df = 1.67). Within this model, findings showed that digital competence improvements predicted quality of life, social isolation, and loneliness (Ad. R. Square = 0.17); the frequency of iPad use influenced social isolation and quality of life (Adj. R Square = 0.29); and loneliness influenced quality of life and social isolation (Adj. R Square = 0.81). There are multiple possible mechanisms through which older participants' lives can be impacted by intergenerational technology programs. Understanding the complex interplay of factors helps us understand the potential benefits of these programs on quality of life.

Abstract citation ID: igad104.1720

### LESSONS LEARNED FROM DIGITAL EQUALITY RESEARCH WITH COMMUNITY PARTNERS

Eleanor Batista-Malat[1], Sindy Lomeli[1], Alexandria Capolino[2], Skye Leedahl[2], and Kathleen Wilber[1], *1. University of Southern California, Los Angeles, California, United States*, 2. *University of Rhode Island, Kingston, Rhode Island, United States*

Despite strong interest among policymakers and service providers and a pipeline of Federal funding for programs that address digital inequalities in older adults, there is limited research on the challenges and outcomes of programs designed to reduce the digital divide. To better understand these issues, we compared and contrasted challenges and lessons learned administering digital inclusion projects in California and Rhode Island. In California, challenges included the complexity of state and local interface, managing public-private partnerships, and deciding on and communicating roles and responsibilities through the required formal channels. In Rhode Island, timing and communication were challenging because universities and community partners operate on different calendar systems and cater to different demands. For both programs, ensuring that training and technical support were available, accessible, and appropriate for diverse populations of older adults was a challenge. However, the training and support approaches were different as California leveraged private and non-profit partnerships while Rhode Island used university student mentors. Further, the rollout and data collection needed to evaluate the programs created barriers and slowed the process, including partners' language capabilities not matching the participants' and the time-consuming nature of data collection via telephone. Nevertheless, in both states, research opportunities afforded by collaborating with state and local agencies and private sector providers were invaluable. Partnerships allowed older adults to participate in research via a trusted organization with linguistic and cultural sensitivity, and researchers accessed underserved populations less likely to participate in research.

### SESSION 4380 (PAPER)

### ASSISTED LIVING AND HOME CARE OF OLDER ADULTS

Abstract citation ID: igad104.1721

### CLINICAL NOTES AND CARE COORDINATION CALLS AS IMPORTANT PREDICTORS OF EMERGENCY CARE UTILIZATION IN HOME HEALTH CARE

Lauren Evans, *VNS Health, New York City, New York, United States*

Home care patients experience a high illness burden and a heightened risk of negative health outcomes. There is a need to understand how clinician behaviors, including notetaking and care coordination activities, can be better integrated into risk modelling in home care settings. This study predicted hospitalization/ED visits using data from 86,866 home care episodes from an agency in the Northeastern United States. We began our analysis with nearly 200 variables, including patient-related factors (e.g., comorbidities, types of medications taken, functional status), and factors relating to clinician behavior, such as problems documented in clinical notes, and calling patterns to the patient or medical care providers. We used least absolute shrinkage and selection operator (LASSO) to aid in the selection of the best subset of variables. In our preliminary analysis, we found that two clinician behavior-related factors were the strongest risk factors for experiencing hospitalization/ED visits, specifically the proportion of concerning notes generated during the care episode (OR 8.97, 95% CI 7.64, 10.52), and a measure reflecting frequent calls to the patient's family (OR 4.51, 95% CI 3.80, 5.37). Clinician documentation of specific problems, and the clinician's decision to conduct a medication review were also risk factors. Patient risk factors included prior hospitalization, health conditions such as cancer and renal disease), functional limitations, certain medication classes (e.g., anticoagulants, diuretics), the use of oxygen, and the use of a urinary catheter. This study demonstrates that clinician behaviors and documentation patterns can be incorporated in risk models to yield important insights.

Abstract citation ID: igad104.1722

### ENGAGEMENT OF FAMILY IN ASSISTED LIVING: SECONDARY QUALITATIVE ANALYSIS USING THE ADAPTIVE LEADERSHIP FRAMEWORK

Youngmin Cho[1], Victoria Crowder[1], Ayomide Bankole[2], Lixin Song[3], Bei Wu[4], Ruth Anderson[1], and Anna Beeber[5], *1. University of North Carolina at Chapel Hill, Chapel Hill, North Carolina, United States*, *2. UNC-Chapel Hill, Chapel Hill, North Carolina, United States*, *3. University of Texas Health Science Center at San Antonio, San Antonio, Texas, United States*, *4. New York University, New York City, New York, United States*, *5. Johns Hopkins University, Baltimore, Maryland, United States*

Assisted living (AL) residents are at high risk for safety issues (e.g., falls) and poor quality of life (QoL) because they can lose their personal preferences and autonomy in personal care or social activities once they enter AL. Family engagement (FE) plays a significant role in addressing these challenges while considering residents' needs and preferences. The purpose of this study was to explore family caregivers' perspectives about FE strategies in AL and contextual factors influencing FE. This study used a secondary analysis of the interview data from a larger study investigating 34 family caregivers' opinions on AL safety and strategies to increase FE. The Adaptive Leadership Framework guided our directed content analyses to identify how family caregivers address the challenges with AL residents and staff. Family caregivers described adaptive leadership strategies pertaining to getting the residents to recognize the seriousness of their health status, finding available resources to support them, and planning for potential challenges. Family caregivers also described adaptive and collaborative work (with AL residents or staff) strategies such as advocating for resident needs, involvement in direct care or social activities, shared understanding of care plan, and making decisions together. We also found that contextual factors such as fear of speaking up and retaliation, previous experiences in FE, influenced family caregivers' engagement in AL safety. These findings highlight the important role of family caregivers in addressing AL resident safety challenges. Also, our study provides the rationale for developing practical FE strategies to enhance residents' safety and QoL.

Abstract citation ID: igad104.1723

### PATIENT-CLINICIAN RELATIONSHIPS IN HOME HEALTH CARE

Ayomide Bankole[1], Tyra Girdwood[2], Dorothy Addo-Mensah[3], and Mark Toles[1], *1. UNC-Chapel Hill, Chapel Hill, North Carolina, United States*, *2. Duke University, Durham, North Carolina, United States*, *3. University of North Carolina at Chapel Hill, Chapel Hill, North Carolina, United States*

Patient-clinician relationships are fundamental attributes of high-quality home health care (HHC); yet little is known about patient-clinician relationships in HHC (where 5 million Medicare beneficiaries receive care annually). The objective of the study was to describe perspectives of HHC patients and HHC clinicians about patient-clinician relationships in HHC. We conducted a secondary qualitative analysis of semi-structured interviews (n=34) from a completed qualitative study investigating perspectives of older adult HHC patients (or caregivers as proxy) and their HHC clinicians (17 pairs) on discharge preparedness in a large HHC organization in North-Carolina. A conceptual model of patient-clinician relationships guided content analysis of the interview data. HHC patients identified as White (65%) and black (35%). Most HHC patients reported female sex (53%) and average age was 83 years (range= 69-93). Clinicians were registered nurses and physical therapists. Across the patient-clinician pairs, HHC patients valued relationships with clinicians with shared commonalities and those who provided reciprocal informational exchange and respected their autonomy. HHC clinicians valued relationships in which they felt helpful and respected as a healthcare professional. Relational conflicts arose when there was discordance in expectations of care (e.g., type of HHC services provided). Conflicts also arose when organizational constraints, such as disruptive scheduling, limited HHC visit time, and when limited continuity of care prevented the formation of patient-clinician relationships. Relational conflicts and organizational constraints influence patient-clinician relationships in HHC. Multi-level interventions (targeting modifiable factors at the patient, clinician, and organizational levels) are needed to improve the patient-clinician relationships and the quality of HHC.

Abstract citation ID: igad104.1724

### PERCEIVED FEASIBILITY TO IMPLEMENT A TASK-ORIENTED EXERCISE PROGRAM BY FITNESS STAFF AT SENIOR LIVING COMMUNITIES

Chiung-ju Liu[1], and Lauren Mansuy[2], *1. University of Florida, Gainesville, Florida, United States*, *2. University of Central Florida, Orlando, Florida, United States*

Older adults who relocate to senior living communities have a desire to maintain their independence. The fitness infrastructure within these communities is a great platform to deliver evidence-based exercise programs to support residents' independence, especially for those who have experienced activity limitations. Task-oriented exercise incorporates daily activities into exercise strategies and has been recommended as an approach to reduce late-life disability. The current study used an interview research design to determine the feasibility of implementing 3-Step Workout for Life, a task-oriented program, in senior living communities. The 3-Step Workout for Life program consists of gym-based group resistance exercise and home-based one-on-one activity exercise. Fourteen fitness instructors from different communities, with independent living units, in the state of Florida completed the study. Interviews were transcribed, coded, and underwent thematic analysis. Instructors perceived the screening procedure to select and enroll residents in this program could help match residents to the right fitness program. The gym-based group resistance exercise is compatible with existing instructor-led fitness programs. Although instructors perceived the home-based one-on-one activity exercise positively, such as the potential benefits for frail residents who do not attend the gym, they acknowledged several barriers to implementing this exercise. Major barriers include their job responsibility restricting them from working in the resident's home, their belief that activity exercise should be better delivered by a rehabilitation professional, and limited staff to support the one-on-one format. The next step is to collaborate with community stakeholders to address these barriers and build capacity to implement task-oriented exercise programs.

Abstract citation ID: igad104.1725

### UNCOVERING THE HIDDEN WORKFORCE: A STUDY OF DIRECT CARE WORKERS IN ASSISTED LIVING HOMES

Christina Wyles[1], and Kimberly Shea[2], *1. Roudebush VA Medical Center, Indianapolis, Indiana, United States*, *2. College of Nursing, The University of Arizona, Tucson, Arizona, United States*

A growing number of 4.5 million U.S. direct care workers (DCWs) provide hands-on care to older adult residents in assisted living homes (ALHs) -- the smallest type of assisted living residences that require employment of DCWs. These homes typically have capacity for 8-10 residents, and they no longer resemble the original non-medical, home-like environment. With an aging population, increasing complexity of residents' medical and functional needs, and increasing closure of nursing homes, demand for DCWs in ALHs continues to grow. Better information on this growing workforce segment, federal oversight, and consistent licensure are needed. The few available studies indicate high resident acuity and quality of care issues. To address this knowledge gap, a study explored and described the learning interests and needs and technology use of ALH DCWs. Guided by a human factors framework and an interpersonal perspective, DCWs (N = 14/ median age 41) were interviewed one-on-one using semi-structured questions. Data analysis involved qualitative content analysis of transcribed taped interviews. Findings revealed DCWs are interested in work-related topics and acknowledge the complexity of their residents. They use various health-related technologies in the care they provide, but need more access to educational opportunities that accommodate their long work hours and low salaries. The study also noted the expanded scope of practice, high risk of injury, and underrepresentation of DCWs in the literature. This study highlights learning and technology needs of DCWs in ALHs, emphasizing the need for more recognition and support for this vital segment of the long-term care workforce.

### SESSION 4385 (PAPER)

### ECONOMICS OF AGING

Abstract citation ID: igad104.1726

### DID STRONGER STATE AGE DISCRIMINATION LAWS REDUCE THE ADVERSE IMPACTS OF COVID-19 ON LABOR OUTCOMES OF OLDER WORKERS?

Truc Bui, *Tulane University, New Orleans, Louisiana, United States*

Covid-19 caused tremendous impacts on the health and employment status of older workers, while current literature shows that age discrimination increased during recessions. In this context, I examine whether stronger state age discrimination laws reduced the adverse impacts of Covid-19 on the labor outcomes of older workers. State age discrimination laws are stronger than federal law because they protect workers in smaller firms or require firms to pay harsher penalties if violating the laws. I use these two classifications to identify the strength of state protections and employ the difference-in-difference-in-difference method. Using CPS and QWI data, I compare the labor outcomes of older and younger workers, in states with stronger and weaker laws, before and after the Covid-19 recession. There is very little evidence that stronger state laws protected older workers from the adverse impacts of the Covid-19 recession. I even found that stronger state protections were associated with more negative effects on older workers. Although stronger state laws were associated with higher hiring and separation rates right after the Covid-19 pandemic started, the increases in hiring rates were high enough to offset the growth in separation rates, and those effects were more pronounced among older men. The findings were consistent with the effectiveness of stronger state laws during the Great Recession, implying that severe labor market conditions weakened the effectiveness of stronger state age discrimination laws. The results also question the protection of discrimination laws on intersectional discrimination against age and gender.

Abstract citation ID: igad104.1727

### INTERGENERATIONAL FINANCIAL TRANSFER AND FAMILY STRUCTURE IN INDIA: EVIDENCE FROM LASI, 2017–2018

Varsha Nagargoje, Dilip T R, and K. S. James, *International Institute for Population Sciences, Mumbai, Mumbai, Maharashtra, India*

Understanding patterns of intergenerational support is critical within the context of demographic change, such as changing family structures and population aging. Existing research has analyzed the proportion of direct financial transfers between older parents and adult children and vice-versa. This study also examined the association between family structure and the direction of financial transfer in India. From the first wave of the Longitudinal Ageing Study in India, samples of 30,147 older adults aged 60+ years whose children alive were included. Bivariate analysis and logistic regression methods opted to fulfill the study objectives. Findings showed that the proportion of children-to-parent transfers (13.1%) was triple that of parents-to-children transfers (4.4%). No rural-urban disparity existed in the overall proportion of parents-to-children financial transfers. Conversely, children-to-parent financial transfers have shown marked variation among rural and urban parts of India. Older adults living alone received the most financial support from their children. While controlling sociodemographic and economic characteristics, family characteristics like having children up to eight were significantly associated with higher parents-to-children financial transfers. However, having more than eight children did not show significant effect. Having more children significantly increases the likelihood of financial transfer from children to older parents. Older adults living alone were more likely to receive financial support from children but were less likely to provide it. Filial piety is deeply rooted in India and is reflected through the bond of reciprocity among younger generations to provide financial support to their older parents, which helps older people to maintain better economic well-being.

Abstract citation ID: igad104.1728

### RESOLUTION OR WORK STRESS? WHY DOES RETIREMENT CHANGE HEALTH BEHAVIOR?

Colin McKenzie[1], Shinya Kajitani[2], and Kei Sakata[3], *1. Keio University, Tokyo, Tokyo, Japan*, *2. Kyoto Sangyo University, Kita-ku, Kyoto, Japan*, *3. Australian Institute of Family Studies, Southbank, Victoria, Australia*

The purpose of this paper is to examine the impact of retirement on actual health outcomes and intentions relating to these outcomes for Japanese men and women. The health outcomes investigated relate to alcohol and tobacco consumption, physical exercise, and psychological distress. This paper uses data from the first 14 waves of the Longitudinal Survey of Middle-aged and Elderly Persons (LSMEP) to estimate regression models explaining these health outcomes. An instrumental variable estimator using instruments constructed from the eligibility ages for various aspects of the Japanese pension is used to account for the endogeneity of retirement and income. Individual heterogeneity is dealt with by using a fixed effects estimator. For both men and women, it is found that there is no significant impact of retirement on the extent to which individuals report that they will take care not to drink too much, nor smoke too much, and to engage in a sufficient amount of exercise. While psychological distress declines for both men and women as they retire, the proportion of men drinking alcohol falls while there is no change for women. For both men and women, the proportion who are smoking falls after retirement. The proportion of males and females engaging in moderate amounts of exercise increases following their retirement. Taken together, the results suggest that reductions in stress following retirement are driving the results. Falls in income associated with retirement explain little of the behaviour observed.

Abstract citation ID: igad104.1729

### THE BURDEN OF COGNITIVE IMPAIRMENT

Ritika Chaturvedi[1], Tadeja Gracner[2], Hanke Heun-Johnson[3], Bryan Tysinger[3], and Dana Goldman[3], *1. USC, Los Angeles, California, United States*, *2. RAND Corporation, Santa Monica, California, United States*, *3. University of Southern California, Los Angeles, California, United States*

Over 10% of American seniors (age≥65) experience cognitive impairment (CI) related to Alzheimer's disease and related dementias (ADRD). An additional 15-22% experience mild cognitive impartment (MCI), which often precedes ADRD and occurs earlier in life. However, CI burden estimates inclusive of MCI and early CI onset (age< 65) are limited. Using microsimulation, we estimated the burden of CI (ADRD and/or MCI) over remaining life years above 50 overall and by disease progression, age of onset, and sociodemographic subgroups for a nationally representative sample of Americans (age≥50) with cognitive status information in the Health and Retirement Study (2000-2016). The per capita lifetime CI burden was $124K (95%CI:$101K-$148K) due to $162K (95%CI:$139K-$185K) and $6.40K (95%CI:$5.03K-$7.77K) in lost Quality Adjusted Life Years (QALYs) and earnings, respectively, and $4.07K (95%CI:$1.82K-$6.33K) in informal care costs, offset by $48.6K (95%CI: $42.4K-$54.8K) in lower medical expenditures. The aggregate CI burden was $627B (95%CI: $511B–$743B): 59% due to ADRD and 41% to MCI. Individuals with MCI without progression to ADRD lost healthy life years valued at $119K (95%CI:$99K-$139K). Those with early CI onset were more likely to be from disadvantaged populations and carried the largest per capita burden [$376K (95% CI: $315K-$436K)] cumulating to 60% on aggregate, nearly half of which experienced during MCI. Individuals with lower education, progression to ADRD, or racial/ethnic minorities also carried disproportionate burden. MCI represents 41% of the overall CI burden and disproportionately affects individuals with early CI onset. Characterizing CI-related health and economic disparities at milder stages and younger ages should be prioritized.

Abstract citation ID: igad104.1730

### THE IMPACT OF A NATIONWIDE PHYSICAL ACTIVITY INTERVENTION FOR DIABETES AND HYPERTENSION PREVENTION

Cynthia Chen[1], Gregory Ang[1], Chuen Seng Tan[1], Falk Müller-Riemenschneider[1], and Yot Teerawattananon[2], *1. National University of Singapore, Singapore, Singapore*, *2. Health Intervention and Technology Assessment Program, Muang Nonthaburi, Nonthaburi, Thailand*

Increasing physical inactivity is a primary risk factor for diabetes and hypertension, contributing to rising healthcare expenditure and productivity losses. We examined the cost-effectiveness of the National Steps Challenge (NSC), an annual nationwide mHealth intervention to increase physical activity in Singapore. We used a Markov model to assess the long-term impact of increased physical activity from NSC on adults. Conducting NSC yearly for 10 years on a mean cohort size of 654,500 participants is projected to prevent 6,120 diabetes cases (95% credible interval: 3,690 to 9,040), 10,300 hypertension cases (6,260 to 14,700) hypertension cases and 4,950 death cases (3,280 to 7,040), leading to 78,800 QALYs (56,500 to 102,000) gained. From the health system perspective, assuming no differentiation of cost among different physical activity levels within each health state, the healthcare cost savings from the averted cases is estimated to be SGD674 million (239 million to 1.48 billion), with SGD364 million (57.8 million to 1.04 billion) for diabetes and SGD311 million (95.0 million to 690 million) for hypertension. Using a willingness to pay threshold of SGD10,000, NSC was cost-saving at -SGD4,510 (-14,000 to 1,430) per QALY gained. We project that increasing physical activity from a yearly nationwide physical activity intervention delays the incidence of diabetes and hypertension. Our results suggest that the intervention is cost-saving and improves the quality of life. The estimated cost savings are more significant when indirect costs are considered, hence providing important information for decision-making in countries when considering similar large-scale physical activity programmes.

### SESSION 4390 (PAPER)

### GENDER, ROLES, AND HEALTH

Abstract citation ID: igad104.1731

### GENDER AND HOUSEWORK IN THE POSTRETIREMENT CONTEXT

In Jeong Hwang[1], Joseph Svec[2], and Jeong Eun Lee[3], *1. Harvard University, Boston, Massachusetts, United States*, *2. Saint Joseph's University, New York City, New York, United States*, *3. Iowa State University, Ames, Iowa, United States*

In recent years, social scientists have noted an uptick in “unretirement” of older adults as a large number of retirees have reentered the labor force. Not only do older Americans’ labor-force reentry have implications for our economy as a whole, this also raises important questions regarding the social and interpersonal relationships within households. Specifically, women, compared to men, might be disproportionately subject to work-family conflicts, due to the gendered expectation that women take primary responsibility for household labor. In this study, we examine 758 American couples aged 65 and above from the 2005-2019 Health and Retirement Study (HRS) core survey and the supplemental Consumption and Activities Mail Survey. We examine whether and to what extent couple’s retirement and re-entry into the labor force correspond with changes in the distribution of household labor. Specifically, we expect that while both men and women shift their household labor contributions in accordance to changes in their formal work statuses, changes in men’s contributions are attenuated relative to women’s. The results show that while both male and female retirees spend more time housework when their spouses work, women tend to perform more housework when they re-enter the labor force whereas men’s contributions decline significantly upon their re-entry. These patterns are increasingly evident from the analysis focused on routine tasks, typically done by women. These results highlight how gender ideological scripts can frame relational contributions and expectations as well as their persistence despite shifts in economic contributions to the household.

Abstract citation ID: igad104.1732

### GENDER, SEXUAL ORIENTATION, AND SPOUSAL CAREGIVING FOR THOSE WITH DEMENTIA: A FOCUS ON EMOTION WORK

Toni Calasanti, Sadie Giles, and Jing Geng, *Virginia Tech, Blacksburg, Virginia, United States*

This study draws upon and expands three research streams. The first relates to spousal caregiving for those with dementia, and gender differences in stress. Based on gender divisions of labor, men are more likely to take a task-oriented approach to caregiving; women take a more holistic, empathetic approach. The second strand relates to emotion work, wherein caregivers manipulate their own emotions to produce a desired outcome in another (Hochschild, 2003). Research shows that spouses find performing emotion work “draining” (Umberson, et al., 2020), and that women do it more. Finally, we draw upon research that finds that same-sex couples are more egalitarian in their division of labor than are straight couples; how this impacts caregiving approaches or emotion work is unclear. Weaving these together, our study explores how inequalities of gender and sexuality intersect in shaping caregiving by older adults who care for their demented spouses. Our analysis of in-depth interviews conducted among 69 straight, gay, and lesbian caregivers finds that, first, heterosexual men rarely engage in emotion work, though they do manage their own feelings. Second, heterosexual wives overwhelmingly manipulate their feelings in attempts to gain compliance from their spouses, calm them, and/or make them happy. Finally, both gay men and lesbians perform emotion work, and they tend to combine the task-oriented and holistic approaches to care. These findings make clear that gender differences found in previous caregiving research are not “natural,” and suggest that straight men’s lower stress levels may result from their tendency to skip emotion work.

Abstract citation ID: igad104.1733

### STRUCTURAL AND FUNCTIONAL ASPECTS OF SOCIAL NETWORKS AND EMOTIONAL WELL-BEING AMONG LGBT OLDER ADULTS

Anyah Prasad[1], Jeffrey Burr[1], Edward Miller[1], and Karen Fredriksen-Goldsen[2], *1. University of Massachusetts Boston, Boston, Massachusetts, United States*, *2. University of Washington, Seattle, Washington, United States*

Network size and composition are structural aspects and perceived support is a functional aspect of social networks. How these social network characteristics are related to LGBT older adults’ emotional wellbeing is not well understood. We investigated these relationships using data from The Caring and Aging with Pride study, a cross-sectional survey of 2,560 LGBT Americans aged 50 years and above, employing a series of mediated and moderated-mediated regression analyses. Results indicated that larger social networks were associated with more perceived support and perceived support partially mediated the association between network size and emotional wellbeing. Results also showed that stress was associated with poor emotional wellbeing via depletion of perceived support but less so when LGBT older adults were embedded in larger networks. These findings are in line with the Convoy Model of Social Relationships and The Stress Process Model; further, the results support Kondrat and colleagues’ suggestion that social network size has a moderating role, while social support has a mediating role between stress and health. Also, the Theory of Homophily postulates that shared identity may enhance group cohesion and feeling supported. Accordingly, we observed that perceived support and its mediation role were stronger with LGBT and older network members compared to non-LGBT and younger network members. A larger network of non-LGBT older adults had a more direct beneficial association with LGBT older adults’ emotional wellbeing. Our observations have practical implications for programs that aim to support LGBT older adults’ emotional wellbeing by strengthening social support through their social networks.

Abstract citation ID: igad104.1734

### WOMEN'S STORIES: NARRATIVES ABOUT RELATIONSHIPS AND RESILIENCE

Wendy Watson, Sandra Faulkner, and Charlie Stelle, *Bowling Green State University, Bowling Green, Ohio, United States*

Research has demonstrated that relationships are important in the lives of older women. Thirty-one women between the ages of 64-86 participated in interviews about their lives and relationships across the life course. In the interviews, women talked about their family of origin, friendships, romantic relationships, and their children and grandchildren. They explained how their relationships developed across the life course and how they have maintained their relationships, including how COVID-19 affected their maintenance behaviors. In the interviews, women constructed narratives that explained who they are as individuals and who they are in relationships. Through a poetic analysis of the interviews, resilience stood out as an overarching theme across the stories from women of different ages, marital statuses, economic backgrounds, and current and former occupations. Many women spoke of growing up in poor, rural areas and finding the strength to build a better life for themselves. There were stories of abuse, both in their family of origin and in chosen partners. Narratives included many instances of loss and hardships through caregiving, deaths of friends and relatives, and dissolution of relationships. Throughout it all, these women claimed resilience brought about through their own strength and determination and through relationships with people who supported them, mentored them, and built them up. This paper will discuss the many ways in which these thirty-one women exemplified resilience in their past, in their current lives, and as they looked toward their futures.

### SESSION 4395 (PAPER)

### GLOBAL HEALTH AND MINORITIES ISSUES

Abstract citation ID: igad104.1735

### COMPARING THE ASSOCIATION BETWEEN DIABETES AND MORTALITY IN TWO INDEPENDENT BIRTH COHORT OF OLDER ADULTS IN MEXICO

Brian Downer, and Jose Cabrero Castro, *University of Texas Medical Branch, Galveston, Texas, United States*

The prevalence of diabetes among middle-aged and older adults in Mexico has nearly doubled since the year 2000. This increase is primarily due to healthcare reforms that promoted diabetes screening. Insulin use and other treatments have also increased during the same period. Early diagnosis and treatment can reduce diabetes mortality risk. However, it is unclear if the increased diagnosis and treatment of diabetes in Mexico have reduced the mortality risk associated with diabetes in older adults. We used the Mexican Health and Aging Study to compare the association between diabetes and 5-year mortality in two independent cohorts of adults aged 60-69 in 2001 (1932-1941) and 2012 (1943-1952). The final sample included 3,694 participants in 2001 and 4,769 in 2012. Demographic variables included age, gender, education, living in an urban community, and having health insurance coverage. Self-reported health conditions included diabetes, hypertension, stroke, and respiratory disease. We used cox-proportional hazard regression models that included an interaction term for diabetes by cohort and adjusted for demographic and self-reported health characteristics. Overall, diabetes was associated with 2.56 times higher mortality (95% CI=2.18-3.00). The 2012 cohort did not have significantly different mortality risk compared to the 2001 cohort (HR=0.91, 95% CI=0.77-1.07). The association between diabetes and mortality for participants in the 2001 cohort (HR=2.52, 95% CI=1.95-3.26) was not significantly different from the 2012 cohort (HR=2.58, 95% CI=2.09-3.17). Our results indicate that diabetes continues to be a major risk factor for mortality in Mexico despite the increase in screening and treatment.

Abstract citation ID: igad104.1736

### ETHNIC DIFFERENCES IN DIET QUALITY AND COGNITION AMONG OLDER ADULTS

Isabella Arellanes, Eileen Crimmins, Jennifer Ailshire, and Jung Ki Kim, *University of Southern California, Los Angeles, California, United States*

Research indicates an association between diet quality and cognitive outcomes. However, the association across ethnicities is not well understood. This study identifies differences in diet quality using the Healthy Eating Index (HEI-2015) score by ethnicity and its relationship to cognitive outcomes within ethnic groups. The sample includes 6,845 older adults from the 2014 wave of the Health and Retirement Study and nutrition data from the 2013 supplemental Health Care and Nutrition Survey. Cognitive functioning was measured based on a scale ranging from 0-27. Overall, 11% of respondents had a good quality diet, indicated by a HEI-2015 score of 81-100. Diet quality was significantly better among Hispanics than either non-Hispanic Blacks or Whites ($p < 0.001$ in both comparisons). Diet quality was not significantly different between Whites and Blacks ($p=0.82$). In a regression model controlling for age, gender, and marital status, the association between diet quality and better cognition was significant among Whites ($p < 0.001$) and Blacks ($p < 0.05$), but not significant among Hispanics ($p=0.11$). A one unit increase in the Healthy Eating Index is associated with a 0.05 increase in cognitive score among Whites and a 0.03 increase among Blacks. These findings suggest significant differences in diet quality across ethnicities that may impact cognition among older adults; however, the impact may differ by ethnicity.

Abstract citation ID: igad104.1737

### INCONSISTENT ESTIMATES OF POOR HEALTH BETWEEN EUROPEAN SURVEYS—A MATTER OF SURVEY DESIGN?

Susanne Kelfve[1], and Linda Enroth[2], *1. Linköping University, Norrköping, Ostergotlands Lan, Sweden, 2. Tampere University, Tampere, Pirkanmaa, Finland*

Several studies have reported about inconsistencies in survey estimates on health, both within and between countries. One potential source of inconsistency in results from population surveys is survey quality. In particular, the prevalence of poor health among older people is sensitive for survey design factors and the inclusion of older people with poor health. We aimed to explore the association between survey design factors and within-country differences (between EU-SILC and SHARE) in estimated prevalence of poor health (long-term illness and global activity limitation (GALI)) among older people. The analyses include people 65 years and older from 25 European countries with data collections both in SHARE and EU-SILC 2017. Results show substantial differences in sociodemographic factors and estimates of poor health, both between and within countries. The within-country differences in the prevalence of long-term illness ranged from -18 percentage points for men in Denmark

(lower in EU-SILC compared with SHARE) to 42 percentage points for women in Cyprus (higher in EU-SILC compared with SHARE). The prevalence of GALI showed similar patterns. We conclude that estimates of poor health, measured by long-term illness and GALI, have great variation between surveys within the same country. Survey design factors, such as response rate and the type and number of survey mode seems to explain part of these inconsistencies. Population surveys are widely used for monitoring health and the results from these surveys are utilized in health policy planning. Thus, it is crucial to understand the possible impact of survey design on health estimates.

Abstract citation ID: igad104.1738

### MULTIDIMENSIONAL EVALUATION OF BIOLOGICAL AGE USING NMR METABOLOMICS AND ANTHROPOMETRY IN 35,541 ADULTS IN MEXICO

Omar Yaxmehen Bello-Chavolla[1], Carlos Fermin-Martinez[2], Luisa Fernández-Chirino[1], Daniel Ramírez-García[3], Neftali Antonio-Villa[4], Alejandra Núñez-Luna[1], Paulina Sánchez-Castro[1], and Martin Roberto Basile-Alvarez[1], *1. Instituto Nacional de Geriatría, Mexico City, Distrito Federal, Mexico*, 2. *School of Medicine, National Autonomous University of México (UNAM), Mexico City, Distrito Federal, Mexico*, 3. *Universidad Nacional Autónoma de México, Mexico City, Distrito Federal, Mexico*, 4. *Instituto Nacional de Cardiologia Ignacio Chavez, Mexico City, Distrito Federal, Mexico*

Aging occurs across different domains at the individual level, encompassing distinct physiological systems which can be captured using biological age (BA) metrics. Second generation BA metrics are aimed at proxying BA using 10-year mortality risk and include PhenoAge and AnthropoAge. Here, we explored the complementary application of AnthropoAge and an NMR-Metabolomics derived BA measure to explore aging in body composition and metabolic domains in a subsample of 35,541 participants of the Mexico City Prospective Study. Using LASSO Cox regression, we identified 17 metabolites associated with all-cause mortality, in men and 75 in women. We then fitted age-adjusted Gompertz regression models and identified 7 unique metabolites for men and 15 for women. We transformed the results from these regressions to define MetaboAge. Both AnthropoAge (AUC 0.784, 95%CI 0.778-0.790) and MetaboAge (AUC 0.808, 95%CI 0.802-0.814) showed superior performance for prediction of all-cause mortality compared to chronological age (AUC 0.779, 95%CI 0.773-0.786). By regressing AnthropoAge and MetaboAge onto chronological age we obtained acceleration metrics (≥0 defined accelerated aging). By combining acceleration metrics from anthropometry and NMR-metabolomics we identified distinct trajectories of all-cause mortality. Notably, cases with acceleration only in AnthropoAge (HR 1.43, 95%CI 1.33-1.54) and those with acceleration only in MetaboAge (HR 1.86, 95%CI 1.73-2.01) had higher risk of all-cause mortality compared to non-accelerated cases but lower compared to cases with acceleration in both metrics (HR 2.28, 95%CI 2.12-2.45). Multidimensional evaluation of BA may prove useful to capture heterogeneity of aging in diverse populations by capturing aging at distinct physiological domains.

Abstract citation ID: igad104.1739

### REFERRAL OF ASIANS TO SKILLED NURSING FACILITIES: FINDINGS FROM THE 2019 NATIONAL MEDICARE DATA

Hoang Nguyen[1], Chih-Ying Li[2], Huey-Ming Tzeng[1], Md Ibrahim Tahashilder[3], and Yong-Fang Kuo[2], *1. University of Texas Medical Branch at Galveston, Galveston, Texas, United States*, 2. *University of Texas Medical Branch, Galveston, Texas, United States*, 3. *The University of Texas Medical Branch, Galveston, Texas, United States*

A recent study of skilled nursing facilities (SNF) found Asian/Pacific Islanders are the most physically dependent and have the greatest cognitive impairment among all residents. Whether or not this is due to differential referral pattern to skilled nursing facilities is not known. We conducted a retrospective analysis of the 2019 national Medicare data to compare differences in referral to SNF among Asian, Black, Hispanic, and White adults ages 66 years and older following hospitalization for fracture (n=148,493), heart failure (n=377,398), joint replacement (n=509,347), pneumonia (n=258,614), urinary tract infection (n=195,816), or sepsis (n=518.619). Asians had the second lowest rates of SNF referral for all medical conditions examined, except for joint replacement. Hispanic had the lowest rates. For joint replacement, Asians had the highest rate of referral. The SNF referral rates among Asians compared to whites were 70.1% and 71.6% for fracture, 16.4% and 22.4% for heart failure, 16.8% and 21.2% for pneumonia, 30.1% and 34.6% for urinary tract infection (UTI), 29.3% and 32.5% for sepsis, and 32.1% and 23.4% for joint replacement. Asians had significantly lower odds of SNF referrals after hospitalization for fracture (OR=0.71; 95% CI: 0.65-0.77), heart failure (OR=0.51; 95% CI: 0.48-0.54), pneumonia (OR=0.46; 95% CI: 0.42-0.49), UTI (OR=0.59; 95% CI: 0.54-0.63), and sepsis (OR=0.55; 95% CI: 0.53-0.57), but significantly higher odds after joint replacement (OR=1.19; 95% CI: 1.12-1.26) compared to non-Hispanic White adults. The reason for the lower referral rates is unclear but could be from a lack of culturally appropriate facilities. More culturally appropriate facilities are needed.

### SESSION 4400 (PAPER)

### INTERGENERATIONAL RELATIONSHIPS ACROSS AGE, ROLES, AND TIME

Abstract citation ID: igad104.1740

### EMOTIONAL ADJUSTMENTS OF CUSTODIAL GRANDPARENTS LIVING WITH COVID-19: A NATIONAL PERSPECTIVE

Deborah Whitley[1], Susan Kelley[1], Christine Fruhauf[2], Andrea Smith[3], and Bert Hayslip[4], *1. Georgia State University, Atlanta, Georgia, United States*, 2. *Colorado State University, Fort Collins, Colorado, United States*, 3. *Western Michigan University, Ada, Michigan, United States*, 4. *University of North Texas, Denton, Texas, United States*

Living with COVID-19 demands has created emotional challenges for grandparents raising grandchildren. The aim of this study was to explore the emotional adjustment of custodial grandparents' caregiving under COVID demands (e.g., masking, hand washing, social distancing) (N=145; age range 30-79 years, Mage = 61.4 years, SD = 8.5). A national sample of grandparents were surveyed using a multi-scale index of psychological distress, reliance on intrapersonal resources during the pandemic, as well as sociodemographics. An initial hierarchical regression analysis of caregivers' emotional adjustment was conducted, wherein (1) sociodemographic variables were first entered as a set followed by 2) a 17-item measure of COVID demands (alpha = .73), 3) two indices of grandparent intrapersonal resources [resilience and empowerment/social support and religious coping], and 4) two indices of psychosocial stress [parental distress, health, social/emotional and loneliness/parental distress, parent-child dysfunction, difficult child and parenting challenges]. The overall model was statistically significant (F 11, 123 = 22.98, p < .01), accounting for 64% of the variance in grandparent adjustment difficulties. While sociodemographics and COVID demands failed to predict emotional distress, both categories of grandparent intrapersonal resources (p < .01), and both categories of psychosocial stress (p < .01) did so. Subsequent correlational findings indicated that intrapersonal resources (social support and religious coping) mediated the relationship between COVID demands and emotional adjustment. These findings suggest that while grandparent caregivers experienced significant emotional distress during the pandemic, reliance upon the support of others as well as their own intrapersonal resources may be instrumental in managing such distress.

Abstract citation ID: igad104.1741

### HEALTH AFTER AGE 50: THE ROLE OF ADULT CHILDREN'S EDUCATION FOR OLDER PARENTS' FRAILTY IN EUROPE

Damiano Uccheddu[1], and Marco Tosi[2], *1. University of Louvain (UCLouvain), Ixelles, Brussels Hoofdstedelijk Gewest, Belgium*, *2. University of Padua, Padua, Veneto, Italy*

Social stratification research has shown that family background can significantly impact a child's life chances. However, intergenerational transmission of (dis-)advantages is a two-way process, because parental investment in children may be reciprocated later in life. Particularly, very little is known about the importance of adult children's socio-economic resources on ageing parents' well-being – what has been called the effect of the "social foreground" on later-life health. This study aims to investigate whether and how adult offspring's educational attainments are associated with parents' health. This study uses dyadic panel data from all the available waves of the Survey of Health, Ageing and Retirement in Europe (SHARE) (i.e., from 2004 to 2020). The sample is composed of 89,752 parents (210,999 parent-child dyads, 250,605 observations) aged 50 or older living in 29 different European countries. Our preliminary results from random intercept linear regression models show that child education is negatively associated with the 40-item Frailty Index (FI) among parents. This association is particularly strong among lower-educated parents (especially mothers). These findings highlight the importance of considering the socio-economic resources of adult children when examining the health and well-being of older adults. This suggests that public investments in younger generations' education may benefit older generations as well. In developing future analyses, we plan to focus on identifying the potential generative mechanisms behind this relationship and exploring potential policy interventions to support ageing parents and their families.

Abstract citation ID: igad104.1742

### INTERGENERATIONAL BONDS ACROSS THE EARLY YEARS OF MARRIAGE

Raquael Joiner[1], Jacqueline Perez[2], Thomas Bradbury[2], and Benjamin Karney[2], *1. University of Southern California , Los Angeles, California, United States*, *2. University of California, Los Angeles, Los Angeles, California, United States*

As adults embark upon marriage, forming their own new nuclear families, they often remain connected to their families of origin. Nevertheless, spouses' relationships with their parents and in-laws are likely to change across the early years of marriage. Given that empirical studies to date are typically limited to only a couple of measurement occasions, however, the stability and impact of such changes are unclear. Using Repeated Measures Latent Class Analyses, the present study addresses this gap by identifying classes of spouses (N=375 dyads) experiencing different types of relationships with their parents and in-laws across the first 9 years of marriage. Further, correlates and consequences of spouses' longitudinal sentiment profiles were also examined. Three sentiment profiles (i.e., Mostly Helpful, Mostly Ambivalent, Mostly Indifferent, Mostly Difficult) emerged when looking at spouse's sentiments toward their own parents and for husbands' sentiments towards their in-laws, whereas 4 profiles (i.e., Mostly Helpful, Mostly Ambivalent, Mostly Indifferent, Mostly Difficult) were found for wives' sentiments towards their in-laws. Mostly Ambivalent sentiment profiles showed the least stability, and spouses showed significantly higher odds of being in the Mostly Ambivalent class if they were living in a multigenerational household and if the wife was a 2nd generation immigrant. Spouses whose marriages dissolved showed significantly higher odds of being in the Mostly Indifferent, Mostly Ambivalent, and Mostly Difficult classes, compared to the Mostly Helpful class. These results provide an important starting point for future research aiming to understand how intergenerational bonds may promote or hinder successful marriages.

Abstract citation ID: igad104.1743

### INTERGENERATIONAL RELATIONS OF MARRIED PERSONS WITH THEIR PATRILINEAL AND MATRILINEAL PARENTS IN CHINA

Jin Fan, and Hong Mi, *Zhejiang University, Hangzhou, Zhejiang, China (People's Republic)*

Objective: Guided by modernization theory and intergenerational solidarity theory, this study examined the general patterns of intergenerational relations with patrilineal and matrilineal parents in contemporary China and explored its influencing factors and mechanism.

**Methods:** Data from the 2017 Chinese General Social Survey were examined. Analytic samples consisted of married respondents with at least one patrilineal parent and one matrilineal parent alive (N = 1098). Latent class analysis (14

functional, structural and associational indicators) classified the intergenerational relations and multinomial logistic regression explored how these intergenerational relation patterns were associated with education, and marital and kinship relation by gender. **Results:** Five intergenerational relation types were revealed, three types (close to both parents and support upward more, 35.7%; close to both parents and reciprocal, 26.4%; distant to both parents, 11%) showed balanced patterns and two types (close to patrilineal parent but distant to matrilineal parent, 20%; close to matrilineal parent but distant to patrilineal parent, 6.9%) with unbalanced patterns. Children with higher educational level tended to form more egalitarian and bilateral intergenerational relations, both for husbands and wives. Moreover, good marital relation and more brothers facilitated this tendency for wives only. **Conclusions:** Results provided evidence for the maintenance and changes of patrilineal culture, and the increased importance of marital relation in China. Findings reaffirmed that married children had different interaction patterns with their patrilineal and matrilineal parents, and discussed the changes in social norms governing the responsibilities of husbands and wives. Keywords: intergenerational relations, patrilineal/matrilineal parents, latent class analysis, gender, China

Abstract citation ID: igad104.1744

### TRANSMISSION OF RELIGIOSITY BETWEEN GRANDPARENTS AND GRANDCHILDREN AS A BASIS FOR ELDERCARE RESPONSIBILITY

Merril Silverstein, Seonhwa Lee, RianSimone Harris, and Wencheng Zhang, *Syracuse University, Syracuse, New York, United States*

Different branches of research demonstrate that religiosity is effectively transmitted from grandparents to grandchildren and that religiosity strengthens norms of eldercare responsibility. This paper unites these two branches of inquiry by proposing that eldercare norms in emerging adulthood are directly and indirectly formed through an intergenerational religious pathway. Data derive from 149 grandparent-grandchild dyads from the 2021-22 wave of the Longitudinal Study of Generations. Religiosity is defined as a composite consisting of self-assessments of religious importance, religious intensity, and religious service attendance. Eldercare norms are assessed based on an evaluation of the obligation that adult children should have to provide support and care to older parents in six areas of assistance. Intermediate factors include grandchildren's familistic values and emotional closeness to grandparents. Structural equation modelling was used to examine direct and indirect pathways linking these factors. Results indicate significant continuity in religiosity between grandparents and grandchildren. The impact of grandchildren's religiosity on their eldercare norms is fully mediated by their familistic values and emotional closeness with grandparents. This analysis reveals that the transmission of religiosity to grandchildren begets eldercare norms through an ideational route of strengthening values of familism, and a personal route of promoting closer, and possibly more empathetic, relationships with grandparents who have or may soon have support needs. Results demonstrate the "hidden" impact of religious orientations of older grandparents on grandchildren and serve as a counterpoint to potentially specious reports about the adverse impact of declining religiosity and weakening intergenerational cohesion on aging families in American society.

### SESSION 4405 (PAPER)

Abstract citation ID: igad104.1745

### INTERVENTIONS TO IMPROVE HEALTH AND QUALITY OF LIFE

Presenters in this session describe research related to efforts to promote health and well-being from biological, psychological, and social perspectives.

Abstract citation ID: igad104.1746

### A CLOSER LOOK AT OLDER ADULTS AS PEER EDUCATORS IN LIFELONG LEARNING INSTITUTIONS IN THE US

Giuliana Casanova[1], Joyce Weil[2], and Margarida Cerqueira[3], *1. University of Aveiro, Aveiro, Aveiro, Portugal*, 2. *Towson University, Towson, Maryland, United States*, 3. *Universidade de Aveiro, Aveiro, Aveiro, Portugal*

Productive engagement in later life refers to paid or unpaid activities that can provide beneficial outcomes for individuals and society. This qualitative study examines the experiences of 17 peer educators, aged 61 to 84 and without cognitive deficits, who teach at three institutions of lifelong learning in Florida, USA. Data were collected through field notes, document analysis, a sociodemographic questionnaire, individual in-depth interviews, and brief interviews with each institution´s director. Descriptive statistics were used to examine the profiles of the peer educators, and emerging themes were identified through thematic analysis of the interview data. Preliminary results suggest that volunteerism and altruism played a role in their decision to become peer educators. Descriptive statistics showed that, on average, peer educators have obtained higher education degrees and have past teaching experience. The study identified several emerging themes related to the experiences of peer educators, including the need for purpose, the value of social connections, enjoyment of their role, and the importance of mutual learning. The analysis also revealed some challenges, including the need for environmental modifications to improve teaching conditions and assistance with marketing to promote classes and increase attendance. Overall, this study provides preliminary evidence to aid in understanding older adults in productive roles and fighting ageism. Identify current gaps in each institution´s approach, capacity to promote their participation and enhance their teaching experience while highlighting their contributions to society.

Abstract citation ID: igad104.1747

### EVALUATING A CO-DESIGNED EXERGAMING PLATFORM (MOUVMAT) FOR OLDER ADULTS LIVING IN LONG-TERM CARE HOMES

Charlene Chu[1], Renée Biss[2], Haniya Bharucha[1], and Henrique Matulis[1], *1. University of Toronto, Toronto, Ontario, Canada*, 2. *University of Windsor, Windsor, Ontario, Canada*

MouvMat is an exergaming technology with an interactive digital gaming surface that was co-designed with a user-centered design approach for older adults (OA) living in long term care homes. The purpose of the study was to evaluate the acceptability and efficacy of MouvMat to improve mobility, cognitive function and psychosocial wellbeing among OAs. The study was conducted as a multi-site pilot randomised controlled trial where OAs in the intervention group attended 1-hour group sessions 3 times per week for 6 weeks. The control group received standard recreational programing. All participants underwent assessments (TUG, 2MW test, UCLA Loneliness Scale, Cornell Scale of Depression, digit span, TMT-A, TMT-B, alternating sequences, and verbal fluency) at baseline, mid-intervention, and end of intervention. Participant ratings indicated that the MouvMat was acceptable and OAs with a range of comorbid health conditions were able to attend sessions and engage with the intervention. Efficacy findings were varied and should be interpreted in the context of the heterogeneous physical and cognitive abilities of participants living in a long-term care setting. Future studies will focus on larger sample size and improvement in MouvMat's design and interface.

Abstract citation ID: igad104.1748

**THE CRUCIAL ROLE OF LEPTIN IN MEDIATING VISCERAL ADIPOSITY-ASSOCIATED AGING PROCESS**

Shangang Zhao, *UT Health San Antonio - Barshop Institute, San Antonio, Texas, United States*

Visceral adiposity is a hallmark of aging and an important contributor to reduced healthspan and lifespan. The factors that mediate the detrimental effects of aging-associated visceral adiposity are poorly identified. Here, we have shown that hyperleptinemia, induced by a leptin transgenic cassette in adipose tissue, accelerates diet-induced obesity and glucose intolerance. In addition, hyperleptinemia promotes adipose tissue inflammation. Chronic culture of stromal vascular fraction (SVF) with leptin reduces the capacity of differentiation and increases inflammation. Moreover, chronic hyperleptinemia in fat depots accelerates the development senescent cells to establish a senescent-associated secretory phenotype (SASP), including increased local inflammation. This result indicates hyperleptinemia may be a "unidentified" driver for cellular senescence. Moreover, deletion of leptin in visceral fat depots significantly reduces visceral adiposity and senescent-associated secretory phenotype (SASP). These results highlight the crucial role of leptin in establishing SASP and targeting leptin may be a novel approach to alleviate aging-associated metabolic syndromes.

### SESSION 4410 (SYMPOSIUM)

Abstract citation ID: igad104.1749

**THE BLUES AND OLDER MINORITY MUSICIANS: MORE THAN JUST MUSIC XXX**

Chair: John Migliaccio

That's right! This GSA Annual Scientific Meeting celebrates 30 years of the ever popular "'Bo Diddley' Track" providing insights into the older performers who have pioneered and preserved this uniquely American musical genre and distinctive cultural treasure. Tampa was, and remains, a significant location in its history. When Hudson Woodbridge (Whittaker), a.k.a. "Tampa Red", had learned all he could of the popular "race records" featuring legendary blues performers like Ma Rainey, Bessie Smith, and Ida Cox and moved to Chicago in 1925 in his early 20s, he brought with him a prolific expertise on the slide guitar and helped introduce multiple new forms of this music. He spread the popular bawdy and humorous "hokum" Blues style and created numerous classic tunes that are still covered by the most famous musicians of today. Tampa continues to nurture a thriving music scene, and this year's GSA conference will take full advantage of the magic, music, and musicians of the Suncoast Blues. This year's special celebration will start with the opening of the exhibit hall. Then continue with the popular traditional lecture, oral history, refreshments, photo op and mini-performance by the best and most resilient local older blues musicians. That evening, join the artist and band, and a multitude of fellow GSA members, for a lively music bash at a local club featuring dancing, local food and refreshments, swag, and one of the best parties at GSA. It's a cornucopia of education and enjoyment like none other.

Abstract citation ID: igad104.1750

**EVERY DAY I HAVE THE BLUES**

Elwood Blues, *GSA Bo Diddley Track, Joliet, Illinois, United States*

The Blues Brothers are an American blues and soul revivalist band founded in 1978 by comedians Dan Aykroyd and John Belushi as part of a musical sketch on Saturday Night Live. Belushi and Aykroyd fronted the band, in character, respectively, as lead vocalist 'Joliet' Jake Blues and harmonica player/vocalist Elwood Blues, donning black suits with matching fedoras and sunglasses.

### SESSION 4415 (SYMPOSIUM)

Abstract citation ID: igad104.1751

**EMERGING TECHNOLOGY-BASED INTERVENTIONS TO SUPPORT FAMILY CAREGIVERS**

Chair: Tiffany Washington Co-Chair: Nicole Ruggiano
Discussant: Joseph Gaugler

There are 53 million family caregivers in the U.S., most of whom experience difficulty coordinating care, health and mental health decline, social isolation, and loneliness. Furthermore, accessing community support services remains challenging. This gap in access can create a significant threat to caregivers' wellbeing and quality of life. The need to develop innovative interventions to support family caregivers is urgent. This symposium will describe the potential of leveraging emerging technology-based interventions to support family caregivers. Speaker one will outline considerations for design and development of social engagement interventions for caregivers delivered via video-conferencing platforms. Speaker two will describe the development and initial testing of a touchscreen mobile application that supports communication between people living with dementia and their caregivers to promote person-centered care. Speaker three will describe the human-centered design of a digital health solution to improve symptom management in advanced cancer care for older adults, emphasizing specific strategies to incorporate evidence-based principles of digital inclusivity. Speaker four will present findings from a proof of concept study examining the acceptability of a virtual respite program for caregivers of individuals with dementia. Speaker five will explore the potential for crowdsourced mapping technologies to support caregivers of people living with dementia. The discussant will tie together themes, offer recommendations for future intervention research with family caregivers, and discuss implications of emerging technologies on practice, policy, and research.

Abstract citation ID: igad104.1752

### VIRTUAL RESPITE FOR FAMILY CAREGIVERS

Tiffany Washington, and Jennifer Rapowitz, *University of Georgia, Athens, Georgia, United States*

The COVID-19 pandemic was detrimental to the wellbeing of individuals everywhere, especially family caregivers. Over 20% of older caregivers reported difficulties finding time for self-care during the pandemic Respite is an avenue to self-care; however, virtual respite programs are nearly non-existent. In this proof of concept study, we examined the acceptability of a virtual respite program with eight family caregivers of individuals living with dementia. Virtual respite visits were delivered by graduate students who engaged the person with dementia in a tailored activity, thereby freeing time for the caregiver to have a respite break. Families received up to six weekly virtual respite visits totaling 122 to 295 minutes. Semi-structured interviews with participating caregivers were transcribed and coded using steps of thematic analysis. Caregivers expressed high acceptability for virtual respite because it was easily adaptable to their existing hardware (e.g., smart devices, laptops), accessible, and required little technological knowledge. They further expressed appreciation for tailored activities for care receivers that allowed respite time for their own self-care activity. Given the limited availability of respite services, there is a need to design and test new types of respite care. This virtual respite program is an opportunity to reach more caregivers.

Abstract citation ID: igad104.1753

### THE POTENTIAL OF CROWDSOURCED ASSET MAPPING TECHNOLOGIES FOR SUPPORTING DEMENTIA CAREGIVERS

Nicole Ruggiano[1], Tish Winton[2], Jane Daquin[2], Zhe Jiang[3], Monica Herzog[4], and Jeff Gray[2], *1. University of Alabama, Hoover, Alabama, United States*, *2. University of Alabama, Tuscaloosa, Alabama, United States*, *3. Computer & Information Science & Engineering, Gainsville, Florida, United States*, *4. College of Engineering, Tuscaloosa, Alabama, United States*

It is well-established that caregivers of people living with dementia (PLWD) often report having difficulty finding information about education and support that is available to them in their community. Caregiver support groups have demonstrated to be an effective approach to caregiver education through mutual support, though many caregivers are unable to participate in caregiver support groups due to time and geographic constraints. Crowdsourced technologies that rely on volunteered geographic information (VGI) are a viable way of community members to provide mutual support on a variety of issues, including disaster response, traffic conditions, health, and travel. These technologies often rely on a mobile app where users upload VGI that are of interest to others who use the app. There are benefits and drawbacks of crowdsourced technologies. For example, they may be an easy way for community members to share needed information, though there may be challenges in the quality of data that users provide. This presentation will report findings from a project that examined dementia caregivers' interest in using crowdsourced mapping technologies to share information about services and resources in their community that they have found helpful. Data were collected from a diverse sample of dementia caregivers living in Alabama through an online survey that was administered between June and November of 2022. Overall, it was found that caregivers were interested in using a crowdsourcing technology for support and education. Additional findings provide guidance for developing a crowdsourcing protocol that is based on caregivers' reported information needs.

Abstract citation ID: igad104.1754

### LEVERAGING VIDEO TECHNOLOGY TO DELIVER SOCIAL ENGAGEMENT OPPORTUNITIES FOR CARE PARTNERS OF PERSONS WITH DEMENTIA

George Mois[1], Elizabeth Lydon[2], Sarah Jones[3], Ruchira Rao[2], Wendy Rogers[2], Raksha Mudar[4], and Minakshi Raj[1], *1. University of Illinois Urbana Champaign, Champaign, Illinois, United States*, *2. University of Illinois Urbana-Champaign, Champaign, Illinois, United States*, *3. University of Illinois-Urbana Champaign, Champaign, Illinois, United States*, *4. University of Illinois Urbana-Champaign (UIUC), Champaign, Illinois, United States*

Care partners of persons with dementia provide critical support towards meeting the needs of their care recipients. Despite the increasing number of family care partners and their growing responsibilities related to caregiving activities, resources available to support their personal needs are limited (e.g., respite from caring, social engagement). Limited opportunities for social engagement can impact care partners' sense of social connectivity, and ability to maintain their social network. Video technologies present an opportunity to help facilitate social engagement among care partners from the comfort of their homes, however, this area of research is understudied. The goal of our study was to understand the needs and preferences of care partners related to a video technology-based social engagement program. We conducted two focus groups (N=6) with care partners aged 60 or older with experience supporting an family member with dementia for at least six months. Participants watched a demonstration of a social engagement event and engaged in semi structured focus group interviews. We solicited their insights about their needs and preferences, perspectives on utilizing a video technology platform and their attitudes toward the proposed program. Our findings indicate that they see value in participating and are interested in using video technology as long as the program covers diverse topics of interest, there is flexibility in scheduling, and sufficient technology support is offered. The feedback provided by the focus group participants informed the optimization of the program for implementation in a pragmatic clinical trial.

Abstract citation ID: igad104.1755

### ENVISION: A DIGITAL TOOL TO IMPROVE SYMPTOM MANAGEMENT AMONG OLDER ADULTS WITH ADVANCED CANCER

Karla Washington[1], Allison Donehower[2], Debra Parker Oliver[1], Jacquelyn Benson[1], and George Demiris[3], *1. Washington University in St. Louis, St. Louis, Missouri, United States*, *2. University Of Missouri, Columbia, Missouri, United States*, *3. University of Pennsylvania, Philadelphia, Pennsylvania, United States*

Hospice is a largely community-based healthcare service provided by an interdisciplinary team of professionals who partner with patients and their family members to control pain and other distressing symptoms commonly encountered at end of life. Research has shown that, despite receipt of hospice care, older patients with advanced cancer experience high rates of symptom distress. This presentation will describe the human-centered design of ENVISION (ENgagement and Visualization to Improve Symptoms In ONcology care), a digital health solution designed to improve hospice symptom management by strengthening patients' and families' symptom management self-efficacy and increasing the caregiver-centeredness of communication with hospice teams. Particular emphasis will be placed on design considerations adopted to enhance the digital inclusivity of the tool, resulting from research conducted to address the following research question: To what extent is ENVISION accessible, adoptable, and applicable to the strengths and needs of older hospice patients and their family caregivers? In answering this question, researchers conducted a multimethod qualitative study during which 34 users (including hospice nurses, social workers, physicians, chaplains, and family caregivers) were individually observed using the tool and interviewed about their experiences. Data were analyzed via conventional content analysis. Findings informed recommendations to address both digital and health literacy concerns, simplify data visualizations, and focus more specifically on high-priority symptoms and other wellbeing indicators, resulting in a more digitally inclusive tool with promise to improve the lives of older adults with advanced cancer and the family members who care for them.

Abstract citation ID: igad104.1756

### DEVELOPING A TOUCHSCREEN COMMUNICATION DEVICE TO EMPOWER PEOPLE WITH DEMENTIA AND THEIR CAREGIVERS

Ellen Brown[1], Nicole Ruggiano[2], Sai Allala[3], Gabriel Puche[3], Lisa Roberts[3], C Framil[3], Mariateresa Munoz[3], and Monica Hough[3], *1. Nicole Wertheim College of Nursing and Health Sciences, Florida International University, Miami, Florida, United States*, *2. University of Alabama, Hoover, Alabama, United States*, *3. Florida International University, Miami, Florida, United States*

People living with dementia (PLWD) often experience communication deficits that create frustration and challenges to caregiving and clinical care for this population. It also constrains person-centered care. To address communication challenges, an interdisciplinary team developed a mobile communication app that promotes communication by PLWDs about their daily care preferences and experiences. This app, My Person Assisted Touchscreen Interface (My PATI), empowers PLWDs and their caregivers by supporting communication with a fully-customizable platform. This platform allows the PLWD to express preferences for daily activities, such as preferred foods and clothing options. It also enables the PLWD to express their experiences relevant to symptoms of pain, depression, and sleep quality. This presentation will describe a multi-phase Quality Implementation Framework that the research team has applied in developing My PATI to maximize its potential for being adopted into the home and residential care settings and used by multiple caregivers. The presentation will include the My PATI implementation video and foundational research: qualitative interviews with PLWDs, caregivers of PLWD, alpha and beta testing with dyads of caregivers and PLWDs, and extensive assessment and development planning with clinicians, geriatric researchers, computer scientists, and a graphic artist to inform My PATI functions and design. The presentation will also describe the designs of future pilot testing and randomized control trial studies that are planned for 2024.

### SESSION 4420 (PAPER)

### MINORITY AND DIVERSE POPULATIONS (PAPERS) III

Abstract citation ID: igad104.1757

### A CULTURALLY-TAILORED INTERVENTION INFORMED BY CAREGIVER VOICES

Susanna Mage[1], Farnaz Hazany[2], Niki Cohanim[2], and Donna Benton[1], *1. University of Southern California, Los Angeles, California, United States*, *2. Sinai Adult Day Health Care Center, Los Angeles, California, United States*

The first wave of Iranian immigration to the United States occurred between 1950-1979. Now, as members of this cohort enter their 60's, the risk of age-related dementia will increase, necessitating younger, bicultural/bilingual members of the community, often family, to take on caregiving roles. Iranian caregivers are aware of neglecting their own health due to their role and often feel sad and lonely. Despite this, they are reluctant to access community resources intended to support caregivers. This could be attributed to the desire to protect and preserve the expected family image: by not using services, the family can maintain a sense of normalcy. The Ghofetegou Koneem for Caring Families Intervention helps caregivers in Los Angeles -- home of the largest Iranian community outside of Iran -- taking care of a person with dementia. The goal is to build a network of support that is personalized, easily accessible, and addresses perceptions of available social services. To tailor the intervention for an Iranian population, investigators partnered with a local adult day health care center to conduct 1:1 interviews with caregivers and perform needs assessments to identify needs prior to development. Qualitative analysis guided by a thematic approach revealed three themes of greatest need: 1) respite/self-care, 2) advocacy, and 3) disease-specific education and training. The importance of helping caregivers self-identify was also stressed, as this may lead to increased comfort in accessing resources. Integrating the voices of Iranian caregivers will help develop a culturally-relevant intervention tailored to the needs of Iranian families.

Abstract citation ID: igad104.1758

### EXAMINING SOCIAL AND CULTURAL FACTORS AMONG EAST ASIAN FAMILY CAREGIVERS OF PERSONS WITH DEMENTIA

Jessica Cassidy[1], Kathy Lee[1], Jihui Lee[2], Chang Hyun Seo[3], Alan Kunz Lomelin[1], Hye-Won Shin[4], and Joshua Grill[5], *1. University of Texas at Arlington, Arlington, Texas, United States*, *2. University of Pittsburgh, Pittsburgh, Pennsylvania, United States*, *3. University of Nevada, Reno, Reno, Nevada, United States*, *4. University of California Irvine, Irvine, California, United States*, *5. University California Irvine, Irvine, California, United States*

The purpose of this study was to examine how frequently Korean and Chinese American family caregivers of persons living with dementia utilized home- and community-based formal support services and to assess whether utilization of such services was associated with overall well-being. We collected and analyzed quantitative and qualitative data (N = 62) in effort to understand how cultural and social factors influenced their utilization of home- and community-based services and programs. A total of 36 Korean American and 26 Chinese American family caregivers completed online surveys and individual interviews over the phone. The most frequently used programs by family caregivers of both ethnic groups were in-home care services (33.9%), senior centers (32.3%), family caregiver support programs (24.2%), and transportation services (22.6%). Providing care longer than 108 months ($p = 0.047$), greater knowledge of information and resources available ($p = 0.043$) and being a Medicaid recipient ($p = 0.008$) were associated with utilization of in-home services. Those who utilized nutrition programs and case management were more likely to report higher overall well-being ($p < 0.05$). Four themes emerged from our qualitative analysis: (1) a lack of knowledge of formal services, (2) language barriers, (3) transportation barriers, and (4) a lack of culturally tailored formal supports. This study emphasizes community-level efforts to increase the utilization of formal support services by developing linguistically and culturally tailored programs, and offering transportation services in existing support programs and online-based education and resources that can be available in different languages.

Abstract citation ID: igad104.1759

### HELP-SEEKING IN A FOREIGN COUNTRY: EXPERIENCES OF OLDER LATINO IMMIGRANTS NAVIGATING ASSISTANCE IN AMERICA

Barbara Mendez Campos[1], Mary Waters[2], Saul Ramirez[2], James Hodges[1], and Rocio Calvo[1], *1. Boston College, Boston, Massachusetts, United States*, *2. Harvard University, Boston, Massachusetts, United States*

Access and utilization of assistance programs for older Latino immigrants focus on familismo or heavy reliance on family for help-seeking. The purpose of this paper is to document the different experiences of navigating services, including healthcare, for immigrants without family support. We also investigate the role of immigration status and age at migration on immigrants' strategies for accessing and using social services. We conducted 141 in-depth interviews with immigrants 60 and over from Cuba, Dominican Republic, El Salvador, Mexico, Puerto Rico, and Venezuela. We used a grounded theory approach to code and analyze the data. Three themes shaped the variation we found in the strategies used by older Latino immigrants without family support: formal assistance rooted in state and local policies; age at migration and the resulting patterns of individual agency and resources, and variation by country of origin that captured legal statuses as well as social networks. Our study provides a deeper understanding of a hard-to-reach population's access to and utilization of social services. It highlights the heterogeneity of Latino immigrants and elucidates differences from culture-based frameworks to understand Latino immigrants' underutilization of social services later in life. We argue for a shift to perspectives that address how multiple structural dimensions intersect to impact older immigrants' use of social services.

Abstract citation ID: igad104.1760

### IMPLEMENTATION SCIENCE: A STRATEGY FOR ADDRESSING HEALTH EQUITY AMONG RACIALLY DIVERSE OLDER ADULTS

Karen Bullock, and Barbara Mendez Campos, *Boston College, Boston, Massachusetts, United States*

Objective: Implementation science as a strategy for helping diverse populations of older adults address barriers to mental treatment, has become increasingly important since COVID19. This paper disseminates information about the results of an interdisciplinary intervention that tailored mental health services to racially and ethnically diverse community-dwelling older adults. Method: An interdisciplinary team of researchers (MD, APRN, MSW) performed an intervention that consisted of weekly visits to the community and provided diagnostic, triage, and psychosocial services to older adults. Evaluation of the intervention included data collected at baseline and three-month follow-up visits. **Results:** 350 elderly residents received mental health screening with some assessment of physical health and well-being. The number of visits per resident during the intervention phase, ranged from 1 to 11, with an average of 3. Tailoring the intervention to the specific cultural needs of the participants led to the development of a weekly community engagement group for monolingual Spanish-speaking residents and others. 49% of those receiving the intervention, self-reported their race and/or ethnicity as African American or Caribbean, 43% were Latino, and 8% were Caucasian, all of whom self-referred as participants in this health service outreach program. Initial evaluations resulted in referral to community providers (60%), team case management (20%), or no services needed (20%). **Conclusion:** Research studies designed to have positive impacts on older adults that have been historically underrepresented in health services research may improve its effectiveness by tailoring processes to be community informed and culturally responsive to structural and system barriers to treatment.

Abstract citation ID: igad104.1761

### OLDER MEDICARE BENEFICIARIES WITH KIDNEY FAILURE FROM PUERTO RICO MIGRATED AFTER HURRICANE MARIA

Maricruz Rivera-Hernandez, *Brown University School of Public Health, Providence, Rhode Island, United States*

The vast majority of the literature regarding migration often focuses on younger and healthy migrants. However, recent studies have shown that older adults with complex chronic conditions are migrating to receive services and supports from family members, especially after natural disasters. After Hurricane Maria landed in Puerto Rico in 2017, health care facilities struggled to provide care. Patients with complex chronic conditions faced significant challenges to receive care. The objective of this study is to characterize migration rates among older Medicare beneficiaries with kidney failure after Hurricane Maria. We conducted a cross-sectional analysis of older Medicare beneficiaries (65 and over) with kidney failure from Puerto Rico from 2010 to 2020. We used data from the Master Beneficiary Summary File and the Chronic Conditions Data Warehouse (CCW) Chronic Conditions Segment. Beneficiaries with kidney failure were identified using the End-Stage Renal Disease Indicator from the CCW. Outmigration was defined as changing the state of residence the following year. There were 9450 which contributed 28445 person-years of information. About 537 (1.9%) older adults with kidney failure migrated to the US mainland during the study period. Migration rates before Maria were 1.5 (95% CI, 1.3 – 1.7), which increased by 3.6 percentage points (95% CI, 2.8 – 4.4). However, migration rates dropped to pre-Maria levels after 2017. Older migrants were more likely to be females, fee-for-service beneficiaries and younger compared to non-migrants. These findings suggest that older adults with kidney failure migrated to the US mainland to access care immediately after Hurricane Maria.

### SESSION 4425 (SYMPOSIUM)

Abstract citation ID: igad104.1762

### HEALTH AND WELL-BEING ACROSS ECOLOGICAL SYSTEMS AND TIME IN MID LIFE AND LATE ADULTHOOD

Chair: Kelly Cichy Co-Chair: Robert Stawski Discussant: Robert Stawski

Health and well-being are shaped by peoples' experiences and ecological contexts (Bronfenbrenner, 2005), reflecting the influence of complex person-context relationships. For example, experiences (e.g., daily stressors, early childhood adversity) occur within microlevel (e.g., marital relationships, neighborhoods) and macrolevel (e.g., societal inequalities) contexts, impacting both daily well-being and healthy aging. Further, a robust literature highlights the significance of naturally occurring everyday experiences for a range of health outcomes, underscoring how daily experiences have immediate and lasting effects on individual physical and cognitive health and psychological well-being. Drawing on Bronfenbrenner's Ecological Model, the current symposium features novel research examining health and well-being outcomes across diverse ecological systems and time scales across midlife and old age, with the goal of articulating how innovative approaches for studying person-context interactions can be leveraged to understand both proximal and long-term health and well-being. Polenick and colleagues will examine associations between daily negative and positive events and well-being among heterosexual married couples living with early-stage dementia. Cichy et al. will examine gender differences in the prospective effects of daily stress processes on long-term chronic disease risk in midlife and later life. Next, Turner and Mogle will examine how positive and negative affect mediate the associations between daily memory lapses and psychological well-being. Finally, Choi, Munoz, Scott, and Sliwinski will examine whether current positive (i.e., neighborhood cohesion) and negative (i.e., neighborhood violence) neighborhood contexts moderate the effect of childhood stress exposure on cognitive function in adulthood. Robert Stawski will synthesize the presentations and offer insights for future research.

Abstract citation ID: igad104.1763

### DAILY STRESS CONTRIBUTES TO GENDER DISPARITIES IN LONG-TERM CHRONIC DISEASE RISK

Kelly Cichy[1], Athena Koumoutzis[2], Dakota Witzel[3], and Robert Stawski[4], *1. Kent State University, Kent, Ohio, United States*, *2. Miami University, Oxford, Ohio, United States*, *3. Penn State University, State College, Pennsylvania, United States*, *4. University of Essex, Colchester, England, United Kingdom*

Compared to men, women exhibit worse health outcomes, including increased risk for chronic disease (Carmel, 2019). Exposure and affective responses to daily stressors represent a risk factor for long-term health (Piazza et al., 2013) with research revealing a complex pattern of gender differences in affective responses to stressors (Birditt et al., 2005; Witzel et al. 2022). Together, findings suggest stressor exposure and affective reactivity may represent accessible intervention targets to reduce chronic disease risk and health disparities. Using data from Wave II of the National Study of Daily Experiences and MIDUS Waves II and III, this study explores: 1) gender differences in chronic disease risk; 2) prospective effects of differential exposure and reactivity on chronic disease risk 10 years later; and 3) whether daily stress processes (DSP) partially account for gender differences in chronic disease. Respondents include adults ages 34-84 years (N = 1209), who reported on daily stressors and affect for eight days during NSDE II and on chronic conditions during MIDUS III. Covariate-adjusted multilevel models revealed women reported more chronic conditions compared to men ($p < .0001$). DSP processes, including differential exposure ($p < .01$) and affective reactivity ($p < .05$) are associated with increased chronic conditions. Further, the magnitude of the gender differences in chronic conditions was reduced after accounting for DSP variables. Findings highlight the contribution of DSP for chronic disease risk and gender disparities in physical health. Implications for addressing DSP as accessible intervention targets will be discussed.

Abstract citation ID: igad104.1764

### DAILY NEGATIVE AND POSITIVE EVENTS AND DAILY WELL-BEING: DYADIC LINKS IN COUPLES LIVING WITH EARLY-STAGE DEMENTIA

Courtney Polenick[1], Diarratou Kaba[1], Nikita Daniel[1], Olivia Lambert[1], Shreya Salwi[1], Juhi Valera[1], and Kira Birditt[2], *1. University of Michigan, Ann Arbor, Michigan, United States*, *2. Institute for Social Research, University of Michigan, Ann Arbor, Michigan, United States*

Spouses have powerful influences on well-being in middle and later life, but we know little about how daily events impact well-being in older couples that include persons living with dementia (PLWD). We examined associations between daily negative and positive events and daily well-being (negative mood and positive mood) in 36 heterosexual married couples living with early-stage dementia. Participants included PLWD (M = 71.47 years, SD = 6.90, range = 53-82 years) and their spouses (M = 69.69 years, SD = 7.26, range = 59-87 years) who both completed brief morning and evening phone interviews over 7 consecutive days. Longitudinal actor-partner interdependence models controlled for own and partner reports of daily well-being in the previous day, whether each day was a weekend day, and the gender of PLWD. We estimated separate models for negative mood and positive mood. Both PLWD and their spouses reported higher negative mood when they reported more negative events in the same day and when their partner reported more negative events in the previous day. Over and above these associations, both partners in the couple reported lower negative mood on days when spouses of PLWD reported more positive events. Spouses of PLWD reported higher positive mood on days when they reported more positive events, but lower positive mood on days when PLWD reported more positive events. These findings highlight the importance of understanding dyadic influences on daily well-being among PLWD and their spouses.

Abstract citation ID: igad104.1765

### CHILDHOOD ADVERSITY, NEIGHBORHOOD CONTEXTS, AND COGNITIVE FUNCTION IN ADULTHOOD

Jean Choi[1], Elizabeth Muñoz[1], Stacey Scott[2], and Martin Sliwinski[3], *1. The University of Texas at Austin, Austin, Texas, United States*, *2. Stony Brook University, Stony Brook, New York, United States*, *3. Pennsylvania State University, State College, Pennsylvania, United States*

Childhood adversity may play an important role in cognitive health and aging. Neighborhood contexts in adulthood can serve as risks or resources that may modify the effects of childhood adversity on adult cognitive function, but these effects are not well-understood. We examined whether current positive (i.e., neighborhood cohesion) and negative (i.e., neighborhood violence) neighborhood contexts moderated the effect of childhood stress exposure on cognitive function in adulthood. We used data from 210 adults (Mage = 46.79, SD = 11.02; range: 25–65) residing in a single Zip code in Bronx, New York who completed measures of childhood adversity, current neighborhood contexts, and lab-based cognitive tasks. After adjusting for relevant demographic covariates, childhood adversity was not associated with cognitive function. The interaction between childhood adversity and neighborhood violence was significant, whereby lower childhood adversity was linked with worse performance in perceptual speed for individuals who reported both low (b = 1.19, SE = 0.37), p = .001) and high neighborhood violence (b = 0.75, SE = 0.26, p = .005), with higher scores indicating slower responses and worse performance. Unexpectedly, neighborhood violence was not predictive for those who reported greater childhood adversity. The interaction effect for neighborhood cohesion was not significant. Results indicate that childhood adversity may not have universal effects across all cognitive domains, but rather its effects are complex and may affect cognitive functioning to be adaptive to certain environmental contexts. This study underscores the need to replicate and examine the long-term consequences of these effects on cognitive aging.

Abstract citation ID: igad104.1766

### AFFECTIVE RESPONSES TO DAILY MEMORY LAPSES IMPACT PSYCHOLOGICAL WELL-BEING IN MIDUS

Jennifer Turner[1], Jacqueline Mogle[2], Nikki Hill[3], and David Almeida[4], *1. University of Hawaii at Hilo, Hilo, Hawaii, United States*, *2. Clemson University, Clemson, South Carolina, United States*, *3. The Pennsylvania State University, University Park, Pennsylvania, United States*, *4. The Pennsylvania State University, State College, Pennsylvania, United States*

Daily experiences have broad and far-reaching impacts on physical and mental health. Past work has linked micro-level experiences (e.g., daily stress) to macro-level processes (e.g., anxiety and depression; Charles et al., 2013) with a goal of understanding how day-to-day experiences influence development of physiological and psychological pathology. One mechanistic pathway for the link between daily experiences and longer-term outcomes are affective responses. This study extends previous work by examining mediation effects of positive and negative affect to a common daily experience: daily memory lapses on psychological well-being in a national sample of participants (N = 2,018; 56.6% Women, Mage = 56.92, SD = 13.37) from the MIDUS Refresher (2011-2014) and the MIDUS-3 (2013-2019). Each day participants reported on positive and negative affect and memory lapses. At the start of each wave, a comprehensive well-being assessment included different facets of psychological well-being (e.g., autonomy, environmental mastery). Daily memory lapses were significantly associated with increased negative affect and decreased positive affect on that same day. As expected, positive affect was associated with greater psychological well-being, while negative affect was associated with an inverse relationship. Importantly, significant indirect (mediation) effects were obtained for all models. This suggests that daily memory lapses have an impact on psychological well-being through their impacts on daily affect. This work provides increased evidence for the importance of emotional responses to daily stressors (such as memory lapses) being a determinate of distal health-related outcomes.

### SESSION 4430 (SYMPOSIUM)

Abstract citation ID: igad104.1767

### THE PAID AND FAMILY DEMENTIA WORKFORCE IN THE COVID-19 PANDEMIC ERA

Chair: Karen Donelan

The COVID-19 pandemic has had a disproportionate impact on persons living with dementia and their carers. The early period of the pandemic led to drastic changes in dementia care at home, in healthcare services, community programs, and institutional care settings, with lasting effects (Capstick et al., 2022; Giebel et al., 2022). This symposium describes the impact of the COVID-19 pandemic on a variety of individuals providing care for persons living with dementia through different stages of the pandemic. Our work will be presented within a translational framework and include a discussion of ways of leveraging findings to improve resources for individuals and caregivers living with dementia in the COVID-19 era. Karen Donelan will provide an overview of the dementia workforce and describe study procedures. Michael Vetter will describe findings from a survey study of self-efficacy of dementia caregiving for family caregivers of individuals in community and assisted living facilities (ALF). Inga Antonsdottir will describe survey data on family caregivers in community and ALF – focus on newer experiential members; how lives changed, how COVID impacted them. Esteban Barreto will present findings describing the impact of the COVID-19 pandemic on frontline staff. Finally, Sarah Bannon will present findings from a qualitative study identifying family caregivers' impressions of their psychosocial stressors and enacted coping strategies in the post-vaccine era of the COVID-19 pandemic.

Abstract citation ID: igad104.1768

### THE NEEDS OF FAMILY CAREGIVERS OF PERSONS LIVING WITH DEMENTIA CARED FOR IN PRIMARY CARE PRACTICES

Michael Vetter, *Brandeis University, Waltham, Massachusetts, United States*

**Background** The proportion of persons living with dementia (PLWD) in the US is projected to increase; the majority will be cared for by family caregivers and primary care providers. Purpose: At baseline for intervention studies to support primary care practices caring for persons with dementia, we assessed caregiver self-efficacy to inform our interventions. **Methods:** We conducted a survey of 143 caregivers of persons living with dementia receiving care from 34 primary care practices in a Medicare ACO. The survey was conducted by postal mail and telephone in English and Spanish and used 9-item caregiver self-efficacy scale.4 , analyzing variation by caregiver relationship and location. Results A majority of 143 respondents were female (70%), White Non-Hispanic (80.4%) and lived with person with dementia (65.7%). Caregivers agreed that their doctors understand how memory or behavioral problems complicate health (85.3%) and least likely to agree that they had a health professional who helped them work through dementia care problems (39.2%). Only 45% agreed that they received advice on what problems to expect in the future related to Alzheimer's or dementia. Caregivers who lived with the care recipient were significantly less likely than those who did not to report getting advice about common behavioral problems (43.6% vs. 68.6%, P = .005) and how to access community services (63.8% vs. 81.3%, P = .03). Discussion System innovations to support caregivers should tailor information to help caregivers of and PLWD to prepare for course of illness including treatment and management of behavioral symptoms of dementia.

Abstract citation ID: igad104.1769

### THE IMPACT OF THE COVID-19 PANDEMIC ON FAMILY CARE PARTNERS OF PERSONS WITH DEMENTIA IN ASSISTED LIVING FACILITIES

Inga Antonsdottir, *Johns Hopkins University, Baltimore, Maryland, United States*

**Background:** The Covid-19 pandemic affected persons living with dementia and their family care-partners. Family care-partners are diverse, including spouses, children, and friends. Care-partners may live with the care recipient, or apart from them. Covid-19 policies and priorities isolated care-partners living at home, while banning others from supporting their loved ones in assisted living facilities (ALF). Purpose: The purpose of this study was to examine the impact the covid-19 pandemic had on family care-partners of persons living with dementia. **Methods:** This study used an online survey of 133 family care-partners (89 living in ALF, 44 living in the community). **Results:** Among family care-partners, 34.8% of those who lived in an ALF reported a severe change in routine compared to 38.6% of those living in the community. 78.7% of ALF care-partners reported that they were unable to visit their loved one in a care facility, compared to 31% of care-partners living in the community. 67.4% of ALF care-partners were separated from the person with dementia that they cared for, compared to 22.7% of care-partners living in the community. 70.5% of CPs in the community reported they had to spend a lot more time taking care of a family member with dementia compared to 13.5% of ALF CPs. **Conclusion:** Unique differences exist in how the pandemic impacted the lives of family care-partners of community-based or ALF-based persons living with dementia. Understanding these differences can help tailor policies and healthcare priorities to best support family care-partners living in community or ALF settings.

Abstract citation ID: igad104.1770

### IMPACT OF THE COVID-19 PANDEMIC ON FRONTLINE PAID DEMENTIA WORKFORCE STAFF

Esteban Barreto, *Massachusetts General Hospital, Boston, Massachusetts, United States*

**Background:** Patients, families and the frontline paid workforce caring for persons with dementia faced dramatic change and high risk in the time of COVID-19 due to underlying vulnerability toward the disease as well as isolation, lockdown, and visitation restrictions. Purpose: The purpose of this study was to examine the paid and informal care workforce caring for persons with dementia at home, in assisted living and skilled nursing facilities. This analysis will focus on the impact on paid workers in ALF and SNF. **Methods:** We conducted online and paper surveys of 182 workers caring for persons with dementia during the COVID-19 pandemic in several assisted living facilities in New England (n=82) and a large skilled nursing facility in Florida (n=100) **Results:** A majority of workers were not married and had less than college level education. 66% knew patients who died of COVID-19; 90% or more said they were exposed to the virus personally. Half reported mild, moderate or severe changes due to the pandemic on their personal access to health care, food security and sleep. Workers in ALF were more likely to report only good or fair personal health status. Most workers indicated they had sufficient education about infection control. **Conclusion:** The workforce in long term services and supports has been the slowest to recover from job losses during the pandemic. Further studies are needed to inform the health and social needs of workers caring for persons with dementia.

Abstract citation ID: igad104.1771
**THE IMPACT OF THE COVID-19 PANDEMIC ON INFORMAL CAREGIVERS OF PERSONS WITH DEMENTIAIN THE POST-VACCINATION ERA**
Sarah Bannon, *Mount Sinai Hospital, New York City, New York, United States*

The COVID-19 pandemic disproportionately impacted individuals living with dementia and their family care-partners. Prior to the pandemic, many family care-partners had insufficient support to manage stressors and experienced resultant negative physical and mental consequences. Systematic reviews of studies from the early pandemic suggest that circumstances dramatically worsened for family care-partners', though it is unclear whether and how they were impacted by ongoing fluctuations in COVID-19 risk for infection and safety guidelines following the availability of vaccines. Using qualitative design informed by a stress-and-coping theoretical framework, our objective was to characterize the ongoing impact of the pandemic on family care-partners of persons living with dementia. We used purposive sampling to recruit participants from a larger survey study of dementia caregiving during the COVID-19 pandemic. Study participants (n=18) completed a 1-hour virtual interview with that was transcribed, de-identified, and analyzed using thematic analysis. We identified 4 overarching themes characterizing family caregivers' stressors that included: 1) changes in care, routines, and support; 2) actual or feared COVID-19 infection; 3) changes in the functioning and health of care-partners and care-recipients; 4) experiences with care facilities care transitions. We also identified 4 overarching themes characterizing care-partners' coping strategies, including: 1) care-partner resourcefulness; 2) participation in meaningful, enjoyable, and restorative activities; 3) personal outlook and attitudes to reduce distress; and 4) connection with specific formal and informal supports. Our findings underscore the continued challenges that family care-partners experience and highlight important skills, supports, and resources to mitigate negative health outcomes and other long-term consequences.

### SESSION 4435 (SYMPOSIUM)

Abstract citation ID: igad104.1772
**POLICY SERIES: DOCTORAL PROGRAMS IN GERONTOLOGY: PROVIDING LEADERSHIP IN POLICY AND AGING**
Chair: Edward Miller Discussant: Brian Kaskie

Advancements in education, medicine, wealth, technology, and population health have increased individual life expectancies. Healthy aging is not just a function of healthy genes, however; aging is modified by the broader context, which, in turn, creates opportunities and challenges that must be addressed by society. Lack of organized responses to population aging exacerbate social inequities based on workplace discrimination, displacement, and impoverishment, and contribute to public health inequalities pertaining to disease and disability, isolation, and vulnerability among older adults. Public policy offers one of the most effective ways to address inequities and inequalities such as these. This symposium spotlights four doctoral programs that prepare gerontologists who possess subject matter expertise in aging policy, conduct reliable policy surveillance and sourcing, execute empirical evaluations of policy formation and implementation, and offer well-informed contributions to policy makers who are most involved with supporting and protecting older adults. These well-established academic programs at the University of Massachusetts Boston (Edward Alan Miller, PhD), University of South Florida (Lyndsay Peterson, PhD), University of Southern California (Kathleen Wilbur, PhD), and Miami University (Jennifer M. Kinney, PhD) offer classroom education, research, and service-learning opportunities for students to acquire expertise in aging and public policy. Together, these programs provide models for training a generation of leaders in gerontology who can contribute productively to policy development and debates, illuminate structures and processes of policy making and implementation, and deploy analytic skills needed to evaluate policies relative to multiple and often competing interests.

Abstract citation ID: igad104.1773
**AT THE NEXUS OF PUBLIC POLICY AND AGING: DOCTORAL STUDIES IN GERONTOLOGY AT THE UNIVERSITY OF MASSACHUSETTS BOSTON**
Edward Miller, Jeffrey Burr, Kathrin Boerner, Nina Silverstein, and Jan Mutchler, *University of Massachusetts Boston, Boston, Massachusetts, United States*

One in five PhDs in Gerontology are graduates of the Gerontology doctoral program at the University of Massachusetts (UMass) Boston. The mission of UMass Boston's program is to provide advanced training and research in gerontology within an interdisciplinary framework drawn primarily, but not exclusively, from the social and behavioral sciences. The program is designed to prepare students for leadership roles as educators, researchers, planners, and policy makers. The program has a deep emphasis on policy development, analysis, and impact, an attribute that goes back to the roots of the Gerontology enterprise at UMass Boston in the late-1970s. This presentation provides an overview of the program's history and curriculum, along with coursework, research and service-learning experiences, and career paths in policy research and implementation. The program also is committed to increasing diversity in the field of gerontology, with the aim of strengthening policy impact and reducing disparities in the aging experience.

Abstract citation ID: igad104.1774
**THREE DECADES OF RESEARCH GUIDING POLICY AND POLICY GUIDING RESEARCH IN AGING AT THE UNIVERSITY OF SOUTH FLORIDA**
Lindsay Peterson, *University of South Florida, Tampa, Florida, United States*

The Florida Legislature created the Florida Policy Exchange Center on Aging more than 30 years ago to conduct independent research and policy analyses on critical topics in aging. Housed at the University of South Florida (USF), the center brought together students and faculty to examine public policies that directly affect the health and well-being of older adults (e.g., the role of Medicaid funding in home- and community-based care and the effects of nurse staffing levels in nursing homes). As new policy questions emerge, the foundational work of the policy center continues to guide undergraduate and graduate education at the USF School of Aging Studies. This presentation describes the multiple opportunities to incorporate policy instruction into an interdisciplinary gerontology program. These opportunities depend on having faculty who maintain ties with the organizations that provide direct care and services to the public and who connect the policy lessons they learn through these relationships to their teaching and research. Another key element is access to health services data needed to answer critical policy questions. The goal of such an approach is to enable gerontology students to engage in policy analysis and development and to understand the essential links between research, public policy, and practice that improves the lives of older adults.

Abstract citation ID: igad104.1775

### LESSONS FROM FOUR DECADES IN AGING POLICY AT THE UNIVERSITY OF SOUTHERN CALIFORNIA: WHAT A LONG FINE TRIP IT'S BEEN

Kathleen Wilber, Maria Henke, and Mireille Jacobson, *University of Southern California, Los Angeles, California, United States*

Aging policy has come a long way over the last four decades. Building on the history of leadership in policy and aging at the University of Southern California's Leonard Davis School of Gerontology, we offer six lessons from the intersection of policy, practice, and research for researchers and for those interested in careers as policy gerontologists. Lessons include: 1) Researchers must communicate scientific truths clearly, humbly, often, relevantly, and broadly to a wide array of key stakeholders; 2) To continue to build the bench for the next generation of leaders, interested educators should convene to plan a Policy Leadership Program 2.0. 3) Researchers and practitioners should remember: If everything is going according to plan, something is wrong; 4) Those considering policy research, program development, or program evaluation should recognize that it can be funded by a variety of sources with different strengths and constraints; 5) Given a host of innovative ideas, perspectives, programs and services, in the aging policy space, evaluators and researchers will learn far more from those doing policy and practice work than they can ever hope to repay; 6) Progress is not linear nor does it consistently advance in the hoped for direction. It requires patience, persistence, and stamina.

Abstract citation ID: igad104.1776

### IT WORKS FOR US: BRIDGING THE GAP BETWEEN EDUCATION, PRACTICE, AND POLICY IN GERONTOLOGY AT MIAMI UNIVERSITY

Jennifer Kinney, Katherine Abbott, Sara McLaughlin, Leah Janssen, and Robert Applebaum, *Miami University, Oxford, Ohio, United States*

We describe a unique and successful partnership between the Department of Sociology and Gerontology and the Scripps Gerontology Center at Miami University in Oxford, Ohio. This partnership concurrently facilitates research that informs aging policy and gerontological training for students. After a brief description of the partnership, examples of current research that contributes to public policy at the federal, state, and local/provider levels are presented, along with students' roles in these efforts. Next, we share factors that contribute to our success (e.g., attracting students from diverse backgrounds, using a shared mentorship model) and challenges (e.g., that research and policy move at different paces). We conclude with a look to the future, specifically, the need to engage students in our academic programs, maintain our long-standing relationships, and build new, cross-organizational ties so that future gerontologists will continue our mission of doing work that positively impacts older people.

### SESSION 4440 (SYMPOSIUM)

Abstract citation ID: igad104.1777

### PREPARING FOR ADVANCING ILLNESS AND END OF LIFE WITH DEMENTIA

Chair: Jenny van der Steen

Dementia involves coping with advancing illness that involves cognitive and functional decline. Also decision-making capacity diminishes, which implies that preparing for a future and taking opportunities to exercise individual and relational autonomy is particularly relevant in the case of dementia. Engaging the person with dementia and family caregivers meaningfully in formal or informal advance care planning conversations may require addressing specific challenges. Health care providers often lack time or are reluctant to broach these conversations. Often conversations are delayed even in institutional long-term care settings as residents, their family caregivers and care providers may all view advance care planning as uncomfortable and difficult to initiate. Special support for health care providers to develop skills, confidence and taking initiative is needed. This symposium presents about new, large qualitative and quantitative studies with contributions from the UK and Canada, and international work. It addresses preparing for an uncertain future through advance care planning and decision making about future care and treatment which may include conversations about the end of life. We present and evaluate tools to support the process across settings.

Abstract citation ID: igad104.1778

### ENGAGING PEOPLE WITH DEMENTIA AND THEIR FAMILY IN ADVANCE CARE PLANNING: A 33 COUNTRY DELPHI STUDY

Jenny van der Steen[1], and Ida Korfage[2], *1. Leiden University Medical Center, Leiden, Zuid-Holland, Netherlands*, *2. Erasmus MC, University Medical Center Rotterdam, Rotterdam, Zuid-Holland, Netherlands*

Reaching a consensus about a definitional framework of advance care planning in dementia was a main goal of an international Delphi study conducted by the European Association for Palliative Care. Four rounds with a panel of 107 experts from 33 countries were conducted between September 2021 and June 2022. The framework comprised a definition and three issues that are of particular importance in the case of dementia, which were capacity, family, and engagement and communication. Focusing on the latter, we provide recommendations that achieved a consensus in the Delphi study. We also interviewed persons with dementia and provide their recommendations for health care providers directly offered by them which the panel found eye-opening. The framework fits dementia and is unique in that it focuses on a communication process that continues independent of the level of capacity, beyond a focus on documenting specific current or end-of-life treatment preferences. It evolved in consensus with experts from western and non-western countries and it should promote inclusiveness of persons with dementia and their family caregivers. The more inclusive conceptualization of what advance care planning in dementia is, provides a much-needed basis for research and policy initiatives on advance care planning in dementia. Further, the work also highlights ambiguity such as about when family caregivers can act as proxies and when not, and the methods may be used as a template for developing frameworks on advance care planning with other specific conditions.

Abstract citation ID: igad104.1779

### DISCUSSING THE FUTURE FOLLOWING A DIAGNOSIS OF DEMENTIA. FINDINGS FROM THE PREPARED STUDY (ENGLAND)

Josie Dixon, *London School of Economics and Political Science, London, England, United Kingdom*

The PrepareD study (England) adopts a relational autonomy perspective to examine the various conversations that people with dementia and their families have about the future, with each other and with health and care providers, in the year following a dementia diagnosis. We present survey data gathered from a large cohort of 900 people in England who have been recently diagnosed with dementia and their caregivers about informal conversations about the future and advance care planning conversations with health and care providers. We also present findings from in-depth qualitative research with 30 of these respondents, a purposively selected mix of people with dementia and caregivers. Findings from the qualitative research cover how people with dementia and family members interact; the objectives, hopes and expectations they have for their conversations; and how input from professionals and wider social influences shape these interactions. We use findings from the study to reflect on what effective support for informal conversations between people newly diagnosed with dementia and their families about future care could look like and how, and by whom, it could be provided. We also aim to identify theoretical models of relational autonomy that could be useful for developing these approaches further.

Abstract citation ID: igad104.1780

### DEVELOPMENT OF A DECISION SUPPORT FRAMEWORK TO PROMOTE COMFORT AMONG PATIENTS WITH SEVERE DEMENTIA IN THE HOSPITAL

Nathan Davies[1], Ellen McCoy Smith[2], Malvi Shah[1], and Elizabeth Sampson[1], *1. University College London, London, England, United Kingdom*, *2. South West London and St George's Mental Health NHS Trust, London, England, United Kingdom*

Hospital wards can be challenging settings, particularly for those with dementia, and exacerbate symptoms and behavioural issues. Improving care for persons with dementia and providing a simple decision support framework to help guide staff in person-centred and holistic management of these problems has the potential to improve hospital care. We aimed to co-design an evidence-based, practical way to support hospital staff and family carers in identifying discomfort and distress, maximising comfort and reducing behaviours that challenge or are distressing to people with dementia who are towards the end-of-life. We conducted a co-design study informed by data from 1. Literature review; 2. Cohort study of comfort and discomfort in people with severe dementia admitted to hospitals (n=63); and 3. Interviews with family carers and health care professionals (n=18). These data sources were synthesised and presented to key stakeholders through co-design meetings and workshops. The resulting decision support framework uses a three-stage process of: 1) assessment of comfort/discomfort; 2) consider causes of discomfort; and 3) address needs of the individual to manage the discomfort. The framework is presented in the form of a series of flowcharts that can be used easily on busy wards. We will present examples of the framework along with key findings from the cohort study. This is the first study to develop decision support for hospital staff and families addressing comfort towards the end of life. This toolkit has practical implications for everyday working on hospital wards but also a resource for planning for end-of-life care.

Abstract citation ID: igad104.1781

### IMPROVING THE ENGAGEMENT OF RESIDENTS WITH DEMENTIA IN ADVANCE CARE PLANNING DISCUSSIONS IN LONG-TERM CARE

Sharon Kaasalainen[1], Abigail Wickson-Griffiths[2], Shirin Vellani[1], and Tamara Sussman[3], *1. McMaster University, Hamilton, Ontario, Canada*, *2. University Of Regina, Regina, Saskatchewan, Canada*, *3. McGill University, Montreal, Quebec, Canada*

As part of the Strengthening a Palliative Approach in Long Term Care (SPA-LTC), we have designed and evaluated a number of tools and resources to promote a palliative approach in dementia, including a Canadian version of the Conversation Starter Kit (CSK) booklet. We used participatory action research to design tools for use with care providers based on survey data and interviews. Data was analyzed using thematic analysis and descriptive statistics. We found that residents reported that they engaged in asking more questions to their care provider after completing the CSK booklet. However, care providers stated that they did not feel comfortable addressing questions and concerns raised by residents and their family members. Hence, we co-designed two tools to help build capacity among care providers to support ACP discussions with residents and families, including an e-learning module about ACP discussions for people with dementia and a Conversation Guide. Pilot findings of these tools suggest that staff appreciate the information and tools to enable them to initiate ACP discussions and address difficult conversations. Clearly, residents with dementia and their families need to be supported to engage in ACP discussions early on in their disease trajectory so that residents are afforded the opportunity to voice their values and wishes for end of life care. At the same time, care providers need to have the skills (e.g., listening, probing) and comfort to engage in these discussions to support and help prepare residents and families for important decisions that need to be made later on.

### SESSION 4445 (SYMPOSIUM)

Abstract citation ID: igad104.1782

### COMMUNITY-BASED RESEARCH ON GRIEF SUPPORT FOR OLDER ADULTS: FROM EXPLORATION TO IMPLEMENTATION

Chair: Lisa Stone-Bury Co-Chair: Rachel Weiskittle

Community-based research has initiated a shift from research existing in an ivory tower to being collected and then implemented for the direct benefit of the public. The social sciences continue to struggle with the balance between rigorous science and feasible interventions, creating a common disconnect between research and practice. This is particularly relevant to grief and bereavement, for which services are often under-utilized among older adults. This symposium will discuss a multidisciplinary team's application of the Exploration, Preparation, Implementation, and Sustainment (EPIS) framework, describing the development, findings, and service outcomes of a community-based research initiative within a mid-sized city. First, Lisa E. Stone will describe the challenges and successful strategies in the collaborative process of questionnaire development with multidisciplinary community stakeholders. She will also present on the methodology used to attain large community and grief professional samples, including recruitment strategies for adults across the lifespan. Second, Caroline Collins-Pisano will present key age-related findings from the community study, highlighting the lifespan factors that impact grief experiences and utilization of grief resources among older adults. Lastly, Rachel E. Weiskittle will present on the team's process of implementing data-driven grief interventions, focusing on the translation of community-based findings to multifaceted grief services for older adults. In sum, this symposium presents a community-based application of the EPIS framework that focuses on direct, tailored, and immediate implementation of grief services, a consistent need for the aging population.

Abstract citation ID: igad104.1783

### EXPLORATION OF THE NEED FOR GRIEF SERVICES: METHODOLOGY OF A COMMUNITY-BASED RESEARCH STUDY

Lisa Stone-Bury, *University of Colorado Colorado Springs, Colorado Springs, Colorado, United States*

Within the Exploration, Preparation, Implementation, and Sustainment (EPIS) framework, community-based research (CBR) is a key component of Exploration and Preparation. This presentation describes the process of conducting a CBR study on grief service needs within Colorado Springs. A community-academic partnership was formed, resulting in a multidisciplinary team of clinical geropsychology academics, a chaplain, a mental health professional, and a funeral director, with the goal of launching a non-profit grief center that catered to identified local needs. Challenges and successes in this partnership will be explored. Two surveys were created: (1) community-wide survey targeting adults across the lifespan and (2) professional survey targeting individuals who interface with grief in their profession (e.g., healthcare providers, educators, lawyers, faith leaders). The authors facilitated survey development that balanced practical needs with existing empirical literature on grief services. Qualitative and quantitative measurement focused both on past experiences with grief (to understand the community's history) and use of future grief services (to understand the types of services most desired by the community). The multidisciplinary team pooled resources related to recruitment and dissemination of the surveys, combining traditional research recruitment practices (e.g., undergraduates, research database of older adults) with networks of local community leaders (e.g., places of worship, local healthcare agencies, newspapers). These CBR efforts resulted in large samples of community members (n = 668; age range = 18 to 65+) and professionals (n = 141). Ultimately, this CBR study laid important groundwork to prepare the multidisciplinary team to match their services to the community's expressed needs.

Abstract citation ID: igad104.1784

### UNDERSTANDING AGE DIFFERENCES IN GRIEF SUPPORT NEEDS: RESULTS FROM A COMMUNITY-WIDE SURVEY

Caroline Collins-Pisano, *University of Colorado Colorado Springs, Colorado Springs, Colorado, United States*

Experiences of grief and bereavement differ across the lifespan. As such, needs for community grief services may vary based on age. This presentation outlines age differences found in the community-wide survey conducted during the Exploration and Preparation phases of the EPIS framework. A total of 668 community members within an age range of 18-65+ completed the survey. The majority of participants were 18-29 (n = 358, 54%), 28% were 65 or older (n = 186), 11% ages 50-64 (n = 71), and 8% ages 30-49 (n = 52). Survey results revealed both age differences and similarities in experiences of loss, participation in past grief services, likelihood of participating in future grief services, and barriers to participating in services. Respondents aged 65 or older experienced significantly greater lifetime loss compared to other age groups, with 100% reporting having experienced the death of someone significant to them. Yet, older adults and adults aged 30-64 reported similar rates of participation in past grief services. However, while older adults shared the least barriers to participating in grief services, they reported similar likelihoods of participating in a variety of future grief services as adults aged 30-49 or 50-64. The survey demonstrated that while older adults experienced the greatest amount of loss, they did not report the highest overall likelihood of utilizing grief services. Rather, each age-group presented a distinct and nuanced hierarchy of preferred grief services and needs, the details of which will be explained further in this presentation.

Abstract citation ID: igad104.1785

### IMPLEMENTING GRIEF SERVICES FOR OLDER ADULTS: TRANSLATING SURVEY FINDINGS INTO TARGETED PROGRAMMING

Rachel Weiskittle, *University of Colorado Colorado Springs, Colorado Springs, Colorado, United States*

This presentation will identify and describe the team's ongoing efforts in actuating the Implementation phase of the EPIS Framework. Evidence-based strategies include formulating a multidisciplinary implementation team, organizing purveyor and intermediary support, and developing a Monitoring, Evaluation, and Learning (MEL) plan. We will highlight the ways in which survey findings were disseminated to the general public and organizational leaders, such as presenting result summaries at city council meetings and in local newspaper articles. The challenges of negotiating survey findings into cohesive program implementation will be discussed, as well as management strategies towards age-related gaps in the implementation process that had not been identified previously. Recent program launches and the ways in which survey results directly informed their delivery will also be reviewed, namely, a free educational guest-speaker event for which over 700 community members attended and a grief-support holiday vigil hosted at a local long-term care facility. This presentation will also review considerations for the project's next steps as it continues to hone its program delivery parameters and eventually enters into the last EPIS phase, Sustainment. Specific foci will include deciding the long-term methods of quality assurance evaluation and program adaptations to revolving community bereavement needs.

### SESSION 4450 (SYMPOSIUM)

Abstract citation ID: igad104.1786

### PARTICIPATION IN AND EXCLUSION FROM EXTENDED LATE WORKING LIFE DURING THE DEMOGRAPHIC, DIGITAL, AND GREEN TRANSITION

Chair: Andreas Motel-Klingebiel

Policies aim to extend working lives by investing in the capacity and employability of older workers, changing the regulatory framework, promoting innovative life-course policies, and advancing lifelong learning, which gains relevance under the impact of increasingly aging populations, digitalisation, and the green transition. However, as some national, branch, and company policies are counterproductive, encouraging early exit and perpetuating ageist training and recruitment practices, working longer may not be equally achievable and beneficial for all workers. This symposium presents research on exclusion and inequality in late working life, providing evidence for policy innovation towards inclusive extended work and sustainable working conditions. It combines comparative multi-level research based on registry and survey data as well as qualitative information. Contributions address the opportunities and limitations of equal and inclusive extended work under the impact of population aging, digitalisation, and the green transition. This includes a conceptual introduction to research and policy on extended late work in Europe during the triple demographic, digital and green transition, an analysis of age discrimination in company and branch policies based on expert interview data, an examination of the perceived significance of lifelong learning for exclusion risks, an analysis of risk experiences associated with extended work, and a commentary discussing the findings in the context of gerontological debates on exclusion and precarity in later life. The (co-)authors represent a mix of early career, mid-career, and senior scholars with interdisciplinary backgrounds from Europe and North America. The audience is invited to discuss concepts, analyses, and conclusions with the presenters.

Abstract citation ID: igad104.1787

### LATE WORKING LIFE IN THE TRIPLE TRANSITION AND THE GLOBAL PERMACRISIS

Andreas Motel-Klingebiel[1], Gerd Naegele[2], Gulin Oylu[3], Indre Genelyte[3], and Susanne Kelfve[4], *1. Linkoping University, Norrköping, Ostergotlands Lan, Sweden, 2. Institut fur Gerontologie, Dortmund, Nordrhein-Westfalen, Germany, 3. Linkoping University, Linkoping, Ostergotlands Lan, Sweden, 4. Linköping University, Norrköping, Ostergotlands Lan, Sweden*

Late working life faces multiple challenges in the triple transition of demographic, technological and environmental change and the global permacrisis. This contribution provides a conceptual framework for research on the significance of extended late work for the sustainability, equity, inclusion, and productivity of aging societies, based on an understanding of the three major long-term shifts as a complex triple transition with potentials and challenges for societies, economies, employers, and individuals. This triple transition is embedded in an ongoing and even accelerating societal and economic permacrisis with increased social risks and limited predictability, in which resilience – of economies, life-courses and welfare states - gains importance, while the dominant tetrad of sustainability, equity, inclusion and productivity remains on the European social and economic policy agenda. The presentation introduces a review of European policy reports on aging and late work and introduces results from the research programme EIWO (www.eiwoproject.org) based on Swedish register information and European survey data to illustrate trends and gaps in European late work, research, and policy. The presentation discusses age integration of life and work courses as a systematic strategy to promote quality of life as well as social sustainability, equity, inclusion and productivity and resilience. Participants will gain an understanding of the conceptualization and implementation of gerontological multi-level research on the macro- and micro-social relevance of late working life in the triple transition and the global permacrisis.

Abstract citation ID: igad104.1788

### THE PERSISTENCE OF AGE DISCRIMINATION IN SHAPING LATE WORKING LIVES

Rachel Crossdale[1], Liam Foster[2], and Alan Walker[2], *1. The University of Sheffield, Sheffield, England, United Kingdom, 2. University of Sheffield, Sheffield, England, United Kingdom*

The aging population and subsequent demographic shift have driven the Extended Working Lives (EWL) agenda to become an important policy goal. However, millions of older workers leave the workforce each year due to unsupportive work environments attributed in part or in whole to ageist policies and practices, raising questions about older workers' perceptions of themselves in the workforce and what this means for the EWL agenda. Using data from 25 qualitative interviews, our research finds that ageism continues to play a part in the decision-making process of older workers when considering their future. These findings fall into two categories: Age barriers within the workplace and Perceptions. Age barriers within the workplace include tasks or events that incite a feeling of separation from younger peers, such as training, social interaction, and digitization. Perceptions are older workers' internalized expectations of themselves and their abilities. Our findings suggest these perceptions do not align with the reality for older workers, thus contributing to internalized ageism. The need to target stereotypical attitudes towards older workers was highlighted as an age barrier to employment before the turn of the century. However, despite the Extended working Lives, Active Aging and Fuller Working Lives Agendas changing the policy context around older workers, this research suggests that the same stereotypes continue to pervade older workers both externally from policy and practice and internally in self-perceptions. A life-course approach to policy is needed to improve conditions for older workers and to challenge ageist stereotypes in a meaningful way.

Abstract citation ID: igad104.1789

### THE IMPORTANCE OF LIFELONG LEARNING FOR AVOIDING EXCLUSION—POLICY FRAMEWORKS AND EXPERIENCES OF OLDER WORKERS

Nehle Penning[1], Rachel Crossdale[2], and Monika Reichert[1], *1. TU Dortmund University, Dortmund, Nordrhein-Westfalen, Germany*, *2. The University of Sheffield, Sheffield, England, United Kingdom*

Qualification requirements in the labour market in Europe have increased across the board. Increasing provision and accessibility of lifelong learning is central to promoting employability among older workers. However, older workers are more likely to lack formal and to hold out of date qualifications. Sweden, Poland, Germany and the UK, despite differences in regime and political approach to lifelong learning, have unequal distribution of access and opportunity. This paper examines how older workers in the four countries assess the role of lifelong learning throughout working life as well as what links to national policies can be identified. The analysis is based on 100 semi-structured and problem-centred interviews in the four countries with older workers (age 55 and older) of different backgrounds (gender, educational level, sector of employment, ethnicity). Content analysis according to Kuckartz (2018) was used as method of analysis. The interview data shows that lifelong learning plays an important role for older workers in the prevention and/or management of exclusion risks throughout working life. However, the potential of lifelong learning for EWL is limited by barriers at the company and regulative level and differences between the public and private sector are noticeable. In late working life, in particular financial constraints as well as ageism lead to inequalities in relation to lifelong learning opportunities. In all four nations a higher priority need to be given to an integral support of lifelong learning throughout the life-course as well as age stereotypes need to be dismantled for a successful EWL agenda.

Abstract citation ID: igad104.1790

### OLDER WORKERS' AND EMPLOYERS' PERSPECTIVE ON EXTENDING WORKING LIFE: THE CASE OF POLAND

Jolanta Perek-Białas[1], Anna Urbaniak[2], Natalia Krygowska-Nowak[2], and Maria Varlamova[2], *1. Jagiellonian University, Kraków, Malopolskie, Poland*, *2. Jagiellonian University Krakow, Krakow, Malopolskie, Poland*

Extending working life is considered an adequate policy response to population aging and workforce shrinkage. However, in some countries, like Poland, there are still no clear mechanisms supporting extending working life. Poland makes an interesting case, as while the eligible retirement age is one of the lowest in Europe, no plans to increase it are set. The aim of this paper is to present perspectives of older workers and employers on extending working life. We identify factors that constitute supportive and unsupportive working environments from the perspective of older Polish workers and show how employers perceive workers over 50 in the workplace. First, the qualitative component of the programme EIWO 'Exclusion and Inequality in Late Working Life' is analysed. We draw from 25 semi-structured interviews conducted between June and November of 2021 with men and women aged 55-75 years old working in private and public sectors in Poland. Secondly, the probability survey among Polish employers (n=1,037) was analysed to show the attitudes and perceptions of employers. Older workers working in the public sector feel less satisfied with the financial aspects of employment, less motivated to extend their working lives, and more often describe practices of pushing older workers out of the labour market. The analyses of employees give matching results. These findings highlight that in the absence of a governmental strategy, extending working life is more dependent on employers and employees that might differ among sectors of employment.

Abstract citation ID: igad104.1791

### ILLUSTRATIONS OF EXTENDED WORKING LIVES: WHAT INSIGHTS DO THE CONCEPTS OF EXCLUSION AND PRECARITY OFFER?

Amanda Grenier, *University of Toronto, Toronto, Ontario, Canada*

Amanda Grenier will act as the discussant for the set of papers in this symposia session, bridging insights from European research with North American perspectives. Situated as an attempt to bridge research findings with a larger critical practice of theory-building theory, she will explore what the concepts of exclusion and precarity offer our interpretations of these papers, as well as how the findings of each contribute to the state of knowledge on exclusion and precarity. She will begin with a brief clarification of the definitions and conceptual boundaries of social exclusion and precarity carried out in Europe and North America and situate key findings of the papers as an effort to theorize older workers lives in contemporary conditions. For example, the well-known concept and domains of social exclusion can be considered to offer ways of understanding processes of exclusion at the mezzo level, as exercised through policies, practices, and/or place. Illustrations from the papers will be linked with existing work on exclusion. She will then draw attention to the concept of precarity as a lens of analysis to connect the micro-level vulnerabilities experienced in the working lives of older people with the shifting social and state structures that form the backdrop for older people's lives (and the systems within which European research is carried out). Specific examples will be drawn from the set of papers, and a moderated question and answer period will follow.

### SESSION 4455 (SYMPOSIUM)

Abstract citation ID: igad104.1792

### EXPLORING INEQUALITIES IN HEALTHY AGING: MANY APPROACHES BUT WHICH IS MOST USEFUL FOR POLICY?

Chair: Barbara Hanratty Discussant: Graciela Muniz-Terrera

Promoting healthy ageing requires an in-depth understanding of health inequalities. There are many different ways in which experiences of health and care may be inequitable, and numerous ways of studying this. In this symposium, we present findings from five disparate studies, united by an intention to shed light on unequal ageing. Our work utilises individual characteristics (frailty), aspects of care received (polypharmacy), access to services (digitalisation), different data sources (social care) and evidence synthesis methods, to characterise inequalities in later life, and inform policy. We start with a multi-method study of digitalisation in health services during the coronavirus pandemic, examining the impact of digital exclusion on inequalities in later life. Analysis of cohort data from the English Longitudinal Study of Ageing investigates associations between socio-economic measures and frailty free life expectancy in the over 50s. The impact of years of education on polypharmacy is explored in the Cognitive Function and Ageing Studies, and the value of information on social care provision exploited in analysis of real-world data. The final presentation will cover the development of a conceptual framework to ensure inequalities are considered within evidence synthesis Discussion will draw together the commonalities and differences of these approaches, and consider which have utility for policy makers, and how methods and approaches can be combined to produce an in-depth understanding of inequalities in healthy ageing.

Abstract citation ID: igad104.1793

### INEQUALITIES IN THE FRAILTY-FREE LIFE EXPECTANCY OF ADULTS IN ENGLAND AGED OVER 50

David Sinclair[1], Asri Maharani[2], and Fiona Matthews[1], *1. Newcastle University, Newcastle upon Tyne, England, United Kingdom*, *2. Manchester Metropolitan University, Manchester, England, United Kingdom*

Understanding how the onset of frailty differs between socio-economic groups will help health and care providers plan for increasing demand from an ageing population and highlight population groups to target interventions to reduce frailty. We aimed to calculate the frailty-free, frail and total life expectancies of adults aged over 50 using data from the English Longitudinal Study of Ageing (ELSA), and analyse associations with socio-economic characteristics. Survey data from ELSA waves 1-9 (2002-2019) was used to follow the frailty trajectories of individuals (n=11,423 at wave 1). Individuals were categorised into non-frail and frail states using a frailty index. A multistate model assessed the risk of individuals transitioning between states or dying. Transitions were associated with the participants' socio-economic characteristics and converted to life expectancies. Increased wealth, reduced deprivation, more education, and marriage are all associated with increased frailty-free and total life expectancies, and reduced frail life expectancies. Wealth is the most important socio-economic indicator of frailty-free life expectancy. At age 50, large inequalities in frailty-free life expectancies exist between the wealthiest, least deprived population (females: 36.1 [35.1-37.0], males: 34.6 [33.7-35.4] years) and the least wealthy, most deprived population (females: 22.1 [21.3-22.7], males: 21.3 [20.4-22.0] years). This is the first study to investigate the associations between frailty-free life expectancies and socio-economic characteristics in Europe. Large inequalities in frailty-free and frail life expectancies exist between those with different socio-economic characteristics. This highlights the people most at risk of early frailty at younger ages.

Abstract citation ID: igad104.1794

### DEVELOPING A HEALTH INEQUALITY/INEQUITY CONCEPTUAL FRAMEWORK TO SUPPORT EVIDENCE SYNTHESIS STUDIES

Tafadzwa Patience Kunonga[1], Peter Bower[2], Barbara Hanratty[1], and Dawn Craig[3], *1. Newcastle University, Newcastle upon Tyne, England, United Kingdom*, *2. Division of Population Health, Health Services Research & Primary Care, National Institute for Health and Care Research (NIHR) Older People and Frailty Policy Research Unit, Manchester, England, United Kingdom*, *3. National Institute for Health and Care Research (NIHR) Older People and Frailty Policy Research Unit, Newcastle-upon-Tyne, England, United Kingdom*

This study aimed to develop a conceptual framework to guide people conducting evidence syntheses, to enable them to visualise links, and intersectionality between various factors that affect health inequality or inequity. Currently, there is no clarity or guidance on how health inequality or inequity should be defined or considered in evidence synthesis, despite recent improvement in reporting standards. In addition, current guides are limited in their ability to provide a roadmap for reviewers that enables them to visualise the links and intersectionality between various factors that affect health inequality or inequity. A series of consensus development workshops were conducted in Spring 2023. Experts in evidence synthesis and health inequality or inequity participated. The workshops were based on methods used previously by researchers developing clinical guidelines. Workshop discussions were centred on (1) important issues/barriers that prevent researchers from having a consensus definition for health inequality or inequity, and (2) how researchers could consider intersectionality of factors affecting health inequality or inequity? Thematic analysis of workshop data and a prioritisation workshop informed the development of a novel conceptual framework. This will be presented and address in particular, ideas around the accumulation of advantage and disadvantage across the life course alongside other dimensions of health and health inequality or inequity.

Abstract citation ID: igad104.1795

### INEQUALITIES IN POLYPHARMACY IN ADULTS AGED 65+ IN ENGLAND

Anum Iqbal[1], Barbara Hanratty[1], Fiona Matthews[1], David Sinclair[1], Connor Richardson[2], and Adam Todd[3], *1. Newcastle University, Newcastle upon Tyne, England, United Kingdom*, *2. Population Health Sciences Institute, Newcastle Upon Tyne, England, United Kingdom*, *3. School*

*of Pharmacy, Newcastle Upon Tyne, England, United King dom*

Understanding the role of socio-economic factors in the number of medications an individual is prescribed, can help healthcare practitioners target medication reviews and safer prescribing, improving quality of life. We aimed to understand the association between socio-economic factors and polypharmacy - defined as the use of five or more medications at any given time. Data was used from the Cognitive Function and Ageing Study (CFAS), focusing on adults aged 65 years and over. Data from CFAS I and CFAS II, was used to understand the relationship between education (a marker of socio-economic status) and polypharmacy. Individuals were categorised on the number of years in full time education, and baseline cohorts (wave 1) from each study were compared, to identify the longitudinal effect. Logistic regression was used to assess the association between polypharmacy and education. Increased number of years of full-time education was associated with a decreased likelihood in polypharmacy, in CFAS II - those with <10 years of full-time education showed higher odds of polypharmacy, when compared to individuals with 10-11 years of full-time education, OR 1.61 (95 % CI 1.44-1.80). However, similar odds were displayed across each education category and polypharmacy in CFAS I. Results have showcased growing inequalities in healthcare, particularly through the influence of education on polypharmacy. Socio-economic factors could be used to trigger for medication related review services.

Abstract citation ID: igad104.1796

### DIGITALIZATION OF HEALTH SERVICES FOR OLDER ADULTS: HEALTH INEQUALITIES AND THE COVID-19 PANDEMIC

Alex Hall[1], Charlotte Eost-Telling[2], Fiona Beyer[3], Abodunrin Aminu[2], David Sinclair[4], Dawn Craig[3], Barbara Hanratty[4], and Chris Todd[2], *1. University of Manchester, Manchester, England, United Kingdom*, *2. National Institute for Health and Care Research (NIHR) Older People and Frailty Policy Research Unit, Manchester, England, United Kingdom*, *3. National Institute for Health and Care Research (NIHR) Older People and Frailty Policy Research Unit, Newcastle-upon-Tyne, England, United Kingdom*, *4. Newcastle University, Newcastle upon Tyne, England, United Kingdom*

There is a growing role for digital technologies in society, but concerns that older adults may be disadvantaged and excluded with the growth of use of these technologies. The COVID-19 pandemic led governments across the world to mandate lockdowns and social restrictions. This was accompanied by rapid implementation of many digitalized services, including to access health and social care. This paper reports on a project to advance our understanding of how health and social care services for older adults (65+) have been digitalized during the COVID-19 pandemic, and to learn what has worked well and what has not, with a specific lens on health inequalities. We report main findings from three workstreams: (i) a mapping review, to identify (a) the types of evidence available on the digitalization of health and social care services for older adults during the COVID-19 pandemic, and (b) the extent to which factors related to health inequalities have been considered in this evidence; (ii) quantitative analysis of health care records to examine the impact of digitalization on access to primary care for older people in England during the COVID-19 pandemic, with a focus on incidence rate of consultation types (face-to-face, telephone and video) examined against age, gender, ethnicity and socioeconomic status; (iii) qualitative work to explore the experiences of UK older adults of South Asian and Black African or Caribbean descent of using digital technologies to access primary care. We consider how digitalization of health and social care services relates to health inequalities for older people.

Abstract citation ID: igad104.1797

### PREFERENCES FOR NEW MODELS OF SOCIAL CARE FOR OLDER PEOPLE IN ENGLAND

Magdalena Walbaum, *London School of Economics and Political Science, London, England, United Kingdom*

Ageing and increasing diversity of the population pose challenges for social care provision. England has implemented several initiatives to improve care quality and make it more personalised, informed, and connected. This study aimed to understand the preferences of older people, including those from diverse backgrounds, for different aspects and models of social care, and how they trade-off different features of care. The study included two workstreams with participants aged 50+: 1) A qualitative study that explored preferences across five themes: housing settings, community assets, technology use, provision of care, and control and dignity; 2) A quantitative survey that analysed variations in preferences by demographic, socioeconomic, and needs-related factors. A discrete choice experiment was also conducted to evaluate the strength of preferences for different attributes. Older people value their independence and control over their lives and prioritise access to community resources and social connections. Participants also emphasised the importance of building a positive relationship with their care provider, respecting their sexual identity and personal beliefs to receive good quality care. In this presentation, differences in preferences among demographic, socioeconomic, and ethnic groups will be highlighted, underscoring the need to ensure that care packages align with individual preferences, beliefs, and values and promote equitable access to care, and outcomes. This approach may ensure that older people from diverse backgrounds receive high-quality care that promotes their independence and dignity and produces equitable care outcomes.

### SESSION 4460 (SYMPOSIUM)

Abstract citation ID: igad104.1798

### NOVEL BEHAVIORAL HEALTH APPROACHES FOR OLDER ADULTS IN UNDERSERVED COMMUNITIES

Chair: Ryan Mace Co-Chair: Ana-Maria Vranceanu Discussant: Kathi Heffner

Chronic conditions and related stressors among older adults are leading drivers of disability, morbidity, and healthcare costs in the US. Underserved communities have the highest rates of chronic conditions and are at the greatest risk for negative health outcomes. Novel behavioral health approaches are needed to address barriers faced by these communities (e.g., stigma, discrimination, structural inequalities) that exacerbate health disparities. Consistent with the GSA 2023 theme, this symposium will showcase multidisciplinary initiatives to build bridges to clinical care, catalyze community-engaged research, and empower underserved older adults. Presenter 1 will present the protocol, preliminary results, and "lessons learned" recruiting ethnoracially diverse older adults for a fully-powered efficacy RCT (target N=260) of Active Brains, a virtual group mind-body walking program aided by Fitbit targeting comorbid chronic pain and early cognitive decline. Presenter 2 will present focus groups with patient and medical provider stakeholders to culturally adapt a mind-body activity program (GetActive) for disadvantaged older adults with chronic pain in community clinics. Presenter 3 will present the rationale, design, and challenges of an RCT testing the effectiveness of a peer support program (Peer Enhanced Depression Care) to alleviate depressive symptoms in ethnoracially diverse and/or low-income older adults (N=160). Presenter 4 will present a stakeholder-partnered approach to equitable home care for older adults and caregivers in diverse communities by eliminating transportation-related barriers. The Discussant will contextualize these initiatives in the broader field of gerontology and share research from their Roybal Center to engage community agencies and ethnoracially diverse older adults.

Abstract citation ID: igad104.1799

### ADDRESSING COMORBID CHRONIC PAIN AND EARLY COGNITIVE DECLINE IN OLDER ADULTS: THE ACTIVE BRAINS EFFICACY TRIAL

Ryan Mace[1], Ana-Maria Vranceanu[2], Nathaniel Choukas[2], Elizabeth Rochon[2], Katherine McDermott[1], Julia Hooker[1], and Christine Ritchie[2], *1. Massachusetts General Hospital/ Harvard Medical School, Boston, Massachusetts, United States*, 2. *Massachusetts General Hospital, Boston, Massachusetts, United States*

Chronic pain (CP) and early cognitive decline (ECD) are costly, highly prevalent, and challenging to treat among community-dwelling older adults. CP and ECD commonly co-occur, exacerbate each other, and can accelerate dementia. Our interdisciplinary team has developed Active Brains (AB), a virtual group mind-body walking program aided by Fitbit, to address this comorbidity. We are conducting a single-blinded, NIH stage 2 RCT to establish the efficacy of AB versus a time- and dose-matched education control (Health Enhancement Program; HEP) in 260 older adults with CP and ECD. In this symposium, we will describe the study design, recruitment numbers, and maximizing the inclusion of racial and ethnic minoritized older adults (38% benchmark). Participants are recruited through providers, social media, and community partnerships. Participants wear an ActiGraph to monitor steps and are randomized to 8 weeks of AB or HEP delivered via Zoom by an MGH psychologist. The AB group receives a Fitbit to reinforce activity goals. Primary (multimodal physical function) and secondary (cognitive and emotional function, pain) outcomes are collected at baseline, post-intervention, and 6 months. We have recruited 37 participants (4 cohorts). All 37 completed the baseline, 26 completed the post-test, and 9 are currently enrolled (total dropout = 5%). Session attendance (92.8%) and watch adherence (93.5%) have been excellent. The trial will establish efficacy, test mechanisms, and inform a future multi-site effectiveness-implementation trial. Consistent with the GSA 2023 theme, AB has the potential to catalyze nonpharmacological interventions for CP–ECD and empower older adults to maintain active and meaningful lives.

Abstract citation ID: igad104.1800

### ADDRESSING THE CHRONIC PAIN EPIDEMIC AMONG OLDER ADULTS IN UNDERSERVED COMMUNITY HEALTH CENTERS

Ana-Maria Vranceanu[1], Jonathan Greenberg[2], Katherine McDermott[2], Madison Ehmann[1], Nadine Lecvy[1], Julie Brewer[1], and Christine Ritchie[1], *1. Massachusetts General Hospital, Boston, Massachusetts, United States, 2. Massachusetts General Hospital/Harvard Medical School, Boston, Massachusetts, United States*

Current nonpharmacological treatments are inadequate for addressing chronic pain among older adults from disadvantaged backgrounds. These treatments have been developed for affluent populations and are not implemented in underserved community health centers (CHCs) where most older adults with chronic pain seek care. We conducted 4 focus groups with 23 providers (e.g., primary care doctors, nurse practitioners, medical interpreters and support staff) and 4 focus groups with 15 English- and Spanish-speaking patients at a CHC. Our goals were to: 1) understand barriers and facilitators to sustained implementation of the GetActive+ mind-body activity program delivered by a nurse practitioner; 2) gather perceptions of program skills to facilitate cultural tailoring; and 3) gather information to support a future hybrid type 1 effectiveness implementation trial of GetActive+ versus usual care to improve pain outcomes. Qualitative analyses were conducted using a deductive-inductive approach and the Framework method. Questions were informed by Aaron's implementation model and the Socioecological model. Results revealed multiple barriers at the individual (e.g., pain misconceptions, perceived bias, stigma, shame, treatment expectations and loneliness, language barriers, food and financial insecurity), health clinic (e.g., provider burnout, understaffed clinic, budget cuts, multiple languages) and environmental levels (e.g., lack of technology, lack of reliable transportation to clinic, billing challenges). Clinic providers felt unprepared to support older adults with chronic pain. Both patients and providers endorsed the need for, and skills of GetActive+. Nuanced differences between patient-provider and English-Spanish patients as well as modifications to the program manual, protocol, and interventionist training will be discussed.

Abstract citation ID: igad104.1801

### INCREASING THE REPERTOIRE FOR DEPRESSION CARE: PEER SUPPORT AS DEPRESSION CARE FOR VULNERABLE OLDER ADULTS

Jinhui Joo[1], Melissa Davey-Rothwell[2], Namkee Choi[3], Joseph Gallo[2], and Alice Xie[4], *1. Masschusetts General Hospital, Boston, Massachusetts, United States*, 2. *Johns*

*Hopkins University, Baltimore, Maryland, United States, 3. University of Texas, Austin, Texas, United States, 4. Massachusetts General Hospital, Boston, Massachusetts, United States*

Despite facing greater risks for poorer health, low-income White and BIPOC older adults underutilize mental health services even when they have indicated need. Increasing the repertoire for depression care that is community-based and uses paraprofessionals has potential to increase access and engagement. We are testing the effectiveness of a peer support intervention called Peer Enhanced Depression Care (PEERS) which is an 8-week community-based intervention that uses trained peer mentors to deliver emotional, appraisal and informational support in addition to encouraging self-care skills to depressed low-income white and BIPOC older adults. Enrolled participants are randomized to either the peer support intervention (PEERS) or to the social interaction control and followed for 12 months. The primary outcome is depression and secondary outcomes include engagement, mental health service use, and social, emotional, and physical functioning. Challenges related to the onset of the COVID-19 pandemic and social isolation required a shift from recruitment initially focused on the health care system to community-based organizations serving older adults. Required contactless recruitment strategies eg. flyers and newspaper ads, led to self-referral of community-dwelling older adults to the study. Challenges to participant enrollment included barriers related to communication, stigma related to help-seeking, distrust and unfamiliarity with research. Recruitment of peer mentors was facilitated by a robust infrastructure supporting the employment of the peer support workforce. Continued PM supervision after initial training, review of skills and evaluation of performance were important in maintaining quality and fidelity to the intervention.

Abstract citation ID: igad104.1802

### A STAKEHOLDER-PARTNERED APPROACH TO INEQUITIES AFFECTING HOME HEALTH CARE WORKERS, OLDER ADULTS, AND CAREGIVERS

Olivia Okereke[1], Vivian Anable Eme[2], Keliane Totten[3], Joseph Locascio[4], Alison Simmons[5], Nancy Carpenter[5], and Anthony Weiner[6], *1. Massachusetts General Hospital/ Harvard Medical School, Boston, Massachusetts, United States, 2. Massachusetts General Hospital, Boston, Massachusetts, United States, 3. Pear Associates, LLC, Wellesley, Massachusetts, United States, 4. Biostatistics, Massachusetts General Hospital, Boston, Massachusetts, United States, 5. Center for Community Health Education Research and Service (CCHERS), Boston, Massachusetts, United States, 6. Geriatric Psychiatry, Massachusetts General Hospital, Boston, Massachusetts, United States*

Home health aides (HHAs) provide on-site support for homebound older adults with cognitive impairments, while lessening strain on familial caregivers. However, HHAs face structural challenges in work-related transportation. Massachusetts General Hospital (MGH) researchers partnered with community and corporate stakeholders to inform a study evaluating whether on-demand transportation for HHAs via Uber rides will: 1) Improve metrics among HHAs (total visits, total unique patients, hours/week and days/week worked, missed visit/no-show rates, work satisfaction); 2) Increase racial, ethnic, socioeconomic and geographic diversity of older adults with cognitive impairments receiving homecare. Community partner CCHERS (Center for Community Health Education Research Services) helps residents of public/publicly-assisted housing in high-need areas of Greater Boston gain training and entry-level employment in healthcare as HHAs. Through its collaborations with other groups (e.g., Mothers for Justice & Equality, HomeCare Aide Council, Mass HomeCare Alliance), CCHERS identified that lack of affordable, accessible, reliable transportation was among the most-cited barriers affecting HHAs and equitable homecare delivery. Corporate partner Uber Health, through work in preliminary social-impact case studies, identified the platform's central ride-coordination approach as preferred by Black and Hispanic women in the 18-45y age group (~80% of the local HHA workforce). MGH researchers' study goals and metrics were informed by input of partners. With its community-engaged approach, innovative partnerships, and focus on achieving equity objectives to benefit patients and homecare workers alike, this project has potential for strong local impacts and future implications for state and/or national policies to provide high-quality, equitable homecare by directly addressing employment transportation-related barriers.

### SESSION 4465 (SYMPOSIUM)

Abstract citation ID: igad104.1803

### BRIDGING RESEARCH TO PRACTICE: PRO-HOME EMPOWERS CARE PARTNERS TO PROMOTE EXERCISE IN HOME CARE

Chair: Naoko Muramatsu

Regular physical activity (PA) benefits people of all ages. Mounting scientific evidence shows PA's health benefits in older persons with chronic conditions. However, there remains a large research-to-practice gap in PA, especially in long-term services and supports (LTSS). Older adults who need help with basic daily activities, such as walking or standing, continue to face barriers at multiple levels of LTSS to become and remain physically active. This symposium will address the much-debated research-to-practice gaps, focusing on frail community-dwelling older adults in the context of a NIA-funded clinical trial. "Promoting Seniors' Health with Home Care Aides (Pro-Home)" tests the effectiveness of a gentle, low-intensity PA program delivered by home care aides (HCAs) for community-dwelling older adults with nursing-home eligible care needs in real-world home care settings. Pro-Home is innovative because it empowers direct care workers (HCAs) with an easy-to-learn toolkit to deliver the program and keep their older clients motivated. The symposium starts with an overview of Pro-Home (Muramatsu), barriers and facilitators of Pro-Home at multiple levels of the LTSS system (Cruz Madrid), followed by Pro-Home's critical elements: of research and practice: client-HCA dyad relationships and partnerships with stakeholders (Hu), in-home assessment of motor function in frail older adults (Yin), and inter-professional co-learning (Skowronski). The symposium concludes with a dialogue with the audience to identify actionable policy and practice strategies for building research-to-practice and practice-to-research bridges to build health promotion into LTSS to promote the well-being of older adults with disabilities and their care partners.

Abstract citation ID: igad104.1804

### PRO-HOME: AN IMPLEMENTATION SCIENCE FRAMEWORK FOR MULTILEVEL BARRIERS AND FACILITATORS IN LTSS

Katya Cruz Madrid, Michael Berbaum, Surrey Walton, Joseph Zanoni, David Marquez, and Naoko Muramatsu, *University of Illinois Chicago, Chicago, Illinois, United Sta tes*

Promoting Seniors' Health with Home Care Aides (Pro-Home) is a randomized controlled trial (RCT) of a gentle physical activity program for frail older adults conducted in a community-based long-term services and supports (LTSS) delivery setting. Pro-Home is a hybrid effectiveness-implementation study, which aims to examine (1) the effectiveness of the program delivered by home care aides (HCAs) in maintaining physical function in older homecare recipients, and (2) how the intervention works in a real-world Medicaid-funded LTSS system. This paper describes barriers and facilitators that have long existed or evolved during the Pro-Home development, using quantitative/qualitative data from the mixed-method study informed by an implementation science framework. Facilitators include the knowledge and partnerships that resulted from multiple preliminary studies that illuminated HCAs' work/life-related stressors, coping resources, and underutilized capacity and motivation to promote the health of clients and HCAs themselves in the context of the LTSS system. Another facilitator has been the physical activity program developed in another state's LTSS service setting (not in an academic setting). The program was easy to learn and adapt into a program to be delivered by HCAs (as opposed to care managers). Barriers included turbulent LTSS environments (e.g., rapidly growing managed care presence, COVID-19). Those barriers have been addressed through partnership with stakeholders at multiple levels. Having completed the intervention and data collection phases, we found the project's planning and implementation challenging but rewarding. Pro-Home has produced rich information that will have many implications for research, practice, and LTSS services delivery and financing systems.

Abstract citation ID: igad104.1805

### HOME CARE DYADS AND STAKEHOLDERS IN THE CONVOY OF SOCIAL RELATIONSHIPS: A BASIS OF COMMUNITY-BASED RCT

Elise Hu[1], Lijuan Yin[2], and Maria Caceres[2], *1. University of Illinois Chicago School of Public Health, Chicago, Illinois, United States*, *2. University of Illinois Chicago, Chicago, Illinois, United States*

Relationships within homecare dyads are the core in both informal and formal caregiving. Home care aides (HCA), a type of formal (paid) caregivers, provide in-home non-medical care to older adults who need assistance with daily activities. The Pro-Home study relied on client-HCA relationships and collaborating homecare stakeholders to recruit dyads in this randomized controlled trial (Randomized N = 128). Participating clients were mostly unmarried and unable to meet with family/friends multiple times a week. More than half lived alone, two thirds were cared for by a HCA who was not a family member, and the majority reported having a good relationship with their HCA. Most participating clients kept the same HCA throughout the 4-month intervention, before and throughout the ongoing COVID-19 pandemic. These characteristics highlight the critical role of homecare workers in caring and health promotion as older clients' social convoy. However, the pandemic has posed challenges to these dyads. A higher proportion of clients participating in the intervention during the pandemic lost or were reassigned a HCA (20%) compared to those completed the intervention prior to the pandemic (10%). Our community collaborators reported increased staff turnovers and nationwide HCA shortages that created difficulties in care coordination. These findings will inform the ongoing debate on future financing and delivery systems of long-term services and supports, which are increasingly provided in home and community settings in the U.S. and other countries.

Abstract citation ID: igad104.1806

### ASSESSING MOTOR FUNCTION IN FRAIL OLDER ADULTS IN THEIR HOME SETTING: CHALLENGES AND OPPORTUNITIES

Lijuan Yin, Woojin Song, Jordan Skowronski, and Maria Caceres, *University of Illinois Chicago, Chicago, Illinois, United States*

Motor function is essential for older adults to perform daily activities and remain in their home as they age. Mounting evidence suggests that physical activity (PA) helps maintain motor function in older adults. However, few evidence-based PA programs exist for home-bound older adults who have difficulties walking or standing. They are typically excluded from randomized controlled trials (RCT). Evidence is difficult to establish, because of the lack of instruments and protocols for assessing motor function safely and effectively in this population. The goal of this paper is to present challenges and opportunities that we encountered in assessing motor function in 119 participants of the Pro-Home study right before the COVID-19 pandemic. As expected, participants had significant physical limitations. One third of the participants did not feel safe doing balance tests or chair stand test included in the Short Physical Performance Battery. Of those who did the balance tests, two thirds had to use compensatory movement or hold on to an object to maintain balance. Interestingly, 30% of participants had cognitive impairment as defined by 1.5 Standard Deviation below the normative mean measured by NIH Toolbox Cognition Battery, although they passed a six-item cognition screener to be eligible for Pro-Home. The challenges of assessing frail older adults have led to opportunities to adapt assessment protocols. We made instructions clear and easy-to-follow and developed a new measure appropriate and safe for our target, frail older participants. Additional safety measures are needed to facilitate motor function assessments in frail older adults.

Abstract citation ID: igad104.1807

### INTERPROFESSIONAL CO-LEARNING AT MULTIPLE LEVELS OF LTSS: PRO-HOME PROGRAMS AND RESEARCH

Jordan Skowronski, Joseph Zanoni, and Maria Caceres, *University of Illinois Chicago, Chicago, Illinois, United States*

Promoting Seniors' Health with Home Care Aides (Pro-Home), a randomized controlled trial (RCT) of a gentle physical activity program delivered by home care aides (HCAs) for their older clients in English or Spanish, provided unprecedented opportunities for collaboration and co-learning across multiple disciplines and professions (public health, health services research, sociology, medicine, physical/occupational therapy, kinesiology, social work, psychology, economics, medicine, nursing; frontline care workers, leaders and staff members of state and home care agencies, , social service agencies, researchers, undergraduate and graduate students). The turbulent long-term services and supports (LTSS) environment, which culminated in the COVID-19 pandemic, provided the study participants (HCAs and their older clients), multiple levels of LTSS stakeholders and the research team with rapidly evolving new contexts for the RCT. This paper presents how the Pro-Home project developed into a learning organization where individuals and organizations learn from each other to pivot at every new turn in the environment, focusing on the development of a novel interactive training for HCAs in-person or remotely. The initial training activities were conducted in-person. After adapting the material for remote delivery, we provided the latter sessions via phone with digital and physical tools. For each training session, we assessed outcome quality using an exit survey and other methods capturing HCAs' program delivery. The presentation concludes with implications for research and practice in LTSS.

### SESSION 4470 (SYMPOSIUM)

Abstract citation ID: igad104.1808

### PALLIATIVE CARE IN RESIDENTIAL CARE HOMES: IMPLEMENTATION CHALLENGES AND EARLY FINDINGS IN THREE CLINICAL TRIALS

Chair: Kathleen Unroe Discussant: Jenny van der Steen

This symposium will describe, compare and contrast three active clinical trials implementing palliative care models for people with dementia in residential care facilities and nursing homes. SPA-LTC is a pragmatic clinical trial in three Canadian provinces, providing training to palliative care teams and a process for triggered palliative care conferences. EMBED-Care, based in the United Kingdom, is testing a digital clinical support tool to improve integrated palliative care for people with dementia using holistic assessment and promoting shared decision-making. UPLIFT-AD is a multi-state U.S. based clinical trial, providing training for in-facility Palliative Care Leads and facilitating specialty palliative care clinician consults for people with moderate to advanced dementia. This symposium provides an opportunity to compare methodologic choices in study design, including intervention design, use of internal (care facility) based resources vs. external supports, and outcome assessment. Presenters will describe implications of these design choices for the implementation of the trial and for longer term sustainability. Implementation experience, including barriers and facilitators to palliative care programs for people with dementia, will be described.

Abstract citation ID: igad104.1809

### UTILIZING PALLIATIVE LEADERS IN FACILITIES TO TRANSFORM CARE: THE UPLIFT CLINICAL TRIAL IMPLEMENTATION

Kathleen Unroe[1], John Cagle[2], and Mary Ersek[3], *1. Indiana University, Indianapolis, Indiana, United States, 2. University of Maryland, Baltimore, Baltimore, Maryland, United States, 3. University of Pennsylvania, Philadelphia, Pennsylvania, United States*

UPLIFT is an NIH funded clinical trial with a goal of improving quality of care for people with moderate to advanced dementia in nursing homes. The UPLIFT intervention consists of specialized training in palliative care and dementia care for two nursing facility staff who are identified as Palliative Care Leads. All staff training on the fundamentals of palliative care is also delivered. In addition to this internal capacity building, UPLIFT integrates specialty medical palliative care consultants into the care team. Palliative Care Leads screen residents for palliative care needs which prompt clinical consultations. Outcomes include surrogate (both nursing facility staff and family) reported symptoms of eligible residents. A total of 16 nursing facilities are enrolled in this stepped wedge trial, which concludes in 2025. Numerous implementation challenges have been identified and navigated throughout the trial. These include Palliative Care Lead turnover, nursing facility leadership turnover, communication with primary medical teams, and integration of palliative care NPs and MDs into the clinical care of the residents. Data on challenges is captured on regular fidelity check-ins with nursing home staff and interviews with Leads and palliative care providers. A description of these challenges and strategies the team is using to navigate them will be presented, including adaptations to study design and the intervention.

Abstract citation ID: igad104.1810

**EMPOWERING BETTER END-OF-LIFE DEMENTIA CARE (EMBED-CARE): A DIGITAL CLINICAL DECISION SUPPORT FRAMEWORK**

Nathan Davies[1], Elizabeth Sampson[1], Tofunmi Aworinde[2], Juliet Gillam[2], Charlotte Kenten[1], Bethan Phillips[1], Catherine Evans[2], and Clare Ellis-Smith[2], *1. University College London, London, England, United Kingdom*, *2. King's College London, London, England, United Kingdom*

People with dementia experience a variety of unmet palliative care needs. There is a need to empower people with dementia, family carers and professionals, to be able to better assess and monitor needs, and manage symptoms. We aimed to co-design and evaluate acceptability, feasibility and implementation of an integrated model of palliative dementia care to support holistic assessment, decision making and management of needs for those living in community and residential care (EMBED-Care Framework).We used a co-design approach, using framework analysis to synthesize data from evidence reviews, routine data, and cohort study, we presented findings to a series of co-design workshops, to produce the Framework. The Framework consists of a holistic assessment; priority setting; and decision support using clinical decision support tools, delivered on an app. The holistic assessment identifies areas of concern and identify priorities of care, through a process of shared decision making. Each concern is linked to a decision support tool to support decision making and to address priorities. App alerts and reminders communicate concerns among teams and carers. We are testing the Framework in a 6-month feasibility study with two residential homes and two community settings in England. We are collecting outcomes including decisional conflict, satisfaction with care, and unmet needs. We are interviewing all users to explore acceptability and implementation. We will present development of the EMBED-Care Framework and preliminary findings from our feasibility study, focusing on the acceptability and lessons for implementation which can be applied to the wider dementia community and similar interventions.

Abstract citation ID: igad104.1811

**EXPLORING THE IMPLEMENTATION OF THE STRENGTHENING A PALLIATIVE APPROACH IN LONG-TERM CARE PROGRAM IN A CLINICAL TRIAL**

Sharon Kaasalainen[1], Paulette Hunter[2], Genevieve Thompson[3], and Abigail Wickson-Griffiths[4], *1. McMaster University, Hamilton, Ontario, Canada*, *2. St. Thomas Moore College, University of Saskatchewan, Saskatoon, Saskatchewan, Canada*, *3. University of Manitoba, Winnipeg, Manitoba, Canada*, *4. University Of Regina, Regina, Saskatchewan, Canada*

Despite the high mortality rates in long-term care (LTC) homes, most do not have a formalized palliative program. Hence, our research team developed the Strengthening a Palliative Approach in Long Term Care (SPA-LTC) program. This presentation will address how the SPA-LTC intervention was designed to be scaled up in a large randomized control trial, related implementation issues and strategies used to address them during the trial. This study used a cross-jurisdictional, effectiveness-implementation type II hybrid cluster design to evaluate the SPA-LTC program in 18 LTC homes across three provinces in Canada. The implementation component was designed as a formative evaluation to explore how to adapt the SPA-LTC program in real time. The SPA-LTC intervention leveraged both internal (palliative champion team) and external supports (palliative consultants) to build capacity within the LTC home to support and prepare residents and families for end-of-life decision making. Due to staff shortages and their inability to attend lengthy education sessions, we held four one-hour education sessions for the palliative champion team that were led by external palliative consultants over three months. Reach of the intervention was compromised as approximately 10% of participating residents died before a palliative care conference could be held. As a result, we prioritized residents who had lower Palliative Performance Scale (PPS) scores when planning a care conference. Clearly, efforts are needed to evaluate both the implementation and effectiveness of complex interventions, such as a palliative program, within a pragmatic approach given the real-life challenges that currently exist in this sector.

### SESSION 4475 (BIOLOGICAL SCIENCES INVITED SYMPOSIUM)

Abstract citation ID: igad104.1812

**ESPO AND BIOLOGICAL SCIENCES SECTION SYMPOSIUM: HIGHLIGHTING THE FUTURE LEADERS OF GEROSCIENCE**

Chair: Matt Yousefzadeh Co-Chair: Bhaswati Ghosh

The Emerging Scholars Symposium (ESPO) is organized by post-docs and PhD students from GSA's BS ESPO Committee. The goal of this symposium is to foster the next generation of aging researchers by recruiting trainees and early career investigators as speakers from contributed abstracts.

Abstract citation ID: igad104.1813

**ANTIDIABETIC THERAPIES METFORMIN AND TRODUSQUEMINE MODULATE MIRNAS INVOLVED WITH LIVER METABOLISM**

Sarah Ashiqueali[1], Augusto Schneider[2], Xiang Zhu[3], Ewelena Juszczyk[4], Yun Zhu[5], Andrzej Bartke[5], Shadab Siddiqi[1], and Michal Masternak[1], *1. University of Central Florida College of Medicine, Orlando, Florida, United States*, *2. Universidade Federal de Pelotas, Pelotas, Rio Grande do Sul, Brazil*, *3. University of Central Florida, Orlando, Florida, United States*, *4. Celon Pahrma S.A., Kazuń Nowy, Mazowieckie, Poland*, *5. Southern Illinois University, Carbondale, Illinois, United States*

Type II diabetes mellitus (T2DM) afflicts approximately 33% of individuals over the age of 65 increasing susceptibility to dyslipidemia, a hallmark of non-alcoholic fatty liver disease (NAFLD). Due to the idiopathic nature of NAFLD, there is no standard of care with only lifestyle intervention strategies available to improve hepatic function. Although dietary modifications and physical activity can prevent the pathogenesis of NAFLD, poor compliance has sparked an urgent need for pharmacological options to achieve glycemic control and prevent the onset of comorbidities. Metformin (MF) is commonly used medication to treat T2DM and has recently gained attention as an anti-aging intervention in non-diabetic individuals. Trodusquemine (MSI-1436) is a drug that induces fat loss by inhibiting the protein tyrosine phosphatase 1B (PTP1B), an enzyme that impairs leptin and insulin signaling. Using miRNA sequencing, we identified a cohort of miRNAs that are influenced by MF and MSI-1436 in the livers of UMHET3 mice. Our in-depth analysis and RT-PCR validation indicated groups of miRNAs regulating the processes of de novo lipogenesis, fatty acid oxidation, very-low-density-lipoprotein and cholesterol transport. Importantly, miR-379-5p, a known modulator of cholesterol metabolism, reported to be inhibited in the livers of patients with NAFLD and in mouse models of diabetes, was found to be upregulated by MSI-1436 in both sexes when comparing with both CTL and MF groups. In summary, our study showed that MSI-1436 more potently inhibits mechanisms involved with liver homeostasis relative to MF yielding insight on its therapeutic potential in concurrent liver diseases resulting from T2DM.

Abstract citation ID: igad104.1814

### INTRAINDIVIDUAL VARIABILITY IN SLEEP DURATION IS ASSOCIATED WITH HIPPOCAMPAL VOLUME IN MID LIFE

Jose Diaz[1], and David Almeida[2], *1. Penn State University, University Park, Pennsylvania, United States*, *2. The Pennsylvania State University, State College, Pennsylvania, United States*

Sleep is associated with global cognition, consolidation of memories, and structural & functional integrity of areas of the brain that support healthy cognition. While there has been research on sleep in older adults that indicates associations between poor sleep, cognitive decline, memory performance, and changes in the brain, there has been comparatively little work assessing the impact of sleep on the structure of the brain during middle adulthood. Changes in the brain begin much earlier than older adulthood, with midlife seeing cortical thinning and a decrease in brain volume in areas that support healthy cognitive functioning and memory, such as the hippocampus. Understanding how sleep may contribute to hippocampal volume in midlife may help inform how changing poor sleep is important for supporting successful aging. The current study examines the associations between 7 days of actigraphy measured sleep and hippocampal volume (MRI) in 82 midlife adults (Mage = 49.13 years, SD = 11.74; 28-76 years old) who completed the Biomarker and Neuroscience projects in the Midlife in the United States Refresher study. Results indicated that bi-lateral hippocampal volume was negatively associated with high (vs low) intraindividual total sleep time variability (>= 60 min; mean average deviation), with a 280 and 252 mm3 (p's = .006 & .035) volume reduction for left and right hemispheres respectively. This relationship was not seen for other measures of sleep quality and the analysis additionally controlled for age, sex, BMI, race, depression status, education, and subjective physical & mental/emotional health.

Abstract citation ID: igad104.1815

### SUPPRESSION OF MICROBIOME SENSING—FREE FATTY ACID RECEPTOR 2 (FFAR2) SIGNALING EXHIBITS EARLY AGING

Sidharth Mishra, Santosh Kumar Prajapati, Shalini Jain, and Hariom Yadav, *University of South Florida, Tampa, Florida, United States*

Abnormalities in gut microbiome contribute to age-related diseases, causal to unhealthy aging, and early mortality, but mechanisms still remain largely unknown. Our recent research shows that old microbiota lacks the capacity to produce beneficial metabolites like short-chain fatty acids (SCFAs), which in turn promote gut permeability and systemic inflammation. SCFAs activates free fatty acid receptor 2 and 3 (FFAR2/3) signaling, at least in part, to exhibit their benefits on host cells; however, the role of these signaling in aging process is not well understood. We discovered that in addition to gut the brains of old mice show lower expression of FFAR2. Further, FFAR2 deletion in the intestine shows a shorter life span compared to wild-type mice. They also show a higher cognitive decline at early age. Similarly, intestine specific FFAR2 KO in APP/PS1 (Alzheimer's disease [AD] model) background shows higher progression of AD including amyloid beta (A-beta) and tau accumulation, linked with higher blood brain barrier (BBB) permeability and neuroinflammation. These changes are associated with increased gut permeability and systemic inflammaging. Interestingly, a newly discovered natural FFAR2 agonist-Fenchol reduces these abnormalities in gut-brain and prevented the progression of cognitive decline and AD pathology. These results show that microbiome sensing FFAR2 signaling deficiency develops an early aging phenotype in mice, while its agonism using natural compound can delay/prevent the progression of early aging.

Abstract citation ID: igad104.1816

### THE ROLE OF CASTRATION BEFORE OR AFTER PUBERTY ON SEX DIFFERENCES IN GROWTH AND LONGEVITY IN UM-HET3 MICE

Nisi Jiang[1], Catherine Cheng[2], and James Nelson[2], *1. UT Health San Antonio, Barshop Institute, San Antonio, Texas, United States*, *2. UT Health San Antonio, San Antonio, Texas, United States*

Sex differences in human aging and longevity are well known. Mortality rates of males are higher throughout life with no definitive mechanisms identified. Previously, we reported that genetically heterogeneous mice have sex differences in longevity like those of humans. Here, we report that prepubertal castration significantly reduced early adulthood mortality and increased median lifespan by 12.6% (n=236/group) to match female levels. It also shifted growth trajectories toward the female pattern, including a reduced rate of body weight gain and a prolonged growth period. Our findings suggest that testicular hormones are critical for these sex differences. We next tested whether a) exposure to gonadal hormones from puberty throughout life is required or b) only hormone exposure during the short period of pubertal development is sufficient to cause these sex differences. We castrated or sham-operated mice (n = 60/group) prepubertally at 20 days, or after puberty at 60 or 240 days. Prepubertal castration prolonged the period of growth as before, with dramatically increased fat mass and reduced lean mass, which reduced the lean/body mass ratio to a level indistinguishable from that of females. Although post-pubertal castrated groups also had reduced lean/body mass ratios, they were significantly higher than prepubertally castrated mice, but were unchanged between 60d and 240d groups. These results suggest that the narrow window of testicular hormone exposure during puberty is necessary for the full expression of sex differences in body composition trajectories. Its effect on lifespan will be presented at the meeting when mice are old enough for assessment.

### SESSION 4480 (SYMPOSIUM)

Abstract citation ID: igad104.1817

### IN THE PRESENT AND IN THE FUTURE: HOW TIME PERCEPTION AND TIME ORIENTATION SHAPE WELL-BEING ACROSS ADULT LIFE

Chair: Yoonseok Choi Co-Chair: Li Chu Discussant: Laura Carstensen

Individuals are able to maintain or even improve their wellbeing well into older adulthood. Socioemotional selectivity theory describes such age-related improvement in terms of motivational shifts toward emotionally meaningful goals with shrinking future time horizons. However, the association between future time perceptions and well-being is in need of being further clarified. In this symposium, four presentations will shed light on three different aspects of future time perceptions — time savoring, future preoccupation, thinking about the future — and their associations with wellbeing. Growney et al will present a study that utilizes a day reconstruction method to examine if time savoring is associated with psychosocial wellbeing and helping behaviors in workplaces. Pauly et al will discuss the potential benefits of time savoring for how solitude is experienced, by examining the buffering role of time savoring in depressive mood and somatic symptoms during solitude. Chu et al offers an alternative conceptualization of future time horizons, future preoccupation, capturing the worries and fixation on future preparation, and discusses its role in wellbeing across the life span. Choi et al discuss the role of thinking about the future for age-related differences in everyday experiences of happiness of varying arousal levels. The symposium will end with a discussion by Laura Carstensen integrating these findings into research on future time perspective and wellbeing across the life span. By elucidating more specific time-related experiences, our findings contribute to a more comprehensive understanding of how age-related changes in future time perspectives shape wellbeing trajectories across the life span.

Abstract citation ID: igad104.1818

### WORKPLACE EMOTIONAL EXPERIENCES, TIME SAVORING, AND HELPING BEHAVIORS IN AN AGE-DIVERSE SAMPLE OF ADULTS

Claire Growney[1], Li Chu[1], and Laura Carstensen[2], *1. Stanford University, Stanford, California, United States*, 2. *Stanford, Stanford, California, United States*

Socioemotional selectivity theory suggests that as future time perspective grows limited with age, older adults place greater priority on emotionally meaningful experiences and social relationships. Older adults are also theorized to savor time spent in emotionally meaningful activities and social interactions. A preliminary study found that age was associated with higher experience of positive emotion at work. In addition, we found that age was positively associated with trait-level reports of time-savoring, which in turn predicted higher engagement in helping behaviors. We will present findings from a study utilizing the day reconstruction method (DRM) that examines associations between time-savoring at work and engagement in positive workplace behaviors in full-time workers. For one full workweek, participants (N = 200, aged 25–80) complete a survey at the end of each day asking about their workdays parsed into three segments. They report on their social and emotional experiences, the extent to which they were savoring their time engaged in different workplace activities, and their helping (e.g., providing advice to a co-worker) and learning (e.g., developing new ideas or solutions to problems) behaviors during each segment of their workday. Findings have implications for workplace productivity in increasingly age-diverse workplaces. Time-savoring at work may be conducive to intergenerational transfers of knowledge.

Abstract citation ID: igad104.1819

### EVERYDAY SOLITUDE, TIME SAVORING, WELL-BEING, AND HEALTH IN OLDER ADULTHOOD

Theresa Pauly[1], Miriam Wallimann[2], and Shira Peleg[1], *1. Simon Fraser University, Vancouver, British Columbia, Canada*, 2. *University of Zurich, Zurich, Zurich, Switzerland*

Despite the significance of social relationships, solitude (being alone and without social interaction) forms an essential part of older adults' lives. The current study aimed to examine the role of time savouring (i.e., the ability to attend to positive experiences and up-regulate positive feelings) for mitigating associations of solitude with indicators of negative well-being and health in old age. For this purpose, a sample of 108 older adults aged 65–92 years took part in a daily diary study. Over 14 days, participants reported daily amount of time spent in solitude, extent of time savouring, levels of depressive mood, and somatic symptoms in an end-of-day diary. Data were analysed using multi-level models controlling for study day, age, gender, education, and living alone. Participants reported higher depressive mood but not more somatic symptoms on days on which they spent more time in solitude than usual. On days on which participants indicated higher time savouring than usual, they reported lower depressive mood and lower levels of somatic symptoms. Importantly, associations of solitude with depressive mood and somatic symptoms were weaker on days with increased time savouring. There were no person-level associations between solitude or time savouring with depressive mood or somatic symptoms. The study's findings highlight the importance of everyday time savouring in attenuating negative health-related aspects of solitude in older adults. Preserving and promoting time savouring in older adulthood may thus promote health and well-being in this age group.

Abstract citation ID: igad104.1820

### FUTURE PREOCCUPATION AND WELL-BEING: EXPANDING CONCEPTUALIZATION OF FUTURE TIME PERSPECTIVE

Li Chu[1], Tyler Matteson[1], and Laura Carstensen[2], *1. Stanford University, Stanford, California, United States*, 2. *Stanford, Stanford, California, United States*

Socioemotional selectivity theory posits that people prioritize emotionally meaningful goals as time horizons grow shorter and this shift in goals contributes to enhanced well-being. However, studies using the future time perspective (FTP) scale have yielded mixed findings regarding the association between time horizons and well-being. The present study aims to explore alternative constructs of time horizons to better understand well-being across the life span. In this preliminary study, a sample of 101 adults (age M = 46.99, SD = 19.20; 33 males, 66 females, and 2 indicated other gender identity) filled out an online questionnaire regarding time horizons and well-being. Exploratory and confirmatory factor analyses resulted in a new time horizon construct - time preoccupation, which captures the worries and fixations associated with future preparation. Serial mediation modeling shows the trend that people experience reduced future preoccupation with age, which contributes to more time savoring, and thus better well-being outcomes. Together, our findings suggest a promising role of future preoccupation in people's well-being across the life span. These findings have important implications for future interventions that aim to improve younger adults' well-being.

Abstract citation ID: igad104.1821

### AGE-RELATED DIFFERENCES IN DAILY EXPERIENCES OF HAPPINESS: THE ROLE OF THINKING ABOUT THE FUTURE

Yoonseok Choi[1], Jennifer Lay[2], Minjie Lu[3], Da Jiang[4], Helene Fung[5], Peter Graf[1], and Christiane Hoppmann[1], *1. University of British Columbia, Vancouver, British Columbia, Canada*, *2. University of Exeter, Exeter, England, United Kingdom*, *3. Beijing Normal University at Zhuhai, Zhuhai, Guangdong, China (People's Republic)*, *4. Education University of Hong Kong, Hong Kong, Hong Kong*, *5. The Chinese University of Hong Kong, Hong Kong, Hong Kong*

We examined the role of future time perspective (thinking about the future) in shaping age-related differences in time-varying experiences of happiness. Older adults' experience of happiness is more strongly associated with low-arousal than with high-arousal positive affect. Low-arousal positive affective states may be conducive to engaging in meaningful social interactions with close others (e.g., listening, adjusting to others) and therefore serve key socio-emotional goals that are prioritized when future time is perceived as limited. We hypothesized that thinking less about the future would be related to stronger associations between happiness and low-arousal positive states in older than younger adults. We used daily life assessments from 258 participants (M = 48.4 years; 68% female; 77% Asian; 73% post-secondary education), which comprised older (M = 63.4 years) and younger (M = 20.1 years) adult samples collected at two locations (Vancouver, Canada; Hong Kong). Participants reported on their momentary affective states and thinking about the future (0-100 scales) up to 30 times over 10 days. Results replicate previous findings by showing that momentary happiness was more strongly associated with momentary calmness and more weakly associated with momentary excitement among older as compared to younger adults. Younger adults reported thinking more about the future than older adults. Thinking less about the future was related to stronger happiness-calmness and weaker happiness-excitement associations in daily life for older participants, only; for younger participants, it was associated with weaker happiness-calmness associations. Age and future thinking-related contours of happiness are discussed in the context of emotional aging theories.

### SESSION 4485 (SYMPOSIUM)

Abstract citation ID: igad104.1822

### METHODOLOGICAL CHALLENGES AND OPPORTUNITIES FOR STUDYING RESILIENCE

Chair: Ravi Varadhan Co-Chair: Karen Bandeen-Roche Discussant: George Kuchel

Many older adults experience a major stressor at some point in their lives. The ability to recover and live well after a major stressor is known as resilience. An important goal of geriatric research is to identify factors which influence resilience to stressors, and ultimately, to devise strategies to enhance the resilience of older adults. In order to accomplish these worthy aims, several methodological challenges need to be addressed. Methodologies for studying resilience in clinical settings are only recently being developed. Investigators at Duke, Johns Hopkins, and Radboud Universities are studying cohorts of older individuals who are undergoing invasive clinical procedures using longitudinal designs. These studies collect a vast array of information on each participant including socio-demographics, health status, physical, cognitive, psychological, and biological functions. Also, large amount of experimental data is collected on the dynamics of select physiological systems as probes of resilience ("deep phenotyping"). Deep phenotyping leads to several challenges in the conduct and analysis of the study including selective participation (selection bias), informatively missing data, characterizing resilient responses based upon trajectories of multidimensional measures before and after stressor, modeling dynamical systems experimental data, and quantification of resilience capacity and its determinants. In this symposium, we will discuss some of the critical methodological challenges of studying resilience and present approaches to address them. At the end of this symposium, the audience members should understand the importance of designs and analytic challenges in studying resilience, and become knowledgeable on the approaches to overcome methodological challenges.

Abstract citation ID: igad104.1823

### IDENTIFYING PREDICTORS OF RESILIENCE IN SINGLE-ARM STUDIES OF PRE–POST CHANGE

Ravi Varadhan[1], Jiafeng Zhu[2], and Karen Bandeen-Roche[3], *1. Johns Hopkins University School of Medicine, Baltimore, Maryland, United States*, *2. Northwestern University, Chicago, Illinois, United States*, *3. Johns Hopkins University, Baltimore, Maryland, United States*

With advancing age, older adults are at a greater risk of experiencing significant stressors. While frailty is the increased vulnerability to stressors, resilience is the ability to respond well to a major stressor. An important goal of geriatric research is to identify factors which influence resilience to stressors. Studies of resilience in older adults are typically conducted using a single-arm design where everyone experiences the stressor. In such designs, resilience is typically quantified as the degree of recovery in physical/cognitive/psychological functions after the stressor. The simplistic approach of regressing change versus baseline yields biased estimates due to mathematical coupling and regression-to-the-mean. Correction is necessary for eliminating the bias and drawing valid inferences regarding the effect of covariates and baseline status on pre-post change. We present a simple method to correct this bias. We extend the method to include covariates. Our approach considers a counterfactual control group and involves sensitivity analyses to evaluate different settings of control group parameters. We illustrate the method using a large, registry of older adults (N=7,239) who underwent total knee replacement (TKR). We demonstrate how external data can be utilized to constrain the sensitivity analysis. Our analysis indicates that baseline (pre-stressor) function was not strongly linked to recovery after TKR. Among the covariates, only age had a consistent effect on post-stressor recovery. A main takeaway of our work is that studies of resilience should consider, either directly or indirectly, the use of an appropriate control group.

Abstract citation ID: igad104.1824

### LATENT VARIABLE MODELING FOR BIOLOGICALLY INFORMED PROGNOSIS IN STUDIES OF PHYSICAL RESILIENCE

Karen Bandeen-Roche[1], Qianli Xue[2], and Ravi Varadhan[3], *1. Johns Hopkins University, Baltimore, Maryland, United States*, *2. Johns Hopkins, Baltimore, Maryland, United States*, *3. Johns Hopkins University School of Medicine, Baltimore, Maryland, United States*

Physical resilience – rebound in relevant functioning and biomarkers following a health stressor – is hypothesized to be rooted in the level of fitness of stress-response physiology defining one's "physiological resilience capacity" (PRC). This physiology forms a dynamical system comprising specific modules (the individual stress response systems and their underlying components) and their dynamic interactions with each other via feedback and other protocols. Such a system can be modeled using differential equations whose parameters may then define the PRC. We do not yet know how to measure these parameters directly, however. Rather, they are conceptually defined "constructs" which must be inferred using indirect measures—ideally, stimulus response data probing multiple aspects of the relevant physiology. Latent variable models are ideally suited to this setting. Two challenges for their application in studies of resilience are presented: (1) Integrating specific scientific knowledge on the dynamical systems in modeling the co-distribution of the indirect measures. (2) Synergizing such models' advantages for construct measurement with advantages of machine learning approaches to optimize accuracy for predicting resilient outcomes by inferred PRC. Challenges and their proposed solutions are illustrated using multifaceted stimulus response data being collected in an ongoing investigation on the physiological basis of resilience to clinical stressors in older adults, the Study of Physical Resilience in agING. The work aims to produce physiologically rooted measures providing effective risk predictors for older adults facing impending stressors as well as intervention candidates by which to promote PRC in the longer term.

Abstract citation ID: igad104.1825

### APPROACHES TO QUANTITATIVELY EXAMINE RESILIENCE IN OLD AGE

Rene Melis[1], Almar Kok[2], and Martijn Huisman[2], *1. Radboud University Medical Center, Nijmegen, Gelderland, Netherlands*, *2. Amsterdam University Medical Center, Amsterdam, Noord-Holland, Netherlands*

Resilience is a complex system's ability to maintain or recover in function following a perturbation. The concept is generic and can be applied to any complex system going from a cell, a person, to their support networks. By applying a resilience approach, we can better understand why people respond differently to a similar exposure. While most people have an intuitive understanding of what resilience is, it's definition and application is not straightforward. Therefore, it is important to carefully define the stressor, system and (functional) outcome(s) and how it is studied using e.g., the TransNIH resilience framework. Quantitative examination of resilience can focus on predictors, mechanisms, trajectories and/or outcomes and each aspect requires a different analytical approach. This talk will review different quantitative approaches to examine resilience in the context of aging. These approaches can be categorized based on the statistical methods that are used to operationalize resilience: the effect modification approach to study how hypothesized resilience factors modify the outcome following perturbation, scale construction in the psychometric approach, comparison of characteristics between groups based on predefined prospective resilience responses in the a priori approach, data-driven subgroup identification based on resilience outcome (trajectories) in the clustering approach, analyzing predictors of adversity-outcome residual values in the residual approach, and analyzing stressor-response patterns in intensive longitudinal data to better understand resilience mechanisms. The approaches are not mutually exclusive. Researchers may choose to combine multiple approaches and may analyze the same data using multiple approaches to compare the findings between them.

Abstract citation ID: igad104.1826

### STATISTICAL MODELS FOR ESTIMATING RESILIENCE

Carl Pieper[1], Cathleen Colon-Emeric[2], Richard Sloane[3], Sarah Peskoe[1], and Heather Whitson[2], *1. Duke University School of Medicine, Durham, North Carolina, United States*, *2. Duke University, Durham, North Carolina, United States*, *3. Duke University Medical Center, Durham, North Carolina, United States*

One important component of the Resilience paradigm is the degree of initial change and rapidity of recovery after a stressor. Resilience is determined by both the degree of response, if any, to an insult and, subsequently, the process of return to the initial state. Modeling the recovery process requires use of longitudinal analysis which can incorporate the underlying non-linear trajectory of change, and the multiple systems impacted by the stressor. We will show the models we have employed for one common stressor, surgery. First, we will define and show findings on the Expected Predicted Differential (ERD), the difference between predicted and observed recovery. Second, we will show multivariate trajectories of recovery to demonstrate methods to (1) define typologies of recovery, using PCA, and (2) typologies of individuals in type of recovery, using latent class analysis of trajectories. Second, given the state of the emerging art in resilience research, we will provide a statistician's view on enhancing the resilience research through design (increasing number and timing of measurements, the number of subjects, and expanding use of the EHR), data structures (incorporating measurements from a greater number of diverse sources, using timing of measures to allow for assessment of dynamic mediation and moderation effects in defining the process), and analysis - extending the ERD to modeling non-linear recovery, incorporating causal analysis, including dynamic mediating and moderating effects into the analytic structure, use Area Under the Curve as a metric for recovery, latent class analysis, and use of multivariate trajectory analysis.

### SESSION 4490 (SYMPOSIUM)

Abstract citation ID: igad104.1827

### MECHANISMS UNDERLYING SLEEP AND DEVELOPMENT OF CHRONIC CONDITIONS IN OLDER ADULTS

Chair: Christopher Kaufmann Co-Chair: Soomi Lee
Discussant: Christina McCrae

Mounting evidence suggests poor sleep is associated with the development of chronic conditions. Numerous mechanisms for these links have been proposed, including inflammation, metabolic function, and brain structure. In this symposium, we will present five studies focused on links between poor sleep and subsequent development of chronic disease across adulthood. Specifically, we will examine this relationship at multiple levels from the cellular to the clinical level with the ultimate goal of identifying potential mechanisms and consequences of links between poor sleep and chronic conditions. Paper 1 will identify within-person patterns of various sleep experiences and link them to incident chronic conditions over a 10-year period. Paper 2 will examine associations between sleep parameters, measured via wrist actigraphy, and levels of adipokines, which are adipose-derived cytokines associated with development of metabolic disease. In Paper 3, we will shift our focus to examine the association between poor sleep and inflammatory biomarkers, specifically C-reactive protein, circulating and lipopolysaccharide-stimulated cytokines. Paper 4 will assess the impact of poor sleep health on fear of falling and actual falls. Paper 5 will focus on the effects of poor sleep and arousal with cognition and brain structure, specifically within a population of patients with chronic pain. As Discussant, Dr. Christina McCrae will integrate these findings and suggest directions for future research. In summary, our symposium will elucidate biological and psychological mechanisms linking poor sleep to manifestation of chronic disease. Better understanding of these mechanisms will inform the development of interventions to promote successful aging.

Abstract citation ID: igad104.1828

### SLEEP TIGHT, STAY HEALTHY? MULTIDIMENSIONAL SLEEP HEALTH AND CHRONIC CONDITION DEVELOPMENT OVER ONE DECADE

Claire Smith[1], and Soomi Lee[2], *1. University of South Florida, Tampa, Florida, United States*, *2. The Pennsylvania State University, University Park, Pennsylvania, United States*

Chronic conditions affect half of adults and are the leading cause of death in the United States. Healthy sleep is a modifiable risk factor, but greater information is needed about specific sleep experiences linked to chronic condition development. The present study aims to (1) identify within-person patterns of various sleep experiences (i.e., sleep health phenotypes describing sleep duration, regularity, sleep onset latency, insomnia symptoms, daytime tiredness, and nap frequency) and (2) understand the connection between transitions in sleep health phenotypes and chronic condition development. We use self-report data from a national sample of adults (N=5,026) taken from the Midlife in the United States (MIDUS) study across two waves (II and III) separated by about ten years. Latent transition analysis revealed four sleep phenotypes: good sleepers (optimal sleep across dimensions), insomnia sleepers (short duration, long sleep onset latency, high daytime tiredness, frequent insomnia symptoms), weekend catch-up sleepers (long sleep duration on weekends compared to weekdays), and nappers (frequent naps). For cardiovascular conditions, consistent insomnia sleepers (i.e., insomnia sleeper -> insomnia sleeper) were at 72% higher risk (CI:[1.04, 2.82]) than consistently good sleepers. For diabetes, consistent insomnia sleepers again were at higher risk (188%; CI:[1.72,4.79]) as were consistent nappers (128%; CI:[1.26,4.09]) and weekend catch-up sleepers -> nappers (137%; CI:[1.21,4.60]). For cancers, only nappers -> insomnia sleepers exhibited higher risk (45%; CI:[1.16,1.83]). We newly identify two sleep health phenotypes (napper and, especially, insomnia sleeper) as risks for developing life-threatening chronic conditions.

Abstract citation ID: igad104.1829

### ASSOCIATIONS OF ACTIGRAPHIC SLEEP WITH ADIPOKINES IN OLDER ADULTS

Yiwei Yue[1], Zhikui Wei[2], Jill Rabinowitz[1], Chee Chia[3], Toshiko Tanaka[3], Eleanor Simonsick[3], Luigi Ferrucci[3], and Adam Spira[1], *1. Johns Hopkins Bloomberg School of Public Health, Baltimore, Maryland, United States*, *2. Johns Hopkins School of Medicine, Baltimore, Maryland, United States*, *3. National Institute on Aging, National Institutes of Health, Baltimore, Maryland, United States*

Adipokines are adipose-derived cytokines that play important roles in metabolism and obesity. Accumulating evidence suggests that sleep disturbance could alter adipokine levels and contribute to systemic metabolic dysfunction in younger and middle-aged populations. Less is known about links between sleep disturbance and adipokines in older adults. We examined cross-sectional associations of actigraphic sleep parameters with adipokine levels in 353 community-dwelling older adults aged 74.8±8.3 years (51.3% male, 26.6% non-white) enrolled in the Baltimore Longitudinal Study of Aging. Participants completed 6.56±1.06 nights of wrist actigraphy and had morning fasting adipokine levels measured in plasma. Predictors included actigraphic total sleep time, sleep onset latency, wake after sleep onset, sleep efficiency, and average wake bout length (WBL), and outcomes were levels of the adipokines, specifically adiponectin and leptin. Adjusting for sex, age, race, years of education, BMI, hypertension, and diabetes, longer WBL was associated with lower adiponectin (B=-1.73, 95%CI: -3.07,-0.38) and leptin (B=-2.93, 95%CI: -4.61,-1.24). WBL interacted with sex (p-value for interaction = 0.018); higher WBL was associated with lower adiponectin in men (B=-4.12, 95%CI: -6.46, -1.79), but not in women (B=-0.34, 95%CI: -2.48,1.80). Findings link sleep fragmentation to lower leptin and adiponectin in older adults, particularly lower adiponectin in older men. Findings provide a potential mechanism by which disturbed sleep may promote metabolic derangements in later life. Prospective studies and sleep intervention trials are needed to evaluate whether these associations are causal, and to elucidate factors contributing to sex differences in these associations.

Abstract citation ID: igad104.1830

### GENDER DIFFERENCES IN THE ASSOCIATION BETWEEN SLEEP AND INFLAMMATORY MARKERS: RESULTS FROM THE EINSTEIN AGING STUDY

Linying Ji[1], Yuqi Shen[2], Jennifer Graham-Engeland[2], Carol Derby[3], Christopher Engeland[4], and Orfeu Buxton[2], *1. Montana State University, Bozeman, Montana, United States*, *2. Pennsylvania State University, State College, Pennsylvania, United States*, *3. Albert Einstein College of Medicine, Bronx, New York, United States*, *4. Pennsylvania State University, University Park, Pennsylvania, United Sta tes*

Prior research links poor sleep and higher systemic inflammation. Gender differences in both sleep and in inflammatory responses have been observed. We investigated gender differences in the association between levels as well as variability of objective sleep health and inflammatory markers (including stimulated inflammatory markers) among a diverse sample of older adults. Participants free of dementia (N=215, Mage=77.5 years, 64% women; 46% White, 39% Black, 15% Hispanic/other) were from The Einstein Aging Study. Actigraphy (2 weeks) yielded wake after sleep onset (WASO), total night sleep time (TST), and midpoint night sleep. Inflammatory markers included circulating C-reactive protein (CRP) and circulating and lipopolysaccharide (LPS)-stimulated levels of interleukin (IL)-1β, IL-4, IL-6, IL-8, IL-10, and tumor necrosis factor (TNF)-$\alpha$. Bayesian variability models evaluated associations of inflammatory markers with mean and variability of sleep measures, adjusted for BMI, gender, age, education, race/ethnicity. Circulating inflammatory markers were not associated with sleep measures, except higher TST variability with higher circulating IL-6 among women. For stimulated cytokines, among men, higher mean WASO, TST, and later midpoint were associated with higher stimulated IL-8 and TNF-$\alpha$. All stimulated cytokines except IL-1β were associated with greater variability in WASO, TST, and sleep timing among men. Among women, the only significant association with stimulated cytokines was between higher mean TST and higher stimulated IL-8. We found gender differences in the associations between sleep and inflammation in older adults, primarily in men and for stimulated (not circulating) cytokines. Interestingly, associations were also more frequently observed for sleep variability than mean levels.

Abstract citation ID: igad104.1831

### POOR SLEEP HEALTH MAY BUILD THE ONSET OF A FEAR OF FALLING AMONG COMMUNITY-DWELLING OLDER ADULTS

Tuo-Yu Chen[1], Soomi Lee[2], Kai-Wen Hsu[1], and Orfeu Buxton[3], *1. Taipei Medical University, Taipei, Taipei, Taiwan (Republic of China)*, *2. The Pennsylvania State University, University Park, Pennsylvania, United States*, *3. Pennsylvania State University, State College, Pennsylvania, United States*

A greater fear of falling (FOF) predicts activity restrictions, falls, disability, and increases the risk of mortality among older adults. Although poor sleep has been identified as a relevant factor for FOF among older adults, evidence is primarily shown in cross-sectional studies using isolated sleep characteristics. Whether multidimensional sleep health contributes to increase in FOF among older adults who are particularly vulnerable to falls is less well understood. We investigated the longitudinal relationship between sleep health and the incidence of FOF among community-dwelling older adults and how the association differed between those with or without prior falls experiences. Data were from individuals who completed the sleep module in the National Health and Aging Trends Study (2013-2014; n=695). FOF was assessed with a single item. Multidimensional sleep health was measured with self-reported sleep items based on the SATED model (i.e., sleep satisfaction, daytime alertness, timing, efficiency, and duration). Covariates included sociodemographics, assistive device usage, health, and sleep medications. Multiple logistic regression was used to analyze the data. Poor sleep health was associated with the onset of FOF at 1-year follow-up (OR=1.20, 95%CI=1.02-1.41). Moreover, poor sleep health increased the odds of having FOF among individuals without prior falls experiences and elevated the already heightened risks of developing FOF among those who fell at baseline. Improving sleep health may potentially prevent older adults from developing FOF.

Abstract citation ID: igad104.1832

### INTERACTIVE ROLE OF SLEEP AND AROUSAL ON COGNITION AND BRAIN STRUCTURE IN CHRONIC PAIN PATIENTS

Ashley Curtis[1], Neetu Nair[2], Jason Craggs[1], Kevin McGovney[2], and Christina McCrae[1], *1. University of South Florida, Tampa, Florida, United States*, *2. University of Missouri, Columbia, Missouri, United States*

Chronic widespread pain (CWP) patients experience heightened arousal, insomnia complaints, poor cognition, and diffuse brain structure alterations, but interactive relationships among symptoms remain unexplored. We assessed whether physiological arousal [assessed via heart rate variability (HRV)] moderated associations between sleep and cognition/brain structure. Adults (N=42, Mage=46.2±13.7) with CWP and insomnia (difficulty falling/staying asleep) completed 14 daily sleep diaries, 5-minute Holter monitoring [HRV root mean square of successive normal heartbeats (RMSSD), low frequency/high frequency (lf/hf) computed], cognitive tasks [Stroop (processing speed/attention/inhibition), Sternberg (working memory)], and magnetic resonance imaging. Moderation analyses examined interactive associations between sleep [total sleep time (TST), wake after sleep onset, sleep onset latency (SOL)] and HRV with cognition/gray matter volume of frontal/temporal regions. SOL and lf/hf interacted in associations with working memory and attention/processing speed. Longer SOL was associated with worse Sternberg/Stroop at lowest sympathetic predominance levels. Conversely, longer SOL was associated with better Stroop at highest lf/hf. SOL and RMSSD interacted in associations with anterior cingulate, showing longer SOL associated with lower volumes at lowest arousal levels. TST and RMSSD interacted in associations with right hippocampus, with shorter TST associated with lower volume at highest/average arousal levels. Shared dynamic physiological arousal and sleep onset hyperarousal mechanisms impact cognition and neural structure in CWP. Longer SOL may only negatively impact cognition/anterior cingulate structure up to a certain arousal threshold, with shared mechanisms potentially benefitting cognition at highest arousal levels. At higher arousal, longer TST may protect against hippocampal volume reduction. Future prospective studies are needed to inform temporal characteristics.

### SESSION 4495 (SYMPOSIUM)

Abstract citation ID: igad104.1833

### TECHNOLOGY-BASED ASSESSMENT OF EMOTIONS AND SOCIAL BEHAVIORS IN OLDER ADULTS' AND COUPLES' HOMES

Chair: Kuan-Hua Chen Discussant: Robert Levenson

As people age, emotions and close social interactions are increasingly important because our social circle tends to shrink. Research on social and emotional aging typically relies on questionnaires and laboratory assessments. However, most emotions and social behaviors occur in naturalistic and interpersonal contexts. Advances in modern technologies, including telecommunications, mobile or embedded devices and sensors, provide researchers with exciting opportunities for naturalistic, long-term, objective, and timely assessment of emotions and social behaviors outside of the lab. Increased accessibility of technologies further allows community-based, large-scale assessment. The proposed symposium highlights recent advances in-home assessment of emotions and social behaviors from four different institutions that leverage various active (e.g., ecological momentary assessment, portable lab) and passive (i.e., motion and door sensors, wearables) technologies to assess multiple aspects of emotions and social behaviors (e.g., physiological responses, emotional experience, behaviors and interpersonal behavioral synchrony, apathy, agitation) in healthy and clinical aging. Four presentations covering four different exemplary studies will be given by researchers at different career stages with diverse ethnic/cultural and training backgrounds (e.g., mechanical engineering, psychology, neuroscience). An integrated discussion will be given by a senior researcher and leading expert in the area. Together, the symposium will provide the general audience with an overview of recent advances in in-home assessment technologies. This symposium will also launch communication and collaborations between researchers from different disciplines who use (or plan to adopt) contemporary technologies to conduct research in naturalistic settings to promote a better understanding of human social and emotional aging.

Abstract citation ID: igad104.1834

### REMOTE ASSESSMENTS OF EMOTIONAL FUNCTIONING FOR INDIVIDUALS WITH DEMENTIA AND THEIR CAREGIVERS

Casey Brown[1], Kuan-Hua Chen[2], Julian Scheffer[2], Deepak Paul[2], and Robert Levenson[2], *1. Georgetown University, Washington, District of Columbia, United States*, *2. University of California, Berkeley, Berkeley, California, United States*

Emotional impairments are a common symptom of neurodegenerative disorders that can be devastating for persons with the dementia (PWDs) and family members who provide care (i.e., caregivers). Emotional functioning can be well characterized and quantified using laboratory assessments of behavioral, physiological, and self-reported responses to emotional stimuli. However, continuous declines in functioning make it especially difficult for PWDs and caregivers to visit a research laboratory repeatedly over time. In order to understand changes in emotional functioning across time and their impact on PWDs and caregivers longitudinally, there is a need for technology to support remote assessments of emotional functioning. We developed a portable version of laboratory assessments—Laboratory in a Suitcase Assessment (LISA)—to collect data from PWDs and their caregivers remotely in their homes. In a shippable suitcase, LISA provides the necessary equipment for: (a) presenting emotional stimuli via laptop screen and headphones; (b) obtaining audio and video recordings of participants' behavior; (c) obtaining continuous ratings of emotional experience via a rating dial; (d) measuring cardiovascular, electrodermal, and somatic responses from PWDs and caregivers via wearable devices; (e) synchronizing behavior and physiology; and (f) maintaining two-way communication with participants via Zoom. We present high quality physiological, behavioral, and self-report data collected using LISA with 17 PWD-caregiver dyads. Data collected with LISA are comparable to data collected in the laboratory, indicating that LISA could enable researchers to continue data collection further into the course of disease when travel to in-person laboratory sessions would no longer be feasible.

Abstract citation ID: igad104.1835

### THINKING ABOUT TIME INFLUENCES EMOTIONAL EXPERIENCE DIFFERENTLY IN OLDER AND YOUNGER ADULTS

Enna Chen[1], Claire Growney[1], and Laura Carstensen[2], *1. Stanford University, Stanford, California, United States*, *2. Stanford, Stanford, California, United States*

According to socioemotional selectivity theory (SST), as future time horizons shorten with age, people come to prioritize present-oriented, emotionally meaningful goals. SST posits that present orientation contributes to emotional well-being. Using experience sampling, the present study examined (1) age differences in momentary time orientations, (2) associations between momentary time orientations and emotional well-being, and (3) age moderation of these associations. A sample of 190 people, aged 18 to 94 years, participated in an ecological momentary assessment study where they simultaneously reported time orientations and experienced emotions at five randomly selected times every day for seven days. . We found that age was not associated with time orientation. However, on occasions when people thought about the past, they experienced less positive affect and more negative affect. On occasions when people thought about the present, they experienced more positive affect. All of these effects were weaker for older as compared to younger participants. However, the association between thinking about the future and positive or negative affect did not vary by age. Findings suggest that younger adults, even more than older adults, benefit emotionally from focusing on the present.

Abstract citation ID: igad104.1836

### PHYSIOLOGICAL LINKAGE WITH CAREGIVERS IS ASSOCIATED WITH EMOTION RECOGNITION IN CARE RECIPIENTS

Kuan-Hua Chen[1], James Casey[2], Casey Brown[3], Jennifer Merrilees[4], and Robert Levenson[2], *1. University of Nebraska Medical Center, Omaha, Nebraska, United States, 2. University of California, Berkeley, Berkeley, California, United States, 3. Georgetown University, Washington, District of Columbia, United States, 4. University of California, San Francisco, San Francisco, California, United States*

Persons with neurodegenerative diseases (PWDs) often develop profound declines in the ability to recognize emotions in others that lead to diminished social connectedness with their loved ones. Physiological linkage, which refers to the degree that people's physiological responses change in coordinated ways, can serve as a proxy measure for social connectedness. Physiological linkage has been found to be diminished during interactions between PWDs (i.e., care recipients) and their family caregivers. However, the association between diminished physiological linkage and PWDs' lower emotion recognition has not been determined. In a sample of 28 PWD-family caregiver dyads, we quantified physiological linkage as the positive ("in-phase") correlations between PWD's and caregiver's somatic activity. Somatic activity was measured remotely via actigraphy using wristwatches worn by both partners in their homes during waking hours for 7 days. PWDs' emotion recognition was quantified as the ability to recognize positive and negative emotions of their caregivers accurately during a 10-minute conflict conversation in our laboratory prior to the 7-day home assessment. Lower emotion recognition by PWDs of their caregivers' negative emotions ($\beta = .49$, $p = .01$), but not positive emotions ($\beta = .12$, $p = .52$) in the laboratory assessment was associated with lower physiological linkage between PWDs and caregivers in their home. Establishing a relationship between PWDs' deficits in emotion recognition and diminished physiological linkage during conversations with their caregivers provides a basis for understanding problems in establishing social connections that are often observed in PWDs.

Abstract citation ID: igad104.1837

### ASSESSMENT OF OLDER ADULTS' BEHAVIORAL SYMPTOMS AND THEIR SOCIAL BEHAVIORS USING SURVEYS AND A TECHNOLOGY PLATFORM

Wan-Tai Au-Yeung, Lyndsey Miller, Allison Lindauer, Nathaniel Rodrigues, Cierra Leon Guerrero, Sarah Gothard, Zach Beattie, and Jeffrey Kaye, *Oregon Health & Science University, Portland, Oregon, United States*

Behavioral symptoms are highly prevalent during the course of cognitive decline associated with dementia. Studies have shown that the social environment may affect these symptoms. Data sources examining the effect of environment come from two ongoing studies: 1) MOnitoring DEmentia-Related Agitation Using Technology Evaluations (MODERATE), focusing on agitation in individuals with later-stage dementia, and 2) Monitoring Apathy, Depression and Anxiety Using Technology Evaluations (ADA), focusing on apathy, depression and anxiety in older adults with or without cognitive decline. To date, a total of 9 dyads have enrolled in MODERATE while a total of 9 participants have enrolled in ADA. Caregivers (MODERATE) and participants with cognitive impairment (ADA) report symptoms via weekly online surveys. MODERATE integrates a modified Cohen-Mansfield Agitation Inventory-Short Form series of questions regarding weekly agitation-related levels/events. The ADA weekly online survey consists of Withdrawal-Apathy-Vigor, a subscale from Geriatric Depression Scale-30, a 4-item depression subscale from Patient-Reported Outcomes Measurement Information System-29 (PROMIS-29), and a 4-item anxiety subscale from PROMIS-29. A multi-sensor array (bed pressure mats, motion sensors, and wearables) is deployed in homes to objectively measure participants' behaviors. With this array, duration of time-out of home, duration of simultaneous activities detected in more than one room, and the total number of occupants and pets within the home are extracted. Additionally, weekly online surveys inquire as to whether participants have been out of town or have overnight visitors. Associations between social activity and behavioral symptoms are obtained combining digital behavioral-activity biomarkers with frequently reported online behavioral data.

### SESSION 4500 (PAPER)

### CHANGE, LOSS, AND LONELINESS

Abstract citation ID: igad104.1838

### GENDER-BASED VIOLENCE IN HOMICIDE–SUICIDE EVENTS: A CALL FOR ADHERENCE TO THE CDC DEFINITION FOR "MERCY KILLING"

Sonia Salari, Carrie Sillito, and Emma Videla, *University of Utah, Salt Lake City, Utah, United States*

Intimate partner homicide suicide (IPHS) events are overwhelmingly male perpetrated (90%). Gender-based violence (GBV) prevention requires 'best practices' for media reporting. Consider the following: In a patient room of a busy hospital, an overwhelmed man (71) suicided after firing shots at his wife (70) who had survived a stroke. The gunfire caused trauma among witnesses. Media reports used the term 'mercy killing,' but critical ingredients were missing. The CDC's NVDRS (2022) defines 'mercy killing' as "…Killed, at the victim's request…to end…pain or distress…Do not assume…murder/suicide by a sick, elderly couple is mercy killing." Evidence of a clear wish to die, is needed "due to a hopeless or terminal condition." Our IPHS, news surveillance study was updated 1999-2022 and used content analysis to identify GBV rationalizations, based upon 2219 events and over 5000 fatalities across young (n=987), middle aged (n=699), and older 60+ (n=533) categories. Only the 60+ group had claims of 'mercy killing,' mostly without adherence to the CDC definition. Older men who are suicidal may also harm an unsuspecting partner. For women, end of life decision-making is lost in 'safe spaces,' like homes or care facilities, which is made worse by rationalized excuses. Women are rarely suicidal and use passive methods (i.e., drugs). When women care for an ailing partner, they don't commit IPHS. Older men are more suicidal and utilize more lethal firearm methods (similar to the IPHS patterns). Media best practices involve valuing persons with disabilities, publicizing services and recognizing violence is a public health problem.

Abstract citation ID: igad104.1839

### MIGRATION AS A PATHWAY TO KINLESSNESS AMONG OLDER ADULTS LEFT BEHIND

Amilcar Matos-Moreno[1], Alexis Santos-Lozada[1], Mara Sheftel[2], and Ashton Verdery[3], *1. Pennsylvania State University, State College, Pennsylvania, United States*, *2. Penn State University, State College, Pennsylvania, United States*, *3. The Pennsylvania State University, University Park, Pennsylvania, United States*

Recent research examines the topic of kinless adults, people over the age of 50 who have neither a living spouse nor living children. Crossnational examinations find that the prevalence of kinlessness in these age groups ranges between 2% and 10% in most countries around the world and that kinless individuals tend to be disadvantaged on health and well-being dimensions. Prior work has not, however, examined migration in the context of kinlessness. Given high rates of migration in numerous sending contexts around the world, this study examines the prevalence of kinlessness in Puerto Rico, Mexico, Costa Rica, and the United States incorporating migration as a pathway to kinlessness and examining its association with well-being. Using surveys from the Health and Retirement family of studies from Puerto Rico, Mexico, Costa Rica, and the United States, we estimate the prevalence of kinlessness focusing on how these estimates change when treating adult family migrants as not available for the provision of social support. We also estimate the association between kinless typologies and health status. Puerto Rico exhibits the highest older adult kinlessness prevalence (Puerto Rico: 8.4%, Costa Rica:6.0%, Mexico:6.5%, United States:7.2%). The incorporation of migration as a pathway to kinlessness increases the prevalence in Puerto Rico (+3.8%) and Mexico (+5.2%) more so than for Costa Rica or United States. Kinlessness is associated with higher levels of depression among Puerto Rican older adults. This study reveals the importance of considering migration as a pathway to kinlessness in high outmigration countries and its impact on health.

Abstract citation ID: igad104.1840

### SOCIAL CONNECTEDNESS ACROSS THE LIFE COURSE: CORE NETWORKS AND EVERYDAY SOCIAL INTERACTIONS OF AMERICAN ADULTS

Siyun Peng[1], Adam Roth[2], and Brea Perry[1], *1. Indiana University, Bloomington, Indiana, United States*, *2. Oklahoma State University, Stillwater, Oklahoma, United States*

Scholars have been monitoring the signs of social isolation and weakening of family ties among Americans. This paper asks: (1) Do Americans socially disengage from society as they age? (2) Are family ties replaced by work or friends as Americans grow out of their original family? Using a state representative sample of egocentric network data (Person to Person Study: P2P) and a national representative sample of time dairy data (American Time Use Survey: ATUS) during 2018-2021, we found that although people spent less social time with others as they age, they maintained a stable sized core network of confidants for discussing important and health matters. This refutes the view that old age has a universal negative influence on social connectedness. We found little evidence that family ties are replaced by non-kins at any age in both core network and daily social contact. By combining core network and time dairy data, we found that although people spent substantial time with coworkers daily when they enter the workforce, they rarely included even a single coworker into their core network. This suggests that people's core network is less influenced by current daily social interactions than many theories on social contact would predict. We speculate that people may organize their core network based on cumulative time they spend with a particular person over the life course rather than current time. The roles of life events and age in shaping changes in daily interactions and core network over the life course are further discussed.

Abstract citation ID: igad104.1841

### THE CO-EVOLUTION OF PERSONAL NETWORKS AND LONELINESS FOLLOWING WIDOWHOOD FOR MEN AND WOMEN

Jina Lee[1], and Markus Schafer[2], *1. University of Toronto, Toronto, Ontario, Canada*, *2. Baylor University, Waco, Texas, United States*

Late-life transitions such as widowhood often re-calibrate people's close personal networks. Some network change—particularly growth in network size and closer geographic access to network members—is assumed to protect against loneliness, but scholars have yet to systematically examine these processes post widowhood. This study uses data from four waves of the German Aging Survey (DEAS), conducting gender-specific hybrid panel modeling to estimate both within- and between-individual effects of (1) network conditions up to seven years past widowhood, and (2) the effects of network change on loneliness. Results reveal that network size takes on a reversed U-shape: Germans becoming widowed tend to see an influx of new core ties from beyond their family, but this trend slows and reverses with time. There was also some evidence of non-linear change related to distance: geographic distance to core network members tended to shrink following widowhood before expanding back outward. Furthermore, moderation analyses an important role for these network characteristics on loneliness. Larger non-kin networks in the aftermath of widowhood partially alleviated the loneliness associated with that transition. The role of geographic proximity was gender-specific, as widowed men with nearby non-kin ties were most protected against loneliness, whereas widowed women fared best if their kin ties were farther away. Altogether, this study presents novel insight into how personal networks evolve after widowhood, revealing the nuanced, gendered ways that networks adapt. Efforts to reduce loneliness after widowhood may consider how gender roles and expectations shape the transmission and meaning of companionship and support.

### SESSION 4505 (PAPER)

### DIET, FRAILTY, AND DISABILITY

Abstract citation ID: igad104.1842

#### ASSOCIATION BETWEEN BALANCED DIET AND FRAILTY IN JAPANESE OLDER ADULTS: THE MODERATING ROLE OF SOCIAL PARTICIPATION

Yaya Li, Yuya Akagi, Natsumi Fujiwara, Hiroko Yoshida, Michiko Kido, and Mai Kabayama, *Osaka University, Suita, Osaka, Japan*

The association between diet and frailty in older adults may be related to social factors. This study aimed to investigate whether there is an interaction effect between balanced diet and social participation on frailty in older Japanese. Balanced diet was defined as the consumption of more than one meal per day that contained carbohydrates, protein, and fiber. Data were collected from 4,832 community-dwelling older adults aged 65-89 years through self-reported questionnaires (response rate 49.0%). Frailty was measured using the Kihon Checklist developed by the Ministry of Health, Labour, and Welfare. We stratified participants by age (65-74 and 75-89 years) and conducted logistic regression models adjusted for economic status, gender, marital status, living alone, disease, and physical activity. The interaction between dietary diversity and social participation in frailty was also assessed. Results showed that in both age groups, consuming balanced diet was associated with a lower risk of frailty (OR=0.56, 95%CI=0.40–0.79). Lack of social participation was found to be a risk factor for frailty (OR=1.51, 95%CI=1.03–2.21). Furthermore, the interaction between balanced diet and social participation revealed that a combination of lack of social participation and balanced diet was also associated with a lower frailty risk in the 75-89 age group (OR=0.63, 95%CI=0.40–0.99). These findings suggest that balanced diet consumed at least once a day may be beneficial for preventing frailty for older adults, however, social participation should also be taken into consideration for those aged 75-89. This study has some limitations as a cross-sectional study. Longitudinal research is needed.

Abstract citation ID: igad104.1843

#### PILOT RCT TO EXAMINE A MOBILE HEALTHY EATING INTERVENTION FOR PERSONS WITH FRAILTY

Oleg Zaslavsky[1], Kuan-Ching Wu[1], Aishwarya Naik[1], and Yan Su[2], *1. University of Washington, Seattle, Washington, United States*, *2. Illinois State University, Normal, Illinois, United States*

New scalable approaches are needed to promote healthy eating and improve health in persons with frailty. We evaluated a new mobile intervention consisting of a patient-facing app and clinician interface to support Mediterranean-style eating in a pilot RCT. Fourteen females and one male, averaging 71 years of age, were randomized to intervention (n=8) or control (n=7) for three months. In the intervention, participants were encouraged to interact with the app at least once a week, whereas, in control, participants received handouts with information and online resources concerning the Mediterranean Diet. Mann-Whitney and Cohen's d estimates were used to compare groups and evaluate effects. All participants completed and consistently adhered to the study protocol throughout the study. At the end of the study, there was a significant improvement in the intervention compared to the control in the following outcomes: SPPB (W=45, p=0.049; d=1.14); Mediterranean Diet Knowledge (W=49.5,p=0.013; d=1.26); consumption of vegetables (W=47, p=0.03; d =1.42); consumption of legumes (W=54, P=0.001; d=1.32), and negative outcome expectations (W=9,p=0.03; d=0.56). Albeit insignificant, intervention group participants improved their inflammatory, lipids, and glycemic blood measures. At the exit interviews, most participants expressed satisfaction with the app and acknowledged that the app was useful and easy to operate. They also provided points for improvement concerning control over the frequency of interactions with the app, more personalized choices for meals, and better visualization of their progress. The study demonstrated the preliminary efficacy of the digital intervention and suggested several mechanistic measures involved in behavioral change.

### SESSION 4510 (PAPER)

### DISPARITIES AND DIFFERENCES FROM BEGINNING TO END

Abstract citation ID: igad104.1844

#### A THREE-TIER EXAMINATION OF RACIAL DISPARITIES IN PAIN AT THE END OF LIFE

Zainab Suntai[1], Susanny Beltran[2], and Elyse McMullen[1], *1. Baylor University, Waco, Texas, United States*, *2. University of Central Florida, Orlando, Florida, United States*

Over the past few decades, research focused on improving end-of-life outcomes has burgeoned within the field of gerontology. Pain is one of the most common symptoms assessed at the end of life and research has shown that Black and Hispanic older adults are significantly more likely to experience pain compared to White older adults. However, few studies have sought to describe racial/ethnic differences in help-seeking behavior for pain and satisfaction with pain management among older adults. This study employed a three-tier approach by investigating 1) differences in the experience of pain at the end of life, 2) differences in help-seeking behavior for pain at the end of life, and 3) differences in satisfaction with pain management at the end of life. Data were derived from the combined 2012 to 2020 last-month-of-life interviews as part of the National Health and Aging Trends Study. Using a multivariate logistic regression model, results showed that Black and Hispanic older adults were more likely to experience pain at the end of life, less likely to have received help for their pain, and less likely to report that they were satisfied with the level of help received. These results point to a disparity in not only the level of pain experienced at the end of life, but the treatment and quality of pain management. Implications for aging research, policy, and practice are discussed.

Abstract citation ID: igad104.1845

### DIFFERENCES IN END-OF-LIFE OUTCOMES BETWEEN IMMIGRANT AND NONIMMIGRANT OLDER ADULTS

Zainab Suntai[1], Edson Chipalo[2], and Susanny Beltran[3], *1. Baylor University, Waco, Texas, United States*, *2. Lewis University, Romeoville, Illinois, United States*, *3. University of Central Florida, Orlando, Florida, United States*

Although the number of immigrants in the United States has continued to grow, few research studies have focused on the experiences of older immigrants at the end life. Research on the immigration experience shows that there are long-lasting physical, mental, and psychological symptoms that continue to affect immigrants even long after arriving in a new country. To better understand how the immigration experience may influence outcomes in late life, this study examined differences between immigrant and non-immigrant older adults in the following end-of-life outcomes: being alone at death, being in pain at the end of life, having anxiety and/or depression in their final months of life, and the quality of care received at the end of life. Data were taken from the combined 2012 to 2020 National Health and Aging Trends study, which is an annual longitudinal study of Medicare beneficiaries aged 65 and older. Chi-square tests were used for bivariate analysis, and multivariate logistic regression models were used to assess differences between immigrant and non-immigrant older adults. The results showed that immigrant older adults were more likely to die alone at the end of life, more likely to be in pain at the end of life, more likely to experience anxiety and/or depression at the end of life, and less likely to receive good quality care at the end of life. Implications for practice, policy, and research are provided.

Abstract citation ID: igad104.1846

### GENDER DISPARITIES IN THE MENTAL HEALTH OF CAREGIVERS BEFORE AND DURING THE COVID-19 PANDEMIC

Lucie Kalousova, and Brina Ratangee, *Vanderbilt University, Nashville, Tennessee, United States*

Prior research has shown that many have experienced worsened mental health during the COVID-19 pandemic. This study uses a large, nationally representative sample of caregivers interviewed by the Behavioral Risk Factor Surveillance System (BRFSS). It implements regression models to examine the changes in mental health among informal caregivers for older adults, who are a high-risk group for COVID-19 infection. We ask: (1) has the average number of poor mental health days among caregivers increased during the pandemic; (2) has the increase varied by gender; (3) the condition of the person receiving care? Our results indicate that caregivers experienced a sharp increase in poor mental health outcomes at the onset of the pandemic, much sharper than non-caregivers. In 2021 and 2022, male caregivers' mental health returned to pre-pandemic levels, while women's caregivers did not. We hypothesize that gender disparity in mental health outcomes could be attributed to differences in the types of caregiving responsibilities, the level of stress experienced, and the social support available. Furthermore, our study also shows that caregivers providing care to Alzheimer's disease (AD) patients have worse mental health outcomes than other caregivers. Women who care for AD patients have worse mental health than male AD caregivers, whose mental health did not significantly differ from other caregivers. The findings could inform policy decisions and support the development of interventions that aim to mitigate the negative impact of the pandemic on caregivers' mental health.

Abstract citation ID: igad104.1847

### GENDER INEQUALITIES IN THE DISRUPTION OF LONG-TERM LIFE SATISFACTION TRAJECTORIES DURING COVID-19

Dario Moreno-Agostino[1], Jayati Das-Munshi[2], and George Ploubidis[3], *1. UCL Centre for Longitudinal Studies and ESRC Centre for Society and Mental Health, London, England, United Kingdom*, *2. IOPPN, KCL, London, England, United Kingdom*, *3. University College London, London, England, United Kingdom*

Evidence suggests that women's mental health has been disproportionately impacted by the COVID-19 pandemic. Time-use differences have been hypothesised as an explanatory factor, as women generally spent more time than men on psychologically taxing activities. We used data on life satisfaction spanning 25 years (1996-2021, age 26-51) from a birth cohort representing adults born in Britain in 1970 (n=6,766). We used piecewise latent growth curve models to study gender differences in the long-term trajectories of life satisfaction and in the impact of the pandemic onset on these trajectories, and whether time-use differences partly explained that differential impact. Women had consistently higher life satisfaction levels prior to the pandemic ($\Delta$intercept,unadjusted=0.204 [95% CI: 0.073, 0.336], p=.002), and then experienced a more accelerated decline with its onset ($\Delta$quad2,unadjusted=-0.019 [-0.026, -0.011], p<.001). This gap remained stable after accounting for time use differences ($\Delta$quad2,adjusted=-0.019 [-0.028, -0.011], p<.001). Nevertheless, time spent working was positively related to life satisfaction in women but negatively in men ($\Delta$working1-8h=0.499 [-0.168, 0.829], p= .003; $\Delta$working>8h=0.543 [0.185, 0.902], p=.003), whereas time spent doing housework was negatively related to life satisfaction in women but positively in men ($\Delta$housework1h=-0.723 [-0.749, -0.045], p=.027). Results were robust to adjustment for the concurrent financial situation. Our study shows gender inequalities in the impact of the pandemic on the long-term life satisfaction trajectories of adults in their 50s and suggests that time-use differences do not explain these inequalities. Further research on the mechanisms underlying the differential impact of the pandemic across genders in positive mental health is needed.

Abstract citation ID: igad104.1848

### HEALTH CARE ACCESS AND USE AMONG ADULTS AGING WITH INTELLECTUAL AND DEVELOPMENTAL DISABILITIES

Jeffrey E. Stokes[1], Danielle A. Waldron[2], Jacquelin Sauer[3], and Emily Hartford[3], *1. University of Massachusetts Boston, Boston, Massachusetts, United States*, *2. Stonehill College, North Easton, Massachusetts, United States*, *3. Stonehill College, Easton, Massachusetts, United States*

Adults with intellectual and developmental disabilities (IDD) are living longer than ever before, a testament to advancements in healthcare. However, adults with IDD face a number of challenges accessing healthcare, and particularly preventative healthcare targeted towards midlife and older adults, which have previously been far less utilized by adults aging with IDD. This study uses longitudinal data from the National Core Indicators In-Person Survey (NCI-IPS) to examine factors associated with adults with IDD's utilization of two preventative healthcare measures specific to mid- and later life: mammograms and colonoscopies. Both mammogram and colonoscopy utilization exhibited curvilinear age trajectories, with usage increasing through ages 60 and 65 respectively, before decline at older ages. For both mammogram and colonoscopy utilization, living with family was associated with a 50% lower likelihood of utilization, while poor health, access to transportation, and community inclusion were associated with increased usage. For mammograms, participating in a community activity was also associated with increased utilization. Women with IDD were also about 25% less likely to receive a colonoscopy than men. Interestingly, the likelihood of receiving a mammogram dropped significantly in 2018-2019 compared to a decade prior, whereas the likelihood of receiving a colonoscopy increased over time. No racial/ethnic differences were found. Results highlight a number of factors associated with preventative healthcare use among the population of adults aging with IDD. These were similar for mammogram and colonoscopy utilization, and highlight the importance of living situation, transportation, and community engagement for adults with IDD's access to preventive medicine.

### SESSION 4515 (PAPER)

### FACTORS IMPACTING RESIDENTS IN LONG-TERM CARE

Abstract citation ID: igad104.1849

#### A SYSTEMATIC REVIEW OF MEASURES OF SOCIAL CONNECTION FOR PEOPLE LIVING IN LONG-TERM CARE HOMES: THE SONNET STUDY

Jennifer Bethell[1], Andrew Sommerlad[2], Hannah Chapman[2], Neha Dewan[1], Madalena Liougas[3], Gill Livingston[2], and Katherine McGilton[1], *1. Toronto Rehabilitation Institute - University Health Network, Toronto, Ontario, Canada*, *2. University College London, London, England, United Kingdom*, *3. University of Toronto, Toronto, Ontario, Canada*

Social connection is important for quality of life and care in long-term care (LTC) homes. However, measuring social connection in LTC residents is challenging and research is limited by a lack of consensus on best approaches to measurement. Our study objective is to systematically review measures of social connection developed for LTC residents. We are following COnsensus-based Standards for the selection of health Measurement INstruments (COSMIN) systematic review methods. We searched multiple bibliographic databases to April 2022 for studies conducted in LTC residents that quantified any aspect(s) of social connection and reported at least one psychometric property for the measure(s) of social connection. We are using COSMIN guidelines to evaluate the properties reported for each measure. We identified 60 studies reporting on 38 measures that assess social connection in LTC homes. Twenty-eight were measures of quality of life, wellbeing or life satisfaction and included a social connection subdomain. Only 10 measures specifically targeted social connection. From our evaluation of 32 measures to date, we found that 16% (n=5) have sufficient structural validity, 9% (n=3) have sufficient internal consistency, 22% (n=7) have sufficient reliability, and 19% (n=6) have sufficient construct validity. Many measures have assessed social connection in LTC homes, but few are designed for this purpose and many have insufficient psychometric properties. This review will provide detailed evidence of the quality of these measures to enable researchers to prioritise valid and reliable tools. Our results will inform our development of a new person-centred measure of social connection for LTC residents.

Abstract citation ID: igad104.1850

#### BLENDED GAMING COVID-19 TRAINING SYSTEM (BGCTS) FOR RESIDENTIAL CARE HOMES: A CLUSTER RANDOMIZED CONTROLLED TRIAL

Angela Y. M. Leung[1], Terence Lau[2], and Doris Y. P. Leung[2], *1. Hong Kong Polytechnic University, Hong Kong, Hong Kong*, *2. The Hong Kong Polytechnic University, Hong Kong, Hong Kong*

Objective: This study aims to assess the effect of the Blended Gaming COVID-19 Training System (BGCTS) on infection control practices, compliance rates and knowledge of standard precautions among all staff in residential care homes (RCHs). Design: A 2-arm single-blinded cluster randomized controlled trial. Setting: Thirty RCHs were recruited and randomized. Due to COVID-19 pandemic and there were infected cases in the homes, 17 RCHs refused or delayed the on-site observations. Participants: 212 staff (from 13 RCHs) were randomized to the intervention group (IG) or the control group (CG) according to their RCHs. Intervention: This is a blended mode of training with contents derived from WHO and local guidelines. **Results:** After using the BGCTS , the use of gloves and personal protective equipment (PPE) was significantly improved in IG than that of CG (F=43.206, p< 0.001). The IG has significantly higher scores in self-reported infection control practices (SICP) than that of CG ($\beta$= 2.619, Wald $\chi2$ 95% CI [4.291, 9.426]). **Discussion.** BGCTS improved RCH staff's performance in infection control practices. BGCTS was shown to be an effective training and it is worth to consider for adopting it as routine training for all staff in residential care homes.

Abstract citation ID: igad104.1851

#### PATTERNS OF ANTIHYPERTENSIVES CHANGE AND MORTALITY IN VA NURSING HOME RESIDENTS

Xiaojuan Liu, and Michelle Odden, *Stanford University, Stanford, California, United States*

Antihypertensive treatment is common in nursing home residents, yet data on the frequency and pattens of treatment changes in this group are lacking. We described the patterns of antihypertensive treatment changes and examined the mortality rate in 24870 Veterans Affairs (VA) nursing home residents ≥65 years with long-term stays (≥180 days) from 2006-2019. The number and dose of antihypertensive use was averaged weekly, and two change events were defined: deprescribing (reduced number of antihypertensives or dose of ≥30% compared to previous week) and intensification (opposite to deprescribing). More than 91% of residents were prescribed antihypertensive and 75% experienced at least one event during their stay. Death occurred in 7881 (32%) residents. We categorized residents into 10 distinct pattern groups: no change (27%), 1 deprescribing event (11%), 1 intensification event (10%), multiple deprescribing events (5%), multiple intensification events (7%), 1 deprescribing followed by 1 intensification (3%), 1 intensification followed by 1 deprescribing (4%), 3 changes with mixed deprescribing/intensification (7%), >3 changes with mixed deprescribing/intensification (10%), and no antihypertensive use (15%). We found treatment changes were more frequent in residents with better mobility and cognitive function. Residents who experienced deprescribing events had higher mortality than those experienced intensification events. The highest mortality was for those without medication (incidence = 344/1000 person-year) and this group also had the worst cognitive function. These findings indicate that complex patterns of medication changes exist in VA nursing home residents. A better understanding of the consequence of antihypertensive treatment changes could inform clinical practice in this population.

Abstract citation ID: igad104.1852

### QUALITY OF WORK LIFE FOR CARE AIDES IN CANADIAN LONG-TERM CARE HOMES BEFORE AND DURING THE PANDEMIC

Yuting Song[1], Janice Keefe[2], Yinfei Duan[3], Brittany Degraves[3], Amber Duynisveld[4], and Carole Estabrooks[3], *1. Qingdao University, Qingdao, Shandong, China (People's Republic)*, *2. Mount Saint Vincent University, Halifax, Nova Scotia, Canada*, *3. University of Alberta, Edmonton, Alberta, Canada*, *4. Mount St. Vincent University, Halifax, Nova Scotia, Canada*

Canadian Long-term care (LTC) homes were profoundly affected by the COVID-19 pandemic influencing work outcomes of care aides (CAs) who provide most direct care in these homes. We compared CAs' demographics and quality of work life (QWL) before and during the pandemic by conducting a repeated cross-sectional analysis of data collected in February 2020 (pre-pandemic) and December 2021 (21 months later) from a stratified random sample of urban LTC homes in western Canada. 2348 and 1116 CAs completed the survey in 2020 and 2021, respectively about their work outcomes (e.g., working short, tasks left undone, burnout) and health. We used three-level mixed-effects regression models to compare CAs' QWL, accounting for repeated-measures and Cas within same care units . Models also adjusted for CA demographics and LTC characteristics. Compared to the 2020 sample, the 2021 sample were 1.56 times more likely to report having worked short-staffed daily-weekly. The 2021 sample of CAs also reported lower levels of professional efficacy and mental health, being less rushed, and experienced fewer responsive behaviors from residents than the 2020 sample. Our prior research has demonstrated stable professional efficacy among CAs over 15 years. In this study, we observed that cracks in CAs' resolve are starting to show (as supported by their decreased efficacy and mental health). Interventions that address long-standing undervaluing of this staff group are needed as are interventions to support improvement in staff mental health.

Abstract citation ID: igad104.1853

### VISITATION SHELTERS FOR THE LONG-TERM CARE SETTING DURING THE PANDEMIC: AN EXPLORATORY STUDY

Michelle Porter[1], and Shauna Mallory-Hill[2], *1. University of Manitoba, Winnipeg, Manitoba, Canada*, *2. Faculty of Architecture, University of Manitoba, Winnipeg, Manitoba, Canada*

In 2020, the province of Manitoba in Canada devised a plan to provide external visitation shelters for long-term care (LTC) facilities. The intent of the shelters was to alleviate the problems with visitor restrictions due to the COVID-19 pandemic. Dozens of visitation shelters, which were re-purposed shipping containers, were installed at facilities across the province. The purpose of this research was to: examine the experiences of the users through online surveys; make field measurements of the conditions ($CO_2$ values, lighting, acoustics); and conduct an environmental scan of documents (government, media, etc.). Media and government documents provided insights into: design considerations; timing of installation/use; policies and procedures; how much they were utilized; as well as constraints. Survey findings from family/friends (n=20), LTC staff (n=9) and a resident (n=1) revealed that while many agreed that the shelters made a difference for their emotional well-being, many felt that the shelters did not support meaningful connections between residents and visitors. Furthermore, many respondents described the shelters as institutional/sterile and suggested that the décor should be improved to make them homier. Field measurements showed that the ventilation system ensured that $CO_2$ values remained low, indicating that substantial fresh air exchange occurred. Acoustic values indicated that there could be challenges with residents hearing visitors. Lighting values demonstrated that color temperature was appropriate, but lux values were relatively low depending on the specific location in the shelter. Overall, this study was able to outline the advantages and disadvantages to using external visitation shelters in a pandemic situation.

### SESSION 4520 (PAPER)

### INTERGENERATIONAL RELATIONSHIPS, CARE VALUES, AND OUTCOMES

Abstract citation ID: igad104.1854

### DIGITAL LITERACY, INTERGENERATIONAL RELATIONSHIPS, AND CARE PREPARATION AMONG CHINESE AGING ADULTS IN HONG KONG

Xue Bai, Wing-Lam Yu, and Chang Liu, *The Hong Kong Polytechnic University, Hong Kong, Hong Kong*

The rapid ageing trend spotlights the need for more eldercare. Care preparation as a form of proactive coping can mitigate the negative effects of potential eldercare needs. Acquisition and preservation of resources are essential for effective proactive coping. The roles of digital literacy and intergenerational relationships as two significant resources in older people's care preparation are still understudied. Integrating social convoy theory, intergenerational solidarity theory, and proactive coping theory, this study compared the levels of digital literacy, intergenerational relationships, and care preparation in son-dominant, daughter-dominant, and daughter & son-balance families; and examined the mediating effects of intergenerational relationships on the relationship between digital literacy and care preparation. Data from 3,626 participants with at least one adult child were drawn from the Panel Study of Active Ageing and Society, a biennial study conducted with a representative sample of people older than 50 years in Hong Kong. ANOVA was used to compare scores of key variables in three types of families. PROCESS was used to examine the mediation effects. Results showed that participants in a son-dominant family had the highest level of digital literacy; those in a daughter-dominant family reported the highest levels of intergenerational relationship quality and care preparation. Intergenerational relationship quality mediated the relationship between digital literacy and care preparation in three types of families while the effect sizes differed. These findings implicate the need to enhance older people's digital literacy and foster their intergenerational relationships, thereby assisting them to be well-prepared for care needs in later life.

Abstract citation ID: igad104.1855

### EARLY PANDEMIC INTERGENERATIONAL RELATIONSHIP TYPOLOGIES AND WELL-BEING AMONG OLDER ADULTS

Athena Chung Yin Chan[1], and Rodlescia Sneed[2], *1. Texas Tech University, Lubbock, Texas, United States*, *2. Wayne State University, Detroit, Michigan, United States*

The COVID-19 pandemic necessitated numerous changes in older adults' social interactions; however, there has been limited inquiry into the structure and function of early pandemic intergenerational relationships and consequent implications for well-being. Informed by intergenerational solidarity theory, we explored typologies of older adults' intergenerational relationships early in the pandemic, examining factors associated with each typology and associations between typologies and well-being. Participants were 7,840 adults aged ≥ 50 from the 2020 and 2021 waves of the Health and Retirement Study. We conducted latent class analysis using 12 indicators: associational (time with pets, frequency of in-person and virtual contact), structural (pet ownership and living arrangement), functional (receiving/providing support), and affectual (better relations). We used analysis of variance and post hoc analyses to determine whether individual differences (i.e., demographics, health status, and pandemic-related stressors), and well-being differed across typologies. Five latent classes were identified: low intergenerational relations (48%), pet owner with low intergenerational relations (15%), high contact and good relationship quality (tightknit, 11%), support provider (15%) and support recipient (12%). Older adults' age, gender, marital status, and health status were related to their relation typologies. Low intergenerational relations and tightknit typologies reported greater well-being. Support recipients reported the most distress, while support providers reported higher growth and resilience. Pet owners with low intergenerational relations reported the lowest positive emotions. Overall, results encourage careful attention to the multifaceted nature of intergenerational relationships when understanding coping of older adults in the pandemic.

Abstract citation ID: igad104.1856

### HOW PATTERNS OF INTERGENERATIONAL SUPPORT RECEIVED PREDICT FUTURE PROVISION OF CARE? EVIDENCE FROM CHINA

Yinkai Zhang, and Yu-Chih Chen, *The University of Hong Kong, Hong Kong, Hong Kong*

Intergenerational support refers to the exchange of financial, emotional, and physical support between generations. The types of support that older adults receive can influence their willingness to provide care in the future. However, there is limited knowledge about the different patterns of support that older adults receive. Examining these patterns can provide insight into how older adults contribute to future financial, emotional, and physical support. Using data from 5,388 older adults aged 60 and older from the China Health and Retirement Longitudinal Study, latent profile analysis was used to explore the patterns of received support. The associations between the patterns and provisions of financial, emotional, and physical support were examined using lagged OLS regression. Results showed that three patterns of received support were identified based on financial and physical aspects: financially and physically disadvantaged (low financial and physical support), engaged but financially disadvantaged (low financial but high physical support), engaged but physically moderate (high financial and medium physical support). Compared to the financially and physically disadvantaged group, those who were engaged but financially disadvantaged offer more financial and emotional support but less physical support, whereas engaged but physically moderate groups are more likely to provide emotional care. Findings suggest that policies and programs should consider the different patterns of support that older adults receive to promote more balanced support across different generations.

Abstract citation ID: igad104.1857

### IS LIFETIME ABUSE FORGIVABLE IN OLD AGE?

Tova Band-Winterstein, Ksenya Shulyaev, and
Zvi Eisikovits, *University of Haifa, Haifa, Hefa, Israel*

Old age is characterized by reflection and retrospective examination of the multiple meanings of various life experiences including lifetime abuse. Forgiveness was found to have salutary effects on older adults' wellbeing. To enhance understanding concerning the place and role of forgiveness in the reflective process in later life we performed a secondary analysis of data from four previous studies using in in-depth, semi-structured interviews (N=78) with older women who survived abuse. Concepts developed deductively from reviewing the literature contributed to an inductive thematic analysis of the interviews. Three major themes emerged from this procedure: (1) The dimensions of forgiving: The victim as subject. Movement towards forgiveness occurs along three dimensions: painful memories with negative emotions, commitment to social obligations and life circumstances. (2) Being forgiven: Between lost forgiveness and hope. This theme related to the boundaries between the roles of being a victim and a victimizer simultaneously and describes the hope and willingness of survivors to be forgiven by closely related persons. (3) Self-forgiveness and the aging self. Forgiving self can be conceptualized from total renunciation due to exhaustion from lifetime abuse to full recognition of self-accepting forgiveness resulting from decision-making based on strength. While recognizing the salutary effects of forgiveness, we cannot disregard that this is not a universally desirable process. The dimension of forgiveness needs to be included in the study of abuse throughout the old person's life-course to better understand its complexity Such view should substitute the either/or approach based on the dichotomy of "forgiveness" or "unforgiveness".

Abstract citation ID: igad104.1858

### LIFE COURSE PATTERNS AND PREDICTORS OF THE RELATIONSHIP BETWEEN ADULT CHILDREN AND THEIR MOTHERS

Mieke Beth Thomeer[1], and Rin Reczek[2], *1. University of Alabama at Birmingham, Birmingham, Alabama, United States*, *2. The Ohio State University, Columbus, Ohio, United States*

Studies demonstrate that the content and quality of the parent-child tie is highly variable across the life course and that multiple social factors predict different aspects of that relationship. Separate research shows that childbirth experiences (e.g., age at birth) matter for the well-being of both the mother and child. However, limited research considers how the mother's childbearing history is associated with adult child relationships at mid-life and beyond—namely contact and emotional closeness. We use Sequence Analysis on two linked datasets—the 1979 National Longitudinal Survey of Youth (NLSY79) and the National Longitudinal Survey of Youth-Young Adults (NLSY79YA) (N=1,953) to identify life course patterns of closeness and contact between adult children and their mothers. We identify six unique sequences: (1) mixed quality/contact (9.9%), (2) distant (10.0%), (3) close with decreasing contact (13.2%), (4) increasing emotional closeness with high contact (16.4%), (5) mostly close with high contact (23.2%), and (6) always close with high contact (27.3%). We use regression analysis to estimate how different aspects of the childbearing biography are associated with each category. Relationships characterized by "always close with high contact" were associated with more siblings/childbirths and older age at birth. Relationships associated with "emotionally distance" were associated with births characterized as "mistimed," being a middle or older child, and younger age at birth. Future analysis will consider selection factors such as family histories. This project demonstrates the need for life course perspectives on the child-mother relationship, recognizing the role of childbearing histories and the important diversity within and between individuals.

### SESSION 4525 (PAPER)

### AGE-FRIENDLY SETTINGS OF CARE FOR OLDER ADULTS

Abstract citation ID: igad104.1859

### ADVANCED-COMFORT: TESTING A CARE PLANNING INTERVENTION FOR NURSING HOME RESIDENTS WITH ADVANCED DEMENTIA

Ruth Palan Lopez[1], Andrea Wei[2], Jenna Locke[2], and Evan Plys[3], *1. MGH Institute of Health Professions, Sharon, Massachusetts, United States*, *2. MGH Institute of Health Professions, Boston, Massachusetts, United States*, *3. Massachusetts General Hospital/ Harvard Medical School, Boston, Massachusetts, United States*

Many nursing home (NH) residents with advanced dementia receive burdensome interventions rather than interventions that promote comfort or quality of life. The purpose of this study was to test the usability of a novel intervention called ADVANCED-Comfort that aims to enhance the provision of personalized, comfort focused care for residents with advanced dementia. The intervention consisted of structured care plan meetings between the NH team and proxies of residents with dementia (e.g., family members). Using the ADVANCED-Comfort workbook, proxies and staff created individualized care plans addressing 6 domains adapted from the Age Friendly Health System Framework (what Matters; Meaningful activities; Mealtime; Mobility; Medications and treatments; and Make comfortable). We evaluated the intervention using surveys, observation, and exit interviews. We found the intervention to be highly usable (acceptable, appropriate, and feasible) and exit interviews identified several promising outcomes. Both families and staff reported that the intervention improved communication and trust. Families reported that they had a newfound trust in staff to provide consistent quality care. Staff reported that the intervention helped them build better relationships with families and allowed families to articulate what they prefer rather than waiting until "something goes wrong." Staff also reported that the intervention improved their excitement to go to work and enhanced their sense of teamwork. Finally, both staff and proxies reported exciting implications for residents such as a reduction in antipsychotic medication, decrease in distressed behaviors, weight stabilization, and initiation of hospice. Based on these preliminary findings, additional testing of the ADVANCED-Comfort intervention is warranted.

Abstract citation ID: igad104.1860

### EVALUATION OF EDUCATION INITIATIVES TO INCREASE DELIVERY OF AGE-FRIENDLY CARE IN CONVENIENT CARE CLINICS

Nicholas Schiltz[1], Anna Bender[2], Grace Armstrong[1], Megan Foradori[1], Evelyn Duffy[1], Anne Pohnert[3], and Mary Dolansky[1], *1. Case Western Reserve University, Cleveland, Ohio, United States*, *2. University of Washingotn, Seattle, Washington, United States*, *3. CVS Health, Bellingham, Massachusetts, United States*

The Age-Friendly Health Systems model of care reliably provides four evidence-based elements of high-quality care, to all older adult patients: What Matters, Medications, Mentation, and Mobility (the 4Ms). MinuteClinic at CVS implemented the 4Ms model in all 1100+ locations nationwide in May 2020. To prepare MinuteClinic providers to deliver 4Ms care, educational modules were developed to provide an understanding of the gerontology that supports the 4Ms model of care: an orientation module with two scenarios illustrating the usual care of an older adult patient followed by the same patient receiving 4Ms care, monthly grand rounds (case studies of older adult conditions with the 4Ms), and ten video vignettes regarding integration of 4Ms care. Our goal was to evaluate the effectiveness of these education initiatives, as measured by changes in the mean number of Ms delivered per visit. Over 70% of 3,188 providers completed at least one education module. The rate of 4Ms delivery was 1.7 times higher (p< 0.001) among those completing at least one education module, and 5.7 times higher (p< 0.001) among those completing ten or more modules compared to those who completed none. Each course was associated with significant within subject increases between 10% and 38% in 4Ms delivery in the month following course completion. The self-directed learning environment (e.g., providers self-select into number and type of courses) reflects real-world variation in engagement; despite this variation, improvements in 4Ms delivery were observed at any educational dose, underscoring the value of prioritizing education time with quality improvement initiatives.

Abstract citation ID: igad104.1861

### PROMOTING PROVIDER ADHERENCE TO THE MEDICATION M OF THE AGE-FRIENDLY 4MS FRAMEWORK

Ugochi Ohuabunwa, and Leela Kantamneni, *Emory University, Atlanta, Georgia, United States*

BACKGROUND: The Age Friendly Health Systems (AFHS) initiative promotes use of the 4Ms Framework: We sought to evaluate provider adherence to protocols addressing the medication M of the framework.

**METHODS:** The project was conducted among patients > 65years treated in the inpatient (IP), outpatient (OP) and emergency departments (ED) of Grady Memorial Hospital (GMH). We implemented 2 approaches to improve provider prescribing among older adults 1) Using a clinical decision support tool via the electronic health record Epic Systems®, a best practice advisory (BPA) offered individual provider feedback regarding potentially inappropriate medications (PIMs) ordered and offered safer alternative medications. 2)A pharmacist-led daily medication review with provider feedback on PIMs in the Acute Care for Elderly (ACE) Unit. Primary outcome: Percentage of PIMs discontinued. Secondary outcomes: Percentage of BPA vs pharmacist recommendations accepted, discharge PIMs, Length of Stay (LOS), mobility, delirium and Katz scores, 30-day readmission rate. RESULTS: 8270 BPA alerts fired for 3291 patients, mean age 73.7, with 5709 BPAs in IP, 1462 in ED & 1083 in OP settings. Providers responded to alerts by overriding BPA (10.2%), canceling BPA (60.4%), removing alerted PIM (20.6%), ordering alternative medication (3.5%).. With the pharmacist led intervention, ACE Unit providers accepted 47.2% recommendations, discontinued 17.9% PIMs . 19% of ACE and 1.1% of GMU patients were discharged without PIMs. 30-day readmission rate was 19.1% (ACE) vs 27% (GMU) (P = 0.31).

**Conclusion:** There was a higher provider acceptance rate with the pharmacist led intervention but no significant difference in PIM discontinuation rate

Abstract citation ID: igad104.1862

### REDUCING FINANCIAL TOXICITY IN THE HEALTH CARE SYSTEM USING COST ESTIMATION TOOLS FOR OLDER ADULTS

Robin Gelburd, *FAIR Health, New York City, New York, United States*

Financial toxicity— the financial, emotional and mental burden patients experience with medical costs that can lead to diminished access to care and reduced quality of life—is a growing issue among older adults, family caregivers and care partners. Research suggests that financial health literacy tools may reduce financial toxicity. With support from The John A. Hartford Foundation, FAIR Health undertook an 18-month initiative to advance shared decision making (SDM) among older adults (aged 65 and older) with serious health conditions, and their family caregivers/care partners, through the development and dissemination of decision aids that combine clinical and cost information with resources and educational content; these tools are offered for free to consumers on fairhealthconsumer.org and to healthcare providers on fairhealthprovider.org. This presentation will discuss salient findings from the initiative, as detailed in the brief, Advancing Shared Decision Making among Older Adults with Serious Health Conditions: Lessons from FAIR Health's Grant-Funded Initiative. Feedback from older patients, caregivers/care partners and providers revealed the demand and need for financial health literacy tools that provide cost information and underscored key insights that included: (1) Healthcare costs, billing and lack of transparency in the healthcare system are sources of frustration; (2) Barriers to SDM include low health literacy and poor access to devices; and (3) SDM is viewed by many as a vehicle for patient empowerment. The session will offer a unique insight into how these findings can be used to usher in a new paradigm of healthcare and retool clinical practice and medical education.

Abstract citation ID: igad104.1863

### WHO, HOW, AND WHEN DOES PRIMARY CARE USE THE 4MS? CLINICIAN PERSPECTIVES OF THE AGE-FRIENDLY FRAMEWORK IN AN FQHC

Jenny Knecht-Fredo[1], Erica Husser[2], Donna Fick[2], Angela Hogue[3], Marie Mulvihill[4], and Lisa Lorelli[5], *1. Pennsylvania State University, State College, Pennsylvania, United States*, *2. Penn State University, State College, Pennsylvania, United States*, *3. Primary Health Network, Sharon, Pennsylvania, United States*, *4. The Primary Health Network, Lewistown, Pennsylvania, United States*, *5. The Primary Health Network, Beaver Falls, Pennsylvania, United States*

Older adults are at higher risk of living with multiple chronic health conditions and confront health care systems ill-prepared to manage the underlying complexities of providing care to older people. The Geriatric Workforce Enhancement Program (GWEP) aims to transform primary care to improve the health of older adults. Our GWEP is a collaboration between the Penn State College of Nursing and Pennsylvania's largest Federally Qualified Health Center (FQHC), the Primary Health Network (covering 16 counties [13 rural]). We aim to disseminate the 4Ms of an Age-Friendly Health System (What Matters, Medication, Mentation, and Mobility). Although AFHS is being disseminated across the nation, there is little research regarding clinicians' perspectives of the 4Ms framework in primary care. After 3 years of program delivery, we administered a PHN system-wide survey to 614 employees with 307 returned (50% response rate) regarding 4Ms knowledge, clinicians' responsibilities, timing of assessments, how results inform care, perceptions on the role of the electronic medical record (EMR), competence, barriers, and facilitators to providing Age-Friendly care. Analysis includes a subset of 40 registered nurses (n=21) and nurse practitioners/physician's assistants/physicians (n=19). Results suggest the medical providers were perceived as most likely to assess What Matters and Medication and multiple team members assessed Mentation and Mobility. 65% either agree or strongly agree that the EMR should prompt them to review the 4Ms with their patients. Survey results provide understanding of the barriers, specific roles, workflows, and EMR alterations needed to implement AFHS care into the busy culture of primary care clinics.

### SESSION 4530 (PAPER)

### OUTCOMES OF COVID-19

Abstract citation ID: igad104.1864

### ANTI-ASIAN RACISM AMONG OLDER KOREAN AMERICANS DURING THE COVID-19 PANDEMIC

Soonhyung Kwon, *Rutgers, the State University of New Jersey, Savoy, Illinois, United States*

**Background:** Hate crimes in the U.S. targeting adults of Asian descent have sharply increased during the COVID-19 pandemic. Korean Americans experience the highest level of everyday racism (37.5%), followed by Chinese (15.2%) and Vietnamese during the pandemic (Jung et al., 2021). Moreover, perceived discrimination in older Korean Americans is significantly related to a greater risk for poor mental health (Chau et al., 2018). However, older Korean Americans were likely to experience barriers to mental healthcare services. With this concern in mind, this study explored how older Korean Americans perceived discrimination related to ethnicity/race and coped with adverse mental health during the pandemic.

**Methods:** Our study used the thematic analysis of textual data to reconstruct and categorize, engaging in six steps: 1) familiarizing the qualitative data, 2) generating initial codes, 3) searching for themes, 4) reviewing themes, 5) defining and naming themes, and 6) producing a final report. **Results:** This study identified that older Korean Americans (N=18) experienced direct and vicarious racism during the pandemic but felt barriers (limited English proficiency and a lack of accessible resources) to mental healthcare services. Active religious activities ameliorated discrimination-related stress. Some participants tried to solve the stress through wrong behaviors that criticized Chinese Americans. As a result, barriers to mental healthcare were likely to cause secondary discrimination and prejudice against Chinese Americans. **Discussion:** To ameliorate adverse mental health and prevent secondary discrimination, it is essential to identify well-accepted, well-timed, sustainable, and cost-effective therapeutic strategies to alleviate the adverse mental health associated with discrimination-related stress.

Abstract citation ID: igad104.1865

### CLOSE KIN TIES AND COVID-19 OUTCOMES AMONG OLDER ADULTS IN EUROPE: THE ROLE OF COVID-19 PRECAUTIONARY BEHAVIORS

Damiano Uccheddu[1], Bruno Arpino[2], and Ester Rizzi[3], *1. University of Louvain (UCLouvain), Ixelles, Brussels Hoofdstedelijk Gewest, Belgium*, *2. University of Florence, Florence, Toscana, Italy*, *3. UCLouvain, Ottignies-Louvain-la-Neuve, Brabant Wallon, Belgium*

The purpose of this study is to examine the relationship between the availability of close kin ties (such as spouses and children) and the risk of COVID-19 contagion among Europeans who are 50 years of age or older. We also investigate the extent to which COVID-19 precautionary behaviors (such as mask-wearing, physical distancing, and frequent handwashing) mediate this relationship. Data were retrieved from the eighth wave of the Survey of Health, Ageing and Retirement in Europe (SHARE) and two waves of the SHARE Corona Survey (SCS). We used logistic regression models and the Karlson-Holm-Breen (KHB) decomposition method to analyze the data. Our preliminary findings indicate that individuals who have close family members are more likely to engage in precautionary behaviors. Furthermore, we found that engaging in such behaviors was associated with a reduced risk of COVID-19 outcomes. These initial results suggest that having close family ties may play an important role in shaping the responses of older adults to the COVID-19 pandemic. Additionally, our study highlights the importance of engaging in self-protective behaviors as a means of reducing the risk of COVID-19 outcomes among older adults.

Abstract citation ID: igad104.1866

### IMPACT OF ENVIRONMENTAL CHARACTERISTICS ON SELF-RATED HEALTH AMONG THE VERY OLD IN GERMANY DURING THE COVID-19 PANDEMIC

Jaroslava Zimmermann, *University of Cologne, Cologne, Nordrhein-Westfalen, Germany*

Current research demonstrates negative impact of COVID-19 pandemic on health. Nevertheless, there is evidence that older population might be more resilient against the effect of pandemic than younger adults. Since very old adults are often underrepresented or excluded from these studies, the aim of this paper was to examine the link between environmental characteristics and self-reported health in very old population in Germany during COVID-19 pandemic. Data from the representative survey "Old Age in Germany" of the population aged 80 and older were used. The study sample included 10,578 community-dwelling as well as institutionalized persons. Multilevel analysis was conducted to investigate the association of environment characteristics on individual, and community level with self-rated health. The results showed that higher perceived walkability of outdoor environment, better housing condition, being closer attached to home environment, higher neighborhood trust, and living in a house increased self-rated health (individual level). On community level, residency in one of the communities in western Germany (compared to eastern Germany) was associated with better self-rated health. 35% of the variance in self-rated health was explained at individual and 21% at community level. The findings revealed that very old adults who lived in better home environment experienced better health during the COVID-19 pandemic in Germany. These findings may indicate that the capacity of the very old to adapt to health losses depends on available environmental resources. Therefore, improvements of home environment inside and outside of houses and apartments (e.g., ensuring barrier-free accessibility) may strengthen the resilience in later life.

Abstract citation ID: igad104.1867

### OLDER-ADULT PERSONAL SUPPORT WORKERS' CONTRIBUTIONS DURING THE COVID-19 EMERGENCY RESPONSE

Haorui Wu, *Dalhousie University, Halifax, Nova Scotia, Canada*

When COVID-19 devastated long-term care facilities across Canada, most public attention was turned to older adult residents/clients, rather than their service providers, especially the personal support workers (PSWs). Some PSWs are over 55 age and above; they featured dual roles in the COVID-19 settings: (1) they were a vulnerable group disproportionately impacted by COVID-19 and (2) they were essential workers providing support in the older-adult care facilities. Since current older-adult-driven research, practice, and policy have primarily focused on generalized assumptions of older adults as a vulnerable, passive, and dependent group rather than recognizing their diversity, expertise, assets, and experiences, this project aims to identify these older-adult PSWs' contributions. This qualitative study invited 13 PSWs from older-adult care organizations in the Greater Toronto area of Canada to participate in in-depth interviews. This study uncovered three major contributions of older-adult PSWs within the individual-work-family triangulation. At the individual level, they bear the potentially high health risks of COVID-19, while supporting people around them. At the workplace level, they took on extra responsibilities to reduce the spread of the virus, dealing with the client's/residents' unfavourable behaviours and stress, and supporting their co-workers to maintain their organizations' operations. At the family level, they worked overtime to compensate for their family members' income loss and kept their family members at home to avoid infections. These outcomes inform the older adult research, practice, and policy by enhancing the appreciation of older adults' diverse strengths and promoting older adults' contributions in disaster settings.

### SESSION 4535 (PAPER)

### SOCIAL RELATIONSHIPS AND HEALTH

Abstract citation ID: igad104.1868

### RECIPROCAL RELATIONSHIP BETWEEN FAMILY, FRIENDS, AND COGNITIVE HEALTH AMONG OLDER ADULTS IN CHINA

Xinfang Yu, *Nanyang Technological University, Singapore, Singapore*

Existing research links perceived social support from family and friends to cognitive health in later life. Yet we know less about how cognitive functioning may also shape social support and whether these reciprocal relationships vary between rural and urban areas, especially in China where rural-urban migration is common. This study investigates: (1) whether family support and friendship support help maintaining cognitive functioning, (2) whether cognitive functioning improves perceived family support and friendship support, and (3) whether and how these relationships differ between rural and urban areas. I estimate cross-lagged panel models with a sample including 9,553 adults aged 60 years and above, from two waves of the China Longitudinal Aging Social Survey (CLASS). Results show that cognitive functioning was positively associated with perceived friendship support, regardless of the area of residence. There was a reciprocal relationship between friendship support and cognitive functioning only for rural older adults, with friendship support predicting lower cognitive functioning for urban older adults. Findings suggest that rural and urban context matter – perceived social support, whether from friends or family, may have different implications for the cognitive health of older Chinese adults depending on where they live.

Abstract citation ID: igad104.1869

### SOCIAL NETWORK AND CAREGIVING EXPERIENCE AMONG SPOUSE AND ADULT-CHILD DEMENTIA CAREGIVERS: A MEDIATION ANALYSIS

Weichu Liu[1], Jun Wang[2], Bing Yang[3], Xuelian Li[1], Yingzhuo Ma[1], and Mingzhao Xiao[1], *1. The First Affiliated Hospital of Chongqing Medical University, Chongqing, China, Chongqing, Chongqing, China (People's Republic)*, *2. Chongqing Medical University, Chongqing, Chongqing, China (People's Republic)*, *3. Stomatological Hospital of Chongqing Medical University, Chongqing, China, Chongqing, Chongqing, China (People's Republic)*

Evidence on differences in social network and their roles in associations between types of family caregivers and caregiving experience is limited. Based on the stress process model (SPM), we aimed to explore the different levels and interactions of social network and caregiving experience among spouses and adult-child caregivers. A questionnaire-based survey was conducted on a total of 146 dementia family caregivers (78 adult-child, and 68 spouses) in China. Data collection comprised four sections: (a) care-related stressors, (b) caregivers' context, (c) social network using Lubben social network scale, and caregiving experience using short-form Zarit burden interview, and nine-item positive aspects of caregiving (PAC) scale. Linear regression, mediation model analysis, and interactive analysis were performed to explore associations between variables. Spouses experienced lower social network (β= -0.294, P=0.008) and higher PAC (β= 0.234, P=0.003) than adult-child caregivers, whereas no significant difference was found in caregiver burden between two groups. Mediation analysis suggested that associations between caregiver type and caregiver burden were indirect-only mediation effects by social network(β=0.140, 95%CI=0.066 to 0.228). The mediation effect of social network on caregiver type and PAC was the suppressing effect, and the interaction effect was significant (P for interaction =0.025). A higher social network was associated with higher positive aspects of caregiving among the spouse subgroup (β= 0.341, P=0.003). Social networks mediate responses to caregiving experiences among different care provider types and are vital intervention targets, especially for spousal caregivers. Our results can serve as references for identifying caregivers for clinical intervention.

Abstract citation ID: igad104.1870

### SOCIAL RELATIONSHIPS AND DUAL FUNCTIONALITY IN LATER LIFE

Patricia Thomas[1], Madison Sauerteig-Rolston[2], Shawn Bauldry[2], Samuel Nemeth[2], and Kenneth Ferraro[2], *1. Purdue University--West Lafayette, West Lafayette, Indiana, United States*, *2. Purdue University, West Lafayette, Indiana, United States*

Longer lives are often accompanied by greater risk of cognitive and physical impairments, but we investigate whether social relationships reduce the risk of those impairments. We examine dual functionality – the absence of both dementia and activities of daily living impairments – and draw upon stress process and social integration theories to examine the impact of positive and negative dimensions of relationship quality with friends and family to better understand how social factors may affect how long people maintain both their physical and cognitive functioning in later life. We use nationally representative panel data from the Health and Retirement Study (2006-2016, N=6,985) and Weibull accelerated failure-time models. Higher strain with family members and with friends was associated with a loss of dual functionality at earlier ages (Time Ratio = .97 [95% CI: .95, .99] and TR = .96 [CI: .94, .98], respectively). Greater social support from family members was beneficial, related to a longer time as dual functional (TR = 1.04, CI: 1.02, 1.06); however, more support from friends was related to loss of dual functionality at earlier ages (TR = .98, CI: .96, .99). Findings reveal the implications of social support and strain in different types of relationships for dual functionality.

### SESSION 4540 (PAPER)

### MENTAL HEALTH

Abstract citation ID: igad104.1871

### ADDRESSING CO-OCCURRING ALCOHOL AND MENTAL HEALTH PROBLEMS IN OLDER ADULTS: CO-DESIGNED DIRECTIONS FOR PRACTICE

Bethany Bareham[1], Deepti John[2], Amy O'Donnell[3], Jennifer Liddle[1], and Barbara Hanratty[1], *1. Newcastle University, Newcastle upon Tyne, England, United Kingdom*, *2. Newcastle University, Newcastle Upon Tyne, England, United Kingdom*, *3. Newcastle Univesrity, Newcastle Upon Tyne, England, United Kingdom*

Older people in the UK experience the highest rates of alcohol-related harm of all age groups, due to reduced tolerance to alcohol with age. Most older people drinking at harmful levels have co-occurring mental health problems. This population fall between services, which are ill-equipped to address their complex and multifaceted needs. This study aimed to co-design directions for integrated services to support this group in North East/North Cumbria, England. Co-design was guided by Intervention Mapping, and was informed by four workshops with older people with co-occurring alcohol/mental health problems; informal caregivers and supporting practitioners. Workshops built on evidence from i) a preceding qualitative study with these groups examining lived experience, support needs, and challenges in supporting this group and ii) a literature review of existing interventions/services for older people with alcohol or mental health problems. Findings highlight the importance of availability, accessibility and flexibility of support for older people, who often present in crisis, and can face additional barriers to accessing services such as hearing/mobility issues. Mental health practitioners should be trained in identifying and addressing harmful drinking in older people. Practitioners must be knowledgeable of relevant, local services and prepared to signpost appropriately. Care should encompass peer support, and support and advocacy to address wider issues that impact mental health and alcohol use including finances, social isolation and illness. This study informs the development of integrated community alcohol/mental health support for this patient group. Applicability of directions to other care systems is considered.

Abstract citation ID: igad104.1872

### ANXIETY AND MEMORY COMPLAINTS IN MIDDLE-AGED AND OLDER ADULTS: THE MODERATING ROLE OF PHYSICAL ACTIVITY

Amy Costa, and Ashley Curtis, *University of South Florida, Tampa, Florida, United States*

The relationship between anxiety and memory complaints is well established. Additionally, physical activity has been shown to improve anxiety symptoms and cognition. Little research, however, has examined if physical activity impacts associations between anxiety and memory complaints in cognitively healthy middle-aged and older adults. Given memory complaints are often the first indicator of subsequent cognitive decline, it is important to understand factors that impact complaints. The present study tested whether physical activity moderated associations between anxiety and memory complaints. Adults aged 50+ (N=271, Mage=64.5, SD=7.8, 124 women) completed the International Physical Activity Questionnaire, Hospital Anxiety and Depression scale (HADS), and the Cognitive Failures questionnaire (CFQ). Multiple linear regressions and simple slope analyses were conducted to examine if physical activity moderated associations between anxiety and memory complaints (CFQ-memory), covarying for age, sex, income, difficulty walking, number of medical conditions, number of medications, body mass index, and depressive symptoms (HADS-depression). Results revealed total physical activity (R2-change=.01, p=.04), vigorous activity (R2-change=.02, p=.02), and walking activity (R2-change=.01, p=.03) moderated associations between anxiety and memory complaints. Specifically, higher anxiety was associated with more memory complaints at all levels of physical activity, with the magnitude of the effect largest at highest levels of physical activity. The present findings suggest higher amounts of physical activity may exacerbate the relationship between anxiety and memory complaints. It is possible aging adults with greater perceived memory disruption experience more anxiety and engage in health enhancing behaviors, such as physical activity. Future studies should explore temporal patterns of this relationship.

Abstract citation ID: igad104.1873

### BUILDING FOUNDATIONAL COMPETENCIES IN OLDER ADULT MENTAL HEALTH: VIRTUAL AND IN-PERSON TRAINING

Erin Emery-Tiburcio, Laurin Mack, Siqi Wang, and Susan Buehler, *Rush University Medical Center, Chicago, Illinois, United States*

Designed to develop foundational knowledge competencies for generalist clinicians, this program was created by a team of geropsychologists, geriatric social workers, geriatric psychiatrists, and neuropsychologists, who reviewed competencies of multiple mental health disciplines, and identified 14 key areas of knowledge for clinicians. Content was created by Rush faculty, externally peer-reviewed, and developed into two versions. The 14-hour online version includes knowledge checks and national expert videos to provide multiple perspectives and voices of experience. Of the first 176 learners in the online version, 96.8% rated the overall program as excellent, with strong agreement (97.4 – 98.3%) that the training was applicable to practice, expectations were met, and learners had intention to apply the knowledge. All learners are required to achieve a minimum of 75% on knowledge post-test to complete each module. The three-day in-person program provided 25 diverse clinicians representing every HHS region with didactic content, interactive exercises, discussion, and a simulated patient experience to practice skills with live expert feedback. Participants demonstrated 14% improvement in knowledge (p<.01). Didactic lectures were rated 100% extremely or very helpful; 82% rated group learning exercises extremely helpful; 100% rated the simulation lab as extremely or very helpful. Balance between activities and match with learning style were rated good to very good. All participants rated the likelihood of recommending the program to a colleague as extremely or very likely. Evaluation of skill implementation is ongoing. Implications for expanding generalist competencies in older adult mental health will be discussed.

Abstract citation ID: igad104.1874

### COGNITION TRAJECTORY AND DISABILITY AMONG OLDER AMERICAN COUPLES: THE MEDIATING ROLE OF DEPRESSIVE SYMPTOMS

Dexia Kong[1], Peiyi Lu[2], Da Jiang[3], and Helen Chan[1], *1. The Chinese University of Hong Kong, Hong Kong, Hong Kong, 2. Columbia University, New York City, New York, United States, 3. Education University of Hong Kong, Hong Kong, Hong Kong*

**Background:** Using a dyadic approach, this study examined the mediating effect of depressive symptoms on the longitudinal relationships between husbands' and wives' memory trajectories and their prospective disability status. Method: Longitudinal data from Health and Retirement Study 2004-2018 were used. Older (aged 50+) heterosexual couples married for 5+ years and had no limitations on activity of daily living at baseline (2004) were included (N=1,310). Latent class growth analysis grouped wives and husbands into distinct memory trajectories in 2004-2014. Structural Equation Model examined the actor and partner effects of memory trajectories on depressive symptoms in 2016 and disability status in 2018. The mediating effect of depressive symptoms was tested. **Results:** Four distinct trajectories were found: persistently high (wives: 11.83%; husbands: 3.83%); high and slow decline (wives: 36.26%; husbands: 33.66%); moderate and slow decline (wives: 41.30%; husband: 47.97%); low and rapid decline (wives: 10.61%; husbands: 14.54%). Only the wife's low and rapid decline memory trajectory predicted her own more depressive symptoms (β=0.588, 95% CI=0.209, 0.967) and her husband's more depressive symptoms (β=0.326, 95% CI=0.004, 0.648). Meanwhile, depressive symptoms had strong and significant actor effects on disability (β=0.046, 95% CI=0.036, 0.057 in the wife's outcome; β=0.060, 95% CI=0.046, 0.074 in the husband's outcome). **Conclusions:** Wife's low and rapid decline trajectory was associated with her own and her husband's more depressive symptoms, which in turn increased the disability risk of both partners. Detection and treatment of cognitive decline among wives have the potential to mitigate the couples' depressive symptoms and ultimately disability risks.

Abstract citation ID: igad104.1875

### SOCIOECONOMIC STATUS, CHANGES IN HEALTH BEHAVIORS, AND DEPRESSIVE SYMPTOMS AMONG OLDER ADULTS DURING THE PANDEMIC

Yujun Liu[1], Heng Wang[2], and M. Courtney Hughes[1], *1. Northern Illinois University, DeKalb, Illinois, United States, 2. Rush University Medical Center, Chicago, Illinois, United States*

Disparities in COVID-19 morbidity and mortality based on race and gender exist among older adults. Few studies have explored the complex relationships between the multiple components of COVID-19 vulnerabilities and the intersectionality across gender and race. This study aims to examine the impact of the COVID-19 pandemic on financial difficulty, health behavior, and depressive symptoms among older adults across the intersectionality of gender and race. Using Round 5 and Round 10 of the National Health and Aging Trends Study (NHATS) linked with the NHATS COVID-19 dataset, our sample included Medicare beneficiaries aged 65 or older in the U.S. (N =3,118). We modeled the interrelationships between financial difficulty, health behaviors, and depressive symptoms using a structural equation model. The results indicate that within the intersectionality framework, female participants reported less walking, more changes in eating habits, less sleeping, and less alcohol consumption during the pandemic than before compared to male counterparts. Compared to Non-Hispanic White, Non-Hispanic Black, Hispanic, and Other races, participants showed higher proportions of experiencing financial difficulty, less walking, less vigorous activity and changes in eating and sleep during than before the pandemic. Financial difficulty was significantly and positively associated with depressive symptoms and sedentary behavior. Active behavior was significantly and negatively associated with depressive symptoms, sedentary behavior was significantly and positively associated with level of depressive symptoms. Health professionals should consider the connection between various sociodemographic and health factors and the impact of COVID-19 on older adults when designing and delivering health and wellness programs post-pandemic.

### SESSION 4545 (PAPER)

### LONG-TERM CARE (BSS)

Abstract citation ID: igad104.1876

### CARING SELF-EFFICACY AND BEHAVIORS OF MIGRANT NURSING ASSISTANTS WORKING IN RESIDENTIAL AGED CARE FACILITIES

Sumina Shrestha, and Christine While, *La Trobe University, Bundoora, Victoria, Australia*

Data show a significant proportion of nursing assistants from migrant backgrounds in the Australian aged care industry. However, compared to locally-born counterparts, they are more likely to be employed as casual staff and offered shorter shifts, irregular hours, and lower pay, often without employment benefits. Previous studies have shown that the working environment of an organization influences employees' self-efficacy, affecting their performance. This study hypothesized that migrant nursing assistants from non-English-speaking countries have lower confidence in caring for older residents and, thus, execute less-satisfactory caring behaviors than their local, English-speaking colleagues. The caring self-efficacy was quantitatively measured from the perspective of nursing assistants in residential aged care facilities (N = 280), and caring behaviors were explored from older residents' perspective (N = 13). Results showed that nursing assistants whose first language was not English had lower levels of caring self-efficacy than native English speakers. However, older residents found that they frequently met residents' individual needs and were more competent, emotionally present, respectful, and amiable, despite communication issues. Older residents insisted that the personalities of nursing assistants have a greater impact on their job performance than their backgrounds. These findings suggest that although migrant nursing assistants from non-English-speaking countries had low self-efficacy to carry out their job, their performance was appreciated by older residents. This study supports the Australian government's strategy of using immigration to prevent or respond to an aged care workforce crisis, but it also advocates for improved working conditions, organizational resources and benefits for those with non-English-speaking backgrounds.

Abstract citation ID: igad104.1877

### CHALLENGES AND STRATEGIES TO IMPLEMENTING THE TRANSITIONAL CARE MODEL: LESSONS LEARNED FROM THE MIRROR-TCM TRIAL

Karen Hirschman[1], Molly McHugh[1], Onome Osokpo[1], Kathleen Mccauley[1], Elizabeth Shaid[1], Alexandra Hanlon[2], Mark Pauly[1], and Mary Naylor[1], *1. University of Pennsylvania, Philadelphia, Pennsylvania, United States*, *2. Virginia Tech, Blacksburg, Virginia, United States*

The Transitional Care Model (TCM) is an advanced practice registered nurse-led, team-based, care management strategy which has repeatedly been shown to improve outcomes for older adults transitioning from hospital to home. The MIRROR-TCM randomized controlled trial was implemented at three healthcare systems (7 hospitals) in four states. Older adults, hospitalized with heart failure, chronic obstructive pulmonary disease, or pneumonia, were enrolled between September 2020 and January 2023. A parallel evaluation study of "what it takes" to implement the TCM was launched to explore the challenges and strategies necessary to implement the Model's 10 components, especially in the context of COVID-19. A qualitative descriptive analysis of 167 meetings (clinical, community, leadership) from June 2020 (Pre-Implementation) through January 2023 (implementation/evaluation) was conducted. Codes were developed inductively over the first year of the study. The most common challenges were patient and caregiver factors (e.g., engagement, preferences, medical complexity), staffing coverage (e.g., time off, absences), and collaboration with multidisciplinary teams (e.g., poor or reluctant collaboration/communication). Common strategies to address challenges included: clarifying and adapting TCM processes, establishing an appropriate plan of care, building and engaging networks and relationships across healthcare and community settings, and trialing alternative strategies (e.g., telehealth in place of in-person visits). While some challenges were more common across sites such as patients refusing visits or no weekend coverage, strategies to resolve them tended to be site-specific. Findings provide important insights into what it takes to successfully implement complex evidence-based interventions such as the TCM.

Abstract citation ID: igad104.1878

### MEASURING IMPLEMENTATION FIDELITY OF THE TRANSITIONAL CARE MODEL IN THE MIRROR-TCM TRIAL

Alexandra Hanlon[1], Monica Ahrens[1], Karen Hirschman[2], Kathleen Mccauley[2], Elizabeth Shaid[2], Onome Osokpo[2], Mark Pauly[2], and Mary Naylor[2], *1. Virginia Tech, Blacksburg, Virginia, United States*, *2. University of Pennsylvania, Philadelphia, Pennsylvania, United States*

Examining the fidelity to the implementation of multicomponent evidence-based interventions is essential but challenging. To capture the application of the Transitional Care Model (TCM) as designed, an advanced practice registered nurse (APRN)-led, team-based, care management strategy in a replication RCT conducted in three diverse health systems, an innovative method was utilized. This session describes the procedures, challenges, and decision-making processes associated with measuring the fidelity of the implementation of TCM's core components over the trial. Standardized data collection forms were created for the APRNs to document the TCM elements addressed during each contact with and on behalf of the hospitalized older adults and their caregivers who had been randomly assigned to the TCM group (N=480). Measurable performance expectations for each element were established a priori (e.g., a minimum of 9 in-person visits). A dichotomous variable was created for each of the 38

elements making up the 10 TCM components which were provided monthly to each site to monitor their intervention implementation. Since not all elements were applicable to all older adults, the overall patient-level fidelity score required scaling to standardize the maximum fidelity score to 38. The individual components were inherently weighted according to their complexity as reflected by their respective number of elements. The average overall patient-level fidelity score was 34.2 (standard deviation: 3.5), median = 34.7. Higher fidelity scores indicated implementation of the minimum dose of the intervention elements. The findings provide critical insight into how to develop measures of implementation fidelity for complex multi-component evidence-based interventions.

Abstract citation ID: igad104.1879

### PARENTAL TRANSITION TO LONG-TERM CARE: IMPLICATIONS FOR PSYCHOLOGICAL WELL-BEING OF ADULT CHILDREN

Viktoryia Kalesnikava[1], Annalise Lane[2], Linh Dang[1], Rodlescia Sneed[3], and Briana Mezuk[1], *1. University of Michigan, Ann Arbor, Michigan, United States*, *2. Department of Veterans Affairs, Ann Arbor, Michigan, United States*, *3. Wayne State University, Detroit, Michigan, United States*

Approximately a quarter of US Baby Boomers provide informal caregiving for their parents. Though the psychological burden of caregiving is well-documented, the extent to which this burden relates to parental transition into long-term care is understudied. This study examined the longitudinal associations between parental transition to long-term care and life satisfaction and depressive symptoms among older adults of the 'Baby Boom' generation. Sample included respondents with at least one living parent from the Health and Retirement Study, 2010 - 2018 (n = 6,519, mean age = 56). Single-item life satisfaction was assessed on a 5-point Likert scale (range: not at all - completely satisfied). Depressive symptoms were indexed by the Composite International Diagnostic Interview (range: 0-8). Multivariate mixed effects poisson regressions of depressive symptoms and life satisfaction were fit, separately, adjusting for demographic characteristics, total wealth, and interview wave. Over the study period, 12% of respondents had a parent transitioning into long-term care. On average, respondents with parents transitioning into long-term care were older, wealthier, had higher depressive symptoms and lower life satisfaction. Longitudinally, parental transitions to long-term care was associated with mild increase in reported depressive symptoms, but there was no association with life satisfaction. Our findings are generally consistent with previous studies, reaffirming that while placement of parents in long-term care may alleviate caregiving burdens, it may not reduce distress in their adult children.

### SESSION 5000 (SYMPOSIUM)

Abstract citation ID: igad104.1880

### RECOGNIZING AND ENGAGING FAMILY CAREGIVERS ACROSS THE CARE CONTINUUM: FULFILLING NATIONAL STRATEGY ACTIONS

Chair: Beth Fields Co-Chair: Lauren Bangerter Discussant: Lauren Bangerter

Millions of family caregivers (relatives, partners, or other support persons) provide support to older adults across the care continuum, but feel unrecognized and disengaged in health care and social service systems. To address this gap, congress passed the Recognize, Assist, Include, Support, and Engage (RAISE) Family Caregiving Act in January 2018. The RAISE Act established a Council to deliver recommendations to the Secretary of Health and Human Services on how to increase recognition and engagement of family caregivers. This Council released the National Strategy to Support Family Caregivers in September 2022, detailing recommended actions for health care and social service systems. In response to the recommended actions, this symposium will present innovative approaches to increase recognition and engagement of family caregivers across the care continuum. This symposium will help bridge the policy and practice gap by offering health care and social service systems examples of how to involve family caregivers across the care continuum. Dr. Riffin will describe a checklist-based intervention to support family caregivers in primary care. Drs Perpezko and Toto will discuss the convergence of goal setting among older adults and their family caregivers in the home setting. Ms. Muntefering will report facilitators and barriers to involving family caregivers in hospital care. Dr. Wyman will identify effective clinician behaviors for including family caregivers in geriatric mental health VA services. Dr. Werner will share an online portal for supporting family caregivers in rural areas.

Abstract citation ID: igad104.1881

### A CHECKLIST-BASED INTERVENTION TO SUPPORT FAMILY CAREGIVERS IN PRIMARY CARE

Catherine Riffin, *Weill Cornell Medicine, New York City, New York, United States*

Family caregivers commonly accompany older adults to their routine medical visits, but caregivers' own needs and concerns are not systematically identified or addressed in clinical practice. Collaborative Healthcare Encounters with Caregivers (CHEC) is a checklist-based intervention that uses a prioritization approach to help caregivers efficiently identify and communicate their most salient concerns to primary care providers. This presentation will report findings from a pilot randomized controlled trial of N=37 patient-caregiver dyads who were randomized to CHEC (N=21) or usual care (n=16). The goals of the pilot trial were to evaluate CHEC's impact on medical visit duration and communication (using time-stamped audio-recordings and a structured coding system) and caregivers' perceptions of CHEC's utility (using questionnaires and interviews). Total visit duration was comparable across treatment groups (CHEC: 29.3 minutes vs. UC: 32.8 minutes; p=0.55), but a greater proportion of the visit time was spent addressing caregivers' concerns among CHEC group participants, as compared to usual care participants (25% vs. 6%; p<.001). In post-visit interviews, caregivers commented that CHEC was useful in helping them to prepare for the visit by thinking about their questions in advance and prompting providers to acknowledge their concerns. Findings from this study explicitly address the RAISE Act's recommendation for integrating evidence-based caregiver assessment instruments into clinical care delivery (Outcome 2.2) and indicate CHEC's potential as an efficient, clinically feasible intervention that can support greater inclusion of caregivers in primary care discussions.

Abstract citation ID: igad104.1882

**GOAL ATTAINMENT SCALING IN CAPABLE: A COMPARISON OF CARE PARTNER AND OLDER ADULT PARTICIPATION**

Kate Perepezko, and Pamela Toto, *University of Pittsburgh, Pittsburgh, Pennsylvania, United States*

Aging in place is an optimal outcome for our maturing population as health systems become increasingly overburdened. One program that facilitates aging in place is the Community Aging in Place, Advancing Better Living for Elders (CAPABLE) program. CAPABLE improves older adult performance in everyday activities and reduces inpatient and long-term service use. Although CAPABLE is designed as a person-centered program aiming to help older adults set and achieve goals, research demonstrates that care partners (i.e. family or friends providing unpaid support) are frequently involved in healthcare decisions and can reinforce intervention goals. This presentation will report findings from the trial stage of a Hybrid Type 1 Study of CAPABLE involving care partners for which recruitment is ongoing (N=100). We aim to describe how older adults and their care partners engage in goal setting using Goal Attainment Scaling, share the frequency of their goal attainment, and present the types of goals that they set. For example, older adult participant goals for our current sample focus on reduced fear when showering, improved mobility, and more efficient dressing. For our care partner participants, goals include finding respite and preventing older adult injury. All participants who have completed CAPABLE were able to achieve their goals by the end of the program. Findings from this study support care partner involvement in interventions to promote aging in place, aligning with the RAISE Act's goal to engage family caregivers as key partners in the delivery of services and support.

Abstract citation ID: igad104.1883

**BECOMING INCLUSIVE: A NECESSARY PURSUIT FOR SUPPORTING FAMILY CAREGIVERS IN HOSPITAL CARE**

Chloe Muntefering, *University of Wisconsin- Madison, Madison, Wisconsin, United States*

Given advances in medicine and technology, shorter hospital stays, and a shrinking health care workforce, an increasing number of older adults are relying on family caregivers for support during and after hospital care. Yet, our healthcare system is often unresponsive to the needs of family caregivers. Recent consensus recommendations including the National Strategy on Family Support encourages health systems to include family caregivers in care processes of older adults. This presentation will report interview and community advisory board findings from a qualitative descriptive study of N=24 stakeholders consisting of hospitalized older adults (n=4), family caregivers (n=6), healthcare providers (n=9), and health policymakers (n=5). The goals of the study were to identify and describe existing barriers and facilitators to the inclusion of family caregivers in older adult's hospital care. Of the participants, 87% were female and from the Midwest region, and all were Caucasian. The average age was 56 years. The resulting themes from this exploration demonstrated that inclusion of family caregivers in hospital care is influenced by personal factors of both the caregiver and the provider (caregiver availability, health literacy, education, values/priorities), interpersonal skills between these two parties (communication style and methods), hospital environment/resources (accessibility, comfortability, staffing, documentation), and social climate/policy (Covid-19 Restrictions, HIPAA, Power of Attorney). Findings from this study can be used to develop or adapt healthcare system policies and practices, fulfilling Outcome 2.1 of the National Strategy on Family Support's recommendation for recognizing family caregivers as essential members of the care team.

Abstract citation ID: igad104.1884

**EFFECTIVE CLINICIAN BEHAVIORS FOR INCLUDING FAMILY CAREGIVERS IN THE GERIATRIC MENTAL HEALTH SETTING**

Mary Wyman[1], Hayley Raj[2], Joseph Perzynski[3], Carey Gleason[4], Ranak Trivedi[5], Amy Byers[6], and Corrine Voils[7], *1. Madison VA/Univ Wisconsin, Madison, Wisconsin, United States*, *2. University of Wisconsin, Madison, Wisconsin, United States*, *3. Madison VA Hospital, Madison, Wisconsin, United States*, *4. UW Madison, Madison, Wisconsin, United States*, *5. Stanford University, Stanford, California, United States*, *6. UCSF / San Francisco VA Health Care System, San Francisco, California, United States*, *7. W.S. Middleton Memorial Veterans Hospital, Madison, Wisconsin, United States*

To improve patient and caregiver outcomes, the 2022 National Strategy to Support Family Caregivers recommends training for professionals on caregiver inclusion and provision of person- and family-centered care. Caregiver inclusion is especially important for older adults with dementia, who experience high rates of comorbid mental health (MH) disorders but are disadvantaged by disparities in care access and quality. Current training options are limited. Our VA-funded project seeks to develop caregiver inclusion training for MH clinicians working with older adults with dementia. We present preliminary findings from in-depth, 45-min qualitative interviews with 9 multidisciplinary MH clinicians (psychiatry, social work, psychology, and clinical pharmacy) on their experiences with engaging family caregivers in MH treatment. A multidisciplinary coding team conducted content analysis to identify facilitators and barriers to effective communication and collaboration with caregivers, with a focus on clinician behaviors. Facilitators of effective caregiver involvement include clinician attention to developing rapport with the caregiver, addressing knowledge gaps about dementia and mental health interventions, and helping to address the challenges of caregiving with supportive resources. Barriers included not tailoring clinician approach to caregiver needs, failing to clarify the caregiver role in healthcare, and failing to address one own's defensiveness or labeling of caregivers in distress. The importance of system factors (e.g., technology, clinic scheduling, and care team dynamics) was noted. Findings will be used to inform the development of strategies to increase clinician skills for caregiver inclusion, supporting improved patient and caregiver outcomes and the goals of the RAISE Act and the National Strategy.

Abstract citation ID: igad104.1885

### PARTICIPATORY DESIGN OF A WEB-BASED COMMUNITY AND RESOURCE CONNECTION TOOL FOR RURAL DEMENTIA FAMILY CAREGIVERS

Nicole Werner, *Indiana University School of Public Health-Bloomington, Bloomington, Indiana, United States*

Rural family caregivers of people living with dementia have higher caregiver burden and experience lower social support than their urban counterparts. Technology has potential to address rural caregiver health disparities, but only if designed to their specific needs. Available technology interventions lack focus on rural caregivers and community influence. This represents a gap in rural caregivers' ability to leverage technology for community supports to overcome their unique barriers. Thus, we are engaging rural caregivers and community resources in a participatory co-design process to design CareVirtue Family Connection, an online community and personalized resource connection tool specific to rural dementia caregivers. We conducted N=19 interviews with dementia caregivers self-identified as living in a rural area. We analyzed transcripts using team-based affinity diagramming to create meaningful clusters related to how rural caregivers get the help they need. We used consensus discussion to refine clusters and applied resulting categories to transcripts to identify confirming/disconfirming evidence. We identified seven dimensions influencing rural caregivers' ability to get the help they need including community attributes, caregiving challenges, community connection, experience with community supports, caregiving goals, attitudes on caregiving and community, and information behavior. Results will guide subsequent co-design in which we will engage rural caregivers and related community organizations to co-design the CareVirtue Family Connection tool. Our participatory approach is aligned with the National Strategy to Support Family Caregivers' goal to advance caregiver engagement, and CareVirtue Family Connection will support the Strategy's vision to "expand access to programs, services, supports, and products to family caregivers."

### SESSION 5005 (PAPER)

### PREDICTING FUTURE OUTCOMES II

Abstract citation ID: igad104.1886

### AGING IN ENVIRONMENTALLY RISKY AREAS: FINDINGS FROM A NATIONALLY REPRESENTATIVE SURVEY

Alexis Merdjanoff, and Katie Lynch, *New York University, New York City, New York, United States*

The Aging in Risky Environmental Areas (AREA) Study is a novel cross-sectional dataset of community-dwelling adults in the U.S. ages 50 and older. The sample of 1,504 participants was collected in November 2022 and is nationally representative. The survey examines several topics related to aging during the climate emergency including, recent climate-related disaster exposure; perspectives on climate adaptation and mitigation strategies; disaster resilience; and, various measures of healthy aging. There is a misconception that a majority of older adults do not believe in climate change or are unwilling to make behavioral changes to decrease their carbon footprint, despite their vulnerability to climate-related disasters. Results from the AREA Study demonstrate that a majority of older adults (50+) are willing to make "a lot" or "some" changes to the way they live or work in order to reduce the impact of climate change and that there are no statistically significant differences among age groups (50-64; 65-74; 75+). Instead, factors such as region, political affiliation, prior disaster exposure, and place attachment are significant predictors of willingness to engage in adaptation strategies. Findings suggest that an overwhelming majority of older adults (74%) believe that climate change is happening and are willing to be active participants in efforts to reduce negative impacts.

Abstract citation ID: igad104.1887

### DEVELOPMENTAL CHANGE IN PROBLEM DRINKING ACROSS THE LIFESPAN: BETWEEN- VERSUS WITHIN-PERSON AGE EFFECTS IN NATIONAL DATA

Matthew Lee[1], and Ellen Yeung[2], *1. Rutgers University New Brunswick, Piscataway, New Jersey, United States, 2. George Washington University, Washington, District of Columbia, United States*

When considering problem drinking from a lifespan developmental perspective, an often-stated underlying premise is that problem drinking peaks around early young adulthood and then declines throughout the remainder of the lifespan. However, relative to adolescence and young adulthood, the supposed later declines in midlife and older adulthood are less firmly established and based primarily on cross-sectional data. Thus, this study contrasted cross-sectional versus longitudinal age effects on problem-drinking changes across the adult lifespan. Analyses used two waves of longitudinal data from a U.S.-representative sample. We generated descriptive "porcupine figures" graphically depicting both cross-sectional and longitudinal age effects simultaneously. In addition, we used multilevel models to partition, test, and contrast cross-sectional versus longitudinal age effects on problem drinking in different age periods of the adult lifespan. While age effects were consistent in young adulthood across the cross-sectional and longitudinal tests, key discrepancies were shown in midlife and older adulthood. Specifically, the supposed continuation of problem-drinking reductions throughout midlife and older adulthood was observed cross-sectionally but not longitudinally. These findings call into question the notion of normative drinking-related declines that continue beyond young adulthood and throughout the remainder of the adult lifespan. We will discuss plausible alternative explanation for how cross-sectional data can lead to spurious conclusion about developmental change (e.g., age-confounded cohort effects). If replicated in future research, our evidence for relative stability or even increases in problem drinking in midlife and older adulthood can contribute to a more lifespan-developmentally-informed understanding of risky behaviors and thereby guide lifespan-developmental tailoring of interventions.

Abstract citation ID: igad104.1888

### EXPLORING CONGRUENCY OF DEATH LOCATION PREFERENCES VERSUS EXPECTATIONS: FINDINGS FROM BRAZIL, ITALY, JAPAN, AND THE US

Sarah Clem[1], Peiyuan Zhang[2], and John Cagle[2], *1. University of Maryland School of Social Work, Baltimore, Maryland, United States*, *2. University of Maryland, Baltimore, Baltimore, Maryland, United States*

Dying in one's preferred death location is a common indicator of a good death. Previous studies exploring the congruency between individuals' death location wishes and their actual death locations relied on samples of patients with terminal diagnoses. Data for this study came from the Four-Country Survey on Aging and End-of-Life Medical Care 2017 from which nationally representative samples of the general public were collected in the U.S., Italy, Japan, and Brazil. Bivariate analyses of the total sample (n=4,239) identified that 45% of participants reported expected death locations that differed from their preferred place of death. Among the majority of participants reporting a preference for a home death, 61% of these individuals in Japan (p<.001) and 43% from Brazil (p<.001) reported they were more likely to die in a hospital. Whereas, in both Italy and the U.S., the majority of participants within each death location category reported the same location for their preferred and most likely death location. Logistic regression models identified unique predictors of congruency for each country. Noteworthy, in the Italy model, females were 43% lower odds of having congruent death location perceptions compared to men (OR=0.57, p=0.02). In the U.S. model, racial differences were also notable, as Black participants had over 2 times greater odds of congruent death location perceptions compared to White participants (OR=2.18, p=0.02). By further understanding the congruency of death location perceptions among the general public, we can gain insight into the perception of whether individuals feel that a quality death experience is attainable.

### SESSION 5010 (PAPER)

### LONELINESS, SOCIAL ISOLATION, AND HEALTH

Abstract citation ID: igad104.1889

### BREATHLESSNESS AND BIOSOCIALITY: AN ETHNOGRAPHY OF LIVING WITH RESPIRATORY DISEASE IN LATER LIFE

Fredrik Nyman, *Mid Sweden University, Jönköping, Jonkopings Lan, Sweden*

This paper is based on multi-sited ethnographic fieldwork conducted predominantly amongst support groups for older people with respiratory disease in the North of England, and sheds light on how communities are formed around chronic breathlessness. By utilising (and reconceptualising) the post-modern framework of biosociality, this paper explores how people living with respiratory disease negotiate and incorporate different kinds of health-related knowledge in their everyday lives—explicitly in support group settings outside of the clinic. People are living longer than ever before according to the World Health Organization, and chronic conditions are now the chief causes of death globally and have surfaced as major causes of disability and functional dependency. More specifically, in the United Kingdom 115,000 people die each year of chronic respiratory disease, which makes it one of the three biggest killer disease areas in the country. These mortality figures have remained stagnant for the past decades. What is more, in the era of neoliberalism respiratory care is individualised. Public health responses now emphasise the responsibility of individuals over collective or institutional responsibility, which is predominantly enforced through self-care and by training (or activating) patients in taking their medications and monitoring their pulmonary performance. By attending to public health responses in neoliberal times where respiratory healthcare regimens are habitually individualised, this paper contributes to understandings of biomedical subjectivities. Explicitly, it examines how support groups—as biosocial gatherings—can be understood as technologies for bridging dialogues between subjective and collective bodily experiences of health, illness, and wellbeing.

Abstract citation ID: igad104.1890

### CONCERNED AND LONELY AMIDST COVID-19: FINANCIAL STRAIN AND LATER-LIFE PSYCHOLOGICAL WELL-BEING IN NEVADA

Tirth Bhatta[1], Dante Miller[2], and Nirmala Lekhak[1], *1. University of Nevada, Las Vegas, Las Vegas, Nevada, United States*, *2. University of Nevada Las Vegas, Las Vegas, Nevada, United States*

**Objectives:** Nevada has some of the nation's highest suicide rates among older adults, which are linked to social isolation, loneliness, and mental health. The COVID-19 pandemic is likely to have exacerbated the loneliness and financial insecurity that older Nevadans have already been experiencing to a greater extent since the 2008 financial crisis. Prior research based on national data has documented the relationship between financial strain and mental health in later life. However, the social and psychological mechanisms that underlie such relationships have not been investigated, particularly in light of the devastating economic and health effects of the pandemic on older adults in Nevada. **Methods:** Based on our web-based survey of adults aged 50 years and older (n=313), we examined the roles of loneliness and concerns about the pandemic in explaining the impact of financial strain on depressive symptoms and anxiety. **Results:** We documented a statistically significant effect of financial strain on depressive symptoms (b=2.41, p-value< 0.001) and anxiety (b=1.74, p-value< 0.001). The inclusion of loneliness and concerns about the pandemic in our regression models led to substantial reduction in the impact of financial strain on depressive symptoms, whereas such impact on anxiety became statistically not significant. We also found significantly higher levels of depressive symptoms and anxiety among our respondents who reported greater feeling loneliness and concerns about the pandemic. **Discussion:** Our findings underscore the need for mental health resources and financial support programs for economically disadvantaged older adults to alleviate their feelings of loneliness and improve mental health in Nevada.

Abstract citation ID: igad104.1891

### NETWORK ANALYSIS OF OBJECTIVE AND SUBJECTIVE INDICATORS OF SOCIAL ISOLATION AND FUNCTIONAL LIMITATIONS

Chuwen Zhong[1], Linh Dang[2], Aparna Ananthasubramaniam[2], and Briana Mezuk[2], *1.*

*School of Public Health, University of Michigan- Ann Arbor, Ann Arbor, Michigan, United States*, 2. *University of Michigan, Ann Arbor, Michigan, United States*

Functional limitations, including decreased physical performance and increased limitations in daily activities, are common among older adults and associated with both objective and subjective social isolation. However, the dynamic interactions between and within different indicators of functional limitations and social isolation are unclear. This study employed network analysis to graphically depict and explore the relationships between various objective and subjective indicators of functional limitations and social isolation. Data came from the 2014/2016 waves of the Health and Retirement Study (n=9,076, mean age =67.25,52.32% female, 80.17% non-Hispanic White). Functional limitations were measured by objective physical performance (grip strength, tandem balance test) and self-reported limitations in daily activities. Objective social isolation was based on marital status, living arrangement, and network composition (children, other family members, and friends); subjective social isolation was indicated by the UCLA loneliness scale. Separate networks were constructed for men and women. Centrality indices, including strength, betweenness centrality, and eigenvector centrality, were computed to quantify the influence of each item. Subjective functioning and isolation items were strongly correlated but had weaker interconnections with one another. Strength and eigenvector centrality indicated that subjective functioning items had high degrees of influential interactions. Subjective social isolation items had high values of betweenness centrality, suggesting important bridging roles of these constructs. Subjective social isolation items had a more influential role in the network of women than men. Findings suggest opportunities for interdisciplinary interventions to address the multifactorial relationship between functional limitations and social isolation among older adults.

### SESSION 5015 (PAPER)

### FACTORS IMPACTING PERSONS WITH DEMENTIA

Abstract citation ID: igad104.1892

### COMPARING SELF-REPORTED MEASURES OF SUBJECTIVE COGNITIVE DECLINE

Benjamin Olivari[1], John Omura[2], John Shean[3], and Lisa McGuire[1], *1. CDC, Atlanta, Georgia, United States*, *2. Centers for Disease Control and Prevention, Atlanta, Georgia, United States*, *3. Alzheimer's Association, Chicago, Illinois, United States*

Symptoms of subjective cognitive decline (SCD) can be early indicators of possible future dementia. Estimates of self-reported SCD prevalence are available from various data sources including the Behavioral Risk Factor Surveillance System's (BRFSS) current Cognitive Decline Optional Module. Recent revisions to the Module were proposed based on input from subject matter experts, including reducing introductory phrasing and refining question and response options. It is unknown how these changes may impact estimates of SCD prevalence. To assess whether prevalence of SCD differs based on Module phrasing and conceptualizations of SCD-related questions, this analysis used data (n=3526) from the 2022 FallStyles survey. Respondents were randomly assigned to one of two groups receiving different sets of Module questions: group A received questions mirroring the former language and group B received the revised phrasing. Estimates were weighted to match the 2021 US Current Population Survey. SCD prevalence among respondents aged ≥18 years was 8.9% in group A versus 16.9% in group B. Among adults aged ≥45 years, SCD prevalence in group A (9.3%) was similar to the 2019-2020 BRFSS estimate (9.8%), while it was nearly double (17.6%) in group B. Within both groups, SCD prevalence was higher among those earning less than $25,000, with lower educational attainment, and who identified as female than their counterparts. Simplified prompts and refined questions may provide higher estimates of SCD prevalence. Given the subjective interpretation of respondents' own memory changes involved in measuring SCD, understanding the impact of question phrasing and language is critical when interpreting resulting data.

Abstract citation ID: igad104.1893

### EXPLORATORY FACTOR ANALYSIS OF THE COMFORT ASSESSMENT IN DYING WITH DEMENTIA SCALE

Peiyuan Zhang[1], Timothy Stump[2], Wanzhu Tu[2], Todd Becker[1], Jessica Orth[1], Alexander Floyd[3], Kathleen Unroe[4], and John Cagle[1], *1. University of Maryland, Baltimore, Baltimore, Maryland, United States*, *2. Indiana University School of Medicine, Indianapolis, Indiana, United States*, *3. IU School of Medicine Regenstrief Institute, Indianapolis, Indiana, United States*, *4. Indiana University, Indianapolis, Indiana, United States*

The 14-item Comfort Assessment in Dying with Dementia (CAD-EOLD) scale is a widely used instrument measuring end-of-life care for people with dementia (PWD). The instrument has been used to evaluate symptom burden among PWD in nursing homes, but the measurement properties are less studied for symptoms reported by family and staff caregivers. We conducted an exploratory factor analysis to evaluate the psychometric properties of the scale using staff and family (N=476) responses to CAD-EOLD at the baseline of an NIH-funded clinical trial. Subjects were long-stay nursing home residents with moderate-to-severe cognitive impairment in Indiana and Maryland. Staff (n=368) and family members (n=108) completed the CAD-EOLD, describing participating residents. We performed separate exploratory factor analyses on family and staff responses. Family and staff data showed similar clustering patterns. Restlessness, anxiety, fear, crying, and moaning had high factor loadings in the first cluster, interpreted as "Physical and Psychological Distress" (loading range = 0.47–0.91). Choking, gurgling, and difficulty swallowing had high loadings in the second cluster that depicted "Dying Symptoms" (loading range = 0.62–1.15). Serenity, calm, and peace had high loadings in the third factor on "Well-Being" (loading range = 0.72–0.93). Three "Physical Distress" items (i.e., discomfort, pain, and shortness of breath) cross-loaded with "Dying Symptoms." Accordingly, "Physical and Psychological Distress," "Dying Symptoms," and "Well-Being" represented important but separate dimensions for measuring end-of-life experiences of

PWD. Findings demonstrated favorable construct validity of CAD-EOLD in PWD with moderate-to-severe cognitive impairment in nursing homes, as reported by staff and family caregivers.

Abstract citation ID: igad104.1894

### FACTORS ASSOCIATED WITH WANDERING AMONG PERSONS WITH DEMENTIA: A RETROSPECTIVE STUDY

Antonio Miguel-Cruz[1], Hector Perez[2], Emily Rutledge[2], Christine Daum[2], and Lili Liu[2], *1. University of Alberta, Edmonton, Alberta, Canada*, *2. University of Waterloo, Waterloo, Ontario, Canada*

Persons living with dementia are at increased risk of getting lost and going missing due to critical wandering. Risk factors associated with critical wandering within this population are underexplored, thus prompting this study. In this retrospective observational study, we examined anonymized data from 25,785 MedicAlert® subscribers. We used a logistic regression model with a self-reported critical wandering incident as the outcome variable ($p< 0.05$). The average age of our sample was 75.42 (SD 14.34). Fifty-one percent (13,064/25,785) of cases had dementia and almost 22% (5,561/25,785) were involved in a critical wandering incident at least once. People living with dementia were two and a half times more likely to be involved in a missing incident compared to people without dementia (OR=2.56, 95% CI [2.39, 2.73], $p< 0.001$). The likelihood of being involved in a lost incident increased with advancing age; people 95-104 years old were seven times more likely to wander than those under age 65 (OR=7.11, 95% CI [5.96, 8.47], $p< 0.001$). Sex at birth, official Canadian languages spoken, ethnic background, population density, living arrangement, and medication were associated with increased dementia-related missing incidents. Numerous risk factors for missing incidents were identified. Our study paves the way for implementing preventative strategies to ultimately decrease the risk of going missing for person living with dementia.

Abstract citation ID: igad104.1895

### FOOD INTAKE WAS TEMPORALLY ASSOCIATED WITH STAFF MEALTIME CARE APPROACHES AND BEHAVIORS OF RESIDENTS WITH DEMENTIA

Wen Liu[1], Yelena Perkhounkova[2], Maria Hein[3], and Roger Bakeman[4], *1. The University Of Iowa, Iowa City, Iowa, United States*, *2. University of Iowa, Iowa City, Iowa, United States*, *3. University of Iowa, College of Nursing, Iowa City, Iowa, United States*, *4. Georgia State University, Atlanta, Georgia, United States*

Nursing home residents with dementia commonly experience low food intake, resulting in functional and nutritional consequences. Prior work supports associative relationships of resident behaviors and staff approaches with food intake, yet research on temporal relationships is limited. This study examined temporal associations of resident behaviors and staff approaches with food intake. Videotaped mealtime observations (N=160) involving 36 staff and 27 residents (53 staff-resident dyads) in 9 nursing homes were analyzed. Sequential analysis was conducted for resident behaviors and staff approaches as antecedents of resident successful food intakes in 5-, 10-, and 15-second time windows. Increased resident-initiated food intake was associated with preceding resident positive verbal behaviors, but not other resident behaviors or staff approaches. Decreased resident-initiated food intake was associated with preceding resident functional impairments and resistive behaviors, but not other behaviors or approaches. Increased staff-initiated food intake was associated with preceding staff person-centered verbal approaches, but not resident behaviors or other staff approaches. Decreased staff-initiated food intake was associated with preceding resident positive/neutral nonverbal behaviors and functional impairments, and staff task-centered care approaches. Findings confirm the importance of certain strategies in promoting food intake, including facilitating resident positive verbal communication, supporting functional impairments, responding to resistive behaviors, maximizing the use of person-centered care, and minimizing the use of task-centered care. Resident positive/neutral nonverbal behaviors which reflect resident active engagement during mealtime, do not facilitate staff-initiated food intake and may be an indicator of resident independence and readiness to initiate food intake. Further investigation is needed using larger diverse samples.

Abstract citation ID: igad104.1896

### GRAVES' DISEASE IS ASSOCIATED WITH INCREASED RISK OF CLINICAL ALZHEIMER'S DISEASE

Arseniy Yashkin[1], Stanislav Kolpakov Nikitin[1], Galina Gorbunova[1], Julia Kravchenko[2], Anatoliy Yashin[3], and Igor Akushevich[1], *1. Duke University, Durham, North Carolina, United States*, *2. Duke University School of Medicine, Durham, North Carolina, United States*, *3. Social Science Research Institute, Duke University, Durham, North Carolina, United States*

Identification of modifiable risk factors for Alzheimer's Disease (AD) onset is an important aspect of controlling the burden imposed by this disease on an increasing number of older U.S. adults. Graves' disease (GD), the most common cause of hyperthyroidism in the U.S., has been hypothesized to be associated with increased AD risk, but there is no consensus. In this study, we explore the link between GD and risk of clinical AD. Cox and Fine-Grey models were applied to a retrospective propensity-score-matched cohort of 15,505 individuals with GD drawn from a nationally representative 5% sample of U.S. Medicare beneficiaries age 65+ over the 1991-2017 period. Results showed that the presence of GD was associated with a higher risk of AD (Hazard Ratio [HR]:1.15; 95% Confidence Interval [CI]:1.07-1.23). Magnitude of associated risk varied across subgroups: Males (HR:1.19; CI:1.01-1.41), Females (HR:1.09; CI:1.02-1.18), Whites (HR:1.13; CI:1.04-1.20), Blacks (HR:1.33; CI:1.04-1.20). Competing risk estimates were consistent with these findings. A potential mechanism connecting GD and AD may involve shared etiological factors between the two diseases. Although replication of our findings is needed, they suggest that GD prevention and treatment may contribute to reducing the burden of AD in U.S. older adults.

### SESSION 5020 (SYMPOSIUM)

Abstract citation ID: igad104.1897

### PROVIDING DEMENTIA CARE ACROSS LOW-RESOURCE COMMUNITIES

Chair: Sarah Holmes Discussant: Kirsten Corazzini

The growing number of persons living with dementia (PLWD) require ongoing care and supportive services, but dementia care access and quality are more limited in communities with fewer health resources and these limitations are exacerbated by current dementia care workforce shortages. Across low-resource communities—including poorer urban and rural settings—health resources tend to be limited and PLWD are predominantly socioeconomically disadvantaged and have few personal financial resources to contribute to care provision. Although there is growing evidence about the consequences of care inequities for PLWD, less is known about key issues and opportunities for improving dementia care in low-resource communities. This symposium features presentations that highlight unique social and economic challenges and opportunities for providing dementia care in low-resource communities. The first presenter reports results from a community-based, participatory research study in four long-term care settings in federally designated medically underserved areas (two rural, two urban) that highlights strategies used by long-term care providers to recruit and retain dementia care staff and describes their perspectives on quality dementia care. The second presenter shares findings from an innovative pilot program of case management services to address challenges of PLWD who are living alone in the community through collaboration with local paramedicine services. The third presenter provides an overview of the Person-Centered Options Counseling Certification program with a discussion on strategies for implementing person-centered dementia care initiatives in low-resource communities. This symposium reveals complexities in providing care for PLWD in diverse low-resource communities and generates directions for addressing dementia care inequities.

Abstract citation ID: igad104.1898

### SUPPORTING PERSONS LIVING ALONE WITH DEMENTIA: FINDINGS FROM A PILOT STUDY

Allison Gibson[1], Natalie Pope[2], and Mohammad Hossain[2], *1. Saint Louis University, St. Louis, Missouri, United States, 2. University of Kentucky, Lexington, Kentucky, United States*

Older adults living alone with suspected cognitive impairment often have little-to-no support for conducting daily activities and may be more isolated from formal sources of support and healthcare services. As a result, many individuals living alone may receive delayed diagnosis of dementia compared to those that reside with others. The purpose of this study was to explore an innovative pilot program of case management services, Services to Age in Your Home (STAY Home), with local paramedicine services (interdisciplinary team of social work, law enforcement, and paramedics) that links this population to healthcare and social services through case management services in an urban area. While many participants were willing to engage in the pilot program, access to healthcare and social services remained challenging due to social service program waitlists, discomfort using formal services, distrust of losing autonomy, and limited financial resources contributed to barriers in pilot implementation. A biopsychosocial survey identified individuals needing cognitive testing (88.9%), support in managing medications and medication adherence (88.9%), unmet financial needs (77.8%), unmet healthcare needs (77.8%), difficulty managing finances (66.7%), challenges accessing transportation (55.6%), and unmet assistance needs for their ADLs (44.4%). Most individuals also reported limited social supports (i.e., neighbors, out-of-town relatives) with most relying on local formal services to address emergency needs. This presentation will include a discussion of ethical challenges for both conducting research as well as providing services to this low-resource population.

Abstract citation ID: igad104.1899

### A FRAMEWORK OF SUPPORT: THE NURSING HOME PERSON-CENTERED OPTIONS COUNSELING CERTIFICATION PROGRAM

Kate Crary, Melissa Mandrell, and Laura Davie, *University of New Hampshire, Durham, New Hampshire, United States*

Person-centered initiatives and approaches are the cornerstone of best practices when it comes to supporting older adults and persons living with dementia. Lack of person-centered care has been associated with higher medical costs and negative health outcomes, particularly in low-resource communities. Implementing person-centered care may help to address disparities and improve care outcomes in these communities, although it is often unclear which approaches are most appropriate. The Person-Centered Options Counseling Certification program attempts to address this gap via a mentoring program and competency framework. Developed and piloted in rural New Hampshire, this framework helps professionals and organizations evaluate the approaches that works best for them, while creating fidelity for ongoing support through a rigorous mentorship program. The framework shows promise of being cost effective, an important consideration for many low-resource communities. Professionals can attain knowledge, skills, and abilities without incurring higher education costs and agencies can utilize pre-developed instructional materials. Pilot data was collected to evaluate the program and demonstrated that professionals found the educational training delivered within the framework was relevant to their community and increased their knowledge about person-centered care, although some expressed hesitancy when applying the skills learned with individuals. Professionals who completed both the training and participated in the mentorship program reported a high level of competency, satisfaction, and confidence in delivering person-centered care. The use of this framework may support staff retention in low-resource communities and approaches can be tailored across different environments to facilitate the delivery of person-centered care.

Abstract citation ID: igad104.1900

### STAKEHOLDER PERSPECTIVES ON QUALITY DEMENTIA CARE IN LOW-RESOURCE LONG-TERM CARE SETTINGS

Alison Rataj[1], Sarah Holmes[2], Nancy Kusmaul[3], Laura Davie[1], Yoon Kim[4], and Michael Lepore[2], *1. University of New Hampshire, Durham, New Hampshire, United States*, *2. University of Maryland School of Nursing, Baltimore, Maryland, United States*, *3. University of Maryland Baltimore County, Baltimore, Maryland, United States*, *4. University of Maryland Baltimore, Baltimore, Maryland, United States*

Having dementia care staff who are knowledgeable about the needs and preferences of residents living with dementia (RLWD) in long-term care (LTC) settings holds promise for supporting quality of care. However, staff who work in low-resource LTC settings tend to have fewer opportunities to get to know the residents, who in turn experience increased risk of adverse outcomes. Low-resource LTC settings can experience barriers in providing quality dementia care due to their limited ability to capture pertinent information about residents' needs and preferences and ensuring that information is known by dementia care staff. We sought to describe stakeholder perspectives regarding the collection and sharing of information about RLWD in four low-resource LTC settings (two nursing home; two assisted living) that serve RLWD. All four settings were in medically underserved areas with two rural settings in New Hampshire and two urban settings in Maryland. Preliminary site visits conducted revealed strategies used by LTC providers to recruit and retain dementia care staff. In-depth semi-structured interviews were conducted with a purposive sample of stakeholders (administrative leaders, direct care staff, RLWD and family members). Interviews were transcribed and thematically analyzed in NVivo12. Themes were identified in four core topic areas: 1) identifying information about RLWD to support quality care; 2) finding and accessing information by the care team; 3) sharing information with RLWD; and 4) describing quality measures most relevant for RLWD. Findings shed light on practical strategies used by low-resource LTC settings and motivate future research on measuring dementia care quality.

### SESSION 5025 (SYMPOSIUM)

Abstract citation ID: igad104.1901

### BRIDGING THE PRACTICE-RESEARCH DIVIDE: BUILDING SUCCESSFUL PARTNERSHIPS WITH STATE GOVERNMENT

Chair: Joan Davitt Co-Chair: Sol Baik Discussant: Joan Dav itt

State governments collect data on LTSS but frequently lack resources to analyze it. Simultaneously, researchers often struggle to translate research to policy and practice realms. This symposium will elucidate strategies and challenges in building successful relationships between researchers and state government to enhance LTSS. Paper 1 explores the growing trend of states developing Multisector Plans for Aging (MPA) and opportunities for researchers to ensure that these state-led planning resources utilize evidence to transform and coordinate aging services across the state. This paper will discuss how states use data to promote accountability and monitor implementation of MPA initiatives. Paper 2 describes the development of a statewide data dashboard for Virginia that can be used by state and local agencies to leverage funding and improve programming. This paper will describe the process of developing and eliciting input from a stakeholder task force and the dashboard design. Paper 3 explores efforts in Minnesota to capitalize on existing data to inform policy advocacy and regulatory reform in assisted living. Data from annual survey inspections is utilized to explore trends, target education, and recommend policy changes. Paper 4 focuses on building relationships with Maryland's Adult Protective Service division to engage collaboratively in logic modelling and continuous quality improvement. Strategies for translating existing administrative data into research ready formats and primary data collection methods will be discussed. Symposium participants will have an opportunity to discuss effective strategies for engaging state government in research.

Abstract citation ID: igad104.1902

### MULTISECTOR PLANS FOR AGING: RESEARCH AND STATE GOVERNMENT PARTNERSHIPS TO TRANSFORM AGING SERVICES

Carrie Graham, *Center for Health Care Strategies, Hamilton, New Jersey, United States*

As the US population becomes older and more ethnically diverse, state leaders are increasingly seeing the need to conduct high-level, cross-sector planning to meet the needs of their aging populations and promote equity. A state Multisector Plan for Aging (MPA)-- also called a master plan or strategic plan-- creates a valuable roadmap that can help states transform the infrastructure and coordination of services for all people who are aging in the state. Developing an MPA requires a state-led process that brings various state agencies (Aging, Medicaid, Public Health, Social Services, Housing, Transportation, etc.) together with stakeholders and researchers to outline a clear framework for addressing the needs of older adults, people with disabilities, and caregivers, for 10 years or more. The growing movement of states developing MPAs offers a unique opportunity for aging researchers to partner with state policymakers and stakeholders to ensure that the state's MPA is grounded in evidence and that implementation progress is evaluated using appropriate data and benchmarks. This paper describes the experience of several states that are developing MPAs and how aging researchers in the state have successfully partnered with policymakers, using their research to help the state garner buy in for developing an MPA, elevate the most pressing issues, project population characteristics and needs in the future to inform the development process. Individual researchers, universities and research subcommittees working with states on MPA development can also partner to mine available state data, identify benchmarks, and create data dashboards to guide implementation and monitoring progress.

Abstract citation ID: igad104.1903

### BRIDGING THE PRACTICE–RESEARCH DIVIDE: BUILDING SUCCESSFUL PARTNERSHIPS WITH STATE GOVERNMENT

Rajean Moone[1], Megan Hakanson[2], and Tetyana Shippee[1], *1. University of Minnesota, Minneapolis, Minnesota, United States*, 2. *UMN School of Public Health, Woodbury, Minnesota, United States*

In 2021 Minnesota implemented systemwide regulatory reform to transform loosely regulated housing with services establishments into licensed assisted living facilities. The new framework aligns assisted living facilities more closely with nursing homes. Today over 2,000 assisted living facilities are licensed in Minnesota ranging in size from two to 377 residents. While considered consensus legislation by consumer and provider groups, small, culturally specific providers were largely absent from negotiations. This absence has resulted in disparities in conforming to new regulatory requirements. The University of Minnesota has partnered with the Minnesota Department of Health to conduct analyses of regulatory inspection surveys. The analysis includes comparisons between small (under 15 residents) and large (over 15 residents) facilities as well as qualitative interviews of licensed directors of assisted living facilities that serve culturally specific communities. This paper focuses on the nature of the partnership between the University and state to organize and obtain survey inspection results and the subsequent development of recommendations to enhance regulatory policy.

Abstract citation ID: igad104.1904

### IMPROVING ACCESS TO DEMOGRAPHIC DATA RESOURCES FOR THE AGING NETWORK IN VIRGINIA

Sol Baik, Qian Cai, and Tolu Odukoya, *University of Virginia, Charlottesville, Virginia, United States*

The statewide aging networks and their partner organizations are committed to data-driven communication and advocacy strategies for the needs of the aging population and betterment of their lives. High quality demographic data helps the professionals in the aging network to thoughtfully plan the allocation of resources and carefully target service delivery to those with greater needs. To promote national, state, and local initiatives, improving access to credible and timely data about aging population is crucial. In this presentation, we will describe the efforts to make data available and accessible to the aging network by offering an example of the demographic data services for the Area Agencies on Aging and affiliated organizations in Virginia. We will also share trials and errors to provide the data in a practical, streamlined, and user-friendly way. Finally, we will discuss the strategies and challenges in building effective and sustainable partnerships with a variety of state stakeholders.

Abstract citation ID: igad104.1905

### BUILDING THE RESEARCH PRACTICE PARTNERSHIP TO PROMOTE CONTINUOUS QUALITY IMPROVEMENT IN ADULT PROTECTIVE SERVICES

Joan Davitt[1], Todd Becker[1], Jocelyn Brown[1], Erin Barry-Dutro[1], Greg Sesek[2], and Peiyuan Zhang[1], *1. University of Maryland, Baltimore, Baltimore, Maryland, United States*, 2. *Department of Human Services, Baltimore, Maryland, United States*

Maryland's Department of Human Services, Office of Adult Services developed a multiyear plan to strengthen Adult Protective Services. This plan included the development of a partnership between OAS and the University of Maryland School of Social Work to develop, implement and sustain a Continuous Quality Improvement (CQI) review process for Adult Services Programs (APS) to improve outcomes for vulnerable, elderly, and disabled adults. This paper focuses on partnership development between state government and university researchers to engage collaboratively in continuous quality improvement and intervention efficacy evaluation. Challenges and strategies for building logic models, developing performance measurement strategies, translating existing administrative data into research ready formats, and engaging stakeholders at the state level and in local departments of social services will be discussed. Key challenges included, staffing issues, technology, access to administrative data, and inadequate system resources to engage in research. Strategies for overcoming these challenges will be discussed.

### SESSION 5030 (SYMPOSIUM)

Abstract citation ID: igad104.1906

### INCLUSION AND INTEGRATION OF COMMUNITY STAKEHOLDER PERSPECTIVES IN RESEARCH: INSIGHT FROM AND FOR GERONTOLOGISTS

Chair: Carrie Leach Discussant: Karen Moss

Engaging diverse stakeholders in research is increasingly required by funding agencies though training, skills and capacity building for engaged approaches have not kept pace. To advance the science of community engaged research (CEnR) we convene leading scholars who will provide insight on the inherent issues of engaging stakeholders in research and strategies for overcoming obstacles. The first presentation will summarize findings from a study of gerontologists and aging trainees to understand the needs and challenges of engaging patient and community stakeholders in research. The second will present strategies for engaging Certified Nursing Assistants (CNAs) in a communication program for people living with dementia in skilled nursing facility. The third will present findings from community discussions that informed adaptations to the development of remote sensing technologies and discuss the continuing role for community engagement in implementation. The fourth presentation will offer insight on integrating diverse perspectives in the development of a dementia-focused intervention in a nursing home. The final presentation will summarize the results of a tobacco exposure study to inform the development of a harm-reduction intervention among long-term renters in extended-stay hotels and the benefits of stakeholder partnerships in building trust and aiding in renter recruitment. We will conclude with an attendee inclusive discussion to envision a path that may empower people of all ages and at all career stages to engage diverse stakeholders as partners to catalyze scientific research that is responsive and relevant to those who have a stake in healthy aging and results in social change. This is a collaborative symposium between the Community-Engaged Research and Patient/Person Engagement in Research Interest Groups.

Abstract citation ID: igad104.1907

### THE PROMISE AND CHALLENGES OF GERONTOLOGISTS WHO ADVANCE THE SCIENCE OF COMMUNITY-ENGAGED RESEARCH

Carrie Leach[1], Daniel Gan[2], Allyson Brothers[3], Lana Sargent[4], and Sarah Dys[5], *1. Wayne State University, Detroit, Michigan, United States*, *2. Thompson Rivers University, Kamloops, British Columbia, Canada*, *3. Colorado State University, Fort Collins, Colorado, United States*, *4. VCU School of Nursing, Ashland, Virginia, United States*, *5. Portland State University, Portland, Oregon, United States*

Our aging population demands more responsive policies and services and calls for a shift from involving older adults as individual participants toward collaborating with or empowering their communities as research partners. Knowing how to build capacity for community-engaged research (CEnR) among gerontologists requires understanding the challenges of such endeavors. A survey was distributed to gerontologists in March 2021 to December 2022, and was supplemented by a brainstorming focus group. We received responses from academic faculty, staff, students, and trainees (n=98) representing multiple academic disciplines from community health and implementation science to behavioral and social sciences. Many identified as a minoritized person (57.7%) and used mixed methods (55.1%). Focus group findings revealed challenges around community and participant recruitment, sustaining relationships, low-resource contexts and recognition. These reflected concerns around time, work, and funding – three most commonly-occurring words in survey responses. Barriers articulated include, "the time it takes to cultivate real relationships with community partners to design and carry out research together," "confusion among academic colleagues on how this work is scholarship," and a "lack of understanding and recognition from universities and funding agencies." To more effectively mobilize knowledge and remain relevant, institutions would do well to heed calls for training on involving community members as co-researchers (78.6%), learning about rapidly developing CEnR methodologies (75.3%), and sustaining successful intervention programs after grant funding has ended (70.4%) among other priorities. This study provides critical insight into the landscape of CEnR among gerontologists and may be used to develop more supportive programs and structures.

Abstract citation ID: igad104.1908

### SMART AGING: ENGAGING OLDER ADULTS TO GUIDE SENSOR DEVELOPMENT TO SUPPORT AGING IN PLACE

Elinor Schoenfeld[1], Tracy Trimboli[2], Givenchy Ayisi-Boahene[2], Patricia Bruckenthal[2], Erez Zadok[2], Shelley Horwitz[2], and Fan Ye[2], *1. Stony Brook School of Medicine, Stony Brook, New York, United States*, *2. Stony Brook University, Stony Brook, New York, United States*

Over 80% of older adults want to live independently in their own homes and communities, maintaining quality of life, autonomy, and dignity as they age. We are using community engaged research methods to aid in developing in-home cost-conscious remote sensing technologies to support older adults age in place. To understand their needs, we engaged older adults in discussions on home-based sensing technologies. We used visuals and demonstrations to facilitate discussions, showing participants our sensor prototypes and a vignette describing the challenges an older adult and the family face managing a chronic condition. Participants voiced their interest in monitoring for select health conditions and situations when either they or the person(s) they care for are home alone. Discussants raised concerns about personal security/privacy, loss of independence, ethics of data collection and sharing, and being overwhelmed by collected data. Discussions have provided valuable feedback to help us develop a sensor system that is flexible enough to accommodate individuals in different life stages and comfort levels, with different home environments, levels of expendable income, and support structures. As a result, we have developed a system that uses nonvisual, non-wearable sensing that measures respiration and heart rates, and indoor location tracking to monitor the health and wellbeing of users. During this session, we will provide detailed results from our community discussions, and discuss the continuing role for community engagement as we move forward with sensor development and testing.

Abstract citation ID: igad104.1909

### ENGAGING CERTIFIED NURSING ASSISTANTS TO SUPPORT COMMUNICATION FOR PEOPLE LIVING WITH DEMENTIA IN NURSING HOMES

Natalie Douglas, *Central Michigan University, Mount Pleasant, Michigan, United States*

Over 2 million Americans live in nursing homes (NHs), and the vast majority of them live with dementia negatively impacting their ability to communicate. Certified nursing assistants (CNAs) provide the majority of direct care to people living with dementia (PLWD) in NHs, yet they do not have access to person-centered, evidence-based tools to support communication with this population. When CNAs do not communicate with PLWD in effective ways, PLWD may demonstrate negative behavioral symptoms that interfere with care, impacting not only the PLWD, but also the CNA, contributing to CNA turnover and burnout. Most interventions designed to support communication between the PLWD and the CNA do not involve CNA engagement which may contribute to the lack of implementation and access of such interventions. The purpose of this presentation is to outline the process taken to engage CNAs in the implementation of an intervention designed to improve communication during care activities. Information about questions asked to CNAs both in formal (e.g., interviews, focus groups) and informal (e.g., hallway conversations) ways will be provided. Although a sample of CNAs from 6 NHs (n=12) initially rated the intervention as acceptable (M = 4.5, SD = .48), appropriate (M = 4.4, SD = .58), and feasible (M = 4.28, SD = .49) where a rating of '5' equals more acceptable, appropriate, and feasible, and '1' equals less acceptable, appropriate, and feasible, this presentation will highlight how additional levels of engagement prompted modifications to the intervention, supporting implementation.

Abstract citation ID: igad104.1910

### ENGAGEMENT OF NURSING HOME COMMUNITY EXPERTS TO MODIFY THE DIGNITY INTERVENTION

Liza Behrens[1], Kalei Kowalchik[2], Marie Boltz[1], and Jennifer Kraschnewski[3], *1. Pennsylvania State University, State College, Pennsylvania, United States*, *2. Pennsylvania State University, University Park, Pennsylvania, United*

*States, 3. Penn State College of Medicine, Hershey, Pennsylvania, United States*

Voices of Nursing Home (NH) residents, family caregivers, staff and leaders (community experts) are underrepresented in research development but integral when developing interventions for implementation these complex organizations. This study aimed to explore opinions of NH community experts in the development of a novel person-centered risk management intervention for residents with dementia. Community engagement studios (CES) engaged 62 community experts to gain their perspectives on the DIGNITY (Decision-making in aging and dementia for autonomy) study procedures and instruments. CESs (N=4) were led by an experienced moderator and co-moderator, recorded, and transcribed verbatim. Content analysis was used to summarize responses in a rapid feedback loop. Descriptive statistics of standard measures describe community experts' ratings of acceptability, appropriateness, and feasibility of DIGNITY intervention. Most community experts identified as female (85%), ranging from 22 - 86 years. Participants were residents with dementia (n=4); family caregivers (n=4); NH administrators (n=16), care manager/ supervisors (n=10), direct-care workers (n=6) licensed healthcare providers (n=6), other NH staff (n=8), ombudsman (n=4) and state surveyors (n=4). Community experts provided suggestions to adapt the study instruments and delivery methods. The study team made changes to all six intervention elements based on expert feedback. Most experts agreed that the DIGNITY intervention was acceptable (M=17.7, SD= 2.08), appropriate (M= 17.24, SD= 2), and feasible (M=16.91, SD= 2.22) to implement in the NH community. Results support practical modifications to the DIGNITY intervention to make it feasible to implement in an upcoming pilot clinical trial.

Abstract citation ID: igad104.1911

### TOBACCO IN HOTELS: A STUDY OF NICOTINE EXPOSURE AND RESIDENT RISK

Terri Lewinson[1], Abhirupta Dasgupta[2], and James Murphey[2], *1. Geisel School of Medicine/ Dartmouth College, Lebanon, New Hampshire, United States*, *2. Geisel School of Medicine, Dartmouth College, Hanover, New Hampshire, United States*

Secondhand smoke (SHS) exposure exacts considerable health and financial impact on thousands of Americans yearly. One in four nonsmokers (about 58 million people) is routinely exposed to SHS, primarily at home. There is wide variability in smoke-free home policy adoption, leading to sociodemographic disparities in tobacco exposure and adverse health effects among low-income renters. Although there is no safe level of tobacco smoke exposure, varying perceptions about exposure effects and risks contribute to inconsistent use of harm-reduction strategies such as smoke-free home policies in private dwellings. Therefore, we surveyed and interviewed low-income extended-stay hotel renters (n=77) to learn about their perceptions of tobacco exposure. We also measured participants' willingness to self-monitor these risks and adopt at-home smoke-free interventions. Most survey participants identified as nonsmokers (56.8%) exposed to tobacco smoke regularly. However, both nonsmokers and smokers preferred smoke-free rooms and indicated they would likely participate in personal health research by sharing air and biosamples to measure their exposure. We will present survey results of tobacco exposure knowledge and attitudes among long-term renters in Metropolitan Atlanta extended-stay hotels, perceptions of exposure risk, and self-efficacy beliefs. From our interview findings, we share renter and hotel manager readiness perceptions for implementing at-home smoke-free interventions. Lastly, we will share the results of integrating community-engaged partnerships with local organizations and respondent-driven sampling methods to build trust and aid renter recruitment.

### SESSION 5035 (SYMPOSIUM)

Abstract citation ID: igad104.1912

### PSYCHOSOCIAL RESOURCES IN THE CONTEXT OF VULNERABILITY AMONG OLDER ADULTS: IMPLICATIONS FOR WELL-BEING

Chair: Aurora Sherman Co-Chair: Kira Birditt

Social ties and mastery may buffer detrimental effects of stressful life circumstances and vulnerability as individuals age. The purpose of this symposium is to understand the role of psychosocial resources for multiple dimensions of well-being across several age-related contexts. Luong et al. examined social ties' effects on moving into senior housing using a burst design, finding that social ties were less helpful in the beginning of the move but regained beneficial effects on well-being later. Kinkade and Fuller examined the role of social integration on the longitudinal effects of the widowhood transition for well-being, concluding that increased social integration had protective impacts on depression and functional health among widows compared to non-widows. Birditt et al. assessed the implications of positive activities and social interactions among dementia caregivers using ecological momentary assessments. Positive activities and positive interactions with care recipients were associated with lower levels of daily burden and greater positive affect especially among caregivers who reported high levels of burden. Ryan examined spouses' psychosocial characteristics on partners' well-being and whether they varied by cognitive status of the partner. She found that spouses' positive support from children amidst other negative psychosocial experiences was protective for their partner's life satisfaction. Sherman found that self-esteem for mid-life and older women was associated with higher mastery and lower constraints beliefs and that both were mediated by frequency of self-objectification. Together, these papers highlight the importance of psychosocial resources for well-being as individuals as they age and face new or on-going challenges.

Abstract citation ID: igad104.1913

### CONTROL BELIEFS MEDIATED BY SELF-OBJECTIFICATION IN RELATION TO ADULT WOMEN'S SELF-ESTEEM

Aurora Sherman, *Oregon State University, Corvallis, Oregon, United States*

Beliefs about mastery of one's environment as well as constraints on one's personal control are predictive of well-being across mid-life and older adulthood. In addition, women's level of self-objectification is predictive of well-being in early adulthood, but less is known about the role of self-objectification and well-being for mid-life or older women. This study investigated the role of control beliefs (mastery and constraints) and self-objectification in their relationship to the self-esteem of a sample of adult women (rage 48-90, Mean age = 67.83 years). We found that, compared to the mid-life women, older adult women reported higher self-esteem as well as lower constraints and self-objectification, but no age group differences in mastery beliefs. Further, using Hayes' PROCESS model 4 we show that both mastery and constraints beliefs are mediated in their relationship with women's self-esteem through self-objectification (R2 = .47 for the model with Mastery and R2= .37 for the model with constraints). Higher mastery predicted lower self-objectification, which, in turn, predicted higher self-esteem, while higher constraints predicted higher self-objectification and lower self-esteem. Additional analyses revealed that age remained a significant independent variable of self-esteem and was not mediated by control beliefs or self-objectification. Results are discussed in light of positive aging and objectification theory.

Abstract citation ID: igad104.1914

### POSITIVE ACTIVITIES AND WELL-BEING AMONG DEMENTIA CAREGIVERS: MODERATING EFFECTS OF CAREGIVER BURDEN

Kira Birditt[1], Angela Turkelson[2], Akari Oya[3], Crystal Ng[3], and Courtney Polenick[3], *1. Institute for Social Research, University of Michigan, Ann Arbor, Michigan, United States*, *2. University of Michigan: Institute for Social Research, Ann Arbor, Michigan, United States*, *3. University of Michigan, Ann Arbor, Michigan, United States*

ADRD caregiving is common and often predicts increased caregiver burden which has negative implications for mental and physical health. Thus, it is important to understand the modifiable factors that may lead to less burden among caregivers. Positive activity theory and research suggest that engaging in positive activities may lead to greater positive and less negative affect and less burden. The purpose of this study was to examine positive activities and positive social interactions among ADRD caregivers and their links with well-being. A total of 98 caregivers completed baselines and EMAS every three hours for 5 days. Greater positive activities and more positive interactions with PLWD predicted greater positive affect, less negative affect, and lower daily caregiver burden. Mediation models indicated that positive and negative affect fully accounted for the links between positive activities and caregiver burden and partially accounted for the links between positive interactions with PLWD and caregiver burden. These effects were greater among caregivers who reported greater overall burden at baseline. There were no such effects when examining positive interactions with social ties other than the PLWD. Thus positive activities and positive social interactions with PLWD may represent an important target for future interventions with an identified mechanism of action (affect) and demonstrated benefit for daily burden among high risk caregivers.

Abstract citation ID: igad104.1915

### INTERPLAY OF COGNITIVE STATUS AND SPOUSAL CAREGIVER PATTERNS ON WELL-BEING

Lindsay Ryan, *University of Michigan, Ann Arbir, Michigan, United States*

The current paper investigated the intersection of cognitive status and caregiver characteristics on older adults' reports of life satisfaction and depressive symptoms. Using a sub-sample from the 2016 Health and Retirement Study Harmonized Cognitive Assessment Protocol (HRS-HCAP), individuals were selected if the HCAP informant was a spouse self-identified caregiver. The sample was trimmed to include those whose spouse caregiver was also a respondent in the main 2016 HRS and completed the measures of interest in the psychosocial leave-behind questionnaire (N = 190). The final analytic sample comprised 109 respondents classified as cognitively normal, 51 with cognitive impairment, not dementia, and 30 with dementia based on the algorithm developed by Manly, Jones, Langa, et al. (2022). The sample was 50% women, 10% racialized as Black, 18% racialized as LatinX, with a mean age of 75.62 and an average 11.75 years of education. Latent Class Analysis identified meaningful caregiver psychosocial classes from spouse reports on purpose in life (Ryff & Keyes, 1995), positive and negative affect (Watson & Clark, 1994), and perceived positive and negative support from one's spouse (in this case the main respondent), children, other family, and friends (Turner, Frankel, & Levin, 1983). The data supported a 4-class solution, which was then used in linear regression analyses to examine associations of cognitive status and caregiver profiles on individual's life satisfaction and depressive symptoms. Interestingly, cognitive status was not associated with well-being in the current study, suggesting associations of spousal psychosocial classes were independent of the care-recipient's dementia/impairment designation.

Abstract citation ID: igad104.1916

### SOCIAL SUPPORT–HEALTH LINKAGES DURING A MAJOR LIFE EVENT: A NEW SPIN ON STRESS-BUFFERING EFFECTS

Gloria Luong, *Colorado State University, Fort Collins, Colorado, United States*

Supportive social relationships are posited to confer benefits to longer-term health and well-being, and may be especially protective in the face of adversity, when people can draw on these resources to cope with stressful events (stress buffering effects). Yet, most previous studies have used retrospective, cross-sectional designs, limiting the ability to delineate how the linkages between social support and health may change prospectively as people anticipate and prepare for an upcoming stressor, such as relocating into a senior housing facility and adjusting to the experiences afterward. The Relocation and Transitional Experiences (RELATE) study tracked 154 older adults using a prospective longitudinal measurement burst design as they transitioned into a senior housing facility. The waves (bursts) of assessments were based on the following schedule: Burst 1 (2 weeks prior to the move), Burst 2 (2 weeks after the move), Burst 3 (1.5 months after the move), and Burst 4 (~3-6 months after the move). At each burst, participants completed questionnaires on their health and well-being, social relationships and social support, and relationship satisfaction. We examined changes in the strength of social support-health linkages across bursts. Results show that social-support health linkages were weakest and least consistent during the early stages of the relocation stressor (Burst 2 and 3) but that by Burst 4, support-health linkages were mostly restored similar to pre-move levels (Burst 1). These findings suggest that stress buffering effects need to be understood within the context of how stressful experiences unfold over time for older adults.

Abstract citation ID: igad104.1917

### TRANSITIONING TO WIDOWHOOD: THE ROLE OF SOCIAL INTEGRATION IN MODERATING WELL-BEING OVER TIME

Emily Kinkade, and Heather Fuller, *North Dakota State University, Fargo, North Dakota, United States*

Prior studies indicate that widowhood may bring declines in both physical and mental health, but that social support may buffer against these declines. Social integration has also been found to alleviate widows' depressive symptoms over time. However, less is known about whether social integration moderates the trajectory of well-being declines over longer periods of time post-widowhood. This study investigates whether social integration moderates the relationship between the transition to widowhood and changes in physical and mental well-being over time. A series of longitudinal multilevel models were run with data from participants (n=244-246) from five waves of the Social Integration and Aging study, collected every two years between 2013 and 2021. Depressive symptoms and subjective happiness were indicators of mental well-being, whereas basic and instrumental activities of daily living and total number of diseases were indicators of physical well-being. Social integration was assessed with the Social Integration in Later Life Scale (SILLS). Social integration moderated the relationship between widowhood and depressive symptoms such that increases in social integration resulted in greater reductions of depression scores over time among widows as compared to non-widows. Social integration similarly buffered against declines in physical functioning (i.e., increased basic activities of daily living) over time after widowhood occurred. No interaction effects of social integration on the relationship between widowhood and happiness or other physical well-being outcomes were found. These findings suggest social integration may be important for maintaining mental well-being and some aspects of physical health as older adults adjust to widowhood.

### SESSION 5040 (SYMPOSIUM)

Abstract citation ID: igad104.1918

### WOMEN AND WORK AT MID LIFE AND BEYOND: STUMBLING, STRIVING, NAVIGATING TRANSITIONS

Chair: Julie Miller

Women at midlife and beyond are making significant and increasing contributions to The Global Longevity Economy, driving increases in Gross Domestic Product (GDP), job creation, and income generation. Indeed, advances in women's life expectancies, statuses, and roles establish women aged 50-plus as a high-powered engine driving economic opportunity. As much as we can and must celebrate the economic contributions of older women, we cannot do so without acknowledging the forces holding them back from contributing even more. Despite the progress women at midlife and beyond have made in the United States relative to many of those who came before them, they still are challenged to reach their ultimate economic potential. This session will explore some of the factors that propel and inhibit women aged 50-plus from reaching their full economic potential, each of which is related in some capacity to paid work. The first presentation in this symposium spotlights a mixed methods study into the economic lives of working women at midlife and beyond across the United States who face one or more barriers, in addition to gender, to building their economic security. The second presentation will focus on financial challenges and experiences of women entrepreneurs aged 40-plus during the Covid-19 pandemic. The final presentation will explore financial and professional impacts of menopause transitions for women. Taken together, implications across all studies point to broad areas of intervention, innovation, and additional areas for scholarship – all as they relate to financial wellbeing and work for women at midlife and beyond.

Abstract citation ID: igad104.1919

### BUILDING AN ECONOMY THAT SUPPORTS WORKING WOMEN TO THRIVE

Heather McCulloch, *The Aspen Institute, San Francisco, California, United States*

Building on previous research about working women's financial wellbeing, a provocative question arises: "What would the economy look like if were designed to work for women?" To answer this question, Heather McCulloch, entrepreneur in residence with the Aspen Institute Financial Security Program, conducted a translational research initiative called Women in the Economy. Intended to take a deep dive into the economic lives of working women across the U.S. who face one or more barriers, in addition to gender, to building their economic security, the project included in-depth interviews with 127 women across urban, suburban, and rural communities in 30 states and D.C. Participants were asked about the economic challenges they face, what they see as the most promising solutions, and their vision for a gender-equitable economy that enables women to strive, thrive, and reach their full potential. Findings revealed that working woman are struggling to make ends meet; they face a financial penalty - a loss of income and savings - due to caregiving; and they are not receiving the education they need to successfully navigate economic and financial systems. Building on qualitative results, a national survey (n=1224) was deployed to understand the scope of emergent topics. Among key findings are that four out of five respondents reported feeling economically insecure and only one in five reported feeling confident they would live comfortably in retirement. Together, findings based on the wisdom, voices, and experiences of working women point to policy, program, and market-based solutions that can build a gender-equitable society.

Abstract citation ID: igad104.1920

### OLDER WOMEN ENTREPRENEURS ARE DRIVEN BY WANT RATHER THAN NEED

Lona Choi-Allum, *AARP, Washington, District of Columbia, United States*

During the COVID-19 pandemic, women ages 50+ pursued their dream of starting a business. This study contacted 278 women ages 50-plus by phone and online, examining the drivers and barriers of women entrepreneurs. Over a quarter (27%) of women said they always wanted to start a business, and 19% said they did it to follow their passion; another 17% were pursuing additional income, and 11% wanted flexible work options. Age, and perhaps the value of experience, has been an advantage in business ownership. Women entrepreneurs age 50-plus were less likely to have faced financial challenges since starting their business, with over two in five (45%) avoiding such challenges, compared to 29% of women entrepreneurs in their 40s. Nearly seven in 10 women (69%) surveyed poured their personal savings into their start-up. In addition, two in three agree that they face unique challenges in trying to access capital for their business that are different from men. Despite these challenges, most women were optimistic about their entrepreneurial path. The majority of women (97%) agreed that they made the right decision in starting their business – with about two in five (39%) saying their business is doing better than expected compared to when they first started. Respondents say they need resources on marketing, recruiting and hiring staff, and financing. And over two in five say they have not taken any type of training. Increasing awareness of business supports, funding sources, and training opportunities will help women as they grow their business.

Abstract citation ID: igad104.1921

### THE ECONOMIC IMPACT OF MENOPAUSE: A MULTIMODE RESEARCH PROJECT

Jennifer Sauer, Laura Mehegan, Alicia Williams, Aisha Bonner-Cozad, Cheryl Lampkin, and Monica Ekman, *AARP, Washington, District of Columbia, United States*

In late 2022, AARP embarked on a robust research program to explore the economic impact of the menopause transition (perimenopause, menopause, and post-menopause) on affected individuals. Other research found that some women incur substantial costs to ameliorate adverse menopausal symptoms such as hot flashes, night sweats, brain fog, and mood swings. Additionally, some women experience symptoms severe enough to interfere with their job causing them to reduce their hours or leave their jobs completely. The first phase of AARP's research included six online focus groups with US women aged 40 and older who were segmented by their stage of menopause (perimenopause, post-menopause). Resulting themes from the focus groups support what previous researchers have concluded that some individuals: (1) experience symptoms that disrupt their lives, (2) have treatments that are not necessarily discussed by a healthcare provider or covered by insurance leading to direct consumer costs, (3) consider leaving their jobs due to the severe and disruptive symptoms, and (4) feel their employers are not equipped to accommodate their needs. These focus group findings informed survey research among a representative sample of women aged 40 and older as well as an employer survey to explore any existing workplace policies that may benefit women experiencing the menopausal transition. Finally, all research results combined inform an information campaign to assist individuals who are navigating this stage of life.

### SESSION 5045 (SYMPOSIUM)

Abstract citation ID: igad104.1922

### DYNAMIC INTERPLAY BETWEEN STRESS AND POOR SLEEP AND THEIR CONSEQUENCES ON DAILY WELL-BEING

Chair: Soomi Lee Discussant: David Almeida

Stress impairs sleep. Poor sleep also increases the likelihood of stressful experiences. Importantly, there is the vicious cycle between stress and poor sleep, such that having either one increases the odds of the other. This is problematic in the context of aging, because midlife and older adults may have physiological vulnerability to stressors and age-related deterioration in sleep. This stalemate wherein aging individuals are exposed to daily stressors and degraded sleep but need to lower stress and improve sleep for long-term health calls for further research describing how stress and poor sleep co-occur in daily life and contribute to daily health and well-being. To respond to this call, this symposium brings together four rigorous studies. Paper 1 examines how recession hardships (financial, job, housing-related) are associated with daily sleep quality in midlife and older adults. Paper 2 focuses on exposure and reactivity to daily interpersonal stressors and reports their associations with daily sleep quality and physical activity in older adults. Paper 3 investigates daily links between worry and rumination and their implications for sleep in older adults. Paper 4 examines how consecutive poor sleep is associated with day-to-day trajectories of rumination and memory lapses in midlife and older adults. These papers use relevant theories and advanced analytic techniques to describe the nature, causes, and consequences of stress and poor sleep in the second half of life. The discussant, Dr. David Almeida will integrate key findings from these studies, discuss their theoretical and methodological contributions, and consider opportunities for future research.

Abstract citation ID: igad104.1923

### CONSECUTIVE POOR SLEEP AND ITS ASSOCIATIONS WITH DAILY RUMINATION AND MEMORY LAPSES

Soomi Lee[1], and Kyoungmin Cho[2], *1. The Pennsylvania State University, University Park, Pennsylvania, United States*, *2. Korea Advanced Institute of Science and Technology, Daejeon, Republic of Korea*

Emerging research has shown that consecutive sleep loss impairs daily affective and physical well-being. Yet, little is known about the effects of consecutive poor sleep (in both quantity and quality) on daily cognitive stress. This study examined whether and how consecutive nights of poor sleep quantity and quality are associated with day-to-day trajectories of rumination and memory lapses. Participants were adults (n=938) from the Midlife in the United States Study III who provided data for eight diary days. Consecutive poor sleep quantity and quality were operationalized as the within-person numbers of consecutive nights with < 6 hours of sleep and with reports of very bad/bad sleep quality, respectively. Multilevel models evaluated the linear, quadratic, and cubic effects of consecutive poor sleep on daily rumination and memory lapses, after controlling for sociodemographic, health, daily covariates, and between-person means of sleep quantity and quality. Daily rumination increased in curvilinear fashion such that it increased (linear), but the rate of increase decelerated (quadratic) and then accelerated again (cubic) as the number of consecutive poor sleep quantity and quality increased. Daily memory lapses increased in linear fashion as the number of consecutive poor sleep quantity (but not sleep quality) increased. Findings show that the day-to-day trajectory of rumination is characterized by high initial reactivity, followed by decrease due to habituation and adaptation, and then increase, as poor sleep is accumulated more consecutive times. The day-to-day trajectory of memory lapses is mostly characterized by a dose-response relationship with the number of consecutive poor sleep quantity.

Abstract citation ID: igad104.1924

### RECESSION EXPERIENCES AND SELF-REPORTED DAILY SLEEP QUALITY IN MID-LIFE AND AGING ADULTS

Aarti Bhat[1], Sun Ah Lee[1], Jose Diaz[2], and David Almeida[3], *1. The Pennsylvania State University, University Park, Pennsylvania, United States*, *2. Penn State University, University Park, Pennsylvania, United States*, *3. The Pennsylvania State University, State College, Pennsylvania, United States*

Adverse economic events can negatively impact various indicators of health, including sleep quality. Sleep quality issues are associated with chronic physical health conditions and poor mental health. While there has been a more established body of work on stress and global indicators of sleep, assessing sleep quality at the daily level can contribute to further understanding how stress can impact everyday life. Given that midlife and older adults are a fast-growing demographic in the United States, who have experienced economic events such as the 2007-2009 Great Recession as well as the COVID-19 pandemic, it becomes critical to study how such economic experiences affect markers of wellbeing, such as daily sleep, for aging adults. This study examines the effect of recession hardships on daily sleep quality in aging adults using the Midlife in the United States Refresher study. This study will combine the MIDUS Refresher Survey project with the MIDUS Refresher Biomarker project (n=863) to assess the contribution of adverse Recession events across financial, job, and housing experiences (86% of participants reported at least one adverse Recession experience) on quality of sleep (e.g., time to fall asleep, wakefulness during night) using the Daily Sleep Diary component of the MIDUS Refresher Biomarker project, in which participants self-reported on their sleep quality for seven days. This study will provide insight into how certain types of Recession events may differentially affect daily sleep patterns for aging adults; and provide information of within and between person differences in how Recession events impact daily sleep.

Abstract citation ID: igad104.1925

### DAILY STRESS PROCESSES, PHYSICAL ACTIVITY, AND SLEEP IN OLDER ADULTHOOD

Kate Leger[1], Susan Charles[2], Colette Brown[3], and Karen Fingerman[4], *1. University of Kentucky, Lexington, Kentucky, United States*, *2. University of California, Irvine, Irvine, California, United States*, *3. UCI, Irvine, California, United States*, *4. The University of Texas at Austin, Austin, Texas, United States*

Older adults who engage in healthy behaviors such as being physically active and sleeping well report lower levels of stress. Less is known about the links between exposure and reactivity to stressful events in daily life and their relationship with physical activity and sleep. The current study examined within-person associations between daily physical activity, sleep, and exposure and affective reactivity to naturally occurring interpersonal stressors. Older adults (N = 180) from the Daily Experiences and Well-being Study completed ecological momentary assessments (EMAs) every 3 hours for 5-6 days where they reported negative affect throughout the day and interpersonal tensions at the end of the day. They also reported their nightly sleep quality and wore Actical accelerometers to capture physical activity. Older adults reported greater numbers of stressors on days when they spent less time being sedentary and engaged in more light physical activity. On days when older adults experienced more stressors, they reported higher levels of negative affect, but this association was attenuated when they were more physically active that day. Additionally, older adults reported lower sleep quality the night after they reported higher numbers of stressors. These findings underscore the importance of assessing physical activity, sleep and stressful events in daily life and have implications for both physical and psychological well-being.

Abstract citation ID: igad104.1926

### DAILY WORRY, RUMINATION, AND SLEEP IN LATE LIFE

Zexi Zhou[1], Kira Birditt[2], Kate Leger[3], and Karen Fingerman[1], *1. The University of Texas at Austin, Austin, Texas, United States*, *2. Institute for Social Research, University of Michigan, Ann Arbor, Michigan, United States*, *3. University of Kentucky, Lexington, Kentucky, United Sta tes*

Perseverative thinking, including worry and rumination, is a common response induced by stress, and can be detrimental to well-being and health especially for older adults. Sleep may represent an important mechanism by which preservative thoughts affect health. Yet, limited research has investigated links between worry and rumination and their implications for sleep in late life. This study examines the associations between older adults' everyday worry, rumination, and sleep. We leveraged the daily diary data over 5 to 6 days from the Daily Experiences and Well-being Study (N = 271, Mage = 73.62). Every evening, older adults reported worry and rumination they experienced that day. Every morning, they indicated how worried they were about something that might happen that day, and sleep the prior night (e.g., duration, quality, disturbances). Multilevel models showed that greater evening rumination was associated with fewer sleep disturbances. More hours of sleep, higher sleep quality, and fewer sleep disturbances were associated with less worry the next morning. Greater evening worry and rumination were both associated with greater next morning's worry, but more hours of sleep buffered the lingering of worry over the night. These results suggest that worry and rumination may tend to persist in older adults' daily life, and highlight the protective role that better sleep may play in reducing older adults' everyday perseverative thinking.

### SESSION 5050 (PAPER)

### AGING IN PLACE (BSS)

Abstract citation ID: igad104.1927

### ACCESS TO LOCAL ESTABLISHMENTS AND NEIGHBORHOOD PERCEPTIONS: A FOCUS ON THE ROLE OF LOCAL BANKING

Alyssa Goldman[1], and Megan Doherty Bea[2], *1. Boston College, Boston, Massachusetts, United States*, *2. University of Wisconsin-Madison, Madison, Wisconsin, United States*

As an increasing number of older adults are aging-in-place, individuals' perceptions of their neighborhood environments have emerged as significant social-contextual issues for healthy aging. Prior studies have focused on how neighborhood characteristics shape perceptions of residential neighborhoods. Far less research has focused on the role of local banking, despite the intersections between local banking establishments and a host of key community social and economic resources, particularly for the older adult population. We use nationally representative data from older adults interviewed at Rounds 2 and 3 of the National Social Life, Health, and Aging Project (N=4,589) linked with the National Establishment Time Series database to examine neighborhood perceptions as a function of local banking establishment density. We find that older adults who live in areas with higher rates of banking establishments report significantly higher levels of neighborhood safety, even when adjusting for a host of individual and neighborhood characteristics. Importantly, other types of establishments also shape perceived neighborhood safety, however, the significance of certain associations only emerges when we adjust for the presence of local banking. We suggest that local banks may be a lynchpin in catalyzing the broader socioeconomic vitality of a place, supporting older residents' favorable perceptions of neighborhood safety. We discuss the implications of our findings for inequality in aging in light of unequal access to local banking across social groups, potentially increasing the vulnerability of older adults who are less likely to adopt mobile banking solutions and who are more likely to be susceptible to financial exploitation.

Abstract citation ID: igad104.1928

### ENDORSEMENT OF DRIVING AVOIDANCE BEHAVIORS AND COGNITIVE, PHYSICAL, AND VISUAL PREDICTORS AMONG OLDER DRIVERS

Alan Mintz[1], Lesley Ross[1], Dustin Souders[1], and Despina Stavrinos[2], *1. Clemson University, Clemson, South Carolina, United States*, *2. University of Alabama at Birmingham, Birmingham, Alabama, United States*

Due to age-related changes in visual, physical, and cognitive abilities, certain driving conditions may become more challenging for older adults. Secondary analyses were conducted to assess driving avoidance habits of 72 healthy drivers (65 – 85 years old). Additionally, the physical function (Turn360, Timed Get Up and Go), cognitive (Useful Field of View, Mazes, Trails B, and Wechsler Memory Scale), and visual (visual acuity, Pelli-Robson contrast sensitivity) performance predictors of these habits were explored using linear regression. 59.7% of participants reported avoiding driving in bad weather, 44.4% of participants reported avoiding driving at night, and 40.3% of participants reported avoiding driving during rush hour, making these the most frequently avoided situations. Only 5.6% of participants reported avoiding driving alone and 15.3% of participants reported avoiding driving on high-speed interstates or expressways, making these the least often avoided situations. Women (β = -.393, p = .001) and poorer physical function (β= -.301, p = .018) were associated with greater driving avoidance. Cognition and vision performance did not predict the driving situations. While these results are limited by the high SES of the sample, the results indicate that driving avoidance interventions should consider gender and physical function. Implications and future directions are discussed.

Abstract citation ID: igad104.1929

### HISTORICAL REDLINING AND COGNITIVE FUNCTIONING AMONG US OLDER ADULTS

Calley Fisk[1], Jennifer Ailshire[1], and Katrina Walsemann[2], *1. University of Southern California, Los Angeles, California, United States*, *2. University of Maryland, College Park, College Park, Maryland, United States*

Historical redlining disproportionately harmed Black and disadvantaged communities by designating them as less desirable for mortgage lending. Residence in neighborhoods historically designated as less desirable is associated with worse health outcomes, though little research exists on older adults. Redlined neighborhoods often have characteristics that increase risk for poor cognitive function among older adults (e.g., greater air pollution and less greenspace). We examine differences in cognitive function trajectories by redlining status over 18 years (1998-2016) using data on 5,527 respondents ages 50 and older from the nationally representative Health and Retirement Study that have been linked to National Community Reinvestment Coalition Historic Redlining Scores Data. Results from multilevel linear regression models - adjusted for individual sociodemographic characteristics - show that baseline level of cognitive function is lower in neighborhoods historically designated as "Declining" (b=-0.61, p<.001) or "Hazardous" (b=-0.95, p<.001) compared with "Best or Desirable". We do not find any association with cognitive change over time. Baseline differences were explained by educational attainment and income for residents of "Declining" neighborhoods, and tract-level affluence and disadvantage for residents of "Hazardous" neighborhoods. Race-stratified analyses showed that the impact of historical redlining on cognitive function was generally similar for Black and White adults, though we found no association between historical redlining and cognitive function for Black residents of "Declining" neighborhoods. Our findings suggest that redlining, as both an historical practice and process affecting the socioeconomic trajectories of individuals and neighborhoods, can shape cognitive health in older adults.

Abstract citation ID: igad104.1930

### NURSING HOME EXPECTATIONS AND MENTAL HEALTH AMONG OLDER ADULTS IN THE HEALTH AND RETIREMENT STUDY

Annalise Lane[1], Linh Dang[2], and Briana Mezuk[2], *1. Department of Veterans Affairs, Ann Arbor, Michigan, United States*, *2. University of Michigan, Ann Arbor, Michigan, United States*

Three million older adults live in long-term care facilities. Transitioning into a nursing home is a major life event that can worsen mental health. Expectedness of nursing home transitions may have implications for mental health; however, this area is understudied. This study examined the relationship between expectations of transitioning into a nursing home in the next five years and depressive symptoms and passive suicidal ideation, and how social integration modified these relationships. Sample included respondents aged 65+ from the 2018 Health and Retirement Study (n=7,944, mean age=73.9, 55.4% female, 80.5% Non-Hispanic White). Nursing home transition expectations were modeled continuously (range: 0-100%). Mental health outcomes were assessed by the Composite International Diagnostic Interview-Short Form. Social integration measured composition, frequency of contact, and closeness with spouses/partners, children, other family members, and friends. Multivariate logistic regressions were fit for depressive symptoms and passive suicidal ideation separately, adjusting for demographic, health characteristics, and health-related reasons for moving. Additional models simultaneously adjusted for all social integration measures. Higher nursing home expectations were associated with elevated depressive symptoms (OR: 1.06, 95% CI: 1.01-1.11), major depressive episode (OR: 1.08, 95% CI: 1.02-1.15), and suicidal ideation (OR: 1.10, 95% CI: 1.03-1.16). These relationships persisted even after accounting for social integration. Future research can explore modifiable factors salient to these relationships, such as perceived social support and family care, to inform mental health-targeted interventions as older adults transition into long-term care.

Abstract citation ID: igad104.1931

### THE IMPACT OF LIVING IN MULTIGENERATIONAL NEIGHBOURHOODS ON LONELINESS IN LATER LIFE

Martin Hyde, and Elizabeth Evans, *Swansea University, Swansea, Wales, United Kingdom*

Intergenerational solidarity has been identified as a key factor for effective social functioning. However, evidence suggests that divisions between younger and older generations within Great Britain (GB) have never been greater. Growing residential age segregation is thought to be driving intergenerational division. This, in turn, is assumed to lead to an increased risk of loneliness among older adults. However, to date, this has not been empirically tested. To examine this issue, we created a new measure of multigenerational neighbourhoods, for all lower super output areas in England and Wales. These were linked to individuals aged 50 and over living in England and Wales in 2018/2019 in the UK Household Longitudinal Survey (N = 14,196). Loneliness was measured using the UCLA-3 scale. Multilevel regression analyses were performed. Covariates included, area level deprivation, urban/rural, residential tax band, home ownership, individual age, sex, marital status and financial situation. The results show that the mix of generations within a neighbourhood has a weak (r = 0.33) and non-significant (p=.493) association with loneliness among older adults. To the best of our knowledge this is the first study to directly examine the impact of neighbourhood age diversity on loneliness in later life. Our findings suggest that the mere presence of different generations within a neighbourhood is not a sufficient condition to combat loneliness in later life

### SESSION 5055 (PAPER)

### CHRONIC DISEASE

Abstract citation ID: igad104.1932

### A PILOT FAMILY COACHING INTERVENTION FOR PEOPLE WITH TYPE 2 DIABETES AND A FAMILY MEMBER

Kristin August[1], Kathleen Jackson[1], and Marsha Rosenthal[2], *1. Rutgers University, Camden, New Jersey, United States*, *2. Rutgers University, New Brunswick, New Jersey, United States*

Type 2 diabetes is a common chronic condition in later life that requires substantial lifestyle changes. Most diabetes management occurs at home with the help – and sometimes hindrance – of family members. Although coaching has become a common strategy in healthcare to supplement diabetes education, few interventions have prepared family members to be coaches, despite the documented benefit of including family members in chronic disease management interventions. The overall goal of our pilot intervention was to develop and test a family coaching approach to enhance patients' diabetes self-management while preserving relationship quality and both individuals' well-being. Participants were recruited from local diabetes education classes and through provider referrals, and included 20 dyads in the intervention group (patient Mage=59.12) and 20 dyads in the control group (patient Mage=57.06). The intervention, involving 5 interactive group sessions (in-person or virtual) led by a facilitator, covered evidence-based material developed by the investigators on diabetes education and coaching strategies. Behavioral, clinical, emotional, and relational metrics were assessed via a survey at baseline, post-intervention, and 3 months later; intervention participants also completed a program evaluation survey and participated in a focus group. Survey findings revealed some positive effects for diabetes self-management behaviors, no effects for short-term clinical markers, negative effects for emotional well-being, and both positive and negative effects for relational well-being. Participants reported being satisfied with the program overall; they indicated learning a lot and having a positive experience. A translation and cultural adaptation of this program for Spanish-speaking populations is currently underway.

Abstract citation ID: igad104.1933

### A SYSTEMATIC REVIEW OF ENABLERS AND BARRIERS TO CANCER SURVIVORSHIP IN NIGERIA

Candidus Nwakasi[1], Kafayat Mahmoud[2], and Darlingtina Esiaka[3], *1. University of Connecticut, Storrs, Connecticut, United States*, *2. Boston University, Boston, Massachusetts, United States*, *3. University of Kentucky, Lexington, Kentucky, United States*

**Background.** Nigeria is benefitting from public health and healthcare advancement which is increasing cancer care in the country. As a result, more people are surviving cancer. However, this should not mask the fact that Nigeria, a rapidly aging country which an increased risk of cancer incidence, bears a heavy health burden of about 72,000 cancer deaths per year — this underscores cancer as a leading cause of death among Nigerians. The purpose of this study was to identify and synthesize factors that are enablers or barriers to cancer survivorship in Nigeria and add to the body of knowledge on cancer survivorship in the global south. Method. Using PRISMA guidelines, we conducted a systematic review across Scopus, PubMed, and databases. Given that cancer survivorship covers periods from diagnoses, to treatment, and end of life, we identified 31 peer-reviewed studies that assessed cancer treatment, management, care, and survivorship in Nigeria.

**Results.** Eight themes emerged from our review of 31 peer-reviewed studies that examined factors that are enablers of barriers to cancer survivorship among Nigerians. Some of the themes include a desire to survive, the availability of treatment options, self-management, and the availability of pseudo-healthcare providers. We later grouped the themes under three categories, healthcare, economic, and psychological factors.

**Conclusion.** Cancer survivors in Nigeria are confronted with several issues that affect their quality of life and health outcomes. This highlights the need for studies on cancer survivorship to inform the design of tailored health improvement interventions for cancer survivors in Nigeria.

Abstract citation ID: igad104.1934

### MULTILEVEL LIFESTYLE INTERVENTIONS ARE NEEDED TO IMPROVE DIABETES SELF-MANAGEMENT IN OLDER ADULTS

Yan Du[1], Lixin Song[2], Mitzi Gonzales[1], Sara Espinoza[3], Carlos Jaen[1], Bei Wu[4], and Erin Finley[1], *1. UT Health San Antonio, San Antonio, Texas, United States*, *2. University of Texas Health Science Center at San Antonio, San Antonio, Texas, United States*, *3. Cedars-Sinai Medical Center, Los Angeles, California, United States*, *4. New York University, New York City, New York, United States*

Type 2 diabetes (T2D) is a major public health concern in older adults. Physical activity (PA) and healthy eating (HE) are two major lifestyle intervention strategies in diabetes self-management for controlling glucose levels, preventing complications, and improving functionality and quality of life. This qualitative study aimed to explore perceived factors influencing PA and HE among predominately Latino older adults with T2D ((age=70.5±4.8; Latinos=73.3%). We conducted semi-structured interviews with 15 community dwelling older adults with T2D at the end of a 12-week pre- and post- study assessing self-monitoring on lifestyles and health outcomes. All interviews were recorded and transcribed. Two researchers analyzed the data independently using a combination of deductive and inductive thematical analysis. Codes were categorized into five themes based on the socio-ecological framework, and sub-themes were identified. The themes included five levels of factors: 1) individual (e.g., knowledge, self-efficacy, self-monitoring, perceived benefits and threats, health status,); 2) interpersonal (e.g., family, friend, health provider); 3) organizational (e.g., availability of physical activity equipment in senior apartments); 4) environment (e.g., culture norms); and 5) policy factors (e.g., health provider, retirement). The factors in one level could influence the factors in another level. Most current lifestyle interventions focus on individual level, and multilevel lifestyle interventions to promote healthy lifestyles, and improve health, independence, and quality of life in older adults with T2D are warranted. The input of additional stakeholders such as family members and community staff need to be accounted when designing multilevel intervention in older adults with T2D.

Abstract citation ID: igad104.1935

### RECOMMENDATIONS REGARDING THE DESIGN OF PAIN ASSESSMENT AND MANAGEMENT TOOL FOR OLDER ADULTS WITH HEART FAILURE

Youjeong Kang[1], Yong Kyung Choi[2], and Seung-Eun Oh[3], *1. University of Utah, Salt Lake City, Utah, United States*, *2. University of Pittsburgh, Pittsburgh, Pennsylvania, United States*, *3. Seoul Women's College of Nursing, Seoul, Republic of Korea*

Nearly 80% of all patients with heart failure (HF) are older adults (≥65 years of age) who are at high risk for frequent hospitalizations. Older adults with HF may delay in responses to pain that could trigger the burden of HF symptoms such as dyspnea due to the increased cardiovascular workload and oxygen demands. However, there is a concern that healthcare providers may be poorly prepared to assess and manage pain when reported by specific populations. This qualitative secondary analysis examined the design recommendations of HF providers for a comprehensive home-based pain assessment and management tool for older adults with HF. The study used in-depth interviews with 20 healthcare providers specializing in HF across different states. Participants provided various recommendations, including a checklist of questions about the patient's past experience with pain, their living environment, and any activities that trigger pain. While some participants preferred electronic tools, such as an mHealth app, for data collection and analysis, they acknowledged the limitations of using technology for older adults. The tool should be straightforward, easy to navigate, and involve patients in the design process to ensure their motivation and buy-in. The tool should also consider the caregiver's input. In conclusion, this study highlights the importance of a comprehensive assessment of pain symptoms and daily activities to provide personalized care for older adults with HF. The design recommendations provided by healthcare providers can aid in the development of a unique home-based pain assessment and management tool for older adults with HF.

Abstract citation ID: igad104.1936

### USING A SOCIOCULTURAL LENS TO EVALUATE CANCER SURVIVORSHIP IN BLACKS AND LATINX IN THE US

Candidus Nwakasi[1], Darlingtina Esiaka[2], Saamantha Pierre-Louis[3], and Onyeka Ekwebene[4], *1. University of Connecticut, Storrs, Connecticut, United States*, *2. University of Kentucky, Lexington, Kentucky, United States*, *3. Providence College, Providence, Rhode Island, United States*, *4. East Tennessee State University, Johnson City, Tennessee, United States*

**Background.** A sociocultural lens such as the PEN-3 Cultural Model offers an opportunity to better understand the extreme psychosocial, physical, emotional, and financial challenges of Black and Latinx cancer survivors in the community experience. By focusing on cultural identity, relationships and expectations, and cultural empowerment, the PEN-3 Cultural Model applied in this study aims to offer a unique insight into cancer survivorship in the Black and Latinx communities in the U.S. Method. A qualitative descriptive approach was used on a purposive sample of 46 Black and Latinx participants recruited. Each of the 19 participants who are cancer survivors including 7 Blacks and 13 Latinx (Mage = 62 years), was interviewed using a semi-structured interviewing style. We also conducted 5 focus group discussions with 27 participants, 2 Latinx groups and 3 Black groups (Mage = 61 years). The focus group participants identified as caregivers of cancer survivors.

**Results.** Thematic analysis resulted in the following themes: culture and dealing with cancer, conflict and control, health care access barriers, and rethinking survivorship in the community. The themes offer insight into sociocultural factors that influence the quality of life of cancer survivors and those in their communities.

**Conclusion.** To better understand and address issues of cancer health inequity in the country, the findings of this study will offer an opportunity to advance knowledge on sociocultural factors influencing cancer survivorship in Black and Latinx communities.

### SESSION 5060 (BIOLOGICAL SCIENCES INVITED SYMPOSIUM)

Abstract citation ID: igad104.1937

### BS GSA AND AGE JOINT SYMPOSIUM

Chair: Blanka Rogina Co-Chair: Rozalyn Anderson

Since 2020, BS GSA and American Aging Association (AGE) recognized the opportunity to present a joint session regularly at each organization's national meeting. Given our unique positions in the field of aging research, we recognize the opportunity to advance each other's missions and strengthen the ecosystem to advance translation of aging research into practice and policy. This symposium has been a resounding success and an ideal forum for early-stage investigators who seek to develop a program at the interface of basic and clinical research.

Abstract citation ID: igad104.1938

### IMPACT OF MITOCHONDRIAL MALFUNCTION IN AGING IS MEDIATED BY IMPAIRED LYSOSOMES AND ENDOPLASMIC RETICULUM

Nuno Raimundo, *Penn State College of Medicine, Hershey, Pennsylvania, United States*

The hallmarks of aging are well established, and include mitochondrial malfunction, deregulated nutrient sensing, loss of proteostasis, and senescence, among others. While these hallmarks are all part of the aging process, they have typically been studied independently. We have found that mitochondrial malfunction can affect lysosomal activity, impacting nutrient sensing, and our that the crosstalk between mitochondria and other organelles such as endoplasmic reticulum and lysosomes affects cellular senescence, proteostasis and intercellular communication. Chronic mitochondrial malfunction causes perturbations in the lysosomal membrane which affect the function of this organelle as well as the recruitment of folliculin (FLCN) to the lysosomes. FLCN regulates the balance between anabolism (stimulating mTORC1) and catabolism (repressing AMPK). Increased recruitment of FLCN to the lysosomal membrane in cells subjected to chronic mitochondrial malfunction results in hyperactive mTORC1 and inhibition of AMPK signaling, thus aberrantly promoting anabolism under conditions that are not optimal. Furthermore, peroxisomal β-oxidation increases, and expression of proteasome subunits decreases, with consequent perturbation of proteostasis. Overall, the impact of chronic mitochondrial malfunction over lysosomes triggers multiple perturbations in organelles and organelle crosstalk that culminate in aberrant nutrient sensing, metabolic rewiring, proteostasis impairment, senescence and release of sterile inflammation-related proteins to the extracellular medium that denote alterations in intercellular communication. We will build on these findings to explore an overall picture of organelle homeostasis in aging and in age-related diseases.

Abstract citation ID: igad104.1939

### THE CELL "SURFACEOME": A STRATEGY TO TARGET SENESCENT CELLS IN VIVO

Reema Banarjee, Amit Dey, Thedoe Nyunt, Jordan Gregg, Dimitrios Tsitsipatis, Allison Herman, Myriam Gorospe, and Nathan Basisty, *National Institute on Aging, National Institutes of Health, Baltimore, Maryland, United States*

Senescent cell accumulation drives aging and related pathologies including metabolic disorders and neurodegeneration, and their selective elimination is a promising therapeutic approach to treat multiple diseases of aging in humans. However, developing drugs to eliminate senescent cells requires specific biomarkers to selectively identify and target these cells in vivo. In this study, we have performed a comprehensive and quantitative proteomic profiling of surface proteomes (surfaceomes) of senescent cells to identify potential senescence biomarker candidates in human tissues. We induced senescence using ionizing radiation in a variety of cell types including lung fibroblasts, monocytes, vascular smooth muscle cells, aortic endothelial cells and preadipocytes. Senescence was validated using a panel of canonical markers including reduced proliferation, increased senescence-associated β-galactosidase activity, and increased expression of p21, p16, IL6, and others. The cell surface proteome was enriched using an optimized surface biotinylation approach (Glyco-cell surface capture) followed by data-independent acquisition (DIA) LC-MS/MS analysis on a Q-Exactive HF Orbitrap mass spectrometer. Our analysis revealed novel cell surface targets that were further validated by flow cytometry and immunohistochemistry. These results provide clinically useful information for establishing biomarkers and therapeutic targets to aid in the translation of senescence-targeted therapies to treat age-associated diseases.

Abstract citation ID: igad104.1940

### THE FOXO3 LONGEVITY GENOTYPE ATTENUATES IMPACT OF HYPERTENSION ON CEREBRAL MICROINFARCT RISK

Richard Allsopp[1], Kazuma Kakagawa[2], Kamal Masaki[3], Brian Morris[3], D. Craig Wilcox[4], and Bradley Willcox[3], *1. University of Hawaii, Honolulu, Hawaii, United States*,

2. *Queen's Hospital, Honolulu, Hawaii, United States*, 3. *Kuakini Medical Center, Honolulu, Hawaii, United States*, 4. *Okinawa International University, Ginowan, Okinawa, Japan*

The G allele of the FOXO3 SNP rs2802292 is associated with human resilience and longevity and has recently been shown to attenuate the impact of hypertension on the risk of intracerebral hemorrhage. We now determined whether FOXO3 G-allele similarly attenuated the impact of hypertension on the risk of cerebral microinfarct (CMI). From a prospective population-based cohort of American men of Japanese ancestry (Kuakini Honolulu Heart Program), age-adjusted prevalence of any (1 or more) CMI on brain autopsies was assessed. Logistic regression models, adjusted for age of death, cardiovascular risk factors, FOXO3 and APOE-ε4 genotypes, were utilized to determine the predictors of any CMI. A potential interaction effect of FOXO3 and hypertension was also analyzed. Among 809 men with complete data, 511 (63.2%) had evidence of any CMI. A full multivariable model demonstrated that body mass index (OR: 1.07, 95% CI: 1.01–1.14; p=0.012) was the only significant predictor of CMI, while hypertension was a borderline predictor (OR: 1.44, 95% CI: 1.00–2.08; p=0.052). A significant interaction between FOXO3 G-allele genotype and hypertension was observed (p=0.02). In the stratified analyses, among the participants without the longevity-associated FOXO3 G-allele, hypertension was a strong predictor of CMI (OR: 2.25, 95% CI: 1.34–3.77;, p=0.0021), while among those with the longevity-associated FOXO3 G-allele, hypertension was not (OR: 0.88, 95% CI: 0.51–1.54; p=0.66). These results suggest that the longevity-associated FOXO3 G-allele attenuated the impact of hypertension on the risk of CMI. More studies of other populations is needed to further validate this interesting relation.

Abstract citation ID: igad104.1941

### A PILOT STUDY OF EPIGENETIC AGING IN THE CONTEXT OF AN INTENSIVE LIFESTYLE INTERVENTION: THE LOOK AHEAD STUDY

Lindsay Reynolds[1], Mark Espeland[1], Timothy Howard[1], Carl Langefeld[1], Mara Vitolins[2], and Lynne Wagenknecht[3], 1. *Wake Forest University School of Medicine, Winston-Salem, North Carolina, United States*, 2. *Wake Forest School of Medicine, Winston Salem, North Carolina, United States*, 3. *Atrium Health/Wake Forest Baptist, Winston-Salem, North Carolina, United States*

Type 2 diabetes and obesity increase the rate of accumulation of age-related health deficits and accelerate biological aging. An intervention promoting weight loss among adults with these conditions, the Action for Health in Diabetes (Look AHEAD) Intensive Lifestyle Intervention (ILI), attenuated health deficit accumulation measured by a frailty index, through a variety of diet and exercise strategies and optional weight loss medications to achieve and maintain ≥7% weight loss. To better understand the long-term effects of the Look AHEAD ILI on aging biology, we leveraged biospecimens from 62 women aged 60-71 years who participated in the Look AHEAD randomized clinical trial of ILI (n=32) and diabetes support and education (control condition, n=30), and tested for associations of ILI vs control condition on the rate of epigenetic aging. Five DNA methylation-based aging measures were generated from blood collected at baseline and 14 years later. Unexpectedly, the intervention was associated with a long-term increase in AgeAccelPheno compared to the control condition (t-test, p< 0.05). ILI was not associated with a change in other epigenetic measures over 14 years (AgeAccelHannum, AgeAccelHorvath, AgeAccelGrim and DunedinPACE), or a change in a frailty index reflecting accumulation of 38 health deficits. Overall, the average change in frailty index was higher among participants with advanced epigenetic age at baseline (AgeAccelHannum>0 and AgeAccelGrim>0,p < 0.05), suggesting that epigenetic aging markers may be useful predictors of frailty risk. Well-powered studies are needed to better understand the long-term impact of intensive lifestyle interventions on epigenetic aging in adults with diabetes and obesity.

Abstract citation ID: igad104.1942

### BIOLOGICAL AGE REDUCTION AND LIFESPAN EXTENSION UPON YOUTHFUL CIRCULATION EXPOSURE

Bohan Zhang, *Brigham and Women's Hospital, Boston, Massachusetts, United States*

We performed extended (3-month) Heterochronic Parabiosis (HPB), followed by a 2-month detachment period of anastomosed pairs. Old detached mice exhibited improved physiological parameters and lived longer than control isochronic mice. HPB drastically reduced the biological age of blood and liver based on epigenetic analyses across several clock models using multiple independent platforms; remarkably, this rejuvenation effect persisted even after 2 months of detachment. Transcriptomic and epigenomic profiles of anastomosed mice showed an intermediate phenotype between old and young, suggesting a global multi-omic rejuvenation effect. In addition, old HPB mice showed gene expression changes opposite to aging, but akin to several lifespan-extending interventions. Altogether, we reveal that long-term Heterochronic Parabiosis can decrease the biological age of mice, in part through long-lasting epigenetic and transcriptome remodeling, culminating in the extension of lifespan and healthspan.

### SESSION 5065 (PAPER)

### ENGAGING STRATEGIES IN CLASSES, COMMUNITIES, AND RESEARCH

Abstract citation ID: igad104.1943

### COMMUNITY ENGAGED LEARNING: COMPETENCIES IN HIGHER EDUCATION FOR EMPOWERING ALL AGES

Betsy Kemeny, *Slippery Rock University, Grove City, Pennsylvania, United States*

Educators can use community engaged learning (CEL), also known as service learning, to empower all ages, bridging both college students and older adults needs. With the wrong approach, service just attached to a course may reinforce negative stereotypes. To assure best practice, educators benefit from CEL competencies to promote positive learning and avoid reinforcing ageism. Competencies, based on a community-engaged taxonomy, include: Reciprocal partnerships, Diversity of interactions and dialogue, Community activities, Civic competencies, Critical reflection, and Assessment (Rathlef, 2022; Kecskes, 2015). A complete explanation of each competency will be explained and illustrated. Using the competencies as a framework, two specific programs with college students and older adults were evaluated using both formative and summative measures. One program, a virtual intergenerational interaction, lasted seven weeks. The second program, an in-person program at the University's equestrian and nature center, spanned five weeks. Pre and post comparisons of civic learning outcomes using CLEO, written reflections, focus group discussion, and partner perspectives were analyzed. Older adults reported some improvement in social interaction and companionship with some expansion in social networks. Students significantly improved civic knowledge outcomes. The students' written reflections revealed a self-reported change in attitude toward older adults. Students also expressed a shift in their perceptions of future work in a health setting with older adults. Community-engaged learning can empower all ages, but an educator's attention to the competencies supporting best practice can make a significant difference to the outcome of these efforts.

Abstract citation ID: igad104.1944

### CONDUCTING COMMUNITY-BASED GERONTOLOGY PROJECTS IN UNDERGRADUATE CLASSROOMS: CAPSTONE CASE STUDIES

Ronald Berkowsky, *California State University Channel Islands, Camarillo, California, United States*

Collaborations between university-level students and community-based organizations have long shown to be mutually beneficial in that they can address pressing community needs, advance the mission of the organization, and enhance the learning of students. In this presentation, I describe project collaborations between a non-profit volunteer caregiving organization based in Ventura County ("Caregivers") and Health Science undergraduate students (N = 76) enrolled in Capstone courses at California State University Channel Islands between 2022-2023. Projects were designed utilizing a community-based approach wherein the community partner ("Caregivers") played an active and central role in determining project goals and outcomes. Four projects, each addressing and/or serving a specific need, were completed by different Capstone sections: (1) the creation of an online governance training program to educate high school and university students on the basics of leadership in nonprofits which serve older adults; (2) the creation of a series of videos to educate on and advocate for volunteer caregiving; (3) the analysis of quantitative and qualitative survey data to evaluate the experiences and satisfaction levels of local volunteer caregivers and care recipients; and (4) the creation of a toolkit to be used by volunteer caregiving organizations implementing community meal delivery programs for older adults. In this presentation, I detail each project along with their major successes and challenges as assessed through personal observation, feedback provided by the community partner, and student reflections. I conclude with discussing best practices in implementing community-based projects in undergraduate settings and describe the value of such projects across all stakeholders.

Abstract citation ID: igad104.1945

### INTERGENERATIONAL HAIKU-MAKING ACTIVITY: CREATING MEANINGFUL CONNECTIONS ACROSS GENERATIONS

Yoshiko Matsumoto[1], Harumi Maeda[1], Emily Wan[2], Deven Bansal[1], and Paolo Tayag[1], *1. Stanford University, Stanford, California, United States*, *2. Waseda University, Tokyo, Tokyo, Japan*

Intergenerational activities rooted in creative arts can improve the social connectedness and quality of life of older people living with cognitive impairment (e.g., Dorris et al., 2022; Ihara et al., 2022). Our study further explores the potential of humanities-based approaches to improve well-being and create meaningful connections across generations. Addressing the increasing concerns of loneliness and social isolation among older people living with cognitive impairment, we carried out a haiku-making activity in which 2-3 older adults with mild cognitive impairment (MCI) interact with 2-3 university students in the creation of haiku poems based on seasonal photos. This Japanese poetic form can be composed using short phrases that describe emotional responses to natural scenes, free from the logical and grammatical constraints of prose. This structure enables a failure-free environment that engages participants in self-expression and imagination. Additionally, haiku's simple but often unfamiliar format allows all participants to interact and contribute on an even playing field (Author et al., in press). Our analysis of the (1) recorded haiku-making activity sessions, (2) recorded post-session interviews, and (3) completed haiku poems reveals positive impacts of the activity on both the older participants with MCI and the younger participants. Our findings illustrate how haiku-making and haiku-based conversations promote emotional well-being and intergenerational understanding among participants. This study presents a model for how incorporating perspectives from the arts and humanities into gerontology/geriatrics programs can improve quality of life and enhance meaningful social/intergenerational connections across life stages.

Abstract citation ID: igad104.1946

### THE THINGS WE KEEP: AN EXAMINATION INTO THE USE OF MATERIAL POSSESSIONS IN QUALITATIVE GERONTOLOGICAL INTERVIEWS

Samuel Van Vleet, *Miami University, Oxford, Ohio, United States*

Qualitative interviews are a useful research method because they allow researchers to gather in-depth data beyond the use of traditional survey methods. In this way, qualitative research is an effective method to understand complex and intricate aging-related concepts because there is room for developing new methodological ways for gathering data. A possible way to expand data collections is through the incorporation of material possessions as research aids. Older adults likely accumulate a series of material objects throughout their life course. These objects can take on a supportive role for older adults by conveying important aspects of meaning and are often attached to their identity.

Some items have direct associations with later life changes (canes, glasses, etc) while other items (photographs and mementos) are linked with an individual's identity and accrue a sense of meaning. While previous researchers have documented the importance of objects in later life through material convoys, little has been done to incorporate these meaningful objects into qualitative interview methodology. This paper assesses how incorporations of material possessions into qualitative interviews can assist researchers in gathering rich data. Through a structured literature review, this paper highlights the key findings, past incorporations, and benefits for qualitative interview methodology. Findings suggest that positive outcomes and enriched data result from incorporating material possessions as aides to qualitative interviews. Future researchers should consider how their studies can benefit from the incorporation of meaningful objects in later life.

### SESSION 5070 (PAPER)

### GERIATRIC AND FUNCTIONAL ASSESSMENT IN OLDER ADULTS

Abstract citation ID: igad104.1947

### DEVELOPMENT AND VALIDATION OF PERSON-CENTERED MEALTIME CARE KNOWLEDGE AND SELF-EFFICACY ASSESSMENTS

Wen Liu[1], Heather Suh[2], Kyu Ri Lee[2], Cameron Coates[2], and Kathleen Buckwalter[2], *1. The University Of Iowa, Iowa City, Iowa, United States*, *2. The University of Iowa, Iowa City, Iowa, United States*

Person-centered mealtime care is individually tailored and directly delivered care that engages individuals in mealtime activities and addresses their needs and preferences. Person-centered mealtime care is associated with positive mealtime behaviors and outcomes in older adults with dementia. Existing tools that assess caregiver knowledge and self-efficacy of mealtime care have low psychometric quality, and few tools focus on person-centered mealtime care. This study developed and tested the Person-Centered Mealtime Care Knowledge and Self-Efficacy Assessments. The 20-item Knowledge Assessment (total score = 0-20, higher score = better knowledge) and 15-item Self-Efficacy Assessment (items are scored from 1 = strongly disagree to 5 = strongly agree; total score = 15 - 75; higher score = better self-efficacy) were developed based on literature and expert reviews and finalized based on content validity. Seventy-two nursing home direct care staff (n=72, age = 45±12.1 years; 8.5% male; 12.3% non-white; 93% provided dementia mealtime care; 89% experienced challenges during mealtime) completed the assessments. The two assessments showed adequate content validity (Scale-CVI = 0.95-1.00; item-CVI = 0.83-1.00) and acceptable-to-good internal consistency (Cronbach's Alpha = .651 and .984 for Knowledge and Self-Efficacy Assessments, respectively). There was a weak and significant correlation between Knowledge and Self-Efficacy Assessments (rs = .262, p = .026). Findings provide preliminary psychometric evidence of the two assessments to measure caregivers' knowledge and self-efficacy in providing person-centered mealtime care. Future validity testing and item-level testing using Item Response Theory is needed among larger populations of diverse caregivers to accumulate rigorous psychometric evidence.

Abstract citation ID: igad104.1948

### EARLY DETECTION AND MANAGEMENT OF COGNITIVE IMPAIRMENT PRACTICES AMONG PRIMARY CARE PROVIDERS

John Omura[1], Roshni Patel[2], Benjamin Olivari[3], and Lisa McGuire[3], *1. Centers for Disease Control and Prevention, Atlanta, Georgia, United States*, *2. Cyberdata Technologies (CDC Contractor), Atlanta, Georgia, United States*, *3. CDC, Atlanta, Georgia, United States*

Primary care providers (PCPs) play a critical role in the early detection and management of cognitive impairment and dementia. Understanding their current practice patterns can help identify potential areas of need for additional support. Using 2022 DocStyles survey data (n=1,182), this study assessed US PCPs' confidence levels in identifying symptoms of cognitive impairment and the actions they take when identifying a patient with cognitive impairment. In terms of their ability to identify symptoms of cognitive impairment, 22.3% of PCPs reported being very confident, 53.2% somewhat confident, 18.3% slightly confident, and 6.2% not at all confident or did not know. PCPs reported taking a variety of actions when they identify a patient with symptoms of cognitive impairment: 70.1% assess for potential underlying causes, 69.4% review medications, 65.7% conduct a cognitive assessment or test, 59.7% refer to a neurologist or other specialist, 43.3% coordinate with a care team, and 20.9% refer to community resources. Compared to PCPs who were very or somewhat confident, those who were slightly or not at all confident were less likely to report taking each action (e.g., 35.1% vs. 75.4% conduct a cognitive assessment or test, respectively), except for being more likely to refer to a neurologist or other specialist (68.8% vs. 56.9%) ($p < 0.001$). PCP practices in early detection and management of cognitive impairment vary by their confidence level. Findings can inform the development of resources to help increase PCPs' confidence and support their practices in early detection and management of cognitive impairment and dementia.

Abstract citation ID: igad104.1949

### NORMATIVE VALUES FOR STRENGTH AND FUNCTION DERIVED FROM THE CANADIAN LONGITUDINAL STUDY ON AGING

Alexandra Mayhew[1], Hon Yiu So[2], Jinhui Ma[1], Marla Beauchamp[1], Lauren Griffith[1], Ayse Kuspinar[1], Justin Lang[3], and Parminder Raina[1], *1. McMaster University, Hamilton, Ontario, Canada*, *2. Oakland University, Rochester, Michigan, United States*, *3. Public Health Agency of Canada, Ottawa, Ontario, Canada*

Decreased muscle strength and physical function often precede disability, nursing home admission, home care use, and mortality in older adults. Normative values for commonly used physical performance-based tests are not widely available for older adults but are required for clinicians and researchers to easily identify individuals with low performance. Our objective was to develop normative values for grip strength, gait speed, Timed Up and Go, single leg balance, and five-repetition chair rise tests in a large population-based sample of Canadians aged 45 to 85 years. Baseline data (2011-2015) from the Canadian Longitudinal Study on Aging was used to estimate age- and sex-specific normative values for each of the physical tests. Participants were without disability or mobility limitation (no assistance with activities of daily living or use of mobility devices). Of the 25,470 participants eligible for the analyses 48.6% (n=12,369) were female with a mean age of 58.6 ±9.5 years. Sex-specific 5th, 10th, 20th, 50th, 80th, 90th, and 95th percentile values for each physical performance-based test were estimated. Cross-validation (n=100 repetitions) with a 30% holdout sample was used to evaluate model fit. The normative values developed in this paper can be used in clinical and research settings to identify individuals with low performance relative to their peers of the same age and sex. Interventions targeting these at-risk individuals including physical activity can prevent or delay mobility disability and the resulting cascade of increasing care requirements, health care costs, and mortality.

Abstract citation ID: igad104.1950

### RELIABILITY AND VALIDITY OF THE MCDR FOR HISPANIC OLDER ADULTS

Lisandra Mendoza[1], Patricia Garcia[2], Dilianna Padron[3], Andres Duarte[4], Michael Marsiske[5], Jacob Fiala[5], Ranjan Duara[6], and Miriam Rodriguez[7], *1. Bay Pines VA Health System, Bay Pines, Florida, United States*, *2. Indiana University School of Medicine, Indianapolis, Indiana, United States*, *3. Central Virginia VA Healthcare System, Richmond, Virginia, United States*, *4. Albizu University, Doral, Florida, United States*, *5. University of Florida, Gainesville, Florida, United States*, *6. Mount Sinai Medical Center, Miami Beach, Miami Beach, Florida, United States*, *7. Indiana University Bloomington, Bloomington, Indiana, United States*

Objective: Effective functional measures can be used among Hispanics to diagnose and evaluate functional dementia progression, especially in the prodromal stages when treatment can be most effective. The current study examined the reliability and validity of the modified version of the modified Clinical Dementia Rating Scale (mCDR) compared to the Alzheimer's Disease Cooperative Study (ADCS)-MCI-ADL. These are quantitative severity measures of functional impairment in dementing conditions. The mCDR uses an itemized informant-based structured interview with multiple-choice responses and does not include cognitive testing (Duara et al., 2010). **Methods:** Functional measures were administered to 101 White non-Hispanic Cognitively Normal (CN, n=20) and MCI (n=81) and 159 Hispanic CN (n=32) and MCI (n=127) participants in the patient's primary language (English or Spanish). Inter-rater reliability and convergent validity for these instruments were examined. Multiple regression analyses were conducted to determine if the administration's language (English vs. Spanish) moderated the raters' scores relationships. **Results:** The results revealed moderate-to-good mCDR inter-rater reliability [$\beta$ =.73, F (40,40) = 6.24, p < .001], even when controlling for language ($\beta$ = .72, p < .001; main effect and interaction were not significant). ADCS-MCI-ADL showed moderate-to-good inter-rater reliability [$\beta$ = .71, F (40,36.6) = 6.34, p < .001] also, when controlling for language ($\beta$ = .72, p < .001; main effect and interaction were not significant). The mCDR and the ADCS-MCI-ADL were correlated at -.75, suggesting a high convergent validity (56%). **Conclusions:** The m-CDR is a valid and reliable quantitative functional measure among Hispanic individuals.

Abstract citation ID: igad104.1951

### SENSITIVITY AND SPECIFICITY OF THE MCDR FOR MCI DIAGNOSIS AMONG HISPANIC AND WHITE NON-HISPANIC OLDER ADULTS

Patricia Garcia[1], Lisandra Mendoza[2], Andres Duarte[3], Dilianna Padron[4], Michael Marsiske[5], Jacob Fiala[5], Ranjan Duara[6], and Miriam Rodriguez[7], *1. Indiana University School of Medicine, Indianapolis, Indiana, United States*, *2. Bay Pines VA Health System, Bay Pines, Florida, United States*, *3. Albizu University, Doral, Florida, United States*, *4. Central Virginia VA Healthcare System, Richmond, Virginia, United States*, *5. University of Florida, Gainesville, Florida, United States*, *6. Mount Sinai Medical Center, Miami Beach, Miami Beach, Florida, United States*, *7. Indiana University Bloomington, Bloomington, Indiana, United States*

Objective: Greater functional impairment has been associated with higher rates of progression to dementia. Identifying sensitive functional measures that can detect early ADRD have immediate implications for prognosis, especially among ethnically diverse groups who are disproportionately affected. The modified Clinical Dementia Rating scale (mCDR) is a multiple-choice format assessment that assesses the five domains of the Clinical Dementia Rating scale. The current study aimed to compare sensitivity and specificity of the mCDR between White non-Hispanic (WNH) and Hispanic participants. **Methods:** The mCDR was administered in the patient's primary language (English or Spanish) to 101 WNH Cognitively Normal (CN, n=20) and with MCI (n=81) and 159 Hispanic CN (n=32) and MCI (n=127) participants. Sensitivity to MCI diagnosis and Specificity to CN, using the mCDR "mean of means" total score, was quantified via Area under the Curve (AUC) calculated from a Receiver Operating Characteristic analysis. **Results:** Among WNH participants, the mCDR score demonstrated 78% Sensitivity and 90% Specificity (AUC = .88; CI95%= .81, .94). Among Hispanics, the mCDR showed 61% Sensitivity and 78% specificity (AUC = .73; CI95%= .64, .83). AUC was significantly worse for Hispanic participants. **Conclusion:** The mCDR is a tool that can be used by a trained psychometrist to facilitate focused answers and reduce administration time when assessing clinical functioning among dementia populations. It demonstrated excellent discrimination between CN and MCI among WNHs and acceptable discrimination among Hispanic individuals. Future research should further examine the utility of this tool for Hispanic older adults in early diagnosis of ADRD.

### SESSION 5075 (PAPER)

### REDUCING FALLS IN THE COMMUNITY

Abstract citation ID: igad104.1952

### BODY COMPOSITION AND FALL RISK IN MUTUALLY EXCLUSIVE CATEGORIES OF PHYSICAL ACTIVITY IN OLDER WOMEN

Renoa Choudhury, Joon-Hyuk Park, Chitra Banarjee, Ladda Thiamwong, Rui Xie, and Jeffrey Stout, *University of Central Florida, Orlando, Florida, United States*

Both physical activity (PA) and sedentary behavior (SB) have been independently linked with health outcomes in older adults. However, limited research has been done to investigate how different combinations of these behaviors influence their body composition and functional balance. This cross-sectional study examined the associations of mutually exclusive categories of PA and SB with body composition and fall risk in older women. Accelerometer-measured PA (ActiGraph GT9X), body composition (Bioelectrical Impedance Analysis) and fall risk data (BTrackS Balance & sit-to-stand) were collected from 91 community-dwelling older women. Participants were grouped into four behavioral categories: active-low sedentary, active-high sedentary, inactive-low sedentary and inactive-high sedentary. Active was defined as ≥150 minutes of moderate-to-vigorous PA (MVPA) per week (inactive: < 150 minutes/week of MVPA) and low sedentary was denoted as residing in the first tertile of sedentary-to-light PA time ratio (high sedentary: residing in the remaining tertiles). Results from linear regression analysis showed that, compared to inactive-high sedentary group, lower body fat mass index (BFMI), higher skeletal muscle mass index (SMMI), lower appendicular fat mass index (AFMI) and higher sit-to-stand (STS) scores were found in both active-low sedentary (BFMI: p=.002; SMMI: p=.017; AFMI: p=.003; STS: p=.014) and inactive-low sedentary participants (BFMI: p=.007; SMMI: p=.014; AFMI: p=.005; STS: p=.031), while active-high sedentary participants only showed lower AFMI (p=.037). Our findings suggest PA interventions integrating approaches for achieving sufficient MVPA and reducing SB concurrently might promote healthy body composition and reduce fall risk among older adults.

Abstract citation ID: igad104.1953

### DIFFERENTIAL ASSOCIATIONS OF HAND GRIP STRENGTH AND CHAIR STAND TIME WITH INDOOR AND OUTDOOR FALLS AMONG OLDER ADULTS

Wenjun Li, *University of Massachusetts Lowell, Lowell, Massachusetts, United States*

Hand grip strength and chair stand time are physical performance measures that are predictive of frailty and muscle strengths in older age. We examined their associations with indoor and outdoor falls among older adults. The Healthy Aging and Neighborhood Study enrolled 397 community-dwelling older adults aged 65-95 years old in Central Massachusetts (2018-2020). Hand grip strength (in kilograms) and chair stand time (in seconds) were measured at baseline by trained research staff. Falls were reported on monthly falls calendar, and when a fall was reported, the circumstances were collected via telephone interview. Negative binomial regression models were used to estimate the associations of hand grip strength and chair stand time with both indoor and outdoor falls, separately. Models were adjusting for sex, age, BMI, bodily pain, comorbidities, ADL and IADL as appropriate. Overall, annual rates (95% CI) of indoor and outdoor falls were 40.2 (33.7-47.6) and 45.3 (38.4-53.1) per 100 person-years respectively. Stronger hand grip strength was associated with lower rate of indoor falls (IRR (95% CI): 0.94 (0.91-0.98) per kilogram). Longer chair stand time was associated with higher rate of indoor falls (IRR (95% CI): 1.06 (1.03-1.09) per second). There was no significant association of outdoor falls with grip strength (p=0.504) or chair stand time (p=0.430). In conclusion, hand grip strength and chair stand time were predictive of indoor falls but not outdoor falls. Future studies should examine the underlying mechanisms of such differential associations indoor vs. outdoor falls.

Abstract citation ID: igad104.1954

### MUSCLE ACTIVATION VARIABILITY AS A DETERMINANT OF GAIT VARIABILITY IN OLDER ADULTS

Dain LaRoche[1], and Purnashree Joshi[2], *1. University of New Hampshire, Durham, New Hampshire, United States, 2. The Plaza Rehabilitation and Nursing Center, Bronx, New York, United States*

Gait variability is the step-to-step fluctuations in spatial, temporal, and kinetic gait parameters that reduces the rhythmicity of walking and places older adults at greater risk for falling. Gait variability is influenced by the senses, balance, executive function, strength, and potentially the consistency of neuromuscular activation. The purpose of this study was to test whether older adults with high muscle activation (HMA) variability during walking have greater kinetic, spatial and temporal gait variability than those with low activation variability (LMA). Twenty-seven men and women 65-85 yr walked at normal and maximal speeds on an instrumented treadmill that recorded gait parameters while the electromyogram (EMG) was recorded from vastus lateralis and gastrocnemius lateralis muscles. EMG was recorded over 10 steps and its coefficient of variation was used to identify HMA and LMA groups. Repeated measures ANOVA tested the effects of group (HMA, LMA) and speed (normal, maximal) on gait variability. A significant main effect of speed existed as weight acceptance peak force variability increased 21% (p = 0.014), push off force variability increased 29% (p = 0.015) and step length variability increased 20% (p = 0.02) at maximal speed. A significant interaction existed between speed and group for weight acceptance peak force variability (p = 0.03) as HMA increased 50% while LMA changed little (-4%). Gait variability increased with speed in older men and women, more so for those who exhibit greater muscle activation variability. This suggests the consistency of neuromuscular activation is a factor that affects the rhythmicity of walking.

Abstract citation ID: igad104.1955

### PERCEIVED BALANCE IS ASSOCIATED WITH SELF-REPORTED FALLS IN COMMUNITY-DWELLING OLDER ADULTS

Hanne Dolan, Janet Pohl, Keenan Pituch, and David Coon, *Arizona State University, Phoenix, Arizona, United States*

Accidental falls are a significant health threat among older adults. Approximately 30% of adults age 65 and older suffer one or more falls each year. Most accidental falls are preventable, and older adults' engagement in fall prevention is imperative. Limited research suggest that older adults do not use the term "fall risk" to describe their risk for falls. Instead, they commonly use the term "balance problems." The aim of this study was to examine how perceived balance problems is associated with self-reported falls in the past month after controlling for known predictors of falls among older adults. The Health Belief Model and the concept of perceived susceptibility served as the theoretical framework. A cross-sectional secondary analysis using data from a subsample of independently living participants (N = 7499) from the National Health and Aging Trends Study from year 2015 was conducted, with 10.3% of the sample reporting a fall. Multiple logistic regression analysis revealed that the odds of reporting a fall in the past month were 3.4 times ($p < .001$) greater for participants who reported having a balance problem compared to those who did not. In contrast, fear of falling and perceived memory problems were not uniquely associated with falls. Using a mobility device, reporting pain, poor self-rated health status, depression, and anxiety scores were also associated with falling. Older adults' perceived balance problem is strongly associated with their fall risk. A focus on balance instead of fall risk may improve older adults' engagement in fall prevention.

Abstract citation ID: igad104.1956

### THE FEASIBILITY AND ACCEPTABILITY OF PERTURBATION BALANCE TRAINING IN RURAL COMMUNITIES: A MIXED METHODS STUDY

Justin Whitten[1], David Graham[1], Michelle Grocke[1], Bryant O'Leary[2], Julie Riley[3], and Dawn Tarabochia[1], *1. Montana State University, Bozeman, Montana, United States*, *2. Montana State University, Missoula, Montana, United States*, *3. Montana State University, Broadus, Montana, United States*

Falls are a leading cause of morbidity and mortality in older adults. Older adults in rural communities have an elevated risk of falling. Perturbation-Based Balance Training (PBT) is an effective fall-prevention paradigm. However, it's acceptability and feasibility in community settings is unknown. The purpose of this study was to evaluate the acceptability and feasibility of a community-based PBT program to local community-dwelling older adults and practitioners. Nineteen healthy older adults (60+ years) completed a 6-week PBT program, implemented in conjunction with three community practitioners. Measures of balance, balance confidence and fear of falling were assessed before and after training. Feasibility and acceptability were assessed prospectively, concurrently, and retrospectively via field notes, surveys, and interviews within the Theoretical Framework of Acceptability. PBT was perceived as effective by older adults. Perceived increases in balance awareness, low burden/opportunity-cost and enjoyment of novel training were identified as facilitators. Notably, participants with poor balance tended to perceive PBT as less acceptable and demonstrated apprehensive and avoidant behavior, reinforcing the importance of comprehensive screening prior to participation in PBT. Practitioners perceived PBT as acceptable, with efficacy, low burden/opportunity-cost, and ethicality being primary facilitators. While the portable nature of the perturbation treadmill increased feasibility in rural communities, cost of equipment and transportation were perceived as substantial barriers to implementation. These results suggest PBT is acceptable in rural communities and demonstrate a need for training modalities that are more feasibly implemented in a community setting. Further investigation of low-tech alternatives such as manual PBT may offer potential solutions.

### SESSION 5080 (PAPER)

### STRESS, DEPRESSION, AND ANXIETY

Abstract citation ID: igad104.1957

### ASSOCIATIONS BETWEEN CONTROL AND RESOLUTION OF DAILY STRESS: RESULTS FROM THE NATIONAL STUDY OF DAILY EXPERIENCES

Eric Cerino[1], Dakota Witzel[2], Chloe Horowitz[1], and David Almeida[3], *1. Northern Arizona University, Flagstaff, Arizona, United States*, *2. Penn State University, State College, Pennsylvania, United States*, *3. The Pennsylvania State University, State College, Pennsylvania, United States*

Literature identifies perceived control as an important psychosocial resource for healthy aging. How perceived control over aspects of daily life influence the daily stress process, however, remains comparatively less clear. Resolution of daily stressors (e.g., an argument resolved, a leaky pipe fixed) is emerging as a characteristic of the daily stress process crucial for emotional downregulation. Using data from the National Study of Daily Experiences (N=1,766, Mage=56.25, SD=12.20, 57% Female), we examined associations between perceived control over daily stress and the likelihood of stressor resolution. In waves conducted in ~2008 and ~2017, participants reported perceived control over stressors they experience across eight consecutive days (arguments, avoided arguments, work overloads, home overloads, network stressors), in addition to whether the stressful experience has been resolved. Generalized multilevel models adjusted for linear trends across days and waves, as well as number of stressors, sex, education, race, and baseline age. People experiencing greater stressor control were more likely to resolve their daily stressors (OR=1.92, 95% CI: 1.74–2.13, p<.001). Further, individuals were more likely to resolve their stressors on days when they perceived more control over their stressors than usual (OR=1.66, 95% CI: 1.57–1.77, p<.001). This within-person association increased in magnitude across assessment waves (OR=1.21, 95% CI: 1.06–1.39, p<.01), resulting in a stronger association between stressor control and resolution 10 years later (OR=1.89, 95% CI: 1.69–2.12, p<.001). Associations did not depend on baseline age (ps>.05). Results indicate perceived control may serve as a psychosocial resource for promoting resolution of daily stressors as people grow older.

Abstract citation ID: igad104.1958

### TRAUMA, PERSONALITY, AND DEPRESSION AMONG OLDER ADULTS DURING THE COVID-19 PANDEMIC

Alejandra Zamora[1], and Richard Zweig[2], *1. Dartmouth College , Lebanon, New Hampshire, United States*, *2. Ferkauf Graduate School of Psychology, Yeshiva University, Bronx, New York, United States*

Older adults who have experienced early trauma are predisposed to develop mental health problems such as depression, though further evidence is needed to examine the roles of personality traits and interpersonal problems. The current study examined the respective influences of personality traits and interpersonal problems in the relationship between early trauma and depressive symptoms among middle and older adults who had experienced a loss during the COVID-19 pandemic (N=214). Participants were 76% female and ranged in age from 55-84 (Age M= 62.7, SD= 5.89). Twelve participants who endorsed at least adverse childhood experience were interviewed regarding their childhood and pandemic experiences. Categorical analyses found significant differences between individuals with high and low levels of ACEs with regard to neuroticism (d=-.32), depression (d=-.28), and interpersonal problems (d=-.55). High neuroticism and low extraversion were significantly predictive of interpersonal problems (ß= .520, p< .001; ß=-.201, p= .004) and depression (ß = .588, p< .001; ß= -.177, p= .006). Early trauma and interpersonal problems were not predictive of depression after accounting for variance due to personality traits. Moderation analyses were non-significant. Qualitative analyses yielded themes of re-encountering problems faced as children and adaptation or perseverance of strategy. These findings suggest that personality appeared to contribute significantly to depressive symptoms among bereaved older adults during the COVID-19 pandemic. Early trauma may influence depression in later life with regard to ease of adaptation to contexts and challenges.

Abstract citation ID: igad104.1959

### TRENDS IN DEPRESSION AND ANXIETY AMONG OLDER ADULTS ACROSS COVID-19 PANDEMIC PHASES

Tiffany Hughes[1], Yueting Wang[1], Ruopu Song[2], Erin Jacobsen[1], Chung-Chou Chang[1], and Mary Ganguli[1], *1. University of Pittsburgh, Pittsburgh, Pennsylvania, United States*, *2. Western Psychiatric Institute & Clinic, UPMC Presbyterian Shadyside, Pittsburgh, Pennsylvania, United States*

The effect of the COVID-19 pandemic on older adults' mental health is especially salient given their increased vulnerability to the pathologic and social effects of both the disease and also the associated mitigation strategies. The purpose of this research is to examine trends in symptoms of depression and anxiety among older adults during two phases of the pandemic (pre- and post-vaccine in study region) compared to before the pandemic began. Data were from a longitudinal cohort study of adults aged 65 and older spanning 26 months prior to the pandemic (phase 1) through 17 months after the pandemic began (9 months pre-vaccine (phase 2) and 8 months post-vaccine (phase 3)). Interrupted time series analyses were used to test for differences in trends over time in depressive symptoms (log-mCES-D) and anxiety symptoms (log-GAD-7) during these phases. Trends in both depressive symptoms and anxiety significantly increased in phase 2 compared to phase 1, then significantly decreased in phase 3 compared to phase 2. The trend for depressive symptoms and anxiety in phase 3 was not significantly different than phase 1. These findings suggest that older adults' mental health significantly worsened during the phase of the pandemic when a vaccine was unavailable, but returned to pre-pandemic estimates after a vaccine was available. Future studies are needed to unpack the contribution of vaccine availability and other factors to changes in mental health observed among older adults during the pandemic.

Abstract citation ID: igad104.1960

### TRENDS IN DESPAIR-RELATED CONDITIONS: A LOSS OF MENTAL HEALTH AND HOPE IN THE US 1995–2014

Anthony Bardo[1], and Takashi Yamashita[2], *1. University of Kentucky, Lexington, Kentucky, United States*, *2. University of Maryland, Baltimore County, Baltimore, Maryland, United States*

The Deaths of Despair (DoD) narrative posits that increased prevalence of pain, psychological distress, and drug/alcohol misuse are linked to increased mortality rates among poor non-Hispanic whites in midlife. While data limitations prevent empirically linking these conditions with DoD mortalities, understanding whether period shifts in mental health and/or hope underly their increased prevalence is needed to inform program and policy directives. The present study used two waves (1995-1996 and 2011-2014) of the Midlife in the United States (MIDUS) study to examine trends in the association between these despair-related conditions with mental health and hope by age, race/ethnicity, and socioeconomic status (SES). Mental health was measured by the Complete Mental Health Continuum, and the negative end of this spectrum (i.e., languishing) was used as a proxy for "despair." SES was based on an innovative index that is comparable across waves. Logit models with a despair x hope interaction term were used to identify demographic-group-specific period shifts. Results show that increased prevalence in these despair-related conditions is concentrated among low-SES groups, and standard decomposition techniques show that the largest drivers of an increased prevalence among these low-SES groups differs by race/ethnicity: among non-Hispanic whites it was a combination of despair and hopelessness, but hopelessness was the primary driver among racial/ethnic minorities. Findings are useful for targeting vulnerable groups in midlife and informing programs and policies to combat rising levels of pain, psychological distress, drug/alcohol misuse and related premature deaths.

Abstract citation ID: igad104.1961

### UNDERLYING CAUSES OF DEMENTIA-RELATED ANXIETY AMONG COGNITIVELY-HEALTHY INDIVIDUALS

Molly Maxfield[1], Allie Peckham[1], and Dara James[2], *1. Arizona State University, Phoenix, Arizona, United States*, *2. Arizona State University, Tempe, Arizona, United States*

Alzheimer's disease and related dementias (ADRD) are prevalent, without cures, and contribute to declining functional independence. Their widespread effects make managing ADRD financially, socially, and psychologically difficult, and present challenges for the diagnosed individual as well as their family, friends, and caregivers. These features represent at least some of the reasons ADRDs are feared diagnoses. Dementia-related anxiety (DRA) is anxiety about a current or potential ADRD diagnosis. In a mixed methods study designed to identify causes of DRA, we used semi-structured interviews to assess participants' thoughts, feelings, and reactions regarding ADRD. Participants included 50 cognitively healthy adults aged 58 to 89 (M = 70.80, SD = 6.02; 64% female). Interviews were analyzed using an inductive iterative thematic approach. All transcripts were coded by two researchers. Relying on this iterative process, we adopted and revised themes as more responses and codes were reviewed. The thematic analysis revealed participants described how the progression of symptoms would destabilize their independence, thereby undermining their identity and relationships in ways that were anxiety provoking. Participants also noted various coping strategies that help manage DRA: making care plans for the future, engaging in healthy, feeling supported by family and friends, and accepting that disappointments, including ADRD diagnoses, are simply a part of life. Results offer insight into factors that contribute to and help reduce DRA, which can be used to inform public health messaging and develop applicable and clinically relevant interventions to meet the needs of individuals experiencing DRA.

Abstract citation ID: igad104.1962

**WHICH MEASURES OF STRESS ARE ASSOCIATED WITH ACCELERATED BIOLOGICAL AGING?**

Kyle Bourassa[1], Avshalom Caspi[2], and Terrie Moffitt[2], *1. Durham VA Medical Center, Durham, North Carolina, United States*, *2. Duke University, Durham, North Carolina, United States*

Stress and stressful events are associated with poorer health. There are multiple approaches to measure stress—from assessing subjective perceptions of stress to assessing the development of posttraumatic stress disorder (PTSD)—and multiple periods that can be assessed across the lifespan. How might stress result in poorer health? One plausible physiological mechanism is accelerated biological aging, which precedes many poor health outcomes. Our study tested which measures of stress were associated with accelerated biological aging in adulthood using 955 participants from the Dunedin Longitudinal Study. The measures of stress included perceived stress, stressful life events, adverse childhood experiences (ACEs), and PTSD assessed between ages 32 to 45 years and accelerated biological aging in midlife was measured using the Pace of Aging. Higher levels of all four stress measures were significantly associated with accelerated aging in separate models (all $\beta$s > 0.14, all ps < .001). In a combined model, more perceived stress and more stressful life events remained associated with faster aging and together the four stress measures accounted for 7% of the variance in biological aging. The magnitudes of the associations between the four measures of stress and biological aging were comparable to associations for smoking and low education, two established risk factors for accelerated aging. Assessing stress, particularly perceived stress, could help identify people at risk of accelerated aging. Stress perceptions could be a potentially modifiable treatment target to slow the rate at which people are aging, with the goal of improving health as people age.

### SESSION 5085 (PAPER)

### MOBILITY AND PHYSICAL FUNCTION IN OLDER ADULTS

Abstract citation ID: igad104.1963

**ASSOCIATIONS BETWEEN WALKING SPEED AND GUT MICROBIOME DIVERSITY IN OLDER MEN FROM THE MROS STUDY**

Samaneh Farsijani[1], Jane Cauley[1], Peggy Cawthon[2], Lisa Langsetmo[3], Eric Orwoll[4], Anne Newman[5], and Deborah Kado[6], *1. University of Pittsburgh, Pittsburgh, Pennsylvania, United States*, *2. California Pacific Medical Center, Research Institute, San Francisco, California, United States*, *3. Minneapolis VA Health Care System, Minneapolis, Minnesota, United States*, *4. OHSU, Portland, Oregon, United States*, *5. University, Pittsburgh, Pennsylvania, United States*, *6. Stanford, Stanford, California, United Sta tes*

While gut dysbiosis has been linked to frailty in aging, its association with early mobility impairments is unclear. Here, our primary goal was to determine the cross-sectional associations between walking speed and gut microbiome in 740 older men (84±4y) from MrOS with available stool samples and 400m walking speed measured in 2014–16. We also analyzed the retrospective longitudinal associations between changes in 6-meter walking speed (from 2005-06 to 2014-16) and gut microbiome composition among participants with available data (702/740). The gut microbiome composition was determined by 16S sequencing (DADA2 and SILVA). We examined diversity, taxa abundance (by ANCOM-BC), and performed network analysis (by NetCoMi) to uncover microbial communities interactions by walking speed levels. Higher walking speed (m/s) was associated with greater microbiome Shannon $\alpha$-diversity (R=0.11; P=0.004). Decline in walking speed was associated with lower Shannon $\alpha$-diversity (R=0.07; P=0.054). Faster walking speed and less decline in walking speed were associated with higher abundance of genus-level bacteria that produce short-chain fatty acids, and possess anti-inflammatory properties, including Paraprevotella, Fusicatenibacter, and Alistipes, adjusting for age, race, site, education, health, marital status, weight, height, physical activity, batch, medications, energy, and fiber intake (P< 0.05). The gut microbiome networks of participants in the first vs. last quartile of walking speed (≤0.9 vs. ≥1.2 m/s) exhibited distinct characteristics, including different cluster numbers, hubs, and centrality measures (P< 0.05). Faster walking speed and its less decline were associated with higher gut microbiome diversity, suggesting potential role of microbiome in preserving mobility in aging.

Abstract citation ID: igad104.1964

### EMBOLDEN: CATALYZING RESEARCH FOR OLDER ADULTS THROUGH CO-DESIGNED MOBILITY-PROMOTING INTERVENTION RESEARCH

Rebecca Ganann, Caroline Moore, Stuart Phillips, Elizabeth Alvarez, Bruce Newbold, Sarah Neil-Sztramko, Maggie MacNeil, and Kathryn Fisher, *McMaster University, Hamilton, Ontario, Canada*

Mobility and social participation are requisites for optimizing health, independence, and quality of life in older populations. Most mobility-enhancing interventions for older adults have been designed by researchers alone, which often experience implementation challenges and limited effectiveness when translated to real-world contexts. The EMBOLDEN research program aims to i) partner with older adults and service providers to co-design, implement, and evaluate a mobility-promoting intervention and ii) enhance physical and mental health, foster social connections, and support system navigation among older adults living in neighbourhoods with health inequities. EMBOLDEN has completed two phases: (1) intervention co-design based on evidence reviews, environmental scan, qualitative study, and strategic guiding council expertise; (2) a mixed-methods pilot randomized controlled trial. Twenty-seven local older adults (≥55y) and 34 service providers contributed to Phase 1, co-designing an integrated health and social care intervention delivered through collaboration with primary care, public health, and recreation. The three-month community-based program includes a weekly interactive group program of physical activity, healthy eating, socialization, and individual system navigation support. The co-design determined priority intervention features (e.g., addressing social determinants of health, assets-based approaches) and implementation strategies. The pilot study evaluated quantitative and qualitative participant outcomes and experiences and intervention team experiences. The pilot study demonstrated intervention feasibility and acceptability to participants and interventionists, informing a larger pragmatic hybrid implementation-effectiveness trial that is currently underway. EMBOLDEN's partnership engagement was critical to successfully co-designing intervention research to address identified health, mobility, and social needs of older adults living in neighbourhoods with significant health inequities.

Abstract citation ID: igad104.1965

### MITOCHONDRIAL DNA COPY NUMBER IS ASSOCIATED WITH PHYSICAL FUNCTION

Qu Tian, David Zweibaum, Yong Qian, Richard Oppong, Jun Ding, and Luigi Ferrucci, *National Institute on Aging, National Institutes of Health, Baltimore, Maryland, United States*

Mitochondria generate the energy required by cells to maintain functionality and muscle health. They may play a key role in age-related physical function decline. Skeletal muscle mitochondrial function is associated with gait speed and muscle strength. Blood-derived mitochondrial DNA copy number (mtDNAcn) is a biomarker of mitochondrial health, but its association with physical function is unclear. We examined cross-sectional associations of mtDNAcn estimated by whole genome sequencing with gait speed and grip strength in participants aged 50+ from two population-based studies: Baltimore Longitudinal Study of Aging(BLSA,n=688) and UK Biobank(n=150,448). Gait speed was measured using 6-meter walk in BLSA and self-reported walking pace in UK Biobank. Handgrip strength was measured using a hand dynamometer. We additionally tested sex differences because of known sex differences in gait speed and grip strength. We found that higher mtDNAcn was associated with faster gait speed and greater grip strength, after adjustment for age,sex,race,and autosomal coverage. Specifically, each standard deviation increase in mtDNAcn was associated with a 0.05 m/sec faster in gait speed and a 19% lower odds of reporting slow walking pace(both $p< 0.0001$). There was no significant sex-by-mtDNAcn interaction for gait (both $p>0.05$). There was a significant sex-by-mtDNAcn interaction for grip strength. In sex-stratified analysis, the association with grip strength was stronger in men ($\beta=0.129,p=0.002;\beta=0.032,p< 2e\text{-}16$,respectively) than in women ($\beta=0.007,p=0.784;\beta=0.015,p< 2e\text{-}16$,respectively). These findings suggest that blood-based estimate of mitochondrial function is related to physical function in community-dwelling middle-aged and older adults. Further research is needed to understand its relationship with physical function decline and underlying mechanisms.

Abstract citation ID: igad104.1966

### MUSCULOSKELETAL PAIN CHARACTERISTICS AND OBJECTIVELY MEASURED PHYSICAL ACTIVITY IN OLDER ADULTS

Yurun Cai[1], Fangyu Liu[2], Amal Wanigatunga[2], Eleanor Simonsick[3], Luigi Ferrucci[3], and Jennifer Schrack[2], *1. University of Pittsburgh, Pittsburgh, Pennsylvania, United States*, *2. Johns Hopkins Bloomberg School of Public Health, Baltimore, Maryland, United States*, *3. National Institute on Aging, National Institutes of Health, Baltimore, Maryland, United States*

Pain is associated with reports of restricted physical activity (PA), yet the associations between multiple musculoskeletal pain characteristics and objectively measured PA quantities and patterns in late life are not well understood. A total of 553 adults (mean age 75.8±8.4 years, 54.4% women) in the Baltimore Longitudinal Study of Aging (BLSA) completed pain assessments and subsequent 7-day wrist-worn accelerometry in the free-living environment between 2015 and 2020. Pain characteristics included pain presence in the shoulders, hands/wrists, low back, hip, knees, and feet, pain laterality in each site (no pain, unilateral pain, and bilateral pain), and pain distribution (no pain, single site pain, and multisite pain). PA metrics were summarized into total daily activity counts (TAC), activity fragmentation, active minutes/day, and diurnal patterns of activity. Linear regression models adjusted for demographics and comorbidities suggested that unilateral knee pain was associated with 184,070 fewer TAC/day ($p=0.04$) and 36.2 fewer active minutes/day ($p=0.03$) while hand/wrist pain was associated with 24.8 more active minutes/day compared to those without pain ($p=0.04$). By time-of-day, participants with hip pain had less activity during the morning (6:00am to 11:59am) while those with unilateral knee pain had fewer activity counts during the afternoon (12:00pm to 5:59pm). Analyses stratified by sex showed that these associations were only significant among women. Our study highlights the importance of assessing pain laterality in addition to traditional pain measures (e.g., pain presence) and suggest that pain interferes with multiple aspects of daily activity. Longitudinal studies are needed to assess the temporality of these findings.

Abstract citation ID: igad104.1967

### RACE AND PLACE: PHYSICAL PERFORMANCE DECLINES IN BLACK AND WHITE OLDER ADULTS IN FOUR US COMMUNITIES

B. Gwen Windham[1], William Windham[2], Hunter Sylvester[1], A. Richey Sharrett[3], Laura Skow[3], Priya Palta[4], Anna Kucharska-Newton[4], and Michael Griswold[1], *1. UMMC-The MIND Center, Jackson, Mississippi, United States*, *2. University of Mississippi School of Medicine, Jackson, Mississippi, United States*, *3. Johns Hopkins Bloomberg School of Public Health, Baltimore, Maryland, United States*, *4. UNC Gillings School of Global Public Health, Chapel Hill, North Carolina, United States*

Studies suggest disparities in physical function between Black and White adults, but most lack longitudinal, objective functional measures across race and geographic regions. We examined Short Physical Performance Battery (SPPB) changes among 5,836 Black and White older adults (>65 years, 22% Black, 41% men) with up to three SPPB assessments. Exams were conducted in the Atherosclerosis Risk in Communities Study (Visit 5 (2011-13)-Visit 7 (2017-19), median follow-up 5.8 years (max 8.4)) in four US communities: North Carolina (n=1248; 7% Black), Mississippi (n=1179, 100% Black), Maryland (n=1618; 0.9% Black) and Minnesota (N=1791; n=0.5% Black). A difference of 0.5 SPPB points (scored 0-12) is clinically meaningful. SPPB declines were estimated using marginal standardization following GEE (with log-links, negative binomial distributions, exchangeable covariance, robust SE, adjusting for demographics, BMI, diabetes, hypertension, smoking, drinking status, heart failure, heart disease, and stroke). SPPB declines were 3.4 times greater among Black versus White participants overall; -1.99 versus -0.59 points; absolute difference=-1.41 (95% CI: -1.67,-1.14), relative difference=3.40 (2.65, 4.15). However, some within-race, between-site differences were similar; Maryland-White SPPB declines were 3.8 (2.3, 6.3) times greater than North Carolina-White SPPB declines, and Mississippi-Black declines were 2.5 (1.10, 5.90) times greater than North Carolina-Black declines. Furthermore, between-race (Black versus White) differences were less supported statistically within the same site; SPPB declines were 2.2 (0.85, 5.80) greater in Black than White participants in North Carolina. This suggests that regional variations in physical function declines may be at least as strong as differences attributed to race; both are important to consider.

### SESSION 5090 (SYMPOSIUM)

Abstract citation ID: igad104.1968

### TRANSCRIPTOMIC SIGNATURES OF AGING, GENETICS, AND MORTALITY RISK: FINDINGS FROM THE LONG LIFE FAMILY STUDY

Chair: Mary Wojczynski Co-Chair: Nancy Glynn
Discussant: Nalini Raghavachari

The Long Life Family Study (LLFS), funded by the National Institute on Aging, is an international collaborative study of the genetics and familial components of exceptional longevity and healthy aging. We phenotyped 4,953 individuals from 539 two-generational families (1,727 proband; 3,226 offspring) at baseline (2006-2009). A second visit (2014-2017) was conducted for 2,904 (478 proband; 2,426 offspring) participants, with a third visit ongoing. The longitudinal, comprehensive in-person visits measured domains of healthy aging, including physical performance, cognition, and blood markers. Extensive genetic analyses were performed using the baseline blood draw, including genotyping with the Illumina 2.5M Human Omni array, linkage analyses with the families, whole genome sequencing using the TopMED protocol, metabolomic assays, and trascriptomics (RNA-seq). Collectively, this symposium will present novel findings that examined differences in gene expression between those of extreme old age compared to younger participants, elucidate potential new genomic regions and variants associated with ankle brachial index, examine rare variants under linkage peaks for perceived physical fatigability, and compare mortality and cause of death between LLFS and the US population. Specifically, Ms. Li will share results on gene expression profiles in aging. Then, Ms. Ressler will share findings on the genetics of ankle brachial index with linkage and association results. Next, Ms. Gay will discuss associations under the linkage peak for perceived physical fatigability. Lastly, Ms. Yao will present mortality and patterns of death in the LLFS. As Discussant, Dr. Nalini Raghavachari from the NIA will provide insights and propose future directions for LLFS.

Abstract citation ID: igad104.1969

### TRANSCRIPTOMIC SIGNATURES FOR AGING AND EXTREME OLD AGE IDENTIFIED IN THE LONG LIFE FAMILY STUDY

Mengze Li[1], Stefano Monti[1], Paola Sebastiani[2], Zeyuan Song[1], Tanya Karagiannis[2], Thomas Perls[1], and Michael Brent[3], *1. Boston University, Boston, Massachusetts, United States*, *2. Tufts Medical Center, Boston, Massachusetts, United States*, *3. Washington University in St. Louis, St. Louis, Missouri, United States*

Transcriptional studies of blood have previously identified changes in gene expression profiles associated with age. Centenarians, a rare population of individuals that reach 100 years of age, experience delays in aging-related diseases and mortality. Thus, the analysis of their blood transcriptome may help shed light on mechanisms contributing to healthy aging and extreme longevity, as well as enable the discovery of candidate molecular therapeutic targets. In this project, we analyzed RNAseq-based blood transcriptomic data and genetic data from 1,286 participants in the Long Life Family Study age ranged from 25 to 108. To identify transcripts with different abundances at different ages, we used linear mixed-effect models and included relevant covariates to control for genetic, socioeconomic, and technical confounders. We also estimated a genetic relationship matrix (GRM) from the genotype data to account for familial relatedness. We used the same models and covariates to compare transcripts' abundance between the extreme old and younger participants to discover markers of extreme old age. Among the 11k RNA transcripts tested, we identified 5,249 aging markers (FDR ≤ 0.01) and 761 extreme old age markers (FDR ≤ 0.01). We successfully replicated our aging signature with previously published aging signature in another cohort with comparable Z-scores. Currently, further investigation is ongoing to distinguish markers that contribute to extreme longevity from markers that represent extreme old age. This study found large signatures of age and extreme old age and will serve as a valuable reference for future research to identify molecular mechanisms of healthy aging.

Abstract citation ID: igad104.1970

### GENETIC EPIDEMIOLOGY OF ANKLE BRACHIAL INDEX IN THE LONG LIFE FAMILY STUDY

Deidra Ressler[1], Ryan Cvejkus[1], Warwick Daw[2], Mary Feitosa[2], Joanne Murabito[3], Ryan Minster[1], Joseph Zmuda[1], and Allison Kuipers[1], *1. University of Pittsburgh School of Public Health, Pittsburgh, Pennsylvania, United States*, *2. Washington University School of Medicine, St. Louis, Missouri, United States*, *3. Boston University Chobanian & Avedisian School of Medicine, Boston, Massachusetts, United States*

The genetic influence on peripheral vascular health in exceptional aging has not been well-studied. Thus, we performed heritability, genome-wide linkage analyses (GWLA) and genome-wide association studies (GWAS) of ankle-brachial index (ABI) and peripheral artery disease (PAD) in 3006 individuals from the Long Life Family Study (LLFS). The LLFS is a longitudinal, family-based study including long-lived siblings and their families. ABI was measured in-home, and the minimum from both sides was used, with exclusions for ABI≥1.4 or non-compressible arteries. PAD was defined as ABI<0.9. We performed heritability and GWLA using SOLAR and GWAS using R accounting for familial relationships. Our full model was adjusted for age, sex, site, significant PCs, blood pressure, and relevant risk factors including smoking, body size, and lipids. We considered significance at a LOD≥3.0 for GWLA and at a p<5x10-8 for GWAS. The proband generation was 53.2% female, aged 88.7 and had a mean ABI of 1.06. The offspring generation was 60.1% female, aged 60.1 and had a mean ABI of 1.19. 18.2% of probands and 1.0% of offspring had PAD (7.4% overall). Residual genetic heritability was 0.12 (p=8.7x10-4) for ABI and 0.23 for PAD (p=0.0662). We found linkage with chromosome 15q12 (LOD=3.65) for ABI and association on chromosome 4q25 (rs12512857; p=6.25x10-8; ANK2; maf=0.021) for PAD. ANK2 is a gene that has been implicated in cardiac dysfunction though there have been no previous associations with ABI or PAD. This suggests that unique genetic variation is associated with peripheral vascular health in long-lived families.

Abstract citation ID: igad104.1971

### LINKAGE ANALYSIS OF PERCEIVED PHYSICAL FATIGABILITY IN THE LONG LIFE FAMILY STUDY

Emma Gay[1], Adam Santanasto[1], Shiow (Rosa) Lin[2], Mary Wojczynski[2], and Nancy Glynn[1], *1. University of Pittsburgh School of Public Health, Pittsburgh, Pennsylvania, United States*, *2. Washington University School of Medicine in St. Louis, St. Louis, Missouri, United States*

Fatigability, whole-body fatigue anchored to specific tasks, is a highly prevalent characteristic in older adults, but the biological mechanisms underlying perceived physical fatigability are unclear. Genetic studies may identify potential biological pathways contributing to physical fatigability. The family study design of the LLFS provides a unique opportunity to explore variants associated with fatigability using whole-genome linkage analysis. Whole genome sequencing (WGS) was available for N=2462, 496 families (age 73.6±10.5 years, 54.8% women, 99% white. Perceived physical fatigability was measured with the Pittsburgh Fatigability Scale (PFS, 0-50, lower=less severe). Prevalence of lower PFS scores (<15) was 57.9%. Multipoint linkage analysis employing likelihood-based variance components analyses were performed to identify families and genetic loci showing strong evidence for linkage. Based on linkage analysis results, a series of association analyses were performed in the subsets of linked families for PFS Physical scores. Association analyses were conducted using linear mixed models adjusted for age, field center, principal components and familial relatedness. For all families the LOD was 2.13 at chr17:79,549,108. The HLOD of the top 27 families (n=225) was 8.97. There were 8 single nucleotide polymorphisms (SNPs) identified under the linkage peak on chromosome 17. These variants were mapped to 8 different genes involved in regulation of transcription and metabolism, cellular proliferation, signal transduction, protein trafficking, and membrane structure; all important in maintenance of cellular health. Results suggest fatigability in older adults may have multiple molecular etiologies and identified variants may allude to molecular mechanisms to target in future interventions to promote healthy aging.

Abstract citation ID: igad104.1972

### ALL-CAUSE MORTALITY AND CAUSE OF DEATH IN US LONG-LIVED SIBLINGS: DATA FROM THE LONG LIFE FAMILY STUDY

Shanshan Yao[1], Robert Boudreau[1], Angéline Galvin[2], Joanne Murabito[3], Lawrence Honig[4], Kaare Christensen[2], and Anne Newman[1], *1. University of Pittsburgh School of Public Health, Pittsburgh, Pennsylvania, United States*, *2. University of Southern Denmark, Odense, Syddanmark, Denmark*, *3. Boston University Chobanian & Avedisian School of Medicine, Boston, Massachusetts, United States*, *4. Columbia University, New York City, New York, United States*

This study compared the mortality risk of long-lived siblings with the general population in the US and investigated the leading causes of death and the correspondence in age at death and causes of death between long-lived siblings. In the Long Life Family Study (LLFS), 1,264 siblings (Mean age 90.1, SD 6.4) were recruited from the US and followed over 12 years. Their survival function, based on the Kaplan-Meier estimator, was compared with an age-, race-, and sex-matched sample from US census data. To examine underlying and contributing causes, we examined in detail 338 deaths with complete death adjudication using death certificates, family interview, hospital and nursing home records at the Pittsburgh field center through the year 2018. Concordance between siblings was examined using correlation coefficients, Bland-Altman plots, percent agreement, and Kappa statistics. The LLFS proband siblings had better survival compared with US general population matched on birth cohort and age at enrollment. Age at death ranged from 75-104, mean 91.4 (SD 4.8). The leading causes of death were cardiovascular disease (33.1%), dementia (22.2%), and cancer (10.7%). In addition, we did not find significant correspondence in the age at death, the underlying cause of death, or conditions directly contributing to death between siblings. Our findings demonstrate further exceptional longevity in the LLFS proband generation compared to the general population, but do not show significant correspondence in age at death and causes of death between siblings.

### SESSION 5095 (SYMPOSIUM)

Abstract citation ID: igad104.1973

### YES! YOU CAN DO DAILY DIARY RESEARCH WITH DEMENTIA CAREGIVERS!

Chair: Vicki Winstead Co-Chair: Carolyn Pickering
Discussant: Vicki Winstead

Successful recruitment and retention of dementia caregivers for intensive research protocols such as daily diary research is thought to be hindered by factors that include having limited time and the stress in dementia caregiving. Knowing why caregivers participate and what keeps them motivated to comply in daily diary research is crucial in understanding the experiences of dementia caregiving for the purpose of developing and ensuring successful interventions. The purpose of this symposium is to present data from three online micro-longitudinal daily diary studies on caregiving and dementia that inform successful practices in recruitment, compliance and reducing missing data in dementia caregivers. Each of the studies required daily participation ranging from 1) four daily diaries each day over 8 consecutive days, 2) 21 consecutive days of diaries once per day, and 3) three different data collection points of 21 consecutive days over one year. The symposium includes three papers that address the following areas: 1) Identifying factors that motivate dementia caregivers to participate in research for the purpose of developing relevant and meaningful recruitment materials. 2) Describing and predicting “missingness” within diary research, and 3) Effective strategies that are intentional in promoting high levels of compliance in completing diaries. Discussion will include ways to enhance recruitment materials that are congruent with caregivers’ motivations to participate, how strategies were used in each of the three studies for compliance in completion of daily surveys and retention and provide evidence that caregivers do take the time to participate in intensive research protocols.

Abstract citation ID: igad104.1974

### THE ROLE OF MISSINGNESS IN DAILY DIARY DATA

Mustafa Yildiz, Vicki Winstead, and Carolyn Pickering, *University of Alabama at Birmingham, Birmingham, Alabama, United States*

Objections are often raised to using intensive daily diary research methods with family caregivers of persons with dementia. These objections include the daily lack of time, stress associated with dementia caregiving, in particular, and depression and anxiety associated with the demands of the caregiving process. It is argued that these factors may deter caregivers from consistent compliance and lead to bias in the data. The aim of this paper is to describe and predict missingness in daily diaries using the data from three micro-longitudinal studies that include 10,680 days of diaries across all three studies. Key indicators and demographic variables were used to predict missing days, which was quantified from compliance data using a latent variable within the framework of a multiple indicators-multiple causes model. Missingness was random and not predicted by demographics or key outcome variables (e.g. stress, depression, race, ethnicity). Findings suggest despite the time demands and stressors of caregiving, caregivers do complete diaries without systematic patterns of missingness. Researchers should not be deterred from doing intensive daily diary protocols with caregivers experiencing psychopathology who may benefit the most from real-time assessment of symptoms.

Abstract citation ID: igad104.1975

### SUCCESSFUL COMPLIANCE OF FAMILY CAREGIVERS IN DEMENTIA RESEARCH

Henrietta Armah, Vicki Winstead, Mustafa Yildiz, Jasmine Vickers, and Carolyn Pickering, *University of Alabama at Birmingham, Birmingham, Alabama, United States*

Attrition is an issue for all longitudinal studies. One concern with intensive protocols with many repeated measures is that participants will drop out before completing the protocol, or will not complete enough of the surveys to provide meaningful data. Features of the study design can be important in increasing compliance with study participations. These features include incentives, survey windows, clear direction, ease of use of the data collection platform, and reminder protocols. This presentation examines compliance, defined as completion of >50% of all surveys, among 3 different micro-longitudinal daily diary studies. Compliance was high for all three studies (78%, 88%, 89%). The lowest compliance rate of 78% was an 8-day protocol with four diaries each day; three diaries were sent out randomly each of the eight days and the one nightly recap was sent at the same time each night. This suggests having multiple surveys in a single day reduce compliance with the study protocol. Compliance did not vary by other study features such as incentives, reminder method or length of the study. For daily diary studies, findings suggest that by doing once-a-day surveys, data collection protocols can be longer and total incentives ranging from $82-$91 are acceptable for maintaining high compliance.

Abstract citation ID: igad104.1976

### DEMENTIA FAMILY CAREGIVERS' REASONS FOR PARTICIPATION IN RESEARCH

Jasmine Vickers, Vicki Winstead, Henrietta Armah, and Carolyn Pickering, *University of Alabama at Birmingham, Birmingham, Alabama, United States*

Recruitment of participants with Alzheimer’s and Related Dementias (ADRD) and their family caregivers can pose significant challenges to the successful completion of research, particularly for intensive protocols like daily diary research. Understanding the reasons ADRD family caregivers are willing to participate in research can provide researchers with the tools for successful recruitment efforts. In this study, we assessed the reasons ADRD family caregivers (N=123) participated in a 21-day diary study and the translatability of the findings for informing the development of recruitment materials to address recruitment challenges. We conducted a thematic analysis of responses to the question "Tell us in a few words why you are interested in participating in this study" as an addendum to the consent form. Thematic analysis was conducted by two independent coders. Participants were mainly women (86%) and biological children of the care recipient (46%). Findings revealed four predominate motivations for participation in the research project: 1) finding meaning for their caregiving experiences by sharing them through the study, 2) altruistic motivations for helping others, 3) personal benefits including helping their care recipient or learning more about caregiving, 4) and the meaningfulness of the study's focus. Caregivers were motivated to participate in this intensive protocol for a variety of meaning-making reasons. Recruitment materials should focus on language that emphasizes opportunities for caregivers to share their experiences and practical relevance of potential study findings.

### SESSION 5100 (PAPER)

### PHYSICAL ACTIVITY AND FALL PREVENTION

Abstract citation ID: igad104.1977

### A LIVE ONLINE EXERCISE INTERVENTION FOR OLDER ADULTS: A MIXED METHODS RANDOMIZED CONTROLLED TRIAL

Giulia Coletta, Kenneth Noguchi, Kayla Beaudoin, Angelica McQuarrie, Meridith Griffin, Ada Tang, and Stuart Phillips, *McMaster University, Hamilton, Ontario, Canada*

Regular physical activity (PA) and exercise alleviate mental health and age-related physical declines in older adults; however, strategies are needed to help older adults engage in PA and exercise. Live online video may be a viable mode of delivery for exercise programs. Thirty-one sedentary community-dwelling older adults (65-80 years) were randomized to an 8-week live online exercise program (ACTIVE, n=16; age 70±4 years; 69% women) or waitlist control (CON, n=15; age 72±5 years; 87% women) group. Quantitative effectiveness data were collected pre-and post-intervention and included accelerometry-derived PA levels and mental health (depression, anxiety, and loneliness). Feasibility outcomes included attendance and participant satisfaction. Semi-structured interviews were optional for all participants after completing the online exercise program (n=22; ACTIVE=12; CON=10; 73% women). The ACTIVE group attended 97% of the online classes, and 98% reported satisfaction with the program. Post-intervention, there were no differences between groups on daily steps (ACTIVE=4336 ± 2638 steps/d; CON=3125 ± 1250 steps/d), daily activity energy expenditure (ACTIVE=227 ± 249 kcal/d; CON=119 ± 166 kcal/d), and moderate-to-vigorous physical activity (ACTIVE=42 ± 44 min/d; CON=23 ± 30 min/d). An effect of the intervention was observed in the ACTIVE group for symptoms of depression (3.9±2.4 to 2.0±1.7; p=0.015). There was no intervention effect compared to CON on anxiety or loneliness. Common themes identified from qualitative interviews included satisfaction with the program and perceived health improvements. A live online exercise program was a feasible approach to delivering physical activity to older adults.

Abstract citation ID: igad104.1978

### EFFECT OF A HOME-BASED EXERCISE PROGRAM ON SUBSEQUENT FALLS IN OLDER ADULTS WITH COGNITIVE FRAILTY

Teresa Liu-Ambrose[1], Ryan Falck[2], Jennifer Davis[3], Karim Khan[1], and Kenneth Madden[1], *1. University of British Columbia, Vancouver, British Columbia, Canada, 2. The University of British Columbia, Vancouver, British Columbia, Canada, 3. University of British Columbia - Okanagan, Kelowna, British Columbia, Canada*

Cognitive frailty is characterized by concurrent physical frailty and mild cognitive impairment. and increases the risk for falls. Whether exercise can reduce falls in older adults with cognitive frailty is unknown. We examined the effects of a home-based exercise intervention on subsequent falls among community-dwelling older adults with cognitive frailty and a history of falls. A secondary of a 12-month randomized controlled trial among older adults aged ≥70 years with a fall in the last 12 months. Participants were randomized to either 12 months of home-based exercise (EX; n=172) or usual care (UC; n=172). For this analysis, we only included participants who were classified as cognitively frail based on a Short Physical Performance Battery (SPPB) score ≤ 9/12 and a Montreal Cognitive Assessment score < 26/30. Our primary analysis examined the effect of EX on self-reported falls over 12 months. The secondary analysis examined whether higher exercise adherence, or dose, benefits physical frailty among the EX participants. At baseline, 192 participants were classified as cognitively frail (EX=93; UC=99). Falls rates were lower in EX participants vs. UC participants (IRR=0.65; p=0.042). At 12 months, in the EX group, SPPB score was significantly higher among participants with high adherence vs. those with low adherence (estimated mean difference: 1.22; p=0.004). Exercise is a promising strategy for reducing subsequent falls in older adults with cognitive frailty and a history of falls. Greater exercise adherence, or dose, may reduce physical frailty in this population at high risk for disability.

Abstract citation ID: igad104.1979

### EFFECTS OF INTERPERSONAL AND INTRAPERSONAL PROMOTION STRATEGIES ON FALLS: A PHYSICAL ACTIVITY TRIAL

Siobhan McMahon[1], Jean Wyman[2], Weihua Guan[1], Elizabeth Choma[3], Shannon Hayes[4], Qi Wang[1], Alexander Rothman[1], and Beth Lewis[1], *1. University of Minnesota, Minneapolis, Minnesota, United States, 2. School of Nursing, University of Minnesota, Minneapolis, Minnesota, United States, 3. Whitworth University, Spokane, Washington, United States, 4. University of*

*Minnesota - Twin Cities, Minneapolis, Minnesota, United States*

Although fall-reducing physical activity (PA) interventions have promising effects on falls, little is known about the relative effects of different PA promotion strategies within such interventions. This trial assessed the effects of two promotion components within a PA intervention on participants' falls 12 months post-intervention. The components included interpersonally-oriented (e.g., social support, social comparison) or intrapersonally-oriented (e.g., goal-setting, action planning) behavior change strategies informed by empirical evidence and theory. In this 2x2 factorial trial (Ready-Steady3 [RS3]),309 community-dwelling older adults (mean age 77+/-5) with > 1 fall risk factor were randomized to 1 of 4 intervention conditions. Factors represented random assignment (No, Yes) to interpersonal or intrapersonal components. All intervention conditions included the Otago Exercise Program adapted for small groups and a PA monitor (Fitbit) and were delivered in community settings over 8 weekly meetings, 90 minutes each. We assessed fall rates using participants' documentation of falls on prospective monthly calendars. The main and interaction effects of interpersonal and intrapersonal components on fall rates were examined using negative binomial regression. The estimated fall incidence rates among participants assigned vs. those not assigned to the interpersonal component or the intrapersonal components, respectively, were:1.0 (+/-1.4) and 0.9 (+/-1.3), and 1.1 (+/-1.6) and 0.9 (+/-1.1) per person-year. Neither the main nor the interaction effects of either component were statistically significant, suggesting that interpersonal and intrapersonal components in RS3 PA interventions did not affect fall rates. Further investigation is warranted to identify which promotion strategies and dosages reduce falls and injuries in community-dwelling older adults.

Abstract citation ID: igad104.1980

### VIGOROUS PHYSICAL ACTIVITY, DEPRESSION, PRO-INFLAMMATORY CYTOKINE AND RACIAL/ETHNIC VARIATIONS IN FALLS

Ayse Malatyali[1], Tom Cidav[2], Rui Xie[1], and Ladda Thiamwong[1], *1. University of Central Florida, Orlando, Florida, United States*, *2. The Johns Hopkins University School of Medicine, Baltimore, Maryland, United States*

Physical activity plays a key role in preventing falls among older adults as it supports muscle strength and improves balance and postural control. Recent studies have revealed that physical activity modulates Interleukin-6 (IL-6) levels and reduces the incidence of falls among older adults. This study describes the relationships between vigorous physical activity, IL-6 plasma levels, depression, and falls among older adults. We conducted a cross-sectional study on Health and Retirement Study participants aged 65 and above (n= 9,942) using datasets from the 2016 interviews. Of the sample, 37.7% reported falling in the last 24 months. Mean ages were: 77.4 (8.14) years in participants who had a fall and 75 (7.31) years in participants without a fall. Participants engaging in regular vigorous physical activity were 24% less likely to have a fall than those not engaging in vigorous activity. We quartiled the IL-6 levels and presented quartile-4 as the most elevated IL-6 levels. Compared to quartile-1 (lowest), participants in quartile-3 were 24%, and those in quartile-4 were 45% more likely to fall. The effect of IL-6 was insignificant in quartile-2. Having depression was also significantly associated with falls (OR=2.40). We observed a significant inverse relationship between ethnic/racial minorities and odds of falling: Hispanics were 16%, and African Americans were 36% less likely to fall than non-Hispanic whites. The likelihood of falling among people living in rural was significantly higher than among those living in urban (OR= 1.15). Interleukin-6 may be incorporated to fall prevention interventions as an indicator of fall risk.

Abstract citation ID: igad104.1981

### VIRTUAL GERAS DANCING FOR COGNITION EXERCISE (DANCE) FOR OLDER ADULTS: A FEASIBILITY RANDOMIZED CONTROL TRIAL

Patricia Hewston[1], George Ioannidis[2], Courtney Kennedy[1], and Alexandra Papaioannou[1], *1. McMaster University, Hamilton, Ontario, Canada*, *2. GERAS Centre for Aging Research, Hamilton, Ontario, Canada*

**Background:** GERAS DANcing for Cognition and Exercise (DANCE) was developed with rehabilitation and geriatric medicine expertise for older adults (age 60+) looking to improve brain health or mobility. Our primary aim of this study was to assess the feasibility of virtual GERAS DANCE. **Methods:** This study utilized a single-center, prospective, parallel-group randomized controlled trial (RCT) feasibility approach. We recruited 50 older adults. Participants were randomized to receive 6-weeks (1-hour class twice weekly) of virtual GERAS DANCE or usual care. Feasibility was assessed using pre-defined criteria for process, outcomes, and acceptability, and the effect of GERAS DANCE on mood, balance confidence, and fear of falling. **Results:** Our study recruitment period occurred over 8 weeks to recruit 50 older adults (mean age = 75.02(5.89) years, range: 63-92, 92% female). The enrollment-to-screening ratio was calculated as 25:103 and the retention rate of participants was 84%. The average class attendance of the study cohort was 77%. One adverse event was reported unrelated to the study intervention. The program had a high-fidelity score and adhered to the standardized curriculum. Both the intervention and usual care groups improved mood (Depression, Anxiety and Stress Scale – 21). The intervention-group had higher balance confidence and lower fear of falling. **Discussion:** Pre-determined thresholds for feasibility were met for all outcomes providing evidence that virtual GERAS DANCE is feasible, well-accepted, and safe for older adults. Improved balance confidence and reduced fear of falling through dance can have significant implications for fall prevention, healthcare costs, and overall quality of life.

### SESSION 5105 (PAPER)

### TECHNOLOGY (PAPERS)

Abstract citation ID: igad104.1982

### EFFECTS OF IMPLEMENTING AGETECH TO SUPPORT DEMENTIA CARE IN A RURAL AND NORTHERN SETTING

Shannon Freeman[1], Sarah Sousa[2], Davina Banner-Lukaris[1], and Kelly Skinner[2], *1. University of Northern British Columbia, Prince George, British Columbia, Canada*, *2. University of Waterloo, Waterloo, Ontario, Canada*

Older adults wishing to age in northern and rural communities deserve equitable access to technologies that support optimal health, well-being, and quality of life. A new dementia care home opened in a rural and northern community in 2022 with consciously embedded multiple AgeTech solutions to enhance residents quality of life and care. The AgeTech solutions including hydroponic gardening, circadian lighting, and virtual exercise programming, were implementated into the 10-bed facility through a partnership between the Center for Technology Adoption for Aging in the North (CTAAN), health systems leaders, and community partners. The purposeful implementation of AgeTech within the facility through this partnership aimed to enhance the life-skills and living model of care to maximize independence, function, and resident well-being. We conducted a process evaluation assessing data from interviews with facility staff, health systems leaders, and secondary analysis of existing documentation to objectively measure the implementation process and inform a framework for ongoing assessment of AgeTech. Results highlight areas for ongoing technology innovation and inform policy decisions for future initiatives. Informants described the benefits in use of non-invasive circadian lighting technology to support resident well-being by enhancing sleep, rest/activity, reducing behaviours associated with dementia. Technology enhanced environmental well-being and expanded opportunities for residents to engage in meaningful activities. Participants also described the physical and social benefits for residents using individual and social multiplayer modes of exercise technologies. Providing equitable access to services across dispersed and sparse populations is challenging, yet with implementation of AgeTech quality of care can be enhanced.

Abstract citation ID: igad104.1983

### EXPLORING THE ROLE OF SOCIAL FACTORS AND CHANGES IN RIDE-HAIL (UBER/LYFT) USE AMONG OLDER ADULTS

Jonathon Vivoda, and Rosa Grippa, *Miami University, Oxford, Ohio, United States*

Previous research has observed low ride-hail (e.g., Uber) use among older adults and identified some factors related to use. However, uptake of technology changes quickly and pandemic-related factors (e.g., social distancing) may have affected ride-hail use. The Pew Research Center's American Trends Panel (ATP) datasets include variables related to political and social views, behavior, religion, geography, internet use, and other factors that have not been previously assessed in relation to ride-hailing among older adults. ATP waves from 2015, 2018, and 2021 included questions about ride-hail use, and were assessed in this study. In 2015, 15.4% of all respondents and 4.5% of older adults reported having ever used ride-hailing. In 2018, the numbers had increased to 36.1% overall and 18.1% for older adults. In 2021, participants were only asked about ride-hail use during the past year, with 22.4% overall and 8.7% of older adults reporting ride-hail use. Separate logistic regression models were used to determine factors associated with reporting ever using ride-hail services (2018) and having used ride-hailing during the past year (2021), among older adults. Higher odds of reporting any use (2018) were related to living in a metropolitan area, living in the US's West, attending religious service monthly vs. never, high income, more liberal ideology, female gender, and volunteering. Higher odds of use during the past year (2021) were related to metropolitan area, male gender, more education, Hispanic ethnicity, divorced and single vs. married, unaffiliated vs Catholic religion, less frequent religious attendance, high income, and frequent internet use.

Abstract citation ID: igad104.1984

### IMPLEMENTATION OF A DIGITAL COGNITIVE SCREENING PROGRAM FOR DEMENTIA IN PRIMARY CARE

Nicole Fowler[1], Judy Mullavey[2], Kristen Swartzell[3], Dustin Hammers[4], Jared Brosch[4], James Murray[5], and Deanna Willis[1], *1. Indiana University School of Medicine, Indianapolis, Indiana, United States*, *2. Linus Health, Boston, Massachusetts, United States*, *3. Indiana University School of Medicine, IU Center for Aging Research, Indianapolis, Indiana, United States*, *4. Indiana University, Indianapolis, Indiana, United States*, *5. Davos Alzheimer's Collaborative, Wayne, Pennsylvania, United States*

Early identification of Alzheimer's disease and related dementias (ADRD) has become paramount given the emergence of disease modifying therapies. Integration of rapid, scalable tools to identify early cognitive impairment in primary care is essential because most at-risk individuals have limited access to specialty care. Yet, competing demands on primary care practices can make integration challenging. This demonstration project is being conducted to understand the feasibility, acceptability, and implementation of digital cognitive screening in primary care. Patients ≥65 years presenting to one of six primary care sites between 06/01/2022 and 06/30/2023 are asked to complete the Linus Health Digital Clock and Recall (DCR™) cognitive screening. Data regarding number of screening attempts that were refused, incomplete, or completed was collected. Results of the first completed screening-results were analyzed using descriptive statistics. As of 2/15/23, there are 3,920 screening attempts. DCR™ screenings were completed 40.8% of the time, refused 57.4%, and attempted but incomplete 1.8%. Thirteen-percent of attempts were positive for cognitive impairment, 37% were borderline, 44% were unimpaired, and 6% were unanalyzable. Average patient age is 73.2±6.0 years, 58% female, and 6% report less than high school education. Patients with positive screenings are older, slightly more female, and reported less education. Cognitive screening via DCR™ is ongoing and has been completed in nearly half of those approached, with half scoring cognitively impaired or borderline. This approach appears to have utility in early detection of cognitive impairment in primary care. By November 2023 we will have follow-up data for patients who screened positive.

Abstract citation ID: igad104.1985

### OLDER ADULTS' UNDERSTANDING OF SIGNALS COMMUNICATED BY ROBOT COMPANIONS FOR CAREGIVING

Bertram Malle[1], Vivienne Chi[1], and Claudia B. Rebola[2], *1. Brown University, Providence, Rhode Island, United States*, *2. University of Cincinnati, Cincinnati, Ohio, United States*

Robotic animal-like companions for older adults are promising technologies to address companionship and new models of care. Our project, Affordable Robotic Intelligence for Elderly Support, aims to design smart communication capabilities in an Ageless Innovation Joy for All™ robotic cat. The cat robot with enhanced capabilities uses intuitive digital cues, including display screen, sound, and LED to create affordances to communicate various interaction intents to engage users into desirable actions. We conducted a study with 315 participants to understand the efficacy of designed affordances. A total of 8 affordances for 8 task scenarios, each with 3 variations of complete or incomplete information, simulating possible experiences were tested with users. Participants were randomly assigned to the conditions "full" (=display+sound+LED), "no_display" (=sound+LED), and "no_sound" (=display+LED) and were asked to provide a response interpretation of the experience signals and rank a list of potential interpretations in order of possibility. Results indicate that participants in the full experience group liked the robot the most (M=6.95 on a scale of 1-10) and gave the highest probability of the robot being a pleasant companion to older adults (M=7.17), compared to the no_display group (M_likability=6.01; M_companionship=6.58) and the no_sound group (M_likability=6.35; M_companionship=6.96). Participants in "no_display" had the greatest difficulty at inferring the robot's intent. However, the no_sound group had less difficulty understanding the robot than the "full" experience group. Sound (e.g., meowing) appeared to be ambiguous, and removing it improved the communicability of the designed interface of this robot companion.

Abstract citation ID: igad104.1986

### USER EXPERIENCES OF OLDER ADULTS NAVIGATING AN ONLINE DATABASE OF COMMUNITY-BASED PHYSICAL ACTIVITY PROGRAMS

Denise Connelly[1], and Ashley Lowndes[2], *1. Western University, London, Ontario, Canada*, *2. University of Western Ontario, London, Ontario, Canada*

As cataloguing health care services becomes more internet-based, older adults are increasingly expected to navigate services online. Previous studies have outlined the needs, barriers, and facilitators of older adults using eHealth, however, none included user experiences in navigating websites. Connecting community-living older adults with local, high quality, community-based physical activity programs is a gap in efforts to promote physical activity by older adults. This study aimed to: 1) explore older adult user experiences navigating an online health database of local physical activity programs; 2) compare navigational feedback with age-friendly website design guidelines; and 3) assess online database completeness. Focus groups, including guided navigation tasks and a semi-structured interview script, gathered user experiences of fifteen older adults (≥65). A literature review produced three age-friendly best practice website design guidelines. A website search for local physical activity programs was completed. The design of the online database website was challenging for older adult participants to navigate and was not 'intuitive'. In navigating the online database, the older adults identified multiple discrepancies with established guidelines for designing age-friendly websites. A total of 187 local physical activity programs were missing from the database. Findings provide novel insight into user experiences of older adults navigating online health and physical activity program websites. Redesign following age-friendly website recommendations would empower older adults in use of online databases and promote awareness of local physical activity programs. Health care providers need reliable and age-friendly online resources to link their patients with local physical activity programs to promote healthy aging.

### SESSION 5110 (PAPER)

### POLICY

Abstract citation ID: igad104.1987

### AGE-BASED ENTITLEMENT: AN AGEIST PRACTICE OR A TOOL FOR COMBATTING AGEISM?

Tove Harnett[1], and Håkan Jönson[2], *1. Lund University, Lund, Skane Lan, Sweden*, *2. Lunds Universitet, Lund, Skane Lan, Sweden*

Researchers in gerontology have addressed the way age-based arrangements may communicate stereotypical and devaluing images of older people, thereby linking high age to frailty and dependence. The present article considers proposed reforms to the Swedish eldercare system designed to guarantee people over 85 the right to move into a nursing home regardless of their needs. The purpose of the article is to investigate older people's views on age-based entitlement in light of this proposal. What might the consequences of implementing the proposal be? Does it communicate devaluing images? Do the respondents consider it a case of ageism? The data consists of 11 peer group interviews with 34 older individuals. Bradshaw's taxonomy of needs was used to code and analyze data. Four positions on the proposed guarantee were identified: care should be arranged: (1) according to needs, not age; (2) according to age as a proxy for needs; (3) according to age, as a right; and (4) according to age, to combat "fourth ageism", meaning ageism directed towards frail older persons with care needs, i.e. persons in the fourth age . The notion that such a guarantee might constitute ageism was dismissed as irrelevant, while difficulties in getting access to care were presented as the real discrimination. It is theorized that some forms of ageism posited as theoretically relevant may not be experienced as such by older people themselves.

Abstract citation ID: igad104.1988

### BIAS AND GENERALIZABILITY IN MEDICARE-BASED ANALYSES ACROSS THREE POLICY PERIODS

Galina Gorbunova[1], Arseniy Yashkin[1], Julia Kravchenko[2], Anatoliy Yashin[3], Igor Akushevich[1], and Eric Stallard[1], *1. Duke University, Durham, North Carolina, United States*, *2. Duke University School of Medicine, Durham, North Carolina, United States*, *3. Social Science Research Institute, Duke University, Durham, North Carolina, United States*

Administrative Big Health data such as that drawn from the Medicare program is an invaluable resource for the study of population health. Although the Medicare program provides near universal health coverage for older U.S. adults age 65+, for most types of care data is collected only for individuals enrolled in traditional Medicare (TM) plans, with those enrolled in private Medicare Advantage (MA) plans being effectively censored in terms of the extent of available data. This presents two potential problems for health outcomes research: bias and generalizability. In this study we use Health and Retirement Study data (1998-2015) to assess the presence and magnitude of group-specific differences between MA and TM beneficiaries as well as plan switchers across three policy-specific periods (Balanced Budget Act; Medicare Modernization Act; Affordable Care Act). We found that the MA population was characterized by higher diversity, adverse behavioral habits, lower education, and economic disadvantage. Initially, lower morbidity levels converged with the TM average over time. Individuals, switching into MA after TM had much larger morbidity levels and mortality risk than any other Medicare group. We found significant loss of generalizability with respect to the Hispanic population; such estimates could be further biased to the extent that MA Hispanics differ from TM Hispanics. Of great concern is the combination of low education and low economic status in MA beneficiaries – a combination also found in many disadvantaged minorities. Care is needed in the design of TM-based studies, especially those focused on ethnic differences.

Abstract citation ID: igad104.1989

### INTERROGATING AGEISM

Douglas Hanes, *Stony Brook University, Stony Brook, New York, United States*

This paper identifies and addresses a puzzle within the conceptualization of ageism. According to the Reframing Aging Initiative, “Ageism refers to stereotypes, prejudice, and discrimination directed toward people on the basis of age”; these adversely affect older people’s lives in daily lives, including in employment and healthcare. Yet, unlike other marginalized groups, like people of color and women, older people are not underrepresented in powerful positions nor are they worse off. Indeed, they tend to fare better than the population overall: the mean age of US CEOs is 59 years; US Representatives and Senators are 58.4 and 64.3, respectively. Older Americans are more likely to be homeowners and less likely to be unemployed or food-insecure than the overall population. This paper considers several possible explanations. First, unlike racism or sexism, ageism may not be structural, but personal: present in individuals’ attitudes and interactions, but without population-level effects, codification, or institutionalization. Second, ageism may only hold intersectionally, becoming operational under conditions of structural oppression like racism and sexism. For example, while older US whites have less risk of food insecurity than younger whites, older Black Americans are at greater risk than younger Blacks. Finally, ageism may be mitigated by selective mortality: while older people face ageism daily, not everyone reaches older age. Wealthier people and whites are more likely to live to older age; older people’s greater power and welfare reflect their earlier status. Each option presents benefits and drawbacks; together they allow us to address those whom ageism most harms.

Abstract citation ID: igad104.1990

### RETIRING TO POVERTY: VETERANS’ PROTESTS FOR NONPAYMENT OF PENSIONS IN NIGERIA

Runcie Chidebe[1], Agha A. Agha[2], Ejike Ugwu[2], Donnette Narine[3], Takashi Yamashita[4], and Phyllis Cummins[1], *1. Miami University, Oxford, Ohio, United States*, *2. University of Nigeria Nsukka, Nsukka, Enugu, Nigeria*, *3. University of Maryland Baltimore, Baltimore, Maryland, United States*, *4. University of Maryland, Baltimore County, Baltimore, Maryland, United States*

The lack of satisfactory retirement plans coupled with poor retirees’ well-being has led to the introduction of different pension and social security policies in Nigeria. Of all the public servants impacted by the weak pension system, Nigerian veterans are the most vulnerable. Veterans spent their youthful years serving the nation and defending its territory; however, when they retire, they retire to poverty. Since 2015, Nigerian veterans have been staging protests and blocking access roads for non-payment of pensions, gratuities, and other allowances. We adopted a qualitative research approach and used content analysis to examine 45 news articles (2015-2022) related to veterans’ protests for non-payment of pensions in Nigeria. A semi-structured interview was conducted with ten veterans. Our findings show the protests were perceived as the last resort to ‘save their lives’ from poverty and ill health. Non-payment of pensions to veterans in Nigeria has a negative socio-psychological impact on the veterans, their families, and the country. Some of the protest placards read: “We have sacrificed our youthful years fighting for Nigeria, we should be celebrated’, "When we remember the dead, we should also remember the living”. Some quotes from the interviews are: “We’re here, alongside our wives and children, and the widows of late military personnel and veterans who died in service, some of whom died fighting Boko Haram terrorists. We’ll be sleeping over at this place until they…accede to our demands.” Veterans organize themselves in diverse groups. Our findings provide evidence to support improved pensions for veterans in Nigeria.

Abstract citation ID: igad104.1991

### SOCIAL PRESCRIBING FOR THE ARTS: LESSONS FROM ABROAD FOR THE HEALTH CARE OF OLDER AMERICANS

Shayna Gleason[1], Sudha Shreeniwas[2], and Joy Birabwa[3], *1. University of Massachusetts, Boston Gerontology, Boston, Massachusetts, United States*, *2. University of North Carolina Greensboro, Greensboro, North Carolina, United States*, *3. University of North Carolina at Greensboro, Greensboro, North Carolina, United States*

Evidence shows the dramatically positive influence of participation in the arts on the health and well-being of older adults (OA). Social Prescribing for the Arts (also called Arts on Prescription or AoP) — in which a professional in a clinical or community setting refers a client to the arts and supports them in accessing arts programming — constitutes one mechanism by which OA may participate in health-promoting arts activities. AoP has been incorporated in other high-income nations including Canada, the UK, and some EU nations. The UK especially has embedded AoP into its healthcare system; however, its universal health insurance program makes its context substantially different from the United States'. This study examined models of AoP abroad and how they could be adapted to the U.S. context for OA. We conducted 25 semi-structured, in-depth interviews with professionals involved in AoP overseas, including physicians, link workers, arts providers, policy experts, and others, as well as 10 interviews with their U.S. counterparts (N=35). Participants were recruited through referral sampling and we intentionally cultivated diversity of role in AoP schemes during recruitment. Transcripts were coded thematically by three team members. Resulting themes included challenges abroad with funding for arts, uneven training among referrers, strain on referrers and arts providers, difficulty reaching the most disadvantaged, skepticism from various stakeholders, the importance of patient involvement in developing a care plan, and a need for open communication between arts providers and referrers. Recommendations for the U.S. include designing and evaluating AoP programs that address these concerns.

Abstract citation ID: igad104.1992
### THE SPILLOVER EFFECTS OF COMPREHENSIVE CARE FOR JOINT REPLACEMENT (CJR) MODEL: A STUDY FROM CALIFORNIA

Narae Kim, and Mireille Jacobson, *University of Southern California, Los Angeles, California, United States*

In 2016, Medicare implemented the Comprehensive Care for Joint Replacement (CJR) program to test whether paying hospitals a bundled payment for 90 days of an episode of care for lower extremity joint replacement (LEJR), the most common surgery for Medicare beneficiaries, can improve care coordination and quality in traditional Medicare (TM). Unlike most Medicare alternative payment models, CJR participation was randomly assigned across Metropolitan Statistical Areas (MSAs). Hospitals in selected MSAs were required to participate in the program, while hospitals in other MSAs were paid in the usual fee-for-service manner. Taking advantage of this random assignment, we examined CJR's direct effects on TM patients and spillover effects on Medicare Advantage (MA) and non-Medicare patients in California. Using California's Patient Discharge Data (PDD) from 2014 to 2017and event study and difference-in-differences models, we studied changes in adjusted length of stay and home discharge rates before and after program implementation in hospitals in treated versus control MSAs. We found that the CJR program affected not only TM patients, but also untargeted MA and non-Medicare patients. Both TM and non-Medicare patients in treated hospitals experienced shortened length of stay (-3.9% & -1.3%, $p<0.05$) and increased likelihood of discharge home (3.4%, 2.3%, $p< 0.001$) relative to those in untreated hospitals after program implementation. MA patients experienced an increase in not only home discharge rates (4.7%, $p< 0.001$) but also length of stay (2.5%, $p< 0.01$). Programs designed to affect Medicare costs have the potential to affect the care of patients not covered by the program.

### SESSION 5115 (PAPER)

### SOCIAL SUPPORT AND HEALTH

Abstract citation ID: igad104.1993
### LONELINESS AND QUALITY OF LIFE IN OLDER ADULT PRIMARY CARE PATIENTS

Monica Williams-Farrelly[1], Matthew Schroeder[2], Claudia Li[1], and Nicole Fowler[1], *1. Indiana University School of Medicine, Indianapolis, Indiana, United States*, *2. Regenstrief Institute, Inc., Indianapolis, Indiana, United States*

Loneliness, defined as the perceived discrepancy in an individual's desired and actual social relationships, is common among older adults. Loneliness among older adult primary care patients is lacking, considering the implications it has on physical and mental health. Our objective was to determine the relationship between loneliness and quality of life (QOL) in older adult primary care patients. Data come from the Caregiver Outcomes of Alzheimer's Disease Screening (COADS) study, an ongoing randomized controlled trial evaluating benefits and risks of Alzheimer's disease and related dementias screening among older primary care patients and their family members. Loneliness (5-item NIH Toolbox), quality of life (QOL)—as measured by physical and mental health component scores—and depression (PHQ-9) and anxiety symptomatology (GAD-7) were measured among primary care patients aged 65 and older from April 2020 to September 2021. Spearman correlation analyses reveal that loneliness was moderately correlated with mental health ($r(601) = -.43$, $p< 0.001$), anxiety ($r(601) =.44$, $p< 0.001$), and depression ($r(601) = .42$, $p< 0.001$), while weakly correlated with physical health ($r(601) = -.15$, $p< 0.001$). After conducting unadjusted and adjusted linear regression models, we found that loneliness was associated with both lower mental ($p< 0.001$) and physical health component scores ($p< 0.001$). Furthermore, loneliness remained significantly associated with worse mental health when adjusting for depression, anxiety, sociodemographic characteristics, and comorbidity. Primary care providers should discuss loneliness with their older adult patients and provide resources to help patients develop and maintain meaningful social relationships.

Abstract citation ID: igad104.1994
### LONELINESS IN OLDER ADULTS LIVING IN PUERTO RICO DURING THE COVID-19 PANDEMIC: AN UNDEREXAMINED PUBLIC HEALTH CONCERN

David Camacho[1], Matthew Morgan[2], and Denise Burnette[2], *1. University of Illinois, Chicago, Chicago, Illinois, United States*, *2. Virginia Commonwealth University, Richmond, Virginia, United States*

Loneliness is a critical public health problem, yet it is under-examined among older adults in Puerto Rico. We explored sociodemographic and health-related risks for loneliness using data from a Knowledge, Attitudes and Practices COVID-19 study of Older Adults in Puerto Rico study. We conducted telephone and face-to-face interviews (January - December 2021) with a nonprobability sample of adults aged 60+ in Puerto Rico (n= 213). Data collection included measures of objective and subjective physical and mental health. We used generalized linear regression analyses to explore main effects of salient demographic and health factors on loneliness. Missing data were treated with Multiple Imputation. Prevalence of loneliness (3-item TILS) was 36.3% . Widowhood (β=.832, SE=.38, p=.029), low household income (<$12,500; β=.691, SE=.23, p=.003), psychological distress (β=.141, SE=.03, p<.001) were positively associated with greater loneliness. Living alone (β=-.664, SE=.27, p=.015) and higher sense of community (β=-.067, SE=.01, p<.001) were negatively associated with loneliness. Pandemic specific challenges (e.g., someone close diagnosed or hospitalized with COVID-19) and other common predictors (e.g., age, female, self-rated health) were not significant. We consider that contextual (e.g., outmigration, under-resourced aging and healthcare infrastructure) and cultural factors (e.g., familism) may help shape predictors and their effects on loneliness. Mainland US and Puerto Rican researchers and practitioners should collaborate on examining loneliness narratives, their impact on health, and implementation of culturally appropriate and contextually feasible interventions in Puerto Rico.

Abstract citation ID: igad104.1995

### SENSE OF CONTROL AND DIGITAL LITERACY AMONG OLDER ADULTS: THE ROLE OF PERSONAL MASTERY AND PERCEIVED CONSTRAINTS

Zhiyu Fan, Huamao Peng, Heng Zhao, and Jingxuan Wu, *Beijing Normal University, Beijing, Beijing, China (People's Republic)*

Digital literacy (DL) is necessary for older adults to fully participate in society in the era of digital technology. This study investigated how the sense of control (SOC) and its two internal dimensions (personal mastery (PM) and perceived constraints (PC)) interact with DL among older Chinese adults. A total of 163 older netizens aged 60 to 76 (Mean age 66.0, SD = 3.57, 72.4% female) were recruited from Beijing in 2021 through convenience sampling and followed for 1 year. DL was measured by a self-designed Digital Literacy Scale among Older Adults (Range 1-5; Cronbach's α = 0.96) and SOC was measured by the Sense of Control Scale (Range 1-7; Cronbach's α = 0.88). Cross-lagged analysis and half-longitudinal mediation analysis were used after adjusting for age, gender, and education. The DL were 3.51 and 3.52 at baseline and year-1 on average. Higher baseline SOC (Adjusted beta = 0.23, 95% Confidence Interval = [0.08, 0.38]) and PM (0.26 [0.10, 0.42]) were associated with higher year-1 DL. Higher baseline DL was related to reduced year-1 PC (-0.16 [-0.30, -0.02]). In addition, half-longitudinal mediation analysis showed that higher PM at baseline predicted an increase in DL from baseline to year-1 (0.29 [0.13, 0.43]), whereas higher baseline DL was related to a decrease in PC from baseline to year-1 (-0.16 [-0.30, -0.03]). Our findings indicate that the DL of older netizens is closely related to their internal factors. Older netizens might benefit from better SOC, especially personal mastery, thus narrowing the secondary digital divide.

Abstract citation ID: igad104.1996

### SOCIAL ENGAGEMENT IN OLDER PEOPLE LIVING WITH HIV

Atami De Main[1], Marshall Glesby[1], Carrie Johnston[1], Mark Brennan-Ing[2], and Eugenia Siegler[1], *1. Weill Cornell Medicine, New York City, New York, United States*, *2. Hunter College, CUNY, New York City, New York, United States*

More than 50% of people with HIV (PWH) are at least 50 years old. PWH experience a high prevalence of multiple chronic conditions and fragile social structures. Although social engagement may be protective against detrimental physical, cognitive, and mental health outcomes, there is limited evidence on older PWH's social engagement and associated factors in engaging with family, friends, and community. This study sought to examine social engagement in a population of older PWH, using the 13-item Frequency of Leisure Activities Scale from the Research on Older Adults with HIV (ROAH 2.0) study at the Weill Cornell Campus in New York City. We conducted factor and regression analyses to characterize social engagement activities and examine their relationships with AIDS diagnosis, social vulnerability index (SVI), financial strain, loneliness, HIV-related stigma, and depressive symptoms, controlling for demographic factors. PWH (n=349, age range=50-84) were less engaged in social (Mean=1.31; SD=0.71) and cultural-physical (e.g. attending cultural events) (Mean=1.55; SD=0.78) than media-based activities (Mean=3.09; SD=0.72), with Omega reliability estimated at 0.716, 0.651, and 0.620 respectively. Having AIDS, experiencing increased HIV-related stigma, loneliness, and depressive symptoms were associated with low social activity engagement ($F(6, 316) = 9.87$, $p < .001$). High SVI score and financial strain were negatively associated with engagement in cultural-physical activities ($F(5, 317) = 2.61$, $p < .001$). Increased depressive symptoms were negatively associated with engagement in media-based activities ($F(4, 318) = 5.88$, $p < .001$). The findings provide foundations for future research to improve our understanding of social engagement in older PWH.

Abstract citation ID: igad104.1997

### THE ROLE OF SOCIAL SUPPORT AND SELF-EFFICACY ON HOPEFULNESS IN LOW-INCOME OLDER ADULTS AGED 50 AND OVER

Soonhyung Kwon[1], Ellen Benoit[2], and Liliane Windsor[3], *1. Rutgers, the State University of New Jersey, Savoy, Illinois, United States*, *2. Center for Drug Use and HIV/HCV Research, NYU School of Global Public Health, New York City, New York, United States*, *3. The University of Illinois at Urbana-Champaign, Champaign, Illinois, United States*

**Background:** Social support and self-efficacy play a significant role in improving positive psychological well-being in marginalized older adults. However, to date, there are few studies on identifying the relationships during the COVID-19 pandemic. We examined the effect of social support and self-efficacy on hopefulness in marginalized low-income older adults during the COVID-19 pandemic. **Methods:** This study used baseline data from a clinical trial designed to increase COVID-19 testing in Essex County, NJ. The dataset involved participants aged 50 and over. We conducted: 1) cross-sectional descriptive/frequency statistics to understand the sociodemographic characteristics, 2) multivariate linear regression to investigate the direct relationships between social support subscales or self-efficacy and hopefulness, and 3) mediation analyses to examine the mediating role of self-efficacy in the relationship between social support and hopefulness. **Results:** After adjusting for covariate variables, social support subscales (emotional/informational social support: b = 0.20, $p < 0.05$; tangible social support: b = 0.13, $p < 0.05$; affectionate social support: b = 0.17, $p < 0.05$; positive social interaction social support: b = 0.15, $p < 0.05$) and self-efficacy (b = 0.55, $p < 0.001$) were significantly associated with hopefulness. The indirect effect of social support via self-efficacy was positive and statistically significant (Effect = 0.14, Bootse = 0.04, BootLLCI - BootULCI = 0.06 - 0.23) **Conclusion:** Self-efficacy mediated the relationship between social support and hopefulness in marginalized older adults aged 50 and over. Further research needs to identify the various facets of positive psychological well-being using longitudinal data and larger sample size.

### SESSION 5120 (SYMPOSIUM)

Abstract citation ID: igad104.1998

### THE CONFIDENCE FINANCIAL WELL-BEING INTERVENTION FOR LATINO FAMILY CAREGIVERS: PILOT STUDY RESULTS

Chair: Kylie Meyer Co-Chair: Susanna Mage Discussant: Donna Benton

Family caregivers to people living with dementia encounter an average of $10,697 per year in out-of-pocket caregiving costs that exert long-term material and psychological consequences. Latino caregivers encounter higher out-of-pocket caregiving costs relative to their income than non-Latino caregivers. There is an unmet need for programs to alleviate financial hardship among caregivers, particularly those culturally tailored to Latino caregivers. The Confidently Navigating Financial Decisions and Enhancing Financial Wellbeing in Dementia Caregiving (CONFIDENCE) program draws on successful intervention approaches identified within nursing and gerontological research to manage out-of-pocket caregiving costs and prevent financial hardship among caregivers to persons living with dementia. Between May 2022 and February 2023, CONFIDENCE was delivered to six cohorts of Latino caregivers at an academic-service center. This symposium will discuss preliminary pilot study results. Donna Benton, PhD, will describe how the intervention was developed with a community advisory council to ensure a culturally-tailored program. Kylie Meyer, PhD, will share the preliminary efficacy of the intervention based on interim analyses of outcomes, including caregiver resourcefulness and financial strain. Alexander Gonzales, BSG, will describe challenges related to recruitment and retention, and considers registration and fidelity data to understand possible causes of low attendance. Lastly, Susanna Mage, PhD(c), MA, will present the findings from the preliminary evaluation of the program to examine the acceptability, including participant satisfaction surveys and one-on-one interviews. Initial success of the pilot phase suggests opportunities for future efficacy testing. If efficacious, CONFIDENCE could help alleviate financial hardship experienced by family caregivers to persons living with dementia.

Abstract citation ID: igad104.1999

### DEVELOPMENT OF A CULTURALLY TAILORED COST MANAGEMENT INTERVENTION INFORMED BY CAREGIVER VOICES

Donna Benton[1], Susanna Mage[1], Kathleen Wilber[1], and Kylie Meyer[2], *1. University of Southern California, Los Angeles, California, United States*, *2. Case Western Reserve University, Cleveland, Ohio, United States*

Caregivers spend an average of 20% of their annual household income on out-of-pocket caregiving costs. Yet, no evidence-based interventions help family caregivers manage these expenses and financial stressors. Responses to financial strain must be culturally tailored to Latino family caregivers, whose out-of-pocket care costs comprise nearly half their annual household income. This presentation describes the development of the CONFIDENCE Financial Education Intervention, a 5-week psychoeducational intervention informed by the Resourcefulness and Quality of Life Theory and made to address the needs of Latino family caregivers. To ensure CONFIDENCE was responsive to the needs of Latino caregivers, the investigators 1) conducted 11 qualitative in-depth interviews with caregivers to learn about their experiences managing care costs and 2) convened a community advisory council of Latino family caregivers to guide intervention components, content, and delivery. Council recommendations guided multiple intervention modifications. For example, based on these recommendations from the council, the investigator team expanded the intervention's target audience from focusing on women to including caregivers of all genders so families could participate together (familialism). Findings from the interviews and the council overlapped. During interviews, caregivers reported feeling worried about the growth of future caregiving costs increasing; similarly, the council described the importance of helping caregivers understand how costs would change throughout the disease trajectory. Caregiver interviews and the advisory council also emphasized the importance of addressing the needs of middle-income caregivers to prevent future financial adversity. By integrating the caregiver voices, we developed an intervention tailored to meet the needs of Latino families.

Abstract citation ID: igad104.2000

### PRELIMINARY RESULTS OF THE CONFIDENCE FINANCIAL INTERVENTION FOR CAREGIVERS TO PERSONS LIVING WITH DEMENTIA

Kylie Meyer[1], Jaclene Zauszniewski[1], Lixin Song[2], and Donna Benton[3], *1. Case Western Reserve University, Cleveland, Ohio, United States*, *2. University of Texas Health Science Center at San Antonio, San Antonio, Texas, United States*, *3. University of Southern California, Los Angeles, California, United States*

Family caregivers to persons living with dementia experience high out-of-pocket costs of care that contribute to material and psychological hardship and can undermine current and future financial security. Latino/Hispanic family caregivers pay nearly double the out-of-pocket costs relative to non-Latino/Hispanic caregivers. CONFIDENCE is a 5-week psychoeducational intervention designed to help caregivers manage out-of-pocket caregiving costs and psychological strain by equipping caregivers with resourcefulness skills and building their self-efficacy in managing care costs. The intervention is culturally tailored to meet the needs of Latino/Hispanic family caregivers. The investigators are conducting a single-arm pre-and post-test trial to determine the preliminary efficacy of the CONFIDENCE intervention among Latino/Hispanic family caregivers to persons living with dementia (NCT05292248). Preliminary results from the first ten study participants, completed as a part of planned interim analyses, indicate a positive change in the hypothesized direction for each study outcome from baseline survey to follow-up surveys, completed within two weeks of program completion. Caregivers who participated in CONFIDENCE reported increased levels of resourcefulness (M=85.1[SD=7.0] vs. M=97.5[SD=5.6], p-value=0.02). We also observed higher levels of caregiver self-efficacy post-intervention (M=40.4[SD=6.0] vs. M=57.2[SD=6.6], p-value<0.001). Lastly, preliminary analyses suggest reduced levels of psychological financial strain following intervention (M=18.3[SD=4.2] vs. M=8.6[SD=2.7], p-value=0.04). Initial results suggest that psychoeducational intervention may prepare caregivers to manage high out-of-pocket care costs. After completing data collection, the study team will examine how intervention participation may affect out-of-pocket caregiving costs. In future studies, we will consider the potential mediating roles of resourcefulness and self-efficacy on study outcomes.

Abstract citation ID: igad104.2001

### WE BUILT IT, BUT THEY DIDN'T COME: CHALLENGES WITH RECRUITMENT AND RETENTION IN THE CONFIDENCE PILOT STUDY

Alexander Gonzalez[1], Shane Rhodes[2], Donna Benton[1], and Kylie Meyer[3], *1. University of Southern California, Los Angeles, California, United States*, *2. University of Texas Health Science Center at San Antonio, San Antonio, Texas, United States*, *3. Case Western Reserve University, Cleveland, Ohio, United States*

This presentation considers reasons for low attendance in a psychoeducational intervention developed to reduce financial strain and manage out-of-pocket caregiving costs. From May 2022 until February 2023, the CONFIDENCE Financial Education Intervention was delivered to six cohorts of family caregivers to persons living with dementia. Delivery of the 5-week, group-based Zoom intervention occurred at an academic service center in Los Angeles, CA that regularly provides caregiver education. Whereas the program team anticipated 192 caregivers to attend over 10 months, 72 caregivers registered in this time, among whom just 38 attended at least one session. The average number of sessions attended was 3.0 (SD=1.5) of five. To understand possible reasons for low participation, the investigators conducted bivariate comparisons of the demographic characteristics of attendees versus non-attendees from registration forms and reviewed fidelity reports. Fidelity reports were completed using a standard form using Likert-scale responses ranging from 0 (Strongly Disagree) to 5 (Strongly Agree). Forms were completed by facilitators after delivering sessions and by an independent observer who reviewed a lesson recording. Evaluation of registration forms showed no statistically significant difference among caregivers who attended at least one session versus those who did not attend according to caregiver age, ethnicity, race, or income. Further, fidelity reports revealed high overall quality of delivery (M=4.0, SD=0.86) and that caregivers were, on average, engaged across lessons (M=4.0, SD=0.4). Based on initial findings and facilitator input, the study team is considering additional reasons for non-participation by surveying those who did not attend after registering.

Abstract citation ID: igad104.2002

### ACCEPTABILITY OF THE CONFIDENCE INTERVENTION: INITIAL RESULTS FROM SATISFACTION SURVEYS AND QUALITATIVE INTERVIEWS

Susanna Mage[1], Donna Benton[1], and Kylie Meyer[2], *1. University of Southern California, Los Angeles, California, United States*, *2. Case Western Reserve University, Cleveland, Ohio, United States*

Evaluation of an intervention's acceptability with end-users during the pilot stage can identify barriers to future implementation. Determination of acceptability is particularly important for the CONFIDENCE intervention, as there are no rigorously tested financial wellbeing interventions among family caregivers to persons living with dementia. As a part of the CONFIDENCE pilot study, investigators are using survey techniques and qualitative interviews with participants to assess intervention acceptability. Thus far, N=9 caregivers have completed satisfaction surveys and N=3 participated in a Zoom-based, one-on-one interview. Descriptive statistics were applied to evaluate preliminary satisfaction survey data. Interview transcripts underwent preliminary thematic analysis by the first author. The codebook was guided by the Theoretical Framework of Acceptability (TFA), which posits seven domains of acceptability (e.g., self-efficacy). Results suggest overall acceptability of the intervention. All caregivers who completed the satisfaction survey "Agreed" (3; 33%) or "Strongly agreed" (6; 67%) that they would recommend the CONFIDENCE program to other caregivers. Within specific domains, most caregivers found content easy to understand (8; 89%), easy to access (7; 88%), and convenient to attend (7;78%). The thematic analyses reinforced these findings of acceptability. ("I came into the program right when I needed it.") Four aspects of the TFA emerged: 1) clear understanding of the intervention and how it works, 2) perception that the intervention was effective in achieving its purpose, 3) confidence in the ability to perform the program requirements, and 4) overall positive affective attitude about the intervention experience. "Every piece of information was helpful," shared one caregiver.

### SESSION 5125 (PAPER)

### HEALTH AND SOCIAL INTERVENTIONS II

Abstract citation ID: igad104.2003

**A BIOPSYCHOSOCIAL APPROACH TOWARD PREDICTING PHYSICAL ACTIVITY AMONG OLDER ADULTS WITH KNEE OSTEOARTHRITIS**

Navin Kaushal[1], Donya Nemati[2], and Thomas Best[3], *1. Indiana University, Indianapolis, Indiana, United States*, *2. The Ohio state University, College of Nursing, Columbus, Ohio, United States*, *3. University of Miami, Miami, Florida, United States*

Regular physical activity (PA) is imperative for preventing disability among older adults with knee osteoarthritis (KOA). However, PA is a complex behavior that relies on determinants from biological, psychological and social factors. Identifying determinants that predict PA would be informative for providing support for patients with KOA who are physically inactive. This study aimed to employ a biopsychosocial (BPS) approach to identify the strongest PA determinants among older adults with KOA.

**Methods.** Adults (age 65+) diagnosed with KOA (n=1,343) from the Osteoarthritis Initiative dataset were included to test the BPS model. Putative BPS determinants from baseline were identified by testing if they correlated with two-year follow-up change scores of accelerometry PA data. Each model factor was tested using ordinary least-squared regressions, and significant determinants were then used for the biopsychosocial model test, which employed hierarchical multiple regression.

**Results.** Bivariate correlations revealed 21 variables representing biological (n=13), psychological (n=4), and social (n=4) variables that correlated with change in levels of PA. Four significant variables were identified in separate regression models. Testing these predictors using hierarchical regression revealed the strongest determinant to be the 400-meter walk ($\beta$ =-.26, p<.001), followed by age ($\beta$=-.23, p<.001), general health ($\beta$=-.15, p<.001), and BMI ($\beta$=-.21, p<.001).

**Conclusion:** The BPS model test revealed 50% of determinants to be mutable (fitness and BMI), and the remaining to be non/less mutable. The 400-meter walk test was the strongest indicator of future PA, suggesting that individuals with KOA who score low on this test could benefit from supported PA programs.

Abstract citation ID: igad104.2004

### AGING WELL, AGING DIFFERENTLY: A COMPARISON BETWEEN LIFE PLAN COMMUNITY RESIDENTS AND THE COMMUNITY AT LARGE

Laurel Mertz[1], Jennifer Smith[1], Nicole Lehpamer[2], Ajla Basic[1], and Catherine O'Brien[1], *1. Mather, Evanston, Illinois, United States*, *2. Mather Institute, Evanston, Illinois, United States*

Residing in Life Plan Communities (or Continuing Care Retirement Communities) may have benefits to well-being due to greater access to wellness programs and services. The purpose of this research was to examine changes in health and wellness between Life Plan Community residents and older adults residing in the community at large over five years. Data included 1,729 residents (69% female, mean baseline age = 81.64) who participated in Years 1 and 5 of the Age Well Study, and 427 older adults (50% female, mean baseline age = 80.78) residing in the community at large who participated in multiple waves of the Health and Retirement Study. The community-at-large group was proportionally sampled to be similar to the residents on age, income, and race/ethnicity, and both groups completed the same measures of health and wellness at each time point. Data were analyzed using multilevel models controlling for age, gender, income, education, and marital status. Compared to the community-at-large older adults, residents tended to exhibit better physical, emotional, social, intellectual, and vocational wellness in Years 1 and 5, but lower spiritual wellness in Year 5. When comparing Year 1 and Year 5, both groups reported similar changes over time on physical and vocational wellness measures. Community-at-large older adults tended to report more favorable changes in emotional and spiritual wellness, and residents showed more favorable changes in social and intellectual wellness. Although effects of the pandemic should be explored further, these findings have implications for supporting aging well in different types of residential settings.

Abstract citation ID: igad104.2005

### ASSOCIATION BETWEEN PET OWNERS' AND PETS' PHYSICAL ACTIVITY LEVELS: A SYSTEMATIC REVIEW

Nanna Notthoff, *Leipzig University, Leipzig, Sachsen, Germany*

Social support can promote physical activity (PA) participation. According to socioemotional selectivity theory, engaging in PA with or for important social partners may be particularly motivating for older adults. Recently, pets have been recognized as important social partners. Although studies and literature syntheses have compared PA levels of people with vs. without pets, less is known about whether humans and pets are actually active together, i.e., whether their activity levels are associated. We examined this question in a systematic literature review. Through searches in Web of Science and PubMed with a priori specified inclusion and exclusion criteria, we identified 17,565 potential records. A two-step screening process yielded 9 studies for inclusion in the systematic review. Using a modified version of the STROBE checklist, we rated their quality as M = 21.89 (SD = 2.93) out of 30 possible points. Of the 9 included studies, only 5 explicitly calculated the association between human and pet PA; 4 found a positive association and 1 found no association. Of the 9 included studies, 4 studies explicitly included older adults 60+ years. However, variations in the association between human and pet PA by age were not examined. It seems that humans and their pets are active together. A more thorough examination of this question could provide a basis for an intervention aimed at increasing human PA by incorporating pets. Such an intervention may be particularly suitable for older adults who have lost important human social partners or who have difficulty accessing PA group programs.

Abstract citation ID: igad104.2006

### RURAL OLDER ADULTS' VIEWS OF DEPRESCRIBING AND NONPHARMACOLOGICAL METHODS IN PAIN TREATMENT

Hyunjin Noh[1], Haelim Jeong[2], Denise Kan[1], and Lewis Lee[3], *1. University of Alabama, Tuscaloosa, Alabama, United States*, *2. The University of Alabama, Tuscaloosa, Alabama, United States*, *3. School of Social Work, The University of Alabama, Tuscalossa, Alabama, United States*

Proper pain management is essential to quality of life. Due to the risks of harmful drug interactions, there has been an increasing effort to reduce medication that may be harmful or ineffective (i.e., deprescribing) and to promote non-medication based (i.e., non-pharmacological) strategies in pain management. The purpose of this qualitative study is to understand the views of deprescribing and non-pharmacological methods in pain management among community-dwelling older adults with multiple chronic health conditions. Eligibility criteria included: 65+, Alabama resident living outside of nursing homes, cognitively intact, have two or more chronic health conditions and chronic pain, and take medications for their health conditions including pain medications. Participants were recruited through the Area Agency on Aging across the state of Alabama as well as other community venues serving older adults. Individual, open-ended interviews were conducted by phone to explore their concerns about medications, their views of deprescribing and using non-pharmacological pain management as well as their needs in doing so. Thematic analysis of the interview data revealed various barriers and needs: concerns about various side effects while also worrying about missing out benefits from the medications if reduced or stopped, uncertainty or skepticism toward non-pharmacological pain treatment, lacking financial and logistical access to non-pharmacological options, and lack of knowledge about available non-pharmacological options and their benefits. These findings have implications for future education and advocacy efforts to promote older adults' knowledge and self-efficacy so that they can consider deprescribing and non-pharmacological methods in managing their pain.

### SESSION 5130 (SYMPOSIUM)

Abstract citation ID: igad104.2007

### MULTILEVEL SOCIAL FACTORS OF HEALTH AND AGING AMONG DIVERSE POPULATIONS

Chair: Agus Surachman Co-Chair: Jourdyn Lawrence
Discussant: Rose Ann DiMaria-Ghalili

While a large body of evidence increasingly documents how individual-level modifiable risk factors shape where and how people age, the aspects of the social environment in which people grow older deserve more focus. Here, we aim to identify examples of multilevel social factors that shape health and aging among samples from diverse populations and datasets. The overarching goal of this work is to yield evidence of the impact of social factors on healthy aging, specifically focusing on cognitive functioning, physical activity, unmet needs, and psychological distress for middle-aged and older adults. Our panel includes four studies focusing on distinct aspects of the social environment and outcomes that affect healthy aging. Specifically, the first presentation uses instrumental variable analysis to estimate a causal association between racial discrimination within institutional settings (e.g., housing, employment) and cognitive function measures. The second study considers neighborhood deprivation (measured using the area deprivation index and personal belief in the neighborhood) as a structural context that may strongly limit adults' engagement in daily physical health. The third presentation identifies an association between the home environment (i.e., home disorder and disrepair) and unmet care needs (e.g., going without needed assistance with everyday activities such as eating or bathing). Our last presentation shows relationships between measures of economic status (e.g., food insecurity), social support impact, and psychological distress. We close by integrating critical findings from each study and consider the implications of each study on future research and interventions.

Abstract citation ID: igad104.2008

### INSTITUTIONAL EXPERIENCES OF RACIAL DISCRIMINATION AND COGNITIVE FUNCTION: INSTRUMENTAL VARIABLE ANALYSIS

Jourdyn Lawrence[1], and Ichiro Kawachi[2], *1. Dornsife School of Public Health, Drexel University, Philadelphia, Pennsylvania, United States*, *2. Harvard University, Boston, Massachusetts, United States*

In addition to enduring the ongoing and persistent legacy of structural racism in the United States, Black adults often encounter sources of social stress, like racial discrimination, that have documented associations with health outcomes. As inequities in cognitive aging persist for Black adults, research has worked to examine modifiable factors, like racial discrimination, that may serve as the basis for intervention development. The dysregulation of inflammatory biomarkers has been proposed as one pathway through which racial discrimination becomes embodied to contribute to racial inequities in cognitive function. We assessed how racial discrimination occurring in institutional settings affects cognitive function among a sample of Black and white adults from the Coronary Artery Risk Development in Young Adults (CARDIA) study. To mitigate the influences of measurement error and unmeasured or residual confounding on our findings, we used an instrumental variable analysis to estimate the unbiased, causal association between racial discrimination in institutional settings and a battery of standardized cognitive function measures. We found that increased experiences of racial discrimination had adverse impacts on cognitive function. Understanding how racial discrimination in these settings influences cognitive outcomes supplies insight into points of potential intervention at institutional and policy levels.

Abstract citation ID: igad104.2009

### NEIGHBORHOOD QUALITY AND WEEKLY PATTERNS OF PHYSICAL ACTIVITY AMONG BLACK AND WHITE ADULTS

Agus Surachman[1], August Jenkins[2], and Marina Armendariz[3], *1. Dornsife School of Public Health, Drexel University, Philadelphia, Pennsylvania, United States*, *2. University of Illinois at Urbana-Champaign, Urbana, Illinois, United States*, *3. University of Texas at San Antonio, San Antonio, Texas, United States*

Engaging in regular moderate to vigorous physical activity across the week is recommended for maintaining health. This analysis examined whether objective and subjective neighborhood quality was associated with different patterns of weekly physical activity across the week among middle and older Black and white adults. We leveraged data from the National Study of Daily Experiences (NSDE), in which 833 Black and white American adults (Mage = 62.65; n Black = 167) reported their daily moderate to vigorous physical activity across eight consecutive days. The objective and subjective measures of neighborhood quality were the area deprivation index (ADI) and personal belief in the neighborhood, respectively. Hypotheses were tested using the latent class analysis (LCA) framework. Weekly patterns of daily physical activity were classified into four different groups: Active Every Day (group size = 35.75%), Active on Weekdays (26.63%), Active on the Weekend (11.76%), and Less Active (15.85%). Lower ADI rankings, but not personal beliefs about one's neighborhood, were associated with a higher probability of being in the three more active relative to the Less Active group, an association stronger among white adults. Finally, the three more active groups showed higher self-rated physical health than the Less Active group. Within-race analyses showed that this association was only evident among white adults. Neighborhood deprivation is an important structural context that may strongly limit adults' engagement in daily physical health.

Abstract citation ID: igad104.2010

### ASSOCIATIONS OF HOME ENVIRONMENT AND UNMET CARE NEEDS AMONG LOW- AND MODERATE-INCOME OLDER ADULTS

Safiyyah Okoye[1], Laura Samuel[2], Chanee Fabius[3], Craig Pollack[4], Laura Gitlin[5], Sarah L. Szanton[3], and Jennifer Wolff[3], *1. College of Nursing and Health Professions, Drexel University, Philadelphia, Pennsylvania, United States*, *2. Johns Hopkins University School of Nursing, Baltimore, Maryland, United States*, *3. Johns Hopkins University, Baltimore, Maryland, United States*, *4. Johns Hopkins, Baltimore, Maryland, United States*, *5. Drexel University, Philadelphia, Pennsylvania, United States*

The home environment is an increasingly important setting for long-term services and support. However, more information is needed about the relationship between the home environment and adverse consequences due to unmet needs (i.e., going without assistance with everyday activities such as eating or bathing) among older adults. We draw on information from N=4,898 low and moderate-income (annual income <$60,000) community-living respondents to the National Health and Aging Trends Study (NHATS). Two aspects of the home environment were directly observed by

NHATS interviewers: home disorder (six items, including flooring problems, tripping hazards, pests) and home disrepair (five items, including missing siding and broken steps). NHATS asked participants who reported a disability whether they experienced one of seven adverse consequences (e.g., going without eating) due to no one being there to provide help or the activity is too difficult for them to complete on their own. About half of our sample (48.5%) reported a disability. We found home disorder and disrepair more prevalent among older adults with disabilities than those without (36.2% v. 28.1% and 18.2% v. 15.0%, respectively). Among older adults with disabilities, the presence of any home disorder and any home disrepair were associated with higher odds of experiencing an adverse consequence of an unmet care need in unadjusted analyses (home disorder: OR 1.33; 95% CI 1.11, 1.60; home disrepair: OR 1.16; 95% CI 0.90, 1.50). Findings show an association between home environment and unmet care needs and can signal the need to target housing-related risk factors in future interventions.

Abstract citation ID: igad104.2011

### SOCIOECONOMIC STATUS, SOCIAL SUPPORT, AND PSYCHOLOGICAL DISTRESS IN AGING KENYANS

James Muruthi, *College of Nursing and Health Professions, Drexel University, Philadelphia, Pennsylvania, United States*

The incidence of psychological distress is on the rise among aging Africans. Studies have indicated that in the absence of public support, relying on informal social networks might benefit the psychological health of aging Africans. However, the associations between social support, economic status, and psychological distress remain unclear in this population. This study investigated the prevalence of psychological distress among aging Kenyans and examined its relationships with economic status (indicated by standard measures for low- and mid-income populations) and perceived social support (multidimensional and provided by family, neighbors, and religious communities). Participants from rural and urban areas of Kenya completed computer-assisted personal interviews in May 2022 (N = 376). Overall, 61% reported high psychological distress. Structural equation modeling results indicated that food insecurity ($\beta = .236$, SE = .074, $p < .001$), sex ($\beta = .146$, SE = .056, $p < .05$) and self-reported physical health ($\beta = .386$, SE = .051, $p < .001$) positively predicted psychological distress while educational status ($\beta = .146$, SE = .058, $p < .05$), and flooring material ($\beta = .107$, SE = .056, $p < .05$) were negative predictors. The dimensions of social support were not significantly associated with the outcome variable. The findings illuminate that psychological distress is a critical health concern for the sample and needs targeted health interventions. They also underline the critical role of economic status on the psychological distress of aging Kenyans. Future studies should further explore these relationships, especially using longitudinal and representative data.

### SESSION 5135 (SYMPOSIUM)

Abstract citation ID: igad104.2012

### STAR CAREGIVERS: IMPLEMENTATION OF AN EVIDENCE-BASED PROGRAM IN REAL-WORLD SETTINGS

Chair: Susan McCurry Co-Chair: Robert Penfold
Discussant: Michele Karel

Evidence-based non-pharmacological community programs to improve care of older adults with dementia are growing. However, many programs have historically been tested in research studies that fail to reach medically underserved populations or communities with unique service needs. STAR-Caregivers (STAR-C, also known as STAR-Community Consultants) is an evidence-based psychosocial support and skill training program designed to teach family caregivers how to identify and increase pleasant events, improve communication, and use behavioral problem-solving skills to reduce the problems experienced by their family member with dementia while improving quality of life for both persons in the caregiver-care receiver dyad. Since its original randomized controlled trial in 2005, STAR-C has been translated in a variety of real-world settings, including in Area Agencies on Aging throughout Oregon and Washington state. This symposium describes three recent new translation efforts: (1) implementation of STAR-C as a virtual training program (STAR-VTF) for caregivers of persons with dementia who are part of the Kaiser Permanente Washington health care system; (2) cultural adaptation of STAR-VTF for Latino caregivers, and (3) incorporation of STAR-C into a largely rural community care setting in Illinois during the COVID-19 pandemic using Amazon Echo technology to conduct telehealth community dyad STAR-C sessions and connect clients to supplemental services. Each presenter will discuss the dementia care needs in their respective populations, the challenges encountered in translation of STAR-C to serve target caregiving dyads, and lessons learned regarding treatment effectiveness and program sustainability.

Abstract citation ID: igad104.2013

### VIRTUAL TRAINING FOR MANAGING SYMPTOMS OF DEMENTIA: A CULTURAL ADAPTATION FOR LATINO CAREGIVERS

Maggie Ramirez[1], and Robert Penfold[2], *1. University of Washington School of Public Health, Seattle, Washington, United States*, *2. Kaiser Permanente Washington, Seattle, Washington, United States*

STAR-Caregivers Virtual Training and Follow-up (STAR-VTF) is an evidence-based intervention that teaches family caregivers how to manage behavioral and psychological symptoms of dementia. The study objective was to identify what adaptations to STAR-VTF are needed to improve cultural relevance for Latino caregivers. Thirty caregivers of people living with dementia who self-identify as Hispanic/Latino and 14 providers of healthcare and social services were interviewed. Interview transcripts were analyzed using thematic analysis methods. The Cultural Treatment Adaptation Framework guided data collection and analysis. Three themes were identified: (1) there was a need to increase awareness about dementia and decrease stigma; (2) semantics mattered as certain words and phrases could be stigmatizing, offensive, or culturally inappropriate; and (3) there was a need to incorporate into program materials the traditional family structure and nature of caregiving in Latino families. Adaptations were performed on STAR-VTF, including expanding content to improve understanding of dementia, revising language that was viewed as problematic, and adding cultural examples to reflect the range of family involvement in caring for people living with dementia and multigenerational living. Findings from this study advance understanding of the Latino caregiver experience and how to modify programs to better serve their needs.

Abstract citation ID: igad104.2014

### RECRUITMENT AND BASELINE RESULTS FOR THE STAR CAREGIVERS VIRTUAL TRAINING AND FOLLOW-UP PRAGMATIC TRIAL

Robert Penfold[1], Eric Johnson[2], Maggie Ramirez[3], James Ralston[4], Susan McCurry[5], and Linda Teri[5], *1. Kaiser Permanente Washington, Seattle, Washington, United States*, *2. KPWHRI, Seattle, Washington, United States*, *3. University of Washington School of Public Health, Seattle, Washington, United States*, *4. Kaiser Permanente Washington Health Research Institute, Seattle, Washington, United States*, *5. University of Washington, Seattle, Washington, United States*

The STAR-Caregivers program is efficacious in reducing the behavioral and psychological symptoms of dementia (BPSD). In this pragmatic trial, we are testing a 6-session, self-directed, online version of the program that leverages secure email, an online learning management system, and brief coaching telephone calls to expand access to training. This paper reports on issues related to recruitment and baseline levels of psychosocial measures collected. Caregivers and persons living with dementia (PLWD) were randomized to intervention or usual care. Baseline measures were collected via REDCap and included standardized measures for PLWD dementia severity and functioning and caregiver measures for anxiety, stress, depression, and caregiving mastery. The study recruited 67 dyads. Recruitment increased greatly when we removed the inclusion that subjects be on antipsychotic medications. Baseline clinical showed the average score on the Dementia Severity Rating Scale was 22.2 (mild) and 28.8 on the Revised Memory and Problem Behavior Checklist (mild). The average Functional Activities Questionnaire was 22/30. Caregivers reported low levels of stress (KCSS), depression (PHQ8), and anxiety (GAD2) with mean and standard deviation: KCSS = 22.8 (6.6), PHQ8 = 4.9 (4.65), and GAD2 = 1.3 (1.4). The mean Caregiver Mastery Score was moderate at 20.4 (3.28). Virtual delivery of the STAR Caregiver training program is feasible. Caregivers of PLWD with mild to moderate cognitive and functional decline seem most amenable to intervention. We are currently analyzing outcome date to determine the efficacy of this program and plan to investigate the optimal timing of caregiver outreach and support programs.

Abstract citation ID: igad104.2015

### UTILIZING TECHNOLOGY AND INTEGRATED SERVICES TO SUPPORT FAMILY CAREGIVERS IN RURAL COMMUNITIES

Skye Satz[1], Quinn White[2], and Laura Gallagher Watkin[1], *1. CRIS Healthy Aging, Champaign, Illinois, United States*, *2. CRIS Healthy Aging, Danville, Illinois, United States*

CRIS Healthy Aging is a non-profit organization in East Central Illinois that serves older adults who are often lower-income and/or living in a rural community. CRIS' mission is to keep older adults safe, healthy, and independent in their own homes for as long as possible. CRIS' Memory Care Program (MCP) expanded upon the evidence-based STAR-Caregivers (STAR-C) program. In addition to administering traditional STAR-C training, CRIS provides caregiving dyads with more integrated services, including (1) implementation of technology like the Amazon Alexa Echo Show devices and OneClick video conferencing for older adults, (2) peer support and education opportunities including Memory Café, Stress-Busting Program for Family Caregivers, and the NCOA Aging Mastery Program, as well as (3) providing financial assistance, respite, and additional aging services. In the past 4 years, the CRIS MCP, funded by a federal ADPI grant through ACL, has served approximately 200 caregiving dyads, and collected pre- and post-treatment survey data from a cohort of 52. Those served consistently reported that the STAR-C program was extremely helpful for them. Most importantly, caregivers reported significant decreases in behavior frequency ($t=14.75$, $p<0.01$) and severity of personal stress response (t-21.51, $p<0.01$). Compared to baseline, caregivers also reported feeling less resentful ($p=0.005$), less socially isolated ($p>0.05$, however qualitative data supports downward trend), and saw improvements to quality of life ($t=-1.77$, $p<0.042$). Ongoing barriers to service in these rural and low-income communities include a global pandemic, misinformation, fear of the unknown, family unwillingness to participate, and financial concerns.

### SESSION 5140 (SYMPOSIUM)

Abstract citation ID: igad104.2016

### IDENTIFYING NEW INTERVENTION TARGETS TO REDUCE RISK OF ELDER ABUSE AND NEGLECT IN DEMENTIA FAMILY CAREGIVING

Chair: Carolyn Pickering Co-Chair: Vicki Winstead Discussant: Melba Hernandez-Tejada

Persons living with dementia experience higher rates of abuse and neglect than their cognitively intact peers. Caregiver burden, including stress related to management of behavioral symptoms, has been repeatedly shown as a risk factor for family caregivers' use of abusive and neglectful behavior. However, interventions intended to reduce caregiver burden and stress and improve management of behavioral symptoms often produce only small effect sizes and are not successful in translation. Therefore, it is important to identify other modifiable risk factors to serve as intervention targets to reduce and prevent elder abuse and neglect in dementia family caregivers. The purpose of this symposium is to understand the role of three potential targets for which there are efficacious interventions available: alcohol consumption, family conflict and self-compassion. The first paper presents the differential impact of screening positive for alcohol use disorder and daily drinking patterns on risk of neglect, physical and psychological abuse. The second paper examines the role of family conflict on a caregiver's use of abusive and neglectful behaviors, including how risk varies by the relationship type (e.g., adult child caregiver vs spouse). The third paper discusses the protective effect of self-compassion on daily risk of abuse and neglect, including the buffering effect it has on daily stress appraisal. The discussant will highlight implications and future directions for intervention research, including how the field may shift to a personalized approach to intervention delivery to match the right intervention to the right person.

Abstract citation ID: igad104.2017

### DYNAMIC IMPACTS OF FAMILY CONFLICT ON ELDER ABUSE AND NEGLECT AMONG ADRD CAREGIVERS

Wesley Browning[1], Jessica Hernandez Chilatra[2], Mustafa Yildiz[3], Vicki Winstead[3], and Carolyn Pickering[3], *1. The University of Texas Health Science Center - Houston, Houston, Texas, United States*, *2. The University of Alabama at Birmingham, Birmingham, Alabama, United States*, *3. University of Alabama at Birmingham, Birmingham, Alabama, United States*

Conflict with other family members impacts dementia caregivers' well-being and stress-appraisals and may also influence their use of elder abuse and neglect (EAN). To test this, we utilized latent class analysis to classify spouse, adult child, grandchild, and nontraditional (step- and in-laws) caregivers (N = 453) based on family conflict levels at baseline. Caregivers were classified into four groups: "low," "moderate," "high," and "EAN accusations." Spouses were significantly more likely to be in the "low" class compared to other classes. Nontraditional caregivers were more likely to be in the "EAN accusations" class versus the "moderate" class ($\beta$ = 1.73, SE = .70, p < .05). Belonging to the EAN accusations class predicted EAN at baseline (t = 3.728, p < .001). Next, dynamic structural equation modeling was used to test whether stress from family conflict on a given day predicts use of EAN behaviors. Family conflict stress predicted EAN on the same day ($\beta$ = 0.05, 95% CI = 0.03-0.07), but not the subsequent day (CL= -0.01, 95% CI = 0.03-0.02). Similar models were conducted for each relationship type. The effect of family conflict stress on EAN was not significant for spouse and grandchildren but remained significant for adult child and non-traditional caregivers. Findings suggest interventions related to conflict management and communication among caregiving families may be beneficial new intervention targets for reducing EAN in ADRD. Addressing family conflict may be most important when the caregiver is an adult child or nontraditional relationship.

Abstract citation ID: igad104.2018

### ALCOHOL CONSUMPTION AND ABUSIVE/NEGLECTFUL BEHAVIORS AMONG FAMILY CAREGIVERS OF PEOPLE LIVING WITH DEMENTIA

Jessica Hernandez Chilatra[1], Wesley Browning[2], Mustafa Yildiz[3], Vicki Winstead[3], and Carolyn Pickering[3], *1. The University of Alabama at Birmingham, Birmingham, Alabama, United States*, *2. The University of Texas Health Science Center - Houston, Houston, Texas, United States*, *3. University of Alabama at Birmingham, Birmingham, Alabama, United States*

Despite its suspected role in elder abuse and neglect (EAN), little is known about alcohol use disorders (AUD) among family caregivers of people with Alzheimer's disease and related dementia (ADRD). As part of a micro-longitudinal study in which caregivers completed 21 daily surveys about their caregiving experiences, we examined the effects of daily drinking behaviors and screening positive for hazardous drinking (based on the Alcohol Use Disorder Identification Test (AUDIT-C)) on the daily risk of EAN. A generalized linear mixed model with days (n = 9513) nested within caregivers (N=453) was used to evaluate the relationships between caregivers' alcohol consumption patterns and the odds of using an abusive and/or neglectful behavior. Caregivers' alcohol consumption on a given day increases the odds of using physical abuse (OR=2.19; 95% CI=1.12–4.28; p=.022), psychological abuse (OR=1.40; 95% CI=1.01–1.94; p=.041) and neglect (OR=1.48; 95% CI=1.17–1.86; p=.001) that same day. Screening positive for AUD significantly increases the odds of a caregiver's use of neglect on a given day (OR=2.43, 95% CI=1.52–3.88, p=<.001), but not physical or psychological abuse. There were no interaction effects between AUD and daily drinking, meaning AUD increases the risk of neglect independent of daily drinking behavior. Interventions to manage AUD may reduce the risk of neglect among ADRD caregivers experiencing hazardous drinking. Interventions focused on daily drinking behaviors are needed to reduce the general risk of EAN among ADRD caregivers. Findings show that subpopulations exist within ADRD caregivers who engage in EAN behaviors that would benefit from different targeted interventions.

Abstract citation ID: igad104.2019

### SELF-COMPASSION, DAILY STRESS APPRAISALS, AND ODDS OF ELDER ABUSE AND NEGLECT IN DEMENTIA FAMILY CAREGIVING

Carolyn Pickering[1], Mustafa Yildiz[1], and Maria Yefimova[2], *1. University of Alabama at Birmingham, Birmingham, Alabama, United States*, *2. UCSF Center for Nursing Excellence & Innovation, San Franscico, California, United States*

Stress related to behavioral symptoms is often found to be a risk factor for elder abuse and neglect in dementia family caregiving. The stress-process model suggests that stressful events alone do not determine caregiving outcomes, rather, they are impacted by personal factors which influence the response to stress. Self-compassion represents how one responds to themselves in moments of distress, with compassionate self-responding consisting of treating oneself with self-kindness (vs. judgement), feelings of common humanity (vs. isolation), and mindful awareness (vs. overidentification). Self-compassion is a modifiable intervention target with efficacious interventions available. We hypothesize that self-compassion can buffer the effects of daily stress on a caregiver's likelihood of using abusive and neglectful behaviors. To test this hypothesis, a generalized linear mixed model with days (n=9513) nested in dementia family caregivers (N=453) was used. Self-compassion was measured at baseline with the Self-Compassion Scale – Short Form, and daily stress appraisal of behavioral symptoms and occurrences of elder abuse and neglect were measured on 21 sequential daily diary surveys. Having higher self-compassion reduced the likelihood of a caregiver using abusive and neglectful behaviors on a given day by 40% (p=0.001, CI 0.45-0.80). Daily stress appraisal of behavioral symptoms significantly increased the odds of abuse and neglect (OR 1.05, CI 1.02-1.09, p=0.002), while the interaction term showed self-compassion significantly lowered the effect of daily stress (OR 1.01, CI 1-1.02, p=0.029). Given the large effect size, self-compassion should be a priority area for future interventions to reduce elder abuse and neglect in dementia family caregiving.

### SESSION 5145 (SYMPOSIUM)

Abstract citation ID: igad104.2020

### BUILDING BRIDGES AND CATALYZING RESEARCH FOR EVIDENCE-BASED INFECTION CONTROL: PROJECT ECHO FOR NURSING HOMES

Chair: Liza Behrens Co-Chair: Emily Heilbrunn Discussant: Marie Boltz

Nursing homes were ill-equipped for the pandemic; though facilities are required to have infection control staff, only 3% have taken a basic infection control course. Significant research has focused on infection control in the acute care setting; however, little is known about the implementation of infection control practices in long-term care communities. In this symposium, we will describe findings from the Project ECHO for Nursing Homes- a national, Patient-Centered Outcomes Research Institute (PCORI) funded study. First, Dr. Kraschnewski will present over all study findings from the stratified, cluster randomized controlled trial of 136 nursing homes that participated in up to 33 weeks of best-practice training to improve COVID-19 infection control practices. Next, Dr. Calo will present on implementation outcomes at 6-months including readiness for change and the implementation climate in the NH. Next, Ms. Heilbrunn will discuss the impacts of implementing evidence-based infection control practices through COVID-19 on NH residents' well-being. Finally, Dr. Behrens will describe NH leadership perspectives (n=14) on preparing and maintaining care quality during times of diminishing resources. Data presented in this symposium can guide NHs in strategies to implement evidence-based infection control in their homes and informs researchers who are looking to implement similar implementation studies in the NH setting.

Abstract citation ID: igad104.2021

### FROM CRISIS TO CONTROL THROUGH COVID-19: NURSING HOME LEADERSHIP PERSPECTIVES ON DELIVERING QUALITY CARE

Liza Behrens[1], Emily Heilbrunn[2], Laruen Van Scoy[3], Ashmita Grewal[3], Nicole Osevala[3], Janice Whitaker[4], William Calo[2], and Jennifer Kraschnewski[2], *1. Pennsylvania State University, State College, Pennsylvania, United States*, *2. Penn State College of Medicine, Hershey, Pennsylvania, United States*, *3. Pennsylvania State University, Hershey, Pennsylvania, United States*, *4. Nese College of Nursing, Penn State University, State College, Pennsylvania, United States*

A year after the US Government announced that the COVID-19 pandemic has transitioned to an endemic phase, nursing home (NH) leaders are still challenged to deliver quality care. This study aimed to describe NH leadership perspectives on preparing and maintaining quality care during times of diminishing resources as experienced through the COVID-19 pandemic to gain a perspective on how best to support NHs moving forward. We used convergent mixed methods to explore experiences of NH leaders (N=15) participating in the Project ECHO (Extension for Community Health Outcomes) study. Data were collected through publicly reported survey data and individual interviews conducted at 12-month follow-up. Quantitative data were analyzed using descriptive statistics. Qualitative data were analyzed using verbatim transcripts and inductive thematic coding subsequently organized by a guiding framework. Convergent analysis indicates that participating NHs (N=136) align with national trends in infection rates of residents, staff, access to resources such as personal protective equipment and testing supplies. Leadership from a sub-sample of these homes (N=14; 43% rural; 71% not-for-profit) perceived their NH to be in varied states of operations needed so support quality infection control at the transition to endemic COVID-19 phase. Leadership reported continued challenges in addressing resident & staff vaccinations, securing testing supplies, obtaining financial resources to maintain acceptable levels of personal protective equipment, and isolation practices. Leadership valued providing person-centered care even in times of crisis. These results indicate socio-cultural reasons behind why NHs continue to struggle delivering quality care post-pandemic and offers areas to focus support efforts.

Abstract citation ID: igad104.2022

### WELL-BEING IN NURSING HOME RESIDENTS THROUGH THE COVID-19 PANDEMIC

Emily Heilbrunn[1], Liza Behrens[2], Laura Felix[1], Erik Lehman[1], Nicole Osevala[1], Janice Whitaker[2], William Calo[1], and Jennifer Kraschnewski[1], *1. Penn State College of Medicine, Hershey, Pennsylvania, United States*, *2. Penn State Ross and Carol Nese College of Nursing, State College, Pennsylvania, United States*

The SARS-CoV-2 (COVID-19) pandemic has disproportionately impacted residents in nursing homes. As a result of the vulnerable nature of congregate living, and to reduce resident morbidity and mortality, the Centers for Medicare and Medicaid Services (CMS) published Quality, Safety, and Oversight (QSO) guidance. Unfortunately, these restrictions may have had unintended negative consequences on the well-being of residents. Significant evidence illustrates that social isolation and loneliness have serious adverse health implications for older adults. We used GEE ordinal logistic regression modeling to examine the trends of depressive symptoms in nursing homes through the COVID-19 pandemic using the CMS Minimum Data Set (MDS). The PHQ-9 was leveraged to measure depressive symptoms, which was then transformed into a categorical variable (no depression = scores 0-4, mild depression = scores 5-9, moderate-severe depression = 10-27). We compared this with the 4-week incidence of COVID-19, transformed into a categorical variable (0 cases, 1-10 cases, 11-40 cases, >40 cases). The odds of depressive symptoms were elevated when comparing some cases to none (1-10 cases vs. 0 cases, OR 1.33 (1.31, 1.36), p-value < 0.001), 11-40 cases vs. 0 cases, OR 1.16 (1.14, 1.18), p-value < 0.001, >40 cases vs. 0 cases, OR 1.19 (1.18, 1.21), p-value < 0.001). While it is important to acknowledge that isolation-based practices are an effective means to reduce disease transmission, it is vital to recognize that they are in conflict with many quality-of-life goals. With this being said, nursing homes worked dutifully to protect residents' health and well-being.

Abstract citation ID: igad104.2023

**LEVERAGING PROJECT ECHO FOR EVIDENCE-BASED INFECTION CONTROL IN NURSING HOMES: A RANDOMIZED CONTROLLED TRIAL**

Jennifer Kraschnewski[1], Lan Kong[1], Erik Lehman[1], Emily Heilbrunn[1], Liza Behrens[2], and William Calo[1], *1. Penn State College of Medicine, Hershey, Pennsylvania, United States*, *2. Penn State Ross and Carol Nese College of Nursing, State College, Pennsylvania, United States*

Nursing homes were ill-equipped for the pandemic; though facilities are required to have infection control staff, only 3% have taken a basic infection control course. Significant research has focused on infection control in the acute care setting; however, little is known about the implementation of practices and effective interventions. We proposed an intervention utilizing Project ECHO to connect Penn State University experts with remote nursing home staff and administrators to support evidence-based infection control guideline implementation. This study sought to answer the critical research question of how evidence-based infection control guidelines can be implemented effectively in nursing homes. A stratified cluster randomized design was used to randomize nursing homes from the Northeast and Midwest to either: 1) AHRQ-funded COVID-19 ECHO that included 16 weekly tele-mentoring sessions addressing COVID-19 guidelines and best practices plus 9 optional, weekly 60-minute office hour sessions, or 2) AHRQ-funded COVID-19 ECHO+, which included 16 weekly tele-mentoring sessions addressing COVID-19 guidelines and best practices, plus 17 weekly 60-minute sessions with a focus on stakeholder-identified needs and CDC infection control training. A total of 136 nursing homes were recruited. There were no statistically significant differences in COVID-19 infection rate, hospitalization, deaths, or flu-like illness. A multipronged approach to improving infection control and emergency preparedness in nursing homes is important. The ECHO model has significant strengths when compared to traditional training, as it allows for remote learning delivered by a multidisciplinary team of experts and utilizes case discussions that match the context and capacity of nursing homes.

Abstract citation ID: igad104.2024

**ASSESSING THE IMPLEMENTATION OF COVID-19 INFECTION CONTROL BEST PRACTICES IN NURSING HOMES**

Emily Heilbrunn[1], Gail D'Souza[1], Erica Francis[1], Jennifer Kraschnewski[1], Liza Behrens[2], and William Calo[1], *1. Penn State College of Medicine, Hershey, Pennsylvania, United States*, *2. Penn State Ross and Carol Nese College of Nursing, State College, Pennsylvania, United States*

The COVID-19 pandemic has claimed over a million lives in the United States, with nursing homes suffering over 158,000 deaths through 2022. In a cluster randomized controlled trial (RCT) with 136 nursing homes, we delivered best-practice training to improve COVID-19 infection control practices. We sought to assess the implementation of infection control practices in participating nursing homes using Project ECHO (Extension for Community Health Outcomes) as an implementation strategy. We delivered a 16-week infectious disease and quality improvement training via real-time, interactive videoconferencing. We surveyed nursing home administrators (NHAs) and nursing staff at baseline and 6-month follow-up. Using validated items from the Centers for Disease Control and Prevention (CDC), the surveys inquired about 80 infection control best-practice activities (yes/no) related to preparedness, visitation policies, communication, protocols, management of deceased residents, education, staffing, personal protective equipment (PPE), and hygiene practices. We also assessed readiness for change and implementation climate in nursing homes with validated scales. We assessed changes in infection control practices and implementation scale scores between baseline and 6-month follow-up. A total of 197 unique participants from 126 nursing homes responded to our surveys. We found that visitation decisions were less likely made on a case-by-case basis by the end of 6 months (91% vs.76%, p=0.025) and an increase in informational materials developed for residents and families at the appropriate reading level (88% vs. 100%, p=0.021) was observed. We also saw changes in some individual items, e.g., an increase in delegating to non-physician staff

### SESSION 5150 (SYMPOSIUM)

Abstract citation ID: igad104.2025

**EMANCIPATORY PEDAGOGY IN GERONTOLOGICAL SPACES**

Chair: Brittney Pond Co-Chair: Nicholas DiCarlo
Discussant: Brittney Pond

Awareness of the field of gerontology has been bolstered by increased training and education programs and other critical pedagogies in several disciplines in higher education. Public and philanthropic initiatives have also supported the development of scholars and researchers in this field (Gross & Eshbaugh, 2011; Merz et al., 2017; Snyder, Wesley, Lin & May, 2008; Wesley, 2005). Furthermore, research notes that incorporating experiences of LGBTQ+ older adults in gerontological spaces can help to address a lack of representation in higher education and provide critical training to support LGBTQ+ older adults (Lipinski, Wilson, Kortes-Miller & Stinchcombe, 2022; Smith, Altman, Meeks & Hinrichs, 2019). This symposium aims to discuss critical pedagogies and emancipatory gerontology in higher education through an intersectional lens. We aim to address the question, where do critical pedagogies flourish, where are they needed, and in what ways? Presenters discuss multiple aspects of this question, describing the ways in which access is a critical component of emancipatory pedagogies, including 1) how we learn and teach abolitionist gerontology in interdisciplinary spaces 2) confronting ableism and ageism in gerontological spaces as part of intergenerational pedagogical praxis and 3) highlighting the need for LGBTQ+ aging education in curricula by discussing a student-led “queering gerontology” campaign with tangible outcomes.

Abstract citation ID: igad104.2026

**CRONE PEDAGOGIES: EMANCIPATORY PRAXIS**

Nicholas DiCarlo, *UCSF / Hunter College / Routledge, New York City, New York, United States*

Confronting ableism and ageism in gerontological spaces promotes an intergenerational praxis rooted in an ethics of care. "Crone pedagogies" signal teaching and learning that raises consciousness collectively across generations by confronting the coloniality of everyday life and challenging the ways in which people are forced to cope with unjust systems (Philips, Adams, & Salter, 2015). This framework extends from the critical gerontological work and mentorship of Carroll Estes. The emancipatory potential flourishes from the restoration of historical memory (Martín-Baró, 1994) and the futurity that intergenerational work embodies. This presentation features examples from teaching practice and scholarship, within and outside the academy, while encouraging real-time experiential learning through reflective awareness about intergenerational access within the conference space.

Abstract citation ID: igad104.2027

### TEACHING ABOLITIONIST GERONTOLOGY IN INTERDISCIPLINARY LEARNING SPACES

Ian Johnson, *University of Texas- San Antonio, San Antonio, Texas, United States*

The discourse surrounding population aging has now infiltrated prisons, jails, and the 'institutional circuit' of homeless services, mental health systems, substance abuse treatment, and safety-net healthcare. Students of gerontology must be prepared to consider the experiences of people most affected by the criminal-legal apparatus. Conversely, those training to be social service and healthcare professionals within and in opposition to these systems must be prepared to. Through a conceptual review of the literature and experiential knowledge gathered through curriculum design and implementation, this presentation will outline a pedagogical framework for understanding the effects of transcarceration across the life course and in later life. This framework will be presented in conjunction with tools and strategies for guiding and enhancing interdisciplinary dialogue about the intersections of eldercare and punishment. This presentation will prompt discussion on the responsibilities of aging care providers and gerontological researchers in realizing an abolitionist future.

Abstract citation ID: igad104.2028

### IMPROVING KNOWLEDGE OF LGBTQ+ AGING THROUGH A QUEERING GERONTOLOGY CAMPAIGN

Austin Oswald[1], Lujira Cooper[2], Leo O'Driscoll[3], and Nancy Giunta[3], *1. University of Washington, Seattle, Washington, United States*, *2. Services and Advocacy for GLBT Elders, New York City, New York, United States*, *3. Silberman School of Social Work, Hunter College, CUNY, New York City, New York, United States*

The marginalization of lesbian, gay, bisexual, transgender, and queer plus (LGBTQ+) content in health and human services education is well documented and varies considerably due to instructors' attitudes as well as government and institutional policies intended to censor conversations about sexual orientation and gender identity. Lack of LGBTQ+ content in the curriculum may require creative solutions to fill the gaps in mainstream education which often goes unchallenged due to dominant heteronormative theory, literature, and teaching. Without adequate training, service providers may not have sufficient knowledge and skills for competent practice with LGBTQ+ older adults. This presentation bridges critical pedagogy with emancipatory gerontology to reflect upon a student led "queering gerontology" campaign to raise awareness about LGBTQ+ aging. Applying principles from participatory action research, the presenters analyze personal narratives in combination with program evaluation data to describe our process conceptualizing, implementing, and evaluating the campaign. We found that students are eager to learn about LGBTQ+ aging experiences and have innovative ideas to close the gaps in knowledge caused by heteronormative educational contexts. Supporting student led learning, intersectional coalition building, and intergenerational wisdom sharing were central to combatting programmatic and institutional constraints that limit LGBTQ+ content in the curriculum. The presentation concludes by highlighting future teaching and research directions that support students in their pursuit of knowledge while challenging heteronormative structures of higher education.

### SESSION 5155 (SYMPOSIUM)

Abstract citation ID: igad104.2029

### HEALTH AND FUNCTION FROM MID LIFE TO LATE ADULTHOOD: FINDINGS FROM THE STUDY OF WOMEN'S HEALTH ACROSS THE NATION (SWAN)

Chair: Carrie Karvonen-Gutierrez Discussant: Nancy Fugate Woods

Midlife (age 40-64) is characterized by physiologic, psychosocial and situational change, and a time when women experience the seminal endocrinological event of menopause. From a lifecourse perspective, understanding the relationship between midlife and older adult health is critical, particularly for identifying modifiable factors in midlife that impact later life health and functioning. Previous research has demonstrated that midlife health and functioning is dynamic and thus potentially amenable to intervention, though there remain crucial gaps in our understanding of this period. The Study of Women's Health Across the Nation (SWAN) is an observational multi-racial/ethnic (White, Black, Chinese, Japanese, Hispanic) longitudinal study of 3,302 women recruited in 1996 from 7 sites in the United States (Boston, MA; Newark, NJ; Pittsburgh, PA; Detroit-area, MI; Chicago, IL; Oakland, CA; Los Angeles, CA). Over the past 27 years, women have participated in up to 17 near-annual study visits, spanning from midlife (age 42-52 years at baseline) to later life (age 69-79 years). This symposium features new science from SWAN including: an examination of rate of change in depressive symptoms across the menopausal transition (Avis); the impact of impaired glucose levels in midlife on muscle quality and power (Leis); the importance of sleep regularity for cognitive function (Derby); and the association of urinary incontinence with disability (Dugan). The symposium provides information regarding data and biospecimen access for investigators interested in using SWAN resources (Brooks). The Discussant will consider the implications of studying midlife health and function in relation to health and well-being in late adulthood.

Abstract citation ID: igad104.2030

### RATE OF CHANGE IN DEPRESSIVE SYMPTOMS OVER THE FINAL MENSTRUAL PERIOD

Nancy Avis[1], Alicia Colvin[2], Yuqing Chen[2], Hadine Joffe[3], and Howard Kravitz[4], *1. Wake Forest University School of Medicine, Winston-Salem, North Carolina, United States*, *2. University of Pittsburgh School of Public Health, Pittsburgh, Pennsylvania, United States*, *3. Harvard Medical School at Brigham and Women's Hospital, Boston, Massachusetts, United States*, *4. Rush University Medical Center, Chicago, Illinois, United States*

Studies have shown that women are more vulnerable to elevated depressive symptoms during the menopause transition (MT) then before. This study evaluates change in depressive symptoms, as measured by the Center for Epidemiological Studies-Depression (CES-D) score, over the final menstrual period (FMP), a more precise measure of menopause timing compared to MT stages. The sample consisted of 1,783 women in the SWAN cohort with an observed FMP and at least one CES-D assessment from baseline through follow-up (1996 – 2017). Piecewise linear growth curves were fit to repeated measures of CES-D scores as a function of time from FMP (+/- 10 years). Results showed two places with a change in slope. CES-D scores decreased by 0.22 annually (95% CI: -0.41, -0.04) until 36 months prior to FMP (Period 1). From -36 months until the FMP (Period 2), there was a non-statistically significant increase in CES-D, followed by a decline (-0.19 annually, 95% CI: -0.34, -0.03) after FMP (Period 3). We examined a range of predictors (sociodemographic, health-related, psychosocial, hormonal) of change in CES-D score during each period. Upsetting life events were related to CES-D change at periods 1 and 2, social support at periods 1 and 3, and smoking at periods 2 and 3. Baseline CES-D was related at period 1, and anxiety was related to CES-D change at period 2. These results provide a more nuanced picture of change in depressive symptoms over the FMP and the variables related to depressive symptoms at different time points over the FMP.

Abstract citation ID: igad104.2031

### PERCENT TIME SPENT IN AN IMPAIRED GLUCOSE STATE AND ADVERSE MUSCLE OUTCOMES: THE SWAN STUDY

Aleda Leis[1], Sidonie Kilpatrick[2], Elsa Strotmeyer[3], and Carrie Karvonen-Gutierrez[4], *1. University of Michigan, Ann Arbor, Michigan, United States*, *2. University College London, London, England, United Kingdom*, *3. University of Pittsburgh, Pittsburgh, Pennsylvania, United States*, *4. University of Michigan, School of Public Health, Ann Arbor, Michigan, United States*

Sarcopenia is common among older adults, but associations of sarcopenia and diabetes are mixed. Beyond sarcopenia, relationships between glucose levels and muscle quality and power are understudied. We examined the association of time spent in an impaired glucose state and muscle outcomes. Muscle quality and power were assessed in 2015-2016 in 1,614 women (aged 66 + 2.7 years) without diabetes at baseline. Muscle quality was the average grip strength (measured in three trials using a Jamar dynamometer) per kilogram whole-body skeletal muscle mass (measured using bioelectrical-impedance analysis). Muscle power (Watts/kilogram body weight) was assessed using a 3-cycle timed stair climb: [(body weight (N)*stair height (m))/average ascent time (sec)]/body weight (kg). Fasting blood was collected at 11 visits; glucose ≥100mg/dL was considered impaired, with percent time spent in an impaired glucose state (%IGS) calculated as:(number visits with impaired glucose)/(number visits with glucose)*100 and categorized as ≤24%(n=1,203), 25-49%(n=184), 50-74%(n=136), ≥75%(n=91). Cross-sectional multivariable linear regression models were constructed for muscle outcomes with the independent predictor %IGS, adjusting for age, menopausal status, race/ethnicity, hormone use, and height. Compared to the lowest %IGS, all other groups had significantly lower mean muscle quality (β range: -0.091, -0.110 and -0.135, respectively) with the largest difference vs. highest %IGS (β= -0.135, 95%CI:-0.192,-0.077). Similar results were observed for muscle power (β range: -0.022, -0.024 and -0.020, respectively), though the highest %IGS was not significantly different. Results suggest that a longer time in an impaired glucose state from middle to older age may be associated with lower muscle function in older women.

Abstract citation ID: igad104.2032

### SLEEP REGULARITY, SLEEP TIMING, AND COGNITIVE PERFORMANCE AMONG WOMEN ENTERING EARLY OLDER AGE

Carol Derby[1], Michelle Hood[2], Martica Hall[3], Hadine Joffe[4], Rebecca Thurston[3], Howard Kravitz[5], and Leslie Swanson[2], *1. Albert Einstein College of Medicine, Bronx, New York, United States*, *2. University of Michigan, Ann Arbor, Michigan, United States*, *3. University of Pittsburgh, Pittsburgh, Pennsylvania, United States*, *4. Harvard Medical School at Brigham and Women's Hospital, Boston, Massachusetts, United States*, *5. Rush University Medical Center, Chicago, Illinois, United States*

Poor sleep health is a modifiable behavior linked to cognitive impairment risk. While prior work has focused primarily on sleep quantity and quality, emerging evidence suggests the importance of sleep timing and regularity. We examined whether sleep timing and regularity were associated with cognitive performance within a diverse cohort of women ages 60.5-72.2 years, and tested whether associations were independent of sleep duration and quality (wake after sleep onset). The sample included 1178 SWAN women who completed 4-7 nights of actigraphy and cognitive testing at the 15th SWAN follow-up in 2015-2016, [Mean age 65.5 (± 2.63 years); 25% Black, 12% Chinese, 6% Hispanic White, 11% Japanese, 46% Non-Hispanic White]. Sleep timing was defined as the midpoint time between sleep onset and waking, averaged across nights and was dichotomized as healthy (2am – 4am) or unhealthy (outside 2am – 4am). Sleep regularity was defined as the standard deviation of the sleep midpoint across nights. Associations of sleep regularity and timing with verbal episodic memory (East Boston Memory immediate (EBMT-I) and delayed (EBMT-D)), and processing speed (Symbol Digit Modalities) were examined using linear regression. Covariates included sleep duration and quality, age, race/ethnicity, education, economic hardship, depression, comorbidities, medications, alcohol use, vasomotor symptoms and testing language. Greater sleep irregularity was associated with worse verbal memory (EBMT-I β= -0.29; p=0.02; EBMT-D β= -0.36; p=0.007). Sleep midpoint outside the healthy range was associated with slower processing speed (β= -1.48; p=0.017). Sleep timing and regularity may be important targets for interventions aimed at preserving cognitive health.

Abstract citation ID: igad104.2033

### THE ASSOCIATION OF URINARY INCONTINENCE AND DISABILITY AMONG A DIVERSE SAMPLE OF MID-LIFE SWAN WOMEN

Sheila Dugan[1], Sybil Crawford[2], Karla Wente[3], L Waetjen[4], Carrie Karvonen-Gutierrez[5], and Sioban Harlow[6], *1. Rush University System for Health, Chicago, Illinois, United States*, 2. *U Mass Chan Medical School, Worcester, Massachusetts, United States*, 3. *Rush University, Chicago, Illinois, United States*, 4. *University of California, Davis, Sacramento, California, United States*, 5. *University of Michigan, School of Public Health, Ann Arbor, Michigan, United States*, 6. *University of Michigan, Ann Arbor, Michigan, United States*

Few studies have examined the prospective association between urinary incontinence (UI) and disability in midlife women. We assessed associations between UI type, frequency and amount with reported disablity in a racially/ethnically diverse cohort of community dwelling midlife women. Data was from longitudinal analyses of questionnaires from the multi-center, prospective cohort Study of Women's Health Across the Nation. We used multivariable ordinal logistic regression to examine if UI type, frequency and amount at the 13th follow-up were associated with the World Health Organization Disability Assessment Schedule (WHODAS) at the 15th follow-up (controlling for other factors, e.g. menopause status, body mass index, lifestyle and psychosocial factors) and disability at Follow-up 13. UI was associated with subsequent reports of disability in participants, particularly in the WHODAS domains of mobility, communication, and societal participation. Associations were strongest for mixed UI type compared to stress UI or urge UI, daily frequency of UI compared to monthly or weekly, and larger amounts of urine leakage compared to drops of leakage. UI appears had a strong association with multiple domains of disability in midlife women after two years of follow-up. It is important for clinicians to address UI earlier in symptom onset. Screening for mixed UI and UI that occurs more frequently and it larger amounts, more specifically, may yield better information regarding a patient's future disability risk.

Abstract citation ID: igad104.2034

### ACCESSING AND UTILIZING SWAN DATA AND BIOSPECIMENS FOR NEW RESEARCH QUESTIONS

Samar El Khoudary[1], Nanette Santoro[2], Sioban Harlow[3], Alicia Colvin[1], Nancy Avis[4], Carrie Karvonen-Gutierrez[2], and Maria Brooks[1], 1. *University of Pittsburgh, Pittsburgh, Pennsylvania, United States*, 2. *University of Colorado, Aurora, Colorado, United States*, 3. *University of Michigan School of Public Health, Ann Arbor, Michigan, United States*, 4. *Wake Forest University School of Medicine, Winston-Salem, North Carolina, United States*

Sharing scientific resources is a critical component of contemporary research. Data and specimens from established studies can be used for manuscripts on special topics and research projects from experienced investigators and advanced trainees. Researchers external to a study, however, may find it challenging to identify available resources and to determine the steps required to access them. This presentation will describe the SWAN datasets, specimens and documents that are housed at the NIA Aging Research Biobank. It will review the process for requesting the archived resources, provide tips for successful applications, and explain the data and/or material transfer agreements created for research projects. The SWAN longitudinal cohort study aims to understand variations in the experience of reproductive aging and its impact on midlife and early old age outcomes across multiple racial and ethnic groups. SWAN includes 3302 women aged 42-54 years from 7 sites in the United States and consists of a baseline and 17 follow-up visits. The study has repeated measures of reproductive hormones, menopausal symptoms, sleep, genitourinary and sexual function, physical and cognitive function, cardiometabolic and bone health, psychological well-being and quality of life. Serum, plasma, and urine specimens were collected at each clinic visit, and a substudy obtained daily urine for ≤50 days annually for ≤10 years. An expansive collection of SWAN biospecimens and datasets are available to external investigators and trainees. Understanding the existing resources and processes required to access them provides researchers the opportunity to use these valuable resources to advance academic research.

### SESSION 5160 (SYMPOSIUM)

Abstract citation ID: igad104.2035

### CONTEXTUAL FACTORS IN AGING AND LONG-TERM SERVICES AND SUPPORTS RESEARCH

Chair: Chanee Fabius Discussant: Katherine Ornstein

In the coming years, there will be more older Americans living in the community with extensive medical and long-term care needs increasing the need for long-term services and supports (LTSS) that assist with routine daily activities (e.g., bathing, dressing, medication management). Increasingly research studies are examining factors associated with receipt of LTSS including care preference, family availability and financial resources. Yet LTSS care experiences are likely a result of factors that extend beyond personal preference or individual factors, such as neighborhood quality. availability of home care workers, and state policy characteristics (e.g., state Medicaid generosity). Little research to date has examined how the LTSS environmental context affects the health and well-being of older adults with disabilities and their caregivers. In order to improve LTSS research we need to expand analyses to consider relevant contextual factors. This symposium will feature five presentations that provide novel insight regarding social and physical contextual factors contributing to LTSS using multiple data sources. We will describe (1) a conceptual framework of LTSS-relevant environmental domains and associations between select LTSS-relevant characteristics and older adult care experiences, (2) the relationship between neighborhood deprivation and place of death, (3) care experiences of older Medicare-Medicaid enrollees living in counties with Medicaid Managed Long-Term Services and Supports, (4) trends in paid home care employment and state public financing, and (5) associations between adverse consequences due to unmet, LTSS use, and social isolation.

Abstract citation ID: igad104.2036

### THE ROLE OF PERSON- AND FAMILY-ORIENTED LONG-TERM SERVICES AND SUPPORTS

Chanee Fabius[1], Safiyyah Okoye[2], Mingche Wu[1], Andrew Jopson[1], Julia Burdgorf[3], Jeromie Ballreich[4], Daniel Scerpella[5], and Jennifer Wolff[1], *1. Johns Hopkins University, Baltimore, Maryland, United States*, *2. College of Nursing and Health Professions, Drexel University, Philadelphia, Pennsylvania, United States*, *3. Center for Home Care Policy & Research, New York, New York, United States*, *4. Johns Hopkins Bloomberg School of public health, Baltimore, Maryland, United States*, *5. Johns Hopkins Bloomberg School of Public Health, Baltimore, Maryland, United States*

Long-term services and supports (LTSS) in the United States are defined by its patchwork and unequal nature. Data constraints obscure our understanding of inequities in LTSS care experiences and factors that may attenuate them. We advance a conceptual framework of LTSS-relevant environmental domains. We then link measures from public use datasets to person-reported care experiences from the 2015 National Health and Aging Trends Study. We assess relationships between domains of LTSS-relevant environmental characteristics and three LTSS care experiences: 1) adverse consequences due to unmet need, 2) participation restrictions in valued activities, and 3) subjective well-being. We examine 2,411 older adults with disabilities, and key subgroups by race, dementia, and Medicaid enrollment status. We find that for the roughly 10.4 million older adults with disabilities in the community, the LTSS environment is associated with care experiences, but the direction of the relationship varies by domain. Measures of neighborhood social and economic deprivation (e.g., poverty, social cohesion) are associated with experiencing adverse consequences due to unmet needs. Measures of the health care and social services delivery environment (e.g., Medicaid Home and Community-Based Service Generosity, average direct care worker wage, paid family leave) are inversely associated with experiencing participation restrictions. Select measures of the built and natural environment (e.g., housing affordability) are associated with participation restrictions and low subjective well-being. Relationships between measures of LTSS-relevant environmental characteristics and care experiences generally held in directionality but were attenuated for key subpopulations. Results reinforce the importance of strengthening environmental policies that support aging in place.

Abstract citation ID: igad104.2037

### ASSOCIATION BETWEEN NEIGHBORHOOD DEPRIVATION AND PLACE OF DEATH: A POPULATION STUDY

Harriet Mather, *Icahn School of Medicine at Mount Sinai, New York City, New York, United States*

While recent decades have witnessed an overall trend away from deaths in hospitals and facilities and towards deaths at home, socioeconomic disparities persist. Minoritized individuals while less likely to die in a nursing facility are more likely to die in hospital and (except for dementia) less likely to die at home; more educated individuals are also less likely to die in a nursing facility but conversely less likely to die in hospital and more likely to die at home. Despite growing interest in the role of neighborhood-level factors in health disparities, no studies have examined the impact of neighborhood deprivation on place of death. Using a representative sample of Medicare beneficiaries (n = 3043) drawn from the National Health and Aging Trends Study, we examined the association between census-tract level social deprivation index (quartiles) and place of death (home vs all else, hospital vs all else, nursing facility vs all else, hospice residence vs all else) in a series of multivariable regression models, adjusting for relevant sociodemographic (age, sex, race, education, income) and clinical covariates (dementia, heart disease, lung disease, cancer). Compared with residence in the least deprived quartile, residence in a neighborhood in the most deprived quartile was associated with reduced odds of home death (aOR: 0.66 (0.45, 0.99)) and increased odds of death in a nursing facility (aOR: 1.68 (1.00, 2.80)). Future studies should confirm these findings and examine mechanisms through which neighborhood deprivation impacts place of death (including availability and quality of home-based clinical care.

Abstract citation ID: igad104.2038

### CHARACTERISTICS AND CARE EXPERIENCES OF OLDER DUAL-ENROLLEES LIVING IN AREAS WITH MEDICAID MANAGED LTSS

Andrew Jopson, Chanee Fabius, and Jennifer Wolff, *Johns Hopkins University, Baltimore, Maryland, United States*

Medicaid-funded long-term services and supports (LTSS) are foundational in helping older dual-enrollees with disabilities remain in the community. Over the past decade, nearly half of U.S. states have transformed LTSS delivery through managed LTSS (MLTSS) with the goal of improving care coordination and avoiding unnecessary health care utilization. Despite this rapid growth, no prior studies describe whether characteristics and care experiences of older dual-enrollees differ between counties with and without MLTSS. We draw on the nationally representative 2015 National Health and Aging Trends Study (NHATS) linked to the census tract of participants' place of residence to examine differences in demographic characteristics, health, and care experiences of older dual-enrollees with LTSS need (n=462) by county-level presence of MLTSS. We broadly defined LTSS need as receiving assistance with one or more self-care or mobility activity or having probable dementia. More than half (56.9%) of older dual-enrollees with LTSS need lived in counties with MLTSS. Hispanic/other individuals were three times more likely to live in counties with (versus without) MLTSS presence (54.2% vs. 15.8%, $p<0.0001$) while individuals living in rural regions less likely to live in counties with MLTSS presence (8.8% vs. 31.8%, $p<0.05$). There were no differences in age, gender, self-reported health, dementia status, or number of chronic conditions of the sample population by MLTSS presence. Similarly, no differences were observed in experiencing adverse consequences from unmet care needs or social participation restrictions by MLTSS presence. These results are a foundational step toward building evidence and understanding who MLTSS programs are serving.

Abstract citation ID: igad104.2039

### THE GROWTH OF THE HOME CARE WORKFORCE FROM 2000 TO 2019 AND THE ROLE OF PUBLIC FINANCING

Karen Shen[1], Jeromie Ballreich[2], Chanee Fabius[3], Katherine Ornstein[3], and Jennifer Reckrey[4], *1. Johns Hopkins Bloomberg School of Public Health, Baltimore, Maryland, United States*, *2. Johns Hopkins Bloomberg School of public health, Baltimore, Maryland, United States*, *3. Johns Hopkins University, Baltimore, Maryland, United States*, *4. Icahn School of Medicine at Mount Sinai, New York City, New York, United States*

Between 2000 and 2019, employment in home care industries grew from under one million workers to more than 3.5 million workers in 2019. However, there is significant variation across states in this growth, with some states experiencing over 300% growth in the share of the population employed in home care, and some states experiencing growth of less than 30%. This study uses state-level data from a variety of sources to explain this variation in growth across states and to quantify the contribution of different demand- and supply-side factors to the growth in the home care workforce. On the demand side, we consider changes in public financing (Medicaid HCBS spending, Medicare home health and hospice reimbursements), population aging and chronic disease burden, and nursing home capacity. On the supply side, we examine changes in the share of the non-elderly population in a state that is foreign-born, and the share without a college education, as these populations are significantly more likely than average to work in the home care industry. We find that demand-side factors can explain almost all of the variation in growth across states. In particular, the growth of public financing in Medicaid and Medicare–which nationally increased by 222% and 140%, respectively, over this period–can explain the majority of the variation in growth across states. These results have implications for understanding differences in the size of the workforce across states, which may impact older adults and persons with disabilities' access to paid home care.

Abstract citation ID: igad104.2040

### SOCIAL ISOLATION, UNMET NEEDS, AND LONG-TERM SERVICES AND SUPPORTS IN COMMUNITY-DWELLING OLDER ADULTS

Mary Louise Pomeroy[1], Andrew Jopson[1], Jenni Reiff[1], MJ Wu[1], Katherine Ornstein[1], Thomas Cudjoe[2], and Chanee Fabius[1], *1. Johns Hopkins University, Baltimore, Maryland, United States*, *2. Johns Hopkins University School of Medicine, Baltimore, Maryland, United States*

Social isolation is associated with adverse health outcomes among older adults but can be attenuated through increased social contact and support. Long-term services and supports (LTSS) are a potential mechanism to increase social contact and provide socially isolated older adults with the help needed to perform routine activities. Using a nationally representative sample of 6,705 community-dwelling older adults from the 2015 National Health and Aging Trends Study (NHATS), we examined associations between social isolation, functional difficulty, adverse consequences due to unmet needs, and indicators of the LTSS environment (e.g., Meals on Wheels). About 22% of older adults were socially isolated. Socially isolated older adults had greater functional difficulty compared to those who were not socially isolated (45.3% vs 38.3%; p<0.0001). Socially isolated older adults were more likely to be Medicaid enrollees (17.5% vs 8.4%; p<0.0001) and use LTSS including SNAP benefits (12.7% vs 5.5%; p<0.0001), Meals on Wheels (4.1% vs 1.6%; p<0.0001), energy/gas financial assistance (7.5% vs 4.2%; p<0.0001), and transportation services for older adults or people with disabilities (4.6% vs 2.7%; p=0.001). Despite greater LTSS use, socially isolated older adults more often experienced adverse consequences due to unmet needs compared to those who were not socially isolated (12.5% vs 7.7%; p<0.0001). Findings suggest that LTSS in the form of social and senior services are critical in supporting those lacking social connection, but they may not be adequate to support aging in place in the absence of informal support.

### SESSION 5165 (SYMPOSIUM)

Abstract citation ID: igad104.2041

### COGNITION ON THE GO: UNIQUE INSIGHTS INTO BRAIN HEALTH FROM HIGH-FREQUENCY COGNITIVE APPROACHES

Chair: Zita Oravecz Discussant: Laura Germine

High-frequency ambulatory cognitive assessments offer the opportunity to examine cognitive change at multiple timescales. When deployed multiple times per day via pervasive mobile technology (e.g., smartphones), ambulatory cognitive assessments allow us to conduct semi-continuous monitoring of cognitive changes while individuals go about their daily lives. Variation in performance that occurs over these short timescales may be associated with a wide range of contextual factors (e.g., our thoughts, emotions, and behaviors) including the way we respond to challenges presented by our everyday environments. Short-term change and variation may also reveal information about overall brain health and risk for cognitive decline over longer timescales. This symposium focuses on how high-frequency, ambulatory cognitive assessments can generate new insights into cognitive aging, psychological processes, and risk for neurocognitive decline by revealing detailed patterns of cognitive change and variation over short timescales (hours to weeks). The talk by Sliwinski et al. gives an overview of high-frequency cognitive assessment approaches. The talk by Harrington et al. will show how dimensions of experienced affect may influence short-term changes in multiple domains of cognition. The talk by Hakun et al. will show how short-term cognitive variation may predict how we respond to everyday stressful experiences. The talk by Oravecz et al. will show how to extract substantively meaningful predictors of long-term cognitive change from short-term processes such as retest learning.

Abstract citation ID: igad104.2042

### OPPORTUNITIES FOR USING HIGH-FREQUENCY ASSESSMENT METHODS TO ADVANCE THE STUDY OF COGNITIVE CHANGE

Martin Sliwinski, *Pennsylvania State University, State College, Pennsylvania, United States*

Embedding mobile cognitive assessments into high frequency assessment (HFA) designs, such as ecological momentary assessments or measurement bursts, affords novel opportunities for the study of both short-term and long-term change. By collecting frequent measurements across short periods of time, it is possible to model short-term within-person trends and fluctuations in cognitive performance, and to relate those features to changes over longer time periods, clinical phenotypes, and neurodegenerative processes. In this presentation, we will review contemporary approaches for HFA of cognitive function and evaluate prior studies utilizing these approaches to examine the short-term coupling among naturally occurring variations in psychosocial variables such as social activity and stress with cognitive function. The challenges and potential for using high frequency mobile cognitive assessments to discover and characterize proximal contextual influences on cognitive functioning and performance will be discussed.

Abstract citation ID: igad104.2043

### FEATURES OF LEARNING FROM HIGH-FREQUENCY COGNITIVE ASSESSMENTS AS DIGITAL BIOMARKERS OF COGNITIVE CHANGE

Zita Oravecz[1], Joachim Vandekerckhove[2], Cuiling Wang[3], Mindy Katz[3], Jonathan Hakun[1], and Martin Sliwinski[1], *1. Pennsylvania State University, State College, Pennsylvania, United States*, *2. Cognitive Sciences, Irvine, California, United States*, *3. Albert Einstein College of Medicine, Bronx, New York, United States*

In measurement burst designs, participants' cognitive performance is measured multiple times per day, for several days, forming a measurement burst. Ideally, these are repeated once or twice a year as people age. Such rich longitudinal data are generated by multiple processes (e.g., aging and learning) that operate on multiple timescales. We propose a Bayesian process model that can extract person-specific, substantively meaningful features of learning and change from such data. We show how to model retest gains across measurement bursts, as well as warm-up effects within a burst, while quantifying change across bursts in peak performance and accounting for short-term within-person variability. Individual differences in these features are also linked with psychosocial variables and biomarkers of cognitive decline in a one-step analysis. We also highlight how this approach allows for drawing intuitive inferences on cognitive decline with Bayesian posterior probabilities.

Abstract citation ID: igad104.2044

### AGE-VARIANT PATTERNS OF WITHIN-PERSON VARIATION IN SELF-REGULATORY CAPACITY REVEALED VIA AMBULATORY ASSESSMENTS

Jonathan Hakun[1], Daniel Elbich[2], Jessie Alwerdt[3], Lizbeth Benson[4], and Allison Fleming[3], *1. Pennsylvania State University, State College, Pennsylvania, United States*, *2. The Pennsylvania State University, Hershey, Pennsylvania, United States*, *3. Pennsylvania State University, University Park, Pennsylvania, United States*, *4. University of Oklahoma Health Sciences Center, Oklahoma City, Oklahoma, United States*

Stressful experiences often evoke changes in experienced negative affect. Individual differences in the degree of stressor-related reactivity may reflect the action of self-regulatory strategies that evolve over the adult lifespan. The executive hypothesis of self-regulation suggests that our ability to exert control over information processing (i.e. cognitive executive control) plays an important role in determining our effective capacity to regulate our emotions and behavior. We recently found evidence in younger adults that stressor reactivity is greater during moments when working memory capacity is lower than usual. Critically, aging is associated with normative declines in executive control and it remains unknown whether this impacts self-regulation in older adults. We'll present the results of a recent ambulatory assessment study involving a sample of older adults (N = 80; 65-87 yrs; 79% female) where we sampled ratings of negative affect, exposure to everyday stressors, and working memory capacity up to 6 times per day for a period of 14 days. Results related to frequency of stressor exposures, mean levels of negative affect, and degree of negative affect reactivity to stressor exposures in this sample were largely consistent previous findings. However, when modeling stressor reactivity as a function of momentary variation in performance on ambulatory assessments of working memory capacity, we found evidence suggesting that older adults may react more to everyday stressors during moments with higher than average working memory capacity. We interpret these findings in terms of the limits of the executive hypothesis and other well-established theories of cognitive and psychological aging.

Abstract citation ID: igad104.2045

### USING HIGH-FREQUENCY ASSESSMENTS TO EXPLORE WITHIN-PERSON COUPLING OF PSYCHOLOGICAL STATES AND COGNITION

John Felt[1], Riki Slayday[2], Stacey Scott[3], Martin Sliwinski[4], and Karra Harrington[5], *1. Center for Healthy Aging, Penn State, University Park, Pennsylvania, United States*, *2. The Pennsylvania State University, State College, Pennsylvania, United States*, *3. Stony Brook University, Stony Brook, New York, United States*, *4. Pennsylvania State University, State College, Pennsylvania, United States*, *5. The Pennsylvania State University, University Park, Pennsylvania, United Sta tes*

From the perspective of traditional assessment approaches, variation in cognitive performance due to participant factors (e.g. mood, fatigue) are treated as part of measurement error. This talk will discuss how high-frequency assessment methods, such as ecological momentary assessment (EMA), can be leveraged to capture meaningful signal from variation in cognitive performance. This approach will be used to explore fluctuations in thought valence and levels of arousal as short-term mechanisms for change in working memory and processing speed performance. Participants (n = 265, aged 25-65, 64.9% female) completed a 14-day measurement burst that included five EMAs per day. Each EMA included self-report measures of current thought valence (pleasant-unpleasant) and level of arousal (sleepy/tired-alert/active), as well as two brief cognitive tasks. We use multi-level modelling to disaggregate between- and within-person effects of thought valence and arousal, and to examine within-person coupling with performance on each of the cognitive tasks. Models also include demographic and contextual covariates (e.g. current location), as well as linear and quadratic functions of time and session number to account for time of day and retest effects. We found that at moments when individuals were experiencing more unpleasant thoughts than usual for them, they also made more errors on the working memory task. Additionally, at moments where individuals were feeling more sleepy/tired than usual for them, they performed more slowly on the processing speed task. These results will be discussed from the perspective of using models of within-person coupling to understand short-term mechanisms for cognitive change.

### SESSION 5170 (PAPER)

### ASSISTED LIVING

Abstract citation ID: igad104.2046

### ASSISTED LIVING ADMINISTRATORS' PERSPECTIVES OF HOW COVID-19 AFFECTED DIRECT CARE STAFF

Carlyn Vogel, Debra Dobbs, Lindsay Peterson, Victor Molinari, and Hongdao Meng, *University of South Florida, Tampa, Florida, United States*

**Background:** Coronavirus disease 2019 (COVID-19) presented many new challenges within assisted living communities (ALCs) and exacerbated preexisting challenges. With high quality of care in ALCs as the continuous goal to achieve, it is important to recognize challenges faced in these communities, especially those related to staffing and COVID-19. Objective: The objective was to investigate issues related to staffing and the effect of COVID-19 in ALCs, including staff absence, leadership, impacts on staff, and impacts on staff retention. **Methods:** Participants were ALC administrators (N=129) from the parent study of the Strategic Approach to Facilitating Evacuation by Health Assessment of Vulnerable Elderly in Nursing Homes II (SAFEHAVEN II) funded by the National Institute on Aging. Quantitative analysis was used to determine structure and process characteristics of ALCs during COVID-19 that impacted a process outcome. Qualitative analyses were used to gain perspectives of administrators about the impact of COVID-19 within their ALCs. **Results:** Provision of memory care services (structure characteristic), challenge with staff sent home to comply with COVID-19 precautions, challenge paying staff for time off due to COVID-19, and staff anxiety (process characteristics) were significant predictors of staff absence from work (process outcome). Three key themes interpreted from administrators' interviews were Leadership Shown Toward Staff, Impact of COVID-19 on Staff, and Impact of COVID-19 on Staff Retention. Implications: This dissertation discusses implications for research, policy, and practice to inform continual efforts to improve aspects of staffing and the quality of resident care in ALCs.

Abstract citation ID: igad104.2047

### DEVELOPMENT AND VALIDATION OF THE MINNESOTA AL REPORT CARD FOR RESIDENT QUALITY OF LIFE DOMAINS

Taylor Bucy[1], Tetyana Shippee[2], and Nidhi Kohli[2], *1. University of Minnesota School of Public Health, Minneapolis, Minnesota, United States*, *2. University of Minnesota, Minneapolis, Minnesota, United States*

Assisted living (AL) describes long-term residential care settings that emphasize person-centered services and choice in a "home-like" environment. There is considerable variability in how AL is defined, what services are provided, and how quality is measured. Minnesota serves as a unique policy environment in which to study AL. In May 2019, Minnesota funded the development of an AL Report Card to measure and report on the quality of AL from resident and family perspectives, created in partnership with the University of Minnesota. Literature review, stakeholder feedback, and pilot testing were completed in 2020-21 and the survey was implemented statewide in 2021-22. Using 2021 AL resident quality of life (QoL) survey pilot data (N=1,392), the goal of this work was to assess the reliability and psychometric properties of the survey in measuring 7 QoL domains: food, staffing, environment, engagement, autonomy, culture, and security. The 7 domains were measured using 6, 10, 3, 6, 5, 3, and 6 items, respectively. We found acceptable internal consistency for four QoL domains: food, staff, security, and engagement (McDonald's omega and Cronbach's alpha ≥0.6). Confirmatory factor analysis was performed for each individual factor using diagonally weighted least squares for ordinal data. We found high interfactor correlations between the three domains with poor internal consistency (environment, culture, and autonomy). We collapsed these three domains into one overarching domain – climate – and found satisfactory data-model fit. Findings of stability and validity of QoL report card domains have important implications for adoption outside of Minnesota.

Abstract citation ID: igad104.2048

### LEADERSHIP IN LONG-TERM CARE LEADERSHIP: THE NURSING HOME ADMINISTRATOR'S JOURNEY DURING THE COVID-19 PANDEMIC

Lydia Manning[1], and Carey Peerman[2], *1. Chaffey College, Rancho Cucamonga, California, United States*, *2. Radford University, Roanoke, Virginia, United States*

The COVID-19 pandemic has impacted long-term care (LTC) leadership and has made us all aware of the preciousness of life and the resilience it takes to keep moving forward during challenging times. This study aimed to understand how long-term care administrators have experienced changes in their leadership abilities and explore what resources they have drawn on to manage the COVID-19 pandemic. A qualitative research design included individual interviews (n = 20) conducted with LTC Administrators serving in leadership roles during the COVID-19 pandemic. The data were subjected to thematic analysis. Three categories related to long-term care administrators' experiences of leading during the COVID-19 pandemic were identified: (1) changes in leadership behavior and decision-making styles, (2) transformational experiences of leadership during COVID-19, and (c) important lessons learned during a pandemic. We found that LTC leaders have responded to the COVID-19 pandemic by adapting, navigating, and creating new ways to be leaders in an environment of rapidly changing regulations. Findings illustrate how LTC leadership has experienced nursing home and assisted living administrators during the COVID-19 pandemic. There have been innovative changes in care processes, workflows, and responses to regulation. Long-term care leaders have been forced to create new ways to lead, resulting in new leadership styles emerging.

Abstract citation ID: igad104.2049

### SOCIAL WORKERS' AND NURSES' VIEWS ON COLLABORATIVE WORK IN A SETTING WITH SKILLED NURSING CARE

Tina Kilaberia[1], Dawn Apgar[2], Deborah Padgett[1], Bei Wu[1], and Teri Kennedy[3], *1. New York University, New York City, New York, United States*, *2. Seton Hall University, South Orange, New Jersey, United States*, *3. University of Kansas Medical Center, Kansas City, Kansas, United States*

This study compares social workers' and nurses' views on collaborative work using descriptive and thematic analysis of semi-structured in-depth interviews conducted at an urban retirement and assisted living community including independent living, assisted living, and skilled nursing care in a United States Midwestern city. Social workers (N=11) and nurses (N=12) responded to questions about how they spent their time, with whom (job category) they worked most and least closely, and what constrained collaborative work. Descriptive analysis showed that social workers exercised greater collaborative awareness than nurses and viewed nurses as partners with whom they worked most closely, whereas nurses did not view social workers and non-clinical peers as such. Qualitative analysis showed that social workers were more focused on resident self-determination, advocacy, and requisite care whereas nurses' orientation was directed toward safety, tasks, and clinical outcomes. In terms of collaborative work, the devalued professional identity of social workers was a threat to collaborative work whereas nurses portrayed failures in collaborative work as stemming from individual actions and attributes. Different approaches to collaborative work between social workers and nurses can impede teamwork. Interprofessional training focused on long-term services and supports may help to reconcile disparate approaches and strengthen collaborative work between social workers and nurses.

Abstract citation ID: igad104.2050

### THE ROLE OF LEADERSHIP IN DISASTER DECISION-MAKING IN ASSISTED LIVING

Lindsay Peterson[1], Debra Dobbs[1], and David Dosa[2], *1. University of South Florida, Tampa, Florida, United States*, *2. Brown University, Providence, Rhode Island, United Sta tes*

Assisted Living Communities (ALCs) make up a growing segment in long-term care for adults with impairments. Research indicates that functional and cognitively impaired older adults who are evacuated for hurricanes are more vulnerable to harm than those who shelter in place. A high proportion of assisted living residents require help with bathing (63.6%), dressing (48.2%), and other activities of daily living. At least 40% have Alzheimer's disease or other dementias. Despite residents' vulnerabilities, we know little about ALC disaster preparedness. In this study, we conducted a secondary analysis of data from interviews with ALC administrative staff after Hurricane Irma to better understand factors influencing the decision to evacuate or shelter in place. Initially we conducted individual interviews and focus groups with stakeholders of 70 Florida-based ALCs concerning a range of preparedness and response issues. We analyzed transcripts using a deductive approach, with research team members meeting regularly to resolve differences, develop/update codes, and identify themes and subthemes. Next, we separately analyzed codes concerning the decision to evacuate or shelter in place. Three overall themes were identified: 1) response to evacuation orders; 2) differing views of how to ensure safety; and 3) characteristics of ALC buildings. Examining our results overall, an overarching theme of leadership emerged, with effective leadership being preparedness based on comprehensive knowledge of residents' needs, the nature of the threat at hand, and the ALC's strengths and vulnerabilities. Our finding show the need for training incorporating "lessons learned" concerning both evacuation and sheltering in place for disasters.

### SESSION 5175 (PAPER)

### COVID-19 AND THE AGING POPULATION

Abstract citation ID: igad104.2051

### GRIEF SYMPTOM PROFILES OF OLDER SPOUSES BEREAVED BY COVID-19

Sarah Stahl[1], Joseph Kazan[1], Robert Krafty[2], Charles Reynolds[3], Bruce Rollman[3], Stephen Smagula[4], and Marie Anne Gebara[1], *1. University of Pittsburgh, Pittsburgh, Pennsylvania, United States*, *2. Emory University, Atlanta, Georgia, United States*, *3. University of Pittsrbugh, Pittsburgh, Pennsylvania, United States*, *4. Pitt SOM, Pittsburgh, Pennsylvania, United States*

Many older adults lost their spouse or life partner to COVID-19. Despite the anticipated rise in the prevalence of prolonged grief disorder, the association between COVID-19 bereavement and symptoms of complicated (or pathological) grief in community-dwelling older adults remains unclear. This study compared six clinically meaningful complicated grief symptom profiles between older spouses who were and were not bereaved by COVID-19. Participants included 80 adults aged 60+ years (20% men, 10% Black/minority status) who lost their spouse or life partner within the previous 12 months (M time since loss=6.6 months [SD=3.4 months]) and were enrolled in a randomized controlled trial for depression prevention. Grief symptom profiles were assessed with the Inventory of Complicated Grief. At the time of enrollment, older spouses bereaved by COVID-19 (n=18) reported more shock and disbelief (M = 7.11 [SD=3.16] versus M=3.83 [SD3.30], F=13.84, p<.001) and more hallucinations (M=1.56 [SD=1.29] versus M=0.45 [SD=0.88], F=17.12, p<.001) compared to older spouses bereaved by other causes of death (n=62). The two groups were similar in terms of yearning, anger and bitterness, estrangement from others, and avoidance behaviors. In addition, older spouses bereaved by COVID-19 had over 3 and a half times the odds of screening positive for probable prolonged grief disorder (OR=3.84 (95% CI = 1.28 -11.53, p = 0.016). These symptom clusters, particularly hallucinations, map to the highest level of prolonged grief disorder severity. These findings suggest that clinical treatment for prolonged grief disorder may be warranted for surviving spouses who lost their loved one to COVID-19.

Abstract citation ID: igad104.2052

### LATENT CLASSES OF RESILIENCE IN A NATIONWIDE SAMPLE OF AMERICAN ADULTS DURING COVID-19 PANDEMIC: THE ROLE OF AGE

Joyline Chepkorir[1], Melissa Hladek[1], Jing Huang[1], Noelle Pavlovic[2], Phillip Phan[1], and Alden Gross[3], *1. Johns Hopkins University, Baltimore, Maryland, United States*, *2. Johns Hopkins School of Nursing, Baltimore, Maryland, United States*, *3. Johns Hopkins University Bloomberg School of Public Health, Baltimore, Maryland, United Sta tes*

Intra-individual coping and responses to stressors vary across people, influencing health outcomes. We aimed to identify distinct subgroups, using latent class analysis, of resilience based on coping resources and responses to COVID-19 using data from a nationally representative sample of American adults (N=3,640), including 2,507 younger (age< 65 years) and 833 older adults (age≥65 years). Data were collected in June 2020, after the first three months of COVID-19 lockdown. We identified sub-groups of resilience based on 11 self-reported coping resources and responses: pain, trauma, depressive and anxiety symptoms, alcohol, drug and food usage, coping resources, social networks, perceived cognitive deficit, and loneliness. Using conventional fit criteria (AIC, BIC, LMR-LRT, BLRT), we identified four latent classes: Low mental/physical resilience (N=187, 5.6%), Low mental resilience (N=432, 13.0%), Low social resilience (N=832, 25.0%), and High resilience (N=1888, 57%). While the first resilience class showed the poorest coping in all measures, the fourth class displayed good resilience qualities across all responses. Low mental resilience was characterized by high anxiety and depression, while the Low social resilience class was characterized by relatively poor social networks. Adults in the High Resilience class were on average 54 years old while adults in the low resilience class were considerably younger (average 33 years old, p< 0.001). Understanding patterns of resilience, especially by age strata, may help identify and support those who have an increased risk for poorer outcomes and may guide the development of individual- and population-level interventions to promote adaptive coping in the event of such a global stressor.

Abstract citation ID: igad104.2053

### LONG COVID: IT'S A BIG DEAL

Laura Mehegan[1], Rohini Khillan[1], Erwin Tan[1], Olivia Dean[2], Teresa Keenan[1], Lona Choi-Allum[1], and Jim Palmieri[1], *1. AARP, Washington, District of Columbia, United States*, *2. AARP, Oakland, California, United States*

AARP fielded two nationally representative surveys of adults aged 50 and older to explore their experiences with COVID and long COVID (lingering symptoms for ≥ 4-weeks). The October 2022 survey (N=1,795) was a re-fielding of the May 2022 survey (N=1,018). Survey results showed that 40% of participants were infected with COVID at least once and, among those, 27% experienced long COVID. Among those who experienced long COVID, 33% said it impacted their job. To explore long COVID in greater depth and to understand the financial, health, and social impacts, seven virtual focus groups were held with either long COVID patients (N=27) or partners of long COVID patients (N=20). Focus group participants reported extensive suffering due to debilitating symptoms, most notably brain fog, severe fatigue, depression, and anxiety. Partners reported caregiving duties that impacted their jobs and patients often altered their work schedule or quit their jobs completely. Social isolation was reported in all focus groups. Most participants reported adapting to the loss of income and increased medical expenses; however, the extent of financial consequences is yet to be observed. When queried on the support they needed, most patients and caregivers said they needed more reliable information, and they needed their healthcare providers to share their medical information. It was difficult for patients to understand their symptoms or to get a diagnosis or symptom relief. This research suggests the need to develop and disseminate consistent information on long COVID symptoms and remedies, and to encourage information sharing within the healthcare community.

Abstract citation ID: igad104.2054

### POSTTRAUMATIC GROWTH DURING COVID PANDEMIC AMONG OLDER ADULTS: A MIXED METHODS STUDY

Lenny Chiang-Hanisko, and Patricia Liehr, *Florida Atlantic University, Boca Raton, Florida, United States*

COVID has impacted life for the older adult population living in residential settings affecting psycho-social well-being. Risk for COVID is associated with increasing age and pre-existing health conditions. The purpose of the study was to investigate the post-traumatic growth (PTG) experience among older adults, and the impact of COVID and associated regulatory precautions, on well-being for older adults living in a continue care community (CCC). A parallel mixed-methods approach was used to explore the experience of older adults with PTG. Quantitative data were analyzed using descriptive statistics. Qualitative data were analyzed using content analysis to identify emergent themes most frequently reported from open-ended responses. Older adults (N=98) living in a single CCC completed the survey. Age ranged from 67-99 years (mean = 86) with over 90% identifying as non-Hispanic White; and female (75%). Over half (51%) reported social-psychological issues, including anxiety (32%) and depression (31%); 41% reported knowing residents who died during COVID, attributed to will to live (35%), social isolation (30%) and coexisting health conditions (75%). Qualitative analysis revealed 13 subthemes related to 5 major PTG concepts: New possibilities, relating to others, personal strength, spiritual growth and appreciation for life. This study provides a deeper understanding of how older adults residing in a CCC coped with COVID and their experiences. Results indicated that older adults experienced positive changes and PTG despite challenges. Healthcare providers should recognize these potential positive changes and growth and use them as a source for improving older adult well-being when facing pandemic challenges.

Abstract citation ID: igad104.2055

### UNMET CARE NEEDS DURING THE COVID-19 PANDEMIC

Angela Curl, and Amy Roberts, *Miami University, Oxford, Ohio, United States*

**Background:** Few studies have examined the characteristics of individuals who experienced unmet care needs during the COVID-19 pandemic (a time when more needs may have gone unmet). **Methods:** Using 2020 data from the Health and Retirement Study, this study selected a sample of respondents over the age of 50 who reported difficulty with completing activities of daily living (ADL; eating, bathing, dressing, toileting, toileting; N=685). Unmet ADL need (outcome) was defined as having difficulty performing one or more ADLs but not receiving help performing that ADL. A logistic regression analyzed predictors of unmet ADL need, using the predictors of cognitive ability (range 0-27; Crimmins et al., 2011), self-rated eyesight, self-rated hearing, education, age, gender, race, marital status, and having a child living within 10 miles. Findings: Females were less likely to report an unmet ADL need (OR=0.66, p=.03), as were married/partnered respondents (OR=.52, p<.01). In contrast, respondents with higher cognitive ability were more likely to report unmet ADL needs (OR=1.04, p=.02). The remaining predictors were not statistically significant. **Discussion:** Having difficulty with basic ADLs and lacking assistance through formal or informal caregiving can increase the risk for a serious adverse event or nursing home placement. While those with higher cognitive ability could make use of creative workarounds (e.g., assistive devices); they may also have been perceived as having less need of assistance from others. By identifying groups who are more likely to have unmet ADL needs, outreach, assessment, and intervention outreach efforts can be targeted to those at greatest risk.

### SESSION 5180 (BIOLOGICAL SCIENCES INVITED SYMPOSIUM)

Abstract citation ID: igad104.2056

### METABOLOMICS OF LONGEVITY AND LIFESPAN

Chair: Paola Sebastiani Discussant: Nalini Raghavachari

Serum metabolomics has been an important source of biomarkers of aging and longevity for years. This symposium will bring together investigators from large studies of human longevity to provide an overview of recent discoveries on serum metabolomics of aging and extreme human longevity, their connections to genetic variations, and highlight the challenges of correlating metabolomic profiles of aging in human studies and across multiple species. Dr. Sebastiani will describe results from analyses of serum metabolomics of participants enrolled in the Long Life Family Study, highlight similarities and differences between metabolomic profiles of old age and extreme old age, and some connections with genetics of extreme human longevity. Dr. Rappaport will connect specific variations of the APOE alleles to metabolomic profiles and describe a possible role of bioenergetics pathways in mediating the effect of APOE to longevity and resistance to Alzheimer's disease. Dr. Monti will expand the characterization of metabolomics of aging and extreme human longevity in a large metabolomic study of very old centenarians by using traditional statistical analyses and novel machine learning techniques. His analysis identifies rich signatures of aging and longevity that include well known metabolites and point to bile acids and several classes of steroids as important marker of longevity. Analytical innovations will be taken further by Dr. Schork who will introduce a novel approach based on distance of profiles to analyze multiple metabolites simultaneously and show the value of this approach to analyze metabolomic profiles of maximum lifespan across multiple species. This is a Geroscience Interest Group Sponsored Symposium.

Abstract citation ID: igad104.2057

### METABOLOMIC SIGNATURES OF AGE, EXTREME OLD AGE, AND LONGEVITY IN LONG LIFE FAMILY STUDY PARTICIPANTS

Paola Sebastiani[1], Zeyuan Sog[2], Michael Lustgarten[3], Thomas Perls[2], Stefano Monti[2], Stacy Andersen[4], Michaela Schwaiger-Haber[5], and Gary Patti[5], *1. Tufts Medical Center, Boston, Massachusetts, United States*, *2. Boston University, Boston, Massachusetts, United States*, *3. Tufts University, Boston, Massachusetts, United States*, *4. Boston University Chobanian & Avedisian School of Medicine, Boston, Massachusetts, United States*, *5. Washington University St Louis, St. Louis, Missouri, United States*

Serum metabolomics of aging has been a growing area of interest and several studies have identified metabolites that correlate with chronological age. The Long Life Family Study (LLFS) has generated serum metabolomics of individuals aged 30 to 110 years and several years of follow up, and offers a unique opportunity to identify (1) a robust signature of age; (2) a signature of extreme old age that differs from the age-related signature; and (3) a signature that predict survival. We analyzed 409 general metabolites and lipid species in approximately 2700 LLFS participants and used a state-of-the-art computational approach with mixed effect models and whole genome sequence data to model within family relation. The analysis identified 305 metabolites that correlate with age at 5% false discovery rate (FDR), 30 metabolites that are not age related and differ in centenarians compared to younger individuals, and 144 metabolites that predict survival at 5%FDR. The aging signature included well known markers: eg ergothioneine and tryptophan that decreased with older age, and was enriched for carbohydrates, organic acids, and several lipid classes. The extreme old age signature was enriched for glycerolipids and glycerophospholipids. The metabolomics signature of survival was enriched for nucleic and organic acids. Comparison with other studies showed strong agreement of results and also highlighted unique finding in extreme old individuals. The analysis also showed substantial variability of serum metabolomics at different ages, thus confirming the heterogeneity of molecular signatures of age and the opportunity to discover specific molecular profiles that promote heathy aging.

Abstract citation ID: igad104.2058

### BIOLOGICAL AGING AND APOE STATUS REWIRE INTER-OMIC ASSOCIATIONS RELATED TO BIOENERGETICS IN HUMANS

Noa Rappaport[1], Dylan Ellis[1], Kengo Watanabe[1], Tomasz Wilmanski[1], Leroy Hood[1], Nathan Price[2], Cory Funk[1], and Priyanka Baloni[3], *1. Institute for Systems Biology, Seattle, Washington, United States*, *2. Thorne HealthTech, New York City, New York, United States*, *3. Purdue University, West Lafayette, Indiana, United States*

Apolipoprotein E (APOE) modifies human aging, with the ε2 and ε4 alleles being among the strongest genetic predictors of longevity and Alzheimer's disease, respectively. However, the mechanisms of APOE's impact on aging and cognition remain largely uncharacterized. In this study, we analyzed interomic context-dependent association patterns across APOE genotype, sex, and health in an undiagnosed cohort of 1950 individuals. We hypothesized that APOE genotypes would show variation in energy metabolites tied to previously-validated metrics of 'biological aging', a modifiable health metric based on blood biomarkers. Our analysis identified top APOE-associated metabolites as diacylglycerols, including oleoyl- and linoleoyl-arachidonoyl-glycerols, similarly increased in APOE ε2- and ε4- carriers compared to ε3-homozygotes. Male ε2-carriers and biologically-older males displayed a similar increase in associations between insulin resistance and bioenergetic metabolites including pyruvate, glucose, gluconate, and lactate, a trend which was validated in an independent cohort of TwinsUK females. These results provide an atlas of APOE allele-rewired associations and support the involvement of bioenergetic pathways in mediating APOE impact on longevity and AD risk, suggesting targets for enhancing healthspan via lifestyle-modifications or drug-repurposing.

Abstract citation ID: igad104.2059

### SERUM METABOLOMICS SIGNATURES OF EXTREME OLD AGE AND LONGEVITY

Stefano Monti[1], Paola Sebastiani[2], Zeyuan Song[1], Ziwei Huang[3], Stacy Andersen[4], and Thomas Perls[1], *1. Boston University, Boston, Massachusetts, United States*, *2. Tufts Medical Center, Boston, Massachusetts, United States*, *3. Physics Department, Boston University, Boston, Massachusetts, United States*, *4. Boston University Chobanian & Avedisian School of Medicine, Boston, Massachusetts, United States*

A total of 1,495 chemicals including 1,213 compounds of known identity and 282 compounds of unknown structural identity were profiled in serum samples collected at enrollment at Metabolon, Inc. from the blood serum of 213 subjects, including centenarians (n=80), offspring (n=70), and controls (n=63), mean ages of 105, 70, and 70, respectively, enrolled in the New England Centenarian Study (NECS). We performed metabolite- and metabolite module-based regression analyses on age, differential analyses comparing centenarian to offspring and control, and Cox proportional hazard-based survival analyses. We annotated the derived signatures by enrichment analysis using metabolite sets curated by Metabolon, RefMet, and Pathbank, among others. We identified 323 (436) metabolites that change with age at an FDR-corrected q-value of 0.01 (0.05), and 298 (407) metabolites differentially abundant between centenarians, their younger offspring, and controls. Our results confirm and expand upon previous metabolomics-based studies of aging, including decreased abundance with age of Tryptophan, DHEA-S, Pregnenolone, Glutathione, and Androgen, among others, and increased abundance of Amino acids (Tyrosines), Phenylacetylglutamine, Ornithine, Urea, Kynurenine, and Creatine, among others. To distinguish metabolites associated with longevity from those associated with extreme old age, we performed age-adjusted survival analysis, and rank-based enrichment analysis identified several metabolites significantly associated with survival independent of age, including secondary and primary (C 24) bile acids, and multiple classes of steroids (androgens, pregnenolone, progestin), among others. Additional analyses are ongoing to further annotate and characterize these findings. If validated, these results could contribute to the identification of novel healthy aging therapeutics.

Abstract citation ID: igad104.2060

### DISTANCE-BASED ANALYSIS OF LONGEVITY-RELATED METABOLOMIC PROFILES

Nicholas Schork[1], Avijit Podder[2], Nivedita Bhadra[2], Janith Don[2], and Anish Raju[2], *1. The Translational Genomics Research Institute (TGen), Phoenix, Arizona, United States*, *2. TGen, Phoenix, Arizona, United States*

Longevity is a complex multifactorial trait with many interacting determinants. Identifying each determinant is complicated given that the marginal effect of any one of them may be small. Contemporary high-throughput biomedical assays, such as transcriptomics, proteomics, and metabolomics assays, generate large amounts of very appropriate data for identifying the determinants of longevity, but can be problematic from an analysis viewpoint. For example, the analytes interrogated by these assays are often correlated, include missing data, are often noisy, and typically reflect their contributions to broader underlying, yet often unknown, pathways, processes, and networks. Standard methods for analyzing such data, which focus on each individual analyte's association with a condition of interest (e.g., long life, health status, etc.) and then exploring commonalities among any associated analytes, can be complemented by multivariate analysis methods that consider the association between different groups of metabolites (e.g., defined by a pathway) and the condition of interest. Distance-based multivariate analysis methods can be used in such settings. We showcase the utility of novel distance-based techniques in the analysis of human and cross-species metabolite data and longevity-related phenotypes collected from the Longevity Consortium (LC). These methods consider the overall similarity of metabolomic profiles among individuals or species with and without, e.g., a long life or a particular age-related disease, or deviations from an optimal longevity associated profile, and have much greater power than aggregated univariate methods when certain assumptions are upheld. We find a number of multivariate patterns in the LC data that could motivate further research.

### SESSION 5185 (PAPER)

### HEALTH PROMOTION AND HEALTH CARE IN CLINICAL PRACTICE

Abstract citation ID: igad104.2061

### DEVELOPING A GERIATRIC EMERGENCY DEPARTMENT BY FOCUSING ON THE THREE PS: PEOPLE, PROCESSES, AND PLACE

John Schumacher[1], and Don Melady[2], *1. University of Maryland, Baltimore County, Baltimore, Maryland, United States*, *2. University of Toronto, Toronto, Ontario, Canada*

Older patients' emergency department (ED) visits in the U.S. rose a surprising 30% during the period 2015-2019 to about 28 million visits annually. Many EDs are now examining emerging geriatric emergency department (GED) models of care to address the care needs of this population. This paper provides a practical overview of how to develop a GED and introduces the 3 Ps model focusing attention on the ED's People, Processes, and the Place thereby providing a clear framework for practical GED development. Key resources facilitating GED development includes: the national GED Collaborative (GEDC) organization, the nationally endorsed GED Guidelines, and resources associated with the Geriatric Emergency Department Accreditation process. Core clinical elements needed to create a GED's operations are found in its care processes including: 1) General approaches to the patient; 2) Screening for high-risk conditions; 3) Enhanced assessment for older patients; 4) potential workflow alterations; and 5) Attention to transitions into and out of the ED. The paper concludes by provides additional practical guidance to EDs seeking to enhance the ED experience of older people and improve the quality of their outcomes.

Abstract citation ID: igad104.2062

### EFFECTS OF MINDFULNESS ON COGNITION IN ADULTS WITH MILD COGNITIVE IMPAIRMENT: A META-ANALYSIS

Rose Lin[1], Jingjing Su[2], and Kathi Heffner[3], *1. University of Rochester, Rochester, New York, United States*, *2. Hong Kong Polytechnic University, Hong Kong, Hong Kong*, *3. University of Rochester Medical Center, Rochester, New York, United States*

Mindfulness, or present-moment awareness, has been linked to greater psychological well-being and cognitive health in older adults. Recent studies on mindfulness-based intervention further explored its cognitive benefits in adults at the preclinical stage of dementia, yet showing mixed results. Therefore, this meta-analysis synthesizes the effects of mindfulness-based intervention on cognitive function for adults with mild cognitive impairment (MCI). Literature search was conducted in MEDLINE, EMBASE, PubMed, CINAHL, Web of Science, and Scopus. Randomized controlled trials that examined the effects of a mindfulness-based intervention on cognitive function in adults with MCI were included. Study effects were calculated by standard mean difference (SMD) and its 95% confidence interval using random effects model. Meta-regression was conducted to test the effects of methodological quality and intervention characteristics (qualification of interveners, types of mindfulness approach and control treatment) on trial effectiveness. Twelve trials involving 530 participants (female=64.26%, mean age=71.21 years) were included. Half of the reviewed studies were pilot trials with a mean attrition rate of 18.7%. Pooled evidence suggested that mindfulness-based intervention exerts a small-to-moderate effect on global cognition (SMD=0.40;95%CI[0.14,0.66]), visuospatial ability (SMD=0.25;95%CI[0.03,0.48]) and long-term memory (SMD=0.21; 95%CI[0.01,0.41]). No significant effect was detected for short-term memory, executive function, and attention. Meta-regression showed that none of the covariates moderate the treatment effect. This meta-analysis suggested that mindfulness-based intervention might improve cognitive function and its more specific domains in adults with MCI. More research with larger sample is warranted to confirm the effectiveness and examine the underlying mechanism to explain the improved outcomes.

Abstract citation ID: igad104.2063

### EVALUATE THE IMPACT OF INTERDISCIPLINARY EDUCATION AND TELEHEALTH PRIMARY CARE: QUALITY AND EFFICIENCY

Ji Yoo[1], and Jay Shen[2], *1. Kirk Kerkorian School of Medicine at UNLV, Las Vegas, Nevada, United States*, *2. School of Public Health, UNLV, Las Vegas, Nevada, United States*

In a provider shortage area, an Interprofessional Education and Telehealth Primary Care (IPETC) for geriatrics workforce was developed in 2020. To analyze the impact of IPETC on quality and efficiency of care, we measured quality outcomes defined by the CMS defined quality measures - dementia caregiver education/referral and advance care plan, and efficiency outcomes by estimating healthcare cost savings from reducing hospitalization. Two-hundred forty-four community-dwelling adults aged 60 to 97 with mild to moderate dementia were selected in an urban safety-net primary care clinic. Propensity - demographics and comorbidity was matched. Main outcomes were (A) number of hospitalization and 90-day rehospitalization rate, hospitalization-related healthcare cost estimates from the State Inpatient Dataset using the ICD-10 codes of principal diagnosis and hospital length of stay between January-December 2021. One-hundred twenty-two patients were cared by primary care providers who received the IPETC;122 patients were by those who did not have the IPETC; (2) CMS quality measures were compared between 2019 (baseline) and 2021 of patients cared by providers with IPETC. Number of hospitalization with IPETC was fewer than those without IPETC (0.72 vs. 1.38, $p < .01$). Number of 90-day rehospitalization with IPETC was fewer than those without IPETC (0.14 vs. 0.30, $p < .01$). An average cost-saving of $20,289 per patient was observed among those with IPETC than those without IPETC ($p < .001$). CMS quality measures improved from 15.6% to 51.0% in dementia caregiver education/referral; 10.2% to 24.7% in documented advance care plans.

Abstract citation ID: igad104.2064

### THE HOSPITAL TO HOME TRANSITION: EFFECTS OF LIFE CHANCES AND LIFE CHOICES ON UNPLANNED HOSPITAL READMISSIONS

Dale Yeatts, and Cynthia Cready, *University of North Texas, Denton, Texas, United States*

Roughly 18% of all patients discharged from hospitals in the United States experience an unplanned hospital readmission (UR) within 30 days which can be life-threatening to patients and extremely costly for the health care system. Medicare alone paid $900.8 billion in 2021, or 21 percent of total national hospital expenditures. A promising theory for adding to our understanding of the causes for URs is Health Lifestyle Theory (HLT). It proposes that structural factors (e.g., SES, hospital procedures) affect one's life chances and that socialization and personal experiences affect one's life choices and the two merge to produce a person's health lifestyle. The purpose of our study was to consider the ability of HLT to explain why some patients recover after hospital discharge while others experience URs. To accomplish our purpose, we interviewed 37 patients in their homes who had been discharged from a regional hospital in north Texas roughly 30 days prior to being interviewed. All the patients had been admitted for various serious illnesses. Fourteen of the patients had experienced an UR. Qualitative analyses included open and focused coding. Data from those experiencing an UR and those avoiding an UR were analyzed separately. We found the HLT to provide sound reasons for patients experiencing recovery or URs. Those who experienced an UR were found to have similar life chances caused by structural factors and similar life choices based on their socialization and personal experiences. Specific structural factors and personal experiences associated with life chances and life choices are presented.

Abstract citation ID: igad104.2065

### WHEN OLDER ADULTS GOT TRAUMATIC INJURY: EFFECTS OF CASE MANAGEMENT

Hsueh-Fen Kao[1], and Sarah Jimenez[2], *1. The University of Texas at El Paso, El Paso, Texas, United States*, *2. University of Texas at El Paso, El Paso, Texas, United States*

Case management can improve patient outcomes from the acute to rehabilitation phase after traumatic injury. However, a lack of evidence on effects of case management in older patients with traumatic injury makes it difficult to translate research findings into practice. The study was to examine the effects of a case management intervention on illness perception, coping strategies used, and quality of life in older patients with traumatic injury across a 9-month span after hospital discharge. A 4-wave longitudinal, experimental design was used in older patients with traumatic injury hospitalized at a regional hospital in Taiwan. Subjects were randomly assigned to case management (experimental) or usual care (control) group. The intervention started during hospitalization and ended with a phone follow-up two weeks post discharge. Illness perception and/or coping strategies used along with health-related quality of life were measured at baseline, 3, 6 and 9 months after discharge. Generalized estimating equations were used for analysis. Findings showed significant differences of illness perceptions at 3 and 6 months as well as coping strategies used at 6 and 9 months after discharge between the two groups. However, no significant difference on quality of life over time between the two groups was found. While case management appears to help older patients with traumatic injury decrease illness perception and better cope with their injury, it does not significantly improve their quality of life nine months after discharge. It is recommended that healthcare professionals develop a comprehensive long-term case management program for older patients with traumatic injury.

### SESSION 5190 (PAPER)

Abstract citation ID: igad104.2066

### INTERGENERATIONAL HEALTH PROMOTION PROJECTS WITH COLLEGE STUDENTS

Presenters in this session describe intergenerational projects that bring college students, older adults, and others together to foster health and well-being.

Abstract citation ID: igad104.2067

### GERIATRIC WORKFORCE ENHANCEMENT PROGRAMS AS AAA AGE-FRIENDLY INITIATIVES PARTNER

Jennifer Severance[1], Linda Edelman[2], Barbara Gordon[3], Susanna Luk-Jones[4], Jacqueline Telonidis[2], and Jennifer Morgan[5], *1. University of North Texas Health Science Center, Fort Worth, Texas, United States*, *2. University of Utah, Salt Lake City, Utah, United States*, *3. University of Louisville, Louisville, Kentucky, United States*, *4. University of North Texas Health Science Center at Fort Worth, Fort Worth, Texas, United States*, *5. Utah State University - Institute for Disability Research, Policy and Practice, Logan, Utah, United States*

The Health Resources and Services Administration funded 48 Geriatric Workforce Enhancement Programs (GWEPs) to partner with community-based organizations in addressing gaps in health care for older adults, promoting Age-Friendly ecosystems, and addressing social determinants of health. This paper discusses three GWEPs collaborative projects with Area Agencies on Aging (AAAs) that deliver community-based programs . The University of Utah's GWEP, a rural AAA, the Alzheimer's Association, and the Utah Department of Veterans and Military Affairs provided a hybrid Dementia Caregiver Conference to six counties, reaching 65 in-person attendees and 10 senior centers online. Attendees agreed the content met their educational needs, would positively influence care, and was motivation to form a caregiver coalition. The University of Louisville Trager Institute (Trager), AAA, and Kentucky Coalition for Healthy Communities provided focused regional training and sharing of community resources utilizing the Project ECHO training model for 11 AAA regions, reaching 412 home and community-based services professionals. The University of North Texas Health Science Center, AAA, and emergency management services expanded evidence-based programs in falls prevention, medication safety, and dementia caregiving, serving an additional 23 urban postal codes, rural counties, and over 1,900 individuals. These examples utilize unique community relationships and partnership strategies to identify local needs, maximize older adult and family caregiver engagement, measure outcomes, and achieve GWEP goals to educate diverse, underserved and rural communities.

Abstract citation ID: igad104.2068

### IMPLEMENTATION AND RESULTS: THE ZESTFUL LIVING PROGRAM FOR OLDER ADULTS RESIDING IN CNY ASSISTED LIVING FACILITIES

Minjung Seo, and Mary Theresa Pagan, *SUNY Oswego, Oswego, New York, United States*

Studies indicate that older adults experiencing more interactions with younger ones through health promotion programs perceive better health and life satisfaction than their counterparts. Positive attitudes toward aging and self-efficacy working with older adults are essential attributes for professionals entering gerontology fields. The aim of this study is two-fold, to examine the changes in attitude toward aging and self-efficacy to work with older adults among young adults and to investigate changes in perceived physical and mental health among older adults participating in the Zestful Living Health Promotion Program in an assisted living facility in Upstate New York. A mixed method design

(questionnaire and interview) was used on seventy-five older adults (Male: 15, Female: 49) and eighty-two college students. The five-week program theoretical framework utilized tenets from social learning theory and the behavioral change model. This evidenced-based program included exercises, memory training, stress reduction activities, and interactive games using implementation strategies such as health communication, health information, behavioral modification, and positive reinforcement. Results indicate attitudes toward aging and self-efficacy to working with older adults significantly differed before and after program implementation (P <.004, P <.0001). The potential benefits of this approach include improved perceived mental and physical health among older adults during five weeks with low resource allocation as a university supported. Sustainable programs like the Zestful Living Program provide research and outreach opportunities within assisted living facilities.

Abstract citation ID: igad104.2069

### LIGHTS! CAMERA! REPRESENTATION!: EXAMINING AN INTERGENERATIONAL FILM COURSE FOCUSED ON MEDIA REPRESENTATION

Samuel Van Vleet, *Miami University, Oxford, Ohio, United States*

This paper explores the humanities-based approach to an intergenerational course focused on providing older adults with a guided course in filmmaking. The goals for this course were to provide opportunities for intergenerational lifelong learning, insight into older adult led media representation, and intergenerational community engagement. This course was hosted through an Institute for Learning in Retirement. Throughout the course, students were taught the fundamentals of filmmaking and storytelling. The class worked together to create an original short film screenplay that focused on topics related to their perspectives as older adults. After the script was completed, older adult students were introduced to undergraduate students from a local university that assisted in the creation of their short film. The film equipment and resources were provided to the students which allowed the students to focus on working together to create an original short film. After the film was completed, it was premiered to the community which included a post-film Q&A session that allowed the students to describe their experience working on this intergenerational project. The Q&A session also focused on how important it was to the older adult students to have a role in creating media representation of an older population. This paper highlights the impact of providing opportunities for older adult representation through a creative process while also promoting opportunities for intergenerational learning related to narrative work.

Abstract citation ID: igad104.2070

### SUCCESSFUL LEARNING EXPERIENCES OF OT AND PT STUDENTS: ENGAGING OLDER ADULTS IN THE USE OF ASSISTIVE DEVICES

Tracey Zeiner[1], Jennifer Moore[2], LaVona Traywick[3], Krista Cox[4], and Robin McAtee[5], *1. Arkansas Colleges of Health Education School of Occupational Therapy, Fort Smith, Arkansas, United States*, *2. AR Colleges of Health Education, Fort Smith, Arkansas, United States*, *3. Arkansas Colleges of Health Education, Fort Smith, Arkansas, United States*, *4. A. T. Still University, Fort Smith, Arkansas, United States*, *5. University of Arkansas for Medical Sciences, Little Rock, Arkansas, United States*

The Arkansas Colleges of Health Education and the Arkansas Geriatric Education Collaborative collaborated on a project to use an authentic teaching/learning approach with occupational and physical therapy students about the use of assistive technologies (AT) through educating older adults. Twenty occupational and physical therapy students were mentored by faculty/staff to collaborate with community-dwelling older adults to research, develop, and disseminate 27 video vignettes shared via Facebook. The vignettes included demonstrations using AT for self-care, home management, community engagement, and leisure participation to help older adults live safely and independently. Through this authentic learning approach, the students learned about the use and benefits of AT, interprofessional practice, and the importance of collaborating with older adults. Viewers of the videos were encouraged to seek further information, providing yet another opportunity for students to acquire additional information about how to secure AT and engage with older adults. Focus groups of faculty & staff involved in the project were held to determine student learning themes arising from this project. Through these focus groups, faculty/staff expressed that students • conveyed AT recommendations without prompts for clients served in the ACHE on-campus clinic; • demonstrated a greater understanding of available non-medical AT; • revealed greater comfort levels working with older adults; • gained an appreciation and understanding of each other's disciplines; • attained professional development goals. It was also noted that faculty involved in the project appreciated the authentic nature of the teaching approach as opposed to more traditional teaching styles such as lectures.

### SESSION 5195 (PAPER)

### SERVICE

Abstract citation ID: igad104.2071

### HOW INTERNET USE MODERATES THE EFFECT OF PHYSICAL LIMITATIONS ON SUBJECTIVE WELL-BEING IN OLDER ADULTS

Wan Wen[1], and Shannon Mejia[2], *1. University of Illinois Urbana-Champaign, Urbana, Illinois, United States*, *2. University of Illinois Urbana-Champaign, Chanpaign, Illinois, United States*

Due to geographic barriers and limitations in mobility, older adults with functional limitations are at greater risk of social isolation and lower well-being. The Internet has the potential to help mitigate the negative effects of functional limitations by connecting older adults to health services and meaningful social networks, and thus contribute to their subjective well-being (SWB). This study examined how Internet use could increase access to health behaviors and close friendships and moderate the impact of physical limitations on older adults' SWB. Using data from 2008 to 2018 U.S. Health and Retirement Study (HRS) (n = 14,178, 76% white, 55% female), this paper used life satisfaction and emotional indicators (positive and negative affect) as measurements of SWB and adopted multilevel random effect models to conduct empirical analysis. The results showed that older adults with physical limitations experienced significant reductions in life satisfaction and positive affect and increases in negative emotions. However, the Internet mitigated the adverse emotional impacts of physical limitations by facilitating the maintenance of older adults' social networks and health behaviors. These findings support the development of actions to enhance the well-being of older adults with physical limitations and put them as the primary beneficiaries of information and communication technology.

Abstract citation ID: igad104.2072

### OPPORTUNITIES AND CONSIDERATIONS WHEN WORKING WITH AGING PET OWNERS: THE DEVELOPMENT OF GUIDES FOR PROFESSIONALS

Jessica Bibbo, Sarah Nicolay, and Justin Johnson, *Benjamin Rose Institute on Aging, Cleveland, Ohio, United States*

Pets can be an important relationship in the lives of older adults and are often a factor in older adults' behaviors and decisions. Guided by the stress process model, the aim of this study was to understand the benefits, challenges, and resources of pet ownership geriatric professionals encountered in their work. An interdisciplinary sample (N=462, 89.13% female, Mage=53.02, SDage=12.18) of geriatric professionals (i.e., people working with older adults [OA], persons with dementia [PWD], and/or caregivers [CG]) working in healthcare, social services, and private and community-based services (e.g., long-term care, housing) completed an online survey on pet ownership issues they had encountered in their work. A convergent parallel approach was used in data analysis. Quantitative data were analyzed with descriptive statistics, t-tests, and repeated measures ANOVAs. Qualitative data were analyzed using a directed content analysis to create codes and themes. The benefits of pet ownership were wellbeing (e.g., positive mental and physical health) and connections. Pet care was the most common challenge (e.g., basic care, financial), caregiver strain was unique to CGs. Other challenges were planning, safety (e.g., falls, pet behavior), and the pet being a barrier to care. The companionship directly experienced by OA and PWD was the central resource of pet ownership. The results were synthesized to develop guides for professionals on the benefits, challenges, opportunities, and considerations when working with older adult pet owners (including PWD) and another was created for professionals working with caregivers aging pet owners (living with and without dementia).

Abstract citation ID: igad104.2073

### PHYSICAL AND MENTAL HEALTH INFORMATION-SEEKING BEHAVIORS AMONG US-BASED LATINO ADULTS AGES 50–86

Melissa DuPont-Reyes[1], Alice Villatoro[2], Elizabeth Vasquez[3], and Lu Tang[4], *1. Columbia University, New York City, New York, United States*, *2. Santa Clara University, Santa Clara, California, United States*, *3. University at Albany, Rensselaer, New York, United States*, *4. Texas A&M University, College Station, Texas, United States*

Introduction: As U.S.-based Latino populations disproportionately experience chronic disease and age earlier than their non-Latino white counterparts, attentiveness to knowledge gaps in health communication is critical to address health disparities among Latino older adults. **Methods:** We conducted a cross-sectional survey among U.S.-based Latinos ages 50-86 (N=274; 2021) that measured physical and mental health information seeking across four sources—TV/radio/print-media, Internet/social-media, family/friends, and healthcare providers. Linear regression models examined patterns of personal, family, and mental health factors on each information seeking outcome. **Results:** Foreign-born Latinos had lower odds of mental health info-seeking on Internet/social media ($p < 0.05$) than U.S.-born Latinos. Compared to higher educational attainment, low education reduced odds of physical/mental health info-seeking in all media sources ($p < 0.05$). Family mental health history vs none saw 3-5 times the odds of physical/mental health info-seeking to all sources ($p < 0.01$). Additionally, moderate/severe mental health vs mild/none was associated with 2-3 times the odds of physical/mental health info-seeking among family/friends and healthcare providers ($p < 0.01$). English media use increased odds of physical health info-seeking in TV/radio/print-media and family/friends ($p < 0.05$), while Spanish/English media use increased odds of mental health info-seeking to healthcare providers ($p < 0.05$). **Conclusions:** Latino older adults report diverse yet unique patterns in health information acquisition. Those with self/familial mental illness especially seek health information more often than Latinos without a lived experience. Findings demonstrate the need to apply culturally appropriate health communication strategies to address the needs of older Latino adults, with special focus on families coping with mental illnesses.

Abstract citation ID: igad104.2074

### RISKS AND HEALTH OUTCOMES OF NEW ONSET OF UNMET NEEDS FOR HELP WITH ACTIVITIES OF DAILY LIVING

Laura Sands, Maham Khan, and Pang Du, *Virginia Tech, Blacksburg, Virginia, United States*

Twenty percent of community-living older adults who have difficulty completing mobility and self-care daily activities have unmet need for help with one or more daily activities. Prior research described correlates of existing unmet needs but did not consider which health characteristics and events increase risks for new onset of unmet needs. Data are from the 2011-2019 annual interviews of the National Health and Aging Trends Study. Onset of unmet need was calculated as having more unmet needs in the subsequent year compared to the prior year. Linear Mixed models were computed to assess which health characteristics and events increased risk for new onset of unmet need across 15,334 observations from persons who needed help with mobility tasks and 12,895 observations from persons who needed help with self-care tasks. Characteristics associated with new onset of mobility unmet needs were: age 80+, minority race, female sex, 2+ mobility limitations, 2+ chronic conditions, and dementia. Hospitalization during the yearly interval increased risk for new onset of unmet need for those with dementia (OR=3.01, 95%CI=2.62-3.44) and for those without dementia (OR=2.27, 95%CI=2.03-2.53). New onset of unmet need increased risk for nursing home or residential care placement (OR=1.63, 95%CI=1.31-2.03) and mortality (OR=2.10 95% CI =1.84-2.39) in the year following new onset of mobility unmet need. Results were similar for self-care daily activities. The findings inform the need for regularly assessing care needs, particularly after hospital discharge, with the goal of addressing new care needs to prevent new onset of unmet needs and its health consequences.

### SESSION 5200 (PAPER)

### TEACHING AND LEARNING THROUGH INTERGENERATIONAL EFFORTS

Abstract citation ID: igad104.2075

### EMERITI PROFESSORS' INTERGENERATIONAL TEAM TALK: REDUCING STUDENTS' AGING ANXIETY ADDRESSES AFU PRINCIPLES

Ramraj Gautam, Karen Devereaux Melillo, Lisa Abdallah, and Patcharee Wangwun, *University of Massachusetts Lowell, Lowell, Massachusetts, United States*

Intergenerational learning is one of the six pillars of the Age-Friendly University (AFU) institutional activity. This study was guided by the AFU principles 9 (involving university's own retired community) and 4 (promote intergenerational learning between learners of all ages) and Positive Education about Aging and Contact Experience (PEACE) Model (Levy, 2018). A team of 4-5 students (12 teams total) in an undergraduate Introduction to Gerontology course in Fall 2022 conducted two, one-hour in-depth Zoom Intergenerational Team Talk (ITT) interviews with emeriti professors. The objective was to assess the change in students' aging anxiety (measured using Bousfield and Hutchison's, 2010, 4-item Aging Anxiety Scale), expectations about aging (measured using Sarkisian et al, 2005, 12-item Expectations Regarding Aging survey), and career and academic trajectories before and after the implementation of the ITT interview. The University IRB approved study sample included 57 students, where 31completed both pre and post surveys. A paired sample t-test showed a significant reduction in students' aging anxiety [$t(29) = 2.45$, $p = 0.020$] and increased positive expectations regarding aging [$t(26) = -2.94$, $p = 0.007$], after the ITT interview activity. The results support the PEACE model that included emeriti professors as positive role models in an intergenerational contact. Emeriti were a valuable resource for lowering aging anxiety and increasing positive expectations regarding aging among university students. Engaging emeriti professors can create a more inclusive and age-friendly environment. Future studies should also explore the impact of intergenerational contacts on emeriti professors' perception of younger adults.

Abstract citation ID: igad104.2076

### ENGAGING COLLEGE STUDENTS AND LIFE-LONG LEARNERS WITH INTERGENERATIONAL JUSTICE ISSUES

Leslie Hasche, Carson De Fries, Ashley Taeckens-Seabaugh, Briana Kohlbrenner, Hannah Decker, and Asia Cutforth, *University of Denver, Denver, Colorado, United States*

Generational conflict and equity issues are often hot topics in popular media that expand across issues of politics, government budgets, environment, community-planning, health care, and employment. While increasing media attention highlights the need to reframe the way we talk about these topics to combat ageism, explicit academic exploration of the ways theories of social justice that consider impacts across generations and time are also needed. With the aim to provide an overview of an innovative course on intergenerational justice, we will showcase how different theories and educational activities are used to engage both graduate students and life-long learners. For over nine years, versions of this course have been offered in graduate programs, in intergenerational learning courses, and in life-long learning trainings and public events for older adults. The course frames these contemporary issues (i.e., politics, ecological justice, health care) by exploring the issue of justice between age groups, which is called intergenerational justice. Additionally, the contemporary issues will also show how considerations for intragenerational justice (i.e., injustices experienced within age groups), need to be considered within the debates of generational equity and conflict. Examples of learning activities and strategies to integrate intergenerational learning opportunities to combat attitudes about aging will be shared from both instructor and former student perspectives. Thus, we hope to demonstrate how intergenerational course topics may be a fruitful focus of gerontological education efforts to engage students, to disrupt generational conflict and ageist narratives, and to offer opportunities for intergenerational connections for problem-solving on contemporary issues.

Abstract citation ID: igad104.2077

### STUDENT EXPERIENCES TEACHING AN INTERPROFESSIONAL COMMUNITY-BASED HEALTH PROMOTION PROGRAM FOR OLDER ADULTS

Allexis Mahanna, Marin Livingston, Cara Chapman, and Britteny Howell, *University of Alaska Anchorage, Anchorage, Alaska, United States*

Nine (N=9) undergraduate and graduate students collaborated on an interprofessional health promotion program in Spring 2023. Funded by the National Institute on Aging, this student-led program utilized Persuasive Hope Theory with older adult participants to increase their fruit and vegetable intake and exercise patterns in 1-hour weekly sessions. At four community locations for 15 weeks, students gained skills in team-teaching, facilitating group discussions and activities, and engaging in physical activity instruction with older adult participants. At the end of the program, students completed a modified Undergraduate Research Student Self-Assessment (URSSA). As a result of participating in this project, students report understanding and familiarity with the research process, following scientific protocols, problem-solving, and working collaboratively. Students also reported their motivations for joining a research study, satisfaction with working relationships with faculty members and opportunities to present their work, and how these experiences with gerontological research have influenced their career goals. The presenters of this paper are the students themselves, who will lend personal experiences and stories to these survey results. The factors contributing to successful student engagement in this project can be applied by other researchers working in community-based contexts.

### SESSION 5205 (PAPER)

### THE FACTS ON FALLS

Abstract citation ID: igad104.2078

### SLEEP MEDICATION USE AND FALLS IN OLDER ADULTS WITH OSTEOPOROSIS

Loretta Anderson[1], Stina Ek[2], Denise Orwig[3], and Alexandra Wennberg[4], *1. University of Maryland Baltimore, Baltimore, Maryland, United States*, *2. Karolinska Institutet, Solna, Stockholms Lan, Sweden*, *3. University of Maryland School of Medicine, Baltimore, Maryland, United States*, *4. Karolinska Institutet, Stockholm, Stockholms Lan, Sweden*

One third of older adults take sleep medications, but they are associated with fall risk. One in four older adults experiences a fall each year, costing the U.S. $50 billion dollars annually. Osteoporosis is linked to falls, but little research has examined sleep medication use and falls risk among older adults with osteoporosis. We examined the 2011 National Health and Aging Trends Study (NHATS). Outcomes were falls in the last month, falls in the last year, multiple falls in the last year, and fear of falling. Self-reported sleep medication use dichotomized for analysis (0= once/week or less; 1= 2 or more times/week) was the exposure of interest. Osteoporosis was self-reported (yes/no). Of 8,245 participants, 58% female, 68% white, and 20.5% had osteoporosis. Multivariable logistic regression showed, sleep medication users, compared to rare- or non-users, had higher odds of a fall in the last month (O.R. = 1.64, 95% CI: 1.10, 2.47), in the last 12 months (O.R. = 1.60, 95% CI: 1.20, 2.12), multiple falls in the last 12 months (O.R. = 1.64, 95% CI: 1.08, 2.50), and fear of falling (O.R. = 1.58, 95% CI: 1.18, 2.11). Osteoporotic sleep medication users show increased risk of falls, multiple falls per year, and fear of falling. As older adults with osteoporosis are at increased risk, reducing sleep medication use may decrease this risk, thus decreasing the need for costly medical care. This study contributes to clinicians' efforts to reduce falls in vulnerable older adults and may reduce overall burden to Medicare.

Abstract citation ID: igad104.2079

### WHAT IT MEANS TO ENGAGE IN FALL PREVENTION: QUALITATIVE ANALYSIS OF MOTIVATIONAL INTERVIEWING SESSIONS

Hiroko Kiyoshi-Teo[1], Siobhan McMahon[2], and Kathlynn Northrup-Snyder[3], *1. Oregon Health & Science University, Portland, Oregon, United States*, *2. University of Minnesota, Minneapolis, Minnesota, United States*, *3. Consultant, Ilwaco, Washington, United States*

Evidence-based strategies to decrease fall risks and rates are well established. However, little is understood about how older people engage in fall prevention strategies. Participants' statements in the motivational interviewing (MI) sessions conducted as part of Motivational Interviewing for Fall Prevention (MI-FP) study were analyzed. The goal was to describe what it means for older adults to engage in fall prevention; specifically, what and how they engage. Participants (n = 16) were purposefully selected from the MI-FP study for maximum variation in age, sex, fall risks, and assigned MI specialist. Participants were community-dwelling, ≥ 65 years old, and high fall risk for which they received fall prevention recommendations from their primary care provider. Baseline MI sessions were recorded, transcribed and analyzed using qualitative content analysis by three researchers. We first coded fall prevention strategies that participants described. Second, we analyzed the characteristics of these strategies and identified themes. Third, we examined how participants engaged in these strategies. Theoretical frameworks were not used for analysis; however, we did use the Capability, Opportunity, and Motivation Behavior model to organize findings. We found participants 1) used evidence-based and personal strategies; 2) engaged in multiple, personally meaningful strategies and engaged at intensities that worked for them; and 3) were influenced by factors such as their capability to engage in fall prevention, their motivation to engage, and access to opportunities. This study identified what matters for older people to engage in fall prevention.

### SESSION 5210 (SYMPOSIUM)

Abstract citation ID: igad104.2080

### THE STUDY OF MUSCLE, MOBILITY AND AGING (SOMMA): MITOCHONDRIAL ENERGETICS OF SKELETAL MUSCLE

Chair: Paul Coen Co-Chair: Russell Hepple Discussant: Bret Goodpaster

Aging leads to a decline in mitochondrial energetics of skeletal muscle. We hypothesize that worse mitochondrial energetics is associated with greater comorbidity burden and worse fatiguability. Furthermore, sex differences in mitochondrial decline with aging are poorly understood. Prior human muscle biopsy studies have tended to be small and did not account for confounding factors including physical activity and adiposity levels. The Study of Muscle, Mobility, and Aging (SOMMA) is an observational longitudinal cohort study of 879 older adults designed to discover the biological basis of mobility disability and to collect tissues and measurements for future studies of the biology of human aging. The multidisciplinary SOMMA team includes two clinical centers (University of Pittsburgh and Wake Forest University), a biorepository (Translational Research Institute at AdventHealth), and the San Francisco Coordinating Center (California Pacific Medical Center Research Institute). At baseline, SOMMA collected biospecimens (muscle and adipose tissue, blood, urine, fecal samples); a variety of questionnaires; physical and cognitive assessments; whole-body imaging (magnetic resonance and computed tomography); accelerometry; and cardiopulmonary exercise testing. Primary outcomes include change in walking speed and change in fitness (VO2 peak). In this symposium, we will present new findings from SOMMA baseline data that link skeletal muscle mitochondrial energetics to age-associated comorbidities, including diabetes, and performance fatiguability. We also highlight the critical role that sex differences in mitochondrial energetics plays in mobility limitation. SOMMA is supported by the National Institutes of Aging (R01AG059416).

Abstract citation ID: igad104.2081

### CARDIORESPIRATORY FITNESS AND MITOCHONDRIAL ENERGETICS IN THE OLDEST OLD: THE STUDY OF MUSCLE, MOBILITY AND AGING

Giovanna Distefano[1], John Noone[1], Sofhia Ramos[1], Philip Kramer[2], Peggy Cawthon[3], Anne Newman[4], David Marcinek[5], and Bret Goodpaster[1], *1. AdventHealth, Orlando, Florida, United States*, *2. Wake Forest University School of Medicine, Winston-Salem, North Carolina, United States*, *3. California Pacific Medical Center, Research Institute, San Francisco, California, United States*, *4. University, Pittburgh, Pennsylvania, United States*, *5. University of Washington, Seattle, Washington, United Sta tes*

Cardiorespiratory fitness (CRF) and mitochondrial energetics are known to decrease with aging, albeit with a great degree of heterogeneity. Studies reporting these declines typically include individuals younger than 85 years of age. We investigated CRF and mitochondrial energetics in a cohort of old (O: 70-84yrs.; n=698) and oldest old (OO: 85+yrs.; n=70) adults enrolled in SOMMA. Cardiorespiratory fitness (VO2peak) was measured by modified Balke treadmill protocol, and mitochondrial energetics was assessed by 31P Magnetic Resonance Spectroscopy (ATPmax) and high-resolution respirometry of myofibers (oxidative phosphorylation, OXPHOS). Bivariate associations and group differences were assessed using unadjusted and adjusted regression models with clinical site/technician, gender, race, body mass index and physical activity (steps per day) as covariates. Old and oldest old groups showed an association between VO2peak and OXPHOS (R2=0.282, p<0.001; R2=0279, p=0.0083; respectively, adjusted for site/technician), but only the old group showed associations between VO2peak and ATPmax (R2=0.201, p<0.001; R2=0.017, p=0.609; respectively, adjusted for site/technician). Group comparison adjusted for clinical site, gender, and race, showed lower VO2peak (p < 0.001), OXPHOS (p=0.026), and ATPmax (p=0.0014) in the OO compared to O group. However, no group differences in OXPHOS (p=0.08) and ATPmax (p=0.09) are observed when adjusting for body composition and physical activity, factors known to affect mitochondria. In conclusion, CRF associates with different measures of mitochondrial energetics in older adults, but only with mitochondrial respiration in oldest old. Additionally, we suggest that decreases in mitochondrial energetics observed in the oldest old are related to age, changes in body composition and physical activity.

Abstract citation ID: igad104.2082

### GENDER DIFFERENCES IN SKELETAL MUSCLE ENERGETICS EXPLAIN THE MOBILITY IMPAIRMENT DISPARITY IN SOMMA

Philip Kramer[1], Giovanna Distefano[2], Sofhia Ramos[2], Bret Goodpaster[2], Anne Newman[3], David Marcinek[4], Peggy Cawthon[5], and Anthony Molina[6], *1. Wake Forest University School of Medicine, Winston-Salem, North Carolina, United States*, *2. AdventHealth, Orlando, Florida, United States*, *3. University, Pittburgh, Pennsylvania, United States*, *4. University of Washington, Seattle, Washington, United States*, *5. California Pacific Medical Center, Research Institute, San Francisco, California, United States*, *6. University of California, San Diego, San Diego, California, United States*

An age-related decline in muscle mitochondrial energetics is known to contribute to the loss of muscle function in older adults. Women experience a higher prevalence of mobility impairment compared to men, but it is unknown whether gender-specific differences in muscle energetics underlie this disparity. In the Study of Muscle, Mobility and Aging (SOMMA), muscle energetics were characterized using in vivo 31-Phosphorus Magnetic Resonance Spectroscopy and High-Resolution Respirometry of the vastus lateralis. A Short Physical Performance Battery score ≤ 8 was used to define lower-extremity mobility impairment. In this analysis of 773 participants age 70-94, women had greater odds (OR=1.83, p=0.03) of mobility impairment compared to men, which was largely explained by significantly lower muscle energetics (Maximal OXPHOS in Women= 55.06 +/- 15.95; Men= 65.80 +/- 19.74; p<0.0001) using mediation modeling (adjusted OR=1.25, p=0.45). The mobility impairment disparity could be further explained by BMI, race, physical activity, and number of comorbidities (adjusted OR=0.98, p=0.96). However, none of these covariates, including age, fully explained gender differences in muscle mitochondrial energetics (e.g. Adjusted Maximal OXPHOS in Women= 56.5, 95%CI=54.8, 58.1; Men= 64.4, 95%CI=62.5, 66.2; p<0.0001). Notably, high BMI and low gait speed were associated with lower muscle energetics, though BMI slightly more so in women (ATPmax BMI/gender interaction, p= 0.02) than men, and age was only significantly negatively associated with muscle energetics in men (e.g. Maximal ETS capacity age/gender interaction, p=0.04). In conclusion, women had lower muscle mitochondrial energetics compared to men, which largely explained their greater odds of lower-extremity mobility impairment.

Abstract citation ID: igad104.2083

### SKELETAL MUSCLE ENERGETICS ARE ASSOCIATED WITH PERFORMANCE FATIGABILITY

Yujia (Susanna) Qiao[1], Adam Santanasto[1], Peggy Cawthon[2], Daniel Forman[1], Stephen Kritchevsky[3], Barb Nicklas[3], Anne Newman[4], and Nancy Glynn[5], *1. University of Pittsburgh, Pittsburgh, Pennsylvania, United States*, *2. California Pacific Medical Center, Research Institute, San Francisco, California, United States*, *3. Wake Forest School of Medicine, Winston Salem, North Carolina, United States*, *4. University, Pittburgh, Pennsylvania, United States*, *5. University of Pittsburgh School of Public Health, Pittsburgh, Pennsylvania, United States*

Performance fatigability manifests as insufficient energy to complete daily physical tasks and worsens with aging, exacerbating vulnerability to disability. Skeletal muscle energetics also declines with aging. Thus, we hypothesized muscle energetics may be an important contributor to performance fatigability. In the Study of Muscle, Mobility and Aging (SOMMA), participants completed a usual-paced 400m walk while wearing a wrist-worn ActiGraph, from which raw data were used to derive the Pittsburgh Performance Fatigability Index (PPFI, higher=more severe fatigability) that quantifies percent decline in the entire individual cadence-versus-time trajectory. Maximal oxidative phosphorylation

(maxOXPHOS) in skeletal muscle mitochondria was quantified in vitro using high-resolution respirometry in permeabilized fiber bundles from vastus lateralis muscle biopsies. Maximal adenosine triphosphate production (ATPmax) was assessed in vivo by 31P magnetic resonance spectroscopy. We conducted separate tobit regressions to examine associations of maxOXPHOS and ATPmax with PPFI, adjusting for technician/site, age, sex, race, height, weight, and mins/day moderate-to-vigorous physical activity measured by ActiGraph in free-living, in N=795 participants with complete PPFI scores and >1 energetics measure (70-94 yrs, 58% women). Median PPFI scores were 1.4% [IQR: 0-2.9%]. After adjustment, each SD (18.4 pmol/(s*mg)) lower maxOXPHOS was associated with 0.55% (95% CI: 0.26, 0.85) higher PPFI scores, while each SD (0.2 mM/sec) lower ATPmax was associated with 0.54% (95% CI: 0.27, 0.81) higher PPFI scores. Our results indicate that lower skeletal muscle energetics were associated with more severe performance fatigability. This suggests that therapeutics targeting muscle energetics may thereby potentially mitigate fatigability and lessen susceptibility to disability among older adults.

Abstract citation ID: igad104.2084

### DECONSTRUCTING MULTIMORBIDITY AND MUSCLE MITOCHONDRIAL RESPIRATION AND ENERGETICS IN OLDER ADULTS

Theresa Mau[1], Sofhia Ramos[2], Philip Kramer[3], Anne Newman[4], Steven Cummings[5], Russell Hepple[6], Paul Coen[7], and Peggy Cawthon[8], *1. San Francisco Coordinating Center, San Francisco, California, United States*, *2. AdventHealth, Orlando, Florida, United States*, *3. Wake Forest University School of Medicine, Winston-Salem, North Carolina, United States*, *4. University, Pittburgh, Pennsylvania, United States*, *5. California Pacific Medical Center Research Institute, San Francisco, California, United States*, *6. University of Florida, Gainesville, Florida, United States*, *7. AdventHealth Research Institute, Orlando, Florida, United States*, *8. California Pacific Medical Center, Research Institute, San Francisco, California, United States*

Lower cardiorespiratory fitness among older adults is associated with greater multimorbidity burden, including cardiovascular disease, cancer, and dementia. Those with poorer muscle mitochondrial energetics also have lower cardiorespiratory fitness. We have previously shown lower mitochondrial respiration and energetics (OXPHOS and ATPmax) have higher morbidity burden using a summary measure, but the associations among specific conditions have not been examined. We leverage data from the SOMMA cohort (N=856, 76.4yrs, 59% women, 85.5% white); participants self-reported physician diagnosis of 11 age-related conditions (i.e., cancer, cardiac arrhythmia, chronic kidney disease (CKD), chronic obstructive pulmonary disease, coronary heart disease (CHD), depression, diabetes). Mitochondrial respiration was assessed by high-resolution respirometry of vastus lateralis using permeabilized fiber bundles. 31P magnetic resonance spectroscopy of the quadriceps measured ATPmax. In adjusted logistic models with beta-coefficients reported per SD decrement in each mitochondrial measure, fatty-acid-supported Leak respiration was associated with CKD (OR: 2.3(95%CI=1.2,4.3)), fatty acid and complex I&II supported oxidative phosphorylation (OXPHOS) with depression (OR: 1.8(95%CI=1.1,2.7)). Many respirometry measures and ATPmax associated with diabetes, with maximal electron transport system (ETS) capacity having the highest odds (OR: 1.5(95%CI=1.1,2.0)). No other associations were statistically significant for conditions listed above. These results indicate that mitochondrial respiration and energetics are more impaired in depression, kidney disease, and diabetes. This raises new questions about the role of mitochondrial respiration and energetics as cause or consequences of these conditions. Overall, our analyses suggest compromised mitochondrial function may have implications for specific aging-related conditions, and global measures of morbidity burden may not reflect these distinctions.

Abstract citation ID: igad104.2085

### ASSOCIATION OF DIABETES WITH BODY COMPOSITION, CARDIORESPIRATORY FITNESS, AND MITOCHONDRIAL ENERGETICS

Sofhia Ramos[1], Giovanna Distefano[1], Philip Kramer[2], Peggy Cawthon[3], Frederico Toledo[4], Steven Cummings[5], Bret Goodpaster[1], and Paul Coen[6], *1. AdventHealth, Orlando, Florida, United States*, *2. Wake Forest University School of Medicine, Winston-Salem, North Carolina, United States*, *3. California Pacific Medical Center, Research Institute, San Francisco, California, United States*, *4. University of Pittsburgh, School of Medicine, Pittsburgh, Pennsylvania, United States*, *5. California Pacific Medical Center Research Institute, San Francisco, California, United States*, *6. AdventHealth Research Institute, Orlando, Florida, United States*

We investigated the association of diabetes with cardiorespiratory fitness and skeletal muscle mitochondrial energetics in older adults (N=876, mean age ± SD: 76.3 ± 5.0 yrs.; 59% females) from the Study of Muscle, Mobility and Aging (SOMMA). Participants were grouped by self-reported diabetes status (N=131, with diabetes). Cardiorespiratory fitness (modified Balke protocol), mitochondrial energetics (31P magnetic resonance spectroscopy [31P-MRS] and high resolution respirometry from skeletal muscle biopsy), physical activity (wearable accelerometer) and body composition (magnetic resonance imaging) were measured. Generalized linear regression models were used to calculate means adjusted by age, race, gender, BMI, and co-morbidities. Thigh fat-free muscle volume (p=0.12) and abdominal subcutaneous adipose tissue depot (p=0.15) were similar between those with and without diabetes. However, visceral (18.84%, p>0.01) and intramuscular adipose tissue (6.57%, p=0.02) were higher in older adults with diabetes. VO2peak (-4.79%, p>0.01), and mitochondria energetics measured by ATPmax (-5.10%, p=0.04), maximum complex I+II carbohydrate (maxOXPHOSCHO) (-6.67%, p=0.02) and fatty acid (maxOXPHOSFAO) (-8.70%, p=0.01) stimulated respiration were lower in those with diabetes compared to those without. Following further adjustments for both physical activity and visceral adiposity, revealed that only VO2peak (-4.65%, p>0.01) and maxOXPHOSFAO (-7.38%, p=0.02) remained significantly lower in older adults with diabetes. Overall, our data suggests that cardiorespiratory fitness and skeletal muscle mitochondria energetics are reduced in older adults with diabetes, independent of visceral adiposity and physical activity.

### SESSION 5215 (PAPER)

### SOCIAL SERVICES: POLICY, FINANCING, AND DELIVERY SYSTEMS (PAPERS)

Abstract citation ID: igad104.2086

### AGE MATTERS IN FINANCIAL STRAIN AND SUBJECTIVE HEALTH AND WELL-BEING FOR PEOPLE WITH LTSS NEEDS IN CALIFORNIA

Lei Chen, *UCSF Philip R. Lee Institute for Health Policy Study, San Francisco, California, United States*

Long-Term Services and Supports (LTSS) are a big concern for older adults and people with disabilities. It is important to reset aging and disability silos and create coordinated LTSS networks for people with disabilities of all ages, including those with cognitive impairments, difficulties performing activities of daily living (ADLs) and/or instrumental activities of daily living (IADLs). This study examined how age differentiates the associations among disability status, financial strain, and subjective health and well-being for people with LTSS needs in California. This study used the first cycle of data (2019-2020) from the California Long-Term Services and Supports (CA-LTSS) survey, merged with select data from the California Health Interview Survey (CHIS) (N = 2,030). Multivariate Regressions and Conditional Process Analysis (CPA) were applied to test the hypothesized relationships. Findings show that young and middle-aged participants with LTSS needs struggled to make financial ends meet. Middle-aged and older participants with LTSS needs reported worse self-rated health, and young participants with LTSS needs had severer psychological distress. Participants with difficulty in IADLs or more types of disability difficulties tended to have more financial strain, and these associations were significantly stronger for young participants than older participants. Similarly, participants with difficulty in IADLs or more types of disability difficulties tended to report severer psychological distress, and these associations were significantly stronger for young participants than older participants. The findings suggest the value of approaching younger people with disabilities and target policies and service programs to meet their financial and mental health needs.

Abstract citation ID: igad104.2087

### ASSOCIATION BETWEEN PROVIDER DISCONTINUITY AND QUALITY OF CARE FOR NURSING HOME RESIDENTS

Hyunkyung Yun[1], Mark Unruh[2], and Hye-Young Jung[3], *1. Brown University, Providence, Rhode Island, United States, 2. Weill Cornell Medical College, Ithaca, New York, United States, 3. Weill Cornell Medical College, New York City, New York, United States*

Discontinuity in outpatient care has been associated with lower quality care in community settings, but little is known about discontinuity of care in nursing homes (NHs). We examined discontinuity in clinician care and care quality for NH residents using Medicare fee-for-service claims for a 20% national sample of beneficiaries (January 1, 2012 to December 31, 2019). For residents with NH stays of ≥2 years, we examined changes in attributed clinicians during the first two years of their stays. Residents were attributed to the physician or advanced practitioner with a plurality of visits provided to them in the NH in a given year. Estimates were adjusted for resident, clinician, and NH characteristics. The determinant of interest reflected whether there was a change in the attributed clinician from the first to the second year. Among 280,831 residents in our sample, 37.6% (105,551) were attributed to a different clinician in their second year. On a quarterly basis, these residents had a 0.2 percentage point [pp] (95%CI, 0.2-0.3; p-value <.001) higher likelihood of an ambulatory care sensitive (ACS) hospitalization, a 0.2 pp (95% CI, 0.1-0.3; p-value <.001) higher likelihood of an ACS emergency department (ED) visit, and $609 (95% CI, $548.4-$669.5; p-value <.001) higher health care costs in their second year compared to residents without a clinician change. Compared to baseline rates, these represent relative increases of 14.2% in ACS hospitalizations, 11.5% in ACS ED visits, and 8% in costs. Discontinuity in care is associated with lower-quality care for NH residents.

Abstract citation ID: igad104.2088

### DESIGNING FOR PROGRAM SUSTAINABILITY: AN EVALUATION OF BC'S PROVINCIAL BETTER AT HOME PROGRAM

Camille Hannah, Bobbi Symes, and Kahir Lalji, *United Way British Columbia, Burnaby, British Columbia, Canada*

Better at Home programs help older adults in communities across British Columbia with non-medical tasks such as light housekeeping, yard work, transportation to appointments, grocery and meal delivery, and friendly visiting, helping them to remain at home and stay connected to their communities. Better at Home is funded by the Province of British Columbia and managed by the United Way. An evaluation of 81 Better at Home programs took place over 2022/23 focusing on the delivery, design, and sustainability of programs. A multipronged evaluation approach was utilized, including site visits; document and administrative data review; interviews with program staff, older adults, and caregivers (n=87); and a survey of program stakeholders (n=399). Over 2021/22, a total of 244,875 services were provided through Better at Home. The most common services provided were light housekeeping, friendly visiting, prepared meal delivery, and grocery services. The evaluation identified design and delivery best practices (e.g., building trust and relationships), program opportunities (e.g., leveraging communities of practice), and lessons learned (e.g., increasing awareness of burnout, capacity, and duplication). Some challenges to program efficiency were also identified (e.g., increased demand for services and complexity of client needs, staff and volunteer turnover). Six program improvements were recommended to ensure the long-term sustainability of Better at Home programs: 1) Securing multi-year funding that accounts for inflation, 2) Ensuring sufficient human resources for service delivery, 3) Offering strategic training opportunities, 4) Continuing to invest in existing programs, 5) Growing partnerships and community networks, and 6) Providing comprehensive supports to program staff.

Abstract citation ID: igad104.2089

### LOWERING THE AGE FOR MEDICARE ELIGIBILITY: WHO BENEFITS?

Rodlescia Sneed, *Wayne State University, Detroit, Michigan, United States*

Advocacy groups and government officials have recommended lowering the Medicare eligibility age to increase health equity and promote utilization of preventive services. Consequently, policy analysts have begun to evaluate the impact of such a policy change. The purpose of this study was to determine who would benefit most from reducing the Medicare eligibility age. Using data from the 2018 Health and Retirement Study, we calculated the number of individuals who would become insured if the Medicare eligibility age was reduced to 60, 55, or 51. We also examined health profiles for such individuals, comparing them to their insured counterparts. Complex survey procedures were used to generate population estimates. We estimate that 1.43 million Americans would become insured if the Medicare eligibility age were reduced to 60. Lowering the age to 55 or 51 would insure 3.45 million and 4.59 million people, respectively. Evaluating data for those ages 51-64, those currently uninsured reported less preventive health screening than their insured counterparts, including lower screening for high cholesterol, cervical cancer, and prostate cancer. They also had lower vaccination rates for influenza and shingles. Of those currently uninsured, 36.5% reported having no usual place where they received care. Hypertension prevalence was similar among insured and uninsured individuals, but the uninsured were less likely to be on antihypertensive medications. Taken together, our findings suggest that lowering the Medicare eligibility age would result in new healthcare coverage for individuals who underutilize the healthcare system, which could have important implications for health.

Abstract citation ID: igad104.2090

### THE RELATIONSHIP BETWEEN LONG-TERM CARE OMBUDSMAN PROGRAM SPENDING AND NURSING HOME OUTCOMES

Katherine Kennedy[1], Cyrus Kosar[2], and Kali Thomas[3], *1. Providence VA Medical Center, Providence, Rhode Island, United States*, *2. Brown University, Providence, Rhode Island, United States*, *3. Johns Hopkins University, Baltimore, Maryland, United States*

As part of the Older Americans Act, State Long-term Care Ombudsman Programs (LTCOP) serve older adults in nursing homes (NHs) and board and care facilities. The National Academies of Science, Engineering, and Medicine called for increased LTCOP funding to improve NH quality. We hypothesized that increases in spending on LTCOP would be associated with a lower prevalence of NH residents with low-care needs who could be cared for in the community and fewer residents taking antipsychotics. We conducted a secondary analysis using the National Ombudsman Reporting System, NH facility-level data from LTCfocUS.org for 15,592 US NHs (2011-2018), and the Area Health Resource File. Using a two-way fixed effects model with standard errors clustered at the facility-level, we examined the relationship between LTCOP spending per LTC bed at the state-level and outcomes, controlling for year, state, facility, and market characteristics. Increased LTCOP spending was associated with a lower proportion of residents in NHs with low-care needs and a lower percent of residents receiving antipsychotics ($p < .0001$). In 2011, the average share of low-care NH residents (weighted by bed size) was 13.34%, and the share of residents on antipsychotics was 25.95%. For every $25 annual change in LTCOP funds, there was a .84 percentage point decrease in the prevalence of low-care residents for the average NH, and a .80 percentage point decrease in the share of residents receiving antipsychotics. States that have increased funding for their LTCOP observe better outcomes. This documents the need to increase funding to support LTCOP.

### SESSION 5220 (PAPER)

### RESEARCH IN CHRONIC DISEASES AND CONDITIONS

Abstract citation ID: igad104.2091

### AN INTEGRATED TRANSITIONAL CARE INTERVENTION IMPROVES PATIENT-REPORTED OUTCOMES IN OLDER ADULTS WITH STROKE

Maureen Markle-Reid[1], Kathryn Fisher[1], Rebecca Ganann[1], Chris Pollard[2], and Carly Whitmore[3], *1. McMaster University, Hamilton, Ontario, Canada*, *2. Hotel Dieu Shaver Health & Rehabilitation Centre, St. Catherines, Ontario, Canada*, *3. Centre for Addiction and Mental Health, Hamilton, Ontario, Canada*

The transition between hospital and home is associated with significant challenges resulting in hospital readmissions and poor health outcomes. This study aimed to test, in real-world clinical practice, the effectiveness of a Transitional Care Stroke Intervention (TCSI) compared to usual care on patient-reported outcomes and health and social service use costs in older adults (> 55 years) with stroke and multimorbidity (> 2 chronic conditions). The TCSI was delivered virtually by an interprofessional (IP) team, and included care coordination/system navigation support, phone/video visits, monthly IP team conferences, and an online resource co-designed with patient partners to support self-management and system navigation. The primary outcome was risk of hospital readmission after six-months. Secondary outcomes included physical and mental functioning, stroke self-management, patient experience, and health and social service costs. Ninety participants were enrolled (44 intervention, 46 control); 11 (12%) participants were lost to follow-up, leaving 79 (39 intervention, 40 control). Differences favoring the intervention group were seen in physical functioning (SF-12 PCS mean difference: 5.10; 95% CI: 1.58-8.62, p=0.005), self-management (Southampton Stroke Self-Management Questionnaire mean difference: 6.00; 95% CI: 0.51—11.50, p=0.03), and patient experience (Person-Centred Coordinated Care Experiences Questionnaire mean difference: 2.64, 95% CI: 0.81, 4.47, p=0.005). No significant between-group differences were seen for baseline to six-month risk of hospital readmission or total health and social service costs. The TCSI produced greater gains in patient-reported physical health, self-management and experience compared with usual stroke care. These improvements were achieved without increasing total healthcare costs.

Abstract citation ID: igad104.2092

### ELEVATED PREVALENCE AND RISK OF KIDNEY DISEASE AMONG SEXUAL MINORITY OLDER ADULTS IN THE US

Sean Cahill[1], Meghana Chandra[2], Mollie Hertel[2], Saumya Khanna[2], Shimontini Mitra[3], Jack Mirabella[4], and Avareena Cropper[5], *1. The Fenway Institute, Boston, Massachusetts, United States*, *2. NORC at the University of Chicago, Chicago, Illinois, United States*, *3. Fenway Health, Boston, Massachusetts, United States*, *4. Centers for Medicare & Medicaid Services, Baltimore, Maryland, United States*, *5. Centers for Medicare and Medicaid Services, Woodlawn, Maryland, United States*

Pooled data from the Behavioral Risk Factor Surveillance System (2014–2019) were used for 22,114 lesbian, gay, bisexual adults and adults who said that their sexual orientation was "other" or "something else" aged 50 and over, and 748,963 heterosexual older adults. Logistic regressions were used to compare prevalence of self-reported kidney disease among older adults by sexual orientation. After adjusting for age, race and ethnicity, and BMI, GB+ men had increased odds of self-reported kidney disease (AOR 1.42; 95% CI, 1.21–1.68). LGB+ women also had higher odds of self-reported kidney disease (AOR 1.29; 95% CI, 1.09–1.52). After controlling for sociodemographic factors, health behaviors, access to healthcare, and self-reported coronary heart disease, HIV, and diabetes, older gay, bisexual and other men (GB+) men (AOR 1.3; 95% CI, 1.09-1.54) were more likely than their heterosexual counterparts to report kidney disease. LGB+ men and women also reported high incidences of known risk factors for CKD. For example, both GB+ men (OR 1.39; [95% CI], 1.26-1.54) and LGB+ women (OR 1.39; [95% CI], 1.25-1.55) were more likely to be smokers and have a higher incidence of activity limitations, adverse health outcomes, and limited access to health care, housing and employment. We did not find any statistically significant differences in kidney disease between transgender and cisgender respondents. There is an increased need for screening for CKD risk factors, for preventative education and culturally responsive and affirming care, and for interventions to address social drivers of marginalization and vulnerability in older LGBT+ adults.

Abstract citation ID: igad104.2093

### PREVALENCE OF SINGLE AND MULTIPLE CHRONIC CONDITIONS AMONG RESETTLED BHUTANESE OLDER ADULTS IN OHIO

Isha Karmacharya, Aman Shrestha, Janardan Subedi, and Saruna Ghimire, *Miami University, Oxford, Ohio, United States*

**Background:** Migrants and refugees are more prone to chronic health conditions, which can worsen with age and accumulated stressors. Bhutanese older adults with refugee backgrounds in the US have not been well-studied. This study aimed to assess the prevalence of chronic diseases (single and multiple conditions) and to identify factors associated with multimorbidity among resettled Bhutanese older adults in Ohio. Additionally, the study compared the prevalence of chronic diseases with state-level data for other racial/ethnic groups. **Methods:** Structured interview was conducted among 276 resettled Bhutanese, aged 55 years and above, in four major Ohio cities (Columbus, Akron, Cleveland, and Cincinnati), selected through snowball sampling with local Bhutanese organizations. State-level data of people, aged 55 years and above in Ohio, from the 2021 Behavioral Risk Factor Surveillance System for Non-Hispanic White, Non-Hispanic Black, Hispanic, and Asian populations were used for comparison. Binary logistic regression was used to identify factors associated with multimorbidity. **Results:** Only 14.1% of participants were free of chronic diseases, while 62.3% had multimorbidity. Depression (31.8%), diabetes (42.8%), and respiratory diseases (26.4%) in older Bhutanese were considerably higher than in other racial/ethnic groups. Hypertension prevalence (63.0%) was higher than all other race/ethnic groups, except Non-Hispanic Black individuals (72.3%). Poor self-reported health and the presence of depressive symptoms had higher odds of having multimorbidity. Study Implications: This study underscores valuable insight into the disease burden among resettled Bhutanese older adults and can inform stakeholders to develop targeted public health programs and practices to meet the needs of minority populations.

Abstract citation ID: igad104.2094

### THE ROLE OF FRAILTY ON THE IMPACT OF SOCIAL RELATIONSHIPS ON HEALTH OUTCOMES: RESULT FROM THE FRÉLE LONGITUDINAL STUDY

Fereshteh Mehrabi, and François Béland, *University of Montreal, Montreal, Quebec, Canada*

The link between social relationships and health is well-established as per Berkman and Krishna's theory. However, the biological mechanisms by which social relationships impact health, such as frailty, remain unknown. This study aimed to examine whether the effects of changes in social relationships on changes in physical, mental, and cognitive health outcomes varied among frail older adults compared to robust peers. Data were from three waves of the FRéLE study among 1643 Canadian community-dwelling older adults aged 65 years and over. We performed latent growth curve models (LGMs) to test our objectives with the assumption of missing not at random. We measured social isolation through social participation, social networks, and support from different social ties (e.g., children, friends). We assessed frailty using the phenotype of frailty. Health outcomes include disability, chronic diseases, depression, and cognitive decline. The results revealed that increasing changes in social participation, social contact with friends, and social support from different social ties were associated with greater changes in cognitive and mental health, but not physical health, among frailer older adults compared to those who were more robust. This longitudinal study suggests that social support has a protective and compensatory role in enhancing mental health among frail older adults, but not among robust peers. Public health policies and interventions should focus on ameliorating social connectedness among physically frail older adults to enhance mental health outcomes. Future research studies could explore other risk factors that impact the relationships between social connectedness and health among older populations.

Abstract citation ID: igad104.2095

### USING NETWORK ANALYSIS METHODS TO STUDY MULTIMORBIDITY PATTERNS

Lauren Griffith[1], Alberto Brini[2], Edwin van den Heuvel[2], Philip St. John[3], Lucy Stirland[4], Alexandra Mayhew[1], and Graciela Muniz-Terrera[5], *1. McMaster University, Hamilton, Ontario, Canada*, *2. Eindhoven University of Technology, Eindhoven, Noord-Brabant, Netherlands*,

*3. Max Rady College of Medicine, Winnipeg, Manitoba, Canada*, *4. Global Brain Health Institute, San Francisco, California, United States*, *5. Ohio University, Athens, Ohio, United States*

Multimorbidity is a risk factor for patient-important outcomes including quality of life and functional decline. Multimorbidity research has focused mainly on disease counts, with less attention to patterns among chronic conditions. Network analysis has been increasingly used to examine multimorbidity clusters, but there are no guidelines for its conduct. In 12 recent studies using network analysis, we found heterogeneity in association measures (10 different measures) and clustering algorithms (5 different methods) used to identify multimorbidity clusters. Using self-reported data on 24 diseases in community-living adults aged 45-85 from the Canadian Longitudinal Study on Aging, we conducted network analyses using the 10 association measures and 5 clustering algorithms to better understand how these choices impact the number and types of clusters identified. We compared the similarity among clusters using the adjusted Rand index (ARI); an ARI of 0 is equivalent to the diseases being randomly assigned to clusters and 1 indicates perfect agreement. Two clinicians independently identified potential disease clusters which we compared to network analyses results. We found results differed greatly across combinations of association measures and cluster algorithms. The number of clusters identified ranged from 1 to 12 and their similarity was generally very low. Compared to clinician-derived clusters, the ARIs ranged from 0 to 0.23 indicating little similarity. These analyses demonstrate the need for a systematic evaluation of the performance of network analysis methods on binary clustered data like diseases. Moreover, diseases may not cluster, and a personalized approach to the care of older adults may be needed.

### SESSION 5225 (PAPER)

### ISSUES IMPACTING COGNITIVE IMPAIRMENT

Abstract citation ID: igad104.2096

#### BIOLOGICAL AGE AND COGNITIVE PERFORMANCE AMONG LATE MID-LIFE WOMEN

Rebecca Thurston[1], Cynthia Kusters[2], Yuefang Chang[1], Pauline Maki[3], and Judith Carroll[2], *1. University of Pittsburgh, Pittsburgh, Pennsylvania, United States*, *2. University of California, Los Angeles, Los Angeles, California, United States*, *3. University of Illinois at Chicago, Chicago, Illinois, United States*

Older biologic age, indicated by accelerated epigenetic aging, may place women at risk for poorer cognitive performance as they age. However, findings are inconsistent and based upon limited neuropsychological batteries. Studies of midlife women are few, yet women comprise two thirds of those with Alzheimer's disease, and midlife is a critical transition and potential window for early intervention. We tested whether greater epigenetic age would be associated with poorer cognitive performance among late midlife women. 224 MsBrain study participants (average age=59 years, 82% White, 14% Black, 4% other ethnicities) underwent phlebotomy, including epigenetic aging clock estimates (GrimAge, DunedinPACE, PCPhenoAGE), and a neuropsychological battery assessing global cognitive function (Montreal Cognitive Assessment), verbal learning and memory (California Verbal Learning Test), working memory and attention (Letter Number Sequencing), letter fluency (P, R, W); semantic fluency (animal task), spatial ability (Card Rotations), processing speed (Symbol Digit Modalities Test), and perceptual speed (Finding A's). In linear regression models controlling for age, race, and education, older PCPhenoAge was associated with poorer global cognitive performance [B(SE)=-.07(.03), p=.01], verbal memory [B(SE)=-.09(.03), p=.006], working memory [B(SE)=-.12(.03), p=.0001], attention [B(SE)=-.10(.04), p=.02], spatial ability [B(SE)=-.77(.34), p=.02], processing speed [B(SE)=-.24(.09), p=.007], and somewhat lower letter fluency [B(SE)=-.23(.13), p=.07]. GrimAge and DunedinPACE showed similar yet less consistent patterns. Interactions by race (p's<.05) indicate that associations between older biologic age and poorer cognitive performance (verbal learning, verbal memory, processing speed) was particularly pronounced among Black women in the sample. Older biologic age is associated with poorer cognitive performance across multiple domains among late midlife women, particularly for Black women. Biologic age may serve as an early indicator of risk that could help identify women warranting early intervention to help maintain cognitive function. Supported by NIH RF1AG053504, R01AG053504, K24HL123565

Abstract citation ID: igad104.2097

#### DENTAL-RELATED DECISION-MAKING CAPACITY IN OLDER ADULTS WITH VARIED COGNITIVE FUNCTIONS

Xi Chen[1], Jirakate Madiloggovit-Lower[2], and Carissa Comnick[3], *1. The Ohio State University, Columbus, Ohio, United States*, *2. Thammasat University, Klong Laung, Pathum Thani, Thailand*, *3. University of Iowa College of Dentistry, Iowa City, Iowa, United States*

**Background:** Decision-making is often impaired in individuals with cognitive impairments. However, how cognitive impairment affects dental treatment decision-making remains unclear. We sought to describe dental-related decision-making capacity in older adults with varied cognitive functions. **Methods:** Sixty-five participants, including 14 healthy controls and 51 with documented cognitive impairment, were categorized into healthy controls, mild cognitive impairment (MCI), mild dementia, moderate dementia and severe dementia. A hyperthetical dental problem along with three treatment options were presented. Participants were first asked to identify the problem, related treatment options and the pros and cons of each option. They were then asked to choose a treatment using the given funds. Visual and verbal cues were provided if participants failed to complete the task independently. Participants were scored based on their performances. Data was analyzed using the Kruskal- Wallis test and Holm method for multiple comparisons and a linear regression model for the multivariate analysis. **Results:** Ninety-six percent of the participants with cognitive impairment showed some sorts of deficits in treatment decision-making. Sixty percent of those with MCI and 70% of participants with mild dementia could not explain pros and cons of the treatment choices, significantly higher than that in the healthy controls (10%). The decision-making capacity significantly differed across groups (p< 000.1). Compared to the health controls, decision-making capacity decreased 18%, 33%, 71% and 86% in the MCI, mild dementia, moderate dementia and severe dementia groups, respectively. **Conclusion:** Dental-related decision-making deficits was highly prevalent in older adults with cognitive impairment and occurred as early as MCI.

Abstract citation ID: igad104.2098

### EFFECTS OF GAMIFIED COGNITIVE TRAINING TO DETER THE PROGRESSION OF MILD COGNITIVE IMPAIRMENT

Doris S.F. Yu[1], Polly Li[1], Wung Wai Two[2], and Mary Waye[3], *1. The University of Hong Kong, Hong Kong, Hong Kong*, *2. Hong Kong Society for Sudoku Advancement, Hong Kong, Hong Kong*, *3. The Chinese University of Hong Kong, Hong Kong, Hong Kong*

Mild cognitive impairment (MCI) is highly prevalent condition among older adults, and has a greater risk of progression to dementia. Even cognitive training has great efficacy to delay the onset of dementia, its time-limited offer by professional members reduces its availability. Whereas 'gamification' evolves as important concept to enhance treatment engagement, we developed an innovative Sudoku solving algorithm titled 'T-code' to assimilates the key domains of cognitive training on attention, logical and deductive reasoning. A single-blinded RCT randomized 288 older adults with MCI to receive the 24-week Sudoku Mind Activation and Revitalizing Training (SMART) Program (n=157) or the usual care (n = 131). A battery of cognitive assessments, the Memory Inventory for Chinese, the Short Form-36 were evaluated at baseline, immediately after the 12-week Sudoku training and after the 12-week home-based self-practice. Focus group interview was conducted to explore subjects' experience and reasons for its therapeutic effects if any. The results indicated that participants in the SMART Program had greater improvements in global cognitive function, working memory, language, short- and long-term delay recalls, recognition memory, CTT 1 and subjective memory complaints immediately after the 12-week training than the control. These effects, together with executive function, were sustained till the end of the programme. The qualitative findings converged with the outcome evaluation, and improved self-confidence, more active life engagement, and enhanced mood status might sustain the cognitive benefits. This game-based cognitive training is a treatment option of high accessibility, availability and acceptance to prevent MCI from progressing to dementia.

Abstract citation ID: igad104.2099

### IN-VEHICLE SENSORS TO DETECT CHANGES IN DRIVING BEHAVIORS WITH EMERGENCE OF MCI

Ruth Tappen[1], Jinwoo Jang[1], Sonia Moshfeghi[2], KwangSoo Yang[2], Monica Rosselli[2], Joshua Coniff[2], Borko Furht[2], and David Newman[1], *1. Florida Atlantic University, Boca Raton, Florida, United States*, *2. Florida Atlantic University (FAU), Boca Raton, Florida, United States*

Driving is a complex behavior requiring attention, judgment, orientation, memory, processing speed, visual acuity, adequate limb strength, and other functional abilities. It has been suggested that a change in driving behavior may be an early indicator of cognitive decline in the aging brain. The literature suggests that older drivers will drive less and more slowly as their cognitive skills begin to decline. In the "In-Vehicle Sensors to Detect Cognitive Change in Older Drivers" study, sensors are installed in participant vehicles and data is downloaded every three months at which time a carefully curated battery of cognitive tests is also administered. Records of those suggesting mild cognitive impairment (MCI) are reviewed by a Consensus Diagnostic Panel for confirmation. In the first 100 participants who have been followed over a year, nine participants evidenced existing or emerging MCI. Of these, six have experienced a decline in cognition in the last year. A comparison of driving behaviors over the year evidenced the previously reported slower, less frequent driving but also a previously unreported increase in hard braking and hard turns. The uniqueness of our longitudinal study is the continuous tracking of vehicle acceleration patterns over time, revealing significant relationships between g-force acceleration and cognition.

Abstract citation ID: igad104.2100

### SUBJECTIVE COGNITIVE DECLINE PHENOTYPES, SOCIAL ENGAGEMENT, AND CARDIOVASCULAR HEALTH

Maritza Dowling[1], and Laurie Theeke[2], *1. George Washington University, Washington, District of COlumbia, United States*, *2. The George Washington University, Washington, District of Columbia, United States*

Epidemiological studies report subjective cognitive decline (SCD) as a risk factor for incident dementia. The co-occurrence of SCD, chronic conditions, and loneliness may contribute to functional limitations and decline. SCD etiologies are multifaceted, suggesting possible underlying phenotypes defined by clustering factors that characterize decline. Using pooled data (2015-2021) from the Behavioral Risk Factor Surveillance System (BRFSS) we investigated SCD phenotypes based on the six-item self-reported Cognitive Decline Module that measured challenges in daily life due to memory loss and confusion over the prior twelve months. We hypothesized that SCD phenotypes would be associated with socio-demographic, cardiovascular health and social engagement variables. Mixture modeling was used to determine unobserved SCD phenotypes (latent classes) based on item response patterns. Latent class membership was predicted from socio-demographic variables using multinomial logistic regression. The resulting classes predicted a six-item cardiovascular risk index (CVRI) and a measure of aloneness. SCD symptoms were reported by 65,217 (45-80+ years old). Mixture models produced four-latent SCD classes labeled as mild (43%), mild-moderate (23%), moderate (24%), and severe (10%). Mean CVRI scores were significant across classes ($p < 0.001$) and highest in the severe subgroup (n=6,491) which was more likely to be non-Hispanic Blacks, female, younger (45-64), low-income, non-homeowners, and report depression, and poor/fair health status. Class membership also significantly predicted aloneness. Studies are needed to examine how SCD phenotypes may be used in the design and development of interventions that are more precise to the cluster of modifiable risk factors which could, subsequently, prevent or delay dementia pathogenesis.

### SESSION 5230 (PAPER)

### ISSUES IN AGING

Abstract citation ID: igad104.2101

### ANTIHYPERTENSIVE DEPRESCRIBING AND COGNITIVE FUNCTION IN VA NURSING HOME RESIDENTS: A TARGET TRIAL EMULATION

Bocheng Jing[1], Xiaojuan Liu[2], Michelle Odden[2], Chintan Dave[3], Laura Graham[4], Yongmei Li[2], Kathy Fung[5], and Michael Steinman[6], *1. NCIRE, San Francisco VA Medical Center, UCSF, San Francisco, California, United States*, *2. Stanford University, Stanford, California, United States*, *3. Rutgers University, New Brunswick, New Jersey, United States*, *4. VA Palo Alto Health Care System, Palo Alto, California, United States*, *5. San Francisco VA Medical Center, San Francisco, California, United States*, *6. University of California, San Francisco, San Francisco, California, United States*

Deprescribing is the practice of discontinuing or reducing medication that no longer provides benefits or causes harm. While deprescribing antihypertensives for frail elderly patients has gained interest, its impact on patient-centered outcomes such as cognitive function remains unknown. We emulated a target trial involving 45,183 Veteran Affairs nursing home residents aged ≥65 with stays ≥12 weeks between 2006-2019. Eligible residents must have a 4-week stable medication use period; they were allocated to the deprescribing/control arms, and censored at death, discharge, or two-year follow-up for intention-to-treat analysis. We used the Cognitive Function Scale (CFS) to assess cognitive outcomes, categorizing residents into cognitively intact, mild-, moderate-, and severely-impaired groups. We used an ordinal generalized linear-mixed model to analyze the effect of deprescribing on CFS, adjusting for confounders with 47 baseline covariates using inverse probability of the treatment weights. 1,290 and 11,354 patients were in deprescribing/control arms. At baseline, deprescribing was associated with an increased risk of worse cognitive function (OR=1.168 for being in a higher CFS category [95% CI:1.115-1.224]). Overall, cognition worsened in both arms over time (OR=1.007 per week for progression to higher CFS category, 95% CI:1.006-1.008). However, deprescribing mitigated the time trend, and the residents in the deprescribing arm had significantly decreased odds (improved CFS) at 0.996/week (95% CI: 0.995-0.997), 0.900 (95%CI: 0.881-0.919) over 26-weeks, and 0.811 (95% CI: 0.777-0.845) over 52-weeks. Our findings suggest that deprescribing is associated with a slower decline in cognitive function and can help inform future deprescribing interventions for BP management in nursing home residents.

Abstract citation ID: igad104.2102

### CONTRAST SENSITIVITY AND BRAIN NETWORK COMMUNITY STRUCTURE IN THE BRAIN NETWORKS AND MOBILITY STUDY

Atalie Thompson[1], Alexis Tanase[1], Michael Miller[2], Jeff Williamson[3], Christina Hugenschmidt[1], Haiying Chen[1], Stephen Kritchevsky[3], and Paul Laurienti[1], *1. Wake Forest University School of Medicine, Winston-Salem, North Carolina, United States*, *2. Wake Forest University School of Medicine, Winston Salem, North Carolina, United States*, *3. Wake Forest School of Medicine, Winston Salem, North Carolina, United States*

Contrast sensitivity (CS) is the ability to perceive differences in shades of light and dark, which is important for pattern recognition and depth perception. We have previously shown that impaired CS is associated with poor performance on the expanded short physical performance battery (eSPPB), and that performance on the eSPPB is associated with sensory motor cortex (SMC) connectivity at rest. We hypothesized that brain networks are important to the relationship of CS with eSPPB. Participants (n=192) were cognitively unimpaired older adults (mean age=76.5±4.7 years; 56.5% female, 9.4% black) with good visual acuity who completed CS testing. We applied graph theory to characterize the cross-sectional relationship of binocular CS to functional brain networks generated from fMRI both at rest and during a motor imagery (MI) task and determined the spatial patterns of SMC, visual (VIS) and default mode (DMN) network connectivity. Regression analyses were used to assess associations of CS with brain network connectivity without and with adjustment for sex and eSPPB. Lower CS (p=0.009) was significantly associated with degraded SMC connectivity at rest even in models adjusted for eSPPB and sex. Similarly, lower CS was significantly associated with degraded VIS and DMN connectivity during the MI task (all p< 0.005) when controlling for eSPPB and sex. These results indicate that low CS and poor eSPPB are both associated with connectivity in the SMC at rest and with VIS and DMN during MI in older adults. These findings may be important for understanding the relationship between age-related visual and mobility dysfunction.

Abstract citation ID: igad104.2103

### FACTORS ASSOCIATED WITH HELP-SEEKING AMONG US CHINESE OLDER ADULTS WHO REPORTED ELDER MISTREATMENT

Ying-Yu Chao[1], Jin Young Seo[2], and Yu-Ping Chang[3], *1. Rutgers, the State University of New Jersey, Somerset, New Jersey, United States*, *2. Hunter College, CUNY, New York City, New York, United States*, *3. University at Buffalo, The State University of New York, Buffalo, New York, United States*

Purpose: Elder mistreatment (EM) is an important public health issue. Guided by the Andersen's Behavioral Model of Health Services Use, this study aimed to examine factors that impede or facilitate help-seeking intentions and behaviors among U.S. Chinese older adults who reported EM. **Methods:** Data were from the Population Study of Chinese Elderly in Chicago (PINE). Different types of mistreatments, informal and formal help-seeking intentions and behaviors were assessed. Descriptive statistics, linear regression, and multinominal logistic regression were used. **Results:** Among 450 participants who reported EM, 46.55% experienced psychological mistreatment, 29.91% had financial exploitation, 14.92% had caregiver neglect, 4.68% had physical mistreatment, 1.11% had sexual mistreatment, and 17.78% had multiple victimizations. Intentions of informal help-seeking was higher among victims who were female and married, and those who experienced financial exploitation and caregiver neglect. Formal help-seeking intentions were lower among victims who had a larger social network size. EM victims who were female, married, perceived more social support, experienced financial exploitation, and experienced caregiver neglect were more likely to seek help from informal resources. EM victims who had higher income, perceived more social support, and had multiple victimizations were more likely to seek formal resources. Conclusion/implication: Our findings suggest that the predisposing factors, enabling resources, and need factors that impacted seeking help from informal and formal resources among older Chinese Americans who reported mistreatment. Findings provided important insights for developing early screening programs and interventions, such as increasing help-seeking channels and improving access to care services.

Abstract citation ID: igad104.2104

### NUTRITION THERAPY IN DISCHARGED OLDER ADULTS REDUCES HOSPITAL READMISSIONS AND LENGTH OF STAY

Alfons Ramel, *University of Iceland, Reykjavik, Hofuoborgarsvaoio, Iceland*

The aim was to investigate effects of six-month nutrition therapy on hospital readmissions, LOS, mortality and need for long-term care residence up to 18-months post-discharge in older Icelandic adults. Participants (>65 years) were randomised into intervention (n=53) and control (n=53) before discharge from a geriatric unit. The intervention group received nutrition therapy based on the Nutrition Care Process, including home visits, phone calls, freely delivered energy- and protein-rich foods and supplements for six months after hospital discharge. The Icelandic electronic hospital registry was accessed to gain information on emergency room visits (ER), hospital readmissions, LOS, mortality and need for long-term care residence. One subject from each arm dropped out during the intervention period. The intervention increased body weight (+5.2kg, 95%CI: 3.9-6.5kg) and energy intake (1696kcal, 95%CI: 1557-1834kcal) compared to control. The intervention group had a lower proportion of participants with at least one readmission compared to control (1 month: 1.9% vs 15.8%, P=0.033; 6 months: 25.0% vs 46.2%, P=0.021; 12 months: 38.5% vs 55.8%, P=0.051; and 18 months: 51.9% vs 65.4%, P=0.107). There was also a lower total number of readmissions (significant at 1, 6 and 12 months) and a shorter LOS (significant at all time points) in the intervention group. However, there were no differences between groups in ER visits, mortality and need for long-term care residence. It is of great clinical relevance that a six-month nutrition therapy in older Icelandic adults discharged from hospital reduced hospital readmissions and shortens LOS at the hospital up to 18-months post-discharge.

Abstract citation ID: igad104.2105

### SELF-PERCEPTION OF AGING AND THE SELF-REPORTED HEALTH/FRAILTY PARADOX

Brian Buta[1], Melissa Hladek[1], Mallak Alzahrani[1], and Qianli Xue[2], *1. Johns Hopkins University, Baltimore, Maryland, United States*, *2. Johns Hopkins, Baltimore, Maryland, United States*

We aimed to study the cross-sectional association between self-perception of aging (SPA) and frailty paradox, defined as self-reporting excellent/very good health despite being frail or fair/poor health despite being robust. The study sample included 2,950 participants from the Health and Retirement Study (2008 wave) who were 65 years and older, completed the Psychosocial and Lifestyle Questionnaire, and had no missing data on the variables included in the models. Frailty was measured by the physical frailty phenotype. Self-reported health (SRH) was rated as either excellent, very good, good, fair, or poor. SPA was measured using an 8-item questionnaire spanning subjective well-being, positive affect, and life satisfaction; respondents rated their agreement with statements such as "Things keep getting worse as I get older" and "So far, I am satisfied with the way I am aging." Negative statements were reverse-coded and the average of all items was calculated; higher scores indicated more positive SPA. 181 (6%) participants met the criteria for the frailty paradox: 54 reported being in excellent/very good health despite being classified as frail, while 127 reported fair/poor health despite being classified as robust. After adjusting for potential confounding variables, including age, sex, race, education, and comorbidity burden, we found that individuals with higher SPA scores had greater odds of excellent/very good SRH despite frailty. Specifically, the odds nearly doubled for every one unit increase in SPA score (p-value< 0.01). Positive self-perception of aging may be a potential interventional buffer against negative impact of physical frailty on self-assessment of overall health status.

### SESSION 5235 (PAPER)

### TECHNOLOGY, MENTAL HEALTH, AND COGNITIVE IMPAIRMENT

Abstract citation ID: igad104.2106

### ASSESSING THE QUALITY OF CHATGPT RESPONSES TO DEMENTIA CAREGIVERS' QUESTIONS POSTED ON SOCIAL MEDIA

Alyssa Aguirre[1], Robin Hilsabeck[2], Tawny Smith[1], Zhendong Wang[3], Ning Zou[3], Bo Xie[1], and Daqing He[3], *1. The University of Texas at Austin, Austin, Texas, United States*, *2. The University of Texas at Austin Dell Medical School, Austin, Texas, United States*, *3. University of Pittsburgh, Pittsburgh, Pennsylvania, United States*

ChatGPT, an innovative, dialogue-based large-scale artificial intelligent model that responds to complex natural language inquiries, holds great promise to improve dementia caregivers' quality of life by providing high-quality responses to meet their information needs. However, limited evidence exists on the quality of ChatGPT responses. This exploratory study aimed to address this gap. We first selected 25 social media posts, verified in our NIH-funded project (R56AG075770) as being representative of dementia caregivers' needs for information about daily care for memory loss and confusion. We then collected ChatGPT responses in March 2023 and assessed their quality using a 4-item rating scale (1 point for each item; scoring range: 0-4; higher score indicates higher quality). This scale includes (1) Factual: Response contained no inaccurate or false information; (2) Interpretation: Response adequately interpreted the poster's main need and correctly disregarded non-priority details; (3) Application: Response included both educational information and tangible actions; and (4) Synthesis: Response included follow-up actions (e.g., referrals). Three clinicians, each with 15+ years of experience with dementia caregivers, independently rated the responses. All raters agreed on the ratings for 22 (88%) responses initially; disagreements centered on responses' comprehensiveness and specificity. After reconciliation, all raters agreed on the ratings: 3 (12%) responses received 3 points and 22 (88%) received 4 points, suggesting high quality. Study limitations included small sample, posts representing only one type of information wanted by caregivers, and potential ceiling effect of the rating scale. In our larger project, we will address these limitations and systematically assess ChatGPT responses.

Abstract citation ID: igad104.2107

### REDUCING HTN AMONG BLACK ELDERS THROUGH AN IMMERSIVE VIRTUAL FOREST

Maureen Mickus, *Western Michigan University, Kalamazoo, Michigan, United States*

Rates of uncontrolled hypertension (HTN) among Black individuals is higher than for any other racial or ethnic group in the U.S. The prevalence of HTN in this minority population, coupled with arterial aging, portend serious risks for cerebrovascular and cardiovascular diseases among Black elders. Past research has indicated that exposure to nature, specifically immersion within a forest setting, can be effective for reducing HTN. But deriving health benefits derived from time in a forest may be difficult for older persons from historically disadvantaged groups. This purpose of this study was to assess whether access to calm and immersive forest images delivered using virtual reality (VR) can modify HTN among community-dwelling Black elders. The study involved a quasi-experimental design and a sample of individuals ages 60-98 recruited from a senior center primarily serving minorities. Each of the 22 participants completed 4 biweekly VR trials using software with a soothing audiovisual simulation of a forest meadow including trees, birds, flowers, and wildlife. Results indicated an average decline of 10 mm HG (SD=7.0) in systolic blood pressure with Hedges g effect sizes ranging from .38 to .96 across sessions. The elders expressed a more relaxed mood and viewed VR as a pleasant distraction overall. Given that none of the participants reported any adverse effects from using this technology, further exploration of forest-based VR as a promising method for decreasing HTN in a high-risk population of older adults is warranted.

Abstract citation ID: igad104.2108

### REFINING AN ICT-ENRICHED NONPHARMACOLOGICAL INTERVENTION FOR PEOPLE WITH DEMENTIA: A PARTICIPATORY DESIGN

Jacky C.P. Choy, Gloria Hoi-Yan Wong, and Kayla Wong, *The University of Hong Kong, Hong Kong, Hong Kong*

Information and communication technology (ICT) has enabled the rapid development of remote non-pharmacological intervention for people living with dementia (PLwD). Yet, PLwD were seldom actively involved in the design process. We developed a prototype of Virtual Cognitive Stimulation Therapy (vCST) by adapting CST, an evidence-based group activity intervention, for remote delivery and tested among PLwD in the community. Following a participatory design approach, the current study aims to understand the needs of refinement from the users' and providers' perspectives for improving person-centred engagement and accessibility of vCST. Participants were 15 PLwD who received fourteen vCST sessions, 15 family carers who assisted the PLwD's participation, and 15 trained volunteers who delivered the sessions. We observed the video-recordings of vCST sessions, and then conducted semi-structured interviews with dyads of PLwD and carers, and focus groups with the providers. Thematic analysis was conducted to understand their views on refinement needs. Two themes and 7 subthemes representing a two-level refinement needs emerged. At intervention operation level, 3 subthemes were 1) pre-assessment of personal characteristics, 2) consistent and comfortable environment and equipment setup, and 3) optimal level of carer involvement. At activity delivery level, 4 subthemes were 5) facilitating skills for positive relationships and active participation, 5) tailoring activity content to PLwD biography, interest, and ability, 6) interactive and collaborative activity design for rapport building and empowerment, and 7) materials use for enhancing stimulation and engagement. With appropriate research design and interview prompts, PLwD could comprehend novel ideas and actively contribute to intervention design.

Abstract citation ID: igad104.2109

### WEB-BASED COGNITIVE BEHAVIORAL THERAPY FOR DEPRESSION AMONG OLDER ADULTS: DEVELOPMENT AND USABILITY STUDY

Jay Kayser, Samson Ash, Chuxuan Zheng, Skyla Turner, and Xiaoling Xiang, *University of Michigan, Ann Arbor, Michigan, United States*

Digital Mental Health Interventions (DMHIs) can potentially improve treatment access for older adults experiencing depression, but few tailored web-based interventions exist. This paper describes the design and development process of a web-based cognitive-behavioral therapy program called Empower@Home and reports outcomes from a usability evaluation. The program was developed in collaboration with community agencies, stakeholders, and older adults. Development was guided by user-centered design principles and informed by multiple data sources. A comparative usability evaluation with ten older adults was conducted to assess the usability of Empower@Home. Paired t-tests were used to compare scores on the System Usability Scale (SUS) between three interventions. In addition, field testing was conducted with 4 end-users to detect any further usability issues. The content and design of the intervention was heavily influenced by feedback and recommendations provided by our community partners. Empower@Home involves nine sessions, completed over ten weeks, and a printed workbook. The sessions involve psychoeducation, short video clips, interactive content, an animated storyline, and weekly out-of-session home practices. In the comparative usability evaluation, Empower@Home received statistically significantly higher usability scores than both comparison interventions, and 80% of users preferred Empower@Home over other interventions. In field testing, all participants "agreed" or "strongly agreed" that they liked the procedures used in Empower@Home and felt confident in performing program-related tasks. Empower@Home is highly responsive to end user needs and the implementation setting's characteristics. Interventions that work with community stakeholders and thoughtfully consider implementation barriers in the design process will likely produce more usable and effective DMHIs.

### SESSION 7000 (POSTER)

### GERONTOLOGY AND GERIATRICS EDUCATION AND TRAINING

Abstract citation ID: igad104.2110

### A COLLABORATIVE AUTOETHNOGRAPHY OF FOREIGN-BORN FACULTY IN THE FIELD OF AGING

Chih-ling Liou[1], and Karel Kalaw[2], *1. Kent State University, Kent, Ohio, United States*, *2. University of Central Oklahoma, Edmond, Oklahoma, United States*

We draw from our personal insights as foreign-born female faculty in the U.S. to explore how our minority status in the U.S. has affected our experiences/identities in the field of aging. We practiced intersectionality by considering how our understandings of "foreignness" in academia and the aging field are intertwined with other markers of difference, including race, national origin, language, and academic programs. We also draw from tenets of collaborative autoethnography to engage two autoethnographies from two countries in Asia to pool their lived experiences and collaboratively analyze and interpret them for commonalities and differences. We begin by sharing stories about graduate study pursuits and becoming faculty in the U.S. We note how our foreignness shapes our lives as aging scholars in the U.S. academy and our personal views on aging. After exchanging our first set of writings, we identified key experiences to focus on in the subsequent writing periods. Our minority backgrounds, teaching aging subjects, and using qualitative methodology are shared identification. Moreover, the themes that deal directly with identity development and the perception of aging of non-white women can be added to promote a deep understanding of aging among the non-native-born population. This study highlights the value of collaborative autoethnography as a method of inquiry and reflection. Findings demonstrate that non-native-born female faculty members in the field of aging faced multi-faceted challenges in both professional and personal realms. Implications for supporting foreign-born female aging scholars are discussed.

Abstract citation ID: igad104.2111

### ADDRESSING CURRICULUM DESIGN CHALLENGES: THREE DELIVERY DESIGN OPTIONS FOR UNDERGRADUATE AGING AND HEALTH COURSE

Mary Theresa Pagan, and Minjung Seo, *SUNY Oswego, Oswego, New York, United States*

Providing a comprehensive gerontology course to diverse academic majors is a curriculum design challenge. Current research indicates students seek out the delivery option best suited to their needs, requiring educators to design or modify courses to provide face-to-face, hybrid, and online opportunities. Further, gerontologists are challenged with initial student knowledge and views on aging, aging processes, and health disparities shaped primarily by personal/life experiences with older adults and media representations of aging. Significant design and quality assurance were minimized by utilizing the recognized work of established gerontologists providing the overall framework for a comprehensive aging course Segmenting the course into two sections, social determinants of health utilizing Deborah Carr's Golden Years? Social Inequalities in Later Life and target population research, Grandmothers at Work, Juggling Families and Jobs by Madonna Harrington Meyer. As a research design, a mixed method design (questionnaire and interview was used on seventy-eight students (Male: 27, Female: 51) enrolled in face-to-face, hybrid, and online delivery options. As a result of attending the three-credit course, significant knowledge-base increases were found in four key content areas: 1) social determinants of health (83%), 2) theories (sociological and biological)-(90%), 3) socioeconomic (SES) variations (80), and 4) mixed methods research design (62%). A curated library of online resources (youtube, Ted Talks, documentaries, news reports) supplemented the course textbooks for each learning module, resulting in a ninety-six percent resource satisfaction evaluation average for the three delivery methods.

Abstract citation ID: igad104.2112

### CONTRIBUTIONS TO AGING RESEARCH BY DOCTORATES IN GERONTOLOGY AND GERIATRICS: A PORTUGUESE CASE STUDY

Oscar Ribeiro[1], Soraia Teles[2], and Constanca Paul[3], *1. University of Aveiro, Aveiro, Aveiro, Portugal*, *2. University of Porto, Institute of Biomedical Sciences Abel Salazar, Porto, Porto, Portugal*, *3. ICBAS, Portugal, Porto, Porto, Portugal*

Portugal is home to one of the world's oldest populations, ranking in the top five OECD countries with the highest share of people aged 65 and over. The PhD in Gerontology and Geriatrics (PDGG) is the first and one-of-its-kind response in Portugal to the need of advanced training in the field. This work describes the contributions of PDGG doctorates from 2009 to 2022. Electronic/paper-based academic records were retrieved to characterize the doctorates' entry profiles. Documentary analysis of dissertations was performed to describe their research. Related publications and its impact were identified from searches on academic databases/engines. Doctorates (N=36; 88.9% female) were aged between 23 and 54 (M 33.0). The background is in health and social sciences, with more representation from nurses (30.6%) and psychologists (22.2%). Approximately thirty percent (n=10) had a Masters in Gerontology, and all were professionally experienced with older adults (1-29 years). Doctoral research topics included cognitive impairment and mental health, healthcare/social services/interventions; intergenerationally/family relations/informal care; frailty; nutrition and physical activity, minority populations, elder abuse and spirituality. The research of the 36 doctorates involved 12374 participants (9499 older persons). Outputs comprised 153 publications in scientific journals, receiving 1640 citations, 87782 usages (e.g., views, downloads), 9538 captures (e.g., exports) and 541 in social media or news articles (PlumX metrics). Other contributes included 8 original intervention programs, 3 new and 11 translated/culturally-adapted assessment scales. This case study shows the intergenerational and lifelong learning environment of the PDGG, and its many contributions to the the aging field.

Abstract citation ID: igad104.2113

### DEVELOPMENT OF TWO GERIATRIC CASES TO PREPARE STUDENTS FOR THE NEXT-GENERATION RN LICENSING EXAM

Mary DiBartolo, *Salisbury University, Salisbury, Maryland, United States*

With the implementation of the Next Generation NCLEX-RN (NGN) national licensing examination in April of 2023, faculty in state of Maryland nursing schools were solicited for participation in a Maryland Nursing Workforce Center grant initiative to develop a repository of NGN case studies. A major change in the exam was the integration of a six-step clinical judgment model using a case-based format with client information presented in an electronic medical record using new question types designated for each of the steps presented sequentially. Twenty faculty “champions” from 13 Maryland schools attended several training sessions and were to develop two peer-reviewed cases for inclusion in the Qualtrics database. Once approved, these cases could then be accessed online via a secure link or QR code by Maryland-based nursing faculty and shared with students for instructional and practice purposes. Two gero-focused cases were developed. The first case focused on an older adult in a long-term care facility admitted to the hospital for a urinary tract infection with potential complication of urosepsis. Another featured a hospitalized older adult client with Alzheimer’s disease admitted from home with dehydration with potential for delirium superimposed on dementia. While a major objective of this innovative and proactive state-wide initiative is to optimize success on the modified NCLEX-RN, it is also hoped that these case examples, when appropriately integrated into the nursing curriculum, will enhance understanding of common geriatric scenarios and better prepare graduates for safe, effective nursing care of older adults.

Abstract citation ID: igad104.2114

### FORETHOUGHT OR AFTERTHOUGHT? THE FUTURE OF GERONTOLOGICAL EDUCATION

Julie Masters[1], Mark Staley[2], and Rona Karasik[3], *1. University of Nebraska Omaha, Omaha, Nebraska, United States*, *2. University of Nebraska - Omaha, Omaha, Nebraska, United States*, *3. Saint Cloud State University, Saint Cloud, Minnesota, United States*

The increasing population of older adults would suggest a comparable increase in the demand for gerontological education. The current workforce shortage and number of Gerontology programs that are struggling and/or closing, however, suggest this is not necessarily the case. Bass and Ferraro (2000) reported over 1,000 gerontology/aging or related programs in operation. Today, there are 537 programs in gerontology and/or aging including 31 Associate degrees; 185 Minors; 79 Undergraduate and 92 Graduate Certificates; 43 Majors; 57 Masters; and 16 doctoral programs (University of Nebraska Omaha, 2023). Many interrelated factors have been suggested to account for the decline. From an economic point of view, these factors are thought to include: decreasing enrollments in and revenue generation from gerontology programs; demographic shifts away from traditional age college students; and decreasing interest and value of higher education overall. From a pedagogical point of view, this might reflect a return to individual courses and programs ensconced in more traditional disciplinary approaches (e.g., family studies, psychology, and sociology). Pervasive ageism has also been thought to be an underlying societal factor. While none of these factors are easily remedied, understanding their impact is an important first step. GSA programming such as Ageism First Aid and Reframing Aging Initiative are taking the next Herculean step in tackling societal ageism. This presentation focuses on reframing the value of gerontological education to students, employers, and policymakers– moving it from an afterthought to a prioritized strategy for addressing the mounting demand for aging services and service providers.

Abstract citation ID: igad104.2115

### IMPACT OF AN INTERDISCIPLINARY CLASS ON FIRST-YEAR UNDERGRADUATE STUDENT KNOWLEDGE AND ATTITUDES TOWARD AGING

Matthew Picchiello, Dylan Agran, Tim Chung, Brian Carpenter, Susy Stark, and Nancy Morrow-Howell, *Washington University in St. Louis, St. Louis, Missouri, United States*

Undergraduate classes on aging have the potential to challenge ageist beliefs and alter students’ academic and career trajectories. For the past eight years, we have led an introductory, interdisciplinary class on aging, bringing first-year students and older adults together to discuss various facets of aging and share their perceptions and experiences. Both prior to and after the class, students in the class ($n = 494$), and a group of their age-matched peers not enrolled in the class ($n = 620$), completed an online survey measuring attitudes and knowledge about aging and older adults. Repeated measure analyses revealed significant group-by-time interactions across several measures. While students who were not in the class did not show statistically significant changes across measures, students in the class displayed statistically significant increases in knowledge about aging, $F(1, 760) = 108.133$, $\eta p2 = 0.125$; decreases in aging related anxiety $F(1, 766) = 51.947$, $\eta p2 = 0.064$; and more favorable expectations about their own aging, $F(1, 627) = 45.598$, $\eta p2 = 0.068$. Moreover, students in the class endorsed fewer ageist beliefs over time, $F(1,768) = 135.313$, $\eta p2 = 0.15$, all p’s $< 0.05$. Despite these promising changes, overall intent to engage in an aging-related career did not statistically change across groups, $F(1, 113) = 2.948$, $p > 0.05$. Results suggest that exposing students to information about aging in an intergenerational classroom has the potential to mitigate biases against older adults, though challenges remain in maintaining student interest in aging-related careers.

Abstract citation ID: igad104.2116

### INTERPROFESSIONAL EDUCATION: STRATEGIES TO REACH RURAL STUDENTS THROUGH GERIATRIC EDUCATION

Elaine Jurkowski, *Southern Illinois University at Carbondale, Cabondale, Illinois, United States*

Inter-professional collaboration across disciplines such as primary care medical practice, physician assistants, social workers, and nutritionists does not occur naturally, since educational programs are often taught independently of each other, but these disciplines are required to work collaboratively with each other. The objective was to promote communication across disciplines and help each discipline understand the roles played in promoting mental health and general health for older adults. Three specific strategies to address Interprofessional Education were devised and implemented within a rural context to address the goal of improving collaboration and communication. An educational seminar was conducted using cases and guide questions focused on identifying strategies for care. A second strategy was through "Student hot-spotting" where students worked with a high-need complex case over the course of a school year weekly, as a team-based approach. A third strategy made use of a weekly medical clinic for team-based patient care. The teams consisted of Medical Residents, Social Work students, Physician Assistant students, and nutrition students. Findings suggest professionals were surprised at what they learned from the other disciplines they were collaborating with. They also learned about community-based resources available as well as strategies to promote health outcomes. All participants felt that the opportunity to collaborate outside of their disciplines would strengthen their impact when working with older adults and their families. In conclusion, a problem-based learning approach coupled with the opportunity to collaborate with other disciplines through (IPE) improves overall collaboration among professionals. All strategies had positive learning outcomes.

Abstract citation ID: igad104.2117

### MAKING AN IMPACT: AN INTEGRATED MODEL TO REDUCE AGEISM AND SUPPORT PROFESSIONAL IDENTITIES

Amy Powell-Versteeg, Rebekah Perkins, R. Scott Ward, Linda Edelman, and Katherine Supiano, *University of Utah, Salt Lake City, Utah, United States*

Nearly one-million people in the U.S. are living with Parkinson's disease (PD) and require skilled care. Despite this, only 20% of educational programs offer a geriatric rotation. Ageism is a major factor in healthcare students' lack of clinical exposure, decreased self-confidence, and desire to specialize in geriatrics. Research shows positive exposure to older adults improves students' knowledge, attitudes, and interest in this population. Yet, limited geriatric curriculum exists that integrates specialized knowledge combined with positive contact experiences to shape students' professional identities as capable providers. This project combined two experience-based learning (ExBL) models to form the Intrinsically-Motivated learning, Professional identity, Awareness, Capacity, and Timing (IMPACT) model—to support specialized knowledge acquisition and positive, repeated contact experiences with older adults. Third-year Doctor of Physical Therapy (DPT) students who completed both didactic training and a 2-week PD Boot Camp were interviewed about their experiences in the program. Nine students participated in the PD Boot Camp and interviews. Qualitative thematic analysis revealed the following: 1) unexpected insights gained about clients' unique abilities, 2) therapeutic alliances formed and mutually created functional goals, 3) improved decision-making through practice with mentorship, and 4) increased self-confidence to provide care to clients with complex conditions. The IMPACT model provided students specialized knowledge and practical skills to capably care for older adults with PD. This model can serve as a guiding educational framework to reduce ageism among healthcare students across a variety of settings, support students in developing their professional identities, and increase interest in caring for older adults.

Abstract citation ID: igad104.2118

### MEDICAL STUDENT MOTIVATIONS TO STUDY GERIATRICS AS MEDICAL SPECIALTY IN ECUADOR: A QUALITATIVE APPROACH

Daniela Belen Sosa Cifuentes, and Jonathan Guillemot, *Universidad San Francisco de Quito, Quito, Pichincha, Ecuador*

Due to a lack of interest in becoming geriatricians in the medical student community and despite studies showing geriatrics as one of the most fulfilling medical specialties, Ecuador, like many countries globally, lacks geriatricians. Although there are insufficient well-established institutions training geriatricians, the issue essentially lies in the lack of student vocations. We conducted a qualitative and participatory study to identify and describe the motivations and barriers associated with medical students' interests in becoming geriatricians in Quito, Ecuador. Qualitative interviews between medical students after interviewing technique training were conducted. Audio recordings were transcribed and coded, and then analyzed for patterns. Thirty-two students were interviewed, none of which considered geriatrics as their first medical specialty option, and two of which considered the possibility of such a choice, but not as a first option. Among the most significant patterns associated with disinterest in geriatrics was the lack of exposure as well as a general ignorance of the lives of older adults beyond direct relatives. While most participants recognized the fundamental importance of the specialty, barriers appearing unsurmountable emerged: patterns of gerontophobia as well as thanatophobia were strong hurdles, combined with the perception of an emotional toll associated with the care of older adults. This joined with the view that physicians could not be fulfilling their purpose of "saving lives" in the context of geriatrics. As the Global South ages, universities must improve student exposure to older adults and the professions associated with their care as a first step towards promoting new vocations.

Abstract citation ID: igad104.2119

### NOW SHOWING: THE USE OF FILMS IN ADVANCED GERONTOLOGY COURSES

Jessica VanderWerf[1], Phyllis Greenberg[2], and Rona Karasik[3], *1. University of South Florida, Tampa, Florida, United States, 2. St. Cloud State University, St. Cloud, Minnesota, United States, 3. Saint Cloud State University, Saint Cloud, Minnesota, United States*

While the use of films to teach about aging and older adults is not new, current movies and the students who view them are ever-changing. So too are the learning goals and practices for incorporating media into the advanced gerontological classroom. The current undertaking considers the unique aspects of employing movies (including documentaries and international films) and builds on previous works

(Cohen-Shalev & Marcus, 2007; Karasik & Hamon, 2014; Markson & Taylor 1993) with an eye toward the more advanced pedagogical goals of upper level undergraduate and graduate courses. This poster focuses on challenges associated with including film in both traditional and emerging classroom delivery formats (e.g., face to face, hybrid, on-line). A matrix of current aging-themed films is provided, along with strategies for successful applications in upper level and graduate courses. Karasik and Hamon (2014) explored the use of popular film in introductory gerontology courses, creating a detailed matrix of films spanning 1951 - 2012. In addition to film descriptions and ratings, the matrix identified content in the following topical areas in (ageism/stereotypes; cognitive impairment; death & dying; diversity; family relationships; health & wellness; sexuality & intimacy; and work & retirement). The current presentation builds on Karasik & Hamon's work by developing a companion matrix of films including feature films from 2012- 2023 , as well as documentaries and international films across the same time period. In addition to the newly created companion matrix, there are examples of activities and assignments that incorporate movies into the gerontological classroom.

Abstract citation ID: igad104.2120

### NURSING STUDENTS' RESPONSE TO THE AGE-FRIENDLY HEALTH SYSTEMS 4MS FRAMEWORK CURRICULUM

Timothy O'Connor[1], Naushira Pandya[2], Sweta Tewary[1], and Oksana Shnayder[1], *1. Nova Southeastern University, Davie, Florida, United States*, *2. Nova Southeastern University, Kiran Patel College of Osteopathic Medicine, Davie, Florida, United States*

Problem: There is a shortage of registered nurses, especially those choosing to work in long-term care. Providing interdisciplinary education on age-friendly health care to nursing students offers long-term care as a career option post-graduation. To evaluate the effectiveness of teaching nursing students about age-friendly health care, the students must provide adequate feedback on the educational content they receive. Purpose: This project is part of the Age-Friendly Health Systems 4Ms project and identified why nursing students did not complete a comprehensive survey after receiving an interdisciplinary educational session based on the Age-Friendly Health Systems 4Ms Framework. Research design: A focus group design was used, and subjects were recruited from nursing students who received the educational session but did not complete the comprehensive survey. Questions for the focus group discussion were derived from the content received during the academic session and from the comprehensive survey. The focus group interview was audio-recorded, and the recordings were transcribed verbatim. Data analysis: Focus group transcripts were analyzed using descriptive content analysis to determine themes. **Results:** The outcome of this study provided researchers with insight into why nursing students did not complete the comprehensive survey. The results of this study will help improve future comprehensive survey response rates for the remainder of the Age-Friendly Health Systems 4Ms project. Significance: The findings of this study enhances the validity and reliability of the comprehensive survey results and informs researchers across disciplines on how to avoid low survey response rates.

Abstract citation ID: igad104.2121

### SIMULATION IN AGE SUIT: AN INNOVATIVE NURSING EDUCATION INTERVENTION TO INCREASE THE UNDERSTANDING OF OLDER ADULTS

Catharina Gillsjö (Gillsjo), Björn Bouwmeester Stjernetun, and Jenny Hallgren, *University of Skövde, Skövde, Vastra Gotaland, Sweden*

**Background:** Negative attitudes towards older adults are often recognized in society, especially among health care providers. Lack of education in gerontology and geriatrics and limited practical training in nursing education have been identified as influencing aspects. Simulation in education through age suit has been recognized to increase empathy and reduce negative attitudes towards older adults. An innovative education intervention with simulation in age suit with a follow up reflection seminar was developed to counteract ageism and increase the understanding of aging, age related health problems, older adults and to influence the willingness to provide care to older adults. **Methods:** The intervention with simulation in age suit, grounded in the pedagogy experiential learning, was implemented in the fourth semester in the three-year nursing education program. The simulation was conducted in a highly accessible home environment equipped with welfare technology and other technical aids. The nursing students were assigned a persona, a person with different health problems, and performed scenarios consisting of daily chores. Research on nurses' experiences of the intervention and its sustainability through the education program and on perceptions of providing care for older adults, is conducted qualitatively and quantitatively in a doctoral project. **Conclusion:** The learning acquainted through simulation in age suit became embodied both physically and psychologically. The simulation in age suit conveyed new insights as need of time and patience. The technology in the environment both hindered and facilitated activities in daily life. Implications suggested the need for all health and social care providers to carry out this simulation.

Abstract citation ID: igad104.2122

### THE AMBIVALENCE OF BACHELOR OF NURSING LECTURERS TOWARD MALNUTRITION EDUCATION: A QUALITATIVE STUDY

Debbie ten Cate[1], Iris Van den Boomgaard[2], and Canan Ziylan[3], *1. Utrecht University of Applied Sciences, Utrecht, Utrecht, Netherlands*, *2. The Hague University of Applied Sciences, The Hague, Zuid-Holland, Netherlands*, *3. Rotterdam University of Applied Sciences, Rotterdam, Zuid-Holland, Netherlands*

Malnutrition in older adults occurs multifactorially and should be prevented interdisciplinary. This requires an improvement in the competencies regarding the integral prevention of malnutrition in students of different Bachelor's programs, such as dietetics, social work and nursing, who are the future professionals. A precondition is that this education is taught by lecturers who have a positive attitude toward malnutrition education. However, it is unknown what this attitude is. We started our exploration within Bachelor of Nursing lecturers. Therefore, the aim of this study was to gain insight into the attitude of these lecturers regarding malnutrition education. A qualitative study, where semi-structured, in-depth interviews were held with nine Bachelor of Nursing lecturers from four different educational institutes. The interviews were transcribed and thematically analyzed. Two main themes emerged from the data: (1) 'malnutrition is important', subdivided in three sub-themes: 'personal experiences', 'nursing experiences' and 'theoretical knowledge', and (2) 'not acting consequently', subdivided in four sub-themes: 'unclear responsibilities', 'insecurity regarding own abilities', 'depending on experts' and 'problems with prioritization'. The results showed that lecturers have a positive perception regarding malnutrition education, but that there is a discrepancy between their attitude and actual behavior. This means that this may lead to difficulties in embedding and providing qualitative malnutrition education in the curriculum. It is advised that lecturers follow train-the-trainer courses to develop and enhance their competencies in providing malnutrition education. Also, an efficient integration of and interdisciplinary approach regarding malnutrition education is advised.

Abstract citation ID: igad104.2123

### THE IMPACT OF LEARNING MOTIVATION ON LEARNING WILLINGNESS AMONG OLDER CHINESE ADULTS

Zhiyu Fan, and Huamao Peng, *Beijing Normal University, Beijing, Beijing, China (People's Republic)*

As life expectancy increases in China, lifelong learning has become an important approach to successful aging during a long retirement period. In the present study, we examined the association between learning motivation and learning willingness among older Chinese adults. A nationwide sample of 5582 older Chinese adults aged 60 to 93 (Mean age 66.5, SD = 4.67) participated in this questionnaire-based study in 2022. The results indicated that the average willingness to learn of older Chinese adults was 3.14 (range 1-5), with 61.7% of older adults showing an active or neutral willingness for learning in old age. These 3444 older adults with an active or neutral learning willingness were most willing to learn to keep healthy (70.3%) and least willing to learn re-employment skills (19.2%). In terms of learning motivation, these older adults were most likely to regard "make life convenient" as the most important motivation (27.6%). Linear regression showed that after controlling for age, education, social support, physical and cognitive health, stress, self-care ability, and daily activities, motivation could predict learning willingness. Comparing with "make life convenient", older adults who valued "social comparison" most tended to have less willingness to learn (i.e. choose fewer learning topics, beta = -0.14, 95% Confidence Interval = [-0.23, -0.05]). In addition, older adults who valued "personal growth" (0.30 [0.21, 0.39]) or "alleviate cognitive decline" (0.14 [0.04, 0.24]) most tend to choose various learning topics. Our findings promote an understanding of how motivation is related to the learning willingness of older adults.

Abstract citation ID: igad104.2124

### TRAINING INTERPROFESSIONAL STUDENTS TO LEAD AND COLLABORATE IN THE FIELD OF GERONTOLOGY AND OLDER ADULT CARE

Jennifer Wong, and Chun-Ya Belinda Kang, *Wallis Annenberg GenSpace, Los Angeles, California, United Sta tes*

With the historic demographic transition of older populations outnumbering younger ones comes an urgent need for services and support for older adults, which entails an integration of various fields. Simultaneously, there has been an alarming decline in the amount of professionals entering older adult care. Furthermore, colleges and universities provide limited education on the service of older adults, and even less fieldwork exposure to older demographics. As such, academia and fieldwork teams must prioritize the training of students in the field of gerontology and of equal importance, students in adjacent fields in order to recruit and equip the incoming workforce in older adult care. Moreover, the training must be integrated and emphasize leadership, as the complex care of older adults requires an interprofessional team engaged in multi-level collaboration. Wallis Annenberg GenSpace is an innovative community space dedicated to older adults and is invested in combating ageism and promoting lifelong learning. GenSpace serves as a fieldwork placement for gerontology, occupational therapy, communication, and public health students. Through supporting community programs and larger policy level work, students are immersed in various aspects of gerontology. Students discover a diversity of crucial ways that their respective professions can contribute to serving older adults. Additionally, GenSpace's team-based environment allows students to understand the various roles of their peers and prepare to build cross-sectoral partnerships when they enter the workforce. The orientation, curriculum, and outcomes of the student fieldwork completed thus far will be presented.

Abstract citation ID: igad104.2125

### CONNECTING THE HEALTH CARE WORKFORCE THROUGH A VIRTUAL LEARNING COMMUNITY

Judy Howe[1], Eve Gottesman[2], Eugenia Dorisca[3], Yuka Shichijo[1], and Fred Ko[3], *1. Icahn School of Medicine at Mount Sinai, New York City, New York, United States*, *2. US Department of Veterans Affairs/Zicklin School of Business, New York, New York, United States*, *3. James J. Peters VA Medical Center, Bronx, New York, United States*

The COVID-19 pandemic highlighted the need for online and virtual educational opportunities and resources. While many healthcare organizations, hospitals and academic medical institutions were forced to quickly pivot from face-to-face training modalities, the Virtual Learning Community (VLC) of the Geriatric Scholars Program, was ahead of the curve. Established in 2014 by the VA Geriatric Scholars Program and the VHA's Office of Rural Health, the VLC is the primary outlet for information on geriatrics and gerontology for healthcare professionals and staff. The VLC provides webinars, discussion forums, case studies, continuing education accredited programs, and resource materials on a myriad of topics related to aging. During the pandemic, the VLC provided up-to-date information on COVID and continued to augment its virtual offerings, which was vital to providers and staff working remotely. The VLC currently has approximately 5,000 registered users, with 1700 users registering since the pandemic began in March 2020. Many of these users come from rural and highly rural areas, where there is often a lack of geriatric knowledge and expertise. The presentation will present findings about the connections and impact made possible by a pre-existing web-based virtual learning community.

### SESSION 7010 (POSTER)

### ABUSE AND AGGRESSION TOWARD OLDER ADULTS

Abstract citation ID: igad104.2126

### A 13-YEAR TREND OF THE ABUSE OF OLDER PEOPLE IN SOUTH KOREA

Jenny Kwon, *Miami University, Oxford, Ohio, United Sta tes*

Compared to the growing number of the older population in South Korea, there is a scarcity of literature investigating issues about the abuse of older people, as well as effective prevention programs. To fill this gap, this study aimed to identify annual changes in patterns of the abuse of older people including the number of reported abuse cases; the type of abuse; and the characteristics of the subject. Data were drawn from the Korean Statistical Information Service (KOSIS), the public national statistical database. The present study used 13-year data during the period of 2008-2020. Descriptive analyses were conducted via SPSS. The number of abuse cases has increased every year from 2,369 in 2008 to 6,259 in 2020 and 10% of the reported cases in 2020 were repeated cases. The most common type of abuse reported was psychological abuse followed by physical abuse and neglect over 13 years. Physical abuse increased substantially from 22.4% to 40.0%. The majority of subjects are consistently women, accounting for 68.4% in 2008 and 75.3% in 2020. One in fourth of the subjects are older people living with dementia in 2020. Considering the serious impact of abuse including depression and suicide, abuse of older people is no longer a single individual or family problem. To protect the older population and improve their rights in society, collaborative efforts are critical. Micro-level movements such as increasing social awareness and national-level initiatives such as implementing policies and developing relevant legislation should be necessary.

Abstract citation ID: igad104.2127

### AGE-RELATED MICROAGGRESSIONS: A FOLLOW-UP DESCRIPTIVE STUDY

Hannah Lewis[1], Stephanie Patt[2], Luke Gietzen[3], and Jeffrey Buchanan[2], *1. University of Minnesota, Mankato, Minnesota, United States*, *2. Minnesota State University, Mankato, Mankato, Minnesota, United States*, *3. University of North Dakota, Fargo, North Dakota, United States*

Age-related microaggressions are forms of ageist discrimination that occur during day-to-day interactions. The aim of this study was to identify common types of age-related microaggressions as well as to determine how negative affect influences emotional reactions to microaggressions. Using an online survey, participants ($n = 200$) were asked if they had experienced any of the 20 most common examples of age-related microaggressions reported in previous research (Gietzen et al, 2022). Follow up questions inquired about the frequency, emotional reactions, and behavioral responses to these microaggressions. Participants also rated their physical health and completed the Positive and Negative Affect Scale (PANAS; Watson et al., 1988). The results indicated that participants were familiar with these microaggressions 53% of the time. Participants also reported having negative reactions to 43% of these microaggressions. The frequency of negative emotional responses to microaggressions was significantly correlated with scores on the negative affect subscale of the PANAS ($r = .34$, $p < .001$) and with ratings of perceived physical health ($r = -.32$, $p = .002$). Finally, an analysis of the 20 survey items revealed that two items were "highly impactful" microaggressions, defined as microaggressions that older adults reported experiencing often and reported having a negative emotional reaction to at least one-third of the time. The results of the study provide further insight into what age-related microaggressions look like, and how older adults experience these interactions.

Abstract citation ID: igad104.2128

### AGING AND MENTAL HEALTH DURING AND AFTER THE PANDEMIC. AND OLDER ADULT SPECIALTY PSYCHOTHERAPY CLINIC FINDINGS

Melba Hernandez-Tejada[1], Deborah Little[2], Madeleine Desrochers[3], Sarly Butte[1], and Ron Acierno[1], *1. University of Texas Health Science Center at Houston, Houston, Texas, United States*, *2. University of Texas Health Science Center, Houston, Texas, United States*, *3. University of Houston, Houston, Texas, United States*

A specialty clinic addressing psychological trauma secondary to elder abuse opened a few months before the pandemic began. The aim was to provide evidence-based psychotherapy for post-trauma psychological sequalae. We leveraged technology in the form of home-based telemedicine, to deliver effective treatment directly into older adults' homes. We here present psychotherapy retention rates and clinical outcomes of this endeavor. Method: single arm, repeated measures, clinical series case study design. Outcomes: treatment initiation and completion rates; standardized measures of PTSD (PCL5), depression (PHQ9), anxiety (GAD7) and sleep (PSQI) collected at pre- and post-treatment. **Results:** Prior to implementing this specialty clinic, the larger clinic within which it was housed saw approximately 2 older adults per year. Tailoring the program to older adults resulted in an annual census of over 100 per year. Analysis of current data indicates excellent completion rates for evidence-based psychotherapy (above 75% compared to 60% of younger patients) and clinical improvements across symptom areas in excess of one standard deviation. **Discussion:** The success of this specialty trauma clinic for older adults experiencing violence was enhanced by a pre-pandemic decision to use telemedicine to increase reach and ease of accessing care. Initial results indicate that televideo technology was well received, was used by older adults and implemented before and during the pandemic with no issues, even for cases of severe trauma reactions such as PTSD and depression in older adults.

Abstract citation ID: igad104.2129

### PREVALENCE OF ELDER ABUSE AND NEGLECT OF PERSONS WITH DEMENTIA: A SYSTEMATIC REVIEW AND META-ANALYSIS

Fei Sun[1], Xuehan Zhang[1], Yan Shen[2], Yali Feng[3], and Teri Kennedy[4], *1. Michigan State University, East Lansing, Michigan, United States*, *2. Hubei Normal University, Huangshi, Hubei, China (People's Republic)*, *3. University*

*of Illinois, Urbana, Illinois, United States*, *4. University of Kansas Medical Center, Kansas City, Kansas, United States*

People with dementia (PWD) are at high risk for abuse and neglect. Little is known about the synthesized prevalence rate of abuse among this population though. This study reviewed articles on abuse and neglect among PWD to learn about the pooled prevalence estimates of elder abuse among this population with a meta-analytic approach and examined the heterogeneity associated with the prevalence estimates through meta-regression and subgroup analyses. A total of 27 studies were selected from 479 relevant studies after careful identification using eight academic databases (e.g., ProQuest, CINAHL Plus). All 27 studies received acceptable scores on study quality and demonstrated no significant publication biases. All the studies included a total sample of 11,246 care recipient and caregiver dyads. In addition to overall abuse, three subtypes of elder abuse including physical abuse, emotional abuse, and neglect were examined. Results showed that the pooled prevalence of overall elder abuse was 41% (95%CI [0.31,0.49], $p < 0.001$). The pooled prevalence of abuse subtypes were 43%(95% CI [0.35, 0.52], $p < .001$) for emotional abuse, followed by neglect at 16%, and physical abuse at10% (95% CI [0.07, 0.13], $p < 0.01$). Overall, studies with caregiver average age below 60. reported higher abuse rates than those with caregivers' age over 60. These results call for more policy and practice efforts to assist PWD who are victims of abuse and neglect, and more so, to prevent caregivers from exerting abuse and neglect behaviors toward PWD via education, skills training and support.

Abstract citation ID: igad104.2130

### THROUGH THE LOOKING-GLASS: THE FAMILY SHAME OF ELDER ABUSE

Jessica Hsieh, and Raza Mirza, *University of Toronto, Toronto, Ontario, Canada*

Elder abuse (EA) has been recognized as a serious public health concern. Recent studies have found that approximately 10% of community-dwelling, cognitively intact older adults experience some form of EA each year. Although EA research has made substantial progress, EA is often under-reported, with only an estimated 15% of cases being reported to formal support services. One of the main reasons for the under-reporting of EA is the victims' feelings of shame, which have been shown to be exacerbated when the perpetrator is a family member and/or an individual with whom the older adults have close trusting relationships. Qualitative interviews conducted with 12 older adult (parent) and adult child caregiver dyads (n=24) revealed that older adults who experience EA by their adult children experience intense shame. Thematic analysis focused on what led to these feelings of shame, and this resulted in four main themes: (1) Failure in their role as a parent; (2) Adult children viewing them as powerless and unworthy; (3) Experiencing negative psychological effects; and (4) Self-blame. With a sense of responsibility to protect their family, older adults tend to keep 'family shame' to themselves, leading to a reluctance to disclose EA. As spouses and adult children, the two most common caregiving types, have also been reported to be the two most common groups of EA perpetrators, gaining a better understanding of the root causes of shame can help to support older adults in living safely in their homes and communities for as long as possible.

### SESSION 7020 (POSTER)

### COGNITION (BSS POSTERS)

Abstract citation ID: igad104.2131

### CHILDHOOD FAMILY ENVIRONMENT, GENETIC RISK FOR ALZHEIMER'S DISEASE, AND LATE-LIFE COGNITIVE DECLINE

Shiyang Zhang[1], and Sae Hwang Han[2], *1. The University of Texas at Austin, Austin, Texas, United States*, *2. University of Texas at Austin, Austin, Texas, United States*

How genetic risks for Alzheimer's disease (AD) interact with environmental factors to shape individuals' cognitive trajectories across the lifespan is a burgeoning area of research. However, relatively little is known about the role childhood family environment plays in the relationship between genetic risk factors for AD and cognitive decline in later life. This study examined whether and how early-life family environments may modify cognitive decline associated with genetic AD risks. Using longitudinal data from the Health and Retirement Study (HRS; 1998-2018), we followed over 9,000 individuals aged 51 and older during the observation period spanning nearly two decades. Cognitive functioning was assessed with the full cognitive battery from the HRS. Childhood family environment was evaluated separately with the relationship quality with one's mother and the number of household adults before the age of 16. We estimated a series of multilevel models to assess the association between polygenic risk score for AD (PGS-AD) and changes in cognitive functioning, and whether the association was moderated by the two measures of childhood social capital. Findings indicated that childhood maternal relationship quality, but not the number of household adults, protected against cognitive decline accelerated by PGS-AD. That is, the associations between higher PGS-AD and accelerated cognitive decline were attenuated for individuals who reported a better childhood maternal relationship. Findings are in line with the biopsychosocial model of health and suggest that a better quality of maternal relationships in childhood may have a protective effect on cognitive functioning across the lifespan.

Abstract citation ID: igad104.2132

### COVARIATE-ADJUSTED ASSOCIATIONS OF COGNITION, DEPRESSIVE SYMPTOMS, AND SOCIAL SUPPORT WITH LIFE SPACE MOBILITY

Henrietta Armah, and Olivio Clay, *University of Alabama at Birmingham, Birmingham, Alabama, United States*

Diabetes mellitus is one of the most common chronic diseases among older adults and can affect their movement, participation, and all aspects of daily living, thus restricting life space mobility. Life space mobility reflects individuals' ability to move independently and engage in social activities. This study examines covariate-adjusted associations of cognition, depressive symptoms, amount of support received and satisfaction with support with life space mobility. The study included 247 older adults aged 65 and above from the University of Alabama at Birmingham (UAB) Diabetes and Aging Study of Health (DASH). Average age was 73, 45% of the sample were Black/African American, 53% were female, and 47% were married. Results from multiple covariate-adjusted regression analyses revealed that being Black/African American, older, female, and higher depressive symptoms significantly predicted lower life space mobility (all p's < .05) while being married, educated, and reporting better health significantly predicted greater life space mobility. Similarly, higher cognitive function was a significant predictor of greater life space mobility (B = .140, p < .05). Results remained significant even when adjusted for covariates. Amount of support received and satisfaction with support did not predict life space mobility. Findings from this investigation identify individuals who are at risk for restricted life space mobility and suggest protective factors. Establishing these associations with life space mobility within a health disparities framework would be important as it would draw attention to functioning in later life for socially disadvantaged groups and help inform interventions.

Abstract citation ID: igad104.2133

### FACTORS ASSOCIATED WITH THE USE OF A PERSONALIZED MUSIC INTERVENTION FOR NURSING HOME RESIDENTS WITH DEMENTIA

Ryan Conard[1], Ellen McCreedy[2], Laura Dionne[2], and Vincent Mor[2], *1. Brown University, Providence, Rhode Island, United States*, *2. Brown University School of Public Health, Providence, Rhode Island, United States*

Music offers a promising non-pharmacological alternative for managing behavioral dysregulations in people with Alzheimer's disease and other dementias (ADRD). Using data from an embedded, pragmatic trial (ePCT) of a personalized music intervention for nursing home (NH) residents with ADRD, we examined resident and NH characteristics associated with exposure to the intervention and dose of music received. Participants were enrolled from 54 NHs (27 treatment, 27 control) between June 2019 and February 2020. The intervention was resident-preferred music delivered at early signs of agitation. Intervention dose was calculated by multiplying song duration and number of plays, averaged over days exposed. Facility and resident-level characteristics were identified using the Minimum Data Set and the Certification and Survey Provider Enhanced Reports. A mixed-effects hurdle model was used. 483 residents participated (67.7% female, mean age 79.8±12.2 years). Female residents (p=0.04) taking antipsychotic medications (p=0.06) were more likely to receive the intervention, as were residents from NHs with greater nursing involvement (p=0.02). Residents with greater health instability received a greater dose (p=0.04). In this ePCT of a personalized music intervention, NHs with more nursing engagement had greater use of the intervention and appropriately chose residents with antipsychotic use to participate. After adjusting for initial selection, staff used the intervention more frequently with residents who had a higher likelihood of death in the next six months, potentially indicating the beneficial use for comfort at the end of life. Our findings offer insights into future tailoring of personalized music interventions to increase the likelihood of successful implementation.

Abstract citation ID: igad104.2134

### OLDER AGE IS ASSOCIATED WITH TEMPORAL DISCOUNTING OF TIME USE BUT NOT MONETARY REWARDS

Yochai Shavit[1], Brett Anderson[2], and Laura Carstensen[3], *1. Stanford University, Stanford, California, United States*, *2. MemorialCare Long Beach Medical Center, Anaheim, California, United States*, *3. Stanford, Stanford, California, United States*

Age differences in temporal discounting have long puzzled researchers. Although older adults tend to prioritize the present over the future due to more limited time horizons compared to younger adults there is no evidence for an age association with temporal discounting of monetary rewards. Socioemotional selectivity theory posits that as time horizons become more limited goals related to emotional meaning are prioritized over future-oriented goals because they are realized in the present, leading older adults to place more value on experiences than younger adults. A small body of evidence showing age-related preferences for small, immediate, rewards in emotionally meaningful domains is consistent with SST. However, prior studies used hypothetical tasks with vague trade-offs, limiting their interpretability and generalizability. In the present study, we developed a novel paradigm to examine age differences in temporal discounting of rewards related to emotional experience in a controlled environment. 120 participants, aged 22-96 years, came to the lab and made a series of choices indicating if they would prefer to replace 5 minutes of boring tasks with an emotionally meaningful variant of the task today, or a larger amount of time in their next visit in six months. Participants then made similar choices about the timing of receiving comparable monetary rewards. We hypothesized that in contrast to monetary rewards, older adults are less likely than younger adults to wait for rewards related to emotional experiences because time becomes increasingly valued as it grows scarce. Findings support the hypothesis and highlight the role of perceived time-horizons.

Abstract citation ID: igad104.2135

### RELATING THE ORGANIZATIONAL CULTURE ASSESSMENT INSTRUMENT TO ADHERENCE IN A PRAGMATIC TRIAL OF A MUSIC INTERVENTION

Enya Zhu, Ellen McCreedy, Laura Dionne, and Vincent Mor, *Brown University School of Public Health, Providence, Rhode Island, United States*

Embedded pragmatic trials encourage the translation of evidence-based interventions to "real-world" settings. Most pragmatic trials of behavioral interventions for people with dementia suffer from low adherence. Understanding how organizational values and structure may increase adherence is important. We report findings from an embedded, pragmatic trial (ePCT) of a personalized music intervention for managing behaviors in residents with dementia, conducted in 54 nursing homes (NHs) from four corporations between June 2019 and February 2020. Before the trial began, the administrator and a nursing staff member from each NH completed the Organizational Culture Assessment Instrument (OCAI). Using the OCAI, respondents rated their organizational culture by allocating a total of 100 points across four competing domains: Clan, Adhocracy, Hierarchy, and Market. Results were aggregated to understand how differences in culture impacted corporate-level adoption of the intervention. All four corporations allocated the majority of their points to Clan culture, which is focused on collaboration and staff engagement. However, corporations differed in their scoring of the secondary culture type. The two corporations that rated Hierarchical culture, which prioritizes consistency and efficiency, highly were more likely to adhere to the intervention protocols. The corporation with Market highly rated had the lowest adherence to the protocols. After controlling for other corporate characteristics, including for-profit status, size, and overall quality, hierarchical culture was associated with greater numbers of exposed residents and a higher dose of the music, compared to other culture types. Understanding the role of organizational culture on pragmatic implementation is an understudied area for research.

Abstract citation ID: igad104.2136

### THE ASSOCIATION BETWEEN COGNITION AND UPPER EXTREMITY MOTOR REACTION TIME IN OLDER ADULTS: A NARRATIVE REVIEW

Alexandria Jones, Natalie Weaver, Mardon So, Abbis Jaffri, and Rosalind Heckman, *Creighton University, Omaha, Nebraska, United States*

Response timing is essential to optimal sensorimotor control across the lifespan. While it is broadly assumed that reaction time increases as cognition declines with age, it is unclear if this assumption is supported by the literature. The purpose of this narrative review was to determine the association between cognition and upper extremity reaction time in older adults. Cognitive domains of sensation and perception, motor construction, perceptual motor function, executive function, attention, learning and memory, and language were considered. We conducted a systematic search using Scopus database. The search strategy was designed to meet four inclusion criteria: 1) community-dwelling adults >60 years, 2) upper extremity motor task, 3) at least one cognitive assessment, 4) simple reaction time measure. 1154 articles were screened. Two articles met the full inclusion criteria, but the studies did not associate the cognitive assessment and simple reaction time measures. Nine articles that met three inclusion criteria were reviewed. We found that executive function and learning and memory have been associated with complex and choice reaction time measures. Language, perceptual motor function, and attention have been studied with mixed evidence for an association with reaction time; whereas, sensation and perception and motor construction have not been assessed. Overall, limited research has compared cognitive domain function and simple reaction time to determine if age-related changes are associated. While the complex interplay between cognition and motor function is of substantial interest, these measures are often interdependent and additional knowledge is needed to understand their influence on sensorimotor control with age.

Abstract citation ID: igad104.2137

### THE EFFECT OF DUAL-TASKS ON WALKING SPEED IN HEALTHY OLDER ADULTS: A SYSTEMATIC REVIEW AND META-ANALYSIS

Hyeon Jung Heselton[1], Adam Rosen[2], and Julie Blaskewicz Boron[3], *1. University of Nebraska Omaha, Omaha, Nebraska, United States*, *2. University of Nebraska at Omaha, Omaha, Nebraska, United States*, *3. UNO, Omaha, Nebraska, United States*

Normative age-related changes occur in both walking and cognitive performance; non-normative changes could lead to decreased mobility, loss of autonomy in daily life, and increased fall risk. This systematic review and meta-analysis aimed to assess the effects of completing a dual-task on overground walking speed in healthy older adults. Database searches were carried out in three electronic databases (PubMed, PsycINFO, ProQuest) from inception through October 2022 to find studies that assessed healthy older adults who completed a walking test and a walking task combined with a neurocognitive assessment (dual-task). Methodological quality and risk of bias was assessed via the Strengthening the Reporting of Observational Studies in Epidemiology (STROBE). Cohen's d effect size with a 95% confidence intervals, Cochrane's Q, I2, and Fail-safe N statistics were calculated between the single- and dual-task walking speeds. The initial search yielded 459 articles; six were included in the final review after completing all screening procedures. Included studies had relatively high STROBE scores (19.97±0.82). Eight individual effects across the six articles (total sample size=5627) were calculated. Walking speed significantly decreased in dual-task conditions with cognitive tasks (walking speed Δ=1.03; 95%CI 0.65 to 1.40; p<.001); individual effects were higher during high load cognitive tasks (i.e., serial 7's subtraction, TMT part B). Dual-tasking with a cognitive component clearly affected walking speed in aging adults. Studying different age groups will help create a model of the aging process to understand when changes begin, as well as the rate of change; this could be useful for earlier fall-prevention interventions.

Abstract citation ID: igad104.2138

### THE LONG-TERM RELATIONSHIP BETWEEN BMI AND COGNITIVE PERFORMANCE: A CROSS-LAGGED PANEL ANALYSIS

Andrew Fiscella[1], and Ross Andel[2], *1. University of South Florida, Tampa, Florida, United States*, *2. Arizona State University, Tempe, Arizona, United States*

While obesity has traditionally been associated with negative outcomes, an obesity paradox has been observed which suggests that older adults may show some benefit from having a higher weight. This longitudinal study examined the association between body mass index (BMI) and episodic memory performance. Data from 14,639 participants collected as part of the Health and Retirement Study were used in a 10-year random intercept cross-lagged panel analysis. BMI was used to measure obesity while T-scores from tests of immediate and delayed recall were used to represent episodic memory performance. Initially, a higher BMI was associated with lower episodic memory scores in the following wave (β= -.132, p<.001), but this association became positive over time (β=.142, p< .001) with higher BMI related to better episodic memory scores, even after accounting for demographic and health covariates. The association between BMI and episodic memory over time was stronger in older adults (β=.068, p<.05) than middle-aged adults (β=.020, p=.533).

Additionally, while episodic memory scores were not significantly associated with BMI at the next wave for middle-aged adults (p=.283), they were for older adults (p<.05). These findings provide support for the obesity paradox and are consistent with previous studies employing more traditional longitudinal designs. The results of this study raise questions as to what is behind the association between episodic memory and BMI and why it appears to trend positively in older adulthood. Further studies are needed to examine potential causal mechanisms.

Abstract citation ID: igad104.2139

### ULTRAPROCESSED FOODS AND COGNITIVE FUNCTIONING IN OLDER ADULTS

Elayna Seago, and Benjamin Katz, *Virginia Tech, Blacksburg, Virginia, United States*

Consumption of ultra-processed foods (UPFs) is increasing globally. Previous research has explored the psychological effects of this dietary shift, including associations between diet and cognition. While this previous work shows that neuroprotective dietary patterns in general, and UPF consumption in particular, may have a deleterious effect on cognition, less research has explored the specific cognitive domains most closely associated with UPF consumption. The present study draws from the 2016 Harmonized Cognitive Assessment Protocol and the 2012 Health Care and Nutrition Study (HCNS) from the Health and Retirement Study to examine the relationship between UPF consumption and a wide a wide array of fluid and memory-based cognitive measures in older adults. Additionally, we used the food frequency questionnaire from the HCNS to categorize participants' foods as ultra-processed using the NOVA system. Initial analyses using analysis of covariance with UPF bins, controlling for age, gender, and self-rated health, indicates that measures of processing speed, but not other fluid measures nor most measures of delayed recall, were most closely associated with UPF consumption. These initial findings provide more specific detail regarding the cognitive correlates of UPF-heavy diets and provide a pathway to future work that more closely examines the longitudinal effects and underlying physiological mechanisms of UPF consumption.

Abstract citation ID: igad104.2140

### WITHIN-PERSON ASSOCIATIONS BETWEEN MARITAL DISSOLUTION AND SUBSEQUENT COGNITIVE FUNCTION IN MIDDLE AND LATER LIFE

Julia Tucker, and Sae Hwang Han, *University of Texas at Austin, Austin, Texas, United States*

In light of the growing burden associated with cognitive impairment and dementias on families and society accompanied by the dramatic shifts in late-life family demography, there is growing literature focusing on the relationship between marital biography and cognitive outcomes in middle and late adulthood. This study aimed to contribute to the literature by examining within-person linkages between cognitive outcomes associated with two forms of marital dissolutions experienced in later life—divorce and widowhood. Framed around the key concepts of the life course perspective, we examined within-person changes in cognitive function associated with divorce and widowhood transitions in middle and late adulthood. Drawing from 11 waves of longitudinal data from the Health and Retirement Study (1998-2018), we followed over twenty-three thousand married individuals 50 and older over the study period that spanned nearly two decades (person-wave observations N=128,033). Cognitive function at each wave was evaluated using a modified version of the Telephone Interview for Cognitive Status (m-TICS). Research aims were addressed using within-between random effects models that yielded within-person estimates unconfounded by person-level characteristics. Approximately 4.4% and 17.7% of the study sample experienced divorce and widowhood transitions during the observation period. The model results indicated that divorce transitions were unrelated to changes in cognitive function. Cognitive outcomes associated with widowhood transitions were heterogeneous for women and men, where women who transitioned to widowhood experienced increases in cognitive function, whereas men experienced reductions in cognitive function. These findings are discussed in the context of implications for practice and policy.

### SESSION 7030 (POSTER)

### DEPRESSION AND ANXIETY

Abstract citation ID: igad104.2141

### A LONGITUDINAL EXAMINATION OF THE EFFECT OF RESILIENCE AGAINST ANXIETY DURING COVID-19

Dawn Carr[1], Natalie Sachs-Ericsson[1], Julia Sheffler[2], Melissa Meynadasy[1], Brad Schmidt[1], and Greg Hajcak[1], *1. Florida State University, Tallahassee, Florida, United States, 2. Florida State University College of Medicine, Tallahassee, Florida, United States*

This longitudinal study of community dwelling older adults (N = 453) examined consequences of COVID-related worries on changes in anxiety symptoms before relative to during the pandemic. We further evaluated if pre-COVID psychological resilience (PR) buffered the impact of COVID-related worry. Pre-COVID data were collected in September 2018. COVID-related worry and COVID anxiety symptoms were collected in October 2020 (Wave 2). Controlling for pre-COVID anxiety symptoms, we examined if COVID-related worries (e.g., I'm worried that I might die from COVID-19) were associated with increased anxiety symptoms, and whether pre-COVID PR moderated the association between COVID-related worries and prospective increases in anxiety symptoms. COVID-related worries were associated with increased anxiety symptoms ($\beta = 0.005$, $p < .01$), whereas pre-COVID PR was associated with a decrease in anxiety symptoms ($\beta = -0.029$, $p < .05$). PR moderated the association; COVID-related worries were associated with greater increases in anxiety symptoms among those with low pre-COVID PR (Model $\eta 2 = 0.35$). Thus, the extent to which COVID-related worries influenced psychological health was dependent on pre-COVID levels of PR. We conclude the combined vulnerabilities of low pre-COVID PR and high COVID-related worries significantly increased the psychological consequences of COVID-19 for our sample of older adults.

Abstract citation ID: igad104.2142

### A RANDOMIZED CONTROLLED TRIAL OF SELF-HELP GUIDED BY LAY PROVIDERS FOR GENERALIZED ANXIETY DISORDER IN OLDER ADULTS

Philippe Landreville[1], Sébastien Grenier[2], Patrick Gosselin[3], and Pierre-Hugues Carmichael[4], *1. Université Laval, Quebec City, Quebec, Canada*, *2. Université de Montréal, Montreal, Quebec, Canada*, *3. Université de Sherbrooke, Sherbrooke, Quebec, Canada*, *4. Centre d'Excellence sur le Vieillissement de Quebec, Quebec, Quebec, Canada*

Low use of professional help for anxiety disorders in older adults may be due in part to limited access to mental health services. Self-help guided by lay providers (LPs) could help improve access to treatment. This study evaluated the efficacy and long-term outcomes of self-help guided by LPs for generalized anxiety disorder (GAD) in older adults. Participants (≥ 60 years) with threshold or subthreshold GAD were randomly assigned to an intervention group (n = 75) or a wait-list control group (n = 75). Intervention group participants used a manual presenting readings and exercises based on principles of cognitive-behavioral therapy and received weekly support calls by LPs. Groups were similar in terms of sociodemographic characteristics and initially did not differ significantly on outcomes. At post-treatment, the intervention group had decreased significantly on various measures while the wait-list group remained stable: Generalized Anxiety Disorder 7-item (GAD-7): -4.8 vs -.03; Penn State Worry Questionnaire (PSWQ): -11.7 vs .07; Geriatric Anxiety Inventory (GAI): -5.3 vs -.07. Probability of having a diagnosis of threshold GAD decreased significantly more in the intervention group (79% to 16% vs 74% to 53%). Intervention group scores on the GAD-7, the PSWQ, and the GAI as well as probability of having a diagnosis of threshold GAD remained stable at 6- and 12-month follow-ups. Results provide evidence of the efficacy of self-help guided by LPs for GAD in older adults as well as of the maintenance of improvement following this treatment.

Abstract citation ID: igad104.2143

### A STUDY ON THE TYPES OF CHANGES IN DEPRESSION AND COVID-19 AMONG OLDER ADULTS IN KOREA

Kyu-Hyoung Jeong[1], Ju Hyun Ryu[2], Seoyoon Lee[2], and Sunghee Kim[2], *1. Semyung University, Jecheon, Republic of Korea*, *2. Yonsei University, Seoul, Republic of Korea*

This study focuses on the long-term trends in depression levels in older adults, with a particular focus on the period following the onset of COVID-19. While previous studies have examined depression levels in older adults, this study aims to complement those studies by providing a longitudinal perspective. This study used data from the Korea Welfare Panel Study(KoWePS), which included 2,716 data of older adults aged 65 and above, from 2017 to 2021. The study variables included gender, age, income, educational background, residential area, living alone, and disability status. The results of this study identified two types of changes in depression levels in older adults: the 'rapidly rising' type and the 'steadily increasing' type. The study found that those who were more likely to belong to the 'rapidly rising' type were those with lower equalized annual income, women, those with lower education, those living in urban areas, and those living alone. These findings suggest that those who are already at risk for depression are at even higher risk during and after the COVID-19 pandemic. Given these findings, it is crucial to provide timely and effective guidelines to prevent depression in older adults during outbreaks of infectious diseases such as COVID-19. Policymakers should consider the findings of this study when implementing policies aimed at preventing depression in older adults. Overall, this study provides important insights into the long-term trends in depression levels in older adults and highlights the importance of addressing this issue in response to the ongoing COVID-19 pandemic.

Abstract citation ID: igad104.2144

### CHANGE IN ENGAGEMENT WITH PRODUCTIVE SOCIAL ACTIVITIES: HOW IT PREDICTS LATE-LIFE DEPRESSION OVER TIME

Chin-Yi Su[1], and Shiau Fang Chao[2], *1. Taiwan Association of Family Caregivers, Taipei, Taipei, Taiwan (Republic of China)*, *2. National Taiwan University, Taipei City, Taipei, Taiwan (Republic of China)*

Research has identified that engaging in activities is associated with lower levels of depression among older adults. However, there is less discussion about how changes in participation in different types of productive social activities (PSAs) affect depression in later life. This study examined data from Taiwan Longitudinal Study on Aging, including 2,865 participants aged 55 or above, from 2011 (T1) to 2015 (T2). The relationship between changes in PSAs and depression was tested through six regression models. Results indicated that: 1. Older adults who dropped out from work or were continuously unemployed experienced higher levels of T2 depression compared to those who remained continuously employed. 2. Older adults who never volunteered experienced higher levels of T2 depression compared to those who continuously engaged in volunteering. 3. Engaging in informal caregiving for relatives did not reduce T2 depression. In contrast, older adults who dropped out of providing care for other adults experienced lower levels of T2 depression compared to those who continuously offered care. This study shows that older people tend to gradually decrease their engagement in PSAs in later life, but those who continue to engage in PSAs tend to have lower levels of depression over time. Employment and volunteering appear to predict depression. It is worth noting that while informal caregiving is also considered a PSA, people who stopped providing care for other adults experience lower levels of depression. This research calls for further investigation into mechanisms of varied type of PSAs affect wellbeing in later life.

Abstract citation ID: igad104.2145

### COVID-19 AND DEMOGRAPHIC PREDICTORS OF CAREGIVER BURDEN AND DEPRESSION

Julian Montoro-Rodriguez[1], Jennifer Ramsey[2], and Dolores Gallagther-Thompson[3], *1. University of North Carolina Charlotte, Charlotte, North Carolina, United States*, *2. Case Western Reserve University, Cleveland, Ohio, United States*, *3. Stanford, Stanford, California, United Sta tes*

Family caregivers are at increased risk for negative impacts on their health including higher depression compared to non-caregivers. The COVID19 pandemic presented challenges to adults with health conditions and exacerbated the demands that caregivers faced to continue providing care, support, services, and managing negative emotions. Our team in North Carolina adapted an evidence-based psychoeducational program "Coping with Caregiving" developed from the REACH II studies, and created the "Caregiver Thrive, Learn & Connect" to assist caregivers to manage stress, learn skills and connect with others. Caregivers attended workshops early in 2021 up to 2023 (https://caregivertlc.org/). COVID-19 research shows that burden, social support, education, income and age are predictors of depression. Using the initial data (pre-program intervention, Time 1) from the Caregiver TLC intervention we examined the impact of COVID-19 on the caregivers' level of burden and depression. Our sample includes 107 informal caregivers (age mean=63; female=84%; White=66%; Black 33%; living with spouse=68%; and median income of $75-$89K). Predictors of depression were regressed on the caregiver burden, the COVID19 severity exposure (2021 vs. 2022/23), the caregiver age, race (Blacks), education, income and perceived health. We also explored predictors of burden. Results indicated moderate adjusted R2 for depression (.42%) and burden (.16%). COVID-19 was a significant predictor for depression (-.33), along with the caregivers' health (-.20). Burden was negatively impacted by the caregivers' health (-.22), age (-.20) and for Blacks (-.20). Caregivers' depression was impacted by the early stage of COVID19 in addition to their level of burden.

Abstract citation ID: igad104.2146

### DEPRESSIVE COGNITIONS AND SYMPTOMS IN CAREGIVING GRANDMOTHERS: TESTING GROWTH CURVE MODEL STABILITY

Christopher Burant, Carol Musil, Jaclene Zauszniewski, and Alexandra Jeanblanc, *Case Western Reserve University, Cleveland, Ohio, United States*

Grandmothers caring for grandchildren have elevated depressive symptoms levels compared to grandmothers who do not provide care. Depressive symptoms are state-like in nature and describe recent depressive symptoms. The Depressive Cognition Scale© captures changes in negative thinking patterns that often precede depressive symptoms. Depressive cognitions, according to Beck's theory of depression, are the first depressive symptoms to appear and typically lead to more serious symptoms of depression. Previously diagnosed depression may also contribute to current levels of depressive cognitions and depressive symptoms. Data were collected on 342 participants in a longitudinal nationwide online research study of caregiving grandmothers. A latent growth curve model of how previously diagnosed depression and depressive cognitions impacted the trajectory of depressive symptoms at four time points (baseline, 2 weeks, 12 weeks, and 24 weeks) was tested. The model fit the data well (Chi Square=24.301.; df=12; p=.019; TLI=.977; CFI=.987; RMSEA=.055). This model was submitted to 6 separate multigroup nested model tests (levels of income, education, age, employment, number of children, and primary vs. multigeneration families) to provide evidence supporting generalizability of the model. All models fit the data well (TLI=.960-.991; CFI=.973-.992; RMSEA=.051-.025). Total invariance/stability, based on nonsignificant changes in chi square and CFI<.01, was supported in the multigroup analyses across all levels testing equality in measurement and regression weights and residual variances. This lack of change in the model across 6 different grandmother scenarios provides further evidence supporting the need for interventions to address diagnosed depression and depressive cognitions for reducing depressive symptoms in grandmother caregivers.

Abstract citation ID: igad104.2147

### EVALUATING THE ACCEPTABILITY AND PRELIMINARY EFFECTS OF EMPOWER@HOME FOR GERIATRIC DEPRESSION

Jay Kayser, Skyla Turner, Chuxuan Zheng, Samson Ash, and Xiaoling Xiang, *University of Michigan, Ann Arbor, Michigan, United States*

Internet-based cognitive behavioral therapy is a cost-effective strategy for meeting older adults' increased mental health needs, but few existing programs are designed for older adults. Our team developed Empower@Home using user-centered design and community-engaged approaches. This poster presents results from year 1 of our ongoing uncontrolled trial (NCT05384704). Participants were recruited from a research volunteer registry, and community agency referrals. 104 adults 50+ (M=63) consented and started the intervention in 2022, of which 96 completed the post-test and a 10-week follow-up (93% retention rate). Items from the Modified Treatment Evaluation Inventory showed high satisfaction and acceptability. For example, 95.5% agreed that the program was "an acceptable way of dealing with depressed moods," and 96.8% agreed that they "would recommend this program to others who experience depressed moods." Adherence was high, with an average of 8.5 sessions (out of 9 total) completed (n=103). Among those who completed the post-test (n=96), all but one finished the entire program. Intention to treat analysis showed a significant within-group moderate reduction in depressive symptoms as measured by the PHQ-9 from pre to post-test (cohen's d=.75). This effect size was large among those with a pre-test PHQ-9 score of ≥10 (cohen's d=1.31). The treatment effect was largely maintained at the 10-week follow-up. Significant improvements in post-test were also observed for secondary clinical outcomes, including anxiety (GAD-7), social isolation (DSSI), loneliness (PROMIS), Anger (PROMIS), and sleep quality. Empower@Home is an acceptable and potentially effective program for treating depression in older adults.

Abstract citation ID: igad104.2148

### FACTORS AFFECTING SUICIDAL IDEATION IN COMMUNITY-DWELLING OLDER ADULTS WITH DEPRESSION

Shelby Smith[1], Lisa Barry[2], David Steffens[3], and Richard Fortinsky[1], *1. University of Connecticut, Farmington, Connecticut, United States*, *2. UConn Center on Aging, Farmington, Connecticut, United States*, *3. University of Connecticut School of Medicine, Farmington, Connecticut, United States*

Suicidal ideation (SI) within the older adult population represents a continuing public health crisis. The previously published 5D Framework utilizes a set of five domains (depression, disability, disconnectedness, disease, and deadly means) to explain multifaceted influences on late-life suicide. This study explored factors associated with SI among community-dwelling older adults with depression by including measures of selected 5D Framework domains and additional factors potentially associated with SI in an explanatory analytic model. We utilized baseline data from an ongoing clinical trial to assemble a cohort of older adults (age 65+) who screened positive for depression via provider diagnosis or scoring ≥16 on the Center for Epidemiologic Studies Depression scale (CES-D). Individuals joining the clinical trial between May 2017-March 2020 completed baseline assessments evaluating levels of depression, cognitive impairment, psychological resilience, dispositional optimism, social engagement, level of financial strain, and educational attainment. Sl was defined as endorsement of Question 9 of the PHQ-9. In the study cohort (N=171), mean age was 73.5 [SD: 5.76], 67.3% female, 92.4% non-Hispanic White, and 18.1% endorsed SI. In a multivariate logistic regression analysis, increased severity of depression was positively associated with SI (AOR 1.18 [95% CI:1.06-1.31]) while higher level of optimism was inversely related with SI (AOR 0.913 [95% CI:0.84-0.96]). No statistical significance was noted for age, gender, education, financial strain, resilience, cognitive impairment, or social engagement. We conclude that optimism is a protective factor against SI, and reporting more severe depressive symptoms represent an important risk factor for SI, among older adults with depression.

Abstract citation ID: igad104.2149

### HEALTH-RELATED ANXIETY, DOCTOR–PATIENT RELATIONSHIP, AND COMMUNICATIONS IN LATE LIFE

Poshan Dahal, Eva Kahana, Nan Zhou, Yuanchang Zhao, and Dyanna Burnham, *Case Western Reserve University, Cleveland, Ohio, United States*

The relationship and communication between doctor and patient have important implications for the quality of health care received in old age. However, their role in health-related anxiety among older patients has received limited attention. In this paper, we explored the association between doctor-patient communication and doctor-patient relationship with health-related anxiety among 661 independent, retirement community-dwelling older adults. The mean age of the respondents was 77.81 years, and the majority (60.54 percent) were women. Our findings show a negative association between health–related anxiety and the doctor-patient relationship (b=-11, p<.05). We also found a negative association between satisfaction with communication and health-related anxiety (b=-.09, p<.05). The relationship between health-related anxiety and doctor-patient relationship and doctor-patient communication remains even after controlling for covariates. Older adults who reported better communication and relationship with their physician had lower health-related anxiety. Compared to Blacks, Whites had significantly lower health-related anxiety (b=-.80, p<.05). For every additional chronic condition, health-related anxiety increased by 0.22 (p< 05). Gender, education, and age, however, were not associated with health anxiety. Overall, our findings support the hypothesis that better doctor-patient relationships and communication are associated with lower health-related anxiety among community-dwelling older adults.

Abstract citation ID: igad104.2150

### IT BOTHERS ME TO ASK FOR HELP: MODERATION OF SELF RELIANCE ON MASCULINE DEPRESSION AND SUICIDAL IDEATION IN OLDER MALES

Sabine Lohmar, Jeongwi An, Montgomery Owsiany, Erika Fenstermacher, and Amy Fiske, *West Virginia University, Morgantown, West Virginia, United States*

Compared to females, males are less likely to seek help and less likely to be diagnosed with depression despite having depressive symptoms. Masculine depression, a ‘masked’ depression is associated with greater endorsement of masculine norms such as self-reliance. Self-reliance, or a preference to not seek help from others, is related to suicidal ideation in males. Although death by suicide remains the highest among adult males over the age of 75, research has yet to examine the role that self-reliance has on suicidal ideation in older males with masculine depression. This study examined the moderating effect of the self-reliance subscale of the Conformity to Masculine Norms Inventory (CMNI-30) on the relation between the Male Depression Risk Scale-22 (MDRS-22) and the Suicide Behaviors Questionnaires-Revised (SBQR). This study included 214 males aged 65 and older surveyed on Mechanical Turk. A multiple regression analysis indicated that self-reliance moderated the relation between the MDRS-22 and the SBQR (β =.220, t = 2.174, p=.031). Graphical representations of these data indicated that the relation between the MDRS-22 and SBQR was stronger in those with higher self-reliance. Thus, older males with masculine depression may be more likely to endorse suicidal ideation if they endorse self-reliance norms, or are less likely to seek help from others. This study illustrates the importance of self-reliance when understanding older males with elevated suicide risk. Future research should address how other masculine norms influence or explain the relation between masculine depression and suicidal ideation.

Abstract citation ID: igad104.2151

### MIGRATION OVER THE LIFE COURSE AND LATER-LIFE DEPRESSION IN CONTEMPORARY CHINA

Nan Zhang, *University of Manchester, Manchester, England, United Kingdom*

Migrating between rural and urban areas over the life course profoundly shapes the conditions of later life. In the Chinese context, living in urban areas with an urban Hukou is associated with socioeconomic advantage. This study is among the first attempt to investigate how migration into urban areas in China is related to these processes and the association with risk of depression in later life by focusing on the timing and the type of migration (rural-urban residential mobility and/or institutional transition of Hukou status) of migration. Using data from China Health and Retirement Longitudinal Study, we found strong associations between migration over the life course and risk of depression in later life in China. The timing and type of migration appears to play an important role. In-situ urbanisation is associated with lower depression scores in later life, and these effects are greater for in-situ urbanisation occurring in middle age compared with young adulthood. Forced urban-rural migration is associated with improved mental wellbeing. Formal social protection, particularly having a private pension, contributes substantially to the mental health advantage of social groups with an urban Hukou. Having an urban Hukou origin has an independent protective role in shaping mental wellbeing in later life in China, potentially partly due to the entitlement to a private pension attached to this status. When informal support has weakened in contemporary China, enhanced formal social protection in the form of adequate pensions should be put in place to mitigate structural inequalities associated with migration in old age.

Abstract citation ID: igad104.2152

**RELATIONSHIP BETWEEN MEDIA IMAGE, PERCEIVED AGE STEREOTYPE, AND AGING ANXIETY IN KOREAN OLDER ADULTS**

Miri Kim, and Soondool Chung, *Ewha Womans University, Seoul, Republic of Korea*

This study aimed to investigate the relationship between media image, perceived age stereotype, and aging anxiety in older adults in South Korea, in order to gain a better understanding of the psychosocial and financial-related anxieties faced by older adults in later life. Given that South Korea is projected to become the most aged nation in the world within 50 years, studying aging anxiety in this context is particularly important. A total of 600 older adults from 65 to 84 years of age were analysed using structural equation modelling and bootstrapping method, using a cross-sectional, secondary data from OO Research Institute of South Korea. The findings indicated that media image was positively correlated with perceived age stereotype (β=.380,p<.001), aging anxiety on financial factors (β=.145,p<.001), and aging anxiety on psychosocial factors (β=.136,p<.01), while perceived age stereotype was correlated with aging anxiety on psychosocial factors (β=.205,p<.001). In terms of indirect effects, perceived age stereotype mediated the relationship between media image and aging anxiety on psychosocial factors (95% CI: 0.034 to 0.121). The results suggest that older adults who encounter more negative images of old age and aging in the media are more likely to develop perceptions of age stereotype, which in turn increases their aging anxiety on psychosocial factors. Practical implementations are suggested to reduce aging anxiety. The study's findings have implications for reducing aging anxiety and understanding the experiences of older adults in the era of population aging.

Abstract citation ID: igad104.2153

**THE EFFECT OF MEDIA USAGE DURING THE COVID-19 PANDEMIC ON DEPRESSIVE SYMPTOMS IN KOREAN OLDER ADULTS**

Miri Kim, and Soondool Chung, *Ewha Womans University, Seoul, Republic of Korea*

The current study was conducted to examine the structural relationship between change in the media usage during the COVID-19 pandemic, media use frequency, perception of social connectivity and depressive symptoms among older people in South Korea. The study sought to address the increased use of media among older adults during the pandemic. A cross-sectional dataset with 398 older adults aged 65 to 94 living in Seoul was analyzed using a structural equation model with the bootstrapping method. The findings are as follows. In terms of direct effects, older adults who used more media during the COVID-19 pandemic showed lower level of depressive symptoms (β=-.141,p<.001), older adults who used media more frequently showed an increase in the perception of social connectivity (β=.123,p<.001), and older adults with higher perception of social connectivity showed lower level of depressive symptoms (β=-.371,p<.001). In terms of the indirect effects, the perception of social connectivity had a significant mediating effect in the relationship between change in the media usage during the COVID-19 pandemic and depressive symptoms (95% CI: -0.044, -.001), as well as in the relationship between media use frequency and depressive symptoms (95% CI: -.059, -.033). The findings of the study underscore the importance of social connectivity in promoting mental health among older adults during periods of social isolation and highlight the potential benefits of media usage.

Abstract citation ID: igad104.2154

**THE EFFECTS OF LATE-LIFE RELOCATION ON ANXIETY AND PERSONALITY TRAITS: EVIDENCE FROM CHINA AND EUROPE**

Yang Sun[1], Cornelia Wrzus[2], and Shao-bo Lv[3], *1. Ruprecht Karl University of Heidelber, Heidelberg, Baden-Wurttemberg, Germany*, *2. Heidelberg University, Heidelberg, Baden-Wurttemberg, Germany*, *3. North China University of Technology, Tangshan, Hebei, China (People's Republic)*

Later-life relocation has been identified as an important life event that affects the living environment, social relationships, and daily routines of older adults with subsequent effects on mental health and personality. We explored the impact of relocation on anxiety and personality traits in older adults from China and Europe in two separate studies., In study 1, we administered the questionnaire to 301 Chinese older adults (Mage=69.51, SD=5.02) who experienced involuntary rural-urban relocation, with 61.8% of them reported severe to extremely severe anxiety. Excessive reassurance-seeking and concomitant negative information bias were processes that contributed to increased anxiety among relocated older adults, which was particularly detrimental for those adults who were otherwise resilient. In study 2, we investigated the differences in personality traits between older adults who moved to nursing homes and those who lived in private housing using data from Wave 7 of the European Health, Ageing and Retirement Survey (SHARE). Reports from a total of 4829 older people living in nursing homes (NNH= 360) and private housing (NPH=4469) were included in the finally analysis. Our results showed that older people who relocated to nursing homes had lower extraversion and conscientiousness, and higher neuroticism. Additionally, older adults' age, gender, health problems, physical activity, community size, and national socio-economic status partly explained personality differences between older adults who relocated and who stayed in their homes. Our findings underscore the importance and necessity of studying advantageous and detrimental effects of late-life relocation, and provide new directions and ideas for future research.

Abstract citation ID: igad104.2155

**THE ROLE OF SOCIAL MEDIA COMMUNICATION IN THE ASSOCIATION BETWEEN WIDOWHOOD AND DEPRESSIVE SYMPTOMS**

Nahyun Kim, Youjin Kim, Hyeonji Cho, and Kyungmin Kim, *Seoul National University, Seoul, Republic of Korea*

Losing a spouse is one of most painful experiences in adulthood; widowed adults often suffer from psychological difficulties. Scholars have examined how social support from close relationships such as family members and friends plays a protective role in the context of spousal loss. Given that the use of mobile technologies and internet became an important way to engage with social relationships, it is crucial to investigate the role of social media communication in the bereavement process. This study examined the moderating effects of social media communication in the association between widowhood and depressive symptoms, comparing different types of social media communication—chatting/messaging (e.g., iMessage), social media (e.g., Facebook), video-sharing (e.g., YouTube), and online community platforms (e.g., forums). From the 2021 Korea Social Integration Survey, we analyzed a sample of 5,238 respondents (aged 40+) who were ever-married. Our regression models showed that widowed respondents (9.79%) were likely to have more depressive symptoms (B = 0.19, p < .001) than non-widow(er)s; all types of social media communication were associated with fewer depressive symptoms, regardless of widowhood. We also found that two types of social media communication moderated the association between widowhood and depressive symptoms; such that widowed respondents who used social media (B = -0.08. p =.002) and online community platforms (B = -0.05. p = .038) more frequently had fewer depressive symptoms, compared to their counterparts. Our findings highlight the importance of examining social engagement via information and communication technologies and the potential mechanism contributing to widowed adults' well-being.

Abstract citation ID: igad104.2156

### WECHAT SOCIAL MEDIA USE AND DEPRESSION AMONG CHINESE OLDER ADULTS

Wan Wen[1], and Shannon Mejia[2], *1. University of Illinois Urbana-Champaign, Urbana, Illinois, United States*, *2. University of Illinois Urbana-Champaign, Chanpaign, Illinois, United States*

Impairments in health and mobility, the loss of a partner, and geographic relocation increase the risk of social isolation and depression in older adulthood. As the world's most populous country, China faces a rapidly aging population, and Chinese society recognizes the potential of the Internet as a measure to reduce depression and promote well-being among older adults. WeChat is the most used social media among Chinese older adults, who use it to connect with social partners and exchange information by reading and posting messages. However, previous research only identified a correlation between WeChat use and lower depressive symptoms but failed to uncover the causality between them. This paper used data from the 2018 China Health and Retirement Longitudinal Study and adopted an instrumental variable (IV) and the two-stage least squares model to strengthen causal inference. The principle is using an IV, which is correlated with WeChat use but not influenced by depressive symptoms, to replace WeChat use in regression. There were 1,680 participants (mean age 73.03, 55% male), 3% of whom reported use of WeChat in the past two years. Regression results suggested that WeChat use did not lead to lower levels of depression among older adults; instead, only non-depressed older adults would use WeChat. Further analysis showed WeChat use could promote older adults' communication with family members and social activity engagement. Although there is no solid evidence of WeChat's effect on depression, it does provide some benefits and still holds the potential to promote the well-being of older adults.

### SESSION 7040 (POSTER)

### EMOTIONS, EMPLOYMENT, AND WELL-BEING

Abstract citation ID: igad104.2157

### AGE DIFFERENCES IN EMOTION UTILITY KNOWLEDGE: COMPARING DISCRETE VERSUS DIMENSIONAL MODELS

Nikolina Kravljaca[1], Jennifer Stanley[2], and Eric Allard[3], *1. The University of Akron, Barberton, Ohio, United States*, *2. University of Akron, Akron, Ohio, United States*, *3. Cleveland State University, Cleveland, Ohio, United States*

Do young and older adults have the same metacognitive schema for emotional utility? We examined young and older adults' knowledge of which emotion would be best for achieving a goal (e.g., anger for confrontation). We captured responses using both a discrete emotions framework (e.g., happy; Ekman 1992) and a dimensional/circumplex model approach (e.g., arousal and valence; Russell, 1980) to determine which framework is most useful for understanding age differences in utilitarian emotion knowledge. Seventy-five young and 66 older adults indicated the most useful emotion when playing video games associated with different goals (Approach, Avoid, Confront, Analyze). Participants were asked to rate discrete emotions as well as identify the best combination of preferred valence and arousal on an affect grid. Two separate mixed-design ANOVAs were conducted for discrete emotion and dimensional ratings. Young and older adults exhibited similar preferences for both the discrete and circumplex model and chose the emotion with the most expected utility for each goal (e.g., happy for approach). One interesting age difference emerged where young adults endorsed the expected utilitarian emotion of sadness for the analyze goal to a greater extent than older adults. For the affect grid ratings, age differences were driven by arousal but not valence. We discuss how a discrete emotions approach may allow some clarity and a common language for interrogating the role of aging in utilitarian emotionality but may lack the nuance provided by the circumplex model.

Abstract citation ID: igad104.2158

### DIGITAL SKILLS, STEM OCCUPATION, AND JOB AUTOMATION RISK AMONG THE OLDER WORKERS IN THE US

Takashi Yamashita[1], Donnette Narine[2], Runcie Chidebe[3], Phyllis Cummins[3], Jenna Kramer[4], and Rita Karam[5], *1. University of Maryland, Baltimore County, Baltimore, Maryland, United States*, *2. University of Maryland Baltimore, Baltimore, Maryland, United States*, *3. Miami University, Oxford, Ohio, United States*, *4. RAND*

*Corporation, Arlington, Virginia, United States*, *5. RAND Corporation, Santa Monica, California, United States*

Rapidly advancing technologies are replacing human labor. Particularly, occupations with more routine tasks, such as sales and simple food preparations, are known to be at a higher risk of job automation than those with non-routine tasks, such as STEM occupations (e.g., engineering, health care). Additionally, an aging workforce generally faces higher job automation risks due to skill obsolescence and lower digital skills than younger counterparts. A better understanding of the associations between job automation risk, digital skills, and STEM (vs. non-STEM) occupations can facilitate preparations for continuing job automation and population aging. We analyzed a nationally representative sample of middle-aged and older U.S. workers aged 50 to 74 (n = 1,570) from the 2012/2014/2017 Program for International Assessment of Adult Competencies (PIAAC) restricted-use-file data. The estimated job automation risks (i.e., % of jobs to be automated in the next decades) by occupation were derived from the previous studies. PIAAC digital problem-solving skills proficiency (0-500 points) was assessed based on a series of practical digital tasks (e.g., sorting emails by responses in an application). Results of the survey-weighted linear regression showed that the greater digital skill proficiency ($b = -0.04$, $p < 0.05$) and STEM occupations ($b = -17.18$, $p < 0.05$) are associated with lower job automation risks, even after adjusting for a series of demographic, socioeconomic and civic engagement characteristics. Education and labor policy interventions to promote digital skills among older workers and non-STEM workers may better prepare the aging workforce for the future labor market in the U.S.

Abstract citation ID: igad104.2159

### EMPLOYMENT AND LIFE SATISFACTION IN LATER LIFE: THE MEDIATING ROLE OF SUBJECTIVE EXPECTATION

Si Young Song[1], Bomi Choi[2], Hayoung Park[2], and Miseon Kang[2], *1. Kyung Hee University, Seoul, Republic of Korea*, *2. Yonsei University, Seoul, Republic of Korea*

Although the productive aging framework has been emphasized to promote active roles such as employment to improve psychological well-being in later life, the mechanisms that explain the association between employment and life satisfaction remain unclear. Therefore, this study sought to examine the mediation role of subjective expectation about future living standards in the association between employment and life satisfaction among older Korean adults. The data was the 8th wave (2020) of the Korean National Longitudinal Study on Aging, and the sample included 4,238 older Korean adults aged 65 or above. Employment was coded as 0 when they are not working and 1 when working. Life satisfaction was measured by overall quality of life on a ten-point scale. Subjective expectation, the mediating variable, was measured by the subjective evaluation of future living standard on a ten-point scale (e.g., "I think my standard of living will get better in the future"). PROCESS MACRO model 4 in SPSS 26.0 were utilized to test indirect and direct effects while gender, education level, household income, and presence of spouse were controlled for. As a result, employment had a direct effect on life satisfaction and indirect effects were also significant, indicating employment contributes to a higher level of life satisfaction through higher subjective expectation. The findings revealed that the mechanism between employment and life satisfaction among older adults can be explained through subjective expectation, indicating the importance of considering beneficial effects of employment that enhance psychological well-being when developing intervention programs for older adults.

Abstract citation ID: igad104.2160

### EXPLORING EMOTION CONTROL BELIEFS ACROSS THE LIFESPAN: ROLES OF AGE AND CHRONIC ILLNESS

Jocelyn Rutledge, and Meaghan Barlow, *Wilfrid Laurier University, Waterloo, Ontario, Canada*

Existing theory and research highlights emotion control beliefs have important implications for emotion regulation and psychological well-being. Emotion control beliefs can be subdivided into four categories: the extent to which people believe 1) they can control their emotion (self-can beliefs), they should control their emotions (self-should beliefs), 3) others can control their emotions (others-can beliefs), and 4) other should control their emotions (others-should beliefs). To our knowledge, research has yet to examine how developmental factors are associated with emotion control beliefs in adults (i.e., age and non-normative experience of chronic illness). Past research highlights age related differences in emotional processes, and the influence of non-normative chronic illness on emotional well-being. Therefore, the present study utilized data from a lifespan sample of 47 participants (younger adults: n = 21, age range = 19-35, M = 27.55; older adults: n = 26, age range = 68-81, M = 72.40) to examine differences in emotion control beliefs among younger and older adults, people with and without chronic illness, and the interaction between these factors. Our analyses revealed levels of self-can beliefs were lower in people with chronic illness. In particular, younger adults with chronic illness reported the lowest levels of self-can beliefs. By contrast, among people without chronic illness, older adults reported lower levels of others-should beliefs, relative to younger adults. These findings inform theories of emotional aging, development, and health by suggesting emotion control beliefs vary with age and chronic illness.

Abstract citation ID: igad104.2161

### EXPLORING THE WITHIN-PERSON RELATIONSHIP BETWEEN DAILY DISCRIMINATION AND DAILY STRESS REACTIVITY

Jessica Maras, and Kate Leger, *University of Kentucky, Lexington, Kentucky, United States*

Experiencing discrimination has been associated with a variety of negative impacts such as poorer health and well-being. Some research has shown that those who experienced discrimination at an initial timepoint were more likely to have poorer health and well-being outcomes 20 years later through the mediating role of threat appraisals and stress reactivity. Research has demonstrated a link between experiencing discrimination and affective reactions to everyday stressful experiences, however, these associations have only been studied at the between-person level. For the current study, we examined the within-person relationship in daily stress reactivity in relation to experiencing discrimination on a daily basis over the course of 20 years. Participants (n = 1,315) completed waves 1 to 3 of the Midlife in the United States (MIDUS) survey with each wave about 10 years apart in which participants were asked to self-report their daily discrimination. Participants also completed the National Study of Daily Experiences (NSDE) which was a daily diary design over 8 consecutive days asking about their daily stress reactivity. Results found that during timepoints when people experienced greater than their average level of discrimination, they also reacted more negatively to daily stressful events (b = .002, 95% CI [0.001, 0.002]). These results are particularly striking because they show that within-person changes in reported discrimination are related to changes in affective responses to daily stressors. These findings suggest that experiencing discrimination is related to greater reactivity to daily stressors, which may lead to worse physical and mental health over time.

Abstract citation ID: igad104.2162

### WHAT IS IMPORTANT FOR THE FEELING OF MEANINGFULNESS? A STUDY OF SWEDES AGED 76–101 YEARS

Ingemar Kåreholt[1], and Amelie Bergström Af Ekenstam[2], *1. Jönköping University, School of Health and Welfare, Jönköping, Jonkopings Lan, Sweden*, *2. School of Health and Welfare, Jönköping, Jonkopings Lan, Sweden*

Sense of coherence (SOC) reflects the coping capacity to deal with everyday life stressors and consists of comprehensibility, manageability and meaningfulness. Meaningfulness is the motivational component and sometimes considered the most important part in SOC. Meaningfulness is about the extent to which a person feels that life has an emotional meaning. Meaningfulness is important in aging since many old people suffer from health problems and meaningfulness is important for coping. We examine which factors that are important for the feeling of meaningfulness in a nationally representative random sample of 777 Swedes aged 76-101 years. Meaningfulness is measured with a single item question "Do you usually feel that your daily life is a source of personal satisfaction?". 11% answered "no", 20% "yes, sometimes", 59% "yes, often". Analyzed with ordered logistic regressions. Control variables: sex, age, education, frailty index, mobility, and psychological wellbeing. Mobility and psychological wellbeing were significant and kept in the analyses, The main independent variables were having a faith (or philosophy), cultural, intellectual, outdoor activities and the social variables loneliness, social contacts outside home, social relations to relatives and friends, contact with children/grandchildren, and phone contacts with children/grandchildren. The social variables were analyzed simultaneously. Loneliness, social relations to relatives and friends, phone contacts with children/grandchildren were significant as well as all other main independent variables. Analyzed simultaneously, controlled for each other, mobility and psychological wellbeing, faith, loneliness, social relations to relatives/friends, and contacts with children/grandchildren were significant. This indicates that these factors are important for meaningfulness in old age.

Abstract citation ID: igad104.2163

### WORKPLACE AND PERSONAL ATTRIBUTES AFFECTING WORKPLACE PSYCHOLOGICAL BURDEN OF MATURE AND AGING WORKERS IN HONG KONG

Daniel W.L. Lai[1], Xue Bai[2], Vincent W.P. Lee[1], Kin Cheung[2], Calvin W. Luk[2], and Chi-Ko Lee[2], *1. Hong Kong Baptist University, Hong Kong, Hong Kong*, *2. The Hong Kong Polytechnic University, Kowloon, Hong Kong*

Older employees are valuable human resources for society. However, changes in cognitive abilities and mental health in older persons may affect their ability to work, as well as their safety and health at work. The purpose of this study is to investigate the effects of older workers' personal attributes such as demographics and lifestyle, physical health status, mental health status, labour intensity of employment, and experience of occupational and health risks on their psychological burden at work. A quantitative telephone survey was conducted with a stratified random sample of 1,225 workers aged 40 or above. The results of multiple linear regression analysis indicated that the men and workers with better sleep quality tended to suffer less from psychological burden at work. Older workers with longer weekly work hours and incidents of work-related injuries tended to have a higher level of psychological burden, whereas a safer work environment perceived by the older workers was associated with a lower psychological burden. To accommodate their needs, employers are recommended to allow the older employees choices of reducing work hours and flexible work arrangements. The application of assistive devices and relevant technologies for minimizing the threats to safety and health for the sake of the employees' long-term psychological well-being is important for institutions and employers to consider.

### SESSION 7050 (POSTER)

### FAMILY AND RELATIONSHIP TIES

Abstract citation ID: igad104.2164

### A CROSS-CULTURAL EXAMINATION OF SOCIAL PARTICIPATION, FAMILY INTERACTION, AND DEPRESSIVE SYMPTOMS

Aruhan Mu, *Huazhong University of Science and Technology, Wuhan, Hubei, China (People's Republic)*

Social participation and family interaction are core components of later life. Less is known about cross-cultural differences regarding the relationships between social participation, financial support, caregiving, and depressive symptoms among older adults. This study aimed to estimate the association between these factors and depressive symptoms among older adults in Europe and China. We selected individuals aged 60 years and above from the 2017 Harmonized Survey of Health, Ageing, and Retirement in Europe (SHARE) (n = 11,676) and the 2018 Harmonized China Health and Retirement Longitudinal Study (CHARLS) (n = 5,559). Stepwise linear regression was used to analyze the data. Participation in social activities was found to be associated with lower levels of depressive symptoms among both European and Chinese older adults. Among Chinese older adults, providing financial transfers to adult children was associated with lower levels of depressive symptoms, whereas transfers to older parents was not significant. Care for older parents was associated with lower levels of depressive symptoms, while caregiving for children or grandchildren was not significant. Among European older adults, providing financial transfers to others was not significant. Care for grandchildren was associated with lower levels of depressive symptoms, while caring for other family members was associated with higher levels of depressive symptoms. In conclusion, participation in social activities, providing financial transfers to adult children, and caring for older parents have positive effect on psychological well-being of Chinese older adults. For European older adults, social activities and caring for grandchildren prove to be more beneficial.

Abstract citation ID: igad104.2165

### A LONGITUDINAL EXAMINATION OF SEXUAL SATISFACTION ACROSS ADULTHOOD IN MIDUS

Riki Slayday[1], Martin Sliwinski[2], and David Almeida[1], *1. The Pennsylvania State University, State College, Pennsylvania, United States*, *2. Pennsylvania State University, State College, Pennsylvania, United States*

Sexuality is fundamental to the human experience and midlife is a transition period in adulthood where sexual changes often occur. Sexual satisfaction (SS) in midlife and older adulthood is still under studied. However, researchers have found a more pronounced decline in SS among women compared to men beginning in late midlife or early old age. The purpose of the present study was to examine how sexual satisfaction changes across adulthood (20-92 years) and at what ages women and men differ on their ratings of sexual satisfaction. Participants were from waves 1, 2, and 3 of MIDUS (n=2,348; Mage=54.34 years). SS was operationalized using "How would you rate the sexual aspect of your life these days," with a scale from 0 to 10 where 0 means "the worst possible situation" and 10 means "the best possible situation". Time-varying effect modeling (TVEM) revealed that there was an overall linear decline until the early 60s, then the decline steepened. Men and women did not differ from ages 20 to 44. However, around age 45, men ($\beta$=5.95; CI:5.83,6.07) began reporting significantly greater SS than women ($\beta$=5.62; CI:5.45,5.74), which lasted almost four decades. At age 84, women ($\beta$=3.36; CI:2.94,3.78) reported significantly greater satisfaction then men ($\beta$=3.32; CI:2.89,3.74). On average, SS declined with age. Women reported lower SS than men for almost four decades (ages 40-80); however, women reported higher satisfaction than men starting in their early eighties. No studies have examined SS across adulthood or gender differences in SS using TVEM.

Abstract citation ID: igad104.2166

### CONNECTING LIFETIME EXPERIENCE OF MISCARRIAGE TO POSITIVE LIFE EVALUATION AMONG WOMEN AGING-IN-CUSTODY

Brianna Barnett[1], Hailey Minet[1], Alexander Bishop[1], and Kevin Randall[2], *1. Oklahoma State University, Stillwater, Oklahoma, United States*, *2. Sam Houston State University, Huntsville, Texas, United States*

This study assessed the interconnection between miscarriage, forgiveness, and social provisions on positive valuation of life among N = 157 older women (45 years and older) under correctional custody in Oklahoma. Based on the Model of Developmental Adaptation, path analysis was conducted positing valuation of life, the developmental outcome, regressed on the proximal influence of socio-emotional support and forgiveness and the distal lifetime report of miscarriage. Endogenous measures were controlled for age, race, marital status, education, and crime type. Using Mplus 8.8 and an estimator (MLR) that provides parameter estimates and a chi-square test statistic robust to non-normality, the model fit well with a non-significant chi square test statistics and significant parameter estimates. For these women, 40.5% of the variation in positive valuation of life was explained, primarily by socio-emotional support. Miscarriage had a significant positive association with socio-emotional provisions ($\beta$ = .15, $p < .05$) but not forgiveness. Meanwhile, forgiveness had a significant direct association with positive valuation of life ($\beta$ = .46, $p < .001$). A significant indirect effect emerged for miscarriage on positive valuation of life through socio-emotional support (.047, p =.035, one-tail test). Results suggest that older incarcerated women with a reported history of miscarriage achieve a positive life outlook to the extent they feel emotionally supported. Greater engagement in forgiveness also appears to contribute to a positive life perspective. This has implications relative to how correctional counselors, social workers, and case managers offer services to support older incarcerated women with a reported history of miscarriage.

Abstract citation ID: igad104.2167

### FACTORS ASSOCIATED WITH THE PSYCHOLOGICAL WELL-BEING OF OLDER MEN WHO HAVE SEX WITH MEN IN THE CHINESE POPULATION

Alex Siu Wing Chan[1], and Elsie Yan[2], *1. The Hong Kong Polytechnic University, Hong Kong, Hong Kong*, *2. THe Hong Kong Polytechnic University, Hong Kong, Hong Kong*

Older men who have sex with men (OMSM) experience discrimination based on their age and sexual orientation, which presents major challenges to their health. Existing studies have examined discrimination experienced by younger MSM. This study aims to examine the discrimination experienced by OMSM in the People's Republic of China (PRC), Hong Kong, and Taiwan. The eligible participants for this research comprised OMSM aged over 60 years. The demographic information of the participants was collected. Particularly, the General Health Questionnaire-12 (GHQ-12), Personal Social Capital Scale (PSCS), Perceived Acceptance Scale (PAS), and Global Index on Legal Recognition of Homosexual Orientation (GIRLHO) scales were used to assess psychological well-being, discrimination, social capital, perceived acceptance, and legal inclusion. Discrimination differs across PRC, Hong Kong, and Taiwan ($F(2, 450) = 112.07$, $p < .001$), and it also has a negative impact on psychological well-being (B = -1.16, $p <.001$). Social capital was found to have a negative impact on psychological well-being (B = -3.38, $p < 0.001$), and a positive impact on legal inclusion (B = 1.55, $p< 0.001$). Lastly, social acceptance has a positive impact on psychological well-being (B = 0.52, p = .035). This study provides new insights into the negative impact of discrimination on the psychological health of OMSM and mitigating factors representing the positive effects. However, future studies are necessary to bridge the literature gap regarding the associations between the different factors described in this study.

Abstract citation ID: igad104.2168

### IN-HOSPITAL INSTRUMENTAL CARE PROVIDED BY FAMILY AND STAFF AND HOSPITALIZATION OUTCOMES OF OLDER ADULTS

Ksenya Shulyaev[1], Nurit Gur-Yaish[2], Efrat Shadmi[1], and Anna Zisberg[1], *1. University of Haifa, Haifa, Hefa, Israel, 2. Oranim Academic College of Education, Tivon, HaZafon, Israel*

Most of hospitalized older adults receive instrumental care (such as help with dressing, bathing, eating) from hospital staff and family members. Current study explored effect of family and staff involvement in this kind of care on hospitalization outcomes. Study sample included 615 acute hospitalized older adults (Age 77.6± 6.7, females 44.3%). Data about patients' independence in Basic Activity of Daily Living (BADL), level of depression and anxiety was collected twice: at admission to hospital and at discharge. Sociodemographic (age, sex, family status and number of children) and health (severity of illness, comorbidity, length of stay and cognitive status) characteristics, were collected at admission and from medical records. Involvement of family and staff in instrumental care during hospitalization was reported by patients at discharge. The multivariate regressions, which included all the covariates mentioned above, revealed that instrumental care provided by family and by hospital staff significantly predicted hospitalization outcomes, yet in opposite directions. Specifically, family involvement in instrumental support was associated with lower BADL recovery ($\beta$=-.36, p<.001), as well as increased depression levels ($\beta$=.13, p=.002) and anxiety ($\beta$=.17, p<.001). In contrast, instrumental care provided by staff was associated with better BADL recovery ($\beta$=.24, p<.001), as well as decreased depression ($\beta$=-.21, p<.001) and anxiety ($\beta$=-.11, p=.013). These findings highlight the importance of effectively balancing family and staff involvement in instrumental care to optimize patient recovery, improve patients' outcomes and ensure that they receive the best possible care during their hospital stay.

Abstract citation ID: igad104.2169

### LINKING ACES TO LIFE APPRAISAL AMONG OLDER WOMEN IN CORRECTIONAL CUSTODY

Brianna Barnett[1], Alexander Bishop[1], and Kevin Randall[2], *1. Oklahoma State University, Stillwater, Oklahoma, United States, 2. Sam Houston State University, Huntsville, Texas, United States*

Adverse Childhood Experiences (ACEs), forgiveness, social support, and valuation of life were assessed for older women (45 years of age and older; N = 157) and younger women (those less than 45 years of age: N = 386) in custody in Oklahoma. The Model of Developmental Adaptation led to a path analysis with positive valuation of life or the developmental outcome regressed on proximal influences assessed by mediating variables, social provisions, and forgiveness, and these on the distal predictor, adverse childhood experiences. All endogenous measures were controlled for age, race, marital status, education, and crime type. Using Mplus 8.8 and an estimator that provides parameter estimates and a chi-square test statistic robust to non-normality, the study found that the model fit well for both age group. For the older women, 41% of the developmental outcome, positive valuation of life was explained; whereas for the younger women, 30% was explained. In addition, the standardized path from ACEs to Forgiveness not significant for either the younger or older women. However, for the younger women, ACEs was negatively associated with social provisions ($\beta$ = -.109, p = .035). In addition, a significant indirect effect was found for the path from ACEs to valuation of life through social provisions (-.03, p = .026, one-tailed). Results have implications relative to how forensic psychologists, correctional case managers, and social workers support the mental health needs of women aging-in-custody. Further discussion will highlight strategies to reduce the impact of ACEs and improve quality-of-life.

Abstract citation ID: igad104.2170

### LONGITUDINAL STUDY OF ADULT SIBLING RELATIONSHIP AND PSYCHOLOGICAL WELL-BEING: THE ROLE OF CHILDHOOD MALTREATMENT

Jooyoung Kong[1], Kristin Homan[2], and Jaime Goldberg[3], *1. University of Wisconsin-Madison, Madison, Wisconsin, United States*, *2. Grove City College, Grove City, Pennsylvania, United States*, *3. University of Wisconsin-Madison , Madison, Wisconsin, United States*

**Objectives:** For many older adults, siblings serve as a source of companionship and support. This study examined a) the longitudinal association between sibling relationship quality and psychological well-being among older adults and b) whether the association would differ by childhood maltreatment. **Methods:** We used three-wave data from the Wisconsin Longitudinal Study. Latent growth curve modeling was conducted to examine the associations between perceived sibling closeness and the psychological well-being of 4,736 older adults. **Results:** Our findings showed that the trajectory of perceived sibling closeness differed between older adults who experienced childhood maltreatment and those who did not. Perceived sibling closeness was lower at baseline for those who experienced maltreatment and did not change over time, whereas perceived sibling closeness increased over time for those who did not experience maltreatment. Further, older adults who reported greater sibling closeness over time showed less of a decline in psychological well-being. The longitudinal associations between perceived sibling closeness and psychological well-being did not differ between the maltreated and non-maltreated groups. **Discussion:** Childhood maltreatment was found to have long-term negative effects on the emotional quality of sibling relationships in late adulthood. More research is needed to explore potential mechanisms that explain these lifelong associations.

Abstract citation ID: igad104.2171

### MIGRATE OR STAY? PSYCHOLOGICAL WELL-BEING AMONG MIGRANT AND LEFT-BEHIND OLDER ADULTS IN CHINA.

Xiaoyuan Chen[1], Minjie Lu[2], and Helene Fung[3], *1. Beijing Normal University at Zhuhai, Guangzhou, Guangdong, China (People's Republic)*, *2. Beijing Normal University at Zhuhai, Zhuhai, Guangdong, China (People's Republic)*, *3.*

*The Chinese University of Hong Kong, Hong Kong, Hong Kong*

When adult children migrate from households, older parents face two options: migrate with children or remain in place ("left behind"). However, it remains unclear which option is better for older adults' psychological well-being. With the data from China Health and Retirement Longitudinal Survey (CHARLS) at the wave of 2015 and 2018 (N = 4989), this study examined the longitudinal changes in depression and life satisfaction among older locals (those who did not migrate and lived with their children), migrants and left-behinds, and the impacts of children support and social support on the well-being of older parents. Results showed that there was an increase in depression level among the left-behind older adults from 2015 to 2018, but this was not found among the migration group or the local group. Furthermore, the left-behind group reported a higher level of depression than the local group and migration group, and this difference was accounted by the time of living with children, but not social activities. In addition, the time of living with children was positively associated with life satisfaction among the left-behind group, and this association was not observed among the migration group or the local group, suggesting the salutary effect of children's support among the left-behind group. These results highlight the adverse effect of being left behind among older adults and the importance of children's support for left-behind older adults.

### SESSION 7060 (POSTER)

### NEW DIRECTIONS IN GERONTOLOGICAL THEORY, METHODOLOGY, AND TRANSLATIONAL RESEARCH

Abstract citation ID: igad104.2172

### ACCEPTABILITY OF A REMINISCENCE THERAPY TABLET APP: IMPACT ON RESIDENTIAL CARE STAFF OF PERSONS LIVING WITH DEMENTIA

Silvia Orsulic-Jeras[1], Ashlee Cordell[1], Sara Powers[1], Farida Ejaz[1], and Lisbeth Sanders[2], *1. Benjamin Rose Institute on Aging, Cleveland, Ohio, United States*, *2. LifeBio, Marysville, Ohio, United States*

With dementia prevalence rapidly on the rise and no viable pharmacological treatments currently available, the development of accessible, efficacious, and low-cost non-pharmacologic interventions for persons living with dementia (PLWD) has become critical. Reminiscence therapy (RT) and life story can be built into residential care provision to provide person-centered solutions that elicit conversation, engagement, and socialization with staff. RT tools can also help inform care staff about residents' lives and preferences to provide higher quality and meaningful interactions. This paper details the development and evaluation of LifeBio Memory, a tablet application designed to record the life stories and care preferences of PLWD in residential care communities. Care staff (n = 60) from 10 communities in Ohio, including memory care, assisted living and nursing homes, were trained to implement LifeBio Memory in their organizations. A pre-post design was used with staff, mainly comprised of life enrichment, administration, social work, rehab, and direct care staff. Outcomes presented include but will not be limited to: 1) acceptability, feasibility, and satisfaction with the tablet app, 2) staff knowledge of resident care preferences and interests, 3) utilization of person-centered care practices, 4) quality of communication between the resident and themselves and other staff, 5) their current understanding of that resident's life history, and 6) level of tension and frustration with the resident. Discussion will focus on the need for sustainable RT interventions that promote person-centered care for PLWD and ways in which technological advancements can be integrated into residential care staff workflow.

Abstract citation ID: igad104.2173

### ADAPTING AUTOETHNOGRAPHY TO EXPLORE CULTURAL AND GENERATIONAL DIFFERENCES ON AGING

Mohammad Hossain, and Natalie Pope, *University of Kentucky, Lexington, Kentucky, United States*

This presentation uses adapted autoethnography to explore cultural and generational differences on aging identity and care for older adults. Our presentation is based on an oral history of Virginia Bell, a gerontological social worker and dementia expert who developed an innovative model of dementia care known as the Best FriendsTM approach (Bell & Troxel, 2012; 2016). Through several in-depth interviews, this oral history project created space for conversation across generations – discussions between two cisgender white women, both social workers and gerontologists. At age 98, Bell reflected on people and experiences that shaped her perspective on caring for people with dementia from a person-centered, relational approach. The interviewer, in her mid-40s, assumed a co-interpreter role with Bell, documenting her childhood and formative years, as well as what informed her approach to caring for people with dementia. In this presentation, we bring a third voice into the conversation. During data analysis, a first-year graduate student was invited to help code and analyze Bell's interview transcripts. As a novice gerontologist and social worker from Bangladesh, this cisgender male in his 40s shares his reactions to Bell's oral history interviews. Specifically, this autoethnography-inspired presentation examines cultural and generational differences on aging identity and cultural norms on care for older adults. We compare and contrast the personal/ professional experiences of Bell and this Bangladeshi student while situating their reflections in extant literature. This project highlights the utility of autoethnography to systematically analyze individuals' experiences with aging embedded in a larger social and cultural context.

Abstract citation ID: igad104.2174

### CHANGES IN DRIVER'S LICENSE STATUS AMONG MID-AGED AND OLDER CANADIANS OVER THREE YEARS

Arne Stinchcombe[1], Shawna Hopper[2], Sylvain Gagnon[1], and Michel Bedard[3], *1. University of Ottawa, Ottawa, Ontario, Canada*, *2. Simon Fraser University, Vancouver, British Columbia, Canada*, *3. Lakehead University, Thunder Bay, Ontario, Canada*

For older people, driving may contribute to health and quality of life. Conversely, driving cessation is associated with negative outcomes, including poor physical and mental health. We examined changes in driving status over a three-year period among participants in the Canadian Longitudinal Study on Aging (CLSA), which includes Canadians aged 45-85 at baseline. At baseline (data collected between 2011 and 2015) and follow-up (data collected between 2015 and 2018), participants reported whether they had a driver's license. Using multiple logistic regression, we examined the relationship between covariates and changes in license status (i.e., having a license at baseline but not at follow-up vs. maintaining license). Of the participants who reported having a driver's license at baseline (n=36,266), 1.19% (n=432) reported no longer having one at follow-up. This change was associated with lower income categories and poorer self-rated health. Age (B=.12, p<.001) and depression symptoms (B =.04, p<.001) were positively associated with no longer having a license. Participants who reported a change had lower scores on a memory task (Rey Auditory Verbal Learning Test; B=-.07, p<.001). Women had greater odds than men to report a change in driver's license at follow-up (OR=1.5, p<.001). The results highlight the salience of health, cognition, and income as correlates of driving cessation in a sample of mid-aged and older adults. These results may help identify individuals who are likely to stop driving and who may need additional supports maintaining mobility, health, and quality of life.

Abstract citation ID: igad104.2175

### DIFFERENT TYPES OF INTERNET USE AND SOCIAL ADAPTATION AMONG OLDER ADULTS

Heng Zhao, Huamao Peng, Zhiyu Fan, and Jingxuan Wu, *Beijing Normal University, Beijing, Beijing, China (People's Republic)*

Adaptation is pivotal in achieving successful aging. However, the surge in Internet use and social distancing measures during COVID-19 exacerbate the existing adaptation difficulties of older adults with insufficient Internet exposure. Despite this, little attention has been paid to older adults' Internet use and related social adaptation. This study aimed to explore how older adults' different types of Internet use influence their social adaptation and determine how to increase their Internet use from the user's perspective to promote social adaptation. We recruited 388 adults aged 60 to 83 and measured their frequency of four principal types of Internet use: information-seeking, social interaction, instrumental use, and leisure & entertainment. Structural equation modeling was employed to compare effects of different types of Internet use on older adults' social adaptation, as well as examine the predictive roles of Internet control belief and involvement. These results revealed instrumental use and leisure & entertainment were significantly and positively associated with the social adaptation of older adults among the four types of Internet use, whereas information-seeking and social interaction were not. Additionally, Internet control belief and involvement positively predicted all four types of Internet use among older adults and were associated with social adaptation through instrumental use and leisure & entertainment. The findings imply that instrumental use and leisure & entertainment are particularly important for promoting social adaptation among older adults. To increase Internet use and promote social adaptation, interventions should prioritize these types of Internet use and focus on enhancing Internet control belief and involvement.

Abstract citation ID: igad104.2176

### GENERATING OLDER ACTIVE LIVES DIGITALLY (GOALD) THROUGH SPORT-BASED REMINISCENCE

Richard Haynes[1], John Ritchie[2], and Simone Tomaz[1], *1. University of Stirling, Stirling, Scotland, United Kingdom*, *2. Communications, Media and Culture, Stirling, Scotland, United Kingdom*

How do digitally-delivered meaningful activities, such as sport-based reminiscence, influence older people's health and well-being and intergenerational relationships? Developing and strengthening older people's 'connectivities' through their links with community, resources and meaningful activities is a key aspect of supporting healthy ageing and reducing health inequalities in later life. Sport-based reminiscence has become an established activity to facilitate social connectivity and increasingly, digital connectivity, which can support an individual's sense of health and well-being. This paper reports on research undertaken within a three-year research programme on 'Connectivity and Digital Design for Promoting Health and Well-being Across Generations, Places and Spaces' focusing on research conducted with four community-based co-production groups in Scotland. The qualitative research was developed with a stakeholder advisory group and co-production groups in care homes and in community. Data was gathered from recording of meetings and researcher observations thematically analysed to document shared experiences of digitally enabled sport reminiscence over a ten month period. We report on the efficacy of live-streamed and digital sport heritage experiences for triggering reminiscence in a hybrid context. We emphasise the need for structured facilitation of hybrid online/in-person reminiscence, which can clearly produce meaningful and enjoyable experiences for older people, and building connectivity.

Abstract citation ID: igad104.2177

### GEROTRANSCENDENCE INTERVENTIONS FOR CAREGIVERS: A SCOPING REVIEW

Oscar Ribeiro[1], Lia Araujo[2], Taiane Abreu[3], and Laetitia Teixeira[4], *1. University of Aveiro, Aveiro, Aveiro, Portugal*, *2. Cintesis, Porto, Porto, Portugal*, *3. ICBAS, Porto, Porto, Portugal*, *4. School of Medicine and Biomedical Sciences, University of Porto, Porto, Porto, Portugal*

According to Tornstam, gerotranscendence is a psychosocial adaptive theory of aging that postulates over a gradual mindset shift that occurs from middle age and progresses during old age, with individuals becoming less materialistic and more transcendental in actions and thoughts. Despite being a natural process, interventions based on gerotranscendence have been associated with improvements in well-being and are starting to receive attention among professionals working with older adults. This study aims to map interventions based on gerotranscendence targeting older adults' caregivers and summarize their main outcomes. Six databases were searched, and three final studies were selected and analyzed based on the Template for Intervention Description and Replication (TIDieR). All studies took place in nursing homes and long-term care (LTC) facilities with staff members. Two studies presented a long-term intervention with a qualitative approach, while one presented a short-term intervention with a quasi-experimental approach. All interventions had a moment of group instruction about the gerotranscendence theory, in which participants were informed about gerotranscendence signs that could be recognized and/or promoted in older adults. All three studies reported positive outcomes suggesting that informing and training caregivers on gerotranscendence can contribute to a higher quality of care and to an improvement of the caregiving relationship.

Abstract citation ID: igad104.2178

### GOLF CARTS AS A TRANSPORTATION MODE: WHO USES THEM?

Anne Barrett, Hope Mimbs, Jessica Noblitt, Cherish Michael, and Brianna Soulie, *Florida State University, Tallahassee, Florida, United States*

Golf carts are rising in popularity among older adults, not only for use on the course but also for traveling short distances. Alongside their rising popularity are increases in related injuries, which have been the focus of the relatively small literature on golf carts. Little is known, however, about the frequency or social patterning of older adults' use of golf carts as a transportation mode. Using data from an online survey of Floridians aged 50 and older that was conducted between December 2020 and April 2021 (n=4,199), we used multivariate regression to examine five sets of predictors of golf cart use: sociodemographics, health, subjective aging experiences, transportation experiences, and social relationships. Sociodemographics predicting more frequent golf cart use included being younger, male, or white. More frequent golf cart use also was associated with lower levels of loneliness but was unrelated to physical health. Other predictors of more frequent golf cart use included reporting older age identities and more frequent walking. Also significant were social relationship measures. Results revealed more frequent golf cart use among married individuals and those interacting more frequently with friends – but less frequently with family members. By examining a variety of factors that may influence golf cart use, our study provides clues about how golf carts may relate to older adults' transitioning from driving.

Abstract citation ID: igad104.2179

### LOCATION MODERATES THE EFFECT OF COGNITIVE TRAINING ON CRASH RISK OVER 20 YEARS: AN ANALYSIS OF THE ACTIVE STUDY

Katie Wheeler[1], Tyler Bell[2], Brooke Carroll[1], Caitlin Pope[3], Karlene Ball[1], Olivio Clay[1], Despina Stavrinos[1], and Sheila Black[4], *1. University of Alabama at Birmingham, Birmingham, Alabama, United States*, *2. University of California, San Diego, San Diego, California, United States*, *3. University of Kentucky, Lexington, Kentucky, United States*, *4. The University of Alabama, Tuscaloosa, Alabama, United States*

Driving is a critical everyday function that helps older adults remain independent. The Advanced Cognitive Training in Vital Elderly (ACTIVE) study has shown cognitive training improves driving safety outcomes for older adults. Few studies have examined the longitudinal effects of cognitive training on crash risk beyond 5 years. To address this empirical gap, we aimed to determine the effects of cognitive training on crash risk over 20 years, and how urbanicity moderates these relationships. Using participants from the no-contact control and intervention (speed of processing, memory, and reasoning training) groups in the ACTIVE study (n=2170) and state-recorded crash reports, cox proportional hazards models were used to examine the effect of three cognitive training interventions as predictors of time to first at-fault crash over 20 years. Participants (mean age 73, 73% White, 23% Male) reported driving 90.6 miles/week on average and 12.6% experienced an at-fault crash. Overall, speed of processing training was the only intervention related to lower crash risk, but this varied by urbanicity. Namely, speed of processing training was related to lower crash risk for individuals living in rural environments (HR=.16, 95% CI [.04-.73], p=.018) but not for individuals in urban environments (HR=.89, 95% CI [.54-1.50], p=.679). Findings highlight the safety benefits of speed of processing training for older drivers living in rural environments where more fatal crashes occur. However, interventions are still needed to improve safety in urban environments where more crashes occur generally. Consideration of location in future interventions may help improve safe driving for older adults.

Abstract citation ID: igad104.2180

### OLDER ADULT PERCEPTIONS OF HEALTHY AGING MEASURES

Payton Rule[1], Mathias Allemand[2], and Patrick Hill[1], *1. Washington University in St. Louis, St. Louis, Missouri, United States*, *2. University of Zurich, Zurich, Zurich, Switzerland*

Little is known about how lay older adults view measures commonly employed to assess healthy aging. This study examined older adults' perceptions of scales capturing psychological wellbeing, psychosocial factors, and physical health. Participants (n = 60 US adults; MAge = 65.49, 41.18% Female, 94.12% White) were asked to rate how easy each measure was to understand and interpret on a scale of 1 (Not easy at all) to 7 (Very easy). In addition, they were asked to identify any items with which they had difficulty responding. Finally, participants provided opinions of whether these scales fully addressed their perspective on healthy aging, or if additional components were needed. Results showed that, on average, participants rated the aging-related outcomes measures as easy to understand and interpret (M = 6.14). Older adults rated measures related to physical health and health behaviors as the easiest to use (the PROMIS Health Measure (M = 6.47) and the Good Health Practices Scale (M = 6.49)), while measures that capture future planning were the most difficult to comprehend and interpret (the Consideration of Future Consequences Scale (CFC; M = 5.19) and the Goals Related to Healthy Aging Scale (M = 5.73)). While participants generally reported satisfaction with the comprehensiveness of the measures, some (22%) felt that these measures did not fully capture the social aspects of aging. Future measurement development may benefit from older adults' input to increase their utility and better capture the experiences of older adults.

Abstract citation ID: igad104.2181

### PROMOTING HONG KONG ADULTS TO AGE WELL: INTERVENTION EFFECTS ON AGING PREPARATION ACROSS FOUR DOMAINS

Hongmei Lin, Verona Ji Ying Leung, Courtney Sen Yee Yue, and Helene Fung, *The Chinese University of Hong Kong, Hong Kong, Hong Kong*

Preparations for old age benefit physical and psychological health in later life. The population in Hong Kong has the longest life expectancy in the world. However, Hong Kong residents prepare less for their old age than their counterparts in the US and Europe. Hence, an evidence-based intervention program was developed to improve old-age preparation in Hong Kong. The program aimed to comprehensively cover the preparation for old age by involving four domains (finance, health, social relationship, and leisure activity). Specifically, the program started with four workshops where participants learned from experts on how to prepare for old age in four domains. Then, participants were grouped according to their domains of interest, and guided planning was provided to empower them to hold their old age-preparation activities for six months. A tracking survey was conducted every two weeks during the period. Finally, 140 adults (31 males; 45-83 years of age, Mage = 65.21, SD = 7.52) finished the intervention program. Findings showed that participants increased their levels of self-control, readiness for, and satisfaction with aging preparation in three domains (all except finance) after joining the workshop. After six months of intervention, a reduction in anxiety and an improvement in meaningfulness were also found in all four domains. These findings suggest that the intervention program motivates individuals to engage more in age-related preparatory behaviors across domains. This has practical implications for training old-age preparation.

Abstract citation ID: igad104.2182

### THE SOCIOLOGY OF AGING AND SOCIAL GERONTOLOGY: CRITICAL TENSIONS AND NECESSARY DISTINCTIONS

Paul Higgs, and Chris Gilleard, *University College London, London, England, United Kingdom*

This paper argues that the sociology of ageing has been diverted from developing a clearly sociological approach to ageing and old age by its entanglement with social gerontology. Dominated by social and health policy and advocacy on behalf of older people, the sociological analysis of later life has taken second place. The rise of critical gerontology and cultural gerontology have put further distance between sociology and the sociology of ageing. The changes to ageing and old age challenge the residualisation of ageing within the wider sociological community and set up new tasks for a sociology of ageing which resonates with contemporary later life. We argue that to achieve those tasks requires not only separating the sociology of ageing from social gerontology, but also employing contemporary sociological theory can enable the sociology of ageing to move beyond the 'problem' of old age. This involves an analysis of the social structural dimensions of contemporary ageing, its symbolic representations and the embodied and agentic experiences of people becoming older. We conclude that a re-envisioned sociology of ageing can also better equip social gerontology with more effective tools to understand the 'new ageing'.

Abstract citation ID: igad104.2183

### USING INERTIAL MEASUREMENT UNITS (IMUS) TO DETECT MOBILITY DECLINES IN MIDDLE-AGED INDIVIDUALS

Elsa Davis[1], Mithran Periassamy[1], Braden Stock[1], Peter Altenburger[2], Satyajit Ambike[1], and Jeffrey Haddad[1], *1. Purdue University, West Lafayette, Indiana, United States, 2. Indiana University, Indianapolis, Indiana, United States*

Valid and reliable assessments of mobility are routinely used in clinics to track patient progress, assess the need for assistive devices, and predict risk of falling. These tests require patients to perform tasks that span varying degrees of difficulty so that therapists can observe changes in mobility and balance that can occur as a function of age and neurological disease. Evidence suggests that subtle gait changes which begin in middle-age can predict the future onset of mobility difficulties. However, standard clinical tests which rely on visual observation are designed to capture easily observable balance and gait problems in patients, rather than small changes that begin in middle-age. In this study we examined mobility changes that occur as a function of age using a common clinical test, the Functional Gait Assessment (FGA). Both middle aged (n=8) and young adult (n=12) participants completed the FGA while wearing small sensors, called inertial measurement units (IMUs). IMUs were placed on each ankle and the trunk to capture subtle differences in gait patterns. Participants also completed a health history questionnaire. Preliminary data suggest gait variability was increased in middle-aged adults (especially towards the end of the gait cycle), in the more difficult tasks. Subtle changes in gait velocity and leg acceleration during the swing phase of gait also appears to emerge by middle-age. Using IMUs to capture changes in gait patterns of middle-aged participants may help to identify those who are at future risk of having mobility difficulties and significant health risk.

Abstract citation ID: igad104.2184

### VIRGINIA BELL: AN ORAL HISTORY OF A GERONTOLOGICAL SOCIAL WORKER AND PIONEER IN DEMENTIA CARE

Natalie Pope[1], Mohammad Hossain[1], and Allison Gibson[2], *1. University of Kentucky, Lexington, Kentucky, United States, 2. Saint Louis University, St. Louis, Missouri, United States*

This presentation highlights the contributions of a gerontological social worker and dementia expert who developed an innovative model of dementia care known as the Best FriendsTM model. Virginia's story is one of a second-chapter career. She spent most of her adult life raising her five children and supporting her husband, who worked in ministry. At age 62, she graduated with her Master of Social Work and completed her practicum at a newly established center on dementia research (the Sanders-Brown Center on Aging at the University of Kentucky). While this practice experience contributed to her professional trajectory as a gerontological social worker, Virginia draws most heavily on lessons learned from her relationships. Her intersecting identities as a daughter, wife, mother, and grandmother informed her pioneering work in dementia care by teaching her the value of mutuality, hard work, and community. Through several in-depth interviews, this oral history project creates a space for conversation across generations –dialogues between two women, both social workers and gerontologists. The narrator, age 98, reflects on the people and experiences that shaped her approach to caring for people with dementia from a relational-centered approach. The interviewer (age 42) assumes a co-interpreter role with Virginia, documenting what shaped her thinking about dementia. This presentation highlights 1) how oral history can be used to document the experiences and knowledge of social service innovators and 2) Virginia Bell's unique contribution via the Best Friends model.

### SESSION 7070 (POSTER)

### SOCIAL SUPPORT AND WELL-BEING

Abstract citation ID: igad104.2185

### BROADBAND ACCESS, SOCIAL CONNECTEDNESS, AND SELF-REPORTED HEALTH IN A RURAL COMMUNITY SAMPLE OF OLDER ADULTS

Tim Killian, and Betsy Garrison, *University of Arkansas, Fayetteville, Arkansas, United States*

Social connections provide older persons with access to important resources supporting their physical and psychological health. People aging in rural areas may have more challenges to maintaining and developing social connectedness because of geographic separation with friends, neighbors, and family members. One way to mitigate the risk of reduced social connectedness is by using internet-enabled technologies to facilitate social connections. However, these solutions are also limited by internet access, stability, and questions about whether or not technology enabled connections are able to meet needs for social connections. The objective of this study was to examine the role of internet access to (a) social connectedness and (b) self-rated health (SRH) and direct and indirect pathways from internet access to SRH. We used data collected in 2020 during the height of COVID-19 pandemic lockdowns from a random sample of about 450 people aged 50 years old or older living in rural counties in Arkansas. Path analyses were used to regress participants' self-reported health on social connectedness and examine direct and indirect pathways from internet access to health. Interestingly, we found that internet access was positively related to self-reported health, but was fully mediated through social connectedness. Moreover, these relationships remained significant even after controlling for variabilities in income. Although more research is needed, these results suggest that maintaining social connections using the internet was an effective approach to support the health of the older rural persons and has policy implications for maintaining and continuing to increase stable broadband technologies for rural areas.

Abstract citation ID: igad104.2186

### DISABILITY CHARACTERISTICS AND ABLEIST MICROAGGRESSION EXPERIENCES AMONG AGING ADULTS WITH DISABILITIES

Dylan Serpas[1], Laura Zettel-Watson[2], Barbara Cherry[2], and Daniel Ignacio[3], *1. University of South Florida, Tampa, Florida, United States*, *2. California State University, Fullerton, Fullerton, California, United States*, *3. UCLA BrainSport, Los Angeles, California, United States*

People with disabilities (PWD) report experiencing overt and subtle interpersonal disability-based discrimination that is related to poorer functional and health outcomes. Moreover, associations between disability-based discrimination and mental and physical health outcomes are stronger among younger middle-aged than older adults. However, differences in this pattern across disability characteristics and mental health have not been explored. This study investigated associations between disability visibility, age, ableist microaggressions, and mental health symptoms among middle-aged and older adults with disabilities. Participants included 236 middle-aged and older adults recruited online using Amazon's Mechanical Turk whose ages ranged from 45 to 70 (M=51.44; SD=5.68). Participants were largely non-Hispanic, white, female, employed full-time, graduate educated, and most reported a mobility/physical disability. Participants provided personal demographics and perceived disability visibility (visible, sometimes visible, not visible) and self-reported ableist microaggression experiences and mental health symptoms. A linear multiple regression model controlling for key demographics examined differences in the association between ableist microaggressions and mental health symptoms moderated by age and disability visibility. Results showed a main effect of microaggressions on mental health symptoms ($b=.16$, $p=.044$, $f2=.02$). An interaction effect was found between age and microaggressions ($b=.02$, $p=.003$) indicating that as age increased, the association between microaggressions and mental health symptoms increased as disability visibility decreased. Findings illustrate the importance of considering disability characteristics when evaluating subtle disability discrimination among aging adults. Disability discrimination impedes health outcomes and equity, and future research is needed to explore stigma-reduction interventions at local and policy levels.

Abstract citation ID: igad104.2187

### EFFECTS OF SOCIAL ENGAGEMENT ON WELL-BEING AND COGNITIVE FUNCTIONING IN OLDER HISPANICS

Hsiao-Wen Liao, and Hyewon Kang, *Georgia Institute of Technology, Atlanta, Georgia, United States*

Research on engaged lifestyles demonstrates the importance of social engagement for aging well. The present study examined this thesis in the context of minority aging. With the theoretical lens that Hispanic cultures value social relationships, we tested whether social engagement was particularly important for maintaining well-being and cognitive functioning in Foreign-born (FB-H) and U.S.-born Hispanics (USB-H) relative to non-Hispanic Whites (NHW). Participants (N = 2677) was drawn from older adults in the Health and Retirement Study who completed the 2008, 2012, 2016, and 2020 module at least once. Two types of social engagement were assessed. Social interactions were based on the frequency of interactions with close partners (i.e., children, family members, and friends). Social activity participation was measured by the frequency of volunteering, doing charity, joining clubs, and attending meetings of non-religious organizations. Results of random intercept linear models controlling for covariates showed that, consistent with the literature, social interactions predicted better life satisfaction and that both aspects of social engagement predicted better immediate and delayed recall. Unexpected patterns emerged when interaction effects of race/nativity and social engagement were tested. While the general pattern held for NHWs, we found that greater social interactions predicted lower life satisfaction in USB-H. For FB-H, greater participation in social activities correlated with lower performance on immediate recall. Echoing a recent study that reveals mixed evidence for the well-documented paradox that Hispanic immigrants hold health advantages (Tarraf et al., 2020), we call for more research on lifestyle factors and aging trajectories in racially diverse populations.

Abstract citation ID: igad104.2188

### ETHICAL IMPUTATION AND THE WILLINGNESS TO HELP PLOCS AMONG THE PUBLIC: THE MODERATING ROLE OF SOCIAL TRUST

Jinfeng Li, and Dahua Wang, *Institute of Developmental Psychology, Beijing, Beijing, China (People's Republic)*

In China, the population of older parents who lost their only child (PLOCs) is increasing and will keep growing for about 30 years. The PLOCs are thought to undertake the ethical imputation that they have not taken good care of the child, for the tradition emphasizes children as the center/fate of a family. This study aimed to investigate the public's ethical imputation concerning PLOCs and its relationships to the willingness to help PLOCs. A nation-wide sample of 2029 (Mage = 36.62 years; SD = 10.06; 50.1% males) Chinses adults aged from 18 to 71 years provided demographic information and responded to the measures of ethical imputation, predicament awareness about PLOCs, social trust, and willingness to help PLOCs. We utilized Hayes's (2018) PROCESS macro for SPSS (Model 1) to conduct analyses. The results indicated that the public had a relatively low level of predicament awareness about PLOCs (M = 4.28, SD = 2.09, range 1-12) and an above-average level of help willingness (M = 3.58, SD = 0.51, range 1-5). More importantly, the relationship between ethical imputation and help willingness was moderated by social trust. Specifically, ethical imputation negatively predicted help willingness among the public with higher levels of social trust; in contrast, this impact was not significant among the public with lower levels of social trust. These findings promote an understanding of how the public's ethical imputation toward PLOCs and social trust can jointly influence their willingness to help PLOCs from the macro-system (i.e., the public attitude) perspective.

Abstract citation ID: igad104.2189

### FEELING VALUED IN OLD AGE: A QUALITATIVE EXPLORATION OF THE SOCIAL STATUS IN LATER LIFE

Tim Kuball, Chiara Hohaus, and Georg Jahn, *Chemnitz University of Technology, Chemnitz, Sachsen, Germany*

Subjective social status describes a person's self-assessed position in a social hierarchy and is an important determinant for mental and physical health. One's perceived position is dynamically changing across the life-span. Individuals attempt to achieve and preserve status in their social environment. Negative age stereotypes are fueled by and contribute to the attribution of lower social status to older adults. However, little research relates to how status changes and potential status losses are subjectively perceived in older adulthood. In the present study, we used semi-structured interviews to qualitatively identify factors contributing to older adults' experienced and anticipated status developments. In addition, resources and strategies that are used to maintain relative social status in old age were openly explored. Our sample of 14 East German citizens (mean age = 73.85, range: 67–81 years) reported a highly differentiated understanding of status that included not only socioeconomic factors but also social participation. Experienced changes in status were connected to societal upheavals and retirement. Autonomy and health were reported as important prerequisites for relative status-level preservation. Furthermore, new personal freedoms after retirement entailed empowering opportunities via engagement and other leisure activities for many respondents. Findings implicate that status development in old age may diverge from societal and loss-oriented status expectations if structural support for individual social participation is enabled. In our discussion, we reflect on suitable methodology for studying the construct of social status in old age.

Abstract citation ID: igad104.2190

### GRATITUDE, RELATIONSHIP QUALITIES, AND LIFE SATISFACTION IN LATE LIFE

Nicole Richards[1], Zexi Zhou[2], and Karen Fingerman[2], *1. University of Texas at Austin, Austin, Texas, United States, 2. The University of Texas at Austin, Austin, Texas, United States*

Gratitude is associated with better well-being. The Find, Remind, and Bind Theory suggests gratitude may support relationships with greater affection and less conflict, contributing to well-being. This study evaluated gratitude, relationship qualities with social partners, and their association with life satisfaction in late life. We leveraged data from the Daily Experiences and Well-being Study. Older adults aged 65-92 (N = 316, Mean age = 74.03) completed measures of trait gratitude and life satisfaction. They also listed their close social partners and rated their affection and conflict with the 10 closest partners. Linear regression models indicated that older adults who reported higher gratitude also experienced higher levels of life satisfaction. Older adults who rated greater affection in ties to close social partners also experienced higher levels of life satisfaction; conflict was not associated with life satisfaction. We also found moderating effects; less affection with social partners was associated with lower life satisfaction only among older adults with lower gratitude levels. Older adults with higher gratitude levels reported high life satisfaction regardless of affection. Low conflict with social partners was associated with greater life satisfaction for older adults with higher gratitude levels, but participants who experienced high conflict with social partners reported less life satisfaction, regardless of gratitude level. In sum, associations between gratitude and well-being are complex. Gratitude may play a protective role for life satisfaction when older adults navigate their social world without much affection from partners but may be less effective in high conflict contexts.

Abstract citation ID: igad104.2191

### LIFE PROBLEMS, PROVIDING ADVICE, AND DAILY MOOD IN LATE LIFE

Sibo Gao, Shiyang Zhang, and Karen Fingerman, *The University of Texas at Austin, Austin, Texas, United States*

Older adults often experience life problems (e.g., health conditions, financial crises) that may be detrimental to their well-being. Yet, meaningful social connections and support exchanges may mitigate the negative effect of life problems on older adults' emotional health. This study used data from the Daily Experiences and Well-being Study to investigate how providing and receiving advice influence the association between life problems and mood. Participants aged 65+ (Mage = 74.15, N = 293) completed an initial interview in which they indicated whether they had different types of life problems (health, financial, legal). Participants then completed an ecological momentary assessment in which they reported their negative and positive mood every 3 hours for 5 to 6 consecutive days. They also reported whether they provided or received advice from any social partner at the end of each day. Results showed that participants with more life problems reported higher negative mood throughout the day. Multilevel models revealed a significant interaction between life problems and providing advice on mood, such that the number of life problems was positively associated with negative mood on days that participants did not give advice, whereas life problems were not associated with negative mood on days that participants gave advice. Receiving advice did not reduce the adverse effect of life problems on negative mood. Findings underline that providing (but not receiving) advice may promote generativity among older adults and thus benefit their well-being. The study provides important implications for improving older adults' well-being especially when they face life problems.

Abstract citation ID: igad104.2192

### LIFELONG LEARNING AND SELF-EFFICACY OF PHYSICAL ACTIVITY ENGAGEMENT IN SEDENTARY OLDER ADULTS: A QUALITATIVE STUDY

Claire Wang[1], Mengchi Li[2], Jing Huang[1], Yeabsira Tufa[1], and Junxin LI[1], *1. Johns Hopkins University, Baltimore, Maryland, United States*, *2. Johns Hopkins, Baltimore, Maryland, United States*

Societal-level norms play an essential role in later-life sentiments surrounding learning. While there is a wealth of literature on the benefits of learning new skills in improving health and physical capacity, there is a gap in how learning perceptions can influence willingness to learn in contemplation, action, and outcome. With sedentary lifestyles increasing, improving learning and self-efficacy will be crucial for physical activity engagement. In a randomized-controlled trial study, we explore the interface between lifelong learning and aging self-efficacy in the "third age," where participants were asked to learn about wearable activity trackers and strengthening exercises. Twenty-nine older adults (mean age 71.1 ± 4.5, sleep problems, exercising ≤ 2x per week, sedentary activity > 6h/day) were included in semi-structured post-study interviews after participating in the 24-week intervention. Qualitative data was analyzed through NVivo, applying a grounded theory approach, while coding categories were produced inductively. Topics of diminished later-life learning and self-efficacy were tied to age-related social norms like retirement, value in age rather than wisdom, and a decline ideology of age. Ambivalence in action was another critical finding related to how participants felt conflicting emotions regarding whether they could complete or learn in the intervention (self-efficacy). However, given both opportunity and encouragement, these same participants learned new skills and thrived in the intervention. This research calls for inclusive approaches to learning that combat ageism-related societal norms and provide conducive environments for hedonic learning motivation. It provides insight into intervention adherence, capacity-building, and manifestations of internalized ageism on later-life learning outcomes.

Abstract citation ID: igad104.2193

### OLDER AGE IS ASSOCIATED WITH MORE COMPLEX PERCEPTIONS OF ROMANTIC RELATIONSHIP QUALITY DURING THE COVID-19 LOCKDOWN

Yochai Shavit[1], Roi Estlein[2], Roni Elran-Barak[3], and Dikla Segel-Karpas[4], *1. Stanford University, Stanford, California, United States*, *2. University of Haifia, Haifa, Hefa, Israel*, *3. University of Haifa, Haifa, HaZafon, Israel*, *4. University of Haifa, Haifa, Hefa, Israel*

The COVID-19 pandemic and the measures taken to mitigate its devastating effects posed unparalleled challenges to romantic relationships. National lockdowns forcing couples to spend long hours in closed quarters were especially straining, yet evidence points to considerable variability in the effect of the pandemic on relationship quality. Drawing on socioemotional selectivity theory, we examined age differences in perceptions of romantic relationship quality as one source of insight into this variability. During the first lockdown in Israel, 280 adults aged 25-81 reported positive and negative qualities of their romantic relationships. Of these, 105 participants completed the survey again once lockdown restrictions were lifted. Contrary to our hypotheses based on socioemotional selectivity theory, we found no evidence for age differences in the effect of the lockdown on perceptions of relationship quality. In addition, the lockdown did not influence participants' positive and negative perceptions of their romantic partners. However, we found that whereas people of all ages represent positive and negative qualities of their romantic partners as separate constructs, the negative association between the two is weaker for older adults compared to younger adults during (but not after) the lockdown. This finding suggests that in stressful times, older adults are better able to avoid negative perceptions clouding positive perceptions and see positive aspects of relationships with romantic partners in the face of negative ones. Findings extend evidence for age associations with complex emotional experiences to emotional aspects of interpersonal relationships and may inform potential interventions for improving romantic relationships during stressful times.

Abstract citation ID: igad104.2194

### SOCIAL CONNECTIONS AND SUICIDE DESIRE AMONG COMMUNITY-DWELLING OLDER ADULTS

Mary Chase Mize, *Agnes Scott College, Atlanta, Georgia, United States*

As the population of older adults in the U.S. continues to increase, so will the prevalence of critical mental health concerns such as suicide (CDC, 2020). The Interpersonal Theory of Suicide posits desire for suicide develops from the simultaneous presence of thwarted belongingness and perceived burdensomeness (Joiner, 2005). Low social connection and perceptions of low social support can result in feelings of thwarted belongingness and perceived burdensomeness among older adults (Van Orden et al., 2012). The purpose of this study was to understand the relationship between social connections (frequency of contact with others, both synchronous or "real-time" and/or asynchronous) and suicide desire among community-dwelling older adults who received home-delivered meals (HDM) during Covid-19. This cross-sectional study was part of a grant-funded project by the Administration for Community Living. Among a sample of 320 older adults in a large southeast metropolitan area, we identified 25 participants who had no social connections of any type within the past week. These individuals had the highest levels of suicide desire and loneliness, and had the lowest levels of perceived social support. Implications for practice and future research include: a) recognition of the social connections HDM volunteers provide to older adults who may be lonely and at risk of suicide, b) an understanding of suicide desire among older adults during the Covid-19 pandemic, and c) opportunities for nutrition service providers to invest in equipping HDM volunteers to respond to suicide.

Abstract citation ID: igad104.2195

### SOCIAL NETWORK BARRIERS AND FACILITATORS TO RHEUMATOID ARTHRITIS MANAGEMENT IN HISPANIC/LATINX FAMILIES

Kamryn Wilson, Sara Dalpe, Zoe Waldman, Laura Koehly, and Jielu Lin, *National Human Genome Research Institute, Bethesda, Maryland, United States*

Compared to their non-Hispanic White counterparts, individuals of Hispanic/Latinx heritage are more likely to develop disabilities from comorbidity and remain disabled for longer periods over the life course. Among chronic conditions, rheumatoid arthritis (RA) is very common, affecting at least 1% of the U.S. population. Evidence suggests that RA is more active and severe among Hispanic/Latinx individuals, characterized by more flare-ups, greater inflammation, higher tender/swollen joint counts, and more self-reported pain, due in part to their underuse of health services, delay in initiating treatment, and difficulty in adhering to treatment plan. In this study, we sought to identify social network factors relevant to managing RA pain and RA-attributable functional limitations, in a sample of Hispanic/Latinx families residing in the Washington DC region. We analyzed network structure and patterns of interpersonal health communication and assessed how these factors may facilitate or impede proactive healthcare, independent of individual and system-level barriers. The findings are three-fold: First, there is substantial heterogeneity in social network structure in the families examined, likely due to different immigration experiences. Second, severely contracted personal networks (e.g., isolate or singe-edge) were observed among older, foreign-born individuals who spoke Spanish and had less schooling. This was a major barrier to RA management. Finally, more health communication, closeness, and advice-seeking ties in one's personal network facilitated use of health services which was positively correlated with health outcomes. We discuss the implications of the findings in the context of developing family network-based health education interventions.

Abstract citation ID: igad104.2196

### SOCIAL RELATIONSHIP TRAJECTORIES AND HEALTH OUTCOMES IN LATER LIFE

Yingzhi Xu, *Duke University, Durham, North Carolina, United States*

Many studies have demonstrated the strong causal association between social relationships and health. Research acknowledges social relationships as a "double-edged" phenomenon—social support favors longevity, while social strain fosters disadvantages in health. However, little research has investigated how social support and social strain develop over the life course. The growth mixture model (GMM) and four waves of the Health and Retirement Study (HRS) were used to identify group-based trajectories of perceived social support and social strain from four categories of social connections: spouse, children, family, and friends. Ordinal logistic regression was employed to examine the degree of exposure to social support and social strain and their effects on physical and psychological health among older populations in the U.S. Four classes of support trajectories involving children, family, and friends, three classes of spousal support trajectories, four classes of strain trajectories involving spouses, children, and friends, and two classes of friend strain trajectories were identified. The majority of older adults have experienced stable high social support and low social strain from four categories of social connections. As an emotionally closer tie, the perceived social support and social strain from spouses are highest among the four categories. Friends provide the second highest social support, while they are the lowest in terms of social strain as a weaker tie. People perceived stable high social support, and low social strain have better psychological health compared to other groups. The findings revealed the social support and strain patterns of different social connections and related health consequences.

Abstract citation ID: igad104.2197

### SOCIALLY ACTIVE OR ISOLATED? OLDER COMMUNITY-DWELLING ITALIANS' EXPERIENCES DURING THE COVID-19 PANDEMIC

Anne Barrett[1], Eliana Fattorini[2], and Monica Consolandi[3], *1. Florida State University, Tallahassee, Florida, United States*, *2. University of Trento, Trento, Trentino-Alto Adige, Italy*, *3. Bruno Kessler Foundation, Trento, Trentino-Alto Adige, Italy*

Media coverage of older adults during the COVID-19 pandemic emphasized their experiences of social isolation – a risk that pre-dated the pandemic but was magnified by it. A focus on older adults' pandemic-induced isolation also is evident in gerontological research. This depiction, however, may not capture the complexity of older adults' experiences during the pandemic. Our study examined this topic using semi-structured interviews that were conducted with participants in a senior center in a mid-sized northern Italian city in May 2022 (n=23). Our analysis revealed that the pandemic had some negative effects on older adults' social relationships; however, it also identified strategies that older adults used to maintain and even enhance their connections with others. Participants described reduced in-person interaction with family and friends, stemming from less daily interaction and the cancellation of family gatherings. They compensated for these losses, to some degree, with regular phone and video calls. Participants also described permanently strained relationships with family members and friends, often the result of differing views of COVID-19 vaccines. The pandemic's challenges, however, enhanced other social relationships through exchanges of moral and practical support. In contrast with the frequent depiction of older adults' isolation during the COVID-19 pandemic, our data reveal strategies that allowed them to remain socially engaged and active in their communities.

Abstract citation ID: igad104.2198
### SPOUSAL SUPPORT AND PERCEIVED RESPONSIVENESS IN THE LINK BETWEEN DAILY RUMINATION AND SLEEP: FINDINGS FROM THE NSDE

Sam Molli, and Stephanie Wilson, *Southern Methodist University, Dallas, Texas, United States*

The Cognitive Model of Insomnia suggests that increased cognitive arousal may negatively influence sleep. Prior work has supported this theory, indicating that both negative affect and rumination may be negatively related to sleep. However, recent empirical evidence suggests that social support may buffer this impact. Yet, much of the current literature focuses on rumination as a trait characteristic. We set out to investigate both between and within-person rumination and its relation to sleep and negative affect. We also tested whether support, both perceived and received, may buffer the relation between rumination and outcome variables. The sample consisted of partnered participants (n=612, Mage= 62, SD=9.73) from the National Study of Daily Experiences in the MIDUS project. Participants reported daily levels of sleep, rumination, negative affect, and received support; as well as perceived partner responsiveness (PPR) and depressive symptoms at baseline. In multilevel models that controlled for sex, age, race, education, and depressive symptoms and included random person-level intercepts, a significant interaction arose between daily rumination and PPR on daily negative affect (b = -0.0061, SE = 0.0021, p = .004), such that participants reporting higher PPR reported a weaker association between daily rumination and negative affect. Trait or daily received support was not a significant moderator of any outcome and PPR did not significantly moderate the relation between rumination and sleep. These findings further add to our understanding of the distinctions in type of support from a partner. Moreover, this suggests that knowing support is available may buffer distress from rumination.

Abstract citation ID: igad104.2199
### STAYING SINGLE INTO VERY LATE LIFE: LONGITUDINAL ANALYSIS OF NEVER-MARRIED ADULTS

Natasha Nemmers[1], Peter Martin[2], and Barbra Brottman[2], *1. Wayne State University Institute of Gerontology, Detroit, Michigan, United States*, 2. *Iowa State University, Ames, Iowa, United States*

With the rise in life expectancy, the phenomenon of singlehood has also increased. However, most studies group unmarried individuals together (i.e., divorced, separated, widowed, never-married), and less is known about lifelong singlehood. This study aims to understand gender and never-married/married status differences in cognition, difficulty in activities of daily living (ADLs), and survival rates. Using the Health and Retirement Study (HRS) of older adults 69 years and older, we conducted longitudinal analyses of health from waves 2-5 and a survival analysis with waves 2-14. The never-married group (n=243) had a higher proportion of racial/ethnic minorities and women than the married group (n=7328) at baseline. Survivorship trajectories by marital group were significantly different: $\chi2(1)$ =13.26, p < .001, such that married persons had a better chance of survival than never-married persons. We found significant gender differences in physical activities of daily living (PADLs, F=6.75, p<.001) and instrumental activities of daily living (IADLs, F=9.03, p<.001), indicating that women had greater functional difficulty than men. Regarding marital status, never-married persons had significantly more difficulty in ADLs than married persons only in waves 4 (F=10.22, p<.01) and 5 (F=4.66, p<.05). No cognition differences were found by gender or marital status. Consistent with prior work, these findings demonstrate the protective effect of marriage on mortality, though IADL-difficulty and cognition did not differ. This study will add to the sparse literature on lifelong singlehood. Future studies should consider other relevant covariates, like social network and personality, to inform tailored health interventions and policies for this subpopulation.

Abstract citation ID: igad104.2200
### THE EFFECT OF ENACTED AND PERCEIVED SOCIAL SUPPORT ON THE WELL-BEING OF KOREAN OLDER ADULTS BY GENDER

Meeryoung Kim, *Daegu University, Seoul, Republic of Korea*

Social support is important for the well-being of older adults. However, different kinds of social support have different effects on well-being. This study investigated enacted social support which is “the actual use of social support,” and perceived social support, in which people think they can get help when they need it. In short, enacted social support is action-oriented. Perceived social support is experience-oriented. For the effects, we looked at whether there were gender differences in the well-being of older adults. This study used the seventh additional wave (2018) and eighth wave (2019) of the Korean Retirement and Income Study. The subjects of this study were 60 and older. The sample size of this study is 4,338. Multiple regressions were used for data analysis. Demographic variables were controlled. This study used enacted social support and perceived social support as independent variables. Health and life satisfaction were used as well-being variables. Research shows that enacted emotional support is important for health. However, perceived emotional support is only significant for men, and perceived instrumental support is only important for women. All kinds of social support affected older women's life satisfaction. For men, however, instrumental support did not affect life satisfaction. These findings imply that emotional support, whether enacted or perceived, is important for the health of older adults. Except enacted instrumental support, every support was significant for older adults regarding life satisfaction. Therefore, when providing social support, it is important to consider what type of support is effective for each gender.

### SESSION 7080 (POSTER)

### TECHNOLOGY: OLDER ADULT INTERFACE AND USE

Abstract citation ID: igad104.2201
### A NOVEL MULTIFREQUENCY GPS APP FOR STUDYING OLDER ADULTS: IMPROVING ACCURACY AND RELIABILITY OF DATA

Dinesh Mendhe[1], Stephanie Bergren[2], and Yanping Jiang[3], *1. Rutgers University New Brunswick, Piscataway, New Jersey, United States*, 2. *Rutgers, the State University of New Jersey,*

*New Brunswick, New Jersey, United States*, 3. *Rutgers University, New Brunswick, New Jersey, United States*

Acquiring global positioning system (GPS) data can help researchers understand the relationship between older adult's location/activity and their health, but existing GPS data collection modes suffer from issues with precision, consistency, and acceptability. Some limitations of current GPS mobile apps are satellite service disruption, power saving mode interference, miscalibration of device's compass, and user-error during data collection which result in missing or inaccurate data. We present a novel multilingual GPS mobile app and live administrative dashboard to address these limitations. The app performs triangulation between GPS satellite data, nearest cellular tower, and WiFi router location data which are processed through a unique GPS algorithm with a mean error of only 1.5 meters. Since GPS data results in coordinates commonly reverse geo-coded to place-based datasets, the accuracy is critical in determining place type or other characteristics. The app is additionally designed with redundancy and fault tolerance in mind to run continuously in the background of a user's smartphone, so there is no data loss from accidentally closing the application or restarting the phone. Moreover, this new application has a flexible sampling rate ranging from 1 second to 20 minutes. Sampling frequency flexibility can adjust the battery life and reduce the need to charge as frequently. Last, application programming interface (API) integrations allow for live data stream, which can improve monitoring and troubleshooting in the field. For research with older adult populations, these features can not only improve the accuracy of collected data but also increase the acceptability of collecting GPS data.

Abstract citation ID: igad104.2202

### ASSOCIATION BETWEEN MOTOR ACTIVITY AND TOTAL NEUROPSYCHIATRIC INVENTORY NURSING HOME VERSION SCORES

Elena Guseva[1], Andrea Iaboni[2], Nathan Herrmann[2], Zahinoor Ismail[3], Krista Lanctot[4], Dallas Seitz[5], Amer Burhan[6], and Machelle Wilchesky[1], 1. *McGill University, Montreal, Quebec, Canada*, 2. *University of Toronto, Toronto, Ontario, Canada*, 3. *Cumming School of Medicine, University of Calgary, Calgary, Ontario, Canada*, 4. *Sunnybrook Health Sciences Centre, Toronto, Ontario, Canada*, 5. *University of Calgary, Calgary, Alberta, Canada*, 6. *Ontario Shores Centre for Mental Health Sciences/Temerty Faculty of Medicine University of Toronto, Whitby, Ontario, Canada*

Accurate neuropsychiatric symptoms (NPS) assessment monitoring is crucial for person-centered dementia care management. Doing so, however, is challenging, since current assessment tools rely on clinical observations, are time consuming, and are somewhat subjective in nature. The Neuropsychiatric Inventory Nursing Home Version (NPI-NH) is an informant-based assessment of 10 sub-domains of behavioral functioning where total NPI-NH score represents an overall behavioral disturbance level. We investigated the evidence pertaining to the diagnostic test accuracy (DTA) of motor activity tracking obtained via wearable sensor technology (WST) using total NPI-NH score as the gold standard in persons living with dementia (PLWD). This was part of a larger systematic review assessing the use of WST for NPS detection and monitoring carried out from inception until September 2022 (https://www.crd.york.ac.uk/prospero/display_record.php?RecordID=219917). A systematic literature search carried out in 7 library databases produced 12,928 articles from which 84 titles were retained for analysis. In total, 8 articles examined the validity of WST for assessing and monitoring of overall behavioral disturbance in PLWD, among which 5 studies used motor activity trackers. Dementia participants predominantly had Alzheimer's, vascular or mixed dementia (40%, 20%, and 40% respectively), with mild-moderate severity. Three studies reported correlations between motor activity and total NPI score that ranged from 0.35 to 0.38. A random effects model indicated that the pooled correlation across studies was 0.37 (0.22-0.51), with no heterogeneity (I2=0%). While our sample reveals WST test accuracy as being consistently moderate, more research is necessary for confirmation.

Abstract citation ID: igad104.2203

### DEVELOPMENT AND ACCEPTABILITY OF A MOBILE APP FOR MONITORING EVERYDAY FUNCTIONAL ABILITIES

Ava Mcvey, Kalvry Cooper, Abigail Stephan, Tatum Steele, Lesley Ross, and Christine Phillips, *Clemson University, Clemson, South Carolina, United States*

Subtle changes in instrumental activities of daily living (IADL), also termed everyday functional abilities, may be early markers of dementia. However, there are no widely available tools for tracking IADL function, and it is unclear whether such a tool would be used by older adults. The present study investigates older adults' adherence to a novel mobile app designed to conveniently and cost-effectively monitor IADL function. We also examine participants' acceptability of the app, including their overall satisfaction, perceived ease of use, benefits and limitations, and suggestions for improvement. Community-dwelling older adults (n=24; Mage=71.63 years) were randomly assigned three different simulated IADL tasks, to complete at specified times, each day for two weeks. Adherence was assessed using app usage data. App perceptions were assessed via survey and individual interview at the end of the two-week study period. Results indicate high adherence, with an average total task completion of 94.2%. Results from follow-up interviews revealed the app was generally perceived as "easy to use" and the assigned tasks could be completed quickly. Participants expressed frustration over app glitches that prevented them from successfully completing tasks and noted the assigned tasks and associated steps to complete were too simplified to be realistic. High adherence rates and general acceptance indicate the app's potential usefulness. Further research should assess long-term effectiveness and user experience, especially among a more diverse sample. If reliable and valid, such apps could enable early dementia detection, but only if their use is feasible and accepted.

Abstract citation ID: igad104.2204

### EVERYDAY DIGITAL LITERACY QUESTIONNAIRE (EDLQ): DEVELOPMENT AND VALIDATION FOR KOREAN OLDER ADULTS

Jiyoung Shin, Jiyeon Choi, Seongmi Choi, Kijun Song, Heejung Kim, Mona Choi, and Yesol Kim, *Yonsei University, Seoul, Republic of Korea*

Aging in the digitalizing society demands digital literacy, a core competency that involves identifying, evaluating, and communicating information using various digital devices or relevant programs. This study aimed to develop the Everyday Digital Literacy Questionnaire [EDLQ], a scale to assess digital literacy, and to evaluate its psychometric properties among community-dwelling older adults in South Korea. The EDLQ was developed using an instrument development design. A nationwide survey was conducted in 1,016 community-dwelling older adults aged 60 to 94 years. To evaluate psychometric properties, following analyses were conducted in two randomly selected subsamples (n=508 per each): internal consistency (Cronbach's alpha), construct validity (exploratory factor analysis [EFA] and confirmatory factor analysis [CFA]), and concurrent validity with the eHealth Literacy Scale (e-HEALS). Among initial 30 items of the EDLQ, 22 items with a 3-factor solution showed 77% of explained variance in total. The subscales includes "Information and Communication" (9 items), "Contents Creation and Management" (4 items) and "Safety and Security" (9 items). CFA was conducted with this 3-factor solution ($\chi^2$/df=1.675, CFI=0.997, TLI=0.997, RMSEA=0.036, SRMR=0.050, CR=0.903-0.959, AVE=0.699–0.724, $r^2$=0.616–0.773). Concurrent validity with the e-HEALS demonstrated a strong correlation (r=0.75). The Cronbach's α coefficient was .98. Our study findings suggest psychometric properties of the 22-item EDLQ is valid and reliable to assess digital literacy for everyday technology use in Korean older adults. The EDLQ will be useful while developing technology-based interventions for community-dwelling older adults.

Abstract citation ID: igad104.2205

### HAS THE RACIAL/ETHNIC DIGITAL DIVIDE AMONG OLDER COMMUNITY DWELLERS DEEPENED OR IMPROVED IN THE 2010S?

Xiayu Chen[1], J. Zak Peet[2], Danan Gu[3], and Kun Wang[4], *1. University of Illinois at Urbana-Champaign, Champaign, Illinois, United States*, *2. Binghamton University, Binghamton, New York, United States*, *3. The United Nations, New York City, New York, United States*, *4. Binghamton University, State University of New York, Binghamton, New York, United States*

After the rapid societal and technological changes that occurred during the 2010s, it is unclear whether the racial/ethnic digital divide has deepened or improved. Guided by critical race theory, this study aims to examine trends of the racial/ethnic effect on the first- and second-level digital divide and how race/ethnicity interacts with gender and education. Data from three rounds (2011, 2015, and 2019) of the National Health and Ageing Trend Study were used in this study. Older community dwellers with normal cognition were included (2011: n = 5214; 2015: n = 5543; and 2019: n = 3242). The first-level digital divide was measured by Internet access. The second-level digital divide was measured by the usage of six online activities. Using weighted multiple logistic regressions, we found that race/ethnicity became not significantly associated with the first-level digital divide in 2019 but was consistently associated with the second-level divide. Neither gender nor education moderated the first-level digital divide association across the three years. For the second-level digital divide, education moderated the racial/ethnic digital divide on online grocery shopping and banking in 2011 and 2015 but not in 2019; by contrast, education's moderation effects on buying prescriptions, contacting health providers, and getting health information online were significant in 2015 and 2019. No general trends were found for the moderation effect of gender on the second-level digital divide. Racial/ethnic gaps in the first-level but not the second-level digital divide were alleviated. More interventions should be provided for racial/ethnic minorities, especially those with low education.

Abstract citation ID: igad104.2206

### HEALTH CONDITIONS, CONTROL BELIEFS, AND ATTITUDINAL BELIEFS OF USING TECHNOLOGY IN LOW-INCOME OLDER ADULTS

Eunice Oladepe Ojo, Ladda Thiamwong, Rui Xie, Nichole Lighthall, Joon-Hyuk Park, Victoria Loerzel, and

Jeffrey Stout, *University of Central Florida, Orlando, Florida, United States*

Integrating assistive technologies into fall prevention interventions could improve health outcomes, but older adults in low-income settings face greater challenges with technology use due to factors such as cost, access, and location. We examined the acceptance of technology in low-income older adults and the factors associated with their acceptance. In this cross-sectional study, 126 adults aged 60 years and older agreed to participate in the study. Three technologies, including a BTrack balance system (BBS), bioimpedance measurement device (InBody), and activity monitoring device (Actigraphs) were used to assess fall risk. The Senior Technology Acceptance (STA) was used to assess the acceptance of technology. STA consists of four domains with 14 items (1-10 rating scale). The Pearson correlation test was used to determine the correlation between STA scores, fall risk, and demographic factors. We found a significant correlation between STA and the CDC's STEADI fall risk score ($r = 0.3$, $p = 0.00075$); between STA and education ($r = 0.26$, $p = 0.0042$); and between STA and age ($r = -0.32$, $p = 0.00031$). Participants had the highest mean STA scores to the lowest mean scores as follows: health conditions (41.9 ± 5.8), control beliefs (30.1 ± 8.1), attitudinal beliefs (20.9 ± 8.2), and gerontological anxiety (8.3 ± 5.7). The use of technology is acceptable to low-income older adults but fall risk, education, and age were associated with acceptance. They may benefit from technologically based fall prevention intervention since they have the potential to use technology with low fear expressed toward it.

Abstract citation ID: igad104.2207

### MIXED-METHODS EVALUATION OF A NOVEL MULTIMODAL COGNITIVE TRAINING "COGMUSE" IN OLDER ADULTS WITH INSOMNIA

Ashley Curtis[1], Amy Costa[1], Madison Musich[2], Chloe Rzeppa[3], Kevin Zemon[4], Isaac Schroeder[5], Charles Sielert[3], and Fang Wang[3], *1. University of South Florida, Tampa, Florida, United States*, *2. University of Missouri-Columbia, Columbia, Missouri, United States*, *3. University of Missouri, Columbia, Missouri, United States*, *4. University of Missouri - Columbia, Columbia, Missouri, United States*, *5. University of Missouri, Columbia, Columbia, Missouri, United States*

Lack of pharmacological agents to improve cognition has prompted research on behavioral interventions, such as cognitive training (CT). CT benefit is inconsistent across non-patient populations. However, our recent work reveals multimodal commercialized CT shows promise for improving cognition and sleep in older adults with insomnia. Evaluation of non-commercialized CT allows for easy implementation of test scenarios/variables, monitoring and iterative optimization. This study: 1) created a novel non-commercialized CT for older adults with insomnia ("COGMUSE") and 2) conducted usability testing/feature evaluation. COGMUSE is a computerized multimodal CT (Star Snatcher: targets spatial attention, perceptual motor skill, processing speed; Worse for Wire: targets spatial attention, inhibition). Ten older adults with insomnia complaints (Mage=69.3±3.3) completed 20 minutes of COGMUSE and the Game User Experience Satisfaction Scale (GUESS-18; rating 1-strongly disagree-7-strongly agree across game features). Open-ended feedback was solicited. Compiled transcripts were analyzed independently (A.F.C./F.W.) through deductive content analysis. Frequent topics/themes were identified. GUESS-18 average scores ranged from 3.5/7.0 (enjoyment) to 5.9/7.0 (personal gratification) for Star Snatcher, and from 3.1/7.0 (audio aesthetics) to 5.5/7 (play engrossment) for Worse for Wire. Feedback themes were largely positive (Star Snatcher: good graphics/sounds, easy/enjoyable; Worse for Wire: enjoyable, good instructions). Negative themes included quick movement and lackluster graphics/gameplay "story" (Worse for Wire) and lack of error consequences and practice trials (both games). Older adults with insomnia evaluated COGMUSE as easy and enjoyable to use with acceptable aesthetics. Improvement themes largely focused on instructions and navigation. Next steps include updating COGMUSE and evaluating its preliminary efficacy on cognition and sleep.

Abstract citation ID: igad104.2208

### OTC SENIOR STATION: CONSUMER-FACING TECHNOLOGY TO PROMOTE MEDICATION SAFETY

Jordan Hill[1], Aaron Ganci[2], Himalaya Patel[3], Noll Campbell[4], Michelle Chui[5], Ephrem Abebe[4], and Richard Holden[1], *1. Indiana University School of Public Health, Bloomington, Indiana, United States*, *2. Indiana University-Purdue University Indianapolis Herron School of Art + Design, Indianapolis, Indiana, United States*, *3. Richard L Roudebush VA Medical Center, Indianapolis, Indiana, United States*, *4. Purdue University College of Pharmacy, West Lafayette, Indiana, United States*, *5. University of Wisconsin-Madison, Madison, Wisconsin, United States*

Over-the-counter (OTC) medications with anticholinergic effects (e.g. doxylamine, diphenhydramine) are widely used by older adults, despite their association with an increased risk of Alzheimer's disease and related dementias (ADRD). Authoritative bodies, such as the American Geriatrics Society and National Academy of Medicine, recommend discouraging the use of both prescription and OTC anticholinergics. However, most interventions previously developed and tested have targeted only prescription anticholinergics despite the use rate of OTC anticholinergics being higher in older adult populations. Consumer-facing technology may be ideal for addressing OTC anticholinergic use as it is cost effective, scalable, and can address the constraints of relying on busy pharmacists to intervene during older adult OTC medication purchases; this has become especially relevant as pharmacies face staffing shortages, increasing pharmacist workload and requiring pharmacies adjust their hours of operation. Our interdisciplinary team of pharmacists, engineers, and designers engaged both older adults and pharmacy staff in the co-design of "OTC Senior Station": a user-centered, consumer-facing kiosk to be placed in community pharmacies to promote reduced anticholinergic use by older adults. Kiosk users are prompted to input brief information, which is used to present safer alternatives to OTC anticholinergics (e.g., for insomnia management). This poster will present OTC Senior Station--what we believe to be the first consumer-facing technology to target OTC anticholinergic use—the participatory design methods used to create it, and the results of studies to assess its usability with older adults.

Abstract citation ID: igad104.2209
### USABILITY OF THE SMARTWATCH AS A REMINDER AID FOR OLDER ADULTS WITH DIVERSE COGNITIVE ABILITIES
Edie Sanders, and Walter Boot, *Florida State University, Tallahassee, Florida, United States*

Smartwatches have potential to provide support for prospective memory, the ability to remember and carry out an intention in the future, crucial for maintaining health, independence, and social connections. Little is known about how older adults, particularly those with cognitive impairment, might interact with smartwatches. This study aimed to understand 1) the types of challenges older adults with and without cognitive impairment experience when using a smartwatch, and 2) the potential for smartwatches to serve as reminder aids for this population. Participants were twenty-seven older adults (age 60+) with normal cognitive functioning or cognitive impairment due to mild cognitive impairment, traumatic brain injury, or stroke. Participants were asked to use a smartwatch's reminder notifications to remember to complete a daily survey for 10 days and completed demographic, neuropsychological, technology acceptance, and usability measures. Half were first asked to use their usual memory strategies without the smartwatch to remember to complete the daily survey for 10 days. Participants gave relatively low usability ratings for the smartwatches overall and reported a variety of challenges. The smartwatches did not appear to significantly help or hurt participants' abilities to remember to complete the daily survey. Perceived usefulness of the smartwatches was significantly associated with subjective memory, suggesting that perceived memory challenges may play an important role in the adoption of smartwatches. Results suggest that the smartwatches used in this study may not be the best fit for supporting prospective memory and can inform the development of future efficacy tests and interventions involving smartwatches.

Abstract citation ID: igad104.2210
### VESTA: A SMARTPHONE-BASED ASSESSMENT OF MODERN FINANCIAL CAPACITY FOR OLDER ADULTS
Samuel Van Vleet[1], Rebekah Griffin[2], William Mallender[2], Matthew Fontanese[2], Haru Sakai[3], Blake Savage[2], Allyson Coldiron[2], and Michael Barnett[2], *1. Miami University, Oxford, Ohio, United States*, *2. University of Texas at Tyler, Tyler, Texas, United States*, *3. The University of Texas at Tyler, Tyler, Texas, United States*

Older adults are often targeted by financial scammers who exploit people unfamiliar with financial technologies and who may struggle to identifying fraudulent activity. Financial technologies have undergone significant changes in recent decades, yet financial capacity measures often do not reflect this shift. There is a clear need for innovating the measurement of financial capacity, particularly among older adults. VESTA (Virtual Economic Simulation and Trust Assessment) is a novel smartphone application that measures older adults' susceptibility to different forms of financial exploitation. VESTA plays out like a smartphone game in which the user furnishes a virtual household. As the user conducts these virtual transactions, they are tasked with spending within a budget, monitoring bank charges, disclosing personal information securely, and determining whether incoming messages (i.e., texts, emails, and voicemail messages) are fraudulent or legitimate. The fraudulent charges and messages include contextual clues that mirror real-life scams. VESTA was designed to assess the user's ability to manage money, identify incorrect information, and judge who to trust independent of memory ability. VESTA therefore purposefully targets severely overlooked pitfalls that impact older adults' ability to identify fraudulent activity. Furthermore, VESTA utilizes a user-friendly, accessible design with large font and complete navigation with the simple press of a finger. As the migration of financial management and communication continues towards predominantly digital landscapes, this research and diagnostic tool represents an evolution of existing measures of financial capacity for an at-risk aging population.

Abstract citation ID: igad104.2211
### WEARABLES IN DEMENTIA RESEARCH: A SYSTEMATIC REVIEW OF BARRIERS AND FACILITATORS TO ADHERENCE
Colleen Peterson, and Carol Flannagan, *University of Michigan, Ann Arbor, Michigan, United States*

With the rapid expansion of wearable technologies, there is increased interest in their utility for passive data collection applications in aging research. Wearables can be specifically beneficial to research featuring people with dementia and their families who have burdens that can make both study participation and reliable data collection more difficult, especially as dementia progresses. For example, population-specific issues may include remembering to wear or charge a device, fluctuating acceptance/consent, and reliance on strained caregivers with other care priorities. Better understanding of how wearables can address these and other issues to enhance participant buy-in and sustained wearable use is necessary to enhance dementia research quality. Therefore, we undertook a systematic evaluation of contemporary wearables research to describe this population's study- and non-study based wearables experiences, desired qualities, and protocol needs. We searched three databases for papers published since 2016 featuring the use or discussion of wearable devices for persons with dementia and related stakeholders (e.g., caregivers, residential care staff). Following removal of duplicates and inclusion of additional references identified through a reference/literature scan, we screened 1436 abstracts and retained 70 studies after a full-text review. We present synthesized preferences, barriers, and facilitators to wearables use and adherence in dementia research from study process analyses, interviews, or other survey measures, highlighting commonalities amongst different devices and study types. These findings inform researcher guidelines for wearable device selection and protocol design to enhance buy-in and adherence amongst persons with dementia and their caregivers.

Abstract citation ID: igad104.2212
### WHAT ABOUT HOBFOLL'S RESOURCES CONSERVATION MODEL IN A DIGITAL SOCIETY?
Angélique Roquet[1], Paolo Martinelli[2], Charikleia Lampraki[3], and Daniela Jopp[4], *1. Lausanne University, Lausanne,*

*Vaud, Switzerland*, 2. *Lausanne University, Lugaggia, Ticino, Switzerland*, 3. *Geneva University, Geneve, Geneve, Switzerland*, 4. *University of Lausanne, Lausanne, Vaud, Switzerland*

Internet use has dramatically increased worldwide, with over two-thirds of the world's population using it, including the elderly population. Technological resources, such as internet use, have been shown to influence psychological variables, such as stress. According to Hobfoll's theory, stress perception depends on individual's resources and their changes. While resources and stress are negatively associated, we ignore the role of technological resources on the relationship between personal resources and stress. This study aims at investigating the moderating role of technological resources (internet use) on the relationship between personal resources and stress in young and older adults. A total of 275 young (18-30 years) and 224 older adults (65 years or older) indicated their levels of stress, personal resources changes (i.e., cognitive, social, and self-efficacy resources loss and gain), and internet use. Results showed that the relationship between resource loss, resource gain, and stress in older adults was moderated by their level of internet use. Specifically, older adults who used internet more frequently were less stressed when they experienced both high levels of loss and gain, compared to their counterparts who used less internet in the same conditions. Furthermore, older adults with low resource gain and high resource loss expressed less stress when they used more internet compared to those who had low internet use. These findings highlight the important role of internet use in mitigating stress among older adults experiencing resource loss and gain, emphasizing the potential of digital interventions to promote mental health and well-being in this population.

Abstract citation ID: igad104.2213

### WHAT FACTORS ARE ASSOCIATED WITH CRITICAL MEDIA LITERACY IN LATER LIFE?

Hayoung Park[1], Bomi Choi[1], Si Young Song[2], and Miseon Kang[1], *1. Yonsei University, Seoul, Republic of Korea*, 2. *Kyung Hee University, Seoul, Republic of Korea*

Who consumes new information from the media more critically? This study sought to empirically explore relevant factors associated with critical media literacy in later life. For the analyses, the 2021 Korean Media Panel Survey was utilized and the sample included 2,352 older Korean adults aged 65 and above (M=75.48, SD=7.34, range=65-106). Critical media literacy was measured by the mean of ten questions asking how critically evaluate the facticity, validity, intentions, and usefulness of new information from the media. Possible relevant factors include age, gender, education level, employment status, marital status, income level, and the ability to use digital technology (PC and smartphone). The results from the linear regression analyses showed that age, income level, and the ability to use digital technology were significantly associated with critical media literacy in later life. Older adults who are younger and have higher levels of income and the ability to use digital technology tend to evaluate new information from the media more critically. The findings from this study imply the importance of specialized media literacy education as social policy for older adults, especially those who are older and have lower levels of income and the ability to use digital technology. This study is significant as it empirically examined various factors related to critical media literacy in later life to inform intervention efforts to reduce misperceptions induced by misinformation that is rampant in this "information era". Future studies are needed to investigate significant factors associated with critical media literacy in later life via various contexts.

Abstract citation ID: igad104.2214

### WHAT LINKS DIGITAL SKILLS AND LIFE SATISFACTION? ROLES OF DIGITAL BENEFITS AND LIVING ARRANGEMENTS

Miseon Kang[1], Seolah Lee[1], Jungup Lee[2], and Hyo Jung Lee[1], *1. Yonsei University, Seoul, Republic of Korea*, 2. *National University of Singapore, Singapore, Singapore*

The benefits of equipping enhanced digital skills in late life have been reported on many occasions. Yet, there have been few attempts to understand the association between digital skills and better psychological outcomes for older adults by integrating factors with multiple domains such as social and digital-related factors. This study investigated the causal structure of the association between 'digital skills' and 'life satisfaction' by specifying a mediator 'self-reported benefits of digital use' and a moderated mediator 'living arrangements'. Using data from '2020 Digital Information Gap Survey' by National Information Society Agency of Korea, the final sample included 1,680 older Koreans aged 55 and over. Analysis was performed by PROCESS macro model 4 and 8 in SPSS. We found a significant positive effect of digital skills on life satisfaction via self-reported benefits of digital use and moderated mediation effect of living arrangements. Specifically, digital skills positively affected self-reported benefits of digital use, and the effect was stronger for older adults living alone. However, we found no moderating effect on the direct path between digital skills and life satisfaction in late life. The conditional direct effect of digital skills on life satisfaction was only significant for older adults living with others. Findings indicate that digital skills can compensate for older adults living alone, who may have fewer sources within a family context. Heterogeneity in family structure needs to be considered when exploring the impact of digital technology use on older adults' lives.

Abstract citation ID: igad104.2215

### WITHIN- AND BETWEEN-PERSON DIFFERENCES IN ACTIVITY FACTOR STRUCTURE: RESULTS FROM AN EMA STUDY

Allison Bielak[1], Jacqueline Mogle[2], and Martin Sliwinski[3], *1. Colorado State University, Fort Collins, Colorado, United States*, 2. *Clemson University, Clemson, South Carolina, United States*, 3. *Pennsylvania State University, State College, Pennsylvania, United States*

Analyses of activity participation scales are typically based on single time points and resulting factors focus on differences between participants. However, as real-world activity engagement varies at more micro-timescales, these analyses provide little insight into how activities cluster together within a person across moments or days. Ecological momentary assessment (EMA) studies capture within-person fluctuations in activity engagement and allow us to compare within-person variability to that observed across persons. Using multilevel factor analysis we examined the factor structure of activity participation within-persons (i.e., across days) and between participants. Using tablet-based assessments, 81 adults aged 41 to 94 years (M=61.26, SD=12.12) reported the activities they completed in the past 3-4 hours 5 times per day (4 at semi-randomly scheduled intervals and 1 at bedtime) for 14 days. Multilevel factor analysis simultaneously computed both intra-individual factors (within-person structure of activity in daily life) and inter-individual factors (between-person structure of activity engagement). A solution of 4 within-person and 4 between-person factors provided the best model fit, with three common factors across levels: 1) cognitive (e.g., read, write, computer tasks); 2) social (e.g., events, mentoring, providing care); and 3) passive (e.g., TV, games) factors. There were notable differences in the fourth factor however. Although there are similarities, the factor structure of activity participation between individuals is different than factors describing activity participation within persons from day to day. Researchers should be aware that common between-person activity factors will not unilaterally fit EMA within-person data and should conduct additional preparatory factor analyses.

### SESSION 7090 (POSTER)

### THE MENTAL HEALTH TAX OF FAMILY CAREGIVING

Abstract citation ID: igad104.2216

### AN INTERSECTIONAL APPROACH TO UNDERSTANDING COMBINING WORK AND CAREGIVING ON MENTAL HEALTH

Samantha Brady, Taylor Patskanick, and Joseph Coughlin, *Massachusetts Institute of Technology, Cambridge, Massachusetts, United States*

While an increasing number of Americans are providing unpaid care to an adult family member, the majority are balancing family care needs with employment responsibilities. Role conflict theory suggests the experience of combining family caregiving and paid work can detrimentally impact mental health, while role enhancement theory suggests that occupying both social roles can alleviate the burden of caregiving. Prior work finds support for both theories, however, there is currently a lack of causal evidence examining how mental health is affected by combining caregiving and employment roles. Additionally, while gendered differences and differences across racial identities in the family caregiving experience are well-documented, even fewer studies have taken an intersectional approach in understanding shifts in mental health when caregiving while employed. The current study uses a causal methodology to examine how the mental health of working adults is impacted when they transition into a family caregiving role and how these impacts vary at the intersections of racial and gender identity. Data are from the Health and Retirement Study (2004-2018), a nationally representative survey of U.S. adults aged 50+. Results from fixed effects models support role conflict theory, suggesting when working adults begin family caregiving they experience an increase in depressive symptoms. Additional analysis using an intersectional lens finds this result applied only to White working women who transitioned into a parental caregiving role. Combining care with paid work did not appear to impact depressive symptoms for employed Black women, Black men, and White men when they began caregiving.

Abstract citation ID: igad104.2217

### APPLYING TIME-VARYING EFFECT MODELING TO EXAMINE DAILY VARIATION IN SUICIDAL IDEATION AMONG DEMENTIA CAREGIVERS

Frank Puga[1], Abigail Poe[1], and Danny Wang[2], *1. University of Alabama at Birmingham, Birmingham, Alabama, United States*, *2. The Pennsylvania State University, University Park, Pennsylvania, United States*

Emerging evidence suggests that caregivers may experience high rates of suicidal ideation. However, relatively little is known about daily contextual factors, including day-to-day depression and anxiety-related symptoms, that increase the severity of suicidal ideation over time. The purpose of this study was to apply time-varying effect modeling (TVEM) to examine daily depression and anxiety symptoms leading up to an individual's highest suicidal ideation score within a span of 14 days in a sample of community-dwelling ADRD caregivers. The intercept-only model revealed an increase in the mean scores of daily suicidal ideation four days leading up to an individual's highest score. Additionally, there was a significant decrease four days after the highest suicidal ideation point. The relationship between depression and daily suicidal ideation was significant 1-day leading up to the highest suicidal ideation score. Similar relationships were observed with daily anxiety as a time-varying predictor in a separate model. The results from this study suggest that suicidal ideation can vary between days, with depression and anxiety influencing the intensity of the slope leading to and following an individual's highest suicidal ideation score. Examining suicidal ideation trajectories over time and factors associated with varying severity can help inform targeted interventions to support dementia caregivers and mitigate the risk of severe psychological distress associated with dementia caregiving.

Abstract citation ID: igad104.2218

### EFFECT OF A COVID-19-RELATED LOCKDOWN ON MIDDLE-AGED AND OLDER FAMILY CAREGIVERS IN SINGAPORE

Rahul Malhotra[1], Vicky Qin[2], and Abhijit Visaria[1], *1. Duke-NUS Medical School, Singapore, Singapore*, *2. Nanyang Technological University, Singapore, Singapore*

Lockdowns, while limiting COVID-19 transmission, can affect care provision by family caregivers and their caregiving experience. We assessed, among 1094 family caregivers aged 50-79 years in Singapore, the (1) perceived effect of nationwide lockdown on their care provision, (2) correlates of different perceptions, and (3) association of the perceptions with negative and positive caregiving experiences. Caregivers reported whether their care provision became harder, easier, or remained the same during versus before the lockdown. Multinomial logistic regression assessed the association of caregiver, care-recipient, and caregiving-context characteristics with their perceptions.

Linear regression models examined the association of their perceptions with subsequent negative and positive caregiving experiences. Care provision became harder for 36%, easier for 18% and remained the same for 46% caregivers. Care provision becoming harder (versus same) was more likely for caregivers who were male, Chinese, in worse health, caring for care-recipients with functional limitations, without caregiving support from cohabiting family before the lockdown, and with caregiving support from non-cohabiting family before the lockdown. Care provision becoming easier (versus same) was less likely among caregivers who were of higher age, unemployed, socially isolated and whose care-recipients had functional limitations. Caregivers for whom care provision became harder were worse off in negative caregiving experiences. A nationwide lockdown did not make care provision harder for all caregivers. However, those for whom it did had greater negative caregiving experiences. Heterogeneity of the effect of lockdowns and offering flexibility to non-cohabiting family who support caregiving should be considerations when implementing such disruptions.

Abstract citation ID: igad104.2219

### EFFECT OF SELF-GUIDED INTERVENTIONS ON MENTAL HEALTH OF INFORMAL DEMENTIA CAREGIVERS: A SYSTEMATIC REVIEW

Eunjung Ko, Thanchanok Wongvibul, Karen Rose, and Jin Jun, *The Ohio State University, Columbus, Ohio, United States*

Informal caregivers of people living with dementia often experience emotional and mental distress due to the demands of caregiving. Self-guided interventions aimed at improving the mental health of these caregivers have emerged, but their effectiveness remains understudied. We systematically examined the effects of self-guided interventions on stress, burden, and mental health of informal caregivers of people living with dementia. PubMed, CINAHL, PsycINFO, Scopus, and Embase databases were searched using relevant search terms for the study aims. Included articles focused on informal caregivers of people living with dementia and self-guided interventions to improve psychological or mental health. Sixteen articles and 1,182 samples were included in this review. Self-guided interventions covered topics on caregiving skills, emotional self-care, and/or information on social or financial resources related to caregiving. Outcomes of each study included stress, burden, depressive symptoms, anxiety, distress, quality of life, self-efficacy, positive aspects of caregiving, and loneliness. Depressive symptoms and burden were the most frequently measured outcomes. Stress was generally reduced after the interventions, whereas the results of burden and mental health from each intervention were inconsistent. Interventions lasting shorter than three months were more likely to be effective compared to longer interventions. Self-guided interventions may be useful for improving mental health of informal caregivers of people living with dementia due to its low time burden, ease-to-access, and affordability. Future research is needed to determine the optimal length and components of self-guided interventions and to collaborate with clinicians for wider distribution to informal caregivers of people living with dementia.

Abstract citation ID: igad104.2220

### FINANCIAL DIFFICULTY, HEALTH BEHAVIORS, AND MENTAL HEALTH OF FAMILY CAREGIVERS DURING THE COVID-19 PANDEMIC

Yujun Liu[1], M. Courtney Hughes[1], and Heng Wang[2], *1. Northern Illinois University, DeKalb, Illinois, United States*, *2. Rush University Medical Center, Chicago, Illinois, United States*

The COVID-19 pandemic greatly burdened family caregivers in the United States and across the globe as they and their family members often suffered from the COVID-19 virus and its societal implications, like social isolation and a shift to remote healthcare. This study examines the impact of the COVID-19 pandemic on financial difficulty, health behavior, and mental health outcomes among family caregivers for older adults across the intersectionality of gender and race. Using the 2020 National Health and Aging Trends Study (NHATS) COVID-19 supplement for Family Members and Friends, our sample included adult family or friend caregivers of Medicare beneficiaries aged 65 or older in the US (N = 2,062). We modeled the interrelationships between financial difficulty, health behaviors, and mental health outcomes using a structural equation model. The results indicate that within the intersectionality framework, during the pandemic, female caregivers reported less vigorous activity, more changes in walking and sleeping patterns, and more eating and watching TV than before the pandemic compared to male counterparts. Compared to white caregivers, during the pandemic, non-white caregivers showed less vigorous activity and more changes in walking, sleeping, I eating, and TV watching patterns than before the pandemic. Financial difficulty was significantly and positively associated with negative mental health. Active behavior was significantly and negatively associated with negative mental health. Health professionals should consider the interrelationships between sociodemographic factors, physical activity levels, and financial difficulties that have come to light during the pandemic when designing future programs and policies to improve family caregiver health.

Abstract citation ID: igad104.2221

### GENDER DIFFERENCES IN PSYCHOLOGICAL OUTCOMES OF DEMENTIA CAREGIVERS

Elena Roden[1], and Francesca Falzarano[2], *1. Fordham University, New York City, New York, United States*, *2. Weill Cornell Medicine, New York City, New York, United States*

Substantial research has been conducted on the high prevalence of anxiety and depression among dementia family caregivers. Stressors, such as financial hardships related to costs of care and feelings of role captivity, can exacerbate adverse mental health outcomes. However, there is limited literature focused specifically on gender differences in the caregiving stress process. Using data from a mixed-methods study of 46 dementia family caregivers between the ages of 23-96, the purpose of this study was to examine gender differences in self-reported caregiving stressors (e.g., cost of care, care intensity) and psychosocial outcomes including depression and anxiety. Results showed that compared to females, male caregivers reported greater costs of care, greater feelings of role captivity, higher frequency of assistance with medical/nursing tasks (p=.01) and activities of daily living (ps range from .01-.04). Male caregivers also reported significantly greater distress related to care tasks (p=.04), as well as higher levels of depression (p=.02) and anxiety (p=.05). These findings provide insight into how the informal caregiving experience can vary by caregiver gender and potential factors contributing to these differential outcomes. The data establishes the importance of considering demographic characteristics in providing needed resources to dementia family caregivers.

Abstract citation ID: igad104.2222

### LIFELONG CAREGIVERS: INFLUENCES OF AGE AND INCOME ON DEPRESSION AMONG PARENTS OF CHILDREN WITH DISABILITIES

Grace Udah, Carly Pullen, and Julie Hicks Patrick, *West Virginia University, Morgantown, West Virginia, United States*

More than 20% of Americans have an intellectual or developmental disability (IDD). Of these, 70% are currently children and 30% are adults. Understanding wellbeing among their family caregivers provides an important avenue to supporting families. However, little research has included a wide range of family caregivers, focusing primarily of caregivers of young children or older caregivers of adults. Thus, it may be important to examine stressors among caregivers as they move across the lifespan. We used the 2020 BRFSS data to examine depression among a group of 522 family caregivers (M age = 56 years; range 24 – 80+) to children and grandchildren. Among the caregivers, 64% assisted with BADLs and 89% performed household chores related to the person with IDD. Among this age-diverse group, our 3-step hierarchical logistic regression revealed nonsignificant influences of caregiving tasks on depression, with Odds Ratios ranging from 1.11 to 1.22. Income was a significant contributor to the model, with ORs greater than 4.8 for the lower ends of income. After allowing caregiving tasks and income to account for their unique and shared variance, age still emerged as significant in Step 3, with younger caregivers somewhat less likely to report depression (OR = .98). Our results suggest the ongoing need to examine areas in which family caregivers need support across the lifespan.

Abstract citation ID: igad104.2223

### PREDICTORS OF DEPRESSION IN KOREAN OLDER ADULT SPOUSAL CAREGIVERS: 2017 NATIONAL SURVEY OF OLDER KOREANS

Stacy Yun[1], and Sara Qualls[2], *1. VA Palo Alto Health Care System, Palo Alto, California, United States*, *2. University of Colorado Colorado Springs, Colorado Springs, Colorado, United States*

Based on the sociocultural stress coping model (Knight & Sayegh, 2010), this study was aimed at examine how factors such as familism, caregiver burden, social support, and coping strategies impact Korean older adult spousal caregivers' depression. 369 caregiver (aged 69 to 96; M = 80.68) and care recipient (aged 69 to 102; M = 81.86) spouse dyads from the 2017 National Survey of Older Koreans was used for analysis. The model demonstrated excellent model fit indices, $\chi2(15) = 16.29$, p = .36, model $\chi2$ to df ratio = 1.09, TLI = .99, CFI = .99, RMSEA = .02 (95% CI: .00 to .05), and SRMR = .02. Higher levels of depression was significantly predicted by lower perceived social support, β = -.30, Z = -6.35, p < .001, being older, β = .16, Z = 3.12, p = .002, less education, β = -.18, Z = -3.81, p < .001, and having fewer living family members, β= -.12, Z = -2.29, p = .02. Additionally, the relationship between caregiver burden and depression was fully mediated by perceived social support (β = .05, t = 3.23; 95% CI: .02 to .09). Results suggest that perceived social support is a salient factor related to depression experienced by older spousal caregivers in Korea. Findings imply the need for clinicians to devise creative interventions to increase caregiver's sense of social support, particularly those who may reside in rural areas of Korea who are primarily older, received less education, and have fewer living family members.

Abstract citation ID: igad104.2224

### RACIAL DIFFERENCES IN CAREGIVER BURDEN AMONG BLACK/AFRICAN AMERICAN AND WHITE ALL-CAUSE CAREGIVERS

Maizonne Fields[1], and Olivio Clay[2], *1. The University of Alabama at Birmingham, Birmingham, Alabama, United States*, *2. University of Alabama at Birmingham, Birmingham, Alabama, United States*

Introduction: As life expectancy and the numbers of older adults in our society continue to rise, serving as a caregiver is more common. Caregivers assist with a variety of daily tasks as changes in functional status occur. The Stress Process Model informs that there are potential buffers to negative caregiver outcomes including religious commitment or involvement and cultural justifications for caregiving. Previously, research has shown that caregivers may turn to religion and cultural practices to cope with or explain their roles. **Methods:** This investigation examines racial differences in caregiver burden and the potential roles of caregiver religiosity and cultural justifications for caregiving as moderators of this effect. Individuals providing care for an adult age 45 and older for at least five hours per week were recruited. **Results:** The sample size for this study was 80 and included 49 Black/African American caregivers (61.25%) and 31 White caregivers (38.75%) between the ages of 23 and 78. Multiple regression models with age, gender, income difficulty, and race as predictors revealed that Black/African American caregivers reported lower levels of caregiver burden compared to White caregivers, p < .05. Additional interaction terms added to the model revealed that religious commitment or involvement and cultural justifications for caregiving did not moderate the effect of race on caregiver burden. **Conclusion:** The initial findings of this investigation may be the result of the study's small sample size and insufficient power. Recruitment is ongoing to help identify factors to potentially explain why Black/African American caregivers report less caregiver burden.

Abstract citation ID: igad104.2225

### RELATIONSHIPS BETWEEN DEMENTIA CAREGIVER PERSONALITY, OUTCOMES, AND MANAGEMENT AND COPING STRATEGIES

Darby Simon[1], Amanda Leggett[2], Emilee Ertle[1], and Benjamin Mast[1], *1. University of Louisville, Louisville, Kentucky, United States*, *2. Wayne State University, Detroit, Michigan, United States*

Family caregivers of people living with dementia are at higher risk for negative outcomes such as depression and burden. However, the experiences of family caregivers are widely variable due to individual differences. Five-factor model personality traits have previously been connected to depression and burden. The current study seeks to expand this area by investigating the links between personality, caregiver management and coping, and outcomes (burden and depression). The sample consisted of 100 dementia family caregivers ranging from 20-90 years old ($\bar{X}$=63.7, SD=16.1) who participated in the STYLE study on caregiving. Sample demographics consisted of 76% females, 80% White, 12% Black, 6% other, and 2% declined to report. Multiple regression models revealed significant relationships while controlling for caregiver age, gender, hours of care provided per week, months of providing care, and relationship to care receiver, though burden did not reveal any significant relationships. Higher conscientiousness (β=.226, p=.014) and higher neuroticism (β=.446, p<.001) were associated with higher depression. Higher agreeableness (β=-.367, p<.001) was associated with lower use of criticism-based management strategies, while higher neuroticism (β=.211, p=.023) was associated with higher use of criticism-based management strategies. High openness (β=.230, p=.020) was associated with higher problem-focused coping. Higher agreeableness (β=.201, p=.048) was associated with higher acceptance-focused coping, while higher neuroticism (β=-.254, p=.013) was associated with lower use of acceptance-focused coping. Higher neuroticism (β=.247, p=.020) was associated with higher emotion-focused coping. These analyses demonstrate links between dementia family caregiver personality, the management and coping strategies they use, and caregiver mental health.

Abstract citation ID: igad104.2226

### SOLO AND PARENT–CHILD DYAD CAREGIVING FOR OLDER ADULTS: IMPLICATIONS FOR CAREGIVER WELL-BEING

Maria Kurth[1], and Kelly Chandler[2], *1. Penn State University, University Park, Pennsylvania, United States*, *2. Oregon State University, Corvallis, Oregon, United States*

Family caregiving for an older adult is predominantly studied as a solo endeavor (NIA, 2022), overlooking care by family subsystems (see Keith, 1995) and youth (Armstrong-Carter et al., 2021). Informed by family systems theory (Cox & Paley, 1997), we analyzed data from the 2019 AARP caregiver survey (NAC & AARP, 2021) to examine demographics, experiences, and outcomes between solo (96%) and parent-child ( < 18) dyad (4%) caregivers of an adult 65+ (N = 921). Adult caregivers were mostly female (59%), non-Hispanic White (63%), and cared for an old-old recipient (Mage = 81. 97, SD = 8.93). On average, caregivers were in midlife (Mage = 60.98, SD = 14.86, range: 19-93). Caregivers with child assistance were more likely to be Hispanic, X2(1) = 6.34, p < .05, employed, X2(1) = 10.87, p < .01, younger, t(46) = 8.79, p < .001, and cared for a younger recipient, t(45) = 3.40, p <.01. They were more likely to receive unpaid help, X2(1) = 28.02, p < .001, care for their father, X2(1) = 5.15, p < .05, and less likely to provide care to a spouse, X2(1) = 5.91, p <. 05, and identify as the primary caregiver, X2(1) = 6.42, p < .05. Preliminary ANCOVA results suggested parent-child dyad caregivers had less emotional stress F(1) = 5.64, p < .05; groups were comparable for financial strain and sense of purpose. Results add to the family caregiving literature by highlighting the experiences of parent-child caregiving dyads, which might buffer against caregiving stress.

Abstract citation ID: igad104.2227

### STRESSORS OF FILIAL ANXIETY REGARDING ANTICIPATED CARE OF OLDER PARENTS: EVIDENCE FROM CHINESE ADULT CHILDREN

Chang Liu, and Xue Bai, *The Hong Kong Polytechnic University, Hong Kong, Hong Kong*

Evidence has shown that the anticipation of providing care for a parent in the future can be anxiety-provoking for adult children, especially for one-child generations in China. Filial anxiety may influence adult children's transition to the caregiver role and may bring about heightened caregiving stress. However, this phenomenon remains insufficiently studied among Chinese adult children. Drawing on the Stress Process Model and proactive coping theory, this study examined how primary stressors (i.e., parent's declining health, adverse psychological health, and lack of eldercare resources), anticipatory stressor (i.e., anticipated parental care needs), and psychosocial resources (i.e., sibling number, the value of filial obligation, intergenerational relationship, work stress, and family stress) influenced the multiple domains of filial anxiety (i.e., Filial Anxiety-Ability, Filial Anxiety-Responsibility, and Filial Anxiety-Welfare). A face-to-face questionnaire survey was conducted with 530 Chinese adult children aged between 26 and 40 years in Shenzhen, China. Descriptive analyses and regression analyses were conducted. Results demonstrated that Chinese adult children experienced a higher level of overall filial anxiety than their Western counterparts and reported a particularly high level of filial anxiety about their parent's welfare. Adult children with higher work and family stress, with fewer siblings, and whose parents lack eldercare resources were more likely to experience a high level of filial anxiety. This study systematically investigated the multiple stressors of filial anxiety. The findings have implications for the development of effective interventions to reduce adult children's future caregiving concerns and better prepare them for their older parents' future care needs.

Abstract citation ID: igad104.2228

### THE RELATIONSHIP BETWEEN ROLE CAPTIVITY, COPING STRATEGIE,S AND DEMENTIA FAMILY CAREGIVER MENTAL HEALTH

Abigail Poe[1], Loreli Alvarez[2], Mallory Wood[1], and Frank Puga[1], *1. University of Alabama at Birmingham, Birmingham, Alabama, United States*, *2. The University of Alabama at Birmingham, Birmingham, Alabama, United States*

Informal caregivers of individuals living with dementia have an increased risk for experiencing depressive and anxiety symptoms. The stress-process model posits that situational stressors and stress moderators, such as role captivity and coping strategies, predict worse mental health outcomes; however, relatively little is known about the relationship between role captivity and specific coping strategies dementia caregivers use to manage caregiving-related stress. The purpose of this study was to examine relationships between role captivity, coping strategies, and depressive and anxiety symptoms. A preliminary analysis was conducted on a sample of community-dwelling family caregivers (N=30) from an ongoing national study on daily caregiving experiences. Data were analyzed using regression analysis. Dementia family caregivers reported moderate anxiety symptoms, measured by the GAD-7 (M= 11.86, SD= 4.39) and moderate depressive symptoms, measured by the CESD, (M=19.59, SD= 12.67). The sample's mean age was 54.7 years old and 76.7% female. Role captivity was positively associated with depression ($\beta$=2.98, t(28) = 3.94, p< 0.001) and anxiety ($\beta$= 0.92, t(28) = 3.31, p< 0.01) among dementia caregivers. Avoidant coping was positively associated role captivity ($\beta$=0.31, t(28) = 4.27, p< 0.001). The results of this study suggest role captivity is associated with an increase in depression and anxiety among dementia caregivers. Further, avoidant coping is associated with an increase in role captivity reported by caregivers. The findings from this study support the benefit of interventions that help dementia family caregivers in their role and promote the use of positive coping strategies.

### SESSION 7100 (POSTER)

### COGNITION AND ALZHEIMERS' DISEASE

Abstract citation ID: igad104.2229

### P. GINGIVALIS–LPS-INDUCED NLRP3 INFLAMMASOME ACTIVATION DRIVES AD PATHOLOGIES

Ambika Verma, Pankaj Patyal, Gohar Azhar, and Jeanne Wei, *University of Arkansas for Medical Sciences, Little Rock, Arkansas, United States*

Alzheimer's disease (AD) is the leading cause of dementia among older adults. AD is characterized by amyloid-$\beta$ deposition, abnormal hyperphosphorylation of tau leading to the formation of neurofibrillary tangles, and neuroinflammation. Porphyromonas gingivalis, the keystone-pathogen of periodontitis, is being increasingly linked with AD and Alzheimer's disease-related dementias (ADRD). Poor oral health is common in older populations, and literature suggests that oral health correlates with prevalence of ADRD. NOD-like receptor family pyrin domain containing 3 (NLRP3) inflammasome-mediated IL-1$\beta$ is significantly involved in periodontal diseases. However, the exact mechanism by which NLRP3 inflammasome is regulated in response to pathogenic bacteria remains unclear. We hypothesized that P. gingivalis-LPS may be responsible for the neuroinflammation via TLR4 activation and its downstream caspase-1, caspase-3 or caspase-4/5/11 dependent cell signaling. Our results suggest that P. gingivalis accelerated the induction of NLRP3. Furthermore, NLRP3/Caspase-1/Caspase-4/5/11 dependent IL-1$\beta$ production may contribute to the dysregulated neuroinflammatory response in AD pathogenesis.

### A MECHANISM OF ACTION OF HUMAN-ORIGIN PROBIOTIC COCKTAIL IN ATTENUATING ALZHEIMER'S DISEASE PATHOLOGY

Santosh Kumar Prajapati[1], Shaohua Wang[2], Sidharth Mishra[1], Shalini Jain[1], and Hariom Yadav[1], *1. University of South Florida, Tampa, Florida, United States, 2. Ohio University, Athens, Ohio, United States*

Alzheimer's disease (AD) is a detrimental public health problem affecting millions of older adults in the USA alone, with no or limited treatment options. Emerging evidence indicates the pivotal role of gut microbiota in AD pathology, but mechanisms remain largely unknown. Also, microbiome modulators like probiotics are proven beneficial in preventing AD with little understanding of their mechanisms. Therefore, we investigated how a microbiota metabolite-short-chain free fatty acid receptor-2 (FFAR2) signaling in the intestine affects AD pathology in brain using intestinal FFAR2 knockout APP/PS-1 mice; as well as determined if a human-origin-probiotic cocktail exhibits its actions via FFAR2 signaling. We measured changes in the microbiome, permeability in gut and blood-brain barriers (BBB), neuroinflammation, AD pathology, and cognitive behaviors. Results showed, the mice lacking FFAR2 in their intestine exacerbated AD pathology by developing abnormal changes in gut microbiome and intestinal permeability, which in turn promotes permeability in BBB and neuroinflammation. It's also promoting microglia activation linked with increased A$\beta$ accumulation and cognitive decline. It's interesting to note that probiotic cocktail feeding reversed these abnormalities in AD mouse models. Further, this probiotic cocktail showed its mechanism in both FFAR2-dependent and independent manner. Overall, our findings indicate that the microbiome sensing mechanism FFAR2 signaling participates in regulating the gut-brain in AD pathology, and a human-origin probiotic cocktail improves the abnormal changes in the microbiome-intestinal & BBB permeability-neuroinflammation axis to ameliorate AD pathology. Thus, FFAR2 agonism and probiotic therapy can be viable options to prevent/ delay the risk of AD progression in older adults.

Abstract citation ID: igad104.2230

### AGE EFFECTS ON WHITE MATTER TOPOLOGY IN OLDER ADULTS AT HIGH RISK OF ALZHEIMER'S DISEASE

Meishan Ai[1], Emma Tinney[1], Charles Hillman[1], Nathan Spreng[2], Arthur Kramer[1], Maiya Geddes[2], and PREVENT-AD Research Group[2], *1. Northeastern University, Boston, Massachusetts, United States, 2. McGill University, Montreal, Quebec, Canada*

White matter integrity shows age-related declines in later life. Studies have found altered topological changes of white matter networks in healthy aging populations. We investigated cross-sectional association between white matter network topology, age, education, and cognition in older adults at high-risk for Alzheimer's disease (AD). A total of 153 cognitively healthy participants (age=67.78±5.03, 111 female, 54 APOE-4 carriers) who have at least one close family member diagnosed with AD from the Pre-symptomatic Evaluation of Experimental or Novel Treatments for Alzheimer's Disease (PREVENT-AD) cohort were included in the study. Whole brain diffusion images were obtained and submitted to preprocessing, fiber tracking and connectome creation in MRtrix. Test scores from the Trail Making task, Stroop task, and Rey Auditory Verbal Learning Test (RAVLT) were included as cognitive function measures. We found that older age was significantly associated with greater modularity (r2=0.48, p corrected< 0.001), lower density (r2=0.32, p corrected< 0.001), greater betweenness centrality (r2=0.39, p corrected=0.003), and greater clustering coefficient (r2=0.05, p corrected=0.042). Older age was also significantly associated with longer Trail Making time series B (r2=0.11, p corrected=0.012) and fewer recalled words (r2=0.18, p corrected=0.007). We further found that participants with higher educational attainment showed less age-related changes in modularity (t=2.42, p=0.017) and density (t=2.40, p=0.018). However, these graph theory measures were not associated with cognitive performance. In conclusion, age related to whole-brain white matter network topology in high-risk healthy older adults, and education may attenuate this relationship. The current study deepens our understanding of white matter changes in high-risk aging.

Abstract citation ID: igad104.2231

### CHANGES IN ADIPOSITY AND COGNITION AMONG EARLY POSTMENOPAUSAL WOMEN FOLLOWING HORMONE TREATMENT

Maritza Dowling[1], JoAnn Manson[2], Frederick Naftolin[3], Sherman Harman[4], Dustin Hammers[5], Marcelle Cedars[6], kejal Kantarci[7], and Carey Gleason[8], *1. George Washington University, Washington, District of COlumbia, United States*, *2. Brigham & Women's Hospital/Harvard University, Boston, Massachusetts, United States*, *3. NYU School of Medicine, New York City, New York, United States*, *4. Phoenix VA Health Care System, Phoenix, Arizona, United States*, *5. Indiana University, Indianapolis, Indiana, United States*, *6. University of California, San Francisco, San Francisco, California, United States*, *7. Mayo Clinics, Rochester, Minnesota, United States*, *8. University of Wisconsin, Madison, Wisconsin, United States*

Evidence suggests that early postmenopausal hormone treatment (MHT) may attenuate metabolic effects on dementia pathogenesis. Using Kronos Early Estrogen Prevention Study (KEEPS) and KEEPS-Continuation data, we investigated heterogeneity in the associations between longitudinal central adiposity (CA), MHT, and cognition, hypothesizing that CA would be related to cognition and that MHT would favorably alter this relationship. KEEPS participants (previously randomized to 48 months treatment with placebo or HT with oral conjugated equine estrogens + progesterone or transdermal 17-β-estradiol+ progesterone) were recruited for KEEPS-Continuation ~10 years post-randomization. Cognitive tests from both studies were analyzed as four-factor scores. Using the original KEEPS data (n=662), growth mixture modeling identified distinct CA trajectories (classes) across 48 months of MHT, which predicted cognition at 48 months and ~10 years, adjusting for covariates. Adiposity trajectories included "low-CA" and "high-CA" classes. Low-CA women exhibited stable low-CA throughout MHT. High-CA women's adiposity was initially elevated but declined significantly after 48 months of MHT. After adjusting for covariates, the high (versus low) CA class predicted significantly lower cognitive factors scores at month 48 with one exception – Verbal-Learning & Memory. Ten years later, high-CA women (n=299) scored significantly lower on two factors. MHT predicted neither CA trajectories nor cognitive performance at 48 months or 10 years later. Adiposity levels significantly declined in the high-CA class on MHT but not placebo and was associated with diminished cognitive function. CA influenced short-and long-term cognition. Organ/tissue studies should examine how heterogeneity in adiposity levels interacts with MHT and other factors driving dementia pathogenesis.

Abstract citation ID: igad104.2232

### COMPARISON OF STEREOLOGY METHODS FOR ASSESSING AGE-RELATED EFFECTS ON IMMUNOSTAINED BRAIN CELLS

Grant Denham[1], Saeed Alahmari[2], Aiden Anderson[1], Krystal Sanchez[1], Dominick Dag[1], Lawrence Hall[3], Dmitry Goldgof[3], and Peter Mouton[1], *1. Stereology Resource Center Biosciences, Tampa, Florida, United States*, *2. Najran University, Najran, Najran, Saudi Arabia*, *3. University of South Florida, Tampa, Florida, United States*

The primary benefit of stereology methods is quantification of well-stained biological objects in tissue sections with the ability to adjust sampling intensity to achieve desired levels of precision. The advent of hand-crafted algorithms and artificial intelligence-based deep learning (DL) provides an opportunity for more standardized collection of stereology data with enhanced efficiency and higher reproducibility compared to state-of-the-art manual stereology. We contrasted and compared the performance of four manual, semi-automatic, and fully automatic approaches for generating data for total number of Neu-N immunostained neurons in neocortex (NCTX) in the mouse brain. The gold standard for these studies was manual counts using the state-of-the-art optical fractionator method on 3-D reconstructed serial z-axis image stacks through a known tissue volume (disector stacks). To allow for direct methodological comparisons on the same images, disector stacks were automatically converted into extended depth of field (EDF) images in which all neurons in the disector stack were imaged at each cell's maximal plane of focus. Total number of Neu-N neurons on the same EDF images were counted by a fully automatic hand-crafted method [automatic segmentation algorithm (ASA)] and a semi-automatic method [ASA counts manually corrected for false positives and negatives]. All comparison counts were done using unbiased frames and counting rules with total counts of NeuN-immunostained neurons by the optical fractionator method. The results were comparable across methods with wide variations in throughput efficiency and inter-rater agreement. These results are discussed with respect to applications to experimental studies of brain aging, neuroinflammation and neurodegenerative disease.

Abstract citation ID: igad104.2233

### DIGITAL CLOCK DRAWING PERFORMANCE ASSOCIATIONS WITH ALZHEIMER'S DISEASE GENETIC RISK SCORE AND APOE IN OLDER ADULTS

Louisa Thompson[1], Michael Cummings[2], Sheina Emrani[3], David Libon[4], Alvin Ang[5], Cody Karjadi[6], Rhoda Au[7], and Chunyu Liu Chunyu Liu[5], *1. Brown University Medical School, Providence, Rhode Island, United States*, *2. Boston University Chobanian & Avedisian School of Medicine, Boston, Massachusetts, United States*, *3. Brown University, Providence, Rhode Island, United States*, *4. Rowan University, Stratford, New Jersey, United States*, *5. Boston University, Boston, Massachusetts, United States*, *6. Framingham Heart Study, Framingham, Massachusetts, United States*, *7. Boston University Chobian & Avedisian School of Medicine, Boston, Massachusetts, United States*

Alzheimer's disease (AD) is the leading cause of dementia in older adults, but most people are not diagnosed until clinical signs and symptoms are present. Non-invasive and cost-effective digital biomarkers for AD have the potential to improve early detection. We examined the validity of DCTclockTM (a digitized clock drawing task) as an AD susceptibility biomarker. We used two primary independent variables, Apolipoprotein E (APOE) ε4 allele carrier status and polygenic risk score (PRS). We examined APOE and PRS associations with DCTclockTM composite scores as dependent measures. We used existing data from the Framingham Heart Study (FHS), a community-based study with the largest dataset of digital clock drawing data to date. The sample consisted of 2,398 older adults ages 60-94 with DCTclockTM data (mean age of 72.3, 55% female and 92% White). PRS was calculated using 38 variants identified in a recent large genome-wide association study (GWAS) and meta-analysis of late-onset AD (LOAD). Results showed that DCTclockTM performance decreased with advancing age, lower education, and the presence of one or more copies of APOE ε4. Lower DCTclockTM Total Score as well as lower composite scores for Information Processing Speed and Drawing Efficiency were significantly associated with higher PRS levels and more copies of APOE ε4. Associations displayed similar effect sizes in men and women. This is the first study to demonstrate significant differences in clock drawing performance on the basis of APOE status or PRS.

Abstract citation ID: igad104.2234

### GWAS OF BRAIN ENDOPHENOTYPES: ASSOCIATION OF AUTOPSY-BASED LEWY BODY COUNTS WITH MOVEMENT DISORDER RISK LOCI

Steven Edland[1], Fernanado Hernandez[1], Chanond Nasamran[1], Kathleen Fisch[2], and Lon White[3], *1. University of California San Diego, San Diego, California, United States*, *2. University of California, San Diego, San Diego, California, United States*, *3. Pacific Health Research and Education Institute, Honolulu, Hawaii, United States*

We performed a genome wide association study (GWAS) of neocortical Lewy body density. Case material were 308 autopsies of Japanese-American men (mean age at death 87.5 years; 11% with mild, and 30% with severe cognitive impairment prior to death). Lewy bodies were assessed by manual count of alpha-synuclein stained neocortical sections from the frontal, temporal, parietal, and occipital lobes. Genetic association was tested by linear models controlling for age at death assuming an additive genotypic effect. Top hits include a locus on chromosome 1q32 previously associated with amyotrophic lateral sclerosis (p=5.8e-8), intronic variants on the dopaminergic neuron axonal guidance gene EPHB1 previously associated with Parkinson's disease (PD) (p=8.4e-6), and a locus containing two independent intron 1 variants on CSMD1, a known risk factor for autosomal dominant PD (p=1.5e-6). Complement inhibitor protein CSMD1 is a type 1 transmembrane cell adhesion molecule implicated in axonal guidance and the development, maintenance, and pruning of synapses in the adult brain. Intermediate hits include genes encoding cell adhesion molecules and a second complement inhibitor implicated in the regulation of synapses (TRAPPC9). This converging evidence points to cell adhesion proteins and complement mediated synaptic regulation in the etiology of Lewy body lesions.

Abstract citation ID: igad104.2235

### IDENTIFYING MARKERS OF NEURODEGENERATION FOR MOTORIC COGNITIVE RISK SYNDROME

Edward Ofori[1], Madeline Hooten[2], Marcus Ortega[2], Jordan Barajas[2], and Dara James[3], *1. Arizona State University, Tempe, Arizona, United States*, *2. Arizona State University, Phoenix, Arizona, United States*, *3. University of South Alabama, Mobile, Alabama, United States*

Cognitive disorders, particularly dementia, are a major cause of disability in the elderly population. Gait disturbances, decreased grip strength and memory complaints are early signs of dementia that can occur well before the disease manifests. To better understand the close interactions between the physical and cognitive domains, the concept of Motoric Cognitive Risk Syndrome (MCRS) has been proposed. We had 25 older adults (10 cognitively unimpaired and 15 with MCRS) generate multiple force pulses at 15% of their maximum voluntary contraction with a fixed duration of 2 seconds for each pulse in which participants must rely on visual spatial abilities to gauge the amount force required in real-time to reach the criterion target. We also assessed neurofilament light chain levels also a blood-based marker of neuronal loss. Our results indicate the MCRS group demonstrated blood oxygenation level dependent (BOLD) hyperactivity in visual cortical/hippocampal regions, parahippocampal regions, and dorsolateral prefrontal cortex. The MCRS group also demonstrated BOLD hypoactivity in primarily involved with movement, such as cerebellar lobules, supplementary motor areas, middle frontal gyrus, and prefrontal cortex areas.be useful in tracking disease progression. We also found a significant strong positive correlation (0.83) between serum NFL levels and Right BOLD coefficients only for the CI group. This finding suggests that these motor task fMRI measures may be specific in tracking neurodegeneration in MCRS group. Overall, the current preliminary findings provide novel insights into the neurobiological mechanisms underlying early changes in Alzheimer's disease and related dementias.

Abstract citation ID: igad104.2236

### LOCALIZATION OF Β-AMYLOID PLAQUES IN THE BRAINS OF WAR VETERANS, PARTICIPANTS OF THE DOD-ADNI STUDY

Stanislav Kolpakov Nikitin, Arseniy Yashkin, and Igor Akushevich, *Duke University, Durham, North Carolina, United States*

We examined the allocation of β-amyloid (Aβ) plaques in the brains of Veterans, long-time survivors of traumatic brain injury (TBI), and those, who did not report brain trauma, and compared it with a spatial distribution of Aβ plaques in the brains of participants of the ADNI program as well as with the pattern of the spatial distribution of Aβ in the brains of participants of ADNI with Alzheimer's disease. This study included 675 community-dwelling male participants from the ADNI and DoD-ADNI databases (137 veterans, 131 cases of TBI, and 123 AD cases) 62 years old or older. We performed regression analysis, using a pseudo-randomization algorithm, and propensity-score inverse-probability weighting to equalize the subsamples for fourteen outcomes, 12 standardized uptake value ratio variables, and 0.79 and 1.11 cutoffs. Race, educational level, geriatric depression score, age when florbetapir-18 (18F) PET scans were performed, APOE genotype, and Modified Hachinski Ischemic Score were used as predictors. The pattern common for AD showed the highest levels of β-amyloid in the cingulate cortex as well as in the frontal, parietal, and temporal lobes. Veterans have shown a statistically significant increase of 18F concentration in the cerebellum gray matter along with a lower concentration of it in the whole cerebrum as well as in neocortical regions than patients, who did not participate in combats. The continuous exposition to micro-TBI events, which does not necessarily suppose the loss of consciousness or severe contusion likely can explain the high concentration of 18F in cerebellum gray matter in ex-combatants.

Abstract citation ID: igad104.2237

### PLASMA BDNF/IRISIN RATIO ASSOCIATES WITH COGNITIVE FUNCTION IN OLDER ADULTS

Qiaoqin Wan[1], Xiuxiu Huang[2], and Shifang Zhang[2], *1. Peking University Health Science Center, Beijing, Beijing, China (People's Republic)*, *2. Peking University, Beijing, Beijing, China (People's Republic)*

**Objectives:** To examine whether plasma biomarkers brain-derived neurotrophic factor (BDNF), irisin, clusterin and BDNF/irisin ratio (BIR) could differentiate people with mild cognitive impairment (MCI) from cognitively normal (CN) individuals, and to explore their relations with cognitive performance. **Methods:** We included 124 participants with MCI and 126 CN participants from a community-based aged and cognitive health cohort. Plasma BDNF, irisin and clusterin were measured, and BIR was calculated. Global cognition was evaluated with Montreal Cognitive Assessment. T-tests, logistic regressions, and linear regressions were used to explore the relations between plasma biomarkers and cognitive function. **Results:** The plasma levels of irisin, but not BDNF, was significantly different between MCI and CN groups. Higher irisin concentration was associated with increased probability for MCI (OR: 1.06, $p = 0.004$) after adjusting for covariates. By contrast, BDNF, but not irisin, was linearly correlated with cognitive performance ($\beta = 0.14$, $p = 0.033$). BIR values were positively correlated with cognitive performance ($\beta= 0.14$, $p = 0.036$), and significant differences on BIR values existed between MCI and CN groups. The MCI risk decreased by 53% (OR=0.47, $p = 0.043$) with each unit increase in BIR values after adjusting for covariates. Plasma BDNF and irisin concentrations increased with aging, whereas BIR values remained stable across the ages. No significant results of clusterin were observed in the above analyses. **Conclusion:** Plasma BIR is a potentially reliable indicator which not only reflects the odds of the presence of MCI but also directly associates with cognitive performance in the aged population.

Abstract citation ID: igad104.2238

### RACE DIFFERENCES IN THE RELATIONSHIP BETWEEN HIGH AND VARIABLE BLOOD PRESSURE AND DOMAIN-SPECIFIC COGNITIVE CHANGE

Michael Oliver[1], Cassandra Morrison[2], and Sondos El-Hulu[1], *1. Belmont University, Nashville, Tennessee, United States*, *2. Montreal Neurological Institute, McGill University, Montréal, Quebec, Canada*

Elevated blood pressure (BP), or hypertension, is a risk factor for several health conditions including Alzheimer's disease. High BP in early- and mid-life is associated with cognitive decline, whereas research is mixed regarding BP and cognition in late-life. Moreover, hypertension disproportionately affects minority populations. Consequently, the effects of hypertension on cognition may differ by race. The present study investigates the relationship between BP and cognition. 4419 older adults (Black, n=1189; White, n= 3230), with 32116 follow-ups for a maximum of 10-years were included from the Rush Alzheimer's Disease Center. Results reveal individuals with high BP exhibit significantly greater declines in global cognition compared to normal BP, regardless of race. When stratifying high BP by race, Blacks exhibit greater declines in working memory and perceptual speed, whereas Whites exhibit greater declines in episodic memory, semantic memory, and visuospatial ability compared to normal BP. Blacks with high BP exhibit greater decline in perceptual speed compared to variable BP, whereas no cognitive domain was worse in Whites with high BP compared to variable. When stratifying variable BP by race, Blacks exhibited greater decline in working memory compared to normal BP. Alternatively, Whites exhibited greater decline in episodic memory, semantic memory, working memory, and global cognition compared to normal and high BP counterparts. Our findings reveal racial differences in the relationship between BP and cognitive decline. Specifically, Blacks and Whites with high and variable BP experience cognitive decline in different cognitive domains, which may give insight into differences in disease trajectory and cognitive outcomes.

Abstract citation ID: igad104.2239

### THE EFFECT OF EARLY SCHOOLING EXPERIENCES AND ADHD POLYGENIC RISK ON COGNITIVE PERFORMANCE IN LATE LIFE

Marrium Mansoor, and Benjamin Katz, *Virginia Tech, Blacksburg, Virginia, United States*

Attention-Deficit/Hyperactivity Disorder (ADHD) is a neurodevelopmental disorder characterized by executive function (EF) deficits that may impact academic performance, workplace achievement, and other outcomes throughout adulthood. Although 6.76% of adults worldwide may be affected by ADHD, the interplay of early life factors and genetic risk is still not fully understood, especially in the context of cognitive function in later life. While literature suggests that early life experiences may moderate the links between genetic risk for ADHD and later life cognition, few studies have examined these links quantitatively. This research included ~7000 participants from the Health and Retirement Study, a nationally-representative longitudinal study of 37,000 Americans. A moderation analysis was conducted to examine the effect of repeating a year of school before the age of 18 on the relationship between ADHD risk and EF performance as measured by scores on Number Series and Serial 7s tests. Results showed that ADHD genetic risk significantly predicted performance on EF measures; furthermore, the interaction term was significant while controlling for demographics, self-rated health, and maternal education. However, only older adults with low polygenic risk for ADHD displayed differential cognitive performance as a function of early-life academic adversity, while those with high polygenic risk displayed reduced performance on EF-related measures regardless of whether they repeated a grade. This finding suggests that the effect of genetic risk for ADHD on later life EF performance may remain stable across earlier educational experiences and informs future work examining the impact of gene-environment interactions on cognition in later life.

Abstract citation ID: igad104.2240

### UNIQUE MICROBIOME SIGNATURES RELATED TO CANCER SURVIVORSHIP AND COGNITIVE DECLINE IN OLDER ADULTS

Jayden Haggler, Brandi Miller, Diptaraj Chaudhari, Hariom Yadav, and Shalini Jain, *University of South Florida, Tampa, Florida, United States*

Significant scientific advancements have resulted in new and effective therapies for patients with cancer. Although this leads to an increased cancer survivor population, there are several long-term side effects of cancer treatments, including a high risk of developing dementia. Emerging evidence shows that the gut microbiota significantly contributes to brain health and dementia pathology; however, its role in cancer treatment-related side effects, specifically in older adults, is not well known. Here, we used 110 participants from our MiaGB (Microbiome in aging Gut and Brain) consortium aged 60 years and older and analyzed their gut microbiome (using whole genome sequencing) with cognitive function (using Montreal Cognitive Assessment (MoCA) and MiniCog). We observed an 8% increase in cognitive impairment (CI) in cancer survivors compared to non-cancer participants (46% vs 38%, respectively). When only comparing cognitively healthy versus CI, the abundance of Escherichia coli was significantly higher in the CI group, while Streptococcus thermophilus was higher in the cognitively healthy group. Additionally, the abundances of Dorea sp. CAG 317, Ruminococcus gnavus, Roseburia faecis, and Eggerthella lenta were significantly increased in the gut of cancer survivors compared to non-cancer participants. Specifically, the abundance of Dorea sp. CAG 317 was increased in cancer survivors with CI. These findings suggest that unique microbiome signatures induced by cancer treatments may impair cognitive function. Furthermore, understanding the associations between the microbiota and cognitive function in cancer survivors will aid the development of therapeutics to combat cancer treatment-related side effects, which are a growing public health concern.

### SESSION 7110 (POSTER)

### GEROSCIENCE

Abstract citation ID: igad104.2241

### CIRCULATING MICRORNA PROFILE IN OKINAWAN CENTENARIANS

Sarah Noureddine[1], Sarah Ashiqueali[2], Augusto Schneider[3], Sydney Strader[4], Richard Allsopp[5], D. Craig Willcox[6], Bradley Willcox[7], and Michal Masternak[2], *1. University of Central Florida, Oviedo, Florida, United States*, *2. University of Central Florida College of Medicine, Orlando, Florida, United States*, *3. Universidade Federal de Pelotas, Pelotas, Rio Grande do Sul, Brazil*, *4. University of Central Florida, Orlando, Florida, United States*, *5. University of Hawaii, Honolulu, Hawaii, United States*, *6. Okinawa International University, Ginowan, Okinawa, Japan*, *7. Kuakini Medical Center, Honolulu, Hawaii, United States*

Centenarians are a successful example of aging and extended longevity, exhibiting advanced regulation of biological mechanisms and homeostasis. Since human longevity is a complex field of study that navigates molecular and biological mechanisms that influence aging, we hypothesized that microRNAs, a class of small non-coding RNAs implicated in regulating gene expression at the post-transcriptional level, demonstrate altered expression profiles in circulation across young, middle-aged, and centenarian cohorts. We sequenced circulating microRNAs in Okinawan males and females < 40, 40-80, and >90 years of age accounting for FOXO3A genetic variations (TT and TG alleles) and validated the findings through RT-qPCR. We report 5 microRNAs exclusively upregulated in both male and female centenarians demonstrating predictive functional roles in TGF-β, FoxO, AMPK, Pi3K-Akt, and MAPK signaling pathways. Our findings suggest that these microRNAs upregulated in centenarians may provide novel insight into their enhanced longevity and warrant further exploration into their roles in human aging and longevity.

Abstract citation ID: igad104.2242

### LATE-LIFE PLASMA PROTEINS ASSOCIATE WITH PREVALENT AND INCIDENT FRAILTY: A PROTEOMIC ANALYSIS

Fangyu Liu[1], Thomas Austin[2], Jennifer Schrack[3], Jeremy Walston[3], Rasika Mathias[3], Josef Coresh[3], and Keenan Walker[4], *1. Johns Hopkins Bloomberg School of Public Health, Baltimore, Maryland, United States*, *2. University of Washington, Seattle, Washington, United States*, *3. Johns Hopkins University, Baltimore, Maryland, United States*, *4. National Institute on Aging, National Institutes of Health, Baltimore, Maryland, United States*

Proteomic approaches have unique advantages in the identification of biological pathways that influence physical frailty, a multifactorial geriatric syndrome predictive of adverse health outcomes in older adults. To date, few proteomic studies included prefrailty as a separate state or follow the participants for incident frailty. We evaluated 4955 plasma proteins (log 2-transformed and standardized) measured using SomaScan in the Atherosclerosis Risk in Community (ARIC) study. We used multinomial logistic regression models for the cross-sectional associations of the proteins with prefrailty and frailty as two distinct states (N=3838), and logistic regression models for the longitudinal associations with incident frailty over the 6-year follow-up (N=1708). After adjusting for demographics, health indicators, and comorbidities, 136 and 186 proteins were cross-sectionally associated with prefrailty and frailty, respectively, after Bonferroni correction (p< 1.01x10-5); 24 and 84 of which were replicated in the Cardiovascular Health Study (CHS) using the same models (FDR p< 0.05). Notably, higher odds of prefrailty and frailty were observed with higher concentrations of growth differentiation factor 15 (GDF15; OR[discovery]=1.50 and 2.17, respectively), transgelin (TAGLN; OR[discovery]=1.43 and 2.43, respectively), and insulin-like growth factor-binding protein 2 (IGFBP2; OR[discovery]=1.43 and 2.08, respectively), and lower concentration of growth hormone receptor (GHR, OR[discovery]=0.70 and 0.47, respectively). Longitudinally, triggering receptor expressed on myeloid cells 1 (TREM1) predicted incident frailty (OR[discovery]=1.64). Pathway and upstream regulator analyses revealed that mechanisms related to lipid metabolism, axonal guidance, inflammation and cell senescence became dysregulated in the prefrail stage and persisted into frail stage, offering new insights into frailty etiology and targets for early intervention.

Abstract citation ID: igad104.2243

### MID-LIFE PLASMA PROTEINS ASSOCIATE WITH PREFRAILTY AND FRAILTY: A PROTEOMIC ANALYSIS

Fangyu Liu[1], Jennifer Schrack[2], Jeremy Walston[2], Rasika Mathias[2], Josef Coresh[2], and Keenan Walker[3], *1. Johns Hopkins Bloomberg School of Public Health, Baltimore, Maryland, United States*, *2. Johns Hopkins University, Baltimore, Maryland, United States*, *3. National Institute on Aging, National Institutes of Health, Baltimore, Maryland, United States*

Physical frailty is a syndrome that typically manifests in later life, although the pathogenic process causing physical frailty likely begins decades earlier. To date, few studies have examined the biological signatures in mid-life associated with frailty in late life. We evaluated 4955 plasma proteins (log 2-transformed and standardized) measured using the SomaScan platform among 3810 participants (57.8+/-5.0 years, 58.2% women) at Visit 3 of the Atherosclerosis Risk in Community (ARIC) study, and their frailty status across up to 3 study visits in late-life (median follow-up 19 years). We used multinomial logistic regression models to examine the associations between the protein levels and the frailty status, adjusting for demographics, health indicators, comorbidities, and follow-up time. Two sets of 2 proteins were identified to be associated with prefrailty and frailty, respectively, after Bonferroni correction (p< 1.01×10-5). Higher concentration of hydroxymethylglutaryl-CoA synthase (HMGCS1; OR=1.22), a regulator of cholesterol synthesis, and lower concentration of dual specificity protein phosphatase 13 isoform A (DUSP13A; OR=0.82), a protein involved in cell apoptosis, were associated with higher odds of prefrailty. Higher concentration of growth differentiation factor 15 (GDF15; OR=1.39), a protein related to inflammation and cell senescence, and lower concentration of contactin-1 (CNTN1; OR=0.72), a protein involved in nervous system development and signalling, were associated with higher odds of frailty. The pathway and upstream regulator analyses of the physical frailty- or prefrailty-associated proteins (p< 0.01) revealed the dysregulation of apoptosis, inflammation, and neuromuscular communication in mid-life. Our findings provides new insights into frailty etiology earlier in the life course.

Abstract citation ID: igad104.2244

### MULTIPLE ENRICHMENT TOOLS POINT TO A CENTRAL ROLE OF CARDIAC MUSCLE IN EXTREME LONGEVITY

Svetlana Ukraintseva[1], Deqing Wu[2], Hongzhe Duan[2], Konstantin Arbeev[2], and Anatoliy Yashin[2], *1. Duke University, Durham, North Carolina, United States*, *2. Social Science Research Institute, Duke University, Durham, North Carolina, United States*

Few genes showed consistent positive associations with extreme longevity in humans. We conducted genome-wide association study of various parental lifespans, LS (85+, 90+, 95+, 100+ vs. 75-), using UK Biobank data. At ages 100+, the negative effect of APOE4 on LS diminished, while SNPs with positive associations with LS emerged among the top results, including rare variants. One of the top SNPs (rs756319938) was in CDKN1A (P21) gene involved in cell senescence, and its rare variant (protein truncating) was significantly (P-value< 10-7) overrepresented in extreme longevity cases. Such nonsense mutations generally result in non-functional protein. In case of P21, this might contribute to longevity via better regenerative response. The latter was previously demonstrated in mice that lacked the p21 expression. For SNPs that were associated with LS>=100 with P-value < 10-4, we identified ~400 corresponding genes and run multiple pathway/process enrichment analyses using different tools utilizing various resources (GO, KEGG, OMIM, MSigDB, and others), to identify pathways/processes that show up across most analyses. We found that cardiac muscle function was among the top pathways/processes yielded by majority of the enrichment tools. In conclusion, we found new rare and common SNPs that were positively associated with extreme longevity of mothers and fathers of UKB participants, and investigated relevant biological pathways. Results of multiple enrichment tools converged on a central role of cardiac muscle function in extreme longevity, while separate enrichment tools did not allow to see this picture. Our results imply that cardiac regeneration is a plausible target for interventions into longevity limits.

Abstract citation ID: igad104.2245

### PROSPECTIVE MULTI-OMIC ANALYSIS OF HUMAN LONGEVITY COHORTS IDENTIFIES ANALYTE NETWORKS ASSOCIATED WITH LONGEVITY

Lance Pflieger[1], Kengo Watanabe[2], Max Robinson[2], Gustavo Glusman[2], Jodi Lapidus[3], Oliver Fiehn[4], Robert Moritz[2], and Noa Rappaport[2], *1. Phenome Health, Seattle, Washington, United States*, *2. Institute for Systems Biology, Seattle, Washington, United States*, *3. Oregon Health & Science University, Portland, Oregon, United States*, *4. UC Davis, Davis, California, United States*

Serum based biomarkers of longevity have long been sought to explain the mechanisms of healthy aging and longevity. Using a 1:3 case cohort design, the Longevity Consortium has produced untargeted mass spectrometry based proteomic and metabolomic datasets from serum of four cohorts with longevity status, defined as those that reached the age corresponding to the 98th percentile of survival using sex specific and birth cohort specific survival percentiles. The cohorts are the Osteoporotic Fractures in Men study, the Study of Osteoporotic Fractures, the Health, Aging, and Body Composition Study, and the Cardiovascular Health Study. In this study, we integrate metabolomics and proteomics using machine learning and system biology approaches to construct multi-omic signatures predictive of longevity and healthy aging. We identify networks enriched for biomarkers previously shown to be associated with longevity such as apolipoproteins, along with novel associations, and we further compare with our findings in a mouse omics LC dataset of molecular changes induced by life-extending interventions. We show substantial differences between male and female longevity networks. The study highlights the effectiveness of using integrative systems biology methods to capture the heterogeneity of underlying molecular aging phenotypes, in order to generate a robust signature of longevity. The identified biomarker signatures may have significant implications for the development of personalized interventions aimed at promoting healthy aging and preventing age-related diseases.

Abstract citation ID: igad104.2246

### PROTOCOL OF A RANDOMIZED, PARALLEL, DOUBLE-BLIND, PLACEBO-CONTROLLED PILOT TRIAL OF A KETONE ESTER IN AGING

Brianna Stubbs[1], Sawyer Peralta[1], Stephanie Roa Diaz[1], Gabriela Alvarez Azanedo[1], Laura Alexander[1], Wendie Silverman-Martin[1], James Johnson[2], and John Newman[1], *1. Buck Institute, Novato, California, United States*, *2. NA, Greenbrae, California, United States*

**Background:** Ketone bodies function as an energy and signaling metabolite and are hypothesized to interact with several geroscience pathways. Ketone esters (KE) are a nutritional tool that induce ketosis without other dietary changes; no studies have addressed KE in an aging population. **Objectives:** The primary objective of this randomized, placebo-controlled, double-blinded, parallel-group, pilot study is to determine the tolerability of 12-weeks of KE ingestion in older adults (>65y). Secondary outcomes include safety and acute ketone kinetics. Exploratory outcomes include physical function, cognitive function, quality of life, general and inflammatory aging biomarkers and gut microbiome. **Methods:** Community dwelling older adults who are independent in activities of daily living, and with no unstable acute medical conditions (n=30) will be recruited. The study intervention is a KE (n=15) or a placebo (PLA). Firstly, ketone kinetics after 12.5 or 25g of KE consumption will be assessed over four hours. Secondly, after collection of baseline safety, functional and biological measurements, participants will be randomly allocated to consume KE or PLA daily for 12-weeks. A questionnaire will assess tolerance daily for 2-weeks, and then via recall at 2-week intervals. Safety assessments will be repeated at week 4. All measures will be repeated at week 12. **Conclusion:** This study will provide data demonstrating the feasibility, tolerance, and safety of 12-weeks of KE consumption in older adults as well as exploratory data across a range of geroscience-related endpoints. This will facilitate our long-term goal: use of KE to study geroscience mechanisms and clinical outcomes relevant to frailty.

### SESSION 7120 (POSTER)

### AGING IN PLACE (HS)

Abstract citation ID: igad104.2247

### ENGAGING OLDER ADULTS TO GUIDE LOW-COST NONWEARABLE SENSOR TECHNOLOGY DEVELOPMENT TO AGE IN PLACE: SURVEY FINDINGS

Elinor Schoenfeld[1], Tracy Trimboli[2], Kaylyn Finnerty[3], Givenchy Ayisi-Boahene[2], Patricia Bruckenthal[2], Erez Zadok[2], Shelley Horwitz[2], and Fan Ye[2], *1. Stony Brook School of Medicine, Stony Brook, New York, United States*, *2. Stony Brook University, Stony Brook, New York, United States*, *3. Mather Hospital Northwell Health, Port Jefferson, New York, United States*

As Americans live longer, a dynamic opportunity has arisen to provide enhanced resources to sustain their well-being. Cost-conscious, convenient in-home sensing will assist with chronic disease management, and become part of a long-term plan to support our aging population and shrinking healthcare workforce. The purpose of this study was to obtain input from older adults about (i) their comfort level and willingness to adopt different sensor technologies, and (ii) opinions on data sharing, security, and privacy to guide our sensor development. Over 4 different survey timeframes (2018-2022), adults aged 60 and older (N=112) completed our survey either in-person (n=77) or via a REDCap online survey (n=35) (53% female; 30% age >80; 78% college graduates; 19% living alone). Though there were significant differences ($p< 0.05$) in demographics based upon recruitment source, no differences in attitudes towards sensor use were found by age, gender, education, or marital status. Opinions and preferences for sensor type/number/install location, and data sharing preferences significantly differed ($p< 0.05$) by home living arrangements (independent, 55+ or continuous care communities). Similar to national surveys, changes in technology use were observed pre- versus post COVID. Respondents living in 55+ and continuous-care housing were more comfortable with having sensors installed in their homes than those in community dwelling independent housing. This study highlights the need to include end users throughout the lifecycle of product development and provides insights into preferences by older adults for sensor use and data sharing.

Abstract citation ID: igad104.2248

### FRAILTY ASSESSMENT FROM PAPER TO ONLINE: THE E-FI-CGA WEB APP

Xiaowei Song, Kiarash Kianpoor, Katayoun Sepehri, Stanley Kwok, Margit Glashutter, Robert McDermid, and Grace Park, *Fraser Health, Surrey, British Columbia, Can ada*

**Background:** Early frailty assessment is crucial for improving care for older adults through enhancing frailty-informed care planning and execution. To increase the access and use of the established electronic Frailty Index based on the Comprehensive Geriatric Assessment tool, we developed an online software application: the web-based eFI-CGA. **Methods:** End-user requirements primarily adapted the standalone version of the eFI-CGA and covered assessment competency, interface familiarity, accessibility and convenient use by front-line health professionals. Web app-bounded features included user-account authentication and authorization, user-space management, record-search and retrieval, and functions for data management, analysis and display. The web app development used the Microsoft Azure web server with the ASP.NET framework, combined Webforms and Model-View-Controller architecture, and C# programming language with standard client-side libraries incorporating scripting and mark-up languages. **Results:** The web-based eFI-CGA was technically released and accessible online (efi-cga). Tests on the web pages for all the web pages (e.g., Home, Signup Login, Assessment, Search, and Analysis) using large sizes of systematically designed test data and mocked patient cohorts showed that the web software functions meet the requirements with 100% accuracy. **Conclusion:** The web-based eFI-CGA provides a valuable remote frailty assessment and information retrieval method for healthcare professionals, promoting effective multidisciplinary integrated care of older adults. Further work is needed to allow the safe use of the web app. We are also planning a patient-oriented frailty assessment tool for community-dwelling older adults.

Abstract citation ID: igad104.2249

### IMPLEMENTING REABLEMENT IN COMMUNITY CARE: HOW DO WE SUCCEED?

Ines Mouchaers[1], Lise Buma[1], Hilde Verbeek[1], Sandra Zwakhalen[1], Ellen Vlaeyen[2], Geert Goderis[2], and Silke Metzelthin[3], *1. Maastricht University, Maastricht, Limburg, Netherlands*, *2. KU Leuven, Leuven, Vlaams-Brabant, Belgium*, *3. Maastricht University, Care and Public Health Research Institute, Maastricht, Limburg, Netherlands*

In the Netherlands, reablement is high on the agenda for inclusion in future health care policy, resulting in more and more care providers wanting to implement reablement into their everyday practice. So far, it has been implemented, resourced and organized in multiple ways. To support the implementation on an (inter)national level, insight is needed into what preconditions and influencing factors are regarding the successful implementation of reablement. Within a qualitative exploratory research design, four focus group interviews were conducted with stakeholders from three Dutch care organizations that have been providing reablement services for at least 6 months prior. Care staff, as well as project leaders, managers, directors, policymakers, and health insurance company representatives, were represented in the interviews. Data were analyzed using the framework method guided by the Consolidated Framework for Implementation Research. In total, 34 stakeholders, distributed over the four focus group interviews, participated in the study. Facilitators included interdisciplinary collaboration, organizational support in terms of leadership engagement and implementation climate, innovation design, and adaptability of the innovation. Barriers included costs and funding of the innovation, time investment (i.e. reporting, number of care visits), and communication. Some factors were listed as both hindering and facilitating such as knowledge and beliefs of care staff, staff's and clients' self-efficacy to change, and motivation of clients and family caregivers. These results enhance the understanding of factors influencing the implementation of reablement in community care. The next step is to identify strategies to overcome the identified barriers.

Abstract citation ID: igad104.2250

### INTERRUPTED TIME SERIES ANALYSIS OF THE 2015 DUTCH LONG-TERM CARE: ACUTE HOSPITAL UTILIZATION OF OLDER ADULTS

Joost Wammes[1], Pieter Bakx[2], Bram Wouterse[3], Bianca Buurman[1], Terrence Murphy[4], and Janet MacNeil Vroomen[1], *1. Amsterdam Medical Centers, Amsterdam, Noord-Holland, Netherlands*, *2. Erasmus University Rotterdam, Rotterdam, Noord-Holland, Netherlands*, *3. Erasmus University Rotterdam, Rotterdam, Zuid-Holland, Netherlands*, *4. Penn State University, State College, Pennsylvania, United States*

In 2015, the Dutch government implemented a long-term care (LTC) reform primarily designed to promote older adults (≥65 years old) to age-in-place. Increased proportions of older adults living in the community may have resulted in more and longer acute hospitalizations. We evaluated the reform's association with the monthly rate of acute clinical hospitalization and monthly average hospital length of stay (LOS), using an interrupted time series design of national hospital data (2009 to 2018) that controlled for population growth and seasonality, and calculated adjusted incident rate ratios (IRR). The pre-reform trend of hospitalization rate was increasing (IRR 1.002, 95% CI 1.001-1.002). A positive average reform effect was observed (IRR 1.116, 95% CI 1.070-1.165), accompanied by a negative change in trend (IRR 0.996, 95% CI 0.995-0.998). This resulted in a decreasing trend over the post-reform period (IRR 0.997, 95% CI 0.997-0.999). The pre-reform trend of LOS was decreasing (IRR 0.998, 95% CI 0.997-0.998), and the 2015 reform exhibited a positive change in trend (IRR 1.002, 95% CI 1.002-1.003). This resulted in a stabilization of LOS in the post-reform period (IRR 0.999, 95% CI 0.999-1.00). Our findings suggest that the increase in the rate of acute hospitalization after the reform implementation was temporary, whereas the LOS appeared to be longer than expected.

Abstract citation ID: igad104.2251

### SUPPORTING OLDER ADULTS WHO LIVE ALONE: UNDERSTANDING THEIR UNMET NEEDS USING NHATS SURVEY DATA

Marissa Bergh[1], Aasha Raval[2], and Jasmine Travers[1], *1. New York University, New York City, New York, United States*, *2. NYU School of Global Public Health, New York City, New York, United States*

Research suggests that older adults living alone in the community are at higher risk for poor health-related outcomes due to lack of cohabitants to support their health needs. Using National Health and Aging Trends Study (NHATS) data, we identified community-residing older adults (≥65 years) with assistance needs for Instrumental Activities of Daily Living

(IADLs), Activities of Daily Living (ADLs), or mobility. We used bivariate analyses to compare the unmet needs related to IADL, ADL, and mobility tasks between those living alone and those living with others. Of the 3,458 individuals identified, 28.9% (n=1001) lived alone. Those living alone were more likely to be women, unpartnered, and lacking informal caregivers. There was no difference in perceived overall health scores between the groups. Individuals living alone experienced more mobility and IADL-related unmet needs. In the month prior to survey completion, those living alone were more likely to forgo getting out of bed (p=0.031), grocery shopping (p< 0.001), cooking (p< 0.001), and paying bills (p=0.025) due to lack of available assistance. Our findings suggest that older adults living alone may need higher levels of formal assistance with IADLs to remain living in the community. Given the key role IADLs play in promoting quality of life and a person's ability to remain independent and engaged in the community, it is important to consider IADLs in addition to ADLs when assessing formal care needs for those living alone. Policy makers should prioritize the specific needs of those living alone when developing national aging strategies.

Abstract citation ID: igad104.2252

### THE ASSOCIATIONS OF GRIP STRENGTH WITH CO-RESIDENCE WITH ADULT CHILDREN AND MORTALITY CHINESE OLDER ADULTS

Long Chen[1], Shiying Gao[1], Yan Du[2], and Rumei Yang[1], *1. Nanjing Medical University School of Nursing, Nanjing, Jiangsu, China (People's Republic)*, *2. UT Health San Antonio, San Antonio, Texas, United States*

Aging in place is a key to coping with population aging in China. Grip strength (GS) is one of the key determinants of aging in place. Under such a context, whether living with adult children would affect older adults' GS and mortality is worth exploring. The objective of this study was to assess the associations of GS with adult children co-residence and mortality, and examine whether the association between GS and mortality would be moderated by adult children co-residence status. We used a nationally representative sample of Chinese older adults who completed three-wave (2011, 2013, and 2015 years) of the China Health and Retirement Longitudinal Study (N=1,088, aged 50-100 years). Mortality status was determined by in-person interviews in waves 2 and 3. GS was treated as a continuous variable in mixed-effect models and a binary variable in Cox regression models (poor GS if GS< 18kg for women and GS< 28kg for men). On average, 52.2% (n=535) participants co-resided with adult children. Adult-children co-residence was longitudinally related to better GS over time (β=2.96, 95% CI=0.53-3.60, p=0.009). Overall, poor GS was an independent risk factor for mortality (HR=1.33, 95% CI=1.04-1.70, p=0.022) after controlling for covariates (e.g., age, sex), but this association was not modified by co-residence status (HR=1.02, 95% CI=0.64-1.64, p=0.900). These results suggest that co-residence with adult children might be beneficial to better GS, which might contribute to less mortality. Future studies are needed to explore the underlying mechanisms through which the co-residence affects GS, thus, contributes to mortality.

### SESSION 7130 (POSTER)

### CLINICAL PRACTICE AND HEALTH

Abstract citation ID: igad104.2253

### A MOBILE HOME HEALTH CARE PHYSICIAN FOR OLDER PERSONS WITH EXTENSIVE HEALTH CARE NEEDS

Lina Emmesjö, Jenny Hallgren, Anna Dahl Aslan, and Catharina Gillsjö (Gillsjo), *University of Skövde, Skövde, Vastra Gotaland, Sweden*

**Background:** The rapidly increasing older population with extensive care needs has shifted health care from institutions to the older person's home. A cross-organisational integrated care model was created by health care authorities to meet these challenges, the Mobile integrated care model. The Mobile integrated care model with a home health care physician is a collaboration between regional and municipal health care, working in the patients' home. **Methods:** Semi-structured interviews with patients, next of kin and health care professionals. **Results:** The home was described by all as the best place to provide health care to these patients, creating safety and increasing autonomy for the patients. The health care professionals found trust in working together as a team, but struggled because of the divided organizations. Patients and next of kin found the Mobile integrated care model to be hierarchic, where the structure sometimes improved participation, and at other times prevented it. **Conclusion:** All participant groups emphasized that there was a need for more time for the health care personnel to spend with the patients. Furthermore, the patients and next of kin longed for a personal contact and being able to form a relationship with the health care personnel. The health care professionals found being employed by separate organizations as a challenge, where divided documentation systems and lack of equipment hindered the work.

Abstract citation ID: igad104.2254

### "IT CAN BE A CHAMELEON": LEWY BODY DEMENTIA PRESENTATION WITH AN ELUSIVE CLINICAL PROFILE—CASE REPORT AND DISCUSSION

Javier Malcolm Barney[1], Mario Moya[1], Jessica Martin[2], and Andrew Dentino[2], *1. DHR Health, Edinburg, Texas, United States*, *2. Rio Grande Valley Graduate Medical Education Consortium, Edinburg, Texas, United States*

Lewy body dementia is a progressive neurodegenerative disease and is considered to be the second most common cause of dementia in the population above 65 years of age. It remains a challenging and complicated disease process to profile clinically with an elusive diagnosis that shares similarities with Alzheimer's, leading to patients being misdiagnosed. The decline is compounded by inappropriate coordinated care efforts, and therefore ill preparing the patient and negatively affecting their relatives and support groups. We describe the case of an 83-year-old Hispanic male who over the course of 32 months sustained multiple injuries due to falls landing him in the emergency department; receiving numerous consultations from hospitalists, surgeons, infectious disease specialists, neurologists, psychiatrists, and radiologists. A combined blend of inpatient and outpatient visits was not enough to circumvent the recurrent theme that plagues similar cases worldwide with non-reliable criteria that can identify Lewy Body Dementia early in the presentation of the disease. The first mention of DLB was almost at the 32 months mark into the patient's journey at this particular Hospital System. A multidisciplinary approach with enhanced communications and round table discussions should be a quality of care improvement initiative to help better serve this population of patients. DLB's rapid progression and sensitive drug therapy parameters should place this as equally scalable as other multi-specialty collaborations such as tumor board, limb salvage committee, ethics, and Trauma review.

Abstract citation ID: igad104.2255

### DOES HAND GRIP STRENGTH CORRELATE WITH SHOULDER DYSFUNCTION AMONG OLDER WOMEN?

Ranyah Almardawi[1], Derik Davis[2], and Denise Orwig[3], *1. University of Maryland Baltimore/School of Medicine, Baltimore, Maryland, United States*, *2. University of Maryland School of Maryland, Baltimore, Maryland, United States*, *3. University of Maryland School of Medicine, Baltimore, Maryland, United States*

**Background:** Shoulder dysfunction and pain are common problems among older adults. Nearly 20% of those aged 65 and older reported shoulder pain in the 2011 National Health and Aging Trends Study. Shoulder pain and dysfunction may lead to the inability to carry out essential household and daily activities, burdening both patients and society. HGS is commonly tested and considered a reliable measure to assess overall muscle strength and function. Objective: The aim of this cross-sectional study is to determine the association between hand grip strength (HGS) and shoulder dysfunction. We hypothesize that HGS is correlated with shoulder dysfunction among older women. **Methods:** A total of 36 older women volunteers with an age range of 62 to 84 years were included. We assessed the HGS for dominant hands using Jamar Plus Digital Hand Dynamometer. We measured both hands twice and used the strongest measure for the dominant hand. Degree of shoulder dysfunction was self-reported using the American Shoulder and Elbow Surgeon survey (ASES) [0, worst; 100, best]. **Results:** Participants had a mean age of 70.9 years (SD:5.7), mean grip strength of 18.2kg (SD:5.9), and mean ASES score of 86.8(SD: 17.2). A significant and positive correlation was found between HGS and ASES score (r=0.612, P=0.001).**Conclusion:** The moderate correlation of HSG with ASES score supports the hypothesis. The results suggest that future research is warranted to test the feasibility of HSG as a clinical screening tooling for shoulder dysfunction in larger diverse samples of older adults, including men and women.

Abstract citation ID: igad104.2256

### GLP-1 AGONISTS: EFFECT IN MANAGEMENT OF DIABETES AND WEIGHT LOSS IN OLDER ADULTS WITH OVERWEIGHT AND OBESITY

Anna Pendrey, *Indiana University, Indianapolis, Indiana, United States*

Prevalence of Diabetes Mellitus Type 2 in older adults in the US is 33%, incidence of newly diagnosed diabetes is highest among those aged 65-79 years. Obesity in older adults impacts not only the morbidity and mortality, but importantly impacts quality of life and risk of institutionalization. Treatment for diabetes with glucagon like peptide 1 (GLP-1) agonists have demonstrated to reduce weight, control glucose, and reduce major adverse cardiovascular events in older adults. This study aims to describe outcomes in the management of older adults with diabetes and obesity with GLP-1 agonists. A retrospective study of 30 older adults with uncontrolled diabetes, overweight and obesity at a local practice that were started on treatment with GLP-1 agonists, since 2022, with ages 65-84 with diabetes mellitus type 2 with initial HbA1C 9.6%-12.6%, and BMI 27-48.2 that were started on GLP-1 agonists with improved results during treatment with current reduction of HbA1C 5.8%-7.7% and BMI reduction 23-39.8. In conclusion GLP-1 agonists have demonstrated significantly improved management of uncontrolled Diabetes Mellitus in older adults as well as a significant reduction in weight improving outcomes.

Abstract citation ID: igad104.2257

### INTEGRAL ROLE OF SOCIAL WORKERS WITHIN A COMPREHENSIVE GERIATRIC ASSESSMENT

Ashley Schwartzkopf[1], Jennifer Myers[1], Cathy Schubert[1], Lauren Penney[2], Teresa Damush[1], Aliana Preddie[1], and Dawn Bravata[1], *1. Richard L Roudebush VA Medical Center, Indianapolis, Indiana, United States*, *2. National Institutes of Health, Bethesda, Maryland, United States*

Comprehensive geriatric assessment (CGA) programs are expanding to support the wellbeing of community-dwelling older adults. Although a variety of CGA models have been described, the specific contributions of social work in CGA programs has not been rigorously evaluated. We examined the role of social work within a well-established, interdisciplinary, home-based geriatric program at one large, midwestern VA Medical Center. Semi-structured interviews were conducted with the VA-Geriatric Resources for Assessment and Care of Elders (GRACE) team to understand their perspective on social work expertise within a geriatric interdisciplinary team. Nine VA-GRACE team members were interviewed: 1 geriatrician, 1 pharmacist, 4 advanced practice nurses, and 3 social workers. VA-GRACE social workers participated in home-based CGAs by identifying psychosocial barriers that affected patients' medical care and quality of life, and engaging in ongoing case management. VA-GRACE team members valued the social workers problem-solving skills which were critical to ensuring that: patients received needed home services; caregiver needs were met; home safety and equipment issues were addressed; and patients received appropriate cognitive services. The VA-GRACE team members valued social workers for their perceived positive work ethic, as well as their critical knowledgebase which they apply to training new staff, supporting nurses (especially during periods of staff shortage), promoting patient enrollment, and developing and implementing cohesive care plans. In conclusion, these results support the integral role of social work in an interdisciplinary team providing comprehensive geriatric assessments and highlight unique contributions that social workers make to promote the health and wellbeing of older adults.

Abstract citation ID: igad104.2258

### MEDICATION DE-ESCALATION AND GLYCATED HEMOGLOBIN AMONG OLDER ADULTS WITH DIABETES AND DEMENTIA

Oluwaseun Adeyemi[1], Mauricio Arcila-Mesa[2], Crystalinda Rapozo[3], and Joshua Chodosh[3], *1. New York University Grossman School of Medicine, New York City, New York, United States*, *2. NYU Langone, New York City, New York, United States*, *3. NYU Grossman School of Medicine, New York City, New York, United States*

Patients with co-occurring diabetes and dementia and low (< 7.0) glycated hemoglobin (HbA1c) are at risk for adverse events and may require antihyperglycemic medication de-escalation. Whether de-escalation leads to a desired HbA1c is unknown. We assessed the relationship between antihyperglycemic de-escalation and attainment of a desired HbA1c. We selected older adults with diabetes and dementia with low HbA1c enrolled in the Enhanced Quality in Primary Care for Elders with Diabetes and Dementia, a multicenter cluster-randomized pragmatic trial. We determined whether antihyperglycemic medications were de-escalated during the twenty-four-month intervention. We classified a positive outcome as a subsequent desired HbA1c range for those who entered the study with HbA1cs < 7.0. We controlled for age, sex, race/ethnicity, Charlson comorbidity index, antihyperglycemic medications, and the COVID-19 pandemic. We performed a multivariable generalized linear binomial regression analysis and report the likelihood (adjusted risk ratio; aRR) (plus 95% confidence interval (CI)) of achieving the desired range HbA1c. The predominant population was older adults 85 years and older (42%), females (60%), and non-Hispanic Whites (64%). A tenth of the population had de-escalation and 19% of the population achieved the desired HbA1c range at the end of the study period. Metformin was the most frequently retained medication after de-escalation (66.7%). After adjusting for potential confounders, de-escalation was associated with 2.2 times the likelihood of attaining the desired HbA1c range (aRR: 2.20; 95% CI: 1.34 – 3.55). In conclusion, antihyperglycemic medication de-escalation improves the glycemic control of older adults with DM/ADRD.

Abstract citation ID: igad104.2259

### RELIGIOSITY AND THE CONTROL OF DIABETES MELLITUS TREATMENT IN THE ELDERLY

Juarez Sales[1], Cris Renata Volpe[1], Luciano Lima[1], Marina Stival[1], and Silvana Funghetto[2], *1. University of Brasilia, Brasilia, Distrito Federal, Brazil*, *2. University of Brasilia- Campus Ceilândia- UNB-Brasil, Brasília, Distrito Federal, Brazil*

Introduction: Diabetes Mellitus (DM) is an important health problem that affects thousands of Brazilians. Patients affected by the disease have many difficulties in following the correct treatment. Many studies point to religiosity as a factor that influences the health conditions of individuals. Objective: The study aimed to evaluate the influence of religiosity on diabetes mellitus treatment control in the elderly. Method: This is an outpatient-based explanatory cross-sectional study, in which a quantitative approach was taken. The study was conducted with 72 elderly people treated in primary health care in the Federal District. The instrument to evaluate the religiosity of the elderly was constructed through the defining characteristics and related factors of the NANDA-I nursing 6 diagnosis of impaired religiosity. **Results:** Environmental barrier to the practice of religion was found to be a risk factor for DM treatment uncontrolled. **Conclusion:** Religiosity plays an important role in the lives of the elderly, so it is becoming increasingly important to conduct research that assesses the importance of religion in the health-disease process of individuals. Nevertheless, nursing professionals must be attentive to this field of life attribute of the elderly and always encourage better relations of the patient with his religiosity.

Abstract citation ID: igad104.2260

### THE PICTURE WITHOUT THE THOUSAND WORDS—PHYSICIAN AND JUDGE COMMUNICATION IN GUARDIANSHIP CASES

Ronan Factora[1], and Matthew Pawlicki[2], *1. Cleveland Clinic, Cleveland, Ohio, United States*, *2. University Hospitals, Chardon, Ohio, United States*

In Ohio, physicians conducting capacity evaluations in guardianship cases are also asked to complete a Statement of Expert Evaluation (SEE) used by Probate Court to aid in decisions determining that person's competence. In a prior survey, physicians voiced reluctance to conduct such evaluations because they were time consuming and judges stated that information provided by physicians in guardianship cases was inadequate or incomplete. Communication between the two groups of what information would be most useful in this process would help optimize the time required by physicians to complete their evaluation and improve the quality of information judges receive when making their decision. A survey was conducted of legal professionals (at an Ohio Probate Court Pre-conference session specifically focused on capacity evaluation and SEE completion) which sought to identify elements these legal professionals felt were most and least useful from SEEs provided by completing physicians. With 52 attendees (including judges (active and retired), magistrates, court personnel, and public attendees), 38-42 persons answered the questions that were provided. By far the least valuable information was identified as the medication list, but the most useful was any detailed information/opinion provided by the completing physician explaining the other information included on the form (e.g. cognitive testing, diagnosis). Though potentially more time consuming, highlighting the importance of this information to completing physicians may help them communicate more effectively to legal authorities and facilitate greater understanding of the physician's opinion in guardianship cases.

### SESSION 7140 (POSTER)

### COVID-19 PANDEMIC IMPACT ON OLDER ADULTS

Abstract citation ID: igad104.2261

### ADDRESSING BENZODIAZEPINE PRESCRIBING PRACTICES AMONGST ELDERLY PTSD VETERANS DURING THE COVID-19 PANDEMIC

Deanna Fernandes[1], and Josepha Cheong[2], *1. University of Florida, Gainesville, Florida, United States*, *2. North Florida/South Georgia Veterans Affairs Medical Center, Gainesville, Florida, United States*

Psychotropic medication prescribing amongst elderly veterans has increased over the course of the COVID-19 pandemic despite the fact that psychotropics such as benzodiazepines, when being prescribed for a period over 2 weeks, are considered as relatively contraindicated amongst such patients with an additional Post-Traumatic Stress Disorder (PTSD) diagnosis due to a worsening of disease state symptoms. The objectives of my study are to inform clinicians and healthcare providers regarding the true extent of current benzodiazepine prescribing practices amongst elderly Veterans with PTSD and to provide a clinical aid/tool for education and improvement of such practices within the setting of the Veterans Affairs Medical Center. A one-page summarized aid will be drafted based upon provider feedback as well as from current Pharmacy Benefits Management Network resources to improve clinical practices with regards to the topics of benzodiazepine recommended length of use, risks of use amongst elderly Veterans, and risks amongst PTSD populations. While benzodiazepine prescribing practices have increased recently during the time of the COVID-10 pandemic, clinical tools may be drafted to improve such practices. Further studies are needed to determine the true impacts of such a tool on the national level as well as upon direct patient care.

Abstract citation ID: igad104.2262

### AN OUTBREAK OF COVID-19 INFECTION WITH NO MORTALITY IN A GERIATRIC INSTITUTION IN COLOMBIA

Carlos Reyes-Ortiz[1], and Jose Ocampo-Chaparro[2], *1. Florida A & M University, Tallahassee, Florida, United States*, *2. Universidad del Valle, Cali, Valle del Cauca, Colombia*

Unusually, an outbreak of Covid-19 during the first year of the pandemic in an underserved geriatric institution had no mortality. This cross-sectional study aimed to determine factors associated with COVID-19 infection among 252 institutionalized older adults in Cali, Colombia's larger geriatric hospital and nursing home, Geriatric Hospital and Nursing Home San Miguel assisting multiethnic, impoverished, and low-educated residents. This population had a median age of 80 years (range 55 to 103) and a median Barthel of 90.0; 50% were women, 19% had dementia, and 2% were obese. 84 (33.3%) were infected, and none died and did not go to the UCI or were referred to a higher-level hospital. In a multivariate logistic regression model, being infected was associated with age 80 and older, a lower Barthel score, and malnutrition. Due to collinearity, separated models showed an association between infection and higher scores on the clinical frailty scale or lower grip strength. All institutionalized older adults with Covid-19 infection in this geriatric institution survived. It was likely related to appropriate general medical and nursing management, including well-controlled comorbidities, and individualized interdisciplinary rehabilitation, attention, and support.

Abstract citation ID: igad104.2263

### EFFECT OF COVID-19 PANDEMIC ON A BODY MASS INDEX OF OLDER AMERICANS: A REPEATED CROSS-SECTIONAL STUDY

Krishna Sapkota[1], J. Scott Brown[2], and Jennifer Kinney[2], *1. Miami University, Oxford, Ohio, Oxford, Ohio, United States*, *2. Miami University, Oxford, Ohio, United States*

Introduction: The COVID-19 pandemic resulted in greater social and physical isolation, potentially leading to changes in eating, physical activity, and health behaviors, which may increase BMI. This study explored whether body mass index (BMI) among middle-aged and older Americans has been affected by the pandemic. **Methods:** This study uses data from the Behavioral Risk Factor Surveillance System to assess the effects of COVID-19 on BMI changes among middle-aged (50-64 years) and older (65 years and above) Americans. Changes in BMI over time are measured for 2019 and 2021. Data from 2013, 2015, and 2017 are used to note the trend of BMI, and sex and racial differences in BMI trajectories are explored. **Results:** Initial results show that the BMI of older adults increases over time but does so at a decreasing rate (2013 to 2105 BMI difference = 0.19, 0.16 from 2015 to 2017, 0.13 from 2017 to 2019, and 0.09 from 2019 to 2021). On the other hand, among middle-aged adults, BMI significantly increased over time and accelerated during COVID-19. The difference in BMI was 0.20 from 2013 to 2015, 0.16 from 2015 to 2017, 0.22 from 2017 to 2019, and 0.28 from 2019 to 2021. BMI increases consistently over the period for middle-aged men and older adult women, whereas BMI growth accelerates for middle-aged women and decelerates for older adult men. Similarly, racial differences are noted in the BMI changes of middle-aged and older adults. **Conclusion:** The COVID-19 pandemic accelerated the accumulation of body mass among middle-aged Americans.

Abstract citation ID: igad104.2264

### EMOTIONAL RESPONSE TO THE COVID-19 PANDEMIC: NURSING HOME RESIDENTS' PERSPECTIVES

Ashley Woods, Amy Vogelsmeier, Lori Popejoy, Steven Miller, Alisha Johnson, Roy Thompson, and Amy Trueblood, *University of Missouri, Columbia, Missouri, United States*

Nursing home residents were at the forefront of challenges imposed by the COVID-19 pandemic, including being forced into extended periods of isolation, yet little is known about their emotional response. Therefore, the purpose of this study is to describe residents' emotional responses to the COVID-19 pandemic, including their response to isolation. Twenty-eight residents residing in 22 Missouri nursing homes during the pandemic participated in semi-structured interviews as part of a larger mixed-methods study to understand nursing homes' response to COVID-19. Interviews were transcribed and analyzed by a team of PhD researchers and doctoral students using a qualitative content analysis approach. Of the 28 resident interviews, most were more concerned about their families, other residents, and staff becoming infected rather than themselves. Many cited their lack of concern was due to trust in nursing home staff to keep them safe. Regarding isolation, a few described being angry about separation from family and friends. Nevertheless, most understood that the restrictions were about "doing what had to be done" and that isolation was "for the greater good." The majority also responded positively about their relationships with staff during isolation and happiness at the return of visitors after isolation restrictions were lifted. Overall, residents' emotional responses during the COVID-19 pandemic varied but primarily reflected their concern for others rather than themselves. Their concern for themselves may have been allayed because of their trust in nursing home staff. Their response to isolation seemed rooted in their understanding of the need to protect themselves and others.

Abstract citation ID: igad104.2265

### MORE ADULT AMERICANS ARE AT NUTRITIONAL RISK SINCE THE COVID-19 PANDEMIC

Lillie Monroe-Lord[1], Azam Ardekani[2], and Cassidy Whitman[2], *1. University of the Dustrict of Columbia, Washington, District of Columbia, United States*, *2. University of the District of Columbia, Washington, District of Columbia, United States*

The nutritional status of individuals has changed since COVID-19 pandemic due to disruptions in social, economic, and psychological factors. This study determines how nutritional status has changed since COVID-19 among American adults. The second aim is to examine which age range group was at a higher risk of nutritional status due to the pandemic. The study design was cross-sectional, with 10,035 participants aged 40-100 who answered a survey distributed by Qualtrics. Nutritional status was measured twice before and since the COVID-19 pandemic by the Dietary Screening Tool (DST). The total DST score was calculated for each participant, which categorized them into three different groups, including “at risk,” “possible risk,” and “not at risk.” The results revealed that the mean DST score before the pandemic was 56.04 (IQR: 48.00–65.00); this score significantly decreased since COVID-19 to 55.54 (IQR: 47.00–64.00) (p <.001). Of those participants who were not at risk before COVID-19, 28.5% became either at risk or at possible risk since COVID-19. Of those participants who were at possible risk before COVID-19, 21% are now at risk since COVID-19. Almost 60% of the younger group (40-60) were at risk before COVID-19 compared to 54.8% of participants aged 61-80 and 45.8% of older participants (81-100). These percentages increased by about 2% since COVID-19 among all three age groups. As a good nutritional status reduce the risk of severe illness, these findings can help policymakers to develop heath-protecting behavior sessions against future pandemics to manage crises.

Abstract citation ID: igad104.2266

### NETWORK ANALYSIS OF PHYSICAL SYMPTOMS OF OLDER ADULTS WITH COVID-19

Junwen Yu[1], Zhongfang Yang[1], Jiamin He[1], Zheng Zhu[2], and Hongzhou Lu[3], *1. Fudan University, Shanghai, Shanghai, China (People's Republic)*, *2. New York University, New York City, New York, United States*, *3. National Clinical Research Center for Infectious Diseases, Shenzhen Third People's Hospital, Shenzhen, Guizhou, China (People's Republic)*

**Background:** COVID-19 can affect multiple organ systems in older adults, making it challenging to manage symptoms that involve different parts of the body. The objectives of our study were to generate symptom networks of COVID-19 physical symptoms in older adults and to explore the core symptoms and the relationships between complex symptoms from a mechanism-based perspective. **Methods:** Data from 470 older adults (≥65 yrs) with COVID-19 were obtained from the Shenzhen Third People’s Hospital. Networks were constructed among 16 physical symptoms to describe relationships among symptoms. Subgroup network analyses were performed based on latent class analysis. We also conducted further analysis of the bridge symptoms among the symptom clusters. **Results:** Fever (rs=4.37) had the highest strength value, followed by chills (rs=4.20) and muscle aches (rs=3.91). Based on the values of bridge strength centrality, dizziness (rbs=0.79), runny nose (rbs=0.71), and fatigue (rbs=0.62) were identified as bridge symptoms. Among the three subgroups of symptom networks, cough had the highest strength values in the high and low symptom severity groups (rs=3.93 and 2.82), while fever had the highest strength value in the moderate symptom severity group (rs=3.50). **Conclusion:** We found that the core symptom was different in the high/low groups and moderate group, suggesting that personalized symptom management plans are essential to optimize the effectiveness of interventions. Keywords: COVID-19; Older adults; Physical symptom; Symptom network

Abstract citation ID: igad104.2267

### NON-COVID-19 UPPER RESPIRATORY INFECTION DECREASES DURING THE COVID-19 PANDEMIC

Alec Pruchnicki, *Vista on 5. th ALF, New York City, New York, United States*

During the Covid 19 pandemic, the Vista on 5th Assisted Living Facility in New York City instituted a wide variety of pharmacological and non-pharmacological interventions (NPIs) to control the spread of the disease. Before and after vaccines were available for Covid 19, this combination of approaches reduced the usual seasonal variation of non-Covid upper respiratory infections (URIs). Almost all residents of the 127-bed facility were seen by the on-site physician with the onset of URI symptoms. Almost all those with symptoms were give an anti-histamine and cough syrup for relief. The single off-site pharmacy which supplied these medications tabulated the total amount of medications dispensed over the four years of 2019 to 2022. During December of 2019, normally the peak month for URIs at the facility, and the last month before significant Covid infections occurred, 67 prescriptions of these two types were dispensed. During 2020 NPIs and these medications kept new Covid 19 infections to about one dozen over the year. Medications being dispensed for all URIs in December of 2020 decreased by 69% to 21. Similar low rates of URI symptoms were seen in December 2021 and 2022 with only 18 prescriptions written in both months. Although some variation from month to month occurred because of other syndromes, the overall burden of URIs during the normally peak respiratory infection season was greatly reduced probably from the strict imposition of NPIs.

### SESSION 7150 (POSTER)

### FALLS

Abstract citation ID: igad104.2268

### A CALL TO ACTION FOR FALL INJURY PREVENTION: QUALITY IMPROVEMENT IN THE DEPARTMENT OF VETERANS HEALTH ADMINISTRATION

Blake Barrett, Margeaux Chavez, Sarah Bradley, Nora Arriola, Vianna Broderick, Anita Ramrattan, Tatjana Bulat, and Linda Cowan, *VISN 8. Patient Safety Center of Inquiry, James A. Haley Veterans' Hospital and Clinics, Department of Veterans Health Administration, Tampa, Florida, United States*

The rates of inpatient falls in the Department of Veterans Health Administration (VHA) have been declining steadily over the last 10 years, but the rates of major fall-related injuries have remained stagnant. This poster describes quality improvement (QI) efforts in the VHA to evaluate and improve implementation of fall injury prevention practices. It also describes the importance of focusing on fall injury prevention, in addition to fall prevention. QI efforts were led by an interdisciplinary team using mixed methods. Objectives included: 1) determine best practices in fall injury prevention in VHA inpatient units, 2) determine the utility of current methods for evaluating implementation of fall injury prevention practices, and 3) improve fall injury prevention practice implementation by co-designing an evaluation tool with VHA staff who are its intended users. Findings suggest the need to raise awareness of and use of fall injury prevention practices, that is distinct from fall prevention practices. Staff do not typically separate fall injury prevention practices from general fall prevention, making it challenging to implement tailored prevention practices (e.g., hip protectors or helmets directed at fall injury prevention vs. fall prevention). Furthermore, staff reported difficulty implementing and evaluating fall injury prevention practices. Staff preferred tools using a formal evaluation process with interdisciplinary teams to outline standards and document barriers and facilitators to reducing fall injuries. Our findings support need for a validated tool to evaluate and improve implementation of fall injury prevention practices (above and beyond fall prevention) to improve care and reduce Veteran fall-related injuries.

Abstract citation ID: igad104.2269

### A CONSULTANT PHARMACIST INTERVENTION TO DEPRESCRIBE OPIOIDS AND BENZODIAZEPINES FOR OLDER ADULTS IN PRIMARY CARE

Joshua Niznik[1], Jan Busby-Whitehead[2], Lori Armistead[3], Benjamin Urick[4], Liang Zhao[5], and Stefanie Ferreri[3], *1. University of North Carolina at Chapel Hill, School of Medicine, Chapel Hill, North Carolina, United States*, *2. UNC Chapel Hill, Chapel Hill, North Carolina, United States*, *3. UNC Chapel Hill Eshelman School of Pharmacy, Chapel Hill, North Carolina, United States*, *4. Prime Therapeutics, Eagan, Minnesota, United States*, *5. UNC Health, Chapel Hill, North Carolina, United States*

Long-term use of opioids and benzodiazepines (BZDs) by older adults may cause harm. We evaluated the impact of a targeted consultant pharmacist opioid and BZD deprescribing intervention for older adults. We conducted a pragmatic study in which fifteen primary care clinics were randomized to receive standard care (n=7) or a consultant pharmacist service (n=8) to provide targeted medication review and recommendations for deprescribing opioids and BZDs. The primary outcome was opioid and BZD prescribing in the following year, using average daily morphine milligram equivalents (MMEs) and diazepam milligram equivalents (DMEs). We used generalized linear models to evaluate the association of the intervention with average daily MMEs and DMEs, adjusted for medication exposures in the prior year and demographics. Incident falls were examined as a secondary outcome. We included 961 opioid and 1107 BZD users, with 15.6% and 13.6% being prescribed both medications, respectively. Average daily MMEs and DMEs prescribed at baseline were 23.6 and 7.67. Deprescribing recommendations were made for 71% of opioid and BZD users seen in intervention clinics. We observed non-statistically significant reductions in average daily MMEs (Intervention: -2.42 MMEs, Control: -1.02 MMEs, p=0.71) and DMEs (Intervention: -1.0 DMEs, Control: -0.1 DMEs, p=0.06). There was no significant reduction in incident falls. A consultant pharmacist-driven intervention is a feasible approach to deliver targeted deprescribing recommendations, but did not significantly impact medication use or falls. Future studies should explore whether multimodal interventions would be more effective in achieving deprescribing and reducing medication-related harms.

Abstract citation ID: igad104.2270

### A SCOPING REVIEW FOR PERSON-FOCUSED FALL PREVENTIVE INTERVENTIONS IN LONG-TERM CARE FACILITIES

Wonkyung Jung[1], Sungwon Lim[2], and Hilaire Thompson[2], *1. Johns Hopkins University, Baltimore, Maryland, United States*, *2. University of Washington, Seattle, Washington, United States*

Falls are a significant concern in long-term care facilities (LTCFs) as fall-related injuries (FRI) can result in functional impairment, disability, and death. Older adults living in LTCFs are at greater risk for falls than those in the community. Using scoping review methodology, we aimed to synthesize evidence examining intervention effects of person-focused interventions for risk assessment and prevention in LTCFs to identify evidence-based practices. Databases searched included Medline, CINAHL, and EMBASE to identify original research from 2017 to April 2022 in accordance with the Preferred Reporting Items for Systematic reviews and Meta-Analyses extension for Scoping Reviews (PRISMA-ScR) guideline. Following the removal of duplicates, 888 articles were identified for possible review, following title and abstract screening 84 studies underwent full-text review against inclusion/exclusion criteria, with 30 studies remaining for analysis. We conducted a narrative synthesis to summarize the included studies. Evidence-based interventions for fall prevention identified include (1) exercise programs (e.g., high-intensity functional exercise, aerobic exercise, short stick exercises, etc.); (2) balance training programs (e.g., Wii Fit); (3) multifaceted, nurse-led education; and (4) others (e.g., whole body vibration, and lavender olfactory stimulation). Outcomes of included studies included the number of falls, fall rate, risk of falls, and fear of falls before and after interventions. Overall, most studies reported significant effects of person-focused interventions in LTCFs. Available evidence supports that well-designed person-focused interventions can reduce falls and fear of falls for individuals in LTCFs.

Abstract citation ID: igad104.2271

### ACCELEROMETRY-BASED PHYSICAL ACTIVITY (MIMS/DAY) AND FALL RISK APPRAISAL MATRIX IN OLDER ADULTS

Renoa Choudhury, Joon-Hyuk Park, Chitra Banarjee, David Fukuda, Rui Xie, Jeffrey Stout, and Ladda Thiamwong, *University of Central Florida, Orlando, Florida, United States*

Fall Risk Appraisal Matrix (FRAM) is vital for understanding the discrepancy between perceived fall risk (i.e., Fear of Falling, FOF) and physiological fall risk (i.e., balance performance) in older adults. However, little is known about how habitual physical activity (PA) levels, expressed in Monitor Independent Movement Summary (MIMS) units, differ across FRAM categories. This cross-sectional study examines the associations of FRAM and wrist-worn accelerometer-based daily PA in community-dwelling older adults. FOF (Short FES-I scale), balance performance (BTrackS Balance) and PA data (ActiGraph GT9X) were collected from 156 older adults (Mean age = 76.74±9.62 years). FRAM categorizes the participants into four groups: i) Rational (low FOF-normal balance), ii) Incongruent (low FOF-poor balance), iii) Irrational (high FOF-normal balance), and iv) Congruent (high FOF-poor balance). PA data were summarized as MIMS/day (the average of daily MIMS) and peak 30-minute MIMS (the average of highest 30 non-consecutive MIMS minutes/day, representing a PA index of higher intensity epochs). Differences across groups were examined using one-way analysis of variance (ANOVA). Results indicate that, total accumulated PA (i.e., average daily MIMS) was 17% lower in Congruent FRA group than Rational FRA group (p=.02). The peak PA effort (i.e., peak 30-minute MIMS) decreased in Incongruent (9%, p=.027), Irrational (12%, p=.002) and Congruent (16%, p=.003) groups as compared to the Rational FRA group. Our results suggest that fall intervention strategies should integrate both cognitive behavioral therapy (for reducing FOF) and balance exercises to promote high-intensity PA in older adults.

Abstract citation ID: igad104.2272

### ADHERENCE TO INTERVENTIONS AT A FALL CLINIC: PRELIMINARY DESCRIPTIVE DATA FROM A SINGLE ACADEMIC INSTITUTION

Yuka Shichijo[1], Fred Ko[2], Renee Slon[3], Nisha Rughwani[2], and Yuji Yamada[2], *1. Mount Sinai Beth Israel, New York City, New York, United States, 2. Icahn School of Medicine at Mount Sinai, New York City, New York, United States, 3. Mount Sinai Morningside, New York City, New York, United States*

**Background:** Falls and fall-related injuries are major public health concerns. Approximately 50 billion dollars are spent on annual medical costs related to falls. Fall prevention is a priority for promoting active and healthy aging in older adults. Fall prevention clinics enable focused evaluation, identification, and treatment of modifiable risk factors, specifically for falls; however, their effectiveness is dependent upon patient adherence to preventive interventions. **Methods:** We analyzed patients aged > 65 years who visited our fall clinic between February 2021 and February 2023. We took an interdisciplinary approach to improve adherence to recommendations, including follow-up phone calls by a dedicated nurse to confirm adherence, nurse-directed interventions such as Tai Chi sessions, and home environment assessments by a social worker. **Results:** 48 patients were included in this analysis, with a mean age of 84 (65-99), and 85% were female. Fall-specific risk factors identified included impairment of strength, gait, or balance (96%); use of fall-risk-increasing medications (54%); problems with feet affecting gait (40%); visual impairment (29%); and postural hypotension (6%). The adherence rates for our interventions were 48% for physical therapy referrals, 28% for podiatry referrals, 36% for ophthalmology referrals, 47% for other referrals, 75% for vitamin D prescriptions, and 50% for assistive device prescriptions. **Conclusions:** There is room for improvement in adherence to referrals. A larger sample size and longer follow-up period are required to investigate factors related to non-adherence. The delivery of both verbal and written recommendations, the identification of each patient's barriers, and same-day referrals may help improve adherence.

Abstract citation ID: igad104.2273

### ARE COMPLETING ONLINE ASSIGNMENTS CORRELATED TO KIRKPATRICK'S LEVEL 2 AND 3 LEARNING OUTCOMES?

Huai Cheng, *University of Pittsburgh, Pittsburgh, Pennsylvania, United States*

**Background:** Pre-class assignments such as online assignments are critical to a flipped classroom-based curriculum. However, it is unknown whether medical students are following online assignments and whether such compliance is associated with learning outcomes. The aim of this study was 1) to examine the compliance of the online assignments and 2) explore whether such compliance was associated with Kirkpatrick's Level 2 and 3 learning outcomes. Summary of Works: Prior to classroom teaching, fourth-year medical students (MS4) were recommended (not required) to complete six online assignments. A simulated patient (SP) with chief complaint of fall was developed to assess students' skills in interview, physical examination, and communication (Kirkpatrick's level 2 learning outcome). Students' clinical practice behavior was based on chart review (Kirkpatrick's level 3 learning outcome). Pearson/Fisher correlations were used to examine the association of completing online assignments with SP performance and clinical practice behavior. Summary of **Results:** 158 from Class 2017 participated in this study. The complete rate of 1-6 online assignment was 88%, 86%, 82%, 61%, 51%, 34%, respectively. Completing three of six online assignments was significantly associated with higher SP performance, but not clinical practice behavior. Discussion and **Conclusions:** Majority of MS4 didn't complete online assignments. Online assignments should be required. Completing online assignments was associated with improving Kirkpatrick's level 2, but not 3 learning outcomes. **Take-home Messages:** Online assignments need to be required. Improving Kirkpatrick's level 2 learning outcome via a flip-based curriculum for MS4 might not improve Kirkpatrick's level 3 learning outcomes.

Abstract citation ID: igad104.2274

### ASSESSING BALANCE INTERVENTION OUTCOMES IN OLDER ADULTS WITH HEARING LOSS

Caitlin Price[1], Holland Hardison[1], and Laura Spradley[2], *1. University of Arkansas for Medical Sciences, Little Rock, Arkansas, United States*, *2. UAMS, Little Rock, Arkansas, United States*

Previous studies reveal individuals with hearing loss are more likely to experience falls and fall-related injury. Fall risk exponentially increases with more severe degrees of hearing loss. Because both the risk of falls and the incidence of hearing loss increase with age, investigating the efficacy of balance intervention programs in older adults with hearing loss is vital to enhancing clinical outcomes and reducing heightened risk of falls in these individuals. Various balance intervention and physical exercise programs have been shown to reduce fall rates. However, which individuals achieve the greatest benefit from these programs remains unclear. Do those at greater risk of falls demonstrate more or less benefit from balance intervention? The current study aimed to (1) assess the impact of the A Matter of Balance (AMOB) program on older adults' fall risk and balance measures and (2) evaluate which measures (e.g., postural stability, balance confidence, fall risk, physical activity, degree of hearing loss) best predict balance intervention outcomes. Pre- and post-intervention balance and fall risk in older adult participants (60-90 years) with hearing loss were assessed via measures of postural sway and subjective questionnaires. Results revealed balance-related benefits following the completion of the AMOB program. Older adults initially identified as having higher fall risk demonstrated the most significant benefits as observed in fall risk reduction. Our results inform clinical practice by clarifying which individuals may benefit most from balance intervention programs and thus improve targeted intervention approaches for older adults at greater risk for falls and fall-related injury.

Abstract citation ID: igad104.2275

### ASSOCIATION BETWEEN COGNITIVE FRAILTY AND THE RISK OF FALLS AMONG OLDER ADULTS: A SYSTEMATIC REVIEW

Shanshan Wang[1], Yueheng Yin[2], and Isaac Sze Him Leung[3], *1. The Hong Kong Polytechnic University, Hong Kong, Hong Kong*, *2. Nanjing Medical University, Nanjing, Jiangsu, China (People's Republic)*, *3. The Chinese University of Hong Kong, Hong Kong, Hong Kong*

**Background:** Falls lead to numerous negative health outcomes and jeopardize the physical function and quality of life in older adults. Cognitive impairment and physical frailty were found to be associated with the risk of falls, but there was no systematic review that estimated the association between cognitive frailty and the risk of falls. **Methods:** A systematic literature search of the cross-sectional, cohort, and case-control studies in Cochrane library, Scopus, CINAHL, EMBASE, and PsycINFO was conducted. Study quality was assessed by using the Joanna Briggs Institute (JBI) critical appraisal tool. A random effects meta-analysis was performed to estimate the odds ratio (OR) of the incidence of falls in older adults with cognitive frailty. **Results:** Seven studies were included. The overall quality of the studies was acceptable. The meta-analysis of cohort studies showed older adults aged 60 and above with cognitive frailty had a pooled OR of 1.45 (95%CI 1.30, 1.61) for at least one fall compared with those without cognitive frailty. The meta-analysis of cross-sectional studies showed that the odds of older adults with cognitive frailty experiencing at least one fall was 1.64 times (95%CI 1.51, 1.79) higher than those without cognitive frailty. **Conclusion:** Cognitive frailty is significantly associated with the risk of falls. Timely detection of cognitive frailty is essential for preventing falls in older adults

Abstract citation ID: igad104.2276

### BENZODIAZEPINE/Z-DRUG DEPRESCRIBING DEFINITIONS AND FALLS OUTCOMES IN ELECTRONIC HEALTH RECORDS

Nicole Schindler[1], Lindsay Zepel[2], Matthew Maciejewski[2], Susan Hastings[3], Amy Clark[2], and Juliessa Pavon[4], *1. Duke University Medical Center, Durham, North Carolina, United States*, *2. Duke University, Durham, North Carolina, United States*, *3. Duke University, Durham VA, Durham, North Carolina, United States*, *4. Duke University School of Medicine, Division of Geriatrics, Durham, North Carolina, United States*

Benzodiazepines and Z-drugs contribute to the burden of falls in older adults; however, there is a lack of data examining the association between deprescribing and falls in real-world settings. We explored this association in a retrospective cohort study of adults aged 65+ with chronic benzodiazepine/z-drug use who received care at an academic health system from January 2017-December 2020. Chronic use of benzodiazepine/z-drugs was defined as ≥ 3 medication dispensings and cumulative days' supply ≥ 45 days within 100 days in 2018 based on electronic health records. Medication deprescribing was defined as having a dispensing gap of either ≥180 days or 90 days within one year of index. Non-deprescribers were matched 4:1 using propensity score methods (180-day gap sample=655; 90-day gap sample=1395). Primary outcome was time until first fall resulting in ED, inpatient, or outpatient visit during 2-year follow-up after meeting deprescriber/non-deprescriber status, modeled using a cox proportional hazards model adjusted for age, gender, and number of comorbidities and chronic medications. Using the 180-day gap deprescribing definition (n=131 deprescribers, and n=524 non-deprescribers), the cumulative incidence of falls-related acute events was 6.9% for deprescribers and 9.7% for non-deprescribers (HR 0.65 [95% CI 0.31, 1.31]). Using the 90-day-gap definition (n=279 deprescribers and n=1116 non-deprescribers), the cumulative incidence was 9.3% for deprescribers and 8.5% for non-deprescribers (HR 1.12 [0.70, 1.77]). In this small single-site sample, neither deprescribing gap definition was associated with falls. There is a need to standardize deprescribing definitions and validate them in larger studies in the context of relevant clinical outcomes.

Abstract citation ID: igad104.2277

### FALL ENCOUNTERS AMONG OLDER ADULTS: IMPACT OF COVID-19

Helen Lach, and Joanne salas, *Saint Louis University, St. Louis, Missouri, United States*

It was suspected that the restrictions in activity and clinical care during the COVID-19 pandemic would result in increased fall risks and falls in older populations. To explore the impact of the pandemic on falls, we examined the trajectory of fall rates among older adults cared for through a large, multi-state, Midwest healthcare system. De-identified medical record data was used to measure falls presenting to emergency, urgent, and hospital care in adults age 50+ from April 2019 through September 2022. We compared the fall rate during the pre-and post-COVID-19 time periods. Differences in fall rates were compared based on age groups, gender, race, and neighborhood socioeconomic status (nSES). The overall fall rate after COVID onset was significantly greater compared to prior to before COVID (11.59; 95%CI:11.50-11.67 vs. 11.20; 95%, CI:11.06-11.33). While White patients had a significantly higher rate of falls post-pandemic, changes in fall rates did not differ based on age, gender, or race. The changes did vary based on nSES, with the lower groups experiencing the largest increases. Findings may be related to differences in access to care and other services both early in the pandemic and as restrictions were lifted. Given the high cost of fall injuries, further work is needed to reduce falls and injuries in older adults.

Abstract citation ID: igad104.2278

### IRRATIONAL FALL RISK APPRAISAL GROUP CHARACTERISTICS IN COMMUNITY-DWELLING OLDER ADULTS

Aleatha Rossler[1], Ladda Thiamwong[2], Jeffrey Stout[2], Victoria Loerzel[2], and Boon Peng Ng[2], *1. UCF, Orlando, Florida, United States*, *2. University of Central Florida, Orlando, Florida, United States*

Approximately 43% of older adults are affected by fear of falling (FOF). A portion of those affected by FOF have irrational fall risk appraisal (FRA) which means they have a high FOF despite normal balance. This secondary analysis aimed to examine the 18% of the 124 study participants living with irrational FRA from the RO3AG06799-0 data source. Descriptive statistics were used to examine characteristics of older adults with Irrational FRA. Spearman Rho and linear regression were used to evaluate factors (age, sex, race, education, financial status, living situation, fall history, depression symptoms, anxiety, hand grip strength, balance, and fear of falling) associated with Irrational FRA in older adults. Of the 22 participants with irrational FRA, 20 were female, 70% were aged 70 or above (average age 74), and 72% were white (25% Asian, AA, and Hispanic). 77% had anxiety symptoms, with 50% having high anxiety scores, and 32% had depression symptoms. 40% lived alone, 45% had no prior falls, and 70% had low hand grip strength. 23% had inadequate financial means, and 72% had a college education or higher. Statistically significant correlations were found between the FES score and age (p=.044), who the participant lived with (p=.033), and balance (p=.042). Regression analysis showed predictors of FES score in this group to be anxiety (p=.042) and who they live with (p=.024). This study provides valuable insights into the demographic and clinical characteristics of older adults with irrational FRA and highlights the role of anxiety and living status in predicting FOF.

Abstract citation ID: igad104.2279

### OLDER WOMEN WITHOUT AND WITH DIABETES MELLITUS: RISK OF FALLS

Alessandro de Oliveira Silva[1], Silvana Funghetto[2], Luciano Ramos de Lima[3], and Marina Morato Stival[4], *1. Center University of Brasilia, Brasília, Distrito Federal, Brazil*, *2. University of Brasilia- Campus Ceilândia- UNB-Brasil, Brasília, Distrito Federal, Brazil*, *3. University of Brasilia, Brasilia, Distrito Federal, Brazil*, *4. University of Brasilia-Campus Ceilândia- UNB-Brasil,, Brasília, Distrito Federal, Brazil*

Evidence points to an association between hyperglycemic state and worsening mobility, with increased risk of falls. The aim of this study was to compare the risk of falls in the women older with and without diabetes mellitus in a sample of women older 60 to 65 years of age (N = 205) care in primary health care in the Federal District - Brazil. The results of the statistical model showed that the highest prevalence of falls was in the group of older women with type 2 diabetes mellitus (52.8%). It was possible to verify in the Time Up and Go performance test that most older women with type 2 diabetes mellitus were at risk of falling (79.2%) (p=0.003). Furthermore, women with type 2 diabetes mellitus showed a higher risk of falls in the Berg Balance test (51.76±5.87). The results suggest that type 2 diabetes mellitus significantly increased the risk of falls in this population. The potential benefits of this approach contribute to the planning of strategies and interventions to prevent falls in this population of older women.

Abstract citation ID: igad104.2280

### PREDICTING FALLS IN OLDER ADULTS

Colleen Brown[1], Marina Oktapodas Feiler[1], and Eleanor Simonsick[2], *1. Temple University, Philadelphia, Pennsylvania, United States*, *2. NIA IRP, Baltimore, Maryland, United States*

Falls are detrimental to older adults causing injury and mortality. Although clinical assessments of balance deficits exist, it is not well understood how well they predict falls. Longitudinal analysis utilized data from 688 men and women aged 60-97 enrolled in the Baltimore Longitudinal Study of Aging with index and subsequent visits approximately 1.7 years apart. Inclusion criteria included objective balance assessment at index visit and answering the question "Have you fallen in the past 12 months?" at both visits. Predictors of interest included thigh muscle strength, progressive static balance test performance, narrow walk, and sway reference with eyes closed/feet together. Tests were parameterized using validated methods. Adjusted logistic regression was modeled to estimate the association between objective measures of balance and fall risk. Adjustment variables included age, sex, race, height, and weight. Results reported those who failed the eyes closed/feet together position had 3.6 (95% CI 1.03, 12.81) times the odds of future falls compared with those who passed. Those who failed the semi-tandem, tandem, and single leg stance, had respectively 3.1 (1.44, 6.75), 3.6 (1.59, 8.06) and 4.8 (1.93, 12.09) times greater odds of history of falls compared with those who did not fail. Progressive standing balance performance did not predict future falls. The eyes closed/feet together test is easily administered, requires no equipment, and takes less than 30 seconds to complete. Failure of eyes closed/feet together may predict future falls, which is clinically relevant as this may suggest shifting the momentum towards proactively preventing an initial fall.

Abstract citation ID: igad104.2281

### PREDICTORS OF MAXIMUM STEP LENGTH IN OLDER ADULTS: ROLES FOR STRENGTH AND ACE GENOTYPE

Allon Goldberg[1], Hayden Norris[2], Johnathon Garber[3], Summer Burkhardt[2], Natalie Little[2], and Joseph Sucic[4], *1. College of Health Sciences, University of Michigan-Flint, Flint, Michigan, United States*, *2. University of Michigan-Flint, Flint, Michigan, United States*, *3. Michigan State University, East Lansing, Michigan, United States*, *4. University of Michigan--Flint, Flint, Michigan, United States*

Deficits in step length, assessed clinically by the maximum step length (MSL) test, are associated with poor balance and falls, in older adults. Determining predictors of MSL would facilitate targeted interventions to improve balance and reduce falls in older adults. Angiotensin-converting enzyme (ACE) insertion/deletion polymorphism genotypes interact with strength to predict stepping speed, but the interplay between ACE genotype, strength and MSL is unclear. Our objective was to determine whether strength and ACE DD genotype, through main effects or interactively, are associated with MSL, in older adults. Adults (N=103, female=58.2%, mean age 72.1 years, range 65-85 years) provided demographic, health and balance confidence information, completed grip strength and MSL testing, and provided saliva for genotyping. Multiple regression determined variance in MSL explained by strength, DD genotype (present/absent), and strength x genotype interaction, while controlling for covariates (age, sex, self-rated health and balance confidence). Covariates (model 1) explained 47.7% of MSL variance, while adding grip strength (model 2) explained additional 9.7% of variance. Adding DD genotype (model 3) explained .2%, while adding a strength x genotype interaction term (model 4) explained additional 3.4%, of MSL variance. The final model explained 61% of the variance in MSL ($p<.001$). DD genotype did not independently predict MSL ($\beta=-.053$; $p=.421$), but was interactively with strength, a significant predictor ($\beta=.216$; $p=.005$) of MSL. Strength was a significant predictor ($\beta=.264$; $p=.003$) of MSL, and as a modifiable physiological factor should be a focus of rehabilitation interventions to improve MSL and reduce falls risk in older adults.

Abstract citation ID: igad104.2282

### COMPARING THE OUTCOMES OF VIRTUAL VERSUS IN-PERSON DELIVERY OF AN EVIDENCE-BASED FALLS PREVENTION PROGRAM

Tamara Herrick[1], Kirsten Dorsey[1], Sarah Hallen[1], and Heidi Wierman[2], *1. MaineHealth, Portland, Maine, United States*, *2. MaineHealth- Maine Medical Partners, Portland, Maine, United States*

Each year one in four older adults has a fall1 and only about half tell their doctor or healthcare provider2. One way to prevent falls is to provide older adults with the evidence-based tools to prevent them. A Matter of Balance: Managing Concerns about Falls (MOB), is a top tier program for grant funding from the Administration for Community Living Falls Prevention Program. MOB has been shown to reduce the fear of falling and increase activity levels among older adults through cognitive restructuring and balance exercises.8 Prior to 2020, MOB was only offered as an in-person, small group (8-12 participants), community based intervention over 8 two-hour sessions led by 2 volunteer facilitators. In response to the global COVID-19 pandemic, MaineHealth's Partnership for Healthy Aging adapted the Lay Leader model of MOB for virtual delivery (MOB-V). Review of MOB-V pilot data suggested that it was also effective in reducing the fear of falling and increasing the activity levels of older adults. Based on this success, the MOB-V was more widely disseminated during 2021 and 2022, but to date no comprehensive program evaluation of the MOB-V has been completed. We will present the preliminary results of our evaluation of participant data collected from the National Falls Prevention database between 2021 through 2022.

### SESSION 7160 (POSTER)

### LONG-TERM CARE (HS)

Abstract citation ID: igad104.2283

### ANTIHYPERTENSIVE DEPRESCRIBING AND DISABILITY IN VA NURSING HOME RESIDENTS WITH AND WITHOUT DEMENTIA

Xiaojuan Liu[1], Laura Graham[2], Bocheng Jing[3], Chintan Dave[4], Yongmei Li[1], Kathy Fung[5], and Michelle Odden[1], *1. Stanford University, Stanford, California, United States*, *2. VA Palo Alto Health Care System, Palo Alto, California, United States*, *3. NCIRE, San Francisco VA Medical Center, UCSF, San Francisco, California, United States*, *4. Rutgers University, New Brunswick, New Jersey, United States*, *5. San Francisco VA Medical Center, San Francisco, California, United States*

Deprescribing antihypertensives is of growing interest in geriatric medicine, yet clinical trial evidence is lacking. We leveraged the Veterans Affairs (VA) electronic health data and emulated a target trial of antihypertensive deprescribing on longitudinal change in disability, measured by activities of daily living (ADL), ranging 0-28. Residents included were ≥65 years with long-term stays ≥12 weeks from 2006-2019 (n=42537). We excluded those not on antihypertensives and with very high blood pressure or heart failure. The eligible residents (n=13041) were divided into newly deprescribed (reduced number of antihypertensives or dose of ≥30% after a 4-week washout) and stable users and followed up for 2 years or censored at death, discharge, or non-adherence. We used linear mixed-effects regressions to estimate per-protocol effects, with inverse probability of treatment weighting (IPTW) to adjust for confounders and additional inverse probability of censoring weighting to account for informative censoring. Analysis was stratified by dementia (yes/no) to examine the potential modification. In non-dementia subgroup (n=5954), newly deprescribed residents (n=561) had a worse ADL score (βtreat [95% CI] = 1.36 [0.82, 1.91]) compared to stable users (n=5393). Over time, ADL scores increased (worsened) 0.13 per 3 months (βtime = 0.13 [0.09, 0.17]) while antihypertensive deprescribing statistically significantly attenuated this increase by 0.25 per 3 months (βtime × treat = -0.25 [-0.48, -0.03]). The dementia subgroup showed imprecise effect (βtime × treat = -0.0003 [-0.13, 0.12]). More evidence is needed on the benefits and harms of deprescribing in long-term care populations and the importance of dementia in these relationships.

Abstract citation ID: igad104.2284

### APATHY IN NURSING HOME RESIDENTS WITH DEMENTIA: ANALYSIS OF WITHIN- AND BETWEEN-PERSON DIFFERENCES

Ying-Ling Jao[1], Yo-Jen Liao[2], Diane Berish[3], Jacqueline Mogle[4], Helen Kales[5], and Marie Boltz[1], *1. Pennsylvania State University, State College, Pennsylvania, United States*, *2. Penn State University, State College, Pennsylvania, United States*, *3. The Pennsylvania State University, University Park, Pennsylvania, United States*, *4. Clemson University, Clemson, South Carolina, United States*, *5. University of California, Davis Health, Davis, California, United States*

Apathy commonly occurs in persons with dementia, yet it is often overlooked in nursing homes. It is unclear how apathy symptoms differ within- and between-individuals. This observational study examined the differences in apathy among nursing home residents with dementia. Three videos, 3-10 minutes each, were recorded for each resident to capture caregiving interactions between caregivers and residents with dementia and apathy. Apathy levels were rated second-by-second by trained researchers via video observations using the Person-Environment Apathy Rating-Apathy subscale (PEAR-Apathy). The total PEAR-Apathy score ranges from 6-24, with higher scores indicating higher apathy levels. A total of 17 residents were enrolled from three nursing homes in Pennsylvania. Correlations and multilevel modeling were used for analyses. PEAR-Apathy score was 16.2 on average. Within-video average variability in apathy was 2.28 (range=0.47-4.24). Across-video within-person results showed average variability was 2.27, and the range between people was 1.36 to 3.31. Residents' baseline apathy and depression, caregiver age, dementia care experience, and experience with the resident were not significantly correlated with apathy variability. Results from multilevel modeling showed that 11.2% of the within-person variance in apathy was accounted for by resident differences. After accounting for the clustering at the resident level, age was significantly associated with apathy variability (t=3.31, p=0.002). Results suggest that residents with dementia have moderate differences in apathy variability. Additionally, variability in apathy tended to increase with age. Future research may further examine factors contributing to individual differences in apathy and develop interventions to minimize apathy in nursing home residents with dementia.

Abstract citation ID: igad104.2285

### CAREGIVING APPROACHES TO RESISTIVE BEHAVIORS AMONG PERSONS LIVING WITH DEMENTIA IN LONG-TERM CARE

Kyung Hee Lee, Ji Yeon Lee, and Ji Yeong Park, *Yonsei University, Seoul, Republic of Korea*

Many older adults living in long-term care have dementia sufficiently severe to interfere with the performance of basic activities of daily living; this number is expected to increase rapidly. Although resistive behaviors are common among long-term care residents with dementia during care, caregivers face difficulty to prevent and manage them. The purpose of this study was to examine how caregivers responded to resistiveness to care of persons living with dementia. We analyzed 107 videos from 24 residents with dementia and 34 caregivers in four long-term care facilities. Each interaction between resident with dementia and caregiver was taken nine times at 0, 3, and 6 months at three care events (i.e., personal care, mealtime, and activity). Resistiveness to care was assessed using the Resistiveness to Care Scale for Dementia of the Alzheimer's Type; caregiving approaches were coded by the Person-Centered Behavior Inventory and the Task-Centered Behavior Inventory. Lag sequential analysis was conducted to examine caregiving approaches right after resistive behaviors of residents with dementia. We analyzed 25.1 hours of care events; 544 episodes of resistiveness to care were observed. When residents with dementia showed resistiveness to care, caregivers provided person-centered behaviors in 34.4% of cases, task-centered behaviors in 35.8% of cases, and nothing in 29.8% of cases. Caregivers used task-centered care approaches or nothing to manage resistiveness to care in more than 65% of cases. It is recommended to provide person-centered caregiving approaches to deal with resistiveness to care among persons living with dementia.

Abstract citation ID: igad104.2286

### COGNITIVE CHANGE AMONG NURSING HOME RESIDENTS; COGRISK-NH SCALE DEVELOPMENT TO PREDICT DECLINE

Elizabeth Howard[1], and John Morris[2], *1. Boston College Connell School of Nursing, Boston, Massachusetts, United States*, *2. Hebrew SeniorLife, The Marcus Institute for Aging Research, Boston, Massachusetts, United States*

**Objectives:** Chronicle cognitive changes in nursing home residents; develop risk model identifying predictors of decline.

**Methods:** Using secondary analysis design with MDS data, cognitive status and change measures were calculated based on Cognitive Performance Scale (CPS). Baseline and quarterly follow-up analyses of US and Canadian interRAI data (n=1,257,832) were completed. Risk model from logistic regression analyses identified predictors of decline. **Results:** Baseline 15% of residents were cognitively intact (CPS = 0); 11.2% borderline intact (CPS=1), 15% mild impairment (CPS = 2). 58.8% of residents fell into more severe CPS categories (3-6). Over time, increased proportion of residents declined – 17.1% at 6 months, 21.6% at 9 months, at 21 months, 34.0%. Over same time, more residents remained stable than declined. Baseline CPS score was strong predictor of decline. CPS categories 0-2 had 3-month decline rates in mid-teens, categories 3-5 had average decline rate of 9%. Two strata risk model construction was employed – one for CPS categories 0-2, second categories 3-5 and both were integrated into 6-category risk scale (CogRisk-NH). Mean decline rates at 3-month assessment ranged from 4.4% to 28.3%. Over time, distinction among risk categories continued – 6.9% to 38.4.% at 6 months, 16.2% to 61.4% at 21 months. Case distribution had 15.9% in category 1, 26.84% category 2, and 36.7% category 3. Three higher risk categories (4-6) represented 20.6% of residents. **Conclusion:** CogRisk-NH scale differentiates among residents and likelihood of decline. Knowledge of risk for cognitive decline enables allocation of resources targeting amenable factors contributing to decline.

Abstract citation ID: igad104.2287

### ENGAGEMENT OF FAMILY/CARE PARTNERS OF OLDER ADULT LONG-TERM CARE RESIDENTS WITH PHYSIOTHERAPISTS: A SCOPING REVIEW

Denise Connelly[1], Alexander Stephen[2], and Nicole Guitar[1], *1. Western University, London, Ontario, Canada*, 2. *University of Western Ontario, London, Ontario, Canada*

The aim of this scoping review was to summarize and critically evaluate literature describing the relationship between physical therapists and family/care partners of older adults living in long-term care homes. A librarian-informed search strategy was applied to Medline, EMBASE, Proquest, PsychINFO, SCOPUS and CINAHL databases. Papers were included if they addressed rehabilitation, physical therapists, family/care partners, and long-term care (LTC) setting or equivalent. Five papers were retrieved; quality was mid-to high. Challenges included minimal integration of family/care partners into the long-term care home 'team', inconsistent resident-centred health goals across staff, and lack of awareness about availability of physical therapy services within long-term care homes. Value for family/care partners as members of the health care team included their historical knowledge of their loved one, ability to perceive and manage behaviour change, signs/symptoms of pain by their loved ones with impaired communication (e.g., dementia diagnosis), and the resource of time and availability to engage in rehabilitation-related care activity. There is little information about partnership with family/care partners of older adults living in long-term care homes. The timing is optimal to explore the engagement of family/care partners in providing care and building rehabilitation capacity within the context of increasing nursing workloads, nurses' experience of burnout, difficulty in attraction and retention of care staff in long-term care homes, and challenges in funding for and standards in provision of optimal rehabilitation for older adult residents of long-term care homes.

Abstract citation ID: igad104.2288

### EXAMINING VETERANS' CHOICES OF HIGH-QUALITY MEDICARE SKILLED HOME HEALTH CARE AGENCIES

Katherine Kennedy[1], Frank DeVone[2], Emily Corneau[3], Portia Cornell[1], Pedro Gozalo[4], Kate Magid[5], Christine Jones[6], and Kali Thomas[7], *1. Providence VA Medical Center, Providence, Rhode Island, United States*, 2. *Department of Veteran Affairs, Providence, Rhode Island, United States*, 3. *Providence VAMC, Providence, Rhode Island, United States*, 4. *Brown University, School of Public Health, Providence, Rhode Island, United States*, 5. *Veterans Health Administration, Aurora, Colorado, United States*, 6. *University of Colorado, Centennial, Colorado, United States*, 7. *Johns Hopkins University, Baltimore, Maryland, United States*

Medicare-certified skilled home health helps Veterans restore function post-hospitalization. Our objective was to understand the quality of VA contracted home health agencies (HHAs) available to Veterans (i.e., had at least 1 VA-paid episode of care in the 12 months) relative to the local market. For comparison, about one-third (30.4%) of traditional Medicare beneficiaries received care from high-quality HHAs (4- or 5-stars on quality of care) in 2015 (Schwartz et al., 2019). We used the Home Health CAHPS (Consumer Assessment of Healthcare Providers and Systems) 2016-2019 quarterly patient experience of care overall ratings (possible values: 1-5) to define a high-quality agency as those with 4- or 5-star ratings. We matched the quarterly ratings with the discharge date from a VA Medical Center to create HHA choice sets per eligible Veteran discharge. A choice set contains all contracted and non-contracted HHAs that serve a Veteran's Zip Code. Among HHAs proximal to Veterans' homes across all years (N= 10,507 unique HHAs), 54.8% were high-quality (n=12,449 HHA-years), 30.1% were 3-star quality (n=6,835 HHA-years), and 15.1% were low-quality (1- or 2- stars) (n=3,444 HHA-years). Among discharges, 53.2% (n=28,081) of the discharges went to high-quality HHAs and 15.7% (n=8,294) went to low-quality HHAs. Veterans tended to live in areas with access to high-quality home health care, and most discharges mapped to high-quality HHAs. However, there is room to improve Veterans' access to high-quality skilled home health and more to understand about VA contracting practices and the HHA selection process.

Abstract citation ID: igad104.2289

### EXPLORING THE IMPACT OF AN EBOOK CLUB PROGRAM ON APATHY AMONG OLDER CANADIANS IN LONG-TERM CARE: A FEASIBILITY STUDY

Aderonke Agboji, and Shannon Freeman, *University of Northern British Columbia, Prince George, British Columbia, Canada*

Apathy is a common and persistent problem among older people in institutional settings. Research has shown that apathy can lead to rapid decline in cognitive status, serious functional impairments and decrease in the life span of those affected, yet it is under-researched, and under-managed. The aim of this study was to explore ways by which apathy can be mitigated in this population using eReader technology during book club program implementation. We recruited participants from various long-term care facilities (N=18) and each participants took part in semi-structured interviews, and self-reported apathy assessment both at the start and end of the program. We tracked engagement in the eBook club and time commitment to reading using a logbook. The findings suggest that older people, including persons with mild to moderate dementia, are open to adopting new technologies and the use of eReaders during book club programs is feasible for long term care facilities. Overall, we found that the use of eReader technology is a safer and more effective way of delivering book club program to older people in long term care facilities particularly during pandemic such as COVID 19 as they are easy to disinfect in comparison to physical books. We recommend that long term care staff should adapt eReaders into their existing book club or literacy programs and consider utilizing it as a strategy to prevent or manage apathy in this population.

Abstract citation ID: igad104.2290

**FEASIBILITY OF THE NUDGELTC CLINICAL DECISION SUPPORT APPLICATION**

Alyce Ashcraft[1], Donna Owen[2], and John Culberson[2], *1. Texas Tech University Health Sciences Center, Lubbock, Texas, United States*, *2. TTUHSC, Lubbock, Texas, United States*

Long-term-care residents are at risk for under reporting of urinary tract infections due to insufficient point-of-care testing and indirect communication. Aim: Examine the feasibility of a Clinical Decision Support application (NudgeLTC) designed to guide Licensed Vocational Nurses (LVNs) in data collection to improve communication confidence and satisfaction between LVN and Primary Care Provider (PCP). Sample size: 4 LVNs and 4 PCPs across 10 events. Qualitative description of interview comments and NudgeLTC output documented data collection and communication between LVN and PCP within the app. Forced choice questions about resident signs, symptoms, and laboratory values guided LVN data collection. Information was collected and stored within NudgeLTC. A summary was relayed directly to the PCP and followed with a PCP response to the LVN. Questionnaires included visual analogue scales for confidence (LVNs M=81.6; PCPs M=100 [0 low, 100 high]), satisfaction (LVNs M=7.8; PCPs M=10 [0 low, 10 high]), and the Systems Usability Scale (LVNs M=85.4; PCPs M=100 [0 poor, 100 high]). The NudgeLTC summary included resident assessment, vital signs, and lab values. Particular signs and symptoms noted included indwelling catheter and confusion. Select Challenges: PCP received LVN message and data, but was unable to respond via app; LVN stated the app did not work as "expected." Transmission issues occurred and were resolved. Asynchronous communication was valued and facilitated confidence in resident data. Difficulties with data collection may, in part, be due to discomfort with technology or infrequent use of electronic charting. Future studies should focus on improving point-of- care data collection.

Abstract citation ID: igad104.2291

**IMPACT OF UNIFORMS IN LONG-TERM CARE FACILITIES: SYNTHESIS OF INTERNATIONAL EVIDENCE AND SURVEY OF UK STAFF VIEWS**

Jennifer Liddle[1], Katie Thomson[1], Abdulwahab Al-Azawi[1], Dawn Esslemont[2], Barbara Hanratty[1], Christine Henderson[3], Louise Jones[4], and Lisa Wilson[5], *1. Newcastle University, Newcastle upon Tyne, England, United Kingdom*, *2. Eothen Care Homes, Cramlington, England, United Kingdom*, *3. Eothen Care Homes, Wallsend, England, United Kingdom*, *4. Northumbria Healthcare NHS Foundation Trust, Ashington, England, United Kingdom*, *5. Eothen Care Homes, Whitley Bay, England, United Kingdom*

Non-uniform-based staff dress codes are beginning to emerge in UK long-term care facilities for older people. However, there is little research evidence on the impact of staff clothing on resident, staff and visitor experiences, wellbeing or quality of life. Uniforms may be reassuring and facilitate identification of staff; a non-uniform approach may create a home-like, relaxed environment. This study aimed to synthesise existing international evidence and explore staff views about work clothing in three not-for-profit long-term care facilities in the UK. Five bibliometric databases were searched from inception. A narrative synthesis of included papers was conducted due to heterogeneity of study designs and outcome reporting. 77 (49%) of 158 staff responded to the survey. 68 staff (88%) did not currently wear a uniform at work. Views about the importance and impact of uniform were mixed: 25 (32%) believed that their role needed a uniform; 60 (78%) thought staff clothing made no difference in resident care quality but 45 (58%) agreed the environment felt more home-like when staff did not wear a uniform. 36 staff (47%) would choose to wear a uniform and 17 staff (22%) reported finding it difficult to afford clothes for work. In the context of limited research evidence, staff clothing is perceived to have little impact on care quality but considerable impact on long-term care facility atmosphere and staff working experiences. There is a need to research the impact of staff clothing to support evidence-based decisions about staff clothing policies in long-term care settings.

Abstract citation ID: igad104.2292

**IMPLEMENTING SMART AMBIENT BRIGHT LIGHT (SABL) IN NURSING HOME RESIDENTS WITH DEMENTIA: CHALLENGES AND STRATEGIES**

Ying-Ling Jao[1], Julian Wang[1], Yo-Jen Liao[1], Diane Berish[1], Marie Boltz[1], and Margaret Calkins[2], *1. Pennsylvania State University, State College, Pennsylvania, United States*, *2. IDEAS Institute, Cleveland Heights, Ohio, United States*

Effective non-pharmacological interventions are critically needed to reduce agitation for nursing home residents with dementia. Lighting interventions (LIs) improve agitation among persons with dementia. However, LIs have not been widely implemented in "real-world" care settings, and a feasible implementation approach to ambient LIs has not been established. This study describes challenges and strategies in implementing the smart ambient bright light (SABL) to provide auto-controlled, consistent indoor lighting that incorporates natural daylight. This SABL includes tunable LED lights, photosensors, and controllers and has a pre-programmed 24-hour control schedule for illuminance. The SABL will be installed in participants' bedrooms, dining areas, and activity rooms for four weeks. Key challenges include nursing home leadership team's concerns about compliance with regulations and increased staff workload. Our implementation strategies include 1) engaging the nursing home stakeholders early on to address their concerns, 2) setting up an advisory board at each facility to involve the administrator, director of maintenance, and frontline healthcare workers to ensure the implementation protocol is congruent with their regulations, policy, and staffing, 3) tailoring lighting schedule based on the care routine and resident schedules, and 4) monitoring the SABL operation on-site and remotely. Moreover, the study incorporates a mixed methods design to interview staff/administrators and evaluate the intervention's acceptability, feasibility, and appropriateness from their perspectives. This is the first study that addresses the implementation strategies of LIs. Findings will establish evidence-based implementation strategies and the best design for SABL to reduce agitation for nursing home residents with dementia.

Abstract citation ID: igad104.2293

### NURSE STAFFING AND VETERAN OUTCOMES IN THE VETERANS HEALTH ADMINISTRATION'S COMMUNITY LIVING CENTERS

Lana Brown[1], C. Heath Gauss[2], Pamela Billings[3], Jade Moore[4], Linda Sawyer[1], Diane Sparks[5], and Sheila Cox Sullivan[6], *1. Central Arkansas Veterans Healthcare System, North Little Rock, Arkansas, United States*, *2. University of Arkansas for Medical Sciences, Little Rock, Arkansas, United States*, *3. Veterans Health Administration, Nashville, Tennessee, United States*, *4. Veterans Health Administration, Washington, District of Columbia, United States*, *5. Veterans Administration, Palo Alto, California, United States*, *6. Department of Veterans Affairs, Washington, District of Columbia, United States*

Higher nurse staffing levels have been associated with better outcomes for patients in the acute care setting and for residents in the long-term care setting. There are several publications available linking nurse staffing to improved quality measures. Conversely, minimal research has occurred examining associations between nurse staffing and Veteran outcomes. The purpose of this research study was to assess for associations between nursing hours per patient day (NHPPD) and Veteran outcome measures in the Veterans Health Administration Community Living Centers. A retrospective data review was completed for NHPPD and quality measures for 134 Community Living Centers. Descriptive statistics were used to analyze the average total NHPPD for each of six Minimum Data Set, version 3.0, quality measures. Linear regression was utilized to assess for a linear association between average total NHPPD and the defined quality measures. This study found no linear association between average total NHPPD and the following Veteran outcome measures: improvement in function ($p=0.15$), ability to move independently worsened ($p=0.13$), catheter in bladder ($p=0.48$), and UTI ($p=0.48$). However, a statistically significant linear association was found between average total NHPPD and the following Veteran outcome measures: falls with major injury ($p=0.02$) and help with ADLs ($p=0.01$). As the average total NHPPD increased, the mean for falls with major injury and the mean for help with ADLs decreased. This study adds to the body of literature regarding the impact of nurse staffing on quality measures in the long-term care setting.

Abstract citation ID: igad104.2294

### PATTERNS OF SOCIAL CARE USE WITHIN THE OLDER POPULATION: WHAT CAN WE LEARN FROM ROUTINELY COLLECTED DATA?

Kelly Brotherhood[1], Barbara Hanratty[1], Gemma Spiers[1], Camila Caiado[2], and Julia Newton[1], *1. Newcastle University, Newcastle upon Tyne, England, United Kingdom*, *2. Durham University, Durham, England, United Kingdom*

Research with routinely collected social care data has untapped potential to inform new care delivery approaches and techniques. To identify opportunities for service improvement and enhance our understanding of care pathways experienced by the older population, we collaborated with a local authority in the North East of England. We set out to characterise the use of social care services and associated outcomes within the local older population (aged 65+). 171,386 records were extracted from the local authority's social care case management system, relating to 38,191 unique individuals across the last 40 years. We identified the care packages provided to the local population, including care provided in care homes (with and without nursing), private households and assisted living facilities. The study population varied in terms of the number of care packages provided to each individual (median 7 packages, IQR 4-11) and the average duration of individual care packages (median 41 days, IQR 14 - 274 days). The care pathways that are most common amongst the older population will be described, including sequencing and outcomes, and grouped by the reason for providing care (e.g., respite, long-term care) and the reason why each care package ended (e.g., death, returning home). The wide range of care pathways experienced demonstrate the heterogeneity in needs and preferences within the older population. This dataset and analyses are an invaluable way of identifying areas of potential unmet need and evaluating the effectiveness of short-term care services.

Abstract citation ID: igad104.2295

### PERSON-CENTERED COMMUNICATION AND APATHY IN NURSING HOME RESIDENTS WITH DEMENTIA

Ying-Ling Jao[1], Yo-Jen Liao[2], Diane Berish[3], Jacqueline Mogle[4], Helen Kales[5], and Marie Boltz[1], *1. Pennsylvania State University, State College, Pennsylvania, United States*, *2. Penn State University, State College, Pennsylvania, United States*, *3. The Pennsylvania State University, University Park, Pennsylvania, United States*, *4. Clemson University, Clemson, South Carolina, United States*, *5. University of California, Davis Health, Davis, California, United States*

Apathy is prevalent in dementia and is associated with poor health outcomes. Communication is fundamental for daily caregiving activities. Person-centered communication has positively impacted some neurobehavioral symptoms, but its impact on apathy remains unclear. This video observation study examined the association between healthcare workers' use of person-centered communication and apathy in nursing home residents with dementia. Three videos, three to ten minutes each, were recorded for each resident to capture daily caregiving interactions. Healthcare workers' communication was transcribed and segmented into phrases. Communication was coded phrase-by-phrase via video observations using the Person-Centered Behavioral Inventory. Communication was categorized as none, person-centered, non-person-centered, or others. Apathy was coded second-by-second via video observations using the Person-Environment Apathy Rating. Concurrent correlations and ANOVA were used for analysis. A total of 17 residents with dementia and apathy and 21 healthcare workers (e.g., certified nurse assistants, activity staff, and nurses) were recruited from three nursing homes.

Results showed that caregiver communication approach had widely mixed correlations with second-by-second apathy scores across all videos (averaged r=0.09, range: r=-0.18 to r=0.54). Further ANOVA analysis showed that using person-centered communication, as compared to other communication approaches, had mixed associations with apathy; 19.6% showed significantly lower apathy, 35.7% showed significantly higher apathy, and 44.6% showed no significant associations. Overall, healthcare workers' communication approach can potentially impact apathy for residents with dementia. Future research that examines the sequential associations between communication and apathy is needed to guide intervention development to reduce apathy and improve dementia care in nursing homes.

Abstract citation ID: igad104.2296

### PRESERVING THE DISCRETENESS OF DEFICITS LEADS TO LOWER FRAILTY INDEX IN INDIVIDUALS LIVING IN LONG-TERM CARE

Brian Greeley[1], Hilary Low[1], Ronald Kelly[2], Robert McDermid[1], and Xiaowei Song[1], *1. Fraser Health, Surrey, British Columbia, Canada*, *2. Fraser Health Authority, Surrey, British Columbia, Canada*

The frailty index (FI), based on the deficit accumulation model, has potential to advance healthcare, but conventional coding of raw scores introduces noise. This study assesses the impact of the two different coding approaches on the FI. Two FI were calculated using 43 variables from 29,758 older (> 65 years old) Canadians adults (84.6 ± 8 years old; 64% female) living in long-term care. Scores were coded as 0, 0.5, or 1 regardless of the number of levels (grouped), or preserved (e.g., a 4 level variable was coded as 0, 0.33, 0.67, or 1; discrete). FI was correlated to age. Each ordinal variable was removed from the FI to further test the impact of the two coding approaches. The median FI for the grouped approach (0.302 (0.221 – 0.372)) was higher relative to the discrete approach (0.237 (0.170 - 0.307)). The discrete (r = .91) and grouped (r = .93) FI showed similar relationships to age. Removal of any ordinal variable reduced the grouped FI it by 0.004 or 0.016, whereas removal lead to both increases (range: 0.003 - 0.001) and reductions (range: 0.002 - 0.008) for the discrete FI. Using a grouped coding approach when quantifying frailty status artificially inflates FI among a large sample of older Canadians adults living in long-term care. The study underscores the importance of future FI research to adopt a discrete coding approach that accurately reflects the true level of impairment, for reducing noise in statistical models and better clinical utility.

Abstract citation ID: igad104.2297

### REDUCING UNPLANNED HOSPITALIZATIONS OF NURSING HOME RESIDENTS: A PROCESS EVALUATION OF INTERCARE

Franziska Zuniga[1], Thekla Brunkert[1], Kornelia Basinska[1], Raphaëlle-Ashley Guerbaai[2], Christine Serdaly[3], and Nathalie Wellens[4], *1. University of Basel, Basel, Basel-Stadt, Switzerland*, *2. The Institute of Nursing Science, Basel, Basel-Stadt, Switzerland*, *3. Serdaly&Ankers snc, Conches (CH), Geneve, Switzerland*, *4. La Source School of Nursing, HES-SO University of Applied Sciences and Arts Western Switzerland, Lausanne, Vaud, Switzerland*

The INTERCARE study provided evidence for a complex intervention to reduce unplanned hospitalizations in 11 Swiss nursing homes. The six intervention components were strengthening of interprofessional collaboration, introduction of a nurse with in-depth geriatric training (INTERCARE nurse), comprehensive geriatric assessment, advanced care planning, evidence-based tools, and data-driven quality improvement. This process evaluation aims to describe the intervention components' use in practice and understand the mechanisms of change to optimize intervention components for scale-up. We used qualitative data from individual and focus group interviews and meetings notes from the research team's conversations with nursing home care workers and leadership, INTERCARE nurses and physicians from five nursing homes participating in the INTERCARE study. Data was extracted into spread sheets before starting inductive coding. Findings were summarized and consolidated in a conceptual model. In connection with the four intervention elements interprofessional collaboration, INTERCARE nurse, advanced care planning and evidence-based tools, we identified four intervention mechanisms, grouped into 1) those changing reasoning of care workers e.g., following a more structured approach to do things and 2) those enabling existing resources e.g., availability of a responsible contact person. We also identified behavior changes in care workers, including faster reaction to changes in resident situations, more comprehensive assessment of resident situations and improved communication which contributed to reductions in hospitalizations. Understanding how interventions work in practice is crucial for informing potential adaptations to the intervention and the implementation strategies. Our findings can help optimize the intervention components for scale-up, ultimately reducing unplanned hospitalizations from nursing homes.

Abstract citation ID: igad104.2298

### RELATED FACTORS ON HOSPITALIZATION OF NURSING HOME RESIDENTS IN KOREA USING QUASI-POISSON REGRESSION

Juh Shin[1], Sun Ok Jung[2], Eun Jeong Min[3], and Jung Eun Kim[4], *1. George Washington University, Washington, District of Columbia, United States*, *2. Ewha Womans University, Seoul, Republic of Korea*, *3. The Catholic University of Korea, Seoul, Republic of Korea*, *4. California State University, Los Angeles, Los Angeles, California, United States*

The appropriateness of hospitalization for nursing home (NH) residents is still debated, with factors such as timeliness, available treatment, healthcare staff, medication options in hospitals, and safety issues determining appropriateness. Although factors impacting hospitalization have been studied expansively, research has been limited to nurse staffing. This study aims to investigate how organizational factors, nurse staffing, and government incentives impact hospitalization due to infection among NH residents in Korea. A cross-sectional design was used, and data were collected via survey from a total of 51 NHs from August 27, 2021 to March 25, 2022 using quota sampling. A total of 35 explanatory variables were included, and the response variable was the count of hospitalized residents due to infection. We analyzed data using quasi-Poisson regression. The average number of residents of hospital transfers due to infection was 5.22 (SD 7.99) per nursing home. The number of residents with Alzheimer's or dementia increased by 1 person, and the number of hospitalized residents due to infection increased by 1.01. Moreover, we found that in NHs that did not receive top-grade incentives due to good performance, the number of residents admitted to the hospital due to infection decreased by Exp (b) 0.5133. More stable, specialized advanced nursing staff and leaders specializing in high-quality care for residents with Alzheimer's or dementia were more effective in decreasing hospitalization. The measure of hospitalization in the future should focus on quality improvement, as hospitalization is universal use for many countries by extricating necessary versus avoidable hospitalization.

Abstract citation ID: igad104.2299

### THE PREVALENCE AND PREDICTORS OF APATHY AMONG CANADIAN LONG-TERM CARE RESIDENTS: A SECONDARY DATA ANALYSIS

Aderonke Agboji, and Shannon Freeman, *University of Northern British Columbia, Prince George, British Columbia, Canada*

Apathy, defined as a reduction in interest or motivation to engage in goal directed activities, is a frequent and persistent health concern among persons residing in long term care facilities. Research showed that apathy is associated with rapid cognitive decline, impaired functional ability, and increased mortality risk. To prevent or mitigate the negative impacts of apathy on persons living in long term care facilities, there is need for better understanding of the risk factors associated with it. Therefore, the aim of this study was to determine the prevalence of apathy and its risk factors in a large sample of Canadian long-term care residents (N=332,454). We performed cross-sectional analysis of data from MDS 2.0 assessments completed between 2015 and 2019 fiscal year. Results from descriptive and inferential analyses showed that apathy was more prevalent among persons aged 65 or younger and speak English. The strongest predictors of apathy were province of residence (OR 4.09 (CI 2.99-5.59) followed by resistance to care (OR 2.43 CI 2.38-2.49), conflict with family (OR 2.30 CI 2.2-2.4), and insomnia (OR 2.16 CI 2.11-2.2). These findings highlight the important role of environmental contextual factors including the physical and social environment as well as personal factors in the development of or recovery from apathy. Strategies for combating apathy in long term care facilities should take into account the individual, institutional and provincial variances.

Abstract citation ID: igad104.2300

### TOOLS FOR MEASURING PERSON-CENTERED CARE FOR OLDER ADULTS IN LONG-TERM SERVICES: A SCOPING REVIEW

Nahida Akter, and Kimberly VanHaitsma, *Penn State University, State College, Pennsylvania, United States*

Person-Centered Care (PCC) involves individuals in their care planning and decision-making. Measuring PCC is crucial in identifying the adequacy of PCC dimensions, which can then be used to promote quality of care and life, yet a comprehensive review of evidence on PCC measurement tools in long-term service and support settings (LTSS) is lacking. Our study aims to identify and evaluate available tools for measuring PCC for older adults in LTSS. This review followed the Arksey and O'Malley (2005) five-stage approach for scoping reviews and search was conducted in PubMed and CINAHL using selected keywords. The inclusion criteria were peer-reviewed journal articles written in English, reporting on PCC measurement tools for older adults (≥ 65 years) living in LTSS. Tools intended for use by family members and gray literature were excluded. Articles (n=23) were chosen from 914 studies after reviewing titles, abstracts, and full texts by two reviewers. They revealed 10 distinct tools for measuring PCC in LTSS, categorized as non-observational (e.g., interview, self-report) and observational (e.g., structured observation). The psychometric properties (reliability and validity) of the tools were reported, and their applicability in different cultural contexts was discussed. Many tools (n=8) demonstrated good reliability and validity, and their applicability was evident in multiple countries (e.g., Spain, Japan, Sweden, China, Italy and Norway). Our study adds to the discussion on PCC measurement tool development as it identifies gaps (e.g., lack of harmony between tools) for future researchers and potential areas for development by policy makers and administrators.

Abstract citation ID: igad104.2301

### USING PROCESS EVALUATION TO IMPROVE DOCUMENTATION OF LIFE-SUSTAINING TREATMENTS FOR VETERANS

Leah Haverhals[1], Kate Magid[2], Jennifer Kononowech[3], Jazmin Beltran[4], Amber Lane[5], and Cari Levy[6], *1. Department of Veterans Affairs Rocky Mountain Regional VA Medical Center, Denver, Colorado, United States*, *2. Veterans Health Administration, Aurora, Colorado, United States*, *3. VA Ann Arbor, Ann Arbor, Michigan, United States*, *4. Department of Veterans Affairs, Aurora, Colorado, United States*, *5. Rocky Mountain Regional VA Medical Center, Aurora, Colorado, United States*, *6. University of Colorado, Denver, Colorado, United States*

The Preferences Elicited and Respected for Seriously Ill Veterans through Enhanced Decision-Making (PERSIVED) intervention began in 2021 to support United States Department of Veterans Affairs (VA) Home Based Primary Care (HBPC) clinicians in completing life-sustaining treatment (LST) templates for Veterans. In PERSIVED, HBPC clinicians partner with the PERSIVED team for 15 months, with clinicians routinely receiving coaching from the PERSIVED team. To assess participation in PERSIVED and to inform coaching sessions, the PERSIVED team conducted process evaluation interviews from August-November 2022 with N=24 clinicians across the country from seven HBPC sites. Interviews were conducted between months six-seven of PERSIVED implementation. Two PERSIVED team members conducted interviews, one acting as interviewer and the other as notetaker. Following interviews, the notetaker cleaned up the notes and the interviewer reviewed them, with the two reaching consensus on summaries on individual interviews and ultimately summarizing interview data by site. Two PERSIVED coaches then reviewed the summaries to brainstorm how to adapt coaching based on process evaluation findings. Coaches noted site-specific barriers and facilitators to improving LST template completion rates; positives of PERSIVED coaching; suggestions to improve PERSIVED; and goals for the remaining nine months of the PERSIVED intervention. Strategies suggested based on the process evaluation analyses included how to integrate checking for the LST template into existing processes, adapting note templates, discussing LST templates during team meetings, and improving education around LST template completion. The rapid integration of process evaluation findings into PERSIVED coaching sessions proved beneficial for participating HBPC teams.

Abstract citation ID: igad104.2302

### WHAT IS THE DIFFERENCE BETWEEN OLDER PERSONS WHO CONTINUE HOME CARE AND THOSE WHO DO NOT?

Yuka Sumikawa, Sameh Eltaybani, Chie Fukui, Ayumi Igarashi, and Noriko Yamamoto-Mitani, *The University of Tokyo, Tokyo, Tokyo, Japan*

Enabling older people to remain in and return to their homes after hospitalization or institutionalization (i.e., admission to care institutions, such as nursing homes), if they wish so, is a key principle of aging-in-place worldwide. This prospective cohort study examined the characteristics of older people who returned to their homes after hospitalization or their stay at other care facilities. The study started with 1,450 older persons (≥75 years) receiving home care services at multiple places in Japan. Data were collected at the baseline and 6-month follow-ups. The study was approved by the Research Ethics Committee of the Graduate School of Medicine, The University of Tokyo (No. 2019087NI). At the 6-month follow-up, 256 clients (female: 152 clients, mean age: 85.1 ± 6.1) were hospitalized or institutionalized at least once, of which 54 clients (female: 38 clients, mean age: 83.6± 6.8) returned home afterward. A multivariate logistic regression showed that females (odds ratio [95% confidence interval]: 2.01 [1.06 to 4.09]), those with more care assistance (1.27 [1.03 to 1.55]) were more likely to return to their home, whereas older clients had a lower likelihood (0. 93 [0.88 to 0.98]). Based on this finding that younger females were more likely to return home if hospitalized or institutionalized, older males may have different needs than females, influencing the type and level of support they require. Further research might identify the support needed for older males with less care assistance.

### SESSION 7170 (POSTER)

### PHYSICAL ACTIVITY AND EXERCISE

Abstract citation ID: igad104.2303

### A SYSTEMATIC REVIEW OF FUNCTIONAL TRAINING EXERCISE IN COMMUNITY-DWELLING OLDER ADULTS WITH COGNITIVE IMPAIRMENT

Yun Chan Shin[1], Chiung-ju Liu[1], Kellen Breton[1], Justin Mason[2], Yi-Ling Hu[3], and Jane Morgan-Daniel[1], *1. University of Florida, Gainesville, Florida, United States, 2. University of Iowa, Coralville, Iowa, United States, 3. Chang Gung University, Kwei-Shan, Taoyuan, Taiwan (Republic of China)*

Older adults with cognitive impairment are at risk of becoming care dependent. Functional training involves movements necessary for daily tasks, which may help maintain or improve independence. As part of a larger systematic review examining the effects of a functional training exercise in community-dwelling older adults, this study aimed to synthesize the functional and cognitive outcomes in those with cognitive impairment. A systematic search was conducted in 10 electronic databases to identify relevant studies published between January 2010 and September 2021. Studies were selected if recruited adults aged > 60 years, participants had a cognitive impairment that was not due to acquired brain injury, and activities of daily living and/or cognitive function were measured. Nine studies (847 participants) were identified. Five studies were conducted on older adults with mild cognitive impairment, all showed an improvement in activities of daily living, and 4 showed an improvement in global cognition. Three were conducted on older adults with dementia, and one showed an improvement in activities of daily living but not global cognition. One was conducted on older adults with mild cognitive impairment or dementia and reported an improvement in both outcomes. Functional training exercise seems effective in improving cognitive function and activities of daily living in older adults with mild cognitive impairment. For those with dementia, although research on functional training is limited, some preliminary evidence suggests that incorporating functional training with other components may be beneficial. Despite inconclusive findings, functional training exercises may hold potential for older adults with cognitive impairment.

Abstract citation ID: igad104.2304

### ACTIVITY LEVEL AND SUCCESSFUL AGING, SUBJECTIVE AGE, AND MENTAL, PHYSICAL, AND GENERAL HEALTH

Isabel Mancilla[1], Lucia Rivera[2], Jaclyn Bergstrom[2], and Anthony Molina[2], *1. University of California San Diego, San Diego, California, United States, 2. University of California, San Diego, San Diego, California, United States*

Perceived well-being refers to an individual's subjective evaluation of their quality of life. Importantly, perceived well-being takes into account an individual's emotional, social, and psychological health, which are all critical components of a fulfilling and satisfying life. The purpose of this study is to evaluate the correlations between activity level from the International Physical Activity Questionnaire (IPAQ) and self-reported outcomes related to perceived well-being (successful aging, subjective age discrepancy), and mental, physical, and general health outcomes from the 36 Item Short Form Survey (SF-36). We utilized existing data from 595 individuals aged 50 years or older enrolled in UC San Diego's Successful Aging Evaluation (SAGE) study. Participants were stratified into low, medium, and high activity levels from the IPAQ. Groups were then compared to self-rated successful aging, subjective age questions, and the SF-36. Our results indicate that increased physical activity is positively associated with subjective successful aging. We observed a significant linear trend in IPAQ physical activity correlated to mental health (p=.0242), physical health (p=<.0001), and general health (p=(<.0001). A possible limitation of this study includes that of the 595 participants, 81% of participants identified as Caucasian. It is critical for future studies to incorporate a more diverse sample size to accurately present future findings. Understanding the factors that influence perceived well-being can lead to strategies for improving the overall outlook to support successful aging.

Abstract citation ID: igad104.2305

### CHANGES IN PHYSICAL ACTIVITY, SEDENTARY TIME, AND SELF-RATED HEALTH ACROSS THE RETIREMENT TRANSITION

Hugo Westerlund[1], Andreas Fröberg[2], Lawrence Sacco[1], Kristin Suorsa[3], Tuija Leskinen[3], Pasan Hettiarachchi[4], Magnus Svartengren[4], and Sari Stenholm[3], *1. Stockholm University, Stockholm, Stockholms Lan, Sweden*, *2. University of Gothenburg, Gothenburg, Vastra Gotaland, Sweden*, *3. University of Turku, Turku, Varsinais-Suomi, Finland*, *4. Uppsala University, Uppsala, Uppsala Lan, Swe den*

The aim was to investigate concurrent changes in accelerometer-measured total physical activity (TPA), moderate-to-vigorous physical activity (MVPA) and sedentary time, and changes in self-rated health across the retirement transition. Data from two longitudinal studies was pooled: the Swedish Retirement Study (SRS) and the Finnish Retirement and Aging (FIREA) study that used similar methods. Data from three waves (each approximately 12 months apart) was included: one pre-retirement (wave 1, W-1) and two post-retirement follow-ups (W-2 and W-3). In total, 245 participants (27% men, mean age 64 years at W-1) were included. Thigh-worn accelerometers were used to collect data for TPA, MVPA and sedentary time at W-1 and W-2 and processed through ActiPASS and harmonized for time spent in different physical activity intensities. To be included in the analyses, a minimum of 4 days with at least 10 hours of accelerometer wear time during waking hours at both W-1 and W-2 was required. Questionnaires were used to collect data for self-rated health at W-1, W-2, and W-3. Ordinal regression models showed that 10 more minutes of MVPA at W-1 was significantly associated with higher odds of reporting better self-rated health at W-1 (OR: 1.12 [1.02-1.22]) in non-manual workers only. The association between MVPA and self-rated health was non-significant in the fully adjusted models. No significant associations were observed between changes in TPA, MVPA or sedentary time during retirement transition, and self-rated health at post-retirement follow-ups. In conclusion, this study showed a significant cross-sectional association between MVPA and better self-rated health before retirement.

Abstract citation ID: igad104.2306

### COMPARISON OF CUT POINTS FOR PREDICTING PHYSICAL ACTIVITY INTENSITY IN OLDER ADULTS

Ameera Chakravarthy[1], Barbara Resnick[2], Elizabeth Galik[1], Kelly Doran[3], Cynthia Renn[1], Eric Lehr[4], and Kasey Knopp[5], *1. University of Maryland School of Nursing, Baltimore, Maryland, United States*, *2. University of Maryland, Baltimore, Maryland, United States*, *3. UMSON, Baltimore, Maryland, United States*, *4. Swedish Heart and Vascular Institute Swedish Medical Center, Seattle, Washington, United States*, *5. University of Maryland School of Medicine, Baltimore, Baltimore, Maryland, United States*

The differences between cut-points, and scarcity of comparative impact studies have prevented researchers from reaching consensus regarding the application of intensity-related accelerometer cut-points for older adults. This study will focus on the measure of physical activity using the MotionWatch 8 in older adults and compare the differences using traditional Freedson cut points versus those developed by Landry for older adults and evaluate the impact of these differences on age, gender, race, cognition, comorbidities as well as function based on the Barthel Index. A total of 177 participants between the ages of 59 to 101 (M 83) from an acute care study were used for the analysis. Physical activity intensity was estimated using 24-hour count data and compared using two independently developed cut-points. Impact on outcomes were evaluated with hierarchical regression and Multivariate analysis of variance. After controlling for age, gender, race, comorbidities, and cognition, Landry sedentary cut-points were all significantly associated with function. Overall, 14% of the variance in function was explained by all the variables in the model. The findings provide support for use of the Landry cut points to quantify physical activity intensity in hospitalized older adults living with dementia to identify low levels of activity.

Abstract citation ID: igad104.2307

### DETERMINANTS OF PHYSICAL ACTIVITY IN COMMUNITY-DWELLING OLDER ADULTS: AN UMBRELLA REVIEW

Cassandra D'Amore, Stephanie Saunders, Neera Bhatnagar, Lauren Griffith, Julie Richardson, and Marla Beauchamp, *McMaster University, Hamilton, Ontario, Canada*

Physical activity (PA) is an important modifiable factor for noncommunicable diseases, mental health, and functional trajectories as we age. The World Health Organization highlights the need to mobilize policies targeted toward modifiable determinants of healthy aging. Therefore, understanding the current evidence for PA determinants is crucial to achieving these goals. This umbrella review aimed to summarize the determinants of PA in older adults. A research librarian searched six databases. Systematic and scoping reviews were included if they investigated community-dwelling people with a mean age of 60 years or older and examined a relationship between any type of PA and a determinant. Two independent reviewers screened 17,277 references and 11 reviews were ultimately included. Of the countries reported, 78% were high-income and zero were lower-income countries. Three of the eleven reviews focused on specific clinical populations living in the community. Only 6% of studies included in all reviews had longitudinal designs. Included studies focused on a variety of types of PA (e.g., total PA, walking, leisure-time PA) and measures of PA, with 76% using self-report, 15% using direct measures (e.g., accelerometry), 3% using both types, and 6% with no outcome measure reported. Physical environment determinants were the most commonly assessed (6/11) with more than 70% of the summarized relationships demonstrating null associations. Three out of four reviews reported a positive relationship between walkability and PA in general community-dwelling older adults. Further prospective longitudinal studies are needed to improve our current understanding of the determinants of PA in community-living older people.

Abstract citation ID: igad104.2308

### SELF-REGULATION AND SELF-EFFICACY OF EXERCISERS DURING COVID-19

Candace Brown[1], and Kira Chiles[2], *1. University of North Carolina Charlotte, Charlotte, North Carolina, United States*, *2. University of North Carolina, Charlotte, Charlotte, North Carolina, United States*

Employment, which was greatly impacted by the COVID-19 pandemic, caused alterations to the 'work-from-home' culture, and resulted in increased time sitting and decreased time in leisure activities, like exercise. This study explored the relationship of full time employment, intrinsic motivation, and self-efficacy to exercise with the understanding that social distancing and quarantining led to increased social isolation among all ages around the world. **Methods:** Guided by the Self-determination theory, online data was collected from U.S. exercisers ages 18 and older who completed the Exercise Self-Regulation Questionnaire and Self-Efficacy for Exercise Scale during the height of the pandemic, in April 2020, and after, in February 2022. **Results:** There were n = 320 participants that completed the surveys at T1 and n = 107 returned for T2 for a total of N = 427 participants. MANOVA results revealed a significance in intrinsic motivation ($p < .001$) in 2020 to exercise when gyms and outdoor spaces were closed. The greatest determinants of not achieving self-efficacy to exercise in 2022, when gyms and outdoor spaces were reopened, included "being bored" (36.1%) and "not enjoying" (29.6%) the activity. **Conclusion:** Results suggest that full time employees, regardless of age, endorse high levels of personal accepted values and goals to exercise that are enjoyable, to them, for continued self-efficacy to exercise.

Abstract citation ID: igad104.2309

### THE VARIATION IN DAILY HABITUAL PHYSICAL ACTIVITY LINKS TO GLOBAL COGNITIVE FUNCTION IN OLDER ADULTS

Levi Ask[1], Brad Manor[2], and On-Yee Lo[3], *1. Hebrew SeniorLife, Boston, Massachusetts, United States*, *2. Hebrew SeniorLife/Harvard Medical School, Amherst, New Hampshire, United States*, *3. Hebrew SeniorLife/ Harvard Medical School, Boston, Massachusetts, United States*

Being physically active is critical to the maintenance of physical and cognitive function into old age. Wearable sensors with the ability to monitor step counts have become popular, making it easier for individuals to track their physical activity levels from day to day. While average daily step count has traditionally been used to quantify physical activity, emerging evidence suggests that the magnitude of day-to-day variation in physical activity or other clinical outcomes may provide additional insight into health. This study aimed to investigate the relationship between the characteristics of habitual physical activity and global cognitive function in older adults. A secondary analysis from a pilot randomized controlled trial was conducted. Thirty-two older adults (79±7 y/o, 29 women) completed the Montreal Cognitive Assessment (MoCA) of global cognitive function at baseline (Total MoCA score: 25.0±2.9). Physical activity was tracked using a Fitbit wrist-worn activity tracker for two consecutive weeks with all participants having 10 complete days. Daily step count mean and standard deviation were calculated to quantify the daily average (4435.12±2768.88 steps) and daily variation (1671.05±1134.03 steps) in physical activity, respectively. After adjusting for age and sex, participants with greater daily variation in step counts exhibited better global cognitive function ($r=0.52$, $p=0.02$). In contrast, average daily steps did not significantly correlate with MoCA performance. Neither of these physical activity outcomes were significantly linked to any MoCA subscore. These results suggest that monitoring day-to-day step count variation may serve as a particularly sensitive indicator of global cognitive health in older adults.

### SESSION 7180 (POSTER)

### TELEHEALTH

Abstract citation ID: igad104.2310

### ACCEPTABILITY OF HIGH-INTENSITY FUNCTIONAL STRENGTH TRAINING AT HOME FOR POSTINJURY OLDER ADULTS

Ashley Morgan, Jennifer Heisz, Ada Tang, Lehana Thabane, and Julie Richardson, *McMaster University, Hamilton, Ontario, Canada*

Regular exercise plays a vital role in optimizing aging and preventing functional decline. Despite the recognized benefit of exercise, adherence is challenging, and acceptability of exercise interventions is crucial. Acceptability is influenced by various factors, including content, context, and delivery. The purpose of this qualitative descriptive study is to determine the acceptability to older adult participants of a home-based high-intensity functional strength training (HIFST) intervention delivered by a physiotherapist via videoconferencing. HIFST involves the use of periods of 'hard' effort using everyday strength-building movements alternating with 'easy' recovery periods. This study accompanies an ongoing pilot randomized controlled trial investigating the feasibility of HIFST (completion: spring 2023). We are conducting semi-structured interviews and a short anonymous survey with participants who have completed HIFST. Data collected is being analyzed using qualitative content analysis. To date, results from 8 participants (2 men, 6 women, 55-81 years) demonstrate a positive overall impression of HIFST with all participants noting enjoyment, a good experience, and/or meeting their expectations. Participants reported certain aspects less/more enjoyable than others (e.g., specific exercises). No technical difficulties have been noted and many participants appreciated the convenience of an online format. Prior knowledge of interval-based exercise was minimal for most participants and the majority commented on a preference for shorter intervals with shorter rest periods. Further data collection and analysis will provide a contextualized understanding of the HIFST intervention and important considerations for exercise prescription in an aging population.

Abstract citation ID: igad104.2311

### INCREASED SUPPORT FOR OLDER VETERANS AND THEIR CAREGIVERS USING TELEHEALTH SERVICES

Bertha Flores[1], Lyda Arevalo-Flechas[2], Rozmin Jiwani[3], Enelda Romo[2], Gabriela Zaragoza[2], Becky Powers[2], Sandra Sanchez-Reilly[2], and Sara Espinoza[2], *1. University of Texas HSC at San Antonio, San Antonio, Texas, United States*, *2. South Texas Veterans Health Care System, San Antonio, Texas, United States*, *3. UT Health San Antonio, San Antonio, Texas, United States*

Five million Veterans live in rural areas in the U.S., and about half of those live in the South. Rural Veterans are more likely to be older and have a disability compared with non-rural Veterans. In Texas, Veterans comprise seven percent of the adult population with a median age of 50 years, of those 17% live in rural areas where access to care is a concern for many Veterans and their families. Technology-based services could help close this gap. The GRECC Connect program is one way to reach rural Veterans and their caregivers. GRECC Connect is a multi-disciplinary virtual geriatrics program that provides support for providers to help manage older, rural Veterans through telemedicine. Patient Tele- consultations follow the Age-Friendly Geriatrics 5 M's framework (Medications, Mentation, Mobility, Multi-complexity, and what Matters most). As a demonstration project at the San Antonio GRECC, we partnered with the Geriatric Extended Care Services (GEC), and the Dementia Caregiver Program (DCP) to educate and support caregivers. We also partnered with the education department to conduct clinic outreach. In Fiscal year 2022, we served 72 Veterans, 66 of those had a caregiver, and the majority (51/66, 77%) were enrolled in the DCP. In Q4 (17/17, 100%) of caregivers were enrolled in the DCP. In addition, Veterans were referred to 248 additional services including palliative care and saved 5,934 miles. The GRECC Connect program decreases barriers experienced by older rural Veterans including transportation, assistance to navigate VA system and access to specialty geriatric and palliative care.

Abstract citation ID: igad104.2312

### OLDER ADULTS' ACCEPTANCE OF TELEGRACE: A HYBRID TELEHEALTH CARE MODEL

Alaina Preddie[1], Lauren Penney[2], Ashley Schwartzkopf[3], Cathy Schubert[1], Dawn Bravata[1], and Teresa Damush[1], *1. Richard L Roudebush VA Medical Center, Indianapolis, Indiana, United States*, *2. National Institutes of Health, Bethesda, Maryland, United States*, *3. Department of Veterans Affairs (VA), Indianapolis, Indiana, United States*

The VA Geriatric Resources for Assessment and Care of Elders (GRACE) at Veterans Health Indiana is an interdisciplinary, home-based program providing comprehensive geriatric assessment and ongoing care management for community-dwelling older Veterans' experiencing geriatric syndromes. TeleGRACE is an extension of the GRACE program and delivers care via a hybrid, telehealth home visit. The TeleGRACE technician operates the audio-visual equipment in the patient's home while the clinical dyad (GRACE nurse practitioner and social worker) conducts the assessment. The specific aim of this study was to evaluate patient acceptability of TeleGRACE. We conducted a mixed methods study which included Likert-scale ratings and open-ended interviews. Participants used a 5-point scale to indicate agreement (5) to disagreement (1) regarding the acceptability of program elements ratings including ease with technician and virtual dyads; access; and clinical telepresence. The sample included 12 Veterans (mean [M] age, 78.7 years; standard deviation [SD] 7.5) who completed both an initial and follow-up TeleGRACE visit. Veterans reported high agreement of TeleGRACE acceptability (M=4.9, SD=0.3). They agreed: TeleGRACE hybrid visits helped them get care that they could not access otherwise (M=4.7, SD=0.65), they were as well taken care of by TeleGRACE virtual clinicians as those met at in-person visits (M=4.8, SD=0.4), they could hear (M=4.8, SD=0.6) and see (M=5.0, SD=0) the clinical dyads through the video visits, and they were able to ask direct questions of the TeleGRACE clinical staff (M=5.0, SD=0). These results indicated that TeleGRACE's key elements of a hybrid telehealth home visit were acceptable to older, community-dwelling Veterans.

Abstract citation ID: igad104.2313

### STRATEGIES USED TO HELP OLDER ADULTS ACCESS VIDEO VISITS DURING COVID-19: A SYSTEMATIC REVIEW

Landon Haller[1], Rena Rodriguez[2], Lindsay (Na Yeon) Park[2], and Kenneth Lam[3], *1. UCSF, San Francisco, California, United States*, *2. University of California, San Francisco, San Francisco, California, United States*, *3. Division of Geriatrics, San Francisco, California, United States*

Many articles have described how older adults struggled to access telemedicine during the COVID-19 pandemic, but few have described how that digital divide was bridged. To comprehensively describe strategies used to help older adults access telemedicine during the pandemic, we conducted a systematic review of 4 databases (PubMed, EMBASE, CINAHL, and PsycInfo) for English articles (search terms: "tele*", "mHealth", "eHealth", "video*", "65 and older") published from March 2020 to July 2022. We included articles that described any effort to help older persons (or a predominantly older population) access synchronous video visits during the pandemic, without requirements for study design. We categorized strategies as micro-, meso-, or macro-level per the Ecological Systems Theory framework to differentiate strategies implemented by clinicians, health systems, and policymakers. From 3799 records, we included 134 articles published in 2020 (23%), 2021 (51%), and 2022 (26%); most (57%) described strategies from the US. The most common specialties were Geriatrics (24%), Other (i.e. Occupational Therapy; 18%), and Medical Subspecialties (i.e. Cardiology, Oncology; 11%). Macro-level interventions included waivers for privacy regulations and increased reimbursement for telemedicine. Meso-level interventions included mobile vans offering devices and internet connection and protocols to test visits in advance. Micro-level interventions included written signs to troubleshoot sound and adapting clinical examinations virtually. We found many interventions at different levels of society to help older adults access video visits during the pandemic. Appraisals of the feasibility of telemedicine in older persons should consider the extent to which these strategies are used.

Abstract citation ID: igad104.2314

### SWEET DREAMS INTERRUPTED: HOW COVID-19 CHANGED THE LANDSCAPE OF SLEEP SERVICES UTILIZATION ON AGING POPULATION

Javad Razjouyan[1], Samuel T Kuna[2], and Amir Sharafkhaneh[1], *1. Baylor College of Medicine, Houston, Texas, United States*, *2. Corporal Michael J. Crescenz VA Medical Center, Philadelphia, Pennsylvania, United States*

We evaluated the impact of the pandemic on sleep test utilization, i.e., home sleep apnea testing (HSAT) and in-laboratory polysomnography (PSG), among aging veterans. We conducted an observational before and during the pandemic study using the national VHA. Electronic medical records from 01/2019 to 09/2022 were gathered for all veterans born with age 50+ by 10/2022. HSAT and PSG utilization were defined based on Current Procedural Terminology codes. Following intervals defined as pre-pandemic (PRE-P, 01/2019-02/2021, 14months), Pandemic-moratorium (PMor, 4/2020-6/2020, 3months), pandemic-pre-vaccination opening (PnoVax, 7/2020-12/2020, 6months), pandemic vaccination (P-Vax, 1/2021-5/2021, 5months), and pandemic post vaccination (P-PVax, 06/2021-09/2022, 15months). We compared the means using univariant analysis. Over the 45 months, 247,817 HSAT and 123,456 PSG were performed. In HSAT, compared to PRE-P (5,669visit/month) as a reference, we observed a significant drop in P-Mor (-70%,P< 0.001), P-noVax (-7%,P=0.166), P-Vax (+9%,P=0.067). However, P-PVax did not differ significantly in average monthly HSAT compared to PRE-P (+6%,P=0.103). In PSG, compared to PRE-P (3,994visit/month) as a reference, we observed a significant drop in P-Mor (-92%,P< 0.001), P-noVax (-61%,P< 0.001), P-Vax (-44%,P< 0.001), and P-PVax (-32%,P< 0.001). PSG per month did not recover to pre-pandemic levels, while the HSAT per month increased, but not to a level that compensated for the reduction in PSG. The pandemic markedly impacted the sleep testing service usage in aging veterans. While HSAT was resilient and increased during the pandemic, the increase did not offset the shift away from PSG. Future research is needed to better understand the reasons for this change in clinical practice among aging populations.

Abstract citation ID: igad104.2315

### TELEMEDICINE CONSULTATIONS IN SWEDEN 2020–2022: EXPLORING AGE DIFFERENCES

Felicia Gabrielsson-Järhult[1], and Ingemar Kåreholt[2], *1. Jönköping University, Jönköping, Jonkopings Lan, Sweden*, *2. Jönköping University, School of Health and Welfare, Jönköping, Jonkopings Lan, Sweden*

Health care in Sweden is heavily subsidized and costs for the user are small, both for physical consultation and telemedicine, and for public and private health care. It has been argued that telemedicine consultations will increase care consumption, lead to higher costs, and create inequalities. As older people often are less skilled in digital technology, increased digitalization of care might increase inequalities. Telehealth is mainly conducted by private companies. This study is based on all private telemedicine consultations in two regions in southern Sweden 2020-2022, with approx. 656,000 inhabitants. 106,000 persons had 343,000 telemedicine consultations, 3.2 consultations p/p. The number of consultations differed neglectable between age groups, for women median was 2.0 for the age groups (< 65/65-79/80+), and for men it was 1.0. The proportion of people WITH telemedicine consultations differed heavily between the age group as well as months. Total number of telemedicine consultations per month varied 4-8.5 thousand among women, and 2-4 thousand among men. The months with most consultations were March-June 2021. Proportion of telehealth consultations was low in the age group 65-79, and even lower among 80+. The proportion of consultations by people 65-79 years increased steadily and reached its peak July-September 2021 with >20%. After that, it increased rapidly down to < 3% among women and < 4% among men. The pattern was similar among people 80+ with peaks at approx. 2% among women and close to 2% among men. The conclusion is that there is still a huge age difference in the use of telehealth consultations.

Abstract citation ID: igad104.2316

### VIRTUAL COMPREHENSIVE GERIATRIC ASSESSMENT FOR VETERANS: AN INTERDISCIPLINARY QUALITY IMPROVEMENT PILOT PROJECT

Maria Cervantes[1], Janette Dunlap[2], Robynn Scott[2], Teresa Howell[3], Amy Neal[2], Heather Bieber[4], and Jacob Tillmann[5], *1. Veterans Health Administration, Saint Johns, Florida, United States*, *2. The North Florida/South Georgia Veterans Health System, Jacksonville, Florida, United States*, *3. North Florida/South Georgia Veterans Health System, Gainesville, Florida, United States*, *4. Department of Veterans Affairs, Lake City, Florida, United States*, *5. Department of Veterans Affairs, Gainesville, Florida, United States*

The Veterans Millennium Health Care and Benefits Act requires comprehensive geriatric assessments (CGA) to be provided to all older Veterans as part of the Veterans Medical Benefits Package. Access to a CGA may be difficult for Veterans with limited ability to travel. Currently, in the North Florida/South Georgia (NFSG) Veterans Health System, approximately 11,500 older Veterans are recognized to be at high risk for hospitalization or placement in a long-term care facility, yet only 0.09% of them have received a CGA. NFSG Geriatrics and Extended Care (GEC) developed a standardized virtual CGA process to increase access to geriatric specialty care utilizing VA Video Connect (VVC). The project design and implementation included: the development of a standard operating procedure using evidence-based screening tools, templates, clinic grids and flowcharts, communication channels, and the development of a dashboard for data collection. In addition, the VHA National CGA Template was piloted. This novel Tele-Geriatrics virtual interdisciplinary project allows older Veterans and/or caregivers to remain at their homes for all telehealth/virtual sessions completed by the CGA interdisciplinary team, including pharmacy, nursing, social work, occupational therapy, and geriatric providers. The project demonstrates increased access to comprehensive assessments, consequently closing the gap between current practice and the Veterans Millennium Health Care and Benefits Act requirements. The pilot also decreased the health disparities of rural Veterans. Furthermore, this project helped to increase Veteran knowledge of geriatric syndromes, VA resources, participation in telehealth, and access to durable medical equipment (DME) to help Veterans age in place.

Abstract citation ID: igad104.2317

### VIRTUAL TAI CHI FOR OLDER VETERANS IMPACTED BY COVID-19

Bonnie Dawson[1], Hallie Keller[2], Linda Sawyer[1], Shannon Gorman[3], Jerome Sabangan[4], Adam McPartlin[5], Karl Brown[6], and Dennis Sullivan[1], *1. Central Arkansas Veterans Healthcare System, North Little Rock, Arkansas, United States*, *2. Central Arkansas VA Healthcare System, North Little Rock, Arkansas, United States*, *3. VA Palo Alto Health Care System, Palo Alto, California, United States*, *4. VA Palo Alto Healthcare System, Palo Alto, California, United States*, *5. Veteran's Affairs Puget Sound, Seattle, Washington, United States*, *6. Puget Sound VA, Seattle, Washington, United States*

As older Veterans (OV) were at risk for loneliness and deconditioning during the COVID pandemic, they were invited to join a Tai Chi program due to its known benefits in these areas. The objectives of this presentation are to examine outcomes, program adherence and feedback of OV enrolled in a 12-week Tai Chi course conducted virtually twice weekly at three VA sites during the COVID-19 pandemic. Participants completed the 30-Second Chair Stand (30CST) and the De Jong Gierveld Loneliness Scale (DJGS) at baseline and again when they finished the course. Attendance was recorded and end-course evaluations were also obtained. Univariate and multivariate statistics were used to summarize results. One hundred eleven OV aged 60 to 94 enrolled. Most were non-Hispanic (84%), white (72%), males (80%), living independently (92%) with a partner (43%). Of these, 86 (77.5%) completed a final assessment after attending a mean of 18.7 out of 24 sessions. Retention did not differ by site nor other demographics. Among the completers, there was not a significant change in DJGS (n=46, p=0.2748), but only 26.1% showed substantive loneliness at baseline. The 30CST improved significantly (n=72, p=0.0150) after controlling for site and baseline value. Most participants described the virtual course as rewarding, beneficial to their health and balance, and conducive to social connection. They also expressed desire to continue the program. Our results suggest OV achieve objective and subjective benefits from, and can feasibly participate in, a virtual Tai Chi program, which appears to be a valid alternative to in-person courses.

### SESSION 7190 (POSTER)

### ATTITUDES ABOUT AGING AND AGEISM

Abstract citation ID: igad104.2318

### "IT'S NOT THE DESTINATION BUT THE JOURNEY": PERSPECTIVES AND ADVICE ON WISDOM

Nicole Lehpamer, *Mather Institute, Evanston, Illinois, United States*

While wisdom has been a common topic of contemplation for hundreds of years, a common theme in the wisdom literature relates to how wisdom is acquired through experiences. However, others have found that it can also be developed through individual factors, such as cognition, emotional intelligence, and personality, and cultivated through social interactions and self-reflection. Older adults have more experience than those of any other generation, giving them the time to develop the individual factors associated with wisdom and the skills to cultivate it. Given their unique social position, older adults are thus most qualified to share wisdom. In attempts to capitalize on the wisdom of older adults, the study uses survey data from adults aged 55 and better to examine how older adults characterize wisdom, it's association with age, and how it can be achieved. Data was collected using surveys administered through the Survey Monkey Platform and the Mather Institute Research Panel. Findings indicate that most older adults believe that wisdom increases with age. They further believe that wise individuals accept their limitations, are realists, have developed strong relationships with others, and seem in control of their lives. Older adults believe that wisdom can primarily be achieved by making mistakes, interacting with people who are different, experiencing adversity, and trying new things. Understanding the ways in which older adults perceive wisdom can be used to enhance intergenerational interactions that are mutually beneficial.

Abstract citation ID: igad104.2319

### AGE LOGICS IN SOCIAL WORK: THE CASE OF HARM REDUCTION FOR 50+ WITH LONG-TERM SUBSTANCE MISUSE

Håkan Jönson[1], and Tove Harnett[2], *1. Lunds Universitet, Lund, Skane Lan, Sweden*, *2. Lund University, Lund, Skane Lan, Sweden*

Age is a commonly used criterion in social work: for entry and exit and in decisions on what actions being appropriate for different clients. This study introduces the concept of age logics in social work and do so by investigating the use of age in wet eldercare facilities, a type of harm reduction arrangements for older people. These settings are for people over 50 years of age with long term substance misuse. No treatment is provided, and residents can continue to consume alcohol and other substances at the facilities for the rest of their lives. Using wet eldercare facilities as a case, the aim of this article is: 1) to introduce age logics as an analytical tool for critical studies on age in social work and 2) to propose a method for how to reveal ageism embedded in locally used age logics. The empirical data consists of interviews with eleven caseworkers, 12 staff-members and 31 residents at two Swedish wet eldercare facilities. The analysis identified four age logics linking chronological age with four meanings: 1) Logic on changeability, 2) Logic on lifestyle 3) Logic on function and 4) Logic on administrative fit. Jointly these logics constructed an ideal type version of the "older addict" that justified existing arrangements. The analysis also served as a basis for a proposal of a "four-question method" that may be used to reveal and challenge ageism hidden in locally used age logics.

Abstract citation ID: igad104.2320

**AGEISM IN SUB-SAHARAN AFRICA: PROFESSIONALS' PERSPECTIVES**

Dolapo Adeniji[1], Abraham Teshome[2], Gifty Ashirifi[2], and Margaret Adamek[2], *1. Indiana State University, Avon, Indiana, United States*, *2. Indiana University, Indianapolis, Indiana, United States*

While ageism is increasing across the globe, older adults in Sub-Saharan Africa are more vulnerable to ageism and are often denied a stable means of livelihood leading to poor health and a decreased quality of life. Scholars report that ageism can be experienced in multiple forms including economic, employment, and healthcare, with the intersectionality of gender and other factors complicating ageism. As little is known about ageism in Sub-Saharan Africa, this study explored the perspectives of scholars and professionals about the levels of ageism and age discrimination against older Africans. A cross-sectional study was conducted with scholars and professionals from sub-Saharan Africa. The 78 respondents ranged in age from 24-75 and over half (55.2%) were female. Using descriptive analysis, our findings show that the perceived level of discrimination against older adults is moderate with over half of the respondents (55.7%) indicating that ageism is getting worse. Respondents indicated that older adults are most discriminated against and face barriers in the areas of economic advantages (72.1%), employment opportunities (60.3%), healthcare access (49.4%), and housing (35.3%). Social workers, practitioners, and policymakers should directly address the problem of ageism to promote the healthy living of older adults in sub-Saharan Africa. To turn the tide of ageism and age discrimination in Sub-Saharan Africa, governments need to provide basic social protection to older adults so that they can have a stable means of support in their later years and will be less likely to be perceived as a burden to families and societies.

Abstract citation ID: igad104.2321

**AGEISM IN THE LABOR MARKET: LEARNING AND EDUCATION FOR OLDER ADULT JOB SEEKERS**

Bora Jin[1], and Lisa Baumgartner[2], *1. Winston Salem State University, Winston-Salem, North Carolina, United States*, *2. Texas State University, San Marcos, Texas, United States*

Despite older workers' value to an organization and no differences in job performance or productivity from younger workers (Rego et al., 2018), older job seekers encounter ageism in employment during their job search (Zaniboni et al., 2019). Ageism in employment and the workplace have detrimental effects on older job seekers, organizations, and society. Therefore, this conceptual paper explores the financial, emotional, psychological, and physical factors that should be considered when supporting older job seekers in navigating reeducation and learning to obtain employment. We collected and reviewed extant literature on this topic by consulting the following databases: EBSCOhost, PsycINFO, Google Scholar, and PubMed and using search terms such as "older adult," "workplace," "ageism," "employment." Ageist practices and stereotypes can occur at any stage of the employment process, including application, interview, hiring, training, job duties, benefits, downsizing, or termination. Older job seekers or employees may experience age discrimination in the workplace directly (e.g., if employers consider candidates cannot project the youthful image that the business desires, they may decline to offer the position) or indirectly (employers reject older applicants for a job because of their overqualification). We provide practical implications for older job seekers by exploring workplace learning strategies for older adults, including peer mentoring, intergenerational learning (Arthanat, 2019), proactive personality, and effective job search activity (Zacher, 2013). In addition, we discuss how older workers need to see the relevance of tasks to be individually motivated to learn and explore the need for personalized learning programs (Seo et al., 2019).

Abstract citation ID: igad104.2322

**DEPICTION OF OLDER ADULTS IN AN INTERNATIONAL PACIFIC RING STUDENT COMPETITION ON OLDER ADULT CARE**

Jonathan Guillemot[1], Raudah Mohd Yunus[2], Carlos Joaquin Rueda Ordoñez[1], Mellissa Withers[3], Meiqi Xin[4], Mary Rozelle[3], Ksenia Kubasova[5], and Noran Naqiah Hairi[6], *1. Universidad San Francisco de Quito, Quito, Pichincha, Ecuador*, *2. Universiti Teknologi MARA, Shah Alom, Selangor, Malaysia*, *3. University of Southern California, Los Angeles, California, United States*, *4. Hong Kong Polytechnic University, Hong Kong, Hong Kong*, *5. NA, NA, Moskva, Russia*, *6. Universiti Malaya, Kuala Lumpur, Kuala Lumpur, Malaysia*

The Global Health Program of the Association of Pacific Rim Universities (APRU), a consortium of universities in countries surrounding the Pacific Ocean, organized a competition in 2020 named "2020 Case Competition Challenge "Improving Elderly Care in the Asia-Pacific"". It yielded proposals from 28 teams from nine countries in Asia and the Americas in the form of 10-minute videos. Our study aimed to analyze the depiction of older adults to identify patterns of portrayal of older adults among university students. We coded the videos according to a two-step process, whereby we first filtered the videos to identify extracts related to the portrayal of older adults and subsequently coded the dataset according to a list of 10 codes, which were elaborated iteratively during the first step of coding. We analyzed the coded data and identified patterns, which served as a basis for analytical axes. Across included economies, we identified strong patterns of discriminatory discourse and depiction. Older adults were often assumed to be systematically sick, disconnected from the social and technological realities as well as incapable of being active contributors to society. Teams often proposed solutions medicalizing socialization, whereby social interactions were represented as clinical interventions void of meaning. In this unusual approach to studying portrayal of older adults among university students, we found concerning patterns of bias, therefore calling for immediate action towards tackling discrimination at university levels. Considering the pace of global aging, such intervention would contribute the essential view that demographic aging, as such, is not a problem.

Abstract citation ID: igad104.2323

### HOW ORGANIZATIONS CAN ADDRESS AGEISM AS PART OF THEIR DIVERSITY, EQUITY, AND INCLUSION INITIATIVES AND PROGRAMS

Sasha Perez, Noelle Marie Javier, Katherine Brown, Martine Sanon, Stephanie Chow, Allen Andrade, and Nathan Goldstein, *Icahn School of Medicine at Mount Sinai, New York City, New York, United States*

The Brookdale Department of Geriatrics and Palliative Medicine at Mount Sinai Health System created a diverse inter-disciplinary committee to advance and promote DEI activities centered in advocating for a socially just, diverse, equitable, inclusive, and anti-racist workplace. Research shows that anti-ageism is a largely neglected component of diversity, equity, and inclusion programs. Thus, many institutions are not learning how age biases and discrimination show up in the workplace and the intersection of age across other dimensions of diversity. Ageism is a vital workplace issue as over a third (37.3%) of the U.S. workforce are aged 50 and older. We aimed to address this by incorporating the topic of anti-ageism via our various DEI initiatives available to all faculty and staff: "Humanities, Arts, and Books Initiative" on Combatting Ageism - structured workday sessions designed to provide a safe virtual space for reflection, discussion, and learning. We also held "Learning Pathway series" on Reframing Aging as well as Caring for Older Adults with HIV – structured sessions focused on knowledge building, and expert-led discussion. Attendance ranged from 40 to 60 participants at each session. Quantitative and qualitative assessment revealed a steadfast interest to address ageism and bias; appreciation of the semi-structured format for dialogue and reflection on relevant topics and finally ongoing education to create a more informed and collegial department. This type of programming can potentially serve as an additional resource for other institutions that are planning in incorporating anti-ageism activities in their DEI programs and everyday practices.

Abstract citation ID: igad104.2324

### INTERGENERATIONAL CO-PRODUCTION IN DIGITAL DESIGN FOR HEALTH, WELL-BEING, AND SOCIAL CONNECTION

Catherine Hennessy[1], Greg Mannion[1], Richard Haynes[1], Anna Whittaker[1], Hannah Bradwell[2], Simone Tomaz[1], John Ritchie[3], and Leonie Cooper[2], *1. University of Stirling, Stirling, Scotland, United Kingdom, 2. University of Plymouth, Plymouth, England, United Kingdom, 3. Communications, Media and Culture, Stirling, Scotland, United Kingdom*

The benefits of intergenerational practice in bringing different generations together in initiatives and programs to address issues like social isolation and loneliness are well-established. Less frequently employed as a vehicle for using intergenerational contact in the development of interventions and design solutions, intergenerational co-production involving older and younger people with academics and designers, has rarely been tried. Thus, intergenerational co-production is not common and is under-theorised despite holding significant promise for advancing societies for all ages. This paper reports on the experience of intergenerational co-production in the GOALD (Generating Older Active Lives Digitally) project, an interdisciplinary collaboration in Scotland and England using intergenerational co-production methods in the creation of digital tools to support older adults' health, well-being and social connectivity. It presents GOALD's multi-year experience of constituting and running intergenerational co-production groups with geographically, socio-demographically and functionally diverse participants to provide feedback and design ideas to business partners for digital devices and applications for health promotion in later life. Mixed methods findings on sources, timing, locations and numbers of participants recruited and retained, their rates of participation, forms, frequency and modes of engagement, as well as organizational partners' and participants' motivations, incentives, capacity and constraints to take part demonstrate the barriers and facilitators of implementing intergenerational co-production, particularly during the Covid-19 pandemic. Our findings highlight lessons learned about the need for creative adaptation of this approach on the ground, and suggest GOALD's potential contributions to new theorisation through enacting and understanding the features of intergenerational co-production and documenting its effects.

Abstract citation ID: igad104.2325

### STRATEGIES TO MITIGATE MACHINE LEARNING BIAS AFFECTING OLDER ADULTS: RESULTS FROM A SCOPING REVIEW

Charlene Chu[1], Simon Donato-Woodger[1], Shehroz Khan[2], Kathleen Leslie[3], Tianyu Shi[1], Rune Nyrup[4], and Amanda Grenier[1], *1. University of Toronto, Toronto, Ontario, Canada, 2. Toronto Rehabilitation Institute, Toronto, Ontario, Canada, 3. Athabasca University, Athabasca, Alberta, Canada, 4. University of Cambridge, Cambridge, England, United Kingdom*

Digital ageism, defined as age-related bias in artificial intelligence (AI) and technological systems, has emerged as a significant concern for its potential impact on society, health, equity, and older people's well-being. This scoping review aims to identify mitigation strategies used in research studies to address age-related bias in machine learning literature. We conducted a scoping review following Arksey & O'Malley's methodology, and completed a comprehensive search strategy of five databases (Web of Science, CINAHL, EMBASE, IEEE Xplore, and ACM digital library). Articles were included if there was an AI application, age-related bias, and the use of a mitigation strategy. Efforts to mitigate digital ageism were sparse: our search generated 7595 articles, but only a limited number of them met the inclusion criteria. Upon screening, we identified only nine papers which attempted to mitigate digital ageism. Of these, eight involved computer vision models (facial, age prediction, brain age) while one predicted activity based on accelerometer and vital sign measurements. Three broad categories of approaches to mitigating bias in AI were identified: i) sample modification: creating a smaller, more balanced sample from the existing dataset; ii) data augmentation: modifying images to create more training data from the existing datasets without adding additional images; and iii) application of statistical or algorithmic techniques to reduce bias. Digital ageism is a newly-established topic of research, and can affect machine learning models through multiple pathways.

Our results advance research on digital ageism by presenting the challenges and possibilities for mitigating digital ageism in machine learning models.

### SESSION 7200 (POSTER)

### GLOBAL AGING (SRPP)

Abstract citation ID: igad104.2326

### DEFINING OLD AGE IN SUB-SAHARAN AFRICA: THE PERSPECTIVE OF AFRICAN SCHOLARS AND PROFESSIONALS.

Gifty Ashirifi[1], Abraham Teshome[1], Margaret Adamek[1], and Dolapo Adeniji[2], *1. Indiana University, Indianapolis, Indiana, United States*, *2. Indiana State University, Avon, Indiana, United States*

The older adult population in Africa is projected to experience the fastest growth rate among regions in the world. Although in most Global North nations, 60-65 is considered old, the issue of what age should be accepted as “old” in Sub-Saharan Africa has remained a controversial issue with governments and scholars. The imminent increase in aging in Africa is a call for action since age definition is directly linked to access to programs and policies necessary to ensure older adults’ well-being. Through an online survey, this study investigated the perspectives of Gerontology and Social Work professionals from Sub-Saharan Africa (n=78, 55% female, age range 24-75) on what age should be accepted as “old” or “elderly” for policy development and research purposes. A descriptive analysis indicated that the most endorsed old age marker in Sub-Saharan Africa was 50 years old (33.3%) ---a full decade lower than the United Nation’s designation of old age. The primary factors respondents indicated should be considered when defining old age in Sub-Saharan Africa are health status (73.6%), retirement age (61.1%), and quality of life (56.9%). Age 60 was identified as the most appropriate age to determine eligibility for aging programs (45.1%) and for conducting research on later life (43.5%). We recommend that age guidelines used in Global South regions such as Sub-Saharan Africa should not necessarily mirror the guidelines for age used in the Global North but should fit the context of aging in those regions.

Abstract citation ID: igad104.2327

### IS MIDDLE-AGED INEQUITY LIKELY TO PERSIST INTO OLD AGE?: TRAJECTORIES OF SOCIAL EXCLUSION IN KOREA

Jae Yeon Jung, and Seok In Nam, *Yonsei University, Seoul, Republic of Korea*

Korea has the highest rate of older adult poverty among the OECD countries. The poverty of the older adult continues even with the government's policies, as the loss of social roles, the reduction of the network, and the difficulty of escaping exclusion are complex. The goal is to find out how the trajectory of social exclusion changes as people get older. We used the data of the Korean Longitudinal Study of Ageing conducted by the Korea Employment Information Service. The target population is middle-aged people aged 45 to 52 (based on the first year), mainly the baby boom generation in Korea, and the total number of cases included in the analysis is 1,348. The trajectory of social exclusion change for 14 years from 2006 to 2020 was confirmed by composing on into economics, health, employment, housing, and social relationships. The group-based multi-trajectory model analysis was performed using Stata 16.1. The groups were identified as four: the low exclusion continuous maintenance group, the employment high exclusion maintenance group, the economic/housing high exclusion maintenance group, and the multi-domain high exclusion continuous maintenance group. All four types showed a tendency to maintain social exclusion from middle age until old age. Factors that caused differences between groups were gender, residential area, marital status, education, and income. These findings suggest that as people get older, the cumulative inequity in their lives becomes more pronounced. As a result, it will provide important clues for establishing policies for the welfare of older adults and alleviating social inequality.

Abstract citation ID: igad104.2328

### LIVED EXPERIENCES OF OLDER FEMALE SEX WORKERS AT SONAGACHI, KOLKATA: A CASE STUDY

Esha Chakravarty[1], and Indrani Chakravarty[2], *1. Calcutta Metropolitan Institute of Gerontology, Mitcham, England, United Kingdom*, *2. Calcutta Metropolitan Institute of Gerontology, Kolkata, West Bengal, India*

Older sex workers are marginalised because of their former professional engagement and old age. This study captured the experiences of older female sex workers (FSWs), aged sixty and above, who delivered sexual services at Sonagachi, Asia’s largest pleasure market. Exploratory research design was used to understand their lived experiences through a qualitative intervention. The study was content driven, not hypothesis driven. Six older FSWs were selected through purposive sampling, primary data was collected through case study method. Their experiences were recorded starting from childhood, entry into sex trade, daily professional challenges and experiences during old age. Data collected was thematically analysed. Older Sonagachi FSWs are a heterogeneous group in terms of socio-demographic variables, health condition, place and nature of work, average load of clients they entertained per day. This study found that economic adversities and poverty during growing years have been the foremost reasons for choosing sex trade. Many of them remained in the sex industry because of lack of alternative professional opportunities. They experienced various forms of community perpetrated exploitation and some had acquired sexually transmitted infections. Other reasons for them to be in this trade were childhood poverty, early marriage, early motherhood, lack of education, lack of employment, neglect and abuse. They were destitute, had no caregivers, struggled to be part of mainstream society. The study suggests the need to develop policies for vocationally training them to generate alternative income sources and to initiate welfare schemes including pension plans and developing separate old age homes for older FSWs.

Abstract citation ID: igad104.2329

### POPULATION AGING IN INDIA: A MICROLEVEL ESTIMATE USING GRIDDED POPULATION DATA

Nawaj Sarif, and Sruthi Anilkumar Hemalatha, *International Institute for Population Sciences, Mumbai, Maharashtra, India*

Population aging is a significant demographic trend that is transforming societies worldwide. Aging populations pose significant challenges to governments and societies, particularly in developing countries such as India, where a large segment is still impoverished and lacks access to adequate health care and social security systems. A lack of accurate data on older populations has been a significant impediment to developing effective policies to address the needs of this vulnerable group. To address this gap, this study tried to examine the micro-level trends and patterns of population aging in India using gridded population data. The study also attempted to estimate the share of the older population in India for the years 2030 and 2040. The study found that India has undergone a dramatic shift in population aging trends, with large intra-state variability. The share of older populations is predicted to rise considerably over the next two decades, highlighting the urgent need for effective policies to address their needs. The study also revealed that population aging patterns differ across India, suggesting that national and state-level measures alone will not suffice to address the needs of older people. Instead, policies directed at smaller units, such as districts and neighborhoods, are critical to ensuring the well-being of older populations. Furthermore, the study highlights the need for accurate and timely data on older populations in India. Such data can enhance policy decisions and help identify areas of need, enabling governments to develop targeted policies that can improve the well-being of older people.

Abstract citation ID: igad104.2330

### WHO ARE THE PORTUGUESE WHO REACH 100 YEARS OF AGE? A DEMOGRAPHIC ANALYSIS USING 2021 CENSUS DATA

Oscar Ribeiro[1], Lia Araujo[2], and Laetitia Teixeira[3], *1. University of Aveiro, Aveiro, Aveiro, Portugal*, *2. Cintesis, Porto, Porto, Portugal*, *3. School of Medicine and Biomedical Sciences, University of Porto, Porto, Porto, Portugal*

Portugal is considered one of the oldest countries of Europe as a conjugation of low birth rates and high life expectancy. The age group of 100+ is one of the fastest growing, with 270 centenarians in 1990 to 1961 centenarians in 2020 (Teixeira et al., 2020). In this study we present the profile of centenarians based on real data of the last Portuguese Census 2021. There are 2801 centenarians, showing a significant increase in relation to the previous censuses (589 centenarians in 2001, and 1526 centenarians in 2011). Most centenarians are women (82.0%), widowed (84.6%), attended school (61.0%), have social pension as main income source (96.3%), are catholic (93.9%) and were born in Portugal (98.6%). The average number of reported difficulties in vision, hearing, mobility, self-care, memory/concentration, and communication is of 5.12, meaning that the current cohort of centenarians face high constrains in their daily living and most probably depend on any sort of care support. Overall, Portugal follows the longevity phenomenon of a growing number of centenarians as verified in USA, Japan, and other European countries (United Nations, 2022). Profiling centenarians' main characteristics with reliable data is a starting point for outlining the specific social, medical, and financial needs of those reaching very advanced ages, and for defining government policies and age-sensitive interventions.

### SESSION 7210 (POSTER)

### HEALTH CARE

Abstract citation ID: igad104.2331

### ANALYSIS OF REPORTING GUIDELINE AND TRIAL REGISTRATION POLICIES AMONG GERIATRIC JOURNALS: A CROSS-SECTIONAL STUDY

Shaelyn Ward[1], Logan Corwin[2], Jacob Duncan[3], Caleb Smith[3], Danya Brewer[3], Griffin Hughes[2], and Matt Vassar[2], *1. Oklahoma State College of Osteopathic Medicine at the Cherokee Nation, Tahlequah, Oklahoma, United States*, *2. Oklahoma Center for Health Sciences, Tulsa, Oklahoma, United States*, *3. Oklahoma State College of Osteopathic Medicine at the Cherokee Nation, Tulsa, Oklahoma, United States*

The use of reporting guidelines and clinical trial registration policies by academic journals reduces bias and improves transparency in clinical research. It is unknown whether geriatric and gerontology journals mention, recommend, or require their use for the studies they may potentially publish. The purpose of this study is to assess the submission guidelines of the top geriatric and gerontology journals for their editorial recommendation or requirement of predetermined reporting guidelines and clinical trial registration. We identified the top 100 journals in the "Geriatrics and Gerontology" subcategory using the 2021 Scopus CiteScore tool. We probed each journal's "Instructions to Authors" for statements regarding reporting guidelines for popular study designs and extracted them as "Not Mentioned", "Recommended", "Does Not Require", or "Required". Further, we classified each journal's reference to clinical trial registration in a similar manner. Of the 100 examined journals included in our analysis, the QUOROM statement was not mentioned by any journals, whereas the CONSORT statement (44/100, 44%) was mentioned and recommended/required the most often. PRISMA guidelines were not mentioned by 57 journals (57/100,57%). Furthermore, 45 journals (45/100,45%) did not mention study registration. Therefore, the recommendation or requirement of reporting guidelines and clinical trial registration in the top 100 geriatric and gerontology journals is inconsistent. Journal editors should strongly recommend that author's follow validated reporting guidelines to reduce potential bias and improve transparency in the articles they publish.

Abstract citation ID: igad104.2332

### APPLICATION OF A NEW TOOL FOR PLANNING IMPLEMENTATION EVALUATIONS IN CLINICAL TRIALS

Whitney Mills[1], Kali Thomas[2], Yuan Huang[3], Michael Wininger[3], and Scott Hummel[4], *1. VA Providence Healthcare System, Providence, Rhode Island, United States*, *2. Johns Hopkins University, Baltimore, Maryland, United States*, *3. VA CT Healthcare System, West Haven,*

*Connecticut, United States*, *4. VA Ann Arbor Healthcare System, Ann Arbor, Michigan, United States*

The VA Cooperative Studies Program (CSP) study "Geriatric OUt-of-hospital Randomized MEal Trial in heart failure – Veterans Affairs" (GOURMET-VA) is a randomized, single-blind, multi-center, clinical trial investigating the effects of home-delivered meals in Veterans discharged from hospitalization for heart failure. GOURMET-VA is one of the first CSP studies to integrate implementation scientists during protocol planning. We describe how the three phases of the new Implementation Planning Assessment (IPA) Tool was used proactively to guide planning of the implementation evaluation for the trial, oriented towards future adoption and sustainment of the intervention. Phase 1 ("Planning, Framing, & Aligning Interested Parties"): During early protocol development, the implementation team helped identify and facilitated meetings with relevant VA national leadership. These meetings resulted in plans for engagement throughout the trial and input on trial design. Phase 2 ("Implementation Process Data Collection"): Guided by IPA, the implementation evaluation uses mixed methods to understand the context into which the intervention is being implemented, experiences of Veterans and clinicians with the intervention, and barriers and facilitators to implementation at the patient, healthcare provider, and leadership levels. Phase 3 ("Planning for Sustainment of Effective Trials"): A preliminary dissemination and sustainability plan was drafted, including how the implementation evaluation data may be used to inform adaptations of the GOURMET-VA intervention to improve effectiveness and uptake. We found that the structured guides that comprise the IPA were valuable in helping the implementation scientists and the trial team to find common ground in terms of rationale, methods, and language.

Abstract citation ID: igad104.2333

### CONSIDERING HOSPITAL CLOSURES IN ALABAMA: NATIONAL POLICY IMPLICATIONS

Avani Shah[1], Joseph Swain[2], and Hailey Lewy[1], *1. University of Alabama, Tuscaloosa, Alabama, United States*, *2. Arkansas Tech University, Russellville, Arkansas, United Sta tes*

Hospital closures have increased over the past 10 years nationally, particularly in rural areas and states that did not receive Medicaid expansion funds (American Hospital Association). These closures can impact older adults health care, employment opportunities, and mortality rates. Recently, the Alabama Hospital Association reported that 12 hospitals are at risk of closing. For this study, we evaluated the locations of hospital closures since 2009 in Alabama to understand gaps geographically in healthcare. We obtained information about current hospitals through Alabama Department of Public Health (ADPH). We searched for news articles to obtain a list of hospitals, which closed between 2009 and 2020. Next, we obtained data from the ADPH on the mortality rates by county and year. We also obtained information about the finances of hospitals in Alabama and classified hospitals with 20% losses as potentially at risk. Using GIS, we mapped areas of hospital closures and at risk hospitals as well as the location of current hospitals as of 2020. We chose to average mortality rates from 2009 to 2019 (pre-COVID) and map these by county. Since 2009, Alabama has closed 13 hospitals: with two reopening at a later date. Based on GIS, we found potential health care gaps in several rural areas and in Southwest Alabama, or the Black Belt area, which has a high rate of poverty, and poor health outcomes. The mean mortality rate for rural counties was significantly higher than urban populations between 2009-2019 $t(54)=4.8$, $p<.0001$. Policy implications will be discussed.

Abstract citation ID: igad104.2334

### ENGAGING OLDER ADULTS AND CAREGIVERS AS STAKEHOLDERS IN THE ARTIFICIAL INTELLIGENCE AND TECHNOLOGY COLLABORATORY

Paavani Jain, Nancy Schoenborn, and Thomas Cudjoe, *Johns Hopkins University School of Medicine, Baltimore, Maryland, United States*

Artificial Intelligence (AI) holds the unique potential to capture older adult functional and behavioral diversity and improve geriatric care. Yet a gap exists between new technologies and the real-world needs of their target populations. Currently in the United States, three Collaboratories support novel AI projects in Aging Research. As the Stakeholder Engagement Core of the Johns Hopkins AI Collaboratory, in this poster, we share early lessons of forming a 'Council' of stakeholders – a group with the charge of informing pilot AI projects longitudinally. The Council was envisioned to include 10-15 persons: older adults, caregivers, and clinicians. We adopted different outreach techniques for clinicians versus non-clinicians; leveraged existing relationships with affiliated Memory and Social Clinics; individually approached community dwelling older adults and caregivers including homebound older adults and older adults with disabilities; and reached out to clinicians and advocates known to be committed to geriatric care. We developed plain language materials and explained the meaning of 'AI' and 'Stakeholder Engagement'. Five out of 5 outreached clinicians, and 10 out of 20 older adults/caregivers agreed to serve on the Council. Many older adults on our Council had difficulty using their email and computer and required support. Several caregivers declined due to high caregiving demands and many older adults with multiple chronic conditions declined due to high personal needs; all reiterated their interest in the topic area despite declining participation. Future novel-technology projects should ensure inclusion of diverse stakeholders; incorporating a flexible plan of engaging high-needs older adults and caregivers is required.

Abstract citation ID: igad104.2335

### NURSING HOMES' FIVE-STAR QUALITY RATING SYSTEM: FORECASTING THE FUTURE STABILITY AND VOLATILITY

Shivani Gupta[1], Ashay Kadam[2], and Neeraj Bhandari[3], *1. University of Houston - Clear Lake, Houston, Texas, United States*, *2. Goa Institute of Management, Sattari, Goa, India*, *3. University of Nevada, Las Vegas, Las Vegas, Nevada, United States*

The utilization of publicly reported quality measures such as nursing homes' five-star quality rating system to hold providers accountable for their quality of care has increased over the years. Therefore, nursing homes have invested significant resources into maintaining or improving the ratings awarded to them by CMS. This study utilizes statistical models to analyze the past rating histories to predict future evolution of ratings of nursing homes. Data on all U.S. nursing homes for the years 2017 - 2021 was derived from CMS Nursing Home Compare. Primary variable represents the overall five-star quality rating of nursing homes. A Markov chain model and a Mover Stayer model are used to predict changes in ratings of nursing homes. The one-year rating transition probabilities are estimated using maximum likelihood estimation. The key findings indicate the likelihoods of future upgrades and downgrades in nursing homes' ratings. Nursing home administrators are under constant pressure to strategically allocate resources for maintaining or improving the five-star ratings awarded to them by CMS. The findings of this study could help nursing home administrators in forecasting future change in their rating and making better resource allocation decisions such as those related to preventing a rating downgrade. The study findings could also be used by policymakers to focus on nursing homes facing downgrades and assisting them in improving their quality of service based on the potential change in their rating. The same results could also be utilized to assess the stability of ratings given to nursing homes.

Abstract citation ID: igad104.2336

### REPEAT AND SERIAL EMERGENCY DEPARTMENT (ED) OLDER ADULT USERS—A STATE-LEVEL ANALYSIS USING THE NYU-JHU ALGORITHM

Uma Kelekar[1], Debasree Das Gupta[2], Amel Ben Abdesslem[3], Diep Tran[4], Jewel Shepherd[5], and Sidney Turner[6], *1. Marymount University, Arlington, Virginia, United States*, *2. Utah State University, Logan, Utah, United States*, *3. Marymount University, Arglington, Virginia, United States*, *4. Johns Hopkins University School of Medicine, Baltimore, Maryland, United States*, *5. University of South Dakota, Vermillion, South Dakota, United States*, *6. Fors Marsh Group LLC, Falls Church, Virginia, United States*

Older adults account for 12-24% of all Emergency Department (ED) visits and studies have shown a 25-35% increase in older adult ED visits over time. While some studies have analyzed ED encounters over a single healthcare system, a year or a specific condition, research comparing the long-term frequent use of the ED across cohorts of older adults in the United States is limited. We address this gap by identifying characteristics associated with repeat (>1 visit during the time period) and serial/frequent (>=4/year) ED utilization for Maryland. Using the 2017-2019 State Emergency Department Databases (SEDD), we extracted ED visits among the elderly (>=60 years) population. By applying the modified New York University Algorithm by John Hopkins University (NYU-JHU-EDA), we classified ED visits into 11 categories, and we conducted bivariate and multivariate analyses to identify inter-age variations in the type of visits to the ED across single, repeat and serial users, after controlling for covariates. Of the total 5,331,843 ED visits between 2017 and 2019, 1,244,878 (~23%) were older adult visits. Relative to the 60–69-year-olds, those 80 and above had higher odds of being repeat users for emergent issues [OR=1.34, CI:1.07-1.67], primary-care treatable condition [OR=1.29, CI:1.03-1.62], and severe [1.30, CI: 1.03-1.64] and non-severe injuries [1.61, CI:1.28-2.01]. Relative to 60-69 year olds non-serial users, the oldest cohort demonstrated lower odds for psychiatric/alcohol and drug-related diagnoses [OR=0.63, CI:0.42, 0.95]. Our findings highlight the need to design age-group specific interventions to reduce the frequent ED utilization among the elderly in Maryland.

### SESSION 7220 (POSTER)

### MINORITY AND DIVERSE POPULATIONS (POSTERS)

Abstract citation ID: igad104.2337

### CHALLENGES AND OPPORTUNITIES IN RECRUITING DIVERSE OLDER ADULTS WHO ARE FRAIL FROM THE MAPS-B STUDY

Suleman Tariq[1], Alexa Kouroukis[1], Courtney Kennedy[1], Jonathan Adachi[1], Carolyn Leckie[2], Alexandra Papaioannou[1], and Isabel Rodrigues[1], *1. McMaster University, Hamilton, Ontario, Canada*, *2. St. Joseph's Healthcare Hamilton, Hamilton, Ontario, Canada*

Including diverse individuals at the research and participant levels are essential to improve the effectiveness of real-world interventions; however, there are challenges when including such individuals. Our study purpose was to report the challenges of recruiting diverse older adults for the Mapping Sedentary Behaviour study. Our methods were guided by Step 1 ("Establish Partnerships") in the Knowledge-to-Action-Ethics Framework. We assembled a diverse team of eleven researchers, clinicians, and patient partners. To recruit a broad group of participants, we partnered with City Housing Hamilton, which provides subsidized housing for older adults. We met with the organization's partnership development advisor who organized two recruitment orientations; 80 potential participants and returning attendees were present for both sessions. The organization provided coffee and donuts. Most attendees were from visible minorities and had visible disabilities (i.e., used a walker or cane). To build rapport, we met with attendees in groups of 5 to 6 to introduce the research team and explain the study. We recruited 13 participants (seven female, one transgender man; Morley FRAIL score≥3). Before their scheduled study visit, twelve participants dropped out citing medical mistrust (i.e., fearing unintentional medical tracking). The last participant dropped out after the initial study visit due to their family's skepticism in research. Additionally, some individuals may have enrolled for financial incentives as they were interested in receiving immediate monetary compensation. We faced challenges when recruiting frail older adults from diverse backgrounds. Future studies should focus on developing methods to target medical mistrust with older adults and their families.

Abstract citation ID: igad104.2338

### HAS YOUR SMILE BEEN CHECKED?: CHARACTERISTICS OF DENTAL CARE UTILIZATION OF MEDICARE BENEFICIARIES

Lillian Agyemang[1], and Louanne Bakk[2], *1. University at Buffalo School of Social Work, Buffalo, New York, United States*, *2. University at Buffalo, Buffalo, New York, United States*

**Background:** An area under researched is oral health disparities in the population of older adults residing in the United States. The current dental benefits offered through health insurance plans that older adults are likely to utilize, particularly Medicare and Medicaid, are inadequate. The deficiency harms this population's physical and mental health and well-being. Additionally, insufficient oral health care has adverse outcomes, including tooth loss, poor self-esteem, malnutrition, and cardiovascular complications. While evidence suggests race and ethnicity can be predisposing factors impacting access to dental care, it is unclear the extent to which disparities in utilization exist. **Methods:** This exploratory, cross-sectional study examined the relationship between dental care utilization and Medicare beneficiary characteristics. Data from the 2018 wave of the Health and Retirement Study were used. The sample consisted of 8,357 respondents aged 65 and older enrolled in Medicare. **Results:** There was an association between dental care utilization and race, ethnicity, health status, education, and age. A much lower proportion of Black and Hispanic older adults reported seeing a dentist in two years than non-Hispanic White beneficiaries (p<.001). Dental care utilization was also less among respondents who were older (p<.01), in poorer health (p<.001), and had less education (p<.001). **Conclusions and Implications:** Findings suggest disparities in dental care utilization exist and disproportionally impact vulnerable Medicare beneficiaries. This is concerning, particularly given racial and ethnic differences in health. The conclusions accentuate areas such as access to dental care services, coverage, and policy implementation concerning and impacting older adults.

Abstract citation ID: igad104.2339

### MENOPAUSAL SYMPTOM EXPERIENCE OF MID-LIFE WOMEN FAMILY CAREGIVERS OF PERSONS LIVING WITH ALZHEIMER'S DISEASE

Hyein Kim[1], Heejung Kim[1], Glenna Brewster[2], Wonshik Chee[2], and Eun-Ok Im[2], *1. Yonsei University, Seoul, Republic of Korea*, *2. Emory University, Atlanta, Georgia, United States*

Midlife women experience a wide range of menopausal symptoms. Such symptoms may be different in midlife women who are family caregivers of persons living with chronic disease, particularly Alzheimer's disease. Because the cultural stigma of dementia differs among racial/ethnic minority groups, midlife minority women tend to feel burdened with family caregiving and report negative health outcomes compared with White women. This study aimed to explore the racial/ethnic differences in menopausal symptom experienced by midlife women who were family caregivers of persons living with Alzheimer's disease (MWPLAD). A secondary data analysis was conducted using the data of a cross-sectional online survey among 172 MWPLAD. Self-report questionnaires including the Midlife Women's Symptom Index were used to gather the data on the women's sociodemographic characteristics, health-related information, and menopausal symptom experience. Descriptive statistics, one-way analysis of variance and multiple linear regression were adopted for the data analysis. There were significant racial/ethnic differences in midlife women's symptoms in the total scores (total frequencies and severity scores) and individual sub-scale scores. Psychological symptoms were reported to be more prevalent in Asian MWPLAD than in other racial/ethnic groups. After controlling for race/ethnicity, several factors were associated with the menopausal symptoms experienced by MWPLAD, such as household income, history of obstetrics and gynecology diseases, underweight, and restriction to perform daily activities. Our study findings support the racial/ethnic differences in menopausal symptoms experienced by MWPLAD. More culturally competent interventions should be established considering the significant factors that differently affect the women's menopausal symptom experience in different racial/ethnic groups.

Abstract citation ID: igad104.2340

### SOCIOCULTURAL BARRIERS TO EQUITY IN SOCIAL SUPPORT FOR DEMENTIA CAREGIVING IN MINORITY WOMEN

Heejung Kim[1], Hyein Kim[1], Glenna Brewster[2], Wonshik Chee[2], and Eun-Ok Im[2], *1. Yonsei University, Seoul, Republic of Korea*, *2. Emory University, Atlanta, Georgia, United States*

Racial and ethnic minority is growing in dementia caregiving population. This study aimed to identify racial/ethnic differences in social support and sociocultural factors related to caregiving among minority midlife women who were family caregivers of persons living with Alzheimer's disease (MWPLAD). A secondary data analysis was conducted using the data from a cross-sectional online survey of 172 MWPLADs. Self-report questionnaires were used to collect the data on the caregivers' sociodemographic and caregiving-related characteristics, social support for caregiving, and sociocultural factors for caregiving. Descriptive statistics, one-way analysis of variance, and hierarchical linear regression were adopted for the data analysis. Non-Hispanic Asian caregivers reported lower social support and greater perceived barrier during dementia caregiving than non-Hispanic African-American and Hispanic caregivers (p ≦ .001). Furthermore, non-Hispanic Asian caregivers reported a higher level of discrimination during caregiving, a lower level of cultural justification for reciprocity, and a lower level of cultural justification for duty than non-Hispanic African-American caregivers (p < .001). After controlling for race/ethnicity and sociodemographic factors, those who received more social support were younger, experienced a lower level of discrimination, and had a higher level of cultural justification for caregiving duties (p < .05). Our study findings support that racial/ethnic differences exist in caregivers' social support. When enhancing social support among racial/ethnic minority caregivers for dementia, it is necessary to develop specific strategies for encouraging each race/ethnicity group in further large-scale studies and clinical practice in the future.

Abstract citation ID: igad104.2341

### THE EFFECT OF RESILIENCE ON USING COMMUNITY ORGANIZATIONS AMONG KOREAN AMERICAN OLDER ADULTS DURING COVID-19

Eunhye Kim[1], Hyesu Yeo[2], and Y.Joon Choi[3], *1. Augusta University, Evans, Georgia, United States*, *2. University of Georgia, Athens, Georgia, United States*, *3. Georgia State University, Atlanta, Georgia, United States*

**Objectives:** This study aimed to examine how resilience and perceived racial discrimination are associated with receiving support from ethnic community organizations (ECO) among Korean American older adults during COVID-19. **Background:** With the vulnerability of Korean American older adults and increasing racial discrimination, ethnic community organizations' roles are important during COVID-19. Although previous studies have proven the association of resilience and racial discrimination with older adults' well-being, there is little research on how those factors impact using ECO. **Methods:** A cross-sectional survey was conducted with 132 first-generation Korean American adults aged 50 or older. A logistic regression analysis was conducted to determine which factors, including resilience, discrimination, age, gender, marriage, health, residence years, Language, and income, within the model made them more likely to receive support from ECO. **Results:** Results showed that a high level of resilience was negatively associated with the use of ECO[OR 0.93, CI 0.87, 0.99]. Korean American older adults who were female [OR 5.60, CI 1.44, 21.50] and lower income [OR 4.42, CI 1.35, 14.48] were more likely to receive support. Those who were healthy [OR 2.15, CI 1.08, 4.26] and had lived longer in the U.S.[OR 1.07, CI 1.00, 1.14] were more likely to use ECO. **Conclusion:** Findings from the study highlight the importance of improving and expanding support for vulnerable ethnic minority older adults in order for them to access services and develop effective coping strategies during the health crisis.

### SESSION 7230 (POSTER)

### SOCIAL DETERMINANTS OF HEALTH, EQUITY, AND AGING

Abstract citation ID: igad104.2342

### A GREEN ACTIVITY PRESCRIPTION PROGRAM FOR CHINESE AMERICANS LIVING WITH DEMENTIA

Hayley Belli[1], Rebecca Lassell[1], Jun Luo[1], Lorna Thorpe[1], Jennifer Wong[2], Stella Yi[2], and Abraham Brody[3], *1. New York University, New York City, New York, United States, 2. New York University School of Medicine, New York City, New York, United States, 3. NYU Rory Meyers College of Nursing, New York City, New York, United States*

Asian Americans account for approximately 161,000 cases of Alzheimer's disease and related dementias (AD/ADRD) among adults ages 65 and older in the United States. Chinese Americans have among the highest incidences of AD/ADRD, and have experienced underdiagnosis, cultural stigmas, and less access to dementia care. In New York City specifically, 28.6% of Chinese American seniors live in poverty, leading to environmental racism, a lower likelihood of engagement in activities, decreased quality of life, and an increased risk of cognitive decline and developing dementia. Recommendations to support activity engagement among Chinese Americans include fostering social cohesion and linking individuals to local activity resources. Green activity prescriptions (GAPs) are one approach to achieving these objectives, and combine nature, social contexts, and meaningful activities to improve health and well-being. GAPs are co-designed with participants based on their interests, needs, and available community resources. Examples include: animal-assisted interventions, gardening, green exercise (walking), and conservation projects (composting). Past studies have provided evidence that these components have improved well-being for PLWD, supported function, and provided meaningful engagement. Yet, these early studies neglected Chinese Americans. GAPs have potential to support the health and well-being of Chinese American PLWD by addressing health inequalities and providing accessible, low-cost care, all while sustaining local green resources. Here we present the study design and implementation outcomes, including recruitment and engagement strategies, of a GAP intervention utilizing a community-based participatory approach aimed at supporting the health and well-being of Chinese American PLWD residing in lower-socioeconomic households within Brooklyn, NY.

Abstract citation ID: igad104.2343

### "THE ONLY WAY FOR US TO STAY SAFE IS TO HIDE": THE EXPERIENCES OF OLDER CHINESE IMMIGRANTS DURING COVID-19 IN NYC

Yiyi Wu[1], Mark Brennan-Ing[2], and Ruth Finkelstein[1], *1. Hunter College, City University of New York, New York City, New York, United States, 2. Hunter College, CUNY, New York City, New York, United States*

The COVID-19 pandemic disproportionately affected older adults, who were at heightened risk for COVID-19 morbidity and mortality, as well as social isolation. Challenges to accessing health information and vaccine resources were amplified among older immigrants due to language barriers, lack of established social networks, and experiences of systemic discrimination. Considering the surge of anti-Asian violence across the country and the high-profile violent incidents targeting Asian older adults, our analysis focuses on the lived experiences of older Chinese immigrants during the pandemic in New York City (NYC). To describe the support needed during the pandemic to inform service delivery, we conducted in-depth interviews with 9 low-income Chinese adults over 65 about their experiences in coping with COVID-19. Interviews were transcribed and subjected to inductive thematic analysis. Results show that driven by the pro-vaccination and science norms in China, participants shared an overall positive attitude towards vaccines and exhibited extraordinary resilience to COVID-misinformation. However, COVID-19 and anti-Asian violence amplified each other. Fear of contracting COVID-19 due to perceived age-related vulnerability and fear of anti-Asian violence were prevalent, leading to self-protective withdrawal and deferred health care. Many expressed concerns about traveling to public places alone. Deeply frustrated with the government's role in controlling violent crimes, some participants decided to stay home indefinitely to evade trouble. Mechanisms to draw out people who have socially withdrawn are needed. Senior service providers in NYC should initiate discussions about perceived safety and implement peer buddy systems to enhance post-pandemic quality of life for Asian older adults.

Abstract citation ID: igad104.2344

### BEHAVIORAL HEALTH PREDICTORS OF PREMATURE MORTALITY IN A BLACK AMERICAN LONGITUDINAL COHORT

Kerry Green[1], Brittany Bugbee[2], and Elaine Doherty[3], *1. University of Maryland at College Park, College Park, Maryland, United States*, 2. *University of Maryland School of Public Health, College Park, Maryland, United States*, 3. *University of Maryland, College Park, Maryland, United Sta tes*

Life expectancy among Americans is now the lowest it has been in 15 years. While startling, more alarming are the persistent racial disparities in mortality. Black Americans continue to die at significantly younger ages with Black men living, on average, 7.0 fewer years and Black women living 4.4 fewer years than their White counterparts. To inform intervention strategies and resource allocation, we need a complete understanding of the role that malleable factors, such as substance use and depression early in the life course, play in premature mortality for aging Black Americans. This study illuminates behavioral health factors that can be targeted to eliminate mortality disparities among a longitudinal cohort followed from childhood into their 60s. Utilizing data from the Woodlawn Study, a well-defined neighborhood cohort study of 1,242 Black Americans that began in 1966 (age 6), we examined the predictive value of family history of substance use, adolescent substance use and depression, and young adult substance use and depression. Through 2021 (modal age 61), based on National Death Index reports, 21.6% of the original cohort (n=268) has passed away. The poster will present cause and timing of death by gender and adjusted logistic regression findings showing that depression symptoms as early as adolescence (age 16) predict midlife mortality occurring between ages 32 and 60 (p=.003) and that those diagnosed with depression or a substance use disorder in young adulthood are 61% (p=.025) and 85% (p=.001) more likely to die by age 60. Policy and prevention implications will be presented.

Abstract citation ID: igad104.2345

### CULTIVATING CULTURAL COMPETENCE WITH INTERDISCIPLINARY TEAMS IN SERIOUS ILLNESS CARE

Karen Bullock[1], and Kim Stansbury[2], *1. Boston College, Boston, Massachusetts, United States*, 2. *NC State University, Raleigh, North Carolina, United States*

A workforce of gerontologists that is prepared to go beyond cultural humility to achieve baseline levels of awareness, skills, and transferrable knowledge to address racial disparities is necessary for creating equity in serious illness care with older adults. Cultural competence, the complex integration of awareness, knowledge, and skills enhances one's capacity to engage effectively across cultural and racial groups to achieve equitable outcomes. **Research Objectives.** The goal of this research was to design and assess outcomes of an innovative curriculum using simulation and role-play to train practitioners how to deliver patient-centered, goal concordant care with patients and families of racial groups that have been historically excluded from equitable healthcare in the U.S. **Procedures:** Interdisciplinary competency-based curriculum was developed and facilitated by an interdisciplinary team of educators and researchers. Advanced practice social workers were key members of serious illness care teams. With specialty training, they were prepared to be leaders in teaching learners how to conduct psychosocial assessments and interventions that beyond superficial levels of curiosity to applying competency-based practices with racially and culturally diverse populations. **Implications:** Addressing the impacts of the COVID19 pandemic in gerontology research and practice with racially diverse patients and families warrants a social justice framework for designing healthcare interventions that are inclusive of populations that have been most adversely impacted due inequities woven into the fabric of our society. **Conclusion:** This cultural competence training (CCT) program was effective in preparing learners to address racial inequities in healthcare for patients and families living with serious illness.

Abstract citation ID: igad104.2346

### FALL PREVENTION MODEL AT LOCAL NON-PROFIT EQUIPMENT EXCHANGE PROGRAM UTILIZING THE STEADI: A COMPILATION OF TWO PILOT STUDIES

Constance Lewis, Karen Probst, Rachel Tanilli, and Alix Schoenfeld, *Gannon University, Erie, Pennsylvania, United States*

Individuals picking up gently used durable medical equipment (DME) from a local non-profit equipment exchange program in Erie, Pennsylvania were prompted with the CDC's Stopping Elderly Accidents, Deaths & Injuries (STEADI) 3-item abbreviated questionnaire and contacted by telephone 6 months later to assess if this survey tool can accurately predict falls. Individuals were also given a STEADI pamphlet upon equipment pickup to educate community members on fall risk. Ninety individuals, who ranged from caregivers to the individual themselves, chose to participate in the follow-up research phone call. Our researchers found that while the abbreviated 3-item STEADI suggested that 80% of community members (in need of DME by way of an equipment exchange program) were predicted to be at risk for a fall, only 22.5% suffered a fall within a six-month follow-up period. This questions the validity of the 3-item abbreviated STEADI, though additional studies are recommended to improve the participant percentage (only 90 out of 255 individuals, or 35%, answered the follow-up call questions) as well as increasing the length of wait time to assess for subsequent fall incident beyond six months. Additionally, only 34% of individuals recalled receiving a pamphlet when picking up equipment, and of those 31 individuals, only 10 of them (32%) remembered what the pamphlet said. This suggests that the simple use of a brochure handout to decrease fall risk amongst community-dwelling adults does not seem to be an effective educational strategy within the Erie, PA community.

Abstract citation ID: igad104.2347

### INTERSECTION OF RACE AND PLACE-BASED DISPARITIES AMONG 65+ IN MISSISSIPPI

Taylor Jansen[1], Yan-Jhu Su[1], Chae Man Lee[1], Nina Silverstein[1], Elizabeth Dugan[2], and Kina White[3], *1. University of Massachusetts Boston, Boston, Massachusetts, United States*, 2. *University of Massachusetts, Boston, Boston, Massachusetts, United States*, 3. *Mississippi State Department of Health, Ridgeland, Mississippi, United States*

Disparities in healthy aging have been attributed to race, gender, SES, and zipcode. The Mississippi Healthy Aging Data Report (HADR) is a new resource in the state to advance age-friendly progress reporting 150 indicators related to healthy aging for 82 counties. The present study explored how race and place may be associated with community health outcomes. The main data sources include the American Community Survey, the Behavioral Risk Factor Surveillance System, and the Centers for Medicare and Medicaid Services. Bivariate statistical analyses and mapping were conducted to compare the 65+ population in Mississippi living in rural (N=65) and urban areas (N=17); counties with high (N=44) and low (N=38) proportions of 65+ black residents; and high and low black populations by rural (high: N= 38; low: N=27) and urban counties (high: N=6; low: N=11). Bivariate analyses found that the rural counties with high proportions of 65+ black residents reported consistently worse rates for 26 out of 28 indicators of healthy aging. ArcGIS Mapping demonstrated that the Mississippi rural, black population tended to be centralized to the Delta region, a historically impoverished area. This population reported the highest rates of chronic conditions and poverty, lowest rates of education and life expectancy, and the lowest access to primary care providers and hospitals. This study underscores the importance of data tools like HADR to improve healthy aging for everyone. Understanding the distribution of community rates makes disparities evident and may help practitioners and policymakers to allocate resources to areas of highest need.

Abstract citation ID: igad104.2348

### LIVING ARRANGEMENTS, HOUSING CHARACTERISTICS, AND PSYCHOLOGICAL WELL-BEING AMONG OLDER ADULTS IN CHINA

Rong Fu, *Siena College, Loudonville, New York, United States*

**Objectives.** Living arrangements is an essential component of social environments for older adults. This study examines the impact of living arrangements and housing characteristics on the psychological well-being of older adults in China. **Methods.** Data were derived from the 2011-2012 wave of the Chinese Longitudinal Healthy Longevity Survey. The final sample consisted of 4,746 Chinese older adults aged 80–105 years. Multivariate logistic regression models were used to estimate the odds of negative psychological outcomes, predicted by living arrangements, housing characteristics and other covariates. **Results.** Overall, older adults who lived in a three-person household were less likely to feel lonely than their counterparts living in other household sizes. Participants who reported at least one housing problem (e.g., exposure to mold, minor home damage) were more likely to feel anxious and depressed than those who did not report any housing problems. Having a private bedroom was associated with better psychological well-being for both older men and women.

**Discussion.** This study demonstrates that both household sizes and housing conditions may influence health and well-being in advanced age. Living with family members and having adequate personal space at home have protective health benefits for older adults. This study has policy implications regarding household sizes and improvement in housing conditions.

Abstract citation ID: igad104.2349

### NEIGHBORHOOD IMPACT ON OLDER ADULTS' HEALTH OUTCOMES IN KOREA

Seon Kim, *Virginia Commonwealth University, Richmond, Virginia, United States*

**Background:** The health of older adults is not only affected by biological factors but also strongly influenced by their social determinants. Neighborhood characteristics have been identified as structural contexts that significantly impact the health outcomes of older adults. As older adults tend to spend more time in their homes and neighborhoods than young adults, it is important to understand the neighborhood factors that affect their health outcomes. **Objectives:** This study aims to explore the neighborhood characteristics that impact the health outcomes of older adults in Korea. Method: This study used the 2020 National Survey of Older Koreans data, which was collected from 10,097 Korean older adults aged 65 and older. Neighborhood characteristics considered in this study included satisfaction with neighborhood characteristics (e.g., safety, transportation, social cohesion) and proximity to infrastructure in the neighborhood (e.g., senior centers, hospitals, supermarkets). The health outcomes considered in this study were self-rated health, the number of chronic diseases, and mental health among older adults. **Results:** The regression results showed that satisfaction with the neighborhood was related to older adults' better self-rated health (B=.014, p<.001), lower number of chronic diseases (B=-.007, p<.001), and better mental health (B=.034, p<.001). On the other hand, proximity to infrastructure in the neighborhood was only related to older adults' better mental health (B=-.018, p<.001) among older adults. Implications: These results suggest that policymakers should consider neighborhood characteristics when addressing older adults' health outcomes. Especially, for the better mental health, policymakers should consider both proximities to infrastructures and satisfaction with the neighborhood.

Abstract citation ID: igad104.2350

### RECRUITMENT AND RETENTION AMONG A LONGITUDINAL COHORT OF AGING ADULTS: EXPERIENCES OF THE WOODLAWN STUDY

Brittany Bugbee[1], Elaine Doherty[2], and Kerry Green[3], *1. University of Maryland School of Public Health, College Park, Maryland, United States*, *2. University of Maryland, College Park, Maryland, United States*, *3. University of Maryland at College Park, College Park, Maryland, United States*

Minimizing participant attrition is critical to the success of longitudinal studies, but locating and retention is challenging when assessments occur decades apart. This poster will describe efforts to locate participants from the Woodlawn Study, a longitudinal cohort of 1,242 Black Americans followed since 1966 who were assessed at ages 6, 16, 32, and 42. In 2021-2023, two decades since the last assessment, we attempted to locate 1038 participants still thought to be alive to complete an Age 62 interview. Key sources for locating were online contact information databases, genealogical records, and public property records. Deceased participants were identified via National Death Index records. Strategies to encourage participation included monetary incentives, flexible scheduling options, and sharing results from previous assessments. To date, 90.8% have been located, 2.5% are unlocatable, and 6.6% have been identified as newly deceased. Although the cohort originated in Chicago, participants were located in more than 25 states. Interviews were completed with 45.7% of located participants. Approximately one-third (37.7%) did not respond to contact attempts, 6.4% were hard refusals, and 10.3% were soft refusals. Mailed letters and phone calls were the most successful contact methods; social media and email were not fruitful. Interview completion rates were highest for participants who participated at Age 42 (51.4%) and lowest among participants not reached since Age 6 (5.3%). Recruitment challenges included non-response to phone calls, suspicion about scams, and unknown accuracy of locating information. Implications for locating and maintaining aging cohort members will be presented.

Abstract citation ID: igad104.2351

### RHYTHM AND NATURE: APPLYING TRAUMA-INFORMED APPROACHES TO RESEARCH TO PRIORITIZE CHOICE, EXPRESSION, AND JUSTICE

Emily Ihara, and Michelle Hand, *George Mason University, Fairfax, Virginia, United States*

Trauma-informed approaches are widespread in direct clinical and particularly in social work practice, and have gained momentum in other areas such as research, organizational culture, supervision, and leadership. Given the recent collective global trauma of the pandemic and the cumulative effects of historic and ongoing racial injustice, a trauma-informed approach to research with older adults, especially with those who have disabilities, such as relating to dementia, can contribute to the movement for access, justice, equity, and inclusion for all. Closely aligned with practices of community-based participatory action research, trauma-informed research pays particular attention to the consideration of the design of studies, the analytical and theoretical frameworks, collaboration among researchers and participants, data collection methods, research questions that capture the knowledge and experiences of participants' resiliency, strengths, and challenges, and the processes of reflexivity. Rhythm & Nature was a 10-week pilot study designed to incorporate trauma-informed research approaches to promote principles of safety, trustworthiness, collaboration, choice, self-expression, and justice. We created a modifiable drumming and nature-based intervention to promote opportunities for self-expression and confidence. Before and after each drumming and nature-based session, we collected data using emojis (e.g., to increase access for people with limited language or literacy) to indicate how participants felt in the moment and used their ongoing feedback to continuously tailor the program to their unique abilities and needs. Both quantitative and qualitative results of this pilot study and implications for future research, policy, practice and education with older adults, particularly with disabilities, will be discussed.

Abstract citation ID: igad104.2352

### SOCIAL DETERMINANTS AND HEALTH EQUITY AMONG NYC OLDER ADULTS

Duy Nguyen[1], Carson De Fries[2], and Ethan Siu Leung Cheung[3], *1. Sacred Heart University, Fairfield, Connecticut, United States*, *2. University of Denver, Denver, Colorado, United States*, *3. University of Utah, Salt Lake City, Utah, United States*

Of the vast opportunities in New York City, older adults from racial/ethnic minority groups face the challenges of social determinants on their health. This study applies the WHO's Commission on Social Determinants of Health model to examine health inequities among older adults in New York City. A deeper understanding of interrelationship between structural and intermediary determinants is crucial to promoting successful aging in an urban environment. Data were extracted from the NYC Community Health Survey to focus on older adults, aged 65 and over (N=1,921). Weighted, limited information regression models were used to test the contributions of structural and intermediary determinants on self-reported daily activity difficulties and self-reported health. The sample comprised of 46% Non-Hispanic White older adults, 20% Black/African American, and another 20% Hispanic. One third of the sample endorsed being in poor health. One in 5 older adults reported having a daily activity difficulty. Multivariate analyses showed Black older adults reported more difficulties with daily activities compared to non-Hispanic Whites. Meanwhile, Hispanic older adults reported being in poor health less frequently than non-Hispanic Whites. Further limited information modeling shows the centrality of daily activity limitations on poor health such that the indicator partially mediates the relationship between race/ethnicity and poor health. Overall, the results reveal a fuller picture of the impact of social determinants on health equity among older adults in New York City. Future research needs to specify the sociocultural and economic dimensions of health equity to provide empirical results that can inform practice and policy.

Abstract citation ID: igad104.2353

### SOCIAL PARTICIPATION AMONG OLDER ADULTS WITH UNMET NEED CONSEQUENCES: FROM A DISABILITY PERSPECTIVE

Natalie Turner, and Brittany Jones, *University of Washington, Seattle, Washington, United States*

This study examines the relationship between social participation and consequences arising from unmet ADL, IADL, and mobility needs using disability studies frameworks. The aging field has yet to fully incorporate theoretical perspectives from disability scholarship into our understanding of health, wellbeing, accessibility and inclusion. By situating the experience of aging, aging with care needs, and having unmet care needs within a disability studies lens we gain insight into the ways we are called to redesign the provision of supportive services to ensure meaningful inclusion in community. To illustrate this idea, we use Wave 5 from the National Health and Aging Trends Study (NHATS) (n=7,070) to examine the relationship between ADL, IADL, and mobility unmet need consequences and social participation. Both social participation and unmet need consequences among older adults have been explored from healthcare, quality of life, and economic perspectives. However, there is a lack of scholarship linking these two concepts using a disability lens. Preliminary results from a series of linear regressions show a significant, negative relationship between presence of unmet ADL and mobility need consequences and social participation (p < .001). These findings indicate that experiencing unmet care needs may not only have detrimental effects on health, but on overall adult wellbeing and may be infringing on the ability of older adults to engage with community to the extent they wish. By applying disability frameworks, aging researchers are called to reexamine the ways in which we consider accessibility and full inclusion of older adults with care needs.

Abstract citation ID: igad104.2354

### SOCIAL RISKS AMONG PREDOMINANTLY AFRICAN AMERICAN/BLACK CAREGIVERS OF PERSONS LIVING WITH DEMENTIA

Victoria Winslow[1], Christina Bernhardy[2], Soo Borson[3], Stacy Tessler Lindau[1], Jennifer Makelarski[1], and Katherine Thompson[1], *1. University of Chicago, Chicago, Illinois, United States*, *2. UChicago Medicine, Chicago, Illinois, United States*, *3. University of Southern California, Los Angeles, California, United States*

Health-related social risk factors (HRSRs) are associated with poorer quality of life and higher caregiving burden and are more prevalent among women and in African American/Black communities due to structural inequities. Little is known about gender differences in HRSRs among dementia caregivers, especially AA/B dementia caregivers. Baseline data were obtained from 345 caregivers of PWD enrolled (17% at their own and 83% at their care recipient's point of care) in a single-blind, randomized controlled trial of CommunityRx-Caregiver, a social care intervention delivered at the point of health care. Gender differences in HRSR rates (food, housing, safety, utilities, transportation, financial, and social support) were tested using chi-square tests. Most caregivers were women (78%) and non-Hispanic AA/B (80%). Female caregivers had significantly higher rates of having HRSRs (64% of women vs. 51% of men, p=0.04, 62% overall). Lack of social support (47% of women vs. 38% of men, 45% overall), financial strain (26% vs. 23%, 25% overall), and housing insecurity (23% vs. 14%, 21% overall) were also most prevalent among women. Thirty-one percent of women compared to 23% of men reported 2 or more HRSRs. Health-related social risks, especially lack of social support, were prevalent among women and men dementia caregivers in a predominantly AA/B community. By 2030, more than 9 million Americans and their families will be affected by dementia and dementia caregiving. Social care interventions should be designed to identify and support caregivers at the point of their own or their care recipient's healthcare.

Abstract citation ID: igad104.2355

### SOCIOECONOMIC DISPARITIES IN PHYSICAL AND OCCUPATIONAL THERAPY HOME HEALTH CARE VISITS FOR PEOPLE WITH DEMENTIA

Ashwath Muruganand[1], Rebecca Lassell[2], Shih-Yin Lin[3], Kimberly Convery[3], Jason Fletcher[3], and Abraham Brody[3], *1. New York University Grossman School of Medicine, New York City, New York, United States*, *2. New York University, New York City, New York, United States*, *3. NYU Rory Meyers College of Nursing, New York City, New York, United States*

People living with Dementia (PLWD) utilize Home Health Care (HHC) services such as Physical Therapy (PT) and Occupational Therapy (OT), but little is known about service utilization patterns among PLWD from different socioeconomic backgrounds. We aimed to identify disparities in service utilization in PLWD in a HHC setting across socioeconomic backgrounds using a measure of geographic socioeconomic deprivation, the Area Deprivation Index (ADI). ADI is a composite measure of census variables describing socioeconomic disadvantage, reported as national percentile rank. This secondary analysis utilized data collected February 2017–May 2022 in a dementia symptom management clinical trial at a large, urban, Medicare certified nonprofit HHC agency in New Jersey. Demographic and service utilization data from 153 dyads of PLWD and caregivers was collected. PLWD's area codes were used to calculate ADI. ADI national percentile was divided into quartiles, and PT and OT utilization for the highest quartile was compared to the three lowest quartiles using Poisson regression, consistent with previous literature. The highest quartile group was defined as ADI above the 36.5th percentile. ADI varied from 2nd to 73rd percentile, with a median of 24th percentile. Analysis controlled for pre-existing comorbidities using pre-trial Charlson Comorbidity Index scores, classified as high (>5), medium (3-4), or low (1-2). High ADI classification was associated with a 50.2% decrease in OT utilization (IRR=.498, CI=.348-.713), and 39.4% decrease in PT utilization (IRR=.606, CI=.485-.759). Further investigation of disparities in PT and OT utilization in HHC is needed, given the disproportionate burden of disease on socioeconomically distinct populations.

Abstract citation ID: igad104.2356

### WORK PRECARITY AND THE AGING WORKFORCE: TRENDS IN HEALTH DISPARITY AMONG OLDER SERVICE SECTOR WORKERS

Renada Goldberg[1], Janette Dill[2], and Jiyeon Kim[3], *1. Simmons University, Boston, Massachusetts, United States*, *2. University of Minnesota, Minneapolis, Minnesota, United States*, *3. University of North Carolina at Chapel Hill, Chapel Hill, North Carolina, United States*

Work precarity may affect workers' physical, mental, and social well-being as navigating uncertain and insecure work conditions interacts with factors such as housing, family caretaking, and personal relationships. In particular, the service sector has long been characterized by work precarity as compared to other industries in the US, where noncollege workers are far more vulnerable to non-standard and low-wage work and Black, Indigenous, and workers of color are overrepresented. For older adults, structural and systemic inequities may exacerbate health conditions and accelerates biological aging. In this study, we measure work precarity among noncollege older workers (55-75 years old) in three large and growing service sector industries in the US: health care, retail, and food service, to access the impact of work precarity on older low-wage worker's health outcomes, exits from the formal wage labor workforce, and linkages between work precarity and health disability.

### SESSION 7250 (POSTER)

### AGE FRIENDLY

Abstract citation ID: igad104.2357

### A GERIATRIC EDUCATION COURSE SPANNING COMMUNITY AND HEALTH CARE ENVIRONMENTS

Michele Gareri[1], Susan Hazelett[2], Denise Kropp[3], Margaret Sanders[3], Jennifer Drost[2], Lori Kidd[4], and Susan Fosnight[5], *1. Summa Health , Akron, Ohio, United States*, *2. Summa Health Systems, Akron, Ohio, United States*, *3. Northeast Ohio Medical University, Rootstown, Ohio, United States*, *4. University of Akron, Akron, Ohio, United States*, *5. Summa Health System, Akron, Ohio, United States*

To address gaps in the geriatrics-trained workforce, we implemented a Geriatric Resource Certificate (GRC) for interprofessional learners from healthcare and community provider settings. This program combines online self-paced didactics, real-time virtual lectures, and a real-world virtual team care planning experience. The education incorporates the 4Ms of the Age Friendly Framework and spans the geriatric experience from healthy aging through end-of-life. It includes Dementia Friends and ECHO sessions. The goal is to training people from a variety of care settings to be resource people regarding geriatric issues, much like the NICHE program in acute care. We had 37 learners who completed evaluations. Practice settings included ACO, Hospice, Emergency Department, nursing homes, hospitals, primary care, Area Agencies on Aging, and other community providers. Overall, the online didactics were rated an average of 4.53 on a 5 point Likert scale and the real-time lectures were rated an average of 4.41. The average score for “the GRC will help me provide care over the continuum” was 4.59. The average for the real team was 4.07, 4.26 for the ECHO, and 4.45 for Dementia Friends. All learners indicated they would refer others to the GRC. These results indicate that the GRC is an effective method to impart basic geriatric training into communities and raise awareness of resources available to assist older adults in maintaining their independence. In this era of high healthcare workforce turnover and staffing shortages, improving geriatric knowledge by training on-site geriatric resource staff is one way to improve care for older adults.

Abstract citation ID: igad104.2358

### AGE-FRIENDLY COMMUNITIES IN THE ANDES: CONSIDERATIONS TO IMPROVE UPTAKE

Jonathan Guillemot[1], and Mildred Warner[2], *1. Universidad San Francisco de Quito, Quito, Pichincha, Ecuador*, *2. Cornell University, Ithaca, New York, United States*

Despite the demographic aging of the Global South, the uptake of WHO’s age-friendly cities framework remains extremely low. The Andean region of Latin America is currently represented by only four cities, out of more than 1,400 globally. Causes may include the partial inadequacy of the eight WHO domains for communities in the Andean region. We argue for a broader human ecological framework to address the macro, meso and micro levels to better address the context, challenges and opportunities for age-friendly cities in the Andean region. WHO’s age-friendly city domains are focused primarily at the meso (community) scale – on built environment, services and participation. We call for more attention to the macro policy scale, in societies where robust social security systems cannot be assumed. We also call for more attention to the micro scale, to recognize the critical role of family and informal care supports. We question whether the WHO domains are the result of a design bias, with Global North settings in mind for its development. We find the domains of UNICEF’s child-friendly cities initiative, which give more attention to the realities of the Global South, helpful to broaden WHO’s age friendly framework.

Abstract citation ID: igad104.2359

### AGE-FRIENDLY PARTNER EVALUATION: REVIEWING FLORIDA ACTION PLANS

Helena Swanson, *Central Connecticut State University, Bristol, Connecticut, United States*

Understanding community collaboration is crucial to advance age-friendly community change (Greenfield, et al., 2022). This poster’s purpose is to provide evidence about age-friendly partner involvement to bring awareness to the extent that partnerships are collaborative and holistic at the age-friendly action plan stage. We conducted a qualitative, thematic analysis by reviewing Age-Friendly Florida Action Plans (N = 13) to code for partner involvement. The evaluation used the World Health Organization’s Age-Friendly Environments framework, which includes 4 cyclical steps: Engage and Understand, Plan, Act, and Measure. The evaluation questions were (1) How many partners are typically involved in action plans? and (2) Which types of partners are typically involved in action plans? The Florida Age-Friendly Communities evaluated have diverse characteristics in community type (e.g., rural, urban, and suburban), population size, and the percentage of the population that is 65+. After an iterative process was conducted between coders, a partner coding structure emerged. The partner coding structure included codes for government, businesses, non-profits, communities, and other partners. Partner themes for each sector (government, businesses, non-profit, community, and other partners) were identified and will be discussed. Overall, results concluded that there were an average of 16 partners involved in age-friendly action plans. On average, age-friendly partners were most involved in the Act step of the World Health Organization’s Age-Friendly Environments framework. Lastly, government sectors were the most frequent partner identified in age-friendly action plans. Considerations and recommendations for age-friendly partner involvement for creating collaborative and holistic age-friendly partners will be included.

Abstract citation ID: igad104.2360

### DEVELOPING AN AGE-FRIENDLY ECOSYSTEM: A CASE STUDY FROM MINNESOTA’S MULTISECTOR BLUEPRINT ON AGING

Farah Baig[1], Rajean Moone[1], and Carrie Graham[2], *1. University of Minnesota, Minneapolis, Minnesota, United States*, *2. Center for Health Care Strategies, Hamilton, New Jersey, United States*

As age demographics continue to shift, age-friendly policy initiatives at the global, national, state, and local levels are rapidly evolving. These emerging age-friendly initiatives underscore a changing perception of aging and older adulthood. Many states, communities, academic institutions, health systems, and other organizations are embracing the demographic shift and leveraging age-friendly frameworks that seek to improve later life experiences. However, there is limited scholarly work that explores contextualized age-friendly policy development. Using qualitative case study and historiography methods, this study provides an overview of various age-friendly frameworks and outlines work in the State of Minnesota where intersections of the various frameworks are coalescing in an age-friendly ecosystem. Minnesota, rather than being a 'best practices' exemplar, serves as a model of an evolving system, led by the Age-friendly Minnesota Council in partnership with organizations such as the Minnesota Board on Aging and the Minnesota Department of Human Services, that are developing impactful policies to address the needs of a growing heterogeneous older adult population. The nuances and realities found within the Minnesota context underscore the need to develop fluid age-friendly ecosystems that can adapt to the uniqueness and complexity found in each state, region, or community. Going forward, it will be critical for Minnesota to evaluate their programs to determine their long term impact on the entire population — including today's older adults as well as future older Minnesotans.

Abstract citation ID: igad104.2361

### DISCRIMINATION AND PSYCHOLOGICAL WELL-BEING IN LATER LIFE: LONGITUDINAL EVIDENCE FROM CHINA

Shuai Zhou, and Xue Bai, *The Hong Kong Polytechnic University, Kowloon, Hong Kong*

Although ample evidence shows a negative association between discrimination and well-being outcomes, yet few longitudinal studies have explored the prevalence and consequences of multiple forms of discrimination among older adults. This study investigated the effects of interpersonal and institutional discrimination on psychological well-being among Chinese older adults and whether such effects were conditioned on subjective socioeconomic status. Data were retrieved from the China Family Panel Studies, consisting of 9,356 older respondents aged 45 and older in 2010 with their follow-up observations during 2012–2016. Random-effects panel regression analyses were performed to examine the relationships between multiple forms of discrimination on psychological distress and life satisfaction, separately, as well as the moderating effects of subjective socioeconomic status. Results revealed that one in four older adults experienced at least one form of discrimination during the past 12 months. Most prevalent discriminatory experiences encompassed wealth-based discrimination, delay and stalling at government agencies, and unfair treatment by government officials. Experiences of discrimination were negatively associated with life satisfaction and positively associated with psychological distress. Subjective socioeconomic status ameliorated the negative effect of interpersonal discrimination on life satisfaction. Findings reveal the detrimental effects of discrimination on psychological well-being that both interpersonal and institutional discrimination could reduce satisfaction with life and exaggerate psychological distress. Findings imply that subjective socioeconomic status could protect older adults from harmful effects of interpersonal discrimination, but not institutional discrimination.

Abstract citation ID: igad104.2362

### IMPORTANT COMMUNITY FEATURES AND SERVICES IN DIVERSE OLDER ADULTS

Kyeongmo Kim[1], and Andy Hong[2], *1. Virginia Commonwealth University, Richmond, Virginia, United States*, *2. University of Utah, Salt Lake City, Utah, United States*

Many cities and towns have tried to create neighborhood environments favorable to older adults. However, it raises the question of whether community aging initiatives reflect the diverse needs of older adults. This study examined how much different types of neighborhood physical and social environments were essential to the life of diverse older adults. This study used data from the 2018 AARP Home and Community Preferences Survey with 762 adults aged 50 and older in the USA (female: 50%; White: 70%, Black: 17%, Hispanic: 12%, other race: 5%). The outcome variables were participants' rates on the relative importance of seven community features (i.e., outdoor space, housing, transportation, street, social participation, civic engagement, and job). We ran multiple regression analyses to examine the relationships between the importance rating of community features and diverse older populations. Women thought all community features were important in their daily life compared to men, except for transportation. Black and Hispanic older adults valued all seven community features more highly than White older adults. People with disabilities thought housing and civic engagement were essential daily; people who experienced loneliness thought housing, transportation, social participation, and civic engagement were important. This study adds to the growing literature on identifying the diverse needs of older adults in developing and creating age-friendly environments. Community features and services were essential to women and racial and ethnic minority adults. Depending on older adults' demographic and health-related characteristics, policymakers and practitioners should develop a plan to reflect the diverse needs of older adults.

### SESSION 7260 (POSTER)

### END OF LIFE

Abstract citation ID: igad104.2363

### AMERICANS AND TAIWANESE: A COMPARISON OF BELIEFS IN AND PREFERENCES FOR ADVANCE DIRECTIVES BY SOCIODEMOGRAPHICS

Yuchi Young[1], Duan-Rung Chen[2], Ashley Shayya[3], Taylor Perre[4], Virgile Barnes[5], and Thomas O'Grady[6], *1. University at Albany, Rensselaer, New York, United States*, *2. College of Public Health, Taipei City, Taipei, Taiwan (Republic of China)*, *3. University at Albany School of Public Health, Rensselaer, New York, United States*, *4. Home Care Association of New York State, Albany, New York, United States*, *5. SUNY Albany, School of Public Health, Albany, New York, United States*, *6. State University of New York at Albany, Albany, New York, United States*

**Introduction.** Advance directives (ADs) are legal documents that allow individuals to express their end-of-life wishes. However, cultural differences related to collectivism and family values may impact AD beliefs and preferences.

This study compares AD beliefs and preferences between American and Taiwanese adults. **Methods.** A survey was used to collect data from adults in the US (n=166) and Taiwan (n=2000) to assess AD beliefs and preferences. Propensity score matching was used to compare the populations, and bivariate analyses were conducted to identify AD-specific differences between the two groups. **Results.** Compared to the US, Taiwanese participants were younger (mean age = 45 vs. 50), higher percentage female (80% vs. 63%), and higher percentage married (50% vs. 48%). Taiwanese adults had a higher belief in preparing an AD (89.3% vs. 80.7%; p=0.02) and were more willing to complete one than American adults (85.6% vs. 63.1%; p< 0.01). 59.4% of Taiwanese adults preferred their family/loved ones make healthcare decisions, compared to 46.0% of Americans (p=0.01). However, only 50.8% of Taiwanese adults believed their loved ones' decisions aligned with their wishes, while 79.5% of Americans held this belief (p< 0.001). **Conclusion.** This study reveals noteworthy differences in AD beliefs and preferences between the two groups, with Taiwanese adults valuing AD preparation more and being more willing to complete them. Taiwanese adults also rely on their loved ones to make AD decisions, even though they believe these wishes may not align with their own. These differences likely stem from cultural norms of filial piety and collectivism in Taiwan.

Abstract citation ID: igad104.2364

### BARRIERS TO HEALTH CARE ACCESS AMONG UNDOCUMENTED IMMIGRANTS: PERSPECTIVE OF PALLIATIVE CARE HEALTH CARE PROVIDERS

Valeria Cardenas[1], Lindsey Hulsebus[2], and Susan Enguidanos[1], *1. University of Southern California, Los Angeles, California, United States*, *2. USC, Los Angeles, California, United States*

Undocumented immigrants face numerous barriers to obtaining healthcare services, largely due to fear of deportation and limited access to health care. The purpose of this study was to learn about challenges and facilitators palliative care healthcare providers experience in caring for seriously ill undocumented immigrants and the factors affecting access to care in this seriously ill population. From November 2022 to January 2023, we conducted semi-structured, individual telephone interviews with 11 palliative care healthcare providers from two inner city hospitals in California. Our interview protocol elicited providers' experience caring for undocumented immigrants, including the patients' stage of illness when providers encounter them, referrals made by the healthcare team, and gaps in care they have witnessed for these undocumented immigrants. Transcripts were analyzed using thematic analysis. Themes related to provider perceived patient care-seeking behaviors and challenges include complications of postponing care, financial burden, fear of deportation, transportation, health literacy, lack of family support, lack of awareness and misperceptions of palliative care. Themes related to provider care challenges include language barriers, longer care process, and cultural barriers. Facilitators and recommendations include awareness of care options and reassurance, importance of social and community resources, expanded palliative care awareness, knowledge, and access, and improved cultural competence. Our findings underscore the need for intervention studies that focus on alleviating identified barriers for undocumented immigrants. Future studies are needed to elicit the perspectives of seriously ill undocumented patients.

Abstract citation ID: igad104.2365

### CLINICIAN EXPRESSIONS OF CONDOLENCE AFTER THE DEATH OF A PATIENT: AN INTERNATIONAL SURVEY

Katlynn Van Ogtrop[1], Sofie Nelson[2], Christian Nouryan[1], Kevin Carratu[3], and Maria Carney, MD[1], *1. Northwell Health, Manhasset, New York, United States*, *2. Northwell Health Physician Partners Geriatrics and Palliative Medicine at New Hyde Park, New Hyde Park, New York, United States*, *3. Feinstein Institute for Medical Research, Manhasset, New York, United States*

There is little literature about expressions of condolence from providers to family members of those that have died, and no known literature reported during the COVID-19 pandemic. This study utilized an investigator-developed survey of healthcare clinicians about contacting families of patients who have died. It conducted via email and online from October to December 2021. Of 131 respondents, 67% were female, from 23 states, Canada, and UK. Half (49%) had >15 years experience, and most (85%) were attending physicians. The majority (99%) reported that a patient had died in their care within last year, while 18% reported lost >10 patients per month. Methods of condolences were phone calls, and personal letters. Most (67%) reported no change in contacting families, while 23% increased. When asked if they would be interested in education on expressing condolences, 47% responded "yes." Barriers to condolences were time (64%), unsure of what to say (20%), afraid family will be upset (19%), medical/legal reasons (10%), no training (8%), and no personal relationship (7%). Most, (85%) reported that most calls "went well or better than expected." Females (vs. male) reported often/always sending letters (45% vs 20%, p=0.13), and often/always calling by phone significantly more (71% vs 63%, p=0.04). Younger (< 40) clinicians (vs. older) reported being very/moderately comfortable talking to families (72% vs 78%, p=0.79), phone calls (64% vs 69%, p=0.68) and personal letters (27% vs 42%, p=0.91). Nearly half of respondents requested training. This is a practice that should be further studied for clinician and family experience purposes.

Abstract citation ID: igad104.2366

### HETEROGENOUS EFFECTS OF MEDICARE BENEFIT FOR ADVANCE CARE PLANNING CONVERSATION BY SOCIODEMOGRAPHIC FACTORS

Yifan Lou, *Yale University, New Haven, Connecticut, United States*

**Background:** Although Medicare makes the option of physician-patient advance care planning (ACP) discussion available to everyone starting in 2016, older adults from disadvantaged backgrounds may be less likely to benefit from the policy. Motivated by Fundamental Cause Theory, this study examines the heterogenous policy effect, as a natural experiment, on older adults' ACP uptake. **Methods:** Data were from Health and Retirement Study 2012-2020 with 15,647 individuals. I used two quasi-experimental models – interrupted time series (ITS) analysis and difference-in-difference

(DID) – to study ACP outcomes before and after the policy implementation between older adults who were Medicare-eligible and those who weren't. I studied the heterogenous effects in stratified samples by race/ethnicity, immigration background, and socioeconomic status. I used coarsened exact matching to address the potential bias that the comparison group is not the ideal counterfactual control unit. **Results:** DID reveals that, in the total population, Medicare expansion was associated with a significant increase (0.05 percentage point) in the proportion of older adults who completed advance directives, but not for having informal conversations. Furthermore, I find no evidence that the Medicare expansion was effective in improving the rates of ACP among Black, Hispanic, Asian or Native American, or non-US-born older adults. Lastly, based on ITS results, the policy only has significant immediate policy effects (level change) on ACP uptake, but not for sustained effects (slope change). Discussions: The current policy reproduced the disparities in ACP. I discuss health policy recommendations to improve the ACP rate among older adults with disadvantaged backgrounds.

Abstract citation ID: igad104.2367

### MAINTAINING PERSONHOOD AND AUTONOMY: A GROUNDED THEORY OF COMMUNITY-BASED HOSPICE CARE TRANSITIONS

Catherine Mann[1], Suzanne Sullivan[2], and Yu-Ping Chang[3], *1. State University of New York at Buffalo, School of Nursing, Buffalo, New York, United States*, *2. SUNY Upstate Medical University, Syracuse, New York, United States*, *3. University at Buffalo, The State University of New York, Buffalo, New York, United States*

Supporting care transitions as decline occurs for patients facing serious illness and their families necessitates ongoing identification of person-centered, family-oriented goals. However, little is known about the process patients and caregivers undergo when making decisions while transitioning to home hospice care. Using a Straussian grounded theory approach, we explored processes patients and caregivers use when transitioning from cure- to comfort-focused care. Data were collected through eight interviews of patients, patient and caregiver dyads, and hospice nurses (n=11). Participants were recruited from hospice and palliative care agencies serving three counties in Western New York. Interviews were recorded and transcribed verbatim. Data were analyzed using constant comparative method. Our results generated a theory grounded in the process of maintaining personhood and autonomy (e.g., changing roles, losing sense of self, wanting to be heard, wanting privacy) while making decisions to transition to hospice care. This process includes five contemporaneous steps: Shopping for the right services (e.g., recognizing existing care limitations, overcoming systemic barriers), Involving caregivers (e.g., acting as an advocate, filling other family members in, accepting the patient's decisions), Navigating the business of dying while living (e.g., dealing with legalities, planning for the end), Processing and expressing emotions (e.g., fear, anger, trauma, loss), and Seeking input as health declines/recognizing futility (e.g., pursuing a diagnosis, validating what is happening with the nurse and physician, recognizing options). The process identified in the study reveals important mechanistic targets for the development of interventions that promote patient-centered hospice care transitions in the home setting.

Abstract citation ID: igad104.2368

### NEIGHBORHOOD COHESION, NEIGHBORHOOD PHYSICAL DISADVANTAGE, AND END-OF-LIFE CARE: HOW NEIGHBORHOOD MATTERS

Kedong Ding[1], and Yifan Lou[2], *1. Case Western Reserve University, Cleveland, Ohio, United States*, *2. Yale University, New Haven, Connecticut, United States*

**Background:** Many studies have focused on the association between individual-level factors and end-of-life care. Yet neighborhood-level factors and end-of-life care are understudied and thus remain unclear. This study aims to examine the association between neighborhood characteristics and U.S. older adults' end-of-life care to fulfill the existing research gaps. Method: Data were from the 2008 to 2016 Health and Retirement Study exit interview (n = 1,561) who answered left behind questionnaires. Participants included community-dwelling adults aged 55 years or older who died between waves. End-of-life care is measured from three dimensions: health service utilization, quality of life, and advance care planning; Neighborhood characteristics include neighborhood cohesion, which was assessed with 4 items to measure the extent to which respondents feel about the neighborhoods and neighbors' trust; neighborhood physical disadvantage, which was assessed with 4 items to measure the extent to which respondents feel about the neighborhoods' physical problems. Logistic regressions with sociodemographic and health controls were conducted. **Results:** A higher level of perceived neighborhood cohesion and lower level of perceived neighborhood physical disadvantages are significantly associated with a lower likelihood of being admitted to the ICU, getting life support, and having trouble with pain. Higher neighborhood cohesion is also associated with increased odds of having a living will (OR= 1.10, $p < 0.05$). Neighborhood characteristics are not associated with end-of-life hospitalization, use of hospice, depression, and having end-of-life care conversations. **Conclusion:** This study demonstrated how neighborhood characteristics will influence end-of-life care service utilization, quality of care, and advance care planning.

Abstract citation ID: igad104.2369

### SERVICE PROVIDER PERSPECTIVES ON ADVANCE CARE PLANNING USE IN RURAL DEMENTIA PATIENTS AND CAREGIVERS

Peiyuan Zhang[1], Ebow Nketsiah[2], and Hyunjin Noh[3], *1. University of Maryland, Baltimore, Baltimore, Maryland, United States*, *2. Saint Louis University, St. Louis, Missouri, United States*, *3. University of Alabama, Tuscaloosa, Alabama, United States*

Compared to urban areas, advance care planning (ACP) utilization remains very limited in rural communities. ACP earlier in the disease trajectory is particularly important for people with dementia (PWD) due to its progressive nature affecting their decision-making ability. Considering well-documented benefits of ACP in improving quality of end-of-life (EOL) care, the rural vs. urban disparity may indicate poorer EOL quality for rural PWD. Moreover, research on factors affecting ACP use among PWD has been primarily based in metropolitan/urban areas. To address the knowledge gap, this study aimed to explore barriers and current resources for ACP of PWD from perspectives of health and social service providers in rural Alabama. Using a qualitative approach, semi-structured face-to-face interviews were conducted with 10 health and social service professionals serving older adults and their caregivers in rural communities of Alabama. Thematic analysis was used to find recurrent themes and patterns from transcribed interview data. Our analysis revealed four areas of barriers to rural PWD's ACP: (1) PWD's and caregivers' lack of knowledge about ACP, dementia, EOL care options, and available resources, (2) misconceptions of completing formal documentations, (3) emotional barriers, and (4) limited access to existing resources. Elder law clinic and local Area Agencies on Aging were the most prominent, existing resources for ACP in rural Alabama. Participants also showed a type of misconception that a lawyer and/or a notary is required for ACP. The study highlighted an urgent need for social policy in ACP education for both caregivers and service providers in rural settings.

Abstract citation ID: igad104.2370

### SOCIODEMOGRAPHIC DIFFERENCES IN ADVANCE CARE PLANNING AMONG OLDER LESBIAN AND GAY ADULTS

Meki Singleton[1], and Susan Enguidanos[2], *1. University of Southern California, Leonard Davis S, Los Angeles, California, United States*, *2. University of Southern California, Los Angeles, California, United States*

Advance care planning (ACP) is a process that supports adults at any stage of health in ensuring that their values, goals, and wishes are met regarding future medical care. Factors associated with completing ACP include older age, being White, higher education, and higher socioeconomic status. However, scant research has investigated if these same characteristics are associated with ACP among lesbian and gay adults. Using data from an online survey (N=139), bivariate analyses were conducted to investigate predictors of ACP outcome measures. Being White ($p<.01$), older ($p<.01$), and employed ($p<.05$) were associated with having a higher level of comfort with medical decision making. Having a higher income ($p<.01$), being employed ($p<.01$), and having a health condition ($p<.05$) were associated with having completed a living will. Having a higher income ($p<.01$) and being employed ($p<.05$) were associated with having a durable power of attorney for healthcare. These outcomes demonstrate that while some variables were similar to previous findings, additional sociodemographic characteristics were found to have an influence on ACP among older lesbian and gay adults. Given that completing ACP has shown to improve quality of care at end of life, it is imperative to understand what factors influence ACP for older lesbian and gay adults. Understanding these within group differences will lead to future research and outreach efforts developed to inform and equip healthcare workers and community organizations with the means to better serve older gay and lesbian adults, a group that has been historically discriminated against by the healthcare system.

Abstract citation ID: igad104.2371

### UNDERUTILIZATION OF HOSPICE CARE IN OLDER BLACK ADULTS

Channing Tate, *University of Colorado Anschutz Medical Campus, Aurora, Colorado, United States*

Hospice is underutilized in Black Americans despite evidence that Black Americans benefit from hospice services. There are several potential reasons hospice is underutilized in Black Americans including lack of knowledge of hospice, poor opinions of hospice, and low self-efficacy in end-of-life decision-making. Patient decision aids are one technique used to improve patient knowledge and agency in making medical decisions. This research project endeavored to addresses potential barriers to hospice enrollment in Black adults and evaluates if a hospice specific patient decision aid could improve hospice knowledge, opinions of hospice, and decision-making self-efficacy. The study was a pilot randomized controlled trial that enrolled Black adults aged ≥ 65 years. The three primary outcomes for the study included hospice knowledge measured by the Hospice Knowledge Scale, opinions of hospice measured by the Hospice Beliefs and Attitudes Scale, and confidence in making decisions measured by the Decision Self-Efficacy Scale. Additionally acceptability and usability outcomes of the decision aid were collected. All participants completed the three baseline surveys prior to randomization. Those randomized to the intervention were provided with the hospice patient decision aid while those in the control did not receive the decision aid. Hospice knowledge, opinions of hospice, and decision self-efficacy improved from baseline to one-month follow-up in the intervention group, but between group (intervention vs control) differences were not statistically significant. Overall acceptability outcomes were favorable, and participants stated the patient decision aid would be beneficial in facilitating hospice decision making.

### SESSION 7270 (POSTER)

### ENVIRONMENT AND AGING (SRPP)

Abstract citation ID: igad104.2372

### DOES NEIGHBORHOOD GENTRIFICATION AFFECT FOOD INSECURITY AMONG URBAN OLDER ADULTS? EVIDENCE FROM NEW YORK CITY

Ethan Siu Leung Cheung, *University of Utah, Salt Lake City, Utah, United States*

Emerging studies have established that food insecurity is a prevalent public health problem among older adults, including those in urban areas. However, the relationship between neighborhood gentrification, an increasing trend in urban development, and food insecurity remains unclear. Using cross-sectional data from the Poverty Tracker Study, this study aimed to answer two research questions: 1) what is the association between living in gentrifying neighborhoods and food insecurity? 2) Do the food environment, public transportation, and social cohesion mediate the association? The sample consisted of 710 New York City residents aged 65 or older. Based on respondents' census tracts, individual-level data were merged with neighborhood data obtained from the American Community Survey. local statistics were estimated by GeoDa 1.20 to examine the spatial distribution and significant clusters of generifying neighborhoods across New York City. ArcGIS 10.1 was then used to produce maps. To address both research questions, logistic regressions and mediation analyses were conducted. Regressions revealed that living in generifying neighborhoods was significantly associated with a greater risk of food insecurity, even after adjusting for demographic characteristics and socioeconomic status, such as income. Additionally, mediation analyses suggested that the association was significantly mediated by community social cohesion and the number of healthy food outlets. Findings suggest that neighborhood gentrification presents a salient risk factor for late-life food insecurity in urban areas. Community programs that foster social connections among neighbors and local food assistance programs may be particularly needed in gentrifying neighborhoods to mitigate and reduce the risk of late-life food insecurity.

Abstract citation ID: igad104.2373

### HEALTH STATUS AND ENVIRONMENTAL FACTORS ASSOCIATED WITH OLDER ADULTS' MOBILITY IN 2021 COMPARED TO 2020

Namkee Choi, *University of Texas, Austin, Texas, United States*

The COVID-19 pandemic has had significant negative impact on life-space mobility of people of all ages around the world. Using the 2019-2021 National Health and Aging Trend Study (N=3,063, age 70+) and the socioecological mobility framework, we examined the changes in the frequency of going outside among U.S. older adults between 2020 (during the pandemic) and 2021 (post-COVID vaccine). Respondents self-reported changes in their frequency of going outside: no change (=about the same), increased frequency (=more often), and decreased frequency (=less often). We then fit multinomial logistic regression to examine the associations of the changes in the frequency of going outside with physical, psychosocial, and cognitive health, environmental (COVID concerns and transportation) factors, and social media use, controlling for the 2020 frequency of going outside. We found that in 2021 compared to 2020, 13% and 16% of those age 70+ reported increased and decreased frequencies, respectively. Increased frequency was associated with social media use. Decreased frequency was associated with poor physical health, depression/anxiety, and perceived memory decline. COVID concerns and transportation problems, as well as female gender, age 90+, and being non-Hispanic Black, were also significant correlates of decreased frequency. In conclusion, most U.S. adults age 70+ appear to have resumed their 2019 level of frequency of going outside in 2021 after the COVID vaccines became available; however, 16% reported decreased frequency of going outside in 2021 compared to 2020. Older adults with physical, mental, and cognitive health challenges need help to increase their frequency of going outside.

Abstract citation ID: igad104.2374

### NEIGHBORHOOD CHARACTERISTICS AND SLEEP PROBLEMS: MEDIATING ROLES OF HEALTH BEHAVIORS AND MENTAL HEALTH

Ethan Siu Leung Cheung, *University of Utah, Salt Lake City, Utah, United States*

Existing studies have well-documented the relationship between the neighborhood environment and health among older adults. Yet very few have focused on how the neighborhood environment predicts late-life sleep quality. Using longitudinal data (Rounds 7-9) from the National Health and Aging Trends Study, this study examined the cross-sectional and longitudinal associations between neighborhood characteristics (physical disorder and social cohesion) and sleep problems among Americans aged 65 or older, as well as whether health behaviors and mental health mediated these associations. The analytical sample included 4,029 adults aged 65 or older. Logistic regressions with a lagged dependent variable were adopted to test longitudinal associations. Longitudinal mediation analyses were also used to examine the mediation effects of health behaviors and mental health. The results suggested that neighborhood physical disorder was a significant predictor of sleep problems cross-sectionally, whereas social cohesion was both cross-sectionally and longitudinally associated with sleep problems. Moreover, mediation analysis revealed that health behaviors and mental health significantly mediated the longitudinal relationship between social cohesion and sleep problems, though the strength of the indirect effect diminished over time. These findings suggest that neighborhood physical disorder and social cohesion are significant predictors of late-life sleep problems among older Americans. The study also highlighted older adults' resilience and adaptability to adverse neighborhood environments and the long-term health benefits of social cohesion in late-life sleep quality. Therefore, community health interventions that facilitate older adults' physical and social activity, along with mental health programs, may help community-dwelling older adults improve their sleep quality.

Abstract citation ID: igad104.2375

### THE DIRECT EFFECT OF COMMUNITY-LEVEL RISK FACTORS ON LUNG CANCER INCIDENCE IN FOUR NEW ENGLAND STATES

Taylor Jansen[1], Qian Song[1], Kristie Long-Foley[2], and Elizabeth Dugan[3], *1. University of Massachusetts Boston, Boston, Massachusetts, United States*, *2. Wake Forest University School of Medicine, Winston-Salem, North Carolina, United States*, *3. University of Massachusetts, Boston, Boston, Massachusetts, United States*

Lung cancer is the most common cancer globally and 70% of diagnoses are among the 65+ population. This study identifies what town-level risk factors are contributing to geographic disparities in 65+ lung cancer in N=796 towns in Connecticut, Massachusetts, New Hampshire, and Rhode Island. Multiple spatial-based analyses (e.g., Moran's I, Getis-Ord Gi*, mapping) were conducted before running a series of spatial regression models to determine the association between town-level risk factors (i.e., environmental exposures, respiratory conditions prevalence, education rates, smoke free legislation) and lung cancer incidence. Mapping and Moran's I test suggested the 65+ rates of lung cancer in our sample were non-randomly distributed and clusters of high and low rates were present. A hot spot analysis was conducted in ArcMap 10.8 which identified several significant clusters of 65+ lung cancer. Spatial regression models showed that community rates of comorbid respiratory conditions (65+ asthma, COPD, and tobacco use disorder (TUD)) increased lung cancer incidence by 7%, while increasing ozone emissions were directly associated with a 10% increase in incidence. Comprehensive smoke free legislation coverage was significantly associated with lung cancer incidence, but no increasing or decreasing relationship was found. No effects were found for the percentage of 65+ with low education, or 60+ who were current or former smokers. Spatial regression models allow for results which are specific to the geographic sample used and this study suggests that comorbid respiratory conditions and ozone emissions are directly associated with increased rates of 65+ lung cancer incidence in our sample.

Abstract citation ID: igad104.2376

### THE INFLUENCE OF POPULATION AGING ON ENERGY CONSUMPTION QUANTITY MECHANISM RESEARCH

Jing Ma, and Hong Mi, *Zhejiang University, Hangzhou, Zhejiang, China (People's Republic)*

The adjustment of population structure and household size is one of the important factors affecting the amount of household energy consumption. Influenced by the deepening of aging degree and the acceleration of aging development, the amount of energy consumption has also produced a relatively obvious change. Based on the provincial panel data from 2008 to 2017 in China, we used fixed effects and random effects models for regression analysis to further analyze the spatial differences and trends of energy consumption quantity distribution, and explore the mechanism of population aging's influence on energy consumption quantity. In addition, we explore the path of population aging affecting energy consumption through intermediary effect analysis. Besides, we also analyzes the aging rate of population, energy consumption and environmental protection policies in different countries. It is found that, under the premise of controlling the influence of other variables, population aging rate has a negative impact on the amount of energy consumption, which is different between urban and rural areas. The increase of population aging rate in urban areas may lead to the increase of energy consumption, while the increase of population aging rate in rural areas may lead to the decrease of energy consumption. Household size and household consumption level are important intermediary variables between population aging and energy consumption. Population aging can indirectly affect energy consumption through affecting household size and household consumption level.

### SESSION 7280 (POSTER)

### SOCIAL NETWORKS, SOCIAL SUPPORT, SOCIAL ISOLATION, AND LONELINESS

Abstract citation ID: igad104.2377

### A SCOPING REVIEW OF LGBTQ+ MULTIGENERATIONAL PROGRAMMING: GAPS AND OPPORTUNITIES

Leyi Zhou, and Angela Perone, *University of California, Berkeley, Berkeley, California, United States*

Extensive research has revealed disparities in health, housing, employment, and economics among LGBTQ+ older adults (Emlet, 2016). However, the LGBTQ+ community has a long-standing tradition of multigenerational support that can potentially alleviate some of these challenges. Innovative caregiving models and non-traditional support systems involving multigenerational networks have emerged during crises such as the AIDS epidemic of the 1980s and 1990s and the recent COVID-19 pandemic (Cahill & Valadéz, 2013; Hafford-Letchfield et al., 2022). Nevertheless, despite the growing attention to LGBTQ+ multigenerational programs, the current state of scholarly research in this area remains uncertain. To address this gap, this scoping review aims to map the literature on intergenerational interventions for the LGBTQ+ community. This study includes peer-reviewed research in English with various study designs related to different categories of intervention, program, or activity that involve LGBTQ+ participants from more than one generation. To ensure accurate and reliable results, we conducted a comprehensive search on seven databases: PsycINFO, PubMed, Web of Science, Scopus, Sociological Abstract, Embase, and CINAHL. After deleting duplicates, 130 peer-reviewed articles were identified. Preliminary findings from the scoping review reveal historical moments when research on multigenerational programming surged, which aligns with pivotal crisis moments in the LGBTQ+ community. It also underscores emerging trends in multigenerational programming, including critical inquiry about what constitutes "multigenerational" and deeper commitment to inclusion of communities of color and transgender communities. These findings provide important insights for practitioners, policymakers, and researchers to strengthen services and support for LGBTQ+ older adults.

Abstract citation ID: igad104.2378

### CHILDLESSNESS AND LONELINESS IN LATER LIFE? THE MODERATING ROLE OF REASONS FOR CHILDLESSNESS

Hannelore Stegen[1], Daan Duppen[2], Liesbeth De Donder[2], and Eva Dierckx[2], *1. Research Foundation Flanders (FWO) & Society and Ageing Research Lab (SARLab), Vrije Universiteit Brussel (VUB), Brussels, Brussels Hoofdstedelijk Gewest, Belgium, 2. Vrije Universiteit Brussel (VUB), Brussels, Brussels Hoofdstedelijk Gewest, Belgium*

Having children is a significant life event that causes major changes in social networks. Despite the fact that percentages of childlessness are on the rise worldwide, the connection between childlessness and loneliness in old age is still underresearched. Additionally, the scientific literature frequently draws a line between childlessness that is voluntary and involuntary, while a considerably gray area of reasons for childlessness is present between these two extremes of the continuum. The hypothesis of this study is that there is a moderating effect of reasons for childlessness on the link between childlessness (i.e., having children or not) and loneliness. A cross-sectional survey was created for this, containing questions on topics like social relationships, childlessness, and reasons for childlessness, as well as several validated measurement instruments, such as the De Jong Gierveld 11-item Loneliness Scale and the 3-item UCLA Loneliness Scale. A total of 500 older adults, at least 60 years old, participated in this study, with half of them being childless. The findings describe 1) the degree of social, emotional, and overall loneliness among older adults with and without children; 2) the reasons of childlessness among older adults; 3) the relationship between childlessness and current loneliness; and 4) whether the relationship between childlessness and loneliness is moderated by the reasons for childlessness. This study increases understanding of various aspects of loneliness among older persons in relation to childlessness, as well as the accompanying changes in the dynamics of social relations in later life.

Abstract citation ID: igad104.2379

### COVID-19 SYMPTOMS AND RISK OF COGNITIVE FUNCTION IMPAIRMENT/DEMENTIA AMONG COMMUNITY-DWELLING OLDER ADULTS

Reza Amini, *University of Michigan-Flint, Flint, Michigan, United States*

Most studies investigating the correlation between cognitive function (CF) and COVID-19 are cross-sectional, with less than one-year intervals post-contracting COVID-19. Using three waves of the National Health and Aging Trends Study (NHATS), this study aims to investigate the correlation between COVID-19 and CF impairment/dementia among community-dwelling older adults. After excluding known cases of dementia, we used before (2019 N=3,292), during (2020 N = 2,756) and one year after COVID-19 pandemic (2021 N=2,379) data. The severity of the disease and COVID-19 preventing contact with family and friends (CFF) were self-reported (in 2020), executive function (EF) was measured by Clock Drawing Test (CDT), memory using delay 10-words recall test (DWRT), and NHATS dementia classification (in 2021). Multivariable ordered logistic regression results showed no significant correlation between dementia (i.e., normal, probable, and possible) and self-reported COVID-19 symptoms or preventing CFF. COVID-19 symptoms did not predict the risk of EF impairment. However, controlling for race/ethnicity interaction with CFF showed Blacks with limited CFF were 74% and 136% more likely to have dementia and EF impairment than their White counterparts, respectively. Experiencing COVID symptoms in 2020 increased the risk of delayed memory impairment in 2021 by 63%, while limited CFF increased this risk by 39%, which was 120% higher for Blacks than Whites. In conclusion, experiencing COVID-19 symptoms can affect delayed memory when this effect cannot be specified as dementia. The impact of social isolation caused by the COVID-19 pandemic on CF and increasing the risk of dementia is more harmful among Blacks than Whites.

Abstract citation ID: igad104.2380

### DID COVID-19 CHANGE REPORTS OF LONELINESS AND SOCIAL ISOLATION AMONG OLDER AMERICANS ACT CLIENTS?

Heather Menne[1], and Claire Pendergrast[2], *1. Miami University, Oxford, Ohio, United States*, *2. Syracuse University, Syracuse, New York, United States*

COVID-19 raised immediate concern about the exacerbation of already high levels of loneliness and social isolation of older adults. Using the cross-sectional 2019 and 2021 National Survey of Older Americans Act Participants, we explored changes among selected populations of OAA clients – family caregivers (CGs), home-delivered meal clients (HDMs), and congregate meal clients (CMs). Descriptive statistics, t-tests, and review of 95% confidence intervals between years were conducted to understand rates of loneliness (UCLA 3-items) and social isolation (1-item). Results were not statistically significant but yield important findings. First, there were more cases of loneliness (48-70%) and social isolation (31-50%) than was known for the general older adult population before COVID-19. Second, the changes between 2019 and 2021 varied by service and gender. Among CGs, there was little difference in the cases of social isolation and loneliness from 2019 to 2021; however, male CGs reported a 20.3% increase in cases of social isolation. For HDMs there was a little difference over time in cases of social isolation and loneliness. There was a 23.3% increase in the number of CMs reporting social isolation and a small increase (9.3%) in CMs reporting loneliness; however, there was an 18.4% increase in cases of loneliness among male CMs. These results warrant further investigation to understand whether OAA services are a protective factor for social connections and further implementation of innovative approaches to lower overall rates of social isolation and loneliness among OAA clients.

Abstract citation ID: igad104.2381

### SOCIAL CONNECTEDNESS RESOURCING OF OLDER PEOPLE IN ASSISTED LIVING IN THE UNITED STATES OF AMERICA: A SCOPING REVIEW

Rekha Pudur, Elias Mpofu, and Gayle Prybutok, *University of North Texas, Denton, Texas, United States*

Abstract This PRISMA scoping review identifies the emerging evidence on social connectedness resources preferences and priorities of older adults in assisted living facilities. The searches in this scoping review were conducted in EBSCOhost. Eight studies were included from 134 articles published in English from January 2000 – September 2022. There are 2482 older adults from 233 assisted living facilities in the USA. Results organized by themes framed in the World Health Organization's International Classification of Functioning, Disability, and Health (WHO-ICF) 1 provided evidence that older adults with or aging with or into chronic health conditions preferred facility based on community-based resources. Those from the larger enrollment assisted living facilities and limited physical mobility preferred facility-based resources more than community resources. Residents' preferences and priorities for social connectedness in facility and community-based activities appear to serve different needs, suggesting that resident facilities should deliberately provide for social needs resources by the context of participation. Further research is needed to profile what residents preferred/prioritized in socially inclusive activities, either facility provided or community-based.

Abstract citation ID: igad104.2382

### SOCIAL ENGAGEMENT AMONG COMMUNITY-DWELLING OLDER ADULTS: A QUALITY-OF-LIFE ANALYSIS

Alisha Thompson[1], Scott Wilks[2], and Rhonda Bardales[2], *1. LSU, Baton Rouge, Louisiana, United States*, *2. Louisiana State University, Baton Rouge, Louisiana, United States*

Older adults are living longer without sufficient efforts from health and social service systems to improve their quality of life (QoL). QoL is a nuanced, dynamic aspect to analyze. Literature shows that higher levels of social engagement provide a range of benefits to physical, mental, and cognitive health, all broad indicators of overall QoL. Social engagement comprises the person's subjective experience of the richness of their relationships. The purpose of this study was to examine empirically the connection among this richness of relationships — specifically social engagement and perceived quality of relationships — and overall QoL among older adults. The study used a large sample (N=746) of non-institutionalized adults, ages 65 and older, from the nationally representative, AARP Social Engagement and Brain Health Survey. Two primary independent variables (IVs) were quantifiable measures of social engagement and perceived quality of social relationships; covariance controlled for several demographic factors (e.g., sex, ethnicity, income) and general health measures of physical, mental, and emotional health. The outcome variable was a composite score from a popular QoL measure. Analyses involved bivariate regression for the primary IVs, then multivariate OLS regression with each IV to predict quality of life. Results showed that QoL significantly regressed with predictors of social engagement (positively); each health variable (positively); unmarried status; ethnicity of Black/African American or Asian; and male gender. These results enhance our understanding of the relationship between social engagement, relationship quality, and QoL in older adults, particularly those who identify within the aforesaid demographic brackets. Implications are discussed.

Abstract citation ID: igad104.2383

### SOCIAL INTEGRATION, COGNITION, AND FUNCTIONAL OUTCOME IN OLDER ADULTS FOLLOWING TRAUMATIC BRAIN INJURY

Wonkyung Jung[1], Jeanne Hoffman[2], Eeeseung Byun[2], and Hilaire Thompson[2], *1. Johns Hopkins University, Baltimore, Maryland, United States*, *2. University of Washington, Seattle, Washington, United States*

Persons aged 65 and older have the highest rates of traumatic brain injury (TBI), and this injury is known to be related to significant, persistent functional changes. What is not known is what factors may mediate these changes. The current study aimed 1) to examine interrelationships among social integration, functional outcome, and cognition in older adults at years 1, 2, and 5 post-traumatic brain injury (TBI) and 2) to examine if functional outcome mediates the concurrent and longitudinal relationships between social integration and cognitive function post-TBI in older adults. This is a secondary analysis of data obtained from Traumatic Brain Injury Model System National Database (TBIMS-NDB). Included subjects from the database were persons aged 65 years and older at the time of injury (N=1469). We used longitudinal mediation analysis to address study aims. We found that social integration had a direct effect on functional outcome and cognition at 1, 2, and 5-years post-TBI. Functional outcome had a positive direct effect on concurrent cognitive function. Regarding mediation, social integration and cognition were mediated by concurrent functional outcome. However, there were no significant mediating effects of concurrent functional outcome on concurrent social integration or subsequent cognitive function. Our findings suggest there are positive relationships between social integration, functional outcome, and cognition over time. With a better understanding of these interrelationships, tailored interventions that target social integration, such as activities fostering a sense of belonging in the community and cultural diversity programs may promote improved functional and cognitive outcomes.

Abstract citation ID: igad104.2384

### SOCIAL ISOLATION OF OLDER ADULTS WITH AND WITHOUT PHYSICAL HEALTH CONDITIONS DURING COVID-19

Tina Kilaberia[1], Yuanyuan Hu[1], Edward Ratner[2], and Janice Bell[3], *1. New York University, New York City, New York, United States*, *2. Minneapolis VA Health Care System, Minneapolis, Minnesota, United States*, *3. University of California, Davis, Davis, California, United States*

Older adults have been disproportionately affected by COVID-19. This study examined health care utilization and social isolation during the pandemic among older adults using written surveys and zoom and phone interviews. We compared two subgroups, those reporting physical health problems (N=12) and those reporting no health problems (N=6). Half of those with physical health problems reported postponing routine or elected healthcare as compared to four (66%) in the subgroup without physical health problems. Seven of 12 (58%) older adults with physical health problems reported isolation specifically from decreased participation in church even though as many lived either in a retirement community or with family, suggesting greater environmental or social supports. This compares to two (33%) in the subgroup without physical health problems reporting isolation from decreased participation in church with only one older adult living in a retirement community. Interviews identified three themes that complement these data: avoiding danger, compound stress (reported only among those with health problems (N=4)), and church as the largest event. These findings suggest that those with physical health problems were less able to delay seeking healthcare, were more likely to feel isolated when unable to attend church services, and suffered from compound stress due to the combination of public health directives and social unrest, such as the George Floyd protests. Religious, faith, or spiritual supports may be important buffers against social isolation during public health emergency, especially for older adults with physical health conditions and when there is concurrent social unrest.

Abstract citation ID: igad104.2385

### THE COGNITIVE DECLINE–MORTALITY ASSOCIATION IS MODIFIED BY LIVING ALONE AND SOCIAL NETWORK: A PARADOX OF ISOLATION

Hiroshi Murayama, Mika Sugiyama, Hiroki Inagaki, Chiaki Ura, Fumiko Miyamae, Ayako Edahiro,

Tsuyoshi Okamura, and Shuichi Awata, *Tokyo Metropolitan Institute of Gerontology, Tokyo, Tokyo, Japan*

Cognitive decline (CD) is a well-known mortality risk. To date, modifiers of this relationship have been insufficiently investigated. This study explored the factors modifying the association between CD and all-cause mortality among older Japanese people. A baseline questionnaire was completed with all 132,005 residents, aged ≥65 years, without long-term care insurance certification in Adachi Ward of the Tokyo Metropolitan area in July 2015 (valid response: 75,358). The final analytic sample was 74,872, excluding 486 who reported a history of dementia (mean age = 73.7 ± 6.0 years; 44.9% males). We assessed CD using a self-administered dementia checklist that was validated via the Clinical Dementia Rating Scale. The survival analysis (average follow-up of 1,657 days) showed that CD was associated with a higher mortality risk (hazard ratio [95% confidence interval] = 1.40 [1.28–1.53]). We examined the interaction between sociodemographics, health behaviors/conditions, and CD on mortality. Only household composition and social network (SN; frequency of contact with others) demonstrated significant associations with CD. The stratified analyses showed that the CD-mortality association was stronger among those with a small SN (1.62 [1.40–1.87]) than a large SN (1.26 [1.12–1.41]). Conversely, the association was weaker in living-alone individuals (1.11 [0.90–1.38]) than in cohabiting individuals (1.46 [1.33–1.61]). Although living alone and having a small SN represented "isolated" status, their modifying effects on the CD-mortality relationship were the exact opposite. Our findings highlight that the isolation type needs to be considered when implementing dementia prevention and support for older people with CD/dementia.

Abstract citation ID: igad104.2386

### TRANSPORTATION-RELATED FACTORS AND THE LIKELIHOOD OF COMPANIONSHIPS AMONG SENIOR CENTER MEMBERS

Rebecca Mauldin[1], Rupal Parekh[2], and Stephen Mattingly[3], *1. The University of Texas at Arlington, Arlington, Texas, United States*, *2. University of Connecticut, Hartford, Connecticut, United States*, *3. University of Texas at Arlington, Arlington, Texas, United States*

Throughout the United States, a variety of programs focus on social engagement to reduce social isolation and loneliness for older adults. An ideal site for this is the senior center. This research takes place in a senior center serving older Hispanic/Latinx adults in an urban community in the northeast United States. Data were collected 9/21-12/21 from 38 of approximately 42 regular members. Cross-sectional social network analysis data were analyzed using exponential random graph modeling (ERGM) of the likelihood of companionship ties among 42 senior center members. Several transportation related factors were significantly associated with the likelihood of companionship ties. Controlling for demographics and frequency of attendance, having missed going to the senior center due to a lack of transportation was associated with a significantly lower likelihood of a companionship tie (p = .004). Companionships were also less likely for members who were transportation cost-burdened (p = .016). Companionships were less likely for those whose primary mode of transportation was driving compared to those who primarily relied on others for rides (p = .016). Results suggest that transportation experiences are significant factors in the social relationships among senior center members. Finding ways to reduce transportation cost burden and the frequency of missing going to the senior center due to lack of transportation should be considered in interventions to enhance social integration for senior center members. Understanding the role of driving oneself in having companionships for senior center members is an area to explore in future research.

Abstract citation ID: igad104.2387

### WHY ARE SOCIALLY ISOLATED OLDER ADULTS UNABLE TO ACCESS SERVICES IN JAPAN? A PRELIMINARY REVIEW OF FOUR FACTORS

Tomoko Ikeuchi[1], Kazunori Kikuchi[1], Yu Taniguchi[2], Cullen Hayashida[3], and Kae Ito[1], *1. Tokyo Metropolitan Institute for Geriatrics and Gerontology, Tokyo, Tokyo, Japan*, *2. National Institute for Environmental Studies, Tsukuba, Ibaraki, Japan*, *3. University of Hawaii, Honolulu, Hawaii, United States*

The COVID-19 pandemic has made it difficult for health and social service agencies to provide the necessary support to community-based older adults. It is believed that the number of socially isolated older adults without the necessary support has increased, but the actual situation is unclear. In this study, we conducted a self-administered mail survey targeting all local municipal governments and social welfare councils throughout Japan from September to October 2022 to examine the actual situation. The response rate was 18.7% (682/3654). Nearly 52% of respondents reported an increase in the number of socially isolated older adults during the pandemic, and 43.6% reported that providing support services to them was difficult. We specifically asked about the difficulties encountered in providing support and coded the open-ended responses (n=275). Four primary themes were identified: (1) generalized refusal by isolated older adults (n=79); (2) difficulty in locating (n=72); (3) difficulty in face-to-face communication (n=40); and (4) difficulty in supporting (n=73). Among the reasons for the difficulty in supporting isolated older adults were their diverse needs and tendency to not recognize their need for support, which may be due to mental illness or dementia. Our findings suggest that a better attempt at classifying refusals among social isolates is a first step to improving the impact of social and health services intervention. Further research is needed to clarify the reasons for refusal of the support provided and to identify the specific types of support that isolated older adults require.

### SESSION 7290 (POSTER)

### WORKFORCE (SRPP POSTERS)

Abstract citation ID: igad104.2388

### ADVANCED CARE PLANNING: LEVERAGING PARTNERSHIPS TO IMPROVE STATE-WIDE DISSEMINATION

Lisa Bodenheimer[1], Jennifer Rutberg[2], Pat Tadel[3], and Jennifer DeGennaro[4], *1. Rowan University, Stratford, New Jersey, United States*, *2. NJ Department of Human Services, Division of Aging Services, Trenton, New Jersey, United*

*States*, *3. Respecting Choices, Oregon, Wisconsin, United States*, *4. Rowan-Virtua SOM / NJISA, Stratford, New Jersey, United States*

Rowan-Virtua School of Osteopathic Medicine, The New Jersey Institute for Successful Aging (NJISA), in collaboration with the New Jersey Division of Aging Services (DoAS) and Respecting Choices, implemented a state-wide project training workforce professionals in Advanced Care Planning (ACP) conversations with older adults. The concept: proactively leverage the long-term care management relationships in community-based, high-touch programs. NJISA identified Respecting Choices, an evidenced-based program approved by the Administration for Community Living. Respecting Choices offers standardized certification for ACP facilitators and instructors, paving a pathway for workforce sustainability. To achieve state-wide penetration, NJISA partnered with NJ DoAS to target staff and professionals serving the state aging network. NJ DoAS provided access to county-level case managers serving the Jersey Assistance for Community Caregiving (JACC) program, Program of All-Inclusive Care for the Elderly (PACE) nurses and social workers, and NJ DoAS staff. Also included in training efforts were staff from select senior affordable housing sites. With funding support through the New Jersey Health Foundation, NJISA and Respecting Choices offered virtual, live training course for certification of First Steps ACP facilitators and instructors and Design and Implementation for state aging networks, and ACP Instructors. NJ DoAS modified the electronic documentation system utilized by JACC staff to capture on-going ACP conversations and outcomes. Despite barriers and mid-course corrections, training totals include (30) certified ACP Facilitators, (3) leaders for Design and Implementation, and (5) certified ACP Instructors. Response to curriculum was positive; participants indicating greater confidence navigating ACP conversations. Future plans to continue dissemination have been discussed.

Abstract citation ID: igad104.2389

### APPLYING A TRAUMA-INFORMED DEI PRACTICE MODEL TO TRANSFORM CARE CULTURE

Jennifer Morgan[1], Kendra Jason[2], and Leigh-Anne Royster[3], *1. Georgia State University, Atlanta, Georgia, United States*, *2. University of North Carolina at Charlotte, Charlotte, North Carolina, United States*, *3. Duke, Durham, North Carolina, United States*

Collective trauma is a psychological distress experienced by large numbers of people or communities in response to shared trauma. It has been documented that the traumatic impacts of COVID-19 in concert with discrimination based on any marginalized identity (e.g., racialized, gender, and sexual minorities; poor, chronically-ill) leads to poorer health, work, and life outcomes. Marginalized older adults who have experienced discrimination over the life course are at increased risks for these challenges as they are more likely than any other group to experience infection, hospitalization, or death due to COVID-19. For older workers, they are more likely to remain unemployed or face more challenges at work than younger workers. Additionally, long-term care services and supports serving marginalized populations are more likely to have staff that have marginalized identities themselves and deal with similar challenges. We offer a conceptual model designed to transform care culture in ways that explicitly foster diversity, equity, inclusion and ultimately belonging and empowerment of individual community members. This conceptual model is guided by three founding principles: DEI (diversity, equity and inclusion), intersectionality, and trauma-informed care. This approach allows for care practitioners to address the intersecting structural and compounding challenges of COVID-19 and discrimination, instead of treating them as separate non-competing factors. By mapping out the evidence of collective trauma experienced by many minoritized and older workers; and those in aging services and long-term care sectors, we explain the principles of a trauma-informed approach and how retention and empowerment practices can support transformation of care culture.

Abstract citation ID: igad104.2390

### COMPARING VARIED EFFECTS OF WORK AND PARTICIPATION ON DEVELOPMENT OF OLDER JAPANESE ACROSS GENDERS

Keiko Katagiri, *Kobe University, Kobe, Hyogo, Japan*

Recently, the physical and cognitive status of older Japanese adults has improved on previous generations. Half of 60-year-old Japanese remain in the labor force. With extended life expectancy, improved health, and ability to remain productive into older age, some gerontologists now regard generativity as a developmental task for older adults before becoming frail, rather than for middle-aged people, as previously thought. These changes assume that older adults continue to develop, and this study examines the effects of work and active participation on development of older adults as a potential human resource for society. A 2019 random sampling survey was conducted with Tokyo and Hyogo Prefecture residents; the response rate was 43.0% (N = 1,063, aged 50-74). ANCOVA tests were conducted to gauge sense of community, interest in politics and generativity (all were positively affected by social and civic participation). Older people exhibited a higher sense of community than younger people. Females showed higher generativity and sense of community than males, while males scored higher than females for interest in politics. We observed interaction effects of work and civic participation. Working males in engaging civic activity exhibited greater positive change compared to non-working males with civic activity. Non-working females with active participation demonstrated greater positive change in generativity and interest in politics than working females with activity. This suggests older males enriched their experience by participating in civic activity while older females developed it through work. Both cases suggested that older adults can develop when they experience an unfamiliar diverse world.

Abstract citation ID: igad104.2391

### DEVELOPMENT AND VALIDATION OF A U.S. AND GERMAN SHORT VERSION OF THE LATER LIFE WORKPLACE INDEX (LLWI-S)

Juergen Deller[1], Julia Finsel[2], Anne Wöhrmann[3], Eduardo Oliveira[4], Hila Axelrad[5], Michela Vignoli[6], Xiuzhu Gu[7], and Beatrice Van der Heijden[8], *1. Leuphana University of Lueneburg, Lueneburg, Niedersachsen, Germany*, *2. Leuphana University Lüneburg, Lüneburg, Niedersachsen, Germany*, *3. Federal Institute for*

*Occupational Safety and Health (BAuA), Dortmund, Nordrhein-Westfalen, Germany*, 4. *University of Porto, Porto, Porto, Portugal*, 5. *Interdisciplinary Center Herzliya – IDC, Herzliya, Tel Aviv, Israel*, 6. *University of Trento, Trento, Trentino-Alto Adige, Italy*, 7. *Tokyo Institute of Technology, Tokyo, Tokyo, Japan*, 8. *Radboud University Nijmegen, Nijmegen, Gelderland, Netherlands*

Employment of older individuals becomes more important in demographic change. To identify organizational practices that foster the motivation, health, and performance of older employees in particular, an efficient holistic assessment of relevant organizational factors is needed. The 80-item Later Life Workplace Index (LLWI) provides such a measure for organizational practices for older employees by differentiating nine domains. The LLWI has been applied in emerging research in various research contexts and countries. However, it is quite a lengthy measure. Therefore, this paper describes the development and validation of the LLWI-S, a 29-item short measure covering all nine domains in order to achieve a more efficient instrument. The development of the short version was both, judgemental- and statistics-driven. A focus group consisting of the original LLWI-authors and several international scholars, whose research expertise lies in the field of employment and older employees, has developed the LLWI-S. A German- and an English-language version of the LLWI-S have been validated in Germany and the U.S. Reliability and model fit for the U.S. and the German version as well as multigroup CFA for measurement invariance will be presented. Results show that the measures have good internal reliability and model fit. We provide researchers and practitioners with a validated short measure to efficiently assess all nine relevant organizational domains for older employees. Researchers can utilize the LLWI-S to gain a comprehensive understanding of all relevant areas of organizational influences on later life work, while practitioners are able to assess their organizational readiness for an aging workforce.

Abstract citation ID: igad104.2392

### EVALUATION OF DEMENTIA EDUCATION TRAINING IN GEORGIA NURSING HOMES

Yun-Zih Chen[1], Jennifer Morgan[2], and Lynnete Okwaro[2], 1. *University of Maryland, Baltimore County, Baltimore, Maryland, United States*, 2. *Georgia State University, Atlanta, Georgia, United States*

The increased use and misuse of antipsychotics and psychotropics in nursing homes have been well documented. Person-centered dementia care training that addresses skill development and practice change around communication strategies and non-pharmacologic approaches has the potential to impact the misuse of these drugs as chemical restraints. The goal of this study is to implement and evaluate an online team-based dementia education (Dementia Beyond Drugs: Changing the Culture of Care) program aimed at skill development and practice change. The program was delivered online over six weeks to teams of nursing home staff for a total of 12 hours of synchronous interactive learning. Teams were also assigned application exercises between meetings. Topics included “dementia, dementia beyond drugs, interpersonal approaches, and understanding personal expressions.” This study implemented the training with staff teams from 22 nursing homes in Georgia (N=97). Pre-post data analysis reveals a significant improvement in the nursing home environment and relationships as measured by the Kansas Culture Change Instrument, and increased attention to the use of antipsychotic drugs in staff meetings. This study indicates that team-based online learning has the potential to improve person-centered dementia care practices and attention to the use of non-pharmacological approaches to address behavioral expressions of residents living with dementia. Future research is needed to expand person-centered dementia care education programs to more nursing homes and evaluate their short and long-term effectiveness.

Abstract citation ID: igad104.2393

### FOSTERING PRACTICE CHANGE BY INTEGRATING INTERPROFESSIONAL EDUCATION IN A COMMUNITY HEALTH COLLABORATION

Jennifer DeGennaro[1], Lisa Bodenheimer[2], Elyse Perweiler[3], Margaret Avallone[4], Marilyn Mock[5], and Sreelekha Prakash[6], 1. *Rowan-Virtua SOM / NJISA, Stratford, New Jersey, United States*, 2. *Rowan University, Stratford, New Jersey, United States*, 3. *Virtual Health College of Medicine & Life Sciences of Rowan University - Rowan-Virtua School of Osteopathic Medicine, Stratford, New Jersey, United States*, 4. *Rutgers University-Camden School of Nursing, Camden, New Jersey, United States*, 5. *Fair Share Support Services, Inc., Camden, New Jersey, United States*, 6. *Stockton University, Galloway, New Jersey, United States*

NJGWEP undertook an interprofessional community/public health initiative collaborating with Rutgers University School of Nursing-Camden, Stockton University, NJ Housing and Mortgage Finance Agency, and four affordable housing sites located in underserved communities, with a potential to reach approximately 600 older residents. An IPE curriculum delivered to faculty, students, and staff from multiple health professions disciplines prepares them for assessing residents with chronic health problems using the 4Ms framework (What Matters, Mind, Medications, Mobility), adding the Geriatric 5th M for multi-complexity. Using a Resident Health Risk Assessment (RHRA) to assess residents’ health/biopsychosocial needs by capturing critical information needed to support “aging in place,” students and staff develop person-centered plans of care that address maintenance of independence, health, and referral to needed services/supports to delay or prevent institutional placement. From 2019-2022, 84 students from multiple disciplines (i.e., nursing, social work, counseling, OT) participated in community-based rotations, completed 279 RHRAs, and developed 101 IPE case reviews and person-centered plans of care. Evaluating residents’ co-morbid conditions (Hypertension 46%, Diabetes Mellitus 42%, Mental Health 40%; 8.6 medications per/resident, 44% Mini-Cog < 3; 19% PHQ+, 51% + Falls Screen; 48% TUG>12; 58% ADL/IADL deficits) highlighted the 5th M, Multi-Complexity. IPE is a sustainable venue for training a health care workforce that acknowledges/values the person-centered approach and encourages learning the roles and responsibilities of other disciplines. Implementation of a customized database in 2022 facilitates longitudinal tracking of outcomes related to resident needs, referrals, interventions, access to services to support aging in place, and informs policy and practice change.

Abstract citation ID: igad104.2394

**JOB AVAILABILITY FOR OLDER WORKERS IN A COMMUNITY: ANALYSIS ON JOB POSTINGS**

Hyesu Yeo, *University of Georgia, Athens, Georgia, United States*

**Objectives:** This study examined the current job availability in a local job market for older workers and investigated the required skills the older workers to be competitive. **Background:** Most studies approached older workers' employment transition from the supply side of the labor market, examining individual-level factors for a successful employment transition. However, there is a lack of knowledge on the demand side, such as job opportunities, regarding older workers' employment transition. **Methods:** The study uses combined data with job postings collected in the 4th quarter of 2021 from three engines and the O*NET database with occupational information to identify job opportunities and required skills. Additionally, the 2020 and 2021 American Community Survey were used to explore the characteristics of the older workforce in the community. **Results:** A total of 5,160 job postings divided into 468 occupations were found in the community. The most frequently available jobs were healthcare (14%), transportation and material moving (13 %), and sales (13%) occupations, which required some work experience needed. The older labor force mostly had a college or associate degree (45.8%) and was in educational (16%), office (11%), and sales (11%) occupations. All occupations require technological skills. **Conclusion:** The study found a mismatch between the available jobs and the actual older labor force, and those who are in employment transition, even downward movement, would be required some related experience. These suggest that a pre-employment transition program providing job training and age-friendly employment support services would be helpful for older workers in all occupational classes.

Abstract citation ID: igad104.2395

**LGBITQ+ INCLUSIVITY IN GERIATRIC CARE IN NORWAY: ATTITUDES AND PRACTICES AMONG HEALTH CARE PERSONNEL**

Janne Bromseth, *Høgskolen i Innlandet, Elverum, Ostfold, Norway*

Person-centered care is the dominating care philosophical framework being used in geriatric care services in Norway today, as a point of departure for good practice. It implies that all care-receivers should be met from an empathic and individual perspective- and an increased focus on basic psychological needs. To be positively confirmed in relation to ones´ identities and to feel included in communities are central parts of this (Kitwood 1997). But is this also the case for older LGBTIQ+-adults? And which competence and knowledge on LGBTIQ+-adults´ life course experiences and aging conditions exist amongst health care staff working in long-term care facilities, home care services and day care activities? This paper is based on results from the interview study "Queer perspectives on geriatric care in Norway", investigating experiences of person-centered care and inclusive practices from two perspectives; non-heterosexual care receivers and care providers in the Oslo-area. The analyzed material consists of: 1) interviews with 11 LGBQ-people between 61- 78 years old about their experiences with health care services over the life course 2) 3 focus groups and 2 individual interviews with health care staff working in a range of health and care services targeting 65+ adults. A majority of the interviewees had received training on LGBTIQ+-inclusive health care. The paper will mainly present results from the second study. What are leaders´ attitudes and knowledge regarding active work with gender and sexual diversity and equal access to care, and which experiences do they have from implementing LGBTIQ-awareness in policy and care practices?

Abstract citation ID: igad104.2396

**PERSPECTIVES OF THE HOME HEALTH WORKFORCE ON TRAINING**

Elise Valdes, *Relias Inc., Tampa, Florida, United States*

As more older adults desire to age in place, home health workers are becoming a vital component of the healthcare workforce. The last large scale nationwide survey among home health workers was conducted in 2007. There has been a variety of research in this area since then; however, the majority of studies are small and qualitative in nature which may limit their generalizability. Understanding the opinions and perspectives of home health workers is vital to retaining this important part of the healthcare workforce. For the current study a broad sample of home health workers (n=1446) including home health aides, hospice health aides, personal care aides, CNAs, LVN/LPNs, and RNs completed an online self-report survey on how well their training prepared them for practice. Results indicated that most hospice health aides felt their training well prepared them for practice (80.3%), while only 56.6% of RNs reported that they felt well prepared. We also explored how prepared respondents felt on a variety of topics from patient care skills to dementia care to end of life skills. Again, hospice health aides reported the highest average scores for receiving excellent training across all topics (61.5%) and RNs reported the lowest average scores (42.6%). Feeling unprepared for a job, may lead to lower job satisfaction and higher turnover. Therefore, understanding how well-prepared home health workers feel is key to retaining this segment of the workforce. Future research should explore how to improve this training and preparation across all home health workers but particularly among RNs.

Abstract citation ID: igad104.2397

**TRAINING FOR CAREGIVER OF OLDER ADULTS IN ECUADOR: REPORT ON PRESENTIAL, DISTANCE, AND HYBRID MODALITIES**

Jonathan Guillemot, Samantha Saenz, and Maria Augusta Vélez Landazuri, *Universidad San Francisco de Quito, Quito, Pichincha, Ecuador*

Caregiving for older adults in Ecuador is provided by family members, professional caregivers as well as untrained domestic workers. All three groups lack access to theoretical and technical training. The accelerating care drain towards Europe and North America, the fast-paced aging in the region as well as increasing trends of North American older adults migration to Ecuador, increase the need for large scale, quality and affordable caregiver training. We report the methods and outcomes of three modalities of training for caregivers of older adults combining over 100 individuals trained over the course of four years: a traditional presential training, which was offered prior to the

COVID-19 pandemic; an online modality via Zoom during the pandemic; and the early outcomes of a novel hybrid modality, combining presential and asynchronous video-based learning. We found that social interaction, including the sharing of personal experiences, was an essential part of the training experience, which translated into long-term social bonds and early stages of solidarity networks between participants. Online synchronous training allowed a broader geographical coverage but failed to contribute to socialization as well as generated challenges related to the learning experience and efficacy. The novel modality offered the broad geographical coverage, lowered the cost and allowed socialization. It however created technical and quality control challenges. Training of caregivers is a key component to promote aging in place and age-friendliness. Our experience and conclusion aim to contribute to the future of training in the Global South as well as in the Global North.

Abstract citation ID: igad104.2398

### TRAUMA AND RESILIENCE AMONG DIRECT CARE WORKERS IN NURSING HOMES

Alfred Boakye[1], Jennifer Morgan[2], Antonius Skipper[2], and Candace Kemp[2], *1. University of Maryland, Baltimore, Baltimore, Maryland, United States*, *2. Georgia State University, Atlanta, Georgia, United States*

Direct Care Workers (DCWs) face persistent challenges with low pay, few benefits, heavy workloads, and limited access to paid leave. Experiences associated with COVID-19 have increased the precarity of the long-term care system and have increased DCW's vulnerability. Against the backdrop of systemic racism and political turmoil, DCWs experience high rates of health risks (e.g., burnout, anxiety disorders, depression, and post-traumatic stress disorders) leading to a reduced quality of life. Building resilience within this workforce requires understanding the protective factors, at the community, organizational, and individual levels, to manage demanding work and personal lives. Yet, existing scholarship on how to build trauma-resilient organizations and identify and cultivate individual DCW strategies to support workers remains limited. Using a socio-ecological framework, the aim of this exploratory study was two-fold: (1) examine DCWs understanding of trauma and how it affects their ability to provide care, and (2) understand the strategies and supports DCWs use to cope with trauma and what it means to be resilient. Using purposive and convenience sampling techniques, participants were recruited into the study. Semi-structured interviews with nursing assistants in nursing centers (n=25) were used to gather data and analyzed using modified grounded theory. Findings demonstrate that the impact of COVID-19, work stress, and systemic inefficiencies negatively impact care work and DCWs' experiences. The study identified strategies that long-term care employers and government agencies can adopt to support resilience-building and ultimately empowerment practices. Implications for retention and support for staff from diverse backgrounds are also discussed.

### SESSION 7300 (POSTER)

### DEMENTIA CARE

Abstract citation ID: igad104.2399

### BUDDY-UP DYADIC PHYSICAL ACTIVITY (BUDPA) PROGRAM ON HEALTH OUTCOMES OF DEMENTIA CARE DYADS: A FEASIBILITY TRIAL

Doris S.F. Yu, and Polly Li, *The University of Hong Kong, Hong Kong, Hong Kong*

Dementia is a challenging disease experience not only jeopardizes the health of the persons with the condition (PWD) and their family caregivers. In views of the health promoting effects of exercise and its high relevance to promote a meaningful encounter between the dementia care dyads, a pilot feasibility was conducted to examine the effects of a 12-week multi-component Buddy-Up Dyadic Physical Activity (BUDPA) Program. Thirty care dyads were randomized to receive the BUDPA program or wait-list control. Outcome evaluation at baseline, 6 weeks and 12 weeks showed greater improvement in most of the cognitive functional domains (Coden's d = 0.30-0.87), neuro-psychiatric symptoms (Coden's d = 0.22), overall mood status (Coden's d = 0.30) and positive aspects of caregiving (Coden's d=0.32) among the BUDPA group, though the limited statistical power did not identify significant difference. The qualitative findings from eight care dyads engaged in BUDPA program converged with the outcome evaluation, and BUDPA was described as a 'physical-based social-cognitive training'. The PwDs were more engaging and attentive in daily life, with less irritability, delusive thoughts and repetitive behaviors. Caregivers reported feelings of vitality and enhanced mood. Converge with the 3-Phase Dyadic Adaptation Model, the care dyads felt more connected and their feeling of 'being cared for by the PwD is very powerful to reward their effort in caregiving. This finding implies the dyadic exercise is of high fidelity, and a full-scale study is needed to evaluate its effect from a health-promoting, social-interactional and existential paradigms.

Abstract citation ID: igad104.2400

### CARDIOVASCULAR DISEASE RISK SCORES AND INCIDENT DEMENTIA AND COGNITIVE DECLINE IN OLDER MEN AND WOMEN

Swarna Vishwanath[1], Ingrid Hopper[1], Andrew Tonkin[1], Anne Murray[2], Raj Shah[3], Robyn Woods[1], John McNeil[1], and Joanne Ryan[1], *1. Monash University, Melbourne, Victoria, Australia*, *2. Hennepin HealthCare and University of Minnesota, Minneapolis, Minnesota, United States*, *3. Rush University Medical Center, Chicago, Illinois, United States*

**Background:** Risk factors for cardiovascular disease (CVD) also increase the risk of dementia. However, whether CVD risk scores are associated with dementia risk in older adults remains unclear. The aim of this study was to determine whether CVD risk scores are associated with dementia and cognitive decline in older men and women. **Methods:** 19,114 participants from a prospective cohort of individuals aged 65+ years. The Atherosclerotic Cardiovascular Disease risk score (ASCVDRS), Systematic Coronary Risk Evaluation 2 – Older Persons (SCORE2-OP) and the Framingham risk score (FRS) were calculated at baseline. Risk of dementia (DSM-IV criteria) and cognitive decline (>1.5 standard deviation (SD) decline from previous assessment in cognitive function) were assessed using hazard ratio. **Results:** Over a median follow-up of 6.4 years, 850 individuals developed dementia and 4352 cognitive decline. Men and women in the highest ASCVDRS tertile had a 41% (95%CI 1.08,1.85) and 45% (1.11,1.89) increased risk of dementia compared to the lowest tertile respectively. Likewise, men and women in the highest SCORE2-OP tertile had a 64% (1.24,2.16) and 60% (1.22,2.11) increased risk of dementia. Findings were similar but the risk was slightly lesser when examining risk of cognitive decline for both ASCVDRS and SCORE2-OP. FRS was only associated with the risk of cognitive decline among women (highest vs. lowest tertiles: 1.13 [1.01-1.26]). **Conclusion:** These findings suggest the utility of CVD risk scores in clinical practice, to not only assess future risk of CVD, but also as potential early indicators of cognitive impairment in older men and women.

Abstract citation ID: igad104.2401

### CHALLENGES OF E-INTERVENTION RESEARCH: LESSONS FROM THE ISUPPORT FOR DEMENTIA-PT FEASIBILITY STUDIES

Soraia Teles, and Constança Paúl, *University of Porto, Institute of Biomedical Sciences Abel Salazar, Porto, Porto, Portugal*

E-interventions have become popular in delivering healthcare services, offering benefits such as increased accessibility and scalability. However, they also present ethical, technological, and research-related challenges. This poster aims to identify challenges of e-intervention research by analyzing feasibility studies of "iSupport for Dementia-PT", an online training and support program for informal dementia caregivers (IDC) culturally adapted to Portugal from the original version by the Word Health Organization. A mixed-methods study collected data on user satisfaction and requirements for the program's contents and interface through focus groups and usability test sessions with IDC (N=17) and health/social support professionals (N=13). A subsequent pilot study followed a mixed-methods experimental parallel between-group design with two arms (iSupport, N=21 and e-book, N=21). Ethical challenges unearthed by both studies included data privacy, e-consent reliability, equity in accessing e-health interventions, and e-health literacy. Challenging methodological decisions for an RCT protocol included defining criteria for a per protocol analysis in the context of a self-help e-intervention, timing for post-test or follow-up assessments, managing attrition, and the implications of resorting to online outcome measurements. The feasibility studies suggest that iSupport for Dementia-PT is a promising resource to support informal dementia caregivers. However, innovation is required in e-intervention research as replicating conventional procedures and methods have shown to be inadequate. Guidelines are needed on how to address the ethical, technological, and research-related challenges in e-intervention research.

Abstract citation ID: igad104.2402

### CLINICIAN AND STAFF PERSPECTIVES IN TRANSITIONS OF CARE FOR DUAL-USE VETERANS LIVING WITH DEMENTIA

Roman Ayele[1], Karen Albright[2], Catherine Battaglia[1], and Cari Levy[3], *1. VA Eastern Colorado Health Care System, Aurora, Colorado, United States*, *2. VA Eastern Colorado Healthcare System, Aurora, Colorado, United States*, *3. University of Colorado, Denver, Colorado, United States*

The Veterans Administration (VA) has partnered with non-VA hospitals to improve access to care. However, older Veterans living with dementia (VLWD) who use both VA and non-VA care may experience gaps in care, placing them at risk of adverse health outcomes during transitions. This research aims to understand the barriers and facilitators in transitions of care for VLWD following non-VA hospitalization. We conducted 23 interviews with VA and non-VA clinicians involved in transitions of care for VLWD. Snowball sampling was used to identify participants, and rapid analysis identified themes. VA Participants faced significant challenges in receiving timely discharge communication from non-VA stakeholders, relying on VLWD, who are poor historians, for information about their non-VA hospitalization. Inadequate staffing for care coordination was also a barrier to timely care, resulting in frustration for VLWD and their families when tests had to be redone due to a lack of appropriate documentation from non-VA hospitals. Both VA and non-VA stakeholders experienced similar care coordination challenges for VLWD, their families, and clinicians. Non-VA stakeholders described the VA as a "black box" due to a lack of communication and collaboration. However, stakeholders expressed a desire to provide quality care, improve care coordination, and give timely care to veterans. These findings highlight the barriers experienced by VA and non-VA stakeholders and provide opportunities for improvement in coordinating care for VLWD. Implementing care coordination programs and improving communication and collaboration between VA and non-VA stakeholders can improve the quality of care for VLWD and their families during transitions.

Abstract citation ID: igad104.2403

### COMMUNICATION PREFERENCES FOR DEPRESCRIBING BISPHOSPHONATES IN NURSING HOME RESIDENTS WITH DEMENTIA

Lauren Fasth[1], Casey Kelley[2], Cathleen Colón-Emeric[3], Carolyn Thorpe[4], Meredith Gilliam[2], Jennifer Lund[5], Laura Hanson[2], and Joshua Niznik[2], *1. UNC Eshelman School of Pharmacy, Chapel Hill, North Carolina, United States*, *2. University of North Carolina at Chapel Hill, School of Medicine, Chapel Hill, North Carolina, United States*, *3. Duke University School of Medicine, Durham, North Carolina, United States*, *4. University of North Carolina at Chapel Hill, Eshelman School of Pharmacy, Chapel Hill, North Carolina, United States*, *5. University of North Carolina at Chapel Hill, Gillings School of Global Public Health, Chapel Hill, North Carolina, United States*

Bisphosphonates are potential targets for deprescribing in nursing home residents with dementia as goals of care change from preventive to palliative. Yet, prescribers lack communication guidance to address deprescribing. We sought to characterize and compare communication preferences of prescribers and family/informal caregivers regarding bisphosphonate deprescribing. We conducted 23 semi-structured interviews with prescribers (12) and caregivers (11). Prescribers and caregivers were asked to provide their impressions of 7 conversation starters for discussing deprescribing bisphosphonates. A qualitative framework analysis was used to identify themes throughout the interviews across the following domains: content of communication, relationship between communicators, and preferred communication styles. Among prescribers, there were 10 physicians and 2 nurse practitioners; most (9) were female and White. Among caregivers, 8 were female, 7 were White, and 5 were Latino/a; most (9) had a post-secondary degree. The most preferred deprescribing conversation starter for prescribers focused on increased risk of adverse effects, while caregivers most preferred a discussion weighing risks versus benefits. The least preferred conversation starter for deprescribing across both groups focused on the extra effort and cost of continuing bisphosphonates. Similarities and differences between prescribers and caregivers regarding important topics for deprescribing conversations were identified, including quality of life, risk versus benefit, and shared decision-making. While caregivers and prescribers agreed on some communication preferences, this study highlights nuanced differences in preferred communication about deprescribing bisphosphonates. The results of this study may contribute to clinical guidance for prescribers and education materials for caregivers to facilitate more effective conversations about deprescribing bisphosphonates.

Abstract citation ID: igad104.2404

### CO-PRODUCTION TO TAILOR A DIGITAL TOOL FOR MONITORING SYMPTOMS OF DEMENTIA IN NURSING HOME CARE IN SWEDEN

Johannes Malm[1], Therése Bielsten[2], Elzana Odzakovic[2], Deborah Finkel[3], Charlotta Nilsen[2], and Ingemar Kåreholt[4], *1. Institute for Gerontology, Jönköping, Jonkopings Lan, Sweden*, *2. Institute of Gerontology, Jönköping, Jonkopings Lan, Sweden*, *3. Jönköping University, Jönköping, Jonkopings Lan, Sweden*, *4. Jönköping University, School of Health and Welfare, Jönköping, Jonkopings Lan, Sweden*

Symptoms of dementia change over time, resulting in complex situations that can negatively impact the person with dementia, as well as their relatives, and create challenges for staff members. Behavioral and Psychological Symptoms of Dementia (BPSD), such as delusions, hallucinations, agitation, depression, anxiety, apathy, irritability, aberrant motor behavior, and sleep disturbances, occur in approximately 90% of older people with dementia. The purpose of the study was to identify potential barriers and facilitators prior to introducing a web-based digital tool, the Daily-BPSD. Daily-BPSD is going to be used by staff members for daily registrations of severity levels of BPSD for persons with dementia in nursing home care in Sweden. Qualitative data collection was conducted in co-production with previous users of a similar digital tool in disability care (n = 11) and future users in dementia care (n = 32). The participants were assistant nurses, care managers, nurses, occupational therapists, and relatives. The responses highlight the importance of an accessible and time-effective registration procedure, a manageable number of variables and registrations occasions per day, and ensuring that the same information does not need to be documented in different systems. The findings will be used to tailor Daily-BPSD and adequately prepare staff members for large-scale data collection in the next step of the research project. Daily-BPSD could provide an extended foundation of knowledge of the person with BPSD, which could be used to provide more person-centered and appropriate care.

Abstract citation ID: igad104.2405

### DEMENTIA AND MILD COGNITIVE IMPAIRMENT: PREVALENCE, CONVERSION RATES, AND LONGEVITY BETWEEN AGES 70 AND 95

Jochanan Stessman[1], and Jeremy Jacobs[2], *1. Hebrew University of Jerusalem, and Hadassah Medical Center, Israel, Jerusalem, Yerushalayim, Israel*, *2. Faculty of Medicine, Hebrew University of Jerusalem, and Hadassah Medical Center, Israel, Jerusalem, Yerushalayim, Israel*

This study describes the prevalence and conversion rates of Mild Cognitive Impairment (MCI) and dementia between age 70-95, and the association between cognitive status and mortality. The Jerusalem Longitudinal Study (1990-2023) prospectively follows a representative community-dwelling cohort born 1920-21, assessed at home visit during 1990,1998, 2005, 2010, 2015 at ages 70, 78, 85, 90, and 95 (n=437, 806, 1170, 584, 468) respectively. Comprehensive assessment included Mini Mental State Examination (MMSE), defining normal cognition (29-30/30), MCI (24-28/30) and dementia (≤23/30). Mortality data were collected from the Ministry of Interior. Cox proportional hazards ratios (HR) were determined, adjusting for gender, education, self-rated health, physical activity, BADL status, diabetes, hypertension, ischemic heart disease. At ages 70, 78, 85, 85, 90, and 95 the prevalence of MCI was 13.5%, 21.5%, 38.7%, 37.0%, and 50%; prevalence of dementia was 3.2%, 4.2%, 24.3%, 31.3%, 35.7%. Between age 70-78, 78-85, 85-90, 90-95, conversion rates from MCI back to normal were 74.2%, 27.4%, 18.2%, 7.3%; MCI declining to dementia were 6.5%, 29.2%, 37.0%, 50.9%; MCI remaining MCI were 19.4%, 43.4%, 44.8%, 41.8%. Dementia was significantly associated with increased mortality throughout the entire study period: at age 70-78 Hazards Ratio=4.36 (CI 95%: 2.09-9.10), age 90-95 HR=1.5 (CI 95%: 1.01-2.21). In contrast, MCI was not associated with increased mortality, apart from at age 85: HR=1.38 (CI 95%: 1.04-1.82). Our findings show that conversion rates from MCI to dementia are age-dependent, with less than half of MCI subjects progressing to dementia. Unlike dementia, MCI was largely unassociated with mortality.

Abstract citation ID: igad104.2406

### EARLY DETECTION OF DEMENTIA AND MILD COGNITIVE IMPAIRMENT WITH BRAINCHECK

Bin Huang, Duong Huynh, and Reza Hosseini Ghomi, *BrainCheck, Austin, Texas, United States*

Dementia is projected to affect 14 million people in the United States and 152 million people globally in the coming decades. Early detection of dementia, which involves diagnosis of mild cognitive impairment (MCI), a possible prestage of dementia, is critical for intervention and care planning but remains a challenge with current clinical protocols. While brief screening tools are narrow in scope, comprehensive neuropsychological tests are time-consuming and require specialized training and expertise to conduct and interpret results. BrainCheck, a computerized cognitive assessment tool, provides an accessible and promising solution to address these current challenges. The aim of this study was to evaluate BrainCheck for its diagnostic accuracy and ability to distinguish the severity of cognitive impairment. A total of 99 participants clinically diagnosed with dementia (DEM; n=42), MCI (n=22), or normal cognition (NC; n=35)

completed the BrainCheck battery. Statistical analyses compared participant performances on BrainCheck based on their diagnostic group. BrainCheck battery performance showed significant differences between the three groups, achieving 88% or higher sensitivity and specificity for separating DEM from NC, and 77% or higher sensitivity and specificity in separating the MCI group from the NC and DEM groups. Three-group classification found true positive rates of 80% or higher for the NC and dementia groups and true positive rates of 64% or higher for the MCI group. These results show that BrainCheck was able to distinguish between diagnoses of DEM, MCI, and NC, providing a potentially reliable tool for early detection of cognitive impairment.

Abstract citation ID: igad104.2407

### EMBEDDING A PHYSICIAN REFERRAL PATHWAY TO LINK PEOPLE WITH DEMENTIA TO COMMUNITY SERVICE PROGRAMS

Michael Lepore[1], Stephani Shivers[2], and Deanna Curbeam[3], *1. University of Maryland School of Nursing, Baltimore, Maryland, United States*, *2. CaringKind, New York, New York, United States*, *3. University of Maryland, Baltimore School of Nursing, Baltimore, Maryland, United States*

Dementia care quality and value can be enhanced by lateral integration between health systems (HS) and community-based organizations (CBOs), however clinical providers are often unaware of CBO programs. HS could heighten access to CBO programs among people living with dementia (PLWD) by recognizing CBOs as extended care teams, streamlining referrals, and facilitating information exchange through electronic health records (EHRs). Catalyzing CBO and HS integration, we piloted physician referral to a CBO for virtual delivery of Cognitive Stimulation Therapy (CST) in an embedded pragmatic trial. CST, a 14-session group intervention for PLWD, has been shown to benefit participant cognition and other outcomes, yet CST access is limited partially due to the risks and costs of in-person gathering. To enhance access, we trialed a version of virtual CST (vCST) and assessed patient follow-through on physician vCST referrals. Participating sites–one primary care clinic and one neurological specialty clinic—identified eligible PLWD via EHR (e.g., cognitive score indicating mild cognitive impairment to moderate dementia) and clinicians verified eligible PLWD appropriateness for vCST. Clinician-verified PLWD were randomized to intervention (referral, n=56) and control (no referral) groups, and clinicians provided vCST referrals. Results show the percentage of PLWD who followed through on ('accepted') the vCST referral. Similarities and differences—across demographics, cognitive scores, and referral sources—between PLWD who accepted versus declined referral are described. Future research will assess conversion rates from vCST referral to program completion, the completeness of outcome measures captured from EHRs, and the impact of vCST on cognitive scores.

Abstract citation ID: igad104.2408

### EMOTIONAL RESPONSES OF ADULTS LIVING WITH DEMENTIA TO AN INTERGENERATIONAL MUSIC INTERVENTION

Jennie Dorris[1], Ketki Raina[1], Stephen Neely[2], and Juleen Rodakowski[1], *1. University of Pittsburgh, Pittsburgh, Pennsylvania, United States*, *2. Carnegie Mellon University, Pittsburgh, Pennsylvania, United States*

Music interventions have shown promise to support emotional well-being in older adults living with dementia. Bolstering emotional well-being could improve the quality of life of the millions of older adults living with dementia. We implemented an intergenerational music intervention at Dementia360, a community program that serves older adults living with dementia. The music intervention featured adolescent musicians performing the preferred music of the older participants and engaging them in interactive musical activities via Zoom. 14 older adult participants attended an average of four sessions, 54 minutes each, of the intervention. We described their positive affect and pleasure as they engaged in the intervention. We compared participants' scores on the Positive and Negative Affect Schedule (PANAS) pre- and post-intervention. We also assessed the participants' emotions by rating them using the Observed Emotion Rating Scale (OERS). On average, the participants' scores increased three points on the Positive Affect subscale of the PANAS. Our computed cohen's d effect size was 0.5, a moderate effect size that correlates with self-report measures of feeling "a little more" to "much more" positive affect. On the OERS Pleasure subscale, we found that pleasure was shown for more than one minute of the ten-minute sample in over 87% of the observations. Intergenerational music interventions employ the safe, pleasurable, and scalable modality of music. These findings suggest that participation in such an intervention has the potential to influence positive affect and pleasure, critical supports for the emotional well-being of older adults living with dementia.

Abstract citation ID: igad104.2409

### EMPOWERING STAFF TO MANAGE MEALTIME DIFFICULTIES AMONG RESIDENTS WITH DEMENTIA: A PILOT STUDY

Lígia Passos[1], João Tavares[1], Melissa Batchelor[2], and Daniela Figueiredo[1], *1. University of Aveiro, Aveiro, Aveiro, Portugal*, *2. George Washington University, Washington, District of Columbia, United States*

People in advanced stages of dementia may experience mealtimes difficulties and depend on others for feeding. This pilot study aimed to evaluate the feasibility, acceptability, and preliminary effects of an educational intervention on mealtime difficulties of people with dementia, in a sample of 16 direct care workers and 9 people with dementia and was conducted in a nursing home. The sessions provided information about dementia, mealtime difficulties, and training on strategies and techniques. Feasibility was assessed through screening, eligibility, consent, retention, completion, and intervention adherence rates; and acceptability through a satisfaction questionnaire. The staff assessment included: burnout, job satisfaction, and knowledge about dementia and mealtime difficulties. In people with dementia, eating and feeding problems, and food intake were assessed. The main findings show that all feasibility rates, except for screening (89%), were excellent (100%). Direct care workers appreciated the intervention and perceived educational and practical benefits, although they reported needing more time to provide better assistance. Burnout levels did not change (mean 58.9 versus 58.6), but job satisfaction showed a positive trend (mean 71 versus 74.5) at the end of the intervention. Knowledge increased 37.5% compared to baseline. EdFED's score presented slight improvement, while the oral intake of people with dementia increased after the intervention (91.1% versus 98.1%). The intervention was found to be feasible and acceptable. However, the educational intervention content needs improvement, and further studies with robust designs and larger samples are needed to better understand its effects on both direct care workers and people with dementia.

Abstract citation ID: igad104.2410

### IDEA-EXECUTION ABILITY, SELF-DETERMINATION, AND CHEERFULNESS PREDICT DEMENTIA ONSET OVER NINE YEARS

Yufang Tu, Teri Undem, and Melissa O'Connor, *North Dakota State University, Fargo, North Dakota, United States*

The prevalence of dementia and its negative effect on well-being has made it a global public health concern. It is important to understand factors that predict the onset of dementia above and beyond age and physical health, to aid in preventative efforts. The current study investigated idea-execution ability, self-determination, and cheerfulness as predictors of the onset of dementia across a nine-year period. Longitudinal data from the first nine waves of the National Health and Aging Trends Study (NHATS) were used. NHATS participants are nationally representative of Medicare recipients in the United States. Data were initially collected in 2011 and annually thereafter. The current study included 6780 participants; 678 participants reported having a diagnosis of dementia at either baseline or a follow-up visit. Cox regression was used to examine the following self-report items as predictors of months-to-dementia onset: idea execution ability ("when I really want to do something, I usually find a way to do it"); self-determination ("other people determine most of what I can and cannot do"); and cheerfulness ("how often I feel cheerful"). Each predictor was time-invariant and scored on a 3-point scale so that higher scores were more positive. Covariates included baseline age group (five-year groups), sex (dichotomized), years of education, and self-rated overall health. Statistically significant results were shown for idea-execution (HR=0.79, p=0.008), self-determination (HR=0.69, p< 0.001), and cheerfulness (HR=0.91, p=0.047). Each of these potentially modifiable variables was associated with reduced odds of developing dementia, above and beyond demographic and health factors.

Abstract citation ID: igad104.2411

### MOTIVATIONS AND ROLE CAPTIVITY AMONG INFORMAL CAREGIVERS TO COMMUNITY-DWELLING PERSONS WITH DEMENTIA

Rachel Bloom[1], Francesco Osso[2], Megan McCarthy[2], and Francesca Falzarano[2], *1. Fordham University, New York City, New York, United States*, *2. Weill Cornell Medicine, New York City, New York, United States*

There are 11 million informal (unpaid) caregivers currently assisting a person with dementia in the United States. Motivations for taking on this challenging role vary, with many citing their responsibility to family or a shared interpersonal history. Many caregivers do not feel as if they have a choice in taking on care responsibilities for relational and/or financial reasons and are held "captive" in this role. Prior research indicates role captivity as a key mediating force in the development of depression among dementia caregivers, but limited work has directly linked quantitative measures of role captivity with qualitative data surrounding motivations for providing care. The current mixed-methods study uses quantitative and qualitative data to explore this connection in a sample of 46 participants providing care to a community-dwelling person with dementia. Study findings show that role captivity was significantly associated with greater costs of care, feelings of grief, and depressive symptoms, as well as lower levels of self-efficacy and mutuality. These relationships were assessed alongside thematic analyses of interview data centered on motivations for providing care, familial and cultural expectations, and financial necessity. This research paints a detailed picture of the way in which dementia caregivers understand and relate to their role and can inform the design of effective psychological supports for this population.

Abstract citation ID: igad104.2412

### NEUROPSYCHIATRIC SYMPTOMS OF DEMENTIA AND CAREGIVERS' BURDEN

Constanca Paul[1], Susana Sousa[2], and Laetitia Teixeira[3], *1. ICBAS, Portugal, Porto, Porto, Portugal*, *2. ICBAS, University of Porto, Porto, Porto, Portugal*, *3. School of Medicine and Biomedical Sciences, University of Porto, Porto, Porto, Portugal*

Informal caregiving (IC) is a major health and social asset although simultaneously a potential burden and depression risk factor for those who care. Neuropsychiatric symptoms of dementia are disturbing and may contribute to a negative experience of care. To understand the neuropsychiatric symptoms of dementia in caregiving we have two objectives: to know the prevalence of neuropsychiatric symptoms perceived by ICs and if there is an association of neuropsychiatric symptoms and burden and depression of caregiver. We study a community-based sample (N=101) of primary care users with mental health concerns, referred by GPs. Instruments include sociodemographic information; Neuropsychiatric Inventory (NPI-Q), Caregiver Burden Scale (CBS) and Geriatric Depression Scale (ABCDS). The sample mean age was 79.4 (sd=7.7) years, 54 (53.5%) were female and 34 (33.7%) had no formal education. Half of the sample were married and 41 (41.90%) were widowed. The NPI-Q total score and the Distress dimension score were 10.1 (sd=5.6) and 11.9 (sd=9.1), respectively. The more reported symptoms were agitation/aggression (69.3%), and apathy/indifference (65.3%). We found a significant positive association between NPI-Q and caregivers' burden (0.296) and depression (0.227) and between Distress and caregivers' burden (0.417) and depression, 0.416.

**Conclusion:** Neuropsychiatric symptoms of dementia appear associated with the burden and depression of informal caregivers. Training ICs to cope with care receiver symptoms is a priority to increase the quality of care and reduce burden and depression of caregivers.

Abstract citation ID: igad104.2413
### NURSING HOME WORKFORCE EDUCATION: DOES ONE SIZE FIT ALL? EVIDENCE FROM THE CHATO NATIONAL TRIAL

Kristine Williams[1], Yelena Perkhounkova[2], Carissa Coleman[3], Maria Hein[4], Frances Yang[3], and Clarissa Shaw[5], *1. University of Kansas Medical Center, Kansas City, Kansas, United States*, *2. University of Iowa, Iowa City, Iowa, United States*, *3. University of Kansas, Kansas City, Kansas, United States*, *4. University of Iowa, College of Nursing, Iowa City, Iowa, United States*, *5. University of Iowa, Iowa City, Iowa, United States*

Educating the nursing home workforce to provide quality dementia care is a national priority. Staff education is usually provided to all staff in communities that vary in size, location, ownership, and resources with little consideration for diversity. To evaluate suitability for diverse staff, we compared learning outcomes for White, non-Hispanic staff and staff of diverse races and ethnicities who completed CHATO in year one. White non-Hispanic staff (N=402) scored higher on knowledge assessments than staff self-reporting other races and ethnicities (N=201) at baseline (mean=57.1% vs. 49.1%, p<.001). Both groups improved on post-assessment (by 19.0 and 23.1 percentage points, respectively, p<.001), Communication ratings indicated that at baseline White, non-Hispanic staff were more knowledgeable about effective (p<.001), appropriate (p=.008), and person-centered communication (p<.001) than diverse staff; while the latter recognized elderspeak better (p=.002). Both groups improved their communication ratings post-CHATO (p from .001 to <.001). While confidence in providing dementia care was similar between groups at baseline (p=.754), it improved only for White, non-Hispanic staff (p<.001). Intention to use learned skills in practice was higher for staff in the diverse group (p<.001). Diverse staff rated their satisfaction with the intervention lower (p <.001), suggesting that it was not tailored to their needs. These findings support the value of CHATO in increasing communication knowledge among diverse NH staff. However, lower satisfaction suggests the need to adapt CHATO to be more inclusive and culturally appropriate. Tailoring education may increase impact on outcomes and overcome disparities in dementia care common in NHs serving diverse populations.

Abstract citation ID: igad104.2414
### PERSON-CENTERED DEMENTIA CARE IN SOUTH KOREA: AN INTEGRATIVE REVIEW

Eun-Hi Kong[1], Hyunju Park[2], and Juh Shin[3], *1. Gachon University, Incheon, Republic of Korea*, *2. Kangwon National University, Chunchon, Republic of Korea*, *3. George Washington University, Washington, District of Columbia, United States*

Person-centered dementia care is a globally recognized best practice; however research on this practice is lacking. In Korea, the number of people living with dementia is rapidly increasing, expanding the need for person-centered care. Thus, we reviewed published studies regarding person-centered dementia care in Korea. We searched for studies in four databases (RISS, KISS, KoreaMED, and DBpia) and selected 14 primary studies published between 2007 and 2023. Two reviewers extracted data and three reviewers analyzed the data. Search keywords included person-centered care, person-centred care, person-centered dementia care, person-centred dementia care, dementia, demented, Alzheimer, or cognitive impairment. A total of 14 articles met inclusion criteria. The primary studies were published between 2007 and 2023, and most studies were conducted in long-term care settings and targeted formal care staff. The authors' disciplines included nursing, social welfare, humanity, and human services. Many studies did not report theory or theoretical models. Research designs included non-experimental study (8), qualitative study (4), and experimental study (2), and randomized clinical trial (1). Many studies did not report on theory or theoretical models. Most quantitative studies included variables such as personhood in dementia, person-directed care, person-centered climate, dementia knowledge, or attitude toward dementia. Most quantitative studies reported that person-centered dementia care education programs enhanced personhood in dementia in long-term care settings. The qualitative studies explored the meaning of humanitude, readiness to adapt, effective communication, or education programs related to person-centered dementia care. Future scholars should conduct experimental studies or mixed-method studies to report on theory or theoretical models.

Abstract citation ID: igad104.2415
### PROTOCOLS FOR COLLECTING CONVERSATIONAL LANGUAGE IN PERSONS WITH DEMENTIA

Oleg Zaslavsky[1], Trevor Cohen[1], Kuan-Ching Wu[1], Jessica Jin[1], Shao-Yun Chien[1], and Serguei Pakhomov[2], *1. University of Washington, Seattle, Washington, United States*, *2. University of Minnesota, Minneapolis, Minnesota, United States*

We describe first-of-its-kind protocols for collecting spontaneous conversations in persons with dementia living in a community through conversation elicitation tools twice a month over six months. We also describe the preliminary feasibility of the protocol deployment in the first enrolled participants over the first month of data collection. Participants were recruited from the University of Washington ADRC registry. The audio was captured using RODE wireless microphone system. Four conversational elicitation techniques were used: 1) reminiscence using personal photographs; 2) reminiscence using generic historical images; 3) picture description test from neurocognitive batteries; 4) open-ended questions about daily activities. Health measures included Cornell Scale for Depression in Dementia (CSDD), Clinical Dementia Rating (CDR), and Clinical Frailty Scale (CFS). Four male and two female participants (average age 75) were recruited. Average health measures at baseline were CDR = 2, suggesting moderate dementia; CSDD=7, suggesting depressive symptoms, CFS=5, suggesting mild frailty. At baseline, the tasks' average conversation time in seconds was as follows: daily activities =528; personal reminiscence=443; historical reminiscence =227; picture description =192. The corresponding average times for the subsequent home visits were 917; 518; 474, and 280 at visit 1 and 525; 767; 629, and 311 at visit 2. We established the feasibility of collecting conversational speech in this population. Participants seemed more interested in conversations focused on daily activities and personal pictures than general topics. There was an increase in the duration of conversations over time, which might be related to participants' greater familiarity with the study protocol and data collectors.

Abstract citation ID: igad104.2416

### RACIAL/ETHNIC AND MULTIMORBIDITY DIFFERENCES IN DEMENTIA ONSET AMONG MIDDLE-AGED AND OLDER ADULTS

Siting Chen[1], Corey Nagel[2], Anda Botoseneanu[3], Heather Allore[4], Jason Newsom[5], Stephen Thielke[6], Jeffrey Kaye[1], and Ana Quiñones[1], *1. Oregon Health & Science University, Portland, Oregon, United States*, *2. University of Arkansas for Medical Sciences, Little Rock, Arkansas, United States*, *3. University of Michigan, Ann Arbor, Michigan, United States*, *4. Yale University, New Haven, Connecticut, United States*, *5. Portland State University, Portland, Oregon, United States*, *6. University of Washington, Seattle, Washington, United States*

Racial/ethnic minoritized older adults in the U.S. have higher prevalence of multimorbidity and experience higher risk of dementia relative to non-Hispanic White older adults. This study examined the relationship between race/ethnicity and dementia onset within the context of multimorbidity during middle and later life in the U.S. Data from the Health & Retirement Study (1998-2018, HRS and War Babies cohorts, N=8,031) were applied to discrete-time survival models to estimate differences in the risk of dementia onset using the Langa-Weir Classification for up to ten time intervals (1998-2018). Models were adjusted for race/ethnicity (non-Hispanic White, non-Hispanic Black, Hispanic), multimorbidity (no/one disease, advanced cardiovascular multimorbidity, metabolic multimorbidity, advanced cardiovascular-metabolic multimorbidity, and neither advanced cardiovascular nor metabolic multimorbidity), age at baseline, sex, education, and whether a proxy provided the interview. Over the 20 years, 9.8% of the respondents (n=785) developed dementia. In covariate-adjusted models, non-Hispanic Black (adjusted Odds Ratio (aOR) =2.27, 95%CI: 1.90, 2.72) and Hispanic (aOR=1.59, 95%CI: 1.26, 2.01) respondents had higher risk of developing dementia compared with non-Hispanic White respondents. Regarding multimorbidity categories, respondents with advanced cardiovascular-metabolic multimorbidity (aOR=1.80, 95%CI: 1.45, 2.24) had significantly higher odds of dementia onset relative to the neither advanced cardiovascular nor metabolic multimorbidity category. Significant differences were observed in dementia onset for up to 20 years between racial/ethnic minoritized and White adults, as well as among multimorbidity combinations. Our findings highlight the need to intervene earlier on modifiable risk factors, such as multimorbidity, to reduce the risk of developing dementia among racial/ethnic minoritized adults.

Abstract citation ID: igad104.2417

### THE ASSOCIATION BETWEEN PSYCHOTROPIC DRUGS AND PHYSICAL FUNCTION IN HOSPITALIZED OLDER ADULTS WITH DEMENTIA

Nayeon Kim[1], and Barbara Resnick[2], *1. University of Maryland Baltimore, Baltimore, Maryland, United States*, *2. University of Maryland, Baltimore, Maryland, United States*

The aim of this study was to test if the use of psychotropic medications (antipsychotics, sedatives, antiseizure, antidepressants, and anxiolytics) was associated with physical function in hospitalized older adults living with dementia. This study was a secondary data analysis using baseline data from the Function Focused Care for Acute Care Using the Evidence Integration Triangle (FFC-AC-EIT) study. FFC-AC-EIT is an ongoing randomized controlled trial and this study included the first 290 participants. A path analysis was conducted to test the relationship between psychotropic drugs and physical function while controlling for age, gender, race, comorbidities, cognitive function, neuropsychiatric symptoms of dementia, and pain. Overall, 63.4% of participants took one or more psychotropic drugs, with the most used psychotropic drugs being antidepressants (39%), followed by antiseizure drugs (23.4%), antipsychotics (16.6%), anxiolytics (13.8%), and sedatives (4.1%). No significant association was found between the use of psychotropic medications and physical function. Cognitive function ($\beta$=1.489, SE 0.265, $p<$ 0.001), comorbidities ($\beta$=-2.680, SE 1.093, p=0.014), and pain ($\beta$=-2.837, SE 0.865, p=0.001) were directly associated with physical function. Cognitive function was also indirectly associated with function through pain. The findings support prior research showing a lack of association between the use of psychotropic medications and physical function. Additional research may be needed to investigate the mediating effects of pain between cognition and physical function in older adults with dementia.

Abstract citation ID: igad104.2418

### THE DREAM TOOLKIT: CO-DEVELOPED TRAINING AND RESOURCES TO PROMOTE WELL-BEING OF PERSONS WITH DEMENTIA

Shannon Freeman[1], Laura Middleton[2], Chelsea Pelletier[1], Kelly Skinner[2], Kayla Regan[2], Rachael Donnelly[2], Heather Keller[2], and DREAM Research Team[2], *1. University of Northern British Columbia, Prince George, British Columbia, Canada*, *2. University of Waterloo, Waterloo, Ontario, Canada*

Promoting wellbeing of persons with dementia is a priority. Engaging multi-perspective partners to co-develop interventions creates impactful solutions. We will describe the process, output, and lessons from the Dementia Resources for Eating, Activity, and Meaningful inclusion (DREAM) project, which co-developed tools/resources with persons with dementia, care partners, community service providers, health care professionals, and researchers. We aimed to increase supports for physical activity, healthy eating, and wellbeing of persons with dementia. Our process included: 1) Engaging/maintaining the DREAM Steering Team; 2) Setting/navigating ways of engagement; 3) Prioritizing audience and content of the toolkit; 4) Drafting content & format of toolkit; 5) Iterative co-development of tools/resources; 6) Usability testing; 7) Implementation and evaluation. In virtual meetings, the DREAM Steering Team confirmed toolkit audiences (primary: community service providers; secondary: persons with dementia and care partners) and discussed and evolved content areas. An environmental scan identified existing, high-quality resources aligned with content areas. The DREAM Steering Team alongside additional community partners and external contractors iteratively co-developed new resources/tools to meet gaps. The DREAM toolkit includes a website, seven learning modules about dementia, healthy eating, and physical activity, a learning manual, six videos, nine handouts, and four wallet cards (www.dementiawellness.ca). Co-development participants rated the virtual co-development process highly in relation to the principles and enablers of Authentic Partnership in a process evaluation. Through the co-developed DREAM toolkit, we anticipate community service providers will learn to provide inclusive wellness programs and services to benefit persons with dementia and their families.

Abstract citation ID: igad104.2419

**UNDERSTANDING CHALLENGES IN DEMENTIA CARE AMONG AFRICAN AMERICAN AND LATINX POPULATIONS**

Rifat Alam[1], Brianna Sirdich[2], and Andiara Schwingel[3], *1. University of Illinois at Urbana-Champaign, Urbana, Illinois, United States*, *2. University of Illinois at Urbana-Champaign, Champaign, Illinois, United States*, *3. University of Illinois, Urbana Champaign, Champaign, Illinois, United States*

Addressing disparities in dementia care among minoritized populations in the US is obligatory. This qualitative study explores perspectives of healthcare providers and administrators about challenges in delivering dementia care among African American and Latinx populations. 12 healthcare providers and administrators from 5 organizations in Illinois and Indiana were interviewed virtually between October 4, 2022, and March 7, 2023, and transcribed verbatim. Interviews were coded and three emerging themes were identified inductively describing participants' perspectives. Delays in treatment-seeking: Includes challenges of the communities to understand normal aging and dementia and trust healthcare system, lack of knowledge of available resources and perceived cost, tradition of informal caregiving and diminished insights of patients. Lack of a comprehensive support system: Describes areas hindering providers and administrators becoming a support for the patients, includes- communication barriers to understand language and vernaculars, lack of access to healthcare, insurance, and resources by the minoritized communities and needs for an efficient primary healthcare team. Difficulties in patient evaluation: Time constraints of the primary care providers and shortage of specialists affect proper evaluation of patients. Engaging patients from low-income groups and immigrants in the system is complex. Non-compliance due to unavailability of transportation and telemedicine and comorbidities of older patients leads to a failure of complete evaluation of patients' cognitive function that needs multiple visits. Our study highlights prevailing challenges and areas of advancement of dementia care among African American and Latinx populations. Developing a roadmap for a policy-level change and cross-culturally valid interventions will help address dementia inequities.

### SESSION 7310 (POSTER)

### EDUCATION AND TRAINING

Abstract citation ID: igad104.2420

**ADAPTING A DEMENTIA CAREGIVER CURRICULUM ACROSS THREE HEALTH PROFESSIONS**

Edmund Duthie[1], Kathryn Denson[1], Deborah Simpson[2], Stacy Barnes[3], Jennifer McAlister[4], Amanda Szymkowski[1], Rachel Kavanaugh[1], and Wendy Betley[5], *1. Medical College of Wisconsin, Milwaukee, Wisconsin, United States*, *2. Advocate Aurora Health, Milwaukee, Wisconsin, United States*, *3. Marquette University, Milwaukee, Wisconsin, United States*, *4. Alzheimer's Association, Milwaukee, Wisconsin, United States*, *5. Alzheimer's Association,, Milwaukee, Wisconsin, United States*

Unpaid caregivers provide >80% of the care for the 6.2 million Americans living with dementia, yet healthcare professionals rarely refer them to community services. The Alzheimer's Association's "Direct Connect" (DC) referral program is provider-initiated connecting patients/caregivers with resources to improve care and quality of life. Our interprofessional team created a 15-minute curriculum to educate students about DC and how to advocate for DC use at clinical sites. Champion faculty, from each profession, utilized a common presentation, with minor modifications for professional relevance. Delivery was both synchronous/asynchronous to learners from 2 different universities with knowledgeable clinician(s) available to answer questions. A Qualtrics survey collected feedback (N=218; 105 medicine, 57 nursing, 56 pharmacy). Relevance of the curriculum to each of the 3 professions was high (1-5 scale; mean range 4.46 – 5.00), with nursing rating it as most relevant. Feedback was also quite positive across the professions (78%-92% rated the curriculum quality as excellent/very good overall), with nursing giving the highest ratings. Most learners indicated a strong likelihood of using DC in clinical settings; 64-100% are very likely or somewhat likely to do so, with nursing being most likely to make referrals. In summary, a common dementia DC curriculum was successfully implemented for learners in 3 health professions; it was highly relevant to each profession, well accepted, and perceived to be clinically actionable. Nurses tended to be the most positive on these measures. While these results are promising, DC remains underutilized and initiatives to further increase referrals from providers must be expanded.

Abstract citation ID: igad104.2421

**COMPETENCY-BASED UNDERGRADUATE PALLIATIVE NURSING EDUCATION USING AN INTERPROFESSIONAL TEACHING APPROACH**

Brandi Felderhoff[1], and Catherine Mbango[2], *1. Texas Woman's University, Denton, Texas, United States*, *2. Texas Woman's University, Houston, Texas, United States*

The nursing workforce is expected to be "work ready" to address healthcare needs in diverse communities. Health inequities continue to persist, and access to healthcare services that promote well-being and improve quality of life is imperative. Nursing curricula that integrate competencies in palliative supportive care for patients with chronic complex illnesses are needed to improve health equity and promote global citizenship (AACN, 2021). The shift to competency-based learning is both an accreditation requirement, and essential to prepare nursing students for the NextGen NCLEX (Wolf, 2022). Through review of the AACN's (2021) The Essentials: Core Competencies for Professional Nursing Education core competency of palliative and supportive care in the domain of population health for entry-level professional nurses, and framework from The Interprofessional Education Collaborative (IPEC) for team-based competencies to improve population health (IPEC, 2016), a nursing-social work education partnership is well-suited to address

AACN's Essentials utilizing IPEC's four core competencies. This approach to teaching allows students to develop knowledge and competence in caring for patients with palliative or supportive care needs, effectively bridging the competency gap (Jones & West, 2017) with curricula developed through interprofessional collaboration (Gravina, 2017). Primary palliative nursing care is an interdisciplinary patient-centered approach that focuses on the management of physical, psychological, social, and spiritual care of patients and their families. The opportunity to learn through an interdisciplinary teaching approach better prepares students to examine their place within the team, and fully understand the related roles of their allied colleagues.

Abstract citation ID: igad104.2422

### EFFECTS OF AN AGE SUIT SIMULATION ON NURSING STUDENTS' PERSPECTIVES ON PROVIDING CARE TO OLDER PERSONS

Jenny Hallgren, Björn Bouwmeester Stjernetun, and Catharina Gillsjö (Gillsjo), *University of Skövde, Skövde, Vastra Gotaland, Sweden*

**Background:** Nursing students are important future health care providers to the growing number of older persons in society. However, two barriers are their common ageist attitudes and lack of interest in geriatrics. This is a concern in light of the global demand for more nurses. Method: This study investigated the effects of ageing simulation with an age suit as a part of experiential learning in a nursing programme among 471 nursing students. The simulation allowed the students to experience specific and common health problems from the patient's point of view in a controlled environment and a relevant context of care provision for future nurses: a home with welfare technology and other aids. The learning process involves a continuous cycle of doing and reflecting resulting in knowledge that also becomes "ingrained" in the body of the learner. Data on the Perspectives on Caring for Older Patients – Short Form (PCOP-SF) scale were collected using a quasi-experimental pretest–posttest design with a control group. **Results:** The results showed that the intervention had a positive effect on various aspects of nursing students' perspectives on caring for older persons. Work experience was associated with more positive attitudes. The control group was more negative towards geriatrics as a career choice than the intervention group. **Conclusion:** Age suit simulation can be an innovative intervention in nurse education as it raises awareness and understanding of aging and the health challenges of older persons, which are important in combatting ageism among future nurses.

Abstract citation ID: igad104.2423

### MAP GERIATRICS CLERKSHIP LEARNING OBJECTIVES WITH GERIATRICS 5MS

Huai Cheng, *University of Pittsburgh, Pittsburgh, Pennsylvania, United States*

**Background** Minimum Geriatrics Competencies (MGC) are used to guide teaching geriatrics for medical students in a required geriatrics clerkship since 2011. These learning objectives can be mapped to Entrustable Professional Activities (EPA). Since 2017, Geriatrics 5Ms (mind, mobility, medication, multi-complexities, and what matters most) was developed to guide teaching geriatrics for medical students and to promote a Geriatrics 4Ms Age-friendly health care system as well. The aim of this study is to examine whether an MGC-based geriatrics clerkship is consistent with geriatrics 5Ms by mapping its learning objectives with geriatrics 5Ms. **Methods** This geriatrics clerkship is a two-weeks required rotation for all fourth-year medical students. It includes didactic classroom teaching and clinical rotation in different geriatrics practice, palliative care, and physical medicine rehabilitation. This clerkship has a total of 50 learning objectives with corresponding MGC teaching methods/strategies, assessment, and evaluation. The author will map each learning objective with Geriatrics 5Ms. Results 13 learning objectives are mapped to "Mind". 9 learning objectives are mapped to "Mobility". 10 learning objectives are mapped to "Medications". 14 learning objectives are mapped to "multi-complexity". 6 learning objectives are mapped to "Matters most". 12 learning objectives are not mapped to any of geriatric 5Ms. Conclusion and discussion The majority of 50 learning objectives in a required geriatrics clerkship can be mapped to geriatrics 5Ms. This MGC-based learning objective can be adopted to teach medical students geriatric 5Ms and geriatric 4M Age-friendly health care system. **Take-home message** MGC-based learning objectives are aligned with geriatrics 5Ms.

Abstract citation ID: igad104.2424

### ORAL HEALTH: THE MOST UNMET HEALTH CARE NEED FOR MEDICALLY VULNERABLE ELDERLY WITH OR WITHOUT DEMENTIA

maryam Tabrizi[1], and Wei-Chen Lee[2], *1. University of Nevada in Las Vegas, Las Vegas, Nevada, United States, 2. University of Texas Medical Branch, Galveston, Texas, United States*

Individuals aged 65 and older are living longer with multiple chronic conditions, leading to a larger population of medically vulnerable elderly with or without dementia. Consequently, only an interdisciplinary health system can meet the comprehensive health needs for older adults. Historically, Oral Health (OH) is the most unmet need in healthcare, particularly in older adults. This is due to several barriers including Interprofessional Education (IPE) and Interprofessional Collaboration (IPC). Therefore, there is a growing gap between OH and overall health. We conducted two studies in the past 4 years. The first study included predoctorate healthcare students (Medical, Nursing, Dental and Pharmacy) evaluating their IPE during their educational training and the influence of their education later as practitioners. The result of this study revealed an unsatisfactory IPE that would lead to minimum or no interdisciplinary collaboration. The second study (2019 to 2022) included dental residents in a one year post graduate program "Advanced Education in General Dentistry" (AEGD). In each year we collected a survey at the beginning of the residency program asking the level of comfort treating medically vulnerable elderly as a provider in a health team. We then exposed them to a comprehensive geriatric oral health both didactics and clinical skills. The same survey was again conducted at the end of the residency. The study revealed adequate training increased the level of residents' ability to provide care within a health team. Interdisciplinary health services require interdisciplinary educational training to close the gap between oral health and overall health.

### SESSION 7320 (POSTER)

### GLOBAL AGING AND HEALTH (HS)

Abstract citation ID: igad104.2425

### MEDIATING EFFECTS OF QUALITY OF LIFE IN THE ASSOCIATION OF DEBTS AND LATER MEMORY FUNCTION IN CHINA, ENGLAND, AND THE US

Tsai-Chin Cho[1], HwaJung Choi[2], Kenneth Langa[2], Sara Adar[1], and Lindsay Kobayashi[1], *1. University of Michigan School of Public Health, Ann Arbor, Michigan, United States*, *2. University of Michigan, Ann Arbor, Michigan, United States*

Non-mortgage debts were linked to negative cognitive health outcomes in later-life older adults. It is unclear how they may be associated with later-life memory function through psychosocial pathways, and whether the associations vary by health care system and policy environment. We examined the mediating effects of depressive symptoms and dissatisfaction with life in the association between non-mortgage debts and subsequent memory function among adults aged 65-101 years in the US, China, and England. Data were from harmonized, nationally representative longitudinal studies of aging in the US (Health and Retirement Study; n=8,388), China (China Health and Retirement Longitudinal Study; n=1,180), and England (English Longitudinal Study of Aging; n=3,390). Non-mortgage debt burden was defined as any pre-existing or new non-mortgage debts in two years (2010-2012 for the US and England; 2011-2013 for China). Memory function was measured by 20-point immediate and delayed word recall summary scores over a subsequent 5- or 6-year follow-up period. Within each country, we used sampling-weighted, multivariable-adjusted causal mediation analysis (product method) to estimate the total and direct effects of non-mortgage debts on memory function, and the indirect effects mediated by depressive symptoms and dissatisfaction with life. We observed significant mediating effects of depressive symptoms on the association between non-mortgage debts and memory function in China only. Dissatisfaction with life had a significant mediating effect on this association in US and China, not in England. Although more investigation is needed, the relationships between non-mortgage debts and later-life memory function through psychosocial pathways may differ across macro-level socioeconomic structures.

Abstract citation ID: igad104.2426

### NETWORK ANALYSIS OF HEALTH PROBLEMS AMONG OLDER WORKERS AGED 65–75 IN KOREA

Layoung Kim[1], Gwang Suk Kim[2], Min Kyung Park[1], and Jae Jun Lee[2], *1. Graduate School of Yonsei University, Seoul, Republic of Korea*, *2. Yonsei University College of Nursing, Seoul, Republic of Korea*

Korea's effective age of labor market exit is 72.3 years the longest age among the Organisation for Economic Co-operation and Development (OECD). The increase in the number and age of older workers can result in the burden of workplace safety and health management. Health policies and programs can be efficient only when they are established based on a specific understanding of health problems by occupational groups. In this study, we identified older workers' central health problems. Six health problems were reported by the 6th Korean Working Conditions Survey. The occupational characteristics were distributed into three groups: white-, blue-, and pink-collar. The data of older workers aged 65-75 who worked over 40 hours per week (N=1,086) was used for network analysis through the bootnet and qgraph packages in the R software. The health problems reported by older workers were: backache (39.9%); muscular pains in the shoulder, neck, and upper limb (39.7%); muscular pains in the lower limb (24.1%); fatigue (31.2%); headaches and eyestrain (17.3%); and anxiety (6.5%). The most central node of network was muscular pains in upper, which had significant relationships with backache, pains in lower, and fatigue. The correlation stability coefficient of strength was 0.672, which was preferable. In the subdivide, the pink-collar group showed muscular pains in the lower limb as the highest centrality. Since older people have simultaneous health problems, healthcare management should consider the relationships between problems. Additionally, centrality differed from occupational groups. These findings can be applicated to provide customized programs for the older workers.

Abstract citation ID: igad104.2427

### NONCOMMUNICABLE DISEASES AND HEALTH CARE UTILIZATION: A STUDY OF OLDER ADULTS IN INDIA

Jitender Prasad, and T V Sekher, *International Institute for Population Sciences (IIPS), Mumbai, Maharashtra, India*

Non-communicable diseases (NCDs) have become one of the leading causes of death in India, and it's more prevalent among aged population. Access to healthcare facilities (HCFs) can reduce this mortality burden considerably. This study addressed the rural-urban disparity in accessing HCFs for the treatment of NCDs among older adults. Using data from LASI wave-1 (2017-18), 10,865 older adults (aged 60+) who accessed HCFs for NCD treatment were analyzed. Bivariate and multivariate analysis was applied to calculate the distance to avail HCFs through public and private transportation for the last outpatient visit. The findings indicated that NCD patients highly preferred to visit private HCFs for their treatment in India. Sixty-eight percent of the rural NCD patients residing up-to 5 kilometres (km) distance from HCFs were utilizing private transport, whereas it was around four-fifths of urban NCD patients. As the distance to HCFs increases from 5km and more, the use of public transportation by NCD patients in rural areas were significantly higher compared to their urban counterparts. However, private transport use for HCFs located between 5-to-50km was not varied much by place of residence. Findings showed that there was no significant variation in health expenditure at public HCFs among rural-urban patients. Nearly one-fourth of urban and one-fifth of rural older adults were utilizing public HCFs free-of-cost. Urban residents who visited private HCFs had to spend eight times more than those who opted for public HCFs. Only nineteen percentage older adults have health insurance coverage in India, indicating considerable burden of out-of-pocket expenditure.

Abstract citation ID: igad104.2428

### RADIOTHERAPY NEEDS BY PORTUGUESE OLDER ADULTS IN 2040—A GLOBOCAN ANALYSIS

Edna Darlene Rodrigues[1], Paulo Almeida[2], Escarlata Lopez[3], and Laetitia Teixeira[1], *1. School of Medicine and Biomedical Sciences, University of Porto, Porto, Porto, Portugal*, *2. Centro Hospitalar Universitário São João, Porto, Porto, Portugal*, *3. GenesisCare Spain, Madrid, Madrid, Spain*

In Portugal, cancer is the second leading cause of death, accounting 28,544 deaths in 2018 (around 25% of all deaths). Radiotherapy (RT) is an effective cancer treatment. Around half of all cancer patients will require RT as part of their treatment. The European SocieTy for Radiotherapy and Oncology - Health Economics in Radiation Oncology (ESTRO-HERO) project data states that Portuguese actual utilisation of RT relative to the evidence-based optimal use is between 50-60%. The aim of this study was to project RT use and resources to maintain RT accessibility for Portuguese older adults in 2040 for the most common types of cancers. The authors examined GLOBOCAN data to determine expected incidence of prostate, colon, rectum, lung, breast, and stomach cancers for those aged 70 and over. Results show that in 2040, new cancer cases in older adults are expected to account for around 60% of total cancer cases. Cancer incidence is estimated to increase between 30 to 40% in this population. In accordance with this projection, the authors used the optimal utilisation percentage of RT to calculate the number of RT courses anticipated to be needed for each cancer type and an increase of around 38% (8878 to 12729) is expected by 2040 to address the needs of this population. The ESTRO-HERO guidelines state that each RT machine should treat 400 to 450 patients per year. So, to cover the older patients' demand, we estimate the need to install 8 to 10 additional RT machines by 2040.

Abstract citation ID: igad104.2429

### SPOUSAL MULTIMORBIDITY AND DEPRESSIVE SYMPTOMS AMONG OLDER INDIAN COUPLES: DOES ONE'S OWN HEALTH AND GENDER MATTER?

Soohyeon Ko, and Rockli Kim, *Korea University, Seoul, Republic of Korea*

With the aging population, increases in non-communicable diseases that require chronic management pose economic and social burdens to individuals with multimorbid conditions and their spousal caregivers. However, little is known about the crossover effect of spousal multimorbidity on mental health outcomes in the context of low-and middle-income countries, and whether it depends on one's own health status and gender. We used data on 6,158 older couples (12,316 individuals aged 60 years or above) from the Longitudinal Aging Study in India (LASI) 2017-18 to examine the association between spousal multimorbidity and depressive symptoms. Overall, 23.4% of the sample were multimorbid and 27.0% reported having depressive symptoms in the past week. Multivariate logistic regression models showed that spousal multimorbidity was associated with depressive symptoms, even after accounting for one's own multimorbidity status (adjusted odds ratio(aOR)=1.23; 95% confidence interval (CI)=1.06-1.44). However, this association was gender-specific. Among men, when jointly considered, spousal multimorbidity was no longer statistically significant whereas their own multimorbidity was associated with 60% higher odds of having depressive symptoms (aOR=1.60; 95% CI=1.28-2.00). Furthermore, the association between spousal multimorbidity and depressive symptoms was contingent upon the presence of their own multimorbidity for men. Among women, spousal multimorbidity was significantly associated with depressive symptoms, regardless of their own multimorbidity status. Our findings indicate that interventions to promote healthy aging should expand the formal caregiving system and consider family-based approaches to minimize the crossover health consequences of morbidity in conjugal relationships, especially for women.

Abstract citation ID: igad104.2430

### THE RADIOTHERAPY USE BY OLDER CANCER PATIENTS IN PORTUGAL—2018 NATIONAL CANCER REGISTRY DATA

Edna Darlene Rodrigues[1], Maria José Bento[2], Rita Calisto[2], Jessica Rodrigues[2], Paulo Almeida[3], Escarlata Lopez[4], and Laetitia Teixeira[1], *1. School of Medicine and Biomedical Sciences, University of Porto, Porto, Porto, Portugal*, *2. Portuguese Oncology Institute of Porto (IPO Porto), Porto, Porto, Portugal*, *3. Centro Hospitalar Universitário São João, Porto, Porto, Portugal*, *4. GenesisCare Spain, Madrid, Madrid, Spain*

In Portugal, cancer is the second leading cause of death, accounting for 28544 in 2018, around 25% of all deaths. Radiotherapy (RT) will be required by around half of the cancer patients. Older patients should be treated with ultrahipofractionation and highly precise/accurate technology to reduce the treatment sessions and to avoid side effects which are important for their quality of life. Nowadays, there is a global lack of RT infrastructure/investment that compromises cancer outcomes. The aim of this study was to evaluate the RT coverage by older patients in Portugal. The authors examined the 2018 national cancer registry data regarding the RT use stratified by cancer site and age groups. A total of 14448 cancer registries regarding patients aged 70 y/o or over were included. We calculated the optimal use through the application of the optimal utilisation percentage of RT for each cancer site, published by European SocieTy for Radiotherapy and Oncology - Health Economics in Radiation Oncology (ESTRO-HERO) project. The ratio between optimal use and real RT use was 56%, 51%, 41%, and 36% for the patients aged 70-74, 75-79, 80-84, and 85+, respectively. We should interpret these results considering the complex older patients' setting of multi-morbidity and geriatric syndromes. Furthermore, radiation oncologists face a lack of evidence that supports optimal treatment decisions for older patients. There is an urgent need for older adults' inclusive clinical trials, measures to reduce ageism, improve access, and provide the cornerstone of quality cancer care for older adults - the geriatric assessment.

Abstract citation ID: igad104.2431

### TOBACCO USE AND COGNITIVE FUNCTION AMONG OLDER ADULTS IN MEXICO AND BRAZIL

Jaqueline Avila[1], Natalia Gomes Goncalves[2], Joseph Saenz[3], Claudia Suemoto[4], and Rebeca Wong[5], *1. University of Massachusetts Boston, Boston, Massachusetts, United*

*States*, *2. University of Sao Paulo Medical School, Sao Paulo, Sao Paulo, Brazil*, *3. Arizona State University, Phoenix, Arizona, United States*, *4. Universidade de São Paulo, São Paulo, Sao Paulo, Brazil*, *5. UTMB, Galveston, Texas, United States*

Older adults in Brazil and Mexico have high lifetime tobacco exposure, which is an important modifiable dementia risk factor. They also have lower levels of education, which is associated with lower cognitive reserve. This study examines how tobacco use is associated with cognition and how this association differs by education among older adults in Brazil and Mexico. Data included adults 50 and older in the 2015 waves of the Mexican Health and Aging Study (MHAS, n=14,018) and the Brazilian Longitudinal Study of Aging (ELSI, n=9,409). The outcome was a global cognitive score, calculated with standardized cognitive tasks. Tobacco use was defined as current, former, and never smoker. Linear regression models were used to test differences in cognitive function by tobacco use, adjusted by sex, educational level, rural/urban area, chronic disease and included the interaction between smoking and education. Current and former smokers represented 17% and 37% of the sample in Brazil and 13.4% and 27.4% in Mexico, respectively. Current smokers in both countries were more likely to be men and be younger. However, current smokers in Brazil were more likely to have lower educational levels whereas those in Mexico were more likely to have 8+ years of education. In Brazil, current smokers had lower cognitive scores than never smokers (B= -0.06; 95%CI: -0.11; -0.01). In Mexico, current and former smokers had higher cognitive scores than never smokers (B=0.09 ; 95%CI: [0.05; 0.13] and B=0.10 [0.07;0.13], respectively). Cognitive scores for current smokers were significantly lower as education increased in Mexico.

### SESSION 7330 (POSTER)

### MENTAL AND BIOBEHAVIORAL HEALTH

Abstract citation ID: igad104.2432

### ASSESSING SYMPTOM NETWORKS OF DEPRESSIVE SYMPTOMS IN OLDER ADULTS: A CROSS-NATIONAL POPULATION-BASED STUDY

Fanghong Dong[1], and Nancy Hodgson[2], *1. University of Pennsylvania, Philadelphia, Pennsylvania, United States*, *2. University of Pennsylvania School of Nursing, Philadelphia, Pennsylvania, United States*

**Introduction** Older adults often experience elevated sleep disturbances, depressive and anxiety symptoms. However, the role of sleep disturbances in explaining individual variability in depressive and anxiety symptoms among older adults is poorly understood. **Methods** The sample was derived from the National Social Life, Health, and Aging Project, a nationally representative longitudinal study among American older adults. MCI was defined as Montreal Cognitive Assessment scored less than 23. Subjective insomnia symptoms and objective sleep measures (total sleep time, wake after sleep onset, percentage sleep) obtained from actigraphy were used. Validated measures on depressive and anxiety symptoms were collected both at round 2(N=645) and round 3(N=456). Multiple regressions were conducted to establish cross-sectional and longitudinal associations between sleep disturbances, depressive and anxiety symptoms. Results Cross-sectionally, compared to cognitively intact older adults, severe insomnia symptoms were associated with poorer depressive symptoms(B=0.40, p< 0.01) in older adults with MCI. Severe insomnia symptoms were associated with poorer anxiety symptoms(B=0.13, p< 0.01) in older adults, and no interaction effects were found by MCI groups. Longitudinally, insomnia symptoms at round 2 were associated with poorer depressive symptoms and poorer anxiety symptoms in older adults at round 3, but no interaction effects were found by MCI groups. No significant relationships were found between objective sleep disturbances and depressive/anxiety symptoms both cross-sectionally and longitudinally. **Conclusions** The findings provide further insight into insomnia symptoms that may be associated with increased risks for developmental depressive and anxiety symptoms. These data suggest that targeting insomnia treatment may confer long-term mental health benefits.

Abstract citation ID: igad104.2433

### ASSESSING SYMPTOM NETWORKS OF DEPRESSIVE SYMPTOMS IN OLDER ADULTS: A CROSS-NATIONAL POPULATION-BASED STUDY

Junwen Yu[1], Zhongfang Yang[1], Jiamin He[1], Zheng Zhu[2], and Hongzhou Lu[3], *1. Fudan University, Shanghai, Shanghai, China (People's Republic)*, *2. New York University, New York City, New York, United States*, *3. National Clinical Research Center for Infectious Diseases, Shenzhen Third People's Hospital, Shenzhen, Guizhou, China (People's Republic)*

**Background:** The complex relationships across depressive symptoms in older adults may differ across countries. We aimed to generate symptom networks of depressive symptoms in older adults and explore different patterns of depressive symptoms across countries from a mechanism perspective. **Methods:** We used harmonized data on 126,025 individuals ≥65 yrs from the Health and Retirement Study (HRS) family data from US, Mexico, UK, China, Israel, European countries, and Korea. We constructed dynamic networks with eight depressive symptoms using cross-lagged panel network analysis. **Results:** We identified two patterns in the networks of depressive symptoms: sadness-centered pattern and non-sadness-centered pattern. In the sadness-centered pattern, "felt sad" was the strongest predictor of other depressive symptoms in the United State (rs=4.38), Mexico (rs=4.96), and European and Israel (rs=4.61). Moreover, "felt depression" was found to have strong relationship with "felt sad" (r=0.586, 0.599, and 0.598, respectively). In the non-sadness-centered pattern, "bothered by little things" had the highest node strength in England (rs=8.16) and "could not get going" had the highest node strength in Korea (rs=10.18). **Discussion:** Our study demonstrated two patterns in the networks of depressive symptoms in older adults. In the sadness-centered pattern, a combined intervention for analyzing the cause of sadness and alleviating it should be considered. Keywords: Global health; Depression; Older adults; Symptom network

Abstract citation ID: igad104.2434
### DEPRESSION AMONG OLDER ADULTS WITH ACUTE CORONARY SYNDROME ASKING FOR EMERGENCY HELP
Audai Hayajneh, *Jordan University of Science and Technology, Irbid, Irbid, Jordan*

Acute coronary syndrome (ACS) has been associated with urgent cardiac symptoms, which require an emergent medical care by the healthcare providers (HCPs), including nurses. These cardiac emergency events may cause disturbance to daily life for older adults with ACS. The purpose of this study was to identify the occurrence of depression and examine its correlates among those patients with ACS asking for emergency help. This is secondary data analysis of data collected from a convenient sample of 300 older adults with ACS asking for emergency help. Correlation and multivariate analyses were utilized in this study. Depression was found to be significantly, positively associated with each of hospital length of stay (LOS), intensive care unit (ICU) LOS, and frailty. The level of depression among older adults with ACS asking for emergency help was 65.7%. The level of depression among older adults asking for emergency help had significant predictors, as follows: Age (t=3.06), troponin (t=2.98), hemoglobin alpha 1C (HbA1c) (t=3.18), and frailty (t=5.77). These factors utilized in the linear regression model explained 56.6% (Adjusted $R^2$=0.566, p=0.017) of the variation in the level of depression. The rate of depression is high among older adults asking for emergency help. Thus, the HCPs, including nurses, should pay a high attention to those at-risk patients for the occurrence of depression, as well as be well-prepared to manage correspondingly. Depression among older adults with ACS asking for emergency help requires emergency intervention protocol to combat depression among those patients by HCPs, including nurses.

Abstract citation ID: igad104.2435
### DEPRESSION AND LONELINESS IN BLACK WOMEN WITH HYPERTENSION: A SYSTEMATIC LITERATURE REVIEW
Lashawn Hutto[1], Michelle Odlum[2], Maritza Dowling[3], and Laurie Theeke[2], *1. George Washington University, Washington, District of Columbia, United States*, *2. The George Washington University, Washington, District of Columbia, United States*, *3. George Washington University, Washington, District of COlumbia, United States*

Hypertension occurs more frequently in Black women than men. Over 50% of Black women have hypertension, compared to 39% of non-Hispanic white women and 38% of Hispanic women. Black women with hypertension are more likely to report depressive symptoms which are associated with hypertension, a known risk for cardiovascular diseases including myocardial infarction, heart failure, and stroke. This paper presents a synthesis of findings from quantitative studies that measured depression and loneliness in black women with hypertension. The literature search used EbscoHost with the following databases: Medline Complete, Academic Search Complete, Medline, Pub-Med, Scopus, APA PsycArticles, APA PsycINFO, and CINAHL. Twelve articles were included. Loneliness and social isolation are predictive of depression in black women ages 40 and older. The issue is further confounded by cultural norms that include not seeking therapy, discussing symptoms, and displaying vulnerability, leading to loneliness and disconnection. Loneliness is increased in middle age black women who've experienced traumatic events. In an intervention trial, the associations between blood pressure control and living arrangements were examined. Middle- aged black women living alone were reported to have a higher prevalence of uncontrolled blood pressure, cardiovascular events, and mortality compared to those living with others. There are limited studies of loneliness and depression in black women with hypertension. Additional studies are needed that include variables reflective of cultural norms. A culturally sensitive clinical environment that facilitates discussion about feelings of depression and loneliness could be foundational to the development of new more precise interventions for black women with hypertension.

Abstract citation ID: igad104.2436
### DOES HEARING LOSS MODERATE THE RELATIONSHIP BETWEEN COGNITION AND DEPRESSION?
Jennifer Lister, Aryn Harrison Bush, Jennifer O'Brien, Laura Conover, and Nasreen Sadeq, *University of South Florida, Tampa, Florida, United States*

Hearing loss, depression, and cognitive impairment are common, co-occurring conditions experienced by older adults, but the nature of the relationship among these conditions remains unclear. Both depression and hearing loss have been identified as risk factors for dementia, but for different stages of life (e.g., Singh-Manoux et al., 2017; Livingston et al., 2020). Hearing loss is described as the strongest modifiable risk factor in mid-life, and depression is second only to smoking as the strongest modifiable risk factor in late-life (Livingston et al., 2020). The association between hearing loss and depression is well documented in the literature (e.g., Lawrence et al., 2015). It is possible that hearing loss, as an earlier risk factor, moderates the relationship between depression and cognition. Therefore, as part of an ongoing longitudinal study of early indicators of cognitive decline, we examined the moderating effect of two measures of hearing (pure-tone hearing threshold average, Dichotic Sentence Identification test) on the relationship between depression (Geriatric Depression Scale) and cognition (Montreal Cognitive Assessment, Digit Symbol Substitution test) for a group of 65 older adults (mean age 74). Although none of the hearing or cognitive measures were significant predictors of depression scores on their own, there was a significant negative interaction between the Montreal Cognitive Assessment and pure-tone hearing threshold average, p = 0.048. These results may be interpreted to suggest that individuals with higher (poorer) hearing thresholds show a decrease in depression as their cognitive scores improve, whereas individuals with lower (better) hearing thresholds show the opposite pattern.

Abstract citation ID: igad104.2437
### DYADS' POSITIVE AND NEGATIVE AFFECT ASSOCIATION WITH MENTAL HEALTH AND PERCEIVED GENERAL HEALTH
Cristina de Rosa, Weijun Wang, and Yu-Ping Chang, *University at Buffalo, The State University of New York, Buffalo, New York, United States*

Positive and negative affect may impact health in older adults and their family caregivers. However, how members of a caregiving dyad may influence each other's health outcomes is not well understood. The purpose of this analysis was to simultaneously examine whether both dyad members' positive and negative affect were associated with their own and each other's mental health (depression and anxiety) and perceived general health. Dyadic data were obtained from the National Health and Aging Trends Study (NHATS) (2021) linked to the National Study of Caregiving (NSOC) IV (2021). The sample consisted of 1,209 pairs of adults aged 71 and older and their respective primary caregivers, defined as the family member or friend who provided the greatest number of hours of care in the preceding month. Actor-partner interdependence models were used to examine associations between positive and negative affect, mental health, and perceived general health, controlled for dyad members' own gender, age, and race/ethnicity. Results indicated that positive and negative affect significantly predicted caregivers' and older adults' own mental health and perceived general health. Furthermore, older adults' negative affect significantly predicted caregivers' expressing little interest or pleasure in doing things ($b = 0.033$ (0.016), $p = .046$). Our results suggest that an individual's mood influences their mental health and perceived general health even when considering partner effects. Caregivers' interest or pleasure is particularly impacted by older adults' negative affect. Dyadic supportive strategies to improve mood could be beneficial for both older adults and their family caregivers.

Abstract citation ID: igad104.2438

### ELECTROCONVULSIVE THERAPY FOR TREATMENT OF TACTILE HALLUCINATIONS

Megan Kummerlowe, Kierstin Utter, and Lapid Maria, *Mayo Clinic, Rochester, Minnesota, United States*

Tactile hallucinations can be highly distressing and lead to psychiatric morbidities and functional impairments. There are no established treatments for tactile hallucinations that occur outside of primary psychotic disorders, and available pharmacologic and nonpharmacologic therapies may have limited effectiveness. We present a case of a 75-year-old female with treatment-refractory tactile hallucinations successfully treated with electroconvulsive therapy (ECT). She endorsed 13 months of distressing tactile hallucinations described as slimly substances on her skin. Symptoms started insidiously, became more severe with time and refractory to multiple antipsychotic medications. She became so severely distressed from the tactile hallucinations that she developed a major depressive episode and became suicidal. Relevant medical history included one prior depressive episode, occipital lobe stroke, restless legs syndrome, and obstructive sleep apnea. She was hospitalized in an inpatient psychogeriatric unit and treated with ECT for 12 sessions over 3.5 weeks. Hallucinations responded well to ECT. Patient experienced some mild memory impairment from treatment. Treatment was optimized with duloxetine titration, addition of mirtazapine and repletion of B6 vitamin. Following the acute course of ECT, she reported an 80 % improvement in tactile hallucinations. Depressive symptoms improved, and suicidal thoughts resolved. She was able to discharge from the hospital and continued with maintenance ECT. Our case illustrates the potential therapeutic role of brain stimulation in the treatment of refractory cases of tactile hallucinations. Further studies are needed to determine effective treatment strategies for treatment-refractory tactile hallucinations to prevent progression to severe psychiatric disturbances and improve quality of life.

Abstract citation ID: igad104.2439

### GENDERED TRAJECTORIES OF DEPRESSIVE SYMPTOMS IN OLDER KOREAN PARENTS BEREAVED OF AN ADULT CHILD

Sujeong Park, and Jinho Kim, *Korea University, Seoul, Republic of Korea*

Despite the existing body of research on the impact of child bereavement, little is known about whether time to the death of an adult child is associated with changes in depressive symptoms among older Korean parents. This study examines (a) trajectories of depressive symptoms before and after the loss of an adult child and (b) whether these trajectories differ across parent-child pairs (father-son, father-daughter, mother-son, and mother-daughter). Using eight waves of the Korean Longitudinal Study of Ageing (KLoSA), the study employs fixed effects models to mitigate potential bias due to unobserved individual-level heterogeneity. The result of this study revealed that depressive symptoms increased within the first year following the loss of an adult child among bereaved parents. Considering the gender of both the parent and the deceased child, differences in psychological adjustment to bereavement were observed in different dyads. Depressive symptoms surged within the first year and persisted even beyond the fourth year of loss among daughter-bereaved fathers, while only an immediate rise in depressive symptoms within the first year of loss occurred for other pairs. Given prevailing gender role socialization in the Korean context, men's conformity to masculinity norms and the traditional expectation for daughters to provide emotional support may contribute to the long-term increase in depressive symptoms for fathers who have lost a daughter. Thus, in societies where Confucian gender culture is ingrained as a social norm, policies for the psychological support of older parents who have lost adult children must consider the different trajectories of parent-child relationships.

Abstract citation ID: igad104.2440

### INTEGRATING GEROPSYCHOLOGY SERVICES INTO MEDICAL SETTINGS

Ann Pearman, *MetroHealth Medical System, Cleveland, Ohio, United States*

Integrating behavioral health into medical settings, such as primary care clinics, has been shown to have a positive impact on the well-being and health of patients. Increased access to mental health services as well as increased identification of psychological issues is an important goal for all patients but may be of particular importance for geriatric patients who often have multiple chronic conditions along with an increased risk of neurocognitive disorders. This paper is intended to describe the process of starting an integrated geriatric behavioral health service line into geriatric and internal medicine clinics at a large urban hospital that serves a primarily underserved population of patients. MetroHealth Medical Centers serves over 300,000 patients, 75% of whom are uninsured or covered exclusively by Medicare and/or Medicaid. The system has one centralized geriatric medicine clinic as well as geriatric providers throughout the system. In 2021, a geropsychologist was brought on board to create a geriatric mental health service for the system. Three different models of integrated care are being used – within-clinic collaborative care, co-location of services, and referrals – and will be described further. There have been over 1200 referrals to the service since its inception, many of whom are receiving mental health care for the first time. This presentation will highlight the types of patients being referred and the efforts put forth to reach as many patients as possible. Finally, the development of Geropsychology-specific training opportunities for psychology and medical residents and fellows will be described.

Abstract citation ID: igad104.2441

### INTEROCEPTION ACROSS DOMAINS: ASSESSING THE ROLE OF AGE

Pietra Bruni, *VA Connecticut / Yale School of Medicine, New Haven, Connecticut, United States*

Interoception plays a prominent role in homeostasis, providing information about the internal state and condition of the human body. It is most typically measured using heartbeat tasks, though there are mounting criticisms against focusing exclusively on signals that originate from the cardiac domain. While prior research has shown that variability in interoceptive ability exists when examined in relationship to numerous health, biological, and psychiatric factors, the role age holds remains largely unexamined. The primary goal of this study was to assess interoception in two distinct domains, cardiac and respiratory, across a community sample (n = 40) of twenty younger (mean age = 24.9 years) and twenty older (mean age = 69.3 years) adults. Two laboratory tasks measured interoception: a Heartbeat Tracking Task and a Respiratory Discrimination Task. Three measures of interoception were extracted: Interoceptive accuracy (IAcc), Interoceptive awareness (IAwe), and Interoceptive sensibility (ISen). Results from this experiment indicated that IAcc and IAwe were not correlated across domains. Of note, a significant difference was found between the two estimates of IAcc, indicating that participants were more accurate at assessing interoception in the respiratory than in the cardiac domain. Furthermore, younger adults (M= 0.49, SD = 0.29) exhibited significantly higher IAcc than older adults (M= 0.67, SD = 0.23); (t(36) = -2.04, p < 0.02) in the cardiac domain. These results indicate dissociable outcomes for two methods of assessing interoception, as well as illustrate the potentially prominent role age may hold when examining variability in interoceptive ability across domains.

Abstract citation ID: igad104.2442

### METABOLIC SYNDROME, LIFESTYLE FACTORS, AND SLEEP-DISORDERED BREATHING IN US HISPANICS: IMPLICATIONS FOR MID LIFE

Shannon Richard, Du Feng, Dieu-My Tran, Brenna Renn, and Jinyoung Kim, *University of Nevada, Las Vegas, Las Vegas, Nevada, United States*

US Hispanics/Latinos are disproportionately susceptible to metabolic syndrome (MetS), attributed in part to health and lifestyle factors such as low physical activity levels, diet quality, alcohol use, tobacco use, and sleep-disordered breathing. The risk of MetS and MetS-related complications increases with age. We examined the relationships between select health and lifestyle factors and MetS among Hispanic gender-heritage subgroups and determined whether gender and heritage moderate those relationships. Participants included 14,155 Hispanic Americans aged 18–76 (59% women, mean age 45.92 ± 13.97) from seven heritage subgroups. This secondary analysis of cross-sectional data from the observational Hispanic Community Health Study / Study of Latinos dataset used multinomial logistic regression and Hayes' PROCESS macro to test these relationships; the dependent variable, MetS, included four categories delineating MetS and related medication use. Results include low physical activity (p< .001) and sleep-disordered breathing (p< .001) were associated with the MetS with medication use group. High alcohol use decreased the probability of being in this group (p< .001). Cigarette pack years were not significantly associated with MetS outcomes. Gender moderated the association between MetS and diet quality (p< .001), alcohol use (p< .001), cigarette pack-years (p< .001), and sleep-disordered breathing (p< .001). Low physical activity and sleep-disordered breathing was significantly associated with MetS group membership, whereas low and high alcohol use were associated with decreased MetS risk. Gender-heritage differences were prominent in each of the study variables. Midlife may be an important target for prevention and intervention; other considerations for aging will be discussed.

Abstract citation ID: igad104.2443

### MYOGENIC CONTRIBUTORS TO PHYSICAL FUNCTION IN OLDER ADULTS WITH SYMPTOMATIC KNEE OSTEOARTHRITIS

Alisa Johnson[1], Jennifer Nichols[1], Heather Vincent[1], Roger Fillingim[2], and Yenisel Cruz-Almeida[2], *1. University of Florida, Gainesville, Florida, United States*, 2. *The University of Florida, Gainesville, Florida, United States*

Knee osteoarthritis (OA) is a leading cause of mobility disability among middle-aged and older adults, and contributes to significant global disease burden. The impact of knee OA on physical function varies widely across individuals, and the factors contributing to this heterogeneity are poorly understood. Recent compelling research suggests that myogenic factors, including muscle quality (MQ), may underly symptoms in knee OA. While MQ is gaining attention in aging research, the role of MQ on functional outcomes in persons with knee OA remains unknown. This pilot study examined morphological and neuromuscular biomarkers of MQ among adults aged 50 years and older (N=44) who met clinical criteria for symptomatic knee OA. Physical performance was assessed using the Short Physical Performance Battery (SPPB). MQ was quantified using B-mode ultrasound (echo intensity) and isokinetic strength measures (force/body mass). Associations between MQ and physical performance were determined, with consideration for clinical pain, age, sex, and body mass index. Pearson correlation analysis indicated SPPB scores were associated with clinical pain, strength, and MQ (r values ranged -.42 – 0.60, p's< 0.05). Multivariable backward stepwise linear regression analysis demonstrated that MQ biomarkers contributed 39% to variance for SPPB score independent of clinical pain and strength, after adjustment for age, sex, and BMI (b's = -0.32 – 0.45; p's < 0.05). MQ is an important consideration for physical function in older adults with chronic knee OA pain, regardless of pain severity, and represents a potentially potent treatment target for disability prevention.

Abstract citation ID: igad104.2444

### PERCEIVED CONTROL, FUNCTION, AND QUALITY OF LIFE AMONG CHRONIC STROKE SURVIVORS: THE MEDIATING EFFECT OF DEPRESSION

Zhijian Liu[1], Jing Wang[2], Shengyong Wu[1], Xiangjing Kong[3], Qin Wang[4], Bei Wu[5], and Juan Li[6], *1. Naval Medical University, Shanghai, Shanghai, China (People's Republic)*, *2. Fudan University, Shanghai, Shanghai, China (People's Republic)*, *3. Air Force Hospital of PLA Eastern Theater Command, Nanjing, Jiangsu, China (People's Republic)*, *4. Outpatient Department, Linyi, Shandong, China (People's Republic)*, *5. New York University, New York City, New York, United States*, *6. Huashan Hospital, Fudan University, Shanghai, Shanghai, China (People's Republic)*

This study examined the association between perceived control and function, quality of life (QoL) among Chinese chronic stroke survivors, and explored the mediating effect of depressive symptoms on the relationship between perceived control and ADL, QoL. We conducted a cross-sectional study and assessed depressive symptoms (by the Center for Epidemiological Studies Depression Scale, CES-D), perceived control (by the Perceived Control in Health Care Questionnaire), function (by the Barthel Index, BI), and QoL (by the Short Version of the Stroke Specific Quality of Life Scale, SV-SS-QoL) of 245 stroke survivors at 6 months after onset from three stroke centers in Shanghai, Linyi, and Nanjing from June to December 2021. Model-based causal mediation analysis was used to analyze the mediating effect. Multiple linear regressions showed that higher level of perceived control was significantly associated with better function and QoL ($\beta = 0.22$, $p< 0.001$; $\beta = 0.22$, $p< 0.001$, respectively), and depression symptoms partially mediated the effect of perceived control on function and QoL ($\beta = 0.13$, $p< 0.001$; $\beta = 0.15$, $p< 0.001$, respectively), accounting for 36.07% and 40.60% of the total effect ($\beta = 0.35$, $p < 0.001$, $\beta = 0.37$, $p < 0.001$, respectively). Our study indicates that depressive symptoms shape the association between perceived control and function and QoLamong chronic stroke survivors. Intervention focusing on depressive symptoms may have the potential to improve function and QoL of chronic stroke survivors.

Abstract citation ID: igad104.2445

### PSYCHOSOCIAL CHARACTERISTICS BY PAIN PRESENCE AND LIMITATIONS AMONG OLDER ADULTS

Ashleigh Holmes, Weijun Wang, and Yu-Ping Chang, *University at Buffalo, The State University of New York, Buffalo, New York, United States*

Older adults experience high rates of pain, which is linked to physical disability, poorer mental health, reduced social engagement, cognitive impairment, and increased health care costs. Due to the heterogeneous nature of pain, further research is needed to identify psychosocial characteristics in older adults grouped by pain experience. Using the nationally representative National Health and Aging Trends Study Round 11 (2021) dataset, we performed ten multiple regression models (controlling for age, gender, and race/ethnicity) examining various psychosocial variables in 3,381 older adults divided into three pain groups: no pain within the last month, pain without activity limitations, and activity-limiting pain. The results showed that older adults with activity-limiting pain compared to those without pain had significantly higher depression (b=0.724, p<.001), anxiety (b=0.675, p<.001), fear of falling (b=1.078, p<.001), difficulty initiating and maintaining sleep (b=0.906, p<.001), and use of sleep medications (b=0.448, p<.001), as well as reduced positive affect (b=-1.812, p<.001), self-realization (b=-0.204, p<.001), self-efficacy (b=-0.041, p=.012), resilience (b=-0.101, p<.001), and social participation (b=-0.205, p<.001). In older adults with non-activity-limiting pain compared to those without pain, all relationships remained significant except for self-realization (p=.366), self-efficacy (p=.122) and resilience (p=.123). Furthermore, older adults with non-activity-limiting pain reported higher levels of self-realization (b=0.173, p<.001) and self-efficacy (b=0.068, p<.001) than those with activity-limiting pain. The findings suggest that pain is strongly associated with all psychosocial outcomes, especially in older adults with activity-limiting pain. Future research should examine the impact of self-realization and self-efficacy on pain experiences in older adults with activity-limiting pain.

Abstract citation ID: igad104.2446

### SELF-REPORTED MENTAL AND PHYSICAL HEALTH IN OLDER PATIENTS WITH RHEUMATOID ARTHRITIS

Comfort Anim-Koranteng[1], Yong Eun[2], and Christfelly Opoku-Agyemang[2], *1. NYCHH-Harlem Hospital Center, Bronx, New York, United States*, *2. Harlem Hospital Center, New York City, New York, United States*

Rheumatoid arthritis (RA) is the most common autoimmune arthritis with a prevalence of 1% among the American population. RA significantly impacts mortality and is associated with worse quality of life among older adults. Management of RA in older patients is challenging due to comorbidities and frailty. This study compared RA patients' self-reported mental and physical health between older (65 and over) and younger (18-64) individuals. In the study, we included participants from the All of Us dataset version 6, which includes survey data collected between May 6, 2018, and January 1, 2022, who had one or more diagnosis codes (ICD9/ICD10) of RA and at least one medication exposure for RA. Total of 2904 patients with RA - 1577 older and 1327 younger - were included in the analysis. PROMIS-PH (Patient-Reported Outcomes Measurement System - Physical Health) and MH (Mental Health) score were calculated from the survey result, from 2 to 10: higher score representing better outcome. Average PROMIS-PH score was 6.5 in older patients and 5.9 in younger patients (P< 0.05). Average PROMIS-MH score was 7.2 in older patient and 6.4 in younger patients (P< 0.05). The findings of this study suggest that older patients with RA may have better self-reported mental and physical health than younger individuals. This could be attributed to better disease management, increased resilience, and adaptability. Further research is warranted to understand the underlying causes of the study's findings.

Abstract citation ID: igad104.2447

### TAILORED INTERVENTION PROGRAM USING HUMANOID AI MINI-ROBOT FOR OLDER ADULTS WITH DEPRESSION IN RURAL AREAS

Young Mi Lim[1], Jungsug Kim[2], Junghee Lim[3], and Minjung, Sun[4], *1. Yonsei University Wonju College of Nursing, Wonju, Republic of Korea*, *2. Yeoju Institute of Technology, Yeonju, Republic of Korea*, *3. Yeoju Community Mental Health Welfare Center &Suicide Prevention Center, Yeoju, Republic of Korea*, *4. Kyung Hee University, Seoul, Republic of Korea*

Loneliness, and social isolation have worsened because most mental health centers were forced to suspend elderly-centric programs due to COVID-19. Recently, with the demand for a frail elderly-care model that enables non face-to-face interactions, strategies to utilize various technologies using robots for depression prevention are emerging. This pilot study aimed to develop and analyze a tailored intervention program using humanoid AI mini-robot for older adults with depression in rural areas. An experimental group of eight depressed elders participated daily in a tailored intervention program using humanoid AI mini-robot involving physical (stretching exercise) and emotional activities (listening to music) for ten weeks. A control group of ten depressed older adults received routine case management with non face-to-face at a mental health center. We compared the grip strengths and levels of depression and mental well-being between the experimental and control groups before and after the intervention, using Mann-Whitney U test and Wilcoxon signed rank test Statistically significant difference ($U = 12.0$, $p < 0.05$) was observed between the mental well-being levels of the two groups; by contrast, grip strengths and depression levels did not differ significantly. The experimental group exhibited a significant difference ($Z = -2.038$, $p < 0.05$) between pre- and post-intervention grip strengths. The number of subjects was too small and the intervention period was not long enough to draw a meaningful conclusion. However, the findings have implications for the need of potential tailored interventions using humanoid AI mini-robot for mental well-being of community dwelling frail older adults.

Abstract citation ID: igad104.2448

### THE FACTORS RELATED TO KENDO PRACTICE THAT AFFECT IKIGAI-KAN OF MIDDLE-AGED AND OLDER KENDO PRACTITIONERS

Daisuke Matsumoto[1], and Hayato Uchida[2], *1. University of Hyogo, Osaka-city, Osaka, Japan*, *2. University of Hyogo, Himeji-city, Hyogo, Japan*

The purpose of this study was to clarify the factors related to kendo practice that affect Ikigai-Kan and actual conditions of generativity, mental health of middle-aged and elderly Kendo practitioners. We selected 97 middle-aged and older Kendo practitioners who are practicing at Dojos in Osaka and Hyogo prefectures as research participants. Items that are basic attributes were grouped into two groups, and the values of each survey item were compared between groups. In addition, correlation analysis was performed to grasp the mutual relationship between the basic attributes and each survey item value. Furthermore, in order to clarify the factors related to the practice of kendo that affect Ikigai-Kan, multiple regression analysis was performed. Each item value indicating the practice status of kendo was used as independent variable. As a result, it was confirmed that the frequency of practice with young people ($\beta=0.384$, $p< 0.01$) affected Ikigai-Kan. These results show that the collaboration between the school and the community, the creation of an environment where the skills and experience of middle-aged and elderly people such as club activity instructors and local sports organizers can be enhanced, and opportunities to interact with or practice with young people would lead to Ikigai.

Abstract citation ID: igad104.2449

### THE MEDIATING ROLE OF PAIN SEVERITY IN THE ASSOCIATION BETWEEN DEPRESSIVE SYMPTOMS AND GAIT SPEED

Rhea Mehta[1], Michelle Shardell[2], Alice Ryan[3], Yu Dong[3], Brock Beamer[3], Marc Hochberg[3], and Alan Rathbun[3], *1. University of Maryland Baltimore, Baltimore, Maryland, United States*, *2. University of Maryland School of Medicine, Baltimore, Maryland, United States*, *3. University of Maryland, Baltimore, Baltimore, Maryland, United States*

Knee osteoarthritis (OA) and comorbid depression adversely impact gait speed over time. Depression can alter pain tolerance and perception; therefore, self-reported knee pain severity may mediate the relationship between depressive symptoms and gait speed, but this has not been evaluated longitudinally using repeated measures data. Thus, we assessed whether knee pain severity mediates the association between depressive symptoms and gait speed over time. Participants (n=2,222) from the Osteoarthritis Initiative with radiographic knee OA (Kellgren-Lawrence grade $\geq 2$) in at least one knee were included. Depressive symptoms were assessed using the Center for Epidemiologic Studies Depression Scale (CES-D; range 0-60) at baseline and first two annual follow-up visits. The twenty-meter gait speed (meters per second) outcome was assessed at the second through fourth annual follow-up visits. Knee pain severity was assessed as a mediator and measured using the Western Ontario and McMaster Universities Osteoarthritis (WOMAC) pain subscale at the first through third annual follow-up visits. Linear regression mediation using a version of the Sobel-Goodman approach was the primary method of analysis. Indirect and direct effects of depressive symptoms on gait speed for each one-unit CES-D score were -0.001 ($p < 0.001$) and -0.003 ($p = 0.020$) standard deviations, respectively. Thus, approximately 24% of the association between depressive symptoms and gait speed was mediated by knee pain severity across time. The current results support the need for simultaneous, multidisciplinary interventions for both depressive symptoms and knee OA pain to prevent future decline in physical performance.

Abstract citation ID: igad104.2450

### USE OF PSYCHIATRIC RISK FACTORS IN PREDICTING 10 YEAR CARDIOVASCULAR RISK FOR CHINESE PATIENTS WITH SCHIZOPHRENIA

Jennifer Yee-man Tang[1], Hao Luo[2], Gary K.K. Lau[2], Edwin H.M. Lee[2], Pengcheng Wang[2], Ian C.K. Wong[2], Terry Lum[2], and Gloria Hoi-Yan Wong[2], *1. The Chinese University of Hong Kong, Hong Kong, Hong Kong*, *2. The University of Hong Kong, Hong Kong, Hong Kong*

Patients with schizophrenia are known to have excessive cardiovascular morbidity. Traditional Framingham risk prediction models do not consider additional risk attributable to factors related to psychiatric illness and treatment. The Framingham risk scores also tend to overestimate cardiovascular risk in Chinese cohorts. This study aimed to develop a 10-year cardiovascular risk prediction model using routine clinical data from an electronic health database in Hong Kong. We identified a cohort of 39,449 patients who were diagnosed with schizophrenia between Year 2005 and 2015 and had no history of cardiovascular disease (CVD). Cox proportional hazards regression models were employed to determine psychiatric and clinical risk factors influencing the timing of incident CVD over 10 years. Psychiatric variables of interest included comorbid psychiatric disorders, duration of schizophrenia, use of antidepressant, use of first- or second-generation antipsychotics (FGA & SGA) at baseline and duration of exposure to FGA and SGA. The main analysis showed that comorbid substance use-related disorders (HR = 1.52; 95% CI 1.22 – 1.90; P < 0.001) increased CVD risk and each year of cumulative exposure to FGA increased CVD risk by 2% (HR = 1.02; 95% CI 1.01 – 1.04; P = 0.001). The use of antidepressant predicted a higher CVD risk in female patients (HR 1.19; 95% CI 1.02 – 1.38; P = 0.028). Our risk model predicted that 20.6% of patients had a CVD risk ≥ 20% over 10 years. Our risk models may offer an accessible clinical tool to help monitor cardiovascular risk in patients with schizophrenia.

Abstract citation ID: igad104.2451

### USER PERCEPTION OF TECHNOLOGICAL INTERVENTIONS FOR PARKINSON'S DISEASE: A SYSTEMATIC REVIEW

Terence Lau[1], and Angela Y. M. Leung[2], *1. The Hong Kong Polytechnic University, Hong Kong, Hong Kong*, *2. Hong Kong Polytechnic University, Hong Kong, Hong Kong*

With the growing number of Parkinson's disease technological intervention studies available, it is uncertain what and how people with Parkinson's disease (PWP) respond to these interventions. This systematic review aims to study the user perception and perceived psychosocial gain of technological interventions among the Parkinson's disease population, and it included 27 eligible studies from 6 databases. Through thematic synthesis, qualitative evidence suggests technological interventions could benefit the psychosocial well-being of people with Parkinson's disease (PWP) under appropriate implementation. Coping with technological interventions was identified as the overarching theme. The theme is closely related to participants' technological literacy and could lead to perceived psychosocial gain. The discrepancy between the implemented technology and PWP's technological literacy level dictated how well PWP coped with the interventions. Ineffective coping attempts could induce undesirable emotions within PWP and tamper with their psychosocial health. In contrast, successful coping attempts reduced initial adverse effects, resulting in psychosocial growth such as increased autonomy and interpersonal relationships. To facilitate PWP's coping process and design a Parkinson's disease-friendly technological intervention. Scholars should include an adjustable, progressive module design and provide sufficient training and technical support to participants during implementation.

Abstract citation ID: igad104.2452

### VASOMOTOR SYMPTOMS AND DIET QUALITY

Pilar Thangwaritorn[1], Jaime Perales-Puchalt[2], Melanie Meister[3], Tiffany Schwasinger-Schmidt[4], Sandra Billinger[5], Briana Bright[6], and Amber Watts[1], *1. University of Kansas, Lawrence, Kansas, United States*, *2. University of Kansas Alzheimer's Disease Research Center, Fairway, Kansas, United States*, *3. University of Kansas School of Medicine, Kansas City, Kansas, United States*, *4. University of Kansas School of Medicine-Wichita, Wichita, Kansas, United States*, *5. University of Kansas Medical Center, Kansas City, Kansas, United States*, *6. Kansas City, Kansas, United States*

During the menopausal transition, women report a variety of physical changes including hot flashes and nights sweats, known as vasomotor symptoms, which are associated with later life health outcomes including vascular disease and Alzheimer's risk. Previous studies suggest that maintaining a healthy diet may alleviate vasomotor symptoms, however, few studies have investigated this association. We examined the potential association between diet quality and vasomotor symptoms in women ages 40-60 years. Recruitment is ongoing and currently consists of N = 44 women (Mage = 50.59, SD = 5.96) from the Kansas City and Wichita areas. We assessed vasomotor symptoms using two questions from the Greene Climacteric Scale and measured diet quality with the Rapid Eating Assessment for Participants-shortened version. We evaluated the association between diet quality and vasomotor symptoms using linear regression. Overall, women in our sample reported moderate diet quality and were moderately bothered by hot flashes and night sweats. The association of diet and vasomotor symptoms was not statistically significant accounting for covariates, possibly related to sample size ($\beta = 6.024$, $p = .307$). However, lower education levels are associated with a greater degree of vasomotor symptoms ($\beta = -0.876$, $p = .011$). Future research could investigate specific types of foods (e.g., vegetables, proteins, etc.) that influence vasomotor symptoms and the associated health outcomes in women who are post-menopausal, including vascular disease and cognitive decline. Lifestyle factors such as diet may be an intervention strategy to reduce disease that emerges during the menopausal transition.

### SESSION 7340 (POSTER)

### MOBILITY AND FUNCTION IN OLDER ADULTS

Abstract citation ID: igad104.2453

### ASSOCIATION OF BIOCHEMICAL, CLINICAL, AND ANTHROPOMETRIC PARAMETERS WITH FUNCTIONAL PERFORMANCE OF OLDER WOMEN

Silvana Funghetto[1], Erika Ferreira[2], Mateus Leite[2], Alessandro de Oliveira Silva[3], Marina Morato Stival Lima[4], Luciano Ramos de Lima[4], Yuri Barbalho Sousa Barbalho[4], and Cris Renata Volpe[4], *1. University of Brasilia- Campus Ceilândia- UNB-Brasil, Brasília, Distrito Federal, Brazil*, *2. University of Brasilia, Brasília, Distrito Federal, Brazil*,

*3. Center University of Brasilia, Brasília, Distrito Federal, Brazil*, *4. University of Brasilia, Brasilia, Distrito Federal, Brazil*

Increased longevity is a widespread worldwide trend, evidenced by demographic and epidemiological data, especially with regard to females, who live an average of seven years longer than males. The aging process is associated with a progressive decline in muscle mass, strength, power, impaired balance, altered heart function and vascular function. These changes negatively affect exercise capacity, increase the risk of cardiovascular disease, and have implications for physical function and the risk of falling. This is particularly relevant among women diagnosed with impaired functional performance. The aim of this study was to determine an association of biochemical, anthropometric, metabolic and inflammatory parameters according to the functional performance of older women aged 62 to 75 years attended for in primary health care. Statistical results showed that the 116 older women (56.86%) identified with impaired functional performance had a pro-inflammatory marker, high anthropometric indices and body composition, impaired cognitive performance and lower muscle strength as older women ( 88 ) with normal functional performance (43.14%). Age, waist-to-height ratio and elevated IL-6 and muscle strength significantly decreased the risk of impaired functional performance. These important findings demonstrate the need for further research documenting the underlying processes and risk factors for preserving functional capacity, carrying out life activities and decreasing the emergence of illnesses. The potential benefits of this approach provide a basis for developing individualized health promotion interventions in the management of this population of older women.

Abstract citation ID: igad104.2454

### DEALING WITH INJURED OR REPAIRED ANKLES IN OLDER ADULTS: ASSESSING ANKLE IMMOBILIZATION METHODS DURING LOCOMOTION

Clark Dickin, *Ball State University, Muncie, Indiana, United States*

Orthopedic walking boots (OWB) are an alternative to casting and immobilizing an injured ankle. Unfortunately, OWB causes a change in leg-length and pelvis angle which alters gait and postural stability and can lead to chronic back pain. Immobilizing the ankle without changing leg-length would be beneficial, especially for individuals with impaired gait and postural stability. The purpose of this study was to assess level-ground walking and stair negotiation in healthy older adults across three ankle support conditions (1) OWB, (2) TayCo ankle brace (TAB) and (3) athletic footwear (SHOD). Sixteen older adults (69+/-7.0 years) performed 2-3 trials of walking, stair ascent, and stair descent using each of the three ankle support conditions. Ankle motion in all three planes was monitored using 3D motion capture cameras to assess differences during walking and stair negotiation. For all tasks, significant differences were revealed between the SHOD and both ankle support methods for all three planes ($p < .001$). Significant differences were also revealed between OWB and TAB during walking in the transverse plane (abd/adduction) ($p = .036$ OWB>TAB) and for stair descent in the sagittal plane (dorsi/plantarflexion) ($p = .001$ TAB>OWB). No other differences were observed between the OWB and TAB for walking or stair negotiation. Ankle motion was similarly controlled with both the OWB and TAB indicating that either method would be appropriate to immobilize an injured ankle, however the TAB does not alter leg length or pelvic tilt since it fits over the individual's footwear.

Abstract citation ID: igad104.2455

### DIFFERENCES IN PHYSICAL PERFORMANCE MEASURES BETWEEN BLACK AND WHITE OLDER ADULTS

William Windham[1], B. Gwen Windham[2], Hunter Sylvester[2], Priya Palta[3], Laura Skow[4], A. Richey Sharrett[4], Anna Kucharska-Newton[3], and Michael Griswold[2], *1. University of Mississippi School of Medicine, Jackson, Mississippi, United States*, *2. UMMC-The MIND Center, Jackson, Mississippi, United States*, *3. UNC Gillings School of Global Public Health, Chapel Hill, North Carolina, United States*, *4. Johns Hopkins Bloomberg School of Public Health, Baltimore, Maryland, United States*

Older Black adults are reported to have poorer physical function relative to White older adults. Cardiovascular risk factors and disease are associated with physical function and are more prevalent in Black adults. We examined objective physical function cross-sectionally using the Short Physical Performance Battery (SPPB, scored 0-12; 0.5 is considered clinically meaningful) in the Atherosclerosis Risk in Communities Study among Black and White participants during Visit 5 (2011-2013). Marginal standardization following generalized linear modelling (log-link, negative-binomial) was used to investigate whether cardiovascular features (hypertension, diabetes, BMI, smoking, heart disease, heart failure, stroke) helped explain race differences by comparing unadjusted (total effect) versus adjusted (direct effect) results. Among 5,836 participants (>65 years, mean age=75.9, 42% men, 22% Black), Black participants scored 1.3 points lower than White participants; unadjusted: 8.2 versus 9.5; relative difference of 0.86 (0.84, 0.88). After accounting for cardiovascular features, Black participants scored 1.4 points lower than White participants; adjusted: 8.2 versus 9.6; relative difference of 0.85 (0.84, 0.87). Cardiovascular risk factors and disease alone did not appear to account for substantial cross-sectional physical performance race differences in this population. Future research should examine additional factors potentially contributing to observed functional differences typically attributed to race such as cumulative vascular risk factors, geographic, social, cultural, and other systemic influences.

Abstract citation ID: igad104.2456

### EFFICACY OF COMBINED THERAPEUTIC EXERCISE AND PSYCHOLOGICAL INTERVENTIONS ON DISABILITY OUTCOMES: A SCOPING REVIEW

Grace Rose[1], Pietra Bruni[2], Mariana Wingood[3], Selmi Kallmi[4], Elizabeth Finer[5], and Patricia Bamonti[1], *1. VA Boston Healthcare System, Boston, Massachusetts, United States*, *2. VA Boston, Boston, Massachusetts, United States*, *3. Wake Forest University, Winston-Salem, North Carolina, United States*, *4. Hines VA, Hines, Illinois, United States*, *5. Hofstra University, Hempstead, New York, United States*

The goal of therapeutic exercise (i.e., physical activity/exercise programs, physical therapy, rehabilitation), restoring and maintaining function in older adults (≥60 years), is hindered by personal factors (e.g., low self-efficacy) impacting exercise engagement. Combining therapeutic exercise with psychological interventions is a promising solution for maximizing treatment benefits. The purpose of this scoping review is to systematically synthesize the knowledge of randomized controlled trials (RCTs) testing combined therapeutic exercise and psychological interventions targeting disability outcomes in older adults. A comprehensive review of the literature, searching four databases, was performed following PRISMA-ScR guidelines. Articles (n=4,995) underwent iterative screening, resulting in 31 RCTs. The World Health Organization's International Classification of Functioning, Disability, and Health (ICF) model was utilized as a conceptual framework. Studies assessed at least one major domain of the ICF model: Body Function and Structure was an outcome in 67.74% of RCTs and both Activity and Participation were an outcome in 77.42% of RCTs. Personal factors were assessed in 64.52% of RCTs. Psychological interventions utilized were cognitive-behavioral (32.26%), motivational interviewing (29.03%), meditation (12.90%), or other (32.26%). Combined interventions compared to either therapeutic exercise alone (25.81%) or control (74.19%) demonstrated greater efficacy on at least one major ICF outcome in 77.42% of RCTs. This review indicates that incorporating psychological interventions within therapeutic exercise can maximize treatment benefits in older adults across diagnoses and setting. Future research is needed to better understand which psychological interventions demonstrate greatest efficacy when paired with therapeutic exercise within certain patient subgroups, in order to guide best practice guidelines.

Abstract citation ID: igad104.2457

### FEASIBILITY OF HIGH-INTENSITY FUNCTIONAL STRENGTH TRAINING AT HOME FOR POSTINJURY OLDER ADULTS

Ashley Morgan, Jennifer Heisz, Ada Tang, Lehana Thabane, and Julie Richardson, *McMaster University, Hamilton, Ontario, Canada*

Older adults who experience a slip, trip, or fall may experience preclinical mobility limitation (PCML), where individuals report modifications but not difficulty in mobility tasks and are at increased risk for future functional decline. Functional decline may be prevented with exercise. The purpose of this pilot trial was to determine the feasibility (adherence, recruitment, retention, safety) and preliminary outcomes (physical functioning, cognitive functioning, enjoyment) of home-based 12-week high-intensity functional strength training (HIFST) for community-dwelling older adults (≥ 55 years) with PCML who have had an injury from a slip, trip, or fall in the previous year. The trial is currently underway (target completion spring 2023). Participants are stratified by sex and randomized (1:1) to HIFST or a stretching program, both delivered by a physiotherapist via videoconferencing. Feasibility will be assessed based on predetermined criteria and reported using descriptive statistics. Preliminary effects will be reported as between group differences. To date, 20 participants (11 HIFST, 9 stretch) have enrolled (target n=24). Six participants have completed the HIFST intervention, and 2 have withdrawn before completion (reasons: mental health crisis and acute knee pain episode) with a total adherence rate of 85.8% of sessions completed (97.7% for the 6 participants who did not withdraw) which exceeds our threshold for feasibility (≥ 70%). No serious intervention-related adverse events have been reported. The results of this pilot will provide essential information for future research regarding the process, resources, and potential effects of home-based HIFST in a PCML post-injury older adult population.

Abstract citation ID: igad104.2458

### GAIT ANALYSIS OF ELDERLY PEOPLE IN ANGOLA: COMPARISON BETWEEN ESTABLISHED ROUTE AND VIRTUAL REALITY AND TIME UP AND GO

Rita Barbara[1], and Natália Luanda[2], *1. Universidade de Aveiro, Sao Paulo, Sao Paulo, Brazil*, *2. Upra, Luanda, Luanda, Angola*

**Background:** Aging limits physical and mental abilities, particularly gait, compromising quality of life and increasing the risk of falls, which is a public health challenge in Angola. Technologies, such as virtual reality, may offer a promising alternative for treatment and prevention of these limitations. Objective: This study aimed to analyze the gait of elderly individuals in Angola by comparing gait on an established course and in a virtual environment, using motion analysis software and SPSS for statistical analysis with a $p>0.05$. **Methods:** Seven elderly individuals, aged 60-80, participated in the study. Gait was evaluated using the Timed Up and Go (TUG) test, an established course, and gait in a virtual environment. **Results:** Statistically significant differences were found between gait on the established course and in virtual reality, with elderly individuals showing slower and more variable gait in the virtual environment. There were also significant correlations between gait and TUG, highlighting the importance of motor coordination and balance in elderly gait. **Conclusion:** Gait analysis in elderly individuals can be performed precisely and stably through virtual reality technology. The results of this study suggest that virtual reality may be an interesting alternative for the treatment and prevention of falls in the elderly. The findings also emphasize the importance of motor coordination and balance in gait and the need for further research on the use of virtual reality technology in this population.

Abstract citation ID: igad104.2459

### MEASURING ANKLE FLEXIBILITY IN OLDER HOME CARE RECIPIENTS WITH DISABILITIES IN THEIR HOME SETTING

Jordan Skowronski, *University of Illinois Chicago, Chicago, Illinois, United States*

Ankle flexibility is critical for daily activities, such as climbing stairs. Ankle range of motion (AROM), or the range through which the ankle joint can bend up and down, decreases with age. Thus, interventions to improve or maintain AROM is an important goal in geriatric rehabilitation. Evaluating the outcome of such interventions requires AROM measures. However, existing measures either require large equipment or for the participant to stand and lunge, and so are not appropriate nor practical for homebound older adults. This paper aims to (1) describe how we developed an AROM measure for frail, older adults as part of the Promoting Seniors' Health with Home Care Aides (Pro-Home) Study, and (2) examine the convergent and discriminant validity of the AROM measure. Data came from the baseline assessment of older home care participants' physical function (N=115). AROM was moderately correlated with lower extremity function as measured by the Short Physical Performance Battery, r(113) = 0.30, p< 0.01 and the gait speed component scores, r(113) = 0.33, p< 0.01, and AROM was not correlated with self-reported upper extremity function. Results from the robust regression analyses are that 1 standard error unit greater averaged AROM is associated with 0.06 points higher SPPB score (p< 0.00) and 0.06 greater Gait Speed Score (p< 0.00), after adjusting for age, gender, and ambulatory device use. The significant associations between AROM and SPPB and AROM and gait speed indicate that the AROM measure has convergent validity, while the nonsignificant associations among AROM and upper extremity function show its discriminant validity.

Abstract citation ID: igad104.2460

### SOCIAL ISOLATION AND DIET QUALITY AMONG US ADULTS WITH A DISABILITY IN NHANES, 2013–2018

Elizabeth Parker[1], Nadia Saif[1], Kathryn Barry[1], Jason Falvey[2], and Odessa Addison[2], *1. UMSOM, Baltimore, Maryland, United States*, *2. University of Maryland School of Medicine, Baltimore, Maryland, United States*

Social isolation and disability are established risk factors for poor nutrition. There is limited understanding of how social isolation affects diet quality specifically among adults with disability. This cross-sectional analysis used NHANES 2013-2018 data to assess whether social isolation is associated with diet quality among adults with disabilities. Adults with a disability, not pregnant/breastfeeding, and not missing dietary intake data were included (n=5,167). Disability was defined based on reporting difficulty with any activities of daily living, instrumental activities of daily living, lower extremity mobility activities, or general physical activities. Diet quality was assessed using the Healthy Eating Index (HEI); higher scores corresponded to higher diet quality. We computed a social isolation index by summing single status, living alone, and two social engagement difficulty measures (1 point for each component met; 4 points maximum). Multivariable linear regression, controlling for demographic and health covariates, estimated differences in HEI scores for dietary intake, by social isolation score. The highest social isolation score category was associated with lower total HEI score (β = -1.71 points, 95% CI -3.08, -0.34), and lower intake of vegetables (β = -0.39 points, 95% CI -0.59, -0.20) and seafood/plant proteins (β = -0.37 points, 95% CI -0.60, -0.13). Single status and both social engagement measures were associated with lower scores on certain adequacy components. There was little evidence of effect modification by age or gender (p-interaction >0.05). Among socially isolated adults with disabilities, increasing access to fresh foods, particularly vegetables and seafood/plant proteins, should be a priority.

### SESSION 7350 (POSTER)

### NUTRITION IN OLDER ADULTS

Abstract citation ID: igad104.2461

### FROM MISFIT TO FIT: THE IMPACT OF NURSING PRACTICE ON COMPLEX NURSING INTERVENTION DEVELOPMENT

Debbie ten Cate[1], Marieke Schuurmans[2], Lisette Schoonhoven[3], and Roelof Ettema[1], *1. Utrecht University of Applied Sciences, Utrecht, Utrecht, Netherlands*, *2. Dutch Healthcare Authority, Utrecht, Utrecht, Netherlands*, *3. University Medical Center Utrecht, Utrecht University, Utrecht, Utrecht, Netherlands*

Nurses have a key role in providing nutritional care to older adults to prevent and treat malnutrition, and stimulate health and well-being. However, evidence for nursing activities regarding nutritional care is often lacking. Therefore, intervention development is necessary. From earlier studies, an evidence-based nutritional intervention carried out by nurses appeared the best solution. The aim of this study was to outline the steps taken to develop a complex nursing intervention to prevent and treat malnutrition in older adults and the challenges faced during this stage. Following the phase of intervention development of the Medical Research Council (MRC) Framework, a systematic review, a questionnaire survey, semi-structured interviews, focus groups and participant observation were carried out. The purpose was to gather information about current nursing practice and context, the needs of future users and providers, and gain insight into the problem. The studies showed that nurses had moderate knowledge of (mal)nutrition. They gave nutritional care low prioritization during daily nursing activities. These results provided insight that the intended nursing nutritional intervention would most likely lead to a misfit with the context. To fit the intervention properly into nursing practice, it was decided to develop an educational intervention targeting nurses instead of a nutritional intervention carried out by nurses. Building proofs in context leads to challenges but is essential to prevent a misfit between complex nursing interventions and nursing practice. As an element of rigorous intervention development following systematic steps, it increases the chance of successful implementation.

Abstract citation ID: igad104.2462

### MEASUREMENTS OF SKIN CAROTENOID STATUS BY VEGGIE METER® IN URBAN ADULTS

Amy Schweitzer[1], Ricardo Brown[2], and Lillie Monroe-Lord[3], *1. University of the District of Columbia, Washington, District of Columbia, United States*, *2. UDC, Washington, District of Columbia, United States*, *3. University of the Dustrict of Columbia, Washington, District of Columbia, United States*

Recent advances in technology related to objective measures of dietary intake have led to tools such as the Veggie Meter®. The Veggie Meter uses pressure-mediated reflection spectroscopy to determine skin carotenoid status as an objective proxy of fruit and vegetable intake. This project is part of a larger study to determine seasonal changes in skin carotenoids of residents in an urban setting. The purpose of this project is to report baseline measures of skin carotenoids in a subset of older urban adults. Adults frequenting community events (senior center, food pantries, health fairs) consented to triplicate Veggie Meter readings. Adults (n=54) ages >60 years old who provided consent completed a survey and a baseline Veggie Meter reading. Participants were 68% female with a mean ± standard deviation age of 68 ± 7.2 years. Mean BMI was 29.9 ± 7.47 kg/m² . Most were non-smokers (75%). Participants identified as African American (78%), Caucasian (7%), two or more races (7%), or other race (6%). Mean Veggie Meter score was 275.6 ± 98.88. Smokers had a significantly lower Veggie Meter® score (216±73.4) than non-smokers (295 ± 98.7) p=0.006. **Conclusion:** For this sample of older adults in the DC area, differences in skin carotenoid status was seen by smoking status. Additional data is pending for Winter, Spring and Summer readings to determine whether seasonal variations exist in skin carotenoid status as measured by the Veggie Meter.

Abstract citation ID: igad104.2463

### POOR ACTIVITIES OF DAILY LIVING PREDICT FUTURE WEIGHT LOSS IN OLDER ADULTS AFTER HOSPITAL DISCHARGE.

Alfons Ramel, *University of Iceland, Reykjavik, Hofuoborgarsvaoio, Iceland*

The aim was to investigate whether older adults with poor activities of daily living (ADL) at hospital discharge had increased weight loss after six months of follow-up and whether nutrition therapy can prevent this weight loss. This was a secondary analysis of the HOMEFOOD dietary intervention trial (N=104) investigating community dwelling older adults (66-95 years) discharged from hospital and at risk for malnutrition, receiving either 6 months nutrition therapy or standard care without any further nutrition intervention. ADL were assessed using seven questions on self-care including mobility.Of the participants, 45 (43%) had good ADL at discharge, 36 (35%) had medium ADL and 23 (22%) had poor ADL with no differences between control and intervention group. During the study period, 79% of the control group lost more than 1 kg body weight with a mean weight loss of -3.5 ± 3.9 kg, whereas only 2% of the intervention group lost more than 1 kg with a mean weight gain of 1.7 kg ± 2.5 kg. According to general linear models, participants in the control roup with poor ADL had significantly higher weight loss than participants with good ADL (estimated and adjusted: 3.6 kg (95%CI: 1.0-6.1 kg, P=0.007). No such difference was observed in the intervention group. In our study, participants with poor ADL at hospital discharge develop lower body weight by around 3.5 kg six months later when compared to participants with good ADL. Receiving nutrition therapy post discharge can nullify these differences in body weight between ADL categories.

Abstract citation ID: igad104.2464

### PREDICTIVE VALUE OF NUTRITIONAL INDICES FOR SARCOPENIA TRANSITIONS IN MIDDLE-AGED AND OLDER ADULTS

Qiao Xiang, Yuxiao Li, Jirong Yue, and Ning Ge, *West China Hospital of Sichuan University, Chengdu, Sichuan, China (People's Republic)*

Since malnutrition plays an important role in sarcopenia development, we aimed to investigate sarcopenia's transitional dynamics and explore value of nutritional indices for predicting state transitions in middle-aged and older Asian adults. We analyzed data from 1910 participants in the WCHAT study who had complete information at baseline (2018) and at least one follow-up between 2019-2022. We defined the normal, sarcopenia, and intermediate subclinical states. Using a continuous-time multistate Markov model, we calculated transition probabilities and examined associations between nutritional indices and transitions. The probabilities of remaining stable versus progressing to the subclinical state in normal individuals were 53.4% versus 42.1% and 40.6% versus 49.0% at 2 and 4 years. In the subclinical population, their 2- and 4-year chances were 60.2% and 51.2% for keeping stable, 11.8% and 16.2% for developing sarcopenia, 28.0% and 32.6% for reverting to normal. For sarcopenic individuals, their likelihood of staying stable versus retrogressing to the subclinical state was 67.0% versus 26.3% and 48.3% versus 36.3% at 2 and 4 years. Increased albumin, BMI, calf circumference (CC), geriatric nutrition risk index (GNRI), mid-arm circumference (MAC), prealbumin, and triceps skinfold thickness (TST) was predictive for reversion to earlier states. Conversely, decreased BMI, CC, GNRI, MAC, and TST was associated with progression to sarcopenia. Vitamin D was not significantly associated with any transitions. This study reveals how sarcopenia changes over time and highlights usefulness of simple and cost-effective nutritional indices for predicting state transitions, which can help identify individuals at risk of sarcopenia and guide targeted interventions.

Abstract citation ID: igad104.2465

### PREVALENCE AND SOCIOECONOMIC DETERMINATES OF FOOD INSECURITY IN VETERANS: FINDINGS FROM NHANES

Ronna Robbins[1], Odessa Addison[2], Elizabeth Parker[3], Sarah Wherry[4], and Monica Serra[5], *1. South Texas Veterans Health Care System, Flower Mound, Texas, United States, 2. University of Maryland School of Medicine, Baltimore, Maryland, United States, 3. UMSOM, Baltimore, Maryland, United States, 4. VA Eastern Colorado Health Care System, Aurora, Colorado, United States, 5. UT Health San Antonio, San Antonio, Texas, United States*

Objective: Determine predictors of the association between being a Veteran and adult food security, as well as to examine the relation of potential covariates to this relationship. **Methods:** Data collected during 2011-2012, 2013-2014, and 2015-2016 National Health and Nutrition Examination Survey (NHANES) were pooled for analyses. 1,227 self-reported Veterans (mean age: 57 years) were matched to 2,432 non-Veterans (mean age: 53 years) on age, race/ethnicity, sex, and education. Adjusted logistic regression was used to determine the odds of Veterans having high food security vs. the combination of marginal, low, and very low food security compared to non-Veterans. **Results:** Veteran-status had no effect on the proportion of food insecurities between Veterans and non-Veterans reporting high (Veterans vs. non-Veteran: 79% vs. 80%), marginal (9% vs. 8%), low (5% vs 6%), and very low (8% vs. 6%) food security (p=0.11). However, after controlling for covariates

(age, race, education, diabetes, cardiovascular disease, hypertension, and depression), Veterans tended to be less likely to have high food security (OR: 0.82 (95% CI: 0.66, 1.02), p=0.07). Further, non-Hispanic Veterans (OR: 0.72 (95% CI: 0.55, 0.95), p=0.02) and Veterans completing some college (OR: 0.71 (95% CI: 0.50, 0.99), p< 0.05), were significantly less likely to experience high food security compared to non-Veterans. **Conclusion:** This study supports previous research findings that after controlling for covariates, Veterans tend to be less likely to have high food security. It also highlights ethnicity and level of education as important socioeconomic determinates of food security status in Veterans.

### SESSION 7360 (POSTER)

### SOCIAL AND HEALTH EQUITY, DIVERSITY, AND INCLUSION

Abstract citation ID: igad104.2466

### BLACK CAREGIVERS' CULTURAL, RELIGIOUS, AND SYMPTOM MANAGEMENT EXPERIENCES WITH HOME HOSPICE CARE

Taeyoung Park[1], Danetta Sloan[2], Dulce Maria Cruz-Oliver[3], M. Cary Reid[1], Sara Czaja[1], Ronald Adelman[1], Ritchell Dignam[4], and Veerawat Phongtankuel[5], *1. Weill Cornell Medicine, New York City, New York, United States*, *2. Johns Hopkins Bloomberg School of Public Health, Baltimore, Maryland, United States*, *3. Johns Hopkins Hospital, Baltimore, Maryland, United States*, *4. VNS Health, New York City, New York, United States*, *5. Weill Medical College of Cornell University, New York City, New York, United States*

Informal Black or African American (Black/AA) caregivers are at high risk for caregiver burden due to both greater caregiving responsibilities and unmet needs. However, there has been minimal research on the challenges Black/AA caregivers face after hospice enrollment. This study seeks to address this knowledge gap by applying qualitative methods to understand Black/AA caregivers' experiences around cultural, religious, and symptom management challenges during home hospice care. Data from semi-structured interviews with 11 bereaved Black/AA caregivers of patients who received home hospice care were qualitatively analyzed. Cultural needs (e.g., knowing their language, having familiarity with foods) were perceived as not on top of mind for many Black/AA caregivers. However, there was concern of stigma around mental health preventing care recipients from sharing their mental health concerns and seeking resources. Many caregivers relied on their personal religious networks rather than services provided by hospice chaplains. Regarding symptom management, caregivers struggled most with managing patients' pain, lack of appetite, and decline near End of Life (EoL). Lastly, caregivers reported increased burden during this phase of caregiving but were satisfied with the overall hospice experience. Our results suggest that tailored approaches that target mental health stigma in the Black/AA community and reduce caregiver distress around EoL symptoms may improve hospice outcomes among Black/AA hospice caregivers. Hospice spiritual services should consider offering services complementary to caregivers' existing religious networks. Larger quantitative studies are needed to examine the clinical implications of these results in terms of patient, caregiver and hospice outcomes.

Abstract citation ID: igad104.2467

### BRAIN HEALTH COMMUNITY REGISTRY: DEVELOPMENT OF A MODEL FOR CONNECTING PARTICIPANTS TO COMMUNITY-BASED SUPPORTS

Laura Block[1], Meghan Haas[1], Lilli Kay[1], Clark Benson[2], and Andrea Gilmore-Bykovskyi[1], *1. University of Wisconsin-Madison, Madison, Wisconsin, United States*, *2. University of Wisconsin School of Medici, Madison, Wisconsin, United States*

Growing evidence demonstrates social and economic factors (e.g. socioeconomic status, level of education) contribute to risk for Alzheimer's disease and related dementia (ADRD). These factors also serve as barriers to research participation, contributing to systematic under-inclusion in ADRD research. The Participant and Relationship-Oriented Research Engagement (PRORE) Model outlines mechanisms through which socioeconomic determinants and reciprocal relationships may shape research participation; however, scalable protocols that address these mechanisms are lacking. Our objective was to develop and pilot the feasibility and acceptability of a research-based resource-matching protocol. The Brain Health Community Registry (BHCR) recruits adults ages 40 and above with and without changes in memory, and caregivers of individuals experiencing memory changes. BHCR connects participants with research opportunities and conducts a needs assessment to match individuals with community-based services and supports. Of 168 participants enrolled in BHCR, 33 (20%) report changes in memory, 93 (55%) do not, and 42 (25%) are caregivers. More than half (n=91, 58%) requested resource matching. Resource matching took 1.8 hours per participant (range 0.5-4.5 hours). Challenges included navigating rural counties with limited resources and effective follow-up; strategies included an interdisciplinary approach, offering multiple modes of contact, and a strengths and relation-based approach. Participants who responded to follow-up at two and six weeks (45% of total), and at annual review (100%, N=6) described resource-matching as instrumental for successful connection to supports, general knowledge/preparation, or on behalf of a friend or family member. Future work must ascertain whether needs assessment and resource matching change study participant perceptions of research.

Abstract citation ID: igad104.2468

### CONTRIBUTION OF CHRONIC CONDITIONS TO MORTALITY: DIFFERENCES BY RACE AND ETHNICITY

Ana Quiñones[1], Miriam Elman[1], Gail McAvay[2], Brent Vander Wyk[2], Corey Nagel[3], and Heather Allore[2], *1. OHSU-PSU School of Public Health, Portland, Oregon, United States*, *2. Yale University, New Haven, Connecticut, United States*, *3. University of Arkansas for Medical Sciences, Little Rock, Arkansas, United States*

Health disparities exist in the timing, order, and accumulation of chronic somatic and neurodegenerative conditions, which are associated with increased health care utilization, expenditure, and mortality. We assessed the contribution of coexisting conditions to mortality among Hispanic, non-Hispanic (NH) White, and NH Black older adults in the United States to measure their burden. We used nationally-representative data from the Health and Retirement Study (HRS) from 1998-2016 (n=11,532). This analysis estimates the absolute additive contributions of seven somatic and two mental/neurodegenerative conditions using a longitudinal average attributable fraction derived from a survey-weighted multivariable discrete survival model adjusted for sociodemographic characteristics (sex, education, net worth, body-mass index, coupled, insured, and any of six ADLs lasting three or more months "because of physical, mental, emotional, or memory problems"). Conditions accounted for over 60% of mortality in each racial/ethnic group (64.0%, 62.2%, and 70.5% in Hispanic, NH White, and NH Black adults, respectively). Although we found that cardiovascular diseases (heart disease and hypertension) were the greatest contributor to morality in each racial/ethnic group, the impact of other conditions varied by race/ethnicity. Leading contributors to mortality were diabetes (12.0%) and dementia (7.2%) for Hispanic participants; cancer (13.0%) and lung disease (10.7%) for NH White participants; and diabetes (11.5%), lung disease (7.4%), and dementia (7.3%) in NH Black participants. This study provides insights into racial and ethnic differences in the contribution of chronic somatic and neurodegenerative conditions to mortality and may help target efforts for specific preventative care services to populations most impacted by them.

Abstract citation ID: igad104.2469

### EDUCATION AS A MEDIATOR OF GENDER DIFFERENCES IN COGNITION IN OLDER ADULTS

Judith Rijnhart[1], Matthew Valente[1], Almar Kok[2], and Martijn Huisman[2], *1. University of South Florida, Tampa, Florida, United States*, *2. Amsterdam University Medical Center, Amsterdam, Noord-Holland, Netherlands*

Although dementia is more prevalent in women than in men, studies on the socio-cultural mechanisms that cause and sustain gender differences in dementia remain scarce. Historic gender differences in education may explain why older women are at higher risk of dementia nowadays. This study aimed to investigate education as a mediator of gender differences in cognitive function, as measured with the Mini-Mental State Examination (MMSE). We used data from the Longitudinal Aging Study Amsterdam. The analytical sample consisted of 2,909 older adults aged 55-85, of whom 1,601 were women and 1,506 were men. We used causal mediation analysis to estimate total, indirect, and direct effects of gender on MMSE. Effect estimates were adjusted for confounding by age, parental education, and childhood adversity. The total effect was -0.60 (95% CI: -0.90; -0.32), indicating that women on average had a 0.60 lower MMSE score than men. The indirect effect was -0.84 (95% CI: -1.11; -0.59), indicating that women on average had a 0.84 lower MMSE score compared to men, because women completed fewer years of education. The direct effect was 0.24 (95% CI: 0.04; 0.44), indicating that if women had the same number of education years as men, their MMSE score would on average be 0.24 points higher than the average MMSE score in men. These findings indicate that education is an important mediator of gender differences in cognitive function. Future studies are needed to explore whether gender differences in cognitive function decrease as gender equality in education increases.

Abstract citation ID: igad104.2470

### HOW THE PATIENT-CENTERED MEDICAL HOME MODEL IMPACTS OLDER ADULTS' ACCESS TO HEALTH CARE IN RURAL GEORGIA

Elisa Childs, *University of Iowa, Iowa City, Iowa, United States*

Rural Americans face several healthcare access barriers, including limited providers and driving further distances to receive care. Georgia healthcare access is especially problematic given its high rurality, hospital closure rate, number of medically underserved areas, and older adult population. The government promotes the Patient-Centered Medical Home (PCMH) model to improve healthcare access. This study supports a better understanding of older Georgian's access to care by exploring PCMH's ability to improve healthcare access. Older adults were surveyed using a modified version of the Healthcare Quality Survey (N=746). Analyses included univariate, bivariate (t-test, ANOVA, chi-square, and a crosstabulation), and multivariate (multiple linear regression). Healthcare access was statistically significantly different depending on care source type, with significant associations between PCMH users and income, age, urban/rural status, and health status. Access was also statistically significantly different based on annual household income, educational attainment, age, and health status. Further, a significant relationship was found between access and age, income, and health status. PCMH users were primarily Caucasian and most often had an advanced degree and income of $60,000 or more. PCMH users most often reported living in Three Rivers, Atlanta Region, Northeast Georgia, and the Georgia Mountains. Finally, urban and rural residents with a regular care source reported "always" receiving needed care more often than PCMH users and those with no care source. The National Committee for Quality Assurance and other accreditation agencies should target low-income rural areas with high older adult populations when implementing the PCMH model to increase older adults' healthcare access.

Abstract citation ID: igad104.2471

### MALE BREAST CANCER IN OLDER ADULTS BELONGING TO THE LOWER-INCOME GROUP

Esha Chakravarty, *Calcutta Metropolitan Institute of Gerontology, Mitcham, England, United Kingdom*

Male breast cancer is a rare type of cancer constituting around 1% of male malignancies. Cases of 21 elderly (aged>60) patients diagnosed with breast cancer were studied with respect to age of onset, treatment choices, access to care, ability to perform activities of daily living (ADL) from CMIG, resource centre on ageing in east India. All 21 patients belonged to lower income group in society and the study was conducted over a 5 year period of 2018-2022. Primary symptom leading to diagnosis was self-detection of lump and the median time of diagnosis from onset of symptoms was 6 months. Twenty of the 21 patients were diagnosed with Stage III or Stage IV breast cancer and 1 patient was diagnosed with Stage I cancer. The average age of diagnosis of the patients was found to be 69 years. The main method of treatment was surgery for patient diagnosed in Stage I and surgery followed by chemotherapy and radiation therapy for the rest. None of the patients underwent hormone therapy and information regarding presence of hormone receptors was not available in all cases. Five of the 21 cases reported metastasis- 4 in bone and 1 in lung. Spouse or adult children were found to be caregivers with no access to trained caregivers or counsellors. All patients showed moderate decline in motor function and ADL after surgery alongwith susceptibility to fall or injury. Free hand exercises showed slight improvement. Further studies can attract state funding and influence heath policies for lower income groups.

Abstract citation ID: igad104.2472

### RELATIONSHIP BETWEEN INFORMAL AND FORMAL HELP-SEEKING BEHAVIORS FOR ELDER MISTREATMENT AMONG CHINESE IMMIGRANTS

Ying-Yu Chao[1], Jin Young Seo[2], and Yu-Ping Chang[3], *1. Rutgers, the State University of New Jersey, Somerset, New Jersey, United States*, *2. Hunter College, CUNY, New York City, New York, United States*, *3. University at Buffalo, The State University of New York, Buffalo, New York, United States*

Purpose: Help-seeking minimizes the consequences from elder mistreatment (EM). However, formal help-seeking could be challenging for older immigrants due to cultural and linguistic barriers. Informal supporters have been shown to promote formal help-seeking among EM victims. This study aimed to examine whether informal help facilitates U.S. Chinese EM victims with seeking help from formal sources. **Methods:** Data were derived from the Population Study of Chinese Elderly in Chicago. EM measurements included psychological, physical, sexual mistreatment, financial exploitation, and caregiver neglect. Seeking help from informal and formal sources were evaluated. Descriptive statistics and multinominal logistic regression were conducted. **Results:** Among 450 EM victims, 53.48% of them sought informal help, 31.75% did not seek any help, 11.42% sought both informal and formal help, and 3.34% sought formal help. The most common informal sources were adult children, partners, and friends/neighbors. The most common formal sources were community social services organizations, followed by legal criminal justice systems. Compared to those who did not seek any help, victims who reported more emotional closeness within their social network and experienced financial exploitation were more likely to seek help from formal sources. However, informal help-seeking was not significantly associated with formal help-seeking. Conclusion/implication: The findings suggest that most U.S. Chinese older adults who experienced EM did not seek formal help. Higher levels of contact with social networks could increase seeking help from formal sources. However, future research is needed to explore why informal supporters did not facilitate seeking help from formal sources in Chinese EM victims.

Abstract citation ID: igad104.2473

### SOCIAL CHARACTERISTICS OF RACIAL MINORITY STROKE SURVIVORS AND PERCEIVED DISCRIMINATION: A SECONDARY ANALYSIS

Lilcelia Williams[1], Jordann Antoan[1], Tina Harris[1], Joy Hammel[2], Natalie Leland[1], and Elizabeth Skidmore[3], *1. The University of Pittsburgh, Pittsburgh, Pennsylvania, United States*, *2. University of Illinois at Chicago, Chicago, Illinois, United States*, *3. University of Pittsburgh, Pittsburgh, Pennsylvania, United States*

African American and Black persons experience significant disability after stroke. They also experience high external stressors, including discrimination, which may attenuate stroke recovery. Further understanding of the associations among external stressors, disability severity, and attenuated recovery are important to reduce healthcare disparities and improve patient outcomes. However, persons of the same race may not have the same lived experiences due to variations in social characteristics such as age or education. Associations between these social characteristics and perceived discrimination may offer insights into the recovery of older adult African American and Black stroke survivors. We conducted a secondary analysis of 11 poststroke patients who were recruited to participate in the Black Lived Experiences of Stroke Study (female=10, age=67±11, ≥2 years post-secondary education=63%) to assess the frequency of perceived discrimination. Perceived discrimination was measured with the Everyday Discrimination Scale survey. We examined correlations between age and education and perceived discrimination using Cramer's V tests. Semi-structured participant interviews were also examined for examples of perceived discrimination during poststroke healthcare. Nine participants reported perceived discrimination post-stroke. Analyses revealed a clinically meaningful but not statistically significant relationship between chronological age and perceived discrimination (Cramer's V=0.62, p=0.12). There was no meaningful relationship between educational level and perceived discrimination. All participants reported challenges with communication, missed diagnoses and poor delivery of care poststroke. Findings support the need for additional research to examine the relationships between participants' social characteristics and perceived discrimination among Black and African American stroke survivors, and the impact on stroke recovery.

Abstract citation ID: igad104.2474

### SOCIAL DETERMINANTS OF HEALTH AND AGING: DISPARITIES AND BARRIERS TO ORAL HEALTH CARE AMONG OLDER ADULTS IN ALABAMA

Nathan Smith[1], Stacey Cofield[2], and Raquel Mazer[3], *1. University of Alabama School of Dentistry, Birmingham, Alabama, United States*, *2. University of Alabama at Birmingham, School of Public Health, Birmingham, Alabama, United States*, *3. University of Alabama at Birmingham, School of Dentistry, Birmingham, Alabama, United States*

Limited data exists on the oral health status of the older adult population of Alabama; improved surveillance programs are needed for estimations and planning. Aim: This study compares gender, racial, and economic differences in barriers to oral healthcare among older adults in Alabama. **Methods:** Self-reported, voluntary surveys including sociodemographics, oral care behaviors, and care utilization were completed along with intraoral screenings at senior centers and senior living communities. Univariate analyses were conducted using Chi-Square or Fisher's Exact test, p< 0.05 considered meaningful. **Results:** 357 respondents were assessed: mean age 73.4 (SD 8.4), 70.3% female, 57.1% Black/

African American (AA), 42.9% Caucasian (Ca), 88.8% sole source of income Social Security/Disability. Ca were more likely than AA to have dental insurance (65.0 vs 51.5%, p=0.013), and more likely to have had a cleaning/check-up (20.9 vs 12.3%, p=0.029). Those with income <$1500/month who received dental care were more likely to receive care for dentures compared to those with higher income (17.1 vs 8.0%, p=0.030). Those still driving who received dental care were more likely to have had a cleaning/check-up compared to those not still driving (20.9 vs 9.2%, p=0.025). Untreated tooth decay was detected in 25% of those with remaining dentition and was more common among AA than Ca (57.4 vs 44.4%, p=0.035). **Conclusions:** The preliminary analysis points to the need to further explore access to oral health services for older Alabamians living independently. Further data is needed for planning and policy making. Barriers to care were not explored for those who are homebound.

Abstract citation ID: igad104.2475

### THE GRAYING RAINBOW: TRACING LGBTQI AGING IN SCANDINAVIAN LITERATURE

Mats Christiansen[1], Mika Handelsman-Nielsen[2], and Manijeh Mehdiyar[2], *1. Åbo Akademi University, Solna, Sweden*, *2. The Public Health Agency of Sweden, Solna, Stockholms Lan, Sweden*

**Background:** There have been several international reviews about aging LGBTQI health and living conditions, but where policy, social insurance, and services differ. The Public Health Agency of Sweden was given a government assignment to review the literature on LGBTI, emphasizing Scandinavian literature. Method: This scoping review includes peer-reviewed literature published in English or Scandinavian languages from January 1, 2012, to May 2022. Literature was searched in PsycINFO, PubMed, Web of Science, CINAHL, International Bibliography of the Social Sciences (IBSS), Social Science Database, Applied Social Sciences Index & Abstracts (ASSIA), and Sociological Abstracts, Sociology Database. Initially, 11,428 articles were found. After removing duplicates using Rayyan and reviewing titles and abstracts, 54 articles were read in full. After the final review, 16 articles remained. Thematic analysis was used to produce themes from the reviewed literature. Findings: The following two themes were identified: Aspects of health and Living conditions. Studies were primarily interpretive. There is a lack of studies about some subgroups of older LGBTQ people in this context; for instance, there are insufficient studies on older lesbian women and gay men. Furthermore, there is a lack of studies on somatic health for older LGBTQ people, generally. Comparatively, albeit in small samples, we better understand trans descriptions of aging than lesbian women and gay men. There were no studies found on intersex individuals. Implications: There remains a paucity of literature regarding the life and living conditions for LGBTQI older adults in Nordic countries.

Abstract citation ID: igad104.2476

### THE INFLUENCE OF RURALITY AND FRAILTY ON HEALTH OUTCOMES IN OLDER ADULTS

Hillary Spangler[1], Emma Mitchell[2], David Lynch[1], Perry Haaland[2], and John Batsis[1], *1. UNC School of Medicine, Chapel Hill, North Carolina, United States*, 2. *UNC Chapel Hill, Chapel Hill, North Carolina, United States*

**Background:** Frailty is a syndrome representing a decline in physical function and an increased vulnerability to stressors. Older adults in rural areas may be at increased risk for frailty and adverse outcomes due to worse overall health. Our aim was to identify how rural/urban residence can influence frailty status. **Methods:** We used National Health and Aging Trend Study (2011-2020) data, a cohort of Medicare beneficiaries. Participants were categorized as robust, pre-frail, and frail (Fried's frailty phenotype). Rural residence included counties outside of a metropolitan statistical area (Office of Management and Budget). Participants were excluded if frailty components or geographical status were incomplete. We used logistic regression analyses for the relationship between adverse outcomes (death, nursing home placement over 2011-2020), rural/urban, and frailty status. **Results:** Of 7,393 participants (57.2% female), median age range was 75-80 years and 19.4% were rural residents. Rates of robust, pre-frailty, and frailty were 41.4%, 48.4%, and 10.2%, respectively. There was no difference of frailty status by rural status nor was there a frailty x rural interaction. Older adults with pre-frailty (OR=1.18; 1.05-1.34) and frailty (OR=1.01; 0.82-1.24) had higher risk of residing in rural than urban areas. Rural residence (OR=1.75;1.33-2.24), pre-frailty (OR=1.87;1.54-2.28) and frailty (OR=3.62; 2.79-4.71) had higher risk of adverse outcomes. **Conclusion:** Participants with pre-frailty had higher odds of living in a rural area. Similarly, participants in rural areas had higher odds of adverse outcomes. These findings may highlight health care disparities in rural areas and opportunities for system- and individual-level interventions to prevent frailty development.

### SESSION 7370 (POSTER)

### BIOLOGY OF AGING

Abstract citation ID: igad104.2477

### A BAYESIAN NETWORK-BASED APPROACH FOR MULTI-OMICS INTEGRATION TO REVEAL UNDERLYING MECHANISMS OF HEALTHY AGING

Anastasia Leshchyk[1], Stefano Monti[1], and Paola Sebastiani[2], *1. Boston University, Boston, Massachusetts, United States*, *2. Tufts Medical Center, Boston, Massachusetts, United States*

Previous research on long-lived individuals showed that centenarians significantly delay the onset of the disability and aging-related diseases such as Alzheimer's, dementia, and cardiovascular diseases to the very end of their lives compared to the general population. Genetic studies of centenarians and healthy agers showed that carriers of the APOE e2 allele had increased odds of reaching longevity compared to the non-e2 allele carriers. In addition, the APOE e2 allele is characterized by distinct serum proteomics and metabolomics profiles that could be useful to understand the mechanism of propagation of the genetic effect of APOE to the molecular level and eventually to phenotypes. We are developing a novel network-based approach of multi-layer data integration to identify shared molecular profiles among the subjects with familial longevity that lead to compression of morbidity, disability, and mortality. This Bayesian network-based approach integrates proteomics, metabolomics, genetics, and multiple phenotype data to decipher the risk factors and pathways contributing to prolonged health span. For example, the preliminary analysis shows that the centenarians with cognitive performance above the average in their age and sex group have lower levels of IL6, GDF15, and PTN proteins than those with below-average cognitive performance. These known inflammation factors might impair the cognitive function of elderly adults contributing to their disability and morbidity, thus affecting their aging.

Abstract citation ID: igad104.2478

### A GENETICALLY INFORMED STUDY ON THE ETIOLOGICAL ASSOCIATIONS AMONG EPIGENETIC CLOCKS

Eric Penichet[1], Christopher Beam[1], Kelly Bakulski[2], Eric Turkheimer[3], Kristin Higdon[4], Lesa Ryan[5], Kendra Sikes[6], and Deborah Davis[5], *1. University of Southern California, Los Angeles, California, United States*, *2. University of Michigan, Ann Arbor, Michigan, United States*, *3. University of Virginia, Charlottesville, Virginia, United States*, *4. Norton Children's Research Institute, Louisville, Kentucky, United States*, *5. University of Louisville School of Medicine, Louisville, Kentucky, United States*, *6. Norton Healthcare, Louisville, Kentucky, United States*

Epigenetic clocks are believed to capture the molecular footprints of biological programs that subserve innate aging processes. Although epigenetic clocks can predict disease phenotypes and mortality with a high degree of accuracy, little is known about the extent to which they capture the same (or different) developmental and maintenance mechanisms. Quantitative genetic studies may provide useful information on the etiological links between epigenetic clocks, particularly pertaining to the question of whether they are influenced by a common set of genetic and environmental factors. Using preliminary data from the Louisville Twin Study, this study used a multivariate independent pathway model to examine the shared and unique genetic and environmental influences of three epigenetic clocks (GrimAge, HannumAge, PhenoAge). Participants consisted of 43 monozygotic pairs and 30 (18 same-sex) dizygotic twin pairs with a mean age of 54.5 years (sd = 5.27). Phenotypic analyses revealed significant correlations among the clocks (r = 0.63 - 0.78). Subsequent multivariate biometric analyses showed that there were common genetic and nonshared environmental influences across all three clocks ($p < 0.001$), with unique genetic and nonshared environmental influences specific to GrimAge and HannumAge ($p < 0.001$). To examine the sources of covariation between these variables, biometric regression models were used to decompose the covariance between each of the clocks (GrimAge and Hannum, Hannum and PhenoAge, PhenoAge and GrimAge) into genetic and environmental components. These analyses revealed large, significant genetic correlations (rg = 0.80-0.86) and medium to large, significant nonshared environmental correlations (re = 0.37-0.73) among the clocks.

Abstract citation ID: igad104.2479

### ALTERED GENE EXPRESSION PROFILE OF AGING BONE MARROW MESENCHYMAL STEM CELLS

Yun Su, Jie Chen, Carlos Isales, and Xingming Shi, *Augusta University, Augusta, Georgia, United States*

Aging is a complex process that affects many physiological systems, including bone metabolism. To gain insight into the molecular mechanisms underlying age-related bone loss, we conducted RNA-seq analysis of mesenchymal stem cells (MSCs) isolated from the bone marrow of 6-month and 24-month-old C57Bl/6 mice. We identified 5857 differentially expressed genes, out of 19,324 genes, with an absolute fold change greater than 1.4 and FDR q-value less than 0.05. These genes are involved in a variety of biological processes, including metabolism, immune response, and signal transduction. KEGG pathway enrichment analysis revealed 28 significantly enriched pathways, suggesting that multiple pathways contribute to age-related bone loss. Our findings provide a foundation for future studies aimed at elucidating the molecular mechanisms underlying age-related bone loss and identifying potential therapeutic targets for the prevention and treatment of osteoporosis.

Abstract citation ID: igad104.2480

### ANALYSIS OF CIRCADIAN TRANSCRIPTION ACTIVATOR BMAL1 AND CLOCK BINDING TARGETS IN YOUNG AND OLD MOUSE SKELETAL MUSCLE

Xiping Zhang, Mark Viggars, and Karyn Esser, *University of Florida, Gainesville, Florida, United States*

Circadian rhythms play important roles in many physiological processes and circadian rhythm disturbances are often observed in older people. Circadian rhythms are regulated by a molecular clock mechanism that is found in virtually all cells of the body. The core clock mechanism consists of transcriptional activators (BMAL1 and CLOCK) and the feedback negative regulators (PER and CRY). Beyond keeping daily time, the core clock factors regulate a daily gene expression program and this daily program is diminished with age. Skeletal muscle is critical in supporting systemic metabolism and mobility. Loss of muscle strength in older people are related to increased morbidity and mortality. Our previous studies have shown a significant decline of circadian genes with age in skeletal muscle and there are very few circadian genes in common between young and old mouse muscle. To understand the mechanism of the diminished circadian gene expression with age, we performed ChIPseq to determine BMAL1 and CLOCK binding and ATACseq to determine chromatin accessibility with muscle from young (6 months) and old (27 months) mouse. The ChIPseq result showed that BMAL1 and CLOCK shared more than 8,000 binding peaks in young muscle and less than 200 binding peaks in old muscle. The ATACseq identified that chromatin accessibility altered in more than 7000 sites with age and BMAL1:CLOCK binding sites were less accessible with age. In conclusion, our research has demonstrated that muscle chromatin accessibility changes with age and the core clock factors are important regulators of gene expression in skeletal muscle.

Abstract citation ID: igad104.2481

### CAN ACUTE MUSCLE DAMAGE LEAD TO OSTEOARTHRITIS?

Kamal Awad[1], Logan Moore[2], Jian Huang[3], Leticia Brotto[2], and Marco Brotto[4], *1. University of Texas at Arlington, Arlington, Texas, United States*, *2. University of Texas Arlington, Arlington, Texas, United States*, *3. University of Texas At Arlington, Arlington, Texas, United States*, *4. The University of Texas at Arlington, Arlington, Texas, United States*

Skeletal muscles are crucial for maintaining musculoskeletal homeostasis, as they provide mechanical support, joint stability, and biochemical functions via myokine secretion. We investigated whether acute muscle damage or weakness could contribute to the development of osteoarthritis (OA). Using a chemical damage model, we induced muscle damage by injecting 1.2% barium chloride into the tibialis anterior muscle, resulting in an immune response characterized by a significant increase in the concentration of Prostaglandin E2 (PGE2) and other lipid mediators derived from the EPA and DHA signaling pathways, as quantified using our innovative lipidomics methods. The muscle damage was confirmed and visualized by muscle histomorphometry. The damage resulted in muscle weakness, detected by a significant decrease in grip strength at 4-days post-injury, but without changes in mice activity measured with force-plate actimeter. After 1-month post-injury, grip strength and muscle histology returned to normal, but histological changes were observed in the articulating cartilage, indicating surface erosion and cartilage matrix loss (OARSI grade 4). We used Raman Spectroscopy and micro-CT to confirm the joint alterations observed in the changes in subchondral bone and increased bone volume fraction in the cortical shell of the subchondral bone, respectively, features characteristic of OA in rodents and humans. These findings suggest that muscle weakness or imbalance due to injury can contribute to joint degeneration. This study highlights the significance of muscles in preventing OA and provides a novel approach for exploring the relationship between muscle damage and OA development. This new model could prove valuable for aging research.

Abstract citation ID: igad104.2482

### CHARACTERIZATION OF AGE-RELATED BIOENERGETIC DECLINE IN HUMAN DERMAL FIBROBLASTS

Howard Phang[1], Stephanie Heimler[2], and Anthony Molina[1], *1. University of California, San Diego, San Diego, California, United States*, *2. UC San Diego School of Medicine, San Diego, California, United States*

Progressive mitochondrial dysfunction is a widely recognized hallmark of the human aging process. However, efforts to model bioenergetic decline in vitro largely fail to recapitulate the context of human aging and metabolic physiology, making investigation of age-related bioenergetic decline and targeted interventions difficult. We sought to model human bioenergetic decline in vitro by profiling the respiratory capacity of cultured human dermal fibroblasts from donors across the adult human lifespan. Primary human dermal fibroblasts (pHDF) are an attractive in vitro model to assess age-related bioenergetic decline due to retention of key genotypic and phenotypic features of the donor during cell culturing, including age-related phenotypes. We hypothesized that respiratory capacity of pHDF is associated with the chronological age of the donor. We leveraged the San Diego Nathan Shock Center of Excellence (SD-NSC) study which is collecting pHDF from healthy adult (aged 20-85+) participants representative of "normal" aging based on strict inclusion and exclusion criteria. We profiled the mitochondrial function of eleven SD-NSC pHDF lines using the Seahorse XFe96 Mito Stress Test. The Pearson correlation identified a strong, negative correlation between maximal respiration and age (r= –0.69, p=0.02) and between spare respiratory capacity and age (r= –0.68, p=0.02). This suggests that human age-related mitochondrial dysfunction can be recapitulated in vitro using primary cell culture. These findings serve as the starting point for deeper analysis involving a larger sample of pHDF lines and other bioenergetic parameters to accurately quantify the effect of human aging on mitochondrial function and bioenergetics.

Abstract citation ID: igad104.2483

### DNA METHYLATION-BASED BIOLOGICAL AGING, PLASMA PROTEINS, AND INCIDENT FRAILTY AND PHYSICAL DISABILITY

Aung Zaw Zaw Phyo[1], Sara Espinoza[2], Anne Murray[3], Peter Fransquet[4], Jo Wrigglesworth[4], Robyn Woods[4], and Joanne Ryan[4], *1. Monash University, Melbourne, Australia. , Melbourne, Victoria, Australia*, *2. Cedars-Sinai Medical Center, Los Angeles, California, United States*, *3. Hennepin HealthCare and University of Minnesota, Minneapolis, Minnesota, United States*, *4. Monash University, Melbourne, Victoria, Australia*

Decline in physical function occurs with advanced age, and biological aging may be a useful marker of decline in physical function, beyond chronological age. This study investigated the associations between DNA methylation-based biological aging (DNAm-based age acceleration (AA)) and plasma proteins and the risk of frailty (based on Fried phenotype and a frailty deficit accumulation index (FI)), and persistent physical disability (defined as loss of ability to perform one or more Katz ADL), among community-dwelling people aged ≥70 years enrolled in the ASPREE study. Five AA indices (Horvath, Hannum, Pheno, Grim, and DunedinPACE) and seven plasma proteins (including plasminogen activation inhibitor-1 (PAI-1), growth differentiation factor-15 (GDF-15), and leptin) were measured in blood from 560 participants (50.7% women). Incident health events were captured over a mean of 5.2 years. Cox proportional-hazard regression adjusted for gender, education, and cell counts. One-standard deviation (SD) increase in AA was associated with an increased risk of frailty (FI) (GrimAA: HR=1.48 (95% CI, 1.19 – 1.84), and DunedinPACE: HR=1.39 (95% CI, 1.11 – 1.73)) and physical disability (GrimAA: HR=1.78 (95% CI, 1.15 – 2.77)). One-SD increase in plasma proteins except for leptin was associated with a higher risk of frailty (both Fried phenotype and FI) (range HR: 1.26 to 2.14). In conclusion, accelerated Grim age and DunedinPACE appear as markers of physical impairments, even in initially healthy individuals. Variations in plasma protein levels might be helpful for the identification of pathophysiological pathways associated with the increased risk of physical impairment in later life.

Abstract citation ID: igad104.2484
### EPIGENETIC AGE ACCELERATION AND IMMUNE CELL COUNT: A TWIN STUDY
Eric Penichet[1], Christopher Beam[1], Kelly Bakulski[2], Eric Turkheimer[3], Kristin Higdon[4], Lesa Ryan[5], Kendra Sikes[6], and Deborah Davis[5], *1. University of Southern California, Los Angeles, California, United States*, *2. University of Michigan, Ann Arbor, Michigan, United States*, *3. University of Virginia, Charlottesville, Virginia, United States*, *4. Norton Children's Research Institute, Louisville, Kentucky, United States*, *5. University of Louisville School of Medicine, Louisville, Kentucky, United States*, *6. Norton Healthcare, Louisville, Kentucky, United States*

Accelerated aging, as measured by epigenetic clocks, is associated with declines in immune system functioning. Investigating the causal basis of this association may provide mechanistic insight into the link between DNA methylation, on which epigenetic clocks are based, and age-related changes in immune cell composition. Using preliminary data from the Louisville Twin Study, this study examined the genetic and environmental sources of covariation between immune cell count (neutrophils, lymphocytes) and GrimAge Acceleration. GrimAge is a composite biomarker derived from 7 DNA methylation-based surrogates associated with mortality and smoking pack years. GrimAge Acceleration is the residual of GrimAge regressed onto chronological age. Participants consisted of 43 monozygotic twin pairs and 30 (18 same-sex) dizygotic twin pairs with a mean age of 54.5 years (sd = 5.27). Phenotypic analyses revealed that GrimAge Acceleration was significantly associated with neutrophil and lymphocyte count (r = 0.45 and -0.49, respectively). To examine the sources of covariation between these variables, biometric regression models were used to decompose the covariance between GrimAge Acceleration and neutrophils (and lymphocytes) into genetic and environmental components. Bivariate analyses revealed moderate, marginally significant genetic correlations between GrimAge Acceleration and immune cell count (rg = 0.32 and -0.38 for neutrophils and lymphocytes, respectively) and large, significant nonshared environmental correlations (re = .58 and -.54 for neutrophils and lymphocytes, respectively). Common genetic factors accounted for 39% of the phenotypic correlation in neutrophils and 43% in lymphocytes, whereas common nonshared environmental factors accounted for 61% and 57% of the correlation in these cell types, respectively.

Abstract citation ID: igad104.2485
### EPIGENETIC AGE DYNAMICS AND EMBRYONIC MORTALITY POINT TO GASTRULATION AS AGING GROUND ZERO IN XENOPUS LAEVIS
Bohan Zhang[1], and Vadim Gladyshev[2], *1. Brigham and Women's Hospital, Boston, Massachusetts, United States*, *2. Harvard Medical School, Boston, Massachusetts, United States*

Recent studies on embryonic development revealed a natural rejuvenation event followed by epigenetic aging. Here, we developed a frog aging clock based on the DNA methylation profile in the African clawed frog Xenopus laevis. Application of this clock to Xenopus laevis early embryonic development identified a decrease in epigenetic age during early embryogenesis, with the minimal epigenetic age reached around gastrulation. We further examined the individual developmental trajectories of 6,457 embryos and found that this minimum age is also accompanied by a higher incidence of disrupted development. Taken together, our data point to gastrulation as a critical stage for aging and natural rejuvenation, characterized by the lowest epigenetic age and increased mortality, defining aging ground zero as the basepoint where rejuvenation ends and the aging process begins.

Abstract citation ID: igad104.2486
### EPIGENETIC AGING AND RHEUMATOID ARTHRITIS
Nandini Mukherjee, and Tracie Harrison, *The University of Arkansas for Medical Sciences, Little Rock, Arkansas, United States*

Indicators of biological aging with rheumatoid arthritis (RA), a chronic autoimmune disease, are not yet fully known. In this cross-sectional study, we tested associations between plasma indicators of epigenetic aging using online data (GSE42861) regarding RA and DNA methylation from the Swedish Epidemiological Investigation of Rheumatoid Arthritis (EIRA). Peripheral blood DNA methylation of participants 18-70 years old was assessed using Illumina HumanMethylation450 arrays. Horvath's online DNA Methylation Age Calculator determined DNAm estimate of Telomere length (DNAmTL), Hannum's, Horvath's 2013 and 2018 epigenetic ages, PhenoAge and GrimAge and the respective age accelerations. Association of RA prevalence with epigenetic age were determined using linear regression, correcting for false discovery rate. We identified significant associations of RA with age acceleration measures of Horvath 2013 (Estimate:-1.34 ; FDR p-value: 1.0 × 10-2) and Horvath 2018 (Estimate:-1.32; FDR p-value: 4.0 × 10-5), extrinsic age acceleration (Estimate: 1.34 ; FDR p-value: 1.0 × 10-2), PhenoAge acceleration (Estimate: 2.31; FDR p-value: 1.1 × 10-5), GrimAge (Estimate: 2.54; FDR p-value: 1.0 × 10-2) and GrimAge acceleration (Estimate: 3.15 ; FDR p-value: 1.7 × 10-17), adjusting for sex and smoking (FDR p-value<=0.05). In sex stratified analyses, first-generation clocks were associated only in women, but no sex specific effects for PhenoAge or GrimAge accelerations were identified. The second generation 'biological' clocks PhenoAge, GrimAge and its components showed higher epigenetic age acceleration in RA cases compared to healthy controls. We conclude that second generation indicators of epigenetic aging provide promise as markers of biological aging in people with RA.

Abstract citation ID: igad104.2487
### EVIDENCE THAT CONSERVED ESSENTIAL GENES ARE ENRICHED FOR PRO-LONGEVITY FACTORS
Alaattin Kaya, *Virginia Commonwealth University, Richmond, Virginia, United States*

At the cellular level, many aspects of aging are conserved across species. This has been demonstrated by numerous studies in simple model organisms like Saccharomyces cerevisiae, Caenorhabdits elegans, and Drosophila melanogaster. Functional genomic studies in these models have contributed enormously to our understanding of conserved genetic pathways influencing aging in evolutionarily divergent organisms. From this perspective, essential genes that are evolutionarily constrained and functionally conserved should be more relevant to the regulation and evolution of aging. However, because most genetic screens examine loss of function mutations or decreased expression of genes through reverse genetics, essential genes have often been overlooked as potential modulators of the aging process. By taking the approach of increasing the expression level of conserved essential genes, we found that 23% of these genes resulted in increased replicative lifespan in S. cerevisiae. This is greater than the ~3.5% of genes found to affect lifespan upon deletion, suggesting that activation of essential genes may have a relatively disproportionate effect on increasing lifespan. These essential prolongevity genes function in a variety of pathways and complexes highlighting both known and previously unknown longevity pathways and their previously unknown essential components. The results of our experiments demonstrate that essential gene overexpression is a rich, relatively unexplored means of increasing eukaryotic lifespan.

Abstract citation ID: igad104.2488

### EXPLORING THE BIOLOGICAL AGING DYNAMICS IN A RENAL DEVELOPMENT ORGANOID MODEL

Tomoko Kasahara[1], Bohan Zhang[2], Alexander Tyshkovskiy[3], Yoshiyasu Tongu[4], Takaaki Abe[4], and Vadim Gladyshev[5], *1. Tohoku University Graduate School of Medicine, Sendai, Miyagi, Japan*, *2. Brigham and Women's Hospital, Boston, Massachusetts, United States*, *3. Brigham and Women's Hospital and Harvard Medical School, Boston, Massachusetts, United States*, *4. Tohoku University, Sendai, Miyagi, Japan*, *5. Harvard Medical School, Boston, Massachusetts, United States*

Aging clocks emerge have emerged as promising molecular estimators of biological age based on DNA methylation or transcriptomic profiles. Among other insights, they revealed "ground zero" of aging during mouse embryogenesis characterized by the lowest biological age. To model the biological age dynamics during human embryogenesis, we established a differentiation culture system that mimics embryonic kidney development from human iPSCs. We then applied multiple aging clocks to the samples from hiPSC to kidney organoid stages. This analyses revealed the lowest biological age around the intermediate mesoderm stage, indicating a potential rejuvenation event at an early stage of renal development. A principal component analysis suggested that development in this system may comprise two processes with distinct contributions to aging, with one process increasing monotonically during development, while the other increasing at primitive streak and decreasing after the mesoderm phase. We further examined the relationship between these two processes and our previously-established transcriptomic signatures of aging and reprogramming. Interestingly, the former process may represent mostly age-related genes, while the latter process reprogramming-related genes. These findings suggest the occurrence of "ground zero" in a cell culture model of early embryonic development and characterize it in the context of molecular pathways and transcriptomic signatures.

Abstract citation ID: igad104.2489

### EXPRESSION OF THE TARDIGRADE DAMAGE SUPPRESSOR PROTEIN IN THE YEAST SACCHAROMYCES CEREVISIAE

Wei-Hsuan Su[1], Jessica Smith[2], Evien Cheng[2], and Samuel Schriner[2], *1. University of California Irvine, Irvine, California, United States*, *2. UCI, Irvine, California, United States*

Tardigrades are resilient organisms that can tolerate extremes in temperature, pH, pressure, and salinity. They are also exceptionally resistant to starvation, dehydration, and irradiation. The damage suppressor (Dsup) protein is thought to be unique to tardigrades and may act by coating and protecting nuclear DNA, particularly the nucleosomes. Nuclear DNA damage and mutation may be one of the driving forces of the aging process. In addition, there has been demonstrated a clear relationship between stress tolerance and aging. In this study, we are asking whether Dsup, when expressed in the yeast Saccharomyces cerevisiae, can afford yeast with an enhanced tolerance to environmental stresses, lowered spontaneous and induced mutation rates, and extended chronological lifespan. Yeast were constructed expressing Dsup with the strong promoter TEF on a high copy plasmid containing the 2 micron origin of replication. Preliminary results show that Dsup is able to enhance yeast survival against hydrogen peroxide, but surprisingly appeared to compromise survival to the DNA damaging agent ethyl methanesulfonate (EMS). Excessive expression of Dsup may explain the unexpected toxicity to EMS. We are therefore planning to test the expression of Dsup with a lower copy plasmid. We then plan to challenge the Dsup-expressing yeast to a variety of stresses, including extremes in temperature, high salt, and heavy metals. Induced mutant frequencies and spontaneous mutations rates at the CAN1 locus will be evaluated. Ultimately, we intend to determine whether Dsup can extend yeast chronological lifespan as a direct test of nuclear DNA damage in the regulation of lifespan.

Abstract citation ID: igad104.2490

### GENES FROM PEROXISOME AND INTEGRATED STRESS RESPONSE IN REGULATION OF HUMAN LONGEVITY

Anatoliy Yashin[1], Deqing Wu[1], Konstantin Arbeev[1], Hongzhe Duan[1], Igor Akushevich[2], Arseniy Yashkin[2], Rachel Holmes[1], and Svetlana Ukraintseva[2], *1. Social Science Research Institute, Duke University, Durham, North Carolina, United States*, *2. Duke University, Durham, North Carolina, United States*

Dietary/caloric restriction shares pathways with peroxisome, a cellular organelle that regulates the synthesis and turnover of complex lipids. PEX3, a peroxisomal biogenesis factor involved in selective type of autophagy. EIF2AK4, a sensor of amino acid deprivation in the integrated stress response (ISR) involved in lifespan regulation. We hypothesize that interplay between EIF2AK4 and PEX3 is associated with human survival. We applied logistic regression model with the interaction term to the analysis of Health and Retirement Study (HRS) and UK Biobank (UKB) parents data to test whether interactions of SNPs from the EIF2AK4 and SNPs from the PEX3 are associated with the dichotomous survival trait (ST) with case group defined as: lifespan ≥ 85; and control as: 75 ≤ (lifespan or age at last follow-up) < 85. Analyses of HRS data showed that interactions between SNPs rs4924405, rs2250402, rs8031319, rs16970049, rs3816899, rs16970112, rs2412459 from the EIF2AK4 gene and SNP rs161070 from the PEX3 gene are significantly associated with ST. In the UKB data, interactions of rs161070 from the PEX3 gene and rs7176881 and rs117778159 SNPs from the EIF2AK4 gene showed nominally significant associations with ST. Analyses of linkage disequilibrium (LD) between the EIF2AK4 SNPs detected in HRS and UKB data showed that their LD are close to the highest values for SNPs with allele frequencies specific for detected variants. Our results confirm association of interplay between EIF2AK4 and PEX3 with human longevity. They also show how information from experimental studies could be transformed to the form useful for human applications.

Abstract citation ID: igad104.2491

### MULTIMORBIDITY PREDICTS REMAINING LIFESPAN IN NONHUMAN PRIMATE MODEL OF ACCELERATED AGING

Ellen Quillen[1], Jamie Justice[2], George Schaaf[3], John Olson[1], and J. Mark Cline[1], *1. Wake Forest School of Medicine, Winston Salem, North Carolina, United States*, *2. Wake Forest University School of Medicine, Winston-Salem, North Carolina, United States*, *3. Wake Forest School of Medicine, Winston-Salem, North Carolina, United States*

Multimorbidity is common among older adults and linked to shorter lifespans, but it is unclear how naturally-occurring multimorbidity impacts lifespan in non-human primate models of aging. We developed a clinical multimorbidity index based on standardized veterinary diagnoses in 288 rhesus macaques (Macaca mulatta) from the Wake Forest Radiation Late Effects Cohort, animals that survived an early-life insult from whole-body radiation and controls (73 female, 237 irradiated, ages 2-24). Our index is based on diagnosis of arthritis, low bone density, cataracts, diabetes, gastrointestinal disorders, heart disease, hepatic dysfunction, hypertension, kidney disease, lung fibrosis, skin disorders, tumors, and overweight/underweight. All diseases were seen in both control and irradiated animals and there was a broad distribution of multimorbidity trajectories across the lifespan both in number and combinations of diseases. On average, the first diagnosis occurred at age 9.0 (equal to 35% of median lifespan) and the second at age 10.7. However, 20% of animals that lived to age 12 were without any diagnoses while others had as many as 9 comorbidities, highlighting broad variability in multimorbidity with aging. Cox proportional-hazard ratios (HR) show significant negative associations ($p < 0.05$) between survival and multimorbidity. Ages 3-6 years, each additional morbidity doubles likelihood of death (HR > 2) with HR falling from 1.9 to 1.4 from ages 6-15. Based on receiver operating characteristic (ROC) curves, multimorbidity predicts 2-year mortality with AUC > 0.65 from ages 7-14. Our findings suggest multimorbidity is prevalent and predicts lifespan in NHP survivors of radiation. Research was funded by NIHU01AI150578.

Abstract citation ID: igad104.2492

### PLACENTAL MUTATIONAL BURDEN NEGATIVELY CORRELATES WITH GESTATIONAL AGE IN EARLY-ONSET PREECLAMPSIA

Hector Chavez[1], Nicholas Guo[2], Molly Huang[2], Morgan Meads[3], Omonigho Aisagbonhi[2], Mana Parast[1], Mariko Horii[1], and Kathleen Fisch[2], *1. University of California San Diego, San Diego, California, United States*, *2. University of California, San Diego, San Diego, California, United States*, *3. UCSD, San Diego, California, United States*

The placenta exists in a hypoxic environment during early pregnancy, making it susceptible to somatic genetic mutations caused by oxidative stress and rapid cell proliferation. Oxidative stress is one of the leading factors involved in cellular aging causing genetic mutations, cellular senescence, and mitochondria dysfunction over time. While it has been demonstrated that healthy placentas accumulate somatic mutations as they develop, we hypothesize that diseased placentas display an accelerated aging phenotype through the accumulation of somatic mutations. To address this, we analyzed RNA-seq data from 84 human placentas throughout gestation from healthy (N=33) and preeclamptic pregnancies (N=51). We identified rare single nucleotide variants, insertions and deletions using GATK Best practices Workflow for RNA-seq short variant discovery using bcbio-nextgen and annotated with VEP and LOFTEE. Mutational burden, defined as the total number of rare mutations per megabase, was calculated after strict quality control and filtering for rare (gnomADg< 0.05) variants using maftools in R. Linear regression models were employed to determine the association between mutational burden and gestational age. Differences in mean values were computed with a pairwise t-test . This study revealed that mutational burden in early-onset preeclampsia placentas is negatively correlated to gestational age (R^2=0.256; p-value=0.032). The early-onset preeclampsia group had a higher mutational burden relative to the other groups (p-value< 0.05). This study revealed a significant correlation of mutational burden with gestational age in early-onset preeclampsia, paving the way for additional investigation of accelerated aging in placentas and its contribution to the development of preeclampsia.

Abstract citation ID: igad104.2493

### SEX DIFFERENCES IN THE LINK BETWEEN DNA METHYLATION–BASED BIOLOGICAL AGING AND HEALTH IN OLDER INDIVIDUALS

Aung Zaw Zaw Phyo[1], Peter Fransquet[2], Jo Wrigglesworth[3], Robyn Woods[3], Sara Espinoza[4], and Joanne Ryan[3], *1. Monash University, Melbourne, Australia. , Melbourne, Victoria, Australia*, *2. Deakin University, Burwood East, Victoria, Australia*, *3. Monash University, Melbourne, Victoria, Australia*, *4. Cedars-Sinai Medical Center, Los Angeles, California, United States*

It is well-established that women live longer than men, however, sex differences in biological aging have not been consistently reported, and may differ depending on the measure used. This study aimed to determine the correlation between DNA methylation (DNAm) age acceleration (AA) measures, with system-wide frailty-index (FI) aging and brain-predicted age difference (brain-PAD), in an initially healthy population of men and women aged ≥70 years. We additionally explored the extent to which these AA measures were associated with clinical measures, and chronic conditions, separately in men and women. DNAm age (Horvath, Hannum, PhenoAge, GrimAge, and DunedinPACE) were estimated in blood from 560 Australians (women, 50.7%) enrolled in the ASPREE study. FI was a deficit accumulation of 67 items including cognition and morbidities. Brain age was estimated from T1-weighted MRI. Clinical measurements, laboratory tests, and self-reports were used to identify chronic illnesses. FI had the strongest correlation with GrimAA and DunedinPACE (range r: 0.20 to 0.24 in both sexes) but was not associated with HorvathAA. Brain-PAD was not correlated with any biological aging measure. Correlations between AA and clinical measures were more commonly found in women than men and were the strongest for DunedinPACE and clinical measures including grip strength, gait speed, and self-rated health (r: -0.23 to 0.24). AA measures, especially GrimAA and DunedinPACE, were significantly associated with hypertension, diabetes, chronic kidney disease in men, and obesity and depression in women. Our findings highlight the importance of considering sex differences when investigating the link between biological age and clinical measures.

Abstract citation ID: igad104.2494

### SEX STEROID HORMONES AND AGE AT DEATH—A MENDELIAN APPROACH

Cynthia Kusters, Kimberly Paul, and Beate Ritz, *University of California, Los Angeles, Los Angeles, California, United States*

Lower concentrations of testosterone have been associated with increased mortality and shorter lifespan in observational studies. An association between androgens and decreased epigenetic age was identified among men. Here, we assessed the association between sex steroid hormones and parental age at death using a Mendelian randomization (MR) approach. An inverse-variance weighting (IVW) MR analysis was performed using effect estimates from external GWAS summary statistics. We included independent variants (linkage disequilibrium R2< 0.001) and a P-value threshold of 5x10-8. Sensitivity analysis included changing the P-value thresholds, MR method and multivariable MR analysis. An increase in androsterone sulfate and DHEAS were associated with a lower age at death for both fathers and mothers (-0.04/-0.05 years, P-value: 7e-4/7e-4), as well as the combined parental age at death (-0.06 years, P-value:5e-4). In addition, an increase in SHBG was associated with a higher age at death (0.06 years, P-value:7e-5). We did not find an association with estradiol or testosterone and age at death. Sensitivity analysis showed consistent findings. Our study could suggest that a higher concentration of androgens, specifically androsterone sulfate and DHEAS, decrease lifespan. This is contradictory to observational studies and deserves further attention. However, the SNPs associated with these androgens are also strongly associated with body mass index (BMI). Adjusting for BMI in the MR models removes the association between sex hormones and age at death. We are currently performing more analyses to shed light on this association.

Abstract citation ID: igad104.2495

### SIRTUIN 1 ISOFORMS DIFFERENTIALLY IMPACT MITOCHONDRIAL GENE EXPRESSION AND FUNCTION IN MUSCLE CELLS

Xiaomin Zhang, Pankaj Patyal, Ambika Verma, Shakshi Sharma, Gohar Azhar, Fathima Ameer, Yingni Che, and Jeanne Wei, *University of Arkansas for Medical Sciences, Little Rock, Arkansas, United States*

**Background** Alternative splicing (AS) is a mechanism that generates multiple mRNA transcripts and protein isoforms from a single gene, thereby increasing transcriptomic and proteomic diversity. The spliced isoforms commonly lack one or more exons, which may have similar or even opposite functions. Alternatively spliced isoforms tend to increase during aging. The sirtuin-1 gene contains multiple exons and undergoes alternative splicing, which generates multiple isoforms. In this study, we assessed the impact of three sirtuin-1 isoforms on mitochondrial gene expression and function in muscle cells. **Methods** Three sirtuin-1 isoforms (V1, V2, V3) were subcloned into expression vectors. Muscle cell lines (H9C2 and C2C12) were transfected with sirtuin-1 isoforms, respectively. Microscopic images were obtained using a Nikon microscope. Gene expression was determined by quantitative RT-PCR and Western blotting. The mitochondrial function was determined with a Seahorse XFe96 Analyzer. Results and conclusions The sirtuin-1 V1 isoform significantly increased the oxygen consumption rate (OCR) and decreased glycolysis (ECAR) in muscle cells, while V2 and V3 isoforms had slight or no significant effect on OCR and ECAR. V1 isoform was localized in the nucleus, whereas V2 and V3 were localized in the cytoplasm. Sirtuin-1 isoforms differentially impacted mitochondrial complex genes, including NDUFS1, NDUFV1, NDUFV2, NDUFA5. Our data indicate that the domain loss changed sirtuin-1 isoform subcellular localization, differentially impacted mitochondrial gene expression, and affected mitochondrial function. The age-related change in the expression of sirtuin-1 isoforms could affect cardiac and skeletal muscle function in aging and senescence. Further exploration of the sirtuin-1 isoforms' functions is warranted.

Abstract citation ID: igad104.2496

### TRANSCRIPTOMIC SIGNATURES FOR AGING AND EXTREME OLD AGE IDENTIFIED IN LONG LIFE FAMILY STUDY

Mengze Li[1], Zeyuan Song[1], Tanya Karagiannis[2], Stefano Monti[1], Paola Sebastiani[2], Thomas Perls[1], and Michael Brent[3], *1. Boston University, Boston, Massachusetts, United States*, *2. Tufts Medical Center, Boston, Massachusetts, United States*, *3. Washington University in St. Louis, St. Louis, Missouri, United States*

Transcriptional studies of blood have previously identified changes in gene expression profiles associated with age. Centenarians, a rare population of individuals that reach 100 years of age, experience delays in aging-related diseases and mortality. Thus, the analysis of their blood transcriptome may help shed light on mechanisms contributing to healthy aging and extreme longevity, as well as enable the discovery of candidate molecular therapeutic targets. In this project, we analyzed RNAseq-based blood transcriptomic data and genetic data from 1,286 participants in the Long Life Family Study age ranged from 25 to 108. To identify transcripts with different abundances at different ages, we used linear mixed-effect models and included relevant covariates to control for genetic, socioeconomic, and technical confounders. We also estimated a genetic relationship matrix (GRM) from the genotype data to account for familial relatedness. We used the same models and covariates to compare transcripts' abundance between the extreme old and younger participants to discover markers of extreme old age. Among the 11k RNA transcripts tested, we identified 5,249 aging markers (FDR ≤ 0.01) and 761 extreme old age markers (FDR ≤ 0.01). We successfully replicated our aging signature with previously published aging signature in another cohort with comparable Z-scores. Currently, further investigation is ongoing to distinguish markers that contribute to extreme longevity from markers that represent extreme old age. This study found large signatures of age and extreme old age and will serve as a valuable reference for future research to identify molecular mechanisms of healthy aging.

Abstract citation ID: igad104.2497

### UNRAVELING NONGENOMIC MECHANISMS BY WHICH 17A-ESTRADIOL EXTENDS HEALTHSPAN AND LONGEVITY

Helena Zomer, Vijay Sirohi, Theresa Medrano, and Paul Cooke, *University of Florida, Gainesville, Florida, United States*

Recent work indicates that exogenous 17α-estradiol (17α-E2), a stereoisomer of 17β-E2, produces unprecedented longevity increases (~20%) in male mice, challenging prevailing assumptions that 17α-E2 is a weak estrogen. However, is unclear how 17α-E2 acts. Therefore, this study aimed to obtain mechanistic insights into 17α-E2 actions in the uterus, the most widely used vivo model system for estrogen action. We hypothesized that 17α-E2 signals through membrane (m)ESR1 to affect target tissues and promote longevity. To test this, we treated pre-pubertal (postnatal day 20) female CD-1 mice with 3 daily IP injections of vehicle or 10 mg/kg of 17α-E2 or 17β-E2 (n=4/group). 17α-E2 treatment did not significantly increase uterine weight – a marker of nuclear (n)ESR1 signaling (20.3 ± 2.1 mg vs. vehicle 15.7 ± 2.0 mg, p>0.05), while 17β-E2 strongly increased it to 41.4 ± 1.1 mg (p< 0.05). Accordingly, histological analysis revealed reduced cell proliferation in 17α-E2- vs. 17β-E2-treated uteri. Both 17α-E2 and 17β-E2 robustly stimulated epithelial p-ERK and p-AKT expression, the active phosphorylated forms of these protein kinases, and western blotting showed a strong and comparable stimulation of EZH2, a critical mediator of estrogen's epigenetic effects, which are linked to mESR1 signaling. In conclusion, our results indicate that despite limited actions through nESR1, 17α-E2 robustly signals through mESR1 to activate uterine protein kinase cascades and produce epigenetic effects. These findings may cause a significant reappraisal of 17α-E2 biological effects and role in normal estrogen physiology and provide critical insights into its unique ability to stimulate longevity.

### SESSION 7380 (POSTER)

### AGING AND THE LIFECOURSE

Abstract citation ID: igad104.2498

### AGING ACROSS THE LIFE COURSE: RESEARCH COLLECTIONS AVAILABLE FROM THE NATIONAL ARCHIVE OF COMPUTERIZED DATA ON AGING

James McNally, and Kathryn Lavender, *University of Michigan, Ann Arbor, Michigan, United States*

Creating and maintaining sustainable data archives can be challenging but offers clear advantages. Properly curated data can be used by multiple researchers, testing a variety of hypotheses and increasing the return on investment to the expensive data collection process. An internally managed archival system also provides greater control and autonomy in the equitable distribution of data resources. This process ensures all researchers will fully use the data for original research, teaching, and new directions once the data leaves the control of the local investigator. This poster reviews the advantages of a local strategy for preserving and sharing gerontological research data. Using the National Archive of Computerized Data on Aging (NACDA) as a working example, the poster offers an overview of collections at NACDA. Using our metadata tools and variable search database, NACDA can identify studies in its collections that examine aspects of aging and health among adults during their lifecourse. Many of the studies are longitudinal or repeat-measure cross-sectional studies. We can also identify studies that focus on aging not maintained by NACDA that are available to interested researchers. Using a strategy of archival preservation combined with a strong focus on productive research Innovation in Aging, NACDA has amassed data and metadata covering a wide array of studies worldwide that address the aging lifecourse. Because our collections are multinational, we share these data at no cost to interested users worldwide.

Abstract citation ID: igad104.2499

### BEREAVED PARENTS OF THE HEALTH AND RETIREMENT STUDY

Stephanie Rubinstein, Gillian Fennell, and Elizabeth Zelinski, *University of Southern California, Los Angeles, California, United States*

The loss of a child—no matter their age—is often regarded as one the most tragic forms of loss. Here, we sought to identify sociodemographic, psychosocial, and health profiles of older adults who have experienced offspring death in the Health and Retirement Study (HRS). Our sample was composed of 2,035 respondents from the 2018 wave of HRS who experienced (n =381) or did not experience (n = 1,654) the loss of a child at any point in their lifetime. In an adjusted logistic regression, we found that both women (OR=1.53, [1.13, 2.09]) and non-Hispanic Black respondents (OR = 1.85; [1.31, 2.61]) had significantly higher odds of experiencing the death of a child. Education was also significantly associated with offspring death: relative to individuals with less than a high school education, high school and college-educated respondents had a 34% and 64% lower likelihood of reporting offspring death, respectively (OR=0.66, [.47, .91]; OR=0.36, [.25, .51]). Lastly, every one unit increase in cognition was associated with a 3% lower likelihood of experiencing the offspring death (OR=0.97, [.94, .99]). Interestingly, bereaved parents were no more likely to experience depression nor report multimorbidity than individuals who had not grieved the loss of a child. These findings highlight a grim picture of premature and untimely death among Black Americans, the power of education, and potential psychological and physiological resilience among this older subpopulation of bereaved parents.

Abstract citation ID: igad104.2500

### CROSS-COUNTRY COMPARISONS OF FUNCTIONING AND CARDIOVASCULAR RISKS IN OLDER ADULTS

Mutian Zhang[1], Yuan Zhang[2], Jennifer Ailshire[3], and Eileen Crimmins[3], *1. University of Southern California, Los Angele, California, United States*, *2. Columbia University, New York City, New York, United States*, *3. University of Southern California, Los Angeles, California, United States*

Life expectancy at birth in China has increased more than 20 years over the past 50 years and is one year higher than in the US in 2021. Whether the rapid improvement in Chinese life expectancy has been accompanied by similar changes in other aspects of the aging process compared to the US remains unclear. In this study, we focus on measured physical functioning and cardiovascular risk factors which signify disability and disease processes, and address how age and gender differences and change with age in these indicators are similar for the two countries. We use data from the 2015 China Health and Retirement Longitudinal Study (n=8,303) and the 2016/18 Health and Retirement Study (n=10,804) on performance-based functioning (e.g., walking speed) and cardiovascular biomarkers (e.g., systolic blood pressure) for those over age 60. We first examine the distribution of each measure by gender and age and conduct t-tests on mean differences. Then we conducted regression analyses to examine cross-country differences. In both countries functioning worsens with age (e.g., mean scores of walking speed for CHARLS females decreased from 0.76 to 0.47 m/s). In contrast, only among older Chinese does cardiovascular risk increase with age while in the US risk is flat (e.g., mean scores of systolic blood pressure for CHARLS females increased from 129.59 to 142.39 mm hg). Although life expectancy is nearly equivalent in these two countries, there are large gaps in health and functioning between China and the US, especially in cardiovascular disease risk.

Abstract citation ID: igad104.2501

### DESCRIPTIVE EPIDEMIOLOGY ON THE TRENDS OF SUICIDAL ATTEMPTS AMONG OLDER ADULTS IN KOREA FROM 2007–2020

Seoyoon Lee[1], Hyung Eun Shin[2], SeungCheor Lee[2], and In-Hwan Oh[2], *1. Yonsei University, Seoul, Republic of Korea*, *2. Kyung Hee University, Seoul, Republic of Korea*

When a suicide attempt results in the act of suicide, personal and social losses increase to a degree that is difficult to quantify. It is urgent to evaluate the status of suicide attempts in Korea accurately. This study is to examine a 14-year time series of older adults who have engaged in suicidal behavior or attempted suicide. We used claim data from Health Insurance Review and Assessment Service. A total of 3,440 individuals with a diagnosis code, 768 individuals with a primary diagnosis, and 2,672 individuals with a secondary diagnosis, were included in the analysis. Suicide attempts were defined by intentional self-harm(X60-X84), harmful biological control agent(X87), and personal history of self-harm(Z91.5). To analyze the frequency of suicide attempts by age, a chi-squared test was conducted. Among those over 60 years with a primary diagnosis of suicide attempt, personal history of self-harm was the most common, accounting for 92.4%, followed by intentional self-harm with a rate of 7.5%. No subjects had a primary diagnosis as code of X87. A similar trend was observed for those under the age of 60. In the case of those with a secondary diagnosis of suicide attempt, personal history of self-harm was the highest at 93.5%. Subsequently, intentional self-harm accounted for about 6.4%, and each code was evenly distributed except for alcohol addiction and exposure(X65) in 2007 and X87 in 2020. Conclusively, this study reported the trend of suicide attempts in Korea, which can provide the basis for preparing effective short- and long-term health policies.

Abstract citation ID: igad104.2502

### DIGNITY THERAPY FOR OLDER ADULTS WITH CANCER: COMMUNION FACILITATES DIGNITY

Susan Bluck[1], Mary Kate Koch[1], Sophia Maggiore[1], Diana Wilkie[1], Harvey Chocninov[2], and Carma Bylund[1], *1. University of Florida, Gainesville, Florida, United States*, *2. University of Manitoba, Winnipeg, Manitoba, Canada*

Nearly 600,000 older Americans die a cancer-related death annually. Maintaining dignity is central to their quality of life. Dignity Therapy (DT; Chochinov, 2005) was designed to preserve dignity despite health declines. Patients report benefits of DT but mechanisms have not been empirically investigated. Grounded in gerontological life review research, DT involves therapists guiding patients to tell their unique life story and leave a legacy. This study investigates level of communion in the life story told in DT as a mechanism for DT efficacy. Communion reflects personal strivings for warm and close relationships (McAdams, 1986). Participants were 204 older cancer patients receiving outpatient palliative care (Mage= 66 years, SD = 7.43, 65% female) who participated in DT. Session narratives were audio-recorded and reliably (κ=.71–1) content-analyzed for communion themes (McAdams, 2001). Participants completed pre-post measures, including the Dignity Impact Scale. Mediation analyses showed significance for the direct effect of Dignity Impact from pre- to post-test, B = .50(.06), p < .001 as well as for paths from pre-test Dignity to Communion, B = .20(.08), p <.01 and from Communion to post-test Dignity, B = .20(.06), p = .001. Sobel test for the mediation effect of Communion between pre- to post-test Dignity was z = 2.06(.02), p = .039; point effect = .04. Participating in DT is shown to increase older cancer patients' feelings of dignity. That is partially explained by the extent to which these older adults tell a life story rich in communion, reflecting on loving, caring relationships, during DT.

Abstract citation ID: igad104.2503

### ECONOMIC BOOM AND FINANCIAL RECESSION—WHAT ARE THE EFFECTS ON RELOCATION PATTERNS FOR OLDER ADULTS?

David Dahlgren, Asa Hansson, Susanne Iwarsson, Steven Schmidt, and Maya Kylén, *Lund University, Lund, Skane Lan, Sweden*

While previous research shows that older adults choose to relocate to a different home for numerous reasons (e.g., changes in the household composition, health decline), little is known about the influence of economic factors. The economic situation on aggregated level can influence the individual in more ways than by consumer demand and income, for example, financial crisis, fluctuating housing prices, and more general uncertainties about the future. Representing a novel approach, the aim of this study was to investigate the impact of macroeconomic events on relocation rates among older adults in Sweden. We used high quality panel data from Swedish registers, based on a random sample made 1994 with 300,000 individuals. Data was analyzed from 1970-2016 using a repeated cross-sectional design. Preliminary results show that the number of relocations was volatile the first year of a financial crisis. Depending on the characteristics of the crisis, the number of relocations decreased or increased; crises that are less connected to the property market had a reduction in number of relocations. This effect was observed for 1-2 years and stabilized to "normal levels" in the subsequent years. Especially high volatility was observed for the older age groups (75-84 and 85-90), which could be because they have a shorter time horizon for investment planning and great need to transfer housing wealth to liquidity. Knowledge in this field enables policymakers to plan housing investment, and negative shocks to the economy may lead to short term impacts on the housing market for older adults.

Abstract citation ID: igad104.2504

### EDUCATION, ADULT LITERACY SKILLS, AND JOB AUTOMATION RISK IN THE OLDER US WORKFORCE

Donnette Narine[1], Takashi Yamashita[2], Runcie Chidebe[3], Phyllis Cummins[3], Jenna Kramer[4], and Rita Karam[5], *1. University of Maryland Baltimore, Baltimore, Maryland, United States*, *2. University of Maryland, Baltimore County, Baltimore, Maryland, United States*, *3. Miami University, Oxford, Ohio, United States*, *4. RAND Corporation, Arlington, Virginia, United States*, *5. RAND Corporation, Santa Monica, California, United States*

Despite the overall benefits of technological advancement, ongoing job automation has disrupted the U.S. workforce. Of particular concern is that increasing levels of job replacement—driven by job automation—is likely to be a risk factor for economic uncertainty, both for workers and the labor market. Older workers with lower educational attainment and basic adult literacy are known to face challenges in learning new knowledge and skills, which are currently required to be competitive in a technology-driven labor market. Thus, this study aimed to examine the relationship between educational attainment, literacy proficiency, and previously published job automation risk by occupations. Using the 2012/2014 Program for International Assessment of Adult Competencies (PIAAC) data, we analyzed a nationally representative sample of middle-aged and older workers (age 45-74 years; n = 1,484). The results of the linear regression indicated that higher educational attainment (college or higher vs. less than high school: $b = -14.11$, $p < 0.05$) and greater literacy proficiency (score 0-500 points: $b = -0.06$, $p < 0.05$) were associated with lower job automation risks (0-100 % replacement in the next decades) among middle-aged and older U.S. workers, accounting for the covariates (R-squared = 0.18). In alignment with the federal Workforce Innovation and Opportunity Act, these findings offer empirical support for policies and interventions that are purposefully designed to boost the human capital (i.e., knowledge and skills) of middle-aged and older workers through adult education and literacy training to lower their job automation risk, thereby increasing their level of job security.

Abstract citation ID: igad104.2505

### FORGING COMMUNITY–ACADEMIC PARTNERSHIPS FOR HEALTHY AGING: A COMMUNITY-LED TOOLKIT TO COMBAT AGEISM

Dana Urbanski[1], Elma Johnson[1], Maby Almiron[2], Steve Chapman[2], Amina Keinan[2], Phyllis Thomas[2], Mayla Yang[2], and Robbin Frazier[3], *1. University of Minnesota, Minneapolis, Minnesota, United States*, *2. Center for Healthy Aging and Innovation, University of Minnesota, Minneapolis, Minnesota, United States*, *3. University of Minnesota, Center for Healthy Aging and Innovation, Minneapolis, Minnesota, United States*

There is growing recognition that community-engaged research approaches are essential for bridging the evidence-to-practice gap, especially for minoritized communities underrepresented among academic researchers. Such approaches often adopt a consultative model in which community members provide insights, perspectives, and feedback to support academic-driven projects. This strategy can be mutually beneficial when paired with cultural sensitivity and humility; however, there is a pressing need to form community-academic partnerships that flip this consultative relationship—placing university researchers as advisers on community-led projects that reflect local needs, priorities, and values. Here, we highlight the work of one such community-academic partnership led by the Center for Healthy Aging and Innovation's (CHAI's) Community Advisory Board with support from University of Minnesota faculty, staff, and graduate students. First, we describe the vision and formation of our Community Advisory Board (CAB), along with recruitment of its diverse membership representing eight cultural communities of the Twin Cities. We then outline the collaborative needs assessment process by which the CAB identified and prioritized action areas to advance healthy aging and health equity. Finally, we present preliminary results and early lessons learned from the CAB's first project initiative—creation of a community-led, culturally responsive toolkit to address and reframe community-based sources of age-related stigma and discrimination. Findings from this project: 1) offer a plausible model and process for community-academic engagement which may be adapted and refined to fit local contexts; 2) underscore the value of community-led academic partnerships for addressing age-related stigma and other social factors associated with poor aging outcomes.

Abstract citation ID: igad104.2506

### HOW NOT TO DATA ARCHIVE IN 10 WAYS

Ashley Millenbah[1], Elizabeth Albers[2], and Katie Louwagie[1], *1. University of Minnesota, Minneapolis, Minnesota, United States*, *2. University of Minnesota School of Public Health, Minneapolis, Minnesota, United States*

Data sharing offers opportunities to replicate published findings, improves transparency in science, and increases the impact of the research by allowing other researchers to conduct new analyses. Effective January 25, 2023, the National Institute on Aging has new mandates for data sharing. However, many researchers are not experienced in data sharing and curation. Researchers will need to become more familiar with data sharing, specifically archiving data in data repositories. With these new requirements, many teams are attempting data sharing or archiving for the first time. We present 10 lessons learned the hard way, from real experience, prohibiting successful data archiving. Many of the challenges encountered could be avoided by effective planning, such as securing funding for curation and/or de-identification, adding data archiving details into the grant, and finding multiple repositories to potentially use. Other problems can be avoided by writing a consent form that allows for data archiving, such as by not limiting access to data, or limiting the type or format of data to be shared. Consulting with a legal team with experience in data archiving, such as at a data repository, is also recommended. By avoiding these pitfalls, researchers can successfully archive their data, ensuring their data are maintained and usable for other researchers in the future.

Abstract citation ID: igad104.2507

### INTENTION TO USE HOSPICE CARE FOR FAMILY AMONG OLDER RURAL BLACK AMERICANS IN ALABAMA

Ellen Csikai[1], and Hee Yun Lee[2], *1. University of Alabama, Tuscaloosa, Alabama, United States*, 2. *The University of Alabama, Tuscaloosa, Alabama, United States*

Hospice usage overall continues to rise however, remains low among people of color, including by Black/African Americans. In 2020, 50.8% of White Medicare decedents and only 35.5% of Black decedents used hospice (NHPCO, 2022). Often reasons are connected to poor knowledge of and access to hospice resources that provide holistic support to those near end of life and their caregivers (Adrien, 2017). Little is known about the intention of older rural Black Americans' to use hospice when caring for family members at home. In this study, older African Americans in Black Belt communities in Alabama were recruited to participate in a survey that addressed health literacy of problems with high prevalence in this rural area (diabetes, cardiovascular disease, stroke and cancer) (CDC Interactive Atlas, 2022). Guided by the Andersen's Behavioral Model of Health Services Use, regression analysis was conducted to identify predisposing, enabling, and need factors influential in older rural African Americans' intention to use hospice for family members. Among non-caregivers, who had heard of hospice (n=118 of 180), education, gathering with friends and social club participation, and PHQ-9 were found influential in predicting intention to use hospice for family members. However, among caregivers (n=107), female gender, education, health literacy, gathering with friends, social club participation, and PHQ-9 were influential. Results point to interventions to bolster health literacy regarding hospice among older rural Black Americans and that social clubs and other gathering places could be targeted with educational programs to increase intention to use hospice care for family members.

Abstract citation ID: igad104.2508

### PATHWAYS TO HEALTHY AGING IN THE UNITED STATES AND JAPAN: THE ROLE OF PSYCHOSOCIAL FACTORS

Ruoying Zhang, and Margie Lachman, *Brandeis University, Waltham, Massachusetts, United States*

The dynamics of aging vary across cultures, with Japan showing a more benign picture of aging compared to the United States. While Japanese longevity and healthier older adulthood are well known, of interest is what mechanisms lead to such results. In American society, there is a strong emphasis on being independent, while Japanese society focuses on interdependence and social harmony. This study explored possible behavioral mechanisms linking these psychosocial factors to health and examined whether the pathways differ by culture. We used the second wave (2004) of the Midlife in the United States (MIDUS) (N = 1045) and the second wave (2012) of the Midlife Development in Japan (MIDJA) studies (N = 317). Participants' ages ranged from 30 to 83. Behavioral mechanisms were operationalized as diet, smoking, and exercise. Psychosocial factors included sense of control, positive relations with others, independence, and interdependence. We computed a health measure by combining two inflammation biomarkers (IL-6 and CRP) and two cardiovascular biomarkers (systolic blood pressure and the ratio of total-to-HDL cholesterol). We expected sense of control and independence would make significant contributions to Americans' health while positive relations with others and interdependence would be significant for Japanese's health. The results supported the predictions, confirming different psychosocial pathways to health for the two samples, with diet and exercise as behavioral mechanisms in both cultures. The results provide new insights into the mechanisms for healthy aging in different cultures, and offer a deeper understanding of the cultural impacts on healthy behaviors and the aging process.

Abstract citation ID: igad104.2509

### SOCIAL CONTEXTS OF FINANCIAL EXPLOITATION OF OLDER ADULTS IN MONGOLIA: AN EXPLORATORY STUDY

Ariunsanaa Bagaajav, *City University of New York, New York City, New York, United States*

Financial exploitation (FE) is a type of elder abuse and is the illegal or improper use of funds and resources of older persons. Approximately one in 15 older adults are victimized by FE globally. Due to its devastating consequences, FE has been identified as an emerging health, financial, and social crisis. Evidence suggests exploring cultural values is crucial to understanding the nature of FE in a specific context. While conceptualization of FE varies significantly across cultures, little is known about FE in Mongolia given its potential for increased vulnerability. We present findings of a secondary analysis of previously conducted exploratory study in Ulaanbaatar, Mongolia. A total of 24 physicians participated in semi-structured interviews, participants who were uniquely positioned to encounter incidences of FE due to requent contact with older patients. All interviews were audiotaped and transcribed verbatim. Grounded theory methods were used for the data analysis. This study found that older Mongolians are at increased vulnerability to financial exploitation due to mounting financial hardships their families experience. To assist, older adults take out pension loans with high interest fees. Due to a lack of personal fund, essential needs of older adults are often ignored. In some instances, older adults' pensions could be the most reliable source of income for poor families. Physicians believe while some older adults act upon willingly to assist, some might be forced under the collective decision made by family members. The findings of this study advanced the knowledge of FE in Mongolia which was formerly understudied.

Abstract citation ID: igad104.2510

### THE ROLE OF LATE-LIFE WORK AMONG KOREAN WIDOWED ADULTS

Hyeonji Cho, Nahyun Kim, and Kyungmin Kim, *Seoul National University, Seoul, Republic of Korea*

According to the Dual Process Model, oscillation between two types of stressors and coping processes—loss-oriented and restoration-oriented leads bereaved individuals toward the adaptive coping. Previous studies have focused on the buffering effects of social engagement such as volunteering or leisure as restoring activities, but little is known about the role of work. In Korea, employment rates of older adults are rising, and many people wish to keep working after the retirement age. This study examines the restoring effects of work among Korean widowed adults. Utilizing five waves of the Korean Retirement and Income Study (2011–2019; conducted biennially), we analyzed 481 middle-aged and older adults (aged 52–95) who experienced spousal loss during the study period. The widowed participants provided data on their employment status and perceived health status, including sociodemographic characteristics at both pre-loss (T1) and post-loss (T2). On average, participants' perceived physical health (MT1 = 2.75, MT2 = 2.61) and mental health (MT1 = 3.20, MT2 = 3.07) deteriorated after the bereavement. Residualized change models found that employed participants (29.7%) showed a smaller decrease in perceived physical health (B = 0.35, $p < .001$) and mental health (B = 0.18, p = .046) compared to their counterparts, which supports the buffering effect of late-life work in the bereavement process. We also found a significant interaction between employment status and net wealth for perceived physical health (B = -0.04, p = .037), showing that the effect of employment stood out more among economically vulnerable people.

Abstract citation ID: igad104.2511

### UTILIZING CARE PREFERENCE ASSESSMENT TO UNDERSTAND THE NEEDS OF OLDER ADULTS AT ADULT DAY SERVICES (ADS) IN TAIWAN

Chih-ling Liou, *Kent State University, Kent, Ohio, United States*

Understanding individuals' preferences and honoring them throughout the care delivery process is essential for care services. When preferences are understood and honored, care recipients perceive a greater sense of autonomy, which improves their quality of life and emotional well-being. Adult Day Services (ADS), a fast-growing community service offers daytime programs for those with physical and/or cognitive impairments. In order to better understand ADS attendees' care preferences and to help ADS staff to deliver person-centered care in Taiwan, this study adapted the Preferences for Everyday Living Inventory for ADS in Chinese (PELI-ADS-C) to assess preferences for activities and lifestyle experiences. Forty-three interviews were conducted with older adults recruited from three different ADS centers in Taiwan. The majority of participants were females (85%) with ages ranging from 67 to 93 years old. Among the 43 preference items, "do things with groups of people" and "participate in religious services or practices" were rated as most important followed by "reminisce about the past" and "exercise." "Volunteer your time" and "gender of the staff taking care of you" were rated as least important. Participants shared that "It is not good for us to be alone." "It is more fun for me to be with a group of people." "I had no time to be a volunteer when I was younger. Now, I have to attend the center every day with no time to be a volunteer." Results can be shared with ADS staff to develop care and activities that corresponded with attendees' preferences.

Abstract citation ID: igad104.2512

### WORKING TOGETHER TO PROVIDE GERIATRIC CARE: INTERDISCIPLINARY TEAM CREATION AND EVALUATION IN CANADA

Jessica Strong, and Rachel Kerzner, *University of Prince Edward Island, Charlottetown, Prince Edward Island, Canada*

Prince Edward Island is Canada's smallest province (pop. 160,000) and has one of the oldest populations in Canada. While there is a robust and growing geriatrics program through provincial healthcare, it consists exclusively of medical providers (i.e., geriatricians and nurse practitioners). An opportunity for formal interprofessional collaboration presented itself when the University of Prince Edward Island began a Doctor of Psychology (PsyD) program in 2019. Soon thereafter, we established an interdisciplinary team between the PsyD and the provincial geriatrics programs. The goals of the team included: 1) enhancing care for older adults on the island, 2) providing training for PsyD students, and 3) increasing interprofessional collaboration and learning. In an evaluation of the first year of the collaboration, we collected data on the types of referrals and characteristics of the patients referred (e.g., number of medications), and asked all providers on the team (N=4 geriatricians, N=9 nurse practitioners, and N=4 psychology student clinicians) to answer open ended questions on the highlights and challenges of establishing the team, the organization of formal team meetings, and provision of patient care. Of the 74 patients seen by psychology, reasons included cognitive or psychodiagnostic assessment (N=22, 30%), psychotherapy (N=20, 27%), caregiver support (N=25, 34%), or multiple services (i.e., both assessment and intervention; n=7, 10%). Additional patient characteristics will be shared, as well as themes from provider survey responses. Our program has implications for other areas in rural North America, particularly training opportunities built into our team functioning.

### SESSION 7390 (POSTER)

### ALCOHOL AND ADDICTIONS

Abstract citation ID: igad104.2513
### ALCOHOL CONSUMPTION AMONG ADULTS IN WISCONSIN

Amy Knepple Carney[1], Imagine Manders[2], Allison Hammond[2], and Leslie Wessel[2], *1. University of Wisconsin- Oshkosh, Oshkosh, Wisconsin, United States, 2. University of Wisconsin-Oshkosh, Oshkosh, Wisconsin, United States*

Alcohol usage as we age can lead to worsening health conditions and higher rates of accidents. There have been mixed results on alcohol use in adulthood, and many of these studies use only one metric of alcohol consumption. With higher-than-average (55.1%) drinking rates in the United States, Wisconsin currently ranks third highest for adult alcohol consumption (64.4%), it is essential to understand age trends for nondrinking, binge drinking, and heavy drinking. Using 2021 data from the CDC, we examined a series of logistic regressions to determine age differences in alcohol consumption. First, whether a person had at least one drink in the past 30 days, odds ratios show that relative to older adults (65+), 45-to-64-year-olds are 1.35 times more likely, 25-to-44-year-olds are 1.72 times more likely, and 18–to-24-year-olds are 1.09 times more likely to have reported having at least one drink in the last 30 days. Second, binge drinking, odds ratios show that relative to older adults (65+), 45-to-64-year-olds are 2.95 more likely, 25-to-44-year-olds are 5.41 times more likely, and 18-to-24-year-olds are 3.86 times more likely to binge drink. Last, heavy drinking, odds ratios show that relative to older adults (65+), 45-to-64-year-olds are 1.84 more likely, 25-to-44-year-olds are 1.94 times more likely, and 18-to-24-year-olds are 1.71 times more likely to drink heavily. Implications of these results are examined more fully but include the adverse effects on health for young-to-middle-aged adults, the potential to die younger for heavy drinkers, and the positive effects of lower drinking rates in later adulthood.

Abstract citation ID: igad104.2514
### ASSOCIATIONS BETWEEN DAILY STRESSORS AND ALCOHOL USE IN THE MIDUS STUDY

Sara Miller[1], and David Almeida[2], *1. Pennsylvania State University, State College, Pennsylvania, United States, 2. The Pennsylvania State University, State College, Pennsylvania, United States*

Daily stressors are minor but frequent challenges of everyday life (e.g., arguments). Although daily stressors are typically less severe than stressful life events, daily stressors can have more immediate effects on wellbeing. Despite this relevance, there is limited research evaluating the influence of daily stressors on alcohol use in older populations. This study examined daily associations between stressor characteristics (exposure, type, and severity) and alcohol use and evaluated differences in associations for younger/middle-aged (< 65 years) vs older adults (65+ years). Participants (n=1,603; Mage=52.82) were non-abstaining adults from the Midlife in the United States (MIDUS) study and the MIDUS Refresher who participated in 8-day diary projects. Survey items included stressor exposure (1=yes), stressor types (interpersonal, work, home, network), stressor severity (0=not at all; 1=not very; 2=somewhat; 3=very), and number of drinks. Covariates included the day of the week, sociodemographic characteristics, personality traits, psychological factors, and health behaviors. Multilevel linear regression analysis revealed that neither daily stressor exposure nor stressor severity was associated with reported alcohol use (p>0.05), but there were differences in alcohol use by stressor type. Specifically, days with interpersonal stressors were associated with reporting more drinks (p=.049), while days with work stressors were associated with reporting fewer drinks (p=.001). Further, age significantly moderated the relationship between any stressor exposure and reported alcohol use (p=.035), such that stronger relationships were observed for older adults than younger/middle-aged adults. Study findings highlight the importance of considering stressor characteristics when evaluating the covariation of daily stressors and alcohol use.

Abstract citation ID: igad104.2515
### EMERGENCE OF ADDICTION SUBSTITUTION: SUBSTANCE USE RESULTING FROM ALCOHOL USE DISORDER RECOVERY IN A US SAMPLE

Yixuan Sun[1], Thomas Kwan[1], Douglas Bowlby[2], and Matthew Lee[1], *1. Rutgers University New Brunswick, Piscataway, New Jersey, United States, 2. Rutgers University, New Brunswick, New Jersey, United States*

Addiction substitution refers to the increased use of other addictive substances after recovery from a prior addiction. Previous research shows that addiction substitution may lead to a poorer recovery prognosis, and an increased chance of relapse with the primary substance. If health concerns affect the likelihood of substance switching, then this concerns aging segments of the U.S. population. The aim of this study was (1) to examine the relationship between recovery from alcohol use disorder (AUD) and the subsequent use of other substances and (2) to identify covariates that affect the relationship between recovery and addiction substitution. This study used two-waves of longitudinal data from NESARC (National Epidemiologic Survey on Alcohol and Related Conditions), a U.S. representative sample aged 18-90+. We used 2*2 Chi-square analyses to examine the likelihood of the emergence of Wave 2 substance use (e.g., opioids) among those who had recovered from AUD between Waves 1 and 2. We ran a subsequent analysis to determine if various health outcomes predicted substance switching. The results showed that there was a significant decrease in the likelihood of becoming an opioid user by wave 2 among those who recovered from AUD in wave 1. Analysis of potential confounds identified that those who recovered from AUD but reported poor health were more likely than other recoveries to increase opioid use. Results suggest that recovery from AUD reduces the use of other substances. However, health issues increase the likelihood of substance switching occurring among those recovering from AUD.

Abstract citation ID: igad104.2516
### EMPOWERING ADULTS SEEKING RECOVERY FROM SUBSTANCE AND OPIOID USE DISORDER IN THE DEEP SOUTH

Michael Buxton[1], Rebecca Allen[2], M. Lindsey Jacobs[1], Brian Cox[3], and Sarah Holditch[2], *1. University of Alabama, Tuscaloosa, Alabama, United States, 2. The University of Alabama, Tuscaloosa, Alabama, United States, 3. Alabama Research Institute on Aging, Tuscaloosa, Alabama, United States*

Substance and opioid use disorder may be missed due to lack of consideration of physical, social, and emotional changes in midlife and later adulthood. This presentation uses data from a Graduate Psychology Education grant to examine predictors of alcohol use and misuse among treatment-seeking and primary care patients. Across three sites, 237 individuals completed behavioral health assessments; a rural (n = 54) and an urban federally qualified health center (n = 59), and a state-certified residential rehabilitation facility (n = 124). Participants ranged from 18 to 70 years of age (M = 40.05, SD = 1.75). Approximately 49.4% were female and 72.2% were non-Hispanic White, 23.3% African American, and 4.6% other. More than 28% of individuals had less than a high school education, and 38.4% reported a high school education or GED. Using the TAPS-II, 34.2% of participants reported alcohol use, and 13.5% reported prescription opioid misuse. Logistic regression analyses revealed that older age, prescription opioid misuse, and depression were significantly associated with alcohol use, $\chi 2$ (7) = 18.04, p = 0.12, while gender, report of adverse childhood experiences, and anxiety were not. Holding age, gender, and experiences constant, prescription opioid misuse had a significant effect on the probability of alcohol use, B = 1.092, SE = .429; Wald = 6.496, p = 0.01. The odds of alcohol use increased by a factor of 2.98 for individuals misusing prescription opioids. Treatment for problematic alcohol use must consider contextual influences including age, prescription opioids, and depression to implement effective evidence-based treatment.

Abstract citation ID: igad104.2517

### IMPROVED NALOXONE PRESCRIBING IN RURAL PRIMARY CARE FOLLOWING ACADEMIC DETAILING FOR OPIOID OVERDOSE PREVENTION

Leah Tobey, Meghan Breckling, Mohab Ali, and Robin McAtee, *University of Arkansas for Medical Sciences, Little Rock, Arkansas, United States*

Performing opioid misuse screenings for older adults (OA) and reviewing high-risk medications are reliable screenings in preventing harm, especially accidental overdose. As one of the HRSA funded Geriatric Workforce Enhancement recipients, the AR Geriatric Education Collaborative (AGEC) worked with a rural federally qualified healthcare clinic system (ARcare) to reduce the likelihood of patient's accidental overdose by co-prescribing Naloxone, when indicated. An Academic Detailing (AD) team of a PT, PharmD and MD, led this interactive training. AD is clinician-to-clinician support customized with the purpose to connect primary care providers to the best available evidence and most up-to-date clinical practice guidelines. A conversational needs assessment was stimulated where the provider shared their perspectives, knowledge deficits, and needs surrounding the AD topic of Naloxone. These AD interactions encouraged safer prescribing of high-risk medications while considering the use of hard reduction resources for patients, such as prescribing Naloxone. ARcare providers (n=22) attended AD on Preventing Opioid Overdose with Naloxone over 11 months. Of those, 9 had previously prescribed Naloxone 40 times pre-training then 107 times post-training; five providers had never prescribed Naloxone but prescribed it 46 times post-training. Eight providers, made no change, some of whom work in schools. Post-training data gathered from the 14 Naloxone prescribing providers showed a total of 153 Naloxone prescriptions after AD, a 283% increase. For primary care settings, age-friendly medication practices are critically important for OA safety, aging in place, and for OA and their caregivers to understand and utilize Naloxone if accidental overdose is suspected.

Abstract citation ID: igad104.2518

### OLDER ADULT SUBSTANCE USE DISORDER TREATMENT LANDSCAPE 2020: CLIENT-LEVEL AND FACILITY-LEVEL FACTORS

Keith Morgen, *Centenary University, Hackettstown, New Jersey, United States*

Older adult substance use disorders (SUD) care requires specialized clinical practice. This poster reviews two datasets to emphasize client-level and facility-level factors (overall and state-by-state) relevant to older adult treatment programming, co-occurring disorders care, and days waiting for admission to treatment. The 2020 data from the National Survey of Substance Abuse Treatment Services (N-SSATS), administered by The Center for Behavioral Health Statistics and Quality (CBHSQ) of the Substance Abuse and Mental Health Services Administration (SAMHSA), U.S. Department of Health and Human Services, collects data from public/private SUD treatment facilities in the United States. Data from 16,066 facilities demonstrated only 24.7% of all facilities reported programming tailored for older adult care, with state-by-state analysis showing a range of 4.6% to 61.4% of treatment programs reporting older adult tailored care. The deficits of older adult programming availability are explained against the context of the 2020 Treatment Episode Dataset-Admissions (TEDS-A), administered by the CBHSQ, that examines admissions data from all 50 states, as well as the District of Columbia, U.S. territories, and Compact of Free Association (COFA) partners. The TEDS-A data consisted of 1,416,357 admissions, of which 11.8% (n=167,200) were aged 55 years-old or older. Of this older adult sample, 32.8% reported a co-occurring SUD and mental health disorder and 13.9% reported some number of days waiting to enter treatment. These (and other) client-level and facility-level factors (overall and state-by-state) will be used to emphasize specific needs for older adult SUD care.

Abstract citation ID: igad104.2519

### PATTERNS OF ALCOHOL USE OVER TIME AMONG OLDER COUPLES: IMPLICATIONS FOR SELF-RATED HEALTH

Angela Turkelson[1], Kira Birditt[2], Courtney Polenick[3], James Cranford[3], and Fred Blow[3], *1. University of Michigan: Institute for Social Research, Ann Arbor, Michigan, United States*, *2. Institute for Social Research, University of Michigan, Ann Arbor, Michigan, United States*, *3. University of Michigan, Ann Arbor, Michigan, United States*

Alcohol use in older adults is increasing which can have implications for health outcomes. Research has examined longitudinal patterns of drinking for individuals over time, but not couples. Couples often engage in concordant drinking behaviors but it is unknown if couples become more or less concordant or discordant in their drinking over time or how increased concordance or discordance influences health.

The purpose of this study was to examine patterns of alcohol use over time among older couples and their links with self-reported health. A total of 8,570 husbands and wives completed the main interview of the Health and Retirement Study (HRS) for at least three consecutive biennial waves between 1996 and 2016. Latent class growth analysis revealed five trajectories of couple drinking: 1. Concordant - husband and wife light (n = 6398, 75%); 2. Concordant - husband and wife light/moderate (n = 781, 9%); 3. Concordant - husband and wife moderate (n = 296, 3.5%); 4. Discordant - husband heavy and wife light (n = 293, 3.5%); 5. Discordant - husband moderate and wife light (n = 802, 9%). Discordant couples and concordant light couples reported worse self-rated health over time compared with concordant light/moderate and concordant moderate couples. The most common pattern of drinking was concordant drinking with husbands showing light decreasing drinking and wives showing light stable drinking over time. Findings are consistent with literature and suggest that discordant drinking couples have poorer health.

Abstract citation ID: igad104.2520

### RISKY BEHAVIORS WITH NONALCOHOLIC FATTY LIVER (NAFLD) AND NONALCOHOLIC STEATOHEPATITIS (NASH) AMONG OLDER ADULTS

Tung-Sung Tseng[1], Wei-Ting Lin[2], Peng-sheng Ting[3], Chiung-Kuei Huang[3], Po-Hung Chen[4], and Hui-Yi Lin[1], *1. LSUHSC, New Orleans, Louisiana, United States, 2. Tulane University, New Orleans, Louisiana, United States, 3. Tulane University School of Medicine, New Orleans, Louisiana, United States, 4. Medicine, Johns Hopkins University, Baltimore, Maryland, United States*

**Background:** Nonalcoholic fatty liver disease (NAFLD) and nonalcoholic steatohepatitis (NASH) are emerging public health concerns among older adults, particularly those with risk behaviors. **Objectives:** We evaluate the association between risk behavior and the prevalence of NAFLD and NASH in older adults. **Methods:** A total of 1444 US individuals aged 60 years and older were included from the 2017-2020 NHANES survey. Dietary, Sugar-sweetened beverages, smoking, physical activities, stiffness, controlled attenuation parameters, alanine aminotransferase (ALT), and aspartate aminotransferase (AST) were measured. We performed multivariable logistic regression models with interaction terms and stratified analysis to assess the interplay effect between soda intake and body mass index (BMI) on liver stiffness measurements. All statistical analyses were under survey modules with an appropriate sampling weight. **Results:** The prevalence of NAFLD/NASH varied significantly by age, gender, race, alcohol use, BMI, total energy consumption, ALT, and (all $p \leq 0.001$). The results showed that several risk behaviors, including smoking, physical inactivity, and sweetened beverage consumption, were not significantly associated with developing and worsening NAFLD and NASH among elderly individuals. However, higher total energy consumption, obesity, and alcohol use were risk factors for NAFLD/NASH. **Conclusion:** Our study findings highlight the importance of promoting some healthy lifestyle behaviors among elderly individuals to prevent the development and progression of NAFLD and NASH. Further research is needed to investigate optimal strategies for promoting healthy beh

Abstract citation ID: igad104.2521

### VIEWS ON HELPFUL SUBSTANCE USE RECOVERY PATHWAYS: DO OLDER AMERICANS DIFFER FROM THEIR YOUNGER COUNTERPARTS?

Naomi Meier[1] Rachita Sharma[1], Paula Garland[1], and Ami Moore[2], *1. University of North Texas: Denton, Denton, Texas, United States, 2. University of North Texas, Denton, Texas, United States*

Recovery from substance use is generally a lifelong process with different pathways. This study used data from the Justice Community Opioid Innovation Network (JCOIN), 2021, a nationally representative survey of over 6,000 American adults, to examine factors that influence the views on most helpful pathways for recovery among older Americans (60 years and older) compared to their younger counterparts (less than 60 years old). Multiple linear regression was used to determine differences across age, political affiliation, cultural identity, and regional location. A factor analysis of 9 questions that asked respondents to rate the most helpful pathways for recovery generated three domains: 1. Medical services enabling environment (PCP, intensive inpatient program, and rehab program); 2. Direct conventional treatment (Prescription medication and therapist); and 3. Sociocultural factors/non-conventional treatment (Family/friends, spiritual/natural healer, self-help group, and faith-based organization). Older and younger Democrats, Independents, and residents of the Northeast and Midwest regions agreed that the medical services were helpful for recovery. While younger and older Democrats believed that direct conventional treatment was helpful, younger and older Republics as well as younger married people believed it to be less helpful. While older Democrats agreed that non-conventional treatments are most helpful, younger Democrats and Independents believed them to be less helpful. However, younger Republicans, Blacks and Hispanics believed that they are most helpful. Given the significant differences in the views of older and younger Americans on helpful pathways to recovery, policymakers and stakeholders are encouraged to be mindful of the several policy recommendations presented in the paper.

### SESSION 7400 (POSTER)

### CAREGIVING, HEALTH PROMOTION, AND WELL-BEING

Abstract citation ID: igad104.2522

### CULTURAL FACTORS PREDICT POSITIVE CAREGIVING APPRAISAL AMONG DIVERSE DEMENTIA FAMILY CAREGIVERS

Michael McCarthy, Mark Remiker, Y. Evie Garcia, Heather Williamson, and Julie Baldwin, *Northern Arizona University, Flagstaff, Arizona, United States*

**Background and Objectives:** Positive caregiving appraisal is strongly linked to health among caregivers of persons with Alzheimer's Disease and Related Dementias (ADRD). Few studies have identified predictors of positive caregiving appraisal, although research suggests that cultural factors may play a role. This study hypothesized that self-identified race and ethnicity, as well as more nuanced cultural factors such as language spoken at home, culture-based values surrounding caregiving, and perceptions of provider cultural competence would predict positive caregiving appraisal. **Research Design and Methods:** A cross-sectional survey was administered to 156 diverse, community dwelling ADRD caregivers. Data were analyzed according to a modified Sociocultural Model of Coping using sequential regression. **Results:** In the final model, self-identified race and ethnicity did not predict positive appraisal. However, culture-based values around caregiving and provider cultural competence contributed significant variance to positive appraisal, after controlling for other factors in the model. Caregiving burden and perceptions that help was available were also linked to positive appraisal. Discussion and Implications: This study is among the first to identify cultural factors that contribute to positive appraisal in a diverse sample of ADRD caregivers, beyond race and ethnicity. Findings reinforce the need to assess and incorporate culture-based values in interventions for ADRD caregivers, as well as the importance of provider cultural competence when working with diverse ADRD family caregivers.

Abstract citation ID: igad104.2523

### EFFECTS OF ACTIVITIES ON THE PSYCHOLOGICAL WELL-BEING OF CAREGIVERS OF OLDER ADULTS

Sarah Won, and Natalie Regier, *Johns Hopkins University School of Nursing, Baltimore, Maryland, United States*

It is well-known that the caregiving role is perceived as stressful and is often associated in the literature with decreased well-being. Given that approximately 41.8 million Americans provide unpaid care to an older adult, it is critical to identify strategies or interventions to maximize their well-being. Activity engagement is one such promising intervention. Consequently, the goal of this study was to investigate which activities of caregivers of older adults significantly improve their well-being. To that end, a three-stage literature review (title/abstract review, full-text review, reference list review) was conducted, resulting in a total of 2,959 articles identified in PsycINFO. Inclusion/exclusion criteria narrowed this down to 24 relevant full-text research articles. Results showed a total of eight cohesive categories of activities for caregivers: Physical (n=5), Social (n=5), Arts/entertainment in the home (n=2), Arts/entertainment outside of the home (n=2), Religious/Spiritual (n=4), Psychotherapy (n=5), Psychoeducation (n=3), and Multimodal (n=4). Indicators of caregiver well-being in the outcome variables of studies included depressive symptoms, overall mental health, stress-induced cellular aging, life satisfaction, distress, quality of life, caregiver burden, satisfaction with the caregiving role, salivary cortisol, caregiver mastery, and stress. Activity categories that had the greatest frequency of positively impacting caregiver well-being were physical activities, social activities, religious/spiritual activities, and psychoeducational activities. All other activity categories except for arts/entertainment activities outside of the home were effective in some studies in improving caregiver well-being. Findings suggest that caregivers of older adults should seek opportunities for engagement in meaningful activity, particularly physical, social, religious/spiritual, and psychoeducational activities.

Abstract citation ID: igad104.2524

### HEALTH INFORMATION–SEEKING BEHAVIORS, CONFIDENCE, AND CHALLENGES AMONG FAMILY CAREGIVERS OF PERSONS WITH DEMENTIA

Jane Chung[1], and Rebecca Davidsson[2], *1. Virginia Commonwealth University, Richmond, Virginia, United States*, *2. University of New Orleans, New Orleans, Louisiana, United States*

Family caregivers play a significant role in providing care for their loved ones with dementia, including communication with healthcare providers (HCP), treatment decision-making, and care coordination. These tasks require family caregivers' ability to seek, manage, and interpret health information they have found or received during and outside clinical encounters. This study examined which sources family caregivers use for seeking dementia care information, how trustworthy and reliable they find these sources, and information seeking confidence and challenges. A survey designed to understand family caregivers' information seeking was completed by 36 participants who provided at least 10 hours of dementia care weekly (mean age 63.4; 83% female). About 80% reported using the Internet to look for dementia information, and less than 20% used a smartphone app to exchange their loved one's medical information with HCP through a protected portal. While family caregivers entrusted HCP, pharmacists, and dementia-related organizations as sources of dementia information, they were skeptical of radio and TV as sources of credible information. Other information sources were identified, including Alzheimer's Association, support groups, books/documentaries, and research journals. Approximately 50% reported concerns about the quality of dementia-related information; 45% experienced difficulties accessing the information they needed; 44% sensed frustration while searching for dementia-related information; and 40% had difficulty understanding the information found. There should be efforts to develop strategies that help dementia caregivers better identify credible information sources to increase confidence and reduce the stress and burden of acquiring reliable information necessary to care for their loved ones.

Abstract citation ID: igad104.2525

### PERCEPTIONS ON A DIGITAL HEALTH PHYSICAL ACTIVITY PROGRAM IN OLDER FAMILY CARE PARTNERS OF PERSONS WITH HEART FAILURE

Dawon Baik, *University of Colorado, Aurora, Colorado, United States*

Many family care partners (FCPs) neglect managing their own health and wellness when providing care to others. Older FCPs are more vulnerable in managing their health because of physical and psychological distress, age-related health needs, and less social support. We developed a digital health physical activity (PA) program, TPA4You, to begin to address these issues. We elicited perceptions of the integrated components of TPA4You (i.e., Zoom PA with a health coach, Fitbit, motivational text messaging) among older FCPs of persons with heart failure (HF-FCPs) using scenario-based design methods. Participants were recruited at the University of Colorado Hospital between September and December 2022. Fifteen qualitative interviews were conducted. Mean age was 65.9±4.4 years, 73% female, 93% White, and

13/15 were spouses. The perceived value of TPA4You was reported as (1) convenience because travel not required, (2) increased motivation from being held accountable by the separate technology components, and (3) consideration of improving FCPs' health and wellness. Challenges described were (1) scheduling, finding time dedicated to exercising with the health coach by Zoom and (2) feeling overwhelmed, integrating the technology components in the FCP's life. The keys to motivating PA and exercise through TPA4You were (1) usefulness of content in text messages and a good relationship with the health coach, (2) encouragement provided by TPA4You engagement, and (3) desire to improve their health and quality of life. Future studies should conduct field-testing of TPA4You in older HF-FCPs by providing one-on-one, hands-on sessions using the integrated technology components to optimize usability and acceptability.

Abstract citation ID: igad104.2526

### PROFILE OF CAREGIVING ACTIVITIES AND ASSOCIATION WITH PHYSICAL HEALTH AMONG DEMENTIA SPOUSAL CAREGIVERS

Jinmyoung Cho[1], Laura Sands[2], Alan Stevens[3], Heather Allore[4], Molly Horstman[5], Rose Milius[6], and Amber Hickey[6], *1. Saint Louis University, St. Louis, Missouri, United States*, *2. Virginia Tech, Blacksburg, Virginia, United States*, *3. Baylor Scott and White Health, Dallas, Texas, United States*, *4. Yale University, New Haven, Connecticut, United States*, *5. Michael E. DeBakey VA Medical Center, Houston, Texas, United States*, *6. Baylor Scott & White Research Institute, Temple, Texas, United States*

The daily care of a person living with Alzheimer's disease and related dementia (ADRD) is associated with threats to the health and well-being of family caregivers. Although more is known about the relationship between caregiving and psychosocial distress, far less is known about the relationship between caregiving and physical health and health behaviors. This study aims to identify intensity of caregiving experience profiles and compare the profiles to multidimensional physical health indicators and health behaviors among spousal caregivers for persons with ADRD. Using data from 152 spousal caregivers aged 65 and over, the intensity of caregiving experience (ICE) was measured as the number and frequency of health- and medical-related helping activities for his/her care recipient. Multidimensional health indicators included fatigue, sleep disturbance, physical functioning, pain interference, and general health from self-reports, and the number of chronic conditions from the electronic health records. Health promotion behaviors were assessed as: health responsibility, physical activity, nutrition, interpersonal relations, and stress management. Cluster analysis was performed to determine the number of ICE profiles. T-tests and chi2 tests were to assess differences in health indicators and behaviors between clusters. We identified two distinct caregiving experience patterns: high-intensity (37.5%) and low-intensity (62.5%) caregiving. Caregivers from the high-intensity caregiving cluster reported feeling more tired ($t=2.254$, $p< .05$), perceiving more sleep disturbance ($t=3.06$, $p< .001$), and performing less physical activity ($t=2.05$, $p< .05$). Future studies are needed to develop effective interventions to reduce sleep disturbance and fatigue among family caregivers who are heavily involved in caregiving activities.

Abstract citation ID: igad104.2527

### UNDERSTANDING THE MEANING OF CARE: FROM THE PERSPECTIVE OF SPOUSE CAREGIVERS OF PEOPLE WITH PARKINSON'S DISEASE

JuHee Lee[1], Subin Yoo[1], Eunyoung Kim[1], Hyeran Park[1], Yun Joong Kim[2], Seok Jong Chung[2], and Jee-Hye Yoo[3], *1. Yonsei University, Seoul, Republic of Korea*, *2. Yonsei University College of Medicine, Seoul, Republic of Korea*, *3. CHA University, Pocheon, Republic of Korea*

Parkinson's disease is a highly prevalent geriatric disease in people aged 60 years and over. Parkinson's disease can largely affect an individual's life because it causes diverse motor and non-motor symptoms. As symptoms worsen, people with Parkinson's disease are likely to become dependent, which can lead to caregiver burden. Therefore, the aim of this study is to understand the meaning of care in spouse caregivers who care for people with Parkinson's disease. This study is a qualitative study using face-to-face in-depth interviews. A total of 15 spouse caregivers (10 husbands and 5 wives) were recruited from a neurological outpatient clinic at a tertiary hospital in South Korea. Two trained nurses interviewed spousal caregivers, and thematic analysis was used for data analysis. In this study, four themes were identified: (a) symptom management, (b) struggling to care, (c) intimacy with one's spouse, and (d) uncertainty of the future illness progress. People with Parkinson's disease experienced at least more than one motor or non-motor symptoms. Physical function and lifestyle modification of people with Parkinson's disease, caregivers' positive attitude towards the care role, and intimate relationships were key factors of self-care. For caregivers, the progression of disease was the major concern of care. Given that the number of older households is increasing in Asia, it is important to understand older spouse caregivers' perspective for care of people with Parkinson's disease. By understanding their needs and difficulties, healthcare providers can build further plans for them.

Abstract citation ID: igad104.2528

### YOGA, TAI CHI, AND PILATES AND FAMILY CAREGIVERS' SUBJECTIVE WELL-BEING: FINDINGS FROM MIDUS

Kallol Kumar Bhattacharyya, Yin Liu, and Elizabeth Fauth, *Utah State University, Logan, Utah, United States*

Being an informal caregiver has been associated with stress and lower levels of subjective well-being. Exercise/movement therapy techniques of yoga, tai chi, and Pilates are physical activities that also incorporate stress reducing practices. The current study aimed to examine the association of exercise/movement therapy techniques on family caregivers' subjective well-being assessed by life satisfaction and mindfulness. Participants were part of the Midlife in the United States (MIDUS) study (wave 2, N = 507 aged 35-86 years; Mage = 56±11, 68% women) who provided informal care in the past 12 months. We recoded the aforementioned exercise/movement therapy activities into three categories, based on the distributions in MIDUS, including regular practice (defined as participating in one or more of them "a lot" or "often"), irregular practice (participating "sometimes" and "rarely"), and no practice ("never"). Subjective well-being was measured using the 5-item global life satisfaction scale and the

9-item mindfulness scale. We considered sociodemographic factors and health as covariates. We used multivariate linear regression models to examine associations between practice and caregivers' subjective well-being. After controlling for covariates, the findings indicated that regular practice was associated with both better mindfulness-related well-being (b = 2.26, p < .05) and better life satisfaction (b = 0.43, p < .05). Future research should examine whether there is a selection effect of caregivers with higher well-being being more likely to choose these activities, and/or if exercise/movement therapies are effective non-pharmacological interventions to improve family caregivers' overall quality of life.

### SESSION 7410 (POSTER)

### CHRONIC DISEASE AND MANAGEMENT

Abstract citation ID: igad104.2529

### ANXIETY IN PATIENTS UNDERGOING OPEN-HEART SURGERY: THE ROLE OF CHARACTER, STRENGTH, AND HOPE IN A STRUCTURE EQUATION MODEL

Veronika Nash, and Amy Ai, *Florida State University, Tallahassee, Florida, United States*

In an era of patient-centered care, more research is needed to examine the protective function of patients' internal strengths against the damage of poor mental health. Mental health plays a significant role in cardiac health. Anxiety is a mortality risk for cardiac patients, the evidence has indicated high prevalence (13-59.5%) post cardiac events. However, anxiety hasn't been extensively investigated as depression in cardiac patients, especially after open-heart surgery (OHS). Even less is known about protective function of Character Strength Hope in cardiac anxiety during stressful OHS, also a life-altering intervention for advanced heart conditions. This interdisciplinary study evaluated a structural equation model (SEM) on the relationship of Hope with pre- and post-OHS anxiety. Two weeks prior to the scheduled operation, research assistants conducted a face-to-face psychosocial interview with 481 patients (male, 58%; mean age=62 years, range=35-89) at the hospital, followed by a second telephone interview to assess an indicator of Hope two-days prior to surgery. Postoperative measures were assessed 36 days after OHS. Female and older patients reported higher levels of anxiety preoperatively. The SEM demonstrated adequate fit of the final solution in which pre- and post-OHS anxiety levels were related. Higher levels of pre-OHS anxiety were related to lower levels of optimism and hope right before OHS. Higher levels of optimism, indicating Hope predicted lower levels of post-OHS anxiety. Because patients with high anxiety would be disadvantaged in postoperative recovery, our finding suggests enhancing preoperative strength, Hope, might have some psychological benefits for optimal outcomes in patients undergoing OHS.

Abstract citation ID: igad104.2530

### ASSOCIATIONS BETWEEN FRAILTY AND SLEEP IN OLDER ADULTS WITH CANCER TREATED WITH CHEMOTHERAPY

Aasha Hoogland[1], Sylvia Crowder[1], Martine Extermann[1], Daneng Li[2], Kristen Carpenter[3], Stacy Fischer[4], Anita Kinney[5], and Heather Jim[1], *1. Moffitt Cancer Center, Tampa, Florida, United States*, *2. City of Hope, Duarte, California, United States*, *3. The Ohio State University College of Medicine, Columbus, Ohio, United States*, *4. UCHealth University of Colorado Hospital (UCH), Aurora, Colorado, United States*, *5. Rutgers Cancer Institute of New Jersey, New Brunswick, New Jersey, United States*

Little is known about the association between frailty and sleep after starting chemotherapy. The goal of this study was to examine frailty and sleep in older adults (65+ years old) with cancer. Patients newly diagnosed with cancer and starting chemotherapy were recruited to a multi-site study. Participants completed questionnaires before chemotherapy (i.e., baseline) and 5 days after their first infusion. A pre-treatment frailty score was derived using a 59-item deficit accumulation index and dichotomized (frail vs. robust). Sleep was assessed with the PROMIS Sleep Disturbance 8a (difficulty sleeping) and Sleep Impairment 8a (daytime sleepiness). Repeated measures ANOVAs adjusted for age, sex, cancer stage, and cancer type evaluated change in sleep over time. Participants were 402 older adults with cancer (M age=72, SD=5). Most were female (65%), non-Hispanic (96%), and married (67%). Difficulty sleeping was higher in frail participants (26%) than robust participants at baseline (p< 0.001). Over time, difficulty sleeping decreased in frail participants but increased in robust participants (p=0.02). Difficulty sleeping remained higher in frail participants post-infusion (p=0.01). Daytime sleepiness at baseline did not differ by frailty status (p=0.86) and was higher than population norms. Frail participants reported smaller reductions in daytime sleepiness over time (p< 0.01), and more daytime sleepiness after infusion (p< 0.0001) than robust participants. Despite improvements in sleep from pre-treatment to 5 days after infusion, frail participants reported more difficulty sleeping and daytime sleepiness than robust participants after infusion. Additional research is needed to better understand the association of frailty with health outcomes in older adults with cancer.

Abstract citation ID: igad104.2531

### BARRIERS TO DISEASE SELF-CARE AMONG OLDER NON-HISPANIC BLACK AND HISPANIC MEN WITH CHRONIC CONDITIONS

Matthew Smith[1], Caroline Bergeron[2], Ledric Sherman[3], Oluyomi Oloruntoba[1], and Ashley Merianos[4], *1. Texas A&M University, College Station, Texas, United States*, *2. University of Ottawa, Ottawa, Ontario, Canada*, *3. Texas A&M University School of Public Health, College Station, Texas, United States*, *4. University of Cincinnati, Cincinnati, Ohio, United States*

Disease self-care is multi-faceted and influenced by physical, mental, social, and environmental factors. Because males traditionally underutilize preventive healthcare services, self-care is increasingly important to manage disease symptomatology and slow disease progression. This study examines factors associated with barriers to disease self-care among Hispanic and non-Hispanic Black men ages ≥65 years with ≥1 chronic condition. Data were analyzed from a national sample of 470 Hispanic (43.2%) and non-Hispanic Black (56.8%) men and collected with an internet-delivered questionnaire. The 5-item Barriers to Self-Care Scale served as the dependent variable (range=5-20). A least squares regression model was fitted to identify factors associated with barriers to disease self-care. The model adjusted for sociodemographics, disease characteristics, health status, and social engagement and support. On average, participants were age 70.1 (±4.5) years and reported 3.9 (±2.6) chronic conditions. Men with higher body mass index (β=0.13, P=0.001), more depressive symptomatology (β=0.09, P=0.046), and lower self-rated quality of life (β=-0.23, P< 0.001) reported higher barriers to disease self-care. Further, men with poorer access to healthcare (β=-0.17, P< 0.001) and higher healthcare frustrations (β=0.17, P< 0.001) reported higher barriers to disease self-care. Barriers to self-care were positively associated with being more socially disconnected (β=0.09, P=0.049) and higher reliance on others to manage health problems (β=0.14, P< 0.001). Findings reinforce the importance of mental health, healthcare access, and social support for disease self-management among Hispanic and non-Hispanic Black men. Efforts are needed in clinical and community-based settings to reduce self-care barriers through culturally appropriate self-management education, interventions, and services.

Abstract citation ID: igad104.2532

### CLINICAL VERSUS SUBJECTIVE PREDICTORS OF DISTRESS AND HEALTH SATISFACTION AND THE MODERATING ROLE OF PERCEIVED CONTROL

Gul Seckin, *University of North Texas, Denton, Texas, United States*

Coping with cancer in old age and a general decline in physical health may result in a lack of control over health. Cancer diagnosis tends to be associated with fear, uncertainty, and loss. It also challenges one's assumptions about the sense of the self and bodily image. This paper explores health perceptions (distress with somatic symptoms, treatment side effects, and satisfaction with health status) in a sample (N=350) of older individuals who reported being treated with cancer (M age=50.23, S.D. =10.91). Three models of hierarchical multiple regressions were performed to estimate the impact of sociodemographic, medical, and psychosocial variables and perceived control over health on subjective health indicators. The results indicated that lower income was associated with greater distress with physical symptoms (Slope = -.12; B= -.17, p =.002), older age was associated with less distress with cancer treatment side effects (Slope = -.01; B= -.12, p =.019) and gender (female) were negatively associated with satisfaction with health status (Slope = -.36; B= -.15, p =.002). Even though the higher stage of cancer was a significant covariate for greater distress with somatic symptoms (Slope = .12; B= .17, p =.001), it was not a significant covariate for distress with treatment side effects and satisfaction with health status. Perceived control over health significantly moderated the association between research covariates and satisfaction with health. These findings suggest that assessing differential aspects of self-rated health may provide health professionals with important information to supplement their diagnosis to understand the patient's illness experience better.

Abstract citation ID: igad104.2533

### DEVELOPING A GERIATRIC ONCOLOGY FELLOWSHIP SUBSPECIALTY TRACK FOR ADVANCED PRACTICE PROVIDERS

Sincere McMillan, Beatriz Korc-Grodzicki, Amelia Cataldo, Nicole Zakak, and Kelly Haviland, *Memorial Sloan Kettering Cancer Center, New York City, New York, United States*

Although a relatively young subspeciality, geriatric oncology is a fast-growing discipline. As the population ages, clinicians skilled in geriatric oncology principles and management are needed to care for an increasingly complex older adult population. Educating Advanced Practice Providers (APP) in geriatric oncology may offer innovative ways of increasing access to care, supporting patient health, and improving quality of life. The need for trained clinicians represented a unique opportunity for the development of a geriatric oncology focused APP fellowship subspecialty track at our institution. In 2019, with support from the Samuels Foundation, an electronic geriatric oncology needs assessment was completed among the APPs at our institution. Data from this needs assessment and from a thorough literature review were used to develop the geriatric oncology fellowship track curriculum. With support from APP leadership at our institution, the fellowship track launched in September, 2021. It is a 12-month postgraduate academic program that focuses on geriatric assessment, risk stratification prior to cancer directed therapies, management of geriatric syndromes, and geriatric co-management. Additionally in 2023, the fellowship track will include an elective in a long-term care facility. We believe this unique elective rotation will be a strong addition to the clinical experience of the fellow. Our current program has one position per fellowship year with the goal of increasing the number of positions in the future. This is a major step forward in the education of APPs in the care of older adults with cancer that we hope will be replicated across the country.

Abstract citation ID: igad104.2534

### FACTORS ASSOCIATED WITH LOW-DOSE COMPUTED TOMOGRAPHY LUNG CANCER SCREENING IN THE UNITED STATES

Tung-Sung Tseng[1], Chien-Ching Li[2], Hui-Yi Lin[1], and Yu-wen Chiu[1], *1. LSUHSC, New Orleans, Louisiana, United States*, *2. Rush University, Chicago, Illinois, United States*

Low-dose computed tomography (LDCT) screening is recommended for high-risk smokers to decrease lung cancer-related mortality. In the US, the uptake of LDCT screening among eligible smokers is suboptimal. The impacts of social-environmental and individual factors on LDCT screening uptake are not clear. The current study investigated multi-level factors associated with the utilization of LDCT screening using national data. **Methods:** The 2017-2021 Behavioral Risk Factor Surveillance System (BRFSS) data and other state-level variables (Medicaid expansion status and the number of screening facilities) were applied. Our study outcome is the utilization of LDCT screening among study participants who met the US Preventive Services Task Force guidelines for lung cancer screening. The final study sample consisted of 15640 respondents from 29 states. All analyses were weighted to account for the complex sampling design applied in BRFSS. **Results:** The overall utilization rate of LDCT screening is only 18.4%. The LDCT utilization rate varied by state (6.2 -31.1%). Among the respondents, individuals who were employed, never married, had good health status, didn't have personal physician, experienced cost problems, never had routine checkups, and did not experience certain chronic conditions (cancer, asthma, COPD) had a lower utilization rate of LDCT screening compare with their counterpart. **Conclusion:** Utilization of LDCT screening among eligible smokers remains low. Risk factors associated with LDCT screening identified from this study can be used to guide future lung cancer screening programs or revise the LDCT screening guidelines to meet the needs of high-risk smokers.

Abstract citation ID: igad104.2535

### FAITH AND HEALTH: THE INTRICATE INTERPLAY OF MARITAL QUALITY, RELIGIOSITY, AND DIABETES

Rebekah Case, and Jeremy Yorgason, *Brigham Young University, Provo, Utah, United States*

Although chronic illness in later life can negatively affect marital quality and life satisfaction through increased stress and burden, religiosity and spirituality are known robust protective factors in relational and psychological outcomes. Stress theory suggests that religious behaviors can help moderate this negative relationship by providing patients with a sense of meaning in illness, external social support, and healthy coping mechanisms. Using data from the Life and Family Legacies Study, we analyzed cross-sectional data from 1,210 married participants around age 70 in 2016. Using an OLS linear regression model, we found main effects which showed that type 2 diabetes among older adults was predictive of lower positive marital quality and lower life satisfaction. When religious behaviors were included in the model as a moderator, religious behaviors interacted significantly with diabetes such that individuals with type 2 diabetes who reported high religiosity had high positive marital quality, while those with low religiosity reported significantly lower positive marital quality. Findings suggest that when persons with type 2 diabetes participate regularly in church services, prayer, and scripture study they experience more positive marital quality. This indicates that religiosity may be a significant protective factor and coping tool for marital quality when chronic illness is present.

Abstract citation ID: igad104.2536

### PAIN INTENSITY AND PAIN CATASTROPHIZING ARE RELATED TO PHYSICAL PERFORMANCE IN OLDER ADULTS WITH BACK PAIN

Corey Simon[1], Patrick Knox[2], Adam Goode[1], Trevor Lentz[1], Steven George[1], Gregory Hicks[2], Cathleen Colón-Emeric[3], and Francis Keefe[1], *1. Duke University, Durham, North Carolina, United States*, *2. University of Delaware, Newark, Delaware, United States*, *3. Duke University School of Medicine, Durham, North Carolina, United States*

Back pain is highly prevalent in older adults and a risk factor for poor/declining physical performance. Back pain intensity and pain catastrophizing are two factors known to be associated with physical performance in younger adults; though a paucity of similar research exists in older adults. Thirty-nine older adults with back pain completed a battery of four standardized physical performance tests: 30-second chair rise, repetitive seated trunk rotation, repetitive standing functional reaching, and six-minute walk. Test scores were z-transformed and summated for a standardized physical performance score. We measured resting pain intensity and dispositional pain catastrophizing before the battery and movement-evoked pain intensity and situational pain catastrophizing during/after the battery. After accounting for age and biological sex, resting and movement-evoked pain intensity explained 14% and 39% unique variance in physical performance. In multivariable models, movement-evoked pain was the stronger factor. Similar modeling showed dispositional and situational pain catastrophizing explained 14% and 28% unique variance in physical performance. In multivariable models, situational pain catastrophizing was the stronger factor. Finally, path modeling showed situational pain catastrophizing mediated the total effect of movement-evoked pain on physical performance (21%). Findings suggest: 1) Factors with movement (i.e., movement-evoked pain, situational pain catastrophizing) are more strongly associated with physical performance than factors measured independent of movement (i.e., resting pain, dispositional pain catastrophizing); and 2) The impact of pain on physical performance is mediated by psychological distress. Importantly, neither movement-evoked pain nor situational pain catastrophizing are part of current standard of care for older adults with back pain.

Abstract citation ID: igad104.2537

### PATTERNS OF USE, RISK FACTORS, AND ATTITUDES OF OLDER ADULT CANNABIS USERS

Elizabeth Anquillare, Adrianna Gallegos, and Rachel Thayer, *University of Colorado Colorado Springs, Colorado Springs, Colorado, United States*

While cannabis use continues to increase among older adults (OA), research within this population is limited. This study aimed to describe OA patterns of use, risk factors, and subjective effects of cannabis use. OA cannabis users ages 60-79 (N=17, Mage=68.29, SD=5.61) were 94% White and 59% women, with an average education of 15.94 years (SD=2.38). Participants completed online questionnaires and semi-structured interviews regarding medical and mental health, subjective cognitive functioning, and cannabis use patterns and attitudes. Participants reported M=33.41 years of cannabis use (SD=20.15) with an average age of first use of 20.11 years (SD=10.40). Current use was reported as 4.29 days/week (SD=3.14). Current reasons for use were recreational (n=6), medical (n=2), or both (n=8). The most common medical conditions for which participants reported using cannabis were arthritis/joint pain (n=4) and disordered sleep (n=4). The most commonly reported benefits of cannabis use were relaxation (n=8), improved sleep (n=6), improved mood/anxiety (n=6), cognitive enhancement (n=5), and pain management (n=4). Common drawbacks included subjective cognitive decline (n=7), social hindrance (n=6), and health complications (n=5), whereas 5 participants reported no drawbacks. Most participants (76%) reported discussing cannabis use with their physician, although only 38% of those reported that discussion was helpful. Disconnect between OA cannabis users and their physicians could result in cannabis use that is contraindicated by preexisting medical conditions or medications. Emphasis should be placed on individualized psychoeducation regarding the potential risks/benefits of cannabis use as well as continuing education for physicians regarding cannabis use in OA.

Abstract citation ID: igad104.2538

### PHYSICAL ACTIVITY PROFILE AND PREFERENCE AMONG OLDER ADULT PATIENTS WITH CANCER: A CROSS-SECTIONAL STUDY

Donya Nemati[1], and Tarah Ballinger[2], *1. The Ohio state University, College of Nursing, Columbus, Ohio, United States*, *2. Indiana University School of Medicine, Indianapolis, Indiana, United States*

Physical activity (PA) is beneficial for older adults (OAs) with cancer by improving physical function, mental health, and lowering the risk of recurrence in some cancer types. However, limited knowledge on OAs preferences may impede effective interventions. This study aimed to identify the PA profiles and preferences of OAs (age≥ 65) compared to younger adult (YA;18-64 years old) cancer patients. Data were collected from 372 adult patients with non-metastatic cancers visiting Indiana University Simon Comprehensive Cancer Center between July 2020-June 2021. Patients completed a cross-sectional survey comprising disease characteristics, PA habits, and PA preferences. A total of 132 OA patients (age 71 ± 5.2 years) were predominantly male (56.1%) and Caucasian (92.4%). Of these, 33.8% reported a decrease in PA after their cancer diagnosis. Fatigue (22%) and lack of motivation (16%) were the most reported PA barriers. While 43% of patients received exercise recommendations from their oncologists, 75% of those did not receive specific guidance. The majority of OAs preferred home-based workouts (65.9%) and exercise motivation packages (52.7%) over exercising at a cancer center facility (26%). Compared to YA patients (n=237), a smaller percentage of OAs (26.5% vs. 38.4%: p<.05) believed that PA could reduce the risk of cancer recurrence, and time constraints were less of a barrier for OAs (7.6% vs. 21.5%: p<.05). A home-based PA program that is supported with motivation packages may appeal to OAs with cancer. These findings are informative for designing tailored PA programs that suit the interests and preferences of OA patients with cancer.

Abstract citation ID: igad104.2539

### PHYSICAL SYMPTOMS REACTIVITY TO DAILY STRESSORS AND CARDIOVASCULAR DISEASE: A COMPARISON OF BLACK AND WHITE ADULTS

Sebastiana Moore[1], Claire Smith[1], Christina Mu[1], and Soomi Lee[2], *1. University of South Florida, Tampa, Florida, United States*, *2. The Pennsylvania State University, University Park, Pennsylvania, United States*

Black adults have higher risk for cardiovascular disease (CVD) compared to their White counterparts. Biopsychosocial frameworks suggest that this racial difference may not only be due to higher stressor exposure (e.g., discrimination) but also to stressor reactivity (i.e., heightened automatic reactions to stressors). This study examined if racial disparities in CVD is explained by elevated physical symptoms in response to daily stressors ("physical symptom reactivity to stressors"). Data came from 1,727 adults (non-Hispanic Black=38%, non-Hispanic White=62%) who participated in the Midlife in the United States Study at Time-1 (T1: 2004-2009). A subset (n=1,482) was reassessed at Time-2 (T2: 2013-2019). Race, daily physical symptoms, and daily stressors were measured at T1. Physical symptom reactivity to stressors (i.e., individual slope of physical symptoms as a function of daily stressor severity) was calculated using 8-day diary data. Physician-diagnosed CVD was captured at T1 and T2. Indirect pathways (A path: race->stressor physical reactivity, B path: race->CVD) were tested, adjusting for health and sociodemographic covariates. At T1, Black adults exhibited a higher risk of CVD compared to White individuals through higher physical symptom reactivity to stressors (A path: B=0.004, SE=0.0006, p<.001; B path: B=-3.56, SE=1.32, p=.007; indirect effect=0.12, SE=0.03, 95% CI=[0.06, 0.18]). These indirect pathways were also supported when predicting the risk of CVD at T2 after controlling for T1 CVD (B path: B=-3.23, SE=1.56, p=.038; indirect effect=0.07, SE=0.04, 95% CI=[0.02, 0.16]). Findings newly suggest that racial differences in physical manifestations of stress may contribute to disproportionate development of CVD, the deadliest disease in America.

Abstract citation ID: igad104.2540

### PREVALENCE AND PREDICTORS OF DECISIONAL CONFLICT AMONG OLDER AFRICAN AMERICANS WITH ADVANCED KIDNEY DISEASE

Tyrone Hamler, *University of Denver, Denver, Colorado, United States*

Chronic kidney disease (CKD) is a major public health concern in the U.S., particularly among African Americans. African Americans are just under four more times likely to develop CKD than White individuals. Older African Americans are a vulnerable and understudied population in CKD literature, despite future projections of an increasingly diverse population of older adults. This study's objectives were: (1) to determine the prevalence of decisional conflict for predialysis older African Americans (2) whether perceived CKD knowledge, clinical characteristics (self-rated health, depression, anxiety, and number of comorbidities), and personal characteristics predicted decisional conflict. Study participants (N =125) were recruited from an outpatient nephrology clinic in a midwestern hospital and administered a telephone survey. Participants were African American, ≥ 50 years of age, pre-dialysis and diagnosed with Stage 4 or 5 CKD. A hierarchical ordinary least-squares (OLS) regression analysis was conducted to examine the relationship between variables. Participants reported mean scores of DC in the moderate range (M = 36.69). Approximately 42% (n =53) of patients' decisional conflict scores were above 37.5, which is the cut-point for scores indicating uncertainty (O'Connor, 1995). Multivariate analyses indicated that DC was only significantly associated with perceived chronic kidney disease knowledge. Lower levels of perceived chronic kidney disease knowledge were associated with higher levels of DC. Findings indicated that DC is a critical area to continue to investigate among older African Americans with advanced CKD. Social workers are well-positioned to impact levels of knowledge in this population through working on interdisciplinary teams in medical settings.

Abstract citation ID: igad104.2541

### PSYCHOSOCIAL RISK, SUBCLINICAL CVD, AND BRAIN VOLUMES: EXAMINING SEX DISPARITIES IN DEMENTIA RISK

Regina Wright[1], and Desiree Bygrave[2], *1. University of Delaware, Newark, Delaware, United States*, *2. Benedict College, Columbia, South Carolina, United States*

The majority of the sex disparity in dementia remains unexplained. One potential risk factor for women's higher incidence of dementia is subclinical cardiovascular disease (SCVD), including carotid atherosclerosis and arterial stiffness. To date, there is a significant gap in what is known about sex differences in the impact of psychosocial risk on pre-dementia risk factors both directly and via SCVD. Therefore, this study aimed to examine the mediating influence of SCVD on the relationship between psychosocial risk and brain volumes, as a proxy for pre-dementia risk, and whether the relationship is moderated by sex. The analysis included 136 older adults with a mean age of 68.04y who underwent neuropsychological, psychosocial, and vascular assessment (pulse wave velocity [PWV], carotid intima media thickness [IMT]), and 3T cranial magnetic resonance imaging. Moderated mediation analyses, adjusted for age and education, showed a mediating role of PWV in the relation of loneliness to total brain volumes (global measure of atrophy) was more pronounced among women (average causal mediation effect [ACME]=.44; p=.11). Unadjusted analyses trended toward moderated mediation, such that the mediating role of carotid IMT in the relation of anxiety to total WMLV (ACME=.001; p=.06) and total frontal WML volumes (ACME=.001; p=.05) was more pronounced among women than men. Findings suggest that in women, but not men, psychosocial risk factors may influence brain volumes via subclinical CVD. Additional research is needed to determine if these interrelations help to explain some of the sex disparity in dementia risk.

Abstract citation ID: igad104.2542

### URBAN–RURAL DISPARITIES IN CHRONIC DISEASE PROGRESSION AMONG CHINESE OLDER ADULTS

Yuanchang Zhao, and Jessica Kelley, *Case Western Reserve University, Cleveland, Ohio, United States*

Over the last thirty years, China has experienced rapid increases in the prevalence of hypertension and diabetes, across urban and rural areas. Studies do indicate that the prevalence rates both hypertension and diabetes are higher in urban areas than in rural areas, seeming to indicate higher chronic disease burden among urban older adults. Few studies, however, focus on the progression of hypertension and diabetes once diagnosed. Given the likelihood of under-diagnosis in rural areas, the aim of our study is to examine urban-rural disparities in the progression of hypertension and diabetes among older Chinese who have been diagnosed with either or both conditions. We utilize two waves of the CHARLS, examining adults over age 50 (n=5,067). Our findings confirm that rural older adults are less likely to be diagnosed with diabetes or hypertension than urban older adults, net of other risk factors. Among those with a diagnosis, and controlling for whether they received treatment, the nested logistic regression models indicate that rural older adults report 32% higher odds of worsening hypertension compared to their urban counterparts. Likewise, the odds of worsening diabetes are 52% higher in rural older adults than in urban older adults. Our findings indicate that although rural older adults are less likely to have a diagnosis of diabetes or hypertension, among those with a diagnosis, rural older adults tend to be sicker and worsen more quickly. We discuss the implications of rural-urban disparities in access to health care and treatment.

### SESSION 7420 (POSTER)

### DEMENTIA

Abstract citation ID: igad104.2543

### ACCELERATING DEMENTIA RESEARCH AND EARLY-CAREER DEVELOPMENT THROUGH THE SUMMER RESEARCH INSTITUTE

Chelsea Kline[1], Matthew Baumgart[2], Julie Bynum[3], Sheryl Zimmerman[4], and Sam Fazio[1], *1. Alzheimer's Association, Chicago, Illinois, United States*, *2. Alzheimer's Association, New York, New York, United States*, *3. University of Michigan, Ann Arbor, Michigan, United States*, *4. University of North Carolina at Chapel Hill, Chapel Hill, North Carolina, United States*

Today, there are more than six million Americans living with Alzheimer's disease and more than 11 million unpaid caregivers. By 2060, the number of Americans living with Alzheimer's could reach 14 million. These statistics highlight the need for early career investigators to join the dementia research workforce and accelerate breakthroughs in this field. In 2020, the National Institutes on Aging (NIA) funded the Alzheimer's Association Interdisciplinary Summer Research Institute (AA-ISRI) focused on early career investigators interested in psychosocial and public health research related to dementia. AA-ISRI is a five-day immersive experience in which 24 early career investigators are selected to come to Chicago, attend didactic lectures led by expert faculty, develop research proposals in small groups, and receive one-to-one mentoring. Participants leave AA-ISRI with a greater understanding of the tools needed to succeed in dementia research and an extensive network of colleagues and mentors. Based on evaluations collected from participants from the first two years of the program, 97% of respondents indicated they were satisfied with the program, 94% felt the program increased their understanding of Alzheimer's, and 90% said the program improved their independent research skills. Participants have shared that AA-ISRI is a highly unique opportunity that every early career investigator in the field should attend. Alumni from the first two years of AA-ISRI have gone on to publish 40+ peer-reviewed publications and have been awarded 15+ grants. This poster will review the development of AA-ISRI, key outcomes, and its impact on the future of dementia research.

Abstract citation ID: igad104.2544

### AN UMBRELLA REVIEW OF SYMPTOM-SPECIFIC NONPHARMACOLOGICAL INTERVENTIONS FOR DEMENTIA

Eunhee Cho[1], Min Jung Kim[2], Ji Yeon Lee[2], Minhee Yang[2], Jiyoon Jang[2], and Jungwon Cho[1], *1. Yonsei University*

*College of Nursing, Seoul, Republic of Korea*, 2. *Yonsei University, Seoul, Republic of Korea*

A variety of non-pharmacological interventions (NPI) have been used for managing behavioral and psychological symptoms of dementia (BPSD), but only a small effect size has been reported. Also, due to a lack of clinical guidelines, individualized BPSD interventions considering each person's preferences and unique symptom profiles have not been available. This umbrella review aimed to summarize the current evidence of NPI for managing BPSD and to suggest an evidence-based algorithm that can guide future clinical practices on choosing an NPI targeting an individual or cluster of BPSD. We used the Cochrane methodology for umbrella reviews following a predetermined protocol registered. A total of five electronic databases (PubMed, CINAHL, Embase, PsychINFO, and Cochrane Database) was searched based on the PICO formulation. The eligibility was independently screened by two reviewers. The quality of the individual review was evaluated by the two reviewers using the A Measurement Tool to Assess Systematic Reviews 2 (AMSTAR2). A total of 32 reviews were included. The reviews collectively indicated that NPI have shown mixed results in managing an individual or cluster of BPSD. As to the NPI, a wide variety was observed in which a music therapy was most commonly used. Of the various BPSD symptoms, affective/hyperactivity symptoms such as depression, anxiety, and agitation have been most investigated. Based on the effect sizes and quality appraisal results, we created an evidence-based algorithm that can provide several NPI options for each sub-category of BPSD. Future studies are required to provide a more comprehensive clinical practice algorithm.

Abstract citation ID: igad104.2545

### CAREGIVER EXPERIENCE WITH FRONTOTEMPORAL DEGENERATION (FTD)

Esther Kane[1], Shana Dodge[2], Sweatha Reddy[3], and Penny Dacks[1], *1. AFTD, King of Prussia, Pennsylvania, United States*, 2. *The Association for Frontotemporal Degeneration, King of Prussia, Pennsylvania, United States*, 3. *FTD Disorders Registry, King of Prussia, Pennsylvania, United States*

Frontotemporal degeneration (FTD) is the umbrella term for a group of young onset neurodegenerative disorders affecting the frontal and temporal lobes of the brain. Symptoms include changes in cognition, language, behavior, motor functioning, and personality. The FTD Insights Survey was designed to learn more about the lived experience of this often misunderstood set of disorders. A total of 1,246 past and current caregivers of people with FTD responded to questions regarding their and their loved one's experiences with FTD. Caregivers most often provided care for more than 80 hours per week and 10% of respondents provided care for more than one person diagnosed with FTD. The majority were caring for someone or had cared for someone who was diagnosed between ages 50 and 69. Only 36% of caregivers reported the person diagnosed was aware or mostly aware of their symptoms and they noted being most distressed by changes in the diagnosed person's language, personality, mood, and behavior. The caregivers described the impact of their loved one's symptoms on activities of daily living at home, in the community, and on interpersonal functioning. The caregivers also provided insight into issues such as the length of time they have provided care, the level of functioning of the person diagnosed, and the impact of FTD symptoms on quality of life. It is critical that healthcare professionals and policy makers understand FTD caregiver strain in order to provide wraparound support and make policy decisions with these features in mind.

Abstract citation ID: igad104.2546

### CHINESE AMERICAN FAMILY CAREGIVERS' PERCEPTIONS OF HOME CARE AND FACILITY CARE FOR LOVED ONES WITH ADRD

Chien-Ching Li[1], Olimpia Paun[1], Xiaojun Gao[2], and Xiao Ying Lei[1], *1. Rush University, Chicago, Illinois, United States*, 2. *Rush University Medical Center, Chicago, Illinois, United States*

Cognitive decline and impairment among older adults are significant public health concerns because they lead to increased disability, caregiver burden, and healthcare costs associated with the treatment of declining cognitive function. The Chinese American population, the largest Asian group in the US, is aging rapidly, and it is expected that older Chinese Americans and their caregivers will increasingly need ADRD care planning for home care and facility care. Therefore, the goal of this study is to examine the beliefs and attitudes of Chinese American family caregivers regarding home care and facility care for their loved ones with ADRD. In our study, N=17 Chinese American caregivers were recruited to participate in the study interview through a partnership with the Alzheimer's Program at the Chinese American Service League. The mean age of the caregivers was 49.6 years, and the majority were female, married, had college degrees or higher, and reported being in good health. Results indicated that most ADRD patients needed medium or high levels of care and were cared for at home. Chinese American caregivers were reluctant to place their loved ones in facilities, such as nursing homes or memory care centers, due to high costs, poor quality of care, language barriers, and Chinese filial piety. Additionally, most Chinese caregivers lacked knowledge regarding the types of home care and facility care and the services provided. Therefore, future development of culturally and linguistically targeted interventions for ADRD care planning is necessary for Chinese American patients with ADRD and family caregivers.

Abstract citation ID: igad104.2547

### DIFFERENTIAL MRI ATROPHY PROFILES IN OLDER ADULTS WITHOUT DEMENTIA FROM AGING STUDIES IN THE US AND CZECH REPUBLIC

Monica Nelson[1], Brent Small[2], Zuzana Nedelska[3], Ondrej Lerch[3], Martin Vyhnalek[3], Ross Andel[4], and Jakub Hort[3], *1. University of Michigan, Ann Arbor, Michigan, United States*, 2. *University of North Carolina-Chapel Hill, Chapel Hill, North Carolina, United States*, 3. *Charles University and Motol University Hospital, Praha 5., Hlavni mesto Praha, Czech Republic*, 4. *Arizona State University, Tempe, Arizona, United States*

Alzheimer's disease is a progressive neurodegenerative disorder with extensive neuropathological heterogeneity. We aimed to identify distinct latent subgroups of neuropathology and differentiate them using sociodemographic, contextual, and cognitive factors. Participants without dementia from the Alzheimer's Disease Neuroimaging Initiative (ADNI; n=1703) and the Czech Brain Aging Study (CBAS; n=385) were included. Latent profile analysis was used to detect profiles using 10 volumetric structural magnetic resonance imaging regions of interest derived using Freesurfer algorithm (hippocampus, middle temporal, superior temporal, precuneus, anterior cingulate, medial orbitofrontal, pericalcarine, precentral, lingual, and caudate regions). Four profiles emerged in ADNI: Minimal Atrophy (n=192), Mild Atrophy (n=691), Moderate Atrophy (n=567), Severe Atrophy (n=253). Two profiles emerged in CBAS: Mild Atrophy (n=208) and Severe Atrophy (n=177). For all but the caudate region, there were significant pairwise differences in volumetry between each profile. Further, Minimal Atrophy was the youngest, had the highest proportion of participants with normal cognition, had the lowest progression to dementia, and the best cognition compared to all other groups – the remaining pairwise comparisons suggested that participants were older, more likely to have mild cognitive impairment, progress to dementia, and have poorer cognition, transitioning from Mild to Moderate to Severe Atrophy. Findings were consistent in CBAS. In ADNI, Minimal and Mild Atrophy had higher education and premorbid abilities compared to Moderate and Severe Atrophy. In CBAS, Mild Atrophy had better premorbid abilities compared to Severe Atrophy. Biological heterogeneity among older adults without dementia should be considered to understand who may be at risk of cognitive decline.

Abstract citation ID: igad104.2548

### INITIAL EFFICACY OF THE SAVVY CAREGIVER PROGRAM IN KOREAN AMERICAN DEMENTIA CAREGIVERS

Yuri Jang[1], Kenneth Hepburn[2], Juyoung Park[1], William Haley[3], and Miyong Kim[4], *1. University of Southern California, Los Angeles, California, United States*, *2. Emory University, Atlanta, Georgia, United States*, *3. University of South Florida, Tampa, Florida, United States*, *4. University of Texas at Austin, Austin, Texas, United Sta tes*

Dementia caregivers in ethnic minority communities have often been excluded from access to caregiver interventions and their benefits because of linguistic and cultural barriers. In pursuit of equal treatments for all, it is imperative to make evidence-based interventions available and accessible to ethnic minority dementia caregivers, and linguistic and cultural adaptation is indispensable for reaching out to them. In the present study, we adapted the Savvy Caregiver Program for Korean American dementia caregivers and tested its initial efficacy for reducing depressive symptoms. This pilot intervention was delivered to 13 Korean American dementia caregivers by two Savvy-certified Korean-speaking trainers. Over the 6-week period of program implementation, there was no attrition (session completion rate = 98%). All participants' depressive symptoms were reduced at completion of the program, and the level of reduction was statistically significant, with a large effect size ($t = 8.64$, $p < .001$, Cohen's $d = .89$). Although patterns of change did vary among participants, the score difference between post-intervention and 6-month follow-up was not statistically significant ($t = .74$, $p = .47$). The program played a significant role in reducing depressive symptoms in this pilot sample of Korean American dementia caregivers, and this benefit was sustained at the 6-month follow-up. Along with the program's notably high retention rate, its efficacy suggests strong unmet needs for psychoeducational programs among Korean American dementia caregivers. The study offers promise for sharing the therapeutic benefit of the Savvy program with linguistic minorities when delivered in a linguistically, culturally appropriate manner.

Abstract citation ID: igad104.2549

### INVESTIGATING THE NEURAL CORRELATES OF SOCIAL AND INDIVIDUAL SINGING IN PERSONS WITH DEMENTIA

Nicholas Tamburri, Debra Sheets, Andre Smith, and Stuart MacDonald, *University of Victoria, Victoria, British Columbia, Canada*

Music interventions for persons with Alzheimer's Disease and related dementias (PwADRD) have documented psychological benefits; however, the neurological correlates underlying these advantages remain less certain. Using functional near infrared spectroscopy (fNIRS), the present study i) investigated within-person patterns of cortical oxygenation during choral vs. individual singing; ii) explored how singing context (choral vs. individual) modulated patterns of functional connectivity (FC) within and across frontal and parietal cortices; and iii) leveraged these functional activations as predictors of cognitive status (degree of impairment) in a series of discriminant function analyses (DFA). Participants were 13 PwADRD who volunteered from a larger, ongoing social-cognitive choir intervention. fNIRS data were collected using a TechEn Cw6 system during both choral and individual singing conditions. Paired sample t-tests evaluated differences in activation patterns between singing conditions, with DFA used to determine whether these activations and neuropsychological function were predictive of between-person differences in cognitive impairment. Significant differences in cerebral oxygenation were identified in the right anterior PFC, with individual singing associated with significantly greater cortical oxygenation relative to group singing. Although not significant, individual singing was also associated with bilateral increases in cortical oxygenation across the majority of fNIRS channels, as well as increased FC, relative to group singing. The DFA analyses were not significant. This novel study is the first to examine differences in music-cognition correlates across environmental contexts for PwADRD. Patterns of functional activation suggest that choral singing in particular may represent an optimal lifestyle activity, placing comparatively fewer demands on cognitive function.

Abstract citation ID: igad104.2550

### PAIN ASSESSMENT AND MANAGEMENT FOR OLDER ADULTS WITH DEMENTIA IN LONG-TERM CARE: A SCOPING REVIEW

Minhee Yang, and Jeongwon Shin, *Yonsei University, Seoul, Republic of Korea*

Self-reporting is essential for assessing pain. However, owing to their cognitive impairment, older adults with dementia may not always express pain. As such, older adults with dementia are at risk of living with undertreated pain. This scoping review aimed to explore and identify current publication trends and strategies regarding pain assessment and management for older adults with dementia in long-term care. Using the scoping review method of Arksey and O'Malley, two reviewers conducted structured searches for articles published from 2012 to 2022 on four databases (CINAHL, Cochrane, PubMed, RISS) and then independently reviewed the full text for eligibility criteria. The review included 40 articles for analysis and identified 20 observational pain tools used in the studies. A few studies assessed pain in older adults with dementia using facial expression recognition technology and electroencephalography. A common pain management strategy identified for older adults with dementia was the application of protocols or clinical guidelines that included a pain assessment and treatment phase. In addition to pharmacological and non-pharmacological treatment, a therapeutic robot (PARO) and play activities were investigated to determine whether the pain was alleviated in older adults with dementia. Although studies have been conducted to assess and manage pain in older adults with dementia in long-term care, the review highlighted the need for education and training on pain tools and treatment approaches, as well as for the development of pain management strategies and non-pharmacological interventions.

Abstract citation ID: igad104.2551

### PREDICTORS OF RESILIENCE AMONGST INFORMAL DEMENTIA CAREGIVERS

Kaitlin Grelle[1], Sarah Vacek[1], Kara Rayha[1], Willie Hale[1], and Rebecca Deason[2], *1. University of Texas at San Antonio, San Antonio, Texas, United States, 2. Texas State University, San Marcos, Texas, United States*

Informal caregivers of People with Alzheimer's Disease (PwAD) report greater levels of burden compared to non-caregivers, which is associated with an increase of impaired physical and mental health (Pinquart & Sorensen, 2003; Cheng, 2017; Zhou et al., 2021). Recent studies focusing on resilience (a person's adaption to the physical and psychological demands of providing care) showed higher levels of resilience in caregivers was inversely related to depression, burden, and anxiety (Lopes Da Rosa et al., 2018; Zhou et al., 2021). With resilience being a protective factor against burden in caregivers, it is imperative to understand what psychological characteristics act as predictors of resilience. Participants (N = 59) completed questionnaires about resilience, mental health, and knowledge of Alzheimer's disease. A classification and regression tree (CART) analysis was conducted to evaluate caregiver psychological characteristics as possible predictors of resilience. The analysis produced a tree with four terminal nodes, with one characterized by having scores greater than 152 for self-efficacy, followed by scores greater than 15 for knowledge of Alzheimer's Disease. The overall model is 79.7% correct in predicted resilience, with a specificity of 95.7% and a sensitivity of 69.4%. These findings provide evidence that caregivers with greater confidence in their abilities are more likely to be resilient, especially for those with increased knowledge about Alzheimer's disease (Choi et al., 2018; Kim & Bolkan, 2022). Interventions that focus on bolstering knowledge of Alzheimer's disease and increasing self-efficacy may be critical in enhancing resilience in caregivers, in turn reducing depression and burden.

Abstract citation ID: igad104.2552

### PRELIMINARY FEASIBILITY, UTILITY, AND ACCEPTABILITY OF THE TBI-AD/ADRD CAREGIVER SUPPORT INTERVENTION (TACSI)

Robyn Birkeland[1], Elizabeth Albers[2], Katie Louwagie[1], Edward Ratner[3], Jacob Finn[4], Sherry Chesak[5], and Joseph Gaugler[1], *1. University of Minnesota, Minneapolis, Minnesota, United States, 2. University of Minnesota School of Public Health, Minneapolis, Minnesota, United States, 3. Minneapolis VA Health Care System, Minneapolis, Minnesota, United States, 4. Minneapolis VAHCS, Minneapolis, Minnesota, United States, 5. Mayo Clinic, Rochester, Minnesota, United States*

Research has established that dementia care partners may encounter socio-emotional and physical health concerns as a consequence of providing dementia care. Similarly, care partners for people living with Traumatic Brain Injury (TBI) often experience a range of stressors and negative mental health outcomes due to their care demands. This study aims to support the unique subset of family care partners facing the challenge of caring for relatives who have both diseases, Alzheimer's Disease/Alzheimer's Disease related dementia (AD/ADRD) and a history of TBI. Phase I of this study focused on the design and subsequent refinement of the TBI-AD/ADRD Caregiver Support Intervention (TACSI). TACSI is a six-session telehealth intervention providing tailored psychosocial and psychoeducational coaching. Session topics include education on TBI and dementia, stress management, lifestyle planning, and communication skills. In partnership with the Minneapolis VA and the Mayo Clinic-Rochester, we enrolled 15 care partners for Phase I. Mixed methods data from 3-month follow-up surveys and qualitative data from post-intervention interviews established the feasibility, utility, and acceptance of TACSI. Care partners found the program valuable and would recommend it to others. They reported that TACSI helped with their stress management. Some participants shared that TACSI would provide greater benefit to newer caregivers needing more education on the diseases and/or caregiving. Phase I feedback will be applied to improve TACSI content and delivery for the Phase II randomized controlled trial evaluation. TACSI Phase I utility, acceptance, and feasibility outcomes will be highlighted as well as next steps for evaluation.

Abstract citation ID: igad104.2553

### QUALITY-OF-LIFE INDICATORS IN CAREGIVERS OF OLDER ADULTS WITH DEMENTIA: CATEGORIZATION USING THE WHO-ICF

Idorenyin Udoh, and Elias Mpofu, *University of North Texas, Denton, Texas, United States*

The quality of life of caregivers of persons with dementia greatly influences their health, physical, psychological, and emotional wellbeing. This in turn influences the quality of care they deliver to persons with dementia. This scoping review aimed to map emerging evidence on the quality of life indicators using service and environmental support factors and a subsequent characterization using the WHO-ICF. The WHO-ICF provides a platform for the understanding of this factors and how it influences the overall wellbeing of caregivers. Using a systematic scoping review approach, we identified 14 studies on service and environmental support influencing quality of life of caregivers of persons with dementia. The search included EMBASE, CINAHL, the Cochrane Database of Systematic Reviews, JBI Evidence Synthesis, and Epistemonicos. A narrative synthesis of the data revealed that the quality of life of caregivers is greatly influenced by service and environmental support factors which in turn influences their activities of daily living and that of persons with dementia. The service and environmental services factors include services from government, family, community, healthcare professionals, peer groups, and activities available, Attitudes from individuals especially family, spouse and others , environmental support and support relationships improving quality of life of caregivers of persons with dementia requires an understanding of the prevailing service and environmental support which drive their commitment to quality care for older adults with dementia especially in a pandemic environment. Keywords: Caregiver, dementia, service, environmental support, quality of life.

Abstract citation ID: igad104.2554

### RELATIONSHIP-TYPE DIFFERENCES IN SUPPORT SEEKING AND MENTAL HEALTH FOR ADULT CHILDREN OF PARENTS WITH MEMORY LOSS

Dustin Gad, and Joan Monin, *Yale School of Public Health, New Haven, Connecticut, United States*

Research shows that midlife children still rely on their parents for emotional and tangible support, which has implications for their mental health. Little is known about how these dynamics change when a parent enters the early stage of dementia or memory loss. One characteristic that may be important for these dynamics is the relationship-type (daughter-mother (n= 82), daughter-father (n= 29), son-mother (n=17), son-father (n= 14)). As part of a larger study, adult children 18 years of age and older (n= 142) completed self-report surveys including measures of emotional and tangible support seeking from their parent (Feeney, 2004), the 10-item CESD (Orme et al., 1986), the 12-item Zarit Burden Inventory (Bédard et al., 2001), and the PANAS (Watson et al., 1988). Results showed that tangible and emotional support seeking levels were low across all dyad-types, with no differences between groups. There was a trend for depressive symptoms (F= 2.559, p= .058) showing more in daughter-father than in son-mother dyads. Daughter-father dyads were the only group meeting the depression cut-off score. There were significant differences for burden (F= 3.377, *p= .020) with more burden in daughter-mother compared to son-father dyads. For the PANAS (F= 2.926, *p= .036), there was more negative affect in daughter-father compared to daughter-mother dyads. Results suggest that the relationship-type of the adult child and parent matters for mental health in the early stages of memory loss. This has implications for dyadic interventions helping adult children and their parents cope in the early stages of the dementia caregiving journey.

Abstract citation ID: igad104.2555

### RUNNING AND WALKING IN DEMENTIA AND MILD COGNITIVE IMPAIRMENT: A SCOPING REVIEW

Benjamin Mast[1], Fairouz Gaballa[2], Dylan Gillespie[2], Kaitlyn Graves[2], Darby Simon[1], Emilee Ertle[1], Nina Sanfilippo[3], and Romina Babazadeh[2], *1. University of Louisville, Louisville, Kentucky, United States*, *2. University of Prince Edward Island, Charlottetown, Prince Edward Island, Canada*, *3. William James College, Newton, Massachusetts, United States*

Growing evidence suggests physical exercise may reduce dementia risk, but there has been little investigation of specific forms of exercise for people living with dementia and cognitive impairment. Prior reviews show running and walking have mental health benefits in the general adult population, but no reviews of walking and running among people living with dementia and mild cognitive impairment have been published. A scoping review was conducted to identify and evaluate evidence concerning whether running and walking affect dementia risk, improve/maintain cognitive functioning, and improve quality of life and psychological well-being. PsycInfo, Medline, CINAHL, and SPORTDiscus were searched to identify studies with the target population (people living with dementia or mild cognitive impairment) and target activities (running, jogging, walking). 1153 records were uploaded into Covidence and 821 studies were excluded based on abstract reviews. Full text review yielded 49 papers. Forty-seven studies examined walking and a broad range of outcomes: cognition, ADL functioning, quality of life, behavioral expressions of distress, social participation, and identity. The remaining two studies examined (1) a combination of running and walking among people with mild cognitive impairment and (2) the link between cognitive decline and a history of running, jogging, and walking (past ten years). This review shows growing interest in walking as an intervention with many possible benefits for people living with dementia or mild cognitive impairment. Studies of jogging and running are still relatively rare in this population. Additional contributing authors: Hannah Gardner, Clarissa Geibel, Katherine King, Jessica Strong, Travis Saunders, Christine Wise.

Abstract citation ID: igad104.2556

### SECONDARY ROLE STRAIN AND GRIEF OF DEMENTIA FAMILY CAREGIVING AS PREDICTORS OF BURDEN

Hannah Mason[1], and Francesca Falzarano[2], *1. Fordham University, New York City, New York, United States*, *2. Weill Cornell Medicine, New York City, New York, United States*

Services often aim to improve caregiver experiences for dementia family caregivers by addressing challenges in daily care activities and enhancing care quality for care-recipients. Supporting caregivers should not exclusively focus on supporting activities related to direct care provision (e.g., primary stressors), but should also include services that holistically support the caregiver's well-being. Identifying an individual's unique needs beyond their caregiving role is crucial; the growing phenomenon of caregiver burnout, burden, and psychosocial struggle includes experiencing a loss of identity, increased desire to escape, and intense role captivity. While some services are more individualized, such as one-on-one therapy, there is an overall gap in caregiver support in secondary domains. The current study examined whether primary stressors (e.g., activities of daily living and everyday care tasks) or secondary stressors (e.g., strain on other relationships and responsibilities) were more predictive of burden in a sample of 46 participants who completed a mixed-method study examining the heterogenous experiences and unmet needs of dementia family caregivers. Analysis shows that interference of the care role with responsibilities to other family (i.e., secondary role strain) and feeling pre-loss grief over their loved one's illness were significant predictors of greater burden compared to primary stressors. Results indicate the importance of addressing secondary stressors such as pre-loss grief and deterioration of one's microenvironment (including social support, familial relationships, and identity) in intervention efforts to better enhance quality of life and well-being in dementia care.

Abstract citation ID: igad104.2557

### SELF- AND PROXY-REPORTED MET NEEDS IN PERSONS WITH DEMENTIA ARE ASSOCIATIED WITH A LOWER HEALTH-RELATED QUALITY OF LIFE

Joost Wammes[1], Holly Laws[2], Hein van Hout[1], Janet MacNeil Vroomen[1], and Joan Monin[3], *1. Amsterdam Medical Centers, Amsterdam, Noord-Holland, Netherlands*, 2. *University of Massachusetts, Amherst, Amherst, Massachusetts, United States*, 3. *Yale School of Public Health, New Haven, Connecticut, United States*

This study examined the dyadic association of self and informal caregiver proxy-reported met needs in persons living with dementia on the health-related quality of life (HRQOL). A total of 237 dementia care dyads were included from the Case Management of Persons With Dementia and Their Caregivers Study. HRQOL was assessed by the EuroQol-5D and the number of met needs by the Camberwell Assessment of Needs for the Elderly. The Actor-Partner Interdependence Model framework was used to analyze the effect of an individual's self or proxy-reported met needs on their own HRQOL (actor effects), and an individual's self or proxy-reported met needs on the other dyad member's HRQOL (partner effects). The number of self-reported met needs by persons living with dementia was negatively associated with their own HRQOL (actor effect $b = -0.200$, $p < 0.001$), and the HRQOL of informal caregivers (partner effect $b = -0.114$, $p = 0.001$). The number of proxy-reported met needs by informal caregivers was negatively associated with their own HRQOL (actor effect $b = -0.105$, $p < 0.001$) but not the person living with dementia's HRQOL ($-0.025$, $p = 0.375$). Study findings suggest that both self-reported and informal caregiver proxy-reported met needs in persons living with dementia should be considered in research and practice because they have different implications for each dyad members' HRQOL.

Abstract citation ID: igad104.2558

### SOURCES OF BURDEN IN CAREGIVERS OF PERSONS WITH EARLY- AND LATE-ONSET ALZHEIMER'S DISEASE

Adele Crouch[1], and Lauren Massimo[2], *1. Indiana University School of Nursing, Indianapolis, Indiana, United States*, 2. *University of Pennsylvania, Philadelphia, Pennsylvania, United States*

Functional impairment and neuropsychiatric symptoms (NPS) are common in Alzheimer's disease (AD) and contribute to caregiver burden. Persons with early-onset AD (EOAD) on average experience greater functional impairment and NPS compared to late-onset AD (LOAD), yet their contribution to caregiver burden have not been explored. Objectives were to: 1) compare functional impairment, NPS, and caregiving burden in EOAD and LOAD; 2) identify sources of caregiver burden including functional impairment and NPS in EOAD and LOAD. Caregivers of 85 persons with EOAD (n=63) and LOAD (n=22) completed questionnaires [Functional Activities Questionnaire, Neuropsychiatric Inventory, Zarit Burden Inventory]. T-tests compared group differences in function, NPS, and caregiver burden. Persons with EOAD had greater NPS frequency than LOAD ($t= 2.275$, $p=0.026$). There were no significant differences between groups in function or caregiver burden. Multivariate regression analyses were performed in AD groups, with function and NPS frequency as predictors of caregiver burden covarying for age, caregiver sex, and global cognitive function (MMSE). In the total AD sample, the model explained 12.6% of the variance in caregiver burden [$F(5,52)=2.64$, adjusted $R2=0.126$; $p=0.033$] and poor function was a significant predictor (standardized $B=0.42$, $p=0.003$) of caregiver burden. In EOAD, the model explained 17% of the variance in caregiver burden [$F(5,35)=2.64$, adjusted $R2=0.17$; $p=0.04$], and poor function was a significant (standardized $B=0.42$, $p=0.015$) predictor of caregiver burden; however this was not the case in LOAD. Results suggest poor function predicts caregiver burden in EOAD. Although NPS in EOAD are more frequent, this was not a predictor of caregiver burden.

Abstract citation ID: igad104.2559

### USE OF VIDEO-BASED DATA TO INFORM PSYCHOSOCIAL INTERVENTIONS FOR PEOPLE LIVING WITH DEMENTIA

Gloria Hoi-Yan Wong, and Jacky C.P. Choy, *The University of Hong Kong, Hong Kong, Hong Kong*

The pandemic has accelerated technological developments in care services. Emerging evidence suggests feasibility of delivering evidence-based psychosocial interventions such as group cognitive stimulation therapy using information and communication technology (ICT) platforms. A unique opportunity offered by ICT is the more readily available data at session level to investigate nuanced, theoretically based outcome predictors, to improve efficacy of these complex interventions. Theoretically, the cognitive and wellbeing benefits of group cognitive stimulation therapy is related to the level of person-centred engagement. As part of an ongoing randomised controlled trial involving ICT-delivered group cognitive stimulation therapy, this study examines the use of big data analytics of session behavioural data to develop predictive algorithms of cognitive and wellbeing outcomes. In 78 people living with mild to moderate dementia from 26 groups, we create automatically estimated markers of facial expression and eye movement pattern, and hand-coded markers (using time-sampling method) of engagement level and speech/verbal production information, to autobiographical information presented in the session. These markers are used to predict changes in cognitive and wellbeing outcomes. Pilot analysis using hand-coded speech sample suggested strong correlation between time-sampled engagement level with cognitive benefits, and changes in information content and fluency with cognitive benefits after the group intervention (ranging from r=0.76 to 0.97, p< 0.001). The markers we develop in this study using video-based data from ICT-delivered therapy sessions, including deep learning-based emotion recognition and eye movement analysis with hidden Markov model, allow further application in understanding the mechanisms of action in different psychosocial interventions.

### SESSION 7430 (POSTER)

### DIVERSITY AND FALLS

Abstract citation ID: igad104.2560

### A SYSTEMATIC REVIEW OF CARE PARTNERS' PERCEPTIONS OF FALL RISK AMONG OLDER PEOPLE WITH DEMENTIA

Nirali Thakkar[1], and Yuanjin Zhou[2], *1. University of Texas-Austin, Austin, Texas, United States*, *2. University of Texas at Austin, Austin, Texas, United States*

Community-dwelling older people with dementia (cd-OPWD) experience a high risk of falling. Care partners have varied levels of involvement in fall risk management for cd-OPWD. However, little is known about what factors may shape their involvement. Understanding care partners' perceptions of fall risk, associated factors, and impacts will be critical for service providers to effectively engage care partners in fall risk management for cd-OPWD. Utilizing the Common Sense Model of Illness Perceptions, we conducted a literature search among 6 research databases and identified 23 articles using predefined inclusion/exclusion criteria. Our study found that care partners' perceptions of cd-OPWD's fall risk comprised beliefs about fall risk management behaviors/programs (i.e., mobility aides, exercise, home modification, fall detector system), their understanding of indicators, causes, consequences, timeline, and controllability of cd-OPWD's fall risk, and emotional reactions. These perceptions were influenced by care partners' previous experiences with falls, cd-OPWD's declining health and functions, experienced care burden, relationships with cd-OPWD, and interactions with healthcare providers. Care partners' beliefs about fall risk management behaviors/programs and their understanding of indicators, causes, consequences, and timeline of falls are often associated with their adoption of fall risk management behaviors. Their emotional responses to cd-OPWD's fall risk are associated with increased care burden. These findings point to a need for solution-oriented fall prevention interventions to increase care partners' perceived benefits of fall risk management behaviors/programs and their knowledge about cd-OPWD's falls. Future interventions should also try to mitigate the negative impact of care partners' emotional responses to cd-OPWD's fall risk.

Abstract citation ID: igad104.2561

### BRINGING COMMUNITY-BASED FALLS PREVENTION PROGRAM TO IMPROVE GLOBAL HEALTH (BRIGHT)

Ladda Thiamwong[1], Sirinart Tongsiri[2], Rui Xie[1], Boon Peng Ng[1], Joon-Hyuk Park[1], Wenjun Li[3], and Elizabeth Eckstrom[4], *1. University of Central Florida, Orlando, Florida, United States*, *2. Mahasarakham University, Mahasarakham, Maha Sarakham, Thailand*, *3. University of Massachusetts Lowell, Lowell, Massachusetts, United States*, *4. Oregon Health & Science University, Portland, Oregon, United States*

Older adults in low- and middle-income countries (LMICs) experience a disparate burden of non-communicable diseases (NCDs) and barriers to accessing health care. Unintentional injuries are among the major NCDs, and falls are the second leading cause of these injuries and deaths. Falls were reported in up to 50% of older people living in Asia, including Thailand. There is a lack of community-based multifactorial fall prevention interventions that target both older adults and the primary care systems. We aimed to culturally adapt the CDC's Stopping Elderly Accidents, Deaths, and Injuries (STEADI) for Thai older adults and explore the feasibility, appropriateness, and acceptability of using STEADI by trained community health workers (CHWs) and care managers (CMs). STEADI consists of three steps: screening, assessing, and intervening. In Step a, CHWs screened fall risk using three key questions and found that all of participants had fall risk, then CHWs screened with a Stay Independent questionnaire (range 0-14) and found that 100% have high fall risk (total scores 9.7± 2.4). In Step b, CMs assessed risk factors and CHWs assessed home hazards. They found that 50% had poor balance, 70% took 4+ medications, 75% fell on the walkway, and 70% had no bathroom modifications. In Step c, individual participants received fall prevention interventions and participants attended all sessions. Our results indicated that STEADI delivered by CHWs and CMs is feasible and acceptable for older adults. The mean scores of acceptability, appropriateness, and feasibility were 19.20,18.80, and 18.60 out of 20 totals, respectively.

Abstract citation ID: igad104.2562

### INCIDENT AND RECURRENT FALLS AMONG NON-HISPANIC BLACK AND HISPANIC MEN WITH CHRONIC CONDITIONS

Temitope Olokunlade[1], Ledric Sherman[2], Mark Benden[2], Gang Han[1], and Matthew Smith[1], *1. Texas A&M University, College Station, Texas, United States*, *2. Texas A&M University School of Public Health, College Station, Texas, United States*

While much is known about the complexities of fall-related risks among older adults, less is known about the risk for falls among men, and especially older non-Hispanic Black and Hispanic men with chronic conditions. This study examined factors associated with incident falls (1 fall) and recurrent falling (2+ falls) among non-Hispanic Black and Hispanic men ages ≥60 years with ≥1 chronic condition. Collected with a web-delivered questionnaire, data were analyzed from a national sample of 779 non-Hispanic Black (58.8%) and Hispanic (41.2%) men. To assess incident and recurrent falls, the number of self-reported falls in the past year was trichotomized (0 falls vs. 1 fall vs. 2+ falls) and used as the dependent variable. A multinomial logistic regression was fitted to assess factors associated with incident and recurrent falls. The model adjusted for sociodemographics, disease characteristics, health status, and social support. On average, participants were age 66.8 (±5.4) years and reported

3.8 (±2.7) chronic conditions. Seventy-three percent of men reported 0 falls, 12.6% reported 1 fall, and 14.4% reported 2+ falls in the past year. Relative to men reporting 0 falls, Hispanic men (P< 0.05), men with worse general health status (P< 0.05), and those with clinical depression (P< 0.05) were more likely to report incident and recurrent falls, respectively. Men with more comorbidities (P< 0.05) and those with less help/support to manage health problems (P< 0.05) were more likely to report recurrent falling. Findings highlight the importance of multi-component interventions to prevent falls by strengthening disease self-management, addressing mental health, and introducing social supports.

Abstract citation ID: igad104.2563

### INVESTIGATING PSYCHOLOGICAL FACTORS AS A PREDICTOR OF FEAR OF FALLING AND FALL RISK IN OLDER ADULTS

Amber Blount, Ladda Thiamwong, Nichole Lighthall, Rui Xie, Joon-Hyuk Park, Victoria Loerzel, and Jeffrey Stout, *University of Central Florida, Orlando, Florida, United States*

Psychological factors related to appetitive and avoidant motivation, perceptions of aging, and regulatory focus (promotion focus on gains versus a prevention focus on non-losses) are likely to play an important role in predicting one's fear of falling (FOF) and resulting impact on physical activity engagement. This pilot study aimed to investigate the gap in current research related to these psychological factors as predictors of older adults' Fear of Falling and Fall Risk Appraisal (FRA). The study assessed positive and negative perceptions about aging in regards to control and consequences of aging (Brief Aging Perceptions Questionnaire [B-APQ]), motivation (Behavioral Inhibition System/Behavioral Activation system [BIS/BAS]), regulatory focus in goal achievement (Regulatory Focus Questionnaire [RFQ]), FOF (Fall-Efficacy Scale-International [FES-I]), and static balance levels (BTracks Balance system [BBS]). Participants (n=48) were categorized into four FRA groups: rational (low FOF/normal balance), irrational (high FOF/normal balance), incongruent (low FOF/poor balance), and congruent (high FOF/poor balance). A Spearman Rho correlation found that FOF was positively correlated with two of the BAPQ sub-domains of Control-Positive ($\rho = 0.46$, $p<.001$) and Consequences and Control Negative ($\rho = 0.43$, $p = .002$),. A Kruskal-Wallis test found the relationship between BAPQ Control Positive and the Irrational and Rational FRA group baselines to be significant. The results suggest that among other psychological factors, perceptions about aging may be predictive of FOF and FRA in aging. These results highlight the importance of clinicians addressing not only the physiological factors of fall risk, but also the psychological factors underlying fall risk in older adults.

Abstract citation ID: igad104.2564

### PAIN MEDICATION AND FALL RISKS AMONG COMMUNITY-DWELLING OLDER ADULTS WITH ARTHRITIS

Aya Yoshikawa[1], and Richard Fortinsky[2], *1. Texas Woman's University, Denton, Texas, United States*, *2. University of Connecticut, Farmington, Connecticut, United States*

Pain medication is commonly used among older adults with arthritis. Research indicates an elevated fall risk among older adults with arthritis, yet this association among those who take medication for pain is not fully studied. This population-based study investigated the association of falls with pain medication use by arthritis status. A sample of community-dwelling Medicare beneficiaries in the 2015 National Health and Aging Trends Study, representing older adults who reported arthritis (n = 4,229) and no arthritis (n = 3,258), were analyzed using survey-weighted logistic regression analysis. Sensitivity analysis was performed to investigate differences in associations by the frequency of taking pain medication. All analyses were controlled for sociodemographic characteristics, living arrangements, balance problems, and chronic conditions. The majority of participants was 70 years or older (70.7%), female (55.3%), White (80.1%), had a high school or higher education (58.4%) and had two or more chronic conditions (53.6%). Significantly higher proportions of those with arthritis reported pain (67.9% vs. 32.1%) and pain medication use (63.1% vs. 36.9%) than those with no arthritis. Pain medication use (OR = 1.11, 95% CI: 1.03, 1.20) and balance problems (OR = 4.39, 95% CI 3.45, 5.59) were associated with falls among those with arthritis. Taking medication for pain every day a week (OR = 1.48, 95% CI: 1.05, 2.09) was associated with falls among those with arthritis. Findings suggest that the heavy use of pain medication may increase fall risk. Nonpharmacological pain management education is encouraged for supporting active living among older adults with arthritis.

### SESSION 7440 (POSTER)

### FAMILY CAREGIVING: MEASURES, METHODS, AND INTERVENTIONS

Abstract citation ID: igad104.2565

### CREATION OF A FAMILY CAREGIVER NEGOTIATION TRAINING PROGRAM USING ARTIFICIAL INTELLIGENCE

Alaine Murawski[1], Vanessa Ramirez-Zohfeld[1], Allie Schierer[1], Ana Ramirez[2], Johnathan Mell[3], Jeanne Brett[4], and Lee A Lindquist[1], *1. Northwestern University, Chicago, Illinois, United States*, *2. New York University, New York City, New York, United States*, *3. University of Central Florida, Orlando, Florida, United States*, *4. Northwestern University Kellogg School of Management, Chicago, Illinois, United States*

Family caregivers of people with dementia (PWD) experience conflicts as they navigate the healthcare system but do not have adequate training to resolve these disputes. We sought to develop and pilot test an artificial-intelligence negotiation training program, NegotiAge, for family caregivers. With NIH funding [R01AG068421], we convened a team of negotiation experts, geriatrician, social worker, and community-based family caregivers (Illinois/Florida/New York/California). Content matter experts created informational videos to teach negotiation skills. Family caregivers generated dialogue surrounding caregiver conflicts. Computer science experts input generated dialogue into the Interactive Arbitration Guide Online (IAGO) platform to develop avatar-based agents (e.g. sibling, older adult, physician) for caregivers to practice negotiating. Family caregivers review didactic material then access scenarios to negotiate (e.g. physician recommendations, sibling disagrees with care, PWD refusing support). Caregivers negotiate in real-time with avatars designed to act like humans, including emotional and irrational behaviors. Caregivers can use negotiation tactics until either mutual agreement occurs or time expires. Immediate feedback is generated from the responses chosen and tactics to assist with the negotiation skills training. Pilot testing was conducted with family caregivers of PWD (n=12) and showed that the negotiation and conflict resolution training program was feasible and usable for family caregivers. Subjects found the program to present real-world applicability. NegotiAge is an Artificial Intelligence-based Caregiver Negotiation Program, that is usable and feasible for family caregivers to become familiar with navigating conflicts seen in caring for PWD.

Abstract citation ID: igad104.2566

### DEVELOPMENT OF AN ONLINE DISASTER PREPAREDNESS PROGRAM FOR CAREGIVERS OF PERSONS WITH DEMENTIA

Maria Donohoe, Nicholas Ostrem, Nicholas Rudzianski, Jane Gay, Brigid Smith, Tiffany Wunderlich, and Sato Ashida, *University of Iowa College of Public Health, Iowa City, Iowa, United States*

Individuals with medical conditions, including persons with dementia (PWD), are vulnerable in disaster situations. Disasters limit caregivers' ability to continue with care due to stress, reduced resources and limited social support. We previously developed and tested a disaster preparedness program for older adults, Disaster PrepWise, and showed its impact on improving preparedness and personal emergency support networks. Through community engaged approaches, we adopted this program to specifically support family caregivers of PWD. Contents were revised based on the emergency management and dementia caregiving literature, and input from the stakeholder advisory board (SAB) consisting of caregivers, aging service providers, public health, and emergency management agencies. The SAB also provided guidance on implementation strategies from the lens of over-burdened caregivers. The new program includes a five-step process with an emphasis on the needs of caregivers and caregiving tasks, for example, developing emergency caregiving network to be activated during and after a disaster. The program also informs caregivers of tasks involved in the event of an emergency and provides various information such as how to navigate staying at public shelters with PWD, identify services during and after an emergency, and manage stress. Preliminary data shows high levels of acceptance and perceived usefulness of this caregiver-specific program. The program is perceived to fill a gap in current disaster management approaches that have limited ability to support vulnerable populations. Caregivers and service providers also praised this program's ability to connect caregivers to community programs and resources. Additional pilot data will be presented.

Abstract citation ID: igad104.2567

### IMPROVING REPLICABILITY THROUGH DATA STANDARDIZATION: REFINING TELE-STELLA FIDELITY PROCEDURES

Aimee Mooney, Allison Lindauer, Sarah Gothard, Glenise McKenzie, Ruth Tadesse, Hannah Bernard, Charles Boardman, and Arlene Danschin, *Oregon Health & Science University, Portland, Oregon, United States*

Tele-STELLA (Support via TEchnology: Living and Learning with Advancing Alzheimer's disease and related dementias) is a prospective, non-randomized, mixed methods clinical trial of a multicomponent videoconference-based psychoeducational intervention for family care partners to facilitate effective management of behavioral symptoms of dementia. The intervention is delivered by nurse "Guides" over two 8-week sessions with the goal of reducing care partner burden, assessed before, during and after intervention. To ensure treatment adherence to this complex intervention protocol, Guides complete intervention checklists after each session. Fidelity monitoring early in study implementation revealed inconsistency in Guide adherence to the intervention protocol as evidenced by gaps and barriers with data collection platforms. To optimize fidelity of the protocol, an iterative process of refinement was applied to procedural operations and data capture practices. These refinements included standardization of protocol tasks (procedural fidelity), explicit definition of guide behaviors (theoretical fidelity) for the continuity of fidelity assessments, and implementation of a unified data capture platform, REDCap®, providing immediate visibility and reference for all team members. We found lower, non-significant Zarit Burden Scores after process revisions (pre=9.14, post=8.43, p=0.29). We also found improvement in data capture with a significantly reduced proportion of missingness for required fields (p=2.2e-16). These findings provide meaningful signaling of improved treatment fidelity, which is associated with those mechanisms of action hypothesized to impact study outcomes. These findings further indicate that a more formal architecture was needed to implement this curriculum and standardization of these processes are vital for long-term replicability and downstream adoption.

Abstract citation ID: igad104.2568

### RESULTS OF AN ONLINE CAREGIVING DURING CRISIS COURSE

Carolyn Clevenger[1], Fayron Epps[2], Melinda Higgins[2], Glenna Brewster[2], Kirandeep Raina[3], and Kenneth Hepburn[2], *1. Nell Hodgson Woodruff School of Nursing, Emory University, Atlanta, Georgia, United States*, 2. *Emory University, Atlanta, Georgia, United States*, 3. *Atlanta, Atlanta, Georgia, United States*

Caregiving during Crisis (CDC) is an online, asynchronous, self-paced course for dementia family caregivers. The course was developed to enhance caregivers' sense of mastery for their role as systems navigator with the secondary effects of improving their own and their person's quality of life. CDC was drafted by subject matter experts and finalized by family caregivers. The final version focused on three main topics: Safe Home; Navigating the Healthcare System; and Managing the Day divided into 54 short interactive segments. One hundred caregivers were enrolled in a randomized waitlist control group design, with measures of mastery and quality of life at three timepoints over 16 weeks. We employed multi-level mixed effects models (MLM) with random (participant) and fixed (group) effects to test linear (or possibly non-linear) trajectories of change for the immediate and waitlist groups and model group X time and group*time interactions. Of 100 consented participants (50 in immediate and 50 in waitlisted group), 76 completed the midpoint study activities (38 in each group). For the immediate group, there was an increase in mastery such that by the midpoint at 2 months, the caregivers in the immediate group had significantly higher mastery scores (p=.039) than those in the waitlist group, with a moderate effect size (d=0.501) difference. Fourteen course participants completed semi-structured interviews. Their responses to the course were very positive, noting it was well organized, logical, and not too technical. They also indicated a need to include guidance for interactions with legal, regulatory, social service, and financial systems.

### SESSION 7450 (POSTER)

### LGBTQIA+ AND AGING

Abstract citation ID: igad104.2569

### AGING AND THE LIVED EXPERIENCES OF TRANSGENDER AND GENDER NONCONFORMING OLDER ADULTS

Jeanne Koller, and Paul Urbanski, *Monmouth University, West Long Branch, New Jersey, United States*

Adults identifying as transgender and gender nonconforming (T/GNC) in the United States may experience adversity, victimization, and discrimination across the life span (McDowell, et al., 2019). T/GNC older adults have higher rates of poverty, homelessness, social isolation, depression, and violence compared to older adults in the general population and compared with lesbian, gay, and bisexual (LGB) older adults (Sloan & Benson, 2022). This descriptive phenomenological study utilized a purposive and snowball sampling strategy and sought to increase understanding of this diverse group. Twenty participants aged 50 and older were interviewed. Themes included fear and/or actual loss of relationships after "coming out" as transgender; concerns and complexity of healthcare and anticipated changes in healthcare if faced with needing long-term care; navigating gendered space; living as their authentic self; "passing" as cisgender; finding acceptance within the LGB community; desire but struggles to have intimate relationships; conflict between internal and external sense of self; and histories of depression, and suicidality. Protective factors included having an internal source of strength; support within the T/GNC community; being in committed relationships; and receiving support from others. Age at time of transitioning, sex assigned at birth, and identifying as GNB but not transgender influenced lived experiences. McDowell, M.J., Hughto, J.M.W., & Resiner, S.L. (2019). Risk and protective factors for mental health morbidity in a community sample of female-to- male trans-masculine adults. BMC Psychiatry, 19(16), 1-12. https://doi.org/10.1186/s12888-018-2008-0 Sloan, S., & Benson, J. J. (2022). Toward a conceptual model for successful transgender aging. Qualitative Social Work, 21(2), 455-471. https://doi.org/10.1177%2F1473325021994666

Abstract citation ID: igad104.2570

### HETERONORMATIVE SILENCES AND QUEER LONGINGS IN LGBTQ PEOPLE'S EXPERIENCES OF ELDER CARE AND HOME

Anna Siverskog, *Södertörn University, Stockholm, Stockholms Lan, Sweden*

The meaning of home for queer people have been widely empirically explored as well as theorized. Not least has the home been important for the older generations of queer people, who lived in times where their sexualities and gender identities have been criminalized, pathologized and where there have been few public meeting places historically. However, having care needs may blur the lines between private and public and complicate notions of integrity in one's home. This paper is based on qualitative interviews and aims to explore experiences of LGBTQ people in a Swedish context who have eldercare services; either people who have home-care-services or who are living in care homes. A queer theoretical framework and thematic analysis was used. The results illustrate how there is a silence around gender and sexuality in the everyday life within eldercare. This in turn is caused by material conditions where downsizing and effectivization of the eldercare has created pressed working conditions that leaves little room for small talk between staff and recipients of care. Norms on age, gender, and sexuality with notions on older people as asexual (as well as cisgender and straight) may play into this silence as well. The boundaries between the private (home) and the public (eldercare) become blurred. This in turn conditions which intimacy practices that become im/possible. Simultaneously, there is a presence of queer resistance as well as of longings for other (queer) futures.

Abstract citation ID: igad104.2571

### IDENTIFYING RESILIENT PERSONALITY THROUGH LATENT PROFILE ANALYSIS IN SEXUAL MINORITY MIDDLE-AGED AND OLDER ADULTS

Christi Nelson, *University of Washington, Seattle, Washington, United States*

Research has found evidence of resilience in sexual minority (i.e., lesbian, gay, bisexual) adults, despite experiencing higher rates of poorer health and facing additional stressors such as discrimination and victimization. Yet, very limited research has been conducted on resilience in this marginalized group. Utilizing data from the Midlife in the United States study, this study conducted latent profile analysis (LPA) using the Big Five personality traits (i.e., openness, conscientiousness, extraversion, agreeableness, neuroticism) to identify distinct personality profiles in a sample of sexual minority and propensity-score matched heterosexual middle-aged and older adults (n=477, mean age= 57.3, SD= 10.9 years, age range= 40-83 years). Additionally, the associations between the personality profiles and health-risk/promoting behaviors were assessed. Four profiles were identified: Average (35.2%), Resilient (47%), Overcontrolled (7.3%), and Undercontrolled (10.5%). The results found that Resilient sexual minority participants were significantly more likely to engage in problematic drinking but were also more likely to have a routine physical exam than Resilient heterosexual participants. Both sexual minority and heterosexual participants with a Resilient personality profile were more likely to engage in moderate physical activity than their counterparts with other personality profiles. These results suggest that sexual minority adults have the potential for resilience which is often overlooked in research. This is one of the first studies to use LPA to identify personality profiles in a sample with a focus on sexual minority participants. Most previous research using LPA to identify personality profiles did not include data on sexual orientation.

Abstract citation ID: igad104.2572

### PHYSICAL AND EMOTIONAL WELL-BEING AMONG OLDER TRANSGENDER AND SEXUAL MINORITY ADULTS

Laura Bernstein, and Julie Hicks Patrick, *West Virginia University, Morgantown, West Virginia, United States*

Health disparities exist between heterosexual adults and those in the LGBTQ+ community. These differences may be exacerbated in late life. However, a comparative approach that includes heterosexual adults as a referent group may obscure important within-group differences among sexual and gender minorities. The purpose of the current study was to examine within-group differences in health and well-being among adults ages 65 and over who identified as LGBTQ+. Data are provided by 2673 adults ages 65+ who completed the Sexual Orientation/Gender Identity module of the 2020 CDC Behavior Risk Factor Surveillance System. Age groups included 1020 young-old adults ages 65 to 70 years, 1056 older adults, ages 71 to 79 years, and 597 old-old adults age 80+. Adults identifying as gay/lesbian (n = 856), bisexual (n=675), transgender heterosexual (n = 135) or some other minoritized sexual identity (n = 1007) supplied assessments of physical, mental, and functional health. A series of 3 (age) by 4 (sexual orientation) ANOVAs examined main effects of age, sexual orientation, and their interaction. Age effects, but not sexual orientation effects, emerged for mental health. Significant effects for functional ability emerged for sexual orientation and for gender, although no interaction was observed. No significant effects emerged for physical health and no interactions emerged as significant for any of the health outcomes. These results emphasize that examining within-group heterogeneity among sexual minority status and age is necessary to localize and address potential health disparities.

Abstract citation ID: igad104.2573

### SOCIAL SUPPORT, MENTAL HEALTH, AND SEXUAL ORIENTATION: A STUDY OF THREE TIME POINTS IN THE CLSA

Alexandra Grady, and Arne Stinchcombe, *University of Ottawa, Ottawa, Ontario, Canada*

Lesbian, gay, and bisexual (LGB) older adults exhibit mental health disparities stemming from a lifetime accumulation of minority stress experiences. Psychological resources like social support may buffer against these poor mental health outcomes. The purpose of this study was to examine indicators of depression and social support by sexual orientation over time in a sample of Canadian adults ages 45-85. We used data collected at three time points in the Canadian Longitudinal Study on Aging (CLSA; baseline, 2011-2015; follow-up 1, 2015-2018; and follow-up 2, 2018-2021). Depression was measured using the Center for Epidemiologic Studies Depression scale (CESD-10) and social support was measured using the Medical Outcomes Study Social Support Survey (MOS-SSS). For depression symptoms (n=38,244; 830 LGB), results indicated a significant effect of sexual orientation ($F(2, 38242)=35.93$, $p<.001$) such that LGB participants had more depression symptoms than heterosexual participants. There was also a significant effect of time on depression symptoms ($F(2, 38242)=26.07$, $p<.001$); mean depression scores decreased over time. For social support (n=35,795; 770 LGB), results indicated a significant effect of sexual orientation ($F(2, 35794)=45.31$, $p<.001$) such that LGB participants reported lower levels of social support than heterosexual participants. There was also a significant effect of time ($F(2, 35794)=5.98$, $p=.003$); the mean scores for social support increased over time. The potential role of social support in moderating depression symptoms will be discussed. This study adds to our understanding of potential protective factors influencing the mental health of older LGB people.

Abstract citation ID: igad104.2574

### THE EFFECT OF CHRONIC CONDITIONS ON DEPRESSIVE SYMPTOMS IS MODERATED BY SEXUAL MINORITY STATUS

Em Trubits[1], Jason Newsom[1], Jennifer Saucedo[1], Anda Botoseneanu[2], Heather Allore[3], Corey Nagel[4], David Dorr[5], and Ana Quiñones[5], *1. Portland State University, Portland, Oregon, United States*, *2. University of Michigan, Ann Arbor, Michigan, United States*, *3. Yale University, New Haven, Connecticut, United States*, *4. University of Arkansas for Medical Sciences, Little Rock, Arkansas, United States*, *5. Oregon Health & Science University, Portland, Oregon, United States*

It is important to examine disparities in the effects of physical health on depression among sexual minority (SM) older adults. We examined whether the effects of chronic conditions (CCs) and self-rated health (SRH) on depressive symptoms were moderated by sexual orientation among 3,741 adults aged 50+ (SM = 216) who participated in the 2016 wave of the Health and Retirement Study (HRS) and responded to the recently added sexual orientation item. Structural equation modeling was used to model the effects of SRH and CCs on depressive symptoms, while controlling for participant sex, age, years of education, net-worth, and race/ethnicity. Greater numbers of CCs were associated with greater depressive symptoms ($b= .24$, $SE = .04$, $p <.001$), whereas better SRH was associated with fewer depressive symptoms ($b = -.60$, $SE = .05$, $p <.001$). The direct effect of SM on depressive symptoms was significant, $b = 1.14$, $SE = .48$, $p =.02$, indicating greater depressive symptoms among SM adults. The interaction between SM and SRH on depressive symptoms was significant ($b = -.39$, $SE = .17$, $p = .02$). The same effect was seen for CCs ($b = -.32$, $SE = .15$, $p = .04$). Simple slopes revealed that for heterosexual older adults, greater CCs were associated with greater depressive symptoms ($b = .27$, $SE = .04$, $p <.001$), however this association was not significant for SM older adults ($b = -.05$, $SE = .16$, $p = .75$). Future research should examine health disparities among SMs in relation to mental health.

Abstract citation ID: igad104.2575
### WIDENING SEXUAL ORIENTATION INEQUITIES IN SMOKING AMONG OLDER ADULTS IN THE UNITED STATES, 2015–2019

Jie Yang, *East Carolina University, Greenville, North Carolina, United States*

A recent editorial in Tobacco Control noted the neglect of older adults in the field of tobacco control, arguing that we must do better. This study seeks to answer that call and amplify its message by assessing what is missed in health equity research when older adults are ignored in another area of inequity: differences in smoking by sexual orientation. We used pooled data from the 2015-2019 National Survey on Drug Use and Health. We estimated smoking prevalence for older adults aged ≥50 (N=43,956) by gender and sexual identity. Following the National Center for Health Statistics guidelines for analysis of trends, we used logistic regression to examine the association between continuous year (time) and smoking controlling for sexual identity. We also tested inequities in change using an interaction between year and sexual identities. In Model 1, there was a significant decreasing trend of smoking among all older adults: With each passing year, the odds of smoking went down (aOR 0.98, 95%CI 0.95-1.00). Heterosexual men, gay men, bisexual women), and lesbian women had a higher likelihood of smoking than heterosexual women. In Model 2, there was a significant interaction between year and sexual identity (aOR 1.34, 95%CI 1.02-1.75). That is, the change in likelihood of smoking from 2015 to 2019 for bisexual women was significantly different than the change for heterosexual women. Widening inequities in current smoking across some sexual identities among older adults indicate the importance of ensuring cessation campaigns for LGB adults are not just reaching younger adults.

### SESSION 7460 (POSTER)

### RISK AND PROTECTIVE FACTORS OF HEALTH/HEALTH SERVICE USE I

Abstract citation ID: igad104.2576
### ASSOCIATION BETWEEN EXERCISE AND UNCONTROLLED BLOOD PRESSURE IN HYPERTENSIVE PATIENTS

Sinwoo Hwang, and Eunhee Cho, *Yonsei University College of Nursing, Seoul, Republic of Korea*

Hypertension is closely associated with cerebrovascular and cardiovascular diseases, the most frequent causes of death in Korean adults. Despite its continuously improving treatment rate, it is necessary to consider patients whose blood pressure remains uncontrolled. Walking exercises are strongly recommended to lower blood pressure. This study aimed to examine the level of walking in hypertensive patients and factors that deterred walking. This cross-sectional study was conducted through secondary data analysis using data collected from the Korea National Health and Nutrition Evaluation Survey from 2016 to 2020. Participants included 1,800 hypertensive patients over the age of 19 years, whose blood pressure was not under control despite medication. Walking was defined as exercise lasting for at least 30 minutes more than five times a week. Multiple logistic regression analysis was conducted to evaluate factors that deterred walking as an exercise in hypertensive patients. Of the total sample, 63.8% patients (n=1,149) were not exercising (walking). Job and obesity were two significant factors associated with walking less. Patients who were employed or obese were more likely to not walk, compared to those unemployed (OR, 1.31; 95% CI, 1.04–1.65) or those with normal weight (OR, 1.43; 95% CI, 1.03–1.98). Patients with uncontrolled blood pressure despite medication need lifestyle modifications as well. High medication adherence should not be the reason to overlook the management of hypertensive patients. In fact, detailed management is required to identify vulnerable groups, particularly those who do not engage in even walking exercises regularly, which is a simple treatment of hypertension.

Abstract citation ID: igad104.2577
### BARRIERS TO HEPATITIS C TREATMENT AMONG US BABY BOOMERS: PROVIDER PERSPECTIVES

Elaine Wethington, *Cornell University, Ithaca, New York, United States*

Chronic Hepatitis C (HCV) is a major health problem in the US for the baby boomer cohort (born 1945-65), affecting 1.8 million persons; 75% acquired it through injection drug use in earlier life. About 20% develop cirrhosis, often leading to end stage liver disease. HCV is curable and treatment has become more affordable and available. However, uptake of treatment has lagged. This paper reports on a semi-structured interview study of 36 health providers from 2 states (NY, AL) with experience treating/referring HCV+ patients, part of a mixed-methods study of providers and patients. Interviews were coded using thematic analysis. Providers reported that baby boomers, especially racial/ethnic minority and low income, experience barriers to HCV care, because of the complexity of the treatment cascade (testing, linkage to qualified providers, prescribing treatment, insurance delays, and successful completion of therapy). Low provider awareness of need to test for and treat HCV, limited access to specialists (the primary prescribers of HCV medications until recently), variable effectiveness of health systems in assuring successful treatment, and insurer initial reluctance to support treating all infected persons, have also contributed to disparities in care. Perceived stigma and discrimination are barriers to treatment even among the majority of newly diagnosed HCV+ baby boomers who no longer use injection drugs. Providers report that patients fear that families and workplaces will avoid or isolate them if their past drug use becomes known. Moreover, patients who have yet to experience serious health impact may delay treatment, given fears about discrimination against drug users.

Abstract citation ID: igad104.2578
### CHILDHOOD TRAUMA AMONG US VETERANS ANDTTHEIR NEGATIVE HEALTH AND RISKY BEHAVIORS IN LATER LIFE

Jessika Dayrit[1], Shirley Salom-Bail[1], Cesz Islaya[1], and Eunice Castro[2], *1. Department of Veterans Affairs, Menlo Park, California, United States*, *2. Department of Veterans Affairs, Agana Heights, Guam, United States*

**Background:** Behavioral and health outcomes of childhood traumas are public health issues. Research on adverse childhood experiences (ACEs) have been explored among the general population; however, ACEs among persons with history of military service remains unknown. **Methods:** Current study used secondary dataset obtained from CDC Behavioral Risk Factor Surveillance System (BRFSS) 2011. Study's total sample size was 7,112. Data was weighted to account for the complex sampling design and nonresponse. Logistic regression was used to explore ACEs among US Veterans, and how early trauma correlates to long-term health problems, while controlling for their socioeconomic status. **Results:** Our analyses suggested that compared to participants with 0 ACEs, those who reported 1 ACE (OR=1.38; CI=1.10-1.73), 2 ACEs (OR=2.30; CI=1.80-2.95), 3 ACEs (OR=2.81; CI=2.11-3.73), and 4+ ACEs (OR=4.04; CI=3.20-5.09) were more likely to report depression. Participants who reported for having 4+ ACEs were 4.16 times more likely to report in risky sexual behaviors (OR=4.16; CI= 1.83-9.46). Individuals with 4+ ACEs scores were 30% more likely to report binge drinking (OR=1.30; CI=1.02-1.67), 94% at risk for being a smoker (OR=1.94; CI=1.54-2.45) and had higher odds of developing poor health status (OR=1.67; CI=1.32-2.10). **Conclusion:** Our research suggests that more ACEs score associates to a higher risk of developing health issues in later life. Research on ACEs has traditionally focused on health outcomes using secondary datasets among the general population. More research should be explored in longitudinal studies on ACEs, particularly with persons who have served in the military.

Abstract citation ID: igad104.2579

### CULTIVATING AN ATTITUDE OF GRATITUDE: A BRIEF GRATITUDE INTERVENTION FOR OLDER ADULTS WITH CHRONIC PAIN

Shelley Condon[1], Brian Cox[2], and Patricia Parmelee[1], *1. The University of Alabama, Tuscaloosa, Alabama, United States*, *2. Alabama Research Institute on Aging, Tuscaloosa, Alabama, United States*

Gratitude interventions have emerged as a promising approach to ameliorate the negative impact of pain and enhance quality of life. Despite the high prevalence of older adults with chronic pain, there are very few gratitude intervention studies in this population. The current study examined the feasibility and effectiveness of a two-week daily gratitude journaling intervention in a sample of 38 older adults (M=67.53 years) with chronic knee or hip pain. Participants were randomly assigned to the gratitude group (n=21), who wrote down three things they were grateful for every day, or the attention-matched control group, who did not journal. All participants completed pre- and post-intervention interviews and received 14 nightly phone calls, allowing for "global" (pre/post interviews) and "daily" (nightly calls) analyses of the study aims. Aim 1 examined the effects of gratitude on well-being using regression and multilevel model analyses, while Aim 2 examined the effects of the intervention on changes in well-being using repeated measures analyses of variance and multilevel model analyses. In Aim 1, significant associations were found between trait and state gratitude and well-being, supporting previous research and a multifaceted conceptualization and measurement of gratitude. In Aim 2, significant main effects for time emerged, suggesting that participants' well-being improved regardless of their treatment group. Income level was also a consistent predictor of well-being, demonstrating disparities in chronic pain. The impact of small sample size, COVID-19, and methodological limitations will be discussed. Future research plans and recommendations will also be provided.

Abstract citation ID: igad104.2580

### DIABETES OCCURRENCE, RISK FACTORS, AND LIFESTYLE PATTERNS IN OLDER ADULTS ATTENDING SENIOR CENTERS

Laurie Ruggiero, Elizabeth Orsega-Smith, and Sophia Kayatta, *University of Delaware, Newark, Delaware, United States*

Diabetes risk and rates increase across age with prevalence rates of diagnosed diabetes of 24% and estimated pre-diabetes rates of 49% in adults 65 or older (2017-2020; CDC, 2020) underscoring the importance of understanding diabetes risk factors and lifestyle behaviors in older adults. Senior centers offer a unique opportunity to reach older adults to deliver health promotion interventions. The study purpose was to examine diabetes/pre-diabetes occurrence, risk factors, lifestyle behaviors, and preferences for promoting healthy lifestyles to reduce diabetes risk in older adults (>60). An anonymous cross-sectional self-report survey was administered in senior centers. It included questions regarding occurrence of diabetes, diabetes risk factors, and hypertension; self-reported health status; lifestyle behavior patterns; cognitive function; and social engagement. Open-ended questions obtained information on diabetes risk awareness and preferences for healthy lifestyle interventions delivered in senior centers. The sample included 159 adults: average age of 76 years (range: 60-96), 77% female, 83% White, and 14% African American. Reported health related patterns include: 89% rated health as good-excellent; 74% were at high risk for pre-diabetes; 64% had hypertension; and 20% were told by provider of diabetes and 32% pre-diabetes. Reported lifestyle patterns included: 73% get regular physical activity, 53% eat 5 daily fruits/vegetables, 7% smoke, and average 7 hours of sleep/night. The large proportion at high risk for pre-diabetes underscores the importance of focusing on diabetes prevention in older adults and potential for delivering healthy lifestyle interventions in senior centers. Presentation will describe the quantitative and qualitative results and implications for lifestyle interventions.

Abstract citation ID: igad104.2581

### DIFFERENCES IN HOSPICE AND NONHOSPICE NURSES' PERSONAL AND PROFESSIONAL CHARACTERISTICS

Janie Taylor, and Renee' Zucchero, *Xavier University, Cincinnati, Ohio, United States*

The overextension of nurses due to an aging population, increase in chronic conditions, and the COVID-19 pandemic, places them at risk of negative outcomes. However, less is known about how hospice nurses compare to nurses in other subfields in terms of professional quality of life or factors that may potentially protect against adverse consequences of caregiving. Therefore, this study investigated differences between hospice and non-hospice nurses across several factors, including the work environment, self-awareness (SA), psychological flexibility (PF), palliative care self-efficacy (SE), and three components of professional quality of life – compassion satisfaction (CS), burnout, and secondary traumatic stress (STS). Using a cross-sectional, exploratory, and quantitative design, participants were sampled through snowball and convenience techniques. Participants (N= 72) completed an online survey, 31 (43%) identifying as working in hospice and 41 (57%) as working in non-hospice settings. Independent samples t-tests were conducted. Hospice nurses reported significantly healthier work environments, higher levels of SA, SE, and CS, and lower levels of burnout than non-hospice nurses. However, there was no significant difference in PF or STS. These findings offer a novel comparison between hospice and non-hospice nurses that could inform workplace change to better support nurses as they face increased demands and increased risk for negative outcome.

Abstract citation ID: igad104.2582

### DIFFERENTIAL PAIN SENSATION AND GUT MICROBIOME BETWEEN YOUNG AND OLDER ADULTS

Jie Chen[1], Zequan Wang[2], Angela Starkweather[3], Ming-Hui Chen[2], Hongyu Miao[1], Hyochol Ahn[1], and Xiaomei Cong[4], *1. Florida State University, Tallahassee, Florida, United States*, *2. University of Connecticut, Storrs, Connecticut, United States*, *3. University of Florida, Gainesville, Florida, United States*, *4. Yale University, New Haven, Connecticut, United States*

This study aimed to investigate the differences in pain sensation and gut microbiota between young and older adults. Twenty-one healthy young adults (YAs) and sixteen healthy older adults (OAs) were recruited. Quantitative sensory testing data, and stool samples were collected. The 16S rRNA V4 gene region of stool samples were sequenced and processed by using the Mothur 1.42.3 pipeline. The mean ages of YAs and OAs were 20.14 (SD = 1.39) and 63.19 (SD = 8.00), respectively. Eleven of the YAs and 12 of the OAs were female, and 8 of the YAs and 3 of the OAs reported pain. OAs had significantly higher heat pain threshold (HPT), higher pressure pain threshold (PPT), lower cold detection threshold (CDT), and lower cold pain threshold (CPT) than YAs (all $p < 0.05$). OAs had significantly different a-diversity indices including higher species of observed (Sobs), Chao index and Shannon index, and lower coverage, and significantly different beta diversity indices (Bray-Curtis, Jaccard, and Theta YC) than YAs. The significantly abundant taxa in YAs included Bacteroidaceae, Veillonellaceae, and Alcaligenaceae. Coriobacteriaceae, Streptococcaceae, Lachnospiraceae, Erysipelotrichaceae, and Verrucomicrobiaceae were significantly enriched in OAs. The Spearman correlation analysis showed that the CDT, CPT, and HPT were significantly associated with gut microbiota Sobs, Chao index, Shannon index, and coverage (all $p < 0.05$). But the directions and coefficients of correlations varied. OAs had distinguished peripheral sensation and gut microbiota profiles than YAs. The changed peripheral sensation and gut microbiota profiles among OAs may be biomarkers of aging-related pain processing.

Abstract citation ID: igad104.2583

### OLDER ADULT PERCEPTIONS OF USING IOT TO SUPPORT MEDICATION ADHERENCE AND COMBAT PHYSICAL INACTIVITY

Yong Kyung Choi[1], Shih-Yin Lin[2], Hilaire Thompson[3], and George Demiris[4], *1. University of Pittsburgh, Pittsburgh, Pennsylvania, United States*, *2. NYU Rory Meyers College of Nursing, New York City, New York, United States*, *3. University of Washington, Seattle, Washington, United States*, *4. University of Pennsylvania, Philadelphia, Pennsylvania, United States*

The literature has explored the potential of using the Internet of Things (IoT) to support aging in place, but less is known about its ability to motivate older adults for health behavior change. This study aimed to understand older adults' perception of using IoT to promote medication adherence and combat physical inactivity. We conducted a qualitative secondary analysis of exit interviews of 22 older adults (78% female; mean age: 77.8) who participated in an IoT feasibility evaluation study. In the feasibility study, older adults were asked to choose any combination of the following IoT devices, a door/window sensor, a multi-sensor (motion, light, humidity, temperature), an IP web camera, and a smart speaker, to be installed in their home for 2 months. Thematic analysis revealed that older adults identified creative uses of smart speakers to 1) update, log, and share medication lists/usage and physical activities; 2) set up medication and exercise reminders; 3) support diabetes self-management – taking the guesswork out of the equation (e.g., continuous blood glucose monitoring combined with a smart speaker to recommend the appropriate insulin dosage); 4) read out loud small-print medication labels (when also linked to a smart camera); 5) verbally direct older adults to reach their medications; 6) provide warnings to get up and move when prolonged inactivity is detected by the motion sensor; and 7) bring up music/video for exercise, or serve as a virtual exercise coach. In summary, older adults are receptive to using IoTs to promote medication adherence and reduce physical inactivity.

Abstract citation ID: igad104.2584

### PLANNING AHEAD FOR POSTHOSPITALIZATION DISCHARGE NEEDS AMONG OLDER ADULTS

Raven Relerford, Amber Miller-Winder, Allie Schierer, Charles Olvera, Alaine Murawski, Vanessa Ramirez-Zohfeld, and Lee A Lindquist, *Northwestern University, Chicago, Illinois, United States*

Frequently hospitalized older adults require assistance at discharge – either with transfer to a skilled nursing facility (SNF) or additional support at home. Our research has demonstrated that older adults can effectively plan ahead for their discharge needs (e.g., selecting SNFs) prior to hospitalization but some chose not to. Very little is known about what motivates older adults to plan. We sought to identify what variables impact older adults' post-hospitalization planning. We surveyed a cohort of non-hospitalized adults age 65+ who received PlanYourLifespan.org (PYL), a tool to help older adults plan for their post-hospitalization needs, and then completed follow-up surveys at one month and every 6 months thereafter. Surveys inquire about their post-discharge plans if they were to be hospitalized, specifically rehabilitation/caregiver preferences. Multivariate logistic regression models adjusted for baseline hospitalization decision-making, sex, race, self-efficacy, living status, cognitive impairment, and PYL use. 293 subjects were enrolled (mean 73.5 yrs,40.4% non-White;12 mo. retention rate 94.5%). Subjects were more likely to have plans if they had increased chronic conditions (OR 1.23;p>0.05,1.02-1.47), increased medications (OR 1.15;p< 0.01,1.07-1.25), power of attorney (OR 1.84;p< 0.05,1.01-3.38), and used PYL (OR 2.61;p< 0.01,1.45-4.72). Subjects were more likely to have plans if they had higher medical complexity (OR 1.13 [p< 0.05, 1.03-1.24]), limited health literacy (OR 3.13 [p< 0.05, 1.25-7.81]), and used the PYL website (OR 1.72 [P< 0.05,1.00-2.93]) when asked about planning for future post-hospitalization requiring help in the home. Higher medical complexity, limited health literacy, and use of the planning website were associated with increased hospitalization discharge planning.

Abstract citation ID: igad104.2585

### POTENTIAL PROTECTIVE EFFECTS OF INSTRUMENTAL ACTIVITIES OF DAILY LIVING ON ALZHEIMER'S DISEASE CHARACTERISTICS

Rachel McCray[1], Daisha Oruru[2], Clarence Locklear[2], Omonigho Bubu[3], and Arlener Turner[2], *1. New York University, New York City, New York, United States*, *2. University of Miami, Miller School of Medicine, Miami, Florida, United States*, *3. New York University, Grossman School of Medicine, New York City, New York, United Sta tes*

Previous research has shown the significance of self-efficacy in improving cognitive trajectory. Additionally, it has been demonstrated that impaired cognitive functioning is a predictor for low functionality in both basic and instrumental activities of daily living (iADLs). However, little research has been done on potential protective effects of iADL functionality on markers of Alzheimer's disease. To examine if iADL functionality (possibly through self-efficacy) has a protective effect on Alzheimer's disease characteristics in older adults, we assessed the relationship between functionality and hippocampal atrophy. This study utilized data from the National Alzheimer's Coordinating Center Uniform Dataset (NACC UDS) covering September 2005 to February 2021. Participants included in the analyses (N=2517, 65.7% women, 11.9% Black/African American, mean age 68.7, SD=9.15) were all cognitively normal. The analyses use baseline Functional Activities Questionnaire (FAQ) assessment, measures of independent living, and required assistance, in addition to presence or absence of Hippocampal Atrophy (measured ~5 years later) in two 2x2 contingency tables. While no relationship between baseline FAQ and later hippocampal atrophy emerged, a relationship between independent living status at baseline and hippocampal atrophy was demonstrated (X2{2,446}=11.38, p=0.003) with higher independence in iADL tasks associated with less atrophy. Though further research is needed, these preliminary results suggest living independently and having agency over iADL tasks may confer some neurocognitive protection and indicate the possibility of less hippocampal atrophy over time in individuals with higher levels of independence. Future studies should also explore the potential positive influence of iADLs on other characteristics of Alzheimer's disease like cognition.

Abstract citation ID: igad104.2586

### RECRUITING AND RETAINING RESIDENTIAL CARE COMMUNITIES DURING THE COVID-19 PANDEMIC: LESSONS LEARNED

Silvia Orsulic-Jeras[1], Ashlee Cordell[1], Sara Powers[1], Farida Ejaz[1], and Lisbeth Sanders[2], *1. Benjamin Rose Institute on Aging, Cleveland, Ohio, United States*, *2. LifeBio, Marysville, Ohio, United States*

Demands within residential care communities have escalated throughout the Covid-19 pandemic as meeting basic care needs has become more critical. Non-pharmacological interventions that aim to alleviate care strain and promote person-centered care are even more important now, as residents have been significantly impacted by social isolation and loss. This presentation highlights successes and challenges faced by researchers and residential care communities related to study recruitment and retainment efforts during the Covid-19 pandemic while conducting a fidelity trial of LifeBio MemoryTM: a novel app-based product designed to collect life story information from persons living with dementia (PLWD). The target sample is comprised of residential care staff (n=60) and PLWD (n=242) across ten residential care communities in the state of Ohio. Successes presented include: 1) high enthusiasm for the study and 2) strong relationships built between researchers and site liaisons. Significant challenges faced include: 1) organization-wide staff shortages, 2) low overall resident census, and 3) Covid-19 outbreaks and quarantines. Key lessons learned include: 1) researchers and residential care communities face competing demands as a result of the Covid-19 pandemic and 2) the Covid-19 pandemic has changed the landscape of residential care and research. The importance of efficient field operations, supportive training resources, and contingency plans when conducting studies in residential care will be discussed.

Abstract citation ID: igad104.2587

### THE CURIOUS CASE OF CONSCIENTIOUSNESS AND DEPRESSION: FINDINGS FROM THE IOWA UNMARRIED SURVIVORS STUDY .

Robert Hensley[1], Alexander Bishop[2], and Peter Martin[3], *1. Southeast Community College, Lincoln, Nebraska, United States*, *2. Oklahoma State University, Stillwater, Oklahoma, United States*, *3. Iowa State University, Ames, Iowa, United States*

The purpose of this study was to analyze the association between personality traits, coping, social support, stress, and loneliness relative to depression reported by N = 227 (M = 78.2, SD = 8.1) participants from the Iowa Unmarried Survivors Study. Using the Development Adaptation Model as a conceptual framework, a series of blocked multiple regression analyses were conducted. In the first block, age, gender, ethnicity, total years of education, and current marital status were included. The second block contained total life events and childhood poverty, whereas the third block included activities of daily living and total number of illnesses. The fourth block contained the aforementioned personality variables, and the fifth block included coping, social support, stress, and loneliness. Lifetime illnesses emerged as a significant predictor of depression, $\beta = .158$, $p < .01$. The more illnesses over one's lifetime, the higher the levels of depression.

In addition, both Neuroticism and Conscientiousness were significant predictors of depression, $\beta$ = .453, p < .00, $\beta$ = .139, p < .05 respectively. Finally, stress served as a significant predictor of depression, $\beta$ = .200, p < .01. This model explained 58% of the variance in depression scores. Being unmarried and navigating through late adulthood presents a challenge to sustaining positive mental health. Results from this study have implications relative to how clinically trained counselors, geriatric social workers, and aging service providers develop interventions and programming to improve mental health and quality of life for persons who remain unmarried throughout later life.

Abstract citation ID: igad104.2588

### THE NARRATIVES OF NURSING HOME RESIDENTS WITH PERSISTENT PAIN ABOUT THE PAINCQ-33

Timothy O'Connor, *Nova Southeastern University, Davie, Florida, United States*

Problem: Persistent pain in older adults, often multimodal, remains poorly managed among nursing home residents. The resident's perceptual barriers can interfere with how clinicians treat pain. Current pain instruments do not identify how older nursing home residents perceive interdisciplinary pain management care (IPMC). Purpose: This study explores the use of the Pain Care Quality (PainCQ-33) to measure how older nursing home residents with persistent pain perceive their IPMC and how these individuals communicate their persistent, multimodal pain to caregivers. Furthermore, this research determines how older residents prioritize their pain management expectations for maintaining their quality of life. Research design: A focus group design was used to explore residents' viewpoints about the ability of the PainCQ-33 to accurately measure the perception of the quality of their pain management care. Data analysis: Transcripts and field notes from focus group discussions were analyzed using descriptive content analysis. **Results:** The identified codes illustrate how pain interference affects the ratings an older resident assigns to their pain management care and expectations of whether their pain can be relieved. Additional codes demonstrate an older resident's pain-relieving measures and who they identify as helping to alleviate their pain. A theme from the codes emerged, where the relevance of pain management was based on the caring nature for chronic pain, which is the underpinning of the PainCQ-33 instrument. Significance: Clinicians, including nurses, can benefit from having a pain assessment tool uniquely designed to assess how this population perceives the quality of their pain management care.

Abstract citation ID: igad104.2589

### THE PREDICTIVE VALIDITY OF SUBJECTIVE SURVIVAL PROBABILITIES AMONG OLDER ADULTS WITH PAIN

Gillian Fennell, *University of Southern California, Los Angeles, California, United States*

Chronic pain, which afflicts 20% of US adults, is associated with heightened mortality risk and lower subjective survival probabilities (SSPs) to advanced ages. However, the accuracy of SSPs for individuals with chronic pain is unknown. To assess whether individuals with and without pain and pain-related activity interference report SSPs that are veridical to their actual lifespans, we exploited multi-wave data from the Health and Retirement Study. Baseline SSPs and covariate data were obtained from the 2000 wave among respondents aged 57-89 with and without pain (N=7,075). SSP reports were then compared to the age at which respondents actually died (if they did) using mortality data from exit interviews in subsequent waves. We conducted a multinomial logistic regression using baseline pain and covariates to predict membership into one of four groups summarizing the accuracy and valence of SSP assessments. The groups were: (1)correctly pessimistic, (2)falsely optimistic, (3)falsely pessimistic, and (4)correctly optimistic. Findings suggest that individuals with baseline pain were significantly more likely to be correctly pessimistic about their longevity with low, but accurate SSPs (falsely optimistic (RRR=.56, p<.001), falsely pessimistic (RRR=.65, p<.001), or correctly optimistic (RRR=.44, p<.001)). This study highlights that chronic pain and associated disability are features of health that individuals use to estimate their own lifespans with greater accuracy. Future research should assess the extent to which this connection is due to self-fulfilling prophecy.

### SESSION 7470 (POSTER)

### WHO CARES FOR THE CAREGIVERS?

Abstract citation ID: igad104.2590

### "WE DON'T TAKE CARE OF OURSELVES AS WELL AS WE NEED TO." A QUALITATIVE STUDY OF FAMILY CAREGIVERS' SELF-MANAGEMENT

Dawon Baik, *University of Colorado, Aurora, Colorado, United States*

Despite the importance of enhancing family caregivers' (FCGs) physical and mental health to prevent FCGs from becoming care recipients themselves and provide a better quality of care to their care recipients, FCGs are not likely to take care of themselves. In this study, we examined the physical and mental health status and challenges in self-management among older FCGs of persons with heart failure (HF-FCGs). We administered to 15 older HF-FCGs, a demographic survey, the Charlson Comorbidity Index, a health-related quality of life (HRQoL) instrument (SF-36) and conducted semi-structured interviews. Mean age was 65.9±4.4 years, eleven were female, and thirteen were spouses. Chronic diseases were reported among HF-FCGs as; 47% arthritis, 33% obesity, hearing impairment, and visual impairment, and 20% depression and anxiety. HRQoL ranged from 52.3 (±18.8) to 84.3 (±10.8) across the eight subscales. The Energy/Fatigue subscale had the lowest (poorest) score on the SF-36. The challenges in their own health care management were a lack of time and concerns related to symptoms worsening of persons with HF. Most participants were struggling to manage their mental health. They reported stress, anxiety, difficulty staying positive and balancing their mood. Other challenges included managing pain from surgeries (e.g., hip replacement). Many participants felt that they did not take care of themselves as much as they needed, and should receive education to improve their own mental, physical, and social health. Future studies should design and test tailored, caregiver-centered support interventions that enhance health and quality of life in the older caregiver population.

Abstract citation ID: igad104.2591

### CAREGIVING THROUGH COVID: POSITIVE ASPECTS OF CAREGIVING AND CAREGIVER STRESS OVER TIME

Haley Schneider[1], Maria Donohoe[2], Lena Thompson[3], and Sato Ashida[2], *1. University of Iowa, Iowa City, Iowa, United States*, *2. University of Iowa College of Public Health, Iowa City, Iowa, United States*, *3. University of Minnesota, Duluth, Minnesota, United States*

With an increase of age-related diseases due to population-aging, there are more caregivers in need of support services. Caregivers are known to experience poor health status due to caregiving burden, stress, and psychological impacts. Caregivers often experience care-related negative health consequences, and such experiences can be exacerbated during stressful times such as COVID-19 pandemic. The concepts of positive aspects of caregiving (PAC) help identify protective factors (sense of meaningfulness in role, familial cohesion, etc.) that have been shown to decrease negative health impacts and enhance caregiver satisfaction. This study included 43 caregivers of individuals diagnosed with Alzheimer's Disease or related dementias (ADRDs) who participated in a randomized-controlled trial (2020-2021) to test an intervention that provides caregiver education and referral to community-based services. A regression model was built using the data from baseline and three-month post-intervention surveys. Controlling for baseline COVID-19 distress levels and gender, an increase in COVID-19 distress from baseline to three months was significantly associated with lower levels of PAC at baseline (b=-0.337, p-value=0.029); there was also a trend for a decrease in PAC over three months (b=-0.300, p-value=0.058). Understanding how lower levels of PAC leads caregivers to experience higher stress levels during the pandemic likely will help identify the opportunity to address caregiver stress. Future studies may explore the strategies to enhance caregiver PAC and investigate how changes in PAC may impact caregiver wellbeing. Future interventions may benefit from incorporating the concepts of PAC to support and empower caregivers during stressful events like the pandemic.

Abstract citation ID: igad104.2592

### DEMENTIA CAREGIVER RE-ENTRY INITIATIVE: FINDING NEW DIRECTION AND PURPOSE AFTER CAREGIVING ENDS

Warren Wolfe[1], and Sheryl Fairbanks[2], *1. Freelance Journalist, Roseville, Minnesota, United States*, *2. University of Minnesota, Minneapolis, Minnesota, United States*

Many former dementia caregivers struggle to find a "new normal" when the long and exhausting caregiving journey ends. Grieving eases, the estate is settled, and now what? Who am I after caregiving ends? Following 12 years of intense care for their parents, two with dementia, Sheryl Fairbanks and Warren Wolfe searched nationally for a program that might help them restart their lives. They found none. In 2016, they launched the Former Dementia Caregiver Re-Entry Initiative, which has served more than sixty people. At a kick-off meeting, former caregivers said their deepest needs were reestablishing relationships with family and friends and finding new life purpose. Typically, six to eight former caregivers meet twice a month – online since the COVID pandemic - and focus on moving forward. They share that these meetings give them a renewed purpose, deeper social connections, increased energy and a new sense of self-esteem. "My kids care. My friends care," one member explained. "But nobody who hasn't lived it really knows how hard that time was, and how hard it is to find a new path." Four similar programs have started in Minnesota, The couple has written a book to encourage more. In 2022, Ms. Fairbanks and Mr. Wolfe became Community Leads in a University of Minnesota research project to see if Black former dementia caregivers have unmet needs and whether this model might help. Preliminary findings will be presented at the Addressing the Needs of African American Bereaved Dementia Caregivers symposium at this GSA annual meeting.

Abstract citation ID: igad104.2593

### EFFECTS OF BEING FORGOTTEN ON ADULT CHILDREN OF MOTHERS WITH DEMENTIA

Kristie Wood[1], and Marie-Anne Suizzo[2], *1. Colorado University, Anschutz Medical School, Denver, Colorado, United States*, *2. UT Austin, Austin, Texas, United States*

Mothers with dementia sometimes do not recognize their adult children. Yet, research on the effects of being forgotten by a mother with dementia is scarce. Object relations theorists posit that healthy development of a child's sense of self is linked to being remembered by a primary caretaker. Further, being held in mind by a primary caregiver allows the child to form a cohesive identity. It is unclear how adult children's identities are impacted by being forgotten by their mothers. To investigate this phenomenon, this qualitative study explored the effects of being forgotten on adult children in the context of dementia. The aims were: 1) to develop an in-depth understanding of what it means for adults to be forgotten by their mothers with dementia, and 2) to gain insight on the effect of being forgotten on adults' identities. Data were gathered through 12 interviews. Data was analyzed following an interpretative phenomenological analysis approach. Preliminary findings suggest adult children of mothers with dementia experience being forgotten as both a threat to their identity and forewarning of continued poor disease outcomes. The following themes illustrate this: 1) The sense that one's existence was not important enough to be remembered, 2) Feelings of failure due to the inability to spark recognition of themselves in their mother's mind, and 3) Dread for the disease progression. While research regarding adult children of parents with dementia largely focuses on practical aspects of caregiving, clinical implications of these findings suggest that this population suffers silently from being forgotten.

Abstract citation ID: igad104.2594

### ENGAGEMENT DURING POST–ACUTE CARE TRANSITION: PERCEPTIONS AND EXPERIENCES OF DEMENTIA FAMILY CAREGIVERS

Ruth Tadesse[1], Karen Lyons[2], Glenise McKenzie[1], Sara Hart[3], Lee Ellington[3], and Kristin Cloyes[1], *1. Oregon Health & Science University, Portland, Oregon, United States*, *2. Boston College, Boston, Massachusetts, United States*, *3. University of Utah, Salt Lake City, Utah, United States*

Hospitalized persons with dementia (PwD) are three times more likely to discharge to skilled nursing facilities (SNF) than those without dementia, and family caregivers are essential during post-acute care transitions (PACT). Descriptions of caregiver engagement are largely provider-centric, derived from either acute or residential settings. Little is known about PACT experiences of family caregivers. This study explored meaning, barriers, and facilitators of engagement for family caregivers navigating PACT, and elucidated caregiver support needs. An interpretive descriptive approach, using Meleis's Middle Range Transition Theory was used to interview family caregivers during a PACT. Data were inductively coded and thematically analyzed. A conceptual framework illustrating caregivers' meaning of engagement was developed, and facilitators, barriers, and needs were described. Participants (n=15), mostly white (n=12, 80%), female (n=13, 87%), married (n=7, 47%), adult children (n=11, 73%), average age of 63 (range: 38-81, SD=12.9). The core concept was advocacy. Key related themes were caregivers' sense of being present, communication with providers, and meaningful connections with the PwD. Barriers to engagement centered on poor communication from professional caregivers and being isolated from the PwD. A facilitator to engagement was effective communication with professional caregivers. Similarly, caregivers' support needs included receiving information related to the transition, and the health status of the PwD. Communication was central to both caregiver engagement and advocacy of PACT. Future policy/practice recommendations should include communication training for both providers and caregivers. Interventions that facilitate caregivers' shared access and input into health information during PACT will promote integral roles on the care team.

Abstract citation ID: igad104.2595

### EXPLORING THE IMPACT OF CHORAL SINGING ON CHANGE IN PERCEIVED DISTRESS FOR CARE PARTNERS OF PERSONS WITH DEMENTIA

Stuart MacDonald, Nicholas Tamburri, Cynthia McDowell, Andre Smith, Carren Dujela, and Debra Sheets, *University of Victoria, Victoria, British Columbia, Canada*

Family care partners (CP) of persons with dementia (PwD) report high levels of psychological distress that impact many facets of their lives. Arts-based interventions have shown promise for improving psychological health. Here, we describe findings from the Voices in Motion (ViM) study, a weekly lifestyle intervention for CP-PwD dyads, with specific emphasis on examining the impact of choral singing on level and change in perceived distress for CP. Data from n=29 CP (Mage=67.7 years, 80% female) were used to examine change in caregiver distress, indexed using the Zarit Caregiver Burden Interview (ZBI). ViM employed an intensive longitudinal design characterized by monthly retests embedded within 3.5-month choral seasons (up to 7 assessments per person). Choral seasons were separated by a 4-month summer break, yielding a naturalistic ABA experimental design optimal for evaluating the impact of choral singing on caregiver distress. Multilevel models of change with piecewise splines were fit; REML was employed due to its advantageous properties for small samples. A significant reduction in caregiver distress (-1.14 units per month, $p<.01$) was observed across the 3.3-month span of season 1. Following the 4-month summer break, level of caregiver distress at the outset of season 2 had increased back to initial baseline levels (ZBI =19.9 units – a moderate to borderline-high level), thereby underscoring the importance of the choir intervention. These findings suggest that lifestyle engagement may offer an effective complement for lessening the impact of caregiver distress for CP, with implications for the development of dementia care plans.

Abstract citation ID: igad104.2596

### HOW CAREGIVERS DIVIDE RESPONSIBILITIES: A LATENT CLASS ANALYSIS OF US CAREGIVING ARRANGEMENTS

Erin Ice, *University of Michigan, Ann Arbor, Michigan, United States*

Family and other unpaid caregivers provide the bulk of caregiving for older adults, but we know little about how kin divide responsibilities amongst themselves, and how they share tasks with paid caregivers. This study identifies the common "care configurations" that emerge to support older adults with functional limitations. Studying older adults (age 65+) who received assistance from a caregiver in the National Health and Aging Trends Study Wave 5 (n = 4,182), I generate an extensive battery of caregiving arrangement indicators, including household, physical care, transportation, and administrative assistance provided by spouses, children, other unpaid kin, or paid caregivers. I use latent class analysis and adjust for survey design to identify common configurations. I find four primary care configurations. In the most common configuration (42%), partners provided all assistance and most often helped with household chores. Conversely, children often assisted with transportation while sharing other responsibilities with paid caregivers. Older adults with limited physical capacity, probable dementia, and Medicaid were most likely to belong to the only configuration featuring paid caregiving. By studying the full population of older adults who receive care assistance, this study provides a broad perspective on how older adults rely on unpaid kin caregivers and when they rely on paid caregiving. In particular, it identifies transportation assistance as an overlooked form of caregiving and demonstrates that children are most frequently dividing responsibilities with other kin or paid caregivers.

### SESSION 7480 (POSTER)

### PHYSICAL AND MENTAL HEALTH EDUCATION AND INTERVENTIONS

Abstract citation ID: igad104.2597

### A SCOPING REVIEW OF SYSTEMS-LEVEL INTERVENTIONS FOR FALL PREVENTION IN LONG-TERM CARE

Andrew Ustach[1], Dahee Wi[2], and Hilaire Thompson[1], *1. University of Washington, Seattle, Washington, United States*, *2. University of Illinois Chicago, Chicago, Illinois, United States*

Older adults residing in long-term care facilities (LTCFs) are at high risk for falls. In addition to individual resident-level interventions to prevent falls and fall-related injury (FRI), system-focused interventions are also necessary to adequately address fall prevention. Relatively little attention has been given to research involving systems-focused interventions. We aimed to synthesize studies on the effects of system-focused interventions for fall and FRI prevention in LTCFs with the goal of identifying promising strategies and gaps. We searched Medline, CINAHL, and EMBASE databases from 2017 to 2022 following the Preferred Reporting Items for Systematic reviews and Meta-Analyses extension for Scoping Reviews guideline. We conducted a narrative synthesis to summarize included studies. In the initial review, 301 studies were identified and underwent title and abstract screening resulting in 98 articles were retrieved for full-text review. Fifteen studies were included in data extraction. Fall prevention interventions evaluated in LTCF settings include: (1) multicomponent fall prevention programs, (2) staff education programs, (3) training in safe handling/transfers, (4) environmental adaptations, (5) electronic health record algorithms and prompts, (6) local culture change, (7) video analysis of falls to change local interventions, and (8) facility-level physical activity programs. About half of reported studies reported significant effects of system-focused interventions to reduce fall and FRI in LTCFs. Multicomponent fall prevention programs and physical activity interventions are the most effective systems-level interventions, while electronic health record interventions were the least effective. Overall, little attention has been given in the literature to evaluation of environmental adaptations at the systems level.

Abstract citation ID: igad104.2598

### ARTIFICIAL INTELLIGENCE IN MENTAL HEALTH FOR THE AGING POPULATION: A SCOPING REVIEW

Duo Helen Wei[1], Bianca Hernandez[2], and David Burdick[1], *1. Stockton University, Galloway, New Jersey, United States*, *2. Rutgers University, Galloway, New Jersey, United States*

Artificial intelligence (AI) has significant potential in mental health research and service delivery for the aging population. This poster provides an overview of its evolving utilization, including research methods, data sources, and predictors of mental health. The review of 26 articles from 2015 to 2023 found that surveys/questionnaires, clinical data, non-clinical data, and electronic health records are the most commonly used data sources, with clinical data being the most common, accounting for more than 50% of the articles. The frequently utilized AI techniques in mental health research for older adults were supervised learning (65.4%), deep learning (42.3%), unsupervised learning (7.7%), NLP (7.7%), and semi-supervised learning (3.8%). Mental health factors that received the most attention in the research were anxiety/depression (65.4%), dementia (Alzheimer's) (38.5%), mild cognitive impairment (23.1%), suicidal ideation (15.4%), and trajectory of cognitive decline (7.7%). AI is currently employed in various aspects of mental health research for older adults, including identifying and diagnosing mental health conditions at an early stage, monitoring mental well-being, assessing the severity of mental health conditions, predicting cognitive trajectories, and providing assistance in treatment. The recent application of generative language models, such as ChatGPT, has further advanced the field of AI in mental health research. However, ethical concerns such as bias, privacy, and transparency must be addressed before widespread implementation. In conclusion, AI has great potential as well as risks and ethical concerns for mental health research and service delivery to aging populations, but further research is needed.

Abstract citation ID: igad104.2599

### ATTITUDES TOWARD AGING: EDUCATIONAL INTERVENTIONS FOR MEDICAL STUDENTS

Bronwyn Keefe, *Boston University, Boston, Massachusetts, United States*

Ageism is stereotyping, prejudice, and discrimination based on a person's age, and in healthcare includes the implicit biases of providers. Like many other forms of prejudice, ageism contributes to health disparities and differences in health outcomes. The implicit and explicit biases of clinicians can impact clinical practice, leading to medical decisions based on stereotypes and prejudices. There is inconclusive evidence on undergraduate and graduate medical learners' attitudes towards aging. Some studies have found that learners hold negative associations with aging, while others have found a mix of positive and negative views toward caring for older adults. Educational interventions and intergenerational contact are two important strategies for increasing awareness and reducing ageism. Hence, it is important to discuss ageism with students and create programs and curricula to combat ageism to ensure the best care is provided to all of our patients. This session will present results from an interactive online module designed for 4th-year medical students that explores ageism and bias. Students completed the Expectations Regarding Aging Survey pre-post. Preliminary results at pre-test show that 70% believe that as they get older, they will be more forgetful and 75% believe the human body is like a car: when it's old, it gets worn out. Healthcare professionals need to understand the existing gaps in health equity that our older adult patients experience that contribute to ageist attitudes. By drawing attention to professionals' implicit and explicit biases towards aging throughout medical training, we are able to help older adults receive better care.

Abstract citation ID: igad104.2600

### DEVELOPING AND IMPLEMENTING A NATIONWIDE CURRICULUM FOR AGING STUDIES. WHAT ARE THE KEY CONSIDERATIONS FOR GHANA?

Grace Karikari[1], and Samuel Asante[2], *1. University of North Dakota, Grand Forks, North Dakota, United States*, *2. Northeastern State University, Broken Arrow, Oklahoma, United States*

In 2019, Ghana reported the implementation of a Geriatric Medicine Fellowship Program within the Faculty of Family Medicine at the Ghana College of Physicians and Surgeons (GCPS). Though this is a laudable effort to address the specialized workforce shortage the country is facing, the GCPS program is meant to provide an in-country specialization path for physicians desiring to become geriatricians. There are currently no national standardized aging studies, training programs or curricula available to other students in disciplines such as nursing, psychology, public health, and social work. Students in the public, government-owned tertiary institutions are offered isolated courses related to aging and the needs of older adults. To effectively address the workforce shortage issue and improve the overall health and wellbeing of older adults in Ghana, it is imperative to train students in diverse fields and disciplines with the needed knowledge and skills to provide the various specialized services for the aging population. This presentation highlights a proposal to the government of Ghana to consider some key issues and recommendations related to developing and implementing a nation-wide aging studies programs in the public higher institutions. Five key evidence-based areas are extensively discussed: (1) Aging studies in Ghana should be culturally relevant. (2) Aging studies in Ghana should address the needs of older Ghanaians. (3) Aging studies in Ghana should be competency-based. (4) Aging studies in Ghana should be implemented and evaluated by expert-educators. (5) Aging studies in Ghana should be attractive to potential service providers.

Abstract citation ID: igad104.2601

### DISPARITIES IN MOCA AND MMSE SCORES AMONG DIVERSE OLDER ADULTS: A CASE ANALYSIS OF CREOLE-SPEAKING PARTICIPANTS

Marie Adonis-Rizzo[1], Ruth Tappen[2], Monica Rosselli[3], David Newman[2], Somi Panday[4], and Joshua Conniff[2], *1. Florida Atlantic University, Lake Worth, Florida, United States*, *2. Florida Atlantic University, Boca Raton, Florida, United States*, *3. Florida Atlantic University (FAU), Boca Raton, Florida, United States*, *4. Florida Atlantic, Boca Raton, Florida, United States*

Almost 11% of older adults in the US have Alzheimer's or related dementia. Most studies suggest that those who are Black or Hispanic are at higher risk. A culturally diverse sample, including Haitian older adults, was recruited from South Florida communities to participate in a longitudinal study, "In-Vehicle Sensors to Detect Change in Cognition of Older Drivers." An extensive cognitive battery was administered for comparison with driving behaviors. No validated translations in Creole were available, so three Haitian-born examiners translated and back-translated test items into Creole. Two additional bilingual examiners verified the results. An analysis of variance (ANCOVA) comparing Haitian participants (N=12) and European Americans (N=135) on the MoCA controlling for age and education indicated the European American group scored higher (M = 25.78, SD = 2.57) than the Haitian Group (M =24.17, SD = 3.01), a significant difference in test performance, F (1, 124) = 6.11, P= .015. Results on four subscales of the MoCA evidenced significantly higher scores for the European American group: Visual EX Total, F (1, 124) = 22.52, p < .001; Serial-7, F (1, 124) = 12.09, p <.001; Attention Total, F (1, 124) = 5.10, p <.001; Letter Score F (1, 124) = 13.09, p <.001. No differences in language, abstract, delayed recall, or orientation were found. These preliminary results suggest cultural influences may account for differences in test performance. Further investigation with a larger sample of Haitian older adults is needed to explain differences in performance on this widely used test.

Abstract citation ID: igad104.2602

### HOME CARE AND QUALITY OF LIFE AMONG COMMUNITY-DWELLING FRAIL OLDER ADULTS IN ISRAEL: A MULTIPLE MEDIATOR MODEL

Rabia Khalaila, *Zefat Academic college, Zefat, HaZafon, Israel*

The aim of the current study is to examine the association between two types of home care provision (combined formal and informal home care vs. family-only home care) and quality of life (QoL) of older adults, as well as the mediating effects of loneliness, social isolation and satisfaction with family relationships and support. A convenience sample of 360 Israeli adults aged 65+ responded to questionnaires. Using bootstrapping, we tested the strength and significance of the conditional indirect effects of the four simultaneous mediators. The results showed that combined formal and informal home care (vs. family-only home care) for older adults was associated with lower QoL of care-recipients, and was fully mediated by feelings of loneliness, social isolation, and low satisfaction with family support. Satisfaction with family relationships did not emerge as a mediator but was positively associated with QoL. Formal home care might reduce the QoL of care-recipients by increasing their feelings of loneliness, social isolation and reduced perceived family support. Practitioners should encourage family members to continue with family regular support and contact alongside the use of formal home care in order to maintain satisfaction and QoL of older relatives.

Abstract citation ID: igad104.2603

### HOPE—A UNIQUE MODEL TO PROVIDE ORAL HEALTH SERVICES FOR NURSING HOME RESIDENTS

Sohini Dhar[1], Rochisha Singh Marwaha[2], and Suman Challa[1], *1. UT Health San Antonio School of Dentistry, San Antonio, Texas, United States*, *2. UTHSCSA, San Antonio, Texas, United States*

Optimal oral health care is an essential component of primary health care for all individuals but especially among the older adults due to chronic health issues and polypharmacy. Moreover, there are several barriers to oral health care for the older adults that include but are not limited to cost of dental care, lack of perceived need for care, dearth of transportation, and fear for dental care. To address these barriers to access, UT Health San Antonio School of Dentistry launched Holistic Oral Health Program for Elders (HOPE) program that aims to address oral health needs of nursing home residents living within a 65-mile radius of San Antonio, TX. The HOPE team is formed of trained dental and public health professionals who work together as a group to ensure that preventive, palliative, and clinical oral health services are provided to all eligible residents of the nursing home. For successful implementation of the HOPE program, the team efficaciously completed several critical steps while planning and developing the program, such as- establishing a relationship and procuring contracts with several nursing homes, collaborating with social workers and nurses, and following an interprofessional approach to provide oral health care to the nursing home residents. Since its inception, the HOPE program has successfully signed contract agreements with several nursing homes and has addressed the oral health needs of more than 300 nursing home residents. The HOPE program aims to continue serving this vulnerable and underserved population to address their oral health needs.

Abstract citation ID: igad104.2604

### INTEGRATING PRIMARY CARE AND COMMUNITY-BASED PROVIDERS IN AN INTERPROFESSIONAL TEAM FOR OLDER ADULTS

Denise Kropp[1], Danielle Clay[2], Susan Fosnight[3], Jennifer Drost[4], Michelle Hughes[5], Lorrie Warren[6], and Jess Wilson[7], *1. Northeast Ohio Medical University, Rootstown, Ohio, United States*, *2. Western Reserve Area Agency on Aging, Cleveland, Ohio, United States*, *3. Summa Health System, Akron, Ohio, United States*, *4. Summa Health Systems, Akron, Ohio, United States*, *5. Direction Home Akron Canton Area Agency on Aging & Disabilities, Uniontown, Ohio, United States*, *6. Summit County Probate Court, Akron, Ohio, United States*, *7. Community Health Care, Copley, Ohio, United States*

The integration of healthcare and community-based providers has been long-recognized as the gold standard in the optimal care of older adults. Social determinants of health account for more than 80% of health outcomes. Therefore, optimal care requires meaningful collaboration between medical and community-based care providers. We present here a collaborative model of care integrating community providers and healthcare system. This is a long-standing collaborative approach where our Area Agency on Aging care managers present their complex cases to a team, which includes nursing, social work, geriatric medicine, and geriatric pharmacy. This team meets weekly for case presentations and interprofessional care planning. We have recently expanded these meetings to extend the reach of the team to remote Area Agencies on Aging, to ACO care managers, to a local FQHC, and to a local Habitat for Humanity affiliate for the Aging in Place initiative. Since September 2022 this expanded team reviewed 12 consumers. On average there were 4.25 medication issues identified (maximum=13, minimum=0). The team addressed the following in at least half of consumers: falls (58%), depression/anxiety (50%), advance care planning (67%), function (50%), caregiver support (58%), and dementia (50%). Additional issues included mobility (42%), finances (42%), pain, (33%), elder abuse (25%), safety (17%), behavioral health (17%), sleep (17%), spiritual care (17%), language barriers (17%), and substance abuse (8%). This team model has been shown to be replicable across provider settings. Expansion of this model extends the limited resources of geriatricians and geriatric pharmacists.

Abstract citation ID: igad104.2605

### INTERVENTIONS FOR SENSE OF COHERENCE AMONG OLDER ADULTS WITH CHRONIC DISEASE: A SYSTEMATIC REVIEW AND META-ANALYSIS

Yaqian Liu, Angela Y. M. Leung, and Jed Montayre, *1. The Hong Kong Polytechnic University, Hong Kong, Hong Kong*

**Background:** Sense of Coherence (SoC) is a core concept of "salutogenesis" in positive psychology, correlated with emotional distress and disease development in older adults with chronic disease (OACD). A diversity of interventions has been developed to enhance SOC, but research findings are conflicting and only a few studies have reviewed the effectiveness. Objective: This paper aimed to explore appropriate interventions and evaluate the effectiveness of these SoC interventions on OACD. **Methods:** Search terms including 'sense of coherence', 'older adult' and 'randomized controlled trial' were performed in nine electronic databases and two grey literature sources. Publications were written in English from January 1979 to January 2023. A narrative synthesis was performed to determine intervention details, and classical meta-analysis was used to analyze available data on SoC using RevMan. **Results:** 27 RCT studies were identified, involving salutogenic-based program, cognitive behavior treatment, and self-management intervention. Meta-analysis was carried out with 21 RCTs involving 1,687 patients. It showed significant effects of all interventions on SoC, compared to usual care, among OACD (SMD=0.19, 95%CI [0.09, 0.28], p<0.001) and at 3-month follow-up (SMD=0.14, 95%CI [0.03, 0.24], p<0.001), but no significant difference was observed at 6-month or 12-month follow-up (SMD=0.12, 95%CI [-0.13, 0.43], p=0.07; SMD=0.15, 95%CI [-0.27, 0.47], p=0.09). **Conclusions:** Interventions for SoC were shown to be effective right after the intervention and at 3 months. However, its effects were not sustained over a longer period. Further research is needed to explore optimal duration and components of interventions for SoC in this population.

Abstract citation ID: igad104.2606

### PERSPECTIVES OF SPEECH LANGUAGE PATHOLOGISTS AND STUDENTS ON PROVIDING CARE TO PEOPLE WITH DEMENTIA

Makenna Green[1], Jamie Mayer[1], and Laura White[2], *1. Northern Illinois University, DeKalb, Illinois, United States*, *2. University of South Alabama, Mobile, Alabama, United States*

This scoping review aimed to explore the extant literature on the experiences and views of speech-language pathologists (SLPs) and SLP students regarding the provision of care to people living with dementia (PLWD). A systematic search was conducted using 10 databases. Sources were included if participants were practicing SLPs and/or students enrolled in undergraduate communicative disorders or graduate SLP programs, and if the concepts of experiences or views on the provision of SLP services to PLWD were explored in the context of any clinical or educational setting. Included sources were systematically extracted for pertinent study characteristics, including SLP roles and settings, concept domains, measures utilized, and facilitators/barriers to effective dementia care. The majority of the 29 included sources were published in either academic journals (n=20) or professional organization publications (n=5) and used a cross-sectional study design (n=19). Participants included SLPs (n=22), and masters (n=6), undergraduate (n=3 studies), and doctoral students (n=1). The included studies addressed five primary conceptual domains: experiences, attitudes, roles, knowledge, and confidence. The most commonly addressed barriers and facilitators of effective dementia care were education and training. Mapping and analysis of the current body of knowledge within this scoping review illuminated several knowledge gaps that we propose need to be addressed to meet the education and training needs of SLPs to provide optimal care to PLWD. These include addressing core competencies, systematically measuring educational intervention outcomes, and developing more evidence-based education and training programs both within and outside of an interprofessional collaborative context.

Abstract citation ID: igad104.2607

### THE IMPACT OF COVID-19: IDENTIFYING WELLNESS STRATEGIES FOR ACADEMIC GERONTOLOGY AND NURSING FACULTY AND STAFF

Katarina Friberg-Felsted, Cheryl Armstrong, Jennifer Macali, Sara Simonsen, and Jennifer Clifton, *University of Utah, Salt Lake City, Utah, United States*

The continually evolving nature of the COVID-19 pandemic has presented challenges for individuals working in all sectors of the population, and academia is no exception. The nursing workforce is aging: the average age of a nurse is 52; 55% of the RN workforce is age 50 or older, and the average age of an employed nursing faculty is 46 years old. To date, little is known about the impact of the pandemic on individuals working in gerontology or nursing academia. The purpose of this study was to determine the impact of the COVID-19 pandemic on faculty and staff in a College of Nursing (CON) with a Gerontology program and identify practical strategies to promote health and wellness. A mixed-method survey was administered to faculty (n=140) and staff (n=139) in a College of Nursing in the Intermountain West. Quantitative and qualitative data were collected through a convenience sample. The researchers asked respondents to rate their experiences working from home, including the effects on work-related factors and social-lifestyle factors. Descriptive statistics were used to analyze the data. A total of 139 respondents completed the survey (faculty, n=86; staff, n=53). Respondents identified areas that were better and worse with regard to both work-related and social-lifestyle factors. Further, practical strategies to promote health and wellness were also identified. Finally, themes regarding support from leadership were recognized. This presentation will discuss these three results. These findings highlight the pandemic impact on aging nursing faculty and staff and introduce valuable strategies for promoting health and wellness.

Abstract citation ID: igad104.2608

### THE NET ON THE RIVER WHERE VERY LITTLE HAS BEEN CAUGHT: DIFFERENCES IN MEMORY FOR DISCOURSE IN YOUNGER AND OLDER ADULTS

Marjorie Getz, *Methodist College, Peoria, Illinois, United States*

Experiential learning is key for courses in a gerontology certificate program at a midwestern nursing and health science college. One curricular emphasis is the uniqueness of every older adult that the student will encounter as they move from a novice student role to professional role mastery several years into their careers. To assist in this transition, one course has a unit on memory for discourse, especially memory for printed materials—essential in support of understanding medical conditions and treatment follow-through. Our project had college students compare their memory for short stories with memories of "students" from an OLLI Program who attended this class. The project, in an adult development course, provides students with community service, direct contact with older adults and reflective practices to integrate course content with this activity. Qualitative analyses were used. Content analyses of reflective essays identified four themes (a) older adult focus on key story propositions as well as incidental propositions with tie-in to acquired knowledge on attention/memory from course work (b) frequent use of situational schema based on greater world knowledge held by older adults and implications of this type of discourse in clinical settings (c) differences, that favored older adults, between groups in memory for details in structured versus unstructured stories, and (d) differences in style of recall for stories between age groups. Reflections were encouraged to tie new age-related knowledge about memory processes into better client follow through. Students also reflected on ways to make memory more optimal in clinical settings.

### SESSION 7500 (POSTER)

### FACTORS THAT INFLUENCE AGE FRIENDLINESS

Abstract citation ID: igad104.2609

### A MODEL FOR TEACHING STUDENTS AGE-FRIENDLY CONCEPTS USING VIRTUAL INTERPROFESSIONAL SIMULATION

Diane Brown[1], Cynthia Hovland[2], Susan Fosnight[3], Brandi Chrzanowski[4], Mary Gergis[2], Rikki Patton[5], and Darcia Simpson[6], *1. University of Akron, Akron, Ohio, United States*, *2. Cleveland State University, Cleveland, Ohio, United States*, *3. Summa Health System, Akron, Ohio, United States*, *4. Direction Home Akron Canton Area Agency on Aging & Disabilities, Uniontown, Ohio, United States*, *5. Drexel University, Philadelphia, Pennsylvania, United States*, *6. Hospice of the Western Reserve, Cleveland, Ohio, United States*

Achieving effective Age-Friendly Health Systems requires teaching the future healthcare workforce how to consistently use the 4Ms framework in the care of older adults: What Matters, Medication, Mentation, and Mobility. Effective interprofessional teamwork is key to operationalizing this model. We describe here a virtual interprofessional student simulation training that demonstrates the importance of teamwork in addressing each of the 4Ms concepts and, importantly, how inattention to one 'M' can lead to ineffective care. Our training prepares learners with online didactic pre-work followed by a virtual simulation experience where interprofessional groups of students simulate a team care planning meeting. The case demonstrates how 'what matters most' to the patient (i.e., providing care for her husband who has dementia) impacts each of the other Ms. Specifically, she is taking medications inappropriately, has symptoms of depression, and will not undergo recommended surgery to correct mobility issues due to concerns about who will care for her husband. We have conducted this training with 548 undergraduate and graduate students representing medicine, nursing, pharmacy, social work, nutrition/dietetics, counseling, physical, occupational and speech therapy. 95% of students rated the simulation experience positively. There was statistically significant improvement in appreciation for teamwork ($p< 0.001$), evidence-based practice ($p< 0.001$), and inclusion of the patient and caregiver perspectives ($p< 0.001$) comparing pre and post simulation survey results. These results indicate a positive impact on student's preparedness to incorporate 4Ms principles into their future practice, thus advancing both Age Friendly and Dementia Friendly Health System initiatives.

Abstract citation ID: igad104.2610

### ASSESSING "AGE-FRIENDLINESS" ON CAMPUS: LESSONS FROM GEORGIA STATE UNIVERSITY 50+ STUDENTS

Grace da Rosa, Mariam Qreshi, Jacqueline Laures-Gore, Hyunji Kim, and Jennifer Morgan, *Georgia State University, Atlanta, Georgia, United States*

Older students (aged 50+) are an important, yet underrecognized group on today's college campuses. Many campuses lack age-friendliness, which deters older students from enrolling and creates barriers for these students. In an effort to better understand age-friendliness of Georgia State University (GSU) campuses, we conducted a survey in Fall 2019 (n=411; 39% response rate). The survey assessed motivations for attending school, challenges on campus, perceptions of how the university is currently addressing their needs, factors/resources that helped adjustment to school, and the extent to which they experienced age discrimination on campus. Of the respondents, 45% were undergraduates (50% identified as Black; 38% identified as White); 41% were graduate students (29% identified as Black; 62% identified as White), and 14% were non-degree students (24% identified as Black; 62% identified as White). Thematic analysis of the responses suggest that the main challenges experienced by GSU older students are course-related (e.g., course load, bureaucracy getting enrolled, limited class options, scheduled course times, course modalities) and time-related (e.g., balance between school, work, and family). For example, students noted, "difficulties finding study groups/lab partners due to age differences" and "administrators not taking older students serious." Technology barriers were pervasive in the open responses. Respondents further identified faculty/instructors as key resources for overcoming barriers. The survey served as an initial needs assessment to answer institutional questions informing the journey to becoming designated as an Age Friendly University. Implications for increasing age inclusivity and supporting older students will be discussed.

Abstract citation ID: igad104.2611

### BUILDING AN AGE-FRIENDLY ECOSYSTEM THROUGH NEIGHBORHOODS, HEALTH SYSTEMS, AND CAMPUSES

Valerie Greer[1], Terry Fulmer[2], Ashley Cadiz[1], Christopher Hernandez[1], and Linda Edelman[1], *1. University of Utah, Salt Lake City, Utah, United States*, *2. The John A. Hartford Foundation, New York City, New York, United States*

The age-friendly movement grew from an idea into a social movement recognizing the importance of adapting multiple facets of society to meet the needs of older adults. The movement integrates multidisciplinary work in research, policy, planning and design to build age-friendly ecosystems. Age-friendly ecosystems comprised of communities, health systems and campuses can become platforms for equity, health, and wellness for older adults. We designed a virtual symposium to examine age friendliness from a place-based approach by examining neighborhoods, campuses and health environments as sites uniquely positioned to catalyze equity in aging. The symposium was convened by national experts and regional aging stakeholders to consider how age friendly environments can address urgent social, environmental and health challenges, and create measurable outcomes that promote aging well for all. Knowledge was generated through lectures, panel discussions, a world café, focus group discussions, and a student-ideas competition. There were 113 attendees, including 27 health professions students; 78% of attendees indicated their work focused on aging/older adults and 52% reported specialized training in geriatrics/gerontology. Attendees reported increased familiarity with age-friendly concepts post-symposium. Key findings indicated the benefits of shared language and metrics across place-types that can collectively transform the social, physical, and economic landscape of aging. Symposium outcomes include a website, graphic facilitation, a collaboration resource guide, and a forthcoming book. Outcomes are serving as a foundation to radically re-imagine cross-sector collaborations between communities, health systems and campuses to create an ecosystem that embraces longevity as an asset and advances equity in aging.

Abstract citation ID: igad104.2612

### DESIGN AND DEVELOPMENT OF VIRTUAL REALITY APPLICATION: AGE-FRIENDLY HEALTH SYSTEM SCENARIO

Samrah Mitha[1], Marie Dezine[1], Sweta Tewary[1], Vincent Guida[1], Hadi Masri[1], Naushira Pandya[2], and Elizabeth Oviawe[1], *1. Nova Southeastern University, Davie, Florida, United States*, *2. Nova Southeastern University, Kiran Patel College of Osteopathic Medicine, Davie, Florida, United States*

With an increase in the aging population, evidence-based practice education can help strengthen knowledge, skills, and clinical practice for healthcare students caring for older adults. Age Friendly Health Systems (AFHS) is one of the frameworks founded on providing evidence-based and low-risk care centered on what matters most to older adults, their families, and caregivers. While didactic education is still the primary force in teaching medical students, innovative methods are being included in the medical curriculum to practice competency. Virtual Reality (VR) platforms are popular due to their ability to provide an immersive hands-on learning experience resembling an actual medical practice. The immersion abilities engage the students' sensory organs promoting an innovative way of learning concepts that are difficult to teach in real life. The project's objective was to develop and publish a case highlighting AFHS that can help educate medical students on the 4Ms (Mobility, Mentation, Medication Management, and What Matters). The case was completed in multiple phases 1) using a design document modified through multiple edits and reviews by our interprofessional team; 2) technical development including environment, objects, and interfaces of the application; and 3) publication. The VR case displays four scenarios where an elderly patient is admitted for a hip fracture. Students learned how to triage and treat patients from admission to discharge while demonstrating their knowledge of AFHS. The case is integrated in the geriatric curriculum for medical students, and the education is ongoing. Future studies will evaluate the learning outcomes of medical students.

Abstract citation ID: igad104.2613
### HOW FILIAL PIETY INFLUENCES THE LINKS BETWEEN ELDER ABUSE, SOCIAL WELL-BEING, AND DEPRESSION AMONG CHINESE SENIORS

Xin Sun[1], and Zi Yan[2], *1. Fudan University, Shanghai, Shanghai, China (People's Republic)*, *2. Waseda University, Tokyo, Tokyo, Japan*

**Objectives:** Based on Interactive Biopsychological Model, this study examined how multi-dimensional social well-being and filial piety influence the association between elder abuse and depression among older adults in China. **Methods:** The participants were 7,700 older adults (aged 60 years or older) enrolled in the 2018 China Longitudinal Aging Social Survey, a national population-based study of older adults. Moderated mediation models were applied to test the mediating effects of multidimensional social well-being (social isolation, loneliness, social network, and social participation) and the moderating effect of filial piety. All mediation and moderated mediation effects were estimated using SPSS.26. **Results:** Different dimensions of social well-being have a partial mediating effect on the association between elder abuse and depression. Traditional attitudes towards filial piety exacerbated the effects of elder abuse on depression by improving social isolation and loneliness while reversing the negative effects of elder abuse on depression through improving the social network. The interaction of elder abuse and filial piety has no significant influence on social participation. **Conclusions:** Although particular sociodemographic factors are associated with a greater risk of depression for elder abuse victims, our findings suggested that victims with traditional attitudes towards filial piety are more likely to feel isolated subjectively (lonely, worse social adaptation) and more depressed. This should be taken into consideration when developing culturally sensitive identification, prevention, and intervention services for older adults.

Abstract citation ID: igad104.2614
### INTEGRATING AGE-FRIENDLY CARE INTO THE ONCOLOGY CONTINUUM

Bianca Alvarez[1], Elana Plotkin[2], Leigh Boehmer[2], Kristine Swartz[3], Samuel Sestito[4], and Ginah Nightingale[3], *1. Association of Community Cancer Centers (ACCC), Chicago, Illinois, United States*, *2. Association of Community Cancer Centers (ACCC), Rockville, Maryland, United States*, *3. Thomas Jefferson Univserity, Philadelphia, Pennsylvania, United States*, *4. Vanderbilt University Medical Center, Nashville, Tennessee, United States*

The number of older adults diagnosed with cancer is steadily rising, and measures for equitable, high-quality care in oncology are relatively undefined. The Association of Community Cancer Centers (ACCC), in collaboration with the Institute for Healthcare Improvement (IHI), led the first oncology-focused cohort guided by the 4Ms Framework for Age-Friendly Health Systems. Representatives from 22 cancer programs across the United States participated in a learning and action period to collaborate on building a program that provides optimal care for the older adult population. The Action Community consisted of a six-part didactic webinar series led by subject matter experts in geriatric oncology to apply the 4Ms model: What Matters, Mobility, Mentation, and Medication. ACCC asked sites to evaluate their program's alignment with current standards using the ACCC Geriatric Gap Assessment tool and IHI's 4Ms Care Description. Throughout the webinar series, participating sites in hospital and ambulatory settings identified a multidisciplinary team to improve workflows. The respondents answered questions regarding the frequency of assessments, documentation, and addressing inequities in their cancer program. Through a webinar poll, 90% of the participants from the multidisciplinary cancer team stated that medication was the most challenging "M" to implement and that further guidance from faculty experts was needed. More than half of the sites are progressing to earn level one (participant) or level two (committed to care excellence) recognition in the Age-Friendly Health Systems movement. ACCC has provided and will continue to provide health systems with resources for scale-up in their Age-Friendly journey.

Abstract citation ID: igad104.2615
### PROMOTING AFHS FRAMEWORK WITHIN NURSING STAFF TRAINING CURRICULUM: NURSING HOME QUALITY IMPROVEMENT INITIATIVE

Oksana Shnayder[1], Sweta Tewary[1], and Naushira Pandya[2], *1. Nova Southeastern University, Davie, Florida, United States*, *2. Nova Southeastern University, Kiran Patel College of Osteopathic Medicine, Davie, Florida, United States*

The recent report by the Committee on the Quality of Care in Nursing Homes highlighted the limitations within nursing home facilities including neglect and abuse of their residents, high living costs and poor quality of life, lack of oversight, as well as nursing staff shortages and burnouts. With multiple challenges in sustaining the nursing home workforce, it is imperative to provide support and continued education to nursing home staff and nursing students on recent developments in evidence-based approaches to geriatric care including Age-Friendly Health Systems (AFHS) and dementia-friendly care. NSU SFGWEP conceptualized, designed, and delivered a modified geriatric curriculum to the nursing workforce at three distinct partner sites. The curriculum integrated eight training modules within the framework of AFHS and covers topics on each of the 4Ms: Matters Most, Mobility, Mentation, and Medication. The trainings are currently being delivered to the nursing home staff, nursing students, and to CNA students. The data on increase in education on AFHS and the 4Ms is being collected via a specifically designed survey which is administered electronically through mobile devices or via paper questionnaires. The survey responses are managed through REDCapTM, a secure web application for administering online surveys. An estimated 160 trainees have completed at least one training session and 38 trainees have submitted the feedback survey. Preliminary data on pre/post-training evaluations suggests an improvement in participants' knowledge of the geriatric topic within AFHS and the 4Ms, proposing a sustainable training program for the nursing workforce to promote age-friendly care for older adults

Abstract citation ID: igad104.2616
### PROMOTING INTERGENERATIONAL EXCHANGES WITHIN RURAL AGE-FRIENDLY COMMUNITIES: A CBPR APPROACH

Victoria Pumilio, and Bernard Steinman, *University of Wyoming, Laramie, Wyoming, United States*

The World Health Organization (WHO) Global Age-Friendly Cities (AFC) guide identifies eight interconnected domains for promoting the well-being, engagement, and participation of older adults in community activities and events. It defines an inclusive society as one that actively encourages older adults to participate in their community's social, civic, and economic life. Research often prioritizes addressing the physical challenges many older adults face, frequently overlooking societal engagement aspects that promote active aging and inclusion. This research outlines how a Community-Based Participatory Research (CBPR) approach was used to uncover and address the societal stigmas and counterproductive attitudes experienced by older adults in a rural age-friendly designated community. Our CBPR approach is characterized as a process encountered in three stages: (i) conducting a multigenerational survey on age-friendliness, (ii) engaging community members in planning intergenerational opportunities, and (iii) implementing intergenerational engagement activities identified in earlier stages. By participating in methodologies from both CBPR and grounded theory approaches, older adults acquired agency and voice in the research process, thus directly impacting age-friendly outcomes in their communities. Our findings detail the importance of utilizing a CBPR approach when researching core AFC fundamentals that are instrumental in creating inclusive policies and promoting healthful, mutually beneficial intergenerational opportunities.

### SESSION 7510 (POSTER)

### AGING AND COGNITION (POSTERS)

Abstract citation ID: igad104.2617

### A DYADIC ASSESSMENT OF COGNITIVE RESOURCES AND COGNITIVE FUNCTION AMONG OLDER KOREAN AMERICAN COUPLES

Nan Park[1], Yuri Jang[2], Kyungmin Kim[3], Soondool Chung[4], William Haley[1], David Chiriboga[1], and Miyong Kim[5], *1. University of South Florida, Tampa, Florida, United States*, *2. University of Southern California, Los Angeles, California, United States*, *3. Seoul National University, Seoul, Republic of Korea*, *4. Ewha Womans University, Seoul, Republic of Korea*, *5. University of Texas at Austin, Austin, Texas, United States*

Individuals' health and well-being are often shaped by significant others in their lives in that spousal relationships are particularly important. In dyadic studies among older couples, few studies have examined cognitive health as an outcome variable. Considering the lack of couple research on cognition especially among older immigrant population, the purpose of this study was to examine the cognitive function of community-dwelling older Korean American couples in the U.S. Data were drawn from a survey with older Korean Americans aged 60 and older, collected during 2017–2018 in multiple locations. Using a subset of 252 couples of the survey (individual N = 504), we examined the extent to which one's cognitive function was associated with the cognitive resources (education, acculturation, friend network, and activity participation) of their own and of their spouse. The analyses with the Actor-Partner Interdependence Model showed the differences in the actor- and partner-effects between spouses. For husbands, acculturation was the only significant actor-level predictor to cognitive function. Wives' cognitive function was associated with a wider array of their own cognitive resources, including education, friend network, and activity participation. The partner effect was only observed in education for husbands, indicating that wives' higher education was beneficial for their husbands' cognitive function. Further, the cognitive benefit of higher education was found to be maximized for husbands who also had greater levels of acculturation or activity participation. For practice implications, cognitive resources both in individual and spousal levels could help promote the cognitive health and well-being of older married couples.

Abstract citation ID: igad104.2618

### A RANDOMIZED CONTROLLED TRIAL OF THE EVERYDAY MEMORY AND METACOGNITIVE INTERVENTION IN OLDER ADULTS

Ann Pearman[1], MacKenzie Hughes[2], Emily Giannotto[3], Clara Coblenz[2], Ethan Flurry[4], Taylor Curley[5], and Christopher Hertzog[2], *1. MetroHealth Medical System, Cleveland, Ohio, United States*, *2. Georgia Institute of Technology, Atlanta, Georgia, United States*, *3. Emory University School of Medicine, Atlanta, Georgia, United States*, *4. Mississippi State University, Starkville, Mississippi, United States*, *5. Air Force Research Laboratory, Dayton, Ohio, United States*

This study evaluated an intervention to improve remembering in everyday life, the Everyday Memory and Metacognitive Intervention (EMMI). EMMI uses strategies designed to enhance everyday remembering and self-regulatory approaches to monitor goal pursuit, anticipate, and address memory demands. A randomized controlled trial compared a group assigned to EMMI with a traditional memory strategy training control (MSC) group. Expanding on earlier work (Pearman et al., 2020) we created a new event-based ecological momentary assessment (EB-EMA) smartphone application for reporting everyday memory errors when they occurred, synched to a nightly diary for collecting additional information about each event. The pretest-posttest design assessed changes in subjective memory beliefs and episodic memory performance and also measured posttest-only performance measures, including EB-EMA frequency of memory errors and a laboratory contact task assessing everyday prospective memory. The protocol was reduced in scope to accommodate fully remote participation due to the pandemic. The final sample included 30 EMMI and 32 MSC participants. Memory strategy training differentially improved MSC associative memory test performance. There were no group differences in EB-EMA reported memory errors or successful prospective memory contacts. The EMMI group showed more improvement in memory self-efficacy than the MSC group; both groups showed increases in perceived control over everyday memory. Individual differences in EMMI intervention benefits suggest that the program worked well in reducing memory errors for some but not all older adults. The EMMI shows promise but may require more extensive in-person training and behavioral shaping than this study provided to improve everyday memory.

Abstract citation ID: igad104.2619

### ASSOCIATIVE AND EXECUTIVE INFLUENCES ON MEMORY FOR EVENTS AS REVEALED BY AGING AND DISTRACTION

Alan Kersten[1], Julie Earles[1], Megan Smithwick[1], and Carlos Vaca Angus[2], *1. Florida Atlantic University, Boca Raton, Florida, United States*, *2. Florida Atlantic University Wilkes Honors College, Boca Raton, Florida, United States*

Diagnostic tests of memory functioning typically employ verbal material, potentially encouraging mnemonic strategies ranging from rehearsal to visual imagery. Poor performance could thus reflect either impairments in basic associative mechanisms or impairments in executive functions underlying strategy deployment. Kersten et al. (2018) developed a more ecologically valid test of episodic memory, the Person-Action Conjunction (PAC) Test, involving memory for who did what in events. They found that when many different actors performed the actions, memory for who performed each action was related to tests of associative functioning, whereas when only two actors performed all the actions, memory performance was related to executive functioning. The present research tested the ensuing prediction that manipulating the availability of executive resources through distraction should only impact performance when a small number of actors perform the actions. The PAC Test was administered to 207 young adults (M age = 20.39) and 163 healthy older adults (M age = 70.80), either with no distraction or while performing a tone counting distractor task. Young adults performed better than older adults at remembering who did what, consistent with an age-related associative deficit. When two actors performed all the actions, distraction impacted both age groups' memory for which actor performed each action. When many different actors performed the actions, distraction had no specific impact on memory for who did what. Implications will be discussed for the development of diagnostic tests to differentiate impairments in basic memory mechanisms (e.g., early-stage Alzheimer's disease) from impairments of executive functioning (e.g., vascular dementia).

Abstract citation ID: igad104.2620

### BLOOD PRESSURE MEASURES ARE NOT CORRELATED WITH NIH TOOLBOX COGNITIVE BATTERY PERFORMANCE IN BLACK OLDER ADULTS

Allison Moll[1], Colt Halter[1], Hannah Adams[1], Loraine DiCerbo[1], Voyko Kavcic[1], Ana Daugherty[1], John Woodard[1], and Bruno Giordani[2], *1. Wayne State University, Detroit, Michigan, United States*, *2. University of Michigan, Ann Arbor, Michigan, United States*

Elevated blood pressure has been inconsistently associated with cognitive performance in older adults. Black adults experience higher rates of elevated blood pressure and cognitive impairment than White adults. This study investigated whether blood pressure measurements were associated with cognitive performance on the NIH Toolbox Cognition Battery (NIHTB-CB) in older Black adults. 77 older Black adults 65+ years of age (M=72.6, SD=4.9) with subjective cognitive decline were administered the NIHTB-CB, a battery of computer-based cognitive tests. Systolic blood pressure (SBP) and diastolic blood pressure (DBP) were measured before and after cognitive testing and a cognitive task-based EEG/ERP. Average SBP, DBP, and pulse pressure (PP) were calculated. Difference scores from pre- to post-EEG/ERP served as a reactivity index following cognitive task engagement. A ratio of the difference scores to the baseline measures controlled for resting blood pressure. Bayesian correlations examined the relations between blood pressure measurements and NIHTB-CB performance. Data analysis used fully corrected T scores from the NIHTB-CB subtests. Average SBP, SBP difference scores, and baseline SBP were not associated with NIHTB-CB performance on any measurement (all BF10 < 3.0). Similarly, DBP and PP were unrelated to NIHTB-CB performance. Systolic and diastolic blood pressure were not associated with any NIHTB-CB measure. Additionally, blood pressure reactivity was not related to cognitive performance. Future research may investigate the relationship between SBP reactivity and cognitive performance across multiple measurement periods. Antihypertensive medication and duration of hypertension among hypertensive individuals may also be an important consideration in future studies.

Abstract citation ID: igad104.2621

### COGNITIVE FUNCTION AND LIFE SATISFACTION IN LATER LIFE: IS EMOTIONAL WELL-BEING A PATHWAY?

Brittany McFeeley[1], Changmin Peng[2], and Jeffrey Burr[1], *1. University of Massachusetts Boston, Boston, Massachusetts, United States*, *2. University of Massachusetts, Boston, Boston, Massachusetts, United States*

Cognitive impairment and dementia represent major public health concerns globally. Life satisfaction measures capture an important element of subjective well-being in later life. This study examines the association between cognitive function and life satisfaction along with the mediating effects of loneliness and depression among older adults. While the study of life satisfaction and cognitive function have received considerable attention among gerontologists, we know relatively little about whether emotional well-being represents a pathway between cognitive function and life satisfaction. To address our research questions, we employed nationally representative data from the 2018 Health and Retirement Study, including information from the Leave Behind Questionnaire, focusing on community dwelling respondents aged 65 and older (N=3,173). Cognitive function is measured with the Telephone Interview for Cognition Status instrument and life satisfaction is based on the Diener Life Satisfaction Scale. Depression is measured by the number of depressive symptoms (CESD-8) and loneliness is based on the UCLA Loneliness Scale. Results from our linear regression models show that cognitive function is positively associated with life satisfaction (B=0.012, p=.028), while depressive symptoms (B=-0.261, p<.001) and loneliness are negatively associated with life satisfaction (B=-1.290, p<.001). Each measure of emotional well-being mediates the cognitive function-life satisfaction relationship, supporting our hypothesis. Future research based on longitudinal designs should evaluate whether other mediators help explain this relationship, including the structural and functional domains of social networks. Social network characteristics may combine with emotional well-being to provide an improved understanding of cognitive function and subject well-being.

Abstract citation ID: igad104.2622

### EXPLORING LINKS BETWEEN COGNITIVE AGING AND FEATURES OF WORKING MEMORY VIA COMPUTATIONAL MODELING

Sharon H. Kim[1], Yanling Li[1], Karra Harrington[1], Jonathan Hakun[2], Daniel Elbich[3], Martin Sliwinski[2], and Zita Oravecz[2], *1. The Pennsylvania State University, University Park, Pennsylvania, United States*, *2. Pennsylvania State University, State College, Pennsylvania, United States*, *3. The Pennsylvania State University, Hershey, Pennsylvania, United States*

With an aging population, understanding cognitive aging is essential for developing interventions to support independence and well-being. Additionally, there is a need for sensitive measures that can differentiate subtle cognitive decline related to neurodegenerative disease from age-related changes. In particular, Alzheimer's Disease and Related Dementia (ADRD) increases in prevalence with age and involves progressive cognitive decline. The Color Shapes task (CST) is a visual working memory task that has been shown to be sensitive to early indicators for ADRD. CST involves feature binding (e.g., mental representation of features into a unified whole) that is impaired in people with ADRD. In an experiment, we manipulated different CST properties to study whether drift diffusion modeling (DDM) can account for individual differences in latent decision-making processes generating manifest CST performance scores. DDM allows us to quantify theoretically meaningful cognitive parameters that underlie task performance captured in reaction times and accuracies. 68 U.S. community-residing adults (28-80 years) completed daily smartphone assessments across 8 days with experimentally manipulated versions of the CST. We implemented a single-step Bayesian estimation of experimental conditions contrasts, latent DDM parameters, and their associations. We show how individual differences in experimental contrasts were related to age in terms of cognitive (drift rate), non-cognitive (non-decision time), and meta-cognitive (boundary separation) parameters. The results highlight the benefits of using cognitive process models with optimized cognitive tasks to delineate features of cognitive aging for ADRD research.

Abstract citation ID: igad104.2623

### FACTOR STRUCTURES OF THE KOREAN VERSION OF NEED FOR COGNITION SCALE SHORT FORM (K-NFC-S) AMONG KOREAN OLDER ADULTS

Juhyeong Lee, Gaeun Han, Yeonsoo Shin, and Giyeon Kim, *Chung-Ang University, Seoul, Republic of Korea*

This study aimed to examine the underlying factor structures of the Korean Version of Need for Cognition Scale Short Form (K-NfC-S) among older adults in South Korea. A nationally representative sample of Korean older adults aged 65 or older (N=2,281) was drawn from the 2020 Korean Media Panel Survey. After dividing the sample into two groups at random, both exploratory (N=1,117) and confirmatory (N=1,164) factor analyses were conducted respectively using SPSS 26.0 and AMOS 26. A model fit was assessed with the Tucker-Lewis Index (TLI), the comparative fit index (CFI), and the root-mean-square error of approximation (RMSEA). Results from the exploratory factor analysis revealed that K-NfC-S were divided into two domains. Factor I consisted of eleven positively worded items and Factor II consisted of four negatively worded items. However, in the confirmatory factor analysis, a single-factor model with correlated errors among the negatively worded items showed the best fit among the postulated models of K-NfC-S (TLI=.917, CFI=.934, RMSEA=.074). Consistent with previous findings, the results from the present study support a single dominant factor structure underlying responses to the K-NfC-S items among older adults in South Korea. The findings suggest that the two-factor structure identified from exploratory factor analysis may be an artifact of method effects associated with the negatively worded items included in the K-NfC-S.

Abstract citation ID: igad104.2624

### FACTORS INFLUENCING COGNITIVE FUNCTION AMONG THE US OLDEST-OLDER ADULTS

Jung Yoen Son, and Janet Larson, *University of Michigan, Ann Arbor, Michigan, United States*

Previous studies have identified factors that influence cognition in older adults (aged ≥ 65); however, the findings cannot be generalized to the oldest-old adults (aged ≥ 80), who face different physical and functional challenges. This study aimed to examine the association between potential influencing factors and cognitive function in community-dwelling oldest-older adults using the National Health Aging Trends Study (NHATS) dataset, a nationally representative sample from 2015. We conducted linear multilevel modeling (MLM) to examine the association between participation in physical activity (PA) and social activity, everyday technology use, mental health, and self-reported health with cognitive function (immediate and delayed word recall), adjusting for five sociodemographic characteristics (age, sex, race/ethnicity, and education). A total of 2779 community-dwelling oldest-old adults from the NHATS were included. Most of study participants were 80-84 years old (47%), female (61%), Non-Hispanic White (70%), not married (63%), and did not obtain a college degree (75%). In the MLM, participating in physical and social activities, using technology, and poorer mental health were significantly associated with higher word-recall scores (better cognitive function). Self-reported health was not associated with word recall. Results from our study suggest that interventions aimed at promoting physical and social activities and improving mental health may help maintain cognitive function among the oldest-old population. Our findings also suggest the potential positive impact of technology use on cognition, although further study is needed to support the evidence.

Abstract citation ID: igad104.2625

### MECHANISMS OF ATTENTIONAL CONTROL AND SUPPRESSION IN AGING

Josh Senior, Andy Kim, and Mara Mather, *University of Southern California, Los Angeles, California, United States*

Attentional control plays a crucial role in our daily lives by allowing our brains to selectively process relevant information while filtering out task-irrelevant distractions. Due to aging, older adults have demonstrated a heightened propensity to be more distractible. This decreased ability to regulate attention has been attributed to an inability to inhibit task-irrelevant salient stimuli. However, the specific mechanisms that underlie this age-related alteration remain unclear. In this study, we utilized a visual search paradigm that directly measures mechanisms of attentional suppression to test our hypothesis that older adults demonstrate reduced capabilities to suppress task-irrelevant information. Young adults (aged 18-35) and older adults (aged 50-80) were tasked to utilize goal-directed attentional control by fixating at a specific target shape. Critically, in some trials, individuals were exposed to a salient color shape that acted as a singleton distractor. While young adults show the ability to suppress attentional allocation to this salient distractor in this paradigm, we hypothesized that older adults would demonstrate attentional capture by this salient distractor. Our findings revealed that older adults made significantly fewer first-fixations towards the target, demonstrating a marked impairment with goal-directed attentional control. Surprisingly, older adults still made fewer fixations toward the distractor in comparison to non-targets, demonstrating that older adults sustained the ability to suppress irrelevant distractors. These findings raise important questions on the mechanisms that underlie increased distractibility in aging and oculomotor attentional control.

Abstract citation ID: igad104.2626

### PROSPECTIVE EXPLORATION OF COMBINED INTERNALIZING SYMPTOMS ON SELF-REPORTED MEMORY DURING COVID-19

Dawn Carr, Natalie Sachs-Ericsson, Brad Schmidt, and Frederick Schubert, *Florida State University, Tallahassee, Florida, United States*

A growing literature suggests depression and anxiety increase risk of cognitive decline. However, few studies have examined their combined effects on cognition, among older adults, especially during periods of high stress. Based on a sample of community dwelling older adults (N=576), we evaluated the effects of pre-pandemic anxiety and depressive symptoms, obtained in September 2018, to changes in self-reported memory (SRM) assessed 3 months into the COVID-19 pandemic. In separate models we found participants with depression scores at least 1-SD above the mean and participants with anxiety scores at least 2-SD above the mean to report a significant decline in SRM. Moderation analyses revealed those with high depressive symptoms (at or above the mean) showed a decrease in SRM regardless of anxiety. The extent to which high pre-pandemic anxiety symptoms influenced SRM is dependent on whether pre-pandemic depression was at or above the mean. Pre-pandemic depression predicted a decline in SRM regardless of anxiety. Moderation analyses revealed that the extent to which anxiety symptoms influenced SRM was dependent on depression being at or above the mean. Those with high anxiety and depression are at highest risk of experiencing cognitive consequences related to stressful exposures like COVID-19.

Abstract citation ID: igad104.2627

### RESILIENCE AND AGE MODIFY INFLUENCES OF STRESS AND POSITIVE EXPERIENCES ON SUBJECTIVE COGNITION

Dakota Witzel[1], Suzanne Segerstrom[2], Maria Kurth[3], Soyoung Choun[2], Paris Crosby[4], and Carolyn Aldwin[2], *1. Penn State University, State College, Pennsylvania, United States*, *2. Oregon State University, Corvallis, Oregon, United States*, *3. Penn State University, University Park, Pennsylvania, United States*, *4. University of Kentucky, Lexington, Kentucky, United States*

The COVID-19 pandemic is a major, chronic, worldwide stressor related to poorer health and well-being, especially among older adults, but less is known about risk and protective factors related to cognitive functioning during this stressful time. This eight-week study of 229 community-dwelling middle-aged and older adults (Mage=71.1; 74% women) examined associations between the intensities of weekly stress and positive experiences on subjective cognitive functioning. We also examined how age and trait resilience modified these associations. Zero-inflated Poisson models revealed a positive association between the intensity of stress and self-reported cognitive difficulties (γ=0.34, SE=0.07), whereas the intensity of positive experiences was associated with fewer difficulties (γ=-0.09, SE=0.03). Age moderated the effect of stress (γ=-0.01, SE=0.002), with the old-old having more subjective cognitive difficulties under more intense stress than the young-old. Resilience modified effects of stress and positive experiences, respectively, on cognition (γ=0.04, SE=0.01 and γ=-0.01, SE=0.01). Those with lower resilience reported more cognitive difficulties under more intense compared to less intense stress; this association was slightly attenuated for people with more resilience. Similar associations emerged between resilience and positive experience intensity. Older age was a risk factor for subjective cognitive difficulties during the COVID-19 pandemic, especially under conditions of high weekly stress, whereas resilience was a protective factor. Although those high in resilience appeared to be more sensitive to cognitive difficulties during more intense positive experiences, they still reported fewer difficulties than those low in resilience. Older adults' cognitive resilience during the COVID-19 pandemic was heterogeneous and multifactorial.

Abstract citation ID: igad104.2628

### SELF-PERCEIVED COGNITIVE CHANGES IN OLDER BLACK ADULTS DURING COVID-19

Nancy Martens[1], Sophie Hanna[1], Hannah Adams[1], Loraine DiCerbo[1], Bruno Giordani[2], and Voyko Kavcic[1], *1. Wayne State University, Detroit, Michigan, United States*, *2. University of Michigan, Ann Arbor, Michigan, United States*

The COVID-19 pandemic has had myriad effects on health and well-being. Contracting the COVID-19 virus and concerns of social and physical consequences during pandemic-related restrictions have been proven to negatively impact health. To better understand possible effects of COVID-19 on Black community-dwelling older adults' perceived cognitive changes, we conducted a pilot study with 48 community-dwelling African Americans with measures obtained over 24 months from 10/2020 to 10/2022. Participants were recruited from the Wayne State University Institute of Gerontology Healthier Black Elders Center, and Michigan Alzheimer's Disease Research Center, and the general Detroit Area (Mage=73.07, Range=65-87 years). The Cognitive Change Index (CCI) was used as a self-report measure of perceived change over the last five years. The COVID Impact Survey (CIS) was used to assess the burden of COVID-19 on daily living. To evaluate emotional-being, the Perceived Stress Scale (PSS responses referred to the last month) and the Geriatric Depression Scale (GDS, within the last week) were administered. Stepwise regression models showed CIS outcome measures did not predict CCI. GDS outcome measures predicted CCI to some degree (R2adj = .15, p = .008), whereas PSS was the strongest predictor (R2adj = .50, p < .001). Results suggest that for older community-dwelling African Americans, past COVID-19 pandemic experiences did not affect their self-evaluated cognitive changes. Higher levels of self-perceived stress within the last month best predicted their subjective cognitive decline. Future research should involve interventions focused on stress reduction, and clinicians should consider stress level when diagnosing cognitive status in older adults.

Abstract citation ID: igad104.2629

### SEMANTIC RETRIEVAL PATTERNS IN COGNITIVELY HEALTHY OLDER ADULTS

Vaishali Phatak, Elizabeth Vlock, Yunju Im, Jennifer Merickel, Erica Aflagah, Daniel Murman, Jun Ha Chang, and Matthew Rizzo, *University of Nebraska Medical Center, Omaha, Nebraska, United States*

Word finding difficulties can be a normative change in aging. The purpose of this descriptive study is to establish metrics of word retrieval patterns in older adults using a semantic fluency task. Forty-five healthy aging, primary English speakers (mean age 75.0; 69% female, mean MoCA = 26.5) participated. Participants were asked “I want you to tell me all the animals you can think of in one minute.” The responses were scored using two novel normalized coefficients. (1) Archetypal Coefficient (AC): frequency proportional to the 60-second frequency. (2) Seeding Coefficient (SC): frequency proportional to both the 60-second frequency and the first-word frequency. Cluster coefficients (CC) also measured adjacency of responses. Participants generated mean of 19.9 words with a low correlation to MoCA (r=0.32). A total of 204 unique animal names including developmental stages (e.g., puppy), sex differences (e.g., rooster), and nicknames (e.g., bunny) were produced. In descending order, the most frequent responses were cat (AC=1.00), dog (AC=0.99), elephant (AC=0.89), horse (AC=0.83), and lion (AC=0.78). SC showed high ranking for archetypal animals (e.g., dog (SC=1.00), cat (SC=0.72)) and animals beginning with the letter “a” (e.g., anteater (SC=0.18), aardvark (SC=0.17)). CC showed greater adjacency for semantic subcategories such as pets (cat-dog) or farm animals (cow-horse), closely related species (mouse-rat) and superordinate labels (bird-fish). Our novel indices and CC revealed semantic retrieval in healthy aging occurs in an organized fashion that includes linguistic (i.e., alphabetical, word frequency) and semantic characteristics of animals, which adds to the current literature on semantic networks.

Abstract citation ID: igad104.2630

### SPEECH-LANGUAGE PATHOLOGISTS’ PRACTICE PATTERNS IN THE MANAGEMENT OF COGNITIVE IMPAIRMENT IN PARKINSON'S DISEASE

Vanessa Neal[1], and Nan Musson[2], *1. Department of Veteran Affairs, Orlando, Florida, United States*, *2. Department of Veteran Affairs, Washington, District of Columbia, United States*

Cognitive impairment is common in patients with Parkinson’s disease (PD) and can have significant impact on quality of life. Speech-language pathologists (SLPs) are qualified to treat cognitive impairment in PD, yet previous studies have shown SLPs tend to place greater focus on other impairment areas (e.g., dysarthria) and may lack confidence treating cognition. This study sought to better understand practice patterns in PD cognitive treatment among Veteran Affairs SLPs (N=14) participating in a national quality improvement project. Using a mixed-methods design, we examined SLPs’ use of formal assessments (e.g., standardized screening) and patient-reported outcome measures for cognition. We also examined SLPs’ clinical recommendations and perspectives toward providing cognitive services. Data was obtained using REDCap and semi-structured interviews. Results showed formal cognitive assessments were completed on 45% of patients (N=191) and patient-reported outcome measures were completed with 36% of patients. Of the patients formally assessed (n=86), 55 scored as having some degree of cognitive impairment and 17 were ultimately enrolled in cognitive treatment. The most common reasons SLPs provided for not treating cognition included: patient declining treatment, supportive home environment, needing further testing, or other medical needs (e.g., sleep management). Overall, results suggest cognitive management was a low priority among clinicians and patients. SLPs reported barriers to providing cognitive treatment included patient desire to participate and therapeutic time constraints when also providing speech and/or swallowing services. Further research is needed to increase integration of cognitive services into SLP treatment of PD and identify strategies to increase patient buy-in of cognitive treatment.

Abstract citation ID: igad104.2631

### THE RELATIONSHIP OF COGNITIVE CONTROL BELIEFS TO 10 YEAR CHANGES IN COGNITIVE PERFORMANCE

Kylie Schiloski, and Margie Lachman, *Brandeis University, Waltham, Massachusetts, United States*

Past work has shown that older adults who believe they have control over their cognitive performance (i.e., higher cognitive control beliefs) have better cognitive performance than those with lower cognitive control beliefs. However, it is unclear whether cognitive control beliefs are related to individual differences in cognitive change among middle-aged and older adults. The current study examined longitudinal associations between cognitive control beliefs and 10-year cognitive changes using data from waves 2 and 3 of the Midlife in the United States study (MIDUS; N=2,509, Ages 33 to 83 at wave 2). We predicted that higher baseline cognitive control beliefs would be related to greater maintenance of episodic memory and executive functioning over 10 years. Age was tested as a moderator of these relationships and each analysis controlled for baseline performance, age, sex, education, and self-rated physical health. Multiple linear regression models revealed that higher baseline control beliefs were related to less decline in episodic memory (p<.001) and executive functioning (p<.05); however, age did not moderate these relationships. Taken together, these results suggest that across age, cognitive control beliefs are associated with greater maintenance of episodic memory and executive functioning over 10 years in middle-aged and older adults. As such, cognitive control beliefs may represent a promising modifiable mechanism to help minimize cognitive declines starting in midlife.

Abstract citation ID: igad104.2632

### THE ROLE OF VERBATIM VERSUS GIST FORMATTING IN AGE-RELATED DIFFERENCES IN INFORMATION AVOIDANCE

Lauren Mei[1], Julia Nolte[2], and Corinna Löckenhoff[3], *1. Weill Cornell Medicine, New York City, New York, United States*, *2. Tilburg University, Tilburg, Noord-Brabant, Netherlands*, *3. Cornell University, Ithaca, New York, United States*

Older adults are more likely to underuse and actively avoid decision-relevant information. Although the mechanisms underlying age-related differences in information avoidance are still unknown, Fuzzy-Trace Theory (Reyna, 2012) suggests they may be due to age-related differences in information processing preferences: According to this framework, information is stored using both gist (subjective meaning) and verbatim representations (objective details/numbers). Older adults prefer gist information and gist-based information processing, whereas younger adults favor verbatim information and verbatim processing. To examine whether age-related differences in information avoidance are affected by age-related information and processing preferences, an adult lifespan sample (N = 432) completed a pre-registered online study. Participants responded to scenarios containing potentially aversive information and chose between accepting or avoiding the information. Information formatting was experimentally manipulated, with participants either being faced with receiving gist or verbatim-formatted information. Across age groups, participants were more likely to avoid verbatim numbers than qualitative gist information (p=.026); no associations were found between avoidance and information or processing preferences. At odds with prior research, age was associated with lower information avoidance (p=.010). Follow-up analyses revealed that older adults were less likely to avoid information in health-related contexts but not in contexts unrelated to health (e.g., consumer information; p=.010). Overall, participants were more likely to avoid non-health information than health information (p<.001). In sum, our findings revealed that older adults show a decreased likelihood of avoiding potentially aversive health information, but that age-related differences in information avoidance cannot be linked to age-related information or processing preferences.

Abstract citation ID: igad104.2633

### WHAT'S ON THE CALENDAR THIS WEEK? EVERYDAY COLLABORATIVE STRATEGY USE AMONG MIDDLE-AGED AND OLDER COUPLES

Celinda Reese-Melancon[1], Rachael Turner[1], Riley Wyatt[1], Erin Harrington[2], Tina Savla[3], Peter Rendell[4], and Jennifer Margrett[5], *1. Oklahoma State University, Stillwater, Oklahoma, United States*, *2. University of Wyoming, Laramie, Wyoming, United States*, *3. Virginia Tech, Blacksburg, Virginia, United States*, *4. Australian Catholic University, Melbourne, Victoria, Australia*, *5. Iowa State University, Ames, Iowa, United States*

Mnemonic strategies support individual memory performance in a variety of domains, including school, work, and home. Less is known about how people collaborate to remember information in their daily lives. We asked married and/or cohabitating middle-aged and older adults (118 individuals, M age = 62 years, Range = 40-88 years, 50% female) about their individual strategy use and whether they engaged in any collaborative behaviors to support their everyday memory success. We employed a mixed methods approach to data analysis. We found that, as a group, husbands did not statistically differ from wives in their reports of individual internal, external, or technology-based strategy use. Most participants did report collaborating with their partner to remember the things they need to do in a given week. Themes regarding collaborative strategy use included weekly or daily calendar planning, making joint To-Do lists or making such a list for one's partner, conferring about upcoming events and appointments, and providing reminders to partners. These findings lay the groundwork for future work examining how partners may support each other cognitively as they age and whether these supports change over time.

### SESSION 7520 (POSTER)

### AGING IN COMMUNITIES

Abstract citation ID: igad104.2634

### CONCORDANCE IN SPOUSES' INTENTION TO MOVE AFTER RETIREMENT AMONG KOREAN MIDDLE-AGED COUPLES

Eunju Lee, and Kyungmin Kim, *Seoul National University, Seoul, Republic of Korea*

Midlife adults often envision life after retirement and start planning for retirement, forming their own expectations of where they live after retirement. Migration after retirement is an important decision in that physical and social environments of housing and communities affect health and well-being in later life. Research has emphasized that moving decisions are made at the household level, but less is known about whether middle-aged couples are concordant in their desire to move after retirement and whether concordant desires are associated with couples' marital characteristics, such as marital satisfaction, dyadic coping styles, and communication. We analyzed a sample of 1,285 middle-aged couples (age 49–64) from the 2012 Korean Baby Boomer Panel Study and Korean Forgotten Generation Study. The majority of couples (83%) were concordant in their moving desires—by either agreeing to move (29%) or to stay (54%), whereas 17% of couples were not in agreement—either only husband desires to move (9%) or only wife desires to move (8%). Logistic/multinomial regression results showed that couples who had a communal problem-solving style and where the wife perceived higher marital satisfaction were more likely to be concordant in their moving desires (i.e., agreeing to move or to stay), after controlling for other individual and family characteristics. Further, couples were more likely to agree to move—when they had discussion about life after retirement and shared individual stress between spouses. Our findings suggest the importance of marital dynamics in understanding concordance in spouses' desire to move after retirement.

Abstract citation ID: igad104.2635

### ETHNIC VARIATIONS IN SOCIAL CAPITAL IN SOUTH ASIAN AND CHINESE OLDER ADULTS IN HONG KONG

Daniel W.L. Lai[1], Alison X.T. Ou[1], Vincent W.P. Lee[1], Doris S.F. Yu[2], Jia Li[3], Shireen Surood[4], and Gary K.K. Lau[2], *1. Hong Kong Baptist University, Hong Kong, Hong Kong, 2. The University of Hong Kong, Hong Kong, Hong Kong, 3. Chinese University of Hong Kong, Hong Kong, Hong Kong, 4. Alberta Health Services, Edmonton, Alberta, Canada*

The role of social capital has gained a lot of attention as one of the determinants of wellbeing of aging adults. It serves the functions of connecting people with vital resources essential to improvement of health, promotion of social cohesion and networks, and enhancement of support received. Social capital could be influenced by a variety of individual and socio-cultural factors among racialized groups. This study aims to examine the variations in social capital in Chinese and South Asian aging adults in Hong Kong. A sample of 1,015 people aged 55 and above, consisting of Chinese (n=800) and South Asian participants (n=215), participated in a survey via telephone (Chinese) and face-to-face (South Asian) interview respectively. A 25-item World Bank's Social Capital Assessment Tool was used for measuring six variables related to social capital including social participation, social support, social connection, trust, cohesion, and reciprocity, covering the structural and cognitive dimensions of social capital. A generalized linear model was performed to detect differences between the two groups after controlling for demographics. The aging Chinese people exhibited a stronger cohesion level than their South Asian counterparts, whereas the aging South Asians demonstrated higher levels of social participation, social support, social connection, trust, and reciprocity. The identified gaps further illustrate the socio-cultural differences between the groups, highlighting the importance of strategies for enhancing social inclusion and equity for racialized older people in the Chinese context.

Abstract citation ID: igad104.2636

### IDENTIFYING CONFLICTS EXPERIENCED BY OLDER ADULTS WHILE AGING IN PLACE

Alaine Murawski[1], Marianne Tschoe[2], Amber Miller-Winder[1], Allie Schierer[1], Raven Relerford[1], Vanessa Ramirez-Zohfeld[1], and Lee A Lindquist[1], *1. Northwestern University, Chicago, Illinois, United States, 2. Northwestern Medicine, Chicago, Illinois, United States*

Older adults are faced with making decisions about aging-in-place as their health changes. These decisions create conflicts with others causing stress and frustration. We sought to characterize the conflicts older adults experience. As part of a larger study on decision making about aging-in-place (R01AG058777), we are longitudinally following a cohort of older adults with surveys every 6 months. Subjects are asked to describe conflicts that have occurred regarding their living situation. Responses were coded using constant comparative analysis examining type, content, and whether statements were interests, rights, or power-based. We enrolled 293 subjects (mean age 73.5 yrs, SD 5 yrs, 40.4% non-White; 94.5% retention rate) and 124 conflicts were reported over 18 months. Thematic analysis revealed three types of conflicts: Interpersonal (subcategories: spouse, intergeneration, other), task, and value-based. Content was coded into 6 themes: Location change, home maintenance issues (e.g., My son insisted I put in a new lower tub, and I didn't want to), different ways of completing tasks (e.g., it is about my children and the way we do things), safety (e.g., Neighbor's boyfriend kicked in the door and destroyed our property), financial, and health-related. Most statements were either interests or rights-based. Older adults experience a range of conflicts involving aging-in-place decision making. Most often older adults experience interpersonal conflicts with supporters about moving from their home or maintaining/adapting their home. Training in negotiation may help alleviate these conflicts between older adults and their care supporters.

Abstract citation ID: igad104.2637

### INDECISIVENESS ABOUT LONG-TERM CARE AMONG OLDER ADULTS

Allie Schierer, Amber Miller-Winder, Raven Relerford, Charles Olvera, Vanessa Ramirez-Zohfeld, Alaine Murawski, and Lee A Lindquist, *Northwestern University, Chicago, Illinois, United States*

As cognition and function worsen, older adults often make decisions about living in long term care (LTC) or accepting support in the home. We are longitudinally studying LTC decision-making among a cohort of older adults (65+), who have viewed PlanYourLifespan.org (PYL), a proven-effective LTC planning tool. Subjects were surveyed at baseline (BL), administered PYL, then followed with surveys every 6 months. Participants were asked about LTC decisions in the event of worsening cognition and to describe those decisions. Responses were analyzed using a mixed-methods approach with open-ended responses coded using constant comparative analysis. Of the 293 subjects, mean age was 73 years, 72.7% (213) female, 40.4% (118) under-represented minority. Between baseline-18 months, 66.5% of subjects changed their LTC plans (28.7% one change, 24.23% two changes, 9.2% three changes) At 1 and 6 mos., the proportion of respondents who changed their decision from their prior timepoint was 44%. By 12 mos., this proportion has declined to 39%, and by 18 mos., the proportion further decreased to 34%. Higher indecision (Yes-No-Yes-No) was seen with changes in family caregivers, timing around COVID-19, and personal health. Decision permanence increased at 12-and 18-month time points. LTC decision-making fluctuates; however, over time, the decision-making progresses from circling to more permanence. Understanding LTC decision making is longitudinally important to understand and should be revisited every 6-12 months in clinical practice.

Abstract citation ID: igad104.2638

### MEASURING MULTIGENERATIONAL NEIGHBORHOODS

Martin Hyde, and Elizabeth Evans, *Swansea University, Swansea, Wales, United Kingdom*

There are growing concerns that societies are becoming more generationally divided and that this is having negative social consequences, such as increasing loneliness and social isolation. Residential age segregation as a key driver of this generational divide and there is worrying evidence that levels of residential age segregation have increased in the USA, UK and Europe. However, measures of age segregation have a number of methodological limitations: i) they only compare younger and older age groups and therefore overlook the wider generational mix in the neighbourhood; ii) different measures of young/older age groups are used in different studies, iii) different geographical scales, e.g. wards, districts, etc., are used in different studies and iv) measures are often at a high level of geographical aggregation, e.g. local authorities. To address these issues we have developed a new measure of multigenerational neighbourhoods, based on an ecological measure used to calculate biodiversity, for all neighbourhoods in England and Wales from 2011-2020. Our analyses shows that i) there is a low correlation between measures of age segregation and neighbourhood multigenerationality and ii) contrary to some findings on age segregation, the vast majority of people in England and Wales live in multigenerational neighbourhoods. However, some areas have seen a drop in multigenerationality, especially those in inner city and costal areas.

Abstract citation ID: igad104.2639

### NEIGHBORHOOD DISORDER MODERATES THE EFFECT OF SLEEP DIFFICULTIES ON COGNITIVE DECLINE AMONG OLDER AMERICANS

Darlingtina Esiaka[1], and Elizabeth Luth[2], *1. University of Kentucky, Lexington, Kentucky, United States*, *2. Rutgers University, New Brunswick, New Jersey, United States*

Research suggests that the neighborhood where people live can be a risk or protective factor for various health outcomes, including cognitive decline. Similar to the impact of neighborhood on health outcomes, sleep quality has been linked to cognitive functions in older adults. However, limited studies have examined how neighborhood disorders moderate the effect of sleep on cognitive decline. Data were obtained from 2,494 respondents (1,065 men and 1,429 women) from wave 11 of National Health and Aging Trends (NHATS) data. Approximately 55% of the respondents were age 80+, and 74% identified as non-Hispanic White Americans. Sleep quality was operationalized as the absence of difficulties in falling and staying asleep. Neighborhood disorder (e.g., vandalism, graffiti) was based on observations by interviewers. Cognitive decline was operationalized as subjective reports of increasing or worse memory loss in the past 12 months. Moderated regression analysis using PROCESS macro (model 1) was performed to determine the associations between sleep, neighborhood, and cognitive decline. Results showed a significant main effect between neighborhood and cognitive decline (higher levels of neighborhood disorder result in an increase in cognitive decline, B= 0.369, p =.014), but no significant main effect between sleep and cognitive decline. There was a significant interaction between sleep and neighborhood on cognitive decline. Participants who reported higher-than-average sleep difficulties experienced a greater effect of neighborhood disorder on cognitive decline. Our findings could add to inform future health interventions and policy recommendations that can address modifiable sources of cognitive decline.

Abstract citation ID: igad104.2640

### PREDICTORS OF DECISION MAKING FOR AGING-IN-PLACE SUPPORT IN ALZHEIMER'S DISEASE

Amber Miller-Winder, Raven Relerford, Allie Schierer, Charles Olvera, Alaine Murawski, Vanessa Ramirez-Zohfeld, and Lee A Lindquist, *Northwestern University, Chicago, Illinois, United States*

Older adults with Alzheimer's Disease (AD) need additional support in their lifetime, yet little is known about how the decision to accept help is made. Our goal is to better understand how older adults' aging-in-place (AIP) decision making is impacted by worsening cognition, functional loss, social influences, and environmental factors. This longitudinal study consists of older adults from an NIA-funded cohort (LitCog) with extensive cognitive testing. The intervention, PlanYourLifespan.org (PYL), facilitates making decisions about AIP needs, including AD. After viewing PYL, subjects are surveyed every 6 months with cognitive, social, functional, health literacy, environmental and decision-making variables. Of 293 subjects, (mean age 73.5, 72.7% female, 40.4% under-represented minority) almost half (47.4%) experienced cognitive decline with 10.3% identifying worsening memory loss from the prior timepoint. Subjects were asked: If you developed AD, have you decided your living/support preferences? At 1-mo, subjects were significantly more likely to have made decisions if they had: limited health literacy (OR 4.36 [p< 0.01, 1.69-11.24]), larger social networks (OR 1.08 [p< 0.05, 1.01-1.15]), completion of a living will (OR 2.43 [p< 0.05,1.11-5.33]. At 6-mo, sufficient social support (OR 3.39 [p< 0.05, 1.19-9.70]. At 12-mo, higher social isolation (OR 1.05 [p< 0.05, 1.01-1.08]) and self-efficacy (OR 1.07 [p< 0.01, 1.04-1.11]). In the event of worsening cognition, the likelihood of making care decision is associated with external (social support) and internal (self-efficacy, health literacy) variables, which change in significance over time. Catastrophic factors (COVID-19) impacted comfortability of utilizing long-term care facilities.

Abstract citation ID: igad104.2641

### READ TO SUCCEED: HOW BOOK CLUBS CAN IMPROVE INTERGENERATIONAL RELATIONS AND FOSTER AGE FRIENDLINESS IN COMMUNITIES

Katelyn Frey[1], Jennifer Sublett[1], Nikolina Kravljaca[2], and Jennifer Stanley[1], *1. University of Akron, Akron, Ohio, United States*, *2. The University of Akron, Barberton, Ohio, United States*

Intergenerational programming is recommended to improve age friendliness in communities, but there is little empirical research to support its effectiveness (Canedo-García et al., 2017). This project examined whether a multimodal intergenerational book club challenged age stereotypes and reduced ageism among participants (N=41). Twenty-one older adults (61-82) and 20 younger adults (18-22) read A Man Called Ove and met three times to introduce themselves (Time 1) and discuss the book (Time 2 and 3). Before the first meeting and after subsequent meetings, participants completed a survey to assess ageist beliefs and stereotypes. We expected ageist beliefs and stereotypes to decrease across time during the book club program. There was a statistically significant interaction between age group and time on benevolent ageism $F(2, 52)= 5.23$, $p= .009$ , partial $\eta2= .167$, such that benevolent ageism was more endorsed in younger adults ($M=6.64$, $SE= 2.48$, $p= .011$) compared to older adults at Time 1. This difference was not observed at Time 2 or Time 3 due to younger adults' decreased overall endorsement. For positive generational stereotypes about older adults, there was a statistically significant main effect of time, $F(2, 52)=7.095$, $p=.002$, partial $\eta2= .214$, such that both younger and older adults increased their endorsement of these stereotypes from Time 1 to Time 3; similar findings occurred for positive stereotypes about younger adults $F(2, 46)=4.53$, $p=.016$, partial $\eta2= .165$. Results indicate intergenerational book clubs are an effective means to improve intergenerational relations and reduce ageist attitudes in younger adults.

Abstract citation ID: igad104.2642

### SAFEGUARDING IN THE INTERNET OF THINGS AGE: A COMPREHENSIVE REVIEW OF SECURITY AND PRIVACY RISKS FOR OLDER ADULTS

Suleiman Saka, and Sanchari Das, *University of Denver, Denver, Colorado, United States*

The advent of the Internet of Things (IoT) has brought about a technological revolution that has transformed the way we live and interact with the world. IoT technology has the potential to enhance the quality of life for individuals, particularly older adults, by enabling them to access and control devices remotely. However, IoT technology also raises significant security and privacy concerns, especially for older adults, who are often less technologically savvy and more susceptible to cyber threats. This paper provides a systematic review of the literature related to IoT technology for older adults and identifies key gaps in the security and privacy of these technologies. Our review analyzed a total of 116 papers related to IoT for older adults and focused on 25 that met our criteria. We found that most of the research in this area focuses on the health and medical sector, where IoT technology can be used to monitor the health and well-being of older adults. However, our analysis revealed that there are significant gaps in the security and privacy of IoT technology for older adults. To address these gaps, we provide actionable recommendations for enhancing the security and privacy of IoT technology designed for older adults. Our recommendations include the development of user-friendly interfaces, the use of secure and reliable communication protocols, the incorporation of data encryption and anonymization, and the establishment of regulatory frameworks to ensure the protection of personal data.

### SESSION 7530 (POSTER)

### CHILDREN, SPOUSES, AND PARENT, OH MY! DIVERSITY IN FAMILY CAREGIVING

Abstract citation ID: igad104.2643

### CAREGIVERS' INTERACTIONS WITH HEALTH CARE PROVIDERS AND THEIR WELL-BEING BY RACE AND ETHNICITY AND GENERATION

Heehyul Moon[1], Sunshine Rote[1], and Sol Baik[2], *1. University of Louisville, Louisville, Kentucky, United States, 2. University of Virginia, Charlottesville, Virginia, United States*

Black and Hispanic family caregivers often face higher levels of care demands than White caregivers, due to factors such as limited resources, cultural differences, and systemic and structural inequalities. Family caregivers from different generations (Baby Boomer vs. Generation X & Millennial ) may approach and experience caregiving process and its health effects to a different extent. Caregivers often interact with healthcare providers on behalf of their care recipients, and these experiences can vary based on caregiver background (race and ethnicity and generation). The current study aimed to explore these interactions and their impact on caregivers' well-being using nationally representative data from the National Study of Caregiving (NSOC, round 11 (N=1,455; 418 Black caregivers, 926 Non-Hispanic White caregivers, and 111 Hispanic caregivers)). Most caregivers were adult children (70%) and female (70%), with approximately 68% were from the baby boomer generation. While Black and Hispanic caregivers were significantly less likely than White caregivers to communicate with medical providers ($\chi2(df=2)=12$, $p<.001$), baby boomer caregivers provided more assistance with speaking to medical providers than caregivers from younger generations ($\chi2(df=1)=4$, $p<.05$). Our regression results indicate that Hispanic caregivers who had more frequent communication with medical professionals reported more depressive symptoms and poorer health, while Black caregivers who rarely communicated with providers reported worse outcomes. Future research should examine the nature and quality of these interactions with healthcare providers to improve caregivers' well-being.

Abstract citation ID: igad104.2644

### EFFECTS OF CHILDCARE, WORK, AND CAREGIVING INTENSITY ON MALE AND FEMALE FAMILY CAREGIVERS

Hansol Kim[1], Scott Beach[1], Esther Friedman[2], Heidi Donovan[1], and Richard Schulz[1], *1. University of Pittsburgh, Pittsburgh, Pennsylvania, United States, 2. University of Michigan, Ann Arbor, Michigan, United States*

**Objectives:** The Behavioral Risk Factor Surveillance System sampled 54,076 caregivers between 2015 and 2017 providing an opportunity to evaluate risk factors for poor mental and physical health among a representative sample of U.S. adult caregivers. This study aimed to evaluate the impact of childcare, work status, and intensity of caregiving among men and women caring for older adults (n = 17,271). **Methods:** Controlling for sociodemographic factors, separate logistic regression analysis for women and men were carried out to assess the main and interaction effects of childcare, work status, and intensity of caregiving on number of poor mental and physical health days in the last month. **Results:** Intensive caregiving demands had adverse effects on both women and men, but being in the workforce was beneficial to both men and women. Women with children at home reported adverse mental health effects but better physical health, while men with children at home reported adverse physical health effects. For women, the combination of not working, children in the household, and high-intensity caregiving were most detrimental to their mental health. Among men, those not working with children in the household, regardless of caregiving intensity, were at highest risk of adverse mental health effects. **Discussion:** Our findings identify caregivers at high risk of adverse outcomes but also point to the need for more fine-grained analyses of how families negotiate the allocation of childcare, work, and caregiving responsibilities over time.

Abstract citation ID: igad104.2645

### FACTORS ASSOCIATED WITH CUSTODIAL GRANDCHILDREN RECEIVING MENTAL HEALTH CARE ASSISTANCE

Julian Montoro-Rodriguez[1], and Bert Hayslip[2], *1. University of North Carolina Charlotte, Charlotte, North Carolina, United States*, *2. University of North Texas, Denton, Texas, United States*

The number of grandparents caring for grandchildren has increased substantially over the past two decades. Grandparent caregivers experience strain and family vulnerabilities as they face challenging child-care responsibilities, less social support, and more adverse emotional and lifestyle changes. These challenges may influence the type of support they provide to their grandchildren, affecting their grandchildren's health and access to supportive services. In this light, research suggests that rates of service use among grandchildren are low (37% and 51% for community and school-based services) with the grandchild's externalizing symptoms and medical/psychiatric diagnoses being the strongest predictors of such use. This paper investigates the influence of grandparent and grandchild-related factors associated with a grandchild's receiving mental health care assistance. We examine how grandchildren's use of counseling services affects grandparent well-being and relationship quality with the grandchild. Guided by the Pearlin stress-outcome paradigm (1990), we used data from 239 grandparent caregivers (Mage = 58.06, SD = 8.17), wherein 80 grandchildren were receiving counseling mental care and 159 were not. Predictors of well-being and quality of grandchild/grandparent relationship outcomes were regressed on the use of the counseling services, stressors (health of grandparent and problem behaviors of grandchildren), and coping resources (resiliency, self-efficacy, social support) using a SEM model with AMOS. Results indicate good model fit (CFI = 0.968; TLI= 0.959; RMSE = 0.04). Findings suggested that grandchildren's use of mental health services influences both the quality of the relationship and the well-being of the grandparent albeit through different coping resources (grandparent's resilience and social support).

Abstract citation ID: igad104.2646

### SPOUSAL CAREGIVING AND PARALLEL COGNITIVE TRAJECTORIES AMONG OLDER ADULT CAREGIVERS AND RECIPIENTS IN CHINA

Guo Yu[1], Zhenmei Zhang[2], and Quanbao Jiang[3], *1. Xi'an Jiaotong University, Lansing, Michigan, United States*, *2. Michigan State University, East Lansing, Michigan, United States*, *3. Xi'an jiaotong university, Xi'an, Shaanxi, China (People's Republic)*

Prior studies have paid much attention to the physical and mental health outcomes of spouse caregivers. However, few have looked at the cognitive trajectory of caregivers and how it was associated with the characteristics of caregivers and care recipients. Based on four waves (2011, 2013, 2015, and 2018) of the Chinese Health and Retirement Longitudinal Survey (CHARLS) (N=3137 dyad couples aged 45 and above), cognitive function was measured by Mini-Mental State Examination (MMSE). A parallel process growth model was conducted to explore the association between spouse caregiving and the joint trajectories of caregivers and their recipients' cognitive decline during the eight years. Preliminary results show that 1) couples' trajectories are significantly related to each other, although men had a higher initial level of cognitive function than women, their cognitive function declined at a faster rate than women did; 2) The relationship between caregiving and cognitive trajectory varies by gender. Specifically, providing care is positively correlated with slower cognitive decline in men, but not in women. Whereas receiving the care was associated with a faster cognitive decline for both gender. These findings emphasize the need of paying attention to both family caregivers and recipients and the need for more targeted intervention strategies based on the nature of care.

### SESSION 7540 (POSTER)

### DEMENTIA: CHARACTERISTICS AND CARE

Abstract citation ID: igad104.2647

### A LATENT VARIABLE APPROACH TO ASSESS DEMENTIA IN MEXICO AND THE UNITED STATES: MEASUREMENT INVARIANCE AND VALIDATION

Joseph Saenz[1], Christopher Beam[2], and Silvia Mejía-Arango[3], *1. Arizona State University, Phoenix, Arizona, United States*, *2. University of Southern California, Los Angeles, California, United States*, *3. University of Texas Rio Grande Valley, Edinburg, Texas, United States*

Latent dementia indices (LDI) use cognitive and functional data to approximate dementia. Few evaluate the LDI's utility in cross-national work. This study tests metric measurement invariance of an LDI in the United States and Mexico and evaluates its validity in Mexico. Data included the United States Harmonized Cognitive Assessment Protocol (HCAP, n=3,267), MexCog (n=2,042), and a Mexican clinical validation sample with diagnosed cognitive status (51 cognitively normal, 49 mild cognitive impairment (MCI), and 50 dementia) who received the MexCog battery. The LDI was measured using highly comparable items (13 cognitive and 10 functional). We tested metric measurement invariance of the LDI in MexCog/HCAP samples and evaluated validity by regressing cognitive status on LDI factor scores in the Mexican validation sample using multinomial regression and evaluating how model-implied cognitive status aligned with diagnosis. Full metric invariance of the LDI in the MexCog and HCAP was supported, suggesting that the LDI may be used in cross-national work to validly compare dementia risk factors in the studies. In the Mexican validation sample, LDI factor scores significantly predicted MCI (β=-2.62, p< 0.001) and dementia (β=-5.93, p< 0.001) versus cognitively normal in multinomial regressions. LDI model-implied cognitive status showed moderately high dementia (84.0%) and MCI (88.9%) sensitivity and moderately high dementia (92.0%) and MCI (88.2%) specificity. The LDI may be used to cross-nationally compare risk/protective factors for dementia in MexCog/HCAP studies. Alignment between the LDI and diagnosed cognitive status in the Mexican validation sample supports its validity as a proxy for dementia in Mexico.

Abstract citation ID: igad104.2648

### CARE-RECEIVING CHARACTERISTICS AMONG OLDER ADULTS WITH DIFFERENT MENTAL–SOMATIC MULTIMORBIDITY COMBINATIONS

Mona Liu[1], Corey Nagel[2], Siting Chen[1], Heather Allore[3], Jeffrey Kaye[1], and Ana Quiñones[1], *1. Oregon Health & Science University, Portland, Oregon, United States*, *2. University of Arkansas for Medical Sciences, Little Rock, Arkansas, United States*, *3. Yale University, New Haven, Connecticut, United States*

Older adults with distinct multimorbidity combinations require different care with varying intensities. This study aimed to examine sociodemographic, health, and care-receiving characteristics among older adults with varying multimorbidity patterns. A total of 4,719 participants from the National Health and Aging Trends Study in 2017 were included, and five clinically-informed multimorbidity categories were assessed: 169 (3.58%) had no conditions, 3031 (64.23%) had somatic conditions only, 347 (7.35%) had depression without cognitive impairment (depression group), 852 (18.05%) had cognitive impairment without depression (cognitive impairment group), and 320 (6.78%) had both depression and cognitive impairment. Compared with the depression group and cognitive impairment group, those with both depression and cognitive impairment were significantly older, more likely to be non-Hispanic Black or Hispanic, widowed, U.S. born, Medicaid recipients, underweight BMI, had lower levels of education and more functional limitations. Compared to the depression group, those with both depression and cognitive impairment had lower self-rated health, less likely to be a smoker, received 118 more hours of care per month on average with more caregivers in ADLs, banking, and money management. Compared with the cognitive impairment group, those with both depression and cognitive impairment had more somatic conditions, received an average of 70 more hours of care per month with more caregivers in ADLs. Results highlighted the substantially greater caregiving hours and intensities pertaining to the combination of depression and/or cognitive impairment, and suggested the potential of addressing mental health among older adults with or without dementia to improve their own and caregivers' health outcomes.

Abstract citation ID: igad104.2649

### DEMENTIA FRIENDS AWARENESS BUSINESS TRAINING PROGRAM: MAKING A POSITIVE IMPACT IN SMALL ARKANSAS BUSINESSES

Laura Spradley[1], Whitney Thomasson[2], and Robin McAtee[3], *1. UAMS, Little Rock, Arkansas, United States*, *2. University of Arkansas for Medical Sciences (UAMS), Little Rock, Arkansas, United States*, *3. University of Arkansas for Medical Sciences, Little Rock, Arkansas, United States*

The Arkansas Geriatric Education Collaborative, a HRSA-funded Geriatric Workforce Enhancement Program grant, and a Dementia Friends USA sub-licensee, provides a program to businesses called Dementia Friends at Work. This program utilizes the tenants of the "Become a Dementia Friend" program created by Dementia Friendly America©, a multi-sector movement that aims to foster "dementia friendly communities". A one-hour training was conducted for 222 business employees, focusing on recognizing the signs of dementia, challenging the stigma about dementia, how to best communicate with individuals with dementia and their caregivers, and practical tips to make themselves and their environment more Dementia-Friendly. During the program, management and staff were asked to self-assess the physical business environment for "dementia friendliness" and review possible changes toward improvement. Pre-program and post-program evaluations were completed. The results showed that 100% of participants agreed the training program made an impact on their business or job and 75% claimed to use the knowledge gained from the program. Qualitative data gathered 90-days post training supported these results with themes emerging such as staff using new ways to navigate conversations with those expected to have dementia, being more aware of the signs of dementia, and utilization of skills learned to more effectively interact with these customers. Findings suggest that many employees were unaware of the common signs of dementia or how to recognize behaviors of those with dementia. They had also not considered learning about or using proven techniques to assist with interactions and conversations; therefore, this training was well-received and effective.

Abstract citation ID: igad104.2650

### DEVELOPING AND TESTING A TWO-PART INTERVENTION: ENHANCING DEMENTIA INSTRUCTION AND TOOL IN HOME HOSPICE CARE

Sophia Geisser[1], Abigail Aamponsah[2], Felix Vasquez[3], and Elizabeth Luth[4], *1. The University of Alabama, Tuscaloosa, Alabama, United States*, *2. Rutgers, The State University of New Jersey, New Brunswick, New Jersey, United States*, *3. VNS Health, New York City, New York, United States*, *4. Rutgers University, New Brunswick, New Jersey, United Sta tes*

Family care partners of persons living with dementia provide extensive care and experience significant stress and strain, particularly near the person living with dementia's end of life. Hospice nurses and social workers (clinicians) are positioned to support family care partners, but seldom receive training to do so. This article describes the approach to developing and pilot testing a two-part intervention, Enhancing Dementia Instruction and Tool in Home Hospice Care (EDITH-HC). This intervention provides 1) educational videos for hospice nurses and social workers about dementia-specific end-of-life care and 2) a worksheet for clinicians to use as part of their clinical practice to use with family care partners to identify and address their stressors and concerns. EDITH-HC development involved: (1) a systematic review of literature and analysis of interview data from 58 family care partners (n=40) of home hospice patients living with dementia and clinicians (physicians, nurses, and social workers, n=18); (2) analysis of primary data collected from two rounds of structured, key stakeholder input from 19 family care partners, hospice clinicians, and research/content experts; and (3) single-arm pilot of the intervention to test initial feasibility and acceptability. The intervention development will lead to a randomized pilot study to determine the feasibility and acceptability of implementing the training and worksheet in clinical practice compared to usual care.

Abstract citation ID: igad104.2651

### EXPLORING "LIVING WELL" FROM THE PERSPECTIVE OF PEOPLE LIVING WITH ALZHEIMER'S DISEASE AND RELATED DEMENTIAS

Sam Fazio, and Lauren Stratton, *Alzheimer's Association, Chicago, Illinois, United States*

Alzheimer's disease is a progressive, degenerative disease; however, at the same time individuals living with dementia experience symptoms, they also want to live well with the disease. Although living well with dementia holds different meaning to different people, it generally includes some commonalities, such as maintaining autonomy and independence. To explore the concept of living well with Alzheimer's disease and related dementia (ADRD), the Alzheimer's Association gathered data from nearly 100 individuals living with dementia through focus groups and online surveys. Themes from the focus groups were revealed on living well, including being viewed as an individual (person-centered care), staying involved, and receiving multiple types of support to assist their well-being. Open-ended questions from the surveys illustrated additional themes related to living well, as well as on personal meaningfulness of disease modifying therapies including more time with loved ones and opportunities to be part of significant family events. Within these findings, an underlying theme emphasizing the importance of person-centered care and well-being arose. Further discussion will explore these interconnected concepts of living well, person-centered care, and well-being, specifically including the perspectives of individuals living with ADRD. In addition, these concepts can be utilized to tailor resources and support to better support individuals living with ADRD to live well. Lastly, the concept of living well in relation to the emergence of disease modifying therapies and how personal meaningfulness is just as important as clinical meaningfulness will also be explored.

Abstract citation ID: igad104.2652

### FORTY WINKS: PILOT MULTISITE PRAGMATIC CLINICAL TRIAL TO IMPROVE SLEEP FOR NURSING HOME RESIDENTS WITH DEMENTIA

Kate Smith[1], A Lynn Snow[1], Robert Morgan[2], Megan McCullough[3], Rosa Baier[4], Ellen McCreedy[4], Kathy Richards[5], and Christine Hartmann[6], *1. University of Alabama, Tuscaloosa, Alabama, United States*, *2. University Of Texas Health Science Center, Houston, Texas, United States*, *3. University of Massachusetts Lowell, Lowell, Massachusetts, United States*, *4. Brown University School of Public Health, Providence, Rhode Island, United States*, *5. University of Texas at Austin, Austin, Texas, United States*, *6. VA Bedford Healthcare System, Bedford, Massachusetts, United States*

Sleep disruption in nursing home (NH) residents can lead to difficulties with activities of daily living, falls, depression, and decreases in memory, and mobility. Staff-delivered interventions are the most logical approach to improving NH resident sleep. But it can be difficult for frontline NH staff to implement quality improvement (QI) due to chronic understaffing, limited staff training, and challenges with data collection. We conducted a pilot multisite pragmatic clinical trial of the "LOCK" sleep program in three NHs. LOCK is an evidence-based framework using frontline huddling for QI uptake: "Learn from the bright spots" (local evidence of positive change), "Observe" (collect data through observation), "Collaborate in huddles" (conduct short frontline huddles), and "Keep it bite-size" (limit activities to 5-15 minutes). We used actiwatches to measure day and night activity and conducted 35 group interviews with leadership/champion implementation teams. We used an iterative, rapid-content analytic approach for qualitative analyses, examining factors associated with implementation variation using the Consolidated Framework for Implementation Research. In qualitative analyses, staff indicated the program was highly feasible and acceptable. From actiwatch data, for enrolled NH residents with ≥9 weeks of intervention exposure (n=11), total daytime activity increased an average of 19 minutes per day (SD=95) and total night-time sleep increased an average of 37 minutes per night (SD=39). The LOCK program is a promising approach and framework for NH staff pursuing QI related to sleep or other topics.

Abstract citation ID: igad104.2653

### INITIAL FINDINGS ON THE BENEFITS OF THE TOVERTAFEL: REDUCING BEHAVIORS IN PERSONS WITH DEMENTIA

Victoria Steiner[1], Jennifer Perion[2], Meggan Hartzog[1], Safa Ibrahim[1], Alexa Lopez[1], Beanna Martinez[1], Barbara Saltzman[1], and Jennifer Kinney[3], *1. University of Toledo, Toledo, Ohio, United States*, *2. The University of Tennessee, Knoxville, Knoxville, Tennessee, United States*, *3. Miami University, Oxford, Ohio, United States*

Technologies have been developed to reduce behaviors in persons with dementia by addressing unmet needs through activities that stimulate multiple senses. The Tovertafel ("magic table") is a novel device from the Netherlands that enables purposeful play using interactive games designed to promote social, physical, and cognitive engagement in people with dementia. This study is investigating whether an intervention using the Tovertafel improves quality of life for residents with moderate to severe dementia living in long-term care communities in the Midwest. Twelve white women with an average BIM score of 6 completed the study at the initial facility. The 4-week intervention consisted of 30-minute game sessions with 2 to 6 individuals each weekday.

Participants attended 2.5 sessions per week on average and displayed increased engagement during the games per facilitator reports. Direct care staff completed the Revised Memory and Behavior Problems Checklist–Nursing Home and the Apathy Evaluation Scale–10 for all participants. Data collected eight times over six consecutive weeks during the control (2 weeks) and intervention (4 weeks) periods were analyzed. After checking assumptions, paired-sample t-tests were conducted that indicated significantly fewer memory-related behaviors (p=.047) (e.g., repetitive behaviors and questions) and instances of apathy (p=.027), during the intervention period than the control. Despite reductions in depressive and disruptive behaviors, no statistically significant differences were found. Initial findings suggest that the Tovertafel could offer innovative ways to provide activities to people with moderate to severe dementia in long-term care settings, increase their quality of life, and improve interactions with staff.

Abstract citation ID: igad104.2654

### LATENT CLASSES OF MULTIMORBIDITY AND ASSOCIATED CARE-RECEIVING CHARACTERISTICS FOR US OLDER ADULTS

Mona Liu[1], Corey Nagel[2], Siting Chen[1], Jeffrey Kaye[1], Heather Allore[3], and Ana Quiñones[1], *1. Oregon Health & Science University, Portland, Oregon, United States*, *2. University of Arkansas for Medical Sciences, Little Rock, Arkansas, United States*, *3. Yale University, New Haven, Connecticut, United States*

Older adults with different multimorbidity combinations require different intensities and types of care. This study aimed to identify subgroups of older adults based on their empirically-determined multimorbidity patterns, and used these to compare their care-receiving characteristics. Data from the National Health and Aging Trends Study (NHATS) in 2011 were utilized and ten chronic conditions were included to estimate latent classes among 7,532 individuals. A four-class solution was selected based on fit indices resulting in a relatively healthy (30%), cardiometabolic (25%), musculoskeletal (24%), and multisystem (21%) classes. The median number of caregivers was 2 for the multisystem class and 1 for the remaining three classes. Average total hours of care per month were 109.07 (SD=209.09), ranging from 94.43 (SD=168.71) for the relatively healthy class to 171.87 (SD=225.72) for the multisystem class. In assessing ADL and IADL domains of care received, the musculoskeletal class had the smallest proportion of people requiring care in all domains except for getting outside, dressing, and money management, even compared with the relatively healthy class. Substantially more people in the multisystem class required care in all domains except for money management. Sensitivity analysis using data from the refreshment cohort (NHATS 2015) corroborated the results. Results highlighted different care needs among persons with distinct combinations of multimorbidity, especially the vulnerability among people with multisystem multimorbidity and their caregivers. In addition, the findings called for further investigation into older adults characterized by musculoskeletal conditions to discern whether they had fewer care needs or more unmet needs.

Abstract citation ID: igad104.2655

### NEIGHBORHOOD COHESION MODERATES THE ASSOCIATION BETWEEN DEMENTIA STATUS AND SUBJECTIVE WELL-BEING

Dokyung Yoon[1], Eileen Crimmins[2], and Jennifer Ailshire[2], *1. University of Southern California, Leonard Davis School of Gerontology, Los Angeles, California, United States*, *2. University of Southern California, Los Angeles, California, United States*

Neighborhood social cohesion may act as a protective factor against the negative effects of certain stressors associated with dementia status on subjective well-being. The current study aimed to investigate the moderating effect of neighborhood social cohesion on the relationship between dementia status and subjective well-being among older adults. The status of dementia was classified as either probable or possible, or as not having dementia. Data were drawn from the 1st round (2011) of the National Health and Aging Trends Study (NHATS), a panel study of nationally representative Medicare beneficiaries aged 65 and older. The sample consisted of 5,407 community-dwelling older adults. A linear regression model indicated that dementia status was associated with a lower level of subjective well-being (b = -2.34, p < .001), while neighborhood social cohesion was associated with a higher level of subjective well-being (b = 1.04, p < .001). Furthermore, neighborhood social cohesion moderated the association between dementia status and subjective well-being (b = .67, p = .003). In older adults with probable or possible dementia, the relationship between neighborhood social cohesion and subjective well-being was observed to be stronger, as evidenced by a steeper slope. The findings of this study contribute to the growing body of evidence that emphasizes the crucial role of ties within one's neighborhood in influencing subjective well-being. Building strong communities of individuals who rely on and trust one another may help alleviate the negative impact of probable or possible dementia on subjective well-being.

Abstract citation ID: igad104.2656

### RE-IMAGINING DEMENTIA FAMILY CAREGIVER ENGAGEMENT FOR POST–ACUTE CARE TRANSITIONS: LESSONS LEARNED FROM COVID-19

Ruth Tadesse[1], Karen Lyons[2], Glenise McKenzie[1], Sara Hart[3], Lee Ellington[3], and Kristin Cloyes[1], *1. Oregon Health & Science University, Portland, Oregon, United States*, *2. Boston College, Boston, Massachusetts, United States*, *3. University of Utah, Salt Lake City, Utah, United States*

Family caregivers of persons with dementia (PwD) experience three-times more post-acute care transitions (PACT) to skilled nursing facilities than other caregivers. During Covid-19, visitation policies drastically changed, limiting family caregiver engagement in the skilled nursing facility. This was a widespread experience; yet, few studies have examined dementia family caregivers' perceptions of PACT engagement during Covid-19 restrictions. This qualitative study focused on the findings and lessons learned about the meaning of engagement, barriers, facilitators, and support needs during Covid-19 using an interpretive descriptive approach. Participants (n=15) were mostly female (n=13), adult children (n=8), and the majority (n=9) had a PACT experience during the Covid-19 pandemic. Family caregivers reported improved engagement with the PwD and healthcare team in the acute care setting, because hospital policies on Covid-19 allowed one visitor which gave family caregivers the option to be physically present and advocate for the PwD. However, after the PACT, only essential workers were allowed at the skilled nursing facility making it impossible for family caregivers to visit the PwD and communicate with professional caregivers. Lack of perceived support negatively affected family caregivers' ability to communicate, share knowledge, advocate, and participate in critical decision making. These Covid-19 challenges highlight the importance of being physically present as a necessary component to engagement which was a form of communication compensation that improves outcomes for the PwD. Future practice policies should view family caregivers as essential to the healthcare team and develop innovative solutions to connect family caregivers to the PwD during Covid-19 or future pandemics.

Abstract citation ID: igad104.2657

### SINGING WITH DEMENTIA: CREATING AND PILOT TESTING A VIDEO TO CHANGE ATTITUDES OF MEDICAL RESIDENTS

Jody Sharninghausen[1], Carrie Ann Davison[1], and Richard Marottoli[2], *1. Yale School of Medicine, New Haven, Connecticut, United States*, *2. Yale Medical School, New Haven, Connecticut, United States*

As part of the "Creativity in Aging" project at Yale Geriatrics, we are making a series of short videos highlighting individuals and programs that transcend dementia through music. The target audience includes health trainees and professionals who care for older adults. The first video, made by a PhD candidate and an internal medicine resident physician, features The Recollections: a chorus of community dwelling people with dementia, their caregivers, and college students. Video learning objectives are to 1) change dementia attitudes; 2) demonstrate preserved skills and abilities of persons with dementia; and 3) raise awareness about local initiatives for music in dementia and how to refer patients. We interviewed the chorus founder, a geriatric psychiatrist, who described the creative process, including logistics and vision, and provided advice to caregivers and medical residents. Live clips from chorus rehearsals were shown, including singing and dancing, with voiceovers describing participant stories. Slides reinforced key points. The video was pilot tested on a group of residents from the Internal Medicine and Psychiatry programs. All respondents (N=6) used positive words such as "optimistic" and "inspired" to describe how the video made them feel. Most indicated it was moderately relevant to their care of patients with dementia and several provided suggestions on how to increase relevance. Constructive feedback included parts to compress or expand, overall length, visual aspects, and desire for more 'how to' guidance. Future plans include sharing a revised version with the broader residency program with pre-and-post dementia attitudes surveys.

Abstract citation ID: igad104.2658

### SOCIOECONOMIC FACTORS AND LATER LIFE OUTCOMES IN UNEXPECTED DEMENTIA TRAJECTORIES

Yulin Yang[1], and Elizabeth Luth[2], *1. University of California, San Francisco, San Francsico, California, United States*, *2. Rutgers University, New Brunswick, New Jersey, United States*

Dementia's uncertain trajectory may complicate its relationship with social determinants of health—race, ethnicity, socioeconomic status—and put individuals who experience unexpected dementia trajectories (e.g., probable dementia at one point, no dementia at a future point) at increased risk of poorer outcomes. This study identifies dementia trajectories in longitudinal survey data, their association with socio-economic factors, and their relationship with later life outcomes. We analyzed weighted data for 9740 adults aged 65+ participating in ≥3 waves of the 2004-2016 Health and Retirement Study. After identifying 5 dementia trajectory patterns, we analyzed the association between sociodemographic factors and dementia trajectories using multinomial logistic regression. We analyzed the relationship between dementia trajectories, socio-economic factors, and three later life outcomes (hospitalization, nursing home admission, and advance care planning) using logistic and multinomial logistic regression. A small percentage (2.2%) of respondents reported an unexpected dementia trajectory. African American and Hispanic respondents and those with lower socioeconomic status had increased risk of unexpected dementia trajectories (vs. no dementia) (e.g., African American RRR: 3.67, 95%CI: 2.30-5.86, p= 0.000; Hispanic RRR: 1.89, 95%CI 1.08-3.30, p=0.26). Older adults with unexpected dementia trajectories had increased risk of nursing home admission (RRR: 4.13, 95%CI: 1.53-11.16, p=0.005), but not hospitalization or advance care planning. Policy and measurement development should proactively address systemic inequities linked to race, ethnicity and socioeconomic status that emerge in and result from dementia trajectory assessments and lived experiences. Interventions should attend to the needs of individuals with unexpected dementia trajectories with respect to institutionalization.

Abstract citation ID: igad104.2659

### THE IMPACT OF ADS CLOSURES DURING COVID-19 ON PERSONS WITH DEMENTIA AND THEIR CAREGIVER

Laura Girling[1], and Gretchen Tucker[2], *1. Towson, Towson, Maryland, United States*, *2. University of Maryland, Baltimore, Baltimore, Maryland, United States*

Due to the COVID-19 pandemic, many Adult Day Services (ADS), community-based programs that provide supervision, social support, and/or assistance with daily activities for clients, either temporarily or permanently terminated in-person services. Such closures left vulnerable older adults with dementia without vital services. To date, there is limited information exploring the nuances and impact of ADS closures on individuals with dementia and their care providers. To address this knowledge gap, data were drawn from an interview-based NIH-funded protocol that evaluated the experiences of informal and formal dementia care providers during the height of the COVID-19 pandemic. Data were filtered by the subsample (n=25) who provided care to an individual with dementia who participated in an ADS program. Analysis focused on all data coded "ADS," which encompassed discussion pertaining to adult day programming. Content analyses indicated a range of themes including 1.accelerated cognitive and psychological decline, 2.engaging is precarious conduct, 3.increased caregiver burden (financial, physical, emotional), and 4.premature transitions to institutional care settings. Despite well intentioned efforts to shield the older adult population from harmful exposures during the height of the pandemic, ADS closures (both permanent and temporary) negatively impacted the health and welfare of an already vulnerable population and their caregivers. Findings add to the ongoing literature suggesting ADS and analogous supports should be classified as essential services and not face closure mandates during similar public health emergencies.

Abstract citation ID: igad104.2660

### THE RELATIONSHIP BETWEEN MOOD OF CAREGIVERS AND CARE RECIPIENTS WITH DEMENTIA

Yeji Hwang, *Seoul National University, Seoul, Republic of Korea*

Anxiety and depression are common behavioral and psychological symptoms of dementia. Although there are many individual level factors related to their anxiety and depression, it is unknown whether caregivers' mood influence anxiety and depression of their care recipients with dementia. Therefore, this study was conducted to examine the relationship between anxiety and depression of caregivers and that of care recipients with dementia. This study was a secondary data analysis using the National Health and Aging Trends Study (NHATS) Round 7 data and National Study of Caregiving (NSOC) III. This study used 553 dyads of older adults aged 65 years and older who had probable dementia at the time of the interview and their family caregivers. Anxiety and depression of both the caregivers and care recipients of dementia were measured with the Generalized Anxiety Disorder-2 (GAD-2) and Patient Health Questionnaire-2 (PHQ-2) respectively. Descriptive statistical analyses, chi-square tests, and logistic regression analyses were used. There were significant relationships between the caregivers' anxiety and the care recipients' anxiety presentation (Chi2=10.06, p< 0.01). Similar relationships were also found for depression (Chi2=5.22, p=0.02). Logistic regression analyses showed that when caregivers had anxiety or depression, it was more likely that their care recipients show anxiety (OR=2.25, p=0.002, 95% CI=1.35-3.74) or depression (OR=1.78, p=0.023, 95% CI=1.08-2.94). Because dementia caregivers' negative mood can influence their care recipients' mood, health care professionals should conduct early interventions for caregivers with negative moods.

### SESSION 7550 (POSTER)

### DISASTERS, EMERGENCIES, AND COVID-19

Abstract citation ID: igad104.2661

### AGE DIFFERENCES IN PSYCHOLOGICAL DISTRESS AFTER MULTIPLE DISASTER EXPOSURES: EFFECT OF NEGATIVE COVID-19 IMPACTS

Zhirui Chen[1], and Zhen Cong[1], *1. The University of Alabama at Birmingham, Birmingham, Alabama, United States*

This study examined how older adults differed from their younger counterparts in psychological distress following exposures to tornadoes and the COVID-19 pandemic; and how the multidimensional negative COVID-19 impacts contextualized the above age differences. Data used were from a two-wave panel study of tornado victims during the COVID-19 pandemic (N = 554). Latent class analysis was first conducted to explore the underlying patterns of negative COVID-19 impacts at T1 (October 2020-August 2021). Negative binomial regressions were performed to examine the age differences in psychological distress at T2 (May-August 2022), as well as the moderating effect of the identified latent classes, with baseline psychological distress and covariates controlled. Three latent classes were identified: class 1 "low overall impacts" (39.24%), class 2 "moderate overall impacts with high emotional distress" (47.71%), and class 3 "severe overall impacts" (13.05%). Individuals aged 65+ reported lower psychological distress at T1 relative to those aged 18-34, 35-49, and 50-64, and their advantages in mental health over people under 50 can be maintained over time. However, compared to people aged 18-34, 35-49, and 50-64, those aged 65+ reported the greatest increases in T2 psychological distress if they had experienced moderate or severe overall COVID-19 impacts at T1. These findings highlighted the resilience and vulnerability of older adults in post-disaster recovery. As the frequency and intensity of cumulative disasters increase across the globe, there is a pressing need for mental health interventions that are tailored to multi-disaster scenarios and age-related differences in long-term disaster recovery.

Abstract citation ID: igad104.2662

### DYNAMIC BENEFITS OF FINDING THE SILVER LINING: COUPLED CHANGES IN SECONDARY CONTROL AND WELL-BEING DURING COVID-19

Matthew Pierce, Jeremy Hamm, Katherine Duggan, and Odalis Garcia, *North Dakota State University, Fargo, North Dakota, United States*

Secondary control beliefs (e.g., "negative experiences can be blessings in disguise") reflect a psychological resource that can protect well-being for individuals who encounter uncontrollable life circumstances. However, previous research has focused on between-person differences in secondary control; little is known about the role of dynamic, within-person shifts in secondary control during major life stressors such as the COVID-19 pandemic. Our study examined whether within-person changes in secondary control beliefs were associated with corresponding within-person shifts in mental health and well-being during the pandemic. We analyzed 1-year data from the NDSU National COVID Study which contains 4 waves of data from a nationally representative sample of U.S. adults aged 18-80 (n = 293). Multilevel models assessed the extent to which within-person shifts in secondary control predicted corresponding changes in mental health (perceived stress, depressive symptoms), hedonic well-being (positive and negative affect, life satisfaction), and eudemonic well-being (personal growth, meaning and purpose). All models controlled for age, sex, education, income, and between-person differences in secondary control. Within-person changes in secondary control predicted corresponding shifts in mental health and well-being outcomes (bs = |.10-.25|, $p < .005$) except life satisfaction ($b = .10$, $p = .061$). In other words, participants reported increased mental health and well-being on occasions during the pandemic when they experienced increased secondary control. Findings advance the literature by documenting that secondary control exhibits dynamic shifts during a major life stressor. Additionally, these findings point to the adaptive role of such shifts (within-person increases) in supporting well-being during the pandemic.

Abstract citation ID: igad104.2663

### EMOTIONAL DISTRESS, ECONOMIC PRESSURE, AND WELL-BEING DURING COVID-19: THE ROLE OF A WARM ROMANTIC PARTNER

Abigail Hart, Jeenkyoung Lee, Tricia Neppl, and M. Heather West Greenlee, *Iowa State University, Ames, Iowa, United States*

The COVID-19 pandemic caused health threats especially for rural older adults. We know less about how characteristics prior to the pandemic impact how older adults fare during the pandemic. Guided by the Family Stress Model, the current study examined midlife economic pressure (age 39) to emotional distress (ages 40-42) and older adult (age 70) COVID-19-related well-being (financial strain, anxiety, general health), and how these associations differ by a warm spouse. Data come from the Family Transitions Project (1989, 1990-1992, 2017, 2020) and included rural white older adults from enduring marriages (N = 265). Multiple group analysis (structural equation modeling, full information maximum likelihood) revealed significant differences between two groups divided by low and high warmth from a spouse in 2017 (low = 135, high = 130). Chi-square difference test between fixed and free model was significant ($\Delta X2 = 9.48$, $\Delta df = 2$, $p < .01$), and free model had an acceptable fit to the data ($X2 = 90.616$, $df = 56$, $p < .01$, $CFI = .97$, $RMSEA = .07$). For low warmth group (but not high), emotional distress from economic pressure in midlife significantly predicted financial strain ($b = 1.02$, $SE = .35$, $p < .01$) and anxiety ($b = 1.03$, $SE = .20$, $p < .001$) during COVID-19. General health during COVID-19 was associated with emotional distress in midlife but did not differ by group. Our findings help understand the role of marital warmth on how earlier family stressors impact older adult well-being during historical events like COVID-19.

Abstract citation ID: igad104.2664

### FACTORS RELATED TO GENERATIVITY OF OLDER READING ALOUD VOLUNTEERS DURING THE COVID-19 PANDEMIC: REPRINTS STUDY

Tomoya Takahashi[1], Kyoko Fujihira[2], Tomoya Sagara[1], Koji Fujita[1], Hiroyuki Suzuki[2], Hiroko Matsunaga[3], Hiroshi Murayama[3], and Yoshinori Fujiwara[1], *1. Tokyo Metropolitan Institute for Geriatrics and Gerontology, Tokyo, Tokyo, Japan*, *2. Tokyo Metropolitan Institute of Geriatrics and Gerontology, Tokyo, Tokyo, Japan*, *3. Tokyo Metropolitan Institute of Gerontology, Tokyo, Tokyo, Japan*

Volunteer activities was hampered by the COVID-19 pandemic. This study focused on generativity of older volunteers' activities during the COVID-19 pandemic and examined the factors associated with one. A self-administered questionnaire survey was conducted among 453 older adults belonging to REPRINTS, a volunteer picture book reading organization from October 2021 to January 2022. 404 respondents were obtained, and 277 respondents (mean age: 73.4, SD: 6.1, female: 91.2%) with no missing responses were included in the analysis. Analysis items were Revised Japanese Version of Generativity Scale (JGS-R) composed of 3 factors (generational interest, behavior and achievement), satisfaction and burden of activities, frequency of activities in the last month, Child Image Scale for Older Adults composed of 5 factors (protection, purity, self-centeredness, creativity and self-reliance), a shortened version of the Help Seeking Scale, physical and mental health (subjective health, TMIG-13, J-WHO-5), living conditions, gender, and age. Multiple regression analysis was used with each subscale score in JGS-R as the dependent variable and others as independent variables. The results showed there was a significant association between generational interest and image of creativity, desire for help, mental health, and social roles; between generational behavior and image of purity (-), creativity image, desire for help, resistance to help (-), and social roles; between generational achievement and desire for help, social roles, and gender (male), respectively. In conclusion, physical and mental health, image of the child, and desire for help were associated with older volunteers' generativity even during the COVID-19 pandemic even during the COVID-19 Pandemic.

Abstract citation ID: igad104.2665

### GRIEF FOLLOWING A COVID-19 DEATH

Karuna Thomas[1], Nancy Pachana[2], Hannah Silverstein[1], Chayna Kanofsky[1], Hyun Jin Cho[3], and Brian Carpenter[1], *1. Washington University in St. Louis, St. Louis, Missouri, United States*, *2. The University of Queensland, Brisbane, Queensland, Australia*, *3. Washington University in St Louis, St. Louis, Missouri, United States*

The large number of deaths due to COVID-19 has resulted in a similarly high number of people experiencing grief. Attending to the needs of grieving individuals will be a critical public health issue for years to come. Moreover, the circumstances surrounding many COVID-19 deaths may contribute to more severe and protracted grief. The aim of this qualitative study was to learn about the social and psychological experiences of pandemic-related grief. Semi-structured interviews were conducted with individuals who had lost someone close to them to COVID-19 (N = 25). Interview questions addressed circumstances surrounding the death, rituals and memorializations undertaken, and subsequent emotional consequences. Thematic analysis was used to identify themes and patterns. Participants described their grief as being highly personal and unique and only understood by others who had experienced a similar loss from COVID-19. They expressed significant distress at the circumstances surrounding the death, with many not having an opportunity to say goodbye. Their distress was amplified by a lack of closure due to limited rituals allowed during the pandemic. Participants also reported feeling invisible in their experience of loss and grief as they reflected on the overwhelmed healthcare systems during the height of the pandemic, comparison to the type of experience they would have had were it not for the pandemic, as well as feeling the world had moved on once pandemic restrictions were lifted. Taken together, these findings suggest unique grief experiences with COVID-19 and the need for targeted interventions from mental health practitioners.

Abstract citation ID: igad104.2666

### HEALTH-RELATED QUALITY OF LIFE IN OLDER ADULTS AFTER A FLOOD

Piper Bordes[1], Katie Cherry[1], Matthew Calamia[1], Emily Elliott[1], Laura Sampson[2], and Sandro Galea[3], *1. Louisiana State University, Baton Rouge, Louisiana, United States*, *2. Harvard University, Boston, Massachusetts, United States*, *3. Boston University, Boston, Massachusetts, United States*

Exposure to multiple back-to-back disasters may threaten physical and mental health for people who live in geographic areas that are prone to severe weather events. In 2005, coastal residents from south Louisiana lost homes in Hurricane Katrina and many moved inland to presumably higher and safer ground. In August of 2016, historic flooding brought widespread destruction, creating a second round of catastrophic losses for those who had permanently relocated to Baton Rouge, Louisiana after Katrina. The present research is part of a larger longitudinal study on health and well-being after multiple disaster exposures. In this study, we examined associations among current and prior flood experience and health-related quality indexed by the SF-36 Health Survey (Ware & Sherbourne, 1992). Three flood exposure groups were compared across two waves of testing: non-flooded (controls), single disaster (flooded in 2016) and double disaster (flooded in 2005 and again in 2016). Results indicated that the flood exposure groups did not differ in physical health, although mental health was poorer for those whose homes flooded relative to the non-flooded controls at both waves of testing. Correlation analyses revealed that age was negatively associated with intolerance of uncertainty and physical health, but positively correlated with mental health at both waves of testing, consistent with the inoculation view of post-disaster psychological reactions. Implications of these data for understanding health-related quality of life after multiple disaster exposures are discussed.

Abstract citation ID: igad104.2667

### HURRICANE KATRINA'S LASTING IMPACT ON YOUNGER AND OLDER ADULTS: THE ROLE OF RELIGIOSITY AND POSTTRAUMATIC GROWTH

Katie Cherry[1], Marlene Friis[2], Piper Bordes[1], and Kennedy Simon[1], *1. Louisiana State University, Baton Rouge, Louisiana, United States*, *2. Tulane University, New Orleans, Louisiana, United States*

In 2005, Hurricane Katrina devastated the U.S. Gulf Coast with catastrophic damage and substantial loss of life. In this study, we examined associations among psychosocial variables and mental health indicators in younger and older adults who lived in south Louisiana in August of 2005 when Katrina made landfall (M age = 51.7 years, SD = 13.03 years, age range: 32 to 78 years). All self-identified as African American / Black women. They were recruited through social media, flyers sent to churches and schools, and word of mouth referrals. On the first day of testing, participants completed sociodemographic, religiosity, and post-traumatic growth surveys and an adapted structured storm questionnaire (Cherry et al., 2011). On the second day of testing one week later, participants responded to open-ended questions on their Katrina experience, and they completed measures of post-traumatic stress disorder (PTSD) and depression. Correlation analyses revealed that age was inversely related to PTSD symptoms, which is compatible with an inoculation perspective on post-disaster psychological reactions. In addition, post-traumatic growth was positively correlated with religious beliefs and practices and religious coping, and negatively correlated with symptoms of PTSD and depression. Religious beliefs and practices were inversely correlated with PTSD symptoms and depression. Qualitative analyses of participants' narrative responses indicated that some reflected on scripture and utilized prayer as a way to navigate their new lives in the years after Katrina. Implications of these data for understanding individual and community-level challenges in the years after exposure to devastating severe weather events are considered.

Abstract citation ID: igad104.2668

### IMPLICATIONS OF THE COVID-19 PANDEMIC ON ADULT DAY SERVICES

Katherine Marx[1], Lauren Parker[1], and Laura Gitlin[2], *1. Johns Hopkins University, Baltimore, Maryland, United States*, *2. Drexel University, Philadelphia, Pennsylvania, United States*

Covid-19 hit Adult Day Services (ADS) hard with sites across the country forced to close. As part of the ADS-Plus study, two focus groups were conducted to understand the impact of Covid-19 on ADS sites, their participants, and staff. Six sites across the country, represented by nine staff and administration (females=n, males=1) participated in focus groups. Focus groups were recorded, transcribed verbatim, and coded by the authors. Four themes emerged, (1) Ways to stay in touch with families; (2) Issues reopening; (3) Family concerns; and (4) Future of the ADS Industry. Sites stayed in touch with families through a variety of measures, including virtual daycare or activities via zoom, house calls, and help obtaining food. Upon reopening, there were multiple issues with getting the census back for both participants and staff. Families were reluctant to return due to fear of Covid-19, placing their family member in LTC due to decline in functioning or due to work, and using more home services. Staff were reluctant to return due to fear of Covid-19 and salary, some reporting they made more on unemployment. There was a decline in both physical and cognitive health of ADS participants, attributed to the lack of socialization. In looking towards the future, focus group participants felt that the old approach of doing ADS is not sustainable and that the industry needs to expand to include more home options. Also, funding of ADS needs to be addressed as many facilities are not able to keep up with increasing salaries.

Abstract citation ID: igad104.2669

### IN-PERSON AND TECHNOLOGY-BASED SOCIAL INTERACTION IN OLDER ADULTS DURING THE COVID-19 PANDEMIC 2022

Jennifer Peszka[1], Anne Goldberg[1], Peter Gess[1], Noura Mussallam[1], Luke Simpson[1], and Laura Spradley[2],

*1. Hendrix College, Conway, Arkansas, United States*, *2. UAMS, Little Rock, Arkansas, United States*

Social support is an important factor in the health and well-being of older adults. Early COVID-19 lockdowns disrupted in-person social interactions but presumably increased technology-based social connection. This work examines social interactions during Spring of 2022 in older Arkansans with widely available vaccines and waning lockdowns to investigate current in-person and online social interactions. During Spring 2022, 603 older Arkansans (65+) (86.5% White, non-Hispanic, 65.9% women) completed the 20 question self-report automated survey (text or phone). Results indicated 27.6% continued to curtail in-person interactions significantly; however, 63% engaged in in-person social interactions outside their home more than once a week. Video chatting pre-pandemic was prevalent (40% video chatting multiple times a week); still, 20.6% never did. 22.8% reported increased video chatting during the pandemic, but surprisingly, 37.3% reported a decline. 70% felt socially connected during online interactions. Only 78% reported being satisfied with their social connection compared to 93% pre-pandemic. These findings suggest that while many have re-established in-person social interactions, around one-quarter of participants continue to curtail in-person interaction and report social connection dissatisfaction. Social technology alternatives were increased by about one quarter of participants and were reported to lead to feelings of social connection when used, and yet a decline in this usage was reported across 40% of these participants during the pandemic. These data support access to technology-based social interactions through expansion and improvement of older adult friendly virtual platforms and internet access which could help improve quality and quantity of social connection and reduce social isolation.

Abstract citation ID: igad104.2670

### RACIAL/ETHNIC DIFFERENCES IN IMPACTS OF COVID-19 ON PSYCHOLOGICAL WELL-BEING AMONG OLDER ADULTS

BoRin Kim[1], Esther Shin[2], and Jihye Baek[3], *1. University of New Hampshire, Durham, New Hampshire, United States*, *2. Illinois State University, Normal, Illinois, United States*, *3. Washington University in St. Louis, St. Louis, Missouri, United States*

Covid-19 caused challenges to psychological well-being among older adults, but the impact may differ across various populations. The purpose of this study is to investigate racial/ethnic differences in psychological well-being among older adults during the first year of the pandemic, particularly focusing on mediating effects of different types of social support. Data came from the 2021 Health and Retirement Study (HRS), which includes a nationally representative sample of older Americans. Our sample was restricted to the respondents aged 65+ who participated in the 2021 HRS Perspectives on the Pandemic that focuses on the first-year pandemic experiences (N=4,656). We compared four racial/ethnic groups including white, black, Hispanic, and others. Psychological well-being was measured by the 12 questions on participants' feeling during the first year of the pandemic (e.g., distressed, afraid, worried). Three distinct groups of help-receiving patterns were identified by latent class analysis: 1) no help, 2) intense help from kids, and 3) moderate help from diverse social relationships. Frequencies of in-person and not-in-person contacts with children or family were also included in our analysis. We found that Hispanic's psychological well-being was the most negatively impacted by the pandemic (Coef.=0.687, p<.05) even after controlling for socio-demographic characteristics, physical and mental health. Our mediation analysis demonstrated that intense help from kids and less in-person contacts negatively mediated the association between Hispanics and their psychological well-being. This study shows that older Hispanics who had been known for strong family ties and support are at risk due to restricted social contacts during the pandemic.

Abstract citation ID: igad104.2671

### RUMINATION IN DYADS DURING TIME OF UNCERTAINTY—DAILY LIFE ASSESSMENTS AFTER THE ONSET OF THE COVID-19 PANDEMIC

Elizabeth Zambrano Garza[1], Rachel Murphy[1], Maureen Ashe[1], Kenneth Madden[1], Anita DeLongis[1], Denis Gerstorf[2], and Christiane Hoppmann[1], *1. University of British Columbia, Vancouver, British Columbia, Canada*, *2. Humboldt University of Berlin, Berlin, Berlin, Germany*

Rumination involves repetitive, self-oriented, negative thinking and is known to be detrimental to psychological well-being and health. However, little is known about the extent to which rumination is associated with well-being and health in close relationship partners in older age. Additionally, the pandemic was a time that was characterized by high stress, making it an important context to study rumination. Using daily diary data from 140 Canadian older adults plus a close other of their choice (59% spouses, M = 66.72 years, SD = 13.01 range: 18-87 years, 88% White, 62% women), this project builds on past evidence examining daily life rumination dynamics and extends it to a dyadic perspective beyond couples. For ten days, both dyad members provided evening ratings of daily rumination and affect quality. Multilevel models replicate individual level evidence that higher daily rumination was associated with more daily negative affect and less daily positive affect. Importantly, we also found partner effects such that more close others' rumination was associated with elevated actors' negative affect (b = 0.03, p = .038) and reduced actors' positive affect (b = -0.04, p = .023), suggesting that it is not only one's own rumination that relates to daily well-being, but also that of a close tie. Findings demonstrate the utility of taking a dyadic perspective on what is typically conceived as an individual-level phenomenon.

Abstract citation ID: igad104.2672

### SENSE OF PURPOSE AMONG OLDER ADULT VOLUNTEERS DURING THE COVID-19 PANDEMIC

Lindsay Wilkinson[1], Mollie George[1], Julie Masters[1], and Julie Blaskewicz Boron[2], *1. University of Nebraska Omaha, Omaha, Nebraska, United States*, *2. UNO, Omaha, Nebraska, United States*

The psychological benefits of volunteering among older adults are well established, but little is known about the influence of the pandemic on this relationship. Indeed, the COVID-19 pandemic led to social distancing, closures, and other restrictions that hampered the volunteer efforts of many older adults. Utilizing data from a survey of older adult volunteers participating in Foster Grandparent and Senior

Companion Programs in a metropolitan area of Nebraska, this study investigates sense of purpose during the COVID-19 pandemic. The analytic sample was comprised of 65 adults ages 57 to 89 years. Linear regression models adjusting for demographic characteristics were used to examine the association between the perceived impact of the pandemic and disruptions to volunteering, and whether disruptions to volunteering were subsequently linked to a reduced sense of purpose. The results reveal that 1 in 4 respondents somewhat or strongly agreed that their sense of purpose had declined during the pandemic. Compared to older adults who perceived that the pandemic had little effect on them, those who perceived that the pandemic had been either difficult or a hardship more strongly agreed that it had been challenging to visit with their clients (both ps < 0.05). Older adults who agreed that there were new challenges in their volunteer role, in turn, more strongly agreed that their sense of purpose had declined (p < 0.01). These findings highlight the importance of volunteering for sense of purpose during the pandemic and suggest a possible pathway through which the pandemic may influence future health.

Abstract citation ID: igad104.2673

### SPINNING YOUR WHEELS: AGE DIFFERENCE IN DAILY RUMINATIONS AND COVID-19 STRESS EXPERIENCES

Ann Pearman[1], Shevaun Neupert[2], MacKenzie Hughes[3], and Emily Lefebvre[4], *1. MetroHealth Medical System, Cleveland, Ohio, United States*, *2. North Carolina State University, Raleigh, North Carolina, United States*, *3. Georgia Institute of Technology, Atlanta, Georgia, United States*, *4. Vermont Department of Health, Colchester, Vermont, United States*

How individuals think about stress affects the impact that stress has on their daily life, however, the nature of this relationship may differ by age and be unique within the context of COVID-19. This study uses a daily diary approach to examine age differences in predictors of daily COVID-19-related stress and appraisals as they unfold. A U.S. sample of 535 adults (white [54%] and Black or African American [46%]) ages 21-79 submitted online diaries for 21 days in 2020. Daily measures included appraisals about how COVID-19 impacted several areas of functioning (e.g. routines, finances), stress due to COVID-19, and amount of rumination about COVID-19. Multilevel modeling showed a significant effect of knowledge on appraisals, where people with more knowledge about the pandemic generally reported that COVID-19 had less impact on their lives. A significant interaction emerged between Age x Daily COVID-19 Stress x Daily COVID-19 Rumination predicting daily appraisals. Whereas the strength of the positive relationship between stress and appraisals remained strong across varying levels of rumination for younger adults, the positive relationship between stress and appraisals was weaker at lower levels of rumination compared to higher levels of rumination for older adults. That is, COVID-19-related stress had less of a perceived impact on older adults' lives on days they engaged in less rumination about the pandemic. These findings have implications for understanding the role of ruminative thinking on stress and appraisals across the adult lifespan. Different processes may be in play with younger and older adults.

Abstract citation ID: igad104.2674

### THE IMPACTS OF AGE AND PREPARATION INFORMATION ON PREPAREDNESS FOR PANDEMIC: A LATENT CLASS ANALYSIS

Zhirui Chen[1], and Zhen Cong[1], *1. The University of Alabama at Birmingham, Birmingham, Alabama, United States*

This study examined the age differences in perceived and actual preparedness for a pandemic, as well as the moderating effect of preparation information. Data used was from 2021 FEMA National Household Survey (N = 6,305). Latent class analysis was first conducted to explore the typologies of the information respondents received about how to prepare for a pandemic based on eight indicators (e.g., basic survival, planning/preparation, testing, vaccines). With the adjustment of sample weights, four latent classes were identified: class 1 "insufficient information", class 2 "information emphasizing disaster preparedness", class 3 "information emphasizing disease preparedness", and class 4 "comprehensive information". Subsequently, the results of logistic regression and Poisson regression using sample weights showed that compared to those aged 60 and older, people aged 18-39 and aged 40-59 tended to perceive a lower level of preparedness but were more likely to engage in actual preparedness activities for a pandemic. Relative to those in "insufficient information" class, people in "information emphasizing disease preparedness" class and "comprehensive information" class were more likely to perceive a higher level of preparedness and take actual preparations for a pandemic. In terms of the interactions between age and preparation information, the gap narrowed between people aged over 60 and their younger counterparts in taking actual preparations if they were in "comprehensive information" class. These findings highlighted the age patterns in pandemic preparedness and the impacts of different preparation information strategies, which provided imperative implications for disaster preparedness education and communication campaigns.

Abstract citation ID: igad104.2675

### UNDERSTANDING THE IMPACT OF COVID-19 ON THE USE OF TRANSPORTATION SERVICES BY OREGON ASSISTED LIVING RESIDENTS

Minju Kim[1], Ozcan Tunalilar[1], and Paula Carder[2], *1. Portland State University, Portland, Oregon, United States*, *2. Portland State University Institute on Aging, Portland, Oregon, United States*

COVID-19 has affected many aspects of daily life, including transportation. This study aims to understand the impact of the COVID-19 pandemic on the use of transportation services by residents of assisted living communities in Oregon. Primary data about transportation service availability and use were collected from 721 AL communities using mailed questionnaires, before (winter of 2020) and during the pandemic (winter of 2022). To identify the impact of the pandemic on transportation use, we estimated a series of logistic regression models for trip purposes: medical appointments and social activities. Results show that the pandemic profoundly impacted transportation services, especially provided transportation services for access to social activities decreased from 77% to 68%, whereas access to medical appointments had not changed. The use of virtual visits allowed residents to remain connected with their loved ones while minimizing the risk of exposure to the virus. The findings of this study can help policymakers and transportation providers understand the impact of the pandemic on the mobility of individuals in community-based care settings. The results can also inform the development of strategies to improve transportation services and ensure the mobility of residents after the pandemic. Ultimately, the study can contribute to enhancing the overall quality of life for individuals in community-based care settings in Oregon.

### SESSION 7560 (POSTER)

### ENVIRONMENT AND AGING (BSS)

Abstract citation ID: igad104.2676

### AGE DIFFERENCES IN CLIMATE CHANGE ATTITUDES AND BEHAVIORS

Yeonsoo Shin, and Giyeon Kim, *Chung-Ang University, Seoul, Republic of Korea*

Although previous studies show that older adults are vulnerable to the effects of climate change, the actions of older adults to address climate change have not received much attention. The purpose of this study is to investigate age differences in the perception of climate change and the characteristics of pro-environmental behavior. We analyzed cross-sectional data from a nationally representative sample drawn from the 2022 Korea Social Survey. The sample consisted of 15,252 adults, including younger adults (aged 19-34, n=6,132) and older adults (aged 65+, n=9,120). Pro-environmental behavior included the following actions: using public transportation, recycling, reducing the use of disposable products, and saving water and power. T-test and moderation analysis were conducted. Results showed that the younger group was more likely to be aware of climate change than the older group (F=68.022, p< 0.01). However, older adults were less likely than younger adults to make food waste and save water and electricity more (p< 0.01). There were no age differences in recycling, which is a representative environmental protection behavior. Results from a moderation analysis revealed that younger adults made more effort to recycle in the lower level of climate change awareness whereas older adults made more effort to recycle in the higher level (B = .031, p< 0.01). The findings indicate that older adults may play a crucial role in taking action to respond to climate change and suggest that promoting awareness of climate change among older adults could be an effective strategy to encourage pro-environmental behavior.

Abstract citation ID: igad104.2677

### APPLYING THE THERAPEUTIC LANDSCAPE MODEL TO IRISH MEN'S SHEDS

Melinda Heinz[1], and Frank Houghton[2], *1. Upper Iowa University, Fayette, Iowa, United States*, *2. Technological University of the Shannon, Limerick, Limerick, Ireland*

Gesler's (1993) introduction of the therapeutic landscape model considers whether environments positively contribute to the wellbeing of users of the space. The model includes both inner/meaning components that consist of 1) natural setting, 2) built environment, 3) sense of place, 4) symbolic landscape and 5) everyday activities and outer/society components of 1) beliefs and philosophies, 2) social relations/inequality and 3) territoriality. The purpose of this study was to explore how environments that older adults are engaged in (e.g., Irish Men's Sheds) exemplify characteristics found in Gesler's therapeutic landscape model. Interviews with Men's Sheds participants aged 65+ (N = 37) were conducted in County Limerick, Ireland. After transcription, Braun and Clarke's (2020) intuitive 6-step process for thematic analysis was used to interpret the data. Interviewee transcripts revealed that the social relations/inequality component of the therapeutic landscape model was most evident in the narratives. The men explained that it was important to create an inclusive space for all men to feel welcome and valued. For example, a participant said, "Some [members] are living on their own, maybe they're lonely and they feel kind of unwanted… so, the shed has made a difference to them." Another participant commented, "Every time someone walks into the gate, you're enthusiastic that they're here and you try to welcome them, but then you see what they have to offer. Everybody has something." Future research should investigate how other environments that older adults engage in, embody components of Gesler's therapeutic landscape model.

Abstract citation ID: igad104.2678

### LONGITUDINAL REPORTS OF NEIGHBORHOOD ENVIRONMENT ON COGNITIVE PERFORMANCE AMONG BLACK ADULTS

Alexa Allan[1], Alyssa Gamaldo[2], Roland J. Thorpe[3], Jennifer D. Roberts[4], Michele K. Evans[5], and Alan B. Zonderman[5], *1. The Pennsylvania State University, University Park, Pennsylvania, United States*, *2. Clemson University, Clemson, South Carolina, United States*, *3. Johns Hopkins University, Baltimore, Maryland, United States*, *4. University of Maryland, College Park, Maryland, United States*, *5. National Institute on Aging, National Institutes of Health, Baltimore, Maryland, United States*

Limited research has explored the association between neighborhood environments and cognitive functioning using longitudinal reports of neighborhood quality. The current study examined the longitudinal association between perceived neighborhood characteristics and computerized cognitive performance among Black adults. This study included 584 community-dwelling socioeconomically diverse Black adults (Mage = 56.27, SDage = 8.94; 58% female) from waves 3 & 4 of the Healthy Aging in Neighborhood of Diversity across the Life Span (HANDLS) study. Neighborhood quality was assessed using three scales about built environment disorder (e.g., litter), social cohesion (e.g., close-knit neighborhood), and social control (e.g., neighbors act if fight in front of house). Cognitive functioning was assessed using the computerized Joggle battery. Multivariable linear regression analyses were conducted with each cognitive measure as the outcome measure. Models were adjusted for age, sex, education, reading literacy, poverty status, and depressive symptoms. In the adjusted models, living in a neighborhood with increasing built environment disorder was associated with slower performance (p <.05) on an attention measure. Increasing social cohesion was associated with faster performance (p <.05) on memory, attention, and visual orientation measures. Similarly, increasing social control was associated with faster performance (p <.05) on attention, executive function/reasoning, visual orientation, and speed measures. Additionally, increasing social control was associated with greater number of correct responses (p <.05) on an attention and executive function/reasoning measure. Significant interactions were observed between changes in neighborhood quality and covariates. These findings amplify the importance of understanding how changes in neighborhood quality impact cognitive performance among Black adults.

Abstract citation ID: igad104.2679

### LONG-TERM EXPOSURE TO FINE PARTICULATE MATTER AND DEPRESSIVE SYMPTOMS AMONG CHINESE OLDER ADULTS

Jiaan Zhang, *Fudan University, Shanghai, Shanghai, China (People's Republic)*

There is a growing body of evidence suggesting that air pollution, especially PM2.5 (particulate matter with a diameter of 2.5 micrometers or less), has been associated with adverse mental health outcomes, but the long-term association with depressive symptoms remains unclear. The purpose of this study is to examine the long-term effect of cumulative exposure to PM2.5 on depressive symptoms among Chinese older adults. Using a nationally representative sample of older adults in China (N=16,064), we tracked their depressive symptoms over a 16-year period (2002-2018). Air pollution data were linked to respondents using provincial identifiers. Three-level random intercept linear regression models that account for the nesting of repeated observations (level-1) within individuals (level-2) and individuals within provinces (level-3) were used to examine the long-term cumulative effects of PM2.5 exposure on depressive symptoms. We found that every 10-μg/m3 increase in cumulative exposure to PM2.5 increased the levels of depressive symptoms by 0.28 points (β = .28, p < .01), controlling for individual-level sociodemographic characteristics and social support, health-related behaviors, and health status. Furthermore, the association between cumulative PM2.5 exposure and more depressive symptoms is more pronounced for urban residents than for rural residents. Our analysis shows that air pollution is a risk factor for depression in older Chinese adults. Findings suggest that stronger PM2.5 regulations, especially in urban areas, may enhance mental health.

Abstract citation ID: igad104.2680

### THE ASSOCIATIONS OF ENERGY CONSUMPTION AND EXPENDITURE WITH SELF-PERCEIVED WELL-BEING AMONG CHINESE ADULTS

Qiyini Ma[1], Fei Sun[2], and Hong Mi[1], *1. Zhejiang University, Hangzhou, Zhejiang, China (People's Republic)*, *2. Michigan State University, East Lansing, Michigan, United States*

**Background and Objectives:** Households in China have gradually moved to use of cleaner fuels, but the proportion using solid fuels in daily life remains as high as 30%, resulting in approximately 420,000 premature deaths annually. Energy use and its impact on health may differ by age, as less active lifestyle in later life may lead to increased household energy consumption aggravating poor health consequences. The twofold aims of this study are: 1) to examine energy consumption patterns and expenditure between young Chinese (aged 18-59) and older Chinese (aged 60 or above), and 2) to identify how energy consumption patterns and expenditure are associated with self-perceived wellbeing in the two respective age groups. **Methods:** A total of 2,303 valid samples (M age = 51, SD = 16.2) were obtained from Chinese General Social Survey (CGSS) in 2015. Hierarchical linear modelling analyses were employed using HLMs 6.08 for each age group to examine the influence of individual-level factors and cluster-level covariates (i.e., environmental pollution, health care resources, and economic development at provincial level). **Results:** Among the young adults, those using oils, gas, and electricity reported better self-perceived wellbeing than those using solid fuels. Among the older adults, reliance on fuel gas and energy expenditure were associated with better self-perceived wellbeing. **Conclusions:** The harnessing of cleaner energy has the potential to increase subjective wellbeing across age groups. For older adults, the higher energy expenditure might indicate a dormant or isolated lifestyle, which calls for practice interventions to improve social engagement and promote wellbeing.

### SESSION 7570 (POSTER)

### GENDER AND AGING

Abstract citation ID: igad104.2681

### BACKWARD WALKING SPEED: A NOVEL MOBILITY MEASURE FOR YOUNGER AND OLDER ADULTS

Braden Popelsky, Kelly Ylitalo, and Samuel Hockett, *Baylor University, Waco, Texas, United States*

Mobility is fundamental to healthy aging. Forward walking speed (FWS) is a well-established measure of mobility, physical functioning, and fall risk but fewer studies have assessed backward walking speed (BWS). This study explored the relationship between FWS, BWS, and fall-related factors. Independently ambulatory, community-dwelling young adults (n=20; age: 22.9 years ±1.6) and older adults (n=19; age: 70.2 years ±6.2) completed forward and backward walking assessments on a 3.9 meter long instrumented pressure mat. Paper surveys assessed sociodemographic characteristics, fall history, and fear of falling using the Falls Efficacy Scale – International (FES). FWS, BWS, and directional difference percent change ((FWS-BWS/FWS)*100) were compared using independent sample t-tests, and simple linear regression was used to model the relationship between mobility measures and fear of falling. In the total sample (n=39), 23% of adults reported a fall in the previous 12 months. Compared to older adults, young adults exhibited significantly faster FWS (138.3 cm/sec ± 16.5 vs. 119.3 cm/sec ±23.4; p=0.006) and BWS (82.6 cm/sec ±18.2 vs. 63.8 cm/sec ±20.7; p=0.005). Males displayed a significantly lower mean directional difference percent change (39.4 ±11.4 vs. 47.4 ±11.1; p=0.034), than females. Among older adults, BWS was strongly and negatively correlated with FES scores (r=-0.58; p=0.009). BWS explained a greater proportion of variance in fear of falling compared to FWS (33.93% vs. 24.84%). BWS and directional difference in walking speed may be novel measures of physical functioning. Prospective studies should consider assessing BWS with future fall risk among older adults.

Abstract citation ID: igad104.2682

### DIFFERENCES IN CONFORMITY TO MASCULINE NORMS ACROSS AGE COHORTS

Montgomery Owsiany, Erika Fenstermacher, Jeongwi An, Sabine Lohmar, and Amy Fiske, *West Virginia University, Morgantown, West Virginia, United States*

Conformity to masculine norms is associated with worse mental health among men, but little is known about conformity to masculine norms across the lifespan. Much of the literature on this topic focuses on early adulthood, rarely extending past middle age. Research indicates that attaining hegemonic masculinity becomes more difficult as one ages. One study with Australian young and middle-aged men found that conformity to masculine norms was lower in middle-aged cohorts than in younger cohorts. A study of Canadian men aged 55 years and up found that conformity to masculine norms did not significantly differ between those aged 55-69 and those aged 70+ years. The present study assessed differences in conformity to masculine norms across the adult lifespan. It was hypothesized that scores on the conformity to masculine norms inventory (CMNI) would significantly differ across six age groups and would be lower in older cohorts. The mean CMNI score for males ($N = 447$) was 60.96 ($SD = 19.09$) and for females ($N = 431$) was 47.19 ($SD = 15.11$). In a one-way ANOVA, scores significantly differed between age cohorts for the male ($F(5, 441) = 20.83$, $p < .001$) and female samples ($F(5,425) = 14.85$, $p < .001$). Tukey's HSD test for multiple comparisons revealed that CMNI scores were significantly lower in older cohorts compared to younger cohorts in both males and females. Findings highlight the importance of considering age-related differences in the presentation of masculinity. Future studies should investigate whether differences are related to aging or cohort effects.

Abstract citation ID: igad104.2683

### GENDER DIFFERENCE IN USING DIGITAL HEALTH TECHNOLOGIES AMONG OLDER ADULTS WITH CANCER: A PILOT QUALITATIVE STUDY

Misun Hwang[1], Youmin Cho[2], Katie Gahn[3], Milisa Manojlovich[3], and Yun Jiang[3], *1. University of Michigan School of Nursing, Ann Arbor, Michigan, United States*, *2. The University of Texas Health Science Center at Houston, Houtston, Texas, United States*, *3. University of Michigan, Ann Arbor, Michigan, United States*

Digital technologies hold promises to improve health and care delivery for various populations, including older adults with cancer. However, older adults' adoption of digital health technology is less optimal. While age and gender can interfere with people's technology behavioral intention, it is unknown how gender differences may influence older adults with cancer's technology use experiences. This pilot qualitative study investigated gender differences in older adults' experiences using digital technologies to manage their cancer and care. Semi-structured interviews were conducted with 16 older adults with breast, colorectal, lung, or prostate cancer (men=7 and women=9) aged 65 to 78 years. Thematic data analysis revealed that older men favored the adjustability and efficiency of using technology to save time and cost compared to in-person visits. Also, they perceived that technology facilitated their adherence to treatment plans. Older women, however, preferred technology-supported individualized care and perceived being able to review their medical notes as beneficial. Women were comfortable using technology to timely communicate with their trusted care providers. However, they preferred to see their providers in person if they experienced severe condition changes or unanticipated symptoms. Despite all these different preferences, both genders were comfortable using video calls with their providers, using the portal to keep track of their medical records and share decision-making with clinicians. These pilot results demonstrated gender differences in experiences and perceptions of using digital technology among older adults with cancer and suggested integrating gender preferences into technology design and development to facilitate personalized user experience and improve patient engagement.

Abstract citation ID: igad104.2684

### WHY DO OLDER WOMEN LIMIT THEIR DRIVING MORE THAN OLDER MEN?

Anne Barrett, Hope Mimbs, Brianna Soulie, Jessica Noblitt, and Cherish Michael, *Florida State University, Tallahassee, Florida, United States*

Older adults tend to avoid driving in situations like heavy traffic or bad weather, but women do so more than men. Explanations for this gender difference, however, have received limited research attention. Using data from an online survey of Floridians aged 50 and older that was conducted between December 2020 and April 2021 (n=3,726), we examined four potential explanations for women's greater self-regulated driving: their worse health, their more frequent rides from others, their more negative attitudes about driving, and their less favorable assessments of their driving ability. The results of multivariate regression models predicting a scale of self-regulated driving revealed support for two of the explanations – rides from others and self-assessed driving ability. Women's more frequent rides from family members accounted for approximately 10 percent of the association between gender and self-regulated driving, while women's less favorable assessments of their driving ability accounted for nearly a third of it. In contrast, health and attitudes about driving did not operate as mediators. They did, however, predict self-regulated driving; having worse health or more negative driving attitudes was associated with more self-regulated driving. Our study identifies women's less favorable assessments of their abilities as a primary determinant of their more rapid transition from driving. Older women's transportation mobility could be better maintained through programs focused on enhancing their skills, as well as driving confidence.

### SESSION 7580 (POSTER)

### HELPFUL AND HARMFUL COPING BEHAVIORS

Abstract citation ID: igad104.2685

### ACKNOWLEDGING ILLNESS WHEN LIFE IS "CUT SHORT": INCREASED COMMUNION IN LEGACIES

Mary Kate Koch, Sophia Maggiore, Carma Bylund, and Susan Bluck, *University of Florida, Gainesville, Florida, United States*

Crafting a legacy is meaningful activity in later life (McAdams et al., 1993). Threat of dying affects how individuals frame their life legacy (Hunter, 2007). We argue feeling one's life is being "cut short" may promote greater communion in legacies. Older individuals with cancer may, however, need to acknowledge their illness to create a communion-focused legacy. To assess being 'cut short, we calculated expected years of life lost (EYLL): difference between participant age and average U.S. life expectancy for men and women (NCHS, 2022). EYLL and self-reported Acknowledgement of Illness were used to predict extent of communion shared in personal legacies. Participants were 204 older adults with cancer receiving outpatient palliative care (M=65.78 years, Range= 55-88 years, 65.69% women) who narrated their legacy during Dignity Therapy (Chochinov, 2005). Narratives were audio-recorded, transcribed and reliably (κ=.71–1) content-analyzed for communion themes (McAdams, 2001). Hierarchical regression analyses (adjusted for gender and cancer stage) examined EYLL, Illness Acknowledgement and their interaction. Interaction between EYLL and illness acknowledgment showed participants with more expected years to lose narrated more total communion but this was particularly true for those acknowledging they were terminally ill, $F(7, 173)=2.42$, $p=.02$. Overall, older adults with cancer appear to value communion as an important aspect of legacy. Note, however, that individuals whose life is 'cut short' were especially likely to weave communion into their legacy. This may indicate a need to cement their legacy during DT, given they have had less time to do so in daily life or formal documents.

Abstract citation ID: igad104.2686

### AGE DIFFERENCES IN PRIOR EXPERIENCES USED TO COPE WITH COVID-19 STRESSORS

Maria Kurth[1], Heidi Igarashi[2], and Carolyn Aldwin[2], *1. Penn State University, University Park, Pennsylvania, United States*, *2. Oregon State University, Corvallis, Oregon, United States*

Despite increased physiological vulnerability to COVID-19 (CDC, 2022), older adults reported fewer mental health consequences than younger adults (APA, 2022). The ability to draw upon prior experiences may be a source for enhanced well-being in late life (Aldwin & Igarashi, 2016; Charles, 2010). Older adults reported various prior experiences and resources for coping with the COVID-19 pandemic (Galcia et al., 2021; Herron et al., 2021), but age differences were not examined. We collected online surveys from adults 50+ (N = 235; Mage = 71.35, SD = 7.39; 74% female; 92% White) between 04/28–05/04/2020. A directed content analysis guided application of pre-existing (Aldwin et al., 1996b) and novel codes to the 60% who responded to an open-ended question of which prior experiences informed coping with COVID-19 pandemic problems. The final coding scheme had 13 codes (Kurth et al., 2022; Krippdendrof ∝ = .82) grouped into three categories: similar experiences, adversity across the lifespan, and personal resources. No age differences emerged in likelihood of providing any of these responses between the midlife (51 – 64; n = 35), young-old (65 – 74; n = 141), and old-old groups (75+; n = 57). Further, there were no age differences in providing a response that fell into the similar experience, adversity across the lifespan, or personal resource categories, supporting the age and well-being hypothesis. Findings suggest that even when exposed to a chronic and uncontrollable stressor, older adults leveraged their advantage of past experiences and accumulated resources to inform coping strategies.

Abstract citation ID: igad104.2687

### DEPRESSIVE TENDENCIES AND NEGATIVE LIFE EVENTS AS PREDICTORS OF HOMEBOUND STATUS AMONG OLDER ADULTS IN JAPAN

Koji Fujita[1], Sachiko Yamazaki[2], Hiromi Imuta[3], Roseline Yong[4], Hiroko Matsunaga[5], and Yoshinori Fujiwara[1], *1. Tokyo Metropolitan Institute for Geriatrics and Gerontology, Tokyo, Tokyo, Japan*, *2. Bunkyo Gakuin University, Fujimino, Saitama, Japan*, *3. Tokyo Metropolitan University, Arakawa, Tokyo, Japan*, *4. Akita University, Akita, Akita, Japan*, *5. Tokyo Metropolitan Institute of Gerontology, Tokyo, Tokyo, Japan*

This study aimed to examine negative life events (NLEs) and depressive tendencies as predictors of homebound status, which poses a risk of long-term frailty in older adults. A population-based cohort study (2018-2020) using self-administered questionnaires was conducted with older adults (65-94 years) in the rural community of Akita Prefecture, Japan. The response rate at the baseline survey in 2018(T1) was 61.5%. About 81.1% (1,048 out of 1,291) of the participants at T1 responded to the follow-up survey in 2020 (T2). Among them, 77.3% (810) were not homebound at T1. After data cleaning, 790 participants' data were analyzed. A frequency of going out less than once a week was considered a homebound status. In this study, NLEs included 1) loss of a close relative, 2) major illness or injury to oneself, 3) major illness or injury to a family member, 4) financial difficulties, and 5) loss of one's role (family, workplace, society, others). Depressive tendencies were evaluated using the 6-item Kessler Psychological Distress Scale (0-24 scale). A K6 score ≥ 9 indicates depressive tendencies. Logistic regression analysis was adjusted for potential confounders. The incidence of being homebound at two years was 10.1% for men and 17.1% for women, with a significant gender difference ($p < 0.01$). The adjusted odds ratio for depressive tendencies was 3.37 (95%CI= 1.62-7.01) among homebound (vs. non-homebound) individuals. However, the five NLEs were not statistically significant. Our results suggest that depressive tendencies, but not NLEs, can cause older adults to remain housebound.

Abstract citation ID: igad104.2688

### GENERATIVITY, COGNITIVE REAPPRAISAL, AND OPTIMIZING SELF-ACCEPTANCE ACROSS MID LIFE AND OLD AGE

Madeline Nichols[1], John Geldhof[1], and Robert Stawski[2], *1. Oregon State University, Corvallis, Oregon, United States*, *2. University of Essex, Colchester, England, United Kingdom*

Midlife is an opportune time during which individuals can establish patterns of behavior (e.g., generativity) and self-regulatory processes (e.g., emotion regulation) that will optimize their psychological wellbeing and aging. We sought to test whether: 1.) generativity is directly associated with better self-acceptance, 2.) the effect of generativity is indirect and mediated by emotion regulation (i.e., cognitive reappraisal), and 3.) age moderates associations among generativity, cognitive reappraisal, and self-acceptance. Using data from the second and third waves of the Midlife in the United States (MIDUS) study (N=935, Mage=54.36, SD=11.05, Range = 34-83, 55.08% Female), results revealed that cognitive reappraisal (β= 0.07, p< .01) and older age (β= 0.06, p= .02) were associated with higher self-acceptance; however, generativity was not, and age did not predict generativity nor cognitive reappraisal. Generativity was associated with more frequent use of cognitive reappraisal (β= 0.16, p< .001), with cognitive reappraisal then significantly mediating the association between generativity and self-acceptance (Natural Indirect Effect= 0.02, SE= .01, p= .02). Age significantly interacted with generativity when predicting self-acceptance (β= 0.39, p= .02), such that the effect of generativity increased with age. These findings are important for reframing midlife as a critical developmental stage during which individuals can engage in behaviors and self-regulatory processes that optimize their self-acceptance throughout the aging process. As age did not moderate most associations, interventions could target generativity to promote effective emotion regulation and cognitive reappraisal to enhance self-acceptance across adulthood and into later life given its positive implications for wellbeing, health, and longevity.

Abstract citation ID: igad104.2689

### INSOMNIA AND UNHEALTHY ALCOHOL USE DURING THE COVID-19 PANDEMIC AMONG WOMEN VETERANS AGE 50 YEARS AND OLDER

Caitlan Tighe[1], Deirdre Quinn[2], Karley Atchison[3], and Rachel Bachrach[2], *1. Providence College, Providence , Rhode Island, United States*, *2. VA Pittsburgh Healthcare System, Pittsburgh, Pennsylvania, United States*, *3. PIttsburgh VA Medical Center, Pittsburgh, Pennsylvania, United States*

Older women Veterans have a high burden of comorbid medical and mental health conditions and may be particularly at risk for co-occurring insomnia and unhealthy alcohol use. Some research suggests rates of both increased during the COVID-19 pandemic; however, few studies extended beyond pandemic year one or examined administrative data. Using Department of Veterans Affairs (VA) electronic health record data, we examined rates of insomnia (diagnoses, prescription insomnia medications) and unhealthy alcohol use (diagnoses, positive alcohol screens) in a national sample of women Veterans aged 50 years and older (N=239,638) prior to and during the pandemic (March 2018-2022). During this timeframe, insomnia rates increased across age groups. Annual insomnia rates were highest among women ages 50-59 (12.8-14.9%) and lower for women age 60 and older (6.2-12.1%). The annual rate of unhealthy alcohol use was also highest among women ages 50-59 (13.9-15.3%) and lower for women age 60 and older (5.4-12.9%). For the youngest and oldest age groups, rates of unhealthy alcohol use decreased annually until 2021 and then increased; for women aged 60-79, rates increased except in 2020, when rates declined. The proportion of women with both conditions in a given year ranged from .5-2.9% and was highest at younger ages and lowest at older ages. Together, results indicate annual rates of insomnia and unhealthy alcohol use during COVID were highest in midlife women but increased over time across ages, signaling a potential need for intervention and prevention efforts aimed at addressing sleep health and alcohol use across mid-late adulthood.

Abstract citation ID: igad104.2690

### THE ASSOCIATIONS OF OPIOID USE IN GUN-RELATED FATALITIES AMONG OLDER DECEDENTS IN FLORIDA

Armiel Suriaga, Ruth Tappen, and David Newman, *Florida Atlantic University, Boca Raton, Florida, United States*

In 2020, the Center for Disease Control and Prevention reported the highest firearm deaths in the U.S. at 45, 222. Homicides and suicides accounted for most fatalities. However, the role of opioids in firearm-related deaths (FRD) among older adults was less explored. Our study aimed to investigate the associations between opioid use and FRD among people >65 years with opioids detected in their system at time of death. We used descriptive statistics to describe decedents' characteristics using the 2021 Florida Department of Law Enforcement de-identified data. We used Pearson's chi-square and logistic regression to examine the associations of opioids to FRD. All data were analyzed using Stata 17. A total of 1982 older decedents were included in the analysis. Age ranged from 65-100 years, mean 72.97 (SD=7.331). Three hundred thirty of 1982 deaths (16.65%) were gun-related; 121 of 330 (14.79%) had opioid involvement. More males (n=274) (83.03%) died than females; 316 (95.76%) were non-Hispanic whites, followed by black (n=10, 3.03%). Suicides accounted for most FRDs (n=330, or 90.91%), and homicide second (n=28 or 7.88%). The association between opioid use to FRD was statistically significant (p<.001). The odds of dying from FRD among older adults with any opioid use increased by .57 compared to those who never used any opioid, controlling for opioids combined with other substances, age, gender, and population density, OR=.57 (95% CI .41-.79). Our results have safety clinical implications as more older adults use opioids, e.g. for chronic pain, which calls for tailored interventions to protect older population.

### SESSION 7590 (POSTER)

### PERCEPTIONS OF AND ATTITUDES TOWARD AGING

Abstract citation ID: igad104.2691

### AGEISM IN THE USE AND DESIGN OF DIGITAL TECHNOLOGY: A THEORETICAL MODEL

Ittay Mannheim, *Ben Gurion University, Beer-Sheva, HaDarom, Israel*

Ageism is currently defined as a main societal challenge for successful and healthy aging. Concurrently, the vast development of digital technology in the last decades highlights a newly under-researched intersection of ageism and the use and design of digital technology. However, a theoretical model explicating how ageism may influence the use and design of technology is currently missing. A proposed model was created based on a mixed-methods approach combining findings from 5 studies of a doctoral dissertation. The integrated analysis identified manifestations of ageism in stereotypes, prejudice and discrimination regarding the use and design of technology, appearing explicitly and implicitly, mainly in a negative context and on different levels (micro-individual, meso-social interaction, and macro-design and policy). Use and design of technology were found to be entangled together. Importantly, two main paradoxes are highlighted. First, the fixation on designing care and healthcare technologies, stereotypically disregarding the diversity of other needs of older persons. A second paradox was found in the discrepancy between acknowledging the "ideal" practice of involving older persons throughout the whole design process, and the actual practice of limited involvement. Ultimately leading to biases in the design process and exclusion of older persons. Consequently, emerging technologies often have low adoption rates, which are further (stereotypically) attributed to the incompetence of older persons to use digital technology, and are contrary to the increase in actual use and motivations older persons have to use technologies that can promote their well-being. Implications of the model for policy and future research are discussed.

Abstract citation ID: igad104.2692

### AN EVALUATION OF REIMAGINE AGING: A NEW THEORY-BASED PROGRAM TO REDUCE INTERNALIZED AGEISM

Dallas Murphy, Michelle Porter, and Corey Mackenzie, *University of Manitoba, Winnipeg, Manitoba, Canada*

Over the lifespan individuals may internalize ageist beliefs and as they enter older age, direct them towards themselves. This internalized ageism has many deleterious effects. Despite this, few theory-based interventions have attempted to decrease internalized ageism. As such, a six-week online program was developed including education, acceptance and commitment therapy, and attributional retraining to target mechanisms of change (psychological flexibility, mindfulness, perceived control, and empowerment). The six 90-minute sessions consisted of recorded videos, and discussion groups, with during and between-session activities also being part of the program. To evaluate the feasibility of this intervention, a sub-sample of 81 program participants (58 – 85 years old, 92% female) were sent an online questionnaire following each session. Each session received between 77 and 80 responses. Results were overwhelmingly positive. On a scale of 1 (not very useful) to 5 (very useful), roughly two-thirds (65%) rated the program as a whole very useful. Items participants felt the most important to learn included ageism information, acceptance and commitment therapy tools, and reimagining what it means to age well. Participant's opinion on what they liked most about the program varied. Among others, common aspects identified were the informational videos, the activities, and the discussion groups. Roughly 81% of participants indicated that they completed the between-session activities, and 79% completed bonus activities. The vast majority indicated the program changed their views on ageism and/or internalized ageism. Going forward, we will evaluate this program's ability to decrease internalized ageism, and the processes by which it achieves this.

Abstract citation ID: igad104.2693

### AN EXAMINATION OF GENDER DIFFERENCES IN THE PERCEPTION OF ELDERSPEAK

Abby Teply[1], Hanna Schultz[2], and Jeffrey Buchanan[2], *1. University of Wyoming, Laramie, Wyoming, United States, 2. Minnesota State University, Mankato, Mankato, Minnesota, United States*

This study aimed to examine how gender impacts perceptions of elderspeak. Participants (n = 81; ages 65-93) were presented with one of two vignettes set in an assisted living facility to simulate the experience of being a recipient of elderspeak. The vignettes were identical except that the gender of the nursing assistant (NA) differed across vignettes. After reading the vignette, participants were asked to rate their emotional reactions using an adapted version of the Positive and Negative Affect Schedule (Watson et al., 1988) and rate their perceptions of the NA using the Emotional Tone Rating Scale (Williams et al., 2012). Because the goal of the study was to determine possible interactions between the gender of participants and the gender of the NA in the vignette, a two-way MANOVA was utilized. Results indicated a difference in positive and negative affect scores across vignette conditions. Specifically, there was evidence that participants reported lower positive affect and greater negative affect when elderspeak was used by a male NA compared to a female NA. Results suggest that both older men and women perceive elderspeak more negatively when it is used by a male compared to a female. However, elderspeak is not received well, regardless of who uses it, suggesting a need for a nursing assistant training curriculum focused on person-centered communication. Future research should include a more diverse sample in terms of ethnic identity, cultural background, sexual orientation, and gender identity to determine whether these results generalize to a larger population of older adults.

Abstract citation ID: igad104.2694

### ASSOCIATION BETWEEN ANXIETY ABOUT AGING AND AGEISM TOWARD OLDER ADULTS AMONG KOREAN DENTAL HYGIENE WORKFORCE

Jenny Kwon, Michael Hughes, and Anh Vo, *Miami University, Oxford, Ohio, United States*

Ageism toward older people is prevalent in Korean healthcare settings, where older adults are likely to encounter other age groups of people. However, not many studies took close attention to ageism in the dental hygiene field. Considering the increase in dental care demand among the older population, this study aims to investigate the level of ageism and the factors associated with ageism among Korean dental hygiene undergraduates and dental hygienists. Based on the convenience sampling strategy, a total of 146 online surveys were collected from July to September 2022 in Seoul and Daejeon, Korea. Aging anxiety, ageist attitude, intergenerational contact measures, demographic characteristics, and geriatric-related experiences of participants were asked. Higher scores indicate lower aging anxiety and higher ageism. The mean (±SD) age of participants was 24 (±6.05) years. The mean score of aging anxiety was 2.97 (±.52) out of 5 and the mean score of ageism was 2.10 (±.31) out of 7. In the multiple regression model, aging anxiety is significantly associated with ageism (β = −0.257, p < 0.001). Participants feeling more anxious about aging have a more ageist attitude toward older people. The results suggest that well-designed gerontological education and intergenerational programs are necessary. In the short term, these efforts will decrease the fear of aging among dental hygiene field workers. In the long term, these will help them have the right understanding of aging and a positive attitude toward older people which can affect the quality of dental care service.

Abstract citation ID: igad104.2695

### BETWEEN-PERSON AND DAILY WITHIN-PERSON VARIATION PATTERNS IN MEASURES OF VIEWS OF AGING

Erica O'Brien, and Joshua Smyth, *Pennsylvania State University, State College, Pennsylvania, United States*

Despite increasing evidence of daily associations between individuals' health and their age-related thoughts, beliefs, feelings and experiences (collectively referred to as views of aging, VOA), we know little about daily variation in VOA more generally. We used data from an online sample of 122 adults (aged 26 to 78 years) who completed multiple measures of VOA (subjective age, age group identity, aging attitudes, implicit theories of aging, awareness of age-related change) on each of 7 consecutive days, partitioning variance in responses to each measure at the person and day levels to assess between- and within-person variability, respectively. Results showed that, in all measures, between-person variability accounted for most of the total observed variation (62% to 92%) whereas within-person variability accounted for a significant but smaller amount (8% to 38%). The ratio of between- to within-person variation, however, depended on the specific measure and age group assessed, with the highest ratios observed for subjective age (versus other measures) and older adults (versus young adults). Findings suggest that VOA are relatively stable in daily life and may become more stable with advanced age. Further study of measures and age groups that exhibit the most within-person variability can yield insights that help increase understanding about the dynamics of VOA (i.e., which VOA constructs are most sensitive to fluctuating contexts and for whom?) and therefore inform future work linking VOA to other phenomenon in daily life.

Abstract citation ID: igad104.2696

### CHALLENGING AGEISM IN PSYCHOLOGY EDUCATION: THE ROLE OF GEROPSYCHOLOGY COURSES AMONG PSYCHOLOGY STUDENTS

Verena Kleissner, Tim Kuball, and Georg Jahn, *Chemnitz University of Technology, Chemnitz, Sachsen, Germany*

Ageism includes negative attitudes, stereotyping, and discriminatory behavior toward older adults and is associated with negative consequences for health and well-being of the growing older population. In addition, ageism affects structural health care including psychological treatment of older individuals. Since educational interventions have been shown to be effective in reducing ageist prejudice and improving knowledge about older people, courses in gerontology taught as part of the psychology curriculum might help future psychologists to be better equipped to challenge ageism and to provide more effective and compassionate care to older adults. In the present study, we compared psychology students (n = 50) that chose courses in applied geropsychology with students of other specialization of choice (n = 26) on multiple scales including affective (attitudes), cognitive (stereotypes) and behavioral (discrimination) dimensions. Questionnaires were completed at the beginning and the end of one semester to conduct a within pre- and post-treatment assessment and analysis with a comparison group. Results showed that geropsychology students improved in self-reported knowledge about aging and older adults (d = 0.71). However, other tests did not show a statistically reliable improvement on the three assessed ageism dimensions within one semester. Findings implicate that gained knowledge about aging does not necessarily lower prejudice. In our discussion we highlight possible interventions targeting psychology students and reflect on methods to assess attitudes, stereotypes, and behavioral aspects of ageism in participants with high education level.

Abstract citation ID: igad104.2697

### CHANGES AMONG COLLEGE STUDENTS' ATTITUDES, KNOWLEDGE, AND INTENTION TO WORK WITH OLDER ADULTS

Christopher O'Brien[1], and Jessica Strong[2], *1. Providence College, Providence, Rhode Island, United States*, 2. *University of Prince Edward Island, Charlottetown, Prince Edward Island, Canada*

Curricular intervention studies have examined if instruction in aging and gerontology affects undergraduates' attitudes, knowledge, and perceptions toward older adults. However, less is known about the curricular impact on undergraduates' intentions to work with older adults and attitudes and knowledge about sexuality and aging. The current longitudinal study examined the impact of an upper-level adult development psychology course on student attitudes, knowledge, and intentions toward working with older adults. Participants were 8 undergraduate students enrolled in upper-level undergraduate psychology courses. Participants completed validated, self-report questionnaires at the beginning of the semester and at the end of the semester related to their attitudes towards working with older adults, ageism attitudes, aging sexual knowledge and attitudes, and attitudes and knowledge about aging. Paired samples t-tests were used to examine changes in main outcome variables. Students endorsed significantly greater attitudes toward working with older adults at the end of the semester (t = -2.57, p = .037). Additionally, knowledge about sexual aging significantly increased (t = 2.62, p = 0.34). These findings suggest curricular interventions may positively impact attitudes toward working with older adults and sexual aging knowledge. Future work will evaluate predictors of change in attitudes, knowledge, and intentions. By identifying factors that increase undergraduates' intentions to work with older adults and improve attitudes and knowledge regarding sexual aging, we may elucidate meaningful points of intervention to enhance the pursuit of careers in the geriatric workforce.

Abstract citation ID: igad104.2698

### CONTROL BELIEFS MEDIATE THE WITHIN-PERSON RELATIONSHIP BETWEEN EVERYDAY CONTEXT AND SUBJECTIVE AGE

Shenghao Zhang[1], and Shevaun Neupert[2], *1. Florida State University, Tallahassee, Florida, United States*, *2. North Carolina State University, Raleigh, North Carolina, United States*

Appraisal and interpretation of personal experiences resulting from interaction with situational contexts might play an important role in shaping subjective age at within-person level, but it is unclear how this process unfolds. We propose that older adults evaluate situational contexts and reflect on their general psychological resources when determining their subjective age, and tested this proposal with volition of daily activities as proxy for appraisal of situational contexts and control beliefs as proxy for psychological resources. We hypothesize that appraising daily activities one engaged in as obligatory would deplete one's perceived control and concomitantly make one feel older. Older adults (n=116) ranging in age from 60 to 90 (M=64.71) completed a nine-day daily diary study online, resulting in 743 total days. Participants reported their sociodemographic characteristics on Day 1 and major daily activities, volition of every reported activity, felt age, and control beliefs on Days 2-9. Results showed that on days when older adults felt that activities they engaged in were more of their own volition, they also felt more in control. Lower-level mediation result suggests that within-person control beliefs mediated the relationship between volition of daily activities and subjective age. Our findings suggest that older adults evaluate situational contexts and reflect on their control beliefs as a general psychological resource when determining their subjective age. These findings show the important role psychological resources play in determining subjective age from a within-person perspective and extended the within-person process proposed in previous theoretical models.

Abstract citation ID: igad104.2699

### DO INTERNALIZED AGE STEREOTYPES MEDIATE THE RELATIONSHIP BETWEEN VOLUNTEERING AND PURPOSE IN LIFE FOR ADULTS 50+?

Andrew Steward[1], and Yating Zhu[2], *1. University of Wisconsin-Milwaukee, Milwaukee, Wisconsin, United States*, *2. University of Denver, Denver, Colorado, United States*

The productive aging literature suggests that volunteering plays a crucial role in enhancing psychosocial well-being among older adults. A growing body of research based in stereotype embodiment theory also demonstrates significant negative health impacts of internalized age stereotypes. Yet, limited research explores which social engagement activities may both reduce internalized ageism and enhance psychosocial health as people age. This cross-sectional study investigated whether internalized age stereotypes mediate the relationship between volunteering and a sense of purpose in life for adults 50+. A convenience sample of volunteers (n = 154) 50+ years of age living in the U.S. Mountain West completed a 15-minute, online survey. The independent variable was number of volunteer hours per week (mean = 6.39, SD = 5.47). The dependent variable was purpose in life measured by six items from the positively worded, five-point purpose in life subscale of the Ryff psychological well-being scale ($\alpha$ = .75; mean = 3.93, SD = 0.61). Drawing from the self-stereotypes of aging scale, the indirect effects of five internalized positive (e.g., "wise" and "capable") and five negative (e.g., "grumpy" and "helpless") age stereotypes were tested. Results indicate that increased internalized positive, not negative, age stereotypes partially mediated the relationship between volunteer hours and purpose in life while holding constant age, gender, race, functional limitation, education, employment, self-rated health, and previous volunteer experience. The findings suggest that internalizing positive age stereotypes may function as a form of esteem and contribute to an enhanced sense of purpose in later life.

Abstract citation ID: igad104.2700

### DOES INTERNET USE PROMOTE OLDER ADULTS' SELF-PERCEPTION OF AGING: SEPARATING WITHIN- AND BETWEEN-PERSON EFFECTS

Kun Wang[1], and Danan Gu[2], *1. Binghamton University, State University of New York, Binghamton, New York, United States*, *2. The United Nations, New York City, New York, United States*

Previous longitudinal studies indicate a positive causal association between older adults' Internet use and self-perception of aging (SPA). However, it is unclear whether this positive effect is truly due to the lagged within-person changes in Internet use or due to some stable, trait-like between-person differences. Thus, with between-person differences controlled, this study aimed to 1) examine reciprocal associations of within-person SPA and Internet use and 2) investigate the potential age, gender, race/ethnicity, and education group differences. Data used in this study were from the Health and Retirement Study (T1: 2008; T2: 2012; T3: 2016). Older adults aged 65 and older with normal cognition were included in the analysis (n = 6,666). Random-intercept cross-lagged panel models (RI-CLPM) were applied to separate the within- and between-person effects regarding the associations between SPA and Internet use. Multiple-group analyses were used for testing group differences. Results showed positive carry-over stability of both Internet use and SPA across time. At the within-person level, only one cross-lagged path, Internet use (T1) ◇ SPA (T2), was significant ($b = .08$, $p < .05$), indicating that the within-person Internet use change was predictive of within-person SPA change at T2. At the between-person level, SPA and ICT use were positively correlated ($r = .16$, $p < .001$). No group differences were identified. This study indicates that the positive effects of Internet use on SPA may be largely due to between-person differences. Internet use may promote SPA to some extent, but effects may plateau after a certain period of time.

Abstract citation ID: igad104.2701

### DOES SELF-REPORTED DISCRIMINATION INCREASE AS COGNITION DECLINES?

Douglas Hanes, and Sean Clouston, *Stony Brook University, Stony Brook, New York, United States*

Progressing cognitive decline causes limitations on daily life and emotional changes, including increased aggression, agitation, and restlessness that might occasion greater scrutiny, limitations, discrimination, and disrespect due to a combination of care-provision and ageism. While there are a number of studies of discrimination as a cause of cognitive decline, there are no studies of how real and perceived discrimination during cognitive decline might affect the aging person and their caregivers. This study analyzes whether people currently experiencing cognitive decline report greater frequency of discrimination. Using the Health and Retirement Study (HRS; 2006–18; N = 37,059), we used multilevel ordered logistic regression to test the role of changes in cognitive performance in predicting self-reported frequency of six forms of discrimination, adjusting for age, socioeconomic, and demographic covariates. We found that lower cognitive scores were associated with increased discrimination frequency in three forms of treatment: less respect (β=-0.010; p=0.022), being treated as "not smart" (β=-0.026; p< 0.001), and worse medical treatment (β=-0.014; p=0.027) though no differences were evident in service quality (β=-0.002, p=0.736), evidence of being threatened (β=-0.11, p=0.069), or having people report being afraid of you (β=-0.004, p=0.455). Results suggest that perceptions of discrimination could emerge as a result of declining cognitive performance and highlight the need for greater sensitivity to discrimination among older adults in both everyday and medical settings. Further investigation will enable us to distinguish between experienced discrimination due to changes in treatment versus ageism.

Abstract citation ID: igad104.2702

### DOES THE FACTS ON AGING QUIZ ASSESS AGING STEREOTYPES?

Savannah Schultz[1], Sarah Francis[1], Mack Shelley[1], Aaron Vincent[1], Cori Hyde[1], and Alexandra Bauman[2], *1. Iowa State University, Ames, Iowa, United States*, 2. *Iowa Department on Aging, National Resource Center on Nutrition & Aging, Des Moines, Iowa, United States*

The World Health Organization (WHO) Global Report on Ageism urges researchers to develop and validate assessment tools, thus enabling the field to better address ageism. This study assesses the internal consistency and construct validity of a short Facts on Aging Quiz (SFAQ, 10-items). We hypothesize that the SFAQ is a valid and reliable tool for assessing aging stereotypes. Data from the National Resource Center on Nutrition and Aging 2022 Needs Assessment were utilized. Data were collected via an online survey through a convenience sample of the National Aging Network (n=1,910). Three aging perspective tools were included: SFAQ, Expectations Regarding Aging (ERA, 12-items), and the WHO Ageism Quiz (AQ, 8-items). An exploratory factor analysis assessed whether negative (NEG) and positive (POS)-stereotype SFAQ items emerged as constructs. The factor analysis showed that NEG and POS-SFAQ accounted for 31.39% and 19.63% of the total variance, respectively. Cronbach alpha evaluated the internal consistency of the tools. Internal consistency for ERA (α=.81) and AQ (α=.57) were acceptable but low for total SFAQ (α=.28); reliability was acceptable for NEG-SFAQ (α=.71) and lower for POS-SFAQ (α=.40). Pearson correlation assessed convergent validity, with a significance level of p<.0001 (df=1,908) for all correlations. A negative correlation was detected between POS-SFAQ and ERA (r=-.31) and positive correlations between NEG-SFAQ and both ERA (r=.55) and AQ (r=.65), confirming convergent validity. POS-SFAQ was negatively correlated with NEG-SFAQ (r=-.19), suggesting the SFAQ may contain conflicting items. In conclusion, versions of the FAQ may perform better as a tool to assess aging stereotypes.

Abstract citation ID: igad104.2703

### EFFECTS OF CONSERVATIVE VALUES AND AGING KNOWLEDGE ON YOUNG ADULTS' AGEISM AGAINST OLDER ADULTS

Joseph Muraira, Erin Hulsman, Christian López, Derek Killingsworth, Kellee Bishop, Jenna Lao, Allyson Coldiron, and Michael Barnett, *University of Texas at Tyler, Tyler, Texas, United States*

Ageism – the stereotypes, prejudice, and discrimination of individuals solely based on age – is an unfortunately prevalent phenomenon for older adults to experience from young adults, and increased ageism among young adults has been associated with more conservative values and beliefs. Previous interventions to reduce ageism in young adults have focused on increasing knowledge about aging and contact between generations, since greater aging knowledge is associated with decreased ageism. However, recent studies identify multiple forms of ageism, including negative ageism (e.g., beliefs about older adults' lack of cognitive and physical ability) and positive ageism (e.g., stereotypes of older people as warm and reliable). This study therefore examined the relationships between young adults' conservative values, aging knowledge, and both negative and positive ageism about older adults. Participants were young adults (N = 459), ages 18 to 25 years (M = 20.10, SD = 1.76), who completed the Relating to Older People Evaluation, Social and Economic Conservatism Scale, and Palmore's Facts on Aging Quiz in an online survey. Results showed that conservatism was positively associated with negative and positive ageism, while aging knowledge was only negatively associated with negative ageism. Hierarchical regressions found that aging knowledge explained significant variance in negative ageism when controlling for conservatism, but only conservatism explained variance in positive ageism. These findings suggest that interventions including education on aging for young adults may be able to overcome the negative ageism associated with conservative values, but that positive ageism may not be meaningfully addressed by such education-based interventions.

Abstract citation ID: igad104.2704

### EVALUATION OF A PROVIDER-DRIVEN STRATEGY TO ENSURE AGE-FRIENDLY CARE

Heba AlDossary[1], Anna Bender[2], Anne Pohnert[3], and Mary Dolansky[4], *1. Frances Payne Bolton, Case Western Reserve University, Cleveland, Ohio, United States*, 2. *University of Washingotn, Seattle, Washington, United States*, 3. *CVS Health, Bellingham, Massachusetts, United States*, 4. *Case Western Reserve University, Cleveland, Ohio, United States*

Implementation of evidence-based care requires innovation. The Age-Friendly Health Systems Questionnaire (AFHS-Q) is an example of a front-line innovation that increases efficiency of implementation by having the patient complete eight 4Ms questions while waiting for their visit. We evaluated the usability, acceptability, feasibility, and effectiveness of the AFHS-Q by surveying nurse practitioners

(NPs) across a retail clinic system and found that the innovative practice-based tool was effective and feasible. The AFHS-Q kit was sent to over 1,100 retail clinic sites and an online survey was sent four weeks later to the 3,300 NPs at these sites to measure their experiences. NPs (N = 100) completed the survey and reported overall positive feedback (scale range 1-5) about the AFHS-Q relating to comfort of use (M = 4.18, SD = .99), usefulness (M = 3.97, SD = 1.10), acceptability (M = 4.12, SD = .83), and effort for use (M = 2.75, SD = 1.17). NP's confidence in completing the 4Ms (before AFHS-Q: M = 3.92, SD = 1.90; after AFHS-Q: M = 4.18, SD = .97) and documenting the 4Ms (before AFHS-Q: M = 3.84, SD = .99; after AFHS-Q: M = 4.17, SD = .91) was significantly higher following AFHS-Q use (completion: t = 3.58, p < .001; documentation: t = 4.33, p < .001). NPs reported a significant reduction in time for visits after using the AFHS-Q. Participants also provided open ended feedback indicating the AFHS-Q was overall easy to use as well as highlighting areas for other improvement.

Abstract citation ID: igad104.2705

### GRAY FOR A DAY: A SIMULATION ACTIVITY IMPACTS PARTICIPANTS IN COMMUNITY-BASED HEALTH EDUCATION

David Buys[1], Jasmine Harris-Speight[2], Pamela Redwine[2], Ann Sansing[2], and Erin Yelland[3], *1. Mississippi State University, Mississippi State, Mississippi, United States*, *2. Mississippi State University, Starkville, Mississippi, United States*, *3. Kansas State University, Manhattan, Kansas, United States*

Nearly 80% of older adults own their own homes; approximately two-thirds live within 25 miles of relatives; and most prefer to age-in-place, without access to geriatric-specific care. They rely on primary care clinics and family, friends, and other local support to meet their health needs. The healthcare workforce and lay public need to better understand age-related functional decline. Simulation activities can increase one's understanding of new concepts or perspectives. One such activity, "Gray for a Day," offers a didactic presentation and simulations of functional decline. In 2022, 10 Mississippi State University Cooperative Extension agents offered the "Gray for a Day" training to 550 individuals across 35 sessions in community colleges, high school career and technical education classes, and hospitals and nursing home food service departments. Participant evaluations were collected to assess learning outcomes and empathy development. Process evaluation data and descriptive statistics are reported. Nearly all (98%) of the participants reported increases in knowledge of age-related sensory and functional decline and increases in understanding how age-related sensory and functional decline can impact one's daily life; and 95% reported an increase in considering how they can better interact with older adults experiencing functional decline. Understanding challenges facing older adults is essential to empathy and important both in a home environment and in the workplace. The "Gray for a Day" simulation activity is feasible for implementation in multiple settings and has the potential to increase knowledge, understanding, and possibly empathy for older adults experiencing functional decline.

Abstract citation ID: igad104.2706

### GRAY HAIR AND PINK SLIPS: AN ANALYSIS OF TWITTER RESPONSES TO GENDERED AGEISM

Anne Barrett, Skyler Bastow, Rachael Dominguez, Melissa Frost, Cherish Michael, Hope Mimbs, and Brianna Soulie, *Florida State University, Tallahassee, Florida, United States*

Popular, long-time Canadian broadcaster, Lisa LaFlamme, was fired in August 2022, with some observers suggesting that CTV National News did not renew her contract because she had "let her hair go gray" during the pandemic. Over the next several months, an international public outcry ensued on Twitter. Our study involved an analysis of over 400 of these tweets. Analyses revealed that over two-thirds of tweets indicated support for LaFlamme's position, with nearly all the remaining tweets indicating neutral rather than negative positions. Frequent themes in the supportive tweets included assessments of her firing as undeserved and the actions of the news corporation as unjust. Calls to boycott the corporation also appeared. Other themes found in supportive tweets included ageism and sexism; however, references to sexism were somewhat more frequent than those to ageism. A theme appearing not only in supportive but also in neutral or oppositional tweets centered on beauty standards, with supportive tweets more likely to critique these standards' sexist and ageist underpinnings. Our study extends the literature on gendered ageism by attending to collective responses to it. In contrast, prior studies have focused on individual-level experiences of and responses to gendered ageism. Our study also contributes to the hashtag activism literature, which has given little attention to efforts to dismantle age inequality.

Abstract citation ID: igad104.2707

### HOW DO PEOPLE PERCEIVE THEIR OWN AGE?—SELF-REFLECTION ON HEALTH STATUS

Hyojin Choi[1], and Kristin Litzelman[2], *1. UW-Madison Institute on Aging, Madison, Wisconsin, United States*, *2. University of Wisconsin-Madison, Madison, Wisconsin, United States*

Subjective age -- how old you feel -- is a simple, but inclusive indicator of how people experience their own aging. This hypothesis-generating study sought to assess how objective and subjective indicators of well-being differ by subjective age, in order to progress our understanding of how this metric internalizes physical and psychological information. Data were drawn from the Midlife in the United States in 2004-2006 (MIDUS 2). The sample represented adults aged 50 years and over (n=612) and was divided into two groups: 1) felt younger than chronological age, and 2) felt the same or older than chronological age. Nearly four in five older adults reported feeling younger than their chronological age. Chi-square statistics showed that subjective indicators differ by subjective age. Specifically, those that felt the same/older than their chronological age were more likely to have depression or functional limitations, and report poor health status than were feeling younger group. Interestingly, bio-marker (i.e., C-reactive protein), physical and cognitive ability (i.e., gaits and episodic memory), and chronic conditions did not differ by subjective age. The findings indicate that reports of subjective age correlate with perceived rather than objective health status. Subjective age may therefore function as an indicator that health problems or life stressors are becoming internalized and overcoming an individual's capacity to cope. Future research is needed to understand how subjective age can act as a bellweather of subjective and objective health decline.

Abstract citation ID: igad104.2708

### RELATION OF SPECIFIC DOMAINS OF AGING KNOWLEDGE AND CONTACT TO AGEIST ATTITUDES AMONG DOCTORAL PSYCHOLOGY STUDENTS

Grace Caskie, Benjamin Johnson, and Eve Root, *Lehigh University, Bethlehem, Pennsylvania, United States*

Knowledge about aging and intergenerational contact were identified in Levy's (2018) Positive Education about Aging and Contact Experiences (PEACE) model as the two elements that are key to reducing ageist attitudes. Existing empirical literature examining these relationships has primarily focused on undergraduate, rather than graduate, students and has typically operationalized knowledge about aging as a unidimensional construct (i.e., total score). Thus, in this study, we examined how knowledge within three specific domains of aging (psychological, social, biological) as well as quantity of contact with older adults related to ageist attitudes among doctoral psychology students (N=192; age=21-58 years). Participants completed the Contact with Older Adults Scale, Facts about Aging Quiz, Fraboni Scale of Ageism (subscales: Stereotypes, Separation, Affective Attitudes), and Ambivalent Aging Scale (subscales: Hostile Ageism, Benevolent Ageism). Controlling for students' age and aging coursework, the three knowledge domains and contact together explained between 8%-24% of the variance in the five measures of ageist attitudes. As expected, greater knowledge of psychological aging related to less endorsement of ageist stereotypes and less separation from older adults; however, greater knowledge of biological aging related to more ageist attitudes across all five measures, and greater knowledge of social aging related to more benevolent ageist attitudes. More contact with older adults related to less separation and less negative affect about older adults, but not to the other ageist attitudes measures. Differentiating between domains of aging knowledge produced unique relations with both explicitly negative and ambivalent ageist attitudes, which ageism intervention designers may need to consider.

Abstract citation ID: igad104.2709

### SELF-DIRECTED AGEISM OUTCOMES ASSOCIATED WITH AGEISM-REDUCTION INTERVENTIONS: A SCOPING REVIEW

Rachel Scrivano[1], and Shannon Jarrott[2], *1. Boston College, Boston, Massachusetts, United States*, *2. The Ohio State University, Columbus, Ohio, United States*

Self-directed ageism refers to stereotyping, prejudice, and discrimination toward oneself based on age or perceived age. Self-directed ageism research is limited but growing as the conceptualization of ageism evolves and the implementation of ageism-reduction interventions increases. A scoping review was conducted to explore self-directed ageism outcomes associated with ageism-reduction interventions among populations measured. The PRISMA-ScR checklist guided study methods. A systematic search for relevant peer-reviewed journal articles was conducted in 2022 using eight EBSCOhost databases. Fifty-two studies were included after screening was completed on 19,309 articles. Ageism-reduction interventions that assessed self-directed ageism outcomes were primarily implemented among college students (k = 20), older adults (k = 18), and children (k = 8). Despite methodological variation among included studies, such as measured domains of self-directed ageism, measures used, duration, dosage, and specific intervention characteristics, results suggest that purposeful in person intergenerational contact across age groups and in person intergenerational contact with aging education among college students and children are commonly effective in reducing self-directed ageism. Other interventions were found to be effective for certain age groups. Results provide initial support to suggest that intergenerational contact and aging education interventions can be similarly effective at reducing self-directed ageism as they are at reducing other-directed ageism. This scoping review also highlights several gaps in the literature. Notably, a universal definition for self-directed ageism is needed, a lifespan perspective of self-directed ageism should be taken, and research is needed to identify mechanisms of change responsible for significant outcomes achieved, especially within multifaceted interventions.

Abstract citation ID: igad104.2710

### SELF-PERCEPTIONS OF AGING AS A PSYCHOLOGICAL RESOURCE FOR ENRICHMENT SEEKING

Faith-Christina Washington[1], Shannon Mejia[2], Wan Wen[3], and Jessie Chin[4], *1. University of Illinois, Urbana, Illinois, United States*, *2. University of Illinois Urbana-Champaign, Chanpaign, Illinois, United States*, *3. University of Illinois Urbana-Champaign, Urbana, Illinois, United States*, *4. University of Illinois at Urbana-Champaign, Champaign, Illinois, United States*

In older adulthood, positive perceptions of one's own aging can facilitate enrichment seeking–the underlying motivation to seek out new experiences and perform intellectually challenging activities–by diminishing and buffering reactions to perceived threats to one's sense of self and perceived capabilities. In this study, we examined the extent to which self-perceptions of aging were associated with cognitive performance and affective experience while completing a series of challenging foraging tasks in the form of word puzzle games within the presence and absence of resources in the environment, such as a fireplace or an observing neurologist. The study employed a 2x2 within-person design (fireplace on vs. not; neurologist present vs. not) and took place within the home office of a home simulation environment. In each of the four conditions, 61 participants (age 50-82, 57% female) played the foraging task and then endorsed adjectives representing their feelings while playing that game. Multilevel models, which nested study stages within participants, showed individuals with higher self-perceptions of aging benefited significantly from both environmental manipulations, with the fireplace acting as environmental support and the observing neurologist raising the stakes of the task. However, despite providing additional motivation and being associated with stronger cognitive performance, the neurologist's presence also provoked a higher negative affect response. The relationship between self-perceptions of aging and receptiveness to environmental resources merits further investigation to assess how certain environmental resources might act as a moderator of both cognition and affect.

Abstract citation ID: igad104.2711

### THE RELATIONSHIP BETWEEN MEDIA PORTRAYALS OF OLDER PEOPLE AND PERCEPTIONS OF INTERGENERATIONAL CONFLICT

Soondool Chung[1], Ahyoung Lee[1], and Ju-Hyun Kim[2], *1. Ewha Womans University, Seoul, Republic of Korea, 2. Chungnam National University, Daejeon, Republic of Korea*

As South Korea experienced rapid social change in several decades, there are lots of gaps in historical and cultural experiences of younger people and older people. In addition, with rapid population aging, generational tension became stronger due to limited resources and opportunities. This study examined the relationship between media portrayals of older people and intergenerational conflict and the mediating role of prejudice against older people in the relationship in South Korea. Also, the relationship between trust and intergenerational conflict was examined. Finally, the buffering effect of trust in the relationship between media portrayals of older adults and intergenerational conflict was examined. Secondary data analyses were conducted using Age Integration and Intergenerational Solidarity survey data of Korean adults aged 20 and over with a total of 1,000 cases. The findings revealed that prejudice against older people mediated the relationship between the media's portrayal of older people and perceptions of intergenerational conflict. Trust showed a significant negative relationship with intergenerational conflict. Finally, trust had a buffering effect on the media's negative portrayal of older adults and intergenerational conflict. For people with lower levels of trust, there was a significant positive relationship between negative media portrayals of older adults and intergenerational conflict. Further implications for practice to reduce intergenerational conflict were discussed.

### SESSION 7600 (POSTER)

### RACE, ETHNICITY, NATIVITY, CULTURE, AND HEALTH

Abstract citation ID: igad104.2712

### DIMINISHED RETURNS ACROSS THE LIFE COURSE FOR BLACK INDIVIDUALS WITH TYPE 1 DIABETES

Mallory Bell, *Purdue University, West Lafayette, Indiana, United States*

Prior literature suggests that Black individuals of high socioeconomic status experience diminished returns on an array of health outcomes. However, few studies have examined whether the association between income and glycemic control varies by race among people with Type 1 diabetes (T1D) across the life course. In the current study, we extend prior literature by using a life course framework to examine the ways in which income interacts with race on glycemic control. Using data from the 2017-2018 wave of the T1D Exchange Registry (N=12,855), our results reveal that black individuals with T1D experience diminished returns on health: Black individuals with an annual income of $100,000 had worse glycemic control, operationalized by HbA1c level, compared to White individuals with an annual household income of $25,000. These racial disparities narrow among older adults. The current study suggests the need for a better understanding of the interpersonal and structural barriers associated with suboptimal glycemic control among Black individuals with T1D, especially as it relates to different stages across the life course.

Abstract citation ID: igad104.2713

### BREAST CANCER SCREENING AMONG OLDER NON-HISPANIC CARIBBEAN BLACK WOMEN IN THE UNITED STATES

Donnette Narine[1], and Takashi Yamashita[2], *1. University of Maryland Baltimore, Baltimore, Maryland, United States, 2. University of Maryland, Baltimore County, Baltimore, Maryland, United States*

Breast cancer is one of the most common forms of the second leading cause of death—cancer, which impacts racial and ethnic groups differently. The mortality rate of breast cancer among Black women is 40% higher than that of non-Hispanic White women. Early detection is critical for improving survival rates and reducing health disparities by race and ethnicity over the life course. However, the heterogeneity (i.e., within-group differences) among Black women in the United States is often overlooked, and relatively little is known about specific sub-populations of Black women and breast cancer screening. Of those sub-populations, Afro-Caribbean women in the United States tend to have different demographic and cultural backgrounds (e.g., a higher % of immigrants), for example, from African/African American women. Thus, this study examined factors associated with breast cancer screening among non-Hispanic Caribbean Black women. Using seven waves (2011-2017) of the National Health Interview Surveys data, we analyzed a sample of non-Hispanic Caribbean Black women living in the United States (age 40-75 years; n = 552). The results of the binary logistic regression indicated that the years in the United States were positively associated with breast cancer screening in the past 12 months among non-Hispanic Caribbean Black women (odds-ratio = 1.39, standard-error = 0.15, $p < 0.05$), after adjusting for the selected covariates. However, age was not associated with breast cancer screening behaviors. Our findings support breast cancer prevention efforts specifically targeting middle-aged and older non-Hispanic Caribbean Black women who have newly immigrated to the United States.

Abstract citation ID: igad104.2714

### CULTURALLY TAILORED INTERVENTION EFFICACY TO PROMOTE BREAST CANCER SCREENING AMONG AMERICAN INDIAN WOMEN

Soonhee Roh[1], Yeon-Shim Lee[2], DenYelle Kenyon[1], Amy Elliott[3], Daniel Petereit[4], and Anu Gaba[5], *1. University of South Dakota, Sioux Falls, South Dakota, United States, 2. San Francisco State University, San Francisco, California, United States, 3. Avera Research Institute - Sioux Falls, Sioux Falls, South Dakota, United States, 4. Rapid City*

*Regional Hospital, Rapid City, South Dakota, United States*, *5. Sanford Roger Maris Cancer Center, Fargo, North Dakota, United States*

Purpose: The purpose of this project was to develop and evaluate the feasibility and effectiveness of the Mobile Web App Breast Cancer Screening (wMammogram) intervention among American Indian (AI) women. Method: Using a randomized controlled trial design, 122 AI women (40-70 years old) were recruited and randomly assigned to the wMammogram group (n=62) to receive culturally and personally tailored multimedia messages through a mobile web app along with health navigator services or the control group (n=60) to receive a printed educational brochure. Outcome measures included mammogram receipt, intention to receive breast cancer screening after the intervention, and satisfaction with and perceived effectiveness of the intervention. Result: A significantly higher proportion of women who received the intervention (42%, 26/62; p<.01) completed mammograms by the 6-month follow-up compared with the control group (20%, 12/60). The wMammogram group, compared with the control group, reported significantly higher ratings on perceived effectiveness of the intervention (t120=-5.22, p<.001), increase in knowledge (t120=-4.75, p<.001), and satisfaction with the intervention (t120=-3.61, p<.001). The wMammogram group also expressed greater intention to receive a mammogram in the future when it is due (100%, 62/62 vs. 85%, 51/60) and were more willing to recommend the intervention they received to their friends (98.4%, 61/62 vs. 90%, 54/60) compared with the brochure group. These differences were statistically significant.

**Conclusion:** Based on our results, a mobile web app-based intervention combined with health navigator service was a feasible, acceptable, and effective intervention mechanism aimed at promoting breast cancer screening in AI women.

Abstract citation ID: igad104.2715

### FOOD TALK AS AGING TALK: LISTENING TO OLDER ITALIANS

Anne Barrett[1], Monica Consolandi[2], and Eliana Fattorini[3], *1. Florida State University, Tallahassee, Florida, United States*, *2. Bruno Kessler Foundation, Trento, Trentino-Alto Adige, Italy*, *3. University of Trento, Trento, Trentino-Alto Adige, Italy*

An extensive literature in gerontology examines older adults' diets and their effects on health and longevity. Much less research examines how food, including experiences of its scarcity and plentitude across the life course, shape older adults' constructions of their own aging. Our study addressed this topic using semi-structured interviews conducted in a mid-sized northern Italian city in 2018-2019 (n=28) and 2022 (n=23). Analyses revealed that participants used vivid references to food – a topic that was not the focus of any interview questions – to illustrate their experiences of aging. Participants' food talk referenced different tenses of time, including durable food memories from the distant past and more ephemeral food activities and concerns of the present. Participants also referred to food when making different comparisons that shaped assessments of their own aging experiences, including comparisons with older family members in the past and age peers in the present. Food figured into participants' discussions of aging in four ways: food as anchors for childhood memories, intergenerational comparisons of food's plentitude versus scarcity in later life, food choices to support aging well, and food sharing to sustain social relationships. Our study illustrates the use of food a lens for understanding individuals' aging experiences, as embedded in historical times that give them meaning.

Abstract citation ID: igad104.2716

### GAMBLING AND PSYCHOLOGICAL WELL-BEING AMONG OLDER CHINESE ADULTS IN THE US

Yuyang Zhu[1], Yiming Ma[2], Fengyan Tang[3], and Yanping Jiang[2], *1. Rutgers University, Piscataway, New Jersey, United States*, *2. Rutgers University, New Brunswick, New Jersey, United States*, *3. University of Pittsburgh, Pittsburgh, Pennsylvania, United States*

Gambling is a common but not harm-free social practice. Few studies have investigated the long-term impact of gambling activities on psychological well-being among racial/ethnic minority groups in the U.S. This study aimed to close this gap by examining the associations between gambling, problem gambling, and psychological well-being among older Chinese immigrants. We drew data from 2,811 (58% female) participants aged 60 years or older in the Population Study of Chinese Elderly in Chicago Study who completed the baseline assessment and at least one of the three follow-ups that were conducted every two years. Gambling participation was assessed using Modified South Oaks Gambling Screen and Problem Gambling Severity Index. The responses were used to categorize participants: never gambled (the reference group), participated in gambling, and problem gambling. Psychological well-being was assessed using Patient Health Questionnaire-9 and Hospital Anxiety and Depression Scale. Of all participants, 761 reported ever participating in gambling activities, and 52 reported problem gambling experiences. Participants who participated in gambling reported the lowest baseline scores of depression and anxiety compared to the other two groups. However, gambling participation predicted faster increases in depression ($\beta$=0.076, p=0.01) and anxiety ($\beta$=0.103, p=0.02) over time. Also, problem gambling was associated with a faster increase in anxiety ($\beta$=0.083, p=0.02). These results show a faster increase in psychological distress related to gambling participation over time. Culturally responsive interventions to reduce gambling activities and their impacts on psychological well-being are needed to promote health among this population.

Abstract citation ID: igad104.2717

### PERSONALITY FACTORS AND ELEVATED DEPRESSIVE SYMPTOMS AMONG MEXICAN ADULTS AGED 50 AND OLDER WITH PAIN

Sarah Navid[1], Sirena Gutierrez[2], Joseph Saenz[3], Michael Antonietti[4], and Sadaf Milani[1], *1. University of Texas Medical Branch, Galveston, Texas, United States*, *2. University of California San Francisco, San Francisco, California, United States*, *3. Arizona State University, Phoenix, Arizona, United States*, *4. University of Miami, Miami, Florida, United States*

Older adults with pain are at risk for developing elevated depressive symptoms. Personality factors, including internal locus of control (LOC) and conscientiousness, have been associated with fewer depressive symptoms in older adults.

Our objective was to examine LOC and conscientiousness as potential buffers of depressive symptoms among Mexican adults aged 50 and older with pain. We used data from the Mexican Health and Aging Study (2015-2018). We included participants who reported frequent pain at both waves (n=2,211). LOC and conscientiousness were measured continuously, with higher values indicating a more internal LOC or conscientious personality. We incorporated depressive symptoms from both waves to create a 4-level depressive symptoms variable: stable low/ no depression (< 5/< 5), recently remitted (5+/< 5), recent onset (< 5/5+), and stable high (5+/5+). We used multinomial logistic regression models to assess the association between personality and depressive symptoms, adjusting for sociodemographic variables. At baseline, participants (73.2% women) were on average 67.6 years old. A more internal LOC was associated with a lower risk of stable high depressive symptoms compared to those with stable low/no depression symptoms [Relative Risk Ratio: 0.82, 95% confidence interval: 0.72, 0.92). Both personality factors were not associated with recently remitted or recent onset depressive symptoms. In a sample of older adults with pain, those with a more internal LOC had decreased risk of experiencing stable high depressive symptoms. Internal LOC may alter the perception of pain. Interventions on orienting individuals to a higher internal LOC may be helpful in pain management by mitigating comorbid depressive symptoms.

Abstract citation ID: igad104.2718

### THE JOINT EFFECTS OF PAIN AND ELEVATED DEPRESSIVE SYMPTOMS AMONG MEXICAN AMERICANS AGED 80 AND OLDER

Claudia Sanchez[1], Phillip Cantu[2], Kyriakos Markides[1], and Sadaf Milani[1], *1. University of Texas Medical Branch, Galveston, Texas, United States*, *2. UTMB, Galveston, Texas, United States*

Pain and depression are common among older Hispanic adults and their combined effects may increase mortality. We examined the joint effects of pain and depressive symptoms on mortality. We used data from the Hispanic Established Population for the Epidemiologic Study of the Elderly (2010-2016), which included Mexican Americans aged 80 and older in the Southwestern US. Participants were categorized into four groups based on self-reported pain on weight-bearing and depressive symptoms: no pain or elevated depressive symptoms, pain only, elevated depressive symptoms only, or both pain and elevated depressive symptoms. Depressive symptoms were measured using the Center for Epidemiologic Studies Depression scale. A score of 16 or more was considered elevated depressive symptoms. Cox proportional hazards models were used to estimate the joint relationship of pain and depressive symptoms with mortality. We examined social support as a potential effect modifier. At baseline, 35.9% reported pain only, 8.6% of participants reported elevated depressive symptoms only, and 16.9% reported pain and elevated depressive symptoms (n=836). Over 6 years of follow-up, 46.2% of the sample died. Those with pain and elevated depressive symptoms had 1.41 times the risk of mortality compared to those without pain or elevated depressive symptoms (95% confidence interval: 1.05, 1.09). Having pain or elevated depressive symptoms only was not significantly associated with mortality. Social support was not a significant effect modifier. Our findings highlight the importance of screening for pain and elevated depressive symptoms among older Mexican Americans.

### SESSION 7610 (POSTER)

### SOCIAL CONNECTEDNESS AND ISOLATION

Abstract citation ID: igad104.2719

### ACTIVE USE OF ONLINE COMMUNITY REDUCES LONELINESS AMONG OLDER ADULTS THROUGH WEAK TIES FORMATION

Nahyun Kim, and Keiko Katagiri, *Kobe University, Kobe, Hyogo, Japan*

Online communities could aid older adults in expanding their social connections and developing weak ties, which may help alleviate their loneliness. However, research investigating the impact of online community participation on the formation of weak ties among older adults remains lacking. This study aimed to investigate 1) the relationship between active use of an online community designed for older adults and the formation of weak ties within the platform, and 2) the potential for these weak ties to alleviate loneliness among members. The online community platform for older adults allowed users to post diary entries and photos, join interest-based communities, and organize virtual and in-person events. An online survey was conducted among members aged 60 to 79 years (N=862). The survey measured active use, weak ties (measured by the number of mutual followers), and loneliness. Path analysis showed that active engagement in the online community was positively associated with the formation of weak ties, which, in turn, were negatively associated with loneliness. Thus, older adults who frequently posted own content and participated in community activities were more likely to form many weak ties in the platform, and these weak ties may help reduce their loneliness. However, this study only examined online community use, meaning that further research is needed to explore the relationship between general social networking services use and weak ties, as well as examine the context of the passive use of these services.

Abstract citation ID: igad104.2720

### ASSOCIATION OF EVERYDAY LIFE INFORMATION ACQUISITION METHODS AND DEPRESSION AMONG US OLDER ADULTS

Wuyi Dong, Yan-Jhu Su, and Andrew Alberth, *University of Massachusetts Boston, Boston, Massachusetts, United States*

Everyday life information acquisition refers to the incidental ways people obtain information through daily activities such as reading, watching television, or communicating with friends rather than through intentional or purposive information seeking behaviors. It has been identified as a fundamental way to obtain informational resources and is a potential means to promote social connections and engagement among older adults. Research has shown that social engagement and connections are protective factors of depression for older adults. However, little research has explored how information seeking behaviors effect risks of depression among older adults. To fill this gap in the literature and provide insights into the mechanism of information access impacts the mental health of older adults, this study examines the relationships between different ways for obtaining daily information (reading, using computer, and watching television) and depression among older adults in United States. The data retrieved from 2018's Health and Retirement Study (HRS). We multiple linear regression analyses on HRS data including adults age 50 years and older (n=3,179). After adjusting for age, gender, race, marital status, education, and chronic diseases, all three different ways to obtaining daily information (reading, using computer, and watching television) were negatively associated with depression (β=-0.19, p< 0.001; β=-0.15, p=0.002; β=-0.15, p< 0.001, respectively). Findings suggest that the association between everyday life information acquisition and depression among older adults varies depending on the specific information acquisition methods used. These findings highlight the importance of implementing targeted interventions to promote effective information access and enhance social inclusion among older adults.

Abstract citation ID: igad104.2721

### CONNECTING THROUGH TECHNOLOGY: EXAMINING THE RELATIONSHIP BETWEEN SOCIAL NETWORKING SITES AND MENTAL HEALTH

Joseph Svec[1], Yeon Ji Ryou[2], and Jeong Eun Lee[2], *1. Saint Joseph's University, New York City, New York, United States*, *2. Iowa State University, Ames, Iowa, United States*

Social isolation and loneliness among older adults correspond with numerous mental and physical health consequences including but not limited to depression, anxiety, declines in cognitive functioning, and poor health behaviors. More recent research highlights the potential benefits of digital-social communication technology through social networking sites (SNS), communicative technology, and computer or tablet use (Khosravi et al. 2016; Jutai and Tuazon 2022; Neil-Sztramko et al. 2020). However, the efficacy of technology on mental and physical well-being remains obscure, particularly given the often interchangeability of isolation and loneliness concepts. In this research, we examine the extent to which conditions of isolation and feelings of loneliness correspond with depression among older adults. Using data from two waves of Health and Retirement Study (HRS) data in 2018 and 2020 (N = 8,994), we conduct a series of linear regressions of depression scales on both isolation and loneliness as well as their interaction with the use of social technology in communication. Preliminary results suggest that that the use of virtual communications by social networking sites (SNS) moderates the associations between isolation and depression levels, but no such effect is observed in terms of self-perception of loneliness. Moreover, the magnitude of SNS-Isolation and depression linkages differs significantly for family and friends. This research contributes to the growing body of literature on the intersection of technology and psychological well-being among isolated older adults, highlighting the both the potential benefits and limitations of virtual communication for improving health outcomes in older adults.

Abstract citation ID: igad104.2722

### EFFECTIVENESS OF TELEDELIVERED BEHAVIORAL ACTIVATION AND MINDFULNESS INTERVENTIONS IN REDUCING LONELINESS

Da Jiang[1], Dannii Yeung[2], Namkee Choi[3], Rainbow Tin Hung Ho[4], Jojo Yan Yan Kwok[4], Youqiang Song[4], Lisa M. Warner[5], and Kee Lee Chou[1], *1. Education University of Hong Kong, Hong Kong, Hong Kong*, *2. City University of Hong Kong, Kowloon Tong, Hong Kong*, *3. University of Texas, Austin, Texas, United States*, *4. The University of Hong Kong, Hong Kong, Hong Kong*, *5. MSB Medical School Berlin, Berlin, Germany*

Loneliness is one of the most prevalent mental health problems in older adults, especially among those who live alone and are digitally excluded. The phenomenon has become more conspicuous during the COVID-19 pandemic because of the constraints on physical contact and travel. In this three-armed randomized controlled trial, we examined the effectiveness of two telephone-delivered loneliness interventions (i.e., behavioral activation [Tele-BA], mindfulness [Tele-MF]) against a telephone-delivered active control group (i.e., befriending [Tele-BF]) in reducing loneliness, based on the behavioral theory of depression and monitor acceptance theory. We trained retirees as lay counselors to deliver the interventions to enhance the scalability and sustainability of the interventions. Older adults who lived alone and under the poverty line in Hong Kong, did not have Internet at home, and felt lonely were randomly assigned to one of the three groups (N = 1045, Mage = 76.59, SDage = 7.83, 78% female). Participants in each group received two 30-minute weekly intervention sessions for four weeks. They completed assessments at the baseline (T1), four weeks (T2) and six months (T3) following the intervention, respectively. Compared with their counterparts in the Tele-BF group, participants in the Tele-BA and Tele-MF groups reported lower levels of loneliness and perceived stress and higher levels of psychological well-being and perceived social support at T2 and T3. These findings provide scientific understandings on the effectiveness of Tele-BA and Tele-MF interventions for reducing loneliness among older adults in Hong Kong, and demonstrate the feasibility of conducting lay counselor-delivered telephone-based interventions for older adults.

Abstract citation ID: igad104.2723

### EMOTIONAL CLOSENESS, SOCIAL NETWORK, NEGATIVE AND POSITIVE AFFECTS, AND AGITATION IN OLDER ADULTS WITH DEMENTIA

Mohammad Rababa, *Jordan University Of Science And Technology, Irbid, Irbid, Jordan*

**Background:** If given a choice, people with dementia (PWD) would prefer to live in their homes instead of nursing homes. Living in older adults' own houses would positively impact their quality of life and be associated with more privacy, security, and social support. In addition, living in a nursing home is not culturally and religiously accepted, especially in a religious and conservative country like Jordan. However, with increased financial obligations and hardships in Jordan, women are forced to work outsides their houses. Therefore, PWD stay alone in their homes without care or attention. This neglect would put PWD at significant risk for adverse physical and psychosocial health consequences.

Method: This descriptive cross-sectional study was conducted on a convenience sample of 102 Jordanian PWD (51 Community-dwelling older adults and 51 NH residents) to examine the association of their emotional closeness and social networks with agitation and positive and negative affects. Result: The finding of this study based on an independent samples t-test showed that NH residents were significantly more likely to have physically non-aggressive behaviors than community-dwelling older adults. The study showed that the PWD's sex (male), higher educational levels, and patients with a family member in the healthcare field are associated significantly with their mean positive affects score. **Conclusion:** Future research would consider exploring potential factors associated with agitation and affect in PWD. Experimental studies that intend to increase the levels of social support and emotional closeness, thus alleviating negative affect and levels of agitation, are recommended in the future.

Abstract citation ID: igad104.2724

### EXPLORING THE PHENOMENON OF BOREDOM IN THE CONTEXT OF LEISURE AMONG OLDER ADULTS

Jaesung An[1], Laura Payne[2], and Megan Janke[3], *1. California State University East Bay, Hayward, California, United States*, *2. University of Illinois Urbana-Champaign, Champaign, Illinois, United States*, *3. Berry College, Mt Berry, Georgia, United States*

Boredom is a concept that seems at odds with leisure, yet research on this topic indicates these constructs are often connected. Leisure boredom has been studied primarily among adolescents (Donati et al., 2019; Weybright et al., 2015). However, boredom is prevalent among older adults. Social isolation and loneliness often intertwine with boredom, and research suggests that prolonged boredom can negatively affect health and well-being (An et al, 2022; Goldberg et al., 2011). Yet, there is a dearth of research on this topic and existing literature typically examines adults' functional status and disengagement or selective investment in leisure activities (Kleiber et al., 2008) rather than examining boredom more broadly. Thus, the purpose of this study was to examine the concept of boredom holistically in the context of leisure among community-dwelling older adults. The study used a multi-method approach utilizing focus groups and projective techniques of sentence completions and drawings. Forty-two older adults were recruited from senior centers and senior communities located in four western US cities. Thematic analysis with a six-step process outlined by Braun and Clarke (2006) was employed to analyze the data. Boredom was described as a personal state (e.g., negative self-perception, low energy and sensation/arousal, lacking motivation) and as activity-related emotions derived from unsatisfied/disengaged leisure participation. Participants also highlighted how life events and circumstances shaped their identity and contributed to boredom. Findings offered meaningful insights into how participants defined boredom, articulated causes of boredom, and strategies they use to alleviate boredom.

Abstract citation ID: igad104.2725

### FEELING YOU BELONG: THE EFFECTS OF COMMUNITY INVOLVEMENT ON WELL-BEING IN LATER LIFE

Emily Briggs, and Jacqui Smith, *University of Michigan, Ann Arbor, Michigan, United States*

The area where someone lives is an important, yet neglected, contextual factor for the health of older adults. Previous literature suggests that an individual may develop an attachment to their neighborhood through a feeling of "rootedness," which may contribute to feelings of belonging (Connerly & Marans, 1985). This sense of belonging is an essential component of harmonious communities with actively engaged residents. Together, feeling that you belong (perceived social cohesion) and active engagement in a community may be related to a higher overall sense of subjective well-being. Perceived social cohesion within a neighborhood also influences an individual's level of community engagement. Using data from the Health and Retirement Study (HRS), this study investigates the impact of perceived social cohesion and community engagement on subjective well-being. The sample (N=5581, Mean Age (SD)= 68.34 (10.35), Range 50-98 years) included participants from the 2018 wave of data collection, who completed the Psychosocial Leave Behind Questionnaire. Multiple regression revealed that community engagement ($\beta$=.19, p=<.001) and social cohesion ($\beta$ =.26, p=<.001) significantly predicted life satisfaction (R2=.096). These relationships remained significant after adjusting for age, gender, and years of education (Adj R2=.111, p<.001). These results suggest a sense of belonging may be more predictive than actual engagement on subjective well-being, this finding also interacts with age. Future work in this area should investigate the other possible influences that impact perceived feelings of belonging.

Abstract citation ID: igad104.2726

### HOW CAN WE SOLVE THE LONELINESS PROBLEMS WITH INTERGENERATIONAL DIGITAL DIVIDE?

Yeseul Lee[1], Si Young Song[2], Susanna Joo[3], and Hyoun Kyoung Kim[1], *1. Yonsei University, Seoul, Republic of Korea*, *2. Kyung Hee University, Seoul, Republic of Korea*, *3. BK21. Symbiotic Society and Design, Seoul, Republic of Korea*

As Korean society is rapidly digitalizing and aging, 'the intergenerational digital divide' due to a large gap in digital skills between the younger and older generations is rising social issues. Lack of digital skills among older adults is likely to make them feel isolated with limited opportunities for social integration in a highly digitalized society. This study examined the extent to which digital skills and intergenerational integration were differently associated with loneliness in Korea between the younger and older generations with the aim of informing efforts to reduce loneliness. The data was collected through an online survey from December 2021 through January 2022. The sample consisted of 315 Korean older adults aged 65 or above and 322 young adults in their 20s. The loneliness with 11 items from Loneliness Scale, digital skills with 4 items from Digital Citizenship Scale, Intergenerational Integration Scale with 30 items and a binary generation variable (young adults=0, older adults=1) were measured. Results from a serial mediation analysis showed that, compared to younger adults, older adults had lower levels of digital skills, which led to low intergenerational integration, which in turn affected loneliness. Findings from two simple mediation paths showed that only digital skills had a significant single mediating role in the association between generation and loneliness but intergenerational integration did not. This study suggests that enhancing digital skills with providing opportunities for online intergenerational interaction can decrease loneliness and associated issues, especially for older adults.

Abstract citation ID: igad104.2727

### IMPACT OF DIGITAL AND SOCIAL ENGAGEMENT ON QUALITY OF LIFE AMONG RURAL OLDER ADULTS

Jeehoon Kim[1], Gesine Hearn[1], and Anna Hall[2], *1. Idaho State University, Pocatello, Idaho, United States*, *2. Idaho National Laboratory, Idaho Falls, Idaho, United States*

**Objectives:** In the current digitalized society, digital and social engagement are important elements in the well-being of older adults. This study examines how different types of digital and social engagement are associated with different aspects of quality of life among rural healthy older adults. **Methods:** Older adults without cognitive impairments, residing in the community in Southeast Idaho (n=154) participated in the 'Aging in Idaho study' in 2018. Internet use for communication, instrumental purposes, and health information searches were measured. Informal and formal social participation were measured. Five aspects of quality of life (QOL) were measured using the WHOQOL-BREF. Multiple regression analyses were performed. **Results:** Well-educated (M=16 years) rural older adults used the Internet for various purposes, actively engaged in social activities, and reported higher level of QOL for overall and life environment (e.g., M=4.6, Range=1-5). Internet use for communication was positively associated with QOL general health (β=.20, P <.05). Online health information search was negatively associated with QOL general health (β= -.42, P <.05). Informal social participation was positively associated with QOL overall (β=.17, P <.05), psychological health (β=.13, P <.05), and life environment (β=.20, P <.05). Formal social participation was positively associated with QOL social relationships (β=.16, P <.05). **Discussion:** Digital and social engagement relate in distinct ways with different aspects of QOL. Older adults perceived QOL to be better when they actively adopted digital technology whereas maintaining social engagement well in the community. Promoting digital engagement which enables to facilitate social engagement in later life will be discussed.

Abstract citation ID: igad104.2728

### PUBLIC BATH USE AT A JAPANESE SENIOR APARTMENT ASSOCIATED WITH NEIGHBORHOOD RELATIONSHIPS AND SOCIAL SUPPORT

Mai Takase[1], Isuzu Nakamoto[2], and Hiroshi Murayama[3], *1. Tokyo Metropolitan Institute for Geriatrics and Gerontology, Tokyo, Tokyo, Japan*, *2. Health Sciences, Graduate School of Medicine, Tohoku University, Aoba-ku, Sendai, Miyagi, Japan*, *3. Tokyo Metropolitan Institute of Gerontology, Tokyo, Tokyo, Japan*

The number of older adults who choose to move into senior apartments is increasing in Japan. The development of quality relationships within the senior apartment is important for the healthy aging of the residents. A "public bath" is a unique facility in Japanese senior apartment where residents can interact with each other. Historically, Japan has had a culture of bathing with family and friends in public baths, and the use of this facility is expected to lead to socialization. This study aimed to examine the association between the frequency of public bath use, and neighborhood relationship and social support within a senior apartment. A cross-sectional survey was conducted in a senior apartment in February 2022 (Saitama Prefecture, Japan) towards the entire residents (n=185). Frequency of public bath use (once a week or more vs. less than once a week) was used as the independent variable. Neighborhood relationship (can support or talk to each other vs. only light greetings or no relationship) and social support (have vs. do not have social support) within the senior apartment were used as dependent variable. A binary regression analysis (n=117, female=60.7%) revealed that a higher frequency of public bath use associated with deep neighborhood relationship (OR: 7.14, 95% CI: 1.39-36.63) and social support (OR: 3.44, 95% CI: 1.19-9.99). These results suggest the importance of having a public bath in Japanese senior apartment setting. Furthermore, integrating facilities that possess cultural significance at senior apartments could be important for fostering social relationships.

Abstract citation ID: igad104.2729

### QUALITY OF LIFE OF OLDER ADULTS DURING THE COVID-19 PANDEMIC: RACIAL DISPARITIES AND SOCIAL CONNECTEDNESS

Babatope Ogunjesa[1], Sadia Anjum Ashrafi[2], Rafael Guimaraes[3], and Andiara Schwingel[4], *1. University of Illinois--Urbana-Champaign, Champaign, Illinois, United States*, *2. University of Illinois Urbana Champaign, Urbana, Illinois, United States*, *3. University of Illinois at Urbana-Champaign, Champaign, Illinois, United States*, *4. University of Illinois, Urbana Champaign, Champaign, Illinois, United States*

Social connectedness is crucial for well-being, especially among older adults. The Covid-19 pandemic has worsened mental distress in society due to prolonged social isolation practices aimed at curbing the virus's spread. This study examined disparities in older adults' self-reported quality of life during the pandemic and the influence of family and friends interaction. This study utilizes secondary data from the coronavirus worries and social isolation among older adults study by AP-NORC Center for Public Affairs Research on individuals aged 50 years and above (N=1,051). We aim to observe the effect of social interactions on the self-reported quality of life (QoL) of adults. Logistic regression analysis and marginal effect post-estimation analysis were utilized in this study, with adjustments made for race, education, gender, and income. The result showed that individuals who meet with family and friends more often (AOR=17.19, 95% CI=3.75-78.88, p< 0.05), or about as often (AOR=2.24, 95% CI=1.15-4.36, p< 0.05), are more likely to report a high quality of life than those who meet with them less frequently. A marginal effect analysis revealed that White adults had an 89.5% probability of reporting a high quality of life, non-Hispanic Black adults had an 83.4% probability of high quality of life, Hispanic adults had an 88.3% probability of high quality of life, and individuals from other ethnic groups had an 83.3% probability of high quality of life. These findings underscore the need for targeted interventions that address social isolation, especially among older adults, particularly those from racial and ethnic minority groups.

Abstract citation ID: igad104.2730

### RELIGIOSITY/SPIRITUALITY PROMOTES POSITIVE WELL-BEING FOR MIDDLE-AGED ADULTS IN THE CONTEXT OF MONTHLY ADVERSITY

Nutifafa Dey, Frank Infurna, and Kevin Grimm, *Arizona State University, Tempe, Arizona, United States*

Religiosity/Spirituality (R/S) are long established in aging research to protect against adversity and promote positive development. However, little about this is known regarding whether and which components of R/S (interpersonal and intrapersonal) in the context of longitudinal research in middle-aged adults. Using data from an intensive longitudinal study of 362 middle-aged adults (ages 50–65) assessed monthly over a period of two years, we applied multilevel models to examine whether R/S at the between- and within-person levels are a protective resource against the detrimental effects of monthly adversity on psychological well-being. Reporting more R/S at monthly assessments (within-person) where adversity was reported was associated with reporting fewer depressive symptoms and negative affect, but was not protective for the outcomes of life satisfaction and positive affect. Considered separately, the two domains of R/S protected against adversity's impact across each of the outcomes. Reporting more between-person levels of intrapersonal R/S (mapping strongly onto spirituality) did not protect against adversity, but instead exacerbated the detrimental effects of adversity on each outcome. The effects observed here imply that on monthly occasions where individuals report more R/S, this will lead to less of a negative impact of monthly adversity on each outcome. Also, at the between-person level, individuals who, on average, report more involvement in R/S will show less detrimental effects of monthly adversity on each outcome; yet this may not be true for intrapersonal R/S. We further elaborate R/S's protection and offer some explanations for why between-person changes in intrapersonal R/S exacerbates adversity.

Abstract citation ID: igad104.2731

### REMOTE COMPASSION- AND EMPATHY-BASED INTERVENTION TARGETING LONELINESS AND STRESS IN OLDER ADULTS

Molly Patapoff[1], Dylan Jester[2], Brent Mausbach[3], Barton Palmer[3], and Danielle Glorioso[3], *1. University of California San Diego, San Diego, California, United States*, *2. VA Palo Alto Health Care System, Palo Alto, California, United States*, *3. University of California, San Diego, San Diego, California, United States*

Loneliness and chronic stress are prevalent issues for older adults in the United States and have been linked to decrements in mental and physical health. Prior research has suggested that increasing compassion and empathy may reduce loneliness and chronic stress in older individuals. The present study is an efficacy pilot of a remote empathy- and compassion-based intervention targeting loneliness and chronic stress in older adults, consisting of a 6-week control and 6-week intervention period. Repeated measures ANOVAs were used to compare changes in ratings and biomarkers over the 12-week period from baseline to completion of the intervention. Bonferroni-adjusted post-hoc analyses were conducted to compare changes during control and intervention periods. The full sample consisted of 43 older adults (Mean Age=76.9 years, SD=7.5), and 12 completed additional biomarker assessments. Over the 12-week period, there were significant decreases in loneliness ($p=.001$) and perceived stress ($p=.03$). Post-hoc analyses found that perceived stress significantly decreased during the intervention period (Mdifference=1.59, $p=.04$), but not the control period. Loneliness significantly decreased during the control period (Mdifference=2.44, $p=.003$) and stayed lower during the intervention period. Preliminary results revealed reduced inflammation as well as improvements in sleep quality and variability over the 12-week period. These results support the efficacy of a remote empathy- and compassion-based intervention at reducing loneliness and stress in older adults. Further investigation in a larger sample is needed to verify whether changes in biomarkers are clinically meaningful.

Abstract citation ID: igad104.2732

### SELF-NEGLECT IN OLDER ADULTS: CONVERSATIONS ABOUT SOCIAL CONNECTION, MEANING AND PURPOSE, AND INTERVENTIONS

Jason Burnett[1], Garielle Hoyumpa[2], Ashwin Kotwal[3], Sylvia Ayieko[4], and Carla Perissinotto[3], *1. University of Texas Health Science Center at Houston, Houston, Texas, United States*, *2. UTHealth, McGovern Medical School, Houston, Texas, United States*, *3. University of California San Francisco, San Francisco, California, United States*, *4. UTHealth, School of Public Health, Houston, Texas, United States*

Social isolation and loneliness are pervasive health threatening conditions increasing the morbidity and mortality of older adults worldwide. Older adults experiencing self-neglect (SN) represent some of the most isolated and socially disconnected members of our communities living with high morbidity and mortality risks. We know very little about the interplay of SN, social isolation, loneliness, and related factors that could inform scalable social interventions for this population. We completed mixed methods structured conversations with N=10 older adults with Adult Protective Services validated SN, focusing on social isolation, loneliness, meaning and purpose, and intervention approaches. The average age was 77 years, 50% African American, 66% male, 83% lived alone with an average of 4 medical conditions. On standardized assessments, 66% reported high ability to engage in social activities, but all were socially isolated, with limited mobility. Eighty-three percent reported moderate to high loneliness, 50% with generalized anxiety and 17% with depression. Only 33% lacked meaning and purpose in their lives. The conversations suggest higher rates of depression. Loneliness was linked to living in limited life spaces and few meaningful and quality social interactions reflecting loss and personal impairments. Meaning and purpose was maintained through spirituality. All participants were willing to be engaged meaningfully and socially, in the future, by someone new to their life, in-person or via telephone, to increase social connection. These preliminary findings suggest that low-cost social connection interventions are acceptable options for improving the social lives and possibly the health of isolated and lonely older adults experiencing SN.

Abstract citation ID: igad104.2733

### SOCIAL ISOLATION AND COGNITIVE FUNCTION: FOCUSING ON RACE/ETHNIC DIFFERENCES FROM THE NHATS STUDY

Murad Taani[1], Yura Lee[1], Julie Ellis[2], Chi Cho[2], and Ammar Hammouri[2], *1. University of Wisconsin-Milwaukee, Milwaukee, Wisconsin, United States*, *2. University of Wisconsin Milwaukee, Milwaukee, Wisconsin, United States*

Social isolation has negative associations with various health outcomes including cognitive function. However, limited research exists delineating the effect of social isolation on cognition using population-based longitudinal data. Moreover, little is known whether there are any race/ethnic differences in this social isolation-cognition relationship. We examined the effects of social isolation on cognitive function and explored the moderating role of race/ethnicity using longitudinal data of 4381 older adults from two waves of NHATS (2015 and 2018). Social isolation was measured by addressing four domains of network and integration: (a) marriage or partnership, (b) family and friends, (c) church participation, and (d) club participation (score range: 0 - 6). A higher score indicates more social isolation. Cognitive function was measured using the composite score of three domains: memory, orientation, and executive function (range: 0 - 33), with higher scores indicating better cognitive function. Repeated-measures ANOVA showed a significant relationship between social isolation and cognitive function. Specifically, the greater the social isolation, the lower the cognitive function. We found significant differences in cognitive function scores across race/ethnic groups. Non-Hispanic White showed higher cognitive function than non-Hispanic black, Hispanics, and other races. Non-Hispanic black and other races also showed significantly higher scores than Hispanics. The findings also showed that the cognitive function score significantly decreased over a 4- year period. There was no moderating effect of race/ethnicity found between social isolation and cognitive function. Interventions are needed to reduce social isolation by facilitating social participation in community activities, thereby protecting against cognitive decline.

Abstract citation ID: igad104.2734

### SOCIAL ISOLATION AND COGNITIVE IMPAIRMENT IN OLDER ADULTS WITH DIABETES

Bohyun Kim, and Jie Hu, *The Ohio State University, Columbus, Ohio, United States*

Social isolation is a notable influencing factor contributing to cognitive impairment in older adults with diabetes, leading to an increased risk of dementia. As the specific mechanisms by which social isolation affect cognitive health have not been fully understood, it is important to identify the underlying mechanisms. This study aims to examine the pathways between social isolation and the psychological mediators of cognitive impairment in older adults with type 2 diabetes. A secondary data analysis was conducted using the National Social Life, Health, and Aging Project Wave 3 Database (2015-2016) of 725 older adults with diabetes aged 60 and over. The Social Disconnectedness Scale, Perceived Isolation Scale, Center for Epidemiological Studies Depression Scale, Hospital Anxiety and Depression Scale, and Perceived Stress Scale were used. Structural equation modeling was employed to investigate the complex interplay of social isolation and psychological mediators with cognitive impairment. Bootstrapping procedures were used to assess significance of indirect effects through psychological mediators. The hypothesized model was supported with adequate fit to the data (CFI=0.90; RMSEA=0.05; SRMR=0.08). Social disconnectedness had an indirect effect on cognitive health through perceived isolation and perceived stress ($\gamma$= -0.122, p< 0.01). However, there was no significant indirect effect through anxiety and depressive symptoms. The model provides a framework for developing interventions to reduce cognitive impairment in socially isolated older adults with diabetes. Understanding the pathways between social isolation and cognitive impairment will provide a foundation for controlling modifiable factors to prevent the progression of dementia in older adults with diabetes.

Abstract citation ID: igad104.2735

### SOCIAL PARTICIPATION, LONELINESS, AND CIRCADIAN RHYTHMS OF MIDDLE-AGED AND OLDER ADULTS IN TAIWAN

Yi-Hua Tsai, and Ching-Ju Chiu, *National Cheng Kung University, Tainan City, Tainan, Taiwan (Republic of China)*

Loneliness among middle-aged and older adults is a growing public health concern in the aging society. While social participation is known as a protective factor against loneliness, not all older adults enjoy social participation. The purpose of this study is to investigate the social participation and loneliness characteristics in communities and their association with the demographic data, health-related variables, objective sleep parameters and rest-activity circadian rhythms with a sample of 242 Taiwan community-dwelling middle-aged and older adults aged 45 to 89 years. Loneliness and social participation levels were grouped in different clusters via Two-Step Cluster Analysis. Subsequently, Chi-square test and ANOVA were performed to identify the differences between clusters with relevant variables. Results showed that five social participation-loneliness clusters were grouped: Average(40.1%), Active-Lonely(3.8%), Not Active-Lonely(13.1%),Active -Not Lonely(5.9%),Not Active -Not Lonely(37.1%). Age, employment status, property management, self-perceived health, depression, life satisfaction and relative amplitude were related to clusters. The Active-Not Lonely cluster was older, had a higher retirement percentage, and higher life satisfaction. Compared with the Not Active-Lonely cluster, the Not Active -Not Lonely cluster had more power to manage their property, felt healthier and less depressive. The Active-Lonely cluster had a high percentage of unemployment and felt unhealthier. The Not Active-Lonely cluster had the lowest relative amplitude, which meant they had weaker rest-activity circadian rhythms. Objective sleep parameters were no difference between each cluster. These findings provide the potential benefits of developing appropriate social care programs to reduce loneliness for middle-aged and older adults via objective data.

Abstract citation ID: igad104.2736

### THE GENERATIONAL COMPARISONS OF SOCIAL NETWORK SITE USE SUBTYPES AND ITS ASSOCIATIONS WITH SELF-ESTEEM

Bomi Choi[1], Hayoung Park[1], Si Young Song[2], and Miseon Kang[1], *1. Yonsei University, Seoul, Republic of Korea*, *2. Kyung Hee University, Seoul, Republic of Korea*

This study aimed to identify subtypes of Social Network Service (SNS) use that affect self-esteem by applying a person-centered approach. The study also aimed to compare the SNS use subtypes and their associations with self-esteem between young and middle-aged adults in Korea. The sample included 2,572 young adults (18-44 years old) and 1,790 middle-aged adults (45-64 years old) drawn from the Korea Media Panel, a nationally representative dataset. Self-esteem was measured by the sum of ten questions from Rosenberg's Self-Esteem Scale. Four indicators of SNS use (e.g., "How often do you check other people's posts (feed)?") were used to perform a 3-step Latent Profile Analysis, which accounts for classification errors. The results of the Latent Profile Analysis showed 5 and 4 subtypes of SNS use for young and middle-aged adults, respectively, depending on the frequency of active and passive usage. Overlapping subtypes include 'passive-low', 'active-low', 'moderate', and 'high-active' users. Young adults had one distinct subtype with a very high frequency of passive usage (checking others' posts and liking them) but a very low frequency of active usage (sharing content with others). The results also revealed that those with more frequent use in both active and passive use showed the lowest self-esteem in both young and middle-aged groups. This study supports previous findings suggesting that frequent SNS use has negative impacts on self-esteem, even for middle-aged adults. Based on these results, digital literacy programs for middle-aged adults should cover healthy ways to use SNS.

Abstract citation ID: igad104.2737

### THE IMPACT OF THE PROGRAM OF ALL-INCLUSIVE CARE FOR THE ELDERLY (PACE) CLOSURE ON SOCIAL ISOLATION AND LONELINESS

Barbara Dabrowski, Elizabeth Punke, Abby Teply, Katy Richardson, Christine McKibbin, and Catherine Carrico, *University of Wyoming, Laramie, Wyoming, United States*

Social isolation and loneliness are public health problems seen in late adulthood that are associated with health risks (e.g., premature mortality, depression, and suicidality) and these issues are even more common among older adults with chronic illnesses (Nicholson, 2012). The Program of All-Inclusive Care for the Elderly (PACE) was developed to serve medical and behavioral needs and provide opportunities for social engagement for older adults with chronic illnesses. Budget cuts shut down Wyoming's PACE program. This study aimed to (1) examine the impact of this closure on social isolation and loneliness, (2) identify how individuals have adapted to the loss of services that address social isolation, and (3) identify needs and preferences for future intervention to address social isolation. A concurrent mixed-methods design was used to examine these aims. Participants (n = 12; M = 74 years old, SD = 9.5) were predominantly White (n = 8, 66%), female (n = 6, 50%), and lived alone (n = 6, 50%). Caregivers of participants (n = 5; M = 63 years old, SD = 11) were predominantly Hispanic, Latino, or of Spanish origin (n = 3, 60%), female (n = 4, 80%), and lived with a partner (n = 4, 80%). Four central themes emerged: social isolation, emotional concerns, transportation loss, and care loss. Data showed decreases in social support and increases in loneliness. Results suggest programs like PACE greatly benefit older adults. Future directions may include developing community-based interventions to address service needs and social isolation concerns in Wyoming.

Abstract citation ID: igad104.2738

### THE MODERATING ROLE OF SOCIAL SUPPORT IN THE RELATION BETWEEN FUNCTIONAL LIMITATIONS AND DEPRESSIVE SYMPTOMS

Junyub Lim, and Giyeon Kim, *Chung-Ang University, Seoul, Republic of Korea*

**Background and Objectives:** Prior research has demonstrated that social support from friends and neighbors can effectively reduce depressive symptoms. However, few studies have investigated how the characteristics of these relationships can alleviate depressive symptoms among older adults with functional limitations. Therefore, the present study aims to investigate the moderating effects of characteristics of friends and neighbors relationships on the link between functional limitations and depressive symptoms among Korean older adults. **Methods:** The data used for analyses were obtained from the 2020 Survey of Living Conditions and Welfare Needs of Korean Older Persons, a nationally representative survey. The sample included a total of 9,985 individuals who were 65 years of age or older residing in Korea. **Results:** Results from the moderator analyses revealed that the link between functional limitations and depressive symptoms was influenced by both the number of close friends and neighbors (b = -0.085, p < .01) and the level of satisfaction with relationships with friends and communities (b = 2.052, p < .05). **Discussion:** Satisfaction with relationships with friends and communities may act as a protective factor that mitigates the impact of functional limitations on depressive symptoms. In particular, for older adults with functional limitations, having a larger network of friends and neighbors network is expected to help reduce the risk of depressive symptoms.

Abstract citation ID: igad104.2739

### UNDERSTANDING LONELINESS PATTERNS AMONG WIDOWED OLDER ADULTS OVER TIME AND THE PROTECTIVE EFFECT OF SOCIAL SUPPORT

Gina Lee, and Daniel Russell, *Iowa State University, Ames, Iowa, United States*

The present study aimed to investigate loneliness among widowed and non-widowed older adults. Additionally, the study sought to identify classes with different loneliness patterns among widowed older adults over time and to determine social resources that impact loneliness patterns after spousal loss. Data from the Health and Retirement Study were utilized to compare loneliness levels between widowed (n = 137) and non-widowed (n = 2,361). Those who were married at T1 (2008/2010), widowed at T2 (2012/2014), and remained widowed at T3 (2016/2018) were defined as widowed. T-tests were conducted to compare loneliness between the two groups, revealing that widowed individuals reported significantly higher levels of loneliness at T2 only. Using growth mixture models, three distinct loneliness patterns were identified among widowed individuals: Group 1, displaying mid-level loneliness at T1 and increasing over time (n=32); Group 2, exhibiting the lowest loneliness at T1 and leveling off (n=88); and Group 3, experiencing the highest loneliness at T1 and decreasing over time (n=17). Finally, analysis of variance tests were conducted to determine whether social support and engagement as measured at T1 differed among the three identified groups. Results indicated that social support from friends and children was significantly higher among group 2 compared to the other two groups. This study provides evidence of the protective effect of social support before widowhood on the psychological well-being of older adults after spousal loss. The findings may have implications for the development of interventions aimed at supporting widowed older adults during this difficult period of transition.

Abstract citation ID: igad104.2740

### WELL-BEING IN MIDDLE-AGED ADULTS: THE MEDIATING ROLE OF SOCIAL MEDIA USE

jingchuan wu[1], Xin Yao Lin[2], Tian Lin[3], and David Almeida[4], *1. Penn State University, State College, Pennsylvania, United States*, *2. Weill Cornell Medicine, New York City, New York, United States*, *3. University of Florida, Gainesville, Florida, United States*, *4. The Pennsylvania State University, State College, Pennsylvania, United States*

As individuals age, social connection becomes increasingly important, and social media has the potential to facilitate social connections. This study aimed to investigate the relationship between age and well-being, focusing on the mediating role of social media use. While previous research has found social connection to be an important predictor of well-being, the specific implications of social media use for social connection on various aspects of well-being remain unclear. To explore this relationship, the study used publicly available data from the Midlife in the United States (MIDUS) study wave 3 and refresher 1, with a sample of 6871 participants (M = 56.80 yrs, SD = 14.58 yrs). Results showed that social media use played a significant mediating role in well-being, specifically in loneliness ($b = 0.001$, $p=0.003$), positive affect ($b = -0.002$, $p< 0.001$), and eudaimonic well-being ($b = -0.020$, $p< 0.001$). However, the indirect pathway from age to well-being through social media use was negative. That is, as individuals aged, they tended to use social media less frequently, which in turn was associated with worse well-being outcomes. These findings add to the expanding body of research on the complicated relationship between social media use and well-being, emphasizing the significance of focused interventions to encourage social media use for social connection in middle-aged adult populations. The study has implications for health professionals working with middle-aged adults, underlining the importance of considering social media use as a potential method of enhancing well-being.

### SESSION 7620 (POSTER)

### ALZHEIMER'S DISEASE AND DEMENTIA (HS)

Abstract citation ID: igad104.2741

### A CONCEPT ANALYSIS OF SHARED DECISION MAKING FOR OLDER PATIENTS WITH DEMENTIA IN ACUTE CARE HOSPITALS

Taeko Saito[1], and Harue Masaki[2], *1. Chiba University, Inzai, Chiba, Japan*, *2. Chiba University, Chiba, Chiba, Japan*

**Background:** Dementia patients often have comorbidities like Diabetes Mellitus, kidney disease, and cardiovascular disease that require patient decision-making about care, despite the challenges in judgment that stem from dementia. The study aimed to determine what Shared Decision-making is for older patients with dementia in acute care hospitals. **Methods:** The study used the Hybrid Model of concept analysis method. This comprises a theoretical phase, a fieldwork phase, and an analytical phase. In the initial phase, the concept is selected. The fieldwork phase collects qualitative data to further analyze the selected concept. The third phase analyzes the empirical observations and writes up the findings. We searched Pub-Med, CINAHL, and PsycINFO for the keywords: "shared decision-making" and "patients with dementia." **Results:** We identified 14 articles that gave rise to over 100 codes. We defined shared decision-making for older patients with dementia in acute care hospitals as "The older person with dementia can be involved in collaborative activities in the care network, including delegation of authority as active decision-making. Open-minded communication based on person-centered care can be the foundation for considering feasible options. Clarify the role of family caregivers and identify decision-making participants in the care network to view the best alternative for the patient's subsequent life". **Conclusions:** We developed a working definition of shared decision-making for patients with dementia. We clarified critical elements of the decision-making process, like based as person-centered care, open-minded communication, and collaborative decision-making activities between the person and the care network members, including delegation of authority.

Abstract citation ID: igad104.2742

### ANALYSIS OF CONVERSATIONAL ENTRAINMENT AMONG OLDER PERSONS WITH ADRD AND UNIMPAIRED ADULTS

Christine Williams[1], Joseph McKinley[1], Charles Cooper[1], Nurgun Erdol[1], and Christopher Beetle[2], *1. Florida Atlantic University, Boca Raton, Florida, United States*, *2. Florida Atlantic university, Boca Raton, Florida, United States*

Social isolation threatens the health of older adults, particularly those with Alzheimer's disease and related dementias (ADRD). Our research uses measures of behavioral coordination to quantify the entrainment of an individual's behavior to the collective dynamics of a group to which they belong, aiming to identify useful strategies to reduce social isolation for persons with ADRD in therapeutic group contexts. The present study is based on data collected during 27,

30-minute group therapy sessions conducted biweekly via WebEx by staff facilitators at a university-affiliated Memory and Wellness Center (MWC) in 2021, when CoViD-19 restrictions limited patients' access to existing, in-person day programs. We study conversational entrainment among facilitators and participants in the recorded sessions using previously identified measures such as speech duration; voiced and voiceless interval durations; variations in pitch, intensity, and syllabic rate; and turn breaks. We present preliminary results identifying dynamical characteristics of sessions showing greater and lesser degrees of group social engagement before discussing possible strategies facilitators could use to enhance social coordination within therapy groups. The mental health benefits of professionally facilitated supportive group discussions for older adults with ADRD are well-accepted. Measuring conversational entrainment has the potential to provide quantitative evidence of cognitive benefits for persons with ADRD, and to help shape a therapeutic environment tailored to the needs of this vulnerable population.

Abstract citation ID: igad104.2743

### ASPECTS OF AMYLOID SCAN DISCLOSURE WHICH INFLUENCE PATIENT AND CARE PARTNER SATISFACTION DURING DEMENTIA DIAGNOSIS

Elyse Couch[1], Wenhan Zhang[2], Emmanuelle Belanger[1], Nicole DePasquale[2], Emily Gabois[3], Megan Shepherd-Banigan[2], Courtney Van Houtven[2], and Terrie Wetle[3], *1. Brown University, Providence, Rhode Island, United States*, *2. Duke University School of Medicine, Durham, North Carolina, United States*, *3. Brown University School of Public Health, Providence, Rhode Island, United States*

Amyloid PET scans can be used to make a differential diagnosis of dementia or indicate a person's risk of developing dementia in the future. The use of biomarkers for diagnosing dementia is not yet standard in clinical practice. Research is needed to better understand how to disclose the results from amyloid PET scans to patients and care partners. We interviewed 38 patients with MCI or dementia and 68 care partners from the CARE-IDEAS study to explore their experiences of receiving the results from amyloid PET scans as part of the diagnostic process for dementia. We were specifically interested in identifying aspects of amyloid disclosure which affected patient and care partner satisfaction. Patients had an average age of 73.5, were mostly male (65.8%), and Non-Hispanic White (67.6%). Care partners were mostly female (75.8%), caring for their spouse (87.1), with a mean age of 69.5. We identified 5 factors affecting patient and care partner satisfaction: 1) clarity of the explanation of the scan result and its implications, 2) discussion of potential options for treatment, research opportunities or follow-up support, 3) disclosure of scan results in person, 4) involvement of a care partner during disclosure, and 5) provision of a written summary of the scan result after disclosure. As diagnostic procedures for dementia evolve, it is important to consider their effect on patients and care partners. These findings can be used to inform the development of disclosure procedures for amyloid PET scans.

Abstract citation ID: igad104.2744

### COLLABORATION IN CARING FOR OLDER ADULTS WITH DEMENTIA LIVING IN LONG-TERM CARE FACILITIES

Phatt Thaitrong[1], Joy Douglas[1], Linda L. Knol[1], Amy Ellis[1], Hyunjin Noh[2], and Shena Sanchez[1], *1. The University of Alabama, Tuscaloosa, Alabama, United States*, *2. University of Alabama, Tuscaloosa, Alabama, United States*

Current evidence calls for an interdisciplinary, team approach to caring for older adults with dementia. Registered dietitian nutritionists (RDNs) are interdisciplinary team members who play an important role in nutrition and dementia care. Our study explored how RDNs collaborate with licensed healthcare providers and direct care staff to provide nutrition care for older adults with dementia living in long-term care (LTC) facilities. RDNs who work with residents with dementia in the U.S. (n = 18) were recruited to participate in one of five focus groups, where they described the various disciplines they collaborate with. Focus groups were led by a trained moderator using a semi-structured interview guide, transcribed verbatim, and audited by the research team for accuracy. Transcripts underwent descriptive content analyses using NVivo© software. Descriptive content analyses revealed the following diverse groups who work collaboratively with RDNs: 1) nursing staff, 2) foodservice staff, 3) therapists, 4) physicians, and 5) other non-clinical staff, including activities staff and nursing home administrators. Additionally, RDNs reported they worked closely with residents' family members and resident members of the food committee. In conclusion, in LTC settings, RDNs collaborate with various groups, encompassing clinical, non-clinical, and even residents/family to care for older adults with dementia. However, collaboration is not limited to clinical staff. To provide optimal nutrition care for older adults with dementia, it is important to recognize the value of collaborating with both clinical and non-clinical stakeholders to meet resident needs.

Abstract citation ID: igad104.2745

### DEVELOPMENT AND EVALUATION OF COGNITIVE IMPAIRMENT SCREENING TOOL BASED ON EYE-TRACKING TECHNOLOGY

Qiaoqin Wan[1], and Shifang Zhang[2], *1. Peking University Health Science Center, Beijing, Beijing, China (People's Republic)*, *2. Peking University, Beijing, Beijing, China (People's Republic)*

**Objectives:** This study aimed to develop a cognitive impairment screening tool based on eye tracking technology (ET-CIS), and apply ET-CIS to community-dwelling older adults to evaluated its screening performance. **Methods:** ET-CIS were developed based on through systematic review, pre-experiments and expert consultation. We recruited older people in the community from July 2022 to November 2022 and collected data including demographics data, Montreal cognitive assessment, the mini-mental state examination, ET-CIS. The t-test and correlation analysis were conducted to screen out statistically significant parameters with of ET-CIS. The screening models were constructed using standardized score assignment, binary logistics regression analysis, and decision tree model in the training set, and were applied in the validation to evaluate the screening performance. The best model was selected as the final scoring model. **Results:** ET-CIS was constructed including applicable objects, pre-assessment preparation, screening dimensions, specific experiments and parameters. A total of 301 subjects were included, including 163 in the cognitively normal group and 138 in the cognitive impairment group. The results showed that the decision tree model results showed that the sensitivity of the training set was 0.752 and the sensitivity of the validation set was 0.818. We use the decision tree model as the final model. **Conclusions:** In this study, ET-CIS was developed including the evaluation of memory function, executive function, visuospatial function and abstract function. The screening model of ET-CIS in community-dwelling older people showed good discrimination, which demonstrated it could be used to effectively screen cognitive impairment in the community in the future.

Abstract citation ID: igad104.2746

### DIFFERENCES IN COGNITIVE DECLINE DUE TO ALZHEIMER'S DISEASE, NEURODEGENERATIVE DISORDERS, AND TRAUMA

Larry Tupler[1], Arseniy Yashkin[2], Masudul Hoque[3], Anatoliy Yashin[4], Igor Akushevich[2], and Eric Stallard[2], *1. Duke University Medical Center, Durham, North Carolina, United States*, *2. Duke University, Durham, North Carolina, United States*, *3. SSRI, Duke University, Durham, North Carolina, United States*, *4. Social Science Research Institute, Duke University, Durham, North Carolina, United States*

Alzheimer's disease (AD) is a form of progressive dementia associated with significant personal, social, and economic burdens, making targeted interventions aimed at mitigating the effects of this condition on cognitive decline highly desirable. However, AD is but one of a range Neurodegenerative Disorders (ND) classified as dementias of aging. Furthermore, neither AD/ ND nor cognitive decline itself are monolithic: each individual disorder degrades specific aspects of cognition at differing rates. Isolating the most vulnerable facet of good cognitive health associated with each condition can lead the way to more personalized health interventions and an overall reduction of the burdens posed by AD /ND. In this paper we assess how the presence of AD and/or other ND degrade immediate (IR) and delayed (DR) recall over an extended period of time vs. the effect of non-degenerative brain trauma and AD/ND-free aging. Using Medicare-linked data from the Health and Retirement Study we found that, when compared to AD/ND-free aging, the onset of AD accelerates the decrease of IR by an overage of 0.30-0.37 points bi-annually, with similar effects on DR. Vascular dementia was associated with a 0.24-point loss of IR, but only for individuals with initially low recall capacity (≤5); those with high initial recall capacity (6+) did not experience loss of IR but lost 1.01 points of DR bi-annually. For comparison, traumatic brain injury was associated with 0.12-0.23 points decline in IR and 0.15 in DR bi-annually.

Abstract citation ID: igad104.2747

### DIRECT AND INDIRECT CAUSAL EFFECTS OF AIR POLLUTANTS ON ALZHEIMER'S DISEASE RISK AND DISPARITIES

Igor Akushevich[1], Arseniy Yashkin[1], Vito Di Bona[1], and Julia Kravchenko[2], *1. Duke University, Durham, North Carolina, United States*, *2. Duke University School of Medicine, Durham, North Carolina, United States*

Ambient air contaminants have been associated with higher risk of Alzheimer's disease (AD), related dementia, and cognitive impairment; however, mechanisms that underlay these effects remain unclear. Pre-existing arterial hypertension, diabetes mellitus, cerebrovascular disease, and renal disease have a strong impact on AD risk and disparities. These diseases, in turn, are associated with exposure to air contaminants, including particulate matter (PM2.5). We applied causal mediation analysis in the formulation of VanderWeele which provides causal estimates of the direct and indirect (through the effects on pre-existing diseases) effects of PM2.5 exposure on the risk of AD. We used Administrative Health Insurance Claims from 5%-Medicare (N=6,042,239; 1991-2020) to identify the date of onset for AD and risk related diseases such as arterial hypertension, diabetes, cerebrovascular, and kidney diseases that were considered mediators in these analyses. Information on ambient PM2.5 at the zip code level were derived from the Socioeconomic Data and Applications Center (SEDAC) that incorporates air monitoring data, satellite aerosol optical depth, meteorological conditions, chemical transport model simulations, and land-use variables. The estimates supported the hypotheses on the linear dependence of the dose-response function with higher effects for Males vs. Females, White vs. Black subpopulations, and stroke-belt states vs. other U.S. regions. We found that the contributions of indirect effects were stronger for younger older adults and reached up to 13% for cerebrovascular disease, 10% for arterial hypertension, 5% for diabetes, and 4% for chronic kidney disease. Analysis in subpopulations demonstrated health disparities in the effects of PM2.5 on AD risk.

Abstract citation ID: igad104.2748

### EARLY DETECTION IN ALZHEIMER'S DISEASE AT ADVENTHEALTH: A DAVOS ALZHEIMER'S COLLABORATIVE FLAGSHIP SITE

Steven Smith[1], Richard Pratley[1], Valeria Baldivieso[2], Magda Baksh[1], Gayle Shepherd[3], Janice Lopez[3], Katherine Selzler[4], and James Murray[5], *1. AdventHealth, Orlando, Florida, United States*, *2. Adventhealth, Orlando, Florida, United States*, *3. AdventHealth, Altamonte Springs, Florida, United States*, *4. Davos Alzheimer Collaborative, Wayne, Pennsylvania, United States*, *5. Davos Alzheimer's Collaborative, Wayne, Pennsylvania, United States*

**Background:** The emergence of new therapeutics highlights the need for more efficient and equitable early identification of people at risk of developing Alzheimer's disease. The Davos Alzheimer's Collaborative flagship program was designed to test feasibility, acceptability, and implementation of a digital cognitive assessment (DCA) followed by a blood-based biomarker (BBM) as part of primary care workflows. **Methods:** Patients ≥65 years were contacted via their AdventHealth primary care provider or through social media to complete the Cogstate Brief Battery (CBB) DCA virtually. The CBB assesses four cognitive domains: processing speed, attention, visual learning, and working memory. Patients with an abnormal or borderline composite score were offered the PrecivityAD® blood test from C2N Diagnostics, an Alzheimer's disease screening tool. **Results:** Approximately 2387 patients expressed interest to participate and 1077 e-consented: 96% social media, 4 % PCP. Of those, 742 patients completed the DCA. 28% were positive, 15% were borderline, and 56% were negative for cognitive issues. Additionally, 192 patients received the blood test results where, 17.7% had high likelihood, 67.7% low likelihood, and 14.6% could not distinguish presence or absence of amyloid plaques. Utilization of digital tools varied across race and ethnicity. **Conclusion:** Both PCP referrals and social media approaches demonstrated opportunities and challenges for early detection of Alzheimer's. Implementation of both the CBB cognitive assessment and PrecivityAD® blood test was successful. Both DCAs and BBMs have value in early detection for cognitive impairment and a first step in identifying patients who could benefit from disease-modifying treatments.

Abstract citation ID: igad104.2749

### EVALUATING POPULATION ATTRIBUTABLE FRACTIONS ASSOCIATED WITH ALZHEIMER'S DISEASE RISK FACTORS

Igor Akushevich[1], Arseniy Yashkin[1], Svetlana Ukraintseva[1], Anatoliy Yashin[2], and Julia Kravchenko[3], *1. Duke University, Durham, North Carolina, United States*, *2. Social Science Research Institute, Duke University, Durham, North Carolina, United States*, *3. Duke University School of Medicine, Durham, North Carolina, United States*

Although many risk factors for Alzheimer's disease (AD) and related dementias (ADRD) have been identified, there is no consensus about their joint role in risk of these disorders. The extent to which joint variation in the effects of behavior, socioeconomic status, comorbidities and genetic variants may affect risk of AD/ADRD is underexplored. In this study, we used Health and Retirement Study (HRS) data linked to Medicare diagnoses and genetic data to evaluate and compare the effects of three groups of AD/ADRD risk factors: i) Medicare diagnoses for AD risk-related diseases, ii) survey-based self-reported health, behavior, physical function, education, and socioeconomic status, and iii) individual SNPs and polygenic risk scores incorporating genetic risk factors for AD/ADRD. We applied univariable and multivariable Cox models for estimating AD and ADRD risks, identified most powerful predictors, evaluated their population attributable fractions, and constructed predictive multivariable models for AD/ADRD risks. The results showed that Medicare-based diagnoses are superior in terms of predictive power of AD/ADRD risk than their self-reported counterparts. Cerebrovascular and heart diseases, depression, and brain injury had the largest effect on AD/ADRD risk. Depression and arterial hypertension were the largest contributors to AD risk in population. These two factors and APOE4 proxy SNP rs769449, together explained approximately half of the population attributable fraction of AD risk. The effect of PRS on AD/ADRD was significant for genes involved in inflammatory pathway. Our study demonstrated that AD/ADRD risk is multifactorial in nature and its variation is tied to multiple demographic, socioeconomic and health-related risk factors.

Abstract citation ID: igad104.2750

### EVALUATING THE IMPLEMENTATION OF A SELF-CARE INTERVENTION FOR CAREGIVERS OF PERSONS WITH DEMENTIA

Lauren Fisher, Lauren Massimo, and Karen Hirschman, *1. University of Pennsylvania, Philadelphia, Pennsylvania, United States*

In this study, we evaluated the implementation of the Virtual Caregiver Coach for You (ViCCY) intervention delivered as part of the "iCare4Me for FTD" (Frontotemporal Degeneration) Study (NCT04686266). This randomized controlled trial was designed to evaluate the efficacy of the evidence-based ViCCY intervention designed to increase self-care in caregivers of persons living with FTD. All caregivers received health information (HI) which included targeted caregiver and FTD websites on an iPad. The intervention group received ViCCY, 10 health coaching sessions focused on self-care and delivered via an iPad over 6 months. Guided by the Consolidated Framework for Intervention Fidelity (CFIF), the goal of this sub-study was to evaluate intervention implementation by measuring key CFIF components: coverage, exposure, and content delivered. Thirty-one caregivers were randomly assigned to ViCCY+HI (n=15) or HI only (n=16). One caregiver withdrew from the HI group. For exposure, at Month 1, a higher number of HI participants (87%) reported reviewing the websites 30 minutes a week compared to ViCCY group (67%). By Months 3 and 6 the groups were identical (3M: 100%, 6M: 93%). Thirteen out of 15 ViCCY caregivers completed all 10 health coaching sessions. On average, these sessions lasted 52 minutes (range: 24.8-113.5). For content adherence, 28% (40/142) of the completed sessions were examined for adherence to the protocol across the 10 sessions. Overall, content adherence across all sessions was 96% (range: 92-100%). This evaluation demonstrated that a virtual intervention targeting self-care is feasible and acceptable in this population. Implications for future research will be discussed.

Abstract citation ID: igad104.2751

### EXAMINING THE PRELIMINARY EFFICACY OF AN ONLINE ASYNCHRONOUS PSYCHOEDUCATION COURSE FOR BLACK DEMENTIA CAREGIVERS

Sloan Oliver, Karah Alexander, Nkosi Cave, and Fayron Epps, *Emory University, Atlanta, Georgia, United States*

Caregiving While Black (CWB) is an online asynchronous psychoeducation course designed to facilitate the growth of caregiver mastery in Black caregivers of persons living with dementia (PLWD), while increasing the PLWD's quality of life and providing resources. CWB was co-developed by Black caregivers, PLWD, and healthcare professionals who identified the need for a responsive, culturally tailored course to combat specific burdens faced by Black caregivers. Course topics included navigating the healthcare system, managing home, and self-care competencies. This pilot study tested the preliminary efficacy of CWB, which included 8-10 hours of content delivered in 37 short interactive segments. Thirty-two Black caregivers completed the course over a 10-week time frame. Socio-demographic information and measures of psychological well-being and self-efficacy were collected at baseline, midpoint, and end of study. Data were analyzed using descriptive statistics and t-tests. Results confirmed the course's ability to improve caregivers' emotional well-being and sense of confidence and mastery in their caregiving role. Pre- and post-course data revealed a significant improvement in caregiver depression (p=.009), burden (p=.034), and role strain (p=.029) within 30 days of course completion. All statistically significant improvements had moderate effect sizes > 0.38 (Cohen's d). The frequency of behavioral problems in PLWD decreased (p=.033), as did caregiver reactions to those behaviors (p=.0054). Further, caregiver mastery from baseline to course completion increased 0.42 points, on average, with an effect size of 0.26 (Cohen's d). These results exemplify the need for novel, culturally tailored and responsive courses like CWB.

Abstract citation ID: igad104.2752

### INFLAMMATORY CYTOKINE, SOCIAL SUPPORT, AND HEALTH DISPARITIES IN PERSONS WITH COGNITIVE IMPAIRMENT AND DEMENTIA

Ayse Malatyali[1], Tom Cidav[2], Rui Xie[1], and Ladda Thiamwong[1], *1. University of Central Florida, Orlando, Florida, United States*, *2. The Johns Hopkins University School of Medicine, Baltimore, Maryland, United States*

The protective role of social support on cognitive functioning is extensively documented. A pro-inflammatory cytokine, Tumor necrosis factor-alpha (Tnf-alpha), might have a role in the connection between social support and dementia as a marker of systemic inflammation. This study describes the influence of lack of friends' support and Tnf-alpha on cognitive functioning. We conducted a cross-sectional study on Health and Retirement Study participants aged 65 and above, using datasets from the 2016 interviews. Of the 9,262 participants, 72.4% had normal cognition. 21.3% had cognitive impairment, and 6.3% had dementia. Our regression model controlled with depression scores revealed that participants with a lack of friends' support were 51% more likely to have cognitive impairment and 54% more likely to have dementia. The effect of Tnf-alpha was comparably higher on participants with dementia and cognitive impairment than on participants with normal cognition. Compared to quartile-1 (lowest Tnf-alpha levels), Quartile-4 (highest Tnf-alpha levels) was significantly associated with cognitive impairment (OR=1.60) and dementia (OR=2.37). Quartile-3 was only a significant predictor of dementia (OR=2.08). The effect of Tnf-alpha was insignificant in quartile-2 for both groups. Hispanics were 65% more, and African Americans were 67% more likely to have cognitive impairment than non-Hispanic whites. The likelihood of having dementia was even higher in racial/ethnic minorities: Hispanics were 3.3 times more, and African Americans were 2.3 times more likely to have dementia than non-Hispanic whites. Future research can focus on racial and ethnic variations in pro-inflammatory cytokines and social support among people with dementia.

Abstract citation ID: igad104.2753

### NURSES' PAIN ASSESSMENT AND TREATMENT OUTCOMES IN NURSING HOME RESIDENTS WITH DEMENTIA AND APATHY

Yo-Jen Liao[1], Ying-Ling Jao[2], Diane Berish[3], and Marie Boltz[2], *1. Penn State University, State College, Pennsylvania, United States*, *2. Pennsylvania State University, State College, Pennsylvania, United States*, *3. The Pennsylvania State University, University Park, Pennsylvania, United States*

Pain is often undermanaged in nursing home (NH) residents with dementia-related apathy; an understanding of related nursing practices is warranted. This descriptive study examined nurses' pain assessment and treatment outcomes in NH residents with dementia and apathy. A two-week chart review of nursing progress notes was extracted. Residents' apathy measured by the Apathy Evaluation Scale (AES) of at least 40 was required. Descriptive and correlation statistical analyses were performed. Seventeen residents were recruited from three NHs; only one NH required daily pain assessment. All residents had pain-related diagnoses (mean=3), with Gastro-esophageal reflux disease and arthritis being the two most common diagnoses. Thirty-four pain-related documentations were identified in progress notes, with 19 pain-related events (e.g., falls, tooth extraction). Among the 19 events, 4 had follow-up documentation of pain management, while the other 15 did not. Among the 34 pain-related documentations, only six described residents' behaviors, and three used a pain assessment tool (Numeric Rating Scale). More frequent as-needed analgesic administration was associated with lower agitation (-0.56, p< 0.05), yet this association was not found in scheduled analgesic use. Residents who reported pain more frequently received more as-needed analgesics (0.63, p< 0.01). Resident apathy levels were not correlated with analgesic prescriptions. Altogether, nurses recognized residents' pain reports. As-needed analgesic use correlated with better pain management outcomes; however, pain-related events were not adequately followed up and/or documented. More research is needed to examine the association between nursing pain management practices and resident outcomes in this population.

Abstract citation ID: igad104.2754

### OLDER ADULTS WHO SCREEN POSITIVE FOR COGNITIVE IMPAIRMENT: PREDICTORS OF FOLLOW-UP

Matthew Cohen[1], Alyssa Lanzi[1], Anna Saylor[1], Aaron Boulton[1], Jennifer Rittereiser[2], Michelle Ritona[2], and James Ellison[2], *1. University of Delaware, Newark, Delaware, United States*, *2. ChristianaCare, Wilmington, Delaware, United States*

Interventions for cognitive decline are most likely to be effective when they are initiated as early as possible. Here, we present data from a community-based program for older adults that provides brain wellness education and cognitive screening. Individuals who screen positive are given the option to have results sent to their primary care provider (PCP). We have compared the characteristics of those who do with those who do not elect to send positive results to identify meaningful differences that might suggest program enhancements. 812 community-dwelling older adults (mean age = 73, SD = 10.7) were screened with the Mini-Cog and AD8 either in person (n = 534; 66%) or by Zoom (n = 278, 34 %). 367 (45.2%) participants screened positive. Of these, 279 (76%) elected to send their results while 82 (23%) declined. A logistic regression revealed that the decision to send results was not systematically related to age, sex, race, ethnicity, Mini-Cog score or AD8 score. It was, however, associated with the participant's number of medical conditions (e.g., high blood pressure): OR = 1.293, p=.005. This indicates that people with greater awareness of their medical status, or those more actively engaged with a PCP are more comfortable communicating with their provider. Future research should investigate these and additional factors that might contribute to older adults' decision to act on positive screening results. For example, participants may be more willing to share results if the screener emphasizes that (like blood pressure) some causes of cognitive impairment are modifiable.

Abstract citation ID: igad104.2755

### ONLINE COGNITIVE MONITORING IN A SIMULATED ANTI-AMYLOID TRIAL: ADHERENCE AND COGNITIVE CHANGE

Jeffrey Kaye[1], Nora Mattek[1], Jennifer Marcoe[1], Amanda Mar[1], Yanan Shang[1], Aimee Pierce[1], Kevin Duff[1], and Adriana Hughes[2], *1. Oregon Health & Science University, Portland, Oregon, United States*, *2. University of Minnesota, Minneapolis, Minnesota, United States*

Remote assessment of cognitive function is increasingly of interest for monitoring cognitive decline. Additional advantages of remote monitoring are more frequent assessments and the examination of change over time, which may yield more sensitive indicators of cognitive status. This concept was tested in DETECT-AD (Digital Evaluations and Technologies Enabling Clinical Translation for Alzheimer's Disease), an ongoing simulated anti-amyloid trial using digital biomarkers as outcome measures. Adherence to testing, relationships to age, global cognition, and SUVR, and trajectories of change were analyzed in the SMART (Survey for Memory, Attention and Reaction Time) test, a brief measure of visual-grid memory and executive function (Trails A/B, Stroop Color-Word), self-administered online every four weeks. To date (02/28/2023), 46/100 have been enrolled with mean(SD): follow-up=31.0(7.9) weeks (no dropout); age=78(6.6); 63% women; MoCABaseline=25.5(2.3); PET SUVR=1.09 (0.20; range=0.850-2.07). SMART surveys (n=184) completed online used a desktop (26%), laptop (48%), tablet (20%), or smartphone (7%). A mean of 4(2.0; range=1-8) SMART surveys/participant were completed with 96% completed tests. Older age significantly correlated with longer overall completion times (p=0.02), Trails-B (p< 0.01) and Stroop (p=0.02) times, and more click counts on Trails-B (p=0.03). MoCA total score significantly correlated with shorter completion times on overall SMART (p=0.02), Trails-A (p< 0.01), and Trails-B (p=0.03). There were no relationships between SUVR and SMART. Total SMART completion time decreased over multiple administrations (p=0.03), driven by shorter Trails-B time (p=0.01). Monthly online screening of cognitive function in a clinical trial setting is well maintained yielding novel information over time.

Abstract citation ID: igad104.2756

### PREVALENCE AND PAYMENTS PER CAPITA OF ADRD IN PUERTO RICO AND THE US: ESTIMATES FROM MEDICARE DATA

Jose Carrion-Baralt, *University of Puerto Rico Medical Sciences Campus, San Juan, Puerto Rico, United States*

Puerto Rico is one of the oldest US jurisdictions (22.7% aged 65 and over – 65+), where over 40% of the 65+ population lives under federal poverty levels. However, very little epidemiological and health economics research on Alzheimer's Disease and Related Dementias (ADRD) has been conducted on this population. This work compares prevalence of ADRD and several of its risk factors, as well as Medicare Payments-Per-Capita (PpC) between the Puerto Rico (PR) and US National populations aged 65+, and between males and females 65+ in PR. We compared prevalence and PpC data for PR and US for ADRD and Diabetes, Hypertension, Hyperlipidemia and Depression from the publicly available CMS Multiple Chronic Conditions Data Warehouse 2018 file. All fee-for-service beneficiaries were included. Prevalence of ADRD in PR was 33% higher than in the US, while Medicare PpC were 146% higher in the US. Prevalence of Diabetes in PR was 77% higher than in the US, while Medicare PpC were 80% higher in the US. Prevalences of hypertension and hyperlipidemia were not significantly different, but PpC were almost double in the US. Prevalence of depression was 30% lower in PR. Prevalences of ADRD and all other conditions were higher among PR women than among PR men. Significant health disparities exist between PR and the US (and between PR males and females), both in terms of prevalence and PpC of ADRD and several associated risk factors. These findings are important evidence for the formulation of public policies that can help eliminate these disparities.

Abstract citation ID: igad104.2757

### PROMOTING PARTICIPATION IN OCCUPATIONS AMONG OLDER ADULTS LIVING WITH NEUROCOGNITIVE DISORDERS AND CAREGIVERS

Patricia Belchior[1], Melanie Levasseur[2], Johanne Filiatrault[3], Meena Ramachandran[1], and Nathalie Bier[4], *1. McGill University, Montreal, Quebec, Canada*, *2. Université de Sherbrooke, Sherbrooke, Quebec, Canada*, *3. Université de Montréal, Montreal, Quebec, Canada*, *4. Université de Montréal, Montréal, Quebec, Canada*

Participation in meaningful occupations promotes self-confidence, self-esteem, overall health, and healthier life transitions. Due to significant life changes brought by neurocognitive disorders older adults living with neurocognitive disorders as well as caregivers may undergo some disruption in their occupational participation. Few interventions have focused on their ability to engage in meaningful occupations independently and together to successfully orchestrate their life to meet their own occupational needs. The goal of this study was to document the priority needs of older adults living with neurocognitive disorders and caregivers to promote occupational participation, to guide us in the development of an intervention. To this end, a rapid literature review was conducted. Three databases (Medline, PsycInfo and CINAHL) were searched. Studies were included if they used qualitative methods, included community-based individuals and were available in English or French. A total of 20 studies met the inclusion criteria. Two needs were prioritized in the literature by both older adults living with neurocognitive disorders and caregivers: 1) to continue participating in daily activities and social roles (e.g., maintain the connection with the community, use everyday technology), 2) to educate the public about the disease (e.g., bring awareness, knowledge, and understanding of dementia among the community at large). In the presentation we will discuss how the results will inform the development of an occupational-based intervention to promote meaningful occupations.

Abstract citation ID: igad104.2758

### QUALITY OF LIFE ASSOCIATED WITH HOSPITAL DEATH AMONG PERSONS LIVING WITH DEMENTIA

Suzanne Sullivan[1], Chin-Shang Li[2], and Yu-Ping Chang[3], *1. SUNY Upstate Medical University, Syracuse, New York, United States*, *2. The State University of New York, University at Buffalo, Buffalo, New York, United States*, *3. University at Buffalo, The State University of New York, Buffalo, New York, United States*

Dementia is a progressive, terminal health condition impacting quality-of-life. Hospice programs supporting family caregivers in managing end-of-life care for persons living with dementia (PLWD) and preventing non-beneficial hospitalizations are underutilized. This univariable logistic regression of the National Health and Aging Trends Study rounds 1-9 (n=719) aimed to identify associations between quality-of-life of PLWD and hospital death (yes/no). Results indicate that PLWD going outside most days (OR=3.34, p=0.0004) were more likely to die in a hospital compared to those never going outside. Those whose health did not prevent them from enjoying life (OR=2.5, p< 0.0001) were more likely to die in a hospital. PLWD needing >30 minutes to fall asleep (OR=2.43, p=0.002) compared to those never needing >30 minutes to fall asleep, PLWD without problems speaking (OR=1.69, p=0.007), not needing help to eat (OR=1.82, p=0.001), without problems chewing or swallowing (OR=1.54, p=0.039), and having possible dementia (OR=2.35, p< 0.0001) were more likely to die in the hospital. African Americans (OR=1.80, p=0.003) were more likely to die in the hospital compared to Whites, PLWD who received gas energy assistance (OR=1.95, p=0.048), and those living in the Pacific West compared to the Midwest or Northeast (OR=2.57, p=0.004) were more likely to die in the hospital. These findings suggest that quality-of-life, availability of support, financial and psychosocial well-being, and social disparities were associated with higher risk of hospital death among PLWD. Quality-of-life and psycho-social demographic features may serve as mechanistic targets for interventions supporting hospice transitions and prevention of non-beneficial end-of-life hospitalizations.

Abstract citation ID: igad104.2759

### SLEEP DISTURBANCES SHOW FEASIBILITY TO MONITOR DISABILITY PROGRESSION IN EARLY ALZHEIMER'S DISEASE

Jun Ha Chang, Ruiqian Wu, Ying Zhang, Matthew Rizzo, and Jennifer Merickel, *University of Nebraska Medical Center, Omaha, Nebraska, United States*

Our objective is to assess the utility of real-world sleep to discriminate activities of daily living (ADL) decline in Alzheimer's disease (AD). ADL decline warns of dementia and disability. Improved understanding of how sleep indexes ADL decline provides a platform for early monitoring and intervention to slow AD disability and progression. Fifty-seven impaired (mean age =76.7 years; mild cognitive impairment [MCI]: N=50, AD: N=7) and 30 unimpaired (mean age = 73.6 years) subjects participated. Demographics and the Dependence Scale (DS) were collected. The DS evaluated ADL decline from partner (e.g., spouse) report. The subject's sleep was monitored for 3-months (N = 6,336 total days) using wrist-worn actigraphy and validated with sleep diaries. Sleep dysfunction was estimated using means and standard deviations (SD) of total sleep time, sleep latency, sleep efficiency, wakefulness after sleep onset (WASO), and awakening count. Logistic regression—adjusting for age, gender, and education—ranked the utility of sleep dysfunctions to discriminate ADL decline. Forty-six subjects (impaired: N=38, unimpaired: N=8) showed ADL decline (DS > 0). WASO variability (SD; b = 1.66, p < 0.001) and mean awakening count (b = -0.98, p = 0.003) best discriminated ADL decline. Sleep duration did not discriminate ADL decline. In line with prior literature, results underscore the role of sleep disturbances over duration in ADL decline and AD progression. Nightly awakenings show key clinical importance for real-world monitoring of AD-related disability risk. Further research may investigate the specificity and longitudinal reliability of sleep dysfunction to discriminate individual AD progression profiles.

Abstract citation ID: igad104.2760

### THE VIRGINIA MEMORY PROJECT: AN INNOVATIVE SERVICE AND EPIDEMIOLOGICAL BRAIN HEALTH REGISTRY

Ann Rhodes[1], Rachel Coney[2], and Melicent Miller[3], *1. Virginia Commonwealth University, Richmond, Virginia, United States*, *2. Virginia Department of Health, Richmond, Virginia, United States*, *3. Division of Prevention and Health Promotion: Chronic Disease Unit, Richmond, Virginia, United States*

The Virginia Memory Project (VMP) is a statewide memory loss and caregiving needs registry in Virginia, United States. The VMP was launched in June of 2022 and supports statewide surveillance efforts via expanding the BRFSS (Behavioral Risk Factor Surveillance System) memory loss and caregiving modules via a digitized, mobile-capable online survey. In addition to collecting BRFSS data, the VMP partners with regional community brain health organizations to support respondents in getting connected to brain health and caregiving services. The VMP model is innovative because it combines a service and epidemiological registry to support improved resource allocation and decision-making in Statewide dementia care planning. It also provides a pathway for caregiving and memory loss services. In the pilot period of the VMP, there were 138 respondents, with a median age of 61, mean=58, range=18-100, and most respondents identified as White 65% (n=99), with smaller proportions of Black/AA, 16.7% (n=22), and Asian 5.3% (n=7). 96.2% of respondents were non-Hispanic. Most respondents were married (n=66, 50.4%) with no children in the home

(77.9%), and 26.7% (n=35) provided contact information for potential brain health or caregiving services. For respondents who completed the memory loss module, 33% (n=26) reported memory loss or confusion worsening over the last year, but only 60% (n=15) had discussed symptoms with a healthcare professional. 33.3% (n=24) reported having caregiving responsibilities. The VMP increases brain health surveillance capacity in Virginia and represents an innovative pathway between a state department of health, a university data registry, and a community service provider.

Abstract citation ID: igad104.2761

### TRAJECTORIES OF MOTORIC COGNITIVE RISK SYNDROME AND INCIDENT DEMENTIA IN SCOTLAND

Donncha Mullin[1], Danni Gadd[1], Michelle Luciano[1], Tom Russ[1], and Graciela Muniz-Terrera[2], *1. University of Edinburgh, Edinburgh, Scotland, United Kingdom*, *2. Ohio University, Athens, Ohio, United States*

**Background:** Motoric cognitive risk syndrome (MCR) - objective slow gait and subjective cognitive complaints - is quick and easy to measure, making it a potentially useful clinical tool for identifying those at high risk of developing dementia. This is the first exploration of MCR syndrome's predictive ability for incident dementia in a Scottish cohort (Lothian Birth Cohort 1936) and the first examination of the various trajectories of MCR. Method: We classified a total of 680 community-dwelling participants (mean [SD] age 76.3 [0.8] years) free from dementia into non-MCR or MCR groups. We used cox proportional hazard methods to evaluate the risk of developing all-cause dementia in the years following MCR diagnosis, adjusting for age, sex, comorbidity index, apolipoprotein E status, physical activity level, and years of education. We analysed the trajectories for everyone with MCR by tracking their outcomes over 9 years. Result: The prevalence of MCR at baseline was 5.6% (n=38/680). After 9 years of follow-up, 11.6% (n=79/680) of the total cohort developed dementia. The presence of MCR increased the risk of dementia (adjusted hazard ratio 2.37 [95%CI 1.06-5.32; p=0.0359]). The trajectories of those with MCR at baseline were: i) reverted to healthy (n=6/38), ii) ongoing MCR (n=13/38), iii) progressed to dementia (n=9/38), or iv) died (n=10/38). **Conclusion:** MCR could identify a target group for early interventions of modifiable risk factors to prevent incident dementia. Individuals with MCR follow similar trajectories to the related predementia syndrome, Mild Cognitive Impairment.

Abstract citation ID: igad104.2762

### USABILITY OF A TABLET-BASED HOME MEMORY TEST IN OLDER ADULTS WITH AND WITHOUT COGNITIVE IMPAIRMENT

Yanchen Liu[1], Lisbeth Sanders[2], Igor Vishnepolskiy[3], Rebecca Williams[2], Jeff Sanders[2], Anthony Serpico[2], Stefan Gravenstein[1], and Thomas Bayer[1], *1. Brown University, Providence, Rhode Island, United States*, *2. LifeBio, Marysville, Ohio, United States*, *3. Lifespan, Providence, Rhode Island, United States*

Increasing Alzheimer's and related disorders prevalence, highlights the need for accurate and reliable cognitive screening and diagnostic tools. We studied usability of LifeBio Brain, a prototype cognitive testing mobile application. We established baseline cognitive performance in volunteers from Brown University's Geriatric Clinic using the Saint Louis University Mental Status (SLUMS). We asked volunteers to use the LifeBio Brain app at home on an iPad twice daily for 10 times, for about 15 minutes each time, followed by completion of a 10-question System Usability Scale on satisfaction that assesses the app and its ease of use. The LifeBio Brain app contains a mix of adapted cognitive tests such as clock draw and Trails A, and game-like experiences, presented in a randomized order. We analyzed a white non-hispanic sample (n=51) with a balanced distribution of age [79.6 (7.2) years, range 66-95] and sex (58.82% women). The median SLUMS score of 25, indicates cognitive impairment in most participants. Similarly, the median usability score on the 50-point System Usability Scale was 41. We found that 80% of participants had a usability score of 35 or greater, with a 95% CI of 31-38, and 67% had a usability score of 40 or greater. SLUMS score ≤14 correctly predicted study completion in 90% of participants. Most volunteers from an outpatient geriatrics clinic found LifeBio Brain usable. Volunteers leaving the study early tended to have lower SLUMS Scores. The LifeBio Brain app may have utility for measuring memory and thinking abilities in a white outpatient geriatrics population.

Abstract citation ID: igad104.2763

### USABILITY TESTING OF THE PACE APP TO SUPPORT FAMILY CAREGIVERS IN MANAGING PAIN FOR PEOPLE WITH DEMENTIA

Nai-Ching Chi[1], Angela Shanahan[1], Kristy Nguyen[2], Ibrahim Demir[1], Ying-Kai Fu[1], and Keela Herr[1], *1. University of Iowa, Iowa City, Iowa, United States*, *2. University of Iowa College of Nursing, Iowa City, Iowa, United States*

Pain is a common symptom in older adults with Alzheimer's Disease and Related Dementias (ADRD). It is difficult to assess and is often undertreated in people with ADRD. The PACE-app (Pain Control Enhancement application, was developed to enhance self-efficacy of family caregivers when controlling the pain of their care partner. The objective of this study was to test the usability of the PACE-app with family caregivers and clinicians. We evaluated the usability of the PACE-app. Participants (17 family caregivers, 6 clinicians) took part in a mixed-methods study that included a one-time recorded zoom meeting with standardized semi-structured guided review, interview, and a survey specific to their role. The Post-Study System Usability Questionnaire (PSSUQ) was used to measure usability. The overall PSSUQ score we found was 1.9. The range of scores can be between 1 and 7 with the lower numbers meaning favorable usability. The qualitative interviews showed a favorable user experience with 75% of all participants feeling the overall design was effective; 48% felt the tailored pain management strategies were useful; 24% suggested improving the app by having direct coaching information for the family caregiver. This study demonstrated favorable usability with those who tested the PACE-app. Suggestions for improvement were given and using the interview results in conjunction with the PSSUQ scores, we are able to make changes to the app interface to impact the overall design to enhance user experience.

### SESSION 7630 (POSTER)

### BIOLOGY OF AGING AND GEROSCIENCE

Abstract citation ID: igad104.2764

### A COMPREHENSIVE COMPOSITE FOR ALLOSTATIC LOAD SCORING AND RISK CATEGORIZATION FOR PREDICTIVE COMPARISONS

Ingrid Buller-Peralta[1], Sarah Gregory[1], Katie Wells[1], Georgios Ntailianis[1], John O'Brien[2], Craig Ritchie[1], and Graciela Muniz-Terrera[3], *1. University of Edinburgh, Edinburgh, Scotland, United Kingdom*, *2. University of Cambridge, Cambridge, England, United Kingdom*, *3. Ohio University, Athens, Ohio, United States*

Introduced three decades ago, the concept of Allostatic Load (AL) describes the wear and tear of the physiological response to chronic stress, and how sustained demand overloads the systems involved, deplete their resources, and leads them to fail. Current evidence shows positive relationship between AL and risk of developing conditions such as depression, fibromyalgia, cardiovascular disease, diabetes, cancer, early cognitive decline, and dementia. However, there is only partial consensus regarding which biomarkers can compose an AL index or how to accurately score them. Current proposals debate between purely statistical cut-off points, inclusion of gender and ethnicity differences, use of clinical reference values, and scoring ongoing medication. The result is a considerable heterogeneity of methods for AL calculation and a lack of universally agreed values, leaving researchers to subjectively choose which framework suits better to their aims. Using data from the PREVENT-Dementia study (cognitively normal mid-life participants, n=700), we developed a comprehensive composite for AL scoring to generate a more accurate and biologically relevant index, that considers both previous and recent clinical evidence. By including gender and age differences, high quartile approach, and up-to-date clinical reference values, individuals were categorized as no-risk, at-risk and high-risk of AL, which will be associated with MRI brain volume measurements. Comparison to serum Thyroid-stimulating hormone (TSH) levels will serve as proxy of the neuroendocrine biomarkers originally proposed as AL primary mediators, due to its association to increased cortisol. Additional stress-related biomarkers, such as sleep quality and the neutrophil-to-lymphocyte ratio will also be assessed for associations.

Abstract citation ID: igad104.2765

### ALLOSTATIC LOAD AND STATUS IN MID LIFE AND BEYOND

Micah Bailey-Arafah, *Case Western Reserve University, Cleveland, Ohio, United States*

Objective: This study explores how status and processes of cumulative dis/advantage shape allostatic load across the life course.

**Methods:** Data from these analyses are from a national sample of adults from the Biomarker Substudy of the Study of Midlife in the US (MIDUS), a longitudinal study of health and well-being in the United States (N=1186) aged 35-86 years old. Education level, multiple indicators of financial stability, and demographic data were used to construct a measure of status. Those in the Biomarker substudy completed a physiological assessment that included blood, urine, and saliva assays to assess functioning of the sympathetic and parasympathetic nervous systems, hypothalamic-pituitary-adrenal axis, cardiovascular, inflammatory immune activity, glucose metabolic system, and lipid metabolic system. These data were used to create an allostatic load index. An alternate allostatic load index was developed using reported medication to affect three of seven biological systems: cardiovascular and the glucose and lipid metabolic systems. Nested regression models were estimated to explain differences in allostatic load. **Results:** Reported financial instability across the life course was associated with higher levels of allostatic load, and higher levels of education were associated with lower levels of allostatic load, both with and without reported medication usage. **Conclusion:** These findings demonstrate that structural forces impact allostatic load, both with and without reported medication usage, and expand previous work on allostatic load and social status.

Abstract citation ID: igad104.2766

### ASSOCIATIONS THAT FITNESS AND BODY MASS INDEX HAVE WITH DEFICIT ACCUMULATION FRAILTY

Mark Espeland[1], Denise Houston[2], Rena Wing[3], Felicia Simpson[4], and KayLoni Olson[5], *1. Wake Forest University School of Medicine, Winston-Salem, North Carolina, United States*, *2. Wake Forest University School of Medicine, Winston Salem, North Carolina, United States*, *3. Miriam Hospital, Providence, Rhode Island, United States*, *4. Winston-Salem State University, Winston-Salem, North Carolina, United States*, *5. Brown Medical School, Providence, Rhode Island, United States*

Lower cardiorespiratory fitness and obesity may accelerate aging processes. The degree to which changes in fitness and body mass index (BMI) may alter the rate of aging may be important for promoting lifestyle changes. We assessed cross-sectional and longitudinal associations that cardiorespiratory fitness and BMI had with a deficit accumulation frailty index (FI). Fitness (based on standardized graded exercise tests) and weight (to calculate BMI) were collected at baseline and year 4 from 3,944 participants, ages 45-76 years, in the Action for Health in Diabetes (Look AHEAD) randomized controlled clinical trial. A validated 38-item FI was used as a marker of aging. Associations between baseline levels and changes in fitness and BMI with changes in FI were assessed using linear models. Both baseline and 4-year changes in fitness and BMI were independently associated with 4-year changes in frailty (all p< 0.001). Participants with the greatest fitness increase and BMI loss tended to have improvements (i.e., decreases) in FI: mean [95% confidence interval] changes -0.019 [-0.024,-0.013]. Those with the greatest fitness loss and increases in BMI tended to have worsening (increased) FI: 0.029 [0.024,0.034]. Associations of 4-year changes in fitness and BMI with FI changes were similar across subgroups based on age, gender, baseline BMI, diabetes duration, and cardiovascular disease history. Increased fitness across 4 years was associated with less FI accumulation independent of baseline fitness. Adults with type 2 diabetes and overweight or obesity may slow aging processes by increasing their cardiorespiratory fitness and losing weight.

Abstract citation ID: igad104.2767

### EPIGENETIC DIFFERENCE BETWEEN INSOMNIA GROUP AND NONINSOMNIA GROUP IN PARTICIPANTS WITH KNEE PAIN

Soamy Montesino Goicolea[1], Lingsong Meng[2], Zhiguang Huo[2], Thomas C Foster[1], Burel R. Goodin[3], Joseph L Riley III[1], Roger Fillingim[2], and Yenisel Cruz-Almeida[2], *1. University of Florida, Gainesville, Florida, United States*, *2. The University of Florida, Gainesville, Florida, United States*, *3. Washington University, St. Louis, Missouri, United States*

Chronic knee pain is a common condition that often co-occurs with insomnia. Epigenetic modifications, such as DNA methylation, may play a role in the sleep-pain relationship. In this study, we aimed to identify differentially methylated CpGs/regions and enriched genomic pathways associated with insomnia in participants with knee pain (KP). We recruited 140 cognitively healthy middle to older-aged adults with self-reported KP and extracted DNA from peripheral blood. MethylationEPIC arrays were used for methylation data quantification, R package minfi was used for data preprocessing and quality control, and Ingenuity Pathway Analysis (IPA) was used to explore functional implications of differential methylation in pathways related to insomnia. Annotated genes within ±5kb of the putative differentially methylated regions (DMRs, p< 0.05) were subjected to the IPA analysis. There were no significant differences in sex or race between the two groups (p>0.05). We observed an over-representation of younger participants in the insomnia group (p=0.005) and also from the University of Florida (p=0.008). At raw p < 0.05 cutoff, we identified a total of 20,006 CpG probes, including 12,674 hypermethylated, and 7,332 hypomethylated CpG probes. Pathway analysis using IPA identified multiple immune-related pathways enriched with insomnia-related differentially methylated regions (DMRs), including the antigen presentation pathway (p=3.39E-08). Verapamil was identified as the most significant upstream regulator (p=8.8E-07). Our findings suggest the importance of epigenetic regulation of the immune system in the sleep-pain relationship and highlight the need for further research to understand the epigenetic contributions to sleep disorders in individuals with chronic pain.

Abstract citation ID: igad104.2768

### FRAILTY TRAJECTORY AMONG VETERANS WITH HEART FAILURE AGED 50+: 20-YEAR LONGITUDINAL STUDY

Javad Razjouyan[1], and Aanand Naik[2], *1. Baylor College of Medicine, Houston, Texas, United States*, *2. University of Texas Health Science Center at Houston, Houston, Texas, United States*

A few studies used large longitudinal cohort of heart failure (HF) patients to report the shape of frailty trajectory (FT) and its importance in predicting death. We assessed the additive predictive power of FT to the cross-sectional frailty index (FI). A retrospective cohort of veterans aged 50+ by 10/2022 were constructed. We included patients admitted with HF for the first time as the principal diagnosis; had two primary care physician visits in for each year in past three years; admitted after 2001 and had reported ejection fraction at the time of admission. FI constructed based on the previously validated FI for veterans. The three FIs for each year before admission were used to fit a linear line model. The slope and the intercept were used to characterize the FT. We used logistic regression to compare the predictivity of FI versus FT by reporting area under the curve (AUC) for 30-day mortality and all-cause mortality. 111,426 included with median follow-up of 3.0 (IQR,1.4,5.3) years. 82% died within the first 5 years (age,72±10;BMI,31±8; EF≤40%,48%). On average 1.8 per year deficits accumulated annually and 78% of patients had FI≥0.2 at the time of admission versus 60% in a year prior to admission. FT improve the AUC of 30-day and all-cause mortality by 6% compared to FI. FT is a better predictor of mortality than cross-sectional FI for HF population. FT provided additional information beyond the cross-sectional FI by enhancing mortality prediction, identifying individuals who are at high risk of mortality with low FI.

Abstract citation ID: igad104.2769

### LINKING PLASMA PROTEOMICS TO BRAIN ACCELERATED/RESILIENT AGING IN THE ATHEROSCLEROSIS RISK IN COMMUNITY STUDY

Ramon Casanova[1], Jamie Justice[2], Keenan Walker[3], Andrea Anderson[2], Ryan Barnard[2], Tim Hughes[2], Stephen Kritchevsky[2], and Lynne Wagenknecht[4], *1. Wake Forest University, Winston Salem, North Carolina, United States*, *2. Wake Forest University School of Medicine, Winston-Salem, North Carolina, United States*, *3. National Institute on Aging, National Institutes of Health, Baltimore, Maryland, United States*, *4. Atrium Health/Wake Forest Baptist, Winston-Salem, North Carolina, United States*

Machine learning models are increasingly being used to estimate ‘brain age’ from neuroimaging data. The gap between chronological age and the estimated brain age (BAG) is potentially a measure of accelerated/resilient brain aging, and we estimated BAG based on an elastic net regression approach. Here, we report associations between this brain age measure and plasma protein levels (measured using an aptamer-based proteomic platform) in the Atherosclerosis Risk in Communities (ARIC) Study to determine whether BAG was associated with proteins linked to biologic aging. We used brain MRI scans from 1507 ARIC visit 5 participants: 938 were Cognitively Normal (CN), 495 had mild cognitive impairment (MCI) and 74 had dementia. The proteomic dataset contains 4877 plasma proteins in these same individuals. We fitted univariate linear regression models adjusting for age, race, smoking, education, sex, diabetes, hypertension and intra-cranial volume. A Bonferroni correction was applied to account for multiple testing to maintain an analysis-wise $\alpha$=0.05. In total 31 proteins were significantly associated with BAG. Among these SEVP1, GDF-15, pleiotrophin, MMP7, Natriuretic-peptides and Hsp70 were found to be associated with accelerated BAG, whereas EGF-receptor, mast/stem-cell-growth-factor-receptor (KIT), Coagulation-factor-VII and cGMP-dependent-protein-kinase-1 were associated to resilient BAG. The analysis based only on CN participants produced 3 significant proteins: retinoblastoma-2 and SEVP1, which were associated with accelerated BAG and Coagulation-factor-VII, which was associated to resilient BAG. These results suggest circulating proteins implicated in biological aging, cellular senescence, angiogenesis and coagulation are associated with a neuroimaging measure of accelerated brain aging.

Abstract citation ID: igad104.2770

### METABOLOMIC SIGNATURES OF HEALTHY AGING AMONG COMMUNITY-DWELLING OLDER ADULTS

Shanshan Yao[1], Megan Marron[1], Anne Newman[1], Ravi Shah[2], and Venkatesh Murthy[3], *1. University of Pittsburgh School of Public Health, Pittsburgh, Pennsylvania, United States*, *2. Vanderbilt University Medical Center, Nashville, Tennessee, United States*, *3. University of Michigan, Ann Arbor, Michigan, United States*

Metabolomic signatures of healthy aging were created by relating comprehensive mass-spectrometry-based metabolomic profiles with the Healthy Aging Index (HAI), a composite of cardiovascular, lung, cognitive, metabolic, and renal function, scored 0-10. Black and white older adults (N=2017, mean age 75 years; 35% Black) in the Health, Aging, and Body Composition Study were randomly divided into a training (70%) and test (30%) sample. In the training sample, metabolites associated with baseline HAI were identified. Penalized regression was used to generate a parsimonious metabolomic risk score for baseline HAI, which was entered into linear regression models for Year 10 HAI and Cox models for mortality adjusting for baseline age, race, gender, and study site. We identified 12 baseline HAI-related metabolites associated with Year 10 HAI after adjusting for baseline HAI. One standard deviation (SD) higher metabolomic risk score was associated with 0.25 [95%confidence interval: 0.10-0.40] higher Year 10 HAI (training adjusted beta: 0.25 [0.10-0.40]; test: 0.42 [0.15-0.68]). Baseline HAI was associated with mortality (training adjusted hazard ratio: 1.54 [1.43-1.66] per SD; test: 1.33 [1.18-1.50]). Adjusting additionally for metabolomic risk score attenuated the association between baseline HAI and mortality by 41% (training) and 67% (test). In this model, the metabolomic risk score was associated with higher mortality (training: 1.28 [1.18-1.40]; test: 1.42 [1.22-1.65]). Pathway analysis suggested that metabolic pathways of Aminoacyl-tRNA biosynthesis, Arginine biosynthesis, and Citrate cycle may contribute to healthy aging. Metabolomic signatures of baseline healthy aging are associated with long-term healthy aging and mortality independent of lifestyle and traditional risk factors.

Abstract citation ID: igad104.2771

### METABOLOMIC SIGNATURES PREDICT HEALTHY AGING IN COMMUNITY-DWELLING OLDER ADULTS

Shanshan Yao[1], Megan Marron[1], Robert Boudreau[1], Anne Newman[1], Ravi Shah[2], and Venkatesh Murthy[3], *1. University of Pittsburgh School of Public Health, Pittsburgh, Pennsylvania, United States*, *2. Vanderbilt University Medical Center, Nashville, Tennessee, United States*, *3. University of Michigan, Ann Arbor, Michigan, United States*

This study sought to identify metabolomic signatures of multi-system aging by relating comprehensive mass-spectrometry-based metabolomic profiles with the aging phenome. We studied 2469 Black and White older adults (mean age, 75 years; 38% Black) in the Health, Aging, and Body Composition Study (Health ABC) with detailed characterization of the aging phenome across 20 aging-related phenotypes spanning 7 domains (physical function, neurocognitive/executive function, adiposity/sarcopenia, vascular aging, dietary pattern, psychosocial stress, and physical activity). Regression was used to identify metabolites associated with the aging phenome for pathway analytics. Penalized regression and principal components analysis were used to generate parsimonious, metabolomic scores, which were entered into Cox models for the endpoint of exceptional healthy aging, defined as surviving free of cardiovascular disease, cancer, dementia, or major disability over 9 years. We identified 4 distinct metabolomic scores independently linked to body composition, physical performance/mental health, muscle strength, and physical activity. The body composition score (hazard ratio (HR)=0.80 per SD [95% Confidence Interval: 0.70–0.90]), mental/physical performance score (0.77 [0.69–0.87]), and muscle strength score (0.82 [0.73–0.93]) were associated with failure to achieve “exceptionally healthy aging” after adjusting for age, race, gender, and lifestyle and traditional risk factors. Metabolomic signatures of different aspects of the aging phenome are associated with long-term healthy aging processes independent of lifestyle and traditional risk factors. Efforts to include precision measures of metabolomic health in risk stratification to achieve healthy aging are warranted. External validation in a diverse cohort of younger adults is underway.

Abstract citation ID: igad104.2772

### METHYLATION RISK SCORES ARE ASSOCIATED WITH CARDIOVASCULAR RISK FACTORS IN OLDER US ADULTS

Lisha Lin[1], Wei Zhao[2], Kira Birditt[2], and Jennifer Smith[1], *1. University of Michigan School of Public Health, Ann Arbor, Michigan, United States*, 2. *Institute for Social Research, University of Michigan, Ann Arbor, Michigan, United States*

DNA methylation risk scores (MRS) are biomarkers that may prove clinically useful by enabling earlier prevention and individualized treatment of cardiovascular diseases (CVD). However, it is unclear whether relationships between methylation and CVD risk factors differ by demographics or health behaviors. Using existing epigenome-wide association studies, we created MRSs for eight CVD risk factors: systolic and diastolic blood pressure, body mass index (BMI), C-reactive protein (CRP), low- and high-density lipoprotein cholesterols (LDL-C, HDL-C), triglycerides (TG), and fasting glucose. Methylation was measured in whole blood samples from Health and Retirement Study participants (N=3,996, mean age=69.5 years). In linear regression, each MRS was positively associated with its corresponding CVD risk factor after controlling for demographics and health behaviors (all p< 0.001). Adding interaction terms between each MRS and age, gender, or alcohol use revealed differences in MRS associations with their corresponding CVD risk factors across groups: higher MRSs were associated with greater increases in BMI, HDL-C, and TG in younger participants than older participants, greater increases in BMI and HDL-C in females than males, and a greater increase in CRP in non-drinkers than heavy drinkers (≤65 years old: >2 per day for males and >1 per day for females; >65 years old: >1 per day for both genders). In conclusion, some MRSs for CVD risk factors have stronger associations in younger adults, women, and non-drinkers. Additional research is needed to better understand the potential clinical utility of MRSs for early CVD prevention and intervention.

Abstract citation ID: igad104.2773

### PREDICTION OF SARCOPENIA USING A MULTIPLE BIOMARKER RISK SCORE OVER TWO-YEAR FOLLOW-UP

Hyung Eun Shin[1], Miji Kim[1], and Chang Won Won[2], *1. Kyung Hee University, Seoul, Republic of Korea*, 2. *Kyung Hee University Hospital, Seoul, Republic of Korea*

It is necessary to consider multiple biomarkers simultaneously to detect sarcopenia due to its multifactorial etiology. This study is to compare the AUC of multiple biomarker risk score (MBS) that combines multiple biomarkers with that of individual biomarkers and to examine the association of MBS with incidence of sarcopenia in older adults. A total of 1,021 older adults were selected from the Korean Frailty and Aging Cohort Study. Sarcopenia was defined using the Asian Working Group for Sarcopenia 2019 criteria. The MBS was calculated from eight biomarkers (Growth differentiation factor-15, Cystatin-C, Dehydroepiandrosterone, Hemoglobin, Myostatin, Aspartate aminotransferase/Alanine aminotransferase, Estimated glomerular filtration rate, and Blood urea nitrogen) using ridge regression. Receiver operating characteristic (ROC) analysis was conducted to identify an utility of MBS to discriminate sarcopenia. The association of MBS with incidence of sarcopenia were examined by multivariate logistic regression. MBS has an area under the ROC curve (AUC) of 0.71 with an optimal cutoff of 1.76, which is higher than all other individual biomarkers (all, p< 0.01). In prospective study, continuous MBS was positively associated with incidence of sarcopenia after adjusting for confounders (odds ratio[OR]=1.63; 95% CI=1.23–2.17). Participants with high risk (>1.76 score) had a higher odds of sarcopenia than those with low risk (≤ 1.76) (OR=1.82; 95% CI=1.04–3.19). In conclusion, MBS better discriminated the presence of sarcopenia than did individual biomarkers, which can further predict sarcopenia after 2 years in older adults. Thus, combination of multiple biomarkers into single risk score can be helpful for earlier detection of sarcopenia.

Abstract citation ID: igad104.2774

### THE RELATIONSHIP BETWEEN CROSS-TISSUE AND INTRA-TISSUE MEASURES OF AGING BIOLOGY IN OLDER ADULTS

Reem Waziry, *Columbia University, New York City, New York, United States*

The present study included individuals (N=2406) aged 50 and older who had complete data for saliva-based telomere length and blood-based biological aging measures in the Health and Retirement Venous blood and Telomere length studies. Average telomere length was assayed using quantitative PCR (qPCR). DNA methylation clocks included: Horvath (353 CpG sites), Hannum (71 CpG sites), Levine (referred to as phenoAge, (513 CpG sites)), GrimAge, (epigenetic surrogate markers for select plasma proteins), Horvath Skin and Blood (391 CpG sites), Lin (99 CpG sites), Weidner (3 CpG sites), and VidalBralo (8 CpG sites). Physiology measures included albumin, creatinine, glucose, [log] C-reactive protein, lymphocyte percent, mean cell volume, red blood cell distribution width, alkaline phosphatase, and white blood cell count. Relationships between aging biology measures in blood and saliva and variations according to gender were assessed. Blood-based measures showed an inverse relationship with saliva-based telomere length. Increase in saliva-based telomere length was associated with 1 to 4 years slower biological aging based on blood-based measures with the highest magnitude being Weidner (Coefficient= -3.97, P = 0.005), GrimAge (-3.33, P < 0.001), and Lin (-3.45, P = 0.008). Variations in DNA methylation and physiology measures of aging biology with changes in telomere length provide new avenues for integration of these measures in clinical care of vulnerable and clinically difficult to reach individuals.

Abstract citation ID: igad104.2775

### TRANSITIONS BETWEEN SARCOPENIA STATES AND ITS DETERMINANTS IN COMMUNITY-DWELLING OLDER ADULTS

Miji Kim[1], Hyung Eun Shin[1], and Chang Won Won[2], *1. Kyung Hee University, Seoul, Republic of Korea*, *2. Kyung Hee University Hospital, Seoul, Republic of Korea*

Sarcopenia is characterized by an age-related progressive reduction in muscle mass and function. Few observational studies have characterized the dynamic nature of sarcopenia, which is potentially reversible. However, little is known about the determinants of sarcopenia reversal among community-dwelling older adults. We aimed to explore transitions in sarcopenia status and identify factors associated with the reversibility of sarcopenia over a 2-year follow-up period. We conducted prospective analyses (n=1,992) among participants (mean age, 76.3±3.9 years; 47.6% men) who underwent measurement of sarcopenia status at baseline and 2-year follow-up from the Korean Frailty and Aging Cohort Study. Sarcopenia status was diagnosed using the criteria of the 2019 Asian Working Group for Sarcopenia. After 2 years of follow-up, 224 (11.2%) individuals developed sarcopenia, 176 (8.8%) had reversed their sarcopenia, 276 (13.9%) remained in a sarcopenic state, and 1,317 (66.1%) had maintained a non-sarcopenic state. For men, moderate to high levels of physical activity (odds ratio [OR]=2.50 [95% confidence interval (CI)]: 1.31–4.56), resistance training more than 2–3 times per week (OR=2.03, 95% CI: 1.04–3.97), and lower body mass index (OR=0.67, 95% CI: 0.55–0.82) were associated with greater odds reversing sarcopenia. For women, cognitive function (OR=1.12, 95% CI: 1.05–1.26 per 1-point increase in the Mini-Mental State Examination) was associated with higher rates of reversing sarcopenia. To conclude, our results support the dynamic nature of sarcopenia and potentially reversible factors such as higher levels of physical activity and cognitive function, which could be beneficial in the treatment of community-dwelling older adults with sarcopenia.

### SESSION 7640 (POSTER)

### COMMUNITY-BASED RESEARCH

Abstract citation ID: igad104.2776

### AGING-RELATED IMMUNE CELLS PHENOTYPES AND ALL-CAUSE MORTALITY IN THE FRAMINGHAM HEART STUDY

Ahmed A.Y. Ragab[1], Margaret Doyle[2], Jiachen Chen[1], Yuan Fang[3], Kathryn Lunetta[1], and Joanne Murabito[4], *1. Boston University School of Public Health, Boston, Massachusetts, United States*, *2. University of Vermont, Burlington, Vermont, United States*, *3. Binghamton University, Binghamton, New York, United States*, *4. Boston University Chobanian & Avedisian School of Medicine, Boston, Massachusetts, United States*

Almost 2 billion people will likely be above the age of 60 by 2050. Aging is a process that is intrinsically complicated. One of the measures for immune system senescence is aging related immune cells phenotypes (ARIP)s. Besides CD8/CD4 ratio, new immunosenescence phenotypes have been proposed. The associations between ARIPs and all-cause mortality during long-term follow up is understudied. We profiled immune cells using flow cytometry and prospectively investigated ten different ARIPs, namely, CD4+CD27-, CD4+CD28-CD27-, CD8+CD27-, CD8+CD28-CD27-, CD4+/CD8+ ratio, CD4+(Tnaive/(T Central Memory (Tcm)+T Effector Memory (Tem)+T Effector (Teff)), CD8+ (Tnaive/(Tcm+Tem+Teff)), Granzyme B+CD8/Granzyme B+CD4 ratio, CD8+ Tc17/Treg ratio and CD4+ Th17/Treg ratio in relation to survival outcome among 990 dementia-free Framingham Heart Study (FHS) Offspring cohort participants who attended the seventh exam (1998-2001, mean age 62 years, range 40-88, 52% female). Cox proportional hazards regression models adjusting for age and sex with robust variance to account for family correlation were used to test for association between the ARIPs and hazard of death. During up to 20 years of follow up, the survival rate was 66%. Higher CD8+ Tc17/Treg ratio was significantly associated with better survival (HR:0.82 [0.7-0.94], p< 0.001). Higher CD4+ Th17/Treg ratio and CD4/CD8 ratio were also nominally associated with lower risk of death (HR:0.87, [0.75-0.97], p=0.01 and HR 0.9, [0.79-0.99], p=0.04, respectively). Other ARIPs were unassociated with all-cause mortality. We conclude that further investigation of the CD8+Tc17/Treg and CD4+ Th17/Treg ratios as ARIP biomarkers for risk of all-cause mortality is justified.

Abstract citation ID: igad104.2777

### BASIC SCREENING SURVEY IN DETROIT SENIOR CENTERS

April Koternamski, Divesh Byrappagari, and Judith Jones, *University of Detroit Mercy School of Dentistry, Detroit, Michigan, United States*

The objective of the Senior Oral Health Equity Project (SOHEP) Basic Screening Survey (BSS) was to determine the oral health status of seniors 55yrs and older in Detroit senior centers. The ASTDD BSS tool was used to collect the data. The screenings were conducted in two local senior centers to determine the prevalence of dental disease as well as dental care needs. The data were collected using Qualtrics© and analyzed using SPSS©. Majority of the seniors (n=67) screened were African American (88.9%), female (79.1%) and between the ages of 65 and 85 yrs. (89.6%). Results showed that there is a high prevalence of tooth loss (6 or more teeth – 59.6%), untreated decay (38.8%), need for periodontal care (52.2%) and need for dental care (43.3%). The results suggest that the senior center participants have limited access to dental care. There is a need to develop preventive dental programs for seniors where they congregate and live.

Abstract citation ID: igad104.2778

### COMMUNITY–CLINICAL LINKAGE MODELS FOR COMMUNITY-DWELLING OLDER ADULTS: A RAPID SCOPING REVIEW

Miriam Gofine, Gregory Laynor, Rachel Massar, and Antoinette Schoenthaler, *NYU Grossman School of Medicine, New York City, New York, United States*

About 10% of older adults (OA) have unmet healthcare needs. Community-clinic linkage models (CCLM) aim to facilitate healthcare access by linking the community and health sectors. However, research on community-based linkages for community-dwelling OA into the healthcare sector is nascent. CCLM implemented for the general adult population are not necessarily age-friendly. This scoping review aims to characterize programs that are designed to facilitate community-dwelling OA's access to healthcare through community sector-based linkages. In order to comprehensively survey CCLM designed for community-dwelling OA, the AgeLine (EBSCO) database was searched to review the academic and grey literature regarding programs that link community-dwelling OA from community to clinical sectors. AgeLine focuses on OA, indexing academic and grey literature from disciplines including health sciences, social work, and public policy. 470 publications were included for title/abstract screening; 24 were assessed for full-text eligibility by two independent reviewers. Data were extracted from two publications; exclusions were due to ineligible intervention (n=9) or setting (n=2); insufficient detail (n=6); full text unavailable (n=5). The sole empirical publication (2021) found that pilot implementation of a pilot linkage program between OA and clinicians was significantly associated with improved medication self-management. A final report (1993) on implementation of a supportive service pilot program in New Hampshire public housing found that OA residents and managers benefitted from the program. Results suggest the benefits of age-friendly CCLM for OA but highlight an existing gap in the literature. The results of this review underscore the need for age-friendly CCLM research, practice and innovation.

Abstract citation ID: igad104.2779

### IMPROVING HAND FUNCTION AMONG OLDER ADULTS THROUGH AN INTERGENERATIONAL SERVICE-LEARNING PROGRAM

Rachel Logue Cook[1], Anna Schwartz[1], Courtney Vanderlaan[2], and Susan Brown[1], *1. University of Michigan, Ann Arbor, Michigan, United States*, *2. Michigan Medicine, Ann Arbor, Michigan, United States*

Adequate hand function is essential for performing daily activities such as eating and dressing, but declines with age. Homebound older adults in particular are at greater risk for losing hand function necessary to remain independent. However, hand function in this population remains largely understudied. The purpose of this study is to determine whether Hands and Health at Home, an intergenerational upper limb-based intervention, improves hand function among homebound Meals on Wheels (MOW) clients. In this program, pre-health college students are trained to deliver a variety of hand strength, dexterity, and sensory exercises to a MOW client twice a week for 8 weeks. In addition, clients are instructed to complete exercises twice a week on their own. Measures of strength, dexterity, and sensation, as well as self-reported hand function are measured before and after the intervention. Preliminary results indicate MOW clients improved their grip strength an average of 42% in the dominant hand and 26% in the nondominant. Approximately 50% of clients improved dexterity (measured via the Purdue Pegboard) in their nondominant hand, and over 65% self-reported increased upper limb mobility. All clients and students valued this hands-on experience with several requesting continued involvement in the program. Results from Hands and Health at Home suggest this intergenerational approach to delivering hand therapy is an effective and mutually beneficial way to serve the needs of homebound older adults in the community and promote student interest in aging careers.

Abstract citation ID: igad104.2780

### PARTNER ENGAGEMENT STRATEGIES FOR AN EMBEDDED PRAGMATIC CLINICAL TRIAL: A CASE STUDY

Ann Reddy[1], Ellen McCreedy[1], Susan Mitchell[2], and Rosa Baier[1], *1. Brown University School of Public Health, Providence, Rhode Island, United States*, *2. Hebrew SeniorLife, Boston, Massachusetts, United States*

Embedded pragmatic clinical trials (ePCTs) rely on providers, rather than research staff, to implement study protocols. Partnerships need to be carefully managed to ensure a sustainable relationship. This case study describes strategies used by our research team while partnering with a large physician practice on an ePCT to improve advance care planning for people with dementia living in 160 assisted living centers. We focus on engagement between the research and provider teams co-designing and leading the implementation. Communication and project management tools held the teams accountable to decisions and set expectations for time and resources. At project initiation, we discussed the commitment of resources through different phases of the research (planning, executing, and monitoring) and determined the frequency and expected attendance for meetings during each phase. We focused on ensuring the intervention and protocols were feasible and integrated into existing clinical workflows. Regular meetings and continuous communication enabled us to make iterative modifications driven by provider needs and feedback. We used a project management register to track deliverables and document key decisions and next steps. A single point of contact on the research team fielded all inquiries and ensured timely responses to provider partners. ePCTs require a unique and active partnership between researchers and providers. In this case study, communication and project management tools enabled researchers and provider partners to identify and make changes to the ePCT's intervention design and implementation protocol in real time. This case study provides best practices for management of stakeholder relationships in the ePCT context.

Abstract citation ID: igad104.2781

### SELF-ASSESSED ORAL HEALTH IN DETROIT SENIOR CENTERS

Divesh Byrappagari, and Judith Jones, *University of Detroit Mercy School of Dentistry, Detroit, Michigan, United States*

The objective of the Detroit Mercy Senior Oral Health Equity Project (SOHEP) survey was to gather information from seniors about their opinion regarding their oral health status and satisfaction with their past dental care experience. The questionnaire was developed based on previously tested questions from other national senior oral health surveys. Data were collected using Qualtrics© and analyzed using SPSS©. The majority of the seniors screened were African American (88.9%), female (79.1%) and between the ages of 65 and 85 yrs. Over half (58.2%) reported their oral as Fair or Poor. Over one third (37.3%) reported avoiding eating certain foods due to poor oral health, and 43.3% have not been to a dentist in over 2 years. Although most participants indicated that they have insurance and have good experiences with their past dental care, a significant number of seniors have not used dental care. A community-based program that is accessible to seniors, especially in places where they congregate frequently might be helpful and improve access to preventive dental care for this population.

Abstract citation ID: igad104.2782

### SOCIAL ISOLATION AS A RISK FACTOR FOR GRIP STRENGTH DECLINE: FINDINGS FROM THE NHATS STUDY

Murad Taani[1], Julie Ellis[2], Yura Lee[1], Chi Cho[2], Ammar Hammouri[2], and Amy Schlueter[3], *1. University of Wisconsin-Milwaukee, Milwaukee, Wisconsin, United States*, *2. University of Wisconsin Milwaukee, Milwaukee, Wisconsin, United States*, *3. Ovation Communities, Milwaukee, Wisconsin, United States*

Low grip strength is associated with negative health outcomes among older adults, including poor physical performance, sarcopenia, and all-cause mortality. Social isolation has been also linked with decreased physical function and mortality. Limited studies examined whether social isolation is a risk factor for grip strength decline among older adults and whether race differences exist in this association using population-based longitudinal data. The aim of this study was to evaluate the relationship between social isolation and grip strength among older adults and whether the relationship varies by race and over time. We analyzed National Health and Aging Trends Study (NHATS) data (2015 - 2018) and the sample included 4877 adults > 65 years old. Grip strength was measured using a digital hand dynamometer. Social isolation was measured using six NHATS items from the literature and the composite score ranged from 0 to 6, with a higher score indicating more isolation. The sample mean age was 75 (SD = 22; 68 - 110 years). Repeated-measures analysis of variance showed significant differences in grip strength across social isolation scores (p = .001) indicating that socially isolated older adults have significantly lower grip strength. We also found that grip strength significantly decreased by 2.046 kg over the four-year period (p = < .001). The findings showed no significant difference in grip strength across race and change in grip strength across time did not differ by race and social isolation. Interventions to improve muscle strength and reduce social isolation among older adults are needed.

Abstract citation ID: igad104.2783

### TRAJECTORIES OF INTRINSIC CAPACITY AND IMPACT ON ADVERSE OUTCOMES IN COMMUNITY OLDER ADULTS: A LONGITUDINAL STUDY

Hui Feng[1], and Yinan Zhao[2], *1. Central South University, Changsha, Hunan, China (People's Republic)*, *2. Central south university, Changsha, Hunan, China (People's Republic)*

**Background:** Maintaining and delaying Intrinsic capacity(IC) in older adults is key to healthy ageing. Determining the point at which IC occurs and declines to delay its progression and intervene in a timely manner is critical. This study aims to explore the trajectories and the critical point of the trajectory changes among the elderly in the Chinese community. **Methods:** The data used in this study are from the ongoing national longitudinal study CHARLS, which surveys the elderly population in China. A total of 3901 older adults from the cohort study were included. Logistic regression, group-based trajectory models (GBTM) were used to identify potential heterogeneity in longitudinal changes over time and investigate associations between baseline predictors and trajectories for different cohort members. Results Four different trajectories of IC in older people, namely, Increase (15.7%), Stable (52.7%), and Decline (31.6%) were found. We found several predictors that predict the trends in IC. Age, gender, living area, BMI, self-reported health states, the number of chronic diseases and initial IC scores significantly impact the IC change rate over time. Decreased cognitive function, psychology status and locomotion at baseline predicted new ADL and IADL dependence after 5 years. **Conclusion:** The nature of genetics and environmental factors were associated with different trajectory groups of intrinsic capacity, which could help to guide the timing and focus of prevention strategies. Early prevention or intervention of the determinants of these trajectories can maintain or delay the rate of decline in intrinsic capacity and improve healthy ageing.

### SESSION 7650 (POSTER)

### EPIDEMIOLOGY

Abstract citation ID: igad104.2784

### COGNITIVE TRAJECTORIES IN MIDDLE-AGED AMERICANS, 1995–2018

Rebecca Brown[1], Xi Zuo[1], Corey McMillan[1], Shana Stites[1], Irma Elo[1], Kenneth Langa[2], Jason Karlawish[1], and Dawei Xie[1], *1. University of Pennsylvania, Philadelphia, Pennsylvania, United States*, *2. University of Michigan, Ann Arbor, Michigan, United States*

Given evidence that Alzheimer's disease and related dementias (ADRD) develop over decades, middle age is increasingly recognized as a key life course period to target modifiable risk factors for cognitive decline and ADRD. Epidemiological trends show that the prevalence of chronic conditions that are risk factors for ADRD has increased in middle-aged adults over the past several decades, but it is unknown if cognitive trajectories have worsened in middle-aged people over this time period. We conducted a retrospective cohort study using data from 19,660 adults ages 50-64 enrolled from 1995-2018 in the longitudinal nationally representative Health and Retirement Study, followed through 2018. We fit group trajectory models to describe changes in cognition over time based on Telephone Interview for Cognitive Status scores and used mixed effects models to investigate change in slope adjusted for key risk factors (age, gender, race/ethnicity, education, net worth, chronic medical conditions, body mass index, age cohort). We identified four distinct groups of participants with differing start points in their cognitive trajectories. In adjusted analyses, trajectories of cognitive decline were steeper among racial/ethnic minorities, persons with lower education, lower net worth, history of stroke, and earlier age cohort. The effect of age cohort on the rate of decline was similar across racial/ethnic groups but differed across education and net worth groups. These findings suggest that cognitive trajectories among middle-aged adults have improved since the mid-1990s. However, disparities in cognitive trajectories persist among racial/ethnic minorities and those with lower education and net worth.

Abstract citation ID: igad104.2785

### DETERMINING THE ASSOCIATION BETWEEN THE 24 HOUR COMPOSITION OF WAKING BEHAVIORS AND SLEEP WITH RECURRENT FALLS

Lauren Roe[1], Elsa Strotmeyer[1], Peggy Cawthon[2], Nancy Glynn[3], Kristine Ensrud[4], Katie Stone[5], Kelley Pettee Gabriel[6], and Jane Cauley[1], *1. University of Pittsburgh, Pittsburgh, Pennsylvania, United States*, *2. California Pacific Medical Center, Research Institute, San Francisco, California, United States*, *3. University of Pittsburgh School of Public Health, Pittsburgh, Pennsylvania, United States*, *4. University of Minnesota, Minneapolis, Minnesota, United States*, *5. California Pacific Medical Center, San Francisco, California, United States*, *6. School of Public Health, Birmingham, Alabama, United Sta tes*

Physical activity (PA), sleep, and sedentary behavior are individually associated with falls, but analyses examining one activity omit information about all other activities. We examined the combination of PA, sedentary behavior, and sleep on recurrent fall risk (2+ falls/year in any year) in older men using compositional data analysis. Data were from 2,705 men aged 79.0±5.1 years in the Osteoporotic Fractures in Men Study (MrOS). Twenty-four-hour accelerometer-measured time sedentary (< 1.5 METs), physically active (PA; >1.5 METs), and sleeping were used to calculate isometric logratios (ILR). ILRs were predictors in logistic regression models estimating the proportion of time in one activity relative to time in all other activities simultaneously, associated with recurrent falls. A total of 1,025 (37.9%) men experienced recurrent falls after four years of follow-up. In minimally adjusted models (age, race, clinic site, season of accelerometer wear), more PA relative to sedentary behaviors and sleep equated to lower odds of recurrent falls (OR: 0.70, 95%CI: 0.60, 0.79). More sedentary behavior relative to PA and sleep resulted in higher odds of recurrent falls (OR: 1.54, 95%CI: 1.14, 2.09). Associations were substantially attenuated after adjustment for height, weight, diabetes, health and smoking status, comorbidities, and instrumental activities of daily living impairments (MVPA vs. sedentary and sleep – OR: 0.88, 95%CI: 0.75, 1.02; sedentary vs. PA and sleep – OR: 1.33, 95%CI: 0.97, 1.82) and not significant. More PA and less sedentary behavior relative to other behaviors were associated with recurrent falls, with associations explained by poor health and comorbidities.

Abstract citation ID: igad104.2786

### HOME BUT HOMEBOUND AFTER TBI: PREVALENCE AND CORRELATES OF HOMEBOUNDNESS AFTER TBI

Raj Kumar[1], Katherine Ornstein[2], Shannon Juengst[3], Jennifer Reckrey[1], Amy Wagner[4], Laura Dreer[5], Kirk Lercher[6], and Kristen Dams-O'Connor[7], *1. Icahn School of Medicine at Mount Sinai, New York City, New York, United States*, *2. Johns Hopkins University, Baltimore, Maryland, United States*, *3. TIRR Memorial Hermann, Houston, Texas, United States*, *4. University of Pittsburgh, Pittsburgh, Pennsylvania, United States*, *5. University of Alabama at Birmingham, Birmingham, Alabama, United States*, *6. Kessler Institute for Rehabilitation, West Orange, New Jersey, United States*, *7. Icahn School Of Medicine At Mount Sinai, New York City, New York, United States*

Discharge home following post-acute care for traumatic brain injury (TBI) has historically been viewed as an unequivocally positive post-treatment outcome. Payors incentivize hospitals to discharge patients home and minimize readmissions. A historically overlooked topic in post-acute TBI care is homeboundness, which is the state where an individual rarely or never leaves home. Homeboundness is associated with premature mortality and poor physical and mental health. Our study investigates the prevalence and correlates of homeboundness among 6,595 non-institutionalized adults (mean age=42, range 16-99) who received inpatient rehabilitation for TBI in the TBI Model Systems National Database, a multicenter prospective cohort study. Our definition of homebound was based on self-reported number of days participants get out of the house in a typical week. We found that 14.2% of participants were completely homebound (never left the home) or partially homebound (left the home 1-2 times/week) 1-year after TBI. Using multivariable regression, we found the following variables were significantly associated with being completely/partially homebound at 1-year post-injury: older age, < college degree, Medicaid insurance, living alone and rural settings, not driving independently, and having functional needs for walking, upper body dressing, bowel and bladder, problem solving, and social interactions. These variables together had an area-under-the-curve of 0.81 distinguishing homeboundness. Our study elucidates an invisible subgroup of non-institutionalized adults living with TBI in the community who require ongoing support services or other follow-up care to improve functional status and participation. Implications for community integration will be discussed.

Abstract citation ID: igad104.2787

### PERSONALITY, SOCIAL ISOLATION, AND LONELINESS: CROSS-SECTIONAL DATA FROM THE CANADIAN LONGITUDINAL STUDY ON AGING

Jennifer Bethell[1], Melissa Andrew[2], Debra Morgan[3], Megan O'Connell[3], Natalie A. Phillips[4], Walter Wittich[5], and Katherine McGilton[1], *1. Toronto Rehabilitation Institute - University Health Network, Toronto, Ontario, Canada*, *2. Dalhousie University, Halifax, Nova Scotia, Canada*, *3. University of Saskatchewan, Saskatoon, Saskatchewan, Canada*, *4. Concordia University, Montreal, Quebec, Canada*, *5. Université de Montréal, Montreal, Quebec, Canada*

Good social connection is associated with better health. However, the relationships between distinct aspects of objective and subjective social connection, and how personality traits may impact these relationships, are not well-understood. For example, in predicting loneliness, personality could moderate the impact of objective social connection by influencing the way social relationships are evaluated. We used cross-sectional Canadian Longitudinal Study on Aging data (n=27,765), collected between 2015 and 2018, to explore the potential confounding or modifying effects of personality traits on the associations between social isolation and loneliness score among 46-90 year-olds. We estimated associations using ordered logistic regression models, whereby the outcome was the loneliness score and exposures were social isolation and each of five personality traits (extraversion, agreeableness, conscientiousness, emotional stability, and openness). We produced three sets of models (social isolation only; personality trait only; and social isolation, personality trait and their interaction) and conducted all analyses stratified by sex and adjusting for potential confounders. We found that, for both males and females, social isolation and each of the five personality traits were associated with loneliness scores (p< 0.001), however, with only one exception (openness among females), there was no statistical evidence that personality modified the association between social isolation and loneliness. Our findings have potential implications for research assessing the health effects of social connection and personality as well as intervention studies that target social isolation as means of addressing loneliness.

Abstract citation ID: igad104.2788

### POPULATION REFERENCE VALUES FOR PEAK EXPIRATORY FLOW IN OLDER US ADULTS

Patrick Donahue[1], Aparna Balasubramanian[1], Anis Davoudi[1], Jennifer Schrack[1], Amal Wanigatunga[2], and Michelle Carlson[3], *1. Johns Hopkins University, Baltimore, Maryland, United States*, 2. *Johns Hopkins School of Public Health, Baltimore, Maryland, United States*, 3. *Johns Hopkins Bloomberg School of Public Health, Baltimore, Maryland, United States*

Respiratory function predicts physical and cognitive health outcomes in older adults. Peak expiratory flow (PEF) is simple, inexpensive, and has been validated against traditional respiratory function measures (FEV1, FVC). PEF has additional utility because it measures maximal respiratory effort without relying on spirometry equipment. However, there is a lack of epidemiological data to support PEF reference values for aging populations, especially those ≥80 years. The National Health and Aging Trends Study (NHATS) is a large, nationally representative sample of US adults ages ≥65 that offers a unique opportunity to develop PEF population reference values. Using a healthy subsample from NHATS Round 1 (N=2,082), excluding those with history of smoking, respiratory, or cardiovascular diseases, we applied NHATS sampling weights to estimate age- and sex-specific PEF percentiles. The sample was stratified by sex (68.9% female) and categorized into six, 5-year age groups: 65-69, 70-74, … ≥90 (36.6% age ≥80 years). In a secondary analysis, we estimated age- and sex-specific PEF/height3 percentiles, per current recommendations. We calculated 5th, 95th, and 10th, 20th, … 90th percentiles, by age and sex, for both absolute and height-adjusted PEF. PEF population estimates were higher in healthy males vs. females (456.8±130.1 vs. 293.9±89.9 liters/minute [mean±SD]). After adjusting for height, estimates remained higher for males (83.1±22.4 vs. 70.9±21.5 liters/m3/minute) and higher for younger (65-79 years) vs. older (≥80 years) participants (78.9±19.7 vs. 62.9±26.6 liters/m3/minute). These results may serve as PEF population reference values for healthy older adults in the US.

Abstract citation ID: igad104.2789

### U-SHAPED ASSOCIATION OF Α-KLOTHO WITH ALL AND CAUSE-SPECIFIC MORTALITY IN US ADULTS: A SECOND ANALYSIS OF NHANES

Kai Wei, and Qi Chen, *Guizhou Provincial People's Hospital, Guiyang, Guizhou, China (People's Republic)*

Laboratory studies have shown that mice deficient in the klotho gene have a shortened lifespan, but the epidemiological evidence linking circulating α-klotho to mortality is inconsistent. Our aim was to clarify the association between serum α-klotho and mortality using a representative population. From the National Health and Nutrition Examination Survey (2007-2016), 13748 participants aged 40-79 years with serum α-klotho test data were selected and followed up to 31 December 2019. Participants were equally divided into 5 groups (Q1 < 622.8pg/ml, Q2: 622.8-739.1pg/ml, Q3: 739.1-857.7pg/ml, Q4: 857.7-1033.1pg/ml, Q5 >1033.1pg/ml) based on quantiles of serum α-klotho concentrations, with the Q3 serving as the reference group. Multivariate Cox proportional hazards regression analysis showed that both participants with low and high serum α-klotho levels had a higher risk of all-cause mortality, compared to the reference group (HR[95%CI], Q1:1.38[1.13,1.69], Q5:HR 1.23[1.02,1.47]). We further found a non-linear and U-shaped association between α-klotho and all-cause, cancer-cause, and diabetes-cause mortality, but an L-shaped association between α-klotho and CVD-cause mortality by using cubic spline function model and smooth curve fitting (penalized spline method). We observed a significant negative association between α-klotho (log-transformed) and all-cause mortality (HR[95%CI], 0.70[0.55,0.91]) in the subgroup of participants with normal liver function. In conclusion, the U-shaped association was found between serum α-klotho and all-cause mortality among US adults, and the reasons for the increased risk of death associated with high levels of α-klotho require further investigation.

### SESSION 7660 (POSTER)

### FRAILTY IN AGING

Abstract citation ID: igad104.2790

### ASSESSMENT OF THE LINK BETWEEN FRAILTY AND DEMENTIA IN THE OLDEST-OLDS USING JOINT MODELS.

Franklin Massa[1], Alejandra Marroig[2], Graciela Muniz-Terrera[3], and Boo Johansson[4], *1. Universidad de la República, Montevideo, Montevideo, Uruguay*, 2. *Universidad de la Republica Uruguay, Montevideo, Montevideo, Uruguay*, 3. *Ohio University, Athens, Ohio, United States*, 4. *University of Gothenburg, Sweden, Gothenburg, Gothenburg, Sweden*

Frailty and dementia affect many older adults worldwide. As the world's population ages, the number of people experiencing frailty and dementia is increasing, posing significant challenges for individuals, health care systems and caregivers. Here, we aim to assess the link between the evolution of frailty and the time to dementia diagnosis in the oldest-old. We derived the frailty index (FI) using data from 5 waves of the OCTO-Twin Study: Origins of Variance in the old-old, and fitted joint longitudinal-survival models to repeat measures of the index and time to dementia diagnosis to understand the association between the trajectory of the FI and time to diagnosis. We accounted for possible effects of sex, education and the presence of the APOE e4 gene. A nonlinear trajectory of the frailty index (p < 0.001) and an effect of education attenuating the levels of the frailty index

(p = 0.032) were observed. Additionally, the results agree with the literature regarding the effect of the APOE e4 as a greater risk for dementia (HR = 2.034, p < 0.001). Moreover, an increasing frailty trajectory is associated with an earlier time to diagnosis of dementia (HR = 1.096, p < 0.001). Our findings advance our understanding of the relation between frailty and dementia in the oldest old. Our approach accounts for the non-linearity of the trajectory of frailty and deliver a dynamic tool that updates dementia prognosis when a new measurement of frailty is available.

Abstract citation ID: igad104.2791

### DEVELOPMENT AND VALIDATION OF A RISK PREDICTION MODEL FOR SARCOPENIA IN COMMUNITY-DWELLING OLDER ADULTS

Taiping Lin[1], and Jirong Yue[2], *1. West China Hospital, Chengdu, Sichuan, China (People's Republic)*, *2. West China Hospital of Sichuan University, Chengdu, Sichuan, China (People's Republic)*

Reliable identification of older populations at high risk of sarcopenia is essential for initiating targeted preventive measures and follow-up, but no sarcopenia risk prediction model is available. Our objective was to develop and validate a prediction model for calculating the 1-year absolute risk of sarcopenia occurrence for older people in the community. The analytic samples for the prediction model derivation were conducted on data from one prospective population-based cohort. The main outcome was the development of sarcopenia within 1 year (defined by 2019 Asian Working Group for Sarcopenia consensus). Multivariate logistic regression analysis was conducted to examine those independent risk factors for sarcopenia and the sarcopenia risk-predicting model was developed using the R software. In the development cohort, 1042 non-sarcopenia residents were enrolled, of whom the mean age was 66.6±5.0 years, 631 participants were women, and 87 participants developed sarcopenia at the 12-month follow-up. The final model contains 7 risk factors-age, gender, BMI, low physical activity, malnutrition, pain, and calf circumference. The model showed excellent discrimination (AUROC 87%, 95% CI 0.83–0.90), calibration capability (slope 0.93) and clinical utility. Internal validation was conducted using 200 times bootstrapping technique and showed a stable discriminative ability (corrected AUROC of 0.85). High risk thresholds of 10% or more were able to identify high-risk individuals of sarcopenia, and low risk thresholds less than 2.5% were useful to rule out the likelihood of sarcopenia. In summary, the sarcopenia risk-predicting model for community-dwelling older people consists of 7 readily available predictors and has an excellent predictive ability.

Abstract citation ID: igad104.2792

### EVALUATING A TRANSITIONAL CARE PROGRAM FOR OLDER ADULTS WITH FRAILTY BETWEEN HOSPITAL AND HOME

Ji Yeon Lee[1], Eunhee Cho[2], Sue Kim[1], Gwang Suk Kim[2], Kyung Hee Lee[1], and Chang Oh Kim[1], *1. Yonsei University, Seoul, Republic of Korea*, *2. Yonsei University College of Nursing, Seoul, Republic of Korea*

Older adults with frailty experience numerous health problems even after hospital discharge, highlighting the necessity of continued intervention until their recovery and return to daily routines. This mixed methods research (i.e., quantitative and qualitative studies) aimed to evaluate the effectiveness of a transitional care program developed for older adults with frailty during the transition from hospital to home and how it was helpful. From June to December 2021, 32 older adults with frailty were enrolled in a randomized controlled trial at a university-affiliated hospital in Korea. They were allocated to either an intervention group (n=16) or a control group (n=16) according to a random number sequence. The intervention group received a 12-week transitional care program that included inpatient care, structured discharge, and follow-up interventions (e.g., home visits and phone follow-ups); the control group received usual care only. At 12 weeks, program participants (eight frail older adults and eight family caregivers) had interviews for the qualitative research. The results showed that the intervention group had a higher score in knowledge readiness (p=.046) at discharge, empowerment (p=.032), and connectedness at four weeks (p=.037); community resource utilization (p <.001) and emergency room visit rate (p=.049) at 12 weeks; and improvement in depression (p <.001), family interaction (p=.045), and subjective health status (p=.002) over time. Participants described the program as a supportive, connected, timely, and personalized intervention. Overall, the study demonstrated that the transitional care program effectively improved the health outcomes of older adults with frailty and facilitated a safe transition between hospital and home.

Abstract citation ID: igad104.2793

### EXAMINING FRAILTY SUBDOMAINS (PHYSICAL, PSYCHOLOGICAL, COGNITIVE, SOCIAL) IN COMMUNITY-LIVING ADULTS 45–85

Lauren Griffith[1], Graciela Muniz-Terrera[2], Edwin van den Heuvel[3], Jayati Khattar[1], David Hogan[4], Megan O'Connell[5], Melanie Levasseur[6], and Parminder Raina[1], *1. McMaster University, Hamilton, Ontario, Canada*, *2. Ohio University, Athens, Ohio, United States*, *3. Eindhoven University of Technology, Eindhoven, Noord-Brabant, Netherlands*, *4. Univeresity of Calgary, Calgary, Alberta, Canada*, *5. University of Saskatchewan, Saskatoon, Saskatchewan, Canada*, *6. Université de Sherbrooke, Sherbrooke, Quebec, Canada*

Frailty prevalence estimates vary considerably (4.0% to 59.1% in one review). Understanding these differences and what drives frailty among individuals could lead to more focused interventions. Breaking frailty into subdomains and exploring their relationships is one approach to this. A 127-item overall Frailty Index (FI) based on data collected on Canadian Longitudinal Study on Aging (CLSA) Comprehensive Cohort participants (n = 30,097 community-living adults aged 45-85 at baseline) was used to create physical, psychological, cognitive, and social subdomain-specific FIs. Each FI was divided into quintiles with the highest 20% (Q5) considered the frailest. We assessed subdomain FI quintile concordance (Goodman and Kruskal's gamma), described the joint distribution of those most frail (Q5), and estimated the association between sex and age with Q5 subdomain FIs membership (logistic regression). The concordance among FI subdomain quartiles was low. The highest gammas

(0.25-0.36) were between the physical/psychological and psychological/cognition subdomains. Concordance was generally higher in females who had higher odds of Q5 membership for overall (1.5), physical (1.7), psychological (1.6) and social frailty (1.1), and lower odds for cognitive frailty (0.7). The odds of Q5 membership increased with age for overall frailty and all subdomains except psychological frailty, which decreased with age. Our results suggest low concordance among frailty subdomains and the relationship between Q5 membership with sex and age differed by subdomain. These data may help to better target frailty interventions, but longitudinal data are needed to explore both the time course and interrelationships across subdomains.

Abstract citation ID: igad104.2794

### FACTORS RELATED TO THE FRAILTY OF OLDER PEOPLE WITH STROKE IN TEHRAN

Ali Darvishpoor Kakhki, and Elham Aghaali, *Shahid Beheshti University of Medical Sciences, Tehran, Tehran, Iran*

Older people are the fastest growing segment of the population particularly in developing countries such as Iran. Older people encounter not only decreasing physical function but also diseases such as stroke which can lead to their frailty. This study was conducted to describe factors related to frailty of older people with stroke in Tehran. This descriptive, correlational study was conducted on a sample of 170 older people with stroke above age 60 in ten educational hospitals in 2020. A socio-demographic questionnaire, the Zarit Scale of Caregiver Burden, and the Frailty Index for Elders were used for data collection. Content validity and Cronbach's alpha were used for evaluating the validity and reliability of questionnaires. Data were analyzed with SPSS 25. 55.9% of participants were female and 54.1% lived with their spouse; mean age was 77.18 (± 8.57). 92.4% were at risk for severe frailty, and 7.6% were at risk of frailty. The scores of frailty were associated significantly with increasing family caregiver burden and hospital admission rates, not having treatment insurance, and not having a reliable income source. The best predictors of frailty were family caregiver burden, hospital admission rates, and treatment insurance. The study showed that frailty is very common in older people with stroke and is related to a number of factors. Reducing family caregiver burden and admission rates to hospital, and having treatment insurance, will help prevent or reduce frailty in older people with stroke.

Abstract citation ID: igad104.2795

### GERIATRIC MEASURES OF PHYSICAL FUNCTION HAVE THEIR ROOTS IN EARLY CHILDHOOD BRAIN HEALTH

J. Kathy Xie, Lauren Winslow, Avshalom Caspi, and Terrie Moffitt, *Duke University, Durham, North Carolina, United States*

Midlife is a pivotal period in the life course in terms of investing resources to mitigate challenges that can come in older age. It is important to understand the relationship between childhood and midlife health, as it could have predictive properties for aging. We investigated the association between early childhood brain health and midlife physical function in the Dunedin Study, a longitudinal birth cohort (n=1,037). Brain health was measured when Study members were age 3 using a neurological examination, tests of cognitive functioning, and temperament rating. Physical function was measured at age 45 using gait speed, step-in-place, chair stands, balance, and grip strength tests. Children with worse brain health had significantly worse physical function as adults (β, 0.23; 95% CI, 0.16 to 0.30; P < .001), after controlling for sex and childhood socioeconomic status. This relationship supports a link between cognitive and physical function in the aging and development process, the reconceptualization of aging and development on the same ontological path, and the utility of physical function tests – which can be used in many clinical settings – as reflections of lifelong, integrated health in addition to indicators of musculoskeletal integrity in older adults.

Abstract citation ID: igad104.2796

### RECENT DEVELOPMENTS IN FRAILTY IDENTIFICATION, TREATMENT, AND PREVENTION IN OLDER ADULTS: A REVIEW

Emiel Hoogendijk[1], and Elsa Dent[2], *1. Amsterdam University Medical Centers, Amsterdam, Noord-Holland, Netherlands*, *2. Torrens University Australia, Adelaide, South Australia, Australia*

Frailty is a clinical condition characterized by an increased susceptibility to stressors and an elevated risk of adverse outcomes such as mortality. In the light of global population aging, the prevalence of frailty is expected to soar in coming decades. The current study provides a review of novel developments and emerging practices in frailty research regarding prevention, identification, and management of the condition in older adults. We searched geriatric journals in the top two quartiles (based on 2021 Impact Factors from Clarivate Journal Citation Reports) for articles published between January 2018 and December 2022. We screened 1519 articles for eligibility and assessed the full text of 278 articles. Popular research trends included the study of frailty transitions and trajectories, analyses of life-course risk factors for frailty, identifying frailty using biomarkers (and biomarker panels), and investigating the altered response to medications by older adults with frailty. Other areas with novel developments included: artificial intelligence (machine learning) for frailty identification; exercise and physical activity for the management of frailty; medication management, gerontechnology; education of healthcare professionals; integrated care; person-centered care; guidelines; cost-effectiveness of management strategies; and COVID-19. This review identified a strong need for the implementation and evaluation of cost-effective and community-based interventions to prevent and manage frailty. Our findings highlight the need to strengthen healthcare systems to better support older adults with frailty and involve those with frailty in shared decision-making regarding their care.

Abstract citation ID: igad104.2797

### THE ASSOCIATION OF FRAILTY AND MORTALITY IN OLDER ADULTS: A SYSTEMATIC REVIEW AND META-ANALYSIS OF COHORT STUDY

Xianqi Gao, Rhayun Song, Moonkyoung Park, Yuelin Li, Linyu Lyu, Eunna Oh, and Xing Fan, *Chungnam National University, Daejeon, Republic of Korea*

Frailty is associated with poor health outcomes in later life, improving lifespan and death through different mechanisms. This study aimed to systematically review the epidemiological evidence from cohort studies investigating the association between frailty and all-cause mortality, and to determine if frailty affects mortality in older adults. We searched for studies published and indexed in 4 electronic databases (EMBASE, PubMed, Cochrane Library, and Web of Science) and manual search and identified 28 cohort studies before Dec 31, 2022. We include studies if their design was longitudinal; the exposure of interest was measured frailty; the endpoint of interest was mortality; they provided a risk estimate (i.e., hazard ratio [HR]) and the corresponding 95% CI for the association between frailty and mortality. They used different indexes as their frailty as exposed definitions. Two investigators independently screened the full-text articles for inclusion. We used a random-effects model to obtain pooled HRs. Thirty-one studies were included for qualitative review, of which twenty-eight were eligible for meta-analysis, and included 67,114 individuals from 16 countries. Compared with healthy adults, frail adults had a significantly higher mortality risk. The pooled HR for mortality per increment of 0.1 frailty of community-dwelling adults was 2.33 (95% CI 2.05-2.65; I2 76.9%). The present study demonstrated that frailty was not only significantly related to an increased mortality risk but was also a strong predictor of mortality in community-dwelling adults.

Abstract citation ID: igad104.2798

### THE RELATIONSHIP OF SELF-MONITORING OF LIFESTYLES WITH PHYSICAL FUNCTION AND METABOLITES IN DIABETIC OLDER ADULTS

Yan Du[1], Lixin Song[2], Mitzi Gonzales[1], Sara Espinoza[3], Carlos Jaen[1], Jing Wang[4], and Kumar Sharma[1], *1. UT Health San Antonio, San Antonio, Texas, United States*, *2. University of Texas Health Science Center at San Antonio, San Antonio, Texas, United States*, *3. Cedars-Sinai Medical Center, Los Angeles, California, United States*, *4. Florida State University, Tallahassee, Florida, United States*

Type 2 diabetes (T2D) is a major risk factor for functional decline in older adults. Mobile/wearable devices are evidenced to improve lifestyles for diabetes management and improve functionality. This study assessed the associations of technology tracked lifestyle and self-monitoring behaviors with glycemic control, physical function and mitochondrial function related metabolites in older adults with T2D. Fitbit fitness trackers were used to track physical activity and food intake over 12-week in 15 older adults with T2D (age=70.5±4.8). Physical function was assessed by Short Physical Performance Battery (SPPB) and grip strength. First void urine was used to assess metabolites. The associations of percentage of days with tracked steps (PDWTS), percentage of days with food log (PDWFL), and 7-day mean steps with HbA1c, SPPB, grip strength and metabolites were examined using Pearson Correlation analysis. At week 12, PDWTS was negatively associated with 3-Hydroxyisovaleric acid and Hydroxyhippuric acid ($P< 0.05$). PDWFL was negatively associated with HbA1c, Hydroxyisovaleric acid, Hydroxyhippuric acid, and Hydroxybutyric acid ($p< 0.05$); SPPB was not related to any self-monitoring and lifestyle behaviors, but was negatively related to Hydroxyisovaleric acid, Glycolic acid and Hydroxyisobutyric acid ($p< 0.05$). In older adults with T2D, self-monitoring might improve glycemic control. Self-monitoring and SPPB were related to several urine metabolites, but the underlying mechanisms is unclear in this study with small sample size. Future research with sufficient power is needed to investigate the underlying mechanisms of self-monitoring and lifestyle behaviors on these metabolites, and how these metabolites might be applied to improve physical function in older adults with T2D.

Abstract citation ID: igad104.2799

### USING HOSPITAL ELECTRONIC HEALTH RECORDS TO DETERMINE FRAILTY WITH THE PICTORIAL FIT-FRAIL SCALE

Alanna Bohnsack[1], Karla Faig[1], Allyson Cook[2], Cameron MacLellan[2], Susan Benjamin[3], Josh Shanks[2], Sherry Gionet[1], and Pamela Jarrett[1], *1. Horizon Health Network, Saint John, New Brunswick, Canada*, *2. Dalhousie University, Saint John, New Brunswick, Canada*, *3. Trauma New Brunswick, Saint John, New Brunswick, Canada*

Hip fractures are common in older adults, particularly those that are frail. There is no “gold standard” tool to measure frailty retrospectively from hospital Electronic Health Records (EHRs). The aim of this research was to explore the utility of the Pictorial Fit-Frail Scale (PFFS) in determining frailty retrospectively in older adults admitted to hospital with a hip fracture. A random sample of 200 patients who had a hip fracture was extracted from a larger sample of 682 hip fractures that occurred in older adults (65 years and older) admitted to a Level 1 Trauma Center (April 2015-March 2019). Each EHR was reviewed to determine the availability of data and the score for each of the 14 PFFS domains. The majority, 189 (94.5%) of the EHRs, had data to complete 11-14 of the domains. Patient information was often unavailable to complete the domains of daytime tiredness and pain. The average age was 83.2 (SD 8.2), and 73.0% were female. The mean Raw PFFS score prior to admission was 9.7 (SD 6.6), and 45.5% of patients were classified as moderately or severely frail. Using the Standardized PFFS score (adjusted for up to three missing domains), the mean score was 11.8 (SD 7.9), and 58.2% of patients were classified as moderately or severely frail. The hospital EHR can be used to complete the PFFS. This finding provides researchers and healthcare professionals with a way to stratify older hospitalized patients by frailty, which may be a useful tool for evaluating health outcomes.

### SESSION 7670 (POSTER)

### PAIN MANAGEMENT AND PALLIATIVE CARE

Abstract citation ID: igad104.2800

### CO-PRESCRIPTION OF OPIOIDS AND BENZODIAZEPINES AND 30 DAY HOSPITAL RETURNS IN OLDER ADULTS

JuYoung Park, Gabriella Engstrom, and Joseph Ouslander, *Florida Atlantic University, Boca Raton, Florida, United States*

With the current opioid epidemic, greater attention has been focused on polypharmacy, in particular use of benzodiazepine (BZD). This study compared prescription of opioids or BZD, co-prescription of both, and prescription of neither at discharge with returns to the hospital within 30 days of discharge of older patients (75+ years). This is a secondary analysis of a quality improvement database developed during implementation of a care transition intervention for hospitalized patients age 75+. The database includes all non-ICU admissions over a 2-year period (24,784 admission cases). Returns to the hospital included Emergency Department visits, inpatient hospital stays, and observation admissions. Among the 24,262 discharges that had medication data, 1,594 (6.6%) were prescribed opioids only, 3,594 (15.0%) BZD only, 544 (2 %) opioids and BZD together, and 18,530 (76.4%) neither opioid or BZD. Each of the four prescription groups had a > 20% return rate within 30 days of discharge: 22.1% opioids only, 23.8% BZDs only, 23.7% opioids and BZDs, and 20.2% neither. Opioids were not associated with a significantly higher incidence of hospital returns. The data highlight the importance of educating healthcare providers about appropriate use of BZD and opioids in hospitalized and post-discharge older patients. Individualized care by interdisciplinary team members and support for family caregivers, as well as careful use of opioids for pain that cannot be managed by other modalities, may reduce hospital return rates and their associated morbidity and costs in the older population.

Abstract citation ID: igad104.2801

### CULTIVATION OF PROGNOSTIC AWARENESS IS A TEAM TASK: PALLIATIVE PHYSICIAN AND SOCIAL WORKER INSIGHTS

Arden ODonnell, and Judith Gonyea, *Boston University, Boston, Massachusetts, United States*

For patients experiencing serious or life-limiting illnesses, informed decision-making depends on understanding prognosis and illness trajectory. Although physicians are viewed as the most appropriate group to share medical information around prognosis, there is growing recognition that other clinicians can contribute to these discussions. Indeed, cultivation of prognostic awareness (CPA) does not happen in a single conversation; it is a series of conversations in which patients continue to redefine medical treatments and broader life goals. The reframing of CPA as an iterative process, coupled with growing palliative care demands, has led to social workers' (SWs) and nurses' engagement, and even leadership, in goals of care (GOC) conversations. Using role theory, this qualitative study therefore explored aligned and/or disparate views on professional role boundaries in CPA through interviews with 17 SWs and 14 physicians. Our data analysis was inductive and iterative with the culminating phases being theme generation and interpretation. Key findings revealed a shared perspective by SWs and physicians that CPA is a "team task" and that SWs have essential roles as experts in assessing and supporting patients' emotional and psychological capacity for processing prognostic information, and as boundary spanners to patients/families and physicians (i.e., translating medical information, increasing effective communication, and reinforcing a holistic perspective). Both professions identified managing patients'/families' expectations as a shared role. SWs experienced role conflict, however, in their advocacy for patient autonomy. Factors enhancing role consensus were communication, collaboration, professional respect, experience, and trust. Findings support the trend of expanding SWs role in serious illness conversations.

Abstract citation ID: igad104.2802

### FACTOR STRUCTURE OF THE BRIEF PAIN INVENTORY–SHORT FORM IN AFRICAN AMERICAN OLDER ADULTS WITH OSTEOARTHRITIS

Jianli Wu, Ann Horgas, and Staja Booker, *University of Florida, Gainesville, Florida, United States*

The Brief Pain Inventory-Short Form (BPI-SF) is a widely used questionnaire designed to assess aspects of cancer pain, such as pain intensity and pain interference. Psychometric properties have rarely been evaluated in other pain conditions such as osteoarthritis. Osteoarthritis is a major contributor to pain and physical disability in African Americans (AA) older adults. Thus, it is important to evaluate the psychometric properties of BPI-SF in this health disparity population. Participants were AA aged 50-94 years with self-reported osteoarthritis. The final convenience sample (N=110) completed surveys including a demographics questionnaire and the validated BPI-SF. Confirmatory Factor Analysis (CFA) was used to evaluate the factor structure. Measurement invariance across two age groups [i.e., young-old (50-69); old-old (70-94)] was examined. CFA revealed that a three-factor model (i.e., pain intensity, activity interference, and affective interference) demonstrated the best fit ($\chi2/df=1.595$, CFI=0.949, RMSEA=0.074) to the data. AAs reported pain intensity (M=4.58, SD=2.01), affective interference (M=3.03, SD=2.54), and activity interference (M=4.19, SD=3.25). The change of CFI between configural and metric invariance is below the cutoff point of .01, supporting the full metric (i.e., factor loadings) invariance across two age groups. However, the full scalar (i.e., items intercepts) invariance was not established. The results support a three-factor structure of BPI-SF that is invariance across two age groups at the full metric level. This suggests that the BPI-SF is an appropriate measure to characterize chronic pain intensity, affective interference, and activity interference for older AAs with osteoarthritis.

Abstract citation ID: igad104.2803

### PAIN INTENSITY AND INTERFERENCE PREDICT STAGE OF ENGAGEMENT IN SELF-MANAGEMENT IN OLDER AFRICAN AMERICAN ADULTS

Staja Booker, Ann Horgas, and Jianli Wu, *University of Florida, Gainesville, Florida, United States*

Osteoarthritis in African American older adults often causes a disproportionate burden of severe chronic joint pain that interferes with physical and emotional/cognitive activities. One tool commonly used in clinical research studies to evaluate patterns in pain intensity and pain interference is the Brief Pain Inventory-SF (BPI-SF). Confirmatory factor analysis validated a three-factor structure of the BPI-SF comprising pain intensity, activity-related pain interference, and affective pain interference. However, what is unclear and novel, is whether these three factors predict stage of readiness to engage in osteoarthritis self-management. Stage of readiness to engage (pre-contemplation, preparation, action) were assessed for seven recommended osteoarthritis self-management strategies: land-based exercise, water exercise, strength training/stretching, assistive devices, warm/cool compresses, medications, and self-management education. Using secondary data from 110 African American men and women (Mean Age 68.4, SD= 12.4), we performed ANOVA and ordinal logistic regression in SPSS. Pain intensity, activity-related pain interference, and affective pain interference were not significantly different between stages of engagement in pain self-management strategies except for medication use and assistive devices (p<.05). After controlling for age and employment, higher pain intensity was significantly associated with 27% reduction in the odds of being in preparation phase for water exercise, and higher levels of activity interference was associated with a 42% increase in the odds of being in the action phase for assistive devices. These results help us understand the relationship between behaviors and pain, particularly the impact of pain intensity and interference with physical activities on which strategies African Americans select to manage pain.

Abstract citation ID: igad104.2804

### THE INTERSECTION OF INSOMNIA AND CENTRALIZED PAIN IN OLDER ADULTS

Michael Smith[1], Meagan Dean[2], and Madison Irwin[1], *1. University of Michigan, Ann Arbor, Michigan, United States*, 2. *University of Michigan Health, Ann Arbor, Michigan, United States*

The intersection of insomnia and pain with age has been broadly studied but has not been evaluated specifically in centralized pain. The objective of this study was to estimate the effect of age on rates of centralized pain and insomnia in older adults. This was a single center, retrospective, observational cohort study consisting of older adults (≥55 years old) with a diagnosis of centralized pain, insomnia, or both seen at University of Michigan Health from January 1, 2016 – January 1, 2022. The primary outcome was the proportion of older adults with both insomnia and centralized pain, defined as least one centralized pain diagnosis coded in the subject's electronic health record within one year of insomnia diagnosis. A total of 26,804 patients were included in the analysis. Mean age was 66.84 years (SD 8.79); 66% of patients (n=17,687) were female. Overall, 92.1% of patients (n=24,683) carried ≥1 centralized pain diagnosis, 5.6% of patients (n=1,505) carried a diagnosis of insomnia, and 2.3% (n=616) had both [95% CI 2.1,2.5]. Of older adults with insomnia, 29% (95% CI 27, 31) also carried a centralized pain diagnosis. Of older adults with centralized pain, 2.4% (95% CI 2.3, 2.6) also carried an insomnia diagnosis. Compared to older adults with insomnia alone, those with both insomnia and centralized pain were more likely to be female (p=< 0.001) and older (p=0.014). Insomnia and centralized pain are highly prevalent in older adults and co-occur frequently in those with insomnia. Older adults with insomnia should be screened for centralized pain.

Abstract citation ID: igad104.2805

### TWO SIDES OF THE SAME COIN: OPIOIDS AND CANNABIS FOR PAIN MANAGEMENT IN AFRICAN AMERICAN OLDER ADULTS

Simone Jackson[1], and Staja Booker[2], *1. University of Florida College of Nursing, Gainesville, Florida, United States*, 2. *University of Florida, Gainesville, Florida, United States*

Approximately 50 million U.S. adults live with chronic pain. Example treatments for chronic pain include opioids and the re-emerging cannabinoid pain management (CPM). However, older African Americans (AAs) are more likely to use pain treatments that are considered safe, affordable, and socially acceptable. We examined the perception of opioid and cannabis use for pain management in AA older adults by conducting: (1) secondary data analyses from two studies to quantify patterns of opioid and cannabis use, (2) a concept analysis to delineate the concept of CPM, and (3) developing data-driven model cases for CPM. Study 1 was a cross-sectional survey with 110 AA adults (age 50 and older) in Louisiana, and study 2 was observational with 72 AA adults (age 55 and older) in Florida with self-reported osteoarthritis. Both samples were primarily women, 79-81% respectively. Only 33% and 1.3% reported using opioid analgesics, while less than 4% in both studies reported using medical or recreational cannabis. Using Walker and Avant's concept analysis approach, the (A) attributes of CPM include lower side effect profile and social acceptability; (B) antecedents include chronic pain, motivation to seek alternative treatment, access to cannabis dispensaries, family and professional support, and health literacy; and (C) the consequences are pain relief, lower mortality, self-efficacy, reduced anxiety, and empowerment. Lastly, based on our quantitative and qualitative data, we developed two model cases demonstrating support for and against CPM. These results illuminate the need to further understand the barriers and facilitators to using opioids and CPM in older AAs.

### SESSION 7680 (POSTER)

### TECHNOLOGY: RESEARCH AND USE IN OLDER ADULTS

Abstract citation ID: igad104.2806

### ACCEPTABILITY OF PODCASTS TO ENHANCE WELLNESS SKILLS AMONG OLDER ADULTS IN AN UNDERSERVED COMMUNITY

Mary Janevic[1], Rebecca Lindsay[1], Robin Brewer[2], Yolanda Hill-Ashford[3], Rebeca Guzman[3], Sheria Robinson-Lane[4], Afton Hassett[5], and Susan Murphy[5], *1. University of Michigan School of Public Health, Ann Arbor, Michigan, United States*, 2. *University of Michigan School of Information, Ann Arbor, Michigan, United States*, 3. *Detroit Health Department, Detroit, Michigan, United States*, 4. *University of Michigan School of Nursing, Ann Arbor, Michigan, United States*, 5. *University of Michigan Medical School, Ann Arbor, Michigan, United States*

Podcasts convey information in an accessible, engaging, and memorable way, yet are seldom incorporated into behavioral interventions for older adults. We developed a podcast series as a component of the RESET intervention (Re-Engaging in Self-care, Enjoying Today), the subject of an ongoing NIH-funded randomized controlled trial in Detroit, Michigan. In RESET, community health workers teach self-management and resilience-building skills to small groups of primarily African American older adults, to address challenges to well-being and functioning caused by the COVID-19 pandemic. Participants listen to six podcast episodes, accessible by website, app, or IVR/

dial-in, which feature an older-adult host from the community interviewing other community members and topic experts. Each episode is then discussed in the corresponding intervention session. The goal of the current study was to evaluate the first three podcast episodes (Power of Positivity, Eating for Well-being, and Stability in Strength) in a convenience sample of 8 older adults and agency staff from our trial's Community Advisory Board. Using 5-point Likert scales, all respondents strongly agreed or agreed that the episodes were enjoyable, relevant, and effectively taught key wellness skills. Podcast strengths identified by respondents included: warm interactions between host and guests, motivational tone, and compelling personal stories. Areas for improvement included the need to represent additional perspectives. These data suggest that podcasts are an acceptable way to provide health information to older adults. The ongoing RESET trial will allow us to determine the extent to which podcasts can help bring about behavior change and functional improvement.

Abstract citation ID: igad104.2807

### ASSESSING FRAILTY USING THE FIT-FRAILTY APP IN A NONGERIATRIC PRACTICE: A FEASIBILITY STUDY

Alexa Kouroukis[1], Suleman Tariq[1], Jonathan Adachi[1], George Ioannidis[2], Courtney Kennedy[1], Carolyn Leckie[3], Alexandra Papaioannou[1], and Isabel Rodrigues[1], *1. McMaster University, Hamilton, Ontario, Canada*, *2. GERAS Centre for Aging Research, Hamilton, Ontario, Canada*, *3. St. Joseph's Healthcare Hamilton, Hamilton, Ontario, Canada*

Frailty is a common medical condition with a prevalence of 24% in adults ≥50 years when using the Frailty Index. Thus, assessing frailty is a priority. The Fit-Frailty Application (App) is a user-friendly and validated measure that incorporates disease-related, physical, cognitive, psychosocial, and functional aspects of frailty. The purpose of this study was to determine the feasibility of using the App in a non-geriatric clinic. We conducted a cross-sectional study in a rheumatology clinic in Hamilton, Ontario. We included participants ≥50 years with osteoporosis who understood English or attended with a caregiver. Our primary outcome was feasibility defined by recruitment rate (criteria for success 90%), length of time to complete the App by a non-healthcare professional (≤15 minutes), and safety/challenges of using the App. Our secondary outcome was to conduct an exploratory analysis between osteoporosis management (osteoporosis medication, vitamin D and calcium) and total Fit-Frailty score. Thirty participants were approached during a routine clinic visit and 25 agreed to participate (mean age 72.2±11.2; 88% female; 44% had higher education). The mean Fit-Frailty score was 0.24±0.14; scores ≥0.25 indicate frailty. Five chose not to participate citing other time commitments. The mean time to complete the App was 15.48±6.6 minutes with no adverse events. Challenges included the need for a private room and space to perform the gait assessment. We found no association between osteoporosis management and Fit-Frailty score (p>0.05). Despite not meeting our feasibility criterion for recruitment, the App was a feasible tool to measure frailty in a non-geriatric clinic.

Abstract citation ID: igad104.2808

### COMPARISON OF SENSOR BASED CONTINUOUS ADL ASSESSMENTS TO CARE-GIVER ADCS-ADL SURVEY RESULTS

John Fitch[1], Rachel Williams[1], Dennis Myers[2], and Michael Devin[1], *1. Birkeland Current, Waco, Texas, United States*, *2. Baylor University, Waco, Texas, United States*

This paper presents analysis results between the Birkeland Current (BC) Sovrin IoT in-home sensing system and monthly ADCS-ADL survey data for Care Recipient/Caregiver (CR/CG) pairs (N=117) as part of a Phase II NIA SBIR study (R44AG065118) conducted from 2021 to 2023. The analysis provides correlation analysis between overall BC ADL Scores and ADCS-ADL scores as well as comparisons to IADL/BADL scores. The CR population was screened to represent mild to mid-level cognitive decline and was continuously monitored in home and assisted living environments for up to 18 months. The BC system demonstrates significant correlation with the ADCS-ADL score at the level of sensitivity of the ADCS survey and subfactor results. The BC system is able to demonstrate sensitivity to a single point change in the two-factor BADL score as well as a single point change in the two-factor IADL score. Discussion of limitations of comparison to retroactive surveys is described. Novel ADL measurement dimensions are discussed for further study.

Abstract citation ID: igad104.2809

### COMPAS: AN APP TO PROMOTE PERSON-CENTERED COMMUNICATION BETWEEN PEOPLE LIVING WITH DEMENTIA AND THEIR CAREGIVERS

Barbara Delacourt[1], Michèle Masson-Trottier[2], Jade Dubuc[2], Catherine Dubé[3], and Ana Inés Ansaldo[2], *1. University of Montreal, Montréal, Quebec, Canada*, *2. University of Montréal, Montréal, Quebec, Canada*, *3. IUGM research center, Montréal, Quebec, Canada*

Introduction: People living with dementia (PWD) experience communication deficits as soon as the early stages of the diseases, deficits which significantly increase over time. In the last stages, constant communication breakdowns lead to reduced exchanges with caregivers, resulting in the isolation of both communication partners. These difficulties have negative impacts on the quality of life of PWD and their caregivers, who themselves face increasing burden. While communication difficulties in PWD are a core issue in care, few interventions to address this issue have been developed. Aims: The present study focused on COMPAs, an app designed to sustain communication between PWD and their caregivers. COMPAS has been shown to trigger emotional communication during the short co-viewing of personalized audiovisual material. Method: This was a pre-post intervention study with COMPAs in 2 long-term care centers. Seventeen caregivers used COMPAs in the context of daily routines over eight weeks with seventeen PWD. Data collection included specific questionnaires and semi-structured interviews to measure effects on communication and caregiver burden. Data analyses combined quantitative and qualitative approaches. **Results:** In caregivers, there was a significant improvement in personal achievement at work. Semi-structured interviews showed an improvement in communication in the dyad and a more empathetic approach to caregiving.

**Discussion:** These results indicate that the communication triggered by COMPAs breaks down communication barriers, by creating positive exchanges through personalized emotionally driven exchanges, while stimulating empathy and personalized interventions. Caregivers see COMPAs as an ecological tool to address communication barriers, while facilitating an empathetic caregiving relationship.

Abstract citation ID: igad104.2810

### DATA ANALYTICS FOR HEALTH-RELEVANT EVENTS DETECTION BASED UPON LONGITUDINAL FITBIT HEART RATE DATA

Prathamesh Dharangutte[1], Jie Gao[2], Elinor Schoenfeld[3], Zongxing Xie[4], Yindong Hua[4], and Fan Ye[4], *1. Rutgers University, New Brunswick, New Jersey, United States*, *2. Rutgers University, PIscataway, New Jersey, United States*, *3. Stony Brook School of Medicine, Stony Brook, New York, United States*, *4. Stony Brook University, Stony Brook, New York, United States*

The increasing prevalence of wearable devices enables low-cost, long-term collection of health relevant data such as heart rate, exercise, and sleep signals. Currently these data are used to monitor short term changes with limited interpretation of their relevance to health. These data provide an untapped resource to monitor daily and long-term activity patterns. Changes and trends identified from such data can provide insights and guidance to the management of many chronic conditions that change over time. In this study we conducted a machine learning based analysis of longitudinal heart rate data collected over multiple years from Fitbit devices. We built a multi-resolutional pipeline for time series analysis, using model-free clustering methods inspired by statistical conformal prediction framework. With this method, we were able to detect health relevant events, their interesting patterns (e.g., daily routines, seasonal differences, and anomalies), and correlations to acute and chronic changes in health conditions. We present the results, lessons, and insights learned, and how to address the challenge of lack of labels. The study confirms the value of long-term heart rate data for health monitoring and surveillance, as complementary to extensive yet intermittent examinations by health care providers.

Abstract citation ID: igad104.2811

### EARLY DEVELOPMENT OF BIO-EXPERIENTIAL TECHNOLOGY TO SUPPORT PERSONS WITH DEMENTIA AND CARE PARTNERS AT HOME

Elizabeth Rochon[1], Maimouna Sy[1], Mirelle Phillips[2], Erik Anderson[2], Evan Plys[3], Christine Ritchie[1], and Ana-Maria Vranceanu[1], *1. Massachusetts General Hospital, Boston, Massachusetts, United States*, *2. Studio Elsewhere, Brooklyn, New York, United States*, *3. Massachusetts General Hospital/ Harvard Medical School, Boston, Massachusetts, United States*

Bio-experiential approaches offer an interactive, multisensory, immersive environmental experience using technology and physical design to promote mind-body wellness. These designs have the potential to provide an in-home solution to help persons living with dementia and their care-partners manage challenges including agitation, conflict, stress, and difficult emotions (e.g., grief). This presentation discusses a partnership between an interdisciplinary academic team and a creative design and technology studio that has pioneered bio-experiential designs. This partnership's mission is to develop a free interactive, immersive bio-experiential platform that offers a positive and engaging experience for both persons living with dementia and their care-partner(s). Our team iteratively developed an initial prototype using multiple strategies rooted in user-centered design, including a literature review, design thinking workshop with the full team, and two focus groups with clinicians (N=10; neurologists, geriatric psychiatrists, geriatricians, neuropsychologists, social workers, occupational therapists). Early development emphasized safety to minimize risk of increased agitation or other neuropsychiatric symptoms (e.g., hallucinations, sleep impairments). Key recommendations to enhance safety and usability included simple calming visuals and sounds, personalized experiences, familiar stimuli, and multisensory engagement (e.g., music, tactile stimuli). Increasing accessibility was also discussed related to ease of use and availability of platforms (e.g., laptop, television). We are now preparing for "live" iterative usability testing of our prototype (i.e., beta testing) with a diverse group of persons living with dementia and care-partner(s) before efficacy testing. The larger goal is implementation and wide dissemination as well as adaptations for other neurodegenerative conditions. *Dr. Vranceanu and Ritchie are co-senior authors.

Abstract citation ID: igad104.2812

### ENGAGING OLDER ADULTS VIA SOCIAL MEDIA DURING A PANDEMIC: SUCCESSFUL LEARNING EXPERIENCES FOR STUDENTS AND ADULTS

Krista Cox[1], Tracey Zeiner[2], Jennifer Moore[3], LaVona Traywick[4], and Robin McAtee[5], *1. A. T. Still University, Fort Smith, Arkansas, United States*, *2. Arkansas Colleges of Health Education School of Occupational Therapy, Fort Smith, Arkansas, United States*, *3. AR Colleges of Health Education, Fort Smith, Arkansas, United States*, *4. Arkansas Colleges of Health Education, Fort Smith, Arkansas, United States*, *5. University of Arkansas for Medical Sciences, Little Rock, Arkansas, United States*

The Arkansas Colleges of Health Education and the Arkansas Geriatric Education Collaborative collaborated on a project to educate senior adults about the use of assistive technologies (AT) for self-care, home management, community engagement, and leisure participation to help them live safely and independently. Occupational and physical therapy students collaborated with 10 community dwelling senior adults to research, develop, and disseminate 27 video vignettes shared via Facebook. These vignettes were developed and shown during a time when senior adults were encouraged to isolate during the pandemic. As of January 2023, the outcomes of sharing the 27 video vignettes on Facebook included: a total of 17,204 views, the most viewed was "Lighting and Magnification" at 1,000+ views, followed by "Sewing" at 992 views, the least viewed was "Fishing" at 147 views. Older adult viewers feedback included: contact with ACHE to ask how to purchase the assistive devices; sharing the information gained with their family members and others; purchase of devices; providing a resource for AT assistance for OA. Additionally, the senior adults that participated in the project reported a better understanding of the

AT after presenting in the videos. In conclusion, these results indicate that creating the video vignettes and sharing them on Facebook had an extensive reach beyond the 27 individual sessions. This also demonstrated that the viewers were actively engaged as noted by their questions and comments.

Abstract citation ID: igad104.2813

### EVALUATION OF A TECHNOLOGY-BASED EXERCISE PROGRAM FOR OLDER ADULTS IN THOSE WITH CHRONIC MEDICAL CONDITIONS

Sarah Lieber[1], Jerad Moxley[2], Joseph Sharit[3], Peter Pirolli[4], M. Cary Reid[2], and Sara Czaja[2], *1. Hospital for Special Surgery, New York City, New York, United States*, *2. Weill Cornell Medicine, New York City, New York, United States*, *3. University of Miami, Miami, Florida, United States*, *4. Florida Institute for Human & Machine Cognition, Pensacola, Florida, United States*

Although physical exercise is known to be beneficial, most older adults do not engage in recommended levels of physical activity, particularly those with chronic medical conditions. In an exploratory analysis, we evaluated the efficacy of a technology-based exercise program (Fittle) for sedentary community-dwelling adults ≥60 years of age with social support features in a subset of participants with ≥2 self-report chronic medical conditions. Participants in the Fittle condition received group training and were placed into teams to complete exercises at home via a computer tablet. They also were able to "chat" with other team members. Participants in the control condition were directed to exercise websites designed for older adults. A total of 181 participants with a mean age 70.1 years were randomized to the intervention or control condition. Participants were predominantly women (80.1%) and came from diverse backgrounds, with 13.4% self-identifying as Black or African American and 12.7% as Hispanic or Latino. Among 93 participants with ≥2 self-report chronic conditions, individuals in the intervention (versus control) arm described greater social support ($t(162)=1.97$, $p=0.03$) and less loneliness ($t(163)=-1.97$, $p=0.03$) at 6 months; there was a non-significant trend for higher self-report physical activity in the Fittle arm ($t(166)=1.17$, $p=0.12$). Fittle improved perceptions of social support and loneliness in older adults with chronic medical conditions. Further study is needed in a larger dedicated cohort of older adults with chronic medical conditions to determine Fittle's efficacy in this population.

Abstract citation ID: igad104.2814

### FEASIBILITY AND VALIDITY OF WEARABLE TECHNOLOGY FOR ASSESSING MOBILITY AMONG HOSPITALIZED OLDER ADULTS

Paulo Roberto Carvalho do Nascimento, Renata Kirkwood, Mylinh Duong, Lauren Griffith, and Marla Beauchamp, *McMaster University, Hamilton, Ontario, Canada*

The need for strategies to promote mobility during acute care hospital stays among older adults is increasingly recognized. Wearable technologies show great potential for this purpose. The objective of this study was to determine the most accurate wearable sensor device and location for capturing in-hospital mobility in older patients. Twenty-five older adults (79.6±8.1 years) admitted to a hospital medical care unit volunteered to test the feasibility and concurrent validity of the ActiGraph wGT3X-BT, Mox1, MetaMotionC and Fitbit Versa. All 25 enrolled patients wore the devices simultaneously on the wrist, hip, and ankle while undertaking a mobility activity protocol consisting of supine lying, sitting, and standing tasks. Participants also performed the Timed Up and Go (TUG) and 10-meter walk test (10MWT) wearing the devices. A trained physiotherapist supervised the performance of the tasks. Most participants (58%) preferred to wear the device on the ankle. Overall, the ActiGraph and Mox1 were the easiest to set up and download. The ActiGraph was the most reliable device, retrieving 100% of the collected data. Regarding body posture, the thigh-worn ActiGraph algorithm accurately classified 78% of sitting and lying postures, as well as 84% of standing postures, indicating high overall accuracy. After implementing the recommended low-frequency filter extension for slower gait speeds, the ankle-worn ActiGraph showed the narrowest limits of agreement and observations closer to zero for step count during the TUG and the 10MWT. These findings lay the foundation for subsequent work to validate wearable devices for monitoring "free-living" mobility in this population.

Abstract citation ID: igad104.2815

### FEASIBILITY OF AN AUTOMATED SELF-ADMINISTERED 24 HOUR (ASA-24) DIETARY ASSESSMENT TOOL IN OLDER ADULTS

Hillary Spangler[1], Tiffany Driesse[1], Michael Fowler[2], David Lynch[1], Danae Gross[3], Xiaohui Liang[4], and John Batsis[1], *1. UNC School of Medicine, Chapel Hill, North Carolina, United States*, *2. UNC Chapel Hill, Chapel Hill, North Carolina, United States*, *3. University of North Carolina, Chapel Hill, North Carolina, United States*, *4. University of Massachusetts Boston, Boston, Massachusetts, United States*

**Background:** Certain dietary patterns impact trajectories of healthy aging. However, dietary assessment tools can be challenging to use. With the increased technology use in older adults, we aimed to explore the feasibility of completing an online, Automated Self-Administered 24-hour (ASA-24) dietary assessment tool. **Methods:** We conducted a randomized, crossover study of n=20 community-dwelling older adults' (>65 years) preference for self-administered (SA) versus research assistant-administered (RAA) ASA-24. Participants were recruited via ResearchMatch.com and randomly allocated 1:1 to each group and completed either an SA or RAA ASA-24. One week later, the alternative sequence was completed. After each session, participants completed an online 11-item feasibility survey (Likert-scale;1-5, strongly disagree to strongly agree). Mean and standard deviations were reported for each question. **Results:** Mean age was 69±3.5 years (90% females), and no differences were observed for sex, age, race, ethnicity, education, or income. Neither group felt a need for RA assistance ($p=0.34$). However, both groups felt the system was easier to follow with a research assistant (RAA 4.4±1.3 vs. SA 4.6±0.5, $p=0.65$), particularly when self-administered first ($p=0.04$). When conducting an SA ASA-24 first, there was less confidence the system could be learned quickly (SA 4.5±0.5 vs RAA 3.4 ±1.0, $p=0.001$). **Conclusion:** Older adults can complete dietary recall using a self-administered online assessment; however, use of a research assistant led to better accessibility. Order of administration method may impact perceived ease of use. Next steps include increasing sample diversity and using an RAA ASA-24 platform to identify dietary patterns that impact aging.

Abstract citation ID: igad104.2816

### FEASIBILITY OF CONTINUOUS PASSIVE MONITORING OF DAILY FUNCTION AND SYMPTOMS IN OLDER ADULTS WITH MULTIPLE MYELOMA

Deanne Tibbitts, Rebecca Silbermann, Eric Roeland, Jason Webb, Gabrielle Meyers, Jackilen Shannon, Jeffrey Kaye, and Kerri Winters-Stone, *Oregon Health & Science University, Portland, Oregon, United States*

The impact of cancer and cancer treatment on older adults' day-to-day health and functioning remains poorly characterized. Therefore, we evaluated the feasibility of using home-based, continuous passive monitoring of daily life functioning plus symptom surveys in older adults with multiple myeloma (MM). We assessed accrual, uptake of passive monitoring, retention, survey completion, and data completeness. Passive monitoring included infrared motion sensors (activity), door contact sensors (time out-of-home), watch (activity/sleep), bed mat (sleep), biometric scale (weight), electronic pillbox (medication-taking habits), and computer-use software (computer habits). A weekly online survey assessed health events (i.e., falls, hospitalization/ED visits, pain) and treatment-related symptoms (PRO-CTCAE). We enrolled 20 patients with MM of 55 referred (36% accrual) with a median age of 71 years (35% female); 8 patients enrolled with a care partner. Half of patients agreed to passive monitoring and weekly surveys; half completed surveys only. Refusal of passive monitoring was primarily due to concerns about privacy/intrusiveness. Retention over 17 months was 85% (3 dropouts from the survey-only group). Survey completion was 82% (missing reasons included "poor health" and "too busy") with a median completion time of 4.6 minutes. Data completeness for passive monitoring ranged from 66% for watch-based sleep (n=9 patients) to 93% for home-based activity (n=14 homes). In sum, passive monitoring of daily life functioning plus symptom surveys is feasible among older adults with MM. Future research will integrate daily functioning with symptom data to examine longitudinal variations in health and develop digital biomarkers to inform optimal timing of future interventional studies.

Abstract citation ID: igad104.2817

### FEASIBILITY OF USING SMARTPHONES AS A TOOL TO PROMOTE PHYSICAL ACTIVITY IN OLDER ADULTS

Josephine Elumeze[1], Erika Ortiz Sarmiento[1], Winnie Chu[1], Ciaran Friel[2], Ann Swartz[3], Alyson K Myers[4], Christian Nouryan[5], and Edith Burns[6], *1. Zucker School of Medicine Hofstra Northwell, New Hyde Park, New York, United States*, *2. Feinstein Institutes for Medical Research, Zucker School of Medicine Hofstra Northwell, Manhasset, New York, United States*, *3. University of Wisconsin-Milwaukee, Milwaukee, Wisconsin, United States*, *4. Montefiore/Einstein, Bronx, New York, United States*, *5. Northwell Health, Manhasset, New York, United States*, *6. Zucker School of Medicine at Hofstra/Northwell, Manhasset, New York, United States*

Smartphones have potential to allow asynchronous education, individualized feedback and coaching around physical activity (PA) though it's unclear if older adults make use of these capabilities. We conducted anonymous, semi-structured interviews/surveys on current PA levels, and experience with mobile technology and PA programs with a convenience sample of older adults, and family members/caregivers receiving care at a large geriatrics academic practice over a 3-month period. Data included demographics, experience with smartphone and PA applications. Current levels of PA were measured with the International Physical Activity Questionnaire (IPAQ). Comparisons of PA levels based on mild vs moderate vs vigorous activity. 25 completed surveys, most of whom were female (67%) and identified as non-White (68%), with a mean age of 80.8 years, range 67-96. The majority (84%) reported owning smartphones, 8% reported using PA apps, and 8% used wearable activity trackers (WATs). 44% walked at least 10 minutes per day, but only 32% reported attaining moderate PA, and 12% vigorous PA. Average time/day walking 1.1-hour, average time/day moderate activity 0.74 hours. Both younger (age≤80 years) and older (age>80 years) adults were similar in owning Smartphones (85% vs. 83%) and using WATs (8% vs. 8%). Only the younger group used PA apps (15% vs. 0%). The ubiquity of Smartphones especially among such a demographically heterogeneous group suggests this may be a viable tool for increasing PA among a wide range of older adults. Next steps should address low rates of PA app use, intergenerational differences, and the possibility of incorporating socialization with technology.

Abstract citation ID: igad104.2818

### IMMERSIVE VIRTUAL REALITY INTERVENTIONS FOR OLDER ADULTS: A SERIES OF META-ANALYSES

Allison Walden[1], Christopher Griffith[2], Payton Downey[2], and Leilani Feliciano[1], *1. University of Colorado Colorado Springs, Colorado Springs, Colorado, United States*, *2. University of Colorado at Colorado Springs, Colorado Springs, Colorado, United States*

Many older adults desire to age in place, a term used to describe living independently versus transitioning to a residential care facility. Virtual reality (VR) has the potential to positively impact the functioning of older adults as an assessment or intervention to help compensate for lost or declining functions. VR captures a wide variety of technologies ranging from non-immersive to fully immersive, designed to give the user a feeling of presence. To date, VR interventions have been used with older adults including improving health outcomes, improving functional abilities, and for neuropsychological purposes. A handful of meta-analyses (M-As) attempt to summarize the overall effectiveness of VR interventions with older adults, but many contain methodological issues and cover the gamut of VR technology. The present M-A fills a gap in the literature by assessing the literature on immersive VR interventions for older adults. Sixteen articles met inclusion for meta-analysis, and 41 effect sizes (ES) were calculated. ES were grouped by category, neuropsychological, health/mental health, functional, and eight meta-analyses were performed with one exploratory analysis. Results revealed small, significant ES for VR interventions on measures of global cognition, simple attention tasks, executive functioning tasks, anxiety ratings, and pain ratings. Nonsignificant ES were found for stress ratings, gait speed, depression ratings, and TUG task (exploratory M-A). Continuing to develop affordable, safe interventions to assist with aging in place is important as the number of older adults continues to increase. Immersive VR is a promising avenue to continue exploring to better assist the aging population.

Abstract citation ID: igad104.2819

### INTERACTIVE VOICE ASSISTANT ROUTINES: A USABILITY STUDY

Marcia Shade[1], Changmin Yan[2], Julie Blaskewicz Boron[3], and Valerie Jones[2], *1. University of Nebraska Medical Center, Omaha, Nebraska, United States*, *2. UNL, Lincoln, Nebraska, United States*, *3. UNO, Omaha, Nebraska, United States*

Musculoskeletal pain is prevalent among older adults and is associated with disability and social isolation. This study explored the feasibility of using conversational voice assistant (CVA) routines for proactive interaction and pain reduction with older adults. A 12-week randomized controlled pilot trial was conducted with adults aged 60 years and older who self-reported living alone and chronic musculoskeletal pain. Participants (N=37) were randomly assigned to a standard routine or an enhanced routine group. Data were collected on participants' CVA engagement, self-reported pain, and system usability. Overall, older adults-initiated morning routines more frequently than the evening routines. The enhanced group initiated more morning routines (M=50.01; SD=33.07) than the standard group (M=16.11, Z= -1.70, p<.05, r= -.28). Participants reported significant post-study pain reduction, t(35)=1.84, p<.05, r= .30 (baseline mean=3.60, SD=1.93; post-study mean=3.19, SD=1.97); there were no significant group differences in amount of pain reduction. The enhanced group participants rated the system usability as good (Mean=74.81, SD=9.28); those in the standard condition reported a lower-tier rating of "OK" (Mean=65.88, SD=12.76), t(31)= -2.29, p<.05, r=.37. System usability predicted pain reduction (B= -0.55, t(13)= -2.46, p<.05) in the enhanced condition (R2=.30, F(1,14)=6.07, p<.05), but the association was non-significant in the standard condition (R2=.14, F(1,15)=0.29, p=.60). These results show that the CVA routines were feasible to use with older adults, and resulted in perceived pain reduction. Future creation of tailored routines based on the older adults' pain characteristics, personality, and interaction preferences when alone, may further increase engagement.

Abstract citation ID: igad104.2820

### SMOOTHING SPLINE ANOVA MONITORING PHYSICAL ACTIVITY AND FALL RISK IN COMMUNITY-DWELLING OLDER ADULTS

Rui Xie, Lulu Chen, Joon-Hyuk Park, Jeffrey Stout, and Ladda Thiamwong, *University of Central Florida, Orlando, Florida, United States*

Emerging sensing techniques, which are easily transportable, user-friendly, and cost-effective, may reveal new possibilities in monitoring daily objectively measured physical activity (PA) and fall risk appraisal (FRA) identification. We used a Smoothing Spline Analysis of Variance (SS-ANOVA) to profile the daily PA using accelerometer-based device across FRA groups. Using the BTrackS system (static balance assessment) and a short Fall-Efficacy Scale-International, 124 participants (mean age 75, range 60-96, 77% female, 72% no history of falls) were categorized into four different FRA groups: rational (46.8%, low fear of falling (FOF) and normal balance), irrational (17.7%, high FOF and normal balance), incongruent (19.4%, low FOF and poor balance), and congruent (16.1%, high FOF and poor balance). The vector magnitude (VM) and the number of steps were extracted per second from an average 10 (SD=4.89) consecutive days of PA recording. Daily (0-24h) temporal trend was analyzed from 1.7 million records. Groupwise, PA of the congruent group was significantly lower than other three groups, while the rational group had the highest PA based on 95% Bayesian C.I. It is evident that there is a periodic trend in PA; in particular, inactive during night (12-5am), increasing trend in the morning (5am-noon) and peak at noon, moderately decrease in the afternoon (noon-6pm), and significantly decline in the evening. No significant gender differences in PA were found. These results indicate that FOF associated with poor balance contributes to low PA, highlighting the necessity of balance enhancing intervention and cognitive reframing to promote PA in older populations.

Abstract citation ID: igad104.2821

### SOCIAL ISOLATION IN OLDER ADULTS POST HIP SURGERY IS CORRELATED WITH MOBILITY AND PHYSIOLOGICAL INDICATORS

Faranak Dayyani[1], Charlene Chu[1], Ali Abedi[2], and Shehroz Khan[3], *1. University of Toronto, Toronto, Ontario, Canada*, *2. University Heatlh Network, Toronto, Ontario, Canada*, *3. Toronto Rehabilitation Institute, Toronto, Ontario, Canada*

Hip fracture affect approximately 30,000 people in Canada and more than 300,000 people in U.S., annually. Older adults post hip-fracture surgery experience social isolation along with reduced physical mobility, changes in sleeping patterns, reduced indoor and outdoor activities, and other physiological changes once they are discharged form inpatient rehabilitation. Our team has developed MAISON (Multimodal AI-based Sensor platform for Older iNdividuals), a cloud-based multimodal sensor system that supports the collection of physiological, ambient and contextual data from various smart devices. Currently, MAISON consists of a smart watch, a smart phone, a motion sensor and a sleep mattress. Using MAISON, we have collected 24 weeks of raw acceleration data, heartrate, step count, frequency of indoor motion, GPS and sleep metrics from three older adults post-hip surgery living in the community. Statistical and domain-specific features were extracted from the collected data with a time window of one day, including average heartrate, maximum acceleration, total sleep time, total steps taken, and average number of exits. Additionally, clinical data is collected from the participants on a biweekly basis consisting of three questionnaires (i.e., Social Isolation Scale (SIS)) and two physical tests. The correlation between features from sensor data and clinical data was performed using Spearman coefficient, which showed a strong positive correlation (>0.5) between the SIS and number of exits, variance of acceleration, total sleep time, and heartrate variance. Various correlation values were found between features from all the sensors and SIS, indicating the usefulness of multimodal sensors for this application.

Abstract citation ID: igad104.2822

### THE RELATIONSHIP BETWEEN SELF-REPORTED QUALITY OF LIFE AND SMARTPHONE GPS DATA

Kyungmi Lee[1], Andres Azuero[2], Sidharth Kumar[2], Frank Puga[2], George Demiris[3], Christine Ritchie[4], Alexi A. Wright[5], and J. Nicholas Dionne-Odom[2], *1. Case Western Reserve University, Cleveland, Ohio, United States, 2. University of Alabama at Birmingham, Birmingham, Alabama, United States, 3. University of Pennsylvania, Philadelphia, Pennsylvania, United States, 4. Massachusetts General Hospital, Boston, Massachusetts, United States, 5. Dana-Farber Cancer Institute, Boston, Massachusetts, United States*

Patients with advanced cancer and their family caregivers often experience poor quality of life. Self-reported measures are commonly used to quantify quality of life of family caregivers but may have limitations such as recall and social desirability bias. Variables derived from passively obtained smartphone GPS data are a novel approach to measuring quality of life that may overcome these limitations, and enable detection of early signs of deteriorating mental and physical health. The purpose of this study was to examine the temporal directionality of the correlations between self-reported quality of life (i.e., PROMIS Global 10: physical and mental health domains) at 3 timepoints and passively acquired smartphone GPS data among 7 family caregivers and 4 advanced cancer patients over a 12-week period. We extracted variables (i.e., total distance, time spent at home, transition time, and number of significant locations) with algorithms applied to the raw GPS sensor data. Uncertainty measures for the correlation estimates were computed as uncorrected 95% Bootstrap confidence intervals. We found a medium-to-large correlation between quality of life and the daily smartphone GPS data averaged by week in certain time periods (e.g., temporal correlations between physical health and transition time for 0–6 weeks; r's ranging from 0.30 to 0.53). Additionally, we found a medium-to-large correlation between quality of life and within-person variability (standard deviation) in daily smartphone GPS data. Our findings indicate that variation in within- and between-person behavior patterns captured by GPS data may signal potential changes in individuals' physical and mental health outcomes.

Abstract citation ID: igad104.2823

### THEORETICAL FRAMEWORK FOR AN ARTIFICIAL INTELLIGENCE–BASED COMPREHENSIVE GERIATRIC ASSESSMENT

Adam Golden, *University of Central Florida College of Medicine, Orlando, Florida, United States*

Comprehensive Geriatric Assessment (CGA) is a process where healthcare professionals evaluate and develop a management strategy for older adults who may have multiple multifactorial illnesses and geriatric syndromes. CGA has traditionally been a labor-intensive process that assesses many factors at a specific point in time. Its availability and feasibility in busy clinical settings is remains limited. Artificial Intelligence (AI) can potentially expand the accessibility and effectiveness of this currently limited resource. By integrating large numbers of longitudinal data sets, AI can evaluate in real time a broad array of ever-changing factors that include medical assessments, diagnostic testing/imaging, laboratory analysis, nursing evaluations, behavioral health interventions, social determinants, electronic monitoring, wearables, community resources, and caregiver support. A geriatric AI framework will need develop computational models that integrate changing biologic, sociodemographic, and mental health factors. The framework could also account for the changing availability of local resources and plan eligibility. The integration of large longitudinal datasets would allow for prediction modeling related to diagnosis, management strategies, and outcomes. Treatment protocols can be automated too. Machine learning will allow for the adjustment of medical alert thresholds to minimize alert fatigue that plaques current electronic health record systems. Just-in-time information can identify appropriate home technology equipment and referrals for community services. An AI framework potentially offers a more comprehensive and longitudinal geriatric evaluation that avoids delays in care while waiting for a single physician to "get to see the patient."

Abstract citation ID: igad104.2824

### UNDERSTANDING THE COMPLEXITY AND USE CHALLENGES OF MEDICARE PLAN FINDER FOR OLDER ADULTS

Hye Soo Lee[1], Maurita Harris[2], and Wendy Rogers[3], *1. University of Illinois--Urbana-Champaign, Champaign, Illinois, United States, 2. Wilfrid Laurier University, Brantford, Ontario, Canada, 3. University of Illinois Urbana-Champaign, Champaign, Illinois, United States*

It is critical for older adults to find an affordable health plan that meets their needs. The Medicare.gov site provides a Medicare plan finder (MPF), which is an online tool designed to assist individuals to find a Medicare plan that is most suitable for them. The MPF was updated in 2021 but remains complex and may be especially difficult for older adults to use, given age-related changes in cognitive abilities. To identify potential use challenges for MFP, we documented the complexity of the steps required for accomplishing the goal of finding a plan. We completed a task analysis using two use-case scenarios to illustrate potential paths one could take in using MPF. Both use cases were set to enroll in the same plan to explore how and why their paths could differ, focusing on whether they have prior information and experience with Medicare and MPF. The number of steps taken by these two use cases ranged from 10 steps (experienced user) to 32 steps (novice user), due to the different needs they had for using MPF. The results imply that the one-for-all design of MPF may not be optimal for meeting the different needs of users. We propose two solutions to reduce the number of steps for using MPF. First, users need to be able to access the functions they want from the start (e.g., using quick links from the initial page). Second, users would benefit from learning what they need to know and prepare before they start using MPF.

Abstract citation ID: igad104.2825

### WHAT DO OLDER ADULTS THINK ABOUT ELECTRONIC PAIN APPS? USING A HEALTH LITERACY FRAMEWORK TO MEASURE QUALITY

Mary Milidonis[1], Hayley Rupp[1], Patrick McGinty[1], and Karen Kopiera-Frye[2], *1. Cleveland State University, Cleveland, Ohio, United States, 2. New Mexico State University, Las Cruces, New Mexico, United States*

Mobile pain applications (apps) may improve health communication. Mobile health apps do not always consider the needs of older adults. This project examines perceptions of pain apps on smartphones among older adults. The sample consisted of 23 older adults (M age = 64 years), with majority female (83%), predominantly Caucasian (91%), and majority some college education (65%). Respondents answered questions about 5 free pain apps using the Mobile Application Rating Scale (MARS). The MARS assesses descriptive and technical aspects of an eHealth apps using five categories including four quality scales: engagement, functionality, aesthetics, and information and one subjective quality scale. Participants currently had pain with range of 3 months to 12 years. The Single Item Health Literacy Screener identified 17 people have never asked for help reading health information; all participants scored 100% on the Rapid Estimate of Adult Literacy in Medicine Short Form. Participants self-reported an average of 2.6 comorbidities on the Functional Comorbidity Scale. The participants overall MARS rating ranged from 1-5; 11 participants rated pain apps very high (4-5). Important app characteristics identified by the older adults as critical for useable app included: Clear instructions, good navigation, and easy functionality. The elders reported that difficulty with loading the app and slow times would deter their use of the app. In terms of frequency of use, participants felt using the app was important weekly only if they experienced pain. These are important considerations in designing age-friendly technology for use with the frequent condition of pain among older adults.

### SESSION 7690 (POSTER)

### COGNITION (SRPP)

Abstract citation ID: igad104.2826

### A SYSTEMATIC REVIEW OF EFFECTIVE STRATEGIES OR INTERVENTIONS TO SUPPORT DRIVING CESSATION AMONG OLDER ADULTS

Anne Dickerson[1], Tadhg Stapleton[2], Isabel Margot-Cattin[3], Priscilla Harries[4], Isabelle Gélinas[5], Moon Choi[6], Lizette Swanepoel[7], and Carolyn Unsworth[8], *1. East Carolina University, Greenville, North Carolina, United States*, *2. Trinity College Dublin, Dublin, Dublin, Ireland*, *3. UNiversity of Applied Sciences of Western Switzerland (HES-SO), Lausanne, Vaud, Switzerland*, *4. Kingston University, Kingston Upon Thames, England, United Kingdom*, *5. McGill University, Montreal, Quebec, Canada*, *6. KAIST, Daejeon, Republic of Korea*, *7. Stellenbosch University, Cape Town, Western Cape, South Africa*, *8. Federation University, Churchill, Victoria, Australia*

Driving remains the primary mode of transportation and often the only mode for rural and suburban areas. However, research is clear we all outlive our ability to drive. Nevertheless, as community mobility is essential to health and quality of life, we need to study and invest in understanding how to use and learn alternative means of transportation, especially for the vulnerable aging. A systematic review was designed to examine the literature for evidence-based strategies or interventions to support driving cessation among older adults while maintaining community mobility. The team of 13 international researchers from seven countries used the Covidence software with support of an expert health sciences librarian. Using multiple search terms (e.g., driving retirement; driving cessation; driving transition; interventions; strategies; older adults), 7317 studies were imported for screening with 7059 eliminated; 205 full texts were assessed for eligibility by 2-3 members. With 187 excluded, 19 studies were included and examined by the two primary authors. Results demonstrate low evidence of effectiveness of programmatic interventions, although multiple strategies have been identified. Overwhelmingly, the evidence supports starting very early with discussions about the process of transitioning from driving to being a passenger; particularly true with progressive diagnoses such as dementia. This presentation will highlight the programs currently available, strategies that appear to be successful, and review current toolkits for supporting driving cessation as well as emerging interventions and strategies.

Abstract citation ID: igad104.2827

### DUAL SENSORY LOSS AND COGNITIVE TEST PERFORMANCE IN OLDER ADULTS IN INDIA

Ethan Wang, and Jennifer Deal, *Johns Hopkins University, Baltimore, Maryland, United States*

Dementia is rising in the global population, especially in low- and middle-income countries like India. Hearing and vision loss are risk factors for cognitive decline/dementia, but studies have primarily been in high income countries. This study aims to investigate the association between dual sensory loss and cognitive function with specific domains among the older Indian population. Data was from the 2017-2019 Wave 1 of the population-based Longitudinal Aging Study in India. The analysis sample consists of 27,975 individuals aged 60 years and older in 35 states and union territories. Dual sensory loss was determined by respondents' self-reported, perceived difficulty regarding hearing and vision function. Linear regression was used to model associations of dual sensory loss with global cognitive performance, summarized as a z-score, and in the domains of memory, orientation, arithmetic, and executive function, adjusted for demographics and health characteristics. Overall, 5.9% had hearing loss, 24.1% had vision loss, and 3.4% had both. In fully adjusted models, hearing loss only, vision loss only, and dual sensory loss were associated with a change of -0.08 (95%CI: -0.1, -0.04), -0.11 (95%CI: -0.12, -0.09), and -0.22 (95%CI: -0.27, -0.18) standard deviations, respectively. Associations were also observed for all domains. Poor hearing and/or vision loss is associated with lower cognitive performance across various cognitive domains in older adults in India. The association was strongest for those with dual sensory loss. Rehabilitative interventions for hearing and vision may be an additional resource in reducing dementia risk in India.

Abstract citation ID: igad104.2828

### HOUSING CONCERNS AND TRAJECTORIES OF KINLESS OLDER ADULTS WITH DEMENTIA

Tam Perry[1], Janelle Taylor[2], and Yasir Mehmood[1], *1. Wayne State University, Detroit, Michigan, United States*, *2. University of Toronto, Toronto, Ontario, Canada*

Housing preferences of older adults (e.g., to remain in residence, or relocate), housing options available, and housing needs related to changing physical and cognitive abilities are common concerns of aging. The challenges are possibly more complex for those living with dementia, who may have difficulty articulating housing preferences, and whose housing needs may include supportive care. For those with dementia who lack close kin, these challenges are likely significantly compounded. This paper focuses on the intersection of housing concerns for 64 participants in the Adult Changes in Thought (ACT) study who were kinless (no living spouse or children) when they developed dementia. All the ACT participants were 65+ when recruited. Everyone in this sample had received a research diagnosis, and their mean age at dementia onset (estimated at the midpoint between the research study that triggered the diagnostic evaluation, and the last research study before that -- usually one year before diagnosis) was 87 (Standard Deviation [SD] 7 years), with a median age of 86 and a range of 71-103.We present an analysis of their housing trajectories, as captured in chart notes from their medical records. We highlight multiple themes illustrating how housing intersects with health and social well-being and conclude by commenting on the potential of medical records for understanding and documenting issues related to housing.

Abstract citation ID: igad104.2829

### INCLUDING PEOPLE WITH COGNITIVE IMPAIRMENT IN HEALTH SERVICES RESEARCH: PROMISING PRACTICES AND LESSONS LEARNED

Alice Prendergast Boughrum, and Kristi Fuller, *Georgia State University, Atlanta, Georgia, United States*

Engaging impacted populations, including consumers, in the design of health policies and services is increasingly recognized and prioritized in the United States. However, people with dementia and other forms of cognitive impairment are underrepresented and have often been excluded from health services research. As the number of people worldwide who are living with some form of cognitive impairment is expected to rise dramatically in the coming years, it is critical that researchers across all areas of health services research consider including this population in their work and ensure decisions for exclusion are fully justified. That said, individuals with cognitive impairment constitute a vulnerable population and additional precautions and safeguards are warranted to ensure they are protected throughout and beyond their participation in research studies. The inclusion of people with cognitive impairment also adds complexity to studies, and appropriate research protocols are often challenging to design and implement. This poster will describe two studies conducted by researchers at Georgia State University that included populations with cognitive impairment. The content will cover background about the study populations, which varied in diagnosis and disease progression; methods for recruitment, screening, and consent; and helpful communication strategies to employ throughout the research process. The poster will also share lessons learned and promising practices identified for both studies.

Abstract citation ID: igad104.2830

### PREVALENCE OF DEMENTIA AND COGNITIVE IMPAIRMENT IN PUBLIC SENIOR HOUSINGS: A SYSTEMATIC REVIEW AND META-ANALYSIS

Soobin Park[1], Takashi Amano[2], Sojung Park[3], and BoRin Kim[4], *1. Washington University in St. Louis, St. Louis, Missouri, United States*, *2. Rutgers University, Newark, New Jersey, United States*, *3. Washington University in Saint Louis, St. Louis, Missouri, United States*, *4. University of New Hampshire, Durham, New Hampshire, United States*

Older residents in public housing represent a particularly vulnerable population at risk for cognitive decline due to their disadvantaged socioeconomic and health-related characteristics which are well-known risk factors of dementia. This study aims to synthesize available evidence regarding the prevalence of dementia and cognitive impairment among public senior housing residents. We conducted a literature search for studies published in English in the following databases: PsychInfo, PubMed, MEDLINE, CINAHL, and The Cochrane Library. We focused on observational studies about residents of public senior housings that reported prevalence and/or incidence of dementia and/or cognitive impairment screened by assessment tools for detecting dementia equivalent cognitive impairment. A total of ten studies were included for a meta-analysis to estimate the overall prevalence of cognitive impairment and dementia. Seven of ten studies utilized screening tools to detect dementia equivalent cognitive impairment. Overall pooled prevalence of cognitive impairment or dementia was 20.4% (95%CI: 14.1-27.5). The subgroup analyses revealed the pooled prevalence of cognitive impairment among residents of public senior housing was 25.5% (95%CI: 22.0-29.1) and pooled prevalence of dementia was 13.5% (95%CI: 8.3-19.7). The subgroup findings suggest the prevalence of dementia may be higher among residents of public senior housing compared to their peers in non-public housing. Also, this population may be particularly susceptible to an unrecognized or delayed diagnosis of dementia due to the lack of formal detection strategies in public senior housing. More resources should be allocated for detection to ensure better support the residents.

Abstract citation ID: igad104.2831

### PROJECTION OF MEDICAID SPENDING ON PEOPLE WITH ALZHEIMER'S DISEASE AND OTHER RELATED DEMENTIAS

Howard Degenholtz, *University of Pittsburgh, Pittsburgh, Pennsylvania, United States*

As the US population is aging, the number of people with Alzheimer's Disease and Other Related Dementias (ADRD) is increasing. People with ADRD have high expenditures on medical care and long-term services and supports (LTSS). Medicaid is the largest single payor for LTSS, however, the literature on the demographic trends and epidemiology of people with ADRD does not offer projections of Medicaid spending. The goal of this study was to develop an estimate for future Medicaid spending for people with ADRD for the state of Pennsylvania. The approach taken was to develop a demographic projection of the size of the older population, then estimate the fraction of people with ADRD who are eligible for Medicaid. Next, we used estimates of current spending trends to project future spending through 2025. Nearly all Medicaid participants with ADRD are dually eligible for both Medicaid and Medicare, however, Medicare managed care encounter data were not available for analysis. Thus, the prevalence of ADRD was constructed using Medicaid and Medicare fee-for-service (FFS) claims data. Two alternate estimates of the prevalence of ADRD among Medicare managed care enrollees were used (same as Medicare FFS, or slightly lower). Estimates were generated under several growth rates in per person spending on home and community-based services (HCBS). The results shows that growth in HCBS spending is the largest factor in predicting spending, leading to estimates ranging from $2.8 billion to $3.5 billion. Future research will extend the model to additional years.

### SESSION 7700 (POSTER)

### FAMILY CAREGIVING (SRPP POSTERS)

Abstract citation ID: igad104.2832

### CAREGIVING PUBLIC HEALTH SURVEILLANCE: EXPERT-GUIDED COLLABORATION FOR BRFSS MODULE REVISION

John Shean[1], and Benjamin Olivari[2], *1. Alzheimer's Association, Chicago, Illinois, United States*, *2. CDC, Atlanta, Georgia, United States*

The Behavioral Risk Factor Surveillance System (BRFSS) Caregiver Optional Module collects population-level data on caregiving prevalence, burdens faced by caregivers, and demographic information about care recipients. Developed in 2009, periodic reviews ensure the module remains current, data collected remain actionable, and encourage uptake of the module by state health agencies. In 2022, the Alzheimer's Association and the Centers for Disease Control and Prevention's Alzheimer's Disease Program formed an expert workgroup to examine the ongoing value and relevance of the module and to propose improvements. The thirteen-member workgroup included academic researchers, caregiving experts, health promotion directors, epidemiologists, survey methodologists, policy analysts, and BRFSS coordinators. Over four months, the workgroup used a consensus-building process to identify areas of agreement and disagreement which informed discussion. Among many factors, the workgroup assessed the existing module for accuracy and utility. Discussion centered on utilizing plain language in the module's questions, streamlining response options, and ensuring collection of a full scope of burden faced by caregivers, including caregivers of persons with dementia. Persons living with cognitive impairment and their caregivers were consulted during the process to ensure phrasing reflected their lived experience. The result was consensus on suggested revisions to the Caregiver Module, which was submitted to CDC for review and approval in April 2023. The revised module will be featured in this session. The robust process, involving a variety of stakeholders and perspectives, can serve as an efficient and effective model for ensuring sustainability and data-informed action using population health surveillance.

Abstract citation ID: igad104.2833

### CONDUCTING A COUNTYWIDE ALZHEIMER'S DISEASE AND RELATED DEMENTIAS CAREGIVER LANDSCAPE ANALYSIS

D'Artagnan Robinson[1], Noel Barragan[1], Monique Parrish[2], Donna Benton[3], Mariana Reyes[1], Susanna Mage[3], and Tony Kuo[1], *1. Los Angeles County Department of Public Health, Los Angeles, California, United States*, *2. LifeCourse Strategies, Orinda, California, United States*, *3. University of Southern California, Los Angeles, California, United States*

In Los Angeles County (LAC), approximately 1 in 5 caregivers provide care to older adults with dementia and cognitive impairment. By 2040, a projected 1.5 million Californians will be living with Alzheimer's disease and related dementias, foreshadowing an unprecedented need for family caregivers across the state. In 2021, the LAC Department of Public Health launched the Centers for Disease Control and Prevention-funded LA BOLD initiative with the intent of developing a strategic plan to address dementia awareness and care in LAC, including improving local infrastructure for supporting unpaid family caregivers. To support this effort, LA BOLD commissioned the University of Southern California Family Caregiver Support Center to conduct a landscape analysis of the caregiver population in LAC. The analysis used a multi-method approach that included: primary analysis of community-based organization surveys, stakeholder interviews, focus groups with family caregivers, and secondary analyses of California Health Interview Survey and California Caregiver Resource Center System CareNav™ data and an environmental scan of best-practice caregiving models in use in other states. Results informed the development of three functional recommendations specific to LAC caregivers: 1) Leverage existing infrastructure to strengthen and build family caregiver services and supports; 2) Promote health care payer, provider, and system recognition of and support for family caregivers; and 3) Increase respite services for caregivers. This presentation highlights the methods and the findings from the landscape analysis, providing insights into the barriers and opportunities that a growing caregiver population of persons with dementia may pose to LAC.

Abstract citation ID: igad104.2834

### CONTACT: AN ONLINE COACHING TOOL TO EMPOWER CARE PARTNERS TO FACILITATE CONVERSATIONS IN NURSING HOMES

Gail Towsley, Alexandra Terrill, and Gregory Bayles, *University of Utah, Salt Lake City, Utah, United States*

In the post COVID-19 context, nursing homes struggle with staffing. The role of care partners (CPs; relative, friend) has never been more crucial, including the need to advocate for persons living with dementia (PLWD) and being an integral part of the care team. The online CONversaTion cAre Coaching Tool (CONTACT) empowers CPs of PLWD prepare for conversations that may include communicating what matters to the PLWD, need for a different type of care, or treatment decisions. We engaged CPs to complete and provide feedback on CONTACT, which includes four sections: 1) education about nursing homes to clarify key terms/processes 2) guidance on goal setting to identify what is important; 3) creation of action plan to achieve the goal; and 4) reflection on the impact of achieving the goal. CPs (n=8) completed a survey about using the tool (acceptability (4 items), appropriateness (4 items), feasibility (4 items); scale of 1=strongly disagree to 5=strongly agree), and participated in a focus group to discuss the tool's content and function. CPs favorably rated the tool. Mean ratings for acceptability ranged from 4.13 – 4.25, appropriateness 3.88 – 4.43, and feasibility 4.13 – 4.57. CPs reported that the tool was clear, simple, logically organized, informative, and it provided direction for starting a conversation. They liked the definitions of terms, large response buttons and that instructions were provided via video and in writing. Recommendations included additional videos depicting conversation prompts and scenarios, and utilizing community-based programs such as the Ombudsman office to disseminate CONTACT.

Abstract citation ID: igad104.2835

### FAMILY CAREGIVERS AT WORK: THE RELATIONSHIP BETWEEN WORK PRODUCTIVITY LOSS AND WORKPLACE SUPPORTIVE MEASURES

Mary Milnamow, and Louanne Bakk, *University at Buffalo - SUNY, Buffalo, New York, United States*

**Background:** Employment and economic security remain elusive for the majority of working family caregivers, especially for caregivers for older adults with complex health problems. Family paid leave remains unattainable in the United States requiring caregivers to work while providing critical support to older adults making them vulnerable for unemployment and financial hardships. While caregivers experience higher rates of absenteeism and presenteeism at work compared to their non-caregiving counterparts, research pertaining to the impact of supportive measures in the workplace is relatively unknown. **Methods:** This exploratory, cross-sectional study examined 1) the relationship between supportive workplace measures (i.e., employment stability, flexible work schedules), and productivity loss, absenteeism and presenteeism; and 2) the relationship between caregiver characteristics (i.e., traditional versus non-traditional) and supportive workplace measures, productivity loss, absenteeism, and presenteeism. Data from 2017 (n=2,652) and 2020 (n=1,938) waves of the National Survey of Caregivers (NSOC) were analyzed using the validated WPAI-CG measure. **Results:** Bivariate findings suggest an association between workplace flexibility and productivity loss (p<.001), absenteeism (p<.001), and presenteeism (p<.001); caregivers with flexible hours had greater productivity loss, absenteeism, and presenteeism compared to caregivers without flexible hours. Additionally, caregivers in traditional dyads reported higher work productivity loss (p<.001) and presenteeism (p<.001) compared to non-traditional dyads. **Conclusions:** Findings suggest flexible hours may not adequately address workplace issues experienced by family caregivers for older adults. As the number of older adults increases over the next decade, greater understanding of the best way to decrease work challenges for diverse caregivers is critical.

Abstract citation ID: igad104.2836

### FAMILY INVOLVEMENT IN BRITISH COLUMBIA LTC FACILITIES DURING A PANDEMIC: THE IMPACT OF COVID-19 RESTRICTIONS

Tracy Christianson[1], Evans Appiah-Kusi[1], Jordan Bremner[1], Andrew Filewich[2], Amna Qazi[1], and Colin Reid[2], *1. Thompson Rivers University, Kamloops, British Columbia, Canada*, 2. *University of British Columbia - Okanagan, Kelowna, British Columbia, Canada*

Family involvement in long-term care is linked directly to LTC resident quality of life and family satisfaction with care. The public health restrictions implemented to protect the vulnerable LTC population, the majority of whom have some type and level of dementia, represented a serious breach of families' ability to visit and care for their institutionalized loved ones. While much of the early research on the impact of the restrictions focused on the LTC resident and the paid care staff, the purpose of this study was to explore what impacts COVID-19 restrictions had on family involvement and family well-being in LTC facilities across the province of British Columbia. A mixed-methods design using a survey, interviews, and arts-based focus groups was the approach implemented. Families (direct, friends, legal guardians) of residents living in long-term care settings were invited to participate. Using a nested approach, online and paper-based surveys (N=55) were distributed, one-to-one interviews conducted (N=19), ending with two arts-based focus groups (n=4 each focus group). Qualitative interview data revealed six different but interconnected themes through the thematic analysis of the data. The themes were: 1) Quality of life, 2) Quality of care, 3) Mental Health concerns, 4) Communication, 5) Communication Strategies, and 6) The Rules. Quantitative results indicated positive relationships between satisfaction with care and a series of variables that aligned with the qualitative themes (example: satisfaction and treatment of family by LTC staff). Strategies offered by family participants to improve public health policies for future outbreaks are reviewed.

Abstract citation ID: igad104.2837

### INFORMAL CAREGIVING EXPERIENCES: CHALLENGES AND OPPORTUNITIES FOR AN AGE-FRIENDLY UNIVERSITY

Melissa Cannon, CJ Johnson, and Emily Winters, *Western Oregon University, Monmouth, Oregon, United States*

The growing Age-Friendly University (AFU) global network has been helping its members assess their institutions and identify action items for increasing age-friendliness. This research investigates one of the issues identified through previous assessment of an AFU as a weakness and area of opportunity, which is the lack of resources and information for campus community members who identify as informal or unpaid family caregivers. Data from N = 70 campus members (26% faculty, 18% staff, 56% students; 84% female, 16% male) were collected in early 2023 through a campus-wide online survey and analyzed by a team of researchers using quantitative analysis for responses to Likert-type items and thematic analysis for open-ended responses. Issues around physical demands, time demands, financial stress, and social and emotional health while caregiving were assessed. Results indicated that respondents are experiencing different types of stress and need additional support and resources as caregivers, particularly related to respite care, mental health services, financial support, and assistance with physical tasks such as housework and transportation. The findings from this research will be used to develop and share resources around caregiving broadly across the university campus, and to ensure that resources are culturally inclusive, particularly as nearly 25% of the enrollment of undergraduate full-time equivalent students is composed of students from a Hispanic/Latinx background. Findings will also be used to establish community partnerships to harness resources from outside the university, and ultimately to help support the campus community moving forward and to achieve a piece of the university's AFU vision.

Abstract citation ID: igad104.2838

### ONLINE MEDICAL RECORD (OMR) USE IN CAREGIVERS OF PEOPLE WITH DEMENTIA IN THE UNITED STATES

Megumi Inoue, Kyeung Mi Oh, Krista Beran, Emma Booker, and Naoru Koizumi, *George Mason University, Fairfax, Virginia, United States*

Objective: Studies have found that there are higher levels of stress among informal caregivers of individuals with dementia. As a result, it is important to seek ways to reduce their burden. Online Medical Records (OMRs), often referred to as patient portals, provide effective communication tools to caregivers and the use of OMRs have been found to have the potential to ease their stress levels. This study examined OMR usage in informal caregivers of people with dementia and identified factors that were associated with their usage. **Methods:** Data came from the HINTS, a National Cancer Institute (NCI)-sponsored survey of American adults. We merged data from HINTS 5 cycles 2, 3, and 4 administered between 2018 and 2020 into 1 data set. Our analytical sample included a total of 304 informal caregivers of people with Alzheimer's disease. The outcome variable was OMR use in the last 12 months, and independent variables included age, sex, race and ethnicity, education, household income, marital status, employment status, relationship to care recipient, and the number of chronic conditions. Data was analyzed using multivariate logistic regression. **Results:** 53% of caregivers have used OMRs at least once in the past 12 months. Caregivers with a higher level of education and greater number of chronic conditions are more likely to have used OMRs. **Conclusion:** Being able to access OMRs could reduce caregiver stress. There are certain caregiver characteristics that increase the likelihood of them utilizing this resource. Targeted approaches may be necessary to increase the range of use.

Abstract citation ID: igad104.2839

### PERCEPTIONS AND EXPERIENCES OF FAMILY CAREGIVERS OF OLDER FIRST-GENERATION CHINESE AMERICANS WITH DEMENTIA

Erh-Chi Hsu[1], Jenny Hsin-Chun Tsai[2], Clarence Spigner[2], and Kannie Chim[3], *1. Johns Hopkins University, Baltimore, Maryland, United States*, 2. *University of Washington, Seattle, Washington, United States*, 3. *International Community Health Services, Seattle, Washington, United States*

Family caregivers of individuals with dementia are precious assets whose health is related to the health of their care recipients. First-generation Chinese American older adults are a growing population; the research about their family caregivers' experience is scarce. Guided by the Health Belief Model (HBM) and the National Institute on Minority Health and Health Disparities (NIMHD) framework, this study examined how the Chinese sociocultural background shapes family caregivers' perception of dementia, caring behaviors, and caring experiences during the COVID-19 pandemic. Data were collected from 16 family caregivers through one-on-one interviews in three languages with the thematic analysis applied. Findings indicated most caregivers could not differentiate dementia from natural cognitive degeneration. Despite the unclarity, the cultural value of filial respect essentially obligated caregivers to unconditionally devote their time and efforts to provide care. Individual coping skills toward care burden were associated with their knowledge, attitude, access to supportive families/friends and reliable community-based organizations (CBOs), and access to government social services. The care recipients' motivation and functional status affected the difficulty of providing care. The pandemic disturbed families' care plans and limited community activities. In addition, the heightened Asian xenophobia atmosphere raised concerns for caregivers to be outdoors. Implications for practice include providing culturally appropriate educational resources on dementia care and incorporating CBOs to promote governmental service referral. Larger studies for Chinese American older adults born outside the US and studies of dementia care burden on their family caregivers' health are needed to address social and health inequities for historically marginalized cohorts.

Abstract citation ID: igad104.2840

### PPA TELE-SAVVY: RESULTS OF AN ONLINE PILOT INTERVENTION WITH CAREGIVERS OF PERSONS WITH PRIMARY PROGRESSIVE APHASIA

Darby Morhardt[1], Kathryn Maley[1], Lauren Dowden[1], Kaitlyn O'Neil[1], Deborah Dyslin-Kman[2], Angela Roberts[3], Allison Lindauer[4], and Tamar Gefen[1], *1. Northwestern University Feinberg School of Medicine, Chicago, Illinois, United States*, 2. *Northwestern Medicine, Chicago, Illinois, United States*, 3. *University of Western Ontario, London, Ontario, Canada*, 4. *Oregon Health & Science University, Portland, Oregon, United States*

Primary Progressive Aphasia (PPA) results from a neurodegenerative disease such as frontotemporal lobar degeneration or Alzheimer's disease and is characterized by a progressive loss of specific language functions eventually progressing to widespread cognitive decline consistent with generalized dementia. Most interventions available to dementia caregivers do not match PPA caregiving families' needs for tailored psychosocial support. This pilot study is an adaptation of Tele-Savvy, an evidence-based online psychoeducation caregiver program. Goals: (1) address communication and other cognitive and behavioral challenges facing informal caregivers of those living with PPA throughout disease progression, and (2) help caregivers achieve competence in their role. Nine spousal caregivers were recruited to a pilot mixed methods study, which included seven 90-minute weekly videoconference sessions, mindfulness exercises and homework assignments. Participants provided feedback throughout the intervention. Pre-post effects were assessed on PPA knowledge, mood, caregiver burden, perceived stress, competence, quality of life and dyadic relationship. A focus group was held 4 weeks post-intervention. Pre-post results revealed a post-intervention trend toward decreased depression and reported relationship strain. Thematic analysis of recorded videos and transcribed weekly sessions, in addition to an evaluation focus group revealed four themes: a) Increasing knowledge of PPA; b) Learning communication and connection strategies; c) Initiating care planning; d) Integrating intellectual and emotional aspects of PPA caregiving. Participants were provided findings for their feedback. This study demonstrated the feasibility of offering an adaptation of the Tele-Savvy intervention for caregivers of persons with PPA. An informed second pilot study will be held in Spring 2023.

Abstract citation ID: igad104.2841

### SOCAL SAVVY CAREGIVER: FOCUS GROUP INSIGHTS

Iris Aguilar[1], and Maria Aranda[2], *1. Florida International University, Miami, Florida, United States*, *2. University of Southern California, Los Angeles, California, United States*

Dementia due to Alzheimer's disease (AD) is a public health crisis in our nation. More than 1.6 million Californians provide unpaid care for a person with AD. Qualitative data from focus group discussions were obtained from unpaid/informal/family caregivers in a clinical trial testing the efficacy-effectiveness of Savvy Caregiver Express compared to the Savvy Caregiver Program. **Objectives:** 1) explore class participation motivations, 2) perceived class benefits, and 3) class improvement recommendations. **Methods:** We randomly selected and invited participants who had completed the class intervention cycle. Focus group subsample (n=25) was 84% female, mean age 54.48 years (28 to 80 years); 36% non-Latino white, 20% Latino, 8% African American, 4% Asian and American/Pacific Islander. 60% cared for a parent (44% mother and 16% father), 16% cared for a spouse/partner, 20% other relatives, and 4% friends. Focus group were transcribed, coded, and themes were created using inductive analysis. **Findings:** Themes identified for discussion topic #1: seeking to gain knowledge about AD and stages, peer support, and caregiver competence; #2 knowing AD stages, management of meaning, caregiving mastery, and self-care; and #3 audiovisuals improvements, class structure changes, printed materials, and ongoing virtual sessions. **Implications:** Participation in caregiving classes are critical to the improvement of quality of life for the person with dementia and caregiver. These findings offer actionable changes to improve the transformation of classes and adjust future materials or provide additional resources to help participants fully understand the course and better understand the motivations for increasing engagement with caregivers.

### SESSION 7710 (POSTER)

### HEALTH BEHAVIOR CHANGE

Abstract citation ID: igad104.2842

### "THERE'S A LOT OF FACTORS": UTILIZING I-POETRY TO UNDERSTAND VACCINE-HESITANT DECISION MAKING

Bryce Van Vleet, Heather Fuller, Andrea Huseth-Zosel, and Paul Carson, *North Dakota State University, Fargo, North Dakota, United States*

Despite higher vaccinations rates as compared to other age groups, many US older adults remain under-vaccinated against common, preventable diseases leaving them at increased risk of serious illness, hospitalization, or death posed from diseases including shingles, pneumonia, influenza, and novel coronavirus. This study investigates the underlying reasons older adults have for not getting vaccinated. Eight older adults (Mean Age = 73.86) selected from a larger interview study conducted in North Dakota were identified as being vaccine hesitant as indicated by receiving two or fewer vaccines from a list of five common vaccines including influenza, shingles, pneumococcal, and initial and booster doses of the COVID-19 vaccine. Participants were asked, "How do you decide about whether or not to get a specific vaccine?" Their open-ended responses were analyzed using the I-poetry qualitative methodology which combined participants' "I" statements into a poem for all eight participants that reflected their underlying motivations of vaccine hesitancy. This poem was structured in stanzas to highlight and convey overarching themes including: practical barriers, vaccine-alternatives, and skepticism. Practical barriers included allergies and concerns about side effects. Vaccine-alternatives highlighted a belief in natural immunity, vitamins, and exercise. Skepticism was directed at government agencies and the efficacy of a vaccine. These findings highlight potential benefits to providing medical professionals with first-person narratives from vaccine hesitant older adults which could help clarify sources of unease. Implications can be drawn for better understanding this at-risk population and developing tailored and effective interventions to bolster older adults' vaccine uptake.

Abstract citation ID: igad104.2843

### COMPARISON OF SEDENTARY BEHAVIOR GUIDELINES FOR OLDER ADULTS: A REVIEW OF LITERATURE AND QUALITY APPRAISAL

Amy Huang[1], Ellen Wang[2], Stephanie Sanger[1], Alexandra Papaioannou[1], and Isabel Rodrigues[1], *1. McMaster University, Hamilton, Ontario, Canada*, *2. University of British Columbia, Vancouver, British Columbia, Canada*

Most older adults (≥65 years) accumulate >8.5 hours/day of continuous sedentary time, which is associated with increased risk of metabolic syndromes and falls. The impact of increased sedentary time in older adults globally has prompted the development of national and international sedentary behaviour guidelines. The purpose of our review was to identify and compare national and international sedentary behaviour guidelines for older adults and appraise the quality of the guidelines to promote best practice in guideline development. We conducted our search in Medline, Embase, Global Health and relevant grey literature. We included the most updated guidelines for older adults written in English. AGREE II was used to assess the quality of the recommendations. We identified eight national and international sedentary behaviour guidelines for older adults. The guidelines were developed from reviews, cohort studies, knowledge user opinions, or other guidelines; all guidelines were based on low quality and certainty of evidence. The terms "sedentary behaviour" and "sedentary time" were used interchangeably, and the definitions of both terms were not consistent between guidelines. Six guidelines recommended a reduction in total time with one suggesting limiting sedentary time to < 8 hours/day. Three guidelines suggested reallocating sedentary time to light activity, with one recommending to stand-and-stretch every hour for 5-minutes. Most sedentary behaviour guidelines for older adults are based on low quality and low certainty evidence. Terminology, definitions, and recommendations were not consistent between guidelines. Further work is needed to develop evidenced-based recommendations specific to older adults.

Abstract citation ID: igad104.2844

### FIT AND STRONG! IN ILLINOIS: LESSONS LEARNED

Susan Hughes, Andrew DeMott, and Julie Bobitt, *University of Illinois at Chicago, Chicago, Illinois, United States*

This session will describe lessons learned from a major effort to disseminate Fit & Strong! (F&S) statewide in Illinois, a state with both strong urban and rural counties. F&S is an evidence-based program for older adults who experience lower extremity mobility challenges. It is recommended for dissemination by the Administration on Community Living and the Centers for Disease Control and Prevention and is currently offered in 32 states. We received a substantial ARPA contract from the Illinois Department of Public Health (IDPH) to disseminate F&S sustainably throughout Illinois, between July 2022 and June 2023. We have convened Referral Network and Sustainability Workgroups to guide the work. Our goal is to identify 111 community-based organizations (CBOs) who can enroll 1,667 older adults into the program. We have developed an extensive number of collaborators including the aging network and a large number of healthcare systems and plans. We are also collaborating with the Illinois Extension Program to reach underserved rural areas of the state. This extensive collaborative effort has enabled us to enroll 70 CBOs that plan to offer the program. This collaboration between aging and healthcare networks that is co-sponsored by the Illinois Department on Aging and IDPH appears to be enabling us to reach a considerable number of providers who previously were not aware of or offering F&S. We are collecting pre/post participant outcomes to evaluate program impact and examining state fall rates and uptake of lower extremity joint replacements. Lessons learned and preliminary findings will be discussed.

Abstract citation ID: igad104.2845

### THE RELATIONSHIPS AMONG VOLUNTEERING, EVERYDAY DISCRIMINATION, AND LIFE SATISFACTION IN TWO AGE GROUPS

Huei-wern Shen[1], and Yi Wang[2], *1. University of North Texas: Denton, Denton, Texas, United States*, *2. University of Iowa, Iowa City, Iowa, United States*

Experiencing discrimination is known to bring life dissatisfaction across different life stages. While volunteering benefits older adults in many aspects, including life satisfaction, the interplay among discrimination, volunteering, and life satisfaction in different age groups is unknown. To explore such relationships, the present study included a subsample of 5791 respondents who were 51+ from the 2016 Health and Retirement Study; among them, 3191 were 65 years (older group) and older and 2600 were between 51 and 64 years old (younger group). Volunteering referred to doing unpaid work for religious, educational, health-related, or other charitable organizations. Discrimination was measured by the six-item Everyday Discrimination Scale (e.g. In your day-to-day life, how often are you treated with less courtesy or respect than other people) and was dummy-coded due to skewness (never experienced vs. ever experienced). Controlling for health-related variables (e.g., ADLs/IADLs), SES (e.g., income), contextual features (e.g., urban/rural), and sociodemographics (e.g., gender, race), findings from multiple multivariate linear regression models showed that in the overall sample, discrimination was negatively associated with life satisfaction whereas volunteering promotes it. When considering the role volunteering played in the relationships between discrimination and life satisfaction, volunteering in the younger group did not mediate the negative relationship between discrimination and life satisfaction. Volunteering in the older group, however, significantly mitigated the negative impacts of discrimination on life satisfaction. Results highlight the possible extended benefits of volunteering in old age. Future research considering the health impacts of different types and levels of volunteering will be discussed.

Abstract citation ID: igad104.2846

### THE ROLE OF TRUST FOR DEMOGRAPHIC VARIABILITY IN VACCINE HESITANCY AND BEHAVIORS AMONG OLDER ADULTS

Andrea Huseth-Zosel, Heather Fuller, Melisa Hajdar, Emily Kinkade, and Paul Carson, *North Dakota State University, Fargo, North Dakota, United States*

Because of increased risks of infectious disease, understanding older adults' decisions related to trust and reception of vaccines is important. This study examines the associations between demographic characteristics, vaccine hesitancy, compliance, and the potential mediating role of uncertainty of who to trust. Seven hundred and thirty-nine older adults in North Dakota, oversampled from rural and lower vaccinated regions, completed a mailed survey about vaccine beliefs and behaviors. Participants reported demographic characteristics (age, gender, years of education, rurality, political ideology, and self-perceived physical health) and answered a 7-item scale of vaccine hesitancy. Vaccination behaviors were measured with an assessment of overall vaccine compliance, as well as measures of uptake for influenza, shingles, pneumonia, and SARS-CoV-2 immunizations. Uncertainty who to trust was assessed with the single-item "I am not sure who to trust when it comes to information about vaccines", which was rated from Strongly Agree (4) to Strongly Disagree (1) and used as a mediator. Bootstrapping analyses indicated uncertainty of who to trust partially or fully mediated associations of rurality, education level, and political leaning with vaccine hesitancy. Further, trust partially or fully mediated associations of education level, health, and political leaning with vaccine behaviors; however, trust did not account for the association between vaccine hesitancy and behaviors. Findings highlight demographic variations in older adults' vaccine beliefs and behaviors that may be partially explained due to uncertainty who to trust, indicating that practitioners should implement education and trust-building interventions, especially with rural-dwelling, conservative-leaning, and lower-educated older adults.

### SESSION 7720 (POSTER)

### LONG-TERM CARE (SRPP POSTERS)

Abstract citation ID: igad104.2847

### AN UNEQUAL WORK ENVIRONMENT: NURSING HOME STAFF PERCEPTIONS OF UNIT ORGANIZATIONAL CONTEXT

Alba Iaconi[1], Kaitlyn Tate[1], Tatiana Penconek[1], Yinfei Duan[1], Seyedehtanaz Saeidzadeh[1], Greta Cummings[1], Peter Norton[2], and Carole Estabrooks[1], *1. University of Alberta, Edmonton, Alberta, Canada*, *2. University of Calgary, Calgary, Alberta, Canada*

The organizational context in nursing homes is associated with quality of care and residents' quality of life. Our objective was to examine nursing home staff perceptions of unit organizational context. We conducted a secondary analysis of cross-sectional data. Participants were 3765 unregulated (health care aides (HCAs)) and 1130 regulated staff (managers, registered nurses, registered psychiatric nurses (RN/RPNs) and licensed practical nurses (LPNs)) from 91 nursing homes in Western Canada. We measured nursing home staff's perceptions of organizational context via the Alberta Context Tool (ACT), comprised of ten concepts: leadership, culture, evaluation, social capital, formal interactions, informal interactions, organizational slack (staff, space, time). Differences between groups were examined via one-way ANOVA. Managers reported significantly higher organizational context scores compared to all other staff members in care homes, followed by RN/RPNs. LPNs and HCAs reported the lowest scores. Specifically, LPNs reported the lowest scores on all organizational context measures except for structural resources and organizational slack space, for which HCAs reported the lowest scores. HCAs reported higher scores than LPNs on unit leadership, culture, evaluation, social capital. When compared with managers and RN/RPNs in the nursing home, LPNs reported the lowest SF-8 mental health scores (49.30 (10.05)) and HCAs reported the lowest physical health scores (48.53 (8.16)). Our work demonstrates the need for improvement of staffing and time for staff in nursing homes. Specific attention is needed to focus on modifiable organizational unit context factors for LPNs and HCAs, who are responsible for providing the majority of the direct care for residents.

Abstract citation ID: igad104.2848

### DON'T SAY GAY: LGBTQIA+ INCLUSION ON LONG-TERM CARE AND ASSISTED LIVING FACILITIES' WEBPAGES

Julie Maier[1], and Andrew Pickett[2], *1. Indiana University-Bloomington, Indianapolis, Indiana, United States*, *2. Indiana University-Bloomington, Bloomington, Indiana, United States*

Many LGBTQIA+ older adults experience a variety of health disparities resulting from a lifetime of minority stress. Due to experiences of discrimination, isolation, and stigmatization, LGBTQIA+ populations have higher rates of depressive symptomology and anxiety, as well as substance abuse when compared to heterosexual, cis-gender peers. They are also more likely to experience social isolation and have a lower average socioeconomic status. For older LGBTQIA+ adults who depend on long-term care, relative levels of facility inclusiveness have the potential to mitigate or exacerbate poor health outcomes. This study examines public signals of LGBTQIA+ inclusion by assisted living and long-term care facilities in the state of Indiana. Data was collected from a stratified-random sample of 100 long term care facilities, located in rural (n = 14), mixed (n = 24), and urban (n = 62) counties in Indiana. Each facility's website was reviewed for the presence of LGBTQIA+ inclusion, operationalized as inclusive images, symbols, messages, anti-discrimination policies, and equal opportunity employment statements or logos. The most observed signals were statements or logos indicating non-discrimination, including fair housing policies (59%) and equal opportunity employment messages (32%). These policies were most common among facilities that were part of a larger ownership group. Non-policy LGBTQIA+ inclusion symbols were rare (1%) and included a blog post with tips on finding an LGBTQIA+ inclusive care community. Findings suggest an overall dearth of LGBTQIA+ inclusive signals among older adult care facilities in Indiana.

Abstract citation ID: igad104.2849

### FACTORS ASSOCIATED WITH SERVICE UTILIZATION AMONG OLDER ADULTS WITH FUNCTIONAL OR COGNITIVE IMPAIRMENT

Yeon Jin Choi, Maria Aranda, and Jennifer Ailshire, *University of Southern California, Los Angeles, California, United States*

Social services (e.g., senior centers, adult day care centers) and home health services (e.g., in-home personal care, visiting nurses) have been found to provide numerous benefits, such as opportunities for social interaction, physical activity, cognitive stimulation, as well as personal care and regular medical check-ups for older adults. These services may have greater positive impacts among older adults with disabilities or impairments given their limited mobility and reduced capacities to perform daily tasks. However, as most previous studies have focused on the general population, our understanding of factors associated with service utilization among those with disabilities or impairments is limited. This study examined individual characteristics associated with social and home health service utilization among older adults with functional limitations or cognitive impairments using data from the Health and Retirement Study. Results from logistic regression models suggest that older age and being not married were positively associated with utilization of both social and home health services, while being foreign-born was negatively associated with the outcomes, adjusting for sociodemographic characteristics. Respondents with higher educational attainment tend to use social services more and are less likely to use home health services than those with less than a high school education. Rural residence was negatively associated with the utilization of social services, but not with home health services. Providing information on available services and enhancing access by increasing the number of service providers, transportation options, and financial assistance would promote utilization of supportive services among older adults with functional limitations and cognitive impairments.

Abstract citation ID: igad104.2850

### IDENTIFYING EFFORTS AND CHALLENGES OF MARKETING THE DEPARTMENT OF VETERANS AFFAIRS MEDICAL FOSTER HOME PROGRAM

Leah Haverhals[1], Chelsea Manheim[2], Mary Nunnery[2], and Cari Levy[3], *1. Department of Veterans Affairs Rocky Mountain Regional VA Medical Center, Denver, Colorado, United States*, *2. Rocky Mountain Regional VAMC, Aurora, Colorado, United States*, *3. University of Colorado, Denver, Colorado, United States*

The United States (US) Department of Veterans Affairs (VA) Medical Foster Home (MFH) program is a long-term care (LTC) option for Veterans needing nursing home-level care. In the MFH program, Veterans live in the home of a community-based caregiver who has been recruited and screened by VA staff. Since 2016, VA Offices of Rural Health and Geriatrics and Extended Care has expanded the program, and by 2026 the VA plans to have programs at every VA Medical Center. To describe current efforts to market the MFH program, we conducted a national survey of MFH staff between Dec. 2, 2021-Jan. 6, 2022. We sent invitations via email, including a website link to the survey. N=94 completed the survey. Three team members analyzed open-ended data, using an inductive and deductive approach to thematic analysis. Findings revealed that external marketing strategies primarily focused on recruiting appropriate caregivers. Efforts included in-person presentations to community groups, utilizing local media outlets, manning booths at local health and job fairs, and providing written materials about the program. Internal marketing efforts included approaching many different VA departments including inpatient social workers, case managers, and discharge planners to identify Veterans for the program, and holding in-service presentations. Challenges included MFH coordinators having minimal time to market the program and receiving inconsistent support from the VA Public Affairs Office depending on location. Findings will inform expansion of the MFH program, which is paramount as the demand for LTC increases concurrently with the aging Veteran population.

Abstract citation ID: igad104.2851

### LONG-TERM CARE CONCERNS AMONG CHILDLESS OR SINGLE CAREGIVERS TO PEOPLE LIVING WITH DEMENTIA

Elyse Couch, *Brown University, Providence, Rhode Island, United States*

It is estimated that 77% of people living with dementia receive help with self-care and household activities from informal caregivers (typically family members), meaning there is a significant proportion of the population not receiving informal support. In this qualitative study, we aimed to explore similarities and differences in family caregivers' concerns about organizing their own care should they develop dementia in the future between those who were childless and/or single and those who had a current partner and children. We interviewed 12 British current and former dementia family caregivers, 5 of whom were childless and/or single, 9 were women and 3 were men, with a mean age of 61. All 5 childless and/or single participants were women. Participants perceived a caregiver as vital for ensuring the person with dementia received the care they needed from services. Caregivers who were childless and/or single expressed concern that by not having someone to be their caregiver should they develop dementia in the future, they would be vulnerable to receiving poor care. In these cases, childless and/or single caregivers described the possibility of exploring options for suicide or euthanasia should they be diagnosed with dementia in the future. Similar sentiments were not expressed by caregivers with current partners and children. While this study uses a small sample size, these findings highlight persons without children and/or a partner have serious concerns about their future care, should they develop dementia. More research is needed to understand how to better support people with dementia without caregivers.

Abstract citation ID: igad104.2852

### MEDICAID ADULT DAY SERVICE CENTERS STAFFING AND SERVICE DIFFERENCES BY PROFIT STATUS

Eleanor Batista-Malat, and Kathleen Wilber, *University of Southern California, Los Angeles, California, United States*

Adult day service centers (ADSC) encompass a broad range of services, yet few studies have examined staffing and service differences in for-profit and non-profit centers. Previous long-term services and supports (LTSS) research has found that for-profit nursing homes have lower levels of staffing, however, this research has not extended to ADSCs. Data from the 2018 wave of the National Post-Acute and Long-Term Care Study were used to examine differences in staff hours per patient day (HPPD) and services offered in ADSCs by profit status. Only ADSCs that accept Medicaid were included because the majority of the responding ADSCs accept Medicaid (76.8%) and Medicaid acceptance and profit status are significantly related ($p<.001$). Using t-tests, there was a significant difference ($p=.002$) in total HPPD with higher HPPD in non-profit ADSCs, with a difference of about 13 minutes. Additionally, there were significant differences in the number of transportation ($p<.001$) and medical ($p<.001$) services offered by profit status, with for-profit ADSCs offering a higher number of services, although there was no difference in the number of social/behavioral services ($p=.122$). These findings suggest that ADSCs that accept Medicaid may be subject to similar incentive structures that lead to lower levels of staffing in for-profit LTSS. The higher number of transportation and medical services in for-profit ADSCs that accept Medicaid also suggests different financial models for for-profit and non-profit ADSCs. Future research should more closely examine factors associated with ADSC staffing levels and service provision, and how these measures are associated with quality of care.

Abstract citation ID: igad104.2853

### PERCEIVED WORK ENVIRONMENT AND OUTCOMES AMONG CARE STAFF IN LONG-TERM CARE SETTINGS DURING COVID-19

Eunhee Cho[1], Min Jung Kim[2], Kyung Hee Lee[2], Bada Kang[2], Jiyoon Jang[2], Jinhee Shin[3], Sameh Eltaybani[4], and Noriko Yamamoto-Mitani[4], *1. Yonsei University College of Nursing, Seoul, Republic of Korea*, *2. Yonsei University, Seoul, Republic of Korea*, *3. Woosuk University, Jeonju, Republic of Korea*, *4. The University of Tokyo, Tokyo, Tokyo, Japan*

Coronavirus disease (COVID-19) has severely affected residents in long-term care (LTC) facilities. However, how care staff in LTC facilities perceive their work environment during the pandemic and its potential impact on their work outcome are less understood. This study examined the associations between perceived work environment, staffing levels, and educational status of care staff and their general and COVID-19 specific work outcomes during the pandemic in LTC facilities. The study employed a cross-sectional, observational, correlational design. A total of 207 care staff were conveniently recruited from 30 LTC facilities in South Korea. The perceived work environment, staffing levels, educational status of care staff, and their work outcomes were collected using questionnaires. Multivariable binary logistic regressions were conducted, controlling for the characteristics of care staff and facilities included. Approximately 45% of the participants were either nurse aids or care workers, and 38% were registered nurses. Nearly half reported that their work environment was poor. Care staff who perceived their work environment to be poor were more likely to be dissatisfied with their work (odds ratio [OR] 15.40), experience high burnout (OR6.93), intend to leave the facility within a year (OR7.83), and experience increased overtime work due to COVID-19 (OR4.03). They were also less likely to perceive an immediate response to COVID-19 from their facility (OR0.45). The LTC work environment should be improved from its current state and government-led initiatives for infection control should be implemented to enable a better response to future public health crises in LTC settings.

Abstract citation ID: igad104.2854

### PROMOTING CO-WORKER RELATIONSHIPS IN LONG-TERM CARE FACILITIES: A SYSTEMATIC REVIEW OF THE LITERATURE

Kirsty Haunch, *University of Leeds, Leeds, England, United Kingdom*

Introduction: Co-worker relationships in long-term care settings are important. The work is physically and emotionally demanding. A largely unregistered workforce with variable preparation and training are expected to provide complex care to the most vulnerable members of society, with little external support. Often staff only have each other to rely on. Current evidence lacks insight into how co-worker relationships are established and what influences them. The aim of the review was to synthesise what is known about co-worker relationships more widely and its applicability to long-term care. **Methods:** A systematic review identified, evaluated, and synthesised published evidence associated with co-worker relationships in health and social care settings. PRISMA guidelines were followed. The COM-B model was used to identify key behaviours and associated triggers of co-worker relationships. **Results:** 37 papers were included. The majority of studies were conducted in the US (n=23), used descriptive study designs (n=30) and were in a hospital setting (n=29). Interdependent organisational and individual factors suggest co-worker relationships occur when staff are 'able' (have the social skills), are 'willing' (have the right values) and are 'supported' (effective leadership). **Discussion:** To date the focus has been on the 'willingness' of people and the 'support' they receive. Paying attention, the 'ability' (the social skills), which can be taught, is of equal, if not more importance. Social skills enable staff to navigate difficult and complex situations that arise, which preserves, and in some cases strengthens relationships. Social skills are particularly important when leadership is poor.

Abstract citation ID: igad104.2855

### RACIAL AND ETHNIC DIFFERENCES HOSPITAL DISCHARGE DISPOSITIONS FOR OLDER ADULTS: A PUBLIC POLICY PERSPECTIVE

Sunny Kang, *University of Baltimore, Baltimore, Maryland, United States*

This paper presents two empirical studies regarding racial disparities in older individuals' hospital discharge. The first study targeting factors associated with older Black patients' readmission to ambulatory care, examined 7,619 older patients from the 2016 National Ambulatory Medical Care Survey; the second study focused on race and mental disorders' impact on older patients' nursing home (NH) admissions upon hospital discharge, analyzing 186,646 older patients from 2007 to 2010 National Hospital Discharge Survey. We mainly used Binary Logistic Regression Analyses (1) to examine the impact of racial disparities in ER readmission within one week to two months' discharge and (2) to study the racial disparities in NH admissions upon hospital discharge. Based on the studies, policy efforts are called upon to address the specific needs of racial minorities using Medicaid as a primary payment method; as well as for individuals residing in facilities with higher concentrations of Medicaid beneficiaries. Discharge diagnoses such as lower-limb fractures, chronic ulcers, and mental health conditions, especially among Black individuals with mental health conditions increased the odds of NH admission. Other factors such as the Medicare as the primary payer for the hospitalization, being unmarried, and prior residence in a skilled nursing facility are also important predictors for NH admissions. Black individuals appear to increasingly be disproportionately represented in the NH population. This may relate in part to the greater morbidity they experience related to chronic conditions such as diabetes and heart disease, which warrants further investigation as to the reasons for their high presence.

Abstract citation ID: igad104.2856

### RESILIENCE AND VULNERABILITY IN GERIATRIC MEDICAL CENTERS DURING AN ONGOING HEALTH CRISIS

Rinat Lifshitz[1], Irit Asher[2], Aya Ben Eliezer[3], Tal Prager Geller[4], Eduard Zalyesov[5], Inna Shugaev[3], Gad Mendelson[2], and Adi Sason[6], *1. The Max Stern Academic College of Emek Yezreel, Israel, Yezreel Valley, HaZafon, Israel, 2. Dorot Medical Center for Rehabilitation and Geriatrics, Netanya, HaZafon, Israel, 3. Fliman Geriatric Hospital, Haifa, HaZafon, Israel, 4. Dorot Care Facility, Netanya, Netanya, HaZafon, Israel, 5. Fliman Medical Center, Haifa, Haifa, HaZafon, Israel, 6. Dorot Geriatric and Rehabilitation Hospital, Netanya, HaZafon, Israel*

Coping in stressful times and under cumulative adversities (e.g., health, terror) may lead to emerging vulnerability, diminish resilience and negative psychological consequences. In an organizational framework, this personal vulnerability may also have an impact on organizational resilience. This study examined the resilience and vulnerability indicators during the COVID-19 pandemic and other crises among employees at Geriatric Medical Centers in Israel. Data were collected from a convenience sample of 283 employees from two Geriatric medical centers. The questionnaire included background variables, resilience indicators (personal resilience, morale, wellbeing and a sense of security at home) as well as vulnerability indicators (sense of danger, fear of death and fear of dying, a sense of stress and perception of threats). Administrative workers reported higher vulnerability and lower resilience than workers in other sectors. Longer period of time working with Covid-19 patients was associated with higher resilience and higher vulnerability. After controlling for personal background, death anxiety, sense of danger and health wellbeing significantly correlated with high stress while functional wellbeing and personal morale significantly correlated with low stress. Wellbeing in the workplace was the only indicator associated with high resilience. These findings demonstrate the dialectical experience of employees in geriatrics centers coping with cumulative adversities, combining both vulnerability and resilience. It is recommended for organizations to assist employees in building resilience mechanisms that will help them cope with stressful situations through strengthening their sense of wellbeing, and as a result will help strengthen organizational resilience.

Abstract citation ID: igad104.2857

### SCIENCE AND CARE PARTNERSHIPS: THE ROLE OF THE SCIENTIFIC LINKING PIN RESEARCHER

Reena Devi[1], Irma Everink[2], Judith Urlings[3], Alys Griffiths[4], Hilde Verbeek[2], Kirsty Haunch[1], Karen Spilsbury[1], and Jan Hamers[5], *1. University of Leeds, Leeds, England, United Kingdom*, *2. Maastricht University, Maastricht, Limburg, Netherlands*, *3. Living Lab in Ageing and Long-Term Care, Maastricht, Limburg, Netherlands*, *4. University of Sheffield, Sheffield, England, United Kingdom*, *5. CAPHRI, Maastricht University, Maastricht, Limburg, Netherlands*

**Background:** Science and care partnerships work on promoting quality of life, care and work in long-term-care (LTC) settings. The Scientific Linking Pin (SLP) is an academic employed by a university and works one day per week in a LTC organisation. The SLP role is pivotal to the success of partnerships; the nature of this role has not yet been captured. **Methods:** Fifteen SLPs took part in semi-structured interviews. Data were thematically analysed. **Results:** Data are presented over two themes. The first theme, the SLP experience, outlines that SLPs have rich insights into what matters to, and the challenges experienced in LTC organisations. SLPs are well positioned to impact practice, and develop their professional careers. SLPs do experience doubts and insecurities and require mentorship/supervision. The second theme, navigating in a SLP role, outlines the multifaceted tasks involved: relationship building, raising awareness, identifying priorities, generating research questions, building committees, brokering knowledge, developing research studies, generating academic output, building links and connections, and assisting with internal projects. The challenges SLPs face in their work include mistrust and poor engagement from care staff, working with staff from different professional backgrounds, research not being a priority, multiple and rapidly changing priorities, and differences in expectations. Relationship building, creating a 'safe' space for care staff, building engagement, and expectation management helped to address these challenges. **Conclusion:** Partnership working in the care sector is gaining international recognition and adoption. This study described one key role that contributes to the success of the partnership model: the SLP.

Abstract citation ID: igad104.2858

### SEEKING ANSWERS FOR UNCERTAINTIES: GATHERING AND ORGANIZING CARE HOME UNCERTAINTIES: THE SEARCH U STUDY

Reena Devi[1], Kirsty Haunch[1], Liz Graham[1], Karen Spilsbury[1], Anne Forster[1], Alys Griffiths[2], and Carl Thompson[1], *1. University of Leeds, Leeds, England, United Kingdom*, *2. University of Sheffield, Sheffield, England, United Kingdom*

Introduction and method: Staff providing care to residents must manage uncertainties whilst using their judgement and making decisions about care. Residents and their relatives may also have questions and ideas about how care could be improved. It is important to capture uncertainties/questions and work on addressing these. We gathered uncertainties/questions from residents, relatives and staff before, and during COVID-19 through interviews, analysis of a COVID-19 WhatsApp discussion group for long-term-care (LTC), and a systematic literature review. **Results:** Questions and uncertainties gathered were categorised into 21 themes: 1. Direct care (personal care, sleep, care planning, medication management, nutrition) 2. Physical environment (safety, mobility, infection risk) 3. Mental health/well-being (relationships, socialising and meaningful activities, spirituality) 4. Resident individuality and dignity 5. End-of-life 6. Quality of work for care staff (reward and recognition, training and career opportunities, well-being, shift flexibility, workload) 7. Staff shortages 8. Promoting staff diversity 9. Care home interaction with wider healthcare services 10. Use of technology 11. Family engagement and communication with staff 12. COVID-19-related anxiety 13. Financial support with care home costs 14. Care homes and climate change and future sustainability 15. Valuing the contribution of care homes 16. Infection prevention control 17. Visiting care homes during COVID-19 18. COVID-19 safety for residents and staff 19. COVID-19 and the individual (symptoms and treatment) 20. Organisational impact of COVID-19 21. Information sharing and learning from COVID-19. **Discussion:** With LTC staff, residents and relatives the identified uncertainties/questions will be shortlisted; the prioritised list will direct future research.

### SESSION 7730 (POSTER)

### SOCIAL SERVICES: POLICY, FINANCING, AND DELIVERY SYSTEMS (POSTERS)

Abstract citation ID: igad104.2859

### ADVOCATING FOR ENGINEERING SOLUTIONS TO IMPROVE OLDER ADULT DRIVING LONGEVITY

Anne Dickerson, *East Carolina University, Greenville, North Carolina, United States*

To age-in-place, older adults need to access their community. As most older adults live in suburban/rural areas, driving is the primary and preferred means of mobility. While recent research has shown that drivers in the seventies have fewer crashes than middle-aged drivers, it is likely due to less exposure. However, it demonstrates that driving decisions need to be based on function, not age. Moreover, with the aging population and increase numbers of older adults with cognitive impairment and/or slowed processing speed, other strategies are needed to preserve and lengthen the driving lifetime. While efforts for developing alternative transportation options are working in some places, rural areas lack the infrastructure and potential for public transportation or volunteer programs beyond medical necessity. This presentation will present some roadway engineering proven countermeasures and community strategies that can protect and benefit aging drivers so they can drive longer while reducing crash risk. Stepping outside the typical gerontological community, this presenter became a contributing member of their state's highway safety five-year plan. As an older driver advocate, I was successful in achieving older adults as an "emphasis" area in the present plan. For the next five-year plan, researching engineering solutions was deemed essential to advocate for specific strategies that protect both aging drivers and pedestrians. Since every state is required have a five-year plan for highway safety to receive federal funding, the presenter will outline successful strategies to participate in innovative ways for improving safety such as influencing engineering and department of motor vehicles partners.

Abstract citation ID: igad104.2860

### ASSESSING THE NEEDS OF OLDER ADULTS IN RURAL WEST TEXAS

Suzie Macaluso, and Julia Free, *Abilene Christian University, Abilene, Texas, United States*

With 1 in 5 adults over the age of 65 living in rural areas, understanding their unique concerns and needs becomes increasingly important. This study was carried out in partnership with the West Central Texas Area Agency on Aging (WCT AAA) to identify the top concerns of older adults in rural West Texas. The survey was conducted in person at four locations across the region served by the WCT AAA. A total of 183 respondents completed the survey and their responses to the question "what are the biggest issues facing older adults today" along with demographic information were collected. Participants were between the ages of 56 and 95 years old with a mean age of 76 years old. 60% of the participants were female and 40% were male. Respondents were predominantly White, 80%, with 16% Hispanic or Latino, and 3% Black or African American. The most common concern among respondents was physical health and well being with the increasing cost of living being the second most frequent response. Additionally, residents from more rural counties reported social interaction as more important than either health or cost of living. These findings highlight the need for targeted interventions to address the concerns of older adults in rural areas, particularly related to healthcare access and social isolation. Strategies such as telemedicine and community-based programs to address transportation and social isolation may be particularly effective in addressing these concerns.

Abstract citation ID: igad104.2861

### EFFICACY OF A HOME-BASED EXERCISE PROGRAM "SENIOR FIT" FOR OLDER ADULTS DURING THE COVID-19 PANDEMIC

Kate Hyun, Kathy Lee, Vina Lervisit, Kimberly Vanhoose, Xiangli Gu, Troyee Saha, Boni Jobaidul, and Angela Liegey-Dougall, *University of Texas at Arlington, Arlington, Texas, United States*

With elevated awareness and concerns of infectious diseases such as COVID-19, the importance of overall health and wellbeing has been highlighted. More than 25% of older adults, including individuals with underlying health conditions, did not maintain the same intensity of physical activity during the pandemic. The purpose of this study was to examine the efficacy of a home-based exercise program, Senior Fit, for older adults during the COVID-19 pandemic. Senior Fit employs conventional behavior boosting approaches to promote physical activities and reduce sedentary behaviors. Older adults (N = 58) participated in the program and completed a weekly set of exercises for eight weeks. We conducted in-depth, individual interviews with 53 participants and identified four themes related to the program: (1) positive perceptions on the structure, (2) individual variances in their overall experience, (3) perceived benefits, and (4) feedback on human interactions. The participants appreciated the structure, especially following schedules provided and using paper forms to track their activities. However, some complained the program was too easy while others thought the program was too challenging. Overall, the participants discussed physical benefits (e.g., weight loss, established exercise habits) and emotional benefits (e.g., better mood, motivations). They suggested the importance of creating an inclusive and interactive environment. Many discussed the importance of the interactions with the research team as well as other participants to stay motivated in the program during the pandemic. These findings highlighted the need to involve participant stakeholders in the development of programs to increase physical activity in older adults.

Abstract citation ID: igad104.2862

### LEARNING FROM THE IMPLEMENTATION OF INTEGRATED COMMUNITY-BASED PROGRAMS FOR OLDER ADULTS WITH HIGHER NEEDS

Bobbi Symes, and Kahir Lalji, *United Way British Columbia, Burnaby, British Columbia, Canada*

Three demonstration projects of integrated community-based programs for older adults with higher needs were implemented in British Columbia over 2020 to 2023: 1) Social Prescribing (SP, n=19 programs), 2) Therapeutic Activation Programs for Seniors (TAPS, n=15 programs), and 3) Family and Friend Caregiver Groups (FFCG, n=16 programs). This presentation reports on findings on the programs' design and implementation based on data collected via interviews and surveys of program staff. Since inception, the total number of unique participants programs have served is 1,078 for TAPS, 1,900 for FFCG, and 1,397 for SP. The majority of clients have been long-term clients receiving services for 3 or more months. Due to the COVID-19 pandemic, 80% of programs reported they had shifted their focus during their first year of operations in response to emergent pandemic needs, thus impacting their start-up and initial intake of clients. The flexibility provided to programs to pivot to offering needed supports during the height of the pandemic was considered an important success. Across all types of programs, client who were socially isolated or lacked social supports were perceived as one of the groups who benefited the most from the programs. Programs reported that their relationships with community-based organizations, primary care networks, and home and community care were strengthened and over three-quarters had formed at least three new partnerships. A variety of facilitating (e.g., leveraging pre-existing relationships) and limiting factors (e.g., awareness of programs and obtaining referrals) were identified that have affected the success of program implementation.

Abstract citation ID: igad104.2863

### WHEN POLICY INTENTIONS GO ASTRAY: THE DEPLOYMENT OF HOME CARE AND HOME SUPPORT MARKET TOOLS IN FRANCE AND QUEBEC.

Patrik Marier[1], and Loïc Trabut[2], *1. Concordia University, Montreal, Quebec, Canada*, *2. INED, Paris, Ile-de-France, France*

Using Gingrich's (2011) typology of welfare markets, this contribution studies the selection and enactment of market tools in two different jurisdictions (France and Quebec). Quebec represents a classic example of a managed market with regional health authorities (CISSS/CIUSSS) seeking the lower the cost of long-term care by contracting services to community groups and the private sector. These contractual agents have gradually replaced health and social care professionals within the public system. France has opted to enact a consumer driven market with the introduction of the allocation personnalisée d'autonomie (APA) which provides cash benefits to eligible older adults who can then select the provider of their choice. Both of these market reforms are compared and analyzed across three types of territories : rural (Finistère and Bas-St-Laurent), urban (Paris and Montreal), and in industrial decline (Somme and Mauricie). Despite operating within highly differentiated long-term care policy frameworks and market tools, both jurisdictions face similar difficulties in the enactment of market mechanisms. For instance, rural territories fail to generate sufficient providers to develop a market where a regional health authority (Quebec) or older adults (France) can actually select a provider and negotiate terms of service. Public authorities must then intervene to generate market-like conditions. Regardless of the jurisdiction, the conceptualization of market mechanism has clearly an urban setting in mind making them ill-suited for other environment and there is gradual movement towards the development of austerity market where individuals are forced to seek alternatives beyond the market tools deployed by governments.

### SESSION 7750 (POSTER)

### TECHNOLOGY (POSTERS)

Abstract citation ID: igad104.2864

### DESIGN NEEDS AND PERCEPTIONS OF OLDER ADULTS REGARDING SHARED-USE AUTOMATED VEHICLES

Justin Lam[1], Tarryn O'Rourke[2], Caitlin Bowman[2], and Clive D'Souza[2], *1. Carnegie Mellon University, Pittsburgh, Pennsylvania, United States*, *2. University of Pittsburgh, Pittsburgh, Pennsylvania, United States*

Many communities struggle to provide safe, accessible, and reliable transportation services for older adults due to high demand, rising costs, driver shortages, and other evolving challenges. Innovative transportation solutions are needed to support the current and future populations of older adults. Low-speed, shared-use, driverless shuttles present an exciting development in automated vehicle (AV) technology with potential to meet mobility needs of older adults in their community. Understanding older adults' perceptions about and willingness to consider using these emerging modes of transportation is vital to realizing the full potential of these technologies. This presentation summarizes an in-person study conducted with 12 older (average: 66 +/- 4 years of age, range: 60 to 80 years) and 10 younger (average: 44 +/- 11 years) adults that evaluated a stationary, proof-of-concept shared-use AV retrofitted with accessibility features. We will present findings on perceptions regarding accessibility, safety, and willingness to use driverless AVs along with human factors design recommendations. While questionnaire-based studies have been the dominant approach to understanding older adults' perceptions about shared-use AVs, in-person evaluations even with prototype AVs as described here, provide opportunities to identify goals, needs and preferences of older adults concerning usability and safety in early design stages, and through hands-on exploration help older adults develop good mental models, i.e., understand AV capabilities and limitations, towards building trust and acceptance for these emerging modes of transportation. Research and policy implications will be discussed towards enabling emerging driverless shared-use AV technologies that support safe and independent community mobility for older adults.

Abstract citation ID: igad104.2865

### EXPERIENCES OF TECHNOLOGY USE BY OLDER ADULTS DURING COVID-19

Tina Kilaberia[1], Yuanyuan Hu[1], Edward Ratner[2], and Janice Bell[3], *1. New York University, New York City, New York, United States*, *2. Minneapolis VA Health Care System, Minneapolis, Minnesota, United States*, *3. University of California, Davis, Davis, California, United States*

Computer and communications technology use expanded during the COVID-19 pandemic, but little is known about how this affected older people. Using the Unified Theory of Acceptance and Use of Technology questionnaire and qualitative interviews, we examined 18 older adults' technology use during COVID-19 across two subgroups of nine older adults each: those scoring above ("high") and those scoring below ("low") the median (4.09). Overall, older adults scored fairly high (mean 4.0; SD=0.5; range 3.1-4.8 across 16 items on a scale of 1 to 5). In each group of nine older adults, four (44%) described technology as being helpful during the pandemic. Among high scoring older adults, 100% utilized technology to order either groceries or household items and six (67%) used either telehealth or online health information system (i.e., MyChart) to communicate with health services providers, compared to three (33%) and four (44%) within the low scoring group. No one in the high scoring group described a degree of cognitive difficulty whereas four 44% in the low scoring group did. All but one (94%) older adults described needing some help with technology from family members, friends, and/or paid technology services. Two themes complement these findings by reflecting strengths ("lots of ways to do everything") and challenges ("I just get through it") of using technology. Greater technology mastery may positively influence the ability of older adults to meet their needs, but cognitive challenges may prevent full engagement with technology.

Abstract citation ID: igad104.2866

**PERSPECTIVES ON TECHNOLOGY ADOPTION AMONG SPANISH-SPEAKING HISPANIC ADULTS AGE 60+**

Sheila Salinas Navarro, Sindy Lomeli, Lilly Estenson, and Kathleen Wilber, *University of Southern California, Los Angeles, California, United States*

By 2060, Hispanics aged 65+ are projected to be 21% (19.9 million) of the total US age 65+ population. Despite the benefits of technology use, such as facilitating social engagement and access to health services, few studies have explored attitudes of older Spanish-Speaking Hispanic adults toward technology adoption. In this secondary analysis of a larger diverse study of attitudes toward technology, we reviewed audio recordings and transcripts of Spanish-language interviews and focus groups with adults ages 63-89. Participants (n=21) self-identified as Hispanic, 67% had less than a high school education, and almost half (n=10) lived alone. Connecting with family members abroad was the primary motivator and a "necessity" for using technology; excess use of a device was negative. As one participant stated: "Technology brings you closer to those far away and pushes you away from those near you." Those without relatives living with or near them reported greater difficulty using technology and identified living alone as a barrier to adoption. Finally, although the sample had low levels of English proficiency and low educational attainment, characteristics associated with lower use and knowledge of technology, findings challenge stereotypes that these older adults lack the capacity or interest to adopt the technology. Rather, the study identified multiple social factors that may hinder adoption. Understanding these cultural and linguistic barriers is important to promote digital inclusion; overcoming the digital divide will involve more than providing free tablets or classes. It will also require addressing barriers with adequate, culturally competent understanding, training and support.

Abstract citation ID: igad104.2867

**SMART MUSIC INTERVENTION: PROTOCOL DEVELOPMENT OF A WEARABLE DEVICE FOR COLLECTION OF PHYSIOLOGICAL DATA**

Emily Ihara, Parth Pathak, Megumi Inoue, Catherine Tompkins, and Y. Alicia Hong, *George Mason University, Fairfax, Virginia, United States*

This study tested a protocol of collecting physiological data through sensors on a wearable device in response to favorite music from late adolescence/early adulthood. The benefits of personalized music for individuals living with dementia have been demonstrated in various studies, showing decreases in agitation, anxiety, depression, and other behavioral and psychological symptoms of dementia and improvement in mood, memory, and well-being. Most studies have relied on observational data, standardized clinical scales, and self- or caregiver-reported perceptions of the effects of personalized music. Passively-collected physiological data may minimize researcher bias during data interpretation. We tested the protocol on 15 participants, triangulating the physiological, observational, and self-reported effects of personalized music on stress. Physiological measures were captured by a wearable wristband Empatica E4 and included heart rate, electrodermal activity, and temperature. Self-reported measures included the Perceived Stress Scale, level of stress on a 0-10 scale, and reactions to each song (happy, energized, calm, relaxed, nostalgic, sad, excited). Prior to music listening, we induced stress through three iterations of the Trier Mental Challenge Test, which involves three minutes of math exercises. Participants reported their level of stress after the Trier Test and after each song. Researchers observed participant reactions during each song. Preliminary analysis shows that nine minutes of a stress-inducing activity was enough to capture stress reactions and demonstrated a feasible protocol for collecting physiological, observational, and self-report data. These results inform the development of a novel protocol that responds to a person's stress levels through a personalized music listening intervention.

### SESSION 7760 (POSTER)

### MENTAL HEALTH AND PSYCHOSOCIAL WELL-BEING

Abstract citation ID: igad104.2868

**A LIFESPAN DEVELOPMENTAL ANALYSIS OF RECIPROCAL RELATIONS BETWEEN HEAVY DRINKING AND SLEEP PROBLEMS**

Douglas Bowlby, *Rutgers University, New Brunswick, New Jersey, United States*

In recent years, numerous studies have begun to explore the ways in which problem drinking behavior is affected by different lifespan developmental changes. However, while there currently exists a plethora of research on adolescence and young adulthood, there is far less research with midlife and older adults examining lifespan developmental changes and problem drinking. As part of larger NIAAA-funded project investigating aging, health, and alcohol use, the current study investigated cross-sectional and longitudinal associations among heavy drinking and sleep problems in a sample of midlife and older adults. One particularly noteworthy analysis used path analysis in Mplus to test a longitudinal moderated mediation model involving sleep problems, sleep-related motives for drinking, and heavy drinking. Results showed an interaction between sleep problems and sleep-related drinking motives. Interestingly, sleep problems appear to significantly reduce the risk for heavy drinking among most participants. However, when combined with high sleep-related drinking motives, sleep problems appear to significantly increase risk for heavy drinking. This phenomenon may help explain the occasionally contradictory results found in previous studies examining the impact of sleep disturbances on drinking behavior and may prove to be an area worth investigating in future studies. From a lifespan-developmental perspective, these findings highlight how there are likely important differences across stages of adulthood in the most relevant factors that affect heavy drinking. Health-related factors including sleep problems, coupled with motives to use alcohol to cope with such problems, appear to be a pathway to heavy drinking that increases in importance across the adult lifespan.

Abstract citation ID: igad104.2869

### EFFECTS OF CIVIC BEHAVIOR AND DEPRESSION KNOWLEDGE ON DEPRESSION STIGMA AMONG OLDER CHINESE: A PATH ANALYSIS MODEL

Jessie Ho-Yin Yau, Tianyin Liu, Dara Kiu Yi Leung, Jennifer Yee-man Tang, Gloria Hoi-Yan Wong, and Terry Lum, *The University of Hong Kong, Hong Kong, Hong Kong*

Older adults are at risk of experiencing depression personal stigma because of negative attitudes towards older persons and the creation of popular stereotypes, therefore reducing stigma becomes necessary. By receiving training and acquiring depression knowledge from engaging in civic behavior, such as providing voluntary services to peers in the community, these knowledge helps counter false assumptions and reduce personal stigma. However, previous stigma-related studies mainly conducted in Western countries and in relation to schizophrenia, research on depression stigma among older Chinese remain scarce. The goal of this study was to examine depression knowledge as a moderator to test its influence on the relationship between civic behavior and personal stigma. A cross sectional survey was conducted with 145 older adults in Hong Kong, they completed Older Adult Mental Health First Aid training and engaged in voluntary services related to mental health promotion after the training. Civic behavior, depression knowledge (symptoms, facts, myths), personal stigma (stereotype, prejudice, discrimination), and demographics were collected. Path analysis was performed to determine the structural relationship between the variables. Civic behavior positively affected symptoms ($\beta$=.089,p=.008) and facts ($\beta$=.088,p=.001). Symptoms negatively affected prejudice ($\beta$=-.627,p=.014). Facts negatively affected all dimensions of personal stigma while myths positively affected all dimensions of personal stigma. Results of the measurement showed an excellent fit ($\chi2$ = 148, $\chi2$ /df = 7.048, RMSEA = .029, SRMR = .023, CFI = 0.997, TLI = 0.98, p<.001). Mediating effects were computed by bootstrap method; facts as a significant mediation was confirmed. Findings and implications were discussed.

Abstract citation ID: igad104.2870

### ELDER ABUSE, ANXIETY, AND SOCIAL SUPPORT: DOES GENDER MATTER?

Sukyung Yoon, *University of Wyoming, Laramie, Wyoming, United States*

Elder abuse is a serious social justice violation and a public health concern that impacts victims' mental and physical health worldwide. In the U.S., about 11.7% of older adults have reported elder abuse each year, but the rate is underestimated due to underreporting (Dong, 2015). To date, research on elder abuse has focused mostly on risk factors rather than protective factors (Storey, 2020). Some risk factors are beyond one's control, such as physical and cognitive dysfunctions, but identifying both risk and protective factors will help healthcare professionals intervene more effectively (Yoon & Cummings, 2019). The research aims were to investigate 1) the impact of elder abuse on anxiety among community-dwelling older adults who experienced elder abuse, 2) the effects of social support on anxiety among older adults, and 3) the differences in the impact of elder abuse, anxiety, and social support between older men and women. This study used data from the Wisconsin Longitudinal Study (WLS), Wave 3. Structured equation modeling was used for the analysis. The independent variable was the experience of elder abuse, the dependent variable was anxiety, and the intervening variable was social support. The control variables were income, physical health, and marital status. The experience of elder abuse had a positive association with anxiety, however; social support had a negative association with anxiety. Multi-group analysis was conducted to investigate gender differences. Measurement invariance was examined by comparing unconstrained and fully constrained models. Both models were good fits. Gender differences were identified. Implications will be discussed.

Abstract citation ID: igad104.2871

### GENDER-SPECIFIC ASSOCIATION BETWEEN DEPRESSION AND MORTALITY IN KOREAN OLDER ADULTS: A LONGITUDINAL STUDY

Jungwon Cho, Jae Jun Lee, and Eunhee Cho, *Yonsei University College of Nursing, Seoul, Republic of Korea*

Late-life depression has become a significant public health problem because it adversely affects physical health, social function, and quality of life. Therefore, prevention and treatment of depression are necessary. Previous studies have indicated that depression in older adults is associated with mortality. However, research on mortality according to gender differences has not been actively conducted. Thus, this study aims to investigate gender differences between depression and mortality among Korean older adults. The data were collected from the Korean Longitudinal Study of Aging (KLoSA), which performed a nationwide aging panel survey for adults aged over 45 years between 2006 and 2020. Out of 10,254 participants, this study included 3,689 participants who were aged 65 or older and assessed depression through CESD-10. The Cox proportional-hazards model was used to calculate Hazard Ratios (HR) for mortality stratified by gender. Of the 1,599 male and 2,090 female older adults, depression was significantly higher in females (16.7%) than in males (8.8%). However, the effect of depression on mortality was significant only in males (male: HR = 1.50, 95% CI = 1.19–1.89; female: HR = 1.10, 95% CI = 0.93–1.31). Depression is more common in females, whereas the effect of depression on mortality was only significant in males. This suggests that health providers need to prevent and care for depression to decrease mortality, especially in males. Further studies are needed to explore the reasons for gender differences between depression and mortality and develop interventions to manage depression in older adults.

Abstract citation ID: igad104.2872

### INFLUENCE OF NEGATIVE ATTITUDES TOWARD VOLUNTEER ACTIVITIES ON MENTAL HEALTH STATUS: THE REPRINTS STUDY

Yoshinori Fujiwara[1], Tomoya Takahashi[1], Hiroshi Murayama[2], Kyoko Fujihira[3], Koji Fujita[1], Mariko Yamashita[3], Hiroko Matsunaga[3], and

Hiroyuki Suzuki[3], *1. Tokyo Metropolitan Institute for Geriatrics and Gerontology, Tokyo, Tokyo, Japan*, *2. Tokyo Metropolitan Institute of Gerontology, Tokyo, Tokyo, Japan*, *3. Tokyo Metropolitan Institute of Geriatrics and Gerontology, Tokyo, Tokyo, Japan*

Although social engagement activities can help older adults maintain mental health status, whether negative attitudes toward volunteering influence poor mental health status due to psychological burdens has not been clarified. This study examined negative influence of intergenerational picture-book reading program "REPRINTS" on older volunteers' attitudes to volunteering activities to clarify its impact on their mental health status. A questionnaire mail survey was conducted for 481 volunteers, ≥55 years, in 12 municipalities across Japan in 2020. Of the 423 respondents (87.9%), 266 responded to the follow-up survey, conducted from November 2021 to February 2022. Mental health status was assessed by WHO-5J; a score of less than 13 was regarded as poor mental health status. Multiple logistic regression analysis was performed on the responses of 220 women, aged 65–84 years, with WHO-5J (good ≥13 points; poor < 13 points) at follow-up as the dependent variable. Participants were asked about their positive and negative attitudes toward volunteer activities using a four-point scale (disagree to agree), and the scores were operationally summed up after converting them into a negative attitude score. Subjective health (healthy/unhealthy), age, WHO-5J score, and continuous years of activity at baseline were entered as adjustment variables. Sixty-four (29.1%) participants had a WHO-5J score of < 13 at baseline. Logistic regression analysis with WHO-5J as the dependent variable showed that the adjusted odds ratio for negative awareness scores was 1.06 (95% CI: 1.00-1.12). In conclusion, negative attitudes toward volunteer activities were suggested to have a significant influence on mental health status.

Abstract citation ID: igad104.2873

### INNOVATIVE APPROACHES TO MENTAL HEALTH SERVICES FOR DIVERSE COMMUNITY OLDER ADULTS

Tobi Abramson[1], Jo Anne Sirey[2], and Jacquelin Berman[3], *1. NYC Department for the Aging, Greenvale, New York, United States*, *2. Weill Cornell Medicine, Ithaca, New York, United States*, *3. NYC Department for the Aging (NYC Aging), New York City, New York, United States*

Addressing the triple stigma (age, mental health, race) faced by older adults in need of mental health services has necessitated innovations that move the service provision needle. Co-located services within community settings, mental health interventions addressing those with nutritional needs, and victims of elder abuse and elder crime benefit from innovative services. The following session will highlight research outcomes from innovative treatment models: (a) In a sample of 138 diverse individuals participating in PROTECT (a brief behavioral intervention for late-life depression among elder abuse/crime victims) showed 5.15 point reductions in PhQ-9 scores across the sample and found video-delivery was non-inferior to traditional in-person therapy. Those receiving video therapy completed treatment faster, with more rapid rate of symptom reduction. (b) Do More Feel Better (a 9-week peer-led manualized intervention for those with depression and nutritional needs) found 78% experienced clinically significant reduction in depressive symptoms and over two-thirds (66%) reported satisfaction with the program. (c) DGMH (mental health services co-located in older adult centers) data indicate that of the 4,118 clients assessed, 3,373 endorsed at least one mental health need (81.9%), significantly higher than the mental health literature indicates. Over 2,133 clients have been treated in 67,597 sessions since program inception in 2018. Within 3-months of treatment, 60.8% and 52% showed clinically significant improvements in depression and anxiety, respectively. A third reported decreased loneliness and 39.4% indicated feeling less socially isolated. Further data will be presented (demographic, racial, ethnic differences). Data supported efficacy lend support to innovative mental health treatment approaches.

Abstract citation ID: igad104.2874

### INTERGENERATIONAL REMINISCENCE PROGRAM IN IMPROVING GENERATION CONNECTIONS: A RADAR ANALYSIS

Ling Xu[1], and Minjaal Raval[1], *1. The University of Texas at Arlington, Arlington, Texas, United States*

**Objectives:** Intergenerational solidarity between grandparents and grandchildren has been highlighted in Asian Families and it is beneficial for the health of grandparents. An intergenerational reminiscence program was developed to improve the well-being of Asian American older adults as well as generational bonding. This paper qualitatively reported the weekly reflection from grandchild participants of this program. **Methods:** Older grandparents received 6 sessions of life-review with their grandchildren remotely or in person for approximately 1 hour each week for 6 weeks. Each grandchild (n = 12) provided written reflection each week after talking with grandparent. The qualitative data were organized and analyzed using the five phases of the Rigorous and Accelerated Data Reduction (RADaR) technique. **Results:** Results showed three categories with themes. Category 1: Positive experience. The grandchildren expressed: (1) more connection with grandparent, (2) learn more about grandparent's past life experience, and (3) have more engagement. Category 2: Strategy to lead the discussion. The grandchildren thought the following good ways in facilitating their weekly conversation with grandparents: (1) taking notes, (2) using guiding question in the manual; (3) use translators, (4) spending time together. Category 3: Challenging experience. The grandchildren felt challenges regarding: (1) over explaining things, (2) language or vocabulary barriers, (3) broad topic. **Discussion:** The reflection pages from grandchild participants showed that intergenerational reminiscence program is promising in bonding the grandparent-grandchild relationship and connection. They also gained knowledge and experienced challenges when talking with their grandparents during the program.

Abstract citation ID: igad104.2875

### INTRINSIC CAPACITY OF OLDER ADULTS IN SINGAPORE USING WHO INTEGRATED CARE FOR OLDER PEOPLE (ICOPE) FRAMEWORK

Carol Ma[1], Angela Y. M. Leung[2], Denise Chua[1], Wai Choo Teo[1], Laura Bee Gek Tay[3], and Wai Chong Ng[4], *1. Singapore University of Social Sciences, Singapore,*

*Singapore*, *2. Hong Kong Polytechnic University, Hong Kong, Hong Kong*, *3. Sengkang General Hospital, Singapore, Singapore*, *4. NWC Longevity Practice, Singapore, Singapore*

The World Health Organization (WHO) proposed the Integrated Care for Older People (ICOPE) approach to guide health systems in better supporting the intrinsic capacity and functional ability of older adults to enable healthy aging (Briggs et al., 2018; de Carvalho et al., 2019). This approach is aligned with Singapore's Healthier SG initiative, which aims to build a good healthcare system that promotes better health and quality of life for everyone. Recent studies have shown that older adults experience intrinsic capacity decline in various countries, including Singapore, highlighting the importance of targeted interventions to maintain functionality and quality of life in old age (Beard et al., 2019; Liu et al., 2021; Tay et al., 2022). In this cross-sectional study, 367 participants were assessed by 43 ICOPE assessors, of whom 77.4% (n=284) had impairments in intrinsic capacity. The three most prevalent intrinsic capacity impairments were visual impairment (42%), hearing loss (33.5%), and cognitive decline (31.3%), followed by limited mobility (24.3%), malnutrition (16.1%), and depressive symptoms (16.1%). Furthermore, 22.6% of participants were unaware of any elderly care services available in the community, and 8.2% did not know where to seek help in case of problems. These findings emphasize the need for ICOPE assessments at the community level to promote early detection of intrinsic capacity impairments and interventions. More discussion of the care pathway, together with health professionals, older adults, and caregivers, should be the next step to offer diagnostic assessment and guide self-management of intrinsic capacity impairment for among older adults.

Abstract citation ID: igad104.2876

### LONG-TERM EFFECTS OF EDUCATION ON LATE-LIFE DEPRESSION OF RURAL WOMEN IN CHINA

Lynn Hu, *Pardee RAND Graduate School, Santa Monica, California, United States*

Despite the noted association between educational attainment and mental health, there is considerable debate on whether this relationship is causal. Up to now, there are only a few studies using credible estimation strategies to examine the causal impact of education on mental health, and almost no evidence exists for low and middle-income developing countries from regions like Asia. This is concerning given that mental health problems and late-life depression are growing in the Asian population, especially for women and rural residents. In this paper, I aim to fill the gap by examining the long-term causal effect of additional educational attainment on the depressive symptoms of rural women in China. Using Harmonized CHARLS data, I leveraged the sudden end of the Chinese Cultural Revolution in 1976 as an exogenous shock to education, and employed both event study and regression discontinuity designs to estimate its causal effect on late-life depressive symptoms. Results show a protective effect of education for the rural women who stayed in rural areas, and a negative impact of education on the urbanized rural women. The impacts are likely to be mediated by childhood adversities (being poor and unhealthy), marriage status (being widowed), and disability status (difficulty in performing activities necessary for independent living). The proposed research will be the first study to examine the causal effect of education on the mental health of the Chinese population. It will add to the existing literature by providing evidence from underdeveloped regions in Asian countries and offering insights for future policymaking.

Abstract citation ID: igad104.2877

### MEDICARE ADVANTAGE (MA) PLAN CHARACTERISTICS ON PHYSICAL AND MENTAL HEALTH OUTCOMES OF MA BENEFICIARIES

Rashmita Basu, *East Carolina University, Greenville, North Carolina, United States*

With growing demands for better and cost-effective care delivery by Medicare Advantage (MA) plans, comparison of self-reported health outcomes is more important because those outcomes capture patients' experiences and perspectives. The purpose of the study is to examine the characteristics of MA plans on Medicare beneficiaries' physical and mental health status. **Methods:** I used the Medicare Health Outcomes (HOS) survey, a random sample of Medicare beneficiaries surveyed from each MA plan with a minimum of 500 enrollees conducted by the CMS. The current study included 3 baseline cohorts from 2015 (n=195,800), 2016 (n=178,973), and 2017 (n=172,047). I used physical component summary (PCS) and mental component summary (MCS) scores as two outcomes which include various domains of physical (exercise, pain, activity limitation) and mental health (social functioning, emotional status, depression) status. Plan characteristics include plan ownership status (for-profit (FP) vs. not-for-profit (NFP), plan types (PPO vs. HMO/cost contract), and the number of plan members. I used the linear regression method with clustering at the plan contract level due to the correlation of multiple beneficiaries within the same plan. **Results:** Adjusted for beneficiary level characteristics, beneficiaries in PPO plans experienced greater physical and mental health scores than HMO or cost contract plans. MA plan with less than 5k beneficiaries is associated with a lower physical health score than plans with more than 100k beneficiaries. For-profit ownership was significantly associated with lower mental health scores. **Discussion:** The characteristics of MA plan insurers are important factors for beneficiaries' enrollment decisions and quality improvement efforts.

Abstract citation ID: igad104.2878

### PREDICTORS OF FUNCTIONAL ABILITY VARY BY AGE

Carly Pullen, and Julie Hicks Patrick, *West Virginia University, Morgantown, West Virginia, United States*

Across adulthood, chronic physical and emotional conditions can interfere with wellbeing. We examined the potential moderating effect of age on the process functional ability Using data from 401,958 adults who completed the 2020 Behavioral Risk Factor Surveillance System survey, we tested the associations among age, sex, race, chronic health conditions, poor mental health, and functional ability. The initial model was a poor fit to the data, $\chi$ 2 (DF = 24) = 592039, p >

.001; RMSEA = .25. In order to examine whether this poor fit was related to age differences in the model, we removed age as a predictor and included it as a moderator. Thus, we tested the model among 122,005 younger adults, 140,122 midlife adults, and 139,831 older adults. The model fit improved, $\chi$ 2 (DF = 54) = 567119.79 p > .001; RMSEA = .25. Variance accounted for in functional ability differed, with 22% among younger adults, 33% at midlife, and 20.5% for older adults. Additional post hoc analyses were conducted and suggest alternate pathways to functional wellbeing across the lifespan. Our results are discussed the within the context of expanding investigations of functional wellbeing to include a set of predictors which captures both common and unique pathways across age.

Abstract citation ID: igad104.2879

### RELATIONSHIP BETWEEN SOCIAL PROBLEM-SOLVING SKILLS AND CAREGIVER BURDEN AMONG DEMENTIA CARE PARTNERS

Chung Lin (Novelle) Kew[1], Matthew Smith[1], Gang Han[1], Brittany Wright[2], Alexandra Holland[2], Alka Khera[3], Kristin Wilmoth[4], and Shannon Juengst[5], *1. Texas A&M University, College Station, Texas, United States*, *2. UT Southwestern Medical Center, Dallas, Texas, United States*, *3. University of Texas Southwestern Medical Center, Dallas, Texas, United States*, *4. UT Southwestern, Dallas, Texas, United States*, *5. TIRR Memorial Hermann, Houston, Texas, United States*

**Background.** This study examined the extent to which social problem-solving abilities, care situations, and depressive symptomatology were associated with caregiver burden among care partners of adults with Alzheimer's Disease and Related Dementias (ADRD). **Methods.** Baseline survey data were analyzed from care partners of ADRD who participated in a larger state-funded study. We used validated measures to assess social problem-solving abilities, care situation, positive aspects of caregiving, depressive symptomatology, and caregiver burden. Multivariate regression analysis with backward stepwise entry was used to identify factors associated with caregiver burden. **Results:** Care partners were, on average, age 60.4 (±12.4) and most were female (82.5%) and lived with their care recipient (69.1%). Care partners who reported high caregiver burden were more likely to be male (p< 0.0001) and have a positive problem-solving orientation (p=0.0002), but less likely to have a negative problem-solving orientation (p=0.0376). Higher caregiver burden was also associated with more resentment for caregiving role (p< 0.0001), more anger towards care recipients (p< 0.0001), more depressive symptomology (p< 0.0001), and higher positive aspects of caregiving (p=0.0010). Care partners who reported high caregiver burden also reported less social support (p=0.0076) and less family conflict (p=0.0159). **Discussion:** Findings suggest that factors thought to be protective against caregiver burden may not sufficiently reduce caregiver burden when considering their feelings of resentment and anger towards their care recipient, limited perceived social support, and high depressive symptomology. Interventions to reduce caregiver burden must consider care partners' internal conflict as a caregiver and the relationship between the care partner and their care recipient.

Abstract citation ID: igad104.2880

### THE IMPACT OF CLOSING MEDICARE PART D COVERAGE GAP ON MENTAL HEALTH OF OLDER ADULTS

Junjie Gai, and Kanika Arora, *University of Iowa, Iowa City, Iowa, United States*

The ACA mandated the gradual elimination of the Medicare prescription drug coverage gap (also called the "Doughnut Hole") beginning in 2011. This policy change can impact mental health through mechanisms such as reduction in out-of-pocket (OOP) expenses and increased access to prescription medication. However, no previous study has examined its impact on mental health outcomes of older adults on Medicare. Employing data from the Medical Expenditure Panel Survey (from 2006 to 2016), compare the mental health of Medicare beneficiaries (66-70 years; N=7,664) with that of non-Medicare beneficiaries (60-64 years; N=8,079) before versus after policy implementation. We find that the 2011 phasing out of Medicare drug coverage gap was associated with 0.86-point (p< 0.05) improvement in the Mental Component score of the Short-Form 12 Health Survey among beneficiaries. Our analysis was robust to using alternate measures of mental health outcomes (Kessler Index and PHQ-2) as well as to an alternate construction of treatment and control groups (comparing individuals aged 60-70 years on Medicare and private insurance). We find no detectable differences in the mental health of Medicare beneficiaries who already receive subsidies for costs associated with the coverage gap through the Low Income Subsidy (LIS) program. Further, we find that elimination of the doughnut hole reduced OOP expenses by $32.65 among Medicare beneficiaries in our sample. Overall, our results for mental health and OOP expenses are mainly attributable to women. This evidence highlights indirect effects of drug coverage gap elimination on mental health outcomes among Medicare beneficiaries.

Abstract citation ID: igad104.2881

### THE POTENTIAL BENEFITS OF COMPANION ROBOTIC PETS ON THE QUALITY OF LIFE AMONG COMMUNITY-DWELLING OLDER WOMEN

Suk-Young Kang[1], Jeungkun Kim[2], and Antonina Poplawski[1], *1. Binghamton University, State University of New York, Binghamton, New York, United States*, *2. Kangnam University, Yongin, Republic of Korea*

This project explores whether companion robotic pets improve mental well-being of community-dwelling older women with depression. Depression is a major mental health issue disproportionately impacting women due to lifelong experiences of sexism and limitations in education and employment. Aging exacerbates an already undervalued status, and feelings of powerlessness increase. Women thus experience double jeopardy from discrimination on both gender and age (Hooyman et al., 2017). This project will investigate the efficacy of companion robotic pets and will recommend plans to the local Office for Aging (OFA). Recruitment was done in collaboration with the local OFA, with mail-survey data collected from community-dwelling women over the age of 65. 38 participants were provided with a robotic pet after an initial screening. Following a one-group pretest posttest design, data from 31 participants were used to determine the impact of a robotic pet intervention on older women's quality of life. Paired samples t-tests were used to compare means of the 15-item Geriatric Depression Scale (GDS), 10-item Geriatric Anxiety Scale (GAS), 6-item De Jong Loneliness Scale, and one physical health status question completed by 30 women. There was a significant average difference between before- and after-companion pet scores for depression ($t29 = 6.597$, $p < 0.001$), anxiety ($t29 = 6.728$, $p < 0.001$), loneliness ($t29 = 6.462$, $p < 0.001$) and physical health ($t29 = - 3.496$, $p = 0.002$). Results confirmed companion robotic pets improve mental health in older women with depression and anxiety. Moreover, they are helpful in improving loneliness and physical health.

Abstract citation ID: igad104.2882

### THE STRONGERMEMORY PROGRAM: EXPLORING COGNITIVE BENEFITS AND FOSTERING COMMUNITY

Catherine Tompkins[1], Emily Ihara[1], Francesca Keesee[1], Mckenzie Lauber[1], Catherine Magee[1], Jessica Fredericksen[2], and Robert Liebreich[2], *1. George Mason University, Fairfax, Virginia, United States*, *2. Goodwin Living, Alexandria, Virginia, United States*

**Background:** StrongerMemory is a brain health program centered on spending 30 minutes per day on handwritten journaling, reading aloud, and arithmetic exercises as a method to enhance cognition in older adults. The StrongerMemory research protocol is a 12-week program that includes a weekly meeting. After 104 participants completed the StrongerMemory research protocol, they were invited to participate in a focus group to share their experiences. Six focus groups were conducted by members of the StrongerMemory research team yielding 30 participants. **Methods:** Focus groups were conducted virtually, with a set of pre-determined, open-ended questions centering around participant experiences and attitudes towards the StrongerMemory program. Participants were also asked to reflect on differences in their memory, cognition and well-being before and after StrongerMemory participation. Two researchers independently coded the focus group transcripts, and utilized the grounded theory analysis techniques of memoing and constant comparative analysis to explore the data. Common themes were then discussed. **Results:** Five overarching themes emerged: Motivating, appreciating, challenging, committing, and enhancing. Conceptualizations of these themes focused on participants' experiences and suggestions for strengthening the program. "Fostering community" was an outcome of the program often discussed. **Conclusion:** The participant experience in StrongerMemory revealed unique perspectives on their motivation for participation and provided the researchers with new insights into the program such as fostering community within older adult groups. Further research includes exploring cognitive and social benefits of group participation in the StrongerMemory program.

Abstract citation ID: igad104.2883

### VOLUNTEERING, DISCRIMINATION, AND DEPRESSION IN OLD AGE: DOES THE FORM OF DISCRIMINATION PLAY A ROLE?

Huei-wern Shen, *University of North Texas: Denton, Denton, Texas, United States*

Older adults experiencing discrimination have an increased risk of poor mental health, such as depression. Discrimination could happen based on age, race, gender, sexual orientation, or other factors but the effects of experiencing different forms of discrimination on depression are unknown. Given the evidence that volunteering in old age promotes mental health, this study explores the relationship between volunteering and depressive symptoms among older adults who experience discrimination, and investigates whether such a relationship differs between those experiencing ageism and those experiencing other forms of discrimination. Using a subsample of respondents 65-96 years of age from the 2016 Health and Retirement Study, 1881 older adults who reported experiencing everyday discrimination were included. Discrimination was measured by the six-item Everyday Discrimination Scale. Among them, 955 respondents named age as the reason behind the discrimination experiences, and 926 named other forms (e.g. race). Controlling for health-related variables (e.g., ADLs/IADLs), SES (e.g., income), and sociodemographics (e.g., gender), findings from the weighted OLS regression models showed that volunteering (doing unpaid work for religious, educational, health-related, or other charitable organizations) related to lower depressive symptoms (8-item CES-D, $P<.05$). When looking at the groups who experienced different forms of discrimination, volunteering continued to have a negative relationship with depressive symptoms for older adults who experienced ageism ($p<.01$); however, such a relationship disappeared for the group experiencing other forms of discrimination. Discussions will target the importance of understanding the complicated relationships that may exist in volunteering, mental health, and the forms of discrimination that older adults experience.

### SESSION 7770 (POSTER)

### CARE VALUES, PREFERENCES, AND ETHICS

Abstract citation ID: igad104.2884

### APPLYING A BILL OF RIGHTS FOR PEOPLE UNDER GUARDIANSHIP IN RURAL AREAS

Pamela Teaster, and E. Carlisle Shealy, *Virginia Tech, Blacksburg, Virginia, United States*

In the U.S., 35.2% of people aged 65 and older live with a disability, of which 8.9% have a cognitive disability. For some, cognitive disabilities necessitate the help of a surrogate decision-maker (e.g., a guardian), including adults with serious mental illness, intellectual disability, and traumatic brain injury. Guardians are bound by statutory requirements, case law, and ethical principles to act in the best interests of an adult adjudicated by a court to lack the capacity to make certain decisions. Guardians are charged to act according to the highest standards of care, accountability, trust, honesty, confidentiality, and avoidance of conflict of interest. Powers given to guardians are often immense (e.g., authority to sell a person's home and personal property, consent to medical treatments). Little is known about the characteristics of people with a guardian. Our study in rural Virginia examined all new guardianships of adults in one jurisdiction across three years (89% Caucasian, 54% male). Their most common disabilities were dementia and intellectual disabilities. No guardian's report indicated any improvements in the adults' mental or physical health. The National Guardianship Network, a collaboration of 13 national organizations, drafted a Bill of Rights for Adults with a Guardian, categorizing rights always retained after a guardian is appointed, personal rights that a court may restrict but not delegate to a guardian, and rights a guardian may exercise on behalf of an adult. Characteristics of a population of persons with a guardian living in a rural area are presented alongside considerations of their rights.

Abstract citation ID: igad104.2885

### IDENTIFYING THE PATTERNS OF CAREGIVING DEMANDS: A LATENT CLASS ANALYSIS APPROACH

Sung Hyun Ko[1], Yeonjung Lee[1], and Alex Bierman[2], *1. Chung-Ang University, Seoul, Republic of Korea, 2. University of Calgary, Calgary, Alberta, Canada*

Caregiving demands comprise the care tasks being performed or assistance being provided. Previous studies have assessed the care demands as intensity and types of care provided using various measures such as the number of care tasks provided, the hours of care provided, the number of ADL/IADL they assisted with, and the number of people assisted. However, most caregiving literature has treated caregiving demands as one-dimensional rather than considering the multi-dimensional attributes of care demands. This study examines distinct patterns of caregiving demands, each formed by varied combinations of the hours spent providing care a week, how often care was provided, the location care provided, and whether care was provided as a primary caregiver. Based on the 2022 Caregiving, Aging, and Financial Experiences (CAFE) Study data, the latent class and regression analysis were conducted on a nationally representative sample of Canadian informal caregivers. We identified four distinct patterns of caregiving demands: excessive, moderate, manageable, and minor. Excessive and moderate patterns share the characteristic of providing in-home care as primary caregivers, but the excessive group has higher burden levels than the moderate pattern. Manageable and minor patterns show low levels of burden as secondary caregivers, but the minor group does not provide in-home care. These patterns differ in caregivers' sociodemographic characteristics (caregiver age, gender, minority, education, living with a partner, working status, and relationship to a recipient). The findings provide the basis for further understanding how the multidimensional layers of care demand conjointly shape the patterns of caregiving demands.

Abstract citation ID: igad104.2886

### PERSON-CENTERED CARE WHEN OLDER ADULTS WITH DEMENTIA AND THEIR FAMILY CAREGIVERS HOLD DIFFERENT CARE PRIORITIES

Emma Quach[1], Martina Azar[2], Katherine Kennedy[3], Catherine Dawson[1], and Chelsea Hawley[1], *1. VA Bedford Healthcare System, Bedford, Massachusetts, United States, 2. VA Boston Health Care, Boston, Massachusetts, United States, 3. Providence VA Medical Center, Providence, Rhode Island, United States*

In person-centered care, clinicians develop care plans that incorporate "what matters most" to patients (i.e., their priorities, concerns, and preferences), a process complicated when patients' family caregivers have their unique priorities. To investigate care planning with patients with dementia and their family caregivers, we audio-recorded, transcribed, and thematically analyzed 4 medical visits dually attended in 2022 by older patients with dementia and their spousal/adult child caregiver(s). In these visits clinicians asked both about their priorities/concerns, which mostly complemented but sometimes conflicted with each other. Older adults' priorities related mostly to social activities and minimally to health care, emphasizing continuity. Older adults strived to resume past social activities (farmer's market), continue enjoyable life-long activities (sports), maintain non-problematic medication regimens, and retain the same home health nurse. Caregivers' priorities focused on emergent physical problems (patient's worsening memory and leg pain), service access, and care coordination. When older adult and caregiver priorities did not conflict, clinicians incorporated priorities from both sources into care plans (initiated referrals for farmer's market). When older adults and caregivers' priorities conflicted (exercise), clinicians simultaneously acknowledged family caregiver's concerns, expressed their (clinician) views, honored older adults' preferences, and invited them to re-assess such preferences in future medical visits. By asking "what matters most" to both patients and their family caregivers, clinicians gain rich insights about both parties to guide care planning. Future research should examine care planning with more dementia dyads to identify care planning practices that promote both patient and caregiver outcomes and ultimately enable aging in place.

Abstract citation ID: igad104.2887

### PILOTING COMMUNICATION TOOLS TO ENHANCE RESIDENT/FAMILY ENGAGEMENT IN NURSING HOMES CARE CONFERENCES

Jingxi Li[1], Alexa Roscizewski[2], Terra Warthen[3], Elizabeth Cox[2], and Tonya Roberts[1], *1. University of Wisconsin-Madison, Madison, Wisconsin, United States, 2. University of Wisconsin-Madison, School of Medicine and Public Health, Madison, Wisconsin, United States, 3. University of Wisconsin-Madison, School of Nursing, Madison, Wisconsin, United States*

Nursing home (NH) regulations mandate resident and family engagement in care planning. Additionally, our previous research indicated residents/families want to be involved in care decisions. Care conferences are an important opportunity for residents/families to engage in decision-making. However, there is a lack of guidance or tools to support their engagement and a dearth of research on the most effective approaches to doing so. This study aimed to pilot test a family engagement intervention using resident, family, and staff communication tools that refocus the agenda of care conferences to topics identified as important by residents/families. The feasibility of the intervention was evaluated using a pre/post-implementation design and a preliminary analysis of effects was performed. A total of 16 (n=8 pre; n=8 post-implementation) care conferences (average length ~22min) were observed, audio-recorded, and coded using the Roter Interaction Analysis System and our prior engagement coding schemas. Frequencies of engagement outcomes were compared pre- and post-implementation using Mann-Whitney U and Fisher's exact tests. Results suggest participants found the intervention valuable for care planning. The agenda and focus of care conferences also shifted to topics residents/families deemed important. Levels of engagement in relationship building and information exchange generally trended in a positive direction. Resident/family agenda setting was significantly higher after implementation (p=0.001). This study provides important insights into enhancing resident/family engagement in decision-making and suggests incorporating communication tools into care conferences may promote resident/family engagement. Future studies using randomized controlled trial designs and larger samples should be conducted to further evaluate effects.

Abstract citation ID: igad104.2888

### SHARED EXPERIENCES OF MANAGING HEART FAILURE AND MULTIMORBIDITY: A QUALITATIVE ANALYSIS

Katie Nelson[1], Chitchanok Benjasirisan[2], Arum Lim[2], Lyndsay DeGroot[3], Sarah Badawi[3], Cheryl Dennison Himmelfarb[2], Patricia Davidson[4], and Binu Koirala[3], *1. Johns Hopkins Center for Indigenous Health, Baltimore, Maryland, United States*, *2. Johns Hopkins University, Baltimore, Maryland, United States*, *3. Johns Hopkins University School of Nursing, Baltimore, Maryland, United States*, *4. University of Wollongong, Wollongong, New South Wales, Australia*

Heart failure affects more than 6 million people nationally; most are older adults who have multimorbidity. Navigating symptom management and self-care maintenance can be challenging given heart failure's non-linear trajectory. Therefore, identifying shared priorities is important for guiding steps to enhance care provision. Our study aimed to elicit experiences and perspectives regarding heart failure and multimorbidity management among patients, caregivers, and healthcare workers via semi-structured interviews. We used purposive and convenience sampling to recruit patients 50 years and older living with heart failure and comorbidities, family caregivers, and interdisciplinary healthcare workers from an academic medical center in the Mid-Atlantic region of the U.S. Participants were asked about disease trajectory, recognizing/treating symptoms, and service utilization. We used inductive thematic analysis and presented key findings at virtual co-design events, whereby participants ranked priorities to determine next steps. Forty-six interviews were conducted: 24 patients (mean ± SD: 70.1 ± 9.16 years; 46% female), 7 family caregivers (63.6 ± 14.18 years; 57% female), and 15 healthcare workers (35.6 ± 10.04 years; 80% female). Four themes were emphasized across groups: 1) clear communication, 2) health education for patients and caregivers, 3) care coordination and follow-up post-discharge, and 4) provision of mental and emotional support. Patients, caregivers, and healthcare workers uniquely contribute to heart failure and multimorbidity management. All groups share priorities surrounding clear communication, health education and comprehensive support. These factors can be significant barriers or facilitators in terms of optimizing patients' symptoms and quality of life.

Abstract citation ID: igad104.2889

### SUPPORTING CARE PARTNERS OF PEOPLE LIVING WITH DEMENTIA: EXPLORING CHANGES IN POLICY AND PRACTICE

Walter Dawson, Jenn Hollandsworth Reed, Anaeliz Colon, Devlin Prince, Sofia Chapela, and Sherril Gelmon, *OHSU-PSU School of Public Health, Portland, Oregon, United States*

Most care and support for people living with Alzheimer's Disease and related dementias (ADRD) is provided unpaid at home or in community settings. To better understand the caregiving-related priorities of people with lived experience as care partners (CP) of people living with ADRD, focus groups were convened with CPs who identify with historically and currently underserved communities that may have difficulty accessing culturally specific services for people living with ADRD in and near Portland, Oregon. In-depth interviews (n=20) were conducted with local, state, and national-level organizational leaders who represent or provide ADRD support services to various underserved communities including Black, Indigenous, Asian, and Latinx Americans to gather additional perspectives on ADRD care and support needs. Care partners across all populations reported a need for additional supports that could help them in carrying out their caregiving responsibilities. While the specific needs and types of supports identified varied across populations, the findings point to multiple unmet needs across all populations in terms of ADRD CP-specific supports. Changes to policy and practice are needed to improve the experience of CPs of people living with ADRD. New policies focused on promoting access to services, education and training, and financing of supports such as respite must be developed and implemented in partnership with community-based organizations and ADRD CPs from underserved communities and communities that may have difficulty accessing culturally-specific services and unique supports. The findings also point to universal needs related to caregiving across all populations that could be addressed through new policy initiatives.

### SESSION 7780 (POSTER)

### AGING IN PLACE (SRPP)

Abstract citation ID: igad104.2890

### A MODEL ACCESSORY-DWELLING UNIT BYLAW: INCLUSIVE ZONING FOR ALL

Travis Gagen, *Springfield College, Springfield, Massachusetts, United States*

Adapting the built-environment to include an accessory-dwelling unit (ADU) is one alternative housing solution for older adults to remain in their home and community, avoid institutionalization, and create age-friendly environments. ADUs are one form of the built-environment in which the field of public health law can intervene to improve population aging. A legal mapping analysis was conducted using the ADU Friendliness Score to assess ADU accessibility across the target population (n = 351 Massachusetts municipalities). Municipalities were stratified into four ordinal classifications according to their ranking on the 0-100

ADU Friendliness Score and served as the dependent variable: 0-24 POOR (89 municipalities, 25%); 25-49 FAIR (30 municipalities, 8.5%); 50-74 GOOD (185 municipalities, 53%); and 75-100 EXCELLENT (47 municipalities, 13.5%). Community demographics and social capital proxy measures were used as independent variables to test for the association between aggregate social capital and the dependent variable. A regression analysis demonstrated housing density and educational attainment as statistically significant predictors of ADU score. Findings informed the first model age-friendly ADU bylaw inclusive for older adults, persons with disability, and their caregivers. Reform of zoning laws can help create age-friendly environments. The model bylaw is available for municipals to adopt and can be modified in response to municipal needs.

Abstract citation ID: igad104.2891

### A POLICY COMPARISON OF THE EVOLUTION OF COMMUNITY-BASED LONG-TERM CARE POLICY BETWEEN TAIWAN AND THE UNITED STATES

Allie Peckham[1], Li-Chuan Liu[2], and Laura Schmid[1], *1. Arizona State University, Phoenix, Arizona, United States, 2. National Taitung University, Taitung, Taitung, Taiwan (Republic of China)*

Governments globally have been facing pressure to develop and implement effective policies to support population aging. This approach was historically rooted in a perception that as we age we become increasingly incapacitated. This research seeks to understand how policies in two countries valuing unpaid care differently, developed and implemented Long-term services and supports (LTSS). We compare the federal level efforts of implementing LTSS within the United States (U.S.) and Taiwan. Relying on a pre-established comparative framework developed by Richard Rose, we utilized a deductive analytical approach to assess the differences in policy options and timelines across Taiwan and the U.S.. After attending this session, participants will develop a deeper understanding of how Taiwan and the U.S. are working to offer home and community-based long-term care services. It wasn't until the 1980s for the U.S. and late 1990s for Taiwan that LTSS were introduced with the intention of supporting people to live as independently as possible in their own homes for as long as possible (e.g., Department of Health and Human Services, 3-year Care Service Development Plan). It is likely that the delay in LTSS delivery between the two countries is rooted in the role that unpaid support is expected to play. There is increasing evidence that countries are shifting their policy efforts to focus less on aging as a social dependency and more on policies that encourage healthy longevity.

Abstract citation ID: igad104.2892

### AGING IN PLACE IN JAPAN: CHALLENGES AND FACILITATORS OF OLDER-TO-OLDER CAREGIVING

Mineko Wada, and Hideaki Hanaoka, *Hiroshima University, Hiroshima, Hiroshima, Japan*

The proportion of older adults (age 65+) in Japan has been steadily growing, and reached almost 30% in 2022, its highest-ever level. As the benefits of aging in place have been promoted at individual, community, and policy levels, the number of households where an older adult cares for another older adult has been increasing. However, currently little is known about the challenges and facilitators of older-to-older caregiving. This study aims to review extant literature to identify challenges experienced by older-to-older caregivers and facilitators that could help them. Drawing on a review protocol informed by Arksey and O'Malley's framework and Preferred Reporting Items for Systematic reviews and Meta-Analysis extension for Scoping Reviews (PRISMA-ScR), we searched six databases (MEDLINE, CINAHL, PsycINFO, Cochrane, Scopus, Google Scholar) using combinations of search terms including "older," "aged," "senior," "caregiver," "carer," "care provider," and "care recipient." To be included in the study, the literature must be written in English and based on empirical studies focusing on older caregivers (age 65+) who care for older adults (age 65+) in community settings. The reviewed literature suggests that older caregivers experience substantial distress, caregiver burden, and sleep deprivation, which increases the risk of depression and diminished well-being, particularly if they care for older adults living with dementia. Facilitators of caregiving include strategies for self-care and problem-solving to address challenges. Future research needs to investigate (1) support options that could reduce older caregivers' burden and promote their well-being and (2) older care recipients' experiences of being cared for by older cares.

Abstract citation ID: igad104.2893

### DEMENTIA-FOCUSED ADULT DAY CARE IN AN URBAN SETTING: PROGRAM STAKEHOLDERS' VIEWS OF CRUCIAL COMPONENTS

Joy Ernst, and Faith Hopp, *Wayne State University, Detroit, Michigan, United States*

Dementia focused adult day centers (ADCs) provide socialization, physical activity, and cognitive activities for participants, and respite for caregivers. This study reports findings from a process evaluation of an ADC serving a predominantly African American population in a large midwestern city. The evaluation examined whether the program was being implemented as intended, what was working/not working, and participant engagement. We conducted in-depth interviews of staff, volunteers, and student interns and surveyed caregivers (n=17). Thematic analysis of the interviews revealed themes such as "structured flexibility" in running the daily activities, an ethos of care that mixes encouragement of participation with respect for autonomy, ongoing and positive activity, and individualized attention to participants. Other findings revealed the value of consistent and experienced staff and reliance upon and benefits for students and volunteers. Quantitative and qualitative responses to caregiver surveys supported the interview themes. Caregivers valued the socialization and creative stimuli provided to participants. Guidance from program personnel included modeling communication strategies and being "in the moment" with the participants. The findings suggest that further work to inform the development of quality care and programming to improve the quality of life of older African Americans with dementia and their caregivers is needed. Specifically, there is a need to 1) identify essential components of ADCs; 2) document the ways that key ADC service philosophies are realized and communicated; and 3) explore the key role of volunteers and students in terms of their roles, activities, and motivations for working with older adults with dementia.

Abstract citation ID: igad104.2894

### DEVELOPMENT OF SELF-ASSESSMENT TOOLS FOR ORGANIZATIONS: PREVENTION AND DELAY OF DISABILITY PROGRAMS IN TAIWAN

Wan-Yu Chiu[1], Ya-Mei Chen[1], Hui-Fen Mao[1], and Ling-Hui Chang[2], *1. National Taiwan University, Taipei City, Taipei, Taiwan (Republic of China), 2. National Cheng Kung University, Tainan City, Tainan, Taiwan (Republic of China)*

This study aimed to develop a tool for service organizations to self-assess their characteristics and abilities of providing better disability prevention and function maintenance services. The development of the tool involved two stages. In the first stage, a self-assessment tool with 40 items was developed through literature based on Dunn's policy evaluation framework. The tool was then subjected to two rounds of Delphi method, and pilot test. Six experts were invited, and the Delphi method results showed high consensus. Pilot test invited 170 service organizations to complete the survey and the results showed good internal consistency and validity with 20 items and six dimensions, including internal efficacy, responsiveness, professional, community collaboration, environmental factors, and sustainability ($\chi2$[df=171, N = 170] = 282.88; $p < 0.001$; CFI = 0.923; RMSEA = .071; SRMR=.055). The sustainability (mean:4.20, SD: 0.61) and responsiveness (mean:4.20, SD: 0.59) showed the highest self-assess ratings. Combining internal resources and efficacy as internal efficacy showed good validity. and together with a new domain "internal efficacy" showed strong association with other domains. This tool could provide the program agencies to self-assess their ability to provide better disability prevention and function maintenance services. There were two new concepts, internal efficacy and sustainability, identified through the process which may indicate both of these two concepts are important to organization that provide health promotion services and culture-sensitive in Taiwanese society. Promoting these two concepts for future organizations is recommended.

Abstract citation ID: igad104.2895

### EMERGENCY PREPAREDNESS AMONG CLIENTS OF OLDER AMERICANS ACT PROGRAMS

Joanne Campione[1], and Kristen Robinson[2], *1. Westat, Durham, North Carolina, United States, 2. Administration for Community Living, Washington, District of Columbia, United States*

Older adults who are clients of Older Americans Act (OAA) programs, such as home-delivered meals and transportation services, often have unique needs during an emergency or crisis. Many OAA clients have mobility challenges and/or chronic health conditions, making it difficult to access and respond to emergency directions. For these reasons, in 2022, the Administration for Community Living added to the annual National Survey of Older Americans Act Participants (NSOAAP) a one-time module of questions regarding emergency preparedness. Among 5,136 respondents of the 2022 NSOAAP, 67% said they have ready 3 days' worth of food and water, 69% have a way to charge their phone without electricity, and 51% have a battery-powered radio. Thirty-seven percent of the clients said their household has a medical device that requires electricity. While 74% said they have a list of list of family, friends, and community organizations, only 37% said their household has a specific plan for a disaster or emergency. Seven percent said they would not evacuate if public authorities announced a mandatory evacuation with the top three reasons for not evacuating being: 1) concern about leaving pets, 2) lack of transportation, and 3) could not be moved due to health problems. In summary, support services that an older adult relies on to live at home, such as help from family caregivers and in-home health care, may be unavailable due to a disaster thus increasing a person's vulnerability and possibly leading to nursing home care that may have been otherwise avoidable.

Abstract citation ID: igad104.2896

### EVALUATION OF THE IMPACT OF COMMUNITY CARE IN KOREA: NAMYANGJU CITY'S FOUR-YEAR EXPERIENCE

Mi Sun Choi[1], Sangmi Cho[2], Yoonsei Park[2], Misuk Woo[2], Sumin Park[2], and Youngmin Cho[2], *1. Silla University, Busan, Republic of Korea, 2. Ewha Womans University, Seoul, Republic of Korea*

In 2019, the Korean government launched a community care project in response to the rising need for care brought on by the nation's aging population and state-funded medical expenses. Namyangju, Korea, has implemented community care for the care of older people. However, little is known about how to ensure the sustainability of community care by investigating the impact of community care services. To enhance our knowledge in this domain, we conducted focus group interviews (FGI) with 24 participants, including 18 service providers and six older adult service users, from August to November 2022. In the FGI, service providers and users identified vehicle and meal support services as core needs. They described the beneficial outcomes of these services, such as increased psychological well-being and the desire for independence and decreased depression. On the other hand, users expressed difficulty in accessing information about various services and mentioned restrictions on service usage (period, number of times), while the providers emphasized the difficulty of providing comprehensive services when health and welfare services were separated amid budget constraints. Both groups proposed solutions to these problems through community care services. Individual citizens' perceptions of universal welfare should be changed, while institutions must actively promote this program and recruit professionals. A partnership between private and public institutions must be established at the regional level, and services must be differentiated to reflect the region's distinctive features. Finally, national legislation must be enacted to promote care coordinators and ensure that appropriate budgets are allocated for successfully operating the program.

Abstract citation ID: igad104.2897

### HEALTH-SEEKING BEHAVIOR OF COMMUNITY-LIVING OLDER ADULTS RELATES TO AWARENESS OF COMMUNITY SERVICES

Denise Connelly[1], Navjot Gill[2], Marla Beauchamp[3], and Laura Brunton[1], *1. Western University, London, Ontario, Canada, 2. University of Waterloo, Waterloo, Ontario, Canada, 3. McMaster University, Hamilton, Ontario, Can ada*

Research has shown that older adults desire to age in their homes. To age at home safely and comfortably, there are a variety and number of community support services available, however, previous studies show older adults do not utilize these services fully. There is a need for clarity about this gap between availability and use. Understanding the construct of health-seeking behaviour by community-living older adults may provide insights into this identified gap. Therefore, the objective was to determine whether health-seeking behaviour in community-living older adults is related to: dimensions of wellness; awareness of community support services; living as an older man or woman; or being younger or older than 75 years. A cross-sectional study design involving 99 community-living older adults (≥ 65 years, without impaired executive function) employed validated outcome measures of physical function, fear of falling, personal resilience, social well-being, spiritual well-being, awareness of community support services, and health-seeking behaviour. Relationships were calculated using multiple linear regression. Health-seeking behaviour was positively correlated (R= 0.40, p<.01) with awareness of community support services. Health-seeking behaviour was not correlated with dimensions of wellness (i.e., physical function, fall risk, personal resilience, spiritual wellbeing, or social wellbeing). No difference between age groups (U=986.5, n1=68, n2=31, p=0.61) nor sex (U=931.5, n1=66, n2=33, p=0.24) was found for health-seeking behaviour. The findings indicate that awareness of community support services positively influenced health-seeking behaviour. The healthcare system should ensure equitable, accessible, age-friendly, and relevant information and distribution strategies to increase awareness of older adults for local supportive services.

Abstract citation ID: igad104.2898

### HELPING SENIORS AGING IN PLACE: ADULT GUARDIANSHIP AND ITS ROLE IN GERIATRIC SOCIAL WORK PRACTICE IN CHINA AND JAPAN

Zi Yan, *Waseda University, Tokyo, Tokyo, Japan*

As the most aging society in East Asia, a renewed adult guardianship system for the idea of respect for self-determination, practical use of remaining abilities, and re-socialization have been practiced in Japan for 20 years. While, compared with Japan, in lieu of a comprehensive adult guardianship regime, not until 2017, voluntary guardianship notarization has become the main alternative for supported decision-making and self-determination for incapacitated adults and a tool for mitigating the social risks of the frail elderly in China. Based on secondary data and semi-structured interviews, this study is the first attempt that demonstrates a comprehensive comparison of the recent developments and current forms of adult guardianship initiatives in mainland China and Japan. Besides highlighting the difficulties and challenges faced by these two countries, this article also outlined the types of guardians under their current legal framework, identified the social functions of guardians, the notary office in adult guardianship socialization, and the emerging geriatric social work initiatives adopted. The geriatric social work practices in China and Japan's adult guardianship point in particular toward the potential provocativeness: adult guardianship in the eastern context—a context marked by a strict family system that emphasizes the virtues of filial piety and discourages outside intervention in family matters. As the number of people with dementia is projected to reach 82 million in 2030, China and Japan's experience potentially holds wide significance for countries that lack substantial policies and programs for caring for the frail elderly.

Abstract citation ID: igad104.2899

### PERSONS AGING WITH A PHYSICAL DISABILITY: EVALUATING PARTICIPATION CHANGE USING A TOOL FOR COMMUNITY CARE PROVIDERS

Michelle Putnam[1], Kerri Morgan[2], Holly Hollingsworth[2], Rachel Heeb[2], Szu-Wei Chen[2], and Susan L Stark[2], *1. Simmons University, Boston, Massachusetts, United States, 2. Washington University in St. Louis School of Medicine, St. Louis, Missouri, United States*

Community participation measures for persons aging with disability were developed and evaluated to support community-based organizations (CBOs) with efficient assessment of change in participation and need for supports/services to facilitate participation. This study aimed to evaluate a set of 9 activity domain measures to broadly assess community participation and change in participation over time. A community-based sample (N=323) of persons ages 45-65 responded to a survey with repeated measures three times annually (T1, T2, T3) between 2019-2022. Nine activity domain measures were developed based on extant research and evaluated with assistance from community-based support service providers. Statistical analyses employed T-tests and Chi-square tests to assess change in participation over time, perceptions of participation satisfaction, and assistance needed to facilitate participation. Participants were asked if they thought changes were attributable to aging, the COVID pandemic, or other factors. Findings showed varying levels of participation across the 9 activity domains, with the lowest participation rate for employment and the highest participation rates for personal leisure and managing medications across T1, T2, and T3. Change in participation over the 3-year period was limited; most change was reported as activity reduction. In general, respondents indicated that reduction was due to their aging or the COVID-19 pandemic. Personal assistance, transportation, environmental modifications, and improved health were identified as factors needed to help increase participation levels. Conclusions were that activity domain measures demonstrated efficiency in identifying participation rates and change. CBOs may deem them useful for assessing support and service needs to facilitate participation.

Abstract citation ID: igad104.2900

### RELATIONSHIP BETWEEN SOCIAL ISOLATION AND EXPECTATION FOR PERMANENT LIVING IN A MARGINALIZED COMMUNITY IN JAPAN.

Yuichi Watanabe, *Musashino University, Nishitokyo, Tokyo, Japan*

Social isolation is a predominant issue for the well-being of older adults in Japan. In marginalized communities, there is a high aging ratio of over 50%, and many older adults live alone. If they did not have connections in the community, there would be huge problems, not just loneliness but also the lack of necessities of daily life. This fact could threaten their permanent living. Therefore, This research examines the relationship between social isolation and permanent living expectation. Data were gathered by surveying a marginalized community every two years from 2015 to 2019 (N=435). Logistic regression analysis was conducted. For measuring social isolation, the questionnaire includes two items as independent valuables; "How many contacts do you have with community residents in a week?", "How many contacts do you have with someone living out of this community in a week?." An item measured permanent living expectation as a dependant valuable; "Would you like to be living here permanently when you live alone?" Gender, age, economic status, health status, and living alone were also included in the analysis. The results show that the number of contacts with community residents significantly predicted the permanent living expectation(P<.00, odds ratio=1.099). Health status is also significantly associated with a permanent living expectation. Findings suggest that social isolation in a community is a risk for giving up a permanent living. It could be social exclusion. Our effort to make mutual connections and contacts among the community residents could ease the risk of social exclusion.

Abstract citation ID: igad104.2901

### RESILIENCE IN JAPANESE OLDER IMMIGRANTS AND ROLES OF COMMUNITY SUPPORT DURING THE COVID-19 PANDEMIC

Mineko Wada[1], and Sarah Canham[2], *1. Hiroshima University, Hiroshima, Hiroshima, Japan*, *2. University of Utah, Salt Lake City, Utah, United States*

The objective of this community-based study was to explore how Japanese older immigrants cultivated resilience in overcoming challenges during the COVID-19 pandemic and how Tonari Gumi, a community service agency, supported the process. As Japanese people make up a small proportion of the population in Canada, there are limited resources to meet their distinct needs. Thus, Japanese older adults were particularly affected by disrupted support and service systems when COVID-19 public health orders were implemented. In this qualitative study, seven community-dwelling Japanese older immigrants and five staff from Tonari Gumi participated in semi-structured interviews. The interviews were analyzed thematically using a conceptual lens of resilience, which refers to the ability to survive and thrive in the face of adverse life experiences. Our analysis yielded three themes: Challenges and concerns; Staying active: physically, mentally, and socially; and Creating needs-based services and programs to survive, connect, and enjoy. The initial challenge experienced by Japanese older immigrants was "a feeling of emptiness," followed by hardships associated with digital literacy, English literacy, fear of COVID, and concerns about the future. In response to the challenges, Japanese older immigrants stayed active by rebuilding and sustaining regular exercise habits, nurturing and sustaining positive mindsets, and sustaining and expanding social connections. Tonari Gumi developed and delivered a variety of new services and programs to meet the needs of survival, social connection, and fun. We will discuss key actions that older individuals and service providers took on to facilitate resilience and implications for research, policy, and practice.

Abstract citation ID: igad104.2902

### US HOSPITAL INFRASTRUCTURE: ARE ALZHEIMER'S CENTERS PREPARED TO ADDRESS DEMENTIA NATIONWIDE?

Angela Gutierrez[1], Cory Cronin[1], Berkeley Franz[2], and Graciela Muniz-Terrera[1], *1. Ohio University, Athens, Ohio, United States*, *2. Ohio University Heritage College of Osteopathic Medicine, Athens, Ohio, United States*

Evidence on the U.S. hospital infrastructure to address dementia is scant, obscuring health improvement efforts. This study investigates the availability of Alzheimer's Centers (ACs) in U.S. hospitals. Data come from the: (1) 2010 – 2021 American Hospital Association Annual Survey; (2) 2020 Area Health Resource File; and (3) 2020 U.S. Census. Utilizing data from U.S. general hospitals (n = 3,251), we employed multivariable logistic regression to examine hospital (size, ownership, teaching status), county (metropolitan, poverty, racial/ethnic county composition), and regional (U.S. region) predictors of AC availability (Yes/No). ACs in U.S. hospitals increased from 4.5% in 2010 to 5.9% in 2020. Large hospitals (>399 beds) had approximately 14 times higher odds of having an AC than small hospitals (< 50 beds; OR = 14.0; 95% CI = 6.44 – 30.46). Counties with a higher proportion of Latinx residents, relative to non-Latinx Whites, had lower odds of having an AC (OR = 0.05; 95% CI = 0.01 – 0.41). Northeastern (OR = 1.92; 95% CI = 1.15 – 3.22) and Midwestern (OR = 2.12; 95% CI = 1.34 – 3.37) hospitals had higher odds of having an AC than Southern hospitals. Findings extend our understanding of U.S. hospital capacity to address dementia. Although ACs have increased over time, hospitals in non-metropolitan and Southern counties, and those in counties with a high proportion of Latinx adults are less likely to have an AC. To address dementia needs fully, investment in a national infrastructure is critical.

Abstract citation ID: igad104.2903

### UTILIZATION OF COMMUNITY CARE SERVICE AND SUBJECTIVE WELL-BEING OF OLDER PEOPLE: EVIDENCE FROM WEIFANG, CHINA

Yichao Li, and Hong Mi, *Zhejiang University, Hangzhou, Zhejiang, China (People's Republic)*

Many developed countries have established community-based care systems to address care needs of older people. However, community-based care in China is still in an early stage, some of its shortcomings are highlighted under the COVID-19 pandemic. Studying the wellbeing effect of community care in China could provide policy reference for developing countries. Based on the Elderly Health Survey in Weifang, China, using the two-stage least squares method, the effect and mechanism of the utilization of community care services on the subjective wellbeing of elder people were evaluated. Results show that: first, the utilization of community care services could promote the subjective wellbeing of older people and has a stronger effect on people with a higher level of subjective wellbeing. Second, the utilization of different types of community elderly care services has different effects on the subjective wellbeing of older people. When receiving a single type of community elderly care services, medical care services have the strongest promotion effect, while taking spiritual and cultural services and other types of services together will be a stronger synergistic effect on subjective wellbeing. Third, with the support of various community service subjects, the utilization of community care services could improve the subjective wellbeing by improving their health status, sleep quality and declining feeling of pain. In this regard, this study proposes policy suggestion in optimizing the types of community care service projects, building elderly care service consortiums, and improving community health management service capabilities to coping with deep aging and fewer birth.

### SESSION 7790 (POSTER)

### ADULT PROTECTION AND ELDER ABUSE (POSTERS)

Abstract citation ID: igad104.2904

### A STUDY ON COOPERATION BETWEEN COMMUNITY COMPREHENSIVE SUPPORT CENTERS AND DV SUPPORT AGENCIES TO SOLVE ELDER ABUSE

Asako Katsumata[1], and Noriko Tsukada[2], *1. Japan Lutheran College Graduate School of Social Work, Chiba, Tokyo, Japan*, *2. Nihon University, Setagata-city, Tokyo, Japan*

The purpose of this study was to examine current and future cooperation between Community Comprehensive Support Centers (CCSCs) and Domestic Violence (DV) support agencies in dealing with elder abuse cases among older couples in Japan. The data used in this study are from a nationwide survey from Certified Social Workers (CSWs) at CCSCs in November 2022. A structured questionnaire survey was provided to a randomly selected 3,563 out of 5400 CCSCs, and a total of 1,472 responses from CSWs were obtained (response rate of 41.3%). Preliminary analyses revealed about 70% of the respondents felt that the cooperation with DV support agencies was important (36.8% “important”, 32.6% “somewhat important“). Reasons for why respondents felt the cooperation was “not very important” (2.2%), “not important at all”(0.1%), or “neutral”(28.3%) included “have never cooperated with DV support agencies before” (62.1%), “CCSCs are supposed to deal with abuse cases with people aged more than 65“ (55%), “don’t know about roles of DV support agencies” (42.5%) , followed by “staff members at DV support agencies were not very helpful” (28.1%). Moreover, among 443 respondents (30.1%) who have dealt with elder abuse cases that have continued from their younger age, 25% of CSWs sought for cooperation with DV support agencies, got help and shared information to solve those cases. Findings suggest that professional staff members at both CCSCs and DV support agencies need to be trained to be aware of importance of exchanging their knowledge and resources regarding how to solve elder abuse cases effectively and efficiently.

Abstract citation ID: igad104.2905

### “A FRAIL LITTLE THING, BUT SHE’S WILEY”: POLICE BRUTALITY MEETS DEMENTIA: A NARRATIVE INQUIRY

Kelly Marnfeldt, *University of Southern California, Los Angeles, California, United States*

From 2000 to 2020 overall arrests in the U.S. declined, but arrests of people 65 and older increased by nearly 30 percent. In addition, between 2010 and 2020 approximately 12,000 adults 65 and older were admitted to the emergency room due to injuries sustained during encounters with police or private security. To illuminate the scope of negative consequences that may occur during such encounters, the present study examined the 2020 arrest of a 73-year-old community-dwelling woman living with dementia who walked out of a big-box store with $13.88 worth of merchandise she did not pay for. During her arrest, she sustained multiple injuries at the hands of two officers, including a dislocated shoulder and a broken arm. Despite her injuries, she did not receive medical care for six hours while in custody. The methodological approach to this case draws on elements of interpretative phenomenological analysis, and narrative analysis to explore publicly available archival data sources, including surveillance and police body-worn camera footage. Supporting data sources include police filings at the time of her arrest and legal filings by a civil rights attorney representing the woman’s family. The analysis demonstrated that deeply entrenched ageism, failure to recognize the signs of dementia, and the systemic normalization and defense of police use of force against non-normative behavior when interacting with neuro diverse older adults. This has critical implications for family caregivers and trusted others who struggle to balance the dialectic between safety and independence for people aging in place with dementia

Abstract citation ID: igad104.2906

### DETECTING UNDUE HARM ARISING FROM ELDER ABUSE SCREENING: INNOVATIVE METHODS FROM THE EASI-LTC© PILOT STUDY

Stephanie Ballard[1], and Machelle Wilchesky[2], *1. Donald Berman Maimonides Centre for Research in Aging, Montreal, Quebec, Canada*, *2. McGill University, Montreal, Quebec, Canada*

Screening for elder abuse can cause victims to experience feelings of unpleasantness and/or relive painful memories which can be an ethical concern. Ensuring the safety of all participants/users, in our case long-term care (LTC) residents, is of the utmost importance. Drawing from approaches used in the intimate partner violence and clinical trials literature, we developed a novel typology of harm and a series of procedures to evaluate any negative consequences that might be incurred as a result of participating in the Piloting the Elder Abuse Suspicion Index-long term care©: A Mixed Methods Feasibility Study. Our typology includes subjective and objective reporting from a multitude of sources, including: 1) Interviews with LTC residents using a modified Consequences of Screening Tool, adapted from MacMillan et al, 2009; Opinions of the 2) Study social worker conducting validation assessments and 3) LTC residents and institutional stakeholders; 4) Feedback from study research assistants who administer the tool using a specialized Adverse Events Form; and 5) Chart reviews. Procedures include the creation of an independent interdisciplinary pilot study safety committee, chaired by a geriatric psychiatrist and convened to conduct interim evaluations after the first 5, 10, and 20 residents have participated, the liberation of social work teams, who are to be on standby and available to intervene as necessary, and the creation of participant resource cards displaying pertinent phone numbers for immediate assistance. To our knowledge, this is the first elder abuse study to conduct a thorough analysis of study-induced harm.

Abstract citation ID: igad104.2907

### I WISH I WAS DEAD: HEALTH CONSEQUENCES OF PHYSICALLY TRAUMATIZED OLDER ADULTS: A QUALITATIVE STUDY IN SOUTHERN GHANA

Abigail Appiah[1], Felix Apiribu[2], Veronica Dzomeku[2], Abigail Diji[3], Lilian Kolbila[4], Elizabeth Osei[5], Thomas Agyarko Poku[6], and Peter Donkor[2], *1. Suntreso Government Hospital (Ghana Health Service, Kumasi, Ashanti, Ghana*, *2. KNUST, Kumasi, Ashanti, Ghana*, *3. Kwame Nkrumah University of Science and Technology, Kumasi, Ashanti, Ghana*, *4. Ghana Health Service, Kumasi, Ashanti, Ghana*, *5. Ministry of Health, Accra, Greater Accra, Ghana*, *6. Ghana Health Service ( Suntreso Government Hospital), Kumasi, Ashanti, Ghana*

The transformation from a physically healthy to a physically impaired older adults due to the effects of ageing, disability or injury can be a difficult transition to navigate. Comparatively, research on trauma in the older adult population is less as opposed to the younger adults. Ghana do not have appropriate policies and programs to support the growing numbers of the older adults. This indicates that the prevalence of the hidden problem of abuse will continue to increase, and create major public health problems. For this reason the study seek to explore the physical health consequences of the physically traumatized older adults. The study employed a descriptive phenomenological design and was guided by the Social-Ecological model. A purposive sampling technique was used to recruit participants for the study. A semi-structured interview guide was used to conduct individual in-depth face-to-face interviews. All interviews were audio-taped and transcribed verbatim. Data collection and analysis were done concurrently and data saturation was achieved after interviewing the eighth participant. Data were analyzed manually using thematic analysis and five (5) themes were generated: Lack of self-support ( inability to perform simple tasks) , Lack of family and friends support (neglect) , Lack of community and government support (no policies and programs to empower them. Physically traumatized older adults experienced extreme physical, emotional, mental and psychological discomfort as a result of their inability to do the things they wish to do, the neglect by immediate family, friends and the community as a whole and poor unfriendly transportation system.

Abstract citation ID: igad104.2908

### USING INTERPERSONAL RELATIONSHIPS AND SOCIAL NETWORK TO UNDERSTAND ELDER MISTREATMENT RECURRENCE

Wei- Lin Xue, Zachary Hass, and Pi-Ju Liu, *Purdue University, West Lafayette, Indiana, United States*

The incidence of elder mistreatment are often recurrent rather than one-time. The recurrence of mistreatment contributes to severe physical and psychological problems for victims and negative social consequences. Poor interpersonal relationships and social isolation are the main factors of mistreatment occurrence in the literature. However, very few studies have considered how the quality of interpersonal relationships and the level of social networks affect the likelihood of mistreatment recurrence. We used the National Social Life, Health, and Aging Project waves 1 (2005–06) and 3 (2015–16) data for longitudinal analyses (N=1,592). The quality of interpersonal relationships (i.e., supportive relationship, strained relationship) and the level of social networks (i.e., social engagement, social isolation, and feeling safe in society) were conceptualized using factor analysis to estimate factor scores. Logistic regression models were built to predict the risk of recurrent mistreatment using interpersonal relationship quality and the level of the social networks. Controlling for demographic variables, victims in wave 1 with support from interpersonal relationships are more likely to experience reoccurring mistreatment ($p < 0.05$, OR=1.58). Model interaction effects indicated that older adults with strained interpersonal relationships and low social engagement are most at risk of experiencing mistreatment recurrence. The surprising finding that supportive interpersonal relationships are positively associated with mistreatment recurrence might indicate that those who support older adults are potential perpetrators. Identifying potential perpetrators and assessing the quality of interpersonal relationships and social networks are essential to preventing mistreatment recurrence.

### SESSION 7800 (POSTER)

### METHODS IN GERIATRIC RESEARCH

Abstract citation ID: igad104.2909

### A MENDELIAN RANDOMIZATION STUDY SUPPORTS CAUSAL EFFECT OF OVERWEIGHT ON LONGEVITY

Hongzhe Duan[1], Rachel Holmes[1], Konstantin Arbeev[1], Olivia Bagley[1], Deqing Wu[1], Anatoliy Yashin[1], and Svetlana Ukraintseva[2], *1. Social Science Research Institute, Duke University, Durham, North Carolina, United States*, *2. Duke University, Durham, North Carolina, United States*

Overweight was associated with better survival in some studies. This was termed “obesity paradox”; however, its mechanism is unclear. Mendelian Randomization (MR) is a common approach to uncovering causality in observational data using SNPs as instrumental variables (IVar). However, only few SNPs usually pass all the required assumptions. Here we use a new strategy of selecting IVar for the MR study of causal relationships between overweight and longevity in the Health and Retirement Study (HRS) data. We paired all SNPs in eight candidate obesity-related genes (ADIPOQ, FTO, LEP, LEPR, INSIG2, MC4R, PCSK1, PPARG) available in HRS, and used such ‘composite SNPs’ as IVar, which resulted in significantly more IVar candidates compared to single SNPs. The following variables were used in MR analysis as “exposure”: (1) ‘overweight’ BMI 25-30 vs. (0) ‘normal’ BMI 18.5-25, at ages 75-85; and “outcome”: (1) survived 85+ vs. (0) died before age 85. Inverse-Variance Weighted (IVW), and maximum likelihood (ML) from R-package MendelianRandomization were used to test causality. We found that being overweight at ages 75-85 had significant protective effect on survival at ages 85+ in both sexes, white males, and black males and females. In conclusion, pairing SNPs is a convenient simple approach to increasing numbers of IVar candidates for MR. Results of our MR study suggest that being overweight in late life causally contributes to longevity. One potential mechanism may involve larger energy reserves contributing to a better resilience of overweight people in face of an adverse health event, such as, e.g., major surgery.

Abstract citation ID: igad104.2910

### A SCOPING REVIEW: EXPLORING COLLABORATIVE GOAL SETTING AMONG AGING ADULTS AND CARE PARTNERS

Kate Perepezko[1], Tucker Alchin[1], Bianca Karibian[2], Catherine Still[2], Kayla Valente[1], Pamela Toto[1], and Beth Fields[2], *1. University of Pittsburgh, Pittsburgh, Pennsylvania, United States*, *2. University of Wisconsin-Madison, Madison, Wisconsin, United States*

Evidence suggests that goal setting and care partner support help aging adults improve their health. Less is known about how aging adults and care partners collaboratively participate in goal setting. The purpose of the current review was to describe the scope of the literature on this topic. A search was conducted in PubMed, CINAHL, SocINDEX, and PscyINFO. Reviewers screened 1,231 articles for the following inclusion criteria: 1) Participants included aging adults (50+ years) and care partners, 2) Goal setting was conducted, and 3) Articles were in English. Of the twenty-nine articles that were included, most (n=21) assessed goal setting for care partners and aging adults separately. Goal setting was primarily directed toward patient care, with only a few studies prompting care partner specific goals (n=4). Common goals reported by aging adults were independence, improving or maintaining functioning, addressing symptoms, and remaining socially active. Care partners listed similar goals but also identified accessing services and supports as important. This review is the first to describe goal setting with aging adults and their care partners, revealing concordant and discordant prioritization of goals within dyads. These findings illustrate the importance and potential complexity of including care partners in the goal setting process. We also found that collaborative goal setting and care partner directed goals are scarce, indicating the need for additional work in this area. Collaborative goal setting aligns with patient and family-centered care approaches and can contribute to better care plans that meet the needs of aging adults and their care partners.

Abstract citation ID: igad104.2911

### DATA SCIENCE TECHNIQUES TO GAIN NOVEL INSIGHTS ON QUALITY OF CARE: A SCOPING REVIEW OF LONG-TERM CARE FOR OLDER ADULTS

Sil Aarts, Ard Hendriks, Coen Hacking, and Hilde Verbeek, *Maastricht University, Maastricht, Limburg, Netherlands*

The increase in powerful computers and technological devices as well as new forms of data analysis such as machine learning have resulted in the widespread availability of data science in healthcare. However, its role in organisations providing long-term care for older adults (LTC) has yet to be systematically synthesized. This study provides a state-of-the-art scoping overview of (1) data science techniques that are used with data accumulated in LTC and for what specific purposes, and (2) the results of employing these techniques in researching the study objective. PubMed and CINHAL was searched using keywords on data science techniques and LTC. The screening-and selection process was carried out by two authors and not limited by research design or publication date. A narrative synthesis was conducted based on the two aims. The search strategy yielded 1,277 studies; 18 were included (the majority conducted in the US and in nursing homes). Machine learning models/algorithms were the most used techniques and were primarily used for researching specific adverse outcomes including the identification of risk factors for falls and the prediction of frailty. This review reveals the limited use of data science techniques on data from LTC facilities. The low number of articles indicates the need for strategies aimed at the effective utilization of data science techniques and evidence of their practical benefits. There is a need for a wider adoption of these techniques in order to exploit data to their full potential and improve the quality of care in LTC by making data-informed decisions.

Abstract citation ID: igad104.2912

### HIDING IN PLAIN SITE: LOW BACK PAIN RATES IN OLDER ADULTS WITH LUMBAR SPINAL STENOSIS

Beth Hogans[1], Bernadette Siaton[2], Stuart Goldman[3], and John Sorkin[4], *1. VAMHCS / Johns Hopkins School of Medicine, Baltimore, Maryland, United States*, *2. University of MD School of Medicine, Baltimore, Maryland, United States*, *3. Sinai/Life Bridge, Pikesville, Maryland, United States*, *4. VAMHCS/UMB, Baltimore, Maryland, United Sta tes*

Introduction: Lumbar spinal stenosis (LSS) is highly prevalent in older adults, negatively impacting mobility and quality of life. Typically associated with progressive impairment of walking distance and pain which radiates into the legs, there is persistent controversy regarding the extent to which low back pain (LBP) is associated with LSS. **Objectives:** To evaluate population-level evidence for and strength of an association of LBP with LSS in older adults. Data Source: Medicare Part B Carrier and Outpatient claims data, 5% standard analytical file for 2017. **Methods:** Unadjusted and adjusted analysis of cross-sectional data, combined with condition process analysis **Results:** Several hundred thousand older adults met criteria for inclusion in this study. Lumbar spinal stenosis was common in both older adult males and females, but slightly more commonly diagnosed in females. Low back pain was prevalent in older adults but more so in females. A majority of older adults with LSS were diagnosed with LBP. Unadjusted and adjusted analyses demonstrated that LBP was present at increased rates in older adults with LSS. **Conclusions:** Low back pain is diagnosed in the majority of older adults diagnosed with lumbar spinal stenosis. It is important to recognize that the symptom profile of older adults with spinal stenosis includes low back pain and treatment should be adjusted to respond to this management need.

Abstract citation ID: igad104.2913

### IMPACTS OF CHANGE FROM 27 TO 30 CHRONIC CONDITIONS ALGORITHM ON PREVALENCE OF DIABETES AND DEMENTIA

Hyosin Kim[1], Anum Zafar[2], Haiqun Lin[3], Soko Setoguchi[2], and Olga Jarrín[2], *1. Oregon State University, Corvallis,*

*Oregon, United States*, 2. *Rutgers, The State University of New Jersey, New Brunswick, New Jersey, United States*, 3. *Rutgers, The State University of New Jersey, Newark, New Jersey, United States*

The Centers for Medicare and Medicaid Services (CMS) Chronic Conditions Warehouse (CCW) algorithm used to identify the presence of chronic conditions recently changed. Not only was the number of chronic conditions changed, but also the diagnosis codes and look-back period. Accompanying documentation encourages researchers to empirically assess the change in algorithm for their studies. The purpose of this study is to highlight the impacts of switching to the 30 CCW Chronic Conditions file on the prevalence of selected chronic conditions (diabetes and dementia) in an end-of-life cohort of Medicare beneficiaries (n=2,182,092), with sub-analysis by insurance type. Data sources included the Master Beneficiary Summary File base file and chronic conditions segments (27 vs. 30). The study population was all U.S. Medicare beneficiaries over age 18 who died in 2019. This retrospective observational study summarizes the change in prevalence of diabetes, dementia, and stroke/transient ischemic attack resulting from the new chronic conditions algorithm (27 to 30), stratified by insurance type. Among Medicare beneficiaries who died in 2019, using the 30 vs. 27 Chronic Conditions algorithm decreased the prevalence of diabetes (45% vs. 32%) and dementia (41% vs. 31%). This change may pose challenges for longitudinal and end-of-life research. A decrease in the counting of dementia, diabetes, and stroke cases could lead to an underestimation of their burden on society and be misleading for policymakers and the public. This could result in inadequate policies and resources to address prevention and treatment, which could result in inadequate healthcare services for the affected population.

Abstract citation ID: igad104.2914

### PEER (DYADIC) SUPPORT: A FEASIBILITY STUDY FOR OLDER AFRICAN AMERICAN WOMEN WITH HYPERTENSION

Angela Groves[1], Maria Roche-Dean[2], and Emily Bosak[2], *1. Western Michigan University, Portage, Michigan, United States*, 2. *Western Michigan University, Kalamazoo, Michigan, United States*

African American women have a higher prevalence of hypertension than women of other ethnicities. Interventions have not adequately addressed the higher prevalence of hypertension in this population. Social support networks can be an effective strategy for improving hypertension self-management behaviors. The objective of this study was to evaluate the feasibility of an 8-week peer (dyadic) support intervention to improve diet adherence and reduce systolic blood pressure among older African American women with hypertension and describe the relationship that occurred during the intervention. This mixed methods study used a convergent parallel design. A total of 26 African American women diagnosed with hypertension, aged 60 and older were paired to form 13 dyads. All participants completed a 3-hour training session consisting of home blood pressure monitoring, DASH diet, and communication. Participants completed pre and post DASH diet surveys, and the Social Support Survey (MOS) post intervention. At the end of the 8-week intervention, participants discussed their experiences in a 60-minute focus group session. Content analysis was used to analyze focus group interviews. Preliminary data analysis will be presented. Emerging themes included, accountability, epiphany, challenges with hypertension management, communication with each other, and receiving support. Preliminary findings suggest that peer (dyadic) support is feasible and can be an effective intervention strategy to improve diet and blood pressure management. Future studies should address specific challenges to hypertension management identified by participants. Lastly, this study used random within site pairing for participants without an identified partner. Future studies should develop criteria for pairing partners.

Abstract citation ID: igad104.2915

### RECRUITMENT OF PERSONS WITH COGNITIVE IMPAIRMENT FROM LONG-TERM CARE SETTINGS

Rebecca Davis[1], and Alla Sikorskii[2], *1. Grand Valley State University, Grand Rapids, Michigan, United States*, 2. *Michigan State University, East Lansing, Michigan, United States*

Research that includes persons with cognitive impairment is important to develop evidence-based care to improve outcomes. Long -term care settings have a large proportion of residents with cognitive impairment; but recruitment is known to be difficult. The purpose of this study was to compare the effectiveness of recruitment methods for a randomized controlled clinical trial of persons with cognitive impairment in 15 long-term care communities. This study utilized a retrospective, descriptive design. Sites were recruited and site characteristics recorded. Residents were recruited to participate in the study based on combinations of 12 recruitment strategies. Recruitment data was analyzed at the level of site, research staff member, and participant level. Over the study period, 279 older adults were screened and 172 enrolled. The participants were cognitively impaired. Research staff spent 39-89 hours recruiting at each site and utilized an average of four different recruitment methods per site. The most common and efficacious method was introductions of participants by site contacts. Cost of recruiting averaged $1490 per site and was correlated to the number of resident rooms (r=.60, p=0.17). Those sites with a recruiter enrolled more participants (M=14.67, S.D. 4.55) than those without a recruiter (M=9.3, S.D. 2.55), t(13) = -2.92, p=.012. Recruitment of older adults with cognitive impairment from long term care communities requires multiple methods of recruitment; skilled recruitment staff, and trust with the LTC community staff and potential participants.

Abstract citation ID: igad104.2916

### UTILIZATION OF LASSO AND POISSON REGRESSION DEALING WITH COUNT VARIABLES IN NURSING HOME RESEARCH

Juh Shin[1], Eun Jeong Min[2], Sun Ok Jung[3], and Jung Eun Kim[4], *1. George Washington University, Washington, District of Columbia, United States*, 2. *The Catholic University of Korea, Seoul, Republic of Korea*, 3. *Ewha Womans University, Seoul, Republic of Korea*, 4. *California State University, Los Angeles, Los Angeles, California, United States*

Regression has been used for decades; thus, more empirical studies are necessary to choose appropriate regression methods, as traditional regression has many drawbacks and is limited in analysis and synthesis with large numbers of covariates. This study aims to investigate factors related to pressure ulcers in nursing homes using three regressions: least absolute shrinkage and selection operator (LASSO) Poisson, LASSO linear, and LASSO logistic regression. We represent how each model's estimating, inferencing, and performance measures work. The datasets accrued from 51 nursing homes that operated between 2021 and 2022. A total of 35 independent variables included organizational and nurse staffing variables. The variable of interest was the number of residents in each nursing home who had an occurrence of pressure ulcers within the last 3 months. The significant factors were different; turnover of Certified Nurse Aides and government incentives of more RNs were significant in the LASSO Poison regression, no factors were significant in the LASSO Linear regression, while and the percentage of residents who are completely dependent for activities of daily living in LASSO was significant factor in the Logistic regression. Using the coefficients in the Poisson regression, we interpreted that more nursing staff turnover increased the percentage of residents with pressure ulcers by 3.02 times, which would not be possible with linear regression. The Lasso model uses a technique of shrinking coefficient estimates to zero to improve the fit of a model that includes all explanatory variables and improves model interpretability by identifying most important features.

### SESSION 7810 (POSTER)

### HEALTH AND SOCIAL SERVICES INTERVENTIONS

Abstract citation ID: igad104.2917

### AGING WITH HIV: ASSOCIATION OF ART REGIMEN AND ADHERENCE TO VIRAL SUPPRESSION

Jasmine Manalel[1], Jennifer Kaufman[2], Ethan Fusaris[3], Arlene Correa[4], Jerome Ernst[3], and Mark Brennan-Ing[5], *1. Hunter College, New York City, New York, United States*, *2. Brookdale Center for Healthy Aging, Hunter College, City University of New York, New York City, New York, United States*, *3. Amida Care, New York, New York, United States*, *4. Brown University, Providence, Rhode Island, United States*, *5. Hunter College, CUNY, New York City, New York, United States*

Adherence to antiretroviral therapy (ART) is essential to effective management of HIV among an increasing population of older adults. Newer ART regimens are more "forgiving" of poor adherence allowing for viral suppression at lower adherence levels (i.e., < 90%). This is especially promising for some older adults who may struggle with adherence due to high rates of multimorbidity and polypharmacy. We aimed to determine how ART forgiveness varies depending on ART type and adherence. Data came from 2017-2019 claims and clinical records of 1,044 HIV-positive members of a Medicaid managed care plan who were 50 to 65 years of age. Pharmacy fill data were examined on a quarterly basis to characterize ART medications using latent class analysis (LCA), which captures the complexity of real-world ART usage (i.e., multiple medications, ART switching). LCA yielded five distinct ART utilization patterns over three years, mostly characterized by recent medications and formulations of ART, though they varied in number of tablets and in medication class (e.g., boosted protease inhibitors). Logistic regression models adjusting for demographics and health-related covariates revealed that ART medication patterns did not consistently differ in odds of maintaining viral suppression across adherence levels (< 50%, 50% to < 80%, 80% to < 85%, 85% to < 90%, and >=90%). In contrast, other factors, including nadir CD4 count and behavioral health conditions, were related to viral suppression across models. This highlights the importance of life-course illness trajectories when considering disease management and treatment strategies for older adults living with HIV, especially those with difficulty maintaining near-perfect adherence.

Abstract citation ID: igad104.2918

### DEMOGRAPHIC VARIATION IN BARRIERS TO VACCINATION AMONG OLDER ADULTS: LINKS TO VACCINATION BEHAVIORS

Heather Fuller, Andrea Huseth-Zosel, Bryce Van Vleet, and Paul Carson, *North Dakota State University, Fargo, North Dakota, United States*

As older adults face increased infectious disease risks, understanding barriers to vaccination is vital. The current study examines associations between vaccination barriers, demographics, and vaccination behaviors among older adults (65+) in North Dakota, a rural state with low vaccination rates. A mailed survey, including closed- and open-ended questions regarding vaccine beliefs and behaviors was conducted with 901 older adults. Potential vaccine barriers (Disliking shots, Concerns about cost, Lacking doctor recommendation, Lacking transportation, and Uncertainty how to schedule) were rated on a four-point Likert-type scale (1=Strongly Disagree; 4=Strongly Agree). Vaccine behaviors were assessed with measures of uptake for influenza, shingles, pneumonia, and SARS-CoV-2 immunizations. While endorsement of perceived barriers was relatively low, the greatest perceived barriers were cost and disliking shots. Regarding demographic variation: lower income was associated with cost concerns, lower education and rurality were associated with lacking doctor recommendation, and lower education was associated with uncertainty how to schedule. Disliking shots was significantly associated with uptake for shingles and pneumonia. Cost concerns was associated with lower shingles uptake, whereas lacking doctor recommendation was associated with lower uptake for pneumonia and SARS-CoV-2 booster. No barriers were associated with influenza or SARS-CoV-2 initial immunization uptake. While perceived vaccination barriers were low, certain populations of older adults are more at-risk of experiencing barriers, suggesting intervention opportunities. Moreover, the relevance of barrier type varied by disease, suggesting the need for disease-specific interventions. These findings suggest that practitioners should consider the diverse factors influencing barriers to older adults' vaccination receipt.

Abstract citation ID: igad104.2919

### HOW PERSON-CENTERED SERVICE DELIVERY SUPPORT QUALITY OF LIFE: WHAT NCI-AD TELLS US

Stephanie Giordano[1], and Rosa Plasencia[2], *1. Human Services Research Institute, Cambridge, Massachusetts, United States*, *2. ADvancing States, Arlington, Virginia, United States*

COVID-19 left a broad impact - for many older adults the pandemic led to changes in perceptions of person-centered services and experience with LTSS. Person-centered service delivery is widely accepted as a gold standard to supporting better outcomes, experience, and satisfaction; yet, there remains a gap in understanding person-centeredness from the perspective of people receiving the service. NCI-AD surveyors hear directly from older adults and people with physical disabilities on satisfaction with service delivery and quality of life. Using 2021-22 data, we'll discuss the perception of responsiveness and person-centered service delivery among other important measures. For example, older adults who were satisfied with provider response were more likely than those who were not satisfied to have more positive outcomes related to access to the community, ability to maintain relationships, and fewer unmet service needs. This session will delve further into this relationship between person-centered service delivery and quality of life outcomes, addressing what this means for policy and practice. Data presented come from the 2021-2022 National Core Indicators® Aging and Disabilities (NCI-AD™) Adult Consumer Survey (ACS). The ACS includes two main survey sections. The Background Information provides key data about survey participants, including demographics, service-related characteristics, and clinical diagnoses, typically collected from administrative records about the person. The main survey section includes questions on a range of topics that address the quality of the service delivery system; including responsiveness of services and supports, availability of needed services, and quality of life outcomes.

Abstract citation ID: igad104.2920

### IMPACT OF HOME HEALTH VALUE-BASED PURCHASE MODEL ON OWNERSHIP OF HOME HEALTH AGENCY: A LONGITUDINAL ANALYSIS

Chenjuan Ma, *New York University, New York City, New York, United States*

Home health care serves over 5 million Americans "aging in place". In January 2016, home health value-based purchasing model (HHVBP) was introduced and piloted in 9 states in the US with a purpose of improving care quality and efficiency, and expanded nationwide in 2023. This study aimed to examine the impact of HHVBP on business operation of home health agencies (HHA), including ownership changes. This is a longitudinal study using 7 years (2013-2019) of data from two national data sources. A dummy variable was used to indicate whether an HHA is in a state with HHVBP or not. Ownership measures included both the type and changes of ownership between 2013-2017. A total of 14,334 HHAs (95,137 agency-years) were included in analysis. Of the 14,334 agencies, about 19% were in HHVBP states, 88% continued business in all study years, 1679 were new agencies and 49 agencies discontinued business. In HHVBP states, 363 out of 2781 (13%) were newly added, compared to 1316 out of 11,553 (11%) in non-HHVBP states. Agencies in HHVBP states were more likely to be public agencies (e.g., 6.08% vs. 4.97% in 2013; 5.62% vs. 4.60% in 2018), though it slightly decreased over time; and experience changes in ownership and particularly the years after the implementation of HHVBP (e.g.,2.44% vs. 2.38% in 2013; 7.03% vs. 4.39% in 2018). The HHVBP can have a significant impact on the operation and ownership type of home health agency, which in turn may impact availability and quality of home health care.

Abstract citation ID: igad104.2921

### INCLUSION OF FAMILY MEMBERS INTO CONVERSATIONS ABOUT AGING RESEARCH: IMPORTANCE OF INTERGENERATIONAL INFLUENCE

Trudy Gaillard[1], Donna Neff[2], Cynthia Morton[3], Phildra Swagger[2], and Fern Webb[3], *1. Florida International University, Miami, Florida, United States*, *2. University of Central Florida, Orlando, Florida, United States*, *3. University of Florida, Gainesville, Florida, United States*

Identifying effective strategies to increase recruitment and retention of culturally diverse adults (African Americans [AA], Caribbean [CN] and Hispanic/Latino [HL]) into aging research is a public health priority. Intergenerational influence (IGI), defined as the "influence of one generation on another in terms of the transfer of skills, attitudes, preferences, values, and behaviors" (Shah & Mittal, 1997), was used to engage AA, CN and HL adults ages 25+ into conversations about aging research. We recruited AA, CN, and HL adults ≥ 65 years and a family member/friend between 25-64 years to participate in virtual listening sessions (LS). A semi-structured guide was used to discuss IGI, attitudes, beliefs, and perspectives of research. All LS were recorded and transcribed verbatim. NVivo software was used for data management and analysis. The constant comparative method was used for analysis. A total of 134 LS was conducted with African American [N=131], Caribbean [N= 112] and Hispanic/Latino [118] adults, representing 93 males and 268 females. We found three major themes highlighting the importance of IGI: 1) transfer of cultural knowledge; 2) benefit of future generations and 3) the lived experiences. The importance of IGI and the inclusion of family members and friends into discussions about healthy aging and research participation was a consistent theme among all culturally diverse groups. Findings suggest that discussing research participation with family and friends can enhance research participation of older adults. Researchers should develop IGI strategies that include family and friends (i.e., younger trusted sources) into recruitment design of aging research.

Abstract citation ID: igad104.2922

### ISSUES OF LIFE-SUSTAINING TREATMENT DECISION ACT IN CLINICAL PRACTICE FOR INDIVIDUALS AT THE END OF LIFE

Eunjeong Song, Dongsoon Shin, Jooseon Lee, Minjeong Eom, Suhee Oh, Seon Young Yun, Heejung Lee, and Rhayun Song, *Chungnam National University Hospital, Daejeon, Republic of Korea,*

This study aimed to investigate the status and issues related to the patient's decision-making regarding life-sustaining treatment (LST) in clinical practice and to provide strategic suggestions to improve the hospital policy. The retrospective observational study was conducted with adult patients, admitted between Jan 1 and Dec 31, 2021, who went through the end-of-life process and died in a university hospital in Korea. The electronic data included age, gender, admission date, expiration date, and Do-not-attempt-resuscitation (DNAR) and LST-related data (date to sign, who signed, and clinical state when signed). A total of 916 patients were included. Only 4.1% of the patients decided on LST by themselves, while the decision was made mainly by the family, specifically children (54.2%). The time of signing DNAR consent was before eight days from the date of death (33.7%) and the previous day of the death (27.6%). While early warning score was used to predict the critical state of the patients, the period from the date of sign to the date of death was 8.9 (SD=25.7) days for DNAR and 2.76 (SD=6.95) days for LST. Strategic suggestions were made to facilitate the timing of signing the consent form for LST based on the early warning score, and encourage patient involvement in the decision-making process. Further efforts are required to improve the awareness of patients and medical staff on the implementation of LST and to clarify the ambiguity of the timing of document preparation.

Abstract citation ID: igad104.2923

### NO DIFFERENCE IN SUBJECTIVE SLEEP MEASUREMENTS BETWEEN MIDDLE-AGED AND OLDER ADULTS

Sally Paulson[1], Michelle Gray[2], Megan Jones[2], Anthony Campitelli[2], Cody Diehl[2], Kelsey Bryk[3], Jordan Glenn[3], and Joshua Gills[4], *1. St. Elizabeth Healthcare, Edgewood, Kentucky, United States, 2. University of Arkansas, Fayetteville, Arkansas, United States, 3. Neurotrack, Redwood City, California, United States, 4. Rutgers University, Newark, New Jersey, United States*

With age, sleep quality tends to decline. Poor sleep quality is associated with adverse physical and mental health outcomes, especially in the aging population. The purpose was to compare subjective sleep quality measurements between middle age (45-64 years) and older adults (over 65 years). Participants (N=200) completed the Pittsburgh Sleep Quality Index (PSQI) and were classified by age as either middle age (MID: n=105, 71.4% female, M±SD: age: 55.5±5.8 years; mass: 85.3±17.4 kg, height: 168.4±9.2 cm, education: 18.2±3.6 years) or older adults (OLD: n=95, 76.8% female, M±SD: age: 69.0±2.9 years; mass: 80.6±14.9 kg, height: 166.2±8.9 cm, education: 17.9±3.5 years). The PSQI Component and Global Scores were calculated. Data were analyzed using descriptive statistics, crosstabulations, and independent samples t-tests ($p \leq .05$). The results showed there was no difference ($p < .05$) between the two age groups on subjective sleep measures, including sleep quality, latency, duration, and disruptions. The average Global score for the MID and OLD adults was 6.3±3.2 and 6.0±3.6, respectively. Only 17% of MID adults and 25% of OLD adults were classified as good quality sleepers (PSQI Global score ≤ 4) and 21.9% and 30.5% rated their sleep quality as very good, respectively. While previous research has shown deterioration in subjective sleep quality measures occurs with age, there was not a difference in subjective measures between middle age and older adults in the present investigation. Further analysis is warranted to examine additional factors impacting sleep quality such as physical activity levels, co-morbidities, and living situation.

Abstract citation ID: igad104.2924

### OPIOID PRESCRIBING AND OPIOID USE IN OLDER ADULTS: BUILDING AND REFINING A CHANGE PACKAGE FOR PRIMARY CARE

Ellen Childs[1], Megan Noel[1], Clarissa Hsu[2], Michael Parchman[2], Kendall Hall[1], Sarah Shoemaker-Hunt[3], and Nicole Keane[3], *1. Abt Associates, Rockville, Maryland, United States, 2. Kaiser Permanent Washington Health Research Institute, Seattle, Washington, United States, 3. Abt Associates, Cambridge, Massachusetts, United States*

Opioid use is associated with increased risk of harms in older adults (≥65) due to physiologic changes, drug interactions, and comorbidities. The AHRQ Opioids in Older Adults project developed a change package based on an evidence review and expert recommendations that included nine high-leverage changes (HLCs) and related key activities to address safe opioid use in this population. We used the change package to support fourteen primary care practices participating in two fifteen-month learning collaboratives convened to implement innovations in safer opioid use in older adults. Given the nascent understanding of how practices address opioid safety among older adults, this presentation will describe the HLCs implemented by practices compared to the original change package offered to them as a way to validate and improve the change package for broad use and uptake. Of the original nine HLCs, practices implemented seven, including staff education, risk assessment, opioid use disorder assessment and treatment, processes and workflows, non-pharmacologic pain management, medication management, and using data to inform practice. All activities fit into an original HLC, however the activities that practices implemented varied from the original key activities identified by the literature review and expert panel. For example, under the using data to inform practice HLC, most practices struggled to complete chart reviews, and were unable to "review data regularly" as offered in the original change package. This presentation will describe strategies used to improve opioid safety for older adults in primary care.

Abstract citation ID: igad104.2925

### PERCEPTIONS OF HEALTH PROFESSIONALS, PATIENTS, AND FAMILIES ON DECISION TO DISCONTINUE LIFE-SUSTAINING TREATMENT

Jooseon Lee, Eunjeong Song, Dongsoon Shin, Seon Young Yun, Minjeong Eom, Suhee Oh, Heejung Lee, and Rhayun Song, *Chungnam National University Hospital, Daejeon, Republic of Korea*

Withholding and withdrawing treatment is debated among health professionals who care for individuals at the end of life. This study aimed to compare the perception of life-sustaining treatment decisions by health professionals, patients, and families by providing data to guide medical professionals to support patients in making self-directed decisions about when to limit or continue their life-sustaining treatment. A descriptive survey was conducted with health professionals (220 from general tertiary hospitals) and 110 patients and their families. The majority of participants (85-95%) agreed with the suspension of meaningless life-sustaining treatment. In a clinical situation, the decision was made mainly by the family (84%), followed by the patient (10%) or the primary doctor (6%). However, the patients and their families reported that patients should make decisions (74%). The appropriate timing to discuss the suspension of the life-sustaining treatment was when the patients were still conscious but expecting severe deterioration" consistently reported by participants (45-58%). The most important factor during their decision-making would be "possibility of the patient's recovery" by health professionals (43-55%), but "pain of the patients (58%)" was reported by the patients and their families. In conclusion, there is still a significant discrepancy in the perception of life-sustaining treatment decisions among health professionals, patients, and their families. The ambiguity of the timing for the decision to terminate life-sustaining treatment and the discrepancy between patients and their families' opinions remain obstacles to implementing the Act to respect the patient's right to self-determination and achieve the goal of a dignified end-of-life.

Abstract citation ID: igad104.2926

### REFRAMING AGING AND VISION LOSS AS A PUBLIC HEALTH IMPERATIVE

Priscilla Rogers, *VisionServe Alliance, Mooresburg, Tennessee, United States*

Severe vision impairment and blindness often have profound effects upon older people and those who care for and about them. Recent translational research reveals in the United States, 7.3% of older people report severe vision impairment or blindness. A recent study estimated that the population of people with vision impairment will increase by 118% by 2050. The greatest increases will be among the most senior, women, African Americans, and Hispanics. People with vision impairment report greater prevalence of chronic conditions, poorer health, and poorer quality of life than older people without vision impairment, and they are much more likely to experience multiple disabilities. These factors point to grave disparities in health equity. While the multiple studies have informed social, health, and economic disparities among older people with vision impairment at the national level, virtually nothing is known about the variability of the prevalence of vision impairment, chronic conditions, health-related quality of life, and disability at the state level. The geographic distribution of vision impairment varies among and within states. Often rural and poorer areas have a higher prevalence of vision impairment — areas that generally have a paucity of eye care providers, social support systems, and vision rehabilitation services. As aging issues are addressed at state and federal levels, vision loss and vision rehabilitation are frequently omitted from the conversations and plans (e.g., Master Plans on Aging and/or Age-Friendly Plans) and from policy instruments such as the Older Americans Act, which has no mention of vision or vision loss.

### SESSION 7820 (POSTER)

### GERIATRIC ASSESSMENT AND FUNCTIONAL STATUS

Abstract citation ID: igad104.2927

### ASSOCIATIONS BETWEEN GAIT MEASURES AND QUADRICEPS MUSCLE THICKNESS AS MEASURED BY POINT-OF-CARE ULTRASOUND

Uyanga Ganbat[1], Boris Feldman[1], Shane Arishenkoff[2], Graydon Meneilly[1], and Kenneth Madden[1], *1. University of British Columbia, Vancouver, British Columbia, Canada*, *2. Vancouver General Hospital, Vancouver, British Columbia, Canada*

Gait parameters and sarcopenia predict falls risk which is one of the major causes of both mortality and morbidity among older adults. Our objective was to evaluate whether anterior thigh muscle measured by point-of-care ultrasound is significantly associated with standard gait measures. All subjects were referred from ambulatory geriatric medicine clinics at an academic center. Quadriceps muscle thickness was measured by a portable ultrasound device. Gait variables were measured by the patient walking for 6 minutes. The primary response variables were gait variables, and the predictor variables were age, biological sex, body mass index, and MT. Univariate and multivariate regression analyses were performed. A total of 150 participants were recruited from geriatric medicine clinics. Muscle thickness was measured in 149 participants and the mean (SD) was 1.91 (0.52) (median 1.82 cm, 0.96 to 3.68 cm). Among all the gait variables, average swing time (P = 0.010) and average stance time (P = 0.010) were correlated significantly with muscle thickness. Muscle thickness also showed a negative association with step time variability percent (P = 0.005). Muscle thickness (P = 0.046), age (P = 0.025), and gender (P = 0.047) had a statistically significant association with present step time variability. PoCUS showed a significant association with average swing time, average stance time, and step time variability all of which are predictive of future falls risk. Although more work needs to be done, PoCUS is a reliable and feasible muscle measurement method, which could lead to faster clinical decision-making and falls risk assessment.

Abstract citation ID: igad104.2928

### DECLINE IN OBJECTIVELY MEASURED STATIC BALANCE WITH AGING

Chitra Banarjee, Renoa Choudhury, Joon-Hyuk Park, Rui Xie, Jeffrey Stout, and Ladda Thiamwong, *University of Central Florida, Orlando, Florida, United States*

Balance performance is a key predictor of falls, contributing to an increased risk of chronic disease and future falls in older adults. In this study, we aimed to characterize the effects of age, gender, and body mass index (BMI) on changes in balance over time. BTrackS, a portable and inexpensive technology system, was utilized to objectively measure the changes in static balance with eyes closed. We assessed 70 community-dwelling older adults (Mage=74.8 years, 77% female, MBMI=26.95 kg/m2) over 2-4 longitudinal observations, with an average of 174 days between baseline and follow-up assessments to characterize the balance performance over time. The balance performance measure was computed as the average center of pressure (COP) path length (cm) across three testing trials. The balance performance measure at baseline significantly positively correlated with age (Pearson's r=0.417, p< 0.001) and with BMI (r=0.264, p=0.029), but did not differ by gender (p=0.480). Change in balance (difference in static balance over time, cm) was correlated (r=0.393, p< 0.001) with age, such that older adults experienced a greater decline in static balance over time, however, gender (p=0.620) and BMI (p=0.709) did not show a significant correlation. Older adults with increased BMI were found to be more likely to have limitations in balance. These results suggest that clinicians and researchers need to account for the age-related effects on changes in balance performance over time. Rapid deterioration of balance with increased age also has implications for increased monitoring of balance function and further intervention in older adults as they age.

Abstract citation ID: igad104.2929

### FACTORS ASSOCIATED WITH HAND GRIP STRENGTH AMONG OLDER ADULTS—FINDINGS FROM THE LASI STUDY INDIA

Snehal Kulkarni, *Dr. Vishwanath Karad's MIT-World Peace University, Pune, Maharashtra, India*

Objective: To identify the factors associated with handgrip strength among older adults in India. **Methods:** Data for the study were drawn from the baseline survey of Longitudinal Ageing Study in India (LASI, 2017–18). This study was conducted on respondents aged 60 years and above with a total sample of 27,347 older adults (men-13,241 and women-14,118). The study assessed handgrip strength using a handheld Smedley's Hand Dynamometer. Descriptive statistics and independent t-test was used to measure the mean difference in the handgrip strengths according to presence of risk factors. **Results:** The mean handgrip strength was 29.76 + 11.23 kgs for both the genders. There was statistically significant difference in the mean handgrip strength of men (36.26 + 10.83 kg) and women (23.70 + 7.65 kg respectively). Significantly lower handgrip strength was observed among participants in rural area (29.58 + 11.20 kg), difficulty in activities of daily living (28.24 + 10.97 kg), low level physical activity, balance impairment (21.09 + 8.83 kg) slowness in walking (25.14 + 9.87 kg), history of falls (28.24 + 10.97 kg), and presence of bone and joint disease (27.44 + 10.60 kg) as compared to their counterparts. **Conclusion:** The study contributed to a better understanding of factors associated with HGS, and thus, the HGS is recommended as a section in the health evaluation among high-risk older adults as the strategy of disease control and prevention.

Abstract citation ID: igad104.2930

### GERIATRIC RENAL TRANSPLANT CO-MANAGEMENT: EVALUATION OF FRAIL OLDER ADULTS FOR TRANSPLANT ELIGIBILITY

Jared Doan[1], Stephanie Chow[1], Abigail Baim-Lance[2], Moritz Blum[1], Susan Lerner[1], Greta Rosen[1], William Hung[1], and Fred Ko[1], *1. Icahn School of Medicine at Mount Sinai, New York City, New York, United States*, *2. Veterans Health Administration, Bronx, New York, United States*

Older transplant patients have higher rates of adverse post-transplant outcomes. The Renal Transplant Co-Management (RCOM) program is a collaboration of surgeons, geriatricians, and social workers to medically and psychosocially optimize older renal transplant candidates by providing comprehensive geriatric-centered vulnerability assessments with corresponding medical and psychosocial interventions. The RCOM assessment aids the surgical team in deciding patient transplant eligibility, and greater clarity on its role may be clinically valuable. Fifty-five patients from a single metropolitan hospital were evaluated during the study period (1/1/22 to 6/16/22). Assessments included frailty (Clinical Frailty Scale/CFS), functional status (Karnofsky Score, Katz Index, Lawton-Brody Scale) and cognition (Montreal Cognitive Assessment/MoCA) – factors that the renal transplant team considers in listing a patient for surgery (i.e., listed/transplanted) or not (i.e., ineligible/removed from the list). Twenty patients (36.4%) were female. Mean age of ineligible/removed (n=10) and listed/transplanted (n=45) patients was 74.7 (4.8) and 72.5 (3.8), respectively, with no differences in age, gender, race, education, or health insurance between groups. Patients who were listed/transplanted had a higher Lawton-Brody instrumental activities of daily living (IADL) score than those not listed (p=0.025, t-test). CFS (p=0.118), Karnofsky (p=0.09), Katz (p=0.074), and MoCA (p=0.094) were similar but approached statistical significance despite a small sample size. Functional status as measured by IADL may be a significant factor considered in determining the transplant eligibility of older adults. Ongoing study and a larger sample size may provide greater clarity on the physical and cognitive functions that impact eligibility for transplant listing in frail older adults.

Abstract citation ID: igad104.2931

### IMPACT OF BODY POSITION AND ARM USE ON EYE DROP INSTILLATION IN OLDER ADULTS

Tanner Trenary[1], Andrew Droste[2], Rachel Logue Cook[1], Stephen Cain[3], Alanson Sample[1], David Burke[2], Paula Anne Newman-Casey[2], and Susan Brown[1], *1. University of Michigan, Ann Arbor, Michigan, United States*, *2. Michigan Medicine, Ann Arbor, Michigan, United States*, *3. West Virginia University, Morgantown, West Virginia, United States*

Visual impairments in older adults are associated with declines in physical, cognitive and social functioning, often leading to a loss of independence and quality of life. Increased age is a major risk factor, with conditions such as glaucoma having a 7-fold increase in prevalence from the 50s to the 80s (Klein & Klein, 2013). Treatment in the form of eye drops is commonly prescribed yet suboptimal adherence can significantly impact outcomes (Newman-Casey et al., 2015). The purpose of this preliminary study was to determine the effects of body posture and hand use on eye drop instillation success in a sample of 20 healthy, non-eye drop users (mean age: 70.0 +/- 3.7 y). Participants administered eyes drops without instruction while seated, standing and in a supine position. Video analysis was used to determine the number of attempts before successful instillation occurred (failed attempts) as well as whether participants used one or two hands. Despite published recommendations to use both hands (Davis et al, 2018), only 65% adopted a bimanual approach. The percentage of failed attempts was lowest in the supine (11.6%) compared to the seated (25.7%) or standing (21.2%) positions. Pinch strength was not correlated with successful instillation as has been previously reported (Naito et al., 2021), possibly due to participants' overall level of physical functioning. Based on these results, a supine position is recommended for eye drop instillation where the head and neck are stabilized.

Abstract citation ID: igad104.2932

### IMPLEMENTING AND TESTING THE EFFICACY OF A GERIATRIC GOALS OF CARE TOOL IN AN ACUTE HOSPITAL SETTING

Pallavi Samudrala[1], Brynn Sheehan[1], Erin Smith[2], and Marissa Galicia-Castillo[1], *1. Eastern Virginia Medical School, Norfolk, Virginia, United States*, *2. Sentara Leigh Hospital, Norfolk, Virginia, United States*

Goals of care (GOC) discussions, which focus on patient values and preferences, are associated with improved outcomes for older adults, including lower hospital readmission rates, increased adherence to advanced care plans, and improved patient satisfaction. GOC discussions do not occur consistently and a reliable method of documentation has yet to be established. The Geriatrics Goals of Care (GGOC) tool was developed to easily review and document GOC discussions for geriatric patients (65 years and older) in the electronic medical record, focusing on pre-hospital function and hospitalization goals. The study objective was to evaluate the GGOC tool's impact on 30-day hospital readmission. Using an experimental design, the GGOC tool was implemented in one hospital unit and a second comparative unit served as a control. The GGOC tool was administered to a total of 82 patients and 162 control unit patients received standard care (N= 244). A logistic regression of patient-level data revealed that the odds of having a hospital readmission within 30 days were approximately 65% lower for patients who received the GGOC tool compared to those in the control unit (OR = .344, SE = .40, p = .008, 95% CI [.156, .759]). The readmission rate for the unit utilizing the GGOC tool yielded a cost savings of $185,019.12. Applying the same readmission rate to the control unit, the expected cost savings was $365,625 for the control group. Results suggest that the GGOC tool may be effective for documenting GOC discussions, reducing the burden of readmissions with their associated costs.

Abstract citation ID: igad104.2933

### RELATIONSHIPS BETWEEN PAIN AND OPTIMISM AMONG COMMUNITY DWELLING OLDER ADULTS

Lucia Rivera[1], Isabel Mancilla[2], Jaclyn Bergstrom[1], and Anthony Molina[1], *1. University of California, San Diego, San Diego, California, United States*, *2. University of California San Diego, San Diego, California, United States*

Measures of life outlook in older adults have been investigated in connection to pain, as both pain management and outlook are important factors of successful aging. Positive outlook helps promote healthy aging while depression is associated with higher pain levels and interference, which negatively contribute to the aging process. Thus, we hypothesized that higher pain levels are associated with lower optimism among community-dwelling older adults. We utilized data from the UC San Diego Successful Aging Evaluation (SAGE), a prospective longitudinal cohort study initiated in 2010, to evaluate the relationship between pain and optimism in 378 community-dwelling adults aged ≥50 years. We used the revised Life Orientation Test (LOT-R) to measure optimism and three pain subscales, PROMIS Pain Interference, PROMIS Pain Intensity, and MOS 36-Item Short-Form Health Survey (SF-36), as pain measures. Regression analyses reveal negative relationships between pain and optimism for all three pain scales, with regression coefficients of -0.277 (p< 0.0001), -0.246 (p< 0.0001), and 0.269 (p< 0.0001) respectively. The correlations were strongest among men, Caucasians, and college graduates. The correlations remained significant when adjusting for gender, age, completion of college, marriage status, and household income, but not for race/ethnicity. This study was limited by the underrepresentation of non-Caucasian participants (N=87) and by the self-reported nature of the SAGE data. These findings indicate value in considering physical and psychological elements together when encouraging healthy aging. Future research can investigate whether interventions that increase optimism can decrease pain in older adults or whether pain management interventions can increase optimism in this population.

Abstract citation ID: igad104.2934

### REMOTE CONTROL THE PHYSICAL ACTIVITY AS HUMAN HEALTH ASSESSMENT OF FUNCTIONAL AGING

Olena Tomarevska[1], and Oleksandr Poliakov[2], *1. D.F. Chebotarev Instutute of Gerontology of the NAMSU, Kyiv, Kyyiv, Ukraine*, *2. D.F. Chebotarev Institute of Gerontology of the NAMS of Ukraine, Kyiv, Kyyiv, Ukraine*

The level of physical activity is modified by the functional age of a person in accordance with his chronological age. Most of the evidence comes from the positive impact of physical activity on the musculoskeletal system and cognitive functions, microcirculation and cardiovascular system in the working population and retirees. In the current study, an online method the investigation of physical activity was tested. Functional age was calculated using appropriate indicators according to age standards: systolic and diastolic pressure, standing on the left leg with closed eyes, maximum breath holding time after a deep inhalation, maximum breath holding time after a deep exhalation. The state of health is extrapolated to the need for specialist supervision in 58.96% of older workers. Thus, the study showed the need to determine the functional age of respondents 50 years and older. Thus, a slowed rate of aging observed in 23.5% of cases, physiological - in 18% of cases, accelerated rate of aging - in 58.5% of cases. In the exams 441 persons, age 50-90 years, reliable associative links were observed rate of functional aging with a decrease in physical activity according to the indicators of individual daily mileage (r=-0.273, p< 0.001) and the variability of physical exercises (r=-0.161, p< 0.001). The pace of functional aging and state of health were reliably correlated (r=-0.364, p< 0.001) in individuals, respectively, with the preservation of individual daily mileage and the performance of daily physical exercises. Thus, aerobic activity is an important in maintaining health in terms of functional indicators.

Abstract citation ID: igad104.2935

### THE IMPACT OF HEALTH-RELATED QUALITY OF LIFE ON CLINICAL OUTCOMES IN ATRIAL FIBRILLATION POPULATIONS

Isabelle Pierre-Louis[1], Benita Bamgbade[2], Sara Lopez-Pintado[2], Sarah Forrester[3], Weijia Wang[4], David McManus[5], and Jane Saczynski[2], *1. University of Massachusetts Medical School, Worcester, Massachusetts, United States*, *2. Northeastern University, Boston, Massachusetts, United States*, *3. University of Massachusetts Chan Medical School,*

*Worcester, Massachusetts, United States*, 4. *Johns Hopkins Hospital, Baltimore, Maryland, United States*, 5. *University of Massachusetts Chan Medical School, Worcester, Worcester, Massachusetts, United States*

Although there is a large body of literature linking atrial fibrillation (AF) to adverse clinical outcomes, little is known with regards to the potential links between health-related quality of life (HRQoL) and AF related clinical events. To address this gap, individuals with AF aged ≥ 65 years were recruited from clinics in Massachusetts and Georgia between 2016-18 and followed until 2020. HRQoL was assessed via the Atrial Fibrillation Effect on Quality of Life (AFEQT). The AFEQT is a validated HRQoL measure that quantifies the effect of AF on patients' daily life on a scale of 0 to 100. AFEQT scores < 80 indicated poor HRQoL and ≥ 80 indicated good HRQoL. The clinical events included major bleeding, stroke, and death. Of the 1,244 participants, the mean age was 75.5 (SD: 7.1), 49% identified as female, 13% were non-White, and 57% were married. After 2 years, 105 major bleeding events (49% poor HRQoL), 19 strokes (42% poor HRQoL) and 108 deaths (59% poor HRQoL) occurred. Our composite outcome included 200 participants (53% poor HRQoL). After adjusting for key covariates, poor HRQoL was associated with an increased risk of our composite outcome (adjusted hazard ratio [AHR]= 1.34, 95% CI: 1.00, 1.80) and death (AHR= 1.58, 95% CI: 1.05, 2.38). We found no significant differences between poor and good HRQoL in major bleeding and strokes. HRQoL is important when assessing health of AF patients. Health providers who treat patients with AF should consider HRQoL when evaluating the risk of death and other adverse outcomes.

### SESSION 7830 (POSTER)

### COGNITION AND COGNITIVE IMPAIRMENT

Abstract citation ID: igad104.2936

### "BEYOND JUST A SINGING GROUP": PERCEPTIONS OF PARTICIPANTS, CAREGIVERS, AND LEADERS OF PARKINSON'S SINGING GROUPS

Jessy Brown, Cailie Logue, Maya Bartel, Tera Jordan, and Elizabeth Stegemoller, *Iowa State University, Ames, Iowa, United States*

Parkinson's disease (PD) is the second most prevalent neurodegenerative disease after Alzheimer's disease, and its prevalence is increasing. There is no cure, so symptom treatment is currently the only method to decrease the disease burden for individuals diagnosed with PD. A common treatment modality option that has been shown to improve both motor and non-motor symptoms in PD is therapeutic singing, often in a group setting. Previous literature has explored perceptions of singing groups for persons with PD (PwPD) and their caregivers (CGs) and has found overall positive experiences and outcomes. No study has sought to understand the perspectives of the leaders of the singing groups. The purpose of this study was to understand the perceptions and experiences of participants in singing groups (n=31), their caregivers (n=8), and those leading the groups (n=10) across several different singing groups in the United States. A qualitative phenomenological methodological framework was used with principles of grounded theory to make meaning from the transcripts. Three main themes emerged from the data: peer support, proactive PD management, and the effects of the pandemic. These findings indicate that even during a global pandemic, all three participant groups experienced positive outcomes resulting from peer support and either experiencing or witnessing effective management of PD symptoms. All three participant groups reported similar perceptions and experiences about their involvement which reinforces the rationale to use singing as a treatment modality for PD.

Abstract citation ID: igad104.2937

### ADVERSE EVENTS IN OLDER ADULTS AT RISK FOR DEMENTIA DURING VIRTUAL EXERCISE AND COGNITIVE TRAINING

Karla Faig[1], Molly Gallibois[2], Alanna Bohnsack[1], Chris McGibbon[2], Andrew Sexton[2], Josée Haché[3], Samantha Knill[1], and Pamela Jarrett[1], *1. Horizon Health Network, Saint John, New Brunswick, Canada*, 2. *University of New Brunswick, Fredericton, New Brunswick, Canada*, 3. *Vitalité Health Network, Moncton, New Brunswick, Canada*

Synchronizing Exercises, Remedies in Gait and Cognition at Home (SYNERGIC@Home/SYNERGIE~Chez soi) is a home-based, double-blind, randomized controlled trial (NCT04997681) targeting community-dwelling older adults at risk for dementia. Anglophone and Francophone participants living in an Atlantic Canadian province (New Brunswick) underwent 16 weeks (three sessions/week) of interventions which consisted of virtual cognitive and physical exercise designed to delay or prevent the onset of dementia. This research aims to determine the frequency, severity, and relationship of the adverse events (AEs) that occurred during the intervention phase. All reported AEs were reviewed and managed by members of the research team, including a geriatrician. Participant's (n=60) mean age was 68.9 years (SD 6.58), and the majority (76.7%) were female. Forty percent were living in rural communities, and 60% were urban. Of these participants, there was a total of 88 AEs reported by 42 individuals; 70% having at least one AE. The most common AE (39.8%) was musculoskeletal in nature. The majority (71.6%) of all AEs were unrelated to the intervention, and 69.3% of all AEs were classified as mild. There was only one serious AE, and it was unrelated to the intervention. The intervention had to be discontinued in two individuals, and modifications were required in 59 cases for participants to continue in the study. When delivered virtually with videoconference supervision, physical and cognitive interventions resulted in no serious related AEs and few related, mostly mild AEs, that were safely managed through modifications to the physical interventions.

Abstract citation ID: igad104.2938

### ASSOCIATION BETWEEN CARDIOVASCULAR DISEASE AND COGNITIVE IMPAIRMENT: IS DEPRESSION A MEDIATOR?

Benita Bamgbade[1], James Lovato[2], David Reboussin[2], Stephen Rapp[2], Byron Jaeger[2], and Jeff Williamson[2], *1. Northeastern University, Boston, Massachusetts, United*

*States*, *2. Wake Forest School of Medicine, Winston Salem, North Carolina, United States*0

By 2040, over 9 million US adults will live with dementia. Understanding the relationship between risk factors and cognitive decline is critical to prevent disease and slow progression. Depression and cardiovascular disease (CVD) are independent risk factors for cognitive impairment (CI) which may interact, suggesting depression may play a mediating role in the relationship between CVD and CI, an hypothesis explored in this study. Data from the Systolic Blood Pressure Intervention Trial (SPRINT), a multi-center, randomized, controlled trial were used in this multivariate logistic regression analysis. CI was defined as adjudicated cases of mild CI and probable dementia. Depression was defined as a Patient Health Questionnaire-9 score ≥10 or current antidepressant therapy. We developed a CVD risk score using data from SPRINT participants and discretized the scores into tertiles. Of 9259 SPRINT participants, our analytic sample included 8456 who had adjudicated mild CI or dementia and had baseline depression scores. They were on average 68 years and 36% were female. After adjusting for age, sex, education, race, prior CVD, chronic kidney disease and treatment assignment, participants with high cardiovascular risk were more likely to have CI (OR: 1.55, 95% CI: 1.17-2.05). Participants with depression were more likely to have CI (OR: 1.29, 95% CI: 1.05-1.58) however, the interaction between cardiovascular risk and cognition was not significant. Findings from this study suggest that while depression and CVD are associated with CI, depression may not explain the CVD-CI relationship. More research is needed to understand the relationship between depression, CVD and CI.

Abstract citation ID: igad104.2939

### CHRONIC VASCULAR COMORBIDITIES WITH SUBJECTIVE COGNITIVE DECLINE IN ADULTS WITH CANCER

Renata Komalasari[1], and Adhe S. Ryant[2], *1. Penn State Nursing, State* College, *Pennsylvania, United States*, *2. Dirgahayu School of Health Sciences, Samarinda, Kalimantan Timur, Indonesia*

People with cancer self-reported memory problems nearly twice as likely than their counterparts, increasing difficulties in daily performances. Despite expected relationships between chronic vascular comorbidities and subjective cognitive decline (SCD), studies are yet to map the likely chain mediation of psychological distress and functional difficulties in the relationship of these variables. This study aimed at examining the association between chronic vascular comorbidities and SCD by psychological distress and functional difficulty mediation in adults with cancers. We utilized the 2019 wave of the Behavioral Risk Factor Surveillance System dataset on 263 adults aged 45 to 80 with cancer (41.1% males and 58.9% females). Chain mediation analysis using 5000 bootstrap samples showed that chronic vascular comorbidities significantly and directly affect SCD. Psychological distress and functional difficulties serially mediated the relationship (indirect effect = .14, 95% CI .05 to .26), with a 26.9% overall mediation effect. Psychological distress alone did not mediate the relationship between chronic vascular comorbidities and SCD (indirect effect = .09, 95% CI -.08 to .27). Functional difficulties mediated the relationship between chronic vascular comorbidities and SCD (indirect effect = .29, 95% CI .05 to .57). These findings suggest that greater chronic vascular comorbidities are associated with a higher sense of SCD through psychological distress which may be magnified by greater functional difficulties. These findings extend the prediction of the chronic stress theory that emotional reactivity to daily functional stressors may exacerbate SCD among adult cancer survivors. These findings may inform person-centric intervention for adults with cancer to self-monitor functional difficulties.

Abstract citation ID: igad104.2940
### COGNITIVE FUNCTIONING AMONG GRANDPARENT CAREGIVERS: AN INTERGENERATIONAL PERSPECTIVE

Khushbu Patel[1], Katelyn Singer[2], and Benjamin Katz[1], *1. Virginia Tech, Blacksburg, Virginia, United States*, *2. Virginia Tech Graduate School, Blacksburg, Virginia, United States*

Grandparents have a unique bond with their grandchildren, given their position in the grandchild's life. They may be a historian, mentor, playmate, caregiver, role model, advocate, and friend. These interactions may offer an opportunity for cognitive engagement, but the level of this engagement may in turn be linked to a grandparent's cognitive status. For example, memory issues may challenge one's ability to serve as a historian for a grandchild. To better understand the association of caregiving and engagement in activities with a grandchild on cognition, a secondary analysis was conducted to examine how cognitive functions differ by engagement time. Drawing from approximately 1,200 participants in the Health and Retirement Study who indicated activity engagement with grandchildren and who also completed the Harmonized Cognitive Assessment Protocol (HCAP) regression analyses were conducted examining the link between grandparent engagement with grandchildren and cognitive measures including Constructional Praxis Delayed, Backwards Counting, Raven's Standard Progressive Matrices, Brave Man Delayed, and Digit Symbol Modalities. Results from the analyses revealed that Raven's Matrices (P = 0.007), Backwards Counting (P = 0.041), and Constructional Praxis (P = 0.030), were most closely linked to activity engagement with grandchildren, providing preliminary evidence that working memory is closely linked to caregiving engagement. However, the results also revealed a bimodal distribution, suggesting that too little and too much caregiving were associated with worse cognition. These findings provide preliminary evidence that illustrates how interacting with and caregiving for grandchildren may be both a protective factor and a risk factor for older adults.

Abstract citation ID: igad104.2941
### COGNITIVE IMPAIRMENT IS ASSOCIATED WITH INFERIOR POSTURAL BALANCE AND REDUCED GAIT SPEED

Laura Katharina Schmidt[1], Tim Stuckenschneider[2], Jessica Koschate[3], and Tania Zieschang[3], *1. Carl von Ossietzky University Oldenburg, Oldenburg, Niedersachsen, Germany*, *2. Carl von Ossietzky University, Oldenburg, Nordrhein-Westfalen, Germany*, *3. Carl von Ossietzky University, Oldenburg, Niedersachsen, Germany*

Fall-related sequelae including balance as well as gait impairments are more pronounced in older adults with cognitive impairment (CIOA) compared to cognitively healthy older adults (CHOA). Evidence is scarce about differences in postural balance and gait in CHOA and CIOA after a fall, even though these are major risks for recurrent falls. Especially, there is a dearth of data based on validated sensor technologies. Thus, it is our aim to use sensor-based data to investigate differences between these two groups. Postural balance and gait were measured using three wirelessly synchronized IMU's (APDM, Inc., Portland, USA) in n=50 CHOA, mean age 73.82±8.25 years and n= 56 CIOA, mean age 78.27±8.0 years. Data were collected at participants' homes as part of a comprehensive geriatric assessment in the "SeFallED" study within four weeks after presentation to the emergency department (ED) after a sentinel fall (German Clinical Trials Register ID: 00025949). Differences in postural balance and gait between two groups were analyzed by using a Mann-Whitney-U-Test. Statistical analysis revealed a significantly poorer postural balance in CIOA compared to CHOA for the parameters Jerk (m2/s5; p=0.039) and mean velocity (m/s; p=0.036). Further, CIOA had a significantly lower gait speed (m/s; p=0.035) and stride length (m/s; p=0.028) compared to CHOA. Even though both groups had shortly experienced a fall with presentation to the ED, impairments in gait and balance parameters were more prominent in CIOA. Based on these results, physical exercise interventions should focus on the improvement of impaired gait speed and balance particularly in CIOA.

Abstract citation ID: igad104.2942
### CUMULATIVE EFFECTS OF COGNITIVE IMPAIRMENT AND FRAILTY ON GERIATRIC SYNDROMES IN A GERIATRIC OUTPATIENT SETTING

Kun-Pei Lin[1], Chung-Sheng Lee[2], wei-Jia Huang[1], Ding-Cheng (Derrick) Chan[1], Yi-Chun Chou[1], Feng-Ping Lu[1], Meng-Chen Wu[1], and Chiung-Jung Wen[1], *1. National Taiwan University Hospital, Taipei City, Taipei, Taiwan (Republic of China)*, *2. Kainan University, Taoyuan, Taoyuan, Taiwan (Republic of China)*

Aim: With underlying geriatric syndromes, only few studies emphasized on the aspect of co-occurrence of frailty and cognitive impairment, though they are independently associated with several geriatric syndromes. **Methods:** The participants were recruited from the outpatient clinic of geriatric department in a medical center. Frailty was assessed by a modified version of Fried's criteria, while cognitive impairment was defined by cutoff scores of the Mini Mental State Examination. Demographic characteristics, self-assessed health status, and geriatric assessment were collected. Polytomous logistic regression model was used to evaluate factors correlated with frailty alone, cognitive impairment alone, and co-occurrence of frailty and cognitive impairment. **Results:** The mean age of the 323 participants (60.1% women) was 80.4 ± 7.3 years. The prevalence of non-frailty, frailty alone, cognitive impairment alone and co-occurrence were 34.4%, 16.7%, 23.8% and 25.1%, respectively. When compared to the not-frail group, the odds ratios (OR) of having activity of daily living impairment (aOR 1.44, 95% CI 1.28-1.63), instrumental activities of daily living impairment (aOR 1.61, 95% CI 1.45-1.81), urinary incontinence (aOR 1.61, 95% CI 1.45-1.81) and with lower mini-nutritional assessment score (aOR 148, 95% CI 1.30-1.69) were significantly higher in the co-occurrence group after adjusted with demographic variables and Charlson comorbidity index. **Conclusions:** Co-occurrence of frailty and cognitive impairment was associated with higher prevalence of disability, incontinence and malnutrition. It is crucial to approach frailty and cognitive impairment simultaneously in daily geriatric clinical practice due to the correlations of several geriatric syndromes to the co-occurrence of physical frailty and cognitive impairment.

Abstract citation ID: igad104.2943

### EFFECTS OF MARITAL STATUS AND ETHNICITY ON NEUROCOGNITIVE FUNCTIONING IN A RURAL OLDER POPULATION

Lauren Elliott[1], Jonathan Singer[1], Peter Rerick[2], Carol Fadalla[1], Elisabeth McLean[1], Alayna Jump[1], Veronica Molinar-Lopez[3], and Volker Neugebauer[4], *1. Texas Tech University, Lubbock, Texas, United States*, *2. Oklahoma City University, Oklahoma City, Oklahoma, United States*, *3. Texas Tech University Health Sciences Center, Lubbock, Texas, United States*, *4. Garrison Institute on Aging, Lubbock, Texas, United States*

Research indicates being married is related to better physical and psychological health. Yet, little is known regarding the relationship between marital status and neurocognitive functioning and whether it differs based on ethnicity (Hispanic vs. non-Hispanic). Data from 1864 participants (Mage=59.68, SD=12.21), who were mostly Hispanic (n=1053), women (n=1295), and married (n=1,125) from Project FRONTIER (Facing Rural Obstacles to Healthcare Now Through Intervention, Education, & Research) were analyzed. Neuropsychological testing comprised RBANS, Trails Making Test, and Clock Drawing. Participants were dichotomized into married and unmarried (i.e., widowed, divorced, separated, never married). There was a significant interaction between Hispanic identity and marital status on overall neurocognitive functioning ($F(1,1480) = 4.79$, $p < .05$, $\eta p2 = .003$). For non-Hispanic individuals, married individuals had higher overall neurocognitive functioning compared to the unmarried individuals, whereas neurocognitive functioning for Hispanic individuals did not significantly differ between married and unmarried individuals. There were significant main effects as married individuals (M=84.95, SD=15.56) had greater overall neurocognitive functioning than unmarried individuals (M=83.47, SD=15.86; $F(1,1480) = 14.67$, $p < .001$, $\eta p2 = .01$), and Hispanic individuals (M=78.02, SD=14.25) had lower overall neurocognitive functioning than non-Hispanic individuals (M=91.43, SD=15.07; $F(1,1480) = 284.99$, $p < .001$, $\eta p2 = .16$). Hispanics living in rural areas experience additional stressors that could lead to worse neurocognitive functioning, which is supported by the Lifespan Biopsychosocial Model of Cumulative Vulnerability and Minority Health, which postulates that race/ethnicity/SES-related stressors exacerbate the impact of other life stressors. Reduction of stress on rural Hispanics should be a priority as it could positively affect their neurocognitive functioning.

Abstract citation ID: igad104.2944

### EVALUATION OF ONLINE AEROBIC TRAINING AND RESISTANCE TRAINING IN OLDER ADULTS WITH MCI: A THREE-ARM RCT

Xiuxiu Huang[1], and Qiaoqin Wan[2], *1. Peking University, Beijing, Beijing, China (People's Republic)*, *2. Peking University Health Science Center, Beijing, Beijing, China (People's Republic)*

This study aimed to evaluate the effects of online aerobic and resistance training programs in community-dwelling older adults with mild cognitive impairment. A total of 108 participants (60~87 years) were recruited, and equally randomized to aerobic group (3-5 times/week, 30-50min/section), resistance group (2 times/week, 5 to 6 exercises/section), or control group. Cognitive functions were evaluated at baseline, the 13rd week (T1) and 26th week (T2). Effects were evaluated by linear mixed effects model (LME) after controlling for covariates. Inter-individual differences on the cognitive effects were explored. After six-month intervention, 15 (13.89%) participants dropped out, and the median compliance rates were 80.77% for aerobic group and 96.15 for resistance group. LME revealed that ADAs-cog scores in aerobic group decreased by 2.14 at T1 and 1.68 at T2 more than the control group. And the ADAs-cog scores in resistance group decreased by1.43 more than the control group at T1. For executive function, the Stroop time interference in resistance group at T2 declined 11.12 seconds more than the control group, and 13.85 seconds more than the aerobic group. For memory performance, the RAVLT scores at T1 in aerobic group tended to increase 5.15 ($p = 0.065$) more than the control group. There existed true inter-individual differences in the responses to exercise. Compliance rates, social activity participation, cognitive function and physical function at baseline were the significant factors. Overall, our study confirmed the positive effects of both aerobic and resistance exercise on global cognition, while their effects on specific cognitive domains were different.

Abstract citation ID: igad104.2945

### HIGH-INTENSITY INTERVAL TRAINING, SLEEP, AND COGNITION IN OLDER ADULTS: A SYSTEMATIC REVIEW

Carleara Weiss[1], and Rebecca Lorenz[2], *1. University at Buffalo, Buffalo, New York, United States*, *2. University at Buffalo, Getzville, New York, United States*

Sleep disturbances have been linked to cognitive and physical decline in older adults. High intensity interval training (HIIT) may resynchronize disrupted sleep-wake cycles, improve sleep quality, and potentially delay detrimental impacts of aging on cognition and physical performance. Comprehensive reviews about the impact of HIIT on cognitive and physical decline in older adults are lacking. A systematic review was conducted using the Preferred Reporting Items for Systematic Reviews and Meta-Analyses (PRISMA) approach to evaluate the impact of HIIT upon sleep and cognition. PubMed, Scopus, and Web of Science were searched for articles. Six publications representing four randomized controlled trials met the inclusion criteria. Data from intervention and control groups included 158 participants, predominately female (70.4%) with median age of 69.5 years. Exercise modalities varied across studies; however, duration of 12-weeks was consistent. HIIT reduced objectively measured sleep onset latency and lowered wake after sleep onset compared to no activity and regular physical activity. However, changes in sleep quality with HIIT did not differ from those achieved with stretching exercise and moderate-intensity physical activity. In addition, only one study investigated the impact of HIIT on both sleep and cognition, indicating improvement in cognitive performance and memory but no significant changes in sleep efficiency. Despite the potential for HIIT to improve sleep outcomes, memory, and cognition, more studies are warranted, particularly those that include diverse populations and larger sample sizes, prior to forming recommendations for use in clinical settings and communities.

Abstract citation ID: igad104.2946

### IMPLEMENTATION OF BRAINCHECK FOR COGNITIVE ASSESSMENT AND CARE PLANNING IN THE CLINICAL SETTING

Bin Huang[1], Patrick Zavala[1], Christine Mann[2], Sarah Harlock[2], Kori Dekle[3], Amy Knighton[3], Bela Ajtai[2], and John Hemphill[3], *1. BrainCheck, Austin, Texas, United States*, *2. DENT Neurologic Institute, Amherst, New York, United States*, *3. Savannah Neurology Specialists, Savannah, Georgia, United States*

With the growth of the older adult population (age ≥ 65 years), age-associated neurocognitive disorders, such as Alzheimer's disease and related dementias (ADRD), are becoming more common in America and worldwide. The increasing number of patients has surpassed the capacity of healthcare systems. Thus, there is a need for time-efficient, cost-effective solutions for better allocation of limited clinical resources that can benefit clinicians and patients. Here, we conducted a quality improvement study in collaboration with Savannah Neurology Specialists and Dent Neurologic Institute. We evaluated users' perspectives on the usability and feasibility of implementing BrainCheck, a computerized tool for cognitive assessment and care planning. One neurologist and their team from each institute received 45 minutes of training on BrainCheck and used it on an iPad from mid-November to the end of December 2022. In total, 49 patients have completed BrainCheck Cognitive Assessment, and 7 of them also completed BrainCheck Cognitive Care Planning during the study period. On average, it took 13-18 minutes to administer BrainCheck Cognitive Assessment, interpret the result, and document it in EHR. The majority of patients (96%) reported satisfaction with the testing experience and found the instructions easy to understand. Both providers reported high usability of BrainCheck (6.2/7.0) as measured by the Post-Study System Usability Questionnaire. These results demonstrate the great potential of BrainCheck for clinical use, as it can support providers in assessing the cognitive function of their patients in a timely and efficient manner.

Abstract citation ID: igad104.2947

### INFLAMMATORY BIOMARKERS ASSOCIATED WITH BRAIN MRI MEASURES: FRAMINGHAM HEART STUDY OFFSPRING COHORT

Jiachen Chen[1], Margaret Doyle[2], Yuan Fang[3], Ahmed A.Y. Ragab[1], Joanne Murabito[4], and Kathryn Lunetta[1], *1. Boston University School of Public Health, Boston, Massachusetts, United States*, *2. University of Vermont, Burlington, Vermont, United States*, *3. Binghamton University, Binghamton, New York, United States*, *4. Boston University Chobanian & Avedisian School of Medicine, Boston, Massachusetts, United States*

Brain MRI volumes measuring brain atrophy have been associated with risks for Alzheimer's Disease (AD) and related dementia. Inflammatory biomarkers associated with brain MRI volumes may provide insight into the neuroinflammation associated with these diseases. The study aim is to identify circulating inflammatory biomarkers associated with total and regional brain MRI volumes in the Framingham Heart Study Offspring cohort. Participants (N=662, 52% female, mean age 62 years) free of dementia and stroke at the time of blood draw and who had MRI measures within five years were profiled using the OLINK Proteomics inflammation panel. Pairwise cross-sectional associations between 68 biomarkers and eight brain MRI volumes were investigated using linear mixed-effect models accommodating familial correlations and adjusting for covariates (age, age2, sex, time between blood draw and MRI measurement, age-sex interaction, and number of APOE ε2 and ε4 alleles), using FDR≤0.1 to declare significance. APOE genotype-stratified analyses were performed to explore effect modification. Higher levels of 8 proteins were significantly associated with smaller total brain volumes (TCBV), including CDCP1, HGF, IL6, IL8, MMP10, OPG, VEGFA, and 4E-BP1. Higher levels of SCF and TWEAK were significantly associated with larger TCBV, and higher levels of SCF were also associated with larger parietal gray matter volume. In APOE ε4 carriers, higher levels of IFNγ were associated with greater white matter hyperintensity volumes. Consistent with our findings, SCF has been shown to have neuroprotective effects in animal models. Further studies are needed to confirm these potential risk and protective factors and to elucidate mechanisms.

Abstract citation ID: igad104.2948

### INFLUENCE OF CHANGES IN MEMORY AND THINKING ON PREFERENCES AND SATISFACTION OF HEALTH CARE COMMUNICATIONS

Andrea Sillner[1], Marie Boltz[1], Diane Berish[2], and Nikki Hill[2], *1. Pennsylvania State University, State College, Pennsylvania, United States*, *2. The Pennsylvania State University, University Park, Pennsylvania, United States*

Communication about changes in symptoms or healthcare management strategies is common between older adults and their healthcare providers, such as physicians and registered nurses. Healthcare communication can occur in the context of an in-person visit or using technology through telehealth or a healthcare portal. Understanding factors that influence preferences for healthcare communication and satisfaction are important for patients and providers. For example, changes in memory and thinking may impact older adults' ability to communicate healthcare needs or to understand what is being told to them about their health conditions. The aim of this study was to understand the influence of cognitive changes on preferences and satisfaction with communications in a sample of adults over the age of 50, living in the community, who reported on changes in memory and thinking over the last 5 years (N=289). Results showed that older adults who reported a decline in their memory and thinking over the last 5 years were significantly more likely to report that their healthcare communication needs were not met. They were also significantly less likely to be satisfied with their healthcare communications. These important findings demonstrate the need to further explore relationships between self-reported cognitive changes and communication with healthcare providers. This can inform psycho-social intervention development, ultimately enhancing healthcare communication to meet older patient preferences and improve satisfaction with the overall goal of impacting healthcare outcomes.

Abstract citation ID: igad104.2949

### LONGITUDINAL NORMS OF COGNITIVE TESTS IN THE ENGLISH LONGITUDINAL STUDY OF AGING

Alejandra Marroig[1], Franklin Massa[2], and Graciela Muniz-Terrera[3], *1. Universidad de la Republica Uruguay, Montevideo, Montevideo, Uruguay*, *2. Universidad de la República, Montevideo, Montevideo, Uruguay*, *3. Ohio University, Athens, Ohio, United States*

Longitudinal research on cognitive change has mostly been focused on mean change in cognition and its associated factors. In this paper, we aim to assess the role of sociodemographic characteristics on different percentiles of the distribution of cognitive trajectories considering sample attrition due to death and intermittent missing data. We used data from 9 waves of the English Longitudinal Study of Ageing (ELSA) covering the period 2002-2019. We consider the numeracy and literacy recall to assess cognition of older adults and applied weighted Generalized Estimating Equations to estimate the 10th, 50th (median), and 90th percentiles of the cognitive tests trajectories. We adjust for sample attrition due to death and assess the role of sex and education on the cognitive trajectories. The results show differences in the cognitive trajectories across the percentiles. There is a steeper cognitive decline for individuals with poorer cognitive condition ($p< 0.001$). Sociodemographic characteristics are associated with cognitive decline but there is heterogeneity across cognitive trajectory percentiles. Education is protective against cognitive decline but it is more effective for individuals with lower cognitive impairment ($p< 0.001$). In this approach we advance the understanding of cognitive trajectories of older adults, showing differences across percentiles. We address the process of missing information and the results show new insights. Thus, this may improve the practice and design of interventions aimed at cognitive decline of older adults. Our results provide important insights into longitudinal norms of cognitive decline in a population-representative of older adults.

Abstract citation ID: igad104.2950

### LONGITUDINAL PATTERNS OF COGNITION CHANGES AMONG KOREAN OLDER ADULTS WITH NO PARTNERS: A LATENT PROFILE ANALYSIS

Yeoju Kim, and Giyeon Kim, *Chung-Ang University, Seoul, Republic of Korea*

Cognitive function decline among older adults becomes a hot topic in South Korea, as the population of the older adults has been increasing dramatically. The purpose of this study was to explore the longitudinal patterns of the cognitive function and identify factors influencing these changes among Korean older adults without their partner, using latent profile analysis. The samples included 612 adults aged 60 and older without partner from the Korean Longitudinal Study of Aging (KLoSA) in 2016, 2018, and 2020. The logistical regression was used to identify factors influencing group classification. Result revealed four types of the latent profiles were derived based on the cognitive function: the mid-level group, the high-level maintenance group, the low-level maintenance group, and low-level decline group. Factors such as depressive symptoms, social activity participation, and age were significantly associated with cognitive function decline in the high-level maintenance group, and the age was associated with the cognitive function changes. The findings highlight the importance of interventions to prevent cognitive function changes among older adults, considering depressive symptoms, social activity participation, and age.

Abstract citation ID: igad104.2951

### MARKERS OF GLUCOSE METABOLISM ARE ASSOCIATED WITHCCOGNITIVE DECLINE AMONG FAMILIES WITH EXCEPTIONAL SURVIVAL

Iva Miljkovic[1], Ryan Cvejkus[1], Stacy Andersen[2], Stephanie Cosentino[3], and Joseph Zmuda[1], *1. University of Pittsburgh, Pittsburgh, Pennsylvania, United States*, *2. Boston University Chobanian & Avedisian School of Medicine, Boston, Massachusetts, United States*, *3. Columbia University Medical Center, New York City, New York, United States*

Although the connection between type 2 diabetes (T2D) and dementia is known, it is less clear whether this relationship extends to cognitive decline. Our aim was to examine whether baseline fasting plasma glucose, insulin, HOMA-IR, and HbA1c, and T2D status predict cognitive decline in a multicenter cohort study of families with a clustering of exceptional survival. We analyzed change across five cognitive tests collected over 7.9 years: Mini-Mental State Examination, Digit Span, Logical Memory, Animal Fluency and Digit Symbol Substitution. Among 2,175 family members (mean age 63±11 years (range 32-100); 54% women), 39.8% had prediabetes and 6.8% had T2D. Multiple linear mixed models were adjusted for age, sex, field center, education, APOEε4 status, BMI and family relatedness. A trend was observed for immediate ($p=0.047$) and delayed ($p=0.037$) memory, with normal glucose tolerance (NGT) predicting increased scores, prediabetes showing no change, and T2D predicting decreased scores. Among those without T2D (N=2,026), each standard deviation (SD) higher glucose (11.5 mg/dl) was related to a greater decline in delayed memory (-0.34±0.16mg/dl; $p=0.032$), each SD higher HbA1c (0.32%) was related to a greater decline in immediate (-0.36±0.15 mg/dl; $p=0.016$) and delayed (-0.31±0.16 %; $p=0.05$) memory, while each SD higher HOMA-IR (1.5) was related to a greater decline in Digit Span Backwards (-0.13±0.06; $p=0.047$). Findings suggest that among individuals with a clustering of exceptional survival, T2D, glucose, and HbA1c may predict greater decline in episodic memory, an ability typically affected early in Alzheimer dementia. The possible mechanisms, including genetic and metabolic mediators, warrant further investigation.

Abstract citation ID: igad104.2952

### MODELING HETEROGENEITY IN COGNITIVE TRAJECTORIES IN THE FRAMINGHAM HEART STUDY

Yuan Fang[1], Jiachen Chen[2], Joanne Murabito[3], and Kathryn Lunetta[2], *1. Binghamton University, Binghamton, New York, United States*, *2. Boston University School of Public Health, Boston, Massachusetts, United States*, *3. Boston University Chobanian & Avedisian School of Medicine, Boston, Massachusetts, United States*

The prevalence of cognitive impairment in the population is growing. Maintaining good cognitive health is central to successful aging, independence, and well-being. Although older age is generally associated with worse cognitive function, there are substantial heterogeneities in the rate of cognitive decline across different cognitive domains and in terms of the clinical onset age of dementia. Recently, harmonized factor scores measuring global cognitive function for the memory, executive function, and language domains have been created in the Framingham Heart Study (FHS). In this work, we identified FHS participants with two or more repeated factor scores after the age of 60 (n=2339; 57% female; 17% APOE-ϵ4 carriers; 64% attended colleges) and were dementia-free at the baseline visits. We fitted latent class mixed models (LCMM) to cluster cognitive trajectories from all three domains using the harmonized factor scores. Non-linear trends with age in the trajectories were modeled via piecewise linear LCMM models followed by stepwise selections to select cluster-specific change points. We identified different latent classes of participants, some characterized by an early cognitive decline before age 70 as compared to late decliners, across different domains. Using 10-fold cross-validation, we also showed that the subgroupings of participants are stable. Our findings indicate class-related differential patterns in cognitive aging in the FHS. Future associations between the identified subclasses, cognitive outcomes, and physical or biological markers may advance the knowledge of multiple pathways of cognitive aging.

Abstract citation ID: igad104.2953

### RATES OF VISION AND HEARING IMPAIRMENT RELATED TO COGNITIVE DIFFICULTIES AMONG OLDER ADULTS IN MISSISSIPPI COUNTIES

Yan-Jhu Su[1], Taylor Jansen[1], Chae Man Lee[2], Nina Silverstein[1], Elizabeth Dugan[3], Shu Xu[3], and Kina White[4], *1. University of Massachusetts Boston, Boston, Massachusetts, United States*, *2. Univ of CT Health, Farmington, Connecticut, United States*, *3. University of Massachusetts, Boston, Boston, Massachusetts, United States*, *4. Mississippi State Department of Health, Ridgeland, Mississippi, United States*

Measures of population health can be a tool to advance healthy equity, because they identify disparities that may otherwise go undetected. In this study, we examined if community rates of sensory impairments (vision and/or hearing difficulty) had any association with self-reported cognitive difficulties among older adults. Understanding the distribution of multiple conditions that impact function and independent living may identify geographic hotspots in need of supportive interventions. We describe the prevalence of visual difficulty and hearing difficulty among Mississippi adults age 65 years and older and examine the association of visual difficulty and hearing difficulty with cognitive difficulty in older adults at the community level in 82 counties in Mississippi. Small area estimation techniques were used to calculate age and gender adjusted community rates using three data sources: the American Community Survey (2016-2020), the Behavioral Risk Factor Surveillance System (2017-2020), and CMS Medicare Beneficiary Summary Files (2018). Results show there are 8.7% of older adults 65+ with self-reported vision difficulty and 15.5% with self-reported hearing difficulty. The multiple linear regression models after adjusting for age, gender, race, marital status, education, income, and chronic diseases, counties with higher rates of vision difficulty and hearing difficulty were significantly associated with higher rates of cognitive difficulty compared to those with lower prevalence (vision difficulty: $\beta$=0.36, p< 0.001, hearing difficulty: $\beta$=0.32, p< 0.001). Implications suggest the need for practitioners and policymakers to establish cognitive difficulty-related interventions targeted to service areas with a high prevalence of sensory difficulties.

Abstract citation ID: igad104.2954

### RISK FACTORS OF OSTEOARTHRITIS AMONG OLDER ADULTS WITH COGNITIVE DECLINE IN SOUTH KOREA

Jennifer (Jeehyun) Kim[1], and Bada Kang[2], *1. Yonsei University, College of Nursing, Seoul, Republic of Korea*, *2. Yonsei University, Seoul, Republic of Korea*

Individuals with cognitive decline are vulnerable to developing osteoarthritis owing to reduced physical activity and impaired communicational ability. While the cross-sectional relationship between cognitive decline and chronic diseases has been previously studied, longitudinal studies are needed to identify determinants associated with the onset of osteoarthritis among older adults with cognitive decline. This study was aimed at elucidating the risk factors of osteoarthritis based on cognitive status. 1,142 older adults, including 334 cognitive decline and 808 normal cognition individuals, were selected from the Korean Longitudinal Study of Aging. Kaplan-Meier curves were plotted to estimate the probability of osteoarthritis based on the participants' sex and cognitive status. Cox proportional hazard regression analysis was performed to determine factors associated with the risk of osteoarthritis after the participants were stratified by cognitive status. Female sex and advanced age were associated with a greater risk of osteoarthritis in both the participants with and without cognitive decline. Furthermore, the risk of osteoarthritis was significant among older adults with cognitive decline if they lived alone, were depressed, had social engagement fewer than once per week, or had a high dependency on others for completing activities of daily living. However, there was an increased risk of osteoarthritis among older adults with normal cognition who had a high body mass index and a chronic medical condition. Therefore, comprehensive coping strategies focusing on the socio-environmental characteristics and clinical symptoms of older adults with cognitive decline should be considered to prevent a further increase in the healthcare burden associated with osteoarthritis.

Abstract citation ID: igad104.2955

### SEX DIFFERENCES IN THE ROLE OF SLEEP ON EXECUTIVE FUNCTION IN OLDER ADULTS

Yumiko Wiranto[1], Pilar Thangwaritorn[1], Kayla Meyer[2], and Amber Watts[1], *1. University of Kansas, Lawrence, Kansas, United States*, *2. KU Alzheimer's Disease Research Center, Fairway, Kansas, United States*

Advancing age is associated with a decline in sleep quality and cognitive performance. Previous work has shown that women report a higher number of sleep complaints, possibly due to hormonal and physiologic developmental changes across the lifespan. However, whether the effects of sleep quality on cognitive performance differ between sexes in older populations remains unclear. To address this gap, we analyzed data from 117 cognitively normal participants (44 men and 73 women) aged 60 and older (M = 74.82, SD = 6.52) to compare the effects of objectively measured sleep on executive function. Total sleep time was recorded with the GT9X Link Actigraph worn for seven consecutive days. The composite score of executive function was derived from a confirmatory factor analysis consisting of four separate tests (Verbal Fluency, Trail Making Test Part B, Digit Symbol Substitution Test, and Stroop Interference). We used hierarchical multiple regression to investigate sex differences in the effects of total sleep time on executive function. A quadratic model of total sleep time was a significant predictor of executive function in women ($\beta$ = -.251, p = .034), but not in men adjusting for age and education. Longer sleep duration predicted improved executive function, but the performance decreased with oversleeping. These findings imply that interventions aimed at optimizing sleep duration may be beneficial for executive function in older women. Further research is necessary to identify sex-specific sleep requirements, which can inform the development of personalized interventions for older adults.

Abstract citation ID: igad104.2956

### THE COGNITION IN PRIMARY CARE PROGRAM: A STEP-BY-STEP CLINIC WORKFLOW PUTTING THE GSA KAER TOOLKIT INTO ACTION

Jaqueline Raetz[1], Annette Fitzpatrick[1], Basia Belza[1], Sarah McKiddy[1], Amy Hsu[1], Joshua Liao[1], Christina Park[2], and Barak Gaster[1], *1. University of Washington, Seattle, Washington, United States*, 2. *University of Washington, Seattle, Seattle, Washington, United States*

In 2020 University of Washington (UW) Medicine began developing Cognition in Primary Care (CPC), a quality improvement program adapting the GSA KAER Toolkit to help primary care providers (PCPs) detect patients with cognitive impairment in their 16-clinic primary care network. The CPC consists of a 3-part training program to help PCPs diagnose, manage and counsel patients; a set of tools embedded into the electronic health record to facilitate cognitive evaluation; and an in-clinic workflow providing on-site support for providers within their own clinic setting. During this process we interviewed providers and developed a hybrid remote and in-person orientation for clinics to initiate a workflow to support improved cognitive evaluations. Utilizing pre-implementation input, the CPC in-clinic workflow was pilot-tested at two UW primary care clinics in 2021/2022. This presentation will describe content of the clinic orientation and implementation of CPC to include 1) the typical timeframe and content of orientation; 2) meetings with clinic leadership; 3) overview of provider/staff meetings; 4) in-person walk-through for placement of materials; 5) invitation to training; 6) a 12-page handbook outlining steps in the workflow; 7) other printed CPC materials, e.g., dementia warning signs, MoCA and AD8 forms, Alzheimer Association referral cards; and 8) follow-up focus group feedback. Evaluation of the pilot clinics following implementation found significant use of all tools by PCPs and an increase in diagnoses of mild cognitive impairment. This clinic workflow may be useful as a model for other health care systems to initiate cognitive evaluation programs in primary care.

Abstract citation ID: igad104.2957

### THE RELATIONSHIP BETWEEN ORAL HEALTH AND COGNITIVE FUNCTION: EVIDENCE FROM THE LASI

Xi Pan[1], Uma Kelekar[2], and T Muhammad[3], *1. Texas State University, San Marcos, Texas, United States*, 2. *Marymount University, Arlington, Virginia, United States*, 3. *International Institute for Population Sciences, Mumbai, Maharashtra, India*

The present study aimed to examine the association between oral health and cognitive function among older adults in India. Data for the study were selected from the Longitudinal Aging Survey of India (LASI, 2017-2019) and the LASI Diagnostic Assessment of Dementia (LASI-DAD) and 4,096 respondents aged 60 and above were included. Oral health was measured by self-reported physician-diagnosed oral health problems including presence of painful teeth, ulcers for more than two weeks, bleeding gums, swelling gums, loose teeth, dental cavities or caries, soreness or cracks in the corners of the mouth, or other problems as well as edentulism (i.e., losing all natural teeth, some natural teeth, or no teeth). A higher number of these problems indicated a more complex oral health condition. Cognitive function was measured by the standardized total score of all cognitive tests in the Harmonized Cognitive Assessment Protocol (HCAP). Binomial logistic regression models were used to analyze the relationship between the diagnosis of oral health problems and cognitive function. Results showed that having one or more oral health problems was associated with lower scores on cognitive function ($\beta$=-0.16, p=0.04), after controlling for sociodemographic characteristics and health status. There was no significant association between edentulism and cognitive function. Findings of the study suggest that oral health is integral to long-term overall health, specifically cognitive function. Our findings highlight the importance of educating older individuals about the connection between oral health and cognitive function along with the need for broadening their access to oral health care in India.

Abstract citation ID: igad104.2958

### THE ROLE OF LIFESTYLES IN ETHNIC DIFFERENCES IN COGNITIVE IMPAIRMENT AMONG ADULTS AGED 50 YEARS OR OLDER

Lisha Hou[1], Birong Dong[1], Jirong Yue[2], Chenkai Wu[3], Ning Ge[4], and Yao Ma[1], *1. West China Hospital, Sichuan University, Chengdu, Sichuan, China (People's Republic)*, 2. *West China Hospital of Sichuan University, Chengdu, Sichuan, China (People's Republic)*, 3. *Duke University, Durham, North Carolina, United States*, 4. *Department of Geriatrics and National Clinical Research Center for Geriatrics, West China Hospital of Sichuan University, Chengdu, Sichuan, China (People's Republic)*

**Background:** We examined ethnic differences in the prevalence of cognitive impairment and explored the impact of lifestyles on disparities in cognitive function among adults aged 50 years or above in Western China. **Methods:** 6,840 participants aged 50 years or older from the West China Health and Aging Trend Study (WCHAT) were included. Cognitive impairment was identified by the Short Portable Mental Status Questionnaire (SPMSQ). We used logistic regression to examine the association between ethnicity (seven groups) and cognitive impairment and the contribution of lifestyle factors to ethnic disparities. **Results:** The prevalence of cognitive impairment ranged from 6.2% in Bai to 29.3% in Yi. After multivariable adjustment, Tibetan had the highest odds of cognitive Impairment (OR=3.22, 95% CI 2.55-4.08), followed by Yi (OR=2.76, 95% CI: 2.121-3.59), Uighur (OR=2.59, 95% CI: 1.86-3.61) and Qiang (OR=2.10, 95% CI: 1.67-2.65), compared to Han. Lifestyle factors explained 22.2% of the ethnic difference in cognitive impairment. **Conclusions:** The prevalence of cognitive impairment varied widely across ethnic groups. Interventions promoting healthy lifestyles might help reduce the ethnic disparities in cognitive function among Chinese older adults.

Abstract citation ID: igad104.2959

### THE TYPES AND CONSISTENCY OF PARTICIPANT-SPECIFIED PROXIES DURING THE SPRINT CLINICAL TRIAL

Kat Coschigano[1], Sarah Gaussoin[2], Byron Jaeger[2], Stephen Rapp[2], and Jeff Williamson[2], *1. Somers High School, Katonah, New York, United States*, *2. Wake Forest School of Medicine, Winston Salem, North Carolina, United States*

**Background:** For clinical trials or patient care, reports from a person familiar with the trajectory of a participant's cognition are needed to ascertain incident Mild Cognitive Impairment and dementia. Informants offer a more unbiased evaluation of cognitive status. Recent large clinical trials studies, such as SPRINT, provide information about the contributions and consistency of informants throughout extended follow-up. **Methods:** SPRINT participants named proxies and relationship at study onset. Proxies provided information for the Functional Activities Questionnaire (FAQ) and Dementia Questionnaire (DQ). We collapsed proxy relationships into seven categories and evaluated the proportion of proxies in each category at baseline, how frequently the same proxy was retained to the last visit, and the frequency of successful proxy contact during the trial. **Results:** Of the 9,361 randomized SPRINT participants, 7,586 participants listed a proxy relationship from which we identified 7 categories. Participants were 35.6% female, with baseline mean age of 67.9 years. The proportion of proxies in each category was spouse/partner (51.7%), child (23.6%), sibling (8.7%), other family member (6.2%), and unelated (8.8%). The overall consistency of proxy continuation between the baseline and final SPRINT visit (mean 5.1 years) was 90.11%. Proxy consistency from first visit to last visit for a child was 93.41%, spouse/partner (91.87%), other family member (80.1%), brother/sister (86.2%), non-related (84.65%), and parent (81.73%). **Conclusion:** Immediate family members (spouse, child, or sibling) comprised the majority of proxies in SPRINT. Importantly, more than 90% of identified proxies at baseline continued to be available over the life of a 5-year trial.

### SESSION 7840 (POSTER)

### CHRONIC DISEASE MANAGEMENT

Abstract citation ID: igad104.2960

### ACUTE CARE USE FOR OLDER ADULTS WITH DIABETES AND DEMENTIA IN A PRAGMATIC CLINICAL TRIAL OF PANEL MANAGEMENT

Brianna Morgan[1], Mauricio Arcila-Mesa[2], Crystalinda Rapozo[1], Oluwaseun Adeyemi[3], and Joshua Chodosh[1], *1. NYU Grossman School of Medicine, New York City, New York, United States*, *2. NYU Langone, New York City, New York, United States*, *3. New York University Grossman School of Medicine, New York City, New York, United States*

Individuals with diabetes mellitus and Alzheimer's disease and related disorders (DM-ADRD) face greater self-management challenges, over and under treatment, and more acute care episodes. Enhanced Quality in Primary Care for Elders with Diabetes-ADRD (EQUIPED-ADRD) is a pragmatic randomized controlled trial providing practice guidelines alone (control) compared to practice guidelines plus panel management (intervention) in primary care. Panel management includes patient self-management support and enhanced engagement and support for staff. Thirty-one primary care practices were randomized (14 control versus 17 intervention clinics). We assessed differences in emergency department (ED) use and hospitalizations in individuals with DM-ADRD comparing groups using univariate analysis. Included patients receiving care at randomized clinics were >65 years old, had DM, ADRD, an identified caregiver, and available utilization data. We tracked ED use and hospitalizations over 24 months using electronic health records. Panel management dose was number of phone calls. Among those having utilization data, 177 received panel management versus 349 who did not (total n=526). Patients were predominantly 75-84 years old (41.3%), female (59.9%), and non-Hispanic white (50.9%); 25.9% were Hispanic and 10.7% were non-Hispanic Black. 52.0% (n=92) panel managed patients had ED visits versus 41.8% (n=146) not panel managed [(p=0.027]; Odds Ratio (OR) 1.50; 95%CI: 1.05-2.16). Hospital use did not significantly differ between groups [35.0%; vs. 31.5%, respectively; p=0.418]. Increased panel management dose did not impact acute care use. These findings suggest panel management increases ED use. Whether this is due to greater detection of clinical problems or other issues needing attention requires further investigation.

Abstract citation ID: igad104.2961

### ASSESSING THE SCALABILITY OF A COMMUNITY PROGRAM FOR OLDER ADULTS WITH DIABETES: BRIDGING THE RESEARCH–PRACTICE GAP

Melissa Northwood, Rebecca Ganann, Kathryn Fisher, Maureen Markle-Reid, and Marie-Lee Yous, *McMaster University, Hamilton, Ontario, Canada*

Implementing person-centred, cost-effective, and comprehensive self-management programs for older adults with multimorbidity and their caregivers in the health and social care system is challenging. Innovations that are effective under controlled research conditions often fail to produce similar results when implemented in real world settings to reach larger populations of older adults. Scalability assessment is a promising methodology to reduce this research-practice gap. This study aims to examine the scalability of a self-management intervention for older adults with diabetes and multimorbidity and their caregivers in two Canadian provinces. Provincial working groups, including patient partners, participated in the scalability assessment. The Intervention Scalability Assessment Tool (ISAT) guided data collection and analysis. Multiple methods were used to collect data, including an environmental scan, document review, and interviews with key informants. Provincial workshops were held to review scalability results, determine the program's scale-up readiness, and identify strategies to enhance scalability. Patient partners, health and social care providers and leaders, provincial decision-makers, and researchers gave high ratings to the readiness of the intervention and its alignment with practice and strategic policy initiatives. The evidence of effectiveness, delivery setting and workforce, and sustainability domains were rated lower. Participants recommended: 1) focusing on high-risk patients, which would be cost effective and likely to demonstrate impact and 2) piloting targeted sites to embed the program within existing health care settings and infrastructure to gather more evidence on program effectiveness and implementation. Collaborative and structured scalability assessments are critical to mobilize innovative programs into health and social care practice.

Abstract citation ID: igad104.2962

### BARRIERS AND FACILITATORS TO IMPLEMENTING MEDICARE'S CHRONIC CARE MANAGEMENT IN RURAL PRIMARY CARE

Elizabeth Punke, Barbara Dabrowski, Abby Teply, Katy Richardson, Christine McKibbin, and Catherine Carrico, *University of Wyoming, Laramie, Wyoming, United States*

In 2015, the Centers for Medicare and Medicaid Services introduced Chronic Care Management (CCM) to provide reimbursement to practices for care coordination services necessary to manage chronic conditions among older adults. However, uptake of this program among primary care practices is low, and few Medicare beneficiaries actually receive this service. Implementation of CCM may be even more challenging in rural, low-resourced areas. The purpose of this study was to examine factors associated with more and less successful implementation of CCM within primary care practices located in a rural state. A qualitative study was conducted, which was guided by the Consolidated Framework for Implementation Research (CFIR). Employees of practices (n = 8) that have implemented CCM were approached for study participation. Members (n = 17) of various healthcare teams were included in the study. Participants completed sociodemographic, professional, and practice history measures and completed a qualitative interview. Participants (n = 17) were predominantly White (n = 16; 94%), female (n = 16; 94%), and represented care coordinators, primary care providers, allied health professionals, and administrators. CrossTX records were analyzed to categorize practices by high and low implementation success. Content analysis, guided by CFIR, identified domains associated with implementation success, including Intervention Characteristics (I.e., Relative Advantage), Outer Setting (e.g., Patient Needs and Resources), Inner Setting (e.g., Implementation Climate, Leadership Engagement), and Process (e.g., Champions). Strategies to address barriers include addressing characteristics of the Inner Setting (e.g., Available Resources). Developing training mechanisms to address staff turnover is critical to address barriers.

Abstract citation ID: igad104.2963

### CARDIOVASCULAR DISEASE AND FRAILTY IN OLDER ADULTS FROM COLOMBIA

Paola Sosa-Sarmiento[1], Jose Ocampo-Chaparro[1], and Carlos Reyes-Ortiz[2], *1. Universidad del Valle, Cali, Valle del Cauca, Colombia*, *2. Florida A & M University, Tallahassee, Florida, United States*

Cardiovascular disease (CV) is a leading cause of death worldwide. Frailty is a growing geriatric syndrome. Limited research has explored the relationship between cardiovascular disease and frailty. This study assesses the association between a cardiovascular disease history and older adults' frailty. We used data from the SABE (Salud, Bienestar y Envejecimiento) Colombia Study, a cross-sectional nationwide population-based survey (2015) including community-dwelling adults ≥60 years. A secondary dataset of 5,242 participant responses was analyzed. Frailty was assessed according to the Fried Frailty Phenotype (weakness, low speed, low physical activity, exhaustion, and weight loss) and categorized as frail (3 or more components) vs. not frail or pre-frail. We performed logistic regression analysis to assess the association between a history of cardiovascular disease (hypertension, stroke, heart disease) and frailty, adjusting for socio-demographics, other comorbidities, and functional status. The prevalence of cardiovascular disease was 59.4%, and frailty was 20.0%. Compared to those who do not have CV disease, those having 1 CV disease (OR=1.23, 95% CI 1.04-1.46) or ≥2 CV diseases (OR=1.55, 95% CI 1.24-1.94) were associated with higher odds for frailty. A higher number of CV diseases were independently associated with frailty among older Colombian adults. In future studies, preventing CV disease might help to decrease frailty in older adults.

Abstract citation ID: igad104.2964

### CHANGE IN USE OF REMOTE HEALTH TECHNOLOGIES AMONG OLDER AFRICAN AMERICAN MEN DURING THE COVID-19 PANDEMIC

Ledric Sherman, and Janae Alexander-Bady, *Texas A&M University School of Public Health, College Station, Texas, United States*

It is well known that minority communities are disproportionately affected by disparities present within the US health care system. With the onset of COVID-19, these gaps in health quality continue to be exacerbated. Understanding health technology use differences pre-COVID and post-COVID among African American men is a necessary first step towards improving health outcomes that may be targeted via health technology interventions. The purpose of this study was to explicate and contextualize post-COVID health technology use among African American men in the United States for self-management of health. Of the 54 participants, all between the ages of 65-85, who completed a 30-item technology use questionnaire, a majority reported having type 1 diabetes and high blood pressure and that their current health was good or very good. For technology devices used pre-COVID, the highest reported devices owned were smartphones, desktop computer, laptop, and fitness tracker. Post-COVID, participants purchased fitness tracker CGM, glucose meter, and scale to check weight. As researchers and providers work towards addressing the prevalent health disparities disproportionately affecting members of our communities, these results provide an informative description of pre- and post-COVID.

Abstract citation ID: igad104.2965

**HEALTH PRIORITIES ALIGNED DECISION MAKING FOR DISCRETE DECISIONS**

Jack Banks[1], Suha Soni[1], Vanessa Madrid[1], Rafael Samper-Ternent[2], Salman Arain[1], Mary Tinetti[3], and Aanand Naik[1], *1. University of Texas Health Science Center at Houston, Houston, Texas, United States*, *2. UTHealth Houston, Houston, Texas, United States*, *3. Yale School of Medicine, New Haven, Connecticut, United States*

The outcomes that establish the evidence-base and frame expectations for discrete interventions, primarily survival and disease-specific markers, may not be the outcomes older adults with multiple chronic conditions (MCCs) prioritize. An advisor group consisting of 6 health professionals with expertise in geriatrics, cardiology, vascular surgery and decision-making plus a panel of patient stakeholders created a decision-making framework titled 'health priorities identification – discrete intervention decision' (HPI-DID) process. This study aimed to refine the HPI-DID process using revascularization for peripheral arterial disease (PAD) as an exemplar procedure through eliciting feedback on the HPI-DID process from patients and caregivers. Seven older adults who recently underwent a revascularization procedure for PAD or their caregivers took part in one-to-one interviews with JB and SS. During interviews, participants were asked to verbalize or 'think-aloud' their thoughts while reviewing and performing each component of the health priorities process and probed what could be added or cut to improve the procedure-related communication and decision-making process. Participant feedback resulted in structural changes to the process. Font size was increased and specific words were bolded. Three additional tables detailing expected benefits, tradeoffs and complications from the procedure and their expected durations were added. The mean percentage of patients who experience these outcomes is now also listed. We will conduct a pilot trial to evaluate the feasibility and acceptability of our refined HPI-DID process with clinical interventionalists and older patients with MCCs considering revascularization for PAD.

Abstract citation ID: igad104.2966

**HYPERTENSIVE URGENCY IN OLDER ADULT WITHOUT HYPERTENSION HISTORY: THE DANGERS OF OVERTREATMENT WITH LEVOTHYROXINE**

Vanessa Joyce Evardone[1], Jesssica Gendernalik[1], Ricardo Blondet[2], Sandra DiScala[2], and Michael Silverman[3], *1. Florida Atlantic University - West Palm Beach VA Medical Center, West Palm Beach, Florida, United States*, *2. West Palm Beach VA Medical Center, West Palm Beach, Florida, United States*, *3. West Palm VA Medical Center, West Palm Beach, Florida, United States*

A 72-yr old woman with well-controlled depression and active lifestyle was seen at urgent care for "nervousness." She was found to have borderline tachycardia and blood pressure of 187/114 mmHg. There were no other associated symptoms. Physical examination and labs were unremarkable, with normal renal function. EKG showed sinus tachycardia at 110 bpm. Recheck of BP after 30 minutes was 135/80mmHg, and she was discharged home on Lisinopril. Two days later, she again had "nervousness" and was taken to the Emergency Department. Blood pressure was 200/105 mmHg. Again, she denied any other symptomatology, and had unremarkable physical exam and labs. Repeat BP was improved, and she was discharged home with instruction to follow up with PCP. Medication list included Bupropion for depression which she has been taking and tolerating well for years, and Levothyroxine 25 mcg daily, which was started by her PCP 8 weeks prior, for TSH of 5.43 mIU/L and FT4 of 1.1 ng/dL. Thyroid hormone therapy was stopped during recent ED visit, and she has had no recurrence of her anxiety symptoms. Her BP has been checked daily at home and has been at baseline level.

Abstract citation ID: igad104.2967

**LEVERAGING IOT AND NATURAL LANGUAGE PROCESSING TO IMPROVE WELL-BEING OF CHRONIC PATIENTS**

Balakrishnan Mullachery[1], Samir Chatterjee[2], and Arindam Brahma[3], *1. ESRI, Claremont, California, United States*, *2. Claremont Graduate University, Claremont, California, United States*, *3. Loyola Marymount University, Aliso Viejo, California, United States*

According to the Centers for Disease Control and Prevention, in the United States, about 80% of older adults - individuals aged 65 years and older, have at least one chronic health condition. Chronic diseases require ongoing medical attention or limit activities of daily living, which in turn, affects quality of life. Heart diseases, cancer, and diabetes are the leading chronic diseases. Literature shows that timely and frequent follow-ups by providers significantly reduce readmissions of chronic patients and contribute to their well-being. However, health care provides do not have adequate bandwidth for regular patient follow-ups, which is vital for chronic patients. One solution for this growing problem, is to leverage smart home monitoring technologies facilitating self-care, which in turn can contribute to patients' well-being. The goal of this research is to build and leverage an IoT-based smart home monitoring platform and assess its impact on the well-being of chronic patients. The platform included an mHealth mobile app with daily journaling capability, which allowed patients to blog about their symptoms, daily activities, and feelings. Analyzing patients' daily blog data with Natural Language Processing (NLP) methods, this research revealed a strong correlation between a patients' blog entries and their well-being score. The results also demonstrated that patients' well-being scores can be predicted from such journal entries analyzed at real time, collected remotely from their home environment, leading to timely diagnosis and intervention opportunities. A field trial was conducted involving five heart disease and eight cancer patients for 180 days to validate the conclusions.

Abstract citation ID: igad104.2968

**LIVING WITH HEART FAILURE AND MULTIMORBIDITY: SEX-BASED DIFFERENCES IN DISEASE BURDEN**

Binu Koirala[1], Xiaoyue Liu[2], Arum Lim[2], Chitchanok Benjasirisan[2], Cheryl Dennison Himmelfarb[2], and Patricia Davidson[3], *1. Johns Hopkins University School of Nursing, Baltimore, Maryland, United States*, *2. Johns Hopkins University, Baltimore, Maryland, United States*, *3. University of Wollongong, Wollongong, New South Wales, Australia*

Heart failure (HF) is one of the leading causes of hospitalization and discharge diagnosis. People with HF present an average of six co-morbid conditions and are mostly older women. Comorbidity or multimorbidity in HF, increases the risk for poor health outcomes, significantly affecting the progression, severity, and complexity of management and care outcomes. This study aimed to examine the sex differences in disease burden among individuals living with multimorbidity. The study is an ongoing cohort study collecting sociodemographic, clinical, and outcome data at baseline, 30 days, and 1-year follow-up. Adults aged 50 years and older living with HF and multimorbidity, defined as the presence of HF and one more chronic condition identified as a common list of comorbidities were recruited using an electronic patient portal. Disease burden data were collected using a modified disease burden impact scale consisting of comorbid conditions included in the eligibility criteria. A preliminary analysis of 406 participants (mean [SD] age = 69.6[9.7] years; 49% female) at baseline found that females (67.7 [9.2] years) were significantly younger than males (71.6 [9.9] years) and females (28.1 [15.2]) had significantly higher disease burden scores compared to males (22.0 [13.6]). An adjusted analysis found females had 3 times higher disease burden scores than males (beta=3.32; 95% confidence interval [CI] = 0.44-6.20, p= 0.024), after controlling for sociodemographic characteristics, social support, resilience, and quality of life. Effective multimorbidity management program requires consideration of both sex and gender-based differences, particularly among older women.

Abstract citation ID: igad104.2969

### PERCEIVED COGNITIVE FUNCTION: RELATIONSHIPS WITH DIABETES COMPLICATIONS AND SELF-EFFICACY IN LATINX OLDER ADULTS

Shenell Wood, and Heather Cuevas, *The University of Texas at Austin, Austin, Texas, United States*

Cognitive dysfunction is a concern for individuals with type 2 diabetes (T2DM) and can affect diabetes self-management. However, it is often overlooked clinically and remains relatively understudied in Latinx older adults as a complication of T2DM. This project sought to explore the relationships between perceived cognitive dysfunction and its effects on self-efficacy and complications of T2DM in older Latinx adults. This was part of an analysis of baseline data from a larger randomized controlled trial investigating an cognitive rehabilitation intervention for older adults with T2DM. A total of 87 participants (mean age 67.3 +/- 5.4) completed a survey consisting of the Diabetes Empowerment Scale - short form (self-efficacy); PROMIS Bank v.2 - Cognitive Function (perceived cognitive function); and a self-report of number and type of diabetes complications. Participants had lower perceived cognitive dysfunction than the general population (Mean total score: 36.6 +/- 7.9); low self-efficacy (mean total score: 2.3 +/- 0.7) and reported at least 2 complications related to T2DM. Greater perceived cognitive dysfunction was related to lower self-efficacy (p = 0.04). Diabetes duration was positively associated with self-efficacy (p = 0.03), but not perceived cognitive dysfunction (p = 0.22). Self-efficacy was positively associated with number of diabetes complications (p = 0.03). Irrespective of diabetes duration, patients with more severe perceived cognitive dysfunction reported lower self-efficacy (p = 0.03) than those with lower levels of cognitive dysfunction. Assessment of cognitive function may help inform patient education interventions for Latinx older adults to improve the self-efficacy and potentially reduce complications.

Abstract citation ID: igad104.2970

### WHOLE HEALTH(Y) AGING WITH GEROFIT: THE DEVELOPMENT OF A PILOT WELLNESS PROGRAM FOR OLDER VETERANS

Elizabeth Parker[1], Heidi Ortmeyer[2], Jamie Giffuni[3], Odessa Addison[4], and Katherine Hall[5], *1. UMSOM, Baltimore, Maryland, United States*, *2. VA, Baltimore, Maryland, United States*, *3. Baltimore VA, Baltimore, Maryland, United States*, *4. University of Maryland School of Medicine, Baltimore, Maryland, United States*, *5. Duke University/Veterans Health Administration, Durham, North Carolina, United States*

The Veterans Health Administration (VHA) services over 9 million Veterans; approximately half are older than 65 years of age. Veterans are at a higher risk of comorbidities, including obesity and related chronic diseases, which contributes to higher health care costs, mobility disability, poor quality of life and even death. Gerofit is a VA Best Practice and Whole Health clinical exercise program for Veterans >65yrs that has positively impacted physical functioning, mental health symptoms, medication burden and 10-year mortality. This project describes the development of a comprehensive wellness and health promotion program incorporated into Gerofit, called Whole Healthy Aging Gerofit (WHAG). This novel program implemented wellness components to supplement the Gerofit in-person or virtual exercise options, including health education seminars, mindfulness classes, yoga classes, nutrition education classes, produce pick up service, and the Companion Dog Fostering program. WHAG deliverables included class attendance, individual Personal Health Inventories, observed physical function, self-reported physical activity levels, diet quality, perceived mental health, and Veteran satisfaction. The average Gerofit participant is approximately 73 years old, with obesity and multiple comorbid conditions (n=16), takes 10 medications, and is functionally impaired as assessed by gait speed (1.04 m/s). Programs that target healthy aging are needed to ensure the health of the population as it ages. This pilot program, WHAG, is one example of a comprehensive, patient centered wellness program that supports participants in setting their own goals and definitions of wellness to promote healthy aging.

### SESSION 7850 (POSTER)

### ASPECTS OF FAMILY CAREGIVING

Abstract citation ID: igad104.2971

### A PILOT STUDY OF YIN YOGA AND THE EFFECTS ON BURDEN AND SELF-COMPASSION IN CAREGIVERS OF PERSONS WITH DEMENTIA

Suzanne E Parkman, and Jill Olausson, *University of Southern Maine, Portland, Maine, United States*

Informal caregivers for persons with dementia face tremendous challenges in balancing their own needs and those of the care recipient. The purpose of this pilot study was to test the feasibility and acceptability of a Yin yoga intervention on caregiver burden and self-compassion. Authors explored recruitment, retention, adherence, and satisfaction with the yoga intervention. Using the three elements of the Neff's Model of Self-compassion, this pilot study was a quasi-experimental pre/post comparisons of caregiver burden (Zarit Burden Interview Scale-ZBI) and self-compassion (Neff Self-compassion Scale-SCS) in which the participants served as their own control group. A total of five participants (N=5) were recruited and completed an 8-week Yin yoga intervention via Zoom. Statistical analysis revealed non-significant results for ZBI ($t(4) = .95$, $p= .39$) and SCS ($t(4) = -.23$, $p= .83$). Wilcoxon signed rank test identified two questions on ZBI pre-and post-intervention had differences approaching significance ($p= .059$). Despite the non-significant results, burden was decreased in three participants and self-compassion scores were increased in four participants. The main perceived benefits were ease of survey process, feeling relaxed after intervention, and treatment fidelity. The main barriers identified were recruitment and retention of participants throughout the 8-week intervention. Overall, we found the study protocol to be feasible and acceptable. Possible modifications to the study would include extending the scope of the study nationally in efforts to recruit maximum participants. Future research is needed with larger sample sizes, randomization, control of confounding variables, and efforts to promote recruitment and retention of participants.

Abstract citation ID: igad104.2972

### “I’M INDEPENDENT. SO FAR...”: INVESTIGATING INFORMAL CAREGIVING PATTERNS OF OLDER ADULTS WITH PRIMARY LIVER CANCER

Deborah Watman, Sasha Perez, Yingtong Chen, Sara Lubetsky, and Christopher Woodrell, *Icahn School of Medicine at Mount Sinai, New York City, New York, United States*

Older adults with advanced stage hepatocellular carcinoma (HCC) are living longer given improved treatment effectiveness. Little is known about their unmet supportive care needs and their caregivers' needs, some of which may be addressed by palliative care. Previous studies of palliative care in populations facing liver disease have cited difficulty with recruitment. This is a cross-sectional study at a single academic medical center in NYC that uses validated survey instruments and qualitative interviews to explore caregiver support for those receiving treatments for HCC. We recruited patients with liver disease, and caregivers of patients 60 years or older. Validated survey instruments were used to collect data and a qualitative interview is conducted that focuses on the experience of supporting a loved one with HCC. 65% (13/20) participants were over the age of 60. PDSA framework was used to improve recruitment to four caregivers thus far. Results showed that language matters. Recruitment materials were updated from “palliative” to “supportive” care, to reduce stigma and fear of worsening health status and the perception of burden. Older adults value their independence and are not likely to identify a “caregiver”. They may be more willing to identify a loved one who is there to help. 90% of older adults identified a caregiver using the Duke Social Support Index. Thoughtfulness and adaptability are necessary when recruiting amongst this vulnerable yet increasingly independent population. Furthermore, older adults facing liver disease may be solo agers with no caregiver, increasing the need for supportive care as their disease progresses.

Abstract citation ID: igad104.2973

### BUILDING RESILIENCE USING PHOTOVOICE AMONG FAMILY CAREGIVERS

Mary Dioise Ramos, *Kennesaw State University, Kennesaw, Georgia, United States*

Filipino Americans remain one of the most underrepresented groups in research. It is challenging to identify the healthcare needs related to palliative and end-of-life care, especially among family caregivers. Palliative care is not yet well known in general, including among Filipino immigrants. Most people seem to adjust to the caregiver role and recover from Trauma/Emotional strain. However, some experience a long-lasting decline in their mental and physical health. This study will fill the gap in our understanding of the experiences of bereaved Filipino American family caregivers who have provided palliative and end-of-life care to their loved ones. This study aimed to explore the opportunities and challenges faced by Filipino American family caregivers who provided palliative and end-of-life care to their loved ones in the United States. The study also examined the impact of Photovoice as an intervention to build resilience, social support, and health-related quality of life among caregivers. The findings revealed that caregivers engaged in self-care activities, sought support from their social networks and healthcare providers, used coping strategies to manage emotional stress, found meaning in their caregiving experiences, and faced physical and emotional demands associated with caregiving. The Photovoice intervention was found to improve caregivers' resilience and HRQOL significantly, but not their social support. Caregivers need assistance to manage the demands of providing care, including preparing for the future, securing help, and navigating agencies and relationships. Healthcare providers need to provide adequate support and resources to help caregivers manage the physical and emotional demands associated with caregiving.

Abstract citation ID: igad104.2974

### CHARACTERIZING END-OF-LIFE COMMUNICATION IN FAMILIES

Melanie Horning, *Towson University, Towson, Maryland, United States*

The chronic disease course can be uncertain, contributing to delayed end-of-life discussion within families resulting in missed opportunity to articulate wishes, increased decisional uncertainty, and delayed hospice care. Consistent with the Family Communication Patterns Theory (FCPT), family communication patterns may increase understanding of end-of-life discussion, hospice utilization, and may affect the experience of “a good death.” This cross-sectional study used a modified Revised Family Communication Pattern instrument

(RFCP) to examine the family communication characteristics of 56 family members of loved ones who died from chronic illnesses while in hospice. Most families (42.9%) were pluralistic, reporting communication styles with high conversation and low conformity orientation; (39.29%) were protective, reporting low conversation and high conformity orientation. Pluralistic families had more end-of-life conversations than did protective families. This study's findings support the idea that using the RFCP to assess familial communication patterns may be useful for end-of-life conversation and decision-making

Abstract citation ID: igad104.2975

### FEAR OF FAILING: PERSPECTIVES ON HOME- AND COMMUNITY-BASED SUPPORT SERVICES FOR RURAL CAREGIVERS

Elizabeth Marfeo[1], Elizabeth Chamberlin[2], Maria Venegas[2], and Lauren Moo[3], *1. Tufts University, Medford, Massachusetts, United States*, *2. US Department of Veterans Affairs, Bedford, Massachusetts, United States*, *3. Veterans Health Administration, Bedford, Massachusetts, United States*

Many older adults rely on family caregivers not only for day-to-day assistance, but also to help navigate the healthcare system and attend medical visits. Finding, scheduling, arranging transportation for, and attending medical visits involves considerable planning, adaptability to a broad array of contextual factors, and can often cause disruption in day-to-day routine of both the family caregiver and the older adult. For Veterans living in rural areas, limited VA and community resources may magnify these challenges leading to higher caregiving burden and negative health and quality of life outcomes for both the Veteran and caregiver. The aim of this study was to conduct in-depth interviews to better understand key contextual factors related to the logistics of managing and attending medical visits among family caregivers of Veterans living in rural communities (N = 19, mean age 65). A clear disconnect emerged between caregiver needs and (1) access to community support services and (2) effective utilization of existing services. Seventy-nine percent of the caregivers reported living with the Veteran and receiving no home services. Interviews revealed both the caregiver and Veteran having significant, complex healthcare needs. Caregivers described fears of their own health negatively affecting their ability to continue providing sufficient supports for their loved one, lack of resources for home management needs, and limited access to transportation assistance. Findings from this study are important for informing continued development and evaluation of caregiver support programs nationwide, especially among those living in rural communities.

Abstract citation ID: igad104.2976

### PERSPECTIVES TOWARD ADVANCE CARE PLANNING IN FAMILY CAREGIVERS OF PERSONS WITH DEMENTIA: Q METHODOLOGY STUDY

KyungAh Cho[1], Myoung Hwan Shin[2], Jung-Ah Lee[3], Seongkum Heo[4], Sang Yi Baek[1], Jisun Yang[1], SeongHu Park[5], and JinShil Kim[1], *1. Gachon university, Incheon, Republic of Korea*, *2. Sookmyung Women's University, Seoul, Republic of Korea*, *3. University of California-Irvine, Irvine, California, United States*, *4. Mercer University, Georgia Baptist College of Nursing, Atlanta, Georgia, United States*, *5. Sungshin Women's University, Seoul, Republic of Korea*

**Background:** Family caregivers' perspectives toward advance care planning (ACP) is critically important to reflect end-of-life wishes of people with dementia (PWD). Purpose: To explore the ACP perspectives among family caregivers of PWD using a Q methodology. Methodology: Q sample with statements regarding the characteristics of caregivers' perspectives toward ACP was constructed. P sample of 25 caregivers completed each grid with a statement for Q sorting table. Data analysis was done using the PQ Method program (Ver. 2.35). **Results:** Three factors emerged through a Varimax rotation and explained 53% of the total variance. Factor 1 (n = 12) (General cases: Planned preparation of pre-care planning (PPP) and understanding and thoughtfulness of the behaviors of PWD) consisted of caregivers who knew the importance of PPP and willingly tried to understand and manage dementia. Factor 2 (n = 7) (Severe cases: Unplanned preparation of PPP and feeling of responsibility and burden for management of PWD) consisted of caregivers who did not have PPP and had concerns and anxiety about management of PWD. Factor 3 (n = 5) (Mild cases: Optimistic preparation of PPP and proudness for caring PWD) consisted of caregivers who were optimistic about dementia care, were proud of their duty of caring PWD, and thought meeting patients' needs and caring them as valuable. **Conclusions:** Caregivers of PWD had different ACP perspectives based on the severity of cognitive impairment in patients. Clinicians should consider the levels of patients' cognitive impairment to provide effective ACP education to caregivers of PWD.

Abstract citation ID: igad104.2977

### RURAL CAREGIVER PERSPECTIVES OF SUPPORTING PATIENT ENGAGEMENT IN VIDEO VISITS

Megan Gately[1], Dylan Waller[2], Matthew Maynard[3], and Lauren Moo[4], *1. VA Bedford Health Care System, Bedford, Massachusetts, United States*, *2. Center to Improve Veteran Involvement in Care (CIVIC), Portland, Oregon, United States*, *3. Veterans Health Administration, Forest City, North Carolina, United States*, *4. Veterans Health Administration, Bedford, Massachusetts, United States*

Rapid integration of telehealth in response to the COVID pandemic highlighted digital divide issues, specifically for video, which is a live, synchronous encounter. Older patients may have difficulty navigating a video session due to age, health-related impairment, or low technical competence. Further, specialty services which involve hands-on care, such as occupational therapy (OT), are more complex to translate to video, potentially necessitating a second person to assist with set-up or delivery of clinical care. Caregivers may aid in bridging the digital divide between patient and clinicians in a video session. However, little is known about caregiver willingness and capacity to assume a support role in video, particularly caregivers of rural patients, who have more complex care needs and face more issues accessing needed technology than urban patients. To understand the caregiver role supporting older rural patient engagement in video sessions, we conducted interviews with caregivers (N=25) of older rural veterans with an OT video visit. Interview topics included caregivers' prior use of and general attitudes toward technology, and experiences of the video visit, including set-up and operation of technology and experience participating and supporting patient engagement. Findings revealed a range of rural caregiver perspectives about the role of technology to facilitate patient access to care, including enabling factors which highlighted caregivers' education and training support needs. This study deepens our understanding of the importance of caregivers to support rural patient access of video sessions, informing development of strategies to optimize caregiver participation.

Abstract citation ID: igad104.2978

### THEORETICALLY BASED FACTORS ASSOCIATED WITH STROKE FAMILY CAREGIVER HEALTH

Cleopatra Kum[1], Holly Jones[2], Elaine Miller[1], Natalie Kreitzer[3], and Tamilyn Bakas[1], *1. University of Cincinnati, Cincinnati, Ohio, United States*, *2. The Ohio State University, Columbus, Ohio, United States*, *3. University of Cincinnati College of Medicine, Cincinnati, Ohio, United States*

**Background:** Most stroke survivors are cared for at home by family members, many of whom experience depressive symptoms and life changes as a result of providing care. While many studies have explored the factors associated with depressive symptoms and quality of life in family caregivers, few have focused on caregiver health. Purpose: To determine theoretically based factors associated with unhealthy days in stroke family caregivers. **Methods:** A conceptual model derived from Lazarus' theory of stress and coping was used to guide analysis. Secondary data analysis was conducted using baseline data from a large randomized controlled clinical trial testing the Telephone Assessment and Skill-building Kit (TASK II) program with 254 family caregivers of stroke survivors within 2 weeks of discharge from acute care and rehabilitation settings in participating Midwestern facilities. Data were analyzed using multiple regression with unhealthy days as the dependent variable, and theoretically based factors (i.e., caregiver task difficulty, level of optimism, threat appraisal, depressive symptoms, and life changes) as independent variables. All scales demonstrated evidence of internal consistency reliability with Cronbach alpha scores above .70. **Results:** Caregivers were mostly female (78%), white (71%), spouses (47%) or adult children (30%). Caregivers reported 9 unhealthy days on average within the past month. Unhealthy days were significantly correlated with caregiver task difficulty (r=.32), level of optimism (r=-.29), threat appraisal (r=.44), depressive symptoms (r=.60), and life changes (r=-.40) (p<.05). 37.8% variance in unhealthy days was explained, with depressive symptoms being the only significant individual predictor due to shared variance.

Abstract citation ID: igad104.2979

### UNMET NEEDS OF CAREGIVERS OF HOSPITALIZED VETERANS LIVING WITH DEMENTIA: A QUALITATIVE STUDY

Molly Horstman[1], Crystal Guo[2], Mandi Sonnenfeld[1], Tracy Evans[2], Alan Stevens[3], and Mark Kunik[2], *1. Michael E. DeBakey VA Medical Center, Houston, Texas, United States*, *2. Baylor College of Medicine, Houston, Texas, United States*, *3. Baylor Scott and White Health, Dallas, Texas, United States*

Family caregivers are essential to safe care transitions for hospitalized adults living with dementia. We aimed to identify resource and training needs of dementia caregivers during care transitions. We conducted semi-structured interviews with caregivers (6 Spouses, 5 Children, 1 Friend) of hospitalized Veterans with dementia at the Michael E. DeBakey Veterans Affairs (VA) Medical Center. Interviews were recorded and transcribed verbatim. Rapid qualitative analysis using structured summaries and matrices were conducted. Although caregivers received information about the Veteran's admitting medical condition, health professionals did not ask about dementia or provide dementia-related health information to the caregiver. Caregivers were willing to assist Veterans with activities of daily living in the hospital; however, caregivers were either prevented from assisting or did not receive training to assist Veterans safely. Caregivers desired information on how to get the Veteran "back on track" and address cognitive and functional declines experienced in the hospital. Although pre-hospital services and supports were easy to restart, caregivers needed earlier and more consistent communication regarding new services and supports starting after discharge, such as rehabilitation at skilled nursing facilities and respite care. Caregivers expressed a need for health professionals to help set "realistic expectations" for post-discharge care as some services and supports did not meet their expectations. Caregivers valued access to support groups for themselves but received inconsistent referrals to the VA Caregiver Support Program. These results will inform the adaptation of a dementia caregiver support intervention for caregivers of hospitalized Veterans living with dementia.

### SESSION 7860 (POSTER)

### ACUTE CARE ISSUES IMPACTING OLDER ADULTS

Abstract citation ID: igad104.2980

### COMPARISON OF DIAGNOSTIC COMPLEXITY AND IMAGING DECISIONS IN YOUNGER, MIDDLE AGE, AND OLDER ADULTS

Varun Saraswathula[1], Aubrey Mwinyogle[2], Lili Schadt[1], Gopal Kowdley[2], Rachel Kelz[1], and Ari Friedman[1], *1. University of Pennsylvania, Philadelphia, Pennsylvania, United States*, *2. Tidal Health, Salisbury, Maryland, United States*

A primary reason for visit of abdominal pain in the emergency department (ED) defines a syndromic presentation that can carry morbidity and mortality comparable to a myocardial infarction. Whether imaging decisions in the ED adequately account for this risk, and whether this risk is mediated in part by greater diagnostic complexity remains unknown. In a prospective cohort of 1,169 patient visits to a US community ED, a surgeon codified each visit according to history, examination, laboratory testing, imaging

(the acuity of CT findings), diagnosis, and disposition. Chi square tests (two-tailed, alpha=.05) tested for differences between age groups (younger: 18-40, middle: 40-60, older adult: 60+). We found that 42.5% of younger adults received a CT, compared to 66.7% and 70.0% of middle age and older adults (P< 0.001). 20.5% of CT findings were acute in younger, 34.0% in middle, and 39.6% in older adults (P< 0.001). The top 3 acute diagnoses accounted for 75.5% of younger, 62.9% of middle, and 62.7% of older adults, with Herfindahl Indices of 0.25 for younger, 0.16 for middle, and 0.15 for older adults. ED clinicians tested modestly more in older adults, but still potentially over-test younger adults and under-test older adults as judged by test positivity. Older adults had greater diagnostic complexity than younger adults. Dissemination of these findings may improve appropriateness of testing.

Abstract citation ID: igad104.2981

### DO OLDER ADULTS APPRAISE ELDERSPEAK COMMUNICATION AS PATRONIZING OR RESPECTFUL IN HOSPITAL DEMENTIA CARE?

Clarissa Shaw[1], Caitlin Ward[2], and Lisa Weimar[1], *1. University of Iowa, Iowa City, Iowa, United States*, *2. University of Minnesota, Minneapolis, Minnesota, United States*

Elderspeak communication is speech that sounds like babytalk to older adults. The Iowa Coding Scheme for Elderspeak (ICodE) contains 11 elderspeak attributes: diminutives, childish terms, collectives, short words, directives, reflectives, minimizers, exaggerated praise, laughter, tag questions, and exaggerated prosody. Thirty-one older adults (aged 74.5±7.8 years) listened to 41 clips of nursing staff providing care to hospitalized patients with dementia and rated each clip using the 5-point Emotional Tone Rating Scale for patronizing and respectful (5=very, 1=not at all). Each clip was classified using the ICodE as either neutral (no elderspeak), containing a single attribute, or containing an attribute with exaggerated prosody. Linear mixed modeling was used to evaluate the differences in patronizing and respectful ratings between neutral clips and each attribute, and clips with each attribute and exaggerated prosody, while controlling for within-subjects correlation and adjusting for multiple comparisons. Mean ratings for neutral clips were 4.0±1.1 for respectful and 1.7±1.0 for patronizing. Ratings for elderspeak attributes ranged from 2.1 to 2.9 for patronizing and 2.8 to 3.8 for respectful. Ten of the 11 attributes, excluding exaggerated praise, were rated as significantly more patronizing and less respectful than neutral. The addition of exaggerated prosody was significant in comparison with the elderspeak attribute alone for exaggerated praise (more patronizing and less respectful), laughter (more patronizing), short words (less patronizing, more respectful), and diminutives (less respectful). This analysis validates that the attributes of the ICodE are considered more patronizing and less respectful by older adults compared to neutral communication in hospital dementia care.

Abstract citation ID: igad104.2982

### EARLY WARNING SYSTEM SCORE FOR URGENT ET TUBE OR UNPLANNED ICU ADMISSION FOR ELDERLY PATIENTS IN GENERAL WARDS

Sunee Suwanpasu[1], Sukanya Poonsap[2], and Werapattra Prapaprpak[2], *1. KCMH, Sao Ching Cha , Krung Thep, Thailand*, *2. King Chulalongkorn Memorial Hospital, Pathumwan, Krung Thep, Thailand*

Early warning system scores are tools used to identify the early signs of physiologic deterioration in order to initiate early intervention and management. This study aims to investigate the efficacy of the National Early Warning Score (NEWS) score and Search out Severity Score (SOS) in predicting urgent ET tube or unplanned ICU admission. A Prospective analytical study of 85 patients aged 60 years and over in general medical wards, was conducted to assess the ability of SOS and NEWS. Prediction ability was assessed using sensitivity, specificity, accuracy, and area under the receiver operating characteristic curve (AUC). The NEWS and SOS systems at 4 hours before the event were the most effective, with the cut-off values of NEWS> 7 and SOS> 4 giving the best accuracy (sensitivity 71.4%, 64.3% specificity 98.2%, 98.2%. Respectively). The area under the ROC curve [AUC]) was 0.90 (95% CI 0.81-0.99) and 0.86 (95% CI 0.75-0.97), respectively. The prediction efficiency decreased over time before the event. However, at 8, 12, and 24 hours before the incident, it was still accurate at a good-fair level (AUC 0.82, 0.82, 0.74, and 0.77, 0.79, 0.72, respectively) The NEWS system provides excellent accuracy, while the SOS system gives a good accuracy level. Therefore, the NEWS system should be used for the early detection of abnormalities and as a guideline for the rapid response to changes in the vulnerable elderly. It keeps patients out of the crisis and is safe and able to predict worsening patients.

Abstract citation ID: igad104.2983

### EMERGENCY DEPARTMENT PRACTICE PATTERNS FOR OLDER ADULTS WITH ABDOMINAL PAIN

Rachel Wu, and Ari Friedman, *University of Pennsylvania, Philadelphia, Pennsylvania, United States*

Abdominal pain is the most common reason for visit (RFV) to the emergency department (ED) for older adults in the United States. Despite high morbidity and mortality, unlike for chest pain, there is no standardized diagnostic pathway. We investigated the rates of computed tomography (CT) scans and X-rays for older adults and compared these to the rates in young and middle-aged adults. We conducted survey-weighted descriptive analyses of the National Hospital Ambulatory Medical Care Survey. We included 27,149 sampled ED chart abstractions for abdominal pain, representing 143.2 million visits nationally; 12.6% were from older adults 65+. CT rates increased over time for all age categories, with 26.2% of all patients receiving a CT scan in 2007 to 42.6% in 2019. The relative percent change in abdominal CT scans was greatest for older adults, with a 30.3% change from 2007 to 2019, compared to 24.0% change for adults aged 46-64 and 15.0% for adults aged 18-45. 13% of older adults received only an X-ray, which is neither sensitive nor specific for acute pathology in older adults in the ED. Test positivity, as defined by receiving an emergency general surgical diagnosis among patients imaged with CT or ultrasound increased from 17.2% in 2007 to 22.9% in years 2019 (p< 0.01), suggesting increasing severity for this high-risk condition among older adults. Our findings suggest potential over-use of ineffective testing (X-rays) as well as the need for better targeted cross-sectional imaging (rising test positivity).

Abstract citation ID: igad104.2984

### GERIATRICS TRAUMA CO-MANAGEMENT: PRELIMINARY DESCRIPTIVE DATA FROM A SINGLE ACADEMIC INSTITUTION

Yuji Yamada[1], Ko Harada[2], Fred Ko[1], Emily Chai[1], Luis Suarez-Rodriguez[1], Raymond Wedderburn[1], and Omar Amir[1], *1. Icahn School of Medicine at Mount Sinai, New York City, New York, United States*, *2. Beth Israel Medical Center, New York, New York, United States*

**Background:** Traumatic injuries cause higher mortality and complications in older patients than in younger individuals. Orthogeriatric co-management programs improve outcomes and quality of care and may be a model for trauma management. We theorized that geriatricians could improve trauma care in older adults with the early identification of geriatric syndromes. **Methods:** A retrospective chart review was conducted for patients aged > 65 years admitted to the trauma surgery service at a Level 2 trauma center between October 2022 and January 2023, who were co-managed by geriatricians as part of a pilot program. **Results:** 67 patients were included in this study. The mean age was 81 (65-99), and 38% were male. The most common injuries were rib fractures (39%), subdural hematomas (32%), and spinal fractures (10%). Geriatric syndromes identified and managed by a geriatrician included frailty (47%), major or minor cognitive impairment (48%), delirium (19%), malnutrition (13%), and polypharmacy (65%), with a mean number of 6.7 home medications. As per Beers criteria, 63% of patients were taking inappropriate medications. Geriatrician-led interventions in advance care planning included identification and documentation of healthcare proxies (i.e., increased to 87% from 11% on admission) and goals of care conversations (54%). **Conclusions:** Given the high prevalence of geriatric syndromes and complex medical and social needs in older patients with trauma, geriatric co-management may enable timely diagnosis and treatment of conditions that cause poor outcomes. Further research is warranted to assess the impact of geriatric co-management models on the long-term clinical outcomes in older individuals after injury.

Abstract citation ID: igad104.2985

### IMPLEMENTING AGE-FRIENDLY HEALTH SYSTEMS: SCALING 4MS CARE ACROSS VA

Laurence Solberg[1], Shivani Jindal[2], Kimberly Church[3], and Andrea Schwartz[2], *1. NF/SG VHS, Newberry, Florida, United States*, *2. VA Boston Healthcare System, Boston, Massachusetts, United States*, *3. VA Central Office, Washington, District of Columbia, United States*

The U.S. Department of Veterans Affairs (VA) provides health care to 9 million enrolled Veterans across a variety of settings. Nearly 50% of enrolled Veterans are age 65 and older, compared to 16% of the U.S. population. The VA has set out to become the largest integrated Age-Friendly Health System (AFHS), using the 4Ms as a framework to improve care for older adults: what Matters, Medication, Mentation and Mobility. When implemented together, the 4Ms allow interprofessional teams and health systems to provide care consistent with what Matters to each older adult. Since joining AFHS movement in 2020, over 190 care settings at 100 VA facilities have earned recognition from the Institute for Healthcare Improvement (IHI). VA has identified key drivers to scale-up and spread 4Ms care at the national, local, and team level. Drivers include active and visible national leadership, building a coalition of local champions, and regular commmunication with teams. VA has accelerated adoption of the 4Ms by following IHI's Action Community (AC) model. At close of registration, 145 teams from 69 VA facilities enrolled in the VA AC. The VA AC offered a series of monthly webinars and coaching calls to empower teams to create a 4Ms plan building on exisiting best practices. In addition to using evidence-based tools identified by IHI, teams may also leverage VA clinical innovations to support their 4Ms plan. Implementation of AFHS provides a framework for interdisciplinary teams to continuously improve Veteran-centered care and ultimately, fulfill VA's mission to honor America's aging Veterans.

Abstract citation ID: igad104.2986

### INVESTIGATING ADVERSE OUTCOMES OF LONG-TERM STEROID IMMUNOSUPPRESSION IN ELDERLY RENAL TRANSPLANT RECIPIENTS

John Johnson, Heather Stevenson, Muhammad Mujtaba, and Michael Kueht, *University of Texas Medical Branch at Galveston, Galveston, Texas, United States*

Steroids are widely used in renal transplantation to prevent allograft rejection; however, long-term steroid use is associated with adverse side-effects such as hyperglycemia and osteoporosis, which elderly patients are especially susceptible to. Our study analyzed the adverse effects of long-term steroid immunosuppression in elderly renal transplant recipients using the TriNetX database. TriNetX was used to perform a propensity-score matched case-control study of 5-year outcomes in elderly renal transplant recipients (≥ 65 yrs) who either underwent an early-steroid withdrawal (ESW) or steroid-continuous (SCI) maintenance immunosuppression regimen. ESW patients were maintained long-term (5 yr) on tacrolimus and mycophenolate and discontinued prednisone 7 days post-transplant. The SCI cohort was maintained on 5yr tacrolimus + mycophenolate + prednisone. Cohorts were matched on age, sex, race/ethnicity, BMI, diabetes, cardiovascular disease, and bone disorders. Primary outcomes included rejection, drug-induced diabetes, bone fractures, and malignancy 5 years post-transplant. There were 197 patients in each cohort after matching (ESW, SCI). SCI patients had significantly higher incidences of 5-year drug-induced diabetes (4.53% vs 15.73%, $p < 0.01$) when compared to ESW. There was no significant difference found for incidences of rejection (33.02% vs 33.91%, $p = 0.86$), bone fractures (12.27% vs 16.29%, $p = 0.19$), or malignancy (11.57% vs 15.78%, $p = 0.26$). Our study found that elderly SCI patients had significantly higher incidences of drug-induced diabetes when compared to ESW. More insight is needed into differences in average glucose, blood pressure, and cholesterol throughout the duration of the study and dosage differences within the SCI cohort.

Abstract citation ID: igad104.2987

### LEVERAGING NURSING ASSESSMENT DATA TO IDENTIFY FRAILTY INDICATORS OF HOSPITAL DISCHARGE DISPOSITION

Sarah Ser[1], Laurence Solberg[2], Ragnhildur Bjarnadottir[1], Robert Lucero[3], Mattia Prosperi[1], Urszula Snigurska[4], and Brian Celso[5], *1. University of Florida, Gainesville, Florida, United States*, *2. NF/SG VHS, Newberry, Florida, United States*, *3. University of California, Los Angeles, Los Angeles, California, United States*, *4. University of Florida College of Nursing, Gainesville, Florida, United States*, *5. University of Florida Jacksonville, Jacksonville, Florida, United States*

Frailty among older adults is associated with higher morbidity and mortality rates and poorer hospital outcomes. Although frailty is not routinely assessed in the hospital setting, elements of frailty are captured in nursing assessments. The purpose of this study was to examine components of the Risk Analysis Index (RAI) captured in electronic health record (EHR) assessment data and their associations with discharge disposition. This was a retrospective observational study of EHR assessment data which included encounters of older adult patients (≥ 65 years) who were admitted from home to a medical/surgical unit of an academic hospital in North Central Florida between January 2012 and May 2021. The components of the RAI included in the study were sex, age, cancer, renal failure, heart failure, cognitive decline, unintentional weight loss, poor appetite, and shortness of breath at rest. Descriptive statistics were generated and compared between patients discharged to home versus those who were discharged to Post-Acute Care (PAC) facilities. Unadjusted associations were assessed using univariate logistic regression. Consistent with existing literature on frailty, all but one of the included RAI components (i.e., male sex) exhibited higher odds of discharge to a PAC facility with strong statistical support. Recognizing functional decline due to frailty is integral to the mission of improving patient safety. This study provides a preliminary proof of concept for leveraging existing assessment data in the EHR to capture frailty in older adults. Future studies of the overall predictive value of RAI with disposition are warranted.

Abstract citation ID: igad104.2988

### PARO ROBOT INTERACTION DECREASES PAIN AND AGITATION SCORES IN HOSPITALIZED OLDER ADULTS WITH ADRD AND/OR DELIRIUM

Pamela Cacchione[1], Ellen Munsterman[2], Lisa Walke[2], Lisa Triantos[3], Michelle Johnson[2], and Jodi Cheeks[3], *1. University of Pennsylvania School of Nursing; Penn Presbyterian Medical Center, Philadelphia, Pennsylvania, United States*, *2. University of Pennsylvania, Philadelphia, Pennsylvania, United States*, *3. Penn Presbyterian Medical Center, Philadelphia, Pennsylvania, United States*

Hospitals are stressful for persons with Alzheimer's Disease and Related Disorders (ADRD) and/or delirium. Therapeutic robotic animals, used in long-term care, are gaining traction in hospitals to decrease agitation. The purpose of this RCT was to 1) test the effectiveness of the PARO Robot in decreasing agitation (CMAI-OT) and pain, (PAINAD); 2) quantify the interactions (SIT) with PARO over 2 visits in persons with ADRD and/or delirium. We enrolled 45 intervention and 42 attention control (2 one-hour PARO interactions vs. research assistant interactions) participants. The CMAI-OT and PAINAD were completed at baseline and every 20 minutes X 3 in both groups. No differences between groups on age (M=83.7), gender (63% female), race (77 % Black) number of antipsychotics prescribed [M=1.2 vs M1.5, p=0.14], or baseline CMAI-OT scores PARO vs control [M=9.3 vs M=9.4, p=0.94]. CMAI-OT decreased at 20 minutes in the PARO group [M=3.9 vs. M=7.0, p= .004] and remained lower for the hour but not significantly. PAINAD scores were similar at baseline [M=2.0 vs M=2.2, p=0.65]. PAINAD scores decreased for PARO vs. control [M= 1.64 vs. M= 2.28, p=0.02; M=0.9 vs. M= 1.8, p=0.03] at 20 & 60 minutes day one without differences day two. Participants held PARO; engaged physically; talked to; smiled; and agreed to have PARO return. We demonstrated improved agitation for first 20 minutes of PARO interaction as well as lower pain over the first 60 minutes day one. Findings did not persist through day two. Next steps are to measure emotional responses to PARO.

### SESSION 7870 (POSTER)

### MICROBIOME AND AGING

Abstract citation ID: igad104.2989

### A NEW ROLE FOR KYNURENINE METABOLISM IN THE AGE-ASSOCIATED DECLINE OF THE BACTERIAL IMMUNE RESPONSE

George Sutphin, and Luis Espejo, *University of Arizona, Tucson, Arizona, United States*

Tryptophan metabolism through the kynurenine pathway becomes dysregulated during normal aging and is implicated in age-associate disease, including chronic inflammation, atherosclerosis, neurodegeneration, and cancer. Kynurenine pathway enzymes and metabolites influence a range of molecular processes critical to healthy aging, including regulation of inflammatory and immune responses. Kynurenine metabolism is active in immune cells and activated in response to proinflammatory cytokine signaling. We previously determined that elevating physiological levels of the kynurenine pathway metabolite 3-hydroxyanthranilic acid (3HAA) via either direct supplementation or inhibition of the enzyme that degrades 3HAA, 3HAA dioxygenase (HAAO), extends lifespan and delays age-associated health decline in Caenorhabditis elegans. Published work suggests that 3HAA is anti-inflammatory in mammals, for example by reducing the ratio of activated to regulatory T cells and inhibiting pathological activation of macrophages. In recent work, we find that elevating physiological 3HAA can beneficially enhance the immune response of C. elegans to bacterial pathogens during aging. 3HAA is sufficient to kill bacterial and inhibit bacterial growth in culture. When HAAO is inhibited in C. elegans, 3HAA accumulates in lysosome related organelles (LROs) in the intestinal cells, the same subcellular compartment that contains engulfed bacteria. LROs are a cellular repository for both iron and zinc, and we further find that iron chelation or zinc supplementation dramatically enhances the bactericidal properties of 3HAA. Here we present a mechanistic model in which age-dependent accumulation of 3HAA in intestinal LROs combines with zinc to enhance bacterial resistance with age in C. elegans.

Abstract citation ID: igad104.2990

### BUTYRATE-PRODUCING BACTERIA INCREASE GUT MICROBIOTA DIVERSITY TO DELAY INTESTINAL INFLAMMAGING

Xiaoang Li, Yiming Zhang, and Liping Duan, *Peking University Third Hospital, Beijing, Beijing, China (People's Republic)*

Intestinal flora dysbiosis is a key link in intestinal inflammaging, and the diversity of gut microbiota plays an important role in maintaining the entire intestinal microecology. Our previous study found that butyrate-producing bacteria reversed aging-induced intestinal inflammation and promoted the expression of intestinal anti-inflammatory factors in 24-month-old mice, but the mechanism was unclear. We found that butyrate-producing bacteria promote the expression of short-chain fatty acid (SCFA) receptors in the intestine. Monocarboxylate transporter 1 (MCT-1) has anti-inflammatory effects on intestinal inflammation. We speculate that butyrate-producing bacteria upregulate MCT-1 expression through the activation of SCFA receptors, thereby promoting gut stability and bacterial diversity. In further experiments, we established inflammation models using cell lines, colon organoids, mouse colonic epithelial cells, and aged mice (24 months old) given different concentrations of LPS and DSS, and then added butyrate-producing bacteria to verify that MCT-1 is involved in the resistance of butyrate-producing bacteria to intestinal inflammation. We found that bacteria up-regulated the expression of MCT-1 by activating SCFA receptors. From the results of 16s rRNA sequencing, it can be seen that the intestinal stability and bacterial diversity of aged mice were improved after the administration of butyrate-producing bacteria. The research results will provide an experimental basis for treating intestinal inflammaging by restoring intestinal stability and diversity of gut microbiota and will help to explore the clinical value of butyrate-producing bacteria, which has important translational significance.

Abstract citation ID: igad104.2991

### COMMON INFECTIONS IN RELATION TO SLEEP DISTURBANCES BY RACE IN A POPULATION-BASED COHORT OF ADULTS

Jill Rabinowitz[1], Yiwei Yue[1], Diefei Chen[2], Robert Yolken[3], Chandra Jackson[4], Brion Maher[1], William Eaton[1], and Adam Spira[1], *1. Johns Hopkins Bloomberg School of Public Health, Baltimore, Maryland, United States*, *2. Johns Hopkins, Baltimore, Maryland, United States*, *3. Johns Hopkins School of Medicine, Baltimore, Maryland, United States*, *4. National Institute of Environmental Health Sciences, Research Triangle Park, North Carolina, United States*

Common infections (e.g., human herpesviruses and Toxoplasma gondii (TOX)), are associated with poor health outcomes, but less is known about associations between these infections and sleep. Due to, for instance, structural racism leading to policies and practices resulting in relative economic disadvantage and subsequently poorer living condition, sleep disturbances and common infections are more prevalent in minoritized populations. We investigated the cross-sectional associations of common infections (i.e., herpes simplex virus type 1 (HSV-1), cytomegalovirus (CMV), varicella zoster virus (VZV), Epstein-Barr virus (EBV), and TOX with sleep disturbances, and determined whether these associations differed by race in 602 adults enrolled in the Baltimore Epidemiologic Catchment Area Study (mean age=59.0±12.8 years, 36.9% male, 35.6% minoritized). Participants donated a blood sample and were asked whether they had the insomnia symptoms “trouble falling asleep, staying asleep, or waking up too early” and reported on their habitual sleep duration. We measured immunoglobin G (IgG) antibodies to HSV-1, CMV, EBV, VZV, and TOX. After adjusting for sociodemographics, body mass index, comorbidities, household income, and household size, there were no associations between common infections and any sleep outcomes ; however, we observed interactions with race. Among White participants only, CMV seropositivity was associated with a lower odds of insomnia symptoms (OR = 0.26, 95% CI 0.10, 0.69) and TOX seropositivity was associated with shorter sleep duration (OR = 0.65, 95% CI 0.09, 1.21). Further research is needed to investigate mechanisms that may account for associations of CMV and TOX with sleep among White but not minoritized participants.

Abstract citation ID: igad104.2992

### LARGE-SCALE ANALYSIS OF HUMAN GUT MICROBIOTA PATTERNS ACROSS GEOGRAPHY AND PEDIATRIC TO GERIATRIC AGES

Sherri Huang[1], Diptaraj Chaudhari[1], Pushti Kanani[1], Wesley Burrow[1], Yi Lin[2], Robert Mankowski[3], Shalini Jain[1], and Hariom Yadav[1], *1. University of South Florida, Tampa, Florida, United States*, *2. University of Florida, Gainesville, Florida, United States*, *3. University of Florida, Gainseville, Florida, United States*

Limited studies to-date have evaluated differences in gut microbiota across a global and temporal scale. Herein, we aim to examine the gut microbiota patterns across geography as defined by continent and countries and how gut microbiota vary across age groups within each geographic region. To approach these questions, we designed a large-scale analysis of gut microbiota across North America, Europe, Africa, Asia and Oceania. Literature across countries within each region was screened to include publications employing human stool samples and 16s rRNA sequencing of gut microbiota;116 publications resulted from this first screen. In the second screen, publications without metadata files or whose metadata files did not clarify sample type, collection timepoint or location were excluded. Raw 16s rRNA sequencing data for included publications were collected, and metadata for corresponding fastq files were screened to include only those of healthy, non-intervention groups. This final screen resulted a total of 25,665 metadata. Corresponding 16s rRNA gut microbiota sequences will be analyzed via the QIIME2 workflow in which we aim to compare and contrast gut microbiota and diversity across continents and countries and evaluate changes in gut microbiota from pediatric to geriatric age. This study will provide insight on biodiversity on a worldwide scale and inform on the changes in gut microbiota across time, allowing further examination of the role of the gut microbiome in the development of chronic disease in human aging. Altogether, our study represents one of the largest scale evaluation of gut microbiota patterns across geography and time.

Abstract citation ID: igad104.2993

### MICROBIOME MODULATORS IN THE PREVENTION AND MITIGATION OF CANCER TREATMENT-INDUCED ACCELERATED AGING BIOLOGY

Brandi Miller, Sidharth Mishra, Diptaraj Chaudhari, Hariom Yadav, andShalini Jain*University of South Florida, Tampa, Florida, United States*

Advancements in oncology research have led to increased survivorship in patients with cancer. However, cancer treatments like chemotherapy cause debilitating side effects including multi-organ dysfunction and increased risk for chronic diseases, which mimic accelerated aging. Abnormalities in the gut microbiome composition (dysbiosis) increase inflammation, resulting in accelerated cognitive impairment with aging and a poor quality of life (QOL), which are all exacerbated by cancer treatments. We have screened, evaluated, and optimized our previously isolated human-origin live bacteria (probiotics) as well as pasteurized probiotics (postbiotics) for their anti-aging potential using Caenorhabditis (C.) elegans and mouse models. Probiotics improved the healthspan in C. elegans when given live and when fermented. Interestingly, PFM and LpD3.5 significantly protected from body weight loss, microbiome dysbiosis, and cognitive impairment in mice receiving 5-fluorouracil chemotherapy and protected from intestinal damage. We are planning a clinical trial where we will (1) cross-sectionally analyze the gut microbiome, leaky gut, and inflammation in cancer survivors with and without brain distress and (2) determine the safety, tolerance, and efficacy of PFM and LpD3.5 on microbiome diversity, inflammation, and cognitive health in cancer survivors with brain distress, using a 60-day open-label, randomized comparison study. These studies will improve our understanding of how cancer treatment-induced changes in the gut microbiome, inflammation, and gut permeability contribute to accelerated aging. Furthermore, they will establish the utilization of probiotics and postbiotics to improve QOL and mitigate cancer treatment-induced accelerated aging, which constitutes a debilitating public health concern among the increasing population of cancer survivors.

Abstract citation ID: igad104.2994

### UNIQUE MICROBIOME-PROTEOME SIGNATURES OF SALIVA IN PREDICTION OF COGNITIVE DECLINE IN OLDER ADULTS OF MIAGB COHORT

Diptaraj Chaudhari[1], Shalini Jain[1], Corinne Labyak[2], Adam Golden[3], Mia GB[1], Peter Holland[4], Michal Masternak[3], and Hariom Yadav[1], *1. University of South Florida, Tampa, Florida, United States*, *2. University of North Florida, Jacksonville, Florida, United States*, *3. University of Central Florida College of Medicine, Orlando, Florida, United States*, *4. Schmidt College of Medicine, Boca Raton, Florida, United States*

The prevalence of age-related cognitive decline and dementia is increasing in older adults, with limited prevention and therapeutic options, increasing tremendous burden on healthcare system. Alzheimer's disease (AD) is the most common age-related cognitive disorder, for which no treatment is available when its pathology is fully developed. Although current FDA-approved therapies are proven to be effective in delaying AD progression, however, inexpensive and non-invasive markers for routine laboratory examination are poorly developed. Studies indicate that poor oral health is associated with a higher risk of dementia via microbiome alteration. In this pilot study, we explored the importance of the multiomics analysis in discriminating cognitively healthy from mild cognitive impaired and dementia by comparing the microbiome and proteomic profiles of saliva from controls (n=37), MCI (n=16) and dementia (n=6) participants of the MiaGB (Microbiome in aging Gut and Brain) consortium. We detected significantly reduced abundance of oral commensals like B. animalis, E. brachy, and F. fastidiosum in MCI and dementia samples than controls with specific increase of P. histicola in MCI and neuronal Aβ1–42 promoting V. parvula in the dementia. In addition, proteome analyses revealed that highly neuronal relevant nitrite-nitrate conversion cycle genes were significantly differed in dementia and MCI than controls. Discriminatory predictive analyses revealed that combination of microbiome and proteome signature are stronger discriminator for dementia/MCI from cognitively healthy controls. Our results indicate the potential links among oral microbiome, proteome and dementia, as well as diagnostic potential for dementia risk.

### SESSION 7880 (POSTER)

### INTERVENTIONS

Abstract citation ID: igad104.2995

### DIETARY RESTRICTION OF ISOLEUCINE INCREASES THE HEALTHSPAN AND LIFESPAN OF GENETICALLY HETEROGENEOUS MICE

Cara Green, *University of Wisconsin-Madison, Madison, Wisconsin, United States*

Low protein (LP) diets promote health and longevity in diverse species, andreducing dietary levels of the branched-chain amino acids (BCAAs) leucine, isoleucine and valine recapitulates these effects in C57BL/6J mice, promoting metabolic health in both sexes, and increases lifespan while reducing frailty in males. Each BCAA has unique metabolic effects, and we recently showed that restriction of isoleucine is both sufficient to promote metabolic health and required for the metabolic benefits of an LP diet in C57BL/6J males. Here, we tested the hypothesis that specifically restricting dietary isoleucine could promote healthy aging in adult genetically heterogenous UM-HET3 mice. We find that isoleucine restriction (IleR) improves the metabolic health of both young and old HET3 mice, promoting leanness and glycemic control in both sexes, and reprograms hepatic metabolism, blunting age-related molecular changes in males and, to a lesser extent, in females. Finally, we find that IleR reduces frailty and extends both the median and maximum lifespan of both male and female HET3 mice, but to a much greater degree in males. Our results demonstrate that restricting dietary isoleucine can increase health span and longevity in a genetically diverse population of mice and suggest that reducing dietary levels of isoleucine, or drug regimens that mimic this effect, may be a novel intervention to promote healthy aging.

Abstract citation ID: igad104.2996

### EAAS AND WHEY PROTEIN MAY IMPROVE PHYSICAL FUNCTION OF OLDER ADULTS INDEPENDENTLY OF GUT MICROBIOME

Gohar Azhar, Ambika Verma, Intawat Nookaew, Pankaj Patyal, Shakshi Sharma, Robert Wolfe, and Jeanne Wei, *University of Arkansas for Medical Sciences, Little Rock, Arkansas, United States*

Essential amino acids (EAAs) and dietary proteins are major nutritional supplements that support the growth and activity of gut microbes, contributing to the wellbeing of their host. We hypothesized that daily ingestion of EAAs or whey proteins for twelve weeks would improve the gut microbiome of older adults and thereby have a positive impact on their physical functioning. The stool samples were collected from the 40 subjects before and after consuming EAAs or whey proteins for twelve weeks and subjected to Illumina based 16S rRNA gene sequencing. In both groups, the most abundant families in order of relative abundance included: Bacteroidaceae, Ruminococcaceae, Lachnospiraceae, Rikenellaceae, and Prevotellaceae, which indicated that these subjects were able to maintain a dominant consortium of microbes in their guts that have been associated with healthy aging. Individual gut microbiome samples analysis with nonmetric multidimensional space (NMDS) showed that each individual was associated with its unique microbiome. The key finding of this study was a significant reduction of plasma Interleukin-18 binding protein (IL-18) in EAAs vs whey group ($p < 0.003$). It is plausible that the reduced level of inflammatory IL-18 contributed to improved intestinal immunity. Moreover, the reduced inflammation might have resulted in better muscle performance in the EAAs group vs whey as evidenced by six-minute walking distance (EAAs $92.55 \pm 4.16$ ft vs whey $47.46 \pm 4.99$ ft, $p< 0.0001$) at the completion of study. Future studies will need to explore the role of EAAs on gut microbiome mediated reduction of immune inflammation on physical function and aging.

Abstract citation ID: igad104.2997

### IDENTIFYING SENOLYTIC TARGETS OF DIETARY FLAVONOIDS IN RENAL CELLS BY THERMAL PROTEOME PROFILING

Quinn Strassheim, Reema Banarjee, Delaney Rutherford, Amit Dey, Dimitrios Tsitsipatis, Ruin Moaddel, Myriam Gorospe, and Nathan Basisty, *National Institute on Aging, National Institutes of Health, Baltimore, Maryland, United States*

Senescent cell accumulation is a known driver of aging and age-related pathologies. Clearance of senescent cells is a promising approach to increase longevity and reduce multiple age-related diseases in humans. Flavonoid compounds are present in fruits and vegetables, of which certain flavonoids (e.g., quercetin and fisetin) have demonstrated senolytic, or 'senescent-cell-killing', activity in culture and in mice. However, the cellular mechanism of senolysis and the efficacy of these compounds are not known in renal epithelial cells. Here, we combine flavonoid drug screening, targeted metabolomics, and thermal proteome profiling (TPP) with analysis by mass spectrometry (MS) to explore protein targets of senolytic drugs in senescent human renal cortical epithelial cells and human renal proximal tubular epithelial cells. Senescence was induced by exposure to ionizing radiation and the senescence phenotype was validated through a rigorous panel of senescence and viability markers. Proliferating and senescent renal epithelial cells were screened with a panel of 8 flavonoids to identify senolytic drugs and their concentrations for selectively killing senescent cells. Targeted MS assays were developed to assess intracellular drug uptake, a potential mechanism of senescent-specific killing. To identify protein targets of the senolytic flavonoids in senescent cells, we performed a variation of TPP in combination with liquid chromatography (LC)-MS/MS proteomics analysis, carefully controlling for non-senolytic interactions by excluding proteins bound by a non-senolytic flavonoid. The results of this study pave the way for the development of a more specific generation of senolytic compounds and identify novel candidate senolytic pathways engaged by dietary flavonoids.

Abstract citation ID: igad104.2998

### LIVING LONGER WITH MODERATE AMOUNTS OF OXYGEN RADICALS IN FRUIT FLIES

Man Su Kim, and Saurav Ghimire, *Inje University, Gimhae, Republic of Korea*

The role of reactive oxygen species (ROS) in aging is complex and paradoxical. While the free radical theory of aging suggests that ROS cause cellular damage and senescence, some studies have shown that moderate ROS levels can enhance cellular adaptation and longevity. In this research, we used Drosophila as an in vivo model to investigate the optimal level of ROS that can promote healthy aging. We exposed flies to different concentrations of paraquat (PQ), a herbicide that generates superoxide anions, and measured their lifespan, stress resistance, and gene expression. We found that intermediate PQ levels (0.1 mM) extended lifespan and improved stress tolerance, while high PQ levels (0.5 mM) shortened lifespan and impaired stress response. We also observed an up-regulation of ROS scavenger genes, such as superoxide dismutase 1 and glutathione peroxidase, in the intermediate PQ group, suggesting a homeostatic mechanism to balance ROS levels. Our findings challenge the conventional view of ROS as harmful agents and provide novel insights into the molecular mechanisms of aging.

Abstract citation ID: igad104.2999

### L-VALINE TREATMENT IMPROVES CELLULAR MITOCHONDRIAL FUNCTION

Shakshi Sharma, Gohar Azhar, Xiaomin Zhang, and Jeanne Wei, *University of Arkansas for Medical Sciences, Little Rock, Arkansas, United States*

Branched-chain amino acids (BCAAs) are pivotal for the health of the human body. Leucine, isoleucine, and valine, which make up the BCAAs, are α-amino acids that promote skeletal muscle growth and development. Valine is one of the essential BCAAs required for the synthesis of muscle proteins and the support growth of skeletal muscles. Valine also plays a favorable role in enhancing insulin sensitivity, preserving intestinal health, and optimizing lipid metabolism. Considering the relevance of valine in promoting muscle growth and metabolism, we utilized C2C12 skeletal muscle cell line to investigate the role of valine in regulating mitochondrial functions. C2C12 cells grown on physiological normal glucose media (100mg/dL) were used for the experiments. Cells were treated with a 1.0 mM concentration of valine for 24 h. The effect of valine treatment on gene expression of important mitochondrial-related genes such as PGC-1α, and PGC-1β and mitofusin (MFN1, MFN), and mitochondrial fission 1 (Fis 1) was determined. Further, Oroboros oxygraph-O2K high-resolution respirometer was used to analyze the mitochondrial respiration in the intact C2C12 cells after valine treatment. The results showed increased gene expression of PGC-1α, PGC-1β, and mitochondrial fission and fusin genes after treatment with valine. The basal respiration, leak respiration, as well as maximal capacity of mitochondrial ETC (Electron Transport Chain), were also significantly improved after valine treatment. The findings from this study enhance our understanding of valine as an important nutrient to improve mitochondrial function to drive cellular function and biological processes.

Abstract citation ID: igad104.3000

### MULTIOMIC SYSTEMS ANALYSIS OF LIFESPAN-EXTENDING INTERVENTIONS IN MOUSE TISSUES

Kengo Watanabe[1], Tomasz Wilmanski[1], Priyanka Baloni[2], Max Robinson[1], Oliver Fiehn[3], Robert Moritz[1], Richard Miller[4], and Noa Rappaport[1], *1. Institute for Systems Biology, Seattle, Washington, United States*, *2. Purdue University, West Lafayette, Indiana, United States*, *3. UC Davis, Davis, California, United States*, *4. University of Michigan, Ann Arbor, Michigan, United States*

Aging is associated with dysregulation of molecular, cellular, and physiological processes. Nutritional and pharmacological interventions with different postulated modes of action have been shown to extend lifespan and delay aging-related diseases in model organisms, suggesting the existence of core cellular processes that mediate the effect on lifespan and healthspan. However, these underlying molecular processes remain largely uncharacterized. In this study, we analyzed multiomic tissue-derived data of lifespan-extending interventions in mice including metabolomics and proteomics from the NIA Longevity Consortium and previously reported transcriptomics. We applied three systems techniques, differential rank conservation (DIRAC) analysis, weighted gene co-expression network analysis (WGCNA), and mouse genome-scale metabolic model (GEM) reconstruction, to the mouse liver proteomic and transcriptomic datasets of lifespan-extending interventions (e.g., acarbose, 17α-estradiol, and rapamycin). We found that these interventions generally strengthen the rank conservation of biological processes and shift metabolic fluxes, with fatty acid metabolism emerging as a common process affected by multiple interventions. We then applied these approaches to an expanded experimental data with additional interventions (e.g., canagliflozin) and tissues (e.g., kidney, muscle, and plasma), confirming our results and further observing varied inter-omic and inter-tissue patterns exerted by lifespan-extending interventions. Our findings highlight the potential of integrative systems analysis to elucidate common and unique cellular and physiological changes relating to aging and lifespan-extending interventions, which have translational potential as preventive and prognostic measures to improve human health.

Abstract citation ID: igad104.3001

### SEROTONIN AND DOPAMINE CIRCUITS MODULATE AGING IN RESPONSE TO FOOD CUES AND AVAILABILITY

Scott Leiser, *University of Michigan, Ann Arbor, Michigan, United States*

An organism's ability to perceive and respond to changes in its environment is crucial for its health and survival. Multiple studies have shown that modifying this perception through genetic or environmental alterations can extend or shorten lifespan cell non autonomously. These results provide the rationale for identifying "longevity circuits" that could be used to extend healthspan and lifespan by using these signaling pathways. Here we reveal how the most well-studied longevity intervention, dietary restriction, acts in-part through a cell non-autonomous signaling pathway that is inhibited by the presence of multiple food cues. Using an intestinal reporter for a key gene induced by dietary restriction but suppressed by attractive food cues, we identify three compounds that block food cue effects in C. elegans, thereby increasing longevity as dietary restriction mimetics. These compounds and our additional data clearly implicate serotonin and dopamine circuits in limiting lifespan in response to food cues. We further identify multiple neurons, receptors and downstream signals within these circuits. Aspects of these pathways are conserved in D. melanogaster, suggesting at least some "longevity circuits" are conserved. Together, our results show that modulating food cue signaling through serotonin or dopamine circuits is a plausible approach to mimic the benefits of dietary restriction.

Abstract citation ID: igad104.3002

### TARGETING MITOCHONDRIAL FUNCTION USING NOVEL RHO/MRTF/SRF INHIBITORS

Pankaj Patyal, Xiaomin Zhang, Gohar Azhar, and Jeanne Wei, *University of Arkansas for Medical Sciences, Little Rock, Arkansas, United States*

Altered mitochondrial metabolism is one of the major hallmarks of aging. Notably, age is the biggest risk factor for numerous cancers. Studies suggest that cancer cells shift from anaerobic respiration to oxidative phosphorylation for increasing energy needs. Therefore, this upregulation is a vulnerability that can be targeted using Rho/MRTF/SRF inhibitors. Recent advancement identified a novel series of oxadiazole-thioether compounds that disrupt the SRF transcription. Among those compounds CCG-203971 and CCG-232601 are most metabolic stable, soluble and have no toxicity. They have been shown efficacious in multiple animal models of acute fibrosis, including scleroderma. However, the direct molecular target of these compounds is unclear. Therefore, we investigated the molecular mechanisms of these inhibitors transcription mediated by RhoA which modulates actin dynamics to induce the nuclear accumulation of Myocardin-Related Transcription Factors and their activation of SRF/p49. Our findings suggest that, CCG-203971 and CCG-232601, inhibit Rho/

MRTF/SRF/p49 mediated transcription in different cell lines. They contribute to H4 hyperacetylation at K12/16 while no change was observed at H4 acetylation at K5 and K8. Moreover, SRF also regulates cell metabolism by maintaining mitochondrial function. Thus, we further investigated the role of these drug molecules in targeting mitochondrial function. We found that these compounds inhibit the genes involved in mitochondrial function and dynamics. Further, these inhibitors repress the mitochondrial function at varying concentrations, investigated using Agilent Seahorse XF Analyzer. Herein, we propose that CCG-203971 and CCG-232601 may prove to be a promising approach therapeutically to target aberrant bioenergetics in aging and cancer.

### SESSION 7890 (POSTER)

### WORK, VOLUNTEERISM, AND RETIREMENT

Abstract citation ID: igad104.3003

### DELAYED RETIREMENT IN ISRAEL: FACTORS THAT CONTRIBUTE TO THE NEW POLICY SUPPORT

Polina Ermoshkina, *Case Western Reserve University, Cleveland, Ohio, United States*

Policies that defer retirement age are fiscally justifiable, but not unanimously supported by the citizens. Studies in other countries (United Kingdom, Sweden, and Norway) show that occupational environment (feeling unappreciated at work, having a physically demanding job) affects workers' support for pension reform. Israel is a unique national context for this question because of the high proportion of immigrants who had not spent their working lives in Israel. This study used The Survey of Health, Ageing and Retirement in Europe (SHARE) Israel (2005-2006) to examine how the current job conditions (having a physically demanding job, having a job that allows workers to develop new skills, receiving support from the supervisors, job satisfaction) affect Israeli workers' attitudes towards delayed retirement age. The analysis was limited to 482 respondents. The average age of the participants was 64.1 (SD=10.1) years. The participants included veteran Jewish-Israelis, Arab-Israelis, and new immigrants from the former Soviet Union. Nearly 40 percent of the respondents were very interested in working until the new retirement age yet 24.73% were not interested at all. Multiple correlation showed that low job satisfaction, having a physically demanding job, and having a job that does not allow one to develop new skills were associated with low interest in prolonged retirement age. Gender, birthplace, and having adequate support at work were not associated with workers' support for delayed retirement. These findings indicate that older adults in demanding, low-reward work most strongly oppose extended working life.

Abstract citation ID: igad104.3004

### EFFECT OF DEPRESSIVE SYMPTOMS AND WORK-RELATED FACTORS ON TRANSITION TO RETIREMENT IN OLDER ADULT

Jennifer Saucedo[1], Jason Newsom[1], Em Trubits[1], Mallory Kroeck[1], Anda Botoseneanu[2], Heather Allore[3], Corey Nagel[4], and Ana Quiñones[5], *1. Portland State University, Portland, Oregon, United States*, *2. University of Michigan, Ann Arbor, Michigan, United States*, *3. Yale University, New Haven, Connecticut, United States*, *4. University of Arkansas for Medical Sciences, Little Rock, Arkansas, United States*, *5. Oregon Health & Science University, Portland, Oregon, United States*

Work demands can spillover to family domains (work-to-family conflict; WFC) and family demands may spillover to work domains (family-to-work conflict; FWC). This spillover between domains may help explain the relationship between depressive symptoms and decision to retire among older adults. This study examined the influence of depressive symptoms on likelihood of retirement transition through WFC and FWC using data from the Health and Retirement Study (HRS 2008-2018; N = 1031). Participants included adults age 51+ employed in 2008 and reported a transition to partial- or full-retirement by 2018. Cox survival models investigated whether WFC and FWC mediated the effects of depressive symptoms (CESD) on the likelihood of retirement transition, controlling for participant sex, age, education, insurance coverage, and race/ethnicity. Higher depressive symptoms were associated with higher FWC ($B = 0.06$, $p < 0.01$) and likelihood of retirement transition ($B = 0.07$, $p < 0.01$). Higher FWC was related to lower likelihood of retirement transition ($B = -0.89$, $p < 0.01$). The indirect effect of FWC was significant ($B = -0.06$, $p < 0.01$). Higher depressive symptoms were associated with higher WFC ($B = 0.37$, $p < 0.01$) and likelihood of retirement transition ($B = 0.09$ $p = 0.04$). However, there was a nonsignificant effect of WFC on retirement transition ($B = -0.19$, $p = 0.07$). WFC had no indirect effect on retirement transition ($B = -0.04$, $p = 0.08$). Results suggest that the experiences outside of work for older adults may explain the relationship between depressive symptoms and retirement decisions.

Abstract citation ID: igad104.3005

### FROM THE WORKROOM TO THE BEDROOM: MEDIATING EFFECTS OF WORK-TO-FAMILY SPILLOVER ON WORK CHARACTERISTICS AND SLEEP

Kian Huang[1], Christina Mu[1], Claire Smith[1], and Soomi Lee[2], *1. University of South Florida, Tampa, Florida, United States*, *2. The Pennsylvania State University, University Park, Pennsylvania, United States*

Negative and positive work experiences may spillover to the home domain and harm one's sleep. This study investigated whether work-to-family spillover mediates the relationship between work characteristics and sleep health. Full-time workers (n=2,106) from the Midlife in the United States Study provided Time-1 data (T1: 2004-2006); a sub-set (n=1,444) was reassessed at Time-2 (T2: 2013-2015). Both negative work characteristics (perceived unfairness at work and job discrimination) and positive work characteristics (coworker support and supervisor support) were assessed. Mediating variables were negative work-to-family spillover (NWFS) and positive work-to-family spillover (PWFS), and outcome was an overall sleep health score that sums across irregularity, dissatisfaction, nap frequency, inefficiency, and suboptimal sleep duration (higher=poorer sleep health). PROCESS Macro evaluated cross-sectional (T1) and longitudinal (T1-T2) mediation pathways, adjusting for sociodemographics, neuroticism, and work hours. NWFS was a consistent mechanism connecting work characteristics and sleep health. Cross-sectionally, higher NWFS mediated the associations of higher unfairness with poorer sleep health and a sub-dimension of greater dissatisfaction (B=0.09, SE=0.04; B=0.12, SE=0.06, respectively). Higher NFWS also mediated the association between higher discrimination and higher sleep dissatisfaction (p=.008). Longitudinally, an increase in unfairness from T1 to T2 was associated with an increase in irregular sleep at T2 (adjusting for T1 unfairness) through increases in NWFS (p= 0.038). PWFS was not a significant mechanism linking positive work experiences and sleep health, although more coworker and supervisor support were associated with better sleep health. Findings suggest that organizations should limit instances of unfairness and discrimination at work to promote employee sleep health.

Abstract citation ID: igad104.3006

**INFLUENCES OF AGE DIVERSITY CLIMATE ON ORG PERFORMANCE: ROLE OF INCLUSION AND ORGANIZATIONAL CITIZENSHIP BEHAVIOR**

Soondool Chung, and Ahyoung Lee, *Ewha Womans University, Seoul, Republic of Korea*

With the rapidly aging population, South Korea's workforce demographics are changing as more older adults remain in the workforce. With limited resources such as work opportunities and pensions, intergenerational conflict may arise in the workplace while employers seek to maximize organizational outcomes. The present study investigated the effects of age diversity climate on organizational performance in private sector employees by focusing on the mediating effects of inclusion and organizational citizenship behavior (OCB). The data for this study was collected online in January 2022 from 900 private sector employees aged 20 to 69 in South Korea. Age-diversity climate, sense of inclusion, OCB, and organizational performance were measured using standardized scales. To test our assumed relationships, we used structural equation modeling and executed bootstrapping procedures to test the significance of the indirect effects. Results indicated that age-diversity climate did not have a significant direct effect on organizational performance. However, the age-diversity climate has an impact on inclusion ($\beta$ = .61, $p < .001$) and OCB ($\beta$ = .13, $p < .01$). Inclusion showed a positive effect on OCB ($\beta$ = .41, $p < .001$) and OCB showed a positive effect on organizational performance ($\beta$ = .10, $p < .05$). Additionally, as hypothesized, inclusion and OCB had a mediating role in the relationship between the age-diversity climate and organizational performance ($\beta$ = .05, s.e. = .02, LLCI = .003, ULCI = .095). The results of this study highlight the importance of the age-diversity climate in the workplace in aging society.

Abstract citation ID: igad104.3007

**OCCUPATIONAL CHARACTERISTICS ASSOCIATED WITH CHRONIC INFLAMMATION: EVIDENCE FROM THE HRS**

Theresa Andrasfay[1], Jennifer Ailshire[2], and Eileen Crimmins[2], *1. California State University San Marcos, San Marcos, California, United States*, *2. University of Southern California, Los Angeles, California, United States*

There is substantial evidence from the occupational health, psychology, and environmental health literatures to suggest that inflammation is a biologically plausible mechanism through which physical, psychosocial, and environmental occupational characteristics influence risks of several health conditions at older ages. However, there is limited empirical evidence of the linkages between occupational characteristics and biomarkers of inflammation in representative studies of older adults, precluding a thorough understanding of how working conditions affect the distribution of inflammation among the older population. We use data from 1,997 individuals who were aged 51-60 and working for pay in the 2010 wave of the Health and Retirement Study (HRS) to assess how occupational characteristics are associated with a summary measure of chronic inflammation constructed from seven inflammatory biomarkers included in the 2016 HRS Venous Blood Study. In addition to HRS respondents' reports of their job characteristics, we use data from the Occupational Information Network (O*NET). We use Poisson models to predict the count of high-risk inflammatory biomarkers from occupational characteristics, controlling for age, sex, race/ethnicity, educational attainment, and use of over-the-counter anti-inflammatory drugs. We find that individuals employed in occupations with higher levels of physical demands, higher levels of harmful workplace exposures, and those working long hours have higher levels of inflammation. Higher levels of autonomy or job control were associated with reduced levels of inflammation. These results suggest that common job conditions may contribute to chronic inflammation at older ages.

Abstract citation ID: igad104.3008

**RETIREMENT TRAJECTORIES AND HEALTH IN JAPAN**

Masaaki Mizuochi[1], and James Raymo[2], *1. Nanzan University, Nagoya, Aichi, Japan*, *2. Princeton University, Princeton, New Jersey, United States*

The relationship between retirement and health is a key issue in aging societies. However, it is unclear whether the nature of retirement from full-time regular employment makes a difference in subsequent health. This study examines the relationship between the trajectory of retirement from full-time regular employment and subsequent health. Using the Longitudinal Survey of Middle-aged and Elderly Persons 2005–2019 collected in Japan, we employ sequence analysis to identify distinct retirement trajectories from full-time regular employment between ages 59 and 66. We then estimate the causal relationship between retirement trajectories and health using instrumental variable methods. Sequence analysis classifies the retirement trajectories into four groups: gradual retirement, abrupt retirement, continued non-standard employment, and continued regular employment. Instrumental variable estimation shows no statistically significant differences in the effects of these retirement trajectories on health, but suggests that gradual retirement may be more beneficial to health than other three retirement trajectories. Several robustness checks also show no statistical difference in the effect of retirement trajectories on health. These findings provide an empirical foundation for understanding the possible implications of heterogenous retirement pathways for health in later life and labor market policies in very old populations.

Abstract citation ID: igad104.3009

**VOLUNTEERISM AND PURPOSE IN LIFE IN A NATIONAL SURVEY OF COMMUNITY-DWELLING MIDDLE-AGED AND OLDER ADULTS.**

Wingyun Mak[1], Orah Burack[1], Margret Howard[1], and Kenneth Boockvar[2], *1. The New Jewish Home Research Institute on Aging, New York City, New York, United States*, *2. University of Alabama Birmingham, Birmingham, Alabama, United States*

Volunteerism has been shown to provide physical, psychological, and functional health benefits as people age. Volunteers are able to participate in meaningful service, develop social connections, and exercise their abilities. In this study, we examined whether engaging in various types of volunteer engagement (e.g. charitable, political, religious, environmental, animal welfare, support group) was associated with self-perceived change in purpose in life (PIL) over a five year lookback period, controlling for demographic and health variables. Data were drawn from the AARP Social Engagement and Brain Health Survey, which included a nationally representative sample of adults aged 40-75+ (mode=55-64 years) across the United States who completed an online survey (N=2,585). Hierarchical linear regression was used. People who were younger ($\beta$=-.06, t=-2.55, p=.01), Black ($\beta$=.23, t=10.50, p<.001) or Hispanic ($\beta$=.12, t=5.20, p<.001), married ($\beta$=.05, t=2.65, p=.01), and had better health ($\beta$=.16, t=7.09, p<.001) and cognition ($\beta$=.18, t=7.69, p<.001) reported greater increase in PIL over the last five years ($\Delta$R2=.13, p<.001). After controlling for demographic and health variables, people who valued PIL ($\beta$=.15, t=7.49, p<.001; $\Delta$R2=.02, p<.001) and participated in volunteer work through a religious organization ($\beta$=.05, t=2.38, p=.02) or support groups ($\beta$=.07, t=3.10, p=.002) were more likely to report greater levels of PIL over the last five years ($\Delta$R2=.02, p<.001). These results suggest that changes in experienced PIL may depend on physical and cognitive health, importance placed on PIL, and type of volunteer engagement, all of which may be points of potential intervention.

### SESSION 7900 (POSTER)

### WHAT A FEELING: EMOTIONS AND END OF LIFE

Abstract citation ID: igad104.3010

**AN AGENTIC PERSPECTIVE ON AGING PREPARATION**

Yaeji Kim-Knauss, and Frieder Lang, *Friedrich-Alexander-Universität Erlangen-Nürnberg, Nuremberg, Bayern, Germany*

When imagining one's future life in old age individuals may respond to an uncertain future with proactive and preparatory activity aiming to maximize possible rewards and minimize risks or costs. The current research applies an agentic perspective on aging preparation that emphasizes the role of individuals' beliefs and attitudes in determining their engagement in actions. The present research aims to investigate the associations between action-related beliefs (perceived utility/risk) and situational cues (experience/appraisals of aging-related life challenges) with individuals' engagement in aging preparation. Data comes from the "Ageing as Future" project, which includes multicultural (i.e., Germany, US, Hong Kong, Taiwan, Czechia), longitudinal (i.e., five measurement points spanning from 2012 to 2020), and lifespan data (i.e., aged 18 to 98). Results from a series of analyses indicate that perceiving more utilities, having experience in caregiving, and having health-related worries at the peak of the COVID-19 pandemic were associated with engagement in aging preparation. However, the magnitude of these associations varied depending on the time perspective and cultural context. Findings suggest that individuals engage in aging preparation to attain desirable or to avoid undesirable outcomes in the future based on personal values and belief systems. The dynamic interplay of personal belief systems with temporal and cultural contexts was also highlighted that differently shapes individual's agentic engagement in aging preparation.

Abstract citation ID: igad104.3011

**BEHAVIORAL HEALTH INTERVENTIONS IN THE HOSPICE SETTING: A SCOPING REVIEW**

Lilla Brody[1], Karolina Sadowska[2], Alexis Holllingsworth[3], Michael Ong[3], Tejas Subramanian[4], Drew Wright[3], M. Cary Reid[2], and Daniel Shalev[2], *1. Weill Cornell Medicine, Washington, District of Columbia, United States*, *2. Weill Cornell Medicine, New York City, New York, United States*, *3. Weill Cornell Medical College, New York City, New York, United States*, *4. Weill Cornell, New York City, New York, United States*

Hospice is a model of care for individuals with a life expectancy of less than six months who choose to focus their treatment on quality-of-life rather than extending life. In 2019, half of all Medicare decedents elected to receive hospice care. Behavioral health comorbidities are prevalent in hospice patients; approximately 40% experience significant depression and/or anxiety. Such comorbidities are associated with poor outcomes including increased physical symptoms, worse function, and lower quality-of-life. Despite the impact of behavioral health comorbidities on hospice patient outcomes, little research has focused on the use of behavioral health interventions in the hospice setting. We conducted a scoping review to characterize the types of behavioral health interventions administered to patients receiving hospice care. We searched four databases, generating 6,842 unique citations over any time period. 70 texts were reviewed and 31 were retained for analysis. Studies examined both psychotherapeutic (n=10) and psychopharmacologic (n=10) interventions, as well as other non-pharmacologic interventions (n=11). Duration of study follow-up was available for 17 of the studies, ranging from six days to six months. 14 studies reported information on interventionists, who were most commonly music (n=3) or massage therapists/acupuncturists (n=3). Average interventions were 47 minutes for six sessions. 18 studies presented statistically significant results related to primary outcomes, most commonly depression (n=9), anxiety (n=6), and pain (n=4). Our findings demonstrate a dearth of behavioral health interventions targeting hospice patients, suggesting the need for increased research and clinical focus on developing, evaluating, and implementing behavioral health interventions in the hospice setting.

Abstract citation ID: igad104.3012

### BLACK/WHITE DIFFERENCES IN LENGTH OF STAY IN HOSPICE AMONG MIDDLE-AGED AND OLDER ADULTS IN THE UNITED STATES

Heshuo Yu[1], and J. Scott Brown[2], *1. Miami University in Oxford, Oxford, Ohio, United States*, *2. Miami University, Oxford, Ohio, United States*

**Objectives:** Hospice length of stay (LOS) is a crucial measure of hospice care. This topic is understudied within a nationally-representative sample across different races/ethnicities, especially among non-White decedents. **Methods:** The study used negative binomial regression to analyze three waves (2014, 2016, and 2018) from HRS (n=1,991). **Results:** African Americans were found to stay longer in hospice than their White counterparts when controlling for all other variables in the model. Not married, having Medicare insurance, cancer, chronic lung disease, and dementia significantly influence length of hospice stay when holding all other variables constant in the model. **Discussion:** The significant Black/White differences in hospice length of stay demonstrate the potential decrease in cumulative inequality in the delivery of appropriate and needed hospice services across the two racial groups. Future research is recommended to explore the potential consequences of longer hospice stays as well as the health and health care mechanisms related to the different disease profiles that lead individuals to hospice care, especially among African Americans.

Abstract citation ID: igad104.3013

### DECISION MAKING ABOUT LIFE-SUSTAINING TREATMENT FOR UNREPRESENTED ADULTS IN KOREAN HOSPITALS

Hyejin Kim[1], Jiyeon Choi[2], Won Lee[1], Ilhak Lee[2], and Mi-Kyung Song[3], *1. Chung-Ang University, Seoul, Republic of Korea*, *2. Yonsei University, Seoul, Republic of Korea*, *3. Emory University, Atlanta, Georgia, United States*

BACKGROUND: In South Korea, unrepresented adults are individuals who lack decision-making capacity, written treatment preferences, and available immediate family. Since the enactment of the Life-Sustaining Treatment Decision Act in 2018, ethical and legal dilemmas surrounding decision-making about life-sustaining treatments for unrepresented patients have emerged among healthcare providers in hospitals. Thus, the objectives of this qualitative descriptive study were to explore decision-making practices about life-sustaining treatments for unrepresented patients.

**METHODS:** We interviewed 37 healthcare providers including physicians, nurses, and social workers from 22 hospitals in South Korea, and analyzed the interview data using a directed content analytic technique. RESULTS: Healthcare providers reported a lack of decision-making guidelines regarding life-sustaining treatments for unrepresented patients in their institutions or being uncertain about the presence of such guidelines. Physicians primarily make decisions about life-sustaining treatments for these patients. Ethics committees' involvement in such decision-making is minimal in most hospitals. Basic life-sustaining treatments are usually initiated if necessary. However, life-sustaining treatments unrepresented patients are currently receiving cannot be discontinued even though those treatments seem pointless. Thus, additional life-sustaining treatments (e.g., extracorporeal membrane oxygenation) tend to be withheld. Important considerations in the decision-making process include the person's reversibility, risk of adverse events, and medical costs. CONCLUSION: Decision-making about life-sustaining treatments for unrepresented patients is currently in legally blind spots in South Korea. Reforms in regulations and policies surrounding such decision-making practices for unrepresented patients and the development of institutional guidelines are urgently necessary to improve decision-making practices about life-sustaining treatments for these individuals.

Abstract citation ID: igad104.3014

### INTOLERANCE FOR UNCERTAINTY MEDIATES DEATH ANXIETY AND HYPOCHONDRIASIS IN OLDER ADULTS

Derek Killingsworth, Joseph Muraira, Matthew Fontanese, Hannah Cornwell, Lance Barela, Karina Kapoor, Allyson Coldiron, and Michael Barnett, *University of Texas at Tyler, Tyler, Texas, United States*

Hypochondriasis – or illness anxiety disorder – is the preoccupation of having an illness, which has been linked with and theorized to be caused by fear of death. While death anxiety tends to decrease with age because of increased exposure to death, the association between illness anxiety and fear of death strengthens with age. Many anxiety disorders, including illness anxiety disorder, have often been explained by an intolerance for uncertainty. The current study therefore investigated whether intolerance of uncertainty mediated the effect of fear of death on hypochondriasis among older adults. Older adults (N = 260), ages 65 to 94 years (M = 75.8, SD = 7.39), responded to an online survey, which included the Death Attitude Profile-Revised questionnaire, the Intolerance of Uncertainty Scale, and the Illness Attitudes Scale. Results showed that fear of death was directly related to hypochondriasis as well as indirectly related through intolerance of uncertainty. Fear of death and intolerance of uncertainty were both positively associated with hypochondriasis. Older adults with more fear about death are more likely to experience illness anxiety and have a higher intolerance for uncertainty. It may be that older adults with higher levels of intolerance to uncertainty are more at risk for anxiety, which then may result in increased fear of death and illness anxiety with the increased exposure to death that is associated with older age. Hypochondriasis in older adulthood may therefore be best addressed by interventions that explore the uncertainty of death and focus on increasing tolerance for uncertainty.

Abstract citation ID: igad104.3015

### PATIENT AND CARE PARTNER QUESTIONS AND CLINICIAN RESPONSES DURING INITIAL OUTPATIENT PALLIATIVE CARE APPOINTMENTS

Meghan McDarby[1], Hannah Silverstein[2], William Rosa[1], Patricia Parker[1], and Brian Carpenter[2], *1. Memorial Sloan Kettering Cancer Center, New York City, New York, United*

*States*, *2. Washington University in St. Louis, St. Louis, Missouri, United States*

Person-centered palliative care (PC) requires person-centered communication. This study characterized questions asked by persons with neurologic illnesses and their care partners and PC clinicians' responses. We analyzed audio recordings from 38 initial outpatient PC appointments to characterize questions stated by patients and care partners. Based on previous research, we identified two question types: direct questions (typically-phrased, stated to obtain or clarify information) and indirect questions (declarative statements, not phrased as a typical question, made with information seeking intent). We rated the completeness of clinicians' responses on a 4-point scale ranging from 1 (completely addressed question and provided additional information) to 4 (no response). We also coded features of clinicians' responses, including offers of support or action and unprompted recommendations. Patients and care partners asked both direct (n = 442; 79.6%) and indirect (n = 113; 20.4%) questions. More than 60% of all questions were about symptoms, treatment, and lifestyle issues. Clinicians responded to 90.9% of direct questions and 90.2% of indirect questions; their responses to both question types were similar in completeness (median = 1, mode = 1). Clinicians' responses to direct questions included more offers of support or action compared to responses to indirect questions (17% v. 11%). Responses to indirect questions included more unprompted recommendations compared to responses to direct questions (21% v. 9%). Persons with neurologic illnesses and their care partners use different question types to obtain information during PC appointments. Implications include the need for clinician training to effectively respond to different question types and topics.

Abstract citation ID: igad104.3016

### PROGNOSTIC ACKNOWLEDGEMENT IN OLDER ADULT CANCER PATIENTS

Sophia Maggiore, Mary Kate Koch, Carma Bylund, and Susan Bluck, *University of Florida, Gainesville, Florida, United States*

Even with improved prognostic transparency (Chochinov et al., 2000) individuals struggle to acknowledge a serious-terminal prognosis. Patients who acknowledge their condition, however, show less distress and increased end-of-life planning (Ray et al., 2006). Aims were to: (1) examine extent older adults with serious cancer acknowledge their illness, and (2) identify factors predicting acknowledgement. Individuals receiving outpatient palliative care (N=204; Mage=65.78 years; SDage=7.43) completed measures of Symptom Severity, the Patient Dignity Inventory (DPI; Chochinov et al., 2008), and Illness Acknowledgement (i.e., healthy, seriously ill, terminally ill). Participants with Stage 4 cancer were more likely to acknowledge serious or terminal illness than Stage 1-3 patients, X2 (2,181)=12.93, p <.01), though only 31% of Stage 4 patients reported being terminally ill. In addition, ANOVA showed Symptom Severity, F(2, 190) = 9.86, p < .001 differed by level of acknowledgement. Those rating themselves as relatively healthy had less severe symptoms than seriously or terminally ill groups. To further predict acknowledgement, DPI subscales were examined. Dependency predicted acknowledgement of serious-terminal illness (Odds Ratio = .75, p <.05). A final model, controlling for age and education, showed Symptom Severity (OR = 1.03, p <.01) and DPI Dependency (OR = .79, p <.05) predicted acknowledging serious-terminal illness versus lack of acknowledgement. Though often relied on in communicating to patients, cancer stage alone may not help older adults with cancer to acknowledge their prognosis. By identifying influences leading to illness acknowledgement, future research can examine positive and negative outcomes during end-of-life coping.

Abstract citation ID: igad104.3017

### THE IMPACT OF DEMENTIA ON SLEEP QUALITY DURING COVID-19

Susanny Beltran[1], Talib Hakim[1], and Zainab Suntai[2], *1. University of Central Florida, Orlando, Florida, United States*, *2. Baylor University, Waco, Texas, United States*

Sleep disturbances are documented among older adults, and it is well-known that COVID-19 disproportionately affected older adults. Yet, studies on sleep quality among older adults with dementia, specifically, tend to focus on caregiver sleep. The purpose of this study was to examine sleep quantity and quality among older adults during 2020. Data were taken from the 2020 National Health and Aging Trends study, an annual study of Medicare beneficiaries aged 65 and older. Chi-square tests were used for bivariate analysis, and multivariate logistic regression models were used to assess differences in sleep quantity and quality between dementia and non-dementia older adults in the national sample. Results revealed that during 2020, the year the COVID-19 pandemic appeared in the U.S., older adults with dementia reported less sleep, and lower quality of sleep compared to those without dementia. This study demonstrates that sleep disturbances are significant among dementia patients. Poor sleep has previously been linked to other risks affecting quality of life, such as falls and caregiver stress, and nonpharmacological approaches are preferred for dementia patients given risks associated with psychotropics. Thus, there is a need for further study to explore changes in sleep quality over time, and to develop non-pharmacological interventions to support dementia patients' sleep quality and quantity.

### SESSION 7910 (POSTER)

### RISK AND PROTECTIVE FACTORS OF HEALTH/ HEALTH SERVICE USE II

Abstract citation ID: igad104.3018

### AGE INVARIANCE IN THE FACTOR STRUCTURE OF ADVERSE CHILDHOOD EXPERIENCES ACROSS THE LIFESPAN

Amber Rusch, and Julie Hicks Patrick, *West Virginia University, Morgantown, West Virginia, United States*

Adverse Childhood Experiences (ACEs) are associated with negative health and well-being across the adult life span. However, it is unclear whether measures of ACEs are equally predictive for adults of differing ages. Using responses from 125,212 adults on the 11-items in the ACEs Module from the 2020 Behavioral Risk Factor Surveillance System (BRFSS), we conducted both an exploratory factor analysis (EFA) and confirmatory factor analysis (CFA). Our EFA identified three interpretable factors, including a 3-item sexual abuse factor ($\alpha$ = .80), a 3-item physical/emotional abuse factor ($\alpha$ = .69) and a 5-item household dysfunction factor ($\alpha$ = .64). Results of our CFA supported the 3-factor solution, X2 (DF = 11) = 14.289, p < .001. A series of 1-way ANOVAS examined age differences in childhood adversity, where middle aged adults reported experiencing more sexual abuse relative to younger and older adults, and younger adults reported experiencing more physical/emotional abuse and household dysfunction compared to middle-aged who reported more than older adults. Additional analyses examined the influence of adverse childhood experiences on physical and mental health outcomes, including depression. Our analyses highlight the persistent effect of ACEs across the lifespan and identify a need for intervention resources for younger and middle-aged adults in order to mitigate effects of ACEs at late life.

Abstract citation ID: igad104.3019

### EVIDENCE OF AGE BIAS ON THE ALTERNATIVE MODEL OF PERSONALITY DISORDERS

Lisa Stone-Bury, Alyssa Premovich, and Daniel Segal, *University of Colorado at Colorado Springs, Colorado Springs, Colorado, United States*

Introduction: Previous research established substantial age bias on personality disorder (PD) diagnostic criteria for older adults. The Alternative Model of Personality Disorders (AMPD) is a new model of PDs proposed in DSM-5's Section III that measures PDs dimensionally along two criteria: personality functioning with four domains (measured by the Levels of Personality Functioning Scale; LPFS) and pathological personality traits with five domains (measured by the Personality Inventory for DSM-5; PID-5). This study examined age bias on the AMPD in a cross-sectional design. Method: Older (n = 200) and younger adults (n = 213) completed the LPFS-Self-Report (LPFS-SR) and the PID-5-Brief Form (PID-5-BF). **Results:** Differential Item Functioning (DIF) analyses were computed on items of the LPFS-SR and PID-5-BF. An item exhibits DIF if two age groups with similar levels of an underlying scale have different probabilities of endorsing an item. Overall, 18 of 80 items on the LPFS-SR demonstrated large DIF. The Empathy (50% of items) and Intimacy (25% of items) domains were most aged biased. For the PID-5-BF, 10 of 25 items showed large DIF. All five domains demonstrated large age bias, with Psychoticism (100% of items) most impacted. Across both DIF models, items showed evidence of being biased for (disproportionately high endorsement of an item) or against (disproportionately low endorsement) older adults. **Discussion:** Findings indicate meaningful age bias on both AMPD diagnostic criteria, indicating that older adults are at-risk of being under- and over-diagnosed with PDs under the AMPD. Revision of the AMPD is warranted to achieve age neutrality.

Abstract citation ID: igad104.3020

### EXPLORING PREDICTORS OF LONGEVITY USING RETROSPECTIVELY CODED AUTOBIOGRAPHICAL DATA

Brianna Barnett, and Alexander Bishop, *Oklahoma State University, Stillwater, Oklahoma, United States*

The aim of this study was to identify predictors of longevity using retrospectively coded autobiographical stories written and recorded from N = 1,858 deceased centenarians (M = 102.79 years; SD = 2.25 years) from the state of Oklahoma. Using the Developmental Adaptation Model as a conceptual framework, total number of years lived, the developmental outcome was regressed on socio-demographic characteristics including sex, race, and education, as well as retrospectively coded variables reflecting parental occupation, total years married, age at retirement, engagement in international travel, and self-attributions of longevity. Results confirmed three key predictors of living to 100 years and beyond. First, race was confirmed as a strong predictor of longevity ($\beta$ = −.65, p < .001). Fatherhood agricultural occupation emerged as second key predictor of living 100 years and longer ($\beta$ = .42, p < .10). Finally, total years spent in a marriage represented a third predictor of longevity ($\beta$ = .47, p < .01). Results suggest being a person of color, being raised by a father who made a livelihood working in the agricultural industry, and remaining within a long-term marital union are contributing variables linked to living 100 years and longer. Further detail regarding descriptive and methodological evaluation of retrospectively coded centenarian biographies will be highlighted. Results have implications relative to how gerontological researchers and aging service professionals may evaluate and link autobiographical information of long-lived adults to developmental outcomes such as longevity.

Abstract citation ID: igad104.3021

### EXTREME WEATHER PREPAREDNESS AND CLIMATE ACTIVISM AT AGE 85+

Taylor Patskanick, Sophia Ashebir, Lisa D'Ambrosio, and Joseph Coughlin, *Massachusetts Institute of Technology, Cambridge, Massachusetts, United States*

Current projected shifts in climate suggest extreme weather events and disasters will become increasingly common and severe. Older adults are a population vulnerable to the impact of extreme weather due to complex mobility, health, and financial situations in later life, affecting their ability to prepare for and respond to extreme weather emergencies. These compound vulnerabilities demonstrate the importance of adequate emergency preparedness among older adults, particularly among the oldest of older adults or the age 85+ demographic. This paper shares findings from a mixed methods study with the MIT AgeLab 85+ Lifestyle Leaders panel, a research panel of U.S. octogenarians and nonagenarians, on climate change, including their perceptions of generational contributions to climate change, extreme weather preparedness, and engagement in climate justice. Utilizing a survey (n=23) and five virtual focus groups (n=19) conducted in July 2022, findings underscore differences in themes related to weather-event-related emergency preparedness among Lifestyle Leaders living in senior housing versus those community-dwelling. Additional themes note a perceived lack of individual agency and control around impacting climate change locally (including a potential life stage effect) and the urgency of climate change as a societal-level and voting issue. The implications of these findings for effective engagement with the over-85 age demographic in conversations about climate change and emergency preparedness will be highlighted.

Abstract citation ID: igad104.3022

**FACILITATORS OF PHYSICAL ACTIVITY IN OLDER ADULTS AND THE IMPACT OF FEAR OF FALLING: WHAT KEEPS US MOVING?**

Bryant O'Leary, Justin Whitten, David Graham, and Dawn Tarabochia, *Montana State University, Missoula, Montana, United States*

Habitual participation in physical activity provides many well-documented health benefits for older adults. It has been suggested that high self-efficacy and internal motivation are facilitators of sustained participation, however, the findings are equivocal. Moreover, research suggests that fear of falling may negatively impact self-efficacy, leading to diminished physical activity levels. Therefore, the purpose of this study was to identify factors associated with sustained participation in physical activity and the potential influence of fear of falling on participation in physical activity for older adults. A phenomenological study was conducted with a convenience sample of 19 community-dwelling older adults aged 55-85. Participants completed an online, open-ended survey to identify potential facilitators influencing habitual physical activity in older adults. Van Manen's (1990) hermeneutical approach was employed to analyze data. Transcripts were analyzed for themes by two members of the research team. Trustworthiness was established by a peer reviewer who assessed theme accuracy. Self-motivation and life-long participation were identified as facilitators of sustained participation in physical activity. This study supports internal motivation as a facilitator; self-efficacy was not identified. Instead, lifelong participation was identified. For this study lifelong participation was defined as participating in physical activity from childhood throughout adulthood and into older adulthood. Furthermore, modification of habitual physical activity was reported due to fear of falling, namely those associated with outdoor activities. All other reported forms of physical activity were maintained. The influence of these modifications on sustained participation in physical activity for older adults is unclear and warrants further investigation.

Abstract citation ID: igad104.3023

**INCONSISTENT AND UNPREPARED: HOW FAMILY CAREGIVERS AND OLDER ADULTS EXPERIENCE HOSPITAL DISCHARGE**

Kathy Gale[1], Leslie Ruffalo[2], and Melinda Kavanaugh[1], *1. University of Wisconsin Milwaukee, Milwaukee, Wisconsin, United States*, *2. Medical College of Wisconsin, Milwaukee, Wisconsin, United States*

Family caregivers and older adult patients report confusion, inconsistency, and a lack of preparation for post-discharge activities after a hospital stay, leading to hospital readmissions. This multi-disciplinary research project investigated how family caregivers, older adult patients, and hospital clinical and administrative staff experienced the hospital discharge process at a midwestern hospital system. The research team brought together expertise in community-engaged research and bridged multiple specializations, including personal family caregiver experience, community service expertise, community engaged research leadership, and instruction of medical students and social work students. Together, we conducted semi-structured interviews with family caregiver, older adult patients, and hospital personnel via video conference and telephone. We analyzed the data using open coding strategies and principles of thematic analysis. Themes included the importance of the timing of discharge planning, communication between all systems and individuals included in the discharge process, a lack of clarity regarding the roles in the process, inconsistent identification of the family caregiver, conflicting priorities, and the impact of medication errors. Implications are (1) each organization within the older adult/family caregiver system must determine their role in the post discharge process including the type of services and resources that support older adults and family caregivers and (2) the current system of healthcare, public sector, and community must determine how to adapt and change to support older adults and their family caregivers during the post-discharge period.

Abstract citation ID: igad104.3024

**LEISURE ACTIVITIES, LIFE SATISFACTION, HAPPINESS, AND HEALTH PERCEPTION OF OLDER KOREAN IMMIGRANTS**

Juwon Lee[1], Yongseop Kim[2], Jaehyun Kim[3], and Junhyoung Kim[2], *1. Henry M Gunn High School, Palo Alto, California, United States*, *2. Indiana University, Bloomington, Indiana, United States*, *3. East Carolina University, Greenville, North Carolina, United States*

The number of Korean immigrants in the United States has been increasing rapidly, with many facing psychological challenges due to cultural differences, limited resources, and language barriers. These challenges can lead to increased stress, which negatively affects mental health. However, research has shown that leisure activities can have positive effects on health outcomes, particularly for older adults. This study aimed to investigate the relationships among leisure activities, life satisfaction, happiness, and health perception in older Korean immigrants. Convenient sampling was used to recruit 51 Korean adults aged 50-85 from community centers and churches. Results showed that participation in leisure time physical activity (LTPA) predicted life satisfaction and happiness, with outdoor physical activity being positively associated with psychological well-being. Furthermore, education was significantly related to life satisfaction and health perception among those who engaged in LTPA. The findings suggest that LTPA may be linked to higher levels of happiness, life satisfaction, and health perception in older Korean immigrants. Therefore, offering recreational programs for Korean adults in the United States could potentially improve their overall well-being. By providing opportunities for older Korean immigrants to engage in leisure activities, such as outdoor physical activity, their mental and physical health may be positively impacted. These findings contribute to the growing body of research on the importance of leisure activities for older adults' well-being and highlight the potential benefits of providing recreational programs for minority populations.

Abstract citation ID: igad104.3025

**RELIGIOSITY, DEATH EXPOSURE, AND HEALTH LITERACY AS PREDICTORS FOR ATTITUDES TOWARD MEDICAL AID IN DYING (MAID)**

Alissa McIntyre, Amy Albright, and Rebecca Allen, *The University of Alabama, Tuscaloosa, Alabama, United States*

Only legal in ten states (Death with Dignity, 2023), Medical aid in dying (MAID) is the current term for requesting lethal medication through a physician to end one's life. Research has explored individuals' characteristics who request MAID and whether they complete the prescribed medication process (Ganzini et al., 2009). However, little research explores relations between health literacy, religiosity, and individuals' attitudes toward MAID. We hypothesized that participants with high health literacy and greater death exposure would hold greater support for MAID, while greater religiosity would be associated with lower support. Having implemented attention checks, participants (N = 265) completed measures via MTurk, assessing for health literacy, religiosity, death exposure, and attitudes toward MAID. Ages ranged from 19 to 83 (M = 37.81, SD = 12.55), with 17.7% of the sample over the age of 50. The majority were self-reported female (62.6%), non-Hispanic white (79.6%), and indicated adequate functional health literacy (82.6%). Significant correlations were observed between religiosity, attitudes toward MAID, and MAID support. Although there was no direct effect on MAID support, health literacy and death exposure remained in the path model to ensure adequate model fit. The results of this study help us understand the effect of individuals' experiences and their attitudes toward MAID, indicating the importance of tailoring health literacy towards supporting autonomous choices in end-of-life care.

Abstract citation ID: igad104.3026

### THE EFFECT OF LIFESPAN TRAUMA EXPOSURE ON PATHOLOGICAL PERSONALITY TRAITS

Lisa Stone-Bury, Julie Hurd, and Daniel Segal, *University of Colorado at Colorado Springs, Colorado Springs, Colorado, United States*

Introduction: The Alternative Model of Personality Disorders (AMPD) is a dimensional model of personality disorder pathology focusing on pathological personality traits, with five trait domains. Limited knowledge exists about relationships between cumulative exposure to traumatic events and the AMPD, a gap particularly relevant for older adults who have had a lifetime of potential exposure. Method: Older adults (n = 200) completed the Trauma Health Questionnaire (THQ) and Personality Inventory for DSM-5 (PID-5). **Results:** Multivariate analysis of covariance (MANCOVA) revealed significant differences in the five PID-5 domains across four levels of cumulative trauma exposure (Pillai's Trace =.14, F(5, 519) =1.73, p =.04, ηp2=.05). Years since first traumatic event was not significant as a covariate (Pillai's Trace =.02, F(4, 173) =0.80, p =.55, ηp2=.02). Significant group differences were found in the PID-5's Psychoticism (F(3, 175) = 5.71, p < .001, ηp2 = .09) and Detachment (F(3, 175) = 2.91, p = .03, ηp2 = .05). According to Bonferroni post-hoc tests, the high trauma exposure group was significantly higher in Psychoticism than low trauma exposure, low to medium trauma exposure, and medium to high trauma exposure groups. For Detachment, the high trauma exposure group was significantly higher than the low trauma exposure and low to medium exposure groups. **Discussion:** Findings suggest that cumulative trauma exposure is associated with specific pathological personality traits in older adults. High levels of trauma exposure may be particularly related to eccentric and detached traits that persist in later life, reflecting the need for clinical assessment in both domains.

Abstract citation ID: igad104.3027

### THE IMPACT OF GROUP-LED YOGA AND MINDFULNESS ON MENTAL AND PHYSICAL HEALTH IN SOUTH ASIAN WOMEN

Rozmin Jiwani[1], Mishel Malik[1], Sara Mithani[1], Sara Espinoza[2], and Monica Serra[1], *1. UT Health San Antonio, San Antonio, Texas, United States*, *2. Cedars-Sinai Medical Center, Los Angeles, California, United States*

One in five South Asians (people from India, Pakistan, and Bangladesh) living in the U.S. report experiencing a mental illness, with women reporting a three-fold greater risk of distress than men. South Asian women commonly interpret mental health symptoms as physical illnesses and resist seeking medical help. Yoga is a multifaceted, effective practice promoting well-being by integrating mind and body. This study aims to determine the feasibility and impact of a 12-week (Jan-April 2023) yoga and mindfulness intervention on mental and physical health outcomes in South Asian women. Thirty community-dwelling South Asian immigrant women, age ≥45 years were recruited to complete weekly 90-minute sessions of Ashtanga yoga including meditation and mindfulness practice. We are currently reporting baseline and 6-week data of mindfulness and physical activity self-reported questionnaires as the intervention is ongoing, but 12-week data will be available for the final presentation. At baseline, participants were 53±6 years old and a majority (80%) were overweight/obese with BMI ≥25kg/m2. At 6 weeks, twenty-eight (93%) participants continued with the study and twenty-five (83%) attended ≥4 of the 6 sessions. By 6 weeks, participants' scores improved on Five Facet Mindfulness by 17% (p< 0.001) and Godin Leisure-Time Exercise by 15% (p>0.05) from baseline. These results suggest the preliminary feasibility, retention, and potential impact of the intervention on mental health outcomes in this population. Future directions include developing a large-scale, randomized lifestyle intervention trial to delay/manage mental health outcomes in South Asian women of all ages.

Abstract citation ID: igad104.3028

### THE ROLE OF HEALTH LITERACY IN HEALTH CARE UTILIZATION AMONG TAIWANESE OLDER ADULTS

Zih-Yong Liao, Pei-Chun Liao, and Fang-Lin Kuo, *National Health Research Institutes, Yunlin, Yunlin, Taiwan (Republic of China)*

Health literacy is one of important capabilities links to health-related behaviours. The extant study provides limited information about the healthcare access of older adults. This study investigated factors determining the healthcare utilisation of 2423 community-dwelling older adults from the Taiwan Longitudinal Study on Ageing between 2011 and 2015. We used linear mixed-effects regression models and cumulative logit models to examine whether personal characteristics are associated with healthcare utilisation. A significant association was found between the level of difficulties in health literacy and healthcare utilisation after controlling for age, comorbidity, depression, mobility and accessibilities. The higher the older adults scored in the difficulty of health literacy, the lower frequencies of clinics (p = .03), inpatient (p = .02) and dentist visits (p = .02). Individuals who access health-related information more easily have a better chance to receive inpatient services when they feel unwell (p = .0004). Better perceived health is less likely to use clinics (p < .0001), emergency (p < .0001) and inpatient services (p = .0007) but not include visits to pharmacy and traditional medicine. People engaging in workouts are less likely to purchase in a pharmacy (p = .02) but visit traditional medicine (p = .02) more often. It echoes that health literacy is an intermediate attribute to self-efficacy in managing personal health matters and suggests a perspective that older adults consider taking traditional medicine as a self-care instead of healthcare treatment. Future intervention programmes can address health literacy promotion as an initiating step to facilitate participants' engagement.

Abstract citation ID: igad104.3029
### UNDERSTANDING HEARING LOSS AMONG COMMUNITY-DWELLING OLDER ADULTS FROM AN ENGAGING WITH AGING PERSPECTIVE
Shaoqing Ge[1], Kuan-Ching Wu[2], Jessica Jin[2], Suah Park[3], Shao-Yun Chien[2], Janet Primomo[3], and Basia Belza[2], *1. University of Texas at Austin School of Nursing, Austin, Texas, United States*, *2. University of Washington, Seattle, Washington, United States*, *3. University of Washington School of Nursing, Seattle, Washington, United States*

Engaging with Aging is an emerging framework proposed by Carnevali which provides a new lens to understand an active, conscious daily living process of coping with age-related changes (ARCs) taken on by older adults. The study aims were to 1) describe the challenges related to hearing loss experienced by community-dwelling older adults, especially during COVID-19; 2) identify the strategies and resources used by older adults to accommodate the daily living challenges caused by hearing loss. We conducted semi-structured interviews with 29 participants aged 64 to 98 online due to COVID-19 restrictions. We used a virtual card sort to assist with data gathering. Hearing loss was mentioned by 18 participants (62%) and their corresponding adaptations were discussed. We found that older adults linked their adaptations to hearing loss based on their changing hearing capacities and needs. Commonly used adaptations included lip reading, volume adjustment, and wearing hearing aids. The challenges caused by COVID-19 in implementing the adaptations were also discussed (e.g., increased difficulty in understanding others due to mask-wearing). While most participants reported making adaptations to hearing loss, some mentioned that hearing loss was "no biggie" to them so no adaptations were needed. This study substantiates the EWA framework by showing the commonality among older adults in linking hearing loss, as one of the common ARCs, with corresponding adaptations. Implications for clinicians and researchers include using EWA to help older adults identify personalized solutions that fit their hearing capacity.

Abstract citation ID: igad104.3030
### URBAN OLDER ADULTS PERSPECTIVES ON NATURE: A DIRECTED CONTENT ANALYSIS
Jessica Cassidy, and Kathy Lee, *University of Texas at Arlington, Arlington, Texas, United States*

Biophilia is a theory which hypothesizes humans have an instinctual desire to interact with nature that in turn, supports health and well-being outcomes through gains in physical and mental functioning. This study aimed to explore how concepts outlined in the theory of Biophilia related to urban older adults' experiences and perceptions of nature. This secondary data analysis examined five focus groups with community-dwelling, urban older adults. All participants were female, and majority were African Americans (83.3%). Using directed content analysis, we developed a codebook from concepts and mechanisms outlined in the theory of Biophilia. Our analysis resulted in six themes, 1) Infinite wonder of nature; 2) Nature activating long-term memories; 3) Nature influencing feelings of well-being; 4) Connecting with nature; 5) Conservation ethic; and 6) Nature across the lifespan. Overall, the data fit the theory well. Through the lens of Biophilia we understood why the sample of participants valued their interactions with nature, as well as how experiences in nature promoted their feelings of well-being. We observed appreciation for nature interactions can deepen in later life suggesting nature-based interventions may be especially well-suited for older adult populations. We suggest the theory of Biophilia can lend well to public health approaches towards addressing the social and economic implications presented by growing numbers of urban dwelling older adults. Moreover, Biophilia can provide a theoretical basis for tailoring nature-based interventions delivered across a variety of settings to match diverse study goals and overall, to promote the health and well-being of older adults.

### SESSION 7920 (POSTER)

### MENTAL HEALTH/SERVICE USE

Abstract citation ID: igad104.3031
### AGING WITH MENTAL HEALTH CONDITONS AND MOTIVATION FOR SELF-CARE: THE HANDWASHING WORKSHOP
Aryane Cazumbá[1], Gabriela Bellizzi[2], Marcia Varricchio[3], Neide Verçosa[3], Felipe Paci Soares[4], Erica Paci Soares[4], and Jaqueline Da Silva[3], *1. UNIFASE, Petropolis, Rio de Janeiro, Brazil*, *2. UNIFASE / FMP, Petropolis, Rio de Janeiro, Brazil*, *3. Federal University of Rio de Janeiro (UFRJ), RIo de Janeiro, Rio de Janeiro, Brazil*, *4. Colégio Bom Jesus São José (CBJSJ), Petropolis, Rio de Janeiro, Brazil*

In Brazil, data from the Influenza Epidemiological Surveillance Information System (SIVEP-Flu), indicate that up to June 2020, about 71% of deaths from Covid19 were of elderly, with and without comorbidities. Since the SARS-Cov2 pandemic, health of the elderly population with mental health problems has been hard hit. The study scenario was the 2023 Summer Edition of the Intergenerational and Inclusion Camp on Health, Science and Technology / Lab Project for the Valorization of Aging (PROVE). The implementation research design was a teaching-research-service action by graduate, undergraduate and two high school students. Supported by the lab faculty and librarian, students searched for evidence-based practices, and in the light of health equity, selected the hand washing demand of underserved older adults to develop a ludic-informative workshop, for motivating self-care practices. In the methodological trajectory, participants (i) were divided into two teams; (ii) had representatives put latex gloves and blindfolds on and washable colored paint poured on the gloves; (iii) received instructions to demonstrate how they washed their hands; (iv) blindfolds were removed and a debate took place on the procedure and its importance; (v) students demonstrated the correct technique using gloves and paint. Older adults reported having had fun and learning significantly more. The approach was evaluated as empowering, noting that the visualization of the inks on the gloves specifically allowed identifying points to be refined. Other topics and subsequent workshops were suggested. In conclusion, light and flexible fun, evidence-based and motivating strategies are protective and promote comprehensive health care.

Abstract citation ID: igad104.3032

### ANXIETY AND DEPRESSION IN OLDER ADULTS POST DEEPWATER HORIZON OIL SPILL

Mickaela Reed, and JoNell Strough, *West Virginia University, Morgantown, West Virginia, United States*

Previous research has investigated challenges such as scarcity in housing, the loss of life, and the onset of mental health disorders such as PTSD that persist once a person has experienced a man-made disaster. This has been analyzed in populations such as older adults and other vulnerable groups of people. However, little to no consideration has been given to how age, in combination with trauma history, relates to indicators of psychological well-being after disasters. Here, using Socioemotional Selectivity Theory (SST) (Carstensen, 2006) and the Strength of Vulnerability Integration Model (SAVI) (Charles, 2013) we investigated age differences in two indicators of psychological well-being—depression and anxiety—among individuals following the 2010 Deepwater Horizon Oil Spill (DHOS). Specifically, trauma history and personal exposure to the DHOS were tested as potential moderators of age differences in psychological well-being. Residents of the US Gulf Coast region (N=2,508, M age= 57.72 yrs; 60.4% Women) were contacted via telephone and asked to complete the Survey of Trauma, Resilience, and Opportunity among Neighborhoods in the Gulf (STRONG). Regression analyses were used to investigate the roles of age, prior trauma, and DHOS exposure on psychological well-being after controlling for income, education, race, and gender. Implications of the findings for disaster preparedness interventions and theories of psychological well-being across the life span are discussed.

Abstract citation ID: igad104.3033

### CHILDHOOD PARENT–CHILD RELATIONSHIP, PARTNER RELATIONSHIP CLOSENESS, AND DEPRESSIVE SYMPTOMS IN LATER LIFE

Zexi Zhou[1], and Yang Qu[2], *1. The University of Texas at Austin, Austin, Texas, United States*, *2. Northwestern University, Chicago, Illinois, United States*

Attachment theory suggests that quality of early-life parent-child relationships may influence how people establish other types of relationships (e.g., romantic relationship, friendship) later in life, and have long-lasting effects on individuals' psychosocial development. Yet, little research has investigated whether such effects may last until later adulthood and the potential moderators in these processes. To address this issue, this study examined the associations among childhood parent-child relationship quality, current partner relationship closeness, and depressive symptoms in late life using a sample of 12,292 community-dwelling adults aged 51 or older (Mage = 63.15, 51% female). We leveraged the seven-wave longitudinal data between 2006–2018 from the Health and Retirement Study. Depressive symptoms were assessed using the CESD score at each wave. Participants also reported closeness with their partner every other wave, and relationship quality with their parents in childhood. Multilevel models showed that better mother-child and father-child relationships during childhood were both associated with greater closeness with partner in late life, especially for female compared to male. Poorer childhood mother-child relationship was associated more depressive symptoms in late life, but this association was attenuated for those who overall reported a greater level of closeness with their partner over the study period. The findings suggest that negative early life parent-child relationship may put people at greater risk for developing depressive symptoms and weaker social connections in late life, and highlight the protective role of positive partner relationship in buffering this association.

Abstract citation ID: igad104.3034

### HOW WIDOWHOOD SHAPES THE EFFECT OF ADULT CHILDREN'S EDUCATION ON MOTHERS' PSYCHOLOGICAL WELL-BEING

Robert Frase[1], Shawn Bauldry[2], J Jill Suitor[2], and Megan Gilligan[3], *1. Southern Illinois University, Carbondale, Illinois, United States*, *2. Purdue University, West Lafayette, Indiana, United States*, *3. University of Missouri, Columbia, Missouri, United States*

Despite a growing interest in the effects of adult children's education on older parents' health and well-being, few studies have focused on how family structure may shape these processes. Drawing on data collected from 160 married and 180 widowed mothers ages 73-85 as part of the Within-Family Differences Study, we explore how mothers' widowhood status moderates the association between their adult children's education and mothers' depressive symptoms. Drawing on past empirical work and Socioemotional Selectivity Theory, we develop and test two competing hypotheses. First, empirical research on depression and financial strain suggests that widowed mothers may be especially reliant on their adult children's resources and, therefore, that adult children's education would have a larger impact on widowed than married mothers' mental health. Alternatively, principles of Socioemotional Selectivity Theory suggest that widows may place less value on their children's material accomplishments, like educational attainment, and instead emphasize the socioemotional aspects of their relationships with their children. Therefore, children's education may exert a smaller influence on the psychological well-being of widowed than married mothers. Results of linear regression models stratified by widowhood status supported our second hypothesis. Specifically, the association between adult children's education and mothers' psychological well-being was significantly larger among married mothers relative to widowed mothers. Our findings highlight the importance of considering family processes and family structure when studying educational attainment and health across generations.

Abstract citation ID: igad104.3035
### LATER LIFE AS A DARING EXPERIENCE: FACTORS ASSOCIATED WITH OLDER ADULTS' RISK PERCEPTION

Rinat Lifshitz[1], Yaacov Bachner[2], and Galit Nimrod[2], *1. The Max Stern Academic College of Emek Yezreel, Israel, Yezreel Valley, HaZafon, Israel*, *2. Ben-Gurion University of the Negev, Beer-Sheva, HaDarom, Israel*

Risk perception refers to people's subjective judgments about the possibility of negative occurrences and the extent to which they are concerned with them. Previous studies have found that older adults who were exposed to ongoing terror threats developed later-life and terror risk perceptions. These studies showed that high risk perception has negative psychological and physiological consequences. This study aimed to identify the factors associated with the development of both later-life risk perception and terror risk perception in later life. Data were collected via an online survey with 306 Internet users aged 50 years and over, half resided in a high-risk zone while the remainder lived in a low-risk zone. The Perceived Risk Scale, measures of depressive symptoms, life satisfaction, social support, spirituality, internet use, and personal background were applied. Low self-rated health was associated with terror and later-life risk perceptions, regardless of the risk zone. After controlling for personal background, only depressive symptoms significantly correlated with high risk perceptions. These findings suggest that older adults with poorer self-rated health, secular beliefs, and elevated depressive symptoms may be susceptible to developing high risk perceptions. Clinicians should encourage older adults to identify preserving resources to improve adjustment to late life stressors.

Abstract citation ID: igad104.3036
### NETWORK ANALYSIS OF ANXIETY AND DEPRESSIVE SYMPTOMS AMONG OLDER ADULTS WITH DIFFERENTIAL MENTAL HEALTH RISKS

Tianyin Liu[1], Wen Zhang[2], Gloria Hoi-Yan Wong[2], and Terry Lum[1], *1. Tthe University of Hong Kong, Hong Kong, Hong Kong*, *2. Hong Kong Metropolitan University, Hong Kong, Hong Kong*

Anxiety and depressive symptoms are common in older people, and network analysis may provide deeper characterization of symptom-symptom interactions to inform personalized care. The aim of this study was to elucidate characteristics of anxiety and depressive symptom networks of older adults with differential risks for depression and anxiety. A total of 4184 older adults (mean age = 77.3±8.8 years, 3245 women) were recruited from the community. Their anxiety and depressive symptom were measured using the Generalized Anxiety Disorder Scale 7-item (GAD-7) and Patient Health Questionnaire 9-item (PHQ-9), respectively. A cut-off score of 5 was used for both scales to differentiate people with mild or above anxiety/depressive symptoms; by this criterion, 1067 (25.5%) of the sample had no anxiety/depressive symptoms, 1690 (40.4%) had symptoms in one domain, and 1427 (34.1%) had symptoms in both domains. Central symptoms and bridge symptoms were identified via centrality indices and bridge centrality indices, respectively. Network stability was examined using the case-dropping procedure. Overall, Restlessness (GAD-7 item 5), Depressed mood (PHQ-9 item 2), and Fear (GAD-7 item 7) had the highest centrality values; two bridge symptoms, Restlessness and Psychomotor disturbances (PHQ-9 item 8), were also identified. Higher risks for depression and/or anxiety were positively associated with global strength; Restlessness and Depressed mood remained to be central symptoms, but in those with both depression and anxiety risks, Restlessness and Guilt/Self-blame (PHQ-9 item 6) bridged the two domains. Interventions designed to target central symptoms and bridge symptoms may be effective in alleviating co-occurring experiences of anxiety and depression.

Abstract citation ID: igad104.3037
### PERSONAL FACTORS AFFECTING OLDER ADULTS' INTERPRETATION OF STRESS: MODERATING ROLE OF DIFFERENT LEISURE TYPES

Jaesung An[1], and Soonhyung Kwon[2], *1. California State University East Bay, Hayward, California, United States*, *2. Rutgers, the State University of New Jersey, New Brunswick, New Jersey, United States*

Holistic stress model (Nelson & Simmons, 2003; 2011) offers the most updated framework to understand how individuals' differences in characteristics (e.g., locus of control, optimism, hardiness) determines their interpretation of the given stressor, whether as eustress or distress. However, dearth of literature exists to further validate this framework, and barely any study has been conducted among older adult population. While daily leisure engagement is one of the most valued activities among older adults, which helps them navigate healthy aging process (An et al., 2022), it also has positive relationships with older adults' sense of control, positive emotions, and resilience (Son et al., 2021). Therefore, the purpose of this study was to examine whether individual differences (sense of control, optimism, social support, spiritual belief) have effects on how older adults respond to given stressor and to investigate whether there is a moderating effect of participating in different types of leisure activities. This study used the Health and Retirement Study (HRS) data and study participants (n=3,038) aged 64.52 years old on average. Multiple linear regression was used for analyses using SPSS. Findings indicated that higher social support, optimism, and sense of control were significantly associated with lower response to chronic stress, whereas higher spiritual belief was related to higher response to chronic stress. In terms of specific moderator effects, physical leisure was a significant moderator between social support, optimism, sense of control, and spiritual belief and response to chronic stress. Further discussion, implication, and future recommendations will be presented.

### SESSION 7930 (POSTER)

### LOSSES AND GAINS: FINANCES, FRAILTY, AND WELL-BEING

Abstract citation ID: igad104.3038
### FINANCIAL DECISION MAKING AND WEALTH LOSS IN OLDER ADULTS WITH EARLY MEMORY LOSS: THE WALLET STUDY

Peter Lichtenberg[1], Emily Flores[1], and Vanessa Rorai[2], *1. Wayne State University, Detroit, Michigan, United States*,

2. *Healthier Black Elders Center, Detroit, Michigan, United States*

Angisani and Lee (2019) used the HRS to determine that wealth loss was related to early memory loss. Using methods described elsewhere (Lichtenberg et al., 2022), we investigated personal finance behaviors and wealth loss. using 12 months of checking account statements and follow up interviews with 114 older adults; 67 of whom had self-reported cognitive impairment (CI), and two-thirds of who were African American. Participants were largely recruited from research registries with the Michigan ADRC and the Michigan Center for Urban African American Aging Research. Mean age was 72 years mean education was 15 years, and 70% of the sample were women. There were no differences between the group on demographic variables, financial literacy (3-item Lusardi and Mitchell scale) or annual income. Bivariate analyses (t tests) indicated that those with CI were significantly more likely to suffer wealth loss across the 12 month period. Those with CI had significantly poorer scores on a person-centered financial decision making scale, and reported significantly lower IADL skills. In addition, those with CI were more likely to financially helping others on a regular basis, and reported a higher rate of financial exploitation. Correlations between wealth loss and CI (r=.22; p<.05), decision-making (r=.54; p<.05) and helping others on a regular basis (r=.51; p<.05). A logistic regression predicting wealth loss resulted in two measures significantly predicting wealth loss; financial decision-making and helping someone else financially on a regular basis. **Conclusions:** (1) early CI is related to wealth loss and (2) decision-making intersects with wealth loss.

Abstract citation ID: igad104.3039

### FINANCIAL PREPARATION FOR RETIREMENT OF WORKING-AGE ADULTS IN HONG KONG: A PERSONALIZED PENSION PROJECTION

Alex Yue Feng Zhu, and Kee Lee Chou, *The Education University of Hong Kong, Hong Kong, Hong Kong*

This study replicated and upgraded the personal pension projections among a sample of middle-aged working adults in Hong Kong (N = 400). We conducted a three-arm randomized controlled trial in which the participants were randomly assigned to either experimental condition A (a personalized pension projection), experimental condition B (a personalized pension projection plus new "identifying feasible plans to achieve the ideal retirement life" component), or the control condition. Analyzing a baseline and two follow-up tests, we found that a personalized pension projection had immediate and short-term positive effects on the participants' financial preparation for retirement. The newly added component strengthened both effects. More specifically, the original personalized pension projection improved the participants' financial preparation for retirement by reducing their temporal discounting. Our findings enhance understanding of the mechanism of a personalized pension projection, clarify its location within the intervention framework of retirement financial planning, and recommend multiple channels to policymakers and social workers to further upgrade and optimize a personalized pension projection and improve its effectiveness.

Abstract citation ID: igad104.3040

### FINANCIAL SATISFACTION AND PERSONALITY IN OLDER ADULTS

Yeon Ji Ryou, Mengya Wang, and Peter Martin, *Iowa State University, Ames, Iowa, United States*

Financial satisfaction is an essential component of the quality of life among adults 65 years and older. However, little is known about how psychosocial factors, such as personality, might influence financial satisfaction in this age group. To address this gap in knowledge, this study explored the relationship between financial satisfaction, the Big-5 personality traits, and social support. For this study, 1,322 participants answering questions, including the Big-5 personality traits, their financial satisfaction, and social activities, were selected from the 2020 Core Health and Retirement Study (HRS) data. Hierarchical regression analyses were conducted to identify the contributors to financial satisfaction, with control variables such as age, gender, and income in the first model. The Big-5 personality traits and social factors, including negative and positive social support, were added to subsequent models. The results revealed that extraversion and conscientiousness were significantly and positively associated with financial satisfaction, whereas neuroticism and agreeableness demonstrated the opposite relationship. Negative social support was also negatively associated with financial satisfaction, whereas positive social support showed the opposite effect. Participating in social activities was found to be positively associated with financial satisfaction among older adults. Older age, being white, female, married or partnered, and reporting better health were also significant predictors of higher financial satisfaction. These findings have significant implications for financial planning and counseling among older adults. Interventions targeting specific personality traits such as extraversion and engaging in more social activities may be particularly effective for improving financial satisfaction among US older adults.

Abstract citation ID: igad104.3041

### ISOTEMPORAL SUBSTITUTION OF 24-HOUR MOVEMENT BEHAVIOR AND FRAILTY STATUS IN OLDER ADULTS

Ting-Fu Lai[1], Yung Liao[1], Ming-Chun Hsueh[2], Chung-Sheng Lee[3], Kun-Pei Lin[4], Ding-Cheng (Derrick) Chan[4], Yung-Ming Chen[5], and Chiung-Jung Wen[4], *1. National Taiwan Normal University, Taipei City, Taipei, Taiwan (Republic of China), 2. University of Taipei, Taipei, Taipei, Taiwan (Republic of China), 3. Kainan University, Taoyuan, Taoyuan, Taiwan (Republic of China), 4. National Taiwan University Hospital, Taipei City, Taipei, Taiwan (Republic of China), 5. National Taiwan University, Taipei City, Taipei, Taiwan (Republic of China)*

**Background:** Properly distributing twenty-four hours (24-h) movement in daily life may provide older individuals chances to prevent frailty. This study aimed to investigate the effects of replacing sedentary behavior (SB) with light physical activity (LPA) and moderate to vigorous physical activity (MVPA) on risks of pre-frailty and frailty in older adults. **Methods:** We recruited 196 older adults (48.5% men; 80.3 ± 7.1 years old) to wear a triaxial accelerometer on the waist. Pre-frailty and frailty were assessed by a modified version of Fried's criteria. Isotemporal substitution analysis was performed to investigate the association between 24-h movement and risks of pre-frailty and frailty. **Results:** After adjusting confounding variables, the results also indicated that only reallocating 60 minutes of SB per day to MVPA was less likely to have pre-frailty (OR = 0.01, 95% CI = 0.01, 0.20) and frailty (OR = 0.02, 95% CI = 0.02, 0.36). In addition, each 5-min increment in MVPA from 20 min as part of total physical activity (40 min in LPA) to replace 60 minutes of SB per day resulted in being less likely to have frailty (OR = 0.546, 95% CI= 0.301, 0.992). **Conclusions:** This study demonstrated that using 60-min MVPA to substitute SB daily may theoretically reduce the risk of being pre-frailty and frailty. Moreover, replacing 60-min SB daily with mixed 40-min LPA and 20-min MVPA was able to reduce frailty risks. Therefore, those findings may provide alternative approaches with acceptable intensity and feasibility when promoting the physical health of older adults.

Abstract citation ID: igad104.3042

### LIFESTYLE CHANGES AFTER FRAILTY ONSET AMONG MIDDLE-AGED AND OLDER ADULTS IN THE UNITED STATES

Hui-wen Xu, Yu-Ming Chen, Zhou Yang, and Bei-bei Xu, *Peking University, Beijing, Beijing, China (People's Republic)*

Frailty could be the teachable moment to promote a lifestyle change for middle-aged and older adults. This study aimed to investigate the impact of sociodemographic characteristics on lifestyle changes among frail adults aged ≥50 years. A total of 5,440 frail individuals aged ≥50 years from the Health and Retirement Study (HRS) 2004-2020 were included. The Paulson–Lichtenberg Frailty Index was used to measure frailty. Lifestyle included smoking status, alcohol consumption, physical activity, and sleep problems. Logistic regression models were used to examine the associations between sociodemographic characteristics and lifestyle change among frail participants. After frailty, a higher proportion of individuals reported maintaining their smoking and drinking status without change. Compared to increased physical activity, more people reported reducing their low, moderate, and high-intensity physical activity. Similarly, a greater proportion of individuals reported worsened sleep problems compared to those who experienced improvements in sleep problems. Age was negatively associated with quitting smoking and increased light, moderate, as well as vigorous physical activity, while was positively associated with improving sleep problems. Males were more likely to quit smoking, quit drinking, and increase light physical activity. Individuals with medium and high education levels were more likely to quit drinking and less likely to increase moderate physical activity. A sensitivity analysis based on the 4-year interval between pre-diagnosis and post-diagnosis of frailty showed that being unmarried was associated with an increased likelihood of quitting smoking. Some people reported lifestyle changes after frailty. Age, gender, marital status, and education levels may affect lifestyle changes after frailty.

Abstract citation ID: igad104.3043

### LOW RESILIENCE FACTORS AND MINORITY RACE PREDICT QUALITY-OF-LIFE DEFICITS AMONG OLDER ADULTS WITH CHRONIC DISEASE

Allan Nkwata[1], Xiao Song[2], Ming Zhang[2], and Amara Ezeamama[3], *1. University of Michigan, Ann Arbor, Michigan, United States*, *2. University of Georgia, Athens, Georgia, United States*, *3. Michigan State University, East Lansing, Michigan, United States*

Resilience-promoting factors (RPF), minority race and their interaction, were evaluated as determinants of quality of life (QOL) decline in a nationally representative sample of US adults ≥50 years old (N=3932) with heart disease and/or type-2 diabetes from 2006-2014 as part of the Health and Retirement Study. RPF included personal mastery and positive social support (PSS). QOL was assessed by self-rated health (SRH) and defined as poor (fair/poor), good, or excellent (very good/ excellent). Repeated measures multinomial regression using generalized estimating equations (GEEs) related RPF, race, and their interaction to SRH declines over 8 years. Odds ratios (OR) and 95% confidence intervals (CIs) were calculated with adjustment for time, sociodemographic and behavioral confounders. Low mastery, low PSS and minority race were each associated with higher odds of QOL declines over 8 years (mastery OR- 2.27, 95% CI: 1.83-2.81; PSS OR- 1.35, 95% CI: 1.10-1.65; African American OR- 1.46, 95% CI: 1.25-1.70, and Other race OR- 1.43, 95% CI: 1.10-1.86). QOL declines related to PSS-friends varied by race and time (race x time x PSS-friends, p=0.093). Among older Caucasians, the association between PSS and QOL declines did not vary over time (p=0.676). However, among African Americans and Other race, low PSS was associated with increased odds of QOL declines over time (p=0.031 and p=0.034 respectively). RPF and minority race predicted QOL decline in older Americans with comorbid conditions. Policies/ interventions to enhance resiliency represent a viable strategy for mitigating racial disparities in overall wellbeing and improving health outcomes in aging Americans.

Abstract citation ID: igad104.3044

### SOCIOECONOMIC DETERMINANTS OF FRAILTY TRAJECTORIES AMONG OLDER ADULTS IN SWITZERLAND (2004–2020)

Mengling Cheng, *Swiss Centre of Expertise in Life Course Research, Lausanne, Vaud, Switzerland*

Frailty in old age is a major public health challenge. Frail older adults are at higher risk of adverse health outcomes and in greater needs for health services as compared to non-frail older adults. An emerging perspective views frailty as a dynamic process and older adults are likely to transition to improved frailty status. However, little is known about the socioeconomic determinants of frailty trajectories over the later life course. This study investigated how frailty evolves and transitions over time and how socioeconomic status impacts frailty trajectories. Data from the Survey of Health, Ageing and Retirement in Europe 2004-2020 (N=4,621 adults aged 50-105 in Switzerland) were analyzed. Fried's phenotype was used to operationalize frailty. Changes in frailty status at a 2-year interval were used to identify frailty trajectories. Growth curve models were performed to investigate how age, sex, education, occupation, and income determined frailty trajectories over the later life course. This study had three findings. First, three frailty states were identified: non-frail, pre-frail, and frail. Second, five frailty trajectories were identified: stable frail, worsening, mixed, improving, and stable non-frail. Third, individuals aged 65 and above, women, and socioeconomically disadvantaged individuals had a higher risk of being stable frail and having a worsening frailty trajectory. Findings indicate that frailty intervention programs in Switzerland need to prioritize individuals in middle age and old age, women, and individuals with lower socioeconomic status to tackle the challenge of frailty.

Abstract citation ID: igad104.3045

### TRENDS IN FRAILTY INCIDENCE AND THE IMPACT OF LIFESTYLE AMONG US ADULTS AGED ≥50 YEARS FROM 2004 TO 2020

Hui-wen Xu, Yu-Ming Chen, Zhou Yang, and Bei-bei Xu, *Peking University, Beijing, Beijing, China (People's Republic)*

Few studies have estimated frailty incidence. The contribution of lifestyle to the incidence of frailty may change over time. It is necessary to quantify the impact of lifestyle on frailty incidence over time. A total of 25,639 subjects aged ≥50 years from the Health and Retirement Study (HRS) 2004-2020 were divided into seven consecutive and overlapping cohorts. Frailty was measured using the Paulson–Lichtenberg Frailty Index. Lifestyle included smoking status, alcohol consumption, physical activity, and sleep problems. The trend of frailty incidence rates was estimated by generalized estimating equation (GEE) models with a natural cubic spline. Population attributable fractions (PAFs) were calculated using hazard ratios from Cox models. Lifestyle-attributable frailty incidence rates were estimated by multiplying PAFs by the overall incidence rate of frailty. The incidence (per 1,000 person-years) of frailty showed a general trend of first decreasing and then increasing, with a peak occurrence of 22.7 (95% CI: 21.0, 24.5) in 2010–2014. Almost 20% of frailty incidence rates were attributed to current smoking, physical inactivity, or sleep problems. The incidence of frailty showed a fluctuating but decreasing trend among middle-aged and older adults in the United States from 2004 to 2020, with a peak occurrence after the Great Recession of 2007–2009. Smoking, physical inactivity, and sleep problems may have contributed importantly to the incidence of frailty. Effective and early frailty prevention strategies should be developed to reduce smoking, physical inactivity, and sleep problems, especially in the context of the global economic crisis caused by the COVID-19 epidemic.

### SESSION 7940 (POSTER)

### GLOBAL AGING AND HEALTH (BSS)

Abstract citation ID: igad104.3046

### ADVERSE CHILDHOOD EXPERIENCES AND DEPRESSIVE SYMPTOMS IN MIDDLE-AGED AND OLDER CHINESE ADULTS

Wenxing Wei, and Aloen Townsend, *Case Western Reserve University, Cleveland, Ohio, United States*

Prior research has shown a significant effect from adverse childhood experiences (ACEs) to depressive symptoms in later adulthood, but there is very limited research on this relationship in China. This study examines the relationship between multiple childhood adversity indicators (childhood abuse, bullying victimization, and witnessing violence) and depressive symptoms in Chinese middle-aged and older adults by gender. The study was based on a nationally representative sample of 10,371 participants aged 45 and older collected through the China Health and Retirement Longitudinal Study (CHARLS). Childhood adversity indicators in the 2014 Life History Survey were merged with the harmonized 2018 CHARLS dataset. Structural equation modeling was conducted separately for males and females because the measurement model was not invariant by gender. Childhood abuse was not a significant predictor for either gender but bullying victimization and witnessing violence were significant. Witnessing violence was a stronger predictor for females (gamma = .15, $p < .001$) than for males (gamma = .08, $p < .05$). For both groups, childhood adversity variables together accounted for 6% variance in depressive symptoms. Experiences of being bullied in school and community and witnessing violence in the family were still influential in middle and late life. Figuring out what role ACEs are playing and how to deal with their effects are vital to providing more effective prevention and interventions. Further investigations are required, for example, regarding the mechanisms behind the significant relationships and why childhood abuse was not a significant predictor among this Chinese sample.

Abstract citation ID: igad104.3047

### AN INTERNATIONAL PERSPECTIVE ON GOAL SETTING AND INTERDISCIPLINARY COLLABORATION WITHIN REABLEMENT PROGRAMS

Lise Buma[1], Hanne Tuntland[2], Sandra Zwakhalen[1], and Silke Metzelthin[3], *1. Maastricht University, Maastricht, Limburg, Netherlands*, 2. *Western Norway University of Applied Sciences, Bergen, Hordaland, Norway*, 3. *Maastricht University, Care and Public Health Research Institute, Maastricht, Limburg, Netherlands*

Over the last two decades, reablement has been studied and implemented in more than 16 countries. It is a person-centred approach aiming to enhance individuals' (physical) functioning and increase or maintain their independence in meaningful activities. Goal setting and interdisciplinary collaboration are identified as important key elements of reablement. Due to limited intervention descriptions in the scientific literature, it is not clear how these two elements are applied in practice. This hinders the uptake of reablement services among healthcare care providers on national and international level. This study aimed to provide insight into: (1) goal setting; and (2) interdisciplinary collaboration within reablement programs. Therefore, a qualitative study in Norway and the Netherlands was conducted. In both countries, reablement staff (n=14) was interviewed. Data was analysed using both deductive and inductive content analysis. To increase rigor in terms of credibility, transferability, dependability and conformability; member checking, uniform data collection methods, note-taking, and direct quotations were used to analyse and present the data in this study. These focus groups resulted in, on the one hand, a clear overview of procedures regarding goal setting, treatment and evaluation methods, and on the other hand, valuable insights into staff experiences regarding interdisciplinary collaboration. In addition, facilitators and barriers for the implementation of goal setting and interdisciplinary collaboration in reablement programs were identified. This study resulted in valuable knowledge for healthcare providers to effectively implement reablement in practice.

Abstract citation ID: igad104.3048

### ASSESSING ADEQUACY OF HEALTH FACILITIES FOR OLDER ADULTS IN INDIAN CITIES

Sruthi Anilkumar Hemalatha, Nawaj Sarif, and Papai Barman, *International Institute for Population Sciences, Mumbai, Maharashtra, India*

1The healthcare system in India is struggling to meet the needs of the current population, and the rising aging population is likely to exacerbate this problem. Therefore, it is essential to plan and prepare for the future. Thus, using a geospatial approach, this study used data from the Census of India to investigate the distribution of older populations' access to improved health facilities across various cities in India. The study found wide variation in the proportion of the aging population across India, with cities in the northern, southern, and eastern parts of India having a higher percentage of older people. The study compared the distribution of older adult populations and health infrastructures, such as hospitals and medical staff, and found considerable regional variations in their availability. Smaller cities tend to have poorer health facilities than larger ones, despite having similar proportions of older populations. The study found that good health facilities were concentrated in specific pockets, particularly in metropolitan cities, while smaller cities lacked them. This infrastructure gap poses a significant challenge in achieving healthy aging in India. Population aging is a significant demographic trend that requires attention from policymakers, researchers, and healthcare professionals. This study highlights the need for improved health facilities in Indian cities, particularly smaller cities, to ensure the well-being and a better quality of life of older adults. By prioritizing the needs of the aging population, we can create a more equitable and sustainable healthcare system that meets the needs of all citizens.

Abstract citation ID: igad104.3049

### COMPREHENSIVE MOBILITY FRAMEWORK VERIFICATION USING CANADIAN LONGITUDINAL STUDY ON AGING DATA

Sandra Webber, Yixiu Liu, Ruth Barclay, Depeng Jiang, Jacquie Ripat, Scott Nowicki, and Robert Tate, *University of Manitoba, Winnipeg, Manitoba, Canada*

Mobility within and between life spaces (one's room, home, local community, and further destinations) positively influences quality of life and health in older adults. Understanding factors that impact mobility is relevant in many contexts (e.g., in clinical and research environments, for city-planning and social programming). The purpose of this study was to verify a comprehensive theoretical framework for mobility (Webber et al., 2010) with structural equation modelling using data from the Canadian Longitudinal Study on Aging (CLSA). We estimated associations between latent factors consisting of physical, psychosocial, environmental, financial, and cognitive attributes, and life space mobility for participants 65-85 years of age (n=11,667, mean age 73 ± 6 years) with age, sex and education as covariates. The model demonstrated good fit (CFI = 0.90, RMSEA (90% CI) = 0.025 (0.024, 0.026)). Physical, psychosocial, and cognitive health were positively associated with life space mobility. Being less afraid to walk after dark (environmental variable) was also associated with greater mobility. Financial status influenced mobility through positive associations with psychosocial health and physical health. Higher education was related to better cognitive function and better financial status. Age was indirectly associated with life space mobility through its negative associations with financial status, cognition, and physical health. The same model can be used for males and females. The comprehensive mobility framework was verified with the large population-based CLSA dataset. Continued use of the framework is supported as it accurately portrays the complexity of factors that influence older adults' life space mobility.

Abstract citation ID: igad104.3050

### CONTACT-FREE SPORTS ACTIVITIES DURING COVID-19: IMPACT ON SUBJECTIVE HEALTH AND STAMINA AMONG KOREAN OLDER ADULTS

Gaeun Han, Juhyeong Lee, and Giyeon Kim, *Chung-Ang University, Seoul, Republic of Korea*

Despite the importance of participation in sports activities among older adults, COVID-19 pandemic has limited access to sports facilities, which may threaten the physical and mental health of older adults. This study examines whether the relationship between age and subjective health and stamina is moderated by participation in contact-free sports activities among Korean older adults. A nationally representative sample of Korean older adults aged 65 or older and doing regular exercise (N=1,118) was drawn from the 2021 National Survey on Sports for All. A moderation analysis was conducted by PROCESS macro using SPSS 26.0. Results showed that age was negatively associated with older adults' subjective health ($B = -.0314$, $p < .001$) and subjective stamina ($B = -.0259$, $p < .001$), respectively. Participation in contact-free sports activities moderated the relationship between age and subjective health ($B = .0266$, $p < .05$) and subjective stamina ($B= .0364$, $p < .01$), indicating that the effect of age on subjective health/stamina varies by participation in contact-free sports activities. However, the conditional effect of age on older adults' subjective health and stamina was significant only for those not participating in contact-free sports activities. The findings suggest that contact-free sports activities may mitigate the association between aging and physical decline in later life, especially during the pandemic and play a vital role in maintaining health and well-being of older adults.

Abstract citation ID: igad104.3051

### FRAILTY AND DEVELOPMENT ACROSS INDIAN STATES

Benjamin Seligman[1], Arunika Agarwal[2], and David Bloom[2], *1. UCLA School of Medicine, Los Angeles, California,*

*United States*, *2. Harvard TH Chan School of Public Health, Boston, Massachusetts, United States*

Introduction Frailty, a common syndrome of vulnerability among older adults, provides insight into the overall health of aging populations. Life-course influences play an important role in late-life health, but the role of social and economic development has not been well-explored. We study these indicators at the state level in the Longitudinal Aging Study in India (LASI). **Methods** Participants in LASI were adults aged 45 years or older from 2017-19 in 35 of 36 states and union territories. Frailty was assessed by a 48-item deficit accumulation frailty index (FI). Robust was defined as FI of 0 to 0.1, pre-frail was >0.1 – 0.2, mildly frail >0.2 – 0.3, moderately frail >0.3 – 0.4, and severely frail was >0.4. Development indicators were GDP per capita, infant mortality rate (IMR), and literacy rate in 1981 and 2018 (2011 for literacy), from the Reserve Bank of India. Associations were assessed using beta regression. Results IMR in 1981 had a small but significant association with frailty (OR 1.00 [1.00-1.00]). For GDP per capita and literacy, 1981 values were associated with greater frailty (1.15 [1.10-1.21] and 1.01 [1.01-1.01] respectively). However, 2018/2011 values were associated with lower frailty (0.94 [0.93-0.95] and 0.99 [0.99-1.00]). These associations were similar after stratifying by age group. Discussion Associations between frailty and state-level development varied both by indicator and by time of assessment. Sensitivity to the environment these indicators measure – such as IMR – may differ by age. Whether that sensitivity leads to protection or vulnerability may also change with age.

Abstract citation ID: igad104.3052

### HOW ELDERLY PEOPLE INTERACT AFTER THEIR RETIREMENT WITH THE COVID-19 ERA IN JAPAN

Fumihiko Yokota[1], Mariko Nishikitani[2], Gretchen Tucker[3], and Dana Bradley[3], *1. Kyushu University, Fukuoka, Fukuoka, Japan*, *2. Kyushu University Hospital, Fukuoka, Fukuoka, Japan*, *3. University of Maryland, Baltimore County, Baltimore, Maryland, United States*

Amid the COVID-19 pandemic, little is still known about how older Japanese people socialize after retirement. This paper aims to describe the dynamics of social interactions in post-retirement older adults during the COVID-19 pandemic. From January to March 2021, we interviewed 17 Japanese elderly people in Fukuoka and Ehime who were over 65 and have already retired. Face-to-face Interviews were conducted using a semi-structured questionnaire. After the initial transcripts were translated from Japanese to English, we checked the transcripts for accuracy and coded for analysis. The participant's mean age was 71 years old while their retirement's mean age was 60 years old. Only one participant had full retirement while the rest were partial retired which they continued to work as part-time workers either with new employers or with the same employers. All participants reported that their social interaction became estranged after retirement, and disrupted during the COVID-19 pandemic. However, participants communicated online and/or interacted face-to-face with (1) neighboring people as a member of community association or volunteers, (2) ex-work colleagues, (3) old schoolmate, (4) their children and/or grandchildren, (5) friends with the same hobbies. Following factors that may affect the interactive behavior of Japanese older people were identified; (1) Japan's healthcare policy and pension system, (2) individual's socio-economic status, (3) family structure and living arrangement, (4) community's characteristics, (5) individual's psychological, cognitive and health status, and (7) individual's personality. Comparisons of social interactions between Japanese and US older adults will be important to find new knowledge for preventing social isolation.

Abstract citation ID: igad104.3053

### LEVEL OF OBSTACLES BETWEEN WORK AND CARE PROVISION AND LIFE SATISFACTION AMONG JAPANESE WORKING FAMILY CAREGIVERS

Ayumi Honda[1], Yin Liu[2], Elizabeth Fauth[2], and Sumihisa Honda[3], *1. St Mary's College, Kurume, Fukuoka, Japan*, *2. Utah State University, Logan, Utah, United States*, *3. Nagasaki University, Nagasaki, Nagasaki, Japan*

**Objectives:** This study aimed to examine whether facing obstacles between work and care provision (work/care obstacles) is a risk factor for life satisfaction and explore whether it moderates the association between care burden and life satisfaction among Japanese working family caregivers. **Methods:** This cross-sectional study was conducted on 141 working family caregivers aged less than 65 years who were living with an older care recipient. We fit a multiple logistic regression model to examine the main and moderating effect of work/care obstacles on life satisfaction, in the context of care burdens. **Results:** Facing work/care obstacles had a main effect on life satisfaction among employed family caregivers. Furthermore, work/care obstacles exacerbated the association between caregiver burden and life satisfaction. As for the family caregivers who did not experience work/care obstacles, the risk of poor life satisfaction did not differ regardless of number of caregiver burdens. In contrary, the family caregivers who experienced work/care obstacles and had 2 or more caregiver burdens had a poor life satisfaction (odds rations = 5.5, 95% confidence intervals = [1.97, 15.43]) compared with those who had 1 or less caregiver burden. **Conclusion:** These findings suggest that obstacles between work and family was a risk factor for poor life satisfaction for employed individuals in Japan caring for an older relative. Mitigating such obstacles may be important for job retention, given the growing number of older adults in Japan. Key words Facing obstacles between work and care provision, family caregiver, life satisfaction, well-being, work–life adaptation

Abstract citation ID: igad104.3054

### SOCIOECONOMIC GRADIENTS IN DEPRESSIVE SYMPTOMS AMONG OLDER ADULTS IN RURAL SOUTH AFRICA

Mansi Verma[1], and Lindsay Kobayashi[2], *1. University of South Carolina Arnold School of Public Health, Columbia, South Carolina, United States*, *2. University of Michigan School of Public Health, Ann Arbor, Michigan, United Sta tes*

Depression is one of the most common mental health disorders throughout the globe with an estimated 300 million people affected worldwide. Socioeconomic status may influence depression risk, due to its status as a critical influencing factor for health-related issues as well as serving as a barrier for healthy aging. The objective of this analysis was to examine the presence of socioeconomic gradients in depressive symptoms among older adults in Agincourt, a low-income rural region of South Africa. We conducted a cross-sectional analysis of baseline interview data from 2014/2015 for 5,059 participants aged ≥40 in the population-representative Health and Aging in Africa: A Longitudinal Study of an INDEPTH Community in South Africa (HAALSI). Household wealth was assessed as quintiles of an asset-based index, including ownership of assets such as housing materials, electricity, piped water, vehicles, and technological goods. Household per capita consumption on food, healthcare, leisure activities, and other goods was also assessed as quintiles. The presence of depressive symptoms was measured and categorized using the 8-item Center for Epidemiologic Studies Depression (CES-D) scale. We used multivariable adjusted logistic regression to examine the association between household wealth and household consumption quintiles and depression. In this study population, the prevalence of depressive symptoms was 17%. We observed no significant trends between household wealth, household consumption and depression (p=0.11 for both exposures). Depressive symptoms are not socioeconomically graded among older, rural South Africa adults. The high prevalence of depressive symptoms within the region should be considered as a public health concern.

Abstract citation ID: igad104.3055

### THE LIFESTYLE OF MEN AND WOMEN AGED 50+ IN TAIWAN: ACTIVITY ON TIME OF DAY AND THEIR CIRCADIAN RHYTHMS

Ching-Ju Chiu, *National Cheng Kung University, Tainan City, Tainan, Taiwan (Republic of China)*

**Background:** To identify the association of circadian rhythms and objective measures of physical activity at a different time-zone of day, as well as examine gender differences. **Methods:** Participants aged 50 years and older living in the community in Taiwan were recruited. Those who suffer from dementia (cognitive dysfunction), mental illness, depression, or those taking drugs that affect daytime activities (e.g. sleeping pills, central nervous system drugs) were excluded. Activity was measured by wearable actigraphy device for at least seven days, diary, and self-reported questionnaires. **Results:** Among the 55 participants enrolled in the beginning, 34 (62%) meet the criteria that wore a wearable actigraphy device at least 7 completed days were analyzed. The activity in the morning was the highest among the participants. Although not statistically significant, as the age increases, the amount of activity decreases (H value=4.943, P=0.084); the amount of activity in the evening is higher for people with higher education(Z=-1.776, P=0.076); people with lower loneliness had higher activity levels (Z=-1.622, P=0.105); people with better nutritional status had higher activity (Z=-1.656, P=0.098). The amount of activity in women is positively and significantly related to regular exercise (Z=-2.653, P=0.007**), and nutrition(Z=-2.367, P=0.017*), while men are significantly related to retired status(Z=-2.132, P=0.033*). **Conclusions:** People who are older, retired, with chronic disease, more depression, and loneliness were associated with lower objective measure of physical activity. Determinant factors exist gender differences: while men are predicted by social variables (retired status), women are predicted by physiological variables (regular physical activity and nutritional status).

Abstract citation ID: igad104.3056

### THE ROLES OF COLATEC SOCIAL DANCING IN HEALTHY AGING AMONG KOREAN OLDER ADULTS

Milae Lee, Xingxing Wu, and Laura Payne, *University of Illinois Urbana-Champaign, Champaign, Illinois, United States*

Leisure activities can contribute to healthy aging (An et al., 2022). However, little research exists on leisure activities with soft stigma (Kraus, 2010). Yet, social dancing in Colatec (i.e., Korean daytime discos for older adults) is becoming more popular among older Koreans. Using qualitative methods, we examined how Colatec social dancing affects Korean older adults' healthy aging. Thirteen participants aged 60 to 75 who regularly social dance at Colatecs voluntarily participated in an in-depth semi-structured interview that lasted between 45 to 75 minutes. The audiotaped interviews were transcribed verbatim, coded, and analyzed following interpretive phenomenological analysis (Smith & Osborn, 2003). Trustworthiness was assessed with member checking and researcher triangulation. Results revealed that although it is still culturally stigmatized, Colatec social dancing was essential for participants' healthy aging. Participants perceived numerous benefits from social dancing that motivated their enduring engagement, despite the risks of the social stigma associated with Colatec dancing. Themes that emerged included: 1) Exercise from social dancing alleviated stress and depressive feelings associated with negative life events; 2) Colatec dancing boosted many participants' self-esteem, enhancing their overall wellbeing; and 3) Meaningful social connections were developed among participants. Findings align with existing literature on how moderate physical activity contributes to older adults' health (Bauman et al., 2016). Moreover, leisure serves as a social lubricant and enhances healthy aging (Mitas et al., 2011). Lastly, perceived by participants as a "playground", Colatec affords older Koreans a space for engaging leisure activity which facilitated their healthy aging process (Heo et al., 2018).

Abstract citation ID: igad104.3057

### TYPOLOGIES OF SOURCE OF CARE AMONG OLDER ADULTS: A COMPARATIVE STUDY OF CHINA AND THE UNITED STATES

Dexia Kong[1], and Peiyi Lu[2], *1. The Chinese University of Hong Kong, Hong Kong, Hong Kong, 2. Columbia University, New York City, New York, United States*

**Background:** We investigated typologies of source of care for older adults in China and the United States and applied Anderson Health Behavior model to examine associated significant determinants. **Methods:** Cross-sectional data from 2014 Health and Retirement Study and 2013 China Health and Retirement Longitudinal Study were used. We included older respondents aged 65+ who had at least one limitation in activities of daily living (ADL) and instrumental ADL (IADL) (NChina=2,482, NUS=3,152). Respondents reported whether they received assistance with ADLs and IADLs from spouse, child/grandchild, relatives, other, and formal helpers, respectively. We employed latent class analysis to characterize the typologies of sources of care and multinomial logistic regression to examine the significant determinants of identified typologies. **Results:** Four classes were identified in China: class 1 (59.91%) minimal care with IADL assistance from child/grandchild; class 2 (8.66%) child/grandchild-based care; class 3 (27.96%) spouse-based care; and class 4 (3.46%) spouse/child/grandchild-based care. Five classes were identified in the US: class 1 (51.97%) minimal care overall with limited spousal support; class 2 (20.88%) child/grandchild-based care; class 3 (8.44%) spouse-based care; class 4 (13.20%) formal care plus child/grandchild support; and class 5 (6.31%) various sources. In both countries, ADL and IADL were significant determinants. **Conclusion:** In the US, sources of care were more diverse and included formal assistance. In contrast, older Chinese relied largely on their spouses and children/grandchildren for support. In both countries, physical circumstances are significant determinants. Policy efforts on supporting family-based care and expanding formal care are needed in both countries, particularly China.

### SESSION 7950 (POSTER)

### FAMILY AND INTERGENERATIONAL RELATIONS: DIVERSE SOURCES AND SPACES

Abstract citation ID: igad104.3058

### A STUDY ON JOB STRESS AS A MEDIATOR BETWEEN DEMOGRAPHIC VARIABLES AND SELF-REPORTED HEALTH AMONG OLDER ADULTS.

Madhyami Deshmukh, and Peter Martin, *Iowa State University, Ames, Iowa, United States*

Job stress among older adults has not been sufficiently researched, and findings balance between work and personal life has become imperative. The aim of this research is to examine a path model that includes demographic variables (age, gender, and education), job stress, and self-reported health. Two recent waves (waves 13 and 14) of the Health and Retirement Study (HRS) were included in this analysis. A total of 8535 participants (3971 males and 4564 females) were part of this study. Bivariate correlations and multiple regression analyses were computed. The process macro in SPSS was used to assess mediation. Results indicated that job stress was associated with age, gender, and education such that women and more highly educated participants had higher job stress levels, whereas older adults had lower job stress levels. The multiple regression analysis showed that only age was significantly associated with stress, whereas gender and education were not. Furthermore, education was positively associated with better health, but health did not correlate with age or gender. As expected, job stress was associated with self-reported health at a later time point, such that more job stress was related to lower self-reported health levels. Finally, job stress mediated between demographic variables and health at a later time point. As the data were collected before the pandemic, it would be important to see if these results would have changed over the course of the pandemic. Future interventions should develop appropriate work-life balance to promote better health in later life.

Abstract citation ID: igad104.3059

### AGING AND SEXUAL RELATIONSHIPS: RECIPROCAL RELATIONS BETWEEN PHYSICAL PLEASURE AND EMOTIONAL SATISFACTION

Getrude Nyang'au, Pei-Shu Chao, and Duane Rudy, *University of Missouri - Columbia, Columbia, Missouri, United States*

Previous research has found significant associations between physical pleasure and emotional satisfaction in sexual relationships. However, much of this research is cross-sectional and has focused on young and middle-aged adults. The present study examined data from the National Social Life, Health, and Aging Project (NSHAP). Physical pleasure and emotional satisfaction were assessed at three points in time (2005/2006; 2010/2011; and 2015/2016). Only data from individuals who were alive at all time points were used in the analysis. Three cohorts were examined: at time 1, the first cohort ranged in age from 57-64, the second cohort from 65-74, and the third cohort from 75-85. We examined reciprocal relations between physical pleasure and emotional satisfaction across time using a Random Intercepts Cross-Lagged Panel Model. The results revealed a positive reciprocal relationship between emotional satisfaction and physical pleasure among the first cohort, with emotional satisfaction at time 1 positively associated with physical pleasure at time 2, which in turn was positively associated with emotional satisfaction at time 3. For the second cohort, physical pleasure at time 2 predicted emotional satisfaction at time 3. Relationships among the two variables were not significant for the third cohort. The implication of this study would be to inform older adults in ongoing sexual relationships on the need to invest in activities that enhance physical pleasure and emotional satisfaction. Future research could examine what variables might account for reciprocal relationships being found for the first as opposed to the third cohort.

Abstract citation ID: igad104.3060

### AGING, MIGRATION, AND GRANDPARENTING IN POST–ONE CHILD POLICY CHINA

Nan Zhang, *University of Manchester, Manchester, England, United Kingdom*

China's rapid rural-urban migration and population ageing are posing significant challenges to traditional patterns of intergenerational familial support. To tackle population ageing, China's one-child policy has been replaced by a two-child policy, subsequently a three-child policy. With limited institutional childcare facilities in place, ageing grandparents from rural areas often move to the cities where their adult migrant children settle to provide care for grandchildren. This group of older, rural people are largely marginalised and are invisible on the current research and policy agenda. This ongoing study, conducted in the Yangtze River delta urban area — one of the most developed, crowded, and the largest migrant-receiving urban regions in China — aims to uncover their experiences during the process of migration and adaptation, and establish the way in which this arrangement of grandparenting has impacted their everyday lives and well-being. Three compelling themes emerged at this stage: 1) intensified caring responsibilities imposed on older women largely owing to patriarchy; 2) intersection of old age, being women and exclusion of social welfare due to having a rural origin contribute to poor well-being; 3) the feeling of a lack of balance in terms of family exchange over the life course is not uncommon. It is possible that urbanisation will reinforce vulnerability and marginalisation of and exacerbate social exclusion of older women from rural origin. This project will form an important evidence base to tackle issues such as social exclusion of disadvantaged communities in China against the backdrop of rapid population ageing and urbanisation.

Abstract citation ID: igad104.3061

### CONNECTING OLDER ADULTS AND DOCTOR OF PHYSICAL THERAPY STUDENTS THROUGH A COMMUNITY-BASED EXERCISE PROGRAM

Gabriele Moriello, Alice Hamilton, Julie Bush, Michelle Nunno-Evans, Shannon Schoellig, and Nancy Hollins, *Utica University, Utica, New York, United States*

The purpose of this qualitative study was to explore the perspectives of older adults regarding their experience participating in an intergenerational Integrated Clinical Experience (ICE) with Doctor of Physical Therapy (DPT) students. This ICE was designed as a community-based exercise program for older adults. DPT students designed individualized one-hour fitness plans for participants every week over the course of a semester. To obtain the perspectives of older adults, a descriptive phenomenological approach was chosen. A purposive sample of 16 older adults involved in the wellness program participated in 6 focus groups and one interview. Results of the thematic analysis revealed six themes; Blending of the Generations, It Motivated Me, I Can Do It Now, Mental and Social Boost, Program Tailored to Me, and Learn and Modify Habits. Participant interviews highlighted how older adults highly valued the relationships with students, and how the intergenerational environment of the program benefited their physical, social, and mental well-being. They also noted how students served as a source of motivation, leading to behavioral change and improvements in functional outcomes. These findings support existing literature on intergenerational programs, but this is one of the few studies that looked specifically at an intergenerational ICE. This ICE provided an innovative manner to meet the physical challenges faced by many older adults, and the intergenerational nature of the experience may have contributed to the well-being of all those involved. Future research using quantitative methods are needed to extend what was learned beyond the specific context of this study.

Abstract citation ID: igad104.3062

### DETERMINANTS OF POSITIVE MARITAL QUALITY AMONG OLDER HISPANIC ADULTS IN THE US

Jaminette Nazario[1], Takashi Yamashita[1], Jennifer Bulanda[2], and J. Scott Brown[2], *1. University of Maryland, Baltimore County, Baltimore, Maryland, United States*, *2. Miami University, Oxford, Ohio, United States*

Marital Quality can significantly influence a variety of health and well-being outcomes in later life. Yet, relatively little is known about how marital quality is determined across subpopulations given their cultural backgrounds. Social networks and familial relationships are culturally important to Hispanic adults over their life course. Also, the unique characteristics (e.g., high % of immigrants) of older Hispanic adults in the U.S. may make their determinants of marital quality different from others. Considering approximately one in three older Hispanic adults is an immigrant in the U.S., this subpopulation may have relatively small social networks as their family members live in their countries of origin. As such, sub-groups of married older Hispanic adults may depend more on their marital relationships for social and cultural support, compared to other racial and ethnic groups. Framed by socioemotional selectivity theory, this study aimed to investigate determinants of marital quality among older Hispanic adults (age 51 and older). Nationally representative samples of older Hispanic adults ($n = 694$) were derived from the 2010 Health and Retirement Study (HRS). Results from linear regression analysis indicated that women ($b = -0.16$, $p < 0.05$) reported lower positive marital quality than men. There was also a statistically significant negative association between depressive symptoms ($b = -0.09$, $p < 0.05$) and positive marital quality. This study further evaluated other marital quality determinants, gender differences, and possible sociocultural explanations.

Abstract citation ID: igad104.3063

### FAMILY GUIDANCE WEAKENS AN EFFECT OF OLD PEOPLE'S EDUCATIONAL ATTAINMENT ON EHEALTH LITERACY BY INTERNET EXPERIENCE

Jingxuan Wu, Huamao Peng, Zhiyu Fan, and Heng Zhao, *Beijing Normal University, Beijing, Beijing, China (People's Republic)*

**Background:** In the emerging era of digitalization, eHealth literacy is of great importance in older adults' health and social adaptation. Objective: Explore the effect of educational attainment on eHealth literacy among older adults and examine the mediating role of Internet experience and the moderating role of guidance on the Internet from family members to reveal the underlying mechanism. Method: Four hundred and ninety-one older adults (aged 59–76 years old, $M_{age} = 65.69 \pm 4.41$) completed the eHealth Literacy Scale (eHEALS) and Internet Experience among Older Adults Scale (IEOAS) and reported their educational attainment and the frequency of family members guiding them on how to use the Internet. **Results:** eHealth literacy of older adults with lower educational attainment did not reach an intermediate level ($M = 2.46 \pm 1.17$). It was significantly lower than that of older adults with higher educational attainment whose scores were beyond the intermediate level ($M = 3.43 \pm 0.90$). Internet experience mediated the effect of older adults' educational attainment on eHealth literacy. This mediation model was moderated by family members' guidance on the Internet. **Discussion:** our results have implications for understanding how educational attainment reinforced the inequality in eHealth literacy among older adults and how to address this gap. Specifically, If older adults with lower educational attainment are given guidance on Internet use by their family members frequently, they will no longer be at a disadvantage in accumulating Internet experience and further cultivate eHealth literacy to search for, distinguish, and take advantage of health information on the Internet well.

Abstract citation ID: igad104.3064

### INTERGENERATIONAL INTERACTIONS AND RELATIONSHIP QUALITY: A DAILY STUDY AMONG MOTHERS AND THEIR ADULT CHILDREN

Da Jiang[1], John Chi Kin Lee[1], and Dexia Kong[2], *1. Education University of Hong Kong, Hong Kong, Hong Kong*, *2. The Chinese University of Hong Kong, Hong Kong, Hong Kong*

The parent–child relationship is one of the longest-lasting and closest relationships in human life. Because of its long-lasting nature, more studies focus on the long-term exchanges between parents and children. Few studies have examined the effects of daily interactions on relationship quality. However, such an understanding is crucial during public health emergencies, when daily support amongst close family members is critical for life quality and even survival. Using data collected during the COVID-19 pandemic, we examined the relationship between daily support and relationship quality among middle-aged and older mothers and their adult children. Seventy-seven dyads of mothers (age range: 44–80 years, Mage = 53.78, SDage = 9.57) and adult children (age range: 18–54 years, Mage = 26.61, SDage = 9.46) reported their daily exchanges with their child/parent (i.e., the support they had received from and provided to the other) and daily relationship quality (i.e., relationship satisfaction and trust) each day for 14 consecutive days. Receiving support was positively associated with relationship satisfaction and trust on both Day N and Day N+1 in mothers, and it was positively associated with relationship satisfaction on Day N and trust on both Day N and Day N+1 in children. Offering support was positively associated with relationship satisfaction on Day N (but not Day N+1) for both mothers and children. Offering support was not associated with daily trust in either mothers or children. The findings highlight the importance of daily support on relationship quality during a public health emergency.

Abstract citation ID: igad104.3065

### INTERGENERATIONAL SOLIDARITY AND FILIAL PIETY PERCEIVED BY OLDER ADULTS IN RURAL CHINA

Wencheng Zhang, Merril Silverstein, and Ying Xu, *Syracuse University, Syracuse, New York, United States*

Filial piety—the obligation of adult children to serve and honor older parents—is a fundamental value in Chinese society. This study explores how dimensions of intergenerational solidarity influence parents' evaluations of their children's filial piety. Data derive from 1473 respondents reporting about 4000 relationships in the 2021 wave of the Longitudinal Study of Older Adults in Anhui Province China. Strength of filial piety was assessed for each adult child along with other aspects of the relationship. Random effects ordinal logistic regression predicted parents' perceptions that their children fulfilled expectations of filial piety based on geographic proximity, financial support, and emotional support. Findings indicate that parents consider children who live with them as most filial, and they view residentially independent children who live farther away as less filial. However, financial support from more distant children raises perceptions that these children are meeting expectations of filial piety. In addition, having more frequent contact with distant children strengthens perceptions of them as being filial. Results suggest that weak filial piety perceived by older parents of their more geographically distant children in rural China—an area characterized by high rates of internal migration—is compensated by financial transfers and frequent communication. As research shows that filial piety perceptions are important to the well-being of older adults in Chinese populations, threats to its decline due to forces of modernization and any adverse consequences may be overcome by forms of intergenerational solidarity that involve contributions of money and time by children to their older parents.

Abstract citation ID: igad104.3066

### NONPARENTAL CAREGIVER DIFFERENCES IN LOCUS OF CONTROL AND GPA OF GRANDCHILDREN AND FOSTER CHILDREN

Maia McLin, and Danielle Nadorff, *Mississippi State University, Mississippi State, Mississippi, United States*

There are approximately 5,950,690 children living with their grandparents, with about half being raised without a parent present. Children raised by non-parental caregivers are at risk of worse academic performance, and locus of control (LOC) is shown to relate to children's academic performance. However, differences between caregiver types and this relation are not well known. The current study hypothesized that caregiver type would affect the strength of the association between LOC and academic performance in children raised by grandparents versus foster parents. Participants (323 caregiving grandparents (GP), m age = 55.66 yr; 105 foster parents (FP), m age = 34.45 yr) were recruited nationwide and surveyed via Qualtrics Panel Service. Measures included an adapted academic locus of control scale from the Child Trend's Student Survey, and a question rating the degree to which the child receives "mostly As and Bs." Controlling for age and socioeconomic status (SES) of caregiver, grandchildren had higher reported LOC, but did not differ in academic performance. Caregiver type was found to differentially affect the relation between LOC and academic performance, with a stronger association for foster children (GP $r = .726$; $p < .001$; FP $r = .831$; $p < .001$). A significant group difference was found ($z = 2.06$; $p < .05$). These results suggest that kincare may buffer the impact of LOC on academic performance in children not raised by their parents. For those raised in foster care, it may be helpful to focus on increasing autonomy and sense of control regarding their grades.

Abstract citation ID: igad104.3067

### OLDER PARENTS' PATTERNS OF AGREEMENT AND INFORMATION SHARING WITH ADULT CHILDREN AND THEIR EMOTIONAL OUTCOMES

Noriko Toyokawa, *Southern Oregon University, Ashland, Oregon, United States*

The current study examined the effect of older parents' pattern of agreement/disagreement with their adult children's perceptions of their functional ability and their information disclosure/non-disclosure to their adult children on their ambivalent, positive, and negative emotions. Older parents, N=263, Mage=75.06, SD=5.49, participated in an online survey. Participants' agreement with adult children's ideas and caregiving practice to support 10 life issues were assessed by five categories: 1=agree, 2=disagree, 3=it depends, 4=it is not my issue, and recorded into 0=agree, 1=fully/partially disagree/not my issue. Participants' information sharing on the same items was assessed by 1=openly share, 2=leave out critical points, 3=avoid talking about, 4=lie or making a story, 5=It is not my issue, and recoded to share 1=openly and 0=not openly share. The median dichotomized the mean scores of 10 items to create four patterns: disagree-do not disclose, disagree-disclose, agree-do not disclose, and agree-disclose. Positive, negative, and ambivalent emotions toward adult children have been assessed by the scales and formula used by Lendon et al. (2013). A series of one-way ANOVA, $F(3, 260)=4.41$, $p=.005$, $\eta=.05$, revealed that participants in agree-do not disclose the pattern, M=3.15, SD=.90, reported the highest level of ambivalence. A post hoc test showed it was significantly higher than that of participants in disagree-did not share, M=2.16, SD=1.10. Disagree-do not disclose group reported the lowest score in positive and negative emotions among groups. Frequency of contact predicted disclosure in auxiliary analysis. The need for within and between differences in older parents' agreement and disclosure patterns was discussed.

Abstract citation ID: igad104.3068

### PREDICTORS OF FAMILY FUNCTIONING IN CUSTODIAL GRANDFAMILIES: A SYSTEMIC PERSPECTIVE

Khushbu Patel, Deneisha Scott-Poe, and Megan Dolbin-MacNab, *Virginia Tech, Blacksburg, Virginia, United States*

According to the U.S. Census, there are approximately 2.5 million grandparents raising grandchildren. Previous research has examined factors associated with grandparent and grandchild well-being; yet, few studies have considered the functioning of the grandfamily unit. Guided by family systems and attachment theories, the purpose of this study was to examine individual and relational predictors of grandfamily functioning, as reported by 95 custodial grandmothers raising adolescent (ages 12 to 18) grandchildren. Survey data were collected in-person or over the telephone from custodial grandmothers residing in the Southern U.S. To analyze the data, we conducted a hierarchical multiple regression. The first model included grandchild gender, the second model added grandmothers' parenting stress and attachment, and the final model further added grandmothers' reports of grandchildren's attachment, emotion regulation, and internalizing and externalizing behavior problems. The first model was not significant, but the second model was significant [$F(3, 91) = 8.85$, $p < .001$; $R2\ \Delta = .22$, $p < .001$], and explained 23% of the variance in grandfamily functioning, with less grandmother parenting stress predicting better grandfamily functioning. More secure grandmother attachment approached significance. The final model accounted for 34% of the variance in grandfamily functioning [$F(7, 87) = 6.35$, $p < .001$; $R2\ \Delta = .11$, $p = .008$], with better grandfamily functioning being predicted by less grandmother parenting stress and better grandchild emotion regulation. More secure grandmother attachment and greater grandchild internalizing problems emerged as trends. Implications for future research and systemic practice with grandfamilies will be discussed.

Abstract citation ID: igad104.3069

### SOCIAL INTELLIGENCE TRAINING AND CUSTODIAL GRANDFAMILIES: A QUALITATIVE ANALYSIS OF RELATIONSHIPS

Megan Dolbin-MacNab[1], Gregory Smith[2], Frank Infurna[3], Britney Webster[4], Saul Castro[3], D. Max Crowley[5], and Carol Musil[6], *1. Virignia Tech, Blacksburg, Virginia, United States*, *2. Kent State University, Kent, Ohio, United States*, *3. Arizona State University, Tempe, Arizona, United States*, *4. Diebold Nixdorf, Louisville, Ohio, United States*, *5. Pennsylvania State University, State College, Pennsylvania, United States*, *6. Case Western Reserve University, Cleveland, Ohio, United States*

Custodial grandfamilies often experience relational challenges related to parenting and the grandparent-grandchild relationship that would benefit from intervention. Social intelligence training (SIT), which focuses on improving socio-emotional skills via online, self-guided modules, holds promise for helping grandfamilies address their relational challenges. The purpose of this qualitative study was to examine SIT skills that custodial grandmothers and grandchildren utilize in their interactions and the impact of SIT on their relationship. The sample consisted of 27 dyads of custodial grandmothers and adolescent (ages 11 to 18) grandchildren enrolled in an RCT examining the effectiveness of SIT. Dyads who completed the SIT were randomly selected and completed individual, open-ended interviews over the telephone, after post-test but prior to the 3-month follow-up. Results of a thematic analysis revealed several themes related to the SIT skills grandmothers and grandchildren were utilizing including taking and understanding the other's perspective, attending to the nonverbal communication, engaging in active listening, reflecting on their treatment of each other, and slowing down communication to think before speaking. Analyses further revealed that grandmothers and grandchildren noted SIT-related improvements in their relationship, with themes reflecting improvements in the quality of communication, the frequency and intensity of conflict, understanding of each other, and sense of emotional connectedness. Those dyads that did not report relational improvements had a strong relationship prior to completing the SIT or the grandchild did not fully engage in the SIT. Directions for research and family-based interventions with grandfamilies will be discussed. [Funded by the National Institute on Aging (R01AG054571)]

Abstract citation ID: igad104.3070

### THE 12 DS: AN UPDATE TO EDWARDS' AND RAY'S REASONS FOR NONPARENTAL CAREGIVING

Acacia Lopez, and Danielle Nadorff, *Mississippi State University, Mississippi State, Mississippi, United States*

This study examined the prevalence of the "Nine D's," a framework developed by Edwards and Ray for understanding the heterogeneity of reasons for which grandparents assume care of grandchildren (i.e., Death, Disease, Detention, Divorce, Departure, Drugs, Desertion, Delivery, Deployment) within a contemporary sample. Using a nationwide sample (N = 427) of custodial grandparents and foster parents, caregivers were asked their reason for assuming care of the grandchild or foster child within their care. The results of the study suggest that the Nine D's are a useful framework, but fail to capture many of the reasons for assuming care. Three new themes—Dollars, Duty, and Daily Grind—were identified and are applicable to both grandfamilies and foster families. These themes represent different motivations for assuming care and provide insight into the social structures which may act as barriers to family formation. This study provides a foundation for future research examining the impact of assumed care by non-parental attachment figures on the health and well-being of both grandchildren and foster children.

Abstract citation ID: igad104.3071

### THE EFFECTS OF GRANDPARENTAL CARE ON MENTAL HEALTH AMONG CHINESE GRANDPARENTS: A LIFE COURSE PERSPECTIVE

Jiahui Lyu, *University of Florida, Gainesville, Florida, United States*

As the population continues to age, grandparental care has become a more prevalent form of childcare, particularly in China. Limited research has investigated the effects of grandparental care on grandparents' mental health and the conclusions are ambiguous with both positive and negative outcomes being found. In this study, a life course perspective is adopted to examine the mechanisms of such effects. Using data from the China Health and Retirement Longitudinal Study (2011–2013), I conducted logistic regression models to examine the association between various stages of grandparental care and depressive symptoms among Chinese grandparents between the ages of 45 and 80 (N = 3946). Specifically, this study examines the impact of transitions into or out of grandparental care on depression and the potential moderating role of gender. Results show that grandparents who stop providing care for grandchildren are less likely to experience depression compared to those who provide no grandparental care (OR = 0.78). Additionally, grandmothers who provide ongoing grandparenting tend to have a higher risk of experiencing depression. The findings presented in this study offer valuable insights into the various mental health implications of grandparental care in the Chinese context, underscoring the significance of accounting for gender differences. This study also highlights the need for evidence-based policies and interventions that promote the well-being of older adults.

Abstract citation ID: igad104.3072

### THE GEOGRAPHY OF FAMILY CAREGIVING IN AN AGING SOCIETY: WHO MOVES CLOSER AND WHY

Stipica Mudrazija[1], Elizabeth Peters[2], and Fernando Hernandez Lepe[2], *1. University of Washington, Seattle, Washington, United States*, *2. Urban Institute, Washington, District of Columbia, United States*

An emerging literature examines the dynamic aspect of the link between parental health and parents-adult children geographic distance, yet who moves when a parent needs care—the parent or the child—and why remains unexplored. Using pooled 2004-2014 data from the Health and Retirement Study on respondents age 65 years or older and their adult children and detailed geographic information, we analyze who moves when parents and children move in together or move closer without coresiding, and who benefits from such moves. Preliminary results reveal that children are roughly four times more likely to move in with parents than parents move in with children, and moving primarily benefits those who move: 89.2 percent of parents who moved reported that either they were the sole beneficiary of the move or that both they and their children benefited, and the same was true of 92.8 percent of children movers. However, as parents' health declines, they increasingly benefit from the proximity-enhancing moves regardless of who moves. For example, about half of all children who move in with parents following the onset of parents' health decline reports doing was to benefit their parents, either exclusively of in conjunction with them. Next, we will fit regression models of who moves for newly coresident dyads and dyads moving closer, respectively, as well as models of who benefited from such moves. This research provides new policy-relevant insights because the caregiver supports needed could be different depending on whether it was the parent or the child that moved.

### SESSION 7960 (POSTER)

### EXAMINING INEQUITIES AND INEQUALITIES IN AGING

Abstract citation ID: igad104.3073

### A PHOTOVOICE STUDY OF THE EXPERIENCES OF BIPOC OLDER ADULTS DURING THE COVID-19 PANDEMIC

Angela Ekwonye, *St. Catherine University, St. Paul, Minnesota, United States*

The coronavirus disease 2019 (COVID-19) pandemic intensified the stressful and already difficult circumstances of communities of color. Yet, no current photovoice research has highlighted the lived experiences of low-income Black, Indigenous, and persons of color (BIPOC) older adults during the pandemic. This qualitative study used photovoice to visually portray the struggles of low-income BIPOC older adults and how they recovered from and adapted to the impact of the pandemic. The data analyzed were drawn from interviews, focus groups, photographs, and written stories exploring their experiences and how they found meaning. Results show that participants faced different challenges during the pandemic, such as the fear of COVID-19 exposure and death, struggles to adopt COVID-19 mitigation strategies, loneliness, and social isolation. Amid this crisis of suffering, isolation, and sadness, participants found meaning through a positive reappraisal of the negative situation and engaging in emotional, spiritual, social, and physical self-care practices. The findings have implications for clinical social workers, mental health counselors, faith communities, nurse managers and administrators, and policymakers.

Abstract citation ID: igad104.3074

### ARE INEQUALITIES IN HEART DISEASE CHARACTERIZED BY INTERGENERATIONAL EDUCATIONAL ATTAINMENT?

Blakelee Kemp[1], Carli Rush[1], and Patricia Morton[2], *1. University of Nebraska, Lincoln, Nebraska, United States*, *2. Wayne State, Detroit, Michigan, United States*

Intergenerational patterns in educational attainment are associated with later-life heart health. Yet, whether the association between intergenerational education patterns and adult heart disease varies by cohort and gender is unclear despite the increasing importance of education for health, especially among women. Informed by a life-course perspective, this study aims to further contextualize the link between education and heart disease in time and across groups. Using data from the Health and Retirement Study, a nationally representative panel survey of adults over the age of 50, we examine how patterns in intergenerational education are associated with heart disease prevalence (n=31,141) and incidence from 1998 to 2018 (person-year observations=253,521) and whether these patterns vary by gender and cohort. Overall, individuals characterized by stable-high education (i.e., parent and respondent have at least some college education) consistently had the lowest prevalence and incidence of heart conditions, whereas those with low adult education, regardless of parent's education, had the highest risk of heart conditions. Findings also indicate that the effects of intergenerational education patterns on adult heart disease were most pronounced for women and the younger cohorts compared to men and older cohorts, respectively. For example, women and the youngest cohort (born 1954-1967) with upward and stably high intergenerational education had the most favorable heart disease outcomes, whereas the same groups with downward intergenerational education experienced the highest heart disease prevalence. Our findings demonstrate the importance of contextualizing how intergenerational educational attainment influences later-life heart conditions by considering variations by gender and cohort.

Abstract citation ID: igad104.3075

### ARE RURAL OLDER ADULTS READY TO ADOPT AUTONOMOUS VEHICLES (AV)? PREDICTORS OF ATTITUDES TOWARD AV IN RURAL AREAS

Mojgan Zoaktafi, Tyler Gandy, and Jong Sung Yoon, *University of South Dakota, Vermillion, South Dakota, United States*

Transportation for older adults residing in rural areas is particularly vital for maintaining their everyday activities including access to health services and community activities. Given unique features of rural areas (e.g., limited public transportation, greater distances to travel but less traffic), autonomous vehicles (AV) can be an innovative solution to rural older adults. Nevertheless, attitudes toward AV can be a significant barrier for older adults to adopt it. In fact, older adults in rural areas tend to hold different attitudes toward and experiences with new technologies. The current study explored how individual difference variables (i.e., age group, gender, technology proficiency, & personality traits) predict attitudes toward AV in rural areas. We first conducted a factor analysis on a 16-item attitude survey (collected from rural areas in USA) which uncovered three factors: concern with AV, willingness to adopt AV, confidence on human driving over AV. ANOVAs on the attitudinal factors showed that older adults (60+ yrs, n = 45) had a lower willingness to adopt AV than young adults (17-21 yrs, n = 47) despite a lower confidence on human driving over AV. There was no age group difference in concern with AV. Multiple regressions supported that technology proficiency can be another significant predictor of willingness to adopt AV over and above other individual difference factors (i.e., age groups, gender, & personality). These results indicate that older adults in rural areas have complex and ambivalent attitudes toward AV, which suggest different approaches to the dissemination of AV technology for them.

Abstract citation ID: igad104.3076

### COGNITIVE AND FUNCTIONAL IMPACTS OF CHILD LABOR IN INDIA: THE MEDIATING ROLE OF EDUCATION

Seolah Lee[1], Kriti Vikram[2], and Hyo Jung Lee[1], *1. Yonsei University, Seoul, Republic of Korea*, *2. National University of Singapore, Singapore, Singapore*

This study aims to examine how entry into the labor market in childhood is associated with deficits in late-life cognitive and functional health. Using the Cumulative Advantage/Disadvantage Theory, it further explores how educational attainment, an indicator of socio-economic status, mediates the associations between child labor and cognitive and functional health among older adults in India. Lastly, it investigates if the results vary by gender. We use the nationally representative World Health Organization Study on global AGEing and Health (SAGE), and select respondents aged 50 years or older (n = 6,215 for Mini-Mental State Examination (MMSE); n=6,408 for the Functional Limitation (FL)). Mediation models were performed for each dependent variable using the sgmediation2 program, bootstrapped 5,000 resamples, and percentile bootstrap confidence interval. The indirect and total effects of child labor on MMSE were significant, while the direct effect of child labor was not. For FL, the indirect effect was significant, but the direct and total effects were not. Gender difference was found in terms of the direct effect, child work was directly associated with a lower MMSE score in older women ($p < .01$), whereas it was not significant for older men. Findings suggest that early entry into the labor market (as a child) has a negative impact on older adults' MMSE and FL through deprivation of educational opportunities. Providing opportunities for completing certain level of education can mitigate the negative impact of child labor on health in old age.

Abstract citation ID: igad104.3077

### DECLINES IN CONTROL BELIEFS OVER TIME: THE ROLE OF PERCEIVED DISCRIMINATION AND INEQUALITY

Stefan Agrigoroaei[1], and Denisa Cristina-Alina Berceanu[2], *1. Universite Catholique de Louvain, Louvain-la-Neuve, Brabant Wallon, Belgium*, *2. University of Bucharest, Bucharest, Bucuresti, Romania*

Higher control beliefs represent a key protective factor for healthy aging, broadly defined. If the benefits of control beliefs are well documented, fewer studies have focused on the antecedents of changes in control. There is empirical evidence that social stress, such as being treated unfairly or discriminated against, may erode personal control. The goal of this study was to examine perceived discrimination and inequality in relation to long-term change in control beliefs, in a national sample, using data from the Midlife in the United States (MIDUS) longitudinal study (Waves II and III, N = 2540, MIDUS II age range: 30-84, Mean=55.53, SD=11.21). Perceived discrimination was quantified as the sum of 9 items capturing experiences of daily discrimination. Perceived inequality was measured in three settings: work, family (relationship with children), and home. Both mastery and perceived constraints were used as indicators of control beliefs. The results revealed a significant decline in control between the two waves. Controlling for age, gender, education, self-rated physical health, and baseline control beliefs, higher daily discrimination and perceived inequality were significantly associated with lower control beliefs, 8 to 10 years later. There were not significant interactions with age and the patterns of results persisted when other relevant factors, such as cognitive performance and physical activity, were considered. The findings suggest that interventional programs should target (sources of) perceived discrimination and inequality and that changes in control beliefs could be considered a potential mechanism of the association between social stress and trajectories of healthy aging.

Abstract citation ID: igad104.3078

### HOUSEHOLD ENVIRONMENTS AND COGNITIVE DECLINE AMONG MIDDLE-AGED AND OLDER ADULTS IN CHINA

Ye Luo[1], Dandan Zhao[1], Xi Pan[2], and Lingling Zhang[3], *1. Clemson University, Clemson, South Carolina, United States*, *2. Texas State University, San Marcos, Texas, United States*, *3. University of Massachusetts Boston, Boston, Massachusetts, United States*

Household conditions play a crucial role in the well-being of older adults, who usually spend much longer time in their homes than younger adults. Few studies have examined the compound effects of multiple household conditions on trajectory of cognition function among older adults. This study examines the relationship between household environments and trajectories of cognitive function among middle-aged and older adults in China. It also examines urban/rural, gender, and age variations in this relationship. Using a representative sample of 16,111 respondents aged 45 years and over from four waves of the China Health and Retirement Longitudinal Study (2011-2018), we estimated multi-level linear growth curve models of household social, economic, and physical conditions on cognitive decline over seven years. The results show that older people who lived with spouse but not with children and those with higher living expenditures, better housing quality, and indoor clean fuels for cooking had slower cognitive decline. Living arrangement more strongly predicted men's cognitive decline while living expenditure, solid fuel use and housing quality significantly predicted only women's cognitive decline. Only for adults aged 60 year and over, living alone or living with spouse and adult children significantly predicted a faster cognitive decline. Only for rural residents, living alone or living with young children and no adult children significantly predicted a faster cognitive decline. These findings underscore the importance of social policies and programs targeting at improving the living conditions of older adults to help mitigate their cognitive decline.

Abstract citation ID: igad104.3079

### INEQUALITY IN AWARENESS, TREATMENT, AND CONTROL OF VISUAL IMPAIRMENTS AMONG OLDER ADULTS AND ELDERLY IN INDIA

Rajeev Singh, *International Institute for Population Sciences, Mumbai, Mumbai, Maharashtra, India*

Globally, 1.1 billion people have some form of visual impairment, and 90 percent of them live in low and middle-income countries. The prevalence of visual impairment is increasing, and the likelihood of increasing this trend is also higher. Yet, there is little evidence on the extent of awareness, treatment, and control (ATC) of visual impairment in India. We estimate the prevalence and ATC of visual impairment among older adults and the elderly across socioeconomic groups. We used the unit data from the first round of the Longitudinal ageing study of India (LASI), 2017-18, bivariate analysis, estimated age sex-adjusted prevalence, logistic regression, concentration index, and concentration curve used in the analyses. Among the 45+ population, 30.18% have any form of visual impairment, around 47.96% have awareness about the visual impairment, 17.21% have taken treatment for vision-related issues, and 12.70% have control vision after treatment. The prevalence was higher among the illiterate poor and females; however, the awareness, treatment, and control were higher among those with higher education who belongs to the richer richest wealth quintile. The concentration index and concentration curve suggest that higher prevalence was concentrated among the poor, and contrary to that, awareness treatment and control were concentrated among richer. To deal with it, public health services from the primary, secondary, and tertiary levels may be integrated for effective care of growing, underdiagnosed, and untreated visual impairments among older adults and the elderly in India.

Abstract citation ID: igad104.3080

### LONGITUDINAL ASSOCIATION OF COGNITIVE IMPAIRMENTS WITH LATE-LIFE WEALTH CHANGES AMONG OLDER AMERICANS

Darlingtina Esiaka[1], Ashly Westrick[2], Ronica Rooks[3], Helen Meier[4], Mark Manning[5], and Wassim Tarraf[6], *1. University of Kentucky, Lexington, Kentucky, United States*, *2. University of Michigan School of Public Health, Ann Arbor, Michigan, United States*, *3. University of Colorado Denver, Denver, Colorado, United States*, *4. University of Michigan, Ann Arbor, Michigan, United States*, *5. Oakland University, Rochester, Michigan, United States*, *6. Wayne State University, Detroit, Michigan, United States*

Disparities in wealth in the US are linked to disadvantageous health outcomes over the lifecourse. Cognitive impairment can widen wealth disparities. We examine how cognitive impairment influences change in wealth starting in mid-life and into older adulthood and the role of social determinants of health in wealth loss due to cognitive impairment. We use biennial longitudinal data (Health and Retirement Study: 2000-2018) on Non-Hispanic White (NH-W), NH-Black (NH-B), and Hispanic (H) middle-aged adults (51-64 years and cognitively healthy in 2000; unweighted n=3,651) to model wealth change (measured as the relative (%) difference in reported wealth between 2000-2018 [in 2018 dollars]) as a function of cognitive status (using three latent phenotypes derived from longitudinal latent class analyses: cognitively healthy [CH; 77%], increasing cognitive impairment [ICI; 19%], and increasing dementia [ID; 4%]). We fit racial/ethnic stratified regression models to examine relationships between cognitive phenotypes and wealth change, adjusting for covariates. Using Blinder-Oaxaca decomposition techniques, we examined the contributions of predisposing (e.g. race/ethnicity), health enabling (e.g. insurance source), and health needs (i.e. chronic conditions) factors in wealth change by cognitive phenotypes. Results show NH-Ws had higher levels of reported wealth in both 2000 and 2018, compared to NH-Bs and Hs. However, the relative rate of change in wealth was not-significantly different across race/ethnic groups. Education, insurance, and health explained most differences in wealth change related to cognitive impairment. Yet, nearly 50% of group differences in wealth changes remained unexplained. Future work should further elucidate sources of these unexplained differences, overall and by race/ethnicity.

Abstract citation ID: igad104.3081

### PERCEIVED SOCIOECONOMIC STATUS, PRODUCTIVE SOCIAL ENGAGEMENT, AND SUBJECTIVE HEALTH: LONGITUDINAL STUDY IN TAIWAN

Chin-Yi Su[1], and Shiau Fang Chao[2], *1. Taiwan Association of Family Caregivers, Taipei, Taipei, Taiwan (Republic of China), 2. National Taiwan University, Taipei City, Taipei, Taiwan (Republic of China)*

Productive social engagement (PSE) has been identified as a crucial factor in promoting subjective health in later life. However, there has been limited focus on how perceived socioeconomic status (SES) among older adults mediates this relationship over the long term. This study employed data from the Taiwan Longitudinal Study on Aging to examine a sample of 2,865 individuals aged 55 or above from 2011 to 2015. By employing a path analysis, this study tested the hypothesized effects developed from active aging theory and social capital theory. Results indicates that : 1. Higher levels of PSE and perceived SES predicted higher subjective health within the same wave, both in T1 and T2. 2. Perceived SES mediated the relationship between PSE and subjective health only in T1. Also, the effect of T1 PSE on T2 PSE operated through T1perceived SES. 3. T1 PSE positively predicted T2 subjective health through T1 perceived SES and T2 perceived SES over the time. This study sheds light on the mechanism linking PSE, perceived SES, and subjective health. The findings suggest that higher perceived SES is associated with more PSE in a four-year interval, which in turn predicts higher subjective health. This indicates that social capital may facilitate older adults' access to PSE over time. Future research should investigate the role of perceived SES and how it interacts with objective and subjective physical well-being in later life. Interventions should target ways to help older adults access opportunities for engaging in productive activities to improve their health.

Abstract citation ID: igad104.3082

### PRIMARY MOTIVATIONS OF AFRICAN AMERICANS AND LATINOS TO PARTICIPATE IN ALZHEIMER'S PREVENTION RESEARCH

Lizbeth Vera Murillo[1], Angel Collie[1], Maria Vander Meulen[1], Monique Villamor[1], and Jody Nicholson-Bell[2], *1. University of North Florida, Jacksonville, Florida, United States*, *2. Clemson University, Clemson, South Carolina, United States*

The prevalence of Alzheimer's Disease and related dementias (ADRD) is higher among racial/ethnic minorities as compared to White/Caucasian individuals, yet there is poor representation in ADRD research for these minoritized groups. It is essential to investigate the motivation to participate in research for individuals from minoritized groups to optimize inclusiveness through recruitment and retention strategies. The current project explores racial/ethnic differences in motivations to participate in an ADRD prevention study using case-controlled matching between White/Caucasians (n=210; M = 71.01 years, SD = 4.41) and African American/Black (n=210; M = 71.26 years, SD = 4.47) participants, and between non-Hispanics (n = 158; M =71.13, SD = 5.33), and Hispanic participants (n = 157; M = 71.48 years, SD = 5.14). Results indicated Latinos/Hispanics were more likely to report concerns about brain health and aging (59.7%; $\chi^2 = 3.99$, $p < .05$, $\varphi=.11$) and improvement of personal brain health (60.9%; $\chi^2 = 4.30$, $p < .05$, $\varphi=.12$) as a motivator compared to non-Hispanics. African American/Blacks were more likely to report concerns about brain health (59.6%; $\chi^2 = 4.66$, $p < .05$, $\varphi=.12$) as a motivator compared to White/Caucasians. Data from the current study about the concerns of cognitive decline and brain health for African Americans/Blacks and Latinos/Hispanic opens an avenue by which participation in clinical trials may be increased by better project marketing/advertisement focusing on the advantages of preventative health behaviors involving non-pharmacological methods and cognitive health resources that might be available within a research environment.

Abstract citation ID: igad104.3083

### RACIAL DIFFERENCES IN CORRESPONDENCE BETWEEN OBJECTIVE HEARING AND SUBJECTIVE HEARING RATINGS

Charity Lewis[1], Joanne Elayoubi[1], Michelle Arnold[1], Yuri Jang[2], Victora Sanchez[1], Julia Toman[1], and William Haley[1], *1. University of South Florida, Tampa, Florida, United States*, *2. University of Southern California, Los Angeles, California, United States*

**Background:** Hearing loss and hearing health are underrecognized public health urgencies affecting over 70% of adults aged 70 and older. Untreated hearing loss has its association with higher prevalence of adverse physical and cognitive health outcomes. Current literature reveals racial disparities in hearing aid utilization. Based on the Health Belief Model suggesting that subjective beliefs are predictive of health behaviors, the present investigation focused on the correspondence between objective and subjective ratings of hearing in older Blacks and Whites. We identified groups that are concordant and discordant (overestimation and underestimation) in their ratings and explored determinants of the group membership. **Methods:** We included participants over age 50 from the Health and Retirement Study (N = 2,495). Subjective and Objective hearing loss relation to race and age was determined via Chi-square analysis. Hierarchical linear regression models were completed to identify odds ratios for overestimation and underestimation of hearing. **Results:** Whites aged 50-64 and 65-75 were significantly more concordant in their ratings than Blacks. Blacks aged 65-75 had significantly higher rates of overestimation of hearing. In a fully adjusted model for overestimation of hearing, Blacks had significantly greater odds of overestimation of hearing than Whites (OR = 1.47), and overestimation was significantly greater with higher age (OR = 1.03) and with tobacco use (OR = 1.29). **Conclusion:** While mechanisms explaining lower hearing aid utilization in Black older adults need further study, perceived severity (consistent with the Health Belief Model) may be one component of reduced hearing aid use in Black older adults.

Abstract citation ID: igad104.3084

### REPORTED HISTORY OF ABORTION AS A CONTRIBUTOR OF RELIGIOSITY AMONG OLDER WOMEN HELD IN CORRECTIONAL CUSTODY

Hailey Minet[1], Brianna Barnett[1], Alexander Bishop[1], and Kevin Randall[2], *1. Oklahoma State University, Stillwater, Oklahoma, United States*, *2. Sam Houston State University, Huntsville, Texas, United States*

This study assessed the interconnection between abortion, forgiveness, and social provisions on religiosity for N = 157 older women (45 years and older) under correctional custody in Oklahoma. Guided by the Model of Developmental Adaptation, path analysis was conducted positing positive religiosity as the developmental outcome, regressed on proximal influences assessed by social provisions and forgiveness and the distal influence, the independent predictor, whether or not a woman had ever had an elective abortion in their lifetime. Endogenous measures were controlled for age, race, marital status, education, and crime type. Using Mplus 8.8 and an estimator that provides parameter estimates and a chi-square test statistic robust to non-normality, the model fit well with a non-significant chi square test statistics and significant parameter estimates as expected. Reported history of abortion was significantly associated with forgiveness ($\beta = .25$, $p < .05$) and social provisions ($\beta = .31$, $p < .05$). Forgiveness was also significantly associated with religiosity ($\beta = .32$, $p < .01$). Mediating paths were not significant. Among these women, 37% of the developmental outcome, religiosity was explained primarily by forgiveness. It appears that older women in correctional custody who have received an elective abortion have a greater disposition to engage in forgiveness and seek social support. A greater disposition to forgive also directly contributes to increased religious activity among these same women. Results have implications relative to how forensic counselors, correctional social workers, and prison chaplains provide intervening services and rehabilitation programming to sustain quality-of-life for older women aging-in-custody.

Abstract citation ID: igad104.3085

### SLEEP DISTURBANCE AND DEPRESSIVE SYMPTOMS AMONG OLDER ADULTS: DO GENDER DIFFERENCES EXIST?

Suha You, and Giyeon Kim, *Chung-Ang University, Seoul, Republic of Korea*

Sleep is a key part of a healthy lifestyle, but aging causes sleep problems by decreased melatonin levels. Sleep disturbance includes problems falling or staying asleep, too much sleep, or obstructive sleep apnea (OSA), the most common sleep-related breathing disorder in older adults. Thus, aging-related changes in sleep should be studied in various aspects of elderly's health such as physical, mental, and cognitive health. As part of that, this study aims to (1) examine the connection between sleep disturbance and depressive symptoms and (2) investigate whether gender differences exist. Participants were Korean adults aged 65 and older (N=1,316) from the 2020 Korean National Health and Nutrition Examination Survey. The survey measured sleep disturbances (OSA, insomnia, hypersomnia), depressive symptoms, and sociodemographic characteristics. Hierarchical multiple regression analysis was conducted to examine whether sleep disturbances influence depressive symptoms and test the interaction between sleep disturbance and gender. Results revealed that after adjusting for covariates, those with more risk factors for OSA (B=1.056, p=.000) or with insomnia (B=1.527, p=.000) were more likely to have greater depressive symptoms than their counterparts. Additionally, the relative influences of OSA (B=1.295, p=.000) or insomnia (B=.965, p=.023) on depressive symptoms were contingent upon gender. However, no correlation was observed between hypersomnia and depressive symptoms. Although both OSA and insomnia are risk factors for depressive symptoms, older women with OSA or insomnia showed more severe depressive symptoms than older men with OSA or insomnia. Findings suggest the need to make sophisticated approaches considering homogeneity and heterogeneity within older adults.

Abstract citation ID: igad104.3086

### SLEEP PROBLEMS MEDIATE THE ASSOCIATION BETWEEN CHILDHOOD ADVERSITY AND LATE-LIFE COGNITIVE FUNCTIONING

SangNam Ahn[1], Hong Xian[1], Jeffrey Scherrer[1], Asos Mahmood[2], Eunhye Ahn[3], Seonghoon Kim[4], McKenzie Beck[1], and George Slavich[5], *1. Saint Louis University, St. Louis, Missouri, United States*, *2. The University of Tennessee Health Science Center, Memphis, Tennessee, United States*, *3. Washington University in St. Louis, St. Louis, Missouri, United States*, *4. Singapore Management University, Singapore, Singapore*, *5. University of California, Los Angeles, Los Angeles, California, United States*

The negative health impacts of childhood adversity have been well documented. However, little is known about the pathways linking childhood adversity with cognitive functioning later in life. We addressed this issue by examining the extent to which lifetime sleep problems mediated the effects of childhood adversity on late-life cognitive functioning. We used the 2002-2018 waves of the Health and Retirement Study in which participants were 20,607 adults (58.1% female), aged ≥50 years (Mage=68.9±9.5 years). Cognitive functioning was based on: 1) a 10-word immediate and delayed recall test of memory; 2) a serial 7s subtraction test of working memory; and 3) counting backwards to assess attention and processing speed. Sleep problems (e.g., difficulty initiating sleep) were assessed with the Jenkins Sleep Scale, and seven items of childhood adversity (e.g., physical abuse) were included from the list of lifetime potentially traumatic events. Path analysis revealed that there was a small but significant indirect relationship between childhood adversity and poor cognitive functioning through sleep problems (coefficient = -.014; p=.001). Indeed, about 3% of the effect of childhood adversity on cognitive functioning is mediated by sleep problems (i.e., 0.014 [indirect effect] / 0.476 [total effect]). Our findings can help policymakers and public health practitioners better understand the risk and protective factors of cognitive functioning later in life in relation to sleep problems among those individuals with childhood adverse events. Further research is warranted to determine if childhood adversity contributes to cognitive decline in those with trauma and PTSD compared to those with trauma alone.

Abstract citation ID: igad104.3087

### SOCIAL DETERMINANTS OF HEALTH AND EMERGENCY DEPARTMENT VISITS AMONG OLDER ADULTS WITH MULTIMORBIDITY

Arum Lim[1], Chitchanok Benjasirisan[1], Xiaoyue Liu[1], Oluwabunmi Ogungbe[1], Cheryl Dennison Himmelfarb[1], Patricia Davidson[2], and Binu Koirala[3], *1. Johns Hopkins University, Baltimore, Maryland, United States*, *2. University of Wollongong, Wollongong, New South Wales, Australia*, *3. Johns Hopkins University School of Nursing, Baltimore, Maryland, United States*

Multimorbidity is prevalent in older adults and is related to various adverse health outcomes, including high emergency department (ED) visits. Evidence suggests that social determinants of health (SDoH) are associated with many health outcomes, but the association between SDoH and ED visits among older adults with multimorbidity has received limited attention. This study aimed to examine the association between SDoH and ED visits among older adults with multimorbidity. A cross-sectional analysis was conducted among 25,078 adults aged 50 years and older from the 2010 to 2018 National Health Interview Survey. Multimorbidity was defined as the presence of two or more self-reported diseases among nine common chronic conditions, such as hypertension, diabetes, asthma, stroke, and cancer. The SDoH assessed included race, education, poverty, marital status, employment status, insurance status, and having a usual place for medical care. Participants' mean (±SD) age was 68.06 (±10.16) years, and 55.13% were female. After adjusting for age, sex, and categories of comorbidities in the logistic regression model, high school or less education (AOR=1.14, 95%CI=1.05-1.24), poverty income ratio < 1 (AOR=1.50, 95%CI=1.35-1.66), unmarried (AOR=1.23, 95%CI=1.15-1.32), and unemployment status (AOR=1.36, 95%CI=1.25-1.48) had higher odds of having 1 or more ED visits. Non-Hispanic Blacks had higher odds (AOR=1.28, 95%CI=1.18-1.40), while non-Hispanic Asians had lower odds (AOR=0.69, 95% CI=0.56-0.84) of 1 or more ED visits than non-Hispanic Whites. SDoH are associated with ED visits among older adults with multimorbidity; providers should consider SDoH in health-seeking behaviors and to promote health equity.

Abstract citation ID: igad104.3088

### SOCIAL DETERMINANTS OF HEALTH RELATED TO GENREAL PHYSICIAN VISITS IN OLDER ADULTS LIVING WITH MULTIMORBIDITY

Chitchanok Benjasirisan[1], Arum Lim[1], Xiaoyue Liu[1], Oluwabunmi Ogungbe[1], Cheryl Dennison Himmelfarb[1], Patricia Davidson[2], and Binu Koirala[3], *1. Johns Hopkins University, Baltimore, Maryland, United States*, *2. University of Wollongong, Wollongong, New South Wales, Australia*, *3. Johns Hopkins University School of Nursing, Baltimore, Maryland, United States*

Multimorbidity- the presence of two or more chronic conditions- is a global concern and is commonly associated with aging. Multimorbidity results in diminished physical function, higher healthcare utilization, and increased mortality rates. To understand healthcare challenges and increase prevention and continuity of care among older adults living with multimorbidity, the social determinants of health (SDoH) affecting general physician visits need to be explored. This study aimed to examine SDoH associated with general physician visits among 50 years and older adults living with multimorbidity using cross-sectional data from the 2010 to 2018 National Health Interview Survey. The SDoH, including marital status, race/ethnicity, education, poverty-income ratio, employment, health insurance, and having a usual place for medical care, were explored using logistic regression. The outcome was at least one visit to the general doctor's office, a clinic, or elsewhere in the last 12 months. Among 25,108 participants (mean age ± SD was 68.1±10.16 years, 55.5% female, with 2.7±0.95 average number of self-reported morbidities) adjusted analysis controlling for age, sex, and categories of comorbidities found that Hispanic (OR=0.78, 95%CI=0.66-0.91), living below the poverty level (OR=0.79, 95%CI=0.68-0.92), and uninsured (OR=0.52, 95%CI=0.42-0.63) had lower odds of having one or more physician visits. Being unemployed (OR=1.26, 95%CI=1.11-1.42) and having a usual place for medical care (OR=7.94, 95%CI=6.39-9.86) had higher odds of having one or more physician visits. The result suggested that SDoH should be considered to navigate targeted health programs and policies to improve prevention and management of multimorbidity as well as to promote health equity.

Abstract citation ID: igad104.3089

### THE PREVALENCE AND CHARACTERISTICS OF UNREPRESENTED ADULTS ADMITTED TO KOREAN HOSPITAL SETTINGS

Hyejin Kim[1], Jiyeon Choi[2], Won Lee[1], Ilhak Lee[2], and Mi-Kyung Song[3], *1. Chung-Ang University, Seoul, Republic of Korea*, *2. Yonsei University, Seoul, Republic of Korea*, *3. Emory University, Atlanta, Georgia, United States*

BACKGROUND: Unrepresented adults refer to persons who have lost their decision-making capacity and have neither available surrogates to make medical decisions for them nor the documentation of their treatment preferences. Recently, concerns about this population have been emerging in the hospital setting in South Korea. However, little is known about this population. Thus, this study aimed to identify the prevalence and demographic and clinical characteristics of unrepresented patients admitted to hospitals in South Korea. **METHODS:** This qualitative descriptive study was conducted using semi-structured interviews with 37 physicians, nurses, social workers, and others from 22 hospitals and directed content analysis. RESULTS: The number of unrepresented patients seemed to be small in hospital settings, but higher in public hospitals than private ones. Participants reported that the reasons unrepresented patients lost their decision-making capacity included loss of consciousness, delirium or alcoholism-related mental change, dementia, and mental retardation. Surrogates were unavailable because no families were found through a rigorous search, or because families were inaccessible, refused to be decision-makers, or were unable to serve as surrogates as they were not a legally-bound immediate family. These patients were mostly in their 50s – 70s, predominantly male, and living alone, and had poor socioeconomic status and poor hygiene. Most of them had no medical records before admission. CONCLUSIONS: Unrepresented patients are recognizable even though the number is small. Based on the common characteristics of unrepresented adults, national and societal efforts are necessary to identify 'at-risk' people and prevent them from becoming unrepresented in healthcare settings.

### SESSION 7970 (POSTER)

### COGNITION: DYSFUNCTION, IMPAIRMENT, AND DECLINE

Abstract citation ID: igad104.3090

### A DIGITAL LIFE STORY BOOK ACTIVITY FOR OLDER ADULTS WITH MEMORY DIFFICULTIES: PROGRAM EVALUATION

Elizabeth Barbour[1], Majse Lind[2], and Susan Bluck[1], *1. University of Florida, Gainesville, Florida, United States*, *2. Aalborg University, Aalborg, Nordjylland, Denmark*

Reminiscence activities are based on maintaining a core set of personal memories to aid self-continuity (Woods, 2012). Some interventions find limited effects (Charlesworth et al, 2016) but with participants reporting high satisfaction with the program. The aims of the current study were to: (i) demonstrate feasibility of intervention delivery to older individuals with memory difficulties, and (ii) provide analyses of program efficacy using a pre-posttest, control group design. Participants (N = 45; Mage = 76.12) were randomized to the Life Story Book intervention or Waitlist Control. A Life Story Book Interview was developed, using five evidence-based principles of autobiographical memory. Personalized multimedia books were created and reviewed by participants for fourteen days. To assess feasibility, participants in the intervention completed a memory quality questionnaire and feedback survey. Those who reviewed Life Story Books reported improved memory quality pre-post intervention, t(22) = -2.39, p = .026. Participants also positively rated their intervention experience at post-test, (M = 4.46/5). All participants completed self-related questionnaires (e.g., self-continuity, self-esteem) at pretest-posttest. No measure showed significant effects. Our results represent a conundrum for researchers. We provide evidence of feasibility of, and participant enthusiasm for, this reminiscence-based intervention. As seen in the literature, however, (Elfrink et al., 2021), sensitivity to detect pretest-postest change remains a challenge. The current study highlights participants positive reactions to reminiscence-based Life Story Books. Findings are discussed in terms of methods for assessing intervention efficacy in individuals with memory decline.

Abstract citation ID: igad104.3091

### CONSEQUENCES OF PERCEIVED STRESS ON COGNITIVE DECLINE AND BLOOD PRESSURE AS A MODERATOR IN OLDER BLACK AMERICANS

DeAnnah Byrd[1], David Coon[1], Roland J. Thorpe[2], and Keith Whitfield[3], *1. Arizona State University, Phoenix, Arizona, United States*, *2. Johns Hopkins University, Baltimore, Maryland, United States*, *3. University of Nevada, Las Vegas, Las Vegas, Nevada, United States*

Previous research suggests that perceptions of stress shape cognitive health outcomes. However, few studies have explored this association longitudinally. The aim of this study was to determine whether perceived stress is associated with cognitive changes over time and if blood pressure (BP) acts as a moderator in a sample of older Blacks 48 to 95 years of age, who were enrolled in the Baltimore Study of Black Aging—Patterns of Cognitive Aging and interviewed at two time points, approximately 3 years apart (N = 450). Cognition included 5 domains: working memory, processing speed, verbal memory, vocabulary, and inductive reasoning. Three readings of orthostatic BP were collected and mean systolic (range = 82-230.3 mmHg) and diastolic BP (range = 51-136.7 mmHg) values were calculated for each participant. Results from the linear regression model showed no direct effect of perceived stress on cognitive decline. Systolic (F(3,426)= 4.37, p= 0.0048) and diastolic (F(3,426) = 3.26 p=0.0215) BP modified the relationship between stress and inductive reasoning at follow-up but was not related to any other cognitive domains, adjusting for age, sex, education, depressive symptoms, comorbidities, and baseline cognition. These important findings demonstrate the joint effects of stress and BP on cognition, highlighting the need for more research documenting the underlying processes and risk factors for domain-specific cognitive decline. The potential benefits of this approach provide a basis for developing cognitive interventions for this population of Black Americans.

Abstract citation ID: igad104.3092

### CORRELATES OF SUBTLE COGNITIVE DYSFUNCTION IN A HIGHLY EDUCATED COHORT OF MID LIFE AND OLDER AFRICAN AMERICANS

Travonia Brown-Hughes[1], Alyssa Gamaldo[2], Corinne Pettigrew[3], and Roland J. Thorpe[3], *1. Hampton University, Hampton, Virginia, United States*, *2. Clemson University, Clemson, South Carolina, United States*, *3. Johns Hopkins University, Baltimore, Maryland, United States*

Remote cognitive screening modalities can be an initial step in assessing cognitive health. This cross-sectional study examined how risk and protective factors relate to cognitive performance in non-demented African Americans aged 50 and older. Participants were part of the African American-United Memory and Aging Project (AA-UMAP), a geographically diverse cohort of college-educated adults recruited and evaluated via telephone and online. Demographic, health variables and activity engagement were self-reported through an online survey. Cognition (global cognition, memory, and psychomotor/attention) was measured remotely using the Telephone Interview for Cognitive Status (TICS-40; N = 143) and the CogState Brief Battery (CBB; N = 51). Health variables included a mental health composite (e.g., depression and/or anxiety) and a metabolic conditions composite (e.g., high cholesterol, hypertension, diabetes, and/or obesity). Activity engagement was assessed with self-reported physical, mental, and social lifestyle activities. Linear regression models indicated that older age was associated with slower psychomotor/attention performance ($p < 0.01$) and marginally lower learning/working memory accuracy ($p = 0.08$) even after adjusting for covariates (e.g., sex and education). Cognitive performance was unrelated to mental health, metabolic conditions, and lifestyle activity engagement. Neither health-related risk factors nor level of engagement activities were significantly associated with cognitive performance in this remotely screened sample of highly educated middle-aged and older African American adults. More studies of midlife and older African Americans are needed to obtain a more in-depth understanding of the degree to which biopsychosocial strengths and deficits in midlife may affect or alter later-life cognitive trajectories.

Abstract citation ID: igad104.3093

### ESTIMATED ARTERIAL STIFFNESS, COGNITIVE DECLINE, AND DEMENTIA AMONG MIDDLE-AGED AND OLDER ADULTS

Kevin Heffernan[1], Janet Wilmoth[1], and Andrew London[2], *1. Syracuse University, Syracuse, New York, United States, 2. Syracuse University, Maxwell School of Citizenship and Public Affairs, Syracuse, New York, United States*

Prior research suggests arterial stiffness may be a useful vascular biomarker that predicts age-related cognitive decline. In this paper, we use the Health and Retirement Study (HRS) to examine the relationship between estimated pulse wave velocity (ePWV), which is an under-analyzed but readily available estimate of arterial stiffness, and cognitive decline. The HRS assesses cognitive function with the Telephone Interview for Cognitive Status (TICS). In this 27-point TICS measure, scores between 7 and 11 indicate cognitively impaired but no dementia (CIND) and scores between 0 and 6 indicate dementia. We modeled the relationship between ePVW and cognitive status change from 2006/2008 through 2018. The findings indicate ePWV increases the odds of having CIND and dementia relative to having no CIND or dementia. In addition, ePWV increases the odds of transitioning into CIND and dementia from a higher cognitive functioning state. The results are robust to controlling for: age, age squared, age cubed, systolic and diastolic blood pressure along with sociodemographic characteristics (gender, race, ethnicity, wealth, income, education), health status (history of cardiovascular disease (CVD,) diabetes, and stroke and related medication use), health behaviors (smoking, physical activity, body mass index), and CVD-related biomarkers (C-reactive protein, cystatin-C, hemoglobin A1c, total cholesterol, high-density lipoprotein (HDL) cholesterol). These findings suggest that ePWV is a simple screening tool and novel biomarker that can be used to monitor vascular cognitive aging and CIND progression in middle-aged and older adults.

Abstract citation ID: igad104.3094

### RACIAL/ETHNIC DIFFERENCES IN NEUROCOGNITIVE PROFILES OF OLDER ADULTS WITH 6-YEAR INCIDENT DEMENTIA

Moyosoreoluwa Jacobs, Carmen Chek, and Wassim Tarraf, *Wayne State University, Detroit, Michigan, United States*

Hispanic and non-Hispanic Black (NHB) older adults have higher dementia rates than non-Hispanic Whites (NHWs). Estimates of dementia prevalence and incidence in community-based cohorts are mostly based on threshold value performance on cognitive assessment batteries. We used longitudinal data from the HRS Harmonized Cognitive Assessment Protocol (HCAP) to (1) generate and compare, across race/ethnicity, neurocognitive profiles for older adults (>65 years) not meeting research criteria for dementia in 2010 and developing dementia by 2016 (using Langa-Weir's dementia classification), (2) assess racial/ethnic differences in these profiles, and (3) test socio-demographic effects on dementia-specific differences in neurocognitive performance. Performance on all HCAP measures was highly sensitive to 6-year incident dementia classification. However, we found notable domain-specific differences in test sensitivity, whereby performance on general screeners (e.g., MMSE) and memory-based tests (e.g., CERAD word list recognition) were significantly lower (≤2 SDs from normed population means). Hispanics and NHBs generally performed lower than NHWs, with more pronounced group differences in general screeners and executive function tests. Sociodemographic characteristics significantly attenuated Hispanic differences, but less so for NHBs, relative to NHWs. Despite these group differences, NHWs with incident dementia had worse neurocognitive profiles than their NHB and Hispanic counterparts. Dementia classification algorithms (e.g., Langa-Weir) sensitively detect cognitive impairment. However, these algorithms more accurately identify higher severity cognitive impairment in NHWs compared to NHBs and Hispanics. Such methods can yield dementia estimates biased by socio-cultural factors. Further research is needed to identify culturally appropriate neurocognitive tests that enhance precise dementia diagnosis, using population-level epidemiological data.

Abstract citation ID: igad104.3095

### SEX AND RACE/ETHNIC DIFFERENCES IN TERMINAL COGNITIVE DECLINE

Iván Mejía-Guevara, *Stanford University, Stanford, California, United States*

**Background:** Cognitive decline has been documented in older adults, but sex and racial/ethnic differences among decedents have not been studied. Here we investigate the relation of mortality to change in the cognitive function of older individuals by sex and race/ethnicity. **Methods:** The study analyzed data from the Health and Retirement Study (HRS) between 1993 and 2018, which included information on total word recall, mental status, and total cognition of individuals aged 65 and older. The study estimated the loss of cognitive function using a multilevel random coefficient model, considering sex and racial/ethnic differences in the risk of death, age, and death status. The study also approximated terminal cognitive decline using cognitive scores reported within two years before death for decedents. **Results:** On average, older women performed better than older men in word recall and overall cognition, while older White Americans performed better than African Americans and Hispanics/Latinos in overall cognition at all ages. Decedents had a similar cognitive function to survivors at age 65, but their cognition declined quickly with age, especially among older women and White Americans. Evidence of terminal cognitive decline within two years before death was significant regardless of age but less conclusive for African Americans and Hispanics/ Latinos due to smaller sample sizes. **Conclusions:** This paper indicated evidence of sex and race/ethnic differences in cognitive decline and terminal cognitive decline among older adults, which may have critical clinical implications directly linked to the onset and duration of dementia risk.

Abstract citation ID: igad104.3096

### THE RELATIONSHIPS OF VOICE PROSODY MEASURES TO COGNITIVE CHANGES OVER 10 YEARS

Elizabeth Mahon, and Margie Lachman, *Brandeis University, Waltham, Massachusetts, United States*

Voice prosody measures, especially jitter and shimmer, can differentiate cognitive impairment from healthy cognition, and may be promising early biomarkers for identifying high risk before later-life disease onset. In our past work, higher jitter and lower pulse assessed during the second cognitive assessment were associated with 10-year cognitive declines in middle-age and older adults from the Midlife in the U.S. (MIDUS) study. The current study assessed voice biomarkers taken at baseline, 10 years before the second cognitive assessment, and measured voice on speech that was independent of the cognitive assessments to address the possible influence of cognitive performance on concurrent voice prosody measures. Participants (ages 34 to 82 at the first occasion) were included if they were in the Boston Longitudinal Study (BOLOS, a subset of MIDUS) and had voice measures collected from the audio recordings of the cognitive interviews. Multilevel analyses controlled for age, sex, education, race, neurological conditions, and depressive symptoms at baseline. Higher jitter, lower shimmer, and lower pulse predicted greater 10-year declines in episodic memory. Higher pitch and lower pulse were associated with greater increases in reaction time on an executive functioning task. Thus, voice biomarkers previously associated with cognitive impairment could be useful as early indicators of later-life cognitive declines. Identifying voice biomarkers that could predict 10-year cognitive change may facilitate earlier intervention for decline through modification of disease risk factors.

### SESSION 7980 (POSTER)

### CAREGIVING ACROSS GENERATIONS AND ROLES

Abstract citation ID: igad104.3097

### CARE TRAJECTORIES AND WELLBEING OUTCOMES OF OLDER CARERS

Norah Keating, Janet Fast, and Choong Kim, *University of Alberta, Edmonton, Alberta, Canada*

The UN Decade of Healthy Ageing states that family carers should not be held responsible for care. Yet families continue to provide the majority of care to members with long term health problems and disabilities. Their contributions to the economy remain unrecognized; their costs noted but relegated to private family matters. The purpose of this presentation is to determine wellbeing outcomes of older Canadians with diverse life course trajectories of care. Data are from the 2018 Statistics Canada nationally representative survey on caregiving. Sample for this study is ≈3000 people 65+ with one or more care episodes across their life course. Based on earlier conceptual and empirical work, we recreated 5 care trajectories. Wellbeing was measured based on material, relational and subjective domains. We conducted multivariate analyses (OLS, logistic and ordered logistic regressions) appropriate to the nature of the dependent variable) to assess whether care trajectory type predicted later life wellbeing. Results show significant differences in wellbeing among trajectory types. Serial carers had longest years of care and most care episodes. They experienced the most negative wellbeing outcomes compared to carers with other trajectory types in material wellbeing (poorer physical and mental health, lower employment income), and subjective wellbeing (higher stress). There were no significant differences across care trajectory types in relational wellbeing (loneliness). We discuss the place of public policy in addressing patterns of cumulative disadvantage in life courses of family care; and call for the development of indicators of wellbeing domains that best reflect these outcomes.

Abstract citation ID: igad104.3098

### CAREGIVER IDENTITY IN EMERGING ADULTHOOD: HOW IDENTITY DISCREPANCY AND SALIENCE RELATE TO DISTRESS

Anastasia Canell[1], and Grace Caskie[2], *1. VA Bedford Healthcare System, Bedford, Massachusetts, United States, 2. Lehigh University, Bethlehem, Pennsylvania, United Sta tes*

As the older adult (age 65+) population grows in the United States, the demographics of caregivers are expanding to include more emerging adults (ages 18-25) who are participating in caregiving tasks. Minimal research has focused on this subgroup of caregivers to older adults, especially with regards to understanding factors that may influence their levels of caregiver distress. Guided by caregiver identity theory, the current study examined 1) the relationship between caregiver identity and caregiver distress, 2) support service utilization as a mediator between caregiver identity and caregiver distress, and 3) the inclusion of ageist attitudes within caregiver identity models. A sample of 259 emerging adult caregivers were recruited through Amazon MTurk. Fit of the hypothesized structural equation model was acceptable and squared multiple correlations (R2) indicated that 73.1% of the variance in caregiver distress, 23.8% of the variance in support service utilization, and 22.7% of the variance in ageist attitudes was explained by this model. Higher caregiver identity salience was significantly related to lower caregiver distress. Support service utilization partially mediated the relationship between identity salience and distress through an inconsistent mediation. The findings of the current study support caregiver identity as a unique, multidimensional process consisting of both identity discrepancy and salience latent constructs. Results showed low-to-moderate caregiver identity discrepancy, possibly due to identity flexibility during this developmental period. High caregiver identity salience was protective against caregiver distress for emerging adults. Ageist attitudes warrant inclusion in future research on caregiving identity development. Implications for support services are discussed.

Abstract citation ID: igad104.3099

### CONTINGENCY PLANNING AND STYLES OF PREPARATION FOR FUTURE CARE BY OLDER GRANDPARENT CAREGIVERS

Tina Peterson, *University of Cincinnati, Cincinnati, Ohio, United States*

The COVID-19 pandemic heightened attention to contingency planning in every area of life including family caregiving. Extended time in the caregiver role can increase problem-solving abilities; yet low rates of traditional contingency planning exist among custodial grandparents. This research applied Steele and colleagues' four styles of preparation for future care (2003)—avoider, thinker, planner, or consenter—to older custodial grandparents raising adolescent grandchildren. Nineteen grandparents participated in face-to-face interviews. Criteria were: primary caregiver for a grandchild 12 or older; grandchild in home at least 3 days weekly; and grandparent 40 or older. Recruitment included word of mouth, newspapers, and flyers. Participants were asked "What is your thinking about caregiving arrangements for your grandchild in case you are no longer able to provide care? What actual steps, if any, have you taken to begin these caregiving arrangements?" Interviews were audiotaped, transcribed verbatim, and analyzed using conventional content analysis. The racial and ethnic composition of the grandparents were white (n=10), Black/African American (n=7), Native American (n=1), and Filipino (n=1). Most respondents were married (58%), white (53%), grandmothers (84%), with some college education or higher (79%), and an average age of 66 years. Grandparents identified as thinkers (42%), planners (32%), consenters (21%), and avoiders (5%). Two married grandfathers reported conflicting preparation styles (thinkers) in comparison to their spouses (planners). Grandparents often relied on oral opposed to formal written plans. Findings suggest research, practice, and education is needed to assess factors inhibiting aspects of contingency planning for future care among thinkers and avoiders, particularly older grandfathers.

Abstract citation ID: igad104.3100

### EFFECTIVE SOLUTIONS FOR CAREGIVERS OF OLDER ADULTS: A RAPID UMBRELLA REVIEW

Molly McHugh, Ellen Munsterman, HanNah Cho, and Mary Naylor, *The University of Pennsylvania, Philadelphia, Pennsylvania, United States*

The negative health impacts confronting over 35 million informal caregivers of older adults in the U.S. are well documented. Although targeted interventions aimed at informal caregivers have demonstrated efficacy in mitigating poor caregiver outcomes, gaps in the literature regarding cost, scalability, and the impact of policy remain. Such knowledge is essential, given the projected growth in the need for caregiving associated with a rapidly aging population coupled with the shrinking of the caregiving workforce. This rapid umbrella review aims to synthesize findings from systematic reviews of randomized clinical trials of interventions that targeted caregivers and specified caregiver outcomes. The National Institute on Minority Health and Health Disparities research framework guided this review to identify gaps in existing intervention research. Databases from diverse disciplinary perspectives, including PubMed, Scopus, APA PsycINFO, ABIM, ATLA, Sociological Abstracts, PAIS, and EconLit, were searched from 2018 to 2023. A total of 52 systematic reviews that met inclusion criteria and were published in English were included in the final review. Reviews described interventions targeting caregivers of older adults with the following conditions: dementia (30), stroke (4), cancer (4), heart disease (1), renal disease (1), mental illness (1), and mixed-diagnoses (11). Major findings reveal varied effects on caregiver burden, quality of life, and mental health, and the growing integration of telehealth interventions. Studies were commonly limited by heterogeneity of interventions and outcomes and small effect sizes. Future intervention development should consider targeting the specific needs of diverse caregivers and scaling effective interventions to broader caregiver populations.

Abstract citation ID: igad104.3101

### EXPERIENCES AND SUPPORTIVE CARE NEEDS OF LATINX MILLENNIAL CAREGIVERS

Anna Welling[1], Megan Thomas Hebdon[1], Galilea Dupree[1], Sharon Horner[1], Michael Thomas[1], Neil Peterson[2], Janice Hernandez[1], and Heather Cuevas[1], *1. The University of Texas at Austin, Austin, Texas, United States*, *2. Brigham Young University, Provo, Utah, United States*

Latinx millennial caregivers are the largest ethnic minority subgroup within the millennial caregiving cohort. Due to age and life stage, members of this understudied group must often manage the competing priorities of career, family, and caregiving. Layered with these challenges are the experiences of being part of a minority group in the United States, with systemic barriers impacting access to healthcare, insurance, a safe built environment, and educational opportunities. The purpose of this qualitative descriptive study was to explore the experiences and needs of Latinx millennial caregivers. Participants were recruited locally and nationally using recruitment flyers and social media advertising. Data were collected from 29 participants using five focus group interviews and one individual interview. Data analysis using interpretive thematic analysis and inductive coding occurred. Twenty female and nine male Latinx millennial caregivers participated in the focus groups. Two meta-themes threaded throughout the analysis included the Latinx experience (immigrant background, Latinx culture, and navigating barriers as a minoritized individual) and being a super-caregiver. Main themes were identified: family well-being, occupational and financial well-being, social support dynamics, challenges and rewards of family caregiving, and coping strategies. Family and community were emphasized in all main themes. In this study, the importance of family and community relationships motivated caregiving and were areas where support was needed for Latinx millennial caregivers. Interventions and clinical support for these caregivers is needed that addresses their cultural background, the value of family and community, and the barriers they experience to accessing care and support.

Abstract citation ID: igad104.3102

### PREPAREDNESS AND NEEDS AMONG YOUNG, MIDDLE-AGED, AND OLDER ADULT LATINX CAREGIVERS

Karen Kopera-Frye, and Refat Srejon, *New Mexico State University, Las Cruces, New Mexico, United States*

Taking care of one's family is a primary obligation among Latinx communities. According to CDC's Behavioral Risk Factor Surveillance System, 22.9% of New Mexican adults reported providing care to a friend or family member in the past 30 days; 10.4 % provided care to an individual with dementia. The southern Border region of New Mexico is remote and desert, with high rates of poverty and inaccessibility to health care and services. Caregivers may feel unprepared for caregiving, lack the knowledge and skills required to provide care, but struggle through because of the importance of La Familia. Adverse physical and psychological consequences have been found; yet those who report greater preparedness exhibit lower levels of strain. Seventy-six primarily female and healthy Latinx caregivers (age range 20-76 years, 30% young, 40% middle-aged, and 30% older adults) were surveyed on caregiving they provide, administered the Preparedness for Caregiving Scale, and asked what types of services are needed in their role. Results indicated the top three provided aspects of caregiving were (in descending order): Food, emotional support, and transportation. Caregivers reported a high degree of preparedness in taking care of the recipient's physical needs, handling emergencies, and proving overall care. Needed resources included emotional support, financial assistance, and respite. Greater caregiving provision was significantly associated with better health (r =. 53, p = .09) and greater resource needs (r = .35, p <.01). Thus, level of preparedness is an important caregiving dimension to address, especially with culturally diverse communities having strong cultural caregiving norms.

### SESSION 7990 (POSTER)

### BIOBEHAVIORAL HEALTH AND AGING

Abstract citation ID: igad104.3103

### A LEAP! FORWARD?: DOES PERSONALIZED EXERCISE HELP MAINTAIN COGNITIVE FUNCTION IN OLDER ADULTS?

Valerie Medina[1], Jeffrey M. Burns[2], James L. Vacek[2], Jonathan Mahnken[2], Jonathan Clutton[2], Angela VanSciver[2], and Amber Watts[1], *1. University of Kansas, Lawrence, Kansas, United States*, *2. KU Medical Center, Kansas City, Kansas, United States*

Maintaining cognitive function is important to health in older adulthood. Physical activity can decrease the risk of cognitive decline in older individuals. We tested whether LEAP! Rx, a personalized exercise, and lifestyle education program would improve cognition in older adults. We randomized 220 participants (Mean age (SD) = 72 (4.83)), into the program or a control group. We used the NIH Toolbox Cognitive Battery to assess cognition. We analyzed change in cognitive performance from baseline to week 12 using repeated measures ANCOVA to compare the intervention and control groups, adjusting for age, gender, and education. Referral source (self- vs. physician-referred), treatment group (intervention vs. control), and measurement time (baseline vs. week 12) had a significant three-way interaction F(8, 212) = 4.57, p< 0.05. That is, clinician-referred participants in the intervention group improved more (vs. the clinician-referred control group), whereas self-referred control group participants improved more (vs. the self-referred intervention group). Older age, male gender, and lower education predicted poorer verbal learning F(1,218 = 18.79, p< 0.05; F(1, 218) = 28.67, p< 0.05; F(1, 219) = 4.13. Overall, cognitive scores improved more over 12 weeks in the intervention group compared to the control group. Thus, we conclude that personalized exercise programs may enhance or maintain cognitive function in older adults. The differences we observed in physician-referred versus self-referred participants has implications for implementation and scalability of health behavior change interventions, as well as implications for recruitment methods and representation in clinical trials research broadly.

Abstract citation ID: igad104.3104

### COPING STYLES AS MEDIATORS BETWEEN PURPOSE IN LIFE AND COGNITIVE FUNCTION: A LONGITUDINAL STUDY

Amanda Sesker[1], Páraic O'Súilleabháin[2], Martina Luchetti[3], Antonio Terracciano[4], and Angelina Sutin[5], *1. University of Minnesota Medical School - Duluth, Duluth, Minnesota, United States*, *2. University of Limerick, Limerick, Limerick, Ireland*, *3. Florida State University, College of Medicine, Tallahassee, Florida, United States*, *4. Florida State University, Tallahassee, Florida, United States*, *5. Florida State University College of Medicine, Tallahassee, Florida, United States*

This study tested the association between purpose in life and coping styles and whether coping styles mediate the association between purpose and cognitive function. Longitudinal data from the Midlife Development in the United States (MIDUS) study were used to investigate associations between purpose assessed at MIDUS I (1996-1997) and coping assessed at MIDUS II (2004-2006) (N=2,386). Emotion-focused and problem-focused coping styles were then tested as mediators of the association between purpose and cognitive function (i.e., memory, executive function, and global cognition) assessed at MIDUS III (2013-2014). Higher purpose was associated with higher problem focused coping (β=.191, p<.001) and lower emotion-focused coping (β=-.227, p<.001). Coping style partially mediated the association between purpose and cognitive function about two decades later. Emotion-focused coping accounted for 14.3% of the total effect of purpose on cognitive function. Problem-focused coping mediated 16.7% of the total effect of the association of purpose on episodic memory, but did not mediate the associations between purpose with either executive function or total cognitive function. These findings provide new evidence for associations between purpose and coping, as well as links between coping and cognitive function in a large, longitudinal sample of adults. The results are consistent with the literature on purpose and better cognitive function in adulthood and suggest that coping is one mechanism linking purpose with better cognitive function.

Abstract citation ID: igad104.3105

### HYDRATION STATUS AND COGNITIVE FUNCTIONING IN OLDER ADULTS

Katelyn Singer[1], and Benjamin Katz[2], *1. Virginia Tech Graduate School, Blacksburg, Virginia, United States*, *2. Virginia Tech, Blacksburg, Virginia, United States*

Hydration status may play a role in chronic behavioral and physiological conditions commonly experienced by older adults (Begum & Johnson, 2010; Lee et al., 2010; Stookey et al., 2020). However, research on the impact of hydration status on cognition specifically, however, has often focused on single outcome tasks or more global measures; furthermore, research is heterogeneous in methodologic approaches. The current study aimed to identify specific cognitive domain(s) most closely associated with hydration status among older adults. Utilizing a wide range of cognitive performance data from the Health and Retirement Study's Harmonized Cognitive Assessment Protocol (2016) and hydration data derived from the physiological data from the Venous Blood Study (2016), curvilinear regression analyses identified a significant relationship between hydration status (measured by serum osmolarity) and processing speed in older adults. Specifically, being in a state of euhydration, compared to being hyper- or hypo- hydrated, was associated with better cognitive performance only on measures of processing speed such as letter cancellation, digit symbol, and trail making A tasks. These associations indicate that hydration is most closely linked to tasks involving mental speed, which include everyday tasks such as doing mental math, decision making, and processing visual information (Owsley et al., 2002). These findings provide complementary evidence to support earlier work suggesting links between processing speed and hydration status (Mantantzis et al., 2020) , but also highlights the need for further studies assessing the causal pathways of this association.

Abstract citation ID: igad104.3106

### IMPACT OF CAREGIVING HOURS ON COGNITIVE OUTCOMES ACROSS ADULTHOOD

Kallol Kumar Bhattacharyya[1], Victor Molinari[2], Yin Liu[1], and Elizabeth Fauth[1], *1. Utah State University, Logan, Utah, United States*, *2. University of South Florida, Tampa, Florida, United States*

Being a caregiver has been associated with poorer physical health and well-being. The current study expands prior work by comparing cognitive functioning among individuals who were caregivers versus non-caregivers, and further examined varying caregiving hours and cognitive functioning among caregivers. Data were from waves 2 (2003-04) and 3 (2013-14) of the Midlife in the United States (MIDUS) study. A total of 2,075 individuals aged 35-86 years (Mage=55±11) were included from wave 2. We identified individuals who served as a caregiver in the past 12 months (N=264) at wave 2. Cognitive functioning was assessed by composite scores for both executive function (inductive reasoning, category verbal fluency, working memory span, processing speed, and attention switching and inhibitory control) and episodic memory (immediate and delayed free recall of 15 words). We used multiple linear regression models to examine associations between wave 2 caregiving versus non-caregiving status on wave 3 cognitive functioning (Model 1); and, among caregivers at wave 2, varying caregiving hours and wave 3 cognitive functioning (Model 2). After controlling for covariates on sociodemographic factors, and health and functional status at baseline in Model 1, caregiving was associated with better performance in episodic memory (b=.13, p<.05), but not executive function (b=-.06, p=.09). Model 2 revealed no significant association for either episodic memory or executive function. The findings supported the healthy caregiver rather than the stress process hypothesis, and that former caregiving may be associated with better memory functioning over time among middle-aged and older adults.

Abstract citation ID: igad104.3107

### LIFESTYLE TRAJECTORIES PREDICT LATER COGNITIVE FUNCTION AMONG OLDER PEOPLE IN TAIWAN

Hui-chuan Hsu, *Taipei Medical University, New Taipei, New Taipei, Taiwan (Republic of China)*

Purpose: The purpose of this study was to identify the group-based trajectories of lifestyles and to predict the later cognitive function among older people in Taiwan. **Methods:** The data were from five waves of the "Taiwan Survey on Aging " (1999-2015). Only those who completed the last wave of cognitive function were used for analysis (n=1314). The cognitive function was measured by 8 items of SPMSQ, reverse numbers and recall 10 words, with score ranged 0-19. Nineteen lifestyle behaviors (healthy behaviors, leisure activities, lifelong learning and social participation) across waves were analysed by group-based trajectory analysis first, and then the effects of lifestyle trajectories on cognitive function were analysed by linear regression. **Results:** Each lifestyle variable was identified as 2 to 6 trajectory groups. Significant lifestyle trajectories on later cognitive function included gardening (high), drinking (increasing), providing instrumental support (increasing), playing chess (high), reading (middle & high), outdoor activities (high), group activities (yes), political participation (no), volunteering (yes), and stress (high vs. lowest). After controlling for covariates, cognitive function was related to gardening (high) (B=0.642), playing chess (high) (B=0.855), reading (high) (B=1.699), group activities (B=0.640), and lower stress (high stress B=−0.806). Other significant variables for better cognitive function included younger age, living with less family members, having less physical function difficulties, having lower depressive symptoms, and being more religious. **Conclusion:** Long-term lifestyle trajectories may affect later cognitive function. Having more social participation, more cognitive-related leisure, having less stress and being physically healthier are protective for cognitive function.

Abstract citation ID: igad104.3108

### MEDIATING ROLE OF REST–ACTIVITY RHYTHM ON THE RELATIONSHIP BETWEEN DEPRESSIVE SYMPTOM AND COGNITION IN OLDER ADULTS

Meina Zhang, Chooza Moon, and Karin Hoth, *University of Iowa, Iowa City, Iowa, United States*

Depressive symptoms and cognitive impairments are common problems in older adults. Rest-Activity Rhythm (RAR) encompasses sleep, physical activity, and rest in 24 hours. Often, individuals with depressive symptoms present altered RAR. The purpose of the study is to investigate the mediating role of RAR on the relationship between depressive symptoms and cognition in older adults using path analysis. Sixty community-dwelling older adults wore the actigraphy for two weeks (mean age ± SD=70 ±7.3, female n =32(53%)). We assessed RAR using nonparametric RAR metrics, such as interdaily stability (IS) (the similarity of one 24-hour cycle to the next), intradaily variability (IV) (the fragmentation of RAR within 24-hour periods), and relative amplitude (RA) (physical activity difference between the least active 5 hours and the most active 10 hours period). Participants completed 90 minutes' neuropsychological assessments (11 tests) across five domains to assess cognition and the Center for Epidemiologic Studies Depression scale to assess depressive symptoms. After adjusting for age, gender, and years of education, greater depressive symptoms were directly associated with reduced cognition (β= -0.406, P= 0.0001) as well as reduced IS (β= -26.9, P= 0.022). We also found direct positive relationships between IS (β=6.68, P=0.013), IV (β= 3.36, P=0.017), RA (β=3.42, P=0.015) and visuospatial memory, as well as IS (β=1.98, P=0.005), IV (β=3.77, P=0.004) and psychomotor speed. The indirect effect of depressive symptoms on cognition through RAR was not significant . Further research should be undertaken using a large, diverse sample, and prospective design.

Abstract citation ID: igad104.3109

### MEDITERRANEAN DIET ADHERENCE PROSPECTIVELY PREDICTS NINE-YEAR CHANGES IN EPISODIC MEMORY

Kelly Parker, Ryan McGrath, Yeong Rhee, and Jeremy Hamm, *North Dakota State University, Fargo, North Dakota, United States*

Mediterranean diet (MD) adherence has been shown to foster cognitive function during aging. **Methods** for measuring adherence to the MD typically provide or withhold points for foods consumed. Few investigations have examined how adherence to a Western adaptation of the MD may influence cognitive function over time in the United States. A modified MD that includes dairy products, whole grains, and plant-based proteins with limited consumption of sugar-sweetened beverages may be more attainable for many Americans than the traditional MD. This secondary analysis examined whether adherence to a modified MD was predictive of episodic memory and executive function over a 9-year study period. The second wave of the Midlife in the United States Study assessed general diet qualities with a food frequency questionnaire, and cognitive function was measured at both waves 2 and 3 (N = 816; age: 46 +/- 12 years; 56% female). Regression analyses were conducted to determine whether MD adherence predicted 9-year regressed change in cognitive function while controlling for age, sex, race, socioeconomic status (SES), and biomarkers of glucose homeostasis (hemoglobin a1c, insulin, and blood glucose). MD predicted changes in episodic memory (β = 0.075, p = 0.013) but not executive functioning (β = -0.028, p = 0.210). Exploratory moderator analyses suggested that MD associations with episodic memory were observed for only younger and middle-aged adults, females, White individuals, and persons with lower SES. Increased MD adherence may serve as a modifiable factor that supports cognitive function and preserved episodic memory during aging.

Abstract citation ID: igad104.3110

### PERCEIVED SOCIO-EMOTIONAL BENEFITS TO GAMEPLAY FOR MILD COGNITIVE IMPAIRMENT–CARE PARTNER DYADS

Kara Mumma[1], Patricia Griffiths[1], Frances Harris[1], Maribeth Gandy[1], and Chantal Kerssens[2], *1. Georgia Institute of Technology, Atlanta, Georgia, United States, 2. care.coach, Millbrae, California, United States*

Mild Cognitive Impairment (MCI) can increase social isolation by making it difficult to engage in once enjoyable activities with others, which in turn, can exacerbate cognitive decline. Games offer stimulating cognitive and social activity, but many are not designed for individuals with MCI. A structured interview was conducted to aid in the co-design of an adaptive game for individuals with MCI and their care partners. Preliminary results on the specific barriers and facilitators affecting game play to inform design and improve accessibility were previously reported (Kerssens et al., 2020). Here we report on the perceived socio-emotional benefits identified by seven dyads who were interviewed regarding game play experience and perceptions. The dyads comprised of an individual with a clinical diagnosis of MCI (85.7% male; age range 62-85 years) and a care partner (85.7% female; age range 41-76 years). The interviews were transcribed and analyzed thematically, independently coded, and reviewed until consensus was reached. The dyads iterated many "social," "emotional," and "impacts" of gameplay, prompting an examination of subjective elements of game play. For example, they enjoy the simplicity and familiarity of playing games – either as a playful diversion from their everyday routine or as part of a routine. Quantitative results are reported across dyads and themes. Descriptive quotes and individual qualia are presented to illustrate specific examples of benefits and detriments to gameplay. Results are discussed in terms of the implications of game play for fostering meaningful, potentially therapeutic social activities for individuals with MCI and their care partners.

Abstract citation ID: igad104.3111

### PHYSICAL ACTIVITY AS A MEDIATOR BETWEEN RACE/ETHNICITY AND CHANGES IN COGNITION

Jason Newsom[1], Mallory Kroeck[1], Em Trubits[1], Jennifer Saucedo[1], Anda Botoseneanu[2], Heather Allore[3], Corey Nagel[4], and Ana Quiñones[5], *1. Portland State University, Portland, Oregon, United States, 2. University of Michigan, Ann Arbor, Michigan, United States, 3. Yale University, New Haven, Connecticut, United States, 4. University of Arkansas for Medical Sciences, Little Rock, Arkansas, United States, 5. Oregon Health & Science University, Portland, Oregon, United States*

Racial/ethnic disparities in the rate of cognitive decline in older age have been established. Studies also report differences in physical activity across racial/ethnic groups. We investigated whether racial/ethnic differences in changes in cognition over a 12-year period (2006-2018) were mediated by physical activity using data from the Health and Retirement Study (N = 16,777, mean baseline age = 66.0 years). Structural equation modeling was used to estimate a latent growth curve model of changes in cognitive scores (27-item TICS) and investigate whether the relationship of race/ethnicity (non-Hispanic Black, Hispanic, non-Hispanic White) to change in cognition was mediated by physical activity after controlling for age, sex, education, marital status, personal wealth, and insurance coverage. Results indicated that Blacks engaged in significantly lower levels of physical activity than Whites (b = -.176, ♌ = -.123, p < .001), but there were no differences between Hispanics and Whites (b = .028, ♌ = .017, ns). Physical activity significantly predicted higher initial cognitive scores (b = 5.205, ♌ = .610, p < .001) and less decline in cognitive scores over time (b = 1.063, ♌ = .796, p < .001). The indirect (mediational) effect for the Black vs. White comparison on changes in cognitive score was significant (b = .187, ♌ = .152, 95% CI [.135,.244]). These results provide important new information for understanding how physical activity, a modifiable lifestyle factor, may help explain racial/ethnic disparities in cognitive decline in middle and later life, suggesting greater need to reduce sedentary behavior and increase activity.

Abstract citation ID: igad104.3112

### PREDICTORS OF EVERYDAY FUNCTIONING, ADLS/IADLS AMONG OLDER ADULTS USING THE ACTIVE STUDY

Deborah Dadson[1], and Karlene Ball[2], *1. University of Alabama, Birmingham, Birmingham, Alabama, United States*, *2. University of Alabama at Birmingham, Birmingham, Alabama, United States*

The increase in life expectancy over the years has triggered both researchers and the aging population to focus more on achieving an active engagement with life. This ability to perform daily living activities (functionality) is a key component in healthy aging which aids older adults live independently with or without disease and disability. Gaining insight into the key elements that enhance physical functioning is necessary as the issue of limited physical functioning affects not only older adults but also, middle aged adults. This study examines the predictors of everyday functioning, Activities of Daily Living (ADLs) and Instrumental Activities of Daily Living (IADLs) among 1920 older adults using data from the Advanced Cognitive Training for Independent and Vital Elderly (ACTIVE) study. ADLs and IADLs were self-reported. Results of the hierarchical linear regression at baseline indicate that, being female and being African American significantly predict lower performance on everyday functioning, after adjusting for age and years of education. Fewer falls in the past 2 months, better cognition and normal weight significantly predict high performance on everyday functioning (all p < .05). For ADLs, being African American and female gender significantly predict higher scores on ADLs while female gender predicted lower scores on IADLs. There were no differences on living arrangements and access to healthcare on everyday functioning, ADLs and IADLs. 43.1% of variability in IADLs were explained by the predictors. Findings from this study identify target populations for interventions to promote active engagement with life.

Abstract citation ID: igad104.3113

### RETIREMENT AND COGNITION: EXAMINING THE MENTAL RETIREMENT CONCEPT

Britney Veal[1], Hongdao Meng[1], and Ross Andel[2], *1. University of South Florida, Tampa, Florida, United States*, *2. Arizona State University, Tempe, Arizona, United States*

The concept of “mental retirement” posits that those approaching retirement experience less incentives to continue to engage with work, which is expressed as the so called “on-the-job” retirement effect, leading to negative changes in cognition. To test this notion, we set out to examine if participants who were about to voluntarily retire would have worse cognitive performance compared to those who continued working. We used members of the Health and Retirement Study who reported voluntarily retirement (n=333) in 2008 compared to those who continued working (n=2424). Cognition was assessed at four and two years prior to 2008 and in 2008. Analyses of covariance (ANCOVAs) were conducted with sociodemographic characteristics, chronic conditions, and baseline (2002) cognition as the covariates. Those who retired in 2008 and those who continued working varied on average age, 62.1±6.1 years vs. 60.7±6.1 years, p<.001, sex, 39% men vs. 45% men, p=.04, and average years of education, 13.3±2.6 years vs. 13.6±2.6, p=.04. ANCOVA results showed no significant between-group differences four years before retirement, F(1, 2669) = 0.00, p=.96, significantly worse cognitive scores among those who were planning to retire in two years compared to those who remained in the workforce, F(1, 2645) = 4.75, p=.03, and smaller, non-significant group differences in 2008, F(1, 2730) = 1.69, p=.19. These results support the existence of the “on-the-job” retirement effect, whereby the expectation of retired is accompanied by reduction in cognitive functioning, although it remains unclear whether this effect carries into retirement and/or under what circumstances.

Abstract citation ID: igad104.3114

### SEDENTARY BEHAVIORS AND COGNITION: DOES THE TYPE OF BEHAVIOR MATTER?

Mariana Wingood[1], Erin Bouldin[2], and Nancy Gell[3], *1. Wake Forest University, Winston-Salem, North Carolina, United States*, *2. University of Utah, Salt Lake City, Utah, United States*, *3. University of Vermont, Burlington, Vermont, United States*

Older adults spend 80% of their waking hours performing sedentary behaviors (SBs) that are either mentally active (e.g., using computers, socializing, and completing hobbies) or passive (e.g., television viewing and resting). Cross-sectional studies report an association between passive SB participation and cognitive impairments. However, longitudinal impact of baseline SB participation on cognition is unclear. We addressed this gap by using the National Health and Aging Trends Study (NHATS) dataset to run competing risks proportional hazards models. In our models, participants were identified as having an event (i.e., outcome) if their cognition scores dropped ≤ 1.5 standard deviations (SD) below the mean during follow-up (rounds 7-11, years 2017-2021). Competing risks included moving to long-term care facilities or dying. Our analyses included 1,574 community-dwelling older adults (mean age 84, SD = 7.1) who completed the sedentary module included in round 6 (year 2016) and had intact orientation, recall, and executive function at baseline. The majority of participants were female (55%) and non-Hispanic white (78%), and reported good or excellent health (80%). We identified that participating in active SBs had a protective effect on orientation, recall, and executive function (subdistribution hazard ratios [SHR]= 0.28-0.46, p< 0.001-0.02). Activities that had significant protective effects across all models included using computers (SHR =0.15-0.46, p=< 0.001-0.001) and socializing (SHR=0.27-0.52, p= < 0.001-0.05). Passive SBs did not have significant SHRs. Our identified protective effects highlight the potential of preventing cognitive impairments through active SB programs. Intervention studies are needed to develop program protocols and identify effectiveness of active SB programs.

Abstract citation ID: igad104.3115
### STRESSOR VARIETY, SEVERITY, AND IRREGULARITY AND THEIR ASSOCIATIONS WITH DAILY MEMORY LAPSES

Kristin Calfee[1], and Soomi Lee[2], *1. University of South Florida, Tampa, Florida, United States*, *2. The Pennsylvania State University, University Park, Pennsylvania, United States*

Long-term negative impacts of chronic stress on memory has been well established; however day-to-day associations between stressors and memory have not been fully examined, with lack of understanding on specific manifestations of stressors that may degrade daily memory. This study conceptualized three patterns of daily stressors: variety, severity, and irregularity. Data came from 1,132 adults who participated in an 8-day diary study as part of the Midlife in the United States study. Participants reported 7 types of stressors (e.g., work-related, home-related, discrimination) they encountered, which was summed daily (variety). On days when a stressor was encountered, they reported how stressful it was (severity). Individual standard deviation of stressor severity across the week (irregularity) was also calculated. Memory lapses were reported with 9 items each day. Results from multi-level modeling revealed that, at the within-person level, participants reported more memory lapses on days when they had greater variety (B= 0.126, SE=0.014, p<.001) or higher severity (B=0.071, SE=0.014, p<.001) of stressors. At the between-person level, participants with greater variety (B=0.717, SE=0.052, p<.001), higher severity (B= 0.168, SE= 0.031, p<.001), or greater irregularity (B= 0.141, SE= 0.047, p=.003) of stressors reported more memory lapses on average. These results were found after controlling for sociodemographic and health covariates. These findings highlight that even short-term stress, particularly severe or inconsistent stress may have a negative impact on day-to-day memory. Potential differences between older (age 65+) and younger (below age 65) adults in the relationship between these daily stressors and memory lapses are currently being explored.

Abstract citation ID: igad104.3116
### THE DYNAMIC AND RECIPROCAL RELATIONSHIP BETWEEN PERCEIVED DISCRIMINATION AND COGNITIVE FUNCTION IN LATER LIFE

Takashi Amano[1], Yuane Jia[1], and Audrey Redding-Raines[2], *1. Rutgers University, Newark, New Jersey, United States*, *2. Rutgers University-Newark, Newark, New Jersey, United States*

Studies have suggested that perceived discrimination is a potentially modifiable risk factor for cognitive health in later life. However, there is a lack of focus in the literature on the changes and directionality of the relationship between these two constructs. This study, based on socioemotional selectivity theory, investigates the dynamic and reciprocal relationship between perceived discrimination and cognitive function in later life. Data were drawn from four waves of the Health and Retirement Study (HRS; 2006, 2010, 2014, and 2018). We utilized a subsample of respondents who completed the psychosocial and lifestyle questionnaire in at least three waves. A total of 4,101 people who were 51 and older were included. Cognitive function was measured by the abbreviated version of the telephone interview for cognitive status (TICS-27). Perceived discrimination was measured using scores of the perceived everyday discrimination scale. Random intercept cross-lagged panel model (RI-CLPM) was utilized. Age was associated with lower perceived discrimination (b=-0.224, p<.0001). While lower cognitive function was cross-sectionally associated with higher perceived discrimination in all racial/ethnic groups, higher everyday discrimination predicted subsequent lower cognitive function only among White respondents. The model fit statistics were favorable: CFI=0.968; SRMR=0.045. Results suggested that a decline in cognitive function may exacerbate perceived discrimination which may result in further reduction of cognitive function in general. Lifetime experience of discrimination was discussed as a possible source of the racial/ethnic variations in the relationship. Further study is needed to examine whether this relationship holds among people with cognitive impairment or dementia.

Abstract citation ID: igad104.3117
### THE IMPACT OF LEISURE ACTIVITIES ON OLDER ADULTS' COGNITIVE FUNCTION

Jungjoo Lee[1], Seokmin Oh[2], Miriam Rodriguez[2], and Junhyoung Kim[2], *1. University of Southern Mississippi, Hattiesburg, Mississippi, United States*, *2. Indiana University Bloomington, Bloomington, Indiana, United States*

The purpose of the present study was to investigate the relationship between types of leisure activities (i.e., Leisure-Time Physical Activity (LTPA), intellectually stimulating activities, and community-based activity and the cognitive functions of older adults. We extracted 3,767 samples from the 2020 Health and Retirement Study (n = 15,723). Cognitive functions: (a) memory was assessed by both immediate and delayed recall tests, b) working memory was measured by a subtraction-by-7 test, and (c) attention and processing speed was assessed by the counting back word test. Hierarchical regression analysis was used to investigate the relationship between types of leisure and cognitive functions. LTPA, intellectual leisure, and community-based leisure were significantly associated with improved memory function. The intellectual activity was significantly associated with improved working memory and improved attention and processing speed. This finding provides practical implications for healthcare providers and therapists to design and implement various types of leisure for older adults.

### LATE BREAKING ABSTRACTS

### SESSION 8900 (POSTER)

### NIA MSTEM: ADVANCING DIVERSITY IN AGING RESEARCH THROUGH UNDERGRADUATE EDUCATION (ADAR)

Abstract citation ID: igad104.3118

### AGE AND EXERCISE DIFFERENTIALLY INFLUENCED MENTAL HEALTH DURING THE PANDEMIC

Zohra Feliachi, and Julie Hicks Patrick, *West Virginia University, Morgantown, West Virginia, United States*

With over 103,436,829 confirmed COVID-19 cases and more than 1.13 million deaths in the USA between January 2020 and August 2023 (WHO, 2023), the pandemic has affected most aspects of life. Regarding emotional wellbeing, most research has shown that older adults fared better than their younger peers (e.g., Ebert et al., 2022), although explanatory mechanisms for this effect have not been examined fully. We sought to fill this gap by examining the influence of age and exercise before and after vaccines were developed for the COVID-19 pandemic in the USA. Using data from the Behavioral Risk Factor Surveillance System (BRFSS), we compared a model in which age and exercise were used to examine the number of poor mental health days experienced. We compared responses from 423367 adults from 2018 and 2019 with responses from 417964 adults interviewed in 2021 and 2022. Results of a 4 (year) by 4 (age group) by 2 (exercise) ANOVA revealed significant effects for for each factor. More importantly, these main effects and two 2-way interactions were moderated by the 3-way interaction, $F(9, 841330) = 2.57$, $p=.006$. Adults who exercised after the pandemic was underway reported fewer poor mental health days relative to those who did not exercise, especially among those younger than age 45 years. Given the ongoing challenges to emotional wellbeing, younger and middle-aged adults may need additional support to age well. Exercise may provide one avenue to greater wellbeing, particularly in the face of non-normative and history-graded events like pandemics.

Abstract citation ID: igad104.3119

### AGE DIFFERENCES IN EMOTION-RELATED OUTCOMES OF COGNITIVE ENGAGEMENT AND MEANINGFULNESS AT WORK

Melanie Johnson[1], Claire Growney[2], Li Chu[2], and Laura Carstensen[2], *1. San Diego State University, San Diego, California, United States*, 2. *Stanford University, Stanford, California, United States*

Research suggests that there are few age differences in the enjoyment of cognitive engagement, but the importance of engagement in emotionally meaningful goals increases with age. In the present study we examined the interplay between cognitive engagement, meaningfulness at work, and related emotional states in older and younger workers. We used a five-day modified day reconstruction method in which 203 employees (aged 25–76) described their work, emotions, and cognitive engagement during the beginning, middle, and end of their workdays. A three-way interaction revealed that older employees who were more cognitively engaged at work reported higher levels of negative emotions than younger employees, and the association was reduced among those who perceived their work as meaningful ($b=-0.006$, $SE=0.003$, $p<.05$). Interestingly, perceived meaningfulness was a stronger predictor of pride at work among younger employees than older employees ($b = -0.015$, $SE = 0.006$, $p = .018$). Cognitive engagement at work was predictive of feeling pride among older employees, and not younger employees ($b=0.02$, $SE=0.01$, $p<.01$). Age also moderated the relationship between cognitive engagement and time-savoring, such that higher levels of cognitive engagement predicted more time-savoring at work among older employees, but not younger employees ($b=0.011$, $SE=0.005$, $p< 05$). Results suggest that the emotional benefits of perceiving one's work as meaningful may be different for younger and older workers. Furthermore, associations between cognitively engaging work and emotional outcomes such as time-savoring and pride may be especially relevant for older employees.

Abstract citation ID: igad104.3120

### AGING IN PLACE FOR OLDER HOMELESS VETERANS LIVING IN VA SUPPORTIVE HOUSING

Medha Maitra[1], and Rebecca Brown[2], *1. University of Georgia, Athens, Georgia, United States*, 2. *University of Pennsylvania, Philadelphia, Pennsylvania, United States*

Aging in place, defined as the ability to live comfortably, safely, and independently in one's own home and community, is a priority for many older adults and a growing focus of research and policy efforts. However, little is known about perspectives on and experiences of aging in place among the growing population of older adults with experiences of homelessness, whose perspectives may differ from adults in the general population. Using qualitative interviews with veterans living in U.S. Department of Housing and Urban Development - VA Supportive Housing (HUD-VASH) apartments and focus groups with HUD-VASH staff members, we explored perspectives on and experiences of aging in place, including barriers and facilitators to aging in place in this setting.

Abstract citation ID: igad104.3121

### DISPARITIES AND TRENDS IN COMPREHENSIVE PAIN MANAGEMENT AMONG THE ADULT POPULATION IN THE POST-OPIOID EPIDEMIC

Sebastián Spataro, and Molly Candon, *University of Pennsylvania, Philadelphia, Pennsylvania, United States*

**Introduction:** Chronic pain is an epidemic, but there are various therapies that are effective at treating it. Disparities in access to chronic back pain management are well documented. However, studies have largely focused on the gap in utilization between Black and White patients, and on the use opioid therapies. The extent of disparities in utilization for other groups and how disparities by demographics interact with socioeconomic characteristics, are unclear. **Methods:** Using insurance claims from Optum Data, we identified patients who had chronic low back pain between 2010- 2020. We measured trends in pharmacological and nonpharmacological therapies, disaggregating measures by sex, age, race/ethnicity, educational attainment, income, and parenthood. We estimated linear probability models to understand which demographic and socioeconomic characteristics were most predictive of utilization. **Results:** There were large differences in the utilization of pharmacological and nonpharmacological pain care, with surprising findings. First, we found that Black patients were the most likely to be prescribed opioids, as well gabapentin, muscle relaxers, and NSAIDs, followed by White patients, Hispanics; Asians were the least likely to engage in pharmacological therapy. Linear probability models indicated that the largest predictor of pharmacological and nonpharmacological pain management was education and income. **Discussion:** We document disparities in pain management, which point to important policy implications. Preferences of pain management warrant further attention, as our findings suggest that income and education are the largest driver. It could be that the time and out-of-pocket costs associated with nonpharmacological pain management reduce the likelihood that patients opt for these therapies.

Abstract citation ID: igad104.3122

### EDITING CDKN1A AND RASD1 IN A CELL CULTURE SYSTEM

Hope Vue, and Kellie Agrimson, *St. Catherine University, St. Paul, Minnesota, United States*

As an individual ages, there is a higher incidence of cancer and infertility. Testicular germ cell cancer rates have been rising year after year. According to the American Cancer Society 1 of every 250 males will develop testicular cancer during their lifetime. In this research project, we aim to develop a molecular toolbox to analyze gene function in male germinal cells using a CRISPR/Cas9 cell culture system. This biotechnology allows for the targeted alteration and cutting of specific DNA strands. To achieve this, we will utilize cancer cell lines (SUSA and NT2/D1 testicular cancer cell lines) and knock out two genes of interest: cyclin-dependent kinase inhibitor 1 (CDKN1A) and ras-related dexamethasone-induced 1 (RASD1). Our objective is to investigate the effects of gene knockout on cell proliferation. For this purpose, we designed primers and single-guide RNAs using bioinformatic databases: Benchling and Chop Chop. The tools we used were employed to amplify human genomic DNA and assess the effectiveness of the guide RNAs through in vitro cutting of our genes of interest, followed by gel electrophoresis observations. Based on the results obtained from these experiments, our next step is to electroporate the guide RNA and Cas9 complex to alter gene function in our cell culture system. Through a better understanding of gene function in testicular cancer cell lines, we hope to inform future therapeutic targets for both testicular cancer and age-related infertility.

Abstract citation ID: igad104.3123

### EXAMINING THE IMPACT OF BURNOUT ON RESEARCH PARTICIPATION IN CLINICIANS TREATING DEMENTIA

Anam Rawoof, Emily Summerhayes, Sonia Talwar, and Nancy Hodgson, *University of Pennsylvania, Philadelphia, Pennsylvania, United States*

Clinicians treating people living with dementia (PLWD) are likely to face burnout due to 1) the significant emotional burden of the job and 2) the increasing number of patients requiring dementia care. While much research has been conducted on the impact of clinician burnout on patient care and outcomes, more research is needed on its effects on clinician participation in research studies. This study will analyze qualitative data to examine the effect of clinician burnout on participation and intervention uptake in a dementia care research study.

Abstract citation ID: igad104.3124

### EXPLORING THE EFFECT OF EXOSOMES AND RADIATION ON GLIOBLASTOMA CANCER CELLS

Hannah Hausman[1], Linda Azizi[2], and Frank Pajonk[2], *1. St. Catherine University, St. Paul, Minnesota, United States*, *2. University of California, Los Angeles, Los Angeles, California, United States*

Glioblastoma is the most common and aggressive type of brain cancer, with higher incidences in aging adults over 75 and little to no long-term survival or cure. The median survival time under the current standard of care, surgical resection and chemoradiation, is 15-18 months. Glioblastoma tumors consist of multiple cell types, including mature brain cells in cell cycle arrest and cancer stem cells (CSCs) that replenish cancer cell populations. However, the cancerous non-stem cells are induced into a cell fate called glioma-initiating cells in response to radiation, where they begin to express stem-like phenotypes. Glioma-initiating cells proliferate at an aggressive rate and are the reason for cancer recurrence, making them a necessary target for new and more effective therapeutics. This research is studying the effect of exposure to exosomes, extracellular vesicles that carry proteins and RNA, on the stem-like phenotype expression of irradiated non-stem cells. Irradiated glioblastoma cells were exposed to varying concentrations of exosomes isolated from glioblastoma CSCs in vitro. The results showed a decrease in ZsGreen expression, a marker for stem-like phenotypes, as exosome concentration increased. Exposure of glioblastoma cancer cells to exosomes post-irradiation decreased the rate of transition to glioma-initiating cells. This suggests that CSC exosomes may signal that stem cells are present, decreasing the need for the formation of glioma-initiating cells. Further research is necessary to better understand the role of exosomes in glioblastoma tumors, however, this result may suggest exosomes as a potential way to delay or reduce the aggressive nature of glioblastoma cancer recurrence.

Abstract citation ID: igad104.3125

### EXPLORING THE POTENTIAL OF PHLPP INHIBITORS FOR OSTEOARTHRITIS TREATMENT

Leslie Victoria-Sanchez[1], Haydee Torres[2], and Jennifer Westendorf[2], *1. St. Catherine University, St. Paul, Minnesota, United States*, *2. Mayo Clinic, Rochester, Minnesota, United States*

Osteoarthritis (OA) is a degenerative joint disease characterized by pain, stiffness, and limited joint motion affecting 7% of the world's population. PH domain leucine-rich repeat protein phosphates (PHLPPs) have been identified as modifiable targets for cartilage regeneration and joint pain. PHLPP1 and PHLPP2 are abnormally expressed in osteoarthritic cartilage and sensory neurons. PHLPP1-/- mice are protected from cartilage degradation and pain-related behaviors

(allodynia and reduced mobility) after surgery that destabilizes the medial meniscus (DMM). Here, we investigate the effects of PHLPP inhibitors (i) on human chondrocytes and the effect of Phlpp1 deletion on cell populations within lumbar dorsal root ganglia (DRGs). Immunohistochemistry was performed on mouse DRGs by cryosectioning fixed L3-5 DRGs and using primary antibodies against Fabp7, Anti tubulin beta 3, and PGP9.5/UCHL1. Human chondrocytes were subjected to Cycloheximide chase experiments and treated with PHLPPi (NSC117079 or NSC45586), followed by Western blot analysis to assess protein levels including Phlpp1, Phlpp2, Sox9, p-AKT (Ser473), total AKT, and B-actin, at various time points. We found that PHLPPi prolongs the phosphorylated AKT response when compared to controls and does not affect degradation of Phlpp1 or Phlpp2; additionally, NSC117079 increased production of cartilage extracellular matrix components (glycosaminoglycans and aggrecan) in over 90% of human articular cartilage explants from OA patients. These data advance our understanding of how PHLPPi function within chondrocytes and provide a basis toward understanding their activity and safety in vivo. Our results indicate that PHLPPi NSC117079 is a novel osteoarthritis disease-modifying drug candidate that may have palliative effects.

Abstract citation ID: igad104.3126

### FUNCTIONAL MRI TO PREDICT RECOVERY FROM DISORDERS OF CONSCIOUSNESS AFTER BRAIN INJURY

Bianca Rodriguez, Christopher Boivin, and David Fischer, *Hospital of the University of Pennsylvania, Philadelphia, Pennsylvania, United States*

Disorders of consciousness (DoC) following brain injury are challenging for families, who often decide about end-of-life care without a clear understanding of a patient's recovery. This has special significance for older and geriatric populations, as they often rely on caregivers, proxies, and family members in making health and life decisions, and now face the added severity of decisions surrounding continuing treatment. In improving neuroprognostication, we hope to decrease this burden in aging-related decision-making. Functional magnetic resonance imaging (fMRI) has proved helpful in identifying brain activity in DoC with implications for recovery of consciousness, function, and quality of life. This project seeks to develop and implement a standardized fMRI protocol to best induce signals of consciousness in participants and predict the likelihood of neurologic recovery. From there, we will measure how the protocol contributes to an accurate outcome prediction following a DoC as compared to standard methods. The study will be conducted in the intensive care unit(s) of the Hospital of the University of Pennsylvania; we anticipate having an initial analysis of results by Fall of 2023. The study will be enrolling and conducting fMRI's with the device over one year under a randomized control trial. This study emphasizes improvements in the practice of neuroprognostication after stroke, anoxic brain injury following cardiac arrest, and other related conditions. The literature on these particular forms of traumatic brain injury and neuroprognostication remains limited, which is of disservice to older and geriatric populations who suffer these events at higher rates.

Abstract citation ID: igad104.3127

### IMPROVING AGING IN PLACE FOR OLDER VETERANS LIVING IN PERMANENT SUPPORTIVE HOUSING

Kelly Su[1], Medha Romee Maitra[2], Amanda Peeples[3], Octavia Goodman[3], and Rebecca Brown[4], *1. Cornell University, Ithaca, New York, United States*, *2. University of Georgia, Athens, Georgia, United States*, *3. Corporal Michael J. Crescenz VA Medical Center, Philadelphia, Pennsylvania, United States*, *4. University of Pennsylvania, Philadelphia, Pennsylvania, United States*

The average age of formerly-homeless Veterans living in HUD-VASH (Housing and Urban Development-Veterans Affairs Supported Housing) aged 50 and older is increasing. Homeless-experienced people experience accelerated aging, with premature onset of geriatric conditions and mortality. However, current supportive services at HUD-VASH do not address these conditions and enhance "aging in place," i.e., the ability to live comfortably and safely in one's own home and community. We conducted qualitative interviews and focus groups to determine barriers, facilitators, and adaptations needed to successfully implement interventions in HUD-VASH. We then used a modified Delphi process to reduce a list of 66 intervention elements based on perceived feasibility and importance. In total, 9 Veteran Affairs HUD-VASH staff members and 1 Veteran participated in 3 conference calls and surveys. The top-rated intervention elements were organized by: who should be involved in delivering the intervention (social workers, physicians, nurse practitioners, physician assistants, nurses), what types of goals/needs they will focus on (geriatrics, mental health needs, general health goals, dementia care, medication management), where and how should the intervention be delivered (in the home, at the VA), how long should the intervention be (when veterans are experiencing functional impairment, memory impairment, mental health problems, trouble caring for themselves, and when they are new to HUD-VASH) and when should the intervention be delivered (regularly scheduled and ongoing as needed). With feedback from expert stakeholders, our findings will be used to develop and implement interventions to improve aging in place for older Veterans living in HUD-VASH.

Abstract citation ID: igad104.3128

### INFLUENCE OF COGNITION AND FUNCTIONAL LIMITATIONS ON HOSPITALIZATIONS AMONG OLDER ADULTS IN THE UNITED STATES.

Augustine Boateng, and Ari Friedman, *University of Pennsylvania, Philadelphia, Pennsylvania, United States*

**Background:** Older adults frequently seek emergency department care, with 45 visits for every 100 older adults. Many health events in later stages of life can be attributed to health conditions and behaviors during middle-age. Yet, while qualitative and quantitative analyses exist, documenting the reasons for emergency department visits among all comers, no study focuses specifically on the determinants of hospitalization among middle-aged and older adults using the emergency department. Additionally, the impact on longer-term outcomes starting in mid-life remains unknown. **Objectives:** To determine whether impaired cognition, functional limitations, and multimorbidity in midlife predict subsequent hospitalizations in older age among older adults in the United

States using core gerontology concepts such as functional limitations, multimorbidity, and cognitive impairment. **Methods:** We performed secondary analysis of 2,221 participants from the Midlife in the United States (MIDUS) Series, a longitudinal study involving US adults aged 45 to 75 years using a balanced Generalized Estimation Equation (GEE) approach. Covariates such as as gender, education, marital status, income, race/ethnicity, social stressors, and social connectedness were adjusted for. **Results:** Findings suggests that impaired cognition (p< 0.05), having two or more health conditions (p< 0.001) and functional limitations (p< 0.05) in midlife increases the number of hospitalizations in old age a decade later. **Conclusions and Implications:** Health behaviors and conditions in midlife can significantly impact the number of hospitalizations among older adults and increase costs associated with hospitalizations. The promotion of preventive care, targeted interventions, and the adoption of healthier lifestyle habits in midlife may reduce hospital admissions in old age.

Abstract citation ID: igad104.3129
### ISOFORM-SPECIFIC FUNCTION OF C. ELEGANS TAU, PTL-1

Charlie Sconiers[1], Su-Hyuk Ko[2], and Lizhen Chen[2], *1. Howard University, Washington, District of Columbia, United States*, *2. University of Texas Health Science Center at San Antonio, San Antonio, Texas, United States*

As life expectancy increases with technological advancements, age-related dementia disorders, such as tauopathies, have become more rampant, impacting over 50 million people worldwide. Tauopathies are neurodegenerative diseases catalyzed by the dysregulation of tau protein in the brain. Alzheimer's disease, the most common tauopathy, has impacted more than 6 million Americans in 2023. It has been speculated that within tauopathies, dysregulation only occurs in specific tau isoforms. After alternative splicing, two types of tau isoforms (one with three microtubule-binding repeats and one with four microtubule-binding repeats) may cause functional differences within the brain. A protein in C. elegans called PTL-1 was discovered to have similar microtubule-binding repeats in its isoforms. In this project, worms with two types of PTL-1 isoforms (PTL-1A and PTL-1B) and wild type worms were aged and consistently tested for their touch sensitivity and speed to document whether certain microtubule-binding repeats negatively impact function. It was found that PTL-1A worms lost speed and touch sensitivity at an expedited rate compared to the other two samples of worms. It was also found that some PTL-1A worms developed abnormal branches throughout their exons. These findings suggest that PTL-1A overexpression might cause morphological defects. These study results will assist in the development of tauopathy research and can be applied in evaluating how isoforms impact the genetic spread of neurodegenerative diseases.

Abstract citation ID: igad104.3130
### RELATIONS BETWEEN MOOD AND DAILY ACTIVITIES: AN ECOLOGICAL MOMENTARY ASSESSMENT

Abel Pichardo[1], and Brent Mausbach[2], *1. San Diego State University, San Diego, California, United States*, *2. University of California, San Diego, San Diego, California, United States*

Behavioral activation (BA) is an evidence-based therapy for depression that has recently been applied for informal caregivers. Investigating the mechanisms of BA could lead to enhanced quality of care by optimizing guidance to patients. We examined concomitant and lagged associations between mood and daily activities in 50 informal caregivers using an Ecological Momentary Assessment approach via smartphone technology. Participants recorded the number and types of activities they did each day in a 14-day period. Activities were rated for enjoyment on a scale of 1 to 5 (5 being most pleasant). Mood for the day was rated on a scale of 1 to 5, (5 being the most positive). Linear mixed-model analyses examined concomitant and lagged (+/- 1 day) associations between activities and moods. Results indicated concomitant associations between mood and both frequency ($t = 2.36$, $p = .019$) and enjoyment of activities ($t = 4.11$, $p < .001$). Previous day's mood was not associated with either frequency or enjoyment of activities the following day. Previous day's enjoyment of activities was associated with mood the following day ($t = 2.34$, $p = .02$). There was no association between frequency of activities the previous day and mood the following day. These findings support BA theory by suggesting that increasing engagement in enjoyable activities may have positive impacts on mood not only in the moment but also have a lasting impact on future mood. Future research should expand this model by including covariates important to the population such as age and activity restriction.

Abstract citation ID: igad104.3131
### SUBJECTIVE COGNITIVE CONCERNS AND 5-YEAR NEUROPSYCHOLOGICAL TRAJECTORIES IN PARKINSON'S DISEASE

Britney Luu[1], Mary Ellen Garcia[2], Katherine Bangen[3], Douglas Galasko[3], and Kelsey Thomas[3], *1. San Diego State University, San Diego, California, United States*, *2. California State University, San Bernardino, San Bernardino, California, United States*, *3. University of California, San Diego School of Medicine, San Diego, California, United States*

Subjective cognitive concerns (SCC) are often associated with Alzheimer's disease (AD)-related cognitive declines in cognitively unimpaired (CU) individuals. However, it is unknown whether the same is true for CU individuals with Parkinson's disease (PD). Therefore, this study examined relationships between SCC and 5-year neuropsychological trajectories in CU individuals with newly-diagnosed PD (mean age=60.45 years; 35.2% female) in the Parkinson's Progression Markers Initiative (PPMI) cohort. Participants were classified as having SCC (n=52) or no SCC (n=204) based on participant or caregiver report for item 1 on the Unified Parkinson's Disease Rating Scale, part 1. First, baseline cerebrospinal fluid (CSF) biomarkers were examined by SCC group. Next, linear mixed effects models, adjusting for age, education, sex/gender, APOE genotype, psychiatric factors, and motor symptoms examined 5-year trajectories of individual neuropsychological measures by SCC status. Relative to participants without SCC, those with SCC had worse levels of CSF tau ($p=.011$), p-tau ($p=.016$), and p-tau/amyloid ratio ($p=.003$), but not amyloid or alpha synuclein. SCC was associated with faster rates of decline in learning ($\beta=-.059$, $p=.018$), memory ($\beta=-.050$, $p=.035$), and processing speed (β=-.052, p=.031), but not in semantic fluency, visuoperceptual abilities, working memory, or on a global cognition screening measure. Overall, SCC was associated with learning/memory and processing speed declines, but not other measures that may be more PD-specific (e.g., working memory, visuoperceptual). Given the elevated AD biomarkers, SCC may be particularly related to early changes due to co-occurring AD and PD pathology. Future work should investigate longitudinal AD and PD-related biomarker trajectories by SCC status.

Abstract citation ID: igad104.3132

### THE DEVELOPMENTAL WINDOWS WHEN EXPOSURE TO ANTIBIOTICS INCREASES THE RISK OF EARLY ONSET COLORECTAL CANCER

Nichelle Tomlin[1], Niti Jani[2], and Marie Pena[3], *1. Claflin University, Summerville, South Carolina, United States*, *2. Columbia, South Carolina, United States*, *3. University of South Carolina, Columbia, South Carolina, United States*

Colorectal cancer is the third most diagnosed cancer and the second leading cause of cancer deaths in the US. About 50,000 people die from colorectal cancer each year. Early onset Colorectal cancer (EOCRC) is a colon and rectum cancer occurring in people under 50. There has been a decline in cases in people over 50 due to healthier lifestyle choices. However, in the last few decades there has been a steady increase in EOCRC cases, and we do not know why. The increase in EOCRC has paralleled the increased use of antibiotics. Antibiotics are especially important for fighting infections, but overuse has become an epidemic. Our study tested the hypothesis that exposure to antibiotics in early life causes dysbiosis and inflammation in the colon that can lead to an increased risk for EOCRC. To test the hypothesis a mice model was used. Female mice who have given birth are giving antibiotic treatment for 7 days, so the children are exposed to the antibiotics through their mother's milk at an early age. The children are weaned from their mothers and at 5 weeks old they are given Azoyxmethane (AOM) treatment once a week for 6 weeks. Fecal pellets were collected before and after the AOM treatment of the kids and antibiotic treatment of the mother. The results show if given multiple dosages of amoxicillin there will be tumor burden in the proximal colon which can increase the risk for EOCRC.

Abstract citation ID: igad104.3133

### THE IMPACT OF FAR-INFRARED TECHNOLOGY ON QUALITY OF LIFE IN OLDER ADULTS

Shavonnye Rath, Joshua Guggenheimer, Melanie Homan, Marcella Myers, Jennifer Hutson, and Ginny Green, *St. Catherine University, St. Paul, Minnesota, United States*

Identifying methods of pain amelioration to meet the unique demands of older adults (OA) may be crucial to increasing quality of life (QOL) for this population. This study aimed to examine the effects of far-infrared heat (FIR) on pain management and QOL in OA. FIR utilizes a long light wavelength that simulates dry sauna-like conditions. Examining the relationship between FIR and pain is important due to the increased prevalence of chronic pain associated with aging and the corresponding impact it has on QOL and physical performance. Seventeen OA completed the study. Participants were randomly assigned to either a convective heat group (CON) or a convective and far-infrared heat (FIR) group, with convective heat set to 60°C (140°F.) Participants received six, 30-minute sessions. Physical assessments were measured (range of motion, gait speed, timed up-and-go, and hand grip strength) as well as pain severity and its interference with daily life, and the impact pain had on overall QOL. T-tests were used to compare pre- and post-assessment measures. Results indicated that pain severity was significantly reduced (from 3.31 to 2.5, $P < 0.05$) in the FIR group and that pain interference was significantly reduced (from 1.26 to 0.43, $P < 0.05$) in the CON group. Findings suggest that heat therapy successfully reduced pain over time, but that FIR heat specifically was not superior to that of convective heat alone. Further research is required to correctly identify the relationship between QOL and the use of FIR.

Abstract citation ID: igad104.3134

### THE ROLE OF NURSES IN DISORDERS OF CONSCIOUSNESS: IMPLEMENTATION OF SENSORY STIMULATION IN THE NEURO-ICU

Rhoodjinie Mentor[1], Bethany Young[2], Leah Coghlan[2], Christopher Boivin[2], and David Fischer[2], *1. University of Pennsylvania, Philadelphia, Pennsylvania, United States*, *2. Hospital of the University of Pennsylvania, Philadelphia, Pennsylvania, United States*

In the Neuro Intensive Care Unit (Neuro-ICU), patients with Disorders of Consciousness (DoC) often experience sensory deprivation. DoC, resulting from brain damage, encompasses varying degrees of wakefulness and awareness, ranging from coma to vegetative state and minimally conscious state. Research highlights a concerning trend in the Neuro-ICU, where older adults with DoC are more susceptible to mortality compared to their younger counterparts. This study emphasizes the indispensable role of nurses in effectively managing DoC within the Neuro-ICU, particularly through the thoughtful application of sensory stimulation interventions. Sensory stimulation is a promising approach that engages patients' various senses—touch, sight, sound, and smell, aiming to awaken awareness and cognitive responses in DoC patients. By fostering neural activity, sensory stimulation holds the potential to contribute to their recovery. Importantly, these techniques can be customized to each patient's specific condition and responses, and nurses, due to their continuous patient interactions, are uniquely positioned to administer these interventions at the bedside. Recognizing this vital role, evidence-based strategies are proposed, encompassing thorough literature analysis, assessment of nurses' attitudes, and exploration of potential interventions within the Neuro-ICU setting. This research highlights the significant impact nursing can have on elevating patient outcomes. It demonstrates how evidence-based interventions like sensory stimulation can be seamlessly woven into patient care, providing a valuable and holistic approach for individuals grappling with the intricate challenges presented by DoC.

Abstract citation ID: igad104.3135

### UNDERSTANDING THE INTERPLAY OF BRAIN HEALTH, COGNITION, AND AUTONOMIC FUNCTION

Evan Simms[1], Sarah Wilson[2], Roger Newman-Norlund[2], Julius Fridriksson[2], and Sarah Newman-Norlund[2], *1. Claflin*

*University, Orangeburg, South Carolina, United States*, 2. *University of South Carolina, Columbia, South Carolina, United States*

Research in clinical populations focused on various age-related diseases report multi-system difficulties in autonomic regulation, motor performance, cognition, and brain health. My research investigated the relationship between brain health, chronological age, and cognition. To assess these domains, I cross analyzed Compass 31, MoCA, and Brain Age Technology. The study population included 158 participants, both healthy and unhealthy. The collected data evaluated the relationship between brain age disparity, chronological age, and autonomic function. Correlation analysis, regression modeling, and subgroup comparisons were employed to explore potential associations, age-related patterns, and autonomic function variations. The findings from this research study contributes to a deeper understanding of the complex interplay between brain health, chronological age, and autonomic function. Age, White Matter Hyperintensities, Brain Age Gap, and Brain Age Variance demonstrate high significance when correlated with the MoCA total score. MoCA and all Compass 31 domains were found to be predictive of total white matter hyperintensity volume. This finding highlights a strong relationship between all three domains and suggests that when participants score high on both assessments, they should follow up with neuroimaging to discover potential brain damage. Future studies should identify risk factors for cognitive impairment and brain health that are also symptoms for autonomic dysregulation.

### SESSION 9000 (POSTER)

### LATE BREAKING POSTER SESSION I

Abstract citation ID: igad104.3136

### 25 YEARS AFTER THE PASSING OF THE NPO LAW: EXAMINING THE ORGANIZATIONAL CAPACITY OF OLDER VOLUNTEER ORGANIZATIONS

Li-Mei Chen, *George Mason University College of Public Health, Fairfax, Virginia, United States*

Japan has experienced a growth in non-profit organizations since the enactment of the NPO Law in 1998. In 2022, there were 175,046 volunteer organizations with approximately 6.7 million volunteers. Many of these non-profits providing community-based social services are disproportionately represented by older volunteers. Tbe benefits of older volunteerism have been well-documented stating that society gains from the older adults' contributions as well as older adults' lives are improved and enhanced through volunteerism. However, evidence is lacking as to how older volunteer organizations have evolved in the past 25 years. This study investigates the types and characteristics of non-profit organizations which recruit older volunteers based on their organizational capacity. Surveys were mailed to 500 volunteer organizations which provide social services in Japan. 155 (Findings from cluster analysis showed that there are types of organizations ranging from "Achiever" which excelled in all dimensions of organizational capacity to "Challenged" which lacked in all dimensions. Other volunteer types include "Government dependent" type which are non-profit organizations which depend on government programs, "Autonomous" type which excel in offering role flexibility to volunteers but lack capacity in all other dimensions, and "Bonding" type which is formed by a tight-knit group of persons and are no longer seeking new volunteers. Bivariate analysis was conducted to examine the relationship between the organization types and volunteer outcomes . "Achiever" type found an increase in volunteers and rarely experienced lack of volunteers while the "Challenged" type seldomly experienced an increase and frequently experienced lack of volunteers.

Abstract citation ID: igad104.3137

### 3-HK SUPPLEMENTATION INCREASES NICOTINATE PHOSPHORIBOSYLTRANSFERASE (NAPRT) EXPRESSION IN DROSOPHILA

Qiao Huang[1], Adam Said[2], Yuqiong Wu[1], Jeremy Walston[3], Reyhan Westbrook[1], and Peter M. Abadir[1], *1. Johns Hopkins University School of Medicine, Baltimore, Maryland, United States*, 2. *Emory College of Arts and Sciences, Atlanta, Georgia, United States*, 3. *Johns Hopkins University, Baltimore, Maryland, United States*

The increasing prevalence of physical frailty and aging related diseases in older adults necessitates the development of novel biological strategies to slow age-related declines. In humans, tryptophan metabolism through the kynurenine pathway (KP), which produces the energy metabolite and cellular cofactor nicotinamide adenine dinucleotide (NAD+), has been linked to various aging-related diseases including metabolic syndrome, neurodegenerative diseases, and physical frailty. Elevated levels of KP metabolites, such as 3-hydroxykinurenine (3-HK), correlate negatively with lifespan and physical function. A profile of downstream KP metabolites in the serum of older adults showed that 3-HK was increased in frail compared to both non-frail and young subjects, and liver kynurenine/tryptophan ratio correlates negatively with maximum lifespan in twenty-six mammalian species. To comprehensively explore the effects of elevated cytotoxic KP metabolites, we fed DGRP_229 Drosophila males diet infused with 3-HK. We found that treatment with 3-HK reduced average lifespan by 17% ($P< 0.0001$) compared against control. To elucidate the biological mechanisms of 3-HK treatment, we performed qRT-PCR on 11 genes involved in tryptophan metabolism. We found that treatment with 3-HK significantly decreased the expression of nicotinate phosphoribosyltransferase (Naprt) ($P=0.0022$), which plays an important role in NAD+ biosynthesis and protects against oxidative stress. Our findings indicate that some of the detrimental effects observed after 3-HK supplementation may be mediated by changes in NAD+ biosynthesis and highlight the potential feasibility of using NAD+ supplementation as an intervention method.

Abstract citation ID: igad104.3138

### A CONTENT ANALYSIS OF NATIONAL AFRICAN AMERICAN REPARATION COMMISSION'S REPARATIONS PLAN FOR OLDER BLACK AMERICANS

Jocelyn Brown, *University of Maryland Baltimore, Baltimore, Maryland, United States*

This policy content analysis critically examined the National African American Reparations Commission's (NAARC) 10-Point Plan for Reparations. As the first comprehensive plan for reparations, the latest revision has gained attention for its potential to rectify socioeconomic inequality. However, the degree to which the plan addresses the nuanced challenges faced by older Black Americans (e.g., higher rates of poverty) is unclear. Therefore, the objectives of this analysis were to: a.) assess the plan's potential to reduce inequality among older Black Americans, and b.) evaluate its capacity to address inequality over the life course and subsequent generations. Before the qualitative analysis began, a coding scheme was developed surrounding cumulative disadvantage theory. For example, codes addressing economic disparities, healthcare barriers, and intergenerational wealth transfer were developed. From there, the 12-page document was deductively coded by the author using this scheme. The findings suggest that the NAARC's 2023 revision is considerably comprehensive, providing strategies to address socioeconomic inequality on structural levels (e.g., funds for entrepreneurial development, criminal justice reform). Even so, the plan needs work to fully address the needs of today's older Black adults. To maximize the plan's impact on older Black Americans, the NAARC could incorporate long-term care provisions, family-centered support systems, and long-term services and supports. This analysis has significant implications for the NAARC's plan and continued fight for reparations. To maximize its impact on older Black Americans and promote intergenerational equity, policymakers should consider inclusive provisions for a demographic most directly haunted by the legacies of slavery and Jim Crow.

Abstract citation ID: igad104.3139

### A KEY DENDRITE DEVELOPMENT REGULATOR PLEXIN-A4 IS ASSOCIATED WITH DENDRITE MAINTENANCE IN AGED MICE

Jiyeon Baek[1], Haniya Naveed[1], Laura Muyingo[1], Angélica Jimenez Romero[2], Carol Eisenberg[1], Jack DeLucia[1], and Tracy Tran[1], *1. Rutgers, The State University of New Jersey, Newark, New Jersey, United States*, *2. Heritage University, Toppenish, Washington, United States*

There is significant loss of dendritic complexity with age, which may account for synaptic loss and cognitive decline in mammals. However, molecular mechanisms underlying these changes are unclear. Key molecular players in dendritic morphogenesis include the guidance cue semaphorin 3A (Sema3A). In developing cortical neurons, Sema3A signals through the Plexin-A4 receptor to induce basal dendritic arborization, while another closely related member, Sema3F, induces apical dendritic spine pruning. Interestingly, both ligands are expressed in adulthood, but their roles in age-associated dendritic remodeling are unknown. To address this, we established a model of aging mouse primary cortical neurons: 5, 15, and 30 days-in-vitro (DIV) representing young, mature, and aged timepoints. Treating with either ligand increases branching at 5-15DIV, but not 30DIV, suggesting age-associated changes in cellular responses to Sema3A/3F. Next, we examined mouse cortices in vivo and found both Plexin-A4 expression and dendritic elaboration decrease with age, suggesting a need for Plexin-A4 to maintain dendritic complexity at older ages. In 6-month-olds, mutant Plexin-A4 mice have less branching than wildtypes, but we found no further decline in 18-month-olds, likely because there can be no age-associated downregulation of Plexin-A4 signaling if the receptor is not present. Finally, we found increased levels of active-RhoA GTPase (a downstream cytoskeleton regulator) in Plexin-A4-/- 6-month-old cortices versus controls. Additionally, our preliminary data trends towards increased active-RhoA in 18-month-olds versus 6-month-olds. Collectively, our study provides novel insights into the diverse cellular functions of semaphorin-plexin, a signaling pathway important for nervous system development, in age-associated dendritic loss and cognitive decline.

Abstract citation ID: igad104.3140

### A KNOWLEDGE-DRIVEN BIO-BEHAVIORAL NETWORK MODEL OF THE INFLAMMASOME AND CALORIC RESTRICTION

Thomas Chacko[1], Gordon Broderick[1], Nadia Moore[1], Daniel Parker[2], William Kraus[3], and Kim Huffman[3], *1. Rochester Regional Health, Rochester, New York, United States*, *2. Duke University School of Medicine, Durham, North Carolina, United States*, *3. Duke University, Durham, North Carolina, United States*

Healthy dynamic equilibrium between immune and endocrine function can be altered not only by illness but by changes in lifestyle including caloric intake. The Comprehensive Assessment of the Long-term Effects of Reducing Intake of Energy Study (CALERIE™) is a large multi-year longitudinal study funded by the National Institute on Aging investigating what metabolic adaptations would result from a 25% caloric restriction sustained for 2 years and how these might positively affect primary aging and inform on aging biomarkers and chronic diseases of aging. A broad range of behavioral and physiological markers were measured at different levels of biology, making comprehensive integration and interpretation of data challenging. Here we propose a knowledge-based framework for integrating data that fully applies our current understanding of biobehavioral regulatory biology. Leveraging the Elsevier Knowledge Graph and the MedScan Natural Language Processing (NLP) engine we extracted from peer-reviewed literature 816 documented regulatory relationships linking a subset of 85 data elements readily available, including ~ 15 immune inflammatory and 12 endocrine markers. With a connection density of ~11%, this network was supported by > 23,000 citations or a median of 7 citations per regulatory interaction. A structural analysis of connectivity patterns identified inflammatory marker CCL2, insulin and testosterone as key mediators of large-scale coordinated response to caloric restriction. Ongoing analysis will identify immune and endocrine decisional logic that drives network dynamics, providing candidate intervention targets potentiating caloric restriction that remain explainable in the context of established pathway biology.

Abstract citation ID: igad104.3141

### A LONGITUDINAL STUDY ON SNAP PARTICIPATION AND SELF-REPORTED HEALTH AMONG DIABETIC AND NON-DIABETIC INDIVIDUALS

Danielle Duran, and Nasim Ferdows, *Northeastern University, Boston, Massachusetts, United States*

Self-reported health is a strong predictor of future health status, new morbidity, mobility decline, illness recovery, and healthcare utilization. Because self-reported health is rooted in social position and social comparison, it dually reflects individual health perceptions and health inequities, especially among those who experience a social struggle for resources. Food insecurity is associated with higher risk of chronic diseases and higher mortality rates. Interventions such as the supplemental nutrition assistance program (SNAP) were created to help address food insecurity. Although morbidity and mortality risks caused by food insecurity is exacerbated for those with nutrition-centered chronic disease, such as diabetes, limited research has assessed the impact of food insecurity and SNAP participation on health among diabetics. Using Health and Retirement Study data, we explore the effect of SNAP participation and food insecurity on the likelihood of reporting fair or poor health comparing 24,446 diabetes to 69,260 non-diabetics individuals aged 65 and above (2004-2020). Our analysis controls for key socioeconomic factors, including age, gender, race/ethnicity, and wealth, accounting for the complex design of HRS. Our key findings reveal that food insecure diabetics have significantly higher odds of reporting fair/poor health, and diabetic SNAP participants also had significantly higher odds of reporting fair/poor health. Our study demonstrates how food insecurity and diabetes concurrently influence health and how, despite the presence of resources, such as SNAP, there was no improvement in self-reported health. This study is important as it highlights the need for future research on the effects of SNAP participation and self-reported health.

Abstract citation ID: igad104.3142

### A META ANALYSIS ON THE ASSOCIATION OF NOVEL PLASMA BIOMARKERS TESTING FOR EARLY DETECTION OF ALZHEIMER'S DISEASE

Charles Dunnam, *Wichita State University, Wichita, Kansas, United States*

The latest research confirms the heterogeneity of Alzheimer's Disease Related Dementia (ADRD). This justifies a meta-analysis study on the association of novel plasma biomarkers with screening assessments for early detection of ADRD. The conceptual framework model will be analyzed from the 2020 edition of the GSA KAER Toolkit with updates. The purpose of the current study is two-fold. First, to determine the need of a universal protocol method for early assessment of ADRD by primary care teams. Second, implementation of Food and Drug Administration (FDA) approved novel plasma biomarkers testing as a follow-up protocol to a screening assessment. The two suggested protocols have recently established Medicare coverage. The study will conclude with an extensive review of the known novel plasma biomarkers that are available for testing, at present and a brief overview of available screening assessments for early detection of ADRD.

Abstract citation ID: igad104.3143

### A MIXED METHODS APPROACH TO UNDERSTANDING SPOUSE AND CHILD CAREGIVERS' EXPERIENCES OF LUCID EPISODES IN DEMENTIA

Kyungmin Kim[1], Lauren Bangerter[2], Dawne Finnie[3], Joseph Gaugler[4], Lapid Maria[3], Theresa Frangiosa[5], and Joan Griffin[3], *1. Seoul National University, Seoul, Republic of Korea*, *2. Medstar Health, Minneapolis, Minnesota, United States*, *3. Mayo Clinic, Rochester, Minnesota, United States*, *4. University of Minnesota, Minneapolis, Minnesota, United States*, *5. Faerge Drinker, Washington, District of Columbia, United States*

Lucid episodes (LE) in people living with late-stages of dementia (PLWD) have been reported anecdotally, but less is known about how this seemingly unexpected phenomenon is experienced by family members. This study aims to examine variability in family caregivers' experiences with LE, focusing on the two most common groups of informal caregivers, spouses and children—whether they may exhibit differential appraisals of and responses to LEs. Using a sample of former and current family caregivers from UsAgainstAlzheimer's A-LIST, we conducted an online survey to spouse and child caregivers (N = 387). Qualitative semi-structured interviews were also conducted with a subset of these caregivers who have witnessed an LE (n = 22). Survey results indicate that child caregivers were more likely to witness a LE. Among "former" caregivers who have witnessed a LE (n = 140), spouses were likely to appraise LEs more negatively and make changes in care decisions (i.e., end-of-life planning and financial decisions) after LE, compared to child caregivers. Among "current" caregivers who have witnessed a LE (n = 80), spouses were more likely to indicate no special circumstances prior to LE, whereas children were more likely to indicate LEs associated with visits from friends and family; there was no difference in positive and negative appraisals of LE between current spouse and child caregivers. Content analysis of qualitative interviews revealed that observed differences between spouses and children were related to their different caregiving contexts, such as relationship history, living arrangement, expectations/motivation, and care resources.

Abstract citation ID: igad104.3144

### A NEW METHOD TO IDENTIFY MEDICARE CLAIMS-BASED FACTORS ASSOCIATED WITH POST-ACUTE RECOVERY IN OLDER ADULTS WITH ADRD

Chixiang Chen[1], Biyi Shen[2], Jason Falvey[1], Michelle Shardell[1], and Haoyu Ren[3], *1. University of Maryland School of Medicine, Baltimore, Maryland, United States*, *2. Regeneron, Ellicott City, Maryland, United States*, *3. University of Maryland, Baltimore County, Baltimore, Maryland, United States*

Nearly 300,000 older adults experience a hip fracture every year, the majority of which occur following a fall. Unfortunately, recovery from hip fracture is poor, where older adults diagnosed with Alzheimer's Disease and Related Dementia (ADRD) spend a particularly long time in hospitals or rehabilitation facilities during the post-operative recuperation period. Because older adults value functional recovery and spending time at home versus facilities as key outcomes after hospitalization, identifying factors that influence days spent at home after hospitalization is imperative. However, few rigorous analytical approaches are available to help overcome potential sources of analysis bias such as hospital-level unmeasured confounders, informative hospital size, and loss to follow-up due to death. To overcome these challenges, we developed a new data science approach equipped with unsupervised learning to simultaneously handle statistical complexities that are often encountered in research using large administrative claims databases. The proposed approach is accessible and performs well in simulation studies. Analysis of 16562 Medicare beneficiaries diagnosed with ADRD who were aged 65 or older and experienced a hip fracture between 2017 and 2019 demonstrated that older age, longer length of stay in the hospital, more chronic health conditions, and less days at home before fracture are significantly associated less days spent at home in the 12-month period after hip fracture ($p<.05$), while existing methods fail to detect some effects. Findings demonstrate that the proposed method can overcome bias in health services research using Medicare data and can lead to findings that may inform care and resource allocation.

Abstract citation ID: igad104.3145

### A PORTABLE TECHNOLOGY-BASED BALANCE ASSESSMENT PREDICTS FALL RISKS IN LOW-INCOME COMMUNITY-DWELLING OLDER ADULTS

Evette Trahan[1], Renata Komalasari[2], and Ladda Thiamwong[1], *1. University of Central Florida, Orlando, Florida, United States*, *2. Penn State Nursing, State College, Pennsylvania, United States*

Despite strong evidence on the association between balance performance and fall risks, there is a need for further studies among racially diverse low-income communities. Sixty-two healthy, low-income community-dwelling older adults (mean 74.11, SD: 6.34, 61-88 years old) in Central Florida were recruited and administered the BTrackS Balance Tracking System (BBS) for measuring balance performance. BBS is a postural sway-based balance performance assessment, where participants were guided to stand still on the BBS plate with hands on hips and eyes closed for about 1-2 minutes. Fall risk was assessed by the Centers for Disease Control and Prevention (CDC)'s twelve-item self-assessment checklist, Stopping Elderly Accidents, Deaths & Injuries (STEADI). About 58% of participants were African Americans, completed high school (54.8%), lived alone (67.7%), and earned just enough financially (54.8%). The simple regression results indicated that the model explained 14.9% of the variance ($R2=.149$, $F(1,60) = 10.47$, $p = .002$). Higher BBS scores ($\beta = .100$, $p = .002$) were significantly associated with higher fall risk scores. As older adults had reduced balance, their fall risks increased. The findings of this study confirmed the benefits of using a combination of a simple fall risk checklist and a portable assessment technology in enhancing the accessibility of balance and fall risk screening. Furthermore, a technology-based balance performance assessment is key in tailoring fall interventions for older adults with poor balance.

Abstract citation ID: igad104.3146

### A QUALITATIVE ANALYSIS OF A DIGITAL FALL PREVENTION EXERCISE PROGRAM FOR OLDER ADULTS WITH INCREASED FALL RISK

Shannon Farrell[1], Nicole Bajdek[1], Mary Dishaw[1], Pamela Garabedian[2], Alisha Williams[3], Naomi Hachen[3], Kieran Reid[4], and Nancy Latham[1], *1. Brigham and Women's Hospital, Boston, Massachusetts, United States*, *2. Mass General Brigham, Somerville, Massachusetts, United States*, *3. Best Buy Health, Richfield, Minnesota, United States*, *4. Brigham and Women's Hospital/Harvard Medical School, Boston, Massachusetts, United States*

Falls are a significant public health problem; one third of individuals aged 65 years or older fall each year. Strength and balance exercises reduce fall risk, but most older adults are inactive. Individuals at risk of falls need clear guidance to ensure exercises performed at home are safe and provide adequate challenge. The aim of this qualitative study was to investigate the experiences and perceptions of older adults with increased fall risk enrolled in a 3-month digitally delivered home-based fall prevention exercise program (DFP). Semi-structured interviews were conducted by an interview specialist on a sample of 16 participants (81% female, age 77.3±5.8 years). Interviews were transcribed, imported, and coded into Dedoose, a tool for qualitative analysis. Codes were refined with each interview and themes were generated from the final codes. Three themes were identified: adherence to a home-based digitally delivered fall prevention exercise program, impact of fall prevention exercises on activities of daily living (ADL), and benefits of home-balance exercises. Participants attributed adherence to the home exercise program with minimal in-person visits. Participants reported fear of falling increased as they aged; upon completion, participants felt reduced fear of falling in their ADL. Balance exercises were the most appealing due to the level of difficulty and motivation to improve balance. Participants recommended changes to improve the DFP exercise program, with majority wanting to continue the program. This qualitative analysis provides guidance to health professionals about the acceptability and recommended changes for a digitally delivered home fall prevention exercise program.

Abstract citation ID: igad104.3147

### A QUALITATIVE ANALYSIS OF UNDERGRADUATE STUDENT AND OLDER ADULT REFLECTIONS ON AN ORAL HISTORY PROJECT

Gabriele Moriello[1], Katie Ehlman[2], and Mary Ligon[3], *1. Utica University, Utica, New York, United States*, *2. University of Southern Indiana, Evansville, Indiana, United States*, *3. York College of Pennsylvania, Grantley, Pennsylvania, United States*

Interaction between generations is diminishing as extended family members live further apart and as neighborhoods, communities, and institutions become increasingly age-segregated. Fostering intergenerational contact can benefit both parties involved. The aim of this qualitative study was to develop a deeper understanding of the meaning of participating in an intergenerational oral history project using a phenomenological approach. Undergraduate students (n=21) interviewed a family member or friend in three, 45-60-minute sessions. After the last interview, each older adult/student pair met to share what the project meant to them. Audio recordings of these final reflections were analyzed using a thematic analysis process. Five themes emerged: Reflection, Learning, Meaningful Connections, Evoked Emotions, and Advice. To the surprise of the researchers, the five themes were common to both students and older adults. The subthemes, however, differed between groups within three of the themes (reflection, learning, and advice). Consistent with previous research, findings support that participating in an oral history project is beneficial for both parties. This study adds to the literature by revealing that both older adults and students reflected on their lives. Older adults tended to accept their lives (or not) after reflecting. Both groups learned as a result of this project and the project evoked emotions. Theoretical study implications include that engaging in reminiscence supports psychological development in late-life, according to Erikson's model. Further, congruent with Mezirow's theory of transformative learning, the experience challenged participants to consider how telling and/or hearing a life story fits or did not fit with prior thoughts.

Abstract citation ID: igad104.3148

### A RECOMMENDATION FOR A UNIFORM DEFINITION OF DISTANCE CAREGIVING: A SCOPING REVIEW

Andrea Budnick, Farina Bünning, and Adelheid Kuhlmey, *Charité - Universitätsmedizin Berlin, Berlin, Berlin, Germany*

Distance caregiving has increasingly attracted the attention of the gerontological research community. This indicates the importance of this caregiving arrangement in the face of progressive demographic change including fewer and fewer individuals providing local care. There have been attempts to define distance caregiving for more than two decades. The literature focuses predominantly on North America and Great Britain (e.g. Cagle & Munn, 2012; Franke et al., 2019). Although Cagle and Munn defined distance caregiving broadly, recent literature continues to focus on individual aspects, such as distance or time. Thus, future research on distance caregiving may benefit from using a uniform definition to compare findings across studies. Accordingly, we reviewed the literature of the last five years by means of a scoping review in 13 English and German databases. Following the systematic review on distance caregiving by Franke et al., we used May 2019 as the starting point of our search. Two independent raters classified the articles using prespecified inclusion and exclusion criteria. We identified a total of N=14,546 articles. After removal of duplicates, and title and abstract screening, we identified a total of n=59 articles for full text screening. We found the broad definition of distance caregiving by Cagle and Munn in 26 articles. The remaining articles referred to aspects like distance in miles or kilometers, time required, or other circumstances. In sum, our findings indicate that gerontological research on distance caregiving does not always build on existing knowledge.

Abstract citation ID: igad104.3149

### A REVIEW OF PAIN MANAGEMENT FOR PEOPLE WITH DEMENTIA: A GENERAL SURGICAL PERSPECTIVE

Emily Neville[1], Ruth Wagstaff[2], Snezana Stolic[3], and Christine Neville[3], *1. University of Notre Dame Australia, Wagga Wagga, New South Wales, Australia*, *2. Charles Darwin University, Darwin, Northern Territory, Australia*, *3. University of Southern Queensland, Toowoomba, Queensland, Australia*

As the global population ages and the prevalence of dementia increases, more people with dementia will present for surgery. Perioperative pain management, particularly for people who are not able to communicate effectively, is challenging for clinicians. Poor surgical outcomes may result if pain is not managed appropriately. The objective of this review was to synthesize the existing research about how pain is managed for people with dementia in the perioperative setting, discuss the implications for general surgery so clinical practice and surgical outcomes are improved. After an extensive database search, the themes identified were pain assessment including commonly used rating scales, the types and amount of pain relief, that people with dementia received less analgesia than people without dementia and the challenges for effective pain management. Findings determined that pain management needs a stronger evidence base, challenges are due to the impaired ability of the person with dementia to communicate pain and that clinicians have difficulty understanding pain behavior in people with dementia. Adequate pain management for people with dementia in the perioperative setting is important for a faster and better recovery and this matter will only become a bigger issue as the ageing population increases.

Abstract citation ID: igad104.3150

### A SCOPING REVIEW OF PHYSICAL ACTIVITY IN ADULTS OVER 50 WITH AUTISM: MANY BARRIERS AND SPARSE DATA

Connie Kartoz, Rebeca Jefferson, Antigone Antonakakis, and Chase Eisenberg, *The College of New Jersey, Ewing, New Jersey, United States*

The population of older adults with autism is growing, yet the literature for this group is still relatively sparse (Mason et al., 2022). Adults with autism are reported to have multiple co-morbidities and a reduced life span (Hand et al., 2020). While physical activity (PA) is known to reduce chronic disease burden, extant research indicates young adults with autism do not engage in physical activity (PA) with the same frequency as neurotypical peers. Little is known about PA for older adults with autism. This scoping review aimed to explore research about PA for older adults with autism. Following the PRISMA-ScR guidelines, 5 databases were searched (2018 and later) with keywords 'autism', 'older adults', 'PA' and age group delimiters. Initially, no articles were found, and the search was expanded to include all adults. After eliminating duplicates, 121 studies were reviewed, with 31 meeting the criteria (PA, autism and no participants less than 18) and 9 having any participants over 50; in each of these 9 studies, the number of participants over 50 was less than 100. Only 2 studies focused on adults over 50. Level of evidence was low. The limited data suggests those aging with autism do not engage in recommended levels of PA, experience barriers to the same, but that interventions improve PA and possibly quality of life. Based on this review, there is a dire need to design interventional research regarding increasing PA in older adults with autism to improve equitable health outcomes for this at-risk group.

Abstract citation ID: igad104.3151

### A SCOPING REVIEW OF SOCIAL DETERMINANTS OF HEALTH IN CHARLES BONNET SYNDROME RESEARCH

Joe Strong[1], Takashi Amano[2], Isabella Hoàng[1], Hannatu Amaza[1], and Rachel Tilsen[3], *1. University of Wisconsin - Madison, Madison, Wisconsin, United States*, *2. Rutgers University, Newark, New Jersey, United States*, *3. University of Wisconsin School of Medicine and Public Health, Madison, Wisconsin, United States*

Charles Bonnet Syndrome (CBS) is a neurological condition featuring pseudohallucinations that affects previously sighted individuals, many of whom are older adults. The CBS literature primarily concerns prevalence estimates, neurological studies, and experiences of people living with the condition; it is not clear what is known concerning social determinants of health (SDoH). We conducted a scoping review guided by Healthy People 2030 SDoH categories including Economic Stability, Education Access & Quality, Health Care Access & Quality, Neighbourhood & Built Environment, and Social & Community Context. Research articles included in this study represent 5 countries on 5 different continents. Sample size ranged from 5 to 88 across all included studies (M = 40.25, Median = 33). Two studies did not report enough info to extract mean age; Mean of mean age across the remaining 6 studies is 63.25 (Range: 27.8-79.47). Across the 8 included articles, 9 different SDoH variables were identified, representing 4 of the 5 Healthy People 2030 SDoH categories, with the exception of Health Care Access & Quality. All studies reported age and gender, most reported education (n = 6, 75%) and living arrangement (n = 5, 62.5%), and half (n = 4) reported marital status. Few studies, however, reported race (n = 2, 25%), loneliness/social life (n = 2, 25%), occupational status (n = 1, 12.5%), and living environment (n = 1, 12.5%). This study highlights the need for expanded inclusion of SDoH variables in CBS research to fill gaps in the knowledge base in this understudied condition.

Abstract citation ID: igad104.3152

### A SCOPING REVIEW ON BARRIERS AND FACILITATORS OF COMMUNITY ENGAGEMENT IN LEARNING HEALTH SYSTEMS

Lillian Hung[1], Karen Lok Yi Wong[1], Annette Berndt[1], Tracy Windsor[1], and Krisztina Vasarhelyi[2], *1. University of British Columbia, Vancouver, British Columbia, Canada, 2. Vancouver Coastal Health, Vancouver, British Columbia, Canada*

This scoping review identifies the challenges and enablers of community engagement in Learning Health Systems (LHS), an area where elder and health care have growing interests on. LHS aims to improve healthcare delivery, translate knowledge into clinical practice, and emphasize patient-centred outcomes. We followed the Joanna Briggs Institute's (JBI) scoping review methodology. First, keywords and index terms were identified from MEDLINE and CINAHL databases. Second, using the keywords and index terms identified in the first step, other databases like Web of Science, PsycINFO, and Google were searched. Third, the reference lists of the chosen literature were searched. The results include 25 papers. The Assessing Community Engagement (ACE) Conceptual Model by Aguilar-Gaxiola et al. was utilized to inform the analysis. Active community engagement enhances the relevancy and applicability of health research and fosters trust and rapport between healthcare providers and their communities. It is essential to have appropriate support, mutual respect, culturally sensitive and locally relevant processes with common-ground language. We identified several barriers to engagement: 1) meaningful motivations, 2) logistical challenges in processes, 3) disparities in power dynamics, 4) cultural misunderstanding, and 5) lack of knowledge and skills. The review underscores the need to move beyond tokenistic engagement towards genuine community collaborations. Future research should investigate the facilitators that enhance such meaningful partnerships.

Abstract citation ID: igad104.3153

### A SIGNAL FOR HEALTH EQUITY: DIFFERENCES IN WHOLE HEALTH AND SOCIAL NEEDS AMONG MEDICARE ADVANTAGE SUBPOPULATIONS

Kelsey McNamara, and Ellen Rudy, *Papa Inc., Miami, Florida, United States*

CMS' 2024 Medicare Advantage (MA) Final Rule introduced the health equity index (HEI) to encourage plans to advance equity and improve outcomes for enrollees with social risk factors. Enrollees of focus include those receiving a low-income subsidy, dually eligible for Medicare and Medicaid, and/or having a disability. These populations historically have higher unmet social needs and poorer health outcomes. The purpose of this epidemiological study was to compare self-reported whole health and social needs among MA subpopulations. Analysis included 28,588 MA members who were screened for a companion care program (January–June 2023). Questionnaire included a social needs screener, UCLA-3 Loneliness Scale, and CDC's Healthy Days Measure. To compare across metrics/domains (mental health, physical health, whole health, social support, loneliness), data was normalized 0 to 100 where higher scores represent better outcomes. Among the cohort, average age was 72, 31% male, and 37 states represented. Subpopulations included 70% traditional MA (TMA), 13% dual-eligible (DE), 17% people with disabilities (PWD), < 1% veterans (V). Average outcomes reported by subpopulations were (TMA; DE; PWD; V): number of unmet social needs (1.6; 2.3; 2.6; 1.4); social support score (63; 54; 53; 70); mental health score (74; 63; 59; 73); physical health score (57; 50; 48; 52); whole health score (65; 56; 53; 65). People with disabilities reported the lowest scores and indicate an important subpopulation in need. Meeting the HEI, and ultimately improving outcomes for some of the most historically marginalized members, will require an individualized and holistic approach to health and social care.

Abstract citation ID: igad104.3154

### A SYSTEMATIC REVIEW OF THE ART-BASED RESEARCH WITH OLD ADULTS IN SOUTH KOREA

Yeojin Yoon, *Chung-Ang University, Seoul, Republic of Korea*

This study aims to review and summarize the literature on the use of Arts-based Research(ABR) with older adults in South Korea. The study was conducted as a systematic literature review. ABR is method that utilizes the arts to expand horizons of awareness. In South Korea, there is a large gap between the literacy levels of young and older adults and the differences in their cultures. Therefore, using art to convey expressions that are difficult to communicate through text and utilizing common values through art can be more effective than using text. This study was designed using Electronic databases. Keywords for older adult and ABR were searched in Electronic databases, and Korean literature published in peer-reviewed journals before December 31, 2022 were identified. After review of the 1515 abstracts yielded from the databases and the reference lists of the associated articles, 5 eligible studies were identified, and relevant findings extracted. The five eligible studies will not have found all of the ABR on older adults published in South Korea. However, this shows the level of research and interest in arts-based research in Korea. These five studies had several commonalities. First, they utilized ABR as an extension of qualitative research methods, with arts-based research as another methodology. Second, the distinction between art-based research and qualitative research was not clear in the literature, so art-based research methods were used, but art-based research was not mentioned. Based on these findings, it was suggested that ABR on older adults needs to be revitalized and diversified.

Abstract citation ID: igad104.3155

**A SYSTEMS APPROACH TO PRACTICE TRANSFORMATION FOR BRAIN HEALTH INTEGRATION INTO COMMUNITY HEALTH CENTER SERVICES**

Cassie Lindholm, and Cheryl Modica, *National Association of Community Health Centers, Bethesda, Maryland, United States*

Community Health Centers are America's largest and most successful primary care system, providing access to quality healthcare services to more than 30 million Americans through more than 14,000 service delivery locations regardless of patients' ability to pay. The National Association of Community Health Centers (NACHC) provides the training and education that health centers need to advance practice transformation and improve care delivery models to succeed in value-based care and meet the needs of patient populations. Through the Value Transformation Framework (VTF), NACHC provides an approach to support health center systems change, organizes and distills evidence-based interventions, provides knowledge, tools and resources, and links performance to the Quintuple Aim – improved health outcomes, patient and staff experience, reduced costs, and equity. NACHC guides health centers in the application of the VTF through a nation-wide learning community, Elevate. Health center participation in Elevate is linked to improved performance on clinical quality measures, improved patient and staff experiences, and reduced costs. Within the Elevate, NACHC successfully implemented a learning track focused on brain health, offering health center-specific guidance on a clinical introduction to Dementia early detection/risk reduction; incorporating Dementia early detection/risk reduction into care management workflows and leveraging reimbursement opportunities; and strengthening community linkages and partnerships to support Dementia early detection/risk reduction. Evaluation data from this learning track indicates health center interest in continuing education related to brain health integration into health center services. This topic is especially relevant considering the increasing population segment of older adult patients that health centers serve.

Abstract citation ID: igad104.3156

**A WAY OF COMMUNICATING...WHICH DOESN'T MAKE THEM FEEL THREATENED: CANCER SURVIVORSHIP IN AGING BLACK/LATINX PEOPLE**

Chizobam Nweke, and Candidus "Candi" Nwakasi, *University of Connecticut, Storrs, Connecticut, United States*

Background. About 2 million people are diagnosed with cancer each year in the US and minoritized groups such as Black and Latinx people share a significant burden of the disease. To better understand how to improve the quality of life of an increasingly diverse population of aging cancer survivors, this study explored the cancer survivorship experiences of older adults in Black and Latinx people in the U.S. Method. A qualitative descriptive approach was used on a sample of 17 participants (Mage 64 years), 6 Black and 12 Latinx cancer survivors, at various stages of cancer treatment. Open-ended, semi-structured interviews were administered to the participants. We used reflexive thematic analysis for data analysis. Results. The following themes were identified from the analysis: cancer is (not) stigmatizing, effects of race/ethnicity on survivorship, and being an immigrant and survivorship. These themes enable a better perception of enablers and barriers to resilience among Black and Latinx cancer survivors. Conclusion. The findings of this study will increase the knowledge on sociocultural factors that influence cancer survivorship experiences in the US. The implications on resilience and access to cancer care will be discussed.

Abstract citation ID: igad104.3157

**"THERE IS A SHORTAGE AND MISUSE OF TERTIARY NURSING": PERSPECTIVES OF NURSING ASSISTANTS IN ACUTE CARE**

Jennifer Wagner, Vivian Miller, Lauren Maziarz, Melissa Burek, Julia Bell, and Kaley Perry, *Bowling Green State University, Bowling Green, Ohio, United States*

The primary role of nursing assistants (CNAs) in the acute care setting is to provide direct care to patients. As frontline workers, they are often the first to report changes in condition to the licensed staff. The U.S. Bureau of Labor Statistics projects an increased employment need for nurse aides over the next ten years. However, the annual rate of nurse aide turnover is over 120 percent. A study conducted in 2015 by Brady suggests that there is a 36% turnover rate nationwide in acute care. Guided by a socio-ecological model, qualitative findings from 17 nurse aides (N=17) reveal interpersonal factors, such as relationships with others at the workplace and relationships with patients, as impacting turnover. Institutional and Community factors were also found to impact turnover, including staffing levels, resources, education, and compensation. Informed by the interview findings, a quantitative study was created. CNAs (N=230) from a variety of health care settings participated. Twenty-five percent were from the acute care system (N=56). More than seventy percent (N= 40) have considered leaving their employment. The top two reasons were pay (N=15) and to pursue other things (N=9). The top two work challenges were providing direct care to bariatric patients (N=28) and not enough supplies (N=13). The favorite part of working in acute care is getting to know the patients (N=26). Little research has been conducted on the turnover of CNAs in the acute care setting. CNAs are vital to caring for patients in the acute care system.

Abstract citation ID: igad104.3158

**"THEY'VE KIND OF SHUT THAT DOWN WITH COVID": HOME-LIKE ENVIRONMENTS FOR RESIDENTS WITH DEMENTIA**

Phatt Thaitrong, Joy Douglas, Linda L. Knol, Amy Ellis, Hyunjin Noh, and Shena Sanchez, *The University of Alabama, Tuscaloosa, Alabama, United States*

Current guidelines recommend providing a home-like dining environment for older adults living in long-term care (LTC) facilities, as this atmosphere has a positive impact on resident independence, social interaction, mealtime experience, and food intake. In LTC settings, registered dietitians (RDs) play a crucial role in providing nutritional care for residents with dementia. Our study explored RDs' perspectives on how they provide home-like mealtime environments for LTC residents with dementia. RDs who work with residents with dementia in the U.S. (n = 18) participated in one of five focus groups. Focus groups were led by a trained moderator using a semi-structured interview guide, transcribed verbatim, and audited by the research team for accuracy. Transcripts underwent descriptive content analyses using NVivo© software. Data analyses revealed a significant barrier, the Coronavirus Diseases of 2019 (COVID-19), in providing home-like environments for LTC residents with dementia. RDs reported the negative impact of COVID-19-related isolation among LTC residents with dementia. To mitigate the spread of COVID-19 among vulnerable residents, strict infection control policies were implemented. Therefore, many LTC facilities suspended communal dining, resulting in increased isolation and a sense of social disconnection, exacerbating the progression of dementia in many residents. Thus, COVID-19 presented a significant barrier for RDs to provide a mealtime environment that is home-like for LTC residents. To provide optimal nutrition care for LTC residents with dementia, having a middle road between the COVID-19 policies and traditional practices has been suggested.

Abstract citation ID: igad104.3159

### "WE DON'T AGE ALONE; WE AGE AS A COMMUNITY": BARRIERS AND ENABLERS OF AGING WELL IN BLACK/AFRICAN AMERICAN OLDER ADULTS

Mushira Khan[1], John Holton[2], and Ajla Basic[1], *1. Mather, Evanston, Illinois, United States*, *2. University of Illinois Chicago, Chicago, Illinois, United States*

Eurocentric understandings of successful aging privilege optimal physical and cognitive health, absence of disease and disability, and active social engagement in later life. This marginalizes the experiences of diverse older adults who may live with chronic conditions but maintain a good quality of life, supported by factors such as spirituality, resilience, and close familial ties. Further, this approach is myopic because it fails to capture social determinants of health (SDOH) that influence the aging process over time. The Perceptions of Aging Well in Diverse Older Adults study is a 5-year series that seeks to identify: 1) micro-, meso- and macro-level factors that shape aging well in racial/ethnic minority older adults, and 2) strategies to support aging well in racial/ethnic minority older adults. This presentation highlights preliminary findings from a sub-study with Black/African American participants. In-depth, semi-structured interviews were conducted with 23 community-dwelling Black/African American adults age 50+ (14 females, 9 males; mean age = 71.2 years). Thematic analysis of the interview data showed that neighborhood safety, food insecurity, pharmacy deserts, adverse childhood experiences, structural racism, and everyday micro-aggressions shaped perceptions of aging well in powerful ways. Strong kinship bonds and a desire to leave a legacy of resilience and empowerment for younger generations motivated participants to engage in prosocial activities such as volunteering in local church communities. These findings suggest that along with addressing key SDOH, fostering generativity through teaching and mentoring, creative arts, and taking on community leadership roles may enhance subjective well-being in Black/African American older adults.

Abstract citation ID: igad104.3160

### ACCULTURATION, ALCOHOL USE, AND SLEEP AMONG LATINX ADULTS AT RISK FOR ALZHEIMER'S DISEASE AND RELATED DEMENTIAS

Daniel Jimenez, Shanna Burke, Timothy Hayes, Adrienne Grudzien, Accacia Russell, Gianinna Munoz Soto, Eriana Peralta, and Eleni Padden, *Florida International University, Miami, Florida, United States*

**Background:** Excessive alcohol use can negatively impact sleep. In the U.S., Latinx people are most likely to engage in problematic drinking when compared to other racial/ethnic groups. Acculturation in U.S. society has been suggested as a contributing factor to problematic drinking. Excessive alcohol use and poor sleep are known risk factors for developing Alzheimer's disease and related dementias (ADRD). Little is known about the interaction between acculturation and alcohol use on sleep outcomes in Latinx adults, especially among those at risk of ADRD. The current study explored whether acculturation to the U.S. moderated the relationship between alcohol use and sleep. **Methods:** A secondary analysis of a community-based sample of 113 Latinx adults, ages 40-60, was conducted. Participants had a parent diagnosed with ADRD. Measures included surveys gauging alcohol use (WHO ASSIST V.30), acculturation (Bicultural Involvement Questionnaire), subjective sleep (Pittsburgh Sleep Quality Index), and objective sleep measures using actigraphy (ActiGraph). A multiple regression analysis was performed to evaluate whether acculturation moderated the relationship between alcohol use and sleep outcomes **Results:** The sample was predominantly female (92%), people born outside the US (74%), with approximately 83% of participants reported lifetime alcohol use. Americanism acculturation (b= 0.30, SE= 0.21, p=0.007) and biculturalism (-b= 0.30, SE= 0.13, p=0.003) significantly moderated the relationship between alcohol use and sleep duration. Implications: Expanded, targeted research and intervention focused on sociocultural factors impacting alcohol use for Latinx people at risk of ADRD must be further explored to identify and intervene in sleep disparities occurring among this population.

Abstract citation ID: igad104.3161

### ACTIVATING THE NEXT GENERATION OF GERONTOLOGY ENTHUSIASTS... ONE SEMESTER AT A TIME

Joanna Culligan, and Ila Schepisi, *Virginia Tech, Blacksburg, Virginia, United States*

At the Engagement Center for Creative Aging at Virginia Tech, we are activating the next generation of gerontology enthusiasts while breaking down stigmas associated with aging and cognitive change. Each semester, we have an opportunity to engage with the undergraduate and graduate student population here on campus through field work, volunteerism and student employment. Here, students engage with adults in our community through a wide range of programs to support aging from community clubs, caregiver wellness and our adult day services program. This paper focuses on Reframing Aging with students who engage in field work at our adult day services program through the Department of Human Development and Family Science at Virginia Tech through strategic application of active learning pedagogical techniques including lecture, class discussion, experiential activities and application exercises. In addition to classroom components, students participate in service delivery at our center. At the beginning of each semester, students are asked (using an anonymous online polling tool), "What words do you associate with older adults" and "… older adults with dementia." The most common words that have appeared, semester after semester, are: stubborn, inflexible, angry, sad, decline, struggling, forgetful and confused. At the end of the semester, sixteen-weeks later, students are asked the same questions. The most common words associated with older adults and those experiencing dementia at the end of the semester are: interesting, unique, wise, lovely, welcoming, insightful, experienced, helpful, normal, honest and human. In one semester, we are helping to Reframe Aging with this approach.

Abstract citation ID: igad104.3162

### ADDRESSING BARRIERS TO A FULL RANGE OF EVIDENCE-BASED OBESITY CARE FOR OLDER ADULTS: A GSA ROUNDTABLE DISCUSSION

John Batsis[1], Jason Lofton[2], Anna Pendrey[3], Kathryn Porter Starr[4], Jennifer Pettis[5], and Karen Tracy[5], *1. University of North Carolina at Chapel Hill, Chapel Hill, North Carolina, United States*, *2. Lofton Family Clinic, De Queen, Arkansas, United States*, *3. Indiana University, Indianapolis, Indiana, United States*, *4. Duke University School of Medicine, Durham, North Carolina, United States*, *5. The Gerontological Society of America, Washington, District of Columbia, United States*

Obesity is identified as a chronic disease by the American Medical Association and several professional societies such as the American College of Cardiology, the American Heart Association, The Obesity Society, and the American Gastroenterological Association. These organizations have all established clinical guidelines on caring for individuals with obesity and overweight. However, there is a critical lack of appropriate and individualized treatment strategies for older adults with obesity. To address this issue, in June 2023 The Gerontological Society of America (GSA) convened 15 multidisciplinary leaders in a roundtable discussion on Addressing Barriers to Accessing a Full Range of Evidence-Based Obesity Care Options by Older Adults. Roundtable participants identified how to overcome obesity/overweight perceptions, communicate appropriately with older adults about their body size, address cultural differences in obesity care, provide care in conjunction with common comorbidities that are faced by older adults, collaborate with an interprofessional team to care for older adults with obesity, overcome barriers to implementation of lifestyle changes including diet and exercise, incorporate medical and surgical interventions, and align needs of older adults with community resources. This poster will illustrate key findings from this roundtable discussion.

Abstract citation ID: igad104.3163

### ADOPTING A STRENGTH-BASED, PERSON-CENTERED RISK ASSESSMENT CLINICAL DECISION SUPPORT TOOL: WHAT ARE THE BENEFITS?

Heather MacLeod[1], Véronique Provencher[2], Dorothy Kessler[3], Mary Egan[4], Dominique Giroux[5], Marie-Jeanne Kergoat[6], Lewis Krystina[4], and Veillette Nathalie[6], *1. Perley Health, Ottawa, Ontario, Canada*, *2. Université de Sherbrooke, Sherbrooke, Quebec, Canada*, *3. Queen's University, Kingston, Ontario, Canada*, *4. University of Ottawa, Ottawa, Ontario, Canada*, *5. Université Laval, Québec, Quebec, Canada*, *6. Université de Montréal, Montréal, Quebec, Canada*

Most older adults want to age in place even if changes in their health results in home safety concerns. A consistent approach to assessing both the physical and psychological risks associated with the decision to remain at home is lacking. The Living with Risk: Decision Support Approach (LwR:DSA) is a recently validated innovative clinical tool that supports a balanced, systematic and person-centered assessment of risks, by analyzing their negative and positive consequences. The aim of this mixed-method study was to understand the barriers and facilitators to using the LwR:DSA during usual care to determine how best to support widespread adoption. Twenty-two hospital- and community-based clinicians used the LwR:DSA for eight weeks. Individual interviews were performed to document the factors that hindered and helped the use of the LwR:DSA in their clinical setting. The interviews were analyzed using Qualitative Description and the positive impact of using the LwR:DSA emerged as one of the facilitator themes. The participants described that using the LwR:DSA in practice improved their clinical decision making, communication and their ability to provide person-centered care. More specifically, the LwR:DSA 1) helped the clinicians understand the risk level and the context, causes, and consequences of the safety concern; 2) guided them to co-create with the older adult, agreeable recommendations to reduce the risk of adverse outcomes; 3) decreased clinician discomfort and 4) supported authentic conversations with the older adult. Understanding the benefits of using the LwR:DSA provides key information for clinicians by challenging the status quo of their current practice.

Abstract citation ID: igad104.3164

### ADRD: A REVIEW OF THE PATIENT-CENTERED OUTCOMES RESEARCH INSTITUTE'S (PCORI) RESEARCH PORTFOLIO AND PRIORITIES

John Audley, Tabassum Majid, Yewande Akinbami, Neeraj Arora, Marissa Coyle, Joanna Philips, Kate Moraras, and Steve Clauser, *Patient Centered Outcomes Research Institute, Washington, District of Columbia, United States*

Given the aging of the U.S. population, the number of individuals living with Alzheimer's Disease and Related Dementias (ADRD) is projected to reach 13 million by 2050. There is an urgent need for research efforts to advance prevention, diagnosis, and treatment strategies for ADRD. Furthermore, caregivers of individuals with ADRD often face distinct challenges and experience significant caregiving burden that has an impact on their well-being. To address unmet needs of individuals with ADRD and their caregivers, PCORI has invested nearly $85 million in patient-centered comparative clinical effectiveness research (CER) focused on ADRD and has initiated several stakeholder convenings to identify future research priorities. PCORI's ADRD portfolio of 15 CER studies includes interventions focused on caregiving, polypharmacy, telehealth, care models, and palliative care. Additionally, PCORI has funded engagement awards, dissemination & implementation initiatives, and research infrastructure projects to help facilitate the conduct and awareness of research on persons living with ADRD and their caregivers. To support continued research in this area, PCORI plans to re-issue a $50 million funding initiative on Healthy Aging with a special area of emphasis on interventions for caregivers of older adults living with ADRD. By systematically synthesizing PCORI's portfolio and themes from stakeholder convenings in ADRD, we can identify the strengths of current investments and understand evidence gaps to guide PCORI's future investments. This will enable us to holistically address future real-world, decisional dilemmas most important to patients, caregivers, and other stakeholders.

Abstract citation ID: igad104.3165

### ADVANCED CARE PLANNING SERVICES BILLING ENCOUNTERS AMONG MEDICARE BENEFICIARIES WHO DIED IN 2019

Fred Kobylarz[1], Hyosin Kim[2], Anum Zafar[3], Haiqun Lin[4], Maria Lopez[3], and Olga Jarrín[3], *1. Rutgers Robert Wood Johnson Medical School, New Brunswick, New Jersey, United States*, *2. Oregon State University, Corvallis, Oregon, United States*, *3. Rutgers, The State University of New Jersey, New Brunswick, New Jersey, United States*, *4. Rutgers, The State University of New Jersey, Newark, New Jersey, United States*

In 2016, the Centers for Medicare & Medicaid Services (CMS) introduced advanced care planning (ACP) services Current Procedural Terminology [CPT] codes 99497 (first 30 minutes) and 99498 (additional 30 minutes). This enabled healthcare providers to seek reimbursement for discussing future medical care preferences when patients are unable to make decisions. In this study, we linked administrative, claims, and encounter files for the 2,238,611 Medicare beneficiaries who died in 2019. Of these, 17.7% (395,258) had a billing code for ACP services in 2018 or 2019. Among fee-for-service (FFS) beneficiaries, 18.8% had ACP services billed compared to 15.7% of Medicare Advantage (MA) beneficiaries. Most ACP encounters were for the first 30 minutes of services (91% FFS, 90% MA). Among FFS beneficiaries, most ACP encounters were attributed to internal medicine (30.8%), family medicine (12.6%), and nurse practitioners (29.9%); and took place in hospitals (47.6%), skilled nursing facilities (18.4%), and office (14.2%) settings. Location of ACP services was available for only 8% of MA encounters: hospitals (40.3%), skilled nursing facilities (18.2%), and office (20.1%). Additional findings will be presented regarding variation in ACP encounters by race/ethnicity and geographic region (state, rural/urban community, area deprivation index) that enhance our understanding of ACP service use at the end-of-life for Medicare fee-for-service and Medicare Advantage beneficiaries.

Abstract citation ID: igad104.3166

### ADVANCING GERONTOLOGY THROUGH EXCEPTIONAL SCHOLARSHIP (AGES): FIRST INSIGHTS FROM A NEW MENTORSHIP MODEL

Juanita-Dawne Bacsu[1], Zachary Baker[2], Darina Petrovsky[3], Zahra Rahemi[4], Justine Sefcik[5], Kris Pui Kwan Ma[6], and Matthew Smith[7], *1. Thompson Rivers University, Kamloops, British Columbia, Canada*, *2. Arizona State University, Tempe, Arizona, United States*, *3. Rutgers University, Jackson, New Jersey, United States*, *4. Clemson University, Clemson, South Carolina, United States*, *5. Drexel University College of Nursing and Health Professions, Philadelphia, Pennsylvania, United States*, *6. University of Washington, Seattle, Washington, United States*, *7. Texas A&M University, College Station, Texas, United States*

Mentorship is vital to supporting professional growth among early career faculty members. Working with the Gerontological Society of America, the Advancing Gerontology through Exceptional Scholarship (AGES) Program was created as a mentorship model to promote productivity and peer support for early career faculty members. This presentation will: 1) describe the AGES Program as a prototype to facilitate peer support, collective learning, productivity, and co-authorship opportunities to advance early career faculty members; and 2) identify effective strategies to facilitate other groups looking to develop high-quality mentorship and training programs to support early career faculty members. After a competitive process, a cohort of 7 AGES members (including 2 program co-leads and 5 participants who all studied dementia) partook in monthly workshops from November 2022 to June 2023. Drawing on our experience with the AGES Program, we identified four strategies that cultivated our AGES Program: 1) being adaptable to address mentorship needs; 2) establishing accountability measures to enhance productivity; 3) fostering collective learning and peer support; and 4) delivering inspirational and educational activities. Following on the completion of the AGES Program, an open discussion was held with the cohort to assess and critically reflect on the program. Discussion results indicate the AGES Program was valuable in supporting early career faculty members. Specifically, findings demonstrate that the program was perceived as successfully supporting team science, collaborative opportunities, and enhancing productivity. Next steps are to conduct an anonymous evaluation survey to identify specific areas to strengthen and enhance the program for future cohorts.

Abstract citation ID: igad104.3167

### AGE AS A FACTOR IN DIVERSITY, EQUITY, AND INCLUSION INITIATIVES IN HIGHER EDUCATION

Natalie Galucia, and Nancy Morrow-Howell, *Washington University in St. Louis, St. Louis, Missouri, United States*

At institutions of higher education, demographic shifts in the population and the growth of diversity, equity, and inclusion (DEI) initiatives are occurring simultaneously. This study seeks to understand their intersection by focusing on age as a diversity factor in DEI initiatives on college campuses. Six focus groups with a total of 42 DEI staff members were convened, including 36 colleges/universities across the country. Thematic data analysis was conducted by three members of the research team who reached consensus on themes, subthemes, and representative quotes. Focus group participants suggest that age as an identity factor is not given much attention in DEI initiatives. Participants acknowledge the issue of age; but in general, they strive to keep other identities, like race and gender, in the forefront, especially in the face of low resources. These findings can be understood in the context of the current political environment around social justice issues and the pervasive ageism at the society level, especially in age-segregated institutions of higher education. While it may be difficult to elevate age in DEI initiatives on campuses, interventions were identified; and advocacy efforts may move us toward advancing age diversity, equity, and inclusion in higher education as well as other organizations.

Abstract citation ID: igad104.3168

### AGE BIAS IN ARTIFICIAL INTELLIGENCE (AI): A VISUAL PROPERTIES ANALYSIS OF AI IMAGES OF OLDER VERSUS YOUNGER PEOPLE

Laura Allen[1], Wenqian Xu[2], Mariko Nishikitani[3], Vaishnavi Atul Patil[1], and Dana Bradley[1], *1. University of Maryland, Baltimore County, Baltimore, Maryland, United States, 2. Lund University, Lund, Skane Lan, Sweden, 3. Kyushu University Hospital, Fukuoka, Fukuoka, Japan*

Concerns about age-related biases in Artificial Intelligence (AI)'s design, development, and data generation raise alarms for potential social inequalities. AI art-generators like MidJourney, known for creating distinctive and artistic images, have gained attention recently. However, the critical examination of their potential biases remains limited. Our study aims to investigate age-related bias in MidJourney by comparing how older and younger individuals are visually depicted. Using 19 keywords (as text prompts) tied to domains of potential social exclusion for older adults, we automated the collection of "old person" and "young person" images in MidJourney. The text-to-image prompts yielded 456 AI-generated images that were analyzed with Amazon Rekognition image detection software to transform them to quantitative data. Data were compared statistically using STATA (version 16). The results show that the images of older people are significantly less bright ($p< 0.001$) and less sharp ($p< 0.001$) than the images of younger people. Additionally, images of older people have significantly more smile expressions (16%; $p< 0.001$) and are more likely to be wearing glasses (56%; $p< 0.001$) compared to images of younger people (6% and 12%, respectively). The findings reveal that MidJourney's algorithm associates older people with a negative sense of antiquity, irrelevance, and dread. The findings also align with the Stereotype Content Model in which older people are seen as having warm personalities but not as competent or capable as younger people. We interpret these results as age bias that the AI art generator MidJourney has learned and in turn, creates, through image generation.

Abstract citation ID: igad104.3169

### AGE DIFFERENCES IN EMOTION REGULATION AND EXECUTIVE FUNCTION

Wei Xing Toh[1], and Hwajin Yang[2], *1. Nanyang Technological University, Singapore, Singapore, 2. Singapore Management University, Singapore, Not Applicable, Singapore*

Age-related improvements in emotion regulation (ER) are thought to account for the relatively healthy levels of well-being observed in late adulthood. However, extant laboratory-based evidence on age differences in the effective implementation of ER strategies—such as reappraisal (i.e., reframing the meaning of an emotion-eliciting event) and distraction (i.e., redirecting attention away from emotional to non-emotional information)—remains equivocal (Isaacowitz, 2022). Moreover, developmental differences in ER monitoring (i.e., adjusting regulatory approach as needed, such as maintaining or switching strategies), and factors that underpin such age-related differences, are poorly understood. Therefore, we investigated how younger and older adults ($N = 335$) differ in the implementation and monitoring aspects of ER, and how executive function (EF)—a collection of higher-order control operations that underlie ER processes (Pruessner et al., 2020; Schmeichel & Tang, 2015)—could explain such age-related differences in ER. Using a comprehensive battery of nine EF and two ER tasks, results indicated age-related decline for the implementation of reappraisal but not distraction. For monitoring, advancing age was associated with a less flexible pattern of strategy maintenance and switching (of reappraisal and distraction) across high- and low-intensity emotional contexts. Importantly, these age-related differences in ER implementation and monitoring were significantly mediated by declines in EF ($\beta s = .25–.29$, $ps < .007$), thereby alluding to the role of EF as a potential mechanism. Our findings suggest that certain ER processes are more cognitively demanding than others, and that EF could be a viable target of intervention to improve such ER processes in late adulthood.

Abstract citation ID: igad104.3170

### AGE-STRATIFIED INVESTIGATION OF SLEEP DISORDERS AND PTSD PREVALENCE AMONG VIETNAM VETERANS

Thomas Chacko[1], Sara Mithani[2], Melissa Bunker[3], Charity Breneman[4], and Gordon Broderick[1], *1. Rochester Regional Health, Rochester, New York, United States, 2. UT Health San Antonio, San Antonio, Texas, United States, 3. The University of Texas at San Antonio, San Antonio, Texas, United States, 4. War Related Illness and Injury Study Center, VA, Washington, District of Columbia, United States*

Military Veterans often experience a range of sleep disorders that significantly impact their physical and psychological wellbeing. Current study describes prevalence and age differences (≥65 to ≤74yrs; n =193)) vs. (≥75 to ≤ 90yrs; n =157) across insomnia, obstructive sleep apnea (OSA), nightmare disorder (ND), and posttraumatic stress disorder (PTSD). Vietnam Veterans (N = 350; AgeMean= 74.4 [SD= 3.6]) completed a survey (online or paper) of their sleep experiences and PTSD as part of a cross-sectional study. Veterans were mostly White (95%), male (97.7%), from the Army (55%), with some or two-year college (43%), and currently married (76%). Insomnia was screened positive in 27.7% of the Veterans, OSA in 58.6%, ND in 5.7%, and PTSD in 18.6%. There were significant mean differences in insomnia and PTSD across age groups ($t(348) = 2.43$, $p = .016$) and ($t(348) = 2.06$, $p =.04$), respectively, with younger age group showing higher insomnia, and PTSD. There were no significant mean differences in OSA and ND. Pearson correlation analyses yielded important associations. Insomnia was significantly positively correlated with PTSD ($r= .60$), OSA ($r= .33$), and ND ($r= .51$). PTSD was significantly positively correlated with OSA ($r= .30$) and ND ($r= .67$). ND was significantly positively correlated with OSA ($r= .22$). There were no significant differences in the associations across the two age groups. Recognizing and addressing various sleep disorders and comorbid PTSD are crucial to improving the quality of life of Veterans. Results emphasize need for comprehensive assessment of sleep disorders and PTSD among Veterans.

Abstract citation ID: igad104.3171

### AGING AND WELL-BEING: TESTING MINDFULNESS AS A MEDIATOR AND RESILIENCE AS A MODERATOR IN A NIGERIAN SAMPLE

Peace Amanambu[1], JohnBosco Chukwuorji[2], and Eric Allard[3], *1. University of Nigeria-Nsukka, Nsukka, Enugu, Nigeria*, *2. Michigan State University, East Lansing, Michigan, United States*, *3. Cleveland State University, Cleveland, Ohio, United States*

The mediating role of mindfulness on the relationship between age and affect (both positive and negative) has been established in Western samples. However, these associations have not been well considered within additional cultural contexts. The present study examined whether mindfulness mediates the relationship between increased age and both positive (PA) and negative (NA) affect. We also took an exploratory approach to assess the strength and direction of the age-affect relationship as a function of resilience. Participants were 242 (95 women, age range = 18-75 years, Mage = 41.79; SDage = 13.58) adults, affiliated with the University of Nigeria-Nsukka, who completed the Positive and Negative Affect Scale, Brief Resilience Scale, and the Mindful Attention Awareness Scale through Qualtrics. Separate moderated-mediation analyses were conducted for PA and NA. For the PA model, the only significant association was between age and mindfulness, with higher age predicting increased self-reported mindfulness. For the NA model, higher self-reported mindfulness predicted lower levels of NA. When NA was simultaneously regressed on age and mindfulness, the direct effect between age and NA was non-significant, but the indirect effect of age on NA through mindfulness was significant, suggesting that the association between age and negative affect was mediated by mindfulness. Resilience did not provide a significant moderating influence in the PA and NA models. The present findings suggest that older adults may experience diminished levels of negative affect due to an increased capacity for mindfulness. Hence, mindfulness-based interventions may be helpful in improving the well-being of older populations.

Abstract citation ID: igad104.3172

### AGING IN JAIL: CHALLENGES AND IMPROVEMENTS

Adriana Hernandez[1], Laura Driscoll[2], Andrea Leverentz[3], and Jan Mutchler[1], *1. University of Massachusetts Boston, Boston, Massachusetts, United States*, *2. Boston University, Boston, Massachusetts, United States*, *3. North Carolina State University, Raleigh, North Carolina, United States*

The increase in incarcerated older people has created challenges in correctional facilities, including settings ranging from large prisons to smaller county jails. Aging while incarcerated brings a new dimension of difficulties for older people, and to the correctional system as a whole. The resources and environments are unsuitable for an aging population and ultimately result in greater health complications. The COVID-19 pandemic exacerbated existing challenges experienced by older adults. To further assess the issues that incarcerated older people face, researchers from UMass Boston partnered with a local jail to determine the current needs and deficiencies experienced by older men. Focus groups were conducted in person with incarcerated older men regarding their experiences, opinions on having a unit specifically for older adults, and institutional changes that occurred during the COVID-19 pandemic. Zoom interviews were also carried out with staff. These conversations suggest that older incarcerated men encounter unique conditions that challenge their well-being. Such challenges include limited access to medical care and proper diets, lack of environmental accommodations, and inadequate exercise opportunities. These challenges exacerbate the burden of being in jail and can lead to higher risks of chronic illness. As well they have potential implications for the communities to which the men are released. Older men and jail staff were receptive to creating a unit specifically for older adults as a means of remedying some of these challenges. This poster offers policy suggestions to improve correctional facilities that better align with the unique needs of incarcerated older people.

Abstract citation ID: igad104.3173

### AGING MODULATION WITH EXERCISE AND MIMETICS

Dennis Lox, *Florida Sports and Regenerative Medicine, Clearwater, Florida, United States*

Understanding the molecular and cellular processes of the Longevity Pathway AMPK-SIRT1, and its activation by exercise and mimetics, plays a vital role in a series of molecular and cellular processes which stimulates optimal healthy cellular efficiency and functioning, ending with improved exercise tolerance. Knowledge of the role of exercise induced activation of the AMPK-SIRT1 pathway, and altering it towards dysfunctional processes and drivers of aging and disease propagation is important to understand to also understand. Cellular dysfunction unfortunately is the norm. This leads to molecular increase of P53 in a binding state not of DNA repair, but upregulation of gene transcription of NF-kB, which increases inflammatory cytokines, over activation of MTOR, and cellular senescence with secretion of the SASP. All these dysfunctions create feedback loops, which makes the process self propagating, resulting in progressive aging, and disease states. The most optimal state is regulation of the optimal molecular and cellular environment, via exercise or a mimetic stimulator of AMPK-SIRT1. Knowledge of the optimal body state for health, and enhancing this AMPK-SIRT process, is low body fat releasing beneficial adiponectin, and no inflammatory cytokines or adipokines. Training muscle releases positive myokines. Various mimetics can stimulate AMPK-SIRT1, such as Metformin, the mitochondrial derived peptide MOTS-C, AICAR, and other compounds intended for people with medical issues that prevents exercise. Some display a synergistic effect with exercise. Understanding, the benefits of exercise and modulation of aging, by encouraging healthy processes, and reducing the deleterious patterns is important to understand.

Abstract citation ID: igad104.3174

### AGING SUSTAINABLY: A PILOT STUDY OF OLDER MIDWESTERN ADULT PERSPECTIVES ON ENVIRONMENTAL BEHAVIOR

Megan Pedersen, Justin Walden, and Melissa O'Connor, *North Dakota State University, Fargo, North Dakota, United States*

Given their life experience and significant political power, the perspectives of older adults on environmental issues are crucial to addressing the climate crisis. Although unsupported by research, the general public tends to believe that older adults do not care about climate change. In seeking to overturn this belief, a qualitative responsive interview study was conducted. With a sample of 5 adults between 50 and 70 from North Dakota and Minnesota, this study is small, but can serve as a pilot study for more intensive research. Interview questions focused on older adults' attitudes, personal behavior, and motivations for engaging (or not) in sustainable behavior. Although the sample size was small, findings showed that most older adults understand the need for sustainability and try to care for the climate in their own ways. Religion, financial incentive, and generativity can be strong motivators for sustainable behavior, but misinformation and distrust can act as de-motivators. Investigating older adults' motivation to engage in sustainable behavior is a necessary step to involving more older adults in environmental advocacy. These interviews demonstrate that many older adults are willing to act on behalf of our shared planet, and future research may uncover more motivations and bring us closer to that goal.

Abstract citation ID: igad104.3175

### ALCOHOL CONSUMPTION AND DECREASED SERUM A-KLOTHO, AN ANTI-AGING HORMONE, LEVELS AMONG US ADULT PARTICIPANTS

Dylan Fromm[1], Jianmin Zhu[2], Wonjun Kim[1], and Yudan Wei[1], *1. Mercer University School of Medicine, Macon, Georgia, United States*, 2. *Fort Valley State University, Fort Valley, Georgia, United States*

**Background:** The Klotho protein is widely accepted to be a prominent anti-aging biomarker for health and longevity. The relationship between alcohol intake and serum levels of soluble α-Klotho is not well understood. **Methods:** In this study, we explored this relationship in a nationally representative sample of US adults. A total of 3970 male and female adults aged 40-79 years from the 2013-2016 National Health and Nutrition Examination Surveys were analyzed using multiple general linear models. Participants were classified as non-drinkers, light drinkers (consumed on average < 4 drinks in males and < 3 drinks in females per day), and heavy drinkers (consumed on average ≥4 drinks in males and ≥3 drinks in females per day), according to a national guideline. **Results:** Significantly decreased serum α-Klotho levels were found in both light drinkers and heavy drinkers, with the weighted geometric mean of 785.18 pg/mL (p = 0.0007) in light drinkers and 750.96 pg/mL (p = <.0001) in heavy drinkers, compared to that in non-drinkers (827.19 pg/mL). After adjusting for covariates, light drinking was associated with a 3.66% (p = 0.0081) decrease in serum α-Klotho levels, and heavy drinking with an 8.07% decrease (p = <.0001) in α-Klotho levels, as compared with non-alcohol drinking. Similarly, binge drinking was also associated in a 5.22% (p = <.0001) decrease in serum α-Klotho, compared to non-binge drinking. **Conclusion:** This study demonstrates a significantly inverse relationship between alcohol consumption and serum α-Klotho levels in middle-aged and older adults. Prospective studies are warranted to elucidate these interactions.

Abstract citation ID: igad104.3176

### ALLEVIATING WORRIES OF DEMENTIA AMONG OLDER ADULTS WHO ATTEND AN AGING BRAIN SEMINAR

Grace Simonson, and Susan Wehry, *University of New England College of Osteopathic Medicine, Biddeford, Maine, United States*

Dementia worry is prevalent among older adults, and these concerns are often not addressed. Research demonstrates the public lacks accurate knowledge of dementia, which contributes to its negative perception. We hypothesized participation in an aging brain seminar would significantly alleviate dementia worry in older adults. The 45-minute in-person talk covered normal and abnormal aging brain changes, and research-based strategies for healthy brain aging. Attendees completed surveys immediately before and after the seminar. Both surveys included the 12-item Dementia Worry Scale (DWS), which uses Likert scales to assess dementia worry. Two additional Likert questions in the post-survey determined the immediate effect on dementia worry and intent to adopt new strategies. 7 sessions garnered 61 viable pre-post surveys from 160 attendees. 69% reported their dementia worry was somewhat or significantly improved; 68% reported they were very likely or definitely going to take action. Collectively, the Dementia Worry scale was reduced (median pre=19, median post=16, range= 12 - 41), but results were insignificant (p=0.058) likely due to the long-term nature of the questions. The aging brain seminar was successful. It resulted in an immediate reduction of dementia worry and intent to change behavior, and it can be effectively delivered by a medical student. Further research is warranted to determine if the seminar has a long-term effect on dementia worry and sustained adoption of healthy behaviors.

Abstract citation ID: igad104.3177

### ALZHEIMER'S DISEASE RISK REDUCTION HEALTH COACHING: COMPARATIVE ANALYSIS

Faika Zanjani, Brian Battle, and Joann Richardson, *Virginia Commonwealth University, Richmond, Virginia, United States*

Recent guidelines point to lifestyle change as a tool for decreasing Alzheimer's disease (AD) risk. To address the limited practice of AD risk reduction and the greater AD prevalence in minority groups, this study aimed to explore the feasibility of a community-level lifestyle intervention targeting high-risk groups. Diverse older adults (60+) living in the Richmond, VA local area, with incomes below $12,000/year and managing diabetes/cardiovascular disease were offered weekly lifestyle telephone-health coaching for 12-weeks in 2019-2020 (Phase 1). The health coaching sessions provided AD lifestyle risk reduction education and goal setting/planning. The intervention sample (n=40, mean age 68 years (range: 60-76 years)) was 90% African American/Black (n=36), 45% males (n=18). Thereafter 2021-2022, n=37 individuals in the same area were recruited as a comparison group and not given health coaching (Phase 2), mean age 65.5 years (range: 57-83 years) was 92% African American/Black (n=34), 50% males (n=18). Demographic differences across groups, indicated that the intervention group was more likely to live alone (p<.01). Repeated measures intervention effects were seen for cognitive ability, indicating greater gains in the intervention group (p<.01). Significant difference score effects indicated greater cognitive ability (p<.01) and physical activity (p<.001) gains in the intervention group; with less physical activity gains (p<.02) for subjective cognitive decline group. This preliminary work creates the impetus for future large-scale AD risk reduction investigations to improve modifiable risk among diverse older adults. Our positive trends in AD risk reduction support the feasibility of telephone-based health coaching AD risk reduction interventions.

Abstract citation ID: igad104.3178

### AN INTERACTION EFFECT OF INTERNET USE AND SOCIAL PARTICIPATION ON LITERACY ABOUT FRAILTY IN OLDER JAPANESE

Natsumi Fujiwara, Yuya Akagi, Hiroko Yoshida, Kei Kamide, Hanayo Koetaka, Michiko Kido, Yaya Li, and Mai Kabayama, *Osaka University, Suita, Osaka, Japan*

**Objectives:** This study aimed to analyze the age difference of the interaction effect of internet use and social participation on literacy about frailty in older Japanese. **Methods:** Data were collected from community-dwelling older adults aged 65-89 years randomly selected from residential registration by self-reported postal questionnaires (n=5005, response rate 49.0%). We asked them about their background, whether they use the internet to obtain health information, and whether they know the term of frailty（know what it meant / don't know what it means but have heard of it or never heard of it and don't know）.Logistic regression stratified by age (65-74 and 75-89 years) was used to evaluate the association of literacy about frailty with internet use and social participation adjusted for gender,education, frailty status, physical activity. The interaction between internet use and social participation on literacy about frailty was also assessed. **Results:** Results showed that in both age groups, the interaction revealed that a combination of social participation and internet use was associated with a high literacy about frailty (65-74 years: OR=1.64,95%CI=1.28-2.11; 75-89 years: OR=1.92,95%CI=1.45-2.56). Only in the 75-89 age group, Internet use alone without social participation was significantly associated with a high literacy about frailty (OR=1.60,95%CI=1.12-2.30). **Conclusion:** Our findings suggest that internet use may be beneficial for improving literacy about frailty especially for the elderly over 75 years old, however, social participation should also be taken into consideration for those. Longitudinal research is needed.

Abstract citation ID: igad104.3179

### ARCHITECTURAL, INTERIOR DESIGN, AND TECHNOLOGICAL INTERVENTIONS TO FACILITATE REHABILITATION AND AGING IN PLACE.

Swati Katyarmal[1], Michelle Wyndham-West[1], and Shehroz Khan[2], *1. OCAD University, Toronto, Toronto, Ontario, Canada*, *2. Toronto Rehabilitation Institute, Toronto, Ontario, Canada*

Most older adults 60 years and above live in the community with either support from their caregivers or independently. Aging in place successfully involves safely carrying out activities of daily living at home without accidents and injuries. However, most traditional homes are not equipped with safe interior designs or support technological interventions, which could be a barrier to aging in place. We performed a narrative review to understand the importance of incorporating architectural, interior design, and technological interventions to support the rehabilitation of older adults with disabilities to successfully age in place. We reviewed 28 research papers from several research databases matching the inclusion criteria on interventions targeting older adults' rehabilitation outcomes. We included studies that focused on various health conditions (e.g., spinal cord injury, stroke) and supporting home modification along with technological interventions. The review shows that the home environment has a significant impact on older adults' overall health, and recovery, including functional capacities, cognitive processes, and emotional states providing autonomy, safety, and accessibility. Several safe design features were found, including barrier-free entrance, mobile stair climber, handrails and grab bars, anti-skid flooring, open layout, and shower seat. The integration of technologies (e.g., sensor lights, voice-activated devices) was identified as a promising avenue for enhancing the quality of life. Co-designing the built space with older adults was found to be effective. Our review highlights the need for collaboration between healthcare professionals, engineers, architects, and policymakers to design existing or new homes that support safe aging in place of older adults.

Abstract citation ID: igad104.3180

### ARE OLDER ADULTS' PERCEPTIONS OF ELDERSPEAK INFLUENCED BY SELF-REPORTED PSYCHOSOCIAL CHARACTERISTICS?

Lisa Weimar, and Clarissa Shaw, *University of Iowa, Iowa City, Iowa, United States*

PROMIS (Patient-Reported Outcomes Measurement Information System) is a system of highly reliable measures of patient-reported health status. Elderspeak is speech that sounds childish to older adults. Use of elderspeak by nursing staff can result in rejection of care. The Iowa Coding Scheme for Elderspeak (ICodE) contains 11 elderspeak attributes: diminutives, childish terms, collectives, short words, directives, reflectives, minimizers, exaggerated praise, laughter, tag questions and exaggerated prosody. Thirty-one older adults (aged 74.5±7.8 years) responded to short form PROMIS instruments measuring self-efficacy, physical function, isolation, cognitive function, depression, anxiety, and pain interference. Participants listened to 41 audio recordings of nursing staff providing care to hospitalized patients with dementia and were asked to rate each audio recording using a 5-point Emotional Tone Rating Scale for patronizing and respectful (5=very, 1=not at all). Using the ICodE, each audio recording was classified as neutral (no elderspeak), containing a single attribute, or containing an attribute combined with exaggerated prosody. Ratings for elderspeak attributes ranged from 2.1 to 2.9 for patronizing and 2.8 to 3.8 for respectful. Using the SPSS statistical package, a Spearman's rho correlation was calculated for each elderspeak rating and health status domain identified above. Among this small, homogenous sample, no statistically significant correlation was found between participants' self-efficacy, isolation, physical or cognitive function, depression, anxiety, or pain interference, as measured by PROMIS tools, and their rating of elderspeak attributes as respectful or patronizing. Future research should continue to explore what factors influence older adults' perceptions of elderspeak as being respectful or patronizing.

Abstract citation ID: igad104.3181

### ARE RURAL OLDER VETERANS SATISFIED WITH VIRTUAL COMPREHENSIVE GERIATRIC ASSESSMENTS?

Linda Sawyer[1], Hallie Keller[1], Teresa Howell[2], Maria Cervantes[3], Janette Dunlap[2], Christine Cigolle[4], and Dennis Sullivan[1], *1. Central Arkansas Veterans Healthcare*

*System, North Little Rock, Arkansas, United States, 2. North Florida/South Georgia Veterans Health System, Gainesville, Florida, United States, 3. Veterans Health Administration, Saint Johns, Florida, United States, 4. Ann Arbor VAMC, Ann Arbor, Michigan, United States*

Rural older Veterans (ROV) have limited access to comprehensive geriatric assessments (CGA). VA Video Connect (VVC) can potentially overcome this access barrier. A standardized process was developed and piloted, to conduct CGAs by VVC. It was found to be feasible and facilitated access to CGAs. The purpose of this study was to assess ROVs' satisfaction with CGA via VVC. The team developed a 10-item questionnaire with good face and content validity to assess satisfaction with the CGA and process. All ROVs who completed a CGA via VVC were telephoned. They were asked to rate each item on a Likert scale. Caregivers were allowed to assist but the final answer came from Veteran themselves. The results are summarized as percentage of respondents who "agree" or "strongly agree". ROVs also provided comments. Of the 79 ROVs who completed the CGA via VVC, 63 OVs completed the questionnaire. Most were white (82.8%) males (95.4%), with mean(±SD) age 75.7(±6.5). Most felt it was easy to communicate with the providers (95.2%) and understood the information they were given (98.4%). Most felt comfortable using the technology (75.8%) and that the visit was private enough (96.8%). Although many commented they would prefer in-person visits, most agreed that they would do virtual visits again if offered (71.4%). Overall, they "agreed that they would not change anything about the CGA process (95.2%). The results suggest that ROVs were satisfied with CGA via VVC. More research is needed to determine the outcomes, benefits, and limitations of CGAs performed by VVC.

Abstract citation ID: igad104.3182

### ASSESSING MODERATED MEDIATION IN THE RELATIONSHIP BETWEEN SYMPTOM COUNTS, FALLS, AND HEALTH OUTCOMES

Michelle McKay, and Margaret Brace, *Villanova University, Villanova, Pennsylvania, United States*

Almost half of older adults experience multiple symptoms that limit function and increase the risk for falls. The study aimed to determine if the relationship between symptoms (pain, endurance, weakness, balance, depression/anxiety) and outcomes (hospitalization, residential care, activity limitations, wellbeing, and mortality) are mediated by falls and whether symptoms further moderate outcomes of falls. Data from community-dwelling older adults (≥ 65 years, n=4921) enrolled in the National Health and Aging Trends Study (NHATS) 2015 cohort were used for analysis. Symptoms were categorized by symptom count (0, 1, 2, 3, 4, ≥5). Using generalized structural equation modeling, 2015 symptom counts were modeled to predict 2016 falls, 2016 health outcomes, and 2017 mortality. Falls partially mediated the association between symptoms in one year and outcomes the following year for all except residential care. In addition, the effect of falls on hospitalization is significantly moderated by symptoms (interaction of symptoms and fall OR=.89, robust SE=.045, p=.025). This dampening effect indicates that the overall effect of falls on hospitalization (OR=2.19, robust SE=.36, p<.001) is stronger at the lower levels of symptom count and weaker at the higher levels of symptom count. When there are ≥5 symptom present, there is no significant difference between the predicted probability of hospitalization for those who have not experienced a fall (.31, 95%CI: .28 to .34) and those who have fallen (.36, 95%CI: .31 to .41). Understanding the complex relationship between symptoms, falls, and outcomes can assist with developing interventions for symptom management and fall prevention.

Abstract citation ID: igad104.3183

### ASSESSING THE UPTAKE OF CORE OUTCOME SETS IN RANDOMIZED CONTROLLED TRIALS FOR PARKINSON'S DISEASE

Joanna George[1], Eli Paul[2], Shaelyn Ward[1], Kyle Fitzgerald[1], Garrett Jones[1], Kimberly Magana[1], Alicia Ford[3], and Matt Vassar[3], *1. Oklahoma State University College of Osteopathic Medicine, Tulsa, Oklahoma, United States, 2. Oklahoma State University, Tulsa, Oklahoma, United States, 3. Oklahoma State University Center for Health Sciences, Tulsa, Oklahoma, United States*

**Background:** Parkinson's Disease (PD) affects 1% of adults aged 60 and older, including 10 million individuals worldwide. As PD's incidence rises, research funding is increasing substantially. PD's core outcome set (COS) provides standardization for PD clinical trial outcomes. Adherence to the PD COS may improve research quality and study comparability. Objective: Analysis of the COS uptake rate before and after the PD COS publication. **Methods:** On June 26th, 2023, we conducted a search of ClinicalTrials.gov to retrieve phase III/IV adult PD trials between 2013 and 2023. Screening for inclusion and data extraction occurred in a masked, duplicate fashion. Trial characteristics and COS uptake rate were extracted from this sample. **Results:** In our 111 included trials, the COS uptake rate was highest for the 'Walking and Balance' outcome and lowest for the 'Hospital Admissions' outcome. Overall, there was a non-significant monthly increase of 0.26% (P = 0.266, CI = [-0.20, 0.72]) in "COS-defined outcome" measurement when comparing pre- and post-COS publication. **Conclusion:** Overall, our study found no significant increase in COS uptake in PD clinical trials. We found multiple outcomes to be vastly unmeasured and heterogeneity among the measurement instruments used. These findings introduce complexity to the standardization and comparability of RCT outcomes. Barriers to COS uptake may be complex; however, overcoming these factors is vital to improving the usefulness of PD research.

Abstract citation ID: igad104.3184

### ASSESSMENT OF DECISIONAL COMPETENCE IN DEMENTIA– A QUALITATIVE STUDY OF 60 EUTHANASIA CASES

Arne van den Bosch, Radboud Marijnissen, Denise Hanssen, and Richard Oude Voshaar, *University Medical Center Groningen, Groningen, Groningen, Netherlands*

The Netherlands allows euthanasia or assisted suicide (EAS) for people with dementia. The number of dementia EAS cases gradually increases every year, up to 288 cases in 2022. This practice remains controversial, as dementia is not directly lethal and may impair decisional competence. The Dutch euthanasia review committees (RTE) refer to Appelbaum and Grisso's criteria for the assessment of decisional competence, but how these criteria are applied in clinical practice remains unknown. This study examined qualitatively which factors, and how, influence the judgment of decisional competence for EAS requests of people with dementia. Thematic analysis of 60 dementia EAS case summaries published online by the RTE between 2012 and 2021. Twenty patients had an advance directive and were decisionally compromised at time of EAS. Forty patients were decisionally competent at time of EAS, of which twenty also had an advance directive (purposive sampling). Two researchers independently coded all text related to decisional competence. A theoretical framework was developed. The four cognitive criteria of Appelbaum and Grisso were dimensional and cut-off points were influenced by six supporting factors that also directly impacted on competence assessment, i.e., level of communication, psychiatric comorbidity, personality, presence of an advance directive, consistency of the request, and the patient-physician relationship. The number of involved physicians and executed investigations were two contextual factors. The multidimensionality and subjectivity of decisional competence assessment may pose ethical and legal challenges. Continuous quality improvement processes may be needed in daily care, including possibilities for reflection.

Abstract citation ID: igad104.3185

### ASSESSMENT OF DEFICITS IN SPECIFIC COGNITIVE DOMAINS IN OLDER ADULTS LIVING WITH HIV (OALWH)

Andrea Reyes-Vega[1], Harideep Samanapally[1], Rishikesh Rijal[1], Stephen Furmanek[2], Christopher B. Shields[3], Brandon C. Dennis[3], Smita Ghare[3], and Shirish Barve[3], *1. University of Louisville, Louisville, Kentucky, United States*, *2. Norton Infectious Diseases Institute, Louisville, Kentucky, United States*, *3. Norton Neuroscience Institute, Louisville, Kentucky, United States*

A significant proportion of people living with HIV (PLWH) have cognitive impairment. Moreover, approximately 70% of PLWH in the United States will be ≥50 years old by 2030, raising concerns of a higher incidence of dementia as they age. Accordingly, there is a clinical need to monitor their cognitive status. The aim of this study was to delineate specific cognition areas impacted in OALWH with a clinical diagnosis of neurocognitive impairment. We used a comprehensive set of tests (paper and NIH Toolbox Cognition Battery), to assess different cognitive domains in a total of 25 OALWH ≥ 50 years. 64% were diagnosed with neurocognitive impairment and 36% were non-impaired. T-scores were compared using t-tests of means. Differences in means and 95% confidence intervals (CI) were reported. Impaired patients scored on average 18.35 T-score points lower on Hopkins Verbal Learning Test (HVLT) retention trial (p 0.016, CI:6.74-29.97) and 9.19 T-score points lower on the NIH Picture Vocabulary Test (PVT) (p 0.033, CI:1.12-17.26). Stroop color word, NIH Card Sort and NIH Picture sequence memory test were trending to be significantly lower in impaired patients (p< 0.07). In impaired OALWH, the HVLT data demonstrated a decreased capacity to learn and an early memory loss, suggesting frontal executive and attention type deficits. Moreover, the decreased PVT scores demonstrated an impact on crystallized intelligence, indicating decline in verbal skills, semantic knowledge, or retrieval. Overall, the observed deficits in different cognitive domains support early neurocognitive screening even when OALWH do not show overt signs of neurocognitive impairment.

Abstract citation ID: igad104.3186

### ASSESSMENT OF ELECTROLYTE ABNORMALITIES IN OLDER ADULTS WITH COVID-19 DELIRIUM

Milenko Petrovic, Esther Park, Amanda Pangle, Jeanne Wei, and Gohar Azhar, *University of Arkansas for Medical Sciences, Little Rock, Arkansas, United States*

This retrospective study explored the impact of COVID-19 on altered sodium and potassium in older adults with delirium. EMRs from the University of Arkansas for Medical Sciences (UAMS) database were reviewed from January 7, 2018 to January 5, 2021 for electrolyte abnormalities and delirium. Inclusion criteria were age 65 or older, all races, genders, and ethnicities with COVID or non-COVID infections. We identified 319 patients with COVID-19 out of which 108 (33.8%) had delirium during COVID. In COVID-delirious patients, hypokalemia was present in 8, hyperkalemia in 10, hyponatremia in 9, and hypernatremia in 20.127/319 (39.8%) COVID patients had an electrolyte disorder at some point with hypokalemia being most represented at 58/127 (45.7%). Out of the patients who experienced hypokalemia at some point, 34/319 (10.6%) had delirium. In another cohort of 306 older adults with non-COVID pneumonias we identified 173 patients with electrolyte disorders and 106 (34.6%) with delirium during pneumonia. Electrolyte disturbances were more present in non-COVID pneumonias vs. those with COVID (p< 0.001). Hypokalemia was more identified in non-COVID, 56/306 (18.3%), vs. COVID patients, 34/319 (10.6%) (p< 0.006). Interestingly, hypernatremia was greater in COVID groups (p< 0.0321). There was no difference in delirium between COVID vs non-COVID patients, but electrolytes disorders and hypokalemia were observed with greater frequency in non-COVID pneumonias. Notably, hypernatremia developed more in patients suffering from COVID and since it can be associated with seizures and cerebral hemorrhage, particular attention should be paid to prevent hypernatremia from developing in order to reduce COVID-related morbidity and mortality.

Abstract citation ID: igad104.3187

### ASSOCIATED FACTORS OF PARTICIPATION IN DIGITAL TECHNOLOGY: A STUDY BASED ON OLDER PEOPLE IN CHINA

Lan Liu[1], Chao Guo[2], Liangshu Qi[3], Yingying Wang[1], and Ruixin Shu[1], *1. Institute of Population Research, Peking University, Beijing, Beijing, China (People's Republic)*, *2. Peking University, Beijing, Beijing, China (People's Republic)*, *3. School of Economics and Management, Beijing, Beijing, China (People's Republic)*

There has been an increasingly important trend that more and more older people take part in activities of digital technology nowadays in China. In this study, the research question associated to determinants of older people's digital engagement was addressed, which has been quantitatively analyzed by statistical models in a sample of older people 60 to 98 years of age from China Longitudinal Aging Social

Survey (N=11207). For dependent variable, digital participation of older people has been measured by the access to the Internet with mobile phones and various digital devices, whereas for independent variables, individual, situational, environmental, and social policy factors which were based on a conceptual model of productive aging framework have been constructed to present academic explanations on dependent variable. Results of Probit and Ordered-Probit regression showed that the older people's participation in digital technology was closely related to individual and situational factors, whereas the effects of environmental factors and social policies were not statistically significant. Older adults who were much younger and physically healthier had higher probability of participation in digital technology. Meanwhile, those who lived in urban area, had higher levels on income, literacy and social support were more likely to be involved in digital activities. Conclusions shed light on the significance of academic research to improve productive ageing and successful ageing which takes a more active role on exploring and grappling with issues related to application of digital technology especially technological difficulties that older people have confronted with in an ageing society.

Abstract citation ID: igad104.3188

### ASSOCIATION AND RISK PREDICTION OF PAIN AND FALL-RELATED INJURIES AMONG COMMUNITY-DWELLING OLDER ADULTS IN CHINA

Xiaodong Chen, Kewei Shi, Lingxiao He, and Ya Fang, *Xiamen University, Xiamen, Fujian, China (People's Republic)*

**Background:** Pain is common in older adults and has also been identified as a fall risk factor, whereas the impact and mechanism of fall-related injuries are still unknown. This study aims to examine the association and risk prediction of pain and fall-related injuries among Chinese older adults. **Method:** This study enrolled 5,721 older adults from the China Health and Retirement Longitudinal Study (wave 2011-2015). Logistic regression was used to analyze the associations between pain characteristics and fall-related injuries. Furthermore, 2022 older adults with pain at baseline were further used to build a fall-related injury prediction model, using five machine learning algorithms (logistic regression, naive bayesian, random forest, adaptive boosting and artificial neural network) with 118 input variables as candidate features, and SHapley Additive exPlanations was used for the prediction model explanation. **Results:** Logistic regression models showed that whether pain (OR=1.35) or pain severity (ORmoderate=1.37, ORsevere =1.48) and multisite pain (OR=1.41) independently predicted fall-related injuries. The Random forest model achieved the best performance among the prediction model with an area under the receiver operating characteristic curve of 0.764. Interpretable results showed that the top 10 significant predictors were: experience of falling, frailty, sex, short physical performance battery, received inpatient care, estimated glomerular filtration rate, lung function, pain quantity, White Blood Cell and Age. **Conclusions:** Pain is associated with fall-related injuries among Chinese older adults. Fall-related injury prevention strategies for older adults with pain should specifically focus on fall history, frailty, and women.

Abstract citation ID: igad104.3189

### ASSOCIATION BETWEEN FRAILTY AND FIVE-YEAR MORTALITY AFTER HIP FRACTURE SURGERY IN OLDER PATIENTS.

Jung-Yeon Choi, and Kwang-il Kim, *Seoul National University Bundang Hospital, Seoul, Republic of Korea*

We aimed to identify the association between frailty and 5-year mortality after hip fracture surgery. The Hip-Multidimensional Frailty Score (Hip-MFS) was calculated using the Comprehensive Geriatric Assessment (CGA). The primary outcome was 5-year mortality. Among 1,363 patients who underwent HF surgery, 598 (44%) patients underwent CGA, and 536 patients were included in the final analysis. The mean age was 80.5 years and 71.3% were females. A total of 223 (41.6%) patients experienced postoperative complications. The median observation time was 1999.5 days, and the overall mortality rate was 60.4% (n = 324), whereas the 1-year mortality and the 5-year mortality rate after HF surgery were 13.8% (n = 74) and 42.8% (n = 235), respectively. In the multivariate regression analysis, after accounting for clinical and demographic factors, the high-risk Hip-MFS group and the group with postoperative complications had hazard ratios of 1.49 (95% confidence interval [CI] 1.090–2.037, p = 0.012) and 1.498 (95% CI 1.139–1.970, p = 0.004), respectively. Patients with postoperative complications with low Hip-MFS showed better 5-year survival than those without postoperative complications with high hip-MFS in the Kaplan-Meier curve (p = 0.013). Compared with the occurrence of postoperative complications, the frailty status evaluated with the Hip-MFS had a more significant impact on long-term mortality after hip fracture surgery.

Abstract citation ID: igad104.3190

### ASSOCIATION BETWEEN LATE LIFE EDUCATION AND COGNITIVE FUNCTION AMONG U.S. OLDER ADULTS

Nan Wang[1], Matthew Dupre[2], and Hanzhang Xu[2], *1. University of California, Davis, Sacramento, California, United States*, *2. Duke University, Durham, North Carolina, United States*

This study assessed whether late-life education (LLE) was associated with better cognitive function and whether the benefits of LLE on cognitive decline differed by gender, race/ethnicity, and education level in a nationally-representative sample of U.S. older adults. We conducted a retrospective cohort study using six waves of data from the Health and Retirement Study ([HRS] 2008-2018) that included adults aged ≥ 65 with no baseline diagnosis of Alzheimer's disease and related dementias (ADRD). Cognitive function was measured at baseline and over time using a summary score that included immediate/delayed word recall, serial 7's test, objective naming test, backwards counting, recall of the current date, and naming the president/vice-president (range=0-35). LLE was measured at every wave and was categorized as "once a month or more," and "not in the last month [or never]." Covariates included participants' demographic background, socioeconomic status, psychosocial and behavioral factors, and health-related factors. Of 12,099 participants (median age 71 [IQR=10]), engaging in LLE at least once a month or more was associated with better cognitive function and was equivalent to a 2-4 year delay in cognitive decline than non-participation in LLE. The association was only partially attenuated after adjusting for a wide range of covariates. In addition, the benefits of LLE on cognitive function did not significantly differ by gender, race/ethnicity, or prior educational attainment. This study provides new insights into the protective role of LLE on cognitive function and underscores the importance of continued learning among older adults.

Abstract citation ID: igad104.3191

### ASSOCIATION BETWEEN LONG-TERM CARE FACILITY STAFF TRUST IN LEADERSHIP AND RECEIVING THE COVID-19 VACCINE

Xinjun Li, David Gifford, and Valerie Williams, *AHCA/NCAL, Washington, District of Columbia, United States*

Trust in the sources recommending COVID-19 vaccine is thought to be associated with COVID-19 vaccine uptake. We compared long-term care (LTC) staff's trust with leadership and public health organizations and their vaccination status. We administered a questionnaire to all staff in 48 long term facilities (39 Skilled Nursing Facilities (SNFs) and 9 Assisted Living (AL) facilities) to assess their overall trust level on a 5-point Likert scale with leadership and public health agencies along with their reported used COVID-19 vaccination status. We received 759 responses, with 34 were excluded due to incomplete data. Overall, 12% were not vaccinated, 25.2% completed initial series, 25.0% received one booster, and 37.8% received bivalent booster. Average trust was lowest with CDC (3.62) & CMS (3.60) and highest with leadership (Supervisor (4.07), Administrator (3.98) and DON (3.89)), which were higher than trust with the LTC organization (3.74). Trust levels increased as the staff's vaccination status increased (p< 0.01) for each category in ANOVA tests. Building overall trust may help increase staff compliance with vaccination recommendations.

Abstract citation ID: igad104.3192

### ASSOCIATION BETWEEN SCHOOL DESEGREGATION AND THE COGNITION OF OLDER BLACK MEN: A CITY-LEVEL ANALYSIS

Collin Perryman[1], Taharka Anderson[2], Roland J. Thorpe[3], and Keith Whitfield[4], *1. Johns Hopkins Bloomberg School of Public Health, Baltimore, Maryland, United States*, *2. University of Texas at Austin, Austin, Texas, United States*, *3. Johns Hopkins University, Baltimore, Maryland, United States*, *4. University of Nevada, Las Vegas, Las Vegas, Nevada, United States*

Objective: We sought to understand the relationship between school setting attendance (desegregated or segregated) and the cognition among older Black men. **Methods:** Data from older Black men (n=153; age range: 50 to 89 years) from the Baltimore Study of Black Aging—Patterns of Cognitive Aging (BSBA-PCA) was used to explore the relationship between type of schooling and cognitive functioning. We conducted ANOVA and OLS regression analyses to find associations between schooling and various cognitive domains. The independent variable, school type, was measured using a variable assessing if participants attended a racially-mixed school in their youth. The outcome variable, cognition, was measured using global cognition, reasoning, memory, immediate recall, working memory, language, and perceptual speed. **Results:** ANOVA analyses found that Black men who attended desegregated schools reported significantly better reasoning and global cognition. OLS regression analyses found that global cognition, reasoning, language, and working memory were significantly better performance in the OLS regression results. The multivariate analysis found that global cognition and reasoning yielded statistically better performance. **Conclusion:** Our study provides insights to how cognition varies in Black men depending on the school settings in which they were educated. Additionally, our study finds evidence that past educational policies represent risk for poor cognitive health of older Black men populations.

Abstract citation ID: igad104.3193

### ASSOCIATION BETWEEN TIME SPENT OUTSIDE THE HOME AND EXECUTIVE FUNCTION IN PATIENTS WITH PAST TRAUMATIC BRAIN INJURY

Emily Richards[1], Patrick Donahue[1], Kyle Moored[2], Dana Eldreth[3], Breanna Crane[2], and Michelle Carlson[2], *1. Johns Hopkins University, Baltimore, Maryland, United States*, *2. Johns Hopkins Bloomberg School of Public Health, Baltimore, Maryland, United States*, *3. University of Illinois Urbana Champaign, Urbana, Illinois, United States*

Time spent out of the home and in the surrounding neighborhood has numerous physical health benefits, but the cognitive benefits are less clear. Evidence suggests that executive functions are important to navigating activity outside the home. This study examined whether time spent outside in participants' immediate neighborhoods was more strongly associated with executive function than memory. Participants were adults with mild cognitive impairment and history of traumatic brain injury (TBI) enrolled in an immersive computer game intervention (N=11; 63.6% male; mean=59.0 years, range: 42-75). At baseline, participants completed the Life Space Questionnaire (LSQ) and cognitive tests of Stroop (executive function), Rey Auditory Verbal Learning Test (RAVLT) (memory), and Pattern Comparison Test (PCT) (processing speed). Cognitive tests were performed on an iPad to precisely measure reaction time. Pearson correlations were used to examine the association between the LSQ and cognitive measures. Although time spent in the neighborhood was not statistically associated with performance on cognitive measures, the magnitude of the correlations was noteworthy. After adjusting for age and education, days spent in the neighborhood were more strongly associated with better executive function (Stroop) (r=-0.58, p=0.10) and processing speed (PCT) (r=-0.27, p=0.49). Additionally, neighborhood exposure explained a larger proportion of the variance in Stroop and PCT rather than in RAVLT. In patients with past TBI, time spent outside of the home in the neighborhood may be associated with better executive functions through engagement with one's environment. We will further describe objective measures of time spent outside the home using GPS.

Abstract citation ID: igad104.3194

### ASSOCIATION OF MASSETER MUSCLE VOLUME WITH RISK OF OSTEOPOROSIS IN OLDER ADULTS: THE BUNKYO HEALTH STUDY

Abulaiti Abudurezake, Saori Kakehi, Shota Sakamoto, Minami Morikawa, Hideyoshi Kaga, Yoshifumi Tamura, Hiroki Tabata, and Ryuzo Kawamori, *Juntendo University Graduate School of Medicine, Bunkyo-Ku, Tokyo, Japan*

Abstract The relationship between bone mineral density (BMD) and oral function has been previously studied. However, the relationship between the masseter muscle, which plays an important role in oral function, and BMD and osteoporosis in older adults has not been investigated. This study investigated the association between masseter muscle volume (MMV) and old age in relation to osteoporosis prevalence and hip joint BMD. Body composition and BMD of 1534 people aged 65–84 years living in Bunkyo-ku, Tokyo, were measured using dual-energy X-ray absorptiometry. MMV was measured using a three-dimensional medical image analysis system. Participants were divided into five groups based on MMV by 20% for each gender (Quintile 1–5: Q1–5, Q1< Q5). Logistic regression models were employed to estimate multivariable-adjusted odds ratios (ORs) for osteoporosis determined by T-score (<–2.5 standard deviation) using the Q5 reference. Subsequently, analysis of covariance was used to investigate the relationship between MMV and BMD. Adjustment for age, body mass index, smoking history, alcohol intake, calcium intake, 25(OH)D, diabetes, and osteoporosis drugs or estrogen. In men, Q1 had higher hip osteoporosis prevalence than Q5 (OR 11.65; 95% confidence interval, 1.48–1.62, p=0.020). Further, hip BMD was significantly higher in Q5 than in Q1 (p=0.001). In women, MMV and old age was not associated with osteoporosis prevalence. However, hip BMD was significantly higher in Q3,4 than in Q1 (p=0.003). Older adults with lower MMV had lower BMD in the hip joint. Additionally, men had higher odds of osteoporosis of the hip joint.

Abstract citation ID: igad104.3195

### ASSOCIATION OF RAPID EYE MOVEMENT SLEEP AND APOE PROTEOTYPE TO COGNITIVE PERFORMANCE IN OLDER ADULTS

Edward Hammel[1], Mitchell Roberts[1], Erica Sappington[1], Paul Dagum[2], Jeffrey Iliff[3], Swati Rane Levendovszky[4], Jeff Lowenkron[5], and Carla VandeWeerd[1], *1. UF Precision Health Research Center - The Villages, The Villages, Florida, United States*, *2. Applied Cognition Inc, Redwood City, California, United States*, *3. University of Washington, Seattle, Washington, United States*, *4. University of Washington Medical Center, Seattle, Washington, United States*, *5. The Villages Health, The Villages, Florida, United States*

Exploring the multifaceted interaction of sleep, disease-related biomarkers and cognitive decline is of particular importance for aging populations. Sleep quality and the APOE E3/E4 allele has been linked to Alzheimer's Disease (AD). This study aims to examine relationships between older adults' level of cognitive performance, proportion of rapid eye movement (REM) sleep, and APOE proteotype. Analysis of sleep study data collected in an active lifestyle retirement community - The Villages, Florida was conducted using a subset of markers collected as part of a larger cognitive health study. Polysomnography, cognitive battery (Montreal Cognitive Assessment(MoCA), Trail Making Tests(TMT), Symbol Digit Modalities Test(SDMT), Psychomotor Vigilance Test(PVT)), and APOE blood-biomarker (E2/E3,E3/E3,E3/E4) data were obtained from healthy participants (n=33) aged 55-65. Descriptive statistics, multilinear regression, and ANOVA were used. Participants' mean age was 61.9 years, with 55% being male. Cognitive test scores did not show a significant relationship with REM sleep percentage (p=0.3096). No consistent dispersion or skew was shown in box plot analysis of APOE proteotype across cognitive performance measures. Unexpectedly, although not statistically significant (p=0.396), average REM sleep percentage was higher in the APOE E3/E4 group (20.04%, Mean:75.44 minutes) as compared to the E3/E3 group (15.99%,Mean: 59.07 minutes). Proteotype E3/E4 was examined specifically due to its potential association with Alzheimer's disease. The REM sleep proportion was lower than the average experienced on a typical night at home, possibly due to study conditions. Further research is needed examining the relationship between older adults with REM sleep insufficiency and cognitive impairment.

Abstract citation ID: igad104.3196

### ASSOCIATIONS BETWEEN COGNITIVE CHANGE AND OBJECTIVE CHANGE IN PHYSICAL ACTIVITY AMONG OLDER ADULTS: THE LIFE STUDY

Erta Cenko, Rachael Seidler, David Cheng, Thomas Pearson, and Todd Manini, *University of Florida, Gainesville, Florida, United States*

Objective: To investigate the associations between domain-specific cognitive change and objectively measured change in physical activity in a sample of community-dwelling older adults. We hypothesized that domain-specific changes in cognition are associated with longitudinal changes in physical activity levels. **Methods:** We used data from 955 participants from The LIFE Study, a multi-center randomized clinical trial. Longitudinal relationships between cognitive domains (i.e., memory, attention, processing speed, executive function) and accelerometer-measured change in daily minutes of physical activity were examined using multivariable linear regression. Cognitive tests included Modified Mini-Mental State Examination (3MSE), Hopkins Verbal Learning Test (HVLT), Digital Symbol Substitution Test (DSST), Task Switching Test, Eriksen Flanker Test and N-Back Test. Physical activity was measured longitudinally using an accelerometer and was categorized to total, light, and moderate to vigorous according to standardized activity count cut points. **Results:** Our results showed an association between cognitive change in the processing speed domain and change in minutes of total ($\beta = 0.47$; 95% CI: 0.04, 0.89; $p = 0.034$) and light ($\beta = 0.42$; 95%CI: 0.03, 0.80; $p = 0.033$) physical activity. Relationships between all other cognitive assessments and physical activity categories were not statistically significant. **Conclusions:** This study demonstrates an association between a decline in processing speed and a decline in the duration of total and light daily physical activity in older adults with low to moderate physical function.

Abstract citation ID: igad104.3197

### ASSOCIATIONS BETWEEN SENSE OF PURPOSE, NEGATIVE AFFECT, AND CORTISOL: EVIDENCE FROM REPEATED DAILY ASSESSMENTS

Nathan Lewis[1], Theresa Pauly[2], Maureen Ashe[1], Kenneth Madden[1], Denis Gerstorf[3], and Christiane Hoppmann[1], *1. The University of British Columbia, Vancouver, British Columbia, Canada*, *2. Simon Fraser University, Vancouver, British Columbia, Canada*, *3. Humboldt University of Berlin, Berlin, Berlin, Germany*

A growing literature suggests that having a sense of purpose in life is key to supporting optimal aging outcomes. One proposed mechanism for such benefits posits that having strong sense of purpose facilitates better management of and less reactivity to daily negative experiences, which over time may impact health. However, there remains a paucity of research investigating sense of purpose, affective experiences, and biological stress markers as individuals engage in their daily life routines. The present study sought to examine everyday associations between sense of purpose in life, negative affect, and salivary cortisol using repeated daily life assessments. A sample of 170 older adults aged 60 to 87 (M =71.18 years, SD=6.06) completed affect ratings and provided concurrent salivary cortisol samples 4 times per day over 7 consecutive days. Multilevel growth modeling was used to examine whether trait sense of purpose in life moderated within-day associations between negative affect and log-transformed cortisol levels (nmol/L), adjusting for relevant demographic information and factors that may impact cortisol (e.g., time since waking, caffeine intake). Analyses revealed that sense of purpose was indeed associated with lower daily negative affect, but it did not predict everyday cortisol and was also not moderating the within-person couplings between everyday negative affect and cortisol. Our results suggest that purpose in life shapes daily affect dynamics of older adults, yet not through modulating the physiological implications of negative affect. Follow-up analyses will explore whether these findings generalize to vulnerable groups of very old adults and those in poor health.

Abstract citation ID: igad104.3198

### ASSOCIATIONS BETWEEN THE GERIATRIC DEPRESSION SCALE AND APNEA-HYPOPNEA INDEX IN OLDER ADULTS

Alyssa Persad[1], Edward Hammel[1], Mitchell Roberts[1], Erica Sappington[1], Paul Dagum[2], Jeffrey Iliff[3], Swati Rane Levendovszky[4], and Carla VandeWeerd[1], *1. UF Health Precision Health Research Center, The Villages, Florida, United States*, *2. Applied Cognition Inc, Redwood City, California, United States*, *3. University of Washington, Seattle, Washington, United States*, *4. University of Washington Medical Center, Seattle, Washington, United States*

The prevalence of sleep apnea increases with age and depression. Sleep plays an important role in memory consolidation, therefore, changes in sleep quality may play a contributing factor to neurodegenerative diseases such as Alzheimer's. Findings from a retrospective analysis of a randomized control sleep study are presented for healthy participants (N=33) with no self-reported mental health conditions between 11/7/2022-6/7/2023. As part of a larger study, this sub-study occurred in The Villages in a partnership between the UFHealth PHRC and Applied Cognition. Participants completed the Geriatric Depression Scale (GDS-15), and Polysomnography (PSG), with the Apnea-Hypopnea Index (AHI) extracted from the PSG data. The relationship between sleep apnea and depression was examined using descriptive statistics and linear regression. The mean age of participants was 61.9, with 55% being male. GDS-15 scores showed a strong negative correlation with AHI ($p<.001$), indicating that as GDS scores increased, AHI decreased. The R2 value (0.06) indicates little variation in the data and is a good fit for the model. Limited data due to the small sample size may contribute to the observed relationship. Although the effect size was small, symptoms related to depression could contribute to decreased AHI. A sample from The Villages may be biased towards a higher social cohesion lifestyle than other communities, showing how elements of depression may differently impact sleep apnea. Further studies are needed to identify further associations and implement appropriate interventions.

Abstract citation ID: igad104.3199

### AVENUES OF SOCIAL ISOLATION AND HEALTH AMONG OLDER CHINESE MIGRANTS: A STRUCTURAL EQUATION MODELING APPROACH

Amber DeJohn[1], Michael Widener[2], Bochu Liu[3], Xinlin Ma[4], and Zhilin Liu[5], *1. Florida State University, Tallahassee, Florida, United States*, *2. University of Toronto, Toronto, Ontario, Canada*, *3. Tongji University, Shanghai, Shanghai, China (People's Republic)*, *4. UNC Chapel Hill, Chapel Hill, North Carolina, United States*, *5. Tsinghua University, Beijing, Beijing, China (People's Republic)*

Social isolation and loneliness have detrimental impacts on health. During the COVID-19 pandemic, access to third places (e.g., coffee shops, libraries) decreased due to the closure of non-essential destinations for fear of serious illness. With reported rises in anti-Asian hate crimes, which primarily impacted older Asian adults, community mobility might have been additionally affected. Older adults reported adopting ICT during pandemic lockdowns, which may have sufficiently replaced previous activities that would require trips out of the home. Understanding different avenues of social connection and their distinct relationships to health for a vulnerable migrant population is critical to supporting equitable, healthy aging in a post-COVID world. Using a survey of older Chinese migrants in the Greater Toronto Area (GTA) during the extended COVID-19 lockdown, we investigate both community mobility and ICT use to understand how either avenue of socializing is related to the built environment and what the impact of community mobility and ICT use has on loneliness (De Jong Gierveld 6-item scale), mental and physical health (SF-12). Specifically, we use a structural equation model to test a theoretical framework of older adult social isolation. Ultimately, our model demonstrates the importance of community mobility in reducing feelings of loneliness, while ICT use is significantly related to better physical health. Both community mobility and ICT use have significant, although opposite, relationships to transit density. Results indicate that ICT use might have a limited ability to reduce loneliness and support mental health when mobility is limited.

Abstract citation ID: igad104.3200

### BARRIERS AND SOCIETAL FACTORS SURROUNDING LONG-STAY RESIDENT DISCHARGE TO THE COMMUNITY IN LOUISIANA

Valerie Williams, *AHCA/NCAL, Washington, District of Columbia, United States*

The focus of this study is to determine the resident characteristics and societal factors that prevent long-stay residents (LSR) from discharging to the community based on the LSR in Louisiana (LA) compared to the United States (US).

This study will also explore solutions that can assist LSR in successfully discharging to the community. Discharge to the community from a skilled nursing facility is influenced by functional status, health of the resident, and the availability of community-based services. About 20% of LSR (>100 days) discharge to the community from skilled nursing facilities (SNF), while that number is more than doubled for short-stay residents. Current data provides little information about the odds of a LSR returning to the community. CMS data shows that LSR in LA have higher rates of Activities of Daily Living Decline (ADL) (LA-18.83%/US-14.68%), Antipsychotic Use (LA-16.9%/US-14.58%), and Pressure Ulcers (LA-9.4%/US-2.4%) than the national average. LSR exhibiting these characteristics are less likely to be discharged to the community. Additional characteristics of this population include high levels of comorbid chronic conditions that are difficult to manage outside of the facility, and having Medicaid (LA-71.5%/US-61.1%) as their payer status, a known barrier to community discharge. States should increase the availability of home health and community-based services that cater to the Medicaid population. Assisted living facilities are a great alternative to living “at home”. Additional research should be conducted to examine affordable assisted living options in more states for long-stay Medicaid residents who desire to live independently in the community.

Abstract citation ID: igad104.3201

### BEING A CAREGIVER DOES NOT INCREASE MORTALITY RISK AT 10-YEAR FOLLOW-UP IN A NATIONAL US SAMPLE

Dominique Cheung[1], Atina Manvelian[2], Erin Bouldin[3], Joseph Wielgosz[1], and Ranak Trivedi[4], *1. VA Palo Alto Health Care System, Palo Alto, California, United States*, *2. Santa Clara University, Santa Clara University, California, United States*, *3. University of Utah, Salt Lake City, Utah, United States*, *4. Stanford University, Stanford, California, United States*

An increasing number of people are expected to become caregivers of relatives with medical or mental health conditions. The chronic stress of family caregiving has been shown to worsen one’s health, leading to concerns that it may also increase mortality risk. Seminal work showed associations between caregiving and increased mortality risk, but recent population-based studies have disputed this. Our objective was to use longitudinal, nationally representative data to rigorously examine whether caregiver status was associated with 10-year mortality. We used data from the National Social Life Health and Aging Project (NSHAP), a nationally representative study of 2,367 older adults (57-85 y) that combines interviews, surveys, and mortality data. We compared 10-year mortality risk between self-reported caregivers and non-caregivers with and without propensity score matching. Baseline sample included 401 caregivers (68.6±7.6 y) and 1,966 non-caregivers (69.2±7.7 y). Among caregivers, 25.0% had 2+ chronic health conditions, compared to 41.1% of non-caregivers. At the 10-year follow-up, 20.6% of caregivers had died versus 29.6% of non-caregivers (p<.0001). After propensity score matching on demographics, health, and limitations in? activities of daily living, there were no differences in mortality between the groups (p>0.05). Our study suggests no difference in mortality risk based on caregiver status. While family caregivers frequently experience negative impacts on well-being because of their role, it is heartening to know that caregiving may not confer an additional risk of death.

Abstract citation ID: igad104.3202

### BETTER DIET QUALITY IS ASSOCIATED WITH REMAINING NON-FRAIL OVER TIME IN A DIVERSE COHORT

Marie Kuczmarski[1], May Beydoun[2], Michael Georgescu[2], Nicole Noren Hooten[2], Nicolle Mode[2], Michele Evans[2], and Alan B. Zonderman[2], *1. University of Delaware, Newark, Maryland, United States*, *2. National Institute on Aging, Baltimore, Maryland, United States*

Frailty is a major public health concern affecting the health and well-being of adults. Poor diet is among several modifiable risk factors for developing frailty. We examined associations of dietary quality assessed by the Healthy Eating Index with frailty status over mean 8.7y follow-up. The sample was comprised of African American (AA) and White participants enrolled in the Healthy Aging in Neighborhoods of Diversity across the Life Span (HANDLS) study (44% male, 44% AA, mean baseline age of 48.5y). Only participants from the baseline study and first 2 follow-up visits with dietary data, 2 recall days per visit, and frailty data were included (n=2901). Frailty was prospectively determined by the modified FRAIL scale. Group-based trajectory modeling identified 3 diet quality groups (high: g3 [5.2% of sample, HEI(X±SD): 57.4±4.1], medium (g2: 48.2%, 46.1±3.7), low (g1: 46.5%, 38.2±2.9) and 2 frailty groups (remaining non-frail [20.2% sample] or being pre-frail or frail over time). Multiple logistic and Cox proportional hazards regressions found high and medium HEI trajectory groups, compared to the low HEI group, were associated with reduced risk of being pre-frail or frail, with a clear dose-response relationship (g2 vs. g1: HR=0.77, 95% CI: 0.64-0.92, p=0.004; g3 vs. g1: HR=0.29, 95% CI: 0.18-0.49, P< 0.001), even after adjusting for sociodemographic factors, smoking, drug use, and allostatic load. These findings were corroborated with Kaplan-Meier curves and log-rank tests, providing longitudinal evidence of the link between better diet quality with lower frailty risk, and the importance of diet in successful aging during middle age.

Abstract citation ID: igad104.3203

### BIOMARKERS IN CNS-ORIGINATING EXTRACELLULAR VESICLES: A DIAGNOSTIC META-ANALYSIS FOR PARKINSONIAN DISORDERS

Hash Brown Taha[1], and Aleks Bogoniewski[2], *1. University of Southern California, Los Angeles, California, United States*, *2. University of California Los Angeles, Los Angeles, California, United States*

Parkinsonian disorders, including Parkinson's disease (PD), multiple system atrophy (MSA), dementia with Lewy bodies (DLB), progressive supranuclear palsy (PSP), and corticobasal syndrome (CBS), present overlapping early symptoms, complicating accurate diagnosis. Predicting the conversion of prodromal conditions like REM behavior disorder (RBD) to PD, MSA, or DLB is also complex. Extracellular vesicles (EVs), small structures released by cells, carry cell-state-specific biomarkers that can cross the blood-brain barrier, providing a window into the brain's biochemistry. Blood-isolated CNS-originating EVs have become a popular diagnostic tool. Yet, challenges remain in replicating and validating these findings. We conducted a PRISMA-guided systematic review and meta-analysis of 15 studies involving 1,455 persons with PD, 206 with MSA, 21 with DLB, 172 with PSP, 152 with CBS, 189 with RBD, and 1,045 healthy controls (HCs). Diagnostic accuracy for distinguishing persons with PD from HCs was moderate but showed high heterogeneity and significant publication bias. This indicates that studies with non-significant or lower effect sizes were less likely to be published. Differentiating persons with PD from those with PSP or CBS is limited due to the small number of involved studies. Our analyses suggest that biomarkers from CNS-originating EVs may not reliably differentiate persons with MSA or RBD from HCs due to variable accuracy and high heterogeneity. Our findings highlight the moderate diagnostic accuracy of EV biomarkers in differentiating Parkinsonian disorders, emphasizing substantial heterogeneity and significant publication bias. The need for larger, more standardized, and unbiased studies is underscored to validate the utility of EV biomarkers as diagnostic tools.

Abstract citation ID: igad104.3204

### BOTH COGNITIVE IMPAIRMENT AND DEPRESSION MAY INCREASE THE RISK OF POOR MUSCLE FUNCTION IN OLDER PEOPLE

I-Ting Liu, *E Da Hospital, Kaohsiung City, Kaohsiung, Taiwan (Republic of China)*

Muscle loss might occur in aging, target organ failure, metabolic and endocrine disorders, etc. The aim of this study was to identify the high-risk group of low grip strength, a key component of sarcopenia. 100 subjects aged 65 or older who received Comprehensive Geriatric Assessment (CGA) and grip strength measurement were enrolled at EDA Hospital, Taiwan, form January 2022 to February, 2023 (IRB: EMRP-110-108). Exclusion criteria: unclear consciousness or unable to cooperate、advanced disease. CGA and grip strength measurement were performed by well-trained nurses and geriatrician. Depressive mood was defined as Geriatric Depression Scale-5 ≧2 or with diagnosed depression. Cognitive impairment was defined as Short Portable Mental State Questionnaire (SPMSQ) < 8 (SPMSQ < 6 in those without education) or with diagnosed dementia. Multiple linear regression was constructed to assess the independent associated factors on increased average grip strength of bilateral hands. Univariate analysis showed male, weight, ADL significantly associated with greater grip strength. Multiple linear regression revealed CKD(ß= -7.70, 95%CI -13.35~-2.05, p=0.011), COPD(ß= -9.15, 95%CI -17.15~-1.17, p=0.027 ), cognitive impairment(ß= -7.67, 95%CI -12.66~-2.65, p=0.005) and depressive mood(ß= -8.39, 95%CI -13.46~-3.32, p=0.003) have detrimental effect on grip strength even after adjustment of age, sex, BMI, socioeconomics, chronic comorbidities, and other geriatric syndromes. Our results resemble another sarcopenia study in which poor muscle function, but not reduced lean muscle mass, was associated with late-life cognitive impairment. Therefore, early screening, education, and intervention of sarcopenia should be applied on high-risk population such as those with CKD, COPD, cognitive impairment, and depressive mood.

Abstract citation ID: igad104.3205

### BRIDGE EMPLOYMENT OR ENCORE CAREER? PREDICTORS THAT DISTINGUISH LATER LIFE CAREER TRANSITIONS

Yun taek Oh, *University of Michigan, Ann Arbor, Michigan, United States*

Moving away from the traditional lock-step retirement to multiple retirement pathways, later-life career transition has been prevalent for about a half-century (Ruhm, 1990). So-called "encore career," many older workers start their second career after leaving their original career jobs. However, leaving one's career job is not a distinct prequel to an encore career; instead, it is an essential step to bridge employment as well. While these two transitions have different purposes, reasons, and outcomes, the predictors distinguishing between them are relatively not well-documented. Using the sample of respondents with career jobs in the Health and Retirement Study, this study distinguished various later-life transitions by the voluntariness of career transition and the duration of labor force participation after the transition and then investigated the predictors that distinguish between bridge employment and encore career. Based on the theoretical predictions from multiple disciplines, such as the resource-based dynamic process model and continuity theory, I stated and tested a set of hypotheses using multinomial logit estimation. The result showed that financial, physical, and mental health resources, and retirement identity when transitioning to new jobs predict whether older workers take bridge employment or encore careers. This result provides a helpful hint to policymakers and employers who want to retain older workers in the labor force. Because the indicators for the voluntariness of career transition are available for those who left their career employers only, further research is needed once the voluntariness measures for other work adjustments are available.

Abstract citation ID: igad104.3206

### CAN YOU HEAR ME? IMPLICATIONS OF HEARING LOSS FOR SOCIAL TIES AMONG OLDER ADULTS

Kira Birditt[1], Angela Turkelson[1], Emily Noyer[1], Jane Stephenson[1], Karen Fingerman[2], and Michael McKee[1], *1. University of Michigan, Ann Arbor, Michigan, United States*, *2. The University of Texas at Austin, Austin, Texas, United States*

Hearing loss is a leading source of disability among older adults; over 65% of those aged 71 and older have hearing loss. Hearing loss may be associated with reduced relationship quality due to greater communication challenges, conflict, and avoidance of social interactions. This study examined links between hearing loss and social ties among older people. Participants included 11,161 respondents from the 2016 and 2018 waves of the Health and Retirement Study (ages 50+) who completed a hearing test and reported on their social ties (contact, quality), social activities (e.g., charity work), and loneliness. Multilevel models controlling for age, gender, and education revealed that having hearing loss was associated with having lower positive quality and higher negative quality relationships with spouse and friends, higher negative relationship quality with children, having fewer close friends, meeting up less with friends, greater loneliness, doing less charity work, and less attendance at social and community events. Older age was associated with more detrimental outcomes. Older aged individuals with hearing loss met up with friends less often, talked on the phone less with friends, did fewer activities with grandchildren/extended children and neighborhood children, did less charity work, and attended fewer social events compared to younger individuals with hearing loss. Overall, older adults with hearing loss are at high risk for relationship problems and social isolation; especially as they grow older. Hearing loss and social ties are important targets for future interventions for older adults with hearing loss as they may be particularly vulnerable.

Abstract citation ID: igad104.3207

### CANCER HISTORY AND FRAILTY LEVEL IN OLDER ADULTS AND THEIR HEALTHCARE UTILIZATION: EVIDENCE FROM THE NHIS IN THE US

Mingzhu Su[1], Siqi Liu[2], Jiahui Lao[3], and Li Liu[1], *1. Shandong University, Jinan, Shandong, China (People's Republic)*, *2. Chinese Academy of Medical Sciences & Peking Union Medical College, Beijing, Beijing, China (People's Republic)*, *3. Shandong First Medical University & Shandong Provincial Qianfoshan Hospital, Jinan, Shandong, Jinan, Shandong, China (People's Republic)*

Previous studies didn't compare effect measures of frailty between cancer survivors and counterparts without a cancer history, and this makes us unable to judge whether the impact of frailty is more substantial in cancer survivors. This study aimed to examine the association between cancer history and frailty level with healthcare utilization among older adults. A descriptive survey design consistent with the STROBE guidelines was used. 14,562 old adults were identified in the 2019 and 2020 National Health Interview Survey (stratified by old cancer survivors [N=3,944] and older adults without a cancer history [N=10,618]). Frailty was assessed by Fatigue, Resistance, Ambulation, Illness, and Low BMI. Healthcare utilization was measured by urgent care, emergency care, hospitalization, delayed care, and needed but did not get care. Multivariable logistic regressions were used to examine the association between frailty-cancer characteristics and healthcare utilization. Participants with cancer (vs without) were older (75+: 49.7% vs 36.8%) and more likely to be frail (23.8% vs 14.5%) and pre-frail (36.0% vs 33.7%). The strength of associations between cancer and health utilization (emergency care and hospitalization) among older adults was significantly modified by their frailty status. Compared with robust older adults, frail cancer survivors were more likely to report emergency care (OR=4.7, 95% CI: 3.9–5.8) and hospitalization (OR=5.6, 95% CI: 4.5–7.0). Our findings are essential for risk stratification and informed decision-making in future healthcare service provision planning.

Abstract citation ID: igad104.3208

### CARE COMPARE STAR RATINGS AND FAMILY SATISFACTION IN MARYLAND NURSING FACILITIES

Roberto Millar[1], Christin Diehl[1], Nancy Kusmaul[1], and Ian Stockwell[2], *1. The Hilltop Institute at UMBC, Baltimore, Maryland, United States*, *2. University of Maryland Baltimore County, Baltimore, Maryland, United States*

Nursing facilities provide critical services and supports to individuals with long-term care needs. The quality of care in nursing facilities varies depending on facility structural characteristics. Moreover, the measurement and perceptions of what constitutes quality of care varies across stakeholders. We used publicly available data to examine the association between family satisfaction with care and the Centers for Medicare & Medicaid Services'(CMS's) Care Compare five-star quality ratings in the context of facility characteristics. Facility-level data of family satisfaction with care were merged with CMS's five-star star ratings of 220 Maryland nursing facilities in 2021. Using univariate and bivariate statistics, we explored differences in family ratings and five-star ratings across facility ownership (for-profit vs. non-profit), geographic location (urban vs. rural), and average resident occupancy (1-60, 61-120, and 121+). Relationships were examined across overall ratings, as well as across subdomain of the two quality rating frameworks (e.g., staffing, autonomy, health inspections). Family members of residents in non-profit, rural, and low-occupancy facilities rated facilities higher. Non-profit and low-occupancy facilities were statistically more likely to be rated four or five stars, while no significant association was observed across geographic location. The association between subdomain-specific family satisfaction and star ratings varied across facilities of different structures. Findings emphasize the need for comprehensive quality of care frameworks that consider views of quality across stakeholders and types of facilities. A clear understanding of nursing facility structure and quality of care is critical to advance data-driven decision making.

Abstract citation ID: igad104.3209

### CARE COORDINATION INTERVENTIONS FOR DEMENTIA CAREGIVERS: AN INTEGRATIVE REVIEW

Diana Layne, Ayaba Logan, and Kathleen Lindell, *Medical University of South Carolina, Charleston, South Carolina, United States*

Alzheimer's disease is a serious illness with an extended caregiving experience however care coordination interventions often lack integration of palliative care. The aim of this integrative review is to identify existing care coordination interventions which integrate palliative care for community dwelling individuals with dementia and their caregivers. An integrative review guided by the Whittemore and Knafl framework was conducted, and the Mixed Methods Appraisal Tool was utilized to assess study quality. The systematic search was initially conducted in June 2021 and repeated in May 2023 to ensure no additional studies were available in collaboration with a medical informationist. Nine interventions across eighteen publications were identified that incorporated informal family caregivers and were aimed at improving care coordination for community dwelling PLWD and their caregivers. Only a single intervention used the term palliative care while the remaining interventions included traditional components of palliative care such as advance care planning, symptom management, and emotional support. Many existing care coordination interventions are not grounded in theory or conceptual models and have been studied in a homogenous sample which is not representative of the population. Further study of the existing interventions in a more heterogenous sample is needed. Additional research is necessary to better understand the lived experience of those persons with dementia and their caregivers to reduce burden associated with care coordination activities.

Abstract citation ID: igad104.3210

### CAREGIVER CHALLENGES: SOCIAL SUPPORT FOR DEMENTIA CARE OF OLDER ADULTS DURING HOSPICE ENROLLMENT

Hannah Cho[1], Justine Sefcik[2], Karla Washington[3], Debra Parker Oliver[3], and George Demiris[1], *1. University of Pennsylvania, Philadelphia, Pennsylvania, United States*, *2. Drexel University College of Nursing and Health Professions, Philadelphia, Pennsylvania, United States*, *3. Washington University in St. Louis, St. Louis, Missouri, United States*

Every year, many ADRD patients rely on informal caregivers for providing care during hospice enrollment. Driven by dedication, these caregivers play a vital role. However, their responsibilities intensify over time, leading to emotional strain. Inadequate support causes burnout and stress. Limited research targets the unique needs of caregivers of ADRD patients enrolled in hospice. The purpose of this study was to describe the caregivers' experiences regarding social support while providing care for older adults with ADRD in hospice. A qualitative descriptive approach was taken with semi-structured interviews of 20 caregivers of ADRD hospice patients (85% female; mean age [SD]: 63.75 [12.1]), including both Caucasian (70%) and African American (30%) individuals. Through content analysis, we identified three themes that described caregivers' experiences with social support: 1) Changes in social life (e.g., homebound, stress, and family conflicts), 2) Opportunities for social connection (e.g., social support systems with family, friends), 3) Communication Modalities. These findings offer crucial insights into the diverse range of experiences caregivers face regarding social support. Additionally, it highlights the significance of caregivers needing assistance to sustain connections, take essential breaks, and adapt to shifts in their social environments. The challenges they confront and the positive elements of the support they receive are brought to light through this study. Further research into effective communication modalities and respite services could significantly enhance caregivers' support.

Abstract citation ID: igad104.3211

### CAREGIVERS & COLONOSCOPIES: A QUANTITATIVE COMPARATIVE STUDY OF CAREGIVERS' PREVENTATIVE HEALTH BEHAVIORS

Claire Quinlan[1], Samantha Brady[2], Sophia Ashebir[2], Alexa Balmuth[2], Lisa D'Ambrosio[2], and Joseph Coughlin[2], *1. Harvard Medical School, Boston, Massachusetts, United States*, *2. Massachusetts Institute of Technology, Cambridge, Massachusetts, United States*

That many caregivers experience some degree of burden is well-established, but little research has investigated the association of burden on caregiver preventative health behaviors. A lack of adherence to these behaviors, including to recommended colonoscopy and mammography screenings, routine vaccinations, annual clinic visits, and HIV testing, may portend worsening health outcomes for family caregivers if diseases are left undetected or untreated; such outcomes could also be caused by and contribute to the crisis of lack of care to meet demand. Survey questionnaires (N=326) were completed by 214 current caregivers and 112 former caregivers from the national MIT AgeLab Caregiver Panel. Respondents predominately identified as white (88%) and female (83%), with a median age of 40-49 years old. Questions assessed caregiving intensity, health status, reported caregiving burden, and adherence to preventative health screenings and vaccinations (per USPTF age- and gender-based recommendations). Among all caregivers, just 17% reported that they were up-to-date with appropriate preventative health screenings. Current caregivers were less likely than previous caregivers to be up-to-date. Controlling for age, income level, and health status, caregiving intensity (as measured by hours per week) was an independent predictor of adherence to preventative health screenings. These findings suggest that current caregivers who are providing high-intensity care are less likely to be engaging in preventative health behaviors for themselves. This finding is concerning for the future health of these caregivers as well as their care recipients and highlights the need for primary care preventative health initiatives specifically targeting family caregivers.

Abstract citation ID: igad104.3212

### CASE STUDY - TREATMENT OF AMIODARONE-INDUCED THYROID DYSFUNCTION IN LONG TERM CARE PATIENT WITH DEMENTIA

John Chen[1], and Ling Ling Cheng[2], *1. Kaiser Permanente Med Group/Southern California, Baldwin Park, California, United States*, *2. Kaiser Permanente Med Group/Southern California, Harbor City, California, United States*

Pharmacodynamic and pharmacokinetic changes in geriatric population warrant a stepwise, patient-focused and interdisciplinary approach for treatment with potent, class III anti-arrhythmic Amiodarone by reviewing 1) benefits of cardiac tachyarrhythmia treatment, 2) risks of adverse drug events (ADE) i.e. pulmonary, hepatic and thyroid dysfunctions, 3) alternative treatment option(s) with consultation1. 81 years old African American female with history of stroke with vascular dementia, non-ST-elevation myocardial infarction (NSTEMI) and Atrial Fibrillation residing in long term care (LTC) was transferred via 911 for intractable supraventricular tachycardia (SVT) stabilized with Amiodarone treatment. The peak level of Thyroid Stimulation Hormone (TSH) 36.67 (reference range 0.35-4.0) reflexed the initiation of Levothyroxine for ADE Amiodarone-induced hypothyroidism, albeit thyrotoxicosis can also result2. Countering the prescribing cascade and polypharmacy, gradual uptitration of Levothyroxine and weaning of Amiodarone resulted in the nadir level of TSH 8.19 (reference range 0.35-4.0)2. Virtual consultation with Cardiology and Endocrinology Teams provided alternative treatment options with recommendations 1) taper off Amiodarone, 2) substitute with beta-blocker Bisoprolol for heart rate control and 3) continue Levothyroxine trending thyroid function tests. Further interdisciplinary research in vulnerable, older patients in LTC settings is imperative to align the risks, benefits, and alternative treatment option(s) of acute and chronic disease with fundamental geriatric principles, including continuous medication reconciliation, shared decision-making, and life care planning.

1 Basaria S, Cooper DS. Amiodarone and the thyroid. Am J Med. 2005;118(7):706-714. doi:10.1016/j.amjmed.2004.11.028
2 Danzi S, Klein I. Amiodarone-induced thyroid dysfunction. J Intensive Care Med. 2015;30(4):179-185. doi:10.1177/0885066613503278

Abstract citation ID: igad104.3213

**CAUSAL EFFECT OF ANTI-DEMENTIA DRUGS ON PATIENTS' ECONOMIC BURDEN USING DOUBLE/ DEBIASED MACHINE LEARNING APPROACH**

Xiangxiang Jiang[1], Gang Lv[2], Minghui Li[3], and Kevin Lu[1], *1. University of South Carolina, Columbia, South Carolina, United States*, *2. First Chinese PLA Hospital, Beijing, Beijing, China (People's Republic)*, *3. University of Tennessee, Memphis, Tennessee, United States*

Alzheimer's disease and related dementia (ADRD) present a significant public health challenge and carry a substantial financial burden. Exploring avenues for treatment, the administration of anti-dementia drugs has emerged as a potential intervention for ADRD. However, prior research has been susceptible to omitted variable bias due to data limitations and struggled to establish causal relationships due to confounding by indication. Our study aims to uncover the causal impact of anti-dementia drug utilization on the economic burden among ADRD patients through Double/ Debiased Machine Learning (DML) method. Leveraging data from the Medicare Current Beneficiary Survey (MCBS) spanning 2015 to 2017, we utilized a nationally representative survey linked to Medicare data, providing comprehensive insights. DML techniques, including Random Forest (RF) regression and Least Absolute Shrinkage and Selection Operator (LASSO) regression algorithms, were employed to investigate the causal effect. After exclusion and inclusion, a total of 218,237 ADRD patients used anti-dementia drugs, with an average total medical cost of $16,695.87; 95.51% (4,646,408 patients) of ADRD patients did not use anti-dementia drug, with an average total medical cost of $27,567.09. Results from generalized linear regression showed that anti-dementia drug use did not have a significant impact on total medial cost (β = -0.29, P = 0.10). However, results from DML demonstrated a significant true effect [RF regression (β = -0.85, P = 6.79×10-3), LASSO regression (β = -0.78, P = 9.95×10-3)]. To conclude, our analysis demonstrated the causal effect of anti-dementia drug utilization in reducing overall medical costs borne by ADRD patients.

Abstract citation ID: igad104.3214

**CELLULAR RESILIENCE AND THE AGING HEART: UNDERSTANDING MITOCHONDRIAL INJURY MECHANISMS USING THE BABOON**

Daniel Adekunbi[1], Elizabeth Reilly[2], Sandra Sanchez-Reilly[3], Cun Li[4], Peter Nathanielsz[4], Laura Cox[5], and Adam Salmon[1], *1. UT Health San Antonio, San Antonio, Texas, United States*, *2. Health Careers High School, San Antonio, Texas, United States*, *3. South Texas Veterans Health Care System, San Antonio, Texas, United States*, *4. Texas Biomedical Research Institute, San Antonio, Texas, United States*, *5. Wake Forest University Health Sciences, Winston Salem, North Carolina, United States*

**Background:** Aging-related cardiovascular disease is deadly worldwide. Cardiac aging in non-human primates (NHP), including baboons, is similar to humans, and intrauterine growth restriction animal models (IUGR) have shown to accelerate cardiac aging and foment cardiomyopathies. Objective: To measure and compare basal and metabolically stressed mitochondrial function among cardiac fibroblasts (CF) isolated from control vs. prematurely older (IUGR) baboons. **Methods:** CF were isolated from IUGR and control baboon hearts under standard conditions (25mM Glucose). Additional subgroups were exposed to Metabolic Stress (MS-exposed to 2-hours of low glucose, 1mM). Mitochondrial Oxygen Consumption Rate (OCR) and extracellular acidification rate (ECAR) were measured via Seahorse XFe96 instrument. **Results:** Donor Age: 13.27 – 17.52 years (elderly baboons), Male/Female data combined, n=4-5. OCR was higher in control relative to IUGR among all groups. ATP demand and non-mitochondrial respiration had similar values between control and IUGR. MS impacted CF bioenergetics similarly in control and IUGR. ECAR was higher in MS cells in both control/IUGR. OCR:ECAR ratio was higher in control vs IUGR while MS lowered OCR:ECAR ratio in control to levels comparable to IUGR. **Conclusion:** Higher OCR indicated increased metabolic demands of aging cells depending on glycolysis for survival. MS significantly impaired mitochondrial function across all cardiac cells regardless of aging. IUGR not only accelerated cardiac aging but impaired basal and metabolic response of CF. CF retain metabolic characteristics of their donor status and represent a cardiac aging model to explore mitochondrial injury mechanisms, understand cellular resilience and subsequently develop protective translational interventions.

Abstract citation ID: igad104.3215

**CENTENARIAN PHYSICAL FUNCTION EVOLUTION AND COVID-19 IMPACT: A STUDY IN JAPAN**

Xinyu Zhang, and Yasuyuki Gondo, *Osaka University, Suita, Osaka, Japan*

Studies show that centenarians' physical function and ADL levels improved in the recent cohort. However, it is unclear whether this positive trend has altered due to the COVID-19 pandemic. Recent studies indicated that physical function decreased even in younger older adults because of the limited daily activity during the pandemic period. Is this true for centenarians? This study has two aims: firstly, to investigate whether the physical function of Japanese centenarians has improved over the years; secondly, to examine the impact of COVID-19. We used the data collected from 2014 to the present day in Kyotango City. The City Hall has been collecting data including the functional status of all centenarians who turned 100 years old every year. In this study, we divided nine years into three periods: 2014-2016 (P1), 2017-2019 (P2), and 2020-2022 (P3). For each period the number of participants was 122, 116, and 153. The results revealed that the prevalence of bedridden centenarians showed an upward trend over the years. Concurrently, the proportion of centenarians capable of unrestricted mobility exhibited an upward trajectory from P1 to P2, followed by a subsequent decrease from P2 to P3. In conclusion, if there was no COVID-19 pandemic, the functional level of Japanese centenarians could be improved over the years. At the same time, the gradually increasing trend of bedridden centenarians might indicate intensive medical and institutional care enables centenarians with fragile conditions to survive.

Abstract citation ID: igad104.3216

### CHALLENGES AND CONSIDERATIONS IN RESEARCH WITH OLDER ADULTS EXPERIENCING HOMELESSNESS

Natalie Pope, Abigail Latimer, Debra Moser, Olivia Sasdi, and Hilda Okeyo*, University of Kentucky, Lexington, Kentucky, United States*

Individuals in their late 50s and 60s constitute the fastest growing cohort of homeless adults; homelessness among the older adult population is expected to triple by 2030 (Culhane et al., 2019). Older adults find themselves homeless due to systemic (e.g., unaffordable housing, structural racism, increasing healthcare costs) and individual factors (e.g., substance misuse, mental health diagnoses). Unhoused older adults have high rates of multiple chronic conditions (Henwood et al., 2018) worsened by unmet healthcare needs (Baggett et al., 2010). Older adults, especially those with serious illness, struggle to find and maintain housing compared to younger adults without illness. Patient and clinician-informed services/programs are needed, but the nature of serious illness and homelessness research comes with unique considerations. Research challenges in this population include inclusive and adequate recruitment, participant screening, and boundary issues (Hanson et al., 2014; Lashley, 2023). We will present lessons learned from completing two successful qualitative studies with unhoused older adults and clinical subject groups. Issues encountered during our research include dealing with recruitment obstacles, evaluating and responding to participants' cognitive impairment and mental health issues, managing emotional labor experienced by the research team, developing stakeholder relationships, and making decisions about adequate and appropriate study compensation. We describe challenges, opportunities, and suggestions for collecting primary data from unhoused older adults. We encourage gerontological researchers not to avoid this hard-to-reach, high-risk population. The lessons learned in designing and implementing studies tailored to the unique challenges of unhoused older adults can promote inclusion of the perspectives and needs this population.

Abstract citation ID: igad104.3217

### CHANGES IN MULTIMORBIDITY AMONG HOSPITALIZED ADULTS IN THE US

Lauren Picken, Yue Zhang, and Christine Loyd*, University of Alabama at Birmingham, Birmingham, Alabama, United States*

Having multiple chronic health conditions, or multimorbidity, is common among adult inpatients and is associated with risk of death, disability, and reduced quality of life. This investigation examines prevalence trends of multimorbidity over time among inpatients based on age, sex, and race and assesses hospitalization rates for individual diseases over time. Retrospective cross-sectional analysis of 2012-2018 US National Inpatient Sample datasets was completed. Participants were hospitalized patients ≥55y from community hospitals. ICD-9 and 10 codes for admitting diagnoses were used to calculate disease burden using the Charlson Comorbidity Index (CCI) and Elixhauser Comorbidity Index (ECI). Unweighted mean index scores and admission rates for diseases were compared. Mean age for the sample was 72y, and inpatients were mostly female (53%) and White (77%). The mean comorbidity scores increased across the sample: mean CCI increased from 1.69 to 1.97 and mean ECI increased from 3.66 to 4.14. Comorbidity scores were higher over time with increasing age until about age 80-84 (for CCI) and age 85-89 (for ECI) and increased similarly among males and females. Black and Native American inpatients had the largest increase in mean CCI and ECI scores. Comorbidities with increased hospitalization rates included congestive heart failure (+5%), dementia (+6%), complicated diabetes (+14%), complicated hypertension (+17%), renal disease/failure (+5%), and obesity (+5%). Growing disease burden among inpatients supports the continued need for programs aiming to prevent and treat chronic diseases and multimorbidity, especially among underrepresented populations including Black and Native American communities.

Abstract citation ID: igad104.3218

### CHARACTERIZING NURSING HOME VISITATION POLICIES AND PROCESSES DURING THE COVID-19 PANDEMIC

Courtney Hawes, Joan Brazier, Amy Meehan, and Emily Gabois*, Brown University School of Public Health, Providence, Rhode Island, United States*

The COVID-19 pandemic drastically changed how nursing homes facilitated visitation between residents and their families. Nursing homes needed to modify visitation approaches in response to changing policies and guidelines from varying governing bodies. Additionally, restrictions were constantly adjusted due to fluctuations in COVID-19 infection rates within facilities and the larger communities in which they exist. Through data from 156 in-depth, semi-structured interviews with nursing home administrators, conducted at three-month intervals throughout the COVID-19 pandemic, administrator experiences are captured as they navigated these changes. Interviews provide valuable insight into strategies used during shut-down of in-person visitation, allowance of compassionate care and essential caregiver visits, and the resumption of in-person visitation, while also demonstrating the challenges of each visitation stage and the second-hand emotional responses of resident families. During the shut-down of in-person visitation, facilities conducted virtual visits and implemented new strategies including parades and window visits. Compassionate care and essential caregiver visits were used for those in hospice or experiencing weight loss and depression resulting from restrictions, though some administrators reported being flexible in their definitions of compassionate and essential caregiver visits. When in-person visitation resumed, interviews highlight the use of screening, social distancing, and monitoring during indoor visitation, as well as the approaches facilities took to respond to the challenges of staffing shortages and outbreak concerns. Study findings provide insight into how facilities may navigate future visitation changes and restrictions to maximize safety, the overall well-being of residents and the efficiency of facilitating visits.

Abstract citation ID: igad104.3219

### CHARACTERIZING SYMPTOM BURDEN AMONG COMMUNITY-DWELLING OLDER ADULTS

Michelle McKay[1], Paul Bernhardt[1], Melissa O'Connor[1], and Suzanne Leveille[2], *1. Villanova University, Villanova, Pennsylvania, United States*, *2. UMass Boston, Boston, Massachusetts, United States*

One-third of symptoms reported by older adults are unexplained and not directly related to a chronic disease diagnosis. This ambiguity contributes to undertreatment and chronic functional burdens in vulnerable older patients. Previous studies have found that older adults often experience multiple co-occurring symptoms that contribute to disablement. Symptom burden in older adults increases healthcare utilization, decrease physical performance, and leads to poorer quality of life. This study aimed to characterize symptom burden in a population-based cohort of community-dwelling older adults enrolled in the MOBILIZE Boston study (n=765). We used descriptive statistics for symptom prevalence including pain, balance, weakness, endurance, sleep difficulty, depression/anxiety and sensory impairments. We used latent class analysis to identify 4 distinct classes based on overall symptom burden: 1) mild (26.6%), 2) moderate (53.8%), 3) moderate-severe (12%) and 4) severe (7.5%). Moderate to high levels of pain, the most prevalent symptom, were reported in all symptom burden classes except the mild class. Older adults with severe symptom burden, group 4, experienced worse levels of all symptoms compared to other classes. They also experienced moderate endurance and weakness symptoms, and mild and/or severe balance symptoms. Older adults in the moderate-severe class tended to also report mild endurance, balance, and weakness symptoms. Understanding overall symptom burden, both in terms of numbers and severity of symptoms, is the first step in determining the impact of symptom burden as a possible new clinical indicator for fall risk and other detrimental health outcomes.

Abstract citation ID: igad104.3220

### CHATGPT, WHAT IS A DEDUCTIBLE? DIGITAL ASSISTANTS AS AN INFORMATION SOURCE FOR MEDICARE QUERIES

Emily Langston, Neil Charness, and Walter Boot, *Florida State University, Tallahassee, Florida, United States*

Since being first introduced to the public in late 2022, chatbots that are based on large language models (LLMs) have attracted a great deal of attention. However, despite their popularity, these chatbots have been shown to give inaccurate information in response to user queries. Several polls conducted in 2022 suggest that the public is generally skeptical about the use of Artificial Intelligence (AI), particularly in the context of healthcare, and older adults are the most distrustful age group, despite the fact that they are the least likely to have interacted with an LLM such as ChatGPT. Meta-analysis on trust in AI has shown that users are most influenced by the accuracy and reliability of the AI trustee. In our study, we assessed the accuracy of speaker-based assistants, Alexa and Google Assistant, as well as two LLMs, Bard and ChatGPT4 on Medicare terminology and knowledge and compared their accuracy to that of a large representative sample of Medicare beneficiaries. Google Assistant was found to perform significantly worse than beneficiaries on both terminology and knowledge questions. Alexa was found to perform significantly worse than beneficiaries on terminology questions. Conversely, both Bard and ChatGPT4 were found to perform significantly better than beneficiaries on both terminology and knowledge questions. We conclude that Medicare beneficiaries should not rely on Google Assistant for terminology help or general knowledge queries, nor should they rely on Alexa for terminology help. ChatGPT4 and Bard are potentially valuable resources for beneficiaries with terminology-based and general knowledge queries.

Abstract citation ID: igad104.3221

### CHILDHOOD ADVERSITY IS ASSOCIATED WITH MENTAL HEALTH BUT NOT COGNITIVE DECLINE IN OLDER ADULTS

James Lian[1], Kim Kiely[2], Bridget Callaghan[3], and Kaarin Anstey[1], *1. University of New South Wales, Sydney, New South Wales, Australia*, *2. University of Wollongong, Sydney, New South Wales, Australia*, *3. University of California, Los Angeles, Los Angeles, California, United States*

**Objective:** The aim of this study was to examine the association between childhood adversity and mental health and cognition in older adults. **Methods:** The sample included older Australian adults from the Personality and Total Health (PATH) Through Life Project (N = 2551). Childhood adversity was measured using a 17-item scale of domestic adversities (e.g., poverty, neglect, physical abuse, verbal abuse) and modelled using cumulative risk analysis (LCA). Mental health was measured using four validated depression and anxiety questionnaires and cognitive impairment was determined by a clinically validated algorithmic diagnostic criteria. The association between childhood adversity and late-life mental health was estimated using generalised additive models, and the association with cognition utilised multiple logistic regressions. Models were adjusted for gender, race, and education. **Results:** Generalised additive models indicated that a greater number of cumulative adversities were associated with poorer scores on all four mental health measures. No notable interactions between ACEs and gender were observed. In contrast, there was no association between childhood adversity and cognitive impairment in any of the tested logistic regression models. No gender differences were observed and no interactions with education or genotype were found. **Conclusion:** Consistent with prevailing literature, our study provides additional evidence for the enduring effects of domestic childhood adversity on anxiety and depression in older adulthood. Furthermore, the absence of associations between early adversity and cognitive impairment indicates that other factors might play a more prominent role in determining late-life cognitive outcomes.

Abstract citation ID: igad104.3222

### CHILDHOOD SOCIOECONOMIC STATUS GRADIENTS IN FUNCTIONAL LIMITATIONS: THE ROLE OF MACRO-ECONOMIC CONTEXT

Thomas Fuller-Rowell[1], Samia Sultana[1], Amy Hudson[1], Eric Kim[2], and Carol Ryff[3], *1. Auburn University, Auburn, Alabama, United States*, *2. University of British Columbia, Vancouver, British Columbia, Canada*, *3. University of Wisconsin--Madison, Madison, Wisconsin, United States*

A large number of studies have documented an association between childhood socioeconomic status (SES) and adult health or illness. However, relatively little is known about how this SES-health gradient varies across macro-economic contexts. The current study examined state-level mean income as a moderator of the association between the education level of a person's parents and functional limitations in the United States (N = 10,685, Mean age = 47.7,

SD = 13.6, 51% female, 86% White; 6% Black; 8% other or missing). Data were from an economic time series database (1917-2018) and the Midlife in the United States Study (MIDUS). Time series data were merged with MIDUS data (1995 core sample and 2012 refresher) by state of residence and year to determine exposure to state economic characteristics during three periods of the life span: prime working years (ages 30-55), early adulthood (20-25) and childhood (0-15). Results indicated that the SES-functional limitations gradient was attenuated for those who lived in a state with a higher mean income from ages 30-55, 20-25, and 0-15 (p's < .05). The moderating role of state-level economic exposures between ages 30 and 55 (but not at earlier life stages) differed by gender such that the attenuation of the SES-functional limitations gradient in more affluent states was more strongly evident among females than males. Findings suggest that the role of childhood SES in later life health varies across state-level economic contexts with notable differences by gender and age group. Future directions and policy implications are discussed.

Abstract citation ID: igad104.3223

### CHOICE TO PROVIDE CARE AND KNOWLEDGE OF DEMENTIA SYMPTOMS: IMPACT ON CAREGIVERS' EXPERIENCES

Sumiyyah Zimami[1], Hala Darwish[1], and Deanna J Marriott[2], *1. University of Michigan, Ann Arbor, Michigan, United States*, *2. University of Michigan School of Nursing, Ann Arbor, Michigan, United States*

As the population ages, understanding the needs of people with dementia and their caregivers becomes increasingly crucial. Caregivers have various experiences related to the rewards and challenges of choosing to provide care. This project investigates how caregivers' choice to provide care for someone with dementia and their knowledge of dementia symptoms influence their caregiving experiences. The study employed a secondary data analysis from the National Alliance for Caregiving and the American Association of Retired Persons. Caregiving experiences were gauged using a 5-point Likert scale ranging from 'completely negative' to 'completely positive'. Statistical analyses were conducted using descriptive statistics, chi-square tests, and ordinal logistic regression adjusting for covariates such as dementia stage, caregiver relationship, mental health, and caregiving attitude. Relative risks and 95% confidence intervals were estimated for interpretation using Stata/SE 18.0 with an a-priori significance level of p< 0.05. The study involves 927 caregivers, predominantly women (66.13%) aged 50 – 74. Caregivers who chose to provide care and recognized dementia symptoms were significantly more likely to report positive experiences (ARR= 1.38, p=< 0.001; ARR= 2.27, p=0.03) respectively. Caregivers who felt fulfilled or optimistic had positive experiences (ARR= 1.92, p< 0.001; ARR= 2.21, p< 0.001). Contrastingly, caregivers dealing with emotional stress or caring for relatives with moderate dementia were more likely to have a negative experience (ARR= 2.80, p< 0.001; ARR= 1.39, p=0.006). These findings highlight the importance of developing tailored nursing interventions to support caregivers' conscious decision to provide care and increase caregivers' knowledge, potentially enhancing the quality of life for caregivers.

Abstract citation ID: igad104.3224

### CHRONIC DISABILITIES AND POLYSUBSTANCE USE IN ADULTS AGED 50 + ACROSS 25 US STATES

John Massey, Mika Thompson, Laura Johnston, and Michael Phillips, *University of Hawaii at Manoa, Honolulu, Hawaii, United States*

Given an aging demographic that reports higher substance use patterns than preceding generations, as well as variability in cannabis legality at the state level, substance use and abuse is an area of concern for older adults experiencing chronic disabilities. Examining trends for polysubstance use for three of the most used substances (nicotine, alcohol, and cannabis) allows for possible insight into use patterns exhibited by older adults, particularly those with disabilities. Using national data across 25 states from 2020-2021 BRFSS, we investigated recent substance use for adults aged 50 and over (N=200,793). We conducted multinomial regression analyses to examine polysubstance use by individual disabilities (including hearing, vision, cognition, mobility, self-care, and independent living) and any disability. Our results indicated that 36.72% reported at least one disability, while 1.46% reported recent polysubstance use of alcohol, nicotine, and cannabis. All but one (mobility) of the individual disabilities were associated with higher odds of polysubstance use. Having any disability was associated with 42% higher odds of polysubstance use (OR: 1.42, 95%CI: 1.30-1.56) compared to no use. Understanding trends associated with polysubstance use for the three most commonly used substances in the U.S. for older adults 50+ with disabilities may influence future policy discussions, public health campaigns, efforts to educate older adults on the potential harms associated with polysubstance use, and at improving health equity and population health outcomes.

Abstract citation ID: igad104.3225

### CIRCADIAN CLOCK DISRUPTION: SEX-SPECIFIC EFFECTS ON METABOLISM & COGNITION IN AN ALZHEIMER'S MODEL

Laila Scroggins[1], Qing Zhang[2], Kevin Koronowski[2], and Juan Palavicini[2], *1. Howard University, Washington, District of Columbia, United States*, *2. The Barshop Institute for Longevity and Aging Studies, San Antonio, Texas, United States*

Preclinical studies have been largely performed on transgenic murine models of early-onset AD, which only accounts for a small fraction of AD cases. A number of confounds have impacted and limited the translatability of these mouse models, including potential off-target genetic effects, artifacts resulting from ultra-physiological overexpression of specific gene products, and overblown pathologies in non-fully developed brains. The objective of this study is to leverage a new microtubule-associated protein tau knockin (MAPT H1-GR) mouse model to assess the effects of an understudied environmental factor associated with AD, a disturbed circadian rhythm, on whole body and brain metabolism, tauopathy, neuroinflammation, neurodegeneration, and cognition. For the experimental design, a cohort of 32 mice was divided into two groups, normal light conditions (NLC) and chronic jet lag (CJL). The CJL mice have a regulated light schedule which shifts 4 hours ahead every 3 days while the NLC mice have a continuous 12 hours of light and 12 hours of dark schedule. Non-fasted and overnight-fasted glucose, ketones, and lactose levels were measured. CJL led to a sex-dependent disruption of fuel utilization during fasting conditions with males displaying more dramatic phenotypes than females. CJL also led to sex-specific changes in body composition, assessed via qMRI testing every other month, specifically at the level of fat mass in males. Cognitive assessment through the Barnes Maze test revealed a disruption of spatial memory in males, while females seemed to be protected from the deleterious effects of CJL on cognition.

Abstract citation ID: igad104.3226

### CIRCULATING IMMUNE CELLS ARE ASSOCIATED WITH BRAIN MRI MEASUREMENTS IN THE FRAMINGHAM OFFSPRING

Yumeng Cao[1], Margaret Doyle[2], Jiachen Chen[3], Ahmed A.Y. Ragab[3], Joanne Murabito[3], and Kathryn Lunetta[3], *1. Boston University, Boston, Massachusetts, United States*, *2. University of Vermont, Colchester, Vermont, United States*, *3. Boston University School of Public Health, Boston, Massachusetts, United States*

Mounting evidence supports the role of the immune system in brain health, yet little is known about the role of circulating immune cells in brain health. We investigated the association of immune cell phenotypes with brain MRI phenotypes in the Framingham Heart Study Offspring participants who attended Exam 7 (mean age 61, 52% women). Immune cells (43 analyzed here) were determined by flow cytometry and previously published. Analysis of the immune cell phenotypes with MRI measurements of 13 brain regions revealed several significant associations, adjusting for age, sex and CMV status (Model 1) in the full sample (n=703) and the CMV+ stratum (n=327). In the full sample, higher senescent/exhausted CD8+ cells (CD8+CD45RO-CCR7-CD27-(Teff), CD8+CD45RA+CD28-CD57+(TEMRA), CD8+CD27-CD28-) associated with higher cerebrum and frontal gray volumes. These associations were strengthened when cardiovascular risk factors were included in the model (Model 2). In the CMV+ stratum, two CD8+ cell types (TEMRA, and Teff) were significantly associated with higher cerebrum gray and frontal gray matter volumes in both models. Also in the CMV+ stratum, higher levels of CD8+CD45RO+CCR7+CD27+ (Central Memory) were associated with lower frontal gray matter, and higher CD3+ (T cells) cells were associated with higher temporal gray matter and higher CD8 Teff associated with lower cerebrum white volumes. There were no associations of CD4 subtypes with brain MRI measures in the full sample or the CMV+ stratum. These associations with brain volumes in adults aged 40-88 may improve understanding of the role of the immune system early in the brain aging process.

Abstract citation ID: igad104.3227

### CLIMATE CHANGE, AIR POLLUTION, AND MENTAL HEALTH AMONG OLDER KOREAN AMERICAN RESIDENTS IN LOW-INCOME SENIOR HOUSING

Juyoung Park, and Yuri Jang, *University of Southern California, Los Angeles, California, United States*

Given the impact of climate change and air pollution on human health, the present study examined how older individuals' perceived disruption by extreme weather and air pollution would be linked with mental health. We focused on older Korean Americans living in low-income senior housing and selected anxiety as an indicator of mental health. Data came from surveys with Korean American residents in five low-income senior housing facilities located in the Greater Los Angeles area (n=343, Mean age = 78.4). Anxiety, measured with the GAD-7, averaged 3.38 (SD = 3.77). Perceived levels of disruption caused by extreme weather and air pollution averaged 1.71 (SD = .69) and 1.75 (SD = .72), respectively, out of a range from 1 (not at all) and 4 (very much). Individuals who reported greater disruptions by extreme weather and air pollution had a higher level of anxiety. Given the high correlation between the two types of disruption ($r = .56$, $p < .001$), we tested separate regression models of anxiety. Each variable of the perceived disruption by extreme weather ($\beta = .18$, $p < .001$) and air pollution ($\beta = .18$, $p < .001$) was found to have a significant impact on anxiety. Although limited by the use of a small sample of convenience restricted to one ethnic group in one geographic location, our findings suggest the potential mental health impact of climate change and air pollution and call attention to intervention strategies.

Abstract citation ID: igad104.3228

### CLINICAL CORRELATION OF MOCA AND TAU IMAGING.

Surya Sunil, Malini Nair, Nicole Byrne, Adriana Rodriguez, and Anil K. Nair, *Boston Neuropsychiatry, Boston, Massachusetts, United States*

Clinical Correlation of MOCA and Tau Imaging **Background:** Tau-PET Scan measures brain tau deposition. Montreal Cognitive Assessment (MOCA) is used for cognitive impairment in (MCI) and Alzheimer Disease (AD). We analyzed the correlation of MOCA to tau-PET imaging in a memory clinic. Hypothesis Tau-PET positivity correlates with lower MOCA scores in memory clinic patients Objectives To assess the correlation of tau-PET positivity to MOCA test scores in memory clinic patients To evaluate an MOCA score cut-off that translates optimally to tau-PET positivity **Method:** Retrospective observational chart review study of 34 patients attending a memory clinic in Boston, MA, with MOCA score and tau-PET scan included for analysis No stratification based on initial or final diagnosis, severity of cognitive impairment, or tracer used. Baseline MOCA and a binary PET scan result were analyzed with covariates age, sex and race. Analysis done on 'R' v. 2.4.0. Pearson correlation estimated MOCA score to tau-PET status. Kruskal Wallis/ Chi square tests for relationship of age, sex, gender and education with MOCA score and tau-PET status. ROC analyses and sensitivity/specificity were obtained. **Results:** Tau PET positivity did not correlate significantly with MOCA score (r= -0.24, 95%CI –0.54 to -0.12, p=0.19). A cut point of 22 on the MOCA estimated tau deposition in the brain by PET scan, with an AUC of 80%, 95% CI –0.6 to –0.99, possibly indicating a ceiling effect on the MOCA. **Conclusion:** Tau PET does not correlate with MOCA scores, possibly due to a ceiling effect in the mild impairment subjects.

Abstract citation ID: igad104.3229

### CLINICIAN PERSPECTIVES ON PRE-VISIT AGENDA-SETTING FOR PERSONS WITH DEMENTIA AND THEIR CARE PARTNERS

Jessica Cassidy[1], Catherine DesRoches[2], Isabel Hurwitz[2], Jennifer Wolff[3], and Hillary Lum[4], *1. University of Texas*

*at Arlington, Arlington, Texas, United States*, *2. Beth Israel Deaconess Medical Center, Boston, Massachusetts, United States*, *3. Johns Hopkins University, Baltimore, Maryland, United States*, *4. Univerisity of Colorado School of Medicine, Aurora, Colorado, United States*

Family care partners often assist persons with dementia in managing their health care, including through healthcare system patient portals. Thus, gaps in communication with family care partners can lead to unmet care needs. OurNotes is an agenda setting intervention included within pre-visit questionnaires that is sent through the patient portal. The intervention aims to encourage patients to share concerns before the visit to enhance communication between patients and clinicians. The objective of this study was to understand clinician perspectives on use of OurNotes in dementia care, including engaging care partners and suggestions for improving the intervention. Semi-structured interviews were conducted with 20 clinicians from two health systems with experience using OurNotes and caring for patients with dementia. Using rapid assessment methods, a codebook was developed and used in a team-based, thematic analysis. Participants were majority White, female, between 30-39 years, worked in primary care, and physicians. Clinicians identified: 1) Benefits of pre-visit preparation that enhanced clinician knowledge of the patient and/or care partner agenda; 2) Improved delivery of family-centered dementia care through greater care partner involvement; 3) Challenges of balancing patient and care partner concerns during clinical visits; and 4) Suggestions for focused efforts on improving care partners' accessibility and use of OurNotes (and the patient portal). We conclude that optimizing use of OurNotes by care partners may support effective agenda setting by patients, care partners, and clinicians, and ultimately improve dementia care.

Abstract citation ID: igad104.3230

### CO-DESIGNING A GREEN ACTIVITY PRESCRIPTION PROGRAM WITH HISPANIC/LATINO PERSONS LIVING WITH MEMORY CHALLENGES

Rebecca Lassell[1], Valeria Tamayo[2], Triana Pena[2], Carlos Tejeda[3], Anderson Torres[4], Jessica Zwerling[3], Laura Gitlin[5], and Abraham Brody[2], *1. Indiana University School of Public Health-Bloomington, Bloomington, Indiana, United States*, *2. New York University, New York City, New York, United States*, *3. Montefiore, Bronx, New York, United States*, *4. R.A.I.N. Total Care, Inc., Bronx, New York, United States*, *5. Drexel University, Philadelphia, Pennsylvania, United States*

Nature activities can improve well-being and may modify risk factors for cognitive decline. There is a gap between nature activities offered within a person's community and their ability to participate in them, including Hispanic/Latino persons living with memory challenges (MCI and mild dementia) in urban lower-resourced areas. An occupational therapist can bridge this gap by tailoring nature activities available in a person's home, neighborhood, and community to their interests and needs. Utilizing a participatory approach, we sought to co-design a 12-week Green Activity Prescription (GAP-H) with Hispanic/Latino individuals living with memory challenges and their care partners. Participants were recruited via convenience and snowball sampling in the Bronx, NY with outdoor activity professionals (n=7), Hispanic/Latino persons living with memory challenges and care partners (n=5), and interdisciplinary healthcare providers/dementia experts (n=7). Co-design occurred iteratively with 5 focus groups and 2 individual interviews lasting 30-90 minutes and focused on intervention and research design. Sessions were recorded and transcribed. Utilizing directed content analysis data was coded using a priori codes intervention design and research design. Participant preferences for intervention design were captured by subcodes session duration (30-90 minutes), frequency (4-8 sessions), and delivery modes (in-person and phone). Participants' preferred nature activities included group exercise and outdoor crafts [crocheting], outcomes of well-being, social participation, decreased loneliness, and stewardship were identified. Preferred language for recruitment materials were memory challenges, Hispanic/Latino, and well-being. Referral pathways were identified including community-based organizations and primary care. Findings can inform the co-design process of other interventions for this population.

Abstract citation ID: igad104.3231

### CO-DESIGNING A HIGH-ACCURACY HOME MONITORING SYSTEM FOR MANAGING FRAILTY IN OLDER ADULTS

Adriana Ríos Rincón[1], Andrew Chan[2], Mathieu Figeys[1], Farnaz Koubasi[1], Yusuf Ahmed[3], Geoffrey Gregson[2], and Antonio Miguel-Cruz[1], *1. University of Alberta, Edmonton, Alberta, Canada*, *2. Glenrose Rehabilitation Hospital, Alberta Health Services, Edmonton, Alberta, Canada*, *3. University of Ilorin, Ilorin, Kwara, Nigeria*

**Background:** Frailty, a condition often affecting older adults, increases vulnerability and diminishes physical abilities across bodily systems. Current non-routine frailty screening in primary care or clinical settings fails to detect "hidden health vulnerabilities" in a timely manner. Smart home technologies offer an affordable and effective solution for continuous frailty tracking and prevention. However, existing home monitoring technologies typically require users to acquire new skills or are invasive, such as camera-based systems. Objective: Our goal is to create a high-accuracy home monitoring system coupled with Internet of Things devices to identify potential frailty indicators. **Methods:** This qualitative description study involves 4 to 8 participants, including older adults with and without Mild Cognitive Impairments/frailty, caregivers, and clinicians, in a group interview. Using card sorting and task mapping, the interview seeks to identify and define the features and challenges of a smart home system to monitor frailty in older adults. **Results:** The study is registered in clinical trials, with data collection commencing soon. At the conference, we will present the research protocol and the findings from the analysis of the interviews. **Conclusion:** Through the early engagement of older adults and caregivers, we strive to design a valuable and meaningful system that (1) uses zero-effort technologies so frail older adults do not need to develop new skills in order to use the system; (2) is a non-camera-based tracking technology preserving autonomy and privacy; (3) generates the frailty data meaningful for older adults, caregivers and the health system.

Abstract citation ID: igad104.3232
### COGNITIVE DECLINE AND FALLS ARE INDEPENDENT RISK FACTORS FOR SWALLOWING DYSFUNCTION IN OLDER PEOPLE

I-Ting Liu, *E Da Hospital, Kaohsiung City, Kaohsiung, Taiwan (Republic of China)*

Aging, oropharyngeal lesion, neuromuscular and progressive neurological disease such as dementia are well-known risk factors of dysphagia. The aim of this study is to evaluate the significant risk factors of swallowing dysfunction in clinical practice. 100 subjects aged 65 or older who received Comprehensive Geriatric Assessment (CGA) were enrolled at EDA Hospital, Taiwan, form January 2022 to February 2023 (IRB: EMRP-110-108). Exclusion criteria: unable to communicate、advanced disease or totally dependent. CGA was performed by well-trained nurses and geriatrician. Swallowing dysfunction was identified by abnormal swallow exam or related complaints. Cognitive impairment was defined as Short Portable Mental State Questionnaire (SPMSQ) < 8 (SPMSQ < 6 in those without education) or with diagnosed dementia. Binary logistic regression was constructed to assess the independent associated factors of dysphagia. Basic demographic analysis showed that patients with dysphagia were older, had poor ADL, higher number of geriatric syndromes and chronic comorbidities. Multivariate analysis revealed that cognitive decline (OR: 82.74, p=0.035) and falls (OR: 45.17, p=0.011) are still independent associated factors of swallow dysfunction after adjustment for age, sex, BMI, socioeconomics, other geriatric syndromes, chronic comorbidities including stoke and Parkinson disease. Dementia was known as one of the risk factors of dysphagia. Another small follow-up study demonstrated that falls risk is predictive of dysphagia in Parkinson's disease. Through this study, participants will be able to identify the high-risk group of dysphagia. Prevention such as pneumococcus vaccination, oral hygiene, and speech therapy can be applied on those who have a fall history and clinical cognitive impairment.

Abstract citation ID: igad104.3233
### COGNITIVE DISPERSION IS ASSOCIATED WITH PROTECTIVE COGNITIVE AND SOCIODEMOGRAPHIC FACTORS IN MIDUS

Laura Klepacz[1], Jeremy Hamm[1], and Eric Cerino[2], *1. North Dakota State University, Fargo, North Dakota, United States*, *2. Northern Arizona University, Flagstaff, Arizona, United States*

Research examining the relationship between an individual's levels of variability across a range of cognitive domains (dispersion) suggests that higher dispersion may be an early indicator of future cognitive impairment. However, this literature is still in its infancy and more research is needed to systematically examine the correlates of dispersion in large-scale, national samples that include individuals across the adult lifespan. Our study thus examined the relationship between cognitive dispersion across 6 tasks, mean cognitive performance, and sociodemographic characteristics in a national sample of adults from the Midlife in the United States (MIDUS) study (n=4,198; age 55±12.45). Dispersion was calculated by generating an intraindividual standard deviation across 6 unique cognitive tasks spanning several neuropsychological domains. In contrast to past research, bivariate correlations indicated that higher levels of cognitive dispersion were associated with higher levels of mean performance across cognitive tasks (r=0.28), higher education (r=0.08), higher income (r=0.04), and younger age (r=-0.07) (all ps<.05). The positive association between cognitive dispersion and mean performance was strongest for the episodic memory and working memory tasks. These results were consistent in both wave 2 and 3 of MIDUS and replicated in regression analyses that also revealed an increase in dispersion across waves 2 and 3 of MIDUS was associated with an increase in mean performance across the two waves (b=0.05, p<.001). Results of our study point to the possibility that, in certain unimpaired adult lifespan populations, dispersion may be associated with protective cognitive and sociodemographic factors linked to healthy cognitive aging.

Abstract citation ID: igad104.3234
### COHORT CHANGE AND LIFE COURSE SOCIOECONOMIC DIFFERENCES IN COGNITIVE HEALTH TRAJECTORIES

Hyungmin Cha, *University of Southern California, Los Angeles, California, United States*

The combination of socioeconomic gains and losses during a lifetime contributes to a large inequality in cognitive capacity in later life. Although studies have examined the connection between an individual's socioeconomic status over the life course and cognitive health trajectories, it is unclear how historical contexts may have influenced these associations. In light of the dynamic macro-social environment in Korea, this study utilizes data from the Korean Longitudinal Study of Aging (KLoSA) from 2006 to 2018. I explore the variation in the socioeconomic patterning of cognitive health trajectories based on the benchmarking of socioeconomic status across the life course. The study reveals three main findings. First, I find cohort-specific cognitive health trajectories based on socioeconomic status across the life course. Notably, childhood socioeconomic status leaves an indelible mark on levels of cognition only among earlier cohorts born during the Japanese Occupation period. Additionally, socioeconomic disparities in the rate of cognitive health decline widen among earlier cohorts, while this trend is not observed among more recent cohorts. Second, compared to childhood and later-life socioeconomic status, education consistently shows a positive association with higher levels of cognition rather than slower cognitive decline, regardless of whether the cohorts are earlier or more recent. Third, there is a notable gender difference in socioeconomic disparities in cognitive decline, with women experiencing more pronounced disparities compared to men, especially among earlier cohorts. These findings underscore the significance of historical and contextual factors when examining the association between socioeconomic status and cognitive health across the life course.

Abstract citation ID: igad104.3235
### COHORT PROFILE FOR THE TONGJI CARDIOVASCULAR HEALTH STUDY: A PROSPECTIVE MULTI-OMICS COHORT STUDY

Ting Xu[1], Yueqi Lu[2], Bangwei Chen[2], Chenxin Deng[1], Yucong Zhang[1], Lei Ruan[1], Tao Li[2], and Cuntai Zhang[3], *1. Tongji Hospital, Wuhan, Hubei, China (People's Republic)*, *2. BGI-Shenzhen, Shenzhen, Guangdong, China*

*(People's Republic)*, *3. Huazhong University of Science and Technology, Wuhan, Hubei, China (People's Republic)*

The Tongji Cardiovascular Health Study aimed to further explore the onset and progression mechanisms of cardiovascular disease (CVD) through the combination of traditional cohort study and multi-omics analysis including genomics, metabolomics and metagenomics. This study included participants aged 20-70 years old from Geriatric Health Management Center of Tongji Hospital of Tongji Medical College of Huazhong University of Science and Technology. After enrollment, each participant underwent a comprehensive series of traditional and novel cardiovascular risk factors assessments at baseline, including questionnaires, physical examinations, laboratory tests, cardiovascular health assessments, and biological samples collection for subsequent multi-omics analysis. A biennial follow-up will be performed for ten years to collect the information above and the outcome data. Totally, 2,601 participants were recruited in this study (73.4% men), with a mean age of 51.5±11.5 years. They were mostly highly educated (college and bachelor's degree or above: 92.8%) and mostly had healthy lifestyle behaviors. A total of 50.5% and 14.9% of men and 23.1% and 3.4% of women were overweight and obese, respectively. The proportion of several comorbidities such as hypertension, diabetes mellitus, and hyperlipidemia increased with age, and the rapid increase in women around the age of 40-50. With the progress of the cohort follow-up work, it is expected to provide unique multidimensional and longitudinal data on cardiovascular health in China.

Abstract citation ID: igad104.3236

### COMMUNITY ENGAGEMENT IN OLDER ADULTS WITH COGNITIVE DECLINE: THE USAGE OF INFORMATION COMMUNICATION TECHNOLOGY

Rong-Fang Zhan[1], Elias Mpofu[1], Sherif Olanrewaju[2], and Laura Sutherland[1], *1. University of North Texas, Denton, Texas, United States*, *2. The Pennsylvania State University, University Park, Pennsylvania, United States*

**Background:** Cognitive decline is typical among older adults, affecting their activities of daily living and community participation. However, the advent of cognitive function support information technologies may improve their community engagement, perhaps more than memory training alone. This study examined information communication technology usage as a mediator of associations of cognitive decline and community engagement in older adults. **Methods:** We sampled older adult cases from Round 11 of the National Health and Aging Trends Study data set (N=3817) with cognitive decline in community dwelling (mean age =81.38, SD=6.266), male(n=1313) and female (n=1771). We utilized multi-level Structural Equation Model (SEM) to examine the mediation of cognitive decline and community connection through the ICTs, as well as direct relationships among memory, orientation, and executive function. **Results:** From SEM analysis, an increase of ICTs usage has statistically significantly effects on older adults' cognitive ability overall (Estimate = 0.103, S.E. = 0.023, C.R. = 4.438, p < 0.001). Cognitive function has a direct impact on the activity orientation function (Estimate=1, p < 0.0011.000). The greater usage of ICTs by older adults is associated with higher levels of community engagement (Estimate = 1.230, S.E. = 0.284, C.R. = 4.338, P < 0.001). **Conclusion:** This finding highlights the potential role of ICTs in fostering older adults' cognitive function for their social interactions and community involvement. Digital age older adults may have greater cognitive function than previous cohort, reducing demand for care services their community participation.

Abstract citation ID: igad104.3237

### COMMUNITY ENGAGEMENT IN THE DEVELOPMENT OF SEE ME: THE STUDENT-ELDER* ENGAGED MENTORING EXPERIENCE

Kimberly Oosterhouse[1], Lisa Skemp[1], Ginger Williams[2], Larry Lesperance[3], Jeanne Heid-Grubman[4], Caesar Hill[5], and Hortense Banks-Wright[5], *1. Loyola University Chicago, Chicago, Illinois, United States*, *2. Edgewater Village Chicago, Chicago, Illinois, United States*, *3. Prime Timers, Chicago, Illinois, United States*, *4. Chicago Methodist Senior Services, Chicago, Illinois, United States*, *5. Hollywood House, Chicago, Illinois, United States*

Loneliness, isolation, and suicide have been exacerbated by COVID-19 and ongoing uncertainties for both older adult and college-age populations. The 2023 Surgeon General's Advisory on loneliness and isolation advocates for the healing effects of social connection, but ageist attitudes and stereotypes hinder social connections across generations. Intergenerational programs aim to address ageism and social connectivity, however, transferability of programs to culturally diverse groups remain challenging. Additionally, nursing curricula tend to focus on frail older adults, emphasizing vulnerability rather than wellness and disconnection rather than connection, contributing to disinterest in gerontological nursing. Prior community engaged ethnographic research identified five diverse older adult subcommunities that prioritized the need to address isolation and loneliness. Collaborating with these community organizations and nursing students, we developed the nursing Student-Elder*-Engaged Mentoring Experience (SEE ME) with the purpose of creating a sustainable and transferable program to address these needs. We will describe the research-based partnership process and resulting program development that focused on mutual wellness through reciprocal social connectivity among older adults in the community and nursing students in a required gerontological nursing course. In partnership, we developed and refined culturally appropriate processes, forms, assignments, activities, schedules, overall course organization, and program evaluation materials. Animated video modules and "Let's Talk" questions were developed to facilitate student - older adult discussion. Dissemination and access to the program materials will be discussed. *The term "elder" was selected by the older adult community partners as their preferred nomenclature.

Abstract citation ID: igad104.3238

### COMPARING ILLNESS PERCEPTIONS OF MULTIMORBIDITY AMONG OLDER ADULT UTILIZERS AND NON-UTILIZERS OF HOME-BASED CARE

Ayomide Bankole, *UNC Chapel Hill, Chapel Hill, North Carolina, United States*

Health behavior interventions targeting illness perceptions improve outcomes for those with chronic illness. Research investigating illness perception in the context of multimorbidity suggests illness-perception of multimorbidity (IP-MM) predicts short-term health status among individuals with multimorbidity. However, less is known about IP-MM among home-based care (HBC) utilizers who receive in-home support for multimorbidity management. This study aimed to compare IP-MM among older adults HBC-utilizers and non-utilizers. Secondary analysis of a cross-sectional study exploring IP-MM in 116 community-dwelling older adults with ≥2 chronic conditions. IP-MM was assessed via MULTIPleS summary and subscale scores (0-78), including causal-links (0-9), prioritization (0-12), treatment-burden (0-18), activity-restrictions (0-9), and emotional-representations (0-30), where higher scores indicate stronger perceptions. HBC-utilizers self-reported receiving nursing, rehabilitative, or medical services at home. One sample t-tests was performed to compare MULTIPLeS subscales between HBC-utilizers and non-utilizers. HBC utilizers comprised of 18% with 82% as non-utilizers. Mean number of chronic conditions was 4.5 (2); Mean age was 73.3 (6.72), 71% female, 35% Hispanic, 33% Black, 27% White, and 5% Asian. Mean (SD) MULTIPleS summary score was 39.39 (21.1). Subscales scores:[activity restriction: 5.19 (2.88); causal relationships: 4.81 (3.07); prioritization:8 .24 (3.52); treatment burden: 7.84 (5.57). HBC utilizers had significantly higher mean treatment-burden subscale scores (10.38 ± 5.51), than non-utilizers (7.28 ± 4.45), p=0.027. No significant differences in other subscales or summary scores. Older adult HBC-utilizers and non-utilizers experienced comparable IP-MM, differing only in treatment burden perceptions. Additional research on IP-MM is needed to develop interventions that improve multimorbidity management and treatment-burden among HBC utilizers.

Abstract citation ID: igad104.3239

### COMPARING THE FEASIBILITY OF A LONELINESS AND SOCIAL ISOLATION GROUP IN A PRIMARY CARE VS COMMUNITY SETTING

Max Zubatsky[1], Marla Berg-Weger[1], Dimitra Tziarli[1], Rachel Livingston[1], Judy Drake[2], and Ashley Taylor[1],
*1. Saint Louis University, St. Louis, Missouri, United States,*
*2. Family Care Health Centers, St. Louis, Missouri, United States*

Social isolation and loneliness is now considered a public health epidemic in the United States for the older adult population. While providers try to address certain health and lifstyle issues in this population through medications and other medical referrals, there are few non-pharmacological group interventions to curb feelings of isolation and loneliness. This study will determine if a psychosocial group intervention in primary care results in decreases of loneliness and health risks compared to a community-based group. The intervention is called "Circle of Friends," which is an evidence-based group to alleviate loneliness and increase socialization through a rehabiliation model of care. Participants must be 60 years and older, report subjective feelings of loneliness, have reported being homebound and/or isolated over the last six months, and have very few social connections. The current study has collected initial pre-group data from eight older adults from the community group, and the research team is recruiting participants in the primary care group setting. It is anticipated that the Primary Care Circle of Friends Group will report higher improvements in loneliness and health indicators compared to the community-based group. The primary group will take place in an underserved Federally Qualified Health Center, and the community-based group will take place in the conference room of a university setting. Early qualitative themes that facilitators have reported around recruitment include: 1.) Challenges with planning transportation, 2.) Building trust in joining a new group, and 3.) requests of extra resources in addition to this group.

Abstract citation ID: igad104.3240

### COMPARISON OF DNA METHYLATION IN SALIVA, BUFFY COAT AND PBMC SAMPLES IN CATSLIFE

Donald Evans, Andrew Smolen, and Chandra Reynolds,
*University of Colorado Boulder, Boulder, Colorado, United States*

The design of the Colorado Adoption/Twin Study of Lifespan behavioral development and cognitive aging (CATSLife) allows for separation of genetic and environmental factors affecting health and cognitive aging. Currently, this sample is transitioning through midlife. One goal of this study is to identify epigenetic changes associated with aging, however methylation profiles differ across tissue types. Therefore, we describe here the analysis of DNA methylation in three CATSLife sample types: saliva, buffy coat, and peripheral blood mononuclear cells (PBMCs). Obtaining saliva is least invasive, although it may be less informative about brain and cognitive aging processes. In contrast, while buffy coats and PBMCs may be more informative, obtaining blood is more invasive. Buffy coats are less expensive to obtain than PBMCs, however they contain more cell types which may complicate analysis. Our analyses of 91 participants, suggested that saliva samples were less likely to pass initial quality control (< 96% CpGs detected). Unsurprisingly, the buffy coat and PBMC DNA methylation signatures are more similar to each other (r=0.97) than either is to saliva (rs=0.88—0.92). In initial analyses, each sample type demonstrated expected familial/heritable patterns of similarity across sites (MZ twins > DZ twins > biological & adoptive siblings). Given the additional cost of isolating PBMCs and computational methods to account for complex cell mixtures, the use of buffy coat DNA increases accessibility to analyze epigenetic changes longitudinally, enabling the early identification of epigenetic biomarkers of cognitive decline and the effect of both genes and environments on this process.

Abstract citation ID: igad104.3241

### COMPARISON OF SELF-RATED HEALTH, FRAILTY-INDEX, AND MULTIMORBIDITY AS HEALTH INDICATORS

Pauliina Halonen[1], Linda Enroth[1], Tuija Jääskeläinen[2], Seppo Koskinen[2], Marja Jylhä[1], and Laura Kananen[1],
*1. Tampere University, Tampere, Pirkanmaa, Finland,*
*2. Finnish Institute for Health and Welfare, Helsinki, Uusimaa, Finland*

Self-rated health (SRH) predicts mortality and other adverse events (e.g. hospitalizations, intensive care), and reflects clinical biomarker levels even in individuals without diseases. We hypothesize SRH captures information relevant to clinical decision-making, with minimal effort, and might be used prior to more intensive and focused health assessments. To explore the clinical utility of SRH, we 1) analysed its overlap with clinical health assessments, multimorbidity, and Rockwood frailty-index (FI), and 2) compared their performances in mortality prediction. Our data came from two population-based cohort studies in Finland: Health 2000 Survey (H2000: n=6,329, aged 30-97 years) and National FinHealth 2017 Study (FH17: n=4,105, aged 30-98 years). Cox regression adjusted for age and sex, and Harrel's C-index were used to assess the performances of continuous SRH, FI, and multimorbidity in 4-years-all-cause mortality prediction. Of those with fair/poor SRH, 85% in H2000 and 91% in FH2017 were also frail (FI≥0.25), and 91% and 81% had at least two diseases, respectively. Baseline SRH (H2000: HR[95CI]=3.14[2.10-4.69], FH17: HR[95CI]=4.57[2.37-8.83]), FI (H2000: HR[95CI]=1.40[1.28-1.53], FH17: HR[95CI]=1.54[1.32-1.80]) and number of diseases (H2000: HR[95CI]=1.18[1.10-1.27], FH17: HR[95CI]=1.18[1.03-1.35]) predicted death, and their C-indices were 0.879, 0.884, 0.877 in H2000 and 0.797, 0.800, 0.779 in FH17, respectively. SRH is concordant with clinical health indicators, such as multimorbidity and FI, and predicts mortality equally well in community-dwelling adults. The analyses are ongoing and we will further assess the utility of SRH in other cohorts, using other outcomes, and clinical indicators from registers, and perform age-, sex- and disease-stratified analyses.

Abstract citation ID: igad104.3242

### COMPARISONS BETWEEN COGNITIVELY-IMPAIRED AND HEALTHY OLDER ADULTS ON NAVIGATION METRICS

Michael Pervratil, Dorota Kossowska-Kuhn, and Neil Charness, *Florida State University, Tallahassee, Florida, United States*

Being able to navigate one's environment is a critical skill that contributes to daily living in a variety of circumstances. Navigation is also a complex skill, composed of multiple subskills that play a part in overall performance. Along with other facets of cognition, navigation ability is expected to decline as a part of normal aging processes. What is of additional interest is how cognitive impairment (CI) relates to navigation in older adults. Throughout the literature, differences in navigation have been demonstrated between CI populations and healthy older adults. The current study sought to probe these components of navigation by comparing CI and healthy older adults on several measures of navigation ability. A sample of CI older adults were collected across three sites in the eastern United States. As a part of a larger project, participants completed objective and subjective measures of navigation, and their results were compared with a large sample of community-dwelling older adults (ages 60-90). The goal of these analyses was to determine where CI and healthy older adults differed in navigation. The CI sample performed significantly worse on the spatial orientation test (SOT) and reported significantly worse outcomes on a general wayfinding scale. However, there was no significant difference in self-assessed need for help when navigating or frequency of feeling lost. This divergence in outcomes is highlighted and deliberated further. Utilization of subjective and objective measures is discussed along with limitations of the data.

Abstract citation ID: igad104.3243

### COMPREHENSIVE MALNUTRITION ASSESSMENT IMPROVES HEALTH OUTCOMES AMONG HOMEBOUND OLDER ADULTS

Susan Saffel-Shrier, Amy Covington, and jing Wang, *University of Utah, Salt Lake City, Utah, United States*

Approximately 30 percent of older adults are malnourished upon hospital admission while others develop malnutrition during hospitalization and subsequent discharge. High hospital readmission rates are associated with malnutrition. Malnutrition is common among older adults discharged to home and receiving home delivered meals. However, there is limited research on the appropriate malnutrition care for this population. The aim of this study was to investigate the effectiveness of a novel social determinants of health (SDOH)-based comprehensive malnutrition assessment (CMA) on malnutrition treatment and functionality. A pilot study of 46 participants recruited from northern Utah Area Agencies on Aging Nutrition Programs was conducted. The SDOH-CMA was performed during home visitations by a registered dietitian nutritionists for both the intervention and control groups over a six-month period. Logistic regression was then conducted to compare the effect of the SDOH-based CMA on malnutrition as assessed by the Nutrition-Focused Physical Exam (NFPE) between intervention and control groups. Odds ratios (OR) with p values were reported. At baseline, 91.4% of participants were white, 54% were female, 76% were unmarried, 67% lived below the federal poverty level, 61% lived alone, 51% received home health, 51% had anxiety, 33% had PHQ-2 screened depression, 22% were unable to cook, and 13% were unable to shop. The intervention group had significant improvements in the NFPE (OR=10.1, p=.02), calorie intake (OR= 19.6, p=.02), protein intake (OR=36.7, p =.007) and the Timed Up and Go test (OR=7.7, p=.05) than the control group. The intervention was significantly effective in improving nutritional status and functionality.

Abstract citation ID: igad104.3244

### CONDITIONS ASSOCIATED WITH SUCCESSFUL IMPLEMENTATION OF AN ADVANCE CARE PLANNING INTERVENTION IN NURSING HOMES

Susan Hickman[1], Edward Miech[1], Timothy Stump[2], Laramie Mack[3], Wanzhu Tu[2], and Kathleen Unroe[1], *1. Indiana University, Indianapolis, Indiana, United States, 2. Indiana University School of Medicine, Indianapolis, Indiana, United States, 3. Regenstrief Institute, Indianapolis, Indiana, United States*

Implementing evidence-based interventions in nursing homes is challenging in part because clinical trials requiring a controlled experimental environment are difficult to sustain. In contrast, pragmatic clinical trials develop and evaluate evidence-based interventions in the "real world" with the goal of streamlining implementation after study completion. However, there is minimal information available identifying conditions associated with successful implementation of pragmatic interventions in the nursing home setting. The "Aligning Patient Preferences - a Role Offering Alzheimer's patients, Caregivers, and Healthcare Providers Education and Support" (APPROACHES) project is a pragmatic trial designed to test and evaluate a staff-led advance care planning (ACP) intervention for residents with ADRD in 131 nursing homes (64

intervention, 67 control) between September 1, 2021 and August 31, 2022. ACP Specialists received training on ACP facilitation and implementation of the ACP program in the facility. Twenty of 65 (31%) sites successfully implemented the APPROACHES intervention and had > 75% of residents with documented ACP conversations. Using configurational analysis, we found two solutions directly linked with successful pragmatic implementation: (1) no executive director turnover during the observation period combined with site participation in monthly intervention support calls with ACP staff at other facilities; and (2) higher rates of hospitalization (3.96 – 7.2 per 1000 resident days) combined with a low number of certified beds. Findings suggest that leadership stability and engagement with peers were essential drivers of successful implementation. Having greater improvement opportunities as well as a more manageable caseload for the ACP Specialist may also help explain successful implementation.

Abstract citation ID: igad104.3245

### CONDUCTING A NEEDS ASSESSMENT TO INFORM AGE-FRIENDLY HEALTH SYSTEMS INITIATIVE FOR UNIVERSITY OF UTAH HEALTH

Ashley Cadiz, Rebekah Perkins, Timothy Farrell, and Linda Edelman, *University of Utah, Salt Lake City, Utah, United States*

Today, people live longer and more productive lives than ever. The number of adults aged 65 and older is projected to nearly double 98 million by 2060 to 24% of the U.S. population (Administration on Aging & Administration for Community Living, 2018). Increased life expectancy, coupled with the exponential growth of the older adult population will create more demand for healthcare professionals to meet the care needs of older adults living with chronic conditions. Chronic conditions can lead to complex care and increase the usage of healthcare systems (Axon & Kamel, 2021). The Age-Friendly Health Systems initiative provides specific evidenced-based best-practice interventions to all older adults in a health system setting (Fulmer et al., 2022). An Age-Friendly Health System is one in which every older adult's care is guided by an essential set of evidence-based practices known as the 4Ms framework: What Matters, Medications, Mentation, and Mobility (Institute for Healthcare Improvement, 2022). This project developed and delivered a Needs Assessment to providers practicing in the U Health Ambulatory Care Clinics to identify baseline knowledge, gaps in knowledge, and current practices related to the AFHS 4Ms Framework. The Needs Assessment consists of using online surveys and semi-structured interviews. Significant findings were that: 1) U Health ambulatory care clinic providers have very little to no baseline knowledge of the AFHS 4Ms Framework, 2) U Health ambulatory care providers are already providing care that aligns with the 4Ms framework, 3) There are barriers and challenges to providing Age-Friendly care.

Abstract citation ID: igad104.3246

### CO-PRODUCING A BOARDGAME TO LEARN AND ENGAGE ABOUT DEMENTIA INEQUALITIES: THE DEMENTIA INEQUALITIES GAME

Clarissa Giebel[1], Kerry Hanna[1], Hilary Tetlow[2], Mark Gabbay[1], and Jacqui Cannon[3], *1. University of Liverpool, Liverpool, England, United Kingdom*, *2. SURF Liverpool, Liverpool, England, United Kingdom*, *3. Lewy Body Society, Wigan, England, United Kingdom*

Receiving a diagnosis and accessing care after a diagnosis of dementia, both for the person and their carer, are fraught with inequalities. Finding different ways to address these inequalities is important. The aim of this public engagement activity was to co-produce a boardgame about dementia inequalities to facilitate learning, dialogue and educate about different barriers, and facilitators, to diagnosis and care. Two virtual and two face-to-face workshops with people with dementia, unpaid carers, health and social care professionals, and Third Sector representatives were held between October 2022 and June 2023. The virtual workshops were split into discussions of inequalities and how a boardgame may feature inequalities. The first face-to-face workshop was split into the same activities, aided by outcomes from workshops 1 and 2. Workshop 4 attendees tested the prototype. Attendees were reimbursed for their time. Nine brief remote interviews with attendees were conducted to evaluate their experiences in the co-production of the boardgame. Forty stakeholders attended four workshops. Workshops provided step-by-step thoughts on how the game could be designed or modified. The final game, prototype tested in Workshop 4, consists of a one-sided, two-half board depicting the pre-diagnosis process (left half) and post-diagnosis process (right half). Interviews with nine attendees reported on their reasons for attending the workshops, experiences of being involved, and the game design and purpose. The game can be used to improve knowledge about dementia inequalities for health and social care professionals, carers, people living with dementia, decision makers, and the general public.

Abstract citation ID: igad104.3247

### CORRELATES OF MILD BEHAVIORAL IMPAIRMENT IN OLDER ADULTS WITH COGNITIVE DECLINE

Seolah Yoon, Innhee Jeong, Jennifer (Jeehyun) Kim, and Bada Kang, *Yonsei University College of Nursing, Seoul, Republic of Korea*

Implementing early interventions in individuals with subjective cognitive decline and mild cognitive impairment during preclinical and prodromal stage of Alzheimer's dementia spectrum, addresses modifiable risk factors thereby reducing potential dementia. Mild behavioral impairment (MBI) is considered as a potential predictor and prognostic marker for dementia. However, comprehensive reviews on how MBI is associated with other factors in these at-risk groups are limited. This scoping review identified and mapped associated factors of MBI on health outcomes among older adults with subjective cognitive decline and mild cognitive impairment. Following Joanna Briggs Institute methodology, we sourced studies from databases including PubMed (MEDLINE), CINAHL, Web of Science, EMBASE, PsycINFO, Cochrane, and SCOPUS, focusing on English-language publications published between January 2003 and July 2023. A total of 36 studies met the inclusion criteria. We classified MBI correlates identified from these studies into three categories: neurocognitive, physical, and psychosocial. Neurocognitive functions such as objective cognitive decline and progression to Alzheimer's disease showed distinct health outcomes related to the MBI. Biomarkers including brain atrophy, beta-amyloid, and p-tau were found to be associated with MBI. Frailty, low gait speed, and hearing loss were physical functional outcomes associated with MBI. Studies on psychosocial aspects with MBI were limited, except one study demonstrating the relationship between caregiver burdens and MBI severity. This review underscores the importance of taking into account MBI and its correlates for developing tailored care approaches to prevent functional decline and improving health outcomes among older adults with subjective cognitive decline and mild cognitive impairment.

Abstract citation ID: igad104.3248

### CORRELATES OF PHYSICIAN TRUST: DEMOGRAPHIC FACTORS AND EXPERIENCES OF MEDICAL CARE DISCRIMINATION

Lindsey Mundy, Suzanne Judd, Olivio Clay, Virginia Howard, Raegan Durant, and Michael Crowe, *University of Alabama at Birmingham, Birmingham, Alabama, United States*

Lack of trust between older adults and their physicians may decrease adherence to medication or limit disclosure of information to healthcare providers. Trust may be shaped in part by demographic factors as well as previous experiences of discrimination in healthcare. We hypothesized that more experiences of discrimination in a medical care setting would be negatively associated with general physician trust. We used cross-sectional data from a follow-up visit (2013-2016) of the national REasons for Geographic and Racial Differences in Stroke (REGARDS) longitudinal cohort study. Our sample included participants who completed an 11-item general physician trust measure (score range=11-55) and a four-point item about discrimination in medical care settings (n=8,235, age 52-98, 31% Black, 45% men). We examined two sequential linear regression models with trust as the outcome, with the first model including demographic factors and the second adding discrimination in a medical setting. Female gender ($\beta$=-0.09, p<.05), Black race ($\beta$=-0.03, p<.05), and higher level of education ($\beta$=-0.8, p<.05) were each related to lower trust. Higher income ($\beta$=0.04, p<.05) and older age ($\beta$=0.12, p<.05) were associated with higher trust. Perceived discrimination had a negative association with physician trust ($\beta$=-0.14, p<.05) and the relationship between Black race and trust reversed direction and was no longer statistically significant ($\beta$=0.20, p=.298) with discrimination in the model. The potential impact of previous discrimination experiences in a medical care setting on physician trust should be considered when providing healthcare services to diverse populations of older adults.

Abstract citation ID: igad104.3249

### CORRELATES OF SLEEP HEALTH AMONG OLDER PEOPLE WITH AND WITHOUT HIV IN UGANDA

Moka Yoo-Jeong[1], Aneeka Ratnayake[1], Brianne Olivieri-Mui[1], Yao Tong[2], Alexander C. Tsai[2], Samson Okello[3], Stephen Asiimwe[4], and Mark Siedner[2], *1. Northeastern University, Boston, Massachusetts, United States, 2. Harvard University, Cambridge, Massachusetts, United States, 3. Harvard T.H. Chan School of Public Health, Boston, Massachusetts, United States, 4. Massachusetts General Hospital, Boston, Massachusetts, United States*

The proportion of older people in Uganda is expected to grow rapidly. Sleep issues disproportionately affect older people and people with HIV (PWH). Identifying determinants of sleep health is essential in the development of interventions. Thus, we assessed sleep-related factors in older Ugandans (aged ≥50 years) using cross-sectional data from the Quality of Life and Aging with HIV in Rural Uganda study. Sleep outcomes were measured by the Pittsburgh Sleep Quality Index (quality, duration, efficiency). The associations between sleep outcomes and potential determinants (loneliness, urban/rural setting, depression, anxiety, alcohol use, physical activity) were evaluated using multivariable linear regressions. Of 556 participants, 271 were PWH and 285 were people without HIV (PWoH). The two groups were similar in age, sex, and education. Depression screening via the 13-item Hopkins Symptoms Checklist was positive in 131 participants (PWH: 28.2%; PWoH: 21.6%; p=.05). There were no differences in sleep outcomes by HIV serostatus. Mean sleep quality was 0.89 (SD=0.78; range=0-3, 0=very good to 3=very bad), mean sleep duration was 6.5 hours (SD=1.7), and mean sleep efficiency was 74% (SD=19.5%; >85% is optimal). Depressive symptoms were associated with worse sleep quality (b=0.58; p<.001), reduced sleep duration (b=-0.94; p<.001), and worse sleep efficiency (b=-0.12; p<.001) regardless of HIV serostatus. Interestingly, older age was related to increased sleep duration (B=0.04, p=.001). Interventions targeting depression among older Ugandans may help with sleep. Longitudinal studies are needed to determine the bi-directionality of this relationship and further elicit potential pathways to support sleep health among older Ugandans.

Abstract citation ID: igad104.3250

### CORRELATES OF SUPERAGING IN TWO REPRESENTATIVE SAMPLES OF HISPANIC OLDER ADULTS

Cassidy Doyle[1], Ross Andel[2], Joseph Saenz[3], and Michael Crowe[4], *1. University of South Florida, Tampa, Florida, United States, 2. Arizona State University, Tempe, Arizona, United States, 3. Arizona State University, Phoenix, Arizona, United States, 4. University of Alabama at Birmingham, Birmingham, Alabama, United States*

'SuperAgers' are generally defined as people 80+ years old with episodic memory performance comparable to those 20 years younger. Understanding what distinguishes SuperAgers from those who age normally is valuable for designing population-wide strategies to promote health with aging. Limited knowledge exists to describe characteristics of SuperAgers, and even less is known about Hispanic SuperAgers. We examined indicators of cognitive, physical, and psychological resilience in relation to the likelihood of being a SuperAger. We used data from two population-based studies including Hispanic older adults [Puerto Rican Elder: Health Conditions (PREHCO) study; Health and Retirement Study (HRS)]. SuperAgers were defined as 1) ≥80 years old, 2) recall scores ≥ the median for Hispanic respondents aged 55-64, and 3) no cognitive impairment at any wave (2002-2003, 2006-2007, 2021-2022 in PREHCO; 2002, 2006, 2018 in HRS). In all, 640 PREHCO participants and 180 HRS participants met criteria, of whom 45 (7%) and 31 (17%) met SuperAging criteria. Logistic regressions controlling for age and sex demonstrated that higher education (PREHCO: odds ratio[OR]=1.20, p<.001; HRS: OR=1.14, p=.044) and fewer IADL limitations (PREHCO: OR=0.79, p=.019; HRS: OR=0.58, p=.077), fewer ADL limitations (PREHCO: OR=0.72, p=.031; HRS: OR=0.67, p=.068) (physical resilience), and fewer depressive symptoms (psychological resilience) (PREHCO: OR=0.84, p=.015; HRS: OR=0.69, p=.007) were associated with SuperAging. In addition, number of chronic conditions, self-rated health, or BMI, known indicators of physical health, did not relate to SuperAging, potentially highlighting resilience in aging rather than resistance to aging. These findings highlight potential pathways to promote aging among older Hispanics.

Abstract citation ID: igad104.3251

### CORRELATION OF SHOULDER RANGE OF MOTION WITH LOWER EXTREMITY PERFORMANCE AMONG COMMUNITY-DWELLING OLDER ADULTS

Ranyah Almardawi, Vicki Gray, Nathan Frakes, Yuanyuan Liang, and Derik Davis, *University of Maryland School of Medicine, Baltimore, Maryland, United States*

More than one-third of older adults have mobility limitations. Yet the impact of shoulder dysfunction on lower extremity performance is only nascent in description. We tested the hypothesis that shoulder range of motion (ROM) correlates with lower extremity performance. Data were collected from 26 community-dwelling older women (80.8%) and men aged ≥ 65 years (mean±standard deviation=71.9±5.3 years) enrolled in a pilot study at one time point. Bilateral shoulder ROM testing for forward flexion (FF), abduction (ABD), and adducted external rotation (Add-ER); and Short Physical Performance Battery (SPPB), gait speed (GS), 6-meter narrow walk test (6M-NWT), and stair climb power test (SCPT) were performed. The sum of bilateral FF, ABD, and Add-ER was determined (Total-ROM) for each participant. Spearman's rank-order correlation (rho) with 95% confidence intervals (CIs) was used to measure the strength and direction of correlation for Total-ROM with the mobility outcome measures. The study sample demonstrated Total-ROM, 709.5° ± 80.1°; SPPB score, 10.9 ± 1.3; GS, 1.2 ± 0.2 m/s, 6M-NWT time, 7.3 ± 3.9 s; and SCPT, 240.7 ± 90.7 watts. Total-ROM correlated with: SPPB 0.56 (0.22,0.78), p=0.003; GS, 0.59 (0.27,0.80), p=0.001; SCPT, 0.54 (0.20,0.77), p=0.004; and 6M-NWT, -0.63 (-0.82,-0.33), p< 0.001. The results provided preliminary evidence that shoulder ROM correlates moderately with measures of lower extremity performance in community-dwelling older adults. Future studies are warranted in older populations to test for the longitudinal association of shoulder ROM with lower extremity performance and to determine if treatment of shoulder dysfunction provides effective prehabilitation to mitigate age-related declines in mobility.

Abstract citation ID: igad104.3252

### COVID-19 IMPACT ON HEALTH JOURNEYS FOR VETERANS WITH CHRONIC CONDITIONS

Sheikh Hossain[1], Kimberly Judon[1], Matthew Augustine[1], Sheldon Brown[1], Kenneth Boockvar[2], Michael Dolamore[3], Madeline Villalba[4], and Abigail Baim-Lance[1], *1. James J. Peters VA Medical Center, Bronx, New York, United States*, *2. University of Alabama Birmingham, Birmingham, Alabama, United States*, *3. VA- Hudson Valley Health Care System, Wappingers Falls, New York, United States*, *4. Icahn School of Medicine at Mount Sinai, New York City, New York, United States*

The majority of Veterans receiving care in the Veterans Administration (VA) are over 65 years, and many have multiple chronic conditions and functional limitations. In this mixed methods pilot study, we sought to understand how these Veterans experienced the pandemic and associated VA health system changes across two contrasting (rural/urban) VA New York medical settings. From December 2021-January 2023, we conducted semi-structured interviews including co-created journey maps and extracted participants' demographic and clinical characteristics and outpatient encounters. Journey maps visualized health and related episodes and associated pain and bright spots calibrated by intensity. 40 Veterans participated. An intense COVID-19 reaction emerged in 25% (10/39) of participant journeys. A majority (80%; 8/10) had a mental health (MH) diagnosis, compared to 55% (21/39) overall. A journey typically began with feeling shocked, afraid, or in extreme distress; subsequently there was divergence with some expressing feeling better and others describing lingering negative effects on daily routines, views of a changed world, and persistent VA service impacts. Some reported new perceptions of vulnerability due to age and decreased well-being. Participants in this group used on average more outpatient care before and during the pandemic than other participants and transitioned to greater consumption of virtual care, reflecting utilization trends across participants with mental health diagnoses. The group had mixed feelings about the benefits of virtual care. Ongoing challenges with COVID-19 may indicate the potential need for additional well-designed supportive services and environments particularly sensitive to the long-term impact of COVID-19.

Abstract citation ID: igad104.3253

### COVID-19 INFORMATION ELICITING STEREOTYPE THREAT CAN HARM OLDER ADULTS' COGNITIVE FUNCTION

Julie Pham[1], Morgan Shumaker[2], and Holly Bowen[1], *1. Southern Methodist University, Dallas, Texas, United States*, *2. Texas Christian University, Fort Worth, Texas, United States*

During the COVID-19 pandemic, older adults were identified as an at-risk group for developing severe physical symptoms. In addition to physical presentations, evidence indicated COVID-19 related changes in cognitive and neurological function; specifically, increased risk of stroke, reduced attention, concentration, and memory problems. Given that older adults remain particularly vulnerable to severe symptoms, exposure to information about COVID-19 and the associated cognitive symptoms may have adverse effects on their cognitive performance. This phenomenon, known as stereotype threat, describes situations in which negative stereotypes about a particular group can impair functioning. The current study aims to examine how exposure to adverse age-related information about the pandemic may impact cognitive performance compared to more neutral age-related information. 484 older adults were recruited February through July 2023 via Amazon Mechanical Turk and Cloud Research Connect. Participants were randomly assigned to a neutral or threat condition and read a paragraph related to aging or COVID-19, respectively. Afterwards, participants completed a memory task followed by a series of questionnaires to assess demographics, mood, depression, and COVID-19 health history. A one-way ANCOVA was conducted to evaluate the effect of COVID-19 threat on memory performance while controlling for history of COVID diagnosis. Findings indicate a significant difference in scores such that individuals in the threat condition had worse memory performance, compared to those in the neutral condition. This suggests that just reading information about COVID-19 may pose a threat to older adults' cognitive abilities and further demonstrates the far-reaching effect the pandemic has had on individuals.

Abstract citation ID: igad104.3254

### CREATIVE ACTIVITIES AND COGNITION AMONG OLDER ADULTS: DOES EDUCATION MODERATE THE BENEFITS OF CREATIVE ACTIVITIES?

Janelle Fassi, *University of Massachusetts Boston, Boston, Massachusetts, United States*

Engagement in leisure activities has been shown to offset age-related cognitive losses. Although activity engagement has a compensatory effect on later life cognition among adults with low educational levels in early life, no studies have examined this effect with creative activity engagement. The aims of the present study were to (1) investigate whether older adults' engagement in any creative leisure activities is related to cognitive functioning, (2) investigate whether the number of creative activities in which older adults engage is related to cognitive functioning, and (3) determine whether education moderates the relationship between creative engagement and cognitive functioning. This present study utilized a nationally representative sample of 2,462 adults aged 65 years and older from the Health and Retirement Study, who completed either the 2016 or 2018 waves of the Leave Behind Questionnaire and the 2015 wave of the Consumption and Activities Mail Survey. Results from linear regression models revealed that creative engagement was associated with better cognitive functioning scores ($\beta=0.74$, $p<.001$). Additionally, more years of education were associated with better cognitive functioning scores ($\beta=0.49$, $p<.001$). Engaging in four creative activities is also associated with better cognitive functioning compared to engaging in no activities ($\beta=1.38$, $p<.01$). In sum, the results were in line with previous research that creative engagement and education are positively associated with cognitive functioning. Contrary to previous findings, creative engagement did not have a compensatory effect on cognitive functioning among individuals with fewer years of education.

Abstract citation ID: igad104.3255

### CROSS-CULTURAL VALIDATION OF THE OARS INSTRUMENT FOR THE ASSESSMENT OF ELDERLY ANGOLANS

Rita Barbara, *Universidade de Aveiro, Sao Paulo, Sao Paulo, Brazil*

**Background:** The Older Americans Resources and Services (OARS) instrument is widely used internationally, but requires validation in specific contexts, such as Angola. This study aims to cross-culturally validate the OARS for assessing elderly Angolans. **Methods:** The cognitive and functional domains of the OARS were cross-culturally adapted, followed by psychometric analysis on a representative sample. Domains of activities of daily living and mental health were evaluated. Internal reliability was assessed using Cronbach's alpha coefficient, resulting in 0.80 for activities of daily living and 0.80 for mental health, with a 95% confidence interval. **Results:** The psychometric analysis demonstrated consistent reliability in the domains of activities of daily living and mental health, with Cronbach's alpha coefficients of 0.80 for both, supported by 95% confidence intervals. These results indicate substantial reliability. **Conclusion:** The cross-cultural validation of the OARS is ongoing, and preliminary results from the psychometric analysis reinforce the reliability of the domains of activities of daily living and mental health. Adapting the OARS could be a valuable tool for assessing elderly Angolans, providing reliable and internationally comparable data. Keywords: OARS, elderly, cross-cultural validation, psychometric analysis.

Abstract citation ID: igad104.3256

### CULTURALLY SENSITIVE STRATEGIES TO RECRUIT FOREIGN EDUCATED NURSES FOR US LONG-TERM CARE RESEARCH

Roy Thompson[1], Deidre Wipke-Tevis[1], Sherif Olanrewaju[2], Olumayowa A. Odemuyiwa[1], and Allison Squires[3], *1. University of Missouri, Columbia, Missouri, United States*, *2. The Pennsylvania State University, University Park, Pennsylvania, United States*, *3. New York University, New York City, New York, United States*

Long-term care (LTC) facilities experience chronic nursing staff instability which negatively impacts resident health outcomes. Recruiting Foreign Educated Nurses (FENs) is one strategy to stabilize the nursing workforce but most studies on FENs, use secondary data from: American Community Survey and the National Sample Survey of Registered Nurses. Few empirical studies have examined FEN experiences, employment outcomes, and impact on US LTC facilities. Since LTC facilities are dependent on FENs to meet minimum staffing requirements, additional research is needed to better understand FEN workforce issues in LTC, how to improve its sustainability, and the links to resident outcomes. Mistrust of researchers, however, may make FENs hard to reach. Therefore, conducting primary research on FENs in LTC requires culturally sensitive and community-engaged recruitment strategies. Drawing from existing literature and lessons learned from primary research conducted with FENs working in LTC facilities in the southeastern US, we will review best practices for recruitment so researchers can understand the cultural and structural issues that may affect research conduct in facilities where FENs are employed and their recruitment as participants. Developing relationships with FEN leaders as well as the Nigerian, Jamaican, Filipino, and/or Asian nurses associations can facilitate access to the population. Key stakeholder relationships also include the American Nurses Association, State Boards of Nursing, and Commission on Graduates of Foreign Nursing Schools as they can facilitate entrée into data sources and networks for recruitment. Understanding recruitment strategies will strengthen research that deepens our understanding of workforce issues among FENs in LTC.

Abstract citation ID: igad104.3257

### DAYS AT DUNROVIN: ANALYSIS OF FIELD NOTES FROM AN ONLINE NATURE-BASED INTERVENTION FOR OLDER ADULTS

Megan Westmore[1], Anna Tulloh[1], Rebecca Mauldin[1], Keith Anderson[2], and Aaron Hagedorn[1], *1. The University*

*of Texas at Arlington, Arlington, Texas, United States, 2. University of Mississippi, Oxford, Mississippi, United States*

Implementing novel interventions for older adults is often challenging, and understanding processes is as important as evaluating outcomes. Days at Dunrovin (D@D) is an online program providing virtual access to a Montana ranch. While originally designed as an individual activity, researchers converted D@D into a group activity for older adults. In this presentation, researchers present an analysis of field notes from 65 group sessions (n = 65) in an assisted living community. Results are organized using the first four elements of the RE-AIM framework (Reach, Effectiveness, Adoption, Implementation, and Maintenance). Reach (i.e., participation) depended on encouraging participation and transporting residents to activity areas – often time-consuming, and diminished by limited resources. Effectiveness (preliminary) was evidenced by participant engagement with the activity, and by staff and family observations of participants' affect and behavior. Laughter, conversations, and reminiscence were consistently observed. Adoption, the level of facility acceptance, was mixed as some staff welcomed the new activity while others found it difficult to support while attending to regular work. Implementation depended on many factors, including the environment (e.g., room arrangement), time of day, fluctuations in participant well-being, and technological capacities such as broadband width. Most notable was a lightning strike on the D@D cameras that resulted in the temporary use of archival content rather than viewing livestreams. Results suggest the feasibility and potential effectiveness of using nature-based livestream broadcasts as a group activity in assisted living and reveal the invaluable role field notes play in understanding implementation and evaluating interventions in applied research.

Abstract citation ID: igad104.3258

### DECISION-MAKING CAPACITY ASSESSMENTS FOR VULNERABLE ADULTS: LESSONS FOR SOCIAL WORK COMPETENCY

Adria Navarro[1], and Eppie Leishman[2], *1. University of Southern California, Los Angeles, California, United States, 2. The University of York, York, England, United Kingdom*

Social work approaches to the assessment of decision-making capacity (DMC) assessment have largely been informed by literature from 25 years ago. Evidence-based practices include the ACED short version and the IDA-CA 3.0. When considering this specific practice by social workers there is the UK policy supporting competent practice through legislation, the Mental Capacity Act (2017) of England and Wales. Nine practitioners practicing through guidance presented by this policy were interviewed to learn what may support practices in the United States. Findings point to the importance of foundational values and efforts to optimize the vulnerable adult's performance. Practical methods used in the assessment are gleaned, along with the training that has informed their work. Training advice included didactic, case review, and role-playing of increasingly complex cases. Experience working with diverse populations of vulnerable adults was also highlighted. This research strives to inform steps that enhance capacity within health and social care settings to adequately assess DMC. Understanding decision-making ability aids in preventing both paternalism and the insufficiency of support and/or protection.

Abstract citation ID: igad104.3259

### DEFINING 'OUTBREAK': ADMINISTRATORS' EXPERIENCES MANAGING COVID-19 AT US NURSING HOMES

Joan Brazier, Amy Meehan, Courtney Hawes, Renee Shield, and Emily Gabois, *Brown University School of Public Health, Providence, Rhode Island, United States*

The CDC defines a COVID-19 outbreak as starting with a single positive case of a staff member or resident in a facility and ending after there have been no new cases for 14 days. According to a report from the U.S. Government Accountability Office, 94% of nursing homes in the U.S. experienced multiple COVID-19 outbreaks, resulting in a reported 1.66 million residents and 1.64 million staff members with confirmed COVID-19 as of July 2023. Research has shown that COVID-19 outbreaks froze new admissions and caused significant financial strain. This qualitative study aimed to examine how COVID-19 outbreaks were managed in U.S. nursing homes. 156 interviews were conducted with nursing home administrators from 40 facilities across the U.S. between July 2020-December 2021. Two major themes emerged from thematic analysis: 1) the definition of "outbreak" as a single individual testing positive with COVID-19 was inconsistently communicated to nursing home administrators at the start of the pandemic; and 2) administrator self-reports of COVID-19 outbreaks at their facilities indicated that many endured multiple outbreaks which were long-lasting (greater than 1 month). As policy makers and industry experts review policies around managing nursing home viral outbreaks, they should consider the emerging infection control strategies from the pandemic which successfully prevented the spread of COVID-19 and update the response to an 'outbreak' such that infection control mitigation measures are effective without causing restrictions that are detrimental to resident well being or nursing home financial hardship.

Abstract citation ID: igad104.3260

### DEMAND-ABILITIES FIT AND PRESTIGE INFLUENCE OLDER WORKERS' INTEREST IN ALTERNATIVE JOBS

Nathan Baker[1], Jenna Van Fossen[2], Amy Schuster[2], Chu-Hsiang Chang[1], and J. Kevin Ford[1], *1. Michigan State University, East Lansing, Michigan, United States, 2. Clemson University, Clemson, South Carolina, United States*

With the increasing displacement of workers due to technological innovations, many older workers may need or want to make career transitions. Therefore, it is critical to understand the factors that influence older workers acceptance of potential alternative careers. In this study, long-distance truck drivers (n=202), a profession at-risk of technological disruption, completed an online survey where they rated six alternative transition occupations (e.g., machine setting, operator and tender, cargo and freight agent) to understand what makes them interested in alternate jobs. Using multilevel modeling (with occupations nested in participants), we evaluated the role of perceptions of demand-abilities fit and prestige, as moderated by age, on interest in alternative careers. Demand-abilities fit and job prestige were positively associated with rated job appeal, which was positively associated with interest in the job as a transition occupation. Older drivers, relative to younger truck drivers, found job prestige to be more appealing, which appeared to make them more interested in transitioning into these jobs. Our results extend person-environment fit and socio-emotional selectivity theory by showcasing how age relates to evaluations of occupations for career transitions, an area that is rising in importance given the increases in an aging workforce and potential disruptions from automation. Results carry practical importance for preparing the workforce for career disruptions given automation by identifying qualities that may shape workers' willingness to transition, and identifying how these qualities differ based on age.

Abstract citation ID: igad104.3261

### DEMENTIA CARE IN ASIAN AND LATINO COMMUNITIES: PERSPECTIVES FROM FAMILY CAREGIVERS AND SERVICE PROFESSIONALS

Arienne Patano[1], Fei Sun[1], Daniel Vélez Ortiz[1], and Ethan Liu[2], *1. Michigan State University, East Lansing, Michigan, United States*, *2. Phillips Exeter Academy, Exeter, New Hampshire, United States*

While community-based support and services are increasingly available to family caregivers (FCGs) of a person with dementia (PWD), those from ethnic minority groups tend to be underserved. This study aimed to identify unaddressed challenges to dementia care and uncover strategies to better assist FCGs within Asian and Latino communities in Michigan. Focus group and individual interviews were conducted with service professionals (SPs) and FCGs. Specifically, we ran two focus groups with SPs (n=11), and one with Asian FCGs (n=8). Four individual telephone interviews were conducted with Latino FCGs. Sessions were audiotaped and conducted in the participants' preferred language, then transcribed and translated into English for analysis. Shared challenges to dementia care included: transportation to services; lack of translation services and language-concordant specialists, daycare/respite workers, and in-home care staff; and feelings of shame in needing support. Latino FCGs reported the emotional burden of witnessing the cognitive decline of their PWD, whereas Asian FCGs focused on challenges related to the behaviors of PWD. SPs and FCGs expressed the desire for more education for FCGs on dementia and self-management of stress. Limited technology literacy impacted their access to digital education. Culturally informed outreach and intervention strategies were recommended, such as partnering with community leaders and adapting cultural practices (e.g., providing family-oriented events, and using social media popular among specific ethnic groups). This study highlights the sociocultural challenges and practical outreach approaches for FCGs in Asian and Latino communities by integrating the perspectives of FCGs and SPs involved in dementia care.

Abstract citation ID: igad104.3262

### DEMENTIA CARE PARTNER ACTIVATION PROGRAM FOR FILIPINO AMERICANS

Mary Dioise Ramos, Nazmus Sakib, and Mehedi Hasan, *Kennesaw State University, Kennesaw, Georgia, United States*

This study aims to enhance the quality of life and health outcomes for individuals with Alzheimer's Disease and Related Dementia (ADRD) and their care partners through a culturally tailored support program. Specifically focusing on the unique challenges Filipino and Filipino American (FA) caregivers face, we seek to address disparities in access to care and develop strategies for effective health system navigation. The research employs a multifaceted approach, combining surveys, focus groups, observational techniques, and interviews to assess the caregiving experience comprehensively. Our primary objectives include evaluating the impact of caregiving burden, socio-emotional support, and health literacy on emergency department visits and hospital admissions for ADRD patients. Preliminary results highlight the significance of culturally sensitive interventions. FA caregivers often encounter difficulties due to cultural beliefs, knowledge gaps about ADRD, and limited support networks. This study outlines the development and testing of a specialized program designed to empower FA caregivers with the skills needed for proficient care management and emergency response. By engaging these caregivers in participatory action research, the program aims to foster active involvement and enhance their caregiving capabilities. The study's outcomes emphasize the potential for improved quality of life and health outcomes for individuals with ADRD and their caregivers. By addressing FA caregivers' unique challenges in the context of disparity populations, our research contributes to enhancing health system navigation behaviors and promoting culturally attuned care practices. This initiative promises to reduce emergency department visits and hospital admissions, benefiting ADRD patients and their dedicated care partners.

Abstract citation ID: igad104.3263

### DEMENTIA PREVENTION: THE COST-EFFECTIVENESS OF PREVENTION SERVICES IN JAPANESE LONG-TERM CARE SYSTEM

Xujing Hu, Yunfan Wu, Miyae Yamakawa, and Reiko Kanaya, *Osaka University, Suita, Osaka, Japan*

**Objectives:** To analyze the cost-effectiveness of prevention services (Named "Support" in Japanese official documents) in Japanese Long-Term Care Insurance system, which draws data from a large volume of 10-year database in Osaka Prefecture. Design: We design an approach to estimate cost-effective prices using aggregate data including costs of dementia in 10 years, time of dementia first diagnosed, changes of care levels, and quality of life. Measurements: The outcomes were average annual expenditure per unit of services. **Results:** In higher risk groups of dementia, prevention services delaying cognitive decline and enhancing quality of life were cost effective at $206 per individual. However, this cost-effectiveness will wear off as symptoms worsen and the care levels increase. **Conclusion:** Prevention services from Long-Term Care Insurance system are possibly to be cost-effective, but this cost benefit is not long-term .

Abstract citation ID: igad104.3264

### RURAL-URBAN DIFFERENCES IN DIGITAL HEALTH USE OF MEDICARE BENEFICIARIES

Shannon Power, and Hyesu Yeo, *University of Georgia, Athens, Georgia, United States*

Growing reliance on digital healthcare appears to marginalize rural older adults with limited technology access and experience. Existing research explores telehealth uptake of Medicare beneficiaries but does not consider the role of locality in digital health use (DHU). Using the 2021 National Health and Aging Trends Study data, this study compares rural-urban Medicare beneficiaries' DHU (weighted N=18,729,829).

Informed by the Senior Technology Acceptance Model, multiple linear regressions were performed on rural and urban models separately, including DHU, social determinant, and technology readiness variables. Participants in this study were predominantly White, female, and in their 70s. In the final model, men were more likely to use DHU than women in both rural-urban samples (β=.13, β=.08, p<.05, respectively). Tablet access was the only significant device for DHU among rural residents (β=.18, p<.05), while computer access (β=.12, p<.05) was the most significant among urban residents. Online grocery shopping was the most predictive prior technology experience of DHU for rural dwellers (β=.24, p<.05), while online banking led for urban dwellers (β=.21, p<.05). These unique differences between rural-urban samples can inform research on how to specifically design and implement digital interventions to increase rural older adult uptake. Healthcare workers can emphasize similarities between DHU and older adults' prior technology experience to build perceived ease of use. Expanding opportunities for rural older adults to interact with technology in daily life builds experience which best predicts DHU. Policymakers should consider the impact of locality and involve aging community stakeholders to advance equitable DHU in the digital age.

### SESSION 9010 (POSTER)

### LATE BREAKING POSTER SESSION II

Abstract citation ID: igad104.3265

### ADAPTING TELEHEALTH EVIDENCED BASED PROTOCOLS FOR VETERANS WITH MOOD AND NEUROCOGNITIVE DISORDERS: A CASE STUDY

Charissa Hosseini, *Veterans Health Administration, Alameda, California, United States*

Aging Veterans with mood and neurocognitive disorders typically have a complex medical and psychiatric presentations leading many providers to exclude them from treatment, expressing that psychotherapy is not appropriate. This form of exclusionary care may decrease help seeking behavior and amplify existing psychological symptoms to an already stigmatized and isolated population. The following case study, outlines a 24-session approach to integrating evidenced based protocols such as Cognitive Behavioral Therapy (CBT) and Narrative Life Review Therapy via telehealth. The presentation will discuss cultural considerations to take when working with Aging Veterans that have co-occurring mood and cognitive difficulties as well as reflect upon the successes and challenges of providing telehealth with this population.

Abstract citation ID: igad104.3266

### DEMENTIA-RELATED STIGMA, KNOWLEDGE, AND WILLINGNESS TO UNDERGO COGNITIVE SCREENING AMONG OLDER ADULTS

Whitney Kasenetz[1], Alex Crisci[1], Wendy Rote[1], and Jennifer O'Brien[2], *1. University of South Florida, St. Petersburg, Florida, United States*, *2. University of South Florida, Tampa, Florida, United States*

Screening for dementia such as Alzheimer's Disease may be most beneficial at the earliest detectable stages of disease presentation, yet there is no definitive recommendation for routine cognitive impairment screening and dementia remains underdiagnosed. Identifying perceptions of cognitive screening, stigmatized beliefs, and misconceptions about dementia could be beneficial for creating interventions that promote dementia knowledge to improve early screening and diagnosis and empower older adults to seek treatment sooner. Thus, we examined the extent to which attitudes, beliefs, and knowledge about cognitive screening and dementia predict willingness to undergo screening and subsequent screening behavior. 295 subjects aged ≥ 60 years completed online surveys containing the Perceptions Regarding Investigational Screening for Memory in Primary Care (PRISM-PC) questionnaire to assess attitudes about dementia screening and diagnosis, as well as the Alzheimer's Disease Knowledge Scale (ADKS). Participants were then invited to complete the Montreal Cognitive Assessment in a university psychology laboratory. 94% of participants reported willingness to undergo screening, but only 69% completed one. Logistic regression analysis revealed that the screening acceptance scale of the PRISM-PC significantly predicted reported willingness to undergo screening but not the pursuit of screening. The other PRISM-PC subscales (perceived benefits of dementia screening, stigma of dementia screening, suffering from dementia screening, and impact of dementia screening on patient's independence) and the ADKS did not predict willingness or pursuit of screening. While most were willing to undergo screening, significantly fewer completed screening within the study. The factors that predict pursuit of cognitive screening require further investigation.

Abstract citation ID: igad104.3267

### DEPRESSION AND ROLE OVERLOAD IN FAMILY CAREGIVERS OF PEOPLE LIVING WITH AND WITHOUT DEMENTIA

Claire McDonald, and Adrian Bravo, *William & Mary, Williamsburg, Virginia, United States*

The present study examined the associations between caregiver assistance, role overload as a stressor, and depression symptoms among caregivers of adults age 65 and older living with and without probable dementia. Specifically, we hypothesized that more time spent caregiving would be associated with higher depressive symptoms via greater role overload and this indirect association would be stronger for those caregiving for an older adult with probable dementia versus no probable dementia. This study analyzed data from round 11 of the National Health and Aging Trends Study (NHATS) and the supplemental National Study of Caregiving (NSOC), which were collected from 2021 through early 2022. The majority of caregivers (207 probable dementia caregivers, 451 no probable dementia caregivers) identified as white (69%), female (70%), and reported an average age of 63.33. A moderated mediation model was tested using the PROCESS Macro (Hayes, 2013) in SPSS. Within our model, dementia classification was found to moderate the effect of the number of days spent caring on role overload (B = .048, SE = .019, t = 2.54, p = .011). Moreover, the overall moderated mediation model was supported with the index of moderated mediation = 0.0094 (95% CI = .0010, .0192). These results indicate that the more days a caregiver spent helping was associated with more symptoms of depression via higher caregiver overload, especially among those caregivers taking care of an older adult with probable dementia. These findings highlight the importance of making healthcare services available and accessible for caregivers.

Abstract citation ID: igad104.3268

### DESIGNING COLLABORATIVE AUGMENTED REALITY ACTIVITIES WITH OLDER ADULTS IN LONG TERM CARE

Cathy Maxwell[1], Mahruhk Tauseef[1], Akshith Ullal[1], Alexandra Watkins[1], Judith Tate[2], Lisa Juckett[2], Lorraine Mion[2], and Nilanjan Sarkar[1], *1. Vanderbilt University, Nashville, Tennessee, United States*, *2. The Ohio State University, Columbus, Ohio, United States*

Loneliness is common among older adults in long term care (LTC) settings. Interactive communication technologies hold great promise in reducing loneliness but require design and evaluation studies. As part of a larger study on use of head mounted display augmented reality (HMD-AR) to connect older LTC adults with family members, we conducted a participatory design study to a) identify and design HMD-AR multi-user activities and b) address barriers found with LTC residents when interacting with HMD-AR.

**Results:** Laboratory testing was conducted with 23 older adults with normal cognition (ages 65 to 93, 6 men) and field testing with iterative feedback cycles with 8 older adults with impaired mobility and cognition (ages 54 to 87, 2 men, 4 with stroke) that resulted in prototype development and testing of three activities: playing checkers, decorating a fireplace, and eating a meal. Challenges successfully addressed through technological innovations included: 1) enhancing perceptions and ease of interacting with photorealistic avatars, 2) providing various techniques to allow older adults to view and move AR objects despite cognitive and functional impairments (e.g., visual-perceptual impairments; limited dexterity), 3) using visual aids and voice commands, 4) developing visual adaptations for central vision and color detection impairments, and 5) creating tutorials and help guides for learnability and remembrance. Sessions lasted for 30 minutes and no adverse effects were noted with prolonged or repeated HMD-AR use. All enjoyed the HMD-AR activities.

**Conclusion:** HMD-AR can be successfully adapted for use by older adults with multiple cognitive and physical impairments.

Abstract citation ID: igad104.3269

### DEVELOPING A PROTOCOL TO ASSESS EXPOSURE TO ENVIRONMENTAL POLLUTANTS AMONG OLDER ADULT WOMEN IN SOUTHEAST MICHIGAN

Jillian Baker, Sung Kyun Park, Kerry Richards-McCullough, Andrew Hoover, and Carrie Karvonen-Gutierrez, *University of Michigan School of Public Health, Ann Arbor, Michigan, United States*

Environmental pollutants, including PFAS, phthalates, and parabens, contribute to the development of age-related diseases like cancer, cardiometabolic disease, and neurodegenerative diseases. Unfortunately, these chemicals are common in everyday exposures, including drinking water, personal care products (PCPs), non-stick cookware, and food packaging. Exposures to these pollutants may be high in communities that have encountered structural disadvantages; neighborhood disinvestment and decaying infrastructure can make exposures to these pollutants more likely, thus increasing the burden of age-related disease in these communities. Interventions that reduce exposures to these chemicals may prevent or delay the onset of age-related disease, especially in groups experiencing poorer health outcomes because of structural racism and discrimination. To explore the feasibility of a behavior change intervention for older adults that reduces exposure to harmful chemicals in PCPs, a protocol was developed to: 1) Assess attitudes, knowledge, and behaviors related to environmental pollutants via questionnaire; 2) Characterize individual-level 'exposomes', the unique mixture of chemicals a person encounters in their everyday lives, by wearing a silicone wristband; 3) Measure environmental exposures in serum. Data collection will begin in September 2023 among older adult women who have participated in the Study of Women's Health Across the Nation (SWAN) at the Michigan site. Older adults have accrued environmental exposures across their lifetimes, likely in a way that reflects patterns of structural disadvantage. While structural solutions are necessary to address issues of health equity, behavior change efforts can work in tandem to reduce harmful environmental exposures to improve the health of older adults.

Abstract citation ID: igad104.3270

### DEVELOPMENT AND APPLICATION OF R1RHO DISPERSION IMAGING OF AGING MUSCLE IN AN ANIMAL MODEL

Fatemeh Adelnia, Feng Wang, and John Gore, *Vanderbilt University Medical Center, Nashville, Tennessee, United States*

Compelling evidence from animal studies demonstrates that microvascular impairment contributes to the etiologies of several age-related chronic diseases, including sarcopenia. For example, capillary hemodynamic profiles are altered in old F344 rat spinotrapezius muscle compared with those of younger counterparts. However, a validated non-invasive measure of microvasculature geometry and structure has not yet been developed. Recently, it has been shown that R1ρ dispersion imaging at weak locking field frequencies (FSL) provides a unique way of characterizing microvessel size and density. Moreover, previous R1ρ images were acquired at strong FSL (≥500Hz) and used to measure the age-related differences linked to macromolecular deposition, such as collagen in the extracellular matrix. Here, we aimed to develop a non-invasive 3D volume R1ρ dispersion imaging method at weak FSLs (< 300Hz), to quantify microvasculature changes in skeletal muscle. We successfully implemented the protocol and applied it to the lower leg of five F334 female rats at age 15 mo. The whole lower leg of each rat was also imaged using conventional multislice T1-FLASH, Diffusion-weighted, and T2-MGE pulse sequences. We found that R1p value measured in calf muscles decreased from 46.92Hz to 41.63Hz by increasing FSL from 5Hz to 240Hz, which we suggest derives from the effects of rephasing some of the signal losses that arise from diffusion in intrinsic magnetic field gradients generated from microvessels. Future direction of this ongoing study will be to compare these results with data collected from young female rats at age 6 months and further validation using ex-vivo histology.

Abstract citation ID: igad104.3271

### DEVELOPMENT AND VALIDATION OF THE COUPLE EFFICACY TO BALANCE WORK AND ELDERCARE SCALE

Eunae Cho[1], Tammy Allen[2], and Grand Cheng[3], *1. National Chengchi University, Taipei City, Taipei, Taiwan (Republic of China), 2. University of South Florida, Tampa, Florida, United States, 3. National University of Singapore, Singapore, Not Applicable, Singapore*

This study developed and validated a measure of couple efficacy to balance work and eldercare (i.e., a couple's shared belief in their conjoint abilities to achieve work-eldercare balance as a couple), a key cognitive factor for work-eldercare arrangements, individual well-being, and work and couple outcomes. Survey data were collected from 408 dual-earner couples in Taiwan who care for an older adult. Exploratory factor analysis identified three factors with eigenvalues greater than 1, but parallel analysis suggested extracting two factors. After considering item contents and factor loadings, a final 2-factor scale comprising 10 items was selected. The first dimension, task-oriented, reflects couple efficacy concerning the execution of various tasks to balance paid work and eldercare (e.g., "I am confident that my spouse/partner and I can work together as a team to devote enough attention to important job and eldercare activities"; $\alpha$=.81). The second dimension, relationship-oriented, reflects couple efficacy concerning the maintenance of intimate relationship while balancing paid work and eldercare ("I am confident that my spouse/partner and I can work together as a team to support each other in times of stress in trying to balance paid work and eldercare"; $\alpha$=.87). Confirmatory factor analysis indicated that a hypothesized 2-factor outperforms an alternative 1-factor model. The nomological network involving generalized self-efficacy and life/job/marital satisfaction supported the validity of the new scale. This study contributes to the literature by introducing a new construct and offering a psychometrically sound measure that propels more empirical research on the important population of working informal caregivers.

Abstract citation ID: igad104.3272

### DEVELOPMENT AND VALIDATION OF THE NOVEL MEDICATION TAKING INSTRUMENT - FOUR FACETS OF ADHERENCE (MTI-4)

Candace Ryan, Debra Schutte, and Ana Daugherty, *Wayne State University, Detroit, Michigan, United States*

Medication adherence is critical for disease management among aging African-Americans who are at greatest risk for poor adherence and affliction with chronic diseases. However, medication adherence is a complex behavior with distinct facets (frequency, pattern of adherence, barriers, and beliefs related to adherence), which current instruments cannot comprehensively measure in this underrepresented population. The Medication Taking Instrument – Four Facets of Adherence is an instrument developed to measure all facets of adherence among diverse, community-dwelling aging adults using the innovative application of the "Nursing Rights" of medication administration. Items were developed following a scoping review of the literature. Validation was obtained from medical professionals in Canada and the United States who prescribe medications to diverse patient populations. 12 of 13 medical professionals responded to the survey request (67% Nurse Practitioners, 33% Physicians) and rated each item (0=Low Agreement to 5=High Agreement) for clarity and validity of each item representing the specific facet. Most instrument items obtained high agreement (response rating > 3) for both validity and clarity from 83% or more of respondents. Experts' feedback augmented items for two facets (barriers and beliefs), improved clarity, minimized jargon, and supported instrument use in the clinical setting. The resulting instrument will comprehensively characterize medication adherence, direct more targeted research aims, and guide interventions to augment adherence among aging African Americans. Ongoing data collection in the Detroit Aging Brain Study will provide additional tests of factor structure, internal scale reliability, and convergent validity using other indicators of medication history and symptom control.

Abstract citation ID: igad104.3273

### DEVELOPMENT OF A PHYSICAL PERFORMANCE SUMMARY SCORE IN MIDDLE AGE: FINDINGS FROM THE CARDIA FUNCTION ANCILLARY STUDY

Brett Burrows[1], Richard Sloane[2], Carl Pieper[1], Cora Lewis[3], Kelley Pettee Gabriel[3], David Jacobs[4], and C. Barrett Bowling[5], *1. Duke University School of Medicine, Durham, North Carolina, United States, 2. Duke University Medical Center, Durham, North Carolina, United States, 3. University of Alabama at Birmingham, Birmingham, Alabama, United States, 4. University of Minnesota, Minneapolis, Minnesota, United States, 5. Duke University, Durham, North Carolina, United States*

Recent evidence suggests that ~20% of middle-aged adults report mobility limitations, supporting the need for assessment strategies to detect early-onset limitations, appropriate for this population. Coronary Artery Risk Development in Young Adults (CARDIA) enrolled 5,115 Black and White men and women ages 18-30 years from four sites (Birmingham, AL; Oakland, CA; Minneapolis, MN; and Chicago, IL) in 1985-86. The CARDIA Function Study added self-reported function (PROMIS SF20a) and 5 physical performance measures (gait speed, single-leg balance, lower extremity strength, grip strength, and 6-minute walk test) to the Year 35 in-person follow-up exam conducted in 2020-22. The CARDIA Physical Performance (CAPP) score was developed by calculating sex-specific quartiles separately for each performance measure, assigning points based on these quartiles (0 for not attempted, 1-4 for each quartile), and summing points across all 5 performance measures (range 0-20, higher scores = greater performance). Among 2,021 CARDIA Function participants [mean age: 60.1 (±3.6) years; 57% female; 45% Black] the mean CAPP score was 12.3 (±4.1; range 0-20). Mean CAPP scores differed by age < 60 vs. ≥60 years old (12.7 vs.12.1, $p< 0.05$) and self-reported function above vs. below the median SF20a score (14.0 vs. 10.6, $p< 0.001$), but as expected, not sex (12.3 vs. 12.3, $p=0.99$). Among middle-aged adults, the newly developed CAPP score captured a wide range of physical performance. Lower CAPP score was associated with lower than median self-reported function suggesting that the CAPP score may be helpful assessment tool for detecting early-onset limitations to attenuate functional declines with aging.

Abstract citation ID: igad104.3274

**DEVELOPMENT OF AN AI-POWERED EMOTIONALLY INTELLIGENT, INTERACTIVE AGENT PROTOTYPE TO SUPPORT INFORMAL CAREGIVERS**

Yu-Ping Chang, Aditya Kumar Dwibedi, Suryakanth Joshi, and Sreyasee Das Bhattacharjee, *University at Buffalo, The State University of New York, Buffalo, New York, United States*

Long-term informal caregiving for older adults with dementia is exhausting and poses risks to caregivers' health outcomes. Most informal caregivers are not well prepared for their new role, facing various challenges and receiving limited support. With the advent of artificial intelligence (AI) and information and communication technologies (ICTs), assistive technology is increasingly useful for supporting caregivers. However, limited research assesses the potential for AI to facilitate a privacy-preserving computation environment to provide proactive assistance to caregivers. This project aims to fill this gap by leveraging the tremendous potential of Multimodal Machine Learning and Large Language Models (LLMs) to develop a prototype of an AI-powered emotionally intelligent, interactive agent for informal caregivers. We introduce an effective context-driven instruction tuning technique that enables LLMs to finetune various caregiving-related tasks (tips generation, answering questions, elaborating a specific training module) with instructions. The project will also deliver a first-of-its-kind custom dataset augmented with manualized instructions for relevant caregiving tasks, which is used for finetuning several state-of-the-art LLM models. Our intelligent interactive agent prototype currently has the ability to evaluate caregivers' unique affective states at query times to provide personalized assistance, addressing various types of caregivers' situation-specific queries (e.g., managing agitation, wandering, or medications). The prototype is capable of understanding and responding to the user's natural language queries and designing short, machine-driven persuasive conversations on a pre-defined set of topics. Our prototype has the potential to provide individualized and contextualized support for caregiving tasks which in turn may greatly reduce caregiver burden.

Abstract citation ID: igad104.3275

**DEVELOPMENT OF AN EVIDENCE-BASED FRAMEWORK FOR INNOVATION READINESS OF LONG-TERM CARE ORGANIZATIONS**

Monique van den Hoed, Ramona Backhaus, Audrey Beaulen, Jan Hamers, and Ramon Daniels, *Maastricht University, Maastricht, Limburg, Netherlands*

Increasing innovation readiness of long-term care organizations is vital to ensure future provision and affordability of care delivery. Against this background, there is a need to obtain more insight into the factors that may lead to innovation readiness of long-term care organizations. Therefore, we conducted a scoping review and an interview study. The scoping review was based on the framework from Arksey and O'Malley and included 44 studies. The interview study was conducted with 16 stakeholders having a professional role in long-term care for older adults in the Netherlands: academics, (top)management, innovation managers and consultants. Based on the results of both studies, we propose a framework of factors contributing to innovation readiness. The framework consists of five main factors enabling innovation readiness of long-term care organizations for older adults: 1) strategic course for innovation 2) innovation journey 3) leadership for innovation 4) learning for innovation and 5) innovative organizational culture. The collective findings support the notion that for innovation readiness the interplay of main factors is vital and benefits from an approach at the individual, team, organizational and inter-organizational levels. Furthermore, our findings indicate that some factors might be more conditional and other factors might play a more supportive role. Research into innovation readiness of health care organizations is a rather new field. Further research directed toward a framework for innovation readiness might deliver a structured approach for managers of long-term care organizations to embed and assess innovation readiness.

Abstract citation ID: igad104.3276

**DIETARY IMPACT ON GUT MICROBIOTA, COGNITIVE PERFORMANCE, AND HIPPOCAMPAL MARKERS IN AGING RATS**

Rebecca Solch-Ottaiano, Colin Harper, Elizabeth Engler-Chiurazzi, Saifudeen Ismael, Madison Prats, Milla Volic, Gregory Bix, and Demetrius Maraganore, *Tulane University School of Medicine, New Orleans, Louisiana, United States*

Many patients with mild cognitive impairment have alterations in gut microbiota composition and dietary consumption directly modulates gut microbiota; therefore, the quality of a diet may impact cognitive function. Twelve-month-old male Fischer 344 rats (n=10/group) were assigned to a Mediterranean diet (MeDi), Western diet (WD), or Chow for three months to determine the effect of diet in aged animals. Animals underwent neurobehavioral assessments including the Morris water maze (MWM), and radial arm water maze (RAWM). Fecal samples were analyzed via 16S rRNA sequencing. Markers of barrier and immune function in the hippocampus were measured via qPCR. The MeDi ($p=0.06$) and Chow ($p=0.003$) groups had a trend to or swam a shorter distance compared to the WD in the MWM. There was no difference in RAWM total errors between Diets ($p=0.43$). Microbiota analyses exhibited differences in alpha- and beta-diversity. Chow diet had a higher Simpson and Shannon index compared to MeDi ($p< 0.02$) and WD ($p< 0.001$). The MeDi ($p=0.03$) and Chow ($p< 0.001$) groups had a higher Chao1 index compared to the WD group. Beta-diversity differed by Diet for weighted UniFrac ($p< 0.03$), and unweighted UniFrac ($p< 0.001$). For barrier integrity, there was a trend for Occludin expression to differ by Diet ($p=0.07$) but Claudin-5 did not differ ($p=0.81$). For immune function, the WD group had a trend to have a higher expression of GFAP compared to the MeDi ($p=0.05$) but a lower Iba-1 expression ($p=0.009$). A short-term diet had a subtle effect on cognitive performance but modulated microbiota compositions and hippocampal immune marker expression.

Abstract citation ID: igad104.3277

**DIETARY NITRATE SUPPLEMENTS AND AEROBIC EXERCISE MODIFY FRAILTY IN AN AGE-DEPENDENT MANNER IN FEMALE C57BL/6 MICE**

Elise Bisset, and Susan Howlett, *Dalhousie University, Halifax, Nova Scotia, Canada*

We previously demonstrated that aerobic exercise attenuates the development of frailty in older female mice. Here we combine the common dietary supplement, sodium nitrate, with aerobic exercise to determine if this combination will work to attenuate frailty across lifespan. Adult (7-9 months) and aged (24-25 months) female mice were given free access to a running wheel and/or sodium nitrate (1mM, drinking water), or neither for 3 months. We measured blood pressure (tail cuff), running volume, frailty (frailty index), and body composition (DEXA). Blood pressure was unaffected by nitrates or exercise at both ages. While young mice ran more than older mice (3.5±0.4 vs 1.2±0.2 km/day), running was unaffected by nitrates. Older mice were frailer at baseline than younger mice (0.13±0.04 vs 0.03±0.004; p< 0.001). In older sedentary controls, frailty increased over time (0.15±0.02 to 0.19±0.03: p=0.03), but this was prevented by nitrates (0.13± 0.02 to 0.12 ± 0.01), exercise (0.12±0.03 to 0.18±0.03), and both (0.12±0.02 to 0.12±0.01). Older sedentary controls saw age-related weight loss (32.5±3.0g to 29.7±2.3g: p=0.02) which was prevented by exercise or nitrates. In older mice, mortality was highest in sedentary controls (46%) and lowest in exercised mice fed nitrates (0%). In contrast, while exercise attenuated frailty in young mice, nitrates did not. Young mice also had few changes in body composition; none died. While nitrates with or without exercise are beneficial for older mice, they had little effect in younger mice. These results suggest that nitrates alone or with exercise, may help prevent frailty in older females.

Abstract citation ID: igad104.3278

### DIFFERENCES BETWEEN TRAINEE AND SUPERVISOR PERSPECTIVES ON THE IMPACT OF COVID-19 ON GEROPSYCHOLOGY TRAINING

Valerie Luskey[1], M. Lindsey Jacobs[1], Michelle Mlinac[2], Hannah Bashian[2], Candice Reel[1], and Julia Boyle[2], *1. University of Alabama, Tuscaloosa, Alabama, United States*, *2. VA Boston Healthcare System, Boston, Massachusetts, United States*

The COVID-19 pandemic majorly impacted geriatric specialty training. To better understand this impact, a survey study was conducted to examine the pandemic's unique impact on geropsychology training. Participants (N=62) included psychology trainees specializing in geropsychology and psychologists providing geropsychology training. The majority of participants identified as white (90.3%), female (87.1%), and 30-39 years old. Using a 5-point Likert scale ranging from "not at all impactful" to "extremely impactful", participants rated their subjective experience on how the pandemic positively and negatively affected geropsychology training in three functional competency areas (assessment, intervention, and consultation) consistent with the Pikes Peak Model for Geropsychology Training. A 2x3 mixed design repeated measures ANOVA was conducted to examine similarities and differences in trainees' and supervisors' perceptions of the pandemic's impact on the competency domains. Results indicate that supervisors' and trainees' perception of the pandemic's negative impacts aligned, F(1,43)=.01, p=.931. Results were consistent across all competency domains, F(2,86)=1.39, p=.26; however, perceptions of the positive impacts significantly differed, F(2,84)=6.459, p=.002. Overall, both trainees and supervisors perceived more positive impacts on intervention and consultation compared to assessment, t(43)=3.54, p≤.001. However, trainees (M=1.8, SD=.112) reported more positive impacts overall compared to supervisors (M=1.3, SD=.09), F(1,42)=9.08, p=.004. These findings have significant implications for the level of competence in emerging geropsychologists', particularly in the areas of cognitive and capacity assessments. Recommendations for additional assessment training and supervision will be provided.

Abstract citation ID: igad104.3279

### DIFFERENCES IN FRAILTY BETWEEN TRADITIONAL MEDICARE AND MEDICARE ADVANTAGE BENEFICIARIES

Sandra Shi[1], Brianne Olivieri-Mui[2], Ellen McCarthy[1], and Dae Hyun Kim[3], *1. Hebrew SeniorLife, Boston, Massachusetts, United States*, *2. Northeastern University, Boston, Massachusetts, United States*, *3. Harvard Medical School, Boston, Massachusetts, United States*

Medicare Advantage (MA) plans cover at a minimum the same services as fee-for-service Medicare (FFS), but may target patient populations that differ in health and frailty. Leveraging 2011 and 2015 National Health and Aging Trends Study (NHATS) linked to Medicare claims, we classified participants as FFS or MA beneficiaries based on 12 months of Medicare enrollment. We calculated a deficit accumulation-based frailty index (FI) and phenotypic frailty from the survey assessment. All analyses accounted for the complex sampling design and weighted to reflect national estimates. In 2011, MA beneficiaries were more likely to be female (MA vs FFS: 59.1% vs 55.6%), Black (9.6% vs 7.4%) or other race (9.8% vs 5.2%), and to have an estimated income <$25,000 (44.5% vs 39.4%). Although the mean FI was similar between MA and FFS (MA vs FFS: 0.25 vs 0.25), MA beneficiaries were more likely to have mild frailty by FI (20.1% vs 18.6%) but less likely to have severe frailty by FI (10.0% vs 11.7%) or phenotypic frailty (13.5% vs 14.0%). MA beneficiaries had a higher prevalence of some comorbidities, such as hypertension (MA vs FFS: 64.9% vs 63.3%) and diabetes (24.4% vs 23.3%), but a lower mean number of ADL disability (0.32 vs 0.39). In 2015 the results were similar, although MA beneficiaries had a higher prevalence of pre-frailty (MA 46.0% vs 43.3%). In conclusion, MA beneficiaries seem to have a lower level of deficit-accumulation frailty and phenotypic frailty. This difference is driven by comorbidities, but not functional disability.

Abstract citation ID: igad104.3280

### DIFFERENCES IN RESILIENCE AMONG MIDLIFE AND OLDER ADULTS BY SEXUAL AND GENDER IDENTITY AND BY GENERATION

Christi Nelson, and Karen Fredriksen-Goldsen, *University of Washington, Seattle, Washington, United States*

Sexual and gender minority (SGM) (i.e., lesbian, gay, bisexual, sexual diverse, and transgender) midlife and older adults face specific health, social, and structural inequities in addition to the late-life challenges experienced by older adults in general. Extant research had largely examined health disparities and related risk and protective factors among SGM individuals as a homogeneous group, without attending to the heterogeneity among the subgroups and differing generations. Most research has also focused on stress with limited attention to resilience, even though higher resilience is associated with better health when facing adversity. Using baseline data from Aging with Pride: The National Health, Aging, and Sexuality/Gender Study, we identify historical/environmental, psychological, and social risk and protective factors that are associated with resilience in midlife and older SGM adults (n=2,450), aged 50-102, and examine differences in the associations between the factors and resilience by SGM and generational subgroups (i.e., Invisible, Silenced, and Pride). The results revealed differences in significant factors associated with resilience by SGM and generational subgroups. Identity affirmation was positively associated with resilience in all but the bisexual and transgender groups. Day-to-day discrimination was negatively associated with resilience for all but bisexual women. Community belongingness was positively associated with resilience for gay and bisexual men and the younger generations (Silenced and Pride). These results suggest that factors associated with fostering resilience, and in turn better health, may differ by SGM subgroup as well as by generation. Assessing differences in factors that promote resilience can help develop responsive interventions for targeted subgroups.

Abstract citation ID: igad104.3281

### DIFFERENCES IN SEX-RELATED PERSONALITY TRAITS AND POLYPHARMACY AMONG OLDER ADULTS WITH DEPRESSIVE DISORDERS

Tsung-Yu Tsai[1], Tzu-Yun Wang[1], Yi-Hsin Wang[2], Kao Chin Chen[1], Huai-Hsuan Tseng[1], and Ching-Ju Chiu[3], *1. National Cheng Kung University Hospital, Tainan, Tainan, Taiwan (Republic of China)*, *2. Tri-Service General Hospital, Taipei, Taipei, Taiwan (Republic of China)*, *3. National Cheng Kung University, Tainan, Tainan, Taiwan (Republic of China)*

This cross-sectional study aimed to explore the potential correlations between personality traits and the occurrence of polypharmacy (defined as the use of five or more medications) in older adults diagnosed with depressive disorders. In this study, 90 participants (30 males, 60 females) with an average age of 71.3±5.0 years were included. Personality traits were evaluated using the short version of the 50-item International Personality Item Pool, while medication usage data were retrieved from the National Health Insurance Pharma Cloud Database. Hierarchical regression models were employed for data analysis to examine the associations between personality traits and polypharmacy. The results showed that the mean number of medications taken was 10.9 ± 6.8 and that 84.4% of the participants satisfied the criterion for polypharmacy. Notably, low levels of openness significantly correlated with a higher number of medications taken (B=-0.38, p< 0.01). Gender-specific analyses demonstrated that low levels of conscientiousness (B=-0.07, p=0.01) and openness (B=-0.49, p=0.03) correlated with polypharmacy among males, whereas these personality traits displayed no significant correlations with polypharmacy in females (B=0.05, p=0.77, and B=-0.28, p=0.07, respectively). The findings underscore the elevated risk of polypharmacy among older adults diagnosed with depressive disorder. Additionally, the study highlights the necessity of considering gender differences in the interplay between personality traits and medication utilization patterns.

Abstract citation ID: igad104.3282

### DIFFERENCES IN WHITE MATTER MICROSTRUCTURE IN MIDDLE-TO-OLDER AGE ADULTS WITH CHRONIC KNEE PAIN

Kristina Bell, Larissa Strath, Chavier Laffitte Nodarse, Pedro Valdes-Hernandez, Roger Fillingim, and Yenisel Cruz-Almeida, *The University of Florida, Gainesville, Florida, United States*

Abundant research suggests aberrant white matter microstructure in patients with chronic pain as well as in older adults independently, where pain can significantly impact daily function. However, most studies fail to consider pain and aging interactions, particularly in middle and older-age. We aimed to explore differences in white matter microstructure (i.e.,Fractional Anisotropy (FA)) by pain's impact on daily function (i.e., high (n=60) versus low pain impact (n=111) in middle-(45-59 years old, n=129) versus older adults (60-XX years old, n=77) using diffusion tensor imaging. Participants (n=171) underwent laboratory visits to collect multi-modal pain and neuroimaging measures. We employed a linear mixed model approach to test main effects of pain impact and age groups as well as their interaction with Bonferroni-corrected multiple comparisons. Findings demonstrated a significant pain impact and age interaction on FA (p's≤0.05) in the left caudal anterior cingulate, left caudal middle frontal, left and right rostral middle frontal and left and right insular regions. Specifically, middle-aged adults with high impact pain had significantly worse brain microstructure across these regions compared to the older adults reporting high impact pain (p's≤0.05). These findings highlight the need to study pain across the lifespan. Given the higher prevalence of chronic pain in middle-age compared to older age, future studies are urgently needed to test whether interventions in middle-age could halt pain's impact on brain and function.

Abstract citation ID: igad104.3283

### DIFFERENT PATTERNS OF VACCINE HESITANCY AMONG OLDER ADULTS BY RACE/ETHNICITY, INCOME, AND EDUCATION

BoRin Kim[1], Jehoon Jeon[2], and Chung Hyeon Jeong[1], *1. University of New Hampshire, Durham, New Hampshire, United States*, *2. Eastern Connecticut State University, Willimantic, Connecticut, United States*

Despite significant efforts to increase COVID-19 vaccination rates, a substantial number of people exhibit vaccine hesitancy. Vaccine hesitancy is considered as one of the greatest threats to global health but also poses substantial risks to individuals. Moreover, vaccine hesitancy patterns may reflect and perpetuate existing health disparities. This study aims to investigate if specific patterns of vaccine hesitancy exist among older adults across racial/ethnic and socioeconomic groups. Our sample included 4,656 respondents aged 65+ who participated in the 2021 Health and Retirement Study Perspectives on the Pandemic that focuses on the first-year pandemic experiences. Latent class analysis and multinomial regression was used to identify vaccine hesitancy patterns and examine the associated factors, adjusting for age, gender, marital status, and self-rated health. Four vaccine hesitancy patterns were identified: no hesitancy (56.7%), mild hesitancy-uncertainty (19.4%), strong hesitancy-personal concerns (12.0%), and strong hesitancy-lack of trust/conspiracy (11.9%). White (RRR=1.659, p<.01), higher income (RRR=1.005, p<.05) and higher education (RRR=1.078, p<.001) were positively associated with no hesitancy. Respondents with higher education (RRR=0.573, p<.05) were less likely to be in the group of mild hesitancy with uncertainty. Hispanics (RRR=0.583, p<.05) and higher education (RRR=0.332, p<.001) were negatively associated with strong hesitancy with personal concerns. African Americans (RRR=2.426, p<.001) and those with lower education (RRR=1.324, p<.001) were more likely to be in the group of strong hesitancy with lack of trust/conspiracy. Our findings show that vaccine hesitancy is not random, which is closely related to social stratification impacting health disparities in this society.

Abstract citation ID: igad104.3284

### DISRUPTED SOCIAL LIFE MEDIATES ASSOCIATION BETWEEN PANDEMIC WORRIES AND RISK OF ELDER ABUSE

Louis To, Lok Man Leung, Debby Wan, Ronald Tsui, and Elsie Yan, *The Hong Kong Polytechnic University, Hong Kong, Hong Kong*

Social isolation is an important risk factor for elder abuse. This study investigated the mediating effects of pandemic-related disruptions of social life between worry about the pandemic and risk of elder abuse. A total of 1078 community dwelling older persons participated in this community survey. They provided information on their demographics, worry about the pandemic, pandemic related disruptions of social life, and risk of elder abuse as measured by a 10-item scale derived from Dong et al. (2015). Participants age ranged from 60 to 100 (mean= 76.2, SD=7.9), majority of them were female (81.6%), single, divorced or widowed (52.8%). Prevalence of risk of abuse was high (21.5%), with the most prevalent type being family conflicts at home (16.6%), followed by feeling uncomfortable with someone in the family (8.1%). Participants who were not in a marital relationship, of larger household, reported feeling worried about the pandemic and felt their social life disrupted were at greater risk of abuse. Logistic regression results show that pandemic related disruptions of social life partially mediated the association between worry about the pandemic and risk of abuse. In the final model, participants who reported feeling worry about the pandemic and those who reported that the pandemic has caused disruptions in their social life were at greater odds of reporting risk of abuse (OR=1.14 and 1.13 respectively, p<.05). The effects of the former diminished after the latter was entered into the model. This finding highlights the importance of social life in protecting older persons against abuse.

Abstract citation ID: igad104.3285

### DISRUPTING THE NEGATIVE AFFECT AND PAIN CONNECTION IN OLDER ADULTS: THE ROLE OF SOCIAL INTERACTIONS AND ENJOYMENT

Jennifer Graham-Engeland, Dakota Witzel, Erin Harrington, Christopher Engeland, Lynn Martir e, and Karina Van Bogart, *Pennsylvania State University, University Park, Pennsylvania, United States*

Although it is well-established that negative affect is bi-directionally linked with pain, little research has examined their relationship in daily life among older adults. Even less is known about what social factors may help attenuate the affect-pain connection. We examined whether social interactions and reported enjoyment in daily life were associated with an attenuated link between negative affect and two important pain outcomes (pain intensity and perceived interference from pain). This was examined among a socioeconomically and racially diverse sample of 317 older adults aged 70+ (Mage = 77.45 ; 67% women; 40% Black; 13% Hispanic) who were recruited from the Bronx, NY as part of the Einstein Aging Study and who completed ecological momentary assessments five times daily for 14 days. Three-level multilevel models were estimated, controlling for mild cognitive impairment (MCI) status, gender, age, education, body mass index, and average level positive affect for models that did not include enjoyment. In given moments, higher negative affect and lower enjoyment were associated with higher pain intensity and pain interference (ps<.05). Moment-level negative affect and enjoyment significantly interacted to predict both higher pain intensity (p=.005) and pain interference (p<.0001), with patterns suggesting a buffering effect of enjoyment. In addition, a three-way interaction emerged such that during moments when no interactions occurred and negative affect was lower than a person's average, there was a buffering effect of momentary enjoyment on pain intensity. Findings extend understanding of the affect-pain connection and the potential mitigating impact of social interactions and moments of enjoyment.

Abstract citation ID: igad104.3286

### DO OLDER ADULTS BELIEVE THEY ARE AGING SUCCESSFULLY IN THE CIRCUMPOLAR NORTH?

Britteny Howell[1], Amber Worthington[1], Dale Golden[1], and Leslie Redmond[2], *1. University of Alaska Anchorage, Anchorage, Alaska, United States*, *2. University of Manitoba, Winnipeg, Manitoba, Canada*

People's perspectives on successful aging may impact their own aging outcomes. To gain more insight, we surveyed a convenience sample of 58 adults aged 55+ years in urban Alaska about their perceptions of their own aging using Fowler's successful aging scale. Overall, older adults rated their aging in the middle of the 1-7 scale across all three questions: how well they are aging, how "successfully" they have aged up to now, and how they rate their lives these days (M=4.17, SD=1.16, range=1-7 where 1 = not at all successful and 7 = extremely successful). Correlational analyses indicated that as perceptions of successful aging increase, self-reported good general health (M=2.90, SD=1.00, range=1=5) increases, r(56)=.499, p<.001, and self-reported disability (M=0.71, SD=0.92, range=0-3) decreases, r(56)=-.27, p<.05. Despite a small sample size, these findings that perceptions of successful aging increased as general heath increased and self-reported disability decreased, suggest that participants may perceive successful aging and good general health to be positively correlated. Perceptions of successful aging were not related to age in this sample (M=71.98, SD=8.74), r(56)=.125, p=.349. This is promising, as it may indicate that older adults recognize good health and absence of disability as integral components for successful aging regardless of chronological age providing opportunities for targeted messaging in future interventions. Future work will explore opportunities to promote successful aging and overcome some of the challenges related to healthy aging in this urban location, including the Circumpolar environment, historical trauma of Indigenous peoples, and a lack of accessible healthcare across Alaska.

Abstract citation ID: igad104.3287

### DO PHYSICAL DISABILITY, FOOD INSECURITY, AND NEIGHBORHOOD COHESION MATTER IN DEPRESSION?

Jihyeong Jeong, and Paul Sacco, *University of Maryland Baltimore, Baltimore, Maryland, United States*

Although previous studies have explained the association between food insecurity and depression, there has been a dearth of studies that have examined this relationship with older adults with physical disabilities and explored other variables that could have potentially impacted this relationship. Given the social capital theory, recognizing community-level factors provides broader contexts about the physical and social environments of communities where older adults with physical disabilities live. Thus, this study aimed to explain how physical disability and food insecurity are related to depression and examine the buffering effect of neighborhood cohesion on the relationship between food insecurity and depression. We analyzed 5,668 older adults aged 60 years or over from the Health and Retirement Study 2020 Core Final Release. This study conducted logistic regressions to examine the buffering effects of neighborhood cohesion on the association between food insecurity and depression, including covariates. The results showed that physical disability and food insecurity were statistically significantly associated with depression. Interestingly, the hypothesis of the buffering effects of neighborhood cohesion was not supported. This result can be explained by characteristics of mental health that different factors beyond food insecurity and neighborhood cohesion may influence. In other words, neighborhood cohesion alone may not be able to explain its buffering effects on the relationship between food insecurity and depression. While the results of this study may seem contrary to social capital theory, future studies need to consider various moderating variables to understand how community and stressors such as food insecurity interact with mental health.

Abstract citation ID: igad104.3288

### DO RELIGION, CULTURE, RACE, OR ETHNICITY PLAY A ROLE IN CHOOSING PEG TUBE AT THE END OF LIFE?

Isabella Park[1], Guisela Hernandez[2], Frederick Lambert[3], Elisabeth Loccisano[2], Christian Nouryan[4], Catherine Cho[4], Asha Gajraj[4], and Edith Burns[5], *1. Northwell Long Island Jewish Forest Hills, Forest Hills, New York, United States*, *2. Northwell Health, Valley Stream, New York, United States*, *3. Northwell Health, Forest Hills, New York, United States*, *4. Northwell Health, Manhasset, New York, United States*, *5. Zucker School of Medicine at Hofstra/Northwell, Manhasset, New York, United States*

Although there is substantial evidence that Percutaneous Endoscopic Gastrostomy (PEG) causes significant morbidity and does not change mortality for patients with advanced dementia it continues to be used. We describe a quality initiative of Palliative Care Consultation (PCC) in conjunction with any new PEG evaluation. EMR review and interview with patient (and/or proxy) being evaluated for first-time PEG in two community hospitals within large health system in demographically diverse major metropolitan area. PCC teams provided consistent, culturally/religiously, language-sensitive education regarding PEG, conducted interviews about subsequent decision. Post-initiative PEG placement dropped from >75% to 17.6%, 76 patients received PCC with PEG evaluation. Average age 83.5 (range: 56-108), 55% female. Religion: 54% Christian, 7% Muslim, 10% Jewish, 11% non-religious, 18% other; 46% White, 37% Black, 8% Asian, 8% Hispanic. Common evaluation reasons: difficulty swallowing (30%), low PO intake (30%). Majority of patients had dementia (71%), stroke (18%), Parkinson's (8%). Most decision-makers (66%) hadn't received prior counseling regarding alternate nutrition options. After PCC, 17.6% chose PEG, 79.7% careful hand feeding, 2.7% remained unsure. Those choosing PEG mostly desired "guaranteed" nutrition despite risks (10/13), and/or a trial seeking patient recovery (5/13). There were no apparent differences in race/ethnicity, age, sex, or religion that distinguished those electing PEG. PCC initiative to support patients and families "choosing wisely" around PEG placement for those with advanced illness has resulted in significant reduction. Decision-makers mostly cited wish to guarantee nutrition while some hoped for patient recovery. Religious affiliation and sex were not cited as reasons in this group.

Abstract citation ID: igad104.3289

### DOES CRIMINAL VICTIMIZATION RAISE THE RISK OF IADL LIMITATIONS AMONG OLDER ADULTS?

Jinnan Ren, Madison Sauerteig-Rolston, Mallory Bell, and Kenneth Ferraro, *Purdue University, West Lafayette, Indiana, United States*

Many studies have analyzed the effect of victimization on children and young adults, but relatively few have explored its impact on older adults' well-being. Drawing from cumulative inequality theory, this study analyzed a sample of 11,860 respondents aged 50 and older from the Health and Retirement Study (HRS) to examine whether victimization experiences, measured by physical attack/assault, robbery/home burglary, and fraud, raise the risk of limitations in performing instrumental activities of daily living (IADLs). The analysis, which used zero-inflated negative binomial models to examine the onset and severity of IADL limitations, revealed that criminal victimization was associated with a higher likelihood of IADL limitations ($IRR=0.735$, $p< 0.01$), even after adjusting for demographic and health-related factors. Additional analyses examining the influence of each type of victimization showed that the experiences of a physical attack and robbery were related to IADL limitations. These findings demonstrate that criminal victimization experiences are associated with compromised IADL function, especially when the encounter involves an intentional act of immediate harm. There is a need for (a) additional research on the consequences of criminal victimization on the health and well-being of older adults and (b) interventions to help older adults who experience a personal or property crime.

Abstract citation ID: igad104.3290

### DOES POSTOPERATIVE GABAPENTIN ANALGESIA ADD TO DELIRIUM IN OLDER COLORECTAL SURGERY PATIENTS?

Ashna Rajan[1], Joao Filipe G Monteiro[1], Nadia Mujahid[1], Matthew Vrees[2], Steven Schechter[2], Lynn McNicoll[1], Stefan Gravenstein[1], and Mriganka Singh[1], *1. Brown University, Providence, Rhode Island, United States, 2. Brown Surgical Associates, Providence, Rhode Island, United States*

Our health system adopted a frailty adjusted low-(100 mg) or high-dose(300 mg) gabapentin regimen for opioid sparing postoperative analgesia for older colorectal surgery (CRS) patients to hasten return of bowel function and discharge. But, was it also associated with delirium? 1,306 elective CRS patients ≥ 65 years admitted from 4/2015-6/2023 were retrospectively studied, after excluding those receiving critical care, or hospitalized >3 months. Delirium was defined by ICD coding or postoperative antipsychotic use. Delirium rates were compared using multivariate logistic regression between no- (NG), low- (LG) and high- (HG) dose gabapentin, controlling for age, gender, race, Charlson Comorbidity Index (CCI), depression, preoperative gabapentin use and length of hospital stay. Bivariate, chi-square and t-tests were also used for comparison, as appropriate. NG recipients were found to be older (75.8±7.5) than LG (74.3±6.6, p-value=0.0041) or HG (73.4±6.8, p-value=0.0040), but similar by CCI (mean CCI:6.5±3.9) and depression (17.5%). While overall delirium rates were similar between all three groups, preoperative exposure to gabapentin (as a home medication) was associated with a 3-fold increase in delirium in the HG vs LG (OR 2.76 95% CI [1.25-6.08]) as well as HG vs NG (3.02 [1.38-6.59]) groups. Without gabapentin exposure preoperatively, the NG group was 3 times more likely to develop delirium compared to HG (3.09 [1.36-6.98]). Gabapentin may contribute to postoperative delirium but the unexpected greater delirium incidence among the NG compared to LG may reflect unmeasured confounders of greater underlying delirium risk, or a LG delirium sparing effect.

Abstract citation ID: igad104.3291

### EARLY DETECTION OF ALZHEIMER'S DISEASE AND RELATED DEMENTIAS FROM VOICE RECORDINGS: THE FRAMINGHAM HEART STUDY

Huitong Ding[1], Adrian Lister[2], Cody Karjadi[3], Rhoda Au[1], Honghuang Lin[4], Brian Bischoff[2], and Phillip Hwang[5], *1. Boston University, Chobanian & Avedisian School of Medicine, Boston, Massachusetts, United States, 2. Headwaters Innovation, Inc., Inver Grove Heights, Minnesota, United States, 3. Framingham Heart Study, Framingham, Massachusetts, United States, 4. UMass Chan Medical School, Boston, Massachusetts, United States, 5. Boston University School of Public Health, Boston, Massachusetts, United States*

With the aging global population and the increasing prevalence of dementia, there is a growing focus on identifying mild cognitive impairment (MCI), a pre-dementia state, to enable timely interventions that could potentially slow down neurodegeneration. Producing speech is a cognitively complex task that engages various cognitive domains, while the ease of audio data collection underscores the potential cost-effectiveness and noninvasiveness that voice may offer. This study aims to construct a machine learning pipeline that incorporates speaker diarization, feature extraction, feature selection, and classification, to identify a set of acoustic features that exhibit strong MCI detection capability. The study included 100 MCI cases and 100 healthy controls (HC) matched for age, sex, and education from the Framingham Heart Study. Participants' speech responses during cognitive tests were recorded, and the recorded audio was processed to identify segments of each participant's voice from recordings that included voices of both testers and participants. A comprehensive set of 6385 acoustic features was then extracted from these voice segments using the OpenSMILE and Praat softwares. Subsequently, we constructed a random forest model using the features that exhibited significant differences between the MCI and HC groups. We identified an optimal subset of 29 features that resulted in an AUC of 0.87, with a 90% confidence interval ranging from 0.82 to 0.93. This study showcases the potential of human voice as a valuable resource for improving early detection of ADRD and motivates future opportunities to use passive voice collection tools, such as hearing aids, to measure brain health.

Abstract citation ID: igad104.3292

### EARLY LIFE LEARNING DISABILITY, PREMATURE PARENTAL DEATH PREDICTS LATE LIFE COGNITIVE IMPAIRMENT

Allan Nkwata, and Jacqui Smith, *University of Michigan, Ann Arbor, Michigan, United States*

Recent studies have increasingly identified links between early learning difficulties and later life cognitive impairment. Additionally, the death of a parent adversely affects various outcomes across the life course, particularly when the death occurs earlier than expected. This study evaluated the impact of early learning problems and death of a parent on change in cognitive function over 10 years among older Americans enrolled in the Health and Retirement Study. Data were from respondents' early educational experiences collected in the 2015 and 2017 Life History Mail Survey and childhood stress measures were from the aggregated childhood histories (N = 10,775). Cognitive impairment was assessed per Langa-Weir's classification, which includes normal, cognitively impaired but not demented (CIND) and demented groups. Generalized Estimating Equations (GEE) regressions estimated odds ratios (ORs) and 95% confidence intervals (CI) with adjustment for socio-demographic and lifestyle factors. Having at least one early learning problem, and death of a parent before the age of 16 were each associated with increased risk of later life cognitive impairment over time (OR 1.60, 95% CI 1.45-1.76 and OR 2.02, 95%CI 1.83-2.24 respectively). However, learning problem-related differences in risk of cognitive impairment varied by parental death status over time (learning problems x death x time, p=0.05). The risk was highest, amongst those that reported both experiencing early learning problems and parental death relative to those without learning problems. Policies and interventions that enhance diagnosis of early learning problems and improve childhood social contexts are needed to promote healthy cognitive aging amongst older Americans.

Abstract citation ID: igad104.3293

### EFFECTS OF HIGH-INTENSITY AQUATIC EXERCISE ON INFLAMMATION AND COGNITION IN OLDER ADULTS WITH MCI

Peter Louras[1], Adriana Savettiere[2], Windy McNerney[1], and Kaci Fairchild[1], *1. VA Palo Alto Healthcare System, Palo Alto, California, United States*, *2. Palo Alto University, Palo Alto, California, United States*

Exercise shows immune benefits in older adults and can reduce the risk of late-life cognitive impairment and dementia. This study sought to explore the effects of high-intensity aquatic-based exercise on older adults with mild cognitive impairment (MCI), specifically by examining changes in inflammatory plasma protein concentrations and cognitive performance. A secondary data analysis was conducted on 20 healthy volunteers (mean age 68.2 ±7.75 years) who participated in 6 months of supervised aquatic exercise training. Participants completed a battery of neuropsychological assessments and whole blood draws at pre- and post-intervention. Plasma protein concentration levels were attained using the Olink Target96 Inflammation panel [Uppsala, Sweden]. Mixed effects models assessed the effects of protein concentration change scores on selected cognitive outcomes, while controlling for age. Results indicated that following the exercise intervention, significant changes in two inflammatory-related proteins (Extracellular newly identified receptor for advanced glycation end-products binding protein [EN-RAGE]; Interleukin-20 receptor subunit alpha [IL-20RA]) were associated with improved verbal memory and processing speed endpoints. Specifically, decreased EN-RAGE concentrations resulted in significantly improved delayed verbal memory scores (parameter estimate= -0.893, p= .023), while increased IL-20RA concentrations led to better task completion (parameter estimate= 2.145, p= .030). These findings demonstrate the cognitive benefits of aquatic-based exercise for older adults and those with MCI, and suggest these effects are mediated by alterations in pro- and anti-inflammatory protein markers such as EN-RAGE and IL-20RA. Larger studies are warranted to replicate these findings, and to further explore the role of activity-induced inflammatory changes on late-life brain health.

Abstract citation ID: igad104.3294

### EFFICACY OF MALNUTRITION SCREENING AMONG OLDER VETERANS AT AN URBAN VA HOSPITAL

Sarah Cassatt[1], and Elizabeth Parker[2], *1. UMBC, Baltimore, Maryland, United States*, *2. UMSOM, Baltimore, Maryland, United States*

Nearly half of the Veteran population is >65 years. Older Veterans are at increased risk of malnutrition associated with increased mortality, morbidity, length of stay, and readmissions. Malnutrition-focused quality measures are needed to combat this public health issue. The purpose of this study was to identify gaps in malnutrition screening at an urban VA facility. Chart auditing was conducted for all medical admissions during 72 hour periods across 14 months. Auditing determined if nutrition screening was completed, with an admission weight, and if nutrition was consulted for nutritionally compromised Veterans. Screening was conducted using the Malnutrition Screening Tool (MST). Additionally, estimated prevalence of nutritionally compromised Veterans was assessed using auditing data from 30 days. Audit data showed that on average only 57.4% (n=458) of Veterans admitted for inpatient care were screened correctly. Of those screened incorrectly, the three most common errors were 1) not collecting a weight on admission (N=82 (42.1%)); 2) inaccurate weight change interpretations (N=41 (21%)); 3) failure to refer nutritionally at-risk Veterans to nutrition services (N=38 19.5%)). Prevalence auditing demonstrated over 1/3 of Veterans were nutritionally compromised at the start of their hospitalization (36.43%, (N=295)). With an increasingly aging population in the United States there is a focus on shifting to value based payment systems. The new CMS Global Composite Malnutrition Score will include evaluation of hospitals malnutrition screening processes. There is a significant opportunity to close these gap areas and to adopt clinically relevant best practices to provide nutrition care earlier to at risk Veterans.

Abstract citation ID: igad104.3295

### EGG CONSUMPTION AND INCIDENT CARDIOVASCULAR DISEASE: VARIATION BY DIETARY PATTERN

Donna Kritz-Silverstein, and Blake Blake Anuskiewicz, *University of California San Diego, San Diego, California, United States*

Some studies link egg consumption with cardiovascular disease, but none considers modification by dietary pattern. This study examines effects of egg consumption on incident cardiovascular disease (CVD), and variations of associations within the context of dietary pattern. Participants were 4,930 men and women from the Rancho Bernardo Study without prevalent CVD in 1972-74, who were followed through 2021 via mailed surveys, follow-up visits, and death certificates. Dietary data from 1988-91 enabled calculation of the alternate Mediterranean diet (aMed) score for 1632 individuals. Comparisons showed those with incident CVD (N=2066, 49.1%)were older (p< 0.001), had higher BMI (p=0.004), cholesterol, triglycerides, systolic and diastolic blood pressures (p's< 0.001) than those without incident CVD, but did not differ in egg consumption. Those consuming more eggs/week were older (p< 0.001) and had higher BMI (p=0.002). Men consumed more eggs/week than women (means=4.2 vs. 3.2, p< 0.001). Adjusted Cox proportional hazards models showed there were no significant associations of egg consumption with incident CVD in men or women (HR=1.00, 95%CI=0.98-1.02; HR=1.01, 95%CI=0.99-1.03, respectively). However,analyses by dietary patterns showed that in men, but not women, increased egg consumption was associated with a 7% reduced risk of incident CVD (HR=0.93; 95%CI=0.88-0.98) among those with high aMed scores, but a 6% increased risk (HR=1.06; 95%CI=1.01-1.11) among those with low aMed scores. In conclusion, for men, dietary pattern modified associations such that among those with a healthier dietary pattern, egg intake was associated with lower incident CVD. For women, egg intake was unassociated with incident CVD regardless of dietary pattern.

Abstract citation ID: igad104.3296

### EGG CONSUMPTION: TRENDS OVER TIME, PATTERNS ACROSS THE LIFESPAN, AND PREDICTORS OF INTAKE

Donna Kritz-Silverstein, and Ricki Bettencourt, *University of California San Diego, San Diego, California, United States*

Eggs are an excellent nutritional source. However, the historical association of dietary cholesterol with serum cholesterol and thus, cardiovascular disease, along with restrictive dietary guidelines may have served as a barrier to egg consumption. This study examines trends over time, and predictors and patterns of egg consumption in older individuals followed for over 45 years. Participants were 6326 men and women enrolled in the Rancho Bernardo Study in 1972-74 when they were asked about the number of eggs consumed/week. Subsequent egg intake information was collected with food frequency questionnaires from subsamples attending clinic visits in 1988-91 (N=1627) and 1992-96 (N=1385), and with the original question on a 2021 mailed survey (N=710). Mean(□SD) eggs consumed was 3.6□3.0 in 1972-74, 1.8□2.1 in 1988-91, 1.8□2.2 in 1992-96, and 3.4□3.5 in 2021. Comparisons within 5-year categories of baseline age (< 20, 20-24, 25-29, 30-34, 35-39, >40) showed no differences in egg intake between 1972-74 and when older in 2021. Men consumed more eggs than women at all timepoints (p's< 0.0001). Those with higher education (p< 0.0001) and diabetes (p=0.0009) consumed more eggs; those with high cholesterol or taking cholesterol lowering medication consumed fewer eggs in 1972-74 (p's< 0.0001), but there were no differences by these factors by 2021. Egg consumption appears responsive to dietary guidelines, decreasing over time then increasing by 2021 to levels similar to the early 1970s. Among those young and middle-aged at baseline, egg intake patterns were similar 45 years later, when older. Cholesterol and cholesterol-lowering medication use are no longer factors in egg intake.

Abstract citation ID: igad104.3297

### ELDER ABUSE RISK ASSESSMENT MODEL FOR ALZHEIMER'S DISEASE WITH SOCIAL DETERMINANTS OF HEALTH AND MEDICAL FACTORS

Monique Pappadis, Leila Wood, Jordan Westra, Karen Schlag, Rebecca Czyz, Yong-Fang Kuo, Mukaila Raji, and Charles Mouton, *University of Texas Medical Branch at Galveston, Galveston, Texas, United States*

Older adults with Alzheimer's Disease and Related Dementias (ADRD) are at an increased risk for elder abuse (EA). Using a 20% national Medicare database, we developed an EA risk-assessment model, including older adults with and without ADRD. Beneficiaries aged 66 and older in 2016, without an EA diagnosis in 2015 or 2016, and Medicare Part A/B/D eligible with no HMO through 2018 were included (N=2,261,166; ADRD, n=187,805, 8.3%). Outcome of interest was an EA diagnosis. The cohort was split into three subsets, training (50%), testing (25%), and validation (25%). Predictive models included demographics, comorbidities, symptoms, injury history, claims-based frailty scores (0-1), medical screening and procedures, and social determinants of health (SDoH). Only 0.2% had an EA diagnosis. EA models were conducted using logistic regression and machine learning methods (random forest, gradient-boosted tree classification, and multilayer perceptron classification). The logistic regression model was the best predictive model (AUC=0.73; Sensitivity=0.80; Specificity=0.47; GINI Impurity=0.50; Agreement=46.77%). Many SDoHs (e.g., primary support and marital problems, housing/income problems and dual eligibility, Black Race), STI testing, behavioral health and medical conditions, and greater frailty were associated with increased risk of EA at 2 years for the total sample (with and without ADRD). For those with ADRD, SDoHs (e.g., marital and housing/income problems), male sex, aged 71-75 (vs 66-70), lung cancer, PTSD , and greater frailty were associated with increased EA risk. Coupled with qualitative interviews, our findings will guide the selection of the most pertinent risk and protective factors to incorporate into an EA screening intervention tool.

Abstract citation ID: igad104.3298

### ELDERLY CARE POLICY IN CHINA: ITS EVOLUTIONARY PATH AND IMPLICATIONS ON GOVERNANCE

Qingwei Wang[1], Yu Ma[2], and Jiayu Chen[1], *1. Xi'an Jiaotong-Liverpool University, Suzhou, Jiangsu, China (People's Republic)*, *2. University of Bristol, Bristol, England, United Kingdom*

Over the years, the Chinese elderly care policy has undergone significant changes, leading to the systematic development of a comprehensive elderly care service system. This study explores the pivotal policies formulated by central policy-making bodies and examines the historical and current state of the elderly care system in China. The findings demonstrate a consistent alignment between the system's evolutionary trajectory, the practical expectations of the population, and the Communist Party of China's (CPC) objective of fostering a unified and balanced society. The study also highlights inherent issues concerning governance mechanisms and role-position within the system. Notably, we highlight the successful protection of older adults during the COVID-19 pandemic as an exemplary care of care service provision and draw lessons from international experience.

Abstract citation ID: igad104.3299

### EMPOWER@HOME:CONNECTED - TESTING A PILOT GROUP INTERVENTION FOR DEPRESSION AND LONELINESS IN OLDER ADULTS

Jay Kayser, Skyla Turner, and Xiaoling Xiang, *University of Michigan, Ann Arbor, Michigan, United States*

Homebound older adults experience high rates of depression, social isolation, and loneliness, which adversely impacts physical and mental well-being. Accessing psychotherapy to address these concerns can be logistically challenging and cost prohibitive. This study examined the feasibility and preliminary effect of a novel group-based internet cognitive-behavioral therapy program called Empower@Home:Connected. The intervention was implemented over nine consecutive weekly sessions in small virtual groups (4-6 participants). These sessions were facilitated by trained mental health professionals and incorporated psychoeducational material, cognitive-behavioral therapy exercises, and socialization. Measurements on primary and secondary outcomes were gathered before and after completion of the intervention. In this preliminary pilot trial (NCT05732740), participants averaged 68 years and of those that attended the first session (n = 25), nearly all completed the program (n = 24). Session attendance was high, with an average session attendance rate of 94.70%. Following program completion, participants had statistically significant reductions in depressive symptoms (Cohen's d = 1.01). Participants also had reductions in loneliness that trended towards statistical significance (p = 0.07); changes in social isolation were not statistically significant. Of the participants, 21 out of 23 expressed satisfaction with the program and would recommend it to peers. For secondary outcomes, analyses revealed significant improvements in anxiety and health-related quality-of-life. In this pilot feasibility trial, Empower@Home:Connected reduced depressive symptoms among older adults. The high adherence and satisfaction rates suggest acceptability. While the intervention produced improvements on several measures, further exploration is warranted to determine long-term impact and efficacy relative to other existing interventions.

Abstract citation ID: igad104.3300

### END-OF LIFE CARE PLANNING: THE ROLE OF FAMILY FOR VIETNAMESE AMERICANS AND FILIPINO AMERICANS

Carson De Fries[1], My Ngoc To[1], Fei Sun[2], Bei Wu[3], Sue Levkoff[4], and Kaipeng Wang[1], *1. University of Denver, Denver, Colorado, United States*, *2. Michigan State University, East Lansing, Michigan, United States*, *3. New York University, New York City, New York, United States*, *4. University of South Carolina, Columbia, South Carolina, United States*

Research has demonstrated a lack of understanding around the significant disparities in end-of-life (EOL) care decisions and culturally sensitive EOL care practices among older Southeast Asian Americans (SAA). Little is known regarding the factors related to the experiences and attitudes toward family involvement in EOL care, especially among these populations. This study explored how family relationships shape attitudes towards and experiences of family involvement in EOL care among older Vietnamese American and Filipino Americans. Three focus groups consisting of one Filipino group (in English), and two Vietnamese groups (one in English, one in Vietnamese) were held with 12 participants aged 55 or above. Interview questions centered around EOL care preferences and experiences and the role of family relationships in EOL care. Participants reported they could trust their children the most with their EOL care wishes, and expressed the preferred place for EOL care to be "home" rather than institutional settings. While some reflected that previous experiences with older family members (e.g., parents, aunts and uncles) facilitated EOL care discussions, others acknowledged resistance from some in participating in these conversations. Cultural and language differences led to participants' resistance to formalized care and hindrances to EOL care planning. Finally, financial cost, concern about burdening younger generations who "have their own lives", and difficulty navigating information were cited as barriers to EOL care. Findings from this study can provide improved understanding of the role that family plays in individuals' EOL care experiences and improve culturally responsive EOL care practices for SAA populations.

Abstract citation ID: igad104.3301

### END-OF-LIFE DOULAS: EXPLORATION OF VALUES, GOALS, AND MOTIVATIONS

Amber Thompson, *University of Utah, Salt Lake City, Utah, United States*

End-of-life doulas provide non-medical support to individuals and families during end-of-life. Current research focuses on identifying the doula role and services offered at end-of-life, primarily in relation to existing medical care. The findings highlight the ambiguity of the role and resulting confusion for healthcare providers, individuals, and families. Using data collected from on-line surveys, the aim of this study was to explore the values, goals, and motivation of those identifying as end-of-life doulas (n=232). In addition to questions about the role and services provided, 14 items assessed the doulas' underlying values through goals and motivations. Questions addressed their motivation for becoming a doula and their goals for this role, such as: 1) promoting well-being during end-of-life 2) providing a less medicalized model, including advocating for death reform, and 3) wanting to professionalize and/or commodify the non-medical care they provide. Exploratory factor analysis supports two underlying factors describing the doulas' goals and motivations including social values (i.e., perceived positive impact on society) and functional values (perceived problem solution) (Cronbach's alpha .85 and .75 respectively). Content analysis of open-ended survey responses describing motivations for becoming an end-of-life doula strengthen this perspective and expand it to include emotional motivations (i.e., "called" to the work, or personal experiences with death). These unique findings demonstrate the need for more research documenting the underlying values organizing end-of-life doulas. The potential benefits of investigating values include progress towards clarification about the role of end-of-life doulas, and how they may contribute to providing person-and-family centered care during end-of-life.

Abstract citation ID: igad104.3302

### ENGAGING DIVERSE OLDER ADULTS IN AGING RESEARCH: IDENTIFYING COMMUNICATION AND COMMUNITY ENGAGEMENT STRATEGIES

Trudy Gaillard[1], Dawn Carr[2], Julia Sheffler[3], Ursula Weiss[2], Donna Neff[4], Neil Charness[2], and Fern Webb[5], *1. Florida International University, Miami, Florida, United States*, *2. Florida State University, Tallahassee, Florida, United States*, *3. Florida State University College of Medicine, Tallahassee, Florida, United States*, *4. University of Central Florida, Orlando, Florida, United States*, *5. University of Florida, Gainesville, Florida, United States*

A growing challenge is ensuring that diverse older adults participate in research studies. If studies are not inclusive of diverse populations, it is not possible to determine if interventions are likely to be universally beneficial or reduce health disparities. Smaller and more regional studies are particularly likely to struggle to recruit and retain diverse older adults for their research. This project uses quantitative and qualitative data based on a new collaboration consisting of independent scholars at four research institutions associated with three participant research registries on aging in Florida. The aims of this project are to: 1) describe characteristics of aging-focused research registrants, and 2) share communication strategies for recruitment specifically designed to increase participation of a variety of underrepresented populations, including minority, rural, and under-served. Preliminary results show that it is important to specifically adopt culturally informed recruitment strategies given its direct result in adequate representation from diverse and under-represented groups. All three participating registries are currently enrolling persons and seek a specific diverse demographic which captures the racial, cultural and geographic variation observed in residents throughout Florida. For example, these registries seek individuals who self-identify as African American/Black, Caribbean, Hispanic/Latino adults aged > 55 years; persons living in rural communities, and persons who have survived cancer or are interested in cancer-related research. We describe current infrastructure employed at each institution as well as recommendations in building long-term trust across each collaborating institution. Finally, we share an innovative model leveraging strengths and opportunities for statewide engagement across each registry.

Abstract citation ID: igad104.3303

### ENGAGING WITH AGING: OLDER ADULTS' ADAPTATIONS TO URINARY CONCERNS

Kuan-Ching Wu, Shaoqing Ge, Shao-Yun Chien, Jessica Jin, Suah Park, and Basia Belza, *University of Washington, Seattle, Washington, United States*

Urinary concerns (UC) increase with age impacting health and quality of life. The aims of this study were to describe: 1) UC as an age-related change (ARC); 2) the challenges of UC; 3) adaptation strategies used to manage UC; and 4) the value of engaging with aging (EWA) as a framework to promote self-management of UC. Data was used from semi-structured interviews with 29 older adults (mean age 77 years). An iterative coding process was used. A codebook was developed based on a-priori themes derived from the EWA framework, our previous publication, and a line-by-line coding of one of the transcripts. As the analysis progressed, additional codes emerged, enriching the codebook. Six themes emerged: 1) the participants' experiences of UC; 2) responses to UC, 3) adaptation and management strategies; 4) knowledge and understanding of UC; 5) available capacities and resources; and 6) the impact of the COVID-19 pandemic on UC. Participants tended to address their UC by adjusting routines, medication schedules, or diet patterns. They tried to secure restroom locations or use tools or reminders to resolve their UC. COVID-19 led to increased inconvenience for older adults to engage in outdoor activities due to the closure of public restrooms. Through a comprehensive qualitative study and in-depth analysis of older adults' UC and adaptations, these participants developed personalized adjustments to address their needs and abilities to their UC. These findings offer insights into the individual aging experience, which will further enhance our understanding and advancement of person-centered care.

Abstract citation ID: igad104.3304

### ENHANCING CAPACITY OF SERVICE PROFESSIONALS TO PREVENT FINANCIAL EXPLOITATION IN RURAL OLDER ADULTS WITH DEMENTIA

Fei Sun[1], Zhenmei Zhang[1], Ha Neul Kim[1], Xuehan Zhang[1], Yuan Xiong[1], Huiyu Yang[2], and Xia Wang[3], *1. Michigan State University, East Lansing, Michigan, United States*, *2. Nanjing University of Information, Science &Technology, Nanjing, Jiangsu, China (People's Republic)*, *3. Arizona State University, Phoenix, Arizona, United States*

Cognitive impairment renders older adults more vulnerable to financial exploitation (i.e., financial abuse committed by family members, or someone known to victims, and financial fraud committed by strangers). This study developed and tested the efficacy of a community-based financial exploitation prevention model for older adults living with dementia in rural Michigan. Guided by the routine activity theory, we chose to address external risk factors for financial abuse and fraud by focusing on service professionals (i.e., social and health care providers) who are at stake for identifying and reporting abuse. Using a randomized control design, we assigned 52 service professionals in intervention groups that received training on dementia and financial exploitation and available community resources, and 39 in control groups that received community resources only. Participants were assessed at baseline and 3 months following the intervention, while 26 dropped out at 3-month follow-up. Linear mixed modeling (LMM) was used to examine time and group effects in dementia literacy, understanding of financial exploitation, and competence in handling financial exploitation. Both groups reported an increase in their knowledge of dementia and their competence of handing financial exploitation cases. Moreover, those in the intervention groups reported more understanding of risk factors for financial abuse committed by family members. Community service agencies comprise a strong safety network for vulnerable older adults at risk for financial exploitation. Policies and practice need to be in place to sustain the coordinated efforts among community service agencies and adult protective services to prevent financial exploitation.

Abstract citation ID: igad104.3305

### EPIDEMIOLOGIC PATTERNS OF POTENTIALLY INAPPROPRIATE MEDICATION USE IN OLDER ADULT DRIVERS

Jesse Cotton[1], Beicheng Huang[2], Stanford Chihuri[2], Barbara Lang[2], and Guohua Li[2], *1. Columbia University Vagelos College of Physicians & Surgeons, New York City, New York, United States*, *2. Columbia University, New York City, New York, United States*

Use of potentially inappropriate medications (PIMs) by older adult drivers has been linked to increased crash risk. The objective of this study was to examine the epidemiologic patterns of PIM use in a cohort of 2990 drivers aged 65-79 years at the time enrollment (July 2015 to March 2017). We applied the 2023 Beers Criteria to identify PIMs from medication data collected through the “brown-bag review” method at baseline, year 2 and year 4 of follow-up for older adults participating in the Longitudinal Research on Aging Drivers project. We assessed time trends in the prevalence of PIM use and the composition of PIMs using the chi-square tests. The prevalence of PIM use was 16.2% at baseline, which decreased slightly in year 2 (14.0%) and year 4 (13.2%). The three most frequently used PIMs were nonbenzodiazepine hypnotics, antidepressants, and estrogens, accounting for 19.4%, 17.8% and 14.1% of all PIMs, respectively. Of PIM users, 12.7% were using ≥2 PIMs at baseline, 12.5% in year 2, and 10.0% in year 4. PIM use among older adult drivers is fairly common. Epidemiologic patterns of PIM use among older adult drivers have remained stable during the four years of follow-up.

Abstract citation ID: igad104.3306

### EPISTASIS ASSOCIATION STUDY TO IDENTIFY NETWORK HUB GENES IN ALZHEIMER'S DISEASE AND RELATED DEMENTIAS

Luke Evans, Chandra Reynolds, and Charles Hoeffer, *University of Colorado Boulder, Boulder, Colorado, United States*

Despite recent advances in understanding ADRD genetic risk, the vast majority of the genetic variance of AD/ADRD risk remains unexplained by identified loci such as APOE or the polygenic signal. One unexplored reservoir of genetic effects is interactions among genes (epistasis), which could identify key and novel molecular pathways, networks, and specific cell types in which the ADRD genetic risk is manifest. We tested all pairwise gene-gene interactions using our recently-developed transcriptome-wide interaction study method on ADRD in the UKBiobank. While no individual epistatic association was genome-wide significant ($p< 2.56e-10$), several approached significance ($p< 3e-8$). More importantly, we identified three core genes with >10 suggestive epistatic interactions. These include genes with some limited, prior evidence of a role in ADRD etiology, but none that have been identified in single-locus GWAS. In follow-up enrichment analyses of neuronal annotations underlying the interaction associations, we found genes intolerant to protein truncating mutations (PI), and those expressed specifically in endothelial cells, excitatory neurons in the prefrontal cortex , neuronal stem cells, and PI excitatory dentate gyrus cells to be nominally enriched ($p< 0.05$) in interaction associations with ADRD using prefrontal cortex imputed expression. Together, these suggest that we can uncover novel aspects of AD biology through these interaction associations and identify genes that may play a key role in integrating signals of AD risk. We anticipate identifying additional core genes and pathways as we integrate other larger and more diverse datasets.

Abstract citation ID: igad104.3307

### ETHICAL CONSIDERATION IN HOME MONITORING ASSISTIVE ROBOT FOR COGNITIVE IMPAIRMENT: A SCOPING REVIEW

Jing Wang[1], Dain LaRoche[1], Momotaz Begum[1], Cassidy Choquette[1], Xueting Tang[2], and Sajay Arthanat[1], *1. The University of New Hampshire, Durham, New Hampshire, United States*, *2. Fudan University, Shanghai, Shanghai, China (People's Republic)*

As the aging population continues to grow, the demand for innovative technologies to support individuals with cognitive impairment in their daily lives has risen significantly. Home monitoring assistive robots (HMAR) have emerged as a promising solution, offering assistance with tasks, scheduling and safety monitoring. This scoping review aims to explore and synthesize the ethical considerations surrounding the use of HMARs for persons with cognitive impairment. We adopted the Arksey and O'Malley framework, encompassing five stages: identifying the research question, identifying relevant studies, selecting studies, charting the data, and summarizing and reporting results. A comprehensive search strategy was employed across electronic databases, including PubMed, PsycINFO, Scopus, and CINAHL, using search terms derived from titles, abstracts, and keywords identified in key publications and previous reviews relevant to the scope of this review. The synthesis of the 25 included literature highlights several key ethical dimensions related to HMARs for individuals with cognitive impairment, including autonomy and decision-making, privacy and surveillance, family dynamics and dyadic relationships, human-robot trust, cultural considerations, and equitable access to technology. The review underscores the complexity of ethical dilemmas posed by HMARs, including issues of informed consent, data security, power differentiation between persons with cognitive impairment and their caregivers, and disparities in technology access. The findings of this scoping review contribute to the ongoing discourse on the ethical implications of utilizing HMARs for cognitive impairment and provide insights for the development of responsible and inclusive technological solutions.

Abstract citation ID: igad104.3308

### EVALUATING AGING IN PLACE LONG-TERM CARE REFORMS: A SCOPING REVIEW

Janet MacNeil Vroomen[1], Isabelle Vullings[1], Miranda Langendam[1], Joost Wammes[1], Dionne Kringos[1], Bram Wouterse[2], and Emiel Hoogendijk[1], *1. Amsterdam University Medical Centers, Amsterdam, Noord-Holland, Netherlands*, *2. Erasmus University Rotterdam, Rotterdam, Zuid-Holland, Netherlands*

There is a global trend of reforming long-term care to enable aging in place making it one of the most pressing health policy issues in both high- and low-middle income countries. However, there is no agreement on what is a successful aging in place reform. A holistic perspective that includes clinical, social and economic indicators is required to evaluate these reforms. In this scoping review, we aim to determine the extent of literature on evaluating aging in place reforms. We used the guidance of the JBI Scoping Review Methodology Group to conduct the scoping review. Articles on evaluation of aging in place reform and reporting on any outcome, process or structure quality indicator were eligible for inclusion. The search strategy was performed in Ovid Medline, Ovid Embase, and Ebscohost Academic Search Premier. There were no restrictions on publication year, language, and study design. The search identified 8,107 records. A total of 84 articles met the inclusion criteria and were included in the scoping review. Most studies were quantitative, observational studies from high income countries, using national population level data or larger cohorts. Frequently reported indicators were: total expenditures on long-term care, program spending per capita, hospitalization rates, length of stay, and home care utilization. Less reported were older adult and informal caregiver related indicators like: health, well-being, informal caregiver hours, and sleeping hours. Policy makers can use our findings to develop appropriate strategies to evaluate and monitor their own aging in place reforms.

Abstract citation ID: igad104.3309

### EVALUATING STOCHASTIC EPIGENETIC MUTATIONS: RELIABILITY, SUBTYPES, AND AN ALTERNATIVE DETECTION APPROACH

Yaroslav Markov[1], and Albert Higgins-Chen[2], *1. Yale Graduate School of Arts & Sciences, New Haven, Connecticut, United States*, *2. Yale School of Medicine, New Haven, Connecticut, United States*

Stochastic Epigenetic Mutations (SEMs) have emerged as promising biomarkers for aging and age-related disorders.

Conceptually, a SEM is defined as an outlier in DNA methylation (DNAme) value at a specific genomic site, compared to a given population or cohort. These mutations are seldom shared among subjects, emphasizing their potential to capture the heterogeneity of age-associated DNAme changes, and their cumulative count in individuals rises with age. We hypothesized that the original statistical method for SEM detection is not well suited for DNAme data and demonstrated its reliability constraints at both the individual and aggregate levels, using technical replicates from whole blood samples. In response to these challenges, we explored the factors influencing individual SEM reliability and identified best practices for SEM detection. We also developed an alternative machine learning-based detection method. Importantly, our classifier demonstrated improvements in the proportion of shared SEM and ICC scores when tested in an independent dataset with technical replicates, maintaining the associations of higher SEM loads with age, mortality, and cardiovascular disorders in the FHS study cohort. To support the wider application of our findings and address the need for SEM analysis tools, we have introduced a novel R package, SEMdetectR. Leveraging parallel programming, this package offers rapid SEM identification using both the original and new methods, accompanied by robust downstream analysis features.

Abstract citation ID: igad104.3310

### EVALUATING THE SENIOR EMPLOYMENT AND SOCIAL ACTIVITY SUPPORT PROGRAM IN KOREA: A SYSTEMATIC REVIEW OF SATISFACTION

Young Bin Koh, and Yeonjung Lee, *Chung-Ang University, Seoul, Republic of Korea*

For older people, jobs provide not only income security, but also a social role. Jobs also give them a sense of subjective security in terms of maintaining self-esteem and self-identity, and improve their overall life satisfaction. The Senior Employment and Social Activity Support Program (SESAP) provides for older Koreans for supplemental income security and social participation in old age by creating and providing social jobs that match the aptitudes and abilities of the senior citizens. It has been 20 years since the SESAP was introduced, and many studies have accumulated to evaluate the program in many different aspects. This study aims to summarize the existing studies focusing on satisfaction with program among participants. A systematic literature review was conducted to identify empirical research studies written in Korean and published in a peer-reviewed journal between 2004 and 2023. To this end, we searched through RISS, KISS, and DBpia databases using the selected keywords and obtained 126 articles. After removing duplicates and excluding gray literature, 26 articles were selected and analyzed through a peer-review process. Analysis of the selected literature shows that a majority of the program participants are women, and satisfaction with salary was lower compared to other types of program satisfaction (i.e., job content, work environment, and relationship with co-workers). Lastly, satisfaction with program are associated with other indicators such as better life satisfaction, and quality of life. This systematic review provides suggestions for further research to improve the satisfaction with SESAP among participants.

Abstract citation ID: igad104.3311

### EVALUATION OF AN OFF-THE-SHELF, WEARABLE ACTIGRAPH SYSTEM FOR USE BY OLDER COUPLES

Cynthia Jacelon, Khadijat Adeleye, Raeann LaBlanc, Sara Mamo, and Rebecca Spencer, *University of Massachusetts Amherst, Amherst, Massachusetts, United States*

There are many off-the-shelf wearable devices that provide the user with information regarding their physical activity and sleep patterns. Little is known about how easy it is for older individuals to use these devices. We tested a wrist-worn actigraph on couples both of whom were at least 70 years old. The aim was to evaluate the usability of the device, and to share data within the couples. Mixed methods were used to collect data. Over the three-week trial, 14 couples, nine of which had one member with mild cognitive impairment, used the system. Each person wore a Garmin Vivofit 4 continuously and used the Garmin application installed on an iPad mini to compare and review results over time. At the end of weeks one and three, participants completed the positively scored System Usability Scale (SUS), and each week met with the researcher to discuss the data and system usability. All couples were able to work together to use the system after being trained. Scores on the SUS indicated the system was moderately easy to use. Qualitative findings were that most participants (88%) liked being able to wear the device continuously, without breaks for charging. They compared information about their activity and sleep patterns within the dyad. There were several age-specific usability challenges, but use of off-the-shelf actigraphs by older individuals holds promise if age-related physical and cognitive changes are considered during selection of the device.

Abstract citation ID: igad104.3312

### EVALUATION OF THE ILLINOIS PUBLIC HEALTH ASSOCIATION ALZHEIMER'S DISEASE EARLY DETECTION AND AWARENESS CAMPAIGN

Julie Bobitt[1], Zohaib Zahir[2], and Krissy Roseberry[2], 1. *University of Illinois Chicago, Chicago, Illinois, United States*, 2. *Illinois Public Health Association, Springfield, Illinois, United States*

Of the 55.8 million adults aged 65 and over in the US, nearly 12% are at risk for or have subjective cognitive decline (SCD), yet more than half have not seen a healthcare provider about their SCD. Furthermore, studies show that fewer than 50% of individuals with Alzheimer's or a related dementia (ADRD) have been diagnosed by a physician. Missed diagnoses of dementia are more common among Blacks/African Americans than Whites and both Black/African American and Latinx individuals with cognitive impairment are less likely than Whites to report that a physician has diagnosed them. To adequately address community needs for ADRD early detection and awareness, the Illinois Public Health Association (IPHA) established the Alzheimer's Disease Early Detection and Awareness Campaign and partnered with community-based organizations (CBOs) and their community health workers to educate underserved populations about the risk for ADRD and the importance of early screening, and to assist clients in achieving referral to ADRD care services. To evaluate the campaign, IPHA partnered with the University of Illinois Chicago. We used the RE-AIM (Reach, Effectiveness, Adoption, Implementation,

Maintenance) implementation outcomes framework to guide the evaluation and share quantitative and qualitative results including the number of participants that reached our target audiences, effectiveness of the program on participant knowledge and behaviors, adoption of the campaign elements by the CBOs, implementation barriers and facilitators, and intentions for program maintenance. Results and lessons learned can be used to inform awareness and education efforts in other communities with special emphasis on reaching underserved populations.

Abstract citation ID: igad104.3313

### EXAMINATION OF STAFFING STABILITY IN LONG-TERM CARE

Winnie Sun, Jen Calver, and Farzana Rahman, *Ontario Tech University, Oshawa, Ontario, Canada*

**Background:** Long-term care (LTC) homes in Ontario, Canada face challenges of recruiting and retaining qualified staff prepared to address resident care needs. **Objectives:** To gain knowledge of the barriers and facilitators associated with recruiting and retaining nurses (RPNs/RNs) and personal support workers (PSW) in LTC. **Method:** Online survey questionnaires were distributed to staff working in a municipal home, and to PSW and nursing students. Staff participants (n=93) ranked how they perceived their work in LTC during COVID-19 to inform staffing stability efforts, and were invited to elaborate further on their responses using open-ended questionnaire. Nursing student participants (n=11) ranked how they perceived working in LTC. **Results:** Findings revealed that staff participants (57%) reported intentions to remain in their LTC employment, leaving about 43% of the workforce at risk of leaving. Four themes emerged from this study: (1) Embracing resident centred care as the top priority; (2) Rebuilding a health workplace through enhanced leadership and organizational support; (3) Promoting quality of care through open communication and professional development opportunities; and (4) Transforming work scheduling policies and staffing practices to support workforce retention. **Conclusion:** Senior leaders and LTC organizations play a critical role in refocusing staffing stability efforts. To help make LTC a workplace of choice, major changes must be considered to include greater visibility and presence of leadership, ongoing training and education, and revisit scheduling policies and practice with input from part-time and casual staff.

Abstract citation ID: igad104.3314

### EXAMINING AGING AMONG GUANAJA, HONDURAS ISLANDERS THROUGH THE UTILIZATION OF SOCIAL EXCLUSION FRAMEWORKS

Lee Ann Ferguson, *UMBC Erickson School of Aging Studies, Baltimore, Maryland, United States*

Guanaja is an island municipality of Honduras located approximately 70 miles from its parent island . Of the country's three main island municipalities, it is the second largest. The central municipality of Guanaja is located less than half of a mile from the coast of Guanaja and is a 100-acre floating island city built over two smaller cays which consists of densely compacted, stilt-built homes and houses approximately 3,000 people. It is estimated that 6.6% of the total population are over the age of 65. The current body of both spatial and social exclusion research considers environmental conditions may act in ways which may restrict or deny access or choices for aging inhabitants. Due to its remote geographic location, the island of Guanaja reflects key risk factor for age-related social and spatial exclusion. Framed by two social exclusion frameworks, this study, which was the cornerstone of this author's dissertation, presents the reality of island aging among Guanajan residents as relayed by them during this focused ethnographic study. The results of this work will describe the influences on Guanajan aging from factors including geographic environment and location, neighborhood and community, civic engagement and participation, social relationships, material and financial resources, socio-cultural practices, and governmental policies and practices.

Abstract citation ID: igad104.3315

### EXAMINING DEMENTIA AND NONDEMENTIA CAREGIVERS' NUTRITION AND OVERALL HEALTH

Abigail Nehrkorn-Bailey, Tanner Edmonds, and Alissondra Quatsoe, *University of Wisconsin-Green Bay, Green Bay, Wisconsin, United States*

The literature on dementia caregivers is sparse. A study from Sheehan et al. (2021) concluded that the well-being of dementia caregivers and nondementia caregivers largely did not differ. To note, the sample size in that study was limited and no health behaviors were assessed. This study extends and adds to the Sheehan et al. (2021) study by utilizing the July 2023 Behavioral Risk Factor Surveillance System data (CDC, 2023) to examine nutrition (i.e., fruit and vegetable intake) and overall physical and mental health among (1) dementia caregivers and (2) nondementia caregivers. Two multiple mediation analyses were conducted comparing caregiving group (n = 4,853 dementia caregivers; n = 28,698 nondementia caregivers): physical health was the outcome in Model 1, and mental health was the outcome in Model 2. The direct pathway from caregiving group to (1) physical health in Model 1 was significant ($b = -.05$, $p < .001$) and (2) mental health in Model 2 was significant ($b = -0.11$, $p < .001$). The pathway from caregiving group to vegetable intake was significant in both models, but the caregiving group-fruit intake pathway was not significant. The pathways from vegetable and fruit intake to physical and mental health were all significant. Lastly, vegetable intake significantly mediated the pathway from caregiving group to (1) physical health ($b = 0.001$, CI = [.0003 to .0020]) and (2) mental health ($b = 0.001$, CI = [.0002 to .0015]). These findings indicate important diet and overall health differences between dementia and nondementia caregivers.

Abstract citation ID: igad104.3316

### EXAMINING THE ASSOCIATION AMONG ADLS, DEMOGRAPHICS, WITH FOOD SECURITY ADULTS AT SENIOR CENTERS IN FULTON COUNTY, GA

Kehinde Akintola[1], Sophia Foley[1], Angelique Willis[1], Suhasini Ramisetty-Mikler[1], Miranda Cook[2], and Kellie Mayfield[1], *1. Georgia State University, Atlanta, Georgia, United States*, *2. Open Hand Atlanta, Atlanta, Georgia, United States*

Food security amongst community-dwelling older adults attending senior centers is not often examined. It is an integral determinant of health because of the impact on multiple health conditions, which are further impacted by functional impairments and co-morbidities that can come with increasing age. Additionally, meal and nutrition education providers do not typically collect this information, which can be used to better provide services to their clients. This study examined the relationship between food security and demographic variables of older adults attending senior centers in Georgia served by nutrition provider Open Hand Atlanta (OHA). One hundred sixty-five senior center attendees were recruited from nine senior centers throughout Fulton County. The age of the participants ranged between 52 to 97, with most identifying as female (86.7%) and African American (79%). Food security (outcome of interest) was measured by the USDA 6-item module. Bivariate associations were tested between food security and ADLs and iADLs (instrumental ADLs) measured by a 15-item list, health conditions, shopping habits, transportation, caregiving, and demographics. Results showed that food security differed based on race/ethnicity X2 (n=165) = 8.94 (df=2), p = .011 and type of insurance X2 (n=100) = 5.95, (df=1) p = 0.015). In addition, those who are under public/Govt. insurance experience a higher proportion of food insecurity compared to those under private insurance (p=.015). Results have implications for nutrition providers when offering additional services to senior centers and the older adults they serve.

Abstract citation ID: igad104.3317

### EXAMINING THE ASSOCIATION BETWEEN HYDRATION STATUS AND OBESITY IN OLDER ADULTS

Kworweinski Lafontant, Ladda Thiamwong, Jeffery Stout, Joon-Hyuk Park, Rui Xie, and David Fukuda, *University of Central Florida, Orlando, Florida, United States*

Adequate hydration is an important modifiable determinant of overall physical health within community-dwelling older adults. Research and clinical practice often use urine osmolarity and bioelectrical impedance analysis (BIA) to assess hydration status. Prior evidence links inadequate hydration to increased obesity, typically measured by body mass index (BMI). However, this association has not yet been evidenced in community-dwelling older adults, especially in low-income settings. This cross-sectional study assessed 103 older adults (16 men, 87 women; mean age 75.7 ± 7.19 years; BMI 26.8 ± 5.22 kg/m2). Total body water (TBW), intracellular water (ICW), fat mass (FM) and fat-free mass (FFM) were measured using multi-frequency BIA (InBody S10). Linear regression analysis examined continuous BMI. TBW/FFM and ICW/FFM as hydration indicators showed weak correlation with FM (TBW/FFM: r = 0.357, p < 0.001; ICW/FFM: r = -0.243, p = 0.013). However, only TBW/FFM significantly correlated with BMI (TBW/FFM: r = 0.236, p = 0.016; ICW/FFM: r = -0.143, p = 0.151). These results suggest TBW/FFM and ICW/FFM may not adequately indicate hydration status while maintaining sensitivity to body composition in adults aged 60-96 years. Further research should examine these findings using urine osmolarity as the hydration assessment.

Abstract citation ID: igad104.3318

### EXAMINING THE ASSOCIATION BETWEEN LAND COVER AND PHYSICAL MOBILITY: FINDINGS FROM THE NHATS STUDY (2021)

Rohini Perera, Carina Gronlund, Grace Noppert, and Kate Duchowny, *University of Michigan, Ann Arbor, Michigan, United States*

The vast majority (88%) of adults wish to age in place. An aesthetically pleasing environment has been found to be associated with better physical functioning and reduced disability, however specific land cover features (i.e. blue, green, urban spaces) and their relation to mobility have not been fully investigated. Data come from the National Health and Aging Trends Study (NHATS, 2021), a nationally representative sample of Medicare beneficiaries, and the National Neighborhood Data Archive (NaNDA), a data repository with census-tract measures of land cover. Mobility was assessed using the objective Short Physical Performance Battery (SPPB) and subjective physical capacity (PC) (range = 0-3 for both measures). Higher categories for both measures indicate better mobility. The analytic sample included 2,939 individuals (58.3% women) with mean age 81.8 years (SD = 6.4) from 2,071 census tracts. In fully-adjusted ordinal logistic regression models, compared to those in the lowest SPPB category, each percentage increase of blue space was associated with a 3.6 greater odds of being in the next highest category of SPPB (aOR= 3.61, 95% CI = 1.20-10.84 ). In contrast, each percentage increase of urban space was associated with a 18% decreased odds of being in the next highest category of SPPB (aOR= 0.82, 95% CI = 0.67-0.99). Similar associations were observed with PC, although were not statistically significant. Since decreased mobility is associated with numerous negative health outcomes, results from this study highlight the need to consider land cover as a potential policy lever to promote mobility among older adults.

Abstract citation ID: igad104.3319

### EXAMINING THE ASSOCIATION OF HISTORICAL REDLINING WITH NURSING HOME QUALITY

Narcissa Plummer[1], Aneeka Ratnayake[1], Chelsea Wong[2], and Brianne Olivieri-Mui[1], *1. Northeastern University, Boston, Massachusetts, United States*, *2. HebrewSenior Life, Boston, Massachusetts, United States*

**Aim:** Redlining systemically relegated people of color to lower-resourced areas which are known to have had long-term effects on access to healthcare; however, it is not currently known if access to nursing home (NH) quality was impacted. **Methods:** In this cross-sectional descriptive study, we created a Geographical Information Systems map of the distribution of Massachusetts NH in the year 2021, layered over a 1934 redlining map with neighborhood categories: Best, Still Desirable, Declining, and Hazardous. Our dataset included redlining categories, 2021 NH characteristics (e.g., bed size, 5-star quality rating), and change in median household income from 1930 – 2021 (proxy for resources, in 2021 dollars). Descriptive statistics estimated the association of each variable with NHs' 5-star quality rating. **Results:** The majority of NHs were in historically Declining (n=45,71%) or Hazardous (n=10,16%) neighborhoods. While 3% (n=2)

were in Best neighborhoods. Both NHs in Best neighborhoods were 5-star, while 1 NH was 5-star in Hazardous neighborhoods. There were 63 NH included with a mean change in median household income of $47,684.06 (SD $37,844.01). The greatest increases in mean income were observed in hazardous neighborhoods containing 1-4-star NHs. The highest increase in mean income was $140,000 (n=1), occurring in a neighborhood with a 4-star NH. **Conclusion:** Despite the growth of resources in hazardous neighborhoods, most NHs in these areas were less than 4-stars. Increasing resources may not benefit NH quality in historically redlined/hazardous areas. Future research should examine this phenomenon at a national level.

Abstract citation ID: igad104.3320

### EXAMINING THE NON-LINEAR ASSOCIATION BETWEEN YEARS OF EDUCATION AND LATE-LIFE COGNITION

Sara Robinson[1], Jason Salemi[2], Hongdao Meng[1], and Judith Rijnhart[1], *1. University of South Florida, Tampa, Florida, United States*, *2. University of South Florida College of Public Health, Tampa, Florida, United States*

Dementia, a rising health concern, is expected to impact 13 million adults in the United States by 2050. Low education is a well-established risk-factor for dementia. However, few studies have investigated possible non-linearity of the association between years of education and late-life cognition, nor have investigated gender differences in this association. This study aimed to investigate whether years of education is non-linearly associated with late-life cognition, and whether the size of this association differed across men and women. To address this aim, we used data from the U.S. representative Health and Retirement Study, encompassing a sample of 32,712 respondents with episodic memory data available between 1998 and 2018 and with complete data on self-reported education (median=12, interquartile range=12-15), gender (18,509 women, 14,203 men), and confounders (ethnicity, race, parental education, childhood immigration status, and birth year). Episodic memory was measured as immediate and delayed recall using a 10-word recall test. We used confounder-adjusted linear mixed-effects models to estimate the associations between years of education and immediate recall (model 1) and delayed recall (model 2). In both models, the estimated education-recall association was non-linear based on the statistically significant squared and cubic terms (p< 0.001). Generally, an increase in education was associated with an increase in immediate and delayed recall, and these associations were more pronounced for larger education values. There were no statistically significant gender differences in the education-recall associations in both models. These findings indicate the importance of assessing non-linearity of the association between years of education and late-life cognition.

Abstract citation ID: igad104.3321

### EXAMINING THE PREVALENCE OF UNDIAGNOSED SLEEP APNEA AND ITS ASSOCIATION WITH BLOOD BIOMARKERS FOR ALZHEIMER'S

Mitchell Roberts[1], Erica Sappington[1], Michael Jaffee[2], Jeff Lowenkron[3], Paul Dagum[4], Jeffrey Iliff[5], Swati Rane Levendovszky[6], and Carla VandeWeerd[1], *1. UF Health Precision Health Research Center-The Villages, The Villages, Florida, United States*, *2. University of Florida College of Medicine, Gainesville, Florida, United States*, *3. The Villages Health, The Villages, Florida, United States*, *4. Applied Cognition Inc, Redwood City, California, United States*, *5. University of Washington, Seattle, Washington, United States*, *6. University of Washington Medical Center, Seattle, Washington, United States*

Obstructive sleep apnea (OSA) prevalence increases with age, but is often undiagnosed. Recent findings suggest association between OSA and Alzheimer's Disease (AD). OSA may impact alterations in amyloid-beta (Aß) protein possibly through interactions with the glymphatic system, thereby unraveling underlying AD mechanisms. In a crossover study conducted 06/22-04/23, older adults (55-65, mean 61.9) without diagnosis of OSA or cognitive impairment(N=33) completed MRIs and blood draws for AD-biomarkers across evening, morning, and midday time points. Polysomnography (PSG) using Philips Sleepware measured Apnea-hypopnea-index (AHI). Morning Trails A&B assessed cognitive function, and abeta40-42-42/40 levels were calculated using Immunoprecipitation-Mass-Spectrometry (IP-MS). Descriptive statistics, ANOVA, and linear regression were utilized. AHI scores averaged 10.5(SD:10.09), with 30% meeting 'normal' (0-5) and 52% mild (5-15) criteria. While not significant(p=.113), males(M:12.5,SD:9.7) averaged higher AHI scores than females (M:8.2 SD:10.3). AHI had a linear relationship with Abeta40 values(R2=0.18,F(2,33)=6.7 1,p=0.012). However, no significant association between AHI, Abeta42(p=0.09), and Abeta42.40(p=0.54) was found. Neither AHI sum scores [Trails A(p=0.09), Trails B(p=0.67)], nor severity rankings [TrailsA(p=0.74),Trails B(p=0.14)] were significantly associated with Trails A&B performance. Elevated AHI was associated with higher levels of Aß40 but not cognitive test performance. Though implications are limited by sample size, findings suggest connection between OSA and initiation of AD processes, necessitating additional research.

Abstract citation ID: igad104.3322

### EXAMINING TWO CONSECUTIVE YEARS OF OLDER ADULTS' EVERYDAY PROSPECTIVE MEMORY LAPSES AND INFLAMMATION BY GENDER

Erin Harrington[1], Jennifer Graham-Engeland[2], Martin Sliwinski[2], Jacqueline Mogle[3], Richard Lipton[4], Mindy Katz[4], and Christopher Engeland[2], *1. University of Wyoming, Laramie, Wyoming, United States*, *2. Pennsylvania State University, University Park, Pennsylvania, United States*, *3. Clemson University, Clemson, South Carolina, United States*, *4. Albert Einstein College of Medicine, Bronx, New York, United States*

Prospective memory (PM, i.e., memory for future intention) is vital to daily life. Although PM deficits can distinguish healthy older adults from those in early stages of dementia, limited work has examined PM and biological markers associated with memory decline. Extending past work, we examined self-reported PM lapses in daily life and systemic (circulating) inflammatory markers by gender across two years. Older dementia-free adults (n=90, Mage=76.84 years; 62%women) enrolled in the ongoing Einstein Aging Study completed two-waves of data collection (approximately one year apart), each including two-week measurement bursts of daily self-reported PM lapses which were summed across respective bursts. Averaged levels of systemic inflammatory cytokines (IL-1β, IL-4, IL-6, IL-8, IL-10, TNF-a) and C-reactive protein were derived from two blood samples per wave (pre/post burst). Among men, cross-sectional and cross-lagged correlations between PM and IL-8 were significant, such that reporting more PM lapses correlated with higher levels of IL-8 (ps<.009). Among women, wave-1 PM correlated with wave-2 TNF-a (p=.018), and wave-2 PM correlated with wave-2 IL-10 (p=.041). No other links emerged. Consistent with past research, these preliminary findings suggest that IL-8 may be an important correlate of poor daily PM in men. The neural substrate of this association could include accelerated aging or increases in Alzheimer's or vascular pathology. Among women, lagged relationships between PM and inflammation may be indicative of pathology. Continued investigation into PM and inflammation that account for gender may reveal longitudinal links that could aid in early identification of persons at risk for cognitive decline.

Abstract citation ID: igad104.3323

### EXPANDING CDC'S STILL GOING STRONG CAMPAIGN TO INCLUDE TRIBAL POPULATIONS AND PROMOTE SOCIAL CONNECTEDNESS.

Yara Haddad, Gwen Bergen, and Erin Bruner, *Centers for Disease Control and Prevention, Atlanta, Georgia, United States*

The Centers for Disease Control and Prevention (CDC) launched the Still Going Strong (SGS) campaign in 2021 to promote ways older adults (65+) can age without injury. The goal was to encourage older adult participation in lifelong activities while taking steps to prevent injuries. Based on initial campaign evaluation, CDC plans to expand SGS to include social connectedness content and reflect greater diversity. To inform this expansion, CDC conducted spring 2023 focus groups with Tribal elders (n=8) and differently-abled older adults (n=9) and presented different concepts depicting older adults engaging in activities with others. Tribal elders were affiliated with five tribes: Navajo, Ojibwa, Citizen Potawatomi, Lumbee Indian, and Wailaki. Half were female (n=4), and majority were 63–70 years of age (n=5). This group tested two new radio concepts: (1) Walking and Talking and (2) Sharing Lessons. Themes across Tribal participants included the importance of connecting with loved ones, sharing culture and spirituality through service, and connecting with younger generations. The differently abled focus group was majority female (n=6), white, (n=5), 70–75 years of age (n=5) and reported using a mobility aid (n=6). This group tested two new video concepts: (1) Best Friends and (2) Health in Numbers. Participants identified social connection and being with others as important for their overall health. Using these data, we will create and refine campaign materials targeting both populations, which will be disseminated and further evaluated. By creating culturally relevant and respectful health promotion messages, we can better engage disproportionately affected persons and reduce injuries.

Abstract citation ID: igad104.3324

### EXPERIENCES AND PERCEPTIONS OF FOREIGN NURSES WORKING IN A SUPER-AGED SOCIETY: A QUALITATIVE DESCRIPTIVE STUDY

Derong Zeng[1], Kyoko Asakura[2], Ayae Kinoshita[1], and Bingbing Qi[3], *1. Kyoto University, Kyoto, Kyoto, Japan, 2. Tohoku University, Sendai, Miyagi, Japan, 3. Villanova University, Villanova, Pennsylvania, United States*

This study delves into the experiences of foreign male nurses from China working within Japan's super-aged society, a distinctive environment marked by unique healthcare and elderly care systems. We directed our focus on the Chinese perspective, aiming to gain a detailed understanding of their experiences, challenges, and perceptions within this context. This qualitative research explores experiences, challenges, and perceptions of 16 Chinese male nurses in Japan's super-aged society. Focused on the Chinese perspective, it provides valuable insights via semi-structured interviews. The interviews were conducted in Chinese via Zoom, with each participant being interviewed once for an average duration of 90 minutes. Key findings include: 1) Job Responsibilities and Challenges: Participants reveal high work intensity and difficulties with disease management and scheduling in the Japanese healthcare system. 2) Aging Society Implications: While Japan's eldercare and healthcare systems provide stability, they also result in increased demand and workloads. Some system aspects were criticized as unreasonable. 3) Cultural Adaptation: Challenges included language barriers, understanding Japanese etiquette, and lifestyle and work habits adjustment. 4) Interpersonal Relationships: Narratives about interactions within this new cultural environment highlighted communication issues and feelings of isolation. this study offers an in-depth understanding of the unique challenges faced by Chinese male nurses within Japan's super-aged society. The findings serve to inform future healthcare policies and practices tailored towards foreign healthcare professionals in Japan. By focusing specifically on Chinese male nurses, this study contributes a fresh perspective to the ongoing discourse on cultural diversity within the international healthcare workforce.

Abstract citation ID: igad104.3325

### EXPERIENCES OF RACISM IN HEALTH AND LIFE-COURSE RESEARCH: A MEASUREMENT BOOKLET AND RECOMMENDATIONS

James Iveniuk[1], and Monica Peek[2], *1. NORC at the University of Chicago, Chicago, Illinois, United States, 2. University of Chicago, Chicago, Illinois, United States*

Numerous, interconnected fields of research study racism, and health inequities emerging from systemic racism. Researchers who are interested in strengthening this work may, however, find themselves at a loss as to where to begin. They may not have a conceptual framework that informs their choice of measures and approaches. They may also lack awareness of appropriate measures, that they can choose from to create impactful work. To meet this need, we create a research-community asset for the measurement of racial identity, intrapersonal/internalized racism, interpersonal racism, institutional racism, and structural racism, with an emphasis on measuring these experiences and their impact on health across the life course. Where applicable, we provide guidance on scoring. The asset is prescriptive in what measures it offers to readers, and in terms of the multi-level theoretical framework that we use to inform our selection. However it is also agnostic about health outcomes or research designs, and we provide measures that are applicable to self-report and administrative data sources, in order to be applicable to as many different researchers as possible, both in academic and community-engaged settings. We also provide some general guidance for translation into different languages, to be inclusive of respondents from diverse linguistic backgrounds.

Abstract citation ID: igad104.3326

### EXPERIENCES OF SOCIAL ISOLATION BY MANDARIN AND PUNJABI-SPEAKING OLDER IMMIGRANTS LIVING IN CALGARY

Prince Ekoh[1], Change Zhu[1], Christine Walsh[1], and Sepali Guruge[2], *1. University of Calgary, Calgary, Alberta, Canada*, *2. Toronto Metropolitan University, Toronto, Ontario, Canada*

Loneliness and social isolation have continued to be risk factors for the health and overall quality of life of older adults globally. The impact of loneliness on healthy ageing may even be more profound for older immigrants in Western countries like Canada, where there is a different language and culture from that of their home countries. As part of the Inclusive Communities for Older Immigrants (ICOI), which seeks to create knowledge about social isolation and improve social connectedness among older immigrants across Canada, we examined the experiences of social isolation amongst older Mandarin and Punjabi-speaking immigrants aged 60 years and over living in Calgary. Quantitative data was collected from 64 (33 Mandarin-speaking and 31 Punjabi-speaking) older adults in Calgary, Alberta. The data were cleaned and analysed using descriptive analysis with the aid of Statistical Packages for Social Sciences (SPSS), and findings showed that older immigrants conceptualised social isolation as limited participation in various community activities. Our results also showed that older immigrants perceived physical and mental health status, social conditions and environmental conditions as significant factors predisposing older immigrants to social isolation. The study, therefore, recommends designing interventions that will increase older immigrants' participation in community activities considering their age, language, disabilities, health, and social conditions.

Abstract citation ID: igad104.3327

### EXPLORATION OF FALL RISK OF PARTICIPANTS ACROSS EVIDENCE-BASED FALL PREVENTION PROGRAMS

Rita Wong, Cathy Elrod, Sara Pappa, Uma Kelekar, and Patricia Heyn, *Marymount University, Arlington, Virginia, United States*

The National Council on Aging houses the Administration for Community Living's (ACL) national database on evidence-based, community-delivered fall prevention programs (EBFPP). Each year, cooperative agreement grants are awarded competitively to groups demonstrating readiness to deliver EBFPP in their communities. Participants in these programs completed a standardized pre-participation survey documenting demographic and self-reported fall risk factors that were entered into the national EBFPP database. Marymount University received permission to analyze the survey data entered into the ACL's National Falls Prevention Database between July 2016 and June 2022. An exploratory analysis was conducted of 105,323 older adults who participated in one of eight ACL-approved EBFPP programs. EBFPPs vary in their targeted fall risk level, tailoring intervention components (health education, self-management training, physical exercise) and adjusting intensity and focus to match the risk level. It is unknown if people join the program that best fits their risk level. This study evaluates and compares participant demographic characteristics and fall risk across EBFPPs. We created an index to categorize risk levels, anchored in the CDC's STEADI toolkit for fall risk, finding that 29% were high risk, 51% moderate risk, and 19% low risk. The factors used to establish risk level included 1) falling in the last 3 months, 2) referral from a healthcare professional, 3) fear of falling, 4) self-reported health (not excellent or very good), 5) depression, and 6) age 85 or older. Exploring participant characteristics by risk level and choice of EBFPP adds unique information to guide future EBFPP recruitment strategies.

Abstract citation ID: igad104.3328

### EXPLORATION OF STRESS FLUCTUATIONS IN FAMILY CAREGIVERS OF OLDER PEOPLE WITH MILD COGNITIVE IMPAIRMENT

Sonoko Kabaya, Chieko Greiner, Masahide Nakamura, Yuko Yamaguchi, and Hirochika Ryuno, *Kobe University, Kobe-city, Hyogo, Japan*

Mild cognitive impairment (MCI) is common, affecting 10%-35% of people over 65, and around two-thirds overall develop dementia over their lifetime. Previous studies have reported that family caregivers (FCs) caring for older people with MCI experience considerable burden, which tends to cause various neuropsychiatric symptoms, such as depression, apathy, and irritability. However, it is not clear how FCs' stress, which leads to increased burden, fluctuates within a day through their daily activities. The aim of this study was to explore the characteristics of stress variability in FCs. We recruited one dyad of an older adult with MCI over the age of 65 and a FC. Data collection was performed continuously for 7 consecutive days. We adopted smart watches (GARMIN vívosmart 4) to measure Heart Rate Variability (HRV)-based stress. Moreover, we asked a FC to talk with a Virtual Agent (VA) on a laptop freely to understand when and what kind of daily activities were done and the FC's feelings. Detecting change points in time series data was performed using Python 3.9. Results showed that change points in the FC's daily stress appeared when the FC starts specific activities such as preparing meals and confirming some medicine that an older adult with MCI should take and daily schedules. The FC also expressed anticipatory grief at the prospect of future dementia through the VA. This finding is beneficial for considering how to reduce FCs' stress during daily activities.

Abstract citation ID: igad104.3329

### EXPLORING INTERPROFESSIONAL HEALTH STUDENTS' ATTITUDES AND PERCEPTIONS OF AGING AND CAREERS IN GERONTOLOGY

Brianna Garrison, *Southern Connecticut State University, New Haven, Connecticut, United States*

ageism continues to impact healthcare professional's attitudes toward older adults. Older adults are often assumed to be frail, weak, or a burden on society. Addressing ageist attitudes and myths about ageing, which often lead to discrimination, can impact policy decisions. Knowledge of opportunities available to older adults to increase healthy ageing is vital in any healthcare training program. Recognizing the growing needs to prepare health professionals to work with the increasing numbers of diverse older adults, this research survey conducted for the college of health and human sciences (CHHS) to examine the students' attitudes and perceptions of ageing, interest in ageing careers, knowledge gaps in ageing, and ageing topics of interest. This survey resulted in 141 respondents who helped provide an informed baselines of CHHS student misconceptions about ageing, baseline of learning needs, and topics and practice opportunities of interest. This poster will share the unique results of this survey providing insight into students' attitudes and perceptions on ageing. Additionally, this poster will provide a brief picture of the college's response to the survey including curriculum development and systemic changes.

Abstract citation ID: igad104.3330

### EXPLORING MULTIDIMENSIONAL RELATIONSHIPS OF OLDER ADULTS LIVING WITH CHRONIC PAIN

Taylor Munson[1], Megan Curtis[1], Jennifer Brailsford[2], Phyllis Hendry[1], and Sophia Sheikh[1], *1. University of Florida College of Medicine Jacksonville, Jacksonville, Florida, United States*, *2. University of Florida, Gainesville, Florida, United States*

Older adults who experience chronic pain (CP) face many challenges from their illness, which can affect their personal and healthcare relationships. Research indicates the importance of relationships on health and quality of life outcomes in older adults with CP. However, minimal research has explored their perspectives on the relationships they have with their healthcare providers, healthcare system, their CP, and family/friends/community. Objective: To understand how older adults' CP impacts their relationship with the healthcare system; and how older adults' CP impacts their personal relationships (i.e. self, family, community). **Methods:** Adults >50 years of age with pain were eligible to enroll in virtual focus groups and/or interviews. Audio recordings were transcribed, coded, and analyzed via a mixed inductive-deductive framework approach using ATLAS.ti. Microsoft. Descriptive statistics were performed using Stata 16. **Results:** Sixteen participants participated in a focus group (n=3) or an interview (n=13). Participants identified as White (n=12,75%), female (n=12,75%), and were between the ages of 55-64 years old (n=8,50%). Most participants held an associate's degree or higher (n=15,93%) and reported private medical insurance coverage (n=11,70%). Sessions lasted between 30-90 minutes. Participants described complex relationships that could have positive or negative impacts on their ability to manage their CP and their overall quality of life. Relationships explored included the healthcare system, clinician(s), family, other patients, and CP. **Discussion:** The relationships of older adults living with CP are multidimensional and can support or adversely affect pain management, mental health, and quality of life.

Abstract citation ID: igad104.3331

### EXPLORING PROPERTY TAX BURDENS AND AGING IN PLACE: A GEOSPATIAL INVESTIGATION IN PENNSYLVANIA

Jongwoong Kim, *West Chester University, West Chester, Pennsylvania, United States*

Utilizing Geographic Information System (GIS) techniques and a composite index, this project delves into the intricate geographic patterns of property tax burdens in Pennsylvania, with a specific focus on older residents contemplating aging in place amidst limited financial resources. The study scrutinizes various factors, including property tax rates, home values, income, demographics, and other housing-related variables, to comprehensively grasp their effects on the financial feasibility of aging in place. Pennsylvania displays notable disparities in property taxes, averaging approximately 1.5% of home value, across counties due to distinct assessment systems and varying assessed-to-market ratios (CLRs). Tax rates are also influenced and specified by smaller administrative and geographic entities, including municipalities and school districts. Despite alternatives (e.g., assisted living), aging in place remains the preferred choice for most older Americans. This accentuates the significance of assessing property tax affordability that facilitates potential aging in place, particularly for financially constrained but "healthy" seniors who may not qualify for tax rebates or local freezes. Moreover, escalating housing prices and assessed values further emphasize the urgency. Cartographic and tabular findings from the study unveil challenging situations for seniors in specific county clusters of Pennsylvania, encompassing not only "expensive" urban but also "typically affordable" rural settings. Additionally, areas with the highest tax rates are not always the most financially burdensome (and vice versa) with all other factors taken into account. This study offers practical insights that have the potential to inform adjustments in housing and tax policies aimed at benefiting older adults in Pennsylvania.

Abstract citation ID: igad104.3332

### EXPLORING THE ASSOCIATION OF SLEEP WITH COGNITIVE DOMAINS AMONG PEOPLE LIVING WITH DEMENTIA

Stevie Lewis, Charmi Patel, and Deborah Jehu, *Augusta University, Augusta, Georgia, United States*

People living with dementia (PWD) commonly experience sleep disruptions, which impacts mental alertness, increases daytime sleepiness, and results in poorer quality of life. While sleep preserves cognitive function among PWD, the relationship between sleep and different cognitive domains is unclear. Our objective was to explore the association between sleep and cognition domains among PWD. Twenty-three PWD completed a battery of cognitive tests (global cognition: Montreal Cognitive Assessment; executive function:

Trail-Making Test (TMT), Digit Span Forward Test; perception and orientation: Benton Judgement of Line Orientation Test (BJLO); language: Boston Naming Test; learning and memory: Rey Auditory Verbal Learning Test; complex attention: Digit Symbol Substitution Test) at two residential care facilities. Participants wore a motion watch on their wrist for 7 days to monitor sleep. Participants were primarily male (74%), white (87%), and 48% had unspecified dementia. The average number of hours of sleep per night was 4.6±1.2 hours. The overall regression model for sleep was significant ($R2=0.80$, $F(10,12)=4.68$, RMSE=42.32, $p< 0.05$). More sleep was associated with better perception and orientation (BJLO:$\beta=0.57$, $p=0.03$), greater sleep efficiency ($\beta=0.93$, $p=0.01$), longer wake after sleep onset ($\beta=1.19$, p 0.002), and there was a trend for better executive function (TMT:$\beta=-0.34$, $p=0.09$). PWD have inadequate sleep. Greater amounts of sleep seem to preserve perception and orientation and may play a protective role for executive function, but more evidence is needed. Regularly assessing sleep may be useful in screening and monitoring cognitive changes among PWD in residential care facilities.

Abstract citation ID: igad104.3333

### EXPLORING THE COMPLEXITY OF LUCID EPISODES IN DEMENTIA: PERSPECTIVES FROM MULTIDISCIPLINARY CLINICAL EXPERTS

Lauren Bangerter[1], Joan Griffin[2], Kyungmin Kim[3], Lapid Maria[2], Joseph Gaugler[4], Dawne Finnie[2], and Theresa Frangiosa[5], *1. Medstar Health, Minneapolis, Minnesota, United States*, *2. Mayo Clinic, Rochester, Minnesota, United States*, *3. Seoul National University, Seoul, Republic of Korea*, *4. University of Minnesota, Minneapolis, Minnesota, United States*, *5. Faerge Drinker, Washington, District of Columbia, United States*

Lucid episodes (LE) occur in people living with dementia who have seemingly lost cognitive capacity. Understanding LEs is a relatively new area of scientific inquiry. Most LE studies have utilized different conceptual and operational definitions. Reaching a definition of LE is critical foundation to facilitate more systematic investigation and new knowledge of LEs. The goal of this study was to convene a group of 13 clinical experts to brainstorm on the key components needed to understand LE among people living with dementia (PLWD). We aimed to reach consensus from the group on (1) potential medical or clinical explanations for LEs; (2) necessary medical and clinical questions to understand LE; (3) interpretation of the presence of LE. Thirteen experts from different disciplines (neurology, psychiatry, psychology, pharmacy, palliative care, hospice, nursing, social work, primary care, geriatrics, and professional home caregivers) participated in a virtual Delphi Method study. Experts provided a range of opinions on potential medical and clinical explanations for LE, necessary questions to understand LE, and did not reach consensus on the presence of LE when presented with clinical vignettes. Panelists highlighted key medical and contextual factors as well as a need to know more information about the general demeanor and typical behavior of the PLWD to make a definitive judgement about whether LE occurred. Clinical consensus on LEs is complex. There is great variability in how behaviors and cognitive fluctuations in PLWD are appraised by clinical experts.

Abstract citation ID: igad104.3334

### EXPLORING THE EFFICACY OF ARTIFICIAL INTELLIGENCE IN DELIVERING HOSPICE INFORMATION

Jeanette Ross[1], Alexander Dentchev[2], Elizabeth Reilly[3], and Sandra Sanchez-Reilly[1], *1. South Texas Veterans Health Care System, San Antonio, Texas, United States*, *2. Saint Mary's Hall, San Antonio, Texas, United States*, *3. Health Careers High School, San Antonio, Texas, United States*

**Background:** Artificial Intelligence Chatbots (AIC) are swiftly gaining popularity due to their language model capability to address user queries by analyzing extensive amounts of available data and generating human-like responses. Objective: To analyze the accuracy of AIC in responding to inquiries related to hospice services and benefits. Additionally, the goal is to assess the user-friendliness of AIC in comparison to traditional web search engines. **Methods:** AICs, namely ChatGPT and Bard, were subjected to testing through a series of questions and scenarios pertinent to hospice services (such as its definition, eligibility criteria, types of provided services, and specific care scenarios). Recorded responses from the AICs were subsequently evaluated for accuracy and quality by experts in geriatric palliative care. **Results:** Both ChatGPT and Bard provided mostly accurate responses to fundamental questions and uncomplicated scenarios regarding hospice. These encompassed explanations about prognosis, interdisciplinary teams, and basic services offered. While ChatGPT did not furnish articles or source references, it did offer occasional medical advice. On the other hand, Bard issued medical information warnings and occasionally included visual aids like pictures, diagrams, and website links. When faced with the same set of questions, conventional web search engines often supplied links to webpages that did not invariably contain specific answers to the queries. **Conclusions:** The AICs can deliver predominantly accurate, generic information about hospice in a conversational and comprehensible manner. Future research could delve into how AICs would respond to more personalized and specific inquiries that could be posed by patients or caregivers.

Abstract citation ID: igad104.3335

### EXPLORING THE EXPERIENCES OF PERCEIVED NEIGHBORHOOD SAFETY OF BLACK ADULTS: RESULTS FROM THE THINK PHRESH STUDY

Omar Staben[1], Alina Palimaru[2], Wendy Troxel[1], Tamara Dubowitz[1], and Andrea Rosso[3], *1. RAND Corporation, Pittsburgh, Pennsylvania, United States*, *2. RAND Corporation, Santa Monica, California, United States*, *3. University of Pittsburgh, Pittsburgh, Pennsylvania, United States*

Racial residential segregation is a complex socio-economic phenomenon that affects many communities across the U.S. Such residential segregation is a result of historic policies in the United States including redlining and inequitable urban redevelopment. Black adults who live in segregated areas face challenges and experience psychosocial stressors, including poverty, crime, and feeling unsafe in one's neighborhood which are risk factors for many negative health and cognitive outcomes. To date, few studies have explored perspectives on experiences of racial residential segregation, and specifically, neighborhood safety trajectories that may relate to healthy aging. This qualitative study used data from a sample of participants in adulthood and older adulthood (aged 35-89, Mage= 64 years) from two predominantly black, low-income neighborhoods that have had different histories with regard to disinvestment and systemic racist policies. We used 60 in-depth interviews to explore perceptions around neighborhood safety trajectories and their perceived impact on aging outcomes. The results highlight several dimensions of perceived changes in neighborhood safety over time and how these are perceived to relate to aging outcomes, e.g., psychological stress. The findings also show a few differences between the two neighborhoods concerning policing strategies, individual behaviors, and resource availability, all of which may contribute to perceptions of safety. Implications of this work highlight the importance of developing culturally relevant interventions that target upstream social determinants of healthy aging and health disparities.

Abstract citation ID: igad104.3336

### EXPLORING THE RELATIONSHIP AMONG PLACE ATTACHMENT, SELF-PERCEPTION OF AGING, AND SOCIAL PARTICIPATION

Wei-An Chen, and Li-Fan Liu, *National Cheng Kung University, Tainan, New Taipei, Taiwan (Republic of China)*

In the context of aging in place, the purpose of the study was to explore the relationship between place attachment, self-perception of aging and social participation of middle-aged and older adults living in the community. The study sample consisted of 226 people aged 50 and over that lived in an urban community in Tainan city, Taiwan. The instrument used were questionnaires that included socio-demographics, health status, and scales for the three main variables. The data was analyzed using correlation, linear regression and mediation testing. The result showed a significant positive correlation between place attachment and both positive and negative self-perception of aging. Furthermore, it was found that negative self-perception of aging and place attachment had significant effects on social participation. Specifically, individuals with lower levels of negative self-perception of aging and higher levels of place attachment showed higher levels of social participation. Finally, the mediation analysis indicated that place attachment played a partial mediating role in the relationship between negative self-perception of aging and social participation. This means that under the positive effect of place attachment, social participation of middle-aged and older adults with negative self-perception of aging can still be increased. In conclusion, based on the interaction between aging and the environment, in addition to the importance of reducing negative views on aging, this study sheds light on the power of emotional bonds with living place in promoting active and healthy aging in familiar neighborhoods.

Abstract citation ID: igad104.3337

### EXPLORING THE SOCIO-SPIRITUAL MEANING AND COPING STRATEGIES OF OLDER ADULTS LIVING WITH CANCER IN NIGERIA

Obinna Odo, Kingsly Udeh, Jonathan Okolie, and Runcie Chidebe, *Scripps Centre, Miami University, Oxford, Ohio, United States*

The growing cancer mortality, poor access to palliative care, treatment hesitancy, poor survivorship, and limited oncology healthcare infrastructure have been investigated in Sub-Saharan Africa. In Nigeria, the orthodox treatment and management of cancer is still greatly influenced by superstitious belief and spiritual connotations. Yet, the socio-spiritual and culturally sensitive meanings attached to cancer and treatment are still lacking in the literature. This study explored the socio-spiritual strategies of older adults living with cancer in Nigeria. The study adopted a qualitative approach and data were collected through a semi-structured interview with 15 older adults from the Oncology unit, University of Nigeria Teaching Hospital (UNTH) Enugu State, Nigeria. The data were managed and inductively coded using NVivo 12 and analyzed thematically. The findings of our study show that cancer is traditionally conceptualized as the aftermath of a disconnection with supernatural/ancestral forces. By implication, such misconceptions affect the level of dispositions and steps taken toward diagnosis and treatment. To this end, our study argues that blending spiritual needs, social support, and culturally sensitive cancer education could improve the quality of life and coping capacity of older adults living with cancer. The study recommends the infusion of socio-spiritual interventions to cancer treatment for older adults in Nigeria.

Abstract citation ID: igad104.3338

### EXPLORING THE STRESS-RELATED GROWTH AMONG ELDERLY KOREAN IMMIGRANTS IN THE CONTEXT OF COVID-19 PANDEMIC.

Jooah Lee[1], Yongseop Kim[2], Hyejin Park[3], and Junhyoung Kim[3], *1. Henry M. Gunn High School, Palo Alto, California, United States*, *2. Indiana University, Bloomington, Indiana, United States*, *3. Texas A&M University, College Station, Texas, United States*

While some studies investigated stress-related growth (SRG) during the COVID-19 pandemic, little research has been pursued to explore positive psychological changes associated with COVID-19 among older Asian immigrants. Thus, we aimed to find positive changes resulting from the COVID-19 pandemic based on the SRG framework. Using a purposeful criterion sampling strategy, semi-structured in-depth interviews were conducted with 11 participants. Content mapping and content mining questions were used. Data were qualitatively analyzed using the constant comparative method. Challenges and stressors associated with the pandemic such as fears of physical or verbal attack, COVID-19 infection, strict COVID-19 regulations and mandates, and limited opportunities to interact with others were identified based on the statements of participants. We identified three salient themes that were characterized as components of SRG: (a) increasing leisure-time physical activity participation, (b) developing a closer relationship with others, and (c) improving resilience. The findings of the present study present qualitative evidence that older Korean immigrants experienced positive changes associated with the pandemic such as being physically active, developing closer relationships, and improving resilience. These changes are associated with the main elements of SRG, and it appears that the pandemic did lead to older Korean immigrants experiencing SRG.

Abstract citation ID: igad104.3339
### EXTRACELLULAR VESICLES BIOMARKERS FOR PARKINSON'S DISEASE: A SYSTEMATIC REVIEW AND DIAGNOSTIC META-ANALYSIS

Hash Brown Taha[1], and Aleks Bogoniewski[2], *1. University of Southern California, Los Angeles, California, United States*, *2. University of California Los Angeles, Los Angeles, California, United States*

Parkinson's disease (PD) and related disorders are frequently misdiagnosed due to shared symptoms and a lack of specific biomarkers, especially in early-stages. Moreover, the prediction of progression and conversion of prodromal conditions, such as RBD, is not possible. Extracellular vesicles (EVs) are tiny, lipid-bilayer bound vesicles released by all cells into the extracellular space and bodily fluids that contain cell-state-specific messages. As such, they have become potential diagnostic tools, but inconsistencies and poor reproducibility hinder their clinical translation. This meta-analysis aimed to clarify the diagnostic ability of EVs extracted from different bodily fluids, such as cerebrospinal fluid (CSF), plasma and serum, for distinguishing patients with parkinsonian disorders from one another or from healthy controls (HCs). Following PRISMA guidelines, we analyzed 21 studies using bivariate and HSROC models, focusing mainly on 1,265 patients with PD vs. 783 HCs due to limited research on other comparisons. The results showed a moderate diagnostic ability of the test (DOR = 21.6 and AUC = 0.852), but substantial heterogeneity and publication bias. The trim-and-fill method estimated two missing studies with null or low DOR. Sub-analysis of media showed that CSF-EVs performed the best (DOR = 42.4 and AUC = 0.924), while plasma-EVs showed the lowest effectiveness. Comparison of biomarkers from general EVs vs. central nervous system (CNS)-originating EVs (n = 13) showed that the former approach is more effective with less publication bias. Though promising, the emphasis should be on standardizing the field by rigorous validation to avoid making practical clinical application a reality.

Abstract citation ID: igad104.3340
### FACIAL-TRUSTWORTHINESS AND BEHAVIORAL-EVALUATION FACILITATE DECISION MAKING AMONG YOUNG AND OLDER ADULTS

Shuyao Liao, *Peking University, Beijing, Beijing, China (People's Republic)*

Objectives. Older adults prefer non-diagnostic cues (e.g., facial-appearance) than diagnostic behaviors when making decision in trust games. However, previous research on decision-making heavily focused on memory, requiring older adults to remember multiple faces and corresponding behaviors. Due to cognitive deficits and the rarity of repetitive interactions with partners in real-life, older adults may experience limitations in their memory-based decision making. This study employs multi-trial one-shot trust game to investigate the reliance of younger and older adults on non-diagnostic (facial-trustworthiness) and diagnostic (behavioral-evaluation) cues, and whether older adults would learn the rule of basing decisions on diagnostic cue, when both cues are available. Method. 104 younger adults (Mage = 21.38, SD = 2.41) and 105 older adults (Mage = 65.5, SD = 4.15) completed 96-trial one-shot trust game, in which partner's facial-trustworthiness and behavioral-evaluation were presented simultaneously. Regardless of facial-trustworthiness, decision outcomes were based on behavioral-evaluation. Results. A reinforcement learning model was established, including learning rate and different weights for facial-trustworthiness and behavioral-evaluation. Results reveled that older adults could indeed show rule-based learning, by exhibiting a non-zero learning rate, and an increase in their weights for behavioral-evaluation after learning, although they initially had higher weights for facial-trustworthiness and lower weights for behavioral-evaluation. Discussion. The present study indicated that older adults tended to rely more on facial-appearance initially. However, they could learn the right rule to adjust decision-making resembling those of younger adults. Keywords: decision-making, facial-trustworthiness, behavioral-evaluation, rule-based learning

Abstract citation ID: igad104.3341
### FACING TRUSTWORTHINESS: AGE-GROUP DIFFERENCES IN TRUST-RELATED DECISION-MAKING AND LEARNING

Marilyn Horta[1], Alayna Shoenfelt[1], Nichole Lighthall[2], Eliany Perez[1], Ian Frazier[3], Tian Lin[1], Amber Heemskerk[1], and Natalie Ebner[1], *1. University of Florida, Gainesville, Florida, United States*, *2. University of Central Florida, Orlando, Florida, United States*, *3. Rutgers University, New Brunswick, New Jersey, United States*

Facial impressions contribute to evaluations of trustworthiness. Older adults are especially vulnerable to trust violations, incurring risks for deception and exploitation. Using the newly developed Social Iowa Gambling Task (S-IGT), we examined age-group differences in the impact of facial trustworthiness on decision-making. Advantageous decks were represented by trustworthy (congruent condition, CS-IGT) or untrustworthy (incongruent condition, IS-IGT) faces and disadvantageous decks were represented by untrustworthy faces in the CS-IGT and by trustworthy faces in the IS-IGT. Younger (n = 143) and older (n = 129) participants completed either the standard IGT, CS-IGT, or IS-IGT. Both age groups preferred trustworthy faces in their initial choices. Older adults performed worse than younger adults across all tasks over time. Further, compared to younger adults, older adults performed worse on the IS-IGT, suggesting that incongruent facial cues interfered with older adults' performance, which aligns with reduced sensitivity to negative social reputations in aging. Exploratory multilevel modeling indicated that age-group differences were most pronounced in the last 40 trials across all tasks. Together these findings indicate that differences between younger and older adults in experience-dependent decision-making are magnified in social contexts that involve a "wolf in sheep's clothing," which may reflect age-related difficulties in information integration.

Abstract citation ID: igad104.3342
### FACTORS ASSOCIATED WITH OLDER ADULTS' READINESS FOR DISCHARGE FROM POST ACUTE CARE TO HOME

Susan Barnason, *University of Nebraska Medical Center, Lincoln, Nebraska, United States*

Following acute hospitalization, older adults are often transferred to post-acute care (PAC) for rehabilitation and nursing care. Most PAC admissions are comprised of adults with multimorbidity (>2 chronic diseases). After PAC discharge many patients are rehospitalized within 30 days. The goal of this study was to evaluate factors associated with older adult's readiness for PAC discharge to home. A prospective one-arm descriptive correlation study was conducted. Older adults (N=19) discharged from PAC to home participated. Subjects were recruited over a 3-month period from a large midwestern facility. Participants completed the Patient Readiness for Hospital Discharge -Short Form (PR RHDS-SF) and Patient Activation Measure (PAM). Medication burden, measured by the medication regimen complexity index (MRCI) calculated from discharge medication record. Participants included 12 females and 5 males, mean age of 73 (SD 8.2)) years. The PT RHDS -SF mean score was 49.3 (SD 16.8). The PAM mean score was 54.7 (SD 7.4). Medication burden was high, mean MRCI was 31.5 (SD 11.6). There was a significant correlation [r (17) .48, p=.04] between total PT RHDS-SF score and patient activation levels. Preparing PAC patients to self-manage their multimorbid conditions may enhance their self-confidence with transition from PAC to home. Evaluating self-management preparedness of older adults prior to PAC discharge is vastly understood. Research is needed to prepare multimorbid older adult patients transitioning from PAC to home focused on perceived readiness and self-management confidence.

Abstract citation ID: igad104.3343

### FACTORS ASSOCIATED WITH THE INCIDENCE OF COGNITIVE IMPAIRMENT IN OLDER COSTA RICAN ADULTS.

Ericka Méndez-Chacón[1], and David Camacho[2], *1. Universidad de Costa Rica, San Jose, San Jose, Costa Rica*, *2. University of Illinois, Chicago, Chicago, Illinois, United States*

Cognitive impairment is a critical global public health problem that contributes to lower quality of life and greater medical costs. Despite the growing number of older adults in Costa Rica, few studies have examined cognitive impairment in this population. We drew data on participants (60 years+) with no cognitive impairment (Score of >=12 on MMSE) from wave 1 of the Costa Rican Longevity and Healthy Aging Study (CRELES; n=2,827). Guided by the diathesis-stress-model, we used logistic regression to determine social and health predictors of cognitive impairment incidence (Score < 12 of on MMSE) 2 years later (wave 2). Incidence of cognitive impairment was 5.8% (95% CI, 5.0-7.0). Formal school education (elementary: OR=0.32; 95% CI, 0.17-0.60; high school or more: OR=0.11; 95% CI, 0.02-0.60) and being married or living as married (OR=0.52; 95% CI, 0.28-0.96) were identified as protective against cognitive impairment. Greater age (OR=1.09; 95% CI, 1.06-1.13), consuming more than 400g of carbohydrates per day (OR=2.20; 95% CI, 1.22-3.91), and more challenges with Instrumental Activities of Daily Living (OR=1.70; 95% CI, 1.23-2.28) increased odds of developing cognitive impairment. Common predictors including biological sex, residential zone, smoking, income, bad health perception, depression, and cortisol (ug/dl) were not associated with the onset of cognitive impairment at wave 2. We consider that contextual (e.g., healthcare infrastructure), cultural factors (e.g., familism), as well as diet may help shape predictors and their effects on cognitive functioning. Researchers and practitioners should collaborate on implementation of culturally appropriate and contextually feasible interventions that address predictors of cognitive impairment.

Abstract citation ID: igad104.3344

### FACTORS INFLUENCING OLDER KOREAN AMERICAN IMMIGRANTS' FRAILTY: A QUALITATIVE STUDY

Hae Sagong, Myoung-Gi Chon, and Pao-Feng Tsai, *Auburn University, Auburn, Alabama, United States*

The older immigrant population, characterized by unique attributes such as diverse cultural backgrounds, health beliefs, health behaviors, language barriers, and low health literacy, requires a specific focus on identifying factors that influence frailty. This study aims to explore the influencing factors of frailty among older Korean American immigrants (OKAIs) residing in suburban areas with limited Korean healthcare infrastructure. Semi-structured interviews were conducted with 9 OKAIs residing in Lee County, Alabama, focusing on four factors associated with frailty: health literacy, physical activity, diet, and mental health. Qualitative content analysis was employed to analyze the data. Validated assessment tools for frailty, health literacy, physical activity, nutrition, depression, and cognitive impairment were also used to gather participant characteristics and complement the qualitative findings. Participants exhibited significant differences in health literacy scores between the Korean and English versions. Content analysis yielded seven categories and twenty-two sub-categories were derived from the eighty identified codes. In addition to typical age-related changes observed among older adults in general, their social activities, health behaviors, and healthcare visits were constrained by different environments/healthcare systems compared to Korea, as well as limited English proficiency and healthcare resources. Participants expressed a need for Korean healthcare providers or interpreters during their healthcare visits and emphasized their preference for Korean food and sought ways to maintain vitality. To prevent and manage frailty among OKAIs, interventions need to consider their unique characteristics, facilitators, and barriers. This approach may also benefit other ethnic minority populations facing similar challenges, thus enhancing healthcare for these communities.

Abstract citation ID: igad104.3345

### FAILURE TO RECOVER FROM PROACTIVE INTERFERENCE & TRAJECTORY OF CLINICAL DEMENTIA RATING SUM OF BOXES IN AMNESTIC MCI

D. Diane Zheng[1], Rosie Curiel[2], Alexandra Ortega[2], Elizabeth Crocco[2], Kiresten Crenshaw[1], Sofia Ramirez[1], Juliana Ortiz[1], and David Loewenstein[2], *1. University of Miami, Miami, Florida, United States*, *2. University of Miami Miller School of Medicine, Miami, Florida, United States*

**Background:** The Loewenstein-Acevedo Scale for Semantic Interferences and Learning (LASSI-L) has been shown to be sensitive to Alzheimer's Disease (AD) pathology among those with preclinical AD, however, LASSI-L's ability to predict longitudinal cognitive and functional decline of preclinical AD has not been examined.

**Method:** We evaluated 97 older adults aged 54-98 who were diagnosed with amnestic mild cognitive impairment (aMCI) at 1Florida Alzheimer's Disease Research Center initial visit. The mean age was 71.9 yr, 51% male, average education 15.7 yr, and mean MMSE score was 28.0. Participants had 3 to 5 visits over a follow-up time as long as 76.7 months. We examined the association of LASSI-L measures on the growth curve trajectory of Clinical Dementia Rating sum of Box (CDRSOB).

**Results:** The growth curve model that best fit the CDR sum of box trajectory is a linear form and included the fixed and random effect of intercept and slope of time. After adjusting for age, gender, education, Hopkins Verbal Learning Test (HVLT) delayed recall and LASSI-L maximum storage capacity, worse performance on LASSI-L measures of vulnerability to proactive semantic interference (B2 cued recall, $\beta$=-0.093, se 0.004, p-0.004) and semantic intrusion (B2 cued intrusions, $\beta$=0.053, se 0.023, p=0.02) were statistically significant in predicting a steeper slope on the trajectory of decline in CDRSOB.

**Conclusions:** The LASSI-L measures reflecting failure to recover from proactive interference and associated intrusions predicted the rate of cognitive/functional decline over time in amnestic MCI and demonstrated the utility of LASSI-L's longitudinal prediction of prodromal AD.

Abstract citation ID: igad104.3346

### FAITH-BASED COMMUNITY OUTREACH TO INCREASE ALZHEIMER'S CLINICAL RESEARCH PARTICIPATION IN THE BLACK COMMUNITY

Ethlyn Gibson[1], Bahar Niknejad[2], Hamid Okhravi[2], Ebony Andrews[3], and Travonia Brown-Hughes[3], *1. Norfolk State University - School of Nursing, Yorktown, Virginia, United States*, *2. Eastern Virginia Medical School, Norfolk, Virginia, United States*, *3. Hampton University, Hampton, Virginia, United States*

Issues: Despite the fact that African Americans are twice more likely to develop Alzheimer's disease (AD) compared to non-Hispanic whites, they are under-represented in AD clinical research. Lower representation exists in the AHEAD Study as well, which is an AD prevention clinical trial evaluating the efficacy and safety of Lecanemab in 1400 study participants. We sought to raise awareness about AD and increase clinical research participation of African Americans in the AHEAD Study, through a community-based education and recruitment program (CERP). Description: Our diverse team from Eastern Virginia Medical School and Hampton University, a Historically Black College University (HBCU), established a Community Advisory Board and Ministerial Alliance with representation from faith-based, long-standing civic and fraternal organizations within the Greater Hampton Roads Black community. The alliance guided us to identify prioritization areas for addressing sociocultural barriers and increasing clinical research awareness and participation in traditional African American events, key historic community sites, and non-traditional community centers (i.e., YMCA, independent-living, assisted-living centers, Annual Juneteenth Celebration, Black fraternity, and sorority events.) Community health workers, embedded within the community, of the same cultural background and ethnicity, drove our CERP. Lessons Learned: As of July 2023, we offered our program at 45 community events, including community health fairs, civics events, and Purple Sunday luncheons and talks at Black churches in urban and localities in Hampton Roads, in partnership with fraternal and civic community organizations. Out of over 1700 participants, 177 community members have been prescreened for the AHEAD Study.

Abstract citation ID: igad104.3347

### FALL RISK AND FEAR OF FALLING IN LOW-INCOME COMMUNITY-DWELLING OLDER ADULTS USING A SIMPLE CHECKLIST

Aleeyah Uddeen[1], Renata Komalasari[2], and Ladda Thiamwong[1], *1. University of Central Florida, Orlando, Florida, United States*, *2. Penn State Nursing, State College, Pennsylvania, United States*

Fall risk is common among older adults and can seriously threaten their well-being and increase fear of falling (FOF). FOF can affect the ability of older adults to maintain daily activities and may cause depression. Using a simple checklist to assess the levels of fall risk and FOF can help tailor interventions to prevent falls and promote physical activity. The purpose of the study was to assess fall risk and FOF using simple checklists and examine the association between fall risk and FOF in community-dwelling older adults in low-income settings. A descriptive cross-sectional design was used to assess 103 older adults (men=16, women=87, mean age=75.70, SD±7.19). We used the CDC's Stay Independent checklist with twelve items to measure fall risk and the Short Fall Efficacy Scale International (Short FES-I) with seven items to assess FOF. Scores equal to or greater than four on the Stay Independent and greater than ten on the Short FES-I indicate high fall risk and high FOF, respectively. The data was analyzed using SPSS version 28. Spearman correlation tests showed that fall risk was positively correlated with FOF (r=.606, p<.001). Our finding suggests that using simple checklists to assess both fall risk and FOF may be helpful for older adults in understanding their risk of falls, thus attenuating their FOF. It may be useful to increase accessibility to these two checklists in efforts to prevent falls and FOF among community-dwelling older adults in low-income settings.

Abstract citation ID: igad104.3348

### FAMILY CAREGIVER COMFORT WITH TELEHEALTH TECHNOLOGIES: DIFFERENCES BY RACE/ETHNICITY IN A CROSS SECTIONAL SURVEY

Reed Bratches[1], Wambui Onsando[2], Frank Puga[1], J. Nicholas Odom[1], and Paul Barr[2], *1. University of Alabama at Birmingham, Birmingham, Alabama, United States*, *2. Dartmouth College, Hanover, New Hampshire, United States*

Telehealth has seen widespread use since the onset of the COVID-19 pandemic, and 82% patients required assistance in accessing their telehealth appointments. This assistance commonly comes from a family caregiver who may or may not be comfortable using the technologies associated with telehealth. The objective of our study was to analyze a demographically representative survey of US family caregivers to understand the level of comfort using telehealth technologies among family caregivers through a secondary analysis of survey data collected during the COVID-19 pandemic in 2020. Level of caregiver comfort using computers, smartphones, and tablets was determined through three Likert-style questions. Proportional odds logistic regression was used to understand the associations between demographic variables and level of caregiver comfort using each technology, when adjusting for covariates. A total of 340 caregivers were included in the analysis. Compared to Non-Hispanic White caregivers, Asian caregivers had a higher odds (OR 3.14; 95%CI 1.36, 8.02; p=0.01) of expressing comfort using computers; Black caregivers (OR 0.46; 95%CI 0.21, 0.98; p=0.04) and Hispanic caregivers (OR 0.36; 95%CI 0.17, 0.79 p=0.01) expressed lower odds of comfort using smartphones; and Asian caregivers had a higher odds (OR 4.64; 95%CI 2.05, 11.69; p=0.001) of expressing comfort using tablets. : There are identified disparities in level of technological comfort using computers, smartphones, and tablets by different racial and ethnic groups. Health systems should consider early stakeholder involvement in the design of telehealth technologies and culturally responsive training materials on telehealth technology use to reduce disparities in comfort using telehealth technologies.

Abstract citation ID: igad104.3349

### FEAR OF CANCER RECURRENCE IN OLDER WOMEN IN REMISSION FROM GYNAECOLOGICAL CANCER IN REGIONAL AUSTRALIA

Senara Kulatunga[1], Stacey Rich[2], Christopher Steer[3], Tshepo Rasekaba[4], Nicole Webb[5], and Irene Darmadi Blackberry[2], *1. University of New South Wales, Albury, New South Wales, Australia*, *2. La Trobe University, Wodonga, Victoria, Australia*, *3. Border Medical Oncology, Albury, New South Wales, Australia*, *4. La Trobe University, Melbourne, Victoria, Australia*, *5. Albury Wodonga Health, Albury, New South Wales, Australia*

Fear of cancer recurrence (FCR) can be a constant feature of cancer survivorship and can negatively impact quality of life. Whilst the symptom burden from FCR is higher in younger patients, the impact of FCR in older adults is poorly understood. This project explores FCR, survivorship and the coping strategies associated with FCR in older women in remission after treatment of gynaecological cancer, a cancer type with high recurrence rates. Qualitative interviews were conducted with ten older women (age range 68-87 years) in remission from gynaecological cancer (Ovarian = 5, Uterine = 5). Interviews were designed to explore the experience of FCR in older women who had been treated at a regional cancer centre in Australia. Thematic analysis was undertaken to uncover themes related to the experience of FCR and coping strategies employed. Emergent themes included fear of burdening others, the emotional impact of the death of close others, and positive or negative experience of prior treatment. Emergent themes in coping with FCR in survivorship included helping others, comparison to others, keeping occupied, and support from others. Patients discussed preferring not to dwell on thoughts of recurrence. These findings are novel in FCR literature as they point to an experience of FCR in older women that may not fit the profile of the general population. Whilst further work is needed, these findings support the need for a tailored approach to supporting older women through survivorship to ensure optimal quality of life.

Abstract citation ID: igad104.3350

### FEAR OF FALLING PREDICTS NEGATIVE PERCEPTIONS OF AGING AMONG LOW-INCOME COMMUNITY-DWELLING OLDER ADULTS

Evette Trahan[1], Renata Komalasari[2], Amber Blount[1], Nichole Lighthall[1], and Ladda Thiamwong[1], *1. University of Central Florida, Orlando, Florida, United States*, *2. Penn State Nursing, State College, Pennsylvania, United States*

Negative self-perceptions of aging (NSPA) are associated with various health-related outcomes, including fear of falling (FOF). Despite the leading cause of injury, disability, and hospitalization among racially diverse low-income older adults, FOF's predictive potential effect on NSPA is less studied. This was a cross-sectional study with a sampling of 54 independent and cognitively healthy older adults (4 males, 47 females, mean age 73.91, SD: 6.4; 61-87 years old) in a low-income community. A five-item NSPA Likert scale, part of the Brief Ageing Perceptions Questionnaire (B-APQ), was used to assess the negative self-perceptions of aging, with higher scores indicating fewer negative views. The Short Fall Efficacy Scale International questionnaire was used to evaluate the level of concern about falls for a range of activities of daily living. More than half of the participants were African Americans (27 or 50%), completed high school (29 or 53.7%), lived alone (37 or 68.5%), and earned just enough financially (29 or 53.7%). The simple regression results indicated that the model explained 14.9% of the variance (R2=.149, F(1,52) = 9.13, p =.004). Fear of falling (FOF) (β = -.406, p =.004) was negatively associated with NSPA. As FOF scores increased, NSPA scores decreased, signifying higher FOF, accentuating worse perceived aging. Future longitudinal research should focus on the relationship between FOF and NSPA in increasing fall risk and injurious falls, especially in ethnically diverse low-income older adults, to further clarify this relationship and its consequences. Additionally, assessing FOF and providing interventions may enhance positive perceptions of aging.

Abstract citation ID: igad104.3351

### FEASIBILITY AND ACCEPTABILITY OF A LOVING-KINDNESS MEDITATION AMONG SOCIO-ECONOMICALLY DIVERSE OLDER ADULTS

Nirmala Lekhak, Tirth Bhatta, Carolyn Streb, Karenna Tran, and Joel Snyder, *University of Nevada, Las Vegas, Las Vegas, Nevada, United States*

It has been established that contemplative practices such as meditation can aid in stress management and the prevention of mental health issues. However, whether one of these techniques, such as loving-kindness meditation (LKM), is feasible for promoting later-life self-compassion, reducing loneliness, and maintaining mental health is unclear. This study examined the feasibility and acceptability of a LKM intervention among socioeconomically diverse older adults. We employed a waiting list control design and mixed methods approach to randomize the participants (n = 59) into two groups. The same intervention was administered to the second group at a later time. We oriented and instructed the older adults on how to meditate and perform the LKM intervention. It contained phrases for the self, immediate family, others, and humanity. We gathered outcome data at baseline, post-intervention, four weeks following journaling, and four weeks without journaling. The intervention was feasible and acceptable (n = 54). One hundred percent of participants rated this meditation practice as either good or better. The range for acceptability was between 4 and 20, while the range for feasibility was between 3 and 15. The participants reported that they accepted (M = 16.8, SD = 2.9) and found this meditative practice feasible (M = 12.9, SD = 1.8). Participants who engaged in this intervention daily reported that this meditative practice helped them reduce anxiety and enhance self-awareness. Our findings highlight the need for large-scale contemplative interventions (including more music and silence) that can promote self-compassion and reduce loneliness in older adults.

Abstract citation ID: igad104.3352

### FEASIBILITY AND ACCEPTABILITY OF INSPIRATORY MUSCLE STRENGTH TRAINING AMONG OLDER ADULTS IN MYSORE, INDIA

Raghavi karnam[1], Nagalambika Ningaiah[1], Shivamma Nanjaiah Nanjaiah[1], Nagaveni Bhyra[1], Kiranmayee Muralidhar[1], Purnima Madhivanan[2], and Karl Krupp[3], *1. Public Health Research Institute of India, Mysore, Mysore, Karnataka, India*, *2. University of Arizona, Tucson, Arizona, United States*, *3. Mezcoph, Tucson, Arizona, United States*

Hypertension, a major risk for cardiovascular and cerebrovascular diseases, affects 1.3 billion globally and 229 million in India. While exercise has shown to lower blood pressure, 40% of older Indians report inadequate physical activity. Inspiratory Muscle Strength Training (IMST), a novel breathing exercise, shows promise for controlling hypertension, but little is known about its acceptability among older Indian adults. This study examined feasibility and acceptability of IMST in older adults with hypertension in Mysore, India. Between November-2022 and January-2023, 31 non-smoking adults aged 65 - 80 years, with a blood pressure □140/90 mm Hg were enrolled in a 6-week interventional study after informed-consent. Participants used a Power-breathe Plus breath trainer for 30 breaths a day, 5 days a week, for 6-weeks, weekly increasing resistance to a maximum of 30% of their Maximum Inspiratory Pressure. Participants underwent medical assessment, interviewer-administered survey to collect data on demographics, depression, cognition, physical performance, pulmonary function and acceptability, at baseline and post-intervention. Univariate analysis was conducted. Of 31 participants, 20% were male and 55% were on antihypertensive. Four participants discontinued by week two. Post-intervention, 22% had □10 mm Hg decrease in systolic- pressure and 37% □10 mm Hg decrease in diastolic- pressure. About 59% showed improvement in depression screening scores, 40% had improved cognitive scores and 48% reported improved physical-performance scores; 80% reported IMST was useful and 90% reported device was user-friendly; 45% reported improvements in breathlessness and exercise-tolerance. IMST was acceptable, feasible and safe as a hypertension intervention for older adults in Mysore, India.

Abstract citation ID: igad104.3353

### FEASIBILITY OF AN ONLINE DEMENTIA CAREGIVER EDUCATION CURRICULUM FOR FAITH-COMMUNITY LEADERS

Jeffrey Graupner[1], Katherine Thompson[1], Monica Long[2], Tiffany Smith[2], Jenil Bennett[1], and Shellie Williams[1], *1. University of Chicago, Chicago, Illinois, United States*, *2. University of Chicago/Share Network, Chicago, Illinois, United States*

Dementia caregivers have well-documented needs including dementia education, resources, and emotional support. Our prior work demonstrated the efficacy of a novel dementia education program designed to transform urban faith-community leaders into “Dementia Resource Champions” equipped to start their own support groups. We adapted this program to be delivered virtually. A 6-week curriculum of presentations, resources, and video interviews with caregivers was created by an interprofessional team of geriatrics specialists. Recruitment for “DRC: Online” utilized email blasts and online sign-up forms. While registration for the course was open to all, recruitment emphasized that this program was originally developed for local faith-based communities. Each week, participants received an email with a pre-recorded video didactic presentation, a caregiver interview video, and a link for the weekly videoconference discussion. Fourteen participants completed the pre-training survey, and seven went on to complete the 6-week course and post-training survey. Participants’ reported self-confidence in dementia knowledge increased significantly from pre- to post-training (5-Likert Scale, Paired t-test: Pre (M = 2.1 , SD = 1.1); Post (M = 4, SD = 0.6), t(6) = 7.1, p < .001). Self-reported ability to use the internet to find dementia resources also increased significantly (Pre: (M = 3.9, SD = 1.1); Post: (M = 4.7, SD = 0.5), t(6) = 2.5, p = .045.) Participants’ average rating of the program was 4.7 (5-Point Likert, 5=Excellent). Results indicate that this relatively low-tech approach to virtual dementia education is a feasible way to train community leaders to become resources for dementia caregivers.

Abstract citation ID: igad104.3354

### FEELING CLOSER WITH FUTURE-SELF PROMOTES RETIREMENT PREPARATION OF YOUNGER AND OLDER WORKERS

Dannii Yeung, Esther Y. K. Tang, Gloria Lin, and Alvin Ho, *City University of Hong Kong, Hong Kong, Hong Kong*

Working adults' preparation for retirement and later life remains low despite past research has revealed its benefits on the well-being of retirees. This study utilized an experimental method to test whether the positive effect of exposure to future-self on retirement planning would be retained two weeks after the manipulation. Younger and middle-aged Hong Kong Chinese workers were recruited and were randomly assigned to one of the four conditions: exposure to a future-self photo plus a 5-day writing task (FSW), exposure to a future-self photo only (FS), exposure to a current-self photo plus a 5-day writing task (CSW), and exposure to a current-self photo only (CS). Participants in the FSW and

CSW conditions were further asked to do a 5-day writing task. Intention to make retirement plans was assessed after viewing the future- or current-self photo (Time 1), while actual preparatory activities for retirement were also assessed two weeks after the photo viewing (Time 2). Preliminary analyses on 91 participants [41 younger (M=31.01,SD=5.55) and 51 middle-aged (M=50.04,SD=6.59) workers] showed significant interaction effects between condition and age on T2 overall retirement planning (F=4.06, p=.01, ηp2=.13) and financial planning (F=4.42, p=.006, ηp2=.14). Specifically, younger participants in the two future-self conditions had more preparatory activities than those in the CSW condition. Middle-aged workers in the FSW condition also reported more retirement preparation than those in the FS condition. These findings suggest that exposure to future-self increases retirement preparation in the short run, and such effect was stronger on younger workers than on older workers.

Abstract citation ID: igad104.3355

### FEMALE BREAST CANCER SURVIVAL IN BLACKS VERSUS WHITES ACCORDING TO TUMOR STAGE, AGE, AND MARITAL STATUS AT DIAGNOSIS

Ray Merrill, and Ian Gibbons, *Brigham Young University, Provo, Utah, United States*

Female breast cancer survival is significantly influenced by tumor stage, age, and marital status at diagnosis. While Blacks have historically had lower breast cancer survival than Whites, it is not clear whether this lower survival differs across the levels of tumor stage, age, or marital status at diagnosis. This paper will assess this using five-year relative survival (5-YRS) estimates from the Surveillance, Epidemiology, and End Results (SEER) Program. Cases reflect 2000-2015 and follow-up extends through 2020. 5-YRS was 90.9% in Whites and 81.1% in Blacks, consistently lower by the levels of tumor stage, age, and marital status at diagnosis. In a regression model adjusting for these variables, the difference in 5-YRS was 8.0% for Whites versus Blacks, -15% for regional stage and -67% for distant stage (versus local stage), lower in older ages at diagnosis, and 5.2% for married (or cohabitating) versus otherwise. The lower 5-YRS in Blacks significantly varied by tumor stage at diagnosis (4.2% for local stage, 9.9% for regional stage, and 11.6% for distant stage; p=0.0098) but did not significantly vary by age or marital status at diagnosis. The variation across tumor stage at diagnosis in the lower 5-YRS for Blacks depended on age (p< 0.0001), with greater variability in the younger age groups and less variability in the older age groups. Thus, poorer 5-YRS for Blacks versus Whites persists across tumor stage, age, and marital status at diagnosis and becomes increasingly more pronounced in later stages at diagnosis, especially in younger versus older age at diagnosis.

Abstract citation ID: igad104.3356

### FINANCIAL AND HEALTH INSURANCE STATUSES OF ALZHEIMER'S DISEASE AND RELATED DEMENTIA CAREGIVERS

Jenny Walker[1], Roshni Patel[2], and Lisa McGuire[1], *1. Centers for Disease Control and Prevention, Atlanta, Georgia, United States*, *2. Cyberdata Technologies (CDC Contractor), Atlanta, Georgia, United States*

The number of individuals with Alzheimer's disease and related dementias (ADRD) is rising as the U.S. population ages. Important sources of care for these individuals are the approximately 11 million caregivers who donate upwards of 18 billion hours of unpaid time while incurring many of the daily expenses and out-of-pocket healthcare costs associated with their role (Alzheimer's Association, 2023). Therefore, the financial statuses of these individuals are important to understand as we continue to develop and fortify ADRD programs and health services. Participants from 40 U.S. states and territories who completed the 2021 Behavioral Risk Factor Surveillance System caregiving module were divided into three categories: non-caregivers (n = 184,709), non-ADRD caregivers (n = 36,459), and ADRD caregivers (n = 10,583). Results from a series of Rao-Scott chi-squared tests indicate that household income levels were similar across these three categories, but a significantly higher percentage of caregivers had other people—likely the care recipient—living in their household (87.3% non-ADRD, 86.8% ADRD, 81.1% non-caregivers). Moreover, while significantly fewer caregivers reported that they lacked health coverage (7.3% non-ADRD, 6.4% ADRD) than non-caregivers (8.4%), a larger percentage reported missing a healthcare appointment in the previous year because they could not afford it (12.6% non-ADRD, 12.8% ADRD, 8.8% non-caregivers). These results further highlight the need for focused federal, state, and local interventions for community-dwelling caregiver populations to support the financial toll of caregiving.

Abstract citation ID: igad104.3357

### FINANCIAL CAPABILITY AMONG OLDER ADULT IMMIGRANTS, A SYSTEMATIC REVIEW

Bruna Lopez, Barbara Mendez Campos, and Erika Sabbath, *Boston College, Boston, Massachusetts, United States*

The Financial Capability approach assesses individuals' financial knowledge and behaviors within the context of opportunities in the environment. Scholars and practitioners have documented how immigration status can influence individuals' access to and eligibility for financial products and services, due to federal, state, and community rules and norms. Those who lack immigration status (i.e., undocumented immigrants) often face additional financial hurdles. A separate line of research has shown how financial capability can influence retirement savings, yet this did not focus on immigration status. As such, this systematic review seeks to understand how variations in legal immigration status impact the financial capability and long-term financial wellbeing of older adult immigrants in the United States. The current review utilized standardized procedures and protocols to identify articles related to financial capability, immigration status, and retirement savings, resulting in an initial list of 3,599 articles. Three studies published before May 2023 were included in the final narrative synthesis due to their inclusion of immigrants over 55. Results demonstrate that financial capability studies of older adult immigrants that met eligibility criteria have centralized Asian individuals, particularly Chinese, Taiwanese, and Korean populations. Furthermore, studies indicate that increasing financial capability among older immigrants can strengthen their financial security, yet these studies focus less on the impact of immigration status. Programs, services, or products operating on the financial capability paradigm warrant continued research as older immigrants have unique attributes (e.g., language, culture) and require careful design, implementation, and evaluation of efforts seeking to target long-term financial wellbeing.

Abstract citation ID: igad104.3358

### FINDABILITY OF HEALTH EQUITY DEFINITIONS ON REPUTABLE AGING AND PUBLIC HEALTH WEBSITES

Mahederemariam Dagne, Elizabeth Terhune, and Patricia Heyn, *Marymount University, Arlington, Virginia, United States*

In the current era of electronic health information, ensuring proper access to consistent and accurate information supporting health equity is crucial for older adults. It is imperative that older adults comprehend health terminologies and definitions on websites, enabling them to understand the necessary information effectively. To evaluate how public health websites define, communicate, and display health equity terms and definitions, we developed a tool to review how easy it is to find and understand information on websites that play an integral role in guiding the health of older adults. Thus, we reviewed over 60 credible websites widely used for health information. Many of them included dedicated aging content. Our evaluation revealed that health equity definitions were inconsistent across websites and were often challenging to locate, requiring substantial time and effort to retrieve the information. Additionally, 50% of them lacked definitions. Furthermore, most websites failed to include citations that could substantiate the provided definitions. This study underscores the absence of a systematic, standardized, and regulated approach to how public health websites inform the public, particularly by using consistent terms to define health equity. This study highlights the lack of a universally shared and standardized health equity definition for websites. We recommend that websites structure their content to be easily findable and propose adopting universal standards for displaying health information on websites, especially when communicating with the public. This can mitigate confusion, enhance the understanding of health equity, and ensure important health information findability for older adults.

Abstract citation ID: igad104.3359

### FRAILTY AND COGNITIVE IMPAIRMENT: RESULTS FROM A DIVERSE SAMPLE OF OLDER PERSONS RECEIVING HEMODIALYSIS

Ana Alfaro, Rachel Bryan, Swathi Balantrapu, and Christine Liu, *Stanford School of Medicine, Stanford, California, United States*

Persons who are receiving hemodialysis are at high risk for frailty and cognitive impairment. However, little is known about the overlap of these conditions in a diverse older hemodialysis population. Our goal is to examine frailty and cognition in a diverse sample of older adults receiving hemodialysis. Data was collected as part of a cross-sectional observational study examining mentation, mood, and mobility. Frailty was identified using the Study of Osteoporotic Fractures Index and cognition was assessed with the Montreal Cognitive Assessment (MoCA) (cut off score ≤ 26). Blind MoCAs were administered (cut off score ≤ 22) when appropriate. 35 participants (mean age = 73.9 ± 10.7) enrolled. 60% were males and 20% were Spanish-speaking. 20% identified as Asian, 23% as Black, and 23% as Hispanic/ Latino. Of note, four blind MoCAs in Spanish were administered. 83% had abnormal MoCA scores. 86% were frail or pre-frail. The majority of the sample (69%) had concurrent cognitive impairment and frailty or pre-frailty. In this diverse sample of older adults receiving hemodialysis, we found that both frailty and cognitive impairment are extremely common, with a large portion of the sample exhibiting both. This suggests that interventions targeting cognitive impairment or frailty should account for the frequent overlap of both conditions, particularly among historically underserved populations.

Abstract citation ID: igad104.3360

### FRAILTY AS A PREDICTOR OF MORTALITY: A COMPARATIVE COHORT STUDY IN COSTA RICA AND THE UNITED STATES

Carolina Santamaría-Ulloa[1], Amanda Lehning[2], Mónica Cortés-Ortiz[2], and Ericka Méndez-Chacón[1], *1. Universidad de Costa Rica, San Jose, San Jose, Costa Rica*, *2. University of Maryland Baltimore, Baltimore, Maryland, United States*

Geriatric frailty results from aging-related declines in multiple systems and leads to increased vulnerability for loss of mobility, falls, hospitalization, and mortality. The aim of this study is to compare the association between frailty and mortality in older adults from Costa Rica and the United States. This prospective cohort study uses secondary nationally representative data of community-dwelling older adults from the Costa Rican Longevity and Healthy Aging Study (CRELES, n = 2124) and the National Health & Aging Trends Study (NHATS, n = 6,680). Frailty status was assessed using Physical Frailty Phenotype, which includes shrinking, exhaustion, weakness, slowness, and low physical activity level. We estimated Cox proportional hazard models to examine the association between frailty and all-cause mortality at 8 years from the date at baseline. Survey weights were used. Sociodemographic characteristics and health behaviors were included as covariates. The death hazard for frail compared to non-frail older adults was three-fold in Costa Rica (HR = 3.14, p< 0.001) and four-fold in the white US (HR = 4.02, p< 0.001). Older age, being male, and smoking were death risk factors in both countries. High education was a protective factor in the US, whereas being married or in union was a protective factor in Costa Rica. Results indicate that frailty can have a differential impact on mortality depending on the context of different countries. The identification of behavioral, social, and policy determinants of frailty may serve as an essential component of population interventions that prevent the progression of frailty and its consequences.

Abstract citation ID: igad104.3361

### FRAILTY IN MIDLIFE AS A PREDICTOR OF CHANGES IN BODY COMPOSITION FROM MIDLIFE INTO OLD AGE

Laura Kananen[1], Markus Haapanen[2], Tuija Mikkola[3], Juulia Jylhävä[4], Niko Wasenius[2], Johan Eriksson[2], and Mikaela von Bonsdorff[5], *1. Tampere University, Tampere, Pirkanmaa, Finland*, *2. University of Helsinki, Helsinki, Uusimaa, Finland*, *3. Folkhälsan, Helsinki, Uusimaa,*

*Finland*, *4. Karolinska institute, Stockholm, Stockholms Lan, Sweden*, *5. University of Jyväskylä, Jyväskylä, Keski-Suomi, Finland*

Few studies have investigated the association between frailty and subsequent body composition. We performed separate linear mixed model analyses in 996 adults (mean age at baseline[SD]: 61.5[2.9]) in a Finnish longitudinal birth cohort study to explore the relationships between changes in frailty status assessed by the Rockwood 41-item-frailty-index (FI) and changes in body mass index (BMI), lean mass index (LMI), fat mass index (FMI), and FMI to LMI-ratio values during 17 years of follow-up. With advancing age, LMI and BMI decreased, whereas FMI and FMI to LMI -ratio increased. Those who were frail (FI≥ 0.25) already at baseline, followed by those who became frail during the follow-up, experienced faster decreases in LMI and faster increases in FMI and FMI to LMI -ratio values relative to those who were 'never frail'. Contrastingly, those in the highest third of absolute annual increase in FMI and FMI to LMI-ratio became frailer faster over time relative to those in the lowest third. We found evidence of an adverse health outcome of frailty where lean indices declined faster and fat indices and fat to lean -ratios increased faster from midlife into old age. The changes resembled those that occurred with aging, but at a faster pace. The relationship between body composition and frailty is likely bidirectional, where high or increasing levels of fat are associated with the risk of becoming frailer earlier, but where a longer duration of frailty may increase the risk of faster age-related changes to body composition.

Abstract citation ID: igad104.3362

### FRAILTY-FOCUSED ENHANCEMENTS TO SENIORS' HOSPITAL CARE (FRESH): DOES SPECIALIZED EDUCATION WORK?

Jennifer Peterson[1], Samantha Fowler[1], Karla Faig[1], Kelly Flanagan[2], Olivia Clancy[2], Caitlin Robertson[3], Linda Yetman[2], and Patrick Feltmate[4], *1. Horizon Health Network, Saint John, New Brunswick, Canada*, *2. Horizon Health, Saint John, New Brunswick, Canada*, *3. Horizon Health Network, Moncton, New Brunswick, Canada*, *4. Horizon Health, Fredericton, New Brunswick, Canada*

Research suggests that specialized education for nurses decreases frailty and improves functionality in hospitalized older adults. This study explored the impact of a specialized geriatric education program on care delivery for older adults in acute care in 5 hospitals across the province of New Brunswick. A mixed-methods approach with pre- and post-questionnaires was used to explore facilitators and challenges of caring for hospitalized older adults, the knowledge base and experiences of staff, and the impact of providing specialized education. Acute care staff (N=64) participated in a geriatric education intervention and completed pre and post questionnaires. A sub-set (n=26) participated in semi-structured interviews guided by Kirkpatrick's theoretical framework. Data was collected on mobility, medications, and delirium from patients' charts (N=99) to identify changes in outcomes pre- and post intervention. Findings revealed that staff had positive attitudes toward caring for older adults; however, their understanding and application of geriatric principles were limited and remained unchanged post-education. There was no significant change in the patient-level measures after the intervention. Interview participants shared that their work environment affects their mindset and workflow, limiting their capacity to deliver the type of care presented in the education sessions. Staff identified the need for specialized education; however, there was no impact on care after participation. Environmental constraints hindered staff from implementing best practices, leading to both practical and psychological challenges to care delivery. These results will inform changes to specialized education programming with the aim of improving care for older adults in hospital.

Abstract citation ID: igad104.3363

### FROM FRONT TO BACK SEAT DRIVER: INVESTIGATING DRIVING RETIREMENT DECISION-MAKING WITH THE 85+

Sophia Ashebir, Taylor Patskanick, Lisa D'Ambrosio, and Joseph Coughlin, *Massachusetts Institute of Technology, Cambridge, Massachusetts, United States*

It is impossible to consider personal transportation in the United States without engaging with the topic of driving. Over 90% of U.S. households own cars, while 83% of people report they drive several times a week. A critical but often overlooked component of the driving narrative particularly among older adults is the decision of when or if to forfeit the car keys, a topic which is both complex and, for many individuals and families, a source of stress and conflict. With a rapidly growing population of the oldest of older adults, the 85+ age demographic, it is crucial to understand the attitudes and behaviors of this diverse age group regarding the experience of driving retirement. Utilizing four semi-structured focus groups (n=17) and a 48-item survey of original and adapted measures (n=34) with a panel of the 85+, this study sought to understand people's perspectives on driving, how current drivers think about and plan for driving retirement – or not, and participants' general perceptions and attitudes toward the future of technology and driving. The data suggest four different vignettes or transportation paths with respect to driving among this age group that intersect with themes of autonomy, the experience of physical aging, the role of family, and key inflection points. Implications of this research highlight the need for targeted solutions and materials for people at varying levels of acceptance with their driving stage and underscore the need for more research and greater involvement of different stakeholders in conversations around transportation and aging.

Abstract citation ID: igad104.3364

### FUNCTIONAL IMPACT ON ADULTS AND OLDER PEOPLE AFTER HOSPITALIZATION BY COVID-19

Gabriela Ochiai, Caroline De Godoy, Erika Gouveia E Silva, Celso Ricardo Fernandes De Carvalho, Carolina Fu, Clarice Tanaka, Carlos Roberto Ribeiro De Carvalho, and Jose Pompeu, *University of Sao Paulo, Sao Paulo, Sao Paulo, Brazil*

Cross-sectional study of 159 individuals, one month after hospitalization at the Hospital das Clínicas of the University of São Paulo for Covid-19, divided into groups: 54.7% (n=87) were adults (< 60 years), and 45.3% (n=72) were older people (≥60 years), with 43.4% (n=69) being female. Those who did not accept to participate, without availability, or without ability to understand the questionnaires were excluded. Functional capacity was assessed by the Barthel Index (BI). Statistical analysis was performed using JASP Statistics-0.15.0, considering significance level of alpha=0.05. Wilcoxon and Mann-Whitney tests were applied to compare pre- and post-Covid periods and to compare adults and older people, respectively. Data collection went from June 30, 2020, to August 24, 2021. The median length of hospitalization was 23 (15-34) days. The median total BI scores were lower one month after discharge in older people, 95 [90-100], when compared to adults, 100 [92.5-100] (p=0.001). Bladder control (p=0.009), toilet use (p=0.021), chair/bed transfers (p=0.043), ambulation (p=0.006), dressing (p=0.015), stair climbing (p< 0.001), and bathing self (p=0.006) were the activities of daily living most affected in older people. Hospitalization due to Covid-19 had functional impacts in the post-discharge period, particularly in older people, manifested by limitations in performance of daily activities with higher physical demand. These findings emphasize the need for functional capacity screening after hospitalization for Covid-19, especially in older people.

Abstract citation ID: igad104.3365

### FURTHER EVALUATION OF A MODIFIED VERSION OF THE HOSPICE PHILOSOPHY SCALE: A DIFFERENTIAL ITEM FUNCTIONING ANALYSIS

Todd Becker[1], Sarah Clem[2], Paul Sacco[3], John Cagle[3], Joan Davitt[3], Nancy Kusmaul[4], and Cindy Cain[5], *1. Washington University in St. Louis, St. Louis, Missouri, United States*, *2. University of Maryland School of Social Work, Baltimore, Maryland, United States*, *3. University of Maryland, Baltimore, Baltimore, Maryland, United States*, *4. University of Maryland Baltimore County, Baltimore, Maryland, United States*, *5. University of Alabama at Birmingham, Birmingham, Alabama, United States*

The hospice philosophy of care (HPOC) is a set of values applicable to all interdisciplinary group members that drives organizational and individual approaches to hospice care. As the only established measure of HPOC attitudes, we aimed to extend previous exploratory psychometric evaluation of the eight-item Hospice Philosophy Scale (HPS-8). An interdisciplinary convenience sample of 481 hospice clinicians participated in this cross-sectional study. Participants were recruited through hospice and palliative care membership associations representing the core members of the Medicare Hospice Benefit-designated hospice interdisciplinary group (physicians, nurses, social workers, chaplains). After examining uniformity through confirmatory factor analysis, we tested HPS-8 items for differential item functioning (DIF) by professional discipline using multiple indicators, multiple causes models. We compared models via nested chi-square difference tests. Our final model assessed uniform DIF for the HPS-8 factor, controlling for DIF-indicated items. We also assessed internal consistency reliability. After correlating errors for two similarly worded items, global fit indices met prevailing thresholds, $\chi^2(19)=55.18$, p<.001 (RMSEA=.06, SRMR=.03, CFI=.98, TLI=.97). The uniform DIF model indicated two items with DIF. Chaplains endorsed the need for mental and spiritual preparation for death more than physicians (β=0.21, p<.001). Nurses endorsed involving patients and families in deciding treatments and services more than physicians (β=0.21, p=.006). Despite statistically significant DIF on these items, statistically significant differences among professions were not observed at the factor level (p>.50 for all). Composite reliability estimates satisfied internal consistency thresholds (CR=.76). Results support the HPS-8's use as a valid and reliable measure for assessing HPOC in hospice clinicians.

Abstract citation ID: igad104.3366

### GDF15 AND REDUCED PHYSICAL FUNCTION FOLLOWING TOTAL KNEE REPLACEMENT: A STUDY OF PHYSICAL RESILIENCE AND AGING

William Fountain[1], Nicholas Milcik[1], Nicholas Schmedding[1], Frederick Sieber[1], Julius Oni[1], Ravi Varadhan[2], Karen Bandeen-Roche[1], and Jeremy Walston[1], *1. Johns Hopkins University, Baltimore, Maryland, United States*, *2. Johns Hopkins University School of Medicine, Baltimore, Maryland, United States*

The metabolic and inflammatory cytokine growth-differentiation factor 15 (GDF15) increases with age and negatively associates with physical and cognitive function in older adults. We hypothesized GDF15 also negatively associates with resilient outcomes after surgery. This hypothesis was tested in the SPRING study of physical resiliency after total knee replacement by assessing relationships between pre-operative plasma GDF15 levels and postoperative resilience measures including short physical performance battery (SPPB) scores, fatigability, and grip strength. GDF15 analyses and physical resilience were assessed in 127 SPRING participants (age 70±6 yrs, n=83 women). Baseline GDF15 levels correlated with age (r=0.263, P< 0.05); age-adjusted analyses were applied. In total, pre-operative GDF15 levels did not significantly correlate with functional measures at any timepoint. However, in men (n=44), GDF15 levels correlated with age (r=0.408, P< 0.05) and significant age-adjusted correlations were observed in fatigue (r=0.368, P< 0.05), gait speed (r=-0.428, P< 0.05), and SPPB score (r=-0.329, P< 0.05). Additionally, GDF15 levels correlated with chair stands at six months (r=-0.398, P< 0.05), SPPB score at six months (r=-0.384, P< 0.05), and gait speed after one year (r=-0.487, P< 0.05). GDF15 did not significantly correlate with changes in SPPB score (P>0.05). There were no significant correlations between GDF15 and any of these functional measures in women (r-value range: -0.259 to 0.107; p-value range: 0.112 to 0.969). Elevated pre-operative GDF15 appears to correlate with worsening physical function following knee replacement in men, but not women. Further investigation is necessary to understand the relationship between GDF15 and the biology of physical resiliency.

Abstract citation ID: igad104.3367

### GENDER DIFFERENCES IN COVID-19 EXPERIENCES OF OLDER FEMALE VETERANS USING THE PROSPECTIVE HERO CARE SURVEY DATA

Pranjal Tyagi[1], Sandra Garcia[2], Erin Bouldin[3], Ranak Trivedi[4], Orna Intrator[5], Mary Jo Pugh[6], Luci Leykum[7], and Stuti Dang[2], *1. South Florida VA Foundation for Research and Education (SFVAFRE), Miami, Florida, United States*, *2. University of Miami, Miami, Florida, United States*, *3. Decision-Enhancement and Analytic Sciences Center (IDEAS 2. .0. ), Salt Lake*

*City, Utah, United States*, 4. *Stanford University, Stanford, California, United States*, 5. *Canandaigua VAMC and University of Rochester, Canandaigua, New York, United States*, 6. *VA Salt Lake City Health Care System, Salt Lake City, Utah, United States*, 7. *South Texas Veterans Health Care System, San Antonio, Texas, United States*

Aging female Veterans faced unique experiences during the COVID-19 pandemic. We used self-reported experiences of older female Veterans during this time in comparison with male Veterans. We received responses for the HERO CARE survey from 2,080 (88 (4.2%) female, 1,992 (95.8%) male) community-dwelling Veterans from five geographically diverse Veterans Affairs Medical Centers. Veterans were prompted to self-report the impact of COVID-19 through an open-ended question asking to share anything they would like us to know about their experience. Open-ended responses were classified through inductive coding into emergent themes and chi-square tests. The most common emergent themes from female Veteran responses were social isolation (17.8%), negative effects on physical health (15.6%), and issues with accessing VA healthcare resources (15.6%). The most common emergent themes from male Veterans were social isolation (12.7%) and issues with accessing VA healthcare resources (8.2%). However, female Veterans were more likely to report experiencing a decline in their physical health compared to males (p< 0.01), more likely to report experiencing a negative change in their mental health (p< 0.01) and more likely to experience a decline in their general satisfaction with life (p< 0.002). They were also more likely to experience an issue with accessing VA healthcare resources (p< 0.02), and to experience financial problems (p< 0.0001). Female Veterans were more likely to report negative impacts of the COVID-19 pandemic to their mental and physical health, general satisfaction with life, and accessing VA healthcare resources. Routine assessment may be needed to adequately address their needs.

Abstract citation ID: igad104.3368

### GENETIC PLEIOTROPY OF KIDNEY FUNCTION AND SOLUBLE RECEPTOR FOR AGE: THE LONG LIFE FAMILY STUDY

Mary Feitosa[1], Shiow Jin[2], Sandeep Acharya[2], Mary Wojczynski[1], Kaare Christensen[3], Joseph Zmuda[4], Michael Brent[2], and Michael Province[1], *1. Washington University School of Medicine, St. Louis, Missouri, United States*, 2. *Washington University at St Louis, St. Louis, Missouri, United States*, 3. *University of Southern Denmark, Odense, Syddanmark, Denmark*, 4. *University of Pittsburgh, Pittsburgh, Pennsylvania, United States*

Chronic kidney disease (CKD) patients have increased inflammation and oxidative stress that may escalate the production of advanced glycation end-products (AGEs), a biomarker implicated in aging. Circulate soluble receptor for AGE (sRAGE) is associated with CKD and inversely associated with the estimated glomerular filtration rate (eGFR). In the Long Life Family Study, eGFR measured by creatinine (eGFRcr) and cystatin-C (eGFRcys) correlated with sRAGE (r~ -0.27, p< 1x10-30). We investigated genetic pleiotropy conducting GWAS for eGFRcr, eGFRcys, and sRAGE and correlated meta-analysis (CMA) on GWAS p-values from whole-genome sequence variants in 4,182 subjects (age range: 24–110). Of the 59 loci identified (p< 5x10-8), 42 were novel eGFR discoveries. We performed TWAS and CMA-TWAS for eGFRcr, eGFRcys, and sRAGE on blood RNA-seq in 1,209 subjects and identified 17 genes with pleiotropic expressions for eGFRs and sRAGE (Bonferroni-p< 2.73x10-6). Several CMA-GWAS gene variants (e.g., SH3GLB1/SELENOF, CENPP) were eQTLs in glomeruli and tubule kidney (Human Kidney eQTL Atlas, HK-Atlas) and other tissues (GTEx-v8). The bioinformatics analyses identified (i)genes harboring eQTLs in glomeruli and tubule kidney (HK-Atlas), (ii)enhancer-promoter gene variants associated with kidney function-related phenotypes at genome-wide significance (GeneHancer/GeneCards/GWAS-catalog), and (iii)enhancer regions predicting gene expressions in kidney related-function (EPRI, enhancer-promoter-RNA maps). Some functional genes were found among bioinformatics (e.g., MTX1, TOPORS, SNTB1, LSP1, SNTB1, ATG2A, TP53INP2) and others were unique (e.g., TEC, CCN1, and ERGIC1, GeneHancer/GeneCards/GWAS-catalog). Genomic, transcriptomic, and bioinformatics results demonstrated evidence that some novel variants and genes have functional regulatory features in the kidney and are likely involved in biological and clinical aging.

Abstract citation ID: igad104.3369

### GENETIC VARIANTS ASSOCIATED WITH BLOOD PRESSURE PHENOTYPES IN OLDER ADULTS

Lana Sargent, Dave Dixon, Marissa Mackiewicz, and Elvin Price, *Virginia Commonwealth University, Richmond, Virginia, United States*

African Americans (AAs) suffer from higher rates of uncontrolled HTN even when receiving commonly prescribed classes of antihypertensive medications. Variation in blood pressure (BP) in older adults is known to be associated with a variety of cardiovascular endpoints, yet our understanding of underlying mechanisms is limited. This study aimed to determine if HTN genetic variants influence blood pressure variation within a population of AA older adults taking antihypertensives. Community-dwelling older adults from the Translational Approaches to Personalized Health (TAPH) cohort were recruited to enroll in a 24-hour ambulatory BP monitoring (ABPM) phenotyping study. The Quant Studio 12-K Flex system was used for genotyping participants for three previously established CACNA1C and CACNB2 genetic risk alleles (rs758173, rs2228645, and rs2357790). 69 participants had ABPM data that passed the minimum 80% successful readings threshold: age=68□6, Female=50.3%, Black=73%, White=21%, and Other=6%. ABPM revealed 50.7% controlled HTN, 27.5% Sustained uncontrolled HTN, 14.5 % White coat uncontrolled HTN and 7.2% Masked uncontrolled HTN. ANOVA and Linear regression models found that the CACNB2 genetic risk allele rs2228645 broadly impacts ABPM Phenotypes (C/C SBP= 120.8+/-12.9 mmHg, C/T SBP= 125.73+/-13.5, and T/T SBP= 140+/-34.8 mmHg, p=0.011), mean awake 24-hour SBP (C/C SBP= 124+/-12.7 mmHg, C/T SBP= 133.5+/-14.8, and T/T SBP= 143.7+/-25.6 mmHg, p=0.012), and mean sleep SBP (C/C SBP= 114+/-16 mmHg, C/T SBP= 125.4+/-15, and T/T SBP= 136+/-50 mmHg, p=0.024). CACNA1C and CACNB2 genetic variants influence blood pressure phenotypes in older AAs. Determining genetic variants that affect antihypertensive effectiveness could lead to improved antihypertensive therapy for individuals with HTN/CVD.

Abstract citation ID: igad104.3370

### GERIATRICS IN HEALTH PROFESSIONS EDUCATION: A BIBLIOMETRIC ANALYSIS OF 70 YEARS OF RESEARCH

Renzo Pajuelo-Vasquez, Wendy Gutierrez-Baca, Javier Flores-Cohaila, and Fernando M. Runzer-Colmenares, *Universidad Cientifica del Sur, Lima, Lima, Peru*

To move forward in the field of Geriatrics in Health professions education (HPE), we must understand its conception and recognize current trends and future directions. Therefore, we conducted a bibliometric and network analysis of all studies indexed in the Scopus Database related to Geriatrics and HPE from 1953 to 2022. We retrieved 2940 studies comprising 7344 authors and 618 journals; these were imported to bibliometrics (RStudio) and Vos Viewer. To understand the intellectual structure, we employed a co-citation analysis; we identified 5 clusters that described fundamentals in geriatrics, nursing education, needs assessment, and interprofessional education (IPE). Furthermore, six topics were placed in the keyword co-occurrence, ranging from medical, nursing, dental, social workers, and interprofessional education. Consequently, we identified geriatrics education for medical students and interprofessional education between 2004 and 2016; furthermore, clinical competence was the most recent trending topic, accompanied by end-of-life care and geriatric psychiatry. In the evolution map, three topics emerged in the last ten years: IPE, online learning, and geriatric dentistry. These findings were confirmed in the bibliographic coupling analysis. Four major themes emerged among 13 clusters: IPE, Attitudes and career orientation to geriatrics, innovative educational methods such as gamification, and geriatric undergraduate education. Although we identified several studies, this barely represents the 1% of production in HPE. Furthermore, we identified a trend toward IPE, undergraduate medical education, career orientation, and innovative education. However, there is a lack of themes such as simulation and entrustable professional activities, with an underrepresentation of the developing world.

Abstract citation ID: igad104.3371

### GEROSCIENCE BLOOD-BASED BIOMARKER INDICES AND PHYSICAL PERFORMANCE IN OLDER ADULTS

Denise Houston[1], Deeya Pathak[1], Rebecca Neiberg[1], Jamie Justice[2], Barb Nicklas[1], and Michael Miller[1], *1. Wake Forest University School of Medicine, Winston-Salem, North Carolina, United States*, *2. XPRIZE Foundation, Winston-Salem, North Carolina, United States*

Dysregulation in cellular and molecular aging biomarkers are hypothesized to contribute to worse physical performance. We examined associations between blood-based geroscience biomarker indices and physical performance in 414 older adults (77.4±4.4 years; 69% female). Physical performance measures included the expanded SPPB, 4m and 400m walk, and grip strength. Serum IL-6, CRP, TNFr1, GDF15, and insulin were measured and three biomarker indices created: 1) a quintile index calculated by summing quintile scores for each of the biomarkers; 2) a composite z-score calculated using the standardized mean of individual biomarker z-scores; and 3) a principal component analysis (PCA) to identify biomarker factor(s). The quintile index and composite z-score were highly correlated ($r=0.94$, $p< 0.0001$). PCA identified two factors: TNFr1 and GDF-15 weighted heavily on Factor 1 and IL6 and CRP weighted heavily on Factor 2. The quintile index and composite z-score were correlated with PCA Factors 1 and 2 ($r>0.61$, $p< 0.0001$). Similar standardized beta coefficients were observed for the quintile index, composite z-score, and PCA Factor 1 with 4m and 400m gait speed (all $p< 0.0001$); every 1 SD higher biomarker index score was associated with ~0.25 SD slower gait speed. For expanded SPPB, higher standardized beta coefficients were observed for the quintile index and composite z-score (~-0.32) than PCA Factors 1 and 2 (~-0.24; all $p< 0.0001$). Weak associations between all indices and grip strength were observed ($p\geq0.05$). Geroscience biomarker indices that capture inflammation and poor stress response appear to have similar associations with worse physical performance in older adults.

Abstract citation ID: igad104.3372

### GETTING TO KNOW YOU: USING VIRTUAL REALITY IN VOLUNTEER RESPITE PROGRAM FOR PERSONS WITH MEMORY LOSS

Louanne Bakk[1], Lillian Agyemang[2], Mary Milnamow[1], Jason Dauenhauer[3], and Katy Allen[4], *1. University at Buffalo, Buffalo, New York, United States*, *2. University at Buffalo School of Social Work, Buffalo, New York, United States*, *3. SUNY Brockport, Brockport, New York, United States*, *4. Lifespan of Greater Rochester, Rochester, New York, United States*

Virtual reality (VR) can provide an immersive experience that simulates real or imagined environments and provides an innovative solution to promote engagement and socialization. Interventions to date have not explored the use of trained volunteers to use VR to promote socialization and provide active experiences with community-dwelling persons with memory loss. This presentation will provide an overview of a volunteer-led drop-in respite caregiving program. The program uses Rendever's VR platform, which includes a series of pre-programmed and on-demand immersive experiences delivered to persons with memory loss through a VR headset via a volunteered-controlled tablet. Volunteers conduct tailored individual and group VR sessions as a scheduled activity with persons with memory loss. This study aimed to understand the experiences of volunteers administering VR programming within the drop-in respite center. A total of eight volunteers participated in a focus group. A semi-structured interview guide explored volunteers' motivation for becoming trained to use VR, observations of using VR with persons with memory loss, how they are implementing VR, challenges they experienced using VR, and ideas for improving/expanding implementation. Volunteers reported increased socialization when using VR with persons with memory loss, a desire for continued and ongoing VR training, and suggested larger group VR sessions delivering the same content to foster greater interaction among respite participants. Results suggest that VR programming can be an effective format for increasing socialization and involvement among persons living with memory loss.

Abstract citation ID: igad104.3373

### GRANDPARENTS RAISING GRANDCHILDREN IN NEW YORK STATE: UNDERSTANDING NEEDS OF A VULNERABLE POPULATION

Kate Guastaferro[1], Nusrat E Mozid[1], Jayna Kramsky[1], Matilda Melkonian[1], Kashyah Young[1], and Essence Spears[2], *1. New York University, New York City, New York, United States*, 2. *Clark Atlanta University, Atlanta, Georgia, United States*

Grandparents raising grandchildren are a rapidly growing population in the US – the 2020 census estimated 5.9million children < 18 were living with a grandparent and in 2023 this estimate exceeded 7.1million. The older adult was likely not anticipating raising a child and is subject to the same stressors as other adults of their age and similar socioeconomic status: financial insecurity, health problems, employment issues, and family conflict. The community-based interventions that exist for 'grandfamilies' address a small portion of their needs and there is no universal network of services for this family unit that can sufficiently address the grandparents' need for support, resources, and training. A total of N = 58 grandparents (56% female; 70% White; 40% urban-dwelling; 63% married) raising grandchildren in New York State participated in the GRAND Study which sought to quantify the needs of grandparents raising grandchildren using mixed methods. Survey results, corroborated by qualitative interview and focus group data, suggest areas of need include: financial needs, legal needs (e.g., acquiring custody), social support and parenting. Additional needs were observed in grandparents' self-reported level of depressive symptoms – 46% of responses indicated major depressive disorder – and in managing relationships with their adult child (i.e., parent of grandchild). Overwhelmingly, grandparents spoke about the 'honor' of raising their grandchildren despite the challenges, specifically how the public health systems with whom they interact are not responsive to their needs. Results can be used to inform a multicomponent behavioral intervention to improve the general health and well-being of grandfamilies.

Abstract citation ID: igad104.3374

### GRAY MATTER CORRELATES OF ANXIETY, DEPRESSION, AND GAIT SPEED IN OLDER ADULTS WITH CHRONIC PAIN

Ania Lipat, David Clark, and Yenisel Cruz-Almeida, *The University of Florida, Gainesville, Florida, United States*

Chronic pain and its comorbidities, (e.g., anxiety, depression, slow gait speeds) are a widespread, disabling condition prevalent among older adults. Worse anxiety and depression have been associated with slower gait speeds in individuals with chronic pain, and each comorbidity has been associated with structural gray matter changes; however, results are inconclusive or limited. Further investigation into these relationships may provide insight into neural mechanisms through which anxiety and depression contribute to slow gait speeds in this population. Therefore, the aim of this study was to identify gray matter measures associated with anxiety, depression, and gait speed in older adults with chronic pain. Participants (n = 31) walked across a GAITRite mat to obtain gait speed, and completed the State-Trait Anxiety Inventory (STAI), Center for Epidemiologic Studies Depression Scale (CES-D), and a T1-weighted anatomical scan. STAI score (mean = 7, $\beta$ = - 0.015, p = 0.010) and CES-D score (mean = 30, $\beta$ = - 0.014, p = 0.016) were significantly associated with gait speed. CES-D score was associated with left cuneus gyrus ($\beta$ = 0.017, p = 0.0002) and frontal inferior sulcus surface area ($\beta$ = 0.019, p = 0.002). Gait speed was associated with right inferior temporal gyrus surface area ($\beta$ = 0.436, p = 0.019). STAI score was not associated with any measure. In conclusion, anxiety and depression symptoms contribute to gait slowing in older adults with chronic pain but without clinical depression or anxiety. This relationship, however, is not reflected in each comorbidity's structural neural correlates.

Abstract citation ID: igad104.3375

### GREATER PHYSICAL ACTIVITY AND SOCIAL ENGAGEMENT OFFER PROTECTION AGAINST COGNITIVE DECLINE OVER TIME

Usha Dhakal, Sara McLaughlin, Seonjin Kim, Amy Roberts, Jonathon Vivoda, and J. Scott Brown, *Miami University, Oxford, Ohio, United States*

Evidence suggests that physical activity and social engagement in later life are associated with a less pronounced and reduced risk of cognitive decline over time; however, findings are mixed and knowledge concerning the effect of these factors on cognitive functioning in older adults with mild cognitive impairment (MCI) is underdeveloped. To enhance understanding, we extracted data from eight waves (2004-2018) of the Health and Retirement Study and used hierarchical linear mixed models to investigate the impact of physical activity and social engagement on cognitive functioning in Americans aged 60 years and older with MCI (n=1462). The average cognitive score (sum of immediate and delayed word recall, serial seven subtraction test, counting backwards, range: 0 to 27) at baseline was 9.6, which decreased by 0.14 points each year. Each unit increase in physical activity (weighted sum of light, moderate, vigorous activity; range: 9 to 45) and social engagement (measure capturing social visits, volunteerism, religious participation; range: 0 to 3) reduced the effect of time on cognitive functioning by 0.01 ($\beta$=0.007, p<.0001) and 0.04 ($\beta$=0.040, p<.0001) points, respectively, while simultaneously controlling for APOE genotype and other covariates. This means, for example, that the cognitive score of someone with a physical activity score of 40 was, on average, 3.1 points higher than someone with a physical activity score of 10 after 16 years of follow-up. Findings suggest that greater physical activity and social engagement offer protection against cognitive decline and could minimize MCI progression among older adults experiencing MCI.

Abstract citation ID: igad104.3376

### GROUP RESIDENTIAL TRAINING OF BLIND OLDER ADULTS OFFERED BY VISUALLY IMPAIRED AND SIGHTED PROFESSIONAL TRAINERS

Nancy Miller, Frankie Ann Marcille, Sierra Storm, and Cathleen McGuire, *VISIONS/Services for the Blind and Visually Impaired, New York City, New York, United States*

VISIONS/Services for the Blind and Visually Impaired piloted a 6-day residential group training for 6 legally blind older adults ages 57-80, self identified as Caucasian or African-American. Assessments occurred in five core areas: personal, home, financial and meal management and communication skills. Mobility and family care skills were also assessed. One legally blind and one sighted professional instructor offered the training along with adaptive equipment and training on how to use it. Each participant received follow up visits in their home to measure how well they retained the skills taught and how well they were safely using them. Referrals were made to VISIONS occupational therapists to address other chronic health conditions, social workers for adjustment to blindness counseling or low vision optometrists for additional follow up. Offering intensive residential group training with peers resulted in documented improvement in ADL/IADL skills and increased social connections. Group training, even when participants had varied skill levels, education, age of onset of vision loss, and eye diseases, proved successful. One participant framed his completion certificate and hung it in his living room. The value of learning new skills with a peer group was confirmed as well as the willingness for older adults with legal blindness to attend a 6-day residential program. Providing group training helps to address the long waitlist for services with a national personnel shortage of nationally certified vision rehabilitation therapists to serve the growing older blind. population. Group training also effectively utilizes the limited government funding for training older blind adults.

Abstract citation ID: igad104.3377

### HAPPY LIFE EXPECTANCY AND REGIONAL AND GROUP HETEROGENEITY AMONG CHINESE OLDER ADULTS

Chao Guo, and Peisen Yang, *Peking University, Beijing, Beijing, China (People's Republic)*

A long, healthy and happy life is the aspiration of most people and an essential part of human well-being. However, longer life expectancy does not mean a higher quality of life. The measure of quality of life includes both objective and subjective indicators. Similar to healthy life expectancy, happy life expectancy is a measure that combines life expectancy with happiness indicators and can reflect both life length and quality of life. But at present, there is relatively little research on happy life expectancy. In China, there have been no studies using panel data to calculate happy life expectancy. We obtained longitudinal data from the Chinese Longitudinal Health Longevity Survey (CLHLS). We used the multistate life table approach to calculate the happy life expectancy of Chinese older adults, and analyzed the regional and group heterogeneity. A total of 13,807 participants aged 65 and older were included in this study. We found that the proportion of happy life expectancy to remaining life of Chinese older adults increased with age, which confirmed the "compression of unhappiness" hypothesis. In addition, older adults with more education had a higher proportion of happy life expectancy than those with less education. Finally, in terms of region, older adults in the more economically developed areas of eastern China have a higher proportion of happy life expectancy. Our findings provide a more comprehensive understanding of the quality of life of Chinese older adults and highlight the importance of education for the happiness of older adults in later life.

Abstract citation ID: igad104.3378

### HAPPY TO CHAT – PROMOTING TALKING TO STRANGERS IN PUBLIC PLACES IN THE UK

Christina Victor, and Dorothy Yen, *Brunel University London, Uxbridge, England, United Kingdom*

After the Covid-19 pandemic, many countries are trying to reboot social connections in communities and societies, especially for older people who suffered mental wellbeing due to lockdowns and social distancing. Existing studies show talking to strangers can improve individuals' well-being and reduce social isolations, although only with data from younger participants. This research aims to understand older people's experience of talking to strangers, specifically in discussing how, why and where they feel most comfortable in talking to strangers. Using semi-structured interviews with 23 people (from 51 to 88 years old) based on their participation in a community-based Happy to Chat intervention (Happytochat.uk) from January to April 2023, we discuss how talking to strangers are consumed and practised by older adults. Applying agency and self-efficacy theory, findings show that their experience vary by age, gender and two types of self-efficacy: a) confidence in enjoying conversations with strangers; b) capability in navigating the risk involved in talking to strangers. The findings show that compared to the younger group (51-64 years old), the older participants (65+) are more willing and spend more time talking to strangers. Also, male participants tend to be more confident than females in navigating the risk associated with talking to strangers. Our research findings have practical and policy implications. Insights derived from the research findings are useful in developing training materials or conversation aids to improve current chatty schemes and increase older people's self-efficacy in having conversations with strangers, thus benefiting from better social connections.

Abstract citation ID: igad104.3379

### HEALTH AND SOCIAL CARE TRAINEES' ATTITUDES ABOUT AGING AND OLDER ADULTS

Min-Kyoung Park[1], and Diane Martin[2], *1. University of Maryland Baltimore and Baltimore County, Baltimore, Maryland, United States*, *2. University of Maryland, Baltimore, Baltimore, Maryland, United States*

Ageism is pervasive in healthcare. It costs billions of healthcare dollars and results in earlier patient mortality and decreased quality of life. Existing research highlights the prevalence of ageist attitudes among health and social care practitioners, but there is a dearth of research regarding attitudes of trainees toward older adults. The present study utilized the Fraboni Scale of Ageism to examine attitudes of health and social care trainees participating in geriatric interprofessional training programs. Participants completed an electronic survey prior to the didactic portion of the program. Seventy-seven surveys were completed between November 2022 and June 2023. Results revealed a nuanced understanding of older adults' contributions, particularly regarding creativity, social and political engagement, and the richness of their accumulated life experiences. Positive sentiments were evident regarding interactions and participants acknowledged the self-management capacities of older adults, encompassing personal hygiene and other aspects of independent living. Notably, findings underscore substantial support for creation of inclusive spaces. Overall, a person-centric understanding of older adulthood was evident in the results; less than 20% of trainees expressed a negative perception of older adults/later life to any given question. Recognizing the significance of bridging intergenerational perception disparities to counter ageism, this research contributes to a foundational understanding of younger generations' awareness of aging. Given the contrast to existing research regarding attitudes of practitioners toward older adults, these results emphasize the importance of fostering opportunities for intergenerational dialogue and activities to maintain an attitude of inclusivity and empathy that underscores a person-centered approach to healthcare.

Abstract citation ID: igad104.3380

### HEALTH PROFESSIONAL STUDENT-LED SOCIAL PHONE CALLS TO ADDRESS LONELINESS IN AGING-IN-PLACE ADULTS AFTER STROKE

Jason Burnett, Sean Savitz, Jordan Broussard, and Jennifer Beauchamp, *University of Texas Health Science Center at Houston, Houston, Texas, United States*

Social isolation and loneliness (SIL) increases the risk of stroke and recurrence by 32% and 40%, respectively. The functional and cognitive deficits that often accompany stroke can impair social engagement behaviors and increase SIL. Intergenerational programs have proven effective for addressing SIL through social engagement behavior change in older adults, but the feasibility of these programs is unspecified for aging-in-place adults after stroke. We paired 14 community-living adults after stroke with 14 health professional students (HPS) for 6-weekly unstructured social phone calls. Baseline and follow-up data measuring SIL and other psychosocial metrics were conducted. HPSs journaled following each call. The 13 (93%) participants completing all calls were an average of 57 years old and white non-Hispanic (77%). The calls lasted approximately 49 minutes. Average baseline scores for loneliness and social isolation were 48 and 22, respectively. The average score for loneliness decreased 4 points at week six. The average score for social isolation was unchanged. Average baseline scores for symptoms of depression and anxiety were 20 and 8, respectively, and scores decreased by 1 point at week six. Participants disclosed physical and emotional challenges, previous valued employment, and enjoyment from the social engagement. HPSs learned about the struggles of aging-in-place after stroke and valuing compassionate care and social engagement. An intergenerational social phone call program provided a feasible, acceptable, and encouraging approach for addressing loneliness in community-living adults after stroke and has encouraging effects on mental health. A randomized controlled trial is needed to provide definitive data on intervention effects.

Abstract citation ID: igad104.3381

### HEALTH PROMOTION AS CATALYST: CHALLENGES AND COMPLEXITIES OF HEALTHCARE AMONG OLDER ASIAN IMMIGRANTS

Sabita Shrestha[1], Jae Kim[1], and Mary Hogue[2], *1. Western Kentucky University, Bowling Green, Kentucky, United States*, *2. Primary Health Clinic, Las Vegas, Nevada, United States*

Older adults are the most disadvantaged, vulnerable, and marginalized groups in the world. They often experience various challenges and complexities in navigating healthcare and other social care. Needless to say, these complexities are profound, especially, among older Asian immigrants in navigating healthcare and social care compared to other populations in the US. They often face various sociocultural challenges including structural barriers in search of proper healthcare and accessibility to care. The studies have shown older Asian immigrants are reluctant to seek or access needed care due to structural barriers poised against them. In addition, lack of cultural awareness and understanding by healthcare professionals further impedes accessing timely healthcare. Despite the complexities of healthcare navigation, health education and promotion play an important role as catalysts in disseminating health information relevant to healthcare and structural barriers to older Asian immigrants. Based on ecological framework, this presentation will showcase a holistic approach to health education intervention and promotion aimed to minimize complexities of healthcare navigation and structural barriers towards older Asian immigrants in the US.

Abstract citation ID: igad104.3382

### HEALTH RELATED SOCIAL NEEDS AND TREATMENT PREFERENCES OF OLDER ADULTS WITH SERIOUS ILLNESS

Amy Jackson[1], and Joan Carpenter[2], *1. University of Maryland, College Park, Maryland, United States*, *2. University of Maryland School of Nursing, Baltimore, Maryland, United States*

The Centers for Medicare & Medicaid Services' (CMS) health related social needs (HRSNs) include housing instability, food insecurity, transportation, utility difficulties, and interpersonal violence. HRSNs shape the experiences and outcomes of seriously ill older adults, influencing their access and ability to choose preferred treatments —from the time of diagnosis until the end of life. Little research has explored disparities in healthcare experiences related to unmet HRSNs and treatment choices, including palliative and end of life care. The purpose of this integrative review is to explore the literature regarding unmet HRSNs and provide research, practice, and policy considerations for investigators, clinicians, and payers regarding HRSNs and their impact on palliative and end of life treatment decisions. We searched PubMed, Embase, CINAHL, PsychINFO, Scopus, and Google Scholar using keywords related to HRSNs and palliative and end of life care. We identified 38 articles between 1998 and 2023 within the United States. Of the 38 articles, housing instability was the most often addressed (n=36), followed by transportation (n=1) and interpersonal violence (n=1). None of the articles explored food insecurity and utility difficulties related to serious illness and treatment preferences. Many of the articles describe the perspectives of people experiencing homelessness at the end of life (n =16). The current literature does not comprehensively address unmet HRSNs in relation to palliative and end of life care treatment choices. Future work must address HRSNs of seriously ill older adults to ensure equitable access to compassionate and person-centered palliative and end of life care.

Abstract citation ID: igad104.3383

**HEALTH SYSTEM LEADERSHIP PERSPECTIVES AND PRIORITIES ON CARE FOR OLDER ADULTS**

Emily Franzosa[1], Lynette Kelley[2], Eileen Dryden[3], Lauren Moo[3], and William Hung[1], *1. Icahn School of Medicine at Mount Sinai, New York City, New York, United States*, *2. VA Eastern Colorado Health Care System, Aurora, Colorado, United States*, *3. VA Bedford Healthcare System, Bedford, Massachusetts, United States*

Innovative care models for older adults such as non-institutional and home-based care have the potential to improve quality and efficiency of healthcare, but adoption of these models requires resources from health systems. Health systems leaders can influence the relative priority of geriatric care and associated resource allocation. This influence is especially important for areas with limited resources such as in rural areas. We interviewed recently retired health system leaders about their experience and how priorities in health systems can be aligned to improve the quality and reach of geriatric care. We conducted qualitative interviews with 10 participants (40% female) using a snowball sampling strategy of geographically diverse, retired hospital, regional, and national leaders in the Veteran Healthcare system, the largest single-payer system in the US. Participants described how they managed myriad competing health system priorities by weighing and devoting attention and time to tackle the crises of the day, the numerous national mandates and directives, and the goals for the local hospital or system, as seen from their own perspectives. Alignment with performance metrics provides strong impetus for some health system leaders to determine their priorities. Incentives to encourage health systems leaders to prioritize and allocate resources aimed at long-term improvements in care could be important. Additionally, building relationships and maintaining trust amongst partners across facilities and offices is critical to initiating and growing geriatrics programs. This work highlights that influence must work on multiple levels to effect change in prioritization of care for older adults.

Abstract citation ID: igad104.3384

**HEALTHCARE SERVICES UTILIZATION IN ELDERLY RESIDENTS OF A PERUVIAN HIGHLAND COMMUNITIES: AUNQUI-ANDES STUDY**

Nelson Luis Cahuapaza-Gutierrez, Kamyla M. Olazo-Cárdenas, Diego Urrunaga-Pastor, and Fernando M. Runzer-Colmenares, *Universidad Científica del Sur, Lima, Lima, Peru*

It is estimated that approximately one out of every three Peruvians does not utilize healthcare services when facing an ailment. Nevertheless, this frequency could be higher in highland regions, given the limited availability of healthcare services. The aim was to estimate the prevalence of non-utilization of healthcare services among elderly residents of a Peruvian highland community during 2022. We conducted an analytical cross-sectional study in the district of Totos (average altitude of 3286 meters above sea level) in the Ayacucho region of Peru during 2022. A census sampling method was employed, including residents aged 60 and older in Totos. Non-utilization of healthcare services was defined as a participant not seeking healthcare services when facing a health issue. The current study was approved by the Institutional Research Ethics Committee of Universidad Científica del Sur. We evaluated 272 elderly adults with an average age of 74 years, of which 60.3% were female, 62.5% were cohabiting or married, 82.3% had not completed primary education, and 65.4% reported being beneficiaries of a social program for economic support. We found that 28.3% did not use healthcare services, while 44.9% reported using traditional medicine, and 13.2% practiced self-medication. Participants reported that the nearest health center to their residence was, on average, 55 minutes away. We found that approximately one out of every five elderly adults did not use healthcare services. It is necessary to reduce cultural barriers to increase healthcare service utilization, as well as to enhance their availability for elderly individuals living at high altitudes.

Abstract citation ID: igad104.3385

**HEALTHCARE WORKERS' PERSPECTIVES ON AI-ENABLED ROBOTS USE IN LONG-TERM CARE: A SCOPING REVIEW.**

Lillian Hung[1], Karen Lok Yi Wong[1], Joey Wong[1], JuYoung Park[2], Hadil Alfares[1], Yong Zhao[1], Hossein Mousavi[1], and Hui Zhao[3], *1. University of British Columbia, Vancouver, British Columbia, Canada*, *2. Florida Atlantic University, Boca Raton, Florida, United States*, *3. James Madison University, Harrisonburg, Virginia, United States*

Artificial intelligence (AI) enabled robots are increasingly implemented in long-term care homes (LTC). However, the views of LTC staff on these robots remain largely unexplored. Our scoping review delves into the staff's perceptions, outlining the advantages and challenges of using AI robots in LTC settings. Using the Joanna Briggs Institute's methodology, we screened 86 articles from 2013 to 2023, with 35 fitting our criteria. Our analysis was informed by McCormack's Person-centred Care Practice (PCP) Framework and the Consolidated Framework for Implementation Research (CFIR). We identified five key barriers: 1) the complexity of the robot, 2) potential job losses and increased workload, 3) concerns about safety and efficacy, 4) risk of depersonalized care, and 5) a lack of supportive regulations and resources in LTC facilities. To address these challenges, we recommend strategies: a) staff training, b) clarifying robot benefits to staff, c) demonstrating how robots can fulfill resident needs, d) implementing ethical guidelines, and e) aligning robot use with LTC policies while ensuring resource availability. In conclusion, partnership is required among healthcare workers, organizational leaders, robot developers, and researchers; they should not work in silos. More research is needed to explore how to facilitate effective partnerships.

Abstract citation ID: igad104.3386

**HEALTHY AGING AND OLDER ADULTS WITH AUTISM: A SCOPING REVIEW**

Mary Stringfellow[1], Noelle Fields[1], Kathy Lee[1], and Keith Anderson[2], *1. University of Texas at Arlington, Arlington, Texas, United States*, *2. University of Mississippi, Oxford, Mississippi, United States*

Autism Spectrum Disorder (ASD) is a lifelong diagnosis. While several studies have examined the effects of this disorder throughout childhood, few have considered older adulthood and the differences between allistic (i.e., persons who are not affected by autism) and autistic older adults. Moreover, an examination of the intersections of healthy aging in older adulthood and autism are largely absent from extant research. The purpose of this scoping review was to examine the state of literature regarding autistic older adults and the domains of healthy aging, utilizing the framework developed by Arksey and O'Malley (2005). After developing the research question, the researchers followed following the PRISM-ScR guidelines. The specific domains of healthy aging considered included physical health, mobility, mental health, cognition and memory, and social connectedness. A total of 35 articles were selected for final review. There was no definitive age group for autistic older adulthood, therefore the studies' selection of ages for the older adult population were not homogenous. Fifty-seven percent of these studies considered only one domain of healthy aging, 23% studied two domains, while 14% measured three domains and 6% assessed four domains. No study considered all five domains. Of the five domains considered for this scoping review, mental health and cognition and memory were the most represented domains in the studies. Overall, healthy aging among autistic older adults is largely understudied. More research is needed to better identify and tailor interventions and services for autistic older adults and their families to support healthy aging.

Abstract citation ID: igad104.3387

### HEARING LOSS AND HOSPITALIZATION IN OLDER ADULTS: EVIDENCE FROM THE ATHEROSCLEROSIS RISK IN COMMUNITIES STUDY

Wuyang Zhang[1], Emmanuel Garcia Morales[2], and Nicholas Reed[1], *1. Johns Hopkins Bloomberg School of Public Health, Baltimore, Maryland, United States*, *2. Johns Hopkins University, Baltimore, Maryland, United States*

Hearing loss is highly prevalent among older adults and is associated with increased healthcare utilization. However, the majority of evidence relies upon self-report measures of hearing and hospitalization which are subjective to validity concerns. Using cross-sectional data from the Atherosclerosis Risk in Communities Study and linked Centers for Medicare and Medicaid Services (CMS) records (2016), we aim to investigate the association between objectively measured hearing loss and record-based hospitalization in a population-based sample. Among 1,330 older adults (Mean age: 79.3 years; Female: 57.8%; Black: 17.4%), hearing loss was measured using pure-tone audiometry and hospitalization outcomes, including occurrence of any hospital stays (yes/no); number of hospital stays; and duration of hospital stays, were ascertained from CMS records. In models adjusted for demographic, socioeconomic, and health characteristics, those with mild and moderate/greater hearing loss had 1.71 (95% CI: 1.13-2.58) and 1.87 (95% CI: 1.18-2.98) times higher prevalence of any hospital stay over 12 months; 2.23 (95% CI: 1.47-3.37) and 1.68 (95% CI: 1.04-2.71) times higher Incident Rate of multiple hospital stays, relative to those without hearing loss. Moreover, both groups had significantly longer durations of hospital stay (mild: Incident Rate Ratio [IRR]=2.41; 95% CI: 1.46-3.98; moderate/greater: IRR=2.32; 95% CI: 1.32-4.07). Using criterion exposure and outcome measures, these findings confirm previous work and provide the first insight into the association of hearing loss with increased inpatient service use. Future work should focus on accommodations for hearing to reduce healthcare utilization and design equitable healthcare systems that meet the needs of all older adults.

Abstract citation ID: igad104.3388

### HEAVY DRINKING, MENTAL HEALTH PROBLEMS AND RECOVERY IN OLDER ADULTS: EXPLORING SUPPORT NEEDS

Deepti John[1], Bethany Bareham[1], Amy O'Donnell[1], Jennifer Liddle[1], Carolyn Chew-Graham[2], Eileen Kaner[1], and Barbara Hanratty[1], *1. Newcastle University, Newcastle upon Tyne, England, United Kingdom*, *2. Keele University, Keele, England, United Kingdom*

Many older adults who consume alcohol at harmful levels experience mental health problems. Common life events such as retirement, bereavement, and declining health, can contribute to anxiety and depression, with alcohol consumption being used as a coping mechanism. Tailored support is crucial to address the unique needs of older adults facing both alcohol misuse and mental health problems. These patients often fall into gaps between services (family practice/alcohol/mental health), which are unable to meet their complex support needs. This qualitative study aimed to understand the lived experiences of older adults with co-occurring heavy drinking and mental health problems, support received and unmet needs. We conducted semi-structured interviews with 14 older adults living in the North of England (completed Summer 2023). Thematic analysis and constant comparison were employed in data analysis. Findings highlighted the importance of age-tailored initiatives such as access to mental health support while actively drinking, supervised detoxification, increased peer support and services that are age inclusive and friendly. Risk factors such as grief, loneliness, financial problems and undiagnosed trauma in older adults contributed to development of these conditions. Older adults need services to work together, integrated around patient needs and addressing heavy drinking and mental health concerns at the same time. Future research should focus on improving mental health literacy and, developing holistic and preventive interventions to address risk factors for co-occurring alcohol and mental health problems in older adults.

Abstract citation ID: igad104.3389

### HEMOPEXIN'S RELATIONSHIP WITH MUSCLE QUALITY AND COGNITIVE FUNCTION IN OLDER ADULTS WITH COGNITIVE IMPAIRMENT

Derong Zeng[1], Xiang Qi[2], Bei Wu[2], Teruaki Kawasaki[3], Ichiro Akiguchi[3], and Ayae Kinoshita[1], *1. Kyoto University, Kyoto, Kyoto, Japan*, *2. New York University, New York City, New York, United States*, *3. Kyoto Clinical and Translational Research Center for Neurocognitive Disorders, Kyoto, Kyoto, Japan*

The imminent increase in Alzheimer's disease (AD) and dementia increases the need for effective prevention strategies. Increasig attention has been paid to exercise as a prevention strateg to promote cognitive health. Recently, the link between hemopexin which is secreted by atrophied skeletal muscle and cognitive function maintenance has been studied, but evidence remains inconclusive.This study examined the relationship between hemopexin, muscle mass, muscle quality (Phase Angle, PhA), and cognitive function (measured by Mini-Mental State Examination) among 377 cognitively impaired older adults (mean age = 83.5 years).TANITA (MC-780A-N) was used to measure body composition. The concentration of hemopexin in human plasma samples was measured using the HPX (Human) ELISA Kit (KA0481). In Pearson correlation analysis, hemopexin showed no significant correlation with muscle mass, quality, or cognitive function. Multivariate regression analysis revealed that lower PhA is significantly associated with poorer cognitive function (t-value=2.012, p< 0.05), but hemopexin and muscle mass were not significant. An interaction analysis indicated that the relationship between hemopexin and muscle quality was modified by sex (t-value=2.052, p< 0.05). In the multivariate analysis for females, hemopexin correlated negatively with muscle quality (t-value=-2.128, p< 0.05). This study shows that hemopexin, though not affecting cognitive function or muscle mass directly, may impact muscle quality, especially considering the link between AD in older women and atrophied muscle with hemopexin secretion. Hence, interventions focused on muscle quality might improve cognitive function in older individuals with cognitive impairment, possibly preventing dementia onset. Further studies are warranted to solidify these connections.

Abstract citation ID: igad104.3390

### HISTORICAL CHANGES IN COLLEGE EDUCATION AND EMPLOYMENT: GENDER EFFECTS ON LATE-LIFE COGNITIVE STATUS

Marina Larkina, and Jacqui Smith, *University of Michigan, Ann Arbor, Michigan, United States*

Early-life education and gender are well-known predictors of cognitive status in old age but multiple mechanisms underlie these associations. Recent US research on late-life cognition and dementia prevalence has highlighted the roles of age-cohort and racial/ethnic differences K-12 education. Less attention has been given to historical increases since the 1970s in the completion of college degrees by women and their subsequent employment histories. We examined new data for two cohorts (N = 9926; 50% born before 1948 vs. Baby Boomers, born 1948-1959) in the Health and Retirement Study (HRS) about the quantity (years, degree types) and content (field of study) of their college degrees. College information and employment histories were collected in a Life History Mail Survey (2015-2017). Cognitive status based on the Langa-Weir algorithm was defined as normal, cognitive impairment not dementia or dementia (CIND/D). Compared to men, women were less likely to have post-high school education (p < .002) or to have worked after their education. Cognitive status was predicted by degree type and work history. Gender by degree and gender by work interactions (for Baby Boomers only) revealed that having a BA/BS degree provided more protection against cognitive impairment for women than for men with the same degree, and women who worked had lower odds ratio of having CIND/D (0.36 [CI 0.17-0.77], p < .01). College majors were gendered and significantly accounted for variance in late-life cognitive status among the college educated. These findings provide new insight into the gendered life histories related to late-life cognition.

Abstract citation ID: igad104.3391

### HOLISTIC ASSESSMENT OF NURSING HOME RESIDENTS PREDICTS QUALITY OF LIFE AND PERCEPTION OF CARE

Emilee Ertle, Darby Simon, Benjamin Mast, and Suzanne Meeks, *University of Louisville, Louisville, Kentucky, United States*

The traditional medical model employs a problem-focused approach to assessment and care, but recent research emphasizes holistic assessment to facilitate person-centered care. The current study examines whether holistic assessment of problems and strengths can predict quality of life (QoL) and perception of care (PoC) of long-term nursing home residents. Sixty-six long-term nursing home residents were tested at time of admission, and at three and six month follow-up. Problem-focused measures were cognition, illness severity, depression, frailty, and anxiety. Strength-focused measures were hope, optimism, and social support. QoL at three months was correlated with anxiety (r=-.47, p=.001) and social support (r=.33, p=.047). At six months, QoL was correlated with anxiety (r=-.53, p<.001) and illness severity (r=-.30, p=.042). PoC at three months was not correlated with any problem-focused measures; it was correlated with optimism (r=-.38, p=.014), hope (r=.416, p=.008), and social support (r=.37, p=.036). PoC at six months was correlated with hope (r=.41, p=.009). Variables with significant correlations were selected for regression analysis; hope and optimism were combined into a single positive outlook variable. Anxiety was the only significant predictor in the regression model for QoL at three months. Anxiety and positive outlook were significant predictors of QoL at six months and PoC at three months. Positive outlook was the only significant predictor in the model for PoC at six months. Traditional problem-focused measures alone may provide insufficient information to predict well-being. Older adults' strengths, particularly hope and optimism, may predict QoL and PoC in the months following nursing home admission.

Abstract citation ID: igad104.3392

### HOME MODIFICATION ON FAVORITE ACTIVITY AND SELF-EFFICACY AMONG OLDER ADULTS WITH DEMENTIA

Soobin Park[1], Eunsun Kwon[2], Sojung Park[1], Oejin Shin[3], and Sehyun Baek[1], *1. Washington University in Saint Louis, St. Louis, Missouri, United States*, *2. Fairleigh Dickinson University, Teaneck, New Jersey, United States*, *3. Illinois State University, Normal, Illinois, United States*

Globally, most older people with dementia live at home and want to remain at home as long as possible with a sense of agency, and engagement in preferred everyday activities. To remain at home, a safe and supportive home environment is crucial as dementia progresses. Yet research on environmental modification among people with dementia is focused on neighborhood or urban built environment, and less on home modification. Guided by the ecological model of aging, this study aimed to explore the impact of home modification on both affective (e.g., self-efficacy) and adaptive behavioral (e.g., favorite activities) responses as a result of person-environmental fit among people living with dementia. Using two waves (2018-2019) data from the National Health and Aging Trends Study (NHATS), we examined the extent to which home modification is associated with self-efficacy and engagement in favorite activities. Multi-group structural equation modeling was conducted on 273 older adults living with dementia in the communities, comparing 145 with functional limitations and 129 without. Although home modification did not affect one's self-efficacy in any group, a positive impact of home modification on engaging favorite activities, especially those that require more physical effort (e.g., active leisure), was significant among older people with dementia with functional difficulties ($p< 0.01$). Our finding points to the importance of home modification for the most vulnerable subgroup of older adults with dementia, not only ensuring physical safety at home but their continued engagement in the activities meaningful for them, thereby helping them age in place.

Abstract citation ID: igad104.3393

### HOME TRIAD: A NEW WAY TO UNDERSTAND THE MEANING OF HOME FOR PEOPLE LIVING WITH DEMENTIA

Wenjin Wang, and Hao Huang, *Texas A&M University, College Station, Texas, United States*

Highlighted by the frequent stating “I want to go home,” understanding the meaning of home for people living with dementia (PLWD) is crucial to provide them better care and environment. Our novel theoretical framework, termed 'home triad', abstracts "home" into three interconnected pillars: conceived, perceived, and lived home. We conducted in-depth interviews with five PLWD, who were over 65 years old with MMSE scores between 11-24, received home care, and could understand and consent to the study. Their caregivers were also consulted for member-checking. The findings showed that the early memories of home were more vivid. The lived home experience held paramount significance; while stable housing was vital, family was of utmost importance to PLWD. Additionally, a gender-based distinction in lived home was evident. It was also found that PLWD's perceived daily activities and lived memories expanded their home beyond their immediate residence, encompassing neighborhoods, villages, and cities. Within home triad, we noticed that the conceived home, focusing on the discourses of power and knowledge, could easily be overlooked. Yet, focusing on this pillar revealed the pivotal roles of design and caregivers in shaping PLWD's home, which in turn influenced perceived and lived experiences. Through home triad, we can better appreciate profound home memories for PLWD. This could bolster reminiscence therapy and enable more effective home modifications, or even improve institutional homelike environments. Our future direction aims to refine and apply home triad with more home stories, thereby informing research and practice to enhance dementia-friendly environment and PLWD's well-being.

Abstract citation ID: igad104.3394

### HOME-BASED END-OF-LIFE CARE COOPERATION BETWEEN VISITING NURSES AND CARE MANAGERS IN JAPAN

Keiko Ono, *Aomori University of Health and Welfare, Aomori, Aomori, Japan*

End-of-life care patients use home-visit nursing services under Japanese long-term care insurance; however, they require a care manager to implement the care plan. However, the cooperation procedure of home-based medical care between visiting nurses and care managers has not been adequately researched. Thus, this study aimed to clarify the presence of support between visiting nurses and care managers to be adapted to this situation. This study was conducted in March and April 2023 at home-visit nursing stations located in a prefecture in Japan. Self-administered questionnaires were mailed to 192 visiting nurses in Japan, and 136 response forms (70.8%) were received through postal mails. A total of 130 questionnaires (67.7%) were determined to be valid responses, which also included the participants' written consent for research. The presence or absence of work experience and the importance of collaboration in the preparatory, introductory, stable, and near-death stages of end-of-life care were answered. The presence of work experience at each stage was over 53.1%. The importance of collaboration at each stage was over 76.9%. Query regarding collaborating well with care managers was answered on a scale from 0 to 100 %. Good collaboration with care managers had an average score of 75.4%. The presence or absence of experience and the importance of collaboration items were clarified at each stage. The research findings could contribute to the development of education and training. This work was supported by JSPS KAKENHI Grant Number JP21K02802.

Abstract citation ID: igad104.3395

### HOSPITALIZED AFTER A FALL: EXAMINING THE IMPACT OF INPATIENT GERIATRIC POLYPHARMACY REVIEW AND DEPRESCRIBING

Pratima Gangupantula, and Alissa Cooney, *University of Texas Health Science Center San Antonio, San Antonio, Texas, United States*

Falls are reported in 1 out of 3 older adults (≥65 years old) annually. While there are many modifiable factors contributing to falls in OA, polypharmacy increases risk of falling as well as recurrent falls by 1.5-2x. In an acute inpatient setting, geriatricians can evaluate an OA's medications to ensure these medications are indicated and potential benefits outweigh potential risks. Little is known regarding the impacts of deprescribing (including discontinuing, holding, or tapering medications) in the inpatient setting for OA admitted for falls. A retrospective chart review was conducted at a single academic institution from January 1st to June 30th 2023 to examine associated polypharmacy and deprescribing in OA hospitalized after a fall. The OA's complete home medication list was obtained, geriatric consultation notes were reviewed to determine if deprescribing was recommended, and commonly deprescribed drugs were noted. Of the 283 OA meeting inclusion criteria, 238 (84.45%) OA experienced polypharmacy. Deprescribing recommendations were given to 203 (84.94%) of OA experiencing polypharmacy. 54.39% of OA had ≥1 medications discontinued, 71.13% of OA had ≥1 medications held, and 23.85% of OA had ≥1 medications tapered. Commonly deprescribed drug classes included antihypertensives, non-steroidal anti-inflammatory drugs, proton pump inhibitors, and opioids. Pantoprazole, aspirin, omeprazole, and zolpidem were the most commonly deprescribed Beers criteria medications. Polypharmacy is associated with deprescribing in older adults admitted for falls. Future studies should examine whether deprescribing recommendations were followed by healthcare providers when given in an inpatient setting and whether deprescribing contributes to decreased fall risk over time.

Abstract citation ID: igad104.3396

### HOW DO EDUCATION AND SOCIAL MEDIA USE MATTER FOR ADVANCE CARE PLANNING AMONG CHINESE OLDER ADULTS?

Yaolin Pei[1], Xiang Qi[1], Zheng Zhu[1], Wei Zhang[2], Ruey-Ming Tsay[3], and Bei Wu[1], *1. New York University, New York City, New York, United States*, *2. University of Hawai'i at Mānoa, Honolulu, Hawaii, United States*, *3. Tunghai University, Taichung, Taichung, Taiwan (Republic of China)*

In general, the rate of advance care planning (ACP) engagement is low in Chinese societies globally. Few studies have compared the ACP engagement among Chinese in different regions. This study aims to examine and compare the associations between education, social media use, and advance care planning (ACP) discussion among Chinese older adults in mainland China (Wuhan), Taiwan (Taichung), and United States (Honolulu). Community-dwelling older adults ≥ 55 were recruited from 2017 to 2018. The ACP discussion rate in Wuhan, Taichung, and Honolulu were 15.2%, 19.2%, and 31.3%, respectively. Logistic regression models reveal that education was positively associated with ACP discussion in Taichung and Honolulu. Social media use was positively associated with ACP discussions in Wuhan and Honolulu, and it attenuated the association between education and ACP discussion in Honolulu. The present study contributes to previous research by comparing the association between education, social media use, and ACP discussion in different settings within the same ethnicity. Practice interventions may need use a social media as a tool to promote advance care planning among Chinese older adults where the palliative care is in the initial stage. Form a policy standpoint, it is important to develop palliative care while promoting advance care planning.

Abstract citation ID: igad104.3397

### HOW DOES EXECUTIVE FUNCTION IMPACT ADHERENCE AND COMPLIANCE TO NOVEL PHYSICAL THERAPY INTERVENTION AMONG VETERANS?

Elisa Ogawa[1], Rebekah Harris[1], Rachel Ward[1], Mary-Kate Palleschi[1], William Milberg[1], David Gagnon[2], Ildiko Halasz[1], and Jonathan Bean[1], *1. VA Boston Healthcare System, Boston, Massachusetts, United States*, *2. Boston University, Boston, Massachusetts, United States*

Executive function problems may make it difficult for a person to adhere to and comply with Physical Therapy (PT) treatment. This study examined adherence and compliance to a novel PT intervention among Veterans with and without executive function deficits (EFD+/EFD-). This study was a preplanned secondary analysis of an ongoing Randomized Controlled Trial among middle to older Veterans (≥50yrs) with slow walking speed. Participants' adherence (dropouts) and compliance (attendance, post-treatment exercise) to 8-week PT treatment were evaluated based on their baseline EFD status. Seventy-two participants were included in this study (mean age 72) and 22% (n=16) were EFD+. Veterans with EFD+ at baseline had worse mobility both objectively (Short Physical Performance Battery, 7.7 ± 1.9 vs. 9.5 ± 2.2, p< 0.01) and subjectively (Activity Measure for Post-Acute Care (AM-PAC), 57.6 ± 4.7 vs. 62.2 ± 5.7, p< 0.01). Dropout rates for EFD+ and EFD- were 44% and 25%, respectively. In the multivariable logistic regression, dropouts were associated with pain interference, depressive symptoms, mobility, and living alone. There were no differences in treatment attendance or post-treatment exercise compliance based on EFD status measured by number of attended sessions or number of post-treatment exercise days. In conclusion, EFD+ was associated with poor mobility and higher rates of dropping out. Screening for EFD is critical and may assist in predicting individuals who will need more support to enhance mobility.

Abstract citation ID: igad104.3398

### HOW IS THE NURSING HOME QUALITY OF CARE ALIGNED WITH THE QUALITY OF LIFE?

Jenny Kwon, and John Bowblis, *Miami University, Oxford, Ohio, United States*

The CMS Five-Star Quality Rating System does not currently incorporate nursing home (NH) consumers' opinions. This study aims to investigate the association between star ratings and resident/family satisfaction. A unique, facility-level dataset of Ohio NHs that contains star ratings, consumer satisfaction scores, and NH characteristics was constructed (N=701). The key dependent variables were the overall, health inspection, and quality star ratings, categorized into two groups: 1-3 stars and 4-5 stars. Key independent variables were overall resident and family satisfaction scores, which ranged from 0 (low) to 100 (high). Bivariate and logistic regression analyses were conducted. Bivariate results showed consumer satisfaction and star ratings were correlated, but not perfectly related. Regression results found an increase in resident satisfaction was associated with an increased probability of being a 4-5 star rated facility across all star ratings (p<.05). An increase in family satisfaction was also associated with an increased probability of being a 4-5 star rated facility for the overall and health inspection ratings (p<.01). Early studies found little relationship between star ratings and consumer satisfaction. Due to improvements made to the star ratings, this study found consumer satisfaction is associated, though imperfectly, with star ratings, suggesting that a consumer-driven measure of quality still needs to be publicly reported.

Abstract citation ID: igad104.3399

### HOW REAL IS THIS DEAL? EVALUATING AI ACCURACY IN PROVIDING SUPPORT TO DEMENTIA CAREGIVERS

Jeanette Ross[1], Elizabeth Reilly[2], Sandra Sanchez-Reilly[1], Lyda Arevalo-Flechas[1], and Alexander Dentchev[3], *1. South Texas Veterans Health Care System, San Antonio, Texas, United States*, *2. Health Careers High School, San Antonio, Texas, United States*, *3. Saint Mary's Hall, San Antonio, Texas, United States*

**Background:** Dementia, a devastating and incurable disease, affects millions of older Americans, placing astronomical levels of burden on caregivers, who usually lack resources to learn to provide their loved ones with high-quality care. Artificial Intelligence (AI), a programmed pioneering resource to generate immediate automated responses, is universally available when one looks for potentially accurate answers. Objective: To evaluate accuracy of AI sites pertaining to questions related to dementia caregiving **Methods:** AI sites (ChatGPT and Textero.ai) were asked questions about dementia caregiving aspects such as self-care, patient symptoms/complications, stress control and managing expectations. Dementia caregiving experts reviewed and classified responses using the validated Zarit Caregiver Burden Scale (ZCBS) as framework. Inter-reliability scores were calculated among reviewers' responses. **Results:** AI sites responded in paragraph and list formats. Common themes identified by reviewers from both AI sites included self-care suggestions and seeking/accepting help. Several ZCBS aspects were not addressed by both sites such as guilt, exhaustion, negative emotions, and patient's dependence. Textero.ai obtained lower scores addressing ZCBS, specifically not addressing potential caregiver's unreasonable expectations. Inter-reliability score (reviewers) was 0.91 (highly significant). **Conclusions:** In evaluating varied aspects of caregiving, results showed that AI sites, as it pertains to dementia caregiving are promising resources for accurately emphasizing caregiver's self-help encouraging positive thoughts. However, validation and exploration of negative feelings and emotions within AI sites might not be properly addressed for dementia caregivers. Further studies need to further explore the use of AI in combination with hands-on dementia interventions to validate all aspects of caregiving.

Abstract citation ID: igad104.3400

### HOW SPOUSAL BEREAVEMENT SHAPES LIFE SATISFACTION: STABILITY AND CHANGE ACROSS HISTORICAL TIME

Urmimala Ghose[1], Michael Krämer[2], David Richter[3], Gert Wagner[4], Frank Infurna[5], Nilam Ram[6], and Denis Gerstorf[2], *1. Humboldt Universität zu Berlin, Berlin, Berlin, Germany*, *2. University of Zurich, Zurich, Zurich, Switzerland*, *3. SHARE Berlin, Berliln, Berlin, Germany*, *4. Max Planck Institute for Human Development (MPIB), Berlin, Berlin, Germany*, *5. Arizona State University, Tempe, Arizona, United States*, *6. Stanford University, Stanford, California, United States*

Lifespan psychological and life course sociological research have long shown that spousal bereavement constitutes one of the most stressful life events in older adulthood that is often associated with marked declines in well-being. It is an open question though whether and how the well-being implications of spousal loss have changed over the past decades. To do so, we used multi-year within-person longitudinal change data from 2,042 participants (Mage at event =65.73 years, 71% women) of the German Socio-Economic Panel obtained annually since 1984. We combine latent basis growth models with methods that analyze the Area under the Curve so as to consider the time window from five years prior to five years after spousal loss. Results revealed that having lost one's spouse in the 2000s and 2010s is associated with overall less well-being declines than having lost one's spouse in the 1980s and 1990s. Building on earlier approaches that have distinguished anticipation, reaction, and adaptation phases of bereavement revealed that such improvements are primarily driven by fewer declines in the anticipation phase and faster recovery in the adaptation phases (both by about 0.1 SD per 10 years of historical time), whereas we found partial evidence for steeper declines in the immediate reaction phase. We discuss and in part examine possible explanatory factors at the individual, spousal, social, and societal level for the observed historical improvements in life satisfaction trajectories surrounding spousal loss in Germany over the last three decades.

Abstract citation ID: igad104.3401

### HUMAN FACTORS ISSUES IN THE DESIGN OF VIRTUAL REALITY TO SUPPORT OLDER ADULTS' SOCIAL AND COGNITIVE ENGAGEMENT

Veronica Falcon Borbolla[1], Walter Boot[1], Andrew Dilanchian[1], Ana Sophia Garces Torres[2], Mario Hernandez[2], Bill Tong Xu[3], Armin Mostafavi[3], and Benjamin Kim[3], *1. Florida State University, Tallahassee, Florida, United States*, *2. Weill Cornell Medicine, New York City, New York, United States*, *3. Cornell University, Ithaca, New York, United States*

Emerging technologies such as virtual reality (VR) hold tremendous potential to improve the lives of older adults, including the opportunity to support social and cognitive engagement. However, this potential will only be realized if VR technologies and applications (apps) are well-designed, considering the needs, preferences, and abilities of older people. To explore and understand design barriers ahead of a randomized controlled trial testing the efficacy of a psychosocial VR intervention for older adults, a team of eight evaluators who underwent standardized and comprehensive training on how to perform a heuristic analysis assessed the design of 28 commercial VR apps. Apps were chosen for their potential to support social interactions, enhance cultural opportunities, facilitate new learning, or support leisure activities. Each app was evaluated based on the Nielsen Norman Group's ten usability heuristics. For different VR components (hardware, app menus, VR environment), this analysis highlighted specific heuristic violations, examined the general human factors of these apps, and identified positive design elements while suggesting solutions for observed heuristic violations. The heuristic analysis revealed significant usability challenges, especially in areas like "System Status Visibility," "User Control and Freedom," and "Help and Documentation." The primary system components exhibiting these heuristic violations were the "User Interface" and "Virtual Environment." It was concluded that current virtual reality systems do not adequately address human factors issues, particularly considering the needs and abilities of older adult users. Based on this extensive evaluation, training and design solutions will be presented and VR guidelines for older adults will be introduced.

Abstract citation ID: igad104.3402

### HYPERTENSION SELF-CARE AMONG MEDICARE BENEFICIARIES WITH SELF-REPORTED HEARING TROUBLE

Laura Wallace[1], and Nicholas Reed[2], *1. University of Pennsylvania, Philadelphia, Pennsylvania, United States*, *2. Johns Hopkins Bloomberg School of Public Health, Baltimore, Maryland, United States*

Hearing loss is common in older adults and frequently comorbid with chronic illnesses like hypertension, which requires self-care practices learned through patient-provider health communication. However, hearing loss is a barrier to communication. This cross-sectional analysis used the 2020 Medicare Current Beneficiary Survey of (N=11,587; weighted estimate, 51.8 million) to characterize hypertension self-care outcomes (measuring blood pressure, taking blood pressure medications, reducing alcohol, and confidence in blood pressure control) by hearing status among Medicare beneficiaries ages >65. In the sample, 39% reported a little and 6% reported a lot of trouble hearing while 66% reported having received a hypertension diagnosis. In models adjusted for sociodemographic and health variables, Medicare beneficiaries with a little trouble hearing (OR=0.84 95% CI=0.72-0.97; P=0.02) and a lot of trouble hearing (OR=0.74; CI 0.57-0.95; P=0.02) had 16% and 26% reduced odds of reporting confidence in hypertension control compared to beneficiaries with no hearing trouble. Similar results were found when excluding hearing aid users (a little trouble hearing OR=0.85; CI=0.72-1.01; P=0.06; a lot of trouble hearing OR=0.69; 95% CI=0.52-0.91; P=0.01). No significant difference was found for other self-care outcomes. To the authors' knowledge, this is the first characterization of hearing difficulty and hypertension self-care and findings align with previous work suggesting hearing loss may be a barrier to vital health communication. Future work should focus on examining self-care activities across chronic illnesses, temporal relationships, and designing population-appropriate interventions to improve self-care understanding among adults with hearing loss.

Abstract citation ID: igad104.3403

### IDENTIFYING BRIDGE SYMPTOMS AMONG DEPRESSIVE SYMPTOMS IN CHINESE OLDER ADULTS: THE ROLE OF EATING CULTURE

Zhongfang Yang[1], Junwen Yu[1], Tiantian Hu[1], and Zheng Zhu[2], *1. Fudan University, Shanghai, Shanghai, China (People's Republic)*, *2. New York University, New York City, New York, United States*

**Introduction:** Identifying bridge symptoms among Chinese older adults with depressive symptoms is crucial for enhancing the precision and individualization of interventions. **Aim:** The aim of this study was to identify the bridge symptoms and to explore the intercorrelation between depressive symptoms clusters in Chinese older adults. **Methods:** Depressive symptoms were measured in 2014, 2016, and 2018 using the nine-item version of Center for Epidemiological Studies Depression Scale, and analyzed with cross-lagged panel dynamic network and centrality examination. **Results:** Total of 3,438 participants were included. "D5: Had poor appetite" and "D9: Enjoy life" reported the highest value of outstrength (rnegative=0.063; rpositive=0.048) and the highest value of bridge strength (rnegative=0.029; rpositive=0.024) in negative and positive affects cluster. The strongest path is from D5 to D9 (β=-0.02). **Discussion:** To explore the potential mechanism of depressive symptoms in Chinese older adults, identifying the bridging symptoms between the positive affect clusters and negative symptoms clusters of depressive symptoms may be needed. **Implications for Practice:** More insights into the relationship between eating culture and the potential mechanisms could be provided to aid in the development of culturally sensitive interventions for Chinese older adults with depressive symptoms.

Abstract citation ID: igad104.3404

### IDENTIFYING CARE TRANSITIONS NEEDS EXPERIENCED BY OLDER ADULTS AND CAREGIVERS HOSPITAL TO HOME

Claire Regan, Karen Hirschman, Molly McHugh, Onome Osokpo, Kathleen Mccauley, Elizabeth Shaid, and Mary Naylor, *University of Pennsylvania, Philadelphia, Pennsylvania, United States*

Transitions from hospital to home are a fragile period for older adults, associated with increased risks for hospital readmission and mortality. The Transitional Care Model (TCM), a multi-component, advanced practice registered nurse (APRN) led, team-based care intervention designed to support older adults and their family caregivers during this transition, has consistently demonstrated improvements in both patients' health outcomes while lowering care costs. This secondary analysis of data generated during a large-scale replication trial of the TCM conducted in three U.S. healthcare systems (ClinicalTrials.gov: NCT04494295) was designed to identify the key challenges experienced by APRNs in implementing TCM's intervention protocol as designed. Data from multiple sources (i.e., case studies, case presentations, APRN documentation related to 184 intervention group patients) collected from September 2020 through August 2022 were analyzed using qualitative content analysis. Across sites, the top challenges in implementing the TCM and percentage reporting these issues were: social determinants of health (SDoH, 23%); complexity of care (15%); patient engagement (14%); and communication with patients/families (12%) and with providers (10%). Non-Veteran Affairs (VA) hospitals faced a higher number of SDoH challenges such as delays in accessing care and supporting non-English speaking patients, while VA hospitals had a higher number of patients with housing safety concerns. The VA sites also had a slightly higher percent of complexity of care (15%) challenges compared to non-VA sites (12%). Findings offer insight into enhancing the design of transitional care interventions by targeted challenges associated with SDoH, complexity of care, engagement, and communication.

Abstract citation ID: igad104.3405

### IDENTIFYING FACTORS THAT CONTRIBUTE TO THE FINANCIAL STRAIN OF HISPANIC CAREGIVERS OF OLDER ADULTS

Margret Howard, Wingyun Mak, and Orah Burack, *The New Jewish Home Research Institute on Aging, New York City, New York, United States*

Informal caregiving has been shown to have negative effects on the caregiver's physical, mental, and financial health. To date, studies have mainly focused on the physical and mental health of caregivers; however, finances too are an area of caregiver vulnerability. In this study we examined which types of financial hardships are the greatest predictors of financial strain in Hispanic caregivers (N=184) for persons 50 or older. Data were obtained from the American Association of Retired Persons (AARP) Caregiving in

2020 survey, which oversampled for Hispanic caregivers. A hierarchical regression analysis was conducted to predict caregiver financial strain. Independent variables were entered in three steps: (1) age and gender, (2) emotional strain, physical strain, length of expected caregiving, and attitude towards caregiving (3) taking on more debt, borrowing money, unable to afford basic expenses, late/unpaid bills, using short-term savings, using long-term savings, being unable to save, and moving to a less expensive home. Age and gender were not significant ($\Delta R2=.007$, $p=.531$). Emotional ($\beta=.309$, $t=3.512$, $p<.001$), and physical strain ($\beta=.229$, $t=2.627$, $p=.009$) were both predictors of financial strain ($\Delta R2=.312$, $p<.001$). Additionally, the specific financial hardships related to financial stress above and beyond steps one and two ($\Delta R2=.137$, $p<.001$) were being unable to afford basic expenses ($\beta=.356$, $t=4.161$, $p<.001$), and using long term savings ($\beta=-.308$, $t=-4.372$, $p<.001$). These results have implications for education and interventions concerning financial hardships and options for Hispanic caregivers of older adults.

Abstract citation ID: igad104.3406

### IDENTIFYING NEW ADRD DIAGNOSES IN HOME HEALTH CARE PATIENTS USING NATURAL LANGUAGE PROCESSING OF NURSES' NOTES

Yolanda Barrón[1], Miriam Ryvicker[1], Jiyoun Song[2], Maryam Zolnoori[3], and Maxim Topaz[3], *1. VNS Health, New York City, New York, United States*, *2. University of Pennsylvania School of Nursing, Philadelphia, Pennsylvania, United States*, *3. Columbia University, New York City, New York, United States*

Patients at risk of developing Alzheimer's Disease and Related Dementias (ADRD) admitted to home health care (HHC) may show signs of the disease before receiving a formal diagnosis. HHC nurses are uniquely positioned to observe signs of patients' cognitive symptoms and may record them in free-text notes in the electronic health record (EHR). We applied natural language processing (NLP) to approximately 1.8 million nurses' notes to identify signs and symptoms of ADRD in a retrospective cohort of 56,652 patients receiving care from a large HHC agency. In a subset of 38,032 patients starting HHC without an ADRD diagnosis, we used survival analysis competing risks models to examine associations between NLP-derived indicators of ADRD constructs during the HHC episode and new ADRD diagnoses during a four-year follow-up period, adjusting for patient demographics, clinical characteristics, and functional status derived from structured standard assessment tools. By the end of follow-up, 23% of the patients were newly diagnosed with ADRD, while 35% died without a diagnosis. We observed higher 4-year cumulative incidence rates of ADRD when more ADRD-related symptoms were present in the nurses' notes (e.g., confusion only, 0.35, 95% CI: 0.32; 0.38, memory loss only, 0.38, 95% CI: 0.36, 0.39; both, 0.52, 95% CI: 0.48, 0.56; none, 0.10, 95% CI 0.097, 0.103). These findings indicate that data from free-text notes taken during HHC serve as significant predictors for potential ADRD diagnoses. Such information can facilitate prompt specialist referrals, allowing for early diagnosis and potentially more effective treatments during the disease's initial stages.

Abstract citation ID: igad104.3407

### IDENTIFYING PATTERNS OF CHANGE IN SPIRITUAL WELL-BEING OVER TIME AMONG NEURO-ONCOLOGY CAREGIVERS

Jiayi Zhu, Paula Sherwood, Susan Sereika, and Heidi Donovan, *University of Pittsburgh, Pittsburgh, Pennsylvania, United States*

Spiritual well-being/spirituality has been recognized as a protective factor against caregiver burden, depression, anxiety, and poor quality of life in family caregivers (CG) of patients diagnosed with serious illnesses, such as cancer. However, inconsistent previous findings may be attributed to the limited understanding of spirituality as a dynamic, multidimensional concept. To address this gap, we conducted a secondary analysis using longitudinal data from two independent studies of CGs of patients with primary malignant brain tumors (pooled N=261) to identify trajectories in overall spirituality (FACIT-SP) and its 3 sub-dimensions (faith, meaning, peace) from diagnosis to 10-12 months post-diagnosis, as well as predictors and outcomes associated with trajectory group membership. For overall spirituality, 3 unique trajectories were identified: low-increasing, medium-stable, and high-stable. The faith and meaning sub-dimensions had 3 and 2 stable trajectories, respectively, while the peace sub-dimension showed low-increasing, medium-stable, and high-increasing trajectories. Older age and higher levels of perceived social support at baseline were associated with increased odds (up to 1.06 and 1.20, respectively) of being in the high-stable/high-increasing trajectories for overall spirituality and the sub-dimensions. The trajectories of overall spirituality and sub-dimensions were positively associated with CG's mental well-being ($\eta2=.030-.069$) and self-esteem ($\eta2=.072-.162$), and negatively associated with CG's depression ($\eta2=.053-.319$), anxiety ($\eta2=.055-.271$), and feeling of abandonment ($\eta2=.032-.160$) at 10-12 months. This study supports recognizing spirituality in CGs as a dynamic, multidimensional concept. Future interventions focusing on early screening in younger adults and supportive measures for low spiritual well-being through improving social support might improve important CG outcomes.

Abstract citation ID: igad104.3408

### IDENTIFYING SOLUTIONS TO ADDRESS THE BEHAVIORAL HEALTH OF OLDER ADULTS: A QUALITATIVE ANALYSIS

Allyson Stodola[1], Sheryl Elliott[1], Nathan Parsons[1], Sarah Dys[1], Susan Sotka[2], and Walter Dawson[3], *1. Portland State University, Portland, Oregon, United States*, *2. Institute on Aging, Portland, Oregon, United States*, *3. Oregon Health & Science University, Portland, Oregon, United States*

The Oregon Older Adult Behavioral Health Initiative (OABHI), a program of the Oregon Health Authority, seeks to improve behavioral health (BH) services for older adults and people living with physical disabilities. Twenty-four OABHI Specialists work with BH and Aging services agencies, community-based organizations, and other key partners to strengthen the BH workforce, educate communities, provide complex care consultations, improve intra-agency collaborations, and foster timely access to coordinated, culturally-responsive services. Semi-structured, in-depth interviews with Aging and BH service agency managers and direct service providers (n=29) across Oregon were conducted over 12 weeks in 2023 to evaluate impacts of their involvement with the OABHI and improvements needed to better address the BH needs of these populations. Direct service staff and managers shared suggestions on programs, policies and inter-agency agreements that should be implemented to address the top BH-related challenges in their area. The first-hand experiences of direct service staff and managers who work with these populations provide unique insight into not only the most prevalent systemic issues, but what they perceive to be the key resources needed to help address these issues. Qualitative data were analyzed to develop systems-level recommendations to address gaps in BH resources for older adults, and were presented to policymakers and other decision makers in Oregon. Results indicate key resources and policy changes needed to support OABHI-delivered interventions, promote intra-agency collaboration, increase investment in BH services, and provide resources for an increasing population of older adults and people living with physical disabilities who have BH needs.

Abstract citation ID: igad104.3409

### IDENTIFYING THE RELATIONSHIP BETWEEN ALZHEIMER'S DISEASE PREVALENCE AND CURRENT SERVICE USE IN OHIO'S 88 COUNTIES

Matt Nelson, and Dr. Heather Menne*, Miami University, Oxford, Ohio, United States*

Recently released data reveals that over 235,000 people over the age of 65 have Alzheimer's disease and related dementia (ADRD) in Ohio. Using 2020 data from the National Center for Health Statistics, county-level specific ADRD prevalence estimates for Ohio's 88 counties were also prepared (Range: 9.4-13.4 per 1000 individuals 65+). Researchers, practitioners, and policy-makers can use these estimates to better understand the reach and impact of ADRD in local communities. Nationally and in Ohio, care and support for older adults and specifically older adults with ADRD typically falls to family caregivers, home- and community-based service (HCBS) providers, and residential care providers such as nursing homes. This study pairs the county-level ADRD prevalence data with county-level data on available services (e.g., Memory Care Units, nursing home beds) and service users (e.g., HCBS users). The 10 counties in Ohio with the highest prevalence rates are home to nearly 60% of the 65+ population statewide. Additionally, these counties had a significantly higher proportion of older Black/Hispanic individuals than the other counties (10.8 versus 2.9). When looking at capacity, the top 10 counties contained just 12% of all nursing home beds, but 54% of nursing homes memory care units for the entire state. When considering service use, these 10 counties served a smaller percentage of the state's HCBS users (13%). For Ohio and other states, it is important to explore available services and current service usage when faced with the increasing estimates of people living with ADRD.

Abstract citation ID: igad104.3410

### IMPACT OF A 4-MINUTE STRENGTH TRAINING PROGRAM ON LOWER BODY FUNCTION IN OLDER ADULTS WITH MOBILITY DISABILITY

Jordan Kurth[1], Christopher Sciamanna[1], Jonathan Stine[1], Matthew Ladwig[2], David Conroy[1], Kathryn Schmitz[3], and Cheyenne Herrell[1]*, 1. The Pennsylvania State University College of Medicine, Hershey, Pennsylvania, United States, 2. Purdue University Northwest, Hammond, Indiana, United States, 3. University of Pittsburgh, Pittsburgh, Pennsylvania, United States*

One in four older adults experience mobility disability, often resulting in financial strain and declines in quality of life. Though resistance training (RT) can improve physical function, too few older adults do RT. This study specifically reports on the impact of a short, daily RT program called Functional Activity Strength Training (FAST) on lower extremity function. FAST entailed one 30-second bout each of four exercises (push-ups, chair stands, two-arm resistance band rows, stair-stepping) followed by 30 seconds of rest daily for 12 weeks. Ninety-seven participants were randomly assigned to either intervention (n = 44) or delayed treatment control (n = 53) group. Research staff remotely provided one-on-one coaching relating to modifications and progress every 2 weeks until week 8. Participants received a daily email reminder to complete the exercises and report repetitions completed for each exercise. Outcome measures (5-time sit-to-stand, maximum 30 second chair stand performance, and single-leg stance duration) were evaluated at baseline and week 12. Participants completed exercises on 80% of possible days. Over the 12 weeks, the intervention group improved over and above the control group by +4.2 chair stands (d = 1.21), -2.3 seconds (d = -0.43) on the 5-times sit-to-stand, and +3.58 seconds (d = 0.46) on the single-leg stance. In sum, a four-minute daily, 12-week functional RT program led to improvements in chair stand, 5-times sit-to-stand, and single-leg stance performance. Efficacy trials, using gold-standard assessments should examine whether similar improvements are observed and adherence is maintained over a longer period of time.

Abstract citation ID: igad104.3411

### IMPACT OF DEPRESSIVE SYMPTOMS ON ORAL HEALTH OF OLDER ADULTS WITH CHRONIC CONDITIONS BY GENDER

Dahye Hong, Jennifer (Jeehyun) Kim, and Bada Kang, *Yonsei University, Seoul, Republic of Korea*

Oral health is crucial for the nutrition and well-being of older adults, especially those with chronic conditions. Although recent studies have demonstrated an association between depressive symptoms and oral health, little is known regarding the gender-specific association between symptoms of depression and oral health among older Korean adults with chronic conditions. Therefore, this study examined the gender-specific relationship between depressive symptoms and oral health, using data from the Korean Longitudinal Study of Aging (2018–2020). Study participants aged 65 or older with one or more chronic conditions were analyzed (N = 1,185 women and 1,919 men). The comprehensive aspects of oral health, including functional limitations, pain, discomfort, and psychological and behavioral impacts, were measured. Generalized Estimation Equation analyses were applied to two models presenting depressive symptoms dichotomously and the severity of depression, respectively. When considering depressive symptoms in dichotomous manner, such as present or absent, having three or more chronic conditions and experiencing cognitive decline were both significantly related to deteriorated oral health for both genders. Moreover, the association between depressive symptoms and deteriorated oral health was more robust in men. While categorizing the severity of depressive symptoms, the worse the depressive symptoms, the poorer the oral health in women. Therefore, this study suggests that interventions to alleviate depressive symptoms could benefit the oral health of both men and women with multiple chronic conditions. For women, targeted interventions based on the severity of depressive symptoms could effectively improve oral health.

Abstract citation ID: igad104.3412

### IMPACT OF DUAL-TASKING AND DEMENTIA DISEASE SEVERITY ON POSTURE, GAIT, AND FUNCTIONAL MOBILITY IN RESIDENTIAL CARE

Deborah Jehu[1], Ryan Langston[1], Richard Sams[1], Lufei Young[2], Mark Hamrick[3], Haidong Zhu[3], and Yanbin Dong[3], *1. Augusta University, Augusta, Georgia, United States*, *2. University of North Carolina at Charlotte, Charlotte, North Carolina, United States*, *3. Medical College of Georgia, Augusta, Georgia, United States*

Dual-tasking may be a useful tool for fall-risk screening among people living with dementia (PWD), but more evidence is needed. Our aims were to compare single- and dual-task posture, gait and functional mobility; and examine the relationship between dementia severity and dual-task interference among PWD. Thirty PWD (Age:81.8±7.3years; 35% Female; Montreal Cognitive Assessment:10.5±6.1points) from two residential care facilities wore six APDM inertial sensors and completed two trials of single- (feet apart) and dual-task posture (feet apart while counting backwards by 1's), single- (walk 4m) and dual-task gait (walk 4m while naming words), and single- (timed-up-and-go (TUG)); and dual-task functional mobility (TUG while completing a category task). Results revealed that greater frequency, jerk, and area were observed during dual- than single-task posture (ps< 0.05), with no differences in velocity. Slower gait speed, greater double limb support, shorter stride length, and reduced elevation at mid-swing were detected during dual-relative to single-task gait (ps< 0.05), with no differences in medial-lateral trunk sway. Dual-tasking resulted in a longer duration, reduced turn angle, and slower turn velocity than single-tasking for the TUG (ps< 0.05), with no differences in sit-to-stand lean angle. Dual-task interference (greater jerk, faster gait speed) was related to moderate-to-severe compared to mild dementia (ps< 0.05). Dual-tasking was more sensitive to detect impairments in posture, gait, and functional mobility than single-tasking. Moderate to severe PWD had poorer dynamic stability and a reduced ability to appropriately select cautious gait during dual-tasking than those with mild dementia, which should be considered in fall-risk screening.

Abstract citation ID: igad104.3413

### IMPACT OF GRANDPARENTS' CHRONIC STRESS ON GRANDPARENT-PARENT RELATIONSHIP AND LIFE SATISFACTION BY GENDER AND RACE

Nusrat E Mozid, and Kate Guastaferro, *New York University, New York City, New York, United States*

Grandparents raising grandchildren are a rapidly growing population in the United States—an estimated 7.1 million children under 18 live with a grandparent in 2023. Family system theory suggests raising a grandchild impacts grandparents' immediate and extended family relationships and amplifies stressors in the relationship with the parent of the grandchild. The quality of relationships with the parents negatively impacts grandparents' psychosocial well-being but may also have lasting adverse effects on the grandchild. Using the most recent wave (2020) of the nationally representative Health and Retirement Study, we conducted linear regression analyses to understand the association between grandparents raising grandchildren's (N=4,238) chronic stress (e.g., health, finances, housing) and anxiety symptoms by gender and race. Then, we examined the grandparents' relationship with the parent and satisfaction with life as potential mediators in these associations. Results suggest grandparents' stressors were significantly associated with anxiety symptoms ($\beta$=0.349; p< 0.01); however, there were no significant differences in total chronic stress by either gender or race. Path analysis showed both the grandparent-parent relationship and grandparents' satisfaction with life partially mediated the association between grandparents' chronic stress and anxiety symptoms (p< 0.001). Cultivating a consistent and quality relationship between grandparent and parent as well as focusing on grandparents' perspectives of life suggests innovative intervention targets to improve the health and well-being of both grandparents and grandchildren.

Abstract citation ID: igad104.3414

### IMPACT OF HEALTHY LIFESTYLE FACTORS ON LIFE EXPECTANCY AND LIFETIME HEALTHCARE COST: A NATIONWIDE COHORT STUDY

Wei-Cheng Lo[1], Tsuey-Hwa Hu[2], Cheng-Yu Shih[3], Hsien-Ho Lin[3], and Jing-Shiang Hwang[2], *1. Taipei Medical University, New Taipei City, New Taipei, Taiwan (Republic of China)*, *2. Academia Sinica, Taipei, Taipei, Taiwan (Republic of China)*, *3. National Taiwan University, Taipei, Taipei, Taiwan (Republic of China)*

In an aging society with increasing life expectancy, the significance of healthy behaviors has garnered considerable attention. Research indicates that adopting healthy behaviors not only contributes to a longer life expectancy but also leads to reduced healthcare expenditure. Our study aims to investigate the impact of individual and combined healthy lifestyle factors on life expectancy and lifetime healthcare expenditure in Taiwan. Analyzing data from the National Health Interview Survey cohort, we examined five healthy lifestyle behaviors: nonsmoking, avoiding excessive alcohol consumption, regular physical activity, sufficient fruit and vegetable intake, and maintaining a normal weight. By employing a rolling extrapolation algorithm, we estimated the lifetime survival function of the study cohorts and calculated the life expectancy and lifetime healthcare expenditure of populations with and without healthy lifestyle factors. Our analysis of 19,893 participants aged 30 and above revealed that adopting a healthy lifestyle is associated with longer life expectancy and reduced healthcare expenditure in Taiwanese adults. Participants adhering to all five behaviors gained 11.93 years in life expectancy compared to those with none, with an annual average healthcare expenditure reduction of $7,308. Smoking and diet were the most significant risk factors for shorter life expectancy, with current and former smokers experiencing a 4.02-year reduction and individuals with sufficient fruit and vegetable intake gaining 3.69 years compared to those with insufficient intake. These findings offer a comprehensive understanding of the impact of healthy lifestyle factors on overall health and economic burden, providing evidence-based guidance for policymakers and stakeholders in promoting healthy aging.

Abstract citation ID: igad104.3415

### IMPACT OF THE TEACHING NURSING HOME ON STAFF SATISFACTION AND BURNOUT

Howard Degenholtz[1], Jacqueline Dunbar-Jacob[1], Kim Ratliff[2], Donna Fick[3], Chelsea Dickson[4], Kenna Campbell[1], Erin Kitt-Lewis[3], and Emily Franke[4], *1. University of Pittsburgh, Pittsburgh, Pennsylvania, United States*, 2. *Wesley Enhanced Living, Media, Pennsylvania, United States*, 3. *Penn State University, State College, Pennsylvania, United States*, 4. *Jewish Healthcare Foundation, Pittsburgh, Pennsylvania, United States*

The Teaching Nursing Home (TNH) is designed to improve the long-term care workforce and improve quality of care in nursing homes (NHs). The initiative is guided by an academic-practice partnership with mutual respect and shared learning and has multiple components: enhanced clinical rotations for nursing students with partner schools of nursing, implementation of the Institute for Healthcare Improvement Age-Friendly Health System "4M" quality improvement model, and an online learning network. Following the principles of participatory research, NH staff were engaged in the selection of outcomes and in decisions regarding data collection. The University of Pittsburgh collected staff satisfaction at two nursing homes, and burnout at three NHs. Baseline surveys were conducted in the winter of 2022 using a combination of web-based and in-person paper administration, and follow-up surveys were collected in 2023, one year after implementation. Response rates ranged from 14% to 54%. A total of 190 surveys were available at baseline, and 183 at follow-up. All surveys were anonymous; cross-sectional analyses were conducted to identify trends. Baseline burnout scores varied considerably across the three participating NHs and improved at two of the three NHs (p = .0000). By contrast, job satisfaction was unchanged in one NH and declined in one NH across two dimensions (p= .0058; p = .01216). After the first year of implementation, the TNH program is showing some benefit in terms of reducing burnout, a factor associated with turnover. As the Age-Friendly NH program is disseminated, it is important to track the impact on the workforce.

Abstract citation ID: igad104.3416

### THE IMPACT OF COVID-19 CONCERNS ON OLDER ADULTS' DEPRESSION: THE ROLES OF RACE/ETHNIC STATUS AND EDUCATION

Shan Qu, and Jeffrey Burr, *University of Massachusetts Boston, Boston, Massachusetts, United States*

Framed within the Conservation of Resources Theory and the Cumulative (Dis)advantage Theory, this study examined the association between COVID-19 concerns (worries) and risk of depression among older adults. The study also investigated whether race/ethnic status and education moderated this relationship. We used a nationally representative sample of Americans over age 50 from the 2020 Health and Retirement Study. Depression was defined as having greater than or equal to three depressive symptoms, as derived from the 8-item Center for Epidemiologic Studies-Depression scale. COVID-19 concerns were measured with respect to respondents' own health, the health of family members, respondents' financial situation, the ability to get help from family, friends, or others during the pandemic, and concerns about the future; the study also included a summative index of these concerns. The associations between COVID-19 concerns and older adults' depression were estimated with unadjusted and adjusted logistic regression models. Compared to respondents with lower scores on the COVID-19 concern measures (the index and the five domains), those with higher scores were more likely to be depressed. The moderation models showed the relationship between respondents' concerns about what will happen in the future and depression was not as strong among non-Hispanic Black older adults compared to non-Hispanic White older adults. There was no statistically significant moderation effect for education. The results imply that healthcare providers should focus on vulnerable older adults to reduce disparities in access to resources during public health emergencies like the COVID-19 pandemic.

Abstract citation ID: igad104.3417

### TRANSCRIPTOMIC ANALYSIS OF PROGERIA MODEL, KLOTHO KO MOUSE AND THE THERAPEUTIC EFFECT OF MITOCHONIC ACID 5

Yoshiyasu Tongu[1], Tomoko Kasahara[2], and Takaaki Abe[1], *1. Tohoku University, Sendai, Miyagi, Japan*, 2. *Tohoku University Graduate School of Medicine, Sendai, Miyagi, Japan*

Klotho is recognized as a protein that enhances longevity, and its deficiency leads to symptoms resembling progeria. The Klotho KO mouse displays several aging-related phenotypes similar to those observed in humans and typically dies by 8-10 weeks old. Klotho is expressed primarily in the kidney and the brain, which leads to severe progeria. We've recently developed a novel mitochondria-homing drug called Mitochonic Acid (MA)-5, which can penetrate the blood-brain barrier and is currently in Phase 1 clinical trials. A decline in mitochondrial function is a well-documented characteristic of aging and is considered a major factor in the aging process. To explore the potential benefits of MA-5 on aging-related symptoms, we administered the drug orally to Klotho KO mice from 4 weeks old and studied them until 7 weeks old. We observed that levels of uremic creatinine and BUN were elevated in the Klotho KO mice, but MA-5 treatment brought about significant improvements. Pathological examinations and snRNA-seq data suggest that kidney fibrosis caused by Klotho KO might be behind the renal dysfunction, and this is mitigated by MA-5. Furthermore, the result of snRNA-seq in brains shows the abnormalities occurring in the brains of Klotho KO mice which leads to progeria and the therapeutic effect of MA-5 on them. Given the role of mitochondrial dysfunction in aging symptoms, our drug presents as a promising candidate to treat age-related renal failure, and a range of other aging manifestations.

### SESSION 9020 (POSTER)

### LATE BREAKING POSTER SESSION III

Abstract citation ID: igad104.3418

### EXPLORING INTERSECTIONALITY IN SPIRITUAL EXPERIENCES DURING DISEASE OF OLDER ADULTS AND CAREGIVERS

Sarah Won[1], Natalie Regier[2], Suratsawadee Kruahong[1], and Changhwan Kim[2], *1. Johns Hopkins University, Baltimore, Maryland, United States*, *2. Johns Hopkins University School of Nursing, Baltimore, Maryland, United States*

Spirituality is known to be an important contributor to older adults' and caregivers' psychological and physical well-being. Despite this significance, their spiritual aspects are often overlooked within the context of treating and managing disease. Moreover, limited evidence exists regarding what specific intersectional identities of older adults and caregivers have the potential to cause disparities in their spiritual well-being. Therefore, the purpose of this study was to identify the extent to which intersectionality has been considered within the literature concerning factors associated with the spiritual aspects (defined as spiritual needs, spiritual support, and spiritual well-being) of older adults and their caregivers. Consequently, a five-stage integrative review was conducted using the protocol proposed by Whittemore and Knafl. This process yielded a total of 543 articles identified in PubMed, Embase, Cochrane, CINAHL, PsycINFO, and Web of Science in March 2023. Following the application of inclusion/exclusion criteria, 18 articles were selected for data extraction. Finally, four themes related to intersectional considerations were found: 1) The potential role of intersecting multiple identities in differences in spiritual experiences; 2) Older adults and caregivers, the pre-existing identities, that intensify the likelihood of intersectionality leading to disparities in spiritual experiences; 3) The importance of spiritual perspective in addressing challenges derived from the intersections of multiple identities; 4) Recommendations for professionals to consider potential intersectional factors in research and clinical contexts to improve inclusiveness. Findings highlight the necessity of adopting an intersectional lens within both research and clinical domains to prevent overlooking any resulting variations in spiritual experiences.

Abstract citation ID: igad104.3419

### IMPLEMENTATION & SUSTAINABILITY OF AGETECH TO SUPPORT AGING IN PLACE: PRACTICAL RECOMMENDATIONS FOR POLICYMAKERS

Heather McNeil[1], Courtney Genge[1], Shannon Freeman[2], and Patricia Debergue[3], *1. National Research Council, Ottawa, Ontario, Canada*, *2. University of Northern British Columbia, Prince George, British Columbia, Canada*, *3. National Research Council, Boucherville, Quebec, Canada*

AgeTech, a subset of the health technology industry, uses technology to support healthy aging, and support care partners and health professionals to improve quality of life for aging adults. By enhancing and adapting alternative care approaches through emerging technologies, it is possible to enable and extend the ability for older adults to safely age in place within their own homes, improve care experiences, and/or decrease long-term care costs/needs. With the rapid development and proliferation of AgeTech into the consumer market, it is paramount for policymakers in the health system to ensure that AgeTech solutions can be leveraged to support older adults to age well in place. This poster highlights five key messages for policymakers. First, AgeTech should solve a real problem. Technology must be a solution that complements care of older adults, not complicates it. Second, it is essential to embrace a life course perspective on aging, recognizing the heterogeneity of older adults who experience diverse and evolving needs. AgeTech should adapt as needs and capacities evolve. Third, health related AgeTech should empower, enhance, or support existing health care services, while recognizing the value of human interactions. In-person interactions can provide meaningful connection and important health data which should be enhanced not replaced. Fourth, the establishment and ongoing fostering of authentic partnerships to inform, co-create and co-design AgeTech solutions is key to developing successful products. Finally, policymakers and funders have an important role to play in enabling accelerated design, development and testing to meet current and future needs.

Abstract citation ID: igad104.3420

### IMPLEMENTATION OF VIRTUAL REALITY TO IMPROVE QUALITY OF LIFE FOR VETERANS IN HOSPICE AND PALLIATIVE CARE

Alexandra Laffer[1], Melanie Corle[1], Anastasia Canell[2], Lauren Moo[2], Megan Gately[2], and Kristen Dillon[1], *1. Edith Nourse Rogers Memorial VA Medical Center (VA Bedford), Bedford, Massachusetts, United States*, *2. VA Bedford Healthcare System, Bedford, Massachusetts, United States*

Virtual Reality (VR) is a safe and effective adjunctive care option with multiple clinical applications. An interdisciplinary VR program aimed at improving quality of life for veterans receiving inpatient hospice and palliative care was implemented at the VA Bedford Health Care System. Principles from the 4Ms of Geriatrics (Mentation, Mobility, Medications, and Matters Most) guided evaluation and outcomes. Implementation involved decision-making around hardware and software, learning about processes at other facilities, financing, and creating policies and procedures. Project development began in 2021 and data collection began in March 2023. Veteran residents enrolled in inpatient hospice and palliative care were invited to participate. As of August 2023, 22 VR visits were conducted with 13 veterans. Preliminary data suggests VR can be used with a wide range of individuals receiving hospice and palliative care. Analysis of pre-post measures indicate improvement in mood (62%) and overall day (81%), and reduced pain. Barriers to engagement include staffing (e.g., availability), technology (e.g., VR technical issues), hospital procedures/policies (e.g., infection control, COVID-19), and patient factors (e.g., interest, symptoms). Facilitators include hospital support, interdisciplinary collaboration, and understanding of the setting and patient preferences/ needs. Although conception to implementation took two years, a VR program to improve quality of life for veterans enrolled in hospice and palliative care appears to be feasible and clinically beneficial. Experience has shown that flexibility, adaptability, and an individualized approach facilitate engagement. Best practices in the application of VR within the hospice and palliative care setting will continue to be evaluated and honed.

Abstract citation ID: igad104.3421

### IMPLEMENTATION SCIENCE IN THE DEVELOPMENT OF RECRUITMENT PROCEDURES AND MATERIALS: A CASE STUDY

Cameron Ulmer[1], Johanna Silbersack[1], Lauren Stratton[2], Sam Fazio[2], and Sheryl Zimmerman[1], *1. University of North Carolina at Chapel Hill, Chapel Hill, North Carolina, United States*, *2. Alzheimer's Association, Chicago, Illinois, United States*

Recruiting long-term care organizations, administrators, and direct-care staff to participate in research has become increasingly challenging after the pandemic due to staffing shortages, limited resources, and numerous competing priorities. In light of these challenges, it is especially important to develop recruitment procedures and materials that are fine-tuned to the participant group based on best practices. This presentation will describe the use of implementation science principles and an expert focus group in the design of recruitment procedures and materials for a NIA-funded clinical trial “Evaluating a National Person-Centered Training Program to Strengthen the Dementia Care Workforce.” The project is especially well-suited to employ and critique best practices given that recruitment is at both the organizational-level (assisted living communities) and the person-level (staff within those communities). Recruitment materials were designed in accordance with the Considering Determinants of Diffusion, Dissemination, and Implementation of Innovations in Health Service Delivery and Organization conceptual model, which emphasizes highlighting the advantages of the training, how the training fits into existing systems, the process of training, the endorsement of organizational leaders, and the transfer of the knowledge conveyed by the training into practice. Best practices from the literature were discussed with a focus group of assisted living experts to evaluate the draft procedures and materials. This presentation will share the resulting procedures and materials and explain their evolution to their final form; the process and results are widely applicable to other clinical trials and other studies. Funded by a grant from the National Institute on Aging (R01 AG079124).

Abstract citation ID: igad104.3422

### IMPLEMENTING AN AREA AGENCY ON AGING COMMUNITY CARE TRANSITION INITIATIVE: FACILITATORS AND BARRIERS

Antoinette Coe[1], Hao-Ping Chiang[1], Brigid Rowell[1], and Nathaniel Bergman[2], *1. University of Michigan College of Pharmacy, Ann Arbor, Michigan, United States*, *2. Region VII Area Agency on Aging, Bay City, Michigan, United States*

The Michigan Region VII Area Agency on Aging (AAA) implemented a Community Care Transition Initiative (CCTI) for rural hospitalized older adults at high risk for readmission. After discharge home, AAA CCTI participants received a home visit by an AAA-trained pharmacy technician community health worker (CHW), a telehealth AAA pharmacist medication review, assessments by the CHW and pharmacist, education, primary care follow-up coaching, and referrals. As part of a program evaluation, we conducted six interviews with AAA key informants (e.g., pharmacist, director, CHWs). A semi-structured interview guide was piloted and revised to assess implementation, processes, impact, and future directions. Interviews were conducted via Zoom, recorded, transcribed, and checked for accuracy. Thematic analysis was used to identify implementation facilitators, barriers, and areas for program refinement. Three researchers coded the transcripts, developed a codebook, and resolved discrepancies by consensus. NVivo 14® was used for data management and analysis. Barriers to AAA CCTI implementation included unknowns at project start and need for procedures, anxiety about entering a client’s house, client’s lack of familiarity with technology, lack of perceived pharmacist benefit, limited personnel, and billing or financial reimbursement. Facilitators identified were the AAA’s reputation and backing, relationships built between healthcare providers, referral processes (e.g., community mental health), and pharmacist telehealth visit efficiency. Program refinements included shortened follow-up time and tailored visits to different client situations and needs. The results provide insights into the design of implementation strategies to disseminate the AAA CCTI to meet rural older adults’ health and social needs after hospitalization.

Abstract citation ID: igad104.3423

### IMPLEMENTING CARE COORDINATION WITH HOME DELIVERED MEALS AND HEALTHCARE: CHALLENGES AND LESSONS LEARNED

Elizabeth Orsega-Smith, Julia O'Hanlon, and Allison Karpyn, *University of Delaware, Newark, Delaware, United States*

Increasing lifespans are resulting in frail, isolated and food insecure older adults. Home Delivered meals (HDMs) are gateways into the homes of frail and vulnerable older adult, and this program will explore how HDMs can provide a natural opportunity to identify health issues potentially preventing unnecessary emergency rooms visits and hospitalizations. A daily wellness check and care coordination involved the use of a program coordinator, a nurse advocate, a local and predominately rural senior center (HDM provider and its HDM volunteers), and university evaluation team designed to support innovative health services provided to vulnerable older adults. The purpose was to examine the evaluation results 12 months after program. At baseline, clients reported high physical dependence (91.6%), hypertension (72.13% ), risk for falls (100%), and risk of food insecurity (48%). During the 12 month period, the nurse advocate team made 6,096 phone calls to participants with most phone calls (79.5%) categorized as a general client check-ins. Calls directly impacting quality of life included transportation support (6.1%), housing support (4.3%), and access to medical equipment (6.7%). Approximately 650 calls were service-related calls to outside agencies, organizations, and medical providers. This model exemplifies the increasing importance of clinicians and public health professionals, working collaboratively with community-based resources. The role of the nurse advocate team is critical in connecting resources to individuals based on specific circumstances and conditions, including geographic location and ability to access medical and social services. These efforts helped to avoid expensive treatments, manage living challenges, and facilitate medical care.

Abstract citation ID: igad104.3424

### IMPORTANT IMPROVEMENTS TOWARD EQUITY IN PROSTATE CANCER SURVIVAL BETWEEN WHITE AND BLACK MEN

Ray Merrill, and Ian Gibbons, *Brigham Young University, Provo, Utah, United States*

Prostate cancer survival is significantly influenced by tumor stage, age, and marital status at diagnosis. There is also evidence that cases diagnosed prior to age 40 and after age 80 years have a poorer prognosis. While Blacks have historically had lower prostate cancer survival than whites, it is not clear whether this difference still exists and if it varies across the levels of tumor stage, age, or marital status at diagnosis. These issues are addressed in this study. Analyses are based on Surveillance, Epidemiology, and End Results (SEER) data to obtain 5-year relative survival (5-YRS) estimates. Cases reflect 2000-2015 and follow-up extends through 2020. Over the study period 5-YRS decreased for Whites and increased for blacks, such that by 2015 the 5-YRS was about the same between races. Multiple regression models with race, age, and marital status simultaneously estimated showed that 5-YRS in Whites versus Blacks was not significantly different for local/regional stage or distant stage. 5-YRS in married (or living with a partner) was 3.02 higher for local/regional stage but not significant for distant stage. 5-YRS was significantly lower in the age groups < 40 and ≥80 for local/regional stage but were highest in the age group < 40 and significantly fell with older age for distant stage. Race did not modify the relationship between 5-YRS and tumor stage, age, or marital status at diagnosis. Thus, while tumor stage, age, and marital status at diagnosis are important predictors of 5-YRS for prostate cancer, White and Black inequality no longer exists.

Abstract citation ID: igad104.3425

### IMPROVING HEALTHCARE REGULATIONS FOR RESIDENTS WITH DEMENTIA: PERSPECTIVES OF REGISTERED DIETITIANS

Phatt Thaitrong, Joy Douglas, Linda L. Knol, Amy Ellis, Hyunjin Noh, and Shena Sanchez, *The University of Alabama, Tuscaloosa, Alabama, United States*

In order to admit residents on Medicare or Medicaid, long-term care (LTC) facilities in the United States are required to comply with federal regulations provided by the Centers for Medicare and Medicaid Services (CMS). However, limited research exists regarding the first-hand experiences of Registered Dietitians (RDs) working in LTCs and their perceptions on the extent to which the current healthcare regulations adequately address the nutritional needs of residents with dementia. Exploring the perspectives of RDs is crucial since residents with dementia may face difficulties expressing their nutritional needs. Our study explored RDs' perspectives and identified how menu planning (F803) and mealtime environment (F558) regulations could be improved to better meet the needs of LTC residents with dementia. RDs who work with residents with dementia in the U.S. (n = 18) participated in one of five focus groups. Focus groups were led by a trained moderator using a semi-structured interview guide, transcribed verbatim, and audited by the research team for accuracy. Transcripts underwent descriptive content analyses using NVivo© software. Analyses revealed the RDs' suggestions that F803 and F558 regulations need to 1) be more clear, 2) specify objective criteria to measure adherence, 3) work toward patient-centered care, and 4) give priority to residents with dementia. To meet the needs of LTC residents with dementia, mealtime-related healthcare regulations need to incorporate clear verbiage with measurable criteria. This will allow for easier understanding of the regulations and assessment of compliance with the regulations in LTCs.

Abstract citation ID: igad104.3426

### IMPROVING LONG-TERM CARE THROUGH GERIATRICS EDUCATION: A DEEP DIVE INTO GWEP AND GACA INITIATIVES

Gina Tucker-Roghi[1], Rajean Moone[2], Christopher Hernandez[3], Hannah Moerk[3], Linda Edelman[3], and Gail Towsley[3], *1. Dominican University of California, San Rafael, California, United States*, *2. University of Minnesota, Minneapolis, Minnesota, United States*, *3. University of Utah, Salt Lake City, Utah, United States*

Formal geriatrics education remains limited for many of the numerous health care professionals working with older adults. A well-prepared long-term services and supports (LTSS) workforce is imperative to care for older adults with multiple chronic conditions, especially dementia. This study highlights the role of Geriatric Workforce Enhancement Programs (GWEPs) and Geriatrics Academic Career Awards (GACAs) in addressing this need. An online survey, conducted 5/2023-6/2023, captured data from GWEPs (n=18) and GACAs (n=11) about training offered towards age- and dementia-friendly care in LTSS settings. Results revealed that 13 GWEPS or GACAs provided 36 learning activities that addressed the first recommendation, "Delivering comprehensive, person-centered, equitable care that ensures residents' health, quality of life and safety" of the National Imperative to Improve Nursing Home Quality (NASEM). Twelve GWEPS/GACAs provided 42 learning activities addressing the second NASEM report recommendation, "ensuring a well-prepared, empowered, and appropriately compensated workforce." GWEPS/GACAs are also aiding LTSS settings in achieving Age-Friendly Health Systems Designations: one each supported level 1 or 2 designation, two supported achieving both levels. Additionally, four GWEP/GACAs are working with LTSS entities toward level 1 status, one toward level 2, and one toward achieving both levels. Our findings underscore the pivotal role of GWEPS/GACAs in enhancing geriatrics education, addressing challenges in LTSS, and the pressing need for continued support. Recommendations emphasize strengthening partnerships between LTSS settings and GWEPs/GACAs to bridge educational gaps. Implications indicate GWEPS and GACAs are playing a crucial role in helping LTSS settings achieve age-friendly standards and align with the NASEM report's vision.

Abstract citation ID: igad104.3427

### INCARCERATION AS A RISK FACTOR FOR IMPAIRMENT IN COGNITIVE FUNCTIONING

George Daniel, Cedric Williams, Joel Wilkinson, and Denee Mwendwa, *Howard University, Washington, District of Columbia, United States*

**Background:** Incarceration is a social determinant of health with public health implications, especially among minoritized communities of color. The experiences of incarcerated people have deleterious implications for mental and physical health outcomes. Research has demonstrated an association between incarceration and poorer global cognition in older adults. The current study examines the long-term impact of incarceration on cognitive functioning in older adults and examines whether this relationship is moderated by race, ethnicity, and sex. **Methods:** Data were collected on participants (n=7,557) in the Health and Retirement Study (HRS). They completed a background questionnaire, psychosocial assessments, and a series of neuropsychological tests. T-tests and Chi-Squared analyses determined differences in demographic and health variables by incarceration status. Linear regression analysis determined if incarceration status predicts poorer cognitive functioning in older adults while controlling for age, race, sex, ethnicity, education, hypertension, diabetes status, wealth, depression, and stroke. Subgroup analyses determined whether associations were modified by race, ethnicity, or sex. **Results:** Our sample is on average 74 years old, 13% Black, and 60% female. Formerly incarcerated people were younger, disproportionately Black, less wealthy and educated, and more unhealthy. Analyses indicated that incarceration predicted reduced performance in delayed verbal recall (B = -0.19, p = 0.047) and this relationship may be stronger in Non-Hispanics, African-Americans and Women. **Conclusion:** Incarceration may negatively impact long-term memory in older adults and varies by gender, race, and ethnicity. Future studies should investigate variables that influence this relationship, including SES, depression, and chronic illnesses.

Abstract citation ID: igad104.3428

### INCIDENCE AND DYNAMICS OF MOBILITY DEVICE USE AMONG COMMUNITY-DWELLING OLDER ADULTS IN THE UNITED STATES

Xinran Liu[1], Sara Baumann[1], Andrea Rosso[1], Elizabeth M. Venditti[1], Yao Yao[2], and Steven Albert[1], *1. University of Pittsburgh, Pittsburgh, Pennsylvania, United States*, *2. Peking University Health Science Center, Beijing, Beijing, China (People's Republic)*

Mobility devices significantly enhance the activities and participation for individuals with mobility disability, particularly in the rapidly expanding population of older adults globally. This research reports patterns of mobility device use among community-dwelling older adults in the United States. Using the National Health and Aging Trends Study (NHATS) waves 1-9, data on basic demographics and mobility device use were collected in a nationally representative group of community-dwelling older adults (65+ years old). Mobility device use included canes, walkers, wheelchairs, and scooters. In a total of 47,722 person-years observed in community settings, 2,943 incident mobility device uses over an average of 3.8±3.0 years were identified among 2,591 participants, with an incidence rate of 61.7/1,000 person-year. Participants had an average age of 80.3±7.2 years, with 57.7% female, 70.2% White, 20.1% Black, and 5.7% Hispanic, and 34.6% living alone. The majority (51.3%) of incident mobility device use lasted no longer than one year, and only 18.6% continued for four or more years. The most popular combinations of mobility device types were cane only (44.3%), cane+walker (15.7%), walker only (15.1%), and walker+wheelchair (8.3%). 30.5% of community-dwelling older adults changed their patterns of mobility device use at least once, with the most common changes including cane only to cane+walker (16.4%), cane+walker to cane only (7.97%), walker only to walker+wheelchair (5.2%), and cane+walker to walker only (5.1%). Mobility device use among community-dwelling older adults is dynamic: use of mobility devices involves mostly short bouts and changes frequently, with a preference for a combination of mobility devices.

Abstract citation ID: igad104.3429

### INCREASED HIPPOCAMPAL ATROPHY IN HIGH INTENSITY INTERVAL TRAINING GROUP 4 YEARS AFTER 5-YEAR EXERCISE INTERVENTION

Asta Håberg, Line Reitlo, Jasmine Pani, and Dorthe Stenvold, *Norwegian University of Science and Technology, Trondheim, Sor-Trondelag, Norway*

The Generation 100 Study is a randomized controlled trial (NCT01666340) investigating the effects of a 5-year exercise intervention on several health outcomes, including brain volumes from MRI. Participants (born 1936-42) were living independently at inclusion. They were randomized (1-1-2) to supervised exercising with either high intensity interval (HIIT) or moderate continuous training (MICT) twice a week, or to a control group adhering to the national guidelines of ≥30 min of moderate to vigorous physical activity at least 5 days a week. The intervention lasted for 5 years, during which participants underwent physical and clinical examinations at baseline, 1-, 3-, 5-years. 105 participants (n: HIIT=33, MICT=24, Cont=48) also underwent brain MRI at 3T. Linear mixed models were used and the interaction between group and time on brain volumes was the main outcome. We have previously reported that the HIIT group had significantly greater hippocampal atrophy rate compared to the control group during the intervention. Participants were invited back 4 years after end of intervention and underwent the same examinations including brain MRI . A total 73 participants (n: HIIT24=, MICT=19, Cont=30) took part. The HIIT group trained at a higher intensity than the other groups. There were no associations between objective VO2 or any self-reported exercise measurements and hippocampal volume/atrophy rate at any timepoint. Still, the hippocampal atrophy rate remained greater in the HIIT group than in the controls 4 years after end of the exercise intervention.

Abstract citation ID: igad104.3430

### INCREASING DEPRESSION PREVALENCE AMONG OLD ADULTS DURING THE COVID-19 PANDEMIC

Jiangyuan Zhu[1], Guohua Li[2], Barbara Lang[2], and Stanford Chihuri[2], *1. Columbia University Medical Center, New York City, New York, United States*, *2. Columbia University, New York City, New York, United States*

Previous research has reported worsening mental health outcomes in children and young adults amid the COVID-19 pandemic. The purpose of this study was to assess the impact of the COVID-19 pandemic on the prevalence of depression in older adults. The Longitudinal Research on Aging Drivers (LongRoad) study involved prospectively following (between July 2015 and September 2022) 2990 drivers aged 65-79 years recruited from five sites across the contiguous United States. Study participants were assessed at baseline and annually during the follow-up period using the Patient-Reported Outcomes Measurement Information System (PROMIS). The overall response rate was 69.1%. The prevalence of depression increased from 7.12% during 2015-2019 to 9.89% during 2020-2022 ($p < 0.001$). After adjusting for age, gender, race and ethnicity, marital status, and annual household income, the risk of depression increased by 42% [adjusted odd ratio (aOR) 1.422, 95% confidence interval 1.237-1.635] during the COVID-19 pandemic. The results of this study indicate that the prevalence of depression among older adults increased significantly during the COVID-19 pandemic.

Abstract citation ID: igad104.3431

### INDICATORS OF WELLBEING AND SELF- AND INFORMANT-REPORTED COGNITIVE DECLINE IN OLDER ADULTS

Karysa Britton, Patrick Hill, Ryan Bogdan, Thomas Oltmanns, and Emily Willroth, *Washington University in St. Louis, St. Louis, Missouri, United States*

Wellbeing has been associated with objective cognitive decline and is a promising target for dementia prevention. Less is known about associations between wellbeing and subjective cognitive decline (SCD), an early indicator for progression to Alzheimer's Disease and related dementias (ADRD). Given the importance of early detection and intervention in ADRD, the current study assessed whether positive and negative indicators of wellbeing were associated with SCD via self- and informant-reports using zero-inflated Poisson regressions. A total of 1,014 participants (MAge=71.8, SD=3.1) were included in analyses. The sample was representative of both males (55%) and females (45%) and was racially diverse (65% White, 32% Black/African American, 2% other races). SCD was assessed with the AD8 Dementia Screening Interview (self- and informant-report versions), sense of purpose with the Life Engagement Test, life satisfaction with the Satisfaction with Life Scale, depressive symptoms with the Beck Depression Inventory-II, and loneliness with the UCLA Loneliness scale. Higher positive indicators of wellbeing were associated with lower SCD via self- and informant-reports. Higher levels of depression were associated with greater SCD via self-report. Higher levels of life satisfaction attenuated the association between depressive symptoms and SCD via self-report. These associations were statistically significant ($p < .05$) and held after applying False Discovery Rate correction. Effect sizes ranged from small to medium (ORPositive Wellbeing Indicators = 0.84-0.89, ORDepression = 1.34). These findings extend past wellbeing and objective cognitive decline research by demonstrating that positive and negative indicators of wellbeing are also associated with SCD assessed via self- and informant-reports.

Abstract citation ID: igad104.3432

### INFLAMMATORY CYTOKINES MEDIATE EXECUTIVE FUNCTION AND STRESS AMONG AGING ADULTS

Julie Kircher[1], Susan Charles[1], and David Almeida[2], *1. University of California, Irvine, Irvine, California, United States*, *2. The Pennsylvania State University, State College, Pennsylvania, United States*

Strong and consistent evidence demonstrates that a risk factor for poorer executive function is elevated levels of perceived stress. More recently, researchers have posited that inflammatory processes may contribute to the association between stress and cognitive declines. Similarly, research suggests that greater sustained inflammation can result from higher levels of stress. Furthermore, older adults are at a higher risk of experiencing cognitive decline when under greater stress. We hypothesized that inflammation, as measured by a composite of pro-inflammatory cytokines CRP, IL-6, and TNF-a, would mediate the relationship between perceived stress and executive function. Data were derived from the cognitive and biomarker projects in the second and third waves of the Midlife in the United States Study (MIDUS). The present study included 1,067 participants (Mage = 63.7; Range 39 - 94). The results showed a significant total effect between executive function and perceived stress ($\beta = .05$, $p <.001$). Furthermore, path analyses revealed significant pathways between stress on inflammation ($\beta = 0.29$ $p <.001$) and inflammation on executive function ($\beta = .06$, $p <.001$). Finally, when inflammation was entered into the relationship between perceived stress and executive function its direct effect was significant ($\beta = 0.06$, $p <.001$). These findings suggest that pro-inflammatory cytokines help to explain why perceived stress is related to lower capability in executive functions. Future research should focus on understanding and assessing the interactions between inflammation, stress, and cognitive functioning over time.

Abstract citation ID: igad104.3433

### INFLUENCE OF PHYSICAL THERAPY REHABILITATION ON FUNCTIONAL RECOVERY OF OLDER PEOPLE AFTER COVID-19

Gabriela Ochiai, Erika Gouveia E Silva, Daniella De Melo, Diêgo Araújo Ramos, Fabio Cavalcanti Freitas, Vivian Cintra Sousa, Ana Carolina Schmitt, and Jose Pompeu, *University of Sao Paulo, Sao Paulo, Sao Paulo, Brazil*

Retrospective cohort conducted through an online survey sent to older people (≥60 years) of both sexes, with confirmed diagnosis of Covid-19 registered in the Municipal Health Department of Sao Paulo, Brazil. Individuals with cognitive limitations and those who refused to participate were excluded. Functional capacity was assessed using Katz and Lawton Indices for pre-Covid, immediate post-Covid, and current time points, and characteristics of post-Covid physiotherapeutic rehabilitation were collected. Statistical analysis was performed using JASP-0.17.3 software, and group comparisons were made using repeated measures ANOVA, considering alpha=0.05. From March to August 2023, 86 individuals participated, with median age of 66.5 [62.3-71.0] and 57.0% (n=49) male. Among them, 39.5% (n=34) received recommendations for physiotherapeutic rehabilitation and 88.2% (n=30) of them were rehabilitated, while 11.8% (n=4) did not receive treatment. Older people with recommendation for physiotherapy post-Covid-19 demonstrated worse functional status ($p< 0.001$). Older people who received treatment were functionally equivalent to those who did not attend physiotherapy rehabilitation (p=1.000 for Katz Index and p=0.812 for Lawton Index). The primary indication for post-Covid-19 physiotherapy was muscular fatigue (70.0%, n=21), and domiciliary care was the predominant modality received (46.7%, n=14). Older people who were recommended for physiotherapy showed worse functional status after Covid-19, but there was no difference in long-term functional capacity. This suggests the potential importance of physiotherapeutic rehabilitation for functional recovery after Covid-19.

Abstract citation ID: igad104.3434

### INHIBITION OF AUTOPHAGY RESTORES IMMUNOMODULATORY CAPACITY OF SENESCENT MSCS VIA COX-2/PGE2 PATHWAY

Yang Ma, *Zhejiang university School of Medicine, Hangzhou, Zhejiang, China (People's Republic)*

Mesenchymal stem cells (MSCs) have been increasingly utilized in many therapeutic applications including autoimmune diseases and tissue repair due to their regenerative and immunomodulatory capacity. In vitro expansion will cause cell senescence and compromise their therapeutic effect. Therefore, underlying mechanisms of MSCs senescence during in vitro culture and methods of enhancing their therapeutic capacity need to be explored. Autophagy is a key regulator of biological properties of MSCs and is highly related to aging process. Whether manipulation of autophagy could restore immunomodulatory capacity of senescent MSCs after in vitro culture is still unclear. We demonstrated here that autophagy was significantly increased in aged MSCs compared with young MSCs. Inhibition of autophagy by BafA and CQ enhanced their immunomodulatory capacity of eliciting M2 polarization of macrophages. COX-2/PGE2 pathway was activated by inhibition of autophagy in this process. Inhibition of COX-2/PGE2 pathway of aged MSCs by SC-236 abandoned the enhanced immunomodulatory capacity of aged MSCs. Inhibition of autophagy by CQ of aged MSCs also increased their therapeutic capacity in cutaneous wound models of rats by increasing their capacity of eliciting M2 polarization of macrophages. Taken together, this work indicates that inhibition of autophagy of MSCs after in vitro expansion could ameliorate senescence-related changes in MSCs and enhance their immunomodulatory capacity. This may represent a potential therapeutic avenue to clinical application of MSCs.

Abstract citation ID: igad104.3435

### INNOVATION IN AGING & ACQUIRED BRAIN INJURY REHABILITATION: THE POTENTIAL OF MIXED REALITY TECHNOLOGIES

Mathieu Figeys[1], Farnaz Koubasi[1], Doyeon Hwang[1], Allison Hunder[1], Andrew Chan[2], Antonio Miguel-Cruz[1], and Adriana Ríos Rincón[1], *1. University of Alberta, Edmonton, Alberta, Canada*, *2. Glenrose Rehabilitation Hospital, Alberta Health Services, Edmonton, Alberta, Canada*

**Background:** Every 21 seconds an American experiences a Traumatic Brain Injury (TBI), and every 40 seconds, another endures a stroke. These events fall under Acquired Brain Injuries (ABI) with prevalence rates impacted by an aging population. ABI can lead to various physical, cognitive, and mental impairments, emphasizing the need for innovative rehabilitation strategies, including those specific to older adults. Mixed Reality (MR) technologies offer potential in enhancing ABI rehabilitation, yet face challenges such as methodological inconsistencies, differing clinical populations, and exaggerated efficacy claims. **Objectives:** 1) Review MR's role in ABI rehabilitation among older adults, assessing the implications of aging, clinical, and technological considerations. 2) Present ongoing MR research at the Glenrose Rehabilitation Hospital (GRH, Edmonton, Canada). **Methods:** 1) A systematic review following PRISMA guidelines was performed across seven databases, with two independent reviewers analyzing the data. The analysis emphasized clinical objectives, MR systems, levels of evidence, and technology readiness levels. 2) QR codes that link to videos will highlight the ongoing research and development of MR-delivered ABI rehabilitation at the GRH. **Results:** Twenty-six studies met the inclusion criteria, totalling 453 subjects with ABI (mean age: 60 ± 5.34 years). MR applications mainly targeted upper limb motor rehabilitation, revealing an overall low level of evidence and a median technology readiness level of 6 (prototypes tested in relevant environments). **Conclusion:** Despite existing variability and technological challenges, the promising results stress the importance of ongoing research and innovation in MR rehabilitation. The GRH stands as a key research hub, actively advancing this field.

Abstract citation ID: igad104.3436

### INTEGRATING HOME HEALTH IN AN INTEGRATED CARE SYSTEM: VETERAN EXPERIENCES WITH A VA PILOT PROGRAM

Heather Davila[1], Mary Good[1], Aaron Seaman[2], Tammy Walkner[1], Irene San Roman[1], Heather Benzel[3], and Samantha solimeo[1], *1. Iowa City VA Health Care System, Iowa City, Iowa, United States*, *2. University of Iowa, Iowa City, Iowa, United States*, *3. VA Midwest Healthcare Network (VISN 23. ), Grand Island, Nebraska, United States*

The Veterans Health Administration (VA) serves nearly 3 million Veterans aged 65 and over, 350,000 of whom use skilled home health (e.g., nursing, rehabilitation therapy) or homemaker/home health aide services annually. Despite being the nation's largest integrated healthcare system, VA has traditionally purchased home health services from contract agencies. Coordinating care between VA and contract agencies is an ongoing challenge, and accessing home health has become increasingly difficult, especially in rural areas. To address these challenges, the Iowa City VA launched VA's first "VA Home Health" (VAHH) program in 2021. In 2022, the VA Midwest

Health Care Network (VISN 23) expanded VAHH to three additional sites, with over 700 Veterans served to date. We interviewed Veterans receiving VAHH who had previously used contract services (n=30) to learn about their care experiences. Veterans described multiple benefits of VAHH over contract services: staff's access to VA electronic health records, mechanisms for quickly sharing information with other care providers, understanding of Veterans' unique care needs (e.g., PTSD), and knowledge of the breadth of services VA offers. Most Veterans said they were highly satisfied with VAHH, with many expressing a desire for VAHH to expand and serve more Veterans. Our preliminary findings suggest VAHH may improve Veterans' experiences with care and overall quality of care. Given VA's focus on expanding Veterans' access to home-based services, these preliminary findings could inform national VA decisions surrounding home health delivery. Our findings are also relevant to other healthcare systems as they address similar coordination and access challenges.

Abstract citation ID: igad104.3437

### INTERACTION OF RESILIENCE AND DISTRESS IN THE ELDERLY-CAREGIVER DYADS: AN ACTOR-PARTNER INTERDEPENDENCE MODEL

Anni Wang[1], and zhiyao xiong[2], *1. Fudan University, Shanghai, Shanghai, China (People's Republic), 2. Huaxi Hospital, Chengdu, Sichuan, China (People's Republic)*

Aims: The aim of this study was to explore the interaction between resilience and psychological distress in disabled elderly-caregiver dyads. Design: A cross-sectional descriptive study. **Methods:** A total of 246 homebound disabled elderly individuals and their family caregivers from five provinces of China were investigated by convenience sampling using the Chinese version of the Resilience Scale and the Distress Thermometer and Problem List. Data analysis was conducted using the actor-partner interdependence model (APIM) method and based on structural equation modelling. **Results:** The effect of resilience on psychological distress shows a mixed pattern. With regard to the actor effect, the resilience of disabled elderly individuals and caregivers was negatively related to their own psychological distress. Regarding the partner effect, the resilience of disabled elderly individuals was negatively associated with caregiver psychological distress, and the resilience of the caregiver was negatively associated with the psychological distress of the disabled elderly individual. **Conclusion:** There is a dyadic interaction between disabled elderly individuals and their caregivers, suggesting that resilience can relieve not only one's own psychological stress but also that of others to some extent. Health professionals can design dyadic interventions that focus on resilience and its dyadic interaction and effectively leverage the resilience advantages of the dyad based on the individuals' level of psychological distress to improve the mental health of elderly people with disabilities and their caregivers.

Abstract citation ID: igad104.3438

### INTERPRETING PERSON-CENTEREDNESS IN RELATION TO CARE AND THE WORKFORCE FOR OLDER ADULTS

Antonio Saavedra[1], Philip Sloane[2], Sheryl Zimmerman[2], and Sam Fazio[3], *1. University of North Texas Health Science Center, Fort Worth, Texas, United States*, *2. University of North Carolina, Chapel Hill, North Carolina, United States*, *3. Alzheimer's Association, Chicago, Illinois, United States*

The concept of person-centeredness has been widely referenced and seemingly adopted as a gold standard practice, especially in relation to care for older persons. However, the concept of person-centeredness has evolved over time, resulting in a lack of definitional clarity. As part of a series of studies exploring person-centeredness, we investigated the meaning of person-centeredness as it relates to care and the workforce for older adults. Six think-tank meetings (two in-person; four virtual) were convened, including a national purposive sample of 33 participants selected for diversity and expertise, and including providers, consumers, policy makers, and researchers. Qualitative methods were used to analyze notes and transcripts, resulting in four overarching themes: (1) Different care settings (e.g., hospice, nursing home) tend to vary in the extent and manner of person-centeredness due to their inherent characteristics and goals of care. (2) Leadership tends to assign priority to quality citations and safety protocols, which creates dissonance between the desire to be more person-centered and a demanding organizational culture. (3) Person-centeredness can be conveyed through concrete practices (e.g., care for activities of daily living) or general principles; the former may be easier to implement. (4) The lack of an operationalized person-centeredness definition has created a gap in how to teach it. This presentation will tie together the four themes and address attitudes and approaches within organizations and toward care providers.

Abstract citation ID: igad104.3439

### INTERPROFESSIONAL GERIATRIC ASSESSMENT CLINIC: STUDENT SKILL DEVELOPMENT AND PATIENT SATISFACTION

Ryan Butler, Lisa Phifer, Katie Ehlman, Suzanne Leahy, Ashley Keller, and Haneul Jung, *University of Southern Indiana, Evansville, Indiana, United States*

The University of Southern Indiana Interprofessional Geriatric Assessment Clinic (IGAC) provides interprofessional clinical experience to medical residents and health profession students using the Institute for Healthcare Improvement's Age-Friendly 4 Ms model while addressing the complex health conditions of medically underserved older adult patients. This study examines student skill development and patient satisfaction. A nursing student, medical resident, Area Agencies on Aging (AAA) case manager, and an occupational therapy or physical therapy student utilize evidence-based assessment tools to examine the four elements of Age-Friendly 4 Ms care, including high risk medications, mobility, mentation, and what matters. Students (N=56) completed a retrospective pre-posttest measuring six skills related to IGAC activities. In addition, patients (N=42) completed a posttest measuring satisfaction of the IGAC visit. "Administering standardized health assessments" and "using information about patient social needs to develop healthcare recommendations" were the two IGAC skills wherein students perceived the largest gains, based on paired samples t-tests of significance and Cohen's d measurement of effect size. Students also reported statistically significant, moderately sized gains in "documenting patient measurements in a medical chart", "communicating medical information to other health professionals", and "conducting clinical visits with older adult patients". Ninety-two percent of patients were satisfied with the overall actions of the care team for their visits. This research indicates that the USI IGAC both increases student skill development and supports patient satisfaction.

Abstract citation ID: igad104.3440

### INTERVENTIONS FOR PERSONS WITH YOUNG-ONSET DEMENTIA AND THEIR FAMILIES: A SCOPING REVIEW

Xiaoyan Cui[1], Junqiao Wang[1], Bei Wu[2], Qianhua Zhao[3], Xueting Tang[1], Roy Thompson[4], and Jing Wang[5], *1. Fudan University, Shanghai, Shanghai, China (People's Republic)*, *2. New York University, New York City, New York, United States*, *3. Fudan University Affiliated Huashan Hospital, Shanghai, Shanghai, China (People's Republic)*, *4. University of Missouri, Columbia, Missouri, United States*, *5. University of New Hampshire, Durham, New Hampshire, United States*

Young Onset Dementia (YOD) poses unique challenges for individuals and their families globally by impacting their working-age years and family responsibilities. The paucity of effective treatments emphasizes the need for tailored interventions and support services to enhance quality of life and social engagement. Dementia care services oftentimes fail to meet YOD-specific needs, necessitating customized approaches. This review aims to synthesize non-pharmacological intervention studies for YOD individuals and families to inform future targeted interventions. We conducted a systematic literature search across four databases. Included articles were carefully screened, categorized, and synthesized. This review examined 12 studies focused on persons with YOD. Interventions targeted daily life support, cognitive decline, and social engagement, using quantitative, qualitative, or mixed methods designs. Nine studies addressed interventions for families focused on self-efficacy, burden, anxiety, depression, quality of life, post-traumatic stress, and well-being. This review revealed a dearth of family-centered intervention studies, emphasizing the need for more comprehensive and tailored interventions for families of individuals with YOD. There is urgent need for more large-scale intervention studies, especially from Low and Middle income countries, which should adopt family-centered and life course perspectives. For future interventions , more extensive incorporation of stakeholder involvement is essential for successful implementation. Moreover, the integration of new technologies shows promise as a potential avenue for intervention advancement.

Abstract citation ID: igad104.3441

### INVESTIGATING DREAMING IN COGNITIVELY DIVERSE OLDER ADULTS

Warren Britton, and Jessica Strong, *University of Prince Edward Island, Charlottetown, Prince Edward Island, Canada*

Dreaming changes across the lifespan. While our understanding of how dreaming relates to aging and well-being has developed with respect to early and middle phases of the lifespan, the dreams of older adults remain under-studied. Reviews of the literature suggest decreases in dream recall and the emotionality of dream content that often precede age-related changes in sleep architecture, with few changes in dreaming occurring between ages 45-75, after which dream recall declines steeply. However, these findings are based on relatively few studies. This point is underscored when considering the effects of secondary aging on dreaming, where virtually no literature exists on the dreams of older adults experiencing mild cognitive impairment. Studying the dreams of older adults experiencing cognitive impairment may seem trivial when compared to other processes implicated in secondary aging, but recent studies examining the relationship between frequency of negative dream content and the increased risk of onset and acceleration of cognitive decline beg a closer examination of how dreaming contributes to a broader understanding of health and functioning across the lifespan. The scoping review of sleep, dreams, well-being and cognition across the lifespan, and the subsequent proposed study design seeks to employ the Hall and Van de Castle Coding System of content analysis to contribute to the literature on the dream frequency and content of cognitively typical older adults while also exploring the feasibility and methodological considerations of studying the dream content of older adults with mild cognitive impairment.

Abstract citation ID: igad104.3442

### INVESTIGATING THE EFFECTS OF PM2.5 ON DEMENTIA RISK: A CASE-CONTROL SWEDISH TWIN REGISTRY STUDY

Luisa Parkinson[1], Ida Karlsson[2], Malin Ericsson[2], Sam Tomlinson[3], Finn Lindgren[1], Tom Russ[1], Deborah Finkel[4], and LifeGEIST consortium[4], *1. University of Edinburgh, Edinburgh, Scotland, United Kingdom*, *2. Karolinksa Institutet, Stockhoolm, Stockholms Lan, Sweden*, *3. UK Centre for Ecology and Hydrology, Lancaster, England, United Kingdom*, *4. University of Southern California, Los Angeles, California, United States*

There is growing evidence that air pollution, and particularly PM2.5, is associated with dementia risk around the time of diagnosis. However few studies are able to investigate longer term effects of air pollution due to a lack of historical air pollution data. In this study we use PM2.5 data for Sweden available at 5-yearly intervals to investigate the short and longer term effects of PM2.5 on dementia risk. A case-control data set from the Swedish Twin Registry with dementia ascertained from hospital admissions between 1990 and 2015 and municipality of residence available at time of admission was analysed using hierarchical spatial models with R-INLA. The analyses show a relative risk of dementia per 5µg/m3 PM2.5 of 1.43 (95% confidence interval: 1.13-1.80), 1.36 (1.14-1.64), 1.31 (1.11-1.54) and 1.04 (1.00-1.09) for 0-5, 6-10, 11-15 and 16-20 years prior to diagnosis respectively. The cumulative effect over 0-20 years prior to diagnosis of an annual 5µg/m3 increase in PM2.5 on the relative risk of dementia is 1.19 (1.06-1.34). The clear association between PM2.5 and dementia risk for up to 20 years prior to diagnosis suggests that there are long term effects of air pollution on dementia risk. For these analyses only one residential location was available, so the uncertainty about an individual's residential location increases with time prior to diagnosis. To fully understand the effects of air pollution on dementia risk a life-course approach in a data set with lifetime residential history and access to historical air pollution data will be necessary.

Abstract citation ID: igad104.3443

### INVESTIGATING THE IMPACTS OF METFORMIN ON AGING AND LIFESPAN IN DIVERSITY OUTBRED MICE

Rong Yuan[1], Yun Zhu[1], Monica Frungieri[2], Michal Masternak[3], and Andrzej Bartke[1], *1. Southern Illinois University School of Medicine School of Medicine, Springfield, Illinois, United States*, 2. *Instituto de Biología y Medicina Experimental, Buenos Aires, Buenos Aires, Argentina*, 3. *University of Central Florida College of Medicine, Orlando, Florida, United States*

Metformin, a type 2 diabetes treatment, has profound effects on regulating development, metabolism, and aging acting via the mTOR/autophagy pathway. Nevertheless, its potential to extend lifespan and counteract aging remains an open question. Previous research highlighted the intricate interplay between genetic background, gender, and treatment commencement age in shaping outcomes. To examine the long-term effect of metformin on aging and lifespan, this project utilizes the diversity outbred (DO) mice, initially derived from the interbreeding of eight inbred strains, effectively emulating the intricate genetic complexity found in humans. Initiated at 2 months old, DO mice were subjected to metformin-infused food [0.1%, w/w] (n=15 and 14, females and males respectively) or a metformin-free counterpart (n=10 for both sexes). Starting from 12 months of age, a statistically significant increase in male body weight was observed within the metformin-treated group, while no significant effect was found in females. Indirect calorimetry assays revealed a significantly elevated respiratory ratio in both sexes of metformin-treated mice. Surprisingly, at the age of 12 months, metformin treatment significantly impaired glucose tolerance in females, whereas in males, pyruvate and insulin tolerance exhibited significant reductions. After the first submission deadline, by 16 months of age, two females died in each of the treatment and control groups. Notably, the metformin-treated male cohort lost four individuals, but no mortality in the control male cohort (P=0.07, log-rank test). These preliminary findings underscore a potential deleterious impact of metformin on metabolic traits and lifespan when initiated from a young age, particularly in male mice.

Abstract citation ID: igad104.3444

### INVESTIGATING THE USE OF FORMAL AND INFORMAL SUPPORTS BY OLDER ADULTS LIVING ALONE WITH MULTIPLE CHRONIC CONDITIONS

Morgan Minyo[1], David Bass[2], Katherine Judge[1], and Catherine McCarthy[2], *1. Cleveland State University, Cleveland, Ohio, United States*, 2. *Benjamin Rose Institute on Aging, Cleveland, Ohio, United States*

Older adults living alone with multiple chronic health conditions and/or functional limitations are at an increased risk for experiencing unmet needs and associated negative outcomes. Formal and informal support services benefit older adults by addressing unmet needs, making this population a common target among clinical interventions aiming to increase levels of support. To further our understanding of this vulnerable population, this investigation describes unmet needs and assistance with tasks in a sample of older adults living alone with chronic conditions (n=38). Baseline data collected from a randomized controlled pilot study was used for descriptive analyses. Participants average age was 77.1 (SD=11.6), 42.1% identify as Black, and 68.4% identify as female. On average, participants reported 6.0 (SD=3.7) chronic health conditions and 52.6% reported difficulty with two or more daily living tasks. Nearly 90% of participants reported at least one unmet need, with 60.5% having four or more (M=6.1, SD=5.2). Interestingly, 71.1% of participants reported receiving assistance from family/friends (M=2.8, SD=2.9) and 86.8% reported receiving assistance from service providers (M=3.1, SD=2.4) with at least one task in the past 3 months. Only one participant reported receiving no assistance. Family/friends most frequently assisted with household chores (47.4%), transportation (44.7%), and emotional support (39.5%). Service providers most frequently assisted with transportation (50.0%), emotional support (31.6%), and getting information about a health problem (31.6%). These findings provide insight on the formal and informal support services used by older adults who live alone and the individual characteristics that could make specific sub-groups successful clinical intervention targets.

Abstract citation ID: igad104.3445

### JOB EXPECTATIONS VS. REALITY: WORKPLACE EXPERIENCES FROM THE NURSING HOME FRONTLINES

Vivian Miller, Jennifer Wagner, Lauren Maziarz, Melissa Burek, Julia Bell, and Kaley Perry, *Bowling Green State University, Bowling Green, Ohio, United States*

Nursing assistants have been at the frontlines of nursing home care, providing direct resident care and services to aging adults. The U.S. Bureau of Labor Statistics projects an increased employment need for nurse aides over the next ten years. However, the annual rate of nurse aide turnover is over 120 percent. To better understand the unique factors that contribute to nurse aide turnover in nursing homes and assisted livings, this sequential mixed-methodological study design reports findings of activities, relationships, and job interactions that contribute to nurse aide turnover. Guided by a socio-ecological model, qualitative findings from 17 nurse aides (N=17) reveal interpersonal factors, such as relationships with others at the workplace and relationships with residents, as impacting turnover. Institutional and Community factors were also found to impact turnover, including staffing levels, resources, education, and compensation. Informed by the interview findings, there were 239 nursing home or assisted living nurse aides (N=239) that participated in the quantitative portion of this study. Over one-quarter of nurse aides (27.80%) reveal that getting to know the residents was one of the best things about working in a nursing home or assisted living. Of direct care tasks, nurse aides reported physical injury or strain (20.16%), and the physical demands of the job (20.16%) as the greatest challenges. Taken together, findings from this study reveal that using community-engaged research methods in nursing home research may lead to a more nuanced understanding of the workplace needs and desires of nursing assistants.

Abstract citation ID: igad104.3446

### KINLESSNESS, RACE, AND QUALITY OF CARE AT THE END OF LIFE: FINDINGS FROM NATIONAL HEALTH AND AGING TRENDS STUDY

Yaolin Pei, Xiang Qi, Zheng Zhu, and Bei Wu, *New York University, New York City, New York, United States*

Close family members provided the majority of end-of-life (EOL) caregiving. The number of older adults who are kinless (without close family/partnerships) are increasing. We therefore examined the association between kinlessness and quality of EOL care among older adults, and the moderating role of race in this association. Data were derived from the combined Round 2 to Round 11 of the National Health and Aging Trends Study. 3066 older adults who aged 65 and above and were living in the community or residential care at last interview were included in the working sample. Kinlessness was defined as lacking children and a spouse/partner. The primary outcome was quality of care at the end of life. Logistic regression models ware used to examine the association between kinlessness, race, and quality of care at the end of life. A total of 8.0% of decedents were kinless at the end of life. Kinless older adults were less likely to receive excellent EOL care than those with kin. Having at least one child or spouse/partner weakened race disparities in EOL care quality among older adults. These results point to a significant disadvantage for the minority kinless older adults, who had worse EOL care quality. Practice interventions may include a culture match between older adults and providers. Form a policy standpoint, guardianship is needed to enable kinless older adults to receive high-quality EOL care.

Abstract citation ID: igad104.3447

### KYNURENINE METABOLITES INDUCE MICROGLIAL CELL SENESCENCE AND STIMULATE NEUROINFLAMMATION

Jimeace Bonaparte[1], Maxwell Cook[2], Steven Dixon[2], Cynthia Wright[2], William Hill[2], and Gavin Wang[2], *1. College of Charleston, Charleston, South Carolina, United States*, *2. Medical University of South Carolina, Charleston, South Carolina, United States*

Aging is the greatest known risk factor for Alzheimer's disease (AD) and other forms of dementia. Aging is associated with increased inflammation, which stimulates the activation of the kynurenine pathway and the production of kynurenine metabolites, including kynurenine (KYN), kynurenic acid (KYNA), 3-hydroxykynurenine (3HK), and quinolinic acid (QA). Previous studies have shown an elevated level of KYN and its metabolites in age-related diseases, including AD. Growing evidence suggests that age-associated accumulation of senescent cells plays a critical role in brain aging and AD pathogenesis. However, it remains to be determined how KYN or its metabolites affect neural cell senescence. The goal of this study was to investigate the effects of KYN and its metabolites on senescence induction in HMC3 human microglial cells. HMC3 cells were treated with a range of doses of KYN metabolites over multiple time points (3-48 hrs). Results showed that multiple independent senescent biomarkers were increased in 3HK-treated HMC3 cells, including SA-β-gal, whereas KYN-treated cells exhibited only a low level of SA-β-gal. The senescence induction effect of 3HK was further confirmed by increased expression of the p21 and p19 senescence markers. Furthermore, our data reveals that 3HK is the most potent metabolite to stimulate the secretion of the neuroinflammatory cytokine interleukin-6 (IL-6) by microglia compared to other KYN metabolites, and this result also demonstrates the SASP phenotype of 3HK-treated HMC3 cells. Taken together, these new findings suggest that KYN and its metabolites may contribute to the pathogenesis of AD via stimulating glial cell senescence and SASP-mediated neuroinflammation.

Abstract citation ID: igad104.3448

### KYNURENINE PATHWAY INHIBITORS COMBINED WITH NAD+ PRECURSORS INCREASES LONGEVITY GENE EXPRESSION IN DROSOPHILA

Adam Said[1], Qiao Huang[2], Yuqiong Wu[2], Jeremy Walston[3], Reyhan Westbrook[2], and Peter M. Abadir[2], *1. Emory College of Arts and Sciences, Atlanta, Georgia, United States*, *2. Johns Hopkins University School of Medicine, Baltimore, Maryland, United States*, *3. Johns Hopkins University, Baltimore, Maryland, United States*

With rates of aging-related functional decline rising, the need for novel intervention strategies to extend lifespan has increased. In humans, the kynurenine pathway (KP) metabolizes tryptophan and produces nicotinamide adenine dinucleotide (NAD+). KP is correlated negatively with longevity and increased KP metabolite concentrations decrease the average and maximum lifespans in flies. However, genetic and pharmacological inhibition of tryptophan metabolism and dietary supplementation of NAD+ precursors increase the average and maximum lifespans in flies. To comprehensively explore the effects of KP manipulation, we fed DGRP_229 Drosophila males diets infused with α-MT (KP inhibitor), nicotinamide (NAM, an NAD+ precursor), or the combination of NAM and α-MT. We found that all treatments resulted in increased average and maximum lifespans, with the combination treatment displaying the greatest increase compared to the control of 30% ($P< 0.0001$). The combination treatment resulted in an average lifespan increase of 15% in relation to α-MT alone ($P< 0.0001$) and 18% in comparison to NAM alone ($P< 0.0001$). To gain more insight into the biological mechanisms through which different treatments affect lifespan, we performed qRT-PCR on 11 longevity genes in Drosophila. We found that the combination treatment significantly increased the expression of gigas (Gig) ($P=0.03$), which interacts with Tsc1 to control cellular growth by regulating insulin and TOR signaling pathways, and sirtuin 1 (Sirt1) ($P=0.03$), an NAD+-dependent histone deacetylase. Our results demonstrate that the combined treatment of KP inhibition and NAD+ precursor elicits a transcription response similar to other lifespan-enhancing treatments and could be used as an alternative intervention method.

Abstract citation ID: igad104.3449

### LEVERAGING CONSUMER HEALTH TECHNOLOGIES TO ENGAGE CARE PARTNERS OF OLDER ADULTS WITH DEMENTIA

Jamie Smith[1], Jessica Cassidy[2], Catherine Riffin[3], Jennifer Wolff[1], and Hillary Lum[4], *1. Johns Hopkins University, Baltimore, Maryland, United States*, *2. University of Texas at Arlington, Arlington, Texas, United States*, *3. Weill Cornell Medicine, New York City, New York, United States*, *4. Univeristy of Colorado School of Medicine, Aurora, Colorado, United States*

Care partners are critical in supporting the complex health needs of older adults with dementia; the importance of their role increases with disease progression. Despite their extensive involvement in dementia care, care partners are not systematically identified or supported in healthcare settings. Consumer health information technology (CHIT) affords a largely untapped opportunity to formalize dementia care partner identification and engagement in healthcare delivery. One example is OurNotes, a portal-based agenda-setting questionnaire that allows patients to submit concerns for discussion ahead of medical visits. In a proof-of-concept study, OurNotes was first adapted to elicit the identity of the person completing the form and clarify their relationship to the patient, and subsequently piloted at an academic geriatric practice. Semi-structured interviews were conducted with 15 care partners to elicit their perceptions of the adapted OurNotes (utility, benefits, challenges) and suggestions for refinement. Transcripts were analyzed using deductive, thematic analysis. Care partners' perceptions of the tool were overwhelmingly positive. Major benefits included enhancing their feelings of preparedness for the visit, having a voice to express concerns, and streamlining visit communication. The most commonly endorsed challenge was the time required to complete the adapted OurNotes in addition to routine check-in items. A primary suggestion was to enable the submitted OurNotes responses to be edited/updated. Findings suggest that the adapted OurNotes is an acceptable approach to identifying care partners in the dementia context. Refinements may further facilitate visit efficacy through preparedness. Future research will examine CHIT engagement on patient and care partner outcomes.

Abstract citation ID: igad104.3450

### LGBTQ+ INCLUSIVITY TRAINING AND EDUCATION FOR SKILLED NURSING FACILITIES

Jennifer May[1], Alexis Domeracki[2], Perisa Ashar[3], Foxx Hart[3], Jason Wheeler[4], Glaucia Salgado[5], and Melanie Wang[3], *1. University of South Carolina, Columbia, South Carolina, United States*, *2. Duke School of Medicine, Durham, North Carolina, United States*, *3. Duke University, Durham, North Carolina, United States*, *4. Duke PHMO, Durham, North Carolina, United States*, *5. Duke Clergy Heath, Durham, North Carolina, United States*

The population of older adults is growing in the United States, leading to greater need for specialized care offered by skilled nursing and long-term care facilities. Among older adults that require specialized health care, lesbian, gay, bisexual, transgender, queer, and other sexual and gender minority (LGBTQ+) older adults are more likely to experience health disparities and health care barriers due to lifelong discrimination and exclusion. Healthcare workers within skilled nursing facilities (SNF) have expressed they do not feel prepared to care for LGBTQ+ older adults. The aim of this research was to develop the LGBTQ+ Inclusivity Training and Education (LITE) toolkit to provide information to healthcare workers, staff, residents, and their families in the SNF environment. Phase 1 used a community engaged approach to develop the LITE toolkit through engagement with an LGBTQ+ Community Advisory Board. Phase 2 was the implementation and evaluation of the LITE toolkit with 25 SNFs throughout North Carolina. The majority of SNF healthcare workers and staff (N=20) who responded to the survey indicated that the LITE toolkit was “easy to use,” they were satisfied with the contents, and that it would improve the way they care for patients. Providing LGBTQ+ focused education for members of the SNF community addresses the need for healthcare worker and staff training, thereby contributing to equitable care and inclusive environments for the LGBTQ+ older adult community. Additional work focused on understanding the facilitators and barriers to using the LITE toolkit in the SNF setting is needed.

Abstract citation ID: igad104.3451

### LIFE COURSE TIMING OF ADVERSITY AND DIURNAL CORTISOL IN LATER LIFE

Nadine Sikora[1], Briana Mezuk[2], James Abelson[2], Jane Rafferty[2], Jamie Abelson[2], and Julie Ober Allen[1], *1. The University of Oklahoma, Norman, Oklahoma, United States*, *2. University of Michigan, Ann Arbor, Michigan, United States*

Exposure to adversity is believed to alter stress sensitive biological processes. One manifestation of this, flatter diurnal cortisol slopes, has been linked to cardiometabolic problems. Questions remain about whether the timing of adversity within the life course influences its effect on cortisol slopes in later life. Two longitudinal cohort studies (Cardiac Rehabilitation and The Experience [CREATE], Tracking Risk Identification for Adult Diabetes [TRIAD]) with identical measures were merged for analysis (merged N = 175, Mage 60.8) using multilevel modeling. Measures included retrospective self-report of childhood trauma (CTQ), major life stressors in adulthood (number and recency of stressful events, affected domains, everyday discrimination), and peak-to-bedtime cortisol slopes (≤12 collected over 15 months). Report of any moderate and severe childhood adversity was not associated with adult cortisol slopes (p>.05). When we probed specific types, emotional abuse was associated with cortisol slopes (b=.006, SE=.003, p=.015). For adult adversity, total number of stressful events (b=.001, SE=.0002, p=.009) and recent stressful events (b=.003, SE=.001, p=.041) were associated with cortisol slopes. When simultaneously modeling all measures of adversity over the life course, number of stressful events in adulthood (b=. 001, SE=.0002, p=.018) and childhood emotional abuse (b=.007, SE=.004, p=.064) were the strongest predictors of flatter diurnal cortisol slopes in adulthood. Given that those reporting childhood adversity were also more likely to have experienced adulthood adversity (r=.08-.39), early life adversity may be an important risk factor for flattened cortisol slopes and associated cardiometabolic outcomes in later life via multiple pathways. Implications for intervention will be discussed.

Abstract citation ID: igad104.3452

### LIFE-COURSE AND CONTEXTUAL FACTORS OF ADVANCE CARE PLANNING AMONG OLDER ADULTS WITH LIMITED INCOME

Christine Kimpel[1], Jana Lauderdale[1], David Schlundt[1], Mary Dietrich[1], Amy Ratcliff[2], and Cathy Maxwell[1], *1. Vanderbilt University, Nashville, Tennessee, United States*, *2. Tennessee Valley Healthcare System VA, Nashville, Tennessee, United States*

**Background:** Cumulative disadvantage across the lifespan can lead to income, health, and advance care planning (ACP) disparities, resulting in increased caregiver decisional distress and reduced healthcare equity during times of cognitive incapacity. ACP is the process of patient and familial learning, communication, and documentation of life-sustaining care and quality of life preferences after personal reflection, but ACP rates remain lower among older adults with limited income. To better understand how life-course factors influence these low ACP rates, we aimed to explore participant perceptions of healthcare and ACP barriers and facilitators through different life stages. **Methods:** We sampled older adults (aged 50+) with limited income from six community-based sites (N=20). Qualitative descriptive design was utilized to perform semi-structured, in-person interviews. **Results:** Themes emerged from the inductive inclusion of in-vivo codes and the deductive application of the Cumulative Disadvantage Theory. Mean age: 64.8 years old (SD: 6.8); 11 participants identified as female (55.0%); 16 identified as Black/African American (80.0%). Four themes emerged: 1) structural, life-stage, 2) social stressors and resources, 3) individual stress responses and 4) ACP readiness. Participants' perceptions denoted that proactive planning inequities among older adults with limited income result from the dynamic interplay of multi-dimensional stressors, protective factors, and personal stress responses. Implications: Identified themes serve as a conceptual basis for future intervention development that is responsive to traumatic life-course experiences and supportive of building ACP resilience (i.e., adapting to ACP stress and actively planning for times of cognitive incapacity), as well as policy and practice implications.

Abstract citation ID: igad104.3453

### LONELINESS AMONG UNDER-REPRESENTED OLDER ADULTS IN THE UK: A STUDY OF MINORITY ETHNIC AND LGBTQ+ POPULATIONS

Christina Victor, and Isla Rippon, *Brunel University London, Uxbridge, England, United Kingdom*

Internationally loneliness has been identified as a major public health problem. Although there is a substantial body of research about loneliness in older adults in the UK, there is a significant evidence gap reporting experiences of loneliness of older people from ethnic minorities and those who identify as lesbian, gay, or bisexual and transgender (LGBT). These two groups, under-represented in UK gerontological research, are included in our recently funded project, Socially Inclusive Ageing across the Lifecourse. In this poster we explicitly focus upon the experiences of loneliness for older adults, aged 50+, from the LGBTQ+ and minority ethnic communities. Using wave 9 data from the UK Household Longitudinal Study (UKHLS/Understanding Society) we measured loneliness using the three-item UCLA scale with a score of 6+ out of 9 defining loneliness. Of our total sample of 16,805 who completed the loneliness measure, 1.5% of respondents identified as LGB and 5.4% as Asian, 2.9% as black and 1.5% as other or mixed ethnicity. Overall, 21.7% of the population aged 50+ were lonely. Participants from a black, Asian or other ethnic minority reported higher loneliness than white respondents: 25.8%, 29.6%, 31.0% and 21.0% respectively. Respondents identifying as gay or lesbian (29.1%) or as bisexual (35.2%) reported greater loneliness in comparison to those who identified as heterosexual (21.3%). Our study is novel and timely in demonstrating the higher prevalence of loneliness in two under-represented groups of older adults with the potential consequences this may have for their health and wellbeing in later life.

Abstract citation ID: igad104.3454

### LONELINESS, ANXIETY AND DEPRESSION AMONG CAREGIVERS OF PERSONS WITH DEMENTIA: MEDIATING EFFECT OF SELF-EFFICACY

Alisha Thompson, Catherine Lemieux, Youn Kyoung (Lily) Kim, Laura Ainsworth, Rhonda Bardales, Julia Trahan, and Scott Wilks, *Louisiana State University, Baton Rouge, Louisiana, United States*

A geriatrics enhancement program in southern United States piloted an interdisciplinary telehealth program to improve the health of caregivers of persons with dementia (PWD). Literature indicates that social isolation, loneliness, anxiety, and depression are common among PWD caregivers. When caregivers struggle to manage their own health, they may lose confidence in their health care abilities, i.e., caregiver self-efficacy. Using loneliness as the primary factor, the current study tested the mediating effect of caregiver self-efficacy on distinct outcomes of anxiety and depression. In a hospital setting in southern United States, the team in medicine, nursing, and social work collected data from 95 PWD caregivers on measures of loneliness, anxiety, depression, and caregiver self-efficacy. SEM with bootstrapping tested the two hypothesized mediation models, controlling for age, gender, and relationship to care recipient. The typical respondent was a 62-year-old Caucasian female providing care to a parent. Mediation model 1 direct effects were significant for loneliness on self-efficacy ($\beta$=-.4.24, p<.01); for self-efficacy on depression ($\beta$=-.02, p<.05); and for loneliness on depression ($\beta$=.67, p<.001). Indirect mediation effect of loneliness on depression through self-efficacy was significant ($\beta$=.09, bootstrapping 95% CI[.01, 21]). Model 2 direct effects were significant for loneliness on self-efficacy ($\beta$=-.4.19, p<.01), and for self-efficacy on anxiety ($\beta$=-.02, p<.05); direct effect of loneliness on anxiety was not significant ($\beta$=.04, p=.706). Indirect mediation effect of loneliness on anxiety was significant ($\beta$=.09, bootstrapping 95% CI[.02, 23]). Results underscore the importance of emphasizing social engagement, and including self-efficacy as a mediating factor, when treating caregivers' anxiety and depression.

Abstract citation ID: igad104.3455

### LONGEVITY DETERMINANTS IN AFRICAN AMERICANS: AN INTRA-GROUP ANALYSIS FROM THE HEALTH AND RETIREMENT STUDY

Meneka Johnson Nicholson[1], and Peter Martin[2], *1. Rural Health Medical Program, Inc., Selma, Alabama, United States, 2. Iowa State University, Ames, Iowa, United States*

Utilizing the Health and Retirement Study data, this research exclusively centered on a sample of 2953 African-American participants to discern longevity predictors, emphasizing the importance of intra-group analysis. This targeted approach is essential as it provides a nuanced understanding of factors affecting longevity within a specific demographic group, avoiding broad generalizations derived from diverse populations. Two analytical models were developed in Mplus. The initial fully saturated model was subsequently trimmed to exclude non-significant paths. Key findings include: Education showed a positive correlation with cognition and a negative association with subjective health. Age had a negative impact on cognition and on subjective health. Depression was negatively associated with cognition and subjective health, and positively with age. Notably, age at death was majorly influenced by age, with subjective health, cognition, and education also playing significant roles. The model demonstrated an exemplary fit with Chi^2 (df=2) = 3.12, RMSEA = .01, and CFI = 1.0. Ultimately, age was identified as the primary predictor of age at death. Moreover, subjective health, education, and cognitive abilities emerged as vital contributors to longevity among African Americans. In essence, this study, with its focus on African Americans, underscores the profound importance of demographic-centric research, charting a path for custom-made interventions that resonate deeply with the African-American community's inherent health dynamics.

Abstract citation ID: igad104.3456

### LONGITUDINAL ASSOCIATION OF SERUM CREATININE- AND CYSTATIN-C–BASED INDICES WITH INCIDENT SARCOPENIA

Jae Young Jang[1], Hyung Eun Shin[1], Chang Won Won[2], and Miji Kim[2], *1. Kyung Hee University, Seoul, Republic of Korea*, *2. Kyung Hee University Hospital, Seoul, Republic of Korea*

Although a cross-sectional link between serum creatinine- and cystatin-C-based indices and sarcopenia has been established, the longitudinal association remains unclear. This study explored the longitudinal association between serum creatinine- and cystatin-C-based indices and the incidence of sarcopenia in community-dwelling older adults. Using a 2–4 year follow-up data from the Korean Frailty and Aging Cohort Study, 690 participants (mean age: 75.2±3.7 years; 45.9% men) without sarcopenia at baseline were analyzed. The association between the incidence of sarcopenia during a 4-year follow-up and serum creatinine-to-cystatin-C ratio, estimated glomerular filtration rate (eGFR) ratio (eGFRcystatin-C/eGFRcreatinine), sarcopenia index (serum creatinine×eGFRcystatin-C), predicted skeletal muscle mass index (pSMI), and total-body muscle mass index (TBMM) from baseline were compared. During the 4-year follow-up period, the incidence of sarcopenia was 21.5% in men and 14.7% in women. The pSMI and TBMM showed a higher area under the receiver operating characteristics curves (AUC) for predicting the incidence of sarcopenia in men (pSMI, AUC:0.616, p=0.003; TBMM, AUC:0.613, p=0.001) and women (pSMI, AUC:0.714, p=0.003; TBMM, AUC:0.714, p=0.001) than other indices. After adjusting potential confounders, higher pSMI and TBMM were associated with sarcopenia onset in men (pSMI, odds ratio [OR]: 0.417, 95% confidence interval [CI]: 0.237–0.733; TBMM, OR: 0.937, 95%CI: 0.901–0.975) and women (pSMI, OR: 0.166, 95%CI: 0.072–0.380; TBMM, OR: 0.906, 95%CI: 0.862–0.952). In conclusion, pSMI and TBMM demonstrated accurate predictive capabilities for sarcopenia. Higher pSMI and TBMM values were associated with sarcopenia incidence. These findings suggest pSMI and TBMM could be potential predictive biomarkers of sarcopenia in community-dwelling older adults.

Abstract citation ID: igad104.3457

### LONGITUDINAL ASSOCIATIONS OF LONELINESS AND FRAILTY IN COMMUNITY DWELLING OLDER ADULTS

Chava Pollak[1], Joe Verghese[1], Aron Buchman[2], and Helena Blumen[1], *1. Albert Einstein College of Medicine, Bronx, New York, United States*, *2. Rush University, Chicago, Illinois, United States*

Loneliness is highly prevalent among older adults and is associated with poor health outcomes. This study examined longitudinal associations between loneliness and frailty and the influence of other objective and subjective social measures on these associations. We analyzed data from 1,3111 older adults without dementia at baseline from the Rush Memory and Aging Project (mean age 78.3 ±7.5 years, 72.5% female). Loneliness was assessed using the de Jong Gierveld Loneliness Scale. Frailty was defined using a 39-item cumulative deficit frailty index. Linear mixed effects models were used to examine the association of baseline loneliness and change in frailty over time adjusted for baseline age, gender, education, depressive symptoms, global cognition, social network, marital status, social support, and social, cognitive, and physical activity. Loneliness and frailty both increased significantly over a mean follow-up period of 4.6 years. Baseline loneliness was associated with an additional increase in frailty by 0.3 deficits per year compared to those who were not lonely (p< 0.001, CI 0.2, 0.5). The 0.3 increase in frailty per 1-point increase in loneliness equaled a 27% increase in frailty per year. Loneliness is associated with accelerated frailty and should be considered in the design of interventions to prevent frailty and promote healthy aging.

Abstract citation ID: igad104.3458

### LONGITUDINAL CHANGE IN PERFORMANCE ON THE MONTREAL COGNITIVE ASSESSMENT IN THE PEOPLE WITH DEMENTIA

Kuan-Ching Wu, Jessica Jin, Shao-Yun Chien, and Oleg Zaslavsky, *University of Washington, Seattle, Washington, United States*

The Montreal Cognitive Assessment (MoCA), a widely employed cognitive screening tool, is designed to identify mild cognitive impairment and diverse forms of dementia. Through its multidimensional evaluation of cognitive domains, MoCA supports clinicians in precise assessment and intervention planning for individuals manifesting cognitive deficits. Despite MoCA's prevalence, limited research has explored its temporal dynamics in individuals experiencing memory loss, particularly across different stages of dementia. This study examined the MoCA changes of a longitudinal study analyzing the conversational speech in 8 older adults (5 males 3 females) with mild- to moderate-stage of dementia. Repeated biweekly measures at 12 intervals over six months were employed to gauge changes in total MoCA scores and its sub-tasks. Our findings reveal: 1) a significant negative correlation (r=-0.22, p=0.03) between the total MoCA score and the total time spent in tests; 2) most participants present a positive correlation (r=0.03 to 0.6) in their first memory trial session and a negative correlation (r=-0.1 to -0.8)in the second trial between words recalled and the time spent; 3) over 60% of participants demonstrating a declining trend in naming sessions, while orientation sessions exhibit no clear trend. These outcomes imply MoCA's susceptibility to a "learning effect" with repeated measurements in people with dementia. Future longitudinal studies should consider alternate MoCA versions or extended assessment intervals to mitigate this effect.

Abstract citation ID: igad104.3459

### LONG-TERM NMN TREATMENT INCREASES LIFESPAN AND HEALTHSPAN IN MICE IN A SEX DEPENDENT MANNER

Alice Kane[1], Karthikeya Chellappa[2], Michael Schultz[3], Christian Diener[1], Sean Gibbons[1], Joseph Baur[4], Luis Rajman[3], and David Sinclair[3], *1. Institute for Systems Biology, Seattle, Washington, United States*, *2. Brown University, Providence, Rhode Island, United States*, *3. Harvard Medical School, Boston, Massachusetts, United States*, *4. Perelman School of Medicine at the University of Pennsylvania, Philadelphia, Pennsylvania, United States*

Levels of nicotinamide adenine dinucleotide (NAD+) decline by up to 50% in aging. NAD+ is an essential co-factor for many metabolic processes, including the deacetylase activity of sirtuins, and previous studies have demonstrated that supplementing NAD+ levels has a range of health benefits in both mice and humans. Here we investigate the effect of long-term (from 13 months of age) administration of the NAD+ precursor nicotinamide mononucleotide (NMN) on frailty and lifespan in male and female mice. NMN treatment delayed the onset of frailty in both sexes, improved metabolic health in male mice, and increased median lifespan by 8.5% in female mice. Exploration of the potential mechanisms of this protection showed that NMN treatment prevented age-related gene expression changes in skeletal muscle and led to a large increase in levels of Anaerotruncus colihominis, a microbe associated with reduced inflammation, in the gut. A thorough characterization of NMN metabolism across age, sex and tissues shows context-specific sex differences in metabolic pathways, including greater Preiss-Handler pathway metabolism in females than males, which may contribute to observed sex differences in health and lifespan. Overall, this data provides preclinical evidence that chronic NMN treatment increases lifespan and improves frailty and metabolic health in aging, and highlights the importance of using both sexes for interventional lifespan studies.

Abstract citation ID: igad104.3460

### LOVOT ROBOT AS COMPANIONS FOR OLDER ADULTS IN LONG-TERM CARE

Lillian Hung, Hiro Ito, and Joey Wong, *University of British Columbia, Vancouver, British Columbia, Canada*

This exploratory, mixed-methods study explores how older adults living in Canadian Long-Term Care (LTC) homes experience and perceive LOVOT, an AI-driven social robot from Japan. It is an extended arm of a mixed-methods, three-country study conducted in Singapore, Hong Kong, and Canada. Our Canadian sample consists of 20 older adults and 40 interdisciplinary staff, and 10 leadership team members. The participants join four weekly sessions of interaction with LOVOT. In the quantitative portion of the study, questionnaires are administered before and after interaction with LOVOT to assess participants' experiences of the LOVOT robot. The qualitative portion consists of individual conversational interviews with older adults and focus groups with the LTC staff and leadership. We use thematic analysis to guide our initial conceptual framework, and later use both Chi-square tests and content analysis for our quantitative and qualitative data. This study demonstrates (1) the experiences and perceptions of older adults and their family members regarding their interactions with the LOVOT robot, and (2) the LTC staff and leadership perceptions on having the LOVOT robot in LTC. The study offers insights into the potential role of social robots in LTC homes across eastern and western countries.

Abstract citation ID: igad104.3461

### MALE AND FEMALE PERSPECTIVES: EXPLORING THE USE OF PASSIVE BRAIN HEALTH TECHNOLOGY TO MEASURE GLYMPHATIC FUNCTION

Cyriah Carey[1], Erica Sappington[1], Mitchell Roberts[1], Paul Dagum[2], Jeffrey Iliff[3], Swati Rane Levendovszky[4], Michael Jaffee[5], and Carla VandeWeerd[1], *1. UF Health Precision Health Research Center, The Villages, Florida, United States*, *2. Applied Cognition Inc, Redwood City, California, United States*, *3. University of Washington, Seattle, Washington, United States*, *4. University of Washington Medical Center, Seattle, Washington, United States*, *5. University of Florida College of Medicine, Gainesville, Florida, United States*

Older adults' (OA) research participation is essential to gain insights into the causes, progression, and potential treatments of Alzheimer's Disease (AD). Brain health technology, such as the Glymphatic Function (GF) Monitor, has the potential to measure the progression of AD. The GF Monitor is a multimodal in-ear dynamic impedance spectroscopy and electroencephalogram (EEG) system worn during sleep and records physiological signals: photoplethysmogram (PPG), bioimpedance (BioZ), and acceleration. This study aims to understand OA perceptions and perceived benefits of passive brain health technology such as the GF Monitor. Six focus groups (3 female, 3 male) were conducted with OA (N=48) between June-August 2022 in The Villages, Florida. Domains assessed included concerns for developing AD, health technology perceptions, and preferences for data/outcomes visualization. Focus groups were transcribed verbatim, and content analysis identified salient themes. Across genders, motivation to participate in brain health technology research was influenced by concerns of family history of AD. Females expressed that using the GF monitor "is a small sacrifice to pay" for data-driven interventions and "preventative measures." Males value "receiving easily digestible results" that allow them to monitor their cognitive health. Overall participants desire passive brain health monitoring to "stay active" and "remain independent" while aging in place. Passive technology has the potential to engage OA in research. Participants value prevention, maintaining independence, and receiving comprehensive results. The GF monitor has the potential to harness these wishes to monitor and receive translational brain health findings.

Abstract citation ID: igad104.3462

### MALNUTRITION IN COVID-19 SURVIVORS: PREVALENCE AND RISK FACTORS

Matteo Tosato, Riccardo Calvani, and Francesco Landi, *Università Cattolica del Sacro Cuore, Rome, Lazio, Italy*

Nutritional status is a critical factor throughout COVID-19 disease course. Malnutrition is associated with poor outcomes in hospitalized COVID-19 patients. The aim of our study is to assess the prevalence of malnutrition and identify its risk factors in COVID-19 survivors. Study cohort included 1230 COVID-19 survivors aged 18-86 attending a post-COVID-19 outpatient service. Data on clinical parameters, anthropometry, acute COVID-19 symptoms, lifestyle habits were collected through a comprehensive medical assessment. Malnutrition was assessed according to Global

Leadership Initiative on Malnutrition (GLIM) criteria. Our results showed that the prevalence of malnutrition was 22% at 4-5 months after acute disease. Participants who were not hospitalized during acute COVID-19 showed a higher rate of malnutrition compared to those who needed hospitalization (26% versus 19%, p< 0.01). Malnutrition was diagnosed in 25% COVID-19 survivors over 65 years of age compared to 21% younger participants (p< 0.01). After multivariable adjustment, the likelihood of being malnourished increased progressively and independently with advancing age (Odds ratio [OR] 1.02; 95% CI 1.01–1.03) and in male participants (OR 5.56; 95% CI 3.53–8.74). Malnutrition was associated with loss of appetite (OR 2.50; 95% CI 1.73–3.62), and dysgeusia (OR 4.05; 95% CI 2.30–7.21) during acute COVID-19. In the present investigation we showed that malnutrition was highly prevalent in a large cohort of COVID-19 survivors at 4-5 months from acute illness. Our findings highlight the need to implement comprehensive nutritional assessment and therapy as an integral part of care for COVID-19 patients

Abstract citation ID: igad104.3463

### MAPPING SEDENTARY BEHAVIOUR USING WEARABLE DEVICES AND DIARIES IN OLDER ADULTS WITH FRAILTY: A FEASIBILITY STUDY

Isabel Rodrigues, Jonathan Adachi, Steven Bray, Qiyin Fang, Dylan Kobsar, Alexander Rabinovich, Rong Zheng, and Alexandra Papaioannou, *McMaster University, Hamilton, Ontario, Canada*

Older adults with frailty are sedentary. Prior interventions to reduce sedentary time in older adults have not been successful as there is little research about context (posture, location, purpose, social environment). There is limited evidence on feasible measures to assess context of sedentary behaviour in older adults. Our aim was to determine feasibility of measuring context of sedentary behaviour in older adults with frailty using objective and self-report measures. We defined "feasibility process" using recruitment (20 participants within two-months), retention (85%), and refusal (20%) rates and "feasibility resource" if the measures capture context and can be linked (e.g., sitting-kitchen-eating-alone), and are all participants willing to use the measures. Context was assessed using a wearable sensor for posture, indoor positioning system [IPS] for location, and electronic or hard-copy diary for purpose and social-context over three days in winter and spring. We approached 80 individuals, and 58 expressed interest; of the 58, 37 did not enroll due to lack of interest or medical mistrust (64% refusal). We recruited 21 older adults (72±7.3 years, 13 females, 13 frail) within two months and experienced two dropouts (90% retention). The measures captured one domain of context, but the hard copy was not completed with detail making it challenging to link with the other devices. Not all participants were willing to use the wearable devices or electronic diary; but we linked the measures of those who did. Future studies will need to determine the most feasible and valid method to assess the context of sedentary behaviour.

Abstract citation ID: igad104.3464

### MCI AND CAREGIVING: DYADIC STRESS AND DEPRESSION CONTAGION EFFECTS

Emily Giannotto, *Emory University, Atlanta, Georgia, United States*

Individuals diagnosed with Mild Cognitive Impairment (MCI) remain functionally independent and able to complete instrumental activities of daily living while simultaneously experiencing subjective and objective decline in one or more areas of cognition. Loved ones of individuals with MCI are also impacted and spouses often serve as formal or informal caregivers. Relationships impacted by cognitive impairment experience widespread impacts among cognitive, behavioral, and affective factors within and between individuals. The present study took a novel approach to understanding longitudinal daily parallel patterns of depressive affect, relationship mutuality, sleep, stress, and behavioral problems associated with cognitive impairment. 27 dyads comprised of one spouse with diagnosed Mild Cognitive Impairment (M= 74.6 years, SD= 8.26, 10 female), and the other as a cognitively healthy caregiving spouse (M= 71.4 years, SD= 8.35, 18 female) completed individual nightly reports for two consecutive weeks. Exploratory multilevel modeling revealed significant and complex interactions among spouses' depressive affect, stress, relationship mutuality, and behavioral problems. Results indicate co-occurring daily effects for depressive affect and stress as contagion effects, with mutuality serving as an important moderator. The impact of mutuality was also significant over and above its role in the interactions. Mutuality seems to act as a buffer for negative affect and stress contagion from one partner to the other across days and from one day to the next. These findings highlight the importance of healthy daily relationship dynamics in mitigating stress and depressive affect contagion among older adults impacted by MCI.

Abstract citation ID: igad104.3465

### MEASURING DISTRESS IN DEMENTIA: A PILOT STUDY ON PERCEIVED MEMORY, DYADIC RELATIONSHIPS, AND SELF-EFFICACY

Amanda MacNeil, and Katherine Judge, *Cleveland State University, Cleveland, Ohio, United States*

Prior literature has suggested that some individuals with dementia are capable of self-reporting details of their lived experience. With this, researchers have been increasingly interested in understanding what it is like to live with dementia from the individual's perspective. To capture a more holistic image of the illness experience of individuals with dementia, it is important to consider how they perceive and cope with various aspects of their illness. While some work has suggested that the perceptions that individuals with dementia have about their illness are important to things such as well-being outcomes, no prior work has addressed the distress that results from these perceptions. This pilot study (N = 26) examined perceptions of difficulties and feelings of distress related to three different areas of the illness: one's memory, the relationship with their caregiver, and self-efficacy. Results suggest that perceived memory distress ($\alpha$ = 0.71), dyadic relationship strain distress ($\alpha$ = 0.80), and self-efficacy distress ($\alpha$ = 0.82) are reliable measures when used in a sample of individuals with mild to moderate dementia (MMSE M = 20.5, SD = 5.20). Interestingly, some of these measures may even be related such as perceived memory distress and self-efficacy distress (r (24) = .50, p = .01). Including distress felt because of perceiving difficulties in various areas of one's illness adds a layer of understanding to the illness experience. This can aid in addressing well-being outcomes and serve as intervention targets in future work.

Abstract citation ID: igad104.3466

### MENTAL HEALTH AND CAREGIVING SELF-EFFICACY AS PREDICTORS OF PERCEIVED OVERALL HEALTH AMONG ADRD CARE PARTNERS

Scott Wilks, Laura Ainsworth, Catherine Lemieux, Youn Kyoung (Lily) Kim, Alisha Thompson, Rhonda Bardales, and Julia Trahan, *Louisiana State University, Baton Rouge, Louisiana, United States*

A geriatrics workforce enhancement program piloted interdisciplinary telehealth services to Alzheimer's disease and related dementias (ADRD) care partners. The team in medicine, nursing, and social work noted that consistently mentioned mental health issues and social isolation. The purpose of this study was to evaluate the extent to which the care partner's sense of caregiving self-efficacy, juxtaposed with the aforesaid mental health issues, predict the care partner's sense of their overall health. In a hospital setting in southern United States, the team collected data from 95 ADRD care partners on demographic characteristics and on measures of loneliness, anxiety, depression, caregiving self-efficacy, and perceived overall health. Data were analyzed using OLS multiple regression. Sample characteristics served as covariates. Adjusted R-squared estimated the corrected goodness-of-fit. The sample's average age was approximately 62 years. Most identified as female (74%) and Caucasian/white (66%); about one-third identified as African American/Black. The plurality reported as adult child of the care recipient (47%). An overwhelming majority (94%) scored as clinically anxious; 29% scored as clinically depressed; 33% reported loneliness; 12% reported low caregiving self-efficacy; and 20% reported poor overall health. Depression and loneliness negatively predicted caregivers' overall health (B=-.17, p<.001; B=-.15, p<.05, respectively). Surprisingly, anxiety positively predicted caregivers' overall health (B=.54, p<.001). Self-efficacy was not a significant predictor. Adjusted R-squared value was .63. Results underscore the importance of including mental health and social networks when evaluating and treating ADRD care partners' overall health. Future studies should consider broader, diverse samples to determine the reliability of these findings.

Abstract citation ID: igad104.3467

### MENTAL HEALTH RISKS SIGNIFIED BY THE CO-EXPERIENCE OF ELDER MISTREATMENT AND SOCIAL ISOLATION

Juyoung Park, Yuri Jang, Maria Aranda, Shinyi Wu, Hans Oh, and Kathleen Wilber, *University of Southern California, Los Angeles, California, United States*

Both elder mistreatment (EM) and social isolation are widely known to pose a significant risk to mental health of older individuals. Despite the general agreement that EM should be addressed in consideration of social contexts, there has been limited attention to the potential harm of the co-experience of EM and social isolation. The issue is of particular concern in older Asian immigrants. Using a sample of older Korean Americans (N = 2,150, Mean age = 73.4), we examined the direct and interactive effect of EM and social isolation on mental distress. In a measure including psychological, physical, and financial mistreatment, 32% reported a single EM exposure and 12% multiple EM exposure in the past year. The rate of social isolation, measured with Lubben Social Network Scale-6, was 24%. About 30% were in the category of mental distress measured with Kessler 6. The direct effects of a single EM or multiple EM exposure in reference to no EM and social isolation were significant. In addition, the interaction between multiple EM exposure and social isolation was significant. The odds of mental distress associated with multiple EM exposure were substantially higher when experienced in social isolation (OR = 7.23, [CI: 3.65, 14.3]) than in non-isolation (OR = 2.68, [CI: 1.66, 4.32]). Findings suggest the signified mental health risk of the co-experience of EM and social isolation and call attention to those who might silently suffer from EM in isolation. Comprehensive interventions that encompass both preventive measures and targeted support systems are in need.

Abstract citation ID: igad104.3468

### MIDLIFE MEDIATORS OF EDUCATIONAL DIFFERENCES IN DEMENTIA RISK IN LATER LIFE:PRELIMINARY RESULTS FROM THE HUNT STUDY

Teferi Mekonnen[1], Vegard Skirbekk[1], Hans-Peter Kohler[2], Yaakov Stern[3], Asta Håberg[4], Bo Engdahl[1], Ekaterina Zotcheva[5], and Bjorn Heine Strand[1], *1. Norwegian Institute of Public Health, Oslo, Oslo, Norway*, *2. University of Pennsylvania, University of Pennsylvania, Pennsylvania, United States*, *3. Columbia University, New York City, New York, United States*, *4. Norwegian University of Science and Technology, Trondheim, Sor-Trondelag, Norway*, *5. Norwegian National Centre for Ageing and Health, Tønsberg, Vestfold, Norway*

There is a well-known association between education and dementia risk. However, there is limited knowledge of the mediating mechanisms. Using data from the HUNT study linked with registries (n=9745), we examined the mediating role of occupation complexity, smoking, physical inactivity, systolic blood pressure, body mass index(BMI), cardiovascular diseases, hearing impairment, diabetes, and depression score (only in HUNT2) assessed during HUNT1(age 45years) and HUNT2(age 56years) for the association between education and dementia among 70+ years using causal mediation analysis. Among eligible participants, 15.7% had dementia at age 70+ years, completed compulsory education (25.7%), lower-secondary (41.7%), upper-secondary(12%), undergraduate(16.5%), and postgraduate education(4%). For every one-unit increase in educational level from compulsory education to the following levels, a decrease in the odds of having dementia among 70+ years was found (OR=0.74). Midlife mediators assessed at 45 and 56 years jointly explained 17.3% and 26.8% of the effect of education on dementia among 70+ years, respectively. Smoking and systolic blood pressure (each explained 3.7%) followed by BMI(explained 2.3%) showed a significant independent mediating role for the educational differences in dementia among 70+ years. Whereas, among the mediators assessed at the age of 56 years, systolic blood pressure(explained 6.1%), hearing impairment (explained 4.4%), smoking(explained 3.1%) and depression score(explained 2.7%) showed significant independent mediating effects. Midlife risk factors of dementia considered in this study explained nearly one-fourth of the effect of education on dementia in particular, systolic blood pressure, hearing impairment, smoking, depression score and BMI. If truly causal, these factors could indicate potential targets for interventions.

Abstract citation ID: igad104.3469

### MIGRATION, STRESS AND SUCCESSFUL AGING AMONG GRANDPARENTS: A MIXED METHODS STUDY

Yue Hu, and Helene Fung, *The Chinese University of Hong Kong, Hong Kong, Hong Kong*

**Objectives:** the present work aims to explore the stress and coping related to successful aging among migrant and non-migrant grandparents. **Methods:** both qualitative and quantitative methodologies were employed. Twenty-one grandparents (12 migrants, median age = 59 years old, 14 females) in mainland China were interviewed about the definition of successful aging, as well as stress and coping related to successful aging based on ecological systems theory. Another 303 grandparents (92 migrants, mean age 61.2 ± 4.8, 158 females) were recruited to complete some questionnaires about the intensity of grandparenting, proactive coping, successful aging, as well as some demographic information (e.g., migration, socioeconomic status). **Results:** the interview data revealed that all grandparents valued harmonious family relationships most. Migrant grandparents uniquely faced stressors in different ecological systems, i.e., hardships in their close relationships, inadequate social welfare, and uncertainty in aging preparation. Regarding coping strategies, migrant grandparents uniquely employed avoidance coping strategies when facing intergenerational conflicts and preparing for their later life, as compared with non-migrant grandparents, in the process of perceiving successful aging. The quantitative study found that intensive grandparenting activated more proactive coping thus establishing a successful late life, which only existed among non-migrant grandparents. **Discussion:** Migration has a detrimental effect on older adults' successful aging via imposing extra pressure on grandparents' life, and/or damaging their ability to proactively cope with potential challenges.

Abstract citation ID: igad104.3470

### MIND, BODY, AND SOUL: PURPOSE IN LIFE AS A MEDIATOR BETWEEN PHYSICAL ACTIVITY AND COGNITIVE AGING

Farima Sadeqi, and Ryan Best, *West Virginia University, Morgantown, West Virginia, United States*

Recent guidelines from the WHO stress the benefits of physical activity and harm of sedentary behavior across the lifespan. Physical activity is associated with multiple positive life outcomes, both physical and psychological, across adulthood and into old age. Previous studies have shown engaging in aerobic physical activity buffers cognitive decline and can even enhance cognition in older adulthood. Compared to the growing literature on physiological links between physical activity and cognitive aging, there is a gap in the literature investigating psychological pathways. Empirical research suggests a positive bidirectional association between purpose in life and engagement in physical activity across adulthood. Furthermore, recent research has provided some evidence that purpose in life, both subjectively and objectively, is a positive predictor of cognitive functioning. Merging these areas of research, we investigated purpose in life as a mediator in the relationship between physical activity and cognitive aging. Using the MIDUS study, a representative sample of adults aged 25 to 74 years, a mediation model was fit to the data to test a psychological mechanism explaining the relationship between physical activity and cognition in adults. Physical activity was categorized into three groups based on the intensity of the activity—light, moderate, and vigorous. A small, significant mediation was present across all levels of activity with light physical activity showing a slightly stronger indirect effect when compared to the other two. Results will be discussed considering levels of physical activity and the implication of differential psychological and physiological pathways describing downstream effects on cognitive aging.

Abstract citation ID: igad104.3471

### MINDFULNESS AND DEPRESSION ASSOCIATED WITH FALL RISK IN LOW-INCOME COMMUNITY-DWELLING OLDER ADULTS

Aleeyah Uddeen[1], Renata Komalasari[2], and Ladda Thiamwong[1], *1. University of Central Florida, Orlando, Florida, United States*, *2. Penn State Nursing, State College, Pennsylvania, United States*

Mindfulness is the state of being aware and present using mind-body exercises. Practicing mindfulness and reducing depression can improve balance and reduce fall risk among older adults. The purpose of the study was to examine the correlation between mindfulness and depression with fall risk among community-dwelling older adults in low-income settings. A descriptive cross-sectional survey design was used to assess 103 older adults (males=16, females=87, mean age=75.70, 95% CI±7.19) using questionnaires to measure mindfulness, depression, and fall risk. Mindfulness was measured using the Day-to-Day Experiences, which is a fifteen-item scale with lower scores indicating less awareness. Depression was assessed using the Patient Health Questionnaire-9 (PHQ-9). Fall risk was assessed using the CDC's Stay Independent checklist. Data analysis was run using SPSS version 28. Spearman rank analysis showed that mindfulness was negatively correlated with fall risk ($r=-.437$, $p<.001$), whilst depression was positively correlated with fall risk ($r=.486$, $p<.001$). Using regression analysis, we examined if mindfulness and depression predicted fall risk. The results of the multiple regression indicated that the model explained 23% of the variance ($R^2=.230$, $F(2,100)=14.97$, $p<.001$). Mindfulness ($\beta=-.060$, $p=.010$) and depression ($\beta=.341$, $p=.002$), respectively, were significantly associated with fall risk. It can be concluded that mindfulness and depression are predictors of fall risk in this study. Older adults, particularly in low-income settings, could potentially benefit from improving mindfulness through mind-body exercises that enhance awareness and mindful movement, as well as from improving mental health, thereby reducing fall risk.

Abstract citation ID: igad104.3472

### MINDFULNESS ENHANCES PROACTIVE BENEFITS OF REAPPRAISAL USE FOR YOUNGER BUT NOT OLDER ADULTS

Bruna Martins-Klein[1], Ziyuan Chen[1], Mabel Shanahan[2], Gregory Theos[3], Kristin Heideman[4], Irina Orlovsky[5], Clara DeFontes[5], and Elizabeth Alwan[6], *1. University of Southern California, Los Angeles, California, United States*, *2. Amherst College, Amherst, Massachusetts,*

*United States*, *3. Advanced Science & Math Academy Charter School, Marlborough, Massachusetts, United States*, *4. Yale University, New Haven, Connecticut, United States*, *5. University of Massachusetts Amherst, Amherst, Massachusetts, United States*, *6. National Academy of Public Administration, Washington, District of Columbia, United States*

Reappraisal and mindfulness are associated with enhanced mood and emotion regulation. Cognitive control is key to both processes, and despite age-related declines in cognitive control, older adults report greater use of both compared to younger adults. The Dual mechanisms of control (DMC) framework posits that cognitive control can be engaged, either in anticipation of a task (proactive control) to enhance task performance, or in-the-moment (reactive control). In classic cognitive tasks, older adults show limited benefits from proactive control and intact reactive control, while mindful individuals show similar performance using both control modes. However, the role of trait mindfulness on proactive versus reactive control of reappraisal has never been explored across the lifespan. In this study, 41 younger (18-29) and 40 older adults (60-85) were cued to reappraise or view 80 negative images following a 4-s proactive or a 500-ms reactive delay, tracking pupil response as a measure of physiological arousal and effort. Multilevel models revealed that mindfulness was associated with greater proactive reappraisal benefits in younger adults. In contrast, mindfulness was associated with diminishing benefits of proactive versus reactive reappraisal in older adults; for low mindfulness, proactive benefits were found, and for high mindfulness, reactive control benefitted reappraisal more than proactive control ($p < .001$). For image viewing, proactive control benefitted older but not younger adults. Findings highlight the importance of considering the interaction of mindfulness and age in emotion regulation, and highlight the need of optimizing the temporal dynamics of reappraisal-based strategies in mental health interventions across the lifespan.

Abstract citation ID: igad104.3473

### MIRROR-TCM TRIAL: THE INFLUENCE OF PATIENT-LEVEL FACTORS ON ACUTE CARE RESOURCE USE OF OLDER ADULTS

Onome Osokpo[1], Karen Hirschman[1], Monica Ahrens[2], Wenyan Ji[2], Alexandra Hanlon[2], and Mary Naylor[1], *1. University of Pennsylvania, Philadelphia, Pennsylvania, United States*, *2. Virginia Tech, Blacksburg, Virginia, United States*

Adverse Social Determinants of Health (SDoH) impact post-hospitalization outcomes (e.g., frequent acute care resource use: emergency department [ED] visits, rehospitalizations). The Transitional Care Model Intervention (TCM), an advanced practice registered nurse–led team-based care-management strategy, has demonstrated improved outcomes among older adults throughout transitions from hospital to home. Yet, little is known about the relationship between specific patient-level factors (i.e., demographic, clinical, SDoH) and acute care resource use (i.e., counts of rehospitalizations and ED visits) among hospitalized older adults with multiple chronic conditions who receive the TCM intervention. A total of 480 patients were randomized to receive the TCM in the MIRROR-TCM trial conducted from September 2020 to March 2023 at three diverse health systems in four states. Using multivariable generalized linear mixed effects regression with backward elimination, we examined the relationship between demographic (e.g., biological sex), clinical (e.g., counts of chronic conditions, etc.), SDoH characteristics (e.g., transportation, etc.), and acute care resource use during the intervention. Compared to those without housing concerns, older adults with housing concerns had 2.4 times higher rates of ED visits ($p=0.049$) and 2.2 times higher rates of rehospitalizations ($p=0.005$). Those who lacked transportation for medical appointments had 2.5 times higher ED visit rates ($p=0.021$) and 1.5 times higher rehospitalization rates ($p=0.041$). For each additional comorbidity, the rate of rehospitalization increased by a multiple of 1.08 ($p=0.008$). Understanding the adverse SDoH factors and increasing clinical complexity impacting resource use and tailoring evidence-based transitional care interventions to mitigate these factors to improve posthospitalization outcomes is critical.

Abstract citation ID: igad104.3474

### MISSED INSIGHTS FOR EARLIER MANAGEMENT OF PARKINSON'S DISEASE AND THE VALUE OF DOPAMINE TRANSPORTER (DAT) SCANS

Mohib Hafeez, Amanda Pangle, Jeanne Wei, and Gohar Azhar, *University of Arkansas for Medical Sciences, Little Rock, Arkansas, United States*

This retrospective study focused on the role of Dopamine Transporter (DAT) scans in diagnosing Parkinson's disease (PD) in older adults with cognitive impairment (CI). We reviewed brain imaging of 6,483 individuals, aged 60 and above with CI. 332 had a DAT scan of which 203 were positive and 80/203 (39%) were subsequently started on treatment with carbidopa-levodopa. On the other hand, 243 patients showed Parkinson's (PD) associated structural changes on CT or MRI including mineral deposition, hypodensities, and lacunar infarcts in dopamine-rich regions such as the caudate, putamen, and basal ganglia but none of these patients had a DAT scan, or treatment with carbidopa-levodopa therapy. 71/243 (29%) of these patients had also experienced falls or had gait disorders. This points towards a potential missed diagnosis of PD, which can respond to therapy in the early stages. Our results suggest that providers may overlook subtle signs of Parkinsonism in patients with CI. This could result in symptom worsening and treatment delay. Since CI is often first brought to the attention of PCPs, our findings call for an increased effort to inform PCPs on the role of DAT scans to aid in the diagnosis of dopamine deficiency states. By having a better understanding of PD-related structural changes seen on brain imaging and then using a DAT scan to confirm dopamine deficiency, treatment for PD might be started earlier or timely referral made to a specialist for comprehensive care, reduction of disability, and improvement of the quality of life of patients.

Abstract citation ID: igad104.3475

### MISSED OPPORTUNITIES IN PREVENTING COGNITIVE DECLINE: UNDERDIAGNOSIS OF POLYCYTHEMIA IN OLDER ADULTS

Blake Eddington, Jeanne Wei, Amanda Pangle, and Gohar Azhar, *University of Arkansas for Medical Sciences, Little Rock, Arkansas, United States*

Polycythemia is a hematologic disorder characterized by an abnormally high red blood cell count which can result in serious complications. Microvascular clotting or emboli in polycythemia can reduce brain oxygenation levels with transient ischemic attacks or strokes and lead to subsequent development of vascular dementia. However, the impact of polycythemia on the brain health of older adults may be under-appreciated by primary care providers. The aim of this retrospective study was to investigate the prevalence of missed diagnoses of polycythemia in older adults with cognitive impairment. Electronic medical records (EMR) of patients with cognitive impairment, aged 60-100 years, inclusive of all races, ethnicities, and genders, seen between January 2018-January 2021, were obtained through the University of Arkansas for Medical Sciences database. Cognitive impairment included all subtypes of dementia and delirium with either primary or secondary polycythemia. A total of 9700 patients with cognitive impairment were analyzed for hemoglobin and hematocrit cut-off values for polycythemia. We found 696 instances of comorbid polycythemia of which only 88 (12.6%) had a correct polycythemia diagnosis on record. However, 608 instances (87.4%) had never been identified as having polycythemia either in the problem list or diagnosis. This study highlights the high prevalence of undiagnosed polycythemia in patients with delirium and dementia and underscores the importance of considering polycythemia as a potential etiology, when evaluating cognitive impairment. Early detection and appropriate management of polycythemia in older adults may have implications for reducing the incidence of vascular dementia and improving overall patient outcomes and quality of life.

Abstract citation ID: igad104.3476

### MISSING IN ACTION: STRATEGIES FOR DEMONSTRATING ORGANIZATIONAL COMMUNICATION IN NURSING HOMES

Patsy Smith, and Julie Gordon, *Fran and Earl Ziegler College of Nursing, The University of Oklahoma Health Sciences Center, Oklahoma City, Oklahoma, United States*

A scoping review was conducted to learn more about strategic organizational communication within long-term care. Strategic organizational communication is a purposeful, system-wide, and system-level effort to transform the organization and delivery of care and services to make it easier to navigate, understand, and coordinate care. Care in nursing homes is complex and must consider residents' goals, values, and preferences. Communication strategies enacted at the system-level may include direct care staff, human resources, nursing-to-resident and family interactions, and care planning. Strategies at the institutional or system-wide level also include corporate stakeholders, administration, and employee leadership. The aim of the literature search was to locate evidence of communication strategies that reflect corporate and organizational goals, and that exemplify the processes by which communication influences administrative policy. Key words in the search were entered in varying iterations and combinations with the guidance of a skillful research librarian. Key words were long-term care, communication, feedback, nursing homes, patient-centered care, quality improvement. Returns were greater than 2,000, yet seemingly relevant abstracts were fewer than 300, and selected articles were fewer than 30. The research reports reviewed were explanatory or explorative and just one article was found to explore the role of communication strategies and the impact on organizational outcomes. Further, no research reports were found to discuss strategic organizational communication and a combination of resident and organizational outcomes. Rather, most work described specific communication interventions among key groups, many of which lacked sustainable impact. Study results, limitations, and examples of communication strategies are presented herein.

Abstract citation ID: igad104.3477

### MITIGATION OF MAJOR HIP INJURY FROM FALLS: EARLY RESEARCH RESULTS WITH SMART BELT STUDY

Rebecca Tarbert, *Active Protective Technologies, Inc, Fort Washington, Pennsylvania, United States*

Hip fractures sustained from falling are a devastating outcome for over 300,000 older adults every year in the US. The recovery from this major injury is extremally costly to the healthcare system and results in reduced mobility, increased dependency and up to 30% higher morbidity within 12 months of fracture. Avoidance of falls and injuries from falls is embedded into the standard of care for older adult providers though these standardized measures have not reduced the rate of death from falls as shown in recent studies. The study, Mitigation of Major Hip Injury due to fall in an At-Risk, Older Adult Population with a Wearable Smart Belt, seeks to compare the safety and efficacy of a wearable smartbelt to be worn around the waist of high risk older adults in order to mitigate major hip injuries related to falls through built-in sensors and the deployment of anatomically situated airbags around the hips during the hip impacting fall. The design of the study includes multiple older adult settings where subjects who are identified as being at high risk of fragility fracture and falls wear the study device for 6 months. Comparison of falls with major hip injury, emergency room visits and hospitalizations from falls and hip fractures from falls will be compared to a retrospective control group matched with the same inclusion/exclusion criteria. The poster will share the subject criteria and data gathered into November 2023. This study is listed on clinicaltrials.gov NCT#05245097

Abstract citation ID: igad104.3478

### MODERATING EFFECT OF ANXIETY ON PROACTIVE CONTROL OF EMOTION ACROSS AGE

Ziyuan Chen, Bruna Martins-Klein, and Ava Varu, *University of Southern California, Los Angeles, California, United States*

Anxiety and aging predict difficulties engaging cognitive control and emotion regulation strategies like reappraisal. Dual mechanisms of control (DMC) theory suggests that cognitive control is engaged in anticipation of a task (proactive control) or in-the-moment (reactive control). Both aging and anxiety predict limited benefit from proactive control and greater reliance on reactive control during cognitive tasks. However, this remains unexamined in the emotion regulation context. In this eyetracking study, 41 younger (18-29) and 40 older adults (60-85) were cued to reappraise or view 80 negative images after a proactive (4s) or reactive delay (500 ms) prior to image onset, as regulatory arousal and effort were tracked by pupil responsivity. Multilevel models revealed that anxiety was associated with greater benefit of proactive over reactive control (reduced pupil response) for older adults during reappraisal and viewing (p < .001). For younger adults, anxiety was associated with reduced benefits of proactive compared to reactive reappraisal use. Image viewing in younger adults revealed an interaction, where low anxiety predicted greater benefit from reactive control, and moderate to highly anxiety predicted greater benefit from proactive control. Thus, while older age was associated with greater proactive benefits in both emotional reactivity and reappraisal, younger adults only showed proactive control benefits during image viewing when anxiety was elevated. Future work should explore the clinical benefit of proactive training in late-life anxiety, and how emotional reactivity and reappraisal outcomes differ with cognitive control temporal dynamics in individuals with diagnosed anxiety disorders across the lifespan.

Abstract citation ID: igad104.3479

### MODULATION OF MITOCHONDRIAL THIOREDOXIN IN AGE-RELATE MUSCLE ATROPHY AND INJURY

Morgan Barkley, *University of Alabama at Birmingham, Birmingham, Alabama, United States*

Maintenance of skeletal muscle is vital to health and wellbeing. Over the course of age there is a progressive decline in muscle mass and function. This decline results in frailty, reduced independence, reduced quality of life and elevated risk of mortality. Lean muscle mass contributes approximately 50% of body mass in a young adult while only 25% in an individual in their 80s. Critically, this decline in muscle mass is not continuous but composed of periods of stability punctuated by rapid declines. The chief driver of such events is loss due to injury related inactivity. Bed rest, is an essential part of recovering from injuries, however, long periods of inactivity can lead to skeletal muscle atrophy. A study of just 10 days bed rest in older adults produced a 6% decline in lower extremity lean muscle mass and a 15% decline in muscle function. Importantly much of the muscle mass lost even from short bed rest events are thought to never be recovered and to accelerate age related muscle declines. For this reason, development of interventions which reduce bed rest induced muscular atrophy would have considerable benefits in maintenance of independence for older adults. In my research project I am investigating a novel genetic target to ameliorate muscle atrophy as a consequence of immobility. We have found the protein TXNIP, a critical driver of apoptosis, to be induced under limb. Using TXNIP knockdown and TXNIP inhibitors I'm testing the hypothesis that prevention of TXNIP induction can reduce muscle atrophy.

Abstract citation ID: igad104.3480

### MULTI OMICS LANDSCAPE OF INSULIN SENSITIVITY IN HEALTHY OLDER PEOPLE FROM THE LONG LIFE FAMILY STUDY COHORT

Vaha Akbary Moghaddam[1], Shu Liao[1], Bharat Thyagarajan[2], Kaare Christensen[3], Gary Patti[1], Michael Brent[1], Michael Province[4], and Ping An[1], *1. Washington University in St. Louis, St. Louis, Missouri, United States*, *2. University of Minnesota, Minneapolis, Minnesota, United States*, *3. University of Southern Denmark, Odense, Syddanmark, Denmark*, *4. Washington University School of Medicine, St. Louis, Missouri, United States*

Insulin sensitivity (HOMA-S), which measures the body's response to insulin, is a crucial indicator of glucose homeostasis, particularly in healthy people. The present study utilizes MultiOmics profiling and integration to explore molecular processes crucial for maintaining healthy HOMA-S. To this end, 3783 non-diabetic individuals from 567 pedigrees in Long Life Family Study Cohort were selected (Average age = 70±16). Upon adjustment of HOMA-S for relevant covariates, linear mixed models were employed to analyze the genome sequence, blood transcriptome, and metabolome associations with HOMA-IS. Moreover, using STAAR and Pascal packages, rare and common variants were collapsed to genomic co-ordinates. The results highlight 10 genes, 2 soluble metabolites, and 2 lipids (phosphatidylcholine 35:1 and 40:6), significantly associated with HOMA-S. Integrative module enrichment analysis with PascalX package, identified multiple significant networks for HOMA-S. Notably, immune response in asthma is one of the processes with most genes (57%) displaying a positive correlation with HOMA-S (module p-val = 1.89 × 10E-6). The network contains 2 novel genes from transcriptome analysis, FCER1A and MS4A2 (p-vals = 1.32 × 10E-8 and 1.42 × 10E-6). Interestingly, one of the key metabolites, N-acetylglycine, also has a strong association with MS4A2 (p-val = 0.001034). Furthermore, SNP colocalization for gene expression, metabolome peak intensities, and HOMA-S, revealed multiple significant signals simultaneously associated with PC 35:1 and two members of this network, HLA-DQA1 and HLA-DQB1. The results of the study underscore the intricate interplay of genetic and metabolic factors for healthy HOMA-S with aging and their potential protective role for glycemic implications.

Abstract citation ID: igad104.3481

### MULTIMORBIDITY PATTERNS AND COGNITIVE CHANGE AMONG MEXICAN OLDER ADULTS IN THE MEXICAN HEALTH AND AGING STUDY

Nicholas Bishop[1], Anda Botoseneanu[2], Sheila Markwardt[3], Priscilla Zambrano[1], Kealie Walker[1], and Ana Quiñones[3], *1. University of Arizona, Tucson, Arizona, United States*, *2. University of Michigan, Ann Arbor, Michigan, United States*, *3. Oregon Health & Science University, Portland, Oregon, United States*

Multimorbidity (≥ 2 chronic conditions) increases the risk of non-normative cognitive decline. Among Mexican older adults, little is known about 1) the prevalence of multimorbidity, 2) patterns of multimorbidity, and 3) the relationship between multimorbidity patterns and cognitive decline. We used representative data from the Mexican Health and Aging Study to estimate prevalence of multimorbidity, identify common multimorbidity patterns, and examine associations between multimorbidity patterns and cognitive change from 2012–2015 in Mexican adults aged ≥ 65 (n=5,079; mean age: 72.2 (SD=6.1)). Chronic conditions including hypertension, diabetes, cancer, lung disease, heart attack, stroke, and arthritis were measured in 2012, and cognitive function was assessed in 2012 and 2015 using the Cross Cultural Cognitive Examination (CCCE; range:3–96). We used descriptive analyses to estimate multimorbidity prevalence and identify disease patterns, then used weighted linear regression (adjusted for age, sex, martial/partnership status, education, literacy, and household income and wealth) to estimate associations between multimorbidity patterns and cognitive change (modeled as CCCE-2015 regressed on CCCE-2012). In 2012, 50.7% of respondents reported multimorbidity and average CCCE score was 46.6 (SD=17.2). From 2012–2015, CCCE decreased on average 2.7 units (SD=13.1). Disease patterns with ≥ 2% prevalence included hypertension+depression (6.0%), hypertension+arthritis (5.9%), hypertension+diabetes (5.8%), hypertension+arthritis+depression (5.4%), hypertension+diabetes+depression (2.8%), hypertension+diabetes+arthritis (2.7%), and arthritis+depression (2.1%). Compared to those reporting no conditions, respondents reporting arthritis+depression (b=-3.88, CI=-6.11:-1.66), hypertension+diabetes (b=-3.72, CI=-5.33:-2.11), or hypertension+diabetes+depression (b=-3.41, CI=-5.32:-1.21) experienced greater cognitive decline. Our results suggest diabetes and depression are common elements of multimorbidity associated with cognitive decline in Mexican older adults.

Abstract citation ID: igad104.3482

### MULTI-OMICS INTEGRATION IDENTIFIES GENES INFLUENCING TRAITS ASSOCIATED WITH CARDIOVASCULAR RISKS

Sandeep Acharya[1], Shu Liao[1], Woo Seok Jung[1], Edward Kang[1], Vaha Akbary Moghaddam[1], Mary Feitosa[2], Michael Province[2], and Michael Brent[1], *1. Washington University in St. Louis, St. Louis, Missouri, United States, 2. Washington University School of Medicine, St. Louis, Missouri, United States*

The Long Life Family Study (LLFS) enrolled 4,953 participants in 539 pedigrees displaying exceptional longevity. To identify genetic mechanisms that protect LLFS participants against age-related cardiovascular risks, we developed a freely available multi-omics integration pipeline and applied it to 11 traits associated with cardiovascular risks. Using our pipeline, we aggregated gene-level statistics from Rare-Variant Analysis, GWAS, and gene expression-trait association by Correlated Meta-Analysis (CMA). Across all traits, CMA identified 51 significant genes after Bonferroni correction (P ≤ 2.8×10-7). CETP, NLRC5, SLC45A3, and TOMM40 lie within 50 Kb of a known trait-associated variant (previously associated genes). Analysis of protein-protein interaction (PPI) networks identified another 63 genes (passing genes) that (1) have CMA p-value ≤ 5×10-3, (2) lie in a PPI module (highly connected subnetwork) enriched for genes with low P-values, and (3) are annotated with a biological process that is enriched among module genes, ten of which were previously associated with the same traits. Permutation analysis showed that passing genes have a false positive rate of 1 in 14876 and are more likely to be previously known than non-passing genes with similar p-values. CMA improved on the 3 input analyses by producing the largest number of modules enriched for genes with low P-values and highly enriched for genes participating in shared biological processes. Overall, module analysis identified highly plausible candidate causal genes whose P-values after CMA alone were merely suggestive.

Abstract citation ID: igad104.3483

### MUTUAL INFORMATION OF NEUROIMAGES AND GENETIC VARIANTS TO STUDY THE DETERMINANTS OF BRAIN AGING

Nicholas Kim, Anar Amgalan, Phoebe Imms, Nahian Chowdhury, Nikhil Chaudhari, and Andrei Irimia, *University of Southern California, Los Angeles, California, United States*

Understanding the genetic determinants of brain aging is important because advanced biological age of the brain is a risk factor for Alzheimer's disease (AD). Single-nucleotide polymorphisms (SNPs) are genetic variations with effects on brain aging; mapping their influence on brain structure can elucidate the genetic correlates of AD. We adopted an information theoretic approach to this by computing the mutual information (MI) between (A) 22 AD-risk SNPs and (B) the MRI intensities of the cortex in 6,000 UK Biobank participants (~50% females) aged 54 to 84 years. These cortex-wide calculations quantified how SNPs impact brain structure. As expected, the APOE-ε4 allele, the strongest genetic risk factor for AD, exhibited significantly ($p < 0.025$) higher MI with MRI intensity, when compared to the average MI expected for a null distribution model, in the medial parietal lobe, pars orbitalis, and left inferior occipital lobe, three brain structures responsible for memory recall, language processing, and visual perception. These findings reflect the memory loss, decreased speech, and reduced peripheral vision experienced by individuals with AD. The ABCA7 allele, a risk factor for late- onset AD, had significantly ($p < 0.025$) higher MI in the inferior temporal sulcus and angular gyrus. These structures are responsible for visual recognition and attention, two functions that are severely impaired by AD. Other findings reveal similar relationships between AD-risk SNPs and brain structure. Our findings illustrate how mapping the MI between MRI intensities and SNPs provides insight into genetic influences on brain structures linked to AD symptoms.

Abstract citation ID: igad104.3484

### NARRATIVE EXPLORATIONS OF CARE: THE TRANSFORMATIVE POWER OF CAREGIVING IN THE CONTEXT OF MENTAL HEALTH AND CANCER

Charlotte Weiss[1], Rachel Johnson-Koenke[2], Karen Sousa[2], Connie Ulrich[1], and Karen Hirschman[1], *1. University of Pennsylvania, Philadelphia, Pennsylvania, United States, 2. University of Colorado- Anschutz Medical Campus, Aurora, Colorado, United States*

Embracing the "narrative turn," we used the post-modern and feminist informed research methodologies of narrative inquiry alongside photo elicitation to co-create an in-depth cancer family caregiver narrative with a 50-year-old daughter who cared for her mother (age 80) affected by lifelong schizophrenia and recent metastatic breast cancer. The participant was invited to share personal photos to support her in co-creating a caregiver narrative within the narrative interview environment. While these photos were not analyzed for content, they prompted the participant to share stories about her private caring experiences and make meaning of them. The I-statements of these stories were then used to create a research poem which explores a life lived alongside a parent with mental health issues, while highlighting the transformation and healing potential that life-limiting cancer introduced to their complex relationship affected by mental health barriers. The I-Poem rendering was analyzed for cognitive and emotional meaning resonant of the human experience. Emergent thematic threads of personal humanistic values include altruism, love, and forgiveness. Additionally, this I-Poem analysis contributes to new knowledge situated in human story telling while capturing a transformational personal experience of caring as an exemplar which resists discrimination and disunity among older persons and their family caregivers on the basis of mental health with concomitant serious illness.

Abstract citation ID: igad104.3485

### NARRATIVE-DRIVEN RECRUITMENT MESSAGING TO ENHANCE ETHNORACIAL DIVERSITY IN AD RESEARCH

Jennifer Lingler[1], Melissa Knox[1], Ishan Williams[2], Judy Cameron[3], Rena Robinson[4], Lisa Tamres[1], Melita Terry[3], and Dianxu Ren[1], *1. University of Pittsburgh School of Nursing, Pittsburgh, Pennsylvania, United States*, *2. University of Virginia, Charlottesville, Virginia, United States*, *3. University of Pittsburgh School of Medicine, Pittsburgh, Pennsylvania, United States*, *4. Vanderbilt University, Nashville, Tennessee, United States*

Underrepresentation of persons of color in research on Alzheimer's disease (AD) is a longstanding issue that is receiving unprecedented attention following reports of severely inadequate racial and ethnic diversity in clinical trials of the field's first anti-amyloid therapies to secure Food and Drug Administration approval. The Recruitment Innovations for Diversity Enhancement (RIDE) Study is one of several federally supported initiatives to address key aspects of this complex and multi-faceted challenge facing the AD research community. RIDE is a multi-phase study to determine the effect of a culture-centric narrative campaign on recruitment of African American adults into clinical research on AD and related disorders. This presentation aims to contrast the RIDE study's "narrative driven approach" to "traditional research messaging" with approaches typically found in research recruitment materials. Narrative driven messaging draws on the words, perspectives and stories from members of a culture-sharing group themselves, while traditional approaches aim to educate research candidates about potential benefits and risks of participation using language scripted from investigators' perspectives. We will demonstrate how the narrative driven approach in RIDE focuses on a) prioritizing information that active research participants from a given culture-sharing group identify as most important and b) sharing that information in the form of culturally relevant stories. The potential benefits of this approach include a concrete, replicable strategy that can be readily implemented by other researchers. This is significant because recruiting representative samples in AD research is foundational to conducting the studies necessary to understand and combat race-based cognitive health disparities.

Abstract citation ID: igad104.3486

### NAVIGATING CAREWORKER CURRICULUM REVISION IN SOUTH KOREA: CHARTING COURSES FOR ELEVATING PRACTICE COMPETENCIES

Jihyun Park[1], Jinhak Kim[2], and Mira Sung[3], *1. Pyeongtaek University, Pyeongtaek, Gyeonggi, Republic of Korea*, *2. Korean Human Resource Development Institute for Health and Welfare, Osong, Chungcheong, Republic of Korea*, *3. Yong-In Arts & Science University, Yongin, Gyeonggi, Republic of Korea*

The purpose of this study is to comprehensively investigate and analyze the process of revising the careworker curriculum in South Korea with a primary focus on augmenting practice competencies. This research involved an analysis of the existing 240-hour curriculum, complemented by a comprehensive literature review. Additionally, Focus Group Interviews were conducted with a cohort of 20 present careworkers to effectively identify the prevailing needs within the practice field. This study expanded the curriculum duration from 240 to 320 hours. Subsequently, pilot courses were executed, incorporating enhanced content related to dementia care, humanity in caregiving, and infectious disease management. These modifications were delivered via webinars to 64 active care workers, a method chosen due to the constraints imposed by the COVID-19 pandemic. This study employed a pre-post design to inquire about the perceptions of older adults and their job skills. Fifty-four female care workers (84.2%) and eleven male care workers (15.8%) participated in the study, with an average age of 52 years, experiencing 3 years and 8 months. Several areas exhibited statistical significance; understanding and managing depression and anxiety (2.175*), supporting leisure activities(2.956**), implementing abuse prevention behaviors (3.187**), creating a safe environment(2.496**), assisting with mobility(2.978**), aiding in illness management(2.805**), transferring responsibilities(2.726**), documenting cases(2.238*), and overseeing caregiver health and safety(2.805*). Based on our findings, we have devised a plan to incorporate the following content within the additional 80 hours. In a broader context, this study contributes to the ongoing discourse on caregiver education and training in South Korea.

Abstract citation ID: igad104.3487

### NAVIGATING LIFE'S PHASES: CONTROL PERCEPTIONS AND GOAL ORIENTATION IN YOUNGER AND OLDER AGE GROUPS

Ryan Muskin, Eric Allard, and Sachin Suri, *Cleveland State University, Cleveland, Ohio, United States*

This study examines age-related differences in the perceptions of control and goal orientation among younger adults (ages 18-35) and older adults (aged 60+). A cohort of 57 younger adults and 51 older adults were exposed to a series of emotionally evocative videos portraying diverse scenarios involving varying levels of control and distinct growth or preservation goals. Participants evaluated perceived control, growth orientation, and preservation orientation using 10-point Likert scales. Our findings reveal significant disparities between the two age groups. Older adults exhibited diminished perceptions of control across all videos ($p < .001$) compared to their younger counterparts. Conversely, younger adults consistently displayed a heightened sense of growth orientation across the videos ($p < .001$), without significant age-based variations in perceptions of preservation goals. These outcomes contribute to our comprehension of developmental trajectories in perception and relevance appraisal, along with emotion evaluation. Our hypothesis posits that the disparity in control perceptions stems from age-related losses such as diminished cognitive and physical capabilities, as well as experiences like bereavements. In contrast, the robust growth, competition, and development characteristic of younger adulthood may foster the tendency to perceive the world through these lenses. By shedding light on the intricate interplay between age, control perceptions, and goal orientations, this study advances our understanding of cognitive and emotional dynamics across the lifespan. The implications are far-reaching, offering insights into potential interventions for age-related emotional well-being and adaptive coping strategies.

Abstract citation ID: igad104.3488

### NAVIGATING THE CHALLENGES OF RETIREMENT TRANSITIONS FOR COUPLES IN ARAB SOCIETY

Reem Nashef-Hamuda, *Haifa University, Qalansawa, Hefa, Israel*

Retirement marks the beginning of the third age and is considered one of the most significant transitions in the later stages of life In the aging process, individuals and couples must adapt to many changes that affect their lifestyle and marital relationships. This study investigates post-retirement couples' dynamics in Arab society, focusing on Relational Turbulence Theory (RTT) and its correlation with marital satisfaction and intimacy. Data from 106 retired couples (average male age = 70.3, female age = 65.1) reveal RTT's relevance in explaining relationship uncertainty and interdependence during transitions. Using Confirmatory Factor Analysis, psychometric properties are validated, and gender equivalence is established. Actor-Partner Interdependence Modeling demonstrates that self-uncertainty, partner uncertainty, relationship uncertainty, interference, and facilitation significantly predict marital quality and intimacy. Partners' impact is crucial. emphasizing how a partner's experiences influence marital outcomes. These findings contribute to a deeper understanding of the complexities of post-retirement relationships in Arab society. The main theoretical contribution of the current study will be linking gerontology and couple dynamics in the context of the Arab society in Israel, which is an ethnic minority group and a traditional society in transition (modernization). the Practical implications of this study are helping professionals understand the factors that affect the quality of a couple's relationship after retirement. This understanding will help professionals to provide adequate interventions for couples pre and postretirement, furthermore, the study's findings will contribute to developing retirement planning interventions and culturally sensitive intervention programs for couples from the Arab society in Israel postretirement.

Abstract citation ID: igad104.3489

### NAVIGATING THE DIGITAL DIVIDE: FACTORS INFLUENCING PATIENT PORTAL ADOPTION BY OLDER ADULTS DURING THE PANDEMIC

Chung Hyeon Jeong, and BoRin Kim, *University of New Hampshire, Durham, New Hampshire, United States*

The pandemic rapidly accelerated the adoption of digital healthcare technologies, including patient portals. The patient portals enhance patient-provider communication, streamline medical records management, and provides tailored education materials. Nevertheless, older patients, known as heavy healthcare users, often encounter challenges when adopting patient portals. This digital divide could lead to worsening health and healthcare disparities. Guided by the Senior Technology Acceptance and Adoption Model (STAM), this study explored the factors influencing the adoption of patient portals by older adults during the pandemic. We conducted semi-structured virtual interviews in 2021 with 31 older adults aged 60 or older with healthcare needs. Interviews were thematically coded and analyzed using constant comparative methods. The STAM proposes an orderly phase of technology acceptance among older adults, comprising three phases: 1) objectification, 2) incorporation, and 3) conversion. Our findings showed that some older patients felt compelled to use patient portals during the pandemic, bypassing the objectification phase, which is crucial for building an intention to use the new technology. Older patients who lack a strong intention to use patient portals tended to report challenges in the incorporation phase, rather than focusing on the usefulness of patient portals. From the interviews, we identified three overarching themes: 1) perceived concerns or limited benefits, 2) aging-related and system-related barriers, and 3) limited training opportunities. These themes could pose obstacles to the adoption of patient portals during the conversion phase. The findings of this study suggest areas for improvement to increase the adoption of patient portals among older patients.

Abstract citation ID: igad104.3490

### NEAR-INFRARED SPECTROSCOPY IN VIVO DISTINGUISHES AMYLOID DEPOSITION IN SUBJECTS WITH ALZHEIMER'S DISEASE

Brent Schell[1], and Frank Greco[2], *1. Boston Medical Center/ Boston University School of Medicine, Boston, Massachusetts, United States*, *2. VA Bedford Healthcare System, Bedford, Massachusetts, United States*

Cerebral amyloid angiopathy (CAA), a condition characterized by amyloid beta peptide deposition in cerebral vasculature, is commonly found in patients with Alzheimer's disease (AD). Previous studies have shown that it is possible to distinguish subjects with Alzheimer's disease from age-matched controls in vivo using near-infrared (NIR) spectroscopy. However, correlating neuropathological findings with the spectral features that best distinguish disease from control remains a challenge. Tissue optics predict that pathology in the leptomeningeal vasculature should be distinguishable from that of the adjacent brain parenchyma. In this study, NIR spectra were acquired in vivo from subjects with autopsy confirmed AD (n= 26). Postmortem specimens were histologically classified according to the degree of CAA (0 to 3+) noted in the leptomeningeal vasculature for each patient. A feature selection approach was used to discover spectral features that distinguished patients with high versus minimal degrees of CAA found at autopsy (scored as 3+ vs. 0, respectively). Two distinct spectral regions around 768nm and 891nm were identified as discriminants which could properly classify patients according to their CAA score, suggesting that these regions are correlated with amyloid beta peptide deposition. These results suggest that NIR spectroscopy can be used to detect and possibly inform the burden of amyloid deposition. More research is needed to establish if this technique can be used to monitor disease progression and response to therapeutic interventions, especially in light of emerging new immunotherapies.

Abstract citation ID: igad104.3491

### NEED-BASED INTERVENTION DELIVERY FOR FAMILY CAREGIVERS OF OLDER ADULTS WITH DEMENTIA

Jaclene Zauszniewski, Christopher Burant, Alexandra Jeanblanc, and Evanne Juratovac, *Case Western Reserve University, Cleveland, Ohio, United States*

The literature on family caregivers of persons with Alzheimer's or other dementias abounds with studies of psychoeducational or cognitive-behavioral interventions to reduce stress and promote mental health. However, the determination of the caregiver's need for specific interventions is rarely documented or based on clinical judgment. Researchers typically assume an intervention was needed when positive outcomes emerge. In a sample of 87 family caregivers of persons with dementia, our research examined baseline cut scores on validated measures of resourcefulness and dementia knowledge, which exemplify cognitive-behavioral and psychoeducational interventions, respectively, to determine whether the caregivers needed Dementia Education or Resourcefulness Training.© We found that 66% of the caregivers showed a high need for Dementia Education and 61% showed moderate to high need for Resourcefulness Training.© More specifically, subscales comprising the Dementia Knowledge Assessment Scale revealed the highest need for education concerning the cause or course of dementia (72%) communication and behaviors accompanying the illness (79%), and associated risk factors (80%). The need for knowledge on care and symptoms was lowest (28%). Resourcefulness Scale© scores indicated a higher need for social (help-seeking) skills training (82%) than personal (self-help) skills training (36%). Interestingly, we found that 55% of caregivers showed a moderate to high need for both interventions. The findings suggest further research to investigate the delivery of Dementia Education and Resourcefulness Training© interventions based on documented caregiver need and the additive and sequential effects on stress and mental health when both interventions are provided, particularly when a need for both interventions is established.

Abstract citation ID: igad104.3492

### NEIGHBORHOOD DISADVANTAGE AND PHYSICAL FUNCTIONING: EXPLORING GENDER-SPECIFIC MECHANISMS

Pui Yin Cheung[1], David Curtis[2], and Ming Wen[2], *1. University of Hong Kong, Hong Kong, Hong Kong*, *2. University of Utah, Salt Lake City, Utah, United States*

The relationship between poverty status and functional limitation among late middle age and older adults is well-established. However, the contextual effect of neighborhood disadvantage on physical functioning is less clear. First, the potential pathways linking the two are largely unexplored. Extant evidence shows that perceived neighborhood safety and order are significant predictors of physical functioning. Moreover, stress eating is a common coping strategy for stressful life events and contexts. These three explanations potentially mediate the relationship between poverty rate and physical functioning. Second, even though existing evidence finds stark gender difference in physical functioning, the explanations for the gender difference remains largely unknown. We propose that gender-specific mechanisms can explain the gender difference in physical functioning. The goal of the study was to test the gender-specific mechanism thesis based on a nationally representative sample of adults 55 to 93 years of age in the MIDUS 2 and 3 data (N = 2099). We employed Multilevel Generalized Structural Equation Modeling to test the gender-specific mediating roles of the three mediators. Results showed that county-level poverty rate is significantly associated with instrumental activities of daily living (IADL) among women and men. In contrast, perceived neighborhood safety and stress eating significantly mediate the relationship between poverty rate and physical functioning among women but not men. Furthermore, the indirect effect among women is significantly larger than among men. The important findings support gender-specific mechanisms linking neighborhood disadvantage and health and highlight the role of subjective neighborhood indicators among older women.

Abstract citation ID: igad104.3493

### NEIGHBORHOOD ENVIRONMENT AND DEPRESSIVE SYMPTOMS OF OLDER ADULTS IN URBAN AND RURAL CHINA : A MODEL OF SOCIAL CAPITAL

Yuekang Li, *North China Electric Power University, Beijing, Beijing, China (People's Republic)*

Depression is a significant mental health issue among older adults worldwide. This study aimed to examine the mechanisms linking neighborhood environment to depressive symptoms among Chinese older adults and how other levels of social environment influence this relationship. Using data from the China Family Panel Studies, mediation and moderated mediation models were tested among 3,483 urban and 3,812 rural adults aged 60+ years. Results showed that community-level social capital (trust, reciprocity, belonging) partially mediated the association between built neighborhood environment and depressive symptoms in both urban and rural areas. The indirect pathway linking neighborhood environment to fewer depressive symptoms through community social capital was more evident when older adults reported less society social capital, suggesting a compensatory relationship between these two types of social capital. However, this relationship was significant only for urban older adults. The findings highlight the importance of distinguishing different levels of older adults' social environment. Fostering community social capital through improving neighborhood walkability, safety, and amenities could benefit older adults' mental health. Community development efforts may play a compensatory role for urban older adults lacking societal-level social resources.

Abstract citation ID: igad104.3494

### NEIGHBORHOOD HEALTH CARE FACILITIES AND INCIDENT DEMENTIA: RESULTS FROM THE EINSTEIN AGING STUDY

Jinshil Hyun, Charles Hall, Mindy Katz, Carol Derby, and Richard Lipton, *Albert Einstein College of Medicine, Bronx, New York, United States*

Though access to health care is associated with improved chronic health outcomes, data on dementia risk are limited. From the Ecological Model of Aging, limited access to health care facilities may be associated with increased likelihood of developing dementia through poor management of comorbidities. The current study aims to investigate the association between the density of neighborhood health care facilities and incident dementia. Participants were community-dwelling older adults, systematically recruited from Bronx, NY (N=1,461, mean age=78.4; mean follow-ups=4.6 years; 68% non-Hispanic White, 26% non-Hispanic Black). Density of health care facilities in each participant's Census Tract was derived from the National Establishment Time-Series data. We used Cox proportional hazards models using age as the time scale and adjusted for sex, race, education, income, and neighborhood deprivation. Incident dementia developed in 10% of the sample. Older adults living in the areas with lower density of health care facilities, compared to those living areas with a higher density of health care facilities, were at increased risk for incident dementia (HR=1.79, 95% CI=[1.12, 2.87] for lowest vs. highest tertile; HR=1.65, 95% CI=[1.02, 2.66] for intermediate vs. highest tertile). Further controlling for cardiovascular and other comorbidities did not change the pattern of results. The current results suggest that unequal distribution of health care facilities across geographic locations may help account for cognitive health disparities among older adults. Future studies will need to identify behavioral and individual-level mechanisms (e.g., healthcare use, better treatment before disease occurs) in the association between neighborhood health care facilities and dementia.

Abstract citation ID: igad104.3495

### NEIGHBORHOOD STRESSORS AND EPIGENETIC AGING AMONG OLDER ADULTS IN THE U.S.

Eunyoung Choi, and Jennifer Ailshire, *University of Southern California, Los Angeles, California, United States*

Exposure to stressful neighborhood environments is a well-established risk factor for health deterioration and premature death. However, the underlying biological underpinnings driving these associations are not fully understood. Epigenetics may function as a key molecular pathway to adverse health outcomes among residents of high-stress neighborhoods. This study examines the association of neighborhood stressors (i.e., socioeconomic deprivation, disorder, and low social cohesion) with three epigenetic aging measures (i.e., DunedinPACE and Principal component adjusted (PC) PCPhenoAge and PCGrimAge) based on DNA methylation levels. Further, we identify sub-populations that are particularly vulnerable to the detrimental effects of neighborhood stressors. Data were from the 2016 Health and Retirement Study (HRS) DNA Methylation Sample, merged with neighborhood stressors measured in the HRS 2012/2014 and the 2012-2016 American Community Survey (N=3,201). Multilevel regression models adjusting for confounders showed that perceived high disorder was associated with accelerated DNAm aging for DunedinPACE (β=0.13, 95%CI=0.04-0.23). Perceived low cohesion was associated with accelerated DNAm aging for DunedinPACE (β=0.21, 95%CI=0.08-0.35) and PCGrimAge (β=0.09, 95%CI=0.04-0.14). High deprivation did not have any significant associations. In models of interactions, associations of low cohesion were more pronounced for females in DunedinPACE (β=0.36, 95%CI=0.13-0.60) and PCPhenoAge (β=0.21, 95%CI=0.07-0.35). No other interaction effects were found for race/ethnicity, living arrangement, and education. Our findings indicate that neighborhood stressors can speed up epigenetic aging, with older females being particularly vulnerable to the effects of low cohesion. This study provides insights into the biological foundations of health disparities rooted in neighborhood environments.

Abstract citation ID: igad104.3496

### NOVEL GENE VARIANTS ASSOCIATED WITH HBA1C CHANGES OVER TIME AMONG NONDIABETIC SUBJECTS IN THE LONG LIFE FAMILY STUDY

Siyu Wang[1], Bharat Thyagarajan[2], Joseph Lee[3], Joseph Zmuda[4], Kaare Christensen[5], Michael Province[6], and Ping An[1], *1. Washington University in St. Louis, St. Louis, Missouri, United States*, *2. University of Minnesota, Minneapolis, Minnesota, United States*, *3. Columbia University, New York City, New York, United States*, *4. University of Pittsburgh, Pittsburgh, Pennsylvania, United States*, *5. University of Southern Denmark, Odense, Syddanmark, Denmark*, *6. Washington University School of Medicine, St. Louis, Missouri, United States*

Longitudinal changes in HbA1c (ΔHbA1c) are associated with insulin resistance, aging, cognition, and mortality. To identify novel gene variants underlying ΔHbA1c, we conducted linkage-guided sequence analysis on 17p12 (LODs=3.59) in the Long Life Family Study, a study with familial clustering of exceptional longevity in the US and Denmark. Subjects with clinical diagnosis of diabetes or diabetes treatment and undiagnosed diabetes cases whose fasting glucose ≥ 126 mg/dl or HbA1c ≥ 6.5% were excluded. ΔHbA1c, collected from two exams 7 years apart, was derived using growth curve modeling, adjusting for age, sex, BMI, smoking, field centers and PCs, and was blom-transformed to approximate normality. We identified a significant variant under the linkage peak (ARHGAP44 rs56340929, p=1.77E-06, MAF=6%, accounting for linkage=26.5%). Taking advantage of our currently available RNAseq data, we found a significant association between quantification of ARHGAP44 RNA transcript and adjusted ΔHbA1c using 16 linkage enriched families (n=176, β = -0.24, SE=0.09, p=0.01). We also assessed currently available lipidomics data (188 metabolites, 13 compound classes) and found phosphatidylcholine (p=0.025) and lysophosphatidylcholine (p=0.02) were marginally associated with adjusted ΔHbA1c. ARHGAP44 is reportedly associated with glycemic traits and is mainly expressed in the brain. Further complete omics-data analyses in the LLFS and a replication study using the Framingham Offspring Study are underway.

Abstract citation ID: igad104.3497

### NURSING ASSISTANT TURNOVER IN HOME HEALTH CARE AGENCIES: A SCOPING LITERATURE REVIEW

Jennifer Wagner, Vivian Miller, Lauren Maziarz, Melissa Burek, Julia Bell, and Kaley Perry, *Bowling Green State University, Bowling Green, Ohio, United States*

Certified nursing assistants (CNAs) who work in Home Health Care (HHC) are part of the continuum of care and aid in reducing the burden to the acute care system. CNAs assist patients with their activities of daily living and provide social engagement. Since CNAs see patients more frequently, they are crucial for referral to the nursing staff in times of crisis. The Affordable Care Act was enacted in 2010 which expanded HHC services and provides consumers with more health care options. There is a projected 25 percent increase in CNAs needed in HHC between 2021 and 2031. Despite the growth in HHC, the sector has high turnover rates of CNAs. It is estimated that 35 to 65 percent of CNAs leave HHC employment annually. This scoping review utilized the Job Demands-Resource (JD-R) and the Social-Ecological (SE) models to identify trends in turnover. The JD-R can be used to understand how the demands of one's job as well as the engagement in one's work can affect overall work-related stress, health, and motivation, and is contrasted with available job resources. The SE framework was used to explore levels of influence, and examines how individuals, relationships, communities, and society interact to influence behavior and perceptions. By using both the JD-R and SE models, it is easier to conceptualize the interaction of factors impacting turnover and that many of these factors are unique to CNAs in HHC. The difficult nature of the work, low pay, and low autonomy mean identifying job resources is critical.

Abstract citation ID: igad104.3498

### NURSING HOME INVESTMENT IN HEALTH INFORMATION TECHNOLOGY – AN ORGANIZATIONAL SLACK PERSPECTIVE

John McHugh, and Gregory Alexander, *Columbia University, New York City, New York, United States*

Organizational slack refers to excess resources (e.g., financial, social capital, human capital) an organization accumulates to adapt to changing internal and external contexts. Nursing homes operate in a severely resource-constrained environment, and therefore have limited ability to accumulate organizational slack. However, nursing homes that do accumulate slack may be more likely to invest in health information technology (HIT). To test this, we examine associations between organizational slack and HIT maturity. HIT maturity is the level of IT capabilities, extent of use, and integration in resident care, clinical support, and administration. We linked 2018 data from a national nursing home HIT maturity survey (N=525) to nursing home characteristics from LTCfocus.org, CMS data, National Center for Health Statistics, Medicare cost reports, and other county-level statistics. We incorporated Simple Linear Regression, controlling for facility-level sex, race, case-mix, and urban/rural location. Average 3-year operating income for Stage 0 nursing homes was -$4,387 per bed (n=7) compared to $42,768 per bed in Stage 6 nursing homes (n=17). Higher HIT maturity was associated with greater percentages of Medicare patients and shorter distances to the closest hospital. Our regression model also demonstrated significant associations with operating income per bed, chain affiliation, total beds, occupancy percentage, moderately concentrated counties and case-mix adjusted staffing. In this sample, larger, more financially stable organizations (i.e., those with more slack) may be able to invest more in HIT. As HIT maturity has been shown to be associated with improved patient outcomes, disparities may widen in organizations with lower levels of slack.

Abstract citation ID: igad104.3499

### NURSING STUDENTS' EXPERIENCES REGARDING PHYSICAL RESTRAINTS: AN INTEGRATIVE REVIEW

Juh Hyun Shin[1], and Eun-Hi Kong[2], *1. George Washington University, Ashburn, Virginia, United States*, *2. Gachon University, Incheon, Gyeonggi, Republic of Korea*

Many harmful effects related to the use of physical restraints have been reported and restraint-free care has been advocated for in many countries. However, the prevalence of physical restraint use is still high in many care settings around the world. There has been no systematic review study focused on nursing students regarding physical restraints. This study aimed to critically review the existing studies targeting nursing students related to physical restraint using an integrative review method. This study was registered in PROSPERO(CRD42023414410). Eleven electronic databases were searched by two authors: CINAHL, PubMed, EMBASE, PsycINFO, ERIC, Education Source, SCOPUS, RISS, DBpia, Cochrane, and KoreaMed from the earliest year to 2023. A total of 19 studies including diverse research designs were selected. For quality appraisal, both the Mixed Methods Appraisal Tool (MMAT) and the Joanna Briggs Institutes (JBI) checklist were employed. Most studies were conducted in acute clinical care settings. Many studies used a cross-sectional study design, convenience sampling method, small number of participants, and weak measurements. There was a lack of experimental studies, mixed methods studies, and financially funded studies. The main outcomes were nursing students' attitude, knowledge, perception, or practice regarding physical restraints. This integrative review provided new knowledge and understanding about nursing students' experiences regarding physical restraints. Future studies must use more robust research design, sample size, and measurements with research funding. This study showed that more research and education targeting nursing students are needed to achieve restraint-free care.

Abstract citation ID: igad104.3500

### NUTRITIONAL STATUS AS A RISK FACTOR FOR DEPRESSION AMONG OLDER ADULTS RECEIVING COMMUNITY-BASED AGING SERVICES

Mary Goldsworthy, Timothy Boaz, Amber Gum, and Lawrence Schonfeld, *University of South Florida, Tampa, Florida, United States*

Nutritional status is a significant determinant of overall health and well-being among older adults and is linked to adverse mental health outcomes, such as depression. This study examined nutritional status as a risk factor for depression among 4,469 older adults receiving home- and community-based services from agencies overseen by a five-county Area Agency on Aging in west central Florida. It was hypothesized that older adults with a higher nutritional score (i.e., increased risk of malnourishment) would be more likely to have elevated depressive symptoms compared to those with lower nutritional risk. The cross-sectional study used secondary data from the AAA, including demographic and social variables, a 20-point nutritional risk score, and the Patient Health Questionnaire-9 (PHQ-9; not depressed = 0-9, depressed = >10) from clients in the AAA planning and service area. In logistic regression, the most significant predictor of depression was the client's nutrition score (OR=

1.22, p < 0.0001). This OR suggests that, for every 1-point increase of the nutrition score, the odds of being depressed increases by 22%. The findings suggest that older adults receiving aging services with worse nutritional status are at increased risk of depression, consistent with other studies. The findings suggest that programs focused on improving nutritional status among older adults may reduce depressive symptoms may improve and may potentially improve long-term physical and mental health outcomes and decrease healthcare burden.

Abstract citation ID: igad104.3501

### OBLIGATIONS IN JAPAN: A THREE-YEAR LONGITUDINAL STUDY OF MID-LIFE ADULTS

Jeewon Oh[1], and Han Na Lee[2], *1. Syracuse University, Syracuse, New York, United States*, *2. Loyola University Chicago, Chicago, Illinois, United States*

Obligations embody a sense of responsibility, whether they are directed toward close relationships or the broader public community. Middle-aged adults may find themselves in increased caregiving roles, but research examining the role of their obligation on their well-being and relationships is scarce. Across studies, obligation is linked with both positive (e.g., higher well-being and relationship quality) and negative outcomes (e.g., greater burden). A previous study in the U.S. suggested that these mixed findings may be partially because there are various types of obligations that differ on the level of investment. Given the Japanese collectivistic culture that values intricate social connection, their obligation may take on a different form and play a different role. We used two waves of data from the Midlife in Japan Project (2009-2012). Participants were 371 middle-aged adults (M = 55.47, SD = 14.04, 56.02% women, 43.98% men, 71.73% married) living in Japan. We factor-analyzed the structure of obligation and found a 3-factor solution fit best (e.g., RMSEA = .06, SRMR = .01, CFI = 1.00). Obligation could be characterized by holding light and substantive obligations to close others and obligations to the public community. Results suggest differential links between the types of obligation and well-being outcomes. For instance, light obligation predicted less negative affect ($\beta = -.24$, $p = .035$) after three years, but otherwise, other types of obligation did not predict positive or negative affect. Links with relational outcomes (e.g., support/strain) and implications of midlife obligations will be discussed.

Abstract citation ID: igad104.3502

### OCCUPATIONAL COGNITIVE DEMANDS AND RISK OF MILD COGNITIVE IMPAIRMENT AND DEMENTIA: THE HUNT STUDY

Bjorn Heine Strand[1], Trine Holt Edwin[2], Ekaterina Zotcheva[3], Asta Håberg[4], Bernt Bratsberg[5], Teferi Mekonnen[1], Yaakov Stern[6], and Vegard Skirbekk[1], *1. Norwegian Institute of Public Health, Oslo, Oslo, Norway*, *2. Oslo University Hospital, Oslo, Oslo, Norway*, *3. Norwegian National Centre for Ageing and Health, Tønsberg, Vestfold, Norway*, *4. Norwegian University of Science and Technology, Trondheim, Sor-Trondelag, Norway*, *5. Ragnar Frisch Center for Economic Research, Oslo, Oslo, Norway*, *6. Columbia University, New York City, New York, United States*

The cognitive reserve hypothesis posits that cognitively stimulating work may delay onset of mild cognitive impairment (MCI) and dementia. However, the impact of work histories across midlife on the risk of these conditions is unclear. We evaluated trajectories of work-related cognitive demands from ages 33-65y and their association with later clinical MCI/dementia. The HUNT4 70+ Study was used (N= 7003 participants, 49.8% women, 2017-19). Group-based-trajectory-modeling was used to identify trajectories of work-related cognitive demands, measured by routine task intensity (RTI) index (Lower RTI indicates less routine-oriented work) from online Occupational Information Network (O*NET). Multinomial regression was used to estimate the relative risk ratios (RRR) of MCI/dementia after adjusting for covariates age, sex and education, income, hypertension, obesity, diabetes, psychiatric impairment, hearing impairment, feeling of loneliness, smoking status, and physical inactivity. Four trajectories were identified; low RTI (20.4%), intermediate-low RTI (22.7%), intermediate-high RTI (36.9%), and high RTI (20.1%). Participants in the high RTI group had higher risk of MCI (RRR 1.74, 95% CI: 1.41,2.14) and dementia (RRR 1.37, 95% CI: 1.01, 1.86) after adjusting for age, sex, and education compared to participants in the low RTI group. When accounting for potential mediators, the increased risk remained for MCI but was no longer significant for dementia, indicating these mediators may account for part of the observed relationship. Our results highlight the importance of cognitive stimulation in the workplace for maintaining cognitive function. This should be taken into account, together with other known risk factors to help prevent cognitive decline in older adults.

Abstract citation ID: igad104.3503

### OFFSPRING EDUCATIONAL (DIS)ADVANTAGE, MECHANISMS, AND COGNITIVE FUNCTION IN KOREA

Hyungmin Cha, *University of Southern California, Los Angeles, California, United States*

Adult children's education plays a significant role in parental cognitive well-being later in the life course. However, there is limited evidence in rapidly changing societies, leaving several key questions unanswered, including (1) whether the effects of offspring educational disadvantage can also flow upward, (2) whether these effects operate through similar explanatory mechanisms, such as financial, social, and informational support from their children, as seen in educational advantage, and (3) whether gender moderates these associations. Focusing specifically on Korea, a society where education has expanded rapidly and support from children is increasingly crucial, this study utilizes longitudinal data from the Korean Longitudinal Study of Aging (2006-2018) to demonstrate the role of offspring education in cognitive function during mid-to-late life. First, having adult children with a college degree or higher is associated with elevated levels of cognition and slower cognitive decline, whereas having adult children with less than a high school education is linked to lower levels of cognition and faster cognitive decline during mid-to-late life. Second, the association between adult children's education and cognitive health, regardless of whether they have a college degree or less than a high school education, remains robust even after accounting for various other mediators. Third, while the effect of adult children's education is more pronounced for mothers compared to fathers, children's gender does not moderate the association. Together, I offer a novel explanation for the association between offspring educational advantage and disadvantage, and their connection to parental cognition and its subsequent decline in rapidly changing society.

Abstract citation ID: igad104.3504

### OLDER ADULTS' PERCEPTIONS OF HOPE REGARDING THEIR ABILITY TO MAKE DIET AND EXERCISE CHANGES

Amber Worthington, Dale Golden, and Britteny Howell, *University of Alaska Anchorage, Anchorage, Alaska, United States*

Successful aging is facilitated by healthy diet and exercise levels; however, older adults may face barriers to obtaining these. One key motivator for improving older adults' diet and exercise may be perceptions of hope. Persuasive Hope Theory (PHT) posits that hope motivates behavior by focusing peoples' thoughts on opportunities for actions that lead to future rewards. Research suggests hope is particularly motivational for older adults, yet little work has examined factors that lead older adults to feel hope. In line with PHT, this project examined whether older adults' perceptions that increasing fruit and vegetable intake (FVI) and exercise are important, possible, congruent with their goals (i.e. goal congruence), and will lead to a better future (i.e., future expectation), influence feelings of hope. A convenience sample of participants (N=58) aged 57-87 (M=71.98, SD =8.74) living in urban Alaska completed a questionnaire as part of a larger study. A multiple regression analysis revealed that importance (M=4.40, SD=0.67) and future expectation (M=4.47, SD=0.66) for increasing FVI significantly predicted feeling hopeful about increasing FVI (M=4.40, SD=0.75), but perceptions that increasing FVI is possible (M=4.26, SD=0.74) and goal congruent (M=4.34, SD=0.64) did not ($F(4, 53)=11.29$, $p<.05$, $R2=.46$). The PHT variables for increasing exercise did not significantly predict feeling hopeful about increasing exercise. These results suggest PHT, specifically importance and future expectation, may be used to strategically design messages to increase older adults' hopeful feelings for increasing FVI; however, more research is needed due to the limitations of the small convenience sample.

Abstract citation ID: igad104.3505

### OLDER ADULTS' SAVORING ABILITY IS ASSOCIATED WITH LONGITUDINAL CHANGE IN WELL-BEING

Jacquelyn Stephens, Laurel Mertz, and Jennifer Smith, *Mather Institute, Evanston, Illinois, United States*

Well-being in older adults is an important concern as the aging population grows, as is identifying modifiable factors that contribute to well-being over time. The ability to savor the moment (i.e., to notice and enhance one's emotional response to positive events) has been shown to be associated with greater well-being, though few studies have examined these relationships longitudinally or in older adults. The current study examined whether savoring ability moderated the trajectory of older adults' life satisfaction and depressive symptoms over time. A sample of 5777 older adults (Mage = 83.83, SD = 6.61; 66.6% female), recruited from Life Plan Communities, completed four consecutive annual surveys. Multilevel growth curve models revealed main effects of small but significant declines in well-being over time (life satisfaction: B = -.09, SE = .01, p < .001; depressive symptoms: B = .13, SE = .01, p < .001), after accounting for age, gender, education, life stress, and health. Interactions between time and savoring ability indicated that participants with greater savoring ability were better able to maintain their levels of well-being (i.e., smaller decline in life satisfaction [B = .05, SE = .01, p < .01] and smaller increase in depressive symptoms [B = -.10, SE = .01, p < .01]). In contrast, individuals lower in savoring ability experienced less favorable well-being trajectories. Savoring is a malleable emotion regulation ability that is associated with attenuated declines in well-being over time. Implications for savoring interventions in older adults will be discussed.

Abstract citation ID: igad104.3506

### OLDER US ADULTS PREFER SHORT, FREQUENT RESISTANCE TRAINING PROGRAMS, ESPECIALLY THOSE WITH DIFFICULTY WALKING

Jordan Kurth[1], Christopher Sciamanna[1], Cheyenne Herrell[1], Margaret Danilovich[2], David Conroy[3], Kathryn Schmitz[4], Matthew Silvis[1], and Matthew Ladwig[5], *1. The Pennsylvania State University College of Medicine, Hershey, Pennsylvania, United States*, 2. *CJE SeniorLife, Chicago, Illinois, United States*, 3. *Penn State University, University Park, Pennsylvania, United States*, 4. *University of Pittsburgh, Pittsburgh, Pennsylvania, United States*, 5. *Purdue University Northwest, Hammond, Indiana, United States*

Though resistance training (RT) improves strength and physical function, fewer than 20% of older adults do RT at least twice weekly, as recommended by national guidelines. Even when programs are free, such as Silver Sneakers, fewer than 25% participate and those that do rarely attend (averaging < 20 visits/year). Many individuals cite "lack of time" as the primary reason for not attending. Therefore, one approach to increase participation in RT programs could be making sessions shorter. However, little is known about older adult RT program preferences. Therefore, we conducted a nationwide survey among 611 US adults aged 65 and over. We compared their interest in a traditional (45 minutes, three days/week) RT program to a shorter, more frequent (5-minute, every day) RT program. Overall, 2.2 times as many older adults preferred the shorter vs. the traditional RT program (68.4% v. 31.6%; p< 0.001). Furthermore, barrier self-efficacy for the shorter program was 50% greater than the traditional program. Adults with health concerns, like poorer self-rated health and a history of falls, were significantly more likely to be interested in the shorter program. Notably, 87% of older adults with difficulty walking also preferred the shorter program. These findings suggest strong interest in short RT programs among older adults, especially those with poorer health and mobility disabilities for whom traditional RT programs may be too challenging. Reducing the duration of RT programs may be an appealing option to engage older adults in RT, particularly among those for whom the program would provide the most benefit.

Abstract citation ID: igad104.3507

### ON THE FRONTLINES: TRAINING A DEMENTIA CAREGIVING WORKFORCE IN CALIFORNIA

Brittney Pond[1], Corinne Eldridge[2], Mel Neri[1], Matthew Beld[1], Leslie Ross[1], Suzanna Martinez[1], Brooke Hollister[3], and Jarmin Yeh[1], *1. University of California, San Francisco, San Francisco, California, United States*, *2. Center for Caregiver Advancement, Los Angeles, California, United States*, *3. Universitetet i Agder, Agder, Aust-Agder, Norway*

Direct care workers who provide in-home care – such as caregivers hired by consumers of California's Medicaid-funded In-Home Supportive Services (IHSS) program – are well positioned to observe changes in their consumer's cognition, health, or behaviors. This proximity to consumers better allows for reporting to healthcare partners, which may reduce risk of emergency department visits, hospitalizations, and other adverse outcomes that are disproportionately high among people living with Alzheimer's disease and related dementias (ADRD). Despite their vital role in supporting people living with ADRD, IHSS caregivers receive minimal formal training for their unique position. To address this training gap, the IHSS+ ADRD Training Project is a five-year effort funded by the California Department of Public Health to train 720 IHSS caregivers in Alameda and Los Angeles counties, with classes in English, Spanish, Cantonese, and Mandarin. To date, 582 IHSS caregivers have been trained. Using a pre-posttest design, our preliminary findings indicate statistically significant increases in dementia knowledge and self-efficacy that could bolster caregiver's skills. These results also reveal that IHSS caregivers value training opportunities to better support their consumers. Correlations between caregiver training outcomes and healthcare utilization patterns of their consumers were further examined by evaluating administrative data before and after the training. The results need to be further understood in the context of COVID-19 and the ADRD illness trajectory. Implications include expanding long-term funding for multi-week training programs, which aligns with the California Master Plan for Aging's goal to support and create one million high-quality caregiving jobs by 2030.

Abstract citation ID: igad104.3508

### ONE MINUTE TO RECOGNITION: THE AGITATION IN ALZHEIMER'S SCREENER FOR CAREGIVERS (AASC™)

Carolyn Clevenger[1], Malaak Brubaker[2], Mehul Patel[2], Jeffrey Cummings[3], W. Clay Jackson[4], Jared Stroud[5], Sue Pechin[6], and George Grossberg[7], *1. Emory University, Atlanta, Georgia, United States*, *2. Otsuka Pharmaceutical, Princeton, New Jersey, United States*, *3. School of Integrated Health Sciences, University of Nevada Las Vegas, Las Vegas, Nevada, United States*, *4. University of Tennessee College of Medicine, Germantown, Tennessee, United States*, *5. OhioHealth, Columbus, Ohio, United States*, *6. Alliance for Aging Research, Washington, District of Columbia, United States*, *7. Saint Louis University, St. Louis, Missouri, United States*

Agitation is a common neuropsychiatric symptom of Alzheimer's dementia (AD) and both a complex and burdensome aspect of patient care. Caregivers are often first to encounter agitation-associated behaviors and play a pivotal role in communicating these symptoms to healthcare providers (HCPs). However, most caregivers do not identify the breadth of signs/symptoms of agitation and are hesitant to discuss these behaviors with HCPs. There is, therefore, an unmet need for an easy, pragmatic screener to facilitate caregiver recognition of agitation associated with AD (AAD) and HCP discussions. Here, we report on the development of the Agitation in Alzheimer's Screener for Caregivers (AASC™), a brief screening tool based on the International Psychogeriatric Association (IPA) consensus definition for agitation in cognitive disorders, designed to assist caregivers in recognizing agitation-associated behaviors in patients with AD. Initial draft development involved identification of specific behaviors that were discriminative for AD with/without agitation and representative of the full range of IPA-defined agitation behaviors (i.e., excessive motor activity, verbal aggression, physical aggression) based on current literature and input from 6 opinion leaders. An initial draft was then refined across 2 rounds of cognitive debriefing with caregivers (n=12; mean age=62 years) who described the screening tool as comprehensive yet easy to complete, and perceived it to be valuable, relevant, and easily understood. The AASC™ is a brief, easy-to-use tool supporting the identification and assessment of impact of agitation-associated behaviors. The screener can be completed by a caregiver in < 1 minute at home or in an HCP's office setting.

Abstract citation ID: igad104.3509

### OPTIMIZING MEALTIME (OPTIMAL) IN NURSING HOME RESIDENTS WITH ADRD: A PILOT TRIAL OF A PERSON-CENTERED CARE PROGRAM

Wen Liu[1], Heather Suh[1], Kristine Williams[2], Elizabeth Galik[3], Barbara Resnick[4], Kathleen Buckwalter[1], Kimberly VanHaitsma[4], and Barbara Rakel[1], *1. The University of Iowa, Iowa City, Iowa, United States*, *2. University of Kansas, Lawrence, Kansas, United States*, *3. University of Maryland School of Nursing, Baltimore, Maryland, United States*, *4. University of Maryland, College Park, Maryland, United States*, *5. Penn State University, State College, Pennsylvania, United States*

Nursing home (NH) residents living with dementia (residents) commonly experience behavioral symptoms during mealtime that negatively impact nutrition and function. Residents may not receive optimal, person-centered mealtime care (PCMC) due to multilevel factors. PCMC training that teaches staff to actively engage residents in eating is a prioritized modifiable factor. We developed Optimizing Mealtime (OPTIMAL), a multifaceted, evidence-based, theoretically grounded PCMC program with five components: community stakeholder engagement, environment/policy assessment/modifications, staff education, individualized care planning, and ongoing motivation. OPTIMAL is designed to create and maintain a supportive PCMC culture/environment through appropriate assessments and modifications of factors to optimize mealtime care and outcomes. Our pilot trial (single-group repeated measures design, stakeholders' interviews) demonstrated OPTIMAL was feasible, acceptable, and perceived useful as evidenced by high recruitment (74%, 77%) and retention (94%, 99%) rates for residents and staff, successful completion of data collection/coding and intervention delivery, and positive stakeholders' feedback. We recruited 95 staff (age=45±12.1 years, 8.5% male, 12.3% non-white, 89% experienced mealtime care challenges) and17 residents (age=74±7.9 years, 88.2% male, 11.8% non-white) from 8 memory care units of one NH. Stakeholders perceived OPTIMAL was useful for staff who provide care to residents with and without dementia who need assistance and/or experience behavioral symptoms during mealtime. Videotaped observations of dyadic mealtime interactions (N=265) at baseline and 6- and 12-weeks following OPTIMAL showed that resident functional impairments and resistive behaviors during mealtime decreased during the study period. Findings provide preliminary evidence for feasibility, acceptability, and usefulness of OPTIMAL in NH staff and residents with dementia.

Abstract citation ID: igad104.3510

### ORAL HEALTH AMONG FILIPINO OLDER ADULT IMMIGRANTS

Emerson Ea, Bei Wu, and Jinyu Hu, *New York University, New York City, New York, United States*

Poor oral health has been associated with decreased quality of life, various non-communicable diseases, and a higher risk of mortality. There is also evidence that older immigrants and those who have been in the US for less than 5 years are more likely to have periodontitis, a serious gum disease and a common oral health condition. Despite this evidence, there is a paucity of studies investigating the influence of acculturation, immigration-related experiences, and resilience on oral health among older Filipino immigrants. To address this significant knowledge gap, a mixed methods study using a survey and focus group interviews was conducted among community-dwelling Filipino immigrants aged 60 years and older. This poster will present the results of the survey (N=103), which used a socio-demographic questionnaire, as well as valid and reliable instruments such as the Geriatric Oral Health Assessment Index, A Short Acculturation Scale for Filipino Americans, Everyday Discrimination Scale, and Connor-Davidson Resilience Scale. The results of the regression analysis revealed that the perception of discrimination and resilience were significantly associated with the perception of oral health among the participants surveyed [$F(10,92) = 1.985$, $p < 0.05$]; this regression model accounted for 18.6% of variance in the perception of oral health. The findings support the need for culturally sensitive interventions that could optimize oral health, including the utilization of dental care services for this underserved population.

Abstract citation ID: igad104.3511

### ORGANIZATIONAL COMMUNICATION STRATEGIES IN NURSING HOMES: A SCOPING REVIEW

Julie Gordon, and Patsy Smith, *Fran and Earl Ziegler College of Nursing, The University of Oklahoma Health Sciences Center, Oklahoma City, Oklahoma, United States*

A scoping review was conducted to learn more about strategic organizational communication within long-term care. Strategic organizational communication is a purposeful, system-wide, and system-level effort to transform the organization and delivery of care and services to make it easier to navigate, understand, and coordinate care. Care in nursing homes is complex and must consider residents' goals, values, and preferences. Communication strategies enacted at the system-level may include direct care staff, human resources, nursing-to-resident and family interactions, and care planning. Strategies at the institutional or system-wide level also include corporate stakeholders, administration, and employee leadership. The aim of the literature search was to locate evidence of communication strategies that reflect corporate and organizational goals, and that exemplify the processes by which communication influences administrative policy. Key words in the search were entered in varying iterations and combinations with the guidance of a skillful research librarian. Key words were long-term care, communication, feedback, nursing homes, patient-centered care, quality improvement. Returns were greater than 2,000, yet seemingly relevant abstracts were fewer than 300, and selected articles were fewer than 30. The research reports reviewed were explanatory or explorative and just one article was found to explore the role of communication strategies and the impact on organizational outcomes. Further, no research reports were found to discuss strategic organizational communication and a combination of resident and organizational outcomes. Rather, most work described specific communication interventions among key groups, many of which lacked sustainable impact. Study results, limitations, and examples of communication strategies are presented herein.

Abstract citation ID: igad104.3512

### ORPHANED ELEPHANTS EXHIBIT HIGHER THYROID HORMONE CONCENTRATIONS COMPARED TO CONTROLS

Daniella Chusyd[1], Janine Brown[2], Steve Paris[2], Stephanie Dickinson[1], Tessa Steiniche[1], Steven Austad[3], David Allison[1], and Michael Wasserman[1], *1. Indiana University, Bloomington, Indiana, United States, 2. Smithsonian Conservation Biology Institute, Front Royal, Virginia, United States, 3. University of Alabama at Birmingham, Birmingham, Alabama, United States*

Wild elephant populations provide a natural experiment to study biological effects of early adverse events, such as being orphaned due to poaching. We compared fecal glucocorticoid metabolite (fGCM) and thyroid hormone (T3) concentrations, and body condition between rescued orphaned elephants and similarly age-matched, control wild elephants. In 2021, single fecal samples were collected during the early (n=20 orphans; n=58 controls) and late (n=20 orphans; n=22 controls) dry seasons. Age was known (+/- 3 months) for orphans and estimated by dung diameter (+/- 1 year) for controls. Sex was assigned based on morphology and unknown for some controls. Body condition scores (BCS: 1 to 9) were assigned at time of sample collection. Linear Mixed Models accounting for repeated measures were performed adjusted for age and season. There was no difference in fGCM between orphans (mean 101.48, SD 59.62ng/g) and controls (mean 93.86, SD 49.97ng/g) (p=0.213). Orphans (mean 271.75, SD 123.16ng/g) had higher T3 concentrations compared to controls (mean 98.65, SD 71.89ng/g) ($p< 0.001$). There was no difference in BCS between groups (p=0.629). In orphans, fGCM was associated with age (beta=-0.499, p=0.011). In controls, T3 was correlated with fGCM (beta=0.287, p=0.011) and season was associated with fGCM (p=0.008) and BCS (p=0.044). Either because of living under human care or because of alterations in T3 secretion, seasonal factors do not seem to impact orphans' health status to the same extent as in control elephants. The support of an adopted herd may attenuate the physiological stress associated with experiencing trauma in our study population.

Abstract citation ID: igad104.3513

### PACE AND FFS MEDICARE OUTCOMES BEFORE AND DURING COVID-19: A PROPENSITY SCORE MATCHED ANALYSIS

Morgan Perry[1], Samuel Obbin[2], Stephen McCall[2], Joseph Dorris[2], Michael Nardone[3], Ani Turner[2], and Christine Stanik[4], *1. University of Michigan, Ann Arbor, Michigan, United States*, *2. Altarum, Ann Arbor, Michigan, United States*, *3. The Nardone Group LLC, Philadelphia, Pennsylvania, United States*, *4. Arbor Research Collaborative, Ann Arbor, Michigan, United States*

We compared outcomes of Program of All-Inclusive Care for the Elderly (PACE) participants and fee-for-service (FFS) Medicare, dually eligible individuals using a retrospective cohort study with propensity score matching on demographic, socioeconomic, and participant-specific acuity level variables. PACE participants in 132 PACE centers nationwide who were dually eligible, enrolled during the study period, and did not have end-stage renal disease were included. Over 80% of eligible PACE participants were matched with FFS beneficiaries during each period, bringing many covariates closer between groups. We assessed differences during each period using Wilcoxon signed rank test and over time using differences-in-differences analyses. PACE participants spent significantly more time in the community and less time in nursing homes and hospitals (all p< 0.01). PACE participants had fewer COVID-19 diagnoses and hospitalizations per member per month (all p< 0.01). Due to the blinding of data, we could not determine definitively which performed better in limiting severe pressure ulcers. However, the results indicate PACE performed better when the mean assumption for blinded data is used. For all-cause mortality, PACE participants continued to perform better than FFS both before and during the pandemic (all p< 0.01), but the rates were closer during the pandemic periods. The flexibility in the PACE model and its person-centered care delivery approach may have contributed to the positive outcomes compared to FFS before and during the pandemic. We should consider how best to leverage the PACE model of care to support older adults to remain in their homes and avoid institutional settings.

Abstract citation ID: igad104.3514

### PATHOGEN APATHY RESULTING FROM SKN-1 ACTIVATION: A MARK OF RESILIENCE OR A RATTLED GUT FEELING?

Tripti Nair, Brandy Weathers, Nicole Stuhr, James Nhan, Vandita Gorla, and Sean Curran, *University of Southern California, Los Angeles, California, United States*

Innate immunity is a key driver in promoting survival against xenobiotic stressors and hostile environments and an appropriate immune response is essential to organismal health across lifespan. Caenorhabditis elegans encounter pathogens in their natural environment and initiate cytoprotective and immune responses that can have long-lasting effects on health, even after the pathogen has been neutralized. Pseudomonas aeruginosa (PA14) is an opportunistic pathogen that is lethal to C. elegans with prolonged exposure. C. elegans employ multiple defense mechanisms to ensure survival upon exposure to pathogens to defend against the current infection and to avoid continued exposure, including activation of the key cytoprotective transcription factor SKN-1 (mammalian homologue Nrf2). Although wild type C. elegans quickly learn to avoid pathogens, our current work documents a peculiar apathy to pathogen in animals with constitutive activation of SKN-1. The outcome is surprising as the role of SKN-1 is to protect an animal from intracellular and environmental insults. This behavior is mediated by tissue specific actions of SKN-1 that initiate cell non-autonomous responses for pathogen avoidance, survival, and metabolic adaptation. Based on transcriptional signature of animals with SKN-1 activation upon exposure to PA14 we observed that the apathy behavior is independent of somatic lipid depletion, thus uncoupling these two exceptional physiological responses. Further, we define the role of neurosignalling molecules in the gut-brain axis that drive the apathy response to pathogens. Our work reveals new insights into how animals perceive pathogens in the environment and subsequently alter behavior and cellular programs to promote survival.

Abstract citation ID: igad104.3515

### PATIENT PORTAL USE IN FAMILY CAREGIVERS OF PATIENTS WITH DEMENTIA OR CANCER: THE NATIONAL STUDY OF CAREGIVING

Reed Bratches, Jaclyn Wall, Frank Puga, Giovanna Pilonieta, Marie Bakitas, David Geldmacher, and J. Nicholas Odom, *The University of Alabama at Birmingham, Birmingham, Alabama, United States*

Family caregivers are often inexperienced and require information from clinic visits to effectively provide care for patients. The patient portal is a common way for health systems to engage family caregivers, especially those of patients with dementia or cancer. The objective of our study was to analyze a large, nationally representative sample of family caregivers from the National Study of Caregiving (NSOC) to determine factors associated with patient portal use among family caregivers of persons with dementia and of persons with cancer. We conducted a secondary data analysis using data from the 2020 NSOC, a large nationally-representative survey of family caregivers. Weighted regression analysis was used to examine associations between family caregiver use of the patient portal and demographic variables including age, race/ethnicity, gender, employment status, caregiver health, education, and religiosity. A total of 462 participants (4,589,844 weighted) were analyzed. In the fully adjusted regression model for caregivers of persons living with dementia, Hispanic ethnicity was associated with a higher odds of patient portal use (OR: 2.81; 95%CI 1.05, 7.57; p=0.04) and having less than a college degree was associated with a lower odds of patient portal use (OR 0.36; 95%CI 0.18, 0.71; p=< 0.001). In the fully adjusted regression model for caregivers of persons living with cancer, no variables were associated with patient portal use. As the patient portal is a common method of connecting caregivers with information from clinic visits, future research should focus on understanding how the portal is used by the groups we have identified, and why.

Abstract citation ID: igad104.3516

### PEOPLE NEED PEOPLE: A MIXED METHODS STUDY OF COMPANION CAREGIVERS

Kelsey McNamara[1], Samantha Lang[2], Jill Meadows[1], Emily Hackel[1], and Ellen Rudy[1], *1. Papa Inc., Miami, Florida, United States*, *2. Lifespan Flourishing Group LLC, Miami, Florida, United States*

Researchers have found positive aspects of caregiving (PAC) for both formal and informal caregivers, including social connection and sense of purpose. However, few have explored the experience of a novel source of helping individuals age in place: companion caregivers (CC), that is someone who is paid for providing companionship and assistance with everyday tasks that are non-medical. We conducted a descriptive mixed methods study to understand the experience of being a CC. Sixteen CCs who completed 10+ caregiving visits participated in semi-structured interviews and related questionnaires in 2023. Quantitative and qualitative data on CC's perceptions of the impact of caregiving on their own whole health domains (physical, mental, social, purpose) were analyzed. In addition we examined CC's perceptions of care recipients' (CR) unmet social needs, risks in the home, and barriers to CRs achieving improved health domains. Among the cohort, the average age was 53, 63% White, and 31% lived alone. Majority of CCs indicated that they always feel a sense of belonging when with their CRs (n=15) and felt better about themselves after interacting with CRs (n=16). Common PAC across whole health domains emerged, including increased physical activity, improved social connection, and helping others improve CCs' lives. Common barriers to CRs' improving their whole health included: decreased social connection, physical limitations, and transportation insecurity. These insights highlight potential benefits for those who might engage in companion caregiving arrangements. More research is needed as new models of caregiving are necessary to meet the demands of our aging population.

Abstract citation ID: igad104.3517

### PERCEIVED HOUSING PROBLEMS AND DEPRESSIVE SYMPTOMS AMONG MIDDLE-AGED AND OLDER AMERICANS

Peiyi Lu[1], Dexia Kong[2], Feifan Cao[3], and Mack Shelley[3], *1. Columbia University, New York City, New York, United States*, *2. The Chinese University of Hong Kong, Hong Kong, Hong Kong*, *3. Iowa State University, Ames, Iowa, United States*

**Background:** Housing insecurity has been shown to be associated with worse mental health. However, previous studies mostly examined one dimension of housing insecurity (e.g., affordability) and few focused on older adults. This study examined the relationship of perceived housing problems with depressive symptoms among middle-aged and older Americans. **Method:** Longitudinal data from the Health and Retirement Study 2006-2018 were used. A total of 7,285 respondents (aged 50+ at baseline) were followed-up every 4 years. Respondents self-reported the status, severity, and duration of their housing problems. Depressive symptoms were assessed by the Center for Epidemiological Studies-Depression Scale (CESD). Mixed-effect models were used to examine the association between perceived housing problems and depressive symptoms, adjusting for individual-level, household-level, and housing-related confounders. **Results:** About 5%-7% of respondents had housing problems during every study visit and 5.67% of them experienced persistent housing problems over the study period of 12 years. Having housing problems was associated with higher risk of depressive symptoms (IRR=1.30, 95% CI=1.23, 1.36). A dose-response relationship was observed in the severity and duration of housing problems, with greater increase of depressive symptoms risk among those experiencing more severe or longer duration of housing problems. Results were similar when modelling the risk of elevated depressive symptoms using a binary measure (CESD score ≥ 4). **Conclusion:** Perceived housing problems were associated with worse mental health among middle-aged and older Americans. The dose-response pattern highlighted the importance of early intervention and consistent assistance to those experiencing housing problems.

Abstract citation ID: igad104.3518

### PERCEPTIONS OF OLDER ADULTS ON YOGA-BASED INTERVENTIONS FOR MULTIMORBIDITY IN MYSORE, INDIA

Kiranmayee Muralidhar[1], Purnima Madhivanan[2], Anil Behal[3], Vijaya Srinivas[1], Nagalambika Ningaiah[1], Poornima Jaykrishna[1], Rahul Shidhaye[4], and Karl Krupp[5], *1. Public Health Research Institute of India, Mysore, Karnataka, India*, *2. University of Arizona, Tucson, Arizona, United States*, *3. Philadelphia, Pennsylvania, United States*, *4. Pravara Institute of Medical Sciences, Loni, Maharashtra, India*, *5. Mezcoph, Tucson, Arizona, United States*

Globally, over half of older adults suffer from ≥2 chronic diseases. In India, proven exercise interventions are limited by individual, social, environmental and policy-level factors. We aimed to qualitatively explore perceptions about yoga interventions for healthy aging in Mysore, India. In collaboration with the Community Advisory Board, we developed a qualitative assessment of knowledge, attitudes and behaviors around yoga among seniors. Between August-September 2022, we conducted focus group discussions (FGDs) with older adults, after obtaining informed consent. Interviews were recorded, transcribed, and translated from the local language Kannada. Data were coded manually, and emerging themes were identified. We conducted 4 FGDs of 7-8 participants each; 14 male and 13 female older adults participated, and their average age was 66.1±4.3 years; 88.9% had at least 10 years of education. Most participants expressed interest in yoga for health maintenance, specially mentioning its Indian roots, but did not prioritize it for routine practice. Yoga practice was also a means to reduce social isolation for many. Reported challenges in adopting yoga practice included inaccessibility to instructors and dependence on family members. Cultural barriers especially affected women, including ageism and gender role expectations. Women reported hesitancy to express their desire to learn yoga due to perceived negative reactions from their younger family members and their community. Promoting health promotion practices among older Indian adults is challenging primarily due to cultural norms and changing family dynamics with age. Promoting healthy behaviors among them through yoga will first involve family education and consent.

Abstract citation ID: igad104.3519

**PERCEPTIONS OF THE ROLE PLAYED BY LIVING ALONE IN PROVIDING SERVICES TO PATIENTS WITH COGNITIVE IMPAIRMENT IN THE US**

Elena Portacolone[1], Julene Johnson[2], Ashwin Kotwal[2], and Robyn Stone[3], *1. University of California, Berkeley, California, United States*, *2. University of California, San Francisco, San Francisco, California, United States*, *3. LeadingAge, Washington, District of Columbia, United States*

Given the estimated 4.3 million older adults from diverse racial/ethnic backgrounds who are living alone with cognitive impairment in the US, the objective of this study was to identify the potential moderating role of living alone in the use of healthcare and social services for patients with cognitive impairment by eliciting providers' perceptions of caring for diverse patients with cognitive impairment living alone, in comparison to counterparts living with others. This study used semi-structured interviews conducted between March 2021 and June 2022 with purposively sampled providers of services to patients with cognitive impairment in MI, CA, and TX. An inductive content analysis was used to analyze the data. The majority of the 76 interviewed providers cared for racially/ethnically diverse patientt. Providers represented 20 professions. Providers elucidated specific factors that made serving people living alone with cognitive impairment (PLACI) more challenging, as well as specific factors that increased their concerns when serving PLACI. Providers also elucidated reasons for systematic unmet needs of PLACI for essential healthcare and social services. The vast majority of providers underscored the critical, yet understudied, role of living alone in moderating access to essential healthcare and social services among patients with cognitive impairment. Findings suggest that living arrangement are a social determinant to health among patients with cognitive impairment because PLACI are more likely to experience gaps in services because they are more challenging to serve than counterparts living with others and the healthcare system is not equipped to address these challenges.

Abstract citation ID: igad104.3520

**PERSONALIZED DEMENTIA PREVENTION IN LUXEMBOURG: INSIGHTS INTO THE PROGRAMME DEMENTIA PREVENTION (PDP)**

Valerie Schröder[1], Amna Skrozic[1], Dorothee Erz[1], Anne Kaysen[1], Joëlle Fritz[2], Joao Loureiro[3], Deborah McIntyre[2], and Rejko Krueger[1], *1. University of Luxembourg, Esch-sur-Alzette, Grevenmacher, Luxembourg*, *2. Luxembourg Institute of Health, Luxembourg, Grevenmacher, Luxembourg*, *3. Centre Hospitalier de Luxembourg (CHL), Luxembourg, Grevenmacher, Luxembourg*

**BACKGROUND:** Given that in an increasingly ageing society, the number of people with cognitive impairment is expected to increase, prevention strategies to reduce the high personal and societal burden of neurodegenerative diseases leading to dementia is of essence. **METHODS:** As part of the "Dementia Prevention Programme (pdp)" in Luxembourg, individuals with Mild Cognitive Impairment (MCI) or Subjective Cognitive Decline (SCD) are recruited via referring physicians and other sources. All participants undergo a comprehensive neuropsychological evaluation and a risk factor profile is created. Factors known to increase the risk to develop dementia include hypertension, hypercholesterolaemia, physical or cognitive inactivity, depression, social isolation or hearing loss. Based on the results of this analysis, personalised lifestyle interventions are recommended to each participant, such as cognitive training, physical activities, psychological counselling or nutritional advice. **RESULTS:** To date, 491 participants (mean age = 69.0 years; SD = 11.2) have been recruited at various sites across the country, of whom 424 (86.4%) present with MCI and 42 (8.6%) with SCD. 25 participants (5.1%) showed more advanced cognitive difficulties associated with loss of autonomy or had neither subjective nor objective cognitive difficulties and thus did not belong to the target group. **CONCLUSION:** We provide evidence for the successful implementation of a nationwide dementia prevention programme and efficient recruitment of the target population by building a network of different health care providers. This may serve other countries as an example to rule out similar programmes targeting at promoting healthy ageing among the general population.

Abstract citation ID: igad104.3521

**PERSON-CENTERED DEMENTIA TRAINING ONLINE FOR BSN STUDENTS: A PILOT STUDY**

Diana Sturdevant[1], Kimetha Broussard[1], Keith Kleszynski[2], and Lee Jennings[2], *1. University of Oklahoma HSC, Fran and Earl Ziegler College of Nursing, Oklahoma City, Oklahoma, United States*, *2. University of Oklahoma Health Sciences Center, Oklahoma City, Oklahoma, United States*

We launched a Person-Centered Dementia Training in March 2023, focused on Four interactive modules were offered via an Online Learning Platform. Topics included: Introduction to Dementia, Communication Process and Approach, Environmental and Cultural Implications in Dementia Care, and Best Practices in Responding to Difficult Behaviors. Subtopics included: Introduction to Age-Friendly and Dementia-Friendly Care, High-risk Medications and Dementia Care, Dementia vs Delirium, and Reframing Aging—Understanding and Recognizing Ageism Specific to People Living with Dementia. BSN students were recruited via (3) email blasts and (1) in-person announcement of participation opportunity. A total of (n=33) participants were recruited from 4 nursing programs, Traditional BSN Junior (34%), Traditional BSN Senior (48%), Degree Completion LPN to BSN (3%) and Accelerated BSN (14%). Most participants were female (96%), between the ages of 18-24 (60%). 24 participants completed the pre-/post-test and Final Program Evaluation. Participants rated the course content and structure as 4.5/5 on 5pt-likert scale. Common themes from evaluations included: need for additional content, more interactive activities, and inclusion as a required course for all nursing students. Future steps to include revising course content based on participant feedback, offer revised course over the next 2 academic semesters, and expand reach by offering course to practicing nurses to improve care of people living with dementia (PLWD).

Abstract citation ID: igad104.3522

**PERSON-FIRST LANGUAGE FOR OLDER ADULTS INCARCERATED IN PRISON: A RAPID EVIDENCE ASSESSMENT**

Kristin Merss[1], Laura Block[2], and Stephen Kwas[3], *1. University of Wisconsin - Madison, School of Nursing, Madison, Wisconsin, United States*, *2. University of Wisconsin-Madison, Madison, Wisconsin, United States*, *3. Swarthmore College, Madison, Wisconsin, United States*

Language can influence a person's sense of self, relationship with healthcare providers, healthcare utilization, or their own symptoms and behaviors. The person-first language movement began decades ago and has been widely adopted by multiple fields including nursing, medicine, gerontology, and social and health sciences more broadly. For research involving older adults, non-stigmatizing language has become the standard for researchers. Incarceration is widely considered a dehumanizing experience, with recent calls for attention to the language used by researchers when describing their incarcerated participants. Researchers are also identifying that individuals with multiple stigmatizing identities (e.g., incarcerated and disabled, older adult) warrant specific consideration. This study presents a secondary review of terminology from a Rapid Evidence Assessment (REA) investigating what is known about transitions of older adults in the incarceration system. The REA included PubMED, PscyhMED, and CINHAL. Nineteen articles met inclusion criteria (11 qualitative, 11 quantitative, 3 mixed methods). As a sub-analysis, articles were analyzed to consider the language that was used to describe the currently or formerly incarcerated older adults who were the subject of the studies. The majority of articles considered older adults who were no longer incarcerated. While person-first language (i.e., "formerly incarcerated person", "reintegrating women", "older adult on parole") was common, stigmatizing language such as "prisoner", "offender", "homicide offender", and "ex-convict" were used in the majority of the studies included in the review. Appreciation and adoption of person-first, non-stigmatizing language should transcend not only research literature surrounding older adults in the community, but also incarcerated older adults.

Abstract citation ID: igad104.3523

**PHARMACIST INTERVENTION IS ASSOCIATED WITH MEDICATION ADHERENCE IN OLDER ADULTS WITH CHRONIC CONDITIONS**

Renae Smith-ray, Liang Feng, Tanya Singh, Kristi Rudkin, Stacey Emmons, Erik Groves, and Heather Kirkham, *Walgreens, Deerfield, Illinois, United States*

Hypertension, hyperlipidemia, and type 2 diabetes (T2D) are prevalent chronic diseases among older Americans, but related medication adherence rates are less than optimal. Consequences of poor adherence include increased mortality, healthcare utilization, and costs. Compounding evidence supports the integration of pharmacists in patients' care across the care continuum. This study's purpose was to examine the impact of a large national pharmacy chain's pharmacist-led interventions to improve medication adherence among older adults with hypertension, hyperlipidemia, or T2D. Participants were Medicare D patients who had ≥2 prescription fills in ≥1 of the three therapeutic classes. The primary outcome, optimal adherence (OA), was defined as proportion of days covered (PDC) ≥80%. A difference-in-differences (DID) design with a generalized linear model approach was applied to examine differences between participants exposed to the intervention and controls across the study period (2020-2022). At baseline, intervention participants (n=317,613, age 70.1, female 57.0%) had lower OA for diabetes (76.9% vs. 79.8%), hypertension (79.0% vs. 83.0%), and cholesterol (78.6% vs. 82.1%) compared to controls (n=943.389, age 73.3, female 56.1%). The DID results showed that between 2020 - 2022, OA had significant absolute increases for intervention participants (diabetes: +4.0%, hypertension: +6.3%, cholesterol: +6.1%) versus controls (diabetes: -1.6%, hypertension: -0.4%, cholesterol: -1.4%). All DID models were significant at $p< 0.0001$. The pharmacist-led intervention was significantly associated with increased OA over 2 years. These findings reinforce the potential for pharmacists as care providers among older adults with chronic conditions. Future research should examine the expansion of pharmacist-provided care.

Abstract citation ID: igad104.3524

**PHARMACOLOGIC AMPK ACTIVATION IMPROVES HEALTHSPAN AND LIFESPAN IN NORMAL AND PREMATURE AGING MODELS**

Ozlem Altintas Akin[1], Sarah Mitchell[2], Shana Sturla[1], and Michael MacArthur[2], *1. ETH Zurich, Zurich, Zurich, Switzerland*, *2. Princeton University, Princeton, New Jersey, United States*

Cockayne Syndrome (CS) is a rare autosomal recessive childhood disease, caused by mutations in CSA (ERCC8) or CSB (ERCC6) genes leading to defective DNA damage repair. CS characteristics include UV sensitivity and premature aging. Deficiencies in CSA or CSB may lead to impaired AMP-activated protein kinase (AMPK) activation. Notably, AMPK-activating interventions have shown promise in preclinical CS mouse models. We tested whether MK8722, an AMPK activator, could mitigate CS phenotypes in a C. elegans model. Our preliminary data show that MK-8722 increases lifespan in wild-type and CS worm model. We also tested the effect of MK-8722 on brood size as a fitness parameter and showed that it does not change the total brood size. We also showed that treating aged mice with MK-8722 improves multiple measures of healthspan including body composition, grip strength and age-associated inflammation. These data suggest that AMPK activation may be a promising therapeutic avenue for both normal and CS-associated aging phenotypes

Abstract citation ID: igad104.3525

**PHYSICAL ACTIVITY BARRIERS AND INTENTIONS OF ALZHEIMER'S DISEASE (AD) AND RELATED DEMENTIAS (AD/ADRD) CAREGIVERS**

Andrew Pickett[1], Rebecca Krukowski[2], and Nicole Werner[3], *1. Indiana University-Bloomington, Bloomington, Indiana, United States*, *2. University of Virginia, School of Medicine, Charlottesville, Virginia, United States*, *3. Indiana University School of Public Health-Bloomington, Bloomington, Indiana, United States*

Caregivers of people living with AD/ADRD often experience negative biopsychosocial health outcomes and have reduced capacity to engage in healthy behaviors (e.g., exercise). Physical activity is an established intervention for promoting physical and mental health across many populations. This research explored physical activity intentions and barriers among AD/ADRD caregivers, as well as potential relationships between physical activity intentions, cohabitation status, and caregiver/care recipient relationship. Data were collected via online survey from family caregivers of people living with AD/ADRD (n=117). The sample was predominantly women (n=89; 76.1%), white (n= 89, 76.1%), and averaged 55.3 (SD= 16.45) years old. Participants averaged 56.31 (SD= 49.15) hours per week on care responsibilities. Caregivers expressed strong desire to be more active (M=4.42 / 5; SD= .71) and that caregiving demands were a strong barrier (M=3.85 / 5; SD= 1.15). Significant differences were observed between caregivers who lived, or did not live with, care recipients across both physical activity intention (p= .022) and burden perceptions (p=.002), measured by the short form Burden Scale for Family Caregivers (BSFC-SF). No differences were observed based on caregiver-care recipient relationship (e.g., parent, partner). Primary themes among open-ended responses to physical activity barriers included: lack of time and energy, inability to leave care recipient, and competing responsibilities. Our findings suggest a need for behavioral interventions to facilitate physical activity among AD/ADRD caregivers, particularly those who live with their care recipient. Social network interventions that leverage social connections to support caregivers may increase physical activity by reducing barriers associated with care responsibilities.

Abstract citation ID: igad104.3526

### PHYSICAL ACTIVITY, SEDENTARY BEHAVIORS AND MYOSTEATOSIS IN AFRICAN CARIBBEANS

Lily Bessette[1], Ryan Cvejkus[1], Adrianna Acevedo-Fontanez[1], Allison Kuipers[1], Joseph Zmuda[1], Victor Wheeler[2], and Iva Miljkovic[1], *1. University of Pittsburgh, Pittsburgh, Pennsylvania, United States*, *2. Scarborough General Hospital, Tobago, Tobago, Trinidad and Tobago*

Skeletal muscle fat infiltration (myosteatosis) leads to poorer muscle quality and function with aging, and is emerging as a predictor of skeletal muscle strength, mobility, and metabolic disorders. Few studies investigated the association of physical activity (PA) and sedentary behavior (SB) with myosteatosis, especially in African Caribbeans, a vastly under-studied, rapidly growing population. We examined the associations of objectively measured PA and SB (Bodymedia SenseWear Armbands) worn over 7 days with calf muscle density (mg/cm3), a marker of intra-muscular fat (Stratec XCT-2000), in older adult African Caribbean men (N=355, mean age 62 years) and women (N=682, mean age 59 years) of the Tobago Health Study. Compared to men, women were younger with higher BMI, more SB, less light (LPA) and moderate to vigorous PA (MVPA), and lower muscle density (p< 0.05, age-adjusted). Meeting MVPA guidelines (150 min MVPA/week) was associated with greater muscle density in obese men and women (p=0.03, p=0.09 respectively, age-adjusted). Adjusting for age, BMI, smoking, diabetes, hypertension, and lipid-lowering treatment, we assessed sex stratified partial spearman correlations between PA and SB and muscle density. MVPA was more strongly correlated with greater muscle density in men (r=0.15, p=0.01) than in women (r=0.07, p=0.09), while SB was associated with lower muscle density in women (r=-0.08, p=.044), but not significantly in men (r=-0.10, p=0.12). Our novel findings indicate that the association between PA and SB with myosteatosis may differ by sex. These findings may have important implications for sex specific activity guidelines related to skeletal muscle health in African Caribbeans.

Abstract citation ID: igad104.3527

### PHYSICAL FUNCTION, DEPRESSION, AND COGNITIVE FUNCTION OF OLDER CHINESE AMERICANS: CROSS-LAGGED MODEL

Soonhyung Kwon[1], Yuyang Zhu[2], Fengyan Tang[3], and Yanping Jiang[4], *1. Rutgers, The State University of New Jersey, New Brunswick, New Jersey, United States*, *2. Rutgers University, Piscataway, New Jersey, United States*, *3. University of Pittsburgh, Pittsburgh, Pennsylvania, United States*, *4. Rutgers University, New Brunswick, New Jersey, United States*

**Introduction:** Understanding the complex interactions of physical function, depression, and cognitive function is crucial for understanding healthy aging. However, few studies have empirically tested these relationships. This study examined the directional relationships of physical function, depression, and cognitive function among older Chinese Americans. **Methods:** This study included a subsample of participants (n=2,811) from the Population Study of Chinese Elderly in Chicago who completed the baseline survey during 2011-2013 (W1) and at least one of the three follow-up surveys conducted in 2013-2015 (W2), 2015- 2017 (W3), and 2017-2019 (W4). Physical function, depression, and cognitive function were measured by a battery of physical mobility, the 9-item Patient Health Questionnaire, and cognitive performance tests (executive function, memory, and cognitive impairments). A random intercept cross-lagged panel model was performed to examine the bidirectional relationships among physical function, depression, and cognitive function. **Results:** The cross-lagged panel model showed that higher levels of depression at W1 were associated with poorer physical function at W2. In turn, poorer physical function at W2 was related to higher levels of depression at W3. Higher levels of depression (W2) were linked to poorer physical function at W3. Poorer physical function at W2 was associated with poorer cognitive function at W3. Finally, poorer cognitive function at W3 was associated with poorer physical function and higher levels of depression at W4. **Discussion:** Our results support a bidirectional relationship between physical function and depression. Also, as individuals age, cognitive function plays a key role in predicting physical function and psychological well-being.

Abstract citation ID: igad104.3528

### PLANT PROTEIN BUT NOT ANIMAL PROTEIN CONSUMPTION IS ASSOCIATED WITH FRAILTY THROUGH PLASMA METABOLITES.

Tanaka Toshiko[1], Jayanta Das[2], Qu Tian[2], Ruin Moaddel[2], Ann Zenobia Moore[2], Katherine Tucker[3], Sameera Talegawkar[4], and Luigi Ferrucci[2], *1. NIA/IRP/TGB/LSS, Baltimore, Maryland, United States*, *2. National Institute on Aging, Baltimore, Maryland, United States*, *3. University of Massachusetts at Lowell, Lowell,*

*Massachusetts, United States*, *4. The George Washington University, Washington, District of Columbia, United States*

There is evidence that the association of protein intake and frailty may depend on the source of dietary protein. The mechanism underlying these associations are not clear. In this study, we explore circulating metabolites as mediators of the relationship between dietary protein and of frailty in participants of the Baltimore Longitudinal Study of Aging (BLSA). Cross-sectional analyses in 735 BLSA participants of associations between plant and animal protein intake and frailty. Usual protein intake from plant and animal sources were estimated with a Food Frequency Questionnaire (FFQ) and frailty was assessed with a 44-item Frailty Index (FI). Compared with the lowest quartile, higher quartiles of plant, but not animal, protein were associated with lower FI. Twenty-five plasma metabolites were associated with plant protein intake; of these, 15, including phosphatidylcholines, cholesterol esters, sphingomyelins, and indole metabolites mediated the association between plant protein intake and FI. The protective association between plant protein consumption and FI is mediated by lower abundance of lipid metabolites and higher abundance of tryptophan-related metabolites.

Abstract citation ID: igad104.3529

### POLYMORPHISMS IN INFLAMMATORY CYTOKINES NOT ASSOCIATED WITH COGNITIVE TRAJECTORIES IN OLDER ADULTS

Karen Lawrence[1], Elana Gloger[1], Cristina Pinheiro[1], Frederick Schmitt[1], and Suzanne Segerstrom[2], *1. University of Kentucky, Lexington, Kentucky, United States*, *2. Oregon State University, Corvallis, Oregon, United States*

Inflammation and key polymorphisms in inflammatory cytokine genes have been associated with increased Alzheimer's disease (AD) risk. It is unclear if the same polymorphisms also predict domain-specific cognitive decline, that could eventually lead to AD/dementia, in otherwise healthy older adults. We investigated whether specific single nucleotide polymorphisms (SNPs) in three cytokine genes, IL-1β (rs16944; A or AA at -511), IL-6 (rs1800795; C or CC at -174), and TNFα (rs1800629; A or AA at -308), were associated with differential longitudinal trajectories of global cognitive function, episodic memory, attention and working memory, and executive function. Participants were 324 non-demented older adults (Mage=72.8 years at first visit, SD=6.09, age range 57-92 years) who completed 9 research visits, on average. Slopes were examined after adjustment for baseline age, sex, education, conversion to MCI or dementia during study, and APOE ε4 status. Findings indicated no statistically significant relationships between the SNPs of interest and longitudinal trajectories (spanning up to 16 years) of cognitive functioning in any cognitive domain examined. Other factors related to AD may be necessary for these SNPs to have significant brain-related effects. Attempts were made to further validate the non-significant findings by conducting post-hoc analyses to determine whether these SNPs were associated with plasma biomarker levels in this sample of healthy older adults. However, variability between and within the assays precluded confidence in the results. Further research is needed to determine whether these SNP genotypes are associated with concentrations of their respective blood-plasma biomarkers in non-demented older adults.

Abstract citation ID: igad104.3530

### POST COVID-19 PANDEMIC EMPLOYMENT RECOVERY BY HEALTHCARE SECTOR IN US RACIAL MAJORITY-MINORITY COUNTIES

Lucille Xiang, and Kiran Sreenivas, *American Health Care Association / National Center for Assisted Living, Washington, District of Columbia, United States*

Health care sector employment trends following the COVID-19 pandemic have revealed a labor force participation gap. Racial/ethnic composition differences between counties may contribute to an uneven labor market recovery. This study's objectives were to compare post-pandemic employment recovery between five health care sectors (ambulatory services (AS), home health care (HHC), nursing and residential care (NRC), assisted living (AL), nursing facilities/homes (NH)), and to examine whether health care sector recovery differences exist between racial majority-minority (MM) and non-racial MM counties in the USA. First Quarter 2020 and fourth Quarter 2022 data from the US Bureau of Labour Statistics Quarterly Census of Employment and Wages (QCEW) were used to calculate cumulative percentage change in health care sector employment. The Race Origin Variables from the latest 2021 data from the American Community Survey were used to identify racial MM counties. The sector with the greatest number of counties with positive employment recovery was the AS sector in 1562/3176 (49%) counties, and 252/468 (54%) MM counties. The NH sector had the lowest number of counties with positive employment recovery in 170/2818 (6%) counties, and 34/389 (9%) MM counties. This is the first analysis of post-pandemic health care sector employment recovery examining MM counties. Differences in county and state policies may account for varying degrees of recovery. Further research is necessary for the development of best practice guidelines and policies to improve post-pandemic health care employment recovery and the quality of nursing home care among older adults.

Abstract citation ID: igad104.3531

### PREDICTORS OF FINANCIAL BURDEN IN TWO SAMPLES OF ADULTS WITH ALZHEIMER'S DISEASE AND RELATED DEMENTIAS

Jordana Clayton[1], Eli Iacob[1], Nancy Aruscavage[2], Rebecca Utz[1], Katherine Supiano[1], Sara Bybee[1], and Kara Dassel[2], *1. University of Utah, Salt Lake City, Utah, United States*, *2. University of Utah College of Nursing, Salt Lake City, Utah, United States*

Compared to other older adults with chronic illness, individuals diagnosed with Alzheimer's disease and related dementias (ADRD) incur much higher care costs at the end of life. The financial strain created by these high costs is experienced unevenly, as individuals who are low-income and minorities may have difficulty meeting out-of-pocket costs of care. Our objective was to examine the relationship between reported concern about being a financial burden to others and socioeconomic and demographic variables. To accomplish this, we conducted a mixed-methods study of two samples of individuals diagnosed with ADRD aged 31-89 (mean 63, n = 44) and 50-89 (mean 66, n=38) using two ordinal logistic regression models and qualitative analysis. The ordinal regression models indicate that the probability of reporting higher financial burden increases with lower age, higher education, and being widowed. One model shows income and employment as significant predictors of financial burden, particularly for the "forgotten middle" who have a high enough income not to qualify for social support programs but low enough income to experience strain from high out-of-pocket costs. In the qualitative analysis, we categorized 309 free responses from the second sample using the Financial Hardship Typology into concerns of material conditions / ability to pay (n=40), psychological distress about being a financial burden (n=28), and coping behaviors / limiting care to reduce costs (n=20). These results point to ways that social programs need to be improved to better support individuals with ADRD who are experiencing financial strain during the end-of-life.

Abstract citation ID: igad104.3532

### PRESENCE OF DYSPHAGIA IN A COHORT OF PERSONS LIVING WITH DEMENTIA: OUTCOMES FROM A MULTIDISCIPLINARY CLINIC

Joanne Yee[1], Danielle Brates[1], Meredith Mackowicz-Torres[2], Raele Robison[2], Brian Lewis[1], Lindsay Clark[1], Ellen Wanninger[1], and Nicole Rogus-Pulia[2], *1. William S. Middleton Memorial Veterans Hospital, Madison, Wisconsin, United States*, *2. University of Wisconsin Madison, School of Medicine and Public Health, Madison, Wisconsin, United States*

The Cognitive Care Clinic (CCC) provides evaluation for Veterans living with dementia. Speech-language pathology (SLP) is incorporated to facilitate early identification and proactive management of dysphagia. This project sought to describe dysphagia frequency in persons living with dementia (PWD) in CCC. Multidisciplinary assessments led to consensus diagnosis of dementia subtype. Stage was determined using the Functional Assessment Staging Tool (FAST). Validated dysphagia screening tools included Eating Assessment Tool (EAT-10), water swallow test (WST), and Test of Mastication and Swallowing Solids (TOMASS). Penetration-Aspiration Scale (PAS) scores were determined following videofluoroscopic swallow studies (VFSS); scores were classified as "safe" (PAS< 3) or "unsafe" (PAS>=3). Descriptive statistics were calculated for dementia subtype, stage, and dysphagia measures. 78 Veterans were diagnosed with dementia between 2017 and 2023 (mean age=77 years; 5% female) and completed dysphagia screening. The majority had Alzheimer's disease (43%) and were in the mild disease stage (61%). 37% failed the WST, suggesting impaired safety. 49% failed the TOMASS, indicating masticatory inefficiency. 26% scored 3 or higher on EAT-10, indicating increased dysphagia risk. 22 patients completed VFSS; 55% exhibited unsafe swallows. No significant differences in EAT-10 total scores were observed between those with safe and unsafe PAS scores. Nearly half of this cohort demonstrated signs of swallowing impairment based on clinical testing and/or VFSS. Additionally, EAT-10 scores were low overall with no differences based on dysphagia status on VFSS, which suggests underreporting of dysphagia. These findings highlight the necessity of SLPs in dementia care teams to support early identification and management of dysphagia.

Abstract citation ID: igad104.3533

### PRESSURED TOUCH FOOT CARE TO RELIEVE CHRONIC PAIN AND IMPROVE QUALITY OF LIFE

Mary Clayton-Jones, *University of Massachusetts, Amherst/ FootCare by Nurses, Amherst, Massachusetts, United States*

**Background:** Chronic pain affects many older adults and is a leading cause of disability. It is often treated pharmacologically, but evidence indicates touch therapy modalities are beneficial. Purpose: To implement a therapeutic touch program for adults enrolled in a Program of All-Inclusive Care for the Elderly (PACE) to help reduce chronic pain and improve quality of life for those patients enrolled in this project. **Method:** Twelve participants in an assisted living facility with chronic foot pain were initially selected by the PACE Family Nurse Practitioner (FNP) for home visits with this Doctor of Nursing Practice (DNP) student for a foot care and pressured touch intervention to occur once a month for 3 months. The McGill Pain and Quality of Life Scores questionnaires were administered before each intervention. **Results:** Four participants completed all three interventions. Every participant experienced a decrease in pain pre- and post-intervention and month-to-month of 15% or more. Three participants had experiences of complicated grief and had a decrease in quality-of-life scores over the 3 months. The participant without complicated grief had an improvement in the quality-of-life scores with pain reduction. **Conclusions:** This small home intervention project for chronic foot pain demonstrated that foot care and pressured touch may be a cost-effective adjunct therapy for reducing pain. Chronic pain can be complicated by grief, which must be considered when looking to support elder wellness and quality of life. Keywords: Chronic pain, therapeutic touch

Abstract citation ID: igad104.3534

### PREVALENCE AND CORRELATES OF DEPRESSION IN OLDER ADULTS: FINDINGS FROM THE LONGROAD STUDY

Yitao Xi, *Columbia University, New York City, New York, United States*

As an important public health issue, depression has been studied extensively in children and young adults. Epidemiologic data on depression for older adults are scant. The Longitudinal Research on Aging Drivers (LongROAD) project was a prospective cohort study of 2990 drivers aged 65-79 years at baseline and recruited between July 2015 and March 2017 from five sites (Ann Arbor, Baltimore, Cooperstown, Denver, and San Diego) in the contiguous United States. Study participants were assessed at baseline with the Patient-Reported Outcomes Measurement Information System (PROMIS). The objective of this study was to examine the prevalence and correlates of depression in older adults participating in the LongROAD project. Of the 2990 older adults studied, 186 (6.2%) participants had depression at the time of enrollment, with the lifetime prevalence of depression being 20.3%. Only 21.9% of the older adults with depression were taking antidepressants. A significantly higher prevalence of depression was found among those who were female (7.2%), not currently married (9.1%), with annual household income less than $20000 (11.9%), having diabetes (9.1%), having anxiety disorder (15.5%), with exercising routine (6.9%), and without doing volunteering work (7.9%). No significant difference was found between urban and rural residents, but urban residents had slightly higher depression prevalence (6.4%). About 1 in 16 older adults has depression. The majority (78%) of older adults with depression are not treated with antidepressants. Depression screening and treatment programs are needed for older adults, particularly women, those living alone, and those in poverty. Volunteering work could potentially decrease depression prevalence.

Abstract citation ID: igad104.3535

### PREVALENCE OF METABOLIC COMORBIDITIES AMONG COGNITIVELY NORMAL AND IMPAIRED WHITE AND AFRICAN AMERICANS

Mohammad Turaani[1], Subhamoy Pal[2], Jonathan Reader[3], Bruno Giordani[3], and Voyko Kavcic[1], *1. Wayne State University, Detroit, Michigan, United States*, *2. Michigan Alzheimer's Disease Research Center, Ann Arbor, Michigan, United States*, *3. University of Michigan, Ann Arbor, Michigan, United States*

Assessing comorbidities associated with a MCI diagnosis is crucial for diagnostic accuracy and for understanding the role of comorbidities in cognitive decline. In this study of amnestic (aMCI) and non-amnestic (naMCI) MCI participants and persons with normal cognition (CN), we compared the prevalence of three primary comorbidities: Hypertension (HTN), Hyperlipidemia (HLD), and Diabetes Mellitus (DM) across African American and White populations using Chi-squares. Participant data (N = 9,342, 17% African American) were available through the National Alzheimer's Coordinating Center and included: CN (n = 5,963; MOCA mean=27), aMCI (2,694; MOCA=22), and naMCI (685; MOCA=24) with diagnosis and data per their first Uniform Data Set (Version 3) visit. Significant differences in the distribution of HTN, HLD, and DM were found among the diagnostic groups for the total cohort and racial groups, separately; however, diagnostic differences across races were not always consistent. The relative rates of DM and HLD across the diagnostic groups for both races were generally similar, though higher percentages were seen in African Americans (25% of African Americans, 10% of Whites). As for HTN (52% of African Americans, 39% of Whites), however, the distributions differ across the diagnostic groups and race (%yes for diagnosis; White Americans: CN 35%, aMCI 47%, naMCI 44%; African Americans: CN 66%, aMCI 70%, naMCI 79%, $p< 0.001$). These findings highlight the importance of considering the contributions of both race and diagnosis when evaluating the role of comorbid factors and metabolic disorders in NC and MCI groups, in particular when considering blood pressure-related measures.

Abstract citation ID: igad104.3536

### PROFILES OF DIGITAL LITERACY AMONG COMMUNITY-DWELLING KOREAN OLDER ADULTS: A LATENT PROFILE ANALYSIS

Jiyoung Shin[1], Hun Kang[2], Seongmi Choi[1], and JiYeon Choi[1], *1. Yonsei University, Seoul, Republic of Korea*, *2. Yale University School of Public Health, New Haven, Connecticut, United States*

Digital divide exists among not only between older and younger generations but also within the older adult population. Understanding digital literacy profiles among older adults is important to identifying strategies for reducing digital divide among this population. This study aimed to identify profiles of digital literacy among community-dwelling older adults and examine associated factors. Data were collected from a national survey of 1016 community-dwelling older adults in Korea (average age 68.0±6.5 years, 47.8% male). Digital literacy was evaluated across three domains: 'Information and Communication' (9 items), 'Contents Creation and Management' (4 items), and 'Safety and Security' (9 items). Latent profile analysis and multinomial logistic regression yielded three digital literacy profiles: "Low-level" (35.7%, n=363), "Middle-level" (39.2%, n=398), and "High-level" (25.1%, n=255). Compared to the low-level group, a higher likelihood of belonging to the middle level group was associated with younger age, higher education level, higher dependence on instrumental activities of daily living, longer digital device use, and participating in offline social activities. Similarly, a higher likelihood of belonging to the high-level group was associated with younger age, higher education level, higher dependence on instrumental activities of daily living, longer digital device use, better physical well-being, doing daily physical exercise, and greater social support. These findings suggest characteristics associated with lower digital literacy, including older age, lower education level, and reduced functional independence. To promote digital literacy in older adults, potential strategies encompass tailored access and guidance for digital device use specifically tailored to older adults, alongside efforts to nurture social connectedness.

Abstract citation ID: igad104.3537

### PROGRAM OF ALL INCLUSIVE CARE FOR THE ELDERLY ORGANIZATIONS FLIP THE SCRIPT IN RESPONSE TO COVID-19

Morgan Perry[1], Stephen McCall[2], Joseph Dorris[2], Michael Nardone[3], Ani Turner[2], Samuel Obbin[2], and Christine Stanik[4], *1. University of Michigan, Ann Arbor, Michigan, United States*, *2. Altarum, Ann Arbor, Michigan, United States*, *3. The Nardone Group LLC, Philadelphia, Pennsylvania, United States*, *4. Arbor Research Collaborative, Ann Arbor, Michigan, United States*

The Program of All-Inclusive Care for the Elderly (PACE) is a unique care delivery model providing comprehensive, community-based, person-centered services to older adults. The COVID-19 pandemic posed unprecedented challenges for PACE organizations to continue providing services while mitigating infection risk, and they were agile and creative in their response. This study leveraged two national surveys and six interviews with PACE directors to examine how PACE organizations met these challenges. The response rate was 71.2% for the first survey and 67.8% for the second. Results revealed a rapid transition of the PACE model from primarily center-based to home-based care. To aid this transition, PACE organizations redeployed staff, repurposed day centers, and leveraged technology, among other strategies. Most PACE organizations plan to continue delivering more services in the home, consistent with participant preferences. The findings from this study indicate that PACE

organizations continued to keep participants at the forefront as they transitioned to a home-based model. These findings highlight PACE organizations' ability to empower nursing-home-eligible older adults to remain living in their preferred residential setting and could inform lasting changes in PACE service delivery.

Abstract citation ID: igad104.3538

### PROJECT, PERSUADE, AND PROTECT: ADULT CHILDREN'S PREDICTION OF PARENTS' HEALTH STATE VALUATIONS

Jessica Hahne, Rachel E. Buenger, Tim Chung, Hannah Silverstein, and Brian Carpenter, *Washington University in St. Louis, St. Louis, Missouri, United States*

Serious illness decision making for older adults frequently involves their adult children. Thus, it is important that adult children are familiar with parents' illness preferences. In this mixed methods analysis (total N = 114), we examined concordance between older adults' valuations of illness states, their children's predictions of their valuations, reasoning behind children's predictions, and children's reactions to discordance between their predictions and parents' valuations. Thirty-eight older parents (M age= 75.8) provided valuations for five different illness states. Two adult children (M age= 46.0) independently predicted their parent's answers. Family triads participated in a semi-structured conversation about their responses. One-way random effects, absolute agreement, average measures intraclass correlation coefficients (ICCs) showed that average concordance within families was poor for incontinence (ICC = .172, 95% CI [-.497, .572]), dialysis (ICC = .300, 95% CI [-.264, .639]), tube feeding (ICC = .320, 95% CI [-.228, .649]), and chronic pain (ICC = .438, 95% CI [-.015, .710]), and moderate for Alzheimer disease (ICC = .556, 95% CI [.198,.771]). Results from qualitative thematic analysis indicated that children often projected their own values when attempting to predict parents' valuations. When faced with discordance, many children attempted to persuade parents to change their valuations, while also making assertions to protect the parent's autonomy. In summary, adult children navigating surrogate or shared decision making may face challenges inferring parent values versus their own and balancing their input with parent autonomy. Future research and interventions in late-life families could target these challenges.

Abstract citation ID: igad104.3539

### PROSPECTIVE ASSOCIATION OF INTRINSIC CAPACITY WITH FALLS AND MULTIPLE FALLS IN OLDER ADULTS (NHATS)

Kewei Shi, Lingxiao He, Xiaodong Chen, and Ya Fang, *Xiamen University, Xiamen, Fujian, China (People's Republic)*

**Background:** Intrinsic capacity (IC) is a recently proposed novel metric of evaluating the overall health condition of older people. The IC has been associated with multiple adverse health outcomes while its associations with falls and multiple falls in community-dwelling older adults are not well studied. **Methods:** 2113 community-dwelling older adults (aged 68-97 years) were selected from two waves of the National Health and Aging Trends Study (NHATS). Data of five IC domains (cognitive, sensory, locomotion, vitality, psychological), falls and multiple falls were collected via questionnaires at baseline and a 4-year follow-up. The IC was evaluated as an IC score calculated by averaging the Z-score in each IC domain. Logistic regression was used to analyze the associations between falls and IC scores with controls for age, gender, marital status, comorbidity index and social activity index. **Results:** A higher baseline IC score was associated with a lower risk of falls (OR= 0.459, 95%CI [0.309,0.681]) and multiple falls (OR= 0.278, 95%CI [0.154, 0.502]). Participants with smaller IC score change demonstrated greater risk of fall (OR= 0.512, 95%CI [0.336, 0.777]) and multiple falls (OR= 0.238, 95%CI [0.128, 0.435]). In terms of interactive effects, there were also no significant interactions between baseline IC score and IC score change in their associations with fall outcomes. **Conclusions:** The IC is longitudinally associated with falls and multiple falls in older adults. Our findings emphasize the potential role of IC as a valuable marker for predicting fall risk in the older population.

Abstract citation ID: igad104.3540

### PSYCHOMETRIC PROPERTIES OF THE KOREAN VERSION HOSPITAL SURVEY ON PATIENT SAFETY CULTURE IN HOME HEALTHCARE

Kayoung Lee[1], Soohee Lee[1], and Seoyoon Woo[2], *1. Gachon University, Incheon, Gyeonggi, Republic of Korea, 2. University of North Carolina Wilmington, Wilmington, North Carolina, United States*

Patient safety culture is essential for quality care in healthcare organizations. Instruments for patient safety have been developed and widely implemented in hospital settings. The revised hospital survey on patient safety culture (HSOPSC) is one of the representative instruments for evaluating patient safety culture. In 2021, the Korean version of the HSOPSC 2.0 (K-HSOPSC 2.0) was developed. However, there is a lack of evidence to examine the validity of the instrument in home healthcare settings. This study aimed to test the psychometric analysis of K-HSOPSC 2.0 in home healthcare settings in South Korea. We collected 215 certified advanced practice nurses (APNs) and nurse managers from 163 out of 175 home healthcare institutions in South Korea. We conducted the confirmation factor analysis (CFA) to examine the existing 10 dimensions of K-HSOPSC 2.0. Statistical analyses were performed with Stata 17.0, and the Satorra-Bentler (SB) method was used to adjust the standard error. The construct validity demonstrated an acceptable fit (RMSEA_SB=0.061, CFI_SB=0.912, TLI_SB=0.889). The K-HSOPSC 2.0 demonstrated high reliability (Cronbach's α=0.90), except for teamwork (α=0.52) and staffing and work pace (α=0.39) among 10 dimensions. The validity of the measurement of patient safety culture was supported in home healthcare settings. However, further evaluation is needed for the two dimensions of 'teamwork' and 'staffing and work pace'. In home healthcare settings, it is crucial to consider the role of patients and caregivers in teamwork and different home healthcare working environments in staffing that reflects unique home healthcare settings.

Abstract citation ID: igad104.3541
### PSYCHOSOCIAL PHENOTYPES OF OLDER ADULTS WITH PAIN AND THEIR ASSOCIATED CLINICAL OUTCOMES

Ashleigh Holmes, Weijun Wang, and Yu-Ping Chang, *University at Buffalo, The State University of New York, Buffalo, New York, United States*

Older adults experience higher rates of pain than any other age group, with a domino effect on quality of life and well-being. Existing literature reveals associations between pain and adverse psychosocial, physical, and cognitive outcomes, with psychosocial variables linking all other aspects of well-being in older adults. Therefore, a comprehensive understanding of the associations of psychosocial variables with clinical outcomes in older adults with pain is needed. Grounded in the Biopsychosocial Model of Chronic Pain for Older Adults, we analyzed cross-sectional 2021 data from the nationally representative survey conducted by the National Health and Aging Trends Study. We performed latent class analysis to identify psychosocial clusters (including anxiety, depression, affect, self-realization, resilience, and social participation) of older adults with pain and then used multiple regression analyses to compare pain characteristics, physical health, and cognition among different clusters. We identified four clusters: "Intermediate Psychosocial Phenotype," "Favorable Psychosocial Phenotype," "Adverse Psychosocial Phenotype," and "Compensated Psychosocial Adversity." Clusters with more psychosocial adversity had worse pain, physical, and cognitive outcomes while controlling for race/ethnicity, gender, and age. Increased self-realization and resilience were seen in clusters with better clinical outcomes. Our findings provide important information for precision pain management interventions for older adults based on their psychosocial phenotypes. Further research should analyze longitudinal trajectories of psychosocial phenotypes as older adults' pain experience progresses. Clinically, pain treatment decisions should include a comprehensive evaluation of psychosocial factors, with associated health policy initiatives aimed at assessing differences in care utilization and cost among older adults based on phenotype.

Abstract citation ID: igad104.3542
### PTSD IS ASSOCIATED WITH LONGITUDINAL CHANGES IN DOMAIN-SPECIFIC COGNITIVE FUNCTION IN OLDER ADULTS

Karen Lawrence[1], Hannah Speaks[1], Erin Abner[1], Frederick Schmitt[1], Jennifer Vasterling[2], Brian Smith[2], and Suzanne Segerstrom[3], *1. University of Kentucky, Lexington, Kentucky, United States*, *2. Boston University Chobanian & Avedisian School of Medicine, Boston, Massachusetts, United States*, *3. Oregon State University, Corvallis, Oregon, United States*

Posttraumatic stress disorder (PTSD) is associated with an increased risk for all-type dementia yet whether PTSD is associated with domain-specific cognitive decline, that may lead to dementia, is unknown. Using existing data from the National Alzheimer's Coordinating Center, we tested the hypothesis that PTSD is associated with cognitive decline in episodic memory, working memory and attention, and executive function. After exclusion for history of dementia, stroke, Down syndrome, Parkinson's and Alzheimer's diseases, schizophrenia, and neurodevelopmental disorders, 130 (1.25%) participants with PTSD and 10,261 (98.75%) without PTSD remained. Prior to analysis, inverse probability of treatment weighting was used to adjust for confounding. Inverse probability estimates were based on PTSD status and conditional on covariates for age, sex, race, ethnicity, APOE ε4 status, anxiety, depression, TBI status, diabetes, and hypertension. Generalized estimating equations (GEE)-type linear regression models with robust standard errors were fit to the weighted data for z-scores for each cognitive domain. During the study, unexpectedly, PTSD was associated with an additional .043 standard deviations of improvement in episodic memory (b = .043, 95%CI: .020, .066) compared to the same population without PTSD. Expectedly, PTSD was associated with an additional .143 standard deviations of decline in working memory and attention (b = -.143, 95% CI: -.225, -.062) and an additional .415 standard deviations of decline in executive function (b = -.415, 95%CI: -.563, -.267). Investigations are ongoing to probe the unexpected finding for episodic memory. These findings suggest that PTSD should potentially be a target for early prevention of cognitive decline.

Abstract citation ID: igad104.3543
### PURPOSE PROMOTES MENTAL HEALTH FOR RETIREES, BUT THEY NEED TO HELP FINDING IT

Rachel Best[1], Anthony Ogliore[2], Payton Rule[3], and Patrick Hill[3], *1. Yeshiva University, Bronx, New York, United States*, *2. Saint Louis University, St. Louis, Missouri, United States*, *3. Washington University in St. Louis, St. Louis, Missouri, United States*

Previous research shows that sense of purpose is significantly related to mental health outcomes, including depression and anxiety (Crego et al., 2021; Dixon, 2007). Retirees are at higher risk for having a lower sense of purpose (Hill & Weston, 2019; Pinquart, 2002). Accordingly, helping retirees increase their sense of purpose may be a good way to also improve their mental health. First, research is needed to understand the relationship between sense of purpose and anxiety and depression in retirees. The current study uses cross-sectional data from 1975 adults (516 retirees and 1459 non-retirees; mean age = 49.23, SD = 18.29 ). Pearson r correlations were run between sense of purpose (Life Engagement Test), depression (PHQ-9) and anxiety (GAD-7) in retirees and non-retirees. T-tests were run between retirement status, sense of purpose, depression, and anxiety. As expected, sense of purpose was associated with lower depression (retirees r = -.33, p <.001 ; non-retirees r = -.28, p <.001) and anxiety (retirees r = -.32, p <.001 ; non-retirees r = -.29, p <.001) for both retirees and non-retirees. Results also showed that retired older adults had lower sense of purpose than older adults who are still working (t(1090) = -4.8778, p < .001). These data suggest that targeting sense of purpose in retirees is a worthwhile goal that may also indirectly improve mental health outcomes.

Abstract citation ID: igad104.3544
### QUALITY OF LIFE AND NUTRITION SCREENING: OVERLAPS, OPPORTUNITIES, AND IMPLICATIONS FOR HEALTHY AGING

Mary Beth Arensberg[1], Jaime Gahche[2], Raquel Clapés Pemau[3], Kirk Kerr[1], and Johanna Dwyer[2], *1. Abbott*

*Nutrition, Columbus, Ohio, United States*, 2. *National Institutes of Health, Bethesda, Maryland, United States*, 3. *Abbott, Granada, Andalucia, Spain*

Quality of life (QoL) is important for healthy aging, both for older adults themselves and healthcare providers/systems caring for them. Eating/nutritious food are essential for maintaining good nutrition status, provide pleasure, help older adults remain healthy/independent, and contribute to important QoL domains. Yet research on nutrition and QoL is limited. This study describes connections between QoL and nutrition by examining QoL instruments and nutrition screening tools used with community-living older adults. 20 validated QoL instruments were evaluated to determine if they included nutrition-related items involving 8 previously identified QoL domains. 75% of QoL screening tools included at least 1 nutrition-related item, most commonly in autonomy (n=11), physical health (n=7), social connection (n=3), environment (n=3), emotional state (n=2), mental health (n=2), and personhood (n=1) domains; none in spiritual feeling domain. 16 validated nutrition screening tools were evaluated to determine how their nutrition items related to the same specific QoL domains; all tools included at least 1 nutrition item related to physical health (n=16). Other domains represented by nutrition items were autonomy (n= 9), emotional state (n=5), social connection (n=5), environment (n=4), and mental health (n=3). Although commonalities existed between QoL instruments and nutrition screening tools in types of nutrition-related items included, there were many inconsistencies/gaps. Only 1 QoL instrument and no nutrition screening tools included items in the personhood domain that were related to enjoyment in eating. Nutrition is a potentially modifiable factor benefiting healthy aging and QoL outcomes; these gaps represent opportunities for further research and policy and program development.

Abstract citation ID: igad104.3545

### QUALITY OF LONG-TERM CARE FACILITIES AND COVID-19 OUTCOMES: A SYSTEMATIC REVIEW

Xiaochuan Wang, and Gabriella Eyler, *University of Central Florida, Orlando, Florida, United States*

U.S. long-term care facilities (LTCFs) have been disproportionally and negatively impacted by the COVID-19 pandemic, reporting high shares of COVID-19 cases and deaths. It is critical to understand whether and how quality of these facilities is related to COVID-19 outcomes, to inform policy and practice to better protect the vulnerable LTCF residents and prepare for future outbreaks. Yet, research on this topic has primarily focused on nursing homes which has yielded inconsistent findings, and less is known in the assisted living care setting. To help address this gap, we systematically reviewed existing literature describing quality indicators and examining their impacts on COVID-19 outcomes in LTCFs. Using PRISMA guidelines, we searched electronic databases (e.g., PubMed) for peer-reviewed articles published in English between 2020 and 2023. The search yielded 1,487 unduplicated publications. After screening articles for relevance based on titles and abstracts, 123 were reviewed in full text. A total of 25 articles were included in this review. The included articles examined a range of quality indicators, such as CMS overall and sub-domain 5-star ratings and previous infection prevention and control deficiencies. Overall, the studies revealed mixed results regarding the association between quality and COVID-19 outcomes in LTCFs. Most studies found that higher quality is generally indicative of better COVID-19 outcomes, and some further suggested that the association varied across pandemic phases. Whereas another 7 studies found no significant relationship. This systematic review highlights the need for future research and evidenced-based policy and practice to better protect the at-risk LTCF resident population.

Abstract citation ID: igad104.3546

### RACIAL AND ETHNIC DISPARITIES IN SOCIAL ISOLATION AND 11-YEAR DEMENTIA RISK AMONG OLDER ADULTS IN THE UNITED STATES

Jason Grullon, Daniel Soong, and Roger Wong, *SUNY Upstate Medical University, Syracuse, New York, United States*

Social isolation has been linked to cognitive impairment. Research on this relationship, however, remains limited because validated social isolation measures have been underutilized when examining dementia risk, while also considering differences among racial-ethnic groups. To address this gap, we analyzed 11 years of data (2011–2021) from the National Health and Aging Trends Study to examine the interplay between social isolation, race-ethnicity, and dementia. We constructed a longitudinal score using a validated social isolation variable for our sample of 6,155 community-dwelling U.S. adults aged 65 years and older. After applying sampling weights and imputing missing data, our analysis revealed a higher average longitudinal frequency of social isolation among Black (27.6%), Hispanic (26.6%), and Asian (21.0%) older adults compared to non-Hispanic White (19.1%) respondents during the 11-year period (t=-7.35, p<.001). Cox regression models indicated higher frequency of social isolation was significantly associated with a 45% increased dementia risk after adjusting for sociodemographic covariates (adjusted Hazard Ratio [aHR]=1.45, 95% CI=1.14-1.84, p<.01), but this association was non-significant after further adjusting for health covariates (aHR=1.16, 95% CI=0.92-1.46, p=.20). Race-ethnicity was also not a significant moderator in the association between social isolation and dementia. Our results suggest older adults of color experience higher longitudinal frequency of social isolation. Despite the elevated dementia risk we observed among older adults with higher social isolation, race-ethnicity did not moderate this relationship. Future research is needed to investigate the underlying mechanisms contributing to racial-ethnic disparities for social isolation, and develop targeted interventions to mitigate the associated risk for dementia.

Abstract citation ID: igad104.3547

### RACIAL COMPARISONS OF RECEPTIVITY TO A BLOOD-BASED BIOMARKER TEST FOR ALZHEIMER RISK

Eleanor Skemp, Hyun Jin Cho, and Brian Carpenter, *Washington University in St. Louis, St. Louis, Missouri, United States*

Blood-based biomarker (BBB) tests to assess risk for developing Alzheimer disease (AD) have recently been approved for clinical use. Little is known about people's interest and concerns about these new tests, although the relative simplicity of the tests may help reduce disparities in early diagnosis for AD in historically marginalized groups, such as African Americans. The current report uses data from a national survey of adults in the United States (N = 1,274) conducted in 2022, oversampling African Americans. In addition to demographic questions, respondents rated the importance of different factors they would weigh when deciding whether to take a BBB test and their trust with the healthcare system. The sample was diverse in age (M = 48.6, SD = 17.6, range = 18-91), balanced in gender (55.3% female) and race (48.1% African American), and ranging in education (41.1% < BA). A higher percentage of respondents rated the accuracy (69.2%) and usefulness (64.5%) of the test as very important to their decision, compared to cost (40.8%) and convenience (38.0%). However, African Americans rated cost, convenience, confidentiality, and the potential for learning about other medical problems as more important than White respondents (chi-square p's < .01). Contrary to hypotheses, trust in the healthcare system was not significantly related to decisional factors. Results from this study have implications for how to tailor culturally sensitive messages about new tests for the early detection of dementia risk.

Abstract citation ID: igad104.3548
### RACIAL DIFFERENCES IN HEARING LOSS AMONG MIDDLE-AGED AND OLDER ADULTS
Jessica West, Sherri Smith, and Matthew Dupre, *Duke University, Durham, North Carolina, United States*

Prior studies have documented how social and systemic factors contribute to racial differences in health; however, less is known about the life course and social determinants of racial differences in hearing loss (HL) among middle-aged and older adults. Evidence from U.S. population-based cohorts indicates that Black adults have better pure-tone thresholds and a 40-70% lower prevalence of HL compared to White adults. However, beyond documenting racial differences in general hearing sensitivity (i.e., degree of HL), it is unknown how the characteristics of HL (e.g., type, laterality, etc.) vary by race. Using ICD codes and the AMCLASS classification system, we will evaluate types of HL (e.g., sensorineural, conductive, and mixed HL); symmetry categories (e.g., symmetrical or asymmetrical HL); and severity categories (by classifying pure-tone average according to standard metrics: normal [-10-15 dB HL], slight [16–25 dB HL], mild [26–40 dB HL], moderate [41–55 dB HL], moderately severe [56–70 dB HL], severe [71–90 dB HL], and profound [>90 dB HL]). We will examine audiological data on 22,905 adults aged 50 and older (17.9% Black, 82.1% White) using electronic health records (EHR) and linked area-level data from a large healthcare system. We will also investigate how socioeconomic (e.g., education, income, health insurance), psychosocial (e.g., mental health), behavioral (e.g., smoking), and clinical factors (e.g., disease comorbidities) are associated with HL characteristics in White and Black adults. Findings will provide important and actionable insights into racial differences in HL.

Abstract citation ID: igad104.3549
### RACIAL DIFFERENCES IN THE RELATIONSHIP BETWEEN LONELINESS AND COGNITION AMONG OLDER ADULTS IN THE MIDWEST
Wade Catt[1], Nicole Fowler[1], Brea Perry[2], Siyun Peng[2], and Monica Williams-Farrelly[1], *1. Indiana University School of Medicine, Indianapolis, Indiana, United States*, *2. Indiana University, Bloomington, Indiana, United States*

Findings from various studies have revealed a relationship between loneliness and negative health outcomes, including cognitive decline and dementia. The strength and direction of this relationship has been contested as there is wide variability in definitions and testing criteria for loneliness. If loneliness is risk factor for cognitive decline, it may represent a cost-effective site for interventional design. We used data from the Precision Health Initiative's Person to Person Health Interview Study (P2P), a cross-sectional survey conducted in Indiana from 2020-2021, to investigate the relationship between loneliness (UCLA 3-item loneliness scale) and cognition (The Montreal Cognitive Assessment; MoCA) among older adults and to determine if the strength of the relationship varies for black and white adults. Among our subsample of adults 55 and older, over one quarter (26.7%) reported loneliness, with white respondents reporting more loneliness than black respondents (26.8 and 22.0%, respectively). Being lonely was associated with lower cognition, as was being older, male, black, and having no college education. However, we found that loneliness was associated with worse cognition, for white adults only. Although black respondents in our sample reported more loneliness than older white adults after age 70, we did not have adequate power to determine if advanced age moderated the relationship. Our findings highlight the role of loneliness in cognition for older, white adults and the need for more research to assess this relationship for the "mid-" and "oldest-old" black adults who may be more susceptible to loneliness due, in part, to racial disparities in mortality.

Abstract citation ID: igad104.3550
### RACIAL DISCRIMINATION TRAJECTORIES AND MULTIMORBIDITY BURDEN AND CHANGE AMONG MIDDLE-AGED AND OLDER BLACKS
Kellee White Whilby[1], Kaitlynn Robinson-Ector[1], and Ana Quiñones[2], *1. University of Maryland, College Park, Maryland, United States*, *2. Oregon Health & Science Institute, Portland, Oregon, United States*

Racial discrimination is a chronic psychosocial stressor that can accelerate aging. Characterizing longitudinal patterns of racial discrimination may provide greater precision in predicting health risk particularly for outcomes such as multimorbidity that is considered a proxy of multisystem dysregulation and a marker of cumulative disease burden. However, longitudinal patterns of racial discrimination and its relationship with multimorbidity has received limited attention. We investigate the association between racial discrimination trajectories (RDT) and multimorbidity among a subsample of middle-aged and older Black adults from the Health and Retirement Study (2006–2020, N = 2,167, 50+). RDTs were constructed based on the Everyday Discrimination Scale using repeated measures latent profile analyses. Multimorbidity was operationalized using the multimorbidity weighted index (MWI) score. Linear mixed models, adjusted for sociodemographic, behavioral, and clinical covariates, estimated the association between RDT and MWI score and change in score over time. Average MWI scores varied across RDT classes: low (5.9), moderate (6.5), and high (5.9). Individuals characterized in the moderate

RDT had the highest multimorbidity burden (3.03; 95% confidence interval [CI]: 1.46, 4.58) and was the only group that experienced an accelerated rate of multimorbidity accumulation, as measured by change in MWI score over time. This relationship was not observed among respondents in the persistently high RDT. Our findings suggest that there are differences in the relationship between RDT and multimorbidity burden. Uncovering specific characteristics associated with the profiles of RDT may advance our understanding of healthy aging processes.

Abstract citation ID: igad104.3551

### RACIAL REPRESENTATIVENESS AND AFFORDABLE HOUSING DISPARITIES: A NATIONAL STUDY OF NURSING ASSISTANTS

Sarah Holmes[1], Merve Gurlu[2], Shijun Zhu[1], Briana Murray[1], and Michael Lepore[1], *1. University of Maryland School of Nursing, Baltimore, Maryland, United States*, 2. *University of Maryland, Baltimore, Baltimore, Maryland, United States*

Occupational racial representation is increasingly recognized as a health equity issue. In the US, racial unrepresentativeness in the healthcare workforce, including nursing homes (NHs), is a pervasive aspect of economic inequality: racially minoritized women are disproportionately overrepresented among nursing assistants (NAs) in NHs, and racially minoritized NAs commonly live in poverty and lack affordable housing. Despite evidence of associations between occupational racial representation and affordable housing, there is limited research on these associations among NAs in NHs. We examined how state-level racial representativeness of NAs in NHs relates to housing affordability using data from the PHI Workforce Data Center, LTCFocus, and US Census. Racial representativeness was calculated as the difference between the percentage of racial group (Black, White) in NA workforce in the state and the percentage of racial group in state working-age population. Multivariable linear regression was used to examine associations between housing affordability and racial representation. The national sample included 527,480 NAs. Results showed overrepresentation of Black NAs (M=17.5%, SD=.166) and underrepresentation of White NAs (M=-18.2%, SD=.127) in most states. Controlling for NH primary payer source and NA education level, lack of affordable housing was associated with higher representation of Black NAs in the workforce (b=1.78, p<.001, R2=.66) and lower representation of White NAs in the workforce (b=-1.45, p<.001, R2=.61). These findings indicate racial disparities in NA access to affordable housing and highlight the need for additional research on these associations and related outcomes to develop equity-centered strategies in the NH workforce that will address housing affordability.

Abstract citation ID: igad104.3552

### RACIAL/ETHNIC DIFFERENCES IN SUBJECTIVE COGNITIVE DECLINE (SCD) AND SCD-INDUCED OUTCOMES.

Moyosoreoluwa Jacobs, Peter Lichtenberg, and Wassim Tarraf, *Wayne State University, Detroit, Michigan, United States*

Alzheimer's disease and related dementias (ADRD) prevalence will double by 2060, with significant racial/ethnic (R/E) differences in rates. Understanding R/E differences in risk factors of subjective cognitive decline (SCD)—a precursor to ADRD—is critical for timely premorbid interventions to modify ADRD onset. We used 2015 CDC Behavioral Risk Factor Surveillance System data from non-Hispanic White (NHW), Black (NHB), and Hispanic individuals aged ≥45 years (n=142,657) to examine R/E differences in SCD Status and five SCD-induced functional limitations and healthcare outcomes (e.g., health provider communication). We used generalized linear modeling to assess the contributions of predisposing (e.g., age), enabling (e.g., insurance), and health need (e.g., chronic disease) factors to explaining R/E differences. NHWs were generally older and healthier, with higher educational attainment than NHBs and Hispanics. NHBs (13.5%) were slightly more likely to endorse SCD (11.5% overall). NHBs and Hispanics endorsing SCD were significantly more likely than NHWs to report SCD-induced functional limitations. R/E differences were minimally attenuated after adjusting for the above-mentioned factors. We found no R/E differences in getting access to help, or in communicating with healthcare providers. NHBs and Hispanics (vs. NHWs) endorsing SCD are more likely to also endorse functional limitations despite reporting equivalent access to help and communication with healthcare providers. Clinical cognitive impairment depends on functional status and as such NHBs and Hispanics are potentially more vulnerable to cognitive disease (e.g., ADRD) onset. More research is required to understand gaps in care and how interventions can help mitigate this differential risk.

Abstract citation ID: igad104.3553

### RACISM AND DISCRIMINATION EXPERIENCES AMONG STAFF IN LONG TERM CARE HOMES

Liwam Nerayo[1], Jasmine Travers[2], and Laura Wagner[1], *1. University of California, San Francisco, San Francisco, California, United States*, 2. *New York University, New York City, New York, United States*

In long term care facilities (LTCFs), there is a growing population of people with dementia and an increasingly racially and ethnically minoritized care workforce. Previous research has shown that relationships between residents with dementia and minoritized care workers have been contentious, involving several racial and discriminatory acts (e.g., racial slurs, physical abuse). This has led to an unsupported and burnt out care workforce. The aim of this study is to develop a survey tool, aimed at examining experiences and understanding the prevalence of racism and discrimination in the LTC care workforce. We pilot tested the study in two LTCFs and performed content validity with experts. We then deployed the survey across 560 facilities in racially and ethnically diverse states. When analyzing responses from the first 113 nursing assistants and vocational nurses, we found that many of the respondents reported having positive perceptions of residents with dementia and no experiences with racism. However, among the one-third of care workers who did experience racism, we discovered that respondents primarily experienced racism through verbal insults (48%) and care refusal (23%). Over 87% of victims of this experience felt supported by their manager, yet only 50% reported getting reassigned. Finally, almost 80% responded that it is more challenging to care for residents who exhibit racist behavior. This feedback on the perspectives and experiences of the care workers who did experience racism can inform improvements for dementia care training and offer discussions among stakeholders and families. Additional data from more diverse LTCFs is forthcoming.

Abstract citation ID: igad104.3554

### REAL EXPERIENCE OF FRONT-LINE NURSING HOME STAFF DURING COVID-19: A SYSTEMATIC REVIEW AND META-SYNTHESIS

Xiufen Yang[1], Chaoran Qu[1], Weisi Peng[1], Lile Xiong[1], Hua Guan[1], Weixiang Luo[1], and Yiping Cheng[2], *1. Shenzhen People's Hospital, Shenzhen, Guangdong, China (People's Republic)*, *2. Shenzhen Hospital of Southern Medical University, Shenzhen, Guangdong, China (People's Republic)*

**Objective:** The COVID-19 has become less of a threat around the world. However, the threat to nursing homes and older adult still exists. The study aimed to explore the challenges and coping strategies of front-line nursing home staff during COVID-19 and provide recommendations for coping strategies and measures for public health of emergency in nursing homes in future. Design: Qualitative Meta-synthesis. Date sources: From the beginning until 30 May 2023, searches were conducted in five international databases (CINAHL, PubMed, Web of Science, PsycINFO and Cochrane library). The studies were screened and reviewed separately by two reviewers using the checklist from Joanna Briggs Instifor assessing qualitative research. A pragmatic meta-aggregator approach was applied to synthesise the findings. **Results:** The meta-synthesis included 16 qualitative studies and four mixed studies, which included 642 participants were analyzed to identify 199 findings that were organized into 15 categories and combined into three syntheses. Three synthesised findings were concluded: ①The challenge of the front-line nursing home staff during the COVID-19, ②The coping strategies of the front-line nursing home staff during the COVID-19, and ③Suggestions for future nursing homes to deal with public health of emergency. Conclusion(s): Social/Health organizations and government, especially health organization, should make joint efforts to provide guidance knowledge and practice, and long-term material and mental support for nursing homes. To improve the quality of life of residents and staffs in nursing homes, further research should implement high-quality interventions based on the victory fighting epidemic experience and clinical treatment.

Abstract citation ID: igad104.3555

### RECOGNIZING THE ROLE OF PETS IN HOME ASSESSMENTS FOR OLDER ADULTS

Carlyn Ellison Keeler[1], Linda Struckmeyer[2], and Jennifer Applebaum[2], *1. University of Illinois at Urbana-Champaign, Champaign, Illinois, United States*, *2. University of Florida, Gainesville, Florida, United States*

To support aging in place, health professionals may identify safety and accessibility concerns via home assessments. Although 50% of older adults share their homes with pets, standardized home assessments often overlook pet care, an instrumental activity of daily living (IADL). To bridge this gap in existing knowledge, we conducted four focus group sessions with older adult pet owners to identify potential elements for a novel assessment tool. We utilized directed content and thematic analysis, informed by the International Classification of Functioning, Disability and Health model. Researchers recruited a purposive sample of older adults (65 years or older) who were the primary caretakers of a dog or cat. Participants (N=30) discussed age-related changes to their body's function that hindered their participation in pet care activities (e.g., the strength needed to pick up a pet). Participants explained how the interaction of their pets and housing features (e.g., stairs) contributed to falls and injuries. Further, personal factors (e.g., restricted income) may contribute to home safety issues (e.g., unable to afford needed modifications). The results indicate that pets' behavior, care needs, and caregiving activities may impact home safety, thereby influencing the potential for aging in place. Findings support the development of holistic approaches when evaluating older adults' functioning in their homes and emphasize the importance of pet care within home assessments.

Abstract citation ID: igad104.3556

### RECONSTRUCTING PERSON-CENTEREDNESS FROM A SYSTEMS PERSPECTIVE: IMPLICATIONS FOR THE CARE OF OLDER ADULTS

Ayesha Syed[1], Philip Sloane[1], Sheryl Zimmerman[1], and Sam Fazio[2], *1. University of North Carolina at Chapel Hill, Chapel Hill, North Carolina, United States*, *2. Alzheimer's Association, Chicago, Illinois, United States*

Person-centeredness has become a common, almost hackneyed phrase in the context of care for older adults, despite a lack of consensus regarding its definition, measurement, and application. As part of a series of studies aimed at better understanding person-centeredness, we sought to identify key themes about person-centeredness as it applies to the system (macro) level, as opposed to the setting (meso) and interpersonal (micro) levels. Four think-tank meetings (two in-person; two virtual) were held and results analyzed; the 32 participants included researchers, providers, policy makers, ethicists, and consumers. Think-tank meeting prompts addressed a range of systems, including education, government, workplace, and healthcare. Analyses identified themes associated with variation in person-centeredness. Five key themes emerged: (1) The goals and manifestation of person-centeredness vary depending on the type of system, level within a system, and the role of the individual within the system. (2) Principles of person-centeredness from the hospitality industry can be applied to other settings, including healthcare and long-term care. (3) Some limits on person-centeredness are necessary for system success. (4) The amount of person-centeredness provided in a setting depends on available resources such as time and money. (5) Societal inequities, such as discrimination, bias, and language barriers, hinder person-centeredness. Presentation of results will draw parallels between a variety of systems, demonstrating universal applicability, including to healthcare and long-term care for older adults. The themes identified may help guide future policy and practice regarding realistic and effective application and evaluation of person-centeredness in relation to the care of older adults.

Abstract citation ID: igad104.3557

### RECRUITMENT LESSONS LEARNED FROM A RANDOMIZED PSYCHOEDUCATIONAL TRIAL FOR DEMENTIA FAMILY CAREGIVERS

Maria Aranda, *University of Southern California, Los Angeles, California, United States*

Family caregivers/care partners are the backbone of long-term services and support on behalf of persons living with memory loss such as Alzheimer's and related dementias. Although there has been increased attention on family caregivers overall, documented recruitment strategies to participate in research—and clinical trials in particular—are lacking. This paper addresses the individual and system-level factors addressed in order to reach target recruitment goals in a community-based dementia caregiver trial conducted online with a racially- and ethnically-diverse cohort of mostly spousal/partner and adult children caregivers. Based on a sample of 231 trial participants and 105 focus group interviewees, we drew from our theoretical model—the Micro-Meso-Macro Framework—to elucidate factors that facilitated or impeded diverse cohort recruitment. We analyzed data from two data sources: a) source of referral and reason for contacting the recruitment team; and b) content analysis from focus group interviews. Based on our multi-level analysis we documented the following factors that facilitated trial participation: 1) MICRO: prior encounters with the research enterprise; educational attainment and occupational status; sense of altruism and beneficence; monetary incentivization; comfort with disclosure of private matters; starting from ground zero; and addressing gender issues; and 2) MESO/MACRO: assembling a proactive scientific team; leveraging logos and reputation; training trial staff and community partners; writing project budgets that incentivize diverse trial recruitment; making inroads with community partners; navigating technological challenges; and tapping into resources to alleviate barriers. In our paper, we will discuss lessons learned, and address future research directions including recruiting underrepresented groups.

Abstract citation ID: igad104.3558

### RECURRENT FALLS IN OLDER ADULTS ARE ASSOCIATED WITH MORTALITY AND HOSPITALIZATION

Samita Heslin[1], Elinor Schoenfeld[2], Melanie Keister[3], Sritha Rajupet[1], Kristi Ladowski[1], and Adam Singer[1], *1. Stony Brook University, Stony Brook, New York, United States*, *2. Stony Brook University School of Medicine, Stony Brook, New York, United States*, *3. LINCATS – Associate Director of Technology and Resources, Stony Brook, New York, United States*

Annually, 1 in 4 older adults fall and roughly half have a second fall within 6 months. We studied 57 hospitals in the TriNetX Network. We included all older adults discharged home from the emergency department (ED) after a fall in 2022 for a total of 89,061 patients. Of these, 13,389 (15%) patients returned to the ED for another fall within 6 months. Patients with repeat falls were older (78.7 vs 76.5 years), more likely to have hypertension (76 vs 56%), prior myocardial infarction (14 vs 3%), heart failure (29 vs. 15%), peripheral vascular disease (18 vs 10%), cerebrovascular event (19 vs 10%), chronic obstructive pulmonary disease (COPD) (23 vs 14%), diabetes (40 vs 23%), liver disease (17 vs 11%), chronic kidney disease (30 vs 16%), and cancer (45 vs 32%); P< 0.001. Patients with recurrent falls were more likely to be prescribed cardiovascular medications (84 vs. 66%), central nervous system medications (87 vs 69%), opioids (71 vs 52%), sedatives/hypnotics (59 vs 42%), antipsychotics (23 vs 12%), antidepressants (51 vs 32%), antiparkinsonian agents (9 vs 4%), and antivertigo agents (15 vs 9%); P< 0.001. Patients with a recurrent fall within 6 months had higher hospital admission rate (46.2 vs 15.8%, OR 4.6 [95%CI, 4.4-4.7]) and higher mortality rate (6.2 vs 3.5%, OR 1.83 [95%CI 1.7-2.0]). Older adults discharged home from the ED after falling are at high risk of a second fall and those who have a repeat fall within 6 months are at higher risk of hospital admission and mortality.

Abstract citation ID: igad104.3559

### RED BLOOD CELL DISTRIBUTION WIDTH: WHAT CAN IT TELL US?

Matt Oberdier[1], Marta Zampino[2], Michelle Shardell[3], Majd AlGhatrif[4], Edward Lakatta[5], Eleanor Simonsick[6], and Luigi Ferrucci[7], *1. National Institute on Aging, Bethesda, Maryland, United States*, *2. University of Maryland, Baltimore, Maryland, United States*, *3. University of Maryland School of Medicine, Baltimore, Maryland, United States*, *4. NIA/NIH, Baltimore, Maryland, United States*, *5. NIH/NIA/ LABORATORY OF CARDIOVASCULAR SCIENCE, Baltimore, Maryland, United States*, *6. NIA IRP, Baltimore, Maryland, United States*, *7. National Institute on Aging, Baltimore, Maryland, United States*

Red blood cell distribution width (RDW), a measure of red blood cell size variability used in evaluating anemia, has been found to predict cardiovascular disease (CVD) morbidity and mortality. RDW increases with age, but the determinants of age-associated elevation in RDW independent of CVD and associated risk factors is limited. Therefore, we examined the association of RDW within the normal range (11.5-15.0%) with age, sex, race, other red blood cell properties, major cardiovascular disease risk factors, and other physiologic data. Data were from 606 men and 627 women (aged 50-95) participating in the Baltimore Longitudinal Study of Aging. Candidate correlates of RDW were included in multiple linear regression models; a reduced model was then obtained by backward selection. RDW was higher with age and female sex. Differences in RDW by sex were explained by hemoglobin, mean corpuscular hemoglobin, and mean corpuscular volume. Further, mean platelet volume (STβ=0.0779, P=0.007), interleukin-6 (STβ=0.0774, P=0.011), erythrocyte sedimentation rate (STβ=0.0746, P=0.028), institutional normalized ratio (STβ=0.0836, P=0.004), thyroid stimulating hormone (STβ=0.0653, P=0.023), and body mass index (STβ=0.1725, P=8.98E-8) were all independently associated with RDW. In the final model, age remained a strong correlate of RDW (STβ=0.2412, P=2.91E-12). In summary, inflammation, metabolic rate, and body habitus independently predict RDW, but do not completely explain its association with age. Future investigation of longitudinal change in RDW with age and its association with these and other factors is needed to advance knowledge on the timing and potential mechanisms of age-associated increases in RDW, and its implication for health outcomes.

Abstract citation ID: igad104.3560

### REDEFINING OBESITY: A RATIO OF FAT AND MUSCLE MASS COMPARED TO BODY MASS INDEX IN OLDER ADULTS

Kworweinski Lafontant, Ladda Thiamwong, Jeffery Stout, Joon-Hyuk Park, Rui Xie, and David Fukuda, *University of Central Florida, Orlando, Florida, United States*

Body composition is an important determinant of physical fitness and overall health in older adults. Body mass index (BMI) is currently the standard used to categorize clinical thresholds for body composition (underweight, normal weight, overweight, obese). However, BMI does not account for key aspects of body composition like skeletal muscle mass (SMM) and fat mass (FM). This can lead to misclassification and inaccurate inferences about metabolic health. A FM/SMM ratio may better capture age-related changes in body composition like sarcopenia and obesity, but it is unknown whether FM/SMM correlates more closely with muscularity and adiposity than BMI. This cross-sectional study assessed 103 older adults (16 men, 87 women; mean age 75.7 ± 7.19 years; BMI 26.8 ± 5.22 kg/m2) for SMM, FM, and body fat percentage (BF%) using bioelectrical impedance analysis (InBody S10). BF% was more highly correlated with FM/SMM ($r = 0.979$, $p < 0.001$) than BMI ($r = 0.702$, $p < 0.001$), and a Fisher's R-to-Z analysis indicates that the difference is significant (95% CI [0.826, 0.926], $z = 12.217$, $p < 0.001$). However, SMM showed weak correlation with both FM/SMM ($r = -0.306$, $p = 0.002$) and BMI ($r = 0.349$, $p < 0.001$). FM/SMM may better detect changes in adiposity compared to BMI, suggesting utility as an obesity measure. Further research should examine the longitudinal validity of FM/SMM in older adults and its ability to predict metabolic and musculoskeletal health.

Abstract citation ID: igad104.3561

### REENGAGING AFRICAN AMERICAN AND BLACK OLDER ADULTS DURING THE COVID-19 ERA: A FOCUS GROUP STUDY

Rupal Parekh[1], and Christine Tocchi[2], *1. University of Connecticut, West Hartford, Connecticut, United States*, *2. University of Connecticut, Storrs, Connecticut, United States*

Churches and senior centers are social engagement hubs for older Black and African Americans (BAA) that can positively impact well-being. Although these facilities re-opened after the COVID-19 pandemic, engagement with these facilities has not returned to pre-pandemic levels. Our long-term goal is to develop a behavioral intervention to address the re-engagement needs/concerns of BAA older adults as a means of preventing/reducing depressive symptoms and disability. In the first phase of this work, we used a community-based participatory research approach to determine barriers to, and facilitators of, activity reengagement through the perspectives of leaders of BAA churches and senior centers around Hartford, Connecticut. We conducted three focus groups with key stakeholders (n=4 senior center staff; n=4 senior center staff; n=5 church staff) between May 2023 and July 2023. Using a Framework Method approach and semi-structured interviews, participants were asked to define engagement as it relates to senior center and church involvement since re-opening and to quantify, in their own words, the problem of disengagement. Participants were also asked about the effectiveness of online engagement vs. in-person engagement and members experience(s) with social isolation and loneliness. Line-by-line thematic analysis of the transcriptions was used to identify and group meaningful phrases and responses into major themes. Three organizing themes emerged: "transportation" concerns impacting members during COVID-19 pandemic, increased health issues among members; and issues and concerns related to family support. Leaders from churches and senior centers provided valuable insights into the overarching issues affecting re-engagement among older BAA senior center and church members.

Abstract citation ID: igad104.3562

### RELATIONSHIP BETWEEN LEISURE CONSTRAINTS AND WELL-BEING IN MIDDLE TO OLDER ADULTS

Junyan Tian[1], Angie Sardina[2], Alexa Allan[3], Alyssa Gamaldo[3], and Jacqueline Mogle[4], *1. Penn State University, State College, Pennsylvania, United States*, *2. UNC Wilmington, Wilmington, North Carolina, United States*, *3. The Pennsylvania State University, University Park, Pennsylvania, United States*, *4. Clemson University, Clemson, South Carolina, United States*

Leisure activity engagement is correlated with several health outcomes, such as depression, a lower risk of dementia and better physical health. However, older adults report experiencing leisure constraints in daily lives due to intrapersonal (e.g., stress), interpersonal (e.g., interaction with friends) and structural factors (e.g., availability of opportunity). Since relatively few research investigated the impact of leisure constraints on middle and older adults' eudaimonic well-being, the major aim of this study was to explore whether perceived leisure constraints are uniquely associated with eudaimonic well-being after adjusting for sociodemographic, loneliness, health and neighborhood covariates. This study accessed leisure constraints using Leisure Barriers and Interest Questionnaire, and accessed eudaimonic well-being by 4 items from the Patient-Reported Outcomes Measurement Information System (PROMIS) questionnaire among 378 adults (age range: 40-92, $M = 58.4$). Correlation results indicated that better well-being was correlated with less perceived leisure constraints ($r = -.40$, $p < .01$), greater household income ($r = .14$, $p = .01$), less reported loneliness ($r = -.53$, $p < .01$), greater reported neighborhood safety ($r = -.18$, $p < .01$), and better reported neighborhood quality ($r = .23$, $p < .01$). Linear regressions revealed that individuals who perceived a lower level of leisure constraints associated with better well-being ($\beta = -0.20$, $p < .01$) after adjusting for age, sex, income, health conditions, loneliness, and neighborhood quality and safety. Our results highlight the importance of reducing leisure constraints in intrapersonal, interpersonal and structural levels to help older adults live a meaningful and happy life.

Abstract citation ID: igad104.3563

### RELATIONSHIP BETWEEN QUADRICEPS ECHOGENICITY AND KNEE EXTENSOR STRENGTH IN OLDER ADULTS: A SYSTEMATIC REVIEW

Michael Harris-Love[1], Dustin Oranchuk[2], Katie Boncella[2], Ben Rush[3], and Stephan Bodkin[4], *1. University of Colorado, Centennial, Colorado, United States*, *2. University of Colorado-Anschutz Medical Campus, Aurora, Colorado,*

*United States*, 3. University of Wisconsin-Madison, Madison, Wisconsin, United States, 4. University of Utah, Salt Lake City, Utah, United States

The clinical availability of assessing muscle quality through diagnostic ultrasound provides a viable screening tool for geriatric syndromes that impair mobility. However, relationships between muscle echogenicity and clinical assessments require further inquiry. The authors aimed to systematically identify and synthesize the literature regarding the relationship between quadriceps echogenicity and maximal knee extensor strength in older adults. A total of 12 extracted articles (N=701, 73.6±5.4 years) independently assessed the relationship between quadriceps echogenicity and peak knee extensor torque. Correlations were weak to moderately negative between knee extensor strength and rectus femoris echogenicity in older adults (mean ±standard deviation; r=-0.398±0.159 [range=-0.681 to -0.080]). While the limited number studies did not warrant advanced analyses, but it appears that the echogenicity of the vastus medialis (study n=3, r=-0.426±1.97 [range=-0.640 to -0.253]) and vastus lateralis (n=3, r=-0.411±0.095 [range=-0.511 to -0.322]) may be superior to the vastus intermedius (n=4, r=-0.341±0.166 [range=-0.484 to -0.120]) and rectus femoris (n=7, r=0.290±0.140 [range=-0.460 to -0.080]) for predicting knee extension strength in older adults. Furthermore, averaging across multiple quadriceps muscles generally improved correlations (n=4, r=-0.544±0.154 [range=-0.681 to -0.334]). Overall, correlations with knee extensor strength were stronger when echogenicity was corrected for subcutaneous fat thickness (n=3, r=-0.491±0.04 [range=-0.521 to -0.453]). However, results regarding fat correction were inconsistent within studies (n=2, uncorrected r=-0.120 and -0.527 versus corrected r=-0.500 and -0.453, respectively). Future studies should aim to report correlations for all quadriceps muscles and for both corrected and uncorrected echogenicity values to allow for more thorough analytical approaches.

Abstract citation ID: igad104.3564

### RELATIONSHIPS BETWEEN DISTRESS AND NEIGHBORHOOD CHARACTERISTICS FOR MEXICAN AMERICAN CAREGIVERS

Florence Johnson[1], Stipica Mudrazija[2], Sheria Robinson-Lane[3], Shekinah Fashaw-Walters[4], and Phillip Cantu[5], *1. University of Michigan, Ann Arbor, Michigan, United States*, *2. University of Washington, Seattle, Washington, United States*, *3. University of Michigan School of Nursing, Ann Arbor, Michigan, United States*, *4. University of Minnesota, Minneapolis, Minnesota, United States*, *5. UTMB, Galveston, Texas, United States*

Previous study demonstrates that caring for older relatives with physical or cognitive disability can increase caregiver stress—particularly if neuropsychiatric symptoms are present. The literature suggests that community features such as the percentage of immigrants may improve Mexican-American caregivers' mental health. Using matched 2011/2012 data on caregivers and care recipients from the Hispanic Established Population for the Epidemiological Study of the Elderly(H-EPESE) and the 2010 census tract characteristics from the National Neighborhood Data Archive (NANDA) recently, we fit multilevel regression models to assess how neighborhood characteristics (census tract proportion Latino, proportion foreign-born, and proportion in poverty) affect caregiver distress (caregiver depression score, caregiver general stress, caregiver reports of neuropsychiatric behavioral symptoms of cognitive decline and caregiver report of stress from neuropsychiatric behavioral symptoms of cognitive decline). We found that living in an area with a higher proportion of foreign-born adults is related to lower reports of neuropsychiatric symptoms and lower levels of caregiver distress. Family caregivers residing in ethnic enclaves with high proportions of foreign-born persons reported significantly fewer symptoms of feeling stubborn and resistive, being in low spirits, feeling upset when separated from the care recipient, losing interest in usual activities, or experiencing changes in weight. While residing in these communities may reduce behavioral concerns in care recipients and increase social support availability, cultural factors such as ideas about normative aging may influence symptom reporting. Increasing available support for caregivers residing in ethnic enclaves is an important step toward developing dementia-capable communities and reducing caregiver health disparities.

Abstract citation ID: igad104.3565

### RELIGIOSITY AND SENSE OF PURPOSE IN WHITE AND BLACK OLDER ADULTS

Selin Toprakkiran, Megan Wilson, Thomas Oltmanns, Ryan Bogdan, and Patrick Hill, *Washington University in St. Louis, St. Louis, Missouri, United States*

Religiosity is a key factor in the lives of many older Americans, and it may help people develop a sense of purpose. This association is highly nuanced, across groups and measures. For instance, religiosity plays a different role across racial/ethnic groups in the United States, which may influence its association with sense of purpose. To examine whether the relationship between religiosity and sense of purpose differs across White and Black Americans, we used the St. Louis Personality and Aging Network data (N = 732), collected from older adults (M age = 67.96; SD age = 4.31; 76.37% White, 23.63% Black American). We used data from multiple waves to examine religiosity, sense of purpose, and social engagement. To disentangle different aspects of religiosity, we included three items focusing on the meaning of religion to one's life, participation in religious activities, and how much religion influences one's daily life. Black participants scored higher on all religiosity items compared to White participants. Nevertheless, the relationship between religiosity and sense of purpose did not differ based on one's racial/ethnic identity or the aspect of religiosity. Additionally, social support did not moderate relationships between religiosity and sense of purpose, suggesting that these relationships are similar regardless of whether one perceives more or less social support from others. These findings add to our understanding of when and for whom religiosity correlates with purpose among older adults, and future directions include examining the influence of religiosity on changes in purpose over time.

Abstract citation ID: igad104.3566

### SELF-REPORTED AND OBJECTIVE ASSESSMENT OF HEARING HANDICAP AND COGNITIVE CHALLENGES IN AGE-RELATED HEARING LOSS

Shraddha Shende[1], and Raksha Mudar[2], *1. Illinois State University, Normal, Illinois, United States, 2. University of Illinois Urbana-Champaign (UIUC), Champaign, Illinois, United States*

Age-related hearing loss (ARHL) is a common condition in older adults, with most reporting challenges in recognizing speech-in-noise (SiN). Growing evidence also indicates cognitive alterations in this population. Concomitant examination of hearing and cognitive abilities using a combination of subjective and objective measures would be useful in characterizing the challenges faced by individuals with ARHL. We examined hearing abilities and cognitive functions in 15 individuals with bilateral mild-to-moderate untreated sensorineural ARHL (age: 70.4 ± 7.4 years, pure tone average: 31.0 ± 3.8 dB HL) and 15 age- and education-matched normal hearing controls (age: 65.7 ± 5.8 years, pure tone average: 15.4 ± 5.9 dB HL). We examined subjective ratings of hearing handicap (Hearing Handicap Inventory for Adults) and cognitive ability (Self-rating of Cognition in Everyday Activities), and objective measures of SiN (Quick Speech-In-Noise) and cognition (Montreal Cognitive Assessment [MoCA] and MoCA-memory index score [MoCA-MIS]). Our analysis revealed that individuals with ARHL self-reported a greater number of cognitive issues in everyday activities and had a higher hearing handicap score (p=.044) relative to controls. Additionally, the ARHL group performed significantly worse on SiN (p=.005) and cognitive screening (MoCA, p=.031; MoCA-MIS, p=.039). Taken together, these findings suggest that in addition to experiencing challenges with hearing abilities, individuals with mild-to-moderate ARHL also experience cognitive changes. Furthermore, these challenges are self-perceived as well as noticeable on objective testing. Our work points towards the consideration of self-report and objective assessment of cognition in geriatric hearing care, which can further inform the development of novel interventions.

Abstract citation ID: igad104.3567

### TELEWORKING TO SUPPORT ACCOMMODATION, INCLUSION, AND HEALTH OF OLDER WORKERS: ISSUES FACED BY MANAGERS

Alexandra Lecours, *Université du Québec à Trois-Rivières, Drummondville, Quebec, Canada*

Telework is increasingly present and has the potential to be used as an accommodation modality to facilitate inclusion and healthy participation in the workplace for older workers (i.e. aged 55 and over). However, there is a need for a practical tool to guide the application of telework with this population. This poster presents the first stage of a study aiming to develop a reflective guide for applying telework to support the accommodation, inclusion, and health of older workers. Following a three-stage developmental research design, this first stage consists in conducting individual interviews with older teleworkers and managers to gather qualitative data on their experience. During the second stage, the issues, considerations, and good practices that emerged from the first stage are compiled into a guide. In the third stage, this guide will be validated by workers and managers to ensure its acceptability and applicability. Preliminary results from the first stage allowed to identify issues faced by managers concerning accommodation (n=8, e.g. ensuring that accommodating older workers does not add extra pressure on colleagues), inclusion (n=5, e.g. difficulty to mobilize older teleworkers for face-to-face social gatherings), and health (n=2, e.g. managers feel powerless to manage older teleworkers' emotions). Managers play a key role in implementing practices that promote the accommodation and inclusion of older workers through teleworking, but they have few tools to support them. The reflective application guide that will be created during this study represents an innovative tool likely to have positive impacts at individual, organizational and societal levels.

### SESSION 9030 (POSTER)

### LATE BREAKING POSTER SESSION IV

Abstract citation ID: igad104.3568

### ASSOCIATION BETWEEN LONG-TERM EXPOSURE TO AIR POLLUTION AND LOWER URINARY TRACT SYMPTOMS IN KOREAN OLDER MEN

Youngmin Jee, *Seoul National University, Seoul, Republic of Korea*

Low Urinary Tract Symptoms(LUTS) is one of the common symptoms in older men and is important factor in terms of quality of life. The purpose of this research was to investigate the association between long-term exposure to air pollution and LUTS in korean older men. The study participants were recruited from a health checkup center of Seoul National University Hospital from January 1st, 2015 to December 31st, 2019. A total of 7,979 participants were aged over 65 years and underwent screening health checkup including International prostate symptom score (IPSS) for LUTS. To evaluate the exposure to air pollution, hourly measurements of air pollutants from nationwide air quality monitoring sites were utilized. The associations between exposure to air pollutants and LUTS were analyzed using logistic analysis in a crude model and an adjusted model, the latter of which included covariates. Most air pollutants including PM10, NO2, SO2, and CO were all significantly associated with LUTS in crude models, but the correlations were no longer significant in the adjusted models. PM2.5 exposures showed significantly positive association with voiding symptoms in adjusted model (OR, 1.07; 95% CI, 1.02~1.13; p=0.012). The associations of other air pollutants with voiding and storage symptoms showed negative tendency in unadjusted model, and some of them were significant. However, the associations were no longer significant in adjusted model. This study demonstrated that air pollutants including PM10, NO2, SO2, and CO are associated with LUTS, particularly PM2.5 showed a positive correlation with voiding symptoms of LUTS in the adjusted model.

Abstract citation ID: igad104.3569

### GLYCATION-LOWERING COMBO EXTENDS LIFESPAN, IMPROVES INSULIN, REDUCES CALORIC INTAKE

Pankaj Kapahi, *Buck Institute for Research on Aging, Novato, California, United States*

Non-enzymatic reactions in glycolysis lead to the accumulation of methylglyoxal (MGO), a reactive precursor to advanced glycation end-products (AGEs), which has been suggested to drive obesity- and aging-associated pathologies. We observe that a combination of nicotinamide, lipoic acid, thiamine, pyridoxamine and piperine, which were selected to lower glycation (Gly-Low), reduce toxic glycolytic byproducts, MGO and MGO-derived AGE, MG-H1. Administration of Gly-Low reduced food consumption and body weight, improving insulin sensitivity and survival in both leptin receptor deficient (Lepr db) and wildtype C57 control mouse models. Unlike calorie restriction, Gly-Low inhibited ghrelin-mediated hunger responses and upregulated Tor pathway signaling in the hypothalamus. Gly-Low also extended lifespan when administered as a late life intervention, suggesting its potential benefits in ameliorating age-associated decline by inducing voluntary calorie restriction and reducing glycation.

Abstract citation ID: igad104.3570

### OLDER ADULT MISUSE OF OVER-THE-COUNTER MEDICATIONS: BENEFITS OF A NOVEL PHARMACY-BASED INTERVENTION

Jason Chladek[1], Aaron Gilson[2], Jamie Stone[3], Elin Lehnbom[4], Emily Hoffins[2], Maria Berbakov[2], Jukrin Moon[1], and Michelle Chui[2], *1. University of Wisconsin-Madison School of Pharmacy, Madison, Wisconsin, United States*, *2. University of Wisconsin-Madison, Madison, Wisconsin, United States*, *3. University of Wisconsin - Madison, Madison, Wisconsin, United States*, *4. UiT The Arctic University of Norway, Tromsø, Troms, Norway*

Older adults' inappropriate use of over-the-counter medications (OTCs) is prevalent and comprises: (1) Drug-Age misuse – medication risks due to advanced age, (2) Drug-Drug misuse – medication interactions, (3) Drug-Disease misuse – contraindications with health conditions, and (4) Drug-Label misuse – deviations from label instructions. Since pharmacies are ubiquitous sources of OTC accessibility and medication safety expertise, a structural redesign of pharmacy aisles (Senior SafeTM) was conceived to mitigate older adult OTC misuse. A central feature of Senior Safe is the use of Stop Signs for high-risk OTCs and Behind-the-Counter Signs (BTC) for particularly high-risk OTCs. Given the signage's practical importance, this study' principal aim was to determine whether Senior Safe reduced misuse of Stop Sign/BTC OTCs. Twenty pharmacies from a mid-Western healthcare system were matched and randomly allocated to either control or intervention groups, from which 288 older adults were recruited (ages 65-91). Participants' reported OTC use for a symptom scenario (pain, sleep, cough/cold/allergy) was evaluated to determine whether it fulfilled the four misuse types. Misuse occurrences between intervention/control sites were compared using multivariate modeling. Drug-Age and Drug-Drug misuse frequencies were significantly lower in intervention sites (Coef.=-1.012, p=.004; Coef.=-1.702, p=.004, respectively), while Drug-Disease and Drug-Label misuse had too few occurrences in intervention sites for statistical comparisons. Also, adults aged 85+ had the greatest likelihood of all misuse types. Senior Safe shows substantial benefit reducing high-risk OTC misuse, which is important particularly for vulnerable adults ages 85-91. Future research should document intervention mechanisms contributing to lower older adult OTC misuse.

Abstract citation ID: igad104.3571

### REMOTE FAMILY CAREGIVERS: INVISIBLE YET INDISPENSABLE DURING HOSPITALIZATION OF OLDER ADULTS

Orly Tonkikh, Yuuko Johnson, Heather Young, and Elena O. Siegel, *University of California Davis, Sacramento, California, United States*

Limited research has addressed the interplay between the roles of nursing staff and families of older adults in acute care settings, including those families involved in-person and those who are unable to be physically present (remote). This descriptive qualitative study explores nurses' and family members' perspectives on the roles and engaging family members or friends during an acute hospitalization in medical-surgical units. Semi-structured interviews were conducted via Zoom or phone with a convenience sample of 19 nurses and families between February and August 2023. Thematic analysis inductively captured key patterns in data. Nurses proactively engaged family members who were present in the hospital, while they engaged with remote family members in a more limited way, mostly during discharge planning. Nurses describe making decisions about including families based on their presence (e.g., education about medications, encouraging to engage the patient in the recommended care, ask to support with ADLs). In contrast, families describe providing support regardless of physical presence, including socio-emotional support, help with medical/nursing tasks, encouragement to recover, mediation of information provided by staff, learning to perform medical-nursing tasks and communication and interpretation of the information with other family members. The findings reveal a gap in nurses and family's perspectives about the feasibility and contribution of remote family involvement during an acute hospitalization. This provides direction for further exploration of acute care structures and nursing care processes needed to support the preferred mode for family involvement.

Abstract citation ID: igad104.3572

### REPRODUCIBILITY OF BRAIN AGE SALIENCIES ACROSS DEEP NEURAL NETWORK ARCHITECTURES

Nicholas Kim, Phoebe Imms, Nikhil Chaudhari, Nahian Chowdhury, Anar Amgalan, Paul Bogdan, and Andrei Irimia, *University of Southern California, Los Angeles, California, United States*

The brain's biological age (BA) reflects structural changes related to neuroanatomic senescence. BA is estimated from magnetic resonance images (MRIs) using convolutional neural networks (CNNs), which provide interpretability through saliency maps. By conveying the importance of brain regions to BA estimation, these maps reveal sex differences in brain-aging. We compared two CNN architectures to assess the reproducibility of such differences. Both regression CNNs had T1-weighted MRIs as inputs; outputs were estimated BAs. One CNN was trained on males and females separately, whereas the other was trained on both sexes. The dataset used consisted of T1-weighted MRIs from 5,851 cognitively normal individuals (3,142 females) aged 22 to 95 years, sampled from the Alzheimer's Disease Neuroimaging Initiative (N = 510), Human Connectome Project Aging (N = 508) and Young Adult (N = 1,112), and UK Biobank (N = 3,721). For both models, compared to the average saliency for a null distribution, males' BA estimation relied significantly (p < 0.05) more on the right lateral temporal lobe and superior frontal gyrus. Females' BA estimation depended significantly (p <; 0.05) more on the right posterior and bilateral occipital regions, and medial parietal lobe. Simulated alterations in brain morphometry indicated saliencies correctly revealed regions with aging-related dynamics, which confirm prior findings on sex differences in brain-aging. Variations in CNN models' saliencies did not affect overall anatomic patterns, suggesting that CNNs can capture brain-aging patterns robustly despite architecture differences. Novel statistical models for formal comparison of CNN saliencies should be developed to accommodate their nonlinear nature.

Abstract citation ID: igad104.3573

### RESEARCH BEST PRACTICES TO SUPPORT RESILIENCE IN EYE STROKE VISION REHABILITATION

Kimberly Hreha[1], Nathan Boucher[2], and Heather Whitson[1], *1. Duke University, Durham, North Carolina, United States*, *2. Veterans Health Administration, Durham, North Carolina, United States*

While effective rehabilitation exists to promote independence and community participation for age-related vision impairment, no rehabilitation program has been developed specifically for eye stroke impairment. We are adapting a macular degeneration self-management program using input from key partners to inform future intervention. The pilot phase objective was to inform future study design for eye stroke survivors. We conducted semi-structured interviews, and applied the 5T Framework (Target population identification, Team composition, Time considerations, Tips to accommodate older adults, and Tools for inclusive enrollment of older adults) to develop the interview guide. Twenty-five people recruited from clinical settings were consented/interviewed over six months. This included 10 stroke survivors (M age= 66 years; 70% male--90% visual field defect), 4 care partners (M age= 57 years; 100% female--1 mother, 1 sister and 2 spouses), and 11 providers (M age= 37 years; 54.5% female--3 occupational therapists, 4 ophthalmologists, 2 optical technicians, 1 optometrist, 1 physical therapist). The most meaningful outcomes identified were driving and returning to full independence. The most frequently identified "facilitator of recovery" after eye stroke was family support. The most frequently identified barriers to participation in intervention research were lack of accessibility of the research building and transportation. Optimal lighting and scanning strategies were identified as key elements of any future intervention as well as approaches to support enrollment and retention in study activities. Interview data are now being used to inform phase two: adaptation of the self-management program and design of the study that will evaluate its effectiveness.

Abstract citation ID: igad104.3574

### RESILIENCE AND PSYCHOLOGICAL DISTRESS IN GENETIC TESTING FOR ALZHEIMER'S DISEASE

Alicia Burgei, *Tiffin University, Tiffin, Ohio, United States*

This research project examines the relationship between resilience and psychological distress resulting from genetic testing for Alzheimer's disease among individuals who are non-cognitively impaired. Preventative genetic testing is increasingly important to determine the likelihood of developing or passing on genetic disorders. However, receiving genetic information, especially regarding incurable diseases like Alzheimer's disease, can lead to significant psychological distress. Resilience, the ability to cope with adversity and recover quickly, is considered a protective factor against psychological distress. The purpose of this study is to investigate whether higher levels of resilience are associated with lower psychological distress from genetic testing for Alzheimer's disease. A cross-sectional survey was conducted online using the Impact of Genetic Testing for Alzheimer's Disease (IGT-AD) scale and the Brief Resilience Scale (BRS), along with a question about family history of Alzheimer's disease. The results indicated a significant negative correlation between resilience and psychological distress, suggesting that individuals with higher resilience experienced lower distress related to genetic testing. However, family history did not moderate the relationship, meaning the protective effect of resilience was consistent regardless of familial risk. These findings have implications for developing targeted support services and resilience-based interventions to help individuals cope with the emotional impact of genetic testing for Alzheimer's disease. Further research could explore other potential protective factors and examine the long-term impact of genetic testing results on psychological well-being and behavior.

Abstract citation ID: igad104.3575

### RETIRING AFTER A JOB WELL DONE: HOW SUBJECTIVE CAREER EVALUATIONS IMPACT RETIREMENT ADJUSTMENT.

Orlaith Tunney[1], Kène Henkens[2], and Hanna van Solinge[1], *1. Netherlands Interdisciplinary Demographic Institute-KNAW, The Hague, Zuid-Holland, Netherlands*, *2. Netherlands Interdisciplinary Demographic Institute (NIDI), The Hague, Zuid-Holland, Netherlands*

An individual's past, and how they reflect on it, may influence current and future well-being, with recent qualitative studies suggesting retirees' reflections on their careers may relate to well-being in retirement. Establishing factors that may promote successful transition to retirement is vital. Particularly in light of population ageing and the subsequent increase in both the number of retirees and the length of retirement. The aim of our study was to investigate the impact subjective career evaluations on retirement adjustment at follow-up. Using data from across three waves of the NIDI Pension Panel Study, (conducted in 2015,2018, and 2023) we examined the pre-retirement career evaluations of a sample of Dutch older workers (N= 3566), factors associated with these evaluations, and the impact of subjective career evaluations on retirement at five or eight-year follow-up. The results of our ordinal logistic regression models showed that how individuals evaluated both the work (b= 0.15 p=.001) and family (b= 0.12 p=.001) domain of career was positively associated with retirement adjustment, with those who evaluated their careers more favorably reporting fewer problems adjusting to retirement. Our results demonstrate the long-term impact of subjective career evaluations and highlight the need for additional research evaluating their predictive utility for other important outcomes in older adulthood such as life satisfaction and health. These findings could also be applied to identify and deliver interventions for those at risk of less favorable retirement outcomes.

Abstract citation ID: igad104.3576

### RISK AND PROTECTIVE FACTORS OF COGNITIVE FUNCTIONING AND DEMENTIA: COMPARING MEXICAN AND NON-MEXICAN HISPANICS

Nasim Ferdows, *Northeastern University, Boston, Massachusetts, United States*

This study investigates the cognitive functioning of Hispanic individuals aged 65 and above, focusing on the unique risk and protective factors associated with dementia in Mexican Hispanics and non-Mexican Hispanics. The study utilizes data from the Harmonized Cognitive Assessment Protocol (HCAP) subsample of the Health and Retirement Study (HRS), comprising 388 Hispanic older individuals who participated in the HCAP in 2016 (traced back to 2000), allowing for a comprehensive examination of cognitive health within this population. Cognitive function was measured using the Mini-Mental State Examination (MMSE) score and to classify dementia. The findings reveal distinct patterns in cognitive functioning between Mexican Hispanic and non-Mexican Hispanic populations. Mexican Hispanic males exhibit lower MMSE scores across all age groups, while Mexican Hispanic females show slightly higher scores compared to non-Mexican Hispanic females. Education and wealth levels do not significantly affect cognitive scores in either group. Mexican Hispanics have lower odds of dementia compared to non-Mexican Hispanics, even when accounting for sociodemographic, midlife, and late-life characteristics. Both educational attainment and wealth emerge as protective factors against dementia for both groups. Additionally, midlife obesity, hearing loss, and later-life physical inactivity are identified as specific risk factors for dementia in Mexican Hispanics but have an insignificant impact on non-Mexican Hispanics. These findings have implications for targeted interventions and policies to promote cognitive well-being and address health disparities in older Hispanics. The aim is to improve the overall quality of life and achieve equitable healthcare outcomes.

Abstract citation ID: igad104.3577

### RISK FACTORS FOR FALL-RELATED MILD TRAUMATIC BRAIN INJURIES AMONG OLDER ADULTS: A SYSTEMATIC REVIEW

Prince Okrah, Shafer Tharrington, Isaac Shin, Aaron Wagoner, Katelyn Woodsmall, and Deborah Jehu, *Augusta University, Augusta, Georgia, United States*

Mild traumatic brain injuries (mTBI) are commonly undiagnosed, which delays treatment and recovery. About 80% of older adults' mTBIs stem from falls, yet factors driving fall-related mTBIs are unclear. This study aimed to systematically review risk factors for fall-related mTBIs among older adults. We used the Preferred Reporting Items for Systematic Reviews with Meta-Analysis protocol and the Meta-Analysis of Observational Studies in Epidemiology guidelines (Prospero ID: CRD42023377847). Our scope included prospective studies analyzing risk factors for fall-related mTBI in adults ≥ 60 years. The primary outcome measure was the relative risk for fall-related mTBIs, and secondary outcomes were: fall rate, total falls, and the number of fallers and non-fallers in those with and without a mTBI. On November 4, 2022, we searched: CINAHL Plus, and Health Source: Nursing Academic Edition, Nursing & Allied Health Database, PubMed, SPORTDiscus, and Web of Science. We conducted a grey literature search by examining reference lists of relevant studies. Two authors screened articles, and one author adjudicated any disagreements. Two authors would have assessed the reporting quality using the Strengthening the Reporting of Observational Studies in Epidemiology checklist, and the risk of bias using the Joanna Briggs Institute Prevalence Critical Appraisal tool. The database search yielded 410 articles; after deduplication, 395 titles and abstracts were screened, and 66 articles underwent full-text review. Overall, no studies were eligible. Fall-related mTBIs can have devastating consequences. There is an urgent need to identify risk factors for fall-related mTBIs in older adults to improve screening procedures and therapeutic intervention.

Abstract citation ID: igad104.3578

### ROAD DIETS IMPACT ON DRIVING BEHAVIOR AMONG OLDER ADULTS WITH AND WITHOUT PRECLINICAL ALZHEIMER'S PATHOLOGY

David Carr[1], Julie Wisch[2], Beau Ances[2], and Ganesh Babulal[2], *1. Washington University in St. Louis, St. Louis, Missouri, United States*, *2. Washington University School of Medicine, St. Louis, Missouri, United States*

Road diets (that is, the reallocation of one or more lanes of car traffic to other uses) have been proposed as a modification to increase pedestrian safety, particularly for older adults. We considered the impacts of road diets on aging drivers, and on those with early pathological accumulation of AD. We observed naturalistic driving data for 60 cognitively normal older drivers (Age Range = 62 – 87 years, Median = 75 years) driving across three road segments located in St. Louis, Missouri. We used neuroimaging and lumbar puncture derived biomarker data to determine which of the drivers had preclinical AD. Since previous AD studies identified a variety of changes in driving behavior among older drivers with preclinical AD, we examined driving speed before and after lane repurposing. We found that drivers with preclinical AD drove at lower speeds compared to those without preclinical AD prior to road diet implementation. After lanes were repurposed, there was no statistical difference in the speed between older drivers with and without preclinical AD. We evaluated cognitive performance and found that attentional control had a mediating effect on driver speed, suggesting that an individual's ability to focus on a specific task and filter out distractions was associated with faster driving. Driving speed after lane repurposing is not mediated by attentional control, suggesting that road diets are impervious to individual driver capacity. We conclude that lane repurposing has potential as an important mobility infrastructure solution that could enhance older driver safety and facilitate aging in place.

Abstract citation ID: igad104.3579

### ROLE OF THE METHYLTRANSFERASE METTL21C IN DRIVING MUSCLE ATROPHY WITH AGING AND HIV INFECTION

Jorge Gonzalez Castillo[1], Meghan McGee-Lawrence[2], Taylor Kress[2], Laszlo Kovacs[2], Eric Belin de Chantemele[2], and Mark Hamrick[2], *1. Universidad Central del Caribe, Bayamón, Puerto Rico, United States*, *2. Medical College of Georgia, Augusta, Georgia, United States*

Osteoporosis and sarcopenia are both frequently observed in older people living with HIV. We developed an animal model to investigate the cellular and molecular mechanisms underlying accelerated muscle aging in the setting of HIV infection. Wild-type C57BL/6 mice received bone marrow from Tg26 HIV-1 transgenic mice at 8 weeks of age and tissues were collected 8 weeks later. Tg26 mice express 7 of 9 HIV proteins but lack gag and pol so are non-infectious. RNA was isolated from the tibialis anterior and microarray was performed using the Clariom S Mouse array that contains more than 22,100 well-annotated genes and 150,300 transcripts. Differential expressions were calculated using ANOVA and filtered with a p-value cutoff of 0.05. Results show that the methyltransferase Mettl21c, a methyltransferase previously shown to stimulate muscle protein synthesis and suppress muscle degradation, is one of the most highly downregulated (more than two-fold) genes in muscle from mice receiving Tg26 bone marrow. We then analyzed GEO data sets to identify changes in muscle Mettl21c expression in various disease settings. These data sets reveal that Mettl21c is consistently downregulated in skeletal muscle with inflammatory conditions such as cancer cachexia and sepsis. In addition, GWAS studies have previously shown Mettl21c deficiency in patients with sarcopenia and osteoporosis. Our data suggest that HIV proteins may produce epigenetic modifications in skeletal muscle leading to muscle loss, and that therapies restoring expression of methyltransferases such as Mettl21c might improve muscle health in patients living with HIV.

Abstract citation ID: igad104.3580

### RU AGE-FRIENDLY? THE RUTGERS AGING INITIATIVE

Lauren Snedeker[1], and Tracy Davis[2], *1. Rutgers School of Social Work, New Brunswick, New Jersey, United States*, *2. School of Health Professions, Rutgers, Rutgers, New Jersey, United States*

Ageism is harmful to everyone everywhere, including university settings. Rutgers is among America's highest-ranked, most diverse public research universities. While efforts have been made to increase diversity, equity, and inclusion, few efforts have focused specifically on ageism. Like many individuals and groups experience, ageism is often the last experience identified in the DEI equation (can cite if needed). The objective of this project was to begin to take steps to end ageism at Rutgers University and beyond and move towards becoming an age-friendly university. As a part of this project, we surveyed 109 full or part time faculty members about their understanding of ageism and their experiences with ageism at the university. Furthermore, faculty were asked about the feasibility of including ageism into their courses and whether they would need training or materials to do so. Our survey results indicate that faculty feel prepared to lead discussions on ageism with students, recognize its importance in advancing equity and inclusion, and but largely lack any formalized training on the matter. Our project, supported by a university grant, helped respond to these findings and create a sustainable action plan for the future that ensures ageism and age inclusivity are experiences that continue to be addressed at the university level.

Abstract citation ID: igad104.3581

### SARCOPENIA AND POST-HOSPITAL OUTCOMES FOR OLDER PATIENTS IN AN EMERGENCY DEPARTMENT

Tai-Hung Ho, and Hsien-Hao Huang, *Taipei Veterans General Hospital, Taipei, Taipei, Taiwan (Republic of China)*

**Introduction:** Sarcopenia, an important condition in older adults, is associated with increased adverse outcomes, including falls, functional decline, frailty, and mortality. Sarcopenia is often not diagnosed in emergency department (ED) settings. Therefore, this study aimed to determine the prevalence, risk factors, and clinical outcomes of sarcopenia patients admitted to the ED. **Methods:** This observational study included patients aged ≥ 65 years admitted to the ED of Taipei Veterans General Hospital from September 2019 to November 2021. The patients were divided into two groups according to the definition of the Asian Working Group for Sarcopenia: sarcopenia and non-sarcopenia. **Results:** Among 867 enrolled, 447 (51.6%) were categorized into non-sarcopenia and 420 (48.4%) into sarcopenia groups. Sarcopenia patients were significantly older, shorter, weighed less, and had significantly lower values of body mass index, skeletal muscle mass, skeletal muscle index, and handgrip strength. The sarcopenia group had significantly fewer daily living and instrumental activities. The independent risk factors for sarcopenia were age (odds ratio [OR] 1.07, $P < 0.001$), malnutrition (OR 3.83, $P < 0.001$), and frailty (OR 1.91, $P = 0.001$). Sarcopenia patients had significantly higher admission (66.7% vs. 59.1%, $P = 0.021$) and 1-month mortality rates ($P = 0.002$). **Conclusion:** Sarcopenia patients are at risk of functional decline, hospital admission, and 1-month mortality. The independent risk factors for sarcopenia include age, malnutrition, and frailty. The recognition of sarcopenia as a medical condition in an ED may prompt targeted nutritional interventions, tailored discharge plans, and referrals to reduce disability, admission, and mortality.

Abstract citation ID: igad104.3582

### SCOPING REVIEW OF CLINICAL STUDIES EXPLORING THE ASSOCIATION BETWEEN EPIGENETIC AGE AND FRAILTY

Ebba Hillstedt[1], Rebecka Rubsenson Wahlin[1], Markus Castegren[1], Gabriel Sandblom[1], and Alice Kane[2], *1. Karolinska institutet, Stockholm, Stockholms Lan, Sweden*, *2. Institute for Systems Biology, Seattle, Washington, United States*

An overall increase in life expectancy shines light on the complexity of human aging where some individuals seem to age at a faster pace than others, a phenomenon health care systems are already battling to identify. Multiple frailty scoring systems have been proposed as models for biological age estimations but their implementation within health care systems is time consuming and often deprioritized. Epigenetic clocks based on DNA methylation (DNAm) changes have been proposed as newer estimations of biological age, but there is conflicting data on the correlation between these two measures. We conducted a review of the literature to identify studies that have explored the correlation between frailty and epigenetic age. Our search strategy identified 37 articles, and after further screening we found 15 articles that directly compared frailty scores to epigenetic age. Most studies measured DNAm in whole blood (9/15), although there was great variability in the specific epigenetic clocks applied. Almost all studies measured frailty with either Fried's frailty phenotype (4/15) or a Frailty Index (9/15). The studies were divided in their findings, with 7/15 showing moderate to strong associations and the other 8 studies showing no or weak associations. Interestingly, the associations with frailty were usually seen for epigenetic clocks predicting mortality or other phenotypes (eg GrimAge, PhenoAge), rather than epigenetic clocks predicting chronological age. More work is needed to understand the relationship between these biological age measures and their potential role in clinical risk assessment for older adults.

Abstract citation ID: igad104.3583

### SECONDARY USE OF NIH AD/ADRD DATA RESOURCES FOR BIOMEDICAL RESEARCH

Sharna Tingle, Damali Martin, and Jennie Larkin, *National Institute on Aging, Bethesda, Maryland, United States*

People are living longer than ever before, and the world's population aged 60 and older is expected to increase from 1 billion to 2.1 billion by 2050. As populations get older, they become more susceptible to age-related diseases such as Alzheimer's Disease and Related Dementias (AD/ADRD), which affects more than 55 million individuals worldwide. The National Institutes of Health (NIH) aims to prevent and treat diseases such as AD/ADRD through generalist and disease-specific data resources that foster findable, interconnected, and interoperable data to accelerate biomedical innovations and medical interventions that improve human health. The goal of this assessment was to understand the value of AD/ADRD secondary data use from NIH genomic and multi-omics data resources: NIA Genetics of Alzheimer's Disease Data Storage Site (NIAGADS), database of Genotypes and Phenotypes (dbGaP), AD Knowledge Portal (ADKP), and Gene Expression Omnibus (GEO). List of AD/ADRD datasets were retrieved through database searches or directly from repository staff (N=183). Citation frequency of data accessions were used to determine utilization of AD/ADRD genomic and multi-omics datasets in published literature. iSearch tool was used to assess secondary use drivers in resulting publications. Query of data accessions in Pubmed Central returned 284 unique publications. iSearch clustering by term showed that genomic datasets were used to study genetic risk factors of AD/ADRD while multi-omics datasets were used to functionally characterize genetic risk variants, identify gene regulatory networks, and biomarkers associated with neurodegenerative diseases. These findings indicate the usefulness of AD/ADRD secondary data to the scientific literature.

Abstract citation ID: igad104.3584

### SEDENTARY BEHAVIOR DIFFERENTIALLY INFLUENCES COGNITIVE HEALTH IN OLDER ADULTS AT DIFFERENT TIMESCALES

Tian Qiu[1], Daniel Elbich[2], Jessie Alwerdt[1], and Jonathan Hakun[1], *1. Pennsylvania State University, University Park, Pennsylvania, United States*, *2. The Pennsylvania State University, Hershey, Pennsylvania, United States*

The negative health effects of sedentary behavior have received increased attention over the past decade. Whether sedentary behavior negatively influences cognitive health at any timescale remains poorly understood. Our study sought to determine whether sedentary behavior negatively affects cognitive health throughout the adult lifespan at multiple timescales (acute within-day, day-to-day, cross-sectionally). We examined ambulatory assessment data generated by an adult lifespan sample (N=161; age range:19-88 years; 84% F) where cognition was repeatedly assessed via ultra-brief cognitive assessments of processing speed and working memory in an ecological momentary assessment (EMA) framework (6 assessments/day for 14 days). Activity was measured continuously via thigh-worn ActivPAL monitors. ActivPAL and EMA data were combined and modeled in a multi-level (3-level; moments within days within persons) isotemporal substitution modeling framework. Increased sedentary behavior was associated with acute decreases in processing speed for older participants (65+ years of age) and substitution modeling indicated that exchanging bouts of walking or standing for sedentary time would result in short-term improvements. In contrast, a consistent result across younger and older adults indicated that days with generally more sedentary time (as well as lying and standing) were associated with overall better processing speed and working memory capacity. No cross-sectional associations were observed. A lack of cross-sectional associations between sedentary behavior and cognitive performance was consistent with previous studies. Our current results extend previous findings by showing that sedentary behavior might influence cognitive performance differently at different timescales in younger and older adults.

Abstract citation ID: igad104.3585

### SENIOR TECHNOLOGY ACCEPTANCE, FEAR OF FALLING AND FALL RISK ASSESSMENT IN COMMUNITY-DWELLING OLDER ADULTS.

Eunice Oladepe Ojo, Boon Peng Ng, and Ladda Thiamwong, *University of Central Florida, Orlando, Florida, United States*

The use of technology, such as the BTrack balance system (BBT) for fall risk assessment and the Senior Technology Acceptance (STA) survey is important for understanding technology-based fall prevention efforts for at-risk older adults. The purpose of this study was to examine the relationship between technology-based fall risk assessment and STA among community-dwelling older adults aged 60 years and older (n=124). Descriptive statistics, bivariate analysis, and multiple linear regression adjusted for socio-demographics were performed. The STA tool was used to obtain information about older adults' acceptance of technology. The STA consists of four domains and 14 items. The Short Fall Efficacy Scale International (FES-I) and the BBT were used to measure fear of falling and balance performance, respectively. Of the study population, 77.4% were female, 22.6% were non-white, 8% reported financially inadequate, 46% had low BBT scores and low FES-I scores, whereas 33.9%

had high BBT and high FES-I scores, and 20.2% had low BBT and high FES-I scores. For the bivariate analysis, we found a statistically significant difference in the STA scores based on age (p=0.006) and education (p=0.032). In the adjusted analysis, only age was negatively associated with the STA (p=0.004); fall risk assessment using the BBT and FES-I were not significantly associated with STA. With a small study population, readers should be aware of this limitation to our findings. Further studies are needed to address this issue and to identify effective strategies to encourage older adults to use technology to improve their well-being.

Abstract citation ID: igad104.3586

### SEQUENTIAL ANALYSIS OF DEMENTIA FAMILY DYADIC COMMUNICATION: EFFECT OF CAREGIVER DISABLING COMMUNICATION

Sohyun Kim, Will Chen, and Shan Sun-Mitchell, *University of Texas at Arlington, Arlington, Texas, United States*

This study examined temporal relationships between antecedent family caregiver disabling communication and subsequent care recipient (persons living with dementia) communication during 75 in-home care video observations. Secondary analysis of 5-, 10-, 15-, 20-, 25-, and 30-second timed-window analysis was conducted. 95% confidence intervals, p-values, and Yule’s Q statistics were used. The results showed that care recipient engaging, challenging, and neutral communication occurred at differing frequencies within each time window following disabling communication by family caregivers. Care recipient challenging verbal communication was more likely to occur within all time widows after caregiver challenging verbal communication preceded (OR = 1.51 – 1.77, p <.001, Yule’s Q = 0.204 – 0.277). Care recipient engaging verbal communication was more likely to occur with weaker chance within all time widows except 30-second window after caregiver disabling verbal communication preceded (OR = 1.11 – 1.17, p = .001 - .028, Yule’s Q = 0.151 – 0.080). Care recipient neutral communication was more likely to occur within all time widows after caregiver disabling verbal communication preceded (OR = 1.34 – 1.39, p <.001, Yule’s Q = 0.144 – 0.162). Care recipient neutral communication more likely occurred within all time widows except 5-second and 10-second windows after caregiver disabling nonverbal communication preceded (OR = 1.28 – 1.34, p =.009 - .029, Yule’s Q = 0.123 – 0.144). Caregiver disabling communication was associated with more likely subsequent care recipient communication. Future research can further explore how participant characteristics and types and frequencies of caregiver communication influence care recipient communication.

Abstract citation ID: igad104.3587

### SERUM TRANSTHYRETIN LEVEL IS ASSOCIATED WITH COGNITIVE FUNCTION IN COMMUNITY-DWELLING OLDER ADULTS.

Misa Nakamura, Masakazu Imaoka, Fumie Tazaki, Mitsumasa Hida, Ryota Imai, Takanari Kubo, Keiko Sakai, and Masatoshi Takeda, *Osaka Kawasaki Rehabilitation University, Kaizuka, Osaka, Japan*

Serum transthyretin (TTR) level has been shown in several studies to be associated with mild cognitive impairment (MCI) and dementia. This study aims to clarify the usefulness of TTR as a biomarker of change in cognitive function in people with normal cognitive function (NC) as a phenotype. We therefore investigate the relationship between cognitive scores and TTR in a cross-sectional study. A longitudinal study then examines the involvement of TTR level in the transition from NC to MCI. Cognitive function was evaluated using Addenbrooke's Cognitive Examination-Revised (ACE-R). A cross-sectional study was conducted in community-dwelling older people (n=211) classified into NC, MCI, or dementia by ACE-R score. A 32-month longitudinal study was then conducted (n=29). Multiple regression analysis used sex, age, years of education and TTR as dependent variables and ACE-R scores as an independent variable. TTR was significantly associated with ACE-R in people with NC (β = -0.295, p =0.0008). No association with TTR was found in the MCI or dementia groups. Multiple regression analysis using sex, age and each ACE-R subdomain as dependent variables and TTR level as independent variable showed significant association with memory and language. In the longitudinal study, data analysis revealed that TTR level at baseline in females with MCI was significantly higher than it in females with NC. Our results suggest serum TTR levels might be a useful marker for cognitive function in preclinical AD. Elevated TTR might be a predictor of transition to MCI in old-aged adults with NC.

Abstract citation ID: igad104.3588

### SEVERE ALZHEIMER'S DEMENTIA ALTERS THE RELATIONSHIP BETWEEN EXECUTIVE FUNCTION AND FALLS

Ferdinand Delgado[1], Fang Yu[2], David MacKinnon[3], Daniel Peterson[2], Edward Ofori[3], Thomas Beach[4], Charles Adler[5], and Cheryl Der Ananian[2], *1. University of New Hampshire, Durham, New Hampshire, United States, 2. Arizona State University, Phoenix, Arizona, United States, 3. Arizona State University, Tempe, Arizona, United States, 4. Banner Sun Health Research Institute, Sun City, Arizona, United States, 5. Mayo Clinic, Scottsdale, Arizona, United States*

Falls are a public health concern for older adults, particularly those with cognitive impairments, like mild cognitive impairment (MCI) and Alzheimer's disease (AD) dementia. Executive function (EF) is linked to falls in older adults, but little is known about the relationship between individual EF assessments and falls in individuals with varying cognitive statuses. Using cross-sectional secondary data analyses with binary logistic regression, we examined the associations between EF assessments (Trail Making Test (ΔTMT), Digit Span (DS), Stroop test, Clock Drawing Test, and phonemic/semantic fluency tests) and falls (fall history and subsequent falls) in 466 participants (aged 66-103) with either intact cognition, MCI, or AD dementia. Surprisingly, a significant interaction effect emerged with AD dementia and EF assessments (ΔTMT, DS, and semantic fluency), revealing that individuals with AD dementia with worse EF had a lower probability of prior fall history. These findings were influenced by a low percentage of reported falls in people with severe AD dementia. Further stratified analysis of the interaction effects showed that DS and semantic fluency were significantly associated with fall history in people without AD dementia. While some EF assessments were individually associated with falls, interpretations should be made cautiously, especially considering the appropriateness of the assessments for specific populations. The counterintuitive result may stem from factors specific to severe AD dementia. In conclusion, this study uncovers complex interactions between cognitive status, EF assessments, and fall history, and the potential for further investigation to enhance our understanding of falls in individuals with severe AD dementia.

Abstract citation ID: igad104.3589

### SEX DIFFERENCES IN EMPLOYMENT HISTORY AND LATE-LIFE COGNITION AMONG OLDER CHINESE

Peiyi Lu[1], Hongyu Yin[2], and Chihua Li[3], *1. Columbia University, New York City, New York, United States*, *2. University of California Irvine, Irvine, Irvine, California, United States*, *3. University of Michigan, Ann Arbor, Michigan, United States*

This study characterizes the employment history of older Chinese men and women and examines the associations between employment trajectories and late-life cognition. Individuals aged 60+ in the 2011 China Health and Retirement Longitudinal Study were included (2,733 men and 2,363 women). Multiple waves of surveys were conducted to assess their cognition in 2011, 2013, and 2015. Sequence analysis identified employment trajectories among men and women separately based on self-reported employment history of whole working age (15-60 years). Mixed-effect models examined the relationship between employment trajectories and cognition. Three distinctive employment trajectories were identified for men and women, separately. Among men, 45% men was consistently agriculture employed, 26% were consistently non-agriculture employed and retired at age 60, and 29% had fluctuating employment featuring in agriculture and early-retirement. Among women, 70% women was consistently agriculture employed, 15% were consistently non-agriculture employed and retired at age 55, and 14% had fluctuating employment featuring unemployment, home, and early-retirement. Compared to the consistently agriculture employed, the consistently non-agriculture employed group reported better cognition for both men and women (men: $\beta$=1.26, 95%CI=0.81, 1.66; women: $\beta$=1.63, 95%CI=1.09, 2.30). Men with fluctuating employment trajectory also had 0.62 points higher cognition (95%CI=0.27, 0.96) than men who were consistently agriculture employed. Older Chinese experienced distinct life-course employment trajectories reflecting the economic and historic development in China. The sex difference was evident as women were more involved in agricultural work and working at home. Non-agricultural employment was associated with better cognition, highlighting the disadvantaged health status of Chinese farmers.

Abstract citation ID: igad104.3590

### SHAPING COVID-19 INTERVENTIONS THAT ADDRESS SOCIAL BARRIERS AMONG OLDER LATINOS IN ASSISTED LIVING FACILITIES

Isis Panellas, and Richard Beaulaurier, *Florida International University, Miami, Florida, United States*

COVID-19 has exposed severe gaps in our understanding of preventing infectious diseases, particularly in congregate living facilities. While the medical and public health communities developed clear insights into preventing or limiting the spread of infection, our understanding of barriers to adopting these measures has lagged, particularly in minority communities. The COVID-19 pandemic has disproportionately affected older Latino adults, particularly those living in assisted living facilities. Among this vulnerable population, older Latinos face unique social barriers such as language and communication difficulties, lack of culturally competent care, and socioeconomic disadvantages that contribute to their increased susceptibility to COVID-19 transmission and which can lead to more severe outcomes. While initial intervention strategies focused on medical interventions to mitigate the virus's effects, there has been a gradual recognition that barriers to prevention have increasingly shifted from medical to social domains. This paper aims to document the existing knowledge and identify knowledge gaps concerning effectively managing these social barriers. Culturally sensitive approaches must consider salient features of Latino family dynamics such as allocentrism, familismo, respeto, and expected gender roles. In addition, there are often barriers related to external factors, such as a lack of Spanish language resources, community engagement, stereotypes, and racism. Prevention approaches need to identify and address these barriers to safeguard the well-being of older Latinos in assisted living facilities during the COVID-19 pandemic. This review will explore our current understanding of these social barriers, provide insights into potential strategies to mitigate their impact and identify continuing gaps in our knowledge.

Abstract citation ID: igad104.3591

### SIAH E3 UBIQUITIN LIGASE2 (SIAH2) DELETION MIMICS AGE-ASSOCIATED PHENOTYPES IN MOUSE MACROPHAGES

Bhaswati Ghosh[1], Pradip Panta[2], Matthew Scott[1], and Elizabeth Floyd[1], *1. Pennington Biomedical Research Center, Baton Rouge, Louisiana, United States*, *2. Southeastern University, Southeastern University, Louisiana, United States*

Aging is generally characterized by physiological as well as metabolic decline. Crosstalk between metabolic organs is remarkably altered by aging. For one, adipose tissue exhibits distinct cellular and molecular attributes of aging. Impaired adipogenesis is one of the aging hallmarks. Previously, we demonstrated Siah E3 Ubiquitin Ligase2 (Siah2) is upregulated during adipogenesis. We are interested if and how Siah2 expression is altered by aging. Recently, the age-related decline in immune function was first detected in mouse adipose tissue. To explore the regulatory role of Siah2 in immune response, we generated mice with Siah2 knockout in macrophages (SKM) and observed elevated expression of inflammatory regulators including TNF-$\alpha$ and Trem2 in SKM macrophages compared to control. Additionally, we detected increased lipid accumulation and expression of Plin2 (lipid droplet marker) in the co-culture model of adipose tissue macrophages derived from SKM mice. These data are consistent with previous research showing an age-related increase in TNF-$\alpha$ expression and lipid content. Varying degrees of age-related alterations have been observed in different adipose tissue depots. For example, aging attenuates "beiging" of white adipose tissue (WAT) to brown adipose-like tissue and consequently is associated with lower levels of mitochondrial respiration. Interestingly, oxygen consumption rate, a quantitative measure of mitochondrial function, is lower in the SKM macrophages compared to the control group, hinting that Siah2 may regulate browning of WAT during aging. Altogether, these data suggest that Siah2 KO phenotypes mimic major aging hallmarks and that Siah2 could be a potential therapeutic target for age-related adipose tissue disorders.

Abstract citation ID: igad104.3592

### SLEEP DISORDERS AND MUSCLE STRENGTH IN OLDER MEXICAN AMERICANS: SEX DIFFERENCES

Jamil Okada, and Soham Al Snih, *University of Texas Medical Branch, Galveston, Texas, United States*

We used data from the Hispanic Established Population for the Epidemiological Study for the Elderly to examine sex differences in the relationship between sleep duration and handgrip strength (HGS) among Mexican Americans aged ≥ 77 years with normal or high HGS (≥ 17 in women and ≥ 27 in men) at baseline over a 9-year period (2007/08 to 2012/13; N=1542). Measures included socio-demographics, sleep duration, HGS (Kg), body mass index, medical conditions, depressive symptoms, physical function, cognitive function, disability, and pain. We categorized sleep duration as ≤ 7, 8, 9, and ≥ 10 hours/night and used linear mixed models to estimate the changes in HGS as a function of sleep duration by sex, adjusting for covariates. Both men and women in the highest sleep duration group (≥10 hours/night) had on average the greatest decline in HGS (1.54 kg and 0.95 kg, respectively) compared to those who slept 8 hours/night, after controlling for all covariates over the follow-up period. Older age, time (years), depressive symptoms, one or more falls in the last year, and low physical function were associated with greater decline in HGS among women. Older age and time (years) were associated with greater decline in HGS among men. In conclusion, longer sleep duration was associated with greater loss of HGS over time in older Mexican Americans. Sleep health promotion and sleep behavioral interventions may reduce the risk of decline in HGS, which has been associated with disability, institutionalization, and mortality in this population.

Abstract citation ID: igad104.3593

### SOCIAL DETERMINANTS OF BRIDGE EMPLOYMENT: DO BIRTH COHORTS MATTER?

Gayoung Choi, and Haena Lee, *Sungkyunkwan University, Seoul, Republic of Korea*

A growing number of retirees now engage in bridge employment, a term referring to their continued participation in the workforce post-retirement. The generation in South Korea born between 1945 and 1955, a period marked by leading industrialization and experiencing economic crises during their working age, exhibits unique socioeconomic characteristics distinct from later generations. Consequently, their retirement trajectories diverge from those of later cohorts, including the Baby Boomer generation (born between 1956 and 1965). In this paper, we investigate whether social, demographic, psychological, and health factors are associated with bridge employment and whether this association differs across birth cohorts. Data were drawn from seven waves of the Korean Longitudinal Study of Aging, conducted between 2008 and 2020. We employ an integrative framework that considers socioeconomic status, health status, and psychological factor s as the main variables to explain the odds of continuing to work after retirement. Retirement status includes three categories: employed, bridge, and total retirement. Results from a multinomial logit model reveal birth cohort differences in the relationship between social/psychological predictors and bridge employment. Additionally, higher educational attainment, a private pension, and poor mental health are associated with decisions regarding bridge employment for the industrialization generation, but not for Baby Boomers. Furthermore, females are more likely to participate in bridge employment than males for the industrialization generation, but not for Baby Boomers. Our findings contribute to the current literature on bridge employment by identifying the social determinants of bridge employment and highlighting the importance of cohort differences in bridge employment.

Abstract citation ID: igad104.3594

### SOCIAL DETERMINANTS OF DEPRESSION AMONG OLDER ADULTS IN THE UAB STUDY OF AGING: THE ROLE OF RACE-BASED DISCRIMINATION

Katie Buys[1], David Buys[2], Pamela Bowen[1], and Richard Kennedy[1], *1. University of Alabama at Birmingham, Birmingham, Alabama, United States, 2. Mississippi State University, Mississippi State, Mississippi, United States*

Mental health is known to be influenced by social determinants of health (SDoH). However, little is known about how racial discrimination, in the context of other social factors, impacts the mental health of older adults. The focus of this research is specifically on the different effects of racial discrimination in that broader context on depression diagnosis vs. symptomology. Using the UAB Study of Aging's cohort of 1000 Alabamians 65+ balanced on rural/urban status, sex (men/women), and race (Black/white), we evaluate the impact of SDoH, including race-based discrimination, on clinically verified depression diagnosis and on depressive symptomology using the Geriatric Depression Scale. Other independent variables include environmental factors (eg: neighborhood disadvantage), status and assets, interpersonal factors, and fixed demographic characteristics. Using generalized estimating equations, we found that experience of racial discrimination is associated with a depression diagnosis (OR: 2.33) independent of race and other factors. Other variables that were significant predictors of having a verified depression diagnosis include life-space mobility and race. Factors associated with depressive symptomology include fear of crime, financial difficulty, social support, life-space mobility, race (Black), and sex (female). While demonstrating that experience of race-based discrimination was associated with depression diagnosis is not surprising, finding in an otherwise identical model, that it was not associated with symptomology was unexpected. Authors will discuss some potential reasons for this counterintuitive outcome and future directions for better understanding this inconsistency.

Abstract citation ID: igad104.3595

### SOCIAL ISOLATION AND HOSPITALIZATION IN A NATIONAL SAMPLE OF COMMUNITY-DWELLING OLDER ADULTS BY DEMENTIA STATUS

Mary Louise Pomeroy[1], Katherine Ornstein[1], Gilbert Gimm[2], and Thomas Cudjoe[3], *1. Johns Hopkins University, Baltimore, Maryland, United States*, *2. George Mason University, Fairfax, Virginia, United States*, *3. Johns Hopkins University School of Medicine, Baltimore, Maryland, United States*

Social isolation poses risks for poor health, yet its impact on hospitalization for persons with dementia is unknown. Dementia may result in reduced social contact that increases the risk of hospitalization. This observational cohort study examined longitudinal associations between social isolation and hospitalization among a nationally-representative sample of community-dwelling older adults with and without dementia. Using the National Health and Aging Trends Study, we conducted multivariable analyses for 5,533 Medicare beneficiaries. Social isolation was measured using a multi-domain 4-item typology. Logistic regression tested associations between social isolation in 2015 and self-reported hospitalization over the next twelve months, stratifying by dementia status. Covariates included sociodemographics and health characteristics. About 21.5% of older adults were socially isolated, with a much higher proportion of social isolation among those with dementia (38.0%) versus without (20.7%) ($p < .001$). Overall, the odds of hospitalization were not significantly associated with social isolation (OR 1.13; $p = .184$). While there was no association between social isolation and hospitalization among those without dementia (OR 1.09; $p = .394$), social isolation was significantly associated with hospitalization among older adults with dementia (OR 2.07; $p = .001$). Among older adults with dementia, social isolation increased the predicted probability of hospitalization by about 14.1% over one year ($p = 0.030$). In this population-based study, social isolation was common among community-dwelling older adults with dementia and significantly associated with increased odds of hospitalization over twelve months. Efforts to reduce hospitalization should explore ways to bolster social relationships for older adults with dementia.

Abstract citation ID: igad104.3596

### SOCIAL SANDWICHING AND PAID WORK IN LATER LIFE: CONSEQUENCES ON MENTAL HEALTH

Marco Albertini[1], Noah Lewin-Epstein[2], Merril Silverstein[3], and Aviad Tur-Sinai[4], *1. Alma Mater Studiorum - Università di Bologna, Bologna, Emilia-Romagna, Italy*, *2. Tel Aviv University, Tel Aviv-Yafo, Tel Aviv, Israel*, *3. Syracuse University, Syracuse, New York, United States*, *4. The Max Stern Yezreel Valley College, Yezreel Valley, HaZafon, Israel*

Conflicting demands over time allocation – between family care and paid work – can be harmful to individual's wellbeing. Individuals who are socially sandwiched and in employment experience potential conflict between supporting older and younger family generations, and with respect to the demands from paid work. These competing time demands may have negative effects on individuals' health. Adding to most existing research, this study focuses on the mature sandwich generation – i.e. European individuals aged between 50 to 65 years, who are demographically sandwiched between adult children and older parents. Using data from the Survey of Health, Ageing and Retirement in Europe and fixed effects models we assess the effect of becoming socially sandwiched (a transition and not a status) on respondents' mental health in according to their status on the labor market. Euro-D and CAS12 are utilized as proxies of individuals psychological wellbeing. It is shown that the effect of becoming socially sandwiched varies by in paid status and gender. The results demonstrate that a transition into social sandwiching has a negative effect on the mental health of working women, while the effect on women who are not in paid work is not statistically significant. The mental health of becoming sandwiched for men who are not in paid work for reasons other than being retired is also negatively affected by becoming socially sandwiched. Whereas, for men who are neither in paid work nor retired, the effect of transitioning into social sandwiching on their mental health is not statistically significant.

Abstract citation ID: igad104.3597

### SOURCES OF VACCINE INFORMATION: MISGIVINGS AND TRUST ISSUES AMONG OLDER ADULTS IN NORTH DAKOTA

Andrea Huseth-Zosel, and Heather Fuller, *North Dakota State University, Fargo, North Dakota, United States*

Older adults consistently have relatively high vaccination rates. What is not fully understood is how trust in immunization information sources impacts vaccine decision-making among older adults. This study examines older adults' perceptions and decision-making processes for vaccines such as influenza, pneumonia, shingles, and COVID-19. Older adults (65+) in North Dakota (N=51) who participated in a larger survey study on vaccine behaviors voluntarily agreed to participate in a follow-up phone interview (November 2022-January 2023). Qualitative, semi-structured phone interviews focused on access, knowledge, and decision about vaccines. Interview transcripts were inductively coded for emergent themes related to trust. Three themes were identified: implicit trust in information sources, uncertainty who to trust for vaccine information, and regaining trust in information sources. Implicit trust focused on familiarity with sources of information (such as inherently trusting family members), source expertise (such as doctor recommendations), and reliability of sources (including if the source always provides accurate information). The second theme, uncertainty who to trust, stressed confusion over source inconsistency, such as changing positions on vaccines, or having opposing sources that both seem to be trustworthy. The final theme, regaining trust in information sources, highlighted if and how trust in a source of vaccine information could be recovered, including transparency, sincerity, and verifying immunization information through obtaining a second opinion. These findings contribute to the understanding of vaccine decision-making among older adults and provide focus areas for interventions to address lack of trust in information sources and improve immunization rates for this population.

Abstract citation ID: igad104.3598

### SPATIAL NAVIGATION SKILLS IN MILD COGNITIVE IMPAIRMENT (MCI) – A META-ANALYSIS.

Dorota Kossowska-Kuhn[1], and Neil Charness[2], *1. Florida State University, Tallahassee, Florida, United States*, *2. FSU, Tallahassee, Florida, United States*

**Background:** Dementia has a tremendous impact on societies and individuals world-wide. One of the earliest symptoms of Alzheimer's Disease (AD) is spatial disorientation (Coughlan et al. 2018). Spatial orientation problems commonly manifest in Mild Cognitive Impairment (MCI), which represents a state between cognitively healthy aging and dementia. It appears that spatial navigation skills might be an early indicator of cognitive decline due to MCI. **Methods:** The present meta-analysis examines the difference between the performance of cognitively healthy older adults and older adults with MCI on spatial navigation tests. The standardized mean difference (Hedge's g) is used as the effect size measure. In addition to simple characteristics (year of publication, country of study, age of participants, gender, education), we examined the form of test administration (i.e., real-world, virtual reality), type of measure (time, accuracy) and the additional spatial tasks connected with the main spatial navigation test in moderator analyses. **Results:** Current data consists of 69 effect sizes across 26 studies. These studies represent 1359 participants (682 MCI, 677 healthy older adults). The overall result shows that the navigation skills of healthy cognitively older adults are significantly better ($p < 0.05$) comparing to people with MCI by the standardized mean difference (SMD) = 0.81. **Conclusion:** Given the large effect size, it is worthwhile to examine potential moderators. It would also be useful to assess the effect size for spatial navigation in people with Alzheimer's Disease.

Abstract citation ID: igad104.3599

### SPIRITUAL NEEDS OF ELDERLY IN CHINESE FAITH-BASED NURSING HOME: ADMINISTRATOR VIEWS

Lin Jiang[1], David Hodge[2], Robin Bonifas[3], and Fei Sun[4],
*1. The University of Texas Rio Grande Valley, Edinburg, Texas, United States*, *2. Arizona State University, Phoenix, Arizona, United States*, *3. Indiana State University, Terre Haute, Indiana, United States*, *4. Michigan State University, East Lansing, Michigan, United States*

**Objectives:** Recognizing and addressing the spiritual needs of elderly individuals constitutes a crucial facet of comprehensive caregiving. Despite China's immense aging population, research on the spiritual needs of Chinese seniors remains limited. This study aimed to: 1) delineate prevalent spiritual needs among Chinese nursing home residents, including those with dementia; 2) elucidate the method employed by staff to identify these needs in non-verbal residents; and 3) expound on the strategies utilized by staff to fulfill these identified needs. **Methods:** In this qualitative exploration, semi-structured interviews were conducted with 21 administrators from faith-based nursing homes, chosen for their potential concentration of residents with dementia. Encompassing establishments across 14 Chinese provinces, interviews were conducted in the participants' native language, subsequently translated into English. Utilizing a constant comparative approach, the collected data underwent analysis to uncover recurring themes. **Results:** The analysis yielded five interconnected themes concerning the spiritual needs of older adults: expression of faith, reception of love and care, familial interaction, social engagement, and participation in activities. For individuals with dementia unable to articulate their needs, staff employed careful observation and communication with family members as means of identification. In response to identified needs, staff harnessed local resources and personalized services whenever feasible. **Discussion:** The findings offer practical insights for holistic caregivers catering to the spiritual needs of Chinese elderly. This study fills an existing gap, aiding in the provision of culturally sensitive care that addresses the spiritual aspects of aging Chinese individuals.

Abstract citation ID: igad104.3600

### STABILITY AND INTER-RELATIONSHIPS OF INFLAMMATORY MARKERS ACROSS TWO WEEKS IN OLDER ADULTS

Robert Tennyson[1], Molly Wright[1], Erik Knight[2], Jennifer Graham-Engeland[1], and Christopher Engeland[1],
*1. Pennsylvania State University, University Park, Pennsylvania, United States*, *2. University of Colorado Boulder, Boulder, Colorado, United States*

The temporal stability of basal and stimulated inflammatory cytokines is unclear in older adults, despite often being used to characterize inflammatory status. Further, it is not apparent how the stability of these markers and their multi-dimensional relationships connect to healthy aging. This analysis examines these issues in 227 participants from the Einstein Aging Study [ages: 70-90 (Mean=76.7), 67% women] with blood draws ~2 weeks apart. Temporal stability was assessed by intra-class coefficients (ICCs) obtained from linear mixed models (controlling for days between measurements, age, gender) for a panel of basal and lipopolysaccharide (LPS)-stimulated cytokines (interleukin [IL]-1β, IL-4, IL-6, IL-8, IL-10, tumor necrosis factor [TNF]-α). Sensitivity analyses compared ICCs across genders and individuals reporting high vs. low subjective health. A composite measure of basal cytokines exhibited strong reliability (ICC: 0.854), whereas individual basal cytokines exhibited moderate (>0.5) to very high reliability (>0.9). Conversely, a composite measure of stimulated cytokines demonstrated moderate reliability (ICC: 0.552), with individual stimulated cytokines having only low (< 0.5) to moderate reliability. No appreciable differences were observed by gender, but individuals reporting low (ICC: 0.457) vs. high (ICC: 0.631) subjective health exhibited considerably lower reliability for the stimulated composite measure. In sum, basal cytokines had moderate to high stability, whereas stimulated cytokines exhibited relatively low stability. Given that individuals reporting lower subjective health demonstrated greater variability in stimulated cytokines, higher fluctuations in inflammatory responsivity may reflect suboptimal health in older adults. The inter-relationships of these cytokines, explored via principal component analysis, will also be discussed.

Abstract citation ID: igad104.3601

### STAKEHOLDER INSIGHTS: STANDARDIZING END-OF-LIFE CARE TRANSITIONS FOR NURSING HOME RESIDENTS WITH DEMENTIA

Susanny Beltran, Latarsha Chisholm, and Emily Jaijairam,
*University of Central Florida, Orlando, Florida, United States*

Sixty-seven percent of dementia patients die in nursing homes (NHs), where end-of-life care is often suboptimal, and hospice not accessed in a timely manner. The eligibility criteria for the Medicare Hospice Benefit (< 6 months prognosis) poses challenges for patients with Alzheimer's disease and related dementias given the variable disease progressions that characterize most dementias. This study describes current NH processes used to identify dementia residents to refer to hospice, and stakeholders' input into the design of a standardized process for clinical decision-making, using the Mitchell Index, a dementia mortality prediction tool. Individual interviews and focus groups were conducted with NH administrators, medical directors, directors of nursing, MDS coordinators, and social workers (N=11). Participants completed an online survey including facility (e.g., size, profit status) and individual demographics (e.g., years of experience), then participated in an interview that involved a brief presentation of the proposed standardized process using the Mitchell Index. Qualitative data analysis followed a deductive approach, guided by constructs from the Consolidated Framework for Implementation Research (CFIR). Preliminary analyses indicate providers believe the Mitchell Index can be successfully adapted for NH use. Participants stated the tool added something to current processes (CFIR relative advantage), would be easy to use (CFIR complexity), and pointed to external influences (i.e., regulations; CFIR domain 2: outer setting) which would be important to consult in later stages of intervention development. Overall, nursing home stakeholders endorse the potential for a standardized process using the Mitchell Index to be acceptable and feasible in high-Medicaid nursing homes.

Abstract citation ID: igad104.3602

### STATE-LEVEL DESCRIPTION OF MEDICAID POLICY AND ACCESS TO LICENSED ASSISTED LIVING CARE FOR LOW-INCOME ADULTS

Rachel Gunderson[1], Lindsey Smith[2], Portia Cornell[3], Gauri Gadkari[1], and Kali Thomas[4], *1. Brown University, Providence, Rhode Island, United States*, *2. Oregon Health & Science University, Portland, Oregon, United States*, *3. Providence VA Medical Center, Providence, Rhode Island, United States*, *4. Johns Hopkins University, Baltimore, Maryland, United States*

A growing proportion of Medicaid funds are allocated to care in assisted living (AL) settings. However, a state must have a state plan amendment or waiver to utilize these funds. States issue levels of licensure for AL communities, which govern the care and oversight that can be provided and may determine whether ALs can accept Medicaid funding. However, until now, Medicaid coverage for AL has been analyzed at the state level and is not well-documented for license types. Using Medicaid state plans and waiver applications, we describe the types of Medicaid funding mechanisms that states use to finance care in AL and identify the demographics and health characteristics of residents in Medicaid-eligible ALs. We find that five states do not utilize Medicaid to finance care in AL. Among the 45 states and DC with Medicaid-financed AL, 12 states limit Medicaid funding to specific licenses, whereas 34 states provide access to Medicaid-funded care for all AL license types. Among identified ALs, 86% are licensed to accept Medicaid funds. Although the modal funding mechanism is through 1915c waivers, the largest percentage (40%) of ALs licensed to accept Medicaid funding are in states that utilize multiple mechanisms. In states that fund AL through state plan amendments, 88% of Medicare beneficiaries reside in covered ALs; conversely, in states that use 1115 demonstration waivers, 99% of Medicare beneficiaries reside in covered ALs. This work will benefit future research and policymaking aiming to use Medicaid funding to increase access to appropriate, high-quality care in licensed ALs.

Abstract citation ID: igad104.3603

### STOPPING OLDER PERSON GENDER-BASED VIOLENCE IN WOMEN 55+ THROUGH PROMISING PRACTICES: A SCOPING REVIEW

Macri Melissa[1], Jihan Abbas[2], Sandra Hirst[3], Melanie Santarossa[4], Raza Mirza[5], Jessica Hsieh[5], Margaret MacPherson[6], and Benedicte Schoepflin[7], *1. Regional Geriatric Program of Toronto, Toronto, Ontario, Canada*, *2. Toronto Metropolitan University, Toronto, Ontario, Canada*, *3. University of Calgary, Calgary, Alberta, Canada*, *4. Baycrest Health Sciences, Toronto, Ontario, Canada*, *5. University of Toronto, Toronto, Ontario, Canada*, *6. University of Western Ontario, London, Ontario, Canada*, *7. Canadian Network for the Prevention of Elder Abuse, Vancouver, British Columbia, Canada*

**Background:** Intimate partner and family violence (IPFV) disproportionately affect women of all ages, but are often understudied in older populations. Older women also face numerous barriers to reporting these types of abuse. A scoping review was undertaken to: (1) synthesize current knowledge about the utility and suitability of screening and intervention tools for older women (55+) experiencing IPFV; and (2) identify existing policy, practice, and research gaps with regard to current screening and intervention tools. **Methods:** A comprehensive search of the databases Medline, CINAHL, PsychINFO, AgeLine, ASSIA, and Sociological Abstracts was conducted. In addition, grey literature sources were searched using Google Scholar, ISI Social Sciences Citation Index, ISI Conference Proceedings Citation Index-Social Science & Humanities, Dissertations & Theses: Full Text, Canadian Institute for Health Information (CIHI), and National Institute of Health (NIH). After screening and selection, 42 documents were included for data extraction. **Results:** There were five major themes that emerged: (1) older women were not the specific targets of the studies/tools; (2) screening and intervention tools should address health outcomes; (3) tools identified were used or developed for some diverse populations; (4) two or more tools were used in combination; and (5) intervention tools should focus on social support and empowerment. **Conclusions:** Older women, especially those with intersectional identities, are rarely represented in studies of IPFV, and are even less frequently the primary focus. Screening and intervention tools should address health outcomes, social support, and empowerment. Effective screening and intervention may stem from utilizing multiple tools in combination.

Abstract citation ID: igad104.3604

### STUDENTS GAIN APPRECIATION FOR PATIENT-CENTERED AND TEAM-BASED CARE PARTICIPATING IN A GERIATRIC CASE COMPETITION

Kristine Talley[1], Marla Berg-Weger[2], Max Zubatsky[2], Elaina Osterbur[2], Rhiannon Williams[1], Teresa Schicker[1], and Jean Wyman[1], *1. University of Minnesota, Minneapolis, Minnesota, United States*, *2. Saint Louis University, St. Louis, Missouri, United States*

Health science students must attain interprofessional teamwork and collaboration competencies to provide effective, patient-centered care to older adults requiring complex care. However, students typically learn in disciplinary silos and lack engaging opportunities for interprofessional teamwork. To provide students with an interprofessional learning activity the Geriatric Workforce Enhancement Programs (GWEP) at Saint Louis University and the University of Minnesota implemented a Geriatric Case Competition. We present the students' experience with the 2022 competition. Students assigned to interprofessional teams with a coach had one month to prepare a 20-25 minute presentation of a comprehensive care plan using a simulated complex geriatric patient case. Judges scored team presentations using a rubric of core competencies for interprofessional collaborative practice. Each GWEP held local competitions with winning teams presenting at the inter-GWEP competition held via live videoconferencing. Top scoring teams received prizes. Students completed an eight-item satisfaction survey using a 5 point likert scale (5=poor to 1=excellent). Answers to open ended questions were analyzed qualitatively to find themes about their experience. 132 students joined the competition and 54 completed an evaluation survey (response rate 41%). Students rated their experience as good to excellent with mean ratings on 8 questions ranging from 1.5-2.2 (N=54). Themes included: the importance of patient centered care and the power of multiple perspectives/expertise. Students offered improvements related to student, coach, and judge expectations. In summary, the competition provided a very positive learning activity that helped students value the importance of patient centered care and interprofessional collaboration for older people.

Abstract citation ID: igad104.3605

### SUBJECTIVE COGNITIVE DECLINE MODERATES THE ASSOCIATION BETWEEN AGE AND MEMORY DECLINE IN MIDLIFE

Kyoung Shin Park[1], Jennifer Etnier[1], Samantha DuBois[1], Samuel Kibildis[1], Hadassah Som-Pimpong[1], Jarod Vance[2], and Christopher Wahlheim[1], *1. University of North Carolina at Greensboro, Greensboro, North Carolina, United States*, *2. North Carolina Institute for Medical Research, Inc, Durham, North Carolina, United States*

Subjective cognitive decline (SCD) represents self-perceived worsening of memory and thinking abilities, in the absence of objective cognitive impairment, and serves as a prognostic marker of Alzheimer's disease (AD). However, no study to date has investigated whether SCD moderates the relationship between aging and memory before older ages. We tested the hypothesis that SCD interacts with age to predict memory performance in midlife. A subset of 123 cognitively unimpaired, middle-aged adults (Mage=56.6±6.44 years; 87% females) from an ongoing clinical trial (NIH R01AG058919) were included for analyses. Multiple linear regression models tested the effects of age and SCD in predicting performance of mnemonic discrimination, auditory-verbal memory, visuospatial memory, paired associative memory, and verbal logical memory, while controlling relevant covariates (e.g., education, sex, and APOE ε4). Based on the optimal cutoff of the Everyday Cognition Scale, 28.2% of the sample were considered to have SCD and 71.8% perceived themselves to have normal cognition. After covariate adjustments, there were significant main and interaction effects of SCD and age on mnemonic discrimination performance on the Mnemonic Similarity Task (MST) but not on other memory outcomes. The negative relationship between age and mnemonic discrimination was evident in middle-aged adults who self-report cognitive concerns but not in those without such concerns. Since mnemonic discrimination is highly sensitive to hippocampal function, these findings support the utility of the MST to detect age-related hippocampal changes and ensuing memory declines in midlife as well as the importance of early interventions among middle-aged adults with SCD to prevent AD.

Abstract citation ID: igad104.3606

### SUBJECTIVE SOCIAL STATUS AND DEPRESSION AMONG KOREAN OLDER ADULTS: FORMAL SOCIAL ACTIVITIES AS MEDIATOR

Junmin Park, and Yeonjung Lee, *Chung-Ang University, Seoul, Republic of Korea*

Previous studies have examined an association between depression and socioeconomic factors such as education, economic status, and social support among older adults. It is widely acknowledged that socioeconomic status (SES) plays a pivotal role in the manifestation of depression. However, subjective social status (SSS) representing the subjective perception of the accumulation of life experiences and the associated overall deprivation may hold even greater importance in older adults. Especially, statistics in Korea shows that most people are likely to consider themselves in middle class regardless of their objective financial status. Therefore, the objective of this study is to examine whether subjective social status is associated with depression in later life as well as how participation in formal social activities (i.e., religion/social gathering, leisure/cultural/sport activity, reunion/hometown alumni, volunteering, party/civil society organization/advocacy group) mediate this association. Multiple regression analyses and Baron & Kenny (1986) analytical approach were applied using a panel data from the 2020 Korean Longitudinal Study of Ageing. Selected sample include those who are aged 65 and older (n= 4,405). Results show that there is a negative association between SSS and depression. SSS was positively associated with formal social activities. Additionally, formal social activities have significant impact on mitigating depression. Participation in formal social activities partially mediates the relationship between the SSS and depression. Findings have implications for promoting an opportunity for older adults to participate in some formal types of social participation. Furthermore, additional research investigating the factors that can augment SSS should be warranted.

Abstract citation ID: igad104.3607

### SUBSIDIZED SENIOR HOUSING IN THE U.S.: A SCOPING REVIEW

Sojung Park[1], Soobin Park[1], Byeongju Ryu[2], Jihye Baek[1], Takashi Amano[3], and BoRin Kim[4], *1. Washington University in Saint Louis, St. Louis, Missouri, United States*, *2. Boston College, Boston, Massachusetts, United States*, *3. Rutgers University, Newark, New Jersey, United States*, *4. University of New Hampshire, Durham, New Hampshire, United States*

Although Subsidized Senior Housing (SSH) has been considered an important model to promote healthy aging among vulnerable subgroups of older adults, progress in developing, expanding, and improving SSH has been limited compared to other housing models. A comprehensive review of the recent knowledge base is a critical step toward developing and expanding policies and programs for senior housing for low-income older adults. This scoping review used a five-stage scoping review methodology to answer two research questions: 1) What are the trends in SSH empirical studies? 2) What are the main topics in the existing literature on SSH in the US? This review explores the social, physical, and service environments related to SSH in older adults in the US. Personal and environmental factors were key in examining study outcomes or main focus. We reviewed a total of 61 articles and found several notable trends, including a focus on racial and ethnic minorities, cognitive impairment among SSH residents, and Collaborative partnerships between housing operators and healthcare providers. More than half of the studies (54%) were quantitative, while the rest were qualitative (38%) or mixed-methods (8%). The lack of longitudinal or causal design research reflects the limited empirical knowledge of SSH. Four themes emerged: health and well-being (52.5%), healthcare use and health behaviors (31.1%), social relations (8.2%), and housing relocation (8.2%). We discussed implications for future research and program development efforts in SSH. Future research should explore the fit between individual characteristics and the multiple-factor environmental context.

Abstract citation ID: igad104.3608

### SUCCESSFUL CARE CONFERENCES: NURSING HOME STAFF, PERSONS WITH DEMENTIA AND CAREGIVER PERSPECTIVES

Gail Towsley, Linda Edelman, and Shelby Montez, *University of Utah, Salt Lake City, Utah, United States*

Work demands of the care team, time constraints, and a lack of guidance from the regulatory environment often result in short, perfunctory mandated quarterly care conferences (e.g., care plan meetings) that lack focus on resident preferences. Me & My Wishes is a video recording approach for documenting, viewing, listening, and discussing nursing home (NH) residents' living with dementia preferences for daily and end-of-life in care conferences. As part of a study adapting Me & My Wishes for remote delivery, we used a qualitative descriptive design to conduct focus groups (February – June 2023) with experts representing persons living with dementia (PLWD) (n=6), informal care partners (CPs) (n=8), and NH staff (n=9) to inform us of what comprises a successful care conference. We coded and analyzed audio-recorded transcripts, resolved coding discrepancies in team meetings, and derived domains from finalized codes. All groups discussed three primary domains characterizing a successful care conference: 1) resident- engagement; 2) key staff and CPs identified and engaged to support the resident perspective; and 3) structured meetings led by trained facilitators for resident-centered discussion and problem-solving. NH Experts noted that a facilitator who anticipates and mitigates potential barriers (e.g., conflicting discourse) is key for a successful care plan meeting. PLWD identified providing technology assistance for the resident and CPs as important. Care conferences that allow virtual attendance of staff and CPs who know the resident well, are resident-driven, and are well organized can facilitate communication and problem solving resulting in improved resident-centered care.

Abstract citation ID: igad104.3609

### SUCCESSFUL METHODS FOR ELICITING VIEWS OF DIRECT CARE WORKERS REGARDING NEEDS AND PREFERENCES OF HOME CARE CLIENTS

Joan Hyde, and Alanna Frost, *University of Massachusetts, Boston, Massachusetts, United States*

Over the past decades, as the recruitment and retention of Direct Care Workers (DCWs) has become a critical issue, numerous studies have sought to elicit workers' views, particularly on job satisfaction, stressors, and intent to stay. This project, which includes a review of those studies, uncovered a wide variety of methods and approaches, which we are evaluating for use in future studies that would capture direct care worker's insights regarding the needs and preferences of the people for whom they provide support. In particular, this project, in conjunction with a larger one designed to assess dually-eligible care recipients' experiences with formal home care, explores methodologies best suited to capturing the congruence of DCWs' views with those of recipients' needs and preferences. It would test the hypothesis that DCWs' assessments may supplement recipients' insights, and may strengthen advocacy to improve home care access, policies and practices. The presentation will offer a systematic review of methods and instruments, including use of validated tools and those with face validity that addresses culturally relevant factors, as well as the use of focused and hypothesis-generating open-ended interviews that are sensitive to the power relationships and voices of those participants in the endeavor. The presenters will offer potentially innovative practices, such as observation, documentation analysis, and the use of Delphi techniques which draw on the knowledge and expertise of DCWs. The presentation will also draw on studies that utilized technology and social media to reach and engage this study population.

Abstract citation ID: igad104.3610

### SUPPORTING CHINESE FAMILY CAREGIVERS OF RELATIVES WITH DEMENTIA——A PILOT INTERVENTION STUDY IN SHANGHAI

Ethan Liu[1], Xinyu Zhang[2], Jiahui Huang[2], Ansar Ayrat[2], Qiuling An[2], Fei Sun[3], and Xuehan Zhang[3], *1. Phillips Exeter Academy, Exeter, New Hampshire, United States, 2. East China Normal University, Shanghai, Shanghai, China (People's Republic), 3. Michigan State University, East Lansing, Michigan, United States*

**Background:** Effective interventions are urgently needed to assist dementia family caregivers in China, where limited formal support and services are available. This pilot study aims to test a psychoeducational program that consists of information, skills training, and the use of binaural sound through the Processing Inner Strength toward Actualization Therapy (PISTA) device to alleviate caregiver burden and improve mental health outcomes. **Methods:** Single subject design approach was used. Eight dementia family caregivers in Shanghai, China, were recruited and received a two-stage intervention over 12 weeks: Stage 1 (S1, Six 90-minute weekly sessions: Psychosocial education programs +PISTA) and Stage 2 (S2, Six 90-minute weekly sessions: Psychosocial education only). Caregiver outcomes, including caregiving burden, anxiety, depression, and positive aspects of caregiving (PAC), were assessed through established scales. Paired t-tests and visual analyses were used to compare outcome levels and trends between stages. **Results:** Seven participants completed the entire study, and 1 participant dropped out during S2. Participants showed a decrease in caregiver burden, anxiety, and depression and an increase in PAC during S1. A similar trend was observed during S2 on burden, anxiety, and PAC outcomes, except for depression. Furthermore, the decrease in burden and anxiety scores and the increase in PAC scores were less salient during S2 than during S1, suggesting more effects were observed in S1 with PISTA. **Conclusion:** Evidence is promising that psychoeducational programs combined with PISTA can lead to better psychological outcomes for dementia family caregivers. Findings need to be affirmed in future investigations using an experimental design.

Abstract citation ID: igad104.3611

### SURVEYING ADULTS' DESIRE TO KNOW AND PAY FOR A TEST OF DEMENTIA RISK

Alexandra Reed[1], Andrew Hooyman[1], Elizabeth Fauth[2], Jill Love[3], and Sydney Schaefer[1], *1. Arizona State University, Phoenix, Arizona, United States*, *2. Utah State University, Logan, Utah, United States*, *3. Neurosessments LLC, Tempe, Arizona, United States*

Advances in biomarkers of Alzheimer's disease (AD) and other dementias are promising, but it is also important to characterize the general public's desire for such biomarkers. It is also important to disentangle the desire to know test results from the desire to disclose the test results with a medical provider/insurance provider. This is particularly important for populations who are at higher risk of developing dementia but who either do not regularly engage with the medical system or who are skeptical of it. The purpose of this study was to survey adults' desire to know about their short-term risk of developing dementia, and the factors that impact their desire to know. A survey of 326 participants (mean+SD age: 41.9 + 11.9 yrs; 76% White non-Hispanic; 48% F) was completed via Amazon MTurk. Results indicated that 75% of participants would take such a test, but only 12% of them would want their medical/insurance provider to know. Non-White participants (n=73) also reported less desire to share knowledge with a medical/insurance provider, but still wanted to know their risk as much as White participants. Furthermore, the out-of-pocket price that participants were willing to pay for such a test was $100. These data suggest that a diverse set of biomarkers needs to be developed that would enable affordable screening within and outside of a medical context.

Abstract citation ID: igad104.3612

### SUSTAINABILITY OF CARE COORDINATION FOR PEOPLE WITH DEMENTIA WHO LIVE ALONE: LESSONS FROM A FEDERAL GRANT PROGRAM

Yoon Chung Kim[1], Michael Lepore[2], Kate Gordon[3], and Heather Menne[4], *1. University of Maryland Baltimore, Baltimore, Maryland, United States*, *2. University of Maryland School of Nursing, Baltimore, Maryland, United States*, *3. Splaine Consulting, Columbia, Maryland, United States*, *4. Miami University, Oxford, Ohio, United States*

Approximately one-third of older adults with dementia or cognitive impairment in the US live alone. People living alone with dementia (PLAWD) are at high risk for undesirable outcomes including malnutrition and falls, and the absence of cohabitants—who typically coordinate care—can contribute to unnecessary hospitalization and institutionalization. Since 2014, the Administration for Community Living (ACL) within the US Department of Health and Human Services has required PLAWD-focused services in ACL's Alzheimer's Disease Program Initiative. As part of a larger study of PLAWD-focused service sustainability, we examined ACL project final reports (n=73) to describe care coordination services for PLAWD and their sustainability plans. We excluded projects without services for PLAWD (n=14) or care coordination services (n=29) and where the lead organization closed (n=2) or project director died (n=1). Of 27 project reports indicating PLAWD-focused services and care coordination services, most were community-based organizations (CBOs, 67%); others were health systems/medical centers (15%), universities (11%), and government agencies (7%). PLAWD-focused care coordination services commonly entailed identifying PLAWD, assessing their needs, developing care plans, connecting PLAWD to need-relevant services, and purchasing supportive equipment, and sometimes involved training volunteers. Most organizations (70%) planned to sustain PLAWD services. The primary sustainability barrier faced by CBOs was lack of funding, whereas for health systems and universities partnering with CBOs was key to sustainability. This review of ACL-supported care coordination services for PLAWD and their sustainability highlights the need for research on the effectiveness of care coordination services for PLAWD and of different approaches to service sustainability.

Abstract citation ID: igad104.3613

### SYMPTOM BURDEN, POSITIVE AFFECT AND RESILIENCY IN OLDER ADULTS EXPERIENCING NORMAL AGING

Reid Anctil[1], Angela Chang[2], Calli Mitchell[1], Lara Traeger[1], Elyse Park[1], and Zeba Ahmad[1], *1. Massachusetts General Hospital, Boston, Massachusetts, United States*, *2. Suffolk University, Boston, Massachusetts, United States*

Older adults (age≥65) with multimorbid disease burden may be at chronic risk for bothersome symptoms, which can consequently reduce positive affect (e.g., feelings of excitement and enthusiasm). However, we hypothesized that individuals with more resiliency (the ability to "bounce back" from distress) may experience positive affect despite symptom burden. We conducted secondary analysis of baseline self-report survey data (collected between 8/2023-10/2023) from a prospective study of resiliency in older adults residing in nine continuing care retirement communities (CCRCs) across the U.S. Eligible participants lived independently in CCRCs and scored >11/15 on the five-minute Montreal Cognitive Assessment. Surveys assessed symptom burden (Condensed Memorial Symptom Assessment Scale), resiliency (Current Experience Scale), and positive affect (Positive and Negative Affect Schedule-Positive Affect). Bivariate correlations and multiple linear regression evaluated associations of symptom burden and resiliency with positive affect. Participants (M age =81, SD=5.6) were predominantly White (96%) and female (75%). Greater symptom burden correlated with lower positive affect (r=-.295, p<.001) and lower resiliency (r=-2.56, p<.001). In a multiple regression model, lower symptom burden (β = -.136, p=.003) and more resiliency (β=.626, p<.001) were independently associated with greater positive affect. The overall model accounted for 45.4% of variance in positive affect (F(2, 284) =117.198, p<.001). Lower symptom burden and more resiliency contributed to more positive affect among older adults in senior living communities. Because resiliency and positive affect are both potentially modifiable targets for intervention, longitudinal study and psychosocial interventions should explore the effects of increased resiliency on positive affect and general well-being over time.

Abstract citation ID: igad104.3614

### SYSTEMATIC REVIEW OF REHABILITATION IN SKILLED NURSING FACILITIES FOR PERSONS LIVING WITH DEMENTIA

Erica Frechman[1], Charles Semelka[2], and Valerie Freeman[3], *1. Atrium Health Wake Forest Baptist, Winston-Salem, North Carolina, United States*, *2. Wake Forest University School of Medicine, Winston Salem, North Carolina, United States*, *3. Atrium Health, Charlotte, North Carolina, United States*

We conducted a systematic review of published literature though April 2023 to identify the experiences and outcomes of persons living with dementia (PLWD) undergoing post-acute care rehabilitation in skilled nursing facilities (SNF). A systematic search for studies published in English was carried out in eight databases including PubMed, Scopus, Google Scholar, Embase, PsycINFO, CINAHL, Cochrane Library, and Web of Science. Two reviewers independently screened articles and conducted a quality appraisal of selected studies. Forty-one articles met inclusion criteria. There was heterogeneity of study designs with most articles (n=33) being observational and cross-sectional, while randomized clinical trials (n=3) and qualitative studies (n=5) were less represented. Certain themes emerged from the results regarding patient outcomes and experiences. Measured outcomes demonstrated increased health service utilization, varied functional outcomes, and suboptimal patient care. Patient experiences included significant challenges with health care transitions, gaps in healthcare worker and caregiver knowledge & education, emphasis on alternate models of care, and importance of goal alignment. Overall, this systematic review provides a comprehensive understanding of the outcomes and experiences of post-acute care rehabilitation in SNFs for PLWD. The findings highlight the need for alternative care pathways, improved healthcare transitions, tailored rehabilitation approaches, enhanced dementia training for healthcare professionals, and person-centered goal setting. By addressing these critical aspects, future interventions can be developed to improve the delivery of post-acute care for PLWD and enhance their health outcomes.

Abstract citation ID: igad104.3615

### SYSTEMS BIOLOGY OF HUMAN AGING - NETWORK MODEL 2023

John Furber, *Legendary Pharmaceuticals, Gainesville, Florida, United States*

This network is presented to aid in conceptualizing the many processes of aging, the causal chains of events, the interactions among them, feedback, and vicious cycles. It suggests promising intervention points for therapy development. The signs of human senescence are hypothesized to result from several primary causes. This network includes both intracellular and extracellular processes, ranging from molecular to whole-body. Lifestyle, environment, and proposed interventions are highlighted. Important pathways include: Nuclear mutations, transposon insertions, telomere shortening, chromosome breaks, chromatin alterations, epigenetic DNA adducts. Extracellular protein damage results in stiffness, weakness, inflammation. Altered niches for cells cause transdifferentiation, arrested cell division, cell death, cancer, stem cell depletion, tissue wasting, neurodegeneration, organ malfunction. Stiffer blood vessels promote stroke, heart disease. Lysosomes accumulate reactive, crosslinked Lipofuscin, which impairs autophagic turnover of macromolecules and organelles. Lipofuscin leaking into cytosol triggers apoptosis. Mitochondrial DNA Mutates; Lamin-A progerin, accumulates in nuclear scaffold, impairing mitosis and telomere maintenance. Nuclear Envelope Pore Proteins become oxidized, allowing inappropriate traffic of other proteins into and out of nucleus. Oxidized Aggregates in cytoplasm crosslink, resist turnover, inhibit proteasome activity, increase redox poise, and physically interfere with intracellular transport in axons. Proteasomes inhibited, reduce turnover of damaged molecules and expired molecular signals. Increased Redox Poise alters signaling and enzyme activities, and erodes telomeres. Inflammatory Cascades, promoted by damaged molecules and sick cells, further damage tissues. Neuroendocrine and immune systems degrade. Misfolded Proteins accumulate in Endoplasmic Reticulum. This diagram is maintained on the Web as a reference for researchers and students. www.LegendaryPharma.com/chartbg.html

Abstract citation ID: igad104.3616

### TARGETING HEALTH-PROMOTING ACTIVITY IN OLDER ADULTS WITH MULTIPLE CHRONIC CONDITIONS AND FUNCTIONAL LIMITATIONS

Tara Klinedinst[1], Nicholas Hollman[2], Carrie Ciro[1], and Darla Kendzor[1], *1. University of Oklahoma Health Sciences Center, Oklahoma City, Oklahoma, United States*, *2. OU-TU School of Community Medicine, Tulsa, Oklahoma, United States*

Nearly half of older adults in the U.S. have multiple chronic health conditions in addition to functional limitations (MCC + FL) that restrict health self-management activities. We tested an innovative combination of occupational therapy (OT) and behavioral activation (BA) to improve health self-management using the goal setting, scheduling/monitoring activities, and problem-solving components of the BA approach, as well as skill building, environmental modification, activity adaptation, and focus on daily routines from OT (BA+OT). BA+OT was delivered by occupational therapists over 10 sessions in the homes of participants. We present descriptive statistics from this single-blind randomized control pilot study of 11 older adults with MCC + FL who received BA+OT (n = 6) or enhanced usual care (UC; n = 5). Participants in both groups were given a Fitbit, and the UC group received a handout on managing chronic conditions. The BA+OT group was required to set at least one goal for improving physical activity. On average, participants were 73.1 years old (SD = 8.4), majority female (n = 8), and white (n = 1 Hispanic/Latino). They had an average of 3.9 chronic conditions (SD = 1.5), and 2.0 functional limitations (SD = 1.8). The most common chronic conditions were depression, high blood pressure, and arthritis. Aside from physical activity, the most common participant-identified goal areas were sleep hygiene and positioning, medication routines, and community access. This study is in progress (aiming for n = 40) but shows promise for improving health self-management by targeting meaningful, goal-directed, and health-promoting daily activity.

Abstract citation ID: igad104.3617

### TARGETING OF P21CIP1-HIGHLY-EXPRESSING CELLS ALLEVIATES NEUROPSYCHIATRIC DISORDERS WITH OBESITY

Lichao Wang[1], Binsheng Wang[1], Nathan Gasek[2], and Ming Xu[1], *1. UConn Health, Farmington, Connecticut, United States*, 2. *University of Connecticut, Farmington, Connecticut, United States*

Obesity remains a major factor for diabetes associated with insulin resistance and increases the risk of neuropsychiatric disorders, such as anxiety, depression, and cognitive deficits, resulting in a shortened in lifespan and healthspan. Cellular senescence, a state of permanent cell-cycle arrest, has been recognized as an important biological process underlying obesity and obesity-associated neurological disorders. My project aims to alleviate metabolic and neuropsychiatric dysfunction in obesity by targeting of p21Cip1-highly-expressing (p21high) cells, a rarely examined senescent cell population. By leveraging a novel transgene containing a p21 promoter driving Cre, we demonstrate the accumulation of p21high cells in brain with obesity, whereas, intermittently clearing p21high cells alleviates anxiety- and depression-related behavior and improve the cognitive function for obese mice. Of note, clearance of p21high cells decreased key factors of the senescence transcriptional program in hippocampus and amygdala. A survival study shows the intermittent clearance of p21high cells extends the lifespan of obese mice fed with high-fat diet. Our findings indicate p21high cells serve as a potential therapeutic target for neuropsychiatric disorders.

Abstract citation ID: igad104.3618

### TARGETING P21CIP1-HIGHLY-EXPRESSING SENESCENT CELLS TO IMPROVE HEALTHSPAN IN PROGEROID MICE

Binsheng Wang, Lichao Wang, and Ming Xu, *UConn Health, Farmington, Connecticut, United States*

Aging is the leading risk factor for a variety of chronic diseases. Emerging evidence suggests that modulating fundamental aging processes such as cellular senescence may delay the onset of most chronic conditions as a group and increase healthy lifespan. Cellular senescence refers to the essentially irreversible growth arrest that occurs when cells experience stress. One common feature of senescent cells is the high expression level of p16Ink4a and/or p21Cip1. We and others have demonstrated the causal roles of p16Ink4a-highly-expressing (p16high) senescent cells in various age-related conditions. However, the role of p21high senescent cells in aging remains largely unknown. To address these questions, we have generated and validated a novel p21-Cre mouse model containing p21 promoter driving inducible Cre. By crossing with floxed mice, we managed to monitor, sort, image, eliminate, or modulate p21high cells in vivo. We investigated the maximal walking speed and grip strength of klotho (kl)-/- progeroid mice weekly starting from 5 weeks old until death. We showed that clearance of p21high cells can improve physical function in kl-/- mice for a long time period. Additionally, inactivation of NF-κB specifically in p21high cells also can improve tissue function in kl-/- mice. This study will provide invaluable research tools and pinpoint the role of cellular senescence in age-related tissue dysfunction.

Abstract citation ID: igad104.3619

### TEAM BASED LEARNING, AN OPPORTUNITY TO PROMOTE INTERPROFESSIONALISM IN GERIATRICS EDUCATION: A SYSTEMATIC REVIEW

Renzo Pajuelo-Vasquez, Wendy Gutierrez-Baca, Javier Flores-Cohaila, and Fernando M. Runzer-Colmenares, *Universidad Científica del Sur, Lima, Lima, Peru*

Geriatric care is complex and requires high coordination between healthcare providers. Thus, interprofessional education remains a priority, and innovative teaching methods are needed. Team-Based Learning (TBL) - "an active learning and small group instructional strategy that focuses on higher-cognitive abilities through a sequence of activities including individual work, teamwork and immediate feedback through significant problems." – appears as one of the most valuable solutions. Following BEME recommendations, this systematic review examined the current evidence on TBL in health professions education (HPE). PubMed, EMBASE, WoS, and Scopus were searched from inception till August 2023 using key terms/subject headings related to TBL and Geriatrics. Four studies published between 2013 and 2022 met our inclusion criteria. Two studies included solely nurses and the remaining two – students from different disciplines. One study was quasi-experimental, while others were cross-sectional. Studies involved undergraduate (n=4) and graduate (n=2) students. Geriatric care was the curriculum's focus in two studies, while it was a component in the remaining two (covered in one or two modules). All studies followed the sequence of a TBL session. Although TBL was not preferred over traditional lectures for students (n=1), there was a gain in knowledge and skills in most of the studies (n=3). To analyze the quality of studies, the MERSQI was employed (Mean = 9.87, SD =1.31), but there was a lack of validity in assessment instruments and zero patient-reported outcomes. TBL is an opportunity to promote interprofessional education. However, further studies using better assessment methods and higher-level outcomes are needed.

Abstract citation ID: igad104.3620

### TEAMWORK MAKES THE DREAMWORK: CHANGES IN INTERPROFESSIONAL READINESS

Ann Rhodes, Kristin Zimmerman, Sarah Marrs, and Leland Waters, *Virginia Commonwealth University, Richmond, Virginia, United States*

Creating Interprofessional Readiness for Complex & Aging Adults (CIRCAA) is an evidence-based program grounded in the 4Ms of age-friendly care. CIRCAA aims to improve care for older adults by enhancing Scholars' ability to affect patients, learners, and team members by emphasizing interdisciplinary collaboration in person-centered care. Among other assessments, Scholars assess their interprofessional skills using the interprofessional education competency (IPEC) tool at the beginning and end of the year-long program. Evaluation data from all four CIRCAA cohorts (2020-2023; n = 22) demonstrated significant improvement in interprofessional readiness; $t(34) = -4.72$, $p < .001$. The sample across years was well-balanced; there were no significant differences in size of cohort {$F(1) = 0.516$, $p = .47$}, race {$F(3) = 0.62$, $p = .6$}, or discipline and clinical practice of Scholars {$F(1) = 0.33$, $p = 0.56$}. There was a significant difference in knowledge gained based on years of experience working with older adults {$F(4,26) = 3.308$, $p = .025$}; those who had worked with older adults for less than five years made the largest gains. Scholars working in primary care settings had larger knowledge gains than Scholars who did not work in primary care, with gains of 4.95 points and 7.87 points, respectively. This difference was not statistically significant; $t(16.3) = 1.14$, $p = 0.2$, 95% CI [-2.49-8.32]. Overall, assessment of outcomes across the four years shows substantial gains in interprofessionalism, which are particularly pronounced in new clinicians and those working in primary care settings.

Abstract citation ID: igad104.3621

### TECHNOSOCIAL INNOVATIONS IN THE HEALTHCARE SECTOR: WHAT ETHICAL ISSUES MIGHT WE FACE AS WE AGE?

Marie-Michele Lord, and Marie-Josee Drolet, *Université du Québec à Trois-Rivières, Trois-Rivieres, Quebec, Canada*

Technosocial innovations are designed to transcend current practices by linking technological development to community well-being. Their dazzling development is gradually transforming health services. Despite their recognized potential, a growing number of studies point to the ethical risks associated with their development. We are currently conducting a study to obtain a comprehensive picture of the ethical issues associated with the use of technosocial innovations for older adults and their care partners in the healthcare sector, and to identify key indicators of the presence of those ethical issues. A prospective design is use and combines a systematic literature review with a Delphi method. Of all the studies looking at the ethical issues involved in deploying technosocial innovations in the healthcare sector, a limited number have focused on those we may experience as we age. Still, six empirically-supported ethical issues have emerged : 1) exacerbation of the digital divide; 2) exclusion at multiple levels; 3) epistemic injustices; 4) infringement of dignity and confidentiality; 5) ageism; and 6) increased dependency dynamics. A number of indicators were identified in the early stages of the Delphi method, including a high rate of abandonment of the use of proposed innovations, and performance indicators with little integration of PREMs and PROMs. It is imperative to translate current knowledge into concrete resources that can be used in order to protect our rights and health as we age. This study is a first step towards identifying indicators that can be concretely used by health organizations wishing to act ethically.

Abstract citation ID: igad104.3622

### TEKIBEN --- DIGITAL DISIMPACTION AT HOME CARE IN JAPAN

Shoko Miyagawa[1], and Young Ah Seong[2], *1. Keio University, Fujisawa, Kanagawa, Japan*, *2. Hosei University, Tokyo, Japan*

Though this may not be the case worldwide, in Japan, digital disimpaction (Tekiben in Japanese) is one of the most frequently performed care procedures done by home-visit nurses. This study aimed to clarify the status of digital disimpaction at home care in Japan by interviewing nurses skilled in disimpaction. The interviews were conducted during the process of developing a simulator for digital disimpaction training and were conducted with seven nurses who provide home care. The interviews lasted between 50 and 75 minutes each. The interview data were transcribed, and descriptions of the frequency and target audience for digital disimpaction were extracted. The frequency with which each nurse performed digital disimpaction ranges from 10 to 30 times per month. The clients who require digital disimpaction include those who require the procedure regularly due to conditions such as cerebral infarction or neuropathy, as well those who are in the terminal stages of cancer or other conditions and require the procedure when assessed as necessary. Digital disimpaction techniques are not taught in educational courses at universities or vocational schools but are learned through on-the-job training at hospitals or in-home nursing. The reason for performing routine digital disimpaction is that it is difficult for family members to assist with defecation. The importance of passing stool at the time of digital disimpaction is pointed out, as it is difficult for family members to handle bowel movements in the absence of a nurse.

Abstract citation ID: igad104.3623

### TESTING OF A WEB-BASED DECISION AID TOOL FOR DEMENTIA CARE PLANNING AMONG CHINESE AMERICAN FAMILY CAREGIVERS

Chien-Ching Li, Xiao Ying Lei, and Olimpia Paun, *Rush University, Chicago, Illinois, United States*

In the US, the Chinese American population is aging rapidly. It is anticipated that Chinese American family caregivers of loved ones living with dementia will face increasing needs for health care planning. The study aimed to examine the usability and acceptability of a linguistically and culturally appropriate and wen-based decision aid (DA) tool for home care and long-term care planning among Chinese American family caregivers. The DA tool provides caregivers with information on (1) dementia stages, behaviors, symptoms, and care tips, (2) home care and long-term care choices, and their benefits and risks, (3) what matters to you when making care decisions, (4) what else do you need to make your decision, and (5) a summary to facilitating decision making with family and providers. A total of 16 family caregivers were recruited in the study. About 88% of study participants were very satisfied with the DA tool. Key feedback reported by the majority of participants includes: (1) the length and amount of information presented in the tool was just right, (2) this tool can provide sufficient information to make appropriate care decisions for loved ones with dementia., (3) the tool is easy to use and most people would learn to use this tool very quickly, (4) various functions in this tool are well integrated, and (5) I will frequently use this tool to help me discuss dementia care plans and decisions with family and providers. Further intervention with the DA tool can be developed within the Chinese population.

Abstract citation ID: igad104.3624

**THE 4MS AGE-FRIENDLY FRAMEWORK PROVES SUCCESSFUL TO ENHANCING A CERTIFIED NURSING ASSISTANT TRAINING PROGRAM**

Valerie Bearden[1], and Robin McAtee[2], *1. Oaklawn Center on Aging, Hot Springs, Arkansas, United States*, *2. University of Arkansas for Medical Sciences, Little Rock, Arkansas, United States*

The Arkansas Geriatric Education Collaborative, a HRSA-funded Geriatric Workforce Enhancement Program grantee, partnered with a local Certified Nursing Assistant training program to develop a curriculum utilizing the 4Ms Framework of Age-Friendly Care of older adults. The curriculum was designed to empower CNA students and other direct care staff with knowledge of how to deliver more effective, person-centered care. This was accomplished by synthesizing current content with each of the 4Ms: What Matters, Medication, Mentation, and Mobility. During the introduction to person-centered care, strategies infused included instruction about how to have meaningful conversations about What Matters to the older adult. For Mobility, instruction was provided regarding safety and the use of adaptive equipment. Mentation was addressed by teaching the students ways to prevent and identify conditions such as depression and delirium. To include the "M" of medication, the students were taught about using non-pharmacological strategies for conditions such as insomnia and pain. The way all elements are intertwined and can interfere with What Matters was emphasized and examples provided. Of the 34 CNA students trained, 100% of students achieved both objectives during a clinical experience. Improvement of care was identified by a student as recognizing how to calm a frightened resident by using validation therapy. Another student reported the strategy of providing a bed bath in lieu of a shower to a resident who was unsteady because of a pain medication. Program development was completed in April, 2023, at which time it was put into practice and evaluated.

Abstract citation ID: igad104.3625

**THE "DOUBLE JEOPARDY" OF MIDLIFE AND OLD AGE MORTALITY TRENDS IN THE UNITED STATES**

Leah Abrams[1], Neil Mehta[2], and Mikko Myrskyla[3], *1. Tufts University, Medford, Massachusetts, United States*, *2. University of Texas Medical Branch, Galveston, Texas, United States*, *3. Max Planck Institute, Rostock, Mecklenburg-Vorpommern, Germany*

Since 2010, U.S. life expectancy growth has stagnated. Much research on U.S. mortality has focused on working age adults given adverse trends in drug overdose deaths, other external causes of death, and cardiometabolic deaths in midlife. We show that the adverse mortality trend at retirement ages (65+ years) has in fact been more consequential to the U.S. life expectancy stagnation since 2010, as well as excess deaths and years of life lost in 2019, than adverse mortality trends at working ages. These results reveal that the U.S. is experiencing a "double jeopardy" that is driven by both midlife and older-age mortality trends, but more so by older-age mortality. Understanding and addressing the causes behind the worsening mortality trend in older ages will be essential to returning to the pace of life expectancy improvements that the U.S. had experienced for decades.

Abstract citation ID: igad104.3626

**THE ASSOCIATION BETWEEN APOE Ε4 AND EPISODIC MEMORY IN MIDLIFE: TESTING THE COGNITIVE PHENOTYPE HYPOTHESIS**

Kyoung Shin Park[1], Jennifer Etnier[1], Samantha DuBois[1], Samuel Kibildis[1], Hadassah Som-Pimpong[1], Jarod Vance[2], and Christopher Wahlheim[1], *1. University of North Carolina at Greensboro, Greensboro, North Carolina, United States*, *2. North Carolina Institute for Medical Research, Inc, Durham, North Carolina, United States*

Carrying at least one ε4 allele of Apolipoprotein E (APOE ε4) is a strong genetic risk factor for Alzheimer's disease (AD). It is contentious whether the effect of APOE ε4 on memory decline is because of cognitive phenotype or prodromal AD cases. Inconsistent findings on this topic could be due to the presence of AD pathology in older ages and the lack of sensitivity of memory measures. To exclude potential prodromal AD cases, we tested the association between APOE ε4 and episodic memory among cognitively unimpaired adults in midlife. Data from a subset of 103 participants (Mage=57±6 years; 87.4% females) from an ongoing clinical trial (NIH R01AG058919) were analyzed. APOE genotype was assessed from passive drool saliva samples, and participants completed memory assessments. Analyses of covariance compared 39 carriers of APOE ε4 versus 64 non-carriers of APOE ε4 for their performance of mnemonic discrimination, auditory-verbal and visuospatial short- and long-term memory, paired associative memory, and verbal logical memory, while controlling relevant covariates (e.g., age, education, sex, and physical activity level). After covariate adjustments, significant negative relationships between APOE ε4 and mnemonic discrimination were observed while nearly significant negative relationships were observed for auditory-verbal long-term memory. These findings imply that APOE ε4 could be associated with a cognitive phenotype of decline in mnemonic discrimination and auditory-verbal long-term memory, and the phenotype effects on memory decline could start in midlife. This suggests the importance of early lifestyle interventions for cognitive health in midlife, especially in people at genetic risk for AD.

Abstract citation ID: igad104.3627

**THE ASSOCIATION OF CAREGIVER STRAIN AND NEEDS OF CAREGIVERS AND PEOPLE LIVING WITH DEMENTIA**

Allison Griswold, Miranda Moore, Joanna Jungermann, Molly Perkins, and Alexis Bender, *Emory University, Atlanta, Georgia, United States*

Caregivers play a vital part of dementia care management and are key to keeping people living with dementia at home. Providing this care can result in caregiver strain, especially among those who live in low or under resourced communities, such as rural or impoverished areas. One instrument for measuring caregiver strain among this population is the BRI

caregiver strain instrument, which includes four dimensions of strain including caregiver mastery, relationship strain, health strain and social isolation. To date, little research assessing the validity of the instrument. We assessed the validity using confirmatory factor analysis in a cohort of 930 caregivers providing care to people undergoing assessment for dementia representing four diverse regions in Georgia (Atlanta, Albany, Augusta, and Macon). Then, we examined the associations of the lack of caregiver knowledge of dementia, care recipient function, and challenging behaviors using univariate and multivariate linear regression. We found that the four-factor solution showed good model fit and was appropriate for subsequent analysis with this population. In our regression models, we found that caregiver lack of understanding of dementia significantly predicted caregiver strain (β=2.09, P<.001). When controlling for care recipient function, lack of knowledge decreased in strength, but remained significant (β=1.86, P<.001). When adding challenging behaviors, lack of dementia knowledge lost significance and challenging behaviors became the strongest predictor of caregiver strain (β=2.03, P<.001). Caregiver strain is a multifactorial process and tools to reduce challenging behaviors and increase caregiver knowledge are promising points of intervention early in the diagnostic process.

Abstract citation ID: igad104.3628

### THE ASSOCIATION OF NON-SEVERE COVID-19 INFECTION AND PROGRESSION TO FRAILTY AMONG ROBUST OLDER VETERANS

Natasha Melo Resendes, Fei Tang, Dominique Tosi, and Iriana Hammel, *Miami VA Healthcare System, Miami, Florida, United States*

**Background:** Frailty is a multisystem syndrome characterized by vulnerability to stressors, such as the infection with COVID-19. We set out to examine if mild COVID-19 infection impacted frailty onset among older robust veterans, and whether other factors were associated with developing frailty. **Methods:** Retrospective cohort study of U.S. Veterans 55 years and older who had a SARS-CoV-2 infection between 3-15-2020, and 11-30-2020, were active VHA users/ and had a VA Frailty Index (VA-FI) of 0.0 at time of diagnosis. We used nationwide VHA data from the VA COVID-19 Shared Data Resource database. We excluded Veterans hospitalized after contracting COVID-19, and who died within the 25-month follow-up. Cox proportional hazard model was used to assess the association between COVID-19 infection and developing frailty or (pre-frailty + frailty), after adjusting for age, BMI, race, ethnicity, gender, smoking, and rurality. **Results:** 86061 Veterans aged 55 and older met inclusion criteria. Mean age was 68.4 yrs (SD=7.8) with 91.0% (78347) males. 7561 (8.8%) tested positive for COVID-19, and 78500 (91.2%) tested negative. The COVID-19 positive group had a higher percentage of black and city-dwelling Veterans than the COVID-19 negative group. After adjustments listed above, testing positive for COVID-19 was associated with an increased hazard of becoming frail (adjusted HR=1.63, 95%CI: 1.24-2.13), and pre-frail + frail (adjusted HR=1.77, 95%CI: 1.53-2.06). **Conclusion:** We showed that mild COVID-19 was associated with higher incidence of frailty and pre-frailty. Follow-up post COVID-19 infection should include a comprehensive geriatric assessment for early frailty identification and intervention.

Abstract citation ID: igad104.3629

### THE ASSOCIATIONS BETWEEN LUNG DISEASE, CAREGIVING, AND PSYCHOSOCIAL WELL-BEING IN OLDER US ADULTS

Mariah Malak Bilalaga[1], and Louay Almidani[2], *1. Medstar Health, Baltimore, Maryland, United States*, *2. Wilmer Eye Institute, Baltimore, Maryland, United States*

The aim of this study was to examine the association between lung disease, caregiving for adults with lung disease, and the psychological and social well-being of older adults using data from the National Health and Aging Trends Study 2021, a nationally representative sample of US adults aged ≥ 65 years. Lung disease was determined based on self-reported data regarding diagnoses of emphysema, asthma, or chronic bronchitis. The Patient Health Questionnaire for Depression and Anxiety (PHQ-4) questionnaire was utilized to assess depressive and anxiety symptoms. Social isolation was defined based on responses about living arrangements, frequency of communication with others, and participation in activities. Prevalence ratios adjusted for covariates were calculated using Poisson regression. Among adults with lung disease, the prevalence of depressive symptoms was notably higher (prevalence ratio [PR]: 1.43; 95% CI: 1.05, 1.96) compared to those without. However, the prevalence of anxiety symptoms and social isolation did not significantly differ when compared to their counterparts without lung disease (p< 0.05). Caregivers responsible for adults with lung disease had a greater prevalence of depressive symptoms (PR: 1.61; 95% CI: 1.16-2.25), but there was no notable difference in the prevalence of anxiety symptoms or social isolation (p< 0.05). We highlight that both lung disease and caregiving for adults with lung disease are significantly associated with increased prevalence of depressive symptoms, but not anxiety symptoms or social isolation, among older adults in the United States. Strategies are required to enhance the psychological well-being of individuals with lung disease as well as their caregivers.

Abstract citation ID: igad104.3630

### THE ASSOCIATIONS OF HANDGRIP STRENGTH AND ANTHROPOMETRIC MEASUREMENTS WITH UPPER LIMB BODY FAT MASS IN OLDER ADULTS

Jethro Raphael Suarez, Joon-Hyuk Park, Renoa Choudhury, Jeffrey Stout, Chitra Banarjee, and Ladda Thiamwong, *University of Central Florida, Orlando, Florida, United States*

Body Fat Mass (BFM) is a crucial body composition measurement serving as a predictor for various health conditions and mortality in older adults. However, limited research has been conducted to investigate how upper limb BFM associates with anthropometric measurements and handgrip strength (HGS) in older adults. In this cross-sectional study, 119 older adults, ranging from 60 to 96 years of age (93 women, 26 men; mean age 74.71 ± 7.35 years), were assessed. Body composition was evaluated using the portable Bioelectrical Impedance Analysis (BIA) device, InBody S10, and average HGS was measured on each side using a hand grip dynamometer (JAMAR 5030J1). Multiple linear regression analysis was performed and found a significant, positive association for age (β = 0.048 , p = 0.024), height (β = 0.649, p = 0.024), and BMI (β = 1.371, p < 0.001) with left arm BFM, while weight and left HGS showed a negative association (β = -0.152, p = 0.007; β = -0.070 , p = 0.004, respectively). Similar trends were seen for the right arm. However, height and right HGS became statistically insignificant (β = 0.536, p = 0.069; β = -0.046 , p = 0.054, respectively) for right arm BFM only. These results show that the independent variables may have the ability to predict upper limb BFM in older adults without the need for body composition measurement methods (e.g., BIA). Further research is warranted to examine the true predictive capacity of the independent variables in relation to upper limb BFM.

Abstract citation ID: igad104.3631

**THE DUAL IMPACT OF EDUCATION AND OCCUPATION ON COGNITIVE FUNCTIONING IN OLDER MEXICAN ADULTS**

Jose Cabrero Castro[1], Mariela Gutierrez[1], Theresa Andrasfay[2], Emma Aguila Vega[3], and Brian Downer[1], *1. University of Texas Medical Branch, Galveston, Texas, United States*, *2. California State University San Marcos, San Marcos, California, United States*, *3. University of Southern California, Los Angeles, California, United States*

Our research investigates the relationships among educational attainment, occupational mental demands, and cognitive functioning in Mexican adults aged 50 and older. We expand on prior studies demonstrating education's link to cognitive function by examining how the complexity of one's primary occupation also influences cognitive health. We hypothesize that cognitively demanding work, irrespective of education level, correlates positively with enhanced cognitive performance. Furthermore, we propose an additive effect, wherein high occupational mental demands amplify cognitive functioning beyond education's influence. Our study uses data from 12,939 participants in the 2012 Mexican Health and Aging Study (MHAS) linked to an occupational database (Occupational Information Network O*NET). Employing generalized linear models to predict a summary cognition score, the findings unveil compelling insights. Both mental processes (β=0.17) and cognitive abilities (β=0.16) required in participants' main occupations demonstrate positive correlations with cognitive scores (p<.01). Education emerges as a predictor of cognitive performance and had a stronger relationship with cognitive functioning compared to occupational mental demands (β=0.79, p<.01), highlighting the long-lasting effects of education in cognition emphasizing its role in shaping cognitive abilities throughout life. Adjusting for demographic and health factors reaffirms these associations. Moreover, our research introduces an innovative approach by creating an additive score for education and occupational mental demands. Our results reveal that the combined impact of education and mental demands is associated with higher cognitive scores (β=0.97, p<.01). In conclusion, our study presents a novel perspective by demonstrating that a combination of education and occupational mental demands may yield amplified cognitive benefits.

Abstract citation ID: igad104.3632

**THE EARLY LIFE CONTEXTS OF THE HEALTH AND RETIREMENT STUDY HCAP SAMPLE: NEW INSIGHTS INTO DEMENTIA RISK FACTORS**

Jacqui Smith, Marina Larkina, and Wenshan Yu, *University of Michigan, Ann Arbor, Michigan, United States*

There's increasing interest in identifying potentially modifiable early-life socioenvironmental factors associated with late-life cognitive decline and dementia. To date, much research points to the importance of the number of years spent in education and age. We sought to extend evidence for potential modifiable targets by examining life history data available to date for women and men in the 2016 HRS-HCAP sample (N = 2645; Mean Age = 76; 61% women). The life history data were drawn from three HRS public cross-wave files: the 2015-2017 Life History Mail Surveys, the 1992-2018 Childhood Family and Childhood Health file, and the 1992-2020 Geographical Mobility File. Among the early factors examined were the US Census region of birth and school location at age 10; childhood physical and mental health conditions; childhood family SES; early learning and sensory/motor problems, early life activity engagement (cognitive, physical, social), college majors, and post-education work. The HRS-HCAP algorithm classifications (Dementia, MCI, vs Normal Cognition) served as outcomes. In addition to quantity of education and age/cohort, significant effects (all ps < .001) were revealed for childhood geographical locations, childhood mental health, and early-life access to and engagement in enriching activities. Over time, whereas the impact of changes such as increased access to post-secondary education and enriching high school activities are observed, other modifiable factors emerge. Tracking the impacts on cognitive aging of historical changes and disparities in the life course contexts of people in the HRS-HCAP subsample opens a new window into relevant social-environmental factors to target for intervention

Abstract citation ID: igad104.3633

**THE EFFECT OF A DRUG-BURDEN INDEX ON FALLS RISK INCREASING MEDICATIONS DEPRESCRIBING IN VETERANS AT RISK OF FALLING**

Michelle Paulsen[1], Sunny Chen[1], Erica Martinez[1], and Katherine Ritchey[2], *1. VA Puget Sound, Seattle, Washington, United States*, *2. University of Washington, Seattle, Washington, United States*

Falls risk-increasing medications (FRIMs) have negative effects on balance and psychomotor functioning which increase the risk of falling in older adults. Thus, deprescribing of FRIMs is a recommended fall prevention intervention. The Drug Burden Index (DBI) measures total exposure to anticholinergic and sedative medications and has been associated with falling risk in older adults. We evaluated if inclusion of a DBI score would influence deprescribing of FRIMs in older veterans at risk of falling. A pharmacist conducted a medication review for FRIMs in two cohorts of older veterans participating in a fall prevention group. The primary care prescriber (PCP) was alerted to recommendations through the electronic medical record. Inclusion of the DBI was included and shared with the PCP in the second cohort only. Changes to FRIMs and reduction in DBI score was calculated for all participants three months after initial FRIM review. Nearly all (95%) participants (n=44) were prescribed a FRIM. The most common active prescriptions contributing to DBI score were for anticholinergics (37.9%), antidepressants (33.3%) or anticonvulsants (31.8%) agents. DBI improved in both cohorts, but DBI change was greatest in the second cohort (n=18) (first cohort mean DBI change= 0.168; second cohort mean DBI change= 0.189; p 0.002). Percentage of recommendations accepted and acted upon by PCP was similar between both groups (60% vs 59%). Inclusion of a DBI is a helpful measure of FRIM deprescribing and may further encourage deprescribing behaviors. Feedback from PCPs will clarify the role of DBI in fall prevention medication reviews.

Abstract citation ID: igad104.3634
### THE EFFECT OF FINANCIAL STRAIN AND LANGUAGE OF INTERVIEW ON DEPRESSIVE SYMPTOMS IN MEXICAN AMERICAN OLDER ADULTS

Veronica Hernandez, Lucy Nasser, and Soham Al Snih, *University of Texas Medical Branch, Galveston, Texas, United States*

We used data from the Hispanic Established Population for the Epidemiological Study of the Elderly to examine the relationship of financial strain and interview language to high depressive symptoms among Mexican Americans aged ≥75 years without history of depressive symptoms at baseline over a 12-year period (2004/05-2016; N=1157). Participants scored < 16 in the Center for Epidemiologic Studies Depression Scale at baseline. Measures included age, marital status, years of formal education, body mass index, social isolation, church attendance, self-reported medical conditions, cognitive and physical function, pain, falls, disability, and hand grip strength. We used a generalized estimating equation model to estimate the odds ratio (OR) and 95% confidence interval (CI) of depressive symptoms as a function of financial strain and interview language over time, adjusting for all covariates. We found that participants experiencing financial strain and interviewed in Spanish had greater odds of experiencing high depressive symptoms (OR=2.48, 95% Cl=1.37-4.48) compared to those who were not experiencing financial strain and selected be interviewed in English. No significant association with depressive symptoms was found in participants with financial strain and interviewed in English or those without financial strain and interviewed in Spanish. In conclusion, Mexican American older adults were at a high risk for high depressive symptoms over time if they were experiencing financial strain and preferred be interviewed in Spanish. Early screening for depression may prevent or delay depressive symptoms for individuals in this population at high risk for depression.

Abstract citation ID: igad104.3635
### THE EFFECT OF PANDEMICS ON TRUST IN DOCTOR IMPROVEMENT AMONG CHINESE OLDER ADULTS: LESSONS FROM COVID-19

Chao Guo, Xiyuan Hu, Dianqi Yuan, Huameng Tang, and Peisen Yang, *Peking University, Beijing, Beijing, China (People's Republic)*

The COVID-19 pandemic has had a major impact on the physician-patient relationship. For older adults, trust in doctors during the pandemic affects their lives and health. This study aimed to estimate the effect of the first wave of COVID-19 transmissibility in China on older adults' trust in doctors. We obtained individual data from the Chinese Family Panel Studies (CFPS). A total of 2,225 participants aged 65 and older were included in this study. The COVID-19 pandemic was used as a natural experiment. We obtained difference-in-differences (DID) estimates of the pandemic's effects by exploiting temporal variation in the timing of COVID-19 exposure across participants interviewed from 2018 to 2020 together with the geographical variation in COVID-19 severity at the provincial district level. Overall, the DID estimates showed that the COVID-19 pandemic could significantly increase trust in doctors after controlling for multiple covariates (adjusted β: 0.053, 95% CI: 0.028–0.078). Stratified analyses showed that the effect was only in females and the more educated ones but not in their counterparts. In addition, the COVID-19 pandemic showed a positive effect on trust in doctors among 65-79-year-olds (0.082, 0.056–0.109), but older adults over the age of 80 showed significantly reduced trust in doctors (-0.236, -0.418–-0.055). Our findings confirm the association between the COVID-19 pandemic and trust in doctors among older Chinese people. Hospitals and other medical organizations should pay more attention to improving older adults' trust in doctors and building a better physician-patient relationship to face the future pandemic.

Abstract citation ID: igad104.3636
### THE EFFECT OF THE JIGSAWDIO PROGRAM ON COGNITION AMONG OLDER ADULTS WITH DEMENTIA

Yongseop Kim[1], Amy Young[2], Jungjoo Lee[3], Kathleen Welsh-Bohmer[4], Marcia Ory[5], and Junhyoung Kim[5], *1. Indiana University, Bloomington, Indiana, United States*, *2. Jigsawdio, Bloomington, Indiana, United States*, *3. University of Southern Mississippi, Hattiesburg, Mississippi, United States*, *4. Duke University, Durham, North Carolina, United States*, *5. Texas A&M University, College Station, Texas, United States*

Puzzle activities in dementia patients have been associated with improvements in mood and cognition across a number of studies. Our research team designed and developed Jigsawdio, a digital technology that provides an innovative and interactive multisensory solution which supports memory recall in patients with dementia through the completion of personalized, audio-visual jigsaw puzzles. This device builds on the benefits derived from traditional puzzles by integrating salient audiovisual stimuli aimed at evoking nostalgia and encouraging reminiscence. The purpose of this study was to assess pre- and post-intervention effects of Jigsawdio use after six weeks among older adults with mild to moderate cognitive impairment. Using a single arm, twice weekly intervention, nine persons participated in the Jigsawdio program study. The Montreal Cognitive Assessment (MoCA) was used to measure changes in cognitive function and recall on the first and last days of the intervention. A paired t-test was used to investigate the group mean differences in cognition before and after participation in the intervention program. We found a significant group mean difference between the post-assessment and the baseline assessment MoCA scores of participants. This study presents suggestive evidence of cognitive benefits and feasibility of the technology in older adults with dementia.

Abstract citation ID: igad104.3637
### THE EFFECTIVENESS OF NON-PHARMACOLOGICAL INTERVENTIONS ON SLEEP QUALITY IN RA AND FMS: A SYSTEMATIC REVIEW

Ching-I Chin, *New York University, New York City, New York, United States*

Objective: Sleep disturbances can significantly impact the well-being of individuals with Rheumatoid Arthritis (RA) and Fibromyalgia (FMS), leading to daytime sleepiness, fatigue, and increased pain. This systematic review aims to assess the available evidence on the effectiveness of non-pharmacological interventions in improving sleep quality for individuals with RA and FMS.

**Method:** A comprehensive search was conducted in English electronic databases (CINAHL plus, SPORTDiscus, EMBASE, Medline, PubMed, Web of Science, and Google Scholar) to identify relevant articles. The review includes 15 studies, consisting of 13 randomized controlled trials (RCTs) and 2 non-randomized controlled trials (non-RCTs), published between 2012 and 2022. Primary and secondary outcome measures related to sleep quality were assessed using subjective and objective measures.

**Results:** Interventions in the studies were diverse, involving physical and mind-body approaches, lasting 6 to 16 weeks with session durations of at least 30 minutes. The total intervention hours ranged from 6 to 32 hours across all studies. Follow-up evaluations were conducted in four studies up to 6 months after the last treatment. While the Arthritis Foundation Exercise Program and Yoga did not report statistically significant improvements in sleep quality for RA, non-pharmacological interventions showed a positive impact on sleep outcomes for RA and FMS populations.

**Conclusion:** Non-pharmacological interventions show promise as a potential alternative method to improve sleep quality for individuals with RA and FMS. However, there is no evidence to support the superiority of one intervention over another. Keywords: Rheumatoid Arthritis. Fibromyalgia Syndrome. Sleep quality. Sleep disturbance. Exercise. Non-pharmacological intervention.

Abstract citation ID: igad104.3638

### THE EFFECTS OF EARLY-LIFE ADVERSITY ON WOMEN'S COGNITIVE HEALTH AFTER AGE 50

Kimson Johnson, *University of Michigan, Ann Arbor, Michigan, United States*

According to the Alzheimer's Association, 6 million people age 65 and older are living with Alzheimer's Disease in the United States, and nearly 4 million are women. Few research studies have examined the impact of adverse childhood events (ACEs) on cognitive health among women over 50. Evaluating gender-specific differences in predictors of cognitive health can help clarify mechanisms that hinder or magnify gender-based health differences and provide information on ways to narrow gender disparities in dementia. The study examined the relationship between ACEs, cognitive impairment, and incident dementia using data from the Health and Retirement Study Cross-wave 2015-2017 Life History Mail Survey (N = 4,980; Mage = 67.6). Cognitive status was measured using the 2016 Langa-Weir three category indicator: Normal, Cognitive Impairment but No Dementia (CIND), and Dementia. Compared to women exposed to 0 ACEs, women exposed to 1 ACE had a higher relative risk for CIND and dementia (RRR=1.71, p<.05; RRR=2.44, p<.05). Women exposed to 3 ACEs had a higher relative risk for dementia (RRR=3.43, p<.05). Non-Hispanic Black women exposed to 3 ACEs had a lower relative risk for dementia (RRR=0.18, p<.05) than white women. Women with 12 years of education have a higher relative risk for CIND and dementia (RRR=2.27, p<.05; RRR=3.45, p<.05) than women with >13 years of education. Although women's life expectancy has increased over the past few decades, women remain at risk for delayed identification of cognitive decline. Future research should examine how other critical social determinants of health influence women's cognitive health after 50.

Abstract citation ID: igad104.3639

### THE EVOLUTION OF NURSING HOME COMMUNICATION WITH RESIDENT FAMILIES DURING THE COVID-19 PANDEMIC

Courtney Hawes, Joan Brazier, Amy Meehan, and Emily Gabois, *Brown University School of Public Health, Providence, Rhode Island, United States*

Nursing homes experienced numerous challenges resulting from the COVID-19 pandemic. Among these challenges was ensuring appropriate and effective communication with friends and family members of residents, particularly in light of visitation restrictions. Using qualitative data from 156 in-depth, semi-structured interviews with administrators of 40 nursing homes across the US from July 2020 through December 2021, challenges and solutions to communicating effectively with resident families are characterized. Findings include three themes: 1. Administrators identified best practices regarding the frequency of communicating with families. Particularly early in the pandemic and when visitation was restricted, families expressed a desire for very frequent updates regarding resident well-being and COVID-19. Administrators reported the challenges of balancing family expectations regarding information with their capacity to meet those expectations. 2. Administrators discussed different modes of communication used with families. Existing systems of mass-communication were expanded and new processes were developed. 3. Administrators described challenges in determining the appropriate content to communicate and navigating changing policies. While nursing home staff were required to share data on COVID-19 outbreaks, administrators shared lessons learned regarding specificity of information to be shared, and how they balanced required infection control information with pleasant updates regarding residents and their activities. Strategies identified by administrators may be informative for efforts to promote effective communication for both general day-to-day updates and for future emergency or pandemic crises.

Abstract citation ID: igad104.3640

### THE EXPERIENCES OF OLDER ADULTS IN HAITI AND THE UNITED STATES AT THE ONSET OF COVID-19

Laurie Blackman[1], Kathryn Krase[2], and Donna Wang[3], *1. Yeshiva University, New York City, New York, United States, 2. Krase Consulting, Brooklyn, New York, United States, 3. Springfield College, Springfield, Massachusetts, United States*

From the early onset of the COVID-19 pandemic, there was a disproportionate risk and impact of the disease on older adults and communities of color. This article explores the differences in experiences of older Haitian and older US adults at the onset of the COVID-19 pandemic. Since wealth creates accessibility to needed resources, including the best healthcare and support services, the disproportionate risk and impact of COVID-19 on older adults required a focused look at how older adults in vulnerable countries such as Haiti faired when compared to older adults in the United States. Using data collected from a sample of 240 Haitian older adults in August 2020 and 204 US older adults in June 2020, the findings indicated that Haitian older adults experienced more significant psychological, financial, and emotional distress due to the COVID-19 outbreak than their US older adult counterparts. The results of this study revealed the challenges that older adults faced at the onset of the pandemic, which was more pronounced in developing countries. However, the surge in violence in Haiti in the first quarter of 2023 killed more people than the COVID-19 outbreak. This research provides important insight for developing and framing policies and interventions that are not modeled on the measures taken elsewhere but adapted to a country's reality and the population's actual needs.

Abstract citation ID: igad104.3641

### THE FEASIBILITY OF TAICHI WITH MUSIC FOR HEALTH IN INDIVIDUALS LIVING WITH ADRD AND THEIR CAREGIVERS

Yan Du[1], Emma Blackmon[1], Christina Smith[2], Cindy Robles[2], Sandeep Subramanian[1], Sue Jee[1], Kylie Meyer[3], and Penny Roberts[4], *1. UT Health San Antonio, San Antonio, Texas, United States*, *2. WellMed Charitable Foundation, San Antonio, Texas, United States*, *3. Case Western Reserve University, Cleveland, Ohio, United States*, *4. Loyola University New Orleans, New Orleans, Louisiana, United States*

Caregiving demands associated with cognitive and physical function decline in care recipients (CRs) living with Alzheimer's disease and related dementias (ADRD) contribute to compromised health and quality of life among ADRD caregivers. In turn, the affected health of caregivers affects the care quality of CR and their health. TaiChi or music is evidenced to be beneficial for the generation population and those with various health conditions. This pilot project assessed the feasibility and preliminary efficacy of an 8-week TaiChi with music (TCM) program on individuals with ADRD (e.g., physical and cognitive function) and their family caregivers (e.g., caregiver distress and quality of life). Outcomes were compared between baseline and post-intervention using paired t-tests. Qualitative interview data exploring the perceptions of participating in TCM program were analyzed using content analysis. Five dyads were enrolled in this study (CR age=77.4±6.8; Caregiver age=62.6±6.7). The overall class attendance rate is 65%. Among the small but diverse sample, adult child care dyads had higher class attendance (93%); spousal care and CR with severe cognitive impairment dyads had lower attendance (46%). Improvements were found in assessed health outcomes for both CR and caregivers, and CR's physical function significantly improved at 8-week (p=0.016). Qualitative data revealed perceived balance improvements; Caregivers perceived calmness and peace, and expressed their interest in participating in TCM in the future. TCM may be more feasible in ADRD individuals with mild to moderate cognitive impairment. Future research will evaluate the efficacy of this intervention in a larger sample with a longer follow-up period.

Abstract citation ID: igad104.3642

### THE FINANCIAL AND EMOTIONAL IMPACT OF COVID-19 FOR DEMENTIA CAREGIVERS: A COMPARISON OF THE NHATS AND HISPANIC EPESE

Sunshine Rote[1], Jacqueline Angel[2], Phillip Cantu[3], and Felipe Antequera[2], *1. University of Louisville, Louisville, Kentucky, United States*, *2. University of Texas at Austin LBJ School, Austin, Texas, United States*, *3. The University of Texas Medical Branch, Galveston, Texas, United States*

During the COVID-19 pandemic, older adults and their caregivers/care partners (hereafter referred to as "caregivers") experienced social isolation, reduced autonomy, disruptions in medical care and barriers to community-based services such as adult day centers. We compare the extent to which these disruptions affected white, Black, and Hispanic caregivers. We utilize newly released data on adult children caregivers from the Caregiver Supplement to the Hispanic Establish Population for the Epidemiologic Study of the Elderly (HEPESE CG, 2021) and the National Health and Aging Trends COVID-19 Family Members and Friends Dataset (NHATS FF, 2020/2021) to compare caregiver emotional well-being and financial strain among non-Hispanic White (NH White, n=327), non-Hispanic Black (NH Black, n=57), and Mexican American (n=57) adult children caregivers. Results reveal that a larger fraction of NH Black and Mexican American caregivers reported financial strain than NH White caregivers, and that more Mexican American caregivers reported loneliness and depression than other groups of caregivers. For Mexican American caregivers, those reporting lower financial well-being tended to be younger. For NH Black caregivers, there were no significant predictors of financial strain or wellbeing. Finally, NH White caregivers were younger, and more likely to be female and NH White dementia caregivers reported more financial strain and loneliness than NH White non-dementia caregivers. Taken together, this work provides valuable insight into the role of the COVID-19 pandemic in exacerbating caregiver health disparities among caregivers and provides valuable insight as well as to the high-impact intervention points for caregivers from minoritized populations.

Abstract citation ID: igad104.3643

### THE FOURTH AGE IN PROSPECT: A STUDY FROM FIVE NATIONS

David Ekerdt[1], Jaroslava Hasmanova Marhankova[2], Helene Fung[3], Shyhnan Liou[4], and Stephan Lessenich[5], *1. University of Kansas, Lawrence, Kansas, United States*, *2. Charles University, Prague, Hlavni mesto Praha, Czech Republic*, *3. The Chinese University of Hong Kong, Hong Kong, Hong Kong*, *4. National Cheng Kung University, Tainan, Tainan, Taiwan (Republic of China)*, *5. Goethe University, Frankfurt, Hessen, Germany*

Higgs and Gilleard (2015) have uniquely theorized the fourth age as a "social imaginary" of deep old age that blends notions of frailty, abjection, and the moral relations of care. This report evaluates the power, reach, and coherence of the fourth age imaginary among older adults in relative good health. In a qualitative design and within samples at five sites (in Czechia, Germany, Hong Kong, Taiwan, and the United States), 138 adults aged 70+ and still living independently discussed what it would mean to be "not independent" in later life. Replies referenced other people in general, specific people, or one's own potential experience. Pooled across sites, the views of our participants confirm the theorized features of the social imaginary. Participants spoke readily of gateway infirmities heralding frailty and of frailty's abjection; expressed dread and abhorrence of dependence, some saying that death would be preferable; and were anxious about nursing homes and about burdening others with an obligation to care for them. In all, the bleak but formidable reputation of the fourth age impinges on those living in the third, raising apprehensions about the future. At the same time, perhaps this prospect of unwanted old age can function as adaptive, motivating efforts to maintain bodily and cognitive integrity, to plan for the future, to cultivate support networks, or to savor the present. Fourth-age studies that document the lived experience of frailty and dependence have the potential to undermine the social imaginary and furnish new narratives for facing the future.

Abstract citation ID: igad104.3644

### THE HEALTHY AGING INITIATIVE: LONGITUDINAL RESEARCH EMPOWERING OLDER ADULTS TO LIVE THEIR BEST LIVES

Heather Andrews[1], Talia Gilfix[1], Benjamin Stein[2], Davide Cappon[2], Maggie Syme[2], and Alvaro Pascual-Leone[2], *1. Hebrew SeniorLife, Boston, Massachusetts, United States*, *2. Hebrew Rehabilitation Center for the Aged, Boston, Massachusetts, United States*

The World Health Organization (WHO) has designated this the decade of Healthy Ageing--focused on developing and maintaining functional ability that enables well-being in older age. We developed the Healthy Aging Initiative (HAI), a prospective cohort study of adults 55+ measuring functional ability, going beyond a disease-focused model of population ageing to holistic intrinsic capacity (physical, cognitive, emotional, behavioral, cultural health) and environmental context. A multidisciplinary team of geroscientists developed a protocol of subjective and objective assessments evaluating several known modifiable risk factors for disability and dementia, mobility, strength, functional status, and mental wellness. By tracking these domains annually, we influence participant health trajectories with timely, actionable feedback and gain insight into causal factors that impact functional ability, creating a blueprint for translational clinical research in healthy ageing. A feasibility pilot (N=37; 80.8 avg. age, 67-93 age range; 92% White) was conducted within two affiliated older adult living communities. The revision process included evaluating specificity, sensitivity, and utility of all assessments (results to be presented), and accounting for participant suggestions (results to be presented). The majority of pilot participants perceived benefit from the assessment (81%) and would participate again (86%), despite 43% reporting the length as challenging. Results were used to revise the protocol content and process, including reducing assessment time from 2+ hours to 40 minutes, and completing surveys over the phone. We have begun the full roll out (HAI 2.0) into all affiliated older adult living communities (2023), and will have second annual assessments in the upcoming months.

Abstract citation ID: igad104.3645
### THE HIGH PREVALENCE OF OVER-THE-COUNTER MEDICATION MISUSE IN OLDER ADULTS: RESPONSE TO THE CHPA-GSA NATIONAL SUMMIT

Jason Chladek[1], Aaron Gilson[2], Jamie Stone[2], Elin Lehnbom[3], Emily Hoffins[2], Maria Berbakov[2], Jukrin Moon[1], and Michelle Chui[2], *1. University of Wisconsin-Madison School of Pharmacy, Madison, Wisconsin, United States*, *2. University of Wisconsin-Madison, Madison, Wisconsin, United States*, *3. UiT The Arctic University of Norway, Tromsø, Troms, Norway*

Older adults (65+) are the largest consumer of over-the-counter (OTC) medications and disproportionately prone to adverse drug events (ADEs). Despite perceptions that OTCs are generally safe, OTC misuse can lead to ADEs. However, as identified through the Consumer Health Products Association (CHPA) and Gerontological Society of America (GSA) National Summit, little is known about OTC misuse in older adults. This is the first muti-site study to prospectively quantify how older adults select OTCs in community pharmacies where products are typically purchased. Older adults (n=144) were recruited from 10 community pharmacies. Participants were given hypothetical symptoms and asked to select one or more OTCs. After selection, they were asked to report how they would use the product, their home medications, and their health conditions. OTC selections were evaluated for the following misuse categories: 1) drug-age (Beers Criteria), 2) drug-drug (interactions with home medications), 3) drug-disease (health conditions), and 4) drug-label (product's indications and directions). At least one type of misuse was identified in 114 (79%) older adults, with 57 (40%) demonstrating misuse in >1 category. Overall, 363 total instances of misuse were identified ($\bar{x}$=3.18 per participant). There was high prevalence of drug-drug and drug-label misuse, as 107 (74%) demonstrated misuse in one or both categories. The results highlight OTC misuse frequency and complexity in older adults. Knowing older adults' OTC misuse prevalence is critical for understanding medication-associated risks and developing effective interventions. Importantly, these results are a first step toward addressing the research gaps identified by CHPA and GSA.

Abstract citation ID: igad104.3646
### THE IMPACT OF WORKPLACE DEMANDS AND RESOURCES ON THE JOB SATISFACTION OF MINORITY AND IMMIGRANT CNAS

Frances Hawes[1], and Shuangshuang Wang[2], *1. University of Wisconsin-Eau Claire, Eau Claire, Wisconsin, United States*, *2. Southwest Jiaotong University, Chengdu, Jiangsu, China (People's Republic)*

This study examines the association between occupational stress (job resources and demands) and job satisfaction among nursing assistants (NAs) in the long-term care (LTC) sector, considering differences based on race/ethnicity and immigration status. Data from 2,763 NAs were retrieved from the National Nursing Assistant Survey (NNAS). Guided by the JD-R model, our findings indicate that higher job resources, including organizational support, personal development opportunities, benefits, and peer support, are associated with greater job satisfaction among NAs. Conversely, higher job demands, such as structural, physical, and emotional demands, are linked to lower job satisfaction. Importantly, significant differences in occupational stress were observed based on race/ethnicity and immigration status. Hispanic NAs reported the lowest benefits, Black NAs reported the lowest levels of residents' respect, and White NAs reported the lowest peer support. Naturalized NAs experienced higher job satisfaction with greater job resources, while resident NAs had lower satisfaction associated with higher job demands. These findings highlight the need for tailored support mechanisms to address disparities in job rewards and recognition among different racial/ethnic and immigration groups.

Abstract citation ID: igad104.3647
### THE IMPORTANCE OF ADEQUATE RESOURCES AND EQUIPMENT TO STAFF ENGAGEMENT IN A HEALTHCARE SYSTEM

Orah Burack[1], Wingyun Mak[1], Margret Howard[1], Tanya Isaacs[2], and Joann Reinhardt[2], *1. The New Jewish Home Research Institute on Aging, New York City, New York, United States*, *2. The New Jewish Home, New York City, New York, United States*

During this time of unprecedented staffing challenges, identifying factors impacting staff engagement may be one step towards rebuilding the healthcare workforce. This study was designed to identify work factors differentiating positively engaged employees from others. In May 2023, 350 employees (response rate=30%) from across a healthcare system (including skilled nursing facilities, adult day centers, community services, and assisted living) serving older adults in New York City and Westchester County, completed surveys examining employee engagement and satisfaction with five work factors. Engagement was measured using the employee net promoter scale (eNPS) in which employees rate from 1 (Not At All) to 10 (Very) "How likely is it that you would recommend the organization as a workplace." Those answering 9-10 are classified as "Promoters" (engaged/positive concerning the workplace), 7-8 "Neutrals", and 1-6 are "Detractors" (unengaged/negative). Participants also responded 1 "very unsatisfied" to 5 "very satisfied" on work factors: general work satisfaction, having enough resources/equipment, receiving timely information, supervisor support, and coworker respect. Based on eNPS responses, 160 (45.7%) employees were "Promoters", 65 (18.6%) "Neutrals", and 125 (35.7%) "Detractors." A series of one-way analysis of variance and post-hoc Bonferonni analyses then examined work factor satisfaction by staff engagement level. As expected, "Promoters" and "Neutrals" were significantly more satisfied than "Detractors" on all five work factors however, only having enough resources/equipment separated "Promoters" (M=3.63, SD=1.24) from "Neutrals" (M=4.06, SD=89, p <.05). These findings indicate that providing staff with sufficient resources/equipment should be prioritized in the effort to establish a healthcare workforce of "Promoters."

Abstract citation ID: igad104.3648
### THE INFLUENCE OF ARTIFICIAL INTELLIGENCE ON CLINICAL DECISION MAKING: GERIATRIC REGISTERED NURSES' PERSPECTIVES

Shannon Freeman[1], Mary Henderson-Betkus[1], Leanne Currie[2], Davina Banner[1], and Piper Jackson[3], *1. University of Northern British Columbia, Prince*

*George, British Columbia, Canada*, 2. *University of British Columbia, Vancouver, British Columbia, Canada*, 3. *Thompson Rivers University, Kamloops, British Columbia, Canada*

The emergence of ChatGPT highlights the potential of AI applications used in nursing care. AI in the form of machine learning or deep learning provides the ability to scan through clinical data using various algorithms to predict a clinical outcome for a client/patient and influencing clinical decisions. Registered nurses (RNs) are accountable for their decisions when caring for older adults receiving geriatric services and supports. Therefore, we conducted an online survey online cross-sectional survey from April to July 2023 to evaluate community focused RNs knowledge and awareness of the use of AI within their decision-making process. 233 RNs completed the survey. RNs were aware of AI in healthcare (56.7%) and in nursing (30.9%) with less familiarity with machine learning (ML) in geriatric nursing (16.3%). Source of knowledge of AI and ML came from informal sources such as media and social media and from family and friends whereas formal sources were less common. RNs agreed (69.8%) that automated outcomes to help them prioritize older adult clients and target services would be useful. However, 77.9% were concerned with who would be responsible if AI offered incorrect recommendations. Most RNs (90.2%) identified they should have the competency of how AI can improve processes and outcomes. RNs should be consulted if AI had implications on geriatric practice. Future phases of this research will examine how RNs can be better involved in AI that influences their practice and how they might be better prepared to evaluate AI outputs as part of their decision-making process.

Abstract citation ID: igad104.3649

### THE INFLUENCE OF SPIRITUALITY AND RELIGION ON NURSING HOME SOCIAL WORKERS PROFESSIONAL IDENTITY AND RESILIENCE

Brianna Garrison[1], and Dennis Myers[2], *1. Southern Connecticut State University, New Haven, Connecticut, United States*, 2. *Baylor University, Waco, Texas, United States*

The complexities of social workers' behavioral integration of personal spirituality within nursing homes, where a highly regulated, stressful environment marginalizes professional identity and challenges retention, remains largely unexplored and untested. The paucity of data limits the profession's commitment to resourcing current and future practitioners with evidence-informed integration of spirituality with professional identity, ethical practice, and continuation in the role. This qualitative study addresses this gap by interviewing and analyzing narratives authored by experienced NHSWs who daily navigate the spirituality-at-work opportunity and challenge. Researchers collected a purposive sample of licensed NHSWs working within these settings and consenting to participate. All respondents held a social work license at the baccalaureate or master's level (LBSW, LMSW, or LCSW) and were employed as a social worker full time for an average of 3.2 years in their current nursing facility and their overall experience in nursing facility social work averaged 8.8 years, with a range of 2 to 30 years' experience. Twenty NHSWs were interviewed and 80% (n=16) associated their role, at some level, with a higher power (God, Christ, Holy Spirit, unnamed) and religious beliefs and practices. Fifteen percent (n=3) stated faith and religious ideology had no impact or was irrelevant for practice, and one (5%) did not respond. It became clear that religion, spirituality, and faith practices matter for a majority of the respondents in how they view and enact their practice.

Abstract citation ID: igad104.3650

### THE MEDIATION EFFECT OF SOCIAL SUPPORT BETWEEN DEPRESSIVE SYMPTOMS AND LONELINESS IN OLDER ADULTS WITH DIABETES

Emma Cho, *University of Pennsylvania, Philadelphia, Pennsylvania, United States*

The purpose of this study was to examine the relationship between depressive symptoms and loneliness in older adults with diabetes, and to explore the mediating role of social support in this association. This study was developed as a secondary data analysis using data from the National Social Life, Health, and Aging Project in the USA. The sample consisted of 407 older adults aged 50 to 93 years (mean = 64.76, SD=9.03; 50.9% male; 60% married and white). A multi-item survey questionnaire was used to assess loneliness, social support, and depressive symptoms. Spearman correlation analysis was used to examine the relationship between depressive symptoms, social support, and loneliness; the macro PROCESS on SPSS was used to examine the mediating effects of social support on the association between depressive symptoms and loneliness. Despite the significant correlation between depressive symptoms and social support, social support and loneliness, and depressive symptoms and loneliness, social support did not mediate the influence of depressive symptoms on loneliness. The results of this study support the need for increased awareness of depressive symptoms in older adults with diabetes. Depressive symptoms should be treated to alleviate loneliness, and more research is needed to explore how other social factors influence the relationship between depressive symptoms and loneliness, thereby preventing a synergistic effect of depressive symptoms and loneliness.

Abstract citation ID: igad104.3651

### THE POTENTIAL OF MULTIDOMAIN MOBILE PROGRAMS IN SUPPORTING COGNITIVE HEALTH OF SENIORS IN RESIDENTIAL FACILITIES

Junhyoung Kim[1], Yongseop Kim[2], Jeff Cho[3], Myungjin Ko[4], and Marcia Ory[1], *1. Texas A&M University, College Station, Texas, United States*, 2. *Indiana University, Bloomington, Indiana, United States*, 3. *Korea International School Jeju, Seogwipo, Jeju, Republic of Korea*, 4. *Silvia Health, Seoul, Republic of Korea*

This study pilot tested the efficacy of a mobile-based multidomain application, Silvia Program, on cognitive functioning of residents in assisted living facilities (ALFs). After a 12-week randomized pilot trial using a two-group pretest-posttest design (Silvia group vs. usual care group), we assessed the cognitive functioning using the Montreal Cognitive Assessment (MoCA). Results are that the total MoCA scores of the Silvia group showed significant improvement while the total scores of the control group declined. The present study provides suggestive evidence for an overall positive effect of the use of the Silvia Program on the cognitive functioning among residents in ALFs.

Abstract citation ID: igad104.3652
### THE PROTECTIVE LIFESPAN BENEFITS OF SOCIAL SUPPORT BUFFERS CHILDHOOD ADVERSITY

Meredith Willard, Ryan Best, and Nicholas Turiano, *West Virginia University, Morgantown, West Virginia, United States*

Childhood adversity has negative effects across the lifespan including increased mortality risk. The support we receive from others, social support, has shown to be a protective factor against ACEs. However, little research has investigated the amplifying effects of poor social support, also known as social strain. Individuals with high levels of social strain and low levels of adversity would have the lowest mortality risk, it was hypothesized. Early life adversity was computed using 16 retrospective items assessing emotional and physical abuse, socioeconomic disadvantage, familial instability, and early-life poor health. Social support was computed using four-family, four-friend, and six-spouse items assessing reliability and emotion availability. Social strain was computed using four-family, four-friend, and six-spouse items assessing annoyance and disappointment. Cox proportional hazards models tested whether adversity, social support, and social strain were associated with the 20+year hazard of dying. Adjusting for age, gender, race, education, and marital status, higher ACEs (HR = 1.06) lower social support (HR = 1.07) and greater social strain (HR = 1.06) were all significantly associated with an increased hazard of dying (all ps < .05). A significant ACE x Social Support interaction emerged (HR = 0.95) suggesting the association between ACEs and hazard of dying was strongly positive at low levels of social support, but that the relationship was negative at high levels of support. Thus, high levels of social support buffer the negative effects ACEs have on mortality risk. This provides robust evidence that interventions increasing social support could reduce the harmful effects ACEs.

Abstract citation ID: igad104.3653
### THE RELATED FACTORS ON HEALTH DEFICIENCIES IN NATIONAL NURSING HOMES USING PANEL LARGE DATASET

Juh Hyun Shin[1], Jinhyun Ahn[2], Soo-Kyoung Lee[3], Charlene Harrington[4], Sunwoo Jung[2], Yeonwoo Lee[2], and Sanna Ali[5], *1. George Washington University, Ashburn, Virginia, United States*, *2. Jeju National University, Jeju, Jeju, Republic of Korea*, *3. Seoul National University, Seoul, Republic of Korea*, *4. University of California, San Francisco, San Francisco, California, United States*, *5. The George Washington University, Washington, District of Columbia, United States*

Health deficiencies are considered scientific research outcomes and should be reported as mandatory for all Medicare-Medicaid nursing homes (NHs). However, health deficiencies have been a concern for several decades. Previous studies have focused on regulatory deficiencies or used nurse staffing variables as control variables. This study investigated health deficiencies in relation to nurse staffing, facility size, and ownership across all Center for Medicare and Medicaid NHs in the United States. This study used secondary data analysis using Payroll-based Journal Daily Nurse Staffing data, Area Resource File, and LTCFocus. Data were merged with federal Medicare/Medicaid provider IDs as the basic unit of analysis for the 2019 fiscal year. A total of 7,802 NH data were analyzed using Poisson regression. The average total weighted health deficiencies survey score was 66.12. The average bed size was 114.02, where 74% were profit NHs. The average hours per resident day (HPRD) for each nurse staffing was as follows: 0.57 registered nurses, 0.86 licensed practical nurses, and 2.16 certified nurse aides. As total HPRD increases by 1 unit (exponential 0.83), it is likely that health deficiencies decrease by 17%. The bed size less than 60 (exponential 0.87) and not-for-profit NHs (exponential 0.09) was related with decreased health deficiencies of 13% and 91%. The worsened residents' acuity was related with 2% more health deficiencies (exponential = 0.98). This large dataset analysis shows that professional nurse staffing is associated with superior NH quality of care with fewer deficiencies and supports the need for age-friendly long-term healthcare systems.

Abstract citation ID: igad104.3654
### THE RELATIONSHIP BETWEEN ALLOSTATIC LOAD AND PROGRESS OF MULTIMORBIDITY AMONG OLDER AMERICANS

Rolla Saud Mira, *King's College London, London, England, United Kingdom*

To examine the relationship between allostatic load and progress of multimorbidity over 10 years among older Americans and whether it mediates socioeconomic inequalities in multimorbidity.

**Methods:** Six Waves of Health and Retirement Study (HRS), a longitudinal survey of older American adults from 2006 -2016. Multimorbidity was indicated by 5 chronic conditions: diabetes, heart conditions, lung diseases, cancer, and stroke. Socioeconomic factors were education, total wealth, poverty-income ratio (income), and race/ethnicity. Behavioural factors were smoking, excessive alcohol consumption, physical activity, and body mass index (BMI). Allostatic load was used as a biomarker of stress and was indicated by a combination of total cholesterol, high-density lipoprotein, glycosylated hemoglobin, c-reactive protein, waist circumference, and high blood pressure. Two Multilevel mixed effects generalized linear models were constructed to assess the association between allostatic load and progress of multimorbidity and whether allostatic load explained socioeconomic inequalities in the progress of multimorbidity. All variables included in the analysis were time-varying except gender, race/ethnicity, and education.

**Results:** Allostatic load was significantly associated with the progress of multimorbidity even after adjusting for socioeconomic and behavioural factors with a rate ratio (RR) of 1.12 (95%CI: 1.11, 1.13). The association between socioeconomic factors and multimorbidity was slightly attenuated after adjusting for allostatic load. Socioeconomic factors were also associated with changes in allostatic load.

**Conclusion:** Biological markers of stress indicated by allostatic load were associated with the progress of multimorbidity. Allostatic load appears to be induced by adverse socioeconomic factors and mediates the relationship between socioeconomic factors and progress of multimorbidity.

Abstract citation ID: igad104.3655

**THE RELATIONSHIP BETWEEN INTERNET USE AND LIFE SATISFACTION IN LATER LIFE: A FOCUS ON THIRD-LEVEL DIGITAL DIVIDE**

Ern Chern Khor, and Moon Choi, *KAIST, Daejeon, Republic of Korea*

The third-level digital divide highlights how social factors can shape disparities in real-life experiences derived from digital use. This study aims to examine how different types of internet use are related to the life satisfaction of older adults with varying health and wealth statuses through the lens of the third-level digital divide. The data for this study came from the 2022 Korea Digital Divide Survey. A sample of 1,165 older adults aged between 65 and 96 years old was grouped into four categories based on their health and wealth statuses, considering that these two factors tend to shape the social positions of older adults in Korean society. Life satisfaction was measured using 5 items from the 4-point Satisfaction with Life Scale (SWLS), while different types of internet use were accessed with 13 dichotomous items. The findings showed that a significant positive effect of internet use was observed only among older adults with good health status, indicating a stronger effect for those with good wealth status. Particularly, online public services (d=1.41, p< 0.001), cloud services (d=1.35, p< 0.001), and social networking services (d=1.27, p< 0.001) exhibited significant effects. No significant effect was observed for older adults with poor health status, except for online entertainment content, which was found to be significantly related to the life satisfaction of those with low wealth status (d=0.87, p< 0.01). The study findings imply diversity in terms of internet use and its association with life satisfaction, while highlighting social disparities.

Abstract citation ID: igad104.3656

**THE RELATIONSHIP BETWEEN SEDENTARY BEHAVIOR AND COGNITIVE DOMAINS AMONG PEOPLE LIVING WITH DEMENTIA**

Charmi Patel, Stevie Lewis, and Deborah Jehu, *Augusta University, Augusta, Georgia, United States*

Greater sedentary behavior negatively impacts cognition among older adults without dementia; however, the relationship between sedentary behavior and cognitive domains among people living with dementia (PWD) is unclear. Our aim was to explore the association between sedentary behavior and cognitive domains in PWD in residential care facilities. The testing took place at one nursing home (n=15/23), one memory care unit (n=4/23), and one assisted living facility (n=4/23). Participants completed a battery of cognitive tests (global cognition: Montreal Cognitive Assessment; executive function: Trail Making test, Digit Span Backwards Test; perception and orientation: Benton Judgment of Line Orientation Test; language: Boston Naming Test; learning and memory: Rey Auditory Verbal Learning Test; complex attention: Digit Symbol Substitution Test). We used the Morse Fall Scale to measure fall risk. Participants wore an actigraphy monitor on their wrist over seven days to measure sedentary behavior. Most participants were male (74%), white (87%), and had unspecified dementia (48%). Participants were sedentary for 8.0±2.7 hours/day. The regression model for sedentary behavior was significant (R2=0.82, F(10, 12)=5.39, RMSE=92.47, p< 0.05). Greater sedentary behavior was associated with poorer language function (β=-0.82, p=0.02), poorer learning and memory (β=-0.82, p=0.002), and greater fall risk (β=-0.43, p=0.03). Sedentary behavior was not associated with the other cognitive domains. Greater sedentary behavior may indicate a decline in language function, learning and memory, and increase fall risk in PWD. Sedentary behavior should be considered in routine assessments, as it may play an important role in monitoring cognitive decline in PWD in residential care facilities.

Abstract citation ID: igad104.3657

**THE RELATIONSHIP BETWEEN THE CAREGIVING ENVIRONMENT, BURNOUT AND QUALITY OF LIFE AMONG HOME HEALTH AIDES**

Sehyun Baek[1], Soobin Park[1], Oejin Shin[2], Sojung Park[1], and Ahra Ko[3], *1. Washington University in Saint Louis, St. Louis, Missouri, United States*, *2. Illinois State University, Normal, Illinois, United States*, *3. Yonsei University, Seoul, Republic of Korea*

The well-being of home health aides (HHAs) is affected by the characteristics of their work environment. As South Korea's population rapidly ages, there is an increasing demand for home aides. However, little is known about how the caregiving environment affects HHAs. Guided by the environment comfort model, we examined the association between care recipients' home environment and HHA's quality of life, focusing on how burnout mediates this relationship. Our data came from a national survey of home health aides in 2020 (N=981). We conducted an exploratory factor analysis to identify six factors related to the care environment in three dimensions: physical (1. space; 2. indoor/outdoor conditions), functional (3. home appliances; 4. heating/air conditioning), and psychological (5. satisfaction with the home environment; 6. relationships with care recipients and their families). We then used a path analysis to examine the relationship between these factors, burnout, and quality of life. Our findings show that safe indoor/outdoor conditions and positive relationships with care recipients and their families are associated with lower levels of burnout, leading to a higher quality of life (p< 0.05, respectively). This highlights the importance of considering both physical and psychological aspects of the caregiving environment to prevent burnout and improve the quality of life for HHAs, ultimately contributing to high-quality services for care recipients.

Abstract citation ID: igad104.3658

**THE RELATIONSHIPS BETWEEN INTRINSIC CAPACITY AND FRAILTY AND DISABILITY IN OLDER OUTPATIENTS**

Mei-Ching Chang, Yu-Tai Lo, Wei Lee, and Sheng-Yu Fan, *National Cheng Kung University, Tainan, New Taipei, Taiwan (Republic of China)*

The ICOPE scale is developed by the WHO to assess intrinsic capacity, including cognition, mobility, nutrition, hearing, vision, and depression. However, the relationships between intrinsic capacity and frailty and disability were unknown. The purposes of this study were to explore the cut-off points of the ICOPE scale for frailty and disability in older outpatients, and the significant intrinsic capacities for frailty and disability. This study employed a cross-sectional research design. The participants were patients aged 65 and above attending the geriatric department of a medical center (n=397). The ICOPE scale, Clinical Frailty Scale (CFS), and Activity of Daily Living (ADL) scale were used. ROC curve analysis was used to determine the optimal cut-off points of the ICOPE scale for frailty and disability, and logistic regression for the significant intrinsic capacities related to frailty and disability. For frailty, the AUC value of abnormal intrinsic capacity was 0.79 ($p < 0.001$), with an optimal cut-off point of 2/3. For disability the AUC value of abnormal intrinsic capacity was 0.82 ($p < 0.001$), with an optimal cut-off point of 2/3. Logistic regression showed cognition, mobility, and nutrition related to frailty significantly; whereas cognition, mobility, nutrition, and hearing related to disability significantly. Older adults with three or more abnormal intrinsic capacities have a higher risk of frailty and disability. The problems of cognition, mobility, and nutrition were the risk factors of frailty and disability. Healthcare staff can pay attention to older outpatients with the problems of intrinsic capacities.

Abstract citation ID: igad104.3659

### THE RELEVANCE OF COGNITIVE ABILITIES IN FRAUD DETECTION

Shenghao Zhang, Neil Charness, and Walter Boot, *Florida State University, Tallahassee, Florida, United States*

Some previous studies suggest that cognitive decline might be responsible for older adults being susceptible to phishing attempts and frauds, but results are not consistent. The current study utilized a structural equation modeling approach to examine the role of different cognitive abilities in fraud detection. We used data from the baseline of the Intervention Comparative Effectiveness for Adult Cognitive Training (ICE-ACT, NCT03141281) Trial. The sample included 230 cognitively normal community-dwelling older adults (Mean age = 72). Reasoning was measured by Raven's Advanced Progressive Matrices and letter sets. Memory was measured by Hopkins Verbal Learning Test and Rey Auditory Verbal Learning Test. Processing speed was measured by Digit Symbol Substitution Test and Useful Field of View. Fraud detection was represented by d-prime score calculated from rating of fraudulence in 10 vignettes describing fraud and non-fraud scenarios. Latent factors were created for reasoning, memory, and processing speed. Results showed that reasoning, memory, and processing speed explained 14% of variance in fraud detection, however, none of those cognitive factors uniquely predicted fraud detection. A higher-order general cognitive factor indicated by the reasoning, memory, and processing speed factors significantly predicted fraud detection and explained 13 % of variance. These results showed that general cognitive abilities have a medium effect in fraud detection in cognitively normal community-dwelling older adults. Discussion will focus on implications of those results on training and interventions aiming to prevent fraud in older adults.

Abstract citation ID: igad104.3660

### THE RISK OF MEDICATIONS IMPAIRING ROAD TEST PERFORMANCE AMONG COGNITIVELY INTACT OLDER ADULTS

David Carr[1], Kebede Beyene[2], and Ganesh Babulal[3], *1. Washington University in St. Louis, St. Louis, Missouri, United States*, *2. St. Louis College of Pharmacy, St. Louis, Missouri, United States*, *3. Washington University School of Medicine, St. Louis, Missouri, United States*

Importance: Older adults are increasingly prescribed medications that have adverse side-effects. Prior studies have found higher risks associated with motor vehicle crashes. Objective: This 10-year study determined whether specific medication classes were associated with performance decline assessed by a standardized road test in a community-sample of healthy, cognitively-normal older adults. Design, Setting, and Participants: This was a prospective cohort study of 198 cognitively-normal older adults ≥ 65 years old with a valid driver's license who were followed annually, with rolling enrollment. Exposure: Potentially driver impairing medications. Main outcomes and measures: The primary outcome measure was performance (pass or marginal/fail) based on the Washington University Road Test. Multivariable Cox proportional hazards models evaluated the association between PDI medication use and road test performance. **Results:** Among the 198 older adults (mean [SD] baseline age, 72.6 [4.6] years; 87 females [43.9%]; and 19 Black participants [9.5%]), 70 participants [35%] received a marginal/fail rating on the road test over an average follow up of 5.70 years. Any antidepressant (HR, 2.81; 95% CI, 1.67-4.71), SSRI/SNRI (HR, 2.73; 95% CI, 1.56- 4.77), sedatives/hypnotics (HR, 2.70; 95% CI, 1.40- 5.19), or NSAIDs/Acetaminophen (HR, 2.80; 95% CI, 1.34- 5.83) use had at least a 2.5 greater risk of receiving a marginal/fail rating on a road test compared to controls. Conclusion and relevance: In this prospective cohort study, specific medication classes increased the longitudinal risk of failing a road test. Patients should be counseled about perceived driving difficulty and risk when prescribed these medications by clinicians.

Abstract citation ID: igad104.3661

### THE ROLE OF OUTDOOR LIGHT EXPOSURE ON SLEEP QUALITY IN OLDER INDIVIDUALS WITH FRAILTY OF DIFFERENT LIVING TYPES

Yi Chin Hsieh, and Ching-Ju Chiu, *National Cheng Kung University, Tainan, Tainan, Taiwan (Republic of China)*

The study investigates the relationship between outdoor light exposure and sleep quality in older individuals with frailty in institutions and the community, where sleep disorders are common, and outdoor light exposure is inadequate. The study includes older individuals with frailty in community and institutions in Taiwan. Participants must be aged 65 or above and have a Clinical Frailty Scale score between 4 to 8. We collect sleep quality and outdoor light exposure data using questionnaires and actigraphy.The study includes 77 older individuals: 29 from day-care centers, 22 from conventional long-term care institutions, and 26 from unit care institutions. The average outdoor light exposure is 15 minutes per day, with an average Pittsburgh Sleep Quality Index (PSQI) score of 8, 74% sleep efficiency, and 125.8 minutes of wake after sleep onset. After controlling for multiple variables. Outdoor light exposure shows no relationship with PSQI, sleep efficiency, and wake after sleep onset.The average PSQI score is > 5, indicating poor subjective sleep quality. Additionally, Wake After Sleep Onset > 60 minutes, and sleep efficiency < 75%, suggesting poor objective sleep quality. The average outdoor light exposure time < 20 minutes, indicating a lack of outdoor light exposure. However, our study does not observe the effects of light exposure, possibly because the average daily light exposure is below the 30-minutes threshold. Older individuals with frailty experience poor sleep quality. It is possible that a longer duration of outdoor light exposure may be needed to observe its impact on sleep quality.

Abstract citation ID: igad104.3662

### THE ROLE OF SELF-EFFICACY IN ADVANCE CARE PLANNING: DOES RACE AND ETHNICITY AFFECT THIS RELATIONSHIP?

Jessica Yauk, and Debra Dobbs, *University of South Florida, Tampa, Florida, United States*

Advance care planning (ACP) has been identified as essential in improving end-of-life patient satisfaction and goal-concordant care. Despite the benefits of ACP, rates remain particularly low among marginalized populations. Marginalized populations are also at an increased risk of receiving care incongruent with their wishes and reporting low-quality end-of-life care. ACP is a complex process where individuals must decide to plan and implement those plans. Because the ACP process is complex, an individual's self-efficacy- or belief that they can perform a particular task, may be an important component when predicting ACP completion. Using data from the 2018 wave of the Health and Retirement Study (HRS), the current study aimed to understand how two facets of self-efficacy- mastery and constraint-, related to varying levels of ACP completion (living will, durable power of attorney for healthcare, and advance care planning discussions (DPAHC)). Furthermore, we looked at the possible moderating effects of race/ethnicity between self-efficacy and ACP. Results indicated that Black and Hispanic older adults are significantly less likely to complete all types of ACP. Furthermore, higher self-reported constraint- was significantly related to lower completion of ACP discussions (OR = 0.9, p= .05). Neither mastery or constraint were related to completion of a living will or DPAHC. Race/ethnicity was not found to moderate the relationship between self-efficacy and any level of ACP. Individual variables may be less important than community level variables or life-long discrimination when assessing completion of ACP among marginalized populations.

Abstract citation ID: igad104.3663

### THE ROLE OF SENSE OF PURPOSE IN HEALTH NAVIGATION AMONG MARGINALIZED OLDER ADULTS

Ameesha Narine[1], Emily Mroz[2], and Shubam Sharma[1],
*1. Kennesaw State University, Kennesaw, Georgia, United States,*
*2. Yale University, New Haven, Connecticut, United States*

A sense of purpose is a central, self-organizing life aim that guides choices and behaviors . In older adulthood, having a sense of purpose is linked with positive health. Understanding how older adults who identify as socially marginalized (e.g., racially/ ethnically diverse, LGBTQ+) leverage purpose to navigate their health, for example, when setting health values, making health decision, or adapting to health challenges may support development of tailored resources to combat social disparities embedded in healthcare systems.. This study used qualitative thematic analysis (Braun & Clarke, 2012). to explore the role of purpose in marginalized older adults' current health navigation and including how purpose and health navigation change across adulthood. Nineteen older men and women who identified as socially marginalized (Mage = 66.5) participated in semi-structured interviews Analyses revealed that older adults draw on purpose to guide health by maintaining personally meaningful, health-related (e.g., health education, caregiving) vocations into late life and by modeling positive health behaviors for others . Further, the role of purpose in health navigation changed across their adulthoods: participants described shifts from adversity-driven purpose to wellness-focused purpose, translating early adversity into current health motivation, and finding space for their authentic purpose when health was considered to stabilize. Findings demonstrate that, for marginalize older adults, their relationship with their own health may improve across the lifespan as they overcome adversity and health becomes enmeshed in purpose. Findings support refining health interventions to account for the role of purpose for older adults who have faced undue social hardship.

Abstract citation ID: igad104.3664

### THE SOCIAL RELATIONSHIP OF OLD ADULTS ACCORDING TO THE USE OF ASSISTIVE APPLIANCES IN SOUTH KOREA.

Yeojin Yoon, *Chung-Ang University, Seoul, Republic of Korea*

This study aims to examine the differences in the social relationship of the old adults between the use of assistive appliances. For the analysis, the data were obtained from "2020 National Data on the Elderly" published by the Ministry of Health and Welfare and the Korea Institute for Health and Social Affair. Data was a survey of people aged 65 and older conducted between June and August 2020, which included data from 10,097 people. The variables used for the use of assistive appliances were vision aids, hearing aids, chewing aids. The variables used to measure older adults' social relationship resource are the frequency of visits and contact with non-resident children, grandchildren and children with whom they have the most contact, and the frequency of visits and contact with relatives, friends, neighbors, and acquaintances. The analysis used multiple regression analysis and t-test for comparison of means between groups, and the main findings were as follows. First, there is no statistical correlation between assistive appliances use and older adults' social relationships. Second, there is no significant difference in the mean of the social relationships of older adults with and without assistive appliances use. In this paper, we overlooked the ease with which the elderly can use assistive appliances with support if they have functional difficulties. These findings confirm that there is no direct relationship between the use of assistive appliances and the social relationships of older adults, and suggest the need for further research on the social relationships of older adults.

Abstract citation ID: igad104.3665

### THE SOCIAL STRATIFICATION OF INFORMAL CAREGIVING ARRANGEMENTS IN EUROPE

Marco Albertini, Francesca Zanasi, and Giorgio Piccitto, *Alma Mater Studiorum - Università di Bologna, Bologna, Emilia-Romagna, Italy*

In ageing societies, the increasing quota of older and frail individuals creates unprecedented needs for care. As care is often costly and not adequately covered by the welfare state, care responsibilities for older individuals fall on the shoulders of family members. The study of informal care provision is acquiring centrality both in the social sciences and policy discourse, since the care load can bear negative consequences on a range of outcomes, from health (e.g., the "caregiver burden") to employment. It is important to gain a better understanding of which individuals are the most likely to provide informal caregiving and face its consequences. In the present study, we explore the educational and income differences in the probability of providing informal caregiving to individuals living outside the household in Europe, using the Survey of Health, Aging and Retirement in Europe (SHARE, 2004-2020). As a contribution to the field, we aim at uncovering the mechanisms behind the socio-economic gradient in caregiving, such as differences in health and longevity and time constraints (due to employment and other care responsibilities) that are unequally distributed across social layers. Results show that individuals with tertiary education, and at the top of the income distribution, are more likely to provide care, net of several other factors. The study concludes with attempted explanations of the results, related to the fact that lower educated, and lower income individuals, can more often rely on publicly provided services and means-tested benefits.

Abstract citation ID: igad104.3666

### THE SOUTHEAST FLORIDA VETERANS' COMMUNITY PERSPECTIVES ON THE MEANING OF WELL-BEING: A PHENOMENOLOGICAL INQUIRY

Cheryl Birmingham, *Florida Atlantic University, Fort Lauderdale, Florida, United States*

The Veterans are a unique community shaped by past military experiences that may have positive and negative effects on their well-being as soldiers. With nearly half of the 18.4 million Veterans in the United States living beyond 61 years the physical and mental health conditions may desolate the Veterans' well-being. Promoting Veteran well-being has been studied extensively but scant on how they define it. It is critical to understand how Veterans define well-being to inform future program development addressing mental health needs. The purpose of this study is to discover the meaning and the live experience of well-being among Veterans. A qualitative hermeneutic interpretative phenomenological approach was used to understand the meaning of Veterans' well-being. It allowed the phenomena to surface while exposing the essence of and the experience. Purposive sampling was used to recruit participants, with semi-structured interviews via ZOOM and observations at Veteran Service Organizations (VSOs). Fourteen Veterans participated in this study, 8 males and 6 females with majority greater than 61 years. 64% were White, 21% Hispanic and 15% African American. With the use of Max van Manen's analysis strategy, 3 major themes emerged: (1) A Connection to a Military Sisterhood/Brotherhood; (2) The Lingering Effects of Military Service and (3) Healthy Companionship/Relationships advocating for Veterans' well-being. The lingering effects persisted and challenged the Veterans' emotional well-being; however, they were able express their military camaraderie continued in Veteran life. Programs based on social relationships that allow connections with Veterans and/or their significant others need to be established.

Abstract citation ID: igad104.3667

### THE UNIQUE CHARACTERISTICS OF "FAMILIES OF CHOICE" AND BIOLOGICAL FAMILIES OF LGBT ADULTS

Ella Cohn-Schwartz[1], Sigal Gooldin[2], and Yaacov Bachner[3], *1. Ben Gurion University, Haifa, HaZafon, Israel*, *2. The Association for LGBTQ Equality, Tel Aviv, Tel Aviv, Israel*, *3. Ben-Gurion University of the Negev, Beer-Sheva, HaDarom, Israel*

This study joins a growing body of research on the unique families of aging individuals in sexual minorities. We explore the "families of choice" (who are close enough to be considered as family) and biological families of LGBT adults and their associations with mental health. Data for this study was collected via an online survey with self-identified lesbian, gay, bi-sexual and transgender (LGBT) adults aged 50+ in Israel (n=483). The participants were asked about characteristics of the relationship with their families of choice and biological families and about experiencing depressive symptoms. Descriptive results indicated that most participants had a family of choice, numbering five people on average. These families of choice were mostly composed of partners and friends, but also of the family members of one's partner, and of ex-partners and colleagues. They reported having about four close biological family members, mostly children, parents, siblings, and nieces. Several differences emerged when comparing the two types of families: The relationships with biological families were more stable and their biological family members were contacted more often. On the other hand, families of choice were more likely to accept their sexual orientation and the relationship with them had fewer negative aspects. Regression analyses showed that individuals had more depressive symptoms if the relationship with their families of choice was less stable and if there were more negative relationships with both types of families. These findings shed light on the unique sources of support among LGBT adults and their associations with mental health.

Abstract citation ID: igad104.3668

### THE USABILITY AND ACCEPTABILITY OF THE AUGMENTED REALITY HOME ASSESSMENT TOOL (ARHAT)

Beth Fields, Zachary Skrove, Ross Tredinnick, Bryce Sprecher, Jenny Lee, Rachael Shields, Kevin Ponto, and Jung-hye Shin, *University of Wisconsin-Madison, Madison, Wisconsin, United States*

Gaining access to reliable home assessments for aging in place can be challenging and expensive for urban residents and particularly those living in rural areas. Evidence has shown that there is a substantial increase in the number of older adults nationally and globally, making the availability of home assessments scarce. As individuals continue to age and become more susceptible to disabilities it is important that they have a home that allows them to age in place and is compliant with housing standards. Technology has the potential to make home assessments more accessible to older adults who want to age in place. The aim of this study was to determine the usability and acceptability of a mobile-based app, the Augmented Reality Home Assessment Tool (ARHAT), across different groups interested in home assessment (occupational therapists (OTs), older adults/

family caregivers, and housing professionals) (N=16). Participants were mailed an iPhone with the ARHAT installed. After participants reviewed the ARHAT, they were emailed an electronic survey with closed and open-ended demographic, usability and acceptability questions. The majority of participants were White (94%), female (75%), from the Midwest (63%), and had a Bachelor's degree or higher (81%). Participants reported that the ARHAT is easy to use and acceptable for measuring features of the home environment and can support aging in place. Future research should test the impact of the ARHAT on improving health service utilization (e.g., delaying institutionalization, reducing emergency department visits) rates among older adults.

Abstract citation ID: igad104.3669
### THE VOLUNTEER EXPERIENCE IN THE FAITH CARE FAMILY PROJECT FOR AFRICAN AMERICAN CAREGIVERS OF PERSONS WITH DEMENTIA

Noelle Fields[1], Ling Xu[1], Ishan Williams[2], and Fayron Epps[3], *1. The University of Texas at Arlington, Arlington, Texas, United States*, *2. University of Virginia, Charlottesville, Virginia, United States*, *3. Emory University, Atlanta, Georgia, United States*

African American adults are disproportionately affected by Alzheimer's disease and related dementias (ADRD). Improving knowledge about ADRD in the African American community is also important for dispelling the myths and stigma about the disease. The Faith Care Family (FCF) Project was developed to help bridge gaps in ADRD knowledge by training volunteers from a predominantly African American church (N = 9) to work with ADRD caregivers in a 6-week, telephone-based pilot intervention. The Alzheimer's Disease Knowledge Scale (ADKS) and qualitative interviews were used to examine the church volunteers' knowledge of dementia. Non-parametric McNemar tests and Wilcoxon signed-rank tests were conducted for each item and the sum scores of the ADKS scale, respectively. Qualitative interview data were analyzed using thematic analysis. The results of the quantitative data indicated overall significant improvements in the sum scores of ADKS after the training (p = .008) as well as after the intervention (p = .05). Some specific ADKS items showed a significant increase after the training including items related to high cholesterol, using reminder notes, and mental exercise as well as after the intervention related to prescription medication. Thematic analysis of the interviews revealed six themes: types of ADRD, ADRD symptoms/behaviors of ADRD, early-onset, fatality, dignity, and previous knowledge. Overall findings suggest that the FCF Project is a promising intervention for enhancing knowledge about ADRD for African American church volunteers. Recommendations for future research and strategies for lay provider ADRD interventions in faith communities are offered.

Abstract citation ID: igad104.3670
### THERANAP: AN EDUCATIONAL SLEEP HEALTH INTERVENTION FOR FRAIL OLDER ADULTS

Amy Berkley, *St. Louis University, St. Louis, Missouri, United States*

Napping and other daytime sleep is often overlooked and poorly defined in insomnia research. Studies have shown correlations between older adults' napping habits and increased medical co-morbidities and risks of dementia, but others report napping enhances memory consolidation and broader aspects of cognition in younger adults. Why is napping recommended for younger adults but considered problematic for older adults? In previous a research study, (18 older adults in an independent living facility: 6 men,12 women, mean age 84, SD= 7.62, range 67-96, who self-reported sleep problems) participants reported napping in qualitative interviews but denied daytime sleep on standard sleep assessments. Participants spoke about napping apologetically and expressed dread of its links to functional and cognitive decline. They stigmatized planned napping, which could potentially benefit their cognitive functioning, but seemed accepting of accidental napping, which could well indicate more serious physical or cognitive issues To address these issues, TheraNap teaches intentional napping as a therapy. This sleep health education intervention empowers sleep-deprived older adults to take control of their sleep habits by practicing intentional napping to improve overall sleep health. TheraNap includes: • Short sleep health training session with recommended napping guidelines • Two-week long planned napping routines • A sleep diary • Supplementary sleep meditations • Follow-session to assess perceptions of sleep health More research is needed into the ways napping can improve physical and cognitive function, and more education is needed for older adults, their families and caregivers, and LTC staff about the importance of sleep for older adults.

Abstract citation ID: igad104.3671
### TRANSITIONS IN MEDICATION MANAGEMENT AMONG PATIENTS WITH DEMENTIA: PATIENT-CAREGIVER PERSPECTIVES

Rachel O'Conor[1], Andrea Russell[1], Dianne Oladejo[1], Sarah Filec[2], Darby Morhardt[3], Emily Rogalski[1], Lee A Lindquist[1], and Michael Wolf[1], *1. Northwestern University, Chicago, Illinois, United States*, *2. Feinberg School of Medicine, Northwestern University, Chicago, Illinois, United States*, *3. Northwestern University Feinberg School of Medicine, Chicago, Illinois, United States*

Older adults with Alzheimer's disease and related dementias (ADRD) have higher rates of multimorbidity and polypharmacy. Medication adherence is critical to maintain health but is difficult with complex regimens. Little is known about how older adults with varying levels of cognitive impairment self-manage medications, and how responsibilities transition to caregivers. We conducted qualitative interviews with patient-caregiver dyads exploring how they managed multidrug regimens. Patients were eligible if they had 1) mild cognitive impairment (MCI), mild ADRD or moderate ADRD, 2) ≥3 chronic conditions, 3) ≥5 medicines and 4) a caregiver. Three coders applied a priori codes to transcripts and analyses followed the framework method. We interviewed 60 participants (Patients: mean age 78 years, 63% female, 50% white, 33% Black; Caregivers: mean age 65 years, 73% female, 60% spouse, 33% child). Patients took 9 medicines on average; cognitively, 36% had MCI, 40% mild ADRD and 23% moderate ADRD. Interviews revealed a tension between preservation of patient independence with early caregiver involvement. Patients and caregivers reported a strong preference towards patient autonomy in medication management, and patients with MCI and mild

ADRD managed their medications independently. Among patients with moderate ADRD, caregivers assumed all medication responsibilities except when living separately. In those scenarios, caregivers set up organizers and made reminder calls, but did not observe medication intake. Transitions in medication management followed patient-driven medication errors that resulted in adverse events. These findings have important implications; clinicians should initiate conversations with caregivers and patients about early medication assistance and work to simplify regimens.

Abstract citation ID: igad104.3672

### TRANSITIONS TO LIVING ALONE AND PSYCHOLOGICAL WELL-BEING OF OLDER ADULTS IN THE U.S: ARE THERE ANY RACE DISPARITIES?

Wuyi Dong, and Qian Song, *University of Massachusetts Boston, Boston, Massachusetts, United States*

We extend the existing literature on living alone and psychological well-being in older adults by focusing on the transition process from other living arrangements to living alone. We examine how transitioning to living alone is associated with psychological well-being scores (measured by 21-item Ryff Measures of Psychological Well-being) and how the associations vary across racial and ethnic groups. Using data from 2016 and 2020 Health and Retirement Study, we specify the transitions to living alone from four living arrangements: living with spouse/partner only, living with spouse/partner and older parents, living with a spouse/partner, children, and parents, and living with others. Respondents consistently living alone were the reference group. Our analysis involves adults aged 50 and above (n=3160). After controlling for final psychological well-being scores and adjusting for age, gender, marital status, and education in 2016, results from multiple linear regression models suggest that the transitions from living with spouse/partner only to living alone are associated with significantly lower scores of psychological well-being ($\beta$= -1.71, p<.01) than those consistently living alone. However, no statistically significant differences were observed between individuals transitioning from the other three living arrangements to living alone and those consistently living alone. The moderation effect shows that older Blacks experienced increased psychological well-being when transitioning from living with a spouse/partner to living alone ($\beta$=2.26) while their White counterparts had decreased psychological well-being during the same transition. This underscores the significance of the transition experience and emphasizes race disparities in its association with psychological health outcomes.

Abstract citation ID: igad104.3673

### TRIALS AND TRIBULATIONS OF CONDUCTING A GEROSCIENCE TRIAL

Courtney Millar[1], Kathryn Baldyga[1], Ikechukwu Iloputaife[1], Alex Wolfe[1], Jasmin Kuo[2], and Lewis Lipsitz[3], *1. Hebrew SeniorLife, Marcus Institute, Harvard Medical School, Roslindale, Massachusetts, United States*, *2. Tufts University, Medford, Massachusetts, United States*, *3. Hebrew SeniorLife, Boston, Massachusetts, United States*

Emerging trials of geroscience interventions show great promise in preventing age-related diseases and disabilities. However, there are many challenges that need to be considered when conducting such trials. Our objective is to describe the major challenges when conducting a geroscience intervention, utilizing the STAMINA study as an example. The STAMINA (Senolytics To Alleviate Mobility Issues and Neurological Impairments in Aging) study is a single-arm geroscience-motivated pilot study in which 12 older adults with mild cognitive impairment and slow gait speed are asked to take 12 doses of two senolytic agents (Quercetin and Dasatinib) over 12 weeks. Study challenges were identified and recorded by the study team and categorized into 3 groups: Regulatory, Recruitment, and Unexpected. This is a descriptive analysis and no formal statistical tests were conducted. Eleven major challenges were identified. The Regulatory Challenges included obtaining the IND from the FDA, hiring a clinical research organization (CRO) to monitor compliance with FDA regulations, and establishing FDA compliant data management tools and procedures. The Recruitment Challenges include drug-drug interactions, comorbidities, need for transportation to and from study visits, and fear of drug side effects. Unexpected Challenges included exorbitant drug costs (Dasatinib costs $500 per dose); requirements for drug storage, dispensing, and destruction; multiple safety lab tests; transportation; biomarker processing, transport, and assays; equipment failure; and intercurrent vaccinations and medicines (e.g., COVID-19 and flu vaccines) which may impact biomarker levels. Conducting a geroscience trial has numerous challenges that need to be considered when developing a budget, hiring staff, and assuring compliance.

Abstract citation ID: igad104.3674

### TRYPTOPHAN: A POTENTIAL BIOMARKER DISTINGUISHING CHRONIC VENOUS LEG ULCERS HEALING IN OLDER ADULTS

Jung Lyun Kim[1], Joyce Stechmiller[2], Michael Weaver[2], Debra Lyon[2], Timothy Garrett[2], and Debra Kelly[2], *1. Chungnam National University, Daejeon, Republic of Korea*, *2. University of Florida, Gainesville, Florida, United States*

Chronic venous leg ulcers (CVLUs) affect 2 million persons annually, including 4% of people over age 65 years. Chronic wound healing is a complex process that is still not well understood. Indoleamine 2,3-dioxygenase (IDO) activity is highly active in chronic wounds. The study aims to examine the tryptophan (TRP) L-Kynurenine (KYN) metabolic pathway as a biomarker of CVLUs healing. We collected 60 serum samples from 30 older adult patients with CVLUs receiving weekly sharp debridement at a university wound clinic. Serum samples were collected at baseline, week 4, and week 8 until wound closure. Liquid chromatography-mass spectrometry (LC-MS) metabolomics was utilized to examine targeted metabolites. A robust Bayesian approach was employed to examine correlations between change of metabolites and linear healing slope. The mean age was 71.13 (±9.46); 25 (83.3%) were white; the mean wound area was 2823.54 (±6002.93) mm2; the mean wound duration was 341.67 (±538.29) days. A total of 11 participants had healed wounds during the study period. Those with healing CVLUs had higher levels of mean TRP at baseline and over time compared to individuals with non-healing CVLUs. Change in Kynurenic Acid (r:-0.36, Bayes factor:

3.70, 95% Credibility Interval [-0.62, -0.06]) was associated with a linear healing slope. Serum tryptophan may serve as a candidate biomarker for predicting wound healing trajectories, which can guide clinicians in making treatment decisions. Specifically, decreasing metabolites associated with the downstream activity of the kynurenine pathway, a pathway for tryptophan metabolism may be an indicator of healing under sharp debridement in CVLUs.

Abstract citation ID: igad104.3675

### TUMOR NECROSIS FACTOR-A, INTERLUKIN-6, AND VISFATIN LEVELS IN OLDER PATIENTS WITH MALNUTRITION

Hsien-Hao Huang, *Taipei Veterans General Hospital, Taipei, Taipei, Taiwan (Republic of China)*

**Introduction:** Proinflammatory cytokine is associated with malnutrition status. Tumor necrosis factor-α (TNF-α) and interleukin-6 (IL-6) were pro-inflammatory cytokines stimulated by nutritional deficiency. Visfatin is an adipokine with a strong correlation with inflammation. The relationships of TNF-α, IL-6 and visfatin are not consistent, and no study have investigated them in the older patients with malnutrition. **Methods:** This prospective study included patients aged ≥ 75 years at the emergency department and all patients underwent Mini-Nutritional Assessment-Short Form (MNA-SF) and blood tests for fasting plasma TNF-α, IL-6 and visfatin levels. **Results:** We enrolled 106 older patients with a mean age of 87.3 years, including 43 (40.5%) patients in at risk of malnutrition group (MNA-SF between 8 to 11), 24 (22.6%) patients in the malnutrition group (MNA-SF < 8), and 39 (36.7%) patients in normal group. Compared to the normal group, both at risk of malnutrition and malnutrition group had significantly lower Mini-Mental State Examination (MMSE). Furthermore, TNF-α was significantly higher in at risk of malnutrition group. In contrast, IL-6 was significantly higher in malnutrition group. Visfatin levels was not correlated to the malnutrition status. Both TNF-α and IL-6 negatively correlated with Barthel index and MMSE. Backward and stepwise multiple logistic regression analyses showed that the independent predictor for both at risk of malnutrition and malnutrition was MMSE. **Conclusion:** The nutrition deficit status was correlated with proinflammatory cytokines, serum TNF-α and IL-6. The independent predictor of both at risk of malnutrition and malnutrition group was MMSE in older patients.

Abstract citation ID: igad104.3676

### TWO NATIVE SOUTH AMERICAN MALE POPULATIONS EXHIBIT CROSS-SECTIONAL INCREASE IN REGIONAL BRAIN VOLUME WITH AGE

Phoebe Imms[1], Nikhil Chaudhari[1], Nahian Chowdhury[1], Margaret Gatz[1], Michael D Gurven[2], Caleb Finch[1], Andrei Irimia[1], and The Tsimane Health and Life History Project[1], *1. University of Southern California, Los Angeles, California, United States*, *2. University of California, Santa Barbara, Santa Barbara, California, United States*

Features of industrialized environments such as consumption of processed foods and sedentarism are associated with faster brain atrophy. Studying brain volume trends with age in non-industrialized populations can elucidate environmental correlates of brain health. Indigenous to the Bolivian Amazon, the Tsimane and Moseten (T&M) are highly active forager-horticulturalists with some of the lowest rates of dementia. Tsimane have minimal interaction with mainstream Bolivians; Moseten are semi-acculturated. Age-related trends in regional brain volumes were estimated from T&M's (N = 1,024; age range: 46-83) computed tomography and compared to trends estimated from Britons' magnetic resonance images (UK Biobank, N = 19,973; same age range). In T&M males, parietal and occipital structures mediating visuospatial abilities exhibit small but significant rates of volume increase with age. Structures recruited during visuospatial navigation are important for survival in Amazonian rainforests. Frontal and temporal structures whose volumes decrease with age in Britons do not trend negatively with age in T&M males. T&M females, however, exhibit steeper cross-sectional rates of volume decrease with age compared to their industrialized counterparts. This suggests that brain volume trajectories, which can reflect dementia risk, are impacted by sex-specific aspects of non-industrialization. Despite T&M females' higher rates of regional brain volume decrease with age compared to UK females, dementia rates in T&M females remain extremely low. Trends demonstrating increases in brain volume with age, such as those observed in T&M males, have not been reported in industrialized populations, suggesting that factors associated with being a male forager-horticulturalist may constrain regional brain atrophy.

Abstract citation ID: igad104.3677

### U.S. REMOTE CANCER CARE FOR OLDER ADULTS: A SYSTEMATIC REVIEW AND SOCIAL NETWORK ANALYSIS

Edwin Garces[1], and Maki Karakida[2], *1. Portland State University, Portland, Oregon, United States*, *2. University of Massachusetts, Allston, Massachusetts, United States*

Telemedicine and telehealth services are a promising solution for older cancer patients who are susceptible to infectious diseases, like COVID-19, and travel a longer distance to their closest hospital. To provide all-inclusive oncology care, understanding social disparities in access to remote cancer care is crucial. Yet, little is known about the accessibility of these technologies among US cancer patients in later life. The present study aimed to evaluate the main sociocultural factors associated with older patients of virtual oncology care interventions in the US. This systematic review explored the literature on telemedicine for geriatric oncology and patients' accessibility to remote health technologies. PubMed, CINAHL, Scopus, and Web of Science databases were searched. Using Bibliometrix, Science of Science (Sci2) Tool, and Gephi, Social Network Analysis (SNA) was conducted to identify the central and intermediate factors linked to remote cancer care for older US adults. Among 427 retrieved articles, 19 articles, published between 2003 and 2023, met the eligibility criteria. The selected studies were focused on rural cancer patients (20%) and telehealth during COVID-19 outbreaks (20%), followed by lifestyle interventions (15%), ehealth literacy (10%), prostate or breast cancer (5%), older sexual minority men with prostate cancer (5%), and patients' race, age, and region (5%). Findings also highlighted that gender, digital health literacy (technology readiness), and location are the key determinants of telemedicine access and use among older US cancer patients.

Multiple socio-demographic aspects of older patients need to be considered for implementing equitable access to remote oncology care.

Abstract citation ID: igad104.3678

### UNDERSTANDING "COMMUNITIES" IN COMMUNITY-BASED SENIOR HOUSING MODELS: A SCOPING REVIEW

Sojung Park[1], Jihye Baek[2], Byeongju Ryu[3], Ahra Ko[4], Jeungkun Kim[5], Takashi Amano[6], and Hyeji Kim[7], *1. Washington University in Saint Louis, St. Louis, Missouri, United States*, *2. Washington University in St. Louis, St. Louis, Missouri, United States*, *3. Boston College, Boston, Massachusetts, United States*, *4. Yonsei University, Seoul, Republic of Korea*, *5. Kangnam University, Yongin, Geyonggi, Republic of Korea*, *6. Rutgers University, Newark, New Jersey, United States*, *7. Good Neighbors Mirae Foundation, Seoul, Republic of Korea*

Various community-based senior housing models have been developed to enhance social engagement and communal living among older adults, alongside their pursuit of independence and later life well-being. However, a comprehensive understanding of the efficacy of these housing models in cultivating "communities" which are integral to sustaining social ties in later life, remains limited. This scoping review investigated the conceptualization, establishment, and management of communities within diverse housing models, with an emphasis on community-related constructs. This scoping review examined empirical, peer-reviewed studies sourced from PsycINFO, CINAHL, MEDLINE, Scopus, and PubMed, resulting in the inclusion of twenty-one articles. Through thematic analysis, three predominant themes emerged: 1) residents' well-being and lived experiences within the community (n=13), 2) engagement in communal activities and social programs (n=4), and 3) the spatial design and physical aspects of the community (n=4). We found that the way communities are defined and conceptualized differed across housing models. Also, residents' individual characteristics (e.g., gender, age, income, health status) influenced their community lives and experiences. Community experiences were often conceptualized as a "sense of community" and "place attachment." The majority of research focused on the experiences of residents' communal living and their well-being. Still, some studies focused on how social and physical environments supported and facilitated community lives. This study underscores the need for further research into the dynamics of communities within diverse senior housing facilities. Also, more studies should be conducted on how those communities are formed and maintained to support older adults' successful social lives in the community.

Abstract citation ID: igad104.3679

### UNDERSTANDING AGE-RELATED DISPARITIES AND ENVIRONMENTAL BARRIERS IN OLDER ADULTS WITH MULTIPLE SCLEROSIS

Kyungmi Lee, and Matthew Plow, *Case Western Reserve University, Cleveland, Ohio, United States*

Given the increasing life expectancy of individuals with multiple sclerosis (MS), the challenges faced by older adults with MS in terms of health changes and decreased physical function contribute to a potentially diminished quality of life. Despite this, our understanding of how this condition manifests in the context of aging remains limited. This study aims to examine age-related variations in fatigue, anxiety, depression, community engagement, and environmental barriers in older adults with MS, as well as to explore health disparities among older adults with MS. This secondary analysis involved 288 individuals with MS, divided into two age groups: below 60 years and 60 years and above. Aim 1 used T-tests and Aim 2 employed ANCOVA with disability adjustment. The results show that older adults with MS had higher levels of community engagement, disability, and behavioral-cognitive fatigue, reporting lower levels of anxiety, cognitive-motor fatigue, fatigue severity, and participation barriers than younger adults with MS. Noteworthy health disparities emerge, characterized by reduced community integration/participation ($F=10.622$, $p=.001$) and heightened environmental barriers ($F=4.323$, $p=.039$), even when adjusting for disability levels. The study highlights environmental barriers impeding social participation among older adults with MS, emphasizing the need to address these obstacles to enhance their quality of life. This underscores the imperative for customized interventions targeting reduced community disengagement and heightened environmental barriers faced by older adults with MS.

Abstract citation ID: igad104.3680

### UNDERSTANDING CHALLENGES AND SOLUTIONS FOR DEAF OLDER ADULTS

Shraddha Shende[1], Lyndsie Koon[2], Jenny Singleton[3], and Wendy Rogers[4], *1. Illinois State University, Normal, Illinois, United States*, *2. Kansas University, Lawrence, Kansas, United States*, *3. Stony Brook University, Stony Brook, New York, United States*, *4. University of Illinois Urbana-Champaign, Champaign, Illinois, United States*

The Aging Concerns, Challenges, and Everyday Solution Strategies (ACCESS) project explores everyday activity challenges and solutions experienced by U.S. older adults as they age with a lifelong disability involving vision, motor, or hearing. Here, we report our findings from our community-engaged research with older adults who self-identify as members of the U.S. Deaf Community and who use American Sign Language (ASL) as their primary language. Using trained Deaf interviewers, we conducted semi-structured interviews in ASL with 60 Deaf participants (age 60-79 years) to understand their challenges performing various everyday activities (e.g., doing things around the home, activities outside the home, transportation, and managing health). Overall, our thematic analyses revealed that Deaf older adults navigate everyday tasks quite well. However, they experience particular challenges with technology, communication, and accessibility; specifically, fragile or inaccessible technology systems. For instance, technology alerting systems at home (e.g., smoke alarms) or airports (e.g., gate change announcements) are typically auditory. Accessibility is also poor when healthcare or wellness providers fail to provide an ASL interpreter. When probed about their responses to these challenges, Deaf older adults often reported frustration or resignation when experiencing barriers; the need for constant vigilance and self-advocacy; or reliance on hearing family members when systems fail them. Improving communication access and technologies that Deaf older adults rely on is critical and requires the inclusion of individuals from the Deaf community to identify the challenges and potential avenues for solutions.

Abstract citation ID: igad104.3681

### UNDERSTANDING THE IMPACT OF AGEISM AMONG HEALTHCARE PROFESSIONALS AND STUDENTS

Laura Finn[1], Kavitha Sukumar[1], Serena Fasola[1], Dennis Phan[1], Kirsten Bautista[1], Michelle Hon[2], and Catherine Oswald[2], *1. Philadelphia College of Pharmacy / St Joseph's University, Philadelphia, Pennsylvania, United States*, *2. Roseman University of Health Sciences, Henderson, Nevada, United States*

With our population aging, the need for healthcare practitioners trained in caring for older adults is urgent. The anticipated shortage of geriatric-trained providers may further the impact of age-related discrimination. Little data is published about the understanding of ageism by healthcare professionals and students. The comparison of these different groups became the focus of the conducted survey, and the presenting research analyzed a total of 467 respondents including dental, nursing, pharmacy, occupational therapy, and physical therapy professions across 2 universities. When comparing demographic differences in studied populations, 69% of faculty/preceptors and 12% of students were over 35 years old. Overall, 33.8% reported being unable to define ageism with 27.7% of all students unable to do so. In looking specifically at pharmacy, 34.3% of students and 37.9% of faculty were unable to define ageism. Understanding ageism and its effects on healthcare for older adults is important for pharmacists as 82.1% of pharmacy faculty reported regularly interacting with older adults and 67% said that primarily occurred at work; yet only 37.5% had taken a class or course in geriatrics. Among pharmacy students, 71.4% interacted regularly with older adults with 41.4% saying this occurred primarily at work. Differences were noted in student education with 50% of pharmacy and 86.9% of physical therapy students having taken a geriatric course. Analysis and conclusions interpret the impact of ageism and emphasize a need for education to address age-related disparities and potential for bias in healthcare.

Abstract citation ID: igad104.3682

### UNMET NEEDS OF OLDER FEMALE VETERANS AND GENDER DIFFERENCES USING THE PROSPECTIVE HERO CARE SURVEY DATA

Stuti Dang[1], Pranjal Tyagi[2], Sandra Garcia[1], Erin Bouldin[3], Ranak Trivedi[4], Orna Intrator[5], Mary Jo Pugh[6], and Luci Leykum[7], *1. University of Miami, Miami, Florida, United States*, *2. South Florida VA Foundation for Research and Education (SFVAFRE), Miami, Florida, United States*, *3. Decision-Enhancement and Analytic Sciences Center (IDEAS 2. .0. ), Salt Lake City, Utah, United States*, *4. Stanford University, Stanford, California, United States*, *5. Canandaigua VAMC and University of Rochester, Canandaigua, New York, United States*, *6. VA Salt Lake City Health Care System, Salt Lake City, Utah, United States*, *7. South Texas Veterans Health Care System, San Antonio, Texas, United States*

Aging female Veterans face unique healthcare needs which are yet to be well-described. We analyzed round one data from HERO CARE, a prospective survey, to describe the medical, psychological, and social unmet needs of female Veterans and compare them with those of male Veterans. We received survey responses from 8,056 community-dwelling Veterans (227 (2.8%) females and 7,829 (97.2%) males) from San Antonio, Palo Alto, Miami, Salt Lake City Veterans Affairs (VA) Medical Centers, and Veteran Integrated Service Network 8 in July 2021. We compared difference in needs between the female and male respondents using chi-square tests and difference in age in years using t-tests. Respondents were classified as having an unmet need if they responded that they needed "a little" or "a lot more help" for specific needs. Female respondents were 74.3 (SD: 14.5) years-old on average, and 7.9% Hispanic, 15.4% non-Hispanic Black, 72.7% non-Hispanic White. Male respondents were older 80.3 (SD: 9.8) years-old on average ($p< 0.01$), with no significant difference in race/ethnicity. Female Veterans reported unmet needs in ADLs (14.1%), IADLs (40.1%), nursing tasks (7.1%), pain management (17.6%), communicating with the healthcare team (10.6%), and social needs like legal or housing (16.3%). All unmet needs were like those of male Veterans (all $p>0.05$). Among female Veterans, 20.1% screened positive for anxiety (GAD-2 score >2) and 22.8% for depression (PHQ-2 score >2), compared to male Veterans: 15.2% ($p< 0.01$) and 21.6% ($p=0.41$), respectively. Routine assessment of aging female Veterans, specifically focusing on mental health and IADLs, may better address their needs.

Abstract citation ID: igad104.3683

### UNVEILING TENSIONS: CAREGIVING FOR OLDER ADULTS WITH CANCER IN THE COVID-19 ERA

Charlotte Weiss[1], Rachel Johnson-Koenke[2], Karen Sousa[2], Connie Ulrich[1], and Karen Hirschman[1], *1. University of Pennsylvania, Philadelphia, Pennsylvania, United States*, *2. University of Colorado- Anschutz Medical Campus, Aurora, Colorado, United States*

The COVID-19 pandemic was traumatic for many individuals, including older adults with serious illness and their families. This qualitative study aimed to explore family caregiver narratives for the historical, societal, and institutional context of caring for older adults with advanced cancer during the COVID-19 pandemic. Using narrative inquiry alongside photo elicitation, 5 caregiver narratives were co-created during in-person semi-structured interviews or virtually through Zoom video conferencing. Each of the participant caregivers started caring for an older adult family member with cancer during the COVID-19 pandemic. Narratively derived thematic threads of COVID-19 emerged as short stories of tension woven throughout the cancer caregiver narratives. The themes within and across the narratives included: advocacy of quality healthcare for care recipients, protection of care recipients from COVID-19; protection of care recipients from medical institutional disregard; financial strain and uncertainty; lack of medical, emotional, social, and spiritual support; intention towards self-care; and saying goodbye in isolation. The backdrop of COVID-19 contributed to caregiver fear, anxiety, feelings of being alone, guilt, and emotional and physical exhaustion. Findings from this study highlight that cancer family caregivers experienced multiple layers of tension when caring for older adults with advanced cancer during COVID-19. By acknowledging tensions emergent through storytelling, researchers and clinicians contribute to valuing the diversity of individually and collectively lived human caregiving experiences and the uncertainty and turmoil in their lives.

Abstract citation ID: igad104.3684

### UNVEILING THE COMPLEX REALITIES: UNDERSTANDING SLEEP AND FATIGUE CHARACTERISTICS OF END-OF-LIFE CAREGIVERS

Hyeyoung Park[1], Sunghoon Lee[2], Amelia Bailey[3], Yunda Liu[2], and Cynthia Jacelon[1], *1. University of Massachusetts Amherst College of Nursing, Amherst, Massachusetts, United States*, *2. University of Massachusetts Amherst, Amherst, Massachusetts, United States*, *3. Brown University, Providence, Rhode Island, United States*

This mixed methods multi-case study explores sleep and fatigue characteristics among end-of-life caregivers. The research sample encompasses cases aiding individuals with advanced (n=4) and chronic illnesses (n=1), including various caregiver demographics such as gender, age groups (19-64 and 65+), and health conditions. Data collection involves comprehensive surveys capturing demographics, care recipient symptom intensity, caregiver depressive symptoms, caregiving stress, caregiver sleep quality, and caregiver fatigue level. The study integrates data from a 30-day Personal Sleep Monitoring Device (PSMD) and a 14-day sleep journal, enriched by insights from semi-structured interviews and field notes. The outcomes indicate a PSQI (Pittsburgh Sleep Quality Index) score of 6.1, accompanied by 7.44 hours of sleep recorded by the PSMD. There were approximately 20.5 nocturnal awakenings within 30 days, with average fatigue at 55.28 and daily fatigue at 49.66. Objective sleep outcomes were consistent among caregivers, while their own sleep ratings ranged from "Fairly Bad" to "Very Good." Themes for each case were formulated to delve into the context of these sleep and fatigue characteristics. These themes encompass adaptive leadership, linchpin roles, emotional labor management, self-reliance, and vigilance. A prevailing overarching theme is the perception of "Not having control." A significant finding underscores the dynamic influence of care recipients' daily statuses on caregivers' sleep quality and fatigue levels, leading to unpredictable outcomes. This study offers profound insights into end-of-life caregivers' intricate journeys, highlighting individual differences and the dynamic nature of caregiving roles, while recognizing the importance of tailored interventions for enhancing caregiver's well-being and caregiving effectiveness.

Abstract citation ID: igad104.3685

### USABILITY OF A SMARTPHONE APP DESIGNED TO COLLECT HEALTH INFORMATION IN OLDER ADULTS

Joanne Murabito[1], Jamie Faro[2], Yuankai Zhang[3], Angelo Demalia[4], Alexander Hamel[2], Nakesha Agyapong[2], David McManus[5], and Belinda Borrelli[6], *1. Boston University Chobanian & Avedisian School of Medicine, Boston, Massachusetts, United States*, *2. University of Massachusetts Chan Medical School, Boston, Massachusetts, United States*, *3. Boston University School of Public Health, Boston, Massachusetts, United States*, *4. Boston University, Boston, Massachusetts, United States*, *5. University of Massachusetts Chan Medical School, Worcester, Boston, Massachusetts, United States*, *6. Boston University, Henry M. Goldman School of Dental Medicine, Boston, Massachusetts, United States*

Few studies evaluate the usability of mobile-phone assessments in older adults. The aim of this study was to identify design-based barriers and facilitators to mobile app survey completion among two samples of older adults; those in the Framingham Heart Study (n=15, mean age 72 years, 40% women, 100% non-Hispanic, White) and, to aid with generalization, a more diverse sample (n=15, mean age 71 years, 47% women, 53% non-White, 20% Hispanic). We used mixed methods to ascertain information about challenging or beneficial features and functions of the mobile app. A variety of measures with different response formats were tested including self-reported surveys, pictorial format assessments (image of a body with checkboxes to indicate pain sites), and cognitive testing tasks, including Trail Making Test (TMT) and Stroop. Participants completed each of the measures using a think-aloud task, while being audio- and video-recorded. Recordings were coded for usability errors by two pairs of coders. Participants completed the Mobile App Rating Scale (MARS) to assess functionality and aesthetics of the app (range 1-5). In general, participants were satisfied with the app. In Framingham Heart Study participants, the average MARS functionality score was 3.6 (SD=0.7) and aesthetics score was 3.9 (SD=0.7) with no significant differences in the diverse sample. The majority of older adults did not have difficulty completing the self-report measures, unless there were lengthy instructions or the pictorial measure, but participants had usability issues with the Stroop and TMT. Our methods and results can guide app development and survey construction for older adults.

Abstract citation ID: igad104.3686

### USE OF THE EXERCISE FEATURE OF A HEALTHCARE APP AND CHANGE IN THE PREVALENCE OF LOWER BACK PAIN

Mai Takase[1], Kishimori Odagami[2], Hisashi Eguchi[3], Koji Mori[3], Masako Itabashi[4], Sei Kono[4], Yosuke Shimizu[4], and Katsuya Iijima[1], *1. The University of Tokyo, Tokyo, Tokyo, Japan*, *2. University of Occupational and Environmental Health, Japan, Kitakyushu, Fukuoka, Japan*, *3. University of Occupational and Environmental Health, Yahata Nishi, Fukuoka, Japan*, *4. Haseko Anesis Corporation, Minato-ku, Tokyo, Japan*

Lower back pain among older adults has been associated with serious conditions, such as depleted cognitive function, and can accordingly limit the degree to which older adults can live independently. Therefore, it is extremely important to engage in exercise and conditioning activities from an early age. We developed a healthcare app with an AI engine that recommends personalized stretch, muscle training, and aerobic exercise programs. These programs were constructed by care prevention exercise experts and are personalized based on the user's physical balance and the locations of their body pain. This study examined the association between the use of the exercise feature of this app and the change in the prevalence of lower back pain among users. A three-month longitudinal survey with three model companies in Japan (n=730) was conducted from the fall of 2022 to the winter of 2023. Online questionnaire surveys were administered to track the participants' levels of lower back pain and app logs were retrieved to assess the users' exercise records. Use of the exercise feature (not used vs. used) was the independent variable and change in the level of lower back pain (none to mild vs. mild to severe) was the dependent variable. A binary logistic regression analysis (n=331, female=34.1%, over 50 years old=43.5%) revealed that the use of the exercise feature led to lower level of back pain (OR = 0.37, 95%CI = 0.29-0.67). This result suggests that the use of this kind of exercise app may help alleviate lower back pain.

Abstract citation ID: igad104.3687

### USER-CENTERED DESIGN FOR SOCIALLY ASSISTIVE ROBOTIC ACTIVITIES WITH OLDER ADULTS IN LONG TERM CARE

Cathy Maxwell[1], Ritam Ghosh[1], Nibraas Khan[1], Miroslava Migovich[1], Judith Tate[2], Emily Latshaw[2], Lorraine Mion[2], and Nilanjan Sarkar[1], *1. Vanderbilt University, Nashville, Tennessee, United States*, *2. The Ohio State University, Columbus, Ohio, United States*

Apathy is common among older adults (OAs) in long term care (LTC) and associated with adverse outcomes. Engaging residents in socially interactive multimodal activities is resource intensive. Socially assistive robots (SARs) may supplement LTC staff resources. However, few studies have involved OAs in the design of SAR activity. We conducted a multi-step user-centered design process to design and evaluate SAR activities that a) used different robots (humanoid, puppy), b) a virtual reality interface, and c) required dyadic interaction between residents. Phase 1 (system design) focused on prototype development with input from literature and an advisory panel of LTC adults and staff. Phase 2 focused on determining usability measures. Phase 3 (iterative testing) focused on field testing at two LTC sites. Multiple sessions were conducted that required looping back to earlier stages of prototype development for refinement and improvement of both the activity and the system until end users deemed them usable. **Results:** 12 residents (ages 72-92, 9 women) and 5 LTC staff participated in the design of four SAR activities (music, fishing, painting, and spelling). Perceived comfort and confidence improved from baseline (d=0.708). Interaction between pairs increased from 0.16/min to 1.14/min. LTC staff were able to administer the system. **Conclusion:** Older LTC adults are pivotal in informing technology development and capable of providing essential information in use and acceptance of technology. Next steps include large scale feasibility and effectiveness testing in a clinical trial among diverse older adults residing in LTC facilities.

Abstract citation ID: igad104.3688

### USING HEALTH INFORMATION EXCHANGE DATA FOR SOCIAL RESEARCH: CHALLENGES AND OPPORTUNITIES

Allen Glicksman[1], Ashley Ritter[1], Steven Albert[2], Adam Davey[3], Ari Friedman[4], Lauren Ring[1], Misha Rodriguez[1], and Michael Liebman[5], *1. NewCourtland, Philadelphia, Pennsylvania, United States*, *2. University of Pittsburgh, Pittsburgh, Pennsylvania, United States*, *3. University of Delaware, Newark, Delaware, United States*, *4. University of Pennsylvania, Philadelphia, Pennsylvania, United States*, *5. IPQ Analytics. LLC, Kennett Square, Pennsylvania, United States*

Data collected in encounters between health providers and older adults can provide an important source of information regarding the experience of older persons with limited English Proficiency (LEP) in the health care system further enhancing our knowledge about health access in disadvantaged populations. Using data acquired from the local health information exchange (HIE) our team examined usage patterns of health services by Spanish and Chinese speaking older adults in Philadelphia. The data, which became available in July, represents one of the first opportunities to use HIE data to study these issues. HIE data link EMR records from providers across a defined geographic area to deliver information on patients served through participating organizations. The initial sample included reports on almost 200,000 persons with 7.7 million encounters. Each record contains information on type of encounter, diagnoses, insurance status, and related items. While availability of these data opens the opportunity to explore a variety of questions regarding health service use, methodological challenges to using HIE data are significant. These include (but are not limited to): 1) different coding schemes for key variables (e.g., encounter type, diagnosis); 2) LEP status, geography, and coding schemes may be confounded; 3) coding schemes used by providers may change over time so longitudinal data can include multiple coding types for the same patient; and 4) significant variation in coding race/ethnicity may be introduced for the same patient. We present examples of such challenges and approaches for resolution to enable and facilitate our analyses.

Abstract citation ID: igad104.3689

### USING MULTI-LEVEL MODELING TO EXAMINE RACIAL DISPARITIES IN PALLIATIVE CARE AMONG OLDER ADULTS WITH HEART FAILURE

Ling Han[1], Erica Abel[2], Yan Zhan[3], Kathleen Akgün[2], and Shelli Feder[3], *1. Yale School of Medicine, New Haven, Connecticut, United States*, *2. VA Connecticut Healthcare System, West Haven, Connecticut, United States*, *3. Yale School of Nursing, New Haven, Connecticut, United States*

Studies have found persistent disparities in guideline-concordant cares for heart failure among patients who self-identify as Black. We investigated how the guideline-concordant specialist palliative care (SPC) varied with race among older adults with advanced heart failure (AHF). We identified 16,464 older adults aged ≥65y (median: 74y) with AHF by ICD-9/10 codes seen between 1/1/2018-7/1/2020 in 83 Veterans Affairs Medical Centers (VAMC). In conventional logistic regression without accounting for between-VAMC variation, Black (28%) race was less likely to receive SPC services than White (70%) race [adjusted odds ratio (aOR) of 0.91 (95% Confidence Intervals (CI): 0.84,0.99); predicted probability: 27.6% vs. 29.5%], independent of age, sex and comorbidity. This association diminished to null [aOR: 0.99 (95% CI: 0.90,1.08)] after adding a random intercept for VAMCs (multi-level model (MLM)). Expanding the MLM with specific VAMC characteristics (SPC team staffing ($p< 0.05$), facility complexity and urbanicity) as fixed effects improved overall model fit (area under ROC: 0.68 vs 0.61, $p< 0.05$), and accounted for 17% of the VAMC-level variance (intraclass correlation coefficient:

0.069 vs 0.083). There were no cross-level interactions between race and VAMC characteristics. These findings suggest that variation in the individual probability of receiving SPC between Black and White race in this older AHF cohort may be partially explained by between-VAMC differences in SPC staffing and other facility characteristics. To promote equitable access to SPC for older adults with AHF, further research is needed to examine service delivery characteristics and broader contextual determinants of health, such as area socioeconomic disparity of healthcare facilities

Abstract citation ID: igad104.3690

### UTILIZING ADVANCED DRIVER ASSISTANCE SYSTEMS IN LATER LIFE: INSIGHTS FROM A LONGITUDINAL STUDY ON AGING DRIVERS

Renee St. Louis[1], David Eby[2], Lidia Kostyniuk[1], Lisa Molnar[2], Jennifer Zakrajsek[2], Nicole Zanier[1], Linda Nyquist[3], and Raymond Yung[3], *1. University of Michigan Transportation Research Institute, Ann Arbor, Michigan, United States*, *2. University of Michigan, Ann Arbor, Michigan, United States*, *3. University of Michigan Geriatrics, Ann Arbor, Michigan, United States*

Level 1 and 2 Advanced Driver Assistance System (ADAS) technologies (e.g., adaptive cruise control, blind spot warning, lane keep assist) are predicted to be available in approximately three-fourths of all vehicles worldwide by 2025. To realize the potential of these systems in extending safe mobility for aging adults, it is critical that the driver understands the functionality of the technology and uses the system appropriately. The current study examined demographic and vehicle technology questionnaire data from the Longitudinal Research on Aging Drivers (LongROAD) cohort study which concluded in December 2022. A total of 1,417 participants changed their vehicle throughout the study. There were statistically significant increases in the prevalence of all 15 ADAS technologies examined. Despite increases in prevalence, frequency of using the technologies remained unchanged across 5 years. Frequency of use also varied by functionality of the technology whereby participants reported higher frequency of using technologies that provide alerts, such as blind spot warning, than technologies that take action to assist drivers with vehicle operations, such as adaptive cruise control. Results showed differences in prevalence and use of technologies by income and education, suggesting disparities in access to vehicles with technologies that could help to create a safer driving experience. In consideration of the rapid proliferation of ADAS into the vehicle fleet, increased research into how older drivers learn about and use ADAS technologies will assist in efforts to develop tailored and accessible programs for training older adults to properly utilize ADAS available in their own vehicles.

Abstract citation ID: igad104.3691

### UTILIZING VALID, RELIABLE, AND PRACTICAL MEASURES OF HEALTH STATUS IN PRIMARY GERIATRIC CARE: TRANSLATING RESEARCH INTO USUAL CARE WITH THE SENIOR'S HEALTH ASSESSMENT REPORT AND PLAN (SHARP™)

Ted Rosenberg, *University of British Columbia, Victoria, British Columbia, Canada*

**Introduction:** To better support evidenced-based care, we combined 9 valid, reliable, and clinically useful tests into a single health status and risk assessment tool, performed by a Team nurse, called the Seniors Health Assessment, Report and Plan (SHARP™). These tests include: CFS, EQ5D-5L, EQ-VAS, MoCA, GDS, Months of the Year Backwards, Gait Speed, Grip Strength, Water Swallow Test, and MNA-6. A published study, with 18 months of follow-up for a primary geriatric home-based practice demonstrated that these tests were stronger predictors of death, nursing home transfer (NHT), or hospital admission(HA) compared to any medical diagnosis, multiple comorbidities, or polypharmacy. Hazard Ratios for SHARP™ vs medical diagnoses were: Death (median HR 5.9 vs. 1.6), NHT (median HR 4.6 vs.1.4), and HA (median HR 6.0 vs. 1.6). The research presented here will provide several case-based studies to demonstrate how we have translated this research into "usual care" for a primary home-based interdisciplinary geriatric medical practice. Specifically, we will demonstrate how we: 1. share this data with patients and caregivers to motivate them for Team interventions, 2. share a summary report of an individual's health with the hospital and other community care providers, 3. use these tests to guide and evaluate Team interventions for individual patients (e.g. changes in gait speed), 4. Track changes in health status and risk, and 5. use aggregated data at a program level for benchmarking, defining population needs, and for planning and evaluation. **Conclusions:** Standardized testing is acceptable to patients, efficient, supports evidenced-based care, and is useful for program planning and evaluation.

Abstract citation ID: igad104.3692

### VOICES OF LOW-INCOME LATINO OLDER ADULTS: WHAT ARE THEIR PERCEIVED LEARNING IMPEDIMENTS?

Tania Rodriguez, and Rachel Wu, *University of California Riverside, Riverside, California, United States*

Cognitive and functional health disparities between Latino and White older adults show that Latinos have a higher risk for developing Alzheimer's and related dementias (ADRD) in the United States. Such risk compromises Latino's likelihood of becoming healthy agers, which is of high concern as they are one of the largest and fastest-growing minoritized groups in the United States. Nonetheless, there is a lack of research aiming to understand how to increase their cognitive and functional abilities. One way of stimulating cognitive and functional growth is through learning of new skills. However, learning is a privilege, and this population may face unique barriers to learning that other racial/ethnic groups may not, especially if they have a low income. This qualitative study focused on identifying the barriers to novel skill learning engagement of low-income Latino older adults. A total of 20 low-income, Latino older adults were interviewed in Spanish over the phone utilizing a semi-structured interview protocol. Themes were derived from the data using a thematic analysis approach. Our results showed that the following barriers were most common among our participants: health problems (80%), lack of money (75%), lack of awareness/knowledge of learning opportunities (75%), lack of technology access/literacy (75%), lack of proficiency in the English language (65%), lack of reliable transportation (55%), and lack of motivation due to being "old" (50%). These findings help inform learning program leaders, policymakers, and future learning interventions on the barriers that should be addressed for minoritized populations.

Abstract citation ID: igad104.3693

### WALKING CADENCE AS A MEASURE OF ACTIVITY INTENSITY AND THE IMPACT ON FUNCTIONAL CAPACITY FOR OLDER ADULTS WITH FRAILTY

Daniel Rubin[1], Anthony Hung[1], Donald Hedeker[1], David Conroy[2], Megan Huisingh-Scheetz[1], Jennifer Brach[3], Nancy Glynn[4], and Margaret Danilovich[5], *1. University of Chicago, Chicago, Illinois, United States*, *2. Penn State University, University Park, Pennsylvania, United States*, *3. University of Pittsburgh, Pittsburgh, Pennsylvania, United States*, *4. University of Pittsburgh School of Public Health, Pittsburgh, Pennsylvania, United States*, *5. Northwestern University, Chicago, Illinois, United States*

**Background:** Walking cadence has been suggested as a measure of activity intensity; however, it remains uncertain if prefrail and frail older adults can increase their walking cadence and if doing so leads to improvements in functional capacity. We aimed to determine if cadence can be increased and if this leads to improvement in functional capacity in prefrail and frail older adults. **Methods:** We performed a secondary data analysis of a walking intervention in prefrail and frail older adults living in retirement communities. Patients were randomized to Casual Speed Walking (CSW) and High-Intensity Walking (HIW) groups. Our primary outcome was improvement in 6-minute walk test (6MWT) distance above the minimally clinical important difference (MCID). We performed linear and logistic mixed-effects regressions to analyze our aims. **Results:** 102 participants were included in the final analysis with 56 in the CSW group and 46 in the HIW group. Participants in the HIW group increased their walking cadence as compared to the CSW group during the intervention (HIW 100[88, 111] steps/min vs. CSW 77[65, 86] steps/min; P < 0.001). Participants that increased their walking cadence demonstrated an increased odds of improvement in their 6MWT MCID (OR: 0.11, 95% CI: 0.033, 0.18; p=0.005). **Conclusions:** Older adults can increase their walking cadence and walking cadence can serve as a surrogate measure of activity intensity during walking interventions. An increase of 14 steps/minute from their comfortable walking cadence increased the odds of improvement in 6MWT MCID.

Abstract citation ID: igad104.3694

### WHAT DO NURSING HOMES RECEIVING HIGH COMPLAINTS GOT TO DO WITH RESIDENTS' QUALITY OF LIFE?

Jenny Kwon, and Xiao Qiu, *Miami University, Oxford, Ohio, United States*

Residents' quality of life is important to understand nursing home (NH) quality. Official complaints are another NH quality indicator. However, little research has examined the two quality measures together. To fill this gap, this study aims to investigate the association between resident satisfaction and NH complaints. 2018–2019 complaints data from the Ohio Department of Health, 2017 Ohio Biennial Survey of Long-Term Care Facilities, and 2017 Ohio Nursing Home Resident Satisfaction Survey were merged (N=629). Eight outcome variables were used: overall resident satisfaction score and seven individual domain scores. They range from 0 (low) to 100 (high). For independent variables, we labeled NHs received 6 or more complaints per 100 residents in both 2018 and 2019 as 'high complaint repeaters.' Multiple linear regression was conducted. There is a statistically significant relationship between official complaints and resident satisfaction. High complaint repeaters had lower overall satisfaction score by 1.48 (p < .01); lower moving in score by 2.16 (p < .01); lower spending time score by 1.30 (p < .05); lower caregivers score by 1.20 (p < .05); lower meals and dining score by 2.18 (p < .05); lower environment score by 1.13 (p < .05); and lower facility culture score by 2.14 (p < .01) than non-high complaint repeaters. However, there is no association between complaints and care and services domain score. State policy makers need to consistently monitor high complaint repeaters and support them to reduce complaints. Also, providers should allocate resources and design strategies to improve residents' experiences.

Abstract citation ID: igad104.3695

### WHAT INTERFERES WITH OLDER ADULTS' PARTICIPATION IN A MODIFIED PRESCRIBED EXERCISE PROGRAM?

Justine Sefcik[1], Sean Harrison[2], Marjorie Skubic[3], Therese Richmond[2], Nancy Hodgson[2], and George Demiris[2], *1. Drexel University College of Nursing and Health Professions, Philadelphia, Pennsylvania, United States*, *2. University of Pennsylvania, Philadelphia, Pennsylvania, United States*, *3. University of Missouri, Columbia, Missouri, United States*

Exercise programs have great potential to help keep older adults active and independent in their homes. However, barriers to participation in a virtual coaching exercise program aimed at fall prevention is not well understood. This qualitative descriptive study with a conventional content analysis examined older adults' perceptions of what interfered with their participation in a modified 3-month Otago exercise program. Ten older adults participated in five bi-weekly telephone coaching calls and interviews where they were asked about their last two weeks of exercise participation. Participants were mean age 71.3 (range 66 - 81), and mostly female (90%), Black (70%), and non-Hispanic (90%). While most of the participants routinely participated in their prescribed exercises, a few had difficulty completing the plan for some weeks. We identified two themes of what participants expressed had gotten in their way of routinely exercising: 1) distracted and busy (e.g., prepping for the holidays, death in the family, needing to clean home) and 2) physical problems (e.g., pain, being sick, injury). Only one participant said they lacked motivation, which resulted in them exercising less than their initial intention. These findings suggest that older adults want to participate in a routine exercise program, however, they sometimes have other priorities or physically cannot exercise. Program implementers should consider expanding timelines of physical training to account for weeks when older adults are unable to exercise and provide modifications for participants when ill, experiencing chronic pain, or have a new injury.

Abstract citation ID: igad104.3696
### WHERE IS THE DIGITAL DIVIDE? INTRA-GENERATIONAL DIVIDE ON APP USAGE IN POST-PANDEMIC HONG KONG
Bobo Hi-po Lau[1], Eric Ngai Yin Shum[1], Fan Yin-Fong Lui[1], Gigi Lam[1], Alex Pak-Ki Kwok[2], and Chung-Kin Tsang[1], *1. Hong Kong Shue Yan University, Hong Kong, Hong Kong*, *2. The Chinese University of Hong Kong, Hong Kong, Hong Kong*

The COVID pandemic has evoked extensive digitalization of our daily lives. This paper reports two studies conducted in the June/July 2023 that evaluated smartphone usage among Hong Kong middle-aged and older adults after 13 months of smartphone-based compulsory track-and-trace COVID policy. A focus group study with 145 adults aged 55 years or above (Max = 92) found that smartphones are used primarily for instant but asynchronous communication, thus, WhatsApp and WeChat are the most popular. Divisive usage is the most salient with apps that involve monetary transactions (e.g., mobile banking, online shopping) with participants having more trust towards the security provided by the device, the network, and the financial institutions as well as higher socio-economic status (SES) being more receptive. Higher SES was also related to a broader repertoire of app use. These findings corroborate with those from a concurrent survey with 750 smartphone users aged 45 or older (Max = 78). Using latent class analysis, we found two classes of users based on their usage on 18 categories of apps. Class 1 (64.3%) were more likely to use all categories of apps than Class 2 (35.7%), with the largest Chi-square values reported on apps for small-sum transactions, online shopping, private hire cab/courier, and mobile banking – all related to monetary transactions. Class 1 were younger, with higher SES, owning more smart devices, more proficient with mobile devices, and more optimistic with technologies than Class 2. Our findings elucidate directions for narrowing the digital divide within the older adult population.

Abstract citation ID: igad104.3697
### WHO'S ON YOUR TEAM? DEMENTIA FAMILY NETWORKS AND CAREGIVER WELL-BEING
Amanda Leggett[1], Srabani Haldar[1], Sophia Tsuker[1], and HwaJung Choi[2], *1. Wayne State University, Detroit, Michigan, United States*, *2. University of Michigan, Ann Arbor, Michigan, United States*

While caregivers are typically enmeshed in broad networks of family and friends assisting with care, this network is largely neglected, in favor of examining a "primary" caregiver. This study examines types of family/unpaid friend networks for persons living with dementia and how one's network type relates to caregiver well-being. Data are drawn from the nationally representative 2017 National Health and Aging Trends Study and associated National Study of Caregiving. The sample includes 336 dementia care networks (Network size mean=2.9). We first identified network types using Latent Class Analysis and then examined the extent to which network type is associated with outcomes of caregiver well-being (emotional difficulty, overload) and support (social/care-related support from family and friends) using ANOVA and linear regressions adjusting for caregiver demographics. The three network types identified were: "Specialists"- small networks with little task sharing between members (29.8% of networks), "Small but mighty"- small networks with high task sharing (23.0% of networks), and "Sharers"- large networks with diverse membership and members who share and who specialize in task assistance (47.2%). "Small but mighty" network membership was associated with significantly greater emotional difficulty of caregiving ($F=4.0$, $p<.05$) and marginally greater overload ($F=2.8$, $p=.06$) than "Specialist" and "Sharer" membership. However, in regressions, only social support significantly differed, with "Sharers" ($B=-0.1$, $p<.01$) and "Small but mighty" ($B=-.2$, $p<.05$) networks receiving significantly more support than "Specialists". Networks with more task sharing may require more social support, yet smaller network size, while carrying complex caregiving responsibilities, may take a greater emotional toll.

Abstract citation ID: igad104.3698
### WITHIN TWIN PAIR DIFFERENCES INDICATE GENETIC EFFECTS OF SES INDICATORS ON LONGITUDINAL CHANGES IN PHYSICAL AGING
Deborah Finkel[1], Malin Ericsson[2], and Margaret Gatz[1], *1. University of Southern California, Los Angeles, California, United States*, *2. Karolinka Institutet, Stockholm, Stockholms Lan, Sweden*

Socioeconomic status (SES) predicts change in health status over age, even after accounting for measured confounders such as environmental and biological risk factors. Nonetheless, the source of SES-health associations continues to be heavily debated. Twin studies offer a method for testing causal hypotheses by incorporating within and between twin pair differences in longitudinal latent growth curve models (LGCM) of physical aging on both level of functioning and rate of change with age. Three longitudinal twin studies of aging from the Swedish Twin Registry (N = 2059) included up to 27 years of follow-up on a Functional Aging Index (FAI) consisting of lung function, grip strength, walking speed, and self-report sensory functioning. SES indicators included education, financial strain, and occupation. Pair means (between family effect) and within pair differences (within family effect) were included as covariates of both intercept and slopes in a two-slope LGCM (intercept at age 75); models were corrected for sex and parental SES. LGCM results were compared across full sample, monozygotic twins, and dizygotic twins. For the FAI intercept, MZ within pair effects in all SES variables were strongly attenuated, indicating genetic confounding. For slope 1, only MZ within pair effect for education was attenuated, and no SES indicators were related to rate of change in FAI after age 75. Thus, results suggest shared genetic factors explain the association between education and change in physical aging in early aging processes. In other words, genes that impact educational achievement may also influence health behaviors and physical aging.

Abstract citation ID: igad104.3699
### WORK-RELATED VIOLENCE OR HARASSMENT AND SICKNESS ABSENCE DUE TO COMMON MENTAL DISORDERS: A PROSPECTIVE TWIN STUDY
Linda Magnusson Hanson[1], Maria Wijkander[1], Jurgita Narusyte[2], Iman Alaie[2], Petra Lindfors[1], Tianwei Xu[1], and Pia Svedberg[2], *1. Stockholm University, Stockholm, Stockholms Lan, Sweden*, *2. Karolinska Institutet, Stockholm, Stockholms Lan, Sweden*

The aim of the present study was to investigate the association between exposure to unacceptable behaviors and practices or threats thereof in working life that result in or aim to result in physical, psychological, sexual or economic harm, such as violence and harassment including unwelcome conduct based on e.g. sex or gender (gender-based harassment/discrimination), and future sickness absence due to common mental disorders among Swedish twins. The study included 8795 twins, from the Swedish Study of Twin Adults: Genes and Environment, asked to report exposure to work-related violence or threats of violence and harassment or bullying and whom were followed-up for certified sickness absence (min 12 years of follow-up). Standard logistic regression indicated that exposure increased the odds of sickness absence due to common mental disorders (adjusted OR 2.11; 95% CI 1.52-2.95 for violence/threats, adjusted OR 1.52; 95% CI 1.10-2.11 for harassment/bullying). A co-twin control analyses based on conditional logistic regression restricted to twin pairs discordant for exposure, in which a co-twin control was used as reference, however, resulted in attenuated ORs (2.0; 0.79-5.07 and 1.56; 0.66-3.66, respectively). This indicates that the relationships could be at least partially confounded by familial factors, such as genetics and shared environment. These results suggests that more work is needed to clarify whether there is a causal association between these types of unacceptable behaviors/practices and mental health outcomes considering familial/genetic factors, and whether prevention of such behaviors/practices can keep individuals increasingly healthy, active on the labor market and promote healthy aging.

### SESSION 9100 (PAPER)

### LATE BREAKING: HEALTH EQUITY ACROSS THE LIFE COURSE I

Abstract citation ID: igad104.3700

### CONTEXTUALIZING ATTITUDES TOWARD MEDICAL AID IN DYING IN A NATIONAL SAMPLE OF HOSPICE CLINICIANS

Todd Becker[1], John Cagle[2], Joan Davitt[2], Paul Sacco[2], Nancy Kusmaul[3], and Cindy Cain[4], *1. Washington University in St. Louis, St. Louis, Missouri, United States, 2. University of Maryland, Baltimore, Baltimore, Maryland, United States, 3. University of Maryland Baltimore County, Baltimore, Maryland, United States, 4. University of Alabama at Birmingham, Birmingham, Alabama, United States*

Research on the contextual characteristics informing the care that hospice professionals offer patients interested in medical aid in dying (MAID) is lacking. Thus, we aimed to explore MAID attitudes vis-à-vis hospice values and state law. This cross-sectional study recruited a convenience sample of 450 hospice-based physicians, nurses, social workers, and chaplains from national hospice and palliative care membership associations. Controlling for covariates, we fit a partial proportional odds model with clustered standard errors to examine the associations between participants' professional experience working in a MAID-legal state, orientation toward patient-centered care, and commitment to the hospice philosophy of care, and their MAID attitudes (0 = "oppose," 1 = "neither support nor oppose," 2 = "support"). Results indicated that experience working in MAID-legal states (vs. not; OR = 2.42, p = .002) and increased orientation toward patient-centered care (OR = 1.38, p < .001) were significantly associated with higher odds of greater MAID support across thresholds. Conversely, the direction and statistical significance of commitment to the hospice philosophy of care depended on the threshold of the dependent variable (odds of MAID support higher than "oppose": OR = 0.98, p = .326; odds of MAID support higher than "neither support nor oppose": OR = 1.05, p = .043). Findings provide the first quantitative evaluation of a predominantly theoretical debate concerning perceptions of MAID's concordance with hospice values. Noting legal protections for conscientious objection, higher support from clinicians in states where MAID is legal may suggest training opportunities as levers to improve patient access.

Abstract citation ID: igad104.3701

### EFFECTS OF THE COVID-19 ON MEDICAL USE OF ELDERLY PATIENTS WITH HYPERTENSION: A NATIONWIDE COHORT STUDY IN KOREA.

Eunbyul Cho, *Seoul National University of Bundang Hospital, Seongnam, Gyeonggi, Republic of Korea*

COVID-19 pandemic disrupted healthcare services, including chronic disease management for vulnerable groups like older individuals with hypertension. This study aims to evaluate hypertension management in South Korea's elderly population during the pandemic using treatment consistency indices like Continuity of Care (COC), Modified, Modified Continuity Index (MMCI), Most Frequent Provider Continuity (MFPC). This study used the the Korea Disease Control and Prevention Agency-COVID-19-National Health Insurance Service cohort (K-COV-N cohort) from the National Health Insurance Service (NHIS) between 2017 and 2021 The research included a total of 4,097,299 hypertensive patients aged 65 or older. We defined 2018 and 2019 as baseline period before COVID 19 pandemic, and 2020 and 2021 as COVID 19 period, and calculated the indices of medical continuity (Number of visits, COC, MMCI and MFPC) on a yearly basis were measured. The number of visits were decreased during COVID 19 period compared to baseline period ( 59.64 ±52.75 to 50.49 ± 50.33, p< 0.001) respectively. However COC, MMCI and MFPC were not decreased in baseline period compared to COVID-19 period ( 0.71 ± 0.21 vs. 0.71 ± 0.22, p< 0.001), (0.97 ± 0.05 vs. 0.96 ± 0.05, p< 0.001), and (0.8 ± 0.17 vs. 0.8 ± 0.17, p< 0.001) respectively. In conclusion, COVID-19 had no significant impact on continuity of care, but it did affect the frequency of outpatient visits of older hypertensive patients. However, this study highlights the importance of addressing healthcare inequalities, especially for older hypertensive patients, during pandemics and advocates for policy changes to ensure continued care for vulnerable populations.

Abstract citation ID: igad104.3702

### ENABLING AGING IN PLACE: THE IMPACT OF HOME MODIFICATIONS ON NURSING HOME ADMISSION RISK

Yuliana Levchenko, *Portland State University, Portland, Oregon, United States*

In an era where the built environment profoundly shapes the ability to age in place, this study aims to investigate the relationship between home modifications and the risk of nursing home admission. Using data from the 2000-2018 Health and Retirement Study, Cox proportional hazard models are employed to analyze the impact of various modifications on admission risk for older adults in the United States (N = 31,133). The home modifications considered include the presence of ramps, railings, wheelchair modifications, grab bars/shower seats, and an emergency call system. The results indicate that having a grab bar/shower seat in one's home is consistently associated with a decreased risk of nursing home admission (HR = 0.42, CI = 0.34, 0.52). However, there is mixed evidence for the remaining modification. These findings underscore the potential significance of incorporating grab bars and shower seats into building codes and promoting their installation in homes. Such modifications may play a vital role in promoting independent living and reducing reliance on institutional care. Further research is needed to explore the underlying mechanisms by which home modifications influence the risk of nursing home admission.

Abstract citation ID: igad104.3703

### MAKING OLDER ADULT FALL PREVENTION A ROUTINE PART OF HEALTHCARE IN TRIBAL CLINICAL SETTINGS

Gwen Bergen[1], Leo Egashira[2], Myra Parker[2], Shakiera Causey[3], and Yara Haddad[1], *1. Centers for Disease Control and Prevention, Atlanta, Georgia, United States*, *2. Seven Directions Public Health Institute, Seattle, Washington, United States*, *3. National Network of Public Health Institutes, New Orleans, Louisiana, United States*

Over one-third of American Indian and Alaska Native (AI/AN) elders (age 65+) report falling annually. This percentage is higher compared to other race/ethnic groups except for White non-Hispanic older adults. CDC's Stopping Elderly Accidents, Deaths, and Injuries (STEADI) initiative provides guidance for making elder fall prevention a routine part of clinical care. The presentation will describe a survey of healthcare providers serving AI/AN populations to gauge their readiness to adopt STEADI. Additionally, it will focus on a culturally informed approach to implementing STEADI within tribal settings. The National Network of Public Health Institutes and Seven Directions, A Center for Indigenous Public Health, surveyed a convenient sample of healthcare providers in clinics serving AI/AN patients to assess providers' knowledge and acceptability of fall prevention best practices, efforts to prevent falls, and receptiveness to receiving fall prevention training and support. The final sample included 94 healthcare providers from 27 clinics. Less than half of those surveyed reported screening elders for fall risk. Only 22% were familiar with STEADI. Most (84%) providers were willing to take part in fall prevention with additional training. Based on these findings CDC is funding two tribes to use an indigenous evaluation approach to improve their tribal health center's elder fall prevention capacity through evidence-based clinical and clinical-community linked strategies. Results from these activities will be used to outline a best practices guide focused on coordinated care for fall prevention and tailored to the varied environments, governments, cultures, and capacity for public health action in tribal health centers.

Abstract citation ID: igad104.3704

### SOCIALIZING CARE FOR OLDER PEOPLE ACROSS THE COUNTRIES: A COMPARATIVE STUDY OF THE LOCATION OF CARE

Aeji Jang, Yeonjung Lee, and Young Choi, *Chung-Ang University, Seoul, Republic of Korea*

As more people are likely to prefer to age in their own place, where and how the care in later life is provided become more important. This paper aims to compare the degree of socialization in care for older people among different countries, especially focusing on the location of care (i.e., home care and institutional care). We applied the Fuzzy-set Ideal Type Analysis to classify the countries depending on how the formal care is provided between home and institutional settings. Measures of socialization in home care include the national expenditure on home care and the number of formal care workers at home, whereas formal care workers and the number of beds in residential LTC facilities are indicators for socialization in institutional care. The four different types of formal care will be classified: HI where both home and institutional cares are highly socialized; Hi where home care is highly socialized but institutional care is not; hI where institutional care is highly socialized but not home care; and hi where neither are socialized. Results show that Denmark, Australia, and the Netherlands belongs to HI; Japan to Hi, New Zealand, Switzerland, and Germany to hI; and Korea and the United States to hi. Findings suggest that countries with high level of socialization of home care are likely to have a care management system for coordinating and monitoring services. This study provides implications for considering the unique cultural, institutional, and policy contexts among the countries in regards to the degree of socialization of care.

Abstract citation ID: igad104.3705

### THE VALUE OF TECHNOLOGY IN LGBT OLDER ADULTS' SOCIAL NETWORKS: LESSONS LEARNED FROM COVID-19

Kelseanne Breder[1], Walter Bockting[2], Elvan Ziyalan[2], and Maureen George[3], *1. New York University, New York City, New York, United States*, *2. Columbia University Mailman School of Public Health, New York City, New York, United States*, *3. Columbia University School of Nursing, New York City, New York, United States*

LGBT older adults maintain unique social networks. They are more likely to live alone, more likely to rely on peers for caregiving, and less likely to have intergenerational support to adopt technologies that help maintain relationships. This research explores how LGBT older adults used technology for social support during COVID-19 social distancing and identifies recommendations for improving social isolation and wellbeing in this population through telehealth guidelines and community programs. Semi-structured individual interviews were conducted with a racially and ethnically diverse sample of 15 LGBT older adults during the summer of 2020. Interview guides were designed according to The Convoy Model of Social Relations. Verbatim transcripts were coded using conventional content analysis. Three major themes were identified, highlighting ways LGBT older adults used technology to meet social support needs, and the perceived gaps in technological fluency that hinder social connectedness and telehealth acquisition: (I) Yearning for "The Hug Factor," (II) Navigating

Online Social Boundaries and (III) "Not for My Generation." Findings emphasize resiliency strategies employed by LGBT adults during social distancing and quarantine mandates that may optimize social and psychological wellbeing among the general aging population, as well as other minority geriatric groups. Our finding may hold relevance during future periods of isolation due to aging, mobility limitations, and climate change. Recommendations for improving social support beyond the pandemic include talk therapies, telehealth policy initiatives, and targeted community programs.

### SESSION 9110 (PAPER)

### LATE BREAKING: ALZHEIMER'S DISEASE INCLUDING ALZHEIMER'S DISEASE RELATED DEMENTIAS I

Abstract citation ID: igad104.3706

### DEVELOPMENT OF THE LONGITUDINAL STUDY OF HEALTH AND AGEING IN KENYA (LOSHAK)

Niranjani Nagarajan[1], Joshua Ehrlich[2], Anthony Ngugi[3], Eunice Mwangi[3], Roselyter Riang'a[3], Shaheen Sayed[3], Muthoni Gichu[4], and Kenneth Langa[2], *1. University of Michigan Kellogg Eye Center, Ann Arbor, Michigan, United States*, *2. University or Michigan, Ann Arbor, Michigan, United States*, *3. Aga Khan University, Nairobi, Nairobi Area, Kenya*, *4. Ministry of Health, Nairobi, Nairobi Area, Kenya*

In Kenya, the number of adults aged ≥60 is expected to nearly quadruple by 2050, making it one of the most rapidly aging countries in Sub-Saharan Africa (SSA). Accordingly, we designed the Longitudinal Study of Health and Ageing in Kenya (LOSHAK) to generate novel data to address the health and economic consequences of this demographic transition. Modeled on the U.S. Health and Retirement Study (HRS), LOSHAK joins a network of harmonized studies on aging in >45 countries worldwide; however, LOSHAK will be only the second such study in SSA. LOSHAK will advance population aging research in low- and middle-income countries through the study of: (1) biomarkers and physiological measures; (2) the impacts of air pollution and climate vulnerability; (3) Alzheimer's disease and related dementias, mental health, disability, caregiving, and psychosocial wellbeing; and (4) economic security, including the impact of social welfare. The current feasibility and initial pilot testing phase of LOSHAK is nested within the Kaloleni/Rabai Community Health and Demographic Surveillance System on the coast of Kenya and aims to validate measures and data collection procedures in a purposive sample of Kenyan adults aged ≥ 45 years. Among 205 participants surveyed, the mean age was 64 years, 58% were female, and 37% were currently employed. In the future, a full-scale Wave 1 LOSHAK will collect data from a nationally representative panel of Kenyans aged ≥45 years and will inform future public health and economic policy to address challenges related to rapid aging in Kenya and throughout SSA.

Abstract citation ID: igad104.3707

### MACHINE-LEARNING-BASED INTERPRETABLE PREDICTION FOR MILD COGNITIVE IMPAIRMENT: FROM ADNI DATABASE

Yafei Wu, Maoni Jia, and Ya Fang, *Center for Aging and Health Research, School of Public Health, Xiamen University, Xiamen, China, Xiamen, Fujian, China (People's Republic)*

Objective: An accurate prediction model was still lacking for mild cognitive impairment (MCI). Therefore, this study aimed to predict the 3- and 5-year MCI risk utilizing interpretable machine learning, and to identify possible biomarkers for intervention. **Methods:** A total of 444 participants were obtained from the ADNI database. Sociodemographic, neuropsychological, ApoE gene, and structural MRI imaging information was used as predictors. Data was randomly divided into training (70%) and test sets (30%). Six machine learning methods were constructed to predict the risk of MCI. Model performance was assessed by area under the ROC curve (AUC). SHapley Additive exPlanations (SHAP) was used to select important predictors. We also constructed a simplified MCI prediction model for clinical practice. **Results:** The mean age (standard deviation) of study population was 73.7 (6.1) years old. A total of 40, and 60 participants progressed into MCI within 3, and 5 years, respectively. Logistic regression (AUC: 0.97) and support vector machine (AUC: 0.93) are the optimal model for 3- and 5-year prediction. The simplified models based on top three neuropsychological test scores also showed high prediction accuracy (AUC > 0.90). APOE gene, intracranial volume, and Clinical Dementia Rating Scale Sum of Boxes (CDRSB) score were top three predictors for 3-year prediction, while CDRSB score, modified Preclinical Alzheimer's Cognitive Composite Test with Follow-up Test scale (mPACCtrailsB) score, and Functional Activity Questionnaire (FAQ) score were top three predictors for 5-year prediction. **Conclusion:** Interpretable machine learning showed promise in early identification of MCI, and would be valuable for targeted prevention.

Abstract citation ID: igad104.3708

### MOBILE-BASED COGNITIVE TRAINING IN CHINESE AMERICANS: PERSPECTIVES FROM OLDER ADULTS AND ADULT CHILDREN

Tingzhong (Michelle) Xue[1], Eleanor McConnell[1], Bei Wu[2], and Hanzhang Xu[3], *1. Duke University School of Nursing, Durham, North Carolina, United States*, *2. New York University, New York City, New York, United States*, *3. Duke University, Durham, North Carolina, United States*

The population of older Chinese Americans is growing rapidly. However, their access to culturally responsive dementia prevention services is very limited. Cognitive training may delay cognitive decline in older adults, but such training has not been widely accessible to older Chinese Americans. This study explored perspectives of older Chinese Americans and their adult children on dementia prevention and cognitive stimulating activities to inform co-design of a culturally appropriate, mobile-based cognitive training for this population. We conducted five focus groups in Mandarin using Zoom video conferencing, with four groups of older Chinese Americans (n=21) and one group of adult children (n=6). We applied rapid qualitative analysis, guided by the Health Belief Model. We identified seven themes. Overall, participants had modest knowledge of dementia, and viewed health care professionals as credible information sources for dementia prevention. Some participants thought any social activity would provide cognitive stimulation. Both older adults and adult children expressed strong interest in cognitive training but were concerned about losing interest over time and transportation and language barriers. Both groups noticed similar advantages (e.g. benefits for cognition, interesting exercises), and disadvantages (e.g. eyestrain, addiction) of mobile-based cognitive training but had different perspectives on acquiring health information online. Adult children were eager to provide support for older adults to participate in cognitive training, whereas older adults preferred to engage in cognitive training autonomously. Knowledge generated from this study forms the foundation for designing of a tailored mobile-based intervention to prevent cognitive decline among older Chinese Americans.

Abstract citation ID: igad104.3709

### MUSIC CREATIVITY INTERVENTION FOR OLDER ADULTS WITH AND WITHOUT MCI: COGNITIVE BENEFITS AND NEURAL MECHANISMS

Christopher Fagundes[1], Lydia Wu-Chung[1], Anthony Brandt[1], Melia Bonomo[1], Bryan Denny[1], Jefferson Frazier[2], and Karl Blench[3], *1. Rice University, Houston, Texas, United States*, *2. Houston Methodist, Houston, Texas, United States*, *3. Musica, Houston, Texas, United States*

Alzheimer's and related dementias are debilitating conditions, and at this time, no cure has been identified. Older age is associated with cognitive decline and dementia. Developing and testing innovative non-pharmacological interventions to delay the loss of functional ability and understand the neurobiological mechanisms underlying the intervention's efficacy is imperative. We conducted a semi-randomized clinical trial evaluating the effects of a novel music creativity curriculum on cognitive and neurological outcomes. Eighty older adults with and without a mild cognitive impairment (MCI) diagnosis were randomized to a 6-week music creativity intervention or a 6-week no-treatment control condition. Before and after the six weeks, both groups participated in a series of self-report and cognitive assessments; they also participated in resting-state fMRI scans to quantify whole-brain network patterns of modularity and flexibility. Those randomized to the music group exhibited improved overall cognitive function and working memory relative to the control group (assessed using standard clinical neuropsychological assessments). The association between being randomized to the music group and increased cognitive function was more robust among those who exhibited greater neural flexibility at baseline. The study shows that music creativity interventions may positively affect cognition, especially among those whose neural network structure is more dynamic or flexible. This study is innovative as it is among the first to identify possible neural mechanisms underlying why music creativity interventions confer a more significant cognitive benefit for some older adults than others.

Abstract citation ID: igad104.3710

### NOT ALL CREATED EQUAL: THE IMPACTS OF POSITIVE, NEGATIVE, AND PARTNER RELATIONSHIPS ON COGNITION IN LATELIFE

Nicole Ee[1], Fiona Matthews[2], Ruth Peters[3], and Kaarin Anstey[1], *1. University of New South Wales, Sydney, New South Wales, Australia*, *2. Newcastle University, Newcastle upon Tyne, England, United Kingdom*, *3. The George Institute for Global Health, Newtown, New South Wales, Australia*

**Background:** Social engagement has been linked to higher cognitive function in later life and it is a well evidenced protective factor against dementia and cognitive decline. However, far less is understood about how the type, quality and quantity of social engagement differentially affects cognitive health outcomes in older adults. **Methods:** The sample consisted of 1972 participants aged 68-74 (M = 70.60, SD = 1.49) from a large population-based study in Australia, Personality and Total Health Through Life (PATH). The constructs underlying nine observed measures of social engagement were first identified through exploratory and confirmatory factor analysis (CFA). A Latent Variable Path Model (LVPA) was then employed to examine the relationships between positive, negative and partner social engagement on memory and executive function, as measured by six validated cognitive tests. **Results:** The CFA identified partner relationship as a distinct social engagement construct, separate to positive and negative relationships. The main analysis demonstrated that negative relationships and partner relationship were positively associated with better memory and executive function, with the former twice the strength of the latter. **Discussion:** These findings provide support that more frequent social interactions and larger support structures contribute to better cognitive function in later life, with partner relationship making a significant and distinct contribution. Positive or high-quality social engagement, however, appears to be a less critical factor in bolstering cognitive health. Close and cognitively demanding relationships may promote cognitive health via frequent cognitive stimulation, but more research is required to confirm the mechanisms underpinning observed effects.

Abstract citation ID: igad104.3711

### VISION IMPAIRMENT AND COGNITION IN INDIA: ASSOCIATIONS AFTER ADJUSTMENT FOR POTENTIAL BIAS

Emma Nichols[1], Yizhou Chen[1], Adina Zeki Al Hazzouri[2], Alden Gross[3], Niranjani Nagarajan[4], Jinkook Lee[1], and Joshua Ehrlich[5], *1. University of Southern California, Los Angeles, California, United States*, *2. Columbia University, New York City, New York, United States*, *3. Johns Hopkins University Bloomberg School of Public Health, Baltimore, Maryland, United States*, *4. Michigan Medicine, Ann Arbor, Michigan, United States*, *5. University or Michigan, Ann Arbor, Michigan, United States*

Increasing evidence suggests that vision impairment may be an important modifiable risk factor for dementia, particularly in low- and middle-income settings where the prevalence of uncorrected vision impairment is high. Although prior studies in various settings, including India, have found strong associations between vision impairment and cognition, this work has not considered potential bias in cognitive testing due to vision impairment. We evaluated and adjusted for measurement differences by vision impairment status using data from the Longitudinal Aging Study in India–Diagnostic Assessment of Dementia (LASI-DAD) study (N=3780). We used Multiple Indicators Multiple Causes models to assess differential item functioning (DIF) (e.g. bias) in cognitive testing by objective near and distance vision impairment. We estimated associations between vision impairment and cognition adjusting for hypothesized confounders before and after DIF adjustment. Although there was statistical evidence of DIF (near vision: 3/10 items, distance vision: 4/10 items), differences between DIF-unadjusted and -adjusted scores were small compared to the standard error of measurement, indicating no evidence of clinically meaningful measurement differences. Both near and distance vision impairment were associated with cognition before and after DIF-adjustment; after DIF-adjustment, severe near and distance vision impairment were associated with -0.43 [95% CI -0.53--0.33] and -0.60 [-0.76--0.43] standard deviation units lower cognitive scores compared to those with normal vision, respectively. In well-conducted large-scale surveys, bias in cognitive testing due to vision impairment is likely minimal, even in low- and middle-income settings. Findings strengthen the evidence base on vision impairment as a risk factor for dementia.

### SESSION 9200 (PAPER)

### LATE BREAKING: ALZHEIMER'S DISEASE INCLUDING ALZHEIMER'S DISEASE RELATED DEMENTIAS II

Abstract citation ID: igad104.3712

### AGE-RELATED KYNURENINE PATHWAY METABOLITES DRIVE SENESCENT-LIKE CHANGES IN NEURONS: POTENTIAL NOVEL AD MECHANISM

Dmitry Kondrikov, Galina Kondrikova, Steven Dixon, Carsten Krieg, Gavin Wang, and William Hill, *Medical University of South Carolina, Charleston, South Carolina, United States*

Kynurenine pathway (KP) metabolites are emerging as important factors of aging-related pathologies. Kynurenine (KYN) and its metabolites increase with age and are known to affect multiple organ systems including CNS, musculoskeletal, and vascular systems. Our group has previously shown that KYN and sub-KYN metabolites below the enzyme Kynurenine monooxygenase (KMO) can act to inhibit osteogenesis of bone marrow MSCs via senescence induction. However, inhibition of KMO, blocks KYN induced senescence and restores osteogenic potentials of MSCs. We are now expanding this work to see if KYN metabolites act on cells of the aging CNS in a similar fashion potentially helping to drive cognitive decline and Alzheimer's disease (AD) progression. We report for the first time that treatment of neuroblastoma-like SH-SY5Y cells with KYN, and sub-KMO metabolites 3-hydroxykynurenine (3HK), or quinolinic acid (QA) significantly increased senescence-associate beta-galactosidase activity, and expression of senescence associated secretory phenotype (SASP) markers, p21, PAI-1, TIMP2 as well as Histone 3 K9methylation. Treatment of SH-SY5Y cells with KMO inhibitors prevented elevated expression of SASP proteins and histone methylation. This makes KMO an attractive target to inhibit kynurenine pathway effects. Taken together, these data suggest that KYN and its metabolites may contribute to pathogenesis of AD via inducing senescence-like changes and SASP-mediated neuroinflammation in neuronal cells. Inhibition of KMO may allow rescue of brain microenvironment, limit cognitive decline and AD progression in older persons. Blocking KYN downstream metabolite formation, by inhibiting KMO, presents a novel therapeutic target for a potential novel AD mechanism.

Abstract citation ID: igad104.3713

### FEASIBILITY OF AEROBIC FITNESS TESTING IN AMNESTIC MILD COGNITIVE IMPAIRMENT FROM THE ACT TRIAL

Fang Yu[1], Dereck Salisbury[2], and Feng Lin[3], *1. Arizona State University, Phoenix, Arizona, United States*, *2. University of Minnesota, Minneapolis, Minnesota, United States*, *3. Stanford University, Stanford, California, United States*

Aerobic fitness has been postulated as a physiological mechanism of action for aerobic exercise to modify Alzheimer's disease, but it has not been adequately studied in older adults with (MCI). The purpose of this study is to evaluate the feasibility of a gold-standard, laboratory-based, symptom-limited cycle-ergometer exercise testing (GXT) in older adults with amnestic mild cognitive impairment (aMCI) to assess peak oxygen consumption (VO2max [ml/kg/min]) using baseline data from the ACT Trial. The test was categorized as a submax, near-max, and max test based on if 0, 1, and ≥2 of the American College of Sports Medicine criteria was met: >90% of age-predicted maximum heart rate, plateau in VO2Peak, respiratory exchange ratio >1.10, and rating of perceived exertion >17. VO2max was predicted as 3.5*maximal metabolic equivalents (METs). Among 146 enrolled participants (73.8±5.7 years old with 16.9±2.9 years of education, 51.4% men, and 91.8% Caucasian), 145 completed GXTs. Sixteen GXTs (11.0%) were submax (VO2Peak 13.5±4.4), 53 (36.6%) were near-max (VO2Peak 16.5±4.1), and 76 (52.4%) were max (VO2Peak 18.4±5.1). Predicted VO2max were 21.6±6.8, 28.7±26.3, and 30.0±20.5 from submax, near-max, and max tests, respectively. Among max GXTs, VO2max was 19.5±4.9 in men (n=37) and 17.4±5.1 in women (n=39). In conclusion, GXTs was feasible for ~52% of the participants. METs-based prediction overly inflates VO2max estimates, in comparison to VO2max obtained from max GXTs. VO2max levels are substantially lower in older adults with aMCI than peers (23.1±6.3 in men and 21.1±3.4 in women), suggesting aerobic exercise as an important intervention for this population.

Abstract citation ID: igad104.3714

### INTEROCEPTIVE ACCURACY ENHANCES DECEPTION DETECTION WITH GREATER AGE

Natalie Ebner, Amber Heemskerk, and Tian Lin, *University of Florida, Gainesville, Florida, United States*

Financial exploitation of older adults is a growing concern, with millions of seniors scammed annually, costing billions, and resulting in devastating consequences for health, independence, and well-being. Exploitation risk is exacerbated in Alzheimer's disease and related dementias (AD/ADRD). Detecting deception is challenging, especially for older adults who tend to believe others are truthful. Understanding the neurobiological mechanisms underlying this susceptibility is essential to design effective intervention toward reducing exploitation in aging. In this research we demonstrate the importance of interoceptive awareness—the ability to interpret body signals—in deception detection in older adults. Seventy-six young (18-34 years) and 74 older (53-82 years) adults completed a heartbeat counting task to assess their interoceptive awareness. They also engaged in deception detection paradigms across two distinct, ecologically valid tasks: i) a lie detection task in which they made veracity judgments of genuine and deceptive individuals, and ii) a phishing email detection task to capture accuracy in online deception detection. Greater interoceptive awareness was associated with greater lie detection accuracy. Furthermore, with greater chronological age among older adults, greater interoceptive awareness was associated with better accuracy in both detecting deceptive individuals and phishing emails. These findings support interoceptive awareness as a relevant factor for interventions aimed at enhancing deception detection abilities in aging. Project results will advance insight into the neurobiology of deception detection to inform effective translation into intervention in mitigating exploitation risk in aging and eventually ADRD.

Abstract citation ID: igad104.3715

### LONGITUDINAL CHANGES IN PROTEOMIC BIOMARKERS IN ALZHEIMER'S DISEASE AND CARDIOVASCULAR DISEASE

Laurie Theeke[1], Ying Liu[2], Silas Wang[3], R. Osvaldo Navia[4], Danqing Xiao[5], Chun Xu[6], and Kesheng Wang[7], *1. The George Washington University, Washington, District of Columbia, United States*, *2. East Tennessee State University, Johnson City, Tennessee, United States*, *3. Carnegie Mellon University, Pittsburgh, Pennsylvania, United States*, *4. West Virginia University, Morgantown, West Virginia, United States*, *5. Regis College, Weston, Massachusetts, United States*, *6. University of Texas Rio Grande Valley, Brownsville, Texas, United States*, *7. School of Nursing, Health Sciences Center, West Virginia University, Morgantown, West Virginia, United States*

Alzheimer's disease (AD) and cardiovascular diseases (CVDs) commonly co-occur in older adults. This correlation prompts an investigation into the potential shared risk factors linking AD and CVDs, and the potential direct causative role of cardiovascular risk factors in AD. The cohort comprised 566 older adults including 111 individuals with AD, 58 without AD, 383 with mild cognitive impairment (MCI), 410 with CVD and 156 without CVD from the Alzheimer's Disease Neuroimaging Initiative (ADNI). The multivariable linear mixed model (LMM) was used to investigate the associations of AD and CVD on longitudinal changes of 146 proteomic biomarkers (measured at baseline and 12-month follow-up), while adjusted for age, gender, education, racial groups, and APOE-ε4 alleles. The LMM showed that AD and CVD were associated with longitudinal changes in 48 and 46 proteomic biomarkers, respectively ($p < 0.05$). Interestingly, both AD and CVD were associated with longitudinal changes in 14 proteomic biomarkers (A1Micro, ApoH, B2M, BNP, Complement C3, Cystatin C, KIM1, NGAL, PPP, TIM1, THP, TFF3, TM, and VEGF). Furthermore, the single biggest risk gene for sporadic AD, APOE-ε4 was associated with 22 proteomic biomarkers. Moreover, MCI and CVD were associated with 13 proteomic biomarkers (A1Micro, ApoD, AXL, BNP, Calcitonin, CD40, C-peptide, pM, PPP, THP, TNFR2, TTR, and VEGF), while MCI, CVD and APOE-ε4 were associated with four proteomic biomarkers (A1Micro, Calcitonin, THP, and TNFR2). This study provides valuable insights into the shared and distinct pathophysiological mechanisms at play, offering a stepping-stone towards a deeper comprehension of the interrelationship between AD and CVDs.

Abstract citation ID: igad104.3716

### PLASMA BIOMARKERS FOR ALZHEIMER'S DISEASE AND RELATED NEUROPATHOLOGY IN RELATION TO MCI AND DEMENTIA: THE ARIC STUDY

Kevin Sullivan[1], Chad Blackshear[1], Keenan Walker[2], Michelle Mielke[3], B. Gwen Windham[4], Michael Griswold[4], Thomas Mosley[1], and Priya Palta[5], *1. University of Mississippi Medical Center, Jackson, Mississippi, United States*, *2. National Institute on Aging, NIH, Baltimore, Maryland, United States*, *3. Wake Forest University School of Medicine, Winston-Salem, North Carolina, United States*, *4. UMMC-The MIND Center, Jackson, Mississippi, United States*, *5. UNC Gillings School of Global Public Health, Chapel Hill, North Carolina, United States*

Blood-biomarkers for Alzheimer's Disease and related neuropathological processes, including amyloidosis, tau deposition, and neurodegeneration, may revolutionize the monitoring of risk and progression of neurocognitive outcomes in older adults. At visit 5 (2011-13) of the ARIC study, a multicenter longitudinal cohort study, amyloid-beta (Aβ42:Aβ40), ptau-181, glial fibrillary acidic protein (GFAP), and neurofilament light (NFL) were measured in plasma using an ultrasensitive single-molecule array (Simoa; Quanterix N4PE + p-tau-181 single-plex) in a sample of 1,711 participants (Age=76±5 years, 60% Female, 28% Black). Neurocognitive outcomes included adjudicated dementia and mild cognitive impairment (MCI) through 2019 for a median follow-up of 5.6 years. Multinomial logistic regressions estimated Relative Risk Ratios (RRR) of the four-category outcome of MCI (n=543), dementia (n=371), and death (n=59) vs. cognitively unimpaired (n=736) for each base(2) log-transformed biomarker, adjusted for age, sex, education, site-race, hypertension, diabetes, BMI, APOE, and eGFR. When estimates appeared similar for MCI and dementia, an additional analysis modeled RRR of a three-category outcome of combined MCI/dementia, death, and cognitively unimpaired. Higher plasma Aβ42:Aβ40 ratio was associated with lower MCI/dementia risk (RRR=0.48, 95%CI: 0.32,0.73). Higher levels of all other biomarkers were associated with higher risk of MCI [p-tau181 (RRR=1.57, 95%CI: 1.23,2.00); GFAP (RRR=1.31, 95%CI: 1.03,1.67); NFL (RRR=1.64, 95%CI: 1.26,2.13)] and dementia [p-tau181 (RRR=3.16, 95%CI: 2.43,4.11); GFAP (RRR=2.40, 95%CI: 1.80,3.20); NFL (RRR=3.62, 95%CI: 2.58,5.09)]. These results indicate blood-biomarkers representative of multiple neuropathological processes were strongly associated with both MCI and all-cause dementia outcomes, emphasizing their potential role as tools in risk identification, early diagnosis, and possibly guiding interventions.

### SESSION 9210 (PAPER)

### LATE BREAKING: EFFECTS OF DISCRIMINATION ACROSS THE LIFE COURSE I

Abstract citation ID: igad104.3717

### CONTEXTUALIZING HOW DAILY DISCRIMINATION EXPERIENCES IMPACT EVERYDAY METAMEMORY

Timothy Ly, Rebecca Allen, Jeanne Cundiff, and Jason DeCaro, *The University of Alabama, Tuscaloosa, Alabama, United States*

Examining day-to-day responses to everyday stressors, such as discrimination, contextualizes how cognitive aging trajectories may vary across the lifespan. Furthermore, recent research investigating stress-induced impairment of metamemory may address the relationship between discrimination experiences and cognitive impairment. This study sought to articulate the association between daily experiences of discrimination and impaired metamemory across the lifespan, as defined by the number of subjective cognitive complaints, using data collected from the Midlife in the United States Refresher Study Daily Diary Project (N = 782; ages 20-75; M = 47.91 years, SD = 12.67 years). A two-level linear mixed model was conducted to examine the within and between-persons relationships concerning lifetime and daily experiences of discrimination, daily affect balance, baseline objective cognitive performance, and sociodemographic variables (age, race, ethnicity, and sex) on impaired everyday metamemory. Results from linear mixed model analyses showed significant within-person fixed effects of daily discrimination and daily affect balance on metamemory accuracy, as well as significant between-persons fixed effects of race and ethnicity on metamemory accuracy. Furthermore, significant interactions were found between race and daily discrimination experiences, ethnicity and daily discrimination experiences, daily affect balance and daily discrimination experiences, daily affect balance and age, and endorsement of lifetime experiences of discrimination and daily discrimination experiences on day-to-day metamemory accuracy. These findings extend our understanding of how discrimination experiences may impair metacognitive processes and demonstrate the need for more research into understanding metamemory accuracy as an underlying mechanism by which the psychosocial stressor of discrimination impacts cognition across the lifespan.

Abstract citation ID: igad104.3718

### DO DEPRESSIVE SYMPTOMS MEDIATE SEGREGATION'S IMPACT ON COGNITIVE AGING IN OLDER MEXICAN AMERICANS?

Emily Morris[1], Jeremy Grant[2], Alexandra Clark[3], Darlingtina Esiaka[4], Kristine Ajrouch[5], and Wassim Tarraf[6], *1. University of Michigan, Ann Arbor, Michigan, United States*, *2. University of Florida, Gainseville, Florida, United States*, *3. University of Texas at Austin, Austin, Texas, United States*, *4. University of Kentucky, Lexington, Kentucky, United States*, *5. Eastern Michigan University, Ypsilanti, Michigan, United States*, *6. Wayne State University, Detroit, Michigan, United States*

Segregation in the United States has important implications for cognitive aging among Mexican Americans as a social determinant of health. Because segregation is systemically engrained, identifying more immediately modifiable mechanisms, such as depressive symptoms, that may underly segregation-cognition associations could point to intervention targets to reduce cognitive aging disparities. We used path mediation modeling to test whether depressive symptoms mediated (indirect effect) the segregation-cognition associations. We used H-EPESE data on older Mexican Americans from the first (n=3,050) wave and returning at wave 5 (n=1,167) combined with Census data from the National Neighborhood Data Archive, which provided indicators for county level segregation (isolation, interaction, dissimilarity). Cognition was operationalized as baseline MMSE, MMSE at wave 5, and change between baseline and wave 5. Depressive symptoms were measured by the CESD at baseline. Covariates included age, sex, and years of education. The sample was 58% female with an average age of 73.6 (SD=6.2) and an average 5-years education (SD=3.9). Higher dissimilarity (i.e., less exposure to non-Hispanic Whites) was associated with better baseline (B=.078, SE=.031, p=.012) and wave 5 cognition (B=.130, SE=.037, p<.001), independent of depressive symptoms. More depressive symptoms were associated with worse baseline and wave 5 cognition, independent of segregation indices (CESD B values range from -.063 to -.142, ps <.05). Depressive symptoms did not mediate segregation-cognition associations. Results suggest that there may be an enclave effect that is protective for both depressive symptoms and cognition in older Mexican Americans. Future research should examine additional biopsychosocial mechanisms underlying segregation-cognition associations.

Abstract citation ID: igad104.3719

### EFFECT OF EARLY-LIFE DISCRIMINATION IN PARENTING ON THE PHYSICAL AND MENTAL HEALTH OF MIDDLE-AGED AND OLDER ADULTS

Chao Guo, Lan Liu, and Jie Liu, *Peking University, Beijing, Beijing, China (People's Republic)*

This study aimed to explore the early-life discrimination in parenting on the physical and mental well-being of middle-aged and older adults, while also exploring the potential mediating role of early-life health. By combined data from the China Health and Retirement Longitudinal Study (CHARLS) in 2018 and related life history survey in 2014, 8204 females and 7305 males aged 45-84 were analyzed in this study. Logistic regression was employed to analyze the association between early-life discrimination in parenting and the incidence of chronic diseases as well as depression in middle-aged and older individuals. The KHB method was used to assess the mediating effect of early-life health in these connections. Regarding gender-based discrimination in parenting, parental preference for sons during early life was found escalated the probability of chronic diseases by 18% (OR=1.18, 95%CI: 1.01-1.38) and increased depression risk by 16% (OR=1.16, 95%CI: 1.03-1.31) among middle-aged and older females. In terms of sibling-related discrimination, parental inclination towards specific siblings heightened the likelihood of depression during middle and older years among females (OR=1.17, 95%CI: 1.03-1.34). However, these effects were not significant in males. The study further identified that early-life health partially mediated the influence of gender-based parental preferences on the mental well-being of middle-aged and older females, accounting for 8.603% of the overall effect. These findings underscored the potential for early-life discrimination within parenting to impact both the physical and mental health of middle-aged and older females by shaping their early-life health status, consequently resonating throughout their mental well-being in later life.

Abstract citation ID: igad104.3720

### TECHNOLOGY-SPECIFIC AGEISM AS A PREDICTOR OF NOT OFFERING TECHNOLOGY-BASED PHYSIOTHERAPY FOR OLDER ADULTS

Ittay Mannheim[1], Cynthia Neiertz[2], and Eveline Wouters[2], *1. Ben Gurion University of the Negev, Beer-Sheva,*

*HaDarom, Israel*, 2. *Fontys University of Applied Science, Eindhoven, Noord-Brabant, Netherlands*

Incorporating digital technologies in healthcare can potentially improve the quality and accessibility of older persons' care. However, ageist attitudes among healthcare professionals can lead to discrimination in treatment decisions and deprive older patients of technology-based treatment. This study explores whether technology-specific ageism influenced physiotherapists' decisions to use technology-based healthcare with older patients. Seventy-eight physiotherapists in Luxembourg filled out an online survey. Technology-specific ageism was measured by the Attitudes Towards Older Adults Using Technology (ATOAUT-11) scale. Additional measures included the Expectations Regarding Aging scale, attitudes towards technology use in the work environment, and whether physiotherapists had not offered technology-based treatment in the past because of a patient's age. Thirty-one percent of the participants reported that they had not offered technology-based treatment to a patient in the past because of the patient's older age. Importantly, using logistic regression, it was found that higher levels of technology-specific ageism predicted not offering technology-based treatment, such that participants with more negative attitudes (1 standard deviation) were two times more likely not to offer treatment. Positive attitudes toward using technology in the work environment were also found to be a significant predictor. All other characteristics (gender, age, work experience, and percentage of patients over 50) did not predict not offering treatment. This study provides initial evidence that technology-specific ageism may lead to discrimination and deprive older persons of receiving optimal treatment. More research is needed to identify the magnitude of ageism in using technology-based treatment in healthcare and develop interventions to mitigate it.

### SESSION 9300 (PAPER)

### LATE BREAKING: ALZHEIMER'S DISEASE INCLUDING ALZHEIMER'S DISEASE RELATED DEMENTIAS III

Abstract citation ID: igad104.3721

### COGNITIVE FRAILTY: PREVALENCE, RISK FACTORS, AND THE RISK OF MORTALITY IN CHINESE MIDDLE-AGED AND OLDER ADULTS

Hongting Ning[1], Hui Feng[1], and Junxin LI[2], *1. Central South University, Changsha, Hunan, China (People's Republic)*, *2. Johns Hopkins University, Baltimore, Maryland, United States*

To examine the age-specific prevalence, risk factors, and magnitude of the association between cognitive frailty and all-cause mortality in Chinese middle-aged and older adults. We used baseline (2011) and follow-up waves (2013, 2015, and 2018) data from the China Health and Retirement Longitudinal Study (CHARLS). The age group was categorized as middle-aged, old, and very old. Factors associated with cognitive frailty and the associations between cognitive frailty statuses and mortality were examined using multinomial logistic regression and Cox proportional hazards models, respectively. Totally 7947 participants were included in this study, and the overall prevalence of cognitive frailty was 12% (middle-aged, 11%; old, 10%; very old, 26%; P< 0.0001). Cognitive frailty was independently associated with rural residential status, long sleep duration, lack of social participation, depressive symptoms, and multimorbidity among all the age groups. Over the 7-year follow-up, participants with cognitive frailty were at a higher risk for all-cause mortality than the robust group (HR 1.70, 95%CI 1.12-2.57 for the middle-aged group, HR 1.70, 95%CI 1.02-2.84 for the old group, and HR 2.06, 95%CI 1.17-3.65 for the very old group). Pre-cognitive frailty was associated with a higher mortality risk in the middle-aged group only (HR 1.34, 95%CI 1.01-1.79). The prevalence of cognitive frailty in middle-aged adults was comparable with that in the old group. Cognitive frailty, a significant risk factor for mortality, was associated with multiple factors in middle-aged and older adults, calling for integrated cognitive frailty prevention strategies. Noticeably, pre-cognitive frailty in middle-aged people increases mortality risk and requires special attention.

Abstract citation ID: igad104.3722

### EMPLOYING PHOTOVOICE TO MAP ADOLESCENT LIFE TRAJECTORIES AMID MATERNAL YOUNG-ONSET DEMENTIA: A CASE STUDY

Xueting Tang[1], Junqiao Wang[1], Bei Wu[2], Xiaoyan Cui[1], and Jing Wang[3], *1. Fudan University, Shanghai, Shanghai, China (People's Republic)*, *2. New York University, New York City, New York, United States*, *3. University of New Hampshire, Durham, New Hampshire, United States*

The impact of parental dementia on family dynamics and roles can be substantial, presenting notable challenges for their offspring. Research concerning the life trajectory of adolescents having parents diagnosed with Young-Onset Dementia (YOD) remains limited. This study employs a combination of semi-structured interviews and photovoice through life course lens to delve into the experiences of an adolescent (Alice), exploring how her mother's condition shapes her life journey and providing novel insights for the development of supportive services. Over a three-month period, Alice captures daily-life moments through photography and selects meaningful photos for sharing. Regular bi-weekly sessions are dedicated to photo-sharing, during which these chosen photos are regarded as pivotal subjects for in-depth exploration in subsequent interview sessions. This approach facilitates gaining insight into her life experience from a nuanced perspective. Thematic analysis reveals four key themes: Firstly, the adaptation of her future ambitions in response to her mother's condition and increased family responsibilities, resulting in a curtailment of her educational and career prospects; secondly, building a stronger connection with her father and collaborating in shouldering responsibilities. Thirdly, her mother's prolonged absence during critical phases of personal development impacts her understanding of interpersonal interactions and relationships, prompting a pressing need to establish empathetic connections with others; and lastly, the emergence of Alice's distinct self-identity and resilience shaped by her unique experiences. These findings underscore the importance of establishing a family-centered support system that addresses the emotional, social, and educational challenges faced by adolescents like Alice as they navigate complex family circumstances.

Abstract citation ID: igad104.3723

### METABOLOMIC PROFILES OF DEMENTIA RISK AND MOBILITY DECLINE IN A BIRACIAL COHORT OF OLDER ADULTS

Qu Tian[1], Shanshan Yao[2], Luigi Ferrucci[1], Venkatesh Murthy[3], Ravi Shah[4], and Anne Newman[5], *1. National Institute on Aging, Baltimore, Maryland, United States, 2. University of Pittsburgh School of Public Health, Pittsburgh, Pennsylvania, United States, 3. University of Michigan, Ann Arbor, Michigan, United States, 4. Vanderbilt University Medical Center, Nashville, Tennessee, United States, 5. University of Pittsburgh, Pittsburgh, Pennsylvania, United States*

Age-related mobility decline precedes future cognitive impairment and dementia. Blood biomarkers underlying this connection are less clear. This study aims to examine metabolomic profiles of dementia risk and mobility decline in the Health, Aging and Body Composition Study. Dementia diagnosis was determined by race-specific decline in Modified Mini-Mental State Examination scores, medication use, and hospital records. 2450 participants were free of dementia at year 2 and had 613 targeted plasma metabolites. 534(22%) developed dementia during a mean follow-up of 9.3(SD=4.1) years. 2028 participants had repeated 20-meter usual gait speed up to 10 years follow-up. We performed Cox regression and linear regression with LASSO models to determine which metabolites would predict dementia risk and gait speed decline, respectively. After adjustment for age, sex, race, and education, 30 metabolites were significant predictors of incident dementia. Top-ranked metabolites included glyceric acid, niacinamide, androsterone 3- glucuronide, phosphatidylcholine (P-34:4)/PC(O-34:5), aminobutyric acid, xanthine, sphingomyelin (d18:1/14:0) (hazard ratios< 1), suberic acid, lysophosphatidylethanolamine (18:1), threitol, and andrenic acid (hazard ratios>1). These metabolites were enriched in arginine biosynthesis, alanine, aspartate and glutamate metabolism, glyoxylate and dicarboxylate metabolism and arginine and proline metabolism(p< 0.05). Of these metabolites, threitol, sphingomyelin (d18:1/14:0), adrenic acid, and phosphatidylethanolamine (P-36:0)/PE(O-36:1) were also significant predictors of mobility decline in a consistent direction. This longitudinal study reveals unique and shared metabolomic risk profiles for dementia and mobility decline. Several metabolites in amino-acid pathways and urea may be important in the development of dementia and certain sugar and lipids with long-chain and very long-chain fatty acids also predict mobility decline.

Abstract citation ID: igad104.3724

### SOCIAL BRIDGING NETWORKS AND COGNITIVE AGING AMONG OLDER ADULTS: ORIGINS IN SOCIAL CONTEXTS, ROLES, AND ACTIVITIES

Brea Perry, Siyun Peng, and Anne Krendl, *Indiana University, Bloomington, Indiana, United States*

There is a strong link between social connectedness and cognitive aging, with recent research pointing to social bridging being a primary underlying mechanism. Social bridging may provide cognitive enrichment through interactions with expansive, heterogeneous, and loosely interconnected social networks of weaker, peripheral ties. Drawing on egocentric social network data and cognitive assessments of 436 older adults from the Social Networks and Alzheimer's Disease (SNAD) study, this paper explores where social bridging networks come from. We used multivariate regression to examine whether a latent social bridging factor comprised of six structural variables (network size, density, effective size, diversity, strength of weakest tie, and sole bridging status) was associated with cognitive function and social conditions. Results indicate that social bridging is significantly associated with global cognitive function (B= 0.19; p<.0001), verbal memory (B=0.26; p<.0001), and episodic memory (B=0.21; p<.0001), consistent with prior studies. Older adults who are women (b=0.40; p<.001) and have more years of education (b=0.05; p<.05) have more access to social bridging. Among community and housing characteristics, only perceived community integration is significantly correlated with higher bridging (b=0.22; p<.05). With respect to social roles, being currently married (b=0.39; p<.001) and being a volunteer (b=0.58; p<.0001) were positively associated with bridging, while being a veteran predicted lower bridging (b=-0.86; p<.0001). Among indicators of social engagement, only number of contacts in one's phone (in 100's) was associated with bridging (b=.16; p<.0001). Findings provide insight into social roles and activities that can be leveraged to expand bridging and improve cognitive aging outcomes.

Abstract citation ID: igad104.3725

### STRESSORS & COPING STRATEGIES OF ADMINISTRATORS IN CHINESE FAITH-BASED DEMENTIA CARE FACILITIES

Lin Jiang[1], David Hodge[2], Robin Bonifas[3], and Fei Sun[4], *1. The University of Texas Rio Grande Valley, Edinburg, Texas, United States, 2. Arizona State University, Phoenix, Arizona, United States, 3. Indiana State University, Terre Haute, Indiana, United States, 4. Michigan State University, East Lansing, Michigan, United States*

**Objectives:** Identifying and meeting the spiritual needs of older adults is a central component of holistic service provision. China has more older adults than any other nation, yet a paucity of research exists on the spiritual needs of Chinese older adults. The purpose of this study was to identify: 1) common spiritual needs among Chinese nursing home residents, including the needs of residents with dementia, 2) the process staff use to identify these needs when residents are unable to verbalize them, and 3) the strategies staff use implement to meet the identified needs. **Methods:** To perform this qualitative study, semi-structured interviews were conducted with 21 administrators of faith-based nursing homes, representing facilities that may be disproportionately likely to have residents with dementia. The nursing homes spanned 14 Chinese provinces. Interviews were conducted in the study participants' native language, translated into English, and analyzed using a constant comparative methodology to identify themes. **Results:** Analysis produced five interrelated themes regarding older adults' spiritual needs, which included the need to: express their faith, receive love and care, have contact with their children, interact with others, and participate in activities. Regarding For residents with dementia who are unable to verbalize their concerns, staff used two strategies to identify spiritual needs: careful observation, and communication with family members. To address the identified needs, staff drew from local resources and attempted to personalize services whenever possible. **Discussion:** The findings offer guidance to individuals providing holistic care that addresses Chinese older adults' spiritual needs.

Abstract citation ID: igad104.3726

### THE QUALITY OF DAY SCALE AND THE LIVING WELL INVENTORY: TWO NEW MEASURES FOR INDIVIDUALS WITH DEMENTIA AND CAREGIVERS

Sheila Molony[1], Sam Fazio[2], Kimberly VanHaitsma[3], Rich Feinn[4], and Sheryl Zimmerman[5], *1. Quinnipiac University, Hamden, Connecticut, United States*, *2. Alzheimer's Association, Chicago, Illinois, United States*, *3. Penn State University, State College, Pennsylvania, United States*, *4. Quinnipiac, Hamden, Connecticut, United States*, *5. University of North Carolina at Chapel Hill, Chapel Hill, North Carolina, United States*

**Background:** Differences in adaptive strategies used by individuals living with dementia have the potential to impact wellbeing. Two new measures were developed using human-centered design to capture daily adaptive strategies and outcomes. The Living Well Inventory for Dementia (LWI-D) and the Quality of Day Scale (QODS) capture important aspects of wellbeing that illuminate options for care and support. Purpose: To provide results from the initial evaluation of the LWI-D and the QODS for face validity, content validity and user acceptability. **Methods:** Acceptability and feasibility testing was conducted with a sample of 17 community-dwelling individuals with early stage dementia (MOCA scores 12-30). After measure optimization, a second pilot test was conducted with 30 dyads (persons living with dementia and family caregivers) in community and residential settings. **Results:** Persons with dementia can use and accurately interpret the QODS, preferring a version with graphic icons. Of the 31 items on the development version of the LWI-D, 17 items were acceptable, meaningful, and easily answered. Additional items yielded meaningful qualitative responses but needed minor revision. Some participants disliked the Likert-type response format, and new administration guidelines were used for the second pilot. Additional outcomes include convergent validity between the LWI-D and the QODS with measures of positive affect-balance, quality of life and wellbeing. **Conclusion:** The LWI-D and the QODS are rigorously developed promising measures that warrant further testing and may enhance the ability to a) identify strengths in living well with dementia and b) identify and test news interventions to bolster care and support.

### SESSION 9310 (PAPER)

### LATE BREAKING: CLIMATE CHANGE AND AGING I

Abstract citation ID: igad104.3727

### A SYSTEMS APPROACH ON THE IMPACT OF ENVIRONMENTAL RACISM AND CLIMATE CHANGE ON RESIDENTS OF VINE CITY IN ATLANTA GA

Kellie Mayfield[1], Kehinde Akintola[1], Sophia Foley[1], Angelique Willis[1], and Miranda Cook[2], *1. Georgia State University, Atlanta, Georgia, United States*, *2. Open Hand Atlanta, Atlanta, Georgia, United States*

There are three interlocking factors impacting the health of older-adults. First is food access, the ecologies of their community and the systems they interact with. In the community of this study the local grocery store closed limiting food access. The ecology of the environment is a heat island; which leads to the incidence of higher mortality amongst other health effects. The interacting systems are the economy, ageism, classism, racism, and, sexism. This conceptual model encapsulates the environment impacting one community of older adults in Atlanta, GA from a mixed methods study employing survey and photoelicitation with the purpose of examining the facilitators and barriers to healthy eating by senior-center clients served by Open Hand Atlanta; a meals and nutrition education provider. Descriptive statistics showed food insecurity to be double that of older adults statewide (19%), 7.9 % reporting consuming at least 5 cups of fruit and vegetables per day, and participants were mostly black women (79 %) . Additionally, residents also live in a community where a portion has been deemed a toxic Superfund site because of elevated levels of lead in soil. The model, therefore, contributes to a comprehensive understanding of how the health of older adults is impacted by a multitude of systems and climate change. It can be used as a guide for other communities, community-dwelling older adults, and older-adult service providers. Furthermore, this model illustrates the systems impacting the health of older southern adults.

Abstract citation ID: igad104.3728

### CLIMATE CHANGE AND EMERGENCY PREPAREDNESS FOR HURRICANES AMONG NURSING HOMES IN TEXAS

Brian Downer[1], Alexandra Holland[2], Yejin Kang[1], Briana Lacy[1], and Huiwen Xu[1], *1. University of Texas Medical Branch, Galveston, Texas, United States*, *2. UT Southwestern Medical Center, Dallas, Texas, United States*

Hurricanes are a major threat to nursing homes along the Texas Gulf Coast, and climate change will increase inland nursing homes' risk of catastrophic winds and flooding. The Centers for Medicare & Medicaid Services (CMS) uses twenty-five regulations to assess nursing homes' emergency preparedness. This study compares the prevalence of any deficiencies in emergency preparedness regulations between Texas nursing homes in coastal and non-coastal counties. We also investigate the risk of future flood and wind events for nursing homes in non-coastal counties with any emergency preparedness deficiencies. Information for emergency preparedness deficiencies came from CMS inspection surveys. We used data from the First Street Foundation for the percentage of properties in a census tract with severe-extreme risk of flooding and damaging winds in 30 years. We used quartiles to define our highest risk categories as the census tracts with >7% of properties with severe-extreme risk for flooding and >50% with severe-extreme risk for damaging winds. The final sample included 1,153 nursing homes. The percentage of nursing homes with any deficiencies was significantly higher in non-coastal (13.9%) than coastal (8.7%) counties. This difference remained statistically significant after adjusting for ownership type, overall rating, and number of beds (OR=0.57, 95% CI=0.33-0.94). Among the 131 non-coastal nursing homes with any emergency preparedness deficiencies, 21.4% and 12.2% were in the highest risk categories for future flood and wind damage. Texas nursing homes, especially those in non-coastal counties, must be aware of their increasing hurricane risk and prepare to keep residents safe.

Abstract citation ID: igad104.3729

**EXPERIENCES OF VETERANS, CAREGIVERS, AND VA HOME-BASED CARE PROVIDERS POST-HURRICANE IAN**

Leah Haverhals, Chelsea Manheim, and Deisy Hernandez-Lujan, *Department of Veterans Affairs Rocky Mountain Regional VA Medical Center, Aurora, Colorado, United States*

On September 28, 2022, Hurricane Ian pummeled parts of south Florida. Disaster and climate change research has shown that disasters exacerbate inequalities, especially amongst older and physically vulnerable people. Florida has a large population of Veterans managing multiple chronic health conditions and receiving long-term services and supports in-home. Many such Veterans receive care from the Veterans Health Administration (VA) Home Based Primary Care (HBPC) or Medical Foster Home (MFH) programs. To describe how VA staff provided high quality care during and post-hurricane, and how Veterans and caregivers accessed needed healthcare and supports post-hurricane, we conducted a site visit to the Fort Myers, Florida area in May 2023. We conducted N=25 in-person interviews with VA HBPC and MFH staff, Veterans, and family and VA MFH caregivers. Findings from qualitative thematic analysis showed that Veterans and caregivers felt highly supported by VA care teams who made proactive changes in the days prior to and the months post-hurricane. Staff efforts post-hurricane focused on improving care coordination in anticipation of future disasters, especially around communicating with Veterans and their caregivers, and a VA workgroup formed to implement changes. Some Veterans and caregivers experienced significant challenges during and post-hurricane, with a few displaced and having to relocate, and others having difficulty accessing needed oxygen and suffering injuries related to the hurricane. As climate change causes more severe natural hazard events, lessons learned from this project can inform how to better support healthcare staff, older adults, and their caregivers pre-and post-major disasters.

Abstract citation ID: igad104.3730

**EXTREME WEATHER AND HEALTH AMONG OLDER POPULATIONS: INDIVIDUAL AND CONTEXTUAL-LEVEL VULNERABILITY AND RESILIENCE**

Eunyoung Choi[1], Bei Wu[2], and Jennifer Ailshire[1], *1. University of Southern California, Los Angeles, California, United States*, *2. New York University, New York City, New York, United States*

Intense, frequent extreme weather events pose a significant public health threat, particularly for middle-aged and older adults who may be at elevated health risk. Yet, the social and environmental factors that determine their vulnerability and resilience remain largely unexplored. This study investigates individual and contextual determinants associated with exposure to and the health effects of extreme weather. Data were from the 2021 California Health Interview Survey of adults aged 50+ (N=14,726). Respondents were asked whether they had experienced any hazardous weather events such as heat waves, floods, and wildfires in the past two years and whether mental or physical health was harmed by these events. About 53% were exposed to extreme weather events. Among the exposed group, 18% and 17% reported harm to mental and physical health, respectively. Logistic regression analysis revealed increased exposure risk among middle-aged adults (age 50-64), those with chronic conditions, people living alone, and residents of rural and unsafe neighborhoods. Similarly, mental and physical health harms were more prevalent among these groups, along with females and those with unstable housing conditions. Conversely, living in cohesive neighborhoods was identified as a resilient factor, associated with reduced mental (OR=0.78, $p< 0.05$) and physical health risks (OR=0.70, $p< 0.01$). This study reveals pronounced vulnerabilities to climate change risks among populations identified as socioeconomically disadvantaged. Moreover, the findings emphasize the role of contextual conditions in shaping vulnerability and resilience. Our study contributes valuable insights to develop targeted climate change mitigation strategies, fostering resilience among older populations and supportive communities.

### SESSION 9320 (PAPER)

### LATE BREAKING: ALZHEIMER'S DISEASE INCLUDING ALZHEIMER'S DISEASE RELATED DEMENTIAS IV

Abstract citation ID: igad104.3731

**ENHANCED SUPPORT FOR CHINESE AND KOREAN AMERICAN DEMENTIA CAREGIVERS: INSIGHT FROM SERVICE PROVIDERS**

Jing Wang[1], Min Kyoung Johnson[2], I tek Leong[2], Yaolin Pei[2], Mary Mittelman[2], Cynthia Epstein[2], Kyung Hee Lee[3], and Bei Wu[2], *1. University of New Hampshire, Durham, New Hampshire, United States*, *2. New York University, New York City, New York, United States*, *3. Yonsei University, Seoul, Republic of Korea*

As members of two large Asian immigrant populations in the U.S., Korean and Chinese American family caregivers of persons with dementia (PWD) have unique challenges impacted by their cultural background. Many of them prefer to and are relying on social services providers who speak the same language as they do for support and assistance. As part of the project to inform the cultural adaptation of NYU Caregiver Intervention-Enhanced Support (NYUCI-ES) program for Chinese and Korean American caregivers with multiple chronic conditions, we explored the perspectives of 13 Chinese and 12 Korean services providers (physicians and social service staff). During focus group discussions, services providers shared their perspectives on care challenges facing these populations, including challenges in initiating advanced care planning, family conflict in decision making and caregiving, insufficient community resources, staffing shortage associated with the pandemic and some care aides' stigmatic beliefs about providing dementia care. We identified nuances in the following aspects: utilization of home health aides' help, attitudes towards diagnosis and education, approaches to selfcare, preferences for engagement settings, trust-building, and family involvement. For instance, Korean services providers highlighted Korean caregivers' strong preference for home health aides of the same gender as PWD and having individual rather than group session to learn about dementia. What we found from services providers largely echoed our findings from family caregivers but also provided unique insights from their work experiences. These findings will inform cultural adaptation of the content of the NYUCI to improve its applicability to Chinese and Korean American caregivers.

Abstract citation ID: igad104.3732

### POSITIVE EMOTION LANGUAGE PREDICTS IMPROVING DEPRESSION TRAJECTORIES AFTER DEMENTIA CAREGIVING ENDS

Jenna Wells[1], Julian Scheffer[2], Suzanne Shdo[2], Claire Yee[3], Katherine Possin[4], and Robert Levenson[2], *1. Cornell University, Ithaca, New York, United States*, *2. University of California, Berkeley, Berkeley, California, United States*, *3. Mayo Clinic, Scottsdale, Arizona, United States*, *4. University of California, San Francisco, San Francisco, California, United States*

There are striking differences among caregivers of people with dementia (PWDs) in how their health and well-being changes during active caregiving; however, less is known about these differences after caregiving has ended. One important factor influencing caregiver health is the emotional quality of the caregiver-PWD relationship. The present study evaluated caregivers' emotional connection to the PWD (during caregiving) and associations with caregivers' depression both concurrently (N = 326 active caregivers) and longitudinally (n = 91 former caregivers). Active caregivers described a recent time they felt connected to the PWD, and their responses were transcribed verbatim. Caregivers' emotional connection to the PWD was operationalized by summing the number of positive and negative emotion words from the transcripts using text analysis software and dividing each figure by the total number of words in the response. Results indicated that neither positive nor negative emotional language was significantly correlated with caregivers' concurrent depression. However, two-wave latent change score modeling revealed that caregivers' greater positive emotional language (adjusting for caregivers' baseline depression and negative emotional language) predicted steeper declines in depression (i.e., improvements in depression trajectories) after the death of the PWD (ß = -.11, SE(ß) = 0.04, p = .033). Negative emotional language was not significantly associated with changes in caregivers' depression. This finding highlights positive emotion expressed by caregivers when describing their connection with the PWD as a potential resilience factor and underscores the important role that positive emotional qualities of the caregiving relationship play in improving caregivers' mental health after caregiving ends.

Abstract citation ID: igad104.3733

### SOCIAL PARTICIPATION AND DEPRESSIVE SYMPTOMS ON THE RELATIONSHIP BETWEEN COGNITIVE FRAILTY AND DISABILITY

Hongting Ning[1], Hui Feng[1], Junxin LI[2], and Mengchi Li[3], *1. Central South University, Changsha, Hunan, China (People's Republic)*, *2. Johns Hopkins University, Baltimore, Maryland, United States*, *3. Johns Hopkins, Baltimore, Maryland, United States*

To explore the multiple mediating effects of social participation and depressive symptoms on the relationship between cognitive frailty and functional disability. A total of 6122 participants from four waves of the China Health and Retirement Longitudinal Study (CHARLS) were included. Functional status was assessed by activities of daily living (ADL) and instrumental ADL (IADL). Analyses were restricted to middle-aged and older adults free of functional disability at baseline. Competing-risks regression and multiple mediation effects analysis adjusted for covariates were conducted. Cognitive frailty (hazard ratio [HR] 1.27, 95% confidence interval [CI] 1.09, 1.48), social participation (HR 0.87, 95%CI 0.79, 0.97), and depressive symptoms (HR 1.75, 95%CI 1.56, 1.95) were associated with the incidence of ADL disability. These factors were also found to predict the occurrence of IADL disability. Cognitive frailty was found to be associated with ADL disability, not only through the pathways of social participation (β = 0.009, 95% CI 0.001, 0.018) and depressive symptoms (β = 0.094, 95% CI 0.062, 0.131) separately, but also through social participation and depressive symptoms sequentially (β = 0.002, 95% CI 0.001, 0.003). Similar findings were also observed for the outcome of IADL disability. Cognitive frailty has been associated with functional disability, wherein social participation and depressive symptoms partially mediate the association. To prevent functional disability, it's suggested to implement integrated and comprehensive intervention measures, including preventing and managing cognitive frailty, promoting social participation, and improving depressive symptoms.

Abstract citation ID: igad104.3734

### SUPPORTING A MOTHER WITH DEMENTIA WHO MISTREATED ME: CAREGIVING EXPERIENCES OF STRAINED MOTHER-DAUGHTER DYADS

Mary Nemec, and Laura Girling, *Towson, Towson, Maryland, United States*

Providing care to an aging parent is a normative expectation within most modern societies. Research steadily demonstrates that around two-thirds of caregivers are women, and over one-third of dementia caregivers are adult daughters. Often there is an assumption of natural attachment between mother and daughter—unconditional loyalty and permanent availability. However, overlooked in the literature is the notion that some adult daughters struggle with caregiving decisions regarding a parent with whom they have been maltreated. To date, little research has focused on dementia caregiving dynamics within strained mother-daughter dyads. To address this knowledge gap, data were drawn from the Aging at Home Alone with Alzheimer's Disease study, an interview-based protocol with a purported sample of 120 community dwelling persons with dementia and their study partner. Thematic analyses were conducted on the subsample of daughters and their mothers with dementia (n=10) who reported a history of maltreatment (e.g., childhood neglect, childhood abuse). Thematic analyses were conducted on all case material for the subsample (e.g., interview data, case notes). Findings indicate four overarching themes: 1. Begrudging caregiving, 2. Fragmentary support, 3. Resurfacing childhood trauma, and 4. Post-traumatic growth and forgiveness. These themes stress the importance of how childhood maltreatment can shape emotional bonds early in life and affect dementia caregiving trajectories. Findings will be discussed in relation to support recommendations for caregiving dyads with a history of parental maltreatment.

Abstract citation ID: igad104.3735

### THE IMPACT OF INFORMAL CARE ON FORMAL CARE UTILIZATION AMONG ELDERLY AMERICANS

Yemiao Ke[1], and Yun Zhang[2], *1. Stony Brook University, Stony Brook, New York, United States*, *2. Columbia University, New York City, New York, United States*

**Objectives:** This research aims (1) to examines the impact of informal care on the utilization of formal care among elderly Americans, and (2) to investigate how probable dementia affect this relationship. **Method:** Using cross-sectional data from the National Health and Aging Trends Study (NHATS 2011 & 2015), we build a correlated Tobit model to analyze the quantity of informal and formal care received by elderly individuals, which not only handles the censored nature of the dependent variables, but also allows a more flexible error structure. To address endogeneity concerns, instrumental variables derived from the characteristics of the elderly's children are incorporated. **Result:** Participants who did not live in long-term care facilities (N = 7,090) were included. Our estimation results indicate that when monthly hours of informal care received are below 83 hours, the monthly hours of formal care decrease with an increase in informal care. Conversely, when monthly hours of informal care received exceed 83 hours, the monthly hours of formal care increase with additional informal care. Probable dementia increases the monthly hours of informal care by 31.2 hours and the monthly hours of formal care by 6.4 hours. **Conclusion:** Informal care substitutes for formal care when elderly individuals require only a low to moderate level of informal care. However, informal and formal care become complementary when the elderly requires more intensive care. Probable dementia has a significant impact on both informal care and formal care.

Abstract citation ID: igad104.3736

### EXPLORING PERCEPTIONS OF A SOCIALLY ASSISTIVE ROBOT IN ASSISTED LIVING: RECOMMENDATIONS FROM FAMILY CARE PARTNERS

Shannon Power[1], Kasey Smith[2], Lydia Burton[3], Reuben Abedine[4], Jenay Beer[1], and Anne Adams[5], *1. University of Georgia, Athens, Georgia, United States*, *2. SimpleC, Atlanta, Georgia, United States*, *3. Institute of Gerontology, UGA, Athens, Georgia, United States*, *4. The University of Georgia, Athens, Georgia, United States*, *5. SimpleC, LLC, Atlanta, Georgia, United States*

Research has shown that socially assistive robots are effective in delivering health and behavioral interventions to persons with and at-risk for dementia living in assisted living facilities. However, to gain a more comprehensive understanding on the potential benefits of socially assistive robots, family care partners should also be consulted for design recommendations. Thus, we conducted semi-structured virtual focus groups to investigate family care partners' (N=10) perceived benefits, concerns, and design recommendations of a socially assistive robot for their loved ones with or at-risk for dementia. Robot interactions (e.g., activity reminders, trivia entertainment, health prompts) were presented to participants via video, followed by in-depth questions and discussion. Thematic qualitative analysis (intercoder agreement = 87.5%) resulted in most recommendations on usefulness and function (30%), followed by engagement and entertainment (18%), social attributes (15%), customization (14%), health promotion, education, and behavior (9%), appearance (7%), and other (7%). Family care partners recommended improvements to build interactivity, social connection, and usefulness, such as improvements to robot appearance, customization and adaptability in robot capability that matches disease progression, and considerations for ethical implementation (i.e., the robot as supplement, not replacement, of human caregiving). Our findings represent the personal perspective of care partners intimately acquainted with dementia and caregiving, and from this perspective, yield specific recommendations for useful design of socially assistive robots.

### SESSION 9330 (PAPER)

### LATE BREAKING: TRANSLATIONAL GEROSCIENCE

Abstract citation ID: igad104.3737

### ADDRESSING THE COMPLEX DRIVING NEEDS OF AN AGING POPULATION: THE RISE OF AUTONOMOUS VEHICLES

David Carr[1], Hannes Devos[2], Abiodun Akinwuntan[3], and Ganesh Babulal[4], *1. Washington University in St. Louis, St. Louis, Missouri, United States*, *2. Kansas University Medical Center, Kansas City, Kansas, United States*, *3. KU School of Medicine, Kansas City, Kansas, United States*, *4. Washington University School of Medicine, St. Louis, Missouri, United States*

Eighty-eight million Americans will be age 65 or older by 2050 and 25% of all drivers will be ≥ 65 years old. Technology has the potential to address some issues such as the inability to drive in advanced age. We postulate that continuing to drive with the use of Advanced Driver Assistance Systems (ADAS) and/or maintaining social connectedness with the use of autonomous vehicles (AVs) have the potential to slow down or reduce the impact of age-related diseases and enhance social mobility. ADAS are electronic technologies that assist drivers in safely operating a vehicle by detecting crash risk stimuli and reducing driver error. Older drivers' willingness to use ADAS depends on individual perceived usefulness, safety, ease-of-learning, and anxiety. Based on the SAE levels of automation (0-5) , level 3-5 AVs can detect, evaluate, and respond to the vehicle environment without human involvement. Still, ADAS and AV technology cannot completely supplant all human driving and it cannot independently determine crash risk and safety. Comprehensive studies are needed to prove the effectiveness of ADAS in reducing crash risk among older drivers, especially those who are most vulnerable. In addition, AV manufacturers need to take into account the requirements and needs of providing this type of mobility service to older adults, especially those with chronic neurodegenerative diseases like dementia. If these technologies are eventually shown to be safe and embraced by older adults, they have the potential to delay driving cessation, prolong quality of life and maintain social connectedness.

Abstract citation ID: igad104.3738

### CARDIOVASCULAR AND MORTALITY RISK WITH COEXISTING HYPERTENSION, DYSLIPIDEMIA AND SYSTEMIC INFLAMMATION

Thomas Karadimasand Helen Meier, *University of Michigan, Ann Arbor, Michigan, United States*

Hypertension and dyslipidemia are established risk factors for cardiovascular disease (CVD), but are often insufficient alone in predicting CVD. Inflammation also contributes to CVD, but research on the co-occurrence of inflammation, hypertension, and dyslipidemia and CVD risk is limited. Prospective data from the Health and Retirement Study, a representative cohort of US adults over 50 years of age (n = 1,527) were used. Hypertension, dyslipidemia, and elevated C-reactive protein (CRP) were used to create a CVD risk: low (0-1 factors), medium (2 factors), or high (all 3 factors). Weighted logistic regression models estimated the odds ratio (OR) of 1) prevalent and incident CVD for medium and high-risk groups versus the low-risk group and 2) 4-year mortality adjusting for covariates. Cross-sectionally, high-risk participants had significantly higher odds of CVD prevalence compared to participants with low-risk (adjusted OR = 1.68, 95% CI: [1.12 - 2.53]). Prospectively, medium and high-risk participants had higher odds of 4-year CVD incidence (medium: OR = 1.41, 95% CI: [0.71 - 2.79]; high OR = 2.05, 95% CI: [0.93 - 4.53]) compared to those with low-risk, but not statistically significant. Risk of 4-year mortality was higher in both medium (OR = 2.00 95% CI: [1.21 - 3.30]) and high-risk (OR = 2.11 95% CI: [1.15 - 3.86]) participants vs. low-risk. Co-occurrence of hypertension, dyslipidemia, and elevated CRP was associated with CVD prevalence and 4-year mortality in older US adults, emphasizing the importance of multifactor screening for CVD risk.

Abstract citation ID: igad104.3739

### DETERMINATION OF OPTIMAL VIBRATION DOSE TO TREAT PARKINSON'S DISEASE GAIT SYMPTOMS: A CLINICAL TRIAL

Ingrid Pretzer-Aboff[1], R.K. Elswick[1], Arnaud Gouelle[2], Noah Helm[3], GinaMari Blackwell[4], and Leslie Cloud[5], *1. Virginia Commonwealth University, Richmond, Virginia, United States*, *2. Université Reims Champagne Ardenne, Reims, Champagne-Ardenne, France*, *3. Virginia Commonwealth University, Mechanicsville, Virginia, United States*, *4. Virginia Commonwealth University, Glen Allen, Virginia, United States*, *5. VCU School of Medicine, Henrico, Virginia, United States*

Most people with Parkinson's disease (PD) will experience gait problems. Previous studies demonstrated improved gait and balance after vibration stimulation was applied to the feet of PD patients. However, not all study participants showed improvement, perhaps due to sub-optimal vibration stimulus. Thus far, the optimal frequency and amplitude of vibration for mitigating gait dysfunction in PD have yet to be systematically explored. The PDVibe2TM (Resonate Forward LLC, DE) delivered vibration to the feet of 26 people with PD gait disturbances. We hypothesized that a global frequency, amplitude, and minimum duration of vibration therapy is required to improve gait. This was a phase Ib trial to identify optimal vibration parameters. Thirteen participants were recruited at Hoehn & Yahr (H&Y) stage II and 13 participants at stage III. Each group was randomly assigned to different frequency and amplitude settings prescribed by the central composite design methodology. Each participant received vibration for 18 minutes per walking session, for eight sessions spread over one week. Results showed an optimal dose response to treatment for frequency and amplitude of vibration based on the Functional Ambulation Performance score for stages II and III. In the H&Y stage II group, outcomes were stabilized after the 4th treatment. This stabilization was not seen in stage III participants. Global frequency and vibration amplitudes have been identified for treating PD gait disorders. Patients with more advanced Parkinson's disease may require a longer duration of therapy.

Abstract citation ID: igad104.3740

### OLDER ADULTS' PERSPECTIVES ON USING TECHNOLOGY TO ASSESS SOCIAL ISOLATION AT THE ONSET OF COVID-19

Jarshini Nanthakumar[1], Karishma Patel[1], Shahrose Aratia[1], Priyanka Thakkar[1], Rosalie Wang[1], and Shehroz Khan[2], *1. University of Toronto, Toronto, Ontario, Canada*, *2. Toronto Rehabilitation Institute, Toronto, Ontario, Canada*

Social isolation (SI) among older adults (OA) is a global health issue that was exacerbated by the pandemic. This study examines OA's perspectives on technology use (i.e., home-based ambient sensor or wearable technology) to assess SI, with consideration of understanding these perspectives in relation to the pandemic. Two questions were examined: (i) How familiar are OA with technology that can be used to assess SI? and (ii) Are OA agreeable to using technology to assess SI? Online surveys (n=4379) and semi-structured interviews were completed by community-dwelling OA at the onset of COVID-19. Quantitative data were analyzed using descriptive statistics and qualitative data were analyzed using content analysis. From survey responses, 16.79% indicated they would be agreeable to using technology to assess SI, whereas 40.37% and 42.83% indicated no and maybe/not sure, respectively. Although most participants were familiar with some technologies that could be used as part of an SI assessment tool, interviews suggested they were unfamiliar with this application of technologies. Preliminary analysis of the interviews (n=31) revealed three broad categories addressing the research questions: (1) perceived barriers to sensor-based technology adoption, (2) user experience and acceptance, and (3) changing social norms. Our study is one of the first to examine the views of OA regarding familiarity and openness to technology use for SI assessment that emerged with COVID-19. Technology developers and healthcare providers need to address ethical concerns around sensor-based technology use and increase opportunities to improve OA's digital literacy and accessibility to support technology acceptance for SI assessment.

Abstract citation ID: igad104.3741

### PLASMA METABOLOMICS OF VIGOR TO FRAILTY AMONG OLDER BLACK AND WHITE MEN AND WOMEN

Megan Marron[1], Shanshan Yao[1], Ravi Shah[2], Venkatesh Murthy[3], and Anne Newman[1], *1. University of Pittsburgh, Pittsburgh, Pennsylvania, United States*,

2. *Vanderbilt University Medical Center, Nashville, Tennessee, United States*, 3. *University of Michgan, Ann Arbor, Michigan, United States*

Older women and Black individuals are more likely to experience frailty. Metabolomics can provide a metabolic characterization of frailty that can help inform more effective interventions aimed at improving health, reducing disparities, and preventing frailty with aging. We sought to identify metabolites and pathways associated with vigor to frailty and determine whether these associations differed by gender and/or race among N=2189 older Black and White men and women from the Health, Aging, and Body Composition (Health ABC) study. Fasting plasma metabolites were measured using liquid chromatography-mass spectrometry. Vigor to frailty was based on weight change, physical activity, gait speed, grip strength, and energy level. We used linear regression of a single metabolite on vigor to frailty, adjusting for age, gender, race, study site, and multiple comparisons using a Bonferroni correction. Among 500 metabolites, 113 were associated with vigor to frailty (p< 0.0001). Associations between metabolites and vigor to frailty did not differ significantly by race and/or gender. Lower amino acids, glycerophospholipids, & sphingolipids and higher acylcarnitines, fatty acids, amino acid derivatives, carboximidic acids, benzenoids, nucleosides, organoheterocyclic compounds, carbohydrates, and trimethylamine oxide were associated with frailer scores. Pathway analyses identified the citrate cycle (p=0.00005), where higher levels of measured metabolites involved in the citrate cycle were associated with frailer scores. Daily calories and protein intake did not differ by vigor to frailty. Frailer Health ABC participants may have lower utilization of energy pathways, potentially a result of less efficient utilization of similar amounts of nutrients and energy demand than more vigorous participants.

### SESSION 9400 (PAPER)

### LATE BREAKING: ALZHEIMER'S DISEASE INCLUDING ALZHEIMER'S DISEASE RELATED DEMENTIAS V

Abstract citation ID: igad104.3742

### DAILY ENGAGEMENT IN MEANINGFUL ACTIVITY FOR HOME CARE PATIENTS WITH SUBJECTIVE COGNITIVE DECLINE AND CAREGIVERS

Yvonne Lu[1], Jennifer Ellis[2], Susan Perkins[3], Susan Hickman[4], Pei-Shiun Chang[5], Joan Haase[1], Laurie Otis[2], and Rebecca Winton[6], 1. *Indiana University School of Nursing, Indianapolis, Indiana, United States*, 2. *Aveanna Healthcare, Atlanta, Georgia, United States*, 3. *Indiana University School of Medicine, Indianapolis, Indiana, United States*, 4. *Indiana University, Indianapolis, Indiana, United States*, 5. *Indiana University School of Nursing, Bloomington, Indiana, United States*, 6. *CenterWell Home Health, Atlanta, Georgia, United States*

Patients with subject cognitive decline (SCD) often lose sense of control, disengage from meaningful activities, and have less confidence in their ability to manage daily challenges. The objectives of pragmatic pilot phase study were to evaluate the feasibility and benefits of a Daily Engagement in Meaningful Activity Professional (DEMA-Pro) for patients with SCD; and explore nurses' experience of DEMA-Pro implementation. Forty-nine patients at four home healthcare sites received six weekly DEMA-Pro telephone sessions. The Outcome and Assessment Information Set-D were collected at starting date (pre-intervention) and discharge date (post-intervention). Quantitative surveys and qualitative focus group methods were used to explore the DEMA-Pro nurses (n = 3) experiences. The consent rate was 67.1%, the completed intervention rate 36.7%, and the partial completed intervention rate 25.5%. For 36 subjects with discharge data available, both IADLs and self-care scores improved (d = 3.11 and d = 2.66, respectively). Specifically, those that completed all DEMA-Pro sessions (n=14), partial completers (n=12), and non-completers (n=10), had improved scores on IADLs (d = 4.0, 4.2, and 2.5, respectively) and Self-Care (d = 3.7, 3.1 and 2.0, respectively). Completers had greater improvement than non-Completers for both outcomes and greater improvement on IADLs than partial completers (all p-values < 0.03). Nurses reported high satisfaction with their training, and high confidence that the implementation of the intervention met patient and caregiver needs. The DEMA-Pro has shown benefits and feasibility that will need further testing in a large pragmatic trial in homecare settings.

Abstract citation ID: igad104.3743

### IMPROVING COGNITIVE DECLINE FOR PERSONS LIVING WITH DEMENTIA: A RCT ART THERAPY BASED ON THE ETC FRAMEWORK

Heesu J. Jeon, *Adler University, Vancouver Campus, Vancouver, British Columbia, Canada*

Abstract This randomized controlled trial explored the impact of Expressive Therapies Continuum (ETC)-based art therapy interventions for older adults with dementia living in a complex care home. The trial examined the efficacy of 10 art therapy intervention sessions over 4 months, comparing results with an active control group that participated in 10 art sessions. Pre- and post-assessments, including the Mini-Mental State Examination (MMSE), Clock Drawing Test (CDT), and the Older People's Quality of Life (OPQOL-Brief) questionnaires, were conducted. Results demonstrated that participants displayed similar visual expression elements, shared preferences for specific media types, and equivalent ETC-based intervention entry levels. In addition, participants in the experimental group showed significant increases in OPQOL-Brief scores. Finally, MMSE-Language scores demonstrated statistically significant increases in the experimental group and a decrease in the control group. The current study aligns with previous research demonstrating that art therapy can improve cognitive functioning and enhance the quality of life in older adults living with dementia. It expands on previous research by providing new insights into expressive and stylistic elements, ETC entry-level, and media preferences for engaging in artmaking. Keywords: Art therapy, dementia, older adults, Expressive Therapies Continuum, cognitive functioning, quality of life

Abstract citation ID: igad104.3744

### REVOLUTIONIZING DIGITAL ENGAGEMENT: EFFECTS OF AN APP ENABLING PERSONS WITH DEMENTIA TO LEAD ACTIVITIES FOR PEERS

Michael Skrajner[1], Gregg Gorzelle[2], John Zeisel[3], and Drew Walker[3], 1. *Hopeful Aging, Bolivar, Ohio, United States*, 2. *Hopeful Aging, Hiram, Ohio, United States*, 3. *Hopeful Aging, Winchester, Massachusetts, United States*

A Cluster Randomized Trial was conducted to evaluate the effects of an app that enables long-term care (LTC) residents with early-stage dementia to lead activities for their peers—i.e., other persons living with dementia (PLWD). A total of 72 PLWD completed the study, including 35 Control Group PLWD (Standard Programming) and 37 Experimental Group PLWD (Resident-Led Programming). Resident-Leaders exhibited 99% overall adherence to the steps required to lead activities and required minimal help from staff. A series of repeated measures ANCOVAs, controlling for Mini Mental State Examination (MMSE) scores, were conducted to examine proximal (immediate) and distal (longer-term) effects of the intervention. Regarding proximal effects, there were significantly greater increases from baseline to treatment in Constructive Engagement (p<.01), Passive Engagement (p<.01), and Pleasure (p<.01), as well as significantly greater decreases in Distracted Engagement (p<.01) and Passive Engagement (p<.01), for the Experimental Group, as compared to the Control Group. Regarding distal effects, there were no significant differences between trends observed by Control and Experimental Group residents from baseline to post-treatment for neuropsychiatric symptoms, agitation, depression, or quality of life. However, for a subsample of the Experimental Group (PLWD who resided in Assisted Living), there was a significant decrease in depressive symptoms (p<.05) and increase in quality of life (p<.01). Data suggest that this is a highly engaging intervention that enables PLWD to lead activities for their peers. The intervention has its greatest impact during the session, when positive forms of engagement/affect are increased, and negative forms of engagement are decreased.

Abstract citation ID: igad104.3745

### STATIN USE REDUCES ALL-CAUSE MORTALITY IN NURSING HOME RESIDENTS WITH DEMENTIA

Paul Gellert[1], Julie L. O'Sullivan[2], Raphael Kohl[2], Sonia Lech[3], Laura Romanescu[2], Johanna Schuster[2], Adelheid Kuhlmey[4], and Sevil Yasar[5], *1. Charité, Charité, Berlin, Germany*, *2. Charité, Berlin, Berlin, Germany*, *3. Charité Universitätsmedizin Berlin, Berlin, Berlin, Germany*, *4. Charité-Universitätsmedizin Berlin, Berlin, Berlin, Germany*, *5. Johns Hopkins University School of Medicine, Baltimore, Maryland, United States*

**Background:** Statins are recommended for the prevention of primary and secondary cardio and cerebrovascular events. However, little is known about the benefits of statin therapy in older adults with dementia. Therefore, this retrospective cohort study aims to investigate the association between statin use and all-cause mortality using health insurance claims data of nursing home residents with and also without dementia. **Methods:** Time-variant Cox proportional hazards models were used to evaluate the association of statin use with all-cause mortality and adjusted for potential confounders. **Results:** Overall, N=326,669 individuals were included in the analysis (mean [SD] age 84.02 [8.45] years; 74% women). During a mean observation period of 2.42 years (SD=1.59), a total of 239,321 deaths occurred. The fully adjusted models showed that participants with dementia statin users compared to non-users had a 20% lower risk for all-cause mortality (HR 1.20; 95%CI: 1.19–1.22, p<.001). In a subanalysis, similar trends remained within those with history of atherosclerotic cardiovascular disease (ASCVD) (HR 1.22; 95%CI: 1.21–1.24, p<.001) and without ASCVD disease (HR 1.12; 95%CI: 1.09–1.16, p<.001); and when stratifying by dementia type, Vascular dementia (VaD) (HR 1.27; 95%CI: 1.22-1.32, p< 0.001) and Alzheimer's disease (AD) (HR 1.19; 95%CI: 1.13-1.26, p< 0.001). Trends remained similar when stratifying by statin intensity, age, nursing home care level required, and sex. **Conclusion:** Our study demonstrates the benefits of statin therapy in highly vulnerable nursing home residents with dementia. This contributes to filling the knowledge gap regarding the harms and benefits of statin therapy in older adults.

Abstract citation ID: igad104.3746

### UNLOCKING PAIN MANAGEMENT FOR PEOPLE LIVING WITH DEMENTIA: AN INTERVENTION TO BE TAILORED

Annalisa Na[1], Justine Sefcik[2], Amy Kwok[1], Molly Drazon[1], and Laura Gitlin[1], *1. Drexel University, Philadelphia, Pennsylvania, United States*, *2. Drexel University College of Nursing and Health Professions, Philadelphia, Pennsylvania, United States*

Effective pain management for persons living with dementia (PLWD) is critical to quality of life and could be achieve through rehabilitative care. However, it's not well understood how physical therapists (PTs) and occupational therapists (OTs) develop and deliver treatment plans for PLWD, as most pain studies and evidence-based guidelines explicitly exclude those with cognitive impairment. In this qualitative descriptive study, we explored clinicians' perceptions of pain management in PLWD as recommended by available guidelines. Semi-structured virtual interviews of 6 PTs and 4 OTs from 4 outpatient facilities were all female, with an average age of 36.7 (SD 8.78) years and an average clinical experience of 7.01 (SD 4.81) years. We analyzed transcripts using conventional content analysis and identified a consistent theme that rehabilitative-based interventions for pain management among PLWD need to be "carefully tailored." Clinicians explained that the plan of care they designed for PLWD need to be tailored to the patient's underlying pain mechanism, physical function, and comorbidities that can affect their participation in therapy, to avoid activities that exacerbate pain symptoms, and take into consideration the extent of memory loss that the person is experiencing and their preferences. This study highlights the important factors practicing clinicians take into consideration when developing rehabilitative programs to manage pain among PLWD. These findings can guide the updating of current pain management guidelines so they are more inclusive and tailored to PLWD.

### SESSION 9410 (PAPER)

### LATE BREAKING: CLINICAL PRACTICE INNOVATIONS I

Abstract citation ID: igad104.3747

### A PILOT FEASIBILITY STUDY INVESTIGATING BALANCE DELIVERY TO OLDER ADULTS WITH CHRONIC CONDITIONS IN REHAB SETTINGS

Elizabeth Choma[1], Jackson Peckenpaugh[2], Diane Treat-Jaconson[3], Lauren Martin[3], Julian Wolfson[3],

Manda Keller-Ross[3], and Siobhan McMahon[3], *1. Whitworth University, Spokane, Washington, United States*, *2. Eastern Washington University, Spokane, Washington, United States*, *3. University of Minnesota, Minneapolis, Minnesota, United States*

Little is known about the integration of balance exercise delivery into physical rehabilitation settings for older individuals with chronic conditions and fall risk. We assessed the feasibility, acceptability and limited efficacy of delivering evidence-based balance exercises to older adults with fall risk enrolled in cardiac rehabilitation (CR) for peripheral artery and/or cardiovascular disease through an explanatory sequential mixed methods pilot study. Phase one included a two-arm randomized trial comparing feasibility, acceptability and functional balance among 10 participants receiving an 8-week balance program delivered adjacent to CR and 10 participants receiving CR only. Phase two included semi-structured interviews to elicit perceived barriers/facilitators of program receipt. Data was analyzed using descriptive statistics to summarize feasibility and acceptability indicators and one-way ANCOVA to test balance effects (Short Physical Performance Battery [SPPB], Mini-BESTest). Qualitative data was analyzed through content analysis. Participants had a mean age of 72.3 (7.3). On average, treatment group participants attended 7.2 (1.8) out of 8 meetings and self-reported completion of 4.5 (1.8) exercise sessions/week. Adjusting for baseline values, the treatment group exhibited greater changes in the SPPB than the usual care group after 8 weeks (10.71±0.36 vs. 9.59±0.36; adjusted difference=1.13 [95% CI, 0.3,2.2]; p=0.045). Mini-BESTest scores were not significantly different (p=0.061). Participants expressed satisfaction with program duration and frequency, quality of instructional materials, challenge level, ease of home exercise completion, convenient delivery location, and improved balance self-awareness/skills. These findings could inform the design of future efficacy trials investigating balance integration within CR settings to improve reach of fall prevention efforts.

Abstract citation ID: igad104.3748

### INNOVATING HOME-BASED CARE THROUGH HOME HEALTH DISCHARGE DECISION SUPPORT (HEADS-UP)

Melissa O'Connor[1], Margaret Brace[1], and Kathryn Bowles[2], *1. M. Louise Fitzpatrick College of Nursing, Villanova University, Villanova, Pennsylvania, United States*, *2. University of Pennsylvania, School of Nursing, Pennsylvania, United States*

Medicare and private payers rely upon home health (HH) nurses and physicians to evaluate the needs of older adults and decide when ready for discharge. However, there are no national, empirically derived clinical decision support tools to assist clinicians in making these frequent and important discharge decisions. The purpose of this work was to begin development of a clinical decision support tool to support HH clinician decision-making grounded in the evidence and based in real time. Clinician experts, in teams of three: two nurses and one physician reviewed and judged 500 case studies via a secure, dedicated website, making the decision if the older adults should be discharged from services versus recertified for additional care. Our sample matches the national older adult HH population by gender, race and primary diagnosis. The clinician experts recommended over 44.6% of older adults be recertified for more care when in reality, HH clinicians recertified only 22% for additional care. Regardless of insurance status, Blacks were 1.75 times more likely than Whites to be discharged by HH clinicians even though experts recommended more care (p=0.031). Hispanics trended toward being more likely to be discharged by HH clinicians compared to the experts' recommendation for more care (p=0.094). Our findings indicate that when provided with organized clinical information and ample time to accurately identify the needs of older adults, expert clinicians refer for additional care significantly more often than real-world HH clinicians and that racial disparities exist in current HH discharge decision making.

Abstract citation ID: igad104.3749

### INTRODUCING EFRAILTY: SIMPLIFYING SELECTION OF FRAILTY ASSESSMENT TOOLS

Megan Cheslock[1], Stephanie Sison[2], Lily Zhong[3], Natalie Newmeyer[4], Vaishnavi Raman[5], Ariela Orkaby[6], Andrea Schwartz[6], and Dae Kim[4], *1. Edith Nourse Rogers Memorial Veterans Hospital (VA Bedford), Bedford, Massachusetts, United States*, *2. UMass Memorial Medical Center - University Campus, Worcester, Massachusetts, United States*, *3. University of Connecticut School of Medicine, Farmington, Connecticut, United States*, *4. Hinda and Arthur Marcus Institute for Aging Research, Brookline, Massachusetts, United States*, *5. University of Toronto, Toronto, Ontario, Canada*, *6. VA Boston Healthcare System, Boston, Massachusetts, United States*

Health care providers recognize the importance of frailty assessment for older adults, but they may be unfamiliar with which frailty assessment tool to use. We sought to create an accessible website to assist clinicians in choosing an effective, evidence-based frailty screening tool. We selected commonly used frailty tools based on the literature and worked with a web designer to develop the eFrailty website prototype. A short description of each tool's key features and estimated time for assessment is included for each frailty tool. An algorithm based on differences in patient characteristics, clinical scenarios, available information, and time for assessment was created to guide users. Modeled after the highly popular ePrognosis website, eFrailty is designed to guide clinicians to select the ideal frailty tool for their clinical context. The site prompts clinicians to choose between patients considering stressful treatment (e.g., major surgery), or patients with or without serious illness. Depending on available information, clinicians choose between 'Self reports/records only,' or 'Performance tests available' including cognitive screens or physical performance testing. Alternatively, Clinicians may use the eFrailty comparison table which builds on the work of several systematic reviews of frailty identification tools to easily select the best instrument for their patient. A recent addition to the site is a crosswalk to compare scores between different frailty assessment tools. Future directions for eFrailty include beta testing to gather clinician input from point of care use.

Abstract citation ID: igad104.3750

### IS EDUCATION ENOUGH? SEATED YOGA AND FALLS PREVENTION FOR OLDER ADULTS

Betsy Kemeny, Heather Bright, Leanne Digman, Krista McCormack, Riley Page, and Madison Setto,

*Slippery Rock University, Slippery Rock, Pennsylvania, United States*

Falls impact 25% of older adults 65+ annually (Bergen et al., 2016) with 1 in 5 falls resulting in trauma (CDC, 2020). Standing yoga can reduce falls by 48% (Hamrick et al., 2017), but some older adults cannot participate in these poses/inversions due to pain, lack of balance, and endurance. Seated yoga can reduce pain, fear of falling, mobility, and balance ( Yao & Tseng, 2019). Ong et al. (2021) found that falls education improves intention to engage in prevention. Although CDC (2022) suggests multi-modal interventions , no known research compares a combination of falls prevention education and seated yoga intervention with an education-only control group. This controlled study compared fifteen older adults living in HUD housing who received both an 8-week "Get Fit While You Sit" seated yoga (LVSY, 2021) and falls prevention education to those who only received falls prevention education. Physical testing (Timed Up and Go (TUG), chair stand test, 4-point balance test) and attitude/participation measures were measured in both groups. Wilcoxon signed rank and Kruskal-Wallis were used to compare pre-to-post and between groups. Results suggest that the multi-modal intervention (combined education and chair yoga) resulted in improvements in TUG and chair stand test but other measures (e.g. self-efficacy for falls) did not change over time. The education-alone group did not improve over time. Between groups, a sig. difference was found in TUG and Chair stand. More research is needed but older adults in LTC settings may benefit from combining seated yoga with prevention education.

Abstract citation ID: igad104.3751

### THE MULTI-DIMENSIONAL ROLE OF GRATITUDE IN FAMILY MEMBERS OF PEOPLE WITH EARLY-STAGE ALZHEIMER'S

Jocelyn McGee, Davie Morgan, and Rebecca Meraz, *Baylor University, Waco, Texas, United States*

The interest in and scientific understanding of the multi-faceted construct of gratitude has grown in recent years. However, research on the role gratitude plays in the lives of family members of persons with early-stage Alzheimer's disease is limited. The purpose of this study, therefore, was to uncover the ways gratitude is experienced and expressed in family members of persons with early-stage Alzheimer's. Interpretative phenomenological analysis, a qualitative methodology, was employed to analyze the narratives of 27 family members. Expressions of gratitude were universal in the study but varied in the dispositional gratitude factors of frequency, intensity, span, and density. Relational conceptions of gratitude were expressed across three subthemes: (1) intrapersonal (e.g., family member grateful for how their own personal strengths and resources had aided them and allowed for growth while caring for their loved one with early-stage Alzheimer's; family member grateful for the individual strengths their family member with early-stage Alzheimer's had retained); (2) interpersonal (e.g., family member expressed gratitude for people who had positively influenced them such as prior role models for giving care to a person with Alzheimer's and people who had shown the family compassion); and 3) transpersonal (e.g., gratitude for God or a divine entity or force that was deemed benevolent and helpful when it came to the experience of living with Alzheimer's). Findings indicate that professionals should attend to gratitude in family members of persons with Alzheimer's and leverage this multifaceted resource to improve health and well-being.

### SESSION 9420 (PAPER)

### LATE BREAKING: COMMUNITY-BASED PARTICIPATORY RESEARCH APPROACHES I

Abstract citation ID: igad104.3752

### ACTIVE PARTICIPATION OF OLDER ADULTS AT POLITICAL RALLIES AS A SOURCE OF RESILIENCE: THE CASE OF THE ISRAELI PROTEST

Boaz Ben-David[1], Ortal Shimon-Raz[1], Yuval Palgi[2], lia Ring[3], and Tchelet Bresslet[1], *1. Reichman University, Herzliya, Tel Aviv, Israel*, *2. University of Haifa, Haifa, Hefa, Israel*, *3. Ashkelon Academic College, Ashkelon, HaDarom, Israel*

The governing Israeli coalition suggested on Dec 2022 a reform plan that would fundamentally alter the system of checks and balances within Israeli society. According to law experts, the plan would effectively end liberal democracy in Israel. This turn of events sparked the largest protest movement in the 75year history of Israel. The current situation is unparalleled, and the mental health costs appear to be significant. However, the toll on older adults has not been directly examined yet. The breadth of the protest movement is remarkable. A July 2023 poll conducted by the Israel Democracy Institute estimated that almost a quarter of Israeli citizens had participated at a protest action at least once. It was primarily through participation at large rallies held weekly across the country - one of the main symbols of the protest movement. Interestingly, the survey reported a 37% participation rate among older adults, the highesy participation rate among all tested age groups. Since older Israelis are members of the founding generation of the state, their participation is not surprising. They feel threatened as their way of life and heritage are at risk. In the current study, we conducted a survey that examined mental health indices among older Israelis. Specifically, we wanted to test whether active participation at the protest could serve as a source for resilience in older age. Unfortunately, political turmoil is not unique to Israel. Thus, we hope that our findings could assist practitioners globally.

Abstract citation ID: igad104.3753

### ADJUSTING TECHNOLOGY FOR AN AGEING POPULATION: CO-PRODUCING TECHNOLOGICAL SOLUTIONS WITH OLDER ADULTS.

Deborah Morgan[1], Hannah R. Marston[2], Carol Maddock[1], Aelwyn Williams[1], Gemma Wilson- Menzfeld[3], Elizabeth Jones[1], and Jessica Gates[3], *1. Swansea University, Swansea, Wales, United Kingdom*, *2. Open University, Milton Keynes, England, United Kingdom*, *3. Northumbria University, Newcastle, England, United Kingdom*

Since the onset of Covid 19, there has been a shift towards moving essential services online. This has the potential to further exclude sub-sections of the population from accessing essential services. This is particularly the case for older people who lack basic digital skills or who are not confident using digital technology, amplifying the risk of exclusion, and increasing the risk of social exclusion, loneliness and social isolation among digitally excluded older people. Research suggests that barriers to engagement with digital technologies include cost, lack of support, and technology being perceived as complicated or not for 'people like me (French, Quinn & Yates, 2019). As such, there is an urgent need to develop digital solutions with older people, co-designing technology that meets their needs. The presentation will draw on data and experiences of older people involved in a seed corn project funded by the Research Wales Innovation Fund (April 2023-July 2023). The project aimed to co-design an affordable digital solution with digitally excluded older people to support the development of digital skills and confidence. The presentation will focus on the skills deficit identified by older adults with limited digital skills and explore how this knowledge is being incorporated into a co-designed prototype launcher app. The presentation will highlight the benefits and challenges of co-producing solutions for an ageing population.

Abstract citation ID: igad104.3754

### ADVANCING POSITIVE SOCIAL CONNECTIONS FOR OLDER ADULTS IN SUBSIDIZED HOUSING: A HUMAN-CENTERED DESIGN APPROACH

Sheena Pubien[1], Becky Slogeris[2], Elizabeth Bazurto[2], Kennedy McDaniel[3], Vilde Ulset[2], Carl Latkin[4], Cynthia Boyd[5], and Thomas Cudjoe[6], *1. Nova Southeastern University, North Lauderdale, Florida, United States*, *2. Maryland Institute College of Art, Baltimore, Maryland, United States*, *3. Johns Hopkins University, Baltimore, Maryland, United States*, *4. Johns Hopkins Bloomberg School of Public Health, Baltimore, Maryland, United States*, *5. Johns Hopkins School of Medicine, Baltimore, Maryland, United States*, *6. Johns Hopkins University School of Medicine, Baltimore, Maryland, United States*

Social isolation is a complex problem that impacts many community-dwelling older adults and has detrimental impacts on mental and physical wellbeing of older adults. This research aims to utilize Human-Centered Design (HCD) to develop and implement tools and design ideas that advance positive social connections of older adults residing in subsidized housing. HCD is a collaborative process used to understand and define social problems, identify opportunities, generate ideas, and design interventions to support positive change. We sought to capture the perspectives and insights from older adults living in two federally subsidized housing communities in Baltimore, Maryland. During a five-month period, we held listening and brainstorming sessions with over 50 residents, service coordinators and members of management. Valuable insights and ideas were recorded during each of these facilitated engagements and synthesized. Through our engagements we identified six themes that influence the social connections of older adults living in subsidized housing: feelings of exclusion, lack of community connections, burdensome transitions to communal living, changes to building policies, and concerns about the physical environment. These themes led to the creation of interventions such as providing physical materials to residents, facilitating cultural inclusion and connections, implementing effective engagement strategies for community coordinators and promoting resident leadership. HCD can be employed to develop tools and innovative opportunities that specifically target and address social isolation in older adults living in subsidized housing. HCD can be utilized to drive changes to policies that impact older adults in subsidized housing leading to social transformations to improve health.

Abstract citation ID: igad104.3755

### LINKING HEALTH ATTRIBUTES TO ACUTE-CARE USAGE IN SOUTH FLORIDA VETERANS, A PILOT SURVEY STUDY

Richard Munoz[1], Zoran Bursac[1], Carlos Gomez[2], Jared Hansen[3], Sandra Garcia[4], Diana Ruiz[2], Marianne Desir[2], and Stuti Dang[4], *1. Florida International University, Miami, Florida, United States*, *2. Miami VA Healthcare System, Miami, Florida, United States*, *3. Veterans Affairs, Salt Lake City, Utah, United States*, *4. University of Miami, Miami, Florida, United States*

"High-Need, High-Risk" (HNHR) Veteran patients are identified quarterly by the Department of Veterans Affairs (VA) as having the top 5% predicted probability of hospitalization or mortality. We fielded a pilot survey to 2,543 older "HNHR" Veterans receiving care at the Miami VA Medical Center between October 2017 and September 2018, where 634 replied; mean age 70.61, standard deviation, 8.98, racially/ethnically White (403, 61.24%), Black (240, 36.47%), Hispanic (537, 81.61%) and nineteen women (3%). The survey contained 42 self-reported questions covering physical, mental, and social determinants of health, and access/use of healthcare resources. Two measured prior 6-month acute-care utilization outcomes: emergency room visits (ERV) and inpatient hospital admissions (IHA). Logistic regression was used to find health attributes associated with both outcomes, and latent class analysis was used to group Veterans into clinically relevant latent classes, analyzing health attributes endorsed by each. We found that transportation access issues to receiving healthcare were associated with both outcomes ($OR = 2.980$, $p = 0.014$), underscoring the role of social determinants of health. Self-perceptions of general health ($OR = 0.806$, $p = 0.04$), and a Veteran attending the Miami VA frailty clinic ($OR = 0.435$, $p < 0.001$), were associated with ERV and IHA, respectively. We identified four latent classes distinctly grouping patients by endorsing 1) depression; 2) not having a caregiver and being unmarried; 3) functional issues, and 4) being homebound and receiving home healthcare. These latent classes will help better inform clinical recommendations and program development to better meet needs for "HNHR" Veterans.

Abstract citation ID: igad104.3756

### PROMOTING MENTAL HEALTH LITERACY AMONG OLDER ADULTS IN THE EAST: AN INTEGRATIVE FRAMEWORK

Jessie Ho-Yin Yau[1], Hotinpo Sky Kanagawa[1], Maggie Wai-Shan Lo[1], Stella Lai-Kuen Wong[1], Gloria Hoi-Yan Wong[1], Terry Lum[1], and Tianyin Liu[2], *1. The University of Hong Kong, Hong Kong, Hong Kong*, *2. The Hong Kong Polytechnic University, Hong Kong, Hong Kong*

Although mental health literacy is of considerable importance to advance the welfare of older adults, previous research has shown that the public has a poor understanding of mental health and recognition of mental health symptoms. Traditional mental health literacy promotion programmes utilized a top-down approach; no bottom-up model had been used. Moreover, the existing participatory research paradigms were developed in the West and might not be applicable in the East, given different socioeconomic and cultural contexts. This research aimed to fill the gap by co-creating an integrative framework with stakeholders to promote mental health literacy among older adults in the East using a community-based participatory approach as a case illustration. We conducted a mental health literacy promotion pilot project in five Hong Kong districts from May 2021 to August 2023, spanning over 2 years. Each district formed an action committee, which comprised 5-10 community older adults and 2 social workers, and 2 researchers. Each district had its own promotional activities initiated and designed by older adults, including street booths, art workshops, and publicity materials to promote mental health and introduce relevant information and resources. We conducted six focus groups (n=25) with stakeholders, and we recorded our experiences on reflection notes. Collected data suggested specific elements are important for an integrative framework in the East, including participant empowerment, stakeholders' role and expectation management, and potential community contribution. We advance gerontological research and practice by providing an integrative framework to promote mental health literacy in the East. Implications will be discussed further.

Abstract citation ID: igad104.3757

### THROUGH THEIR LENS: A PHOTOVOICE STUDY OF OLDER ADULTS' EXPERIENCES OF ACCESSIBILITY AND AGING IN COMMUNITY

Yeonjung Lee[1], Lauren Migrino[2], Rochelle Deloria[2], and Kurt Robertson[2], *1. Chung-Ang University, Seoul, Republic of Korea*, *2. University of Calgary, Calgary, Alberta, Canada*

The objective of this research is to explore the lived experiences and perceptions of older adults in relation to aging in community and accessibility to social, physical, and community resources. Photovoice was used as the methodology for this study so that older adults could take pictures of people, places or things that might shed light on what is most important to them and their experiences of accessibility and aging in their respective community. Findings present qualitative themes with thirteen older adults in seven neighbourhoods in Calgary, Canada including planning for the future; convenient access to resources and services; importance of ethno-cultural supports and diversity; affordability; and importance of cultivating connection. These themes demonstrate that aging in place requires older adults to proactively anticipate and plan for the aging process. Moreover, aging in community for older adults means having access to physical resources that are convenient and in close proximity. Additionally, improving access to ethno-cultural resources and language supports enhances older adults' sense of safety and belonging. Older adults highlight an importance of having a diverse group of community members encompassing different ages, ethno-cultural backgrounds, and ability levels. Affordable resources and supports are essential for older adults to maintain a fulfilling and independent lifestyle within their built and social environments. Lastly, older adults seek social connection and belonging, but limited access to social supports can lead to social isolation and disconnection from their community.

### SESSION 9430 (PAPER)

### LATE BREAKING: EFFECTS OF DISCRIMINATION ACROSS THE LIFE COURSE II

Abstract citation ID: igad104.3758

### OLDER LGBTQ+ PEOPLE'S PERSPECTIVES ON DISCRIMINATION AND AGEING IN THE UK: EMERGING FINDINGS FROM A TIMELINE STUDY

Michael Thomas, Amy Prescott, and Christina Victor, *Brunel University London, Uxbridge, England, United Kingdom*

This paper presents emerging findings from a United Kingdom study of socially inclusive ageing, reporting on timeline interviews with a sample of 25 LGBTQ+ people aged forty years and above, carried out during summer and fall of 2023. The research interrogates older LGBTQ+ people's understanding of the impact of minority sexuality and gender identity on their lived experience and the ways in which this impacts their approach to challenges and opportunities linked to ageing. We draw on an innovative methodological approach, adapting Adriansen's (2012) timeline interview method to fit the needs of older participants. Timeline interviews provide an approach to life-story research that structures a conversation around a written timeline, produced on paper during unstructured qualitative interviews. During the paper presentation we share anonymised timeline and narrative data highlighting milestones in individual biographies. We focus on moments and periods where sexuality and gender identity may have been a positive factor in individual life stories (romantic relationships, community membership) or have been seen as more problematic (discrimination, stigma and marginalisation). The timelines and associated interview data also provide opportunities to ground the biographies of older LGBTQ+ people in the context of social, cultural, political and policy contexts. The timelines reveal compelling data about ageing and identity, belonging and exclusion. Our emerging findings reflect C. Wright Mills's concept of the sociological imagination, placing personal troubles and histories in the context of public issues. We gratefully acknowledge the funding this study has received from the United Kingdom Economic and Social Research Council.

Abstract citation ID: igad104.3759

### THE EFFECTIVENESS OF REIMAGINE AGING: AN INTERVENTION TO REDUCE INTERNALIZED AGEISM IN OLDER ADULTS

Dallas Murphy, Michelle Porter, Corey Mackenzie, and Judith Chipperfield, *University of Manitoba, Winnipeg, Manitoba, Canada*

A life time of exposure to ageist messaging and beliefs may lead older adults to internalize this messaging and direct it inwards, or towards their peers. This internalized ageism can have severe multi-faceted consequences on the well-being of older adults. However, little research has addressed reducing internalized ageism. Thus, Reimagine Aging, a six-week process-based intervention to reduce internalized ageism was designed and implemented, using tools of education, acceptance and commitment therapy, and attributional retraining to target theoretically based mechanisms of change. The data from 72 community-dwelling older adults (M = 70.4 years, SD = 6.4 years) were analyzed for changes in perceptions of aging and mechanisms of change. Participants completed questionnaires prior to the intervention, immediately following the intervention, and at a two-month follow-up. Results demonstrated that participants' self-perceptions of aging (partial eta squared = 0.37, $p < .001$) and perceptions of older adults (partial eta squared = 0.27, $p < .001$) became significantly more positive and maintained these gains at follow-up, associated with large effect sizes. Furthermore, the increases in self-perceptions of aging were mediated by increases in the theory-related mechanisms of psychological flexibility, mindfulness, and perceived control. Positive gains in perceptions of older adults were mediated by these process-based factors to a lesser degree. This study provides initial support for the effectiveness of this process-based intervention targeting a reduction of internalized ageism. This has the potential to reduce the harmful consequences of internalized ageism impacting older adults globally.

Abstract citation ID: igad104.3760

### UNDERSTANDING HEALTHCARE UTILIZATION, FRAILTY, AND DELAYS AMONG OLDER SEXUAL AND GENDER MINORITY ADULTS

Chelsea Wong[1], Louisa Smith[2], Robert Cavanaugh[3], and Brianne Olivieri-Mui[3], *1. HebrewSenior Life, Boston, Massachusetts, United States*, *2. Northeastern University, Boston, Maine, United States*, *3. Northeastern University, Boston, Massachusetts, United States*

Older sexual and gender minority adults (OSGM) have a higher burden of frailty, mental health conditions, and healthcare delays compared to non-SGM older adults (non-OSGM). The study aimed to evaluate the impact of health disparities related to frailty and delays on the relationship between OSGM and healthcare utilization, we hypothesized that improving frailty and delays would reduce healthcare utilization. We used marginal structural models in the All of Us version 6 Controlled Tier Dataset to study the mediating role of frailty and delays on healthcare utilization (self-reported general doctor (PCP) and mental health (MH) visits) by SGM status. OSGM (n=4,763) compared to non-OSGM (n=68,146) were younger (mean [SD], 63 [8] vs 66 [8]), had higher frailty (26% vs 19%), reported more delays (30% vs 24%), less excellent mental health (23.2% vs 30.2%), and majority were insured (96% vs 97%). Minimal differences between the total effect and controlled direct effect between SGM status and visits for frailty (PCP -0.01, MH 0.15) and delays (PCP 0, MH 0.16), suggests visits would remain similar if all OSGM were not frail or reported no delays. Contrary to our hypothesis, improving frailty and delays did not impact healthcare utilization among OSGM. Factors other than frailty and healthcare delays may be influencing healthcare utilization among OSGM in All of Us. Further, bias due to self-report/volunteer bias may affect the ability to examine the association of interest. Future work should examine the consensus between survey and EHR reported healthcare utilization within All of Us.

### SESSION 9440 (PAPER)

### LATE BREAKING: HEALTH EQUITY ACROSS THE LIFE COURSE II

Abstract citation ID: igad104.3761

### ACTIVITIES OF DAILY LIVING RECOVERY IN HOME HEALTH PATIENTS WITH DIABETES

Katelyn Webster-Dekker[1], Yvonne Lu[1], Susan Perkins[2], Jennifer Ellis[3], Laurie Otis[3], Rebecca Winton[4], and Eileen Hacker[5], *1. Indiana University School of Nursing, Indianapolis, Indiana, United States*, *2. Indiana University School of Medicine, Indianapolis, Indiana, United States*, *3. Aveanna Healthcare, Atlanta, Georgia, United States*, *4. CenterWell Home Health, Atlanta, Georgia, United States*, *5. The University of Texas MD Anderson Cancer Center, Houston, Texas, United States*

Older adults with diabetes are at high risk for impairments in their ability to perform activities of daily living (ADLs). Home health (HH) services help patients regain their ability to perform ADLs after being hospitalized, but there may be disparities in degree of ADL improvement based on characteristics such as race/ethnicity. We aimed to identify factors associated with improvements in ADLs from the start of HH care to discharge in older adult (age ≥65) patients with diabetes receiving HH. This secondary analysis used Outcome and Assessment Information Set-D data collected between October 1, 2021, and March 31, 2022 in the Southern U.S by a HH agency. We used multiple linear regression to examine factors associated with improvement in ADL performance. The sample (n=1350) was 55% female and 76% White, with a mean age of 76.3 (SD 7.3). Ninety-seven percent of patients improved their ADL score from start of HH care to discharge. Black/African American race (b= -0.33) and having bowel incontinence or an ostomy (b= -0.51) were associated with less ADL improvement. Having a caregiver who needed training/support (b= 0.44) or was unlikely to provide assistance (b= 0.78), the presence of a surgical wound (b= 0.52), pain that interfered with activity (b= 0.46), confusion (b= 0.30), and better scores in prior functioning (b= 0.13) at the start of HH were associated with greater improvement in ADLs upon discharge from HH. These findings require further investigation, but indicate Black patients experienced disparities in ADL improvement which should be addressed.

Abstract citation ID: igad104.3762

### GENDER DIFFERENCES IN COGNITIVE IMPAIRMENT OF AFRICAN AMERICAN AND HISPANIC SEXUAL AND GENDER MINORITY OLDER ADULTS

Austin Oswald, and Karen Fredriksen-Goldsen, *University of Washington, Seattle, Washington, United States*

Cognitive health of sexual and gender minority (SGM) older adults with intersecting racial/ethnic minority identities is an under-researched topic. This research utilizes data from Aging with Pride: National Health Sexuality/Gender study to examine differences in health-promoting and risk factors for cognitive impairment among African American and Hispanic SGM older adults. Informed by the Health Equity Promotion Model, bivariate statistics and linear regression models were applied to better understand subgroup differences in cognitive impairment and risk and protective factors. Daily discrimination and identity stigma were positively associated with cognitive impairment for African American and Hispanic groups. Outdoor leisure was negatively associated with cognitive impairment for African Americans whereas social support was negatively associated with cognitive impairment for Hispanics. Analysis of gender differences revealed that African American men experienced significantly higher rates of identity stigma than African American women; however, the effect of identity stigma on cognitive impairment was stronger for African American women. Among Hispanics, women compared to men were significantly more vulnerable to the effects of daily discrimination and identity stigma on cognitive impairment. Regarding cognitive impairment, engaging in physical activity and outdoor leisure were significantly protective for African American men but not for women, whereas social support was significantly protective for both Hispanic men and women. These findings address a void in SGM aging research by examining racial/ethnic and gender differences in cognitive health. They highlight the need for intersectionality and subgroup differences in research to develop tailored interventions to improve cognitive health among racially/ethnically diverse SGM older adults.

Abstract citation ID: igad104.3763

### RACIAL/ETHNIC DISPARITIES IN MARITAL STATUS AND LIVING ARRANGEMENT TRANSITIONS AMONG US OLDER ADULTS

Xueqing Wang[1], Xuezhixing Zhang[2], and Emma Zang[2], *1. Princeton University, Princeton, New Jersey, United States*, *2. Yale University, New Haven, Connecticut, United States*

US older adults' family life, including marital status and living arrangement, has become increasingly complex and diverse in recent decades. However, there is limited understanding of the average duration each racial/ethnic group typically spends in different marital statuses and living arrangements during older ages. Using data from the Health and Retirement Study 1992-2018, this research note investigates the racial/ethnic differences in the expected years to be spent at age 50 in each of the marital statuses (i.e., married, divorced, widowed, and never married) and living arrangements (i.e., living alone, living with spouse, living with non-spouses only, and nursing home. At age 50, Whites spend significantly more time married compared to Blacks and Hispanics, primarily due to the latter spending longer years divorced or never-married. Regarding living arrangements, Whites also spend notably more time living with spouse than Blacks and Hispanics, with the latter spending more time living only with non-spouses. These findings suggest that Whites have a distinct advantage in maintaining a stable, two-person family structure in late adulthood, while Blacks and Hispanics are more likely to spend their later years without their spouses. These patterns have broad implications for understanding persistent social inequality in late adulthood.

Abstract citation ID: igad104.3764

### THE ASSOCIATION BETWEEN FINANCIAL HARDSHIP AND MARKERS OF INFLAMMATION AMONG CANCER SURVIVORS

Agus Surachman[1], Jingxin Yao[2], Rose Ann DiMaria-Ghalili[2], and Hee-Soon Juon[3], *1. Dornsife School of Public Health, Drexel University, Philadelphia, Pennsylvania, United States*, *2. Drexel University, Philadelphia, Pennsylvania, United States*, *3. Thomas Jefferson University, Philadelphia, Pennsylvania, United States*

This study examined the cross-sectional association between financial hardship and inflammation markers among cancer survivors. Furthermore, we tested if financial hardship mediated the link between education and inflammation. We used data from 304 cancer survivors (Ages = 32-90, 51% female, 81% non-Hispanic white) in the Midlife in the United States (MIDUS) wave 3 and Refresher Biomarker studies. Financial hardship was based on three domains: material (e.g., lower income to poverty line ratio), psychosocial (e.g., difficult paying bills), and behavioral (e.g., cut back on spending). We included interleukin-6 (IL-6) and c-reactive protein (CRP) as inflammation markers. Analyses were adjusted for confounders, including age, race/ethnicity, sex, marital status, body mass index (BMI), and smoking status. Analysis was conducted using the structural equation model framework. The second-order measurement model of financial hardship with three domains (material, psychosocial, and behavioral) in the first order showed a satisfactory model fit. Higher financial hardship was significantly associated with elevated CRP (Est = 0.14, SE = 0.06, $p < .01$) but not IL-6 (Est = -0.07, SE = 0.05, $p = .13$). Furthermore, financial hardship partially mediated the association between education and CRP (indirect effect: Est = -0.03, SE = 0.02, $p < .05$). Inflammation is a major biological pathway associated with socioeconomic disparities in long-term chronic health conditions among cancer survivors. Explicating socioeconomic conditions contributing to this disease process beyond traditional socioeconomic indicators are critical to identifying potential intervention areas to narrow the disparities.

### SESSION 9500 (PAPER)

### LATE BREAKING: ALZHEIMER'S DISEASE INCLUDING ALZHEIMER'S DISEASE RELATED DEMENTIAS VI

Abstract citation ID: igad104.3765

### INVESTIGATING THE RELATIONSHIP BETWEEN HABITUAL ACTIVITY AND COGNITIVE DOMAINS AMONG PEOPLE LIVING WITH DEMENTIA

Faheem Pottayil[1], Yanbin Dong[2], Haidong Zhu[2], Richard Sams[3], Lufei Young[4], and Deborah Jehu[3], *1. Medical College of Georgia, Marietta, Georgia, United States*, *2. Medical College of Georgia, Augusta, Georgia, United States*, *3. Augusta University, Augusta, Georgia, United States*, *4. University of North Carolina at Charlotte, Charlotte, North Carolina, United States*

Habitual activity preserves cognitive function in people without dementia, but the relationship between habitual activity and cognitive domains among people living with dementia is unclear. The purpose of this study was to explore the association between habitual activity and cognition domains among people living with dementia. Participants living with dementia in residential care facilities (complete case analysis: n=24/42) completed a battery of cognitive tests (global cognition: Montreal Cognitive Assessment; executive function: Trail-Making Test, Digit Span Forward Test; perception and orientation: Benton Judgement of Line Orientation Test; language: Boston Naming Test; learning and memory: Rey Auditory Verbal Learning Test; complex attention: Digit Symbol Substitution Test). Participants wore an actigraphy monitor on their non-dominant wrist over seven days. We conducted a linear regression for total habitual activity (independent variable) with race (white/black), fall risk (Morse Fall Scale), and the number of comorbidities (Functional Comorbidities Index) as covariates, and cognitive tests as variables of interest. Participants were primarily male (75%), white (87.5%), and 50% had unspecified dementia (Alzheimer's disease: 33%). Greater habitual activity was associated with poorer global cognition, better executive function, and better learning and memory (ps< 0.05). Habitual activity was not related to visuospatial perception, language, or complex attention. Habitual activity may preserve executive function and learning and memory among people living with dementia. Wandering is more common in later stages of dementia, which may explain greater habitual activity observed with lower global cognition. Regularly assessing habitual activity may be useful in screening and monitoring cognitive changes.

Abstract citation ID: igad104.3766

### LIVING ALONE WITH DEMENTIA AND NAVIGATING ESSENTIAL TRANSPORTATION NEEDS

Mary Nemec[1], and Laura Girling[2], *1. Towson University, Elkridge, Maryland, United States*, *2. Towson, Towson, Maryland, United States*

Transportation serves a basic human need for older adults and is firmly linked to independence, quality of life, and access to essential services (e.g., medical, nutritional, social). The matter of transportation and driving reduction/cessation is especially challenging in regard to people with Alzheimer's and related dementias (ADRD). Many individuals with ADRD rely on cohabitating caregiver(s) (e.g., spouse, partner, adult children) to arrange and execute essential transportation needs. However, little is known about how individuals with ADRD who reside alone in the community navigate required transportation. In order to address this gap in the literature, this paper combines narrative data from two ongoing National Institutes of Health (NIH)-funded interview-based protocols that focus on understanding the needs of community-dwelling older adults with ADRD and a collateral informant (N=84). Interview data underwent thematic analyses. Findings indicate four overarching themes: 1. Nuances of ridesharing apps, 2. Family/fictive kin transportation network(s), 3. Unpredictable public transportation, 4. Unmet needs exacerbated by inadequate transportation, and 5. Balancing driving autonomy and risk of harm to self/others. These thematic illations have implications to assist development of strategies for continued mobility and appropriate access to required services for those who reside alone with ADRD, despite changing needs and capabilities. Historically, the focus on transportation among those with ADRD has centered on those with a cohabitating caregiver, however, our findings provide insights into the nuances of how this understudied subpopulation with ADRD experience and navigate their mobility needs.

Abstract citation ID: igad104.3767

### NEIGHBORHOOD DISADVANTAGE, IMAGING BIOMARKERS OF ALZHEIMER'S DISEASE, AND COGNITION IN DIVERSE OLDER ADULTS

Erica Fan[1], Sarah Royse[2], Beth Snitz[2], Tharick Pascoal[2], C. Shaaban[2], Brian Lopresti[2], Victor Villemagne[2], and Ann Cohen[2], *1. University of Pittsburgh, School of Public Health, Pittsburgh, Pennsylvania, United States*, *2. University of Pittsburgh, Pittsburgh, Pennsylvania, United States*

Neuroimaging biomarkers of Alzheimer's disease (AD) are well-validated in White populations. However, it remains unclear how these biomarkers are associated with cognition in non-White populations disproportionately affected by systemic socioeconomic discrimination. We investigated whether neighborhood disadvantage modifies associations of neuroimaging biomarkers with cognition in a diverse cohort of 198 adults ≥50 years, 50% of whom are Black. Outcomes included cognitive impairment and neuropsychological test scores. Predictors were indices of neurodegeneration, vascular damage, and brain amyloid burden, quantified using MR-based measures of cortical thickness and white matter hyperintensities (WMH) and [11C]PiB PET, respectively. We measured neighborhood disadvantage using area deprivation index (ADI), a composite measurement of socioeconomic conditions in a census tract. In linear models, higher ADI, neurodegeneration, and WMH were separately associated with worse cognition. We found an ADI*neurodegeneration interaction on delayed recall memory (p=0.08). Neurodegeneration-positive participants had worse story delayed recall compared to neurodegeneration-negative regardless of ADI (β=-1.57, p=0.01). In neurodegeneration-negative participants, higher ADI is associated with worse delayed recall (β=-0.04, p=0.004): those with a high ADI exhibited low delayed recall, comparable to neurodegeneration-positive participants. ADI was not associated with cognition in neurodegeneration-positive participants. WMH and ADI interacted in models of impairment, visual memory, and visual copy (interaction p's< 0.10). In those with high ADI, greater WMH was associated with worse cognition (β=-0.30, p=0.02) while participants with low ADI scored well regardless of WMH volume. Taken together, the relationship between neuroimaging biomarkers and cognition differs by neighborhood socioeconomic conditions, highlighting the need for clinical strategies that acknowledge structural disadvantage.

Abstract citation ID: igad104.3768

### PSYCHOSOCIAL WELL-BEING AND COGNITION AMONG OLDER ADULTS IN A LATE LIFE LEARNING PROGRAM IN LEBANON

Peiyi Lu[1], Martine Elbejjani[2], Lara Chehabeddine[2], Hana Arabi[2], Maya Abi Chahine[2], Katrina Kezios[1], Adina Zeki Al Hazzouri[1], and Abla Sibai[2], *1. Columbia University, New York City, New York, United States*, *2. American University of Beirut, Beirut, Aakk, Lebanon*

Growing evidence suggests that engaging in cognitively stimulating activities across the lifecourse is associated with improved health. However, very few studies have evaluated the benefits of late life learning (LLL), especially in the low- and middle-income country setting. We designed a prospective cohort study to evaluate whether LLL is associated with better cognition among older Lebanese and related underlying mechanism. The University for Seniors (UfS) is an established and ongoing LLL program at the American University of Beirut, since 2012, providing cognitively and socially enriching activities (e.g., lectures, study groups, social and cultural events) to older adults, aged 50 and above. Our study will select 700 members (both previous longer-term members and new enrollees) and recruit a comparison group composed of 700 age-, sex-, and education-matched non-UfS-participating community residents. Participants will be interviewed on various topics at baseline and at a two-year follow-up visit. Older Lebanese are exposed to a host of lifecourse psychosocial adversities. We developed a questionnaire to capture their life history such as war-related exposures and economic changes due to recent financial crises. We will also measure participants' psychological well-being including social network and support, ageism, resilience, hopelessness, loneliness. The challenges of adapting psychosocial scales and capturing lifecourse collective and individual adversities in a particularly burdened low-resourced settings and contextual complexity will be presented. With comprehensive data on LLL and lifecourse adversities, we will be able to examine the potential for scalable low-cost LLL programs to mitigate the impacts of earlier and accumulated psychosocial adversities on cognition.

Abstract citation ID: igad104.3769

### THE DIFFERENCE BETWEEN CYSTATIN C- AND CREATININE-BASED EGFR AND ASSOCIATIONS WITH COGNITION IN THE CHS

Lindsay Miller[1], O Potok[1], Dena Rifkin[1], William Longstreth[2], Oscar Lopez[3], and Joachim Ix[1], *1. University of California San Diego, San Diego, California, United States*, *2. University of Washington, Seattle, Washington, United States*, *3. University of Pittsburgh, Pittsburgh, Pennsylvania, United States*

The difference (eGFRDiff) between cystatin C-based estimated glomerular filtration rate (eGFRcys) and creatinine based eGFR (eGFRcr) has been shown to be associated with frailty and mortality, but its association with cognition is unclear. Among 4,764 adults aged ≥ 65 from the Cardiovascular Health Study, we analyzed eGFRDiff per 1-SD lower (18.6 mL/min/1.73m2). Cognitive function was evaluated annually using the Modified Mini Mental State Exam (3MS; range 0-100), and the Digit Symbol Substitution Test (DSST; range 0-90); higher scores mean better performance. We used linear regression for cross-sectional analyses, and mixed models for longitudinal analyses adjusted for demographic and clinical variables. The mean age was 72.2 ±, the mean eGFRDiff was 0.64, and mean follow-up was 4.4 ± years. eGFRDiff was not associated with 3MS at baseline. Each SD lower eGFRDiff at baseline was associated with a 0.82 [95% CI: -1.22, -0.42] lower DSST score. In longitudinal analyses each SD lower eGFRDiff was associated with a significant 0.06 [95% CI: -0.11, -0.01] faster decline in 3MS scores and a 0.04 [95% CI: -0.08, -0.01] faster decline in DSST scores over time. In older adults, we found that lower eGFRDiff was associated with lower cognitive scores at baseline, and associated with more rapid decline over time. This suggests that eGFRDiff could be used clinically as a laboratory-based screen to identify older adults in need for cognitive assessment.

### SESSION 9510 (PAPER)

### LATE BREAKING: APPLYING PRINCIPLES TO REFRAME AGING IN YOUR WORK

Abstract citation ID: igad104.3770

### ADMIRATION, SKEPTICISM, INDECISIVENESS? ATTITUDES TOWARD AI IN CHINESE MIDDLE-AGED AND OLDER ADULTS

Bobo Hi-po Lau[1], Eric Ngai Yin Shum[2], Fan Yin-Fong Lui[2], Gigi Lam[2], Alex Pak-Ki Kwok[3], and Chung-Kin Tsang[1], *1. Hong Kong Shue Yan University, Hong Kong, Hong Kong*, *2. Hong Kong Shue Yan University, Hong Kong, Hong Kong*, *3. The Chinese University of Hong Kong, Hong Kong, Hong Kong*

In recent years, AI is increasingly visible in daily lives from chatbots in banking apps and SIRI, and generative AI such as ChatGPT, to more controversial usage concerning facial recognition, online surveillance, and even self-driving cars. Attitudes toward AI may predict acceptance of AI-facilitated applications and the Web 3.0 at-large. In June 2023, we conducted a survey with 750 smartphone users aged 45 years or older (Max = 78) in Hong Kong as a part of a wider effort to elucidate the digital divide post-pandemic. Using principal component analysis with varimax rotation, we found three components from the General Attitude to Artificial Intelligence Scale - Negative, Positive, and Powerful (compared to humans). Subsequently, with latent profile analysis we revealed three profiles: (i) Admiration (18.4%; high on positive and powerful but low on negative); (ii) Skepticism (12.3%; high on negative but low on positive and powerful), and (iii) Indecisiveness (69.3%; moderate on all three components). Admiration was more likely to be male, with higher income, better self-rated health, and greater mobile device proficiency, optimism, innovativeness, but also insecurity with technology, compared to Indecisiveness, and then Skepticism. Corroborating these quantitative findings, AI, or generative AI in specific, were only sparsely featured in a concurrent focus group study on smartphone usage with a similar cohort of smartphone users. Those who mentioned were appreciative of their superior power to provide information beyond traditional search engines; yet apprehension toward associated fraud and deception was also highlighted.

Abstract citation ID: igad104.3771

### REDUCING BENEVOLENT AGEISM AMONG UNDERGRADUATE HEALTHCARE MAJOR STUDENTS IN A GERONTOLOGY CLASS

Jiawei Cao, and Jialin Cui, *Nanjing University of Chinese Medicine, Nanjing, Jiangsu, China (People's Republic)*

While gerontologists have devoted continuing efforts to combating ageism through education and interventions, benevolent ageism remains to be a more subtle and covert concept to understand and recognize. This study aims to examine the effectiveness of an innovative pedagogy to reduce benevolent ageism among undergraduates majoring in healthcare with an aging focus. Two classes (36 students in the intervention group and 43 in the control group) that were both enrolled in an entry-level gerontology class were recruited. The intervention consisted of a semester-long synchronized lecture with pre-lecture quizzes, videos, examples, and in-class group projects regarding benevolent ageism, while the control group received a regular gerontology class with no additional emphasis on ageism. At the beginning and the end of the semester, all participants completed a questionnaire that was built upon the translated Ambivalent Ageism Scale (AAS) benevolent subscale. Mixed-design ANOVA was conducted to examine the effects of the pedagogy (intervention vs. control) and time (pre-test and post-test) on students' benevolent score. The score for both groups did not differ at pre-test, and the results showed a significant interaction of group by time ($F(1, 77) = 13.24$, $p < .001$). The benevolent subscale score of the intervention group decreased significantly over time, while the control group only showed a slight but insignificant decrease in the score. Findings from our study suggested that this innovative pedagogy may be useful in reducing benevolent ageism among healthcare major undergraduates. However, further randomized studies are needed to confirm our findings.

Abstract citation ID: igad104.3772

### REFRAMING AGING FOR HIGHER EDUCATION IN HEALTH CARE PROFESSIONS

Caitlyn Coyle, Cindy Bui, Setarreh Massihzadegan, Suha Ballout, and Jan Mutchler, *University of Massachusetts Boston, Boston, Massachusetts, United States*

As students in health care professions will serve older patients, it is critical to proactively reframe aging in their education, confront implicit biases, and encourage age-inclusivity before they enter the workforce. We developed a training to address the limited focus on aging and later life among undergraduate curricula in health care professional disciplines (e.g., nursing, health sciences). The 1-hour, online training was piloted among current undergraduate nursing students (N=69). The content uses data and real-person interviews to introduce demographic trends in aging, define and deconstruct ageism concepts, illustrate the impact of ageism on individual health and health care, and highlight the diverse experiences of aging. The training also includes communication tools and interactive activities, such as reflection questions and case studies, to engage students in their own aging experience and to garner empathy for others' aging experiences. Pre- and post-training evaluations revealed that the training impacted students in expressing positive behavioral change towards age-inclusivity and awareness at internal, interpersonal and institutional levels. Evaluation results also showed statically significant evidence that the training increased students' knowledge about facts versus stereotypes about aging, confidence in understanding ageism and its relevance to health care, and consideration of working with older adults in their career. This pilot research project encourages other higher education institutions to incorporate training and education in their curricula that distinctively focuses on later life and reframing aging in a long-term effort towards improving health care for older people and the experiences of health care professionals who serve them.

### SESSION 9520 (PAPER)

### LATE BREAKING: CLINICAL PRACTICE INNOVATIONS II

Abstract citation ID: igad104.3773

### A DEPRESCRIBING CURRICULAR FRAMEWORK USING AN INTER-PROFESSIONAL APPROACH: IMPLICATIONS FOR NURSING EDUCATION

Winnie Sun[1], Cheryl Sadowski[2], Lalitha Raman-Wilms[3], and Camille Gagnon[4], *1. Ontario Tech University, Lindsay, Ontario, Canada*, *2. University of Alberta, Edmonton, Alberta, Canada*, *3. University of Manitoba, Winnipeg, Manitoba, Canada*, *4. Institut Universitaire de Gériatrie de Montréal, Montreal, Quebec, Canada*

Background Deprescribing is an important component of managing polypharmacy and reducing harm from potentially inappropriate medications. Current undergraduate nursing education does not consistently incorporate components of deprescribing into curricula. It is essential to bridge the gap in promoting deprescribing competencies, teach related knowledge and skills and assess learning outcomes. The purpose of this presentation is to engage nursing educators who teach geriatrics to identify and implement a curriculum framework for deprescribing. Methodology The Canadian Medication Appropriateness and Deprescribing Network (CaDeN) Healthcare Professional Committee undertook a consensus approach to developing competencies for deprescribing, along with literature review and analysis of prescribing competencies. The authors outlined the required knowledge and skills related to the competencies, with recommended teaching and assessment strategies. Results The seven deprescribing competencies include: gathering and interpreting patients' medication history and clinical information within their context, using tools that help identify potentially inappropriate medications, weighing potential benefit and harm of continuing or deprescribing medications, using shared decision-making about deprescribing, communicating deprescribing and monitoring plans, and monitoring progress and outcomes. Integrating deprescribing competencies in nursing curricula requires an intentional and structured approach across all years of the program, focusing on interprofessional collaboration. Learning activities should be active and practical, progressing from early to advanced learner skills and include integration of deprescribing through experiential education. Conclusion This presentation provides implications for the nursing profession to apply deprescribing competencies at different learner levels, using appropriate learning and assessment outcomes, and strategies in which deprescribing competencies could be achieved in an interprofessional setting.

Abstract citation ID: igad104.3774

### INNOVATING HOME HEALTH DELIVERY TO IMPROVE VETERANS' ACCESS TO CARE AND CARE COORDINATION

Heather Benzel[1], Jammie Carter[2], Dina Censky[3], Daniel Janssen[4], Rachel Murphy[5], Leann Putz[6],

Heather Davila[5], and Irene San Roman[5], *1. VA Midwest Healthcare Network (VISN 23. ), Grand Island, Nebraska, United States*, *2. VA Iowa City VA Health Care System, Iowa City, Iowa, United States*, *3. Sioux Falls VA Health Care System, Sioux Falls, South Dakota, United States*, *4. VA Black Hills Health Care System, Fort Meade, South Dakota, United States*, *5. Iowa City VA Health Care System, Iowa City, Iowa, United States*, *6. Central Iowa VA Health Care System, Des Moines, Iowa, United States*

Nearly 350,000 Veterans receive home health services through the Veterans Health Administration (VA) annually. Historically, VA has purchased most home health services from contract agencies in the community. VA leaders have expressed growing concerns about Veterans' access to home health, especially in rural areas. Coordinating care between VA and contract agencies is an ongoing challenge. To address these challenges, the VA Midwest Health Care Network (VISN 23) developed the VA Home Health (VAHH) pilot program. The program launched at the Iowa City VA and is currently operating at 4 sites in Iowa and South Dakota. Through VAHH, local VA Medical Centers hire home health staff directly (nurses, aides, physical and occupational therapists), rather than purchase these services through contract agencies. In this presentation, we describe program development, challenges, and early evaluation findings. In fiscal year (FY) 2022, 200 Veterans received VAHH. So far in FY2023, over 700 Veterans have been served. Pre/post comparisons involving Veterans receiving VAHH show modest reductions in emergency room visits (727 vs. 664 visits) and inpatient admissions (227 vs. 192 admissions). Overall hospital lengths of stay have reduced more dramatically (2132 vs. 1390 total days). VAHH staff note close communication with Veterans' primary care teams, and Veterans describe being highly satisfied with the program. Interest in VAHH is growing nationally, particularly as access to home health in many regions is limited. Early lessons from the VAHH pilot program could inform VA decisions about home health delivery and assist other sites in improving care for Veterans.

Abstract citation ID: igad104.3775

### PREDICTORS OF PHYSICAL FUNCTION DECLINE AMONG OLDER ADULTS RECEIVING HOME CARE NURSING: PROSPECTIVE COHORT STUDY

Taisuke Yasaka[1], Ayumi Igarashi[1], Chie Fukui[2], Asa Inagaki[1], Yuka Sumikawa[1], Manami Takaoka[1], Sameh Eltaybani[1], and Noriko Yamamoto-Mitani[1], *1. The University of Tokyo, Tokyo, Tokyo, Japan*, *2. YuPia Inc., Nagoya, Aichi, Japan*

Older adults receiving home care nursing have certain health conditions and face continuous risk of declining physical functions. Identifying those at risk of functional decline based on these factors is essential for developing appropriate strategies for maintaining their conditions. This prospective cohort study in home care nursing agencies across Japan examined ADL decline incidence and its factors among clients aged ≥75. Data were collected from nurses in charge of clients using online questionnaires. Functional status was evaluated using the ADL Hierarchy (ADLH) score, ranging from 0 (independent) to 6 (dependent). A binary logistic regression examined the relationships between clients' characteristics at baseline and ADLH score deterioration after 6 months, controlling for ADLH score at the baseline. Of the 715 clients analyzed, 31% had deteriorated ADLH scores between baseline and 6 months later. Older age (Adjusted odds ratio 1.04 [95% confidence interval 1.01–1.07]), having neurology diseases (2.23 [1.06–4.69]), use of home visiting physicians (1.54 [1.04–2.27]), falls with/without trauma in the previous 6 months (2.77 [1.41–5.44], 1.90 [1.11–3.25]), higher ADLH score at baseline (1.35 [1.18–1.56]), higher severity of dementia (1.23 [1.02–1.47]), occurring dyspnea in the previous 30 days (1.69 [1.05–2.71]), and having BMI lower than 18.5 (between 25 and 30 as a reference) (2.58 [1.12–5.94]) were associated with ADLH score deteriorations. The results suggest that inhibiting functional decline among older adults receiving home care nursing might be possible by optimizing the management of modifiable predictors such as frequent falls, dyspnea, and weight loss.

Abstract citation ID: igad104.3776

### THE PREDICTIVE EFFECT OF DIFFERENT MACHINE LEARNING ALGORITHMS FOR PRESSURE INJURIES: A NETWORK META-ANALYSES

Chaoran Qu, Xiufen Yang, Weisi Peng, Xiujuan Wang, and Weixiang Luo, *1. Shenzhen People's Hospital, Shenzhen, Guangdong, China (People's Republic)*

Objective: This review aims to systematically synthesize existing evidence to determine the effectiveness of applying machine learning algorithms for pressure injury management, to further evaluate and compare pressure injury prediction models constructed by numerous machine learning algorithms, and to derive evidence for the best algorithms for predicting and managing pressure injuries. **Methods:** A systematic electronic search was conducted in the EBSCO, Embase, PubMed, and Web of Science databases. We included all retrospective diagnostic accuracy trials and prospective diagnostic accuracy trials constructing a predictive model by machine learning for pressure injuries up to December 2021. The network meta-analysis was conducted using statistical software R and STATA. The certainty of the evidence was rated using the QUADAS-2 tool. **Result:** Twenty-five clinical diagnostic trials with a total of 237397 participants were identified in this review. The results of our study revealed that pressure injury machine learning models can effectively predict these injuries. Combining the algorithms separately yields the main **results:** decision trees (sensitivity: 0.66, specificity: 0.90, AUC: 0.88), logistic regression (sensitivity: 0.71, specificity: 0.83, AUC: 0.84), neural networks (sensitivity: 0.73, specificity: 0.78, AUC: 0.82), random forests (sensitivity: 0.72, specificity: 0.96, AUC: 0.95), support vector machines (sensitivity: 0.81, specificity: 0.81, AUC: 0.88). According to the analysis of ROC and AUC values, random forest is the best algorithm for the prediction model of pressure injury. **Conclusions:** This review revealed that machine learning algorithms are generally effective in predicting pressure injuries, the random forest algorithm is the best algorithm for pressure injury prediction.

Abstract citation ID: igad104.3777

### USE OF CHRONIC CARE MANAGEMENT SERVICE AMONG MEDICARE BENEFICIARIES IN 2015-2019

Jieun Jang[1], and Dae Hyun Kim[2], *1. Hebrew Senior Life, Boston, Massachusetts, United States*, *2. Harvard Medical School, Boston, Massachusetts, United States*

The Centers for Medicare and Medicaid Services (CMS) introduced Chronic Care Management (CCM) services in 2015 for patients with multiple chronic diseases. Few studies examined the use of CCM services by demographic, geographic, and clinical characteristics. We used 2014-2019 Medicare claims data from a 5% random sample of fee-for-service beneficiaries aged 65 years or over and enrolled Medicare Part B. Of the approximately 1.4 million beneficiaries annually, approximately 75% were potentially eligible beneficiaries of CCM services who had been diagnosed with two or more chronic conditions before the year (2015: 1,073,729; 2016: 1,094,277; 2017: 1,106,929; 2018: 1,118,386; 2019: 1,130,523). The primary outcome was the rate of patients receiving CCM services among potentially eligible beneficiaries using CPT codes 99490, 99491, and 99487 in each year. Among the potentially eligible beneficiaries, the rate of patients receiving CCM services increased from 1.1% in 2015 to 3.4% in 2019 (2015: 11,480; 2016: 22,154; 2017: 31,890; 2018: 37,341; 2019: 38,863). CCM use was concentrated in the South (2015: Georgia: 1.4%; Vermont: 0.1%; 2019: Georgia: 6.1%; Vermont: 0.1%). The increase was higher in beneficiaries with ten or more chronic diseases than in those with two to five chronic diseases (2015: 2-5 chronic diseases: 0.7%; 10 + chronic diseases 2.0%; 2019: 2-5 chronic diseases: 2.1%; 10 chronic diseases: 7.0%), and higher in severely frail beneficiaries than in robust beneficiaries (2015: robust: 0.6%; severely frail: 3.3%; 2019: robust: 1.9%; severely frail: 9.4%). Variations were observed in the utilization of CCM services across demographic, geographic, and clinical characteristics.

### SESSION 9530 (PAPER)

### LATE BREAKING: COMMUNITY-BASED PARTICIPATORY RESEARCH APPROACHES II

Abstract citation ID: igad104.3778

### AGE-ASSOCIATED CHALLENGES AMONG HIV POPULATION IN VIETNAM: PERSPECTIVES FROM MULTIPLE STAKEHOLDERS

Qing Xia[1], Yuan Xiong[1], Fei Sun[1], Giang Le[2], Van Hoang[2], Khanh Nguyen[2], Huong Dang[2], and Ethan Liu[3], *1. Michigan State University, East Lansing, Michigan, United States, 2. Hanoi Medical University, Hanoi, An Giang, Vietnam, 3. Phillips Exeter Academy, Exeter, New Hampshire, United States*

HIV remains a significant global public health concern. The growing population of aging individuals living with HIV in Vietnam presents a multifaceted pressing issue, calling for research, policy, and practice interventions. This study aims to identify the aging-associated healthcare needs among the HIV population from three stakeholder groups: healthcare professionals, family caregivers, and persons living with HIV-AIDs. Qualitative data was collected through five focus group interviews involving 10 people living with HIV (PLWH), 9 caregivers, and 8 healthcare providers in Hanoi, Vietnam, in March 2023. Data analysis followed thematic analysis techniques, uncovering recurring patterns and themes across the three participant groups.PLWH reflected diverse experiences, showing mental health struggles, aging-related concerns, and a lack of preparation due to health challenges or unexpected longevity. Resilience and optimism are also manifested in PLWH. Caregivers demonstrated crucial roles, including bridging care, medication adherence, and financial and emotional support. They highlighted the necessity of training programs to enhance their care capabilities. Both PLWH and caregivers expressed a desire for formalized training programs on HIV and aging. Furthermore, healthcare providers reported mental and physical health changes among aging PLWH, such as insomnia, forgetfulness, and memory deterioration. They noted a lack of resources and called for comprehensive training across medical staff to enhance the well-being of the aging PLWH.This study demonstrated the complicated age-associated needs among PLWH in Vietnam. The findings underscore the need for policy and practice innovations to address aging-related cognitive, mental health, and long-term care concerns.

Abstract citation ID: igad104.3779

### BUILDING LONG-TERM HIV SURVIVOR RESEARCH CHAMPIONS FOR PERSON-CENTERED CARE VIA COMMUNITY DISSEMINATION

Margaret Danilovich[1], Andy Rapoport[2], and Meredith Nicholson[3], *1. Northwestern University, Chicago, Illinois, United States, 2. CJE SeniorLife, Chicago, Illinois, United States, 3. GMHC, New York City, New York, United States*

As HIV becomes a chronic condition, it is critical for research to include the perspectives of long-term HIV survivors who face the challenges of managing both HIV and aging. We created SHARE (Survivors of HIV Advocating for Research Engagement), a nationwide research advisory board for long-term survivors. SHARE's goal is to incorporate long-term HIV survivor voices into research to influence person-centered care for long-term survivors. Through SHARE, board members have built research capacity through research methods training culminating in the board conducting a community needs assessment to learn community-directed priorities for HIV and aging. Following the needs assessment, each board member worked to identify and conduct research dissemination activities within their own communities to engage others in conversations about aging and HIV and educate on opportunities for greater research involvement. In this paper presentation, the speakers describe the process of building capacity among SHARE members so that they could serve as research champions through community dissemination efforts. The presenters will describe the methods of community dissemination including lunch and learns, webinars, infographics, podcasts, photovoice, and advocacy. Mixed-methods analysis of the impact of research dissemination will be presented including changes in community member knowledge about research, willingness to participate in research studies, and understanding of tools to find and use research. The presenters will also share data from SHARE board member self-reflection on the challenges and opportunities for researchers to engage community member in dissemination activities that are tailored to the community and can influence adoption of research among end users.

Abstract citation ID: igad104.3780

**EPIDEMIOLOGY OF FALLS AMONG OLDER ADULTS IN THE COMMUNITY FROM THE HEALTH AND RETIREMENT STUDY**

Lien Quach[1], Elizabeth Dugan[2], Uyen-Sa Nguyen[3], and Jeffrey Burr[3], *1. Massachusetts General Hospital, Boston, Massachusetts, United States*, *2. University of Massachusetts, Boston, Boston, Massachusetts, United States*, *3. University of North Texas Health Science Center School of Public Health, Fort Worth, Texas, United States*

Many older adults experience falls that lead to adverse health consequences. The primary objective of this study is to estimate the associations between risk factors of falls and actual falls. The second objective is to identify whether race modifies these relationships. Panel data from the Health and Retirement Study from 2006-2012 were analyzed. The total sample size of community-dwelling adults age 65+ was 22,153, and respondents were observed at different time points. Independent variables included physiological characteristics and health, lifestyle indicators, social environment, and socio-economic status. At each time point, the outcome was the number of falls reported in the two years prior to the interview. Generalized Estimating Equations for negative binomial regression models adjusted for repeated measures were performed. The mean age of participants in 2006 was 74, and 38% experienced at least one fall every two years. For each additional chronic health condition, the fall risk increased by 13%. Vision impairment increased fall risk by 21%. Conversely, higher cognitive function was correlated with a 3% reduction in fall risk. Every unit increase in ADL limitations escalated fall risk by 17%. Loneliness increased fall risk by 12%. Both vigorous (12%) and moderate (13%) physical activities were linked to a decrease in fall risk. Notably, minority racial group status modified the interplay between loneliness and falls and also bolstered the relationship between vision impairment and fall frequency. Tailoring fall prevention programs to racial and ethnic minority older adults is essential to reduce health disparities at the population level.

Abstract citation ID: igad104.3781

**USING COLLECTIVE IMPACT TO ASSESS HOME AND COMMUNITY-BASED SERVICES WORKER TRAINING AND CREDENTIALING**

Sandi Lane[1], Zavera Basrai[2], Caroline Yoon[2], Trish Farnham[3], Kezia Scales[4], Erin Carson[5], and Nathan Boucher[6], *1. Appalachian State University, Boone, North Carolina, United States*, *2. Duke University, Durham, North Carolina, United States*, *3. NC Coalition on Aging, Raleigh, North Carolina, United States*, *4. PHI, New York City, New York, United States*, *5. National Domestic Workers Alliance, Durham, North Carolina, United States*, *6. Veterans Health Administration, Durham, North Carolina, United States*

Older adults and people living with disabilities receive home- and community-based services (HCBS) from approximately 120,000 often poorly paid and inadequately supported direct care workers in North Carolina (NC). The demand for these workers is increasing rapidly, with NC projected to add almost 23,000 new direct care jobs from 2020 to 2030. With the goal of optimizing the workforce pipeline to prepare for the high need for HCBS across NC, we conducted a Medicaid-funded landscape analysis of direct care training/credentialing using Collective Impact (CI). CI is used for diverse collaborations through forming a common agenda, shared measurement, mutually reinforcing activities, continuous communication, and "backbone" support (dedicated coordination team). Engaging with lived experiences is crucial in CI as it leads to sustainable decision-making informed by those affected by policy change. With input from long-term services and support agencies, licensing bodies, advocates, and those with lived experience, we characterized NC HCBS training/credentialing landscape, generating a first-of-its-kind comprehensive crosswalk resource for the state. Findings reveal the complexity of training/requirements, limited portability, lack of flexibility, and a need for more person-centered and relational core competencies. In addition, funding and incentives for additional training are lacking, which limits opportunities for professional development and career advancement. CI is an effective method to analyze direct care worker training/credentialing, it allowing researchers to harness the wisdom of community partners and incorporate lived experiences of the people most impacted by potential policy changes - workers, HCBS recipients, and families.

### SESSION 9540 (PAPER)

### LATE BREAKING: QUALITY IMPROVEMENT INNOVATIONS IN HEALTH CARE

Abstract citation ID: igad104.3782

**A TECHNOLOGY SUPPORTED FALL PREVENTION INTERVENTION FOR OLDER ADULTS WITH MILD COGNITIVE IMPAIRMENT**

George Demiris[1], Nancy Hodgson[1], Therese Richmond[1], Marjorie Skubic[2], Justine Sefcik[3], and Sean Harrison[1], *1. University of Pennsylvania, Philadelphia, Pennsylvania, United States*, *2. University of Missouri, Columbia, Missouri, United States*, *3. Drexel University College of Nursing and Health Professions, Philadelphia, Pennsylvania, United States*

Falls and fall-related injuries are significant public health issues for adults 65 years of age and older. Over a third of older adults (OA) fall each year and 10-20% of falls result in serious injuries. Cognitive impairment has been identified as a leading risk factor with over 60% of OA living with mild cognitive impairment (MCI) falling annually. Housing conditions are also a risk factor for falls. We have developed Sense4Safety, a technology-enhanced intervention that includes multiple components: 1) in-home assessment and action; 2) education and regular communication with a coach; 3) passive monitoring facilitated by depth sensors in the home; and 4) alerts. The goal of this study was to access the feasibility of Sense4Safety and its acceptability. We recruited 10 low-income OA with MCI; each participant received the intervention for 3 months. All participants completed all study procedures. The Short Physical Performance Battery (SPPB) scores which provide an objective assessment of lower extremity functioning based on clinical experts' observation showed high correlation to the sensor system assessment

(r=0.94, p< 0.01). Two fall alerts were generated during the study period that triggered a timely response. Participants and their family members found the intervention to provide peace of mind and empower OA to proactively address changes in fall risk through exercise and home modifications. The combination of passive sensing and personalized coaching offers an innovative ecological approach to continuously assess functional mobility and gait in the home and captures changes that provide an early warning for fall risk.

Abstract citation ID: igad104.3783

### EVALUATING MHEALTH APPLICATIONS FOR OLDER ADULTS WITH DIABETES: SYSTEMATIC META-ANALYSIS OF CLINICAL TRIALS

Renato Ferreira Leitao Azevedo[1], Michael Varzino[2], Erika Steinman[2], and Wendy Rogers[2], *1. University of Illinois Urbana-Champaign, Urbana, Illinois, United States, 2. University of Illinois Urbana-Champaign, Champaign, Illinois, United States*

Diabetes is a common chronic condition among older adults and health technology interventions for diabetes management have the potential to support their self-management. However there is no systematic review and meta-analysis of the effects of mobile health applications (mHealth apps) for older adults. We conducted a systematic review and meta-analysis of randomized controlled trials (RCTs) to examine the effectiveness of mHealth apps for improving older adults' diabetes outcomes. We searched the EBSCO, Clinical Trials, Cochrane Library, ProQuest, PubMed, ScienceDirect, Scopus, and Web of Science databases for relevant studies. We retrieved 4247 papers and two reviewers completed a full screen review of 257. We excluded papers if the study was not a RCT, did not examine the effect of mHealth apps, or was not conducted with older adults, resulting in 7 papers included in the final analysis. Our results indicate that mHealth apps can be effective for older adults managing diabetes, with significant reductions in glycated hemoglobin (HbA1C) (Hedge's g: -0.40, 95% CI [-0.75 to -0.06]), and fasting blood sugar (FBS) levels (g: -0.29, 95% CI [-1.52 to 0.42]), as well as improvements in medication adherence (g: 0.93, 95% CI [0.53 to 1.28]). These effect sizes are comparable with other meta-analysis conducted across different aging groups. We describe each intervention and evaluate the risk of bias and the app components that could relate to effectiveness to inform the design of other diabetes management RCTs, and more broadly, the design of other mHealth tools for older adults. Pre-registration: http://doi.org/10.17605/OSF.IO/AWVCX

Abstract citation ID: igad104.3784

### FACTORS AFFECTING THE ACCEPTANCE OF CARE ROBOTS BY OLDER ADULTS: FOCUSING ON TECHNICAL FACTORS

Hee Jeong Yoon, Hyun Ju Lee, Si Woo Ban, Ha Yul Kim, Eun Gyo Cha, Jung Wan Lee, Hye Ri Shin, and Young Sun Kim, *Kyung Hee University, Seoul, Republic of Korea*

Care robots have the potential to support the healthy and independent lives of older adults and alleviate the burden on care workers. However, the potential benefits of care robots can only be realized if they are accepted. Given that most older adults do not have experience with care robots and lack digital literacy, an understanding of the factors affecting the acceptance of care robots is important. The goal of this study was to analyze the factors that affect older adults' acceptance of care robots by distinguishing the factors into sociodemographic, health, and technical factors. Data were collected from older adults aged 60 or over living in the community of Korea in 2022 (N= 506, mean age=70.11±7.74, women=54.55%). Hierarchical regression analysis was conducted to identify the factors related to acceptance of care robots. The results showed that factors influencing older adults' acceptance of care robots were significant in sociodemographic factors (education and place of residence), health factors (self-reported health and instrumental activities for daily living), and technical factors (digital literacy and technostress). The findings indicate that interventions are needed to increase the acceptance of care robots by vulnerable groups with low levels of education, living in rural areas, and poor health conditions, and that it is also important to increase their digital literacy and lower their technostress. This study would provide valuable guidelines to stakeholders such as service providers, and policymakers to increase the acceptance of care robots and improve the quality of life of older adults.

Abstract citation ID: igad104.3785

### IMPACT OF THE TEACHING NURSING HOME ON NURSING STUDENT'S COMPETENCIES AND WILLINGNESS TO WORK IN LONG-TERM CARE

Howard Degenholtz[1], Jacqueline Dunbar-Jacob[1], Kim Ratliff[2], Donna Fick[3], Chelsea Dickson[4], Erin Kitt-Lewis[5], Emily Franke[6], and Kenna Campbell[1], *1. University of Pittsburgh, Pittsburgh, Pennsylvania, United States, 2. Wesley Enhanced Living, Media, Pennsylvania, United States, 3. Penn State University, State College, Pennsylvania, United States, 4. Jewish Healthcare Foundation, Pittsburgh, Pennsylvania, United States, 5. The Pennsylvania State University, University Park, Pennsylvania, United States, 6. Jewish Healthcare Association, Pittsburgh, Pennsylvania, United States*

The Teaching Nursing Home (TNH) is designed to improve the long-term care workforce and improve quality of care in nursing homes. The initiative has multiple components: enhanced clinical rotations for nursing students with partner schools of nursing, implementation of the Institute for Healthcare Improvement Age-Friendly Health System "4M" quality improvement model, and an online learning network. Nursing students at three schools of nursing participated in clinical rotations at four regional nursing homes. The experience was limited to students in one specific course at each school of nursing. At the beginning and end of the Spring 2023 semester, students rated their competence in: patient assessment, collaborating with the care team, gathering clinical information, medication review, eliciting resident values, and health promotion. Students also rated their preferences for working in long-term care and with older adults. Data from 165 responses at the start of semester (January 2023) and 155 responses to the end of semester (April 2023) survey were analyzed. Analysis of student responses found that students self-rated competencies improved in all areas. Prior to their clinical experience, students ranked working in long-term care and with older adults lower than other settings or populations. The rankings were unchanged after their clinical experiences. Qualitative analysis found that most student comments (50%) were about skills, while only 1.5% were about potential careers in nursing homes. These findings suggest that the Teaching Nursing Home Program is meeting the pedagogical goals, however attitudinal shifts may require different strategies.

Abstract citation ID: igad104.3786

### SELF-MANAGEMENT SUPPORT STRATEGIES BASED ON THE RNAO GUIDELINES IN AECOPD PATIENTS: A RANDOMISED CONTROLLED TRIAL

Weisi Peng, Weixiang Luo, Xiufen Yang, Chaoran Qu, Di Huang, Yan Liu, Yi Fan, and Weiwei Zhang, *Shenzhen People's Hospital, Shenzhen, Guangdong, China (People's Republic)*

Abstract Chronic disease management is a key intervention for the management of AECOPD patients. We conducted a Chinese-based trial according to RNAO's chronic disease management guidelines.The aim of this study was topply an evidence-based guideline practice protocol for self-management in patients with chronic disease to improve the clinical outcomes, as well as reduce the economic costs for patients discharged with AECOPD.In this study, the "Strategies to Support Self-Management in Chronic Conditions: Collaboration with Clients", published by RNAO, was used as a practice guideline to be applied in the follow-up of patients discharged from COPD to understand patients' post-discharge self-management ability, pulmonary function, and readmission within one year, and to compare the cost-effectiveness for patients after discharge. The follow-up of both groups was conducted over time, and the patients' self-management ability and pulmonary function in the observation group were effectively improved at month 6 and month 12 and was significantly different in comparison with the control group (P< 0.05). The observation group showed significant differences in readmission rates and economic costs at month 12 as compared to the control group (P< 0.05).Application of the guideline's chronic disease self-management support strategies could stabilize patients' blood oxygenation levels, better maintain pulmonary function, significantly improve patients' self-management ability, and reduce patients' medication costs. Keywords: COPD;Chronic disease self-management; RCT; economic analysis

Abstract citation ID: igad104.3787

### UNDERUTILIZATION OF TRANSITIONAL CARE MANAGEMENT SERVICE AMONG MEDICARE BENEFICIARIES AT HIGH RISK OF READMISSION

Chan Mi Park[1], Ellen McCarthy[1], Sandra Shi[1], Brianne Olivieri-Mui[2], Jieun Jang[1], and Dae Hyun Kim[3], *1. Hebrew Senior Life, Boston, Massachusetts, United States*, *2. Northeastern University, Boston, Massachusetts, United States*, *3. Harvard Medical School, Boston, Massachusetts, United States*

The CMS introduced transitional care management (TCM) service in 2013 to improve care transitions from hospital to home and reduce readmission. TCM uptake has been slowly increasing over time; however, it remains uncertain whether TCM is delivered to patients at the highest risk of readmission. We used a 5% random sample of 2015-2019 Medicare fee-for-service claims. TCM service was identified using CPT codes and demographic information, census region, diagnosis codes, and frailty were measured. Of 1,556,347 eligible discharges (mean [standard deviation] age, 78.3 [7.8] years, 56.7% female, 84.2% White), TCM was delivered in 169,536 (10.9%) discharges and 30-day readmission occurred in 123,796 (8.0%) discharges. The 30-day readmission risk was higher among beneficiaries who were < 75 years (8.1%) vs ≥75 years (7.9%), male (8.7%) vs female (7.4%), Black (9.3%) or Hispanic (8.7%) vs White (7.8%) and Asian or Pacific Islander (8.0%), with dementia diagnosis (8.2%) vs without (7.9%), or with greater frailty (robust to severe frailty: 6.1% to 14.1%). TCM use was not necessarily higher among those with high-risk characteristics. Notably, TCM was more likely to be delivered to beneficiaries who were ≥75 years (12.3%) vs < 75 years (8.8%), White (11.3%) and Asian or Pacific Islander (10.8%) vs Black (9.0%) and Hispanic (8.4%), without dementia (11.0%) vs with (10.6%), and pre-frail or mildly frail (11.4%) vs moderately or severely frail (10.0-10.5%). Our study found that TCM is not delivered effectively to Medicare beneficiaries at high risk of readmission, including racial and ethnic minority groups, and those with moderate-to-severe frailty.

### SESSION 9550 (PAPER)

### LATE BREAKING: ALZHEIMER'S DISEASE INCLUDING ALZHEIMER'S DISEASE RELATED DEMENTIAS VII

Abstract citation ID: igad104.3788

### ADVANCING TECHNOLOGY OF A GROUP MUSIC INTERVENTION FOR PERSONS WITH DEMENTIA: FINDINGS FORM A FOCUS GROUP STUDY

Silvia Orsulic-Jeras[1], Gregg Gorzelle[2], Michael Skrajner[3], Sara Powers[1], and Sarah Nicolay[1], *1. Benjamin Rose Institute on Aging, Cleveland, Ohio, United States*, *2. Hopeful Aging, Hiram, Ohio, United States*, *3. Hopeful Aging, Bolivar, Ohio, United States*

The benefits of using music to engage persons living with dementia (PLWD) are widely reported in the literature Music can elicit memories and positive feelings as well as decrease anxiety and agitation in PLWD. Moreover, conducting music interventions in a group setting has been shown to increase social interaction, produce positive mood, and promote feelings of belonging. This paper discusses the adaptation and technological development of a group music intervention for persons living in the mild to moderate stages of dementia, Making Connections through Music (MCTM). Originally, MCTM was comprised of 6 individually themed sessions containing songs and corresponding discussion prompts; materials such as handheld instruments, photos, and props were used to elicit engagement and increase socialization among group members. Session leaders, older adult volunteers, were trained to provide the experience by offering the necessary structure and support PLWD need for successful participation. This paper will focus on the technological adaptation of the original MCTM intervention. Prototypes of a tablet application and online training course for volunteers were evaluated across seven focus groups with LTC staff (n=25) and older adult volunteers (n=25). Results from focus groups demonstrated 95% of volunteers and 100%

of staff were satisfied with both the tablet app and training course. Acceptability and feasibility findings will be presented. Discussion also will highlight the implementation of this program within senior centers, adult day, and residential care as well as next steps in technological advancement of this intervention and associated training.

Abstract citation ID: igad104.3789

### BRIDGING RESEARCH AND POLICY TO EXPAND SUPPORTS TO OLDER ADULTS LIVING ALONE WITH COGNITIVE IMPAIRMENT IN THE US

Elena Portacolone[1], and Robyn Stone[2], *1. University of California, Berkeley, California, United States*, *2. LeadingAge, Washington, District of Columbia, United States*

Bridging research and policy allows research findings to have a lasting impact in the wellbeing of vulnerable population such as the estimated 4.3 million older adults living alone with cognitive impairment in the United States. For example, a lasting impact comes from healthcare and social services provided by Medicare and Medicaid tailored to address unmet needs of these populations identified via rigorous research. Despite the importance of bridging research with policy, researchers seldom collaborate with policy makers, thus missing the opportunity of influencing policies. Reasons for these limited collaborations include limited knowledge on methods for researchers to collaborate with policy makers. This session will draw from the expertise gained by the research team of the 5-year Living Alone with Cognitive Impairment Project to involve policy experts in the design of their mixed-method study. Regular interactions between the research team and the Policy Advisory Group led to: 1) a mixed-method design aligned with policy priorities; 2) a report highlighting the priorities of PAG members with regard to policies to enhance the wellbeing of older adults living alone in the US; 3) a series of policy briefs highlighting key findings and related policy recommendations. The oversight of a Policy Advisory Group allowed the Living Alone with Cognitive Impairment Project to focus research activities that are likely to be translated into policy solutions, thus likely to mostly benefit older adults living alone with cognitive impairment, with emphasis on communities of color. These practices can be replicated by researchers in studies supporting underserved populations.

Abstract citation ID: igad104.3790

### DEMENTIA CAREGIVERS' ETHICAL CONSIDERATIONS OF HOME-MONITORING ASSISTIVE ROBOTS IN DEMENTIA CARE

Jing Wang[1], Dain LaRoche[1], Momotaz Begum[2], Xueting Tang[3], and Sajay Arthanat[2], *1. University of New Hampshire, Durham, New Hampshire, United States*, *2. The University of New Hampshire, Durham, New Hampshire, United States*, *3. Fudan University, Shanghai, Shanghai, China (People's Republic)*

Understanding dementia caregivers' perspectives on the ethical dimensions of socially assistive robot technology is essential for its responsible and sustainable implementation. This study provides comprehensive insights into the ethical considerations of using home-monitoring assistive robots in dementia care, as seen through the perspectives of caregivers. A focus group and a follow-up interview were conducted with seven participant caregivers following their exploration of a home-monitoring assistive robot sharing their insights and experiences related to its use in dementia care. The data were subjected to thematic analysis, identifying recurring patterns and themes within the participants' accounts. Key findings included Responsibility and Safety: Need for safety outweighed privacy concerns of caregivers. They highlighted features such as reminders, danger alerts, and integration with security systems; Autonomy and Informed Consent: our findings emphasized promoting robot technology while respecting the autonomy of individuals with dementia in deciding technology use for their own care, acknowledging the variability in their responses; Technology Maturity and Expectation Management: Caregivers expressed expectations regarding robot functionalities, highlighting the importance of technological reliability, setup processes, and technical support. Dilemma in Monitoring and Privacy: Striking a balance between monitoring and privacy emerged as a crucial concern, sparking discussions on camera usage, sensor placement, and maintaining a suitable equilibrium; Affordability Challenges: Caregivers recognized the affordability challenges associated with robot technology as a significant hurdle. The themes highlight the complexity of integrating Socially assistive robots for dementia care responsibly, thereby informing future development and implementation strategies to ensure its beneficial and ethical use.

Abstract citation ID: igad104.3791

### NATURAL ENVIRONMENTS' INFLUENCE ON RESPONSIVE BEHAVIOUR, WORK-RELATED STRESS, BURNOUT, AND TURNOVER INTENTION

Peggy Chi[1], Adrian Wagg[2], Stephen Verderber[1], Olesya Falenchuk[1], and Whitney Berta[1], *1. University of Toronto, Toronto, Ontario, Canada*, *2. University of Alberta, Edmonton, Alberta, Canada*

Natural environments in healthcare design are thought to promote health. However, its empirical research in long-term care (LTC) has been limited; the degree of rigor is mixed, focusing on older adults with little research on professional caregivers. This study examined the relationships between the natural environment and the mental health and well-being of older adults 65+ living with Alzheimer's disease and related dementias (residents) and of their professional caregivers in LTC homes using a rigorously developed survey to standardize an approach to the evaluation of natural environments for these population groups. A cross-sectional, multiphase mixed method study was conducted that included a scoping review (n=137), a Delphi panel (n=24 experts), cognitive debriefing interviews (n=33), psychometric evaluations (n=83 LTC home areas), and multilevel modeling and regression analyses (n=1599 residents; n=563 professional caregivers). A conceptual framework on the natural environment in healthcare was developed, which informed the development of the Natural Environment Survey for Long-Term Care. This survey evaluates 1) the design of natural environments, 2) residents' usage of and exposure to natural environments, and 3) professional caregivers' usage of, exposure to, and perception of natural environments. Unlocked doors to outdoor spaces, outdoor activities, and indoor activities were significantly associated with residents' responsive behavior in secure home areas (i.e., locked access and egress).

Usage of and exposure to the natural environment during breaks and leisure were associated with their professional caregivers' work-related stress, burnout, and turnover intention in secure and non-secure home areas. These findings have potential utility for decision-makers transforming LTC.

Abstract citation ID: igad104.3792

### PATIENT AGE AND SEX DO NOT APPEAR TO INFLUENCE DECISION MAKING AROUND BIOMARKER TESTING FOR COGNITIVE IMPAIRMENT

Mark Monane[1], Kim Johnson[2], Joy Snider[3], R. Scott Turner[4], Jonathan Drake[5], Julia Ortega[6], Yan Jiang[7], and Joel Braunstein[6], *1. C2. N DIagnostics, St. Louis, Missouri, United States*, *2. Duke University, Durham, North Carolina, United States*, *3. Washington University at St Louis, St. Louis, Missouri, United States*, *4. Georgetown University, Washington, District of Columbia, United States*, *5. The Warren Alpert Medical School of Brown University, Providence, Rhode Island, United States*, *6. C2. N Diagnostics, St. Louis, Missouri, United States*, *7. Stat4. ward, Pittsburgh, PA, Wexford, Pennsylvania, United States*

The QUIP I Study (ClinicalTrials.gov Identifier: NCT05477056) was a prospective, single-arm study which included 347 older persons (average age 74, 56% women) presenting with signs and symptoms of cognitive impairment. In a subgroup analysis, we measured the effect of a person's age and sex on clinical decision making around the PrecivityAD® blood biomarker (BBM) test result. The test result was reported as the Amyloid Probability Score (APS), which measures the likelihood of a positive result on an amyloid PET scan. Clinical decision making was recorded by clinician survey pre- and post-BBM testing. Clinician-reported probability of Alzheimer's disease ("AD") changed pre-test to post-test from 58% to 23% (Low APS group) and from 71% to 89% (High APS group) ($p < 0.0001$ for all APS groups). The relationships between APS and change in diagnostic certainty were not significantly different as analyzed by age ($p=0.344$ for Low APS, $p=0.292$ for High APS) or sex ($p=0.167$ for Low APS, $p=0.213$ for High APS). Overall use of AD drug therapy decreased from 48% to 26% (Low APS group) and increased from 56% to 88% (High APS group) ($p < 0.0001$ for all APS groups). The relationships between APS and change in medication prescribing were not significantly different as analyzed by age ($p=0.4534$ for Low APS, $p=0.9939$ for High APS) or sex ($p=1$ for Low APS, $p=0.931$ for High APS). We believe that the current study results help to underscore the usefulness and generalizability of this BBM among older adults in clinical care pathways.

### SESSION 9560 (PAPER)

### LATE BREAKING: CLIMATE CHANGE AND AGING II

Abstract citation ID: igad104.3793

### THE ASSOCIATION OF AIR POLLUTION AND CLIMATE FACTORS WITH DEPRESSIVE SYMPTOMS AMONG OLDER MEXICAN AMERICAN ADULTS

Diane Saab, Phillip Cantu, Brian Downer, Kyriakos Markides, and Sadaf Milani, *University of Texas Medical Branch, Galveston, Texas, United States*

Air pollution, drought, and extreme heat are associated with elevated depressive symptoms. Older Mexican American adults experience high levels of depressive symptoms compared to other demographic groups, but little is known about the role of environmental factors in depressive symptoms for this population. Mexican American adults are resilient to the harmful health effects of socioeconomic disadvantage, but it is unclear if this resilience extends to air pollution and climate change. This study uses data from the Hispanic Established Populations for the Epidemiologic Study of the Elderly (H-EPESE) linked to air pollution and climate data from the Centers for Disease Control and Prevention, to assess the association of air pollution (PM2.5 and ozone) and climate factors (drought and extreme heat) with elevated depressive symptoms. We used data from the 2005 H-EPESE which includes Mexican American adults aged 75 and older. We used generalized estimation equations to test the association between air pollution and climate factors and depressive symptoms, adjusting for sociodemographic and health characteristics. Our sample (n=1,699) was 62.0% female with a mean age of 81.6 (standard deviation: 5.0). About 19% of participants reported elevated depressive symptoms. PM2.5 concentration was associated with higher odds of depressive symptoms (odds ratio: 1.11; 95% confidence interval: 1.04, 1.19). Ozone concentration, drought, and extreme heat were not associated with depressive symptoms. Our results suggest PM2.5 is a risk factor for elevated depressive symptoms. It is unclear if older Mexican American adults are resilient to the effects of environmental risk factors relative to other demographic groups.

Abstract citation ID: igad104.3794

### UNDERSTANDING THE RELATIONSHIP BETWEEN AIR QUALITY AND FIVE-YEAR SURVIVAL OF PATIENTS IN THE AMERICAN FAMILY COHORT

Divya Periyakoil[1], Ndola Prata[2], and Marie Diener-West[3], *1. Johns Hopkins Bloomberg School of Public Health, Baltimore, Maryland, United States*, *2. University of California, Berkeley, Berkeley, California, United States*, *3. Johns Hopkins, Baltimore, Maryland, United States*

Air pollution is an important social determinant of health and associated with cardiopulmonary disease, cancer, diabetes, and cognitive impairment. This study examined the impact of air pollution on the mortality of patients in the American Family Cohort (AFC), a national dataset of 6.6 million Americans, derived from the American Board of Family Medicine PRIME Registry electronic health record data. The AFC data were merged with the Environmental Protection Agency's Air Quality Index (AQI) measures for the study period (2016-2022). Two Cox-Regression-Models were performed to determine how (1) baseline air quality and (2) air quality measures over time would affect patients' five-year survival. Fixed covariates of interest--gender, race, age, location, and year of entry into the study--were studied as well. The adjusted hazard of death in the group with baseline AQI greater than 50 was 4.02 times higher than that of the group with AQI less than or equal to 50 (95% CI: 3.36, 4.82, $p < 0.05$). Compared to younger patients, the hazard of death was 6.73 times higher in persons older than 80 years of age (95% CI: 5.47, 8.28; $p < 0.05$). Black/African American patients had a 4.27 times higher hazard of death

(95%CI: 3.47, 5.26; $p < 0.05$) compared to other races. Our study shows that vulnerable populations (older adults and persons from minority communities) are disproportionately burdened by poor air quality. Public health initiatives that focus on improving air quality for minority communities will improve their longevity.

Abstract citation ID: igad104.3795

### UNRAVELING COMORBIDITY PREVALENCE AND PM2.5 EXPOSURE AMONG MIDDLE-AGED AND ELDERLY: A SPATIAL-TEMPORAL ANALYSIS

Linjiang Wei, Liangwen Zhang, and Ya Fang, *Xiamen University, Xiamen, Fujian, China (People's Republic)*

Objective: This study describes regional differences and dynamic changes in the prevalence of comorbidities among middle-aged and elderly people with chronic diseases (PCMC) in China from 2011-2018, and explores distribution patterns and the relationship between PM2.5 and PCMC, aiming to provide data support for regional prevention and control measures for chronic disease comorbidities in China.

**Methods:** This study utilized CHARLS follow-up data for ≥45-year-old individuals from 2011, 2013, 2015, and 2018 as research subjects. Missing values were filled using the random forest machine learning method. PCMC spatial clustering investigated using spatial autocorrelation methods. The relationship between macro factors and PCMC was examined using Geographically and Temporally Weighted Regression, Ordinary Linear Regression, and Geographically Weighted Regression.

**Results:** PCMC in China showing a decreasing trend. Hotspots of PCMC appeared mainly in western and northern provinces, while cold spots were in southeastern coastal provinces. PM2.5 content was a risk factor for PCMC, the range of influence expanded from the southeastern coastal areas to inland areas, and the magnitude of influence decreased from the southeastern coastal areas to inland areas.

**Conclusion:** PM2.5 content, as a risk factor, should be given special attention, taking into account regional factors. In the future, policy-makers should develop stricter air pollution control policies based on different regional economic, demographic, and geographic factors, while promoting public education, increasing public transportation, and urban green coverage.

### SESSION 9570 (PAPER)

### LATE BREAKING: EFFECTS OF DISCRIMINATION ACROSS THE LIFE COURSE III

Abstract citation ID: igad104.3796

### ADDRESSING UNMET MENTAL HEALTH NEEDS OF OLDER ADULTS IN TURBO, COLOMBIA: A PILOT INTERVENTION

Clarissa Giebel[1], Gabriel Saldarriaga[2], Erika Montoya[2], Mark Gabbay[1], and Maria Isabel Zuluaga[2], *1. University of Liverpool, Liverpool, England, United Kingdom, 2. Universidad de Antioquia, Medellin, Antioquia, Colombia*

Mental health is often stigmatised in lower- and middle-income countries, with little resources to provide care. Having lived through extreme and stressful live events in Colombia, including armed conflict, and older adults found to lack adequate mental health support, the aim of this pilot intervention is to improve their mental health. Based on a meta-analysis of community-based mental health interventions for older adults in LMICs, qualitative interviews with older adults and local stakeholders, and a mental health needs assessment survey of the older population in Turbo, Colombia, we consulted older adults in the region to co-produce a community-based intervention. The pilot intervention is running for 12 weeks from May to August 2023, with two sessions provided per week in a community centre. The intervention involves four components - social engagement, educational interventions, physical activities (singing, dancing, cooking), and peer support. We will use a mixed-methods approach to evaluate the effects of the intervention on older adults' well-being, social support, and loneliness, involving pre- and post-questionnaires and semi-structured interviews. Thirty-five older adults are actively participating in the intervention. Full results will be available in October. This co-produced and evidence-based intervention for older Colombians has the potential to circumvent heavy healthcare resource costs for providing mental health support, and instead involve the community in providing care and support, with trained facilitation. In light of limited mental health support across LMICs, this intervention, if effective, could provide mental health support for older adults in other countries via cultural adaptation.

Abstract citation ID: igad104.3797

### DEFENSIVENESS AGAINST INTERGROUP BIASES ACROSS ADULTHOOD

Jennifer Beatty, and Patrick Hill, *Washington University in St. Louis, St. Louis, Missouri, United States*

Research has demonstrated the pernicious role of discrimination against marginalized groups. Less work has considered the perspective of the transgressor and how defensive they may be when told they were biased. Such work is critical for understanding which individuals may be more willing to acknowledge and integrate feedback about their personal biases, allowing them to avoid future discriminatory and biased actions. Moreover, older adults may be more defensive in the face of feedback if they are less willing to change well-entrenched personal attitudes. The current study developed a new measure of feedback defensiveness and investigated whether defensiveness levels increase or decrease with age. In a sample of U.S. White adults ($n = 303$; age range: 19 to 85), we found that individuals reported being more defensive in the face of negative feedback about intergroup biases relative to feedback about other aspects of the self. Correlational analyses found that these biases were weaker among individuals who reported a stronger sense of personal identity. Critically, defensiveness against intergroup biases was initially unassociated with age, but when we consider individuals who avoid identity information, there is a modest age effect. This data challenges the assumption that all older adults would be defensive to feedback about their bias and considers the role of one's sense of identity. These findings have clear implications for how clinicians, practitioners, and researchers should focus their efforts to reduce discriminatory events by alerting them to which individuals may be more willing to accept feedback about their biases.

Abstract citation ID: igad104.3798

### DOES RURAL LIVING INCREASE RISK FOR MORTALITY? FINDINGS FROM TWO LONGITUDINAL STUDIES

Olivia Atherton, *University of Houston, Houston, Texas, United States*

Most prior work on rural-urban health disparities has been conducted with population-level data, which is limited in its capacity for causal inferences about individuals and lifespan health. The present study leverages individual-level longitudinal data, spanning up to 29 years, to understand how rurality-urbanicity predicts individuals' risk for all-cause mortality; whether these effects hold above and beyond socioeconomic status; and whether the association between rurality-urbanicity and mortality risk varies by sex, socioeconomic status, race, and partner status. The present pre-registered study uses data from two large longitudinal studies of U.S. Americans (HRS and MIDUS; total N = ~55,000), who reported on their socio-demographic characteristics, had their addresses linked to geographical indicators (i.e., rural-urban continuum codes), and have data from the National Death Index regarding vital status and survival time. Using a series of cox proportional hazards regression models, findings showed that suburban and rural residents were at a 12% and 18% greater risk for earlier all-cause mortality compared to urban residents in HRS, but the effects of rurality-urbanicity on mortality risk were nonsignificant in MIDUS. The longitudinal effects of rurality-urbanicity on mortality risk were largely independent of the effects of socioeconomic status. Finally, the three statistically significant interaction effects suggest that the strength and direction of the association between rurality-urbanicity and mortality risk differed by race (HRS, MIDUS) and partner status (HRS). The discussion elaborates on the theoretical implications for our understanding of social determinants of health, as well as practical implications for health equity in rural areas.

Abstract citation ID: igad104.3799

### THE IMPACT OF 3 GENERATIONS OF VIOLENCE ON THE MENTAL HEALTH OF COLOMBIAN OLDER ADULTS: COMPARATIVE MULTI-SITE SURVEY

Mark Gabbay[1], Maria Isabel Zuluaga[2], gabriel Saldarriaga-ruiz[2], Erika Montoya[2], and Clarissa Giebel[1], *1. University of Liverpool, Liverpool, England, United Kingdom*, *2. Universidad de Antioquia, Medellin, Antioquia, Colombia*

Endemic violence across Colombia for 70 years has displaced generations of the poorest among Colombian society particularly in (semi)rural and peri-urban settings, approximately (10m- 20% total population). In 2022, 12.5% (1.2m) were older adults living with the health and socioeconomic consequences, losses and life-changing disruptions. Our joint Colombian UK-funded project in Turbo explores ways to co-produce and pilot an intervention to help mitigate impacts older-adults' mental-health and wellbeing. This survey explores their burden of ill-health and adversity. We compared our 2022 older-adults survey in Turbo ( n=611) with 2016 (n=~0.5m) survey findings from three other Colombian regions with different exposures to 'La Violencia'. Both surveys measured Cognitive status, Functionality, Autonomy, Depression, Anxiety, Alcohol-use, Well-being, Social support, Loneliness, Nutrition and Maltreatment plus demographics and used probability cluster two-stage sampling to enhance representativeness. Mental health is not a concept used by older adults, 'depression' (5-28%) and 'anxiety' (4-21%) were lower than anticipated. An unexpectedly high proportion in Turbo respondents had at least some cognitive deficit (62%), much higher than elsewhere (1.3-5.1%). Most respondents were living in significant poverty with low educational attainment, low or minimal income, low nutrition, poor housing and social isolation. Reported elder abuse rates ranged from 4-30% across sites. The survey findings confirm high rates of vulnerability among older adults living with the severe consequences of 'La Violencia' upon mental-health. Our project co-producing and delivering affordable and sustainable interventions, seeks to improve mental health and wellbeing, self-esteem and belonging, within a society that largely neglects older adults.

Abstract citation ID: igad104.3800

### SOCIAL DETERMINANTS OF BRIDGE EMPLOYMENT: DO BIRTH COHORTS MATTER?

Gayoung Choi, and Haena Lee, *Sungkyunkwan University, Seoul, Republic of Korea*

A growing number of retirees now engage in bridge employment, a term referring to their continued participation in the workforce post-retirement. The generation in South Korea born between 1945 and 1955, a period marked by leading industrialization and experiencing economic crises during their working age, exhibits unique socioeconomic characteristics distinct from later generations. Consequently, their retirement trajectories diverge significantly from those of later cohorts, including the Baby Boomer generation (born between 1956 and 1965). In this paper, we investigate whether social, demographic, psychological, and health factors are associated with bridge employment and whether this association differs across birth cohorts. Data were drawn from seven waves of the Korean Longitudinal Study of Aging, conducted between 2008 and 2020. We employ an integrative framework that considers socioeconomic status, health status, and psychological factors as the main variables to explain the odds of continuing to work after retirement. Retirement status includes three categories: employed, bridge, and total retirement. Results from a multinomial logit model reveal birth cohort differences in the relationship between social/psychological predictors and bridge employment. Additionally, higher educational attainment, a private pension, and poor mental health are associated with decisions regarding bridge employment for the industrialization generation, but not for Baby Boomers. Furthermore, females are more likely to participate in bridge employment than males for the industrialization generation, but not for Baby Boomers. Our findings contribute to the current literature on bridge employment by identifying the social determinants of bridge employment and highlighting the importance of cohort differences in bridge employment.

### SESSION 9580 (PAPER)

### LATE BREAKING: MOTIVATING PATIENTS FOR HEALTH BEHAVIOR CHANGE

Abstract citation ID: igad104.3801

### FACTORS ASSOCIATED WITH A FOUR-YEAR SARCOPENIA TRANSITION IN CHINESE OLDER ADULTS

Shujing Lin, Lingxiao He, Jinzhu Yang, and Ya Fang, *Xiamen University, Xiamen, Fujian, China (People's Republic)*

**Background:** Previous studies have reported a dynamic change of sarcopenia status while factors associated with such status changes remained unexplored. This study aimed to identify factors associated with sarcopenia status transitions based on Chinese older adults. **Methods:** 6065 older adults (aged 66.8±6.2) with complete data at baseline and at least one follow-up (2 years) were included from the China Health and Retirement Longitudinal Study (CHARLS). Sarcopenia was determined based on AWGS2019 criteria. Multistage Markov model was used to explore potential sociodemographic, behavioral, psychological and disease-related factors associated with sarcopenia transitions. **Results:** At baseline, 59.1% of participants were non-sarcopenic, 24.7% had probable sarcopenia and 16.2% were diagnosed with sarcopenia. Participants with non-probable sarcopenia had 1-year transition probability of developing probable sarcopenia (0.101) or sarcopenia (0.056). Overweight (HR=1.89, 95%CI: 1.55-2.30), obesity (HR=1.85, 95%CI:1.37-2.48) and presence of chronic diseases (HR=1.26, 95%CI: 1.03-1.56) increased the risk of progression from non-sarcopenia to probable sarcopenia. Older age (HR=1.10, 95%CI:1.08-1.13), female sex (HR=1.38, 95%CI: 1.08-1.75), shorter sleep duration (HR=1.42, 95%CI:1.12-1.80), depression (HR=1.27, 95%CI:1.01-1.59) and presence of chronic diseases (HR=1.35, 95%CI: 1.05-1.74) were associated with transition towards sarcopenia from non-sarcopenia. Higher cognitive function (HR=1.03, 95CI:1.01-1.05), rural dwelling(HR=1.25, 95%CI: 1.00-1.55) and alcohol consumption (HR=1.41, 95%CI:1.15-1.73) were associated with reversed status from probable sarcopenia to non-sarcopenia. Higher cognitive function increased the conversion from sarcopenia to probable sarcopenia (HR=1.21, 95%CI:1.06-1.37) and non-sarcopenia (HR=1.03, 95%CI:1.00-1.06) . **Conclusions:** Sarcopenia is a dynamic condition in older adults. Possible interventions should focus on improving cognitive functions, sleep quality and better controlling on chronic conditions.

Abstract citation ID: igad104.3802

### RCT OF A BEHAVIORAL, TELEHEALTH WELLNESS PROMOTION PROGRAM FOR MIDDLE-AGED ADULTS WITH DISABILITIES

Ivan Molton[1], Donovan Rivera[1], Katie Singsank[2], Aaron Flaster[1], Leah Munroe[3], and Andrew Humbert[1], *1. University of Washington, Seattle, Washington, United States*, *2. University of Nevada, Las Vegas, Las Vegas, Nevada, United States*, *3. Louisiana State University, Baton Rouge, Louisiana, United States*

For people aging with physical disabilities, there is a striking need for evidence-based approaches to promote positive health behaviors and to support active community participation. This is especially true in midlife, which is a period of significant health and social vulnerability for this population. This study describes a 3-arm, randomized controlled trial of a telehealth, motivational-interviewing based health promotion program (EnhanceWellness, EW) adapted for people with physical disability. 505 individuals with long-term physical disability were randomized to 8 sessions of individual EW coaching over 6 months, or to one of two control conditions (health education or treatment as usual). Primary outcomes included self-report of ability to participate in social activities, satisfaction with community participation, and mood, using validated NIH PROMIS scales. Participants completed assessments before randomization, at 7 and 12 months post-randomization. Statistical analysis included linear mixed-effects modeling with random intercepts. Participants were on average 56 years old (SD=5.6), were mostly women (77%) and had lived with a medical condition causing ADL impairment (disability) for an average of 23 years. Results demonstrated significant group by time interaction terms (p's < .01), underlying statistically significant improvements in the EW group in both ability to participate and satisfaction with community participation, relative to either control condition. These changes tended to increase or maintain over time. There were no significant intervention effects on mental health outcomes. Results emphasize the applicability of individualized health behavior change coaching in supporting community participation among adults aging with disability.

Abstract citation ID: igad104.3803

### RELATIVE AND ABSOLUTE METABOLIC COST OF DAILY PHYSICAL ACTIVITIES ACROSS THE LIFESPAN

Todd Manini[1], Eileen Handberg[1], Mamoun Mardini[1], Bhanu Sandesara[1], Ramon Casanova[2], Duane Corbett[3], and Amal Wanigatunga[4], *1. University of Florida, Gainesville, Florida, United States*, *2. Wake Forest University, Winston Salem, North Carolina, United States*, *3. Axogen, Gainesville, Florida, United States*, *4. Johns Hopkins School of Public Health, Baltimore, Maryland, United States*

Americans spend over 90% of their activity related energy expenditure performing common daily activities. However, there is a gap in knowledge pertaining to potential age-related differences in the metabolic cost of performing these activities which diminishes proper physical activity prescription. We hypothesized age differences in both the absolute and relative metabolic cost of performing common daily physical. Participants (n=248, age: 20-94 yrs) performed 33 activities (e.g. dressing, walking, making a bed, gardening, computer work) in a standardized setting. A portable indirect calorimeter was worn for approximately 6-10 minutes to achieve steady-state metabolic rate. A graded exercise treadmill test was performed to quantify peak metabolic rate. A metabolic equivalent (MET as a function of 3.5 milliliter•min-1•kg-1) and percentage of peak were used to express absolute and relative metabolic rate, respectively. Watching television (1.0+/-0.36 METs; 15.6+/-5.7% of peak) produced the lowest and climbing stairs (6.2+/-2.2 METs; 84.8+/-17.0% of peak) elicited the highest metabolic rate. Results from mixed effect regression models adjusting for health conditions, demographics and anthropometry showed no association between absolute MET and age. However, when expressed relative to peak, adjusted models showed a strong association with age (0.66+/-0.33% per year, p=0.006). As an example, adults < 40 yrs old worked at 34.1+/-14.8% of peak during a leisurely walk while those 80+ years old worked at 64.2+/-16.3%. Aging is associated with a substantial increase in the relative metabolic cost of performing common daily physical activities. These results could have implications on prescribing physical activity older adults to achieve higher fitness.

Abstract citation ID: igad104.3804

**UNDERSTANDING OLDER ADULTS' MOTIVATIONS TO ADOPT DIGITAL HEALTH PORTALS**

Mimi Trinh, Varitnan Hattakitjamroen, and Wendy Rogers, *University of Illinois--Urbana-Champaign, Champaign, Illinois, United States*

Older adults are likely to develop chronic illnesses (e.g., hypertension or high cholesterol), resulting in the need to manage their symptoms. Self-management often involves tracking symptoms, medication taking, nutrition, or exercise. Digital health portals (DHPs) have the potential to fulfill these tracking needs by viewing records of symptoms, doctor's notes relating to diet or exercise, and reviewing medication regimens. However, DHPs are often not used to their full potential. The goal of this study was to understand what motivates older adults to utilize their DHPs, and what features are needed for health self-management. We conducted a qualitative study consisting of surveys and semi-structured interviews to elicit the thoughts of 26 older participants. Following Self Determination Theory and Unified Theory of Acceptance and Use of Technology Integrated Model, we organized their responses into motivation factors of autonomy, competence, and relatedness as well as facilitators such as perceived usefulness, perceived ease of use, social influence, and facilitating conditions. Using this integrated analysis approach, we identified themes related to health portal experience, motivation, barriers and facilitators, health provider support, and ideal design. These themes provide insights into how the DHPs were used and perceived; why older adults use DHPs; and their overall positive experiences using them. The participants generally found the DHPs to be convenient due to features related to self-management. They also shared their core needs and what could be improved for a better and easier experience. The Integrated Model helps in understanding the foundation needed to motivate the acceptance of DHPs.

### Participant Index

Arabic numbers refer to session numbers.
Duplicate numbers indicate the participant is listed as an author on multiple abstracts within a session.

**A**

Aamponsah, Abigail: 7540
Aarts, Sil: 1260, 7800
Aartsen, Marja J.: 1220
A'Azman, Shamirah: 4025
Abadir, Peter M. M.: 1060, 9000, 9020
Abaei, Elnaz: 2170
Abbas, Jihan: 9030
Abbott, Katherine M.: 4435
Abboud, Anissa: 3360, 4010
Abdallah, Lisa: 1080, 5200
Abdel Magid, Hoda S.: 1015
Abe, Takaaki: 7370, 9010
Abebe, Ephrem: 7080
Abedi, Ali: 7680
Abedine, Reuben: 9320
Abel, Erica A.: 9030
Abelson, James: 9020
Abelson, Jamie: 9020
Abi Chahine, Maya: 9500
Abner, Erin L.: 9020
Abraham, Alison G.: 2240, 2365
Abrahamse, Paul: 1085
Abrams, Leah: 3225, 3440, 9030
Abramson, Tobi A.: 7760
Abrantes, Ana M.: 1030
Abreu, Taiane: 7060
Abshire Saylor, Martha: 1035, 3415, 4170
Abudurezake, Abulaiti: 9000
Abujarad, Fuad: 2085
Acevedo-Fontanez, Adrianna I.: 9020
Acharya, Sandeep: 9010, 9020
Acierno, Ron: 7010
Adachi, Jonathan: 4268, 7220, 7680, 9020
Adamek, Margaret E.: 2010, 7190, 7200
Adams, Anne: 9320
Adams, Hannah: 7510
Adams, Swann Arp: 4005
Adams-Price, Carolyn E.: 1135, 2040, 3425
Adar, Sara: 7320
Addison, Odessa: 7340, 7350, 7840
Addo-Mensah, Dorothy: 4380
Adekoya, Adebusola: 1165
Adekunbi, Daniel: 9000
Adeleye, Khadijat: 9010
Adelman, Ronald: 7360
Adelnia, Fatemeh: 9010
Adeniji, Dolapo O.: 7190, 7200
Adepoju, Omolola E.: 2085
Adesanya, Ikmat: 4040
Adeyemi, Oluwaseun: 7130, 7840
Adjei, Naomi: 4045
Adkins-Jackson, Paris B.: 1345, 2225
Adler, Charles: 9030
Adonis-Rizzo, Marie T.: 4132, 7480
Aflagah, Erica: 7510
Ågård, Pernilla: 4330
Agarwal, Arunika: 7940
Agboji, Aderonke: 7160
Agha, Agha A.: 4130, 5110
Aghaali, Elham: 7660
Agmon, Maayan: 3125, 4265
Agran, Dylan: 4406, 7000
Agrawal, Yuri: 2150, 3390
Agrigoroaei, Stefan: 7960
Agrimson, Kellie: 8900
Aguila Vega, Emma: 9030
Aguilar, Iris: 7700
Aguirre, Alyssa: 5235
Aguirre, Annabella: 3155
Agyapong, Nakesha: 9030
Agyarko Poku, Thomas: 7790
Agyemang, Lillian: 7220, 9010
Ahmad, Nina: 2035
Ahmad, Suzan: 4105
Ahmad, Zeba: 9030
Ahmed, Adeeb: 3295
Ahmed, Ali: 3490
Ahmed, Neelam H.: 4025
Ahmed, Yusuf K.: 9000
Ahn, Eunhye: 7960
Ahn, Hyochol: 7460
Ahn, Jinhyun: 9030
Ahn, SangNam: 7960
Aho, Velma T E.: 1225
Ahrens, Monica: 4545, 9020
Ai, Amy L.: 2280, 7410
Ai, Meishan: 7100
Aichele, Stephen R.: 4185
Ailey, Sarah: 3445
Ailshire, Jennifer A.: 1125, 1165, 2050, 2175, 3160, 3465, 4395, 5050, 7380, 7540, 7720, 7890, 9020, 9310
Ainsworth, Laura A.: 9020
Aisagbonhi, Omonigho: 8900
Aitken, Rebecca: 1130
Ajja, Rahma: 3505, 4350
Ajrouch, Kristine J.: 1355, 2100, 3440, 4285, 9210
Ajtai, Bela: 7830
Akagi, Yuya: 4505, 9000
Akbary Moghaddam, Vaha: 9020
Akgün, Kathleen A.: 9030
Akiguchi, Ichiro: 9010
Akinbami, Yewande: 9000
Akintola, Kehinde G.: 9010, 9310
Akinwuntan, Abiodun: 9330
Akksilp, Katika: 3440
Akter, Nahida: 3020, 7160
Akushevich, Igor: 2015, 2180, 3470, 3510, 5015, 5110, 7100, 7370, 7620
Al Snih, Soham: 9030
Alahmari, Saeed: 7100
Alaie, Iman: 9030
Alain, Gabriel N.: 2260
Alam, Rifat B.: 7300
Alavi, Mohammad Hesam: 4265
Al-Azawi, Abdulwahab: 7160
Albala, Cecilia: 3465
Albers, Elizabeth A.: 2130, 3305, 7380, 7420
Albers, Hannah: 1285
Albert, Steven M.: 2190, 3530, 9020, 9030
Alberth, Andrew: 3385, 7610
Albertini, Marco: 9030
Alberts, Susan: 1195
Albright, Amy: 7910
Albright, Karen: 7300
Alcantar, Sandy: 1135
Alchin, Tucker: 7800
AlDossary, Heba: 7590
Aldrich, Vicky: 2300
Aldridge, Melissa D.: 2235, 3335
Aldwin, Carolyn M.: 1160, 1390, 7510, 7580
Aleksic, Sandra: 1125
Alexander, Gregory L.: 3175, 9020
Alexander, Ivana: 3360
Alexander, Karah: 7620
Alexander, Karen J.: 2315
Alexander, Laura: 7110
Alexander-Bady, Janae: 7840
Alfares, Hadil: 9010
Alfaro, Ana J.: 3250
Alfaro, Ana J.: 9010
AlGhatrif, Majd: 9020
Ali, Imran: 4165
Ali, Mohab: 7390
Ali, Sanna: 9030
Ali, Sumbleen: 1255
Ali, Talha: 3360, 4010
Ali Morshed, Hanan: 3170
Alkabbani, Wajd: 4280
Allala, Sai Chaithra: 4415
Allan, Alexa C.: 7560, 9020
Allard, Eric S.: 7040, 9000, 9020
Allemand, Mathias: 7060
Allen, Julie Ober: 1285, 9020
Allen, Katy: 9010
Allen, Kelli D.: 4165
Allen, Laura D.: 2160, 9000
Allen, Odell: 3560
Allen, Rebecca S.: 1365, 7390, 7910, 9210
Allen, Tammy: 9010
Allison, David: 9020
Allore, Heather: 1305, 2360, 3230, 7300, 7360, 7400, 7450, 7540, 7890, 7990
Allsopp, Richard: 2380, 5060, 7110
Almardawi, Ranyah: 7130, 9000
Almeida, David M.: 1360, 3135, 3230, 3545, 4425, 4475, 5045, 5080, 7050, 7390, 7610, 9020
Almeida, Paulo: 7320
Almidani, Louay: 1345, 2135, 9030

© The Author(s) 2023. Published by Oxford University Press on behalf of The Gerontological Society of America.
This is an Open Access article distributed under the terms of the Creative Commons Attribution License (https://creativecommons.org/licenses/by/4.0/), which permits unrestricted reuse, distribution, and reproduction in any medium, provided the original work is properly cited.

Almiron, Maby: 7380
Alston, Daniel M.: 3350
Altenburger, Peter: 7060
Altintas Akin, Ozlem: 9020
Altona, Janissa: 2370
Alvarez, Bianca: 7500
Alvarez, Elizabeth: 5085
Alvarez, Loreli: 3545, 4145, 7090
Alvarez Azanedo, Gabriela: 7110
Alvear Portaccio, Marcela: 3465
Alwan, Elizabeth: 9020
Alwerdt, Jessie: 5165, 9030
Alyssa, Camp: 3425
Alzahrani, Mallak: 5230
Amanambu, Peace: 9000
Amano, Takashi: 3310, 4210, 4266, 7690, 7990, 9000, 9030
Amaza, Hannatu: 9000
Ambike, Satyajit: 7060
Ambroise, Alexis Z.: 1070
Ambrose, Madison: 3300
Ameer, Fathima: 7370
Amgalan, Anar: 9020, 9030
Amin, Iftekhar: 3205
Amini, Reza: 7280
Aminu, Abodunrin: 4455
Amir, Omar: 7860
Amjad, Halima: 2185
An, Jaesung: 7610, 7920
An, Jeongwi: 7030, 7570
An, Ping: 9020
An, Qiuling: 9030
An, Yang: 4035
Anable Eme, Vivian: 4460
Ananthasubramaniam, Aparna: 5010
Ances, Beau: 9030
Anctil, Reid: 9030
Andel, Ross: 3050
Andel, Ross: 7020, 7420, 7990, 9000
Andersen, Julie K.: 2335
Andersen, Stacy L.: 1040, 5180, 7830
Andersen, Troy C.: 1150
Anderson, Aiden: 7100
Anderson, Andrea M.: 7630
Anderson, Brett E.: 7020
Anderson, Erik: 7680
Anderson, Keith A.: 1095, 2130, 9000, 9010
Anderson, Loretta: 1085, 5205
Anderson, Rozalyn: 5060
Anderson, Ruth: 3540, 4380
Anderson, Taharka: 9000
Anderson-Hanley, Cay: 3480
Andersson, Bjoern: 3490
Andrade, Allen: 4266, 7190
Andrade, Flavia: 3050
Andrasfay, Theresa: 3065, 7890, 9030
Andreoletti, Carrie: 1080
Andrew, Melissa: 7650
Andrew, Simon: 2315
Andrews, Ebony: 9010
Andrews, Heather: 9030
Ang, Alvin: 7100
Ang, Gregory: 4385
Angel, Jacqueline L.: 9030
Anghel, Ionut: 1220
Anilkumar Hemalatha, Sruthi: 7200, 7940
Anim-Koranteng, Comfort: 7330
Anisa Simoni, Anisa: 4300
Ankuda, Claire K.: 3575
Anosike, Chioma A.: 2125
Anquillare, Elizabeth: 7410
Ansaldo, Ana Inés: 7680
Anstey, Kaarin J.: 3570, 9000, 9110
Antequera, Felipe: 9030
Antoan, Jordann: 7360
Antonakakis,, Antigone: 9000
Antonietti, Michael: 7600
Antonio-Villa, Neftali E.: 3160, 4395
Antonoplis, Stephen: 4300
Antonsdottir, Inga M.: 4430
Antonucci, Toni C.: 1255, 2100, 4285, 4325
Apgar, Dawn: 5170
Apiribu, Felix: 7790
Appiah, Abigail Acheampomaa: 7790
Appiah-Kusi, Evans: 7700
Applebaum, Allison J.: 4030
Applebaum, Jennifer W.: 9020
Applebaum, Robert A.: 1115, 2200, 3190, 4435
Appleby, James: 3055
Arabi, Hana: 9500
Arain, Salman: 7840
Aranda, Maria P.: 7700, 7720, 9020
Aratia, Shahrose: 9330
Araujo, Lia: 3130, 7060, 7200
Arbeev, Konstantin: 2015, 7110, 7370, 7800
Arcila-Mesa, Mauricio: 7130, 7840
Ardekani, Azam: 7140
Ardelt, Monika: 1390
Ardisson Korat, Andres Victor: 1290
Arellanes, Isabella C.: 4395
Arensberg, Mary Beth: 9020
Arevalo-Flechas, Lyda: 7180, 9010
Arishenkoff, Shane: 7820
Armah, Henrietta: 7020
Armendariz, Marina: 5130
Armistead, Lori: 7150
Armstrong, Alissa: 1325
Armstrong, Cheryl: 7480
Armstrong, Grace: 4525
Armstrong, Joshua J.: 1280
Arnette, Shannon: 3300
Arnold, Michelle: 1295, 4110, 7960
Arora, Kanika: 7760
Arora, Neeraj: 9000
Arpino, Bruno: 4530
Arriola, Nora B.: 7150
Arthanat, Sajay: 9010, 9550
Artyomov, Maxim: 4065
Aruah, Simeon Chinedu: 4130
Aruscavage, Nancy: 1150, 2035, 9020
Asakura, Kyoko: 9010
Asante, Samuel: 3035, 7480
Ash, Samson: 5235, 7030
Ashar, Perisa: 9020
Ashcraft, Alyce S.: 7160
Ashe, Maureen: 3275, 7550, 9000
Ashebir, Sophia: 7910, 9000, 9010
Asher, Irit: 7720
Asher, Merav: 3125
Ashida, Sato: 2120, 3445, 3540, 7440, 7470
Ashiqueali, Sarah A.: 4475, 7110
Ashirifi, Gifty D.: 7190, 7200
Ashrafi, Sadia Anjum: 7610
Asiimwe, Stephen: 9000
Ask, Levi: 7170
Asquith-Heinz, Zayla: 2120, 3015
Assi, Sahar: 2150
Aswani, Truphosa: 3240
Atchison, Karley: 7580
Atherton, Olivia E.: 4300, 9570
Atkinson, Elizabeth: 1325
Attuquayefio, Tuki: 3570
Atul Patil, Vaishnavi: 9000
Au, Rhoda: 1040, 7100, 9010
Audley, John: 9000
August, Kristin J.: 5055
augustine, Matthew: 9000
Austad, Steven: 1060, 3575, 9020
Austin, Thomas: 7110
Au-Yeung, Wan-Tai M.: 4495
Avallone, Margaret A.: 1130, 7290
Avent, Elizabeth S.: 2220
Avigezer, Avital: 2345
Avila, Jaqueline C.: 3160, 7320
Avis, Nancy E.: 5155
Awad, Kamal: 7370
Awata, Shuichi: 7280
Aworinde, Tofunmi: 4470
Axelrad, Hila: 7290
Ayalon, Liat: 1245, 2105, 3425
Ayele, Roman A.: 7300
Ayieko, Sylvia: 7610
Ayisi-Boahene, Givenchy: 5030, 7120
Ayoob, Carol: 4215
Ayrat, Ansar: 9030
Azar, Martina: 7770
Azhar, Gohar: 1085, 4130, 7100, 7370, 7880, 9000, 9020
Azizi, Linda: 8900
Azizi, Mehrnoosh: 1200
Azuero, Andres: 4340, 7680

B

Baarbé, Julianne: 4280
Babar, Huma: 4030
Babazadeh, Romina: 7420
Babbs, Gray: 4000
Babulal, Ganesh: 9030, 9330
Baby, Eliza: 1260
Bachman, Noa: 3275
Bachner, Yaacov G.: 7920, 9030
Bachrach, Rachel L.: 7580
Backhaus, Ramona: 9010
Backman, Annica: 3180
Bacon, Suzette Mizutani: 1310
Bacsu, Juanita-Dawne: 1200, 9000
Badawi, Sarah: 7770
Baek, Jihye: 7550, 9030
Baek, Jiyeon: 9000
Baek, Sang Yi: 7850
Baek, Sehyun: 9010, 9030
Bagaajav, Ariunsanaa: 7380
Bagley, Olivia: 7800
Bahnson, Judy L.: 4355
Bai, Xue: 1130, 2230, 4020, 4520, 7040, 7090, 7250
Baier, Rosa: 4190, 4290, 7540, 7640
Baig, Farah: 3460, 7250
Baik, Dawon: 7400, 7470
Baik, Sol: 5025, 7530

Bailey, Amelia: 9030
Bailey, Dionne: 1065, 3300
Bailey, Donald E.: 3540
Bailey-Arafah, Micah: 7630
Baillargeon, Emma M.: 2155
Baim-Lance, Abigail: 1375, 7820, 9000
Bajdek, Nicole: 3480, 9000
Bakas, Tamilyn: 7850
Bakeman, Roger: 5015
Baker, Jillian: 9010
Baker, Nathan: 9000
Baker, Tamara A.: 2115
Baker, Zachary G.: 1025, 3460, 9000
Bakerjian, Deb: 3005
Bakitas, Marie: 9020
Bakk, Louanne: 7220, 7700, 9010
Baksh, Magda: 7620
Bakulski, Kelly M.: 7370
Bakx, Pieter: 7120
Balachandran, Sowmya: 2315
Balantrapu, Swathi: 9010
Balasubramanian, Aparna: 7650
Baldivieso, Valeria C.: 7620
Baldwin, Julie: 7400
Baldyga, Kathryn: 9030
Balestra, Dennys P.: 2280
Balkan, Em: 4000
Ball, Karlene K.: 7060, 7990
Ballard, Erin: 3050
Ballard, Stephanie A.: 7790
Ballinger, Tarah: 7410
Ballout, Suha: 9510
Ballreich, Jeromie: 5160
Balmert Bonner, Lauren: 4310
Balmuth, Alexa: 1175, 3090, 9000
Baloni, Priyanka: 5180, 7880
Bamgbade, Benita A.: 4134, 7830
Bamonti, Patricia M.: 4095, 7340
Ban, Si Woo: 9540
Banarjee, Chitra: 5075, 7150, 7820, 9030
Banarjee, Reema: 5060, 7880
Banaszak-Holl, Jane: 2350
Bandeen-Roche, Karen: 1110, 1370, 3240, 4485, 9010
Bandosz, Piotr: 2255
Band-Winterstein, Tova: 4520
Banerjee, Sube: 3335
Bangen, Katherine: 8900
Bangerter, Lauren: 3030, 5000, 9000, 9010
Bankole, Ayomide Okanlawon: 4380
Bankole, Ayomide Okanlawon: 9000
Banks, Jack: 7840
Banks-Wright, Hortense: 9000
Banner, Davina: 9030
Banner-Lukaris, Davina: 5105
Bannon, Sarah: 2035, 4430
Bansal, Deven M.: 5065
Banskota, Nirad: 2195
Bao, Dapeng: 1260
Barajas, Jordan: 7100
Baranyi, Gergő: 1015
Barbara, Rita: 7340, 9000
Barbee, Harry: 4000
Barbour, Elizabeth: 7970
Barclay, Ruth: 7940
Bardales, Rhonda: 7280, 9020
Bardo, Anthony R.: 5080
Bareham, Bethany K.: 4540, 9010
Barela, Lance: 7900
Barger, Kathryn: 1290
Barkley, Morgan: 9020
Barlow, Meaghan A.: 7040
Barman, Papai: 7940
Barnard, Ryan T.: 7630
Barnason, Susan A.: 9010
Barnes, Deborah E.: 1010, 2035, 3490, 4035
Barnes, Haley: 3500
Barnes, Lisa L.: 2120, 2225, 4100
Barnes, Stacy: 7310
Barnes, Virgile P.: 3365, 7260
Barnett, Brianna: 7050, 7910, 7960
Barnett, Michael D.: 7080, 7590, 7900
Barr, Paul: 9010
Barrón, Yolanda: 9010
Barragan, Noel: 7700
Barreto, Esteban A.: 4430
Barrett, Anne E.: 4230, 7060, 7070, 7570, 7590, 7600
Barrett, Blake: 7150
Barrett, Tyler: 1010
Barrie, Helen: 3260
Barry, Kathryn H.: 7340
Barry, Lisa C.: 3525, 4115, 7030
Barry-Dutro, Erin: 5025
Bartel, Maya: 7830
Bartke, Andrzej: 4475, 9020
Barve, Shirish: 9000
Barzilai, Nir: 1125
Basaran Sahin, Duygu: 3080, 4175
Bashian, Hannah M.: 9010
Basic, Ajla: 3035, 5125, 9000
Basile-Alvarez, Martin Roberto: 4395
Basinger, Erin D.: 1235
Basinska, Kornelia: 7160
Basisty, Nathan: 1325, 5060, 7880
Basrai, Zavera K.: 9530
Bass, David M.: 9020
Bastow, Skyler: 7590
Basu, Anirban: 1115
Basu, Koushambhi: 1335
Basu, Rashmita: 7760
Batchelor, Melissa: 7300
Bate, Angela: 2375
Bateman, James: 1320
Batista-Malat, Eleanor: 4375, 7720
Batsis, John A.: 4268, 7360, 7680, 9000
Battaglia, Catherine: 7300
Battar, Saraswathy S.: 3110
Battle, Brian: 9000
Bauguess, Caleb: 4350
Bauldry, Shawn: 4535, 7920
Bauman, Alexandra: 7590
Baumann, Sara: 9020
Baumbusch, Jennifer: 1335
Baumgart, Matthew: 1265, 3020, 7420
Baumgartner, Lisa M.: 7190
Baur, Joseph: 2205, 9020
Bautista, Kirsten: 9030
Bautmans, Ivan: 1125
Baxter, Pamela: 1145
Bayer, Thomas: 7620
Bayles, Gregory N.: 7700
Bayne, Alycia: 1065, 3075
Bazurto, Elizabeth: 9420
Beach, Brian: 3265, 3370, 4015
Beach, Scott: 7530
Beach, Thomas G.: 9030
Beachy, Tim: 3540
Beadle, Janelle N.: 4045
Beam, Christopher R.: 7370, 7540
Beamer, Brock: 7330
Bean, Jonathan F.: 9010
Beard, John: 1110
Bearden, Valerie: 9030
Beattie, Zach: 4100, 4495
Beatty, Jennifer F.: 9570
Beaty, Carissa: 3055
Beauchamp, Jennifer: 9010
Beauchamp, Marla: 4268, 5070, 7170, 7680, 7780
Beaudoin, Kayla D.: 5100
Beaudreau, Sherry A.: 4300
Beaulaurier, Richard L.: 9030
Beaulen, Audrey: 9010
Beck, Emorie D.: 1255
Beck, McKenzie: 7960
Beck, Monica L.: 3140
Becker, Clemens: 3550
Becker, David: 3365, 4190
Becker, Todd D.: 2020, 5015, 5025
Becker, Todd D.: 9010, 9100
Bedard, Michel: 3570, 7060
Beeber, Anna S.: 2290, 4025, 4280, 4380
Beech, Bettina M.: 3450
Beer, Jenay M.: 9320
Beetle, Christopher: 7620
Begum, Momotaz: 9010, 9550
Behal, Anil: 9020
Behar-Zusman, Victoria: 4340
Behrens, Liza: 3395, 5030, 5145
Beitelshees, Amber: 4080
Béland, François: 5220
Belanger, Emmanuelle: 7620
Belchior, Patricia: 7620
Beld, Matthew: 9020
Belfi, Amy: 4045
Belin de Chantemele, Eric: 9030
Bell, Gregg: 3005
Bell, Janice: 7280, 7750
Bell, Julia: 9000, 9020
Bell, Justice: 1325
Bell, Kristina: 9010
Bell, Mallory J.: 7600, 9010
Bell, Sue Anne: 2295
Bell, Tyler R.: 7060
Bellettiere, John: 3390
Belli, Hayley: 7230
Bellizzi, Gabriela: 7920
Bello-Chavolla, Omar Yaxmehen: 3160, 4395
Belsky, Daniel W.: 1110, 1320, 2285
Beltran, Jazmin: 7160
Beltran, Susanny: 4510, 7900, 9030
Belza, Basia: 7830, 7910, 9010
Ben Abdesslem, Amel: 7210
Ben Eliezer, Aya: 7720
Ben-David, Boaz M.: 9420
Benden, Mark E.: 7430

Bender, Alexis A.: 1375, 3285, 3565, 9030
Bender, Anna E.: 4525, 7590
Ben-Ezra, Menachem: 3275
Benjamin, Cynthia: 2035
Benjamin, Susan: 7660
Benjamini, Dan: 3495
Benjasirisan, Chitchanok: 7770, 7840, 7960
Bennett, Jeanette: 1320
Bennett, Jenil: 9010
Benoit, Ellen: 5115
Bensimon, Moshe: 1140
Benson, Clark: 7360
Benson, Jacquelyn J.: 4415
Benson, Lizbeth: 5165
Bentley, Megan: 2085
Bento, Maria José: 7320
Benton, Donna: 4420, 5120, 7700
Benzel, Heather: 9020, 9520
Beran, Krista M.: 7700
Berbakov, Maria E.: 9030
Berbaum, Michael: 4465
Berceanu, Denisa Cristina-Alina: 7960
Berg, Karina: 2300
Berg, Kristen A.: 1170
Bergeman, Cindy S.: 1070, 1075, 3245
Bergen, Gwen S.: 9010, 9100
Bergeron, Caroline D.: 1200, 7410
Bergh, Marissa S.: 7120
Bergman, Elizabeth J.: 2245, 3350
Bergman, Nathaniel: 9020
Bergren, Stephanie: 4050, 7080
Bergström Af Ekenstam, Amelie: 7040
Bergstrom, Jaclyn: 7820, 8900
Berg-Weger, Marla L.: 2250, 9000, 9030
Berhane, Kiros T.: 1110
Berish, Diane: 1300, 4315, 7160, 7620, 7830
Berkley, Amy S.: 9030
Berkman, Lisa F.: 3225
Berkowsky, Ronald W.: 5065
Berman, Jacquelin: 7760
Bernard, Hannah: 7440
Berndt, Annette: 9000
Bernhardt, Paul: 9000
Bernhardy, Christina: 7230
Berning, Michelle: 3460
Bern-Klug, Mercedes: 1100
Bernstein, Laura E.: 7450
Berridge, Clara: 1280
Berry, Katherine: 1130
Berry, Sarah: 2385
Berta, Whitney: 9550
Bertisch, Suzanne: 4355
Bertogg, Ariane: 1225
Bertola, Laiss: 3160
Besser, Lilah: 3490
Bessette, Lily G.: 9020
Best, Rachel D.: 9020
Best, Ryan: 4185, 9020, 9030
Best, Thomas M.: 5125
Bethell, Jennifer: 4515, 7650
Betley, Wendy: 7310
Bettencourt, Ricki: 9010
Beydoun, May: 9000
Beyene, Kebede: 9030
Beyer, Fiona: 4455
Bhadra, Nivedita: 5180
Bhagianadh, Divya: 1030
Bhanbhro, Sadiq: 4245
Bhandari, Neeraj: 7210
Bhansali, Rebekah: 2290
Bharucha, Haniya: 4405
Bhat, Aarti C.: 5045
Bhatnagar, Neera: 7170
Bhatta, Tirth R.: 1070, 3485, 5010, 9010
Bhattacharjya, Sutanuka: 1120
Bhattacharyya, Kallol Kumar: 7400, 7990
Bhookya, Divya: 1120
Bhowmick, Pallabi: 4020
Bhyra, Nagaveni: 9010
Bibbo, Jessica: 2060, 5195
Bibriescas, Natashia: 3545, 4145
Bieber, Heather: 7180
Bielak, Allison A.: 7080
Bielsten, Therése: 7300
Bier, Nathalie: 7620
Bierman, Alex: 7770
Biggar, Virginia: 2180
Bilalaga, Mariah Malak: 9030
Bilecen, Basak: 3530
Bilgel, Murat: 2195
Billinger, Sandra: 7330
Billings, Pamela: 7160
Binder, Carrie: 3090
Birabwa, Joy C.: 5110
Birchander, ellen: 2245
Birditt, Kira: 4230, 4235, 4425, 5035, 5045, 7390, 7630, 9000
Birkeland, Robyn W.: 3570, 7420
Birmingham, Cheryl L.: 9030
Bischoff, Brian: 9010
Bishop, Alexander: 2390, 7050, 7460, 7910, 7960
Bishop, Christine E.: 1115
Bishop, Kellee: 7590
Bishop, Nicholas J.: 2360, 9020
Bishop, Teddy: 4290
Bishop-Royse, Jessica: 3445
Biss, Renée K.: 4405
Bisset, Elise S.: 9010
Bix, Gregory: 9010
Bizon, Jennifer L.: 1030
Bjarnadottir, Ragnhildur I.: 7860
Bjork, Jonas: 2340
Bjornson, Samantha: 4025
Black, Kathy: 4150
Black, Sheila: 7060
Blackman, Laurie: 9030
Blackmon, Emma: 9030
Blackshear, Chad: 9200
Blackwell, GinaMari: 9330
Blackwell, Terri: 3475, 3500
Blain, Hubert: 3550
Blair, Beth: 3045
Blake Anuskiewicz, Blake: 9010
Blanco, Ana: 3260
Blanco-Arroyo, Nancy Angelica: 2290
Blaskewicz Boron, Julie: 4268, 7020, 7550, 7680
Blaxton, Jessica: 1075
Blazer, III, Dan: 1370
Bleijlevens, Michel H.: 3340
Blench, Karl: 9110
Block, Laura: 7360, 9020
Blondet, Ricardo: 7840
Bloom, David E.: 7940
Bloom, Rachel F.: 2075, 7300
Bloomberg, Mikaela: 2015
Blount, Amber: 7430, 9010
Blow, Fred: 7390
Blozis, Shelley A.: 2290
Bluck, Susan: 1390, 7380, 7580, 7900, 7970
Blues, Elwood: 4410
Blum, Moritz: 7820
Blumen, Helena: 3160, 3480, 9020
Bluth, Karen: 3320
Bluthenthal, Ricky: 2175
Boafo, Jonelle: 2235
Boakye, Alfred: 7290
Boardman, Charles: 7440
Boateng, Augustine CO.: 3485, 4145, 8900
Boaz, Timothy: 9020
Bobitt, Julie: 3075, 7710, 9010
Bobry, Melanie D.: 1335
Bockting, Walter: 9100
Bodenheimer, Lisa: 7290
Bodkin, Stephan: 9020
Bodner, Ehud: 3275
Boehmer, Leigh: 7500
Boer, Bram de: 2370
Boerner, Kathrin: 2245, 3130, 4435
Bogdan, Paul: 9030
Bogdan, Ryan: 3520, 4320, 9020
Boger, Jennifer: 3255, 4245
Bogoniewski, Aleks: 9000, 9010
Bohnsack, Alanna: 7660, 7830
Boivin, Christopher: 8900
Bolkan, Cory: 1365, 3250
Boltz, Marie P.: 1300, 2305, 2370, 3330, 3340, 4315, 5030, 5145, 7160, 7620, 7830
Bonaguidi, Michael: 3070
Bonaparte, Jimeace M.: 9020
Boncella, Katie: 9020
Bondi, Mark W.: 4340
Bonds Johnson, Kalisha: 3535, 4010
Bonifas, Robin: 9030, 9300
Bonner, Alice: 3175
Bonner-Cozad, Aisha: 5040
Bonomo, Melia: 9110
Boockvar, Kenneth: 7890, 9000
Booker, Emma: 7700
Booker, Staja Q.: 7670
Boon, Jeffrey T.: 3395
Boot, Walter R.: 1040, 1315, 2085, 3025, 7080, 9000, 9010, 9030
Booth, Debbie: 4355
Booth, Sarah L.: 1290
Bordes, Piper J.: 2105, 7550
Borella, Erika: 4185
Borgogna, Joanna-Lynn: 4080
Boris, Lisa M.: 2030
Borrelli, Belinda: 9030
Borrero, Lisa: 2080, 3350, 3430
Borson, Soo: 1265, 7230
Borth, Laura: 1290
Bós, Angelo Jose G.: 3140
Bosak, Emily: 7800
Boscardin, John: 2300
Bose Brill, Seuli: 3345
Bosma, Hans: 1225
Botoseneanu, Anda: 2360, 7300, 7450, 7890, 7990, 9020

Bouchard, Lauren Michele: 1250, 2215
Boucher, Nathan: 4165, 9030, 9530
Boudreau, Robert M.: 5090, 7630
Bouhrara, Mustapha: 3495
Bouldin, Erin D.: 1005, 1280, 1355, 2020, 4365, 7990, 9000
Bouldin, Erin D.: 9010, 9030
Boulton, Aaron J.: 7620
Bourassa, Kyle J.: 1305, 5080
Bouwmeester Stjernetun, Björn: 7000, 7310
Bowblis, John R.: 1115, 9010
Bowen, Holly J.: 9000
Bowen, Lauren M.: 2250
Bowen, Pamela: 9030
Bower, Peter: 4455
Bowers, Barbara: 3180
Bowlby, Douglas: 7390, 7760
Bowles, Kathryn H.: 9410
Bowling, C. Barrett: 9010
Bowman, Caitlin: 7750
Boyd, Cynthia M.: 1035, 9420
Boyle, Julia T.: 9010
Brace, Margaret: 9000, 9410
Brach, Jennifer: 1015, 2155, 9030
Bradbury, Thomas: 4400
Bradley, Dana: 2160, 3000, 3430, 7940, 9000
Bradley, Sarah E.: 7150
Bradshaw, Tancia: 1325
Bradwell, Hannah: 7190
Brady, Samantha: 7090, 9000
Brahma, Arindam: 7840
Brailsford, Jennifer: 9010
Bramajo, Octavio N.: 3425
Brandt, Anthony: 9110
Brandt, Martina: 1220
Brandt, Nicole: 2210
Brannock, M Daniel: 3075
Brasher, Melanie S.: 4375
Bratches, Reed: 9010, 9020
Brates, Danielle: 9020
Bratsberg, Bernt: 9020
Brauer, Simon G.: 2100
Braunstein, Joel B.: 9550
Bravata, Dawn M.: 7130, 7180
Bravo, Adrian: 9010
Bray, Steven: 9020
Brazier, Joan: 9000, 9030
Breckling, Meghan: 7390
Breder, Kelseanne P.: 9100
Brekke, Bailee: 3190, 3455
Bremner, Jordan: 7700
Breneman, Charity: 9000
Brennan, Grace M.: 1305
Brennan-Ing, Mark: 2055, 3565, 5115, 7230, 7810
Brenowitz, Willa D.: 2240
Brenowitz, Willa: 2240
Brent, Michael R.: 5090, 7370, 9010, 9020
Bresslet, Tchelet: 9420
Breton, Kellen: 7170
Brett, Jeanne: 7440
Brewer, Danya: 7210
Brewer, Julie: 2035, 4460
Brewer, Robin: 7680
Brewster, Glenna S.: 2180, 3290, 3535, 7220, 7440
Brewster, Pete: 2295
Bridgforth, James: 4150
Briggs, Emily K.: 7610
Bright, Briana: 7330
Bright, Heather: 9410
Brini, Alberto: 5220
Britt, Katherine Carroll: 3485, 4145
Britton, Karysa: 9020
Britton, Warren: 9020
Brocklebank, Laura: 2015
Brodaty, Henry: 4035
Broderick, Gordon: 9000
Broderick, Vianna: 7150
Brody, Abraham A.: 2070, 2235, 3345, 7230, 9000
Brody, Lilla: 7900
Brohard, Cheryl: 1280
Bromberg, Dan H.: 2055
Bromseth, Janne: 7290
Brooks, Maria M.: 5155
Brosch, Jared R.: 4210, 5105
Brosens, Dorien: 4330
Brotherhood, Kelly: 7160
Brothers, Allyson: 5030
Brotman, Rebecca M.: 4080
Brottman, Barbra: 7070
Brotto, Leticia: 7370
Brotto, Marco: 7370
Broussard, Jia: 1010
Broussard, Jordan: 9010
Broussard, Kimetha: 9020
Brouwers, Mara: 2370
Brown, Candace: 7170
Brown, Casey L.: 4495
Brown, Colette: 5045
Brown, Colleen: 7150
Brown, Diane K.: 4406, 7500
Brown, Ellen L.: 4415
Brown, J. Scott: 7140, 7900, 7950, 9010
Brown, Janine: 9020
Brown, Jessy: 7830
Brown, Jocelyn L.: 5025, 9000
Brown, Justin: 3245
Brown, Karl: 7180
Brown, Katherine: 4266, 7190
Brown, Lana M.: 7160
Brown, Laura J E.: 1130
Brown, Lauren L.: 1000, 3440
Brown, Lisa M.: 2295, 3050
Brown, Maria T.: 1180
Brown, Monique J.: 1085
Brown, Rebecca: 7650, 8900
Brown, Ricardo: 7350
Brown, Sheldon: 9000
Brown, Susan H.: 7640, 7820
Brown, Terrell: 2115
Brown, Todd T.: 3390
Brown IV, Charles H.: 1370
Brown-Hughes, Travonia B.: 7970, 9010
Browning, Wesley R.: 5140
Brubaker, Malaak: 9020
Bruce, Marino A.: 3450
Bruckenthal, Patricia A.: 5030, 7120
Bruine de Bruin, Wändi: 1090
Bruner, Erin: 9010
Bruni, Pietra T.: 4134, 7330
Bruni, Pietra T.: 7340
Brunkert, Thekla: 7160
Brunner, Eric John: 2255
Brunt, Christopher: 1115
Brunton, Laura K.: 7780
Bryan, Rachel: 9010
Bryant, Ashley Leak: 3540
Bryanton, Olive: 3255
Bryk, Kelsey: 2015, 7810
Bubu, Omonigho M.: 7460
Buch, Elana: 2120
Buchanan, Jeffrey A.: 7010, 7590
Buchman, Aron: 9020
Buchman, Emily: 1120
Buck, Harleah G.: 3345, 3395
Buckwalter, Kathleen: 5070, 9020
Bucy, Taylor I.: 1010, 3010, 4140, 5170
Budnick, Andrea: 9000
Budzynska, Katarzyna: 2225
Bueckle, Andreas D.: 4200
Buehler, Susan K.: 4540
Buenger, Rachel E.: 9020
Buerger-Cole, Haley: 3015
Bugbee, Brittany A.: 4408, 7230
Bugos, Jennifer A.: 3420
Bui, Cindy N.: 9510
Bui, Truc Thi Mai: 4385
Bulanda, Jennifer: 7950
Bulat, Tatjana: 7150
Buller-Peralta, Ingrid: 7630
Bullock, Karen: 3345, 4420, 7230
Bültmann, Ute: 4130
Buma, Lise E.: 7120, 7940
Bunker, Jennifer: 4030, 4290
Bunker, Melissa: 9000
Bünning, Farina: 9000
Burack, Orah: 7890, 9010, 9030
Burant, Christopher J.: 2005, 7030, 9020
Burdgorf, Julia G.: 2185, 5160
Burdick, David: 4132, 7480
Burek, Melissa W.: 9000, 9020
Burg, Emily: 2365
Burgei, Alicia: 9030
Burhan, Amer M.: 7080
Burke, Anne E.: 4080
Burke, David T.: 7820
Burke, Shanna: 9000
Burkewitz, Kristopher: 3355
Burkhardt, Summer: 7150
Burnes, David: 1145, 2060, 3375, 4215
Burnett, Jason: 4205, 7610, 9010
Burnette, Denise: 5115
Burnham, Dyanna: 7030
Burns, Edith A.: 4030, 4268, 7680, 9010
Burns, Jeffrey M.: 7990
Burr, Jeffrey A.: 3165, 3350, 4230, 4390, 4435, 7510, 9010, 9530
Burrow, Wesley: 7870
Burrows, Brett: 9010
Bursac, Zoran: 9420
Burton, Lydia M.: 9320
Busby-Whitehead, Jan: 7150
Bush, Julie: 7950
Bussell, Theodore: 2280
Buta, Brian J.: 1370, 5230
Butler, Ryan: 9020
Buto, Peter: 2240
Butte, Sarly: 7010
Buurman, Bianca: 7120
Buxton, Michael R.: 7390
Buxton, Orfeu: 1180, 3500, 4490

Buys, David R.: 7590, 9030
Buys, Katie C.: 9030
Bybee, Sara: 1155, 1250, 9020
Byers, Amy L.: 2280, 4115, 5000
Bygrave, Desiree C.: 7410
Bylund, Carma: 7380, 7580, 7900
Bynum, Julie: 7420
Byrappagari, Divesh: 4300, 7640
Byrd, DeAnnah R.: 7970
Byrne, Nicole: 9000
Byun, Eeeseung: 7280

C

Cabrero Castro, Jose E.: 4395, 9030
Cacchione, Pamela Z.: 7860
Caceres, Maria: 4465
Cadet, Tamara J.: 2260
Cadiz, Ashley D.: 4132, 7500, 9000
Caffrey, Christine: 1095
Cagle, John: 1035, 2020, 4470, 5005, 5015, 9010, 9100
Cahill, Ewan A.: 1245
Cahill, Kevin E.: 3370
Cahill, Sean R.: 5220
Cahuapaza-Gutierrez, Nelson Luis: 9010
Cai, Qian: 5025
Cai, Shubing: 2145
Cai, Yurun: 3510, 5085
Caiado, Camila: 7160
Cain, Cindy: 1075, 2020, 9010, 9100
Cain, Stephen M.: 7820
Caiola, Courtney: 3540
Calamia, Matthew R.: 2105, 7550
Calasanti, Toni M.: 4390
Caldera, Selena: 2395
Calderon, Russell: 3500
Caldwell, Joseph: 3010
Calfee, Kristin R.: 7990
Calisto, Rita: 7320
Calkins, Margaret: 7160
Callaghan, Bridget: 9000
Callaway-Cole, Larissa: 2390
Calloway, Shayla: 4100
Calo, William: 5145
Calvani, Riccardo: 9020
Calver, Jen: 9010
Calvo, Rocio: 4420
Camacho, David: 5115, 9010
Cameron, Judy L.: 9020
Cammer, Allison: 1200
Camp, Cameron J.: 3365
Campbell, Kenna: 9010, 9540
Campbell, Kimberly C.: 3260
Campbell, Laura: 4340
Campbell, Noll L.: 7080
Campetti, Randi: 2030
Campione, Joanne R.: 2375, 7780
Campitelli, Anthony: 2015, 7810
Campos, Fernando A.: 1195
Campos, Jennifer: 3270
Can, Reyyan: 4180
Candon, Molly: 8900
Canell, Anastasia E.: 7980, 9020
Canham, Sarah L.: 1155, 1205, 7780
Cannon, Jacqui: 9000
Cannon, Melissa L.: 4408, 7700
Cantu, Phillip: 3465, 7600, 9020, 9030, 9560
Cao, Feifan: 9020
Cao, Jiawei: 9510
Cao, Katy (Qiuchang): 1065, 2045, 3095
Cao, Yanpei: 2265
Cao, Yuanyuan: 3085, 3295
Cao, Yumeng: 9000
Capolino, Alexandria C.: 4375
Cappola, Anne R.: 4080
Cappon, Davide: 2155, 9030
Caprio, Thomas V.: 3285
Capuano, Ana W.: 2120
Caraballo, Christina: 3175
Carbone, Elena: 4185
Carden, Keisha D.: 1365
Cardenas, Cynthia: 2160
Cardenas, Valeria L.: 7260
Carder, Paula: 1210, 2105, 3220, 3320, 7550
Carey, Cyriah: 9020
Carey, James: 1195
Carlson, Bryant: 2105, 3220
Carlson, Chalise: 4095
Carlson, Michelle: 1015, 2155, 3390, 3405, 4145, 7650, 9000
Carmichael, Pierre-Hugues: 7030
Carney, Maria Torroella: 1020, 7260
Carpenter, Brian D.: 1365, 4210, 4406, 7000, 7550, 7900, 9020
Carpenter, Joan: 3395, 4005, 9010
Carpenter, Kristen: 7410
Carpenter, Nancy: 4460
Carpenter, Rebekah: 2045, 3095
Carr, David B.: 9030, 9330
Carr, Dawn C.: 1230, 2045, 3095, 7030, 7510, 9010
Carr, Deborah: 1305, 2105
Carranza, Maria: 2270
Carratu, Kevin: 7260
Carrión, Carmen: 1305
Carrico, Catherine: 7610, 7840
Carrion, Iraida V.: 2260, 2330
Carrion-Baralt, Jose R.: 7620
Carroll, Brooke: 7060
Carroll, Judith E.: 1085, 2285, 5225
Carson, Erin: 9530
Carson, Paul: 7710, 7810
Carstensen, Laura Lee: 1320, 4480, 4495, 7020, 8900
Carter, Jammie: 9520
Carter, Kara: 3210, 4050
Carter, Rachel M.: 1335
Carvalho do Nascimento, Paulo Roberto: 4268, 7680
Casanova, Giuliana: 2215, 4405
Casanova, Ramon: 7630, 9580
Case, Donna: 3570
Case, Rebekah: 7410
Caserta, Mike: 1150
Casey, James: 4495
Caskie, Grace IL.: 3250, 7590, 7980
Caspi, Avshalom: 1305, 5080, 7660
Cassatt, Sarah: 9010
Cassidy, Jessica L.: 2330, 2390, 4420, 7910, 9000, 9020
Castegren, Markus: 9030
Castillo-Azofeifa, David: 3070
Castle, Nicholas: 3165
Castro, Eunice: 7460
Castro, Saul: 7950
Cataldo, Amelia: 7410
Catt, Wade: 9020
Caudillo-Ortega, Lucia: 2290
Cauley, Jane A.: 4260, 5085, 7650
Causey, Shakiera T.: 9100
Cavalli, Stefano: 3130
Cavanaugh, Robert: 9430
Cave, Nkosi: 7620
Cawthon, Peggy M.: 2320, 3475, 3500, 4260, 5085, 5210, 7650
Cazumbá, Aryane S.: 7920
Cedars, Marcelle: 7100
Celso, Brian: 7860
Cenko, Erta: 9000
Censky, Dina: 9520
Cenzer, Irena: 4205
Cerino, Eric S.: 1360, 3230, 3545, 5080, 9000
Cerino, Lauren: 1175, 3090
Cerqueira, Margarida: 4405
Ceruso, Marco: 1315
Cervantes, Diana: 4220
Cervantes, Maria: 7180, 9000
Céspedes, Amarilis: 3415
Cha, Eun Gyo: 9540
Cha, Hyungmin: 9000, 9020
Chabala, Peg: 4315
Chacko, Thomas P.: 9000
Chai, Emily: 7860
Chai, Hye Won: 4370
Chakravarthy, Ameera A.: 7170
Chakravarty, Esha: 7200, 7360
Chakravarty, Indrani: 7200
Challa, Suman: 7480
Chamberlain, Stephanie A.: 4025
Chamberlin, Elizabeth S.: 7850
Chambers, Yolima: 1150, 3240
Chan, Alex Siu Wing: 7050
Chan, Andrew: 9000, 9020
Chan, Angelique: 2230, 3435, 4020, 4175
Chan, Athena Chung Yin: 3060, 4520
Chan, Ding-Cheng (Derrick): 4134, 7830, 7930
Chan, Helen Yue-lai: 4540
Chan, Kiera: 1375
Chan, On Fung: 3115, 4255
Chan Carusone, Soo: 4340
Chandler, Kelly D.: 4045, 7090
Chandra, Meghana: 5220
Chang, Angela: 9030
Chang, Catie: 1215
Chang, Chu-Hsiang: 9000
Chang, Chung-Chou H.: 5080

Chang, E-Shien: 1145, 2030, 2060
Chang, Jun Ha: 7510, 7620
Chang, Ling-Hui: 7780
Chang, Mei-Ching: 9030
Chang, Pei-Shiun: 9400
Chang, Yuanqing: 1245
Chang, Yuefang: 5225
Chang, Yu-Ping: 5230, 7260, 7330, 7360, 7620, 9010, 9020
Chao, Linda L.: 1040, 2035, 4035
Chao, Pei-Shu: 7950
Chao, Shiau Fang: 7030, 7960
Chao, Ying-Yu: 5230, 7360
Chapela, Sofia: 7770
Chapman, Brian: 4035
Chapman, Cara: 5200
Chapman, Hannah: 4515
Chapman, Joyce: 2355
Chapman, Steve: 7380
Chappell, Richard J.: 2240
Char, Adarsh: 3235
Charles, Doreek: 1240
Charles, Susan: 1105, 3135, 3195, 3230, 5045, 9020
Charness, Neil: 1315, 9000, 9010, 9030
Chasteen, Alison L.: 1200
Chatterjee, Samir: 7840
Chaturvedi, Ritika: 4385
Chaudhari, Diptaraj S.: 7100, 7870
Chaudhari, Nikhil: 9020, 9030
Chaudhury, Habib: 1145, 2340
Chavez, Arlette ": 2085
Chavez, Hector J.: 8900
Chavez, Margeaux: 7150
Che, Yingni: 7370
Chee, Wonshik: 7220
Cheeks, Jodi: 7860
Chehabeddine, Lara: 9500
Chek, Carmen Jia-Wen: 7970
Chellappa, Karthikeya: 9020
Chen, Allison: 3270
Chen, Bangwei: 9000
Chen, Chen: 2280
Chen, Chixiang: 3550, 4115, 9000
Chen, Cynthia: 3440, 4385
Chen, Diefei: 3335, 3510, 4265, 7870
Chen, Duan-Rung: 7260
Chen, Enna: 4495
Chen, Haiying: 4355, 5230
Chen, Hao-Min: 3400
Chen, Honglei: 2240
Chen, Jiachen: 7640, 7830, 9000
Chen, Jia-Jen: 3400
Chen, Jiayu: 9010
Chen, Jie: 7370
Chen, Jie: 7460
Chen, Jiu-Chiuan: 2320
Chen, John S.: 9000
Chen, Kao Chin: 9010
Chen, Kuan-Hua: 4495
Chen, Kuan-Hua: 4495
Chen, Kuan-Hua: 4495
Chen, Lei: 3210, 5215
Chen, Li-Mei: 9000
Chen, Lin Yee: 3390
Chen, Lingming: 1350, 2385, 3155
Chen, Lizhen: 8900
Chen, Long: 1260, 7120
Chen, Lulu: 7680
Chen, Min-Chia: 4155
Chen, Ming-Hui: 7460
Chen, Nai-Wei: 1180
Chen, Po-Hung: 7390
Chen, Qi: 7650
Chen, Qize: 2045, 3095
Chen, Ruijia: 2300
Chen, Shanquan: 1165
Chen, Shyh-Huei: 3505
Chen, Siting: 7300, 7540
Chen, Sunny: 9030
Chen, Szu-Wei: 7780
Chen, Tuo-Yu: 4490
Chen, Wei-An: 9010
Chen, Will: 9030
Chen, Xi: 1045
Chen, Xi: 1165, 2225, 2300, 3230, 3450
Chen, Xi: 5225
Chen, Xiaodong: 9000, 9020
Chen, Xiaoyuan: 7050
Chen, Xiayu Summer: 7080
Chen, Xirun: 4120
Chen, Ya-Mei: 7780
Chen, Yanjun: 2240
Chen, Yi-An: 1120
Chen, Yingtong: 7850
Chen, Yizhou: 9110
Chen, Yu-Chih: 2160, 4305
Chen, Yu-Ming: 1125, 4310, 7930
Chen, Yung-Ming: 7930
Chen, Yuntao: 2255
Chen, Yun-Zih: 7290
Chen, Yuqing: 5155
Chen, Yuxin: 4155
Chen, Yuxuan: 1305
Chen, Zhirui: 7550
Chen, Ziyuan: 9020
Cheng, Catherine J.: 4475
Cheng, Clio Yuen Man: 3515
Cheng, David: 9000
Cheng, Evien: 7370
Cheng, Grand: 9010
Cheng, Greta Jianjia: 3405
Cheng, Huai Y.: 7150, 7310
Cheng, Jie: 1350, 3155
Cheng, Ling Ling: 9000
Cheng, Mengling: 2285, 7930
Cheng, Sheung Tak: 1140, 1145
Cheng, Yan: 3490
Cheng, Yiping: 9020
Cheng, Zijing (Flora): 4140
Cheong, Josepha: 7140
Chepkorir, Joyline: 5175
Cherry, Barbara J.: 7070
Cherry, Katie E.: 2105, 7550
Chesak, Sherry: 7420
Cheshire-Allen, Maria: 1335
Cheslock, Megan: 9410
Chesney, Margaret A.: 2035, 4035
Chester, Mia Recha: 2180
Cheung, Dominique: 9000
Cheung, Ethan Siu Leung: 3305, 7230, 7270
Cheung, Kin: 7040
Cheung, Pui Yin: 9020
Cheung, Ying: 4310
Chew-Graham, Carolyn: 9010
Chi, Iris: 1185, 3005
Chi, Nai-Ching: 4134, 7620
Chi, Peggy: 9550
Chi, Vivienne: 5105
Chia, Arthur: 3435
Chia, Chee: 4490
Chiang, Hao-Ping: 9020
Chiang-Hanisko, Lenny: 5175
Chiareli, Alexandra: 3260
Chidebe, Runcie CW.: 2010, 2375, 4130, 4345, 5110, 7040, 7380, 9010
Chien, Shao-Yun: 7300, 7910, 9010, 9020
Chihuri, Stanford: 9010, 9020
Childs, Elisa M.: 7360
Childs, Ellen: 7810
Chiles, Kira: 7170
Chim, Kannie: 4408, 7700
Chin, Ching-I.: 9030
Chin, Janecca: 1340
Chin, Jessie: 7590
Chin, Kristi: 2100
Chipalo, Edson: 4510
Chipperfield, Judith G.: 9430
Chiriboga, David: 7510
Chisholm, Latarsha: 9030
Chisolm, Theresa: 1295
Chiu, Ching-Ju: 7610, 7940, 9010, 9030
Chiu, Wan-Yu: 7780
Chiu, Yi-Chen: 4155
Chiu, Yu-wen: 7410
Chladek, Jason S.: 9030
Chmelo Kemp, Elizabeth: 3505, 4350
Cho, Catherine: 9010
Cho, Chi: 7610, 7640
Cho, Christine: 1215
Cho, Emma: 9030
Cho, Eunae: 9010
Cho, Eunbyul: 9100
Cho, Eunhee: 7420, 7460, 7660, 7720, 7760
Cho, Hannah: 7980, 9000
Cho, Hyeonji: 7030, 7380
Cho, Hyun Jin: 7550, 9020
Cho, Jeff: 9030
Cho, Jinmyoung: 7400
Cho, Joonyoung: 1170, 4325
Cho, Jungwon: 7420, 7760
Cho, Kyoungmin: 5045
Cho, KyungAh: 7850
Cho, Sangmi: 7780
Cho, Soyeon: 2090
Cho, Tsai-Chin: 2280, 7320
Cho, Youmin: 7570
Cho, Youngmin: 3540, 4380
Cho, Youngmin: 7780
Chocninov, Harvey M.: 7380
Chodosh, Joshua: 1265, 7130, 7840
Choe, Ian: 4075
Choi, Bomi: 7040, 7080, 7610
Choi, Eunyoung: 9020, 9310
Choi, Gayoung: 9030, 9570
Choi, HwaJung: 1340, 7320, 9030
Choi, Hyojin: 7590
Choi, Jean: 4425
Choi, JiYeon: 7080, 7900, 7960, 9020
Choi, Jung-Yeon: 9000
Choi, Mi Sun: 7780
Choi, Mona: 7080
Choi, Moon: 1260, 7690, 9030
Choi, Namkee G.: 4375, 4460, 7270, 7610
Choi, Seongmi: 7080, 9020

Choi, Shinae L.: 1305
Choi, Soyoung: 2290
Choi, Won: 1320, 4205
Choi, Y.Joon: 7220
Choi, Yeon Jin: 1165, 7720
Choi, Yong K.: 3205, 5055, 7460
Choi, Yoonseok: 4480
Choi, Young: 9100
Choi-Allum, Lona H.: 3095, 5040, 5175
Chok, Jay: 1095
Choma, Elizabeth A.: 5100, 9410
Chon, Myoung-Gi: 9010
Chong, Natalie: 3010
Chopik, William J.: 4145, 4325, 4370
Choquette, Cassidy: 9010
Chou, Kee Lee: 1065, 7610, 7930
Chou, Yi-Chun: 4134, 7830
Choudhury, Renoa: 5075, 7150, 7820, 9030
Choukas, Nathaniel R.: 4460
Choula, Rita B.: 1155, 2395
Choun, Soyoung: 1160, 7510
Chow, Denise: 4010
Chow, Stephanie: 4266, 7190, 7820
Chowdhury, Nahian: 9020, 9030
Choy, Jacky C.P.: 5235, 7420
Christensen, Kaare: 1195, 4260, 5090, 9010, 9020
Christian, Thomas: 3230, 4195
Christiansen, Mats: 7360
Christianson, Tracy: 7700
Chrzanowski, Brandi L.: 4406, 7500
Chu, Charlene H.: 3255, 4245, 4405, 7190, 7680
Chu, Li: 1320, 4480, 8900
Chu, Winnie: 4268, 7680
Chua, Denise Qian Ling: 7760
Chui, Cheryl: 3115, 3515, 4255
Chui, Geoffrey: 1095
Chui, Michelle A.: 7080, 9030
Chukwuorji, JohnBosco: 9000
Chung, Edwin K H.: 3280
Chung, Jane: 3025, 7400
Chung, Seok Jong: 7400
Chung, Soondool: 7030, 7510, 7590, 7890
Chung, Tim: 4406, 7000, 9020
Chungkham, Holendro Singh: 3265
Chunyu Liu, Chunyu Liu: 7100
Church, Kimberly: 7860
Churchill, Linda C.: 1350, 2385, 3155
Chusyd, Daniella E.: 1060, 9020
Cichy, Kelly E.: 3545, 4425
Cidav, Tom: 5100, 7620
Cigolle, Christine: 9000
Ciolfi, Mary Lou: 3175
Cipher, Daisha M.: 3305
Ciro, Carrie: 9030
Clair, Catherine A.: 2290, 3240
Clancy, Olivia: 9010
Clapés Pemau, Raquel: 9020
Claproth, Alycia: 1285
Clare, Linda: 4070
Clark, Alexandra L.: 9210
Clark, Amy G.: 7150
Clark, David: 9010
Clark, Janet M.: 1375
Clark, Kirsty A.: 1180
Clark, Lindsay: 9020
Clarke, Anthony: 1350
Clarke, Philippa: 1170
Classen, Sherrilene: 1030
Clauser, Steve: 9000
Clay, Danielle: 7480
Clay, Olivio J.: 7020, 7060, 7090, 9000
Clayton, Jordana L.: 1150, 2035, 9020
Clayton-Jones, Mary C.: 9020
Clem, Sarah: 5005, 9010
Clevenger, Carolyn K.: 2070, 2300, 7440, 9020
Clifford, Kristen: 3055
Clifton, Jennifer: 7480
Cline, J. Mark: 7370
Cloud, Leslie: 9330
Clouston, Sean AP.: 2230, 7590
Cloyes, Kristin G.: 7470, 7540
Clutton, Jonathan: 7990
Coates, Cameron: 5070
Coates, Martha C.: 4005
Coblenz, Clara W.: 7510
Coco-Ripp, Jo Ann: 4350
Coe, Antoinette B.: 9020
Coe, Norma B.: 1115, 4365
Coen, Paul M.: 5210
Cofield, Stacey: 7360
Cogdill-Richardson, Kiana: 1390
Coghlan, Leah: 8900
Cohanim, Niki: 4420
Cohen, Alan A.: 1110
Cohen, Ann D.: 9500
Cohen, Jonathan E.: 1020, 2175
Cohen, Matthew L.: 7620
Cohen, Pinchas: 2175
Cohen, Steven A.: 4025
Cohen, Trevor: 7300
Cohn-Schwartz, Ella: 4125, 9030
Colón-Emeric, Cathleen: 7300, 7410
Colburn, Jessica: 4170
Coldiron, Allyson M.: 7080, 7590, 7900
Cole, Steve: 1215, 2325
Coleman, Carissa: 2160, 3205, 3335, 7300
Coletta, Giulia: 5100
Coley, Rebecca Yates: 3490
Collins, Stacey: 2325
Collins-Pisano, Caroline: 4445
Colon, Anaeliz: 7770
Colon-Emeric, Cathleen: 1370, 4485
Colvin, Alicia B.: 5155
Comnick, Carissa: 5225
Conard, Ryan: 7020
Condo, Angela: 1375
Condon, Shelley E.: 7460
Cone, Nicholas: 1055
Coney, Rachel: 7620
Cong, Xiaomei: 7460
Cong, Zhen: 7550
Coniff, Joshua: 5225
Connelly, Denise: 5105, 7160, 7780
Conner, Kyaien: 3560
Conniff, Joshua: 4132, 7480
Connolly, M.T.: 3375, 4215
Connor, Lisa Tabor: 1255
Conover, Laura: 7330
Conrow, Elizabeth: 1310
Conroy, David E.: 4310, 9010, 9020, 9030
Consolandi, Monica: 7070, 7600
Consortium, LifeGEIST.: 9020
Convery, Kimberly: 7230
Conway, Cathy: 1280
Cook, Allyson: 7660
Cook, Maxwell: 9020
Cook, Miranda: 9010, 9310
Cook, Penny: 3175
Cook, Robert L.: 1030
Cook Maher, Amanda: 4325
Cooke, Paul S.: 1380, 7370
Coon, David W.: 5075, 7970
Cooney, Alissa: 9010
Cooper, Charles: 7620
Cooper, Dayna: 2145
Cooper, Hannah LF.: 3565
Cooper, Kalvry A.: 7080
Cooper, Leonie A N.: 7190
Cooper, Lujira J.: 1250, 5150
Corazzini, Kirsten: 3145, 5020
Corbett, Duane: 9580
Corbett, Kyle: 4280
Corcos, Daniel: 4310
Cordell, Ashlee: 7060, 7460
Coresh, Josef: 2365, 3510, 7110
Corle, Melanie: 9020
Corley, Samantha S.: 4045
Corneau, Emily: 7160
Cornelison, Laci: 3175
Cornelius, Talea: 2035
Cornell, Portia: 3320, 7160, 9030
Cornwell, Hannah E.: 7900
Correa, Arlene: 3565, 7810
Corrigan, Anne E.: 3405
Cortés-Ortiz, Mónica: 9010
Corwin, Logan: 7210
Coschigano, Kat E.: 7830
Cosentino, Stephanie: 1040, 7830
Costa, Amy N.: 3420, 4540, 7080
Cothran, Fawn: 3030
Cotten, Shelia R.: 3025
Cotter, Valerie T.: 1075, 4170
Cotton, Cecilia J.: 4280
Cotton, Jesse J.: 9010
Cotton, Quinton D.: 1150, 2130, 3240
Cotton, Sam: 4040, 4360
Couch, Elyse: 7620, 7720
Coughlin, Joseph F.: 1175, 3090, 7090, 7910, 9000, 9010
Coulourides Kogan, Alexis M.: 3220
Couve, Amanda: 4040
Covington, Amy: 9000
Cowan, Linda J.: 7150
Cox, Brian S.: 7390, 7460
Cox, Elizabeth: 7770
Cox, Krista M.: 5190, 7680
Cox, Laura: 9000
Cox, Polly Madson: 4215
Cox Sullivan, Sheila: 7160

Coyle, Caitlin: 4150
Coyle, Caitlyn E.: 9510
Coyle, Marissa: 9000
Craggs, Jason G.: 4490
Craig, Dawn: 4455
Crampton, Alexandra: 4325
Crane, Breanna: 1015, 2155, 3405, 4145, 9000
Cranford, James: 7390
Crary, Kate: 5020
Crawford, Nicole O.: 3560, 4010
Crawford, Sybil L.: 5155
Cready, Cynthia: 5185
Crenshaw, Kiresten: 9010
Crettenand Pecorini, Béatrice: 2390
Crews, Deidra C.: 1345
Crimmins, Eileen M.: 1125, 1320, 1325, 2325, 3465, 4395, 7380, 7540, 7890
Crisci, Alex: 9010
Crittenden, Jennifer: 2020, 3075
Crocco, Elizabeth A.: 9010
Croff, Raina: 4100
Cronin, Cory E.: 7780
Cropper, Avareena: 5220
Crosby, Paris: 7510
Cross, Dori: 1010
Crossdale, Rachel: 4450
Crouch, Adele: 7420
Crouter, Scott E.: 2385, 3155
Crowder, Sylvia: 7410
Crowder, Victoria R.: 4380
Crowe, Michael: 3050, 9000
Crowley, D. Max: 7950
Cruitt, Patrick J.: 3520
Cruz Madrid, Katya Y.: 4465
Cruz-Almeida, Yenisel: 4134, 7330, 7630, 9010
Cruz-Carrillo, Yesenia: 1360
Cruz-Oliver, Dulce Maria: 7360
Csikai, Ellen L.: 7380
Cudjoe, Thomas: 4030, 4145, 5160, 9030, 9420
Cuevas, Heather E.: 7840, 7980
Cui, Jialin: 9510
Cui, Xiaoyan: 9020, 9300
Culberson, John W.: 7160
Culligan, Joanna RS.: 9000
Cummings, Greta: 4408, 7720
Cummings, Jeffrey: 9020
Cummings, Michael: 7100
Cummings, Steven R.: 3475, 3500, 5210
Cummins, Phyllis: 2375, 4130, 4345, 5110, 7040, 7380
Cundiff, Jeanne: 9210
Curbeam, Deanna: 7300
Curiel, Rosie: 9010
Curl, Angela L.: 5175
Curley, Taylor M.: 7510
Curran, Sean: 9020
Currie, Leanne: 9030
Curriero, Frank C.: 3405
Curry, Natasha: 2255
Curtis, Ashley F.: 3420, 4490, 4540, 7080
Curtis, David: 9020
Curtis, Elizabeth Heaton: 1310
Curtis, Megan E.: 9010
Curtis, Sarah: 1015
Curyto, Kimberly J.: 4290
Cutchin, Malcolm: 2340
Cutforth, Asia: 3410, 5200
Cvejkus, Ryan K.: 5090, 7830, 9020
Czaja, Sara: 1145, 1315, 2050, 2060, 3170, 3515, 4268, 7360, 7680
Czyz, Rebecca: 9010

### D

Dabelko-Schoeny, Holly: 1095, 1205, 1330, 2130, 3045, 4305
Dabrowski, Barbara S.: 7610, 7840
Dacks, Penny A.: 7420
Dadgostar, Porooshat: 2145
Dadson, Deborah A.: 7990
Dag, Dominick: 7100
Dagne, Mahederemariam: 9010
Dagum, Paul: 9000, 9010, 9020
Dahal, Poshan: 7030
Dahl Aslan, Anna K.: 7130
Dahlgren, David: 2340, 7380
Dalkin, Sonia: 2375
Dallo, Florence: 3440
Dalpe, Sara: 7070
Dalton, Jarrod: 1170
D'Ambrosio, Lisa A.: 7910, 9000, 9010
D'Amore, Cassandra H.: 7170
Dampf, Hana: 4280
Dams-O'Connor, Kristen: 7650
Damush, Teresa M.: 7130, 7180
Dang, Huong: 9530
Dang, Linh: 3085
Dang, Linh: 3380
Dang, Linh: 4545, 5010, 5050
Dang, Patrick: 2085
Dang, Stuti: 1005, 9010, 9030, 9420
Daniel, George: 9020
Daniel, Kathryn M.: 2390, 3305
Daniel, Nikita R.: 4425
Daniels, Ramon: 9010
Danielson, Jodi: 2095
Danilovich, Margaret: 4310, 9020
Danilovich, Margaret: 9030, 9530
Danschin, Arlene: 7440
D'Antonio, Patricia M.: 1285, 2165, 3315, 3455
Daquin, Jane C.: 4415
da Rosa, Grace: 7500
Darby, Timothy: 1125
Darcy, Derek: 2145
Darmadi Blackberry, Irene: 2260, 9010
Darvishpoor Kakhki, Ali: 7660
Da Silva, Jaqueline: 7920
Darwish, Hala: 1355, 9000
Das, Ayushi: 4120
Das, Jayanta K.: 9020
Das, Phebe: 3340
Das, Sanchari: 3075, 7520
Das Bhattacharjee, Sreyasee: 9010
Das Gupta, Debasree: 3220, 7210
Dasanayake, Thakshila: 3020
Dasgupta, Abhirupta: 5030
Dash, Kim: 2030
Das-Munshi, Jayati: 4510
Dassel, Kara Bottiggi: 1150, 2035, 9020
Datcher, Ivree: 1010, 3435
Dauenhauer, Jason: 9010
Daugherty, Ana M.: 7510, 9010
Daum, Christine H.: 1165, 1280, 5015
Dave, Chintan: 5230, 7160
Davey, Adam: 3530, 9030
Davey-Rothwell, Melissa: 4460
David, Daniel: 2070, 2235
Davidson, Patricia M.: 7770, 7840, 7960
Davidsson, Rebecca: 7400
Davie, Laura: 5020
Davies, Abigail: 1335
Davies, Megan: 3180
Davies, Nathan: 4440, 4470
Davila, Heather: 2180, 9020, 9520
Davis, Darlene: 2295
Davis, Deborah W.: 7370
Davis, Derik L.: 7130, 9000
Davis, Elsa: 7060
Davis, Ian: 4355
Davis, Jamaine: 1380
Davis, Jennifer C.: 5100
Davis, Rebecca: 7800
Davis, Tracy EK.: 9030
Davison, Carrie Ann: 7540
Davitt, Joan: 2020, 5025, 9010, 9100
Davlyatov, Gani: 2350
Davlyatov, Ganisher: 2350, 3365, 4190
Davoudi, Anis: 2150, 3390, 7650
Davy, Philip: 2380
Daw, Warwick E.: 5090
Dawson, Bonnie D.: 7180
Dawson, Catherine MP.: 7770
Dawson, Walter: 7770, 9010
Dayama, Neeraj: 2350
Dayrit, Jessika G.: 7460
Dayyani, Faranak: 7680
De, Supriyo: 2195
de Boer, Bram: 2370
de Cabo, Rafael: 2065, 3495, 3575
De Carvalho, Carlos Roberto Ribeiro: 9010
De Carvalho, Celso Ricardo Fernandes: 9010
De Donder, Liesbeth: 1065, 4330, 7280
De Fries, Carson M.: 2090, 2330, 4195, 5200, 7230, 9010
De Godoy, Caroline G.: 9010
De Main, Atami: 5115
de Man-van Ginkel, Janneke M M.: 2280, 3340
de Mederios, Kate: 3200
De Melo, Daniella: 9020
de Munck, Isabel M.: 1370
de Oliveira Silva, Alessandro: 7150, 7340
de Rosa, Cristina: 7330
de Souto Barreto, Philipe: 2320
de Souza, Yolanda A.: 3140
Deac, Alexandra: 2055
Deal, Jennifer: 1295, 2135, 2240, 2365, 3335, 3390, 3510, 4110, 4265, 4266, 7690

Dean, Doug: 3495
Dean, Meagan: 7670
Dean, Olivia: 5175
Deason, Rebecca: 7420
Debergue, Patricia: 9020
deCardi Hladek, Melissa: 3240, 4060
DeCaro, Jason A.: 9210
DeCian, Enrica: 2105
Decker, Autumn: 1240, 1365, 3250
Decker, Hannah: 5200
DeFontes, Clara: 9020
Degen, Nora M.: 3100
Degenholtz, Howard B.: 3175, 4190, 7690, 9010, 9540
DeGennaro, Jennifer: 1130, 7290
Degraves, Brittany S.: 4515
DeGroot, Lyndsay: 7770
Deindl, Christian: 4275
DeJohn, Amber: 9000
Dekle, Kori: 7830
Delacourt, Barbara: 7680
Delgado, Daphne: 1265
Delgado, Ferdinand: 9030
Delgado-Ortiz, Laura: 3550
Delgado-Velandía, Mario: 2320
DeLiema, Marguerite: 3075, 4215
DeLiema, Marti: 3090, 3460
Delk, Michelle: 3435
Deller, Juergen: 7290
DeLongis, Anita: 3135, 3520, 7550
Deloria, Rochelle: 9420
DeLucia, Jack: 9000
Demalia, Angelo: 9030
Demir, Ibrahim: 4134, 7620
Demiris, George: 4415, 7460, 7680, 9000, 9030, 9540
DeMott, Andrew: 7710
Demuth, Ilja: 1060
Deng, Chenxin: 9000
Deng, ZhiDi: 1350
Denham, Grant: 7100
Dennis, Brandon C.: 9000
Dennison Himmelfarb, Cheryl R.: 7770, 7840, 7960
Denno, Ben: 3020
Denny, Bryan: 9110
Denson, Kathryn: 7310
Dent, Elsa: 7660
Dentchev, Alexander: 9010
Dentino, Andrew: 7130
DePasquale, Nicole: 7620
Deputy, Amanda: 4350
Der Ananian, Cheryl: 9030
Derby, Carol A.: 3500, 4490, 5155, 9020
Deshmukh, Madhyami: 7950
Desir, Marianne: 9420
Desjardins, Michael R.: 1015, 3405
Desrochers, Madeleine: 7010
DesRoches, Catherine: 9000
Devi, Reena: 2375, 7720
Devin, Michael: 7680
Devkota, Rashmi: 4280
DeVone, Frank: 7160
Devos, Hannes: 9330
Dewan, Neha: 4515
Dewji, Aleena: 4290
Dey, Amit: 5060, 7880
Dey, Judith: 2375
Dey, Nutifafa E Y.: 7610
Dezine, Marie L.: 7500
Dhakal, Usha: 9010
Dhar, Sohini: 7480
Dharangutte, Prathamesh: 7680
Di, Chongzhi: 3390
Di Bona, Vito: 7620
Di Gessa, Giorgio: 4015, 4275
Diaz, Jose A.: 4475, 5045
Diaz, Miranda H.: 3365
Diaz-Ramirez, Grisell: 2300
DiBartolo, Mary C.: 7000
DiCarlo, Nicholas: 5150
DiCerbo, Loraine: 7510
Dickerson, Anne E.: 7690, 7730
Dickerson, Bradford: 2035
Dickerson, Kimberly: 3110
Dickin, Clark: 7340
Dickinson, Stephanie: 9020
Dickson, Chelsea: 9010, 9540
Dieciuc, Michael A.: 1040
Diederich, Freya: 1115, 3530
Diefenbach, Michael: 4030
Diehl, Christin: 9000
Diehl, Cody L.: 7810
Diehl, Manfred: 2105
Diener, Christian: 9020
Diener-West, Marie: 9560
Dieppa, Emily: 4165
Dierckx, Eva: 1065, 7280
Dietrich, Mary S.: 9020
DiGirolamo, Marissa: 3195
Digman, Leanne: 9410
Dignam, Ritchell: 7360
Diji, Abigail Kusi-Amponsah: 7790
Dikhtyar, Oksana: 3190
Dikken, Jeroen: 4125
Dikmans, Bas: 4330
Dilanchian, Andrew: 1040, 9010
Dill, Janette: 7230
Dillon, Ellis C.: 1240, 3525
Dillon, Kaitlyn E.: 4340
Dillon, Kristen: 9020
DiMaria-Ghalili, Rose Ann: 3395, 4005, 5130, 9440
Ding, Huitong: 9010
Ding, Jingzhong: 4355
Ding, Jun: 5085
Ding, Kedong: 7260
Dionne, Laura: 4290, 7020
Dionne-Odom, J. Nicholas: 7680
DiScala, Sandra: 7840
Dishaw, Mary: 3480, 9000
Distefano, Giovanna: 5210
Dixit, Vishwa Deep: 2065
Dixon, Dave: 9010
Dixon, Josie: 4440
Dixon, Steven: 9020, 9200
Doan, Jared: 7820
Dobalian, Aram: 2295
Dobbs, Debra: 2000, 2245, 3165, 3345, 5170, 9030
Dodge, Shana: 7420
Doherty, Elaine: 4408, 7230
Doherty Bea, Megan: 5050
Dolamore, Michael J.: 9000
Dolan, Hanne R.: 5075
Dolansky, Mary A.: 4525, 7590
Dolbin-MacNab, Megan L.: 7950
Dolsen, Emily: 2280
Domeracki, Alexis: 9020
Dominguez, Rachael: 7590
Don, Janith: 5180
Donahue, Patrick T.: 1015, 3390, 3405, 7650, 9000
Donath, Carolin: 2370
Donato-Woodger, Simon: 7190
Donega, Stefano: 2195
Donehower, Allison K.: 4415
Donelan, Karen: 4430
Dong, Birong: 7830
Dong, Fanghong: 3485
Dong, Fanghong: 3490
Dong, Fanghong: 4134, 7330
Dong, Wuyi: 7610, 9030
Dong, Yanbin: 9010, 9500
Dong, Yu: 7330
Donkers, Hanneke: 3340
Donkor, Peter: 7790
Donnelly, John: 2295
Donnelly, Rachael: 7300
Donohoe, Maria: 3445, 3540, 7440, 7470
Donorfio, Laura KM.: 1080, 2095, 3325, 3350, 4035
Donovan, Heidi: 3030, 7530, 9010
Doolittle, Benjamin: 4145
Doose, Michelle: 3210
Doran, Kelly: 7170
Doran, Patty: 1385, 3260
Dore, Emily: 4045
Dorisca, Eugenia: 7000
Dorr, David: 2360, 7450
Dorris, Jennie L.: 7300
Dorris, Joseph: 9020
Dorsey, Kirsten: 7150
Dosa, David M.: 2295, 5170
Dougherty, Ryan J.: 2150, 3390
Douglas, Joy: 7620, 9000, 9020
Douglas, Natalie: 5030
Douglas, Vanja: 1380
Douma, Gebrich: 3340
Dowden, Lauren: 7700
Dowling, Maritza: 1070, 5225, 7100, 7330
Downer, Brian G.: 4395, 9030, 9310, 9560
Downey, Payton A.: 4268, 7680
Doyle, Cassidy: 9000
Doyle, Margaret F.: 7640, 7830, 9000
Dozier, Mary E.: 2040
Dragomir, Gerry: 3255
Drake, Jonathan D.: 9550
Drake, Judy: 9000
Drazich, Brittany F.: 2305
Drazon, Molly: 9400
Dreer, Laura E.: 7650
Driesse, Tiffany: 4268, 7680
Driscoll, Laura: 9000
Driver, Jane: 4310
Drolet, Marie-Josee: 9030
Drost, Jennifer: 7250, 7480
Droste, Andrew P.: 7820
Dryden, Eileen: 9010
D'Souza, Clive: 7750
D'Souza, Gail: 5145
Du, Pang: 5195
Du, Yan: 5055, 7120, 7660, 9030
Duan, Hongzhe: 2015, 7110, 7370, 7800
Duan, Liping: 7870
Duan, Yinfei: 4408, 4515, 7720
Duangjina, Thitinan: 4025
Duara, Ranjan: 2180, 5070

Duarte, Andres: 2180, 5070
Dubé, Catherine: 7680
Duberstein, Paul R.: 1240, 4105, 4305
Dublin, Sascha: 3490
Dubois, Sacha: 3570
DuBois, Samantha: 9030
Dubowitz, Tamara: 3405, 9010
Dubuc, Jade: 7680
Duchowny, Kate: 3225, 9010
Duck, Angela: 1070
Duff, Kevin: 7620
Duffy, Evelyn G.: 4525
Dugan, Elizabeth: 4266, 7230, 7270, 7830, 9530
Dugan, Sheila A.: 5155
Duggan, Katherine: 1360, 7550
Dujela, Carren: 7470
Dumitrescu, Ramona Gianina: 2270
Dunbar, Annie Zean: 4195
Dunbar-Jacob, Jacqueline: 9010, 9540
Duncan, Carly A.: 2180
Duncan, Francesca E.: 3215
Duncan, Jacob: 7210
Dunkle, Ruth E.: 1170, 4325
Dunlap, Janette: 7180, 9000
Dunlap, Pamela: 1015, 4145
Dunleavy, Daniel: 1030
Dunnam, Charles W.: 9000
Duong, Mylinh: 4268, 7680
Duplàa, Emmanuel: 2390
DuPont-Reyes, Melissa: 5195
Duppen, Daan: 1065, 7280
Dupre, Matthew E.: 2265, 3180, 9000, 9020
Dupree, Galilea D.: 7980
Duque, Gustavo: 1060, 2190
Duran, Danielle: 9000
Durant, Raegan: 9000
Durga, Aditi: 2070
Dury, Sarah: 1385, 4330
Duthie, Edmund H.: 7310
Duynisveld, Amber: 4515
Dwibedi, Aditya Kumar: 9010
Dwyer, Johanna T.: 9020
Dy, Sydney L.: 1035, 4170
Dye, Maya: 1120
Dymchuk, Emily: 3335, 4025
Dys, Sarah: 1210, 3320, 5030, 9010
Dyslin-Kman, Deborah: 7700
Dzomeku, Veronica: 7790

E
Ea, Emerson: 9020
Earles, Julie L.: 7510
Earnshaw, Valerie: 2055
Easley, Mikki: 2245
Easton, Scott: 1130
Eaton, Andrew D.: 4340
Eaton, Jacqueline: 1150, 1250, 2075
Eaton, William W.: 7870
Ebner, Natalie C.: 9010, 9200
Eby, David W.: 9030
Echavarria, Diane: 1035
Eckstrom, Elizabeth: 2385, 7430
Edahiro, Ayako: 7280
Eddington, Blake: 9020
Edelman, Linda S.: 4132, 4406, 5190, 7000, 7500, 9000, 9020, 9030
Edelstein, Offer E.: 1075
Edland, Steven: 7100
Edmonds, Tanner: 9010
Edwards, Chelsea: 2085
Edwin, Trine Holt: 9020
Ee, Nicole A.: 9110
Efird-Green, Lea: 3320
Egan, Mary: 9000
Egashira, Leo: 9100
Eguchi, Hisashi: 9030
Ehlman, Katie: 9000, 9020
Ehmann, Madison: 4460
Ehrlich, Joshua: 2135, 2150, 2365, 9110
Eimicke, Joseph P.: 1145
Eisenberg, Carol: 9000
Eisenberg, Chase: 9000
Eisenstein, Amy R.: 4085
Eisikovits, Zvi: 4520
Ejaz, Farida K.: 2060, 2220, 4215, 7060, 7460
Ejem, Deborah: 4060
Ek, Stina: 5205
Ekerdt, David J.: 1185, 9030
Ekman, Monica: 5040
Ekoh, Prince: 9010
Ekwebene, Onyeka: 5055
Ekwonye, Angela: 7960
El Khoudary, Samar R.: 4120, 5155
El-Abbadi, Naglaa H.: 1290
Elayoubi, Joanne: 7960
Elbejjani, Martine: 9500
Elbich, Daniel B.: 5165, 7510, 9030
Eldadah, Basil: 3040
Eldreth, Dana: 9000
Eldridge, Corinne: 9020
Eldridge, Madeleine R.: 4165
Elfenbein, Pamela: 4125
El-Hulu, Sondos: 7100
Eliazar-Macke, Nathaniel: 2170
Elkjær, Emma: 3195
Ellen, Moriah: 1175, 4255
Ellington, Lee: 1150, 2075, 7470, 7540
Elliott, Amy: 7600
Elliott, Cerise L.: 1355
Elliott, Emily M.: 2105, 7550
Elliott, Lauren: 7830
Elliott, Sheryl: 9010
Ellis, Amy: 7620, 9000, 9020
Ellis, Dylan: 5180
Ellis, Jennifer: 9400, 9440
Ellis, Julie: 7610, 7640
Ellison, James M.: 7620
Ellison Keeler, Carlyn: 9020
Ellis-Smith, Clare: 4470
Elman, Miriam R.: 7360
Elo, Irma: 7650
Elran-Barak, Roni: 7070
Elrod, Cathy: 9010
Elswick, R.K.: 9330
Eltaybani, Sameh: 7160, 7720, 9520
Elumeze, Josephine: 4268, 7680
Emery-Tiburcio, Erin E.: 4540
Emmesjö, Lina: 7130
Emmons, Stacey: 9020
Emrani, Sheina: 7100
Engdahl, Bo: 9020
Engeland, Christopher G.: 3545, 4490, 9010, 9030
Engelman, Michal: 3005, 3105, 3450
Engler-Chiurazzi, Elizabeth: 9010
English, Tammy: 1105
Engstrom, Gabriella: 7670
Enguidanos, Susan: 7260
Ennis, Nicole: 1030
Enroth, Linda: 4395, 9000
Ensrud, Kristine: 4260, 7650
Eom, Minjeong: 7810
Eost-Telling, Charlotte: 4455
Eppich, Kaleb: 1355
Epps, Fayron: 1265, 2070, 2180, 3305, 3485, 7440, 7620, 9030
Epstein, Cynthia: 2090, 9320
Epstein-Lubow, Gary: 2030
Eramudugolla, Ranmalee: 3570
Erdol, Nurgun: 7620
Erickson, Mary Ann: 2245
Erickson, Melissa L.: 3475
Ericsson, Malin: 9020, 9030
Eriksson, Erik: 2340
Eriksson, Johan G.: 9010
Erisman, Morgan: 1205
Ermer, Ashley: 1185
Ermoshkina, Polina: 3360, 7890
Ernst, Jerome: 3565, 7810
Ernst, Joy S.: 4266, 7780
Ersek, Mary: 4005, 4470
Ertle, Emilee: 7090, 7420, 9010
Erving, Christy L.: 2275
Erz, Dorothee: 9020
Escobar, Romeo: 4240
Esiaka, Darlingtina: 1355, 2010, 2110, 4130, 5055, 7520, 7960, 9210
Espejo, Luis: 7870
Espeland, Mark A.: 2320, 4355, 5060, 7630
Espinoza, Sara E.: 4355, 5055
Espinoza, Sara: 7180
Espinoza, Sara E.: 7370, 7660, 7910
Esser, Karyn: 3475, 3500, 4335, 7370
Esslemont, Dawn: 7160
Estabrooks, Carole A.: 3335, 4408, 4515, 7720
Estapé, Jorge: 2260
Estapé, Tania: 2260
Estenson, Lilly: 3220, 7750
Estlein, Roi: 7070
Etnier, Jennifer L.: 9030
Ettema, Roelof GA.: 4125, 7350
Euiler, Elizabeth: 4005
Eun, Yong: 7330
Evangelista, Lorraine S.: 4040
Evans, Catherine: 4470
Evans, Donald: 3080, 9000
Evans, Elizabeth A.: 5050, 7520
Evans, Joni K.: 4355
Evans, Lauren: 4380
Evans, Luke: 9010
Evans, Michele: 9000
Evans, Michele K.: 4130, 7560
Evans, Tracy: 7850
Evans, William J.: 4260
Evardone, Vanessa Joyce M.: 7840

Everink, Irma: 7720
Extermann, Martine: 7410
Eyler, Gabriella: 9020
Ezeamama, Amara: 7930
Ezeokonkwo, Francesca C.: 3485

**F**

Fabbre, Vanessa: 1250, 4195
Fabius, Chanee D.: 2185, 3415, 5130, 5160
Factora, Ronan: 7130
Fadalla, Carol: 7830
Fadel, Meghan: 3055
Fagundes, Christopher P.: 9110
Fahey, Margaret C.: 2175
Faig, Karla: 7660, 7830, 9010
Fairbanks, Sheryl A.: 1025, 7470
Fairchild, Kaci: 9010
Fairman, Ciaran M.: 2260
Falchetta, Giacomo: 2105
Falciola, Justine: 3130
Falck, Ryan S.: 5100
Falcon Borbolla, Veronica: 9010
Falenchuk, Olesya: 9550
Falvey, Jason: 3550, 7340, 9000
Falzarano, Francesca: 1315, 2050, 2075, 3170, 3385, 4010, 7090, 7300, 7420
Fan, Erica K.: 3405, 9500
Fan, Jin: 4400
Fan, Rong: 4200
Fan, Sheng-Yu: 9030
Fan, Xing: 7660
Fan, Yi: 9540
Fan, Zhiyu: 5115, 7000, 7060, 7950
Fan, Zhongqi: 1290
Fang, Mei: 3255
Fang, Mei Lan: 4245
Fang, Qiyin: 9020
Fang, Ya: 9000, 9020, 9110, 9560, 9580
Fang, Yuan: 7640, 7830
Farah, Kimberly: 2080
Farina, Mateo P.: 2050
Farina, Mateo: 2325, 3465
Farley, Jason: 1375
Farmer, Heather R.: 1070, 3080, 4055
Farnham, Trish: 9530
Faro, Jamie M.: 9030
Farrell, Shannon M.: 3480, 9000
Farrell, Timothy: 9000
Farsijani, Samaneh: 3475, 4260, 5085
Fashaw-Walters, Shekinah: 1380, 9020
Fasola, Serena: 9030
Fassi, Janelle E.: 3385, 9000
Fast, Janet: 7980
Fasth, Lauren M.: 7300
Fastring, Danielle: 1070
Fattorini, Eliana: 7070, 7600
Faul, Anna: 4040, 4360
Faul, Jessica: 2320, 2325, 3225
Faulkner, Sandra: 4390
Fauth, Elizabeth B.: 7400, 7940, 7990, 9030
Fazal, Amaanali: 2020
Fazeli, Pariya L.: 4340
Fazio, Elena: 2150
Fazio, Sam: 3205, 7420, 7540, 9020, 9300
Feder, Shelli A.: 9030
Federwitz, Chad F.: 3485
Feinhals, Gretchen: 3305
Feinn, Rich: 9300
Feitosa, Mary F.: 4260, 5090, 9010, 9020
Felderhoff, Brandi J.: 7310
Feldman, Boris: 7820
Feldman, Corinne: 3220
Feldman, Sue S.: 3365, 4190
Feliachi, Zohra: 8900
Feliciano, Leilani: 4268, 7680
Felix, Laura L.: 5145
Felt, John M.: 5165
Feltmate, Patrick: 9010
Felts, Adam: 1175, 3090
Fendrich, Michael: 1390
Feng, Du: 4134, 7330
Feng, Hui: 7640, 9300, 9320
Feng, Liang: 9020
Feng, Yali: 7010
Feng, Zhe: 1390
Fennell, Charles: 4100
Fennell, Gillian: 1165, 7380, 7460
Fenstermacher, Erika A.: 7030, 7570
Fenton, Laura: 4275
Ferdows, Nasim: 9000, 9030
Ferguson, Erin L.: 2240
Ferguson, Giselle A.: 4045
Ferguson, Lee Ann S.: 9010
Ferguson, Melissa M.: 4370
Fermin-Martinez, Carlos A.: 3160, 4395
Fernández-Chirino, Luisa: 4395
Fernandes, Deanna C.: 3500, 7140
Fernandez Cajavilca, Moroni D.: 1150, 2075
Fernandez-Chirino, Luisa: 3160
Ferrari, Michel J.: 1390
Ferraro, Kenneth F.: 3230, 4535, 9010
Ferreira, Erika A.: 7340
Ferreira Leitao Azevedo, Renato: 1260, 9540
Ferreri, Stefanie: 7150
Ferri, Cleusa Pinheiro: 3160
Ferrucci, Luigi: 1295, 2155, 2195, 2240, 2285, 3510, 4035, 4265, 4490, 5085, 9020, 9300
Fetzko, Joanne E.: 2315
Fiala, Jacob A.: 2180, 5070
Fick, Donna: 4315, 4525, 9010, 9540
Fiehn, Oliver: 7110, 7880
Fielding, Roger: 1290
Fields, Beth: 2070, 2170, 3030, 5000, 7800, 9030
Fields, Maizonne: 7090
Fields, Noelle L.: 2390, 3305, 9010, 9030
Figeys, Mathieu: 9000, 9020
Figueiredo, Daniela: 7300
Filec, Sarah: 2355, 9030
Filewich, Andrew: 7700
Filiatrault, Johanne: 7620
Fillingim, Roger B.: 4134, 7330, 7630, 9010
Finch, Caleb: 9030
Finer, Elizabeth: 7340
Fingerman, Karen L.: 4145, 5045, 7070, 9000
Finkel, Deborah: 3470, 7300
Finkel, Deborah: 9020, 9030
Finkelstein, Ruth: 7230
Finley, Erin: 5055
Finley, Margaret A.: 4005
Finn, Jacob A.: 7420
Finn, Laura A.: 9030
Finnerty, Kaylyn: 7120
Finnie, Dawne: 9000, 9010
Finsel, Julia S.: 7290
Fiori, Katherine L.: 1235, 4230
Fiscella, Andrew J.: 7020
Fisch, Kathleen: 7100, 8900
Fischer, David: 8900
Fischer, Stacy: 7410
Fishbein, Ken: 2195
Fisher, Anna: 2295, 3175
Fisher, Kathryn: 5085, 5220, 7840
Fisher, Lauren: 4134, 7620
Fisk, Calley: 5050
Fiske, Amy: 7030, 7570
Fitch, John E.: 7680
Fitzgerald, Kyle: 9000
Fitzpatrick, Annette L.: 7830
Fix, Gemmae M.: 1375
Flanagan, Kelly: 9010
Flannagan, Carol A.: 7080
Flaster, Aaron: 9580
Fleming, Allison: 5165
Fletcher, Jason: 1130
Fletcher, Jason: 7230
Flett, Gordon L.: 3155
Fleysher, Roman: 1125
Flood, Sarah: 2050, 3060
Flores, Bertha E.: 7180
Flores, Elena: 1270
Flores, Emily: 7930
Flores-Cohaila, Javier: 9010, 9030
Floyd, Alexander: 5015
Floyd, Elizabeth: 9030
Flurry, Ethan: 7510
Fogg, Louis: 3445
Foley, Sophia: 9010, 9310
Follis, Shawna: 3210
Fonseca, Carolina: 4060
Fontanese, Matthew S.: 7080, 7900
Foradori, Megan A.: 4525
Ford, Alicia: 9000
Ford, Cassandra: 2125
Ford, J. Kevin: 9000
Ford, Tiffany N.: 2225
Forder, Michael: 3300
Forman, Daniel E.: 3475, 5210
Forster, Anne: 7720
Fortinsky, Richard H.: 2300, 4140, 7030, 7430
Fosnight, Susan: 4406, 7250, 7480, 7500
Foster, Liam: 4450
Foster, Thomas C.: 7630
Fountain, William: 9010
Fowe, Ibukun E.: 1040
Fowler, Mackenzie E.: 2260
Fowler, Michael: 4268, 7680
Fowler, Nicole R.: 4210, 5105, 5115, 9020
Fowler, Samantha A.: 9010
Fowler-Davis, Sally: 4245
Fox, Aimee: 4010
Foxworth, Judy: 4350
Fröberg, Andreas: 7170
Frager, Victoria A.: 1010
Frakes, Nathan: 9000
Framil, C Victoria: 4415
Francis, Erica: 5145
Francis, Sarah L.: 7590
Frangiosa, Theresa: 9000, 9010
Frank, Colleen: 1090
Frank, Jennifer S.: 4340
Frank, Mabel: 1065
Franke, Emily: 9010, 9540

Fransquet, Peter D.: 7370
Fransquet, Peter D.: 7370
Franz, Berkeley: 7780
Franzosa, Emily: 1020, 2145, 2235, 3415, 4165, 9010
Frase, Robert T.: 1050, 7920
Fraser, Sarah: 1200
Frazier, Charlotte: 2080
Frazier, Ian: 9010
Frazier, Jefferson: 9110
Frazier, Robbin H.: 1025, 2180, 7380
Frechman, Erica: 3395, 9030
Freddolino, Paul Peter: 2085, 4020
Frederick, Miranda: 3445
Fredericksen, Jessica: 7760
Fredriksen-Goldsen, Karen: 2310, 4000, 4195, 4390, 9010, 9440
Free, Julia: 7730
Freedman, Jonathan D.: 2225
Freedman, Vicki A.: 1340, 2150, 3390
Freeman, Shannon: 2340, 5105, 7160, 7300, 9020, 9030
Freeman, Valerie: 9030
Freemdan, Vicki: 1340
Frei, Anja: 3550
Freitas, Fabio Cavalcanti: 9020
French, Eric: 2255
French, Molly: 3045
Frenier, Christopher: 3010, 4355
Frey, Katelyn T.: 7520
Freytes, I. Magaly: 2170
Friberg-Felsted, Katarina: 7480
Fridriksson, Julius: 8900
Fried, Linda P.: 1110
Fried, Terri R.: 3360, 4010
Friedman, Ari B.: 3530, 7860, 8900, 9030
Friedman, Daniela B.: 2260
Friedman, Esther M.: 1340, 2100, 7530
Friedmann, Erika: 4040
Friel, Ciaran: 4268, 7680
Friis, Marlene: 7550
Fritschi, Cynthia: 4025
Fritz, Joëlle V.: 9020
Froberg, Ruthann: 2030
Fromm, Dylan: 9000
Frost, Alanna: 9030
Frost, Melissa: 7590
Fruhauf, Christine A.: 3060, 3385, 4010, 4400
Frungieri, Monica: 9020
Fry, Liam: 4290
Fu, Carolina: 9010
Fu, Cheng: 3115
Fu, Rong: 7230
Fu, Xiaoyu: 4085
Fu, Ying-Kai: 4134, 7620
Fuentealba, Matias: 1380
Fugate Woods, Nancy A.: 5155
Fugate-Whitlock, Elizabeth: 2245, 3350
Fujihira, Kyoko: 7550, 7760
Fujita, Koji: 7550, 7580, 7760
Fujiwara, Natsumi: 4505, 9000
Fujiwara, Yoshinori: 7550, 7580, 7760
Fukuda, David H.: 7150, 9010, 9020
Fukui, Chie: 7160
Fukui, Chie: 9520
Fuller, Heather: 1235, 3380, 5035, 7710, 7810, 9030
Fuller, Kristi: 7690
Fuller, Patrice: 4100
Fuller-Rowell, Thomas E.: 9000
Fuller-Thomson, Elysia G.: 1350
Fuller-Thomson, Esme R.: 1350
Fulmer, Terry: 2060, 4132, 7500
Fung, Helene H.: 3100, 3400, 4480, 7050, 7060, 9020, 9030
Fung, Kathy: 5230, 7160
Fung, Nicole Long Ki: 3100
Fung, Victor: 2390
Funghetto, Silvana Schwerz: 7130, 7150, 7340
Funk, Cory: 5180
Furber, John D.: 9030
Furht, Borko: 5225
Furman, David: 1380
Furmanek, Stephen: 9000
Fusaris, Ethan: 3565, 7810

### G

G Monteiro, Joao Filipe: 9010
Gaba, Anu: 7600
Gaballa, Fairouz: 7420
Gabbay, Mark: 9000, 9570
Gabois, Emily: 4290, 7620, 9000, 9030
Gabrielsson-Järhult, Felicia: 7180
Gad, Dustin: 7420
Gadd, Danni A.: 4134, 7620
Gadkari, Gauri: 1210, 9030
Gaeta, Raphael: 2220
Gagen, Travis M.: 7780
Gagnon, Camille: 9520
Gagnon, David R.: 9010
Gagnon, Sylvain: 7060
Gahche, Jaime J.: 9020
Gahn, Katie: 7570
Gai, Junjie: 7760
Gaillard, Trudy: 7810, 9010
Gajraj, Asha: 9010
Galasko, Douglas: 8900
Gale, Kathy S.: 7910
Galea, Sandro: 2105, 7550
Galik, Elizabeth: 2305, 3330, 3340, 7170, 9020
Galladay, Katelyn A.: 3075
Gallagher, Amelia M.: 3140
Gallagher, Elizabeth A.: 3130
Gallagher Watkin, Laura: 5135
Gallagther-Thompson, Dolores: 7030
Gallegos, Adrianna C.: 7410
Gallibois, Molly: 7830
Gallo, Joseph: 3415, 4460
Galucia, Natalie: 9000
Galvin, Angéline: 5090
Galvin, James: 3490
Gamaldo, Alyssa A.: 2275, 3245, 4055, 7560, 7970
Gamaldo, Alyssa A.: 9020
Gamble, Jean-Michael: 4280
Gamble, Laura: 4070
Gammonley, Denise: 1100
Gan, Daniel R Y.: 5030
Ganann, Rebecca: 5085, 5220, 7840
Ganbat, Uyanga: 7820
Ganci, Aaron: 7080
Gandy, Maribeth: 7990
Gandy, Tyler: 7960
Gange, Stephen J.: 3390
Gangnon, Ronald: 2230
Ganguli, Mary: 5080
Gangupantula, Pratima: 9010
Gans, Kim: 4030
Gao, Jie: 7680
Gao, Shiying: 7120
Gao, Sibo: 7070
Gao, Xianqi: 7660
Gao, Xiaojun: 7420
Gao, Yubo: 2180
Gaona, Carlos A.: 4100
Garabedian, Pamela: 9000
Garber, Johnathon D.: 7150
Garces, Edwin: 9030
Garces Torres, Ana Sophia: 9010
Garcia, Catherine: 1000, 3050
Garcia, Emmanuel: 2020
Garcia, Joshua M.: 1165, 3050
Garcia, Linda: 3260
Garcia, Mary Ellen: 8900
Garcia, Odalis G.: 7550
Garcia, Patricia: 2180, 5070
Garcia, Sandra: 9010, 9030, 9420
Garcia, Y. Evie: 7400
Garcia Morales, Emmanuel E.: 9010
Garcia Rogers, Juliet: 1325
Garcia Walker, Veronica: 2290
Garcia-Andres, Adelaido: 4240
Garcia-Aymerich, Judith: 3550
Gareri, Michele: 7250
Garg, Parveen K.: 3405
Garland, Paula: 7390
Garner, Jennifer: 2080
Garrett, Timothy: 9030
Garrison, Betsy: 7070
Garrison, Brianna: 9010, 9030
Garrison, Jennifer: 3215
Gary-Webb, Tiffany L.: 3405
Gasek, Nathan S.: 9030
Gassoumis, Zach: 2030, 2220
Gaster, Barak: 7830
Gately, Megan: 2085, 7850, 9020
Gates, Jessica: 9420
Gatz, Margaret: 2320, 9030
Gaugler, Joseph E.: 1050, 1150, 1265, 2130, 2190, 3020, 3235, 3240, 3300, 3305, 3460, 3570, 4170, 4290, 4415, 7420, 9000, 9010
Gauss, C. Heath: 7160
Gaussoin, Sarah A.: 7830
Gautam, Ramraj: 1080, 5200
Gaveras, Eleni: 4195
Gay, Emma L.: 3475, 5090
Gay, Jane: 7440
Gazarov, Emely: 1030
Gaziano, J. Michael: 4310
GB, Mia: 7870
Ge, Ning: 7350, 7830
Ge, Shaoqing: 7910
Ge, Shaoqing: 9010
Ge, Song: 3490
Gebara, Marie Anne: 5175

Geddes, Maiya: 7100
Gee, Nancy R.: 4040
Gefen, Tamar: 7700
Geiger, Olivia: 4025
Geisinger, Maria L.: 1075
Geisser, Sophia R.: 2110, 7540
Gelburd, Robin: 4525
Geldhof, John: 7580
Geldmacher, David: 9020
Gélinas, Isabelle: 7690
Gell, Nancy: 7990
Gellert, Paul: 9400
Gelmon, Sherril: 7770
Gendernalik, Jesssica: 7840
Gendron, Tracey: 3025, 3285, 3425
Genelyte, Indre: 4450
Geng, Jing: 4390
Genge, Courtney J.: 9020
George, Claudene: 3480
George, Joanna: 9000
George, Maureen: 9100
George, Mollie J.: 7550
George, Steven: 7410
Georgescu, Michael: 9000
Geraets, Anouk FJ.: 1225
Gerald, Melissa S.: 3525
Gergis, Mary: 4406, 7500
Germine, Laura Thi: 5165
Gerstorf, Denis: 1060, 3275, 4225, 4235, 7550, 9000, 9010
Gess, Peter: 7550
Getz, Marjorie A.: 7480
Ghare, Smita: 9000
Ghazali, Nadia: 1115
Ghazi-Saidi, Ladan: 2080
Ghezzi, Elisa: 1045
Ghiasi, Akbar: 2350
Ghimire, Saruna: 3380, 5220
Ghimire, Saurav: 7880
Ghisletta, Paolo: 4185
Ghose, Urmimala: 9010
Ghosh, Bhaswati: 4475, 9030
Ghosh, Nayanika G.: 2385
Ghosh, Ritam: 9030
Ghosh-Dastidar, Bonnie: 3405
Giambrone, Alessandro: 3160
Giandrea, Michael D.: 3370
Giannotto, Emily L.: 7510, 9020
Giannouli, Eleftheria: 3115
Gibbons, Ian: 9010, 9020
Gibbons, Sean M.: 9020
Gibson, Allison: 3305, 5020, 7060
Gibson, Carolyn: 2280
Gibson, Ethlyn: 9010
Gibson, Regina V.: 4130
Gichu, Muthoni: 9110
Giebel, Clarissa: 9000, 9570
Gieniusz, Marzena: 4030
Gietzen, Luke J.: 7010
Gifford, Allen L.: 1375
Gifford, David: 9000
Giffuni, Jamie: 7840
Gigi, Amateau: 3425
Giles, Sadie: 4390
Gilfix, Talia: 9030
Gill, Jessica: 4310
Gill, Navjot: 7780
Gill, Thomas: 1165, 3230, 3450
Gillam, Juliet: 4470
Gilleard, Chris J.: 4245, 7060
Gillespie, Dylan: 7420
Gilliam, Meredith: 7300
Gilligan, Megan: 1050, 2075, 4275, 7920
Gills, Joshua L.: 2015, 7810
Gillsjö (Gillsjo), Catharina: 7000, 7130, 7310
Gilmore-Bykovskyi, Andrea: 7360
Gilson, Aaron M.: 9030
Gimm, Gilbert: 1335, 9030
Gin, June L.: 2295
Gionet, Sherry: 7660
Giordani, Bruno: 7510, 9020
Giordano, Stephanie: 3010, 7810
Giovannetti, Erin: 1035, 4170
Girdwood, Tyra: 4380
Girling, Laura: 7540, 9320, 9500
Giroux, Dominique: 9000
Gitlin, Laura N.: 1035, 2130, 3240, 4005, 5130, 7550, 9000, 9400
Giunta, Nancy: 5150
Gladyshev, Vadim N.: 4065, 7370
Glashutter, Margit: 7120
Gleason, Carey E.: 5000, 7100
Gleason, Kelly T.: 2185
Gleason, Shayna: 4150, 5110
Gleissner, Laura: 2390
Glenn, Jordan M.: 2015, 7810
Glesby, Marshall J.: 5115
Glicksman, Allen: 3530, 4285, 9030
Gloger, Elana M.: 9020
Glorioso, Danielle K.: 7610
Glowinski, Sarah: 1190
Glusman, Gustavo: 7110
Glymour, M Maria: 2240, 2300
Glynn, Nancy W.: 1295, 3390, 3475, 3500, 5090, 5210, 7650, 9030
Gmelin, Theresa: 1295
Gobaud, Ariana: 2225
Goderis, Geert: 7120
Godina, Sara L.: 3405
Gofine, Miriam: 7640
Goh, Dion Hoe Lian: 3295
Goldberg, Allon: 7150
Goldberg, Anne J.: 7550
Goldberg, Jaime: 3170, 7050
Goldberg, Renada M.: 7230
Golden, Adam: 7680, 7870
Golden, Blair: 1190
Golden, Dale M.: 9010, 9020
Golden, Justine: 4260
Goldgof, Dmitry: 7100
Goldman, Alyssa: 1350, 5050
Goldman, Dana P.: 4355, 4385
Goldman, Noreen: 3065
Goldman, Stuart: 7800
Goldstein, Nathan E.: 4266, 7190
Goldsworthy, Mary: 9020
Goler, Timothy D.: 1070
Golonka, Ona: 4100
Golstein, Nathan: 1375
Gomes, Ana: 2065
Gomes da Rocha, Carla: 3130
Gomes Goncalves, Natalia: 3160, 7320
Gomez, Carlos A.: 9420
Gomez, Jennifer: 3090
Gondo, Yasuyuki: 3555, 9000
Gonyea, Judith: 7670
Gonzales, Ernest: 4085
Gonzales, Mitzi: 4100, 5055, 7660
Gonzalez, Alexander: 5120
Gonzalez, John: 4240
Gonzalez, Richard: 3105
Gonzalez Castillo, Jorge: 9030
Gonzalez-Freire, Marta: 2195
Good, Mary: 9020
Goodale, Sarah E.: 1215
Goode, Adam: 7410
Goodman, Claire: 2375
Goodman, Octavia: 8900
Goodpaster, Bret H.: 3475, 5210
Goodwin, Rebecca: 4365
Gooldin, Sigal: 9030
Gorbunova, Galina A.: 5015, 5110
Gorbunova, Vera: 4065
Gordon, Barbara: 4040, 5190
Gordon, Julie A.: 9020
Gordon, Kate: 3235, 9030
Gordon, Michelle: 3505
Gordon-Wexler, Mikayla: 3415
Gore, Janelle E.: 2070, 2180, 3485
Gore, John: 9010
Gorla, Vandita: 9020
Gorman, Shannon: 7180
Gorospe, Myriam: 5060, 7880
Gorzelle, Gregg: 9400, 9550
Gosselin, Patrick: 7030
Gothard, Sarah: 4100, 4495, 7440
Gottesman, Eve M.: 7000
Gottesman, Rebecca F.: 3510
Gouaux, Ben: 4340
Gouelle, Arnaud: 9330
Gould, Christine E.: 3250, 4095
Goulet, Joseph L.: 4300
Gouse, Hetta: 4340
Gouskova, Natalia: 2155
Gouveia E Silva, Erika Christina: 9010, 9020
Gowen, Emma: 1130
Gozalo, Pedro: 7160
Grabowski, David: 1115
Gracner, Tadeja: 4385
Grady, Alexandra: 7450
Grady, James: 4140
Graessel, Elmar: 2370
Graf, Allyson S.: 2080
Graf, Peter: 4480
Graf, Sylvie: 3100
Graff, Maud: 3340
Graham, Carrie L.: 5025, 7250
Graham, David: 5075, 7910
Graham, Eileen K.: 1255, 3520, 4300
Graham, Kirsten L.: 1365
Graham, Laura A.: 5230, 7160
Graham, Liz: 7720
Graham, Lyndsey N.: 3195
Graham, Robert J.: 3045
Graham-Engeland, Jennifer E.: 3500, 3545, 4490, 9010, 9030

Grant, Jeremy G.: 9210
Grant, Shakira J.: 2355
Graupner, Jeffrey P.: 9010
Gravenstein, Stefan: 7620, 9010
Graves, Kaitlyn: 7420
Gravitt, Patti E.: 4080
Gray, Jeff: 4415
Gray, Laura: 2375
Gray, Michelle: 2015, 7810
Gray, Nicholas: 1315
Gray, Vicki: 9000
Greaney, Mary L.: 4025
Greco, Frank: 9020
Greeley, Brian: 7160
Green, Cara L.: 7880
Green, Ginny: 8900
Green, Kerry M.: 4408, 7230
Green, Makenna R.: 4406, 7480
Greenberg, Jonathan: 4460
Greenberg, Phyllis A.: 2095, 4406, 7000
Greenberg, Sherry A.: 4005
Greenblatt Kimron, Lee: 3275
Greendale, Gail A.: 4120
Greenfield, Emily A.: 1330, 2315, 3045, 4150, 4195
Greenwood-Hickman, Mikael Anne: 1010, 3490
Greer, Valerie Chang: 4132, 7500
Gregg, Jeffrey J.: 3250, 4095
Gregg, Jordan: 5060
Gregory, Sarah: 7630
Gregson, Geoffrey: 9000
Greiner, Chieko: 9010
Grelle, Kaitlin: 7420
Grenier, Amanda: 4450, 7190
Grenier, Sébastien: 7030
Grewal, Ashmita J.: 5145
Grewal, Karl: 1200
Griffin, Joan: 9000, 9010
Griffin, Meridith: 5100
Griffin, Rebekah: 7080
Griffith, Christopher X.: 4268, 7680
Griffith, Lauren E.: 4268, 4280, 5070, 5220, 7170, 7660, 7680
Griffiths, Alys: 7720
Griffiths, Jane: 4110
Griffiths, Patricia: 7990
Grill, Joshua: 4420
Grimm, Kevin J.: 7610
Grimmett, Hailey: 2390
Grippa, Rosa: 5105
Griswold, Allison: 9030
Griswold, Michael: 5085, 7340, 9200
Grittner, Alison: 1205
Grocke, Michelle: 5075
Grodstein, Francine: 2120
Groen, Wim: 2370
Gronlund, Carina: 9010
Gross, Alden: 2150, 2240, 2365, 3390, 3510, 5175, 9110
Gross, Danae C.: 4268, 7680
Grossberg, George: 9020
Groulx, Mark: 2340
Groves, Angela: 7800
Groves, Erik: 9020
Growney, Claire M.: 1105, 4480, 4495, 8900
Gruber-Baldini, Ann L.: 3550, 4260
Grudzien, Adrienne: 9000
Gruenewald, Tara L.: 2320
Grullon, Jason Rafael: 9020
Gruneir, Andrea: 4280
Gruss, Valerie: 4025
Gu, Danan: 7080, 7590
Gu, Xiangli: 7730
Gu, Xiuzhu: 7290
Guan, Hua: 9020
Guan, Weihua: 5100
Guastaferro, Kate: 9010
Guerbaai, Raphaëlle-Ashley: 7160
Guerrier, Tami: 4350
Guess, Aundaray: 1250
Guggenheimer, Joshua: 8900
Gugliucci, Marilyn R.: 2095, 2215
Guida, Vincent: 7500
Guillemot, Jonathan: 4266, 4406, 7000, 7190, 7250, 7290
Guimaraes, Rafael: 7610
Guimond, Josée: 1280
Guinn, Victoria LA.: 3050
Guitar, Nicole A.: 7160
Gum, Amber M.: 3560, 4010, 9020
Gunderson, Rachel: 9030
Guo, Chao: 9000, 9010, 9030, 9210
Guo, Crystal: 7850
Guo, Man: 3210, 4050
Guo, Nicholas: 8900
Guo, Yingqqi: 3115
Gupta, Shivani: 7210
Guralnik, Jack: 4260
Gurlu, Merve: 4005, 4040, 9020
Guruge, Sepali: 9010
Gurven, Michael D.: 9030
Gur-Yaish, Nurit: 7050
Guseva, Elena: 7080
Gusmano, Michael K.: 3165
Gustafson, Deborah: 3565
Gutierrez, Angela: 1115, 7780
Gutierrez, Mariela: 9030
Gutierrez, Sirena: 7600
Gutierrez-Baca, Wendy: 9010, 9030
Gutman, Roee: 4290
Guyonnet, Sophie: 2320
Guzman, Rebeca: 7680

**H**

Haaland, Perry: 7360
Haapanen, Markus: 9010
Haardörfer, Regine: 3565
Haas, Meghan: 7360
Haas, Steven A.: 2360
Haase, Claudia M.: 1105
Haase, Joan: 9400
Håberg, Asta K.: 9020
Haché, Josée: 7830
Hachen, Naomi: 3480, 9000
Hackel, Emily: 9020
Hacker, Eileen: 9440
Hackett, Sara E.: 2030
Hacking, Coen: 1260, 7800
Haddad, Jeffrey: 7060
Haddad, Yara L.: 9010, 9100
Hadlandsmyth, Katherine E.: 2180
Hafeez, Mohib: 9020
Hafner, Camryn: 2095
Hagedorn, Aaron: 9000
Haggar, Katherine: 1055
Haggler, Jayden Austin: 7100
Hahn, Sarah J.: 2075
Hahne, Jessica: 9020
Haigh, Sylvia V.: 4290
Hailer-O'Keefe, Laurie: 3285
Hairi, Noran Naqiah: 4266, 7190
Hajcak, Greg: 7030
Hajdar, Melisa: 1235, 7710
Hakanson, Megan: 5025
Hakim, Talib: 7900
Häkkinen, Emilia: 4330
Hakun, Jonathan G.: 5165, 7510, 9030
Halasz, Ildiko: 9010
Halaszynski, Jaime J.: 2030
Haldar, Srabani: 9030
Hale, Timothy M.: 1260
Hale, Willie: 7420
Haley, William E.: 2245, 3345, 7420, 7510, 7960
Hall, Alex: 4455
Hall, Anna: 7610
Hall, Charles: 9020
Hall, Daniel: 2180
Hall, Katherine: 7840
Hall, Kelli S.: 1110
Hall, Kendall: 7810
Hall, LaToya N.: 3090
Hall, Lawrence: 7100
Hall, Martica: 5155
Hallen, Sarah: 7150
Haller, Landon J.: 7180
Hallgren, Jenny: 7000, 7130, 7310
Halonen, Pauliina: 9000
Halpin, Sean N.: 2260
Halter, Colt: 7510
Halvorsen, Cal J.: 3095, 3290, 4210, 4255
Hamada, Fumiko: 3135
Hamama Raz, Yaira: 3275
Hamel, Alexander: 9030
Hamer, Mark: 2015
Hamers, Jan PH.: 1260, 7720, 9010
Hamilton, Alice: 7950
Hamilton, Jill: 3485
Hamler, Tyrone C.: 2050, 3005, 7410
Hamm, Jeremy: 1360, 7550, 7990, 9000
Hammel, Edward W.: 9000
Hammel, Iriana: 9030
Hammel, Joy: 7360
Hammers, Dustin: 4210, 5105, 7100
Hammond, Allison: 7390
Hammouri, Ammar: 7610, 7640
Hampton, Kischa: 1265
Hamrick, Mark: 9010, 9030
Han, Chengming: 4305
Han, Duke: 3090, 4275
Han, Gaeun: 7510, 7940
Han, Gang: 7430, 7760
Han, Gyounghae: 3130
Han, Ling: 2360, 9030
Han, Sae Hwang: 4255, 7020
Hanaoka, Hideaki: 7780
Hancock, Cynthia R.: 3350
Hancock, David W.: 2060
Hancock, Laura M.: 2240
Hand, Michelle D.: 3310, 7230
Handberg, Eileen: 9580
Handelsman-Nielsen, Mika: 7360
Hanes, Douglas W.: 5110, 7590
Hanlon, Alexandra L.: 4545, 9020

Hanna, Kerry: 9000
Hanna, Sophie: 7510
Hanna, Valecia D.: 1035, 4170
Hannah, Camille J.: 5215
Hannan, Marian T.: 2385
Hanratty, Barbara: 4455, 4540, 7160, 9010
Hansen, Abigail: 3570
Hansen, Jared: 9420
Hansmann, Kellia J.: 2230
Hanson, Laura C.: 4005, 7300
Hanssen, Denise: 9000
Hansson, Asa: 7380
Happ, Mary Beth: 3345
Harada, Ann SM.: 4355
Harada, Ko: 7860
Hardern, Sonia: 3270
Harding, Alyson B.: 1015
Hardison, Holland: 7150
Harlock, Sarah: 7830
Harlow, Sioban D.: 5155
Harman, Sherman M.: 7100
Harnett, Tove: 5110, 7190
Harnish, Kathryn: 4215
Harper, Colin: 9010
Harrell, Rodney: 2315
Harries, Priscilla: 7690
Harrington, Charlene: 9030
Harrington, Erin: 7510, 9010
Harrington, Erin E.: 9010
Harrington, Karra D.: 5165, 7510
Harris, Frances: 7990
Harris, Jolie: 4250
Harris, Maurita T.: 7680
Harris, Rebekah: 9010
Harris, RianSimone: 4400
Harris, Tina Marie: 7360
Harris-Love, Michael O.: 9020
Harrison, Sean: 9030, 9540
Harrison, Tracie Culp: 2290, 4155, 7370
Harrison, Zinnia: 4195
Harrison Bush, Aryn L.: 7330
Harris-Speight, Jasmine: 7590
Hart, Abigail: 7550
Hart, Foxx: 9020
Hart, Sara: 7470, 7540
Hartford, Emily G.: 4510
Hartmann, Christine W.: 3365, 3415, 4290, 7540
Hartnell, Lisa M.: 2195
Hartzog, Meggan: 7540
Harvath, Terri A.: 2215
Harvey, Kaitlyn: 3310
Harvey, Marie: 4105
Harvey, Philip: 3515
Harvey, S. Marie: 1240, 4105
Hasan, Mehedi: 9000
Hasche, Leslie: 4195, 5200
Hasmanova Marhankova, Jaroslava: 9030
Hass, Zachary: 4215, 7790
Hassenstab, Jason: 3245
Hassett, Afton: 7680
Hasson, Seana: 1065
Hastings, Susan: 7150
Hathaway, Wendy: 1005
Hathaway, Zachary: 3395
Hattakitjamroen, Varitnan: 9580
Hattori, Robin: 1255
Haunch, Kirsty Jayne: 2375, 7720
Hausman, Hannah: 8900
Haverhals, Leah M.: 1160, 2295, 4408, 7160, 7720, 9310
Havers, Fiona P.: 4130
Haviland, Kelly: 7410
Hawes, Courtney: 9000, 9030
Hawes, Frances M.: 3295, 9030
Hawkley, Louise: 4205
Hawley, Chelsea: 2085, 7770
Hawley, Sarah: 1085
Hayajneh, Audai: 7330
Hayashida, Cullen T.: 7280
Hayden, Kathleen M.: 4355
Hayes, Shannon: 5100
Hayes, Timothy: 9000
Haynes, Richard: 7060, 7190
Hayslip, Bert: 3060, 4400, 7530
Hazany, Farnaz: 4420
Hazelett, Susan: 7250
He, Daqing: 5235
He, Jiamin: 7140, 7330
He, Lingxiao: 2320, 9000, 9020, 9580
He, Mengying: 4190
He, Qianyu: 2255
He, Ranran: 1050
He, Yuelin: 3225
Head, Elizabeth: 1215
Hearn, Gesine: 7610
Heath, Diana: 1305
Heaton Curtis, Elizabeth: 1310
Hebert, James R.: 2260
Heckman, Rosalind: 7020
Hedeker, Donald: 9030
Heeb, Rachel: 7780
Heemskerk, Amber: 9010, 9200
Heffernan, Kevin S.: 7970
Heffner, Kathi L.: 1335, 4460, 5185
Heideman, Kristin: 9020
Heid-Grubman, Jeanne: 9000
Heijmans, Bas: 2285
Heiland, Frank: 3080
Heilbrunn, Emily S.: 5145
Heimler, Stephanie R.: 7370
Hein, Maria: 2160, 3335, 5015, 7300
Heinrich, Allison: 4350
Heinz, Melinda: 2390, 7560
Heisel, Marnin J.: 3155
Heisz, Jennifer: 7180, 7340
Helbostad, Jorunn L.: 3550
Helene Choquet, Tom Hoffmann: 2240
Helm, Noah: 9330
Helmly, Victoria: 4115
Hemmings, Nina: 2255
Hemmy, Laura S.: 3460
Hemphill, John: 7830
Henderson, Christine: 7160
Henderson-Betkus, Mary: 9030
Hendriks, Ard: 7800
Hendry, Phyllis: 9010
Henke, Maria: 4435
Henkens, Kène: 4130, 9030
Henne, Mike: 3355
Henneberry, Kathleen: 4035
Hennessy, Catherine: 7190
Henry, Maureen: 1020
Hensley, Robert B.: 7460
Heo, Seongkum: 7850
Hepburn, Kenneth: 1010, 2070, 2130, 2300, 3235, 3435, 3535, 7420, 7440
Hepple, Russell T.: 3475, 5210
Herbstman, Julie B.: 1110
Herd, Pamela: 1000
Herman, Allison: 5060
Hermelbracht, James: 3350
Hermens, Lotte: 3340
Hernandez, Adriana: 4150, 9000
Hernandez, Alyssa: 3525
Hernandez, Bianca: 4132, 7480
Hernandez, Christopher D.: 4132, 7500, 9020
Hernandez, Fernanado: 7100
Hernandez, Guisela: 9010
Hernandez, Janice F.: 7980
Hernandez, Mario A.: 9010
Hernandez, Veronica: 9030
Hernandez Chilatra, Jessica A.: 5140
Hernandez Lepe, Fernando: 1185, 7950
Hernandez-Bigos, Kizzy: 4010
Hernandez-Lujan, Deisy: 9310
Hernandez-Tejada, Melba A.: 5140, 7010
Herr, Keela: 4134, 7620
Herrell, Cheyenne T.: 9010, 9020
Herrick, Tamara: 7150
Herrmann, Francois R.: 3130
Herrmann, Nathan: 7080
Hersey, Catherine: 4195
Hertel, Mollie: 5220
Hertzog, Christopher K.: 7510
Herzog, Monica Anderson: 4415
Heselton, Hyeon Jung: 7020
Heslin, Samita: 9020
Hess, Thomas M.: 3100
Hetherington-Rauth, Megan: 4260
Hettiarachchi, Pasan: 7170
Heun-Johnson, Hanke: 4385
Hewston, Patricia: 5100
Heyn, Patricia C.: 9010
Hiatt, Shirin O.: 3535
Hickey, Amber: 7400
Hickman, Susan: 9000, 9400
Hicks, Gregory: 7410
Hicks Patrick, Julie: 1365, 3165, 7450, 7760, 7910, 8900
Hida, Mitsumasa: 9030
Higa, Moritake: 2380
Higdon, Kristin: 7370
Higgins, Melinda: 2070, 2300, 7440
Higgins Tejera, César: 2225
Higgins-Chen, Albert: 9010
Higgs, Paul: 4245, 7060
Hilgeman, Michelle M.: 3365, 4290
Hill, Andrea M.: 3285
Hill, Caesar: 9000
Hill, Jordan R.: 7080
Hill, Kaliya: 4350
Hill, Nikki L.: 4425, 7830
Hill, Patrick L.: 3135, 3245, 3520, 7060, 9020, 9570
Hill, William D.: 9020, 9200
Hill-Ashford, Yolanda: 7680
Hill-Jarrett, Tanisha G.: 2225

Hillman, Charles: 7100
Hillstedt, Ebba: 9030
Hilsabeck, Robin C.: 5235
Himes, Dani: 2105, 3220
Hinrichs, Timo: 3115
Hirsch, Jana A.: 1015
Hirsch, Jen: 1280, 2330
Hirsch, Mark: 4350
Hirschman, Karen: 4134, 4545, 7620, 9010, 9020, 9030
Hirst, Sandra: 1145, 9030
Hirvela, Kari: 1190
Hladek, Melissa: 5175, 5230
Ho, Alvin: 3280, 9010
Ho, Hung Chak: 3115
Ho, Rainbow Tin Hung: 7610
Ho, Ringo Moon-Ho: 3085, 3295, 3470
Ho, Tai-Hung: 9030
Ho, Tuong-Vi: 1165
Hoàng, Isabella: 9000
Hoang, Van: 9530
Hobbs, Matthew: 1015
Hobday, John V.: 3235
Hoben, Matthias: 3335, 4025, 4280
Hochberg, Marc: 7330
Hockett, Samuel: 7570
Hodes, Richard: 3040, 3185
Hodge, David R.: 9030, 9300
Hodge, Oliver: 2260
Hodges, James: 4420
Hodgson, Nancy: 3485, 4005, 4134, 7330, 8900, 9030, 9540
Hoeffer, Charles: 9010
Hoffins, Emily L.: 9030
Hoffman, Geoffrey: 1340
Hoffman, Jeanne: 7280
Hoffman, Jessica A.: 3350
Hoffman, Rashelle: 3535
Hoffman, Yaakov: 3275
Hoffmann, Coles: 4035
Hogan, David B.: 4280, 7660
Hogans, Beth B.: 7800
Högnäs, Robin S.: 3265
Hogue, Angela: 4525
Hogue, Mary H.: 9010
Hohaus, Chiara: 7070
Holden, Richard J.: 7080
Holditch, Sarah C.: 7390
Holland, Alexandra: 7760, 9310
Holland, Peter: 7870
Hollandsworth Reed, Jenn: 7770
Holley, Kynleigh: 4350
Holley, Lyn M.: 3325
Hollingshaus, Michael: 4365
Hollingsworth, Holly: 7780
Hollins, Nancy: 7950
Hollis-Sawyer, Lisa: 1080, 3250
Hollister, Brooke: 9020
Holllingsworth, Alexis: 7900
Hollman, Nicholas: 9030
Holmes, Ashleigh: 7330, 9020
Holmes, Chad E.: 2295
Holmes, Rachel: 2015, 7370, 7800
Holmes, Sarah D.: 2210, 5020, 9020
Holt, Heidi: 3055
Holt, Janet: 2125
Holtgrave, Peter: 3055
Holton, John: 9000
Holtzer, Roee: 2155
Homan, Kristin: 7050
Homan, Melanie: 8900
Hon, Michelle: 9030
Honda, Ayumi: 7940
Honda, Sumihisa: 7940
Honfo, Sewanou Hermann: 1110
Hong, Andy: 7250
Hong, Dahye: 9010
Hong, Seunghye: 4025
Hong, Y. Alicia: 7750
Hong, Yinglu: 1260
Honig, Lawrence S.: 5090
Hood, Leroy: 5180
Hood, Michelle: 5155
Hoogendijk, Emiel O.: 7660, 9010
Hoogland, Aasha I.: 7410
Hook, Mary L.: 1190
Hooker, Julia: 4460
Hooker, Steven P.: 3390
Hooten, Madeline: 7100
Hoover, Andrew: 9010
Hooyman, Andrew: 9030
Hooyman, Nancy R.: 1280, 4135
Hopp, Faith P.: 1005, 4266, 7780
Hopp, Sarah: 1325
Hopper, Ingrid: 7300
Hopper, Shawna: 7060
Hoppmann, Christiane: 3275, 4235, 4480, 7550, 9000
Hoque, Masudul: 7620
Horgas, Ann L.: 7670
Horhota, Michelle: 1200
Horii, Mariko: 8900
Horn, Andrea B.: 4225
Horner, Sharon: 7980
Horning, Melanie A.: 7850
Horowitz, Chloe S.: 5080
Horstman, Molly J.: 7400, 7850
Hort, Jakub: 7420
Horta, Marilyn: 9010
Horwitz, Shelley: 5030, 7120
Hoselton, Jill H.: 1205
Hosking, Diane: 3570
Hossain, Mohammad Sajjad: 5020, 7060
Hossain, Sharmin: 4265
Hossain, Sheikh R.: 9000
Hosseini, Charissa: 9010
Hosseini Ghomi, Reza: 7300
Hoth, Karin: 7990
Hou, Anthony: 2220
Hou, Lisha: 7830
Hou, Su-I.: 2345, 3400
Hou, Xiaohui: 1165
Hough, Monica Strauss: 4415
Houghton, Frank: 7560
Hounsell, Cindy: 2025
Houser, Ari: 2395
Houston, Denise K.: 3505, 4355, 7630, 9010
Hovland, Cynthia A.: 4406, 7500
Howard, Elizabeth P.: 3095, 7160
Howard, Margret P.: 7890, 9010, 9030
Howard, Timothy D.: 5060
Howard, Virginia: 9000
Howe, Judy: 7000
Howe, Melissa: 1230
Howell, Britteny M.: 1365, 2245, 5200, 9010, 9020
Howell, Teresa F.: 7180, 9000
Howlett, Susan E.: 1060, 9010
Hoy-Ellis, Charles P.: 2310
Hoyumpa, Garielle: 7610
Hreha, Kimberly: 9030
Hsieh, Jessica: 7010, 9030
Hsieh, Ning: 4230
Hsieh, Yi Chin: 9030
Hsu, Amy P.: 7830
Hsu, Clarissa: 3490, 7810
Hsu, Erh-Chi: 4408, 7070, 7700
Hsu, Hui-chuan: 7990
Hsu, Kai-Wen: 4490
Hsu, Wan-Hsiang: 3365
Hsu, Wen-Chuin: 4155
Hsueh, Ming-Chun: 7930
Hu, Bo: 1130
Hu, Elise Z.: 4465
Hu, Jie: 7610
Hu, Jinyu: 9020
Hu, Lynn: 7760
Hu, Qing: 4155
Hu, Rita X.: 1255, 2100, 3290, 4285, 4325
Hu, Tiantian: 9010
Hu, Tsuey-Hwa: 9010
Hu, Xiyuan: 9030
Hu, Xujing: 9000
Hu, Yi-Ling: 7170
Hu, Yuanyuan: 7280, 7750
Hu, Yue: 9020
Hua, Cassandra L.: 1210, 3320
Hua, Yindong: 7680
Huang, Alison R.: 1295, 2135, 2240, 3335
Huang, Amy: 7710
Huang, Beicheng: 9010
Huang, Bin: 7300, 7830
Huang, Chiung-Kuei: 7390
Huang, Di: 9540
Huang, Hao: 9010
Huang, Hsien-Hao: 9030
Huang, Hsui-Li: 4155
Huang, Jiahui: 9030
Huang, Jian: 7370
Huang, Jing: 3500, 4310, 5175, 7070
Huang, Kian A.: 7890
Huang, Molly: 8900
Huang, Qiao: 9000, 9020
Huang, Sherri: 7870
Huang, wei-Jia: 4134, 7830
Huang, Xiuxiu: 7100, 7830
Huang, Yuan: 7210
Huang, Ziwei: 5180
Hubbard, Erin: 2280
Huber, Lesa: 4020
Huber, Zilla M.: 4225
Hubert, Terrence: 2295
Hucek, Ally: 1085
Huckfeldt, Peter: 3010, 4355
Hudson, Amy E.: 9000
Hueluer, Gizem: 4225
Huffman, Kim: 9000
Hugenschmidt, Christina: 5230
Hughes, Adriana: 7620
Hughes, Griffin: 7210
Hughes, Jaime M.: 3505, 4350
Hughes, Kelly L.: 3365
Hughes, M. Courtney: 4540, 7090
Hughes, MacKenzie L.: 1255, 7510, 7550
Hughes, Michael: 7590
Hughes, Michelle: 7480

Hughes, Susan: 7710
Hughes, Tiffany F.: 5080
Hughes, Tim M.: 7630
Hughto, Jaclyn MW.: 4000
Hui, Ferdinand: 2380
Hui, Jenny: 4340
Huisingh-Scheetz, Megan: 9030
Huisman, Martijn: 4485, 7360
Huisman - De Waal, Getty: 3340
Hulsebus, Lindsey: 7260
Hulsman, Erin: 7590
Humber, Marika B.: 4095
Humbert, Andrew T.: 9580
Hummel, Scott L.: 7210
Hunder, Allison: 9020
Hung, Anthony: 9030
Hung, Lillian: 2340, 3270, 3570, 9000, 9010, 9020
Hung, William W.: 2085
Hung, William: 2145, 7820, 9010
Hunt, Riley: 1375
Hunter, Alistair: 4285
Hunter, Melissa L.: 2375
Hunter, Paulette: 4470
Huntington-Symons, Makeba: 4010
Huo, Meng: 1050, 3425
Huo, Zhiguang: 7630
Huppert, Theodore J.: 2155
Hurd, Julie A.: 7910
Hurley, Jennifer: 4335
Hurwitz, Isabel: 9000
Huseth-Zosel, Andrea: 1235, 7710, 7810, 9030
Hussain, Naaz A.: 1035
Husser, Erica K.: 4315, 4525
Hutson, Jennifer: 8900
Hutto, Lashawn: 1070, 7330
Hutton Johnson, Stacy: 3535
Huynh, Duong: 7300
Hwang, Doyeon: 9020
Hwang, E. Shelley: 1370
Hwang, In Jeong: 4390
Hwang, Jing-Shiang: 9010
Hwang, Misun: 7570
Hwang, Phillip: 9010
Hwang, Sinwoo: 7460
Hwang, Ula: 2085
Hwang, Woosang: 4085
Hwang, Yeji: 7540
Hydén, Lars-Christer: 4190
Hyde, Cori D.: 7590
Hyde, Joan J.: 9030
Hyde, Martin: 5050, 7520
Hyun, Jinshil: 9020
Hyun, Kate: 7730

I

Iaboni, Andrea: 7080
Iacob, Eli: 1150, 2035, 4365, 9020
Iaconi, Alba: 4408, 7720
Ibáñez-Carrasco, Francisco: 4340
Ibrahim, Safa: 7540
Ice, Erin: 7470
Idler, Ellen L.: 4045
Igarashi, Ayumi: 7160, 9520
Igarashi, Heidi: 7580
Ignacio, Daniel: 7070
Ihara, Emily S.: 1335, 3310, 7230, 7750, 7760
Iijima, Katsuya: 3310, 9030
Ikeuchi, Tomoko: 7280
Iktilat, Khalil: 3125
Ilardo, Joan L.: 4155
Iliff, Jeffrey: 9000, 9010, 9020
Iloputaife, Ikechukwu: 9030
Im, Eun-Ok: 7220
Im, Yunju: 7510
Imai, Ryota: 9030
Imai, Shin-ichiro: 2205
Imaoka, Masakazu: 9030
Imms, Phoebe: 9020, 9030
Imuta, Hiromi: 7580
Inagaki, Asa: 9520
Inagaki, Hiroki: 7280
Infurna, Frank J.: 7610, 7950, 9010
Ingle, M. Pilar: 3410
Ingvalson, Stephanie N.: 2130, 3570
Inker, Jenny: 3300, 3425
Inloes, Jennifer: 2295
Inoue, Megumi: 1335, 3310, 7700, 7750
Insel, Kathleen C.: 1260
Intrator, Orna: 1005, 2145
Intrator, Orna: 9010, 9030
Inventor, Ben: 3445
Ioanitoaia-Chaudhry, Iulia: 4075
Ioannidis, George: 4268, 5100, 7680
Ipakchi, Narda: 2200
Iqbal, Anum: 4455
Irimia, Andrei: 9020, 9030
Irwin, Madison: 7670
Isaacowitz, Derek: 1105, 3195
Isaacs, Tanya: 9030
Isales, Carlos M.: 7370
Ishak, Emily: 2365
Islaya, Cesz: 7460
Ismael, Saifudeen: 9010
Ismail, Zahinoor: 7080
Israel, Sarah: 2040
Istvanek, Kathryn: 2170
Itabashi, Masako: 9030
Ito, Hiro: 9020
Ito, Kae: 7280
Iveniuk, James: 1230, 9010
Iwarsson, Susanne: 2340, 7380
Ix, Joachim H.: 9500
Izzetoglu, Meltem: 2155

J

Jääskeläinen, Tuija: 9000
Jablonski, Rita A.: 1075, 3545, 4145
Jaboob, Saida: 4025
Jacelon, Cynthia: 9010, 9030
Jackson, Amy L.: 4005, 9010
Jackson, Casey: 3395
Jackson, Chandra: 7870
Jackson, Eva MJ.: 3300
Jackson, Heide: 3005
Jackson, Kathleen J.: 5055
Jackson, Kathryn L.: 1255
Jackson, Lexie: 3015
Jackson, Piper: 9030
Jackson, Simone: 7670
Jackson, W. Clay: 9020
Jacob, Peter: 3570
Jacobs, David R.: 9010
Jacobs, Jeremy M.: 2015, 7300
Jacobs, Karen: 4125
Jacobs, M. Lindsey: 7390, 9010
Jacobs, Michael A.: 2180
Jacobs, Moyosoreoluwa O.: 7970, 9020
Jacobsen, Erin: 5080
Jacobson, Mireille: 3220, 4435, 5110
Jacques, Paul: 1290
Jaeger, Byron: 4134, 7830
Jaen, Carlos: 5055, 7660
Jaffee, Michael: 9010, 9020
Jaffri, Abbis: 7020
Jahn, Georg: 7070, 7590
Jaijairam, Emily: 9030
Jain, Shalini: 4475, 7100, 7870
James, Bryan D.: 2120, 2365, 3245
James, Dara: 5080
James, Dara: 7100
James, K. S.: 4385
Janevic, Mary R.: 7680
Jang, Aeji: 9100
Jang, Heejung: 1360
Jang, Jae Young: 9020
Jang, Jieun: 9520, 9540
Jang, Jinwoo: 5225
Jang, Jiyoon: 7420, 7720
Jang, Yuri: 2090, 7420, 7510, 7960, 9000, 9020
Jani, Niti: 8900
Janke, Megan: 7610
Jansen, Carl-Philipp: 3550
Jansen, Jacobus FA.: 1225
Jansen, Taylor: 4266, 7230, 7270, 7830
Janssen, Daniel: 9520
Janssen, Leah M.: 3190, 4435
Jao, Ying-Ling: 3020, 7160, 7620
Jarrín, Olga F.: 1240, 4105, 7800, 9000
Jarrett, Pamela: 7660, 7830
Jarrott, Shannon E.: 2140, 3385, 4085, 7590
Jason, Kendra: 7290
Javier, Noelle Marie: 4266, 7190
Jayakody, Oshadi: 3160
Jaykrishna, Poornima: 9020
Jeamjitvibool, Thanakrit: 4025
Jeanblanc, Alexandra: 2005, 3360, 7030, 9020
Jee, Sue: 9030
Jee, Youngmin: 9030
Jefferson, Rebeca C.: 9000
Jehu, Deborah: 9010, 9030, 9500
Jen, Sarah: 4195
Jenkins, August: 5130
Jennings, Lee: 4060, 9020
Jensen, Francine Bench: 3410
Jeon, Heesu J.: 9400
Jeon, Jehoon: 9010
Jeon, Sangha: 3135
Jeong, Chung Hyeon: 9010, 9020
Jeong, Haelim: 5125
Jeong, Innhee: 9000
Jeong, Jihyeong: 9010
Jeong, Kyu-Hyoung: 7030
Jeste, Dilip V.: 1390, 3565
Jester, Dylan J.: 7610
Ji, Linying: 1180, 3500, 4490
Ji, Wenyan: 9020
Jia, Maoni: 9110
Jia, Yuane: 4210, 7990

Jiang, Da: 4480, 4540, 7610, 7950
Jiang, Depeng: 7940
Jiang, Kening: 2135, 2240, 3335, 4265
Jiang, Lin: 4240, 9030, 9300
Jiang, Nisi: 4475
Jiang, Quanbao: 7530
Jiang, Xiangxiang: 9000
Jiang, Yan: 9550
Jiang, Yanping: 2090, 3035, 4050, 7080, 7600, 9020
Jiang, Yun: 1260, 7570
Jiang, Zhe: 4415
Jim, Heather: 7410
Jimenez, Daniel: 4340
Jimenez, Daniel R.: 9000
Jimenez, Sarah Y.: 5185
Jimenez Romero, Angélica: 9000
Jin, Bora: 7190
jin, Haiyu: 4090
Jin, Jessica: 7300, 7910, 9010, 9020
Jin, Shiow J.: 9010
Jindal, Shivani K.: 2145, 7860
Jing, Bocheng: 5230, 7160
Jiwani, Rozmin: 7180, 7910
Jobaidul, Boni: 7730
Joffe, Hadine: 5155
Johansson, Boo: 7660
John, Deepti Anna: 4540, 9010
Johnson, Abree: 3550
Johnson, Alisa J.: 4134, 7330
Johnson, Alisha H.: 7140
Johnson, Benjamin B.: 3250, 7590
Johnson, Christopher: 4155
Johnson, CJ L.: 4408, 7700
Johnson, Elma: 1150, 3240, 3300, 7380
Johnson, Eric: 5135
Johnson, Florence U.: 9020
Johnson, Ian M.: 3410
Johnson, Ian: 3410
Johnson, Ian M.: 5150
Johnson, James: 7110
Johnson, John C.: 7860
Johnson, Julene: 1150, 2075, 9020
Johnson, Justin: 5195
Johnson, Karen C.: 4355
Johnson, Kim G.: 9550
Johnson, Kimberly S.: 4165
Johnson, Kimson E.: 9030
Johnson, Melanie L.: 8900
Johnson, Michelle J.: 7860
Johnson, Min Kyoung: 2090, 9320
Johnson, Roxann H.: 4155
Johnson, Sterling C.: 2240
Johnson, Yuuko M.: 9030
Johnson Nicholson, Meneka C.: 9020
Johnson-Koenke, Rachel A.: 9020, 9030
Johnston, Carrie: 5115
Johnston, Laura: 9000
Joiner, Raquael J.: 1070, 4400
Jones, Alexandria: 7020
Jones, Bobby L.: 3500
Jones, Brittany R.: 7230
Jones, Christine D.: 7160
Jones, Elizabeth J.: 9420
Jones, Floyd A.: 4100
Jones, Garrett: 9000
Jones, Harlan P.: 3450
Jones, Holly: 7850
Jones, Judith: 4300, 7640
Jones, Leanne: 3070
Jones, Liz: 2375
Jones, Louise: 7160
Jones, Megan D.: 2015, 7810
Jones, Patricia: 3040, 1380
Jones, Sarah E.: 1315, 4415
Jones, Valerie: 4268, 7680
Jönson, Håkan: 5110, 7190
Joo, Jinhui: 4460
Joo, Susanna: 4370, 7610
Jopp, Daniela S.: 3130, 7080
Jopson, Andrew D.: 5160
Jordan, Tera R.: 7830
Joshi, Purnashree: 5075
Joshi, Suryakanth: 9010
Ju, Eunae: 4025
Juckett, Lisa: 9010
Judd, Suzanne: 9000
Judge, Katherine: 9020
Judon, Kimberly M.: 9000
Juengst, Shannon B.: 7650, 7760
Jump, Alayna: 7830
Jun, Jin: 7090
June, Andrea: 1080
Jung, Hailey H.: 2310
Jung, Haneul: 9020
Jung, Hye-Young: 5215
Jung, Jae Yeon: 7200
Jung, Sun Ok: 7160, 7800
Jung, Sunwoo: 9030
Jung, Wonkyung: 7150, 7280
Jung, Woo Seok J.: 9020
Jungermann, Joanna: 9030
Juon, Hee-Soon: 9440
Juratovac, Evanne: 9020
Juris, Jill: 3060, 4085
Jurkowski, Elaine T.: 7000
Justice, Jamie: 3290, 7370, 7630, 9010
Juszczyk, Ewelena: 4475
Jutkowitz, Eric: 2130, 3010, 3305, 4140
Jylhä, Marja: 9000
Jylhävä, Juulia: 9010

**K**

K. Nair, Anil: 9000
Kaasalainen, Sharon: 4440, 4470
Kaba, Diarratou: 4425
Kabaya, Sonoko: 9010
Kabayama, Mai: 4505, 9000
Kabeto, Mohammed U.: 1085
Kadam, Ashay: 7210
Kado, Deborah: 5085
Kaga, Hideyoshi: 9000
Kagai, Violet: 2010
Kahana, Eva: 3360, 3485, 7030
Kahana, Shoshana: 2270
Kahsay, Eskira: 3080
Kahya, Melike: 2155
Kaiksow, Farah: 4250
Kaitlin Casaletto, Shea Andrews: 2240
Kajitani, Shinya: 4385
Kakagawa, Kazuma: 5060
Kakehi, Saori: 9000
Kalaw, Karel: 4406, 7000
Kales, Helen: 7160
Kalesnikava, Viktoryia: 4545
Kallestrup, Peter: 3515
Kallmi, Selmi: 7340
Kalousova, Lucie: 4510
Kamath, Vidyulata: 2240
Kamide, Kei: 9000
Kamijo, Mio: 3310
Kan, Denise: 5125
Kan, Winnie: 3270
Kanagawa, Hotinpo Sky: 9420
Kananen, Laura: 9000, 9010
Kanani, Pushti: 7870
Kanaya, Reiko: 9000
Kane, Alice E.: 9020, 9030
Kane, Esther: 7420
Kane, Kevin: 2385
Kaner, Eileen: 9010
Kang, Bada: 7720, 7830, 9000, 9010
Kang, Chun-Ya Belinda: 7000
Kang, Edward: 9020
Kang, Ezer: 3440
Kang, Hun: 9020
Kang, Hyewon: 7070
Kang, Hyun: 3310, 4195
Kang, Jee eun: 1180
Kang, Mingon: 4075
Kang, Miseon: 7040, 7080, 7610
Kang, Suk-Young: 7760
Kang, Sunny: 7720
Kang, Yejin: 9310
Kang, Youjeong: 5055
Kanofsky, Chayna: 7550
Kantamneni, Leela: 4525
Kantarci, Kejal: 7100
Kanter, Jeremy: 1235
Kao, Hsueh-Fen S.: 5185
Kapahi, Pankaj: 4335, 9030
Kapogiannis, Dimitrios: 4260
Kapoor, Karina: 7900
Kappel, Rachel: 1065
Kapteyn, Arie: 3160
Karadimas, Thomas: 2325, 9330
Karagiannis, Tanya: 5090, 7370
Karakida, Maki: 9030
Karakose, Selin: 4055
Karam, Rita: 4345, 7040, 7380
Karasik, Rona J.: 2095, 4406, 7000
Kåreholt, Ingemar: 3470, 7040, 7180, 7300
Karel, Michele J.: 4095, 5135
Karibian, Bianca: 7800
Karikari, Grace: 3035, 7480
Karjadi, Cody: 7100, 9010
Karlamangla, Arun: 4120
Karlawish, Jason: 7650
Karlin, Nancy J.: 2125
Karliner, Leah: 3490
Karlsson, Ida K.: 9020
Karmacharya, Isha: 3045, 3190, 5220
karnam, Raghavi: 9010
Karney, Benjamin: 4400
Karpyn, Allison: 9020
Karraker, Amelia W.: 4205
Kartoz, Connie R.: 9000
Karvonen-Gutierrez, Carrie A.: 5155, 9010
Kasahara, Tomoko: 7370, 9010
Kasenetz, Whitney E.: 9010
Kaskie, Brian: 1030, 2175, 4435
Katagiri, Keiko: 3280, 7290, 7610
Katie, White: 3045
Katsev, Danielle: 4005
Katsumata, Asako: 7790
Katyarmal, Swati: 9000

Katz, Benjamin: 7020, 7100, 7830, 7990
Katz, Mindy: 3480, 5165, 9010, 9020
Katzel, Leslie I.: 4130
Kaufman, Brystana G.: 1020
Kaufman, Jennifer E.: 3565, 7810
Kaufmann, Christopher N.: 3500, 4490
Kaur, Simran: 3270
Kaur, Tripat: 4345
Kaushal, Navin: 5125
Kavalieratos, Dio: 1010, 3435
Kavanaugh, Melinda S.: 3020, 7910
Kavanaugh, Rachel: 7310
Kavcic, Voyko: 7510, 9020
Kawachi, Ichiro: 5130
Kawamori, Ryuzo: 9000
Kawasaki, Teruaki: 9010
Kay, Lilli: 7360
Kaya, Alaattin: 7370
Kayatta, Sophia: 7460
Kaye, Jeffrey A.: 2360, 3115, 3535, 4100, 4495, 7300, 7540, 7620, 7680
Kaysen, Anne: 9020
Kayser, Jay R.: 4325, 5235, 7030, 9010
Kazan, Joseph: 5175
Ke, Yemiao: 9320
Keane, Nicole: 7810
Keating, Norah C.: 7980
Keefe, Bronwyn: 4125, 7480
Keefe, Francis: 7410
Keefe, Janice M.: 2170, 4515
Keenan, Teresa: 5175
Keenoy, Jaclyn E.: 1185
Keesee, Francesca: 7760
Keinan, Amina: 7380
Keister, Melanie: 9020
Kelekar, Uma: 3220, 7210, 7830, 9010
Kelfve, Susanne: 4190, 4395, 4450
Keller, Ashley: 9020
Keller, Hallie E.: 7180, 9000
Keller, Heather: 7300
Keller-Ross, Manda L.: 9410
Kelley, Amy: 3040, 3185, 3415
Kelley, Casey J.: 7300
Kelley, Jessica: 2050, 2190, 7410
Kelley, Lynette: 9010
Kelley, Susan J.: 3060, 4400
Kelly, Debra: 9030
Kelly, Ronald: 7160
Kelz, Rachel: 7860
Kemeny, Betsy E.: 5065, 9410
Kemp, Blakelee: 7960
Kemp, Candace: 3285, 7290
Kendzor, Darla: 9030
Kennedy, Courtney: 4268, 5100, 7220, 7680
Kennedy, David: 2100
Kennedy, Katherine A.: 3320, 4290, 5215, 7160, 7770
Kennedy, Quinn: 1040
Kennedy, Richard: 9030
Kennedy, Teri: 1330, 5170, 7010
Kenten, Charlotte: 4470
Kenyon, DenYelle: 7600
Keogh, Alison: 3550
Kergoat, Marie-Jeanne: 9000
Kerr, Eleanor M.: 1000
Kerr, Kirk W.: 9020
Kerssens, Chantal: 7990
Kersten, Alan W.: 7510
Kervin, Lucy: 1335
Kerzner, Rachel: 7380
Kessler, Dorothy: 9000
Kew, Chung Lin (Novelle): 7760
Key, Kent: 1270
Keyes, Laura M.: 2315, 4150
Keys, Fay: 1005
Keys, Matthew T.: 4260
Kezios, Katrina: 9500
Khalaila, Rabia: 7480
Khalidi, Sarah: 4340
Khambaty, Tasneem: 4130
Khan, Karim M.: 5100
Khan, Maham: 5195
Khan, Mushira M.: 3035, 3440, 4285, 9000
Khan, Nibraas: 9030
Khan, Shehroz: 7190, 7680, 9000, 9330
Khan, Zain: 1110
Khanh Toan, Tran: 2050
Khanna, Saumya: 5220
Khattar, Jayati: 7660
Khavjou, Olga: 2375
Kheirbek, Raya E.: 4115, 4160, 4250
Khera, Alka: 7760
Khillan, Rohini: 5175
Kho, Minjung: 2320
Khong W. H., Andy: 3295
Khor, Ern Chern: 9030
Kianpoor, Kiarash: 7120
Kibildis, Samuel W.: 9030
Kidd, Lori: 7250
Kido, Michiko: 4505, 9000
Kieffer, Elana F.: 2315
Kiely, Kim M.: 3570
Kiely, Kim: 9000
Kikuchi, Kazunori: 7280
Kilaberia, Tina R.: 5170, 7280, 7750
Killian, Tim S.: 7070
Killingsworth, Derek C.: 7590, 7900
Kilpatrick, Sidonie: 5155
Kim, Andy: 7510
Kim, Benjamin: 9010
Kim, Boah: 2230
Kim, Boeun: 1345, 2225
Kim, Bohyun: 7610
Kim, BoRin: 4266, 7550, 7690, 9010, 9020, 9030
Kim, Chang Oh: 7660
Kim, Changhwan: 9020
Kim, Choong: 7980
Kim, Dae H.: 9410
Kim, Dae Hyun: 3565, 9010, 9520, 9540
Kim, Dahee L.: 1390
Kim, Eric S.: 2140, 9000
Kim, Eunbea: 3170, 3440
Kim, Eunhye: 7220
Kim, Eun-Kyeong: 3115
Kim, Eunyoung: 7400
Kim, Giyeon: 3245, 7510, 7560, 7610, 7830, 7940, 7960
Kim, Gwang Suk: 7320, 7660
Kim, Ha Neul: 1280, 2085, 9010
Kim, Ha Yul: 9540
Kim, Hansol: 7530
Kim, Heejung: 7080, 7220
Kim, Hosung: 4265
Kim, Hyein: 7220
Kim, Hyeji: 9030
Kim, Hyejin: 3445
Kim, Hyejin: 7900, 7960
Kim, Hyosin: 1240, 4105, 7800, 9000
Kim, Hyoun Kyoung: 7610
Kim, Hyunji: 7500
Kim, Hyun-Jun: 2310, 4195
Kim, Jae: 9010
Kim, Jaehyun: 7910
Kim, Jeehoon: 7610
Kim, Jennifer (Jeehyun): 7830, 9000, 9010
Kim, Jeungkun: 7760, 9030
Kim, Jinhak: 9020
Kim, Jinho: 7330
Kim, JinShil: 7850
Kim, Jinyoung: 4134, 4265, 7330
Kim, Jiyeon: 7230
Kim, Jongwoong: 9010
Kim, Joohyun: 3130
Kim, Ju-Hyun: 7590
Kim, Julie: 4025
Kim, Jung Eun: 7160, 7800
Kim, Jung Ki: 2325, 4395
Kim, Jung Lyun: 9030
Kim, Jungsug: 7330
Kim, Junhyoung: 7910, 7990
Kim, Junhyoung: 9010, 9030
Kim, Kwang-il: 9000
Kim, Kyeongmo: 7250
Kim, Kyungmin: 1050, 3130, 3165, 3425, 7030, 7380, 7510, 7520, 9000, 9010
Kim, Laurie: 4075
Kim, Layoung: 7320
Kim, Man Su: 7880
Kim, Meeryoung: 7070
Kim, Miji: 7630, 9020
Kim, Min Hee: 2300
Kim, Min Jung: 7420, 7720
Kim, Minju: 7550
Kim, Miri: 7030
Kim, Miyong T.: 7420, 7510
Kim, Nahyun: 7030, 7380
Kim, Nahyun: 7610
Kim, Narae: 5110
Kim, Nayeon: 7300
Kim, Nicholas: 9020, 9030
Kim, Patricia: 2235
Kim, Pearl: 4075
Kim, Rockli: 7320
Kim, Samsuk: 3155
Kim, Seon: 7230
Kim, Seonghoon: 7960
Kim, Seonjin: 9010
Kim, Seoyoun: 4210, 4255
Kim, Sharon H.: 7510
Kim, Sohyun: 3020, 9030
Kim, Steffi M.: 2120, 3015
Kim, Sue: 7660
Kim, Sunghee: 7030
Kim, Wonjun: 9000
Kim, Yeoju: 7830
Kim, Yesol: 7080
Kim, Yijung K.: 3130
Kim, Yongseop: 7910, 9010, 9030
Kim, Yoon Chung: 5020, 9030
Kim, Youjin: 7030
Kim, Youn Kyoung (Lily): 9020
Kim, Young Sun: 9540
Kim, Yulri: 3360
Kim, Yun Joong: 7400
Kimball, Patricia F.: 3375, 4215

Kim-Knauss, Yaeji: 3100, 7900
Kimpel, Christine C.: 9020
Kimzey, Michelle: 2080, 4220
Kincaid, Reilly: 1255
Kindratt, Tiffany B.: 3440
King, Angelique: 3250
King, Barbara: 1190
King, Deborah A.: 3490
King, Jessica: 4165
King, Natalie: 2375
Kinkade, Emily: 1235, 5035, 7710
Kinney, Anita: 7410
Kinney, Jennifer M.: 4435, 7140, 7540
Kinoshita, Ayae: 9010
Kiosses, Dimitris: 3520
Kip, Stephanie: 3560
Kircher, Julie A.: 9020
Kirchner, Jessica: 2375
Kiriazova, Tetiana: 2055
Kirk, Allan D.: 1370
Kirkham, Heather: 9020
Kirkwood, Renata: 4268, 7680
Kissell, Paul: 1150, 3240
Kitaigorodsky, Marcela: 4340
Kitt-Lewis, Erin: 9010, 9540
Kitzman, Dalane W.: 3505
Kiyoshi-Teo, Hiroko: 5205
Klaiber, Patrick: 3135, 3520, 3545, 4320
Klee, Matthias: 1225
Kleijwegt, Hannah: 3415
Kleissner, Verena: 7590
Klenk, Jochen: 3550
Klepacz, Laura M.: 9000
Kleszynski, Keith: 2215, 9020
Kline, Chelsea R.: 1265, 7420
Klinedinst, Jennifer: 2210
Klinedinst, Tara C.: 9030
Klingman, Karen J.: 2110
Klopack, Eric T.: 1325, 2325
Kloss, Katie: 1030
Knapp, David: 3160
Knebl, Janice: 4220, 4360
Knecht-Fredo, Jenny L.: 4525
Knepple Carney, Amy: 7390
Knight, Erik: 9030
Knighton, Amy: 7830
Knill, Samantha: 7830
Knol, Linda L.: 7620, 9000, 9020
Knopp, Kasey: 7170
Knox, Melissa L.: 9020
Knox, Patrick: 7410
Ko, Ahra: 9030
Ko, Eunjung: 7090
Ko, Fred: 7000
Ko, Fred: 7150, 7820, 7860
Ko, Myungjin: 9030
Ko, Soohyeon: 7320
Ko, Su-Hyuk: 8900
Ko, Sung Hyun: 2375, 7770
Kobayashi, Erika: 4225
Kobayashi, Lindsay: 1085, 1170, 2280, 3290, 7320, 7940
Kobsar, Dylan: 9020
Kobylarz, Fred: 4105, 9000
Koch, Mary Kate: 7380, 7580, 7900
Koehler, Sebastian: 1225
Koehly, Laura: 7070
Koetaka, Hanayo: 9000
Koeuth, Sokha: 2130
Koffer, Rachel E.: 3135
Koffski, Maya J.: 3300, 3570
Koh, Young Bin: 9010
Kohanski, Ronald A.: 3040
Kohl, Raphael: 9400
Kohlbrenner, Briana: 5200
Kohler, Hans-Peter: 9020
Kohli, Nidhi: 5170
Kohon, Jacklyn N.: 2105, 3220
Koirala, Binu: 7770, 7840, 7960
Koizumi, Naoru: 7700
Kok, Almar: 4485, 7360
Kolade, Opeyemi R.: 1175, 4255
Kolbila, Lilian: 7790
Koletar, Susan L.: 3390
Kolind, Shannon: 3495
Koller, Jeanne M.: 7450
Kolpakov Nikitin, Stanislav: 5015, 7100
Komalasari, Renata: 7830, 9000, 9010, 9020
Kondrikov, Dmitry: 9200
Kondrikova, Galina: 9200
Konetzka, R.Tamara: 1115, 4140
Kong, Dexia: 4540, 7940, 7950, 9020
Kong, Eun-Hi: 7300, 9020
Kong, Jooyoung: 1130, 3170, 7050
Kong, Lan: 5145
Kong, Xiangjing: 2265, 7330
Kono, Sei: 9030
Kononowech, Jennifer: 7160
Koon, Lyndsie: 9030
Kopera-Frye, Karen F.: 4268, 7680, 7980
Korc-Grodzicki, Beatriz: 7410
Koren, Chaya: 3170
Korfage, Ida J.: 4440
Korfhage, Justin: 1060
Korinek, Kim: 2050
Kornadt, Anna E.: 3100, 4180
Koronowski, Kevin: 9000
Kosar, Cyrus: 5215
Koschate, Jessica: 7830
Koskinen, Seppo: 9000
Kossowska-Kuhn, Dorota: 1315, 9000, 9030
Koster, Annemarie: 1225
Kostyniuk, Lidia: 9030
Koszalinski, Rebecca S.: 2180
Kotarba, Joseph A.: 4040
Kotecho, Messay: 2010
Koternamski, April: 7640
Kotwal, Ashwin: 4205, 7610, 9020
Koubasi, Farnaz: 9000, 9020
Koumoutzis, Athena: 4425
Kouroukis, Alexa: 4268, 7220, 7680
Kovacs, Laszlo: 9030
Kowalchik, Kalei H.: 5030
Kowdley, Gopal: 7860
Kozlov, Elissa: 4305
Krämer, Michael: 9010
Krafty, Robert: 5175
Kramer, Arthur: 7100
Kramer, Jenna: 4345, 7040, 7380
Kramer, Josea: 3250, 4095
Kramer, Philip: 3475, 5210
Kramsky, Jayna: 9010
Kraschnewski, Jennifer: 5030, 5145
Krase, Kathryn: 9030
Kratzer, Andre: 2370
Kraus, William: 9000
Kravchenko, Julia: 3510, 5015, 5110, 7620
Kravitz, Howard M.: 5155
Kravljaca, Nikolina: 7040, 7520
Kreitzer, Natalie: 7850
Krendl, Anne: 9300
Kress, Taylor: 9030
Krieg, Carsten: 9200
Kringos, Dionne S.: 9010
Kristen, Jacobs: 3425
Kritchevsky, Stephen B.: 2155, 3475, 5210, 5230, 7630
Kritz-Silverstein, Donna: 9010
Kroeck, Mallory: 7890, 7990
Krok-Schoen, Jessica L.: 2260
Kropp, Denise J.: 7250, 7480
Kruahong, Suratsawadee: 9020
Krueger, Rejko: 1225, 9020
Krukowski, Rebecca: 9020
Krupp, Karl: 9010, 9020
Krygowska-Nowak, Natalia: 4450
Krystina, Lewis: 9000
Kuball, Tim: 7070, 7590
Kubasova, Ksenia: 4266, 7190
Kubo, Takanari: 9030
Kucharska-Newton, Anna: 5085, 7340
Kuchel, George A.: 4485
Kuczmarski, Marie Fanelli: 9000
Kueht, Michael: 7860
Kuffel, Randall L.: 2280
Kuhlmey, Adelheid: 9000, 9400
Kuipers, Allison L.: 5090, 9020
Kulati, Ellam: 1220
Kulatunga, Senara: 9010
Kulkarni, Snehal S.: 7820
Kulminski, Alexander: 4260
Kum, Cleopatra: 7850
Kumar, Raj: 7650
Kumar, Sidharth: 7680
Kummerlowe, Megan: 7330
Kuna, Samuel T.: 7180
Kung, Claryn S J.: 4015
Kunik, Mark E.: 1280, 7850
Kunkel, Suzanne: 3045
Kunonga, Tafadzwa Patience: 4455
Kunz Lomelin, Alan: 3305, 4420
Kuo, Fang-Lin: 7910
Kuo, Jasmin: 9030
Kuo, Pei-Lun: 2195
Kuo, Tony: 7700
Kuo, Tsuann: 2345
Kuo, Yong-Fang: 4395, 9010
Kurth, Jordan D.: 9010, 9020
Kurth, Maria: 1160, 7090, 7510, 7580
Kusmaul, Nancy: 1020, 1100, 2020, 3150, 5020, 9000, 9010, 9100
Kuspinar, Ayse: 5070

Kusters, Cynthia D.: 5225, 7370
Kuzmik, Ashley: 1300, 2305
Kvale, Elizabeth: 3345
Kwak, Jung: 3345, 4000
Kwan, Thomas: 7390
Kwas, Stephen: 9020
Kwok, Alex Pak-Ki: 9030, 9510
Kwok, Amy: 9400
Kwok, Jojo Yan Yan: 7610
Kwok, Stanley: 7120
Kwok, Timothy: 1145
Kwon, Eunsun: 9010
Kwon, Jenny: 7010, 7590, 9010, 9030
Kwon, Sohyeon: 3390
Kwon, Soonhyung: 4530, 5115, 7920, 9020
Kylén, Maya: 2340, 7380

L
LaBlanc, Raeann: 9010
Labyak, Corinne: 7870
Lach, Helen W.: 7150
Lachman, Margie E.: 1360, 7380, 7510, 7970
Lachs, Mark S.: 1145, 2060
Lackmeyer, Abbe: 3045
LaCroix, Andrea Z.: 3390
Lacy, Briana: 9310
Ladowski, Kristi: 9020
Ladwig, Matthew A.: 9010, 9020
Laffer, Alexandra: 9020
Laffitte Nodarse, Chavier: 9010
Lafontant, Kworweinski: 9010, 9020
Lafountain, Olivia: 4195
Lagacé, Martine: 1200
Lagoo-Deenadayalan, Sandhya: 1370
LaHue, Sara: 1380
Lahvic, Jamie: 2270
Lai, Daniel W.L.: 1145, 7040, 7520
Lai, Francisco: 2280
Lai, Patrick HL.: 3095
Lai, Philip: 3530
Lai, Ting-Fu: 7930
Lai, Wen-Hua "Zoey": 1180
Lakatta, Edward: 9020
Lakshminarayan, Kamakshi: 3460
Lalji, Kahir: 2020, 5215, 7730
Lam, Gigi: 9030, 9510
Lam, Justin W.: 7750
Lam, Kenneth: 7180
Lamb, Raza H.: 3020
Lambert, Frederick: 9010
Lambert, Olivia J.: 4425
Lambrou, Nickolas: 3015
Lamerato, Lois: 2225
LaMonte, Michael J.: 3390
Lampe, Nik M.: 4000
Lampkin, Cheryl: 5040
Lampraki, Charikleia: 7080
Lancaster, Caroline: 3075
Lanctot, Krista: 7080
Landes, Scott: 1160
Landi, Francesco: 9020
Landman, Bennett A.: 2195
Landreville, Philippe: 7030
Lane, Amber: 7160
Lane, Annalise: 4545, 5050
Lane, Sandi J.: 9530
Lang, Barbara: 9010, 9020
Lang, Frieder R.: 3100, 7900
Lang, Justin: 5070
Lang, Samantha: 9020
Langa, Kenneth: 2280, 7320, 7650, 9110
Lange Maia, Brittney S.: 2120
Langefeld, Carl: 5060
Langendam, Miranda: 9010
Langsetmo, Lisa: 5085
Langston, Emily M.: 9000
Langston, Ryan: 9010
Langton, Lynn: 3075
Lanzi, Alyssa M.: 7620
Lao, Jenna: 7590
Lao, Jiahui: 9000
Lapidus, Jodi: 7110
Larkin, Jennie: 3040, 9030
Larkina, Marina: 9010, 9030
LaRoche, Dain P.: 5075, 9010, 9550
Larsen, Pernille S.: 4260
Larson, Janet: 7510
Laskow, Thomas: 1375
Lassell, Rebecca KF.: 7230, 9000
Latham, Nancy: 3480, 9000
Latham-Mintus, Kenzie: 4115, 4250
Lathren, Christine: 3320
Latimer, Abigail: 9000
Latkin, Carl: 9420
Latshaw, Emily: 9030
Lau, Bobo Hi-po: 3555, 9030, 9510
Lau, Gary K.K.: 7330, 7520
Lau, Terence K.: 4515, 7330
Lauber, Mckenzie S.: 7760
Lauderdale, Jana: 9020
Laumann, Edward: 1230
Laura, Bradbury: 1150
Laures-Gore, Jacqueline: 7500
Laurienti, Paul J.: 5230
Lavender, Kathryn: 7380
Lawrence, Jourdyn A.: 5130
Lawrence, Karen A.: 9020
Lawrence, Sacheen: 3015
Laws, Holly: 7420
Lay, Jennifer C.: 3275, 4480
Layman, Shannon: 1335
Layne, Diana: 9000
Laynor, Gregory: 7640
Laz, Mike: 2355
Le, Giang: 9530
Le, Qun: 1350, 2385, 3155
Leach, Carrie: 1270, 5030
Leahy, Suzanne: 9020
LeBrasseur, Nathan: 1325
Lech, Sonia: 9400
Leckie, Carolyn: 4268, 7220, 7680
Lecours, Alexandra: 9020
Lecvy, Nadine: 4460
Ledermann, Thomas: 4055
Lee, Ahyoung: 7590, 7890
Lee, Chae Man: 1240, 4140
Lee, Chae Man: 4266, 7230
Lee, Chae Man: 7830
Lee, Chi-Ko: 2230, 4020, 7040
Lee, Christopher S.: 3535
Lee, Chung-Sheng: 4134, 7830, 7930
Lee, Edwin H.M.: 7330
Lee, Eunju: 7520
Lee, Gina: 3440, 3555, 7610
Lee, Haena: 1230, 1320, 9030, 9570
Lee, Han Na: 9020
Lee, Hee Yun: 3005, 7380
Lee, Heejung: 7810
Lee, Hye Soo: 1120, 7680
Lee, Hyo Jung: 7080, 7960
Lee, Hyun Ju: 9540
Lee, Ilhak: 7900, 7960
Lee, Jae Jun: 7320, 7760
Lee, Jeannie K.: 1260
Lee, Jeenkyoung: 7550
Lee, Jennifer: 1290
Lee, Jennifer A.: 2035
Lee, Jenny: 9030
Lee, Jeong Eun: 1055, 2075, 3170, 3360, 3440, 3445, 4390, 7610
Lee, Ji Hyun: 1170, 4325
Lee, Ji Yeon: 7160, 7420, 7660
Lee, Jihui: 4420
Lee, Jihye: 4370
Lee, Jina: 4020, 4500
Lee, Jinkook: 9110
Lee, John Chi Kin: 7950
Lee, Jooah: 9010
Lee, Jooseon: 7810
Lee, Joowon: 7910
Lee, Joseph H.: 9020
Lee, JuHee: 7400
Lee, Juhyeong: 7510, 7940
Lee, Jung Wan: 9540
Lee, Jung-Ah: 4025, 7850
Lee, Jungjoo: 7990, 9030
Lee, Jungup: 7080
Lee, Kathy: 2330, 4420, 7730, 7910, 9010
Lee, Kayoung: 9020
Lee, Knoo: 1310
Lee, Kyu Ri: 5070
Lee, Kyung Hee: 7160, 7660, 7720, 9320
Lee, Kyungmi: 7680, 9030
Lee, Lewina O.: 3155
Lee, Lewis H.: 3005, 5125
Lee, Matthew R.: 5005, 7390
Lee, Milae: 7940
Lee, Moon: 1345
Lee, Nur 'Atiqah: 3435
Lee, Othelia: 3345
Lee, Sei: 3490
Lee, Seolah: 7080, 7960
Lee, Seonhwa: 4400
Lee, Seoyoon J.: 7030, 7380
Lee, SeungCheor: 7380
Lee, Soohee: 9020
Lee, Soo-Kyoung: 9030
Lee, Soomi: 3135, 3500, 4490, 5045, 7410, 7890, 7990
Lee, Sun Ah: 3135, 5045
Lee, Sunghoon I.: 9030
Lee, Sunmin: 2275
Lee, Susan: 1120
Lee, Tou S.: 3450
Lee, Vincent W.P.: 7040, 7520
Lee, Wei: 9030
Lee, Wei-Chen: 7310
Lee, Won: 7900, 7960
Lee, Yeonjung: 2375, 7770, 9010, 9030, 9100, 9420
Lee, Yeonjung Lee: 2230
Lee, Yeon-Shim: 7600
Lee, Yeonwoo: 9030
Lee, Yeseul: 7610
Lee, Youjung: 1005, 2005, 3060
Lee, Yura: 3035, 7610, 7640
Leedahl, Skye N.: 1350, 3385, 4085, 4375
Lees Haggerty, Kristin: 2030
Lefebvre, Emily L.: 7550
Leger, Kate: 5045, 7040

Leggett, Amanda N.: 1340, 1355, 7090
Leggett, Amanda N.: 9030
Lehman, Erik: 5145
Lehmberg, Lisa J.: 2390
Lehnbom, Elin C.: 9030
Lehning, Amanda J.: 9010
Lehpamer, Nicole: 5125, 7190
Lehr, Eric: 7170
Lei, Xiao Ying: 7420, 9030
Leis, Aleda M.: 5155
Leiser, Scott F.: 7880
Leishman, Eppie: 9000
Leist, Anja K.: 1225
Leite, Mateus Medeiros: 7340
Lekhak, Nirmala: 3485, 5010, 9010
Lekwauwa, Ruby: 4145
Leland, Natalie: 7360
LeLaurin, Jennifer H.: 2170
Lemieux, Catherine: 9020
Lendon, Jessica: 1095
Lengacher, Cecile A.: 3420
Lentz, Trevor: 7410
Leon Guerrero, Cierra: 4495
Leong, I tek: 2090, 9320
Lepore, Michael: 3180, 5020, 7300, 9020, 9030
Lerch, Ondrej: 7420
Lercher, Kirk: 7650
Lerner, Susan: 7820
Lervisit, Vina: 7730
Leshchyk, Anastasia: 7370
Leskinen, Tuija: 7170
Leslie, Kathleen: 7190
Lesperance, Larry: 9000
Lessenich, Stephan: 9030
Letendre, Scott: 4340
Letts, Elyse: 1280
Leung, Angela Y. M.: 4132, 4515, 7330, 7480, 7760
Leung, Dara Kiu Yi: 7760
Leung, Doris Y. P.: 4515
Leung, Isaac Sze Him: 7150
Leung, Lok Man: 9010
Leung, Verona Ji Ying: 7060
Leutwyler, Heather C.: 2280
Levan, Darius T.: 1305
Levasseur, Melanie: 1200, 7620, 7660
Levchenko, Yuliana: 9100
Leveille, Suzanne: 9000
Levenson, Robert W.: 1305, 4495, 9320
Leverentz, Andrea: 9000
Levin, Bonnie: 4340
Levin, Bruce L.: 4010
Levine, Adele: 2145
Levine, Deborah: 1085
Levine Coley, Rebekah: 3095
Levkoff, Sue E.: 2330, 9010
Levy, Becca R.: 3325
Levy, Cari: 1160, 4408, 7160, 7300, 7720
Lewin-Epstein, Noah: 9030
Lewinson, Terri: 5030
Lewis, Beth A.: 5100
Lewis, Brian: 9020
Lewis, Charity: 4110, 7960
Lewis, Constance: 4408, 7230
Lewis, Cora E(: 9010
Lewis, Hannah M.: 7010
Lewis, Jordan P.: 1140, 1270, 2120, 3015, 3200
Lewis, Nathan A.: 9000
Lewis, Penny J.: 4110
Lewis, Stevie: 9010, 9030
Lewis, Stuart: 3375, 4215
Lewis-Thames, Marquita W.: 2355
Lewy, Hailey: 7210
Leykum, Luci: 9010, 9030
Li, Chien-Ching: 3440, 7410, 7420, 9030
Li, Chihua: 2285, 3225, 9030
Li, Chih-Ying: 4395
Li, Chin-Shang: 7620
Li, Claudia: 5115
Li, Cun: 9000
Li, Daisy: 3280
Li, Daneng: 7410
Li, Guohua: 9010, 9020
Li, Jia: 7520
Li, Jiejin: 2145
Li, Jinfeng: 7070
Li, Jingxi: 7770
Li, Juan: 2265, 7330
LI, Junxin: 3500, 4310, 7070, 9300, 9320
Li, Ke: 4050
Li, Lun: 2230
Li, Lydia: 4325
Li, Martina: 3525
Li, Meiyi: 1230
Li, Mengchi: 3500, 7070, 9320
Li, Mengze: 5090, 7370
Li, Minghui: 9000
Li, Polly WC.: 5225, 7300
Li, Tao: 9000
Li, Wenjun: 1350, 2385, 3155, 5075, 7430
Li, Xia: 4240
Li, Xiaoang: 7870
Li, Xiaoli: 3380
Li, Xiaoling: 2205
Li, Xiaoqian: 4185
Li, Xinjun: 9000
Li, Xuelian: 4535
Li, Yanling: 7510
Li, Yawen: 1095
Li, Yaya: 4505, 9000
Li, Yiang: 1230, 4205
Li, Yichao: 7780
Li, Yixia: 2280, 4115
Li, Yongmei: 5230, 7160
Li, Yue: 4140
Li, Yuekang: 9020
Li, Yuelin: 7660
Li, Yuxiao: 7350
Li, Zhigang: 1030
Lian, James: 9000
Liang, Su-Ying: 3525
Liang, Xiaohui: 4268, 7680
Liang, Yuanyuan: 9000
Liao, Hsiao-Wen: 1320, 7070
Liao, Jing: 2255
Liao, Joshua: 7830
Liao, Pei-Chun: 7910
Liao, Shu: 9020
Liao, Shuyao: 9010
Liao, Yo-Jen K.: 3020, 7160, 7620
Liao, Yung: 7930
Liao, Zih-Yong: 7910
Libon, David J.: 7100
Lichtenberg, Peter A.: 3090, 7930, 9020
Liddle, Jennifer: 4540, 7160, 9010
Lieber, Sarah B.: 4268, 7680
Liebman, Michael: 3530, 9030
Liebreich, Robert: 7760
Liebzeit, Daniel J.: 4025
Liegey-Dougall, Angela: 7730
Liehr, Patricia: 5175
Lifshitz, Rinat: 7720, 7920
Light, Michael A.: 3410
Lighthall, Nichole R.: 7080, 7430, 9010
Ligon, Mary: 9000
Lilly, Flavius R W.: 3310
Lim, Aaron: 4275
Lim, Arum: 7770, 7840, 7960
Lim, Crystal: 1070
Lim, Diane: 4265
Lim, Emily: 4230
Lim, Jie Xin: 3085
Lim, Junghee: 7330
Lim, Junyub: 7610
Lim, Sungwon: 7150
Lim, Young Mi: 7330
Lima, Luciano Ramos: 7130
Lim-Soh, Jeremy: 3435
Lin, Feng: 9200
Lin, Frank R.: 2135, 2240, 2365, 4265
Lin, Gloria: 3280, 9010
Lin, Haiqun: 1240, 4105, 7800, 9000
Lin, Honghuang: 9010
Lin, Hongmei: 7060
Lin, Hsien-Ho: 9010
Lin, Hui-Yi: 7390, 7410
Lin, I-Fen: 1340
Lin, Jielu: 7070
Lin, Kun-Pei: 4134, 7830, 7930
Lin, Lisha: 7630
Lin, Rose: 5185
Lin, Shih-Yin: 2070, 3205, 7230, 7460
Lin, Shiow (Rosa): 5090
Lin, Shujing: 9580
Lin, Taiping: 7660
Lin, Tian: 7610, 9010, 9200
Lin, Wei-Ting: 7390
Lin, Xin Yao: 7610
Lin, Yan: 2250
Lin, Yi: 7870
Lin, Zhixuan: 3400
Lin, Zhiyong: 3105
Lin, Zhuoer: 1165, 2225, 2300, 3230, 3450
Lincoln, Karen D.: 2275, 3005
Lind, Majse: 7970
Lindauer, Allison: 4100, 4495, 7440, 7700
Lindberg, Brian W.: 2025, 2165, 2297, 3315, 3455, 4160
Lindell, Kathleen: 9000
Lindfors, Petra: 9030
Lindgren, Finn: 9020
Lindholm, Cassie: 9000
Lindquist, Lee A.: 7440, 7460, 7520, 9030
Lindsay, Rebecca: 7680
Lingler, Jennifer H.: 4210, 9020
Liou, Chih-ling: 4406, 7000, 7380
Liou, Shyhnan: 3100, 9030
Liougas, Madalena P.: 4515
Lipat, Ania: 9010
Lippe, Megan: 3005
Lipsitz, Lewis A.: 2155, 2190, 2320, 9030
Lipton, Michael L.: 1125
Lipton, Richard: 9010, 9020
Lister, Adrian: 9010
Lister, Jennifer J.: 7330
Liston, Dorion: 1040
Little, Deborah M.: 7010
Little, Natalie: 7150

Litzelman, Kristin: 7590
Liu, Bochu: 9000
Liu, Chang: 1130, 4020, 4520, 7090
Liu, Chiung-ju: 4380, 7170
Liu, Christine: 9010
Liu, Ethan J.: 9000, 9030, 9530
Liu, Fangyu: 1370, 5085, 7110
Liu, Hui (Cathy): 3105, 4230, 4370
Liu, I-Ting: 9000
Liu, Jie: 9210
Liu, Jingwen: 3515
Liu, Jinyu: 3305, 4050
Liu, Kathy Y.: 1215
Liu, Kehan: 4310
Liu, Lan: 9000, 9210
Liu, Li: 9000
Liu, Li-Chuan: 7780
Liu, Li-Fan: 2345, 3400, 9010
Liu, Lili: 1165, 1280, 5015
Liu, Minhui: 4310
Liu, Molei: 1110
Liu, Mona: 7540
Liu, Pi-Ju: 4215, 7790
Liu, Ruotong Mona: 3005
Liu, Siqi: 9000
Liu, Tianyin: 7760, 7920, 9420
Liu, Weichu: 4535
Liu, Wen: 3020, 5015, 5070, 9020
Liu, Wenlong: 3510
Liu, Xiaojuan: 4515, 5230, 7160
Liu, Xiaoyue: 7840, 7960
Liu, Xinran: 9020
Liu, Yan: 9540
Liu, Yanchen: 7620
Liu, Yaqian: 4132, 7480
Liu, Yin: 4370, 7400, 7940, 7990
Liu, Ying: 9200
Liu, Yixiu: 7940
Liu, Yuezhong: 3295, 3470
Liu, Yujun: 4540, 7090
Liu, Yunda: 9030
Liu, Yuqi: 3115
Liu, Yuyang: 2255
Liu, Zhijian: 2265, 7330
Liu, Zhilin: 9000
Liu-Ambrose, Teresa: 5100
Livingston, Gill: 4515
Livingston, Marin J.: 5200
Livingston, Rachel: 9000
Lloyd, Althea: 4095
Lo, Maggie Wai-Shan: 9420
Lo, Margaret C.: 1030
Lo, On-Yee: 2155, 7170
Lo, Wei-Cheng: 9010
Lo, Yu-Tai: 9030
Lobanov-Rostovsky, Sophia: 2255
Lobo, Judith D.: 4340
Locascio, Joseph: 4460
Loccisano, Elisabeth: 9010
Lo-Ciganic, Wei-Hsuan: 3500
Locke, Jenna R.: 4525
Löckenhoff, Corinna E.: 1090, 7510
Locklear, Clarence E.: 7460
Loerzel, Victoria: 7080, 7150, 7430
Loewenstein, David: 9010
Lofton, Jason: 9000
Logan, Ayaba: 9000
Logue, Cailie I.: 7830
Logue Cook, Rachel N.: 7640, 7820
Lohmar, Sabine: 7030, 7570
Lombard, David B.: 1060
Lomeli, Sindy: 4375, 7750
London, Andrew S.: 1160, 7970
Long, Monica: 9010
Long-Foley, Kristie: 7270
Longobardi, Isaac: 3175
Longoria, Denise: 4240
Longstreth, William T.: 9500
Lopez, Acacia: 7950
Lopez, Alexa: 7540
Lopez, Bruna: 9010
López, Christian: 7590
Lopez, Escarlata: 7320
Lopez, Janice: 7620
Lopez, Maria J.: 1240, 4105, 9000
Lopez, Oscar L.: 4080, 9500
Lopez, Ruth Palan: 4525
López Anuarbe, Mónika: 1115
LoPilato, Danielle M.: 1350
Lopresti, Brian J.: 9500
Lor, Maichou: 3450
Lord, Justin: 2350, 3365, 4190
Lord, Marie-Michele: 9030
Lorelli, Lisa: 4525
Lorenz, Rebecca A.: 7830
Lou, Vivian Weiqun: 1145, 3515
Lou, Yifan: 3305, 4050, 7260
Louras, Peter: 9010
Loureiro, Joao M.: 9020
Louwagie, Katie W.: 3305, 3570, 7380, 7420
Lovasi, Gina S.: 3405
Lovato, James: 4134, 7830
Love, Jill W.: 9030
Lovegrove, Peter J.: 2220
Lovett, Hephzibah: 3245
Low, Hilary: 7160
Lowe, Enya: 3220
Lowenkron, Jeff: 9000, 9010
Lowensen, Kelly: 1375
Lowers, Jane: 1010, 3435
Lowndes, Ashley: 5105
Lox, Dennis M.: 9000
Loyd, Christine M.: 9000
Lozinski, Tristen: 3520
Lu, Feng-Ping: 4134, 7830
Lu, Hongzhou: 7140, 7330
Lu, Jiehua: 4155
Lu, Kevin: 9000
Lu, Minjie: 4480, 7050
Lu, Peiyi: 4540, 7940, 9020, 9030, 9500
Lu, Shiyu: 3115, 3515, 4255
Lu, Yi: 1090
Lu, Yidan: 2150
Lu, Yueqi: 9000
Lu, Yvonne: 9400, 9440
Lu, Zhaohui: 1095
Luanda, Natália: 7340
Lubben, James: 2135
Lubetsky, Sara: 7850
Luborsky, Mark R.: 4300
Lucas, Alexandre: 2320
Lucero, Robert J.: 7860
Luchetti, Martina: 3245, 4035, 4055, 7990
Luciano, Michelle: 4134, 7620
Lucius-Milliman, Darby: 1315
Ludwig, Lindsay: 4350
Luebke, Matthew: 1145, 2295
Lui, Fan Yin-Fong: 9030, 9510
Lui, Li-Yung: 4260
Luk, Calvin W.: 7040
Luk-Jones, Susanna: 5190
Lum, Hillary: 9000, 9020
Lum, Terry: 3115, 3515, 4255, 7330, 7760, 7920, 9420
Lumey, Lambert: 2285
Lund, Jennifer L.: 7300
Lunetta, Kathryn L.: 7640, 7830, 9000
Luo, Hao: 2280, 3490, 3510, 7330
Luo, Jing: 1255
Luo, Jun: 7230
Luo, Minxia: 3115
Luo, Weixiang: 9020, 9520, 9540
Luo, Yan: 4310
Luo, Ye: 7960
Luo, Yuqian: 4310
Luong, Gloria: 5035
Lüscher, Janina: 4235
Luskey, Valerie E.: 9010
Lustgarten, Michael: 5180
Luth, Elizabeth: 2110, 7520, 7540
Lutsey, Pamela L.: 3510
Lutz, Gabriel: 4115, 4250
Lutz, Julie: 4300
Luu, Britney: 8900
Lv, Gang: 9000
Lv, Shao-bo: 7030
Ly, Timothy K.: 9210
Lydon, Elizabeth A.: 1315, 4415
Lynch, David H.: 4268, 7360, 7680
Lynch, Katie: 5005
Lynch, Scott M.: 3180
Lynn, Freda: 3445
Lyon, Debra: 9030
Lyons, Christopher: 3525
Lyons, Karen S.: 3095, 3535, 4235, 7470, 7540
Lytle, Ashley: 1080
Lyu, Jiahui: 7950
Lyu, Linyu: 7660

**M**

Ma, Carol Hok Ka: 2345, 7760
Ma, Chenjuan: 7810
Ma, Jing: 7270
Ma, Jinhui: 5070
Ma, Kris Pui Kwan: 9000
Ma, Qiyini: 7560
Ma, Xinlin: 9000
Ma, Yang: 9020
Ma, Yao: 7830
Ma, Yiming: 4050, 7600
Ma, Yingzhuo: 4535
Ma, Yu: 9010
Macali, Jennifer: 7480
Macaluso, Suzie: 7730
MacArthur, Michael R.: 9020
MacDonald, Catherine: 3300
MacDonald, Elizabeth: 4095
MacDonald, Stuart: 7420, 7470
Mace, Ryan A.: 4460
Macias Moreno, Isis S.: 4240
Maciejewski, Matthew: 7150
Mack, Laramie: 9000
Mack, Laurin J.: 4540

Mackenstadt, Darby: 2040, 3425
Mackenzie, Corey S.: 7590, 9430
Mackiewicz, Marissa: 9010
Mackin, R. Scott: 2240
MacKinnon, David P.: 9030
Mackowicz-Torres, Meredith: 9020
MacLellan, Cameron: 7660
MacLeod, Heather A.: 9000
MacNeil, Amanda M.: 9020
MacNeil, Andie: 3375
MacNeil, Maggie: 5085
MacNeil Vroomen, Janet: 7120, 7420, 9010
Macomber, Catherine: 2085, 4020
MacPherson, Andrew: 2165
MacPherson, Margaret: 9030
Madden, Kenneth: 3275, 5100, 7550, 7820, 9000
Maddock, Carol: 9420
Madhivanan, Purnima: 9010, 9020
Madiloggovit-Lower, Jirakate: 5225
Madrid, Vanessa: 7840
Madrigal, Caroline: 3365
Maeda, Harumi: 5065
Maeda, Nobuhiro: 3310
Magana, Kimberly: 9000
Magaziner, Jay: 3550
Mage, Susanna M.: 4420, 5120, 7700
Magee, Catherine: 7760
Maggiore, Sophia: 7380, 7580, 7900
Magid, Kate H.: 1160, 7160
Magnusson Hanson, Linda: 9030
Mah, Jasmine: 2170
Mahanna, Allexis: 5200
Maharani, Asri: 4455
Maher, Brion S.: 7870
Mahmood, Asos: 7960
Mahmood, Atiya: 1205
Mahmoud, Kafayat: 1185, 5055
Mahmoudi, Elham: 2300
Mahnken, Jonathan: 7990
Mahon, Elizabeth: 7970
Mahoney, Jeannette R.: 3480
Maier, Julie: 7720
Maina, Lucy: 2010
Maitra, Medha: 8900
Maitra, Medha Romee: 8900
Majid, Tabassum: 9000
Mak, Wingyun: 7890, 9010, 9030
Makarem, Nour: 1110
Makaroun, Lena K.: 2030
Makelarski, Jennifer: 7230
Makhmutov, Rustem: 1175
Maki, Pauline: 5225
Makineni, Rajesh: 2145
Malatyali, Ayse: 5100, 7620
Malcolm Barney, Javier: 7130
Maley, Kathryn: 7700
Malhotra, Rahul: 1055, 3435, 4025, 4175, 7090
Malik, Mishel: 7910
Malinsky, Daniel: 1110
Malle, Bertram F.: 5105
Mallender, William D.: 7080
Mallory-Hill, Shauna: 4515
Malm, Johannes: 7300
Malone, Susan K.: 1320
Mamo, Sara: 9010
Man, Kenneth: 2280
Man - van Ginkel, Janneke de: 3340
Manalel, Jasmine: 2055, 3565, 7810
Mancilla, Isabel: 7820, 8900
Manders, Imagine: 7390
Mandrell, Melissa: 5020
Manheim, Chelsea E.: 1160, 4408, 7720, 9310
Manini, Todd: 9000, 9580
Manis, Derek R.: 1210
Mankowski, Robert: 7870
Manly, Jennifer J.: 2225, 3405
Mann, Catherine M.: 7260
Mann, Christine: 7830
Mann, Jim: 3255, 3270
Mannheim, Ittay: 7590, 9210
Manning, Kevin J.: 2240
Manning, Lydia K.: 5170
Manning, Mark: 7960
Mannion, Greg: 7190
Manojlovich, Milisa: 7570
Manor, Brad: 1260, 2155, 3145, 7170
Mansion, Natalie: 3025
Manson, JoAnn E.: 7100
Mansoor, Marrium: 7100
Mansuy, Lauren: 4380
Manvelian, Atina: 9000
Mao, Hui-Fen: 7780
Mao, Weiyu: 1045, 2265
Mao, Ziling: 3475, 4260
Mar, Amanda B.: 7620
Maraganore, Demetrius: 9010
Maras, Jessica: 7040
Marchini, Leonardo: 3540
Marcille, Frankie Ann: 9010
Marcinek, David J.: 5210
Marcoe, Jennifer L.: 7620
Mardini, Mamoun: 9580
Marfeo, Elizabeth: 7850
Margolick, Joseph B.: 3390
Margot-Cattin, Isabel: 7690
Margrett, Jennifer: 7510
Maria, Lapid: 7330, 9000, 9010
Marier, Patrik: 7730
Marijnissen, Radboud: 9000
Marini, Christina M.: 1235
Marino, Francesca R.: 2150, 3335, 3390
Marino, Victoria R.: 3155
Markides, Kyriakos S.: 1165, 3050, 7600, 9560
Markle-Reid, Maureen: 5220, 7840
Markov, Yaroslav: 9010
Markwardt, Sheila: 9020
Marnfeldt, Kelly A.: 7790
Maroto-Rodriguez, Javier: 2320
Marottoli, Richard: 2085, 7540
Marquez, David X.: 1230, 4465
Marriott, Deanna J.: 9000
Marroig, Alejandra: 7660, 7830
Marron, Megan M.: 7630, 9330
Marrs, Sarah: 3425, 9030
Mars, Lori: 2220
Marshall, Gillian: 1230, 3450
Marsiske, Michael: 2180, 5070
Marston, Hannah R. R.: 9420
Martens, Nancy L.: 7510
Martin, Damali: 9030
Martin, Diane: 9010
Martin, Jessica G.: 7130
Martin, Lauren: 9410
Martin, Mike: 3115, 3130, 4225
Martin, Peter: 2170, 2380, 3555, 7070, 7460, 7930, 7950, 9020
Martinelli, Paolo: 7080
Martinez, Beanna: 7540
Martinez, Brandy S.: 1070
Martinez, Erica: 9030
Martinez, Julia Margaret: 2220
Martinez, Meghan: 3525
Martinez, Suzanna: 9020
Martinez Amezcua, Pablo: 2150
Martinez Tyson, Dinorah: 4010
Martinez-Amezcua, Pablo: 2150
Martin-Matthews, Anne: 3285
Martinos, Fadi: 1030
Martins-Klein, Bruna: 9020
Martire, Lynn M.: 4235, 9010
Martyr, Anthony: 4070
Marwaha, Rochisha Singh: 7480
Marx, Katherine A.: 2130, 7550
Marxen, Lucas J.: 2315
Mary Thoma, Jingxuan Wang: 2240
Marziliano, Allison: 4030
Masaki, Harue: 7620
Masaki, Kamal H.: 5060
Mason, Hannah: 7420
Mason, Justin: 7170
Masood, Zainab: 2325
Masri, Hadi: 7500
Massa, Franklin: 7660, 7830
Massar, Rachel: 7640
Massey, John R.: 9000
Massihzadegan, Setarreh: 9510
Massimo, Lauren: 4134, 4145, 7420, 7620
Masson-Trottier, Michèle: 7680
Mast, Benjamin T.: 3205, 7090, 7420, 9010
Mastel-Smith, Beth: 2080
Masternak, Michal: 4475, 7110, 7870, 9020
Masters, Julie L.: 7000, 7550
Masys, Anthony: 4010
Matatall, Elise K.: 3410
Mather, Harriet: 5160
Mather, Mara: 1215, 7510
Mathias, Rasika: 7110
Matos-Moreno, Amilcar: 3050, 4500
Matsumoto, Daisuke: 7330
Matsumoto, Yoshiko: 5065
Matsunaga, Hiroko: 7550, 7580, 7760
Matta-Singh, Tara D.: 3360, 4010
Mattek, Nora: 7620
Matteson, Tyler J.: 4480
Matthews, Fiona E.: 4070, 4455, 9110

Matthews, Karen: 4120
Matthews, Kathleen: 4095
Mattingly, Stephen: 7280
Matulis, Henrique: 4405
Matuszek, Gregory: 1290
Matz, Christina: 3095
Mau, Theresa: 3475, 5210
Mauldin, Rebecca L.: 7280, 9000
Maulod, Ad: 2230, 3435, 4175
Mausbach, Brent T.: 7610, 8900
Maxfield, Molly: 5080
Maxwell, Cathy A.: 3395, 9010, 9020, 9030
Maxwell, Colleen J.: 4280
Maxwell, Hillary: 3570
Maxwell-Crudup, Kasual: 4350
May, Jennifer T.: 9020
May, Patrick: 1225
Mayahara, Masako: 3345, 3445
Mayer, Jamie: 4406, 7480
Mayfield, Kellie: 9010, 9310
Mayhew, Alexandra: 5070, 5220
Maynard, Matthew C.: 2085, 7850
Mazer, Raquel: 7360
Maziarz, Lauren: 9000, 9020
Mbango, Catherine: 7310
McAlister, Jennifer: 7310
McAndrews, Carolyn: 2230
McAtee, Robin E.: 5190, 7390, 7540, 7680, 9030
McAvay, Gail J.: 1305, 2360, 7360
McCall, Stephen: 9020
McCarthy, Catherine: 9020
McCarthy, Ellen: 3565, 9010, 9540
McCarthy, Megan: 2075, 4010, 7300
McCarthy, Michael J.: 7400
Mccauley, Kathleen: 4545, 9010
McClaskey, Brady N.: 1360
McClellan, Angela: 1165, 1280
McClintock, Martha: 1230
McClure, Leslie A.: 4005
McConnell, Eleanor: 9110
McConnell, William R.: 1055
McCormack, Krista: 9410
McCoy Smith, Ellen: 4440
McCrae, Christina: 3420, 4490
McCray, Rachel: 7460
McCreedy, Ellen: 2035, 4190, 4290, 7020, 7540, 7640
McCulloch, Heather: 5040
McCullough, Megan B.: 2085
McCullough, Megan: 4290, 7540
McCurry, Susan M.: 5135
McDaniel, Kennedy: 1375, 9420
McDarby, Meghan: 7900
McDermid, Robert: 7120, 7160
McDermott, Katherine: 4460
McDonald, Claire: 9010
McDonald, Lynn: 1145
McDonald, Margaret V.: 2235
McDonald, Shelley: 1370
McDowell, Cynthia: 7470
McDuffie, Danielle L.: 4320
McFeeley, Brittany M.: 7510
McGee, Jocelyn S.: 3300, 9410
McGee-Lawrence, Meghan: 9030
McGibbon, Chris: 7830
McGilton, Katherine: 4515, 7650
McGinley, Jacqueline M.: 1005
McGinty, Patrick: 4268, 7680
McGovern, Justine: 3120
McGovney, Kevin: 4490
McGrail, Kim: 4280
McGrath, Ryan: 1360, 7990
McGraw, Meghan: 4315
McGuire, Cathleen: 9010
McGuire, Lisa: 1265, 2070, 3020, 3055, 5015, 5070, 9010
McGuire, Maura: 4170
McHugh, John: 1010, 9020
McHugh, Molly: 4545, 7980, 9010
McIntosh, Roger: 4340
McIntyre, Alissa C.: 7910
McIntyre, Deborah: 9020
McKay, Mary Jo: 3560
McKay, Michelle A.: 9000
McKay, Tara: 1180, 4000
McKee, Michael: 9000
McKelvey, Miechelle: 2080
Mckendrick, Karen: 3335
McKenzie, Colin: 4385
McKenzie, Glenise: 7440, 7470, 7540
McKibbin, Christine: 7610, 7840
McKiddy, Sarah: 7830
McKinley, Joseph: 7620
McLaughlin, Sara J.: 4435, 9010
McLean, Elisabeth: 7830
McLennan, Lauren: 1280
McLin, Maia: 7950
McMahon, Siobhan: 5100, 5205, 9410
McManus, David: 9030
McMillan, Barbara: 2020
McMillan, Corey: 7650
McMillan, Sincere S.: 7410
McMullen, Elyse: 4510
McMullen, Tara L.: 3175
McNally, James: 4175, 7380
McNamara, Kelsey C.: 9000, 9020
McNeil, Heather: 9020
McNeil, John: 7300
McNerney, Windy: 9010
McNicoll, Lynn: 9010
McPartlin, Adam: 7180
McPherson, Rachel: 3330
McPhillips, Miranda Varrasse: 3500
McQuarrie, Angelica: 5100
McReynolds, Melanie: 2205
Mcvey, Ava: 7080
Meadows, Jill: 9020
Meads, Morgan: 8900
Mecca, Marcia C.: 4060
Medders, Laura: 2300
Medina, Luis D.: 3050
Medina, Valerie: 7990
Medlin, Austin: 2070
Medrano, Theresa: 1380, 7370
Meeador, Rhoda: 2030
Meears, Rachel: 2145
Meehan, Amy: 9000, 9030
Meeks, Suzanne: 9010
Mehdiyar, Manijeh: 7360
Mehegan, Laura: 2035, 5040, 5175
Mehling, Wolf: 2035
Mehlsen, Mimi: 3195
Mehmood, Yasir: 7690
Mehrabi, Fereshteh: 5220
Mehta, Neil: 9030
Mehta, Priyanka S.: 3250, 4095
Mehta, Rhea: 7330
Mei, Lauren: 7510
Meier, Helen CS.: 2325, 7960, 9330
Meier, Naomi: 7390
Meier, Tabea: 4225
Meinertz, Naomi R.: 2075, 3445
Meinow, Bettina: 4190
Meiry, Lian: 4125
Meister, Melanie: 7330
Mejía-Arango, Silvia: 7540
Mejía-Guevara, Iván: 7970
Mejia, Shannon: 5195, 7030, 7590
Mekonnen, Teferi: 9020
Melady, Don: 5185
Melekis, Kelly A.: 3200, 3410
Melillo, Karen Devereaux: 1080, 5200
Melis, Rene JF.: 1370, 4485
Melissa, Macri: 9030
Melkonian, Matilda: 9010
Mell, Johnathan: 7440
Melo Resendes, Natasha: 9030
Meltzer, Gabriella: 3050
Mena, Emily: 2370
Mendelson, Gad: 7720
Mendez Campos, Barbara: 4420, 9010
Méndez-Chacón, Ericka: 9010
Mendhe, Dinesh: 4050, 7080
Mendoza, Lisandra: 2180, 5070
Mendoza, Nancy: 2005
Meneilly, Graydon: 7820
Meng, Hongdao: 3345, 5170, 7990, 9010
Meng, Lingsong: 7630
Menne, Dr. Heather: 9010
Menne, Heather L.: 7280, 9030
Mentor, Rhoodjinie: 8900
Meraz, Rebecca: 9410
Merdjanoff, Alexis: 5005
Merianos, Ashley L.: 7410
Merickel, Jennifer: 7510, 7620
Merkel, Emily: 3460
Merrilees, Jennifer: 1305, 4495
Merrill, Ray M.: 9010, 9020
Merss, Kristin: 9020
Merten, Natascha: 2240, 4120
Mertz, Laurel: 5125, 9020
Messier, Steve P.: 3505
Metzelthin, Silke: 3340, 7120, 7940
Meyer, Kayla: 4134, 7830
Meyer, Kylie: 2160, 5120, 9030
Meyer, Oanh L.: 3050
Meyers, David MW.: 4000
Meyers, Gabrielle: 7680
Meynadasy, Melissa J.: 7030
Mezuk, Briana: 3080, 3085, 4545, 5010, 5050, 9020

Mi, Hong: 4240, 4400, 7270, 7560, 7780
Mia, Mullen: 3425
Miao, Hongyu: 7460
Michael, Cherish: 4230, 7060, 7570, 7590
Michael, Yvonne L.: 1015
Mickus, Maureen A.: 5235
Middleton, Laura E.: 7300
Miech, Edward: 9000
Mielke, Michelle M.: 9200
Migliaccio, John N.: 4410
Migneault, Deborah: 1240, 4140
Migovich, Miroslava: 9030
Migrino, Lauren: 9420
Miguel-Cruz, Antonio: 1165, 5015, 9000, 9020
Mihailovic, Aleksandra: 2135
Mikels, Joseph A.: 1105
Mikkelsen, Mai B.: 3195
Mikkola, Tuija M.: 9010
Milani, Sadaf A.: 3295, 7600, 9560
Milberg, William: 9010
Milcik, Nicholas: 9010
Miles, Allyson: 3305
Milidonis, Mary: 4268, 7680
Milius, Rose: 7400
Miljkovic, Iva: 7830, 9020
Millar, Courtney: 9030
Millar, Roberto: 9000
Millenbah, Ashley: 1025, 7380
Miller, Brandi C.: 7100, 7870
Miller, Dante: 5010
Miller, Debra A.: 3015
Miller, Edward A.: 2245, 3165, 3350, 4390, 4435
Miller, Elaine L.: 7850
Miller, Henry: 1325
Miller, Julie: 1175, 3090, 3095, 5040
Miller, Katherine: 1175, 4365
Miller, Lindsay M.: 9500
Miller, Lyndsey M.: 3535, 4495
Miller, Melicent: 7620
Miller, Michael: 3505, 5230, 9010
Miller, Nancy D.: 9010
Miller, Rhonda: 2135
Miller, Richard: 7880
Miller, Ryan: 4080
Miller, Sara: 7390
Miller, Steven: 7140
Miller, Vivian J.: 9000, 9020
Miller-Winder, Amber: 7460, 7520
Mills, Sarah: 3345
Mills, Whitney L.: 3365, 4290, 7210
Milman, Sofiya: 1125
Milnamow, Mary: 7700, 9010
Mimbs, Hope: 4230, 7060, 7570, 7590
Min, Eun Jeong: 7160, 7800
Min, Joohong: 3130
Min, Jungwon: 1215
Mindlis, Irina: 3470
Minet, Hailey: 7050, 7960
Minster, Ryan: 5090
Minton, Alyssa R.: 1105
Mintz, Alan: 5050
Minyo, Morgan J.: 9020
Mion, Lorraine: 9010, 9030
Mira, Rolla Saud: 9030
Mirabella, Jack E.: 5220
Mirza, Raza: 7010, 9030
Mishra, Sidharth Prasad: 4475, 7100, 7870
Misonow, Julia: 2370
Mitchell, Anne B.: 4005
Mitchell, Calli O.: 9030
Mitchell, Colter: 2320, 2325
Mitchell, Emma: 7360
Mitchell, Jamie: 1270
Mitchell, Lori: 2170
Mitchell, Sarah J.: 9020
Mitchell, Susan: 2035, 7640
Mitha, Samrah N.: 7500
Mithani, Sara: 7910, 9000
Mitra, Shimontini: 5220
Mittal, Anuva: 3220
Mittelman, Mary: 2090, 9320
Mitzner, Tracy L.: 1120
Miyagawa, Shoko: 9030
Miyamae, Fumiko: 7280
Miyawaki, Christina E.: 1165, 1280, 2020, 3050, 4365
Mize, Mary Chase: 7070
Mizuochi, Masaaki: 7890
Mizutani Bacon, Suzette: 1310
Mlinac, Michelle E.: 9010
Mo, Cen: 4310
Moïse, Valerie: 1285
Moaddel, Ruin: 7880, 9020
Mochel, Amy: 4290
Mock, Marilyn: 1130, 7290
Mode, Nicolle: 9000
Modica, Cheryl: 9000
Moen, Maria: 3175
Moen, Phyllis: 2050, 3085
Moerk, Hannah R.: 9020
Moffit, Reagan E.: 3475
Moffitt, Terrie E.: 1305, 5080
Moghekar, Abhay R.: 2195, 4035
Mogle, Jacqueline: 1300, 1360, 2275, 3245, 4055, 4425, 7080, 7160, 9010, 9020
Mois, George: 1315, 4415
Molina, Anthony: 5210, 7370, 7820, 8900
Molinari, Alexa: 2130
Molinari, Victor A.: 5170, 7990
Molinar-Lopez, Veronica: 7830
Moll, Allison C.: 7510
Molli, Sam: 7070
Molnar, Lisa J.: 9030
Molony, Sheila L.: 9300
Molton, Ivan R.: 9580
Monane, Mark: 9550
Mondick, Lindsay: 1065
Mongelli, Jessica: 4030
Monin, Joan: 1305, 2055, 3360, 3535, 4010, 7420
Monroe, Stephanie: 1265, 2180
Monroe-Lord, Lillie: 7140, 7350
Monserud, Maria: 3105
Montayre, Jed: 4132, 7480
Montepare, Joann M.: 1330, 2080, 2250, 3150, 3325, 4160
Montero-Odasso, Manuel: 4120
Montesino Goicolea, Soamy: 7630
Montez, Shelby: 9030
Monti, Stefano: 5090, 5180, 7370
Montoro-Rodriguez, Julian: 7030, 7530
Montoya, Erika: 9570
Moo, Lauren R.: 2085, 3415, 7850, 9010, 9020
Moon, Chooza: 7990
Moon, Heehyul: 7530
Moon, Jukrin: 9030
Moone, Rajean: 4000, 5025, 7250, 9020
Mooney, Aimee: 7440
Moore, Ami: 7390
Moore, Ann Zenobia: 2195, 9020
Moore, Caroline: 5085
Moore, David J.: 3565, 4340
Moore, Jade: 7160
Moore, Jennifer: 5190, 7680
Moore, Justin: 3505
Moore, Logan: 7370
Moore, Miranda: 9030
Moore, Nadia: 9000
Moore, Sebastiana O.: 7410
Moored, Kyle D.: 1015, 2155, 3405, 4145, 9000
Moorman, Sara: 1000, 3095, 3105
Mor, Sharon Ost: 3275
Mor, Vincent: 2035, 4290, 7020
Moraras, Kate: 9000
Morato Stival, Marina: 7150
Morato Stival Lima, Marina: 7340
Moreines, Laura T.: 2235
Moreno-Agostino, Dario: 4510
Morgan, Ashley: 7180, 7340
Morgan, Brianna: 1380, 3290, 7840
Morgan, Davie: 9410
Morgan, Deborah: 9420
Morgan, Debra: 7650
Morgan, Jennifer: 3285
Morgan, Jennifer: 5190
Morgan, Jennifer: 7290, 7500
Morgan, Kerri: 7780
Morgan, Matthew R.: 5115
Morgan, Robert O.: 4290, 7540
Morgan-Daniel, Jane: 7170
Morgen, Keith: 3570, 7390
Morhardt, Darby J.: 7700, 9030
Mori, Koji: 9030
Moriello, Gabriele: 7950, 9000
Morikawa, Minami: 9000
Moritz, Robert: 7110, 7880
Morris, Brian J.: 5060
Morris, Emily P.: 1170, 9210
Morris, John: 7160
Morris, Nichole: 3460
Morris, Stephanie: 1390
Morrison, Cassandra: 7100
Morrow-Howell, Nancy: 2000, 3310, 4085, 4406, 7000, 9000
Morton, Cynthia: 3175
Morton, Cynthia: 7810
Morton, Patricia: 7960
Moser, Debra K.: 9000
Moshfeghi, Sonia: 5225
Moskow, Marian: 1335
Mosley, Thomas H.: 9200
Mosqueda, Laura: 1145, 2220, 4275
Moss, Karen O.: 2070, 3345, 4255, 5030

Mostafavi, Armin: 9010
Motel-Klingebiel, Andreas: 4450
Mouchaers, Ines: 7120
Moulton, Marjorie: 3255
Mousavi, Hossein: 9010
Mouton, Charles P.: 9010
Mouton, Peter: 7100
Mowat, Freya: 4120
Moxley, Jerad: 2050, 2060, 4268, 7680
Moy, Marilyn: 4095
Moya, Mario: 7130
Moye, Jennifer: 4095
Mozid, Nusrat E.: 9010
Mpofu, Elias: 7280, 7420, 9000
Mroczek, Daniel: 1255, 3230, 4300
Mroz, Emily L.: 3360, 4010, 9030
Mu, Aruhan: 3450, 7050
Mu, Christina X.: 3455, 3500, 7410, 7890
Muñoz, Elizabeth: 3080, 4185, 4425
Muchenje, Marvelous: 4340
Mudar, Raksha A.: 1260, 1315, 4415, 9020
Mudrazija, Stipica: 1185
Mudrazija, Stipica: 1265, 4325
Mudrazija, Stipica: 7950, 9020
Muhammad, T.: 3085, 7830
Mujahid, Nadia: 9010
Mujtaba, Muhammad: 7860
Mukherjee, Dahlia: 3135
Mukherjee, Namrata: 3410
Mukherjee, Nandini: 7370
Mulcahy, John: 3010, 4140
Mullachery, Balakrishnan: 7840
Mullavey, Judy: 5105
Mullen, Rebecca: 4205
Müller-Riemenschneider, Falk: 4385
Mullin, Donncha S.: 4134, 7620
Mullin, Patricia: 4150
Mullins, Megan: 1085
Mulvey, Michael: 3260
Mulvihill, Marie: 4525
Mumford, Elizabeth: 3075
Mumma, Kara T.: 1120, 7990
Mundy, Lindsey: 9000
Muniz-Terrera, Graciela: 1225, 3520, 4134, 4455, 5220, 7620, 7630, 7660, 7780, 7830
Munoz, Mariateresa H.: 4415
Munoz, Richard A.: 9420
Munoz Soto, Gianinna: 9000
Munroe, Leah A.: 9580
Munroe, Melanie: 1390
Munson, Taylor: 9010
Munsterman, Ellen: 7860, 7980
Muntefering, Chloe: 5000
Mur, Jure: 1225
Murabito, Joanne M.: 5090, 7640, 7830, 9000, 9030
Muraira, Joseph J.: 7590, 7900
Muralidhar, Kiranmayee: 9010, 9020
Muramatsu, Naoko: 4465
Murawski, Alaine: 7440, 7460, 7520
Murayama, Hiroshi: 7280, 7550, 7610, 7760
Murman, Daniel: 7510
Murphey, James M.: 5030
Murphy, Dallas J.: 7590, 9430
Murphy, James: 2100
Murphy, Rachel: 3275, 7550
Murphy, Rachel: 9520
Murphy, Sara: 4220, 4360
Murphy, Susan L.: 7680
Murphy, Terrence: 7120
Murray, Anna: 3215
Murray, Anne: 7300, 7370
Murray, Briana: 9020
Murray, Emily T.: 1015
Murray, James F.: 4210, 5105, 7620
Murthy, Venkatesh L.: 7630, 9300, 9330
Muruganand, Ashwath: 7230
Muruthi, James: 2010, 5130
Muruthi, Ndungu: 2010
Murzin, Kate: 4340
Musich, Madison: 3420, 7080
Musil, Carol: 2005, 3360, 7030, 7950
Muskin, Ryan: 9020
Mussallam, Noura: 7550
Musson, Nan: 7510
Mutambudzi, Miriam: 1180
Mutchie, Heather: 3550
Mutchler, Jan E.: 3350, 4435, 9000, 9510
Muyingo, Laura: 9000
Mwangi, Eunice Muthoni: 9110
Mwega, Elijah: 2010
Mwendwa, Denee: 9020
Mwinyogle, Aubrey: 7860
Myck, Michal: 1220
Myers, Alyson K.: 4268, 7680
Myers, Dennis R.: 7680, 9030
Myers, Jennifer: 7130
Myers, Marcella: 8900
Myrskyla, Mikko: 9030

**N**

Na, Annalisa: 4005, 9400
Na, Hunui: 1315
Na, Muzi: 1085
Naar, Sylvie: 3520
Nachtweih, Emily: 1150
Nadash, Pamela: 1175, 2245, 3165, 4295
Nadorff, Danielle K.: 1070, 2005, 7950
Naegele, Gerd: 4450
Naftolin, Frederick: 7100
Nagarajan, Niranjani: 2365, 9110
Nagarajan, Niranjani: 9110
Nagargoje, Varsha P.: 4385
Nagel, Corey: 2290, 2360, 7300, 7360, 7450, 7540, 7890, 7990
Nah, Suyoung: 4140
Naik, Aanand: 4060, 7630, 7840
Naik, Aishwarya: 4505
Nair, Malini: 9000
Nair, Neetu: 4490
Nair, Tripti: 9020
Nakagawa, Kazuma: 2380
Nakagawa, Takeshi: 4225
Nakamoto, Isuzu: 7610
Nakamura, Masahide: 9010
Nakamura, Misa: 9030
Nakashima, Taeko: 3210
Nakhai, Bita: 2270
Nalawade, Vinit: 3510
Nam, Seok In: 7200
Nam, Stephanie Jungah: 4265
Nanjaiah, , Shivamma Nanjaiah: 9010
Nanthakumar, Jarshini: 9330
Nardone, Michael: 9020
Narine, Ameesha: 9030
Narine, Donnette: 2375, 5110, 7040, 7380, 7600
Narusyte, Jurgita: 9030
Nasamran, Chanond: 7100
Nash, Veronika J.: 2280, 7410
Nashef-Hamuda, Reem: 9020
Nashiro, Kaoru: 1215
Näsman, Marina: 4330
Nasr, Lubna A.: 4240
Nasr, Samer: 1005
Nasser, Lucy: 9030
Nathalie, Veillette: 9000
Nathanielsz, Peter: 9000
Naumova, Elena N.: 1290
Navarro, Adria E.: 9000
Naveed, Haniya: 9000
Navia, R. Osvaldo: 9200
Navid, Sarah: 7600
Naylor, Mary D.: 4545, 7980, 9010, 9020
Nazario, Jaminette M.: 7950
Neal, Amy: 7180
Neal, Vanessa L.: 7510
Nearing, Kathryn A.: 2160
Nedelska, Zuzana: 7420
Neelamegam, Malinee: 2260
Neely, Stephen: 7300
Neff, Donna F.: 7810, 9010
Nehrkorn-Bailey, Abigail: 9010
Neiberg, Rebecca: 3505, 4355, 9010
Neiertz, Cynthia: 9210
Neil-Sztramko, Sarah E.: 5085
Neller, Sarah A.: 1150, 2075
Nelligan, Labrini I.: 2020
Nelson, Christi L.: 2310, 7450, 9010
Nelson, Ian Matt: 3190
Nelson, James F.: 4135, 4475
Nelson, Katie E.: 7770
Nelson, Matt M.: 3320, 9010
Nelson, Monica E.: 7420
Nelson, Niccole A.: 1070, 3245
Nelson, Sofie J.: 7260
Nemati, Donya: 5125, 7410
Nemec, Mary C.: 9320, 9500
Nemeth, Samuel R.: 4535
Nemmers, Natasha L.: 2075, 3360, 7070
Neppl, Tricia K.: 7550
Nerayo, Liwam: 9020
Neri, Mel: 9020
Neugebauer, Volker: 7830
Neugroschl, Judith: 2085
Neupert, Shevaun D.: 1255, 3195, 4180, 7550, 7590
Neville, Christine: 9000
Neville, Emily: 9000
Newbold, Bruce: 5085
Newman, Anne B.: 3475, 3500, 4260, 5085, 5090, 5210, 7630, 9300, 9330

Newman, David: 2180, 4132, 5225, 7480, 7580
Newman, John C.: 1380, 7110
Newman-Casey, Paula Anne: 7820
Newman-Norlund, Roger: 8900
Newman-Norlund, Sarah: 8900
Newmeyer, Natalie: 9410
Newsham, Tina M.: 2245, 3150, 3350
Newsom, Jason T.: 1305, 2360, 7300, 7450, 7890, 7990
Newton, Julia: 7160
Neylan, Thomas C.: 2280
Ng, Boon Peng: 2385, 7150, 7430, 9030
Ng, Crystal: 5035
Ng, Wai Chong: 7760
Ng, Yee To: 4145, 4230
Ngugi, Anthony: 9110
nguyen, Ann: 3005
Nguyen, Annie L.: 4275
Nguyen, Duy: 7230
Nguyen, Hoang T.: 4395
Nguyen, Khanh: 9530
Nguyen, Kim N.: 1165, 3050
Nguyen, Kimberly: 1290
Nguyen, Kristy B.: 4134, 7620
Nguyen, Uyen-Sa: 9530
Nhan, James: 9020
Nicholas, Marjorie L.: 1255
Nichols, Emma: 2365, 3160, 9110
Nichols, Jennifer A.: 4134, 7330
Nichols, Madeline J.: 4045, 7580
Nicholson, Meredith: 9530
Nicklas, Barb J.: 3475, 3505, 4350, 5210, 9010
Nicolay, Sarah: 5195, 9550
Nicosia, Francesca M.: 2035
Nie, Jing: 4240
Nie, Zuoting: 1260
Niedernhofer, Laura J.: 4200
Nielsen, Lisbeth: 3040
Nightingale, Ginah: 7500
Nikitin, Jana: 2105, 3100, 4055
Niknejad, Bahar: 9010
Nikzad-Terhune, Katherina A.: 2080, 3350
Nilsen, Charlotta: 3470, 7300
Nimrod, Galit: 7920
Ning, Hongting: 9300, 9320
Ningaiah, Nagalambika: 9010, 9020
Nishikitani, Mariko: 7940, 9000
Nix, Caitlyn A.: 2040
Niznik, Joshua: 7150, 7300
Nketsiah, Ebow T.: 7260
Nkimbeng, Manka E.: 1150, 3240, 3305
Nkwata, Allan K.: 7930, 9010
Noah, Naheem: 3075
Noblitt, Jessica: 4230, 7060, 7570
Noel, Megan: 7810
Noguchi, Kenneth S.: 5100
Noh, Hyunjin: 3005, 5125, 7260, 7620, 9000, 9020
Nolte, Julia: 1090, 7510
Nookaew, Intawat: 7880
Noone, John: 5210
Noppert, Grace A.: 3225, 9010
Noren Hooten, Nicole: 9000
Norgaard, Martin: 1120
Norris, Grace: 3270
Norris, Hayden S.: 7150
Northrup-Snyder, Kathlynn: 5205
Northwood, Melissa: 7840
Norton, Peter G.: 4408, 7720
Norton, Sally: 1335
Norton, Sara A.: 3520, 4320
Nothelle, Stephanie: 2185
Notthoff, Nanna: 5125
Noureddine, Sarah: 7110
Nouryan, Christian: 4268, 7260, 7680, 9010
Novak, Joshua R.: 4235
Novik, Nuelle: 4340
Nowacek, Nancy: 1080
Nowels, David: 4305
Nowels, Molly: 4305
Nowicki, Scott: 7940
Noyer, Emily: 9000
Ntailianis, Georgios: 7630
Núñez-Luna, Alejandra: 4395
Nunnery, Mary H.: 1160, 4408, 7720
Nunno-Evans, Michelle: 7950
Nwakanma-Akanno, Onyedikachi Chioma: 4130
Nwakasi, Candidus "Candi": 2010, 4130, 5055, 9000
Nweke, Chizobam: 2010
Nweke, Chizobam: 9000
Nyang'au, Getrude K.: 2055, 7950
Nygård, Mikael: 4330
Nyman, Fredrik: 5010
Nyquist, Linda: 9030
Nyqvist, Fredrica: 4330
Nyrup, Rune: 7190
Nyunt, Thedoe: 5060

**O**

Obbin, Samuel: 9020
Oberdier, Matt T.: 9020
Obregon, Alejandra Michaels: 3160
O'Brien, Catherine: 5125
O'Brien, Christopher T.: 7590
O'Brien, Erica: 7590
O'Brien, Jennifer L.: 7330, 9010
O'Brien, John: 7630
Ocampo-Chaparro, Jose M.: 7140, 7840
Ochiai, Gabriela S.: 9010, 9020
Ochoa Scussiatto, Henrique: 1230
O'Connell, Denise: 2020
O'Connell, Megan E.: 1200, 7650, 7660
O’Connor, Melissa: 9000, 9410
O'Connor, Melissa L.: 7300, 9000
O'Connor, Timothy D.: 7000, 7460
O'Conor, Rachel: 2355, 9030
Oczkowska, Monika: 1220
Odagami, Kishimori: 9030
Odden, Michelle C.: 1015, 4080, 4515, 5230, 7160
Odemuyiwa, Olumayowa A.: 9000
Odlum, Michelle: 1070, 1335, 3415, 7330
Odo, Obinna Casmir: 9010
Odom, J. Nicholas: 9010, 9020
ODonnell, Arden: 3410, 7670
O'Donnell, Amy J.: 4540, 9010
O'Donovan, Aoife S.: 2280
O'Driscoll, Leo: 5150
Odukoya, Tolu: 5025
Odzakovic, Elzana: 7300
Ofori, Edward: 7100, 9030
Ofotokun, Ighovwerha: 3565
Ogawa, Elisa F.: 9010
Ogidigo, Oloaigbe (Joyce) O.: 2125
Ogle, Destiny: 1050, 1255
Ogletree, Aaron M.: 3210
Ogliore, Anthony M.: 9020
O'Grady, Thomas: 3365, 7260
Ogungbe, Oluwabunmi: 7960
Ogunjesa, Babatope: 7610
Oh, Esther S.: 2185
Oh, Eunna: 7660
Oh, Gahee: 3565
Oh, Hans: 9020
Oh, Hayoung: 4145
Oh, In-Hwan: 7380
Oh, Jeewon: 9020
Oh, Kyeung Mi: 7700
Oh, Patricia: 4150
Oh, Seokmin: 7990
Oh, Seung-Eun: 5055
Oh, Suhee: 7810
Oh, Yun taek: 3085, 9000
O'Hanlon, Julia: 9020
Ohuabunwa, Ugochi: 4525
Ojo, Eunice Oladepe O.: 7080, 9030
Okada, Jamil: 9030
Okamura, Tsuyoshi: 7280
Okello, Samson: 9000
Okem akwiwu, Chika: 4130
Okereke, Olivia I.: 4460
Okeyo, Hilda: 9000
Okhravi, Hamid: 9010
Okine, Joana: 4275
Okoli, Chizimuzo: 2280
Okolie, Jonathan: 9010
Okoye, Safiyyah M.: 5130, 5160
Okpalefe, Okiemute: 2125
Okrah, Prince A.: 9030
Oktapodas Feiler, Marina: 7150
Okuyan, Canan: 4120
Okwaro, Lynnete: 7290
Oladeinde, Yewande: 3210
Oladejo, Dianne: 2355, 9030
Olanrewaju, Sherif: 9000
Olausson, Jill: 7850
Olazo-Cárdenas, Kamyla M.: 9010
O'Leary, Bryant J.: 5075, 7910
Olivari, Benjamin S.: 2070, 5015, 5070, 7700
Oliveira, Eduardo: 7290
Oliveira, Iara B.: 2375, 3365
Oliver, Joanna T.: 4265
Oliver, Michael D.: 7100

Oliver, Sloan: 7620
Olivieri-Mui, Brianne: 3565, 9000, 9010, 9430, 9540
Olokunlade, Temitope: 7430
Oloruntoba, Oluyomi: 7410
Olson, John D.: 7370
Olson, KayLoni: 7630
Oltmanns, Thomas F.: 3520, 4320, 9020
Olvera, Charles: 7460, 7520
O'Malley, Kelly A.: 1365
O'Malley, Terry: 3175
Omeje, Kingsley U.: 2125
Omura, John D.: 2070, 5015, 5070
O'Neil, Kaitlyn: 7700
Ong, Anthony D.: 1255
Ong, Lydia Q.: 4320
Ong, Michael: 7900
Oni, Julius: 9010
Ono, Keiko: 9010
Onsando, Wambui: 9010
Oosterhouse, Kimberly: 9000
Openo, Kyle P.: 4130
Opoku-Agyemang, Christfelly: 7330
Oppong, Richard F.: 5085
Oranchuk, Dustin J.: 9020
Orange, Joseph B.: 3270
Oravecz, Zita: 5165, 7510
Orewa, Gregory N.: 2350, 3365, 4190
Orkaby, Ariela: 4310
Orkaby, Ariela: 9410
Orlovsky, Irina: 9020
Ornstein, Katherine: 1375, 3415, 4165, 4365, 5160, 7650, 9030
O'Rourke, Tarryn: 7750
Orr, Miranda E.: 2335
Orsega-Smith, Elizabeth: 7460, 9020
Orsulic-Jeras, Silvia: 7060, 7460, 9550
Ortega, Alexandra: 9010
Ortega, Julia L.: 9550
Ortega, Marcus: 7100
Ortega Hernández, María: 1055
Orth, Jessica: 5015
Ortiz, Hector: 2025
Ortiz, Juliana: 9010
Ortiz Sarmiento, Erika: 4268, 7680
Ortmeyer, Heidi: 7840
Ortola, Rosario: 2320
Oruru, Daisha: 7460
Orwig, Denise: 3550, 5205, 7130
Orwoll, Eric: 4260, 5085
Ory, Marcia: 9030
Osakwe, Zainab Toteh: 1335, 3415
O'Súilleabháin, Páraic S.: 7990
Osei, Elizabeth: 7790
Osevala, Nicole J.: 5145
Osika, Jocie: 4300
Osokpo, Onome: 4545, 9010, 9020
Osso, Francesco S.: 2075, 7300
Østbye, Truls: 2265, 3180
Osterbur, Elaina: 9030
Ostrem, Nicholas: 7440
O'Sullivan, Julie L.: 9400
O'Sullivan, Kelly: 1365
O'Sullivan, Roger: 2135, 4160
Osuna, Margarita Maria M.: 3465
Oswald, Austin: 1250, 2310, 5150, 9440
Oswald, Catherine L.: 9030
Oswald, Frank: 2340
Otero, Marcela: 4300
Otis, Laurie: 9400, 9440
O'Toole, Mia S.: 3195
Ou, Alison X.T.: 7520
Ouchi, Kei: 3300
Oude Voshaar, Richard C.: 9000
Ouslander, Joseph: 7670
Oviawe, Elizabeth: 7500
Owen, Donna C.: 7160
Owino, Jonix: 3380
Owsiany, Montgomery T.: 7030, 7570
Oya, Akari: 5035
Oylu, Gulin: 4450
Ozawa, Tabasa: 3160

**P**

P. de Paula Couto, M. Clara: 3100
Paccione, Jane A.: 2160
Pace, Sarah: 3025
Pachana, Nancy A.: 7550
Pachur, Thorsten: 1090
Padakandla, Deepthi: 4190
Padden, Eleni: 9000
Padgett, Deborah: 5170
Padron, Dilianna: 2180, 5070
Pagan, Mary Theresa: 5190, 7000
Pagan, Michael: 2315
Page, Riley: 9410
Pai, Manacy: 3085
Pajewski, Nicholas M.: 4355
Pajonk, Frank: 8900
Pajuelo-Vasquez, Renzo: 9010, 9030
Pakhomov, Serguei: 7300
Pal, Subhamoy: 9020
Palavicini, Juan: 1325, 9000
Palella, Frank J.: 3390
Palen, Ted: 1240
Palermo, Francisco: 3080
Palgi, Yuval: 3275, 4180, 9420
Palimaru, Alina: 9010
Palleschi, Mary-Kate: 9010
Pallis, Maryssa: 1175
Palmer, Allyson K.: 1325
Palmer, Barton W.: 7610
Palmieri, Jim: 5175
Palms, Jordan D.: 1170, 4325
Palovics, Robert: 4065
Palta, Priya: 5085, 7340, 9200
Pan, Xi: 7830, 7960
Panday, Somi: 4132, 7480
Pandya, Naushira: 2297, 7000, 7500
Panellas, Isis O.: 9030
Pangle, Amanda: 4130, 9000, 9020
Pani, Jasmine: 9020
Panta, Pradip: 9030
Papaioannou, Alexandra: 4268, 5100, 7220, 7680, 7710, 9020
Pappa, Sara: 9010
Pappadis, Monique R.: 9010
Parada Jr., Humberto: 3390
Parast, Mana M.: 8900
Parchman, Michael: 7810
Parekh, Rupal: 7280, 9020
Parini, Angelo: 2320
Paris, Steve: 9020
Park, Chan Mi: 9540
Park, Chang: 4025
Park, Christina: 7830
Park, Crystal L.: 1390
Park, Elyse R.: 9030
Park, Esther: 9000
Park, Geunhye: 2055
Park, Grace: 7120
Park, Hayoung: 7040, 7080, 7610
Park, Hyejin: 9010
Park, Hyeran: 7400
Park, Hyeyoung K.: 9030
Park, Hyunju: 7300
Park, Isabella: 9010
Park, Ji Yeong: 7160
Park, Jihyun: 9020
Park, Joon-Hyuk: 2385, 5075, 7080, 7150, 7430, 7680, 7820, 9010, 9020, 9030
Park, Junmin: 9030
Park, JuYoung: 2125
Park, Juyoung: 7420
Park, JuYoung: 7670
Park, Juyoung: 9000
Park, JuYoung: 9010
Park, Juyoung: 9020
Park, Kyoung Shin: 9030
Park, Lindsay (Na Yeon): 7180
Park, Min Kyung: 7320
Park, Min-Kyoung: 9010
Park, Moonkyoung: 7660
Park, Nan S.: 7510
Park, Seho: 1040
Park, SeongHu: 7850
Park, Sojung: 4266, 7690, 9010, 9030
Park, Soobin: 4266, 7690, 9010, 9030
Park, Stella: 4095
Park, Suah: 7910, 9010
Park, Sujeong: 7330
Park, Sumin: 7780
Park, Sung S.: 3065
Park, Sung Kyun: 9010
Park, Sungjae: 2145
Park, Taeyoung: 7360
Park, Van Ta: 1165
Park, Yoonsei: 7780
Parker, Andrew M.: 1090
Parker, Daniel C.: 1370, 9000
Parker, Elizabeth A.: 7340, 7350, 7840, 9010
Parker, Kelly RB.: 1360, 7990
Parker, Lauren: 1265, 2130, 3240, 7550
Parker, Myra: 9100
Parker, Patricia A.: 7900
Parker, Robert: 2035
Parker Oliver, Debra: 4415, 9000
Parkinson, Luisa: 9020
Parkman, Suzanne E.: 7850
Parmelee, Patricia A.: 7460
Parris, James J.: 2145
Parrish, Elise: 1175
Parrish, Monique: 7700
Parsons, Nathan: 9010
Parsons, Pamela L.: 3025
Pascoal, Tharick A.: 9500
Pascoe, Tori: 2300
Pascual-Leone, Alvaro: 2155, 9030
Pascuzzi, Emma N.: 3385
Passegue, Emmanuelle: 3070
Passos, Lígia: 2215, 7300
Patano, Arienne: 9000
Patapoff, Molly A.: 7610
Patel, Charmi S.: 9010, 9030

Patel, Himalaya: 7080
Patel, Juhi: 3520
Patel, Kadam: 4130
Patel, Karishma: 9330
Patel, Khushbu S.: 7830, 7950
Patel, Manali: 3525
Patel, Mehul: 9020
Patel, Priya: 4030
Patel, Roshni: 2070, 5070, 9010
Patel, Snehal: 3345
Pathak, Deeya: 9010
Pathak, Parth: 7750
Patille, Rachelle: 1205
Patricia, Boyle: 1230
Patskanick, Taylor: 7090, 7910, 9010
Patt, Stephanie: 7010
Patterson, Logan: 3250
Patterson, Sarah: 1340, 4055
Patti, Gary J.: 5180, 9020
Patton, Rikki: 4406, 7500
Patyal, Pankaj: 7100, 7370, 7880
Paudel, Anju: 1300, 3020
Paul, Constanca: 3130, 4406, 7000, 7300
Paul, Deepak: 4495
Paul, Eli: 9000
Paul, Kimberly: 7370
Paulsen, Adam J.: 2240
Paulsen, Michelle: 9030
Paulson, Sally: 2015, 7810
Pauly, Mark: 4545
Pauly, Theresa: 3275, 4235, 4480, 9000
Paun, Olimpia: 3445, 7420, 9030
Pavlovic, Noelle: 5175
Pavon, Juliessa M.: 7150
Pawlicki, Matthew: 7130
Payen, Ameante: 1320
Payne, Laura: 7610, 7940
Pearman, Ann: 7330, 7510, 7550
Pearson, Thomas: 9000
Pebley, Anne: 3065
Pechin, Sue: 9020
Peckenpaugh, Jackson W.: 9410
Peckham, Allie: 5080, 7780
Pedersen, Dorthe A.: 4260
Pedersen, Megan: 9000
Peek, Monica: 9010
Peeples, Amanda D.: 4095, 8900
Peerman, Carey: 5170
Peet, J. Zak: 7080
Peeters, Geeske: 1370
Pei, Sen: 1110
Pei, Yaolin: 2090, 2265, 3540, 9010, 9020, 9320
Peiro-Milan, Inma: 4330
Peleg, Shira: 4480
Pelkmans, Jordan: 3535
Pelletier, Chelsea: 7300
Pena, Marie: 8900
Pena, Triana: 9000
Penconek, Tatiana: 4408, 7720
Pendergrast, Claire: 7280
Pendrey, Anna: 7130, 9000
Penfold, Robert B.: 5135
Peng, Changmin: 3165, 7510
Peng, Huamao: 5115, 7000, 7060, 7950
Peng, Li-Min: 4155
Peng, Siyun: 4500, 9020, 9300
Peng, Weisi: 9020, 9520, 9540
Peng, Wenting: 4310
Penichet, Eric N.: 7370
Penney, Lauren S.: 1005, 7130, 7180
Penning, Nehle: 4450
Pentecost, Claire: 4070
Perales-Puchalt, Jaime: 7330
Peralta, Eriana: 9000
Peralta, Sawyer: 7110
Perek-Białas, Jolanta: 4450
Perepezko, Kate: 5000, 7800
Perera, Rohini: 9010
Perez, Eliany: 9010
Perez, Gilberto: 4060
Perez, Hector: 5015
Perez, Jacqueline: 4400
Perez, Sasha: 1335, 3415, 4266, 7190, 7850
Perez Montes, Viviana: 4200
Periassamy, Mithran: 7060
Perion, Jennifer: 7540
Perissinotto, Carla: 4205, 7610
Periyakoil, Divya: 9560
Perkhounkova, Yelena: 2160, 3335, 5015, 7300
Perkins, Molly: 1010, 1375, 3435, 3565, 9030
Perkins, Rebekah: 4406, 7000, 9000
Perkins, Susan: 9400, 9440
Perlman, Saritte: 4255
Perls, Thomas T.: 1040, 5090, 5180, 7370
Perone, Angela K.: 1100, 3200, 7280
Perre, Taylor: 3365, 7260
Perreira, Krista M.: 3320
Perry, Brea: 4500, 9020, 9300
Perry, Kaley: 9000, 9020
Perry, Morgan: 9020
Perry, Tam E.: 1270, 4150, 7690
Perryman, Collin: 9000
Persad, Alyssa: 9000
Pervratil, Michael: 1315, 9000
Perweiler, Elyse: 1130, 7290
Perzynski, Adam T.: 1170
Perzynski, Joseph: 3015, 5000
Peskoe, Sarah: 4485
Pestine-Stevens, Althea R.: 1330, 2315
Peszka, Jennifer: 7550
Petereit, Daniel: 7600
Peters, Elizabeth: 1185, 7950
Peters, Ruth: 9110
Peterson, Colleen M.: 3570, 7080
Peterson, Daniel S.: 9030
Peterson, Jennifer: 9010
Peterson, Lindsay J.: 2295, 4435, 5170
Peterson, Matthew: 3150
Peterson, Neil E.: 7980
Peterson, Tina L.: 7980
Petri, Florence: 3015
Petrovic, Milenko T.: 9000
Petrovskaya, Olga: 3570
Petrovsky, Darina V.: 2110, 4005, 9000
Pettee Gabriel, Kelley: 7650, 9010
Pettigrew, Corinne: 3450, 7970
Pettis, Jennifer: 9000
Pflieger, Lance: 7110
Pfund, Gabrielle N.: 3245, 3520
Pham, Julie Q.: 9000
Phan, Dennis: 9030
Phan, Phillip: 5175
Phang, Howard J.: 7370
Phatak, Vaishali: 7510
Phelan, Elizabeth A.: 1280, 2385
Phifer, Lisa: 9020
Philips, Joanna: 9000
Phillips, Bethan: 4470
Phillips, Christine: 7080
Phillips, Karon: 1330
Phillips, Michael M.: 9000
Phillips, Mirelle: 7680
Phillips, Natalie A.: 7650
Phillips, Nicole: 3450
Phillips, Stuart: 5085, 5100
Phipps, Denham: 4110
Phongtankuel, Veerawat: 7360
Phyo, Aung Zaw Zaw: 7370
Piazza, Jennifer: 3230
Picard, Martin: 1110
Picchiello, Matthew C.: 4210, 4406, 7000
Piccinin, Andrea M.: 3520
Piccitto, Giorgio: 9030
Pichardo, Abel: 8900
Pichora-Fuller, Kathleen: 4110
Picken, Lauren: 9000
Pickering, Carolyn E.: 1075, 5140
Pickett, Andrew C.: 7720, 9020
Piechota, Amanda: 3360, 4010
Piedra, Lissette M.: 1230, 4205
Pieper, Carl F.: 4485, 9010
Pierce, Aimee: 4100, 7620
Pierce, Matthew J.: 7550
Pierre-Louis, Saamantha: 5055
Pierson, Katie R.: 3485
Pieruccini-Faria, Frederico: 4120
Pietruszka, Brittany L.: 2145
Pike, James R.: 3510
Pillemer, Karl: 1145, 2030, 2050, 2060, 2105, 2295, 3375
Pilonieta, Giovanna: 9020
Pimentel, Camilla B.: 3365
Pinheiro, Cristina: 9020
Pinho, Hianca: 3540
Pinto, Jayant M.: 1230, 1350
Pirolli, Peter: 4268, 7680
Pituch, Keenan A.: 5075
Pizzi, Laura T.: 2130
Plasencia, Rosa: 7810
Plassman, Brenda L.: 3540
Platts, Loretta G.: 3370
Player, Nathanael: 1155
Pleasants, Debbie: 4355
Pless Kaiser, Anica: 1160
Pletcher, Scott: 2335
Plotkin, Elana: 7500
Plotzke, Michael R.: 3230, 4195
Ploubidis, George: 4510
Plow, Matthew: 9030
Plummer, Narcissa: 9010
Plys, Evan: 3525, 4525, 7680
Podder, Avijit: 5180
Poe, Abigail: 3545, 4145, 7090

Pohl, Janet S.: 5075
Pohnert, Anne M.: 4525, 7590
Polenick, Courtney A.: 4235, 4425, 5035, 7390
Polhemus, Ashley: 3550
Poliakov, Oleksandr A.: 7820
Pollack, Craig E.: 5130
Pollak, Chava: 9020
Pollard, Chris: 5220
Pomeroy, Mary Louise: 5160, 9030
Pompeu, Jose Eduardo: 9010, 9020
Ponce Gonzalez, Kimberly: 1150
Pond, Brittney: 5150, 9020
Ponto, Kevin: 9030
Poon, Cecilia: 3250
Poonsap, Sukanya: 7860
Popat, Shena: 1065
Pope, Caitlin: 4340, 7060
Pope, Natalie E.: 1330, 4150
Pope, Natalie D.: 5020, 7060, 9000
Popejoy, Lori L.: 1310, 7140
Popelsky, Braden: 7570
Poplawski, Antonina: 7760
Porat-Dahlerbruch, Joshua: 1175, 4255
Port, Cynthia: 3205
Portacolone, Elena: 9020, 9550
Porter, Kristie A.: 2375, 3365
Porter, Michelle M.: 4515, 7590, 9430
Porter Starr, Kathryn N.: 1370, 4350, 9000
Portz, Jennifer D.: 1240
Poss, Jeff: 2170
Possin, Katherine L.: 9320
Potok, O Alison: 9500
Pottayil, Faheem: 9500
Potts, Daniel C.: 1365
Powell, Danielle: 2185
Powell-Versteeg, Amy N.: 4406, 7000
Power, Shannon R.: 9000, 9320
Powers, Becky: 7180
Powers, James: 3120
Powers, John David: 1240
Powers, Sara: 7060, 7460, 9550
Pradhan, Rohit: 2350
Prager Geller, Tal: 7720
Prajapati, Santosh Kumar: 4475, 7100
Prakash, Sreelekha: 7290
Praparpak, Werapattra: 7860
Prasad, Anyah: 4390
Prasad, Jitender: 7320
Prata, Ndola: 9560
Pratley, Richard: 7620
Prats, Madison: 9010
Pratt, Borianna: 3065
Preble, Edward: 3075
Precupetu, Iuliana: 1220
Preddie, Alaina K.: 7130, 7180
Preis, Sarah R.: 4310
Premovich, Alyssa M.: 7910
Prendergast-Boughrum, Alice: 7690
Prescott, Amy: 9430
Pretzer-Aboff, Ingrid A.: 9330
Price, Caitlin N.: 7150
Price, Elvin: 9010
Price, Nathan D.: 5180
Primomo, Janet: 7910
Primossi, Valentina: 3260
Prince, Devlin: 7770
Prioli, Katherine: 2130
Probst, Karen: 4408, 7230
Procter-Gray, Elizabeth: 1350, 2385, 3155
Proffitt, Rachel: 1310
Prosperi, Mattia: 7860
Proulx, Christine M.: 3080, 4235, 4370
Provencher, Véronique: 9000
Province, Michael A.: 9010, 9020
Pruchnicki, Alec D.: 7140
Prybutok, Gayle: 7280
Przkora, Rene: 1030
Pubien, Sheena: 9420
Puche, Gabriel Prieto: 4415
Pudelek, Kelly M.: 1230
Pudur, Rekha: 7280
Puga, Frank: 1075, 3545, 4145, 7090, 7680, 9010, 9020
Pugh, Mary Jo: 9010, 9030
Puhan, Milo A.: 3550
Pulido, Joanne M.: 2125
Pullen, Carly E.: 1365, 7760, 8900
Pumilio, Victoria L.: 7500
Punke, Elizabeth: 7610, 7840
Puterman, Eli: 3520
Putnam, Michelle M.: 7780
Putz, Leann: 9520
Pynoos, Jon: 4270

### Q

Qazi, Amna: 7700
Qazi, Saadia: 4310
Qi, Bingbing: 9010
Qi, Liangshu: 9000
Qi, Meiyuzhen: 4120
Qi, Mingyu: 1115
Qi, Xiang: 1045, 1320, 2090, 2265, 3540, 9010, 9020
Qian, Yong: 5085
Qian, Yuting: 1165
Qiao, Yujia (Susanna): 3475, 5210
Qin, Vicky Mengqi: 7090
Qin, Weidi: 3005
Qin, Yue: 3105
Qiu, Tian: 9030
Qiu, Xiao: 9030
Qreshi, Mariam: 7500
Qu, Chaoran: 9020, 9520, 9540
Qu, Shan: 1175, 9010
Qu, Xiaomin: 1045
Qu, Yang: 7920
Quach, Emma: 3415, 7770
Quach, Ha-Linh: 3435
Quach, Lien T.: 9530
Qualls, Sara H.: 7090
Quashie, Nekehia Tamara: 2115, 3050
Quatsoe, Alissondra: 9010
Quellhorst, Jennifer A.: 3110
Quiñones, Ana R.: 2360, 7300, 7360, 7450, 7540, 7890, 7990, 9020
Quillen, Ellen E.: 7370
Quinlan, Claire: 9000
Quinn, Catherine: 4070
Quinn, Deirdre A.: 7580
Quinn, Joseph F.: 3370
Quinn, Marielle: 2055

### R

R. Goodin, Burel: 7630
Rababa, Mohammad: 7610
Rabinovich, Alexander: 9020
Rabinowitz, Jill A.: 4265, 4490, 7870
Rachmuth, Lisa: 3090
Radpour, Siavash: 3370
Raetz, Jaqueline G.: 7830
Rafferty, Jane: 9020
Ragab, Ahmed A.Y.: 7640, 7830, 9000
Raghavachari, Nalini: 5090, 5180
Rahemi, Zahra: 4005, 9000
Rahman, Farzana: 9010
Raimundo, Nuno: 5060
Raina, Ketki: 7300
Raina, Kirandeep: 7440
Raina, Parminder: 5070, 7660
Rainville, Chuck: 2035
Raj, Hayley: 5000
Raj, Minakshi: 4415
Rajagopal, Shilpa: 3295
Rajan, Ashna: 9010
Raji, Mukaila: 3295, 9010
Rajman, Luis: 9020
Raju, Anish: 5180
Rajupet, Sritha: 9020
Rakel, Barbara: 9020
Ralston, James D.: 5135
Ram, Nilam: 9010
Ramachandran, Meena: 7620
Raman, Vaishnavi: 9410
Raman-Wilms, Lalitha: 9520
Ramírez-García, Daniel: 4395
Ramel, Alfons: 5230, 7350
Ramirez, Ana V.: 7440
Ramirez, Maggie: 5135
Ramirez, Mildred: 1145
Ramirez, Saul: 4420
Ramirez, Sofia: 9010
Ramirez-Garcia, Daniel: 3160
Ramirez-Zohfeld, Vanessa: 7440, 7460, 7520
Ramisetty-Mikler, Suhasini: 9010
Ramos, Diêgo Araújo de Moraes: 9020
Ramos, Mary Dioise: 7850, 9000
Ramos, Sofhia V.: 5210
Ramos de Lima, Luciano: 7150, 7340
Ramrattan, Anita: 7150
Ramsey, Jennifer: 7030
Ramulu, Pradeep: 2135
Randa, Cari: 2340
Randall, Kevin: 7050, 7960
Randazzo, Aliza: 3505
Rane, Manas: 4310
Rane Levendovszky, Swati: 9000, 9010, 9020
Rank, Jenny: 4315
Rao, Ruchira: 4415
Rao, Smitha: 3045
Raper, James L.: 4340
Rapoport, Andy: 9530
Rapowitz, Jennifer: 4415
Rapozo, Crystalinda: 7130, 7840
Rapp, Stephen R.: 4134, 4355, 7830
Rappaport, Noa: 5180, 7110, 7880
Rasekaba, Tshepo: 2260, 9010

Raszewski, Rebecca: 4025
Rataj, Alison C.: 5020
Ratangee, Brina A.: 4510
Ratcliff, Amy C.: 9020
Rath, Shavonnye: 8900
Rathbun, Alan: 4260, 7330
Ratliff, Kim: 9010, 9540
Ratnayake, Aneeka: 9000, 9010
Ratner, Edward R.: 7280, 7420, 7750
Raue, Patrick: 3560
Rauer, Amy: 1235, 3060, 4370
Raulston, Journie: 4100
Raval, Aasha: 7120
Raval, Minjaal: 7760
Ravel, Jacques: 4080
Ravindran, Malcolm S.: 2230
Rawoof, Anam: 8900
Rayha, Kara: 7420
Raymo, James: 7890
Razjouyan, Javad: 7180, 7630
Reader, Jonathan M.: 9020
Reading Turchioe, Meghan: 2070
Rebala, Sri: 4170
Rebok, George W.: 3335, 3510
Rebola, Claudia B.: 5105
Reboussin, David: 4134, 7830
Reckrey, Jennifer: 3335, 3415, 4165, 5160, 7650
Reczek, Rin: 4520
Redd, Tara: 3300
Redding-Raines, Audrey: 7990
Reddy, Ann: 7640
Reddy, Sweatha: 7420
Redline, Susan: 4355
Redmond, Leslie: 9010
Redwine, Pamela: 7590
Reed, Alexandra M.: 9030
Reed, David: 1210
Reed, Lai: 3235
Reed, Mickaela R.: 7920
Reed, Nicholas: 1295, 2020, 2135, 2150, 2185, 2240, 2365, 4110, 4265, 9010
Reed, Peter: 4075
Reeder, Blaine: 1310
Reel, Candice D.: 1365, 9010
Reese-Melancon, Celinda: 7510
Reeves, Alexis: 4080
Regan, Claire: 9010
Regan, Kayla: 7300
Regan, Michael A.: 3570
Regier, Natalie G.: 1075, 7400, 9020
Reich, Richie: 3420
Reichert, Monika: 4450
Reid, Colin: 7700
Reid, Kieran F.: 3480, 9000
Reid, M. Cary: 4268, 7360, 7680, 7900
Reider, Lisa: 3550
Reiff, Jenni Seale: 1035, 5160
Reilly, Elizabeth: 9000, 9010
Reinhard, Susan C.: 2200, 2395, 4295
Reinhardt, Joann p: 9030
Reiter, David A.: 3495
Reitlo, Line S.: 9020
Rejeski, W J.: 3505
Relerford, Raven: 7460, 7520
Remiker, Mark: 7400
Remillard, Elena T.: 1120
Ren, Dianxu: 4210, 9020
Ren, Haopu: 3570
Ren, Haoyu: 3550, 9000
Ren, Jinnan: 9010
Rendell, Peter: 7510
Renn, Brenna N.: 4134, 7330
Renn, Cynthia: 7170
Rerick, Peter: 7830
Research Group, PREVENT-AD.: 7100
Research Team, DREAM.: 7300
Resnick, Barbara: 1300, 2210, 2305, 3330, 3340, 4040, 7170, 7300, 9020
Resnick, Susan M.: 2155, 2195, 3390, 3510, 4035
Ressler, Deidra A.: 5090
Revenson, Tracey A.: 3470
Reyes, Andrew-Thomas: 4040
Reyes, Mariana: 7700
Reyes-Ortiz, Carlos A.: 7140, 7840
Reyes-Vega, Andrea: 9000
Reynolds, Addam S.: 4195, 4210
Reynolds, Caitlin M.: 1080
Reynolds, Chandra A.: 3080, 4185, 9000, 9010
Reynolds, Charles: 5175
Reynolds, Courtney: 2060, 2220, 4215
Reynolds, Lindsay M.: 5060
Rhee, Yeong: 7990
Rhodes, Ann: 3285, 7620, 9030
Rhodes, Shane: 5120
Riang'a, Roselyter M.: 9110
Ribeiro, Oscar: 2215, 3130, 3555, 4406, 7000, 7060, 7200
Rich, Katherine: 2055
Rich, Stacey: 2260, 9010
Richard, Shannon L.: 4134, 7330
Richards, Bradley: 1240
Richards, Emily: 1015, 9000
Richards, Kathy: 4290, 7540
Richards, Nicole E.: 7070
Richards-McCullough, Kerry: 9010
Richardson, Connor: 4455
Richardson, Joann: 9000
Richardson, Julie A.: 7170, 7180, 7340
Richardson, Katy: 7610, 7840
Richmond, Therese: 9030, 9540
Richter, David: 9010
Riffin, Catherine: 3535, 5000, 9020
Rifkin, Dena E.: 9500
Rijal, Rishikesh: 9000
Rijnhart, Judith J M.: 7360, 9010
Riley, Alicia: 1230
Riley, Julie: 5075
Riley III, Joseph L.: 7630
Ring, Lauren: 3530, 9030
Ring, lia: 9420
Ríos Rincón, Adriana: 9000, 9020
Ripat, Jacquie: 7940
Rippon, Isla R.: 4070, 9020
Riser, Tiffany J.: 3240
Ritchey, Katherine C.: 9030
Ritchie, Christine S.: 2035, 4460, 7680
Ritchie, Craig: 7630
Ritchie, John: 7060, 7190
Ritona, Michelle K.: 7620
Ritter, Ashley Z.: 3530, 9030
Rittereiser, Jennifer C.: 7620
Ritz, Beate: 7370
Rivera, Donovan: 9580
Rivera, Lucia: 7820, 8900
Rivera-Hernandez, Maricruz: 4420
Rivera-Leon, Adrianna M.: 3460
Rizzi, Ester L.: 4530
Rizzo, Matthew: 7510, 7620
Roa Diaz, Stephanie: 7110
Robbins, Ronna: 7350
Robbins, Sarah J.: 4080
Roberson, Tonya: 2355
Robert, Stephanie A.: 2230
Roberto, Karen A.: 1050, 4140
Roberts, Amy R.: 1100, 5175, 9010
Roberts, Angela: 7700
Roberts, Ashley: 1310
Roberts, Eric: 3220
Roberts, Jennifer D.: 7560
Roberts, Lisa: 4415
Roberts, Mitchell: 9000, 9010, 9020
Roberts, Penny: 9030
Roberts, Scott: 4210
Roberts, Shelby: 3055, 3300
Roberts, Tonya J.: 3175, 3180, 7770
Robertson, Caitlin: 9010
Robertson, Kurt: 9420
Robinson, D'Artagnan M.: 7700
Robinson, Erin: 1310, 2055, 3565
Robinson, Kristen: 7780
Robinson, Max: 7110, 7880
Robinson, Rena S.: 9020
Robinson, Sara R.: 9010
Robinson-Ector, Kaitlynn: 9020
Robinson-Lane, Sheria G.: 1140, 7680, 9020
Robison, Julie: 1240, 3165, 4140
Robison, Raele: 9020
Robitaille, Annie: 3260
Robles, Cindy: 9030
Robson, Karen: 2030
Roche-Dean, Maria: 3105, 7800
Rochester, Lynn: 3550
Rochon, Elizabeth: 4460, 7680
Rodakowski, Juleen: 7300
Roden, Elena: 7090
Rodniam, Jaroonrat: 2385
Rodrigue-Rouleau, Philippe: 1200
Rodrigues, Edna Darlene: 2215, 7320
Rodrigues, Isabel B.: 4268, 7220, 7680, 7710, 9020
Rodrigues, Jessica: 7320
Rodrigues, Nathaniel: 4495
Rodriguez, Adriana: 9000
Rodriguez, Bianca: 8900
Rodriguez, Juan: 3055
Rodriguez, Miriam J.: 2180, 5070, 7990
Rodriguez, Misha: 3530, 9030

Rodriguez, Rachel L.: 3250, 4095
Rodriguez, Rena M.: 7180
Rodriguez, Tania M.: 9030
Rodriguez-Artalejo, Fernando: 2320
Rodriguez-Fernandez, Imilce A.: 1380
Roe, Lauren S.: 7650
Roecke, Christina: 3115
Roeland, Eric J.: 7680
Roets, Lieve: 3340
Rogalski, Emily: 9030
Rogers, Callie: 4120
Rogers, Gail: 1290
Rogers, Geoff: 3375, 4215
Rogers, Priscilla A.: 7810
Rogers, Wendy A.: 1120, 1260, 1315, 4415, 7680, 9030, 9540, 9580
Rogina, Blanka: 1325, 2065, 5060
Rogus-Pulia, Nicole: 9020
Roh, Soonhee: 7600
Rojas, Julio: 1380
Rolland, Yves: 2320
Rollman, Bruce: 5175
Roman, Anna: 3250
Romanelli, Meghan: 2310
Romanescu, Laura: 9400
Romo, Enelda: 7180
Ronan, Ana B.: 1290
Rooks, Ronica N.: 7960
Rooney, Mary R.: 3390
Roosan, Don: 1095
Root, Eve Z.: 3250, 7590
Roque, Nelson A.: 3480
Roquet, Angélique: 7080
Rorai, Vanessa: 1270, 4150, 7930
Rorick, Leann: 2030
Rosa, William E.: 7900
Rosano, Caterina: 2155, 3475
Roscizewski, Alexa: 7770
Rose, Grace A.: 4095, 7340
Rose, Karen M.: 1300, 4255, 7090
Roseberry, Krissy: 9010
Roseman, Jeffrey: 3490
Rosen, Adam B.: 7020
Rosen, Francesca C.: 2355
Rosen, Greta: 7820
Rosen, Tony: 1145, 2060
Rosenberg, Ted: 9030
Rosenthal, Marsha: 5055
Rosich, Rosellen M.: 2245
Ross, Jeanette: 9010
Ross, Lesley: 3570, 5050, 7080
Ross, Leslie: 9020
Ross, Sarah E.: 4360
Rosselli, Monica: 4132, 5225, 7480
Rossler, Aleatha M.: 7150
Rosso, Andrea L.: 1015, 2155, 3405, 3475, 4050, 4145, 9010, 9020
Rosteius, Katharina: 2370
Rote, Sunshine: 7530, 9030
Rote, Wendy M.: 9010
Roth, Adam R.: 4500
Roth, David L.: 1035, 2130, 3305, 4170
Roth, Nicole: 1040
Rothermund, Klaus: 3100, 3195
Rothman, Alexander: 5100
Rouanet, Jeremy: 1215
Rouse, Sasha: 3435
Rowan, Julia Margaret: 2220
Rowell, Brigid: 9020
Rowland, Julia: 2260
Royse, Sarah K.: 9500
Royster, Leigh-Anne: 7290
Rozanova, Julia: 2055
Rozelle, Mary: 4266, 7190
Ruan, Lei: 9000
Rubin, Daniel: 9030
Rubinstein, Stephanie R.: 7380
Rubio, Enid: 3560
Rubio, Olivia: 4315
Rubsenson Wahlin, Rebecka: 9030
Rubtsova, Anna A.: 2055, 3565
Rudkin, Kristi: 9020
Rudolph, James: 4290
Rudy, Duane: 7950
Rudy, Ellen: 9000, 9020
Rudzianski, Nicholas: 7440
Rueda Ordoñez, Carlos Joaquin: 4266, 7190
Ruffalo, Leslie: 7910
Ruggiano, Nicole: 4415
Ruggiero, Laurie: 7460
Rughwani, Nisha: 7150
Ruiz, Diana I.: 9420
Rule, Payton: 1255, 3245, 7060, 9020
Rumfelt, Kalee: 2320
Runge, Katharina: 4130
Runzer-Colmenares, Fernando M.: 9010, 9030
Rupp, Hayley: 4268, 7680
Rupprecht, Fiona S.: 2105, 4055
Rusch, Amber V.: 1365, 7910
Rush, Ben: 9020
Rush, Carli: 7960
Rush, Jonathan: 3230
Russ, Tom: 4134, 7620, 9020
Russel, Wynfred: 3240
Russell, Accacia: 9000
Russell, Andrea: 9030
Russell, Beth S.: 1390
Russell, Daniel W.: 7610
Russell, David: 4365
Russell, Elizabeth: 4150
Rutberg, Jennifer L.: 7290
Rutherford, Delaney: 7880
Rutledge, Emily: 1165, 5015
Rutledge, Jocelyn: 7040
Ryan, Alice: 7330
Ryan, Calen: 2285
Ryan, Candace E M.: 9010
Ryan, Joanne: 7300, 7370
Ryan, Kathleen A.: 3550
Ryan, Lesa: 7370
Ryan, Lindsay H.: 5035
Ryan, Susan: 3175
Ryant, Adhe S.: 7830
Ryff, Carol D.: 9000
Ryou, Yeon Ji: 7610, 7930
Ryu, Byeongju: 9030
Ryu, Ju Hyun: 7030
Ryuno, Hirochika: 9010
Ryvicker, Miriam: 9010
Rzeppa, Chloe: 3420, 7080

**S**

Saab, Diane: 9560
Saad, Odelyah: 1005
Saavedra, Antonio: 9020
Sabangan, Jerome: 7180
Sabbath, Erika: 9010
Sabino-Laughlin, Eilleen: 4025
Sacco, Lawrence B.: 1125, 3265, 7170
Sacco, Paul: 2020, 9010, 9100
Sachs, Bonnie C.: 4355
Sachs-Ericsson, Natalie J.: 7030, 7510
Sadak, Tatiana: 2385
Sadarangani, Tina: 1095
Sadeq, Nasreen: 2245, 3430, 7330
Sadeqi, Farima: 4185, 9020
Sadowska, Karolina: 7900
sadowski, Cheryl: 9520
Saeidzadeh, Seyedehtanaz: 4408, 7720
Saenz, Joseph: 7320, 7540, 7600, 9000
Saenz, Samantha: 7290
Saffaran, Pouria: 1390
Saffel-Shrier, Susan: 9000
Safford, Monika M.: 2060
Sagara, Tomoya: 7550
Sagi, Doron: 4125
Sagong, Hae: 9010
Sagrak, Adriana: 3270
Saha, Troyee: 7730
Said, Adam: 9000, 9020
Saif, Nadia: 7340
Saint Onge, Jarron: 1185
Sainz, Mayra R.: 2180, 3485
Saito, Taeko: 7620
Saito, Yasuhiko: 3380
Saka, Suleiman: 7520
Sakai, Haru: 7080
Sakai, Keiko: 9030
Sakamoto, Shota: 9000
Sakata, Kei: 4385
Sakib, Nazmus: 9000
Salari, Sonia L.: 4500
Salas, Joanne: 7150
Saldarriaga, Gabriel: 9570
Saldarriaga-ruiz, gabriel: 9570
Salecker, Mariana C.: 3140
Saleem, Zainab: 1200
Salemi, Jason L.: 9010
Sales, Juarez Fayriston Coelho: 7130
Salgado, Glaucia: 9020
Salinas Navarro, Sheila A.: 4375, 7750
Salisbury, Dereck: 9200
Salmon, Adam: 9000
Salom-Bail, Shirley: 7460
Saltzman, Barbara: 7540
Salvo, Erin: 3375
Salwi, Shreya M.: 4425
Salzman, Michele: 4120
Samanapally, Harideep: 9000
Samant, Akanksha: 2145
Samper-Ternent, Rafael: 3525, 4010, 4060, 7840
Sample, Alanson P.: 7820
Sampson, Elizabeth l: 4440, 4470
Sampson, Laura: 2105, 7550
Sams, Richard: 9010, 9500
Samuel, Laura J.: 1345, 5130
San Roman, Irene: 9020, 9520
Sanchez, Claudia A.: 7600
Sanchez, Krystal: 7100
Sanchez, Shena: 7620, 9000, 9020
Sanchez, Victora: 1295, 4110, 7960
Sánchez-Castro, Paulina: 4395
Sanchez-Reilly, Sandra: 7180, 9000, 9010

Sánchez Sánchez, Juan Luis: 2320
Sandberg, Linn: 1250
Sandblom, Gabriel: 9030
Sanders, Edie C.: 7080
Sanders, Jeff: 7620
Sanders, Lisbeth: 7060, 7460, 7620
Sanders, Margaret B.: 7250
Sandesara, Bhanu: 9580
Sandoval, Moises H.: 3465
Sands, Laura: 5195, 7400
Sands, Talyah: 3055
Sanfilippo, Nina A.: 7420
Sanford, Jon a: 1120
Sanger, Stephanie: 7710
Sanon, Martine: 4266, 7190
Sansing, Ann: 7590
Santamaría-Ulloa, Carolina: 9010
Santanasto, Adam J.: 3475, 4260, 5090, 5210
Santarossa, Melanie: 9030
Santilli, Josie N.: 4375
Santora, Kenneth E.: 3040 3185
Santoro, Nanette: 5155
Santos-Lozada, Alexis R.: 4500
Sapkota, Krishna P.: 3380, 7140
Sappington, Erica: 9000, 9010, 9020
Sarango Cancel, Mariana: 4195
Saraswathula, Varun: 7860
Sardina, Angie L.: 2275, 9020
Sargent, Lana: 5030, 9010
Sarid, Orly: 2345
Sarif, Nawaj: 7200, 7940
Sarkar, Nilanjan: 9010, 9030
Sarmiento, Cassandra Vanessa: 4240
Sarquis-Adamson, Yanina: 4120
Sasdi, Olivia: 9000
Sason, Adi: 7720
Sato, Shogo: 4335
Satz, Skye A.: 5135
Saucedo, Jennifer: 7450, 7890, 7990
Sauer, Jacquelin: 4510
Sauer, Jennifer: 5040
Sauerteig-Rolston, Madison R.: 3230, 4535, 9010
Saunders, Gabrielle H.: 4110
Saunders, Michele J.: 3540
Saunders, Pamela: 3200
Saunders, Stephanie: 7170
Sauter, Julia: 1220
Savage, Blake: 7080
Savettiere, Adriana: 9010
Savitz, Sean: 9010
Savla, Tina: 1050, 3445, 4140, 7510
Savundranayagam, Marie Y.: 3270
Sawyer, Linda M.: 7160, 7180, 9000
Sayed, Shaheen: 9110
Saylor, Anna K.: 7620
Scala-Foley, Marisa: 3045
Scales, Kezia: 3285, 3320, 4165, 9530
Scambray, Kiana: 1170
Scerpella, Daniel L.: 1035, 4170, 5160
Schaaf, George W.: 7370
Schadt, Lili: 7860
Schaefer, Sydney: 9030
Schafer, Markus: 1170, 4020, 4500
Schaie, Warner: 4225
Schechter, Gail: 2355
Schechter, Steven: 9010
Scheffer, Julian A.: 1305, 4495, 9320
Schell, Brent R.: 9020
Schepens Niemiec, Stacey L.: 2275
Schepisi, Ila: 9000
Scher, Clara: 2315, 4210
Scherrer, Jeffrey: 7960
Schicker, Teresa: 9030
Schierer, Allie: 7440, 7460, 7520
Schiloski, Kylie: 7510
Schiltz, Nicholas K.: 4525
Schindler, Nicole: 7150
Schlag, Karen: 9010
Schlesinger, Mark: 2300
Schlueter, Amy: 7640
Schlundt, David G.: 9020
Schmedding, Nicholas: 9010
Schmid, Laura: 7780
Schmidt, Annika: 2370
Schmidt, Brad: 7030, 7510
Schmidt, Laura Katharina: 7830
Schmidt, Seigfried O.: 1030
Schmidt, Steven M.: 2340, 7380
Schmidt-Trucksäss, Arno: 3115
Schmitt, Ana Carolina Basso: 9020
Schmitt, Frederick A.: 9020
Schmitz, Alina: 1220
Schmitz, Kathryn H.: 9010, 9020
Schmitz, Lauren L.: 2285
Schneider, Augusto: 4475, 7110
Schneider, Edward: 3000
Schneider, Haley: 3445, 3540, 7470
Schoellig, Shannon: 7950
Schoen, Julie R.: 1285
Schoenfeld, Alix: 4408, 7230
Schoenfeld, Elinor R.: 5030, 7120, 7680, 9020
Schoenthaler, Antoinette: 7640
Schoepflin, Benedicte: 9030
Scholz, Urte: 4235
Schonfeld, Lawrence: 3560, 9020
Schoonhoven, Lisette: 2280, 4125, 7350
Schork, Nicholas: 5180
Schröder, Valerie E.: 9020
Schrack, Jennifer A.: 2150, 3390, 3475, 3510, 5085, 7110, 7650
Schram, Miranda T.: 1225
Schrauf, Robert W.: 3530
Schriner, Samuel E.: 7370
Schroeder, Isaac: 7080
Schroeder, Matthew W.: 5115
Schubert, Carla R.: 2240
Schubert, Cathy C.: 7130, 7180
Schubert, Frederick: 7510
Schuez, Benjamin: 2370
Schulman-Green, Dena: 2235
Schultz, Hanna: 7590
Schultz, Leslie: 2030
Schultz, Michael: 9020
Schultz, Savannah: 7590
Schulz, Richard: 7530
Schumacher, Benjamin: 3390, 3475
Schumacher, John G.: 5185
Schumann, Annette: 3270
Schumm, L Philip: 1230
Schurr, Stephanie: 3570
Schuster, Amy M.: 3025, 9000
Schuster, Johanna: 9400
Schutte, Debra L.: 9010
Schuurmans, Marieke J.: 4125, 7350
Schwaiger-Haber, Michaela: 5180
Schwartz, Andrea: 7860, 9410
Schwartz, Anna: 7640
Schwartzkopf, Ashley L.: 7130, 7180
Schwasinger-Schmidt, Tiffany: 7330
Schweitzer, Amy: 7350
Schwingel, Andiara: 7300, 7610
Sciamanna, Christopher N.: 9010, 9020
Sconiers, Charlie Kira-Nolan: 8900
Scott, Matthew: 9030
Scott, Rachel K.: 2005
Scott, Robynn: 7180
Scott, Stacey B.: 2230, 4045, 4425, 5165
Scott-Poe, Deneisha: 7950
Scrivano, Rachel: 4085, 7590
Scroggins, Laila: 9000
Seago, Elayna: 7020
Seaman, Aaron T.: 2260, 9020
Seaman, Kendra L.: 1090
Sebastiani, Paola: 1040, 1290, 5090, 5180, 7370
Sebu, Joshua: 4145
Seckin, Gul: 7410
Seetharaman, Kishore: 1335, 2340
Sefcik, Justine S.: 3395, 4005, 9000, 9030, 9400, 9540
Segal, Daniel L.: 7910
Segel-Karpas, Dikla: 3275, 7070
Segelman, Micah: 3365
Segerstrom, Suzanne: 1160, 7510, 9020
Seidler, Rachael: 9000
Seidmann, Vered: 3295
Seitz, Dallas: 7080
Sekher, T V.: 7320
Seligman, Benjamin: 4310, 7940
Selovin, Fabio: 4055
Selzler, Katherine J.: 7620
Semelka, Charles: 9030
Sen, Payel: 2065
Sengupta, Manisha: 1095
Senior, Josh: 7510
Seo, Chang Hyun: 4420
Seo, Jin Young: 5230, 7360
Seo, Minjung: 5190, 7000
Seong, Young Ah: 9030
Sepehri, Katayoun: 7120
Ser, Sarah E.: 7860
Serafica, Reimund: 4040
Serdaly, Christine: 7160
Sereika, Susan M.: 9010
Serpas, Dylan G.: 7070
Serpico, Anthony: 7620
Serra, Monica: 7350, 7910

Serrano-Rubio, Luis H.: 4100
Serrat, Rodrigo: 4330
Serre, Marc L.: 2320
Sesek, Greg: 5025
Seshadri, Sudra: 4100
Sesker, Amanda A.: 7990
Sestito, Samuel F.: 7500
Setlow, Barry: 1030
Setoguchi, Soko: 4105, 7800
Setto, Madison: 9410
Severance, Jennifer J.: 4220, 4360, 5190
Sexton, Andrew: 7830
Shaaban, C.: 9500
Shacham, Mayan: 3275
Shade, Marcia: 4268, 7680
Shadmi, Efrat: 7050
Shah, Avani A.: 7210
Shah, Malvi: 4440
Shah, Raj C.: 7300
Shah, Ravi V.: 7630, 9300, 9330
Shaid, Elizabeth C.: 4545, 9010
Shalev, Daniel: 7900
Shamim, Asma: 1390
Shanahan, Angela: 4134, 7620
Shanahan, Mabel: 9020
Shang, Yanan: 7620
Shanks, Josh: 7660
Shanmuganathan, Vasuki: 1145
Shannon, Jackilen: 7680
Shannon, Shen: 3105
Shapira, Stav: 2345
Sharafkhaneh, Amir: 7180
Sharareh, Nasser: 1155
Shardell, Michelle: 3550, 4080, 4260, 7330, 9000, 9020
Sharit, Joseph: 4268, 7680
Sharma, Kumar: 7660
Sharma, Rachita: 7390
Sharma, Shakshi: 4130, 7370, 7880
Sharma, Shubam: 3360, 4010, 9030
Sharninghausen, Jody: 7540
Sharp, Julia L.: 4010
Sharrett, A. Richey: 2365, 3510, 5085, 7340
Shavit, Yochai Z.: 7020, 7070
Shaw, Benjamin: 3370
Shaw, Clarissa: 2160, 3335, 7300, 7860, 9000
Shay, Kimberly: 4300
Shayya, Ashley: 3365, 7260
Shdo, Suzanne M.: 9320
Shea, Kimberly: 4380
Shea, Kyla: 1290
Shealy, E. Carlisle: 7770
Shean, John: 3055, 5015, 7700
Sheehan, Connor: 2275
Sheets, Debra: 7420, 7470
Sheets, Kerry: 3460
Sheffler, Julia L.: 3520, 7030, 9010
Sheftel, Mara: 3065, 4500
Sheikh, Sophia: 9010
Sheldon, Marisa: 1330, 3045
Shelley, Mack: 7590, 9020
Shelton, Kyle: 3460
Shen, Biyi: 9000
Shen, Huei-wern: 7710, 7760
Shen, Jay: 4075, 5185
Shen, Karen: 5160
Shen, Yan: 7010
Shen, Yuqi: 3500, 4490
Shende, Shraddha: 1315, 9020, 9030
Shenoi, Sheela: 2055
Shepherd, Gayle: 7620
Shepherd, Jewel: 7210
Shepherd-Banigan, Megan: 7620
Sheridan-Johnson, Jackie: 3075
Sherman, Aurora M.: 5035
Sherman, Carey Wexler: 3235
Sherman, Ledric D.: 7410, 7430, 7840
Sherwood, Paula R.: 9010
Shi, Kewei: 9000, 9020
Shi, Sandra: 3565, 9010, 9540
Shi, Tianyu: 7190
Shi, Xingming: 7370
Shichijo, Yuka: 7000, 7150
Shidhaye, Rahul: 9020
Shield, Renee: 9000
Shields, Christopher B.: 9000
Shields, Pamela: 4255
Shields, Rachael: 9030
Shih, Cheng-Yu: 9010
Shih, Regina A.: 1265
Shih, Yao-Chi: 1185
Shilbayeh, Mariam: 4300
Shim, Soyeon: 4040
Shimabukuro, Michio: 2380
Shimizu, Yosuke: 9030
Shimon-Raz, Ortal: 9420
Shin, Dongsoon: 7810
Shin, Esther: 7550
Shin, Hye Ri: 9540
Shin, Hye-Won: 2330, 4420
Shin, Hyung Eun: 7380, 7630, 9020
Shin, Isaac: 9030
Shin, Jeongwon: 7420
Shin, Jinhee: 7720
Shin, Jiyoung: 7080, 9020
Shin, Juh Hyun: 7160, 7300, 7800, 9020, 9030
Shin, Jung-hye: 9030
Shin, Marlena: 2145
Shin, Myoung Hwan: 7850
Shin, Oejin: 9010, 9030
Shin, Yeonsoo: 7510, 7560
Shin, Yun Chan: 7170
Shippee, Tetyana: 3010, 3175, 3240, 3305, 3460, 4140, 5025, 5170
Shireman, Theresa: 4000
Shively, Carol: 4160
Shivers, Stephani: 7300
Shnall, Adriana: 1145
Shnayder, Oksana: 7000, 7500
Shochat, Tamar: 4265
Shoemaker-Hunt, Sarah: 7810
Shoenfelt, Alayna: 9010
Shorr, Ronald I.: 3500
Shovali, Tamar E.: 3150
Shreeniwas, Sudha: 5110
Shrestha, Aman: 3380, 5220
Shrestha, Sabita: 9010
Shrestha, Shovana: 4280
Shrestha, Srishti: 2240
Shrestha, Sumina: 4545
Shrira, Amit: 1140, 4180
Shropshire, Jeremey: 3210
Shu, Ruixin: 9000
Shugaev, Inna: 7720
Shugrue, Noreen: 1240
Shultz, Ken: 1095
Shulyaev, Ksenya: 4520, 7050
Shum, Eric Ngai Yin: 9030, 9510
Shumaker, Morgan D.: 9000
Shuman, Stephen K.: 3540
Si, Yafei: 4090
Siaton, Bernadette C.: 7800
Sibai, Abla: 9500
Sibille, Kimberly T.: 1030
Sicinski, Kamil: 1000
Siddiqi, Shadab: 4475
Sieber, Frederick: 1370, 9010
Siedner, Mark: 9000
Siegel, Elena O. O.: 9030
Siegler, Eugenia: 5115
Sielert, Charles A.: 7080
Sikes, Kendra: 7370
Sikora, Nadine: 9020
Sikorskii, Alla: 7800
Silberbogen, Amy: 4095
Silberman, Susan: 2315
Silbermann, Rebecca: 7680
Silbersack, Johanna: 9020
Sillito, Carrie: 4500
Sillner, Andrea: 7830
Silverman, Michael A.: 3110, 7840
Silverman-Martin, Wendie: 7110
Silverstein, Hannah I.: 7550, 7900, 9020
Silverstein, Merril: 4085, 4400, 7950, 9030
Silverstein, Nina M.: 2250, 4266, 4435, 7230, 7830
Silvis, Matthew: 9020
Simmons, Alison: 4460
Simmons, Jabrina: 1010
Simms, Evan: 8900
Simon, Corey: 7410
Simon, Darby M.: 7090, 7420, 9010
Simon, Kennedy: 7550
Simonsen, Sara: 7480
Simonsick, Eleanor M.: 2155, 2195, 2240, 3390, 3510, 4040, 4260, 4265, 4490, 5085, 7150, 9020
Simonson, Grace: 9000
Simpson, Darcia L.: 4406, 7500
Simpson, Deborah: 7310
Simpson, Elizabeth: 3165
Simpson, Felicia: 4355, 7630
Simpson, Luke: 7550
simpson, Ryan B.: 1290
Sin, Mo-kyung: 3005, 3490
Sin, Nancy L.: 3135, 3520, 3545, 4320
Sinclair, David: 4455
Sinclair, David A.: 9020
Sindi, Shireen: 3470
Singer, Adam: 9020
Singer, Jonathan: 7830
Singer, Katelyn J.: 7830, 7990
Singh, Maya: 2085
Singh, Mriganka: 9010
Singh, Priyanka: 1095
Singh, Rajeev Ranjan: 7960
Singh, Revika: 2355
Singh, Tanya: 9020
Singleton, Jenny: 9030
Singleton, Meki: 7260
Singsank, Katie T.: 9580
Sirdich, Brianna: 7300
Sirey, Jo Anne: 3560, 7760
Sirohi, Vijay K.: 1380, 7370

Sison, Maria: 1370
Sison, Stephanie DM.: 9410
Sivananthan, Saskia: 1280
Siverskog, Anna: 1250, 7450
Sixsmith, Andrew: 3255, 4245
Sixsmith, Judith: 3255, 4245
Skalla, Emily: 2130
Skemp, Eleanor: 9020
Skemp, Lisa: 9000
Skidmore, Elizabeth: 7360
Skinner, Kelly: 5105, 7300
Skipper, Antonius: 2115, 7290
Skirbekk, Vegard: 9020
Skoblow, Hanamori F.: 3080, 4370
Skow, Laura: 5085, 7340
Skowronski, Jordan: 4465, 7340
Skrajner, Michael J.: 9400, 9550
Skrove, Zachary: 9030
Skrozic, Amna: 9020
Skubic, Marjorie: 9030, 9540
Slater, Craig: 4125
Slattum, Patricia W.: 4360
Slaug, Björn: 2340
Slavich, George: 7960
Slayday, Riki E.: 5165, 7050
Sliwinski, Martin: 1180, 3080, 3245, 3480, 4055, 4185, 4425, 5165, 7050, 7080, 7510, 9010
Sloan, Danetta L.: 4170, 7360
Sloan, Stef: 4195
Sloane, Philip D.: 1210, 3120, 3320, 9020
Sloane, Richard: 1370, 4485, 9010
Slogeris, Becky: 9420
Slolane, Philip: 1210
Slon, Renee E.: 7150
Smagula, Stephen: 5175
Small, Brent: 1085, 7420
Smeekens, Bridget A.: 3505
Smetcoren, An-Sofie: 1385
Smith, Ambrym S.: 1035
Smith, Andre: 7420, 7470
Smith, Andrea B.: 3060, 4400
Smith, Brian N.: 1160
Smith, Brian N.: 9020
Smith, Brigid: 7440
Smith, Caleb: 7210
Smith, Christina: 2160, 9030
Smith, Claire E.: 4490, 7410, 7890
Smith, Dylan: 2230
Smith, Gregory C.: 3170, 7950
Smith, Jacqui: 1170, 3290, 4325, 7610, 9010, 9030
Smith, Jamie M.: 1375, 3415, 9020
Smith, Jason R.: 2240, 2365, 3335, 3510
Smith, Jennifer A.: 2320
Smith, Jennifer L.: 5125
Smith, Jennifer A.: 7630
Smith, Jennifer L.: 9020
Smith, Jessica J.: 7370
Smith, Kasey N.: 9320
Smith, Kate: 7540
Smith, Kathrine A.: 2030
Smith, Kelly M.: 4170
Smith, Ken R.: 4365
Smith, Kristen: 4030
Smith, Lindsey: 1155, 1210, 3320, 9030
Smith, Louisa H.: 9430
Smith, Matthew Lee: 7410, 7430, 7760, 9000
Smith, Michael: 7670
Smith, Nathan: 7360
Smith, Patsy R.: 9020
Smith, Ryan: 3080
Smith, Shelby M.: 7030
Smith, Sherri L.: 9020
Smith, Steven R.: 7620
Smith, Tawny L.: 5235
Smith, Tiffany: 9010
Smith, Trey: 2325
Smith-ray, Renae L.: 9020
Smithwick, Megan S.: 7510
Smolen, Andrew: 3080, 9000
SM-Rahman, Atiqur: 4190
Smyth, Joshua: 7590
Snedeker, Lauren R.: 9030
Sneed, Rodlescia: 1270, 3080, 4520, 4545, 5215
Snider, Joy: 9550
Snigurska, Urszula A.: 7860
Snitz, Beth E.: 9500
Snow, A Lynn: 3365
Snow, A Lynn: 4290, 7540
Snyder, Jason S.: 1105
Snyder, Joel S.: 3485, 9010
Snyder, Rani E.: 2200, 4295
So, Christine: 1235
So, Hon Yiu: 5070
So, Mardon E.: 7020
Soares, Erica Paci: 7920
Soares, Felipe Paci: 7920
Sofios, Alexandros: 3115
Sog, Zeyuan: 5180
Sol, Ketlyne: 1170
Solberg, Laurence M.: 7860
Solch-Ottaiano, Rebecca: 9010
Soliman, Elsayed Z.: 3390
solimeo, Samantha: 9020
Solway, Erica: 1285
Somerville, Ceara: 4150
Sommerlad, Andrew: 4515
Sommers, Maruska: 3090
Som-Pimpong, Hadassah: 9030
Son, Jung Yoen: 7510
Son, Surim: 4120
Song, Eunjeong: 7810
Song, Jiyoun: 9010
Song, Kijun: 7080
Song, Lixin: 4380, 5055, 5120, 7660
Song, Mi-Kyung: 7900, 7960
Song, Qian: 7270, 9030
Song, Rhayun: 7660, 7810
Song, Richard W.: 1215
Song, Ruopu: 5080
Song, Shijie: 1245
Song, Si Young: 7040, 7080, 7610
Song, Woojin: 4465
Song, Xiao: 7930
Song, Xiaokang: 1245
Song, Xiaowei: 7120, 7160
Song, Yimeng: 3115
Song, Youqiang: 7610
Song, Yuting: 4515
Song, Zeyuan: 5090, 5180, 7370
Soni, Suha: 7840
Sonnega, Amanda: 2045, 3065, 3095
Sonnenfeld, Mandi: 7850
Soong, Daniel: 1010, 9020
Sorkin, John: 7800
Sorra, Joann: 2375
Sosa Cifuentes, Daniela Belen: 4406, 7000
Sosa-Sarmiento, Paola V.: 7840
Sotka, Susan: 9010
Sotos Prieto, Mercedes: 2320
Souders, Dustin J.: 5050
Soulie, Brianna: 4230, 7060, 7570, 7590
Sousa, Karen H.: 9020, 9030
Sousa, Sarah: 5105
Sousa, Susana: 7300
Sousa, Vivian Cintra: 9020
Sousa Barbalho, Yuri Barbalho G.: 7340
Souza, Kristin F.: 3385
Spangler, Hillary B.: 4268, 7360, 7680
Sparks, Cathi: 1150
Sparks, Diane: 7160
Sparks, Lauren M.: 3475
Spataro, Sebastián: 8900
Speaks, Hannah R.: 9020
Spears, Essence: 9010
Spears, Isaiah: 3520, 4320
Spencer, Rebecca: 9010
Spiers, Gemma F.: 7160
Spigner, Clarence: 4408, 7700
Spilsbury, Karen: 2375, 7720
Spira, Adam P.: 2135, 2240, 3500, 4265, 4355, 4490, 7870
Spiro, Avron: 1160, 3155
Spiteri, Raymond J.: 1200
Splain, Ashley: 4130
Spradley, Laura: 7150, 7540, 7550
Sprecher, Bryce: 9030
Spreng, Nathan: 7100
Springgate, Benjamin: 1020
Springstein, Tabea: 1105
Squillace, Marie: 2375
Squires, Allison: 9000
Sreenivas, Kiran: 9020
Srejon, Refat: 7980
Srinivas, Vijaya: 9020
St. John, Philip: 5220
St. Louis, Renee M.: 9030
Staben, Omar: 9010
Stahl, Sarah T.: 5175
Staley, Mark: 7000
Stallard, Eric: 5110, 7620
Stanik, Christine: 9020
Stanley, Jennifer: 7040, 7520
Stansbury, Kim L.: 7230
Stapleton, Tadhg: 7690
Stark, Susan L.: 7780
Stark, Susy: 4406, 7000
Starkweather, Angela: 7460
Staudacher, Sandra: 3180
Stavrinos, Despina: 5050, 7060
Stawski, Robert S.: 4425, 7580
Stearns, Melanie: 3420
Stebbins, Rebecca: 3225
Stechmiller, Joyce: 9030
Steele, Joel S.: 3535
Steele, Maritza V.: 3460
Steele, Tatum P.: 7080
Steer, Christopher: 2260, 9010
Stefancic, Ana: 1335
Steffens, David C.: 4140, 7030
Stegemoller, Elizabeth: 7830
Stegen, Hannelore: 1065, 1385, 7280

Stehouwer, Coen DA.: 1225
Stein, Benjamin: 9030
Steiner, Victoria: 7540
Steinhauser, Karen E.: 4165
Steiniche, Tessa: 9020
Steinman, Bernard A.: 7500
Steinman, Erika: 9540
Steinman, Michael: 5230
Steinmann, Gijs: 2370
Stelle, Charlie: 4390
Stellman, Jeanne M.: 1160
Stellman, Steven D.: 1160
Stenholm, Sari: 7170
Stennett, Christina A.: 4080
Stenvold, Dorthe: 9020
Stephan, Abigail T.: 3060, 7080
Stephen, Alexander J.: 7160
Stephens, Caroline: 4365
Stephens, Jacquelyn E.: 9020
Stephenson, Jane: 9000
Stepick, Lina: 3285
Stepniak, Catherine: 4275
Steptoe, Andrew: 1255, 2015
Stern, Yaakov: 9020
Sterns, Harvey L.: 3000, 3325
Stessman, Jochanan: 2015, 7300
Stessman-Lande, Irit: 2015
Stetten, Nichole: 1030
Stevens, Alan B.: 2060, 7400, 7850
Stevenson, Heather L.: 7860
Steward, Andrew: 4195, 7590
Stewart, Meagan: 1235
Stiefler, Susanne: 2370
Still, Catherine: 2070, 2170, 7800
Stinchcombe, Arne: 3570, 7060, 7450
Stine, Jonathan G.: 9010
Stirland, Lucy E.: 5220
Stites, Shana D.: 3005, 7650
Stival, Marina Morato: 7130
Stock, Braden: 7060
Stockwell, Ian: 9000
Stodola, Allyson: 9010
Stoeckle, Rebecca J.: 2030
Stokes, Jeffrey E.: 2245, 3080, 4055, 4510
Stolic, Snezana: 9000
Stone, Jamie: 9030
Stone, Katie L.: 3500, 7650
Stone, Robyn: 3120, 9020, 9550
Stone-Bury, Lisa E.: 4445, 7910
St-Onge, Marie-Pierre: 4355
Storm, Sierra: 9010
Stout, Jeffery: 9010, 9020
Stout, Jeffrey: 5075, 7080, 7150, 7430, 7680, 7820, 9030
Stoye, George: 2255
Strader, Sydney: 7110
Strand, Bjorn Heine: 9020
Strassheim, Quinn: 7880
Strath, Larissa: 9010
Stratton, Lauren: 1085, 7540, 9020
Strayer, Andrea: 2170, 2180
Streb, Carolyn: 9010
Stringer, Wilma: 4150
Stringfellow, Mary K.: 9010
Strong, Jessica: 7380, 7590, 9020
Strong, Joe: 9000
Strotmeyer, Elsa S.: 5155, 7650
Stroud, Jared: 9020
Strough, JoNell: 1090, 7920
Struckmeyer, Linda R.: 9020
Stuart, Nicole S.: 3520, 4320
Stubbs, Brianna: 7110
Stuckenschneider, Tim: 7830
Stuhr, Nicole: 9020
Stump, Timothy E.: 5015, 9000
Sturdevant, Diana: 9020
Sturla, Shana J.: 9020
Su, Chin-Yi: 7030, 7960
Su, Jingjing: 5185
Su, Kelly: 8900
Su, Mingzhu: 9000
Su, Wei-Hsuan: 7370
Su, Yan: 4505
Su, Yan-Jhu: 3385, 4266, 7230, 7610, 7830
Su, Yun: 7370
Suarez, Jethro Raphael M.: 9030
Suarez-Rodriguez, Luis: 7860
Subedi, Janardan: 5220
Sublett, Jennifer: 7520
Subramanian, Sandeep: 9030
Subramanian, Tejas: 7900
Sucic, Joseph F.: 7150
Sudore, Rebecca: 2035, 4035
Suemoto, Claudia Kimie: 3160, 7320
Sugiyama, Mika: 7280
Suh, Heather: 5070, 9020
Suh, Yousin: 3215
Suitor, J Jill: 1050, 4275, 7920
Suizzo, Marie-Anne P.: 7470
Sukumar, Kavitha: 9030
Sullivan, Dennis H.: 7180, 9000
Sullivan, Jennifer L.: 2145
Sullivan, Kevin J.: 9200
Sullivan, Suzanne S.: 2110, 7260, 7620
Sultana, Munira: 4120
Sultana, Samia: 9000
Sumikawa, Yuka: 7160, 9520
Summerhayes, Emily: 8900
Sun, Chuxuan B.: 1115
Sun, Fei: 1095, 2085, 2330, 4155, 7010, 7560, 9000, 9010, 9030, 9300, 9530
Sun, Haosen: 4020
Sun, Juanjuan: 4155
Sun, Minjung,: 7330
Sun, Peter C.: 4085
Sun, Qi: 1290
Sun, Sicong: 4305
Sun, Winnie: 9010, 9520
Sun, Xin: 4132, 7500
Sun, Yang: 7030
Sun, Yixuan: 7390
Sundaresan, Lakshmi: 2225
Sunderman, Erin E.: 4340
Sung, Mira: 9020
Sung, Pildoo: 3435, 4020, 4025, 4175
Sunil, Surya: 9000
Sun-Mitchell, Shan: 9030
Suntai, Zainab: 3005, 4275, 4510, 7900
Suorsa, Kristin: 7170
Supiano, Katherine P.: 1025, 1150, 2035, 4406, 7000, 9020
Surachman, Agus: 5130, 9440
Suri, Sachin: 9020
Suriaga, Armiel A.: 7580
Surood, Shireen: 7520
Sussman, Tamara: 4440
Sutherland, Laura: 9000
Sutin, Angelina R.: 3245, 4035, 4055, 7990
Sutphin, George L.: 7870
Suwanpasu, Sunee: 7860
Suzuki, Hiroyuki: 7550, 7760
Suzuki, Makoto: 2380
Svartengren, Magnus: 7170
Svec, Joseph: 1055, 2075, 3360, 4390, 7610
Svedberg, Pia: 9030
Swagger, Phildra: 7810
Swain, Joseph: 7210
Swanepoel, Lizette: 7690
Swanson, Helena L.: 7250
Swanson, Leslie: 5155
Swartz, Ann: 4268, 7680
Swartz, Kristine: 7500
Swartzell, Kristen: 4210, 5105
Swenor, Bonnielin K.: 1345, 2365
Swift, Hannah J.: 1200
Swift, Henry O.: 4150
Sy, Maimouna: 7680
Syed, Ayesha: 9020
Sylvester, Hunter: 5085, 7340
Syme, Maggie L.: 9030
Symes, Bobbi: 5215, 7730
Szanton, Sarah L.: 1375
Szanton, Sarah L. L.: 1345, 2290, 3240, 4310, 5130
Szymkowski, Amanda: 7310

### T

T R, Dilip: 4385
Taani, Murad H.: 1190, 7610, 7640
Tabata, Hiroki: 9000
Tabrizi, Maryam: 4075, 7310
Tadel, Pat M.: 7290
Tadesse, Ruth S.: 7440, 7470, 7540
Taeckens-Seabaugh, Ashley: 5200
Taffet, George E.: 4160
Taha, Hash Brown: 9000, 9010
Tahashilder, Md Ibrahim: 4395
Tai, Chiu-Mei: 4155
Takahashi, Tomoya: 7550, 7760
Takaoka, Manami: 9520
Takase, Mai: 7610
Takase, Mai: 9030
Takeda, Masatoshi: 9030
Tal, Noa: 1125
Talbot, H. Keipp: 4130
Talegawkar, Sameera: 9020
Talley, Kristine M.: 9030
Talwar, Sonia: 8900
Tamayo, Valeria: 9000
Tambor, Ellen: 2030
Tamburri, Nicholas: 7420, 7470
Tamres, Lisa: 9020
Tamura, Yoshifumi: 9000
Tan, Alai: 3345
Tan, Chuen Seng: 4385
Tan, Erwin J.: 5175
Tanaka, Clarice: 9010
Tanaka, Katsuya: 2105, 3220

Tanaka, Toshiko: 4490
Tanase, Alexis D.: 5230
Tang, Ada: 5100, 7180, 7340
Tang, Dan: 4155
Tang, Esther Y. K.: 9010
Tang, Fei: 9030
Tang, Fengyan: 2090, 4050, 7600, 9020
Tang, Huameng: 9030
Tang, Jennifer Yee-man: 7330, 7760
Tang, Junhan: 4090
Tang, Lu: 5195
Tang, Paul: 4035
Tang, Vivien Foong Yee: 1065
Tang, Xueting: 9010, 9020, 9300, 9550
Taniguchi, Yu: 7280
Tanilli, Rachel: 4408, 7230
Tappen, Ruth M.: 2180, 4132, 5225, 7480, 7580
Tarabochia, Dawn: 5075, 7910
Tarbert, Rebecca J.: 9020
Tariq, Suleman: 4268, 7220, 7680
Tarraf, Wassim: 7960, 7970, 9020, 9210
Tasyurek, Ezgi: 1200
Tate, Channing E.: 4408, 7260
Tate, Judith: 4255, 9010, 9030
Tate, Kaitlyn: 4408, 7720
Tate, Robert: 7940
Tauseef, Mahruhk: 9010
Tavares, João: 7300
Tavares, Karina L.: 1350
Tay, Djin L.: 4365
Tay, Laura Bee Gek: 7760
Tayag, Paolo: 5065
Taylor, Ashley: 9000
Taylor, Christopher A.: 4130
Taylor, Courtney: 4195
Taylor, Harry: 4030
Taylor, Janelle S.: 7690
Taylor, Janie: 7460
Taylor, Janiece L.: 2290, 3240
Taylor, Kate: 4360
Taylor, Philip: 3285, 4345
Taylor, Simeon: 4080
Taylor, Tonya N.: 3565
Taylor, Zachary E.: 3545
Tazaki, Fumie: 9030
Teaster, Pamela B.: 7770
Teerawattananon, Yot: 3440, 4385
Teerawichitchainan, Bussarawan: 2050
Teixeira, Laetitia: 3130, 7060, 7200, 7300, 7320
Teixeira, Samantha: 3095
Tejeda, Carlos: 9000
Teles, Soraia: 4406, 7000, 7300
Tell, Eileen J.: 1175, 4295
Telonidis, Jacqueline: 5190
ten Cate, Debbie: 4125, 7000, 7350
Tennyson, Robert L.: 9030
Teo, Wai Choo: 7760
Teply, Abby L.: 7590, 7610, 7840
Teran, Dianne: 2160
Teresi, Jeanne A.: 1145, 2030, 2060
Terhune, Elizabeth: 9010
Teri, Linda: 5135
Terracciano, Antonio: 3245, 4035, 4055, 7990
Terrill, Alexandra L.: 1150, 7700
Terry, Melita: 9020
Teshale, Salom M.: 4295
Teshome, Abraham: 7190, 7200
Tessler Lindau, Stacy: 7230
Tetlow, Hilary: 9000
Tewarson, Hemi: 2200
Tewary, Sweta: 7000, 7500
Thabane, Lehana: 7180, 7340
Thaitrong, Phatt: 7620, 9000, 9020
Thakkar, Nirali: 7430
Thakkar, Priyanka: 9330
Thangwaritorn, Pilar: 4134, 7330, 7830
Tharrington, Shafer: 9030
Thayer, Erin K.: 2220
Thayer, Julian F.: 1215
Thayer, Rachel E.: 7410
Theeke, Laurie A.: 1070, 5225, 7330, 9200
Theng, Yin-Leng: 3085, 3295, 3470
Theos, Gregory: 9020
Thiamwong, Ladda: 2385, 5075, 5100, 7080, 7150, 7430, 7620, 7680, 7820, 9000, 9010, 9020, 9030
Thielke, Stephen: 7300
Thierry, Amy: 2115
Thomas, Kali S.: 1210, 3320, 4030, 4290, 5215, 7160, 7210, 9030
Thomas, Karuna S.: 7550
Thomas, Kelsey: 8900
Thomas, Michael: 7980
Thomas, Michael J.: 9430
Thomas, Patricia A.: 4535
Thomas, Phyllis: 7380
Thomas, Shanice: 4110
Thomas Hebdon, Megan C.: 7980
Thomasson, Whitney: 7540
Thomeer, Mieke Beth: 4520
Thompson, Alisha R.: 3455, 7280, 9020
Thompson, Amber: 1150, 9010
Thompson, Atalie: 5230
Thompson, Carl Anthony: 7720
Thompson, Genevieve: 4470
Thompson, Hilaire: 7150, 7280, 7460, 7480
Thompson, Katherine: 7230, 9010
Thompson, LaDora V.: 3150
Thompson, Lena E.: 2120, 3015, 3445, 7470
Thompson, Louisa: 7100
Thompson, Mika D.: 9000
Thompson, Roy A.: 7140, 9000, 9020
Thompson, Sheila M.: 3050
Thompson, Tess: 1255
Thomson, Cynthia A.: 2260
Thomson, Katie: 7160
Thorpe, Anna M.: 3505
Thorpe, Carolyn T.: 7300
Thorpe, Lorna: 7230
Thorpe, Roland J.: 1345, 3240, 3450, 7560, 7970, 9000
Thunell, Johanna: 1175
Thurston, Rebecca C.: 5155, 5225
Thyagarajan, Bharat: 2325, 9020
Tian, Chong: 3490
Tian, Jiayin: 4155
Tian, Junyan: 9020
Tian, Qu: 2155, 2195, 3390, 3510, 5085, 9020, 9300
Tibbitts, Deanne C.: 7680
Tighe, Caitlan: 7580
Tillmann, Jacob J.: 7180
Tilsen, Rachel: 9000
Tinetti, Mary: 4010, 7840
Ting, Peng-sheng: 7390
Tingle, Sharna R.: 9030
Tinney, Emma: 7100
To, Louis: 9010
To, My Ngoc: 2090, 2330, 9010
Tobey, Leah R.: 7390
Tocchi, Christine: 9020
Toda, Eiji: 2105, 3220
Todd, Adam: 4455
Todd, Chris: 4455
Todd, Christopher: 4110
Todorova, Valentina K.: 1085
Toh, Wei Xing: 9000
Tolea, Magdalena I.: 2125, 3490
Toledo, Frederico G.: 3475, 5210
Toles, Mark: 4380
Toman, Julia: 4110, 7960
Tomarevska, Olena S.: 7820
Tomaz, Simone: 7060, 7190
Tomlin, Nichelle: 8900
Tomlinson, Sam: 9020
Tompkins, Catherine J.: 1335, 3310, 7750, 7760
Tong, Yao: 9000
Tongsiri, Sirinart: 2385, 7430
Tongu, Yoshiyasu: 7370, 9010
Tonkikh, Orly: 9030
Tonkin, Andrew: 7300
Tooth, Leigh: 1370
Topaz, Maxim: 9010
Topchishvili, Davit: 2315
Toprakkiran, Selin: 9020
Torigoe, Trevor: 2380
Torres, Anderson: 9000
Torres, Haydee: 8900
Torres, Sandra: 3165, 3530, 4285, 4330
Tosato, Matteo: 9020
Toshiko, Tanaka: 9020
Tosi, Dominique: 9030
Tosi, Marco: 4400
Toto, Pamela E.: 5000, 7800
Totten, Keliane: 4460
Townsend, Aloen: 7940
Towsley, Gail L.: 7700, 9020, 9030
Toyama, Itsuko K.: 3210
Toyokawa, Noriko: 7950
Trabut, Loïc: 7730
Tracy, Karen: 9000
Traeger, Lara: 9030
Trahan, Evette: 9000, 9010
Trahan, Julia: 9020
Tran, Diep Q.: 7210
Tran, Dieu-My T.: 4040, 4134, 7330
Tran, Karenna: 9010
Tran, Nathaniel M.: 1180, 4000
Tran, Tracy S.: 9000
Trautman, Lana: 1180
Traver, Anthony C.: 1205, 3410, 4305
Travers, Jasmine: 7120, 9020

Traywick, LaVona S.: 5190, 7680
Treat-Jaconson, Diane J.: 9410
Tredinnick, Ross: 9030
Trenary, Tanner T.: 7820
Triantos, Lisa: 7860
Trimble, Shawn: 2355
Trimboli, Tracy: 5030, 7120
Trinh, Mimi: 9580
Trinkaus, Eva-Maria: 1005
Triolo, Federico: 2280
Trivedi, Ranak: 3030, 4095, 5000, 9000, 9010, 9030
Troutman, Brooke: 2390, 3305
Troxel, Wendy M.: 3405, 9010
Trubits, Em F.: 7450, 7890, 7990
Trueblood, Amy: 7140
Trujillo, Miguel: 4195
Truskinovsky, Yulya: 1340
Tsai, Alexander C.: 9000
Tsai, Jenny Hsin-Chun: 4408, 7700
Tsai, Pao-Feng: 9010
Tsai, Tsung-Yu: 9010
Tsai, Yi-Hua: 7610
Tsang, Chung-Kin: 9030, 9510
Tsang, Sze Pui: 1110
Tsay, Pei-Kwei: 4155
Tsay, Ruey-Ming: 9010
Tschoe, Marianne: 7520
Tse, Dwight: 1245, 4180
Tseng, Heidi: 3060
Tseng, Huai-Hsuan: 9010
Tseng, Tung-Sung: 7390, 7410
Tsimane Health and Life History Project, The: 9030
Tsitsipatis, Dimitrios: 5060, 7880
Tsotsoros, Cindy E.: 1350, 1355, 4375
Tsotsoros, Jessica D.: 1350
Tsui, Emma: 4165
Tsui, Ronald: 9010
Tsukada, Noriko: 3310, 7790
Tsuker, Sophia: 1340, 9030
Tu, Wanzhu: 5015, 9000
Tu, Yufang: 7300
Tucker, Gretchen G.: 7540, 7940
Tucker, Julia E.: 7020
Tucker, Katherine: 9020
Tucker-Roghi, Gina C.: 9020
Tufa, Yeabsira: 3500, 7070
Tufă, Laura: 1220
Tuft, Samantha E.: 2060, 2220, 4215
Tulloh, Anna: 9000
Tumar, Alisha: 3270
Tunalilar, Ozcan: 3320, 7550
Tung, Yi-Hsuan: 1185
Tunney, Orlaith C.: 9030
Tuntland, Hanne K.: 7940
Tupler, Larry A.: 3470, 7620
Tupper, Emily: 4100
Turaani, Mohammad: 9020
Turiano, Nicholas A.: 3135, 9030
Turkelson, Angela E.: 5035, 7390, 9000
Turkheimer, Eric: 7370
Turner, Ani: 9020
Turner, Arlener D.: 7460
Turner, Jennifer R.: 4425
Turner, Natalie: 7230
Turner, R. Scott: 9550
Turner, Rachael: 7510
Turner, Sidney C.: 3220, 7210
Turner, Skyla: 5235, 7030, 9010
Turo, Patricia: 4035
Tur-Sinai, Aviad: 9030
Twardzik, Erica: 2150
Two, Wung Wai: 5225
Ty, Diane: 3055
Tyagi, Pranjal: 1005, 9010, 9030
Tyler, Denise A.: 2375
Tyshkovskiy, Alexander: 7370
Tysinger, Bryan C.: 4385
Tzemah-Shahar, Roy: 3125, 4265
Tzeng, Huey-Ming: 4395
Tziarli, Dimitra: 9000

U

Ubaida-Mohien, Ceereena: 2195
Uccheddu, Damiano: 4400, 4530
Uchida, Hayato: 7330
Udah, Grace: 8900
Uddeen, Aleeyah: 9010, 9020
Udeh, Kingsly C.: 9010
Udoh, Idorenyin I.: 7420
Ugwu, Ejike s: 5110
Uittenhove, Kim: 3130
Ukraintseva, Svetlana: 2015, 7110, 7370, 7620, 7800
Ullal, Akshith: 9010
Ulmer, Cameron: 9020
Ulrey, Page B.: 3375
Ulrich, Connie: 9020, 9030
Ulset, Vilde: 9420
Umar, Tooba: 1120
Umberson, Debra J.: 4370
Undem, Teri: 7300
Unroe, Kathleen: 4470, 5015, 9000
Unruh, Mark A.: 5215
Unsworth, Carolyn A.: 7690
Uphold, Constance: 2170
Upreti, Mona: 3270
Upshaw, Jenica N.: 3535
Ura, Chiaki: 7280
Urbaniak, Anna: 4450
Urbanski, Dana P.: 3460, 7380
Urbanski, Paul: 7450
Urbina, Ray: 2015
Ureña, Stephanie: 2050
Urick, Benjamin: 7150
Urlings, Judith: 7720
Urrunaga-Pastor, Diego: 9010
Ustach, Andrew: 7480
Utter, Kierstin: 7330
Utz, Rebecca: 1150, 2035, 4365, 9020

V

Vaca Angus, Carlos A.: 7510
Vacek, James L.: 7990
Vacek, Sarah: 7420
Vahedi, Vanessa: 1165
Valdes, Elise: 7290
Valdes-Hernandez, Pedro: 9010
Valdez, Gregorio: 2335
Valdez, Rupa: 2070
Vale, Michael T.: 4320
Valeika, Sarah: 2055
Valente, Kayla: 7800
Valente, Matthew: 7360
Valera, Juhi R.: 4425
Van Vleet, Samuel C.: 5065, 5190, 7080
van Achterberg, Theo: 1175, 4255
Van Bogart, Karina: 3545, 9010
van Boxtel, Martin PJ.: 1225
Van den Boomgaard, Iris: 7000
van den Bosch, Arne: 9000
van den Heuvel, Edwin: 5220, 7660
van den Hoed, Monique W.: 9010
Van der Heijden, Beatrice: 7290
van der Steen, Jenny T.: 4440, 4470
van Es, Cat: 3260
Van Fossen, Jenna A.: 9000
van Hout, Hein: 7420
Van Houtven, Courtney: 1115, 4165, 7620
Van Laethem, Lauren: 4350
van Nimwegen, Dagmar JJ.: 2280
Van Ogtrop, Katlynn: 7260
Van Orden, Kimberly A.: 1335
Van Scoy, Laruen J.: 5145
van Solinge, Hanna: 9030
Van Vleet, Bryce A.: 1235, 7710, 7810
van Zon, Sander KR.: 4130
Vance, David Eugene: 3545, 3565, 4145, 4340
Vance, Jarod C.: 9030
Vandekerckhove, Joachim: 5165
Vander Wyck, Brent: 1305
Vander Wyk, Brent: 2360, 7360
Vanderlaan, Courtney: 7640
VanderWerf, Jessica: 2095, 4406, 7000
VandeVrede, Lawren: 1380
VandeWeerd, Carla: 9000, 9010, 9020
Vang, Casper: 3450
VanHaitsma, Kimberly: 3205, 7160, 9020, 9300
Vanhoose, Kimberly: 7730
VanSciver, Angela: 7990
Varadaraj, Varshini: 1345, 2135
Varadhan, Ravi: 1370, 4485, 9010
Varlamova, Maria: 4450
Varricchio, Marcia: 7920
Varu, Ava: 9020
Varzino, Michael: 9540
Vasarhelyi, Krisztina: 9000
Vasquez, Elizabeth: 5195
Vasquez, Felix A.: 7540
Vassar, Matt: 7210, 9000
Vasterling, Jennifer J.: 9020
Vaughan-Sarrazin, Mary S.: 2180
Vaughn, Ivana A.: 2225
Veal, Britney: 7990
Vega, Irving E.: 3105
Velazquez, Janice: 4345
Velazquez, Nathan: 3570
Vellani, Shirin: 4440
Vellas, Bruno: 2320
Vélez Landazuri, Maria Augusta: 7290

Vélez Ortiz, Daniel: 9000
Venditti, Elizabeth M.: 9020
Venegas, Maria D.: 2085, 7850
Venkatraman, Tishya: 2255
Verçosa, Neide: 7920
Verbeek, Hilde: 1260, 2370, 3180, 7120, 7720, 7800
Vercauteren, Toon: 4330
Verderber, Stephen: 9550
Verdery, Ashton: 4500
Vereijken, Beatrix: 3550
Verghese, Joe: 3145, 3160, 3480, 9020
Verma, Ambika: 7100, 7370, 7880
Verma, Mansi: 7940
Vest, Amanda R.: 3535
Vetrano, Davide: 2280
Vetter, Michael: 4430
Victor, Christina R.: 4030, 4070, 9010, 9020, 9430
Victoria-Sanchez, Leslie: 8900
Videla, Emma: 4500
Viggars, Mark: 7370
Vignoli, Michela: 7290
Vikram, Kriti: 7960
Villalba, Madeline: 9000
Villatoro, Alice P.: 5195
Villemagne, Victor L.: 9500
Vincent, Aaron D.: 7590
Vincent, Heather K.: 4134, 7330
Vincenti, Virginia B.: 3075
Visaria, Abhijit: 1055, 7090
Vishnepolskiy, Igor: 7620
Vishwanath, Swarna: 7300
Visovsky, Constance: 3420
Visser-Meily, Johanna: 2280
Vitolins, Mara Z.: 5060
Vivoda, Jonathon: 5105, 9010
Vizuere, William: 2320
Vlaeyen, Ellen: 7120
Vlock, Elizabeth: 7510
Vluggen, Stan: 3340
Vo, Anh: 7590
Vo, Tina T.: 3080, 4185
Vogel, Carlyn E.: 5170
Vogel, Rosi: 2260
Vogelsmeier, Amy A.: 1310, 7140
Voils, Corrine I.: 5000
Volic, Milla: 9010
Volpe, Cris Renata Grou: 7130, 7340
von Bonsdorff, Mikaela B.: 9010
von Gunten, Armin: 3130
Voss, Andreas: 3195
Vranceanu, Ana-Maria: 2035, 3525, 4460, 7680
Vrees, Matthew: 9010
Vu, Nguyen C.: 3380
Vu, Thi: 2055, 4010
Vue, Hope N.: 8900
Vullings, Isabelle: 9010
Vyhnalek, Martin: 7420

W

Wachholz, Patrick Alexander: 3180
Wada, Mineko: 7780
Wadely, Virgina G.: 4340
Wadsworth, Sally J.: 3080, 4185
Waetjen, L Elaine: 5155
Wagenknecht, Lynne E.: 4355, 5060, 7630
Wagg, Adrian S.: 9550
Wagner, Amy: 7650
Wagner, Gert G.: 9010
Wagner, Jennifer: 9000, 9020
Wagner, Laura M.: 3285, 9020
Wagoner, Aaron: 9030
Wagstaff, Ruth: 9000
Wahl, Hans-Werner PD.: 4225, 4235
Wahlheim, Christopher N.: 9030
Waite, Linda J.: 1230, 4205
Wakefield, Dorothy: 1240, 4140
Walbaum, Magdalena: 4455
Walden, Allison: 4268, 7680
Walden, Justin: 9000
Waldestein, Shari R.: 4130
Waldman, Zoe: 7070
Waldron, Danielle A.: 4510
Walke, Lisa M.: 7860
Walker, Alan: 4450
Walker, Drew: 9400
Walker, Jenny: 9010
Walker, Kathryn A.: 4170
Walker, Kealie J.: 9020
Walker, Keenan: 2195, 2240, 3495, 4035, 7110, 7630, 9200
Walkner, Tammy: 9020
Wall, Jaclyn: 9020
Wallace, Andrea: 1155
Wallace, Cara L.: 3395
Wallace, Laura: 9010
Waller, Dylan E.: 2085, 7850
Wallhagen, Margaret I.: 2135, 2280
Wallimann, Miriam: 4480
Wallner, Lauren P.: 1085
Wallsworth, Christine: 3270
Walmsley, Sharon: 4340
Walsemann, Katrina M.: 1000, 2050, 5050
Walsh, Christine A.: 1205, 9010
Walston, Jeremy D.: 1370, 7110, 9000, 9010, 9020
Walters, Anna: 3535
Walton, Surrey: 4465
Wammes, Joost: 7120, 7420, 9010
Wan, Debby: 9010
Wan, Emily Y.: 5065
Wan, Qiaoqin: 7100, 7620, 7830
Wan, Shaowei: 1240
Wang, Anni: 9020
Wang, Binsheng: 9030
Wang, Claire: 7070
Wang, Cuiling: 5165
Wang, Dahua: 7070
Wang, Danny: 3545, 4145, 7090
Wang, Donna: 9030
Wang, Ellen: 7710
Wang, Ethan B.: 4266, 7690
Wang, Fang: 7080
Wang, Fei: 3005
Wang, Feng: 9010
Wang, Gavin Y.: 9020, 9200
Wang, Grace Hsin-Min: 3500
Wang, Hang: 3390
Wang, Haowei: 1290
Wang, Heng: 4540, 7090
Wang, Janet: 2050
Wang, Jing: 2090, 2265, 3180
Wang, Jing: 7330
Wang, Jing: 7660
Wang, Jing: 9000
Wang, Jing: 9010, 9020, 9300, 9320, 9550
Wang, Julian: 7160
Wang, Jun: 4535
Wang, Junqiao: 9020, 9300
Wang, Kaipeng: 2090, 2330, 9010
Wang, Katherine: 2090
Wang, Kesheng: 9200
Wang, Kun: 7080, 7590
Wang, Lichao: 9030
Wang, Melanie: 9020
Wang, Meng: 3355
Wang, Mengya: 1175, 7930
Wang, Molin: 1290
Wang, Nan: 2265, 9000
Wang, Pengcheng: 2280, 7330
Wang, Qi: 5100
Wang, Qin: 2265, 7330
Wang, Qingwei: 9010
Wang, Rosalie H.: 9330
Wang, Shanshan: 7150
Wang, Shaohua: 7100
Wang, Shiyu: 1215
Wang, Shuang: 2285
Wang, Shuangshuang: 3295, 9030
Wang, Sijiu: 4140
Wang, Silas: 9200
Wang, Siqi: 4540
Wang, Siyu: 9020
Wang, Tzu-Yun: 9010
Wang, Weijun: 7330, 9020
Wang, Wenjin: 9010
Wang, Xia: 9010
Wang, Xiaochuan: 9020
Wang, Xinhui: 2320
Wang, Xiujuan: 9520
Wang, Xueqing: 9440
Wang, Yan: 1030
Wang, Yan: 1110
Wang, Yan: 4210
Wang, Yi: 3210
Wang, Yi: 3450
Wang, Yi: 4050, 7710
Wang, Yi-Hsin: 9010
Wang, Yingying: 9000
Wang, Yixuan: 2330
Wang, Yueting: 5080
Wang, Zequan: 7460
Wang, Zhendong: 5235
Wangwun, Patcharee: 1080, 5200
Wanigatunga, Amal A.: 2150, 3390, 3510, 4265, 5085, 7650, 9580
Wanka, Anna: 2340
Wanninger, Ellen: 9020
Ward, Caitlin: 7860
Ward, R. Scott: 4406, 7000
Ward, Rachel: 9010
Ward, Shaelyn: 7210, 9000
Waring, Stephen: 3460
Warner, Lisa M.: 7610
Warner, Mildred: 7250
Warren, Allison: 4300
Warren, Lorrie: 7480
Warren, Teresa S.: 1260
Warthen, Terra: 7770
Wasenius, Niko S.: 9010
Washington, Faith-Christina I.: 7590
Washington, Karla T.: 1240, 1310, 4415, 9000
Washington, Tiffany R.: 2115, 4270, 4415
Wasserman, Michael: 2295
Wasserman, Michael: 9020
Waszczuk, Daniel: 4310
Watanabe, Kengo: 5180, 7110, 7880
Watanabe, Yuichi: 7780

Watanabe-Galloway, Shinobu: 3535
Waterhouse, Jodi: 2160
Waters, Leland: 3285, 3300, 4360, 9030
Waters, Mary: 4420
Watkins, Alexandra: 9010
Watman, Deborah: 3415, 4165, 7850
Watson, Wendy: 4390
Watts, Amber: 4134, 7330, 7830, 7990
Waugh, Christian E.: 1105
Waye, Mary MY.: 5225
Wazinki, Karla: 2340
Waziry, Reem: 4120, 7630
Weathers, Brandy: 9020
Weaver, Bruce: 3570
Weaver, Michael: 9030
Weaver, Natalie: 7020
Weaver, Raven H.: 1240, 1365, 3250
Webb, Fern: 7810, 9010
Webb, Jason A.: 7680
Webb, Nicole: 2260, 9010
Webber, Sandra: 7940
Webster, Britney A.: 7950
Webster, Noah J.: 1170, 1355, 2100
Webster-Dekker, Katelyn E.: 9440
Wec, Aleksandra: 2185, 3415
Wedderburn, Raymond: 7860
Weech-Maldonado, Robert: 2350, 3365, 4190
Weems, Jacy A.: 1210, 3320, 4190
Wehry, Susan: 9000
Wei, Andrea: 4525
Wei, Duo Helen: 4132, 7480
Wei, Jeanne: 1085, 4130, 7100, 7370, 7880, 9000, 9020
Wei, Jingkai: 1085, 3490
Wei, Kai: 7650
Wei, Linjiang: 9560
Wei, Wenxing: 7940
Wei, Ying: 1110
Wei, Yudan: 9000
Wei, Zhikui: 4490
Weibel, Robert: 3115
Weidenbacher, Hollis J.: 4165
Weil, Joyce: 4405
Weimar, Lisa: 7860, 9000
Weinberg, Gil: 1120
Weinberg, Samuel Jesse: 2225
Weiner, Anthony: 4460
Weinstein, Andrea M.: 3405
Weinstein, Elliott R.: 4340
Weintraub, Susan: 1325
Weiskittle, Rachel E.: 4445
Weiss, Carleara: 7830
Weiss, Charlotte R.: 9020, 9030
Weiss, David: 1245, 3100
Weiss, Erica F.: 1125
Weiss, Joan: 2297
Weiss, Ursula: 9010
Weissberger, Gali: 1140, 4275
Weisse, Carol S.: 3200
Weldrick, Rachel: 1205
Wellens, Nathalie IH.: 7160
Wellenstein, Kerry: 1290
Welling, Anna: 7980
Wells, Jenna L.: 1305
Wells, Jenna L.: 9320
Wells, Katie: 7630
Welsh, Kimberly: 4350
Welsh-Bohmer, Kathleen: 9030
Wen, Chiung-Jung: 4134, 7830, 7930
Wen, Jin: 3520, 3545, 4320
Wen, Ming: 9020
Wen, Wan: 5195, 7030, 7590
Wen, Xinyue: 2330
Wennberg, Alexandra: 5205
Wente, Karla: 5155
Werner, Nicole E.: 2070, 2170, 5000, 9020
Wessel, Leslie: 7390
West, Jessica S.: 2265, 9020
West Greenlee, M. Heather: 7550
Westbrook, Reyhan M.: 9000, 9020
Westendorf, Jennifer: 8900
Westerlund, Hugo: 1125, 3265, 7170
Westmore, Megan R.: 2390, 9000
Westra, Jordan: 9010
Westrick, Ashly C.: 1085, 2280, 7960
Wethington, Elaine: 7460
Wetle, Terrie: 7620
Wheeler, Jason: 9020
Wheeler, Katie: 7060
Wheeler, Victor: 9020
Wherry, Sarah J.: 7350
While, Christine: 4545
Whitaker, Janice: 4315, 5145
Whitbourne, Susan Krauss: 2250
White, C.J.: 1285
White, Elizabeth L.: 3320
White, Katie: 4255
White, Kina: 4266, 7230, 7830
White, Laura: 4406, 7480
White, Lon: 7100
White, Quinn: 5135
White, Rebecca: 4245
White, Thomas: 1325
White Whilby, Kellee: 9020
Whitfield, Keith: 7970, 9000
Whitlatch, Carol J.: 3535
Whitley, Deborah M.: 3060, 4400
Whitman, Cassidy: 7140
Whitmire, Meredith: 1290
Whitmore, Carly: 5220
Whitney, Sarah: 4150
Whitson, Heather: 1370, 2240, 4485, 9030
Whittaker, Anna C.: 7190
Whitten, Justin M.: 5075, 7910
Wi, Dahee: 7480
Wickersham, Karen E.: 2260
Wickson-Griffiths, Abigail: 4440, 4470
Widener, Michael: 9000
Wiegelmann, Henrik: 2370
Wielgosz, Joseph: 9000
Wiemers, Emily: 1340
Wierman, Heidi: 7150
Wiersma Strauss, Anna: 1340
Wiese, Lisa Ann Kirk: 2125
Wijkander, Maria: 9030
Wilber, Kathleen H.: 2220, 3365, 4215, 4375, 4435, 5120, 7720, 7750, 9020
Wilchesky, Machelle: 7080, 7790
Wilcox, D. Craig: 5060
Wild, Tess: 1090
Wilder, Jocelyn: 1230, 4205
Wilkie, Diana: 7380
Wilkinson, Joel: 9020
Wilkinson, Lindsay R.: 7550
Wilks, Scott E.: 7280, 9020
Will, Celia E.: 3345
Willard, Meredith A.: 9030
Willcox, Bradley: 2380, 5060, 7110
Willcox, D. Craig: 2380, 7110
Willett, Walter: 1290
Williams, Aelwyn: 9420
Williams, Alicia R.: 5040
Williams, Alisha: 3480, 9000
Williams, Cedric J.: 9020
Williams, Christine L.: 7620
Williams, Ginger: 9000
Williams, Isaiah: 1325
Williams, Ishan C.: 3305, 9020, 9030
Williams, Kristine: 2160, 3020, 3205, 3270, 3335, 3445, 7300, 9020
Williams, Lilcelia A.: 7360
Williams, Madison S.: 5215
Williams, Rachel: 7680
Williams, Rebecca: 7620
Williams, Rhiannon: 9030
Williams, Shellie: 9010
Williams, Valerie: 9000
Williams, Victoria: 1000
Williams-Farrelly, Monica M.: 5115, 9020
Williamson, Heather: 7400
Williamson, Jeff D.: 4134, 5230, 7830
Willis, Angelique B.: 9010, 9310
Willis, Deanna R.: 4210, 5105
Willis, Sherry L.: 3510, 4225
Willroth, Emily: 1255, 3230, 3245, 3520, 9020
Wilmanski, Tomasz: 5180, 7880
Wilmes, Paul: 1225
Wilmoth, Janet M.: 1160, 7970
Wilmoth, Kristin: 7760
Wilson, Fernando: 1155
Wilson, Haley: 3540
Wilson, Jason: 3560
Wilson, Jess: 7480
Wilson, Kamryn: 7070
Wilson, Lisa: 7160
Wilson, Megan E.: 4320, 9020
Wilson, Paige N.: 3205
Wilson, Sarah: 8900
Wilson, Stephanie J.: 4235, 4370, 7070
Wilson, Traci L.: 3045
Wilson- Menzfeld, Gemma: 9420
Windham, B. Gwen: 3510, 5085, 7340, 9200
Windham, William A.: 5085, 7340
Windsor, Liliane C.: 5115
Windsor, Tracy: 9000
Wing, Ian Sue: 2105
Wing, Rena R.: 4355, 7630
Wingood, Gina: 3565
Wingood, Mariana: 7340, 7990
Wininger, Michael: 7210
Winship, Jodi: 3025

Winslow, Lauren: 7660
Winslow, Victoria: 7230
Winstead, Vicki: 5140
Winters, Emily D.: 4408, 7700
Winters-Stone, Kerri M.: 7680
Winton, Rebecca: 9400, 9440
Winton, Tish: 4415
Wipke-Tevis, Deidre: 9000
Wiranto, Yumiko: 4134, 7830
Wirth, Maria: 3100, 3195
Wisch, Julie: 9030
Wister, Andrew: 2230
Withers, Mellissa: 4266, 7190
Wittenberg, Grace: 4190
Wittich, Walter: 7650
Wittink, Marsha N.: 1335
Wittke, Michael: 3030, 4295
Witzel, Dakota D.: 1160, 3545, 4425, 5080, 7510, 9010
Wöhrmann, Anne M.: 7290
Wojczynski, Mary K.: 4260, 5090, 9010
Wolf, Michael S.: 9030
Wolfe, Alex: 9030
Wolfe, Hannah: 1105, 3195
Wolfe, Megan: 1330
Wolfe, Robert R.: 4130, 7880
Wolfe, Warren: 1025, 7470
Wolff, Jennifer: 1035, 2185, 3415, 4170, 5130, 5160, 9000, 9020
Wolf-Ostermann, Karin: 2370
Wolfson, Julian: 9410
Won, Chang Won: 7630, 9020
Won, Sarah: 7400, 9020
Wong, Bob: 1150, 1250
Wong, Chek Hooi: 3470
Wong, Chelsea N.: 9010, 9430
Wong, Gloria Hoi-Yan: 5235, 7330, 7420, 7760, 7920, 9420
Wong, Ian C.K.: 7330
Wong, Jennifer L.: 7000
Wong, Jennifer A.: 7230
Wong, Joey: 2340, 3270, 9010, 9020
Wong, Karen Lok Yi: 9000, 9010
Wong, Kayla: 5235
Wong, Rebeca: 3160, 7320
Wong, Rita: 9010
Wong, Roger: 1010, 9020
Wong, Stella Lai-Kuen: 9420
Wongvibul, Thanchanok: 7090
Woo, Misuk: 7780
Woo, Seoyoon: 9020
Wood, Kristie A.: 7470
Wood, Leila: 9010
Wood, Mallory L.: 7090
Wood, Rachel E.: 3025
Wood, Shenell T.: 7840
Wood, Sinai: 3300
Woodard, John: 7510
Woodrell, Christopher D.: 7850
Woods, Ashley N.: 7140
Woods, Robyn L.: 7300, 7370
Woodsmall, Katelyn: 9030
Woodward, Amanda T.: 4155
Worthington, Amber K.: 9010, 9020
Wouters, Eveline JM.: 9210
Wouterse, Bram: 7120, 9010
Wretman, Christopher: 1210
Wrigglesworth, Jo: 7370
Wright, Alexi A.: 7680
Wright, Brittany: 7760
Wright, Cynthia F.: 9020
Wright, Drew: 7900
Wright, Kathy D.: 4255
Wright, Molly: 9030
Wright, Regina S.: 7410
Wright, Rhonda: 1345
Wright, Rollin: 4315
Wroblewski, Kristen: 1230
Wrzus, Cornelia: 7030
Wu, Bei: 1045, 1240, 1320, 2090, 2265, 2330, 3180, 3540, 4105, 4380, 5055, 5170, 7330, 9010, 9020, 9110, 9300, 9310, 9320
Wu, Chenkai: 4090, 7830
Wu, Deqing: 2015, 7110, 7370, 7800
Wu, Haorui: 4530
Wu, Hau-Tieng: 3390
Wu, Heng: 3370
Wu, Jianli: 7670
Wu, Jingchuan: 7610
Wu, Jingxuan: 5115, 7060, 7950
Wu, Kuan-Ching: 4505, 7300, 7910, 9010, 9020
Wu, Meng-Chen: 4134, 7830
Wu, Mingche MJ.: 2185, 5160
Wu, MJ Mingche: 2185, 5160
Wu, Qiao: 2325
Wu, Rachel: 7860
Wu, Rachel: 9030
Wu, Ruiqian: 7620
Wu, Shengyong: 7330
Wu, Shinyi: 1185, 3005, 9020
Wu, Xingxing: 7940
Wu, Yafei: 9110
Wu, Yanjuan: 2255
Wu, Yiyi: 3565, 7230
Wu, Yunfan: 9000
Wu, Yuqiong: 9000, 9020
Wu, Yuxia: 4240
Wu-Chung, Lydia: 9110
Wunderlich, Tiffany: 7440
Wyatt, Riley: 7510
Wyles, Christina L.: 4380
Wyman, Jean F.: 5100, 9030
Wyman, Mary F.: 3015, 5000
Wyndham-West, Michelle: 9000
Wynn, Matthew J.: 4210
Wyte-Lake, Tamar: 2295

**X**

Xi, Wanyu: 1245
Xi, Yitao: 9020
Xia, Qing: 9530
Xian, Hong: 7960
Xian, Ying: 2265
Xiang, Lucille: 9020
Xiang, Qiao: 7350
Xiang, Xiaoling: 5235, 7030, 9010
Xiao, Chunhong: 1075
Xiao, Danqing: 9200
Xiao, Mingzhao: 4535
Xie, Alice: 4460
Xie, Bo: 5235
Xie, Dawei: 7650
Xie, Rui: 2385, 5075, 5100, 7080, 7150, 7430, 7620, 7680, 7820, 9010, 9020
Xie, Zongxing: 7680
Xin, Meiqi: 4266, 7190
Xiong, Lile: 9020
Xiong, Mai: 3450
Xiong, Yuan: 9010, 9530
Xiong, Zhiyao: 9020
Xu, Bei-bei: 1125, 4310, 7930
Xu, Bill Tong: 9010
Xu, Bowen: 1110
Xu, Chun: 9200
Xu, Hanzhang: 2265, 3180, 9000, 9110
Xu, Huiwen: 1115, 9310
Xu, Hui-wen: 1125, 4310, 7930
Xu, Ling: 2390, 3305, 4240, 7760, 9030
Xu, Ming: 3575, 9030
Xu, Shu: 2135, 2250, 7830
Xu, Shuo: 2330
Xu, Tianwei: 9030
Xu, Ting: 2330
Xu, Ting: 9000
Xu, Wenqian: 9000
Xu, Ying: 7950
Xu, Yingzhi: 7070
Xue, Qianli: 1370, 4090, 4485, 5230
Xue, Tingzhong (Michelle): 9110
Xue, Wei- Lin: 4215, 7790

**Y**

Yadav, Hariom: 4475, 7100, 7870
Yaffe, Kristine: 2240
Yahr, Andrea: 2170
Yam, Hawking: 3020, 3460
Yamada, Yuji: 7150, 7860
Yamaguchi, Yuko: 9010
Yamakawa, Miyae: 9000
Yamamoto-Mitani, Noriko: 7160, 7720, 9520
Yamashita, Mariko: 7760
Yamashita, Takashi: 2375, 3310, 4345, 5080, 5110, 7040, 7380, 7600, 7950
Yamazaki, Sachiko: 7580
Yan, Changmin: 4268, 7680
Yan, Elsie: 1145, 7050, 9010
Yan, Mengzhao: 3365, 4375
Yan, Zi: 4132, 7500, 7780
Yancura, Loriena: 2005
Yang, Amy: 1290
Yang, Bing: 4535
Yang, Frances M.: 2160, 3335, 7300
Yang, Huiyu: 9010
Yang, Hwajin: 9000
Yang, Jie: 7450
Yang, Jinzhu: 9580
Yang, Jisun: 7850
Yang, KwangSoo: 5225
Yang, Mayla: 7380
Yang, Mia: 4355
Yang, Minhee: 7420
Yang, Peilin: 4205
Yang, Peisen: 9010, 9030
Yang, Qi: 2195
Yang, Rumei: 1260, 7120
Yang, Ruoqi: 1215
Yang, Wei: 1045
Yang, Xiufen: 9020, 9520, 9540
Yang, Yang Claire: 1320
Yang, Yang: 4240

Yang, Yulin: 4115, 4250, 7540
Yang, Zhongfang: 7140, 7330
Yang, Zhongfang: 9010
Yang, Zhou: 1125, 4310, 7930
Yankeelov, Pamela: 4040
Yao, Jingxin: 9440
Yao, Shanshan: 5090, 7630, 9300, 9330
Yao, Yao: 9020
Yaphe, Sean R.: 2225
Yasaka, Taisuke: 9520
Yasar, Sevil: 9400
Yashin, Anatoliy: 2015, 5015, 5110, 7110, 7370, 7620, 7800
Yashkin, Arseniy P.: 2015, 2180, 3510, 5015, 5110, 7100, 7370, 7620
Yau, Jessie Ho-Yin: 7760, 9420
Yauk, Jessica: 3345, 9030
Ye, Fan: 5030, 7120, 7680
Ye, Justin: 3230
Ye, Minzhi: 2005, 2260
Ye, Nina Ni: 4185
Yeatts, Dale E.: 5185
Yee, Claire I.: 1305, 9320
Yee, Joanne: 9020
Yefimova, Maria: 5140
Yeh, Jarmin: 9020
Yelland, Erin: 7590
Yen, Dorothy: 9010
Yeo, Hyesu: 7220, 7290, 9000
Yeoman, Carl J.: 4080
Yeshua-Katz, Daphna: 2345
Yetman, Linda: 9010
Yeung, Dannii Y.: 3280, 7610, 9010
Yeung, Ellen: 5005
Yi, Stella: 7230
Yildiz, Mustafa: 5140
Yin, Hongyu: 9030
Yin, Lijuan: 4465
Yin, Na: 3080
Yin, Yueheng: 7150
Ylitalo, Kelly: 7570
Yokota, Fumihiko: 7940
Yolken, Robert: 7870
Yonashiro-Cho, Jeanine: 2220
Yoneda, Tomiko: 1255, 3520
Yong, Roseline: 7580
Yong, Ting: 1055
Yoo, Hyun Joo: 1215
Yoo, Jee-Hye: 7400
Yoo, Ji: 4075, 5185
Yoo, Subin: 7400
Yoo-Jeong, Moka: 9000
Yook, Soonhyun: 4265
Yoon, Caroline: 9530
Yoon, Dokyung: 7540
Yoon, Hee Jeong: 9540
Yoon, Jong Sung: 7960
Yoon, Seolah: 9000
Yoon, Sukyung: 7760
Yoon, Yeojin: 9000, 9030
Yorgason, Jeremy B.: 4235, 4370, 7410
Yoshida, Hiroko: 4505, 9000
Yoshikawa, Aya: 7430
You, Suha: 7960
Youn, Joy: 1380
Younan, Diana: 2320
Young, Amy: 9030
Young, Bethany: 8900
Young, Heather M.: 9030
Young, Jessica: 1160
Young, Kashyah S.: 9010
Young, Lufei: 9010, 9500
Young, Vanessa: 4100
Young, Yuchi: 3365, 7260
Young, Yvette: 2050
Yount, Naomi: 2375
Yous, Marie-Lee: 7840
Yousefi Nooraie, Reza: 2145
Yousefzadeh, Matt: 4475
Yow, W. Quin: 4185
Yu, Doris S.F. SF.: 1140, 5225, 7300, 7520
Yu, Fang: 9030, 9200
Yu, Guo: 7530
Yu, Heshuo: 7900
Yu, Junwen: 7140, 7330, 9010
Yu, Kexin: 1155, 4100, 4160
Yu, Miao: 2320
Yu, Wenshan: 9030
Yu, Wing-Lam: 4520
Yu, Xinfang: 4535
Yu, Xuexin: 2280
Yuan, Dianqi: 9030
Yuan, Rong: 9020
Yuan, Yiyang: 3105
Yue, Courtney Sen Yee: 7060
Yue, Jirong: 7350, 7660, 7830
Yue, Yiwei: 4265, 4490, 7870
Yun, Hyunkyung: 2035, 5215
Yun, Seon Young: 7810
Yun, Stacy W.: 7090
Yung, Raymond: 9030
Yunus, Raudah Mohd: 4266, 7190

### Z

Zachmeyer, Morgan L.: 2160
Zadok, Erez: 5030, 7120
Zafar, Anum: 1240, 4105, 7800, 9000
Zagorski, William A.: 1095
Zahedikia, Negin: 2280
Zahir, Zohaib: 9010
Zahodne, Laura B.: 1170, 1355, 2100, 3440, 4325
Zakak, Nicole: 7410
Zakrajsek, Jennifer S.: 9030
Zakrzewski, Chris: 3300
Zalyesov, Eduard: 7720
Zambrano, Priscilla L.: 9020
Zambrano Garza, Elizabeth: 3275, 7550
Zamora, Alejandra: 5080
Zampino, Marta: 9020
Zamrini, Edward: 3490
Zanaj, Skerdi: 1225
Zanasi, Francesca: 9030
Zang, Emma: 1305, 9440
Zanier, Nicole: 9030
Zaninotto, Paola: 3265, 4015
Zanjani, Faika: 9000
Zanoni, Joseph P.: 4465
Zaragoza, Gabriela: 7180
Zarling, Amie: 3445
Zaslavsky, Oleg: 4505, 7300, 9020
Zauszniewski, Jaclene A.: 2005, 5120, 7030, 9020
Zavala, Daisy: 1255, 4045
Zavala, Patrick: 7830
Zebrak, Katarzyna: 2375
Zeiner, Tracey: 5190, 7680
Zeisel, John: 9400
Zeki Al Hazzouri, Adina: 9110, 9500
Zelinski, Elizabeth: 7380
Zell, Angela: 4155
Zeller, Andreas: 3115
Zemon, Kevin A.: 7080
Zeng, Derong: 9010
Zenobia-Moore, Ann: 2195
Zepel, Lindsay: 7150
Zequeira, Sabrina: 1030
Zernial, Carol: 2160
Zettel-Watson, Laura: 7070
Zeziulin, Oleksandr: 2055
Zhan, Rong-Fang: 9000
Zhan, Yan: 9030
Zhang, Bohan: 5060, 7370
Zhang, Chen: 2330
Zhang, Cuntai: 9000
Zhang, Jiaan: 7560
Zhang, Liangwen: 9560
Zhang, Lingling: 2225, 7960
Zhang, Meina: 7990
Zhang, Meng: 2385
Zhang, Ming: 7930
Zhang, Mutian: 7380
Zhang, Nan: 7030, 7950
Zhang, Ning Jackie: 4240
Zhang, Peiyuan: 1035, 2330, 5005, 5015, 5025, 7260
Zhang, Qing: 9000
Zhang, Qiuyang: 2260
Zhang, Ruoying: 7380
Zhang, Shenghao: 1040, 3025, 7590, 9030
Zhang, Shifang: 7100, 7620
Zhang, Shiyang: 1255, 7020, 7070
Zhang, Talan L.: 1035, 2150, 4170
Zhang, Wei: 9010
Zhang, Weiwei: 9540
Zhang, Wen: 7920
Zhang, Wencheng: 4400, 7950
Zhang, Wenhan: 7620
Zhang, Wuyang: 2150, 9010
Zhang, Xiaomin: 7370, 7880
Zhang, Xin: 1245
Zhang, Xinyu: 3555, 9000
Zhang, Xinyu: 9030
Zhang, Xiping: 7370
Zhang, Xuehan: 7010, 9010, 9030
Zhang, Xuezhixing: 9440
Zhang, Yalu: 3220
Zhang, Yan: 3105
Zhang, Yao: 2225
Zhang, Yiming: 7870
Zhang, Ying: 7620
Zhang, Yingyang: 2280
Zhang, Yuan: 3225, 3440, 7380
Zhang, Yuankai: 9030
Zhang, Yucong: 9000
Zhang, Yue: 9000
Zhang, Yun: 2230, 9320
Zhang, Zhenmei: 4230, 7530, 9010
Zhao, Dandan: 7960
Zhao, Erfei: 1125
Zhao, Heng: 5115, 7060, 7950
Zhao, Hui: 2260, 3485, 9010
Zhao, Liang: 7150
Zhao, Lingya: 4240

Zhao, Qianhua: 9020
Zhao, Shangang: 4405
Zhao, Wei: 2320, 7630
Zhao, Yinan: 7640
Zhao, Yong: 9010
Zhao, Yuanchang: 7030, 7410
Zhao, Yuxiang: 1245
Zheng, Anqing: 3080, 4185
Zheng, Chuxuan: 5235, 7030
Zheng, D. Diane: 9010
Zheng, Rong: 9020
Zheng, Yaguang: 1320, 2265, 3540
Zhong, Chuwen: 3080, 5010
Zhong, Lily: 9410
Zhong, Selena: 1230
Zhou, Chuankai (Kai): 3355
Zhou, Huiquan: 3510
Zhou, Jiajia: 1130, 2225
Zhou, Jiayi: 2285
Zhou, Jiayi: 3510
Zhou, Junhong: 1260, 2155, 3510
Zhou, Leyi: 7280
Zhou, Nan: 4305, 7030
Zhou, Shuai: 2225, 2230, 7250
Zhou, Yuanjin: 1280, 2385, 7430
Zhou, Yunshu: 2135
Zhou, Zexi: 5045, 7070, 7920
Zhu, Alex Yue Feng: 7930
Zhu, Change: 9010
Zhu, Enya: 7020
Zhu, Haidong: 9010, 9500
Zhu, Jiafeng: 4485
Zhu, Jiangyuan: 9020
Zhu, Jianmin: 9000
Zhu, Jiayi: 9010
Zhu, Jingmin: 4015
Zhu, Shijun: 4005, 9020
Zhu, Xiang: 4475
Zhu, Xiaonan: 1015
Zhu, Yating: 4195, 7590
Zhu, Yujun: 3365
Zhu, Yun: 4475, 9020
Zhu, Yuyang: 7600, 9020
Zhu, Zheng: 2090, 2265, 3540, 7140, 7330, 9010, 9020
Zieschang, Tania: 7830
Ziglor, Candace: 4300
Zimami, Sumiyyah: 9000
Zimmer, Zachary: 2050
Zimmerman, Kristin: 9030
Zimmerman, Lindsey: 2145
Zimmerman, Sheryl: 1210, 3205, 3320, 4135, 7420, 9020, 9300
Zimmermann, Jaroslava: 4530
Zingmark, Magnus: 2340
Zipunnikov, Vadim: 2150
Zisberg, Anna: 7050
Ziyalan, Elvan: 9100
Ziylan, Canan: 7000
Zmuda, Joseph M.: 5090, 7830, 9010, 9020
Zoaktafi, Mojgan: 7960
Zolnoori, Maryam: 9010
Zomer, Helena D.: 1380, 7370
Zonderman, Alan B.: 4130, 7560, 9000
Zotcheva, Ekaterina: 9020
Zou, Ning: 5235
Zubatsky, Max: 4160, 9000, 9030
Zucchelli, Alberto: 2280
Zucchero, Renee' A.: 7460
Zuluaga, Maria Isabel: 9570
Zuniga, Franziska: 3180, 7160
Zuo, Xi: 7650
Zvonkovic, Anisa: 3060
Zwakhalen, Sandra: 3340, 7120, 7940
Zweibaum, David: 5085
Zweig, Richard: 5080
Zwerling, Jessica: 2265, 9000
